# sraX: A Novel Comprehensive Resistome Analysis Tool

**DOI:** 10.3389/fmicb.2020.00052

**Published:** 2020-02-05

**Authors:** Leonardo G. Panunzi

**Affiliations:** ^1^Institut Pasteur, Biodiversity and Epidemiology of Bacterial Pathogens, Paris, France; ^2^Institut Français de Bioinformatique, CNRS UMS 3601, Evry, France

**Keywords:** antimicrobial resistance, antibiotic resistance gene, resistome profiling, stand-alone software, sequence analysis, gene context visualization

## Abstract

The accurate identification of the assortment of antibiotic resistance genes within a collection of genomes enables the discernment of intricate antimicrobial resistance (AMR) patterns while depicting the diversity of resistome profiles of the analyzed samples. The availability of large amount of sequence data, owing to the advancement of novel sequencing technologies, have conceded exciting possibilities for developing suitable AMR exploration tools. However, the level of complexity of bioinformatic analyses has raised as well, since the achievement of desired results involves executing several challenging steps. Here, sraX is proposed as a fully automated analytical pipeline for performing a precise resistome analysis. Our nominated tool is capable of scrutinizing hundreds of bacterial genomes in-parallel for detecting and annotating putative resistant determinants. Particularly, sraX presents unique features: genomic context analysis, validation of known mutations conferring resistance, illustration of drug classes and type of mutated loci proportions and integration of results into a single hyperlinked navigable HTML-formatted file. Furthermore, sraX also exhibits relevant operational features since the complete analysis is accomplished by executing a single-command step. The capacity and efficacy of sraX was demonstrated by re-analyzing 197 strains belonging to *Enterococcus* spp., from which we confirmed 99.15% of all detection events that were reported in the original study. sraX can be downloaded from https://github.com/lgpdevtools/srax.

## 1. Introduction

Antimicrobial resistance (AMR) constitutes a serious menace to global public health, since its rise is being detected in samples from a wide variety of environmental sources (Munk et al., 2018). In addition, a growing number of imputable deaths per year is evidenced and is calculated to surpass the 10 million by 2050 (O'Neill, 2016). Under these circumstances, the accompanying development of novel sequencing technologies—along with continually decreasing costs—have raised the amount of available sequence data, and consequently, have led to devise viable AMR exploration tools. Particularly, whole-genome sequencing (WGS) and whole-metagenome sequencing (WMS) approaches have demonstrated enormous capabilities of epidemiological surveillance, outbreak detection, and infection control of bacterial pathogens (Didelot et al., 2012). In relation to the type of demanded sequence data, two main methodological approaches have materialized: those capable of processing raw reads—read-based methods—and those requiring contig-assembled genome sequences—assembly-based methods. Ultimately, both procedures exhaustively examine for AMR determinants by aligning the input sequence data to curated antibiotic resistance genes (ARG) from custom or public dedicated reference AMR databases (DB). Essentially, read-based methods are faster and less computationally demanding. However, false positives originated from spurious mapping might arise. Moreover, since the genomic context is generally missed, the arrangement of adjacent genes can not be evinced and it constitutes a major drawback (Boolchandani et al., 2019). Quite contrary, assembly-based methods are computationally expensive and time consuming because of the *de novo* assembly step. Nonetheless, when sequencing at adequate genome coverage, known or novel ARGs bearing low sequence similarity with AMR DBs are normally detected, and remarkably, genomic context, and regulatory sequence elements are captured (Boolchandani et al., 2019).

In consequence, applying any of previously indicated techniques, several bioinformatic tools tailored to annotate ARGs have been produced (see Table 1). For instance, considering read-based methods, SRST2 (Inouye et al., 2014) and KmerResistance (Clausen et al., 2016) are not confined to any specific microbial species or AMR type, but both approaches completely neglect resistance conferred by single–nucleotide polymorphisms (SNPs). By contrast, Mykrobe predictor (Bradley et al., 2015) is suitable for detecting sequence variants but, regrettably, is restricted to *Staphylococcus aureus* and *Mycobacterium tuberculosis* and to 12 types of antibiotics. On the contrary, ARIBA (Hunt et al., 2017) can identify not merely ARGs corresponding to AMR from any type but SNPs linked to resistance as well. However, some visualization files (like gene/SNP presence) must be obtained via the Phandango server (Hadfield et al., 2017). Additionally, before mapping the reads, ARIBA performs a clustering procedure for finding a single sequence representative from each ARG locus on the employed reference AMR DBs. Albeit reducing ambiguous alignments, this strategy unaccount for substantial sequence variation existing within gene families (Munk et al., 2017). To circumvent this lack of accuracy, a novel method called GROOT (Rowe and Winn, 2018) has been proposed and it relies on building variation graphs of previously clustered ARGs from AMR DBs, before mapping the reads to them. Despite succeeding at characterizing variation through sequence graphs, GROOT is mainly limited to properly perform the annotation of ARGs on metagenome samples, without any graphical output or further analysis. Other read-based tools include: SEAR (Rowe et al., 2015)—already archived, SSTAR (de Man and Limbago, 2016), PATRIC (Antonopoulos et al., 2017)—not standalone mode, and DeepARG (Arango-Argoty et al., 2018). Regarding assembly-based tools, certain of them are capable of elucidating SNPs developing AMR but, to our knowledge, none of them is suited for providing a genomic context analysis of identified ARGs. In addition, the output information and its visualization is generally limited (see Table 1). Current implementations entirely relying on already assembled sequences include: ResFinder (Zankari et al., 2012), ARG-ANNOT (Gupta et al., 2014), RAST (Davis et al., 2016), RGI (Jia et al., 2016), PointFinder (Zankari et al., 2017), ARGs-OAP (Yin et al., 2018), and NCBI-AMRFinder (Feldgarden et al., 2019).

**Table 1:** Features of different resistome analysis pipelines, in comparison to sraX.

**Bioinformatic**	**Standalone**	**SNP**	**Gene context**	**Batch**	**Single-step**	**Output**	**References**
**tool**	**mode**	**analysis**	**analysis**	**mode**	**command**	**results**	
**CONTIG-ASSEMBLED SEQUENCE DATA**							
ResFinder	Yes	Yes	No	Yes	Yes	Tables	Zankari et al., 2012
ARG-ANNOT	No	Yes	No	No	No	Tables	Gupta et al., 2014
RAST	Yes	No	No	No	Yes	Tables	Davis et al., 2016
RGI	Yes	Yes	No	No	No	Tables/Plots	Jia et al., 2016
PointFinder	No	Yes	No	Yes	Yes	Tables	Zankari et al., 2017
ARGs-OAP	Yes	No	No	Yes	No	Tables/Plots	Yin et al., 2018
NCBI-AMRFinder	Yes	Yes	No	Yes	Yes	Tables	Feldgarden et al., 2019
sraX	**Yes**	**Yes**	**Yes**	**Yes**	**Yes**	**Tables/Plots**	Present study
**RAW-READS SEQUENCE DATA**							
SRST2	Yes	Yes	No	No	Yes	Tables	Inouye et al., 2014
Mykrobe predictor	Yes	Yes	No	No	Yes	Tables	Bradley et al., 2015
SSTAR	Yes	No	No	No	No	Tables	de Man and Limbago, 2016
SEAR	Yes	No	No	No	Yes	Tables/Plots	Rowe et al., 2015
KmerResistance	No	No	No	No	Yes	Tables	Clausen et al., 2016
PATRIC	No	No	Yes	Yes	No	Tables/Plots	Antonopoulos et al., 2017
ARIBA	Yes	Yes	No	No	No	Tables/Plots	Hunt et al., 2017
GROOT	Yes	No	No	No	No	Tables	Rowe and Winn, 2018
DeepARG	Yes	No	No	No	No	Tables/Plots	Arango-Argoty et al., 2018

*Bold characters emphasize the functional features of the proposed tool*.

**Figure 1:**
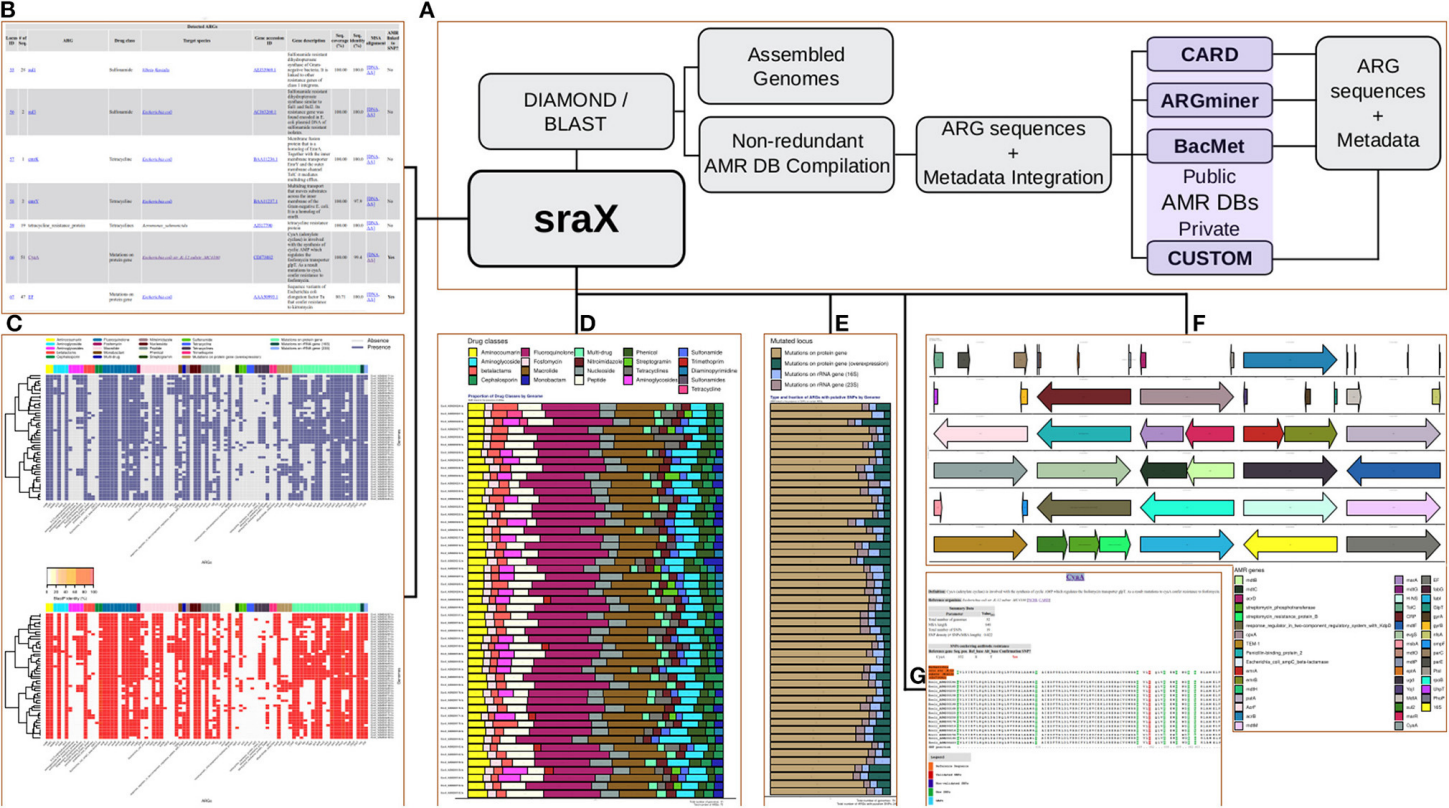
**(A)** sraX workflow. **(B)** ARG repertoire. **(C)** Heat-maps of gene presence and sequence identity. **(D)** Proportion of drug classes. **(E)** Type of mutated loci. **(F)** Spatial distribution of detected ARGs per genome (gene context analysis). **(G)** SNP validation and detection of putative new variants.

In this context, and attempting to address the mentioned limitations, a suit of federated modular functions was developed and integrated into a user-friendly tool named sraX. It has been devised as a fully automated pipeline for performing a systematic resistome profiling analysis through a series of operational steps, which are conveniently concatenated for achieving a greater computational efficiency (see Figure 1 for a schematic diagram). sraX follows this analytical workflow by executing a montage of custom Perl and R scripts that opportunely call external open source software and make use of reference AMR DBs. The main capabilities of sraX and a detailed comparison with other pipelines is shown in Table 1. Apart from the usual strategy of retrieving AMR data from public—or privately owned—repositories, compiling a local DB and detecting resistance determinants in analyzed samples, sraX has unique and noteworthy built-in features, like: gene context exploration, SNP analysis, complete graphical output—including drug classes and type of mutated loci—and integration of results into a fully navigable HTML report file. In addition, sraX is proposed as a single-command tool—envisaging that inexperienced users without any technical or bioinformatic knowledge would run it—and it has been devised for running on desktop computer systems, under limited RAM and processing resources. Wherefore, sraX operates as a standalone tool and a simple and straightforward deployment is achieved from source code—available at https://github.com/lgpdevtools/srax. In addition, easier instances have been produced in the form of a bioconda package—https://anaconda.org/lgpdevtools/srax—and a docker image—https://hub.docker.com/r/lgpdevtools/srax. The complete procedures for properly installing and using sraX are described in the User Manual, Supplementary Information.

## 2. Materials and Methods

### 2.1. Third-Party Data and Software Requirements

#### 2.1.1. Reference Databases

sraX depend upon a locally compiled AMR DB, which is obtained by gathering sequence data and extensive metadata from external reference AMR DBs. When launching the default resistome analysis, CARD v3.0.7 (Jia et al., 2016) constitutes the primary data source of genetic determinants. The reasoning behind our choice is that CARD not only provides regularly updated and curated sequence data, but additional complementary information that is specifically organized into ontology entries. Ultimately, sraX benefits from this level of organization for the straightforward access and retrieval of collected AMR data. Nevertheless, in order to conduct a more extensive and thorough ARG homology search, the ARGminer v1.1.1 (Argoty et al., 2019) and BacMet v2.0 (Pal et al., 2014) DBs—or even custom-provided ARGs—are allowed to be eventually incorporated into the sraX analysis. A noteworthy feature of ARGminer (Argoty et al., 2019) is that it aggregates AMR data from several dedicated repositories, including: ResFinder (Zankari et al., 2012), ARG-ANNOT (Gupta et al., 2014), CARD (Jia et al., 2016), MEGARes (Lakin et al., 2016), ARDB (Liu and Pop, 2008), SARG (Yang et al., 2016), NDARO (https://www.ncbi.nlm.nih.gov/pathogens/antimicrobial-resistance/), and DeepARG (Arango-Argoty et al., 2018). In consequence, a larger collection of resistance determinants is acquired by combining curated AMR data from CARD, ARGminer and BacMet that ensures a massive search through a wider space.

#### 2.1.2. Software Dependencies

Perl v5.26.x and the following complementary Perl libraries are required for having operative sraX modules: LWP::Simple, Data::Dumper, JSON, File::Slurp, FindBin, and Cwd. For aligning the ARGs to the analyzed genomes, sraX makes use of DIAMOND dblastx v0.9.29 (Buchfink et al., 2015) and NCBI blastx/blastn v2.10.0 (Altschul et al., 1990). In addition, prior to validating known polymorphic positions conferring AMR, multiple-sequence alignment (MSA) files are created using MUSCLE v3.8.31 (Edgar, 2004a,b), MAFFT v7.450 (Katoh et al., 2002), or CLUSTAL Ω v1.2.4 (Sievers et al., 2011). The figures generated during sraX analysis are achieved using R v.3.6.1 and the additional following packages: ggplot2 (Wickham, 2016), dplyr (Wickham et al., 2019), and gridExtra (Auguie, 2017). Importantly, these same software versions are currently employed for producing the docker image file.

### 2.2. Systematic Resistome Analysis

sraX accomplishes a series of challenging tasks for completing the resistome profiling analysis (see Figure 1).

The main assignments are fully described as follows:

#### 2.2.1. Creation of the Required Arrangement of Directories

Output results and temporary files are allocated within a defined configuration of specific folders and sub-folders, which is systematically produced at the beginning of sraX analysis (see Figure S1).

#### 2.2.2. Acquisition of Data Sources for AMR DB Compilation

The core collection of reference ARG sequences and their associated metadata is automatically retrieved only from CARD (Jia et al., 2016) when sraX is executed under default parameters. Nevertheless, a more exhaustive search can be performed by selecting the proper option, for including the ARGminer (Argoty et al., 2019) and BacMet (Pal et al., 2014) DBs into the analysis. Moreover, alternative user-provided and curated ARG (nucleotide or protein) sequences in FASTA format can be added (see the User Manual, Supplementary Information). In order to create the corresponding hyperlinks in the final report file, the header should include the following arrayed metadata: gene name, NCBI Accession ID, gene description, type of evinced AMR (protein homolog, protein variant, protein over-expression or rRNA gene variant), drug class, and species name. The added metadata is certainly not mandatory for sraX to incorporate the user-provided ARG sequences, though it allows the pertinent interrelation of acquired results. Ultimately, after appending the user-provided ARGs some redundancy might arise, thus identical sequences are excluded before compiling the final AMR DB.

#### 2.2.3. Detection of ARGs

A directory containing the assembled genome files in FASTA format is the only requirement for accomplishing the sraX analysis. Prokaryote and eukaryote WGS data at any assembly level (e.g., complete genome, chromosome, scaffolds, or contigs) can be utilized. The homologous genomic regions are detected by aligning the genome assemblies to previously compiled AMR DB. DIAMOND dblastx (Buchfink et al., 2015) is applied by default for making the procedure faster. However, for a higher detection accuracy, NCBI blastx (Altschul et al., 1990) can be chosen as well. Importantly, NCBI blastn (Altschul et al., 1990) is regularly used for aligning rRNA gene sequences and subsequently identifying the variants conferring resistance.

#### 2.2.4. Analytical Processing

Genome files are examined in-parallel for optimizing speed processing—up to 100 files are simultaneously queried, while a series of concerted actions are performed simultaneously for accomplishing the systematic resistome analysis. These actions constitute the core of the AMR scanning procedure and includes:

1. Depletion of redundant BLAST hits and compilation of the final ARG inventory. Concurrent matching ARGs—constituting variants of large gene families—are eliminated according to their sequence identity and coverage. Only those hits—or gene variants—revealing the highest values of both parameters are kept.
2. Detection of putative paralog copies of identified ARGs. The selected gene variants—from the previously compiled list of ARGs—which are detected in non-overlapping genome regions, are presumed paralog copies and cataloged on a separate file for further analyses.
3. Production of heatmaps ellucidating the gene presence and its sequence identity with respect to the reference. The devoted modularized function employs binary data (0 = absence, 1 = presence) and sequence similarity (0–100%) values for generating the corresponding heatmaps.
4. Calculation of drug classes and type of mutated locus proportions, for subsequent production of interpretive stacked barplots.
5. Extensive gene context exploration and production of resulting elucidative arrowplots, on every single genome (see Figure S3).
6. Clustering of homologous genomic sequences and their consecutive alignment by running MUSCLE (Edgar, 2004a,b), MAFFT (Katoh et al., 2002), or Clustal Ω (Sievers et al., 2011) on each ARG.
7. Detection of sequence variants on previously obtained MSA files, for the subsequent validation of known SNPs conferring AMR.
8. Creation of the readily navigable HTML-formatted final report, by integrating the corresponding text and plot files.

### 2.3. Genome Collection: Phenotyping and Genotyping Assays

The genome sequence files from 100 *Enterococcus faecium* and 97 *Enterococcus faecalis* strains that were previously obtained (Tyson et al., 2018a,b), and whose AMR profiles were further characterized employing a panel of 9 antibiotic drugs (Tyson et al., 2018b), were downloaded from the NCBI repository (Accession: PRJNA292665 and PRJNA292669) in July 2019, and alternatively, have been made available from a dedicated Zenodo repository (Panunzi, 2019). The main biological and genomic features, including their genome, assembly and BioSample Accession IDs, sequence coverage, N50 values as well as isolation sources and antibiotic drug susceptibility characterization can be found in Table S1.

The authors (Tyson et al., 2018b) appraised the antimicrobial susceptibilities of the selected strains by comparing the minimum inhibitory concentration (MIC) values, which were determined using an automated broth microdilution method (Trek Diagnostics, Independence, OH) with the US National Antimicrobial Resistance Monitoring System (NARMS) CMV3AGPF plates, to the breakpoints defined in the 2015 Clinical and Laboratory Standards Institute (CLSI) guidelines (CLSI, 2015). However, since the analyzed dataset is mainly composed of bacterial isolates derived from animals, the zone diameter interpretations should be made using veterinary standards. Regrettably, this relevant information was not provided by Tyson et al. (2018b). Therefore, in our present study we specify that these reported susceptibilities were employed only for validation purposes. Afterwards, the contrast between the measured and the MIC breakpoint values allowed the determination of the “resistant” and “susceptible” phenotypes. With reference to the ARG determination at genomic level, Tyson et al. (2018b) employed the ResFinder (Zankari et al., 2012) and the NCBI Pathogen Detection (https://www.ncbi.nlm.nih.gov/pathogens/) DBs. Thereafter, Tyson et al. (2018b) added two further categories—“resistant*” and “susceptible*”—when differences between genotype (presence or absence of corresponding ARG) and phenotype (resistant or susceptible) occurred. The “resistant*” label indicated a resistant phenotype but the absence of a verified ARG, while the “susceptible*” label indicated the opposite: a susceptible phenotype but the presence of a verified ARG. Nevertheless, in our study we have replaced this latter category for “silenced resistance.”

### 2.4. Genome Annotation, Pan-Genome Estimation, and Phylogeny Assessment

The automated annotation of genomes from both datasets (Accession: PRJNA292665 and PRJNA292669) was performed using PROKKA v1.11 (Seemann, 2014), while the core-genome and pan-genome were estimated using CD-HIT (Fu et al., 2012) by clustering predicted genes when ortholog loci shared ≥ 95% of amino acid sequence identity and ≥ 95% of alignment coverage. A total of 424 genes existing in all strains were estimated to compose the core set. Afterwards, those 424 genes were aligned using MUSCLE (Edgar, 2004a,b) and concatenated into a final MSA file comprising 327,376 bps of DNA sequence. A phylogenetic tree was built from this core-genome MSA after filtering recombination regions using GUBBINS (Croucher et al., 2014) under default parameters. Visualization of the tree and AMR phenotypes was conducted using the Interactive Tree Of Life (iTOL) v4 online tool (Letunic and Bork, 2019).

## 3. Results

### 3.1. Phylogeny and Distribution of AMR Phenotypes

A total of 100 *E. faecium* and 97 *E. faecalis* isolates constituted our validation set. This collection of genomes had been sequenced and phenotyped–phenotyping is described in *Methods*, while genome samples accession codes and phenotyping data are included in Table S1—in previous studies (Tyson et al., 2018a,b). However, the authors did not include a phylogeny of the analyzed samples.

**Figure 2:**
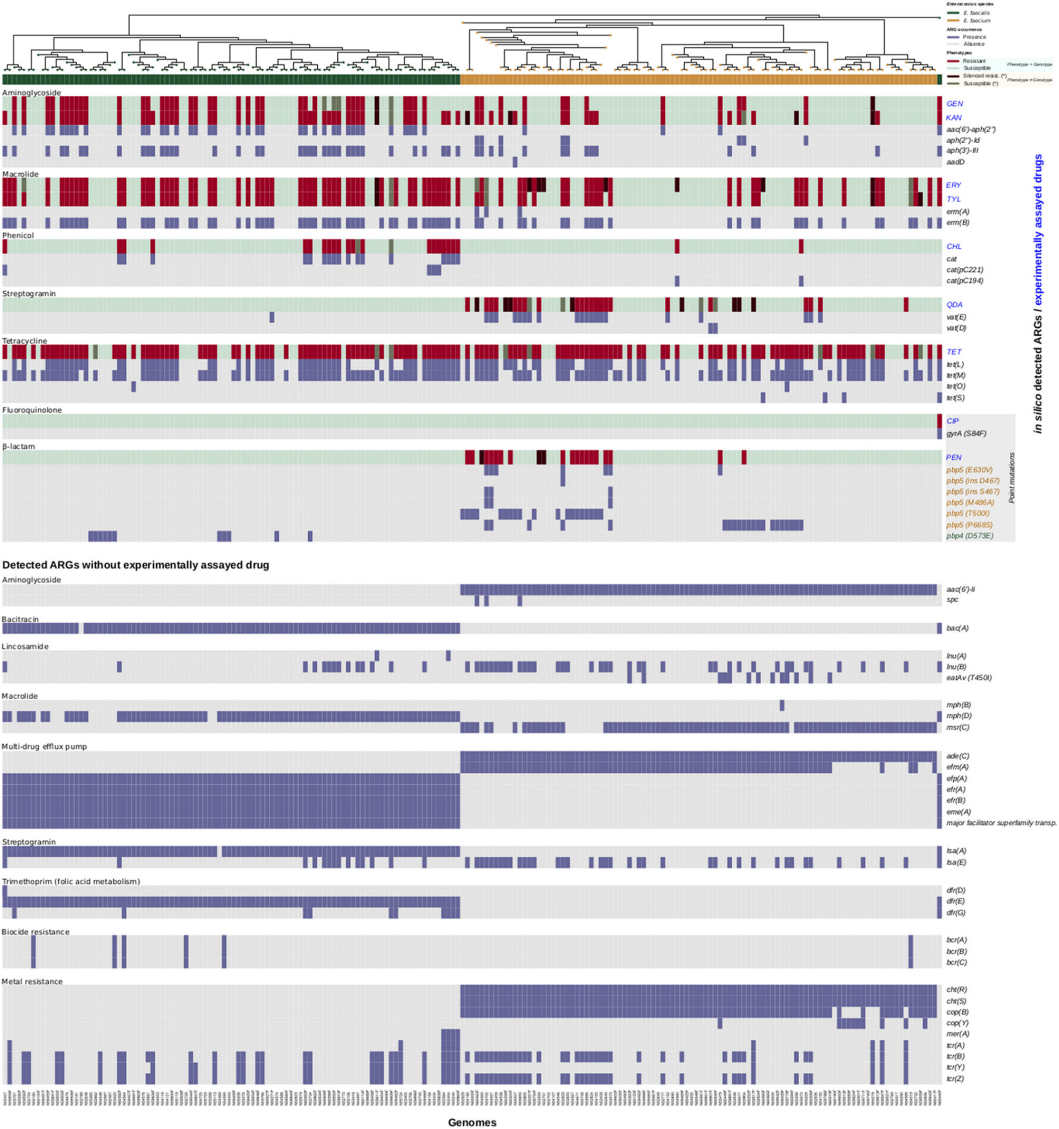
AMR activities of the collection of 197 *Enterococcus* spp. strains. A phylogenetic tree is shown on top of the heatmap. Red and light-green colors indicate resistance and susceptibility to corresponding antibiotics. Dark-red color indicates an exhibited resistance phenotype in the absence of an *in silico* detected ARG, while a dark-green color indicates an exhibited susceptible phenotype in the presence of an *in silico* detected ARG.

In our present study, the annotation of the 197 genome assemblies produced a sum of 531,915 putative protein-coding sequences, that were subsequently grouped into 13,702 clusters of orthologous genes (COGs). The pan-genome calculation evinced that 424 of these COGs were present in all the samples and constituted the core-genome. The successive alignment and catenation of core-genome COGs procured a MSA composed of 327,376 nucleotides that was later employed in the phylogeny reconstruction (illustrated on the uppermost of Figure 2).

The phylogeny showed a large evolutionary distance between both species—faecium and faecalis—that produced, as awaited, a complete break inside the tree. Briefly, in terms of AMR distribution, the original study (Tyson et al., 2018b) found that 46 isolates were pan-susceptible, with 90 isolates resistant to drugs in at least three antimicrobial classes (Table S1). We further compared the number of resistant isolates between both species according to the antimicrobial classes, and found important differences in: aminoglycosides (*E. faecalis*: 50, *E. faecium*: 38), β-lactams (*E. faecalis*: 6, *E. faecium*: 23), fluoroquinolones (*E. faecalis*: 1, *E. faecium*: 0), lipopeptides (*E. faecalis*: 2, *E. faecium*: 12), macrolides (*E. faecalis*: 55, *E. faecium*: 34), nitrofuran (E. faecalis: 1, *E. faecium*: 41), phenicol (*E. faecalis*: 20, *E. faecium*: 2), and streptogramin (*E. faecalis*: 0, *E. faecium*: 30). On the contrary, tetracyclines (*E. faecalis*: 69, *E. faecium*: 64), glycopeptides (all susceptibles) and oxazolidinones (all susceptibles) indicated somewhat similar AMR profiles.

### 3.2. Efficiency of sraX in Resolving AMR Determinants: Concordance With Phenotypes

The automated *in silico* resistome analysis using sraX was performed for establishing the one-to-one correspondence between the phenotyping and genotyping data (see Figure 2).

As previously mentioned, all isolates were vancomycin and linezolid susceptibles, and consistent with this phenotype, neither related ARG nor 23S rRNA mutations were discovered. Regarding ciprofloxacin, only one *E. faecalis* isolate evinced the resistant phenotype. The sraX's SNP analysis detected the *gyrA S84F* mutation, in agreement with previous studies accounting for its association with the resistant phenotype (El Amin et al., 1999) (see the corresponding MSA file inside the “SNPs_conferring_AMR” folder, Supplementary Information).

Considering chloramphenicol resistance, phenotyping data revealed a remarkable species restraint that made *E. faecalis* almost exclusive. The chloramphenicol acetyltransferase genes *cat, cat(pC221)*, and *cat(pC194)* were responsible for the exhibited resistance, although 2 isolates had the genes but were phenotyped as susceptibles (see Table 2).

**Table 2:** Genotype–phenotype concordance of sraX analysis for selected *E. faecalis* and *E. faecium* strains.

		**Phenotype: resistant (R)**		**Phenotype: susceptible (S)**		**Concordance** **)(%)**
**Drug**	**Species**	**Genotype: R**	**Genotype: S**	**Genotype: R**	**Genotype: S**	
GEN	*E. faecium*	12	1	0	87	99
	*E. faecalis*	37	1	4	55	95
KAN	*E. faecium*	18	4	1	77	95
	*E. faecalis*	47	1	1	48	98
ERY	*E. faecium*	25	7	3	65	90
	*E. faecalis*	53	1	2	41	97
TYL	*E. faecium*	26	2	2	70	96
	*E. faecalis*	54	1	1	41	98
CHL	*E. faecium*	2	0	0	98	100
	*E. faecalis*	20	0	2	75	98
TET	*E. faecium*	64	0	5	31	95
	*E. faecalis*	68	0	3	25	97
QDA	*E. faecium*	22	8	4	66	88
CIP	*E. faecalis*	1	0	0	96	100
VAN	*E. faecium*	0	0	0	100	100
	*E. faecalis*	0	0	0	97	100
LZD	*E. faecium*	0	0	0	100	100
	*E. faecalis*	0	0	0	97	100
Total		449	26	28	1269	**97**

*GEN, gentamicin; KAN, kanamycin; ERY, erythromycin; TYL, tylosin; CHL, chloramphenicol; TET, tetracycline; QDA, quinupristin-dalfopristin; CIP, ciprofloxacin; VAN, vancomycin; LZD, linezolid*.

*Bold character indicates the verified global average concordance rate*.

In respect to erythromycin and tylosin resistance, it was revealed that both phenotypes consistently co-occurred: 80 tylosin-resistant isolates out of 86 erythromycin-resistant isolates were the same samples. Among these isolates, 81 harbored the *erm(B)* gene, while 3 of them the *erm(A)* gene. In addition, the *msr(C)* gene was also present in macrolide-resistant isolates.

Regarding streptomycin resistance, many strains were phenotyped as susceptible but harbored the *aadE* and *str* genes. In the original study the authors mentioned to have employed the same resistance cutoff for both species, a decision that probably was the source of the experimental error (Tyson et al., 2018b). For this reason, we had not further considered the streptomycin phenotyping data in our present study.

Concerning kanamycin and gentamicin resistance, the *aac(6')-aph(2”), aph(2”)-Id, aph(3')-III*, and *aadD* genes were responsible for the resulting phenotype in 96.75% of resistant isolates. Remarkably, the co-occurrence of the *aac(6')-aph(2”), aph(3')-III*, and *erm(B)* genes was revealed, indicating their putative transmission on a mobile element.

In view of tetracycline resistance, the *tet(L), tet(M), tet(S)*, and *tet(O)* genes were detected in 96% of 133 resistant strains. On the contrary, eight susceptible isolates held a copy of *tet(L)* or *tet(M)* genes. In respect to streptogramin resistance, *E. faecalis* is considered constitutionally resistant, while the *vat(D)* and/or *vat(E)* genes were found in *E. faecium* in 22 out of 30 resistant strains and in 4 out of 70 susceptible isolates, what produced a 88% of concordance.

Considering penicillin resistance, previous studies have shown that mutations on the *pbp4* (Duez et al., 2001) and *pbp5* (Zorzi et al., 1996; Rice et al., 2004) genes accounted for the observed resistant phenotypes in *E. faecalis* and *E. faecium*, respectively. In accordance with these results, our SNP analysis (see the corresponding MSA file, Supplementary Information) verified the occurrence of the *T500I* and *E630V* mutations in 17 out of 21 resistant *E. faecium* strains. Additionally, the co-occurrence of the serine or aspartate codon insertion at position 467 was detected in 4 resistant isolates harboring the *E630V* mutation. The event of concurrent mutations was detected likewise in the *M486A* and *P668S* mutations, which were found in 3 and 6 resistant isolates harboring the *E630V* mutation, respectively. However, the *T500I* and *P668S* mutations were also found in several susceptible isolates and 2 resistant *E. faecium* strains neither evidenced any recognized mutations on the *pbp5* gene nor revealed alternative genetic determinants, like β-lactamases enzymes. In view of recognized *pbp4* mutations, our SNP analysis has validated the *D573E* amino-acid change that has been recently reported (Conceição et al., 2014; Infante et al., 2016) in 11 *E. faecalis* strains (see the corresponding MSA file, Supplementary Information). Furthermore, we detected an alternative mutation (*I50T*) in the same sequence position where a validated mutation (*I50Y*) was formerly reported (Ono et al., 2005). Nevertheless, none of the *E. faecalis* strains were phenotyped as penicillin-resistant in the original study (Tyson et al., 2018b). For this reason, and because of the apparent inconsistencies in the *pbp5* phenotypes, we did not include the penicillin in the concordance estimation study.

Regarding daptomycin resistance, some mutations occurring on the *lia(F), lia(S), lia(R), YybT, gsh(F), gdp(D)*, and *cls* genes were previously reported (Arias et al., 2011). However, a similar situation was found: our SNP analysis was capable of confirming the E192G mutation on the *lia(S)* gene in 5 *E. faecium* strains (see the corresponding MSA file, Supplementary Information), but none of these samples were phenotyped as resistant in the original study (Tyson et al., 2018b). Still, the putative existence of not yet recognized alternative mutations or unknown mechanisms is a possibility, since several other strains were certainly phenotyped as resistant.

Finally, tigecycline and nitrofurantoin susceptibilities were assayed as well. With regard to tigecycline resistance, only 8 resistant strains were detected and all of them possessed the *tet(L)* and *tet(M)* genes. A previous study linked the upregulation of these tetracycline resistance genes with the only known resistance mechanism (Fiedler et al., 2015). However, regarding nitrofurantoin resistance, the discovery of genomic AMR determinants is impracticable since any clear mechanism has been established so far.

In addition, the *in silico* analysis allowed to identify further ARGs which confer resistance to trimethoprim, lincomycin, spectinomycin, biocides and metals, and some other antimicrobials. However, none of them are currently employed for treating *Enterococcus spp*. infections. Probably, these ARGs are involved in conferring resistance to susceptible organisms via their horizontal transference. Notably, trimethoprim resistance genes *dfr(D), dfr(E)*, and *dfr(G)* were only detected in *E. faecalis* strains, as well as the *efr(A)* and *efr(B)* genes conferring resistance to multiple drugs and the macrolide resistance gene *mph(D)*. Additionally, the bacitracin resistance gene *bac(A)* and the streptogramin resistance gene *lsa(A)*, along with the *efp(A), eme(A)* and *major facilitator superfamily transporter* genes conferring resistance to multiple drugs, were only found in *E. faecalis* strains likewise. Conversely, the macrolide resistance genes *mph(B)* and *msr(C)* were only found in *E. faecium* strains, as well as the *aac(6')-li* and *spc* genes conferring resistance to aminoglycosides. In addition, the *ade(C)* and *efm(A)* genes conferring resistance to multiple drugs were also detected only in *E. faecium* strains. In contrast, the *lsa(E)* and *lnu(B)* genes conferring resistance to streptogramin and lincosamide, respectively, were observed in both *E. faecalis* and *E. faecium* strains. Lastly, the *bcr(A), bcr(B)*, and *bcr(C)* biocide genes, which are involved in benzalkonium chloride—a quaternary ammonium compound—resistance (Dutta et al., 2013), were detected in both *E. faecalis* and *E. faecium* strains as well as the *tcr(A), tcr(B), tcr(Y)*, and *tcr(Z)* copper resistance genes. Additionally, the *cht(R), cht(S), cop(B)*, and *cop(Y)* copper resistance genes were only detected in *E. faecium* strains, while the *mer(A)* mercury resistance gene was only found in *E. faecalis* strains, respectively. For a thorough and comparative analysis of *in silico* detected ARGs (see Figure 2, Figure S2, and Table S1).

### 3.3. Confirmation Rate of Detected AMR Determinants

Our previous results have proven a close correspondence between the *in silico* AMR genotype predictions and the experimentally assessed phenotypes. These findings were in accordance with the original study (Tyson et al., 2018b). Following the fidelity assessment of sraX, we next calculated the proportion of matching ARGs in corresponding genomes (see Table 3) and contrasted with the original values (Tyson et al., 2018b). A total of 25 ARGs conferring resistance to nine antimicrobial classes were distinguished across the samples. Among identified ARGs, 18 of them had a direct connection with phenotyping assays, while the remaining were related to drugs not usually employed for treating *Enterococcus spp*. infections. Comparing to original findings (Tyson et al., 2018b), we globally verified 425 out of 436 detection events that were reported. Our results implied almost an exact correspondence with the original study (validation rate: 99.15%), suggesting a considerable accuracy of our proposed tool.

**Table 3:** List of detected ARGs in isolates.

**Gene**	**Drug class**	**Drug on panel**	**Number of *E*.**	**Number of *E*.**	**Validation (%)**
			***faecium* isolates**	***faecalis* isolates**	
*aac(6)-aph(2)*	Aminoglycoside	GEN, KAN	3 (4)	39 (41)	93.4
*aph(2)-Id*	Aminoglycoside	GEN, KAN	8 (8)	0 (0)	100
*aph(3)-III*	Aminoglycoside	KAN	13 (13)	38 (42)	92.7
*aadD*	Aminoglycoside	KAN	1 (1)	0 (0)	100
*erm(A)*	Macrolide	ERY TYL	3 (3)	0 (0)	100
*erm(B)*	Macrolide	ERY TYL	27 (27)	54 (55)	98.8
*cat*	Phenicol	CHL	0 (0)	17 (18)	94.5
*cat(pC221)*	Phenicol	CHL	0 (0)	4 (4)	100
*cat(pC194)*	Phenicol	CHL	2 (2)	0 (0)	100
*vat(E)*	Streptogramin	QDA	20 (20)	1 (1)	100
*vat(D)*	Streptogramin	QDA	2 (2)	0 (0)	100
*lsa(A)*	Streptogramin	QDA	0 (0)	96 (97)	99
*tet(L)*	Tetracycline	TET	50 (50)	53 (53)	100
*tet(M)*	Tetracycline	TET	59 (59)	69 (70)	99.2
*tet(O)*	Tetracycline	TET	1 (1)	1 (1)	100
*tet(S)*	Tetracycline	TET	3 (3)	1 (1)	100
*gyrA (S84F)*	Fluoroquinolone	CIP	0 (0)	1 (1)	100
*pbp5 (E630V)*	β-lactam	PEN	3 (3)	0 (0)	100
*pbp5 (ins D467)*	β-lactam	PEN	1 (1)	0 (0)	100
*pbp5 (ins S467)*	β-lactam	PEN	3 (3)	0 (0)	100
*spc*	Aminoglycoside	None	3 (3)	0 (0)	100
*lnu(A)*	Lincosamide	None	0 (0)	2 (2)	100
*lnu(B)*	Lincosamide	None	38 (38)	15 (15)	100
*mph(B)*	Macrolide	None	1 (1)	0 (0)	100
*msr(C)*	Macrolide	None	85 (85)	0 (0)	100
*dfrD*	Trimethoprim	None	0 (0)	1 (1)	100
*dfrG*	Trimethoprim	None	0 (0)	7 (7)	100
Total			325 (327)	400 (409)	**99.15**

*The numbers represent their prevalence within the analyzed dataset, that was established by sraX. In parentheses are shown the original values reported by Tyson et al. (2018b). For computing the confirmation rate, only those ARGs and SNPs which were coincidental in both studies were considered*.

*Bold character indicates the corroborated global average validation rate*.

## 4. Discussion

Several studies have proven the significant advantages of WGS approaches over traditional methods for the molecular disease characterization and further epidemiological surveillance of bacterial pathogens. Nevertheless, major technical difficulties are encountered not only with implementing WGS in clinical and reference laboratories, but when analysing the obtained data as well. The amount and complexity of data impose the requirement of specific bioinformatics skills. However, these tough challenges are largely overcome in the laboratories by employing automated and user-friendly bioinformatics pipelines. In addition, the application of standardized analytical methods allows the reproducibility of results that might be subsequently shared among laboratories.

Aiming at fulfilling these technical needs, in the current study we present sraX as a novel bioinformatic tool for detecting, characterizing, plotting and producing a final comprehensive report about the presence of AMR determinants on assembled genomes. It constitutes a comprehensive, automated, and efficient standalone tool for performing diverse complex analyses and producing graphical results embedded in a HTML-based summary. The simplicity of its computational operation allow users to easily download, install, configure and finally complete several difficult assignments, by orchestrating diverse algorithms through a primary single-step command. It can properly be deployed from source code, by running an assistance bash script, or even simpler, as a docker image. Regarding its functionalities, despite apparent similarities with previously developed tools, sraX offers several unique advantages over them as it supplies non-redundant ARG annotations, SNP analysis through multiple-sequence alignment (MSA) files and graphic representation of discovered ARGs illustrating their existence and corresponding sequence identity percentage on each genome, their subsequent genomic context analysis and the global proportions of drug classes and type of mutated locus.

**Figure 3:**
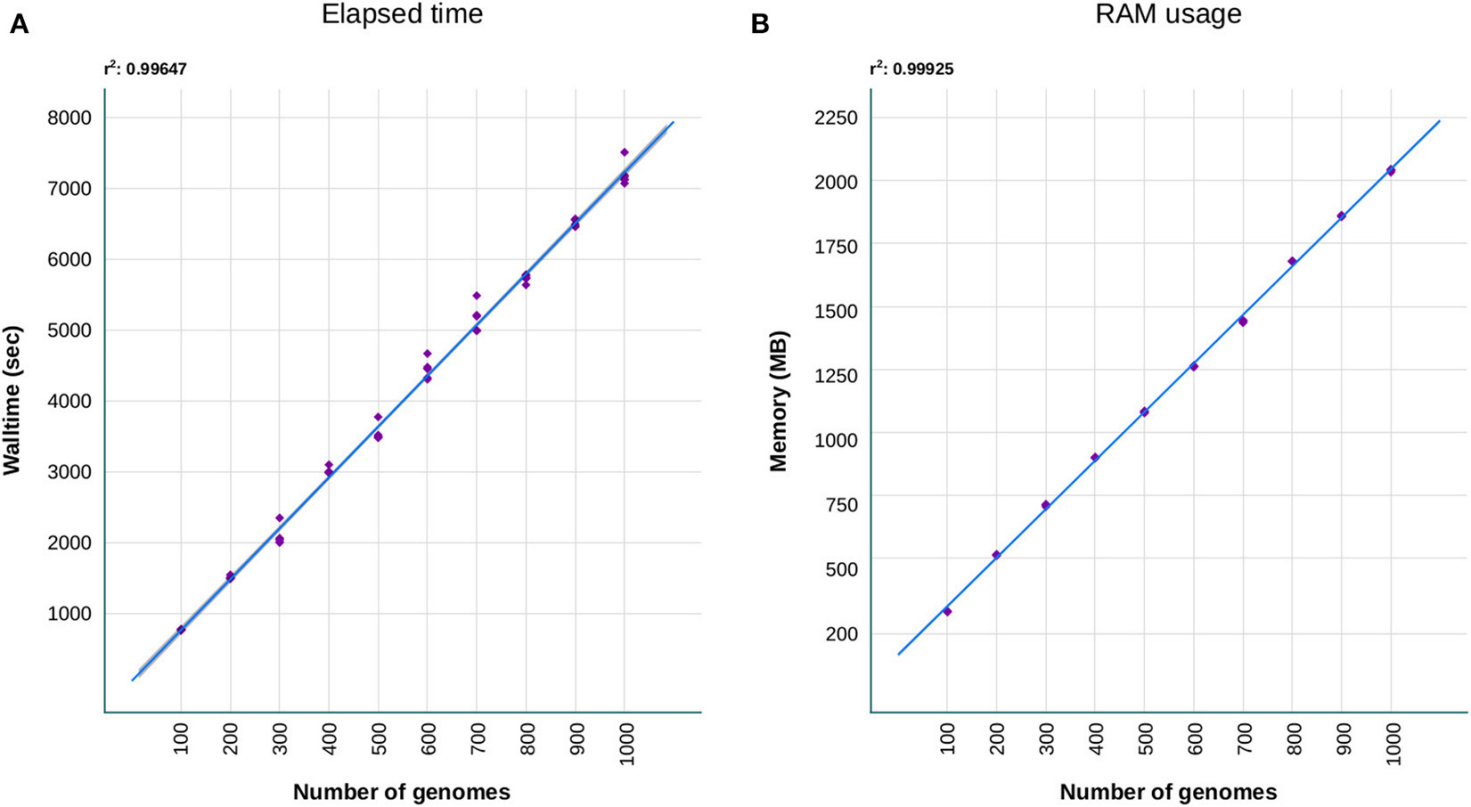
Benchmark comparisons of **(A)** walltime and **(B)** RAM consuming that were required by sraX to complete the resistome analysis. A collection of 5,000 Klebsiella pneumoniae genomes (average size: 5.5 Mbp) were randomly sampled five times at increasing 100-genome intervals and run on a desktop computer (4 cores, 32GB RAM). The processing of additional genomes evidenced a linear scaling of sraX performance, based on running time and RAM usage.

Concerning the performance of our tool, we have evaluated the walltime and memory required to achieve the resistome analysis. Both metrics were obtained after processing with sraX increasing amount of data, that was acquired by randomly sampling intervals of 100 up to 1,000 genome sequence files, in a five replicates bootstrapped operation (see Figure 3). These sequence files were selected from a set composed of 5,000 *Klebsiella pneumoniae* distinct genomes. We have chosen this organism since its genome size (≈5.5 Mbp) is typical from a Gram-negative bacteria. Our results indicated a linear scale of running time (≈12.5 min) and memory consumption (≈250 MB) with the processing of every 100-genome interval (Figures 3A,B, respectively). Defaults parameters were employed to ensure consistency during the testing. Each test was independently performed on a desktop computer Intel^Ⓡ^ Xeon^Ⓡ^ W-2104 processor (4-core, 3.20 GHz) with 32 GB RAM. The fact of employing a simple desktop computer and completing the resistome analysis quite rapidly, envisages a greater performance when running sraX under more powerful computational resources.

Afterwards, in order to assess the functionality and verify the accuracy of our proposed tool, we selected a genome dataset belonging to *Enterococcus* spp. for which AMR has been previously analyzed and genome sequences were available (Tyson et al., 2018b). When examining the genotype-phenotype correspondence for each assayed antimicrobial drug, sraX achieved an overall 97% of success and completely matched original results (Tyson et al., 2018b). Regarding the overall accuracies, our implementation was able to confirm a global 99.15% of all detection events—involving AMR determinants and genomes—reported in the original study (Tyson et al., 2018b). In addition, sraX confirmed the existence of distinct AMR profiles according to the involved species. For example, phenotyping and genotyping data evinced that phenicol resistance was almost exclusively determined in *E. faecalis* strains, while streptogramin resistance was only detected in *E. faecium* strains. However, in both species, tetracycline resistance genes were extremely frequent. Regarding not assayed drugs, the *in silico* AMR profiling analysis showed that trimethoprim resistance genes *dfrD* and *dfrE* were detected only in *E. faecium*, while macrolide and streptogramin resistance genes *mph(B)* and *msr(C)* were only found in *E. faecalis*. Globally, except for a few discrepancies, the results were entirely compatible with published findings (Tyson et al., 2018b). On that account, sraX has demonstrated a proven capability for discovering AMR determinants and has supported the utility of WGS applications in clinical microbiology. In addition, sraX was able to validate known SNPs conferring resistance.

Considering the drawbacks, sraX is wholly dependent on assembled genomic contigs that must be provided. Since the *de novo* assembly is a time consuming and computationally expensive procedure which, in addition, its accuracy is overwhelmingly influenced by the nature of the genome, we have finally decided to omit this step and give priority to AMR detection facets. In general, the tools that exploit read mapping-based methods or generate *de novo* or reference-based assemblies are targeted for metagenomic samples. The main problem with these samples is their high microbial diversity and unbalanced abundance that clearly limit the AMR profiling analysis.

## 5. Conclusions

sraX facilitates the resistome analysis of all-levels assembled genomes through a series of automated procedures. In the end, the obtained results are easily visualized as fully navigable HTML-formatted files containing summarized data and embedded plots. Challenging analysis, such as the SNP validation or gene context examination, can be performed in a one-step systematic and user-friendly manner. Additional tough assignments on ARG multiple-hits depletion and complex graphical representations are effectively completed as well. Lastly, sraX is capable of analyzing a variety of curated data-sets of properly formatted ARG sequences.

## Data Availability Statement

The datasets generated for this study can be found in a purposeful Zenodo repository, available at: http://doi.org/10.5281/zenodo.3571224.

## Author Contributions

LP conceived and designed the tool, created the software, performed all the necessary testing, and wrote the manuscript.

### Conflict of Interest

The author declares that the research was conducted in the absence of any commercial or financial relationships that could be construed as a potential conflict of interest.

## Abbreviations

AMR: antimicrobial resistance
ARG: antibiotic resistance gene
DB: data base.
