# Corrigendum: sraX: A Novel Comprehensive Resistome Analysis Tool

**DOI:** 10.3389/fmicb.2020.594635

**Published:** 2020-09-22

**Authors:** Leonardo G. Panunzi

**Affiliations:** ^1^Institut Pasteur, Biodiversity and Epidemiology of Bacterial Pathogens, Paris, France; ^2^Institut Français de Bioinformatique, CNRS UMS 3601, Evry, France

**Keywords:** antimicrobial resistance, antibiotic resistance gene, resistome profiling, stand-alone software, sequence analysis, gene context visualization

In the original article, there was an error in the affiliation of the corresponding author. Since a substantial part of the work was not carried out at this university, instead of “**ARTbio Bioinformatics Analysis Facility, Sorbonne Université, CNRS FR3631, Institut de Biologie Paris Seine, Paris, France**,” the affiliation should be “**Institut Pasteur, Biodiversity and Epidemiology of Bacterial Pathogens, Paris, France**.” Additionally, the author should also have the following affiliation: “**Institut Français de Bioinformatique, CNRS UMS 3601, Evry, France**.” Furthermore, the **Acknowledgments** section should be amended to recognize supportive institutions. The correct **Acknowledgments** appears below.

