# Supplementary material for: sraX: A Novel Comprehensive Resistome Analysis Tool
Source: Front Microbiol. 2020 Feb 5;11:52. doi: 10.3389/fmicb.2020.00052 (PMC7025521; doi:10.3389/fmicb.2020.00052)
Supplement: Supplementary file 2 [file Data_Sheet_1.pdf]

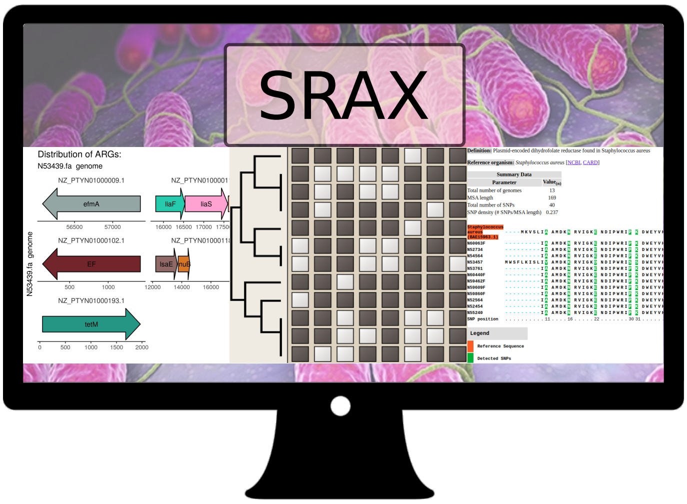

LGPDEVTOOLS

User Manual & Tutorial

---

sraX

Systematic Resistome Analysis Tool

Leonardo G. Panunzi

version: 24th January 2020

The **sraX** pipeline and its documentation are provided for an unlimited free usage. However, whenever **sraX** is employed in your analysis, the acknowledgement should be given by referring its source. Please, cite:

Panunzi, LG. (2020). sraX: a novel comprehensive resistome analysis tool. *Front. Microbiol.* doi:10.3389/fmicb.2020.00052

A sustained effort is made to ensure the proper functioning of our proposed tool and its effective documentation. Nevertheless, we would like to be warned about any problems or bugs that might arise when you launch your queries. All correspondence should be sent to `mailto:lgpanunzi@gmail.com`.

The development website is `https://github.com/lgpdevtools/sraX`. **Please use the Github website 'Issues' tracker to report any bugs, make suggestions or ask questions.**

This document was produced using **sraX** version 1.5.

Copyright © 2020 Leonardo G. Panunzi.

Contents

1 Abbreviations **4**

2 Introduction **5**
- 2.1 AMR context . . . 5
- 2.2 Implementation . . . 6

3 Installation **6**
- 3.1 Dependencies . . . 6
- 3.2 Installation . . . 8
  - 3.2.1 (Bio) conda packages . . . 8
  - 3.2.2 Docker image file . . . 8
  - 3.2.3 Bash script . . . 9

4 Usage **9**
- 4.1 Main parameters . . . 10
- 4.2 Command line . . . 11
- 4.3 Performance . . . 11
- 4.4 Real data examples . . . 12
  - 4.4.1 Define the working environment . . . 12
  - 4.4.2 Download genome data . . . 12
  - 4.4.3 **sraX** resistome analysis . . . 16

5 Creating a custom AMR DB **18**
- 5.1 Curated ARG sequences from users' private repositories . . . 18
- 5.2 Curated ARG sequences from public repositories . . . 20

References **22**

1 Abbreviations

Table 1.1 Main terms used within the text.

Abbreviation	Meaning
"AMR"	Antimicrobial resistance
"ARG"	Antibiotic resistance gene
"DB"	Data Base
"NGS"	Next-Generation Sequencing
"WGS"	Whole-Genome Sequencing
"MSA"	Multiple-sequence alignment

2 Introduction

2.1 AMR context

AMR constitutes a serious global threat to public health that is mainly caused by the extensive prescription of antibiotics in human diseases and agriculture. It has been estimated that $\sim$ 10 million deaths per year might be reached toward 2050 (O'Neill 2016). The arise of pathogenic super-resistant "bugs" elsewhere supposes the acquisition of ARG genes by —primarily— new gene exchange mechanisms among unrelated microbial taxa. This fact clearly indicates the demand of novel analytical methods, based on NGS techniques, for surveilling and further analysing AMR. Particularly, whole-genome sequencing (WGS) and whole-metagenome sequencing (WMS) approaches have demonstrated enormous capabilities of epidemiological surveillance, outbreak detection, and infection control of bacterial pathogens (Didelot et al. 2012). Uncommon AMR phenotypic profiles have led to the discovery of distinct genetic AMR determinants when inspecting peculiar harmful pathogens at genomic level. Nowadays, the `resistome profiling` analysis refers to the discovery of AMR determinants —along with their additional classification and quantification— of an assortment of genomes[a]. According to the type of sequence data that can be handled, two main methods are the basis of current tools for performing the `resistome profiling` examination: read-based and assembly-based methods. Considering read-based approaches, the following tools are distinguished: SRST2 (Inouye et al. 2014), KmerResistance (Clausen et al. 2016), Mykrobe predictor (Bradley et al. 2015), ARIBA (Hunt et al. 2017), SEAR (Rowe et al. 2015), SSTAR (Man and Limbago 2016), PATRIC (Antonopoulos et al. 2017), GROOT (Rowe and Winn 2018) and DeepARG (Arango-Argoty et al. 2018). Regarding assembly-based approaches, the following implementations have been proposed: ResFinder (Zankari et al. 2012), ARG-ANNOT (Gupta et al. 2014) –already archived–, RAST (Davis et al. 2016), RGI (Jia et al. 2016), PointFinder (Zankari et al. 2017), ARGs-OAP (Yin et al. 2018) and NCBI-AMRFinder (Feldgarden et al. 2019).

Here a novel assembly-based method called **sraX** (systematic resistome analysis) is presented for evincing AMR determinants through a genomics profiling analysis. It has been devised as a fully automated tool that performs a serie of catenated steps which are executed with a single primary command. In contrast to existing approaches, sraX has unique and noteworthy built-in features, like: genomic context and SNP analysis, complete graphical output and integration of results into a single hyperlinked HTML file. Furthermore, sraX has been devised for running on desktop computer systems,

[a]generally in the sense of a microbial community

under limited RAM and processing resources. In summary, **sraX** constitutes a simple and straightforward method, envisaging that inexperienced users without any technical or bioinformatic knowledge would run and deploy it. sraX operates as a standalone tool and it can be installed from source code –available at https://github.com/lgpdevtools/srax– or as a docker image –available at https://hub.docker.com/r/lgpdevtools/srax–.

2.2 Implementation

sraX is proposed as a single-command tool for performing a complete resistome profiling analysis of assembled genomes (see Figure 2.1 for a schematic workflow). **sraX** comprises several automated –and catenated– steps, which are modularized to achieve a greater computational efficiency and for acquiring more interpretable and graphical results. **sraX** accomplishes the following tasks:

- Creation and compilation of a local AMR database (DB) using public or proprietary repositories.
- Accurate identification of AMR determinants (ARGs or SNPs presence) in a non-redundant manner
- Detection of putative new variants through the SNP analysis
- Calculation and graphical representation of drug classes and type of mutated loci
- In-depth gene context exploration

The results are presented in fully navigable HTML-formatted files with embedded plots of previously mentioned analysis.

3 Installation

3.1 Dependencies

1. Though **sraX** is fully written in Perl and should work with any OS, it has only been tested with a 64-bit Linux distribution. Finally, for large amounts of sequence data (>= 100 genomes) is important to consider the processing capacity of the employed computer in terms of number of CPUs and RAM memory (see Figure 4.1 for an operational reference).
2. Perl version 5.26.x or higher. You can verify on your own computer by typing the following command in the bash terminal:

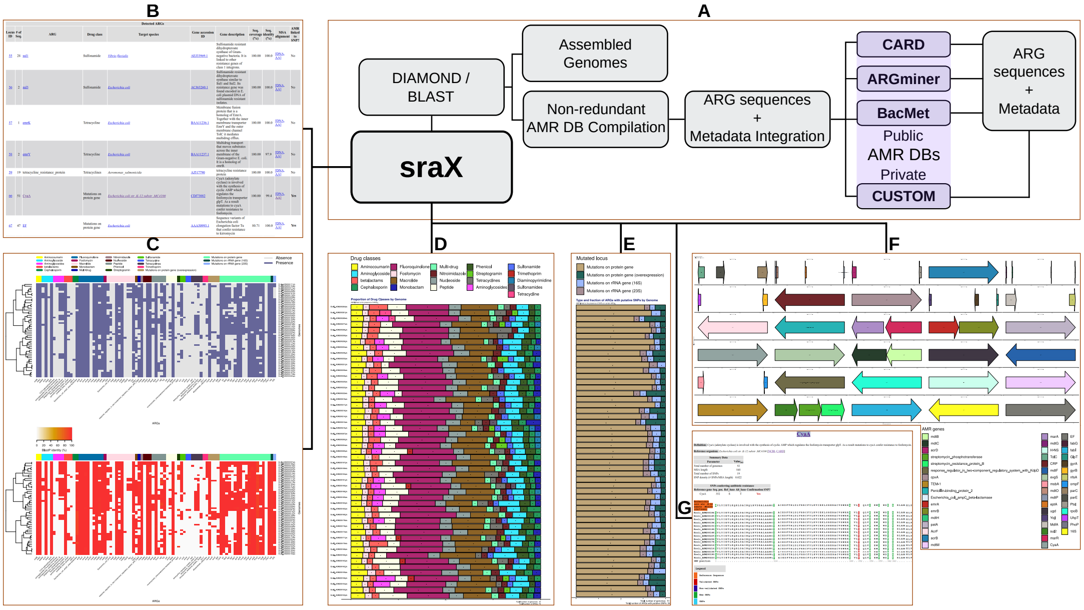

FIGURE 2.1 Starting steps of **sraX** resistome analysis (§2.2).

```
perl -h
```

The latest version of Perl can be obtained from the official website. Consult the installation guide.

The following Perl libraries are also required and can be installed using CPAN:

- `LWP::Simple`
- `Data::Dumper`
- `JSON`
- `File::Slurp`
- `FindBin`
- `Cwd`

3. Third-party software:

- **`BLAST+ v2.10.0`** (Altschul et al. 1990)
- **`DIAMOND v0.9.29`** (Buchfink et al. 2015)
- **`MUSCLE v3.8.31`** (Edgar 2004)
- **`CLUSTAL Ω v1.2.4`** (Sievers et al. 2011)
- **`MAFFT v7.450`** (Katoh et al. 2002)
- **`R v.3.6.1`** (R Core Team 2018)

Besides, we must install the subsequent packages belonging from **R**:

- dplyr
- ggplot2
- gridExtra

NOTE: The bash script "install_srax.sh" is provided, in order to confirm the existence of these dependencies in your computer. If any of them would be missing, the bash script will guide you for a proper installation.

3.2 Installation

To successfully install **sraX**, please see the details provided below. If you encounter an issue during the process, please contact your local system administrator. If you encounter a bug please log it here or email me at mailto:lgpanunzi@gmail.com.

3.2.1 (Bio) conda packages

The conda or bioconda packages constitute straightforward deployment instances. In order to list the available **sraX** versions, run the following command:

```
anaconda search -t conda srax
```

Afterwards, for accomplishing the deployment in your local computer, any of the following commands should be run:

```
conda install -c bioconda srax
```

or (generally the latest version):

```
conda install -c lgpdevtools srax
```

3.2.2 Docker image file

Type the following command in the bash terminal for obtaining the Docker image file (see Figure 3.1):

```
sudo docker pull lgpdevtools/srax
```

If everything has gone well, your computer should looks like Figure 3.2.

In order to check the appropriate running state of the image file:

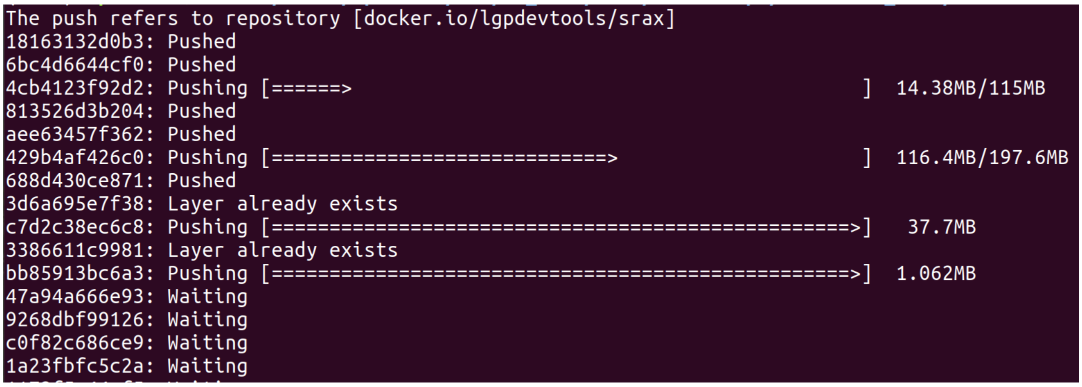

FIGURE 3.1 Start of pulling the dedicated Docker image file (§3.2.2).

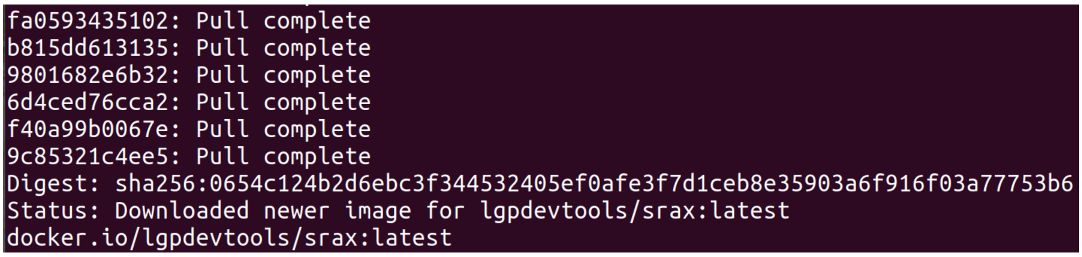

FIGURE 3.2 End of pulling the Docker image file (§3.2.2).

```
sudo docker run -it lgpdevtools/srax -v
sudo docker run -it lgpdevtools/srax
```

3.2.3 Bash script

Open a bash terminal and clone the repository:

```
git clone https://github.com/lgpdevtools/sraX.git
```

To verify the existence of required dependencies and ultimately install the perl modules composing **sraX**, inside the cloned repository run:

```
sudo bash install_srax.sh
```

4 Usage

sraX effectively operates as one-step application. It means that just a single command is required to obtain the totality of results and their visualization.

NOTE: For a detailed explanation and examples from real datasets, please follow the Tutorial.

4.1 Main parameters

-i|input Input directory [/path/to/input_dir] containing the input file(s), which must be in FASTA format and consisting of individual assembled genome sequences.

-o|output Output directory to store obtained results [/path/to/output_dir]. Default: 'input_sraX_id_aln_cov_seqal'

Example:

Input directory (-i): 'Test'

Options: -id 85; -c 95; -p dblastx

Output directory (-o): 'Test_sraX_85_95_dblastx'

-s|seqal The preferred algorithm for aligning the assembled genome(s) to a locally compiled AMR DB. The possible choices are: 'dblastx' (DIAMOND blastx) or 'blastx' (NCBI blastx). In any case, the process is parallelized (up to 100 genome files are run simultaneously) for reducing computing times. [string] Default: dblastx

-a|msa The preferred algorithm for producing the alignment of clustered homologous sequences (multiple-sequence files). The possible choices are: 'muscle', 'clustalo' or 'mafft'. [string] Default: muscle

-e|eval Minimum evalue cut-off to filter false positives. [number] Default: 1e-05

-id Minimum identity cut-off to filter false positives. [number] Default: 85

-c|aln_cov Minimum length of the query which must align to the reference sequence. [number] Default: 60

-db|dbsearch The level of the ARG search, on account of the number and type of employed AMR DB. The possible choices are: 'basic' or 'ext'. The 'basic' option only applies 'CARD', while the 'ext' option utilizes as well the 'ARGminer' (compilation of multiple AMR DBs) and 'BACMET' (biocides and metal resistance) repositories. [string] Default: basic

-u|user_sq Customary AMR DB provided by the user. The sequences must be in FASTA format.

-t|threads Number of threads when running sraX. [number] Default: 6

-h|help Displays this help information and exits.

-v|version Displays version information and exits.

-d|debug Verbose output (for debugging).

4.2 Command line

Basic usage:

```
sraX -i [/path/to/input_genome_directory]
```

Extended options:

```
sraX -a mafft -s blastx -db ext -id 95 -c 90 -t 12 -d -o [/path/
   to/output_results_directory] -i [/path/to/
   input_genome_directory]
```

Using the Docker image file, you have to run the following commands:

```
sudo docker run --rm -v /path/to/input_genome_directory:/IN \
lgpdevtools/srax -i IN
```

```
sudo docker run --rm -v /path/to/input_genome_directory:/IN \
-v /path/to/output_results_directory:/OUT \
lgpdevtools/srax -i IN -o OUT -a mafft -s blastx -db ext -id 95 -
   c 90 -t 12 -d
```

4.3 Performance

We tested the **sraX**'s performance based on the calculation of walltime and memory consumption during the resistome analysis. We randomly sampled an increasing amount of sequence data (100-genome intervals up to 1000 genomes) and performed 5 replicates on each measured interval (see Figure 4.1). These sequence files were selected from a set composed of 5000 *Klebsiella pneumoniae* distinct genomes (genome size ≈ 5.5 Mbp). Our results indicated a linear scale of running time (≈ 12.5 min) and memory consumption (≈ 250 MB) with the processing of every 100-genome interval. Each test was independently performed on a desktop computer Intel R Xeon R W-2104 processor (4-core, 3.20GHz) with 32GB RAM.

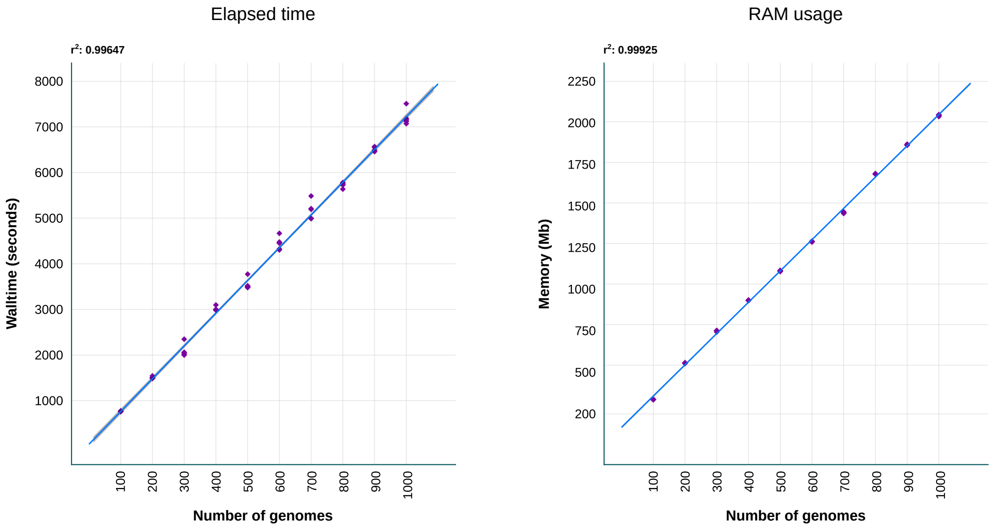

FIGURE 4.1 **sraX** performance: running time and RAM consumption (§3.1 & §4.3).

4.4 Real data examples

4.4.1 Define the working environment

The first task is to define our working directory[b] and create the required sub-directories in order to proceed with the analysis.

```
cd ~/Desktop/
mkdir -p SRAX_analysis/dataset1
mkdir SRAX_analysis/dataset2
mkdir SRAX_analysis/dataset3
mkdir SRAX_analysis/dataset4
mkdir SRAX_analysis/dataset5
mkdir SRAX_analysis/userdb
```

4.4.2 Download genome data

We are going to download from the **NCBI** public repository three different genome datasets that have already been analyzed and further published. Since the genome files cannot be obtained by running a terminal bash command (like “`wget`”), we need to click separately on each of the following links:

[b]The [/absolute_path/to/working_dir] could be, like in this example, “∼/Desktop/”.

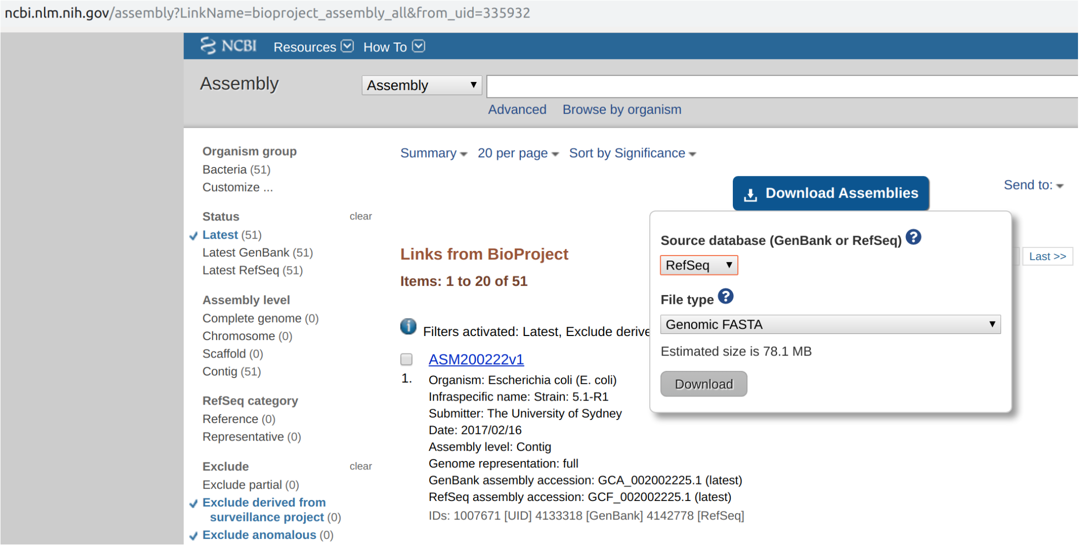

FIGURE 4.2 Procedure for downloading the `genome assemblies` of each dataset.

1. ***Data-set 1:*** 51 genomes belonging to commensal AMR strains of *Escherichia coli* (Moran et al. 2017).
2. ***Data-set 2:*** 76 genomes belonging to multi-drug AMR strains of *Escherichia coli* from farm isolates (Tyson et al. 2015).
3. ***Data-set 3:*** 133 genomes belonging to AMR strains of *Shigella sonnei* with different antibiotic resistance patterns (Holt et al. 2013).
4. ***Data-set 4:*** 390 genomes belonging to AMR strains of *Pseudomonas aeruginosa* with different antibiotic resistance patterns (Kos et al. 2015).
5. ***Data-set 5:*** 616 genomes belonging to AMR strains of *Streptococcus pneumoniae* with different antibiotic resistance patterns (Croucher et al. 2015).

Afterwards, we have to click on the **"Download Assemblies"** field in order to select the **"Refseq"** and **"Genomic FASTA"** parameters from the **"Source database"** and **"File Type"** fields. Your computer screen should looks like Figure 4.2.

A single compressed file (`genome_assemblies.tar`) is downloaded from each dataset. Therefore, each genome data has to be extracted in the right directory. Next, according to the selected dataset, the following recurrent steps should be accomplished. For example, if we would like to start with ***Data-set 1*** (§1), we have to run these commands:

```
tar -xvf genome_assemblies.tar -C SRAX_analysis/dataset1/
rm genome_assemblies.tar
gunzip SRAX_analysis/dataset1/ncbi-genomes-yyyy-mm-dd/*.gz
mv SRAX_analysis/dataset1/ncbi-genomes-yyyy-mm-dd/*.fna\
   SRAX_analysis/dataset1/
```

```
rm -r SRAX_analysis/dataset1/ncbi-genomes-yyyy-mm-dd
rm SRAX_analysis/dataset1/*.txt
```

Where `ncbi-genomes-yyyy-mm-dd` is a subfolder containing the genome FASTA files that is automatically generated by **NCBI**. The ***yyyy-mm-dd*** [c] information represents the actual date the `genome_assemblies.tar` file was retrieved.

NOTE: Alternatively, these datasets we can be downloaded from the following Zenodo repository (Panunzi 2019): DOI 10.5281/zenodo.3571224

Therefore, if we prefer this option, we need to place each dataset on their corresponding folder. In consequence, before performing the **sraX** analysis, we should run the following commands:

Data-set(1)

```
wget -O SRAX_analysis/ds1.tar.gz \
https://zenodo.org/record/3571224/files/
   Escherichia_coli_commensal.tar.gz?download=1
tar -xvf SRAX_analysis/ds1.tar.gz -C SRAX_analysis/dataset1/
mv SRAX_analysis/dataset1/Escherichia_coli_commensal_51/*
   SRAX_analysis/dataset1/
rmdir SRAX_analysis/dataset1/Escherichia_coli_commensal_51
rm SRAX_analysis/ds1.tar.gz
```

Data-set(2)

```
wget -O SRAX_analysis/ds2.tar.gz \
https://zenodo.org/record/3571224/files/Escherichia_coli.tar.gz?
   download=1
tar -xvf SRAX_analysis/ds2.tar.gz -C SRAX_analysis/dataset2/
mv SRAX_analysis/dataset2/Escherichia_coli/* SRAX_analysis/
   dataset2/
rmdir SRAX_analysis/dataset2/Escherichia_coli
rm SRAX_analysis/ds2.tar.gz
```

Data-set(3)

```
wget -O SRAX_analysis/ds3.tar.gz \
https://zenodo.org/record/3571224/files/Shigella_sonnei.tar.gz?
   download=1
tar -xvf SRAX_analysis/ds3.tar.gz -C SRAX_analysis/dataset3/
```

[c] In the format of YEAR (4 digits), MONTH (2 digits) and DAY (2 digits)

```
mv SRAX_analysis/dataset3/Shigella_sonnei/* SRAX_analysis/
   dataset3/
rmdir SRAX_analysis/dataset3/Shigella_sonnei
rm SRAX_analysis/ds3.tar.gz
```

Data-set(4)

```
wget -O SRAX_analysis/ds4.tar.gz \
https://zenodo.org/record/3571224/files/Pseudomonas_aeruginosa.
   tar.gz?download=1
tar -xvf SRAX_analysis/ds4.tar.gz -C SRAX_analysis/dataset4/
mv SRAX_analysis/dataset4/Pseudomonas_aeruginosa/* SRAX_analysis/
   dataset4/
rmdir SRAX_analysis/dataset4/Pseudomonas_aeruginosa
rm SRAX_analysis/ds4.tar.gz
```

Data-set(5)

```
wget -O SRAX_analysis/ds5.tar.gz \
https://zenodo.org/record/3571224/files/Streptococcus_pneumoniae.
   tar.gz?download=1
tar -xvf SRAX_analysis/ds5.tar.gz -C SRAX_analysis/dataset5/
mv SRAX_analysis/dataset5/Streptococcus_pneumoniae/*
   SRAX_analysis/dataset5/
rmdir SRAX_analysis/dataset5/Streptococcus_pneumoniae
rm SRAX_analysis/ds5.tar.gz
```

Alternatively, we can download each separate dataset by clicking on the following links:

Data-set 1, ***Data-set 2***, ***Data-set 3***, ***Data-set 4***, ***Data-set 5***

Afterwards, we have to follow the next procedure:

Data-set(1)

```
tar -xvf Escherichia_coli_commensal.tar.gz -C SRAX_analysis/
   dataset1/
mv SRAX_analysis/dataset1/Escherichia_coli_commensal_51/*
   SRAX_analysis/dataset1/
rmdir SRAX_analysis/dataset1/Escherichia_coli_commensal_51
```

Data-set(2)

```
tar -xvf Escherichia_coli.tar.gz -C SRAX_analysis/dataset2/
mv SRAX_analysis/dataset2/Escherichia_coli/* SRAX_analysis/
   dataset2/
```

```
rmdir SRAX_analysis/dataset2/Escherichia_coli
```

Data-set(3)

```
tar -xvf Shigella_sonnei.tar.gz -C SRAX_analysis/dataset3/
mv SRAX_analysis/dataset3/Shigella_sonnei/* SRAX_analysis/
   dataset3/
rmdir SRAX_analysis/dataset3/Shigella_sonnei
```

Data-set(4)

```
tar -xvf Pseudomonas_aeruginosa.tar.gz -C SRAX_analysis/dataset4/

mv SRAX_analysis/dataset4/Pseudomonas_aeruginosa/* SRAX_analysis/
   dataset4/
rmdir SRAX_analysis/dataset4/Pseudomonas_aeruginosa
```

Data-set(5)

```
tar -xvf Streptococcus_pneumoniae.tar.gz -C SRAX_analysis/
   dataset5/
mv SRAX_analysis/dataset5/Streptococcus_pneumoniae/*
   SRAX_analysis/dataset5/
rmdir SRAX_analysis/dataset5/Streptococcus_pneumoniae
```

4.4.3 **sraX** resistome analysis

For each downloaded dataset we have to run just `a single-step` command in order to perform the complete **sraX** resistome analysis. At the beginning, regardless the chosen run command and considering not error messages, your computer screen should looks like Figure 4.3. Now, we are going to explore the diverse **sraX** parameters and the resulting output:

1. Using default options:

   ```
   sraX -i SRAX_analysis/dataset1
   ```

2. Adding the ARGminer (Argoty et al. 2019) and BacMet (Pal et al. 2014) DBs for a deeper search of AMR determinants:

   ```
   sraX -i SRAX_analysis/dataset1 -db ext
   ```

3. Selecting alternative algorithms for aligning the queries against the reference genome and for producing the MSA files:

```
The 'sraX' analysis started at: Time: 09:51 hs | Date: 2019-12-10


The compilation process of the ARG DB started at:       Time: 09:51 hs | Date: 2019-12-10

        Nucleotide sequence data from 'CARD' was successfully fetched
        Total number of DNA sequences: 2820
        Total number of RNA sequences: 44
        Protein sequence data from 'CARD' was successfully fetched
        Total number of AA sequences: 2773
        The collection of FASTA sequences from CARD took 3.20  wallclock secs


The compilation process of the ARGminer DB started at:  Time: 09:51 hs | Date: 2019-12-10

        Protein sequence data from 'ARGminer' was successfully fetched
        Total number of AA sequences: 14872
        Protein sequence data from 'ARGminer' was successfully gathered
        Total number of AA sequences: 12389
        The collection of FASTA sequences from ARGminer took 5.24  wallclock secs


The compilation process of the BacMet DB started at:    Time: 09:51 hs | Date: 2019-12-10

        Protein sequence data from 'BacMet' was successfully fetched
        Total number of AA sequences: 753
        Protein sequence data from 'BacMet' was successfully gathered
        Total number of AA sequences: 644
        The collection of FASTA sequences from BacMet took 5.59  wallclock secs

        Nucleotide sequence data from 'USER' was successfully gathered
        Total number of DNA sequences: 4
        Protein sequence data from 'USER' was successfully gathered
        Total number of AA sequences: 4
        The collection of FASTA sequences from USER (file: pbp4_pbp5_gyrA_parC.fa) took 0.06  wallclock secs


        The collection of all selected sequence repositories took 5.90  wallclock secs


The compilation process of the ARG DB finished at:      Time: 09:51 hs | Date: 2019-12-10


The final ARG DB is composed of the following FASTA sequences:
        Total number of compiled DNA sequences: 2777
        Total number of compiled RNA sequences: 44
        Total number of compiled AA sequences: 15810
```

FIGURE 4.3 Starting steps of **sraX** resistome analysis (§1).

```
sraX -i SRAX_analysis/dataset1 -a mafft -s blastx
```

4. Increasing the amino-acid identity percentage and alignment coverage for selecting more stringent hits:

```
sraX -i SRAX_analysis/dataset1 -id 95 -c 95
```

5. Setting up other options (output directory, number of threads, debug):

```
sraX -i SRAX_analysis/dataset1 -db ext -id 85 -c 90 -o
    SRAX_analysis/dataset1_my_custom_name -t 12 -d
```

Finally, the corresponding **sraX** report files are available for their examination:
SRAX_analysis/dataset1_sraX_85_60_dblastx/Results/**sraX_analysis.html**
SRAX_analysis/dataset1_sraX_95_95_dblastx/Results/**sraX_analysis.html**
SRAX_analysis/dataset1_my_custom_name/Results/**sraX_analysis.html**

5 Creating a custom AMR DB

In order to increase the AMR DB volume and its detection range, a suitable AMR DB can be added from custom curated ARG sequences. In next examples, we are going to provide ARG sequences belonging to user's own data and as well as from dedicated public repositories.

5.1 Curated ARG sequences from users' private repositories

In order to add a custom AMR DB is mandatory to provide a single file in (multi-)FASTA format containing the curated ARG (DNA or AA) sequences. Each sequence should have a different header for its accurate identification. Considering that **sraX** is able to generate a HTML-formatted report file, the following fields with specific data –each of them separated by a vertical bar– should be included in the header for creating the hyperlinks and fulfilling the summary files:

gene_name | **accession ID** | **gene description** | **AMR determination** | **amino-acid changes** (if apply) | **organism name**

Field description:

gene_name: The corresponding ARG name (e.g: CMY-2).

accession ID: The NCBI accession ID (e.g: CAA62957.1).

gene description: A short description of the gene or AMR mechanism (e.g: Beta-lactamase found in *Klebsiella pneumoniae*).

AMR determination: According to the AMR determination, **sraX** considers three possible choices: 1) protein homolog (AMR linked to ARG presence); 2) protein variant (AMR linked to mutations on ARGs); 3) rRNA gene variant (AMR linked to mutations on rRNAs).

When the AMR is linked to sequence mutations, the following information has to be included for validating their presence in the analyzed samples:

amino-acid changes: Each amino-acid change should be in the format: 'reference amino-acid + sequence position + mutated amino-acid' (e.g: V146F). When several mutations should be analyzed, every amino-acid change has to be separated by an underscore / low dash (e.g: H526Y_V146F_T563P_L533P_P564L_R529C_R529S_S531F_Q513L_Q513P_R687H)

organism name: The name of the bacteria from which the curated ARG has been detected (e.g: *Klebsiella pneumoniae*).

It is important to mention that the order of the fields and, in consequence, the information that they hold is precise. It implies that **sraX** will look for each piece of data on its corresponding position for performing the analysis. However, only the gene name is mandatory and, whether desired, all the remaining fields can be neglected.

Taking into account the previous facts, a complete custom header should have any of these appearances:

AMR linked to ARG presence:
>CMY-2|CAA62957.1|Beta-lactamase found in Klebsiella pneumoniae|protein_homolog|Beta-lactam|Klebsiella pneumoniae

AMR linked to mutations:
>rpoB|BAB38333.1|Mutations on rpoB result in resistance to rifampicin|protein variant|H526Y_V146F_T563P_L533P_P564L_R529C_S531F_Q513L_Q513P_R687H|Escherichia coli O157:H7 str Sakai

In order to have a clear example, please copy the following DNA sequence into a text file and save as "gyrA.fa":

>gyrA | | | protein variant | S84F |

ATGAGTGAAGAAATCAGAGAAAACATCCATGATGTCAATCTAACCAGCGAAATGAAAGACTCCTTCATCG

ATTATGCGATGAGTGTTATTGTCGCACGTGCACTTCCTGATGTCAGAGATGGTCTGAAACCCGTTCATCG

CCGTATTTTATACGGCATGAACGAATTAGGTGTAACACCGGATAAAGCCCATAAAAAATCTGCCAGAATC

GTCGGAGATGTCATGGGTAAATACCATCCTCATGGAGACAGTGCGATCTATGAGTCAATGGTACGGATGG

CACAGCCATTCAGCTACCGTTATATGCTAGTGGATGGTCATGGAAACTTTGGTTCTGTTGACGGTGACGG

TGCCGCCGCTATGCGTTATACCGAAGCACGGATGAGCAAAATCGCTACAGAAATGCTGCGTGATATCAAT

AAGAACACTGTTGATTTCCAAAGCAACTATGATGATACAGAAAAAGAACCAGTTGTACTACCTGCTCGCT

TCCCGAATCTTTTAGTGAACGGAACAACGGGGATCGCCGTAGGTATGGCCACGAATATCCCACCTCATAA

CTTGTCTGAGGTAGTAGCGGCTATCGATTTGCTGATGGATAATCCTGATGTGACGACCAATGAATTGATG

GAAGTATTACCTGGACCAGATTTTCCAACAGGTGGTTTGGTTATGGGGAAATCAGGAATCCGAAGAGCGT

ATGAAACAGGTAAGGGTTCTATTACCGTACGTGCAAAAGTTGAGATTACTGAGATGCCAAATGGTAAAGA

ACGGATCATCGTGACAGAATTGCCTTATATGGTCAATAAAGCAAAATTGATCGAACGGATTTCTGAACTG

CACAGAGACAAGCGTATCGAAGGGATCACAGATTTGCGGGACGAATCTTCCCGTGAAGGTATGCGGATCG

TCATTGACATCCGTCGTGACGCCAGCGCATCCGTTATCTTGAATAACTTATATAAATTGACATCCTTGCA

AAATTCATTTGGATTCAATATGTTGGCGATCGAAAAAGGTGTGCCGAAAGTTTTGAGCTTGAAACAAATC

CTTGAAAACTATATCGAACATCAACGCGAAGTCATCACACGACGGACGGTCTTTGAAAAAGAAAAAGCAG

AAGCAAGAGCACACATCTTAGAAGGTTTGCGTATTGCTTTGGATCATATCGATGAAATCATTGCGATTAT

CCGCGGGTCTAAATCAGATGACGAGGCAAAAGCAACAATGATCGAACGGTTCGATTTATCCGATCGCCAG

TCTCAAGCTATTTTAGATATGCGTTTACGCCGATTGACCGGTTTGGAACGGGAAAAAATCGAAAATGAAT

ACCAAGAACTCTTAAAACTGATTGAAGATTTAACTGACATTCTGGCAAGACCAGAACGTGTTACAGAAAT

CATCAAGAATGAATTAGCAGACTTGAATCAACGATTTGGTGATAAACGACGCACAGAATTACTAGTCGGT

GAGGTCTTAAGTCTGGAAGATGAAGATTTAATCGAAGAAGAAGAAATCGTTATTACATTGACAAACAATG

GTTACATCAAACGTTTGGCAAATAATGAATTCAGAGCTCAACGAAGGGGTGGACGTGGTGTCCAAGGAAT

GGGTATCCACAATGACGACTTCGTCAAAAATCTGGTTTCTTGTTCTACCCATGATACATTGTTATTCTTT

ACGAATAACGGAAAAGTGTATCGGGCAAAAGGATACGAAATTCCAGAATACGGGCGTACAGCCAAAGGAA

TCCCAATCATCAATTTACTAGGTATCGATTCGAGTGAAAGCATCCAGGCAATCATTGCGGTGACGGGAGA

AGCAGAAGAAGGCCACTATCTCTTCTTTACGACGCGTCAAGGTACAGTAAAACGTACTTCTGTCACTGCT

TTTGCTAACATCCGAAGTAATGGTCTGATCGCAATTGGTTTGAAAGAAGACGATGAGCTCGTCAATGTGC

TACTGACAGATGGTCAATCTAATATCATTATTGGAACACATAACGGCTACTCTGTCACTTTCAAAGAAGA

AGCAGTCCGCGATATGGGACGTACAGCTTCCGGGGTCAGAGGGATACGCCTGCGTGAGGAAGATTATGTC

GTTGGGGCAGCCTTAATGAAGGAAAATCAAGAAGTATTAGTGATCACAGAAAAAGGTTACGGAAAACGGA

CAAAAGTATCGGAATATCCAGTGAAAGGCCGCGGAGGTAAAGGGATCAAAACGGCGAACATCACTGAAAA

AAATGGTCCACTAGCTGGATTGACCACTGTTTCTGGAGATGAAGACATCATGCTGATTACAGATAAAGGT

GTCATTATCCGTTTCAACGTATCGACTGTTTCTCAAACTGGACGTTCAACATTAGGCGTACGTTTGATGA

AGATGGAAGCTGATACGAAAGTTGTCACGATGGCAGCAGTCGAACCAGAAGCTGCAGAACAAACAGTTGA

AGAACAAGGATCAGAAGAATAA

Then, execute the following commands:

```
cp gyrA.fa SRAX_analysis/userdb/gyrA.fa
wget -O SRAX_analysis/Enterococci.tar.gz \
https://zenodo.org/record/3571224/files/Enterococcus_spp.tar.gz?
   download=1
tar -zxvf SRAX_analysis/Enterococci.tar.gz \
-C SRAX_analysis/userdb/
rm SRAX_analysis/Enterococci.tar.gz
sraX -i SRAX_analysis/userdb/Enterococcus_spp -db ext \
-u SRAX_analysis/userdb/gyrA.fa
```

Finally, open the corresponding report file and check for the added *gyrA.fa* gene (see Figure 5.1 for verifying the expected file):
SRAX_analysis/userdb/Enterococcus_spp_sraX_85_60_dblastx/Results/**sraX_analysis.html**

5.2 Curated ARG sequences from public repositories

1. **`ARGDIT:`** A recently published work (Chiu and Ong 2018) describes the `ARGDIT toolkit` for creating curated AMR DBs. The authors provided already **pre-compiled AMR DBs** as examples, and this valuable information is going to be employed for demonstrating **sraX**'s practicality and utility for resistome profiling.

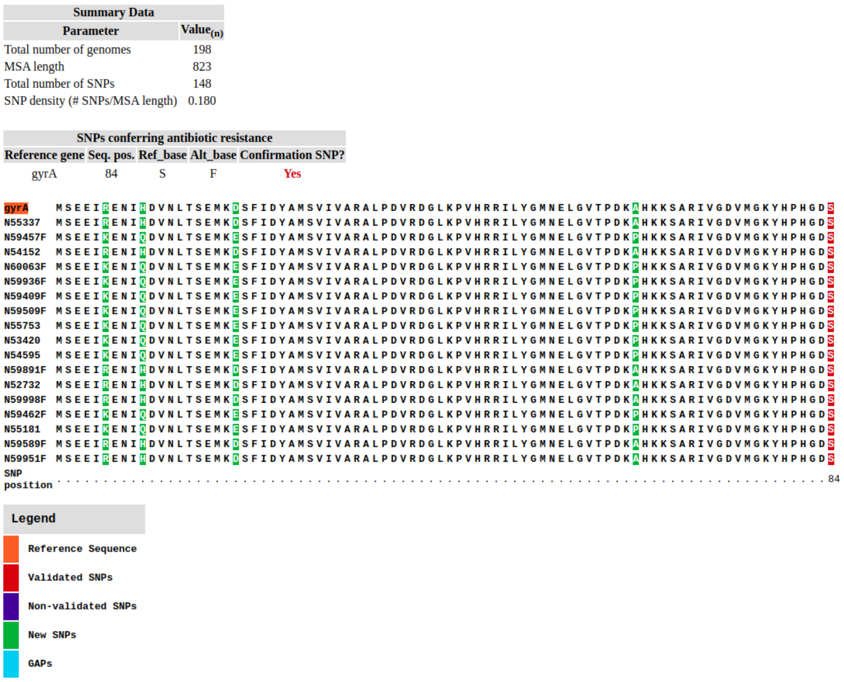

FIGURE 5.1 Expected results from **sraX** SNP analysis using a user provided ARG sequence (§5.1).

Firstly, a the ARG sequences are going to downloaded from the `ARGDIT` repository as a FASTA file named **"argdit_dna.fa"**, that will be created and further located inside the **"SRAX_analysis/userdb/"** folder:

```
wget -O SRAX_analysis/userdb/argdit_dna.fa \
https://github.com/phglab/ARGDIT/blob/master/\
sample_integrated_dbs/argdit_nt_db.fa?raw=true
```

Secondly, a new FASTA file named **"argdit_dna_formatted.fa"** is going to be created and it will contains important meta–data which is currently employed by **sraX** in producing the output results.

```
awk -F \| '/^>/ { print ">"$2"|"$1"|"$3"|protein_homolog|\
"$9"|"$5; next } 1' SRAX_analysis/userdb/argdit_dna.fa \
> SRAX_analysis/userdb/argdit_dna_formatted.fa

sed -i 's/|>/|/g' SRAX_analysis/userdb/argdit_dna_formatted.
    fa

rm -f SRAX_analysis/userdb/argdit_dna.fa
```

Finally, we can contrast previous obtained results with the ones that are achieved by employing our newly added AMR DB:

```
sraX -i SRAX_analysis/dataset1 -u \
```

```
SRAX_analysis/userdb/argdit_dna_formatted.fa \
-id 95 -c 95
```

2. **NCBI Bacterial AMR Reference Gene Database:** The NCBI public repository is comprised by annotated DNA sequences that encode proteins conferring AMR, and it constitutes an aggregation of collections from multiple sources which were previously curated and further expanded by reviewing the dedicated literature.

   Following a similar procedure, we are going to run a few commands for creating an alternative AMR DB, that will contain a distinct arrangement of ARG sequences.

   Firstly, a multi-FASTA file named **"ncbi_aa.fa"** will contain the ARG sequences from the NCBI Bacterial AMR Reference Gene Database repository and it will be located inside the same folder (**"SRAX_analysis/userdb/"**):

```
wget -O SRAX_analysis/userdb/ncbi_aa.fa \
ftp://ftp.ncbi.nlm.nih.gov/pathogen/\
Antimicrobial_resistance/AMRFinder/\
data/latest/AMRProt
```

   Secondly, a formatted FASTA file (**"argdit_dna_formatted.fa"**) containing the relevant meta–data is going to be created:

```
awk -F \| '/^>/ { print ">"$6"|"$2"|"$9"\
|protein_homolog|"$8"|Not_indicated"; next } 1' \
SRAX_analysis/userdb/ncbi_aa.fa > \
SRAX_analysis/userdb/ncbi_aa_formatted.fa

rm -f SRAX_analysis/userdb/ncbi_aa.fa
```

   Finally, we can contrast previous obtained results with the ones that are achieved by employing our newly added AMR DB:

```
sraX -i SRAX_analysis/dataset1 -u \
SRAX_analysis/userdb/ncbi_aa_formatted.fa \
-id 95 -c 95
```

References

Altschul, S. F., W. Gish, W. Miller, E. W. Myers and D. J. Lipman (1990). "Basic local alignment search tool". In: *Journal of Molecular Biology* 215.3, pp. 403–410. DOI: 10.1016/S0022-2836(05)80360-2 (cit. on p. 7).

Antonopoulos, D. A., R. Assaf, R. K. Aziz, T. Brettin, C. Bun, N. Conrad, J. J. Davis, E. M. Dietrich, T. Disz, S. Gerdes, R. W. Kenyon, D. Machi, C. Mao, D. E. Murphy-Olson, E. K. Nordberg, G. J. Olsen, R. Olson, R. Overbeek, B. Parrello, G. D. Pusch, J. Santerre, M. Shukla, R. L. Stevens, M. VanOeffelen, V. Vonstein, A. S. Warren, A. R. Wattam, F. Xia and H. Yoo (2017). "PATRIC as a unique resource for studying antimicrobial resistance." In: *Briefings in bioinformatics*. DOI: 10.1093/bib/bbx083 (cit. on p. 5).

Arango-Argoty, G., E. Garner, A. Pruden, L. S. Heath, P. Vikesland and L. Zhang (2018). "DeepARG: a deep learning approach for predicting antibiotic resistance genes from metagenomic data". In: *Metagenomics* 6. DOI: 10.1186/s40168-018-0401-z (cit. on p. 5).

Argoty, G. A. A., G. K. Guron, E. Garner, M. V. Riquelme, L. Heath, A. Pruden, P. Vikesland and L. Zhang (2019). "ARG-miner: A web platform for crowdsourcing-based curation of antibiotic resistance genes". In: *bioRxiv*, p. 274282 (cit. on p. 16).

Bradley, P., N. C. Gordon, T. M. Walker, L. Dunn, S. Heys, B. Huang, S. Earle, L. J. Pankhurst, L. Anson, M. de Cesare, P. Piazza, A. A. Votintseva, T. Golubchik, D. J. Wilson, D. H. Wyllie, R. Diel, S. Niemann, S. Feuerriegel, T. A. Kohl, N. Ismail, S. V. Omar, E. G. Smith, D. Buck, G. McVean, A. S. Walker, T. E. A. Peto, D. W. Crook and Z. Iqbal (Dec. 2015). "Rapid antibiotic-resistance predictions from genome sequence data for Staphylococcus aureus and Mycobacterium tuberculosis". In: *Nature Communications* 06. DOI: 10.1038/ncomms10063 (cit. on p. 5).

Buchfink, B., C. Xie and D. H. Huson (2015). "Fast and sensitive protein alignment using DIAMOND". In: *Nature methods* 12.1, p. 59. DOI: 10.1038/nmeth.3176 (cit. on p. 7).

Chiu, J. K. H. and R. T.-H. Ong (Dec. 2018). "ARGDIT: a validation and integration toolkit for Antimicrobial Resistance Gene Databases". In: *Bioinformatics*. DOI: 10.1093/bioinformatics/bty987 (cit. on p. 20).

Clausen, P. T. L. C., O. Lund, E. Zankari and F. M. Aarestrup (June 2016). "Benchmarking of methods for identification of antimicrobial resistance genes in bacterial whole genome data". In: *Journal of Antimicrobial Chemotherapy* 71.9, pp. 2484–2488. DOI: 10.1093/jac/dkw184 (cit. on p. 5).

Croucher, N. J., L. Kagedan, C. M. Thompson, J. Parkhill, S. D. Bentley, J. A. Finkelstein, M. Lipsitch and W. P. Hanage (Mar. 2015). "Selective and Genetic Constraints on Pneumococcal Serotype Switching". In: *PLOS Genetics* 11.3, pp. 1–21. DOI: 10.1371/journal.pgen.1005095 (cit. on p. 13).

Davis, J. J., S. Boisvert, T. Brettin, R. W. Kenyon, C. Mao, R. Olson, R. Overbeek, J. Santerre, M. Shukla, A. R. Wattam, R. Will, F. Xia and R. Stevens (June 2016). "Antimicrobial

Resistance Prediction in PATRIC and RAST". In: *Scientific Reports* 6, pp. -. DOI: 10.1038/srep27930 (cit. on p. 5).

Didelot, X., R. Bowden, D. J. Wilson, T. E. Peto and D. W. Crook (2012). "Transforming clinical microbiology with bacterial genome sequencing". In: *Nat Rev Genet* 13. DOI: 10.1038/nrg3226 (cit. on p. 5).

Edgar, R. C. (2004). "MUSCLE: a multiple sequence alignment method with reduced time and space complexity". In: *BMC bioinformatics* 5.1, p. 113 (cit. on p. 7).

Feldgarden, M., V. Brover, D. H. Haft, A. B. Prasad, D. J. Slotta, I. Tolstoy, G. H. Tyson, S. Zhao, C.-H. Hsu, P. F. McDermott et al. (2019). "Using the NCBI AMRFinder Tool to Determine Antimicrobial Resistance Genotype-Phenotype Correlations Within a Collection of NARMS Isolates". In: *BioRxiv*, p. 550707 (cit. on p. 5).

Gupta, S. K., B. R. Padmanabhan, S. M. Diene, R. Lopez-Rojas, M. Kempf, L. Landraud and J.-M. Rolain (2014). "ARG-ANNOT, a New Bioinformatic Tool To Discover Antibiotic Resistance Genes in Bacterial Genomes". In: *Antimicrobial Agents and Chemotherapy* 58.1, pp. 212–220. DOI: 10.1128/AAC.01310-13 (cit. on p. 5).

Holt, K. E., T. V. T. Nga, D. P. Thanh, H. Vinh, D. W. Kim, M. P. V. Tra, J. I. Campbell, N. V. M. Hoang, N. T. Vinh, P. Van Minh et al. (2013). "Tracking the establishment of local endemic populations of an emergent enteric pathogen". In: *Proceedings of the National Academy of Sciences* 110.43, pp. 17522–17527 (cit. on p. 13).

Hunt, M., A. E. Mather, L. Sanchez-Buso, A. J. Page, J. Parkhill, J. A. Keane and S. R. Harris (2017). "ARIBA: rapid antimicrobial resistance genotyping directly from sequencing reads". In: *Microbial Genomics* 3.10, pp. - (cit. on p. 5).

Inouye, M., H. Dashnow, L.-A. Raven, M. B. Schultz, B. J. Pope, T. Tomita, J. Zobel and K. E. Holt (2014). "SRST2: Rapid genomic surveillance for public health and hospital microbiology labs". In: *Genome Medicine* 6.11, p. 90. DOI: 10.1186/s13073-014-0090-6 (cit. on p. 5).

Jia, B., A. R. Raphenya, B. Alcock, N. Waglechner, P. Guo, K. K. Tsang, B. A. Lago, B. M. Dave, S. Pereira, A. N. Sharma, S. Doshi, M. Courtot, R. Lo, L. E. Williams, J. G. Frye, T. Elsayegh, D. Sardar, E. L. Westman, A. C. Pawlowski, T. A. Johnson, F. S. Brinkman, G. D. Wright and A. G. McArthur (Oct. 2016). "CARD 2017: expansion and model-centric curation of the comprehensive antibiotic resistance database". In: *Nucleic Acids Research* 45.D1, pp. D566–D573. DOI: 10.1093/nar/gkw1004. eprint: http://oup.prod.sis.lan/nar/article-pdf/45/D1/D566/8846551/gkw1004.pdf (cit. on p. 5).

Katoh, K., K. Misawa, K.-i. Kuma and T. Miyata (2002). "MAFFT: a novel method for rapid multiple sequence alignment based on fast Fourier transform." In: *Nucleic acids research* 30, pp. 3059–3066. DOI: 10.1093/nar/gkf436 (cit. on p. 7).

Kos, V. N., M. Déraspe, R. E. McLaughlin, J. D. Whiteaker, P. H. Roy, R. A. Alm, J. Corbeil and H. Gardner (2015). "The Resistome of Pseudomonas aeruginosa in Relationship to Phenotypic Susceptibility". In: *Antimicrobial Agents and Chemotherapy* 59.1, pp. 427–436. DOI: 10.1128/AAC.03954-14. eprint: https://aac.asm.org/content/59/1/427.full.pdf (cit. on p. 13).

Man, T. J. B. de and B. M. Limbago (2016). "SSTAR, a Stand-Alone Easy-To-Use Antimicrobial Resistance Gene Predictor". In: *mSphere* 1.1. Ed. by P. Dunman. DOI: 10.1128/mSphere.00050-15 (cit. on p. 5).

Moran, R. A., S. Anantham, K. E. Holt and R. M. Hall (2017). "Prediction of antibiotic resistance from antibiotic resistance genes detected in antibiotic-resistant commensal Escherichia coli using PCR or WGS". In: *Journal of Antimicrobial Chemotherapy* 72.3, pp. 700–704. DOI: 10.1093/jac/dkw511 (cit. on p. 13).

O'Neill (2016). *Tackling drug-resistant infections globally: Final report and recommendations. The review on antimicrobial resistance.* Wellcome Trust and the UK Department of Health. London, UK (cit. on p. 5).

Pal, C., J. Bengtsson-Palme, C. Rensing, E. Kristiansson and D. G. J. Larsson (2014). "BacMet: antibacterial biocide and metal resistance genes database." In: *Nucleic acids research* 42, pp. D737–D743. DOI: 10.1093/nar/gkt1252 (cit. on p. 16).

Panunzi, L. (Oct. 2019). *AMR datasets from diverse spp.* Version v0.1.0. DOI: 10.5281/zenodo.3571224 (cit. on p. 14).

R Core Team (2018). *R: A Language and Environment for Statistical Computing.* R Foundation for Statistical Computing. Vienna, Austria (cit. on p. 7).

Rowe, W. P. M. and M. D. Winn (2018). "Indexed variation graphs for efficient and accurate resistome profiling". In: *Bioinformatics* 34, pp. 3601–3608. DOI: 10.1093/bioinformatics/bty387 (cit. on p. 5).

Rowe, W., K. S. Baker, D. Verner-Jeffreys, C. Baker-Austin, J. J. Ryan, D. Maskell and G. Pearce (July 2015). "Search Engine for Antimicrobial Resistance: A Cloud Compatible Pipeline and Web Interface for Rapidly Detecting Antimicrobial Resistance Genes Directly from Sequence Data". In: *PLOS ONE* 10.7, pp. 1–12. DOI: 10.1371/journal.pone.0133492 (cit. on p. 5).

Sievers, F., A. Wilm, D. Dineen, T. J. Gibson, K. Karplus, W. Li, R. Lopez, H. McWilliam, M. Remmert, J. Soding, J. D. Thompson and D. G. Higgins (Oct. 2011). "Fast, scalable generation of high-quality protein multiple sequence alignments using Clustal Omega." In: *Molecular systems biology* 7, p. 539. DOI: 10.1038/msb.2011.75 (cit. on p. 7).

Tyson, G. H., P. F. McDermott, C. Li, Y. Chen, D. A. Tadesse, S. Mukherjee, S. Bodeis-Jones, C. Kabera, S. A. Gaines, G. H. Loneragan et al. (2015). "WGS accurately predicts antimicrobial resistance in Escherichia coli". In: *Journal of Antimicrobial Chemotherapy* 70.10, pp. 2763–2769 (cit. on p. 13).

Yin, X., X. Jiang, B. Chai, L. Li, Y. Yang, J. R. Cole, J. M. Tiedje and T. Zhang (2018). "ARGs-OAP v2.0 with an expanded SARG database and Hidden Markov Models for enhancement characterization and quantification of antibiotic resistance genes in environmental metagenomes". In: *Bioinformatics* 34.13, pp. 2263–2270. DOI: 10.1093/bioinformatics/bty053 (cit. on p. 5).

Zankari, E., H. Hasman, S. Cosentino, M. Vestergaard, S. Rasmussen, O. Lund, F. M. Aarestrup and M. V. Larsen (2012). "Identification of acquired antimicrobial resistance genes". In: *J Antimicrob Chemother* 67. DOI: 10.1093/jac/dks261 (cit. on p. 5).

Zankari, E., R. Allesøe, K. G. Joensen, L. M. Cavaco, O. Lund and F. M. Aarestrup (2017). "PointFinder: a novel web tool for WGS-based detection of antimicrobial resistance associated with chromosomal point mutations in bacterial pathogens". In: *Journal of Antimicrobial Chemotherapy* 72.10, pp. 2764–2768 (cit. on p. 5).
