# Supplementary material for: sraX: A Novel Comprehensive Resistome Analysis Tool
Source: Front Microbiol. 2020 Feb 5;11:52. doi: 10.3389/fmicb.2020.00052 (PMC7025521; doi:10.3389/fmicb.2020.00052)
Supplement: Supplementary file 3 [file Data_Sheet_2.PDF]

# Supplementary Material

## 1 SUPPLEMENTARY TABLES AND FIGURES

### 1.1 Figures

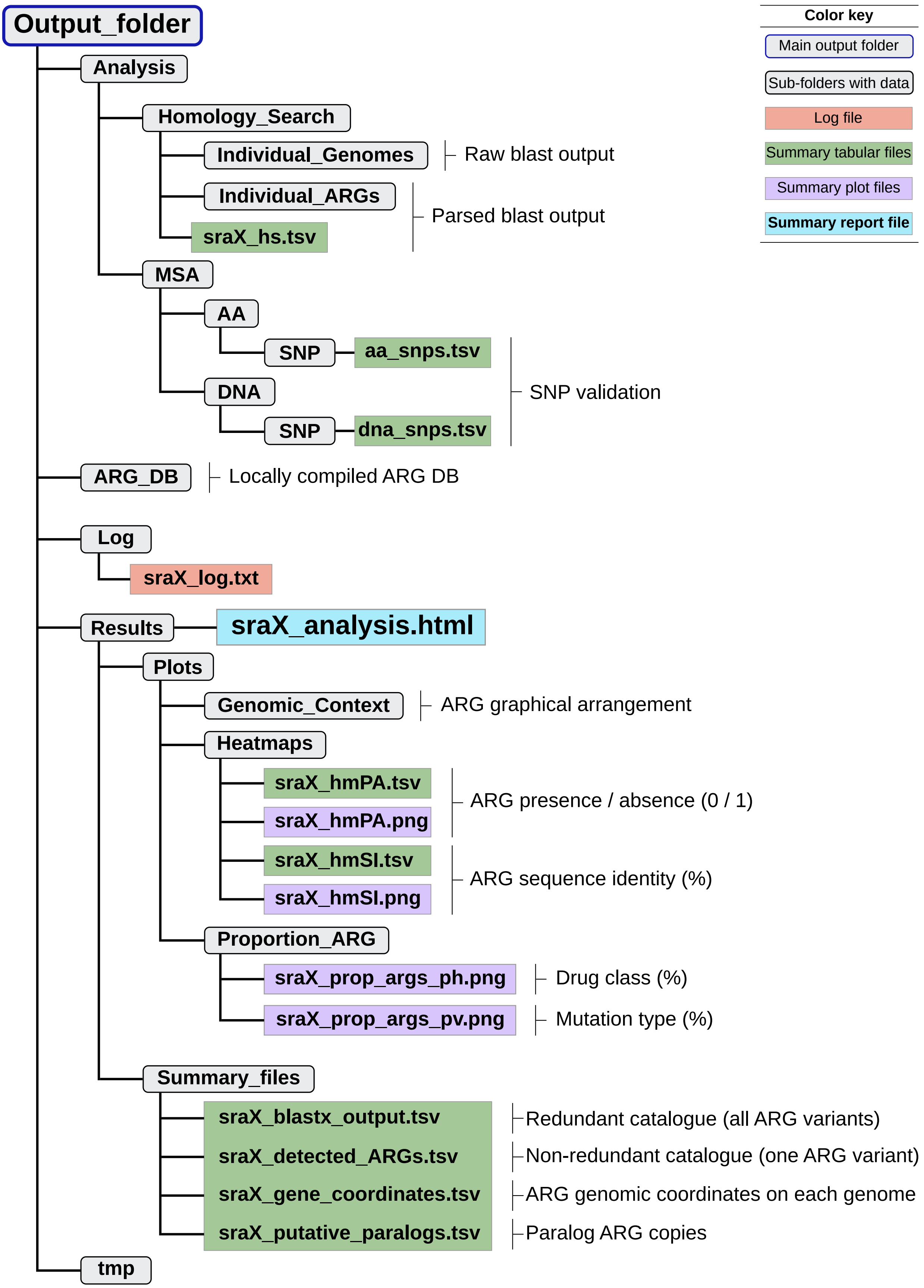




**Figure S1.** Directory structure automatically created by sraX, where all output files are currently stored.

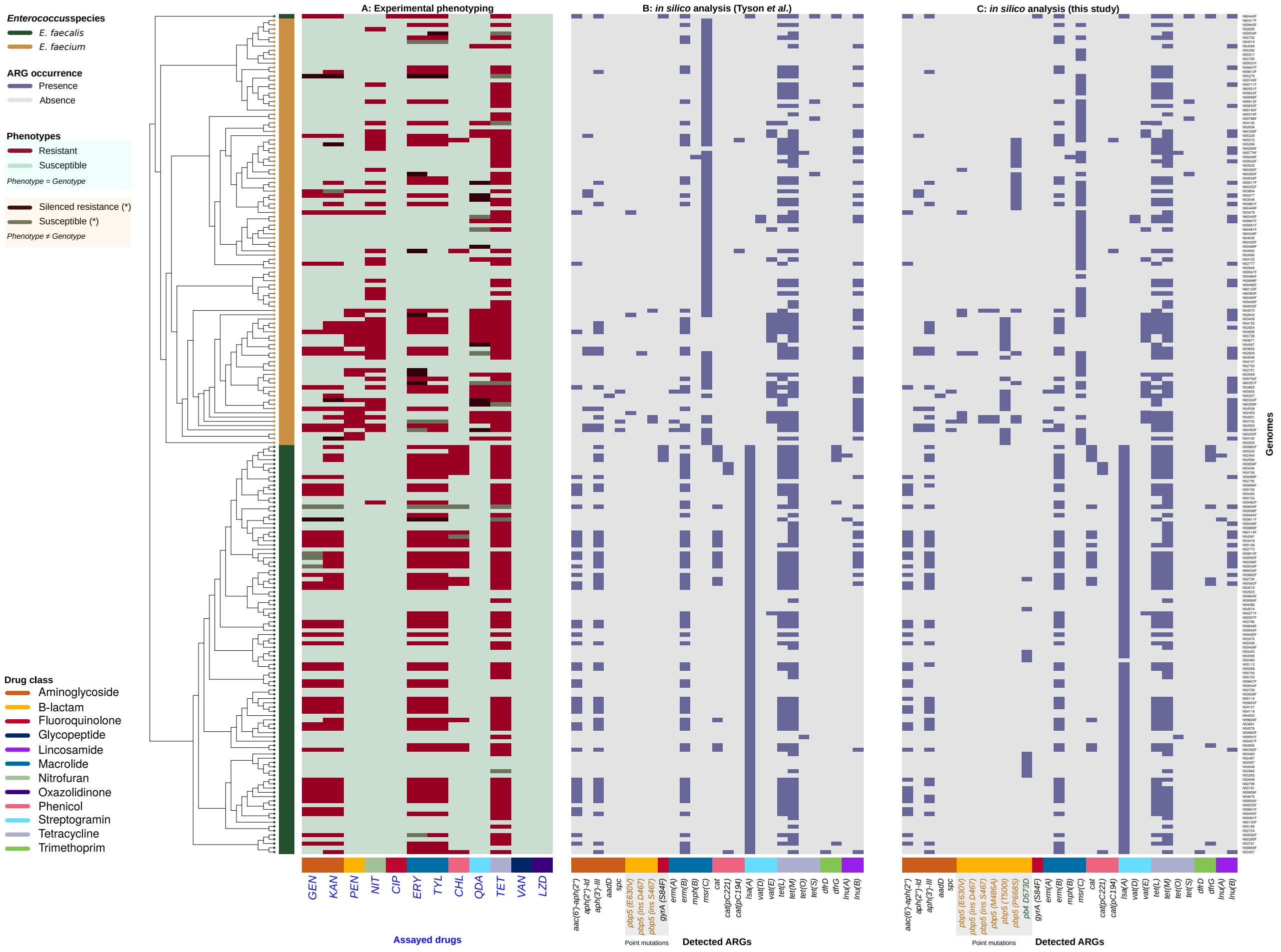


**Figure S2.** Comparative analysis of *in-silico* detected ARGs within the collection of 197 *Enterococcus spp.* strains in the original study (heatmap B) versus our proposed tool (heatmap C). Violet and light-gray colors indicate presence and absence of specific ARG in a given genome. In order to evince their phenotype correspondence, the AMR activities of assayed drugs are as well shown (heatmap A). Red and light-green colors indicate resistance and susceptibility to corresponding antibiotics. Dark-red color indicates an exhibited resistance phenotype in the absence of an *in silico* detected ARG, while a dark-green color indicates an exhibited susceptible phenotype in the presence of an *in silico* detected ARG. A phylogenetic tree is shown on the left side of heatmap A.

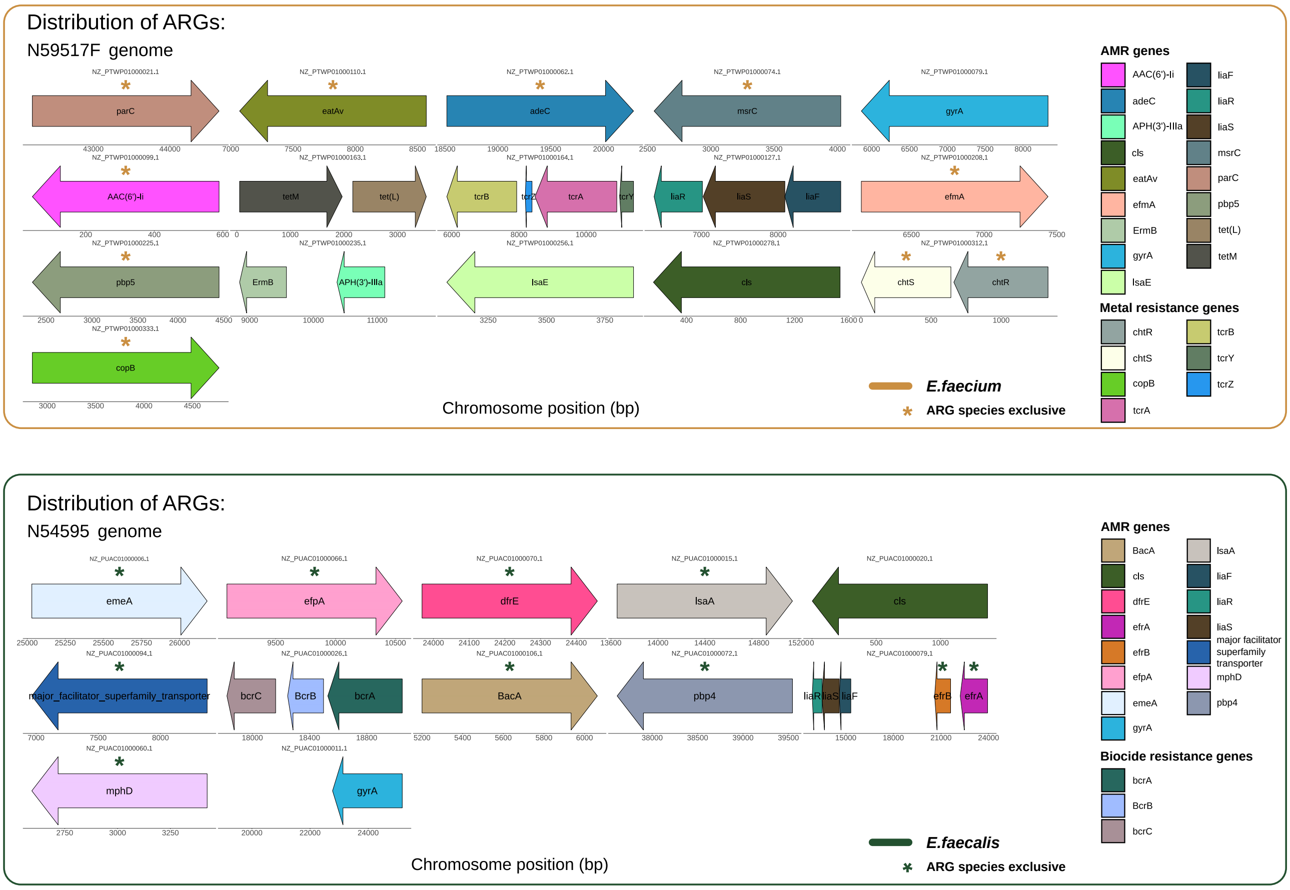


**Figure S3.** ARG context exploration within representatives genomes from *E. faecium* and *E. faecalis* species. As expected, certain related genes like those conferring resistance to biocides (*bcr(A)*, *bcr(B)* and *bcr(C)* benzalkonium chloride resistance genes) or to specific metals (*tcr(A)*, *tcr(B)*, *tcr(Y)* and *tcr(Z)* copper resistance genes) are located contiguously, indicating their putative transmission on mobile elements. In addition, the ARGs which are limited to only one species are marked with an asterisk.
