# Supplementary material for: sraX: A Novel Comprehensive Resistome Analysis Tool
Source: Front Microbiol. 2020 Feb 5;11:52. doi: 10.3389/fmicb.2020.00052 (PMC7025521; doi:10.3389/fmicb.2020.00052)
Supplement: Supplementary file 4 [file Data_Sheet_3.ZIP › pbp4/pbp4_dna.html]

 
 
 
 

   pbp4   

    
    
    Definition:  Not indicated  
    
    
    Reference organism:  Not indicated     
    
     Summary Data     Parameter    Value (n)     Total number of genomes  97    MSA length  2101    Total number of SNPs  149    SNP density (# SNPs/MSA length)  0.071      
    
    
    
 
    Enterococcus_faecalis (sraxID_jCuwG4bHjSL4)    
   A   
   T   
   G   
   G   
   A   
   A   
   C   
   G   
   A   
   A   
   G   
   C   
   A   
   A   
   T   
   C   
   G   
   T   
   A   
   A   
   T   
   A   
   A   
   A   
   A   
   A   
   A   
   T   
   C   
   T   
   T   
   C   
   A   
   A   
   A   
   A   
   A   
   A   
   A   
   C   
   C   
   A   
   C   
   T   
   T   
   A   
   T   
   T   
   C   
   T   
   T   
   G   
   G   
   T   
   G   
   T   
   T   
   T   
   C   
   T   
   G   
   C   
   C   
   T   
   T   
   G   
   G   
   T   
   T   
   C   
   T   
   A   
   A   
   T   
   C   
   G   
   C   
   T   
   G   
   C   
   T   
   G   
   C   
   C   
   G   
   G   
   T   
   G   
   G   
   C   
   G   
   G   
   G   
   T   
   A   
   T   
   T   
   A   
   T   
   G   
   C   
   T   
   T   
   A   
   T   
   A   
   G   
   T   
   C   
   A   
   A   
   T   
   G   
   G   
   C   
   A   
   A   
   G   
   C   
   C   
   A   
   A   
   A   
   C   
   A   
   A   
   G   
   A   
    A    
   T   
   T   
   A   
   G   
   C   
    C    
   G   
   A   
   A   
   G   
   C   
   G   
   A   
   A   
   G   
   A   
   A   
   A   
   A   
    T    
   A   
   G   
   C   
    T    
   A   
    C    
   T   
    A    
    C    
   A   
   T   
   T   
   T   
   T   
   T   
   A   
   A   
   A   
    C    
   G   
   T   
   A   
   T   
   T   
   G   
    T    
   C   
   A   
   A   
   A   
   A   
   C   
   A   
   G   
    G    
   A   
   A   
   T   
   T   
   T   
   G   
   A   
   T   
   A   
   A   
   G   
   T   
   T   
   A   
   C   
   C   
    G    
   T   
   C   
   C   
   G   
   T   
   T   
    G    
   T   
   T   
   C   
   A   
   A   
   G   
   A   
   A   
    G    
   C   
   T   
   A   
   G   
   C   
   T   
   T   
   A   
   A   
   A   
   G   
   A   
   A   
   A   
   A   
   A   
   T   
   G   
   G   
    C    
   T   
   A   
   T   
   G   
   A   
   T   
   A   
   C   
   T   
   A   
   A   
   A   
   T   
   C   
    T    
   G   
   T   
   T   
   G   
   T   
   T   
   G   
   A   
   A   
   A   
   A   
   A   
   T   
   A   
   C   
   C   
   A   
   A   
   G   
   C   
   A   
   A   
   T   
   T   
   T   
   A   
   T   
   T   
   C   
   A   
   G   
   G   
   G   
   A   
   T   
   T   
   C   
   A   
   A   
   G   
   C   
    A    
   G   
   A   
   A   
    G    
   G   
    A    
   G   
   T   
   C   
   A   
   A   
    A    
   G   
   C   
   T   
   A   
   G   
   T   
   G   
   A   
   T   
   G   
   T   
    T    
   C   
   A   
   A   
   G   
   T   
    C    
   A   
   A   
   A   
   A   
   A   
    G    
   G   
    C    
   G   
   A   
   A   
   A   
   G   
   A   
    C    
   A   
   A   
    T    
   C   
   A   
   A   
   T   
   A   
   C   
   A   
   C   
   A   
   T   
   T   
   T   
   A   
    C    
   C   
   T   
   A   
   T   
   A   
   A   
   A   
   T   
   T   
   A   
   T   
   C   
   G   
   A   
   T   
   G   
   A   
   G   
    C    
   A   
   C   
    T    
   C   
   C   
   T   
   T   
   T   
   A   
   G   
   G   
   C   
   G   
   A   
   A   
   A   
   T   
   G   
   A   
   A   
   A   
   G   
   A   
   T   
    T    
   T   
   G   
   T   
   C   
   T   
   T   
   A   
   T   
   C   
   A   
   A   
   T   
   C   
   A   
   A   
   G   
   T   
   A   
   T   
    C    
   G   
    C    
   C   
   A   
   A   
    G    
   A   
   A   
   A   
   G   
   G   
    C    
   G   
   A   
   T   
   A   
    C    
   C   
   T   
   A   
   C   
   C   
   A   
   A   
   A   
   T   
    C    
    G    
   C   
   T   
   T   
   G   
   G   
    A    
    A    
    G    
   C   
   C   
    G    
   T   
   C   
   T   
   T   
   T   
   A   
   A   
   T   
   T   
   T   
   T   
   T   
   C   
   C   
   A   
   G   
   A   
   T   
   A   
   T   
   G   
   T   
   C   
   A   
   G   
   G   
   A   
   A   
   A   
   T   
   G   
   A   
   T   
   A   
   A   
   A   
    A    
   T   
   T   
   T   
   C   
   G   
   A   
   T   
   T   
   C   
   A   
   A   
   G   
   T   
   A   
   G   
   A   
   T   
   A   
   A   
   T   
   G   
   C   
   C   
   A   
   A   
   A   
   C   
   G   
   T   
   G   
   G   
   A   
   G   
   A   
   A   
   A   
   T   
   T   
   G   
   T   
   C   
   G   
   A   
   T   
   C   
   G   
   T   
   A   
   A   
    T    
   G   
   G   
   T   
   A   
   G   
   T   
   G   
   G   
   G   
   C   
   T   
   A   
   G   
    C    
   A   
   A   
   T   
   T   
   A   
   A   
   C   
   A   
   A   
   A   
   G   
   T   
   G   
   T   
   T   
   T   
   G   
   A   
    C    
   G   
   A   
   A   
   G   
   T   
   G   
   G   
   G   
   C   
   G   
   T   
   A   
   G   
   T   
    G    
   C   
   C   
   T   
    G    
   G   
   C   
   A   
   A   
   A   
   C   
   T   
    C    
   G   
   G   
   T   
   T   
   C   
   T   
   G   
   G   
   C   
   G   
   C   
   A   
   G   
   A   
   A   
   A   
   A   
   A   
    A    
   C   
   A   
   G   
   C   
    C    
   A   
   A   
   T   
   A   
   T   
   C   
   A   
   A   
   A   
   G   
   C   
   T   
   T   
   T   
   T   
   A   
   G   
   T   
   G   
   A   
   T   
   A   
   A   
    A    
   T   
   T   
    C    
   G   
   G   
   C   
   G   
   T   
   T   
   T   
   C   
   T   
    G    
   T   
   T   
   G   
   A   
   T   
   G   
   A   
   A   
   A   
   T   
   C   
   A   
   A   
   T   
   C   
    A    
   A   
   A   
   A   
    G    
   T   
   T   
   A   
   A   
   G   
   C   
   C   
   A   
   A   
   G   
   G   
    A    
   T   
   G   
   G   
   G   
   T   
   C   
   C   
   A   
   A   
   G   
   C   
   A   
   G   
   A   
   C   
   T   
   C   
    C    
   T   
   T   
   T   
   G   
   T   
   A   
   C   
   C   
   A   
   A   
   T   
   C   
   A   
   C   
   A   
   G   
   T   
    C    
   G   
   C   
   T   
   T   
   C   
   T   
   G   
   A   
   A   
   C   
   C   
   A   
   G   
   T   
   G   
   A   
   C   
   A   
   G   
   A   
   A   
   T   
   T   
   A   
   C   
   C   
   A   
   A   
   C   
   A   
   G   
   G   
   G   
   G   
   C   
   T   
   G   
   C   
   G   
   A   
   C   
   A   
   A   
   A   
   A   
   G   
    A    
    T    
   A   
   C   
   A   
   G   
   A   
   G   
   T   
   C   
   A   
   C   
   G   
   T   
   T   
   A   
   T   
   T   
   A   
   T   
   C   
   C   
    G    
    C    
   T   
   G   
   G   
   G   
   G   
   G   
   A   
   A   
   G   
   C   
   A   
   G   
   C   
   T   
   G   
   C   
   G   
   C   
   A   
   A   
   T   
   T   
   A   
   A   
   T   
   C   
   G   
   G   
    G    
   T   
   A   
   T   
   A   
   C   
    G    
   G   
   G   
    C    
   A   
   C   
    C    
   A   
   T   
   T   
   A   
   C   
   T   
   G   
   C   
   A   
   G   
   A   
    A    
   G   
   A   
   T   
   A   
   T   
   T   
    G    
   A   
   A   
   A   
   A   
   A   
   A   
   A   
   T   
   C   
   C   
   A   
   G   
   A   
   G   
   T   
   T   
   A   
   A   
   G   
   T   
   A   
   G   
   T   
   A   
   C   
   G   
   G   
   G   
    C    
   G   
   T   
   A   
   A   
   T   
   T   
   G   
   G   
    T    
   A   
   A   
   A   
   A   
   C   
   T   
   G   
   G   
    T    
   T   
   T   
   A   
   G   
   A   
   A   
   C   
   G   
   A   
   G   
   C   
   G   
   T   
   T   
   T   
   G   
   A   
   T   
   A   
   A   
   A   
   G   
   A   
    A    
   T   
   T   
   A   
   A   
   G   
   A   
   G   
   G   
    G    
   C   
   A   
   A   
   G   
   A   
   T   
   G   
   G   
    T    
   G   
   G   
    C    
   T   
   C   
    A    
   C   
   T   
   A   
   G   
   T   
   T   
   A   
   T   
   T   
   T   
   T   
   A   
   G   
   A   
   T   
   G   
   A   
   C   
   A   
   A   
   A   
   G   
   A   
   G   
   A   
   A   
   T   
   G   
   T   
   C   
   A   
   A   
   A   
   A   
   A   
   A   
   G   
   C   
   T   
   T   
   T   
   A   
   C   
   A   
   A   
   A   
   C   
   C   
   A   
   A   
   A   
   G   
   A   
    G    
   A   
   A   
   A   
   A   
   A   
   A   
   G   
   A   
   T   
   G   
   G   
   T   
   C   
   A   
   A   
   A   
   C   
    G    
   A   
   T   
   T   
   A   
   A   
   A   
   T   
   T   
   A   
   A   
   C   
   G   
   A   
   T   
   T   
   G   
   A   
    C    
   A   
   G   
    T    
   G   
   G   
    C    
   G   
   T   
   A   
   C   
   A   
   A   
   C   
   A   
   A   
   C   
   A   
   A   
   G   
   C   
    T    
   T   
   T   
    T    
   G   
   C   
   T   
   A   
   T   
   A   
   T   
   T   
   T   
   G   
   A   
   C   
   A   
   A   
   A   
   C   
   G   
    C    
    C    
   C   
   T   
   G   
   G   
   T   
   A   
   G   
   T   
   G   
   C   
   G   
   G   
   T   
   C   
   A   
   T   
   T   
   A   
   C   
   C   
   G   
   A   
   T   
   C   
   C   
   T   
   C   
   A   
   A   
   A   
   A   
   A   
   G   
   G   
   C   
   G   
   A   
   T   
   T   
   T   
   A   
    T    
   T   
   A   
   G   
   C   
    A    
   A   
   C   
   G   
   G   
   T   
   T   
   A   
   G   
   T   
   T   
   C   
   A   
   C   
   C   
   T   
   T   
   C   
   G   
   T   
   A   
   T   
   G   
   A   
   T   
   C   
   C   
   T   
   A   
   A   
   T   
   A   
   A   
   A   
   A   
   T   
   G   
   G   
   C   
   C   
   A   
   A   
   T   
   G   
   G   
    C    
   A   
   T   
   T   
   T   
   C   
   T   
   C   
   A   
   A   
   A   
   A   
   A   
   G   
   A   
    A    
   T   
   A   
   T   
   G   
   A   
   T   
   G   
   C   
   T   
   T   
   A   
   C   
   A   
   A   
   T   
   A   
   A   
   C   
   A   
   A   
   C   
   A   
   A   
   A   
   G   
   A   
   T   
   T   
   T   
   A   
   C   
   C   
   A   
   T   
   T   
    C    
   A   
   C   
   A   
   G   
   C   
   A   
   C   
   G   
   T   
   T   
   T   
    T    
   G   
   C   
   G   
   A   
   C   
   A   
   G   
   G   
    C    
   T   
   A   
   T   
   G   
   C   
   A   
   C   
   C   
   A   
   G   
   G   
    C    
   T   
   C   
   T   
   A   
   C   
   C   
   T   
   T   
   T   
   A   
   A   
   A   
   A   
   C   
   A   
   A   
   T   
   T   
   A   
   C   
   C   
   G   
   G   
   T   
   G   
   C   
   C   
   A   
   T   
   T   
   G   
   G   
   T   
   T   
   T   
   A   
   G   
   A   
   T   
    G    
   C   
   A   
   G   
   G   
   G   
   A   
   C   
   A   
   T   
   T   
   G   
   A   
   A   
   G   
   C   
   C   
   A   
   G   
   A   
   T   
   G   
   A   
   A   
   G   
   A   
   G   
   C   
   T   
   A   
   G   
   A   
   A   
   A   
   T   
   T   
   A   
   A   
   T   
   G   
   G   
    T    
   T   
   T   
   G   
   A   
   A   
   A   
   T   
   G   
   G   
   C   
   A   
   A   
   A   
   A   
   A   
   G   
   A   
   T   
   A   
   A   
   A   
   T   
   C   
   T   
   T   
   G   
   G   
   G   
   G   
    C    
   G   
   G   
   C   
   T   
   A   
   T   
   T   
   T   
   T   
   G   
   C   
    G    
   A   
   C   
   A   
   C   
   G   
   T   
   G   
   T   
   A   
   A   
   A   
   A   
   G   
   A   
   A   
   G   
   C   
   A   
   A   
   G   
   T   
   C   
   C   
   A   
   G   
   T   
   C   
   A   
   A   
   T   
   C   
   T   
    G    
   C   
   G   
   T   
   A   
   C   
   C   
   G   
   C   
   T   
   T   
   T   
   A   
   G   
   T   
   T   
   A   
   A   
   T   
   T   
   C   
   A   
   G   
   A   
   T   
   A   
   A   
   T   
   A   
   T   
   T   
   T   
   A   
   T   
   T   
   T   
   T   
   G   
   C   
    C    
   C   
   A   
   A   
   C   
   A   
   A   
   A   
   C   
   A   
   T   
   T   
   A   
   C   
   G   
    G    
   A   
   T   
   G   
   G   
   G   
    A    
   G   
   A   
   A   
   G   
   A   
   C   
   A   
   A   
   A   
   T   
   T   
   T   
   A   
   G   
   A   
   G   
   C   
    G    
   G   
   G   
   T   
   C   
   T   
   T   
   A   
   A   
   T   
   A   
   A   
   A   
   T   
   T   
   C   
   A   
   T   
   T   
   T   
   T   
   C   
   G   
   G   
   T   
   G   
   A   
   G   
   G   
   A   
   A   
   C   
   T   
   A   
   G   
   A   
   T   
   T   
   T   
   A   
   C   
   C   
   A   
   A   
   T   
    T    
   G   
   C   
   C   
   A   
   T   
   G   
   A   
   C   
   G   
   C   
   C   
    T    
   G   
   C   
   T   
   C   
   A   
   A   
   A   
   T   
   T   
   T   
   C   
   A   
   A   
   A   
    T    
   G   
   A   
   A   
   G   
   A   
   T   
   A   
   A   
   A   
   T   
   T   
   T   
   A   
   A   
   T   
   T   
   C   
   T   
   G   
   A   
   A   
   A   
   T   
   T   
   C   
   T   
   T   
   T   
   T   
    A    
   G   
   C   
   A   
   G   
   A   
   T   
   A   
   C   
    G    
   G   
   G   
   C   
   T   
   A   
    C    
   G   
   G   
    T    
   C   
   A   
   A   
   G   
   G   
    T    
   C   
   A   
   A   
   T   
   T   
   G   
   T   
   T   
   A   
   A   
   T   
   T   
   T   
   C   
    G    
   C   
   C   
   A   
   A   
   T   
   C   
   C   
   A   
   G   
   C   
   A   
   A   
   G   
   C   
   T   
   A   
   C   
   C   
   A   
   T   
   G   
   T   
   A   
   T   
   A   
   G   
   T   
   G   
   T   
   T   
   T   
   T   
   C   
   C   
   A   
   A   
   A   
   A   
   T   
   A   
   A   
   T   
   G   
   G   
    A    
   A   
   C   
   A   
    T    
   T   
    A    
   G   
   T   
    C    
   T   
   A   
   T   
   C   
   C   
   A   
   A   
   A   
   A   
    C    
   T   
   A   
   G   
   T   
   T   
   T   
   T   
   A   
   G   
   A   
   C   
   A   
   A   
   A   
   G   
   A   
   A   
   A   
   C   
    G    
   A   
   A   
   G   
   A   
   A   
   A   
   A   
   A   
   A   
   G   
   A   
   C   
   A   
   A   
   T   
   G   
   T   
   A   
   A   
   T   
   C   
   A   
   G   
   C   
   G   
   C   
   C   
   A   
   A   
   C   
   G   
   C   
   A   
   G   
   C   
   G   
   A   
   A   
   T   
   A   
   C   
   A   
   A   
   T   
   T   
   G   
   C   
   C   
   A   
   C   
   A   
   G   
   A   
   T   
   C   
   T   
   T   
   T   
   T   
   A   
   G   
   G   
   T   
   A   
   G   
   T   
   G   
   T   
   G   
   G   
   A   
   A   
   G   
   A   
   T   
   C   
   C   
   T   
   T   
   C   
   A   
   G   
   G   
   T   
   T   
   A   
   T   
   G   
   T   
   T   
   T   
   A   
   T   
   A   
   A   
   T   
   A   
   T   
   G   
   T   
   A   
   C   
   A   
   A   
   T   
   C   
   C   
   G   
   A   
   A   
   C   
   T   
   T   
   C   
   T   
   C   
   A   
   T   
   T   
   G   
   G   
   C   
   T   
   G   
   C   
   T   
   A   
   A   
   A   
   A   
   C   
   A   
   G   
   G   
   A   
   A   
   C   
   A   
   G   
   C   
   T   
   G   
   A   
   A   
   A   
   T   
   A   
   A   
   A   
   A   
   G   
   A   
   T   
   A   
   A   
   A   
   C   
   A   
   A   
   G   
   A   
   T   
   A   
   C   
   A   
   G   
   A   
   T   
   G   
   G   
   C   
   A   
   A   
   A   
   G   
   A   
   A   
   A   
   A   
   C   
   A   
   G   
   T   
   T   
   T   
   C   
   C   
   T   
   T   
   C   
   T   
   A   
   A   
   C   
   G   
   T   
   T   
   A   
   G   
   A   
   T   
   C   
   G   
   T   
   A   
   G   
   T   
   A   
   A   
   C   
   A   
   A   
   T   
   A   
   A   
   A   
   T   
   T   
   C   
   T   
   T   
   A   
   A   
   C   
   A   
   A   
   T   
   G   
   A   
   T   
   T   
   A   
   T   
   G   
   G   
   T   
   T   
   G   
   A   
   A   
   A   
   A   
   T   
   T   
   C   
   A   
   G   
   G   
   A   
   G   
   A   
   A   
   A   
   A   
   T   
   G   
   G   
   T   
   T   
   C   
   T   
   G   
   C   
   A   
   A   
   C   
   A   
   G   
   A   
   T   
   A   
   T   
   C   
   A   
   G   
   T   
   A   
   A   
   A   
   C   
   C   
   A   
   T   
   T   
   A   
   A   
   T   
   T   
   G   
   A   
   T   
   T   
   A   
   C   
   T   
   T   
   A   
   G   
   A   
   A   
   G   
   C   
   A   
   A   
   C   
   C   
   A   
   T   
   T   
   A   
   A   
   A   
   T   
   A   
   A   
   A   
   A   
   A   
   G   
   A   
   G   
   A   
   A   
   A   
   A   
   T   
   G   
   A   
   A   
   C   
   G   
   A   
   A   
   A   
   C   
   G   
   A   
   G   
   G   
   T   
   T   
   T   
   C   
   T   
   G   
   T   
   A   
   A   
   A   
   A   
   A   
   G   
   A   
   A   
   A   
   C   
   C   
   T   
   C   
   G   
   T   
   T   
   T   
   T   
   C   
   C   
   C   
   T   
   T   
   T   
   T   
   T   
   A   
 

 
   N52662   
   A   
   T   
   G   
   G   
   A   
   A   
   C   
   G   
   A   
   A   
   G   
   C   
   A   
   A   
   T   
   C   
   G   
   T   
   A   
   A   
   T   
   A   
   A   
   A   
   A   
   A   
   A   
   T   
   C   
   T   
   T   
   C   
   A   
   A   
   A   
   A   
   A   
   A   
   A   
   C   
   C   
   A   
   C   
   T   
   T   
   A   
   T   
   T   
   C   
   T   
   T   
   G   
   G   
   C   
   G   
   T   
   T   
   T   
   C   
   T   
   G   
   C   
   C   
   T   
   T   
   G   
   G   
   T   
   T   
   C   
   T   
   G   
   A   
   T   
   C   
   G   
   C   
   T   
   G   
   C   
   T   
   G   
   T   
   C   
   G   
   G   
   T   
   G   
   G   
   T   
   G   
   G   
   G   
   T   
   A   
   T   
   T   
   A   
   T   
   G   
   C   
   T   
   T   
   A   
   T   
   A   
   G   
   C   
   C   
   A   
   A   
   T   
   G   
   G   
   C   
   A   
   A   
   G   
   C   
   C   
   A   
   A   
   A   
   C   
   A   
   A   
   G   
   A   
    A    
   T   
   T   
   A   
   G   
   C   
    T    
   G   
   A   
   A   
   G   
   C   
   G   
   A   
   A   
   G   
   A   
   A   
   A   
   A   
    C    
   A   
   G   
   C   
    C    
   A   
    A    
   T   
    A    
    C    
   A   
   T   
   T   
   T   
   T   
   T   
   A   
   A   
   A   
    C    
   G   
   T   
   A   
   T   
   T   
   G   
    T    
   C   
   A   
   A   
   A   
   A   
   C   
   A   
   G   
    G    
   A   
   A   
   T   
   T   
   T   
   G   
   A   
   T   
   A   
   A   
   G   
   T   
   T   
   A   
   C   
   C   
    A    
   T   
   C   
   C   
   G   
   T   
   T   
    G    
   T   
   T   
   C   
   A   
   A   
   G   
   A   
   A   
    A    
   C   
   T   
   A   
   G   
   C   
   T   
   T   
   A   
   A   
   A   
   G   
   A   
   A   
   A   
   A   
   A   
   T   
   G   
   G   
    C    
   T   
   A   
   T   
   G   
   A   
   T   
   A   
   C   
   T   
   A   
   A   
   A   
   T   
   C   
    T    
   G   
   T   
   T   
   G   
   T   
   T   
   G   
   A   
   A   
   A   
   A   
   A   
   T   
   A   
   C   
   C   
   A   
   A   
   G   
   C   
   A   
   A   
   T   
   T   
   T   
   A   
   T   
   T   
   C   
   A   
   G   
   G   
   G   
   A   
   T   
   T   
   C   
   A   
   A   
   G   
   C   
    A    
   G   
   A   
   A   
    G    
   G   
    G    
   G   
   T   
   C   
   A   
   A   
    A    
   G   
   C   
   T   
   A   
   G   
   T   
   G   
   A   
   T   
   G   
   T   
    T    
   C   
   A   
   A   
   G   
   T   
    C    
   A   
   A   
   A   
   A   
   A   
    A    
   G   
    G    
   G   
   A   
   A   
   A   
   G   
   A   
    C    
   A   
   A   
    C    
   C   
   A   
   A   
   T   
   A   
   C   
   A   
   C   
   A   
   T   
   T   
   T   
   A   
    C    
   C   
   T   
   A   
   T   
   A   
   A   
   A   
   T   
   T   
   A   
   T   
   C   
   G   
   A   
   T   
   G   
   A   
   G   
    C    
   A   
   C   
    T    
   C   
   C   
   T   
   T   
   T   
   A   
   G   
   G   
   C   
   G   
   A   
   A   
   A   
   T   
   G   
   A   
   A   
   A   
   G   
   A   
   T   
    T    
   T   
   G   
   T   
   C   
   T   
   T   
   A   
   T   
   C   
   A   
   A   
   T   
   C   
   A   
   A   
   G   
   T   
   A   
   T   
    C    
   G   
    C    
   C   
   A   
   A   
    A    
   A   
   A   
   A   
   G   
   G   
    C    
   G   
   A   
   T   
   A   
    A    
   C   
   T   
   A   
   C   
   C   
   A   
   A   
   A   
   T   
    C    
    G    
   C   
   T   
   T   
   G   
   G   
    G    
    C    
    A    
   C   
   C   
    G    
   T   
   C   
   T   
   T   
   T   
   A   
   A   
   T   
   T   
   T   
   T   
   T   
   C   
   C   
   A   
   G   
   A   
   T   
   A   
   T   
   G   
   T   
   C   
   A   
   G   
   G   
   A   
   A   
   A   
   T   
   G   
   A   
   T   
   A   
   A   
   A   
    A    
   T   
   T   
   T   
   C   
   G   
   A   
   T   
   T   
   C   
   A   
   A   
   G   
   T   
   A   
   G   
   A   
   T   
   A   
   A   
   T   
   G   
   C   
   C   
   A   
   A   
   A   
   C   
   G   
   T   
   G   
   G   
   A   
   G   
   A   
   A   
   A   
   T   
   T   
   G   
   T   
   C   
   G   
   A   
   T   
   C   
   G   
   T   
   A   
   A   
    T    
   G   
   G   
   T   
   A   
   G   
   T   
   G   
   G   
   G   
   C   
   T   
   A   
   G   
    C    
   A   
   A   
   T   
   T   
   A   
   A   
   C   
   A   
   A   
   A   
   G   
   T   
   G   
   T   
   T   
   T   
   G   
   A   
    T    
   G   
   A   
   A   
   G   
   T   
   G   
   G   
   G   
   C   
   G   
   T   
   A   
   G   
   T   
    G    
   C   
   C   
   T   
    G    
   G   
   C   
   A   
   A   
   A   
   C   
   T   
    C    
   G   
   G   
   T   
   T   
   C   
   T   
   G   
   G   
   C   
   G   
   C   
   A   
   G   
   A   
   A   
   A   
   A   
   A   
    A    
   C   
   A   
   G   
   C   
    C    
   A   
   A   
   T   
   A   
   T   
   C   
   A   
   A   
   A   
   G   
   C   
   T   
   T   
   T   
   T   
   A   
   G   
   T   
   G   
   A   
   T   
   A   
   A   
    G    
   T   
   T   
    T    
   G   
   G   
   C   
   G   
   T   
   T   
   T   
   C   
   T   
    G    
   T   
   T   
   G   
   A   
   T   
   G   
   A   
   A   
   A   
   T   
   C   
   A   
   A   
   T   
   C   
    A    
   A   
   A   
   A   
    G    
   T   
   T   
   A   
   A   
   G   
   C   
   C   
   A   
   A   
   G   
   G   
    A    
   T   
   G   
   G   
   G   
   T   
   C   
   C   
   A   
   A   
   G   
   C   
   A   
   G   
   A   
   C   
   T   
   C   
    G    
   T   
   T   
   T   
   G   
   T   
   A   
   C   
   C   
   A   
   A   
   T   
   C   
   A   
   C   
   A   
   G   
   T   
    T    
   G   
   C   
   T   
   T   
   C   
   T   
   G   
   A   
   A   
   C   
   C   
   A   
   G   
   T   
   G   
   A   
   C   
   A   
   G   
   A   
   A   
   T   
   T   
   A   
   C   
   C   
   A   
   A   
   C   
   A   
   G   
   G   
   G   
   G   
   C   
   T   
   G   
   C   
   G   
   A   
   C   
   A   
   A   
   A   
   A   
   G   
    A    
    T    
   A   
   C   
   A   
   G   
   A   
   G   
   T   
   C   
   A   
   C   
   G   
   T   
   T   
   A   
   T   
   T   
   A   
   T   
   C   
   C   
    A    
    T    
   T   
   G   
   G   
   G   
   G   
   G   
   A   
   A   
   G   
   C   
   A   
   G   
   C   
   T   
   G   
   C   
   G   
   C   
   A   
   A   
   T   
   T   
   A   
   A   
   T   
   C   
   G   
   G   
    G    
   T   
   A   
   T   
   A   
   C   
    G    
   G   
   G   
    C    
   A   
   C   
    T    
   A   
   T   
   T   
   A   
   C   
   T   
   G   
   C   
   A   
   G   
   A   
    A    
   G   
   A   
   T   
   A   
   T   
   T   
    G    
   A   
   A   
   A   
   A   
   A   
   A   
   A   
   T   
   C   
   C   
   A   
   G   
   A   
   G   
   T   
   T   
   A   
   A   
   G   
   T   
   A   
   G   
   T   
   A   
   C   
   G   
   G   
   G   
    C    
   G   
   T   
   A   
   A   
   T   
   T   
   G   
   G   
    C    
   A   
   A   
   A   
   A   
   C   
   T   
   G   
   G   
    C    
   T   
   T   
   A   
   G   
   A   
   A   
   C   
   G   
   A   
   G   
   C   
   G   
   T   
   T   
   T   
   G   
   A   
   T   
   A   
   A   
   A   
   G   
   A   
    A    
   T   
   T   
   A   
   A   
   G   
   A   
   G   
   G   
    A    
   C   
   A   
   A   
   G   
   A   
   T   
   G   
   G   
    T    
   G   
   G   
    T    
   T   
   C   
    A    
   C   
   T   
   A   
   G   
   T   
   T   
   A   
   T   
   T   
   T   
   T   
   A   
   G   
   A   
   T   
   G   
   A   
   C   
   A   
   A   
   A   
   G   
   A   
   G   
   A   
   A   
   T   
   G   
   T   
   C   
   A   
   A   
   A   
   A   
   A   
   A   
   G   
   C   
   T   
   T   
   T   
   A   
   C   
   A   
   A   
   A   
   C   
   C   
   A   
   A   
   A   
   G   
   A   
    G    
   A   
   A   
   A   
   A   
   A   
   A   
   G   
   A   
   T   
   G   
   G   
   T   
   C   
   A   
   A   
   A   
   C   
    A    
   A   
   T   
   T   
   A   
   A   
   A   
   T   
   T   
   A   
   A   
   C   
   G   
   A   
   T   
   T   
   G   
   A   
    T    
   A   
   G   
    T    
   G   
   G   
    C    
   G   
   T   
   A   
   C   
   A   
   A   
   C   
   A   
   A   
   C   
   A   
   A   
   G   
   C   
    C    
   T   
   T   
    C    
   G   
   C   
   T   
   A   
   T   
   A   
   T   
   T   
   T   
   G   
   A   
   C   
   A   
   A   
   A   
   C   
   G   
    T    
    C    
   C   
   T   
   G   
   G   
   T   
   A   
   G   
   T   
   G   
   C   
   G   
   G   
   T   
   C   
   A   
   T   
   T   
   A   
   C   
   C   
   G   
   A   
   T   
   C   
   C   
   T   
   C   
   A   
   A   
   A   
   A   
   A   
   G   
   G   
   C   
   G   
   A   
   T   
   T   
   T   
   A   
    C    
   T   
   A   
   G   
   C   
    G    
   A   
   C   
   G   
   G   
   T   
   T   
   A   
   G   
   T   
   T   
   C   
   A   
   C   
   C   
   T   
   T   
   C   
   G   
   T   
   A   
   T   
   G   
   A   
   T   
   C   
   C   
   T   
   A   
   A   
   T   
   A   
   A   
   A   
   A   
   T   
   G   
   G   
   C   
   C   
   A   
   A   
   T   
   G   
   G   
    C    
   A   
   T   
   T   
   T   
   C   
   T   
   C   
   A   
   A   
   A   
   A   
   A   
   G   
   A   
    A    
   T   
   A   
   T   
   G   
   A   
   T   
   G   
   C   
   T   
   T   
   A   
   C   
   A   
   A   
   T   
   A   
   A   
   C   
   A   
   A   
   C   
   A   
   A   
   A   
   G   
   A   
   T   
   T   
   T   
   A   
   C   
   C   
   A   
   T   
   T   
    C    
   A   
   C   
   A   
   G   
   C   
   A   
   C   
   G   
   T   
   T   
   T   
    T    
   G   
   C   
   G   
   A   
   C   
   A   
   G   
   G   
    C    
   T   
   A   
   T   
   G   
   C   
   A   
   C   
   C   
   A   
   G   
   G   
    C    
   T   
   C   
   T   
   A   
   C   
   C   
   T   
   T   
   T   
   A   
   A   
   A   
   A   
   C   
   A   
   A   
   T   
   T   
   A   
   C   
   C   
   G   
   G   
   T   
   G   
   C   
   C   
   A   
   T   
   T   
   G   
   G   
   T   
   T   
   T   
   A   
   G   
   A   
   T   
    G    
   C   
   A   
   G   
   G   
   G   
   A   
   C   
   A   
   T   
   T   
   G   
   A   
   A   
   G   
   C   
   C   
   A   
   G   
   A   
   T   
   G   
   A   
   A   
   G   
   A   
   G   
   C   
   T   
   A   
   G   
   A   
   A   
   A   
   T   
   T   
   A   
   A   
   T   
   G   
   G   
    C    
   T   
   T   
   G   
   A   
   A   
   A   
   T   
   G   
   G   
   C   
   A   
   A   
   A   
   A   
   A   
   G   
   A   
   T   
   A   
   A   
   A   
   T   
   C   
   T   
   T   
   G   
   G   
   G   
   G   
    C    
   G   
   G   
   C   
   T   
   A   
   T   
   T   
   T   
   T   
   G   
   C   
    G    
   A   
   C   
   A   
   C   
   G   
   T   
   G   
   T   
   A   
   A   
   A   
   A   
   G   
   A   
   A   
   G   
   C   
   A   
   A   
   G   
   T   
   C   
   C   
   A   
   G   
   T   
   C   
   A   
   A   
   T   
   C   
   T   
    T    
   C   
   G   
   T   
   A   
   C   
   C   
   G   
   C   
   T   
   T   
   T   
   A   
   G   
   T   
   T   
   A   
   A   
   T   
   T   
   C   
   A   
   G   
   A   
   T   
   A   
   A   
   T   
   A   
   T   
   T   
   T   
   A   
   T   
   T   
   T   
   T   
   G   
   C   
    C    
   C   
   A   
   A   
   C   
   A   
   A   
   A   
   C   
   A   
   T   
   T   
   A   
   C   
   G   
    G    
   A   
   T   
   G   
   G   
   G   
    G    
   G   
   A   
   A   
   G   
   A   
   C   
   A   
   A   
   A   
   T   
   T   
   T   
   A   
   G   
   A   
   G   
   C   
    G    
   G   
   G   
   T   
   C   
   T   
   T   
   A   
   A   
   T   
   A   
   A   
   A   
   T   
   T   
   C   
   A   
   T   
   T   
   T   
   T   
   C   
   G   
   G   
   T   
   G   
   A   
   G   
   G   
   A   
   A   
   C   
   T   
   A   
   G   
   A   
   T   
   T   
   T   
   A   
   C   
   C   
   A   
   A   
   T   
    T    
   G   
   C   
   C   
   A   
   T   
   G   
   A   
   C   
   G   
   C   
   C   
    T    
   G   
   C   
   T   
   C   
   A   
   A   
   A   
   T   
   T   
   T   
   C   
   A   
   A   
   A   
    C    
   G   
   A   
   A   
   G   
   A   
   T   
   A   
   A   
   A   
   T   
   T   
   T   
   A   
   A   
   T   
   T   
   C   
   T   
   G   
   A   
   A   
   A   
   T   
   T   
   C   
   T   
   T   
   T   
   T   
    A    
   G   
   C   
   A   
   G   
   A   
   T   
   A   
   C   
    A    
   G   
   G   
   C   
   T   
   A   
    T    
   G   
   G   
    C    
   C   
   A   
   A   
   G   
   G   
    C    
   C   
   A   
   A   
   T   
   T   
   G   
   T   
   T   
   A   
   A   
   T   
   T   
   T   
   C   
    G    
   C   
   C   
   A   
   A   
   T   
   C   
   C   
   A   
   G   
   C   
   A   
   A   
   G   
   C   
   T   
   A   
   C   
   C   
   A   
   T   
   G   
   T   
   A   
   T   
   A   
   G   
   T   
   G   
   T   
   T   
   T   
   T   
   C   
   C   
   A   
   A   
   A   
   A   
   T   
   A   
   A   
   T   
   G   
   G   
    A    
   A   
   C   
   A   
    T    
   T   
    A    
   G   
   T   
    T    
   T   
   A   
   T   
   C   
   C   
   A   
   A   
   A   
   A   
    T    
   T   
   A   
   G   
   T   
   T   
   T   
   T   
   A   
   G   
   A   
   G   
   A   
   A   
   A   
   G   
   A   
   A   
   A   
   C   
    G    
   A   
   A   
   G   
   A   
   A   
   A   
   A   
   A   
   A   
   G   
   A   
   C   
   A   
   A   
   T   
   G   
   T   
   A   
   A   
   T   
   C   
   A   
   G   
   C   
   G   
   C   
   C   
   A   
   A   
   C   
   G   
   C   
   A   
   G   
   C   
   G   
   A   
   A   
   T   
   A   
   C   
   A   
   A   
   T   
   A   
   G   
   C   
   C   
   A   
   C   
   A   
   G   
   A   
   T   
   C   
   T   
   T   
   T   
   T   
   A   
   G   
   G   
   T   
   A   
   G   
   T   
   G   
   T   
   G   
   G   
   A   
   A   
   G   
   A   
   T   
   C   
   C   
   T   
   T   
   C   
   A   
   G   
   G   
   T   
   T   
   A   
   T   
   G   
   T   
   T   
   T   
   A   
   T   
   A   
   A   
   T   
   A   
   T   
   G   
   T   
   A   
   C   
   A   
   A   
   T   
   C   
   C   
   G   
   A   
   A   
   C   
   T   
   T   
   C   
   T   
   C   
   A   
   T   
   T   
   G   
   G   
   C   
   T   
   G   
   C   
   C   
   A   
   A   
   A   
   A   
   C   
   A   
   G   
   G   
   A   
   A   
   C   
   A   
   G   
   C   
   T   
   G   
   A   
   A   
   A   
   T   
   A   
   A   
   A   
   A   
   G   
   A   
   T   
   A   
   A   
   A   
   C   
   A   
   A   
   G   
   A   
   T   
   A   
   C   
   A   
   G   
   A   
   T   
   G   
   G   
   C   
   A   
   A   
   A   
   G   
   A   
   A   
   A   
   A   
   C   
   A   
   G   
   T   
   T   
   T   
   C   
   C   
   T   
   T   
   C   
   T   
   A   
   A   
   C   
   G   
   T   
   T   
   A   
   G   
   A   
   T   
   C   
   G   
   T   
   A   
   G   
   T   
   A   
   A   
   C   
   A   
   A   
   T   
   A   
   A   
   A   
   T   
   T   
   T   
   T   
   T   
   A   
   A   
   C   
   A   
   A   
   T   
   G   
   A   
   T   
   T   
   A   
   T   
   G   
   G   
   T   
   T   
   G   
   A   
   A   
   A   
   A   
   T   
   T   
   C   
   A   
   G   
   G   
   A   
   G   
   A   
   A   
   A   
   A   
   T   
   G   
   G   
   T   
   T   
   C   
   T   
   G   
   C   
   A   
   A   
   C   
   A   
   G   
   A   
   T   
   A   
   T   
   C   
   A   
   G   
   T   
   A   
   A   
   A   
   C   
   C   
   A   
   T   
   T   
   A   
   A   
   T   
   T   
   G   
   A   
   T   
   T   
   A   
   C   
   T   
   T   
   A   
   G   
   A   
   A   
   G   
   C   
   A   
   A   
   C   
   C   
   A   
   T   
   T   
   A   
   A   
   A   
   -   
   -   
   -   
   -   
   -   
   -   
   -   
   -   
   -   
   -   
   -   
   -   
   -   
   -   
   -   
   -   
   -   
   -   
   -   
   -   
   -   
   -   
   -   
   -   
   -   
   -   
   -   
   -   
   -   
   -   
   -   
   -   
   -   
   -   
   -   
   -   
   -   
   -   
   -   
   -   
   -   
   -   
   -   
   -   
   -   
   -   
   -   
   -   
   -   
   -   
   -   
   -   
   -   
   -   
   -   
   -   
   -   
   -   
   -   
   -   
   -   
   A   
   T   
   G   
   G   
   A   
   A   
   C   
   G   
   A   
   A   
   G   
   C   
   A   
   A   
   T   
   C   
   G   
   T   
   A   
   A   
   T   
   A   
   A   
   A   
   A   
   A   
   A   
   T   
   C   
   T   
   T   
   C   
   A   
   A   
   A   
   A   
   A   
   A   
   A   
   C   
   C   
   A   
   C   
   T   
   T   
   A   
   T   
   T   
   C   
   T   
   T   
   G   
   G   
   C   
   G   
   T   
   T   
   T   
   C   
   T   
   G   
   C   
   C   
   T   
   T   
   G   
   G   
   T   
   T   
   C   
   T   
   G   
   A   
   T   
   C   
   G   
   C   
   T   
   G   
   C   
   T   
   G   
   T   
   C   
   G   
   G   
   T   
   G   
   G   
   T   
   G   
   G   
   G   
   T   
   A   
   T   
   T   
   A   
   T   
   G   
   C   
   T   
   T   
   A   
   T   
   A   
   G   
   C   
   C   
   A   
   A   
   T   
   G   
   G   
   C   
   A   
   A   
   G   
   C   
   C   
   A   
   A   
   A   
   C   
   A   
   A   
   G   
   A   
   A   
   T   
   T   
   A   
   G   
   C   
   T   
   G   
   A   
   A   
   G   
   C   
   G   
   A   
   A   
   G   
   A   
   A   
   A   
   A   
   C   
   A   
   G   
   C   
   C   
   A   
   A   
   T   
   A   
   C   
   A   
   T   
   T   
   T   
   T   
   T   
   A   
   A   
   A   
   C   
   G   
   T   
   A   
   T   
   T   
   G   
   T   
   C   
   A   
   A   
   A   
   A   
   C   
   A   
   G   
   G   
   A   
   A   
   T   
   T   
   T   
   G   
   A   
   T   
   A   
   A   
   G   
   T   
   T   
   A   
   C   
   C   
   A   
   T   
   C   
   C   
   G   
   T   
   T   
   G   
   T   
   T   
   C   
   A   
   A   
   G   
   A   
   A   
   A   
   C   
   T   
   A   
   G   
   C   
   T   
   T   
   A   
   A   
   A   
   G   
   A   
   A   
   A   
   A   
   A   
   T   
   G   
   G   
   C   
   T   
   A   
   T   
   G   
   A   
   T   
   A   
   C   
   T   
   A   
   A   
   A   
   T   
   C   
   T   
   G   
   T   
   T   
   G   
   T   
   T   
   G   
   A   
   A   
   A   
   A   
   A   
   T   
   A   
   C   
   C   
   A   
   A   
   G   
   C   
   A   
   A   
   T   
   T   
   T   
   A   
   T   
   T   
   C   
   A   
   G   
   G   
   G   
   A   
   T   
   T   
   C   
   A   
   A   
   G   
   C   
   A   
   G   
   A   
   A   
   G   
   G   
   G   
   G   
   T   
   C   
   A   
   A   
   A   
   G   
   C   
   T   
   A   
   G   
   T   
   G   
   A   
   T   
   G   
   T   
   T   
   C   
   A   
   A   
   G   
   T   
   C   
   A   
   A   
   A   
   A   
   A   
   A   
   G   
   G   
   G   
   A   
   A   
   A   
   G   
   A   
   C   
   A   
   A   
   C   
   C   
   A   
   A   
   T   
   A   
   C   
   A   
   C   
   A   
   T   
   T   
   T   
   A   
   C   
   C   
   T   
   A   
   T   
   A   
   A   
   A   
   T   
   T   
   A   
   T   
   C   
   G   
   A   
   T   
   G   
   A   
   G   
   C   
   A   
   C   
   T   
   C   
   C   
   T   
   T   
   T   
   A   
   G   
   G   
   C   
   G   
   A   
   A   
   A   
   T   
   G   
   A   
   A   
   A   
   G   
   A   
   T   
   T   
   T   
   G   
   T   
   C   
   T   
   T   
   A   
   T   
   C   
   A   
   A   
   T   
   C   
   A   
   A   
   G   
   T   
   A   
   T   
   C   
   G   
   C   
   C   
   A   
   A   
   A   
   A   
   A   
   A   
   G   
   G   
   C   
   G   
   A   
   T   
   A   
   A   
   C   
   T   
   A   
   C   
   C   
   A   
   A   
   A   
   T   
   C   
   G   
   C   
   T   
   T   
   G   
   G   
   G   
   C   
   A   
   C   
   C   
   G   
   T   
   C   
   T   
   T   
   T   
   A   
   A   
   T   
   T   
   T   
   T   
   T   
   C   
   C   
   A   
   G   
   A   
   T   
   A   
   T   
   G   
   T   
   C   
   A   
   G   
   G   
   A   
   A   
   A   
   T   
   G   
   A   
   T   
   A   
   A   
   A   
   A   
   T   
   T   
   T   
   C   
   G   
   A   
   T   
   T   
   C   
   A   
   A   
   G   
   T   
   A   
   G   
   A   
   T   
   A   
   A   
   T   
   G   
   C   
   C   
   A   
   A   
   A   
   C   
   G   
   T   
   G   
   G   
   A   
   G   
   A   
   A   
   A   
   T   
   T   
   G   
   T   
   C   
   G   
   A   
   T   
   C   
   G   
   T   
   A   
   A   
   T   
   G   
   G   
   T   
   A   
   G   
   T   
   G   
   G   
   G   
   C   
   T   
   A   
   G   
   C   
   A   
   A   
   T   
   T   
   A   
   A   
   C   
   A   
   A   
   A   
   G   
   T   
   G   
   T   
   T   
   T   
   G   
   A   
   T   
   G   
   A   
   A   
   G   
   T   
   G   
   G   
   G   
   C   
   G   
   T   
   A   
   G   
   T   
   G   
   C   
   C   
   T   
   G   
   G   
   C   
   A   
   A   
   A   
   C   
   T   
   C   
   G   
   G   
   T   
   T   
   C   
   T   
   G   
   G   
   C   
   G   
   C   
   A   
   G   
   A   
   A   
   A   
   A   
   A   
   A   
   C   
   A   
   G   
   C   
   C   
   A   
   A   
   T   
   A   
   T   
   C   
   A   
   A   
   A   
   G   
   C   
   T   
   T   
   T   
   T   
   A   
   G   
   T   
   G   
   A   
   T   
   A   
   A   
   G   
   T   
   T   
   T   
   G   
   G   
   C   
   G   
   T   
   T   
   T   
   C   
   T   
   G   
   T   
   T   
   G   
   A   
   T   
   G   
   A   
   A   
   A   
   T   
   C   
   A   
   A   
   T   
   C   
   A   
   A   
   A   
   A   
   G   
   T   
   T   
   A   
   A   
   G   
   C   
   C   
   A   
   A   
   G   
   G   
   A   
   T   
   G   
   G   
   G   
   T   
   C   
   C   
   A   
   A   
   G   
   C   
   A   
   G   
   A   
   C   
   T   
   C   
   G   
   T   
   T   
   T   
   G   
   T   
   A   
   C   
   C   
   A   
   A   
   T   
   C   
   A   
   C   
   A   
   G   
   T   
   T   
   G   
   C   
   T   
   T   
   C   
   T   
   G   
   A   
   A   
   C   
   C   
   A   
   G   
   T   
   G   
   A   
   C   
   A   
   G   
   A   
   A   
   T   
   T   
   A   
   C   
   C   
   A   
   A   
   C   
   A   
   G   
   G   
   G   
   G   
   C   
   T   
   G   
   C   
   G   
   A   
   C   
   A   
   A   
   A   
   A   
   G   
   A   
   T   
   A   
   C   
   A   
   G   
   A   
   G   
   T   
   C   
   A   
   C   
   G   
   T   
   T   
   A   
   T   
   T   
   A   
   T   
   C   
   C   
   A   
   T   
   T   
   G   
   G   
   G   
   G   
   G   
   A   
   A   
   G   
   C   
   A   
   G   
   C   
   T   
   G   
   C   
   G   
   C   
   A   
   A   
   T   
   T   
   A   
   A   
   T   
   C   
   G   
   G   
   G   
   T   
   A   
   T   
   A   
   C   
   G   
   G   
   G   
   C   
   A   
   C   
   T   
   A   
   T   
   T   
   A   
   C   
   T   
   G   
   C   
   A   
   G   
   A   
   A   
   G   
   A   
   T   
   A   
   T   
   T   
   G   
   A   
   A   
   A   
   A   
   A   
   A   
   A   
   T   
   C   
   C   
   A   
   G   
   A   
   G   
   T   
   T   
   A   
   A   
   G   
   T   
   A   
   G   
   T   
   A   
   C   
   G   
   G   
   G   
   C   
   G   
   T   
   A   
   A   
   T   
   T   
   G   
   G   
   C   
   A   
   A   
   A   
   A   
   C   
   T   
   G   
   G   
   C   
   T   
   T   
   A   
   G   
   A   
   A   
   C   
   G   
   A   
   G   
   C   
   G   
   T   
   T   
   T   
   G   
   A   
   T   
   A   
   A   
   A   
   G   
   A   
   A   
   T   
   T   
   A   
   A   
   G   
   A   
   G   
   G   
   A   
   C   
   A   
   A   
   G   
   A   
   T   
   G   
   G   
   T   
   G   
   G   
   T   
   T   
   C   
   A   
   C   
   T   
   A   
   G   
   T   
   T   
   A   
   T   
   T   
   T   
   T   
   A   
   G   
   A   
   T   
   G   
   A   
   C   
   A   
   A   
   A   
   G   
   A   
   G   
   A   
   A   
   T   
   G   
   T   
   C   
   A   
   A   
   A   
   A   
   A   
   A   
   G   
   C   
   T   
   T   
   T   
   A   
   C   
   A   
   A   
   A   
   C   
   C   
   A   
   A   
   A   
   G   
   A   
   G   
   A   
   A   
   A   
   A   
   A   
   A   
   G   
   A   
   T   
   G   
   G   
   T   
   C   
   A   
   A   
   A   
   C   
   A   
   A   
   T   
   T   
   A   
   A   
   A   
   T   
   T   
   A   
   A   
   C   
   G   
   A   
   T   
   T   
   G   
   A   
   T   
   A   
   G   
   T   
   G   
   G   
   C   
   G   
   T   
   A   
   C   
   A   
   A   
   C   
   A   
   A   
   C   
   A   
   A   
   G   
   C   
   C   
   T   
   T   
   C   
   G   
   C   
   T   
   A   
   T   
   A   
   T   
   T   
   T   
   G   
   A   
   C   
   A   
   A   
   A   
   C   
   G   
   T   
   C   
   C   
   T   
   G   
   G   
   T   
   A   
   G   
   T   
   G   
   C   
   G   
   G   
   T   
   C   
   A   
   T   
   T   
   A   
   C   
   C   
   G   
   A   
   T   
   C   
   C   
   T   
   C   
   A   
   A   
   A   
   A   
   A   
   G   
   G   
   C   
   G   
   A   
   T   
   T   
   T   
   A   
   C   
   T   
   A   
   G   
   C   
   G   
   A   
   C   
   G   
   G   
   T   
   T   
   A   
   G   
   T   
   T   
   C   
   A   
   C   
   C   
   T   
   T   
   C   
   G   
   T   
   A   
   T   
   G   
   A   
   T   
   C   
   C   
   T   
   A   
   A   
   T   
   A   
   A   
   A   
   A   
   T   
   G   
   G   
   C   
   C   
   A   
   A   
   T   
   G   
   G   
   C   
   A   
   T   
   T   
   T   
   C   
   T   
   C   
   A   
   A   
   A   
   A   
   A   
   G   
   A   
   A   
   T   
   A   
   T   
   G   
   A   
   T   
   G   
   C   
   T   
   T   
   A   
   C   
   A   
   A   
   T   
   A   
   A   
   C   
   A   
   A   
   C   
   A   
   A   
   A   
   G   
   A   
   T   
   T   
   T   
   A   
   C   
   C   
   A   
   T   
   T   
   C   
   A   
   C   
   A   
   G   
   C   
   A   
   C   
   G   
   T   
   T   
   T   
   T   
   G   
   C   
   G   
   A   
   C   
   A   
   G   
   G   
   C   
   T   
   A   
   T   
   G   
   C   
   A   
   C   
   C   
   A   
   G   
   G   
   C   
   T   
   C   
   T   
   A   
   C   
   C   
   T   
   T   
   T   
   A   
   A   
   A   
   A   
   C   
   A   
   A   
   T   
   T   
   A   
   C   
   C   
   G   
   G   
   T   
   G   
   C   
   C   
   A   
   T   
   T   
   G   
   G   
   T   
   T   
   T   
   A   
   G   
   A   
   T   
   G   
   C   
   A   
   G   
   G   
   G   
   A   
   C   
   A   
   T   
   T   
   G   
   A   
   A   
   G   
   C   
   C   
   A   
   G   
   A   
   T   
   G   
   A   
   A   
   G   
   A   
   G   
   C   
   T   
   A   
   G   
   A   
   A   
   A   
   T   
   T   
   A   
   A   
   T   
   G   
   G   
   C   
   T   
   T   
   G   
   A   
   A   
   A   
   T   
   G   
   G   
   C   
   A   
   A   
   A   
   A   
   A   
   G   
   A   
   T   
   A   
   A   
   A   
   T   
   C   
   T   
   T   
   G   
   G   
   G   
   G   
   C   
   G   
   G   
   C   
   T   
   A   
   T   
   T   
   T   
   T   
   G   
   C   
   G   
   A   
   C   
   A   
   C   
   G   
   T   
   G   
   T   
   A   
   A   
   A   
   A   
   G   
   A   
   A   
   G   
   C   
   A   
   A   
   G   
   T   
   C   
   C   
   A   
   G   
   T   
   C   
   A   
   A   
   T   
   C   
   T   
   T   
   C   
   G   
   T   
   A   
   C   
   C   
   G   
   C   
   T   
   T   
   T   
   A   
   G   
   T   
   T   
   A   
   A   
   T   
   T   
   C   
   A   
   G   
   A   
   T   
   A   
   A   
   T   
   A   
   T   
   T   
   T   
   A   
   T   
   T   
   T   
   T   
   G   
   C   
   C   
   C   
   A   
   A   
   C   
   A   
   A   
   A   
   C   
   A   
   T   
   T   
   A   
   C   
   G   
   G   
   A   
   T   
   G   
   G   
   G   
   G   
   G   
   A   
   A   
   G   
   A   
   C   
   A   
   A   
   A   
   T   
   T   
   T   
   A   
   G   
   A   
   G   
   C   
   G   
   G   
   G   
   T   
   C   
   T   
   T   
   A   
   A   
   T   
   A   
   A   
   A   
   T   
   T   
   C   
   A   
   T   
   T   
   T   
   T   
   C   
   G   
   G   
   T   
   G   
   A   
   G   
   G   
   A   
   A   
   C   
   T   
   A   
   G   
   A   
   T   
   T   
   T   
   A   
   C   
   C   
   A   
   A   
   T   
   T   
   G   
   C   
   C   
   A   
   T   
   G   
   A   
   C   
   G   
   C   
   C   
   T   
   G   
   C   
   T   
   C   
   A   
   A   
   A   
   T   
   T   
   T   
   C   
   A   
   A   
   A   
   C   
   G   
   A   
   A   
   G   
   A   
   T   
   A   
   A   
   A   
   T   
   T   
   T   
   A   
   A   
   T   
   T   
   C   
   T   
   G   
   A   
   A   
   A   
   T   
   T   
   C   
   T   
   T   
   T   
   T   
   A   
   G   
   C   
   A   
   G   
   A   
   T   
   A   
   C   
   A   
   G   
   G   
   C   
   T   
   A   
   T   
   G   
   G   
   C   
   C   
   A   
   A   
   G   
   G   
   C   
   C   
   A   
   A   
   T   
   T   
   G   
   T   
   T   
   A   
   A   
   T   
   T   
   T   
   C   
   G   
   C   
   C   
   A   
   A   
   T   
   C   
   C   
   A   
   G   
   C   
   A   
   A   
   G   
   C   
   T   
   A   
   C   
   C   
   A   
   T   
   G   
   T   
   A   
   T   
   A   
   G   
   T   
   G   
   T   
   T   
   T   
   T   
   C   
   C   
   A   
   A   
   A   
   A   
   T   
   A   
   A   
   T   
   G   
   G   
   A   
   A   
   C   
   A   
   T   
   T   
   A   
   G   
   T   
   T   
   T   
   A   
   T   
   C   
   C   
   A   
   A   
   A   
   A   
   T   
   T   
   A   
   G   
   T   
   T   
   T   
   T   
   A   
   G   
   A   
   G   
   A   
   A   
   A   
   G   
   A   
   A   
   A   
   C   
   G   
   A   
   A   
   G   
   A   
   A   
   A   
   A   
   A   
   A   
   G   
   A   
   C   
   A   
   A   
   T   
   G   
   T   
   A   
   A   
   T   
   C   
   A   
   G   
   C   
   G   
   C   
   C   
   A   
   A   
   C   
   G   
   C   
   A   
   G   
   C   
   G   
   A   
   A   
   T   
   A   
   C   
   A   
   A   
   T   
   A   
   G   
   C   
   C   
   A   
   C   
   A   
   G   
   A   
   T   
   C   
   T   
   T   
   T   
   T   
   A   
   G   
   G   
   T   
   A   
   G   
   T   
   G   
   T   
   G   
   G   
   A   
   A   
   G   
   A   
   T   
   C   
   C   
   T   
   T   
   C   
   A   
   G   
   G   
   T   
   T   
   A   
   T   
   G   
   T   
   T   
   T   
   A   
   T   
   A   
   A   
   T   
   A   
   T   
   G   
   T   
   A   
   C   
   A   
   A   
   T   
   C   
   C   
   G   
   A   
   A   
   C   
   T   
   T   
   C   
   T   
   C   
   A   
   T   
   T   
   G   
   G   
   C   
   T   
   G   
   C   
   C   
   A   
   A   
   A   
   A   
   C   
   A   
   G   
   G   
   A   
   A   
   C   
   A   
   G   
   C   
   T   
   G   
   A   
   A   
   A   
   T   
   A   
   A   
   A   
   A   
   G   
   A   
   T   
   A   
   A   
   A   
   C   
   A   
   A   
   G   
   A   
   T   
   A   
   C   
   A   
   G   
   A   
   T   
   G   
   G   
   C   
   A   
   A   
   A   
   G   
   A   
   A   
   A   
   A   
   C   
   A   
   G   
   T   
   T   
   T   
   C   
   C   
   T   
   T   
   C   
   T   
   A   
   A   
   C   
   G   
   T   
   T   
   A   
   G   
   A   
   T   
   C   
   G   
   T   
   A   
   G   
   T   
   A   
   A   
   C   
   A   
   A   
   T   
   A   
   A   
   A   
   T   
   T   
   T   
   T   
   T   
   A   
   A   
   C   
   A   
   A   
   T   
   G   
   A   
   T   
   T   
   A   
   T   
   G   
   G   
   T   
   T   
   G   
   A   
   A   
   A   
   A   
   T   
   T   
   C   
   A   
   G   
   G   
   A   
   G   
   A   
   A   
   A   
   A   
   T   
   G   
   G   
   T   
   T   
   C   
   T   
   G   
   C   
   A   
   A   
   C   
   A   
   G   
   A   
   T   
   A   
   T   
   C   
   A   
   G   
   T   
   A   
   A   
   A   
   C   
   C   
   A   
   T   
   T   
   A   
   A   
   T   
   T   
   G   
   A   
   T   
   T   
   A   
   C   
   T   
   T   
   A   
   G   
   A   
   A   
   G   
   C   
   A   
   A   
   C   
   C   
   A   
   T   
   T   
   A   
   A   
   A   
   -   
   -   
   -   
   -   
   -   
   -   
   -   
   -   
   -   
   -   
   -   
   -   
   -   
   -   
   -   
   -   
   -   
   -   
   -   
   -   
   -   
   -   
   -   
   -   
   -   
   -   
   -   
   -   
   -   
   -   
   -   
   -   
   -   
   -   
   -   
   -   
   -   
   -   
   -   
   -   
   -   
   -   
   -   
   -   
   -   
   -   
   -   
   -   
   -   
   -   
   -   
   -   
   -   
   -   
   -   
   -   
   -   
   -   
   -   
   -   
   -   
 

 
   N54097   
   -   
   -   
   -   
   -   
   -   
   -   
   -   
   -   
   -   
   -   
   -   
   -   
   -   
   -   
   -   
   -   
   -   
   -   
   -   
   -   
   -   
   -   
   -   
   -   
   -   
   -   
   -   
   -   
   -   
   -   
   -   
   -   
   -   
   -   
   -   
   -   
   -   
   -   
   -   
   -   
   -   
   -   
   -   
   -   
   -   
   -   
   -   
   -   
   -   
   -   
   -   
   -   
   -   
   -   
   -   
   -   
   -   
   -   
   -   
   -   
   -   
   -   
   -   
   -   
   -   
   -   
   -   
   -   
   -   
   -   
   -   
   -   
   -   
   -   
   -   
   -   
   -   
   -   
   -   
   -   
   -   
   -   
   -   
   -   
   -   
   -   
   -   
   -   
   -   
   -   
   -   
   -   
   -   
   -   
   -   
   -   
   -   
   -   
   -   
   -   
   -   
   -   
   -   
   -   
   -   
   -   
   -   
   -   
   C   
   A   
   A   
   T   
   G   
   G   
   C   
   A   
   A   
   G   
   C   
   C   
   A   
   A   
   A   
   C   
   A   
   A   
   G   
   A   
    A    
   T   
   T   
   A   
   G   
   C   
    C    
   G   
   A   
   A   
   G   
   C   
   G   
   A   
   A   
   G   
   A   
   A   
   A   
   A   
    C    
   A   
   G   
   C   
    T    
   A   
    C    
   T   
    A    
    C    
   A   
   T   
   T   
   T   
   T   
   T   
   A   
   A   
   A   
    C    
   G   
   T   
   A   
   T   
   T   
   G   
    T    
   C   
   A   
   A   
   A   
   A   
   C   
   A   
   G   
    G    
   A   
   A   
   T   
   T   
   T   
   G   
   A   
   T   
   A   
   A   
   G   
   T   
   T   
   A   
   C   
   C   
    G    
   T   
   C   
   C   
   G   
   T   
   T   
    G    
   T   
   T   
   C   
   A   
   A   
   G   
   A   
   A   
    G    
   C   
   T   
   A   
   G   
   C   
   T   
   T   
   A   
   A   
   A   
   G   
   A   
   A   
   A   
   A   
   A   
   T   
   G   
   G   
    C    
   T   
   A   
   T   
   G   
   A   
   T   
   A   
   C   
   T   
   A   
   A   
   A   
   T   
   C   
    T    
   G   
   T   
   T   
   G   
   T   
   T   
   G   
   A   
   A   
   A   
   A   
   A   
   T   
   A   
   C   
   C   
   A   
   A   
   G   
   C   
   A   
   A   
   T   
   T   
   T   
   A   
   T   
   T   
   C   
   A   
   G   
   G   
   G   
   A   
   T   
   T   
   C   
   A   
   A   
   G   
   C   
    A    
   G   
   A   
   A   
    G    
   G   
    A    
   G   
   T   
   C   
   A   
   A   
    A    
   G   
   C   
   T   
   A   
   G   
   T   
   G   
   A   
   T   
   G   
   T   
    T    
   C   
   A   
   A   
   G   
   T   
    A    
   A   
   A   
   A   
   A   
   A   
    G    
   G   
    C    
   G   
   A   
   A   
   A   
   G   
   A   
    C    
   A   
   A   
    T    
   C   
   A   
   A   
   T   
   A   
   C   
   A   
   C   
   A   
   T   
   T   
   T   
   A   
    C    
   C   
   T   
   A   
   T   
   A   
   A   
   A   
   T   
   T   
   A   
   T   
   C   
   G   
   A   
   T   
   G   
   A   
   G   
    T    
   A   
   C   
    C    
   C   
   C   
   T   
   T   
   T   
   A   
   G   
   G   
   C   
   G   
   A   
   A   
   A   
   T   
   G   
   A   
   A   
   A   
   G   
   A   
   T   
    T    
   T   
   G   
   T   
   C   
   T   
   T   
   A   
   T   
   C   
   A   
   A   
   T   
   C   
   A   
   A   
   G   
   T   
   A   
   T   
    C    
   G   
    C    
   C   
   A   
   A   
    A    
   A   
   A   
   A   
   G   
   G   
    C    
   G   
   A   
   T   
   A   
    C    
   C   
   T   
   A   
   C   
   C   
   A   
   A   
   A   
   T   
    C    
    G    
   C   
   T   
   T   
   G   
   G   
    A    
    A    
    G    
   C   
   C   
    G    
   T   
   C   
   T   
   T   
   T   
   A   
   A   
   T   
   T   
   T   
   T   
   T   
   C   
   C   
   A   
   G   
   A   
   T   
   A   
   T   
   G   
   T   
   C   
   A   
   G   
   G   
   A   
   A   
   A   
   T   
   G   
   A   
   T   
   A   
   A   
   A   
    A    
   T   
   T   
   T   
   C   
   G   
   A   
   T   
   T   
   C   
   A   
   A   
   G   
   T   
   A   
   G   
   A   
   T   
   A   
   A   
   T   
   G   
   C   
   C   
   A   
   A   
   A   
   C   
   G   
   T   
   G   
   G   
   A   
   G   
   A   
   A   
   A   
   T   
   T   
   G   
   T   
   C   
   G   
   A   
   T   
   C   
   G   
   T   
   A   
   A   
    T    
   G   
   G   
   T   
   A   
   G   
   T   
   G   
   G   
   G   
   C   
   T   
   A   
   G   
    C    
   A   
   A   
   T   
   T   
   A   
   A   
   C   
   A   
   A   
   A   
   G   
   T   
   G   
   T   
   T   
   T   
   G   
   A   
    T    
   G   
   A   
   A   
   G   
   T   
   G   
   G   
   G   
   C   
   G   
   T   
   A   
   G   
   T   
    G    
   C   
   C   
   T   
    G    
   G   
   C   
   A   
   A   
   A   
   C   
   T   
    C    
   G   
   G   
   T   
   T   
   C   
   T   
   G   
   G   
   C   
   G   
   C   
   A   
   G   
   A   
   A   
   A   
   A   
   A   
    A    
   C   
   A   
   G   
   C   
    C    
   A   
   A   
   T   
   A   
   T   
   C   
   A   
   A   
   A   
   G   
   C   
   T   
   T   
   T   
   T   
   A   
   G   
   T   
   G   
   A   
   T   
   A   
   A   
    A    
   T   
   T   
    C    
   G   
   G   
   C   
   G   
   T   
   T   
   T   
   C   
   T   
    G    
   T   
   T   
   G   
   A   
   T   
   G   
   A   
   A   
   A   
   T   
   C   
   A   
   A   
   T   
   C   
    A    
   A   
   A   
   A   
    G    
   T   
   T   
   A   
   A   
   G   
   C   
   C   
   A   
   A   
   G   
   G   
    A    
   T   
   G   
   G   
   G   
   T   
   C   
   C   
   A   
   A   
   G   
   C   
   A   
   G   
   A   
   C   
   T   
   C   
    C    
   T   
   T   
   T   
   G   
   T   
   A   
   C   
   C   
   A   
   A   
   T   
   C   
   A   
   C   
   A   
   G   
   T   
    C    
   G   
   C   
   T   
   T   
   C   
   T   
   G   
   A   
   A   
   C   
   C   
   A   
   G   
   T   
   G   
   A   
   C   
   A   
   G   
   A   
   A   
   T   
   T   
   A   
   C   
   C   
   A   
   A   
   C   
   A   
   G   
   G   
   G   
   G   
   C   
   T   
   G   
   C   
   G   
   A   
   C   
   A   
   A   
   A   
   A   
   G   
    A    
    T    
   A   
   C   
   A   
   G   
   A   
   G   
   T   
   C   
   A   
   C   
   G   
   T   
   T   
   A   
   T   
   T   
   A   
   T   
   C   
   C   
    G    
    C    
   T   
   G   
   G   
   G   
   G   
   G   
   A   
   A   
   G   
   C   
   A   
   G   
   C   
   T   
   G   
   C   
   G   
   C   
   A   
   A   
   T   
   T   
   A   
   A   
   T   
   C   
   G   
   G   
    G    
   T   
   A   
   T   
   A   
   C   
    G    
   G   
   G   
    C    
   A   
   C   
    C    
   A   
   T   
   T   
   A   
   C   
   T   
   G   
   C   
   A   
   G   
   A   
    A    
   G   
   A   
   T   
   A   
   T   
   T   
    G    
   A   
   A   
   A   
   A   
   A   
   A   
   A   
   T   
   C   
   C   
   A   
   G   
   A   
   G   
   T   
   T   
   A   
   A   
   G   
   T   
   A   
   G   
   T   
   A   
   C   
   G   
   G   
   G   
    C    
   G   
   T   
   A   
   A   
   T   
   T   
   G   
   G   
    T    
   A   
   A   
   A   
   A   
   C   
   T   
   G   
   G   
    T    
   T   
   T   
   A   
   G   
   A   
   A   
   C   
   G   
   A   
   G   
   C   
   G   
   T   
   T   
   T   
   G   
   A   
   T   
   A   
   A   
   A   
   G   
   A   
    A    
   T   
   T   
   A   
   A   
   G   
   A   
   G   
   G   
    G    
   C   
   A   
   A   
   G   
   A   
   T   
   G   
   G   
    T    
   G   
   G   
    C    
   T   
   C   
    A    
   C   
   T   
   A   
   G   
   T   
   T   
   A   
   T   
   T   
   T   
   T   
   A   
   G   
   A   
   T   
   G   
   A   
   C   
   A   
   A   
   A   
   G   
   A   
   G   
   A   
   A   
   T   
   G   
   T   
   C   
   A   
   A   
   A   
   A   
   A   
   A   
   G   
   C   
   T   
   T   
   T   
   A   
   C   
   A   
   A   
   A   
   C   
   C   
   A   
   A   
   A   
   G   
   A   
    G    
   A   
   A   
   A   
   A   
   A   
   A   
   G   
   A   
   T   
   G   
   G   
   T   
   C   
   A   
   A   
   A   
   C   
    G    
   A   
   T   
   T   
   A   
   A   
   A   
   T   
   T   
   A   
   A   
   C   
   G   
   A   
   T   
   T   
   G   
   A   
    C    
   A   
   G   
    T    
   G   
   G   
    C    
   G   
   T   
   A   
   C   
   A   
   A   
   C   
   A   
   A   
   C   
   A   
   A   
   G   
   C   
    C    
   T   
   T   
    C    
   G   
   C   
   T   
   A   
   T   
   A   
   T   
   T   
   T   
   G   
   A   
   C   
   A   
   A   
   A   
   C   
   G   
    T    
    C    
   C   
   T   
   G   
   G   
   T   
   A   
   G   
   T   
   G   
   C   
   G   
   G   
   T   
   C   
   A   
   T   
   T   
   A   
   C   
   C   
   G   
   A   
   T   
   C   
   C   
   T   
   C   
   A   
   A   
   A   
   A   
   A   
   G   
   G   
   C   
   G   
   A   
   T   
   T   
   T   
   A   
    T    
   T   
   A   
   G   
   C   
    A    
   A   
   C   
   G   
   G   
   T   
   T   
   A   
   G   
   T   
   T   
   C   
   A   
   C   
   C   
   T   
   T   
   C   
   G   
   T   
   A   
   T   
   G   
   A   
   T   
   C   
   C   
   T   
   A   
   A   
   T   
   A   
   A   
   A   
   A   
   T   
   G   
   G   
   C   
   C   
   A   
   A   
   T   
   G   
   G   
    T    
   A   
   T   
   T   
   T   
   C   
   T   
   C   
   A   
   A   
   A   
   A   
   A   
   G   
   A   
    A    
   T   
   A   
   T   
   G   
   A   
   T   
   G   
   C   
   T   
   T   
   A   
   C   
   A   
   A   
   T   
   A   
   A   
   C   
   A   
   A   
   C   
   A   
   A   
   A   
   G   
   A   
   T   
   T   
   T   
   A   
   C   
   C   
   A   
   T   
   T   
    C    
   A   
   C   
   A   
   G   
   C   
   A   
   C   
   G   
   T   
   T   
   T   
    T    
   G   
   C   
   G   
   A   
   C   
   A   
   G   
   G   
    C    
   T   
   A   
   T   
   G   
   C   
   A   
   C   
   C   
   A   
   G   
   G   
    C    
   T   
   C   
   T   
   A   
   C   
   C   
   T   
   T   
   T   
   A   
   A   
   A   
   A   
   C   
   A   
   A   
   T   
   T   
   A   
   C   
   C   
   G   
   G   
   T   
   G   
   C   
   C   
   A   
   T   
   T   
   G   
   G   
   T   
   T   
   T   
   A   
   G   
   A   
   T   
    G    
   C   
   A   
   G   
   G   
   G   
   A   
   C   
   A   
   T   
   T   
   G   
   A   
   A   
   G   
   C   
   C   
   A   
   G   
   A   
   T   
   G   
   A   
   A   
   G   
   A   
   G   
   C   
   T   
   A   
   G   
   A   
   A   
   A   
   T   
   T   
   A   
   A   
   T   
   G   
   G   
    T    
   T   
   T   
   G   
   A   
   A   
   A   
   T   
   G   
   G   
   C   
   A   
   A   
   A   
   A   
   A   
   G   
   A   
   T   
   A   
   A   
   A   
   T   
   C   
   T   
   T   
   G   
   G   
   G   
   G   
    C    
   G   
   G   
   C   
   T   
   A   
   T   
   T   
   T   
   T   
   G   
   C   
    G    
   A   
   C   
   A   
   C   
   G   
   T   
   G   
   T   
   A   
   A   
   A   
   A   
   G   
   A   
   A   
   G   
   C   
   A   
   A   
   G   
   T   
   C   
   C   
   A   
   G   
   T   
   C   
   A   
   A   
   T   
   C   
   T   
    G    
   C   
   G   
   T   
   A   
   C   
   C   
   G   
   C   
   T   
   T   
   T   
   A   
   G   
   T   
   T   
   A   
   A   
   T   
   T   
   C   
   A   
   G   
   A   
   T   
   A   
   A   
   T   
   A   
   T   
   T   
   T   
   A   
   T   
   T   
   T   
   T   
   G   
   C   
    C    
   C   
   A   
   A   
   C   
   A   
   A   
   A   
   C   
   A   
   T   
   T   
   A   
   C   
   G   
    G    
   A   
   T   
   G   
   G   
   G   
    A    
   G   
   A   
   A   
   G   
   A   
   C   
   A   
   A   
   A   
   T   
   T   
   T   
   A   
   G   
   A   
   G   
   C   
    G    
   G   
   G   
   T   
   C   
   T   
   T   
   A   
   A   
   T   
   A   
   A   
   A   
   T   
   T   
   C   
   A   
   T   
   T   
   T   
   T   
   C   
   G   
   G   
   T   
   G   
   A   
   G   
   G   
   A   
   A   
   C   
   T   
   A   
   G   
   A   
   T   
   T   
   T   
   A   
   C   
   C   
   A   
   A   
   T   
    T    
   G   
   C   
   C   
   A   
   T   
   G   
   A   
   C   
   G   
   C   
   C   
    T    
   G   
   C   
   T   
   C   
   A   
   A   
   A   
   T   
   T   
   T   
   C   
   A   
   A   
   A   
    T    
   G   
   A   
   A   
   G   
   A   
   T   
   A   
   A   
   A   
   T   
   T   
   T   
   A   
   A   
   T   
   T   
   C   
   T   
   G   
   A   
   A   
   A   
   T   
   T   
   C   
   T   
   T   
   T   
   T   
    A    
   G   
   C   
   A   
   G   
   A   
   T   
   A   
   C   
    G    
   G   
   G   
   C   
   T   
   A   
    C    
   G   
   G   
    T    
   C   
   A   
   A   
   G   
   G   
    T    
   C   
   A   
   A   
   T   
   T   
   G   
   T   
   T   
   A   
   A   
   T   
   T   
   T   
   C   
    G    
   C   
   C   
   A   
   A   
   T   
   C   
   C   
   A   
   G   
   C   
   A   
   A   
   G   
   C   
   T   
   A   
   C   
   C   
   A   
   T   
   G   
   T   
   A   
   T   
   A   
   G   
   T   
   G   
   T   
   T   
   T   
   T   
   C   
   C   
   A   
   A   
   A   
   A   
   T   
   A   
   A   
   T   
   G   
   G   
    A    
   A   
   C   
   A   
    T    
   T   
    A    
   G   
   T   
    C    
   T   
   A   
   T   
   C   
   C   
   A   
   A   
   A   
   A   
    C    
   T   
   A   
   G   
   T   
   T   
   T   
   T   
   A   
   G   
   A   
   C   
   A   
   A   
   A   
   G   
   A   
   A   
   A   
   C   
    G    
   A   
   A   
   G   
   A   
   A   
   A   
   A   
   A   
   A   
   G   
   A   
   C   
   A   
   A   
   T   
   G   
   T   
   A   
   A   
   T   
   C   
   A   
   G   
   C   
   G   
   C   
   C   
   A   
   A   
   C   
   G   
   C   
   A   
   G   
   C   
   G   
   A   
   A   
   T   
   A   
   C   
   A   
   A   
   T   
   T   
   G   
   C   
   C   
   A   
   C   
   A   
   G   
   A   
   T   
   C   
   T   
   T   
   T   
   T   
   A   
   G   
   G   
   T   
   A   
   G   
   T   
   G   
   T   
   G   
   G   
   A   
   A   
   G   
   A   
   T   
   C   
   C   
   T   
   T   
   C   
   A   
   G   
   G   
   T   
   T   
   A   
   T   
   G   
   T   
   T   
   T   
   A   
   T   
   A   
   A   
   T   
   A   
   T   
   G   
   T   
   A   
   C   
   A   
   A   
   T   
   C   
   C   
   G   
   A   
   A   
   C   
   T   
   T   
   C   
   T   
   C   
   A   
   T   
   T   
   G   
   G   
   C   
   T   
   G   
   C   
   T   
   A   
   A   
   A   
   A   
   C   
   A   
   G   
   G   
   A   
   A   
   C   
   A   
   G   
   C   
   T   
   G   
   A   
   A   
   A   
   T   
   A   
   A   
   A   
   A   
   G   
   A   
   T   
   A   
   A   
   A   
   C   
   A   
   A   
   G   
   A   
   T   
   A   
   C   
   A   
   G   
   A   
   T   
   G   
   G   
   C   
   A   
   A   
   A   
   G   
   A   
   A   
   A   
   A   
   C   
   A   
   G   
   T   
   T   
   T   
   C   
   C   
   T   
   T   
   C   
   T   
   A   
   A   
   C   
   G   
   T   
   T   
   A   
   G   
   A   
   T   
   C   
   G   
   T   
   A   
   G   
   T   
   A   
   A   
   C   
   A   
   A   
   T   
   A   
   A   
   A   
   T   
   T   
   C   
   T   
   T   
   A   
   A   
   C   
   A   
   A   
   T   
   G   
   A   
   T   
   T   
   A   
   T   
   G   
   G   
   T   
   T   
   G   
   A   
   A   
   A   
   A   
   T   
   T   
   C   
   A   
   G   
   G   
   A   
   G   
   A   
   A   
   A   
   A   
   T   
   G   
   G   
   T   
   T   
   C   
   T   
   G   
   C   
   A   
   A   
   C   
   A   
   G   
   A   
   T   
   A   
   T   
   C   
   A   
   G   
   T   
   A   
   A   
   A   
   C   
   C   
   A   
   T   
   T   
   A   
   A   
   T   
   T   
   G   
   A   
   T   
   T   
   A   
   C   
   T   
   T   
   A   
   G   
   A   
   A   
   G   
   C   
   A   
   A   
   C   
   C   
   A   
   T   
   T   
   A   
   A   
   A   
   -   
   -   
   -   
   -   
   -   
   -   
   -   
   -   
   -   
   -   
   -   
   -   
   -   
   -   
   -   
   -   
   -   
   -   
   -   
   -   
   -   
   -   
   -   
   -   
   -   
   -   
   -   
   -   
   -   
   -   
   -   
   -   
   -   
   -   
   -   
   -   
   -   
   -   
   -   
   -   
   -   
   -   
   -   
   -   
   -   
   -   
   -   
   -   
   -   
   -   
   -   
   -   
   -   
   -   
   -   
   -   
   -   
   -   
   -   
   -   
   -   
   -   
   -   
   -   
   -   
   -   
   -   
   -   
   -   
   -   
   -   
   -   
   -   
   -   
   -   
   -   
   -   
   -   
   -   
   -   
   -   
   -   
   -   
   -   
   -   
   -   
   -   
   -   
   -   
   -   
   -   
   -   
   -   
   -   
   -   
   -   
   -   
   -   
   -   
   -   
   -   
   -   
   -   
   -   
   -   
   -   
   -   
   -   
   -   
   -   
   -   
   -   
   -   
   -   
   -   
   -   
   -   
   -   
   -   
   -   
   -   
   -   
   -   
   -   
   -   
   -   
   -   
   -   
   -   
   -   
   -   
   -   
   -   
   -   
   -   
   -   
   -   
   -   
   -   
   -   
   -   
   -   
   -   
   -   
   -   
   -   
   -   
   -   
   -   
   -   
   -   
   -   
   -   
   -   
   -   
   -   
   -   
   -   
   -   
   -   
   -   
   -   
   -   
   -   
   -   
   -   
   -   
   -   
   -   
   C   
   A   
   A   
   T   
   G   
   G   
   C   
   A   
   A   
   G   
   C   
   C   
   A   
   A   
   A   
   C   
   A   
   A   
   G   
   A   
   A   
   T   
   T   
   A   
   G   
   C   
   C   
   G   
   A   
   A   
   G   
   C   
   G   
   A   
   A   
   G   
   A   
   A   
   A   
   A   
   C   
   A   
   G   
   C   
   T   
   A   
   C   
   T   
   A   
   C   
   A   
   T   
   T   
   T   
   T   
   T   
   A   
   A   
   A   
   C   
   G   
   T   
   A   
   T   
   T   
   G   
   T   
   C   
   A   
   A   
   A   
   A   
   C   
   A   
   G   
   G   
   A   
   A   
   T   
   T   
   T   
   G   
   A   
   T   
   A   
   A   
   G   
   T   
   T   
   A   
   C   
   C   
   G   
   T   
   C   
   C   
   G   
   T   
   T   
   G   
   T   
   T   
   C   
   A   
   A   
   G   
   A   
   A   
   G   
   C   
   T   
   A   
   G   
   C   
   T   
   T   
   A   
   A   
   A   
   G   
   A   
   A   
   A   
   A   
   A   
   T   
   G   
   G   
   C   
   T   
   A   
   T   
   G   
   A   
   T   
   A   
   C   
   T   
   A   
   A   
   A   
   T   
   C   
   T   
   G   
   T   
   T   
   G   
   T   
   T   
   G   
   A   
   A   
   A   
   A   
   A   
   T   
   A   
   C   
   C   
   A   
   A   
   G   
   C   
   A   
   A   
   T   
   T   
   T   
   A   
   T   
   T   
   C   
   A   
   G   
   G   
   G   
   A   
   T   
   T   
   C   
   A   
   A   
   G   
   C   
   A   
   G   
   A   
   A   
   G   
   G   
   A   
   G   
   T   
   C   
   A   
   A   
   A   
   G   
   C   
   T   
   A   
   G   
   T   
   G   
   A   
   T   
   G   
   T   
   T   
   C   
   A   
   A   
   G   
   T   
   A   
   A   
   A   
   A   
   A   
   A   
   G   
   G   
   C   
   G   
   A   
   A   
   A   
   G   
   A   
   C   
   A   
   A   
   T   
   C   
   A   
   A   
   T   
   A   
   C   
   A   
   C   
   A   
   T   
   T   
   T   
   A   
   C   
   C   
   T   
   A   
   T   
   A   
   A   
   A   
   T   
   T   
   A   
   T   
   C   
   G   
   A   
   T   
   G   
   A   
   G   
   T   
   A   
   C   
   C   
   C   
   C   
   T   
   T   
   T   
   A   
   G   
   G   
   C   
   G   
   A   
   A   
   A   
   T   
   G   
   A   
   A   
   A   
   G   
   A   
   T   
   T   
   T   
   G   
   T   
   C   
   T   
   T   
   A   
   T   
   C   
   A   
   A   
   T   
   C   
   A   
   A   
   G   
   T   
   A   
   T   
   C   
   G   
   C   
   C   
   A   
   A   
   A   
   A   
   A   
   A   
   G   
   G   
   C   
   G   
   A   
   T   
   A   
   C   
   C   
   T   
   A   
   C   
   C   
   A   
   A   
   A   
   T   
   C   
   G   
   C   
   T   
   T   
   G   
   G   
   A   
   A   
   G   
   C   
   C   
   G   
   T   
   C   
   T   
   T   
   T   
   A   
   A   
   T   
   T   
   T   
   T   
   T   
   C   
   C   
   A   
   G   
   A   
   T   
   A   
   T   
   G   
   T   
   C   
   A   
   G   
   G   
   A   
   A   
   A   
   T   
   G   
   A   
   T   
   A   
   A   
   A   
   A   
   T   
   T   
   T   
   C   
   G   
   A   
   T   
   T   
   C   
   A   
   A   
   G   
   T   
   A   
   G   
   A   
   T   
   A   
   A   
   T   
   G   
   C   
   C   
   A   
   A   
   A   
   C   
   G   
   T   
   G   
   G   
   A   
   G   
   A   
   A   
   A   
   T   
   T   
   G   
   T   
   C   
   G   
   A   
   T   
   C   
   G   
   T   
   A   
   A   
   T   
   G   
   G   
   T   
   A   
   G   
   T   
   G   
   G   
   G   
   C   
   T   
   A   
   G   
   C   
   A   
   A   
   T   
   T   
   A   
   A   
   C   
   A   
   A   
   A   
   G   
   T   
   G   
   T   
   T   
   T   
   G   
   A   
   T   
   G   
   A   
   A   
   G   
   T   
   G   
   G   
   G   
   C   
   G   
   T   
   A   
   G   
   T   
   G   
   C   
   C   
   T   
   G   
   G   
   C   
   A   
   A   
   A   
   C   
   T   
   C   
   G   
   G   
   T   
   T   
   C   
   T   
   G   
   G   
   C   
   G   
   C   
   A   
   G   
   A   
   A   
   A   
   A   
   A   
   A   
   C   
   A   
   G   
   C   
   C   
   A   
   A   
   T   
   A   
   T   
   C   
   A   
   A   
   A   
   G   
   C   
   T   
   T   
   T   
   T   
   A   
   G   
   T   
   G   
   A   
   T   
   A   
   A   
   A   
   T   
   T   
   C   
   G   
   G   
   C   
   G   
   T   
   T   
   T   
   C   
   T   
   G   
   T   
   T   
   G   
   A   
   T   
   G   
   A   
   A   
   A   
   T   
   C   
   A   
   A   
   T   
   C   
   A   
   A   
   A   
   A   
   G   
   T   
   T   
   A   
   A   
   G   
   C   
   C   
   A   
   A   
   G   
   G   
   A   
   T   
   G   
   G   
   G   
   T   
   C   
   C   
   A   
   A   
   G   
   C   
   A   
   G   
   A   
   C   
   T   
   C   
   C   
   T   
   T   
   T   
   G   
   T   
   A   
   C   
   C   
   A   
   A   
   T   
   C   
   A   
   C   
   A   
   G   
   T   
   C   
   G   
   C   
   T   
   T   
   C   
   T   
   G   
   A   
   A   
   C   
   C   
   A   
   G   
   T   
   G   
   A   
   C   
   A   
   G   
   A   
   A   
   T   
   T   
   A   
   C   
   C   
   A   
   A   
   C   
   A   
   G   
   G   
   G   
   G   
   C   
   T   
   G   
   C   
   G   
   A   
   C   
   A   
   A   
   A   
   A   
   G   
   A   
   T   
   A   
   C   
   A   
   G   
   A   
   G   
   T   
   C   
   A   
   C   
   G   
   T   
   T   
   A   
   T   
   T   
   A   
   T   
   C   
   C   
   G   
   C   
   T   
   G   
   G   
   G   
   G   
   G   
   A   
   A   
   G   
   C   
   A   
   G   
   C   
   T   
   G   
   C   
   G   
   C   
   A   
   A   
   T   
   T   
   A   
   A   
   T   
   C   
   G   
   G   
   G   
   T   
   A   
   T   
   A   
   C   
   G   
   G   
   G   
   C   
   A   
   C   
   C   
   A   
   T   
   T   
   A   
   C   
   T   
   G   
   C   
   A   
   G   
   A   
   A   
   G   
   A   
   T   
   A   
   T   
   T   
   G   
   A   
   A   
   A   
   A   
   A   
   A   
   A   
   T   
   C   
   C   
   A   
   G   
   A   
   G   
   T   
   T   
   A   
   A   
   G   
   T   
   A   
   G   
   T   
   A   
   C   
   G   
   G   
   G   
   C   
   G   
   T   
   A   
   A   
   T   
   T   
   G   
   G   
   T   
   A   
   A   
   A   
   A   
   C   
   T   
   G   
   G   
   T   
   T   
   T   
   A   
   G   
   A   
   A   
   C   
   G   
   A   
   G   
   C   
   G   
   T   
   T   
   T   
   G   
   A   
   T   
   A   
   A   
   A   
   G   
   A   
   A   
   T   
   T   
   A   
   A   
   G   
   A   
   G   
   G   
   G   
   C   
   A   
   A   
   G   
   A   
   T   
   G   
   G   
   T   
   G   
   G   
   C   
   T   
   C   
   A   
   C   
   T   
   A   
   G   
   T   
   T   
   A   
   T   
   T   
   T   
   T   
   A   
   G   
   A   
   T   
   G   
   A   
   C   
   A   
   A   
   A   
   G   
   A   
   G   
   A   
   A   
   T   
   G   
   T   
   C   
   A   
   A   
   A   
   A   
   A   
   A   
   G   
   C   
   T   
   T   
   T   
   A   
   C   
   A   
   A   
   A   
   C   
   C   
   A   
   A   
   A   
   G   
   A   
   G   
   A   
   A   
   A   
   A   
   A   
   A   
   G   
   A   
   T   
   G   
   G   
   T   
   C   
   A   
   A   
   A   
   C   
   G   
   A   
   T   
   T   
   A   
   A   
   A   
   T   
   T   
   A   
   A   
   C   
   G   
   A   
   T   
   T   
   G   
   A   
   C   
   A   
   G   
   T   
   G   
   G   
   C   
   G   
   T   
   A   
   C   
   A   
   A   
   C   
   A   
   A   
   C   
   A   
   A   
   G   
   C   
   C   
   T   
   T   
   C   
   G   
   C   
   T   
   A   
   T   
   A   
   T   
   T   
   T   
   G   
   A   
   C   
   A   
   A   
   A   
   C   
   G   
   T   
   C   
   C   
   T   
   G   
   G   
   T   
   A   
   G   
   T   
   G   
   C   
   G   
   G   
   T   
   C   
   A   
   T   
   T   
   A   
   C   
   C   
   G   
   A   
   T   
   C   
   C   
   T   
   C   
   A   
   A   
   A   
   A   
   A   
   G   
   G   
   C   
   G   
   A   
   T   
   T   
   T   
   A   
   T   
   T   
   A   
   G   
   C   
   A   
   A   
   C   
   G   
   G   
   T   
   T   
   A   
   G   
   T   
   T   
   C   
   A   
   C   
   C   
   T   
   T   
   C   
   G   
   T   
   A   
   T   
   G   
   A   
   T   
   C   
   C   
   T   
   A   
   A   
   T   
   A   
   A   
   A   
   A   
   T   
   G   
   G   
   C   
   C   
   A   
   A   
   T   
   G   
   G   
   T   
   A   
   T   
   T   
   T   
   C   
   T   
   C   
   A   
   A   
   A   
   A   
   A   
   G   
   A   
   A   
   T   
   A   
   T   
   G   
   A   
   T   
   G   
   C   
   T   
   T   
   A   
   C   
   A   
   A   
   T   
   A   
   A   
   C   
   A   
   A   
   C   
   A   
   A   
   A   
   G   
   A   
   T   
   T   
   T   
   A   
   C   
   C   
   A   
   T   
   T   
   C   
   A   
   C   
   A   
   G   
   C   
   A   
   C   
   G   
   T   
   T   
   T   
   T   
   G   
   C   
   G   
   A   
   C   
   A   
   G   
   G   
   C   
   T   
   A   
   T   
   G   
   C   
   A   
   C   
   C   
   A   
   G   
   G   
   C   
   T   
   C   
   T   
   A   
   C   
   C   
   T   
   T   
   T   
   A   
   A   
   A   
   A   
   C   
   A   
   A   
   T   
   T   
   A   
   C   
   C   
   G   
   G   
   T   
   G   
   C   
   C   
   A   
   T   
   T   
   G   
   G   
   T   
   T   
   T   
   A   
   G   
   A   
   T   
   G   
   C   
   A   
   G   
   G   
   G   
   A   
   C   
   A   
   T   
   T   
   G   
   A   
   A   
   G   
   C   
   C   
   A   
   G   
   A   
   T   
   G   
   A   
   A   
   G   
   A   
   G   
   C   
   T   
   A   
   G   
   A   
   A   
   A   
   T   
   T   
   A   
   A   
   T   
   G   
   G   
   T   
   T   
   T   
   G   
   A   
   A   
   A   
   T   
   G   
   G   
   C   
   A   
   A   
   A   
   A   
   A   
   G   
   A   
   T   
   A   
   A   
   A   
   T   
   C   
   T   
   T   
   G   
   G   
   G   
   G   
   C   
   G   
   G   
   C   
   T   
   A   
   T   
   T   
   T   
   T   
   G   
   C   
   G   
   A   
   C   
   A   
   C   
   G   
   T   
   G   
   T   
   A   
   A   
   A   
   A   
   G   
   A   
   A   
   G   
   C   
   A   
   A   
   G   
   T   
   C   
   C   
   A   
   G   
   T   
   C   
   A   
   A   
   T   
   C   
   T   
   G   
   C   
   G   
   T   
   A   
   C   
   C   
   G   
   C   
   T   
   T   
   T   
   A   
   G   
   T   
   T   
   A   
   A   
   T   
   T   
   C   
   A   
   G   
   A   
   T   
   A   
   A   
   T   
   A   
   T   
   T   
   T   
   A   
   T   
   T   
   T   
   T   
   G   
   C   
   C   
   C   
   A   
   A   
   C   
   A   
   A   
   A   
   C   
   A   
   T   
   T   
   A   
   C   
   G   
   G   
   A   
   T   
   G   
   G   
   G   
   A   
   G   
   A   
   A   
   G   
   A   
   C   
   A   
   A   
   A   
   T   
   T   
   T   
   A   
   G   
   A   
   G   
   C   
   G   
   G   
   G   
   T   
   C   
   T   
   T   
   A   
   A   
   T   
   A   
   A   
   A   
   T   
   T   
   C   
   A   
   T   
   T   
   T   
   T   
   C   
   G   
   G   
   T   
   G   
   A   
   G   
   G   
   A   
   A   
   C   
   T   
   A   
   G   
   A   
   T   
   T   
   T   
   A   
   C   
   C   
   A   
   A   
   T   
   T   
   G   
   C   
   C   
   A   
   T   
   G   
   A   
   C   
   G   
   C   
   C   
   T   
   G   
   C   
   T   
   C   
   A   
   A   
   A   
   T   
   T   
   T   
   C   
   A   
   A   
   A   
   T   
   G   
   A   
   A   
   G   
   A   
   T   
   A   
   A   
   A   
   T   
   T   
   T   
   A   
   A   
   T   
   T   
   C   
   T   
   G   
   A   
   A   
   A   
   T   
   T   
   C   
   T   
   T   
   T   
   T   
   A   
   G   
   C   
   A   
   G   
   A   
   T   
   A   
   C   
   G   
   G   
   G   
   C   
   T   
   A   
   C   
   G   
   G   
   T   
   C   
   A   
   A   
   G   
   G   
   T   
   C   
   A   
   A   
   T   
   T   
   G   
   T   
   T   
   A   
   A   
   T   
   T   
   T   
   C   
   G   
   C   
   C   
   A   
   A   
   T   
   C   
   C   
   A   
   G   
   C   
   A   
   A   
   G   
   C   
   T   
   A   
   C   
   C   
   A   
   T   
   G   
   T   
   A   
   T   
   A   
   G   
   T   
   G   
   T   
   T   
   T   
   T   
   C   
   C   
   A   
   A   
   A   
   A   
   T   
   A   
   A   
   T   
   G   
   G   
   A   
   A   
   C   
   A   
   T   
   T   
   A   
   G   
   T   
   C   
   T   
   A   
   T   
   C   
   C   
   A   
   A   
   A   
   A   
   C   
   T   
   A   
   G   
   T   
   T   
   T   
   T   
   A   
   G   
   A   
   C   
   A   
   A   
   A   
   G   
   A   
   A   
   A   
   C   
   G   
   A   
   A   
   G   
   A   
   A   
   A   
   A   
   A   
   A   
   G   
   A   
   C   
   A   
   A   
   T   
   G   
   T   
   A   
   A   
   T   
   C   
   A   
   G   
   C   
   G   
   C   
   C   
   A   
   A   
   C   
   G   
   C   
   A   
   G   
   C   
   G   
   A   
   A   
   T   
   A   
   C   
   A   
   A   
   T   
   T   
   G   
   C   
   C   
   A   
   C   
   A   
   G   
   A   
   T   
   C   
   T   
   T   
   T   
   T   
   A   
   G   
   G   
   T   
   A   
   G   
   T   
   G   
   T   
   G   
   G   
   A   
   A   
   G   
   A   
   T   
   C   
   C   
   T   
   T   
   C   
   A   
   G   
   G   
   T   
   T   
   A   
   T   
   G   
   T   
   T   
   T   
   A   
   T   
   A   
   A   
   T   
   A   
   T   
   G   
   T   
   A   
   C   
   A   
   A   
   T   
   C   
   C   
   G   
   A   
   A   
   C   
   T   
   T   
   C   
   T   
   C   
   A   
   T   
   T   
   G   
   G   
   C   
   T   
   G   
   C   
   T   
   A   
   A   
   A   
   A   
   C   
   A   
   G   
   G   
   A   
   A   
   C   
   A   
   G   
   C   
   T   
   G   
   A   
   A   
   A   
   T   
   A   
   A   
   A   
   A   
   G   
   A   
   T   
   A   
   A   
   A   
   C   
   A   
   A   
   G   
   A   
   T   
   A   
   C   
   A   
   G   
   A   
   T   
   G   
   G   
   C   
   A   
   A   
   A   
   G   
   A   
   A   
   A   
   A   
   C   
   A   
   G   
   T   
   T   
   T   
   C   
   C   
   T   
   T   
   C   
   T   
   A   
   A   
   C   
   G   
   T   
   T   
   A   
   G   
   A   
   T   
   C   
   G   
   T   
   A   
   G   
   T   
   A   
   A   
   C   
   A   
   A   
   T   
   A   
   A   
   A   
   T   
   T   
   C   
   T   
   T   
   A   
   A   
   C   
   A   
   A   
   T   
   G   
   A   
   T   
   T   
   A   
   T   
   G   
   G   
   T   
   T   
   G   
   A   
   A   
   A   
   A   
   T   
   T   
   C   
   A   
   G   
   G   
   A   
   G   
   A   
   A   
   A   
   A   
   T   
   G   
   G   
   T   
   T   
   C   
   T   
   G   
   C   
   A   
   A   
   C   
   A   
   G   
   A   
   T   
   A   
   T   
   C   
   A   
   G   
   T   
   A   
   A   
   A   
   C   
   C   
   A   
   T   
   T   
   A   
   A   
   T   
   T   
   G   
   A   
   T   
   T   
   A   
   C   
   T   
   T   
   A   
   G   
   A   
   A   
   G   
   C   
   A   
   A   
   C   
   C   
   A   
   T   
   T   
   A   
   A   
   A   
   -   
   -   
   -   
   -   
   -   
   -   
   -   
   -   
   -   
   -   
   -   
   -   
   -   
   -   
   -   
   -   
   -   
   -   
   -   
   -   
   -   
   -   
   -   
   -   
   -   
   -   
   -   
   -   
   -   
   -   
   -   
   -   
   -   
   -   
   -   
   -   
   -   
   -   
   -   
   -   
   -   
   -   
   -   
   -   
   -   
   -   
   -   
   -   
   -   
   -   
   -   
   -   
   -   
   -   
   -   
   -   
   -   
   -   
   -   
   -   
   -   
 

 
   N55265   
   A   
   T   
   G   
   G   
   A   
   A   
   C   
   G   
   A   
   A   
   G   
   C   
   A   
   A   
   T   
   C   
   G   
   T   
   A   
   A   
   T   
   A   
   A   
   A   
   A   
   A   
   A   
   T   
   C   
   T   
   T   
   C   
   A   
   A   
   A   
   A   
   A   
   A   
   A   
   C   
   C   
   A   
   C   
   T   
   T   
   A   
   T   
   T   
   C   
   T   
   T   
   G   
   G   
   C   
   G   
   T   
   T   
   T   
   C   
   T   
   G   
   C   
   C   
   T   
   T   
   G   
   G   
   T   
   T   
   C   
   T   
   G   
   A   
   T   
   C   
   G   
   C   
   T   
   G   
   C   
   T   
   G   
   T   
   C   
   G   
   G   
   T   
   G   
   G   
   T   
   G   
   G   
   G   
   T   
   A   
   T   
   T   
   A   
   T   
   G   
   C   
   T   
   T   
   A   
   T   
   A   
   G   
   C   
   C   
   A   
   A   
   T   
   G   
   G   
   C   
   A   
   A   
   G   
   C   
   C   
   A   
   A   
   A   
   C   
   A   
   A   
   G   
   A   
    A    
   T   
   T   
   A   
   G   
   C   
    T    
   G   
   A   
   A   
   G   
   C   
   G   
   A   
   A   
   G   
   A   
   A   
   A   
   A   
    C    
   A   
   G   
   C   
    C    
   A   
    A    
   T   
    A    
    C    
   A   
   T   
   T   
   T   
   T   
   T   
   A   
   A   
   A   
    C    
   G   
   T   
   A   
   T   
   T   
   G   
    T    
   C   
   A   
   A   
   A   
   A   
   C   
   A   
   G   
    G    
   A   
   A   
   T   
   T   
   T   
   G   
   A   
   T   
   A   
   A   
   G   
   T   
   T   
   A   
   C   
   C   
    A    
   T   
   C   
   C   
   G   
   T   
   T   
    G    
   T   
   T   
   C   
   A   
   A   
   G   
   A   
   A   
    A    
   C   
   T   
   A   
   G   
   C   
   T   
   T   
   A   
   A   
   A   
   G   
   A   
   A   
   A   
   A   
   A   
   T   
   G   
   G   
    C    
   T   
   A   
   T   
   G   
   A   
   T   
   A   
   C   
   T   
   A   
   A   
   A   
   T   
   C   
    T    
   G   
   T   
   T   
   G   
   T   
   T   
   G   
   A   
   A   
   A   
   A   
   A   
   T   
   A   
   C   
   C   
   A   
   A   
   G   
   C   
   A   
   A   
   T   
   T   
   T   
   A   
   T   
   T   
   C   
   A   
   G   
   G   
   G   
   A   
   T   
   T   
   C   
   A   
   A   
   G   
   C   
    A    
   G   
   A   
   A   
    G    
   G   
    G    
   G   
   T   
   C   
   A   
   A   
    A    
   G   
   C   
   T   
   A   
   G   
   T   
   G   
   A   
   T   
   G   
   T   
    T    
   C   
   A   
   A   
   G   
   T   
    C    
   A   
   A   
   A   
   A   
   A   
    A    
   G   
    G    
   G   
   A   
   A   
   A   
   G   
   A   
    C    
   A   
   A   
    C    
   C   
   A   
   A   
   T   
   A   
   C   
   A   
   C   
   A   
   T   
   T   
   T   
   A   
    C    
   C   
   T   
   A   
   T   
   A   
   A   
   A   
   T   
   T   
   A   
   T   
   C   
   G   
   A   
   T   
   G   
   A   
   G   
    C    
   A   
   C   
    T    
   C   
   C   
   T   
   T   
   T   
   A   
   G   
   G   
   C   
   G   
   A   
   A   
   A   
   T   
   G   
   A   
   A   
   A   
   G   
   A   
   T   
    T    
   T   
   G   
   T   
   C   
   T   
   T   
   A   
   T   
   C   
   A   
   A   
   T   
   C   
   A   
   A   
   G   
   T   
   A   
   T   
    C    
   G   
    C    
   C   
   A   
   A   
    A    
   A   
   A   
   A   
   G   
   G   
    C    
   G   
   A   
   T   
   A   
    A    
   C   
   T   
   A   
   C   
   C   
   A   
   A   
   A   
   T   
    C    
    G    
   C   
   T   
   T   
   G   
   G   
    G    
    C    
    A    
   C   
   C   
    G    
   T   
   C   
   T   
   T   
   T   
   A   
   A   
   T   
   T   
   T   
   T   
   T   
   C   
   C   
   A   
   G   
   A   
   T   
   A   
   T   
   G   
   T   
   C   
   A   
   G   
   G   
   A   
   A   
   A   
   T   
   G   
   A   
   T   
   A   
   A   
   A   
    A    
   T   
   T   
   T   
   C   
   G   
   A   
   T   
   T   
   C   
   A   
   A   
   G   
   T   
   A   
   G   
   A   
   T   
   A   
   A   
   T   
   G   
   C   
   C   
   A   
   A   
   A   
   C   
   G   
   T   
   G   
   G   
   A   
   G   
   A   
   A   
   A   
   T   
   T   
   G   
   T   
   C   
   G   
   A   
   T   
   C   
   G   
   T   
   A   
   A   
    T    
   G   
   G   
   T   
   A   
   G   
   T   
   G   
   G   
   G   
   C   
   T   
   A   
   G   
    C    
   A   
   A   
   T   
   T   
   A   
   A   
   C   
   A   
   A   
   A   
   G   
   T   
   G   
   T   
   T   
   T   
   G   
   A   
    T    
   G   
   A   
   A   
   G   
   T   
   G   
   G   
   G   
   C   
   G   
   T   
   A   
   G   
   T   
    G    
   C   
   C   
   T   
    G    
   G   
   C   
   A   
   A   
   A   
   C   
   T   
    C    
   G   
   G   
   T   
   T   
   C   
   T   
   G   
   G   
   C   
   G   
   C   
   A   
   G   
   A   
   A   
   A   
   A   
   A   
    A    
   C   
   A   
   G   
   C   
    C    
   A   
   A   
   T   
   A   
   T   
   C   
   A   
   A   
   A   
   G   
   C   
   T   
   T   
   T   
   T   
   A   
   G   
   T   
   G   
   A   
   T   
   A   
   A   
    G    
   T   
   T   
    T    
   G   
   G   
   C   
   G   
   T   
   T   
   T   
   C   
   T   
    G    
   T   
   T   
   G   
   A   
   T   
   G   
   A   
   A   
   A   
   T   
   C   
   A   
   A   
   T   
   C   
    A    
   A   
   A   
   A   
    G    
   T   
   T   
   A   
   A   
   G   
   C   
   C   
   A   
   A   
   G   
   G   
    A    
   T   
   G   
   G   
   G   
   T   
   C   
   C   
   A   
   A   
   G   
   C   
   A   
   G   
   A   
   C   
   T   
   C   
    G    
   T   
   T   
   T   
   G   
   T   
   A   
   C   
   C   
   A   
   A   
   T   
   C   
   A   
   C   
   A   
   G   
   T   
    T    
   G   
   C   
   T   
   T   
   C   
   T   
   G   
   A   
   A   
   C   
   C   
   A   
   G   
   T   
   G   
   A   
   C   
   A   
   G   
   A   
   A   
   T   
   T   
   A   
   C   
   C   
   A   
   A   
   C   
   A   
   G   
   G   
   G   
   G   
   C   
   T   
   G   
   C   
   G   
   A   
   C   
   A   
   A   
   A   
   A   
   G   
    A    
    T    
   A   
   C   
   A   
   G   
   A   
   G   
   T   
   C   
   A   
   C   
   G   
   T   
   T   
   A   
   T   
   T   
   A   
   T   
   C   
   C   
    A    
    T    
   T   
   G   
   G   
   G   
   G   
   G   
   A   
   A   
   G   
   C   
   A   
   G   
   C   
   T   
   G   
   C   
   G   
   C   
   A   
   A   
   T   
   T   
   A   
   A   
   T   
   C   
   G   
   G   
    G    
   T   
   A   
   T   
   A   
   C   
    G    
   G   
   G   
    C    
   A   
   C   
    T    
   A   
   T   
   T   
   A   
   C   
   T   
   G   
   C   
   A   
   G   
   A   
    A    
   G   
   A   
   T   
   A   
   T   
   T   
    G    
   A   
   A   
   A   
   A   
   A   
   A   
   A   
   T   
   C   
   C   
   A   
   G   
   A   
   G   
   T   
   T   
   A   
   A   
   G   
   T   
   A   
   G   
   T   
   A   
   C   
   G   
   G   
   G   
    C    
   G   
   T   
   A   
   A   
   T   
   T   
   G   
   G   
    C    
   A   
   A   
   A   
   A   
   C   
   T   
   G   
   G   
    C    
   T   
   T   
   A   
   G   
   A   
   A   
   C   
   G   
   A   
   G   
   C   
   G   
   T   
   T   
   T   
   G   
   A   
   T   
   A   
   A   
   A   
   G   
   A   
    A    
   T   
   T   
   A   
   A   
   G   
   A   
   G   
   G   
    A    
   C   
   A   
   A   
   G   
   A   
   T   
   G   
   G   
    T    
   G   
   G   
    T    
   T   
   C   
    A    
   C   
   T   
   A   
   G   
   T   
   T   
   A   
   T   
   T   
   T   
   T   
   A   
   G   
   A   
   T   
   G   
   A   
   C   
   A   
   A   
   A   
   G   
   A   
   G   
   A   
   A   
   T   
   G   
   T   
   C   
   A   
   A   
   A   
   A   
   A   
   A   
   G   
   C   
   T   
   T   
   T   
   A   
   C   
   A   
   A   
   A   
   C   
   C   
   A   
   A   
   A   
   G   
   A   
    G    
   A   
   A   
   A   
   A   
   A   
   A   
   G   
   A   
   T   
   G   
   G   
   T   
   C   
   A   
   A   
   A   
   C   
    A    
   A   
   T   
   T   
   A   
   A   
   A   
   T   
   T   
   A   
   A   
   C   
   G   
   A   
   T   
   T   
   G   
   A   
    T    
   A   
   G   
    T    
   G   
   G   
    C    
   G   
   T   
   A   
   C   
   A   
   A   
   C   
   A   
   A   
   C   
   A   
   A   
   G   
   C   
    C    
   T   
   T   
    C    
   G   
   C   
   T   
   A   
   T   
   A   
   T   
   T   
   T   
   G   
   A   
   C   
   A   
   A   
   A   
   C   
   G   
    T    
    C    
   C   
   T   
   G   
   G   
   T   
   A   
   G   
   T   
   G   
   C   
   G   
   G   
   T   
   C   
   A   
   T   
   T   
   A   
   C   
   C   
   G   
   A   
   T   
   C   
   C   
   T   
   C   
   A   
   A   
   A   
   A   
   A   
   G   
   G   
   C   
   G   
   A   
   T   
   T   
   T   
   A   
    C    
   T   
   A   
   G   
   C   
    G    
   A   
   C   
   G   
   G   
   T   
   T   
   A   
   G   
   T   
   T   
   C   
   A   
   C   
   C   
   T   
   T   
   C   
   G   
   T   
   A   
   T   
   G   
   A   
   T   
   C   
   C   
   T   
   A   
   A   
   T   
   A   
   A   
   A   
   A   
   T   
   G   
   G   
   C   
   C   
   A   
   A   
   T   
   G   
   G   
    C    
   A   
   T   
   T   
   T   
   C   
   T   
   C   
   A   
   A   
   A   
   A   
   A   
   G   
   A   
    A    
   T   
   A   
   T   
   G   
   A   
   T   
   G   
   C   
   T   
   T   
   A   
   C   
   A   
   A   
   T   
   A   
   A   
   C   
   A   
   A   
   C   
   A   
   A   
   A   
   G   
   A   
   T   
   T   
   T   
   A   
   C   
   C   
   A   
   T   
   T   
    C    
   A   
   C   
   A   
   G   
   C   
   A   
   C   
   G   
   T   
   T   
   T   
    T    
   G   
   C   
   G   
   A   
   C   
   A   
   G   
   G   
    C    
   T   
   A   
   T   
   G   
   C   
   A   
   C   
   C   
   A   
   G   
   G   
    C    
   T   
   C   
   T   
   A   
   C   
   C   
   T   
   T   
   T   
   A   
   A   
   A   
   A   
   C   
   A   
   A   
   T   
   T   
   A   
   C   
   C   
   G   
   G   
   T   
   G   
   C   
   C   
   A   
   T   
   T   
   G   
   G   
   T   
   T   
   T   
   A   
   G   
   A   
   T   
    G    
   C   
   A   
   G   
   G   
   G   
   A   
   C   
   A   
   T   
   T   
   G   
   A   
   A   
   G   
   C   
   C   
   A   
   G   
   A   
   T   
   G   
   A   
   A   
   G   
   A   
   G   
   C   
   T   
   A   
   G   
   A   
   A   
   A   
   T   
   T   
   A   
   A   
   T   
   G   
   G   
    C    
   T   
   T   
   G   
   A   
   A   
   A   
   T   
   G   
   G   
   C   
   A   
   A   
   A   
   A   
   A   
   G   
   A   
   T   
   A   
   A   
   A   
   T   
   C   
   T   
   T   
   G   
   G   
   G   
   G   
    C    
   G   
   G   
   C   
   T   
   A   
   T   
   T   
   T   
   T   
   G   
   C   
    G    
   A   
   C   
   A   
   C   
   G   
   T   
   G   
   T   
   A   
   A   
   A   
   A   
   G   
   A   
   A   
   G   
   C   
   A   
   A   
   G   
   T   
   C   
   C   
   A   
   G   
   T   
   C   
   A   
   A   
   T   
   C   
   T   
    T    
   C   
   G   
   T   
   A   
   C   
   C   
   G   
   C   
   T   
   T   
   T   
   A   
   G   
   T   
   T   
   A   
   A   
   T   
   T   
   C   
   A   
   G   
   A   
   T   
   A   
   A   
   T   
   A   
   T   
   T   
   T   
   A   
   T   
   T   
   T   
   T   
   G   
   C   
    C    
   C   
   A   
   A   
   C   
   A   
   A   
   A   
   C   
   A   
   T   
   T   
   A   
   C   
   G   
    G    
   A   
   T   
   G   
   G   
   G   
    G    
   G   
   A   
   A   
   G   
   A   
   C   
   A   
   A   
   A   
   T   
   T   
   T   
   A   
   G   
   A   
   G   
   C   
    G    
   G   
   G   
   T   
   C   
   T   
   T   
   A   
   A   
   T   
   A   
   A   
   A   
   T   
   T   
   C   
   A   
   T   
   T   
   T   
   T   
   C   
   G   
   G   
   T   
   G   
   A   
   G   
   G   
   A   
   A   
   C   
   T   
   A   
   G   
   A   
   T   
   T   
   T   
   A   
   C   
   C   
   A   
   A   
   T   
    T    
   G   
   C   
   C   
   A   
   T   
   G   
   A   
   C   
   G   
   C   
   C   
    T    
   G   
   C   
   T   
   C   
   A   
   A   
   A   
   T   
   T   
   T   
   C   
   A   
   A   
   A   
    C    
   G   
   A   
   A   
   G   
   A   
   T   
   A   
   A   
   A   
   T   
   T   
   T   
   A   
   A   
   T   
   T   
   C   
   T   
   G   
   A   
   A   
   A   
   T   
   T   
   C   
   T   
   T   
   T   
   T   
    A    
   G   
   C   
   A   
   G   
   A   
   T   
   A   
   C   
    A    
   G   
   G   
   C   
   T   
   A   
    T    
   G   
   G   
    C    
   C   
   A   
   A   
   G   
   G   
    C    
   C   
   A   
   A   
   T   
   T   
   G   
   T   
   T   
   A   
   A   
   T   
   T   
   T   
   C   
    G    
   C   
   C   
   A   
   A   
   T   
   C   
   C   
   A   
   G   
   C   
   A   
   A   
   G   
   C   
   T   
   A   
   C   
   C   
   A   
   T   
   G   
   T   
   A   
   T   
   A   
   G   
   T   
   G   
   T   
   T   
   T   
   T   
   C   
   C   
   A   
   A   
   A   
   A   
   T   
   A   
   A   
   T   
   G   
   G   
    A    
   A   
   C   
   A   
    T    
   T   
    A    
   G   
   T   
    T    
   T   
   A   
   T   
   C   
   C   
   A   
   A   
   A   
   A   
    T    
   T   
   A   
   G   
   T   
   T   
   T   
   T   
   A   
   G   
   A   
   G   
   A   
   A   
   A   
   G   
   A   
   A   
   A   
   C   
    G    
   A   
   A   
   G   
   A   
   A   
   A   
   A   
   A   
   A   
   G   
   A   
   C   
   A   
   A   
   T   
   G   
   T   
   A   
   A   
   T   
   C   
   A   
   G   
   C   
   G   
   C   
   C   
   A   
   A   
   C   
   G   
   C   
   A   
   G   
   C   
   G   
   A   
   A   
   T   
   A   
   C   
   A   
   A   
   T   
   A   
   G   
   C   
   C   
   A   
   C   
   A   
   G   
   A   
   T   
   C   
   T   
   T   
   T   
   T   
   A   
   G   
   G   
   T   
   A   
   G   
   T   
   G   
   T   
   G   
   G   
   A   
   A   
   G   
   A   
   T   
   C   
   C   
   T   
   T   
   C   
   A   
   G   
   G   
   T   
   T   
   A   
   T   
   G   
   T   
   T   
   T   
   A   
   T   
   A   
   A   
   T   
   A   
   T   
   G   
   T   
   A   
   C   
   A   
   A   
   T   
   C   
   C   
   G   
   A   
   A   
   C   
   T   
   T   
   C   
   T   
   C   
   A   
   T   
   T   
   G   
   G   
   C   
   T   
   G   
   C   
   C   
   A   
   A   
   A   
   A   
   C   
   A   
   G   
   G   
   A   
   A   
   C   
   A   
   G   
   C   
   T   
   G   
   A   
   A   
   A   
   T   
   A   
   A   
   A   
   A   
   G   
   A   
   T   
   A   
   A   
   A   
   C   
   A   
   A   
   G   
   A   
   T   
   A   
   C   
   A   
   G   
   A   
   T   
   G   
   G   
   C   
   A   
   A   
   A   
   G   
   A   
   A   
   A   
   A   
   C   
   A   
   G   
   T   
   T   
   T   
   C   
   C   
   T   
   T   
   C   
   T   
   A   
   A   
   C   
   G   
   T   
   T   
   A   
   G   
   A   
   T   
   C   
   G   
   T   
   A   
   G   
   T   
   A   
   A   
   C   
   A   
   A   
   T   
   A   
   A   
   A   
   T   
   T   
   T   
   T   
   T   
   A   
   A   
   C   
   A   
   A   
   T   
   G   
   A   
   T   
   T   
   A   
   T   
   G   
   G   
   T   
   T   
   G   
   A   
   A   
   A   
   A   
   T   
   T   
   C   
   A   
   G   
   G   
   A   
   G   
   A   
   A   
   A   
   A   
   T   
   G   
   G   
   T   
   T   
   C   
   T   
   G   
   C   
   A   
   A   
   C   
   A   
   G   
   A   
   T   
   A   
   T   
   C   
   A   
   G   
   T   
   A   
   A   
   A   
   C   
   C   
   A   
   T   
   T   
   A   
   A   
   T   
   T   
   G   
   A   
   T   
   T   
   A   
   C   
   T   
   T   
   A   
   G   
   A   
   A   
   G   
   C   
   A   
   A   
   C   
   C   
   A   
   T   
   T   
   A   
   A   
   A   
   -   
   -   
   -   
   -   
   -   
   -   
   -   
   -   
   -   
   -   
   -   
   -   
   -   
   -   
   -   
   -   
   -   
   -   
   -   
   -   
   -   
   -   
   -   
   -   
   -   
   -   
   -   
   -   
   -   
   -   
   -   
   -   
   -   
   -   
   -   
   -   
   -   
   -   
   -   
   -   
   -   
   -   
   -   
   -   
   -   
   -   
   -   
   -   
   -   
   -   
   -   
   -   
   -   
   -   
   -   
   -   
   -   
   -   
   -   
   -   
   -   
   A   
   T   
   G   
   G   
   A   
   A   
   C   
   G   
   A   
   A   
   G   
   C   
   A   
   A   
   T   
   C   
   G   
   T   
   A   
   A   
   T   
   A   
   A   
   A   
   A   
   A   
   A   
   T   
   C   
   T   
   T   
   C   
   A   
   A   
   A   
   A   
   A   
   A   
   A   
   C   
   C   
   A   
   C   
   T   
   T   
   A   
   T   
   T   
   C   
   T   
   T   
   G   
   G   
   C   
   G   
   T   
   T   
   T   
   C   
   T   
   G   
   C   
   C   
   T   
   T   
   G   
   G   
   T   
   T   
   C   
   T   
   G   
   A   
   T   
   C   
   G   
   C   
   T   
   G   
   C   
   T   
   G   
   T   
   C   
   G   
   G   
   T   
   G   
   G   
   T   
   G   
   G   
   G   
   T   
   A   
   T   
   T   
   A   
   T   
   G   
   C   
   T   
   T   
   A   
   T   
   A   
   G   
   C   
   C   
   A   
   A   
   T   
   G   
   G   
   C   
   A   
   A   
   G   
   C   
   C   
   A   
   A   
   A   
   C   
   A   
   A   
   G   
   A   
   A   
   T   
   T   
   A   
   G   
   C   
   T   
   G   
   A   
   A   
   G   
   C   
   G   
   A   
   A   
   G   
   A   
   A   
   A   
   A   
   C   
   A   
   G   
   C   
   C   
   A   
   A   
   T   
   A   
   C   
   A   
   T   
   T   
   T   
   T   
   T   
   A   
   A   
   A   
   C   
   G   
   T   
   A   
   T   
   T   
   G   
   T   
   C   
   A   
   A   
   A   
   A   
   C   
   A   
   G   
   G   
   A   
   A   
   T   
   T   
   T   
   G   
   A   
   T   
   A   
   A   
   G   
   T   
   T   
   A   
   C   
   C   
   A   
   T   
   C   
   C   
   G   
   T   
   T   
   G   
   T   
   T   
   C   
   A   
   A   
   G   
   A   
   A   
   A   
   C   
   T   
   A   
   G   
   C   
   T   
   T   
   A   
   A   
   A   
   G   
   A   
   A   
   A   
   A   
   A   
   T   
   G   
   G   
   C   
   T   
   A   
   T   
   G   
   A   
   T   
   A   
   C   
   T   
   A   
   A   
   A   
   T   
   C   
   T   
   G   
   T   
   T   
   G   
   T   
   T   
   G   
   A   
   A   
   A   
   A   
   A   
   T   
   A   
   C   
   C   
   A   
   A   
   G   
   C   
   A   
   A   
   T   
   T   
   T   
   A   
   T   
   T   
   C   
   A   
   G   
   G   
   G   
   A   
   T   
   T   
   C   
   A   
   A   
   G   
   C   
   A   
   G   
   A   
   A   
   G   
   G   
   G   
   G   
   T   
   C   
   A   
   A   
   A   
   G   
   C   
   T   
   A   
   G   
   T   
   G   
   A   
   T   
   G   
   T   
   T   
   C   
   A   
   A   
   G   
   T   
   C   
   A   
   A   
   A   
   A   
   A   
   A   
   G   
   G   
   G   
   A   
   A   
   A   
   G   
   A   
   C   
   A   
   A   
   C   
   C   
   A   
   A   
   T   
   A   
   C   
   A   
   C   
   A   
   T   
   T   
   T   
   A   
   C   
   C   
   T   
   A   
   T   
   A   
   A   
   A   
   T   
   T   
   A   
   T   
   C   
   G   
   A   
   T   
   G   
   A   
   G   
   C   
   A   
   C   
   T   
   C   
   C   
   T   
   T   
   T   
   A   
   G   
   G   
   C   
   G   
   A   
   A   
   A   
   T   
   G   
   A   
   A   
   A   
   G   
   A   
   T   
   T   
   T   
   G   
   T   
   C   
   T   
   T   
   A   
   T   
   C   
   A   
   A   
   T   
   C   
   A   
   A   
   G   
   T   
   A   
   T   
   C   
   G   
   C   
   C   
   A   
   A   
   A   
   A   
   A   
   A   
   G   
   G   
   C   
   G   
   A   
   T   
   A   
   A   
   C   
   T   
   A   
   C   
   C   
   A   
   A   
   A   
   T   
   C   
   G   
   C   
   T   
   T   
   G   
   G   
   G   
   C   
   A   
   C   
   C   
   G   
   T   
   C   
   T   
   T   
   T   
   A   
   A   
   T   
   T   
   T   
   T   
   T   
   C   
   C   
   A   
   G   
   A   
   T   
   A   
   T   
   G   
   T   
   C   
   A   
   G   
   G   
   A   
   A   
   A   
   T   
   G   
   A   
   T   
   A   
   A   
   A   
   A   
   T   
   T   
   T   
   C   
   G   
   A   
   T   
   T   
   C   
   A   
   A   
   G   
   T   
   A   
   G   
   A   
   T   
   A   
   A   
   T   
   G   
   C   
   C   
   A   
   A   
   A   
   C   
   G   
   T   
   G   
   G   
   A   
   G   
   A   
   A   
   A   
   T   
   T   
   G   
   T   
   C   
   G   
   A   
   T   
   C   
   G   
   T   
   A   
   A   
   T   
   G   
   G   
   T   
   A   
   G   
   T   
   G   
   G   
   G   
   C   
   T   
   A   
   G   
   C   
   A   
   A   
   T   
   T   
   A   
   A   
   C   
   A   
   A   
   A   
   G   
   T   
   G   
   T   
   T   
   T   
   G   
   A   
   T   
   G   
   A   
   A   
   G   
   T   
   G   
   G   
   G   
   C   
   G   
   T   
   A   
   G   
   T   
   G   
   C   
   C   
   T   
   G   
   G   
   C   
   A   
   A   
   A   
   C   
   T   
   C   
   G   
   G   
   T   
   T   
   C   
   T   
   G   
   G   
   C   
   G   
   C   
   A   
   G   
   A   
   A   
   A   
   A   
   A   
   A   
   C   
   A   
   G   
   C   
   C   
   A   
   A   
   T   
   A   
   T   
   C   
   A   
   A   
   A   
   G   
   C   
   T   
   T   
   T   
   T   
   A   
   G   
   T   
   G   
   A   
   T   
   A   
   A   
   G   
   T   
   T   
   T   
   G   
   G   
   C   
   G   
   T   
   T   
   T   
   C   
   T   
   G   
   T   
   T   
   G   
   A   
   T   
   G   
   A   
   A   
   A   
   T   
   C   
   A   
   A   
   T   
   C   
   A   
   A   
   A   
   A   
   G   
   T   
   T   
   A   
   A   
   G   
   C   
   C   
   A   
   A   
   G   
   G   
   A   
   T   
   G   
   G   
   G   
   T   
   C   
   C   
   A   
   A   
   G   
   C   
   A   
   G   
   A   
   C   
   T   
   C   
   G   
   T   
   T   
   T   
   G   
   T   
   A   
   C   
   C   
   A   
   A   
   T   
   C   
   A   
   C   
   A   
   G   
   T   
   T   
   G   
   C   
   T   
   T   
   C   
   T   
   G   
   A   
   A   
   C   
   C   
   A   
   G   
   T   
   G   
   A   
   C   
   A   
   G   
   A   
   A   
   T   
   T   
   A   
   C   
   C   
   A   
   A   
   C   
   A   
   G   
   G   
   G   
   G   
   C   
   T   
   G   
   C   
   G   
   A   
   C   
   A   
   A   
   A   
   A   
   G   
   A   
   T   
   A   
   C   
   A   
   G   
   A   
   G   
   T   
   C   
   A   
   C   
   G   
   T   
   T   
   A   
   T   
   T   
   A   
   T   
   C   
   C   
   A   
   T   
   T   
   G   
   G   
   G   
   G   
   G   
   A   
   A   
   G   
   C   
   A   
   G   
   C   
   T   
   G   
   C   
   G   
   C   
   A   
   A   
   T   
   T   
   A   
   A   
   T   
   C   
   G   
   G   
   G   
   T   
   A   
   T   
   A   
   C   
   G   
   G   
   G   
   C   
   A   
   C   
   T   
   A   
   T   
   T   
   A   
   C   
   T   
   G   
   C   
   A   
   G   
   A   
   A   
   G   
   A   
   T   
   A   
   T   
   T   
   G   
   A   
   A   
   A   
   A   
   A   
   A   
   A   
   T   
   C   
   C   
   A   
   G   
   A   
   G   
   T   
   T   
   A   
   A   
   G   
   T   
   A   
   G   
   T   
   A   
   C   
   G   
   G   
   G   
   C   
   G   
   T   
   A   
   A   
   T   
   T   
   G   
   G   
   C   
   A   
   A   
   A   
   A   
   C   
   T   
   G   
   G   
   C   
   T   
   T   
   A   
   G   
   A   
   A   
   C   
   G   
   A   
   G   
   C   
   G   
   T   
   T   
   T   
   G   
   A   
   T   
   A   
   A   
   A   
   G   
   A   
   A   
   T   
   T   
   A   
   A   
   G   
   A   
   G   
   G   
   A   
   C   
   A   
   A   
   G   
   A   
   T   
   G   
   G   
   T   
   G   
   G   
   T   
   T   
   C   
   A   
   C   
   T   
   A   
   G   
   T   
   T   
   A   
   T   
   T   
   T   
   T   
   A   
   G   
   A   
   T   
   G   
   A   
   C   
   A   
   A   
   A   
   G   
   A   
   G   
   A   
   A   
   T   
   G   
   T   
   C   
   A   
   A   
   A   
   A   
   A   
   A   
   G   
   C   
   T   
   T   
   T   
   A   
   C   
   A   
   A   
   A   
   C   
   C   
   A   
   A   
   A   
   G   
   A   
   G   
   A   
   A   
   A   
   A   
   A   
   A   
   G   
   A   
   T   
   G   
   G   
   T   
   C   
   A   
   A   
   A   
   C   
   A   
   A   
   T   
   T   
   A   
   A   
   A   
   T   
   T   
   A   
   A   
   C   
   G   
   A   
   T   
   T   
   G   
   A   
   T   
   A   
   G   
   T   
   G   
   G   
   C   
   G   
   T   
   A   
   C   
   A   
   A   
   C   
   A   
   A   
   C   
   A   
   A   
   G   
   C   
   C   
   T   
   T   
   C   
   G   
   C   
   T   
   A   
   T   
   A   
   T   
   T   
   T   
   G   
   A   
   C   
   A   
   A   
   A   
   C   
   G   
   T   
   C   
   C   
   T   
   G   
   G   
   T   
   A   
   G   
   T   
   G   
   C   
   G   
   G   
   T   
   C   
   A   
   T   
   T   
   A   
   C   
   C   
   G   
   A   
   T   
   C   
   C   
   T   
   C   
   A   
   A   
   A   
   A   
   A   
   G   
   G   
   C   
   G   
   A   
   T   
   T   
   T   
   A   
   C   
   T   
   A   
   G   
   C   
   G   
   A   
   C   
   G   
   G   
   T   
   T   
   A   
   G   
   T   
   T   
   C   
   A   
   C   
   C   
   T   
   T   
   C   
   G   
   T   
   A   
   T   
   G   
   A   
   T   
   C   
   C   
   T   
   A   
   A   
   T   
   A   
   A   
   A   
   A   
   T   
   G   
   G   
   C   
   C   
   A   
   A   
   T   
   G   
   G   
   C   
   A   
   T   
   T   
   T   
   C   
   T   
   C   
   A   
   A   
   A   
   A   
   A   
   G   
   A   
   A   
   T   
   A   
   T   
   G   
   A   
   T   
   G   
   C   
   T   
   T   
   A   
   C   
   A   
   A   
   T   
   A   
   A   
   C   
   A   
   A   
   C   
   A   
   A   
   A   
   G   
   A   
   T   
   T   
   T   
   A   
   C   
   C   
   A   
   T   
   T   
   C   
   A   
   C   
   A   
   G   
   C   
   A   
   C   
   G   
   T   
   T   
   T   
   T   
   G   
   C   
   G   
   A   
   C   
   A   
   G   
   G   
   C   
   T   
   A   
   T   
   G   
   C   
   A   
   C   
   C   
   A   
   G   
   G   
   C   
   T   
   C   
   T   
   A   
   C   
   C   
   T   
   T   
   T   
   A   
   A   
   A   
   A   
   C   
   A   
   A   
   T   
   T   
   A   
   C   
   C   
   G   
   G   
   T   
   G   
   C   
   C   
   A   
   T   
   T   
   G   
   G   
   T   
   T   
   T   
   A   
   G   
   A   
   T   
   G   
   C   
   A   
   G   
   G   
   G   
   A   
   C   
   A   
   T   
   T   
   G   
   A   
   A   
   G   
   C   
   C   
   A   
   G   
   A   
   T   
   G   
   A   
   A   
   G   
   A   
   G   
   C   
   T   
   A   
   G   
   A   
   A   
   A   
   T   
   T   
   A   
   A   
   T   
   G   
   G   
   C   
   T   
   T   
   G   
   A   
   A   
   A   
   T   
   G   
   G   
   C   
   A   
   A   
   A   
   A   
   A   
   G   
   A   
   T   
   A   
   A   
   A   
   T   
   C   
   T   
   T   
   G   
   G   
   G   
   G   
   C   
   G   
   G   
   C   
   T   
   A   
   T   
   T   
   T   
   T   
   G   
   C   
   G   
   A   
   C   
   A   
   C   
   G   
   T   
   G   
   T   
   A   
   A   
   A   
   A   
   G   
   A   
   A   
   G   
   C   
   A   
   A   
   G   
   T   
   C   
   C   
   A   
   G   
   T   
   C   
   A   
   A   
   T   
   C   
   T   
   T   
   C   
   G   
   T   
   A   
   C   
   C   
   G   
   C   
   T   
   T   
   T   
   A   
   G   
   T   
   T   
   A   
   A   
   T   
   T   
   C   
   A   
   G   
   A   
   T   
   A   
   A   
   T   
   A   
   T   
   T   
   T   
   A   
   T   
   T   
   T   
   T   
   G   
   C   
   C   
   C   
   A   
   A   
   C   
   A   
   A   
   A   
   C   
   A   
   T   
   T   
   A   
   C   
   G   
   G   
   A   
   T   
   G   
   G   
   G   
   G   
   G   
   A   
   A   
   G   
   A   
   C   
   A   
   A   
   A   
   T   
   T   
   T   
   A   
   G   
   A   
   G   
   C   
   G   
   G   
   G   
   T   
   C   
   T   
   T   
   A   
   A   
   T   
   A   
   A   
   A   
   T   
   T   
   C   
   A   
   T   
   T   
   T   
   T   
   C   
   G   
   G   
   T   
   G   
   A   
   G   
   G   
   A   
   A   
   C   
   T   
   A   
   G   
   A   
   T   
   T   
   T   
   A   
   C   
   C   
   A   
   A   
   T   
   T   
   G   
   C   
   C   
   A   
   T   
   G   
   A   
   C   
   G   
   C   
   C   
   T   
   G   
   C   
   T   
   C   
   A   
   A   
   A   
   T   
   T   
   T   
   C   
   A   
   A   
   A   
   C   
   G   
   A   
   A   
   G   
   A   
   T   
   A   
   A   
   A   
   T   
   T   
   T   
   A   
   A   
   T   
   T   
   C   
   T   
   G   
   A   
   A   
   A   
   T   
   T   
   C   
   T   
   T   
   T   
   T   
   A   
   G   
   C   
   A   
   G   
   A   
   T   
   A   
   C   
   A   
   G   
   G   
   C   
   T   
   A   
   T   
   G   
   G   
   C   
   C   
   A   
   A   
   G   
   G   
   C   
   C   
   A   
   A   
   T   
   T   
   G   
   T   
   T   
   A   
   A   
   T   
   T   
   T   
   C   
   G   
   C   
   C   
   A   
   A   
   T   
   C   
   C   
   A   
   G   
   C   
   A   
   A   
   G   
   C   
   T   
   A   
   C   
   C   
   A   
   T   
   G   
   T   
   A   
   T   
   A   
   G   
   T   
   G   
   T   
   T   
   T   
   T   
   C   
   C   
   A   
   A   
   A   
   A   
   T   
   A   
   A   
   T   
   G   
   G   
   A   
   A   
   C   
   A   
   T   
   T   
   A   
   G   
   T   
   T   
   T   
   A   
   T   
   C   
   C   
   A   
   A   
   A   
   A   
   T   
   T   
   A   
   G   
   T   
   T   
   T   
   T   
   A   
   G   
   A   
   G   
   A   
   A   
   A   
   G   
   A   
   A   
   A   
   C   
   G   
   A   
   A   
   G   
   A   
   A   
   A   
   A   
   A   
   A   
   G   
   A   
   C   
   A   
   A   
   T   
   G   
   T   
   A   
   A   
   T   
   C   
   A   
   G   
   C   
   G   
   C   
   C   
   A   
   A   
   C   
   G   
   C   
   A   
   G   
   C   
   G   
   A   
   A   
   T   
   A   
   C   
   A   
   A   
   T   
   A   
   G   
   C   
   C   
   A   
   C   
   A   
   G   
   A   
   T   
   C   
   T   
   T   
   T   
   T   
   A   
   G   
   G   
   T   
   A   
   G   
   T   
   G   
   T   
   G   
   G   
   A   
   A   
   G   
   A   
   T   
   C   
   C   
   T   
   T   
   C   
   A   
   G   
   G   
   T   
   T   
   A   
   T   
   G   
   T   
   T   
   T   
   A   
   T   
   A   
   A   
   T   
   A   
   T   
   G   
   T   
   A   
   C   
   A   
   A   
   T   
   C   
   C   
   G   
   A   
   A   
   C   
   T   
   T   
   C   
   T   
   C   
   A   
   T   
   T   
   G   
   G   
   C   
   T   
   G   
   C   
   C   
   A   
   A   
   A   
   A   
   C   
   A   
   G   
   G   
   A   
   A   
   C   
   A   
   G   
   C   
   T   
   G   
   A   
   A   
   A   
   T   
   A   
   A   
   A   
   A   
   G   
   A   
   T   
   A   
   A   
   A   
   C   
   A   
   A   
   G   
   A   
   T   
   A   
   C   
   A   
   G   
   A   
   T   
   G   
   G   
   C   
   A   
   A   
   A   
   G   
   A   
   A   
   A   
   A   
   C   
   A   
   G   
   T   
   T   
   T   
   C   
   C   
   T   
   T   
   C   
   T   
   A   
   A   
   C   
   G   
   T   
   T   
   A   
   G   
   A   
   T   
   C   
   G   
   T   
   A   
   G   
   T   
   A   
   A   
   C   
   A   
   A   
   T   
   A   
   A   
   A   
   T   
   T   
   T   
   T   
   T   
   A   
   A   
   C   
   A   
   A   
   T   
   G   
   A   
   T   
   T   
   A   
   T   
   G   
   G   
   T   
   T   
   G   
   A   
   A   
   A   
   A   
   T   
   T   
   C   
   A   
   G   
   G   
   A   
   G   
   A   
   A   
   A   
   A   
   T   
   G   
   G   
   T   
   T   
   C   
   T   
   G   
   C   
   A   
   A   
   C   
   A   
   G   
   A   
   T   
   A   
   T   
   C   
   A   
   G   
   T   
   A   
   A   
   A   
   C   
   C   
   A   
   T   
   T   
   A   
   A   
   T   
   T   
   G   
   A   
   T   
   T   
   A   
   C   
   T   
   T   
   A   
   G   
   A   
   A   
   G   
   C   
   A   
   A   
   C   
   C   
   A   
   T   
   T   
   A   
   A   
   A   
   -   
   -   
   -   
   -   
   -   
   -   
   -   
   -   
   -   
   -   
   -   
   -   
   -   
   -   
   -   
   -   
   -   
   -   
   -   
   -   
   -   
   -   
   -   
   -   
   -   
   -   
   -   
   -   
   -   
   -   
   -   
   -   
   -   
   -   
   -   
   -   
   -   
   -   
   -   
   -   
   -   
   -   
   -   
   -   
   -   
   -   
   -   
   -   
   -   
   -   
   -   
   -   
   -   
   -   
   -   
   -   
   -   
   -   
   -   
   -   
   -   
 

 
   N55106   
   -   
   -   
   -   
   -   
   -   
   -   
   -   
   -   
   -   
   -   
   -   
   -   
   -   
   -   
   -   
   -   
   -   
   -   
   -   
   -   
   -   
   -   
   -   
   -   
   -   
   -   
   -   
   -   
   -   
   -   
   -   
   -   
   -   
   -   
   -   
   -   
   -   
   -   
   -   
   -   
   -   
   -   
   -   
   -   
   -   
   -   
   -   
   -   
   -   
   -   
   -   
   -   
   -   
   -   
   -   
   -   
   -   
   -   
   -   
   -   
   -   
   -   
   -   
   -   
   -   
   -   
   -   
   -   
   -   
   -   
   -   
   -   
   -   
   -   
   -   
   -   
   -   
   -   
   -   
   -   
   -   
   -   
   -   
   -   
   -   
   -   
   -   
   -   
   -   
   -   
   -   
   -   
   -   
   -   
   -   
   -   
   -   
   -   
   -   
   -   
   -   
   -   
   -   
   -   
   -   
   -   
   -   
   -   
   C   
   A   
   A   
   T   
   G   
   G   
   C   
   A   
   A   
   G   
   C   
   C   
   A   
   A   
   A   
   C   
   A   
   A   
   G   
   A   
    A    
   T   
   T   
   A   
   G   
   C   
    C    
   G   
   A   
   A   
   G   
   C   
   G   
   A   
   A   
   G   
   A   
   A   
   A   
   A   
    C    
   A   
   G   
   C   
    T    
   A   
    C    
   T   
    A    
    C    
   A   
   T   
   T   
   T   
   T   
   T   
   A   
   A   
   A   
    C    
   G   
   T   
   A   
   T   
   T   
   G   
    T    
   C   
   A   
   A   
   A   
   A   
   C   
   A   
   G   
    G    
   A   
   A   
   T   
   T   
   T   
   G   
   A   
   T   
   A   
   A   
   G   
   T   
   T   
   A   
   C   
   C   
    G    
   T   
   C   
   C   
   G   
   T   
   T   
    G    
   T   
   T   
   C   
   A   
   A   
   G   
   A   
   A   
    G    
   C   
   T   
   A   
   G   
   C   
   T   
   T   
   A   
   A   
   A   
   G   
   A   
   A   
   A   
   A   
   A   
   T   
   G   
   G   
    C    
   T   
   A   
   T   
   G   
   A   
   T   
   A   
   C   
   T   
   A   
   A   
   A   
   T   
   C   
    T    
   G   
   T   
   T   
   G   
   T   
   T   
   G   
   A   
   A   
   A   
   A   
   A   
   T   
   A   
   C   
   C   
   A   
   A   
   G   
   C   
   A   
   A   
   T   
   T   
   T   
   A   
   T   
   T   
   C   
   A   
   G   
   G   
   G   
   A   
   T   
   T   
   C   
   A   
   A   
   G   
   C   
    A    
   G   
   A   
   A   
    G    
   G   
    A    
   G   
   T   
   C   
   A   
   A   
    A    
   G   
   C   
   T   
   A   
   G   
   T   
   G   
   A   
   T   
   G   
   T   
    T    
   C   
   A   
   A   
   G   
   T   
    A    
   A   
   A   
   A   
   A   
   A   
    G    
   G   
    C    
   G   
   A   
   A   
   A   
   G   
   A   
    C    
   A   
   A   
    T    
   C   
   A   
   A   
   T   
   A   
   C   
   A   
   C   
   A   
   T   
   T   
   T   
   A   
    C    
   C   
   T   
   A   
   T   
   A   
   A   
   A   
   T   
   T   
   A   
   T   
   C   
   G   
   A   
   T   
   G   
   A   
   G   
    T    
   A   
   C   
    C    
   C   
   C   
   T   
   T   
   T   
   A   
   G   
   G   
   C   
   G   
   A   
   A   
   A   
   T   
   G   
   A   
   A   
   A   
   G   
   A   
   T   
    T    
   T   
   G   
   T   
   C   
   T   
   T   
   A   
   T   
   C   
   A   
   A   
   T   
   C   
   A   
   A   
   G   
   T   
   A   
   T   
    C    
   G   
    C    
   C   
   A   
   A   
    A    
   A   
   A   
   A   
   G   
   G   
    C    
   G   
   A   
   T   
   A   
    C    
   C   
   T   
   A   
   C   
   C   
   A   
   A   
   A   
   T   
    C    
    G    
   C   
   T   
   T   
   G   
   G   
    A    
    A    
    G    
   C   
   C   
    G    
   T   
   C   
   T   
   T   
   T   
   A   
   A   
   T   
   T   
   T   
   T   
   T   
   C   
   C   
   A   
   G   
   A   
   T   
   A   
   T   
   G   
   T   
   C   
   A   
   G   
   G   
   A   
   A   
   A   
   T   
   G   
   A   
   T   
   A   
   A   
   A   
    A    
   T   
   T   
   T   
   C   
   G   
   A   
   T   
   T   
   C   
   A   
   A   
   G   
   T   
   A   
   G   
   A   
   T   
   A   
   A   
   T   
   G   
   C   
   C   
   A   
   A   
   A   
   C   
   G   
   T   
   G   
   G   
   A   
   G   
   A   
   A   
   A   
   T   
   T   
   G   
   T   
   C   
   G   
   A   
   T   
   C   
   G   
   T   
   A   
   A   
    T    
   G   
   G   
   T   
   A   
   G   
   T   
   G   
   G   
   G   
   C   
   T   
   A   
   G   
    C    
   A   
   A   
   T   
   T   
   A   
   A   
   C   
   A   
   A   
   A   
   G   
   T   
   G   
   T   
   T   
   T   
   G   
   A   
    T    
   G   
   A   
   A   
   G   
   T   
   G   
   G   
   G   
   C   
   G   
   T   
   A   
   G   
   T   
    G    
   C   
   C   
   T   
    G    
   G   
   C   
   A   
   A   
   A   
   C   
   T   
    C    
   G   
   G   
   T   
   T   
   C   
   T   
   G   
   G   
   C   
   G   
   C   
   A   
   G   
   A   
   A   
   A   
   A   
   A   
    A    
   C   
   A   
   G   
   C   
    C    
   A   
   A   
   T   
   A   
   T   
   C   
   A   
   A   
   A   
   G   
   C   
   T   
   T   
   T   
   T   
   A   
   G   
   T   
   G   
   A   
   T   
   A   
   A   
    A    
   T   
   T   
    C    
   G   
   G   
   C   
   G   
   T   
   T   
   T   
   C   
   T   
    G    
   T   
   T   
   G   
   A   
   T   
   G   
   A   
   A   
   A   
   T   
   C   
   A   
   A   
   T   
   C   
    A    
   A   
   A   
   A   
    G    
   T   
   T   
   A   
   A   
   G   
   C   
   C   
   A   
   A   
   G   
   G   
    A    
   T   
   G   
   G   
   G   
   T   
   C   
   C   
   A   
   A   
   G   
   C   
   A   
   G   
   A   
   C   
   T   
   C   
    C    
   T   
   T   
   T   
   G   
   T   
   A   
   C   
   C   
   A   
   A   
   T   
   C   
   A   
   C   
   A   
   G   
   T   
    C    
   G   
   C   
   T   
   T   
   C   
   T   
   G   
   A   
   A   
   C   
   C   
   A   
   G   
   T   
   G   
   A   
   C   
   A   
   G   
   A   
   A   
   T   
   T   
   A   
   C   
   C   
   A   
   A   
   C   
   A   
   G   
   G   
   G   
   G   
   C   
   T   
   G   
   C   
   G   
   A   
   C   
   A   
   A   
   A   
   A   
   G   
    A    
    T    
   A   
   C   
   A   
   G   
   A   
   G   
   T   
   C   
   A   
   C   
   G   
   T   
   T   
   A   
   T   
   T   
   A   
   T   
   C   
   C   
    G    
    C    
   T   
   G   
   G   
   G   
   G   
   G   
   A   
   A   
   G   
   C   
   A   
   G   
   C   
   T   
   G   
   C   
   G   
   C   
   A   
   A   
   T   
   T   
   A   
   A   
   T   
   C   
   G   
   G   
    G    
   T   
   A   
   T   
   A   
   C   
    G    
   G   
   G   
    C    
   A   
   C   
    C    
   A   
   T   
   T   
   A   
   C   
   T   
   G   
   C   
   A   
   G   
   A   
    A    
   G   
   A   
   T   
   A   
   T   
   T   
    G    
   A   
   A   
   A   
   A   
   A   
   A   
   A   
   T   
   C   
   C   
   A   
   G   
   A   
   G   
   T   
   T   
   A   
   A   
   G   
   T   
   A   
   G   
   T   
   A   
   C   
   G   
   G   
   G   
    C    
   G   
   T   
   A   
   A   
   T   
   T   
   G   
   G   
    T    
   A   
   A   
   A   
   A   
   C   
   T   
   G   
   G   
    T    
   T   
   T   
   A   
   G   
   A   
   A   
   C   
   G   
   A   
   G   
   C   
   G   
   T   
   T   
   T   
   G   
   A   
   T   
   A   
   A   
   A   
   G   
   A   
    A    
   T   
   T   
   A   
   A   
   G   
   A   
   G   
   G   
    G    
   C   
   A   
   A   
   G   
   A   
   T   
   G   
   G   
    T    
   G   
   G   
    C    
   T   
   C   
    A    
   C   
   T   
   A   
   G   
   T   
   T   
   A   
   T   
   T   
   T   
   T   
   A   
   G   
   A   
   T   
   G   
   A   
   C   
   A   
   A   
   A   
   G   
   A   
   G   
   A   
   A   
   T   
   G   
   T   
   C   
   A   
   A   
   A   
   A   
   A   
   A   
   G   
   C   
   T   
   T   
   T   
   A   
   C   
   A   
   A   
   A   
   C   
   C   
   A   
   A   
   A   
   G   
   A   
    G    
   A   
   A   
   A   
   A   
   A   
   A   
   G   
   A   
   T   
   G   
   G   
   T   
   C   
   A   
   A   
   A   
   C   
    G    
   A   
   T   
   T   
   A   
   A   
   A   
   T   
   T   
   A   
   A   
   C   
   G   
   A   
   T   
   T   
   G   
   A   
    C    
   A   
   G   
    T    
   G   
   G   
    C    
   G   
   T   
   A   
   C   
   A   
   A   
   C   
   A   
   A   
   C   
   A   
   A   
   G   
   C   
    C    
   T   
   T   
    C    
   G   
   C   
   T   
   A   
   T   
   A   
   T   
   T   
   T   
   G   
   A   
   C   
   A   
   A   
   A   
   C   
   G   
    T    
    C    
   C   
   T   
   G   
   G   
   T   
   A   
   G   
   T   
   G   
   C   
   G   
   G   
   T   
   C   
   A   
   T   
   T   
   A   
   C   
   C   
   G   
   A   
   T   
   C   
   C   
   T   
   C   
   A   
   A   
   A   
   A   
   A   
   G   
   G   
   C   
   G   
   A   
   T   
   T   
   T   
   A   
    T    
   T   
   A   
   G   
   C   
    A    
   A   
   C   
   G   
   G   
   T   
   T   
   A   
   G   
   T   
   T   
   C   
   A   
   C   
   C   
   T   
   T   
   C   
   G   
   T   
   A   
   T   
   G   
   A   
   T   
   C   
   C   
   T   
   A   
   A   
   T   
   A   
   A   
   A   
   A   
   T   
   G   
   G   
   C   
   C   
   A   
   A   
   T   
   G   
   G   
    T    
   A   
   T   
   T   
   T   
   C   
   T   
   C   
   A   
   A   
   A   
   A   
   A   
   G   
   A   
    A    
   T   
   A   
   T   
   G   
   A   
   T   
   G   
   C   
   T   
   T   
   A   
   C   
   A   
   A   
   T   
   A   
   A   
   C   
   A   
   A   
   C   
   A   
   A   
   A   
   G   
   A   
   T   
   T   
   T   
   A   
   C   
   C   
   A   
   T   
   T   
    C    
   A   
   C   
   A   
   G   
   C   
   A   
   C   
   G   
   T   
   T   
   T   
    T    
   G   
   C   
   G   
   A   
   C   
   A   
   G   
   G   
    C    
   T   
   A   
   T   
   G   
   C   
   A   
   C   
   C   
   A   
   G   
   G   
    C    
   T   
   C   
   T   
   A   
   C   
   C   
   T   
   T   
   T   
   A   
   A   
   A   
   A   
   C   
   A   
   A   
   T   
   T   
   A   
   C   
   C   
   G   
   G   
   T   
   G   
   C   
   C   
   A   
   T   
   T   
   G   
   G   
   T   
   T   
   T   
   A   
   G   
   A   
   T   
    G    
   C   
   A   
   G   
   G   
   G   
   A   
   C   
   A   
   T   
   T   
   G   
   A   
   A   
   G   
   C   
   C   
   A   
   G   
   A   
   T   
   G   
   A   
   A   
   G   
   A   
   G   
   C   
   T   
   A   
   G   
   A   
   A   
   A   
   T   
   T   
   A   
   A   
   T   
   G   
   G   
    T    
   T   
   T   
   G   
   A   
   A   
   A   
   T   
   G   
   G   
   C   
   A   
   A   
   A   
   A   
   A   
   G   
   A   
   T   
   A   
   A   
   A   
   T   
   C   
   T   
   T   
   G   
   G   
   G   
   G   
    C    
   G   
   G   
   C   
   T   
   A   
   T   
   T   
   T   
   T   
   G   
   C   
    G    
   A   
   C   
   A   
   C   
   G   
   T   
   G   
   T   
   A   
   A   
   A   
   A   
   G   
   A   
   A   
   G   
   C   
   A   
   A   
   G   
   T   
   C   
   C   
   A   
   G   
   T   
   C   
   A   
   A   
   T   
   C   
   T   
    G    
   C   
   G   
   T   
   A   
   C   
   C   
   G   
   C   
   T   
   T   
   T   
   A   
   G   
   T   
   T   
   A   
   A   
   T   
   T   
   C   
   A   
   G   
   A   
   T   
   A   
   A   
   T   
   A   
   T   
   T   
   T   
   A   
   T   
   T   
   T   
   T   
   G   
   C   
    C    
   C   
   A   
   A   
   C   
   A   
   A   
   A   
   C   
   A   
   T   
   T   
   A   
   C   
   G   
    G    
   A   
   T   
   G   
   G   
   G   
    A    
   G   
   A   
   A   
   G   
   A   
   C   
   A   
   A   
   A   
   T   
   T   
   T   
   A   
   G   
   A   
   G   
   C   
    G    
   G   
   G   
   T   
   C   
   T   
   T   
   A   
   A   
   T   
   A   
   A   
   A   
   T   
   T   
   C   
   A   
   T   
   T   
   T   
   T   
   C   
   G   
   G   
   T   
   G   
   A   
   G   
   G   
   A   
   A   
   C   
   T   
   A   
   G   
   A   
   T   
   T   
   T   
   A   
   C   
   C   
   A   
   A   
   T   
    T    
   G   
   C   
   C   
   A   
   T   
   G   
   A   
   C   
   G   
   C   
   C   
    T    
   G   
   C   
   T   
   C   
   A   
   A   
   A   
   T   
   T   
   T   
   C   
   A   
   A   
   A   
    T    
   G   
   A   
   A   
   G   
   A   
   T   
   A   
   A   
   A   
   T   
   T   
   T   
   A   
   A   
   T   
   T   
   C   
   T   
   G   
   A   
   A   
   A   
   T   
   T   
   C   
   T   
   T   
   T   
   T   
    A    
   G   
   C   
   A   
   G   
   A   
   T   
   A   
   C   
    G    
   G   
   G   
   C   
   T   
   A   
    C    
   G   
   G   
    T    
   C   
   A   
   A   
   G   
   G   
    T    
   C   
   A   
   A   
   T   
   T   
   G   
   T   
   T   
   A   
   A   
   T   
   T   
   T   
   C   
    G    
   C   
   C   
   A   
   A   
   T   
   C   
   C   
   A   
   G   
   C   
   A   
   A   
   G   
   C   
   T   
   A   
   C   
   C   
   A   
   T   
   G   
   T   
   A   
   T   
   A   
   G   
   T   
   G   
   T   
   T   
   T   
   T   
   C   
   C   
   A   
   A   
   A   
   A   
   T   
   A   
   A   
   T   
   G   
   G   
    A    
   A   
   C   
   A   
    T    
   T   
    A    
   G   
   T   
    C    
   T   
   A   
   T   
   C   
   C   
   A   
   A   
   A   
   A   
    C    
   T   
   A   
   G   
   T   
   T   
   T   
   T   
   A   
   G   
   A   
   C   
   A   
   A   
   A   
   G   
   A   
   A   
   A   
   C   
    G    
   A   
   A   
   G   
   A   
   A   
   A   
   A   
   A   
   A   
   G   
   A   
   C   
   A   
   A   
   T   
   G   
   T   
   A   
   A   
   T   
   C   
   A   
   G   
   C   
   G   
   C   
   C   
   A   
   A   
   C   
   G   
   C   
   A   
   G   
   C   
   G   
   A   
   A   
   T   
   A   
   C   
   A   
   A   
   T   
   T   
   G   
   C   
   C   
   A   
   C   
   A   
   G   
   A   
   T   
   C   
   T   
   T   
   T   
   T   
   A   
   G   
   G   
   T   
   A   
   G   
   T   
   G   
   T   
   G   
   G   
   A   
   A   
   G   
   A   
   T   
   C   
   C   
   T   
   T   
   C   
   A   
   G   
   G   
   T   
   T   
   A   
   T   
   G   
   T   
   T   
   T   
   A   
   T   
   A   
   A   
   T   
   A   
   T   
   G   
   T   
   A   
   C   
   A   
   A   
   T   
   C   
   C   
   G   
   A   
   A   
   C   
   T   
   T   
   C   
   T   
   C   
   A   
   T   
   T   
   G   
   G   
   C   
   T   
   G   
   C   
   T   
   A   
   A   
   A   
   A   
   C   
   A   
   G   
   G   
   A   
   A   
   C   
   A   
   G   
   C   
   T   
   G   
   A   
   A   
   A   
   T   
   A   
   A   
   A   
   A   
   G   
   A   
   T   
   A   
   A   
   A   
   C   
   A   
   A   
   G   
   A   
   T   
   A   
   C   
   A   
   G   
   A   
   T   
   G   
   G   
   C   
   A   
   A   
   A   
   G   
   A   
   A   
   A   
   A   
   C   
   A   
   G   
   T   
   T   
   T   
   C   
   C   
   T   
   T   
   C   
   T   
   A   
   A   
   C   
   G   
   T   
   T   
   A   
   G   
   A   
   T   
   C   
   G   
   T   
   A   
   G   
   T   
   A   
   A   
   C   
   A   
   A   
   T   
   A   
   A   
   A   
   T   
   T   
   C   
   T   
   T   
   A   
   A   
   C   
   A   
   A   
   T   
   G   
   A   
   T   
   T   
   A   
   T   
   G   
   G   
   T   
   T   
   G   
   A   
   A   
   A   
   A   
   T   
   T   
   C   
   A   
   G   
   G   
   A   
   G   
   A   
   A   
   A   
   A   
   T   
   G   
   G   
   T   
   T   
   C   
   T   
   G   
   C   
   A   
   A   
   C   
   A   
   G   
   A   
   T   
   A   
   T   
   C   
   A   
   G   
   T   
   A   
   A   
   A   
   C   
   C   
   A   
   T   
   T   
   A   
   A   
   T   
   T   
   G   
   A   
   T   
   T   
   A   
   C   
   T   
   T   
   A   
   G   
   A   
   A   
   G   
   C   
   A   
   A   
   C   
   C   
   A   
   T   
   T   
   A   
   A   
   A   
   -   
   -   
   -   
   -   
   -   
   -   
   -   
   -   
   -   
   -   
   -   
   -   
   -   
   -   
   -   
   -   
   -   
   -   
   -   
   -   
   -   
   -   
   -   
   -   
   -   
   -   
   -   
   -   
   -   
   -   
   -   
   -   
   -   
   -   
   -   
   -   
   -   
   -   
   -   
   -   
   -   
   -   
   -   
   -   
   -   
   -   
   -   
   -   
   -   
   -   
   -   
   -   
   -   
   -   
   -   
   -   
   -   
   -   
   -   
   -   
   -   
   -   
   -   
   -   
   -   
   -   
   -   
   -   
   -   
   -   
   -   
   -   
   -   
   -   
   -   
   -   
   -   
   -   
   -   
   -   
   -   
   -   
   -   
   -   
   -   
   -   
   -   
   -   
   -   
   -   
   -   
   -   
   -   
   -   
   -   
   -   
   -   
   -   
   -   
   -   
   -   
   -   
   -   
   -   
   -   
   -   
   -   
   -   
   -   
   -   
   -   
   -   
   -   
   -   
   -   
   -   
   -   
   -   
   -   
   -   
   -   
   -   
   -   
   -   
   -   
   -   
   -   
   -   
   -   
   -   
   -   
   -   
   -   
   -   
   -   
   -   
   -   
   -   
   -   
   -   
   -   
   -   
   -   
   -   
   -   
   -   
   -   
   -   
   -   
   -   
   -   
   -   
   -   
   -   
   -   
   -   
   -   
   -   
   -   
   -   
   -   
   -   
   -   
   -   
   -   
   -   
   -   
   -   
   -   
   C   
   A   
   A   
   T   
   G   
   G   
   C   
   A   
   A   
   G   
   C   
   C   
   A   
   A   
   A   
   C   
   A   
   A   
   G   
   A   
   A   
   T   
   T   
   A   
   G   
   C   
   C   
   G   
   A   
   A   
   G   
   C   
   G   
   A   
   A   
   G   
   A   
   A   
   A   
   A   
   C   
   A   
   G   
   C   
   T   
   A   
   C   
   T   
   A   
   C   
   A   
   T   
   T   
   T   
   T   
   T   
   A   
   A   
   A   
   C   
   G   
   T   
   A   
   T   
   T   
   G   
   T   
   C   
   A   
   A   
   A   
   A   
   C   
   A   
   G   
   G   
   A   
   A   
   T   
   T   
   T   
   G   
   A   
   T   
   A   
   A   
   G   
   T   
   T   
   A   
   C   
   C   
   G   
   T   
   C   
   C   
   G   
   T   
   T   
   G   
   T   
   T   
   C   
   A   
   A   
   G   
   A   
   A   
   G   
   C   
   T   
   A   
   G   
   C   
   T   
   T   
   A   
   A   
   A   
   G   
   A   
   A   
   A   
   A   
   A   
   T   
   G   
   G   
   C   
   T   
   A   
   T   
   G   
   A   
   T   
   A   
   C   
   T   
   A   
   A   
   A   
   T   
   C   
   T   
   G   
   T   
   T   
   G   
   T   
   T   
   G   
   A   
   A   
   A   
   A   
   A   
   T   
   A   
   C   
   C   
   A   
   A   
   G   
   C   
   A   
   A   
   T   
   T   
   T   
   A   
   T   
   T   
   C   
   A   
   G   
   G   
   G   
   A   
   T   
   T   
   C   
   A   
   A   
   G   
   C   
   A   
   G   
   A   
   A   
   G   
   G   
   A   
   G   
   T   
   C   
   A   
   A   
   A   
   G   
   C   
   T   
   A   
   G   
   T   
   G   
   A   
   T   
   G   
   T   
   T   
   C   
   A   
   A   
   G   
   T   
   A   
   A   
   A   
   A   
   A   
   A   
   G   
   G   
   C   
   G   
   A   
   A   
   A   
   G   
   A   
   C   
   A   
   A   
   T   
   C   
   A   
   A   
   T   
   A   
   C   
   A   
   C   
   A   
   T   
   T   
   T   
   A   
   C   
   C   
   T   
   A   
   T   
   A   
   A   
   A   
   T   
   T   
   A   
   T   
   C   
   G   
   A   
   T   
   G   
   A   
   G   
   T   
   A   
   C   
   C   
   C   
   C   
   T   
   T   
   T   
   A   
   G   
   G   
   C   
   G   
   A   
   A   
   A   
   T   
   G   
   A   
   A   
   A   
   G   
   A   
   T   
   T   
   T   
   G   
   T   
   C   
   T   
   T   
   A   
   T   
   C   
   A   
   A   
   T   
   C   
   A   
   A   
   G   
   T   
   A   
   T   
   C   
   G   
   C   
   C   
   A   
   A   
   A   
   A   
   A   
   A   
   G   
   G   
   C   
   G   
   A   
   T   
   A   
   C   
   C   
   T   
   A   
   C   
   C   
   A   
   A   
   A   
   T   
   C   
   G   
   C   
   T   
   T   
   G   
   G   
   A   
   A   
   G   
   C   
   C   
   G   
   T   
   C   
   T   
   T   
   T   
   A   
   A   
   T   
   T   
   T   
   T   
   T   
   C   
   C   
   A   
   G   
   A   
   T   
   A   
   T   
   G   
   T   
   C   
   A   
   G   
   G   
   A   
   A   
   A   
   T   
   G   
   A   
   T   
   A   
   A   
   A   
   A   
   T   
   T   
   T   
   C   
   G   
   A   
   T   
   T   
   C   
   A   
   A   
   G   
   T   
   A   
   G   
   A   
   T   
   A   
   A   
   T   
   G   
   C   
   C   
   A   
   A   
   A   
   C   
   G   
   T   
   G   
   G   
   A   
   G   
   A   
   A   
   A   
   T   
   T   
   G   
   T   
   C   
   G   
   A   
   T   
   C   
   G   
   T   
   A   
   A   
   T   
   G   
   G   
   T   
   A   
   G   
   T   
   G   
   G   
   G   
   C   
   T   
   A   
   G   
   C   
   A   
   A   
   T   
   T   
   A   
   A   
   C   
   A   
   A   
   A   
   G   
   T   
   G   
   T   
   T   
   T   
   G   
   A   
   T   
   G   
   A   
   A   
   G   
   T   
   G   
   G   
   G   
   C   
   G   
   T   
   A   
   G   
   T   
   G   
   C   
   C   
   T   
   G   
   G   
   C   
   A   
   A   
   A   
   C   
   T   
   C   
   G   
   G   
   T   
   T   
   C   
   T   
   G   
   G   
   C   
   G   
   C   
   A   
   G   
   A   
   A   
   A   
   A   
   A   
   A   
   C   
   A   
   G   
   C   
   C   
   A   
   A   
   T   
   A   
   T   
   C   
   A   
   A   
   A   
   G   
   C   
   T   
   T   
   T   
   T   
   A   
   G   
   T   
   G   
   A   
   T   
   A   
   A   
   A   
   T   
   T   
   C   
   G   
   G   
   C   
   G   
   T   
   T   
   T   
   C   
   T   
   G   
   T   
   T   
   G   
   A   
   T   
   G   
   A   
   A   
   A   
   T   
   C   
   A   
   A   
   T   
   C   
   A   
   A   
   A   
   A   
   G   
   T   
   T   
   A   
   A   
   G   
   C   
   C   
   A   
   A   
   G   
   G   
   A   
   T   
   G   
   G   
   G   
   T   
   C   
   C   
   A   
   A   
   G   
   C   
   A   
   G   
   A   
   C   
   T   
   C   
   C   
   T   
   T   
   T   
   G   
   T   
   A   
   C   
   C   
   A   
   A   
   T   
   C   
   A   
   C   
   A   
   G   
   T   
   C   
   G   
   C   
   T   
   T   
   C   
   T   
   G   
   A   
   A   
   C   
   C   
   A   
   G   
   T   
   G   
   A   
   C   
   A   
   G   
   A   
   A   
   T   
   T   
   A   
   C   
   C   
   A   
   A   
   C   
   A   
   G   
   G   
   G   
   G   
   C   
   T   
   G   
   C   
   G   
   A   
   C   
   A   
   A   
   A   
   A   
   G   
   A   
   T   
   A   
   C   
   A   
   G   
   A   
   G   
   T   
   C   
   A   
   C   
   G   
   T   
   T   
   A   
   T   
   T   
   A   
   T   
   C   
   C   
   G   
   C   
   T   
   G   
   G   
   G   
   G   
   G   
   A   
   A   
   G   
   C   
   A   
   G   
   C   
   T   
   G   
   C   
   G   
   C   
   A   
   A   
   T   
   T   
   A   
   A   
   T   
   C   
   G   
   G   
   G   
   T   
   A   
   T   
   A   
   C   
   G   
   G   
   G   
   C   
   A   
   C   
   C   
   A   
   T   
   T   
   A   
   C   
   T   
   G   
   C   
   A   
   G   
   A   
   A   
   G   
   A   
   T   
   A   
   T   
   T   
   G   
   A   
   A   
   A   
   A   
   A   
   A   
   A   
   T   
   C   
   C   
   A   
   G   
   A   
   G   
   T   
   T   
   A   
   A   
   G   
   T   
   A   
   G   
   T   
   A   
   C   
   G   
   G   
   G   
   C   
   G   
   T   
   A   
   A   
   T   
   T   
   G   
   G   
   T   
   A   
   A   
   A   
   A   
   C   
   T   
   G   
   G   
   T   
   T   
   T   
   A   
   G   
   A   
   A   
   C   
   G   
   A   
   G   
   C   
   G   
   T   
   T   
   T   
   G   
   A   
   T   
   A   
   A   
   A   
   G   
   A   
   A   
   T   
   T   
   A   
   A   
   G   
   A   
   G   
   G   
   G   
   C   
   A   
   A   
   G   
   A   
   T   
   G   
   G   
   T   
   G   
   G   
   C   
   T   
   C   
   A   
   C   
   T   
   A   
   G   
   T   
   T   
   A   
   T   
   T   
   T   
   T   
   A   
   G   
   A   
   T   
   G   
   A   
   C   
   A   
   A   
   A   
   G   
   A   
   G   
   A   
   A   
   T   
   G   
   T   
   C   
   A   
   A   
   A   
   A   
   A   
   A   
   G   
   C   
   T   
   T   
   T   
   A   
   C   
   A   
   A   
   A   
   C   
   C   
   A   
   A   
   A   
   G   
   A   
   G   
   A   
   A   
   A   
   A   
   A   
   A   
   G   
   A   
   T   
   G   
   G   
   T   
   C   
   A   
   A   
   A   
   C   
   G   
   A   
   T   
   T   
   A   
   A   
   A   
   T   
   T   
   A   
   A   
   C   
   G   
   A   
   T   
   T   
   G   
   A   
   C   
   A   
   G   
   T   
   G   
   G   
   C   
   G   
   T   
   A   
   C   
   A   
   A   
   C   
   A   
   A   
   C   
   A   
   A   
   G   
   C   
   C   
   T   
   T   
   C   
   G   
   C   
   T   
   A   
   T   
   A   
   T   
   T   
   T   
   G   
   A   
   C   
   A   
   A   
   A   
   C   
   G   
   T   
   C   
   C   
   T   
   G   
   G   
   T   
   A   
   G   
   T   
   G   
   C   
   G   
   G   
   T   
   C   
   A   
   T   
   T   
   A   
   C   
   C   
   G   
   A   
   T   
   C   
   C   
   T   
   C   
   A   
   A   
   A   
   A   
   A   
   G   
   G   
   C   
   G   
   A   
   T   
   T   
   T   
   A   
   T   
   T   
   A   
   G   
   C   
   A   
   A   
   C   
   G   
   G   
   T   
   T   
   A   
   G   
   T   
   T   
   C   
   A   
   C   
   C   
   T   
   T   
   C   
   G   
   T   
   A   
   T   
   G   
   A   
   T   
   C   
   C   
   T   
   A   
   A   
   T   
   A   
   A   
   A   
   A   
   T   
   G   
   G   
   C   
   C   
   A   
   A   
   T   
   G   
   G   
   T   
   A   
   T   
   T   
   T   
   C   
   T   
   C   
   A   
   A   
   A   
   A   
   A   
   G   
   A   
   A   
   T   
   A   
   T   
   G   
   A   
   T   
   G   
   C   
   T   
   T   
   A   
   C   
   A   
   A   
   T   
   A   
   A   
   C   
   A   
   A   
   C   
   A   
   A   
   A   
   G   
   A   
   T   
   T   
   T   
   A   
   C   
   C   
   A   
   T   
   T   
   C   
   A   
   C   
   A   
   G   
   C   
   A   
   C   
   G   
   T   
   T   
   T   
   T   
   G   
   C   
   G   
   A   
   C   
   A   
   G   
   G   
   C   
   T   
   A   
   T   
   G   
   C   
   A   
   C   
   C   
   A   
   G   
   G   
   C   
   T   
   C   
   T   
   A   
   C   
   C   
   T   
   T   
   T   
   A   
   A   
   A   
   A   
   C   
   A   
   A   
   T   
   T   
   A   
   C   
   C   
   G   
   G   
   T   
   G   
   C   
   C   
   A   
   T   
   T   
   G   
   G   
   T   
   T   
   T   
   A   
   G   
   A   
   T   
   G   
   C   
   A   
   G   
   G   
   G   
   A   
   C   
   A   
   T   
   T   
   G   
   A   
   A   
   G   
   C   
   C   
   A   
   G   
   A   
   T   
   G   
   A   
   A   
   G   
   A   
   G   
   C   
   T   
   A   
   G   
   A   
   A   
   A   
   T   
   T   
   A   
   A   
   T   
   G   
   G   
   T   
   T   
   T   
   G   
   A   
   A   
   A   
   T   
   G   
   G   
   C   
   A   
   A   
   A   
   A   
   A   
   G   
   A   
   T   
   A   
   A   
   A   
   T   
   C   
   T   
   T   
   G   
   G   
   G   
   G   
   C   
   G   
   G   
   C   
   T   
   A   
   T   
   T   
   T   
   T   
   G   
   C   
   G   
   A   
   C   
   A   
   C   
   G   
   T   
   G   
   T   
   A   
   A   
   A   
   A   
   G   
   A   
   A   
   G   
   C   
   A   
   A   
   G   
   T   
   C   
   C   
   A   
   G   
   T   
   C   
   A   
   A   
   T   
   C   
   T   
   G   
   C   
   G   
   T   
   A   
   C   
   C   
   G   
   C   
   T   
   T   
   T   
   A   
   G   
   T   
   T   
   A   
   A   
   T   
   T   
   C   
   A   
   G   
   A   
   T   
   A   
   A   
   T   
   A   
   T   
   T   
   T   
   A   
   T   
   T   
   T   
   T   
   G   
   C   
   C   
   C   
   A   
   A   
   C   
   A   
   A   
   A   
   C   
   A   
   T   
   T   
   A   
   C   
   G   
   G   
   A   
   T   
   G   
   G   
   G   
   A   
   G   
   A   
   A   
   G   
   A   
   C   
   A   
   A   
   A   
   T   
   T   
   T   
   A   
   G   
   A   
   G   
   C   
   G   
   G   
   G   
   T   
   C   
   T   
   T   
   A   
   A   
   T   
   A   
   A   
   A   
   T   
   T   
   C   
   A   
   T   
   T   
   T   
   T   
   C   
   G   
   G   
   T   
   G   
   A   
   G   
   G   
   A   
   A   
   C   
   T   
   A   
   G   
   A   
   T   
   T   
   T   
   A   
   C   
   C   
   A   
   A   
   T   
   T   
   G   
   C   
   C   
   A   
   T   
   G   
   A   
   C   
   G   
   C   
   C   
   T   
   G   
   C   
   T   
   C   
   A   
   A   
   A   
   T   
   T   
   T   
   C   
   A   
   A   
   A   
   T   
   G   
   A   
   A   
   G   
   A   
   T   
   A   
   A   
   A   
   T   
   T   
   T   
   A   
   A   
   T   
   T   
   C   
   T   
   G   
   A   
   A   
   A   
   T   
   T   
   C   
   T   
   T   
   T   
   T   
   A   
   G   
   C   
   A   
   G   
   A   
   T   
   A   
   C   
   G   
   G   
   G   
   C   
   T   
   A   
   C   
   G   
   G   
   T   
   C   
   A   
   A   
   G   
   G   
   T   
   C   
   A   
   A   
   T   
   T   
   G   
   T   
   T   
   A   
   A   
   T   
   T   
   T   
   C   
   G   
   C   
   C   
   A   
   A   
   T   
   C   
   C   
   A   
   G   
   C   
   A   
   A   
   G   
   C   
   T   
   A   
   C   
   C   
   A   
   T   
   G   
   T   
   A   
   T   
   A   
   G   
   T   
   G   
   T   
   T   
   T   
   T   
   C   
   C   
   A   
   A   
   A   
   A   
   T   
   A   
   A   
   T   
   G   
   G   
   A   
   A   
   C   
   A   
   T   
   T   
   A   
   G   
   T   
   C   
   T   
   A   
   T   
   C   
   C   
   A   
   A   
   A   
   A   
   C   
   T   
   A   
   G   
   T   
   T   
   T   
   T   
   A   
   G   
   A   
   C   
   A   
   A   
   A   
   G   
   A   
   A   
   A   
   C   
   G   
   A   
   A   
   G   
   A   
   A   
   A   
   A   
   A   
   A   
   G   
   A   
   C   
   A   
   A   
   T   
   G   
   T   
   A   
   A   
   T   
   C   
   A   
   G   
   C   
   G   
   C   
   C   
   A   
   A   
   C   
   G   
   C   
   A   
   G   
   C   
   G   
   A   
   A   
   T   
   A   
   C   
   A   
   A   
   T   
   T   
   G   
   C   
   C   
   A   
   C   
   A   
   G   
   A   
   T   
   C   
   T   
   T   
   T   
   T   
   A   
   G   
   G   
   T   
   A   
   G   
   T   
   G   
   T   
   G   
   G   
   A   
   A   
   G   
   A   
   T   
   C   
   C   
   T   
   T   
   C   
   A   
   G   
   G   
   T   
   T   
   A   
   T   
   G   
   T   
   T   
   T   
   A   
   T   
   A   
   A   
   T   
   A   
   T   
   G   
   T   
   A   
   C   
   A   
   A   
   T   
   C   
   C   
   G   
   A   
   A   
   C   
   T   
   T   
   C   
   T   
   C   
   A   
   T   
   T   
   G   
   G   
   C   
   T   
   G   
   C   
   T   
   A   
   A   
   A   
   A   
   C   
   A   
   G   
   G   
   A   
   A   
   C   
   A   
   G   
   C   
   T   
   G   
   A   
   A   
   A   
   T   
   A   
   A   
   A   
   A   
   G   
   A   
   T   
   A   
   A   
   A   
   C   
   A   
   A   
   G   
   A   
   T   
   A   
   C   
   A   
   G   
   A   
   T   
   G   
   G   
   C   
   A   
   A   
   A   
   G   
   A   
   A   
   A   
   A   
   C   
   A   
   G   
   T   
   T   
   T   
   C   
   C   
   T   
   T   
   C   
   T   
   A   
   A   
   C   
   G   
   T   
   T   
   A   
   G   
   A   
   T   
   C   
   G   
   T   
   A   
   G   
   T   
   A   
   A   
   C   
   A   
   A   
   T   
   A   
   A   
   A   
   T   
   T   
   C   
   T   
   T   
   A   
   A   
   C   
   A   
   A   
   T   
   G   
   A   
   T   
   T   
   A   
   T   
   G   
   G   
   T   
   T   
   G   
   A   
   A   
   A   
   A   
   T   
   T   
   C   
   A   
   G   
   G   
   A   
   G   
   A   
   A   
   A   
   A   
   T   
   G   
   G   
   T   
   T   
   C   
   T   
   G   
   C   
   A   
   A   
   C   
   A   
   G   
   A   
   T   
   A   
   T   
   C   
   A   
   G   
   T   
   A   
   A   
   A   
   C   
   C   
   A   
   T   
   T   
   A   
   A   
   T   
   T   
   G   
   A   
   T   
   T   
   A   
   C   
   T   
   T   
   A   
   G   
   A   
   A   
   G   
   C   
   A   
   A   
   C   
   C   
   A   
   T   
   T   
   A   
   A   
   A   
   -   
   -   
   -   
   -   
   -   
   -   
   -   
   -   
   -   
   -   
   -   
   -   
   -   
   -   
   -   
   -   
   -   
   -   
   -   
   -   
   -   
   -   
   -   
   -   
   -   
   -   
   -   
   -   
   -   
   -   
   -   
   -   
   -   
   -   
   -   
   -   
   -   
   -   
   -   
   -   
   -   
   -   
   -   
   -   
   -   
   -   
   -   
   -   
   -   
   -   
   -   
   -   
   -   
   -   
   -   
   -   
   -   
   -   
   -   
   -   
   -   
 

 
   N53761   
   -   
   -   
   -   
   -   
   -   
   -   
   -   
   -   
   -   
   -   
   -   
   -   
   -   
   -   
   -   
   -   
   -   
   -   
   -   
   -   
   -   
   -   
   -   
   -   
   -   
   -   
   -   
   -   
   -   
   -   
   -   
   -   
   -   
   -   
   -   
   -   
   -   
   -   
   -   
   -   
   -   
   -   
   -   
   -   
   -   
   -   
   -   
   -   
   -   
   -   
   -   
   -   
   -   
   -   
   -   
   -   
   -   
   -   
   -   
   -   
   -   
   -   
   -   
   -   
   -   
   -   
   -   
   -   
   -   
   -   
   -   
   -   
   -   
   -   
   -   
   -   
   -   
   -   
   -   
   -   
   -   
   -   
   -   
   -   
   -   
   -   
   -   
   -   
   -   
   -   
   -   
   -   
   -   
   -   
   -   
   -   
   -   
   -   
   -   
   -   
   -   
   -   
   -   
   -   
   -   
   -   
   -   
   -   
   C   
   A   
   A   
   T   
   G   
   G   
   C   
   A   
   A   
   G   
   C   
   C   
   A   
   A   
   A   
   C   
   A   
   A   
   G   
   A   
    A    
   T   
   T   
   A   
   G   
   C   
    C    
   G   
   A   
   A   
   G   
   C   
   G   
   A   
   A   
   G   
   A   
   A   
   A   
   A   
    C    
   A   
   G   
   C   
    T    
   A   
    C    
   T   
    A    
    C    
   A   
   T   
   T   
   T   
   T   
   T   
   A   
   A   
   A   
    C    
   G   
   T   
   A   
   T   
   T   
   G   
    T    
   C   
   A   
   A   
   A   
   A   
   C   
   A   
   G   
    G    
   A   
   A   
   T   
   T   
   T   
   G   
   A   
   T   
   A   
   A   
   G   
   T   
   T   
   A   
   C   
   C   
    G    
   T   
   C   
   C   
   G   
   T   
   T   
    G    
   T   
   T   
   C   
   A   
   A   
   G   
   A   
   A   
    G    
   C   
   T   
   A   
   G   
   C   
   T   
   T   
   A   
   A   
   A   
   G   
   A   
   A   
   A   
   A   
   A   
   T   
   G   
   G   
    C    
   T   
   A   
   T   
   G   
   A   
   T   
   A   
   C   
   T   
   A   
   A   
   A   
   T   
   C   
    T    
   G   
   T   
   T   
   G   
   T   
   T   
   G   
   A   
   A   
   A   
   A   
   A   
   T   
   A   
   C   
   C   
   A   
   A   
   G   
   C   
   A   
   A   
   T   
   T   
   T   
   A   
   T   
   T   
   C   
   A   
   G   
   G   
   G   
   A   
   T   
   T   
   C   
   A   
   A   
   G   
   C   
    A    
   G   
   A   
   A   
    G    
   G   
    A    
   G   
   T   
   C   
   A   
   A   
    A    
   G   
   C   
   T   
   A   
   G   
   T   
   G   
   A   
   T   
   G   
   T   
    T    
   C   
   A   
   A   
   G   
   T   
    C    
   A   
   A   
   A   
   A   
   A   
    G    
   G   
    C    
   G   
   A   
   A   
   A   
   G   
   A   
    C    
   A   
   A   
    T    
   C   
   A   
   A   
   T   
   A   
   C   
   A   
   C   
   A   
   T   
   T   
   T   
   A   
    C    
   C   
   T   
   A   
   T   
   A   
   A   
   A   
   T   
   T   
   A   
   T   
   C   
   G   
   A   
   T   
   G   
   A   
   G   
    C    
   A   
   C   
    T    
   C   
   C   
   T   
   T   
   T   
   A   
   G   
   G   
   C   
   G   
   A   
   A   
   A   
   T   
   G   
   A   
   A   
   A   
   G   
   A   
   T   
    T    
   T   
   G   
   T   
   C   
   T   
   T   
   A   
   T   
   C   
   A   
   A   
   T   
   C   
   A   
   A   
   G   
   T   
   A   
   T   
    C    
   G   
    C    
   C   
   A   
   A   
    G    
   A   
   A   
   A   
   G   
   G   
    T    
   G   
   A   
   T   
   A   
    C    
   C   
   T   
   A   
   C   
   C   
   A   
   A   
   A   
   T   
    C    
    G    
   C   
   T   
   T   
   G   
   G   
    A    
    A    
    G    
   C   
   C   
    G    
   T   
   C   
   T   
   T   
   T   
   A   
   A   
   T   
   T   
   T   
   T   
   T   
   C   
   C   
   A   
   G   
   A   
   T   
   A   
   T   
   G   
   T   
   C   
   A   
   G   
   G   
   A   
   A   
   A   
   T   
   G   
   A   
   T   
   A   
   A   
   A   
    A    
   T   
   T   
   T   
   C   
   G   
   A   
   T   
   T   
   C   
   A   
   A   
   G   
   T   
   A   
   G   
   A   
   T   
   A   
   A   
   T   
   G   
   C   
   C   
   A   
   A   
   A   
   C   
   G   
   T   
   G   
   G   
   A   
   G   
   A   
   A   
   A   
   T   
   T   
   G   
   T   
   C   
   G   
   A   
   T   
   C   
   G   
   T   
   A   
   A   
    T    
   G   
   G   
   T   
   A   
   G   
   T   
   G   
   G   
   G   
   C   
   T   
   A   
   G   
    C    
   A   
   A   
   T   
   T   
   A   
   A   
   C   
   A   
   A   
   A   
   G   
   T   
   G   
   T   
   T   
   T   
   G   
   A   
    C    
   G   
   A   
   A   
   G   
   T   
   G   
   G   
   G   
   C   
   G   
   T   
   A   
   G   
   T   
    G    
   C   
   C   
   T   
    G    
   G   
   C   
   A   
   A   
   A   
   C   
   T   
    C    
   G   
   G   
   T   
   T   
   C   
   T   
   G   
   G   
   C   
   G   
   C   
   A   
   G   
   A   
   A   
   A   
   A   
   A   
    A    
   C   
   A   
   G   
   C   
    C    
   A   
   A   
   T   
   A   
   T   
   C   
   A   
   A   
   A   
   G   
   C   
   T   
   T   
   T   
   T   
   A   
   G   
   T   
   G   
   A   
   T   
   A   
   A   
    G    
   T   
   T   
    C    
   G   
   G   
   C   
   G   
   T   
   T   
   T   
   C   
   T   
    G    
   T   
   T   
   G   
   A   
   T   
   G   
   A   
   A   
   A   
   T   
   C   
   A   
   A   
   T   
   C   
    A    
   A   
   A   
   A   
    G    
   T   
   T   
   A   
   A   
   G   
   C   
   C   
   A   
   A   
   G   
   G   
    A    
   T   
   G   
   G   
   G   
   T   
   C   
   C   
   A   
   A   
   G   
   C   
   A   
   G   
   A   
   C   
   T   
   C   
    C    
   T   
   T   
   T   
   G   
   T   
   A   
   C   
   C   
   A   
   A   
   T   
   C   
   A   
   C   
   A   
   G   
   T   
    T    
   G   
   C   
   T   
   T   
   C   
   T   
   G   
   A   
   A   
   C   
   C   
   A   
   G   
   T   
   G   
   A   
   C   
   A   
   G   
   A   
   A   
   T   
   T   
   A   
   C   
   C   
   A   
   A   
   C   
   A   
   G   
   G   
   G   
   G   
   C   
   T   
   G   
   C   
   G   
   A   
   C   
   A   
   A   
   A   
   A   
   G   
    A    
    C    
   A   
   C   
   A   
   G   
   A   
   G   
   T   
   C   
   A   
   C   
   G   
   T   
   T   
   A   
   T   
   T   
   A   
   T   
   C   
   C   
    G    
    C    
   T   
   G   
   G   
   G   
   G   
   G   
   A   
   A   
   G   
   C   
   A   
   G   
   C   
   T   
   G   
   C   
   G   
   C   
   A   
   A   
   T   
   T   
   A   
   A   
   T   
   C   
   G   
   G   
    G    
   T   
   A   
   T   
   A   
   C   
    G    
   G   
   G   
    C    
   A   
   C   
    C    
   A   
   T   
   T   
   A   
   C   
   T   
   G   
   C   
   A   
   G   
   A   
    A    
   G   
   A   
   T   
   A   
   T   
   T   
    G    
   A   
   A   
   A   
   A   
   A   
   A   
   A   
   T   
   C   
   C   
   A   
   G   
   A   
   G   
   T   
   T   
   A   
   A   
   G   
   T   
   A   
   G   
   T   
   A   
   C   
   G   
   G   
   G   
    C    
   G   
   T   
   A   
   A   
   T   
   T   
   G   
   G   
    T    
   A   
   A   
   A   
   A   
   C   
   T   
   G   
   G   
    C    
   T   
   T   
   A   
   G   
   A   
   A   
   C   
   G   
   A   
   G   
   C   
   G   
   T   
   T   
   T   
   G   
   A   
   T   
   A   
   A   
   A   
   G   
   A   
    A    
   T   
   T   
   A   
   A   
   G   
   A   
   G   
   G   
    G    
   C   
   A   
   A   
   G   
   A   
   T   
   G   
   G   
    T    
   G   
   G   
    C    
   T   
   C   
    A    
   C   
   T   
   A   
   G   
   T   
   T   
   A   
   T   
   T   
   T   
   T   
   A   
   G   
   A   
   T   
   G   
   A   
   C   
   A   
   A   
   A   
   G   
   A   
   G   
   A   
   A   
   T   
   G   
   T   
   C   
   A   
   A   
   A   
   A   
   A   
   A   
   G   
   C   
   T   
   T   
   T   
   A   
   C   
   A   
   A   
   A   
   C   
   C   
   A   
   A   
   A   
   G   
   A   
    G    
   A   
   A   
   A   
   A   
   A   
   A   
   G   
   A   
   T   
   G   
   G   
   T   
   C   
   A   
   A   
   A   
   C   
    G    
   A   
   T   
   T   
   A   
   A   
   A   
   T   
   T   
   A   
   A   
   C   
   G   
   A   
   T   
   T   
   G   
   A   
    C    
   A   
   G   
    T    
   G   
   G   
    C    
   G   
   T   
   A   
   C   
   A   
   A   
   C   
   A   
   A   
   C   
   A   
   A   
   G   
   C   
    C    
   T   
   T   
    C    
   G   
   C   
   T   
   A   
   T   
   A   
   T   
   T   
   T   
   G   
   A   
   C   
   A   
   A   
   A   
   C   
   G   
    C    
    C    
   C   
   T   
   G   
   G   
   T   
   A   
   G   
   T   
   G   
   C   
   G   
   G   
   T   
   C   
   A   
   T   
   T   
   A   
   C   
   C   
   G   
   A   
   T   
   C   
   C   
   T   
   C   
   A   
   A   
   A   
   A   
   A   
   G   
   G   
   C   
   G   
   A   
   T   
   T   
   T   
   A   
    T    
   T   
   A   
   G   
   C   
    A    
   A   
   C   
   G   
   G   
   T   
   T   
   A   
   G   
   T   
   T   
   C   
   A   
   C   
   C   
   T   
   T   
   C   
   G   
   T   
   A   
   T   
   G   
   A   
   T   
   C   
   C   
   T   
   A   
   A   
   T   
   A   
   A   
   A   
   A   
   T   
   G   
   G   
   C   
   C   
   A   
   A   
   T   
   G   
   G   
    C    
   A   
   T   
   T   
   T   
   C   
   T   
   C   
   A   
   A   
   A   
   A   
   A   
   G   
   A   
    A    
   T   
   A   
   T   
   G   
   A   
   T   
   G   
   C   
   T   
   T   
   A   
   C   
   A   
   A   
   T   
   A   
   A   
   C   
   A   
   A   
   C   
   A   
   A   
   A   
   G   
   A   
   T   
   T   
   T   
   A   
   C   
   C   
   A   
   T   
   T   
    C    
   A   
   C   
   A   
   G   
   C   
   A   
   C   
   G   
   T   
   T   
   T   
    T    
   G   
   C   
   G   
   A   
   C   
   A   
   G   
   G   
    C    
   T   
   A   
   T   
   G   
   C   
   A   
   C   
   C   
   A   
   G   
   G   
    C    
   T   
   C   
   T   
   A   
   C   
   C   
   T   
   T   
   T   
   A   
   A   
   A   
   A   
   C   
   A   
   A   
   T   
   T   
   A   
   C   
   C   
   G   
   G   
   T   
   G   
   C   
   C   
   A   
   T   
   T   
   G   
   G   
   T   
   T   
   T   
   A   
   G   
   A   
   T   
    G    
   C   
   A   
   G   
   G   
   G   
   A   
   C   
   A   
   T   
   T   
   G   
   A   
   A   
   G   
   C   
   C   
   A   
   G   
   A   
   T   
   G   
   A   
   A   
   G   
   A   
   G   
   C   
   T   
   A   
   G   
   A   
   A   
   A   
   T   
   T   
   A   
   A   
   T   
   G   
   G   
    C    
   T   
   T   
   G   
   A   
   A   
   A   
   T   
   G   
   G   
   C   
   A   
   A   
   A   
   A   
   A   
   G   
   A   
   T   
   A   
   A   
   A   
   T   
   C   
   T   
   T   
   G   
   G   
   G   
   G   
    C    
   G   
   G   
   C   
   T   
   A   
   T   
   T   
   T   
   T   
   G   
   C   
    G    
   A   
   C   
   A   
   C   
   G   
   T   
   G   
   T   
   A   
   A   
   A   
   A   
   G   
   A   
   A   
   G   
   C   
   A   
   A   
   G   
   T   
   C   
   C   
   A   
   G   
   T   
   C   
   A   
   A   
   T   
   C   
   T   
    G    
   C   
   G   
   T   
   A   
   C   
   C   
   G   
   C   
   T   
   T   
   T   
   A   
   G   
   T   
   T   
   A   
   A   
   T   
   T   
   C   
   A   
   G   
   A   
   T   
   A   
   A   
   T   
   A   
   T   
   T   
   T   
   A   
   T   
   T   
   T   
   T   
   G   
   C   
    C    
   C   
   A   
   A   
   C   
   A   
   A   
   A   
   C   
   A   
   T   
   T   
   A   
   C   
   G   
    T    
   A   
   T   
   G   
   G   
   G   
    G    
   G   
   A   
   A   
   G   
   A   
   C   
   A   
   A   
   A   
   T   
   T   
   T   
   A   
   G   
   A   
   G   
   C   
    G    
   G   
   G   
   T   
   C   
   T   
   T   
   A   
   A   
   T   
   A   
   A   
   A   
   T   
   T   
   C   
   A   
   T   
   T   
   T   
   T   
   C   
   G   
   G   
   T   
   G   
   A   
   G   
   G   
   A   
   A   
   C   
   T   
   A   
   G   
   A   
   T   
   T   
   T   
   A   
   C   
   C   
   A   
   A   
   T   
    T    
   G   
   C   
   C   
   A   
   T   
   G   
   A   
   C   
   G   
   C   
   C   
    T    
   G   
   C   
   T   
   C   
   A   
   A   
   A   
   T   
   T   
   T   
   C   
   A   
   A   
   A   
    T    
   G   
   A   
   A   
   G   
   A   
   T   
   A   
   A   
   A   
   T   
   T   
   T   
   A   
   A   
   T   
   T   
   C   
   T   
   G   
   A   
   A   
   A   
   T   
   T   
   C   
   T   
   T   
   T   
   T   
    A    
   G   
   C   
   A   
   G   
   A   
   T   
   A   
   C   
    G    
   G   
   G   
   C   
   T   
   A   
    C    
   G   
   G   
    T    
   C   
   A   
   A   
   G   
   G   
    C    
   C   
   A   
   A   
   T   
   T   
   G   
   T   
   T   
   A   
   A   
   T   
   T   
   T   
   C   
    G    
   C   
   C   
   A   
   A   
   T   
   C   
   C   
   A   
   G   
   C   
   A   
   A   
   G   
   C   
   T   
   A   
   C   
   C   
   A   
   T   
   G   
   T   
   A   
   T   
   A   
   G   
   T   
   G   
   T   
   T   
   T   
   T   
   C   
   C   
   A   
   A   
   A   
   A   
   T   
   A   
   A   
   T   
   G   
   G   
    A    
   A   
   C   
   A   
    T    
   T   
    A    
   G   
   T   
    C    
   T   
   A   
   T   
   C   
   C   
   A   
   A   
   A   
   A   
    T    
   T   
   A   
   G   
   T   
   T   
   T   
   T   
   A   
   G   
   A   
   C   
   A   
   A   
   A   
   G   
   A   
   A   
   A   
   C   
    A    
   A   
   A   
   G   
   A   
   A   
   A   
   A   
   A   
   A   
   G   
   A   
   C   
   A   
   A   
   T   
   G   
   T   
   A   
   A   
   T   
   C   
   A   
   G   
   C   
   G   
   C   
   C   
   A   
   A   
   C   
   G   
   C   
   A   
   G   
   C   
   G   
   A   
   A   
   T   
   A   
   C   
   A   
   A   
   T   
   T   
   G   
   C   
   C   
   A   
   C   
   A   
   G   
   A   
   T   
   C   
   T   
   T   
   T   
   T   
   A   
   G   
   G   
   T   
   A   
   G   
   T   
   G   
   T   
   G   
   G   
   A   
   A   
   G   
   A   
   T   
   C   
   C   
   T   
   T   
   C   
   A   
   G   
   G   
   T   
   T   
   A   
   T   
   G   
   T   
   T   
   T   
   A   
   T   
   A   
   A   
   T   
   A   
   T   
   G   
   T   
   A   
   C   
   A   
   A   
   T   
   C   
   C   
   G   
   A   
   A   
   C   
   T   
   T   
   C   
   T   
   C   
   A   
   T   
   T   
   G   
   G   
   C   
   T   
   G   
   C   
   T   
   A   
   A   
   A   
   A   
   C   
   A   
   G   
   G   
   A   
   A   
   C   
   A   
   G   
   C   
   T   
   G   
   A   
   A   
   A   
   T   
   A   
   A   
   A   
   A   
   G   
   A   
   T   
   A   
   A   
   A   
   C   
   A   
   A   
   G   
   A   
   T   
   A   
   C   
   A   
   G   
   A   
   T   
   G   
   G   
   C   
   A   
   A   
   A   
   G   
   A   
   A   
   A   
   A   
   C   
   A   
   G   
   T   
   T   
   T   
   C   
   C   
   T   
   T   
   C   
   T   
   A   
   A   
   C   
   G   
   T   
   T   
   A   
   G   
   A   
   T   
   C   
   G   
   T   
   A   
   G   
   T   
   A   
   A   
   C   
   A   
   A   
   T   
   A   
   A   
   A   
   T   
   T   
   C   
   T   
   T   
   A   
   A   
   C   
   A   
   A   
   T   
   G   
   A   
   T   
   T   
   A   
   T   
   G   
   G   
   T   
   T   
   G   
   A   
   A   
   A   
   A   
   T   
   T   
   C   
   A   
   G   
   G   
   A   
   G   
   A   
   A   
   A   
   A   
   T   
   G   
   G   
   T   
   T   
   C   
   T   
   G   
   C   
   A   
   A   
   C   
   A   
   G   
   A   
   T   
   A   
   T   
   C   
   A   
   G   
   T   
   A   
   A   
   A   
   C   
   C   
   A   
   T   
   T   
   A   
   A   
   T   
   T   
   G   
   A   
   T   
   T   
   A   
   C   
   T   
   T   
   A   
   G   
   A   
   A   
   G   
   C   
   A   
   A   
   C   
   C   
   A   
   T   
   T   
   A   
   A   
   A   
   -   
   -   
   -   
   -   
   -   
   -   
   -   
   -   
   -   
   -   
   -   
   -   
   -   
   -   
   -   
   -   
   -   
   -   
   -   
   -   
   -   
   -   
   -   
   -   
   -   
   -   
   -   
   -   
   -   
   -   
   -   
   -   
   -   
   -   
   -   
   -   
   -   
   -   
   -   
   -   
   -   
   -   
   -   
   -   
   -   
   -   
   -   
   -   
   -   
   -   
   -   
   -   
   -   
   -   
   -   
   -   
   -   
   -   
   -   
   -   
   -   
   -   
   -   
   -   
   -   
   -   
   -   
   -   
   -   
   -   
   -   
   -   
   -   
   -   
   -   
   -   
   -   
   -   
   -   
   -   
   -   
   -   
   -   
   -   
   -   
   -   
   -   
   -   
   -   
   -   
   -   
   -   
   -   
   -   
   -   
   -   
   -   
   -   
   -   
   -   
   -   
   -   
   -   
   -   
   -   
   -   
   -   
   -   
   -   
   -   
   -   
   -   
   -   
   -   
   -   
   -   
   -   
   -   
   -   
   -   
   -   
   -   
   -   
   -   
   -   
   -   
   -   
   -   
   -   
   -   
   -   
   -   
   -   
   -   
   -   
   -   
   -   
   -   
   -   
   -   
   -   
   -   
   -   
   -   
   -   
   -   
   -   
   -   
   -   
   -   
   -   
   -   
   -   
   -   
   -   
   -   
   -   
   -   
   -   
   -   
   -   
   -   
   -   
   -   
   -   
   -   
   -   
   -   
   -   
   C   
   A   
   A   
   T   
   G   
   G   
   C   
   A   
   A   
   G   
   C   
   C   
   A   
   A   
   A   
   C   
   A   
   A   
   G   
   A   
   A   
   T   
   T   
   A   
   G   
   C   
   C   
   G   
   A   
   A   
   G   
   C   
   G   
   A   
   A   
   G   
   A   
   A   
   A   
   A   
   C   
   A   
   G   
   C   
   T   
   A   
   C   
   T   
   A   
   C   
   A   
   T   
   T   
   T   
   T   
   T   
   A   
   A   
   A   
   C   
   G   
   T   
   A   
   T   
   T   
   G   
   T   
   C   
   A   
   A   
   A   
   A   
   C   
   A   
   G   
   G   
   A   
   A   
   T   
   T   
   T   
   G   
   A   
   T   
   A   
   A   
   G   
   T   
   T   
   A   
   C   
   C   
   G   
   T   
   C   
   C   
   G   
   T   
   T   
   G   
   T   
   T   
   C   
   A   
   A   
   G   
   A   
   A   
   G   
   C   
   T   
   A   
   G   
   C   
   T   
   T   
   A   
   A   
   A   
   G   
   A   
   A   
   A   
   A   
   A   
   T   
   G   
   G   
   C   
   T   
   A   
   T   
   G   
   A   
   T   
   A   
   C   
   T   
   A   
   A   
   A   
   T   
   C   
   T   
   G   
   T   
   T   
   G   
   T   
   T   
   G   
   A   
   A   
   A   
   A   
   A   
   T   
   A   
   C   
   C   
   A   
   A   
   G   
   C   
   A   
   A   
   T   
   T   
   T   
   A   
   T   
   T   
   C   
   A   
   G   
   G   
   G   
   A   
   T   
   T   
   C   
   A   
   A   
   G   
   C   
   A   
   G   
   A   
   A   
   G   
   G   
   A   
   G   
   T   
   C   
   A   
   A   
   A   
   G   
   C   
   T   
   A   
   G   
   T   
   G   
   A   
   T   
   G   
   T   
   T   
   C   
   A   
   A   
   G   
   T   
   C   
   A   
   A   
   A   
   A   
   A   
   G   
   G   
   C   
   G   
   A   
   A   
   A   
   G   
   A   
   C   
   A   
   A   
   T   
   C   
   A   
   A   
   T   
   A   
   C   
   A   
   C   
   A   
   T   
   T   
   T   
   A   
   C   
   C   
   T   
   A   
   T   
   A   
   A   
   A   
   T   
   T   
   A   
   T   
   C   
   G   
   A   
   T   
   G   
   A   
   G   
   C   
   A   
   C   
   T   
   C   
   C   
   T   
   T   
   T   
   A   
   G   
   G   
   C   
   G   
   A   
   A   
   A   
   T   
   G   
   A   
   A   
   A   
   G   
   A   
   T   
   T   
   T   
   G   
   T   
   C   
   T   
   T   
   A   
   T   
   C   
   A   
   A   
   T   
   C   
   A   
   A   
   G   
   T   
   A   
   T   
   C   
   G   
   C   
   C   
   A   
   A   
   G   
   A   
   A   
   A   
   G   
   G   
   T   
   G   
   A   
   T   
   A   
   C   
   C   
   T   
   A   
   C   
   C   
   A   
   A   
   A   
   T   
   C   
   G   
   C   
   T   
   T   
   G   
   G   
   A   
   A   
   G   
   C   
   C   
   G   
   T   
   C   
   T   
   T   
   T   
   A   
   A   
   T   
   T   
   T   
   T   
   T   
   C   
   C   
   A   
   G   
   A   
   T   
   A   
   T   
   G   
   T   
   C   
   A   
   G   
   G   
   A   
   A   
   A   
   T   
   G   
   A   
   T   
   A   
   A   
   A   
   A   
   T   
   T   
   T   
   C   
   G   
   A   
   T   
   T   
   C   
   A   
   A   
   G   
   T   
   A   
   G   
   A   
   T   
   A   
   A   
   T   
   G   
   C   
   C   
   A   
   A   
   A   
   C   
   G   
   T   
   G   
   G   
   A   
   G   
   A   
   A   
   A   
   T   
   T   
   G   
   T   
   C   
   G   
   A   
   T   
   C   
   G   
   T   
   A   
   A   
   T   
   G   
   G   
   T   
   A   
   G   
   T   
   G   
   G   
   G   
   C   
   T   
   A   
   G   
   C   
   A   
   A   
   T   
   T   
   A   
   A   
   C   
   A   
   A   
   A   
   G   
   T   
   G   
   T   
   T   
   T   
   G   
   A   
   C   
   G   
   A   
   A   
   G   
   T   
   G   
   G   
   G   
   C   
   G   
   T   
   A   
   G   
   T   
   G   
   C   
   C   
   T   
   G   
   G   
   C   
   A   
   A   
   A   
   C   
   T   
   C   
   G   
   G   
   T   
   T   
   C   
   T   
   G   
   G   
   C   
   G   
   C   
   A   
   G   
   A   
   A   
   A   
   A   
   A   
   A   
   C   
   A   
   G   
   C   
   C   
   A   
   A   
   T   
   A   
   T   
   C   
   A   
   A   
   A   
   G   
   C   
   T   
   T   
   T   
   T   
   A   
   G   
   T   
   G   
   A   
   T   
   A   
   A   
   G   
   T   
   T   
   C   
   G   
   G   
   C   
   G   
   T   
   T   
   T   
   C   
   T   
   G   
   T   
   T   
   G   
   A   
   T   
   G   
   A   
   A   
   A   
   T   
   C   
   A   
   A   
   T   
   C   
   A   
   A   
   A   
   A   
   G   
   T   
   T   
   A   
   A   
   G   
   C   
   C   
   A   
   A   
   G   
   G   
   A   
   T   
   G   
   G   
   G   
   T   
   C   
   C   
   A   
   A   
   G   
   C   
   A   
   G   
   A   
   C   
   T   
   C   
   C   
   T   
   T   
   T   
   G   
   T   
   A   
   C   
   C   
   A   
   A   
   T   
   C   
   A   
   C   
   A   
   G   
   T   
   T   
   G   
   C   
   T   
   T   
   C   
   T   
   G   
   A   
   A   
   C   
   C   
   A   
   G   
   T   
   G   
   A   
   C   
   A   
   G   
   A   
   A   
   T   
   T   
   A   
   C   
   C   
   A   
   A   
   C   
   A   
   G   
   G   
   G   
   G   
   C   
   T   
   G   
   C   
   G   
   A   
   C   
   A   
   A   
   A   
   A   
   G   
   A   
   C   
   A   
   C   
   A   
   G   
   A   
   G   
   T   
   C   
   A   
   C   
   G   
   T   
   T   
   A   
   T   
   T   
   A   
   T   
   C   
   C   
   G   
   C   
   T   
   G   
   G   
   G   
   G   
   G   
   A   
   A   
   G   
   C   
   A   
   G   
   C   
   T   
   G   
   C   
   G   
   C   
   A   
   A   
   T   
   T   
   A   
   A   
   T   
   C   
   G   
   G   
   G   
   T   
   A   
   T   
   A   
   C   
   G   
   G   
   G   
   C   
   A   
   C   
   C   
   A   
   T   
   T   
   A   
   C   
   T   
   G   
   C   
   A   
   G   
   A   
   A   
   G   
   A   
   T   
   A   
   T   
   T   
   G   
   A   
   A   
   A   
   A   
   A   
   A   
   A   
   T   
   C   
   C   
   A   
   G   
   A   
   G   
   T   
   T   
   A   
   A   
   G   
   T   
   A   
   G   
   T   
   A   
   C   
   G   
   G   
   G   
   C   
   G   
   T   
   A   
   A   
   T   
   T   
   G   
   G   
   T   
   A   
   A   
   A   
   A   
   C   
   T   
   G   
   G   
   C   
   T   
   T   
   A   
   G   
   A   
   A   
   C   
   G   
   A   
   G   
   C   
   G   
   T   
   T   
   T   
   G   
   A   
   T   
   A   
   A   
   A   
   G   
   A   
   A   
   T   
   T   
   A   
   A   
   G   
   A   
   G   
   G   
   G   
   C   
   A   
   A   
   G   
   A   
   T   
   G   
   G   
   T   
   G   
   G   
   C   
   T   
   C   
   A   
   C   
   T   
   A   
   G   
   T   
   T   
   A   
   T   
   T   
   T   
   T   
   A   
   G   
   A   
   T   
   G   
   A   
   C   
   A   
   A   
   A   
   G   
   A   
   G   
   A   
   A   
   T   
   G   
   T   
   C   
   A   
   A   
   A   
   A   
   A   
   A   
   G   
   C   
   T   
   T   
   T   
   A   
   C   
   A   
   A   
   A   
   C   
   C   
   A   
   A   
   A   
   G   
   A   
   G   
   A   
   A   
   A   
   A   
   A   
   A   
   G   
   A   
   T   
   G   
   G   
   T   
   C   
   A   
   A   
   A   
   C   
   G   
   A   
   T   
   T   
   A   
   A   
   A   
   T   
   T   
   A   
   A   
   C   
   G   
   A   
   T   
   T   
   G   
   A   
   C   
   A   
   G   
   T   
   G   
   G   
   C   
   G   
   T   
   A   
   C   
   A   
   A   
   C   
   A   
   A   
   C   
   A   
   A   
   G   
   C   
   C   
   T   
   T   
   C   
   G   
   C   
   T   
   A   
   T   
   A   
   T   
   T   
   T   
   G   
   A   
   C   
   A   
   A   
   A   
   C   
   G   
   C   
   C   
   C   
   T   
   G   
   G   
   T   
   A   
   G   
   T   
   G   
   C   
   G   
   G   
   T   
   C   
   A   
   T   
   T   
   A   
   C   
   C   
   G   
   A   
   T   
   C   
   C   
   T   
   C   
   A   
   A   
   A   
   A   
   A   
   G   
   G   
   C   
   G   
   A   
   T   
   T   
   T   
   A   
   T   
   T   
   A   
   G   
   C   
   A   
   A   
   C   
   G   
   G   
   T   
   T   
   A   
   G   
   T   
   T   
   C   
   A   
   C   
   C   
   T   
   T   
   C   
   G   
   T   
   A   
   T   
   G   
   A   
   T   
   C   
   C   
   T   
   A   
   A   
   T   
   A   
   A   
   A   
   A   
   T   
   G   
   G   
   C   
   C   
   A   
   A   
   T   
   G   
   G   
   C   
   A   
   T   
   T   
   T   
   C   
   T   
   C   
   A   
   A   
   A   
   A   
   A   
   G   
   A   
   A   
   T   
   A   
   T   
   G   
   A   
   T   
   G   
   C   
   T   
   T   
   A   
   C   
   A   
   A   
   T   
   A   
   A   
   C   
   A   
   A   
   C   
   A   
   A   
   A   
   G   
   A   
   T   
   T   
   T   
   A   
   C   
   C   
   A   
   T   
   T   
   C   
   A   
   C   
   A   
   G   
   C   
   A   
   C   
   G   
   T   
   T   
   T   
   T   
   G   
   C   
   G   
   A   
   C   
   A   
   G   
   G   
   C   
   T   
   A   
   T   
   G   
   C   
   A   
   C   
   C   
   A   
   G   
   G   
   C   
   T   
   C   
   T   
   A   
   C   
   C   
   T   
   T   
   T   
   A   
   A   
   A   
   A   
   C   
   A   
   A   
   T   
   T   
   A   
   C   
   C   
   G   
   G   
   T   
   G   
   C   
   C   
   A   
   T   
   T   
   G   
   G   
   T   
   T   
   T   
   A   
   G   
   A   
   T   
   G   
   C   
   A   
   G   
   G   
   G   
   A   
   C   
   A   
   T   
   T   
   G   
   A   
   A   
   G   
   C   
   C   
   A   
   G   
   A   
   T   
   G   
   A   
   A   
   G   
   A   
   G   
   C   
   T   
   A   
   G   
   A   
   A   
   A   
   T   
   T   
   A   
   A   
   T   
   G   
   G   
   C   
   T   
   T   
   G   
   A   
   A   
   A   
   T   
   G   
   G   
   C   
   A   
   A   
   A   
   A   
   A   
   G   
   A   
   T   
   A   
   A   
   A   
   T   
   C   
   T   
   T   
   G   
   G   
   G   
   G   
   C   
   G   
   G   
   C   
   T   
   A   
   T   
   T   
   T   
   T   
   G   
   C   
   G   
   A   
   C   
   A   
   C   
   G   
   T   
   G   
   T   
   A   
   A   
   A   
   A   
   G   
   A   
   A   
   G   
   C   
   A   
   A   
   G   
   T   
   C   
   C   
   A   
   G   
   T   
   C   
   A   
   A   
   T   
   C   
   T   
   G   
   C   
   G   
   T   
   A   
   C   
   C   
   G   
   C   
   T   
   T   
   T   
   A   
   G   
   T   
   T   
   A   
   A   
   T   
   T   
   C   
   A   
   G   
   A   
   T   
   A   
   A   
   T   
   A   
   T   
   T   
   T   
   A   
   T   
   T   
   T   
   T   
   G   
   C   
   C   
   C   
   A   
   A   
   C   
   A   
   A   
   A   
   C   
   A   
   T   
   T   
   A   
   C   
   G   
   T   
   A   
   T   
   G   
   G   
   G   
   G   
   G   
   A   
   A   
   G   
   A   
   C   
   A   
   A   
   A   
   T   
   T   
   T   
   A   
   G   
   A   
   G   
   C   
   G   
   G   
   G   
   T   
   C   
   T   
   T   
   A   
   A   
   T   
   A   
   A   
   A   
   T   
   T   
   C   
   A   
   T   
   T   
   T   
   T   
   C   
   G   
   G   
   T   
   G   
   A   
   G   
   G   
   A   
   A   
   C   
   T   
   A   
   G   
   A   
   T   
   T   
   T   
   A   
   C   
   C   
   A   
   A   
   T   
   T   
   G   
   C   
   C   
   A   
   T   
   G   
   A   
   C   
   G   
   C   
   C   
   T   
   G   
   C   
   T   
   C   
   A   
   A   
   A   
   T   
   T   
   T   
   C   
   A   
   A   
   A   
   T   
   G   
   A   
   A   
   G   
   A   
   T   
   A   
   A   
   A   
   T   
   T   
   T   
   A   
   A   
   T   
   T   
   C   
   T   
   G   
   A   
   A   
   A   
   T   
   T   
   C   
   T   
   T   
   T   
   T   
   A   
   G   
   C   
   A   
   G   
   A   
   T   
   A   
   C   
   G   
   G   
   G   
   C   
   T   
   A   
   C   
   G   
   G   
   T   
   C   
   A   
   A   
   G   
   G   
   C   
   C   
   A   
   A   
   T   
   T   
   G   
   T   
   T   
   A   
   A   
   T   
   T   
   T   
   C   
   G   
   C   
   C   
   A   
   A   
   T   
   C   
   C   
   A   
   G   
   C   
   A   
   A   
   G   
   C   
   T   
   A   
   C   
   C   
   A   
   T   
   G   
   T   
   A   
   T   
   A   
   G   
   T   
   G   
   T   
   T   
   T   
   T   
   C   
   C   
   A   
   A   
   A   
   A   
   T   
   A   
   A   
   T   
   G   
   G   
   A   
   A   
   C   
   A   
   T   
   T   
   A   
   G   
   T   
   C   
   T   
   A   
   T   
   C   
   C   
   A   
   A   
   A   
   A   
   T   
   T   
   A   
   G   
   T   
   T   
   T   
   T   
   A   
   G   
   A   
   C   
   A   
   A   
   A   
   G   
   A   
   A   
   A   
   C   
   A   
   A   
   A   
   G   
   A   
   A   
   A   
   A   
   A   
   A   
   G   
   A   
   C   
   A   
   A   
   T   
   G   
   T   
   A   
   A   
   T   
   C   
   A   
   G   
   C   
   G   
   C   
   C   
   A   
   A   
   C   
   G   
   C   
   A   
   G   
   C   
   G   
   A   
   A   
   T   
   A   
   C   
   A   
   A   
   T   
   T   
   G   
   C   
   C   
   A   
   C   
   A   
   G   
   A   
   T   
   C   
   T   
   T   
   T   
   T   
   A   
   G   
   G   
   T   
   A   
   G   
   T   
   G   
   T   
   G   
   G   
   A   
   A   
   G   
   A   
   T   
   C   
   C   
   T   
   T   
   C   
   A   
   G   
   G   
   T   
   T   
   A   
   T   
   G   
   T   
   T   
   T   
   A   
   T   
   A   
   A   
   T   
   A   
   T   
   G   
   T   
   A   
   C   
   A   
   A   
   T   
   C   
   C   
   G   
   A   
   A   
   C   
   T   
   T   
   C   
   T   
   C   
   A   
   T   
   T   
   G   
   G   
   C   
   T   
   G   
   C   
   T   
   A   
   A   
   A   
   A   
   C   
   A   
   G   
   G   
   A   
   A   
   C   
   A   
   G   
   C   
   T   
   G   
   A   
   A   
   A   
   T   
   A   
   A   
   A   
   A   
   G   
   A   
   T   
   A   
   A   
   A   
   C   
   A   
   A   
   G   
   A   
   T   
   A   
   C   
   A   
   G   
   A   
   T   
   G   
   G   
   C   
   A   
   A   
   A   
   G   
   A   
   A   
   A   
   A   
   C   
   A   
   G   
   T   
   T   
   T   
   C   
   C   
   T   
   T   
   C   
   T   
   A   
   A   
   C   
   G   
   T   
   T   
   A   
   G   
   A   
   T   
   C   
   G   
   T   
   A   
   G   
   T   
   A   
   A   
   C   
   A   
   A   
   T   
   A   
   A   
   A   
   T   
   T   
   C   
   T   
   T   
   A   
   A   
   C   
   A   
   A   
   T   
   G   
   A   
   T   
   T   
   A   
   T   
   G   
   G   
   T   
   T   
   G   
   A   
   A   
   A   
   A   
   T   
   T   
   C   
   A   
   G   
   G   
   A   
   G   
   A   
   A   
   A   
   A   
   T   
   G   
   G   
   T   
   T   
   C   
   T   
   G   
   C   
   A   
   A   
   C   
   A   
   G   
   A   
   T   
   A   
   T   
   C   
   A   
   G   
   T   
   A   
   A   
   A   
   C   
   C   
   A   
   T   
   T   
   A   
   A   
   T   
   T   
   G   
   A   
   T   
   T   
   A   
   C   
   T   
   T   
   A   
   G   
   A   
   A   
   G   
   C   
   A   
   A   
   C   
   C   
   A   
   T   
   T   
   A   
   A   
   A   
   -   
   -   
   -   
   -   
   -   
   -   
   -   
   -   
   -   
   -   
   -   
   -   
   -   
   -   
   -   
   -   
   -   
   -   
   -   
   -   
   -   
   -   
   -   
   -   
   -   
   -   
   -   
   -   
   -   
   -   
   -   
   -   
   -   
   -   
   -   
   -   
   -   
   -   
   -   
   -   
   -   
   -   
   -   
   -   
   -   
   -   
   -   
   -   
   -   
   -   
   -   
   -   
   -   
   -   
   -   
   -   
   -   
   -   
   -   
   -   
   -   
 

 
   N54121   
   -   
   -   
   -   
   -   
   -   
   -   
   -   
   -   
   -   
   -   
   -   
   -   
   -   
   -   
   -   
   -   
   -   
   -   
   -   
   -   
   -   
   -   
   -   
   -   
   -   
   -   
   -   
   -   
   -   
   -   
   -   
   -   
   -   
   -   
   -   
   -   
   -   
   -   
   -   
   -   
   -   
   -   
   -   
   -   
   -   
   -   
   -   
   -   
   -   
   -   
   -   
   -   
   -   
   -   
   -   
   -   
   -   
   -   
   -   
   -   
   -   
   -   
   -   
   -   
   -   
   -   
   -   
   -   
   -   
   -   
   -   
   -   
   -   
   -   
   -   
   -   
   -   
   -   
   -   
   -   
   -   
   -   
   -   
   -   
   -   
   -   
   -   
   -   
   -   
   -   
   -   
   -   
   -   
   -   
   -   
   -   
   -   
   -   
   -   
   -   
   -   
   -   
   -   
   -   
   -   
   -   
   -   
   -   
   C   
   A   
   A   
   T   
   G   
   G   
   C   
   A   
   A   
   G   
   C   
   C   
   A   
   A   
   A   
   C   
   A   
   A   
   G   
   A   
    A    
   T   
   T   
   A   
   G   
   C   
    C    
   G   
   A   
   A   
   G   
   C   
   G   
   A   
   A   
   G   
   A   
   A   
   A   
   A   
    C    
   A   
   G   
   C   
    T    
   A   
    C    
   T   
    A    
    C    
   A   
   T   
   T   
   T   
   T   
   T   
   A   
   A   
   A   
    C    
   G   
   T   
   A   
   T   
   T   
   G   
    A    
   C   
   A   
   A   
   A   
   A   
   C   
   A   
   G   
    A    
   A   
   A   
   T   
   T   
   T   
   G   
   A   
   T   
   A   
   A   
   G   
   T   
   T   
   A   
   C   
   C   
    G    
   T   
   C   
   C   
   G   
   T   
   T   
    G    
   T   
   T   
   C   
   A   
   A   
   G   
   A   
   A   
    G    
   C   
   T   
   A   
   G   
   C   
   T   
   T   
   A   
   A   
   A   
   G   
   A   
   A   
   A   
   A   
   A   
   T   
   G   
   G   
    C    
   T   
   A   
   T   
   G   
   A   
   T   
   A   
   C   
   T   
   A   
   A   
   A   
   T   
   C   
    T    
   G   
   T   
   T   
   G   
   T   
   T   
   G   
   A   
   A   
   A   
   A   
   A   
   T   
   A   
   C   
   C   
   A   
   A   
   G   
   C   
   A   
   A   
   T   
   T   
   T   
   A   
   T   
   T   
   C   
   A   
   G   
   G   
   G   
   A   
   T   
   T   
   C   
   A   
   A   
   G   
   C   
    A    
   G   
   A   
   A   
    G    
   G   
    A    
   G   
   T   
   C   
   A   
   A   
    A    
   G   
   C   
   T   
   A   
   G   
   T   
   G   
   A   
   T   
   G   
   T   
    T    
   C   
   A   
   A   
   G   
   T   
    C    
   A   
   A   
   A   
   A   
   A   
    A    
   G   
    C    
   G   
   A   
   A   
   A   
   G   
   A   
    C    
   A   
   A   
    T    
   C   
   A   
   A   
   T   
   A   
   C   
   A   
   C   
   A   
   T   
   T   
   T   
   A   
    C    
   C   
   T   
   A   
   T   
   A   
   A   
   A   
   T   
   T   
   A   
   T   
   C   
   G   
   A   
   T   
   G   
   A   
   G   
    T    
   A   
   C   
    C    
   C   
   C   
   T   
   T   
   T   
   A   
   G   
   G   
   C   
   G   
   A   
   A   
   A   
   T   
   G   
   A   
   A   
   A   
   G   
   A   
   T   
    T    
   T   
   G   
   T   
   C   
   T   
   T   
   A   
   T   
   C   
   A   
   A   
   T   
   C   
   A   
   A   
   G   
   T   
   A   
   T   
    C    
   G   
    C    
   C   
   A   
   A   
    A    
   A   
   A   
   A   
   G   
   G   
    C    
   G   
   A   
   T   
   A   
    C    
   C   
   T   
   A   
   C   
   C   
   A   
   A   
   A   
   T   
    C    
    G    
   C   
   T   
   T   
   G   
   G   
    A    
    A    
    G    
   C   
   C   
    G    
   T   
   C   
   T   
   T   
   T   
   A   
   A   
   T   
   T   
   T   
   T   
   T   
   C   
   C   
   A   
   G   
   A   
   T   
   A   
   T   
   G   
   T   
   C   
   A   
   G   
   G   
   A   
   A   
   A   
   T   
   G   
   A   
   T   
   A   
   A   
   A   
    A    
   T   
   T   
   T   
   C   
   G   
   A   
   T   
   T   
   C   
   A   
   A   
   G   
   T   
   A   
   G   
   A   
   T   
   A   
   A   
   T   
   G   
   C   
   C   
   A   
   A   
   A   
   C   
   G   
   T   
   G   
   G   
   A   
   G   
   A   
   A   
   A   
   T   
   T   
   G   
   T   
   C   
   G   
   A   
   T   
   C   
   G   
   T   
   A   
   A   
    T    
   G   
   G   
   T   
   A   
   G   
   T   
   G   
   G   
   G   
   C   
   T   
   A   
   G   
    C    
   A   
   A   
   T   
   T   
   A   
   A   
   C   
   A   
   A   
   A   
   G   
   T   
   G   
   T   
   T   
   T   
   G   
   A   
    C    
   G   
   A   
   A   
   G   
   T   
   G   
   G   
   G   
   C   
   G   
   T   
   A   
   G   
   T   
    G    
   C   
   C   
   T   
    G    
   G   
   C   
   A   
   A   
   A   
   C   
   T   
    C    
   G   
   G   
   T   
   T   
   C   
   T   
   G   
   G   
   C   
   G   
   C   
   A   
   G   
   A   
   A   
   A   
   A   
   A   
    A    
   C   
   A   
   G   
   C   
    C    
   A   
   A   
   T   
   A   
   T   
   C   
   A   
   A   
   A   
   G   
   C   
   T   
   T   
   T   
   T   
   A   
   G   
   T   
   G   
   A   
   T   
   A   
   A   
    G    
   T   
   T   
    C    
   G   
   G   
   C   
   G   
   T   
   T   
   T   
   C   
   T   
    G    
   T   
   T   
   G   
   A   
   T   
   G   
   A   
   A   
   A   
   T   
   C   
   A   
   A   
   T   
   C   
    A    
   A   
   A   
   A   
    G    
   T   
   T   
   A   
   A   
   G   
   C   
   C   
   A   
   A   
   G   
   G   
    G    
   T   
   G   
   G   
   G   
   T   
   C   
   C   
   A   
   A   
   G   
   C   
   A   
   G   
   A   
   C   
   T   
   C   
    C    
   T   
   T   
   T   
   G   
   T   
   A   
   C   
   C   
   A   
   A   
   T   
   C   
   A   
   C   
   A   
   G   
   T   
    C    
   G   
   C   
   T   
   T   
   C   
   T   
   G   
   A   
   A   
   C   
   C   
   A   
   G   
   T   
   G   
   A   
   C   
   A   
   G   
   A   
   A   
   T   
   T   
   A   
   C   
   C   
   A   
   A   
   C   
   A   
   G   
   G   
   G   
   G   
   C   
   T   
   G   
   C   
   G   
   A   
   C   
   A   
   A   
   A   
   A   
   G   
    A    
    T    
   A   
   C   
   A   
   G   
   A   
   G   
   T   
   C   
   A   
   C   
   G   
   T   
   T   
   A   
   T   
   T   
   A   
   T   
   C   
   C   
    G    
    C    
   T   
   G   
   G   
   G   
   G   
   G   
   A   
   A   
   G   
   C   
   A   
   G   
   C   
   T   
   G   
   C   
   G   
   C   
   A   
   A   
   T   
   T   
   A   
   A   
   T   
   C   
   G   
   G   
    G    
   T   
   A   
   T   
   A   
   C   
    A    
   G   
   G   
    C    
   A   
   C   
    C    
   A   
   T   
   T   
   A   
   C   
   T   
   G   
   C   
   A   
   G   
   A   
    A    
   G   
   A   
   T   
   A   
   T   
   T   
    A    
   A   
   A   
   A   
   A   
   A   
   A   
   A   
   T   
   C   
   C   
   A   
   G   
   A   
   G   
   T   
   T   
   A   
   A   
   G   
   T   
   A   
   G   
   T   
   A   
   C   
   G   
   G   
   G   
    C    
   G   
   T   
   A   
   A   
   T   
   T   
   G   
   G   
    T    
   A   
   A   
   A   
   A   
   C   
   T   
   G   
   G   
    C    
   T   
   T   
   A   
   G   
   A   
   A   
   C   
   G   
   A   
   G   
   C   
   G   
   T   
   T   
   T   
   G   
   A   
   T   
   A   
   A   
   A   
   G   
   A   
    A    
   T   
   T   
   A   
   A   
   G   
   A   
   G   
   G   
    G    
   C   
   A   
   A   
   G   
   A   
   T   
   G   
   G   
    T    
   G   
   G   
    C    
   T   
   C   
    A    
   C   
   T   
   A   
   G   
   T   
   T   
   A   
   T   
   T   
   T   
   T   
   A   
   G   
   A   
   T   
   G   
   A   
   C   
   A   
   A   
   A   
   G   
   A   
   G   
   A   
   A   
   T   
   G   
   T   
   C   
   A   
   A   
   A   
   A   
   A   
   A   
   G   
   C   
   T   
   T   
   T   
   A   
   C   
   A   
   A   
   A   
   C   
   C   
   A   
   A   
   A   
   G   
   A   
    G    
   A   
   A   
   A   
   A   
   A   
   A   
   G   
   A   
   T   
   G   
   G   
   T   
   C   
   A   
   A   
   A   
   C   
    G    
   A   
   T   
   T   
   A   
   A   
   A   
   T   
   T   
   A   
   A   
   C   
   G   
   A   
   T   
   T   
   G   
   A   
    C    
   A   
   G   
    T    
   G   
   G   
    C    
   G   
   T   
   A   
   C   
   A   
   A   
   C   
   A   
   A   
   C   
   A   
   A   
   G   
   C   
    C    
   T   
   T   
    C    
   G   
   C   
   T   
   A   
   T   
   A   
   T   
   T   
   T   
   G   
   A   
   C   
   A   
   A   
   A   
   C   
   G   
    C    
    C    
   C   
   T   
   G   
   G   
   T   
   A   
   G   
   T   
   G   
   C   
   G   
   G   
   T   
   C   
   A   
   T   
   T   
   A   
   C   
   C   
   G   
   A   
   T   
   C   
   C   
   T   
   C   
   A   
   A   
   A   
   A   
   A   
   G   
   G   
   C   
   G   
   A   
   T   
   T   
   T   
   A   
    T    
   T   
   A   
   G   
   C   
    A    
   A   
   C   
   G   
   G   
   T   
   T   
   A   
   G   
   T   
   T   
   C   
   A   
   C   
   C   
   T   
   T   
   C   
   G   
   T   
   A   
   T   
   G   
   A   
   T   
   C   
   C   
   T   
   A   
   A   
   T   
   A   
   A   
   A   
   A   
   T   
   G   
   G   
   C   
   C   
   A   
   A   
   T   
   G   
   G   
    C    
   A   
   T   
   T   
   T   
   C   
   T   
   C   
   A   
   A   
   A   
   A   
   A   
   G   
   A   
    A    
   T   
   A   
   T   
   G   
   A   
   T   
   G   
   C   
   T   
   T   
   A   
   C   
   A   
   A   
   T   
   A   
   A   
   C   
   A   
   A   
   C   
   A   
   A   
   A   
   G   
   A   
   T   
   T   
   T   
   A   
   C   
   C   
   A   
   T   
   T   
    C    
   A   
   C   
   A   
   G   
   C   
   A   
   C   
   G   
   T   
   T   
   T   
    T    
   G   
   C   
   G   
   A   
   C   
   A   
   G   
   G   
    C    
   T   
   A   
   T   
   G   
   C   
   A   
   C   
   C   
   A   
   G   
   G   
    C    
   T   
   C   
   T   
   A   
   C   
   C   
   T   
   T   
   T   
   A   
   A   
   A   
   A   
   C   
   A   
   A   
   T   
   T   
   A   
   C   
   C   
   G   
   G   
   T   
   G   
   C   
   C   
   A   
   T   
   T   
   G   
   G   
   T   
   T   
   T   
   A   
   G   
   A   
   T   
    A    
   C   
   A   
   G   
   G   
   G   
   A   
   C   
   A   
   T   
   T   
   G   
   A   
   A   
   G   
   C   
   C   
   A   
   G   
   A   
   T   
   G   
   A   
   A   
   G   
   A   
   G   
   C   
   T   
   A   
   G   
   A   
   A   
   A   
   T   
   T   
   A   
   A   
   T   
   G   
   G   
    T    
   T   
   T   
   G   
   A   
   A   
   A   
   T   
   G   
   G   
   C   
   A   
   A   
   A   
   A   
   A   
   G   
   A   
   T   
   A   
   A   
   A   
   T   
   C   
   T   
   T   
   G   
   G   
   G   
   G   
    C    
   G   
   G   
   C   
   T   
   A   
   T   
   T   
   T   
   T   
   G   
   C   
    G    
   A   
   C   
   A   
   C   
   G   
   T   
   G   
   T   
   A   
   A   
   A   
   A   
   G   
   A   
   A   
   G   
   C   
   A   
   A   
   G   
   T   
   C   
   C   
   A   
   G   
   T   
   C   
   A   
   A   
   T   
   C   
   T   
    G    
   C   
   G   
   T   
   A   
   C   
   C   
   G   
   C   
   T   
   T   
   T   
   A   
   G   
   T   
   T   
   A   
   A   
   T   
   T   
   C   
   A   
   G   
   A   
   T   
   A   
   A   
   T   
   A   
   T   
   T   
   T   
   A   
   T   
   T   
   T   
   T   
   G   
   C   
    C    
   C   
   A   
   A   
   C   
   A   
   A   
   A   
   C   
   A   
   T   
   T   
   A   
   C   
   G   
    G    
   A   
   T   
   G   
   G   
   G   
    A    
   G   
   A   
   A   
   G   
   A   
   C   
   A   
   A   
   A   
   T   
   T   
   T   
   A   
   G   
   A   
   G   
   C   
    G    
   G   
   G   
   T   
   C   
   T   
   T   
   A   
   A   
   T   
   A   
   A   
   A   
   T   
   T   
   C   
   A   
   T   
   T   
   T   
   T   
   C   
   G   
   G   
   T   
   G   
   A   
   G   
   G   
   A   
   A   
   C   
   T   
   A   
   G   
   A   
   T   
   T   
   T   
   A   
   C   
   C   
   A   
   A   
   T   
    T    
   G   
   C   
   C   
   A   
   T   
   G   
   A   
   C   
   G   
   C   
   C   
    T    
   G   
   C   
   T   
   C   
   A   
   A   
   A   
   T   
   T   
   T   
   C   
   A   
   A   
   A   
    T    
   G   
   A   
   A   
   G   
   A   
   T   
   A   
   A   
   A   
   T   
   T   
   T   
   A   
   A   
   T   
   T   
   C   
   T   
   G   
   A   
   A   
   A   
   T   
   T   
   C   
   T   
   T   
   T   
   T   
    G    
   G   
   C   
   A   
   G   
   A   
   T   
   A   
   C   
    G    
   G   
   G   
   C   
   T   
   A   
    C    
   G   
   G   
    C    
   C   
   A   
   A   
   G   
   G   
    C    
   C   
   A   
   A   
   T   
   T   
   G   
   T   
   T   
   A   
   A   
   T   
   T   
   T   
   C   
    G    
   C   
   C   
   A   
   A   
   T   
   C   
   C   
   A   
   G   
   C   
   A   
   A   
   G   
   C   
   T   
   A   
   C   
   C   
   A   
   T   
   G   
   T   
   A   
   T   
   A   
   G   
   T   
   G   
   T   
   T   
   T   
   T   
   C   
   C   
   A   
   A   
   A   
   A   
   T   
   A   
   A   
   T   
   G   
   G   
    G    
   A   
   C   
   A   
    T    
   T   
    A    
   G   
   T   
    C    
   T   
   A   
   T   
   C   
   C   
   A   
   A   
   A   
   A   
    C    
   T   
   A   
   G   
   T   
   T   
   T   
   T   
   A   
   G   
   A   
   C   
   A   
   A   
   A   
   G   
   A   
   A   
   A   
   C   
    G    
   A   
   A   
   G   
   A   
   A   
   A   
   A   
   A   
   A   
   G   
   A   
   C   
   A   
   A   
   T   
   G   
   T   
   A   
   A   
   T   
   C   
   A   
   G   
   C   
   G   
   C   
   C   
   A   
   A   
   C   
   G   
   C   
   A   
   G   
   C   
   G   
   A   
   A   
   T   
   A   
   C   
   A   
   A   
   T   
   T   
   G   
   C   
   C   
   A   
   C   
   A   
   G   
   A   
   T   
   C   
   T   
   T   
   T   
   T   
   A   
   G   
   G   
   T   
   A   
   G   
   T   
   G   
   T   
   G   
   G   
   A   
   A   
   G   
   A   
   T   
   C   
   C   
   T   
   T   
   C   
   A   
   G   
   G   
   T   
   T   
   A   
   T   
   G   
   T   
   T   
   T   
   A   
   T   
   A   
   A   
   T   
   A   
   T   
   G   
   T   
   A   
   C   
   A   
   A   
   T   
   C   
   C   
   G   
   A   
   A   
   C   
   T   
   T   
   C   
   T   
   C   
   A   
   T   
   T   
   G   
   G   
   C   
   T   
   G   
   C   
   T   
   A   
   A   
   A   
   A   
   C   
   A   
   G   
   G   
   A   
   A   
   C   
   A   
   G   
   C   
   T   
   G   
   A   
   A   
   A   
   T   
   A   
   A   
   A   
   A   
   G   
   A   
   T   
   A   
   A   
   A   
   C   
   A   
   A   
   G   
   A   
   T   
   A   
   C   
   A   
   G   
   A   
   T   
   G   
   G   
   C   
   A   
   A   
   A   
   G   
   A   
   A   
   A   
   A   
   C   
   A   
   G   
   T   
   T   
   T   
   C   
   C   
   T   
   T   
   C   
   T   
   A   
   A   
   C   
   G   
   T   
   T   
   A   
   G   
   A   
   T   
   C   
   G   
   T   
   A   
   G   
   T   
   A   
   A   
   C   
   A   
   A   
   T   
   A   
   A   
   A   
   T   
   T   
   C   
   T   
   T   
   A   
   A   
   C   
   A   
   A   
   T   
   G   
   A   
   T   
   T   
   A   
   T   
   G   
   G   
   T   
   T   
   G   
   A   
   A   
   A   
   A   
   T   
   T   
   C   
   A   
   G   
   G   
   A   
   G   
   A   
   A   
   A   
   A   
   T   
   G   
   G   
   T   
   T   
   C   
   T   
   G   
   C   
   A   
   A   
   C   
   A   
   G   
   A   
   T   
   A   
   T   
   C   
   A   
   G   
   T   
   A   
   A   
   A   
   C   
   C   
   A   
   T   
   T   
   A   
   A   
   T   
   T   
   G   
   A   
   T   
   T   
   A   
   C   
   T   
   T   
   A   
   G   
   A   
   A   
   G   
   C   
   A   
   A   
   C   
   C   
   A   
   T   
   T   
   A   
   A   
   A   
   -   
   -   
   -   
   -   
   -   
   -   
   -   
   -   
   -   
   -   
   -   
   -   
   -   
   -   
   -   
   -   
   -   
   -   
   -   
   -   
   -   
   -   
   -   
   -   
   -   
   -   
   -   
   -   
   -   
   -   
   -   
   -   
   -   
   -   
   -   
   -   
   -   
   -   
   -   
   -   
   -   
   -   
   -   
   -   
   -   
   -   
   -   
   -   
   -   
   -   
   -   
   -   
   -   
   -   
   -   
   -   
   -   
   -   
   -   
   -   
   -   
   -   
   -   
   -   
   -   
   -   
   -   
   -   
   -   
   -   
   -   
   -   
   -   
   -   
   -   
   -   
   -   
   -   
   -   
   -   
   -   
   -   
   -   
   -   
   -   
   -   
   -   
   -   
   -   
   -   
   -   
   -   
   -   
   -   
   -   
   -   
   -   
   -   
   -   
   -   
   -   
   -   
   -   
   -   
   -   
   -   
   -   
   -   
   -   
   -   
   -   
   -   
   -   
   -   
   -   
   -   
   -   
   -   
   -   
   -   
   -   
   -   
   -   
   -   
   -   
   -   
   -   
   -   
   -   
   -   
   -   
   -   
   -   
   -   
   -   
   -   
   -   
   -   
   -   
   -   
   -   
   -   
   -   
   -   
   -   
   -   
   -   
   -   
   -   
   -   
   -   
   -   
   -   
   -   
   -   
   -   
   -   
   -   
   -   
   -   
   -   
   -   
   -   
   -   
   -   
   -   
   -   
   -   
   -   
   C   
   A   
   A   
   T   
   G   
   G   
   C   
   A   
   A   
   G   
   C   
   C   
   A   
   A   
   A   
   C   
   A   
   A   
   G   
   A   
   A   
   T   
   T   
   A   
   G   
   C   
   C   
   G   
   A   
   A   
   G   
   C   
   G   
   A   
   A   
   G   
   A   
   A   
   A   
   A   
   C   
   A   
   G   
   C   
   T   
   A   
   C   
   T   
   A   
   C   
   A   
   T   
   T   
   T   
   T   
   T   
   A   
   A   
   A   
   C   
   G   
   T   
   A   
   T   
   T   
   G   
   A   
   C   
   A   
   A   
   A   
   A   
   C   
   A   
   G   
   A   
   A   
   A   
   T   
   T   
   T   
   G   
   A   
   T   
   A   
   A   
   G   
   T   
   T   
   A   
   C   
   C   
   G   
   T   
   C   
   C   
   G   
   T   
   T   
   G   
   T   
   T   
   C   
   A   
   A   
   G   
   A   
   A   
   G   
   C   
   T   
   A   
   G   
   C   
   T   
   T   
   A   
   A   
   A   
   G   
   A   
   A   
   A   
   A   
   A   
   T   
   G   
   G   
   C   
   T   
   A   
   T   
   G   
   A   
   T   
   A   
   C   
   T   
   A   
   A   
   A   
   T   
   C   
   T   
   G   
   T   
   T   
   G   
   T   
   T   
   G   
   A   
   A   
   A   
   A   
   A   
   T   
   A   
   C   
   C   
   A   
   A   
   G   
   C   
   A   
   A   
   T   
   T   
   T   
   A   
   T   
   T   
   C   
   A   
   G   
   G   
   G   
   A   
   T   
   T   
   C   
   A   
   A   
   G   
   C   
   A   
   G   
   A   
   A   
   G   
   G   
   A   
   G   
   T   
   C   
   A   
   A   
   A   
   G   
   C   
   T   
   A   
   G   
   T   
   G   
   A   
   T   
   G   
   T   
   T   
   C   
   A   
   A   
   G   
   T   
   C   
   A   
   A   
   A   
   A   
   A   
   A   
   G   
   C   
   G   
   A   
   A   
   A   
   G   
   A   
   C   
   A   
   A   
   T   
   C   
   A   
   A   
   T   
   A   
   C   
   A   
   C   
   A   
   T   
   T   
   T   
   A   
   C   
   C   
   T   
   A   
   T   
   A   
   A   
   A   
   T   
   T   
   A   
   T   
   C   
   G   
   A   
   T   
   G   
   A   
   G   
   T   
   A   
   C   
   C   
   C   
   C   
   T   
   T   
   T   
   A   
   G   
   G   
   C   
   G   
   A   
   A   
   A   
   T   
   G   
   A   
   A   
   A   
   G   
   A   
   T   
   T   
   T   
   G   
   T   
   C   
   T   
   T   
   A   
   T   
   C   
   A   
   A   
   T   
   C   
   A   
   A   
   G   
   T   
   A   
   T   
   C   
   G   
   C   
   C   
   A   
   A   
   A   
   A   
   A   
   A   
   G   
   G   
   C   
   G   
   A   
   T   
   A   
   C   
   C   
   T   
   A   
   C   
   C   
   A   
   A   
   A   
   T   
   C   
   G   
   C   
   T   
   T   
   G   
   G   
   A   
   A   
   G   
   C   
   C   
   G   
   T   
   C   
   T   
   T   
   T   
   A   
   A   
   T   
   T   
   T   
   T   
   T   
   C   
   C   
   A   
   G   
   A   
   T   
   A   
   T   
   G   
   T   
   C   
   A   
   G   
   G   
   A   
   A   
   A   
   T   
   G   
   A   
   T   
   A   
   A   
   A   
   A   
   T   
   T   
   T   
   C   
   G   
   A   
   T   
   T   
   C   
   A   
   A   
   G   
   T   
   A   
   G   
   A   
   T   
   A   
   A   
   T   
   G   
   C   
   C   
   A   
   A   
   A   
   C   
   G   
   T   
   G   
   G   
   A   
   G   
   A   
   A   
   A   
   T   
   T   
   G   
   T   
   C   
   G   
   A   
   T   
   C   
   G   
   T   
   A   
   A   
   T   
   G   
   G   
   T   
   A   
   G   
   T   
   G   
   G   
   G   
   C   
   T   
   A   
   G   
   C   
   A   
   A   
   T   
   T   
   A   
   A   
   C   
   A   
   A   
   A   
   G   
   T   
   G   
   T   
   T   
   T   
   G   
   A   
   C   
   G   
   A   
   A   
   G   
   T   
   G   
   G   
   G   
   C   
   G   
   T   
   A   
   G   
   T   
   G   
   C   
   C   
   T   
   G   
   G   
   C   
   A   
   A   
   A   
   C   
   T   
   C   
   G   
   G   
   T   
   T   
   C   
   T   
   G   
   G   
   C   
   G   
   C   
   A   
   G   
   A   
   A   
   A   
   A   
   A   
   A   
   C   
   A   
   G   
   C   
   C   
   A   
   A   
   T   
   A   
   T   
   C   
   A   
   A   
   A   
   G   
   C   
   T   
   T   
   T   
   T   
   A   
   G   
   T   
   G   
   A   
   T   
   A   
   A   
   G   
   T   
   T   
   C   
   G   
   G   
   C   
   G   
   T   
   T   
   T   
   C   
   T   
   G   
   T   
   T   
   G   
   A   
   T   
   G   
   A   
   A   
   A   
   T   
   C   
   A   
   A   
   T   
   C   
   A   
   A   
   A   
   A   
   G   
   T   
   T   
   A   
   A   
   G   
   C   
   C   
   A   
   A   
   G   
   G   
   G   
   T   
   G   
   G   
   G   
   T   
   C   
   C   
   A   
   A   
   G   
   C   
   A   
   G   
   A   
   C   
   T   
   C   
   C   
   T   
   T   
   T   
   G   
   T   
   A   
   C   
   C   
   A   
   A   
   T   
   C   
   A   
   C   
   A   
   G   
   T   
   C   
   G   
   C   
   T   
   T   
   C   
   T   
   G   
   A   
   A   
   C   
   C   
   A   
   G   
   T   
   G   
   A   
   C   
   A   
   G   
   A   
   A   
   T   
   T   
   A   
   C   
   C   
   A   
   A   
   C   
   A   
   G   
   G   
   G   
   G   
   C   
   T   
   G   
   C   
   G   
   A   
   C   
   A   
   A   
   A   
   A   
   G   
   A   
   T   
   A   
   C   
   A   
   G   
   A   
   G   
   T   
   C   
   A   
   C   
   G   
   T   
   T   
   A   
   T   
   T   
   A   
   T   
   C   
   C   
   G   
   C   
   T   
   G   
   G   
   G   
   G   
   G   
   A   
   A   
   G   
   C   
   A   
   G   
   C   
   T   
   G   
   C   
   G   
   C   
   A   
   A   
   T   
   T   
   A   
   A   
   T   
   C   
   G   
   G   
   G   
   T   
   A   
   T   
   A   
   C   
   A   
   G   
   G   
   C   
   A   
   C   
   C   
   A   
   T   
   T   
   A   
   C   
   T   
   G   
   C   
   A   
   G   
   A   
   A   
   G   
   A   
   T   
   A   
   T   
   T   
   A   
   A   
   A   
   A   
   A   
   A   
   A   
   A   
   T   
   C   
   C   
   A   
   G   
   A   
   G   
   T   
   T   
   A   
   A   
   G   
   T   
   A   
   G   
   T   
   A   
   C   
   G   
   G   
   G   
   C   
   G   
   T   
   A   
   A   
   T   
   T   
   G   
   G   
   T   
   A   
   A   
   A   
   A   
   C   
   T   
   G   
   G   
   C   
   T   
   T   
   A   
   G   
   A   
   A   
   C   
   G   
   A   
   G   
   C   
   G   
   T   
   T   
   T   
   G   
   A   
   T   
   A   
   A   
   A   
   G   
   A   
   A   
   T   
   T   
   A   
   A   
   G   
   A   
   G   
   G   
   G   
   C   
   A   
   A   
   G   
   A   
   T   
   G   
   G   
   T   
   G   
   G   
   C   
   T   
   C   
   A   
   C   
   T   
   A   
   G   
   T   
   T   
   A   
   T   
   T   
   T   
   T   
   A   
   G   
   A   
   T   
   G   
   A   
   C   
   A   
   A   
   A   
   G   
   A   
   G   
   A   
   A   
   T   
   G   
   T   
   C   
   A   
   A   
   A   
   A   
   A   
   A   
   G   
   C   
   T   
   T   
   T   
   A   
   C   
   A   
   A   
   A   
   C   
   C   
   A   
   A   
   A   
   G   
   A   
   G   
   A   
   A   
   A   
   A   
   A   
   A   
   G   
   A   
   T   
   G   
   G   
   T   
   C   
   A   
   A   
   A   
   C   
   G   
   A   
   T   
   T   
   A   
   A   
   A   
   T   
   T   
   A   
   A   
   C   
   G   
   A   
   T   
   T   
   G   
   A   
   C   
   A   
   G   
   T   
   G   
   G   
   C   
   G   
   T   
   A   
   C   
   A   
   A   
   C   
   A   
   A   
   C   
   A   
   A   
   G   
   C   
   C   
   T   
   T   
   C   
   G   
   C   
   T   
   A   
   T   
   A   
   T   
   T   
   T   
   G   
   A   
   C   
   A   
   A   
   A   
   C   
   G   
   C   
   C   
   C   
   T   
   G   
   G   
   T   
   A   
   G   
   T   
   G   
   C   
   G   
   G   
   T   
   C   
   A   
   T   
   T   
   A   
   C   
   C   
   G   
   A   
   T   
   C   
   C   
   T   
   C   
   A   
   A   
   A   
   A   
   A   
   G   
   G   
   C   
   G   
   A   
   T   
   T   
   T   
   A   
   T   
   T   
   A   
   G   
   C   
   A   
   A   
   C   
   G   
   G   
   T   
   T   
   A   
   G   
   T   
   T   
   C   
   A   
   C   
   C   
   T   
   T   
   C   
   G   
   T   
   A   
   T   
   G   
   A   
   T   
   C   
   C   
   T   
   A   
   A   
   T   
   A   
   A   
   A   
   A   
   T   
   G   
   G   
   C   
   C   
   A   
   A   
   T   
   G   
   G   
   C   
   A   
   T   
   T   
   T   
   C   
   T   
   C   
   A   
   A   
   A   
   A   
   A   
   G   
   A   
   A   
   T   
   A   
   T   
   G   
   A   
   T   
   G   
   C   
   T   
   T   
   A   
   C   
   A   
   A   
   T   
   A   
   A   
   C   
   A   
   A   
   C   
   A   
   A   
   A   
   G   
   A   
   T   
   T   
   T   
   A   
   C   
   C   
   A   
   T   
   T   
   C   
   A   
   C   
   A   
   G   
   C   
   A   
   C   
   G   
   T   
   T   
   T   
   T   
   G   
   C   
   G   
   A   
   C   
   A   
   G   
   G   
   C   
   T   
   A   
   T   
   G   
   C   
   A   
   C   
   C   
   A   
   G   
   G   
   C   
   T   
   C   
   T   
   A   
   C   
   C   
   T   
   T   
   T   
   A   
   A   
   A   
   A   
   C   
   A   
   A   
   T   
   T   
   A   
   C   
   C   
   G   
   G   
   T   
   G   
   C   
   C   
   A   
   T   
   T   
   G   
   G   
   T   
   T   
   T   
   A   
   G   
   A   
   T   
   A   
   C   
   A   
   G   
   G   
   G   
   A   
   C   
   A   
   T   
   T   
   G   
   A   
   A   
   G   
   C   
   C   
   A   
   G   
   A   
   T   
   G   
   A   
   A   
   G   
   A   
   G   
   C   
   T   
   A   
   G   
   A   
   A   
   A   
   T   
   T   
   A   
   A   
   T   
   G   
   G   
   T   
   T   
   T   
   G   
   A   
   A   
   A   
   T   
   G   
   G   
   C   
   A   
   A   
   A   
   A   
   A   
   G   
   A   
   T   
   A   
   A   
   A   
   T   
   C   
   T   
   T   
   G   
   G   
   G   
   G   
   C   
   G   
   G   
   C   
   T   
   A   
   T   
   T   
   T   
   T   
   G   
   C   
   G   
   A   
   C   
   A   
   C   
   G   
   T   
   G   
   T   
   A   
   A   
   A   
   A   
   G   
   A   
   A   
   G   
   C   
   A   
   A   
   G   
   T   
   C   
   C   
   A   
   G   
   T   
   C   
   A   
   A   
   T   
   C   
   T   
   G   
   C   
   G   
   T   
   A   
   C   
   C   
   G   
   C   
   T   
   T   
   T   
   A   
   G   
   T   
   T   
   A   
   A   
   T   
   T   
   C   
   A   
   G   
   A   
   T   
   A   
   A   
   T   
   A   
   T   
   T   
   T   
   A   
   T   
   T   
   T   
   T   
   G   
   C   
   C   
   C   
   A   
   A   
   C   
   A   
   A   
   A   
   C   
   A   
   T   
   T   
   A   
   C   
   G   
   G   
   A   
   T   
   G   
   G   
   G   
   A   
   G   
   A   
   A   
   G   
   A   
   C   
   A   
   A   
   A   
   T   
   T   
   T   
   A   
   G   
   A   
   G   
   C   
   G   
   G   
   G   
   T   
   C   
   T   
   T   
   A   
   A   
   T   
   A   
   A   
   A   
   T   
   T   
   C   
   A   
   T   
   T   
   T   
   T   
   C   
   G   
   G   
   T   
   G   
   A   
   G   
   G   
   A   
   A   
   C   
   T   
   A   
   G   
   A   
   T   
   T   
   T   
   A   
   C   
   C   
   A   
   A   
   T   
   T   
   G   
   C   
   C   
   A   
   T   
   G   
   A   
   C   
   G   
   C   
   C   
   T   
   G   
   C   
   T   
   C   
   A   
   A   
   A   
   T   
   T   
   T   
   C   
   A   
   A   
   A   
   T   
   G   
   A   
   A   
   G   
   A   
   T   
   A   
   A   
   A   
   T   
   T   
   T   
   A   
   A   
   T   
   T   
   C   
   T   
   G   
   A   
   A   
   A   
   T   
   T   
   C   
   T   
   T   
   T   
   T   
   G   
   G   
   C   
   A   
   G   
   A   
   T   
   A   
   C   
   G   
   G   
   G   
   C   
   T   
   A   
   C   
   G   
   G   
   C   
   C   
   A   
   A   
   G   
   G   
   C   
   C   
   A   
   A   
   T   
   T   
   G   
   T   
   T   
   A   
   A   
   T   
   T   
   T   
   C   
   G   
   C   
   C   
   A   
   A   
   T   
   C   
   C   
   A   
   G   
   C   
   A   
   A   
   G   
   C   
   T   
   A   
   C   
   C   
   A   
   T   
   G   
   T   
   A   
   T   
   A   
   G   
   T   
   G   
   T   
   T   
   T   
   T   
   C   
   C   
   A   
   A   
   A   
   A   
   T   
   A   
   A   
   T   
   G   
   G   
   G   
   A   
   C   
   A   
   T   
   T   
   A   
   G   
   T   
   C   
   T   
   A   
   T   
   C   
   C   
   A   
   A   
   A   
   A   
   C   
   T   
   A   
   G   
   T   
   T   
   T   
   T   
   A   
   G   
   A   
   C   
   A   
   A   
   A   
   G   
   A   
   A   
   A   
   C   
   G   
   A   
   A   
   G   
   A   
   A   
   A   
   A   
   A   
   A   
   G   
   A   
   C   
   A   
   A   
   T   
   G   
   T   
   A   
   A   
   T   
   C   
   A   
   G   
   C   
   G   
   C   
   C   
   A   
   A   
   C   
   G   
   C   
   A   
   G   
   C   
   G   
   A   
   A   
   T   
   A   
   C   
   A   
   A   
   T   
   T   
   G   
   C   
   C   
   A   
   C   
   A   
   G   
   A   
   T   
   C   
   T   
   T   
   T   
   T   
   A   
   G   
   G   
   T   
   A   
   G   
   T   
   G   
   T   
   G   
   G   
   A   
   A   
   G   
   A   
   T   
   C   
   C   
   T   
   T   
   C   
   A   
   G   
   G   
   T   
   T   
   A   
   T   
   G   
   T   
   T   
   T   
   A   
   T   
   A   
   A   
   T   
   A   
   T   
   G   
   T   
   A   
   C   
   A   
   A   
   T   
   C   
   C   
   G   
   A   
   A   
   C   
   T   
   T   
   C   
   T   
   C   
   A   
   T   
   T   
   G   
   G   
   C   
   T   
   G   
   C   
   T   
   A   
   A   
   A   
   A   
   C   
   A   
   G   
   G   
   A   
   A   
   C   
   A   
   G   
   C   
   T   
   G   
   A   
   A   
   A   
   T   
   A   
   A   
   A   
   A   
   G   
   A   
   T   
   A   
   A   
   A   
   C   
   A   
   A   
   G   
   A   
   T   
   A   
   C   
   A   
   G   
   A   
   T   
   G   
   G   
   C   
   A   
   A   
   A   
   G   
   A   
   A   
   A   
   A   
   C   
   A   
   G   
   T   
   T   
   T   
   C   
   C   
   T   
   T   
   C   
   T   
   A   
   A   
   C   
   G   
   T   
   T   
   A   
   G   
   A   
   T   
   C   
   G   
   T   
   A   
   G   
   T   
   A   
   A   
   C   
   A   
   A   
   T   
   A   
   A   
   A   
   T   
   T   
   C   
   T   
   T   
   A   
   A   
   C   
   A   
   A   
   T   
   G   
   A   
   T   
   T   
   A   
   T   
   G   
   G   
   T   
   T   
   G   
   A   
   A   
   A   
   A   
   T   
   T   
   C   
   A   
   G   
   G   
   A   
   G   
   A   
   A   
   A   
   A   
   T   
   G   
   G   
   T   
   T   
   C   
   T   
   G   
   C   
   A   
   A   
   C   
   A   
   G   
   A   
   T   
   A   
   T   
   C   
   A   
   G   
   T   
   A   
   A   
   A   
   C   
   C   
   A   
   T   
   T   
   A   
   A   
   T   
   T   
   G   
   A   
   T   
   T   
   A   
   C   
   T   
   T   
   A   
   G   
   A   
   A   
   G   
   C   
   A   
   A   
   C   
   C   
   A   
   T   
   T   
   A   
   A   
   A   
   -   
   -   
   -   
   -   
   -   
   -   
   -   
   -   
   -   
   -   
   -   
   -   
   -   
   -   
   -   
   -   
   -   
   -   
   -   
   -   
   -   
   -   
   -   
   -   
   -   
   -   
   -   
   -   
   -   
   -   
   -   
   -   
   -   
   -   
   -   
   -   
   -   
   -   
   -   
   -   
   -   
   -   
   -   
   -   
   -   
   -   
   -   
   -   
   -   
   -   
   -   
   -   
   -   
   -   
   -   
   -   
   -   
   -   
   -   
   -   
   -   
 

 
   N52734   
   -   
   -   
   -   
   -   
   -   
   -   
   -   
   -   
   -   
   -   
   -   
   -   
   -   
   -   
   -   
   -   
   -   
   -   
   -   
   -   
   -   
   -   
   -   
   -   
   -   
   -   
   -   
   -   
   -   
   -   
   -   
   -   
   -   
   -   
   -   
   -   
   -   
   -   
   -   
   -   
   -   
   -   
   -   
   -   
   -   
   -   
   -   
   -   
   -   
   -   
   -   
   -   
   -   
   -   
   -   
   -   
   -   
   -   
   -   
   -   
   -   
   -   
   -   
   -   
   -   
   -   
   -   
   -   
   -   
   -   
   -   
   -   
   -   
   -   
   -   
   -   
   -   
   -   
   -   
   -   
   -   
   -   
   -   
   -   
   -   
   -   
   -   
   -   
   -   
   -   
   -   
   -   
   -   
   -   
   -   
   -   
   -   
   -   
   -   
   -   
   -   
   -   
   -   
   -   
   -   
   -   
   -   
   -   
   C   
   A   
   A   
   T   
   G   
   G   
   C   
   A   
   A   
   G   
   C   
   C   
   A   
   A   
   A   
   C   
   A   
   A   
   G   
   A   
    A    
   T   
   T   
   A   
   G   
   C   
    C    
   G   
   A   
   A   
   G   
   C   
   G   
   A   
   A   
   G   
   A   
   A   
   A   
   A   
    C    
   A   
   G   
   C   
    T    
   A   
    C    
   T   
    A    
    C    
   A   
   T   
   T   
   T   
   T   
   T   
   A   
   A   
   A   
    C    
   G   
   T   
   A   
   T   
   T   
   G   
    T    
   C   
   A   
   A   
   A   
   A   
   C   
   A   
   G   
    G    
   A   
   A   
   T   
   T   
   T   
   G   
   A   
   T   
   A   
   A   
   G   
   T   
   T   
   A   
   C   
   C   
    G    
   T   
   C   
   C   
   G   
   T   
   T   
    G    
   T   
   T   
   C   
   A   
   A   
   G   
   A   
   A   
    G    
   C   
   T   
   A   
   G   
   C   
   T   
   T   
   A   
   A   
   A   
   G   
   A   
   A   
   A   
   A   
   A   
   T   
   G   
   G   
    C    
   T   
   A   
   T   
   G   
   A   
   T   
   A   
   C   
   T   
   A   
   A   
   A   
   T   
   C   
    T    
   G   
   T   
   T   
   G   
   T   
   T   
   G   
   A   
   A   
   A   
   A   
   A   
   T   
   A   
   C   
   C   
   A   
   A   
   G   
   C   
   A   
   A   
   T   
   T   
   T   
   A   
   T   
   T   
   C   
   A   
   G   
   G   
   G   
   A   
   T   
   T   
   C   
   A   
   A   
   G   
   C   
    A    
   G   
   A   
   A   
    G    
   G   
    A    
   G   
   T   
   C   
   A   
   A   
    A    
   G   
   C   
   T   
   A   
   G   
   T   
   G   
   A   
   T   
   G   
   T   
    T    
   C   
   A   
   A   
   G   
   T   
    C    
   A   
   A   
   A   
   A   
   A   
    G    
   G   
    C    
   G   
   A   
   A   
   A   
   G   
   A   
    C    
   A   
   A   
    T    
   C   
   A   
   A   
   T   
   A   
   C   
   A   
   C   
   A   
   T   
   T   
   T   
   A   
    C    
   C   
   T   
   A   
   T   
   A   
   A   
   A   
   T   
   T   
   A   
   T   
   C   
   G   
   A   
   T   
   G   
   A   
   G   
    T    
   A   
   C   
    C    
   C   
   C   
   T   
   T   
   T   
   A   
   G   
   G   
   C   
   G   
   A   
   A   
   A   
   T   
   G   
   A   
   A   
   A   
   G   
   A   
   T   
    T    
   T   
   G   
   T   
   C   
   T   
   T   
   A   
   T   
   C   
   A   
   A   
   T   
   C   
   A   
   A   
   G   
   T   
   A   
   T   
    C    
   G   
    C    
   C   
   A   
   A   
    A    
   A   
   A   
   A   
   G   
   G   
    C    
   G   
   A   
   T   
   A   
    C    
   C   
   T   
   A   
   C   
   C   
   A   
   A   
   A   
   T   
    C    
    G    
   C   
   T   
   T   
   G   
   G   
    A    
    A    
    G    
   C   
   C   
    G    
   T   
   C   
   T   
   T   
   T   
   A   
   A   
   T   
   T   
   T   
   T   
   T   
   C   
   C   
   A   
   G   
   A   
   T   
   A   
   T   
   G   
   T   
   C   
   A   
   G   
   G   
   A   
   A   
   A   
   T   
   G   
   A   
   T   
   A   
   A   
   A   
    A    
   T   
   T   
   T   
   C   
   G   
   A   
   T   
   T   
   C   
   A   
   A   
   G   
   T   
   A   
   G   
   A   
   T   
   A   
   A   
   T   
   G   
   C   
   C   
   A   
   A   
   A   
   C   
   G   
   T   
   G   
   G   
   A   
   G   
   A   
   A   
   A   
   T   
   T   
   G   
   T   
   C   
   G   
   A   
   T   
   C   
   G   
   T   
   A   
   A   
    T    
   G   
   G   
   T   
   A   
   G   
   T   
   G   
   G   
   G   
   C   
   T   
   A   
   G   
    C    
   A   
   A   
   T   
   T   
   A   
   A   
   C   
   A   
   A   
   A   
   G   
   T   
   G   
   T   
   T   
   T   
   G   
   A   
    C    
   G   
   A   
   A   
   G   
   T   
   G   
   G   
   G   
   C   
   G   
   T   
   A   
   G   
   T   
    G    
   C   
   C   
   T   
    G    
   G   
   C   
   A   
   A   
   A   
   C   
   T   
    C    
   G   
   G   
   T   
   T   
   C   
   T   
   G   
   G   
   C   
   G   
   C   
   A   
   G   
   A   
   A   
   A   
   A   
   A   
    A    
   C   
   A   
   G   
   C   
    C    
   A   
   A   
   T   
   A   
   T   
   C   
   A   
   A   
   A   
   G   
   C   
   T   
   T   
   T   
   T   
   A   
   G   
   T   
   G   
   A   
   T   
   A   
   A   
    G    
   T   
   T   
    C    
   G   
   G   
   C   
   G   
   T   
   T   
   T   
   C   
   T   
    A    
   T   
   T   
   G   
   A   
   T   
   G   
   A   
   A   
   A   
   T   
   C   
   A   
   A   
   T   
   C   
    A    
   A   
   A   
   A   
    G    
   T   
   T   
   A   
   A   
   G   
   C   
   C   
   A   
   A   
   G   
   G   
    A    
   T   
   G   
   G   
   G   
   T   
   C   
   C   
   A   
   A   
   G   
   C   
   A   
   G   
   A   
   C   
   T   
   C   
    C    
   T   
   T   
   T   
   G   
   T   
   A   
   C   
   C   
   A   
   A   
   T   
   C   
   A   
   C   
   A   
   G   
   T   
    C    
   G   
   C   
   T   
   T   
   C   
   T   
   G   
   A   
   A   
   C   
   C   
   A   
   G   
   T   
   G   
   A   
   C   
   A   
   G   
   A   
   A   
   T   
   T   
   A   
   C   
   C   
   A   
   A   
   C   
   A   
   G   
   G   
   G   
   G   
   C   
   T   
   G   
   C   
   G   
   A   
   C   
   A   
   A   
   A   
   A   
   G   
    A    
    T    
   A   
   C   
   A   
   G   
   A   
   G   
   T   
   C   
   A   
   C   
   G   
   T   
   T   
   A   
   T   
   T   
   A   
   T   
   C   
   C   
    G    
    C    
   T   
   G   
   G   
   G   
   G   
   G   
   A   
   A   
   G   
   C   
   A   
   G   
   C   
   T   
   G   
   C   
   G   
   C   
   A   
   A   
   T   
   T   
   A   
   A   
   T   
   C   
   G   
   G   
    G    
   T   
   A   
   T   
   A   
   C   
    G    
   G   
   G   
    C    
   A   
   C   
    C    
   A   
   T   
   T   
   A   
   C   
   T   
   G   
   C   
   A   
   G   
   A   
    A    
   G   
   A   
   T   
   A   
   T   
   T   
    G    
   A   
   A   
   A   
   A   
   A   
   A   
   A   
   T   
   C   
   C   
   A   
   G   
   A   
   G   
   T   
   T   
   A   
   A   
   G   
   T   
   A   
   G   
   T   
   A   
   C   
   G   
   G   
   G   
    C    
   G   
   T   
   A   
   A   
   T   
   T   
   G   
   G   
    T    
   A   
   A   
   A   
   A   
   C   
   T   
   G   
   G   
    C    
   T   
   T   
   A   
   G   
   A   
   A   
   C   
   G   
   A   
   G   
   C   
   G   
   T   
   T   
   T   
   G   
   A   
   T   
   A   
   A   
   A   
   G   
   A   
    A    
   T   
   T   
   A   
   A   
   G   
   A   
   G   
   G   
    G    
   C   
   A   
   A   
   G   
   A   
   T   
   G   
   G   
    T    
   G   
   G   
    C    
   T   
   C   
    A    
   C   
   T   
   A   
   G   
   T   
   T   
   A   
   T   
   T   
   T   
   T   
   A   
   G   
   A   
   T   
   G   
   A   
   C   
   A   
   A   
   A   
   G   
   A   
   G   
   A   
   A   
   T   
   G   
   T   
   C   
   A   
   A   
   A   
   A   
   A   
   A   
   G   
   C   
   T   
   T   
   T   
   A   
   C   
   A   
   A   
   A   
   C   
   C   
   A   
   A   
   A   
   G   
   A   
    G    
   A   
   A   
   A   
   A   
   A   
   A   
   G   
   A   
   T   
   G   
   G   
   T   
   C   
   A   
   A   
   A   
   C   
    G    
   A   
   T   
   T   
   A   
   A   
   A   
   T   
   T   
   A   
   A   
   C   
   G   
   A   
   T   
   T   
   G   
   A   
    C    
   A   
   G   
    T    
   G   
   G   
    C    
   G   
   T   
   A   
   C   
   A   
   A   
   C   
   A   
   A   
   C   
   A   
   A   
   G   
   C   
    C    
   T   
   T   
    C    
   G   
   C   
   T   
   A   
   T   
   A   
   T   
   T   
   T   
   G   
   A   
   C   
   A   
   A   
   A   
   C   
   G   
    T    
    C    
   C   
   T   
   G   
   G   
   T   
   A   
   G   
   T   
   G   
   C   
   G   
   G   
   T   
   C   
   A   
   T   
   T   
   A   
   C   
   C   
   G   
   A   
   T   
   C   
   C   
   T   
   C   
   A   
   A   
   A   
   A   
   A   
   G   
   G   
   C   
   G   
   A   
   T   
   T   
   T   
   A   
    T    
   T   
   A   
   G   
   C   
    A    
   A   
   C   
   G   
   G   
   T   
   T   
   A   
   G   
   T   
   T   
   C   
   A   
   C   
   C   
   T   
   T   
   C   
   G   
   T   
   A   
   T   
   G   
   A   
   T   
   C   
   C   
   T   
   A   
   A   
   T   
   A   
   A   
   A   
   A   
   T   
   G   
   G   
   C   
   C   
   A   
   A   
   T   
   G   
   G   
    T    
   A   
   T   
   T   
   T   
   C   
   T   
   C   
   A   
   A   
   A   
   A   
   A   
   G   
   A   
    A    
   T   
   A   
   T   
   G   
   A   
   T   
   G   
   C   
   T   
   T   
   A   
   C   
   A   
   A   
   T   
   A   
   A   
   C   
   A   
   A   
   C   
   A   
   A   
   A   
   G   
   A   
   T   
   T   
   T   
   A   
   C   
   C   
   A   
   T   
   T   
    C    
   A   
   C   
   A   
   G   
   C   
   A   
   C   
   G   
   T   
   T   
   T   
    T    
   G   
   C   
   G   
   A   
   C   
   A   
   G   
   G   
    C    
   T   
   A   
   T   
   G   
   C   
   A   
   C   
   C   
   A   
   G   
   G   
    C    
   T   
   C   
   T   
   A   
   C   
   C   
   T   
   T   
   T   
   A   
   A   
   A   
   A   
   C   
   A   
   A   
   T   
   T   
   A   
   C   
   C   
   G   
   G   
   T   
   G   
   C   
   C   
   A   
   T   
   T   
   G   
   G   
   T   
   T   
   T   
   A   
   G   
   A   
   T   
    G    
   C   
   A   
   G   
   G   
   G   
   A   
   C   
   A   
   T   
   T   
   G   
   A   
   A   
   G   
   C   
   C   
   A   
   G   
   A   
   T   
   G   
   A   
   A   
   G   
   A   
   G   
   C   
   T   
   A   
   G   
   A   
   A   
   A   
   T   
   T   
   A   
   A   
   T   
   G   
   G   
    C    
   T   
   T   
   G   
   A   
   A   
   A   
   T   
   G   
   G   
   C   
   A   
   A   
   A   
   A   
   A   
   G   
   A   
   T   
   A   
   A   
   A   
   T   
   C   
   T   
   T   
   G   
   G   
   G   
   G   
    C    
   G   
   G   
   C   
   T   
   A   
   T   
   T   
   T   
   T   
   G   
   C   
    G    
   A   
   C   
   A   
   C   
   G   
   T   
   G   
   T   
   A   
   A   
   A   
   A   
   G   
   A   
   A   
   G   
   C   
   A   
   A   
   G   
   T   
   C   
   C   
   A   
   G   
   T   
   C   
   A   
   A   
   T   
   C   
   T   
    G    
   C   
   G   
   T   
   A   
   C   
   C   
   G   
   C   
   T   
   T   
   T   
   A   
   G   
   T   
   T   
   A   
   A   
   T   
   T   
   C   
   A   
   G   
   A   
   T   
   A   
   A   
   T   
   A   
   T   
   T   
   T   
   A   
   T   
   T   
   T   
   T   
   G   
   C   
    T    
   C   
   A   
   A   
   C   
   A   
   A   
   A   
   C   
   A   
   T   
   T   
   A   
   C   
   G   
    G    
   A   
   T   
   G   
   G   
   G   
    A    
   G   
   A   
   A   
   G   
   A   
   C   
   A   
   A   
   A   
   T   
   T   
   T   
   A   
   G   
   A   
   G   
   C   
    G    
   G   
   G   
   T   
   C   
   T   
   T   
   A   
   A   
   T   
   A   
   A   
   A   
   T   
   T   
   C   
   A   
   T   
   T   
   T   
   T   
   C   
   G   
   G   
   T   
   G   
   A   
   G   
   G   
   A   
   A   
   C   
   T   
   A   
   G   
   A   
   T   
   T   
   T   
   A   
   C   
   C   
   A   
   A   
   T   
    T    
   G   
   C   
   C   
   A   
   T   
   G   
   A   
   C   
   G   
   C   
   C   
    T    
   G   
   C   
   T   
   C   
   A   
   A   
   A   
   T   
   T   
   T   
   C   
   A   
   A   
   A   
    T    
   G   
   A   
   A   
   G   
   A   
   T   
   A   
   A   
   A   
   T   
   T   
   T   
   A   
   A   
   T   
   T   
   C   
   T   
   G   
   A   
   A   
   A   
   T   
   T   
   C   
   T   
   T   
   T   
   T   
    A    
   G   
   C   
   A   
   G   
   A   
   T   
   A   
   C   
    G    
   G   
   G   
   C   
   T   
   A   
    C    
   G   
   G   
    T    
   C   
   A   
   A   
   G   
   G   
    C    
   C   
   A   
   A   
   T   
   T   
   G   
   T   
   T   
   A   
   A   
   T   
   T   
   T   
   C   
    G    
   C   
   C   
   A   
   A   
   T   
   C   
   C   
   A   
   G   
   C   
   A   
   A   
   G   
   C   
   T   
   A   
   C   
   C   
   A   
   T   
   G   
   T   
   A   
   T   
   A   
   G   
   T   
   G   
   T   
   T   
   T   
   T   
   C   
   C   
   A   
   A   
   A   
   A   
   T   
   A   
   A   
   T   
   G   
   G   
    A    
   A   
   C   
   A   
    T    
   T   
    G    
   G   
   T   
    C    
   T   
   A   
   T   
   C   
   C   
   A   
   A   
   A   
   A   
    A    
   T   
   A   
   G   
   T   
   T   
   T   
   T   
   A   
   G   
   A   
   -   
   A   
   A   
   A   
   G   
   A   
   A   
   A   
   C   
    G    
   A   
   A   
   G   
   A   
   A   
   A   
   A   
   A   
   A   
   G   
   -   
   -   
   -   
   -   
   -   
   -   
   -   
   -   
   -   
   -   
   -   
   -   
   -   
   -   
   -   
   -   
   -   
   -   
   -   
   -   
   -   
   -   
   -   
   -   
   -   
   -   
   -   
   -   
   -   
   -   
   -   
   -   
   -   
   -   
   -   
   -   
   -   
   -   
   -   
   -   
   -   
   -   
   -   
   -   
   -   
   -   
   -   
   -   
   -   
   -   
   -   
   -   
   -   
   -   
   -   
   -   
   -   
   -   
   -   
   -   
   -   
   -   
   -   
   -   
   -   
   -   
   -   
   -   
   -   
   -   
   -   
   -   
   -   
   -   
   -   
   -   
   -   
   -   
   -   
   -   
   -   
   -   
   -   
   -   
   -   
   -   
   -   
   -   
   -   
   -   
   -   
   -   
   -   
   -   
   -   
   -   
   -   
   -   
   -   
   -   
   -   
   -   
   -   
   -   
   -   
   -   
   -   
   -   
   -   
   -   
   -   
   -   
   -   
   -   
   -   
   -   
   -   
   -   
   -   
   -   
   -   
   -   
   -   
   -   
   -   
   -   
   -   
   -   
   -   
   -   
   -   
   -   
   -   
   -   
   -   
   -   
   -   
   -   
   -   
   -   
   -   
   -   
   -   
   -   
   -   
   -   
   -   
   -   
   -   
   -   
   -   
   -   
   -   
   -   
   -   
   -   
   -   
   -   
   -   
   -   
   -   
   -   
   -   
   -   
   -   
   -   
   -   
   -   
   -   
   -   
   -   
   -   
   -   
   -   
   -   
   -   
   -   
   -   
   -   
   -   
   -   
   -   
   -   
   -   
   -   
   -   
   -   
   -   
   -   
   -   
   -   
   -   
   -   
   -   
   -   
   -   
   -   
   -   
   -   
   -   
   -   
   -   
   -   
   -   
   -   
   -   
   -   
   -   
   -   
   -   
   -   
   -   
   -   
   -   
   -   
   -   
   -   
   -   
   -   
   -   
   -   
   -   
   -   
   -   
   -   
   -   
   -   
   -   
   -   
   -   
   -   
   -   
   -   
   -   
   -   
   -   
   -   
   -   
   -   
   -   
   -   
   -   
   -   
   -   
   -   
   -   
   -   
   -   
   -   
   -   
   -   
   -   
   -   
   -   
   -   
   -   
   -   
   -   
   -   
   -   
   -   
   -   
   -   
   -   
   -   
   -   
   -   
   -   
   -   
   -   
   -   
   -   
   -   
   -   
   -   
   -   
   -   
   -   
   -   
   -   
   -   
   -   
   -   
   -   
   -   
   -   
   -   
   -   
   -   
   -   
   -   
   -   
   -   
   -   
   -   
   -   
   -   
   -   
   -   
   -   
   -   
   -   
   -   
   -   
   -   
   -   
   -   
   -   
   -   
   -   
   -   
   -   
   -   
   -   
   -   
   -   
   -   
   -   
   -   
   -   
   -   
   -   
   -   
   -   
   -   
   -   
   -   
   -   
   -   
   -   
   -   
   -   
   -   
   -   
   -   
   -   
   -   
   -   
   -   
   -   
   -   
   -   
   -   
   -   
   -   
   -   
   -   
   -   
   -   
   -   
   -   
   -   
   -   
   -   
   -   
   -   
   -   
   -   
   -   
   -   
   -   
   -   
   -   
   -   
   -   
   -   
   -   
   -   
   -   
   -   
   -   
   -   
   -   
   -   
   -   
   -   
   -   
   -   
   -   
   -   
   -   
   -   
   -   
   -   
   -   
   -   
   -   
   -   
   -   
   -   
   -   
   -   
   -   
   -   
   -   
   -   
   -   
   -   
   -   
   -   
   -   
   -   
   -   
   -   
   -   
   -   
   -   
   -   
   -   
   -   
   -   
   -   
   -   
   -   
   -   
   -   
   -   
   -   
   -   
   -   
   -   
   -   
   -   
   -   
   -   
   -   
   -   
   -   
   -   
   -   
   -   
   -   
   -   
   -   
   -   
   -   
   -   
   -   
   -   
   -   
   -   
   -   
   -   
   -   
   -   
   -   
   -   
   -   
   -   
   -   
   -   
   -   
   -   
   -   
   -   
   -   
   -   
   -   
   -   
   -   
   -   
   -   
   -   
   -   
   -   
   -   
   -   
   -   
   -   
   -   
   -   
   C   
   A   
   A   
   T   
   G   
   G   
   C   
   A   
   A   
   G   
   C   
   C   
   A   
   A   
   A   
   C   
   A   
   A   
   G   
   A   
   A   
   T   
   T   
   A   
   G   
   C   
   C   
   G   
   A   
   A   
   G   
   C   
   G   
   A   
   A   
   G   
   A   
   A   
   A   
   A   
   C   
   A   
   G   
   C   
   T   
   A   
   C   
   T   
   A   
   C   
   A   
   T   
   T   
   T   
   T   
   T   
   A   
   A   
   A   
   C   
   G   
   T   
   A   
   T   
   T   
   G   
   T   
   C   
   A   
   A   
   A   
   A   
   C   
   A   
   G   
   G   
   A   
   A   
   T   
   T   
   T   
   G   
   A   
   T   
   A   
   A   
   G   
   T   
   T   
   A   
   C   
   C   
   G   
   T   
   C   
   C   
   G   
   T   
   T   
   G   
   T   
   T   
   C   
   A   
   A   
   G   
   A   
   A   
   G   
   C   
   T   
   A   
   G   
   C   
   T   
   T   
   A   
   A   
   A   
   G   
   A   
   A   
   A   
   A   
   A   
   T   
   G   
   G   
   C   
   T   
   A   
   T   
   G   
   A   
   T   
   A   
   C   
   T   
   A   
   A   
   A   
   T   
   C   
   T   
   G   
   T   
   T   
   G   
   T   
   T   
   G   
   A   
   A   
   A   
   A   
   A   
   T   
   A   
   C   
   C   
   A   
   A   
   G   
   C   
   A   
   A   
   T   
   T   
   T   
   A   
   T   
   T   
   C   
   A   
   G   
   G   
   G   
   A   
   T   
   T   
   C   
   A   
   A   
   G   
   C   
   A   
   G   
   A   
   A   
   G   
   G   
   A   
   G   
   T   
   C   
   A   
   A   
   A   
   G   
   C   
   T   
   A   
   G   
   T   
   G   
   A   
   T   
   G   
   T   
   T   
   C   
   A   
   A   
   G   
   T   
   C   
   A   
   A   
   A   
   A   
   A   
   G   
   G   
   C   
   G   
   A   
   A   
   A   
   G   
   A   
   C   
   A   
   A   
   T   
   C   
   A   
   A   
   T   
   A   
   C   
   A   
   C   
   A   
   T   
   T   
   T   
   A   
   C   
   C   
   T   
   A   
   T   
   A   
   A   
   A   
   T   
   T   
   A   
   T   
   C   
   G   
   A   
   T   
   G   
   A   
   G   
   T   
   A   
   C   
   C   
   C   
   C   
   T   
   T   
   T   
   A   
   G   
   G   
   C   
   G   
   A   
   A   
   A   
   T   
   G   
   A   
   A   
   A   
   G   
   A   
   T   
   T   
   T   
   G   
   T   
   C   
   T   
   T   
   A   
   T   
   C   
   A   
   A   
   T   
   C   
   A   
   A   
   G   
   T   
   A   
   T   
   C   
   G   
   C   
   C   
   A   
   A   
   A   
   A   
   A   
   A   
   G   
   G   
   C   
   G   
   A   
   T   
   A   
   C   
   C   
   T   
   A   
   C   
   C   
   A   
   A   
   A   
   T   
   C   
   G   
   C   
   T   
   T   
   G   
   G   
   A   
   A   
   G   
   C   
   C   
   G   
   T   
   C   
   T   
   T   
   T   
   A   
   A   
   T   
   T   
   T   
   T   
   T   
   C   
   C   
   A   
   G   
   A   
   T   
   A   
   T   
   G   
   T   
   C   
   A   
   G   
   G   
   A   
   A   
   A   
   T   
   G   
   A   
   T   
   A   
   A   
   A   
   A   
   T   
   T   
   T   
   C   
   G   
   A   
   T   
   T   
   C   
   A   
   A   
   G   
   T   
   A   
   G   
   A   
   T   
   A   
   A   
   T   
   G   
   C   
   C   
   A   
   A   
   A   
   C   
   G   
   T   
   G   
   G   
   A   
   G   
   A   
   A   
   A   
   T   
   T   
   G   
   T   
   C   
   G   
   A   
   T   
   C   
   G   
   T   
   A   
   A   
   T   
   G   
   G   
   T   
   A   
   G   
   T   
   G   
   G   
   G   
   C   
   T   
   A   
   G   
   C   
   A   
   A   
   T   
   T   
   A   
   A   
   C   
   A   
   A   
   A   
   G   
   T   
   G   
   T   
   T   
   T   
   G   
   A   
   C   
   G   
   A   
   A   
   G   
   T   
   G   
   G   
   G   
   C   
   G   
   T   
   A   
   G   
   T   
   G   
   C   
   C   
   T   
   G   
   G   
   C   
   A   
   A   
   A   
   C   
   T   
   C   
   G   
   G   
   T   
   T   
   C   
   T   
   G   
   G   
   C   
   G   
   C   
   A   
   G   
   A   
   A   
   A   
   A   
   A   
   A   
   C   
   A   
   G   
   C   
   C   
   A   
   A   
   T   
   A   
   T   
   C   
   A   
   A   
   A   
   G   
   C   
   T   
   T   
   T   
   T   
   A   
   G   
   T   
   G   
   A   
   T   
   A   
   A   
   G   
   T   
   T   
   C   
   G   
   G   
   C   
   G   
   T   
   T   
   T   
   C   
   T   
   A   
   T   
   T   
   G   
   A   
   T   
   G   
   A   
   A   
   A   
   T   
   C   
   A   
   A   
   T   
   C   
   A   
   A   
   A   
   A   
   G   
   T   
   T   
   A   
   A   
   G   
   C   
   C   
   A   
   A   
   G   
   G   
   A   
   T   
   G   
   G   
   G   
   T   
   C   
   C   
   A   
   A   
   G   
   C   
   A   
   G   
   A   
   C   
   T   
   C   
   C   
   T   
   T   
   T   
   G   
   T   
   A   
   C   
   C   
   A   
   A   
   T   
   C   
   A   
   C   
   A   
   G   
   T   
   C   
   G   
   C   
   T   
   T   
   C   
   T   
   G   
   A   
   A   
   C   
   C   
   A   
   G   
   T   
   G   
   A   
   C   
   A   
   G   
   A   
   A   
   T   
   T   
   A   
   C   
   C   
   A   
   A   
   C   
   A   
   G   
   G   
   G   
   G   
   C   
   T   
   G   
   C   
   G   
   A   
   C   
   A   
   A   
   A   
   A   
   G   
   A   
   T   
   A   
   C   
   A   
   G   
   A   
   G   
   T   
   C   
   A   
   C   
   G   
   T   
   T   
   A   
   T   
   T   
   A   
   T   
   C   
   C   
   G   
   C   
   T   
   G   
   G   
   G   
   G   
   G   
   A   
   A   
   G   
   C   
   A   
   G   
   C   
   T   
   G   
   C   
   G   
   C   
   A   
   A   
   T   
   T   
   A   
   A   
   T   
   C   
   G   
   G   
   G   
   T   
   A   
   T   
   A   
   C   
   G   
   G   
   G   
   C   
   A   
   C   
   C   
   A   
   T   
   T   
   A   
   C   
   T   
   G   
   C   
   A   
   G   
   A   
   A   
   G   
   A   
   T   
   A   
   T   
   T   
   G   
   A   
   A   
   A   
   A   
   A   
   A   
   A   
   T   
   C   
   C   
   A   
   G   
   A   
   G   
   T   
   T   
   A   
   A   
   G   
   T   
   A   
   G   
   T   
   A   
   C   
   G   
   G   
   G   
   C   
   G   
   T   
   A   
   A   
   T   
   T   
   G   
   G   
   T   
   A   
   A   
   A   
   A   
   C   
   T   
   G   
   G   
   C   
   T   
   T   
   A   
   G   
   A   
   A   
   C   
   G   
   A   
   G   
   C   
   G   
   T   
   T   
   T   
   G   
   A   
   T   
   A   
   A   
   A   
   G   
   A   
   A   
   T   
   T   
   A   
   A   
   G   
   A   
   G   
   G   
   G   
   C   
   A   
   A   
   G   
   A   
   T   
   G   
   G   
   T   
   G   
   G   
   C   
   T   
   C   
   A   
   C   
   T   
   A   
   G   
   T   
   T   
   A   
   T   
   T   
   T   
   T   
   A   
   G   
   A   
   T   
   G   
   A   
   C   
   A   
   A   
   A   
   G   
   A   
   G   
   A   
   A   
   T   
   G   
   T   
   C   
   A   
   A   
   A   
   A   
   A   
   A   
   G   
   C   
   T   
   T   
   T   
   A   
   C   
   A   
   A   
   A   
   C   
   C   
   A   
   A   
   A   
   G   
   A   
   G   
   A   
   A   
   A   
   A   
   A   
   A   
   G   
   A   
   T   
   G   
   G   
   T   
   C   
   A   
   A   
   A   
   C   
   G   
   A   
   T   
   T   
   A   
   A   
   A   
   T   
   T   
   A   
   A   
   C   
   G   
   A   
   T   
   T   
   G   
   A   
   C   
   A   
   G   
   T   
   G   
   G   
   C   
   G   
   T   
   A   
   C   
   A   
   A   
   C   
   A   
   A   
   C   
   A   
   A   
   G   
   C   
   C   
   T   
   T   
   C   
   G   
   C   
   T   
   A   
   T   
   A   
   T   
   T   
   T   
   G   
   A   
   C   
   A   
   A   
   A   
   C   
   G   
   T   
   C   
   C   
   T   
   G   
   G   
   T   
   A   
   G   
   T   
   G   
   C   
   G   
   G   
   T   
   C   
   A   
   T   
   T   
   A   
   C   
   C   
   G   
   A   
   T   
   C   
   C   
   T   
   C   
   A   
   A   
   A   
   A   
   A   
   G   
   G   
   C   
   G   
   A   
   T   
   T   
   T   
   A   
   T   
   T   
   A   
   G   
   C   
   A   
   A   
   C   
   G   
   G   
   T   
   T   
   A   
   G   
   T   
   T   
   C   
   A   
   C   
   C   
   T   
   T   
   C   
   G   
   T   
   A   
   T   
   G   
   A   
   T   
   C   
   C   
   T   
   A   
   A   
   T   
   A   
   A   
   A   
   A   
   T   
   G   
   G   
   C   
   C   
   A   
   A   
   T   
   G   
   G   
   T   
   A   
   T   
   T   
   T   
   C   
   T   
   C   
   A   
   A   
   A   
   A   
   A   
   G   
   A   
   A   
   T   
   A   
   T   
   G   
   A   
   T   
   G   
   C   
   T   
   T   
   A   
   C   
   A   
   A   
   T   
   A   
   A   
   C   
   A   
   A   
   C   
   A   
   A   
   A   
   G   
   A   
   T   
   T   
   T   
   A   
   C   
   C   
   A   
   T   
   T   
   C   
   A   
   C   
   A   
   G   
   C   
   A   
   C   
   G   
   T   
   T   
   T   
   T   
   G   
   C   
   G   
   A   
   C   
   A   
   G   
   G   
   C   
   T   
   A   
   T   
   G   
   C   
   A   
   C   
   C   
   A   
   G   
   G   
   C   
   T   
   C   
   T   
   A   
   C   
   C   
   T   
   T   
   T   
   A   
   A   
   A   
   A   
   C   
   A   
   A   
   T   
   T   
   A   
   C   
   C   
   G   
   G   
   T   
   G   
   C   
   C   
   A   
   T   
   T   
   G   
   G   
   T   
   T   
   T   
   A   
   G   
   A   
   T   
   G   
   C   
   A   
   G   
   G   
   G   
   A   
   C   
   A   
   T   
   T   
   G   
   A   
   A   
   G   
   C   
   C   
   A   
   G   
   A   
   T   
   G   
   A   
   A   
   G   
   A   
   G   
   C   
   T   
   A   
   G   
   A   
   A   
   A   
   T   
   T   
   A   
   A   
   T   
   G   
   G   
   C   
   T   
   T   
   G   
   A   
   A   
   A   
   T   
   G   
   G   
   C   
   A   
   A   
   A   
   A   
   A   
   G   
   A   
   T   
   A   
   A   
   A   
   T   
   C   
   T   
   T   
   G   
   G   
   G   
   G   
   C   
   G   
   G   
   C   
   T   
   A   
   T   
   T   
   T   
   T   
   G   
   C   
   G   
   A   
   C   
   A   
   C   
   G   
   T   
   G   
   T   
   A   
   A   
   A   
   A   
   G   
   A   
   A   
   G   
   C   
   A   
   A   
   G   
   T   
   C   
   C   
   A   
   G   
   T   
   C   
   A   
   A   
   T   
   C   
   T   
   G   
   C   
   G   
   T   
   A   
   C   
   C   
   G   
   C   
   T   
   T   
   T   
   A   
   G   
   T   
   T   
   A   
   A   
   T   
   T   
   C   
   A   
   G   
   A   
   T   
   A   
   A   
   T   
   A   
   T   
   T   
   T   
   A   
   T   
   T   
   T   
   T   
   G   
   C   
   T   
   C   
   A   
   A   
   C   
   A   
   A   
   A   
   C   
   A   
   T   
   T   
   A   
   C   
   G   
   G   
   A   
   T   
   G   
   G   
   G   
   A   
   G   
   A   
   A   
   G   
   A   
   C   
   A   
   A   
   A   
   T   
   T   
   T   
   A   
   G   
   A   
   G   
   C   
   G   
   G   
   G   
   T   
   C   
   T   
   T   
   A   
   A   
   T   
   A   
   A   
   A   
   T   
   T   
   C   
   A   
   T   
   T   
   T   
   T   
   C   
   G   
   G   
   T   
   G   
   A   
   G   
   G   
   A   
   A   
   C   
   T   
   A   
   G   
   A   
   T   
   T   
   T   
   A   
   C   
   C   
   A   
   A   
   T   
   T   
   G   
   C   
   C   
   A   
   T   
   G   
   A   
   C   
   G   
   C   
   C   
   T   
   G   
   C   
   T   
   C   
   A   
   A   
   A   
   T   
   T   
   T   
   C   
   A   
   A   
   A   
   T   
   G   
   A   
   A   
   G   
   A   
   T   
   A   
   A   
   A   
   T   
   T   
   T   
   A   
   A   
   T   
   T   
   C   
   T   
   G   
   A   
   A   
   A   
   T   
   T   
   C   
   T   
   T   
   T   
   T   
   A   
   G   
   C   
   A   
   G   
   A   
   T   
   A   
   C   
   G   
   G   
   G   
   C   
   T   
   A   
   C   
   G   
   G   
   T   
   C   
   A   
   A   
   G   
   G   
   C   
   C   
   A   
   A   
   T   
   T   
   G   
   T   
   T   
   A   
   A   
   T   
   T   
   T   
   C   
   G   
   C   
   C   
   A   
   A   
   T   
   C   
   C   
   A   
   G   
   C   
   A   
   A   
   G   
   C   
   T   
   A   
   C   
   C   
   A   
   T   
   G   
   T   
   A   
   T   
   A   
   G   
   T   
   G   
   T   
   T   
   T   
   T   
   C   
   C   
   A   
   A   
   A   
   A   
   T   
   A   
   A   
   T   
   G   
   G   
   A   
   A   
   C   
   A   
   T   
   T   
   G   
   G   
   T   
   C   
   T   
   A   
   T   
   C   
   C   
   A   
   A   
   A   
   A   
   A   
   T   
   A   
   G   
   T   
   T   
   T   
   T   
   A   
   G   
   A   
   -   
   A   
   A   
   A   
   G   
   A   
   A   
   A   
   C   
   G   
   A   
   A   
   G   
   A   
   A   
   A   
   A   
   A   
   A   
   G   
   -   
   -   
   -   
   -   
   -   
   -   
   -   
   -   
   -   
   -   
   -   
   -   
   -   
   -   
   -   
   -   
   -   
   -   
   -   
   -   
   -   
   -   
   -   
   -   
   -   
   -   
   -   
   -   
   -   
   -   
   -   
   -   
   -   
   -   
   -   
   -   
   -   
   -   
   -   
   -   
   -   
   -   
   -   
   -   
   -   
   -   
   -   
   -   
   -   
   -   
   -   
   -   
   -   
   -   
   -   
   -   
   -   
   -   
   -   
   -   
   -   
   -   
   -   
   -   
   -   
   -   
   -   
   -   
   -   
   -   
   -   
   -   
   -   
   -   
   -   
   -   
   -   
   -   
   -   
   -   
   -   
   -   
   -   
   -   
   -   
   -   
   -   
   -   
   -   
   -   
   -   
   -   
   -   
   -   
   -   
   -   
   -   
   -   
   -   
   -   
   -   
   -   
   -   
   -   
   -   
   -   
   -   
   -   
   -   
   -   
   -   
   -   
   -   
   -   
   -   
   -   
   -   
   -   
   -   
   -   
   -   
   -   
   -   
   -   
   -   
   -   
   -   
   -   
   -   
   -   
   -   
   -   
   -   
   -   
   -   
   -   
   -   
   -   
   -   
   -   
   -   
   -   
   -   
   -   
   -   
   -   
   -   
   -   
   -   
   -   
   -   
   -   
   -   
   -   
   -   
   -   
   -   
   -   
   -   
   -   
   -   
   -   
   -   
   -   
   -   
   -   
   -   
   -   
   -   
   -   
   -   
   -   
   -   
   -   
   -   
   -   
   -   
   -   
   -   
   -   
   -   
   -   
   -   
   -   
   -   
   -   
   -   
   -   
   -   
   -   
   -   
   -   
   -   
   -   
   -   
   -   
   -   
   -   
   -   
   -   
   -   
   -   
   -   
   -   
   -   
   -   
   -   
   -   
   -   
   -   
   -   
   -   
   -   
   -   
   -   
   -   
   -   
   -   
   -   
   -   
   -   
   -   
   -   
   -   
   -   
   -   
   -   
   -   
   -   
   -   
   -   
   -   
   -   
   -   
   -   
   -   
   -   
   -   
   -   
   -   
   -   
   -   
   -   
   -   
   -   
   -   
   -   
   -   
   -   
   -   
   -   
   -   
   -   
   -   
   -   
   -   
   -   
   -   
   -   
   -   
   -   
   -   
   -   
   -   
   -   
   -   
   -   
   -   
   -   
   -   
   -   
   -   
   -   
   -   
   -   
   -   
   -   
   -   
   -   
   -   
   -   
   -   
   -   
   -   
   -   
   -   
   -   
   -   
   -   
   -   
   -   
   -   
   -   
   -   
   -   
   -   
   -   
   -   
   -   
   -   
   -   
   -   
   -   
   -   
   -   
   -   
   -   
   -   
   -   
   -   
   -   
   -   
   -   
   -   
   -   
   -   
   -   
   -   
   -   
   -   
   -   
   -   
   -   
   -   
   -   
   -   
   -   
   -   
   -   
   -   
   -   
   -   
   -   
   -   
   -   
   -   
   -   
   -   
   -   
   -   
   -   
   -   
   -   
   -   
   -   
   -   
   -   
   -   
   -   
   -   
   -   
   -   
   -   
   -   
   -   
   -   
   -   
   -   
   -   
   -   
   -   
   -   
   -   
 

 
   N59454F   
   -   
   -   
   -   
   -   
   -   
   -   
   -   
   -   
   -   
   -   
   -   
   -   
   -   
   -   
   -   
   -   
   -   
   -   
   -   
   -   
   -   
   -   
   -   
   -   
   -   
   -   
   -   
   -   
   -   
   -   
   -   
   -   
   -   
   -   
   -   
   -   
   -   
   -   
   -   
   -   
   -   
   -   
   -   
   -   
   -   
   -   
   -   
   -   
   -   
   -   
   -   
   -   
   -   
   -   
   -   
   -   
   -   
   -   
   -   
   -   
   -   
   -   
   -   
   -   
   -   
   -   
   -   
   -   
   -   
   -   
   -   
   -   
   -   
   -   
   -   
   -   
   -   
   -   
   -   
   -   
   -   
   -   
   -   
   -   
   -   
   -   
   -   
   -   
   -   
   -   
   -   
   -   
   -   
   -   
   -   
   -   
   -   
   -   
   -   
   -   
   -   
   -   
   -   
   -   
   -   
   -   
   -   
   -   
   C   
   A   
   A   
   T   
   G   
   G   
   C   
   A   
   A   
   G   
   C   
   C   
   A   
   A   
   A   
   C   
   A   
   A   
   G   
   A   
    A    
   T   
   T   
   A   
   G   
   C   
    C    
   G   
   A   
   A   
   G   
   C   
   G   
   A   
   A   
   G   
   A   
   A   
   A   
   A   
    C    
   A   
   G   
   C   
    T    
   A   
    C    
   T   
    G    
    A    
   A   
   T   
   T   
   T   
   T   
   T   
   A   
   A   
   A   
    C    
   G   
   T   
   A   
   T   
   T   
   G   
    T    
   C   
   A   
   A   
   A   
   A   
   C   
   A   
   G   
    G    
   A   
   A   
   T   
   T   
   T   
   G   
   A   
   T   
   A   
   A   
   G   
   T   
   T   
   A   
   C   
   C   
    G    
   T   
   C   
   C   
   G   
   T   
   T   
    G    
   T   
   T   
   C   
   A   
   A   
   G   
   A   
   A   
    G    
   C   
   T   
   A   
   G   
   C   
   T   
   T   
   A   
   A   
   A   
   G   
   A   
   A   
   A   
   A   
   A   
   T   
   G   
   G   
    C    
   T   
   A   
   T   
   G   
   A   
   T   
   A   
   C   
   T   
   A   
   A   
   A   
   T   
   C   
    T    
   G   
   T   
   T   
   G   
   T   
   T   
   G   
   A   
   A   
   A   
   A   
   A   
   T   
   A   
   C   
   C   
   A   
   A   
   G   
   C   
   A   
   A   
   T   
   T   
   T   
   A   
   T   
   T   
   C   
   A   
   G   
   G   
   G   
   A   
   T   
   T   
   C   
   A   
   A   
   G   
   C   
    A    
   G   
   A   
   A   
    G    
   G   
    A    
   G   
   T   
   C   
   A   
   A   
    A    
   G   
   C   
   T   
   A   
   G   
   T   
   G   
   A   
   T   
   G   
   T   
    C    
   C   
   A   
   A   
   G   
   T   
    C    
   A   
   A   
   A   
   A   
   A   
    G    
   G   
    C    
   G   
   A   
   A   
   A   
   G   
   A   
    C    
   A   
   A   
    T    
   C   
   A   
   A   
   T   
   A   
   C   
   A   
   C   
   A   
   T   
   T   
   T   
   A   
    C    
   C   
   T   
   A   
   T   
   A   
   A   
   A   
   T   
   T   
   A   
   T   
   C   
   G   
   A   
   T   
   G   
   A   
   G   
    C    
   A   
   C   
    T    
   C   
   C   
   T   
   T   
   T   
   A   
   G   
   G   
   C   
   G   
   A   
   A   
   A   
   T   
   G   
   A   
   A   
   A   
   G   
   A   
   T   
    T    
   T   
   G   
   T   
   C   
   T   
   T   
   A   
   T   
   C   
   A   
   A   
   T   
   C   
   A   
   A   
   G   
   T   
   A   
   T   
    C    
   G   
    C    
   C   
   A   
   A   
    A    
   A   
   A   
   A   
   G   
   G   
    C    
   G   
   A   
   T   
   A   
    C    
   C   
   T   
   A   
   C   
   C   
   A   
   A   
   A   
   T   
    C    
    G    
   C   
   T   
   T   
   G   
   G   
    A    
    A    
    G    
   C   
   C   
    G    
   T   
   C   
   T   
   T   
   T   
   A   
   A   
   T   
   T   
   T   
   T   
   T   
   C   
   C   
   A   
   G   
   A   
   T   
   A   
   T   
   G   
   T   
   C   
   A   
   G   
   G   
   A   
   A   
   A   
   T   
   G   
   A   
   T   
   A   
   A   
   A   
    A    
   T   
   T   
   T   
   C   
   G   
   A   
   T   
   T   
   C   
   A   
   A   
   G   
   T   
   A   
   G   
   A   
   T   
   A   
   A   
   T   
   G   
   C   
   C   
   A   
   A   
   A   
   C   
   G   
   T   
   G   
   G   
   A   
   G   
   A   
   A   
   A   
   T   
   T   
   G   
   T   
   C   
   G   
   A   
   T   
   C   
   G   
   T   
   A   
   A   
    T    
   G   
   G   
   T   
   A   
   G   
   T   
   G   
   G   
   G   
   C   
   T   
   A   
   G   
    C    
   A   
   A   
   T   
   T   
   A   
   A   
   C   
   A   
   A   
   A   
   G   
   T   
   G   
   T   
   T   
   T   
   G   
   A   
    C    
   G   
   A   
   A   
   G   
   T   
   G   
   G   
   G   
   C   
   G   
   T   
   A   
   G   
   T   
    G    
   C   
   C   
   T   
    G    
   G   
   C   
   A   
   A   
   A   
   C   
   T   
    C    
   G   
   G   
   T   
   T   
   C   
   T   
   G   
   G   
   C   
   G   
   C   
   A   
   G   
   A   
   A   
   A   
   A   
   A   
    A    
   C   
   A   
   G   
   C   
    C    
   A   
   A   
   T   
   A   
   T   
   C   
   A   
   A   
   A   
   G   
   C   
   T   
   T   
   T   
   T   
   A   
   G   
   T   
   G   
   A   
   T   
   A   
   A   
    A    
   T   
   T   
    C    
   G   
   G   
   C   
   G   
   T   
   T   
   T   
   C   
   T   
    G    
   T   
   T   
   G   
   A   
   T   
   G   
   A   
   A   
   A   
   T   
   C   
   A   
   A   
   T   
   C   
    A    
   A   
   A   
   A   
    A    
   T   
   T   
   A   
   A   
   G   
   C   
   C   
   A   
   A   
   G   
   G   
    A    
   T   
   G   
   G   
   G   
   T   
   C   
   C   
   A   
   A   
   G   
   C   
   A   
   G   
   A   
   C   
   T   
   C   
    C    
   T   
   T   
   T   
   G   
   T   
   A   
   C   
   C   
   A   
   A   
   T   
   C   
   A   
   C   
   A   
   G   
   T   
    C    
   G   
   C   
   T   
   T   
   C   
   T   
   G   
   A   
   A   
   C   
   C   
   A   
   G   
   T   
   G   
   A   
   C   
   A   
   G   
   A   
   A   
   T   
   T   
   A   
   C   
   C   
   A   
   A   
   C   
   A   
   G   
   G   
   G   
   G   
   C   
   T   
   G   
   C   
   G   
   A   
   C   
   A   
   A   
   A   
   A   
   G   
    A    
    T    
   A   
   C   
   A   
   G   
   A   
   G   
   T   
   C   
   A   
   C   
   G   
   T   
   T   
   A   
   T   
   T   
   A   
   T   
   C   
   C   
    G    
    C    
   T   
   G   
   G   
   G   
   G   
   G   
   A   
   A   
   G   
   C   
   A   
   G   
   C   
   T   
   G   
   C   
   G   
   C   
   A   
   A   
   T   
   T   
   A   
   A   
   T   
   C   
   G   
   G   
    G    
   T   
   A   
   T   
   A   
   C   
    G    
   G   
   G   
    C    
   A   
   C   
    C    
   A   
   T   
   T   
   A   
   C   
   T   
   G   
   C   
   A   
   G   
   A   
    A    
   G   
   A   
   T   
   A   
   T   
   T   
    G    
   A   
   A   
   A   
   A   
   A   
   A   
   A   
   T   
   C   
   C   
   A   
   G   
   A   
   G   
   T   
   T   
   A   
   A   
   G   
   T   
   A   
   G   
   T   
   A   
   C   
   G   
   G   
   G   
    C    
   G   
   T   
   A   
   A   
   T   
   T   
   G   
   G   
    T    
   A   
   A   
   A   
   A   
   C   
   T   
   G   
   G   
    C    
   T   
   T   
   A   
   G   
   A   
   A   
   C   
   G   
   A   
   G   
   C   
   G   
   T   
   T   
   T   
   G   
   A   
   T   
   A   
   A   
   A   
   G   
   A   
    A    
   T   
   T   
   A   
   A   
   G   
   A   
   G   
   G   
    G    
   C   
   A   
   A   
   G   
   A   
   T   
   G   
   G   
    T    
   G   
   G   
    C    
   T   
   C   
    A    
   C   
   T   
   A   
   G   
   T   
   T   
   A   
   T   
   T   
   T   
   T   
   A   
   G   
   A   
   T   
   G   
   A   
   C   
   A   
   A   
   A   
   G   
   A   
   G   
   A   
   A   
   T   
   G   
   T   
   C   
   A   
   A   
   A   
   A   
   A   
   A   
   G   
   C   
   T   
   T   
   T   
   A   
   C   
   A   
   A   
   A   
   C   
   C   
   A   
   A   
   A   
   G   
   A   
    G    
   A   
   A   
   A   
   A   
   A   
   A   
   G   
   A   
   T   
   G   
   G   
   T   
   C   
   A   
   A   
   A   
   C   
    G    
   A   
   T   
   T   
   A   
   A   
   A   
   T   
   T   
   A   
   A   
   C   
   G   
   A   
   T   
   T   
   G   
   A   
    C    
   A   
   G   
    T    
   G   
   G   
    C    
   G   
   T   
   A   
   C   
   A   
   A   
   C   
   A   
   A   
   C   
   A   
   A   
   G   
   C   
    C    
   T   
   T   
    T    
   G   
   C   
   T   
   A   
   T   
   A   
   T   
   T   
   T   
   G   
   A   
   C   
   A   
   A   
   A   
   C   
   G   
    C    
    C    
   C   
   T   
   G   
   G   
   T   
   A   
   G   
   T   
   G   
   C   
   G   
   G   
   T   
   C   
   A   
   T   
   T   
   A   
   C   
   C   
   G   
   A   
   T   
   C   
   C   
   T   
   C   
   A   
   A   
   A   
   A   
   A   
   G   
   G   
   C   
   G   
   A   
   T   
   T   
   T   
   A   
    T    
   T   
   A   
   G   
   C   
    A    
   A   
   C   
   G   
   G   
   T   
   T   
   A   
   G   
   T   
   T   
   C   
   A   
   C   
   C   
   T   
   T   
   C   
   G   
   T   
   A   
   T   
   G   
   A   
   T   
   C   
   C   
   T   
   A   
   A   
   T   
   A   
   A   
   A   
   A   
   T   
   G   
   G   
   C   
   C   
   A   
   A   
   T   
   G   
   G   
    C    
   A   
   T   
   T   
   T   
   C   
   T   
   C   
   A   
   A   
   A   
   A   
   A   
   G   
   A   
    A    
   T   
   A   
   T   
   G   
   A   
   T   
   G   
   C   
   T   
   T   
   A   
   C   
   A   
   A   
   T   
   A   
   A   
   C   
   A   
   A   
   C   
   A   
   A   
   A   
   G   
   A   
   T   
   T   
   T   
   A   
   C   
   C   
   A   
   T   
   T   
    C    
   A   
   C   
   A   
   G   
   C   
   A   
   C   
   G   
   T   
   T   
   T   
    T    
   G   
   C   
   G   
   A   
   C   
   A   
   G   
   G   
    C    
   T   
   A   
   T   
   G   
   C   
   A   
   C   
   C   
   A   
   G   
   G   
    C    
   T   
   C   
   T   
   A   
   C   
   C   
   T   
   T   
   T   
   A   
   A   
   A   
   A   
   C   
   A   
   A   
   T   
   T   
   A   
   C   
   C   
   G   
   G   
   T   
   G   
   C   
   C   
   A   
   T   
   T   
   G   
   G   
   T   
   T   
   T   
   A   
   G   
   A   
   T   
    G    
   C   
   A   
   G   
   G   
   G   
   A   
   C   
   A   
   T   
   T   
   G   
   A   
   A   
   G   
   C   
   C   
   A   
   G   
   A   
   T   
   G   
   A   
   A   
   G   
   A   
   G   
   C   
   T   
   A   
   G   
   A   
   A   
   A   
   T   
   T   
   A   
   A   
   T   
   G   
   G   
    T    
   T   
   T   
   G   
   A   
   A   
   A   
   T   
   G   
   G   
   C   
   A   
   A   
   A   
   A   
   A   
   G   
   A   
   T   
   A   
   A   
   A   
   T   
   C   
   T   
   T   
   G   
   G   
   G   
   G   
    C    
   G   
   G   
   C   
   T   
   A   
   T   
   T   
   T   
   T   
   G   
   C   
    G    
   A   
   C   
   A   
   C   
   G   
   T   
   G   
   T   
   A   
   A   
   A   
   A   
   G   
   A   
   A   
   G   
   C   
   A   
   A   
   G   
   T   
   C   
   C   
   A   
   G   
   T   
   C   
   A   
   A   
   T   
   C   
   T   
    G    
   C   
   G   
   T   
   A   
   C   
   C   
   G   
   C   
   T   
   T   
   T   
   A   
   G   
   T   
   T   
   A   
   A   
   T   
   T   
   C   
   A   
   G   
   A   
   T   
   A   
   A   
   T   
   A   
   T   
   T   
   T   
   A   
   T   
   T   
   T   
   T   
   G   
   C   
    C    
   C   
   A   
   A   
   C   
   A   
   A   
   A   
   C   
   A   
   T   
   T   
   A   
   C   
   G   
    T    
   A   
   T   
   G   
   G   
   G   
    G    
   G   
   A   
   A   
   G   
   A   
   C   
   A   
   A   
   A   
   T   
   T   
   T   
   A   
   G   
   A   
   G   
   C   
    G    
   G   
   G   
   T   
   C   
   T   
   T   
   A   
   A   
   T   
   A   
   A   
   A   
   T   
   T   
   C   
   A   
   T   
   T   
   T   
   T   
   C   
   G   
   G   
   T   
   G   
   A   
   G   
   G   
   A   
   A   
   C   
   T   
   A   
   G   
   A   
   T   
   T   
   T   
   A   
   C   
   C   
   A   
   A   
   T   
    T    
   G   
   C   
   C   
   A   
   T   
   G   
   A   
   C   
   G   
   C   
   C   
    T    
   G   
   C   
   T   
   C   
   A   
   A   
   A   
   T   
   T   
   T   
   C   
   A   
   A   
   A   
    T    
   G   
   A   
   A   
   G   
   A   
   T   
   A   
   A   
   A   
   T   
   T   
   T   
   A   
   A   
   T   
   T   
   C   
   T   
   G   
   A   
   A   
   A   
   T   
   T   
   C   
   T   
   T   
   T   
   T   
    A    
   G   
   C   
   A   
   G   
   A   
   T   
   A   
   C   
    G    
   G   
   G   
   C   
   T   
   A   
    C    
   G   
   G   
    T    
   C   
   A   
   A   
   G   
   G   
    C    
   C   
   A   
   A   
   T   
   T   
   G   
   T   
   T   
   A   
   A   
   T   
   T   
   T   
   C   
    G    
   C   
   C   
   A   
   A   
   T   
   C   
   C   
   A   
   G   
   C   
   A   
   A   
   G   
   C   
   T   
   A   
   C   
   C   
   A   
   T   
   G   
   T   
   A   
   T   
   A   
   G   
   T   
   G   
   T   
   T   
   T   
   T   
   C   
   C   
   A   
   A   
   A   
   A   
   T   
   A   
   A   
   T   
   G   
   G   
    A    
   A   
   C   
   A   
    T    
   T   
    G    
   G   
   T   
    C    
   T   
   A   
   T   
   C   
   C   
   A   
   A   
   A   
   A   
    A    
   T   
   A   
   G   
   T   
   T   
   T   
   T   
   A   
   G   
   A   
   C   
   A   
   A   
   A   
   G   
   A   
   A   
   A   
   C   
    G    
   A   
   A   
   G   
   A   
   A   
   A   
   A   
   A   
   A   
   G   
   A   
   C   
   A   
   A   
   T   
   G   
   T   
   A   
   A   
   T   
   C   
   A   
   G   
   C   
   G   
   C   
   C   
   A   
   A   
   C   
   G   
   C   
   A   
   G   
   C   
   G   
   A   
   A   
   T   
   A   
   C   
   A   
   A   
   T   
   T   
   G   
   C   
   C   
   A   
   C   
   A   
   G   
   A   
   T   
   C   
   T   
   T   
   T   
   T   
   A   
   G   
   G   
   T   
   A   
   G   
   T   
   G   
   T   
   G   
   G   
   A   
   A   
   G   
   A   
   C   
   C   
   C   
   T   
   T   
   C   
   A   
   G   
   G   
   T   
   T   
   A   
   T   
   G   
   T   
   T   
   T   
   A   
   T   
   A   
   A   
   T   
   A   
   T   
   G   
   T   
   A   
   C   
   A   
   A   
   T   
   C   
   C   
   G   
   A   
   A   
   C   
   T   
   T   
   C   
   T   
   C   
   A   
   T   
   T   
   G   
   G   
   C   
   T   
   G   
   C   
   T   
   A   
   A   
   A   
   A   
   C   
   A   
   G   
   G   
   A   
   A   
   C   
   A   
   G   
   C   
   T   
   G   
   A   
   A   
   A   
   T   
   A   
   A   
   A   
   A   
   G   
   A   
   T   
   A   
   A   
   A   
   C   
   A   
   A   
   G   
   A   
   T   
   A   
   C   
   A   
   G   
   A   
   T   
   G   
   G   
   C   
   A   
   A   
   A   
   G   
   A   
   A   
   A   
   A   
   C   
   A   
   G   
   T   
   T   
   T   
   C   
   C   
   T   
   T   
   C   
   T   
   A   
   A   
   C   
   G   
   T   
   T   
   A   
   G   
   A   
   T   
   C   
   G   
   T   
   A   
   G   
   T   
   A   
   A   
   C   
   A   
   A   
   T   
   A   
   A   
   A   
   T   
   T   
   C   
   T   
   T   
   A   
   A   
   C   
   A   
   A   
   T   
   G   
   A   
   T   
   T   
   A   
   T   
   G   
   G   
   T   
   T   
   G   
   A   
   A   
   A   
   A   
   T   
   T   
   C   
   A   
   G   
   G   
   A   
   G   
   A   
   A   
   A   
   A   
   T   
   G   
   G   
   T   
   T   
   C   
   T   
   G   
   C   
   A   
   A   
   C   
   A   
   G   
   A   
   T   
   A   
   T   
   C   
   A   
   G   
   T   
   A   
   A   
   A   
   C   
   C   
   A   
   T   
   T   
   A   
   A   
   T   
   T   
   G   
   A   
   T   
   T   
   A   
   C   
   T   
   T   
   A   
   G   
   A   
   A   
   G   
   C   
   A   
   A   
   C   
   C   
   A   
   T   
   T   
   A   
   A   
   A   
   -   
   -   
   -   
   -   
   -   
   -   
   -   
   -   
   -   
   -   
   -   
   -   
   -   
   -   
   -   
   -   
   -   
   -   
   -   
   -   
   -   
   -   
   -   
   -   
   -   
   -   
   -   
   -   
   -   
   -   
   -   
   -   
   -   
   -   
   -   
   -   
   -   
   -   
   -   
   -   
   -   
   -   
   -   
   -   
   -   
   -   
   -   
   -   
   -   
   -   
   -   
   -   
   -   
   -   
   -   
   -   
   -   
   -   
   -   
   -   
   -   
   -   
   -   
   -   
   -   
   -   
   -   
   -   
   -   
   -   
   -   
   -   
   -   
   -   
   -   
   -   
   -   
   -   
   -   
   -   
   -   
   -   
   -   
   -   
   -   
   -   
   -   
   -   
   -   
   -   
   -   
   -   
   -   
   -   
   -   
   -   
   -   
   -   
   -   
   -   
   -   
   -   
   -   
   -   
   -   
   -   
   -   
   -   
   -   
   -   
   -   
   -   
   -   
   -   
   -   
   -   
   -   
   -   
   -   
   -   
   -   
   -   
   -   
   -   
   -   
   -   
   -   
   -   
   -   
   -   
   -   
   -   
   -   
   -   
   -   
   -   
   -   
   -   
   -   
   -   
   -   
   -   
   -   
   -   
   -   
   -   
   -   
   -   
   -   
   -   
   -   
   -   
   -   
   -   
   -   
   -   
   -   
   -   
   -   
   -   
   -   
   -   
   -   
   -   
   -   
   -   
   -   
   -   
   -   
   C   
   A   
   A   
   T   
   G   
   G   
   C   
   A   
   A   
   G   
   C   
   C   
   A   
   A   
   A   
   C   
   A   
   A   
   G   
   A   
   A   
   T   
   T   
   A   
   G   
   C   
   C   
   G   
   A   
   A   
   G   
   C   
   G   
   A   
   A   
   G   
   A   
   A   
   A   
   A   
   C   
   A   
   G   
   C   
   T   
   A   
   C   
   T   
   G   
   A   
   A   
   T   
   T   
   T   
   T   
   T   
   A   
   A   
   A   
   C   
   G   
   T   
   A   
   T   
   T   
   G   
   T   
   C   
   A   
   A   
   A   
   A   
   C   
   A   
   G   
   G   
   A   
   A   
   T   
   T   
   T   
   G   
   A   
   T   
   A   
   A   
   G   
   T   
   T   
   A   
   C   
   C   
   G   
   T   
   C   
   C   
   G   
   T   
   T   
   G   
   T   
   T   
   C   
   A   
   A   
   G   
   A   
   A   
   G   
   C   
   T   
   A   
   G   
   C   
   T   
   T   
   A   
   A   
   A   
   G   
   A   
   A   
   A   
   A   
   A   
   T   
   G   
   G   
   C   
   T   
   A   
   T   
   G   
   A   
   T   
   A   
   C   
   T   
   A   
   A   
   A   
   T   
   C   
   T   
   G   
   T   
   T   
   G   
   T   
   T   
   G   
   A   
   A   
   A   
   A   
   A   
   T   
   A   
   C   
   C   
   A   
   A   
   G   
   C   
   A   
   A   
   T   
   T   
   T   
   A   
   T   
   T   
   C   
   A   
   G   
   G   
   G   
   A   
   T   
   T   
   C   
   A   
   A   
   G   
   C   
   A   
   G   
   A   
   A   
   G   
   G   
   A   
   G   
   T   
   C   
   A   
   A   
   A   
   G   
   C   
   T   
   A   
   G   
   T   
   G   
   A   
   T   
   G   
   T   
   C   
   C   
   A   
   A   
   G   
   T   
   C   
   A   
   A   
   A   
   A   
   A   
   G   
   G   
   C   
   G   
   A   
   A   
   A   
   G   
   A   
   C   
   A   
   A   
   T   
   C   
   A   
   A   
   T   
   A   
   C   
   A   
   C   
   A   
   T   
   T   
   T   
   A   
   C   
   C   
   T   
   A   
   T   
   A   
   A   
   A   
   T   
   T   
   A   
   T   
   C   
   G   
   A   
   T   
   G   
   A   
   G   
   C   
   A   
   C   
   T   
   C   
   C   
   T   
   T   
   T   
   A   
   G   
   G   
   C   
   G   
   A   
   A   
   A   
   T   
   G   
   A   
   A   
   A   
   G   
   A   
   T   
   T   
   T   
   G   
   T   
   C   
   T   
   T   
   A   
   T   
   C   
   A   
   A   
   T   
   C   
   A   
   A   
   G   
   T   
   A   
   T   
   C   
   G   
   C   
   C   
   A   
   A   
   A   
   A   
   A   
   A   
   G   
   G   
   C   
   G   
   A   
   T   
   A   
   C   
   C   
   T   
   A   
   C   
   C   
   A   
   A   
   A   
   T   
   C   
   G   
   C   
   T   
   T   
   G   
   G   
   A   
   A   
   G   
   C   
   C   
   G   
   T   
   C   
   T   
   T   
   T   
   A   
   A   
   T   
   T   
   T   
   T   
   T   
   C   
   C   
   A   
   G   
   A   
   T   
   A   
   T   
   G   
   T   
   C   
   A   
   G   
   G   
   A   
   A   
   A   
   T   
   G   
   A   
   T   
   A   
   A   
   A   
   A   
   T   
   T   
   T   
   C   
   G   
   A   
   T   
   T   
   C   
   A   
   A   
   G   
   T   
   A   
   G   
   A   
   T   
   A   
   A   
   T   
   G   
   C   
   C   
   A   
   A   
   A   
   C   
   G   
   T   
   G   
   G   
   A   
   G   
   A   
   A   
   A   
   T   
   T   
   G   
   T   
   C   
   G   
   A   
   T   
   C   
   G   
   T   
   A   
   A   
   T   
   G   
   G   
   T   
   A   
   G   
   T   
   G   
   G   
   G   
   C   
   T   
   A   
   G   
   C   
   A   
   A   
   T   
   T   
   A   
   A   
   C   
   A   
   A   
   A   
   G   
   T   
   G   
   T   
   T   
   T   
   G   
   A   
   C   
   G   
   A   
   A   
   G   
   T   
   G   
   G   
   G   
   C   
   G   
   T   
   A   
   G   
   T   
   G   
   C   
   C   
   T   
   G   
   G   
   C   
   A   
   A   
   A   
   C   
   T   
   C   
   G   
   G   
   T   
   T   
   C   
   T   
   G   
   G   
   C   
   G   
   C   
   A   
   G   
   A   
   A   
   A   
   A   
   A   
   A   
   C   
   A   
   G   
   C   
   C   
   A   
   A   
   T   
   A   
   T   
   C   
   A   
   A   
   A   
   G   
   C   
   T   
   T   
   T   
   T   
   A   
   G   
   T   
   G   
   A   
   T   
   A   
   A   
   A   
   T   
   T   
   C   
   G   
   G   
   C   
   G   
   T   
   T   
   T   
   C   
   T   
   G   
   T   
   T   
   G   
   A   
   T   
   G   
   A   
   A   
   A   
   T   
   C   
   A   
   A   
   T   
   C   
   A   
   A   
   A   
   A   
   A   
   T   
   T   
   A   
   A   
   G   
   C   
   C   
   A   
   A   
   G   
   G   
   A   
   T   
   G   
   G   
   G   
   T   
   C   
   C   
   A   
   A   
   G   
   C   
   A   
   G   
   A   
   C   
   T   
   C   
   C   
   T   
   T   
   T   
   G   
   T   
   A   
   C   
   C   
   A   
   A   
   T   
   C   
   A   
   C   
   A   
   G   
   T   
   C   
   G   
   C   
   T   
   T   
   C   
   T   
   G   
   A   
   A   
   C   
   C   
   A   
   G   
   T   
   G   
   A   
   C   
   A   
   G   
   A   
   A   
   T   
   T   
   A   
   C   
   C   
   A   
   A   
   C   
   A   
   G   
   G   
   G   
   G   
   C   
   T   
   G   
   C   
   G   
   A   
   C   
   A   
   A   
   A   
   A   
   G   
   A   
   T   
   A   
   C   
   A   
   G   
   A   
   G   
   T   
   C   
   A   
   C   
   G   
   T   
   T   
   A   
   T   
   T   
   A   
   T   
   C   
   C   
   G   
   C   
   T   
   G   
   G   
   G   
   G   
   G   
   A   
   A   
   G   
   C   
   A   
   G   
   C   
   T   
   G   
   C   
   G   
   C   
   A   
   A   
   T   
   T   
   A   
   A   
   T   
   C   
   G   
   G   
   G   
   T   
   A   
   T   
   A   
   C   
   G   
   G   
   G   
   C   
   A   
   C   
   C   
   A   
   T   
   T   
   A   
   C   
   T   
   G   
   C   
   A   
   G   
   A   
   A   
   G   
   A   
   T   
   A   
   T   
   T   
   G   
   A   
   A   
   A   
   A   
   A   
   A   
   A   
   T   
   C   
   C   
   A   
   G   
   A   
   G   
   T   
   T   
   A   
   A   
   G   
   T   
   A   
   G   
   T   
   A   
   C   
   G   
   G   
   G   
   C   
   G   
   T   
   A   
   A   
   T   
   T   
   G   
   G   
   T   
   A   
   A   
   A   
   A   
   C   
   T   
   G   
   G   
   C   
   T   
   T   
   A   
   G   
   A   
   A   
   C   
   G   
   A   
   G   
   C   
   G   
   T   
   T   
   T   
   G   
   A   
   T   
   A   
   A   
   A   
   G   
   A   
   A   
   T   
   T   
   A   
   A   
   G   
   A   
   G   
   G   
   G   
   C   
   A   
   A   
   G   
   A   
   T   
   G   
   G   
   T   
   G   
   G   
   C   
   T   
   C   
   A   
   C   
   T   
   A   
   G   
   T   
   T   
   A   
   T   
   T   
   T   
   T   
   A   
   G   
   A   
   T   
   G   
   A   
   C   
   A   
   A   
   A   
   G   
   A   
   G   
   A   
   A   
   T   
   G   
   T   
   C   
   A   
   A   
   A   
   A   
   A   
   A   
   G   
   C   
   T   
   T   
   T   
   A   
   C   
   A   
   A   
   A   
   C   
   C   
   A   
   A   
   A   
   G   
   A   
   G   
   A   
   A   
   A   
   A   
   A   
   A   
   G   
   A   
   T   
   G   
   G   
   T   
   C   
   A   
   A   
   A   
   C   
   G   
   A   
   T   
   T   
   A   
   A   
   A   
   T   
   T   
   A   
   A   
   C   
   G   
   A   
   T   
   T   
   G   
   A   
   C   
   A   
   G   
   T   
   G   
   G   
   C   
   G   
   T   
   A   
   C   
   A   
   A   
   C   
   A   
   A   
   C   
   A   
   A   
   G   
   C   
   C   
   T   
   T   
   T   
   G   
   C   
   T   
   A   
   T   
   A   
   T   
   T   
   T   
   G   
   A   
   C   
   A   
   A   
   A   
   C   
   G   
   C   
   C   
   C   
   T   
   G   
   G   
   T   
   A   
   G   
   T   
   G   
   C   
   G   
   G   
   T   
   C   
   A   
   T   
   T   
   A   
   C   
   C   
   G   
   A   
   T   
   C   
   C   
   T   
   C   
   A   
   A   
   A   
   A   
   A   
   G   
   G   
   C   
   G   
   A   
   T   
   T   
   T   
   A   
   T   
   T   
   A   
   G   
   C   
   A   
   A   
   C   
   G   
   G   
   T   
   T   
   A   
   G   
   T   
   T   
   C   
   A   
   C   
   C   
   T   
   T   
   C   
   G   
   T   
   A   
   T   
   G   
   A   
   T   
   C   
   C   
   T   
   A   
   A   
   T   
   A   
   A   
   A   
   A   
   T   
   G   
   G   
   C   
   C   
   A   
   A   
   T   
   G   
   G   
   C   
   A   
   T   
   T   
   T   
   C   
   T   
   C   
   A   
   A   
   A   
   A   
   A   
   G   
   A   
   A   
   T   
   A   
   T   
   G   
   A   
   T   
   G   
   C   
   T   
   T   
   A   
   C   
   A   
   A   
   T   
   A   
   A   
   C   
   A   
   A   
   C   
   A   
   A   
   A   
   G   
   A   
   T   
   T   
   T   
   A   
   C   
   C   
   A   
   T   
   T   
   C   
   A   
   C   
   A   
   G   
   C   
   A   
   C   
   G   
   T   
   T   
   T   
   T   
   G   
   C   
   G   
   A   
   C   
   A   
   G   
   G   
   C   
   T   
   A   
   T   
   G   
   C   
   A   
   C   
   C   
   A   
   G   
   G   
   C   
   T   
   C   
   T   
   A   
   C   
   C   
   T   
   T   
   T   
   A   
   A   
   A   
   A   
   C   
   A   
   A   
   T   
   T   
   A   
   C   
   C   
   G   
   G   
   T   
   G   
   C   
   C   
   A   
   T   
   T   
   G   
   G   
   T   
   T   
   T   
   A   
   G   
   A   
   T   
   G   
   C   
   A   
   G   
   G   
   G   
   A   
   C   
   A   
   T   
   T   
   G   
   A   
   A   
   G   
   C   
   C   
   A   
   G   
   A   
   T   
   G   
   A   
   A   
   G   
   A   
   G   
   C   
   T   
   A   
   G   
   A   
   A   
   A   
   T   
   T   
   A   
   A   
   T   
   G   
   G   
   T   
   T   
   T   
   G   
   A   
   A   
   A   
   T   
   G   
   G   
   C   
   A   
   A   
   A   
   A   
   A   
   G   
   A   
   T   
   A   
   A   
   A   
   T   
   C   
   T   
   T   
   G   
   G   
   G   
   G   
   C   
   G   
   G   
   C   
   T   
   A   
   T   
   T   
   T   
   T   
   G   
   C   
   G   
   A   
   C   
   A   
   C   
   G   
   T   
   G   
   T   
   A   
   A   
   A   
   A   
   G   
   A   
   A   
   G   
   C   
   A   
   A   
   G   
   T   
   C   
   C   
   A   
   G   
   T   
   C   
   A   
   A   
   T   
   C   
   T   
   G   
   C   
   G   
   T   
   A   
   C   
   C   
   G   
   C   
   T   
   T   
   T   
   A   
   G   
   T   
   T   
   A   
   A   
   T   
   T   
   C   
   A   
   G   
   A   
   T   
   A   
   A   
   T   
   A   
   T   
   T   
   T   
   A   
   T   
   T   
   T   
   T   
   G   
   C   
   C   
   C   
   A   
   A   
   C   
   A   
   A   
   A   
   C   
   A   
   T   
   T   
   A   
   C   
   G   
   T   
   A   
   T   
   G   
   G   
   G   
   G   
   G   
   A   
   A   
   G   
   A   
   C   
   A   
   A   
   A   
   T   
   T   
   T   
   A   
   G   
   A   
   G   
   C   
   G   
   G   
   G   
   T   
   C   
   T   
   T   
   A   
   A   
   T   
   A   
   A   
   A   
   T   
   T   
   C   
   A   
   T   
   T   
   T   
   T   
   C   
   G   
   G   
   T   
   G   
   A   
   G   
   G   
   A   
   A   
   C   
   T   
   A   
   G   
   A   
   T   
   T   
   T   
   A   
   C   
   C   
   A   
   A   
   T   
   T   
   G   
   C   
   C   
   A   
   T   
   G   
   A   
   C   
   G   
   C   
   C   
   T   
   G   
   C   
   T   
   C   
   A   
   A   
   A   
   T   
   T   
   T   
   C   
   A   
   A   
   A   
   T   
   G   
   A   
   A   
   G   
   A   
   T   
   A   
   A   
   A   
   T   
   T   
   T   
   A   
   A   
   T   
   T   
   C   
   T   
   G   
   A   
   A   
   A   
   T   
   T   
   C   
   T   
   T   
   T   
   T   
   A   
   G   
   C   
   A   
   G   
   A   
   T   
   A   
   C   
   G   
   G   
   G   
   C   
   T   
   A   
   C   
   G   
   G   
   T   
   C   
   A   
   A   
   G   
   G   
   C   
   C   
   A   
   A   
   T   
   T   
   G   
   T   
   T   
   A   
   A   
   T   
   T   
   T   
   C   
   G   
   C   
   C   
   A   
   A   
   T   
   C   
   C   
   A   
   G   
   C   
   A   
   A   
   G   
   C   
   T   
   A   
   C   
   C   
   A   
   T   
   G   
   T   
   A   
   T   
   A   
   G   
   T   
   G   
   T   
   T   
   T   
   T   
   C   
   C   
   A   
   A   
   A   
   A   
   T   
   A   
   A   
   T   
   G   
   G   
   A   
   A   
   C   
   A   
   T   
   T   
   G   
   G   
   T   
   C   
   T   
   A   
   T   
   C   
   C   
   A   
   A   
   A   
   A   
   A   
   T   
   A   
   G   
   T   
   T   
   T   
   T   
   A   
   G   
   A   
   C   
   A   
   A   
   A   
   G   
   A   
   A   
   A   
   C   
   G   
   A   
   A   
   G   
   A   
   A   
   A   
   A   
   A   
   A   
   G   
   A   
   C   
   A   
   A   
   T   
   G   
   T   
   A   
   A   
   T   
   C   
   A   
   G   
   C   
   G   
   C   
   C   
   A   
   A   
   C   
   G   
   C   
   A   
   G   
   C   
   G   
   A   
   A   
   T   
   A   
   C   
   A   
   A   
   T   
   T   
   G   
   C   
   C   
   A   
   C   
   A   
   G   
   A   
   T   
   C   
   T   
   T   
   T   
   T   
   A   
   G   
   G   
   T   
   A   
   G   
   T   
   G   
   T   
   G   
   G   
   A   
   A   
   G   
   A   
   C   
   C   
   C   
   T   
   T   
   C   
   A   
   G   
   G   
   T   
   T   
   A   
   T   
   G   
   T   
   T   
   T   
   A   
   T   
   A   
   A   
   T   
   A   
   T   
   G   
   T   
   A   
   C   
   A   
   A   
   T   
   C   
   C   
   G   
   A   
   A   
   C   
   T   
   T   
   C   
   T   
   C   
   A   
   T   
   T   
   G   
   G   
   C   
   T   
   G   
   C   
   T   
   A   
   A   
   A   
   A   
   C   
   A   
   G   
   G   
   A   
   A   
   C   
   A   
   G   
   C   
   T   
   G   
   A   
   A   
   A   
   T   
   A   
   A   
   A   
   A   
   G   
   A   
   T   
   A   
   A   
   A   
   C   
   A   
   A   
   G   
   A   
   T   
   A   
   C   
   A   
   G   
   A   
   T   
   G   
   G   
   C   
   A   
   A   
   A   
   G   
   A   
   A   
   A   
   A   
   C   
   A   
   G   
   T   
   T   
   T   
   C   
   C   
   T   
   T   
   C   
   T   
   A   
   A   
   C   
   G   
   T   
   T   
   A   
   G   
   A   
   T   
   C   
   G   
   T   
   A   
   G   
   T   
   A   
   A   
   C   
   A   
   A   
   T   
   A   
   A   
   A   
   T   
   T   
   C   
   T   
   T   
   A   
   A   
   C   
   A   
   A   
   T   
   G   
   A   
   T   
   T   
   A   
   T   
   G   
   G   
   T   
   T   
   G   
   A   
   A   
   A   
   A   
   T   
   T   
   C   
   A   
   G   
   G   
   A   
   G   
   A   
   A   
   A   
   A   
   T   
   G   
   G   
   T   
   T   
   C   
   T   
   G   
   C   
   A   
   A   
   C   
   A   
   G   
   A   
   T   
   A   
   T   
   C   
   A   
   G   
   T   
   A   
   A   
   A   
   C   
   C   
   A   
   T   
   T   
   A   
   A   
   T   
   T   
   G   
   A   
   T   
   T   
   A   
   C   
   T   
   T   
   A   
   G   
   A   
   A   
   G   
   C   
   A   
   A   
   C   
   C   
   A   
   T   
   T   
   A   
   A   
   A   
   -   
   -   
   -   
   -   
   -   
   -   
   -   
   -   
   -   
   -   
   -   
   -   
   -   
   -   
   -   
   -   
   -   
   -   
   -   
   -   
   -   
   -   
   -   
   -   
   -   
   -   
   -   
   -   
   -   
   -   
   -   
   -   
   -   
   -   
   -   
   -   
   -   
   -   
   -   
   -   
   -   
   -   
   -   
   -   
   -   
   -   
   -   
   -   
   -   
   -   
   -   
   -   
   -   
   -   
   -   
   -   
   -   
   -   
   -   
   -   
   -   
 

 
   N55119   
   -   
   -   
   -   
   -   
   -   
   -   
   -   
   -   
   -   
   -   
   -   
   -   
   -   
   -   
   -   
   -   
   -   
   -   
   -   
   -   
   -   
   -   
   -   
   -   
   -   
   -   
   -   
   -   
   -   
   -   
   -   
   -   
   -   
   -   
   -   
   -   
   -   
   -   
   -   
   -   
   -   
   -   
   -   
   -   
   -   
   -   
   -   
   -   
   -   
   -   
   -   
   -   
   -   
   -   
   -   
   -   
   -   
   -   
   -   
   -   
   -   
   -   
   -   
   -   
   -   
   -   
   -   
   -   
   -   
   -   
   -   
   -   
   -   
   -   
   -   
   -   
   -   
   -   
   -   
   -   
   -   
   -   
   -   
   -   
   -   
   -   
   -   
   -   
   -   
   -   
   -   
   -   
   -   
   -   
   -   
   -   
   -   
   -   
   -   
   -   
   -   
   -   
   -   
   -   
   -   
   -   
   -   
   -   
   C   
   A   
   A   
   T   
   G   
   G   
   C   
   A   
   A   
   G   
   C   
   C   
   A   
   A   
   A   
   C   
   A   
   A   
   G   
   A   
    A    
   T   
   T   
   A   
   G   
   C   
    C    
   G   
   A   
   A   
   G   
   C   
   G   
   A   
   A   
   G   
   A   
   A   
   A   
   A   
    C    
   A   
   G   
   C   
    T    
   A   
    C    
   T   
    A    
    C    
   A   
   T   
   T   
   T   
   T   
   T   
   A   
   A   
   A   
    C    
   G   
   T   
   A   
   T   
   T   
   G   
    A    
   C   
   A   
   A   
   A   
   A   
   C   
   A   
   G   
    A    
   A   
   A   
   T   
   T   
   T   
   G   
   A   
   T   
   A   
   A   
   G   
   T   
   T   
   A   
   C   
   C   
    G    
   T   
   C   
   C   
   G   
   T   
   T   
    G    
   T   
   T   
   C   
   A   
   A   
   G   
   A   
   A   
    G    
   C   
   T   
   A   
   G   
   C   
   T   
   T   
   A   
   A   
   A   
   G   
   A   
   A   
   A   
   A   
   A   
   T   
   G   
   G   
    C    
   T   
   A   
   T   
   G   
   A   
   T   
   A   
   C   
   T   
   A   
   A   
   A   
   T   
   C   
    T    
   G   
   T   
   T   
   G   
   T   
   T   
   G   
   A   
   A   
   A   
   A   
   A   
   T   
   A   
   C   
   C   
   A   
   A   
   G   
   C   
   A   
   A   
   T   
   T   
   T   
   A   
   T   
   T   
   C   
   A   
   G   
   G   
   G   
   A   
   T   
   T   
   C   
   A   
   A   
   G   
   C   
    A    
   G   
   A   
   A   
    G    
   G   
    A    
   G   
   T   
   C   
   A   
   A   
    A    
   G   
   C   
   T   
   A   
   G   
   T   
   G   
   A   
   T   
   G   
   T   
    T    
   C   
   A   
   A   
   G   
   T   
    C    
   A   
   A   
   A   
   A   
   A   
    A    
   G   
    C    
   G   
   A   
   A   
   A   
   G   
   A   
    C    
   A   
   A   
    T    
   C   
   A   
   A   
   T   
   A   
   C   
   A   
   C   
   A   
   T   
   T   
   T   
   A   
    C    
   C   
   T   
   A   
   T   
   A   
   A   
   A   
   T   
   T   
   A   
   T   
   C   
   G   
   A   
   T   
   G   
   A   
   G   
    T    
   A   
   C   
    C    
   C   
   C   
   T   
   T   
   T   
   A   
   G   
   G   
   C   
   G   
   A   
   A   
   A   
   T   
   G   
   A   
   A   
   A   
   G   
   A   
   T   
    T    
   T   
   G   
   T   
   C   
   T   
   T   
   A   
   T   
   C   
   A   
   A   
   T   
   C   
   A   
   A   
   G   
   T   
   A   
   T   
    C    
   G   
    C    
   C   
   A   
   A   
    A    
   A   
   A   
   A   
   G   
   G   
    C    
   G   
   A   
   T   
   A   
    C    
   C   
   T   
   A   
   C   
   C   
   A   
   A   
   A   
   T   
    C    
    G    
   C   
   T   
   T   
   G   
   G   
    A    
    A    
    G    
   C   
   C   
    G    
   T   
   C   
   T   
   T   
   T   
   A   
   A   
   T   
   T   
   T   
   T   
   T   
   C   
   C   
   A   
   G   
   A   
   T   
   A   
   T   
   G   
   T   
   C   
   A   
   G   
   G   
   A   
   A   
   A   
   T   
   G   
   A   
   T   
   A   
   A   
   A   
    A    
   T   
   T   
   T   
   C   
   G   
   A   
   T   
   T   
   C   
   A   
   A   
   G   
   T   
   A   
   G   
   A   
   T   
   A   
   A   
   T   
   G   
   C   
   C   
   A   
   A   
   A   
   C   
   G   
   T   
   G   
   G   
   A   
   G   
   A   
   A   
   A   
   T   
   T   
   G   
   T   
   C   
   G   
   A   
   T   
   C   
   G   
   T   
   A   
   A   
    T    
   G   
   G   
   T   
   A   
   G   
   T   
   G   
   G   
   G   
   C   
   T   
   A   
   G   
    C    
   A   
   A   
   T   
   T   
   A   
   A   
   C   
   A   
   A   
   A   
   G   
   T   
   G   
   T   
   T   
   T   
   G   
   A   
    C    
   G   
   A   
   A   
   G   
   T   
   G   
   G   
   G   
   C   
   G   
   T   
   A   
   G   
   T   
    G    
   C   
   C   
   T   
    G    
   G   
   C   
   A   
   A   
   A   
   C   
   T   
    C    
   G   
   G   
   T   
   T   
   C   
   T   
   G   
   G   
   C   
   G   
   C   
   A   
   G   
   A   
   A   
   A   
   A   
   A   
    A    
   C   
   A   
   G   
   C   
    C    
   A   
   A   
   T   
   A   
   T   
   C   
   A   
   A   
   A   
   G   
   C   
   T   
   T   
   T   
   T   
   A   
   G   
   T   
   G   
   A   
   T   
   A   
   A   
    G    
   T   
   T   
    C    
   G   
   G   
   C   
   G   
   T   
   T   
   T   
   C   
   T   
    G    
   T   
   T   
   G   
   A   
   T   
   G   
   A   
   A   
   A   
   T   
   C   
   A   
   A   
   T   
   C   
    A    
   A   
   A   
   A   
    G    
   T   
   T   
   A   
   A   
   G   
   C   
   C   
   A   
   A   
   G   
   G   
    G    
   T   
   G   
   G   
   G   
   T   
   C   
   C   
   A   
   A   
   G   
   C   
   A   
   G   
   A   
   C   
   T   
   C   
    C    
   T   
   T   
   T   
   G   
   T   
   A   
   C   
   C   
   A   
   A   
   T   
   C   
   A   
   C   
   A   
   G   
   T   
    C    
   G   
   C   
   T   
   T   
   C   
   T   
   G   
   A   
   A   
   C   
   C   
   A   
   G   
   T   
   G   
   A   
   C   
   A   
   G   
   A   
   A   
   T   
   T   
   A   
   C   
   C   
   A   
   A   
   C   
   A   
   G   
   G   
   G   
   G   
   C   
   T   
   G   
   C   
   G   
   A   
   C   
   A   
   A   
   A   
   A   
   G   
    A    
    T    
   A   
   C   
   A   
   G   
   A   
   G   
   T   
   C   
   A   
   C   
   G   
   T   
   T   
   A   
   T   
   T   
   A   
   T   
   C   
   C   
    G    
    C    
   T   
   G   
   G   
   G   
   G   
   G   
   A   
   A   
   G   
   C   
   A   
   G   
   C   
   T   
   G   
   C   
   G   
   C   
   A   
   A   
   T   
   T   
   A   
   A   
   T   
   C   
   G   
   G   
    G    
   T   
   A   
   T   
   A   
   C   
    A    
   G   
   G   
    C    
   A   
   C   
    C    
   A   
   T   
   T   
   A   
   C   
   T   
   G   
   C   
   A   
   G   
   A   
    A    
   G   
   A   
   T   
   A   
   T   
   T   
    A    
   A   
   A   
   A   
   A   
   A   
   A   
   A   
   T   
   C   
   C   
   A   
   G   
   A   
   G   
   T   
   T   
   A   
   A   
   G   
   T   
   A   
   G   
   T   
   A   
   C   
   G   
   G   
   G   
    C    
   G   
   T   
   A   
   A   
   T   
   T   
   G   
   G   
    T    
   A   
   A   
   A   
   A   
   C   
   T   
   G   
   G   
    C    
   T   
   T   
   A   
   G   
   A   
   A   
   C   
   G   
   A   
   G   
   C   
   G   
   T   
   T   
   T   
   G   
   A   
   T   
   A   
   A   
   A   
   G   
   A   
    A    
   T   
   T   
   A   
   A   
   G   
   A   
   G   
   G   
    G    
   C   
   A   
   A   
   G   
   A   
   T   
   G   
   G   
    T    
   G   
   G   
    C    
   T   
   C   
    A    
   C   
   T   
   A   
   G   
   T   
   T   
   A   
   T   
   T   
   T   
   T   
   A   
   G   
   A   
   T   
   G   
   A   
   C   
   A   
   A   
   A   
   G   
   A   
   G   
   A   
   A   
   T   
   G   
   T   
   C   
   A   
   A   
   A   
   A   
   A   
   A   
   G   
   C   
   T   
   T   
   T   
   A   
   C   
   A   
   A   
   A   
   C   
   C   
   A   
   A   
   A   
   G   
   A   
    G    
   A   
   A   
   A   
   A   
   A   
   A   
   G   
   A   
   T   
   G   
   G   
   T   
   C   
   A   
   A   
   A   
   C   
    G    
   A   
   T   
   T   
   A   
   A   
   A   
   T   
   T   
   A   
   A   
   C   
   G   
   A   
   T   
   T   
   G   
   A   
    C    
   A   
   G   
    T    
   G   
   G   
    C    
   G   
   T   
   A   
   C   
   A   
   A   
   C   
   A   
   A   
   C   
   A   
   A   
   G   
   C   
    C    
   T   
   T   
    C    
   G   
   C   
   T   
   A   
   T   
   A   
   T   
   T   
   T   
   G   
   A   
   C   
   A   
   A   
   A   
   C   
   G   
    C    
    C    
   C   
   T   
   G   
   G   
   T   
   A   
   G   
   T   
   G   
   C   
   G   
   G   
   T   
   C   
   A   
   T   
   T   
   A   
   C   
   C   
   G   
   A   
   T   
   C   
   C   
   T   
   C   
   A   
   A   
   A   
   A   
   A   
   G   
   G   
   C   
   G   
   A   
   T   
   T   
   T   
   A   
    T    
   T   
   A   
   G   
   C   
    A    
   A   
   C   
   G   
   G   
   T   
   T   
   A   
   G   
   T   
   T   
   C   
   A   
   C   
   C   
   T   
   T   
   C   
   G   
   T   
   A   
   T   
   G   
   A   
   T   
   C   
   C   
   T   
   A   
   A   
   T   
   A   
   A   
   A   
   A   
   T   
   G   
   G   
   C   
   C   
   A   
   A   
   T   
   G   
   G   
    C    
   A   
   T   
   T   
   T   
   C   
   T   
   C   
   A   
   A   
   A   
   A   
   A   
   G   
   A   
    A    
   T   
   A   
   T   
   G   
   A   
   T   
   G   
   C   
   T   
   T   
   A   
   C   
   A   
   A   
   T   
   A   
   A   
   C   
   A   
   A   
   C   
   A   
   A   
   A   
   G   
   A   
   T   
   T   
   T   
   A   
   C   
   C   
   A   
   T   
   T   
    C    
   A   
   C   
   A   
   G   
   C   
   A   
   C   
   G   
   T   
   T   
   T   
    T    
   G   
   C   
   G   
   A   
   C   
   A   
   G   
   G   
    C    
   T   
   A   
   T   
   G   
   C   
   A   
   C   
   C   
   A   
   G   
   G   
    C    
   T   
   C   
   T   
   A   
   C   
   C   
   T   
   T   
   T   
   A   
   A   
   A   
   A   
   C   
   A   
   A   
   T   
   T   
   A   
   C   
   C   
   G   
   G   
   T   
   G   
   C   
   C   
   A   
   T   
   T   
   G   
   G   
   T   
   T   
   T   
   A   
   G   
   A   
   T   
    A    
   C   
   A   
   G   
   G   
   G   
   A   
   C   
   A   
   T   
   T   
   G   
   A   
   A   
   G   
   C   
   C   
   A   
   G   
   A   
   T   
   G   
   A   
   A   
   G   
   A   
   G   
   C   
   T   
   A   
   G   
   A   
   A   
   A   
   T   
   T   
   A   
   A   
   T   
   G   
   G   
    T    
   T   
   T   
   G   
   A   
   A   
   A   
   T   
   G   
   G   
   C   
   A   
   A   
   A   
   A   
   A   
   G   
   A   
   T   
   A   
   A   
   A   
   T   
   C   
   T   
   T   
   G   
   G   
   G   
   G   
    C    
   G   
   G   
   C   
   T   
   A   
   T   
   T   
   T   
   T   
   G   
   C   
    G    
   A   
   C   
   A   
   C   
   G   
   T   
   G   
   T   
   A   
   A   
   A   
   A   
   G   
   A   
   A   
   G   
   C   
   A   
   A   
   G   
   T   
   C   
   C   
   A   
   G   
   T   
   C   
   A   
   A   
   T   
   C   
   T   
    G    
   C   
   G   
   T   
   A   
   C   
   C   
   G   
   C   
   T   
   T   
   T   
   A   
   G   
   T   
   T   
   A   
   A   
   T   
   T   
   C   
   A   
   G   
   A   
   T   
   A   
   A   
   T   
   A   
   T   
   T   
   T   
   A   
   T   
   T   
   T   
   T   
   G   
   C   
    C    
   C   
   A   
   A   
   C   
   A   
   A   
   A   
   C   
   A   
   T   
   T   
   A   
   C   
   G   
    G    
   A   
   T   
   G   
   G   
   G   
    A    
   G   
   A   
   A   
   G   
   A   
   C   
   A   
   A   
   A   
   T   
   T   
   T   
   A   
   G   
   A   
   G   
   C   
    G    
   G   
   G   
   T   
   C   
   T   
   T   
   A   
   A   
   T   
   A   
   A   
   A   
   T   
   T   
   C   
   A   
   T   
   T   
   T   
   T   
   C   
   G   
   G   
   T   
   G   
   A   
   G   
   G   
   A   
   A   
   C   
   T   
   A   
   G   
   A   
   T   
   T   
   T   
   A   
   C   
   C   
   A   
   A   
   T   
    T    
   G   
   C   
   C   
   A   
   T   
   G   
   A   
   C   
   G   
   C   
   C   
    T    
   G   
   C   
   T   
   C   
   A   
   A   
   A   
   T   
   T   
   T   
   C   
   A   
   A   
   A   
    T    
   G   
   A   
   A   
   G   
   A   
   T   
   A   
   A   
   A   
   T   
   T   
   T   
   A   
   A   
   T   
   T   
   C   
   T   
   G   
   A   
   A   
   A   
   T   
   T   
   C   
   T   
   T   
   T   
   T   
    G    
   G   
   C   
   A   
   G   
   A   
   T   
   A   
   C   
    G    
   G   
   G   
   C   
   T   
   A   
    C    
   G   
   G   
    C    
   C   
   A   
   A   
   G   
   G   
    C    
   C   
   A   
   A   
   T   
   T   
   G   
   T   
   T   
   A   
   A   
   T   
   T   
   T   
   C   
    G    
   C   
   C   
   A   
   A   
   T   
   C   
   C   
   A   
   G   
   C   
   A   
   A   
   G   
   C   
   T   
   A   
   C   
   C   
   A   
   T   
   G   
   T   
   A   
   T   
   A   
   G   
   T   
   G   
   T   
   T   
   T   
   T   
   C   
   C   
   A   
   A   
   A   
   A   
   T   
   A   
   A   
   T   
   G   
   G   
    G    
   A   
   C   
   A   
    T    
   T   
    A    
   G   
   T   
    C    
   T   
   A   
   T   
   C   
   C   
   A   
   A   
   A   
   A   
    C    
   T   
   A   
   G   
   T   
   T   
   T   
   T   
   A   
   G   
   A   
   C   
   A   
   A   
   A   
   G   
   A   
   A   
   A   
   C   
    G    
   A   
   A   
   G   
   A   
   A   
   A   
   A   
   A   
   A   
   G   
   A   
   C   
   A   
   A   
   T   
   G   
   T   
   A   
   A   
   T   
   C   
   A   
   G   
   C   
   G   
   C   
   C   
   A   
   A   
   C   
   G   
   C   
   A   
   G   
   C   
   G   
   A   
   A   
   T   
   A   
   C   
   A   
   A   
   T   
   T   
   G   
   C   
   C   
   A   
   C   
   A   
   G   
   A   
   T   
   C   
   T   
   T   
   T   
   T   
   A   
   G   
   G   
   T   
   A   
   G   
   T   
   G   
   T   
   G   
   G   
   A   
   A   
   G   
   A   
   T   
   C   
   C   
   T   
   T   
   C   
   A   
   G   
   G   
   T   
   T   
   A   
   T   
   G   
   T   
   T   
   T   
   A   
   T   
   A   
   A   
   T   
   A   
   T   
   G   
   T   
   A   
   C   
   A   
   A   
   T   
   C   
   C   
   G   
   A   
   A   
   C   
   T   
   T   
   C   
   T   
   C   
   A   
   T   
   T   
   G   
   G   
   C   
   T   
   G   
   C   
   T   
   A   
   A   
   A   
   A   
   C   
   A   
   G   
   G   
   A   
   A   
   C   
   A   
   G   
   C   
   T   
   G   
   A   
   A   
   A   
   T   
   A   
   A   
   A   
   A   
   G   
   A   
   T   
   A   
   A   
   A   
   C   
   A   
   A   
   G   
   A   
   T   
   A   
   C   
   A   
   G   
   A   
   T   
   G   
   G   
   C   
   A   
   A   
   A   
   G   
   A   
   A   
   A   
   A   
   C   
   A   
   G   
   T   
   T   
   T   
   C   
   C   
   T   
   T   
   C   
   T   
   A   
   A   
   C   
   G   
   T   
   T   
   A   
   G   
   A   
   T   
   C   
   G   
   T   
   A   
   G   
   T   
   A   
   A   
   C   
   A   
   A   
   T   
   A   
   A   
   A   
   T   
   T   
   C   
   T   
   T   
   A   
   A   
   C   
   A   
   A   
   T   
   G   
   A   
   T   
   T   
   A   
   T   
   G   
   G   
   T   
   T   
   G   
   A   
   A   
   A   
   A   
   T   
   T   
   C   
   A   
   G   
   G   
   A   
   G   
   A   
   A   
   A   
   A   
   T   
   G   
   G   
   T   
   T   
   C   
   T   
   G   
   C   
   A   
   A   
   C   
   A   
   G   
   A   
   T   
   A   
   T   
   C   
   A   
   G   
   T   
   A   
   A   
   A   
   C   
   C   
   A   
   T   
   T   
   A   
   A   
   T   
   T   
   G   
   A   
   T   
   T   
   A   
   C   
   T   
   T   
   A   
   G   
   A   
   A   
   G   
   C   
   A   
   A   
   C   
   C   
   A   
   T   
   T   
   A   
   A   
   A   
   -   
   -   
   -   
   -   
   -   
   -   
   -   
   -   
   -   
   -   
   -   
   -   
   -   
   -   
   -   
   -   
   -   
   -   
   -   
   -   
   -   
   -   
   -   
   -   
   -   
   -   
   -   
   -   
   -   
   -   
   -   
   -   
   -   
   -   
   -   
   -   
   -   
   -   
   -   
   -   
   -   
   -   
   -   
   -   
   -   
   -   
   -   
   -   
   -   
   -   
   -   
   -   
   -   
   -   
   -   
   -   
   -   
   -   
   -   
   -   
   -   
   -   
   -   
   -   
   -   
   -   
   -   
   -   
   -   
   -   
   -   
   -   
   -   
   -   
   -   
   -   
   -   
   -   
   -   
   -   
   -   
   -   
   -   
   -   
   -   
   -   
   -   
   -   
   -   
   -   
   -   
   -   
   -   
   -   
   -   
   -   
   -   
   -   
   -   
   -   
   -   
   -   
   -   
   -   
   -   
   -   
   -   
   -   
   -   
   -   
   -   
   -   
   -   
   -   
   -   
   -   
   -   
   -   
   -   
   -   
   -   
   -   
   -   
   -   
   -   
   -   
   -   
   -   
   -   
   -   
   -   
   -   
   -   
   -   
   -   
   -   
   -   
   -   
   -   
   -   
   -   
   -   
   -   
   -   
   -   
   -   
   -   
   -   
   -   
   -   
   -   
   -   
   -   
   -   
   -   
   -   
   -   
   -   
   -   
   -   
   -   
   -   
   -   
   -   
   -   
   -   
   -   
   -   
   -   
   C   
   A   
   A   
   T   
   G   
   G   
   C   
   A   
   A   
   G   
   C   
   C   
   A   
   A   
   A   
   C   
   A   
   A   
   G   
   A   
   A   
   T   
   T   
   A   
   G   
   C   
   C   
   G   
   A   
   A   
   G   
   C   
   G   
   A   
   A   
   G   
   A   
   A   
   A   
   A   
   C   
   A   
   G   
   C   
   T   
   A   
   C   
   T   
   A   
   C   
   A   
   T   
   T   
   T   
   T   
   T   
   A   
   A   
   A   
   C   
   G   
   T   
   A   
   T   
   T   
   G   
   A   
   C   
   A   
   A   
   A   
   A   
   C   
   A   
   G   
   A   
   A   
   A   
   T   
   T   
   T   
   G   
   A   
   T   
   A   
   A   
   G   
   T   
   T   
   A   
   C   
   C   
   G   
   T   
   C   
   C   
   G   
   T   
   T   
   G   
   T   
   T   
   C   
   A   
   A   
   G   
   A   
   A   
   G   
   C   
   T   
   A   
   G   
   C   
   T   
   T   
   A   
   A   
   A   
   G   
   A   
   A   
   A   
   A   
   A   
   T   
   G   
   G   
   C   
   T   
   A   
   T   
   G   
   A   
   T   
   A   
   C   
   T   
   A   
   A   
   A   
   T   
   C   
   T   
   G   
   T   
   T   
   G   
   T   
   T   
   G   
   A   
   A   
   A   
   A   
   A   
   T   
   A   
   C   
   C   
   A   
   A   
   G   
   C   
   A   
   A   
   T   
   T   
   T   
   A   
   T   
   T   
   C   
   A   
   G   
   G   
   G   
   A   
   T   
   T   
   C   
   A   
   A   
   G   
   C   
   A   
   G   
   A   
   A   
   G   
   G   
   A   
   G   
   T   
   C   
   A   
   A   
   A   
   G   
   C   
   T   
   A   
   G   
   T   
   G   
   A   
   T   
   G   
   T   
   T   
   C   
   A   
   A   
   G   
   T   
   C   
   A   
   A   
   A   
   A   
   A   
   A   
   G   
   C   
   G   
   A   
   A   
   A   
   G   
   A   
   C   
   A   
   A   
   T   
   C   
   A   
   A   
   T   
   A   
   C   
   A   
   C   
   A   
   T   
   T   
   T   
   A   
   C   
   C   
   T   
   A   
   T   
   A   
   A   
   A   
   T   
   T   
   A   
   T   
   C   
   G   
   A   
   T   
   G   
   A   
   G   
   T   
   A   
   C   
   C   
   C   
   C   
   T   
   T   
   T   
   A   
   G   
   G   
   C   
   G   
   A   
   A   
   A   
   T   
   G   
   A   
   A   
   A   
   G   
   A   
   T   
   T   
   T   
   G   
   T   
   C   
   T   
   T   
   A   
   T   
   C   
   A   
   A   
   T   
   C   
   A   
   A   
   G   
   T   
   A   
   T   
   C   
   G   
   C   
   C   
   A   
   A   
   A   
   A   
   A   
   A   
   G   
   G   
   C   
   G   
   A   
   T   
   A   
   C   
   C   
   T   
   A   
   C   
   C   
   A   
   A   
   A   
   T   
   C   
   G   
   C   
   T   
   T   
   G   
   G   
   A   
   A   
   G   
   C   
   C   
   G   
   T   
   C   
   T   
   T   
   T   
   A   
   A   
   T   
   T   
   T   
   T   
   T   
   C   
   C   
   A   
   G   
   A   
   T   
   A   
   T   
   G   
   T   
   C   
   A   
   G   
   G   
   A   
   A   
   A   
   T   
   G   
   A   
   T   
   A   
   A   
   A   
   A   
   T   
   T   
   T   
   C   
   G   
   A   
   T   
   T   
   C   
   A   
   A   
   G   
   T   
   A   
   G   
   A   
   T   
   A   
   A   
   T   
   G   
   C   
   C   
   A   
   A   
   A   
   C   
   G   
   T   
   G   
   G   
   A   
   G   
   A   
   A   
   A   
   T   
   T   
   G   
   T   
   C   
   G   
   A   
   T   
   C   
   G   
   T   
   A   
   A   
   T   
   G   
   G   
   T   
   A   
   G   
   T   
   G   
   G   
   G   
   C   
   T   
   A   
   G   
   C   
   A   
   A   
   T   
   T   
   A   
   A   
   C   
   A   
   A   
   A   
   G   
   T   
   G   
   T   
   T   
   T   
   G   
   A   
   C   
   G   
   A   
   A   
   G   
   T   
   G   
   G   
   G   
   C   
   G   
   T   
   A   
   G   
   T   
   G   
   C   
   C   
   T   
   G   
   G   
   C   
   A   
   A   
   A   
   C   
   T   
   C   
   G   
   G   
   T   
   T   
   C   
   T   
   G   
   G   
   C   
   G   
   C   
   A   
   G   
   A   
   A   
   A   
   A   
   A   
   A   
   C   
   A   
   G   
   C   
   C   
   A   
   A   
   T   
   A   
   T   
   C   
   A   
   A   
   A   
   G   
   C   
   T   
   T   
   T   
   T   
   A   
   G   
   T   
   G   
   A   
   T   
   A   
   A   
   G   
   T   
   T   
   C   
   G   
   G   
   C   
   G   
   T   
   T   
   T   
   C   
   T   
   G   
   T   
   T   
   G   
   A   
   T   
   G   
   A   
   A   
   A   
   T   
   C   
   A   
   A   
   T   
   C   
   A   
   A   
   A   
   A   
   G   
   T   
   T   
   A   
   A   
   G   
   C   
   C   
   A   
   A   
   G   
   G   
   G   
   T   
   G   
   G   
   G   
   T   
   C   
   C   
   A   
   A   
   G   
   C   
   A   
   G   
   A   
   C   
   T   
   C   
   C   
   T   
   T   
   T   
   G   
   T   
   A   
   C   
   C   
   A   
   A   
   T   
   C   
   A   
   C   
   A   
   G   
   T   
   C   
   G   
   C   
   T   
   T   
   C   
   T   
   G   
   A   
   A   
   C   
   C   
   A   
   G   
   T   
   G   
   A   
   C   
   A   
   G   
   A   
   A   
   T   
   T   
   A   
   C   
   C   
   A   
   A   
   C   
   A   
   G   
   G   
   G   
   G   
   C   
   T   
   G   
   C   
   G   
   A   
   C   
   A   
   A   
   A   
   A   
   G   
   A   
   T   
   A   
   C   
   A   
   G   
   A   
   G   
   T   
   C   
   A   
   C   
   G   
   T   
   T   
   A   
   T   
   T   
   A   
   T   
   C   
   C   
   G   
   C   
   T   
   G   
   G   
   G   
   G   
   G   
   A   
   A   
   G   
   C   
   A   
   G   
   C   
   T   
   G   
   C   
   G   
   C   
   A   
   A   
   T   
   T   
   A   
   A   
   T   
   C   
   G   
   G   
   G   
   T   
   A   
   T   
   A   
   C   
   A   
   G   
   G   
   C   
   A   
   C   
   C   
   A   
   T   
   T   
   A   
   C   
   T   
   G   
   C   
   A   
   G   
   A   
   A   
   G   
   A   
   T   
   A   
   T   
   T   
   A   
   A   
   A   
   A   
   A   
   A   
   A   
   A   
   T   
   C   
   C   
   A   
   G   
   A   
   G   
   T   
   T   
   A   
   A   
   G   
   T   
   A   
   G   
   T   
   A   
   C   
   G   
   G   
   G   
   C   
   G   
   T   
   A   
   A   
   T   
   T   
   G   
   G   
   T   
   A   
   A   
   A   
   A   
   C   
   T   
   G   
   G   
   C   
   T   
   T   
   A   
   G   
   A   
   A   
   C   
   G   
   A   
   G   
   C   
   G   
   T   
   T   
   T   
   G   
   A   
   T   
   A   
   A   
   A   
   G   
   A   
   A   
   T   
   T   
   A   
   A   
   G   
   A   
   G   
   G   
   G   
   C   
   A   
   A   
   G   
   A   
   T   
   G   
   G   
   T   
   G   
   G   
   C   
   T   
   C   
   A   
   C   
   T   
   A   
   G   
   T   
   T   
   A   
   T   
   T   
   T   
   T   
   A   
   G   
   A   
   T   
   G   
   A   
   C   
   A   
   A   
   A   
   G   
   A   
   G   
   A   
   A   
   T   
   G   
   T   
   C   
   A   
   A   
   A   
   A   
   A   
   A   
   G   
   C   
   T   
   T   
   T   
   A   
   C   
   A   
   A   
   A   
   C   
   C   
   A   
   A   
   A   
   G   
   A   
   G   
   A   
   A   
   A   
   A   
   A   
   A   
   G   
   A   
   T   
   G   
   G   
   T   
   C   
   A   
   A   
   A   
   C   
   G   
   A   
   T   
   T   
   A   
   A   
   A   
   T   
   T   
   A   
   A   
   C   
   G   
   A   
   T   
   T   
   G   
   A   
   C   
   A   
   G   
   T   
   G   
   G   
   C   
   G   
   T   
   A   
   C   
   A   
   A   
   C   
   A   
   A   
   C   
   A   
   A   
   G   
   C   
   C   
   T   
   T   
   C   
   G   
   C   
   T   
   A   
   T   
   A   
   T   
   T   
   T   
   G   
   A   
   C   
   A   
   A   
   A   
   C   
   G   
   C   
   C   
   C   
   T   
   G   
   G   
   T   
   A   
   G   
   T   
   G   
   C   
   G   
   G   
   T   
   C   
   A   
   T   
   T   
   A   
   C   
   C   
   G   
   A   
   T   
   C   
   C   
   T   
   C   
   A   
   A   
   A   
   A   
   A   
   G   
   G   
   C   
   G   
   A   
   T   
   T   
   T   
   A   
   T   
   T   
   A   
   G   
   C   
   A   
   A   
   C   
   G   
   G   
   T   
   T   
   A   
   G   
   T   
   T   
   C   
   A   
   C   
   C   
   T   
   T   
   C   
   G   
   T   
   A   
   T   
   G   
   A   
   T   
   C   
   C   
   T   
   A   
   A   
   T   
   A   
   A   
   A   
   A   
   T   
   G   
   G   
   C   
   C   
   A   
   A   
   T   
   G   
   G   
   C   
   A   
   T   
   T   
   T   
   C   
   T   
   C   
   A   
   A   
   A   
   A   
   A   
   G   
   A   
   A   
   T   
   A   
   T   
   G   
   A   
   T   
   G   
   C   
   T   
   T   
   A   
   C   
   A   
   A   
   T   
   A   
   A   
   C   
   A   
   A   
   C   
   A   
   A   
   A   
   G   
   A   
   T   
   T   
   T   
   A   
   C   
   C   
   A   
   T   
   T   
   C   
   A   
   C   
   A   
   G   
   C   
   A   
   C   
   G   
   T   
   T   
   T   
   T   
   G   
   C   
   G   
   A   
   C   
   A   
   G   
   G   
   C   
   T   
   A   
   T   
   G   
   C   
   A   
   C   
   C   
   A   
   G   
   G   
   C   
   T   
   C   
   T   
   A   
   C   
   C   
   T   
   T   
   T   
   A   
   A   
   A   
   A   
   C   
   A   
   A   
   T   
   T   
   A   
   C   
   C   
   G   
   G   
   T   
   G   
   C   
   C   
   A   
   T   
   T   
   G   
   G   
   T   
   T   
   T   
   A   
   G   
   A   
   T   
   A   
   C   
   A   
   G   
   G   
   G   
   A   
   C   
   A   
   T   
   T   
   G   
   A   
   A   
   G   
   C   
   C   
   A   
   G   
   A   
   T   
   G   
   A   
   A   
   G   
   A   
   G   
   C   
   T   
   A   
   G   
   A   
   A   
   A   
   T   
   T   
   A   
   A   
   T   
   G   
   G   
   T   
   T   
   T   
   G   
   A   
   A   
   A   
   T   
   G   
   G   
   C   
   A   
   A   
   A   
   A   
   A   
   G   
   A   
   T   
   A   
   A   
   A   
   T   
   C   
   T   
   T   
   G   
   G   
   G   
   G   
   C   
   G   
   G   
   C   
   T   
   A   
   T   
   T   
   T   
   T   
   G   
   C   
   G   
   A   
   C   
   A   
   C   
   G   
   T   
   G   
   T   
   A   
   A   
   A   
   A   
   G   
   A   
   A   
   G   
   C   
   A   
   A   
   G   
   T   
   C   
   C   
   A   
   G   
   T   
   C   
   A   
   A   
   T   
   C   
   T   
   G   
   C   
   G   
   T   
   A   
   C   
   C   
   G   
   C   
   T   
   T   
   T   
   A   
   G   
   T   
   T   
   A   
   A   
   T   
   T   
   C   
   A   
   G   
   A   
   T   
   A   
   A   
   T   
   A   
   T   
   T   
   T   
   A   
   T   
   T   
   T   
   T   
   G   
   C   
   C   
   C   
   A   
   A   
   C   
   A   
   A   
   A   
   C   
   A   
   T   
   T   
   A   
   C   
   G   
   G   
   A   
   T   
   G   
   G   
   G   
   A   
   G   
   A   
   A   
   G   
   A   
   C   
   A   
   A   
   A   
   T   
   T   
   T   
   A   
   G   
   A   
   G   
   C   
   G   
   G   
   G   
   T   
   C   
   T   
   T   
   A   
   A   
   T   
   A   
   A   
   A   
   T   
   T   
   C   
   A   
   T   
   T   
   T   
   T   
   C   
   G   
   G   
   T   
   G   
   A   
   G   
   G   
   A   
   A   
   C   
   T   
   A   
   G   
   A   
   T   
   T   
   T   
   A   
   C   
   C   
   A   
   A   
   T   
   T   
   G   
   C   
   C   
   A   
   T   
   G   
   A   
   C   
   G   
   C   
   C   
   T   
   G   
   C   
   T   
   C   
   A   
   A   
   A   
   T   
   T   
   T   
   C   
   A   
   A   
   A   
   T   
   G   
   A   
   A   
   G   
   A   
   T   
   A   
   A   
   A   
   T   
   T   
   T   
   A   
   A   
   T   
   T   
   C   
   T   
   G   
   A   
   A   
   A   
   T   
   T   
   C   
   T   
   T   
   T   
   T   
   G   
   G   
   C   
   A   
   G   
   A   
   T   
   A   
   C   
   G   
   G   
   G   
   C   
   T   
   A   
   C   
   G   
   G   
   C   
   C   
   A   
   A   
   G   
   G   
   C   
   C   
   A   
   A   
   T   
   T   
   G   
   T   
   T   
   A   
   A   
   T   
   T   
   T   
   C   
   G   
   C   
   C   
   A   
   A   
   T   
   C   
   C   
   A   
   G   
   C   
   A   
   A   
   G   
   C   
   T   
   A   
   C   
   C   
   A   
   T   
   G   
   T   
   A   
   T   
   A   
   G   
   T   
   G   
   T   
   T   
   T   
   T   
   C   
   C   
   A   
   A   
   A   
   A   
   T   
   A   
   A   
   T   
   G   
   G   
   G   
   A   
   C   
   A   
   T   
   T   
   A   
   G   
   T   
   C   
   T   
   A   
   T   
   C   
   C   
   A   
   A   
   A   
   A   
   C   
   T   
   A   
   G   
   T   
   T   
   T   
   T   
   A   
   G   
   A   
   C   
   A   
   A   
   A   
   G   
   A   
   A   
   A   
   C   
   G   
   A   
   A   
   G   
   A   
   A   
   A   
   A   
   A   
   A   
   G   
   A   
   C   
   A   
   A   
   T   
   G   
   T   
   A   
   A   
   T   
   C   
   A   
   G   
   C   
   G   
   C   
   C   
   A   
   A   
   C   
   G   
   C   
   A   
   G   
   C   
   G   
   A   
   A   
   T   
   A   
   C   
   A   
   A   
   T   
   T   
   G   
   C   
   C   
   A   
   C   
   A   
   G   
   A   
   T   
   C   
   T   
   T   
   T   
   T   
   A   
   G   
   G   
   T   
   A   
   G   
   T   
   G   
   T   
   G   
   G   
   A   
   A   
   G   
   A   
   T   
   C   
   C   
   T   
   T   
   C   
   A   
   G   
   G   
   T   
   T   
   A   
   T   
   G   
   T   
   T   
   T   
   A   
   T   
   A   
   A   
   T   
   A   
   T   
   G   
   T   
   A   
   C   
   A   
   A   
   T   
   C   
   C   
   G   
   A   
   A   
   C   
   T   
   T   
   C   
   T   
   C   
   A   
   T   
   T   
   G   
   G   
   C   
   T   
   G   
   C   
   T   
   A   
   A   
   A   
   A   
   C   
   A   
   G   
   G   
   A   
   A   
   C   
   A   
   G   
   C   
   T   
   G   
   A   
   A   
   A   
   T   
   A   
   A   
   A   
   A   
   G   
   A   
   T   
   A   
   A   
   A   
   C   
   A   
   A   
   G   
   A   
   T   
   A   
   C   
   A   
   G   
   A   
   T   
   G   
   G   
   C   
   A   
   A   
   A   
   G   
   A   
   A   
   A   
   A   
   C   
   A   
   G   
   T   
   T   
   T   
   C   
   C   
   T   
   T   
   C   
   T   
   A   
   A   
   C   
   G   
   T   
   T   
   A   
   G   
   A   
   T   
   C   
   G   
   T   
   A   
   G   
   T   
   A   
   A   
   C   
   A   
   A   
   T   
   A   
   A   
   A   
   T   
   T   
   C   
   T   
   T   
   A   
   A   
   C   
   A   
   A   
   T   
   G   
   A   
   T   
   T   
   A   
   T   
   G   
   G   
   T   
   T   
   G   
   A   
   A   
   A   
   A   
   T   
   T   
   C   
   A   
   G   
   G   
   A   
   G   
   A   
   A   
   A   
   A   
   T   
   G   
   G   
   T   
   T   
   C   
   T   
   G   
   C   
   A   
   A   
   C   
   A   
   G   
   A   
   T   
   A   
   T   
   C   
   A   
   G   
   T   
   A   
   A   
   A   
   C   
   C   
   A   
   T   
   T   
   A   
   A   
   T   
   T   
   G   
   A   
   T   
   T   
   A   
   C   
   T   
   T   
   A   
   G   
   A   
   A   
   G   
   C   
   A   
   A   
   C   
   C   
   A   
   T   
   T   
   A   
   A   
   A   
   -   
   -   
   -   
   -   
   -   
   -   
   -   
   -   
   -   
   -   
   -   
   -   
   -   
   -   
   -   
   -   
   -   
   -   
   -   
   -   
   -   
   -   
   -   
   -   
   -   
   -   
   -   
   -   
   -   
   -   
   -   
   -   
   -   
   -   
   -   
   -   
   -   
   -   
   -   
   -   
   -   
   -   
   -   
   -   
   -   
   -   
   -   
   -   
   -   
   -   
   -   
   -   
   -   
   -   
   -   
   -   
   -   
   -   
   -   
   -   
   -   
 

 
   N60114F   
   -   
   -   
   -   
   -   
   -   
   -   
   -   
   -   
   -   
   -   
   -   
   -   
   -   
   -   
   -   
   -   
   -   
   -   
   -   
   -   
   -   
   -   
   -   
   -   
   -   
   -   
   -   
   -   
   -   
   -   
   -   
   -   
   -   
   -   
   -   
   -   
   -   
   -   
   -   
   -   
   -   
   -   
   -   
   -   
   -   
   -   
   -   
   -   
   -   
   -   
   -   
   -   
   -   
   -   
   -   
   -   
   -   
   -   
   -   
   -   
   -   
   -   
   -   
   -   
   -   
   -   
   -   
   -   
   -   
   -   
   -   
   -   
   -   
   -   
   -   
   -   
   -   
   -   
   -   
   -   
   -   
   -   
   -   
   -   
   -   
   -   
   -   
   -   
   -   
   -   
   -   
   -   
   -   
   -   
   -   
   -   
   -   
   -   
   -   
   -   
   -   
   -   
   -   
   -   
   -   
   -   
   -   
   -   
   C   
   A   
   A   
   T   
   G   
   G   
   C   
   A   
   A   
   G   
   C   
   C   
   A   
   A   
   A   
   C   
   A   
   A   
   G   
   A   
    A    
   T   
   T   
   A   
   G   
   C   
    C    
   G   
   A   
   A   
   G   
   C   
   G   
   A   
   A   
   G   
   A   
   A   
   A   
   A   
    C    
   A   
   G   
   C   
    T    
   A   
    C    
   T   
    A    
    C    
   A   
   T   
   T   
   T   
   T   
   T   
   A   
   A   
   A   
    C    
   G   
   T   
   A   
   T   
   T   
   G   
    T    
   C   
   A   
   A   
   A   
   A   
   C   
   A   
   G   
    G    
   A   
   A   
   T   
   T   
   T   
   G   
   A   
   T   
   A   
   A   
   G   
   T   
   T   
   A   
   C   
   C   
    G    
   T   
   C   
   C   
   G   
   T   
   T   
    G    
   T   
   T   
   C   
   A   
   A   
   G   
   A   
   A   
    G    
   C   
   T   
   A   
   G   
   C   
   T   
   T   
   A   
   A   
   A   
   G   
   A   
   A   
   A   
   A   
   A   
   T   
   G   
   G   
    C    
   T   
   A   
   T   
   G   
   A   
   T   
   A   
   C   
   T   
   A   
   A   
   A   
   T   
   C   
    T    
   G   
   T   
   T   
   G   
   T   
   T   
   G   
   A   
   A   
   A   
   A   
   A   
   T   
   A   
   C   
   C   
   A   
   A   
   G   
   C   
   A   
   A   
   T   
   T   
   T   
   A   
   T   
   T   
   C   
   A   
   G   
   G   
   G   
   A   
   T   
   T   
   C   
   A   
   A   
   G   
   C   
    A    
   G   
   A   
   A   
    G    
   G   
    A    
   G   
   T   
   C   
   A   
   A   
    A    
   G   
   C   
   T   
   A   
   G   
   T   
   G   
   A   
   T   
   G   
   T   
    T    
   C   
   A   
   A   
   G   
   T   
    A    
   A   
   A   
   A   
   A   
   A   
    G    
   G   
    C    
   G   
   A   
   A   
   A   
   G   
   A   
    C    
   A   
   A   
    T    
   C   
   A   
   A   
   T   
   A   
   C   
   A   
   C   
   A   
   T   
   T   
   T   
   A   
    C    
   C   
   T   
   A   
   T   
   A   
   A   
   A   
   T   
   T   
   A   
   T   
   C   
   G   
   A   
   T   
   G   
   A   
   G   
    T    
   A   
   C   
    C    
   C   
   C   
   T   
   T   
   T   
   A   
   G   
   G   
   C   
   G   
   A   
   A   
   A   
   T   
   G   
   A   
   A   
   A   
   G   
   A   
   T   
    T    
   T   
   G   
   T   
   C   
   T   
   T   
   A   
   T   
   C   
   A   
   A   
   T   
   C   
   A   
   A   
   G   
   T   
   A   
   T   
    C    
   G   
    C    
   C   
   A   
   A   
    A    
   A   
   A   
   A   
   G   
   G   
    C    
   G   
   A   
   T   
   A   
    C    
   C   
   T   
   A   
   C   
   C   
   A   
   A   
   A   
   T   
    C    
    G    
   C   
   T   
   T   
   G   
   G   
    A    
    A    
    G    
   C   
   C   
    G    
   T   
   C   
   T   
   T   
   T   
   A   
   A   
   T   
   T   
   T   
   T   
   T   
   C   
   C   
   A   
   G   
   A   
   T   
   A   
   T   
   G   
   T   
   C   
   A   
   G   
   G   
   A   
   A   
   A   
   T   
   G   
   A   
   T   
   A   
   A   
   A   
    A    
   T   
   T   
   T   
   C   
   G   
   A   
   T   
   T   
   C   
   A   
   A   
   G   
   T   
   A   
   G   
   A   
   T   
   A   
   A   
   T   
   G   
   C   
   C   
   A   
   A   
   A   
   C   
   G   
   T   
   G   
   G   
   A   
   G   
   A   
   A   
   A   
   T   
   T   
   G   
   T   
   C   
   G   
   A   
   T   
   C   
   G   
   T   
   A   
   A   
    T    
   G   
   G   
   T   
   A   
   G   
   T   
   G   
   G   
   G   
   C   
   T   
   A   
   G   
    C    
   A   
   A   
   T   
   T   
   A   
   A   
   C   
   A   
   A   
   A   
   G   
   T   
   G   
   T   
   T   
   T   
   G   
   A   
    T    
   G   
   A   
   A   
   G   
   T   
   G   
   G   
   G   
   C   
   G   
   T   
   A   
   G   
   T   
    G    
   C   
   C   
   T   
    G    
   G   
   C   
   A   
   A   
   A   
   C   
   T   
    C    
   G   
   G   
   T   
   T   
   C   
   T   
   G   
   G   
   C   
   G   
   C   
   A   
   G   
   A   
   A   
   A   
   A   
   A   
    A    
   C   
   A   
   G   
   C   
    C    
   A   
   A   
   T   
   A   
   T   
   C   
   A   
   A   
   A   
   G   
   C   
   T   
   T   
   T   
   T   
   A   
   G   
   T   
   G   
   A   
   T   
   A   
   A   
    A    
   T   
   T   
    C    
   G   
   G   
   C   
   G   
   T   
   T   
   T   
   C   
   T   
    G    
   T   
   T   
   G   
   A   
   T   
   G   
   A   
   A   
   A   
   T   
   C   
   A   
   A   
   T   
   C   
    A    
   A   
   A   
   A   
    G    
   T   
   T   
   A   
   A   
   G   
   C   
   C   
   A   
   A   
   G   
   G   
    A    
   T   
   G   
   G   
   G   
   T   
   C   
   C   
   A   
   A   
   G   
   C   
   A   
   G   
   A   
   C   
   T   
   C   
    C    
   T   
   T   
   T   
   G   
   T   
   A   
   C   
   C   
   A   
   A   
   T   
   C   
   A   
   C   
   A   
   G   
   T   
    C    
   G   
   C   
   T   
   T   
   C   
   T   
   G   
   A   
   A   
   C   
   C   
   A   
   G   
   T   
   G   
   A   
   C   
   A   
   G   
   A   
   A   
   T   
   T   
   A   
   C   
   C   
   A   
   A   
   C   
   A   
   G   
   G   
   G   
   G   
   C   
   T   
   G   
   C   
   G   
   A   
   C   
   A   
   A   
   A   
   A   
   G   
    A    
    T    
   A   
   C   
   A   
   G   
   A   
   G   
   T   
   C   
   A   
   C   
   G   
   T   
   T   
   A   
   T   
   T   
   A   
   T   
   C   
   C   
    G    
    C    
   T   
   G   
   G   
   G   
   G   
   G   
   A   
   A   
   G   
   C   
   A   
   G   
   C   
   T   
   G   
   C   
   G   
   C   
   A   
   A   
   T   
   T   
   A   
   A   
   T   
   C   
   G   
   G   
    G    
   T   
   A   
   T   
   A   
   C   
    G    
   G   
   G   
    C    
   A   
   C   
    C    
   A   
   T   
   T   
   A   
   C   
   T   
   G   
   C   
   A   
   G   
   A   
    A    
   G   
   A   
   T   
   A   
   T   
   T   
    G    
   A   
   A   
   A   
   A   
   A   
   A   
   A   
   T   
   C   
   C   
   A   
   G   
   A   
   G   
   T   
   T   
   A   
   A   
   G   
   T   
   A   
   G   
   T   
   A   
   C   
   G   
   G   
   G   
    C    
   G   
   T   
   A   
   A   
   T   
   T   
   G   
   G   
    T    
   A   
   A   
   A   
   A   
   C   
   T   
   G   
   G   
    T    
   T   
   T   
   A   
   G   
   A   
   A   
   C   
   G   
   A   
   G   
   C   
   G   
   T   
   T   
   T   
   G   
   A   
   T   
   A   
   A   
   A   
   G   
   A   
    A    
   T   
   T   
   A   
   A   
   G   
   A   
   G   
   G   
    G    
   C   
   A   
   A   
   G   
   A   
   T   
   G   
   G   
    T    
   G   
   G   
    C    
   T   
   C   
    A    
   C   
   T   
   A   
   G   
   T   
   T   
   A   
   T   
   T   
   T   
   T   
   A   
   G   
   A   
   T   
   G   
   A   
   C   
   A   
   A   
   A   
   G   
   A   
   G   
   A   
   A   
   T   
   G   
   T   
   C   
   A   
   A   
   A   
   A   
   A   
   A   
   G   
   C   
   T   
   T   
   T   
   A   
   C   
   A   
   A   
   A   
   C   
   C   
   A   
   A   
   A   
   G   
   A   
    G    
   A   
   A   
   A   
   A   
   A   
   A   
   G   
   A   
   T   
   G   
   G   
   T   
   C   
   A   
   A   
   A   
   C   
    G    
   A   
   T   
   T   
   A   
   A   
   A   
   T   
   T   
   A   
   A   
   C   
   G   
   A   
   T   
   T   
   G   
   A   
    C    
   A   
   G   
    T    
   G   
   G   
    C    
   G   
   T   
   A   
   C   
   A   
   A   
   C   
   A   
   A   
   C   
   A   
   A   
   G   
   C   
    C    
   T   
   T   
    C    
   G   
   C   
   T   
   A   
   T   
   A   
   T   
   T   
   T   
   G   
   A   
   C   
   A   
   A   
   A   
   C   
   G   
    T    
    C    
   C   
   T   
   G   
   G   
   T   
   A   
   G   
   T   
   G   
   C   
   G   
   G   
   T   
   C   
   A   
   T   
   T   
   A   
   C   
   C   
   G   
   A   
   T   
   C   
   C   
   T   
   C   
   A   
   A   
   A   
   A   
   A   
   G   
   G   
   C   
   G   
   A   
   T   
   T   
   T   
   A   
    T    
   T   
   A   
   G   
   C   
    A    
   A   
   C   
   G   
   G   
   T   
   T   
   A   
   G   
   T   
   T   
   C   
   A   
   C   
   C   
   T   
   T   
   C   
   G   
   T   
   A   
   T   
   G   
   A   
   T   
   C   
   C   
   T   
   A   
   A   
   T   
   A   
   A   
   A   
   A   
   T   
   G   
   G   
   C   
   C   
   A   
   A   
   T   
   G   
   G   
    T    
   A   
   T   
   T   
   T   
   C   
   T   
   C   
   A   
   A   
   A   
   A   
   A   
   G   
   A   
    A    
   T   
   A   
   T   
   G   
   A   
   T   
   G   
   C   
   T   
   T   
   A   
   C   
   A   
   A   
   T   
   A   
   A   
   C   
   A   
   A   
   C   
   A   
   A   
   A   
   G   
   A   
   T   
   T   
   T   
   A   
   C   
   C   
   A   
   T   
   T   
    C    
   A   
   C   
   A   
   G   
   C   
   A   
   C   
   G   
   T   
   T   
   T   
    T    
   G   
   C   
   G   
   A   
   C   
   A   
   G   
   G   
    C    
   T   
   A   
   T   
   G   
   C   
   A   
   C   
   C   
   A   
   G   
   G   
    C    
   T   
   C   
   T   
   A   
   C   
   C   
   T   
   T   
   T   
   A   
   A   
   A   
   A   
   C   
   A   
   A   
   T   
   T   
   A   
   C   
   C   
   G   
   G   
   T   
   G   
   C   
   C   
   A   
   T   
   T   
   G   
   G   
   T   
   T   
   T   
   A   
   G   
   A   
   T   
    G    
   C   
   A   
   G   
   G   
   G   
   A   
   C   
   A   
   T   
   T   
   G   
   A   
   A   
   G   
   C   
   C   
   A   
   G   
   A   
   T   
   G   
   A   
   A   
   G   
   A   
   G   
   C   
   T   
   A   
   G   
   A   
   A   
   A   
   T   
   T   
   A   
   A   
   T   
   G   
   G   
    T    
   T   
   T   
   G   
   A   
   A   
   A   
   T   
   G   
   G   
   C   
   A   
   A   
   A   
   A   
   A   
   G   
   A   
   T   
   A   
   A   
   A   
   T   
   C   
   T   
   T   
   G   
   G   
   G   
   G   
    C    
   G   
   G   
   C   
   T   
   A   
   T   
   T   
   T   
   T   
   G   
   C   
    G    
   A   
   C   
   A   
   C   
   G   
   T   
   G   
   T   
   A   
   A   
   A   
   A   
   G   
   A   
   A   
   G   
   C   
   A   
   A   
   G   
   T   
   C   
   C   
   A   
   G   
   T   
   C   
   A   
   A   
   T   
   C   
   T   
    G    
   C   
   G   
   T   
   A   
   C   
   C   
   G   
   C   
   T   
   T   
   T   
   A   
   G   
   T   
   T   
   A   
   A   
   T   
   T   
   C   
   A   
   G   
   A   
   T   
   A   
   A   
   T   
   A   
   T   
   T   
   T   
   A   
   T   
   T   
   T   
   T   
   G   
   C   
    C    
   C   
   A   
   A   
   C   
   A   
   A   
   A   
   C   
   A   
   T   
   T   
   A   
   C   
   G   
    G    
   A   
   T   
   G   
   G   
   G   
    A    
   G   
   A   
   A   
   G   
   A   
   C   
   A   
   A   
   A   
   T   
   T   
   T   
   A   
   G   
   A   
   G   
   C   
    G    
   G   
   G   
   T   
   C   
   T   
   T   
   A   
   A   
   T   
   A   
   A   
   A   
   T   
   T   
   C   
   A   
   T   
   T   
   T   
   T   
   C   
   G   
   G   
   T   
   G   
   A   
   G   
   G   
   A   
   A   
   C   
   T   
   A   
   G   
   A   
   T   
   T   
   T   
   A   
   C   
   C   
   A   
   A   
   T   
    T    
   G   
   C   
   C   
   A   
   T   
   G   
   A   
   C   
   G   
   C   
   C   
    T    
   G   
   C   
   T   
   C   
   A   
   A   
   A   
   T   
   T   
   T   
   C   
   A   
   A   
   A   
    T    
   G   
   A   
   A   
   G   
   A   
   T   
   A   
   A   
   A   
   T   
   T   
   T   
   A   
   A   
   T   
   T   
   C   
   T   
   G   
   A   
   A   
   A   
   T   
   T   
   C   
   T   
   T   
   T   
   T   
    A    
   G   
   C   
   A   
   G   
   A   
   T   
   A   
   C   
    G    
   G   
   G   
   C   
   T   
   A   
    C    
   G   
   G   
    T    
   C   
   A   
   A   
   G   
   G   
    T    
   C   
   A   
   A   
   T   
   T   
   G   
   T   
   T   
   A   
   A   
   T   
   T   
   T   
   C   
    G    
   C   
   C   
   A   
   A   
   T   
   C   
   C   
   A   
   G   
   C   
   A   
   A   
   G   
   C   
   T   
   A   
   C   
   C   
   A   
   T   
   G   
   T   
   A   
   T   
   A   
   G   
   T   
   G   
   T   
   T   
   T   
   T   
   C   
   C   
   A   
   A   
   A   
   A   
   T   
   A   
   A   
   T   
   G   
   G   
    A    
   A   
   C   
   A   
    T    
   T   
    A    
   G   
   T   
    C    
   T   
   A   
   T   
   C   
   C   
   A   
   A   
   A   
   A   
    C    
   T   
   A   
   G   
   T   
   T   
   T   
   T   
   A   
   G   
   A   
   C   
   A   
   A   
   A   
   G   
   A   
   A   
   A   
   C   
    G    
   A   
   A   
   G   
   A   
   A   
   A   
   A   
   A   
   A   
   G   
   A   
   C   
   A   
   A   
   T   
   G   
   T   
   A   
   A   
   T   
   C   
   A   
   G   
   C   
   G   
   C   
   C   
   A   
   A   
   C   
   G   
   C   
   A   
   G   
   C   
   G   
   A   
   A   
   T   
   A   
   C   
   A   
   A   
   T   
   T   
   G   
   C   
   C   
   A   
   C   
   A   
   G   
   A   
   T   
   C   
   T   
   T   
   T   
   T   
   A   
   G   
   G   
   T   
   A   
   G   
   T   
   G   
   T   
   G   
   G   
   A   
   A   
   G   
   A   
   T   
   C   
   C   
   T   
   T   
   C   
   A   
   G   
   G   
   T   
   T   
   A   
   T   
   G   
   T   
   T   
   T   
   A   
   T   
   A   
   A   
   T   
   A   
   T   
   G   
   T   
   A   
   C   
   A   
   A   
   T   
   C   
   C   
   G   
   A   
   A   
   C   
   T   
   T   
   C   
   T   
   C   
   A   
   T   
   T   
   G   
   G   
   C   
   T   
   G   
   C   
   T   
   A   
   A   
   A   
   A   
   C   
   A   
   G   
   G   
   A   
   A   
   C   
   A   
   G   
   C   
   T   
   G   
   A   
   A   
   A   
   T   
   A   
   A   
   A   
   A   
   G   
   A   
   T   
   A   
   A   
   A   
   C   
   A   
   A   
   G   
   A   
   T   
   A   
   C   
   A   
   G   
   A   
   T   
   G   
   G   
   C   
   A   
   A   
   A   
   G   
   A   
   A   
   A   
   A   
   C   
   A   
   G   
   T   
   T   
   T   
   C   
   C   
   T   
   T   
   C   
   T   
   A   
   A   
   C   
   G   
   T   
   T   
   A   
   G   
   A   
   T   
   C   
   G   
   T   
   A   
   G   
   T   
   A   
   A   
   C   
   A   
   A   
   T   
   A   
   A   
   A   
   T   
   T   
   C   
   T   
   T   
   A   
   A   
   C   
   A   
   A   
   T   
   G   
   A   
   T   
   T   
   A   
   T   
   G   
   G   
   T   
   T   
   G   
   A   
   A   
   A   
   A   
   T   
   T   
   C   
   A   
   G   
   G   
   A   
   G   
   A   
   A   
   A   
   A   
   T   
   G   
   G   
   T   
   T   
   C   
   T   
   G   
   C   
   A   
   A   
   C   
   A   
   G   
   A   
   T   
   A   
   T   
   C   
   A   
   G   
   T   
   A   
   A   
   A   
   C   
   C   
   A   
   T   
   T   
   A   
   A   
   T   
   T   
   G   
   A   
   T   
   T   
   A   
   C   
   T   
   T   
   A   
   G   
   A   
   A   
   G   
   C   
   A   
   A   
   C   
   C   
   A   
   T   
   T   
   A   
   A   
   A   
   -   
   -   
   -   
   -   
   -   
   -   
   -   
   -   
   -   
   -   
   -   
   -   
   -   
   -   
   -   
   -   
   -   
   -   
   -   
   -   
   -   
   -   
   -   
   -   
   -   
   -   
   -   
   -   
   -   
   -   
   -   
   -   
   -   
   -   
   -   
   -   
   -   
   -   
   -   
   -   
   -   
   -   
   -   
   -   
   -   
   -   
   -   
   -   
   -   
   -   
   -   
   -   
   -   
   -   
   -   
   -   
   -   
   -   
   -   
   -   
   -   
   -   
   -   
   -   
   -   
   -   
   -   
   -   
   -   
   -   
   -   
   -   
   -   
   -   
   -   
   -   
   -   
   -   
   -   
   -   
   -   
   -   
   -   
   -   
   -   
   -   
   -   
   -   
   -   
   -   
   -   
   -   
   -   
   -   
   -   
   -   
   -   
   -   
   -   
   -   
   -   
   -   
   -   
   -   
   -   
   -   
   -   
   -   
   -   
   -   
   -   
   -   
   -   
   -   
   -   
   -   
   -   
   -   
   -   
   -   
   -   
   -   
   -   
   -   
   -   
   -   
   -   
   -   
   -   
   -   
   -   
   -   
   -   
   -   
   -   
   -   
   -   
   -   
   -   
   -   
   -   
   -   
   -   
   -   
   -   
   -   
   -   
   -   
   -   
   -   
   -   
   -   
   -   
   -   
   -   
   -   
   -   
   -   
   -   
   -   
   -   
   -   
   -   
   -   
   -   
   -   
   -   
   -   
   -   
   C   
   A   
   A   
   T   
   G   
   G   
   C   
   A   
   A   
   G   
   C   
   C   
   A   
   A   
   A   
   C   
   A   
   A   
   G   
   A   
   A   
   T   
   T   
   A   
   G   
   C   
   C   
   G   
   A   
   A   
   G   
   C   
   G   
   A   
   A   
   G   
   A   
   A   
   A   
   A   
   C   
   A   
   G   
   C   
   T   
   A   
   C   
   T   
   A   
   C   
   A   
   T   
   T   
   T   
   T   
   T   
   A   
   A   
   A   
   C   
   G   
   T   
   A   
   T   
   T   
   G   
   T   
   C   
   A   
   A   
   A   
   A   
   C   
   A   
   G   
   G   
   A   
   A   
   T   
   T   
   T   
   G   
   A   
   T   
   A   
   A   
   G   
   T   
   T   
   A   
   C   
   C   
   G   
   T   
   C   
   C   
   G   
   T   
   T   
   G   
   T   
   T   
   C   
   A   
   A   
   G   
   A   
   A   
   G   
   C   
   T   
   A   
   G   
   C   
   T   
   T   
   A   
   A   
   A   
   G   
   A   
   A   
   A   
   A   
   A   
   T   
   G   
   G   
   C   
   T   
   A   
   T   
   G   
   A   
   T   
   A   
   C   
   T   
   A   
   A   
   A   
   T   
   C   
   T   
   G   
   T   
   T   
   G   
   T   
   T   
   G   
   A   
   A   
   A   
   A   
   A   
   T   
   A   
   C   
   C   
   A   
   A   
   G   
   C   
   A   
   A   
   T   
   T   
   T   
   A   
   T   
   T   
   C   
   A   
   G   
   G   
   G   
   A   
   T   
   T   
   C   
   A   
   A   
   G   
   C   
   A   
   G   
   A   
   A   
   G   
   G   
   A   
   G   
   T   
   C   
   A   
   A   
   A   
   G   
   C   
   T   
   A   
   G   
   T   
   G   
   A   
   T   
   G   
   T   
   T   
   C   
   A   
   A   
   G   
   T   
   A   
   A   
   A   
   A   
   A   
   A   
   G   
   G   
   C   
   G   
   A   
   A   
   A   
   G   
   A   
   C   
   A   
   A   
   T   
   C   
   A   
   A   
   T   
   A   
   C   
   A   
   C   
   A   
   T   
   T   
   T   
   A   
   C   
   C   
   T   
   A   
   T   
   A   
   A   
   A   
   T   
   T   
   A   
   T   
   C   
   G   
   A   
   T   
   G   
   A   
   G   
   T   
   A   
   C   
   C   
   C   
   C   
   T   
   T   
   T   
   A   
   G   
   G   
   C   
   G   
   A   
   A   
   A   
   T   
   G   
   A   
   A   
   A   
   G   
   A   
   T   
   T   
   T   
   G   
   T   
   C   
   T   
   T   
   A   
   T   
   C   
   A   
   A   
   T   
   C   
   A   
   A   
   G   
   T   
   A   
   T   
   C   
   G   
   C   
   C   
   A   
   A   
   A   
   A   
   A   
   A   
   G   
   G   
   C   
   G   
   A   
   T   
   A   
   C   
   C   
   T   
   A   
   C   
   C   
   A   
   A   
   A   
   T   
   C   
   G   
   C   
   T   
   T   
   G   
   G   
   A   
   A   
   G   
   C   
   C   
   G   
   T   
   C   
   T   
   T   
   T   
   A   
   A   
   T   
   T   
   T   
   T   
   T   
   C   
   C   
   A   
   G   
   A   
   T   
   A   
   T   
   G   
   T   
   C   
   A   
   G   
   G   
   A   
   A   
   A   
   T   
   G   
   A   
   T   
   A   
   A   
   A   
   A   
   T   
   T   
   T   
   C   
   G   
   A   
   T   
   T   
   C   
   A   
   A   
   G   
   T   
   A   
   G   
   A   
   T   
   A   
   A   
   T   
   G   
   C   
   C   
   A   
   A   
   A   
   C   
   G   
   T   
   G   
   G   
   A   
   G   
   A   
   A   
   A   
   T   
   T   
   G   
   T   
   C   
   G   
   A   
   T   
   C   
   G   
   T   
   A   
   A   
   T   
   G   
   G   
   T   
   A   
   G   
   T   
   G   
   G   
   G   
   C   
   T   
   A   
   G   
   C   
   A   
   A   
   T   
   T   
   A   
   A   
   C   
   A   
   A   
   A   
   G   
   T   
   G   
   T   
   T   
   T   
   G   
   A   
   T   
   G   
   A   
   A   
   G   
   T   
   G   
   G   
   G   
   C   
   G   
   T   
   A   
   G   
   T   
   G   
   C   
   C   
   T   
   G   
   G   
   C   
   A   
   A   
   A   
   C   
   T   
   C   
   G   
   G   
   T   
   T   
   C   
   T   
   G   
   G   
   C   
   G   
   C   
   A   
   G   
   A   
   A   
   A   
   A   
   A   
   A   
   C   
   A   
   G   
   C   
   C   
   A   
   A   
   T   
   A   
   T   
   C   
   A   
   A   
   A   
   G   
   C   
   T   
   T   
   T   
   T   
   A   
   G   
   T   
   G   
   A   
   T   
   A   
   A   
   A   
   T   
   T   
   C   
   G   
   G   
   C   
   G   
   T   
   T   
   T   
   C   
   T   
   G   
   T   
   T   
   G   
   A   
   T   
   G   
   A   
   A   
   A   
   T   
   C   
   A   
   A   
   T   
   C   
   A   
   A   
   A   
   A   
   G   
   T   
   T   
   A   
   A   
   G   
   C   
   C   
   A   
   A   
   G   
   G   
   A   
   T   
   G   
   G   
   G   
   T   
   C   
   C   
   A   
   A   
   G   
   C   
   A   
   G   
   A   
   C   
   T   
   C   
   C   
   T   
   T   
   T   
   G   
   T   
   A   
   C   
   C   
   A   
   A   
   T   
   C   
   A   
   C   
   A   
   G   
   T   
   C   
   G   
   C   
   T   
   T   
   C   
   T   
   G   
   A   
   A   
   C   
   C   
   A   
   G   
   T   
   G   
   A   
   C   
   A   
   G   
   A   
   A   
   T   
   T   
   A   
   C   
   C   
   A   
   A   
   C   
   A   
   G   
   G   
   G   
   G   
   C   
   T   
   G   
   C   
   G   
   A   
   C   
   A   
   A   
   A   
   A   
   G   
   A   
   T   
   A   
   C   
   A   
   G   
   A   
   G   
   T   
   C   
   A   
   C   
   G   
   T   
   T   
   A   
   T   
   T   
   A   
   T   
   C   
   C   
   G   
   C   
   T   
   G   
   G   
   G   
   G   
   G   
   A   
   A   
   G   
   C   
   A   
   G   
   C   
   T   
   G   
   C   
   G   
   C   
   A   
   A   
   T   
   T   
   A   
   A   
   T   
   C   
   G   
   G   
   G   
   T   
   A   
   T   
   A   
   C   
   G   
   G   
   G   
   C   
   A   
   C   
   C   
   A   
   T   
   T   
   A   
   C   
   T   
   G   
   C   
   A   
   G   
   A   
   A   
   G   
   A   
   T   
   A   
   T   
   T   
   G   
   A   
   A   
   A   
   A   
   A   
   A   
   A   
   T   
   C   
   C   
   A   
   G   
   A   
   G   
   T   
   T   
   A   
   A   
   G   
   T   
   A   
   G   
   T   
   A   
   C   
   G   
   G   
   G   
   C   
   G   
   T   
   A   
   A   
   T   
   T   
   G   
   G   
   T   
   A   
   A   
   A   
   A   
   C   
   T   
   G   
   G   
   T   
   T   
   T   
   A   
   G   
   A   
   A   
   C   
   G   
   A   
   G   
   C   
   G   
   T   
   T   
   T   
   G   
   A   
   T   
   A   
   A   
   A   
   G   
   A   
   A   
   T   
   T   
   A   
   A   
   G   
   A   
   G   
   G   
   G   
   C   
   A   
   A   
   G   
   A   
   T   
   G   
   G   
   T   
   G   
   G   
   C   
   T   
   C   
   A   
   C   
   T   
   A   
   G   
   T   
   T   
   A   
   T   
   T   
   T   
   T   
   A   
   G   
   A   
   T   
   G   
   A   
   C   
   A   
   A   
   A   
   G   
   A   
   G   
   A   
   A   
   T   
   G   
   T   
   C   
   A   
   A   
   A   
   A   
   A   
   A   
   G   
   C   
   T   
   T   
   T   
   A   
   C   
   A   
   A   
   A   
   C   
   C   
   A   
   A   
   A   
   G   
   A   
   G   
   A   
   A   
   A   
   A   
   A   
   A   
   G   
   A   
   T   
   G   
   G   
   T   
   C   
   A   
   A   
   A   
   C   
   G   
   A   
   T   
   T   
   A   
   A   
   A   
   T   
   T   
   A   
   A   
   C   
   G   
   A   
   T   
   T   
   G   
   A   
   C   
   A   
   G   
   T   
   G   
   G   
   C   
   G   
   T   
   A   
   C   
   A   
   A   
   C   
   A   
   A   
   C   
   A   
   A   
   G   
   C   
   C   
   T   
   T   
   C   
   G   
   C   
   T   
   A   
   T   
   A   
   T   
   T   
   T   
   G   
   A   
   C   
   A   
   A   
   A   
   C   
   G   
   T   
   C   
   C   
   T   
   G   
   G   
   T   
   A   
   G   
   T   
   G   
   C   
   G   
   G   
   T   
   C   
   A   
   T   
   T   
   A   
   C   
   C   
   G   
   A   
   T   
   C   
   C   
   T   
   C   
   A   
   A   
   A   
   A   
   A   
   G   
   G   
   C   
   G   
   A   
   T   
   T   
   T   
   A   
   T   
   T   
   A   
   G   
   C   
   A   
   A   
   C   
   G   
   G   
   T   
   T   
   A   
   G   
   T   
   T   
   C   
   A   
   C   
   C   
   T   
   T   
   C   
   G   
   T   
   A   
   T   
   G   
   A   
   T   
   C   
   C   
   T   
   A   
   A   
   T   
   A   
   A   
   A   
   A   
   T   
   G   
   G   
   C   
   C   
   A   
   A   
   T   
   G   
   G   
   T   
   A   
   T   
   T   
   T   
   C   
   T   
   C   
   A   
   A   
   A   
   A   
   A   
   G   
   A   
   A   
   T   
   A   
   T   
   G   
   A   
   T   
   G   
   C   
   T   
   T   
   A   
   C   
   A   
   A   
   T   
   A   
   A   
   C   
   A   
   A   
   C   
   A   
   A   
   A   
   G   
   A   
   T   
   T   
   T   
   A   
   C   
   C   
   A   
   T   
   T   
   C   
   A   
   C   
   A   
   G   
   C   
   A   
   C   
   G   
   T   
   T   
   T   
   T   
   G   
   C   
   G   
   A   
   C   
   A   
   G   
   G   
   C   
   T   
   A   
   T   
   G   
   C   
   A   
   C   
   C   
   A   
   G   
   G   
   C   
   T   
   C   
   T   
   A   
   C   
   C   
   T   
   T   
   T   
   A   
   A   
   A   
   A   
   C   
   A   
   A   
   T   
   T   
   A   
   C   
   C   
   G   
   G   
   T   
   G   
   C   
   C   
   A   
   T   
   T   
   G   
   G   
   T   
   T   
   T   
   A   
   G   
   A   
   T   
   G   
   C   
   A   
   G   
   G   
   G   
   A   
   C   
   A   
   T   
   T   
   G   
   A   
   A   
   G   
   C   
   C   
   A   
   G   
   A   
   T   
   G   
   A   
   A   
   G   
   A   
   G   
   C   
   T   
   A   
   G   
   A   
   A   
   A   
   T   
   T   
   A   
   A   
   T   
   G   
   G   
   T   
   T   
   T   
   G   
   A   
   A   
   A   
   T   
   G   
   G   
   C   
   A   
   A   
   A   
   A   
   A   
   G   
   A   
   T   
   A   
   A   
   A   
   T   
   C   
   T   
   T   
   G   
   G   
   G   
   G   
   C   
   G   
   G   
   C   
   T   
   A   
   T   
   T   
   T   
   T   
   G   
   C   
   G   
   A   
   C   
   A   
   C   
   G   
   T   
   G   
   T   
   A   
   A   
   A   
   A   
   G   
   A   
   A   
   G   
   C   
   A   
   A   
   G   
   T   
   C   
   C   
   A   
   G   
   T   
   C   
   A   
   A   
   T   
   C   
   T   
   G   
   C   
   G   
   T   
   A   
   C   
   C   
   G   
   C   
   T   
   T   
   T   
   A   
   G   
   T   
   T   
   A   
   A   
   T   
   T   
   C   
   A   
   G   
   A   
   T   
   A   
   A   
   T   
   A   
   T   
   T   
   T   
   A   
   T   
   T   
   T   
   T   
   G   
   C   
   C   
   C   
   A   
   A   
   C   
   A   
   A   
   A   
   C   
   A   
   T   
   T   
   A   
   C   
   G   
   G   
   A   
   T   
   G   
   G   
   G   
   A   
   G   
   A   
   A   
   G   
   A   
   C   
   A   
   A   
   A   
   T   
   T   
   T   
   A   
   G   
   A   
   G   
   C   
   G   
   G   
   G   
   T   
   C   
   T   
   T   
   A   
   A   
   T   
   A   
   A   
   A   
   T   
   T   
   C   
   A   
   T   
   T   
   T   
   T   
   C   
   G   
   G   
   T   
   G   
   A   
   G   
   G   
   A   
   A   
   C   
   T   
   A   
   G   
   A   
   T   
   T   
   T   
   A   
   C   
   C   
   A   
   A   
   T   
   T   
   G   
   C   
   C   
   A   
   T   
   G   
   A   
   C   
   G   
   C   
   C   
   T   
   G   
   C   
   T   
   C   
   A   
   A   
   A   
   T   
   T   
   T   
   C   
   A   
   A   
   A   
   T   
   G   
   A   
   A   
   G   
   A   
   T   
   A   
   A   
   A   
   T   
   T   
   T   
   A   
   A   
   T   
   T   
   C   
   T   
   G   
   A   
   A   
   A   
   T   
   T   
   C   
   T   
   T   
   T   
   T   
   A   
   G   
   C   
   A   
   G   
   A   
   T   
   A   
   C   
   G   
   G   
   G   
   C   
   T   
   A   
   C   
   G   
   G   
   T   
   C   
   A   
   A   
   G   
   G   
   T   
   C   
   A   
   A   
   T   
   T   
   G   
   T   
   T   
   A   
   A   
   T   
   T   
   T   
   C   
   G   
   C   
   C   
   A   
   A   
   T   
   C   
   C   
   A   
   G   
   C   
   A   
   A   
   G   
   C   
   T   
   A   
   C   
   C   
   A   
   T   
   G   
   T   
   A   
   T   
   A   
   G   
   T   
   G   
   T   
   T   
   T   
   T   
   C   
   C   
   A   
   A   
   A   
   A   
   T   
   A   
   A   
   T   
   G   
   G   
   A   
   A   
   C   
   A   
   T   
   T   
   A   
   G   
   T   
   C   
   T   
   A   
   T   
   C   
   C   
   A   
   A   
   A   
   A   
   C   
   T   
   A   
   G   
   T   
   T   
   T   
   T   
   A   
   G   
   A   
   C   
   A   
   A   
   A   
   G   
   A   
   A   
   A   
   C   
   G   
   A   
   A   
   G   
   A   
   A   
   A   
   A   
   A   
   A   
   G   
   A   
   C   
   A   
   A   
   T   
   G   
   T   
   A   
   A   
   T   
   C   
   A   
   G   
   C   
   G   
   C   
   C   
   A   
   A   
   C   
   G   
   C   
   A   
   G   
   C   
   G   
   A   
   A   
   T   
   A   
   C   
   A   
   A   
   T   
   T   
   G   
   C   
   C   
   A   
   C   
   A   
   G   
   A   
   T   
   C   
   T   
   T   
   T   
   T   
   A   
   G   
   G   
   T   
   A   
   G   
   T   
   G   
   T   
   G   
   G   
   A   
   A   
   G   
   A   
   T   
   C   
   C   
   T   
   T   
   C   
   A   
   G   
   G   
   T   
   T   
   A   
   T   
   G   
   T   
   T   
   T   
   A   
   T   
   A   
   A   
   T   
   A   
   T   
   G   
   T   
   A   
   C   
   A   
   A   
   T   
   C   
   C   
   G   
   A   
   A   
   C   
   T   
   T   
   C   
   T   
   C   
   A   
   T   
   T   
   G   
   G   
   C   
   T   
   G   
   C   
   T   
   A   
   A   
   A   
   A   
   C   
   A   
   G   
   G   
   A   
   A   
   C   
   A   
   G   
   C   
   T   
   G   
   A   
   A   
   A   
   T   
   A   
   A   
   A   
   A   
   G   
   A   
   T   
   A   
   A   
   A   
   C   
   A   
   A   
   G   
   A   
   T   
   A   
   C   
   A   
   G   
   A   
   T   
   G   
   G   
   C   
   A   
   A   
   A   
   G   
   A   
   A   
   A   
   A   
   C   
   A   
   G   
   T   
   T   
   T   
   C   
   C   
   T   
   T   
   C   
   T   
   A   
   A   
   C   
   G   
   T   
   T   
   A   
   G   
   A   
   T   
   C   
   G   
   T   
   A   
   G   
   T   
   A   
   A   
   C   
   A   
   A   
   T   
   A   
   A   
   A   
   T   
   T   
   C   
   T   
   T   
   A   
   A   
   C   
   A   
   A   
   T   
   G   
   A   
   T   
   T   
   A   
   T   
   G   
   G   
   T   
   T   
   G   
   A   
   A   
   A   
   A   
   T   
   T   
   C   
   A   
   G   
   G   
   A   
   G   
   A   
   A   
   A   
   A   
   T   
   G   
   G   
   T   
   T   
   C   
   T   
   G   
   C   
   A   
   A   
   C   
   A   
   G   
   A   
   T   
   A   
   T   
   C   
   A   
   G   
   T   
   A   
   A   
   A   
   C   
   C   
   A   
   T   
   T   
   A   
   A   
   T   
   T   
   G   
   A   
   T   
   T   
   A   
   C   
   T   
   T   
   A   
   G   
   A   
   A   
   G   
   C   
   A   
   A   
   C   
   C   
   A   
   T   
   T   
   A   
   A   
   A   
   -   
   -   
   -   
   -   
   -   
   -   
   -   
   -   
   -   
   -   
   -   
   -   
   -   
   -   
   -   
   -   
   -   
   -   
   -   
   -   
   -   
   -   
   -   
   -   
   -   
   -   
   -   
   -   
   -   
   -   
   -   
   -   
   -   
   -   
   -   
   -   
   -   
   -   
   -   
   -   
   -   
   -   
   -   
   -   
   -   
   -   
   -   
   -   
   -   
   -   
   -   
   -   
   -   
   -   
   -   
   -   
   -   
   -   
   -   
   -   
   -   
 

 
   N59462F   
   -   
   -   
   -   
   -   
   -   
   -   
   -   
   -   
   -   
   -   
   -   
   -   
   -   
   -   
   -   
   -   
   -   
   -   
   -   
   -   
   -   
   -   
   -   
   -   
   -   
   -   
   -   
   -   
   -   
   -   
   -   
   -   
   -   
   -   
   -   
   -   
   -   
   -   
   -   
   -   
   -   
   -   
   -   
   -   
   -   
   -   
   -   
   -   
   -   
   -   
   -   
   -   
   -   
   -   
   -   
   -   
   -   
   -   
   -   
   -   
   -   
   -   
   -   
   -   
   -   
   -   
   -   
   -   
   -   
   -   
   -   
   -   
   -   
   -   
   -   
   -   
   -   
   -   
   -   
   -   
   -   
   -   
   -   
   -   
   -   
   -   
   -   
   -   
   -   
   -   
   -   
   -   
   -   
   -   
   -   
   -   
   -   
   -   
   -   
   -   
   -   
   -   
   -   
   -   
   -   
   -   
   -   
   -   
   C   
   A   
   A   
   T   
   G   
   G   
   C   
   A   
   A   
   G   
   C   
   C   
   A   
   A   
   A   
   C   
   A   
   A   
   G   
   A   
    A    
   T   
   T   
   A   
   G   
   C   
    C    
   G   
   A   
   A   
   G   
   C   
   G   
   A   
   A   
   G   
   A   
   A   
   A   
   A   
    C    
   A   
   G   
   C   
    T    
   A   
    C    
   T   
    A    
    C    
   A   
   T   
   T   
   T   
   T   
   T   
   A   
   A   
   A   
    C    
   G   
   T   
   A   
   T   
   T   
   G   
    T    
   C   
   A   
   A   
   A   
   A   
   C   
   A   
   G   
    G    
   A   
   A   
   T   
   T   
   T   
   G   
   A   
   T   
   A   
   A   
   G   
   T   
   T   
   A   
   C   
   C   
    G    
   T   
   C   
   C   
   G   
   T   
   T   
    G    
   T   
   T   
   C   
   A   
   A   
   G   
   A   
   A   
    G    
   C   
   T   
   A   
   G   
   C   
   T   
   T   
   A   
   A   
   A   
   G   
   A   
   A   
   A   
   A   
   A   
   T   
   G   
   G   
    C    
   T   
   A   
   T   
   G   
   A   
   T   
   A   
   C   
   T   
   A   
   A   
   A   
   T   
   C   
    T    
   G   
   T   
   T   
   G   
   T   
   T   
   G   
   A   
   A   
   A   
   A   
   A   
   T   
   A   
   C   
   C   
   A   
   A   
   G   
   C   
   A   
   A   
   T   
   T   
   T   
   A   
   T   
   T   
   C   
   A   
   G   
   G   
   G   
   A   
   T   
   T   
   C   
   A   
   A   
   G   
   C   
    A    
   G   
   A   
   A   
    G    
   G   
    A    
   G   
   T   
   C   
   A   
   A   
    A    
   G   
   C   
   T   
   A   
   G   
   T   
   G   
   A   
   T   
   G   
   T   
    T    
   C   
   A   
   A   
   G   
   T   
    C    
   A   
   A   
   A   
   A   
   A   
    G    
   G   
    C    
   G   
   A   
   A   
   A   
   G   
   A   
    C    
   A   
   A   
    T    
   C   
   A   
   A   
   T   
   A   
   C   
   A   
   C   
   A   
   T   
   T   
   T   
   A   
    C    
   C   
   T   
   A   
   T   
   A   
   A   
   A   
   T   
   T   
   A   
   T   
   C   
   G   
   A   
   T   
   G   
   A   
   G   
    C    
   A   
   C   
    T    
   C   
   C   
   T   
   T   
   T   
   A   
   G   
   G   
   C   
   G   
   A   
   A   
   A   
   T   
   G   
   A   
   A   
   A   
   G   
   A   
   T   
    T    
   T   
   G   
   T   
   C   
   T   
   T   
   A   
   T   
   C   
   A   
   A   
   T   
   C   
   A   
   A   
   G   
   T   
   A   
   T   
    C    
   G   
    C    
   C   
   A   
   A   
    G    
   A   
   A   
   A   
   G   
   G   
    C    
   G   
   A   
   T   
   A   
    C    
   C   
   T   
   A   
   C   
   C   
   A   
   A   
   A   
   T   
    C    
    G    
   C   
   T   
   T   
   G   
   G   
    A    
    A    
    G    
   C   
   C   
    G    
   T   
   C   
   T   
   T   
   T   
   A   
   A   
   T   
   T   
   T   
   T   
   T   
   C   
   C   
   A   
   G   
   A   
   T   
   A   
   T   
   G   
   T   
   C   
   A   
   G   
   G   
   A   
   A   
   A   
   T   
   G   
   A   
   T   
   A   
   A   
   A   
    A    
   T   
   T   
   T   
   C   
   G   
   A   
   T   
   T   
   C   
   A   
   A   
   G   
   T   
   A   
   G   
   A   
   T   
   A   
   A   
   T   
   G   
   C   
   C   
   A   
   A   
   A   
   C   
   G   
   T   
   G   
   G   
   A   
   G   
   A   
   A   
   A   
   T   
   T   
   G   
   T   
   C   
   G   
   A   
   T   
   C   
   G   
   T   
   A   
   A   
    T    
   G   
   G   
   T   
   A   
   G   
   T   
   G   
   G   
   G   
   C   
   T   
   A   
   G   
    C    
   A   
   A   
   T   
   T   
   A   
   A   
   C   
   A   
   A   
   A   
   G   
   T   
   G   
   T   
   T   
   T   
   G   
   A   
    C    
   G   
   A   
   A   
   G   
   T   
   G   
   G   
   G   
   C   
   G   
   T   
   A   
   G   
   T   
    G    
   C   
   C   
   T   
    G    
   G   
   C   
   A   
   A   
   A   
   C   
   T   
    C    
   G   
   G   
   T   
   T   
   C   
   T   
   G   
   G   
   C   
   G   
   C   
   A   
   G   
   A   
   A   
   A   
   A   
   A   
    A    
   C   
   A   
   G   
   C   
    C    
   A   
   A   
   T   
   A   
   T   
   C   
   A   
   A   
   A   
   G   
   C   
   T   
   T   
   T   
   T   
   A   
   G   
   T   
   G   
   A   
   T   
   A   
   A   
    A    
   T   
   T   
    C    
   G   
   G   
   C   
   G   
   T   
   T   
   T   
   C   
   T   
    G    
   T   
   T   
   G   
   A   
   T   
   G   
   A   
   A   
   A   
   T   
   C   
   A   
   A   
   T   
   C   
    A    
   A   
   A   
   A   
    G    
   T   
   T   
   A   
   A   
   G   
   C   
   C   
   A   
   A   
   G   
   G   
    A    
   T   
   G   
   G   
   G   
   T   
   C   
   C   
   A   
   A   
   G   
   C   
   A   
   G   
   A   
   C   
   T   
   C   
    C    
   T   
   T   
   T   
   G   
   T   
   A   
   C   
   C   
   A   
   A   
   T   
   C   
   A   
   C   
   A   
   G   
   T   
    C    
   G   
   C   
   T   
   T   
   C   
   T   
   G   
   A   
   A   
   C   
   C   
   A   
   G   
   T   
   G   
   A   
   C   
   A   
   G   
   A   
   A   
   T   
   T   
   A   
   C   
   C   
   A   
   A   
   C   
   A   
   G   
   G   
   G   
   G   
   C   
   T   
   G   
   C   
   G   
   A   
   C   
   A   
   A   
   A   
   A   
   G   
    A    
    T    
   A   
   C   
   A   
   G   
   A   
   G   
   T   
   C   
   A   
   C   
   G   
   T   
   T   
   A   
   T   
   T   
   A   
   T   
   C   
   C   
    G    
    C    
   T   
   G   
   G   
   G   
   G   
   G   
   A   
   A   
   G   
   C   
   A   
   G   
   C   
   T   
   G   
   C   
   G   
   C   
   A   
   A   
   T   
   T   
   A   
   A   
   T   
   C   
   G   
   G   
    G    
   T   
   A   
   T   
   A   
   C   
    G    
   G   
   G   
    C    
   A   
   C   
    C    
   A   
   T   
   T   
   A   
   C   
   T   
   G   
   C   
   A   
   G   
   A   
    A    
   G   
   A   
   T   
   A   
   T   
   T   
    G    
   A   
   A   
   A   
   A   
   A   
   A   
   A   
   T   
   C   
   C   
   A   
   G   
   A   
   G   
   T   
   T   
   A   
   A   
   G   
   T   
   A   
   G   
   T   
   A   
   C   
   G   
   G   
   G   
    C    
   G   
   T   
   A   
   A   
   T   
   T   
   G   
   G   
    T    
   A   
   A   
   A   
   A   
   C   
   T   
   G   
   G   
    T    
   T   
   T   
   A   
   G   
   A   
   A   
   C   
   G   
   A   
   G   
   C   
   G   
   T   
   T   
   T   
   G   
   A   
   T   
   A   
   A   
   A   
   G   
   A   
    A    
   T   
   T   
   A   
   A   
   G   
   A   
   G   
   G   
    G    
   C   
   A   
   A   
   G   
   A   
   T   
   G   
   G   
    T    
   G   
   G   
    C    
   T   
   C   
    A    
   C   
   T   
   A   
   G   
   T   
   T   
   A   
   T   
   T   
   T   
   T   
   A   
   G   
   A   
   T   
   G   
   A   
   C   
   A   
   A   
   A   
   G   
   A   
   G   
   A   
   A   
   T   
   G   
   T   
   C   
   A   
   A   
   A   
   A   
   A   
   A   
   G   
   C   
   T   
   T   
   T   
   A   
   C   
   A   
   A   
   A   
   C   
   C   
   A   
   A   
   A   
   G   
   A   
    G    
   A   
   A   
   A   
   A   
   A   
   A   
   G   
   A   
   T   
   G   
   G   
   T   
   C   
   A   
   A   
   A   
   C   
    G    
   A   
   T   
   T   
   A   
   A   
   A   
   T   
   T   
   A   
   A   
   C   
   G   
   A   
   T   
   T   
   G   
   A   
    C    
   A   
   G   
    T    
   G   
   G   
    C    
   G   
   T   
   A   
   C   
   A   
   A   
   C   
   A   
   A   
   C   
   A   
   A   
   G   
   C   
    C    
   T   
   T   
    C    
   G   
   C   
   T   
   A   
   T   
   A   
   T   
   T   
   T   
   G   
   A   
   C   
   A   
   A   
   A   
   C   
   G   
    T    
    C    
   C   
   T   
   G   
   G   
   T   
   A   
   G   
   T   
   G   
   C   
   G   
   G   
   T   
   C   
   A   
   T   
   T   
   A   
   C   
   C   
   G   
   A   
   T   
   C   
   C   
   T   
   C   
   A   
   A   
   A   
   A   
   A   
   G   
   G   
   C   
   G   
   A   
   T   
   T   
   T   
   A   
    T    
   T   
   A   
   G   
   C   
    A    
   A   
   C   
   G   
   G   
   T   
   T   
   A   
   G   
   T   
   T   
   C   
   A   
   C   
   C   
   T   
   T   
   C   
   G   
   T   
   A   
   T   
   G   
   A   
   T   
   C   
   C   
   T   
   A   
   A   
   T   
   A   
   A   
   A   
   A   
   T   
   G   
   G   
   C   
   C   
   A   
   A   
   T   
   G   
   G   
    T    
   A   
   T   
   T   
   T   
   C   
   T   
   C   
   A   
   A   
   A   
   A   
   A   
   G   
   A   
    A    
   T   
   A   
   T   
   G   
   A   
   T   
   G   
   C   
   T   
   T   
   A   
   C   
   A   
   A   
   T   
   A   
   A   
   C   
   A   
   A   
   C   
   A   
   A   
   A   
   G   
   A   
   T   
   T   
   T   
   A   
   C   
   C   
   A   
   T   
   T   
    C    
   A   
   C   
   A   
   G   
   C   
   A   
   C   
   G   
   T   
   T   
   T   
    T    
   G   
   C   
   G   
   A   
   C   
   A   
   G   
   G   
    C    
   T   
   A   
   T   
   G   
   C   
   A   
   C   
   C   
   A   
   G   
   G   
    C    
   T   
   C   
   T   
   A   
   C   
   C   
   T   
   T   
   T   
   A   
   A   
   A   
   A   
   C   
   A   
   A   
   T   
   T   
   A   
   C   
   C   
   G   
   G   
   T   
   G   
   C   
   C   
   A   
   T   
   T   
   G   
   G   
   T   
   T   
   T   
   A   
   G   
   A   
   T   
    G    
   C   
   A   
   G   
   G   
   G   
   A   
   C   
   A   
   T   
   T   
   G   
   A   
   A   
   G   
   C   
   C   
   A   
   G   
   A   
   T   
   G   
   A   
   A   
   G   
   A   
   G   
   C   
   T   
   A   
   G   
   A   
   A   
   A   
   T   
   T   
   A   
   A   
   T   
   G   
   G   
    T    
   T   
   T   
   G   
   A   
   A   
   A   
   T   
   G   
   G   
   C   
   A   
   A   
   A   
   A   
   A   
   G   
   A   
   T   
   A   
   A   
   A   
   T   
   C   
   T   
   T   
   G   
   G   
   G   
   G   
    C    
   G   
   G   
   C   
   T   
   A   
   T   
   T   
   T   
   T   
   G   
   C   
    G    
   A   
   C   
   A   
   C   
   G   
   T   
   G   
   T   
   A   
   A   
   A   
   A   
   G   
   A   
   A   
   G   
   C   
   A   
   A   
   G   
   T   
   C   
   C   
   A   
   G   
   T   
   C   
   A   
   A   
   T   
   C   
   T   
    G    
   C   
   G   
   T   
   A   
   C   
   C   
   G   
   C   
   T   
   T   
   T   
   A   
   G   
   T   
   T   
   A   
   A   
   T   
   T   
   C   
   A   
   G   
   A   
   T   
   A   
   A   
   T   
   A   
   T   
   T   
   T   
   A   
   T   
   T   
   T   
   T   
   G   
   C   
    C    
   C   
   A   
   A   
   C   
   A   
   A   
   A   
   C   
   A   
   T   
   T   
   A   
   C   
   G   
    G    
   A   
   T   
   G   
   G   
   G   
    A    
   G   
   A   
   A   
   G   
   A   
   C   
   A   
   A   
   A   
   T   
   T   
   T   
   A   
   G   
   A   
   G   
   C   
    G    
   G   
   G   
   T   
   C   
   T   
   T   
   A   
   A   
   T   
   A   
   A   
   A   
   T   
   T   
   C   
   A   
   T   
   T   
   T   
   T   
   C   
   G   
   G   
   T   
   G   
   A   
   G   
   G   
   A   
   A   
   C   
   T   
   A   
   G   
   A   
   T   
   T   
   T   
   A   
   C   
   C   
   A   
   A   
   T   
    T    
   G   
   C   
   C   
   A   
   T   
   G   
   A   
   C   
   G   
   C   
   C   
    T    
   G   
   C   
   T   
   C   
   A   
   A   
   A   
   T   
   T   
   T   
   C   
   A   
   A   
   A   
    T    
   G   
   A   
   A   
   G   
   A   
   T   
   A   
   A   
   A   
   T   
   T   
   T   
   A   
   A   
   T   
   T   
   C   
   T   
   G   
   A   
   A   
   A   
   T   
   T   
   C   
   T   
   T   
   T   
   T   
    A    
   G   
   C   
   A   
   G   
   A   
   T   
   A   
   C   
    G    
   G   
   G   
   C   
   T   
   A   
    C    
   G   
   G   
    T    
   C   
   A   
   A   
   G   
   G   
    T    
   C   
   A   
   A   
   T   
   T   
   G   
   T   
   T   
   A   
   A   
   T   
   T   
   T   
   C   
    G    
   C   
   C   
   A   
   A   
   T   
   C   
   C   
   A   
   G   
   C   
   A   
   A   
   G   
   C   
   T   
   A   
   C   
   C   
   A   
   T   
   G   
   T   
   A   
   T   
   A   
   G   
   T   
   G   
   T   
   T   
   T   
   T   
   C   
   C   
   A   
   A   
   A   
   A   
   T   
   A   
   A   
   T   
   G   
   G   
    A    
   A   
   C   
   A   
    T    
   T   
    A    
   G   
   T   
    C    
   T   
   A   
   T   
   C   
   C   
   A   
   A   
   A   
   A   
    C    
   T   
   A   
   G   
   T   
   T   
   T   
   T   
   A   
   G   
   A   
   C   
   A   
   A   
   A   
   G   
   A   
   A   
   A   
   C   
    G    
   A   
   A   
   G   
   A   
   A   
   A   
   A   
   A   
   A   
   G   
   A   
   C   
   A   
   A   
   T   
   G   
   T   
   A   
   A   
   T   
   C   
   A   
   G   
   C   
   G   
   C   
   C   
   A   
   A   
   C   
   G   
   C   
   A   
   G   
   C   
   G   
   A   
   A   
   T   
   A   
   C   
   A   
   A   
   T   
   T   
   G   
   C   
   C   
   A   
   C   
   A   
   G   
   A   
   T   
   C   
   T   
   T   
   T   
   T   
   A   
   G   
   G   
   T   
   A   
   G   
   T   
   G   
   T   
   G   
   G   
   A   
   A   
   G   
   A   
   T   
   C   
   C   
   T   
   T   
   C   
   A   
   G   
   G   
   T   
   T   
   A   
   T   
   G   
   T   
   T   
   T   
   A   
   T   
   A   
   A   
   T   
   A   
   T   
   G   
   T   
   A   
   C   
   A   
   A   
   T   
   C   
   C   
   G   
   A   
   A   
   C   
   T   
   T   
   C   
   T   
   C   
   A   
   T   
   T   
   G   
   G   
   C   
   T   
   G   
   C   
   T   
   A   
   A   
   A   
   A   
   C   
   A   
   G   
   G   
   A   
   A   
   C   
   A   
   G   
   C   
   T   
   G   
   A   
   A   
   A   
   T   
   A   
   A   
   A   
   A   
   G   
   A   
   T   
   A   
   A   
   A   
   C   
   A   
   A   
   G   
   A   
   T   
   A   
   C   
   A   
   G   
   A   
   T   
   G   
   G   
   C   
   A   
   A   
   A   
   G   
   A   
   A   
   A   
   A   
   C   
   A   
   G   
   T   
   T   
   T   
   C   
   C   
   T   
   T   
   C   
   T   
   A   
   A   
   C   
   G   
   T   
   T   
   A   
   G   
   A   
   T   
   C   
   G   
   T   
   A   
   G   
   T   
   A   
   A   
   C   
   A   
   A   
   T   
   A   
   A   
   A   
   T   
   T   
   C   
   T   
   T   
   A   
   A   
   C   
   A   
   A   
   T   
   G   
   A   
   T   
   T   
   A   
   T   
   G   
   G   
   T   
   T   
   G   
   A   
   A   
   A   
   A   
   T   
   T   
   C   
   A   
   G   
   G   
   A   
   G   
   A   
   A   
   A   
   A   
   T   
   G   
   G   
   T   
   T   
   C   
   T   
   G   
   C   
   A   
   A   
   C   
   A   
   G   
   A   
   T   
   A   
   T   
   C   
   A   
   G   
   T   
   A   
   A   
   A   
   C   
   C   
   A   
   T   
   T   
   A   
   A   
   T   
   T   
   G   
   A   
   T   
   T   
   A   
   C   
   T   
   T   
   A   
   G   
   A   
   A   
   G   
   C   
   A   
   A   
   C   
   C   
   A   
   T   
   T   
   A   
   A   
   A   
   -   
   -   
   -   
   -   
   -   
   -   
   -   
   -   
   -   
   -   
   -   
   -   
   -   
   -   
   -   
   -   
   -   
   -   
   -   
   -   
   -   
   -   
   -   
   -   
   -   
   -   
   -   
   -   
   -   
   -   
   -   
   -   
   -   
   -   
   -   
   -   
   -   
   -   
   -   
   -   
   -   
   -   
   -   
   -   
   -   
   -   
   -   
   -   
   -   
   -   
   -   
   -   
   -   
   -   
   -   
   -   
   -   
   -   
   -   
   -   
   -   
   -   
   -   
   -   
   -   
   -   
   -   
   -   
   -   
   -   
   -   
   -   
   -   
   -   
   -   
   -   
   -   
   -   
   -   
   -   
   -   
   -   
   -   
   -   
   -   
   -   
   -   
   -   
   -   
   -   
   -   
   -   
   -   
   -   
   -   
   -   
   -   
   -   
   -   
   -   
   -   
   -   
   -   
   -   
   -   
   -   
   -   
   -   
   -   
   -   
   -   
   -   
   -   
   -   
   -   
   -   
   -   
   -   
   -   
   -   
   -   
   -   
   -   
   -   
   -   
   -   
   -   
   -   
   -   
   -   
   -   
   -   
   -   
   -   
   -   
   -   
   -   
   -   
   -   
   -   
   -   
   -   
   -   
   -   
   -   
   -   
   -   
   -   
   -   
   -   
   -   
   -   
   -   
   -   
   -   
   -   
   -   
   -   
   -   
   -   
   -   
   -   
   -   
   -   
   -   
   -   
   -   
   -   
   -   
   C   
   A   
   A   
   T   
   G   
   G   
   C   
   A   
   A   
   G   
   C   
   C   
   A   
   A   
   A   
   C   
   A   
   A   
   G   
   A   
   A   
   T   
   T   
   A   
   G   
   C   
   C   
   G   
   A   
   A   
   G   
   C   
   G   
   A   
   A   
   G   
   A   
   A   
   A   
   A   
   C   
   A   
   G   
   C   
   T   
   A   
   C   
   T   
   A   
   C   
   A   
   T   
   T   
   T   
   T   
   T   
   A   
   A   
   A   
   C   
   G   
   T   
   A   
   T   
   T   
   G   
   T   
   C   
   A   
   A   
   A   
   A   
   C   
   A   
   G   
   G   
   A   
   A   
   T   
   T   
   T   
   G   
   A   
   T   
   A   
   A   
   G   
   T   
   T   
   A   
   C   
   C   
   G   
   T   
   C   
   C   
   G   
   T   
   T   
   G   
   T   
   T   
   C   
   A   
   A   
   G   
   A   
   A   
   G   
   C   
   T   
   A   
   G   
   C   
   T   
   T   
   A   
   A   
   A   
   G   
   A   
   A   
   A   
   A   
   A   
   T   
   G   
   G   
   C   
   T   
   A   
   T   
   G   
   A   
   T   
   A   
   C   
   T   
   A   
   A   
   A   
   T   
   C   
   T   
   G   
   T   
   T   
   G   
   T   
   T   
   G   
   A   
   A   
   A   
   A   
   A   
   T   
   A   
   C   
   C   
   A   
   A   
   G   
   C   
   A   
   A   
   T   
   T   
   T   
   A   
   T   
   T   
   C   
   A   
   G   
   G   
   G   
   A   
   T   
   T   
   C   
   A   
   A   
   G   
   C   
   A   
   G   
   A   
   A   
   G   
   G   
   A   
   G   
   T   
   C   
   A   
   A   
   A   
   G   
   C   
   T   
   A   
   G   
   T   
   G   
   A   
   T   
   G   
   T   
   T   
   C   
   A   
   A   
   G   
   T   
   C   
   A   
   A   
   A   
   A   
   A   
   G   
   G   
   C   
   G   
   A   
   A   
   A   
   G   
   A   
   C   
   A   
   A   
   T   
   C   
   A   
   A   
   T   
   A   
   C   
   A   
   C   
   A   
   T   
   T   
   T   
   A   
   C   
   C   
   T   
   A   
   T   
   A   
   A   
   A   
   T   
   T   
   A   
   T   
   C   
   G   
   A   
   T   
   G   
   A   
   G   
   C   
   A   
   C   
   T   
   C   
   C   
   T   
   T   
   T   
   A   
   G   
   G   
   C   
   G   
   A   
   A   
   A   
   T   
   G   
   A   
   A   
   A   
   G   
   A   
   T   
   T   
   T   
   G   
   T   
   C   
   T   
   T   
   A   
   T   
   C   
   A   
   A   
   T   
   C   
   A   
   A   
   G   
   T   
   A   
   T   
   C   
   G   
   C   
   C   
   A   
   A   
   G   
   A   
   A   
   A   
   G   
   G   
   C   
   G   
   A   
   T   
   A   
   C   
   C   
   T   
   A   
   C   
   C   
   A   
   A   
   A   
   T   
   C   
   G   
   C   
   T   
   T   
   G   
   G   
   A   
   A   
   G   
   C   
   C   
   G   
   T   
   C   
   T   
   T   
   T   
   A   
   A   
   T   
   T   
   T   
   T   
   T   
   C   
   C   
   A   
   G   
   A   
   T   
   A   
   T   
   G   
   T   
   C   
   A   
   G   
   G   
   A   
   A   
   A   
   T   
   G   
   A   
   T   
   A   
   A   
   A   
   A   
   T   
   T   
   T   
   C   
   G   
   A   
   T   
   T   
   C   
   A   
   A   
   G   
   T   
   A   
   G   
   A   
   T   
   A   
   A   
   T   
   G   
   C   
   C   
   A   
   A   
   A   
   C   
   G   
   T   
   G   
   G   
   A   
   G   
   A   
   A   
   A   
   T   
   T   
   G   
   T   
   C   
   G   
   A   
   T   
   C   
   G   
   T   
   A   
   A   
   T   
   G   
   G   
   T   
   A   
   G   
   T   
   G   
   G   
   G   
   C   
   T   
   A   
   G   
   C   
   A   
   A   
   T   
   T   
   A   
   A   
   C   
   A   
   A   
   A   
   G   
   T   
   G   
   T   
   T   
   T   
   G   
   A   
   C   
   G   
   A   
   A   
   G   
   T   
   G   
   G   
   G   
   C   
   G   
   T   
   A   
   G   
   T   
   G   
   C   
   C   
   T   
   G   
   G   
   C   
   A   
   A   
   A   
   C   
   T   
   C   
   G   
   G   
   T   
   T   
   C   
   T   
   G   
   G   
   C   
   G   
   C   
   A   
   G   
   A   
   A   
   A   
   A   
   A   
   A   
   C   
   A   
   G   
   C   
   C   
   A   
   A   
   T   
   A   
   T   
   C   
   A   
   A   
   A   
   G   
   C   
   T   
   T   
   T   
   T   
   A   
   G   
   T   
   G   
   A   
   T   
   A   
   A   
   A   
   T   
   T   
   C   
   G   
   G   
   C   
   G   
   T   
   T   
   T   
   C   
   T   
   G   
   T   
   T   
   G   
   A   
   T   
   G   
   A   
   A   
   A   
   T   
   C   
   A   
   A   
   T   
   C   
   A   
   A   
   A   
   A   
   G   
   T   
   T   
   A   
   A   
   G   
   C   
   C   
   A   
   A   
   G   
   G   
   A   
   T   
   G   
   G   
   G   
   T   
   C   
   C   
   A   
   A   
   G   
   C   
   A   
   G   
   A   
   C   
   T   
   C   
   C   
   T   
   T   
   T   
   G   
   T   
   A   
   C   
   C   
   A   
   A   
   T   
   C   
   A   
   C   
   A   
   G   
   T   
   C   
   G   
   C   
   T   
   T   
   C   
   T   
   G   
   A   
   A   
   C   
   C   
   A   
   G   
   T   
   G   
   A   
   C   
   A   
   G   
   A   
   A   
   T   
   T   
   A   
   C   
   C   
   A   
   A   
   C   
   A   
   G   
   G   
   G   
   G   
   C   
   T   
   G   
   C   
   G   
   A   
   C   
   A   
   A   
   A   
   A   
   G   
   A   
   T   
   A   
   C   
   A   
   G   
   A   
   G   
   T   
   C   
   A   
   C   
   G   
   T   
   T   
   A   
   T   
   T   
   A   
   T   
   C   
   C   
   G   
   C   
   T   
   G   
   G   
   G   
   G   
   G   
   A   
   A   
   G   
   C   
   A   
   G   
   C   
   T   
   G   
   C   
   G   
   C   
   A   
   A   
   T   
   T   
   A   
   A   
   T   
   C   
   G   
   G   
   G   
   T   
   A   
   T   
   A   
   C   
   G   
   G   
   G   
   C   
   A   
   C   
   C   
   A   
   T   
   T   
   A   
   C   
   T   
   G   
   C   
   A   
   G   
   A   
   A   
   G   
   A   
   T   
   A   
   T   
   T   
   G   
   A   
   A   
   A   
   A   
   A   
   A   
   A   
   T   
   C   
   C   
   A   
   G   
   A   
   G   
   T   
   T   
   A   
   A   
   G   
   T   
   A   
   G   
   T   
   A   
   C   
   G   
   G   
   G   
   C   
   G   
   T   
   A   
   A   
   T   
   T   
   G   
   G   
   T   
   A   
   A   
   A   
   A   
   C   
   T   
   G   
   G   
   T   
   T   
   T   
   A   
   G   
   A   
   A   
   C   
   G   
   A   
   G   
   C   
   G   
   T   
   T   
   T   
   G   
   A   
   T   
   A   
   A   
   A   
   G   
   A   
   A   
   T   
   T   
   A   
   A   
   G   
   A   
   G   
   G   
   G   
   C   
   A   
   A   
   G   
   A   
   T   
   G   
   G   
   T   
   G   
   G   
   C   
   T   
   C   
   A   
   C   
   T   
   A   
   G   
   T   
   T   
   A   
   T   
   T   
   T   
   T   
   A   
   G   
   A   
   T   
   G   
   A   
   C   
   A   
   A   
   A   
   G   
   A   
   G   
   A   
   A   
   T   
   G   
   T   
   C   
   A   
   A   
   A   
   A   
   A   
   A   
   G   
   C   
   T   
   T   
   T   
   A   
   C   
   A   
   A   
   A   
   C   
   C   
   A   
   A   
   A   
   G   
   A   
   G   
   A   
   A   
   A   
   A   
   A   
   A   
   G   
   A   
   T   
   G   
   G   
   T   
   C   
   A   
   A   
   A   
   C   
   G   
   A   
   T   
   T   
   A   
   A   
   A   
   T   
   T   
   A   
   A   
   C   
   G   
   A   
   T   
   T   
   G   
   A   
   C   
   A   
   G   
   T   
   G   
   G   
   C   
   G   
   T   
   A   
   C   
   A   
   A   
   C   
   A   
   A   
   C   
   A   
   A   
   G   
   C   
   C   
   T   
   T   
   C   
   G   
   C   
   T   
   A   
   T   
   A   
   T   
   T   
   T   
   G   
   A   
   C   
   A   
   A   
   A   
   C   
   G   
   T   
   C   
   C   
   T   
   G   
   G   
   T   
   A   
   G   
   T   
   G   
   C   
   G   
   G   
   T   
   C   
   A   
   T   
   T   
   A   
   C   
   C   
   G   
   A   
   T   
   C   
   C   
   T   
   C   
   A   
   A   
   A   
   A   
   A   
   G   
   G   
   C   
   G   
   A   
   T   
   T   
   T   
   A   
   T   
   T   
   A   
   G   
   C   
   A   
   A   
   C   
   G   
   G   
   T   
   T   
   A   
   G   
   T   
   T   
   C   
   A   
   C   
   C   
   T   
   T   
   C   
   G   
   T   
   A   
   T   
   G   
   A   
   T   
   C   
   C   
   T   
   A   
   A   
   T   
   A   
   A   
   A   
   A   
   T   
   G   
   G   
   C   
   C   
   A   
   A   
   T   
   G   
   G   
   T   
   A   
   T   
   T   
   T   
   C   
   T   
   C   
   A   
   A   
   A   
   A   
   A   
   G   
   A   
   A   
   T   
   A   
   T   
   G   
   A   
   T   
   G   
   C   
   T   
   T   
   A   
   C   
   A   
   A   
   T   
   A   
   A   
   C   
   A   
   A   
   C   
   A   
   A   
   A   
   G   
   A   
   T   
   T   
   T   
   A   
   C   
   C   
   A   
   T   
   T   
   C   
   A   
   C   
   A   
   G   
   C   
   A   
   C   
   G   
   T   
   T   
   T   
   T   
   G   
   C   
   G   
   A   
   C   
   A   
   G   
   G   
   C   
   T   
   A   
   T   
   G   
   C   
   A   
   C   
   C   
   A   
   G   
   G   
   C   
   T   
   C   
   T   
   A   
   C   
   C   
   T   
   T   
   T   
   A   
   A   
   A   
   A   
   C   
   A   
   A   
   T   
   T   
   A   
   C   
   C   
   G   
   G   
   T   
   G   
   C   
   C   
   A   
   T   
   T   
   G   
   G   
   T   
   T   
   T   
   A   
   G   
   A   
   T   
   G   
   C   
   A   
   G   
   G   
   G   
   A   
   C   
   A   
   T   
   T   
   G   
   A   
   A   
   G   
   C   
   C   
   A   
   G   
   A   
   T   
   G   
   A   
   A   
   G   
   A   
   G   
   C   
   T   
   A   
   G   
   A   
   A   
   A   
   T   
   T   
   A   
   A   
   T   
   G   
   G   
   T   
   T   
   T   
   G   
   A   
   A   
   A   
   T   
   G   
   G   
   C   
   A   
   A   
   A   
   A   
   A   
   G   
   A   
   T   
   A   
   A   
   A   
   T   
   C   
   T   
   T   
   G   
   G   
   G   
   G   
   C   
   G   
   G   
   C   
   T   
   A   
   T   
   T   
   T   
   T   
   G   
   C   
   G   
   A   
   C   
   A   
   C   
   G   
   T   
   G   
   T   
   A   
   A   
   A   
   A   
   G   
   A   
   A   
   G   
   C   
   A   
   A   
   G   
   T   
   C   
   C   
   A   
   G   
   T   
   C   
   A   
   A   
   T   
   C   
   T   
   G   
   C   
   G   
   T   
   A   
   C   
   C   
   G   
   C   
   T   
   T   
   T   
   A   
   G   
   T   
   T   
   A   
   A   
   T   
   T   
   C   
   A   
   G   
   A   
   T   
   A   
   A   
   T   
   A   
   T   
   T   
   T   
   A   
   T   
   T   
   T   
   T   
   G   
   C   
   C   
   C   
   A   
   A   
   C   
   A   
   A   
   A   
   C   
   A   
   T   
   T   
   A   
   C   
   G   
   G   
   A   
   T   
   G   
   G   
   G   
   A   
   G   
   A   
   A   
   G   
   A   
   C   
   A   
   A   
   A   
   T   
   T   
   T   
   A   
   G   
   A   
   G   
   C   
   G   
   G   
   G   
   T   
   C   
   T   
   T   
   A   
   A   
   T   
   A   
   A   
   A   
   T   
   T   
   C   
   A   
   T   
   T   
   T   
   T   
   C   
   G   
   G   
   T   
   G   
   A   
   G   
   G   
   A   
   A   
   C   
   T   
   A   
   G   
   A   
   T   
   T   
   T   
   A   
   C   
   C   
   A   
   A   
   T   
   T   
   G   
   C   
   C   
   A   
   T   
   G   
   A   
   C   
   G   
   C   
   C   
   T   
   G   
   C   
   T   
   C   
   A   
   A   
   A   
   T   
   T   
   T   
   C   
   A   
   A   
   A   
   T   
   G   
   A   
   A   
   G   
   A   
   T   
   A   
   A   
   A   
   T   
   T   
   T   
   A   
   A   
   T   
   T   
   C   
   T   
   G   
   A   
   A   
   A   
   T   
   T   
   C   
   T   
   T   
   T   
   T   
   A   
   G   
   C   
   A   
   G   
   A   
   T   
   A   
   C   
   G   
   G   
   G   
   C   
   T   
   A   
   C   
   G   
   G   
   T   
   C   
   A   
   A   
   G   
   G   
   T   
   C   
   A   
   A   
   T   
   T   
   G   
   T   
   T   
   A   
   A   
   T   
   T   
   T   
   C   
   G   
   C   
   C   
   A   
   A   
   T   
   C   
   C   
   A   
   G   
   C   
   A   
   A   
   G   
   C   
   T   
   A   
   C   
   C   
   A   
   T   
   G   
   T   
   A   
   T   
   A   
   G   
   T   
   G   
   T   
   T   
   T   
   T   
   C   
   C   
   A   
   A   
   A   
   A   
   T   
   A   
   A   
   T   
   G   
   G   
   A   
   A   
   C   
   A   
   T   
   T   
   A   
   G   
   T   
   C   
   T   
   A   
   T   
   C   
   C   
   A   
   A   
   A   
   A   
   C   
   T   
   A   
   G   
   T   
   T   
   T   
   T   
   A   
   G   
   A   
   C   
   A   
   A   
   A   
   G   
   A   
   A   
   A   
   C   
   G   
   A   
   A   
   G   
   A   
   A   
   A   
   A   
   A   
   A   
   G   
   A   
   C   
   A   
   A   
   T   
   G   
   T   
   A   
   A   
   T   
   C   
   A   
   G   
   C   
   G   
   C   
   C   
   A   
   A   
   C   
   G   
   C   
   A   
   G   
   C   
   G   
   A   
   A   
   T   
   A   
   C   
   A   
   A   
   T   
   T   
   G   
   C   
   C   
   A   
   C   
   A   
   G   
   A   
   T   
   C   
   T   
   T   
   T   
   T   
   A   
   G   
   G   
   T   
   A   
   G   
   T   
   G   
   T   
   G   
   G   
   A   
   A   
   G   
   A   
   T   
   C   
   C   
   T   
   T   
   C   
   A   
   G   
   G   
   T   
   T   
   A   
   T   
   G   
   T   
   T   
   T   
   A   
   T   
   A   
   A   
   T   
   A   
   T   
   G   
   T   
   A   
   C   
   A   
   A   
   T   
   C   
   C   
   G   
   A   
   A   
   C   
   T   
   T   
   C   
   T   
   C   
   A   
   T   
   T   
   G   
   G   
   C   
   T   
   G   
   C   
   T   
   A   
   A   
   A   
   A   
   C   
   A   
   G   
   G   
   A   
   A   
   C   
   A   
   G   
   C   
   T   
   G   
   A   
   A   
   A   
   T   
   A   
   A   
   A   
   A   
   G   
   A   
   T   
   A   
   A   
   A   
   C   
   A   
   A   
   G   
   A   
   T   
   A   
   C   
   A   
   G   
   A   
   T   
   G   
   G   
   C   
   A   
   A   
   A   
   G   
   A   
   A   
   A   
   A   
   C   
   A   
   G   
   T   
   T   
   T   
   C   
   C   
   T   
   T   
   C   
   T   
   A   
   A   
   C   
   G   
   T   
   T   
   A   
   G   
   A   
   T   
   C   
   G   
   T   
   A   
   G   
   T   
   A   
   A   
   C   
   A   
   A   
   T   
   A   
   A   
   A   
   T   
   T   
   C   
   T   
   T   
   A   
   A   
   C   
   A   
   A   
   T   
   G   
   A   
   T   
   T   
   A   
   T   
   G   
   G   
   T   
   T   
   G   
   A   
   A   
   A   
   A   
   T   
   T   
   C   
   A   
   G   
   G   
   A   
   G   
   A   
   A   
   A   
   A   
   T   
   G   
   G   
   T   
   T   
   C   
   T   
   G   
   C   
   A   
   A   
   C   
   A   
   G   
   A   
   T   
   A   
   T   
   C   
   A   
   G   
   T   
   A   
   A   
   A   
   C   
   C   
   A   
   T   
   T   
   A   
   A   
   T   
   T   
   G   
   A   
   T   
   T   
   A   
   C   
   T   
   T   
   A   
   G   
   A   
   A   
   G   
   C   
   A   
   A   
   C   
   C   
   A   
   T   
   T   
   A   
   A   
   A   
   -   
   -   
   -   
   -   
   -   
   -   
   -   
   -   
   -   
   -   
   -   
   -   
   -   
   -   
   -   
   -   
   -   
   -   
   -   
   -   
   -   
   -   
   -   
   -   
   -   
   -   
   -   
   -   
   -   
   -   
   -   
   -   
   -   
   -   
   -   
   -   
   -   
   -   
   -   
   -   
   -   
   -   
   -   
   -   
   -   
   -   
   -   
   -   
   -   
   -   
   -   
   -   
   -   
   -   
   -   
   -   
   -   
   -   
   -   
   -   
   -   
 

 
   N59555F   
   -   
   -   
   -   
   -   
   -   
   -   
   -   
   -   
   -   
   -   
   -   
   -   
   -   
   -   
   -   
   -   
   -   
   -   
   -   
   -   
   -   
   -   
   -   
   -   
   -   
   -   
   -   
   -   
   -   
   -   
   -   
   -   
   -   
   -   
   -   
   -   
   -   
   -   
   -   
   -   
   -   
   -   
   -   
   -   
   -   
   -   
   -   
   -   
   -   
   -   
   -   
   -   
   -   
   -   
   -   
   -   
   -   
   -   
   -   
   -   
   -   
   -   
   -   
   -   
   -   
   -   
   -   
   -   
   -   
   -   
   -   
   -   
   -   
   -   
   -   
   -   
   -   
   -   
   -   
   -   
   -   
   -   
   -   
   -   
   -   
   -   
   -   
   -   
   -   
   -   
   -   
   -   
   -   
   -   
   -   
   -   
   -   
   -   
   -   
   -   
   -   
   -   
   -   
   -   
   -   
   -   
   -   
   -   
   C   
   A   
   A   
   T   
   G   
   G   
   C   
   A   
   A   
   G   
   C   
   C   
   A   
   A   
   A   
   C   
   A   
   A   
   G   
   A   
    A    
   T   
   T   
   A   
   G   
   C   
    C    
   G   
   A   
   A   
   G   
   C   
   G   
   A   
   A   
   G   
   A   
   A   
   A   
   A   
    C    
   A   
   G   
   C   
    T    
   A   
    A    
   T   
    A    
    C    
   A   
   T   
   T   
   T   
   T   
   T   
   A   
   A   
   A   
    T    
   G   
   T   
   A   
   T   
   T   
   G   
    T    
   C   
   A   
   A   
   A   
   A   
   C   
   A   
   G   
    G    
   A   
   A   
   T   
   T   
   T   
   G   
   A   
   T   
   A   
   A   
   G   
   T   
   T   
   A   
   C   
   C   
    G    
   T   
   C   
   C   
   G   
   T   
   T   
    G    
   T   
   T   
   C   
   A   
   A   
   G   
   A   
   A   
    T    
   C   
   T   
   A   
   G   
   C   
   T   
   T   
   A   
   A   
   A   
   G   
   A   
   A   
   A   
   A   
   A   
   T   
   G   
   G   
    C    
   T   
   A   
   T   
   G   
   A   
   T   
   A   
   C   
   T   
   A   
   A   
   A   
   T   
   C   
    T    
   G   
   T   
   T   
   G   
   T   
   T   
   G   
   A   
   A   
   A   
   A   
   A   
   T   
   A   
   C   
   C   
   A   
   A   
   G   
   C   
   A   
   A   
   T   
   T   
   T   
   A   
   T   
   T   
   C   
   A   
   G   
   G   
   G   
   A   
   T   
   T   
   C   
   A   
   A   
   G   
   C   
    A    
   G   
   A   
   A   
    G    
   G   
    A    
   G   
   T   
   C   
   A   
   A   
    A    
   G   
   C   
   T   
   A   
   G   
   T   
   G   
   A   
   T   
   G   
   T   
    C    
   C   
   A   
   A   
   G   
   T   
    C    
   A   
   A   
   A   
   A   
   A   
    G    
   G   
    C    
   G   
   A   
   A   
   A   
   G   
   A   
    C    
   A   
   A   
    T    
   C   
   A   
   A   
   T   
   A   
   C   
   A   
   C   
   A   
   T   
   T   
   T   
   A   
    C    
   C   
   T   
   A   
   T   
   A   
   A   
   A   
   T   
   T   
   A   
   T   
   C   
   G   
   A   
   T   
   G   
   A   
   G   
    C    
   A   
   C   
    T    
   C   
   C   
   T   
   T   
   T   
   A   
   G   
   G   
   C   
   G   
   A   
   A   
   A   
   T   
   G   
   A   
   A   
   A   
   G   
   A   
   T   
    C    
   T   
   G   
   T   
   C   
   T   
   T   
   A   
   T   
   C   
   A   
   A   
   T   
   C   
   A   
   A   
   G   
   T   
   A   
   T   
    C    
   G   
    C    
   C   
   A   
   A   
    A    
   A   
   A   
   A   
   G   
   G   
    C    
   G   
   A   
   T   
   A   
    C    
   C   
   T   
   A   
   C   
   C   
   A   
   A   
   A   
   T   
    C    
    T    
   C   
   T   
   T   
   G   
   G   
    A    
    A    
    G    
   C   
   C   
    G    
   T   
   C   
   T   
   T   
   T   
   A   
   A   
   T   
   T   
   T   
   T   
   T   
   C   
   C   
   A   
   G   
   A   
   T   
   A   
   T   
   G   
   T   
   C   
   A   
   G   
   G   
   A   
   A   
   A   
   T   
   G   
   A   
   T   
   A   
   A   
   A   
    A    
   T   
   T   
   T   
   C   
   G   
   A   
   T   
   T   
   C   
   A   
   A   
   G   
   T   
   A   
   G   
   A   
   T   
   A   
   A   
   T   
   G   
   C   
   C   
   A   
   A   
   A   
   C   
   G   
   T   
   G   
   G   
   A   
   G   
   A   
   A   
   A   
   T   
   T   
   G   
   T   
   C   
   G   
   A   
   T   
   C   
   G   
   T   
   A   
   A   
    T    
   G   
   G   
   T   
   A   
   G   
   T   
   G   
   G   
   G   
   C   
   T   
   A   
   G   
    C    
   A   
   A   
   T   
   T   
   A   
   A   
   C   
   A   
   A   
   A   
   G   
   T   
   G   
   T   
   T   
   T   
   G   
   A   
    C    
   G   
   A   
   A   
   G   
   T   
   G   
   G   
   G   
   C   
   G   
   T   
   A   
   G   
   T   
    G    
   C   
   C   
   T   
    G    
   G   
   C   
   A   
   A   
   A   
   C   
   T   
    C    
   G   
   G   
   T   
   T   
   C   
   T   
   G   
   G   
   C   
   G   
   C   
   A   
   G   
   A   
   A   
   A   
   A   
   A   
    A    
   C   
   A   
   G   
   C   
    C    
   A   
   A   
   T   
   A   
   T   
   C   
   A   
   A   
   A   
   G   
   C   
   T   
   T   
   T   
   T   
   A   
   G   
   T   
   G   
   A   
   T   
   A   
   A   
    G    
   T   
   T   
    C    
   G   
   G   
   C   
   G   
   T   
   T   
   T   
   C   
   T   
    G    
   T   
   T   
   G   
   A   
   T   
   G   
   A   
   A   
   A   
   T   
   C   
   A   
   A   
   T   
   C   
    A    
   A   
   A   
   A   
    G    
   T   
   T   
   A   
   A   
   G   
   C   
   C   
   A   
   A   
   G   
   G   
    G    
   T   
   G   
   G   
   G   
   T   
   C   
   C   
   A   
   A   
   G   
   C   
   A   
   G   
   A   
   C   
   T   
   C   
    C    
   T   
   T   
   T   
   G   
   T   
   A   
   C   
   C   
   A   
   A   
   T   
   C   
   A   
   C   
   A   
   G   
   T   
    T    
   G   
   C   
   T   
   T   
   C   
   T   
   G   
   A   
   A   
   C   
   C   
   A   
   G   
   T   
   G   
   A   
   C   
   A   
   G   
   A   
   A   
   T   
   T   
   A   
   C   
   C   
   A   
   A   
   C   
   A   
   G   
   G   
   G   
   G   
   C   
   T   
   G   
   C   
   G   
   A   
   C   
   A   
   A   
   A   
   A   
   G   
    A    
    T    
   A   
   C   
   A   
   G   
   A   
   G   
   T   
   C   
   A   
   C   
   G   
   T   
   T   
   A   
   T   
   T   
   A   
   T   
   C   
   C   
    A    
    C    
   T   
   G   
   G   
   G   
   G   
   G   
   A   
   A   
   G   
   C   
   A   
   G   
   C   
   T   
   G   
   C   
   G   
   C   
   A   
   A   
   T   
   T   
   A   
   A   
   T   
   C   
   G   
   G   
    G    
   T   
   A   
   T   
   A   
   C   
    G    
   G   
   G   
    C    
   A   
   C   
    C    
   A   
   T   
   T   
   A   
   C   
   T   
   G   
   C   
   A   
   G   
   A   
    A    
   G   
   A   
   T   
   A   
   T   
   T   
    G    
   A   
   A   
   A   
   A   
   A   
   A   
   A   
   T   
   C   
   C   
   A   
   G   
   A   
   G   
   T   
   T   
   A   
   A   
   G   
   T   
   A   
   G   
   T   
   A   
   C   
   G   
   G   
   G   
    T    
   G   
   T   
   A   
   A   
   T   
   T   
   G   
   G   
    T    
   A   
   A   
   A   
   A   
   C   
   T   
   G   
   G   
    C    
   T   
   T   
   A   
   G   
   A   
   A   
   C   
   G   
   A   
   G   
   C   
   G   
   T   
   T   
   T   
   G   
   A   
   T   
   A   
   A   
   A   
   G   
   A   
    G    
   T   
   T   
   A   
   A   
   G   
   A   
   G   
   G   
    G    
   C   
   A   
   A   
   G   
   A   
   T   
   G   
   G   
    T    
   G   
   G   
    C    
   T   
   C   
    A    
   C   
   T   
   A   
   G   
   T   
   T   
   A   
   T   
   T   
   T   
   T   
   A   
   G   
   A   
   T   
   G   
   A   
   C   
   A   
   A   
   A   
   G   
   A   
   G   
   A   
   A   
   T   
   G   
   T   
   C   
   A   
   A   
   A   
   A   
   A   
   A   
   G   
   C   
   T   
   T   
   T   
   A   
   C   
   A   
   A   
   A   
   C   
   C   
   A   
   A   
   A   
   G   
   A   
    G    
   A   
   A   
   A   
   A   
   A   
   A   
   G   
   A   
   T   
   G   
   G   
   T   
   C   
   A   
   A   
   A   
   C   
    G    
   A   
   T   
   T   
   A   
   A   
   A   
   T   
   T   
   A   
   A   
   C   
   G   
   A   
   T   
   T   
   G   
   A   
    C    
   A   
   G   
    T    
   G   
   G   
    C    
   G   
   T   
   A   
   C   
   A   
   A   
   C   
   A   
   A   
   C   
   A   
   A   
   G   
   C   
    C    
   T   
   T   
    C    
   G   
   C   
   T   
   A   
   T   
   A   
   T   
   T   
   T   
   G   
   A   
   C   
   A   
   A   
   A   
   C   
   G   
    T    
    C    
   C   
   T   
   G   
   G   
   T   
   A   
   G   
   T   
   G   
   C   
   G   
   G   
   T   
   C   
   A   
   T   
   T   
   A   
   C   
   C   
   G   
   A   
   T   
   C   
   C   
   T   
   C   
   A   
   A   
   A   
   A   
   A   
   G   
   G   
   C   
   G   
   A   
   T   
   T   
   T   
   A   
    T    
   T   
   A   
   G   
   C   
    A    
   A   
   C   
   G   
   G   
   T   
   T   
   A   
   G   
   T   
   T   
   C   
   A   
   C   
   C   
   T   
   T   
   C   
   G   
   T   
   A   
   T   
   G   
   A   
   T   
   C   
   C   
   T   
   A   
   A   
   T   
   A   
   A   
   A   
   A   
   T   
   G   
   G   
   C   
   C   
   A   
   A   
   T   
   G   
   G   
    T    
   A   
   T   
   T   
   T   
   C   
   T   
   C   
   A   
   A   
   A   
   A   
   A   
   G   
   A   
    A    
   T   
   A   
   T   
   G   
   A   
   T   
   G   
   C   
   T   
   T   
   A   
   C   
   A   
   A   
   T   
   A   
   A   
   C   
   A   
   A   
   C   
   A   
   A   
   A   
   G   
   A   
   T   
   T   
   T   
   A   
   C   
   C   
   A   
   T   
   T   
    C    
   A   
   C   
   A   
   G   
   C   
   A   
   C   
   G   
   T   
   T   
   T   
    T    
   G   
   C   
   G   
   A   
   C   
   A   
   G   
   G   
    C    
   T   
   A   
   T   
   G   
   C   
   A   
   C   
   C   
   A   
   G   
   G   
    C    
   T   
   C   
   T   
   A   
   C   
   C   
   T   
   T   
   T   
   A   
   A   
   A   
   A   
   C   
   A   
   A   
   T   
   T   
   A   
   C   
   C   
   G   
   G   
   T   
   G   
   C   
   C   
   A   
   T   
   T   
   G   
   G   
   T   
   T   
   T   
   A   
   G   
   A   
   T   
    G    
   C   
   A   
   G   
   G   
   G   
   A   
   C   
   A   
   T   
   T   
   G   
   A   
   A   
   G   
   C   
   C   
   A   
   G   
   A   
   T   
   G   
   A   
   A   
   G   
   A   
   G   
   C   
   T   
   A   
   G   
   A   
   A   
   A   
   T   
   T   
   A   
   A   
   T   
   G   
   G   
    C    
   T   
   T   
   G   
   A   
   A   
   A   
   T   
   G   
   G   
   C   
   A   
   A   
   A   
   A   
   A   
   G   
   A   
   T   
   A   
   A   
   A   
   T   
   C   
   T   
   T   
   G   
   G   
   G   
   G   
    C    
   G   
   G   
   C   
   T   
   A   
   T   
   T   
   T   
   T   
   G   
   C   
    A    
   A   
   C   
   A   
   C   
   G   
   T   
   G   
   T   
   A   
   A   
   A   
   A   
   G   
   A   
   A   
   G   
   C   
   A   
   A   
   G   
   T   
   C   
   C   
   A   
   G   
   T   
   C   
   A   
   A   
   T   
   C   
   T   
    G    
   C   
   G   
   T   
   A   
   C   
   C   
   G   
   C   
   T   
   T   
   T   
   A   
   G   
   T   
   T   
   A   
   A   
   T   
   T   
   C   
   A   
   G   
   A   
   T   
   A   
   A   
   T   
   A   
   T   
   T   
   T   
   A   
   T   
   T   
   T   
   T   
   G   
   C   
    C    
   C   
   A   
   A   
   C   
   A   
   A   
   A   
   C   
   A   
   T   
   T   
   A   
   C   
   G   
    G    
   A   
   T   
   G   
   G   
   G   
    G    
   G   
   A   
   A   
   G   
   A   
   C   
   A   
   A   
   A   
   T   
   T   
   T   
   A   
   G   
   A   
   G   
   C   
    A    
   G   
   G   
   T   
   C   
   T   
   T   
   A   
   A   
   T   
   A   
   A   
   A   
   T   
   T   
   C   
   A   
   T   
   T   
   T   
   T   
   C   
   G   
   G   
   T   
   G   
   A   
   G   
   G   
   A   
   A   
   C   
   T   
   A   
   G   
   A   
   T   
   T   
   T   
   A   
   C   
   C   
   A   
   A   
   T   
    T    
   G   
   C   
   C   
   A   
   T   
   G   
   A   
   C   
   G   
   C   
   C   
    T    
   G   
   C   
   T   
   C   
   A   
   A   
   A   
   T   
   T   
   T   
   C   
   A   
   A   
   A   
    T    
   G   
   A   
   A   
   G   
   A   
   T   
   A   
   A   
   A   
   T   
   T   
   T   
   A   
   A   
   T   
   T   
   C   
   T   
   G   
   A   
   A   
   A   
   T   
   T   
   C   
   T   
   T   
   T   
   T   
    A    
   G   
   C   
   A   
   G   
   A   
   T   
   A   
   C   
    G    
   G   
   G   
   C   
   T   
   A   
    C    
   G   
   G   
    C    
   C   
   A   
   A   
   G   
   G   
    C    
   C   
   A   
   A   
   T   
   T   
   G   
   T   
   T   
   A   
   A   
   T   
   T   
   T   
   C   
    G    
   C   
   C   
   A   
   A   
   T   
   C   
   C   
   A   
   G   
   C   
   A   
   A   
   G   
   C   
   T   
   A   
   C   
   C   
   A   
   T   
   G   
   T   
   A   
   T   
   A   
   G   
   T   
   G   
   T   
   T   
   T   
   T   
   C   
   C   
   A   
   A   
   A   
   A   
   T   
   A   
   A   
   T   
   G   
   G   
    A    
   A   
   C   
   A   
    T    
   T   
    A    
   G   
   T   
    C    
   T   
   A   
   T   
   C   
   C   
   A   
   A   
   A   
   A   
    T    
   T   
   A   
   G   
   T   
   T   
   T   
   T   
   A   
   G   
   A   
   C   
   A   
   A   
   A   
   G   
   A   
   A   
   A   
   C   
    G    
   A   
   A   
   G   
   A   
   A   
   A   
   A   
   A   
   A   
   G   
   A   
   C   
   A   
   A   
   T   
   G   
   T   
   A   
   A   
   T   
   C   
   A   
   G   
   C   
   G   
   C   
   C   
   A   
   A   
   C   
   G   
   C   
   A   
   G   
   C   
   G   
   A   
   A   
   T   
   A   
   C   
   A   
   A   
   T   
   T   
   G   
   C   
   C   
   A   
   C   
   A   
   G   
   A   
   T   
   C   
   T   
   T   
   T   
   T   
   A   
   G   
   G   
   T   
   A   
   G   
   T   
   G   
   T   
   G   
   G   
   A   
   A   
   G   
   A   
   T   
   C   
   C   
   T   
   T   
   C   
   A   
   G   
   G   
   T   
   T   
   A   
   T   
   G   
   T   
   T   
   T   
   A   
   T   
   A   
   A   
   T   
   A   
   T   
   G   
   T   
   A   
   C   
   A   
   A   
   T   
   C   
   C   
   G   
   A   
   A   
   C   
   T   
   T   
   C   
   T   
   C   
   A   
   T   
   T   
   G   
   G   
   C   
   T   
   G   
   C   
   T   
   A   
   A   
   A   
   A   
   C   
   A   
   G   
   G   
   A   
   A   
   C   
   A   
   G   
   C   
   T   
   G   
   A   
   A   
   A   
   T   
   A   
   A   
   A   
   A   
   G   
   A   
   T   
   A   
   A   
   A   
   C   
   A   
   A   
   G   
   A   
   T   
   A   
   C   
   A   
   G   
   A   
   T   
   G   
   G   
   C   
   A   
   A   
   A   
   G   
   A   
   A   
   A   
   A   
   C   
   A   
   G   
   T   
   T   
   T   
   C   
   C   
   T   
   C   
   C   
   T   
   A   
   A   
   C   
   G   
   T   
   T   
   A   
   G   
   A   
   T   
   C   
   G   
   T   
   A   
   G   
   T   
   A   
   A   
   C   
   A   
   A   
   T   
   A   
   A   
   A   
   T   
   T   
   C   
   T   
   T   
   A   
   A   
   C   
   A   
   A   
   T   
   G   
   A   
   T   
   T   
   A   
   T   
   G   
   G   
   T   
   T   
   G   
   A   
   A   
   A   
   A   
   T   
   T   
   C   
   A   
   G   
   G   
   A   
   G   
   A   
   A   
   A   
   A   
   T   
   G   
   G   
   T   
   T   
   C   
   T   
   G   
   C   
   A   
   A   
   C   
   A   
   G   
   A   
   T   
   A   
   T   
   C   
   A   
   G   
   T   
   A   
   A   
   A   
   C   
   C   
   A   
   T   
   T   
   A   
   A   
   T   
   T   
   G   
   A   
   T   
   T   
   A   
   C   
   T   
   T   
   A   
   G   
   A   
   A   
   G   
   C   
   A   
   A   
   C   
   C   
   A   
   T   
   T   
   A   
   A   
   A   
   -   
   -   
   -   
   -   
   -   
   -   
   -   
   -   
   -   
   -   
   -   
   -   
   -   
   -   
   -   
   -   
   -   
   -   
   -   
   -   
   -   
   -   
   -   
   -   
   -   
   -   
   -   
   -   
   -   
   -   
   -   
   -   
   -   
   -   
   -   
   -   
   -   
   -   
   -   
   -   
   -   
   -   
   -   
   -   
   -   
   -   
   -   
   -   
   -   
   -   
   -   
   -   
   -   
   -   
   -   
   -   
   -   
   -   
   -   
   -   
   -   
   -   
   -   
   -   
   -   
   -   
   -   
   -   
   -   
   -   
   -   
   -   
   -   
   -   
   -   
   -   
   -   
   -   
   -   
   -   
   -   
   -   
   -   
   -   
   -   
   -   
   -   
   -   
   -   
   -   
   -   
   -   
   -   
   -   
   -   
   -   
   -   
   -   
   -   
   -   
   -   
   -   
   -   
   -   
   -   
   -   
   -   
   -   
   -   
   -   
   -   
   -   
   -   
   -   
   -   
   -   
   -   
   -   
   -   
   -   
   -   
   -   
   -   
   -   
   -   
   -   
   -   
   -   
   -   
   -   
   -   
   -   
   -   
   -   
   -   
   -   
   -   
   -   
   -   
   -   
   -   
   -   
   -   
   -   
   -   
   -   
   -   
   -   
   -   
   -   
   -   
   -   
   -   
   -   
   -   
   -   
   -   
   -   
   -   
   -   
   -   
   -   
   -   
   -   
   -   
   -   
   -   
   -   
   -   
   C   
   A   
   A   
   T   
   G   
   G   
   C   
   A   
   A   
   G   
   C   
   C   
   A   
   A   
   A   
   C   
   A   
   A   
   G   
   A   
   A   
   T   
   T   
   A   
   G   
   C   
   C   
   G   
   A   
   A   
   G   
   C   
   G   
   A   
   A   
   G   
   A   
   A   
   A   
   A   
   C   
   A   
   G   
   C   
   T   
   A   
   A   
   T   
   A   
   C   
   A   
   T   
   T   
   T   
   T   
   T   
   A   
   A   
   A   
   T   
   G   
   T   
   A   
   T   
   T   
   G   
   T   
   C   
   A   
   A   
   A   
   A   
   C   
   A   
   G   
   G   
   A   
   A   
   T   
   T   
   T   
   G   
   A   
   T   
   A   
   A   
   G   
   T   
   T   
   A   
   C   
   C   
   G   
   T   
   C   
   C   
   G   
   T   
   T   
   G   
   T   
   T   
   C   
   A   
   A   
   G   
   A   
   A   
   T   
   C   
   T   
   A   
   G   
   C   
   T   
   T   
   A   
   A   
   A   
   G   
   A   
   A   
   A   
   A   
   A   
   T   
   G   
   G   
   C   
   T   
   A   
   T   
   G   
   A   
   T   
   A   
   C   
   T   
   A   
   A   
   A   
   T   
   C   
   T   
   G   
   T   
   T   
   G   
   T   
   T   
   G   
   A   
   A   
   A   
   A   
   A   
   T   
   A   
   C   
   C   
   A   
   A   
   G   
   C   
   A   
   A   
   T   
   T   
   T   
   A   
   T   
   T   
   C   
   A   
   G   
   G   
   G   
   A   
   T   
   T   
   C   
   A   
   A   
   G   
   C   
   A   
   G   
   A   
   A   
   G   
   G   
   A   
   G   
   T   
   C   
   A   
   A   
   A   
   G   
   C   
   T   
   A   
   G   
   T   
   G   
   A   
   T   
   G   
   T   
   C   
   C   
   A   
   A   
   G   
   T   
   C   
   A   
   A   
   A   
   A   
   A   
   G   
   G   
   C   
   G   
   A   
   A   
   A   
   G   
   A   
   C   
   A   
   A   
   T   
   C   
   A   
   A   
   T   
   A   
   C   
   A   
   C   
   A   
   T   
   T   
   T   
   A   
   C   
   C   
   T   
   A   
   T   
   A   
   A   
   A   
   T   
   T   
   A   
   T   
   C   
   G   
   A   
   T   
   G   
   A   
   G   
   C   
   A   
   C   
   T   
   C   
   C   
   T   
   T   
   T   
   A   
   G   
   G   
   C   
   G   
   A   
   A   
   A   
   T   
   G   
   A   
   A   
   A   
   G   
   A   
   T   
   C   
   T   
   G   
   T   
   C   
   T   
   T   
   A   
   T   
   C   
   A   
   A   
   T   
   C   
   A   
   A   
   G   
   T   
   A   
   T   
   C   
   G   
   C   
   C   
   A   
   A   
   A   
   A   
   A   
   A   
   G   
   G   
   C   
   G   
   A   
   T   
   A   
   C   
   C   
   T   
   A   
   C   
   C   
   A   
   A   
   A   
   T   
   C   
   T   
   C   
   T   
   T   
   G   
   G   
   A   
   A   
   G   
   C   
   C   
   G   
   T   
   C   
   T   
   T   
   T   
   A   
   A   
   T   
   T   
   T   
   T   
   T   
   C   
   C   
   A   
   G   
   A   
   T   
   A   
   T   
   G   
   T   
   C   
   A   
   G   
   G   
   A   
   A   
   A   
   T   
   G   
   A   
   T   
   A   
   A   
   A   
   A   
   T   
   T   
   T   
   C   
   G   
   A   
   T   
   T   
   C   
   A   
   A   
   G   
   T   
   A   
   G   
   A   
   T   
   A   
   A   
   T   
   G   
   C   
   C   
   A   
   A   
   A   
   C   
   G   
   T   
   G   
   G   
   A   
   G   
   A   
   A   
   A   
   T   
   T   
   G   
   T   
   C   
   G   
   A   
   T   
   C   
   G   
   T   
   A   
   A   
   T   
   G   
   G   
   T   
   A   
   G   
   T   
   G   
   G   
   G   
   C   
   T   
   A   
   G   
   C   
   A   
   A   
   T   
   T   
   A   
   A   
   C   
   A   
   A   
   A   
   G   
   T   
   G   
   T   
   T   
   T   
   G   
   A   
   C   
   G   
   A   
   A   
   G   
   T   
   G   
   G   
   G   
   C   
   G   
   T   
   A   
   G   
   T   
   G   
   C   
   C   
   T   
   G   
   G   
   C   
   A   
   A   
   A   
   C   
   T   
   C   
   G   
   G   
   T   
   T   
   C   
   T   
   G   
   G   
   C   
   G   
   C   
   A   
   G   
   A   
   A   
   A   
   A   
   A   
   A   
   C   
   A   
   G   
   C   
   C   
   A   
   A   
   T   
   A   
   T   
   C   
   A   
   A   
   A   
   G   
   C   
   T   
   T   
   T   
   T   
   A   
   G   
   T   
   G   
   A   
   T   
   A   
   A   
   G   
   T   
   T   
   C   
   G   
   G   
   C   
   G   
   T   
   T   
   T   
   C   
   T   
   G   
   T   
   T   
   G   
   A   
   T   
   G   
   A   
   A   
   A   
   T   
   C   
   A   
   A   
   T   
   C   
   A   
   A   
   A   
   A   
   G   
   T   
   T   
   A   
   A   
   G   
   C   
   C   
   A   
   A   
   G   
   G   
   G   
   T   
   G   
   G   
   G   
   T   
   C   
   C   
   A   
   A   
   G   
   C   
   A   
   G   
   A   
   C   
   T   
   C   
   C   
   T   
   T   
   T   
   G   
   T   
   A   
   C   
   C   
   A   
   A   
   T   
   C   
   A   
   C   
   A   
   G   
   T   
   T   
   G   
   C   
   T   
   T   
   C   
   T   
   G   
   A   
   A   
   C   
   C   
   A   
   G   
   T   
   G   
   A   
   C   
   A   
   G   
   A   
   A   
   T   
   T   
   A   
   C   
   C   
   A   
   A   
   C   
   A   
   G   
   G   
   G   
   G   
   C   
   T   
   G   
   C   
   G   
   A   
   C   
   A   
   A   
   A   
   A   
   G   
   A   
   T   
   A   
   C   
   A   
   G   
   A   
   G   
   T   
   C   
   A   
   C   
   G   
   T   
   T   
   A   
   T   
   T   
   A   
   T   
   C   
   C   
   A   
   C   
   T   
   G   
   G   
   G   
   G   
   G   
   A   
   A   
   G   
   C   
   A   
   G   
   C   
   T   
   G   
   C   
   G   
   C   
   A   
   A   
   T   
   T   
   A   
   A   
   T   
   C   
   G   
   G   
   G   
   T   
   A   
   T   
   A   
   C   
   G   
   G   
   G   
   C   
   A   
   C   
   C   
   A   
   T   
   T   
   A   
   C   
   T   
   G   
   C   
   A   
   G   
   A   
   A   
   G   
   A   
   T   
   A   
   T   
   T   
   G   
   A   
   A   
   A   
   A   
   A   
   A   
   A   
   T   
   C   
   C   
   A   
   G   
   A   
   G   
   T   
   T   
   A   
   A   
   G   
   T   
   A   
   G   
   T   
   A   
   C   
   G   
   G   
   G   
   T   
   G   
   T   
   A   
   A   
   T   
   T   
   G   
   G   
   T   
   A   
   A   
   A   
   A   
   C   
   T   
   G   
   G   
   C   
   T   
   T   
   A   
   G   
   A   
   A   
   C   
   G   
   A   
   G   
   C   
   G   
   T   
   T   
   T   
   G   
   A   
   T   
   A   
   A   
   A   
   G   
   A   
   G   
   T   
   T   
   A   
   A   
   G   
   A   
   G   
   G   
   G   
   C   
   A   
   A   
   G   
   A   
   T   
   G   
   G   
   T   
   G   
   G   
   C   
   T   
   C   
   A   
   C   
   T   
   A   
   G   
   T   
   T   
   A   
   T   
   T   
   T   
   T   
   A   
   G   
   A   
   T   
   G   
   A   
   C   
   A   
   A   
   A   
   G   
   A   
   G   
   A   
   A   
   T   
   G   
   T   
   C   
   A   
   A   
   A   
   A   
   A   
   A   
   G   
   C   
   T   
   T   
   T   
   A   
   C   
   A   
   A   
   A   
   C   
   C   
   A   
   A   
   A   
   G   
   A   
   G   
   A   
   A   
   A   
   A   
   A   
   A   
   G   
   A   
   T   
   G   
   G   
   T   
   C   
   A   
   A   
   A   
   C   
   G   
   A   
   T   
   T   
   A   
   A   
   A   
   T   
   T   
   A   
   A   
   C   
   G   
   A   
   T   
   T   
   G   
   A   
   C   
   A   
   G   
   T   
   G   
   G   
   C   
   G   
   T   
   A   
   C   
   A   
   A   
   C   
   A   
   A   
   C   
   A   
   A   
   G   
   C   
   C   
   T   
   T   
   C   
   G   
   C   
   T   
   A   
   T   
   A   
   T   
   T   
   T   
   G   
   A   
   C   
   A   
   A   
   A   
   C   
   G   
   T   
   C   
   C   
   T   
   G   
   G   
   T   
   A   
   G   
   T   
   G   
   C   
   G   
   G   
   T   
   C   
   A   
   T   
   T   
   A   
   C   
   C   
   G   
   A   
   T   
   C   
   C   
   T   
   C   
   A   
   A   
   A   
   A   
   A   
   G   
   G   
   C   
   G   
   A   
   T   
   T   
   T   
   A   
   T   
   T   
   A   
   G   
   C   
   A   
   A   
   C   
   G   
   G   
   T   
   T   
   A   
   G   
   T   
   T   
   C   
   A   
   C   
   C   
   T   
   T   
   C   
   G   
   T   
   A   
   T   
   G   
   A   
   T   
   C   
   C   
   T   
   A   
   A   
   T   
   A   
   A   
   A   
   A   
   T   
   G   
   G   
   C   
   C   
   A   
   A   
   T   
   G   
   G   
   T   
   A   
   T   
   T   
   T   
   C   
   T   
   C   
   A   
   A   
   A   
   A   
   A   
   G   
   A   
   A   
   T   
   A   
   T   
   G   
   A   
   T   
   G   
   C   
   T   
   T   
   A   
   C   
   A   
   A   
   T   
   A   
   A   
   C   
   A   
   A   
   C   
   A   
   A   
   A   
   G   
   A   
   T   
   T   
   T   
   A   
   C   
   C   
   A   
   T   
   T   
   C   
   A   
   C   
   A   
   G   
   C   
   A   
   C   
   G   
   T   
   T   
   T   
   T   
   G   
   C   
   G   
   A   
   C   
   A   
   G   
   G   
   C   
   T   
   A   
   T   
   G   
   C   
   A   
   C   
   C   
   A   
   G   
   G   
   C   
   T   
   C   
   T   
   A   
   C   
   C   
   T   
   T   
   T   
   A   
   A   
   A   
   A   
   C   
   A   
   A   
   T   
   T   
   A   
   C   
   C   
   G   
   G   
   T   
   G   
   C   
   C   
   A   
   T   
   T   
   G   
   G   
   T   
   T   
   T   
   A   
   G   
   A   
   T   
   G   
   C   
   A   
   G   
   G   
   G   
   A   
   C   
   A   
   T   
   T   
   G   
   A   
   A   
   G   
   C   
   C   
   A   
   G   
   A   
   T   
   G   
   A   
   A   
   G   
   A   
   G   
   C   
   T   
   A   
   G   
   A   
   A   
   A   
   T   
   T   
   A   
   A   
   T   
   G   
   G   
   C   
   T   
   T   
   G   
   A   
   A   
   A   
   T   
   G   
   G   
   C   
   A   
   A   
   A   
   A   
   A   
   G   
   A   
   T   
   A   
   A   
   A   
   T   
   C   
   T   
   T   
   G   
   G   
   G   
   G   
   C   
   G   
   G   
   C   
   T   
   A   
   T   
   T   
   T   
   T   
   G   
   C   
   A   
   A   
   C   
   A   
   C   
   G   
   T   
   G   
   T   
   A   
   A   
   A   
   A   
   G   
   A   
   A   
   G   
   C   
   A   
   A   
   G   
   T   
   C   
   C   
   A   
   G   
   T   
   C   
   A   
   A   
   T   
   C   
   T   
   G   
   C   
   G   
   T   
   A   
   C   
   C   
   G   
   C   
   T   
   T   
   T   
   A   
   G   
   T   
   T   
   A   
   A   
   T   
   T   
   C   
   A   
   G   
   A   
   T   
   A   
   A   
   T   
   A   
   T   
   T   
   T   
   A   
   T   
   T   
   T   
   T   
   G   
   C   
   C   
   C   
   A   
   A   
   C   
   A   
   A   
   A   
   C   
   A   
   T   
   T   
   A   
   C   
   G   
   G   
   A   
   T   
   G   
   G   
   G   
   G   
   G   
   A   
   A   
   G   
   A   
   C   
   A   
   A   
   A   
   T   
   T   
   T   
   A   
   G   
   A   
   G   
   C   
   A   
   G   
   G   
   T   
   C   
   T   
   T   
   A   
   A   
   T   
   A   
   A   
   A   
   T   
   T   
   C   
   A   
   T   
   T   
   T   
   T   
   C   
   G   
   G   
   T   
   G   
   A   
   G   
   G   
   A   
   A   
   C   
   T   
   A   
   G   
   A   
   T   
   T   
   T   
   A   
   C   
   C   
   A   
   A   
   T   
   T   
   G   
   C   
   C   
   A   
   T   
   G   
   A   
   C   
   G   
   C   
   C   
   T   
   G   
   C   
   T   
   C   
   A   
   A   
   A   
   T   
   T   
   T   
   C   
   A   
   A   
   A   
   T   
   G   
   A   
   A   
   G   
   A   
   T   
   A   
   A   
   A   
   T   
   T   
   T   
   A   
   A   
   T   
   T   
   C   
   T   
   G   
   A   
   A   
   A   
   T   
   T   
   C   
   T   
   T   
   T   
   T   
   A   
   G   
   C   
   A   
   G   
   A   
   T   
   A   
   C   
   G   
   G   
   G   
   C   
   T   
   A   
   C   
   G   
   G   
   C   
   C   
   A   
   A   
   G   
   G   
   C   
   C   
   A   
   A   
   T   
   T   
   G   
   T   
   T   
   A   
   A   
   T   
   T   
   T   
   C   
   G   
   C   
   C   
   A   
   A   
   T   
   C   
   C   
   A   
   G   
   C   
   A   
   A   
   G   
   C   
   T   
   A   
   C   
   C   
   A   
   T   
   G   
   T   
   A   
   T   
   A   
   G   
   T   
   G   
   T   
   T   
   T   
   T   
   C   
   C   
   A   
   A   
   A   
   A   
   T   
   A   
   A   
   T   
   G   
   G   
   A   
   A   
   C   
   A   
   T   
   T   
   A   
   G   
   T   
   C   
   T   
   A   
   T   
   C   
   C   
   A   
   A   
   A   
   A   
   T   
   T   
   A   
   G   
   T   
   T   
   T   
   T   
   A   
   G   
   A   
   C   
   A   
   A   
   A   
   G   
   A   
   A   
   A   
   C   
   G   
   A   
   A   
   G   
   A   
   A   
   A   
   A   
   A   
   A   
   G   
   A   
   C   
   A   
   A   
   T   
   G   
   T   
   A   
   A   
   T   
   C   
   A   
   G   
   C   
   G   
   C   
   C   
   A   
   A   
   C   
   G   
   C   
   A   
   G   
   C   
   G   
   A   
   A   
   T   
   A   
   C   
   A   
   A   
   T   
   T   
   G   
   C   
   C   
   A   
   C   
   A   
   G   
   A   
   T   
   C   
   T   
   T   
   T   
   T   
   A   
   G   
   G   
   T   
   A   
   G   
   T   
   G   
   T   
   G   
   G   
   A   
   A   
   G   
   A   
   T   
   C   
   C   
   T   
   T   
   C   
   A   
   G   
   G   
   T   
   T   
   A   
   T   
   G   
   T   
   T   
   T   
   A   
   T   
   A   
   A   
   T   
   A   
   T   
   G   
   T   
   A   
   C   
   A   
   A   
   T   
   C   
   C   
   G   
   A   
   A   
   C   
   T   
   T   
   C   
   T   
   C   
   A   
   T   
   T   
   G   
   G   
   C   
   T   
   G   
   C   
   T   
   A   
   A   
   A   
   A   
   C   
   A   
   G   
   G   
   A   
   A   
   C   
   A   
   G   
   C   
   T   
   G   
   A   
   A   
   A   
   T   
   A   
   A   
   A   
   A   
   G   
   A   
   T   
   A   
   A   
   A   
   C   
   A   
   A   
   G   
   A   
   T   
   A   
   C   
   A   
   G   
   A   
   T   
   G   
   G   
   C   
   A   
   A   
   A   
   G   
   A   
   A   
   A   
   A   
   C   
   A   
   G   
   T   
   T   
   T   
   C   
   C   
   T   
   C   
   C   
   T   
   A   
   A   
   C   
   G   
   T   
   T   
   A   
   G   
   A   
   T   
   C   
   G   
   T   
   A   
   G   
   T   
   A   
   A   
   C   
   A   
   A   
   T   
   A   
   A   
   A   
   T   
   T   
   C   
   T   
   T   
   A   
   A   
   C   
   A   
   A   
   T   
   G   
   A   
   T   
   T   
   A   
   T   
   G   
   G   
   T   
   T   
   G   
   A   
   A   
   A   
   A   
   T   
   T   
   C   
   A   
   G   
   G   
   A   
   G   
   A   
   A   
   A   
   A   
   T   
   G   
   G   
   T   
   T   
   C   
   T   
   G   
   C   
   A   
   A   
   C   
   A   
   G   
   A   
   T   
   A   
   T   
   C   
   A   
   G   
   T   
   A   
   A   
   A   
   C   
   C   
   A   
   T   
   T   
   A   
   A   
   T   
   T   
   G   
   A   
   T   
   T   
   A   
   C   
   T   
   T   
   A   
   G   
   A   
   A   
   G   
   C   
   A   
   A   
   C   
   C   
   A   
   T   
   T   
   A   
   A   
   A   
   -   
   -   
   -   
   -   
   -   
   -   
   -   
   -   
   -   
   -   
   -   
   -   
   -   
   -   
   -   
   -   
   -   
   -   
   -   
   -   
   -   
   -   
   -   
   -   
   -   
   -   
   -   
   -   
   -   
   -   
   -   
   -   
   -   
   -   
   -   
   -   
   -   
   -   
   -   
   -   
   -   
   -   
   -   
   -   
   -   
   -   
   -   
   -   
   -   
   -   
   -   
   -   
   -   
   -   
   -   
   -   
   -   
   -   
   -   
   -   
   -   
 

 
   N59508F   
   -   
   -   
   -   
   -   
   -   
   -   
   -   
   -   
   -   
   -   
   -   
   -   
   -   
   -   
   -   
   -   
   -   
   -   
   -   
   -   
   -   
   -   
   -   
   -   
   -   
   -   
   -   
   -   
   -   
   -   
   -   
   -   
   -   
   -   
   -   
   -   
   -   
   -   
   -   
   -   
   -   
   -   
   -   
   -   
   -   
   -   
   -   
   -   
   -   
   -   
   -   
   -   
   -   
   -   
   -   
   -   
   -   
   -   
   -   
   -   
   -   
   -   
   -   
   -   
   -   
   -   
   -   
   -   
   -   
   -   
   -   
   -   
   -   
   -   
   -   
   -   
   -   
   -   
   -   
   -   
   -   
   -   
   -   
   -   
   -   
   -   
   -   
   -   
   -   
   -   
   -   
   -   
   -   
   -   
   -   
   -   
   -   
   -   
   -   
   -   
   -   
   -   
   -   
   -   
   -   
   -   
   -   
   -   
   C   
   A   
   A   
   T   
   G   
   G   
   C   
   A   
   A   
   G   
   C   
   C   
   A   
   A   
   A   
   C   
   A   
   A   
   G   
   A   
    A    
   T   
   T   
   A   
   G   
   C   
    C    
   G   
   A   
   A   
   G   
   C   
   G   
   A   
   A   
   G   
   A   
   A   
   A   
   A   
    C    
   A   
   G   
   C   
    T    
   A   
    C    
   T   
    A    
    C    
   A   
   T   
   T   
   T   
   T   
   T   
   A   
   A   
   A   
    C    
   G   
   T   
   A   
   T   
   T   
   G   
    A    
   C   
   A   
   A   
   A   
   A   
   C   
   A   
   G   
    A    
   A   
   A   
   T   
   T   
   T   
   G   
   A   
   T   
   A   
   A   
   G   
   T   
   T   
   A   
   C   
   C   
    G    
   T   
   C   
   C   
   G   
   T   
   T   
    G    
   T   
   T   
   C   
   A   
   A   
   G   
   A   
   A   
    G    
   C   
   T   
   A   
   G   
   C   
   T   
   T   
   A   
   A   
   A   
   G   
   A   
   A   
   A   
   A   
   A   
   T   
   G   
   G   
    C    
   T   
   A   
   T   
   G   
   A   
   T   
   A   
   C   
   T   
   A   
   A   
   A   
   T   
   C   
    T    
   G   
   T   
   T   
   G   
   T   
   T   
   G   
   A   
   A   
   A   
   A   
   A   
   T   
   A   
   C   
   C   
   A   
   A   
   G   
   C   
   A   
   A   
   T   
   T   
   T   
   A   
   T   
   T   
   C   
   A   
   G   
   G   
   G   
   A   
   T   
   T   
   C   
   A   
   A   
   G   
   C   
    A    
   G   
   A   
   A   
    G    
   G   
    A    
   G   
   T   
   C   
   A   
   A   
    A    
   G   
   C   
   T   
   A   
   G   
   T   
   G   
   A   
   T   
   G   
   T   
    T    
   C   
   A   
   A   
   G   
   T   
    C    
   A   
   A   
   A   
   A   
   A   
    A    
   G   
    C    
   G   
   A   
   A   
   A   
   G   
   A   
    C    
   A   
   A   
    T    
   C   
   A   
   A   
   T   
   A   
   C   
   A   
   C   
   A   
   T   
   T   
   T   
   A   
    C    
   C   
   T   
   A   
   T   
   A   
   A   
   A   
   T   
   T   
   A   
   T   
   C   
   G   
   A   
   T   
   G   
   A   
   G   
    T    
   A   
   C   
    C    
   C   
   C   
   T   
   T   
   T   
   A   
   G   
   G   
   C   
   G   
   A   
   A   
   A   
   T   
   G   
   A   
   A   
   A   
   G   
   A   
   T   
    T    
   T   
   G   
   T   
   C   
   T   
   T   
   A   
   T   
   C   
   A   
   A   
   T   
   C   
   A   
   A   
   G   
   T   
   A   
   T   
    C    
   G   
    C    
   C   
   A   
   A   
    A    
   A   
   A   
   A   
   G   
   G   
    C    
   G   
   A   
   T   
   A   
    C    
   C   
   T   
   A   
   C   
   C   
   A   
   A   
   A   
   T   
    C    
    G    
   C   
   T   
   T   
   G   
   G   
    A    
    A    
    G    
   C   
   C   
    G    
   T   
   C   
   T   
   T   
   T   
   A   
   A   
   T   
   T   
   T   
   T   
   T   
   C   
   C   
   A   
   G   
   A   
   T   
   A   
   T   
   G   
   T   
   C   
   A   
   G   
   G   
   A   
   A   
   A   
   T   
   G   
   A   
   T   
   A   
   A   
   A   
    A    
   T   
   T   
   T   
   C   
   G   
   A   
   T   
   T   
   C   
   A   
   A   
   G   
   T   
   A   
   G   
   A   
   T   
   A   
   A   
   T   
   G   
   C   
   C   
   A   
   A   
   A   
   C   
   G   
   T   
   G   
   G   
   A   
   G   
   A   
   A   
   A   
   T   
   T   
   G   
   T   
   C   
   G   
   A   
   T   
   C   
   G   
   T   
   A   
   A   
    T    
   G   
   G   
   T   
   A   
   G   
   T   
   G   
   G   
   G   
   C   
   T   
   A   
   G   
    C    
   A   
   A   
   T   
   T   
   A   
   A   
   C   
   A   
   A   
   A   
   G   
   T   
   G   
   T   
   T   
   T   
   G   
   A   
    C    
   G   
   A   
   A   
   G   
   T   
   G   
   G   
   G   
   C   
   G   
   T   
   A   
   G   
   T   
    G    
   C   
   C   
   T   
    G    
   G   
   C   
   A   
   A   
   A   
   C   
   T   
    C    
   G   
   G   
   T   
   T   
   C   
   T   
   G   
   G   
   C   
   G   
   C   
   A   
   G   
   A   
   A   
   A   
   A   
   A   
    A    
   C   
   A   
   G   
   C   
    C    
   A   
   A   
   T   
   A   
   T   
   C   
   A   
   A   
   A   
   G   
   C   
   T   
   T   
   T   
   T   
   A   
   G   
   T   
   G   
   A   
   T   
   A   
   A   
    G    
   T   
   T   
    C    
   G   
   G   
   C   
   G   
   T   
   T   
   T   
   C   
   T   
    G    
   T   
   T   
   G   
   A   
   T   
   G   
   A   
   A   
   A   
   T   
   C   
   A   
   A   
   T   
   C   
    A    
   A   
   A   
   A   
    G    
   T   
   T   
   A   
   A   
   G   
   C   
   C   
   A   
   A   
   G   
   G   
    G    
   T   
   G   
   G   
   G   
   T   
   C   
   C   
   A   
   A   
   G   
   C   
   A   
   G   
   A   
   C   
   T   
   C   
    C    
   T   
   T   
   T   
   G   
   T   
   A   
   C   
   C   
   A   
   A   
   T   
   C   
   A   
   C   
   A   
   G   
   T   
    C    
   G   
   C   
   T   
   T   
   C   
   T   
   G   
   A   
   A   
   C   
   C   
   A   
   G   
   T   
   G   
   A   
   C   
   A   
   G   
   A   
   A   
   T   
   T   
   A   
   C   
   C   
   A   
   A   
   C   
   A   
   G   
   G   
   G   
   G   
   C   
   T   
   G   
   C   
   G   
   A   
   C   
   A   
   A   
   A   
   A   
   G   
    A    
    T    
   A   
   C   
   A   
   G   
   A   
   G   
   T   
   C   
   A   
   C   
   G   
   T   
   T   
   A   
   T   
   T   
   A   
   T   
   C   
   C   
    G    
    C    
   T   
   G   
   G   
   G   
   G   
   G   
   A   
   A   
   G   
   C   
   A   
   G   
   C   
   T   
   G   
   C   
   G   
   C   
   A   
   A   
   T   
   T   
   A   
   A   
   T   
   C   
   G   
   G   
    G    
   T   
   A   
   T   
   A   
   C   
    A    
   G   
   G   
    C    
   A   
   C   
    C    
   A   
   T   
   T   
   A   
   C   
   T   
   G   
   C   
   A   
   G   
   A   
    A    
   G   
   A   
   T   
   A   
   T   
   T   
    A    
   A   
   A   
   A   
   A   
   A   
   A   
   A   
   T   
   C   
   C   
   A   
   G   
   A   
   G   
   T   
   T   
   A   
   A   
   G   
   T   
   A   
   G   
   T   
   A   
   C   
   G   
   G   
   G   
    C    
   G   
   T   
   A   
   A   
   T   
   T   
   G   
   G   
    T    
   A   
   A   
   A   
   A   
   C   
   T   
   G   
   G   
    C    
   T   
   T   
   A   
   G   
   A   
   A   
   C   
   G   
   A   
   G   
   C   
   G   
   T   
   T   
   T   
   G   
   A   
   T   
   A   
   A   
   A   
   G   
   A   
    A    
   T   
   T   
   A   
   A   
   G   
   A   
   G   
   G   
    G    
   C   
   A   
   A   
   G   
   A   
   T   
   G   
   G   
    T    
   G   
   G   
    C    
   T   
   C   
    A    
   C   
   T   
   A   
   G   
   T   
   T   
   A   
   T   
   T   
   T   
   T   
   A   
   G   
   A   
   T   
   G   
   A   
   C   
   A   
   A   
   A   
   G   
   A   
   G   
   A   
   A   
   T   
   G   
   T   
   C   
   A   
   A   
   A   
   A   
   A   
   A   
   G   
   C   
   T   
   T   
   T   
   A   
   C   
   A   
   A   
   A   
   C   
   C   
   A   
   A   
   A   
   G   
   A   
    G    
   A   
   A   
   A   
   A   
   A   
   A   
   G   
   A   
   T   
   G   
   G   
   T   
   C   
   A   
   A   
   A   
   C   
    G    
   A   
   T   
   T   
   A   
   A   
   A   
   T   
   T   
   A   
   A   
   C   
   G   
   A   
   T   
   T   
   G   
   A   
    C    
   A   
   G   
    T    
   G   
   G   
    C    
   G   
   T   
   A   
   C   
   A   
   A   
   C   
   A   
   A   
   C   
   A   
   A   
   G   
   C   
    C    
   T   
   T   
    C    
   G   
   C   
   T   
   A   
   T   
   A   
   T   
   T   
   T   
   G   
   A   
   C   
   A   
   A   
   A   
   C   
   G   
    C    
    C    
   C   
   T   
   G   
   G   
   T   
   A   
   G   
   T   
   G   
   C   
   G   
   G   
   T   
   C   
   A   
   T   
   T   
   A   
   C   
   C   
   G   
   A   
   T   
   C   
   C   
   T   
   C   
   A   
   A   
   A   
   A   
   A   
   G   
   G   
   C   
   G   
   A   
   T   
   T   
   T   
   A   
    T    
   T   
   A   
   G   
   C   
    A    
   A   
   C   
   G   
   G   
   T   
   T   
   A   
   G   
   T   
   T   
   C   
   A   
   C   
   C   
   T   
   T   
   C   
   G   
   T   
   A   
   T   
   G   
   A   
   T   
   C   
   C   
   T   
   A   
   A   
   T   
   A   
   A   
   A   
   A   
   T   
   G   
   G   
   C   
   C   
   A   
   A   
   T   
   G   
   G   
    C    
   A   
   T   
   T   
   T   
   C   
   T   
   C   
   A   
   A   
   A   
   A   
   A   
   G   
   A   
    A    
   T   
   A   
   T   
   G   
   A   
   T   
   G   
   C   
   T   
   T   
   A   
   C   
   A   
   A   
   T   
   A   
   A   
   C   
   A   
   A   
   C   
   A   
   A   
   A   
   G   
   A   
   T   
   T   
   T   
   A   
   C   
   C   
   A   
   T   
   T   
    C    
   A   
   C   
   A   
   G   
   C   
   A   
   C   
   G   
   T   
   T   
   T   
    T    
   G   
   C   
   G   
   A   
   C   
   A   
   G   
   G   
    C    
   T   
   A   
   T   
   G   
   C   
   A   
   C   
   C   
   A   
   G   
   G   
    C    
   T   
   C   
   T   
   A   
   C   
   C   
   T   
   T   
   T   
   A   
   A   
   A   
   A   
   C   
   A   
   A   
   T   
   T   
   A   
   C   
   C   
   G   
   G   
   T   
   G   
   C   
   C   
   A   
   T   
   T   
   G   
   G   
   T   
   T   
   T   
   A   
   G   
   A   
   T   
    A    
   C   
   A   
   G   
   G   
   G   
   A   
   C   
   A   
   T   
   T   
   G   
   A   
   A   
   G   
   C   
   C   
   A   
   G   
   A   
   T   
   G   
   A   
   A   
   G   
   A   
   G   
   C   
   T   
   A   
   G   
   A   
   A   
   A   
   T   
   T   
   A   
   A   
   T   
   G   
   G   
    T    
   T   
   T   
   G   
   A   
   A   
   A   
   T   
   G   
   G   
   C   
   A   
   A   
   A   
   A   
   A   
   G   
   A   
   T   
   A   
   A   
   A   
   T   
   C   
   T   
   T   
   G   
   G   
   G   
   G   
    C    
   G   
   G   
   C   
   T   
   A   
   T   
   T   
   T   
   T   
   G   
   C   
    G    
   A   
   C   
   A   
   C   
   G   
   T   
   G   
   T   
   A   
   A   
   A   
   A   
   G   
   A   
   A   
   G   
   C   
   A   
   A   
   G   
   T   
   C   
   C   
   A   
   G   
   T   
   C   
   A   
   A   
   T   
   C   
   T   
    G    
   C   
   G   
   T   
   A   
   C   
   C   
   G   
   C   
   T   
   T   
   T   
   A   
   G   
   T   
   T   
   A   
   A   
   T   
   T   
   C   
   A   
   G   
   A   
   T   
   A   
   A   
   T   
   A   
   T   
   T   
   T   
   A   
   T   
   T   
   T   
   T   
   G   
   C   
    C    
   C   
   A   
   A   
   C   
   A   
   A   
   A   
   C   
   A   
   T   
   T   
   A   
   C   
   G   
    G    
   A   
   T   
   G   
   G   
   G   
    A    
   G   
   A   
   A   
   G   
   A   
   C   
   A   
   A   
   A   
   T   
   T   
   T   
   A   
   G   
   A   
   G   
   C   
    G    
   G   
   G   
   T   
   C   
   T   
   T   
   A   
   A   
   T   
   A   
   A   
   A   
   T   
   T   
   C   
   A   
   T   
   T   
   T   
   T   
   C   
   G   
   G   
   T   
   G   
   A   
   G   
   G   
   A   
   A   
   C   
   T   
   A   
   G   
   A   
   T   
   T   
   T   
   A   
   C   
   C   
   A   
   A   
   T   
    T    
   G   
   C   
   C   
   A   
   T   
   G   
   A   
   C   
   G   
   C   
   C   
    T    
   G   
   C   
   T   
   C   
   A   
   A   
   A   
   T   
   T   
   T   
   C   
   A   
   A   
   A   
    T    
   G   
   A   
   A   
   G   
   A   
   T   
   A   
   A   
   A   
   T   
   T   
   T   
   A   
   A   
   T   
   T   
   C   
   T   
   G   
   A   
   A   
   A   
   T   
   T   
   C   
   T   
   T   
   T   
   T   
    G    
   G   
   C   
   A   
   G   
   A   
   T   
   A   
   C   
    G    
   G   
   G   
   C   
   T   
   A   
    C    
   G   
   G   
    C    
   C   
   A   
   A   
   G   
   G   
    C    
   C   
   A   
   A   
   T   
   T   
   G   
   T   
   T   
   A   
   A   
   T   
   T   
   T   
   C   
    G    
   C   
   C   
   A   
   A   
   T   
   C   
   C   
   A   
   G   
   C   
   A   
   A   
   G   
   C   
   T   
   A   
   C   
   C   
   A   
   T   
   G   
   T   
   A   
   T   
   A   
   G   
   T   
   G   
   T   
   T   
   T   
   T   
   C   
   C   
   A   
   A   
   A   
   A   
   T   
   A   
   A   
   T   
   G   
   G   
    G    
   A   
   C   
   A   
    T    
   T   
    A    
   G   
   T   
    C    
   T   
   A   
   T   
   C   
   C   
   A   
   A   
   A   
   A   
    T    
   T   
   A   
   G   
   T   
   T   
   T   
   T   
   A   
   G   
   A   
   C   
   A   
   A   
   A   
   G   
   A   
   A   
   A   
   C   
    G    
   A   
   A   
   G   
   A   
   A   
   A   
   A   
   A   
   A   
   G   
   A   
   C   
   A   
   A   
   T   
   G   
   T   
   A   
   A   
   T   
   C   
   A   
   G   
   C   
   G   
   C   
   C   
   A   
   A   
   C   
   G   
   C   
   A   
   G   
   C   
   G   
   A   
   A   
   T   
   A   
   C   
   A   
   A   
   T   
   T   
   G   
   C   
   C   
   A   
   C   
   A   
   G   
   A   
   T   
   C   
   T   
   T   
   T   
   T   
   A   
   G   
   G   
   T   
   A   
   G   
   T   
   G   
   T   
   G   
   G   
   A   
   A   
   G   
   A   
   T   
   C   
   C   
   T   
   T   
   C   
   A   
   G   
   G   
   T   
   T   
   A   
   T   
   G   
   T   
   T   
   T   
   A   
   T   
   A   
   A   
   T   
   A   
   T   
   G   
   T   
   A   
   C   
   A   
   A   
   T   
   C   
   C   
   G   
   A   
   A   
   C   
   T   
   T   
   C   
   T   
   C   
   A   
   T   
   T   
   G   
   G   
   C   
   T   
   G   
   C   
   C   
   A   
   A   
   A   
   A   
   C   
   A   
   G   
   G   
   A   
   A   
   C   
   A   
   G   
   C   
   T   
   G   
   A   
   A   
   A   
   T   
   A   
   A   
   A   
   A   
   G   
   A   
   T   
   A   
   A   
   A   
   C   
   A   
   A   
   G   
   A   
   T   
   A   
   C   
   A   
   G   
   A   
   T   
   G   
   G   
   C   
   A   
   A   
   A   
   G   
   A   
   A   
   A   
   A   
   C   
   A   
   G   
   T   
   T   
   T   
   C   
   C   
   T   
   T   
   C   
   T   
   A   
   A   
   C   
   G   
   T   
   T   
   A   
   G   
   A   
   T   
   C   
   G   
   T   
   A   
   G   
   T   
   A   
   A   
   C   
   A   
   A   
   T   
   A   
   A   
   A   
   T   
   T   
   C   
   T   
   T   
   A   
   A   
   C   
   A   
   A   
   T   
   G   
   A   
   T   
   T   
   A   
   T   
   G   
   G   
   T   
   T   
   G   
   A   
   A   
   A   
   A   
   T   
   T   
   C   
   A   
   G   
   G   
   A   
   G   
   A   
   A   
   A   
   A   
   T   
   G   
   G   
   T   
   T   
   C   
   T   
   G   
   C   
   A   
   A   
   C   
   A   
   G   
   A   
   T   
   A   
   T   
   C   
   A   
   G   
   T   
   A   
   A   
   A   
   C   
   C   
   A   
   T   
   T   
   A   
   A   
   T   
   T   
   G   
   A   
   T   
   T   
   A   
   C   
   T   
   T   
   A   
   G   
   A   
   A   
   G   
   C   
   A   
   A   
   C   
   C   
   A   
   T   
   T   
   A   
   A   
   A   
   -   
   -   
   -   
   -   
   -   
   -   
   -   
   -   
   -   
   -   
   -   
   -   
   -   
   -   
   -   
   -   
   -   
   -   
   -   
   -   
   -   
   -   
   -   
   -   
   -   
   -   
   -   
   -   
   -   
   -   
   -   
   -   
   -   
   -   
   -   
   -   
   -   
   -   
   -   
   -   
   -   
   -   
   -   
   -   
   -   
   -   
   -   
   -   
   -   
   -   
   -   
   -   
   -   
   -   
   -   
   -   
   -   
   -   
   -   
   -   
   -   
   -   
   -   
   -   
   -   
   -   
   -   
   -   
   -   
   -   
   -   
   -   
   -   
   -   
   -   
   -   
   -   
   -   
   -   
   -   
   -   
   -   
   -   
   -   
   -   
   -   
   -   
   -   
   -   
   -   
   -   
   -   
   -   
   -   
   -   
   -   
   -   
   -   
   -   
   -   
   -   
   -   
   -   
   -   
   -   
   -   
   -   
   -   
   -   
   -   
   -   
   -   
   -   
   -   
   -   
   -   
   -   
   -   
   -   
   -   
   -   
   -   
   -   
   -   
   -   
   -   
   -   
   -   
   -   
   -   
   -   
   -   
   -   
   -   
   -   
   -   
   -   
   -   
   -   
   -   
   -   
   -   
   -   
   -   
   -   
   -   
   -   
   -   
   -   
   -   
   -   
   -   
   -   
   -   
   -   
   -   
   -   
   -   
   -   
   -   
   -   
   -   
   -   
   -   
   -   
   -   
   -   
   -   
   -   
   C   
   A   
   A   
   T   
   G   
   G   
   C   
   A   
   A   
   G   
   C   
   C   
   A   
   A   
   A   
   C   
   A   
   A   
   G   
   A   
   A   
   T   
   T   
   A   
   G   
   C   
   C   
   G   
   A   
   A   
   G   
   C   
   G   
   A   
   A   
   G   
   A   
   A   
   A   
   A   
   C   
   A   
   G   
   C   
   T   
   A   
   C   
   T   
   A   
   C   
   A   
   T   
   T   
   T   
   T   
   T   
   A   
   A   
   A   
   C   
   G   
   T   
   A   
   T   
   T   
   G   
   A   
   C   
   A   
   A   
   A   
   A   
   C   
   A   
   G   
   A   
   A   
   A   
   T   
   T   
   T   
   G   
   A   
   T   
   A   
   A   
   G   
   T   
   T   
   A   
   C   
   C   
   G   
   T   
   C   
   C   
   G   
   T   
   T   
   G   
   T   
   T   
   C   
   A   
   A   
   G   
   A   
   A   
   G   
   C   
   T   
   A   
   G   
   C   
   T   
   T   
   A   
   A   
   A   
   G   
   A   
   A   
   A   
   A   
   A   
   T   
   G   
   G   
   C   
   T   
   A   
   T   
   G   
   A   
   T   
   A   
   C   
   T   
   A   
   A   
   A   
   T   
   C   
   T   
   G   
   T   
   T   
   G   
   T   
   T   
   G   
   A   
   A   
   A   
   A   
   A   
   T   
   A   
   C   
   C   
   A   
   A   
   G   
   C   
   A   
   A   
   T   
   T   
   T   
   A   
   T   
   T   
   C   
   A   
   G   
   G   
   G   
   A   
   T   
   T   
   C   
   A   
   A   
   G   
   C   
   A   
   G   
   A   
   A   
   G   
   G   
   A   
   G   
   T   
   C   
   A   
   A   
   A   
   G   
   C   
   T   
   A   
   G   
   T   
   G   
   A   
   T   
   G   
   T   
   T   
   C   
   A   
   A   
   G   
   T   
   C   
   A   
   A   
   A   
   A   
   A   
   A   
   G   
   C   
   G   
   A   
   A   
   A   
   G   
   A   
   C   
   A   
   A   
   T   
   C   
   A   
   A   
   T   
   A   
   C   
   A   
   C   
   A   
   T   
   T   
   T   
   A   
   C   
   C   
   T   
   A   
   T   
   A   
   A   
   A   
   T   
   T   
   A   
   T   
   C   
   G   
   A   
   T   
   G   
   A   
   G   
   T   
   A   
   C   
   C   
   C   
   C   
   T   
   T   
   T   
   A   
   G   
   G   
   C   
   G   
   A   
   A   
   A   
   T   
   G   
   A   
   A   
   A   
   G   
   A   
   T   
   T   
   T   
   G   
   T   
   C   
   T   
   T   
   A   
   T   
   C   
   A   
   A   
   T   
   C   
   A   
   A   
   G   
   T   
   A   
   T   
   C   
   G   
   C   
   C   
   A   
   A   
   A   
   A   
   A   
   A   
   G   
   G   
   C   
   G   
   A   
   T   
   A   
   C   
   C   
   T   
   A   
   C   
   C   
   A   
   A   
   A   
   T   
   C   
   G   
   C   
   T   
   T   
   G   
   G   
   A   
   A   
   G   
   C   
   C   
   G   
   T   
   C   
   T   
   T   
   T   
   A   
   A   
   T   
   T   
   T   
   T   
   T   
   C   
   C   
   A   
   G   
   A   
   T   
   A   
   T   
   G   
   T   
   C   
   A   
   G   
   G   
   A   
   A   
   A   
   T   
   G   
   A   
   T   
   A   
   A   
   A   
   A   
   T   
   T   
   T   
   C   
   G   
   A   
   T   
   T   
   C   
   A   
   A   
   G   
   T   
   A   
   G   
   A   
   T   
   A   
   A   
   T   
   G   
   C   
   C   
   A   
   A   
   A   
   C   
   G   
   T   
   G   
   G   
   A   
   G   
   A   
   A   
   A   
   T   
   T   
   G   
   T   
   C   
   G   
   A   
   T   
   C   
   G   
   T   
   A   
   A   
   T   
   G   
   G   
   T   
   A   
   G   
   T   
   G   
   G   
   G   
   C   
   T   
   A   
   G   
   C   
   A   
   A   
   T   
   T   
   A   
   A   
   C   
   A   
   A   
   A   
   G   
   T   
   G   
   T   
   T   
   T   
   G   
   A   
   C   
   G   
   A   
   A   
   G   
   T   
   G   
   G   
   G   
   C   
   G   
   T   
   A   
   G   
   T   
   G   
   C   
   C   
   T   
   G   
   G   
   C   
   A   
   A   
   A   
   C   
   T   
   C   
   G   
   G   
   T   
   T   
   C   
   T   
   G   
   G   
   C   
   G   
   C   
   A   
   G   
   A   
   A   
   A   
   A   
   A   
   A   
   C   
   A   
   G   
   C   
   C   
   A   
   A   
   T   
   A   
   T   
   C   
   A   
   A   
   A   
   G   
   C   
   T   
   T   
   T   
   T   
   A   
   G   
   T   
   G   
   A   
   T   
   A   
   A   
   G   
   T   
   T   
   C   
   G   
   G   
   C   
   G   
   T   
   T   
   T   
   C   
   T   
   G   
   T   
   T   
   G   
   A   
   T   
   G   
   A   
   A   
   A   
   T   
   C   
   A   
   A   
   T   
   C   
   A   
   A   
   A   
   A   
   G   
   T   
   T   
   A   
   A   
   G   
   C   
   C   
   A   
   A   
   G   
   G   
   G   
   T   
   G   
   G   
   G   
   T   
   C   
   C   
   A   
   A   
   G   
   C   
   A   
   G   
   A   
   C   
   T   
   C   
   C   
   T   
   T   
   T   
   G   
   T   
   A   
   C   
   C   
   A   
   A   
   T   
   C   
   A   
   C   
   A   
   G   
   T   
   C   
   G   
   C   
   T   
   T   
   C   
   T   
   G   
   A   
   A   
   C   
   C   
   A   
   G   
   T   
   G   
   A   
   C   
   A   
   G   
   A   
   A   
   T   
   T   
   A   
   C   
   C   
   A   
   A   
   C   
   A   
   G   
   G   
   G   
   G   
   C   
   T   
   G   
   C   
   G   
   A   
   C   
   A   
   A   
   A   
   A   
   G   
   A   
   T   
   A   
   C   
   A   
   G   
   A   
   G   
   T   
   C   
   A   
   C   
   G   
   T   
   T   
   A   
   T   
   T   
   A   
   T   
   C   
   C   
   G   
   C   
   T   
   G   
   G   
   G   
   G   
   G   
   A   
   A   
   G   
   C   
   A   
   G   
   C   
   T   
   G   
   C   
   G   
   C   
   A   
   A   
   T   
   T   
   A   
   A   
   T   
   C   
   G   
   G   
   G   
   T   
   A   
   T   
   A   
   C   
   A   
   G   
   G   
   C   
   A   
   C   
   C   
   A   
   T   
   T   
   A   
   C   
   T   
   G   
   C   
   A   
   G   
   A   
   A   
   G   
   A   
   T   
   A   
   T   
   T   
   A   
   A   
   A   
   A   
   A   
   A   
   A   
   A   
   T   
   C   
   C   
   A   
   G   
   A   
   G   
   T   
   T   
   A   
   A   
   G   
   T   
   A   
   G   
   T   
   A   
   C   
   G   
   G   
   G   
   C   
   G   
   T   
   A   
   A   
   T   
   T   
   G   
   G   
   T   
   A   
   A   
   A   
   A   
   C   
   T   
   G   
   G   
   C   
   T   
   T   
   A   
   G   
   A   
   A   
   C   
   G   
   A   
   G   
   C   
   G   
   T   
   T   
   T   
   G   
   A   
   T   
   A   
   A   
   A   
   G   
   A   
   A   
   T   
   T   
   A   
   A   
   G   
   A   
   G   
   G   
   G   
   C   
   A   
   A   
   G   
   A   
   T   
   G   
   G   
   T   
   G   
   G   
   C   
   T   
   C   
   A   
   C   
   T   
   A   
   G   
   T   
   T   
   A   
   T   
   T   
   T   
   T   
   A   
   G   
   A   
   T   
   G   
   A   
   C   
   A   
   A   
   A   
   G   
   A   
   G   
   A   
   A   
   T   
   G   
   T   
   C   
   A   
   A   
   A   
   A   
   A   
   A   
   G   
   C   
   T   
   T   
   T   
   A   
   C   
   A   
   A   
   A   
   C   
   C   
   A   
   A   
   A   
   G   
   A   
   G   
   A   
   A   
   A   
   A   
   A   
   A   
   G   
   A   
   T   
   G   
   G   
   T   
   C   
   A   
   A   
   A   
   C   
   G   
   A   
   T   
   T   
   A   
   A   
   A   
   T   
   T   
   A   
   A   
   C   
   G   
   A   
   T   
   T   
   G   
   A   
   C   
   A   
   G   
   T   
   G   
   G   
   C   
   G   
   T   
   A   
   C   
   A   
   A   
   C   
   A   
   A   
   C   
   A   
   A   
   G   
   C   
   C   
   T   
   T   
   C   
   G   
   C   
   T   
   A   
   T   
   A   
   T   
   T   
   T   
   G   
   A   
   C   
   A   
   A   
   A   
   C   
   G   
   C   
   C   
   C   
   T   
   G   
   G   
   T   
   A   
   G   
   T   
   G   
   C   
   G   
   G   
   T   
   C   
   A   
   T   
   T   
   A   
   C   
   C   
   G   
   A   
   T   
   C   
   C   
   T   
   C   
   A   
   A   
   A   
   A   
   A   
   G   
   G   
   C   
   G   
   A   
   T   
   T   
   T   
   A   
   T   
   T   
   A   
   G   
   C   
   A   
   A   
   C   
   G   
   G   
   T   
   T   
   A   
   G   
   T   
   T   
   C   
   A   
   C   
   C   
   T   
   T   
   C   
   G   
   T   
   A   
   T   
   G   
   A   
   T   
   C   
   C   
   T   
   A   
   A   
   T   
   A   
   A   
   A   
   A   
   T   
   G   
   G   
   C   
   C   
   A   
   A   
   T   
   G   
   G   
   C   
   A   
   T   
   T   
   T   
   C   
   T   
   C   
   A   
   A   
   A   
   A   
   A   
   G   
   A   
   A   
   T   
   A   
   T   
   G   
   A   
   T   
   G   
   C   
   T   
   T   
   A   
   C   
   A   
   A   
   T   
   A   
   A   
   C   
   A   
   A   
   C   
   A   
   A   
   A   
   G   
   A   
   T   
   T   
   T   
   A   
   C   
   C   
   A   
   T   
   T   
   C   
   A   
   C   
   A   
   G   
   C   
   A   
   C   
   G   
   T   
   T   
   T   
   T   
   G   
   C   
   G   
   A   
   C   
   A   
   G   
   G   
   C   
   T   
   A   
   T   
   G   
   C   
   A   
   C   
   C   
   A   
   G   
   G   
   C   
   T   
   C   
   T   
   A   
   C   
   C   
   T   
   T   
   T   
   A   
   A   
   A   
   A   
   C   
   A   
   A   
   T   
   T   
   A   
   C   
   C   
   G   
   G   
   T   
   G   
   C   
   C   
   A   
   T   
   T   
   G   
   G   
   T   
   T   
   T   
   A   
   G   
   A   
   T   
   A   
   C   
   A   
   G   
   G   
   G   
   A   
   C   
   A   
   T   
   T   
   G   
   A   
   A   
   G   
   C   
   C   
   A   
   G   
   A   
   T   
   G   
   A   
   A   
   G   
   A   
   G   
   C   
   T   
   A   
   G   
   A   
   A   
   A   
   T   
   T   
   A   
   A   
   T   
   G   
   G   
   T   
   T   
   T   
   G   
   A   
   A   
   A   
   T   
   G   
   G   
   C   
   A   
   A   
   A   
   A   
   A   
   G   
   A   
   T   
   A   
   A   
   A   
   T   
   C   
   T   
   T   
   G   
   G   
   G   
   G   
   C   
   G   
   G   
   C   
   T   
   A   
   T   
   T   
   T   
   T   
   G   
   C   
   G   
   A   
   C   
   A   
   C   
   G   
   T   
   G   
   T   
   A   
   A   
   A   
   A   
   G   
   A   
   A   
   G   
   C   
   A   
   A   
   G   
   T   
   C   
   C   
   A   
   G   
   T   
   C   
   A   
   A   
   T   
   C   
   T   
   G   
   C   
   G   
   T   
   A   
   C   
   C   
   G   
   C   
   T   
   T   
   T   
   A   
   G   
   T   
   T   
   A   
   A   
   T   
   T   
   C   
   A   
   G   
   A   
   T   
   A   
   A   
   T   
   A   
   T   
   T   
   T   
   A   
   T   
   T   
   T   
   T   
   G   
   C   
   C   
   C   
   A   
   A   
   C   
   A   
   A   
   A   
   C   
   A   
   T   
   T   
   A   
   C   
   G   
   G   
   A   
   T   
   G   
   G   
   G   
   A   
   G   
   A   
   A   
   G   
   A   
   C   
   A   
   A   
   A   
   T   
   T   
   T   
   A   
   G   
   A   
   G   
   C   
   G   
   G   
   G   
   T   
   C   
   T   
   T   
   A   
   A   
   T   
   A   
   A   
   A   
   T   
   T   
   C   
   A   
   T   
   T   
   T   
   T   
   C   
   G   
   G   
   T   
   G   
   A   
   G   
   G   
   A   
   A   
   C   
   T   
   A   
   G   
   A   
   T   
   T   
   T   
   A   
   C   
   C   
   A   
   A   
   T   
   T   
   G   
   C   
   C   
   A   
   T   
   G   
   A   
   C   
   G   
   C   
   C   
   T   
   G   
   C   
   T   
   C   
   A   
   A   
   A   
   T   
   T   
   T   
   C   
   A   
   A   
   A   
   T   
   G   
   A   
   A   
   G   
   A   
   T   
   A   
   A   
   A   
   T   
   T   
   T   
   A   
   A   
   T   
   T   
   C   
   T   
   G   
   A   
   A   
   A   
   T   
   T   
   C   
   T   
   T   
   T   
   T   
   G   
   G   
   C   
   A   
   G   
   A   
   T   
   A   
   C   
   G   
   G   
   G   
   C   
   T   
   A   
   C   
   G   
   G   
   C   
   C   
   A   
   A   
   G   
   G   
   C   
   C   
   A   
   A   
   T   
   T   
   G   
   T   
   T   
   A   
   A   
   T   
   T   
   T   
   C   
   G   
   C   
   C   
   A   
   A   
   T   
   C   
   C   
   A   
   G   
   C   
   A   
   A   
   G   
   C   
   T   
   A   
   C   
   C   
   A   
   T   
   G   
   T   
   A   
   T   
   A   
   G   
   T   
   G   
   T   
   T   
   T   
   T   
   C   
   C   
   A   
   A   
   A   
   A   
   T   
   A   
   A   
   T   
   G   
   G   
   G   
   A   
   C   
   A   
   T   
   T   
   A   
   G   
   T   
   C   
   T   
   A   
   T   
   C   
   C   
   A   
   A   
   A   
   A   
   T   
   T   
   A   
   G   
   T   
   T   
   T   
   T   
   A   
   G   
   A   
   C   
   A   
   A   
   A   
   G   
   A   
   A   
   A   
   C   
   G   
   A   
   A   
   G   
   A   
   A   
   A   
   A   
   A   
   A   
   G   
   A   
   C   
   A   
   A   
   T   
   G   
   T   
   A   
   A   
   T   
   C   
   A   
   G   
   C   
   G   
   C   
   C   
   A   
   A   
   C   
   G   
   C   
   A   
   G   
   C   
   G   
   A   
   A   
   T   
   A   
   C   
   A   
   A   
   T   
   T   
   G   
   C   
   C   
   A   
   C   
   A   
   G   
   A   
   T   
   C   
   T   
   T   
   T   
   T   
   A   
   G   
   G   
   T   
   A   
   G   
   T   
   G   
   T   
   G   
   G   
   A   
   A   
   G   
   A   
   T   
   C   
   C   
   T   
   T   
   C   
   A   
   G   
   G   
   T   
   T   
   A   
   T   
   G   
   T   
   T   
   T   
   A   
   T   
   A   
   A   
   T   
   A   
   T   
   G   
   T   
   A   
   C   
   A   
   A   
   T   
   C   
   C   
   G   
   A   
   A   
   C   
   T   
   T   
   C   
   T   
   C   
   A   
   T   
   T   
   G   
   G   
   C   
   T   
   G   
   C   
   C   
   A   
   A   
   A   
   A   
   C   
   A   
   G   
   G   
   A   
   A   
   C   
   A   
   G   
   C   
   T   
   G   
   A   
   A   
   A   
   T   
   A   
   A   
   A   
   A   
   G   
   A   
   T   
   A   
   A   
   A   
   C   
   A   
   A   
   G   
   A   
   T   
   A   
   C   
   A   
   G   
   A   
   T   
   G   
   G   
   C   
   A   
   A   
   A   
   G   
   A   
   A   
   A   
   A   
   C   
   A   
   G   
   T   
   T   
   T   
   C   
   C   
   T   
   T   
   C   
   T   
   A   
   A   
   C   
   G   
   T   
   T   
   A   
   G   
   A   
   T   
   C   
   G   
   T   
   A   
   G   
   T   
   A   
   A   
   C   
   A   
   A   
   T   
   A   
   A   
   A   
   T   
   T   
   C   
   T   
   T   
   A   
   A   
   C   
   A   
   A   
   T   
   G   
   A   
   T   
   T   
   A   
   T   
   G   
   G   
   T   
   T   
   G   
   A   
   A   
   A   
   A   
   T   
   T   
   C   
   A   
   G   
   G   
   A   
   G   
   A   
   A   
   A   
   A   
   T   
   G   
   G   
   T   
   T   
   C   
   T   
   G   
   C   
   A   
   A   
   C   
   A   
   G   
   A   
   T   
   A   
   T   
   C   
   A   
   G   
   T   
   A   
   A   
   A   
   C   
   C   
   A   
   T   
   T   
   A   
   A   
   T   
   T   
   G   
   A   
   T   
   T   
   A   
   C   
   T   
   T   
   A   
   G   
   A   
   A   
   G   
   C   
   A   
   A   
   C   
   C   
   A   
   T   
   T   
   A   
   A   
   A   
   -   
   -   
   -   
   -   
   -   
   -   
   -   
   -   
   -   
   -   
   -   
   -   
   -   
   -   
   -   
   -   
   -   
   -   
   -   
   -   
   -   
   -   
   -   
   -   
   -   
   -   
   -   
   -   
   -   
   -   
   -   
   -   
   -   
   -   
   -   
   -   
   -   
   -   
   -   
   -   
   -   
   -   
   -   
   -   
   -   
   -   
   -   
   -   
   -   
   -   
   -   
   -   
   -   
   -   
   -   
   -   
   -   
   -   
   -   
   -   
   -   
 

 
   N55753   
   -   
   -   
   -   
   -   
   -   
   -   
   -   
   -   
   -   
   -   
   -   
   -   
   -   
   -   
   -   
   -   
   -   
   -   
   -   
   -   
   -   
   -   
   -   
   -   
   -   
   -   
   -   
   -   
   -   
   -   
   -   
   -   
   -   
   -   
   -   
   -   
   -   
   -   
   -   
   -   
   -   
   -   
   -   
   -   
   -   
   -   
   -   
   -   
   -   
   -   
   -   
   -   
   -   
   -   
   -   
   -   
   -   
   -   
   -   
   -   
   -   
   -   
   -   
   -   
   -   
   -   
   -   
   -   
   -   
   -   
   -   
   -   
   -   
   -   
   -   
   -   
   -   
   -   
   -   
   -   
   -   
   -   
   -   
   -   
   -   
   -   
   -   
   -   
   -   
   -   
   -   
   -   
   -   
   -   
   -   
   -   
   -   
   -   
   -   
   -   
   -   
   -   
   -   
   -   
   -   
   -   
   -   
   -   
   C   
   A   
   A   
   T   
   G   
   G   
   C   
   A   
   A   
   G   
   C   
   C   
   A   
   A   
   A   
   C   
   A   
   A   
   G   
   A   
    A    
   T   
   T   
   A   
   G   
   C   
    C    
   G   
   A   
   A   
   G   
   C   
   G   
   A   
   A   
   G   
   A   
   A   
   A   
   A   
    C    
   A   
   G   
   C   
    T    
   A   
    C    
   T   
    A    
    C    
   A   
   T   
   T   
   T   
   T   
   T   
   A   
   A   
   A   
    C    
   G   
   T   
   A   
   T   
   T   
   G   
    T    
   C   
   A   
   A   
   A   
   A   
   C   
   A   
   G   
    G    
   A   
   A   
   T   
   T   
   T   
   G   
   A   
   T   
   A   
   A   
   G   
   T   
   T   
   A   
   C   
   C   
    G    
   T   
   C   
   C   
   G   
   T   
   T   
    G    
   T   
   T   
   C   
   A   
   A   
   G   
   A   
   A   
    G    
   C   
   T   
   A   
   G   
   C   
   T   
   T   
   A   
   A   
   A   
   G   
   A   
   A   
   A   
   A   
   A   
   T   
   G   
   G   
    C    
   T   
   A   
   T   
   G   
   A   
   T   
   A   
   C   
   T   
   A   
   A   
   A   
   T   
   C   
    T    
   G   
   T   
   T   
   G   
   T   
   T   
   G   
   A   
   A   
   A   
   A   
   A   
   T   
   A   
   C   
   C   
   A   
   A   
   G   
   C   
   A   
   A   
   T   
   T   
   T   
   A   
   T   
   T   
   C   
   A   
   G   
   G   
   G   
   A   
   T   
   T   
   C   
   A   
   A   
   G   
   C   
    T    
   G   
   A   
   A   
    G    
   G   
    A    
   G   
   T   
   C   
   A   
   A   
    A    
   G   
   C   
   T   
   A   
   G   
   T   
   G   
   A   
   T   
   G   
   T   
    T    
   C   
   A   
   A   
   G   
   T   
    C    
   A   
   A   
   A   
   A   
   A   
    A    
   G   
    C    
   G   
   A   
   A   
   A   
   G   
   A   
    C    
   A   
   A   
    T    
   C   
   A   
   A   
   T   
   A   
   C   
   A   
   C   
   A   
   T   
   T   
   T   
   A   
    C    
   C   
   T   
   A   
   T   
   A   
   A   
   A   
   T   
   T   
   A   
   T   
   C   
   G   
   A   
   T   
   G   
   A   
   G   
    C    
   A   
   C   
    G    
   C   
   C   
   T   
   T   
   T   
   A   
   G   
   G   
   C   
   G   
   A   
   A   
   A   
   T   
   G   
   A   
   A   
   A   
   G   
   A   
   T   
    T    
   T   
   G   
   T   
   C   
   T   
   T   
   A   
   T   
   C   
   A   
   A   
   T   
   C   
   A   
   A   
   G   
   T   
   A   
   T   
    C    
   G   
    A    
   C   
   A   
   A   
    A    
   A   
   A   
   A   
   G   
   G   
    C    
   G   
   A   
   T   
   A   
    C    
   C   
   T   
   A   
   C   
   C   
   A   
   A   
   A   
   T   
    C    
    G    
   C   
   T   
   T   
   G   
   G   
    A    
    A    
    G    
   C   
   C   
    A    
   T   
   C   
   T   
   T   
   T   
   A   
   A   
   T   
   T   
   T   
   T   
   T   
   C   
   C   
   A   
   G   
   A   
   T   
   A   
   T   
   G   
   T   
   C   
   A   
   G   
   G   
   A   
   A   
   A   
   T   
   G   
   A   
   T   
   A   
   A   
   A   
    A    
   T   
   T   
   T   
   C   
   G   
   A   
   T   
   T   
   C   
   A   
   A   
   G   
   T   
   A   
   G   
   A   
   T   
   A   
   A   
   T   
   G   
   C   
   C   
   A   
   A   
   A   
   C   
   G   
   T   
   G   
   G   
   A   
   G   
   A   
   A   
   A   
   T   
   T   
   G   
   T   
   C   
   G   
   A   
   T   
   C   
   G   
   T   
   A   
   A   
    T    
   G   
   G   
   T   
   A   
   G   
   T   
   G   
   G   
   G   
   C   
   T   
   A   
   G   
    C    
   A   
   A   
   T   
   T   
   A   
   A   
   C   
   A   
   A   
   A   
   G   
   T   
   G   
   T   
   T   
   T   
   G   
   A   
    C    
   G   
   A   
   A   
   G   
   T   
   G   
   G   
   G   
   C   
   G   
   T   
   A   
   G   
   T   
    G    
   C   
   C   
   T   
    A    
   G   
   C   
   A   
   A   
   A   
   C   
   T   
    C    
   G   
   G   
   T   
   T   
   C   
   T   
   G   
   G   
   C   
   G   
   C   
   A   
   G   
   A   
   A   
   A   
   A   
   A   
    G    
   C   
   A   
   G   
   C   
    C    
   A   
   A   
   T   
   A   
   T   
   C   
   A   
   A   
   A   
   G   
   C   
   T   
   T   
   T   
   T   
   A   
   G   
   T   
   G   
   A   
   T   
   A   
   A   
    G    
   T   
   T   
    C    
   G   
   G   
   C   
   G   
   T   
   T   
   T   
   C   
   T   
    G    
   T   
   T   
   G   
   A   
   T   
   G   
   A   
   A   
   A   
   T   
   C   
   A   
   A   
   T   
   C   
    A    
   A   
   A   
   A   
    G    
   T   
   T   
   A   
   A   
   G   
   C   
   C   
   A   
   A   
   G   
   G   
    A    
   T   
   G   
   G   
   G   
   T   
   C   
   C   
   A   
   A   
   G   
   C   
   A   
   G   
   A   
   C   
   T   
   C   
    C    
   T   
   T   
   T   
   G   
   T   
   A   
   C   
   C   
   A   
   A   
   T   
   C   
   A   
   C   
   A   
   G   
   T   
    T    
   G   
   C   
   T   
   T   
   C   
   T   
   G   
   A   
   A   
   C   
   C   
   A   
   G   
   T   
   G   
   A   
   C   
   A   
   G   
   A   
   A   
   T   
   T   
   A   
   C   
   C   
   A   
   A   
   C   
   A   
   G   
   G   
   G   
   G   
   C   
   T   
   G   
   C   
   G   
   A   
   C   
   A   
   A   
   A   
   A   
   G   
    A    
    C    
   A   
   C   
   A   
   G   
   A   
   G   
   T   
   C   
   A   
   C   
   G   
   T   
   T   
   A   
   T   
   T   
   A   
   T   
   C   
   C   
    G    
    T    
   T   
   G   
   G   
   G   
   G   
   G   
   A   
   A   
   G   
   C   
   A   
   G   
   C   
   T   
   G   
   C   
   G   
   C   
   A   
   A   
   T   
   T   
   A   
   A   
   T   
   C   
   G   
   G   
    G    
   T   
   A   
   T   
   A   
   C   
    G    
   G   
   G   
    C    
   A   
   C   
    C    
   A   
   T   
   T   
   A   
   C   
   T   
   G   
   C   
   A   
   G   
   A   
    A    
   G   
   A   
   T   
   A   
   T   
   T   
    A    
   A   
   A   
   A   
   A   
   A   
   A   
   A   
   T   
   C   
   C   
   A   
   G   
   A   
   G   
   T   
   T   
   A   
   A   
   G   
   T   
   A   
   G   
   T   
   A   
   C   
   G   
   G   
   G   
    C    
   G   
   T   
   A   
   A   
   T   
   T   
   G   
   G   
    T    
   A   
   A   
   A   
   A   
   C   
   T   
   G   
   G   
    C    
   T   
   T   
   A   
   G   
   A   
   A   
   C   
   G   
   A   
   G   
   C   
   G   
   T   
   T   
   T   
   G   
   A   
   T   
   A   
   A   
   A   
   G   
   A   
    A    
   T   
   T   
   A   
   A   
   G   
   A   
   G   
   G   
    G    
   C   
   A   
   A   
   G   
   A   
   T   
   G   
   G   
    T    
   G   
   G   
    C    
   T   
   C   
    A    
   C   
   T   
   A   
   G   
   T   
   T   
   A   
   T   
   T   
   T   
   T   
   A   
   G   
   A   
   T   
   G   
   A   
   C   
   A   
   A   
   A   
   G   
   A   
   G   
   A   
   A   
   T   
   G   
   T   
   C   
   A   
   A   
   A   
   A   
   A   
   A   
   G   
   C   
   T   
   T   
   T   
   A   
   C   
   A   
   A   
   A   
   C   
   C   
   A   
   A   
   A   
   G   
   A   
    G    
   A   
   A   
   A   
   A   
   A   
   A   
   G   
   A   
   T   
   G   
   G   
   T   
   C   
   A   
   A   
   A   
   C   
    G    
   A   
   T   
   T   
   A   
   A   
   A   
   T   
   T   
   A   
   A   
   C   
   G   
   A   
   T   
   T   
   G   
   A   
    C    
   A   
   G   
    C    
   G   
   G   
    G    
   G   
   T   
   A   
   C   
   A   
   A   
   C   
   A   
   A   
   C   
   A   
   A   
   G   
   C   
    T    
   T   
   T   
    T    
   G   
   C   
   T   
   A   
   T   
   A   
   T   
   T   
   T   
   G   
   A   
   C   
   A   
   A   
   A   
   C   
   G   
    T    
    C    
   C   
   T   
   G   
   G   
   T   
   A   
   G   
   T   
   G   
   C   
   G   
   G   
   T   
   C   
   A   
   T   
   T   
   A   
   C   
   C   
   G   
   A   
   T   
   C   
   C   
   T   
   C   
   A   
   A   
   A   
   A   
   A   
   G   
   G   
   C   
   G   
   A   
   T   
   T   
   T   
   A   
    T    
   T   
   A   
   G   
   C   
    A    
   A   
   C   
   G   
   G   
   T   
   T   
   A   
   G   
   T   
   T   
   C   
   A   
   C   
   C   
   T   
   T   
   C   
   G   
   T   
   A   
   T   
   G   
   A   
   T   
   C   
   C   
   T   
   A   
   A   
   T   
   A   
   A   
   A   
   A   
   T   
   G   
   G   
   C   
   C   
   A   
   A   
   T   
   G   
   G   
    C    
   A   
   T   
   T   
   T   
   C   
   T   
   C   
   A   
   A   
   A   
   A   
   A   
   G   
   A   
    A    
   T   
   A   
   T   
   G   
   A   
   T   
   G   
   C   
   T   
   T   
   A   
   C   
   A   
   A   
   T   
   A   
   A   
   C   
   A   
   A   
   C   
   A   
   A   
   A   
   G   
   A   
   T   
   T   
   T   
   A   
   C   
   C   
   A   
   T   
   T   
    C    
   A   
   C   
   A   
   G   
   C   
   A   
   C   
   G   
   T   
   T   
   T   
    C    
   G   
   C   
   G   
   A   
   C   
   A   
   G   
   G   
    C    
   T   
   A   
   T   
   G   
   C   
   A   
   C   
   C   
   A   
   G   
   G   
    C    
   T   
   C   
   T   
   A   
   C   
   C   
   T   
   T   
   T   
   A   
   A   
   A   
   A   
   C   
   A   
   A   
   T   
   T   
   A   
   C   
   C   
   G   
   G   
   T   
   G   
   C   
   C   
   A   
   T   
   T   
   G   
   G   
   T   
   T   
   T   
   A   
   G   
   A   
   T   
    G    
   C   
   A   
   G   
   G   
   G   
   A   
   C   
   A   
   T   
   T   
   G   
   A   
   A   
   G   
   C   
   C   
   A   
   G   
   A   
   T   
   G   
   A   
   A   
   G   
   A   
   G   
   C   
   T   
   A   
   G   
   A   
   A   
   A   
   T   
   T   
   A   
   A   
   T   
   G   
   G   
    C    
   T   
   T   
   G   
   A   
   A   
   A   
   T   
   G   
   G   
   C   
   A   
   A   
   A   
   A   
   A   
   G   
   A   
   T   
   A   
   A   
   A   
   T   
   C   
   T   
   T   
   G   
   G   
   G   
   G   
    C    
   G   
   G   
   C   
   T   
   A   
   T   
   T   
   T   
   T   
   G   
   C   
    G    
   A   
   C   
   A   
   C   
   G   
   T   
   G   
   T   
   A   
   A   
   A   
   A   
   G   
   A   
   A   
   G   
   C   
   A   
   A   
   G   
   T   
   C   
   C   
   A   
   G   
   T   
   C   
   A   
   A   
   T   
   C   
   T   
    G    
   C   
   G   
   T   
   A   
   C   
   C   
   G   
   C   
   T   
   T   
   T   
   A   
   G   
   T   
   T   
   A   
   A   
   T   
   T   
   C   
   A   
   G   
   A   
   T   
   A   
   A   
   T   
   A   
   T   
   T   
   T   
   A   
   T   
   T   
   T   
   T   
   G   
   C   
    C    
   C   
   A   
   A   
   C   
   A   
   A   
   A   
   C   
   A   
   T   
   T   
   A   
   C   
   G   
    G    
   A   
   T   
   G   
   G   
   G   
    G    
   G   
   A   
   A   
   G   
   A   
   C   
   A   
   A   
   A   
   T   
   T   
   T   
   A   
   G   
   A   
   G   
   C   
    G    
   G   
   G   
   T   
   C   
   T   
   T   
   A   
   A   
   T   
   A   
   A   
   A   
   T   
   T   
   C   
   A   
   T   
   T   
   T   
   T   
   C   
   G   
   G   
   T   
   G   
   A   
   G   
   G   
   A   
   A   
   C   
   T   
   A   
   G   
   A   
   T   
   T   
   T   
   A   
   C   
   C   
   A   
   A   
   T   
    T    
   G   
   C   
   C   
   A   
   T   
   G   
   A   
   C   
   G   
   C   
   C   
    T    
   G   
   C   
   T   
   C   
   A   
   A   
   A   
   T   
   T   
   T   
   C   
   A   
   A   
   A   
    T    
   G   
   A   
   A   
   G   
   A   
   T   
   A   
   A   
   A   
   T   
   T   
   T   
   A   
   A   
   T   
   T   
   C   
   T   
   G   
   A   
   A   
   A   
   T   
   T   
   C   
   T   
   T   
   T   
   T   
    A    
   G   
   C   
   A   
   G   
   A   
   T   
   A   
   C   
    G    
   G   
   G   
   C   
   T   
   A   
    T    
   G   
   G   
    T    
   C   
   A   
   A   
   G   
   G   
    T    
   C   
   A   
   A   
   T   
   T   
   G   
   T   
   T   
   A   
   A   
   T   
   T   
   T   
   C   
    G    
   C   
   C   
   A   
   A   
   T   
   C   
   C   
   A   
   G   
   C   
   A   
   A   
   G   
   C   
   T   
   A   
   C   
   C   
   A   
   T   
   G   
   T   
   A   
   T   
   A   
   G   
   T   
   G   
   T   
   T   
   T   
   T   
   C   
   C   
   A   
   A   
   A   
   A   
   T   
   A   
   A   
   T   
   G   
   G   
    A    
   A   
   C   
   A   
    T    
   T   
    A    
   G   
   T   
    C    
   T   
   A   
   T   
   C   
   C   
   A   
   A   
   A   
   A   
    C    
   T   
   A   
   G   
   T   
   T   
   T   
   T   
   A   
   G   
   A   
   C   
   A   
   A   
   A   
   G   
   A   
   A   
   A   
   C   
    G    
   A   
   A   
   G   
   A   
   A   
   A   
   A   
   A   
   A   
   G   
   A   
   C   
   A   
   A   
   T   
   G   
   T   
   A   
   A   
   T   
   C   
   A   
   G   
   C   
   G   
   C   
   C   
   A   
   A   
   T   
   G   
   C   
   A   
   G   
   C   
   G   
   A   
   A   
   T   
   A   
   C   
   A   
   A   
   T   
   T   
   G   
   C   
   C   
   A   
   C   
   A   
   G   
   A   
   T   
   C   
   T   
   T   
   T   
   T   
   A   
   G   
   G   
   T   
   A   
   G   
   T   
   G   
   T   
   G   
   G   
   A   
   A   
   G   
   A   
   T   
   C   
   C   
   T   
   T   
   C   
   A   
   G   
   G   
   T   
   T   
   A   
   T   
   G   
   T   
   T   
   T   
   A   
   T   
   A   
   A   
   T   
   A   
   T   
   G   
   T   
   A   
   C   
   A   
   A   
   T   
   C   
   C   
   G   
   A   
   A   
   C   
   T   
   T   
   C   
   T   
   C   
   A   
   T   
   T   
   G   
   G   
   C   
   T   
   G   
   C   
   T   
   A   
   A   
   A   
   A   
   C   
   A   
   G   
   G   
   A   
   A   
   C   
   A   
   G   
   C   
   T   
   G   
   A   
   A   
   A   
   T   
   A   
   A   
   A   
   A   
   G   
   A   
   T   
   A   
   A   
   A   
   C   
   A   
   A   
   G   
   A   
   T   
   A   
   C   
   A   
   G   
   A   
   T   
   G   
   G   
   C   
   A   
   A   
   A   
   G   
   A   
   A   
   A   
   A   
   C   
   A   
   G   
   T   
   T   
   T   
   C   
   C   
   T   
   T   
   C   
   T   
   A   
   A   
   C   
   G   
   T   
   T   
   A   
   G   
   A   
   T   
   C   
   G   
   T   
   A   
   G   
   T   
   A   
   A   
   C   
   A   
   A   
   T   
   A   
   A   
   A   
   T   
   T   
   C   
   T   
   T   
   A   
   A   
   C   
   A   
   A   
   T   
   G   
   A   
   T   
   T   
   A   
   T   
   G   
   G   
   T   
   T   
   G   
   A   
   A   
   A   
   A   
   T   
   T   
   C   
   A   
   G   
   G   
   A   
   G   
   A   
   A   
   A   
   A   
   T   
   G   
   G   
   T   
   T   
   C   
   T   
   G   
   C   
   A   
   A   
   C   
   A   
   G   
   A   
   T   
   A   
   T   
   C   
   A   
   G   
   T   
   A   
   A   
   A   
   C   
   C   
   A   
   T   
   T   
   A   
   A   
   T   
   T   
   G   
   A   
   T   
   T   
   A   
   C   
   T   
   T   
   A   
   G   
   A   
   A   
   G   
   C   
   A   
   A   
   C   
   C   
   A   
   T   
   T   
   A   
   A   
   A   
   -   
   -   
   -   
   -   
   -   
   -   
   -   
   -   
   -   
   -   
   -   
   -   
   -   
   -   
   -   
   -   
   -   
   -   
   -   
   -   
   -   
   -   
   -   
   -   
   -   
   -   
   -   
   -   
   -   
   -   
   -   
   -   
   -   
   -   
   -   
   -   
   -   
   -   
   -   
   -   
   -   
   -   
   -   
   -   
   -   
   -   
   -   
   -   
   -   
   -   
   -   
   -   
   -   
   -   
   -   
   -   
   -   
   -   
   -   
   -   
   -   
   -   
   -   
   -   
   -   
   -   
   -   
   -   
   -   
   -   
   -   
   -   
   -   
   -   
   -   
   -   
   -   
   -   
   -   
   -   
   -   
   -   
   -   
   -   
   -   
   -   
   -   
   -   
   -   
   -   
   -   
   -   
   -   
   -   
   -   
   -   
   -   
   -   
   -   
   -   
   -   
   -   
   -   
   -   
   -   
   -   
   -   
   -   
   -   
   -   
   -   
   -   
   -   
   -   
   -   
   -   
   -   
   -   
   -   
   -   
   -   
   -   
   -   
   -   
   -   
   -   
   -   
   -   
   -   
   -   
   -   
   -   
   -   
   -   
   -   
   -   
   -   
   -   
   -   
   -   
   -   
   -   
   -   
   -   
   -   
   -   
   -   
   -   
   -   
   -   
   -   
   -   
   -   
   -   
   -   
   -   
   -   
   -   
   -   
   -   
   -   
   -   
   -   
   -   
   -   
   -   
   -   
   -   
   -   
   C   
   A   
   A   
   T   
   G   
   G   
   C   
   A   
   A   
   G   
   C   
   C   
   A   
   A   
   A   
   C   
   A   
   A   
   G   
   A   
   A   
   T   
   T   
   A   
   G   
   C   
   C   
   G   
   A   
   A   
   G   
   C   
   G   
   A   
   A   
   G   
   A   
   A   
   A   
   A   
   C   
   A   
   G   
   C   
   T   
   A   
   C   
   T   
   A   
   C   
   A   
   T   
   T   
   T   
   T   
   T   
   A   
   A   
   A   
   C   
   G   
   T   
   A   
   T   
   T   
   G   
   T   
   C   
   A   
   A   
   A   
   A   
   C   
   A   
   G   
   G   
   A   
   A   
   T   
   T   
   T   
   G   
   A   
   T   
   A   
   A   
   G   
   T   
   T   
   A   
   C   
   C   
   G   
   T   
   C   
   C   
   G   
   T   
   T   
   G   
   T   
   T   
   C   
   A   
   A   
   G   
   A   
   A   
   G   
   C   
   T   
   A   
   G   
   C   
   T   
   T   
   A   
   A   
   A   
   G   
   A   
   A   
   A   
   A   
   A   
   T   
   G   
   G   
   C   
   T   
   A   
   T   
   G   
   A   
   T   
   A   
   C   
   T   
   A   
   A   
   A   
   T   
   C   
   T   
   G   
   T   
   T   
   G   
   T   
   T   
   G   
   A   
   A   
   A   
   A   
   A   
   T   
   A   
   C   
   C   
   A   
   A   
   G   
   C   
   A   
   A   
   T   
   T   
   T   
   A   
   T   
   T   
   C   
   A   
   G   
   G   
   G   
   A   
   T   
   T   
   C   
   A   
   A   
   G   
   C   
   T   
   G   
   A   
   A   
   G   
   G   
   A   
   G   
   T   
   C   
   A   
   A   
   A   
   G   
   C   
   T   
   A   
   G   
   T   
   G   
   A   
   T   
   G   
   T   
   T   
   C   
   A   
   A   
   G   
   T   
   C   
   A   
   A   
   A   
   A   
   A   
   A   
   G   
   C   
   G   
   A   
   A   
   A   
   G   
   A   
   C   
   A   
   A   
   T   
   C   
   A   
   A   
   T   
   A   
   C   
   A   
   C   
   A   
   T   
   T   
   T   
   A   
   C   
   C   
   T   
   A   
   T   
   A   
   A   
   A   
   T   
   T   
   A   
   T   
   C   
   G   
   A   
   T   
   G   
   A   
   G   
   C   
   A   
   C   
   G   
   C   
   C   
   T   
   T   
   T   
   A   
   G   
   G   
   C   
   G   
   A   
   A   
   A   
   T   
   G   
   A   
   A   
   A   
   G   
   A   
   T   
   T   
   T   
   G   
   T   
   C   
   T   
   T   
   A   
   T   
   C   
   A   
   A   
   T   
   C   
   A   
   A   
   G   
   T   
   A   
   T   
   C   
   G   
   A   
   C   
   A   
   A   
   A   
   A   
   A   
   A   
   G   
   G   
   C   
   G   
   A   
   T   
   A   
   C   
   C   
   T   
   A   
   C   
   C   
   A   
   A   
   A   
   T   
   C   
   G   
   C   
   T   
   T   
   G   
   G   
   A   
   A   
   G   
   C   
   C   
   A   
   T   
   C   
   T   
   T   
   T   
   A   
   A   
   T   
   T   
   T   
   T   
   T   
   C   
   C   
   A   
   G   
   A   
   T   
   A   
   T   
   G   
   T   
   C   
   A   
   G   
   G   
   A   
   A   
   A   
   T   
   G   
   A   
   T   
   A   
   A   
   A   
   A   
   T   
   T   
   T   
   C   
   G   
   A   
   T   
   T   
   C   
   A   
   A   
   G   
   T   
   A   
   G   
   A   
   T   
   A   
   A   
   T   
   G   
   C   
   C   
   A   
   A   
   A   
   C   
   G   
   T   
   G   
   G   
   A   
   G   
   A   
   A   
   A   
   T   
   T   
   G   
   T   
   C   
   G   
   A   
   T   
   C   
   G   
   T   
   A   
   A   
   T   
   G   
   G   
   T   
   A   
   G   
   T   
   G   
   G   
   G   
   C   
   T   
   A   
   G   
   C   
   A   
   A   
   T   
   T   
   A   
   A   
   C   
   A   
   A   
   A   
   G   
   T   
   G   
   T   
   T   
   T   
   G   
   A   
   C   
   G   
   A   
   A   
   G   
   T   
   G   
   G   
   G   
   C   
   G   
   T   
   A   
   G   
   T   
   G   
   C   
   C   
   T   
   A   
   G   
   C   
   A   
   A   
   A   
   C   
   T   
   C   
   G   
   G   
   T   
   T   
   C   
   T   
   G   
   G   
   C   
   G   
   C   
   A   
   G   
   A   
   A   
   A   
   A   
   A   
   G   
   C   
   A   
   G   
   C   
   C   
   A   
   A   
   T   
   A   
   T   
   C   
   A   
   A   
   A   
   G   
   C   
   T   
   T   
   T   
   T   
   A   
   G   
   T   
   G   
   A   
   T   
   A   
   A   
   G   
   T   
   T   
   C   
   G   
   G   
   C   
   G   
   T   
   T   
   T   
   C   
   T   
   G   
   T   
   T   
   G   
   A   
   T   
   G   
   A   
   A   
   A   
   T   
   C   
   A   
   A   
   T   
   C   
   A   
   A   
   A   
   A   
   G   
   T   
   T   
   A   
   A   
   G   
   C   
   C   
   A   
   A   
   G   
   G   
   A   
   T   
   G   
   G   
   G   
   T   
   C   
   C   
   A   
   A   
   G   
   C   
   A   
   G   
   A   
   C   
   T   
   C   
   C   
   T   
   T   
   T   
   G   
   T   
   A   
   C   
   C   
   A   
   A   
   T   
   C   
   A   
   C   
   A   
   G   
   T   
   T   
   G   
   C   
   T   
   T   
   C   
   T   
   G   
   A   
   A   
   C   
   C   
   A   
   G   
   T   
   G   
   A   
   C   
   A   
   G   
   A   
   A   
   T   
   T   
   A   
   C   
   C   
   A   
   A   
   C   
   A   
   G   
   G   
   G   
   G   
   C   
   T   
   G   
   C   
   G   
   A   
   C   
   A   
   A   
   A   
   A   
   G   
   A   
   C   
   A   
   C   
   A   
   G   
   A   
   G   
   T   
   C   
   A   
   C   
   G   
   T   
   T   
   A   
   T   
   T   
   A   
   T   
   C   
   C   
   G   
   T   
   T   
   G   
   G   
   G   
   G   
   G   
   A   
   A   
   G   
   C   
   A   
   G   
   C   
   T   
   G   
   C   
   G   
   C   
   A   
   A   
   T   
   T   
   A   
   A   
   T   
   C   
   G   
   G   
   G   
   T   
   A   
   T   
   A   
   C   
   G   
   G   
   G   
   C   
   A   
   C   
   C   
   A   
   T   
   T   
   A   
   C   
   T   
   G   
   C   
   A   
   G   
   A   
   A   
   G   
   A   
   T   
   A   
   T   
   T   
   A   
   A   
   A   
   A   
   A   
   A   
   A   
   A   
   T   
   C   
   C   
   A   
   G   
   A   
   G   
   T   
   T   
   A   
   A   
   G   
   T   
   A   
   G   
   T   
   A   
   C   
   G   
   G   
   G   
   C   
   G   
   T   
   A   
   A   
   T   
   T   
   G   
   G   
   T   
   A   
   A   
   A   
   A   
   C   
   T   
   G   
   G   
   C   
   T   
   T   
   A   
   G   
   A   
   A   
   C   
   G   
   A   
   G   
   C   
   G   
   T   
   T   
   T   
   G   
   A   
   T   
   A   
   A   
   A   
   G   
   A   
   A   
   T   
   T   
   A   
   A   
   G   
   A   
   G   
   G   
   G   
   C   
   A   
   A   
   G   
   A   
   T   
   G   
   G   
   T   
   G   
   G   
   C   
   T   
   C   
   A   
   C   
   T   
   A   
   G   
   T   
   T   
   A   
   T   
   T   
   T   
   T   
   A   
   G   
   A   
   T   
   G   
   A   
   C   
   A   
   A   
   A   
   G   
   A   
   G   
   A   
   A   
   T   
   G   
   T   
   C   
   A   
   A   
   A   
   A   
   A   
   A   
   G   
   C   
   T   
   T   
   T   
   A   
   C   
   A   
   A   
   A   
   C   
   C   
   A   
   A   
   A   
   G   
   A   
   G   
   A   
   A   
   A   
   A   
   A   
   A   
   G   
   A   
   T   
   G   
   G   
   T   
   C   
   A   
   A   
   A   
   C   
   G   
   A   
   T   
   T   
   A   
   A   
   A   
   T   
   T   
   A   
   A   
   C   
   G   
   A   
   T   
   T   
   G   
   A   
   C   
   A   
   G   
   C   
   G   
   G   
   G   
   G   
   T   
   A   
   C   
   A   
   A   
   C   
   A   
   A   
   C   
   A   
   A   
   G   
   C   
   T   
   T   
   T   
   T   
   G   
   C   
   T   
   A   
   T   
   A   
   T   
   T   
   T   
   G   
   A   
   C   
   A   
   A   
   A   
   C   
   G   
   T   
   C   
   C   
   T   
   G   
   G   
   T   
   A   
   G   
   T   
   G   
   C   
   G   
   G   
   T   
   C   
   A   
   T   
   T   
   A   
   C   
   C   
   G   
   A   
   T   
   C   
   C   
   T   
   C   
   A   
   A   
   A   
   A   
   A   
   G   
   G   
   C   
   G   
   A   
   T   
   T   
   T   
   A   
   T   
   T   
   A   
   G   
   C   
   A   
   A   
   C   
   G   
   G   
   T   
   T   
   A   
   G   
   T   
   T   
   C   
   A   
   C   
   C   
   T   
   T   
   C   
   G   
   T   
   A   
   T   
   G   
   A   
   T   
   C   
   C   
   T   
   A   
   A   
   T   
   A   
   A   
   A   
   A   
   T   
   G   
   G   
   C   
   C   
   A   
   A   
   T   
   G   
   G   
   C   
   A   
   T   
   T   
   T   
   C   
   T   
   C   
   A   
   A   
   A   
   A   
   A   
   G   
   A   
   A   
   T   
   A   
   T   
   G   
   A   
   T   
   G   
   C   
   T   
   T   
   A   
   C   
   A   
   A   
   T   
   A   
   A   
   C   
   A   
   A   
   C   
   A   
   A   
   A   
   G   
   A   
   T   
   T   
   T   
   A   
   C   
   C   
   A   
   T   
   T   
   C   
   A   
   C   
   A   
   G   
   C   
   A   
   C   
   G   
   T   
   T   
   T   
   C   
   G   
   C   
   G   
   A   
   C   
   A   
   G   
   G   
   C   
   T   
   A   
   T   
   G   
   C   
   A   
   C   
   C   
   A   
   G   
   G   
   C   
   T   
   C   
   T   
   A   
   C   
   C   
   T   
   T   
   T   
   A   
   A   
   A   
   A   
   C   
   A   
   A   
   T   
   T   
   A   
   C   
   C   
   G   
   G   
   T   
   G   
   C   
   C   
   A   
   T   
   T   
   G   
   G   
   T   
   T   
   T   
   A   
   G   
   A   
   T   
   G   
   C   
   A   
   G   
   G   
   G   
   A   
   C   
   A   
   T   
   T   
   G   
   A   
   A   
   G   
   C   
   C   
   A   
   G   
   A   
   T   
   G   
   A   
   A   
   G   
   A   
   G   
   C   
   T   
   A   
   G   
   A   
   A   
   A   
   T   
   T   
   A   
   A   
   T   
   G   
   G   
   C   
   T   
   T   
   G   
   A   
   A   
   A   
   T   
   G   
   G   
   C   
   A   
   A   
   A   
   A   
   A   
   G   
   A   
   T   
   A   
   A   
   A   
   T   
   C   
   T   
   T   
   G   
   G   
   G   
   G   
   C   
   G   
   G   
   C   
   T   
   A   
   T   
   T   
   T   
   T   
   G   
   C   
   G   
   A   
   C   
   A   
   C   
   G   
   T   
   G   
   T   
   A   
   A   
   A   
   A   
   G   
   A   
   A   
   G   
   C   
   A   
   A   
   G   
   T   
   C   
   C   
   A   
   G   
   T   
   C   
   A   
   A   
   T   
   C   
   T   
   G   
   C   
   G   
   T   
   A   
   C   
   C   
   G   
   C   
   T   
   T   
   T   
   A   
   G   
   T   
   T   
   A   
   A   
   T   
   T   
   C   
   A   
   G   
   A   
   T   
   A   
   A   
   T   
   A   
   T   
   T   
   T   
   A   
   T   
   T   
   T   
   T   
   G   
   C   
   C   
   C   
   A   
   A   
   C   
   A   
   A   
   A   
   C   
   A   
   T   
   T   
   A   
   C   
   G   
   G   
   A   
   T   
   G   
   G   
   G   
   G   
   G   
   A   
   A   
   G   
   A   
   C   
   A   
   A   
   A   
   T   
   T   
   T   
   A   
   G   
   A   
   G   
   C   
   G   
   G   
   G   
   T   
   C   
   T   
   T   
   A   
   A   
   T   
   A   
   A   
   A   
   T   
   T   
   C   
   A   
   T   
   T   
   T   
   T   
   C   
   G   
   G   
   T   
   G   
   A   
   G   
   G   
   A   
   A   
   C   
   T   
   A   
   G   
   A   
   T   
   T   
   T   
   A   
   C   
   C   
   A   
   A   
   T   
   T   
   G   
   C   
   C   
   A   
   T   
   G   
   A   
   C   
   G   
   C   
   C   
   T   
   G   
   C   
   T   
   C   
   A   
   A   
   A   
   T   
   T   
   T   
   C   
   A   
   A   
   A   
   T   
   G   
   A   
   A   
   G   
   A   
   T   
   A   
   A   
   A   
   T   
   T   
   T   
   A   
   A   
   T   
   T   
   C   
   T   
   G   
   A   
   A   
   A   
   T   
   T   
   C   
   T   
   T   
   T   
   T   
   A   
   G   
   C   
   A   
   G   
   A   
   T   
   A   
   C   
   G   
   G   
   G   
   C   
   T   
   A   
   T   
   G   
   G   
   T   
   C   
   A   
   A   
   G   
   G   
   T   
   C   
   A   
   A   
   T   
   T   
   G   
   T   
   T   
   A   
   A   
   T   
   T   
   T   
   C   
   G   
   C   
   C   
   A   
   A   
   T   
   C   
   C   
   A   
   G   
   C   
   A   
   A   
   G   
   C   
   T   
   A   
   C   
   C   
   A   
   T   
   G   
   T   
   A   
   T   
   A   
   G   
   T   
   G   
   T   
   T   
   T   
   T   
   C   
   C   
   A   
   A   
   A   
   A   
   T   
   A   
   A   
   T   
   G   
   G   
   A   
   A   
   C   
   A   
   T   
   T   
   A   
   G   
   T   
   C   
   T   
   A   
   T   
   C   
   C   
   A   
   A   
   A   
   A   
   C   
   T   
   A   
   G   
   T   
   T   
   T   
   T   
   A   
   G   
   A   
   C   
   A   
   A   
   A   
   G   
   A   
   A   
   A   
   C   
   G   
   A   
   A   
   G   
   A   
   A   
   A   
   A   
   A   
   A   
   G   
   A   
   C   
   A   
   A   
   T   
   G   
   T   
   A   
   A   
   T   
   C   
   A   
   G   
   C   
   G   
   C   
   C   
   A   
   A   
   T   
   G   
   C   
   A   
   G   
   C   
   G   
   A   
   A   
   T   
   A   
   C   
   A   
   A   
   T   
   T   
   G   
   C   
   C   
   A   
   C   
   A   
   G   
   A   
   T   
   C   
   T   
   T   
   T   
   T   
   A   
   G   
   G   
   T   
   A   
   G   
   T   
   G   
   T   
   G   
   G   
   A   
   A   
   G   
   A   
   T   
   C   
   C   
   T   
   T   
   C   
   A   
   G   
   G   
   T   
   T   
   A   
   T   
   G   
   T   
   T   
   T   
   A   
   T   
   A   
   A   
   T   
   A   
   T   
   G   
   T   
   A   
   C   
   A   
   A   
   T   
   C   
   C   
   G   
   A   
   A   
   C   
   T   
   T   
   C   
   T   
   C   
   A   
   T   
   T   
   G   
   G   
   C   
   T   
   G   
   C   
   T   
   A   
   A   
   A   
   A   
   C   
   A   
   G   
   G   
   A   
   A   
   C   
   A   
   G   
   C   
   T   
   G   
   A   
   A   
   A   
   T   
   A   
   A   
   A   
   A   
   G   
   A   
   T   
   A   
   A   
   A   
   C   
   A   
   A   
   G   
   A   
   T   
   A   
   C   
   A   
   G   
   A   
   T   
   G   
   G   
   C   
   A   
   A   
   A   
   G   
   A   
   A   
   A   
   A   
   C   
   A   
   G   
   T   
   T   
   T   
   C   
   C   
   T   
   T   
   C   
   T   
   A   
   A   
   C   
   G   
   T   
   T   
   A   
   G   
   A   
   T   
   C   
   G   
   T   
   A   
   G   
   T   
   A   
   A   
   C   
   A   
   A   
   T   
   A   
   A   
   A   
   T   
   T   
   C   
   T   
   T   
   A   
   A   
   C   
   A   
   A   
   T   
   G   
   A   
   T   
   T   
   A   
   T   
   G   
   G   
   T   
   T   
   G   
   A   
   A   
   A   
   A   
   T   
   T   
   C   
   A   
   G   
   G   
   A   
   G   
   A   
   A   
   A   
   A   
   T   
   G   
   G   
   T   
   T   
   C   
   T   
   G   
   C   
   A   
   A   
   C   
   A   
   G   
   A   
   T   
   A   
   T   
   C   
   A   
   G   
   T   
   A   
   A   
   A   
   C   
   C   
   A   
   T   
   T   
   A   
   A   
   T   
   T   
   G   
   A   
   T   
   T   
   A   
   C   
   T   
   T   
   A   
   G   
   A   
   A   
   G   
   C   
   A   
   A   
   C   
   C   
   A   
   T   
   T   
   A   
   A   
   A   
   -   
   -   
   -   
   -   
   -   
   -   
   -   
   -   
   -   
   -   
   -   
   -   
   -   
   -   
   -   
   -   
   -   
   -   
   -   
   -   
   -   
   -   
   -   
   -   
   -   
   -   
   -   
   -   
   -   
   -   
   -   
   -   
   -   
   -   
   -   
   -   
   -   
   -   
   -   
   -   
   -   
   -   
   -   
   -   
   -   
   -   
   -   
   -   
   -   
   -   
   -   
   -   
   -   
   -   
   -   
   -   
   -   
   -   
   -   
   -   
   -   
 

 
   N55268   
   -   
   -   
   -   
   -   
   -   
   -   
   -   
   -   
   -   
   -   
   -   
   -   
   -   
   -   
   -   
   -   
   -   
   -   
   -   
   -   
   -   
   -   
   -   
   -   
   -   
   -   
   -   
   -   
   -   
   -   
   -   
   -   
   -   
   -   
   -   
   -   
   -   
   -   
   -   
   -   
   -   
   -   
   -   
   -   
   -   
   -   
   -   
   -   
   -   
   -   
   -   
   -   
   -   
   -   
   -   
   -   
   -   
   -   
   -   
   -   
   -   
   -   
   -   
   -   
   -   
   -   
   -   
   -   
   -   
   -   
   -   
   -   
   -   
   -   
   -   
   -   
   -   
   -   
   -   
   -   
   -   
   -   
   -   
   -   
   -   
   -   
   -   
   -   
   -   
   -   
   -   
   -   
   -   
   -   
   -   
   -   
   -   
   -   
   -   
   -   
   -   
   -   
   -   
   -   
   -   
   -   
   -   
   -   
   C   
   A   
   A   
   T   
   G   
   G   
   C   
   A   
   A   
   G   
   C   
   C   
   A   
   A   
   A   
   C   
   A   
   A   
   G   
   A   
    A    
   T   
   T   
   A   
   G   
   C   
    C    
   G   
   A   
   A   
   G   
   C   
   G   
   A   
   A   
   G   
   A   
   A   
   A   
   A   
    C    
   A   
   G   
   C   
    T    
   A   
    C    
   T   
    A    
    A    
   A   
   T   
   T   
   T   
   T   
   T   
   A   
   A   
   A   
    C    
   G   
   T   
   A   
   T   
   T   
   G   
    T    
   C   
   A   
   A   
   A   
   A   
   C   
   A   
   G   
    G    
   A   
   A   
   T   
   T   
   T   
   G   
   A   
   T   
   A   
   A   
   G   
   T   
   T   
   A   
   C   
   C   
    G    
   T   
   C   
   C   
   G   
   T   
   T   
    G    
   T   
   T   
   C   
   A   
   A   
   G   
   A   
   A   
    G    
   C   
   T   
   A   
   G   
   C   
   T   
   T   
   A   
   A   
   A   
   G   
   A   
   A   
   A   
   A   
   A   
   T   
   G   
   G   
    C    
   T   
   A   
   T   
   G   
   A   
   T   
   A   
   C   
   T   
   A   
   A   
   A   
   T   
   C   
    T    
   G   
   T   
   T   
   G   
   T   
   T   
   G   
   A   
   A   
   A   
   A   
   A   
   T   
   A   
   C   
   C   
   A   
   A   
   G   
   C   
   A   
   A   
   T   
   T   
   T   
   A   
   T   
   T   
   C   
   A   
   G   
   G   
   G   
   A   
   T   
   T   
   C   
   A   
   A   
   G   
   C   
    A    
   G   
   A   
   A   
    G    
   G   
    A    
   G   
   T   
   C   
   A   
   A   
    A    
   G   
   C   
   T   
   A   
   G   
   T   
   G   
   A   
   T   
   G   
   T   
    C    
   C   
   A   
   A   
   G   
   T   
    C    
   A   
   A   
   A   
   A   
   A   
    G    
   G   
    C    
   G   
   A   
   A   
   A   
   G   
   A   
    C    
   A   
   A   
    T    
   C   
   A   
   A   
   T   
   A   
   C   
   A   
   C   
   A   
   T   
   T   
   T   
   A   
    C    
   C   
   T   
   A   
   T   
   A   
   A   
   A   
   T   
   T   
   A   
   T   
   C   
   G   
   A   
   T   
   G   
   A   
   G   
    C    
   A   
   C   
    T    
   C   
   C   
   T   
   T   
   T   
   A   
   G   
   G   
   C   
   G   
   A   
   A   
   A   
   T   
   G   
   A   
   A   
   A   
   G   
   A   
   T   
    T    
   T   
   G   
   T   
   C   
   T   
   T   
   A   
   T   
   C   
   A   
   A   
   T   
   C   
   A   
   A   
   G   
   T   
   A   
   T   
    C    
   G   
    C    
   C   
   A   
   A   
    G    
   A   
   A   
   A   
   G   
   G   
    C    
   G   
   A   
   T   
   A   
    C    
   C   
   T   
   A   
   C   
   C   
   A   
   A   
   A   
   T   
    C    
    G    
   C   
   T   
   T   
   G   
   G   
    A    
    A    
    G    
   C   
   C   
    G    
   T   
   C   
   T   
   T   
   T   
   A   
   A   
   T   
   T   
   T   
   T   
   T   
   C   
   C   
   A   
   G   
   A   
   T   
   A   
   T   
   G   
   T   
   C   
   A   
   G   
   G   
   A   
   A   
   A   
   T   
   G   
   A   
   T   
   A   
   A   
   A   
    A    
   T   
   T   
   T   
   C   
   G   
   A   
   T   
   T   
   C   
   A   
   A   
   G   
   T   
   A   
   G   
   A   
   T   
   A   
   A   
   T   
   G   
   C   
   C   
   A   
   A   
   A   
   C   
   G   
   T   
   G   
   G   
   A   
   G   
   A   
   A   
   A   
   T   
   T   
   G   
   T   
   C   
   G   
   A   
   T   
   C   
   G   
   T   
   A   
   A   
    T    
   G   
   G   
   T   
   A   
   G   
   T   
   G   
   G   
   G   
   C   
   T   
   A   
   G   
    C    
   A   
   A   
   T   
   T   
   A   
   A   
   C   
   A   
   A   
   A   
   G   
   T   
   G   
   T   
   T   
   T   
   G   
   A   
    T    
   G   
   A   
   A   
   G   
   T   
   G   
   G   
   G   
   C   
   G   
   T   
   A   
   G   
   T   
    G    
   C   
   C   
   T   
    G    
   G   
   C   
   A   
   A   
   A   
   C   
   T   
    C    
   G   
   G   
   T   
   T   
   C   
   T   
   G   
   G   
   C   
   G   
   C   
   A   
   G   
   A   
   A   
   A   
   A   
   A   
    A    
   C   
   A   
   G   
   C   
    C    
   A   
   A   
   T   
   A   
   T   
   C   
   A   
   A   
   A   
   G   
   C   
   T   
   T   
   T   
   T   
   A   
   G   
   T   
   G   
   A   
   T   
   A   
   A   
    G    
   T   
   T   
    C    
   G   
   G   
   C   
   G   
   T   
   T   
   T   
   C   
   T   
    G    
   T   
   T   
   G   
   A   
   T   
   G   
   A   
   A   
   A   
   T   
   C   
   A   
   A   
   T   
   C   
    A    
   A   
   A   
   A   
    G    
   T   
   T   
   A   
   A   
   G   
   C   
   C   
   A   
   A   
   G   
   G   
    G    
   T   
   G   
   G   
   G   
   T   
   C   
   C   
   A   
   A   
   G   
   C   
   A   
   G   
   A   
   C   
   T   
   C   
    C    
   T   
   T   
   T   
   G   
   T   
   A   
   C   
   C   
   A   
   A   
   T   
   C   
   A   
   C   
   A   
   G   
   T   
    C    
   G   
   C   
   T   
   T   
   C   
   T   
   G   
   A   
   A   
   C   
   C   
   A   
   G   
   T   
   G   
   A   
   C   
   A   
   G   
   A   
   A   
   T   
   T   
   A   
   C   
   C   
   A   
   A   
   C   
   A   
   G   
   G   
   G   
   G   
   C   
   T   
   G   
   C   
   G   
   A   
   C   
   A   
   A   
   A   
   A   
   G   
    A    
    T    
   A   
   C   
   A   
   G   
   A   
   G   
   T   
   C   
   A   
   C   
   G   
   T   
   T   
   A   
   T   
   T   
   A   
   T   
   C   
   C   
    G    
    C    
   T   
   G   
   G   
   G   
   G   
   G   
   A   
   A   
   G   
   C   
   A   
   G   
   C   
   T   
   G   
   C   
   G   
   C   
   A   
   A   
   T   
   T   
   A   
   A   
   T   
   C   
   G   
   G   
    G    
   T   
   A   
   T   
   A   
   C   
    G    
   G   
   G   
    C    
   A   
   C   
    C    
   A   
   T   
   T   
   A   
   C   
   T   
   G   
   C   
   A   
   G   
   A   
    A    
   G   
   A   
   T   
   A   
   T   
   T   
    G    
   A   
   A   
   A   
   A   
   A   
   A   
   A   
   T   
   C   
   C   
   A   
   G   
   A   
   G   
   T   
   T   
   A   
   A   
   G   
   T   
   A   
   G   
   T   
   A   
   C   
   G   
   G   
   G   
    C    
   G   
   T   
   A   
   A   
   T   
   T   
   G   
   G   
    T    
   A   
   A   
   A   
   A   
   C   
   T   
   G   
   G   
    C    
   T   
   T   
   A   
   G   
   A   
   A   
   C   
   G   
   A   
   G   
   C   
   G   
   T   
   T   
   T   
   G   
   A   
   T   
   A   
   A   
   A   
   G   
   A   
    A    
   T   
   T   
   A   
   A   
   G   
   A   
   G   
   G   
    G    
   C   
   A   
   A   
   G   
   A   
   T   
   G   
   G   
    T    
   G   
   G   
    C    
   T   
   C   
    A    
   C   
   T   
   A   
   G   
   T   
   T   
   A   
   T   
   T   
   T   
   T   
   A   
   G   
   A   
   T   
   G   
   A   
   C   
   A   
   A   
   A   
   G   
   A   
   G   
   A   
   A   
   T   
   G   
   T   
   C   
   A   
   A   
   A   
   A   
   A   
   A   
   G   
   C   
   T   
   T   
   T   
   A   
   C   
   A   
   A   
   A   
   C   
   C   
   A   
   A   
   A   
   G   
   A   
    G    
   A   
   A   
   A   
   A   
   A   
   A   
   G   
   A   
   T   
   G   
   G   
   T   
   C   
   A   
   A   
   A   
   C   
    G    
   A   
   T   
   T   
   A   
   A   
   A   
   T   
   T   
   A   
   A   
   C   
   G   
   A   
   T   
   T   
   G   
   A   
    C    
   A   
   G   
    C    
   G   
   G   
    G    
   G   
   T   
   A   
   C   
   A   
   A   
   C   
   A   
   A   
   C   
   A   
   A   
   G   
   C   
    T    
   T   
   T   
    T    
   G   
   C   
   T   
   A   
   T   
   A   
   T   
   T   
   T   
   G   
   A   
   C   
   A   
   A   
   A   
   C   
   G   
    T    
    C    
   C   
   T   
   G   
   G   
   T   
   A   
   G   
   T   
   G   
   C   
   G   
   G   
   T   
   C   
   A   
   T   
   T   
   A   
   C   
   C   
   G   
   A   
   T   
   C   
   C   
   T   
   C   
   A   
   A   
   A   
   A   
   A   
   G   
   G   
   C   
   G   
   A   
   T   
   T   
   T   
   A   
    T    
   T   
   A   
   G   
   C   
    A    
   A   
   C   
   G   
   G   
   T   
   T   
   A   
   G   
   T   
   T   
   C   
   A   
   C   
   C   
   T   
   T   
   C   
   G   
   T   
   A   
   T   
   G   
   A   
   T   
   C   
   C   
   T   
   A   
   A   
   T   
   A   
   A   
   A   
   A   
   T   
   G   
   G   
   C   
   C   
   A   
   A   
   T   
   G   
   G   
    C    
   A   
   T   
   T   
   T   
   C   
   T   
   C   
   A   
   A   
   A   
   A   
   A   
   G   
   A   
    A    
   T   
   A   
   T   
   G   
   A   
   T   
   G   
   C   
   T   
   T   
   A   
   C   
   A   
   A   
   T   
   A   
   A   
   C   
   A   
   A   
   C   
   A   
   A   
   A   
   G   
   A   
   T   
   T   
   T   
   A   
   C   
   C   
   A   
   T   
   T   
    C    
   A   
   C   
   A   
   G   
   C   
   A   
   C   
   G   
   T   
   T   
   T   
    T    
   G   
   C   
   G   
   A   
   C   
   A   
   G   
   G   
    C    
   T   
   A   
   T   
   G   
   C   
   A   
   C   
   C   
   A   
   G   
   G   
    C    
   T   
   C   
   T   
   A   
   C   
   C   
   T   
   T   
   T   
   A   
   A   
   A   
   A   
   C   
   A   
   A   
   T   
   T   
   A   
   C   
   C   
   G   
   G   
   T   
   G   
   C   
   C   
   A   
   T   
   T   
   G   
   G   
   T   
   T   
   T   
   A   
   G   
   A   
   T   
    G    
   C   
   A   
   G   
   G   
   G   
   A   
   C   
   A   
   T   
   T   
   G   
   A   
   A   
   G   
   C   
   C   
   A   
   G   
   A   
   T   
   G   
   A   
   A   
   G   
   A   
   G   
   C   
   T   
   A   
   G   
   A   
   A   
   A   
   T   
   T   
   A   
   A   
   T   
   G   
   G   
    C    
   T   
   T   
   G   
   A   
   A   
   A   
   T   
   G   
   G   
   C   
   A   
   A   
   A   
   A   
   A   
   G   
   A   
   T   
   A   
   A   
   A   
   T   
   C   
   T   
   T   
   G   
   G   
   G   
   G   
    C    
   G   
   G   
   C   
   T   
   A   
   T   
   T   
   T   
   T   
   G   
   C   
    G    
   A   
   C   
   A   
   C   
   G   
   T   
   G   
   T   
   A   
   A   
   A   
   A   
   G   
   A   
   A   
   G   
   C   
   A   
   A   
   G   
   T   
   C   
   C   
   A   
   G   
   T   
   C   
   A   
   A   
   T   
   C   
   T   
    G    
   C   
   G   
   T   
   A   
   C   
   C   
   G   
   C   
   T   
   T   
   T   
   A   
   G   
   T   
   T   
   A   
   A   
   T   
   T   
   C   
   A   
   G   
   A   
   T   
   A   
   A   
   T   
   A   
   T   
   T   
   T   
   A   
   T   
   T   
   T   
   T   
   G   
   C   
    C    
   C   
   A   
   A   
   C   
   A   
   A   
   A   
   C   
   A   
   T   
   T   
   A   
   C   
   G   
    G    
   A   
   T   
   G   
   G   
   G   
    A    
   G   
   A   
   A   
   G   
   A   
   C   
   A   
   A   
   A   
   T   
   T   
   T   
   A   
   G   
   A   
   G   
   C   
    G    
   G   
   G   
   T   
   C   
   T   
   T   
   A   
   A   
   T   
   A   
   A   
   A   
   T   
   T   
   C   
   A   
   T   
   T   
   T   
   T   
   C   
   G   
   G   
   T   
   G   
   A   
   G   
   G   
   A   
   A   
   C   
   T   
   A   
   G   
   A   
   T   
   T   
   T   
   A   
   C   
   C   
   A   
   A   
   T   
    T    
   G   
   C   
   C   
   A   
   T   
   G   
   A   
   C   
   G   
   C   
   C   
    C    
   G   
   C   
   T   
   C   
   A   
   A   
   A   
   T   
   T   
   T   
   C   
   A   
   A   
   A   
    T    
   G   
   A   
   A   
   G   
   A   
   T   
   A   
   A   
   A   
   T   
   T   
   T   
   A   
   A   
   T   
   T   
   C   
   T   
   G   
   A   
   A   
   A   
   T   
   T   
   C   
   T   
   T   
   T   
   T   
    A    
   G   
   C   
   A   
   G   
   A   
   T   
   A   
   C   
    G    
   G   
   G   
   C   
   T   
   A   
    C    
   G   
   G   
    T    
   C   
   A   
   A   
   G   
   G   
    C    
   C   
   A   
   A   
   T   
   T   
   G   
   T   
   T   
   A   
   A   
   T   
   T   
   T   
   C   
    A    
   C   
   C   
   A   
   A   
   T   
   C   
   C   
   A   
   G   
   C   
   A   
   A   
   G   
   C   
   T   
   A   
   C   
   C   
   A   
   T   
   G   
   T   
   A   
   T   
   A   
   G   
   T   
   G   
   T   
   T   
   T   
   T   
   C   
   C   
   A   
   A   
   A   
   A   
   T   
   A   
   A   
   T   
   G   
   G   
    A    
   A   
   C   
   A   
    T    
   T   
    A    
   G   
   T   
    C    
   T   
   A   
   T   
   C   
   C   
   A   
   A   
   A   
   A   
    T    
   T   
   A   
   G   
   T   
   T   
   T   
   T   
   A   
   G   
   A   
   C   
   A   
   A   
   A   
   G   
   A   
   A   
   A   
   C   
    G    
   A   
   A   
   G   
   A   
   A   
   A   
   A   
   A   
   A   
   G   
   A   
   C   
   A   
   A   
   T   
   G   
   T   
   A   
   A   
   T   
   C   
   A   
   G   
   C   
   G   
   C   
   C   
   A   
   A   
   C   
   G   
   C   
   A   
   G   
   C   
   G   
   A   
   A   
   T   
   A   
   C   
   A   
   A   
   T   
   T   
   G   
   C   
   C   
   A   
   C   
   A   
   G   
   A   
   T   
   C   
   T   
   T   
   T   
   T   
   A   
   G   
   G   
   T   
   A   
   G   
   T   
   G   
   T   
   G   
   G   
   A   
   A   
   G   
   A   
   T   
   C   
   C   
   T   
   T   
   C   
   A   
   G   
   G   
   T   
   T   
   A   
   T   
   G   
   T   
   T   
   T   
   A   
   T   
   A   
   A   
   T   
   A   
   T   
   G   
   T   
   A   
   C   
   A   
   A   
   T   
   C   
   C   
   G   
   A   
   A   
   C   
   T   
   T   
   C   
   T   
   C   
   A   
   T   
   T   
   G   
   G   
   C   
   T   
   G   
   C   
   T   
   A   
   A   
   A   
   A   
   C   
   A   
   G   
   G   
   A   
   A   
   C   
   A   
   G   
   C   
   T   
   G   
   A   
   A   
   A   
   T   
   A   
   A   
   A   
   A   
   G   
   A   
   T   
   A   
   A   
   A   
   C   
   A   
   A   
   G   
   A   
   T   
   A   
   C   
   A   
   G   
   A   
   T   
   G   
   G   
   C   
   A   
   A   
   A   
   G   
   A   
   A   
   A   
   A   
   C   
   A   
   G   
   T   
   T   
   T   
   C   
   C   
   T   
   T   
   C   
   T   
   A   
   A   
   C   
   G   
   T   
   T   
   A   
   G   
   A   
   T   
   C   
   G   
   T   
   A   
   G   
   T   
   A   
   A   
   C   
   A   
   A   
   T   
   A   
   A   
   A   
   T   
   T   
   C   
   T   
   T   
   A   
   A   
   C   
   A   
   A   
   T   
   G   
   A   
   T   
   T   
   A   
   T   
   G   
   G   
   T   
   T   
   G   
   A   
   A   
   A   
   A   
   T   
   T   
   C   
   A   
   G   
   G   
   A   
   G   
   A   
   A   
   A   
   A   
   T   
   G   
   G   
   T   
   T   
   C   
   T   
   G   
   C   
   A   
   A   
   C   
   A   
   G   
   A   
   T   
   A   
   T   
   C   
   A   
   G   
   T   
   A   
   A   
   A   
   C   
   C   
   A   
   T   
   T   
   A   
   A   
   T   
   T   
   G   
   A   
   T   
   T   
   A   
   C   
   T   
   T   
   A   
   G   
   A   
   A   
   G   
   C   
   A   
   A   
   C   
   C   
   A   
   T   
   T   
   A   
   A   
   A   
   -   
   -   
   -   
   -   
   -   
   -   
   -   
   -   
   -   
   -   
   -   
   -   
   -   
   -   
   -   
   -   
   -   
   -   
   -   
   -   
   -   
   -   
   -   
   -   
   -   
   -   
   -   
   -   
   -   
   -   
   -   
   -   
   -   
   -   
   -   
   -   
   -   
   -   
   -   
   -   
   -   
   -   
   -   
   -   
   -   
   -   
   -   
   -   
   -   
   -   
   -   
   -   
   -   
   -   
   -   
   -   
   -   
   -   
   -   
   -   
   -   
   -   
   -   
   -   
   -   
   -   
   -   
   -   
   -   
   -   
   -   
   -   
   -   
   -   
   -   
   -   
   -   
   -   
   -   
   -   
   -   
   -   
   -   
   -   
   -   
   -   
   -   
   -   
   -   
   -   
   -   
   -   
   -   
   -   
   -   
   -   
   -   
   -   
   -   
   -   
   -   
   -   
   -   
   -   
   -   
   -   
   -   
   -   
   -   
   -   
   -   
   -   
   -   
   -   
   -   
   -   
   -   
   -   
   -   
   -   
   -   
   -   
   -   
   -   
   -   
   -   
   -   
   -   
   -   
   -   
   -   
   -   
   -   
   -   
   -   
   -   
   -   
   -   
   -   
   -   
   -   
   -   
   -   
   -   
   -   
   -   
   -   
   -   
   -   
   -   
   -   
   -   
   -   
   -   
   -   
   -   
   -   
   -   
   -   
   -   
   -   
   -   
   -   
   -   
   -   
   -   
   -   
   -   
   -   
   C   
   A   
   A   
   T   
   G   
   G   
   C   
   A   
   A   
   G   
   C   
   C   
   A   
   A   
   A   
   C   
   A   
   A   
   G   
   A   
   A   
   T   
   T   
   A   
   G   
   C   
   C   
   G   
   A   
   A   
   G   
   C   
   G   
   A   
   A   
   G   
   A   
   A   
   A   
   A   
   C   
   A   
   G   
   C   
   T   
   A   
   C   
   T   
   A   
   A   
   A   
   T   
   T   
   T   
   T   
   T   
   A   
   A   
   A   
   C   
   G   
   T   
   A   
   T   
   T   
   G   
   T   
   C   
   A   
   A   
   A   
   A   
   C   
   A   
   G   
   G   
   A   
   A   
   T   
   T   
   T   
   G   
   A   
   T   
   A   
   A   
   G   
   T   
   T   
   A   
   C   
   C   
   G   
   T   
   C   
   C   
   G   
   T   
   T   
   G   
   T   
   T   
   C   
   A   
   A   
   G   
   A   
   A   
   G   
   C   
   T   
   A   
   G   
   C   
   T   
   T   
   A   
   A   
   A   
   G   
   A   
   A   
   A   
   A   
   A   
   T   
   G   
   G   
   C   
   T   
   A   
   T   
   G   
   A   
   T   
   A   
   C   
   T   
   A   
   A   
   A   
   T   
   C   
   T   
   G   
   T   
   T   
   G   
   T   
   T   
   G   
   A   
   A   
   A   
   A   
   A   
   T   
   A   
   C   
   C   
   A   
   A   
   G   
   C   
   A   
   A   
   T   
   T   
   T   
   A   
   T   
   T   
   C   
   A   
   G   
   G   
   G   
   A   
   T   
   T   
   C   
   A   
   A   
   G   
   C   
   A   
   G   
   A   
   A   
   G   
   G   
   A   
   G   
   T   
   C   
   A   
   A   
   A   
   G   
   C   
   T   
   A   
   G   
   T   
   G   
   A   
   T   
   G   
   T   
   C   
   C   
   A   
   A   
   G   
   T   
   C   
   A   
   A   
   A   
   A   
   A   
   G   
   G   
   C   
   G   
   A   
   A   
   A   
   G   
   A   
   C   
   A   
   A   
   T   
   C   
   A   
   A   
   T   
   A   
   C   
   A   
   C   
   A   
   T   
   T   
   T   
   A   
   C   
   C   
   T   
   A   
   T   
   A   
   A   
   A   
   T   
   T   
   A   
   T   
   C   
   G   
   A   
   T   
   G   
   A   
   G   
   C   
   A   
   C   
   T   
   C   
   C   
   T   
   T   
   T   
   A   
   G   
   G   
   C   
   G   
   A   
   A   
   A   
   T   
   G   
   A   
   A   
   A   
   G   
   A   
   T   
   T   
   T   
   G   
   T   
   C   
   T   
   T   
   A   
   T   
   C   
   A   
   A   
   T   
   C   
   A   
   A   
   G   
   T   
   A   
   T   
   C   
   G   
   C   
   C   
   A   
   A   
   G   
   A   
   A   
   A   
   G   
   G   
   C   
   G   
   A   
   T   
   A   
   C   
   C   
   T   
   A   
   C   
   C   
   A   
   A   
   A   
   T   
   C   
   G   
   C   
   T   
   T   
   G   
   G   
   A   
   A   
   G   
   C   
   C   
   G   
   T   
   C   
   T   
   T   
   T   
   A   
   A   
   T   
   T   
   T   
   T   
   T   
   C   
   C   
   A   
   G   
   A   
   T   
   A   
   T   
   G   
   T   
   C   
   A   
   G   
   G   
   A   
   A   
   A   
   T   
   G   
   A   
   T   
   A   
   A   
   A   
   A   
   T   
   T   
   T   
   C   
   G   
   A   
   T   
   T   
   C   
   A   
   A   
   G   
   T   
   A   
   G   
   A   
   T   
   A   
   A   
   T   
   G   
   C   
   C   
   A   
   A   
   A   
   C   
   G   
   T   
   G   
   G   
   A   
   G   
   A   
   A   
   A   
   T   
   T   
   G   
   T   
   C   
   G   
   A   
   T   
   C   
   G   
   T   
   A   
   A   
   T   
   G   
   G   
   T   
   A   
   G   
   T   
   G   
   G   
   G   
   C   
   T   
   A   
   G   
   C   
   A   
   A   
   T   
   T   
   A   
   A   
   C   
   A   
   A   
   A   
   G   
   T   
   G   
   T   
   T   
   T   
   G   
   A   
   T   
   G   
   A   
   A   
   G   
   T   
   G   
   G   
   G   
   C   
   G   
   T   
   A   
   G   
   T   
   G   
   C   
   C   
   T   
   G   
   G   
   C   
   A   
   A   
   A   
   C   
   T   
   C   
   G   
   G   
   T   
   T   
   C   
   T   
   G   
   G   
   C   
   G   
   C   
   A   
   G   
   A   
   A   
   A   
   A   
   A   
   A   
   C   
   A   
   G   
   C   
   C   
   A   
   A   
   T   
   A   
   T   
   C   
   A   
   A   
   A   
   G   
   C   
   T   
   T   
   T   
   T   
   A   
   G   
   T   
   G   
   A   
   T   
   A   
   A   
   G   
   T   
   T   
   C   
   G   
   G   
   C   
   G   
   T   
   T   
   T   
   C   
   T   
   G   
   T   
   T   
   G   
   A   
   T   
   G   
   A   
   A   
   A   
   T   
   C   
   A   
   A   
   T   
   C   
   A   
   A   
   A   
   A   
   G   
   T   
   T   
   A   
   A   
   G   
   C   
   C   
   A   
   A   
   G   
   G   
   G   
   T   
   G   
   G   
   G   
   T   
   C   
   C   
   A   
   A   
   G   
   C   
   A   
   G   
   A   
   C   
   T   
   C   
   C   
   T   
   T   
   T   
   G   
   T   
   A   
   C   
   C   
   A   
   A   
   T   
   C   
   A   
   C   
   A   
   G   
   T   
   C   
   G   
   C   
   T   
   T   
   C   
   T   
   G   
   A   
   A   
   C   
   C   
   A   
   G   
   T   
   G   
   A   
   C   
   A   
   G   
   A   
   A   
   T   
   T   
   A   
   C   
   C   
   A   
   A   
   C   
   A   
   G   
   G   
   G   
   G   
   C   
   T   
   G   
   C   
   G   
   A   
   C   
   A   
   A   
   A   
   A   
   G   
   A   
   T   
   A   
   C   
   A   
   G   
   A   
   G   
   T   
   C   
   A   
   C   
   G   
   T   
   T   
   A   
   T   
   T   
   A   
   T   
   C   
   C   
   G   
   C   
   T   
   G   
   G   
   G   
   G   
   G   
   A   
   A   
   G   
   C   
   A   
   G   
   C   
   T   
   G   
   C   
   G   
   C   
   A   
   A   
   T   
   T   
   A   
   A   
   T   
   C   
   G   
   G   
   G   
   T   
   A   
   T   
   A   
   C   
   G   
   G   
   G   
   C   
   A   
   C   
   C   
   A   
   T   
   T   
   A   
   C   
   T   
   G   
   C   
   A   
   G   
   A   
   A   
   G   
   A   
   T   
   A   
   T   
   T   
   G   
   A   
   A   
   A   
   A   
   A   
   A   
   A   
   T   
   C   
   C   
   A   
   G   
   A   
   G   
   T   
   T   
   A   
   A   
   G   
   T   
   A   
   G   
   T   
   A   
   C   
   G   
   G   
   G   
   C   
   G   
   T   
   A   
   A   
   T   
   T   
   G   
   G   
   T   
   A   
   A   
   A   
   A   
   C   
   T   
   G   
   G   
   C   
   T   
   T   
   A   
   G   
   A   
   A   
   C   
   G   
   A   
   G   
   C   
   G   
   T   
   T   
   T   
   G   
   A   
   T   
   A   
   A   
   A   
   G   
   A   
   A   
   T   
   T   
   A   
   A   
   G   
   A   
   G   
   G   
   G   
   C   
   A   
   A   
   G   
   A   
   T   
   G   
   G   
   T   
   G   
   G   
   C   
   T   
   C   
   A   
   C   
   T   
   A   
   G   
   T   
   T   
   A   
   T   
   T   
   T   
   T   
   A   
   G   
   A   
   T   
   G   
   A   
   C   
   A   
   A   
   A   
   G   
   A   
   G   
   A   
   A   
   T   
   G   
   T   
   C   
   A   
   A   
   A   
   A   
   A   
   A   
   G   
   C   
   T   
   T   
   T   
   A   
   C   
   A   
   A   
   A   
   C   
   C   
   A   
   A   
   A   
   G   
   A   
   G   
   A   
   A   
   A   
   A   
   A   
   A   
   G   
   A   
   T   
   G   
   G   
   T   
   C   
   A   
   A   
   A   
   C   
   G   
   A   
   T   
   T   
   A   
   A   
   A   
   T   
   T   
   A   
   A   
   C   
   G   
   A   
   T   
   T   
   G   
   A   
   C   
   A   
   G   
   C   
   G   
   G   
   G   
   G   
   T   
   A   
   C   
   A   
   A   
   C   
   A   
   A   
   C   
   A   
   A   
   G   
   C   
   T   
   T   
   T   
   T   
   G   
   C   
   T   
   A   
   T   
   A   
   T   
   T   
   T   
   G   
   A   
   C   
   A   
   A   
   A   
   C   
   G   
   T   
   C   
   C   
   T   
   G   
   G   
   T   
   A   
   G   
   T   
   G   
   C   
   G   
   G   
   T   
   C   
   A   
   T   
   T   
   A   
   C   
   C   
   G   
   A   
   T   
   C   
   C   
   T   
   C   
   A   
   A   
   A   
   A   
   A   
   G   
   G   
   C   
   G   
   A   
   T   
   T   
   T   
   A   
   T   
   T   
   A   
   G   
   C   
   A   
   A   
   C   
   G   
   G   
   T   
   T   
   A   
   G   
   T   
   T   
   C   
   A   
   C   
   C   
   T   
   T   
   C   
   G   
   T   
   A   
   T   
   G   
   A   
   T   
   C   
   C   
   T   
   A   
   A   
   T   
   A   
   A   
   A   
   A   
   T   
   G   
   G   
   C   
   C   
   A   
   A   
   T   
   G   
   G   
   C   
   A   
   T   
   T   
   T   
   C   
   T   
   C   
   A   
   A   
   A   
   A   
   A   
   G   
   A   
   A   
   T   
   A   
   T   
   G   
   A   
   T   
   G   
   C   
   T   
   T   
   A   
   C   
   A   
   A   
   T   
   A   
   A   
   C   
   A   
   A   
   C   
   A   
   A   
   A   
   G   
   A   
   T   
   T   
   T   
   A   
   C   
   C   
   A   
   T   
   T   
   C   
   A   
   C   
   A   
   G   
   C   
   A   
   C   
   G   
   T   
   T   
   T   
   T   
   G   
   C   
   G   
   A   
   C   
   A   
   G   
   G   
   C   
   T   
   A   
   T   
   G   
   C   
   A   
   C   
   C   
   A   
   G   
   G   
   C   
   T   
   C   
   T   
   A   
   C   
   C   
   T   
   T   
   T   
   A   
   A   
   A   
   A   
   C   
   A   
   A   
   T   
   T   
   A   
   C   
   C   
   G   
   G   
   T   
   G   
   C   
   C   
   A   
   T   
   T   
   G   
   G   
   T   
   T   
   T   
   A   
   G   
   A   
   T   
   G   
   C   
   A   
   G   
   G   
   G   
   A   
   C   
   A   
   T   
   T   
   G   
   A   
   A   
   G   
   C   
   C   
   A   
   G   
   A   
   T   
   G   
   A   
   A   
   G   
   A   
   G   
   C   
   T   
   A   
   G   
   A   
   A   
   A   
   T   
   T   
   A   
   A   
   T   
   G   
   G   
   C   
   T   
   T   
   G   
   A   
   A   
   A   
   T   
   G   
   G   
   C   
   A   
   A   
   A   
   A   
   A   
   G   
   A   
   T   
   A   
   A   
   A   
   T   
   C   
   T   
   T   
   G   
   G   
   G   
   G   
   C   
   G   
   G   
   C   
   T   
   A   
   T   
   T   
   T   
   T   
   G   
   C   
   G   
   A   
   C   
   A   
   C   
   G   
   T   
   G   
   T   
   A   
   A   
   A   
   A   
   G   
   A   
   A   
   G   
   C   
   A   
   A   
   G   
   T   
   C   
   C   
   A   
   G   
   T   
   C   
   A   
   A   
   T   
   C   
   T   
   G   
   C   
   G   
   T   
   A   
   C   
   C   
   G   
   C   
   T   
   T   
   T   
   A   
   G   
   T   
   T   
   A   
   A   
   T   
   T   
   C   
   A   
   G   
   A   
   T   
   A   
   A   
   T   
   A   
   T   
   T   
   T   
   A   
   T   
   T   
   T   
   T   
   G   
   C   
   C   
   C   
   A   
   A   
   C   
   A   
   A   
   A   
   C   
   A   
   T   
   T   
   A   
   C   
   G   
   G   
   A   
   T   
   G   
   G   
   G   
   A   
   G   
   A   
   A   
   G   
   A   
   C   
   A   
   A   
   A   
   T   
   T   
   T   
   A   
   G   
   A   
   G   
   C   
   G   
   G   
   G   
   T   
   C   
   T   
   T   
   A   
   A   
   T   
   A   
   A   
   A   
   T   
   T   
   C   
   A   
   T   
   T   
   T   
   T   
   C   
   G   
   G   
   T   
   G   
   A   
   G   
   G   
   A   
   A   
   C   
   T   
   A   
   G   
   A   
   T   
   T   
   T   
   A   
   C   
   C   
   A   
   A   
   T   
   T   
   G   
   C   
   C   
   A   
   T   
   G   
   A   
   C   
   G   
   C   
   C   
   C   
   G   
   C   
   T   
   C   
   A   
   A   
   A   
   T   
   T   
   T   
   C   
   A   
   A   
   A   
   T   
   G   
   A   
   A   
   G   
   A   
   T   
   A   
   A   
   A   
   T   
   T   
   T   
   A   
   A   
   T   
   T   
   C   
   T   
   G   
   A   
   A   
   A   
   T   
   T   
   C   
   T   
   T   
   T   
   T   
   A   
   G   
   C   
   A   
   G   
   A   
   T   
   A   
   C   
   G   
   G   
   G   
   C   
   T   
   A   
   C   
   G   
   G   
   T   
   C   
   A   
   A   
   G   
   G   
   C   
   C   
   A   
   A   
   T   
   T   
   G   
   T   
   T   
   A   
   A   
   T   
   T   
   T   
   C   
   A   
   C   
   C   
   A   
   A   
   T   
   C   
   C   
   A   
   G   
   C   
   A   
   A   
   G   
   C   
   T   
   A   
   C   
   C   
   A   
   T   
   G   
   T   
   A   
   T   
   A   
   G   
   T   
   G   
   T   
   T   
   T   
   T   
   C   
   C   
   A   
   A   
   A   
   A   
   T   
   A   
   A   
   T   
   G   
   G   
   A   
   A   
   C   
   A   
   T   
   T   
   A   
   G   
   T   
   C   
   T   
   A   
   T   
   C   
   C   
   A   
   A   
   A   
   A   
   T   
   T   
   A   
   G   
   T   
   T   
   T   
   T   
   A   
   G   
   A   
   C   
   A   
   A   
   A   
   G   
   A   
   A   
   A   
   C   
   G   
   A   
   A   
   G   
   A   
   A   
   A   
   A   
   A   
   A   
   G   
   A   
   C   
   A   
   A   
   T   
   G   
   T   
   A   
   A   
   T   
   C   
   A   
   G   
   C   
   G   
   C   
   C   
   A   
   A   
   C   
   G   
   C   
   A   
   G   
   C   
   G   
   A   
   A   
   T   
   A   
   C   
   A   
   A   
   T   
   T   
   G   
   C   
   C   
   A   
   C   
   A   
   G   
   A   
   T   
   C   
   T   
   T   
   T   
   T   
   A   
   G   
   G   
   T   
   A   
   G   
   T   
   G   
   T   
   G   
   G   
   A   
   A   
   G   
   A   
   T   
   C   
   C   
   T   
   T   
   C   
   A   
   G   
   G   
   T   
   T   
   A   
   T   
   G   
   T   
   T   
   T   
   A   
   T   
   A   
   A   
   T   
   A   
   T   
   G   
   T   
   A   
   C   
   A   
   A   
   T   
   C   
   C   
   G   
   A   
   A   
   C   
   T   
   T   
   C   
   T   
   C   
   A   
   T   
   T   
   G   
   G   
   C   
   T   
   G   
   C   
   T   
   A   
   A   
   A   
   A   
   C   
   A   
   G   
   G   
   A   
   A   
   C   
   A   
   G   
   C   
   T   
   G   
   A   
   A   
   A   
   T   
   A   
   A   
   A   
   A   
   G   
   A   
   T   
   A   
   A   
   A   
   C   
   A   
   A   
   G   
   A   
   T   
   A   
   C   
   A   
   G   
   A   
   T   
   G   
   G   
   C   
   A   
   A   
   A   
   G   
   A   
   A   
   A   
   A   
   C   
   A   
   G   
   T   
   T   
   T   
   C   
   C   
   T   
   T   
   C   
   T   
   A   
   A   
   C   
   G   
   T   
   T   
   A   
   G   
   A   
   T   
   C   
   G   
   T   
   A   
   G   
   T   
   A   
   A   
   C   
   A   
   A   
   T   
   A   
   A   
   A   
   T   
   T   
   C   
   T   
   T   
   A   
   A   
   C   
   A   
   A   
   T   
   G   
   A   
   T   
   T   
   A   
   T   
   G   
   G   
   T   
   T   
   G   
   A   
   A   
   A   
   A   
   T   
   T   
   C   
   A   
   G   
   G   
   A   
   G   
   A   
   A   
   A   
   A   
   T   
   G   
   G   
   T   
   T   
   C   
   T   
   G   
   C   
   A   
   A   
   C   
   A   
   G   
   A   
   T   
   A   
   T   
   C   
   A   
   G   
   T   
   A   
   A   
   A   
   C   
   C   
   A   
   T   
   T   
   A   
   A   
   T   
   T   
   G   
   A   
   T   
   T   
   A   
   C   
   T   
   T   
   A   
   G   
   A   
   A   
   G   
   C   
   A   
   A   
   C   
   C   
   A   
   T   
   T   
   A   
   A   
   A   
   -   
   -   
   -   
   -   
   -   
   -   
   -   
   -   
   -   
   -   
   -   
   -   
   -   
   -   
   -   
   -   
   -   
   -   
   -   
   -   
   -   
   -   
   -   
   -   
   -   
   -   
   -   
   -   
   -   
   -   
   -   
   -   
   -   
   -   
   -   
   -   
   -   
   -   
   -   
   -   
   -   
   -   
   -   
   -   
   -   
   -   
   -   
   -   
   -   
   -   
   -   
   -   
   -   
   -   
   -   
   -   
   -   
   -   
   -   
   -   
   -   
 

 
   N53418   
   -   
   -   
   -   
   -   
   -   
   -   
   -   
   -   
   -   
   -   
   -   
   -   
   -   
   -   
   -   
   -   
   -   
   -   
   -   
   -   
   -   
   -   
   -   
   -   
   -   
   -   
   -   
   -   
   -   
   -   
   -   
   -   
   -   
   -   
   -   
   -   
   -   
   -   
   -   
   -   
   -   
   -   
   -   
   -   
   -   
   -   
   -   
   -   
   -   
   -   
   -   
   -   
   -   
   -   
   -   
   -   
   -   
   -   
   -   
   -   
   -   
   -   
   -   
   -   
   -   
   -   
   -   
   -   
   -   
   -   
   -   
   -   
   -   
   -   
   -   
   -   
   -   
   -   
   -   
   -   
   -   
   -   
   -   
   -   
   -   
   -   
   -   
   -   
   -   
   -   
   -   
   -   
   -   
   -   
   -   
   -   
   -   
   -   
   -   
   -   
   -   
   -   
   -   
   -   
   -   
   -   
   -   
   -   
   C   
   A   
   A   
   T   
   G   
   G   
   C   
   A   
   A   
   G   
   C   
   C   
   A   
   A   
   A   
   C   
   A   
   A   
   G   
   A   
    A    
   T   
   T   
   A   
   G   
   C   
    C    
   G   
   A   
   A   
   G   
   C   
   G   
   A   
   A   
   G   
   A   
   A   
   A   
   A   
    C    
   A   
   G   
   C   
    T    
   A   
    C    
   T   
    A    
    C    
   A   
   T   
   T   
   T   
   T   
   T   
   A   
   A   
   A   
    C    
   G   
   T   
   A   
   T   
   T   
   G   
    T    
   C   
   A   
   A   
   A   
   A   
   C   
   A   
   G   
    G    
   A   
   A   
   T   
   T   
   T   
   G   
   A   
   T   
   A   
   A   
   G   
   T   
   T   
   A   
   C   
   C   
    G    
   T   
   C   
   C   
   G   
   T   
   T   
    G    
   T   
   T   
   C   
   A   
   A   
   G   
   A   
   A   
    G    
   C   
   T   
   A   
   G   
   C   
   T   
   T   
   A   
   A   
   A   
   G   
   A   
   A   
   A   
   A   
   A   
   T   
   G   
   G   
    C    
   T   
   A   
   T   
   G   
   A   
   T   
   A   
   C   
   T   
   A   
   A   
   A   
   T   
   C   
    T    
   G   
   T   
   T   
   G   
   T   
   T   
   G   
   A   
   A   
   A   
   A   
   A   
   T   
   A   
   C   
   C   
   A   
   A   
   G   
   C   
   A   
   A   
   T   
   T   
   T   
   A   
   T   
   T   
   C   
   A   
   G   
   G   
   G   
   A   
   T   
   T   
   C   
   A   
   A   
   G   
   C   
    A    
   G   
   A   
   A   
    G    
   G   
    A    
   G   
   T   
   C   
   A   
   A   
    A    
   G   
   C   
   T   
   A   
   G   
   T   
   G   
   A   
   T   
   G   
   T   
    T    
   C   
   A   
   A   
   G   
   T   
    A    
   A   
   A   
   A   
   A   
   A   
    G    
   G   
    C    
   G   
   A   
   A   
   A   
   G   
   A   
    C    
   A   
   A   
    T    
   C   
   A   
   A   
   T   
   A   
   C   
   A   
   C   
   A   
   T   
   T   
   T   
   A   
    C    
   C   
   T   
   A   
   T   
   A   
   A   
   A   
   T   
   T   
   A   
   T   
   C   
   G   
   A   
   T   
   G   
   A   
   G   
    T    
   A   
   C   
    C    
   C   
   C   
   T   
   T   
   T   
   A   
   G   
   G   
   C   
   G   
   A   
   A   
   A   
   T   
   G   
   A   
   A   
   A   
   G   
   A   
   T   
    T    
   T   
   G   
   T   
   C   
   T   
   T   
   A   
   T   
   C   
   A   
   A   
   T   
   C   
   A   
   A   
   G   
   T   
   A   
   T   
    C    
   G   
    C    
   C   
   A   
   A   
    A    
   A   
   A   
   A   
   G   
   G   
    C    
   G   
   A   
   T   
   A   
    C    
   C   
   T   
   A   
   C   
   C   
   A   
   A   
   A   
   T   
    C    
    G    
   C   
   T   
   T   
   G   
   G   
    A    
    A    
    G    
   C   
   C   
    G    
   T   
   C   
   T   
   T   
   T   
   A   
   A   
   T   
   T   
   T   
   T   
   T   
   C   
   C   
   A   
   G   
   A   
   T   
   A   
   T   
   G   
   T   
   C   
   A   
   G   
   G   
   A   
   A   
   A   
   T   
   G   
   A   
   T   
   A   
   A   
   A   
    A    
   T   
   T   
   T   
   C   
   G   
   A   
   T   
   T   
   C   
   A   
   A   
   G   
   T   
   A   
   G   
   A   
   T   
   A   
   A   
   T   
   G   
   C   
   C   
   A   
   A   
   A   
   C   
   G   
   T   
   G   
   G   
   A   
   G   
   A   
   A   
   A   
   T   
   T   
   G   
   T   
   C   
   G   
   A   
   T   
   C   
   G   
   T   
   A   
   A   
    T    
   G   
   G   
   T   
   A   
   G   
   T   
   G   
   G   
   G   
   C   
   T   
   A   
   G   
    C    
   A   
   A   
   T   
   T   
   A   
   A   
   C   
   A   
   A   
   A   
   G   
   T   
   G   
   T   
   T   
   T   
   G   
   A   
    T    
   G   
   A   
   A   
   G   
   T   
   G   
   G   
   G   
   C   
   G   
   T   
   A   
   G   
   T   
    G    
   C   
   C   
   T   
    G    
   G   
   C   
   A   
   A   
   A   
   C   
   T   
    C    
   G   
   G   
   T   
   T   
   C   
   T   
   G   
   G   
   C   
   G   
   C   
   A   
   G   
   A   
   A   
   A   
   A   
   A   
    A    
   C   
   A   
   G   
   C   
    C    
   A   
   A   
   T   
   A   
   T   
   C   
   A   
   A   
   A   
   G   
   C   
   T   
   T   
   T   
   T   
   A   
   G   
   T   
   G   
   A   
   T   
   A   
   A   
    A    
   T   
   T   
    C    
   G   
   G   
   C   
   G   
   T   
   T   
   T   
   C   
   T   
    G    
   T   
   T   
   G   
   A   
   T   
   G   
   A   
   A   
   A   
   T   
   C   
   A   
   A   
   T   
   C   
    A    
   A   
   A   
   A   
    G    
   T   
   T   
   A   
   A   
   G   
   C   
   C   
   A   
   A   
   G   
   G   
    A    
   T   
   G   
   G   
   G   
   T   
   C   
   C   
   A   
   A   
   G   
   C   
   A   
   G   
   A   
   C   
   T   
   C   
    C    
   T   
   T   
   T   
   G   
   T   
   A   
   C   
   C   
   A   
   A   
   T   
   C   
   A   
   C   
   A   
   G   
   T   
    C    
   G   
   C   
   T   
   T   
   C   
   T   
   G   
   A   
   A   
   C   
   C   
   A   
   G   
   T   
   G   
   A   
   C   
   A   
   G   
   A   
   A   
   T   
   T   
   A   
   C   
   C   
   A   
   A   
   C   
   A   
   G   
   G   
   G   
   G   
   C   
   T   
   G   
   C   
   G   
   A   
   C   
   A   
   A   
   A   
   A   
   G   
    A    
    T    
   A   
   C   
   A   
   G   
   A   
   G   
   T   
   C   
   A   
   C   
   G   
   T   
   T   
   A   
   T   
   T   
   A   
   T   
   C   
   C   
    G    
    C    
   T   
   G   
   G   
   G   
   G   
   G   
   A   
   A   
   G   
   C   
   A   
   G   
   C   
   T   
   G   
   C   
   G   
   C   
   A   
   A   
   T   
   T   
   A   
   A   
   T   
   C   
   G   
   G   
    G    
   T   
   A   
   T   
   A   
   C   
    G    
   G   
   G   
    C    
   A   
   C   
    C    
   A   
   T   
   T   
   A   
   C   
   T   
   G   
   C   
   A   
   G   
   A   
    A    
   G   
   A   
   T   
   A   
   T   
   T   
    G    
   A   
   A   
   A   
   A   
   A   
   A   
   A   
   T   
   C   
   C   
   A   
   G   
   A   
   G   
   T   
   T   
   A   
   A   
   G   
   T   
   A   
   G   
   T   
   A   
   C   
   G   
   G   
   G   
    C    
   G   
   T   
   A   
   A   
   T   
   T   
   G   
   G   
    T    
   A   
   A   
   A   
   A   
   C   
   T   
   G   
   G   
    T    
   T   
   T   
   A   
   G   
   A   
   A   
   C   
   G   
   A   
   G   
   C   
   G   
   T   
   T   
   T   
   G   
   A   
   T   
   A   
   A   
   A   
   G   
   A   
    A    
   T   
   T   
   A   
   A   
   G   
   A   
   G   
   G   
    G    
   C   
   A   
   A   
   G   
   A   
   T   
   G   
   G   
    T    
   G   
   G   
    C    
   T   
   C   
    A    
   C   
   T   
   A   
   G   
   T   
   T   
   A   
   T   
   T   
   T   
   T   
   A   
   G   
   A   
   T   
   G   
   A   
   C   
   A   
   A   
   A   
   G   
   A   
   G   
   A   
   A   
   T   
   G   
   T   
   C   
   A   
   A   
   A   
   A   
   A   
   A   
   G   
   C   
   T   
   T   
   T   
   A   
   C   
   A   
   A   
   A   
   C   
   C   
   A   
   A   
   A   
   G   
   A   
    G    
   A   
   A   
   A   
   A   
   A   
   A   
   G   
   A   
   T   
   G   
   G   
   T   
   C   
   A   
   A   
   A   
   C   
    G    
   A   
   T   
   T   
   A   
   A   
   A   
   T   
   T   
   A   
   A   
   C   
   G   
   A   
   T   
   T   
   G   
   A   
    C    
   A   
   G   
    T    
   G   
   G   
    C    
   G   
   T   
   A   
   C   
   A   
   A   
   C   
   A   
   A   
   C   
   A   
   A   
   G   
   C   
    C    
   T   
   T   
    C    
   G   
   C   
   T   
   A   
   T   
   A   
   T   
   T   
   T   
   G   
   A   
   C   
   A   
   A   
   A   
   C   
   G   
    T    
    C    
   C   
   T   
   G   
   G   
   T   
   A   
   G   
   T   
   G   
   C   
   G   
   G   
   T   
   C   
   A   
   T   
   T   
   A   
   C   
   C   
   G   
   A   
   T   
   C   
   C   
   T   
   C   
   A   
   A   
   A   
   A   
   A   
   G   
   G   
   C   
   G   
   A   
   T   
   T   
   T   
   A   
    T    
   T   
   A   
   G   
   C   
    A    
   A   
   C   
   G   
   G   
   T   
   T   
   A   
   G   
   T   
   T   
   C   
   A   
   C   
   C   
   T   
   T   
   C   
   G   
   T   
   A   
   T   
   G   
   A   
   T   
   C   
   C   
   T   
   A   
   A   
   T   
   A   
   A   
   A   
   A   
   T   
   G   
   G   
   C   
   C   
   A   
   A   
   T   
   G   
   G   
    T    
   A   
   T   
   T   
   T   
   C   
   T   
   C   
   A   
   A   
   A   
   A   
   A   
   G   
   A   
    A    
   T   
   A   
   T   
   G   
   A   
   T   
   G   
   C   
   T   
   T   
   A   
   C   
   A   
   A   
   T   
   A   
   A   
   C   
   A   
   A   
   C   
   A   
   A   
   A   
   G   
   A   
   T   
   T   
   T   
   A   
   C   
   C   
   A   
   T   
   T   
    C    
   A   
   C   
   A   
   G   
   C   
   A   
   C   
   G   
   T   
   T   
   T   
    T    
   G   
   C   
   G   
   A   
   C   
   A   
   G   
   G   
    C    
   T   
   A   
   T   
   G   
   C   
   A   
   C   
   C   
   A   
   G   
   G   
    C    
   T   
   C   
   T   
   A   
   C   
   C   
   T   
   T   
   T   
   A   
   A   
   A   
   A   
   C   
   A   
   A   
   T   
   T   
   A   
   C   
   C   
   G   
   G   
   T   
   G   
   C   
   C   
   A   
   T   
   T   
   G   
   G   
   T   
   T   
   T   
   A   
   G   
   A   
   T   
    G    
   C   
   A   
   G   
   G   
   G   
   A   
   C   
   A   
   T   
   T   
   G   
   A   
   A   
   G   
   C   
   C   
   A   
   G   
   A   
   T   
   G   
   A   
   A   
   G   
   A   
   G   
   C   
   T   
   A   
   G   
   A   
   A   
   A   
   T   
   T   
   A   
   A   
   T   
   G   
   G   
    T    
   T   
   T   
   G   
   A   
   A   
   A   
   T   
   G   
   G   
   C   
   A   
   A   
   A   
   A   
   A   
   G   
   A   
   T   
   A   
   A   
   A   
   T   
   C   
   T   
   T   
   G   
   G   
   G   
   G   
    C    
   G   
   G   
   C   
   T   
   A   
   T   
   T   
   T   
   T   
   G   
   C   
    G    
   A   
   C   
   A   
   C   
   G   
   T   
   G   
   T   
   A   
   A   
   A   
   A   
   G   
   A   
   A   
   G   
   C   
   A   
   A   
   G   
   T   
   C   
   C   
   A   
   G   
   T   
   C   
   A   
   A   
   T   
   C   
   T   
    G    
   C   
   G   
   T   
   A   
   C   
   C   
   G   
   C   
   T   
   T   
   T   
   A   
   G   
   T   
   T   
   A   
   A   
   T   
   T   
   C   
   A   
   G   
   A   
   T   
   A   
   A   
   T   
   A   
   T   
   T   
   T   
   A   
   T   
   T   
   T   
   T   
   G   
   C   
    C    
   C   
   A   
   A   
   C   
   A   
   A   
   A   
   C   
   A   
   T   
   T   
   A   
   C   
   G   
    G    
   A   
   T   
   G   
   G   
   G   
    A    
   G   
   A   
   A   
   G   
   A   
   C   
   A   
   A   
   A   
   T   
   T   
   T   
   A   
   G   
   A   
   G   
   C   
    G    
   G   
   G   
   T   
   C   
   T   
   T   
   A   
   A   
   T   
   A   
   A   
   A   
   T   
   T   
   C   
   A   
   T   
   T   
   T   
   T   
   C   
   G   
   G   
   T   
   G   
   A   
   G   
   G   
   A   
   A   
   C   
   T   
   A   
   G   
   A   
   T   
   T   
   T   
   A   
   C   
   C   
   A   
   A   
   T   
    T    
   G   
   C   
   C   
   A   
   T   
   G   
   A   
   C   
   G   
   C   
   C   
    T    
   G   
   C   
   T   
   C   
   A   
   A   
   A   
   T   
   T   
   T   
   C   
   A   
   A   
   A   
    T    
   G   
   A   
   A   
   G   
   A   
   T   
   A   
   A   
   A   
   T   
   T   
   T   
   A   
   A   
   T   
   T   
   C   
   T   
   G   
   A   
   A   
   A   
   T   
   T   
   C   
   T   
   T   
   T   
   T   
    A    
   G   
   C   
   A   
   G   
   A   
   T   
   A   
   C   
    G    
   G   
   G   
   C   
   T   
   A   
    C    
   G   
   G   
    T    
   C   
   A   
   A   
   G   
   G   
    T    
   C   
   A   
   A   
   T   
   T   
   G   
   T   
   T   
   A   
   A   
   T   
   T   
   T   
   C   
    G    
   C   
   C   
   A   
   A   
   T   
   C   
   C   
   A   
   G   
   C   
   A   
   A   
   G   
   C   
   T   
   A   
   C   
   C   
   A   
   T   
   G   
   T   
   A   
   T   
   A   
   G   
   T   
   G   
   T   
   T   
   T   
   T   
   C   
   C   
   A   
   A   
   A   
   A   
   T   
   A   
   A   
   T   
   G   
   G   
    A    
   A   
   C   
   A   
    T    
   T   
    A    
   G   
   T   
    C    
   T   
   A   
   T   
   C   
   C   
   A   
   A   
   A   
   A   
    C    
   T   
   A   
   G   
   T   
   T   
   T   
   T   
   A   
   G   
   A   
   C   
   A   
   A   
   A   
   G   
   A   
   A   
   A   
   C   
    G    
   A   
   A   
   G   
   A   
   A   
   A   
   A   
   A   
   A   
   G   
   A   
   C   
   A   
   A   
   T   
   G   
   T   
   A   
   A   
   T   
   C   
   A   
   G   
   C   
   G   
   C   
   C   
   A   
   A   
   C   
   G   
   C   
   A   
   G   
   C   
   G   
   A   
   A   
   T   
   A   
   C   
   A   
   A   
   T   
   T   
   G   
   C   
   C   
   A   
   C   
   A   
   G   
   A   
   T   
   C   
   T   
   T   
   T   
   T   
   A   
   G   
   G   
   T   
   A   
   G   
   T   
   G   
   T   
   G   
   G   
   A   
   A   
   G   
   A   
   T   
   C   
   C   
   T   
   T   
   C   
   A   
   G   
   G   
   T   
   T   
   A   
   T   
   G   
   T   
   T   
   T   
   A   
   T   
   A   
   A   
   T   
   A   
   T   
   G   
   T   
   A   
   C   
   A   
   A   
   T   
   C   
   C   
   G   
   A   
   A   
   C   
   T   
   T   
   C   
   T   
   C   
   A   
   T   
   T   
   G   
   G   
   C   
   T   
   G   
   C   
   T   
   A   
   A   
   A   
   A   
   C   
   A   
   G   
   G   
   A   
   A   
   C   
   A   
   G   
   C   
   T   
   G   
   A   
   A   
   A   
   T   
   A   
   A   
   A   
   A   
   G   
   A   
   T   
   A   
   A   
   A   
   C   
   A   
   A   
   G   
   A   
   T   
   A   
   C   
   A   
   G   
   A   
   T   
   G   
   G   
   C   
   A   
   A   
   A   
   G   
   A   
   A   
   A   
   A   
   C   
   A   
   G   
   T   
   T   
   T   
   C   
   C   
   T   
   T   
   C   
   T   
   A   
   A   
   C   
   G   
   T   
   T   
   A   
   G   
   A   
   T   
   C   
   G   
   T   
   A   
   G   
   T   
   A   
   A   
   C   
   A   
   A   
   T   
   A   
   A   
   A   
   T   
   T   
   C   
   T   
   T   
   A   
   A   
   C   
   A   
   A   
   T   
   G   
   A   
   T   
   T   
   A   
   T   
   G   
   G   
   T   
   T   
   G   
   A   
   A   
   A   
   A   
   T   
   T   
   C   
   A   
   G   
   G   
   A   
   G   
   A   
   A   
   A   
   A   
   T   
   G   
   G   
   T   
   T   
   C   
   T   
   G   
   C   
   A   
   A   
   C   
   A   
   G   
   A   
   T   
   A   
   T   
   C   
   A   
   G   
   T   
   A   
   A   
   A   
   C   
   C   
   A   
   T   
   T   
   A   
   A   
   T   
   T   
   G   
   A   
   T   
   T   
   A   
   C   
   T   
   T   
   A   
   G   
   A   
   A   
   G   
   C   
   A   
   A   
   C   
   C   
   A   
   T   
   T   
   A   
   A   
   A   
   -   
   -   
   -   
   -   
   -   
   -   
   -   
   -   
   -   
   -   
   -   
   -   
   -   
   -   
   -   
   -   
   -   
   -   
   -   
   -   
   -   
   -   
   -   
   -   
   -   
   -   
   -   
   -   
   -   
   -   
   -   
   -   
   -   
   -   
   -   
   -   
   -   
   -   
   -   
   -   
   -   
   -   
   -   
   -   
   -   
   -   
   -   
   -   
   -   
   -   
   -   
   -   
   -   
   -   
   -   
   -   
   -   
   -   
   -   
   -   
   -   
   -   
   -   
   -   
   -   
   -   
   -   
   -   
   -   
   -   
   -   
   -   
   -   
   -   
   -   
   -   
   -   
   -   
   -   
   -   
   -   
   -   
   -   
   -   
   -   
   -   
   -   
   -   
   -   
   -   
   -   
   -   
   -   
   -   
   -   
   -   
   -   
   -   
   -   
   -   
   -   
   -   
   -   
   -   
   -   
   -   
   -   
   -   
   -   
   -   
   -   
   -   
   -   
   -   
   -   
   -   
   -   
   -   
   -   
   -   
   -   
   -   
   -   
   -   
   -   
   -   
   -   
   -   
   -   
   -   
   -   
   -   
   -   
   -   
   -   
   -   
   -   
   -   
   -   
   -   
   -   
   -   
   -   
   -   
   -   
   -   
   -   
   -   
   -   
   -   
   -   
   -   
   -   
   -   
   -   
   -   
   -   
   -   
   -   
   -   
   -   
   -   
   -   
   -   
   -   
   -   
   -   
   -   
   -   
   C   
   A   
   A   
   T   
   G   
   G   
   C   
   A   
   A   
   G   
   C   
   C   
   A   
   A   
   A   
   C   
   A   
   A   
   G   
   A   
   A   
   T   
   T   
   A   
   G   
   C   
   C   
   G   
   A   
   A   
   G   
   C   
   G   
   A   
   A   
   G   
   A   
   A   
   A   
   A   
   C   
   A   
   G   
   C   
   T   
   A   
   C   
   T   
   A   
   C   
   A   
   T   
   T   
   T   
   T   
   T   
   A   
   A   
   A   
   C   
   G   
   T   
   A   
   T   
   T   
   G   
   T   
   C   
   A   
   A   
   A   
   A   
   C   
   A   
   G   
   G   
   A   
   A   
   T   
   T   
   T   
   G   
   A   
   T   
   A   
   A   
   G   
   T   
   T   
   A   
   C   
   C   
   G   
   T   
   C   
   C   
   G   
   T   
   T   
   G   
   T   
   T   
   C   
   A   
   A   
   G   
   A   
   A   
   G   
   C   
   T   
   A   
   G   
   C   
   T   
   T   
   A   
   A   
   A   
   G   
   A   
   A   
   A   
   A   
   A   
   T   
   G   
   G   
   C   
   T   
   A   
   T   
   G   
   A   
   T   
   A   
   C   
   T   
   A   
   A   
   A   
   T   
   C   
   T   
   G   
   T   
   T   
   G   
   T   
   T   
   G   
   A   
   A   
   A   
   A   
   A   
   T   
   A   
   C   
   C   
   A   
   A   
   G   
   C   
   A   
   A   
   T   
   T   
   T   
   A   
   T   
   T   
   C   
   A   
   G   
   G   
   G   
   A   
   T   
   T   
   C   
   A   
   A   
   G   
   C   
   A   
   G   
   A   
   A   
   G   
   G   
   A   
   G   
   T   
   C   
   A   
   A   
   A   
   G   
   C   
   T   
   A   
   G   
   T   
   G   
   A   
   T   
   G   
   T   
   T   
   C   
   A   
   A   
   G   
   T   
   A   
   A   
   A   
   A   
   A   
   A   
   G   
   G   
   C   
   G   
   A   
   A   
   A   
   G   
   A   
   C   
   A   
   A   
   T   
   C   
   A   
   A   
   T   
   A   
   C   
   A   
   C   
   A   
   T   
   T   
   T   
   A   
   C   
   C   
   T   
   A   
   T   
   A   
   A   
   A   
   T   
   T   
   A   
   T   
   C   
   G   
   A   
   T   
   G   
   A   
   G   
   T   
   A   
   C   
   C   
   C   
   C   
   T   
   T   
   T   
   A   
   G   
   G   
   C   
   G   
   A   
   A   
   A   
   T   
   G   
   A   
   A   
   A   
   G   
   A   
   T   
   T   
   T   
   G   
   T   
   C   
   T   
   T   
   A   
   T   
   C   
   A   
   A   
   T   
   C   
   A   
   A   
   G   
   T   
   A   
   T   
   C   
   G   
   C   
   C   
   A   
   A   
   A   
   A   
   A   
   A   
   G   
   G   
   C   
   G   
   A   
   T   
   A   
   C   
   C   
   T   
   A   
   C   
   C   
   A   
   A   
   A   
   T   
   C   
   G   
   C   
   T   
   T   
   G   
   G   
   A   
   A   
   G   
   C   
   C   
   G   
   T   
   C   
   T   
   T   
   T   
   A   
   A   
   T   
   T   
   T   
   T   
   T   
   C   
   C   
   A   
   G   
   A   
   T   
   A   
   T   
   G   
   T   
   C   
   A   
   G   
   G   
   A   
   A   
   A   
   T   
   G   
   A   
   T   
   A   
   A   
   A   
   A   
   T   
   T   
   T   
   C   
   G   
   A   
   T   
   T   
   C   
   A   
   A   
   G   
   T   
   A   
   G   
   A   
   T   
   A   
   A   
   T   
   G   
   C   
   C   
   A   
   A   
   A   
   C   
   G   
   T   
   G   
   G   
   A   
   G   
   A   
   A   
   A   
   T   
   T   
   G   
   T   
   C   
   G   
   A   
   T   
   C   
   G   
   T   
   A   
   A   
   T   
   G   
   G   
   T   
   A   
   G   
   T   
   G   
   G   
   G   
   C   
   T   
   A   
   G   
   C   
   A   
   A   
   T   
   T   
   A   
   A   
   C   
   A   
   A   
   A   
   G   
   T   
   G   
   T   
   T   
   T   
   G   
   A   
   T   
   G   
   A   
   A   
   G   
   T   
   G   
   G   
   G   
   C   
   G   
   T   
   A   
   G   
   T   
   G   
   C   
   C   
   T   
   G   
   G   
   C   
   A   
   A   
   A   
   C   
   T   
   C   
   G   
   G   
   T   
   T   
   C   
   T   
   G   
   G   
   C   
   G   
   C   
   A   
   G   
   A   
   A   
   A   
   A   
   A   
   A   
   C   
   A   
   G   
   C   
   C   
   A   
   A   
   T   
   A   
   T   
   C   
   A   
   A   
   A   
   G   
   C   
   T   
   T   
   T   
   T   
   A   
   G   
   T   
   G   
   A   
   T   
   A   
   A   
   A   
   T   
   T   
   C   
   G   
   G   
   C   
   G   
   T   
   T   
   T   
   C   
   T   
   G   
   T   
   T   
   G   
   A   
   T   
   G   
   A   
   A   
   A   
   T   
   C   
   A   
   A   
   T   
   C   
   A   
   A   
   A   
   A   
   G   
   T   
   T   
   A   
   A   
   G   
   C   
   C   
   A   
   A   
   G   
   G   
   A   
   T   
   G   
   G   
   G   
   T   
   C   
   C   
   A   
   A   
   G   
   C   
   A   
   G   
   A   
   C   
   T   
   C   
   C   
   T   
   T   
   T   
   G   
   T   
   A   
   C   
   C   
   A   
   A   
   T   
   C   
   A   
   C   
   A   
   G   
   T   
   C   
   G   
   C   
   T   
   T   
   C   
   T   
   G   
   A   
   A   
   C   
   C   
   A   
   G   
   T   
   G   
   A   
   C   
   A   
   G   
   A   
   A   
   T   
   T   
   A   
   C   
   C   
   A   
   A   
   C   
   A   
   G   
   G   
   G   
   G   
   C   
   T   
   G   
   C   
   G   
   A   
   C   
   A   
   A   
   A   
   A   
   G   
   A   
   T   
   A   
   C   
   A   
   G   
   A   
   G   
   T   
   C   
   A   
   C   
   G   
   T   
   T   
   A   
   T   
   T   
   A   
   T   
   C   
   C   
   G   
   C   
   T   
   G   
   G   
   G   
   G   
   G   
   A   
   A   
   G   
   C   
   A   
   G   
   C   
   T   
   G   
   C   
   G   
   C   
   A   
   A   
   T   
   T   
   A   
   A   
   T   
   C   
   G   
   G   
   G   
   T   
   A   
   T   
   A   
   C   
   G   
   G   
   G   
   C   
   A   
   C   
   C   
   A   
   T   
   T   
   A   
   C   
   T   
   G   
   C   
   A   
   G   
   A   
   A   
   G   
   A   
   T   
   A   
   T   
   T   
   G   
   A   
   A   
   A   
   A   
   A   
   A   
   A   
   T   
   C   
   C   
   A   
   G   
   A   
   G   
   T   
   T   
   A   
   A   
   G   
   T   
   A   
   G   
   T   
   A   
   C   
   G   
   G   
   G   
   C   
   G   
   T   
   A   
   A   
   T   
   T   
   G   
   G   
   T   
   A   
   A   
   A   
   A   
   C   
   T   
   G   
   G   
   T   
   T   
   T   
   A   
   G   
   A   
   A   
   C   
   G   
   A   
   G   
   C   
   G   
   T   
   T   
   T   
   G   
   A   
   T   
   A   
   A   
   A   
   G   
   A   
   A   
   T   
   T   
   A   
   A   
   G   
   A   
   G   
   G   
   G   
   C   
   A   
   A   
   G   
   A   
   T   
   G   
   G   
   T   
   G   
   G   
   C   
   T   
   C   
   A   
   C   
   T   
   A   
   G   
   T   
   T   
   A   
   T   
   T   
   T   
   T   
   A   
   G   
   A   
   T   
   G   
   A   
   C   
   A   
   A   
   A   
   G   
   A   
   G   
   A   
   A   
   T   
   G   
   T   
   C   
   A   
   A   
   A   
   A   
   A   
   A   
   G   
   C   
   T   
   T   
   T   
   A   
   C   
   A   
   A   
   A   
   C   
   C   
   A   
   A   
   A   
   G   
   A   
   G   
   A   
   A   
   A   
   A   
   A   
   A   
   G   
   A   
   T   
   G   
   G   
   T   
   C   
   A   
   A   
   A   
   C   
   G   
   A   
   T   
   T   
   A   
   A   
   A   
   T   
   T   
   A   
   A   
   C   
   G   
   A   
   T   
   T   
   G   
   A   
   C   
   A   
   G   
   T   
   G   
   G   
   C   
   G   
   T   
   A   
   C   
   A   
   A   
   C   
   A   
   A   
   C   
   A   
   A   
   G   
   C   
   C   
   T   
   T   
   C   
   G   
   C   
   T   
   A   
   T   
   A   
   T   
   T   
   T   
   G   
   A   
   C   
   A   
   A   
   A   
   C   
   G   
   T   
   C   
   C   
   T   
   G   
   G   
   T   
   A   
   G   
   T   
   G   
   C   
   G   
   G   
   T   
   C   
   A   
   T   
   T   
   A   
   C   
   C   
   G   
   A   
   T   
   C   
   C   
   T   
   C   
   A   
   A   
   A   
   A   
   A   
   G   
   G   
   C   
   G   
   A   
   T   
   T   
   T   
   A   
   T   
   T   
   A   
   G   
   C   
   A   
   A   
   C   
   G   
   G   
   T   
   T   
   A   
   G   
   T   
   T   
   C   
   A   
   C   
   C   
   T   
   T   
   C   
   G   
   T   
   A   
   T   
   G   
   A   
   T   
   C   
   C   
   T   
   A   
   A   
   T   
   A   
   A   
   A   
   A   
   T   
   G   
   G   
   C   
   C   
   A   
   A   
   T   
   G   
   G   
   T   
   A   
   T   
   T   
   T   
   C   
   T   
   C   
   A   
   A   
   A   
   A   
   A   
   G   
   A   
   A   
   T   
   A   
   T   
   G   
   A   
   T   
   G   
   C   
   T   
   T   
   A   
   C   
   A   
   A   
   T   
   A   
   A   
   C   
   A   
   A   
   C   
   A   
   A   
   A   
   G   
   A   
   T   
   T   
   T   
   A   
   C   
   C   
   A   
   T   
   T   
   C   
   A   
   C   
   A   
   G   
   C   
   A   
   C   
   G   
   T   
   T   
   T   
   T   
   G   
   C   
   G   
   A   
   C   
   A   
   G   
   G   
   C   
   T   
   A   
   T   
   G   
   C   
   A   
   C   
   C   
   A   
   G   
   G   
   C   
   T   
   C   
   T   
   A   
   C   
   C   
   T   
   T   
   T   
   A   
   A   
   A   
   A   
   C   
   A   
   A   
   T   
   T   
   A   
   C   
   C   
   G   
   G   
   T   
   G   
   C   
   C   
   A   
   T   
   T   
   G   
   G   
   T   
   T   
   T   
   A   
   G   
   A   
   T   
   G   
   C   
   A   
   G   
   G   
   G   
   A   
   C   
   A   
   T   
   T   
   G   
   A   
   A   
   G   
   C   
   C   
   A   
   G   
   A   
   T   
   G   
   A   
   A   
   G   
   A   
   G   
   C   
   T   
   A   
   G   
   A   
   A   
   A   
   T   
   T   
   A   
   A   
   T   
   G   
   G   
   T   
   T   
   T   
   G   
   A   
   A   
   A   
   T   
   G   
   G   
   C   
   A   
   A   
   A   
   A   
   A   
   G   
   A   
   T   
   A   
   A   
   A   
   T   
   C   
   T   
   T   
   G   
   G   
   G   
   G   
   C   
   G   
   G   
   C   
   T   
   A   
   T   
   T   
   T   
   T   
   G   
   C   
   G   
   A   
   C   
   A   
   C   
   G   
   T   
   G   
   T   
   A   
   A   
   A   
   A   
   G   
   A   
   A   
   G   
   C   
   A   
   A   
   G   
   T   
   C   
   C   
   A   
   G   
   T   
   C   
   A   
   A   
   T   
   C   
   T   
   G   
   C   
   G   
   T   
   A   
   C   
   C   
   G   
   C   
   T   
   T   
   T   
   A   
   G   
   T   
   T   
   A   
   A   
   T   
   T   
   C   
   A   
   G   
   A   
   T   
   A   
   A   
   T   
   A   
   T   
   T   
   T   
   A   
   T   
   T   
   T   
   T   
   G   
   C   
   C   
   C   
   A   
   A   
   C   
   A   
   A   
   A   
   C   
   A   
   T   
   T   
   A   
   C   
   G   
   G   
   A   
   T   
   G   
   G   
   G   
   A   
   G   
   A   
   A   
   G   
   A   
   C   
   A   
   A   
   A   
   T   
   T   
   T   
   A   
   G   
   A   
   G   
   C   
   G   
   G   
   G   
   T   
   C   
   T   
   T   
   A   
   A   
   T   
   A   
   A   
   A   
   T   
   T   
   C   
   A   
   T   
   T   
   T   
   T   
   C   
   G   
   G   
   T   
   G   
   A   
   G   
   G   
   A   
   A   
   C   
   T   
   A   
   G   
   A   
   T   
   T   
   T   
   A   
   C   
   C   
   A   
   A   
   T   
   T   
   G   
   C   
   C   
   A   
   T   
   G   
   A   
   C   
   G   
   C   
   C   
   T   
   G   
   C   
   T   
   C   
   A   
   A   
   A   
   T   
   T   
   T   
   C   
   A   
   A   
   A   
   T   
   G   
   A   
   A   
   G   
   A   
   T   
   A   
   A   
   A   
   T   
   T   
   T   
   A   
   A   
   T   
   T   
   C   
   T   
   G   
   A   
   A   
   A   
   T   
   T   
   C   
   T   
   T   
   T   
   T   
   A   
   G   
   C   
   A   
   G   
   A   
   T   
   A   
   C   
   G   
   G   
   G   
   C   
   T   
   A   
   C   
   G   
   G   
   T   
   C   
   A   
   A   
   G   
   G   
   T   
   C   
   A   
   A   
   T   
   T   
   G   
   T   
   T   
   A   
   A   
   T   
   T   
   T   
   C   
   G   
   C   
   C   
   A   
   A   
   T   
   C   
   C   
   A   
   G   
   C   
   A   
   A   
   G   
   C   
   T   
   A   
   C   
   C   
   A   
   T   
   G   
   T   
   A   
   T   
   A   
   G   
   T   
   G   
   T   
   T   
   T   
   T   
   C   
   C   
   A   
   A   
   A   
   A   
   T   
   A   
   A   
   T   
   G   
   G   
   A   
   A   
   C   
   A   
   T   
   T   
   A   
   G   
   T   
   C   
   T   
   A   
   T   
   C   
   C   
   A   
   A   
   A   
   A   
   C   
   T   
   A   
   G   
   T   
   T   
   T   
   T   
   A   
   G   
   A   
   C   
   A   
   A   
   A   
   G   
   A   
   A   
   A   
   C   
   G   
   A   
   A   
   G   
   A   
   A   
   A   
   A   
   A   
   A   
   G   
   A   
   C   
   A   
   A   
   T   
   G   
   T   
   A   
   A   
   T   
   C   
   A   
   G   
   C   
   G   
   C   
   C   
   A   
   A   
   C   
   G   
   C   
   A   
   G   
   C   
   G   
   A   
   A   
   T   
   A   
   C   
   A   
   A   
   T   
   T   
   G   
   C   
   C   
   A   
   C   
   A   
   G   
   A   
   T   
   C   
   T   
   T   
   T   
   T   
   A   
   G   
   G   
   T   
   A   
   G   
   T   
   G   
   T   
   G   
   G   
   A   
   A   
   G   
   A   
   T   
   C   
   C   
   T   
   T   
   C   
   A   
   G   
   G   
   T   
   T   
   A   
   T   
   G   
   T   
   T   
   T   
   A   
   T   
   A   
   A   
   T   
   A   
   T   
   G   
   T   
   A   
   C   
   A   
   A   
   T   
   C   
   C   
   G   
   A   
   A   
   C   
   T   
   T   
   C   
   T   
   C   
   A   
   T   
   T   
   G   
   G   
   C   
   T   
   G   
   C   
   T   
   A   
   A   
   A   
   A   
   C   
   A   
   G   
   G   
   A   
   A   
   C   
   A   
   G   
   C   
   T   
   G   
   A   
   A   
   A   
   T   
   A   
   A   
   A   
   A   
   G   
   A   
   T   
   A   
   A   
   A   
   C   
   A   
   A   
   G   
   A   
   T   
   A   
   C   
   A   
   G   
   A   
   T   
   G   
   G   
   C   
   A   
   A   
   A   
   G   
   A   
   A   
   A   
   A   
   C   
   A   
   G   
   T   
   T   
   T   
   C   
   C   
   T   
   T   
   C   
   T   
   A   
   A   
   C   
   G   
   T   
   T   
   A   
   G   
   A   
   T   
   C   
   G   
   T   
   A   
   G   
   T   
   A   
   A   
   C   
   A   
   A   
   T   
   A   
   A   
   A   
   T   
   T   
   C   
   T   
   T   
   A   
   A   
   C   
   A   
   A   
   T   
   G   
   A   
   T   
   T   
   A   
   T   
   G   
   G   
   T   
   T   
   G   
   A   
   A   
   A   
   A   
   T   
   T   
   C   
   A   
   G   
   G   
   A   
   G   
   A   
   A   
   A   
   A   
   T   
   G   
   G   
   T   
   T   
   C   
   T   
   G   
   C   
   A   
   A   
   C   
   A   
   G   
   A   
   T   
   A   
   T   
   C   
   A   
   G   
   T   
   A   
   A   
   A   
   C   
   C   
   A   
   T   
   T   
   A   
   A   
   T   
   T   
   G   
   A   
   T   
   T   
   A   
   C   
   T   
   T   
   A   
   G   
   A   
   A   
   G   
   C   
   A   
   A   
   C   
   C   
   A   
   T   
   T   
   A   
   A   
   A   
   -   
   -   
   -   
   -   
   -   
   -   
   -   
   -   
   -   
   -   
   -   
   -   
   -   
   -   
   -   
   -   
   -   
   -   
   -   
   -   
   -   
   -   
   -   
   -   
   -   
   -   
   -   
   -   
   -   
   -   
   -   
   -   
   -   
   -   
   -   
   -   
   -   
   -   
   -   
   -   
   -   
   -   
   -   
   -   
   -   
   -   
   -   
   -   
   -   
   -   
   -   
   -   
   -   
   -   
   -   
   -   
   -   
   -   
   -   
   -   
   -   
 

 
   N59458F   
   -   
   -   
   -   
   -   
   -   
   -   
   -   
   -   
   -   
   -   
   -   
   -   
   -   
   -   
   -   
   -   
   -   
   -   
   -   
   -   
   -   
   -   
   -   
   -   
   -   
   -   
   -   
   -   
   -   
   -   
   -   
   -   
   -   
   -   
   -   
   -   
   -   
   -   
   -   
   -   
   -   
   -   
   -   
   -   
   -   
   -   
   -   
   -   
   -   
   -   
   -   
   -   
   -   
   -   
   -   
   -   
   -   
   -   
   -   
   -   
   -   
   -   
   -   
   -   
   -   
   -   
   -   
   -   
   -   
   -   
   -   
   -   
   -   
   -   
   -   
   -   
   -   
   -   
   -   
   -   
   -   
   -   
   -   
   -   
   -   
   -   
   -   
   -   
   -   
   -   
   -   
   -   
   -   
   -   
   -   
   -   
   -   
   -   
   -   
   -   
   -   
   -   
   -   
   -   
   -   
   -   
   -   
   -   
   C   
   A   
   A   
   T   
   G   
   G   
   C   
   A   
   A   
   G   
   C   
   C   
   A   
   A   
   A   
   C   
   A   
   A   
   G   
   A   
    A    
   T   
   T   
   A   
   G   
   C   
    C    
   G   
   A   
   A   
   G   
   C   
   G   
   A   
   A   
   G   
   A   
   A   
   A   
   A   
    C    
   A   
   G   
   C   
    T    
   A   
    C    
   T   
    G    
    A    
   A   
   T   
   T   
   T   
   T   
   T   
   A   
   A   
   A   
    C    
   G   
   T   
   A   
   T   
   T   
   G   
    T    
   C   
   A   
   A   
   A   
   A   
   C   
   A   
   G   
    G    
   A   
   A   
   T   
   T   
   T   
   G   
   A   
   T   
   A   
   A   
   G   
   T   
   T   
   A   
   C   
   C   
    G    
   T   
   C   
   C   
   G   
   T   
   T   
    G    
   T   
   T   
   C   
   A   
   A   
   G   
   A   
   A   
    G    
   C   
   T   
   A   
   G   
   C   
   T   
   T   
   A   
   A   
   A   
   G   
   A   
   A   
   A   
   A   
   A   
   T   
   G   
   G   
    C    
   T   
   A   
   T   
   G   
   A   
   T   
   A   
   C   
   T   
   A   
   A   
   A   
   T   
   C   
    T    
   G   
   T   
   T   
   G   
   T   
   T   
   G   
   A   
   A   
   A   
   A   
   A   
   T   
   A   
   C   
   C   
   A   
   A   
   G   
   C   
   A   
   A   
   T   
   T   
   T   
   A   
   T   
   T   
   C   
   A   
   G   
   G   
   G   
   A   
   T   
   T   
   C   
   A   
   A   
   G   
   C   
    A    
   G   
   A   
   A   
    G    
   G   
    A    
   G   
   T   
   C   
   A   
   A   
    A    
   G   
   C   
   T   
   A   
   G   
   T   
   G   
   A   
   T   
   G   
   T   
    C    
   C   
   A   
   A   
   G   
   T   
    C    
   A   
   A   
   A   
   A   
   A   
    G    
   G   
    C    
   G   
   A   
   A   
   A   
   G   
   A   
    C    
   A   
   A   
    T    
   C   
   A   
   A   
   T   
   A   
   C   
   A   
   C   
   A   
   T   
   T   
   T   
   A   
    C    
   C   
   T   
   A   
   T   
   A   
   A   
   A   
   T   
   T   
   A   
   T   
   C   
   G   
   A   
   T   
   G   
   A   
   G   
    C    
   A   
   C   
    T    
   C   
   C   
   T   
   T   
   T   
   A   
   G   
   G   
   C   
   G   
   A   
   A   
   A   
   T   
   G   
   A   
   A   
   A   
   G   
   A   
   T   
    T    
   T   
   G   
   T   
   C   
   T   
   T   
   A   
   T   
   C   
   A   
   A   
   T   
   C   
   A   
   A   
   G   
   T   
   A   
   T   
    C    
   G   
    C    
   C   
   A   
   A   
    A    
   A   
   A   
   A   
   G   
   G   
    C    
   G   
   A   
   T   
   A   
    C    
   C   
   T   
   A   
   C   
   C   
   A   
   A   
   A   
   T   
    C    
    G    
   C   
   T   
   T   
   G   
   G   
    A    
    A    
    G    
   C   
   C   
    G    
   T   
   C   
   T   
   T   
   T   
   A   
   A   
   T   
   T   
   T   
   T   
   T   
   C   
   C   
   A   
   G   
   A   
   T   
   A   
   T   
   G   
   T   
   C   
   A   
   G   
   G   
   A   
   A   
   A   
   T   
   G   
   A   
   T   
   A   
   A   
   A   
    A    
   T   
   T   
   T   
   C   
   G   
   A   
   T   
   T   
   C   
   A   
   A   
   G   
   T   
   A   
   G   
   A   
   T   
   A   
   A   
   T   
   G   
   C   
   C   
   A   
   A   
   A   
   C   
   G   
   T   
   G   
   G   
   A   
   G   
   A   
   A   
   A   
   T   
   T   
   G   
   T   
   C   
   G   
   A   
   T   
   C   
   G   
   T   
   A   
   A   
    T    
   G   
   G   
   T   
   A   
   G   
   T   
   G   
   G   
   G   
   C   
   T   
   A   
   G   
    C    
   A   
   A   
   T   
   T   
   A   
   A   
   C   
   A   
   A   
   A   
   G   
   T   
   G   
   T   
   T   
   T   
   G   
   A   
    C    
   G   
   A   
   A   
   G   
   T   
   G   
   G   
   G   
   C   
   G   
   T   
   A   
   G   
   T   
    G    
   C   
   C   
   T   
    G    
   G   
   C   
   A   
   A   
   A   
   C   
   T   
    C    
   G   
   G   
   T   
   T   
   C   
   T   
   G   
   G   
   C   
   G   
   C   
   A   
   G   
   A   
   A   
   A   
   A   
   A   
    A    
   C   
   A   
   G   
   C   
    C    
   A   
   A   
   T   
   A   
   T   
   C   
   A   
   A   
   A   
   G   
   C   
   T   
   T   
   T   
   T   
   A   
   G   
   T   
   G   
   A   
   T   
   A   
   A   
    A    
   T   
   T   
    C    
   G   
   G   
   C   
   G   
   T   
   T   
   T   
   C   
   T   
    G    
   T   
   T   
   G   
   A   
   T   
   G   
   A   
   A   
   A   
   T   
   C   
   A   
   A   
   T   
   C   
    A    
   A   
   A   
   A   
    A    
   T   
   T   
   A   
   A   
   G   
   C   
   C   
   A   
   A   
   G   
   G   
    A    
   T   
   G   
   G   
   G   
   T   
   C   
   C   
   A   
   A   
   G   
   C   
   A   
   G   
   A   
   C   
   T   
   C   
    C    
   T   
   T   
   T   
   G   
   T   
   A   
   C   
   C   
   A   
   A   
   T   
   C   
   A   
   C   
   A   
   G   
   T   
    C    
   G   
   C   
   T   
   T   
   C   
   T   
   G   
   A   
   A   
   C   
   C   
   A   
   G   
   T   
   G   
   A   
   C   
   A   
   G   
   A   
   A   
   T   
   T   
   A   
   C   
   C   
   A   
   A   
   C   
   A   
   G   
   G   
   G   
   G   
   C   
   T   
   G   
   C   
   G   
   A   
   C   
   A   
   A   
   A   
   A   
   G   
    A    
    T    
   A   
   C   
   A   
   G   
   A   
   G   
   T   
   C   
   A   
   C   
   G   
   T   
   T   
   A   
   T   
   T   
   A   
   T   
   C   
   C   
    G    
    C    
   T   
   G   
   G   
   G   
   G   
   G   
   A   
   A   
   G   
   C   
   A   
   G   
   C   
   T   
   G   
   C   
   G   
   C   
   A   
   A   
   T   
   T   
   A   
   A   
   T   
   C   
   G   
   G   
    G    
   T   
   A   
   T   
   A   
   C   
    G    
   G   
   G   
    C    
   A   
   C   
    C    
   A   
   T   
   T   
   A   
   C   
   T   
   G   
   C   
   A   
   G   
   A   
    A    
   G   
   A   
   T   
   A   
   T   
   T   
    G    
   A   
   A   
   A   
   A   
   A   
   A   
   A   
   T   
   C   
   C   
   A   
   G   
   A   
   G   
   T   
   T   
   A   
   A   
   G   
   T   
   A   
   G   
   T   
   A   
   C   
   G   
   G   
   G   
    C    
   G   
   T   
   A   
   A   
   T   
   T   
   G   
   G   
    T    
   A   
   A   
   A   
   A   
   C   
   T   
   G   
   G   
    C    
   T   
   T   
   A   
   G   
   A   
   A   
   C   
   G   
   A   
   G   
   C   
   G   
   T   
   T   
   T   
   G   
   A   
   T   
   A   
   A   
   A   
   G   
   A   
    A    
   T   
   T   
   A   
   A   
   G   
   A   
   G   
   G   
    G    
   C   
   A   
   A   
   G   
   A   
   T   
   G   
   G   
    T    
   G   
   G   
    C    
   T   
   C   
    A    
   C   
   T   
   A   
   G   
   T   
   T   
   A   
   T   
   T   
   T   
   T   
   A   
   G   
   A   
   T   
   G   
   A   
   C   
   A   
   A   
   A   
   G   
   A   
   G   
   A   
   A   
   T   
   G   
   T   
   C   
   A   
   A   
   A   
   A   
   A   
   A   
   G   
   C   
   T   
   T   
   T   
   A   
   C   
   A   
   A   
   A   
   C   
   C   
   A   
   A   
   A   
   G   
   A   
    G    
   A   
   A   
   A   
   A   
   A   
   A   
   G   
   A   
   T   
   G   
   G   
   T   
   C   
   A   
   A   
   A   
   C   
    G    
   A   
   T   
   T   
   A   
   A   
   A   
   T   
   T   
   A   
   A   
   C   
   G   
   A   
   T   
   T   
   G   
   A   
    C    
   A   
   G   
    T    
   G   
   G   
    C    
   G   
   T   
   A   
   C   
   A   
   A   
   C   
   A   
   A   
   C   
   A   
   A   
   G   
   C   
    C    
   T   
   T   
    T    
   G   
   C   
   T   
   A   
   T   
   A   
   T   
   T   
   T   
   G   
   A   
   C   
   A   
   A   
   A   
   C   
   G   
    C    
    C    
   C   
   T   
   G   
   G   
   T   
   A   
   G   
   T   
   G   
   C   
   G   
   G   
   T   
   C   
   A   
   T   
   T   
   A   
   C   
   C   
   G   
   A   
   T   
   C   
   C   
   T   
   C   
   A   
   A   
   A   
   A   
   A   
   G   
   G   
   C   
   G   
   A   
   T   
   T   
   T   
   A   
    T    
   T   
   A   
   G   
   C   
    A    
   A   
   C   
   G   
   G   
   T   
   T   
   A   
   G   
   T   
   T   
   C   
   A   
   C   
   C   
   T   
   T   
   C   
   G   
   T   
   A   
   T   
   G   
   A   
   T   
   C   
   C   
   T   
   A   
   A   
   T   
   A   
   A   
   A   
   A   
   T   
   G   
   G   
   C   
   C   
   A   
   A   
   T   
   G   
   G   
    C    
   A   
   T   
   T   
   T   
   C   
   T   
   C   
   A   
   A   
   A   
   A   
   A   
   G   
   A   
    A    
   T   
   A   
   T   
   G   
   A   
   T   
   G   
   C   
   T   
   T   
   A   
   C   
   A   
   A   
   T   
   A   
   A   
   C   
   A   
   A   
   C   
   A   
   A   
   A   
   G   
   A   
   T   
   T   
   T   
   A   
   C   
   C   
   A   
   T   
   T   
    C    
   A   
   C   
   A   
   G   
   C   
   A   
   C   
   G   
   T   
   T   
   T   
    T    
   G   
   C   
   G   
   A   
   C   
   A   
   G   
   G   
    C    
   T   
   A   
   T   
   G   
   C   
   A   
   C   
   C   
   A   
   G   
   G   
    C    
   T   
   C   
   T   
   A   
   C   
   C   
   T   
   T   
   T   
   A   
   A   
   A   
   A   
   C   
   A   
   A   
   T   
   T   
   A   
   C   
   C   
   G   
   G   
   T   
   G   
   C   
   C   
   A   
   T   
   T   
   G   
   G   
   T   
   T   
   T   
   A   
   G   
   A   
   T   
    G    
   C   
   A   
   G   
   G   
   G   
   A   
   C   
   A   
   T   
   T   
   G   
   A   
   A   
   G   
   C   
   C   
   A   
   G   
   A   
   T   
   G   
   A   
   A   
   G   
   A   
   G   
   C   
   T   
   A   
   G   
   A   
   A   
   A   
   T   
   T   
   A   
   A   
   T   
   G   
   G   
    T    
   T   
   T   
   G   
   A   
   A   
   A   
   T   
   G   
   G   
   C   
   A   
   A   
   A   
   A   
   A   
   G   
   A   
   T   
   A   
   A   
   A   
   T   
   C   
   T   
   T   
   G   
   G   
   G   
   G   
    C    
   G   
   G   
   C   
   T   
   A   
   T   
   T   
   T   
   T   
   G   
   C   
    G    
   A   
   C   
   A   
   C   
   G   
   T   
   G   
   T   
   A   
   A   
   A   
   A   
   G   
   A   
   A   
   G   
   C   
   A   
   A   
   G   
   T   
   C   
   C   
   A   
   G   
   T   
   C   
   A   
   A   
   T   
   C   
   T   
    G    
   C   
   G   
   T   
   A   
   C   
   C   
   G   
   C   
   T   
   T   
   T   
   A   
   G   
   T   
   T   
   A   
   A   
   T   
   T   
   C   
   A   
   G   
   A   
   T   
   A   
   A   
   T   
   A   
   T   
   T   
   T   
   A   
   T   
   T   
   T   
   T   
   G   
   C   
    C    
   C   
   A   
   A   
   C   
   A   
   A   
   A   
   C   
   A   
   T   
   T   
   A   
   C   
   G   
    T    
   A   
   T   
   G   
   G   
   G   
    G    
   G   
   A   
   A   
   G   
   A   
   C   
   A   
   A   
   A   
   T   
   T   
   T   
   A   
   G   
   A   
   G   
   C   
    G    
   G   
   G   
   T   
   C   
   T   
   T   
   A   
   A   
   T   
   A   
   A   
   A   
   T   
   T   
   C   
   A   
   T   
   T   
   T   
   T   
   C   
   G   
   G   
   T   
   G   
   A   
   G   
   G   
   A   
   A   
   C   
   T   
   A   
   G   
   A   
   T   
   T   
   T   
   A   
   C   
   C   
   A   
   A   
   T   
    T    
   G   
   C   
   C   
   A   
   T   
   G   
   A   
   C   
   G   
   C   
   C   
    T    
   G   
   C   
   T   
   C   
   A   
   A   
   A   
   T   
   T   
   T   
   C   
   A   
   A   
   A   
    T    
   G   
   A   
   A   
   G   
   A   
   T   
   A   
   A   
   A   
   T   
   T   
   T   
   A   
   A   
   T   
   T   
   C   
   T   
   G   
   A   
   A   
   A   
   T   
   T   
   C   
   T   
   T   
   T   
   T   
    A    
   G   
   C   
   A   
   G   
   A   
   T   
   A   
   C   
    G    
   G   
   G   
   C   
   T   
   A   
    C    
   G   
   G   
    T    
   C   
   A   
   A   
   G   
   G   
    C    
   C   
   A   
   A   
   T   
   T   
   G   
   T   
   T   
   A   
   A   
   T   
   T   
   T   
   C   
    G    
   C   
   C   
   A   
   A   
   T   
   C   
   C   
   A   
   G   
   C   
   A   
   A   
   G   
   C   
   T   
   A   
   C   
   C   
   A   
   T   
   G   
   T   
   A   
   T   
   A   
   G   
   T   
   G   
   T   
   T   
   T   
   T   
   C   
   C   
   A   
   A   
   A   
   A   
   T   
   A   
   A   
   T   
   G   
   G   
    A    
   A   
   C   
   A   
    T    
   T   
    G    
   G   
   T   
    C    
   T   
   A   
   T   
   C   
   C   
   A   
   A   
   A   
   A   
    A    
   T   
   A   
   G   
   T   
   T   
   T   
   T   
   A   
   G   
   A   
   C   
   A   
   A   
   A   
   G   
   A   
   A   
   A   
   C   
    G    
   A   
   A   
   G   
   A   
   A   
   A   
   A   
   A   
   A   
   G   
   A   
   C   
   A   
   A   
   T   
   G   
   T   
   A   
   A   
   T   
   C   
   A   
   G   
   C   
   G   
   C   
   C   
   A   
   A   
   C   
   G   
   C   
   A   
   G   
   C   
   G   
   A   
   A   
   T   
   A   
   C   
   A   
   A   
   T   
   T   
   G   
   C   
   C   
   A   
   C   
   A   
   G   
   A   
   T   
   C   
   T   
   T   
   T   
   T   
   A   
   G   
   G   
   T   
   A   
   G   
   T   
   G   
   T   
   G   
   G   
   A   
   A   
   G   
   A   
   C   
   C   
   C   
   T   
   T   
   C   
   A   
   G   
   G   
   T   
   T   
   A   
   T   
   G   
   T   
   T   
   T   
   A   
   T   
   A   
   A   
   T   
   A   
   T   
   G   
   T   
   A   
   C   
   A   
   A   
   T   
   C   
   C   
   G   
   A   
   A   
   C   
   T   
   T   
   C   
   T   
   C   
   A   
   T   
   T   
   G   
   G   
   C   
   T   
   G   
   C   
   T   
   A   
   A   
   A   
   A   
   C   
   A   
   G   
   G   
   A   
   A   
   C   
   A   
   G   
   C   
   T   
   G   
   A   
   A   
   A   
   T   
   A   
   A   
   A   
   A   
   G   
   A   
   T   
   A   
   A   
   A   
   C   
   A   
   A   
   G   
   A   
   T   
   A   
   C   
   A   
   G   
   A   
   T   
   G   
   G   
   C   
   A   
   A   
   A   
   G   
   A   
   A   
   A   
   A   
   C   
   A   
   G   
   T   
   T   
   T   
   C   
   C   
   T   
   T   
   C   
   T   
   A   
   A   
   C   
   G   
   T   
   T   
   A   
   G   
   A   
   T   
   C   
   G   
   T   
   A   
   G   
   T   
   A   
   A   
   C   
   A   
   A   
   T   
   A   
   A   
   A   
   T   
   T   
   C   
   T   
   T   
   A   
   A   
   C   
   A   
   A   
   T   
   G   
   A   
   T   
   T   
   A   
   T   
   G   
   G   
   T   
   T   
   G   
   A   
   A   
   A   
   A   
   T   
   T   
   C   
   A   
   G   
   G   
   A   
   G   
   A   
   A   
   A   
   A   
   T   
   G   
   G   
   T   
   T   
   C   
   T   
   G   
   C   
   A   
   A   
   C   
   A   
   G   
   A   
   T   
   A   
   T   
   C   
   A   
   G   
   T   
   A   
   A   
   A   
   C   
   C   
   A   
   T   
   T   
   A   
   A   
   T   
   T   
   G   
   A   
   T   
   T   
   A   
   C   
   T   
   T   
   A   
   G   
   A   
   A   
   G   
   C   
   A   
   A   
   C   
   C   
   A   
   T   
   T   
   A   
   A   
   A   
   -   
   -   
   -   
   -   
   -   
   -   
   -   
   -   
   -   
   -   
   -   
   -   
   -   
   -   
   -   
   -   
   -   
   -   
   -   
   -   
   -   
   -   
   -   
   -   
   -   
   -   
   -   
   -   
   -   
   -   
   -   
   -   
   -   
   -   
   -   
   -   
   -   
   -   
   -   
   -   
   -   
   -   
   -   
   -   
   -   
   -   
   -   
   -   
   -   
   -   
   -   
   -   
   -   
   -   
   -   
   -   
   -   
   -   
   -   
   -   
   -   
   -   
   -   
   -   
   -   
   -   
   -   
   -   
   -   
   -   
   -   
   -   
   -   
   -   
   -   
   -   
   -   
   -   
   -   
   -   
   -   
   -   
   -   
   -   
   -   
   -   
   -   
   -   
   -   
   -   
   -   
   -   
   -   
   -   
   -   
   -   
   -   
   -   
   -   
   -   
   -   
   -   
   -   
   -   
   -   
   -   
   -   
   -   
   -   
   -   
   -   
   -   
   -   
   -   
   -   
   -   
   -   
   -   
   -   
   -   
   -   
   -   
   -   
   -   
   -   
   -   
   -   
   -   
   -   
   -   
   -   
   -   
   -   
   -   
   -   
   -   
   -   
   -   
   -   
   -   
   -   
   -   
   -   
   -   
   -   
   -   
   -   
   -   
   -   
   -   
   -   
   -   
   -   
   -   
   -   
   -   
   -   
   -   
   -   
   -   
   -   
   -   
   -   
   -   
   -   
   -   
   -   
   -   
   -   
   C   
   A   
   A   
   T   
   G   
   G   
   C   
   A   
   A   
   G   
   C   
   C   
   A   
   A   
   A   
   C   
   A   
   A   
   G   
   A   
   A   
   T   
   T   
   A   
   G   
   C   
   C   
   G   
   A   
   A   
   G   
   C   
   G   
   A   
   A   
   G   
   A   
   A   
   A   
   A   
   C   
   A   
   G   
   C   
   T   
   A   
   C   
   T   
   G   
   A   
   A   
   T   
   T   
   T   
   T   
   T   
   A   
   A   
   A   
   C   
   G   
   T   
   A   
   T   
   T   
   G   
   T   
   C   
   A   
   A   
   A   
   A   
   C   
   A   
   G   
   G   
   A   
   A   
   T   
   T   
   T   
   G   
   A   
   T   
   A   
   A   
   G   
   T   
   T   
   A   
   C   
   C   
   G   
   T   
   C   
   C   
   G   
   T   
   T   
   G   
   T   
   T   
   C   
   A   
   A   
   G   
   A   
   A   
   G   
   C   
   T   
   A   
   G   
   C   
   T   
   T   
   A   
   A   
   A   
   G   
   A   
   A   
   A   
   A   
   A   
   T   
   G   
   G   
   C   
   T   
   A   
   T   
   G   
   A   
   T   
   A   
   C   
   T   
   A   
   A   
   A   
   T   
   C   
   T   
   G   
   T   
   T   
   G   
   T   
   T   
   G   
   A   
   A   
   A   
   A   
   A   
   T   
   A   
   C   
   C   
   A   
   A   
   G   
   C   
   A   
   A   
   T   
   T   
   T   
   A   
   T   
   T   
   C   
   A   
   G   
   G   
   G   
   A   
   T   
   T   
   C   
   A   
   A   
   G   
   C   
   A   
   G   
   A   
   A   
   G   
   G   
   A   
   G   
   T   
   C   
   A   
   A   
   A   
   G   
   C   
   T   
   A   
   G   
   T   
   G   
   A   
   T   
   G   
   T   
   C   
   C   
   A   
   A   
   G   
   T   
   C   
   A   
   A   
   A   
   A   
   A   
   G   
   G   
   C   
   G   
   A   
   A   
   A   
   G   
   A   
   C   
   A   
   A   
   T   
   C   
   A   
   A   
   T   
   A   
   C   
   A   
   C   
   A   
   T   
   T   
   T   
   A   
   C   
   C   
   T   
   A   
   T   
   A   
   A   
   A   
   T   
   T   
   A   
   T   
   C   
   G   
   A   
   T   
   G   
   A   
   G   
   C   
   A   
   C   
   T   
   C   
   C   
   T   
   T   
   T   
   A   
   G   
   G   
   C   
   G   
   A   
   A   
   A   
   T   
   G   
   A   
   A   
   A   
   G   
   A   
   T   
   T   
   T   
   G   
   T   
   C   
   T   
   T   
   A   
   T   
   C   
   A   
   A   
   T   
   C   
   A   
   A   
   G   
   T   
   A   
   T   
   C   
   G   
   C   
   C   
   A   
   A   
   A   
   A   
   A   
   A   
   G   
   G   
   C   
   G   
   A   
   T   
   A   
   C   
   C   
   T   
   A   
   C   
   C   
   A   
   A   
   A   
   T   
   C   
   G   
   C   
   T   
   T   
   G   
   G   
   A   
   A   
   G   
   C   
   C   
   G   
   T   
   C   
   T   
   T   
   T   
   A   
   A   
   T   
   T   
   T   
   T   
   T   
   C   
   C   
   A   
   G   
   A   
   T   
   A   
   T   
   G   
   T   
   C   
   A   
   G   
   G   
   A   
   A   
   A   
   T   
   G   
   A   
   T   
   A   
   A   
   A   
   A   
   T   
   T   
   T   
   C   
   G   
   A   
   T   
   T   
   C   
   A   
   A   
   G   
   T   
   A   
   G   
   A   
   T   
   A   
   A   
   T   
   G   
   C   
   C   
   A   
   A   
   A   
   C   
   G   
   T   
   G   
   G   
   A   
   G   
   A   
   A   
   A   
   T   
   T   
   G   
   T   
   C   
   G   
   A   
   T   
   C   
   G   
   T   
   A   
   A   
   T   
   G   
   G   
   T   
   A   
   G   
   T   
   G   
   G   
   G   
   C   
   T   
   A   
   G   
   C   
   A   
   A   
   T   
   T   
   A   
   A   
   C   
   A   
   A   
   A   
   G   
   T   
   G   
   T   
   T   
   T   
   G   
   A   
   C   
   G   
   A   
   A   
   G   
   T   
   G   
   G   
   G   
   C   
   G   
   T   
   A   
   G   
   T   
   G   
   C   
   C   
   T   
   G   
   G   
   C   
   A   
   A   
   A   
   C   
   T   
   C   
   G   
   G   
   T   
   T   
   C   
   T   
   G   
   G   
   C   
   G   
   C   
   A   
   G   
   A   
   A   
   A   
   A   
   A   
   A   
   C   
   A   
   G   
   C   
   C   
   A   
   A   
   T   
   A   
   T   
   C   
   A   
   A   
   A   
   G   
   C   
   T   
   T   
   T   
   T   
   A   
   G   
   T   
   G   
   A   
   T   
   A   
   A   
   A   
   T   
   T   
   C   
   G   
   G   
   C   
   G   
   T   
   T   
   T   
   C   
   T   
   G   
   T   
   T   
   G   
   A   
   T   
   G   
   A   
   A   
   A   
   T   
   C   
   A   
   A   
   T   
   C   
   A   
   A   
   A   
   A   
   A   
   T   
   T   
   A   
   A   
   G   
   C   
   C   
   A   
   A   
   G   
   G   
   A   
   T   
   G   
   G   
   G   
   T   
   C   
   C   
   A   
   A   
   G   
   C   
   A   
   G   
   A   
   C   
   T   
   C   
   C   
   T   
   T   
   T   
   G   
   T   
   A   
   C   
   C   
   A   
   A   
   T   
   C   
   A   
   C   
   A   
   G   
   T   
   C   
   G   
   C   
   T   
   T   
   C   
   T   
   G   
   A   
   A   
   C   
   C   
   A   
   G   
   T   
   G   
   A   
   C   
   A   
   G   
   A   
   A   
   T   
   T   
   A   
   C   
   C   
   A   
   A   
   C   
   A   
   G   
   G   
   G   
   G   
   C   
   T   
   G   
   C   
   G   
   A   
   C   
   A   
   A   
   A   
   A   
   G   
   A   
   T   
   A   
   C   
   A   
   G   
   A   
   G   
   T   
   C   
   A   
   C   
   G   
   T   
   T   
   A   
   T   
   T   
   A   
   T   
   C   
   C   
   G   
   C   
   T   
   G   
   G   
   G   
   G   
   G   
   A   
   A   
   G   
   C   
   A   
   G   
   C   
   T   
   G   
   C   
   G   
   C   
   A   
   A   
   T   
   T   
   A   
   A   
   T   
   C   
   G   
   G   
   G   
   T   
   A   
   T   
   A   
   C   
   G   
   G   
   G   
   C   
   A   
   C   
   C   
   A   
   T   
   T   
   A   
   C   
   T   
   G   
   C   
   A   
   G   
   A   
   A   
   G   
   A   
   T   
   A   
   T   
   T   
   G   
   A   
   A   
   A   
   A   
   A   
   A   
   A   
   T   
   C   
   C   
   A   
   G   
   A   
   G   
   T   
   T   
   A   
   A   
   G   
   T   
   A   
   G   
   T   
   A   
   C   
   G   
   G   
   G   
   C   
   G   
   T   
   A   
   A   
   T   
   T   
   G   
   G   
   T   
   A   
   A   
   A   
   A   
   C   
   T   
   G   
   G   
   C   
   T   
   T   
   A   
   G   
   A   
   A   
   C   
   G   
   A   
   G   
   C   
   G   
   T   
   T   
   T   
   G   
   A   
   T   
   A   
   A   
   A   
   G   
   A   
   A   
   T   
   T   
   A   
   A   
   G   
   A   
   G   
   G   
   G   
   C   
   A   
   A   
   G   
   A   
   T   
   G   
   G   
   T   
   G   
   G   
   C   
   T   
   C   
   A   
   C   
   T   
   A   
   G   
   T   
   T   
   A   
   T   
   T   
   T   
   T   
   A   
   G   
   A   
   T   
   G   
   A   
   C   
   A   
   A   
   A   
   G   
   A   
   G   
   A   
   A   
   T   
   G   
   T   
   C   
   A   
   A   
   A   
   A   
   A   
   A   
   G   
   C   
   T   
   T   
   T   
   A   
   C   
   A   
   A   
   A   
   C   
   C   
   A   
   A   
   A   
   G   
   A   
   G   
   A   
   A   
   A   
   A   
   A   
   A   
   G   
   A   
   T   
   G   
   G   
   T   
   C   
   A   
   A   
   A   
   C   
   G   
   A   
   T   
   T   
   A   
   A   
   A   
   T   
   T   
   A   
   A   
   C   
   G   
   A   
   T   
   T   
   G   
   A   
   C   
   A   
   G   
   T   
   G   
   G   
   C   
   G   
   T   
   A   
   C   
   A   
   A   
   C   
   A   
   A   
   C   
   A   
   A   
   G   
   C   
   C   
   T   
   T   
   T   
   G   
   C   
   T   
   A   
   T   
   A   
   T   
   T   
   T   
   G   
   A   
   C   
   A   
   A   
   A   
   C   
   G   
   C   
   C   
   C   
   T   
   G   
   G   
   T   
   A   
   G   
   T   
   G   
   C   
   G   
   G   
   T   
   C   
   A   
   T   
   T   
   A   
   C   
   C   
   G   
   A   
   T   
   C   
   C   
   T   
   C   
   A   
   A   
   A   
   A   
   A   
   G   
   G   
   C   
   G   
   A   
   T   
   T   
   T   
   A   
   T   
   T   
   A   
   G   
   C   
   A   
   A   
   C   
   G   
   G   
   T   
   T   
   A   
   G   
   T   
   T   
   C   
   A   
   C   
   C   
   T   
   T   
   C   
   G   
   T   
   A   
   T   
   G   
   A   
   T   
   C   
   C   
   T   
   A   
   A   
   T   
   A   
   A   
   A   
   A   
   T   
   G   
   G   
   C   
   C   
   A   
   A   
   T   
   G   
   G   
   C   
   A   
   T   
   T   
   T   
   C   
   T   
   C   
   A   
   A   
   A   
   A   
   A   
   G   
   A   
   A   
   T   
   A   
   T   
   G   
   A   
   T   
   G   
   C   
   T   
   T   
   A   
   C   
   A   
   A   
   T   
   A   
   A   
   C   
   A   
   A   
   C   
   A   
   A   
   A   
   G   
   A   
   T   
   T   
   T   
   A   
   C   
   C   
   A   
   T   
   T   
   C   
   A   
   C   
   A   
   G   
   C   
   A   
   C   
   G   
   T   
   T   
   T   
   T   
   G   
   C   
   G   
   A   
   C   
   A   
   G   
   G   
   C   
   T   
   A   
   T   
   G   
   C   
   A   
   C   
   C   
   A   
   G   
   G   
   C   
   T   
   C   
   T   
   A   
   C   
   C   
   T   
   T   
   T   
   A   
   A   
   A   
   A   
   C   
   A   
   A   
   T   
   T   
   A   
   C   
   C   
   G   
   G   
   T   
   G   
   C   
   C   
   A   
   T   
   T   
   G   
   G   
   T   
   T   
   T   
   A   
   G   
   A   
   T   
   G   
   C   
   A   
   G   
   G   
   G   
   A   
   C   
   A   
   T   
   T   
   G   
   A   
   A   
   G   
   C   
   C   
   A   
   G   
   A   
   T   
   G   
   A   
   A   
   G   
   A   
   G   
   C   
   T   
   A   
   G   
   A   
   A   
   A   
   T   
   T   
   A   
   A   
   T   
   G   
   G   
   T   
   T   
   T   
   G   
   A   
   A   
   A   
   T   
   G   
   G   
   C   
   A   
   A   
   A   
   A   
   A   
   G   
   A   
   T   
   A   
   A   
   A   
   T   
   C   
   T   
   T   
   G   
   G   
   G   
   G   
   C   
   G   
   G   
   C   
   T   
   A   
   T   
   T   
   T   
   T   
   G   
   C   
   G   
   A   
   C   
   A   
   C   
   G   
   T   
   G   
   T   
   A   
   A   
   A   
   A   
   G   
   A   
   A   
   G   
   C   
   A   
   A   
   G   
   T   
   C   
   C   
   A   
   G   
   T   
   C   
   A   
   A   
   T   
   C   
   T   
   G   
   C   
   G   
   T   
   A   
   C   
   C   
   G   
   C   
   T   
   T   
   T   
   A   
   G   
   T   
   T   
   A   
   A   
   T   
   T   
   C   
   A   
   G   
   A   
   T   
   A   
   A   
   T   
   A   
   T   
   T   
   T   
   A   
   T   
   T   
   T   
   T   
   G   
   C   
   C   
   C   
   A   
   A   
   C   
   A   
   A   
   A   
   C   
   A   
   T   
   T   
   A   
   C   
   G   
   T   
   A   
   T   
   G   
   G   
   G   
   G   
   G   
   A   
   A   
   G   
   A   
   C   
   A   
   A   
   A   
   T   
   T   
   T   
   A   
   G   
   A   
   G   
   C   
   G   
   G   
   G   
   T   
   C   
   T   
   T   
   A   
   A   
   T   
   A   
   A   
   A   
   T   
   T   
   C   
   A   
   T   
   T   
   T   
   T   
   C   
   G   
   G   
   T   
   G   
   A   
   G   
   G   
   A   
   A   
   C   
   T   
   A   
   G   
   A   
   T   
   T   
   T   
   A   
   C   
   C   
   A   
   A   
   T   
   T   
   G   
   C   
   C   
   A   
   T   
   G   
   A   
   C   
   G   
   C   
   C   
   T   
   G   
   C   
   T   
   C   
   A   
   A   
   A   
   T   
   T   
   T   
   C   
   A   
   A   
   A   
   T   
   G   
   A   
   A   
   G   
   A   
   T   
   A   
   A   
   A   
   T   
   T   
   T   
   A   
   A   
   T   
   T   
   C   
   T   
   G   
   A   
   A   
   A   
   T   
   T   
   C   
   T   
   T   
   T   
   T   
   A   
   G   
   C   
   A   
   G   
   A   
   T   
   A   
   C   
   G   
   G   
   G   
   C   
   T   
   A   
   C   
   G   
   G   
   T   
   C   
   A   
   A   
   G   
   G   
   C   
   C   
   A   
   A   
   T   
   T   
   G   
   T   
   T   
   A   
   A   
   T   
   T   
   T   
   C   
   G   
   C   
   C   
   A   
   A   
   T   
   C   
   C   
   A   
   G   
   C   
   A   
   A   
   G   
   C   
   T   
   A   
   C   
   C   
   A   
   T   
   G   
   T   
   A   
   T   
   A   
   G   
   T   
   G   
   T   
   T   
   T   
   T   
   C   
   C   
   A   
   A   
   A   
   A   
   T   
   A   
   A   
   T   
   G   
   G   
   A   
   A   
   C   
   A   
   T   
   T   
   G   
   G   
   T   
   C   
   T   
   A   
   T   
   C   
   C   
   A   
   A   
   A   
   A   
   A   
   T   
   A   
   G   
   T   
   T   
   T   
   T   
   A   
   G   
   A   
   C   
   A   
   A   
   A   
   G   
   A   
   A   
   A   
   C   
   G   
   A   
   A   
   G   
   A   
   A   
   A   
   A   
   A   
   A   
   G   
   A   
   C   
   A   
   A   
   T   
   G   
   T   
   A   
   A   
   T   
   C   
   A   
   G   
   C   
   G   
   C   
   C   
   A   
   A   
   C   
   G   
   C   
   A   
   G   
   C   
   G   
   A   
   A   
   T   
   A   
   C   
   A   
   A   
   T   
   T   
   G   
   C   
   C   
   A   
   C   
   A   
   G   
   A   
   T   
   C   
   T   
   T   
   T   
   T   
   A   
   G   
   G   
   T   
   A   
   G   
   T   
   G   
   T   
   G   
   G   
   A   
   A   
   G   
   A   
   C   
   C   
   C   
   T   
   T   
   C   
   A   
   G   
   G   
   T   
   T   
   A   
   T   
   G   
   T   
   T   
   T   
   A   
   T   
   A   
   A   
   T   
   A   
   T   
   G   
   T   
   A   
   C   
   A   
   A   
   T   
   C   
   C   
   G   
   A   
   A   
   C   
   T   
   T   
   C   
   T   
   C   
   A   
   T   
   T   
   G   
   G   
   C   
   T   
   G   
   C   
   T   
   A   
   A   
   A   
   A   
   C   
   A   
   G   
   G   
   A   
   A   
   C   
   A   
   G   
   C   
   T   
   G   
   A   
   A   
   A   
   T   
   A   
   A   
   A   
   A   
   G   
   A   
   T   
   A   
   A   
   A   
   C   
   A   
   A   
   G   
   A   
   T   
   A   
   C   
   A   
   G   
   A   
   T   
   G   
   G   
   C   
   A   
   A   
   A   
   G   
   A   
   A   
   A   
   A   
   C   
   A   
   G   
   T   
   T   
   T   
   C   
   C   
   T   
   T   
   C   
   T   
   A   
   A   
   C   
   G   
   T   
   T   
   A   
   G   
   A   
   T   
   C   
   G   
   T   
   A   
   G   
   T   
   A   
   A   
   C   
   A   
   A   
   T   
   A   
   A   
   A   
   T   
   T   
   C   
   T   
   T   
   A   
   A   
   C   
   A   
   A   
   T   
   G   
   A   
   T   
   T   
   A   
   T   
   G   
   G   
   T   
   T   
   G   
   A   
   A   
   A   
   A   
   T   
   T   
   C   
   A   
   G   
   G   
   A   
   G   
   A   
   A   
   A   
   A   
   T   
   G   
   G   
   T   
   T   
   C   
   T   
   G   
   C   
   A   
   A   
   C   
   A   
   G   
   A   
   T   
   A   
   T   
   C   
   A   
   G   
   T   
   A   
   A   
   A   
   C   
   C   
   A   
   T   
   T   
   A   
   A   
   T   
   T   
   G   
   A   
   T   
   T   
   A   
   C   
   T   
   T   
   A   
   G   
   A   
   A   
   G   
   C   
   A   
   A   
   C   
   C   
   A   
   T   
   T   
   A   
   A   
   A   
   -   
   -   
   -   
   -   
   -   
   -   
   -   
   -   
   -   
   -   
   -   
   -   
   -   
   -   
   -   
   -   
   -   
   -   
   -   
   -   
   -   
   -   
   -   
   -   
   -   
   -   
   -   
   -   
   -   
   -   
   -   
   -   
   -   
   -   
   -   
   -   
   -   
   -   
   -   
   -   
   -   
   -   
   -   
   -   
   -   
   -   
   -   
   -   
   -   
   -   
   -   
   -   
   -   
   -   
   -   
   -   
   -   
   -   
   -   
   -   
   -   
 

 
   N53661   
   -   
   -   
   -   
   -   
   -   
   -   
   -   
   -   
   -   
   -   
   -   
   -   
   -   
   -   
   -   
   -   
   -   
   -   
   -   
   -   
   -   
   -   
   -   
   -   
   -   
   -   
   -   
   -   
   -   
   -   
   -   
   -   
   -   
   -   
   -   
   -   
   -   
   -   
   -   
   -   
   -   
   -   
   -   
   -   
   -   
   -   
   -   
   -   
   -   
   -   
   -   
   -   
   -   
   -   
   -   
   -   
   -   
   -   
   -   
   -   
   -   
   -   
   -   
   -   
   -   
   -   
   -   
   -   
   -   
   -   
   -   
   -   
   -   
   -   
   -   
   -   
   -   
   -   
   -   
   -   
   -   
   -   
   -   
   -   
   -   
   -   
   -   
   -   
   -   
   -   
   -   
   -   
   -   
   -   
   -   
   -   
   -   
   -   
   -   
   -   
   -   
   -   
   -   
   -   
   -   
   -   
   -   
   -   
   C   
   A   
   A   
   T   
   G   
   G   
   C   
   A   
   A   
   G   
   C   
   C   
   A   
   A   
   A   
   C   
   A   
   A   
   G   
   A   
    A    
   T   
   T   
   A   
   G   
   C   
    C    
   G   
   A   
   A   
   G   
   C   
   G   
   A   
   A   
   G   
   A   
   A   
   A   
   A   
    C    
   A   
   G   
   C   
    T    
   A   
    C    
   T   
    A    
    C    
   A   
   T   
   T   
   T   
   T   
   T   
   A   
   A   
   A   
    C    
   G   
   T   
   A   
   T   
   T   
   G   
    A    
   C   
   A   
   A   
   A   
   A   
   C   
   A   
   G   
    A    
   A   
   A   
   T   
   T   
   T   
   G   
   A   
   T   
   A   
   A   
   G   
   T   
   T   
   A   
   C   
   C   
    G    
   T   
   C   
   C   
   G   
   T   
   T   
    G    
   T   
   T   
   C   
   A   
   A   
   G   
   A   
   A   
    G    
   C   
   T   
   A   
   G   
   C   
   T   
   T   
   A   
   A   
   A   
   G   
   A   
   A   
   A   
   A   
   A   
   T   
   G   
   G   
    C    
   T   
   A   
   T   
   G   
   A   
   T   
   A   
   C   
   T   
   A   
   A   
   A   
   T   
   C   
    C    
   G   
   T   
   T   
   G   
   T   
   T   
   G   
   A   
   A   
   A   
   A   
   A   
   T   
   A   
   C   
   C   
   A   
   A   
   G   
   C   
   A   
   A   
   T   
   T   
   T   
   A   
   T   
   T   
   C   
   A   
   G   
   G   
   G   
   A   
   T   
   T   
   C   
   A   
   A   
   G   
   C   
    A    
   G   
   A   
   A   
    G    
   G   
    A    
   G   
   T   
   C   
   A   
   A   
    A    
   G   
   C   
   T   
   A   
   G   
   T   
   G   
   A   
   T   
   G   
   T   
    T    
   C   
   A   
   A   
   G   
   T   
    C    
   A   
   A   
   A   
   A   
   A   
    A    
   G   
    C    
   G   
   A   
   A   
   A   
   G   
   A   
    C    
   A   
   A   
    T    
   C   
   A   
   A   
   T   
   A   
   C   
   A   
   C   
   A   
   T   
   T   
   T   
   A   
    A    
   C   
   T   
   A   
   T   
   A   
   A   
   A   
   T   
   T   
   A   
   T   
   C   
   G   
   A   
   T   
   G   
   A   
   G   
    T    
   A   
   C   
    C    
   C   
   C   
   T   
   T   
   T   
   A   
   G   
   G   
   C   
   G   
   A   
   A   
   A   
   T   
   G   
   A   
   A   
   A   
   G   
   A   
   T   
    T    
   T   
   G   
   T   
   C   
   T   
   T   
   A   
   T   
   C   
   A   
   A   
   T   
   C   
   A   
   A   
   G   
   T   
   A   
   T   
    C    
   G   
    C    
   C   
   A   
   A   
    A    
   A   
   A   
   A   
   G   
   G   
    C    
   G   
   A   
   T   
   A   
    C    
   C   
   T   
   A   
   C   
   C   
   A   
   A   
   A   
   T   
    C    
    G    
   C   
   T   
   T   
   G   
   G   
    A    
    A    
    G    
   C   
   C   
    G    
   T   
   C   
   T   
   T   
   T   
   A   
   A   
   T   
   T   
   T   
   T   
   T   
   C   
   C   
   A   
   G   
   A   
   T   
   A   
   T   
   G   
   T   
   C   
   A   
   G   
   G   
   A   
   A   
   A   
   T   
   G   
   A   
   T   
   A   
   A   
   A   
    A    
   T   
   T   
   T   
   C   
   G   
   A   
   T   
   T   
   C   
   A   
   A   
   G   
   T   
   A   
   G   
   A   
   T   
   A   
   A   
   T   
   G   
   C   
   C   
   A   
   A   
   A   
   C   
   G   
   T   
   G   
   G   
   A   
   G   
   A   
   A   
   A   
   T   
   T   
   G   
   T   
   C   
   G   
   A   
   T   
   C   
   G   
   T   
   A   
   A   
    T    
   G   
   G   
   T   
   A   
   G   
   T   
   G   
   G   
   G   
   C   
   T   
   A   
   G   
    C    
   A   
   A   
   T   
   T   
   A   
   A   
   C   
   A   
   A   
   A   
   G   
   T   
   G   
   T   
   T   
   T   
   G   
   A   
    C    
   G   
   A   
   A   
   G   
   T   
   G   
   G   
   G   
   C   
   G   
   T   
   A   
   G   
   T   
    G    
   C   
   C   
   T   
    G    
   G   
   C   
   A   
   A   
   A   
   C   
   T   
    C    
   G   
   G   
   T   
   T   
   C   
   T   
   G   
   G   
   C   
   G   
   C   
   A   
   G   
   A   
   A   
   A   
   A   
   A   
    A    
   C   
   A   
   G   
   C   
    C    
   A   
   A   
   T   
   A   
   T   
   C   
   A   
   A   
   A   
   G   
   C   
   T   
   T   
   T   
   T   
   A   
   G   
   T   
   G   
   A   
   T   
   A   
   A   
    G    
   T   
   T   
    C    
   G   
   G   
   C   
   G   
   T   
   T   
   T   
   C   
   T   
    G    
   T   
   T   
   G   
   A   
   T   
   G   
   A   
   A   
   A   
   T   
   C   
   A   
   A   
   T   
   C   
    A    
   A   
   A   
   A   
    G    
   T   
   T   
   A   
   A   
   G   
   C   
   C   
   A   
   A   
   G   
   G   
    G    
   T   
   G   
   G   
   G   
   T   
   C   
   C   
   A   
   A   
   G   
   C   
   A   
   G   
   A   
   C   
   T   
   C   
    C    
   T   
   T   
   T   
   G   
   T   
   A   
   C   
   C   
   A   
   A   
   T   
   C   
   A   
   C   
   A   
   G   
   T   
    C    
   G   
   C   
   T   
   T   
   C   
   T   
   G   
   A   
   A   
   C   
   C   
   A   
   G   
   T   
   G   
   A   
   C   
   A   
   G   
   A   
   A   
   T   
   T   
   A   
   C   
   C   
   A   
   A   
   C   
   A   
   G   
   G   
   G   
   G   
   C   
   T   
   G   
   C   
   G   
   A   
   C   
   A   
   A   
   A   
   A   
   G   
    A    
    T    
   A   
   C   
   A   
   G   
   A   
   G   
   T   
   C   
   A   
   C   
   G   
   T   
   T   
   A   
   T   
   T   
   A   
   T   
   C   
   C   
    G    
    C    
   T   
   G   
   G   
   G   
   G   
   G   
   A   
   A   
   G   
   C   
   A   
   G   
   C   
   T   
   G   
   C   
   G   
   C   
   A   
   A   
   T   
   T   
   A   
   A   
   T   
   C   
   G   
   G   
    G    
   T   
   A   
   T   
   A   
   C   
    A    
   G   
   G   
    C    
   A   
   C   
    C    
   A   
   T   
   T   
   A   
   C   
   T   
   G   
   C   
   A   
   G   
   A   
    A    
   G   
   A   
   T   
   A   
   T   
   T   
    A    
   A   
   A   
   A   
   A   
   A   
   A   
   A   
   T   
   C   
   C   
   A   
   G   
   A   
   G   
   T   
   T   
   A   
   A   
   G   
   T   
   A   
   G   
   T   
   A   
   C   
   G   
   G   
   G   
    C    
   G   
   T   
   A   
   A   
   T   
   T   
   G   
   G   
    T    
   A   
   A   
   A   
   A   
   C   
   T   
   G   
   G   
    C    
   T   
   T   
   A   
   G   
   A   
   A   
   C   
   G   
   A   
   G   
   C   
   G   
   T   
   T   
   T   
   G   
   A   
   T   
   A   
   A   
   A   
   G   
   A   
    A    
   T   
   T   
   A   
   A   
   G   
   A   
   G   
   G   
    G    
   C   
   A   
   A   
   G   
   A   
   T   
   G   
   G   
    T    
   G   
   G   
    C    
   T   
   C   
    A    
   C   
   T   
   A   
   G   
   T   
   T   
   A   
   T   
   T   
   T   
   T   
   A   
   G   
   A   
   T   
   G   
   A   
   C   
   A   
   A   
   A   
   G   
   A   
   G   
   A   
   A   
   T   
   G   
   T   
   C   
   A   
   A   
   A   
   A   
   A   
   A   
   G   
   C   
   T   
   T   
   T   
   A   
   C   
   A   
   A   
   A   
   C   
   C   
   A   
   A   
   A   
   G   
   A   
    G    
   A   
   A   
   A   
   A   
   A   
   A   
   G   
   A   
   T   
   G   
   G   
   T   
   C   
   A   
   A   
   A   
   C   
    G    
   A   
   T   
   T   
   A   
   A   
   A   
   T   
   T   
   A   
   A   
   C   
   G   
   A   
   T   
   T   
   G   
   A   
    C    
   A   
   G   
    T    
   G   
   G   
    C    
   G   
   T   
   A   
   C   
   A   
   A   
   C   
   A   
   A   
   C   
   A   
   A   
   G   
   C   
    C    
   T   
   T   
    C    
   G   
   C   
   T   
   A   
   T   
   A   
   T   
   T   
   T   
   G   
   A   
   C   
   A   
   A   
   A   
   C   
   G   
    C    
    C    
   C   
   T   
   G   
   G   
   T   
   A   
   G   
   T   
   G   
   C   
   G   
   G   
   T   
   C   
   A   
   T   
   T   
   A   
   C   
   C   
   G   
   A   
   T   
   C   
   C   
   T   
   C   
   A   
   A   
   A   
   A   
   A   
   G   
   G   
   C   
   G   
   A   
   T   
   T   
   T   
   A   
    T    
   T   
   A   
   G   
   C   
    A    
   A   
   C   
   G   
   G   
   T   
   T   
   A   
   G   
   T   
   T   
   C   
   A   
   C   
   C   
   T   
   T   
   C   
   G   
   T   
   A   
   T   
   G   
   A   
   T   
   C   
   C   
   T   
   A   
   A   
   T   
   A   
   A   
   A   
   A   
   T   
   G   
   G   
   C   
   C   
   A   
   A   
   T   
   G   
   G   
    T    
   A   
   T   
   T   
   T   
   C   
   T   
   C   
   A   
   A   
   A   
   A   
   A   
   G   
   A   
    A    
   T   
   A   
   T   
   G   
   A   
   T   
   G   
   C   
   T   
   T   
   A   
   C   
   A   
   A   
   T   
   A   
   A   
   C   
   A   
   A   
   C   
   A   
   A   
   A   
   G   
   A   
   T   
   T   
   T   
   A   
   C   
   C   
   A   
   T   
   T   
    C    
   A   
   C   
   A   
   G   
   C   
   A   
   C   
   G   
   T   
   T   
   T   
    T    
   G   
   C   
   G   
   A   
   C   
   A   
   G   
   G   
    C    
   T   
   A   
   T   
   G   
   C   
   A   
   C   
   C   
   A   
   G   
   G   
    T    
   T   
   C   
   T   
   A   
   C   
   C   
   T   
   T   
   T   
   A   
   A   
   A   
   A   
   C   
   A   
   A   
   T   
   T   
   A   
   C   
   C   
   G   
   G   
   T   
   G   
   C   
   C   
   A   
   T   
   T   
   G   
   G   
   T   
   T   
   T   
   A   
   G   
   A   
   T   
    G    
   C   
   A   
   G   
   G   
   G   
   A   
   C   
   A   
   T   
   T   
   G   
   A   
   A   
   G   
   C   
   C   
   A   
   G   
   A   
   T   
   G   
   A   
   A   
   G   
   A   
   G   
   C   
   T   
   A   
   G   
   A   
   A   
   A   
   T   
   T   
   A   
   A   
   T   
   G   
   G   
    C    
   T   
   T   
   G   
   A   
   A   
   A   
   T   
   G   
   G   
   C   
   A   
   A   
   A   
   A   
   A   
   G   
   A   
   T   
   A   
   A   
   A   
   T   
   C   
   T   
   T   
   G   
   G   
   G   
   G   
    C    
   G   
   G   
   C   
   T   
   A   
   T   
   T   
   T   
   T   
   G   
   C   
    A    
   A   
   C   
   A   
   C   
   G   
   T   
   G   
   T   
   A   
   A   
   A   
   A   
   G   
   A   
   A   
   G   
   C   
   A   
   A   
   G   
   T   
   C   
   C   
   A   
   G   
   T   
   C   
   A   
   A   
   T   
   C   
   T   
    G    
   C   
   G   
   T   
   A   
   C   
   C   
   G   
   C   
   T   
   T   
   T   
   A   
   G   
   T   
   T   
   A   
   A   
   T   
   T   
   C   
   A   
   G   
   A   
   T   
   A   
   A   
   T   
   A   
   T   
   T   
   T   
   A   
   T   
   T   
   T   
   T   
   G   
   C   
    C    
   C   
   A   
   A   
   C   
   A   
   A   
   A   
   C   
   A   
   T   
   T   
   A   
   C   
   G   
    G    
   A   
   T   
   G   
   G   
   G   
    A    
   G   
   A   
   A   
   G   
   A   
   C   
   A   
   A   
   A   
   T   
   T   
   T   
   A   
   G   
   A   
   G   
   C   
    G    
   G   
   G   
   T   
   C   
   T   
   T   
   A   
   A   
   T   
   A   
   A   
   A   
   T   
   T   
   C   
   A   
   T   
   T   
   T   
   T   
   C   
   G   
   G   
   T   
   G   
   A   
   G   
   G   
   A   
   A   
   C   
   T   
   A   
   G   
   A   
   T   
   T   
   T   
   A   
   C   
   C   
   A   
   A   
   T   
    T    
   G   
   C   
   C   
   A   
   T   
   G   
   A   
   C   
   G   
   C   
   C   
    T    
   G   
   C   
   T   
   C   
   A   
   A   
   A   
   T   
   T   
   T   
   C   
   A   
   A   
   A   
    T    
   G   
   A   
   A   
   G   
   A   
   T   
   A   
   A   
   A   
   T   
   T   
   T   
   A   
   A   
   T   
   T   
   C   
   T   
   G   
   A   
   A   
   A   
   T   
   T   
   C   
   T   
   T   
   T   
   T   
    G    
   G   
   C   
   A   
   G   
   A   
   T   
   A   
   C   
    G    
   G   
   G   
   C   
   T   
   A   
    C    
   G   
   G   
    C    
   C   
   A   
   A   
   G   
   G   
    C    
   C   
   A   
   A   
   T   
   T   
   G   
   T   
   T   
   A   
   A   
   T   
   T   
   T   
   C   
    G    
   C   
   C   
   A   
   A   
   T   
   C   
   C   
   A   
   G   
   C   
   A   
   A   
   G   
   C   
   T   
   A   
   C   
   C   
   A   
   T   
   G   
   T   
   A   
   T   
   A   
   G   
   T   
   G   
   T   
   T   
   T   
   T   
   C   
   C   
   A   
   A   
   A   
   A   
   T   
   A   
   A   
   T   
   G   
   G   
    G    
   A   
   C   
   A   
    T    
   T   
    A    
   G   
   T   
    C    
   T   
   A   
   T   
   C   
   C   
   A   
   A   
   A   
   A   
    T    
   T   
   A   
   G   
   T   
   T   
   T   
   T   
   A   
   G   
   A   
   C   
   A   
   A   
   A   
   G   
   A   
   A   
   A   
   C   
    G    
   A   
   A   
   G   
   A   
   A   
   A   
   A   
   A   
   A   
   G   
   A   
   C   
   A   
   A   
   T   
   G   
   T   
   A   
   A   
   T   
   C   
   A   
   G   
   C   
   G   
   C   
   C   
   A   
   A   
   C   
   G   
   C   
   A   
   G   
   C   
   G   
   A   
   A   
   T   
   A   
   C   
   A   
   A   
   T   
   T   
   G   
   C   
   C   
   A   
   C   
   A   
   G   
   A   
   T   
   C   
   T   
   T   
   T   
   T   
   A   
   G   
   G   
   T   
   A   
   G   
   T   
   G   
   T   
   G   
   G   
   A   
   A   
   G   
   A   
   T   
   C   
   C   
   T   
   T   
   C   
   A   
   G   
   G   
   T   
   T   
   A   
   T   
   G   
   T   
   T   
   T   
   A   
   T   
   A   
   A   
   T   
   A   
   T   
   G   
   T   
   A   
   C   
   A   
   A   
   T   
   C   
   C   
   G   
   A   
   A   
   C   
   T   
   T   
   C   
   T   
   C   
   A   
   T   
   T   
   G   
   G   
   C   
   T   
   G   
   C   
   C   
   A   
   A   
   A   
   A   
   C   
   A   
   G   
   G   
   A   
   A   
   C   
   A   
   G   
   C   
   T   
   G   
   A   
   A   
   A   
   T   
   A   
   A   
   A   
   A   
   G   
   A   
   T   
   A   
   A   
   A   
   C   
   A   
   A   
   G   
   A   
   T   
   A   
   C   
   A   
   G   
   A   
   T   
   G   
   G   
   C   
   A   
   A   
   A   
   G   
   A   
   A   
   A   
   A   
   C   
   A   
   G   
   T   
   T   
   T   
   C   
   C   
   T   
   T   
   C   
   T   
   A   
   A   
   C   
   G   
   T   
   T   
   A   
   G   
   A   
   T   
   C   
   G   
   T   
   A   
   G   
   T   
   A   
   A   
   C   
   A   
   A   
   T   
   A   
   A   
   A   
   T   
   T   
   C   
   T   
   T   
   A   
   A   
   C   
   A   
   A   
   T   
   G   
   A   
   T   
   T   
   A   
   T   
   G   
   G   
   T   
   T   
   G   
   A   
   A   
   A   
   A   
   T   
   T   
   C   
   A   
   G   
   G   
   A   
   G   
   A   
   A   
   A   
   A   
   T   
   G   
   G   
   T   
   T   
   C   
   T   
   G   
   C   
   A   
   A   
   C   
   A   
   G   
   A   
   T   
   A   
   T   
   C   
   A   
   G   
   T   
   A   
   A   
   A   
   C   
   C   
   A   
   T   
   T   
   A   
   A   
   T   
   T   
   G   
   A   
   T   
   T   
   A   
   C   
   T   
   T   
   A   
   G   
   A   
   A   
   G   
   C   
   A   
   A   
   C   
   C   
   A   
   T   
   T   
   A   
   A   
   A   
   -   
   -   
   -   
   -   
   -   
   -   
   -   
   -   
   -   
   -   
   -   
   -   
   -   
   -   
   -   
   -   
   -   
   -   
   -   
   -   
   -   
   -   
   -   
   -   
   -   
   -   
   -   
   -   
   -   
   -   
   -   
   -   
   -   
   -   
   -   
   -   
   -   
   -   
   -   
   -   
   -   
   -   
   -   
   -   
   -   
   -   
   -   
   -   
   -   
   -   
   -   
   -   
   -   
   -   
   -   
   -   
   -   
   -   
   -   
   -   
   -   
   -   
   -   
   -   
   -   
   -   
   -   
   -   
   -   
   -   
   -   
   -   
   -   
   -   
   -   
   -   
   -   
   -   
   -   
   -   
   -   
   -   
   -   
   -   
   -   
   -   
   -   
   -   
   -   
   -   
   -   
   -   
   -   
   -   
   -   
   -   
   -   
   -   
   -   
   -   
   -   
   -   
   -   
   -   
   -   
   -   
   -   
   -   
   -   
   -   
   -   
   -   
   -   
   -   
   -   
   -   
   -   
   -   
   -   
   -   
   -   
   -   
   -   
   -   
   -   
   -   
   -   
   -   
   -   
   -   
   -   
   -   
   -   
   -   
   -   
   -   
   -   
   -   
   -   
   -   
   -   
   -   
   -   
   -   
   -   
   -   
   -   
   -   
   -   
   -   
   -   
   -   
   -   
   -   
   -   
   -   
   -   
   -   
   -   
   -   
   -   
   -   
   -   
   -   
   -   
   -   
   -   
   -   
   -   
   C   
   A   
   A   
   T   
   G   
   G   
   C   
   A   
   A   
   G   
   C   
   C   
   A   
   A   
   A   
   C   
   A   
   A   
   G   
   A   
   A   
   T   
   T   
   A   
   G   
   C   
   C   
   G   
   A   
   A   
   G   
   C   
   G   
   A   
   A   
   G   
   A   
   A   
   A   
   A   
   C   
   A   
   G   
   C   
   T   
   A   
   C   
   T   
   A   
   C   
   A   
   T   
   T   
   T   
   T   
   T   
   A   
   A   
   A   
   C   
   G   
   T   
   A   
   T   
   T   
   G   
   A   
   C   
   A   
   A   
   A   
   A   
   C   
   A   
   G   
   A   
   A   
   A   
   T   
   T   
   T   
   G   
   A   
   T   
   A   
   A   
   G   
   T   
   T   
   A   
   C   
   C   
   G   
   T   
   C   
   C   
   G   
   T   
   T   
   G   
   T   
   T   
   C   
   A   
   A   
   G   
   A   
   A   
   G   
   C   
   T   
   A   
   G   
   C   
   T   
   T   
   A   
   A   
   A   
   G   
   A   
   A   
   A   
   A   
   A   
   T   
   G   
   G   
   C   
   T   
   A   
   T   
   G   
   A   
   T   
   A   
   C   
   T   
   A   
   A   
   A   
   T   
   C   
   C   
   G   
   T   
   T   
   G   
   T   
   T   
   G   
   A   
   A   
   A   
   A   
   A   
   T   
   A   
   C   
   C   
   A   
   A   
   G   
   C   
   A   
   A   
   T   
   T   
   T   
   A   
   T   
   T   
   C   
   A   
   G   
   G   
   G   
   A   
   T   
   T   
   C   
   A   
   A   
   G   
   C   
   A   
   G   
   A   
   A   
   G   
   G   
   A   
   G   
   T   
   C   
   A   
   A   
   A   
   G   
   C   
   T   
   A   
   G   
   T   
   G   
   A   
   T   
   G   
   T   
   T   
   C   
   A   
   A   
   G   
   T   
   C   
   A   
   A   
   A   
   A   
   A   
   A   
   G   
   C   
   G   
   A   
   A   
   A   
   G   
   A   
   C   
   A   
   A   
   T   
   C   
   A   
   A   
   T   
   A   
   C   
   A   
   C   
   A   
   T   
   T   
   T   
   A   
   A   
   C   
   T   
   A   
   T   
   A   
   A   
   A   
   T   
   T   
   A   
   T   
   C   
   G   
   A   
   T   
   G   
   A   
   G   
   T   
   A   
   C   
   C   
   C   
   C   
   T   
   T   
   T   
   A   
   G   
   G   
   C   
   G   
   A   
   A   
   A   
   T   
   G   
   A   
   A   
   A   
   G   
   A   
   T   
   T   
   T   
   G   
   T   
   C   
   T   
   T   
   A   
   T   
   C   
   A   
   A   
   T   
   C   
   A   
   A   
   G   
   T   
   A   
   T   
   C   
   G   
   C   
   C   
   A   
   A   
   A   
   A   
   A   
   A   
   G   
   G   
   C   
   G   
   A   
   T   
   A   
   C   
   C   
   T   
   A   
   C   
   C   
   A   
   A   
   A   
   T   
   C   
   G   
   C   
   T   
   T   
   G   
   G   
   A   
   A   
   G   
   C   
   C   
   G   
   T   
   C   
   T   
   T   
   T   
   A   
   A   
   T   
   T   
   T   
   T   
   T   
   C   
   C   
   A   
   G   
   A   
   T   
   A   
   T   
   G   
   T   
   C   
   A   
   G   
   G   
   A   
   A   
   A   
   T   
   G   
   A   
   T   
   A   
   A   
   A   
   A   
   T   
   T   
   T   
   C   
   G   
   A   
   T   
   T   
   C   
   A   
   A   
   G   
   T   
   A   
   G   
   A   
   T   
   A   
   A   
   T   
   G   
   C   
   C   
   A   
   A   
   A   
   C   
   G   
   T   
   G   
   G   
   A   
   G   
   A   
   A   
   A   
   T   
   T   
   G   
   T   
   C   
   G   
   A   
   T   
   C   
   G   
   T   
   A   
   A   
   T   
   G   
   G   
   T   
   A   
   G   
   T   
   G   
   G   
   G   
   C   
   T   
   A   
   G   
   C   
   A   
   A   
   T   
   T   
   A   
   A   
   C   
   A   
   A   
   A   
   G   
   T   
   G   
   T   
   T   
   T   
   G   
   A   
   C   
   G   
   A   
   A   
   G   
   T   
   G   
   G   
   G   
   C   
   G   
   T   
   A   
   G   
   T   
   G   
   C   
   C   
   T   
   G   
   G   
   C   
   A   
   A   
   A   
   C   
   T   
   C   
   G   
   G   
   T   
   T   
   C   
   T   
   G   
   G   
   C   
   G   
   C   
   A   
   G   
   A   
   A   
   A   
   A   
   A   
   A   
   C   
   A   
   G   
   C   
   C   
   A   
   A   
   T   
   A   
   T   
   C   
   A   
   A   
   A   
   G   
   C   
   T   
   T   
   T   
   T   
   A   
   G   
   T   
   G   
   A   
   T   
   A   
   A   
   G   
   T   
   T   
   C   
   G   
   G   
   C   
   G   
   T   
   T   
   T   
   C   
   T   
   G   
   T   
   T   
   G   
   A   
   T   
   G   
   A   
   A   
   A   
   T   
   C   
   A   
   A   
   T   
   C   
   A   
   A   
   A   
   A   
   G   
   T   
   T   
   A   
   A   
   G   
   C   
   C   
   A   
   A   
   G   
   G   
   G   
   T   
   G   
   G   
   G   
   T   
   C   
   C   
   A   
   A   
   G   
   C   
   A   
   G   
   A   
   C   
   T   
   C   
   C   
   T   
   T   
   T   
   G   
   T   
   A   
   C   
   C   
   A   
   A   
   T   
   C   
   A   
   C   
   A   
   G   
   T   
   C   
   G   
   C   
   T   
   T   
   C   
   T   
   G   
   A   
   A   
   C   
   C   
   A   
   G   
   T   
   G   
   A   
   C   
   A   
   G   
   A   
   A   
   T   
   T   
   A   
   C   
   C   
   A   
   A   
   C   
   A   
   G   
   G   
   G   
   G   
   C   
   T   
   G   
   C   
   G   
   A   
   C   
   A   
   A   
   A   
   A   
   G   
   A   
   T   
   A   
   C   
   A   
   G   
   A   
   G   
   T   
   C   
   A   
   C   
   G   
   T   
   T   
   A   
   T   
   T   
   A   
   T   
   C   
   C   
   G   
   C   
   T   
   G   
   G   
   G   
   G   
   G   
   A   
   A   
   G   
   C   
   A   
   G   
   C   
   T   
   G   
   C   
   G   
   C   
   A   
   A   
   T   
   T   
   A   
   A   
   T   
   C   
   G   
   G   
   G   
   T   
   A   
   T   
   A   
   C   
   A   
   G   
   G   
   C   
   A   
   C   
   C   
   A   
   T   
   T   
   A   
   C   
   T   
   G   
   C   
   A   
   G   
   A   
   A   
   G   
   A   
   T   
   A   
   T   
   T   
   A   
   A   
   A   
   A   
   A   
   A   
   A   
   A   
   T   
   C   
   C   
   A   
   G   
   A   
   G   
   T   
   T   
   A   
   A   
   G   
   T   
   A   
   G   
   T   
   A   
   C   
   G   
   G   
   G   
   C   
   G   
   T   
   A   
   A   
   T   
   T   
   G   
   G   
   T   
   A   
   A   
   A   
   A   
   C   
   T   
   G   
   G   
   C   
   T   
   T   
   A   
   G   
   A   
   A   
   C   
   G   
   A   
   G   
   C   
   G   
   T   
   T   
   T   
   G   
   A   
   T   
   A   
   A   
   A   
   G   
   A   
   A   
   T   
   T   
   A   
   A   
   G   
   A   
   G   
   G   
   G   
   C   
   A   
   A   
   G   
   A   
   T   
   G   
   G   
   T   
   G   
   G   
   C   
   T   
   C   
   A   
   C   
   T   
   A   
   G   
   T   
   T   
   A   
   T   
   T   
   T   
   T   
   A   
   G   
   A   
   T   
   G   
   A   
   C   
   A   
   A   
   A   
   G   
   A   
   G   
   A   
   A   
   T   
   G   
   T   
   C   
   A   
   A   
   A   
   A   
   A   
   A   
   G   
   C   
   T   
   T   
   T   
   A   
   C   
   A   
   A   
   A   
   C   
   C   
   A   
   A   
   A   
   G   
   A   
   G   
   A   
   A   
   A   
   A   
   A   
   A   
   G   
   A   
   T   
   G   
   G   
   T   
   C   
   A   
   A   
   A   
   C   
   G   
   A   
   T   
   T   
   A   
   A   
   A   
   T   
   T   
   A   
   A   
   C   
   G   
   A   
   T   
   T   
   G   
   A   
   C   
   A   
   G   
   T   
   G   
   G   
   C   
   G   
   T   
   A   
   C   
   A   
   A   
   C   
   A   
   A   
   C   
   A   
   A   
   G   
   C   
   C   
   T   
   T   
   C   
   G   
   C   
   T   
   A   
   T   
   A   
   T   
   T   
   T   
   G   
   A   
   C   
   A   
   A   
   A   
   C   
   G   
   C   
   C   
   C   
   T   
   G   
   G   
   T   
   A   
   G   
   T   
   G   
   C   
   G   
   G   
   T   
   C   
   A   
   T   
   T   
   A   
   C   
   C   
   G   
   A   
   T   
   C   
   C   
   T   
   C   
   A   
   A   
   A   
   A   
   A   
   G   
   G   
   C   
   G   
   A   
   T   
   T   
   T   
   A   
   T   
   T   
   A   
   G   
   C   
   A   
   A   
   C   
   G   
   G   
   T   
   T   
   A   
   G   
   T   
   T   
   C   
   A   
   C   
   C   
   T   
   T   
   C   
   G   
   T   
   A   
   T   
   G   
   A   
   T   
   C   
   C   
   T   
   A   
   A   
   T   
   A   
   A   
   A   
   A   
   T   
   G   
   G   
   C   
   C   
   A   
   A   
   T   
   G   
   G   
   T   
   A   
   T   
   T   
   T   
   C   
   T   
   C   
   A   
   A   
   A   
   A   
   A   
   G   
   A   
   A   
   T   
   A   
   T   
   G   
   A   
   T   
   G   
   C   
   T   
   T   
   A   
   C   
   A   
   A   
   T   
   A   
   A   
   C   
   A   
   A   
   C   
   A   
   A   
   A   
   G   
   A   
   T   
   T   
   T   
   A   
   C   
   C   
   A   
   T   
   T   
   C   
   A   
   C   
   A   
   G   
   C   
   A   
   C   
   G   
   T   
   T   
   T   
   T   
   G   
   C   
   G   
   A   
   C   
   A   
   G   
   G   
   C   
   T   
   A   
   T   
   G   
   C   
   A   
   C   
   C   
   A   
   G   
   G   
   T   
   T   
   C   
   T   
   A   
   C   
   C   
   T   
   T   
   T   
   A   
   A   
   A   
   A   
   C   
   A   
   A   
   T   
   T   
   A   
   C   
   C   
   G   
   G   
   T   
   G   
   C   
   C   
   A   
   T   
   T   
   G   
   G   
   T   
   T   
   T   
   A   
   G   
   A   
   T   
   G   
   C   
   A   
   G   
   G   
   G   
   A   
   C   
   A   
   T   
   T   
   G   
   A   
   A   
   G   
   C   
   C   
   A   
   G   
   A   
   T   
   G   
   A   
   A   
   G   
   A   
   G   
   C   
   T   
   A   
   G   
   A   
   A   
   A   
   T   
   T   
   A   
   A   
   T   
   G   
   G   
   C   
   T   
   T   
   G   
   A   
   A   
   A   
   T   
   G   
   G   
   C   
   A   
   A   
   A   
   A   
   A   
   G   
   A   
   T   
   A   
   A   
   A   
   T   
   C   
   T   
   T   
   G   
   G   
   G   
   G   
   C   
   G   
   G   
   C   
   T   
   A   
   T   
   T   
   T   
   T   
   G   
   C   
   A   
   A   
   C   
   A   
   C   
   G   
   T   
   G   
   T   
   A   
   A   
   A   
   A   
   G   
   A   
   A   
   G   
   C   
   A   
   A   
   G   
   T   
   C   
   C   
   A   
   G   
   T   
   C   
   A   
   A   
   T   
   C   
   T   
   G   
   C   
   G   
   T   
   A   
   C   
   C   
   G   
   C   
   T   
   T   
   T   
   A   
   G   
   T   
   T   
   A   
   A   
   T   
   T   
   C   
   A   
   G   
   A   
   T   
   A   
   A   
   T   
   A   
   T   
   T   
   T   
   A   
   T   
   T   
   T   
   T   
   G   
   C   
   C   
   C   
   A   
   A   
   C   
   A   
   A   
   A   
   C   
   A   
   T   
   T   
   A   
   C   
   G   
   G   
   A   
   T   
   G   
   G   
   G   
   A   
   G   
   A   
   A   
   G   
   A   
   C   
   A   
   A   
   A   
   T   
   T   
   T   
   A   
   G   
   A   
   G   
   C   
   G   
   G   
   G   
   T   
   C   
   T   
   T   
   A   
   A   
   T   
   A   
   A   
   A   
   T   
   T   
   C   
   A   
   T   
   T   
   T   
   T   
   C   
   G   
   G   
   T   
   G   
   A   
   G   
   G   
   A   
   A   
   C   
   T   
   A   
   G   
   A   
   T   
   T   
   T   
   A   
   C   
   C   
   A   
   A   
   T   
   T   
   G   
   C   
   C   
   A   
   T   
   G   
   A   
   C   
   G   
   C   
   C   
   T   
   G   
   C   
   T   
   C   
   A   
   A   
   A   
   T   
   T   
   T   
   C   
   A   
   A   
   A   
   T   
   G   
   A   
   A   
   G   
   A   
   T   
   A   
   A   
   A   
   T   
   T   
   T   
   A   
   A   
   T   
   T   
   C   
   T   
   G   
   A   
   A   
   A   
   T   
   T   
   C   
   T   
   T   
   T   
   T   
   G   
   G   
   C   
   A   
   G   
   A   
   T   
   A   
   C   
   G   
   G   
   G   
   C   
   T   
   A   
   C   
   G   
   G   
   C   
   C   
   A   
   A   
   G   
   G   
   C   
   C   
   A   
   A   
   T   
   T   
   G   
   T   
   T   
   A   
   A   
   T   
   T   
   T   
   C   
   G   
   C   
   C   
   A   
   A   
   T   
   C   
   C   
   A   
   G   
   C   
   A   
   A   
   G   
   C   
   T   
   A   
   C   
   C   
   A   
   T   
   G   
   T   
   A   
   T   
   A   
   G   
   T   
   G   
   T   
   T   
   T   
   T   
   C   
   C   
   A   
   A   
   A   
   A   
   T   
   A   
   A   
   T   
   G   
   G   
   G   
   A   
   C   
   A   
   T   
   T   
   A   
   G   
   T   
   C   
   T   
   A   
   T   
   C   
   C   
   A   
   A   
   A   
   A   
   T   
   T   
   A   
   G   
   T   
   T   
   T   
   T   
   A   
   G   
   A   
   C   
   A   
   A   
   A   
   G   
   A   
   A   
   A   
   C   
   G   
   A   
   A   
   G   
   A   
   A   
   A   
   A   
   A   
   A   
   G   
   A   
   C   
   A   
   A   
   T   
   G   
   T   
   A   
   A   
   T   
   C   
   A   
   G   
   C   
   G   
   C   
   C   
   A   
   A   
   C   
   G   
   C   
   A   
   G   
   C   
   G   
   A   
   A   
   T   
   A   
   C   
   A   
   A   
   T   
   T   
   G   
   C   
   C   
   A   
   C   
   A   
   G   
   A   
   T   
   C   
   T   
   T   
   T   
   T   
   A   
   G   
   G   
   T   
   A   
   G   
   T   
   G   
   T   
   G   
   G   
   A   
   A   
   G   
   A   
   T   
   C   
   C   
   T   
   T   
   C   
   A   
   G   
   G   
   T   
   T   
   A   
   T   
   G   
   T   
   T   
   T   
   A   
   T   
   A   
   A   
   T   
   A   
   T   
   G   
   T   
   A   
   C   
   A   
   A   
   T   
   C   
   C   
   G   
   A   
   A   
   C   
   T   
   T   
   C   
   T   
   C   
   A   
   T   
   T   
   G   
   G   
   C   
   T   
   G   
   C   
   C   
   A   
   A   
   A   
   A   
   C   
   A   
   G   
   G   
   A   
   A   
   C   
   A   
   G   
   C   
   T   
   G   
   A   
   A   
   A   
   T   
   A   
   A   
   A   
   A   
   G   
   A   
   T   
   A   
   A   
   A   
   C   
   A   
   A   
   G   
   A   
   T   
   A   
   C   
   A   
   G   
   A   
   T   
   G   
   G   
   C   
   A   
   A   
   A   
   G   
   A   
   A   
   A   
   A   
   C   
   A   
   G   
   T   
   T   
   T   
   C   
   C   
   T   
   T   
   C   
   T   
   A   
   A   
   C   
   G   
   T   
   T   
   A   
   G   
   A   
   T   
   C   
   G   
   T   
   A   
   G   
   T   
   A   
   A   
   C   
   A   
   A   
   T   
   A   
   A   
   A   
   T   
   T   
   C   
   T   
   T   
   A   
   A   
   C   
   A   
   A   
   T   
   G   
   A   
   T   
   T   
   A   
   T   
   G   
   G   
   T   
   T   
   G   
   A   
   A   
   A   
   A   
   T   
   T   
   C   
   A   
   G   
   G   
   A   
   G   
   A   
   A   
   A   
   A   
   T   
   G   
   G   
   T   
   T   
   C   
   T   
   G   
   C   
   A   
   A   
   C   
   A   
   G   
   A   
   T   
   A   
   T   
   C   
   A   
   G   
   T   
   A   
   A   
   A   
   C   
   C   
   A   
   T   
   T   
   A   
   A   
   T   
   T   
   G   
   A   
   T   
   T   
   A   
   C   
   T   
   T   
   A   
   G   
   A   
   A   
   G   
   C   
   A   
   A   
   C   
   C   
   A   
   T   
   T   
   A   
   A   
   A   
   -   
   -   
   -   
   -   
   -   
   -   
   -   
   -   
   -   
   -   
   -   
   -   
   -   
   -   
   -   
   -   
   -   
   -   
   -   
   -   
   -   
   -   
   -   
   -   
   -   
   -   
   -   
   -   
   -   
   -   
   -   
   -   
   -   
   -   
   -   
   -   
   -   
   -   
   -   
   -   
   -   
   -   
   -   
   -   
   -   
   -   
   -   
   -   
   -   
   -   
   -   
   -   
   -   
   -   
   -   
   -   
   -   
   -   
   -   
   -   
   -   
 

 
   N59509F   
   -   
   -   
   -   
   -   
   -   
   -   
   -   
   -   
   -   
   -   
   -   
   -   
   -   
   -   
   -   
   -   
   -   
   -   
   -   
   -   
   -   
   -   
   -   
   -   
   -   
   -   
   -   
   -   
   -   
   -   
   -   
   -   
   -   
   -   
   -   
   -   
   -   
   -   
   -   
   -   
   -   
   -   
   -   
   -   
   -   
   -   
   -   
   -   
   -   
   -   
   -   
   -   
   -   
   -   
   -   
   -   
   -   
   -   
   -   
   -   
   -   
   -   
   -   
   -   
   -   
   -   
   -   
   -   
   -   
   -   
   -   
   -   
   -   
   -   
   -   
   -   
   -   
   -   
   -   
   -   
   -   
   -   
   -   
   -   
   -   
   -   
   -   
   -   
   -   
   -   
   -   
   -   
   -   
   -   
   -   
   -   
   -   
   -   
   -   
   -   
   -   
   -   
   -   
   -   
   -   
   -   
   -   
   -   
   C   
   A   
   A   
   T   
   G   
   G   
   C   
   A   
   A   
   G   
   C   
   C   
   A   
   A   
   A   
   C   
   A   
   A   
   G   
   A   
    A    
   T   
   T   
   A   
   G   
   C   
    C    
   G   
   A   
   A   
   G   
   C   
   G   
   A   
   A   
   G   
   A   
   A   
   A   
   A   
    C    
   A   
   G   
   C   
    T    
   A   
    C    
   T   
    A    
    C    
   A   
   T   
   T   
   T   
   T   
   T   
   A   
   A   
   A   
    C    
   G   
   T   
   A   
   T   
   T   
   G   
    A    
   C   
   A   
   A   
   A   
   A   
   C   
   A   
   G   
    A    
   A   
   A   
   T   
   T   
   T   
   G   
   A   
   T   
   A   
   A   
   G   
   T   
   T   
   A   
   C   
   C   
    G    
   T   
   C   
   C   
   G   
   T   
   T   
    G    
   T   
   T   
   C   
   A   
   A   
   G   
   A   
   A   
    G    
   C   
   T   
   A   
   G   
   C   
   T   
   T   
   A   
   A   
   A   
   G   
   A   
   A   
   A   
   A   
   A   
   T   
   G   
   G   
    C    
   T   
   A   
   T   
   G   
   A   
   T   
   A   
   C   
   T   
   A   
   A   
   A   
   T   
   C   
    T    
   G   
   T   
   T   
   G   
   T   
   T   
   G   
   A   
   A   
   A   
   A   
   A   
   T   
   A   
   C   
   C   
   A   
   A   
   G   
   C   
   A   
   A   
   T   
   T   
   T   
   A   
   T   
   T   
   C   
   A   
   G   
   G   
   G   
   A   
   T   
   T   
   C   
   A   
   A   
   G   
   C   
    A    
   G   
   A   
   A   
    G    
   G   
    A    
   G   
   T   
   C   
   A   
   A   
    A    
   G   
   C   
   T   
   A   
   G   
   T   
   G   
   A   
   T   
   G   
   T   
    T    
   C   
   A   
   A   
   G   
   T   
    C    
   A   
   A   
   A   
   A   
   A   
    G    
   G   
    C    
   G   
   A   
   A   
   A   
   G   
   A   
    T    
   A   
   A   
    T    
   C   
   A   
   A   
   T   
   A   
   C   
   A   
   C   
   A   
   T   
   T   
   T   
   A   
    C    
   C   
   T   
   A   
   T   
   A   
   A   
   A   
   T   
   T   
   A   
   T   
   C   
   G   
   A   
   T   
   G   
   A   
   G   
    C    
   A   
   C   
    T    
   C   
   C   
   T   
   T   
   T   
   A   
   G   
   G   
   C   
   G   
   A   
   A   
   A   
   T   
   G   
   A   
   A   
   A   
   G   
   A   
   T   
    T    
   T   
   G   
   T   
   C   
   T   
   T   
   A   
   T   
   C   
   A   
   A   
   T   
   C   
   A   
   A   
   G   
   T   
   A   
   T   
    C    
   G   
    C    
   C   
   A   
   A   
    A    
   A   
   A   
   A   
   G   
   G   
    C    
   G   
   A   
   T   
   A   
    C    
   C   
   T   
   A   
   C   
   C   
   A   
   A   
   A   
   T   
    C    
    G    
   C   
   T   
   T   
   G   
   G   
    A    
    A    
    G    
   C   
   C   
    G    
   T   
   C   
   T   
   T   
   T   
   A   
   A   
   T   
   T   
   T   
   T   
   T   
   C   
   C   
   A   
   G   
   A   
   T   
   A   
   T   
   G   
   T   
   C   
   A   
   G   
   G   
   A   
   A   
   A   
   T   
   G   
   A   
   T   
   A   
   A   
   A   
    A    
   T   
   T   
   T   
   C   
   G   
   A   
   T   
   T   
   C   
   A   
   A   
   G   
   T   
   A   
   G   
   A   
   T   
   A   
   A   
   T   
   G   
   C   
   C   
   A   
   A   
   A   
   C   
   G   
   T   
   G   
   G   
   A   
   G   
   A   
   A   
   A   
   T   
   T   
   G   
   T   
   C   
   G   
   A   
   T   
   C   
   G   
   T   
   A   
   A   
    T    
   G   
   G   
   T   
   A   
   G   
   T   
   G   
   G   
   G   
   C   
   T   
   A   
   G   
    C    
   A   
   A   
   T   
   T   
   A   
   A   
   C   
   A   
   A   
   A   
   G   
   T   
   G   
   T   
   T   
   T   
   G   
   A   
    C    
   G   
   A   
   A   
   G   
   T   
   G   
   G   
   G   
   C   
   G   
   T   
   A   
   G   
   T   
    G    
   C   
   C   
   T   
    G    
   G   
   C   
   A   
   A   
   A   
   C   
   T   
    C    
   G   
   G   
   T   
   T   
   C   
   T   
   G   
   G   
   C   
   G   
   C   
   A   
   G   
   A   
   A   
   A   
   A   
   A   
    A    
   C   
   A   
   G   
   C   
    C    
   A   
   A   
   T   
   A   
   T   
   C   
   A   
   A   
   A   
   G   
   C   
   T   
   T   
   T   
   T   
   A   
   G   
   T   
   G   
   A   
   T   
   A   
   A   
    G    
   T   
   T   
    C    
   G   
   G   
   C   
   G   
   T   
   T   
   T   
   C   
   T   
    G    
   T   
   T   
   G   
   A   
   T   
   G   
   A   
   A   
   A   
   T   
   C   
   A   
   A   
   T   
   C   
    A    
   A   
   A   
   A   
    G    
   T   
   T   
   A   
   A   
   G   
   C   
   C   
   A   
   A   
   G   
   G   
    G    
   T   
   G   
   G   
   G   
   T   
   C   
   C   
   A   
   A   
   G   
   C   
   A   
   G   
   A   
   C   
   T   
   C   
    C    
   T   
   T   
   T   
   G   
   T   
   A   
   C   
   C   
   A   
   A   
   T   
   C   
   A   
   C   
   A   
   G   
   T   
    C    
   G   
   C   
   T   
   T   
   C   
   T   
   G   
   A   
   A   
   C   
   C   
   A   
   G   
   T   
   G   
   A   
   C   
   A   
   G   
   A   
   A   
   T   
   T   
   A   
   C   
   C   
   A   
   A   
   C   
   A   
   G   
   G   
   G   
   G   
   C   
   T   
   G   
   C   
   G   
   A   
   C   
   A   
   A   
   A   
   A   
   G   
    A    
    T    
   A   
   C   
   A   
   G   
   A   
   G   
   T   
   C   
   A   
   C   
   G   
   T   
   T   
   A   
   T   
   T   
   A   
   T   
   C   
   C   
    G    
    C    
   T   
   G   
   G   
   G   
   G   
   G   
   A   
   A   
   G   
   C   
   A   
   G   
   C   
   T   
   G   
   C   
   G   
   C   
   A   
   A   
   T   
   T   
   A   
   A   
   T   
   C   
   G   
   G   
    G    
   T   
   A   
   T   
   A   
   C   
    G    
   G   
   G   
    C    
   A   
   C   
    C    
   A   
   T   
   T   
   A   
   C   
   T   
   G   
   C   
   A   
   G   
   A   
    A    
   G   
   A   
   T   
   A   
   T   
   T   
    A    
   A   
   A   
   A   
   A   
   A   
   A   
   A   
   T   
   C   
   C   
   A   
   G   
   A   
   G   
   T   
   T   
   A   
   A   
   G   
   T   
   A   
   G   
   T   
   A   
   C   
   G   
   G   
   G   
    C    
   G   
   T   
   A   
   A   
   T   
   T   
   G   
   G   
    T    
   A   
   A   
   A   
   A   
   C   
   T   
   G   
   G   
    C    
   T   
   T   
   A   
   G   
   A   
   A   
   C   
   G   
   A   
   G   
   C   
   G   
   T   
   T   
   T   
   G   
   A   
   T   
   A   
   A   
   A   
   G   
   A   
    A    
   T   
   T   
   A   
   A   
   G   
   A   
   G   
   G   
    G    
   C   
   A   
   A   
   G   
   A   
   T   
   G   
   G   
    T    
   G   
   G   
    C    
   T   
   C   
    A    
   C   
   T   
   A   
   G   
   T   
   T   
   A   
   T   
   T   
   T   
   T   
   A   
   G   
   A   
   T   
   G   
   A   
   C   
   A   
   A   
   A   
   G   
   A   
   G   
   A   
   A   
   T   
   G   
   T   
   C   
   A   
   A   
   A   
   A   
   A   
   A   
   G   
   C   
   T   
   T   
   T   
   A   
   C   
   A   
   A   
   A   
   C   
   C   
   A   
   A   
   A   
   G   
   A   
    G    
   A   
   A   
   A   
   A   
   A   
   A   
   G   
   A   
   T   
   G   
   G   
   T   
   C   
   A   
   A   
   A   
   C   
    G    
   A   
   T   
   T   
   A   
   A   
   A   
   T   
   T   
   A   
   A   
   C   
   G   
   A   
   T   
   T   
   G   
   A   
    C    
   A   
   G   
    T    
   G   
   G   
    C    
   G   
   T   
   A   
   C   
   A   
   A   
   C   
   A   
   A   
   C   
   A   
   A   
   G   
   C   
    C    
   T   
   T   
    C    
   G   
   C   
   T   
   A   
   T   
   A   
   T   
   T   
   T   
   G   
   A   
   C   
   A   
   A   
   A   
   C   
   G   
    C    
    C    
   C   
   T   
   G   
   G   
   T   
   A   
   G   
   T   
   G   
   C   
   G   
   G   
   T   
   C   
   A   
   T   
   T   
   A   
   C   
   C   
   G   
   A   
   T   
   C   
   C   
   T   
   C   
   A   
   A   
   A   
   A   
   A   
   G   
   G   
   C   
   G   
   A   
   T   
   T   
   T   
   A   
    T    
   T   
   A   
   G   
   C   
    A    
   A   
   C   
   G   
   G   
   T   
   T   
   A   
   G   
   T   
   T   
   C   
   A   
   C   
   C   
   T   
   T   
   C   
   G   
   T   
   A   
   T   
   G   
   A   
   T   
   C   
   C   
   T   
   A   
   A   
   T   
   A   
   A   
   A   
   A   
   T   
   G   
   G   
   C   
   C   
   A   
   A   
   T   
   G   
   G   
    C    
   A   
   T   
   T   
   T   
   C   
   T   
   C   
   A   
   A   
   A   
   A   
   A   
   G   
   A   
    A    
   T   
   A   
   T   
   G   
   A   
   T   
   G   
   C   
   T   
   T   
   A   
   C   
   A   
   A   
   T   
   A   
   A   
   C   
   A   
   A   
   C   
   A   
   A   
   A   
   G   
   A   
   T   
   T   
   T   
   A   
   C   
   C   
   A   
   T   
   T   
    T    
   A   
   C   
   A   
   G   
   C   
   A   
   C   
   G   
   T   
   T   
   T   
    T    
   G   
   C   
   G   
   A   
   C   
   A   
   G   
   G   
    C    
   T   
   A   
   T   
   G   
   C   
   A   
   C   
   C   
   A   
   G   
   G   
    C    
   T   
   C   
   T   
   A   
   C   
   C   
   T   
   T   
   T   
   A   
   A   
   A   
   A   
   C   
   A   
   A   
   T   
   T   
   A   
   C   
   C   
   G   
   G   
   T   
   G   
   C   
   C   
   A   
   T   
   T   
   G   
   G   
   T   
   T   
   T   
   A   
   G   
   A   
   T   
    G    
   C   
   A   
   G   
   G   
   G   
   A   
   C   
   A   
   T   
   T   
   G   
   A   
   A   
   G   
   C   
   C   
   A   
   G   
   A   
   T   
   G   
   A   
   A   
   G   
   A   
   G   
   C   
   T   
   A   
   G   
   A   
   A   
   A   
   T   
   T   
   A   
   A   
   T   
   G   
   G   
    T    
   T   
   T   
   G   
   A   
   A   
   A   
   T   
   G   
   G   
   C   
   A   
   A   
   A   
   A   
   A   
   G   
   A   
   T   
   A   
   A   
   A   
   T   
   C   
   T   
   T   
   G   
   G   
   G   
   G   
    C    
   G   
   G   
   C   
   T   
   A   
   T   
   T   
   T   
   T   
   G   
   C   
    G    
   A   
   C   
   A   
   C   
   G   
   T   
   G   
   T   
   A   
   A   
   A   
   A   
   G   
   A   
   A   
   G   
   C   
   A   
   A   
   G   
   T   
   C   
   C   
   A   
   G   
   T   
   C   
   A   
   A   
   T   
   C   
   T   
    G    
   C   
   G   
   T   
   A   
   C   
   C   
   G   
   C   
   T   
   T   
   T   
   A   
   G   
   T   
   T   
   A   
   A   
   T   
   T   
   C   
   A   
   G   
   A   
   T   
   A   
   A   
   T   
   A   
   T   
   T   
   T   
   A   
   T   
   T   
   T   
   T   
   G   
   C   
    C    
   C   
   A   
   A   
   C   
   A   
   A   
   A   
   C   
   A   
   T   
   T   
   A   
   C   
   G   
    G    
   A   
   T   
   G   
   G   
   G   
    A    
   G   
   A   
   A   
   G   
   A   
   C   
   A   
   A   
   A   
   T   
   T   
   T   
   A   
   G   
   A   
   G   
   C   
    G    
   G   
   G   
   T   
   C   
   T   
   T   
   A   
   A   
   T   
   A   
   A   
   A   
   T   
   T   
   C   
   A   
   T   
   T   
   T   
   T   
   C   
   G   
   G   
   T   
   G   
   A   
   G   
   G   
   A   
   A   
   C   
   T   
   A   
   G   
   A   
   T   
   T   
   T   
   A   
   C   
   C   
   A   
   A   
   T   
    T    
   G   
   C   
   C   
   A   
   T   
   G   
   A   
   C   
   G   
   C   
   C   
    T    
   G   
   C   
   T   
   C   
   A   
   A   
   A   
   T   
   T   
   T   
   C   
   A   
   A   
   A   
    T    
   G   
   A   
   A   
   G   
   A   
   T   
   A   
   A   
   A   
   T   
   T   
   T   
   A   
   A   
   T   
   T   
   C   
   T   
   G   
   A   
   A   
   A   
   T   
   T   
   C   
   T   
   T   
   T   
   T   
    A    
   G   
   C   
   A   
   G   
   A   
   T   
   A   
   C   
    G    
   G   
   G   
   C   
   T   
   A   
    C    
   G   
   G   
    T    
   C   
   A   
   A   
   G   
   G   
    T    
   C   
   A   
   A   
   T   
   T   
   G   
   T   
   T   
   A   
   A   
   T   
   T   
   T   
   C   
    G    
   C   
   C   
   A   
   A   
   T   
   C   
   C   
   A   
   G   
   C   
   A   
   A   
   G   
   C   
   T   
   A   
   C   
   C   
   A   
   T   
   G   
   T   
   A   
   T   
   A   
   G   
   T   
   G   
   T   
   T   
   T   
   T   
   C   
   C   
   A   
   A   
   A   
   A   
   T   
   A   
   A   
   T   
   G   
   G   
    A    
   A   
   C   
   A   
    T    
   T   
    A    
   G   
   T   
    C    
   T   
   A   
   T   
   C   
   C   
   A   
   A   
   A   
   A   
    C    
   T   
   A   
   G   
   T   
   T   
   T   
   T   
   A   
   G   
   A   
   C   
   A   
   A   
   A   
   G   
   A   
   A   
   A   
   C   
    G    
   A   
   A   
   G   
   A   
   A   
   A   
   A   
   A   
   A   
   G   
   A   
   C   
   A   
   A   
   T   
   G   
   T   
   A   
   A   
   T   
   C   
   A   
   G   
   C   
   G   
   C   
   C   
   A   
   A   
   C   
   G   
   C   
   A   
   G   
   C   
   G   
   A   
   A   
   T   
   A   
   C   
   A   
   A   
   T   
   T   
   G   
   C   
   C   
   A   
   C   
   A   
   G   
   A   
   T   
   C   
   T   
   T   
   T   
   T   
   A   
   G   
   G   
   T   
   A   
   G   
   T   
   G   
   T   
   G   
   G   
   A   
   A   
   G   
   A   
   T   
   C   
   C   
   T   
   T   
   C   
   A   
   G   
   G   
   T   
   T   
   A   
   T   
   G   
   T   
   T   
   T   
   A   
   T   
   A   
   A   
   T   
   A   
   T   
   G   
   T   
   A   
   C   
   A   
   A   
   T   
   C   
   C   
   G   
   A   
   A   
   C   
   T   
   T   
   C   
   T   
   C   
   A   
   T   
   T   
   G   
   G   
   C   
   T   
   G   
   C   
   T   
   A   
   A   
   A   
   A   
   C   
   A   
   G   
   G   
   A   
   A   
   C   
   A   
   G   
   C   
   T   
   G   
   A   
   A   
   A   
   T   
   A   
   A   
   A   
   A   
   G   
   A   
   T   
   A   
   A   
   A   
   C   
   A   
   A   
   G   
   A   
   T   
   A   
   C   
   A   
   G   
   A   
   T   
   G   
   G   
   C   
   A   
   A   
   A   
   G   
   A   
   A   
   A   
   A   
   C   
   A   
   G   
   T   
   T   
   T   
   C   
   C   
   T   
   T   
   C   
   T   
   A   
   A   
   C   
   G   
   T   
   T   
   A   
   G   
   A   
   T   
   C   
   G   
   T   
   A   
   G   
   T   
   A   
   A   
   C   
   A   
   A   
   T   
   A   
   A   
   A   
   T   
   T   
   C   
   T   
   T   
   A   
   A   
   C   
   A   
   A   
   T   
   G   
   A   
   T   
   T   
   A   
   T   
   G   
   G   
   T   
   T   
   G   
   A   
   A   
   A   
   A   
   T   
   T   
   C   
   A   
   G   
   G   
   A   
   G   
   A   
   A   
   A   
   A   
   T   
   G   
   G   
   T   
   T   
   C   
   T   
   G   
   C   
   A   
   A   
   C   
   A   
   G   
   A   
   T   
   A   
   T   
   C   
   A   
   G   
   T   
   A   
   A   
   A   
   C   
   C   
   A   
   T   
   T   
   A   
   A   
   T   
   T   
   G   
   A   
   T   
   T   
   A   
   C   
   T   
   T   
   A   
   G   
   A   
   A   
   G   
   C   
   A   
   A   
   C   
   C   
   A   
   T   
   T   
   A   
   A   
   A   
   -   
   -   
   -   
   -   
   -   
   -   
   -   
   -   
   -   
   -   
   -   
   -   
   -   
   -   
   -   
   -   
   -   
   -   
   -   
   -   
   -   
   -   
   -   
   -   
   -   
   -   
   -   
   -   
   -   
   -   
   -   
   -   
   -   
   -   
   -   
   -   
   -   
   -   
   -   
   -   
   -   
   -   
   -   
   -   
   -   
   -   
   -   
   -   
   -   
   -   
   -   
   -   
   -   
   -   
   -   
   -   
   -   
   -   
   -   
   -   
   -   
   -   
   -   
   -   
   -   
   -   
   -   
   -   
   -   
   -   
   -   
   -   
   -   
   -   
   -   
   -   
   -   
   -   
   -   
   -   
   -   
   -   
   -   
   -   
   -   
   -   
   -   
   -   
   -   
   -   
   -   
   -   
   -   
   -   
   -   
   -   
   -   
   -   
   -   
   -   
   -   
   -   
   -   
   -   
   -   
   -   
   -   
   -   
   -   
   -   
   -   
   -   
   -   
   -   
   -   
   -   
   -   
   -   
   -   
   -   
   -   
   -   
   -   
   -   
   -   
   -   
   -   
   -   
   -   
   -   
   -   
   -   
   -   
   -   
   -   
   -   
   -   
   -   
   -   
   -   
   -   
   -   
   -   
   -   
   -   
   -   
   -   
   -   
   -   
   -   
   -   
   -   
   -   
   -   
   -   
   -   
   -   
   -   
   -   
   -   
   -   
   -   
   -   
   -   
   -   
   -   
   -   
   -   
   -   
   C   
   A   
   A   
   T   
   G   
   G   
   C   
   A   
   A   
   G   
   C   
   C   
   A   
   A   
   A   
   C   
   A   
   A   
   G   
   A   
   A   
   T   
   T   
   A   
   G   
   C   
   C   
   G   
   A   
   A   
   G   
   C   
   G   
   A   
   A   
   G   
   A   
   A   
   A   
   A   
   C   
   A   
   G   
   C   
   T   
   A   
   C   
   T   
   A   
   C   
   A   
   T   
   T   
   T   
   T   
   T   
   A   
   A   
   A   
   C   
   G   
   T   
   A   
   T   
   T   
   G   
   A   
   C   
   A   
   A   
   A   
   A   
   C   
   A   
   G   
   A   
   A   
   A   
   T   
   T   
   T   
   G   
   A   
   T   
   A   
   A   
   G   
   T   
   T   
   A   
   C   
   C   
   G   
   T   
   C   
   C   
   G   
   T   
   T   
   G   
   T   
   T   
   C   
   A   
   A   
   G   
   A   
   A   
   G   
   C   
   T   
   A   
   G   
   C   
   T   
   T   
   A   
   A   
   A   
   G   
   A   
   A   
   A   
   A   
   A   
   T   
   G   
   G   
   C   
   T   
   A   
   T   
   G   
   A   
   T   
   A   
   C   
   T   
   A   
   A   
   A   
   T   
   C   
   T   
   G   
   T   
   T   
   G   
   T   
   T   
   G   
   A   
   A   
   A   
   A   
   A   
   T   
   A   
   C   
   C   
   A   
   A   
   G   
   C   
   A   
   A   
   T   
   T   
   T   
   A   
   T   
   T   
   C   
   A   
   G   
   G   
   G   
   A   
   T   
   T   
   C   
   A   
   A   
   G   
   C   
   A   
   G   
   A   
   A   
   G   
   G   
   A   
   G   
   T   
   C   
   A   
   A   
   A   
   G   
   C   
   T   
   A   
   G   
   T   
   G   
   A   
   T   
   G   
   T   
   T   
   C   
   A   
   A   
   G   
   T   
   C   
   A   
   A   
   A   
   A   
   A   
   G   
   G   
   C   
   G   
   A   
   A   
   A   
   G   
   A   
   T   
   A   
   A   
   T   
   C   
   A   
   A   
   T   
   A   
   C   
   A   
   C   
   A   
   T   
   T   
   T   
   A   
   C   
   C   
   T   
   A   
   T   
   A   
   A   
   A   
   T   
   T   
   A   
   T   
   C   
   G   
   A   
   T   
   G   
   A   
   G   
   C   
   A   
   C   
   T   
   C   
   C   
   T   
   T   
   T   
   A   
   G   
   G   
   C   
   G   
   A   
   A   
   A   
   T   
   G   
   A   
   A   
   A   
   G   
   A   
   T   
   T   
   T   
   G   
   T   
   C   
   T   
   T   
   A   
   T   
   C   
   A   
   A   
   T   
   C   
   A   
   A   
   G   
   T   
   A   
   T   
   C   
   G   
   C   
   C   
   A   
   A   
   A   
   A   
   A   
   A   
   G   
   G   
   C   
   G   
   A   
   T   
   A   
   C   
   C   
   T   
   A   
   C   
   C   
   A   
   A   
   A   
   T   
   C   
   G   
   C   
   T   
   T   
   G   
   G   
   A   
   A   
   G   
   C   
   C   
   G   
   T   
   C   
   T   
   T   
   T   
   A   
   A   
   T   
   T   
   T   
   T   
   T   
   C   
   C   
   A   
   G   
   A   
   T   
   A   
   T   
   G   
   T   
   C   
   A   
   G   
   G   
   A   
   A   
   A   
   T   
   G   
   A   
   T   
   A   
   A   
   A   
   A   
   T   
   T   
   T   
   C   
   G   
   A   
   T   
   T   
   C   
   A   
   A   
   G   
   T   
   A   
   G   
   A   
   T   
   A   
   A   
   T   
   G   
   C   
   C   
   A   
   A   
   A   
   C   
   G   
   T   
   G   
   G   
   A   
   G   
   A   
   A   
   A   
   T   
   T   
   G   
   T   
   C   
   G   
   A   
   T   
   C   
   G   
   T   
   A   
   A   
   T   
   G   
   G   
   T   
   A   
   G   
   T   
   G   
   G   
   G   
   C   
   T   
   A   
   G   
   C   
   A   
   A   
   T   
   T   
   A   
   A   
   C   
   A   
   A   
   A   
   G   
   T   
   G   
   T   
   T   
   T   
   G   
   A   
   C   
   G   
   A   
   A   
   G   
   T   
   G   
   G   
   G   
   C   
   G   
   T   
   A   
   G   
   T   
   G   
   C   
   C   
   T   
   G   
   G   
   C   
   A   
   A   
   A   
   C   
   T   
   C   
   G   
   G   
   T   
   T   
   C   
   T   
   G   
   G   
   C   
   G   
   C   
   A   
   G   
   A   
   A   
   A   
   A   
   A   
   A   
   C   
   A   
   G   
   C   
   C   
   A   
   A   
   T   
   A   
   T   
   C   
   A   
   A   
   A   
   G   
   C   
   T   
   T   
   T   
   T   
   A   
   G   
   T   
   G   
   A   
   T   
   A   
   A   
   G   
   T   
   T   
   C   
   G   
   G   
   C   
   G   
   T   
   T   
   T   
   C   
   T   
   G   
   T   
   T   
   G   
   A   
   T   
   G   
   A   
   A   
   A   
   T   
   C   
   A   
   A   
   T   
   C   
   A   
   A   
   A   
   A   
   G   
   T   
   T   
   A   
   A   
   G   
   C   
   C   
   A   
   A   
   G   
   G   
   G   
   T   
   G   
   G   
   G   
   T   
   C   
   C   
   A   
   A   
   G   
   C   
   A   
   G   
   A   
   C   
   T   
   C   
   C   
   T   
   T   
   T   
   G   
   T   
   A   
   C   
   C   
   A   
   A   
   T   
   C   
   A   
   C   
   A   
   G   
   T   
   C   
   G   
   C   
   T   
   T   
   C   
   T   
   G   
   A   
   A   
   C   
   C   
   A   
   G   
   T   
   G   
   A   
   C   
   A   
   G   
   A   
   A   
   T   
   T   
   A   
   C   
   C   
   A   
   A   
   C   
   A   
   G   
   G   
   G   
   G   
   C   
   T   
   G   
   C   
   G   
   A   
   C   
   A   
   A   
   A   
   A   
   G   
   A   
   T   
   A   
   C   
   A   
   G   
   A   
   G   
   T   
   C   
   A   
   C   
   G   
   T   
   T   
   A   
   T   
   T   
   A   
   T   
   C   
   C   
   G   
   C   
   T   
   G   
   G   
   G   
   G   
   G   
   A   
   A   
   G   
   C   
   A   
   G   
   C   
   T   
   G   
   C   
   G   
   C   
   A   
   A   
   T   
   T   
   A   
   A   
   T   
   C   
   G   
   G   
   G   
   T   
   A   
   T   
   A   
   C   
   G   
   G   
   G   
   C   
   A   
   C   
   C   
   A   
   T   
   T   
   A   
   C   
   T   
   G   
   C   
   A   
   G   
   A   
   A   
   G   
   A   
   T   
   A   
   T   
   T   
   A   
   A   
   A   
   A   
   A   
   A   
   A   
   A   
   T   
   C   
   C   
   A   
   G   
   A   
   G   
   T   
   T   
   A   
   A   
   G   
   T   
   A   
   G   
   T   
   A   
   C   
   G   
   G   
   G   
   C   
   G   
   T   
   A   
   A   
   T   
   T   
   G   
   G   
   T   
   A   
   A   
   A   
   A   
   C   
   T   
   G   
   G   
   C   
   T   
   T   
   A   
   G   
   A   
   A   
   C   
   G   
   A   
   G   
   C   
   G   
   T   
   T   
   T   
   G   
   A   
   T   
   A   
   A   
   A   
   G   
   A   
   A   
   T   
   T   
   A   
   A   
   G   
   A   
   G   
   G   
   G   
   C   
   A   
   A   
   G   
   A   
   T   
   G   
   G   
   T   
   G   
   G   
   C   
   T   
   C   
   A   
   C   
   T   
   A   
   G   
   T   
   T   
   A   
   T   
   T   
   T   
   T   
   A   
   G   
   A   
   T   
   G   
   A   
   C   
   A   
   A   
   A   
   G   
   A   
   G   
   A   
   A   
   T   
   G   
   T   
   C   
   A   
   A   
   A   
   A   
   A   
   A   
   G   
   C   
   T   
   T   
   T   
   A   
   C   
   A   
   A   
   A   
   C   
   C   
   A   
   A   
   A   
   G   
   A   
   G   
   A   
   A   
   A   
   A   
   A   
   A   
   G   
   A   
   T   
   G   
   G   
   T   
   C   
   A   
   A   
   A   
   C   
   G   
   A   
   T   
   T   
   A   
   A   
   A   
   T   
   T   
   A   
   A   
   C   
   G   
   A   
   T   
   T   
   G   
   A   
   C   
   A   
   G   
   T   
   G   
   G   
   C   
   G   
   T   
   A   
   C   
   A   
   A   
   C   
   A   
   A   
   C   
   A   
   A   
   G   
   C   
   C   
   T   
   T   
   C   
   G   
   C   
   T   
   A   
   T   
   A   
   T   
   T   
   T   
   G   
   A   
   C   
   A   
   A   
   A   
   C   
   G   
   C   
   C   
   C   
   T   
   G   
   G   
   T   
   A   
   G   
   T   
   G   
   C   
   G   
   G   
   T   
   C   
   A   
   T   
   T   
   A   
   C   
   C   
   G   
   A   
   T   
   C   
   C   
   T   
   C   
   A   
   A   
   A   
   A   
   A   
   G   
   G   
   C   
   G   
   A   
   T   
   T   
   T   
   A   
   T   
   T   
   A   
   G   
   C   
   A   
   A   
   C   
   G   
   G   
   T   
   T   
   A   
   G   
   T   
   T   
   C   
   A   
   C   
   C   
   T   
   T   
   C   
   G   
   T   
   A   
   T   
   G   
   A   
   T   
   C   
   C   
   T   
   A   
   A   
   T   
   A   
   A   
   A   
   A   
   T   
   G   
   G   
   C   
   C   
   A   
   A   
   T   
   G   
   G   
   C   
   A   
   T   
   T   
   T   
   C   
   T   
   C   
   A   
   A   
   A   
   A   
   A   
   G   
   A   
   A   
   T   
   A   
   T   
   G   
   A   
   T   
   G   
   C   
   T   
   T   
   A   
   C   
   A   
   A   
   T   
   A   
   A   
   C   
   A   
   A   
   C   
   A   
   A   
   A   
   G   
   A   
   T   
   T   
   T   
   A   
   C   
   C   
   A   
   T   
   T   
   T   
   A   
   C   
   A   
   G   
   C   
   A   
   C   
   G   
   T   
   T   
   T   
   T   
   G   
   C   
   G   
   A   
   C   
   A   
   G   
   G   
   C   
   T   
   A   
   T   
   G   
   C   
   A   
   C   
   C   
   A   
   G   
   G   
   C   
   T   
   C   
   T   
   A   
   C   
   C   
   T   
   T   
   T   
   A   
   A   
   A   
   A   
   C   
   A   
   A   
   T   
   T   
   A   
   C   
   C   
   G   
   G   
   T   
   G   
   C   
   C   
   A   
   T   
   T   
   G   
   G   
   T   
   T   
   T   
   A   
   G   
   A   
   T   
   G   
   C   
   A   
   G   
   G   
   G   
   A   
   C   
   A   
   T   
   T   
   G   
   A   
   A   
   G   
   C   
   C   
   A   
   G   
   A   
   T   
   G   
   A   
   A   
   G   
   A   
   G   
   C   
   T   
   A   
   G   
   A   
   A   
   A   
   T   
   T   
   A   
   A   
   T   
   G   
   G   
   T   
   T   
   T   
   G   
   A   
   A   
   A   
   T   
   G   
   G   
   C   
   A   
   A   
   A   
   A   
   A   
   G   
   A   
   T   
   A   
   A   
   A   
   T   
   C   
   T   
   T   
   G   
   G   
   G   
   G   
   C   
   G   
   G   
   C   
   T   
   A   
   T   
   T   
   T   
   T   
   G   
   C   
   G   
   A   
   C   
   A   
   C   
   G   
   T   
   G   
   T   
   A   
   A   
   A   
   A   
   G   
   A   
   A   
   G   
   C   
   A   
   A   
   G   
   T   
   C   
   C   
   A   
   G   
   T   
   C   
   A   
   A   
   T   
   C   
   T   
   G   
   C   
   G   
   T   
   A   
   C   
   C   
   G   
   C   
   T   
   T   
   T   
   A   
   G   
   T   
   T   
   A   
   A   
   T   
   T   
   C   
   A   
   G   
   A   
   T   
   A   
   A   
   T   
   A   
   T   
   T   
   T   
   A   
   T   
   T   
   T   
   T   
   G   
   C   
   C   
   C   
   A   
   A   
   C   
   A   
   A   
   A   
   C   
   A   
   T   
   T   
   A   
   C   
   G   
   G   
   A   
   T   
   G   
   G   
   G   
   A   
   G   
   A   
   A   
   G   
   A   
   C   
   A   
   A   
   A   
   T   
   T   
   T   
   A   
   G   
   A   
   G   
   C   
   G   
   G   
   G   
   T   
   C   
   T   
   T   
   A   
   A   
   T   
   A   
   A   
   A   
   T   
   T   
   C   
   A   
   T   
   T   
   T   
   T   
   C   
   G   
   G   
   T   
   G   
   A   
   G   
   G   
   A   
   A   
   C   
   T   
   A   
   G   
   A   
   T   
   T   
   T   
   A   
   C   
   C   
   A   
   A   
   T   
   T   
   G   
   C   
   C   
   A   
   T   
   G   
   A   
   C   
   G   
   C   
   C   
   T   
   G   
   C   
   T   
   C   
   A   
   A   
   A   
   T   
   T   
   T   
   C   
   A   
   A   
   A   
   T   
   G   
   A   
   A   
   G   
   A   
   T   
   A   
   A   
   A   
   T   
   T   
   T   
   A   
   A   
   T   
   T   
   C   
   T   
   G   
   A   
   A   
   A   
   T   
   T   
   C   
   T   
   T   
   T   
   T   
   A   
   G   
   C   
   A   
   G   
   A   
   T   
   A   
   C   
   G   
   G   
   G   
   C   
   T   
   A   
   C   
   G   
   G   
   T   
   C   
   A   
   A   
   G   
   G   
   T   
   C   
   A   
   A   
   T   
   T   
   G   
   T   
   T   
   A   
   A   
   T   
   T   
   T   
   C   
   G   
   C   
   C   
   A   
   A   
   T   
   C   
   C   
   A   
   G   
   C   
   A   
   A   
   G   
   C   
   T   
   A   
   C   
   C   
   A   
   T   
   G   
   T   
   A   
   T   
   A   
   G   
   T   
   G   
   T   
   T   
   T   
   T   
   C   
   C   
   A   
   A   
   A   
   A   
   T   
   A   
   A   
   T   
   G   
   G   
   A   
   A   
   C   
   A   
   T   
   T   
   A   
   G   
   T   
   C   
   T   
   A   
   T   
   C   
   C   
   A   
   A   
   A   
   A   
   C   
   T   
   A   
   G   
   T   
   T   
   T   
   T   
   A   
   G   
   A   
   C   
   A   
   A   
   A   
   G   
   A   
   A   
   A   
   C   
   G   
   A   
   A   
   G   
   A   
   A   
   A   
   A   
   A   
   A   
   G   
   A   
   C   
   A   
   A   
   T   
   G   
   T   
   A   
   A   
   T   
   C   
   A   
   G   
   C   
   G   
   C   
   C   
   A   
   A   
   C   
   G   
   C   
   A   
   G   
   C   
   G   
   A   
   A   
   T   
   A   
   C   
   A   
   A   
   T   
   T   
   G   
   C   
   C   
   A   
   C   
   A   
   G   
   A   
   T   
   C   
   T   
   T   
   T   
   T   
   A   
   G   
   G   
   T   
   A   
   G   
   T   
   G   
   T   
   G   
   G   
   A   
   A   
   G   
   A   
   T   
   C   
   C   
   T   
   T   
   C   
   A   
   G   
   G   
   T   
   T   
   A   
   T   
   G   
   T   
   T   
   T   
   A   
   T   
   A   
   A   
   T   
   A   
   T   
   G   
   T   
   A   
   C   
   A   
   A   
   T   
   C   
   C   
   G   
   A   
   A   
   C   
   T   
   T   
   C   
   T   
   C   
   A   
   T   
   T   
   G   
   G   
   C   
   T   
   G   
   C   
   T   
   A   
   A   
   A   
   A   
   C   
   A   
   G   
   G   
   A   
   A   
   C   
   A   
   G   
   C   
   T   
   G   
   A   
   A   
   A   
   T   
   A   
   A   
   A   
   A   
   G   
   A   
   T   
   A   
   A   
   A   
   C   
   A   
   A   
   G   
   A   
   T   
   A   
   C   
   A   
   G   
   A   
   T   
   G   
   G   
   C   
   A   
   A   
   A   
   G   
   A   
   A   
   A   
   A   
   C   
   A   
   G   
   T   
   T   
   T   
   C   
   C   
   T   
   T   
   C   
   T   
   A   
   A   
   C   
   G   
   T   
   T   
   A   
   G   
   A   
   T   
   C   
   G   
   T   
   A   
   G   
   T   
   A   
   A   
   C   
   A   
   A   
   T   
   A   
   A   
   A   
   T   
   T   
   C   
   T   
   T   
   A   
   A   
   C   
   A   
   A   
   T   
   G   
   A   
   T   
   T   
   A   
   T   
   G   
   G   
   T   
   T   
   G   
   A   
   A   
   A   
   A   
   T   
   T   
   C   
   A   
   G   
   G   
   A   
   G   
   A   
   A   
   A   
   A   
   T   
   G   
   G   
   T   
   T   
   C   
   T   
   G   
   C   
   A   
   A   
   C   
   A   
   G   
   A   
   T   
   A   
   T   
   C   
   A   
   G   
   T   
   A   
   A   
   A   
   C   
   C   
   A   
   T   
   T   
   A   
   A   
   T   
   T   
   G   
   A   
   T   
   T   
   A   
   C   
   T   
   T   
   A   
   G   
   A   
   A   
   G   
   C   
   A   
   A   
   C   
   C   
   A   
   T   
   T   
   A   
   A   
   A   
   -   
   -   
   -   
   -   
   -   
   -   
   -   
   -   
   -   
   -   
   -   
   -   
   -   
   -   
   -   
   -   
   -   
   -   
   -   
   -   
   -   
   -   
   -   
   -   
   -   
   -   
   -   
   -   
   -   
   -   
   -   
   -   
   -   
   -   
   -   
   -   
   -   
   -   
   -   
   -   
   -   
   -   
   -   
   -   
   -   
   -   
   -   
   -   
   -   
   -   
   -   
   -   
   -   
   -   
   -   
   -   
   -   
   -   
   -   
   -   
   -   
 

 
   N59646F   
   -   
   -   
   -   
   -   
   -   
   -   
   -   
   -   
   -   
   -   
   -   
   -   
   -   
   -   
   -   
   -   
   -   
   -   
   -   
   -   
   -   
   -   
   -   
   -   
   -   
   -   
   -   
   -   
   -   
   -   
   -   
   -   
   -   
   -   
   -   
   -   
   -   
   -   
   -   
   -   
   -   
   -   
   -   
   -   
   -   
   -   
   -   
   -   
   -   
   -   
   -   
   -   
   -   
   -   
   -   
   -   
   -   
   -   
   -   
   -   
   -   
   -   
   -   
   -   
   -   
   -   
   -   
   -   
   -   
   -   
   -   
   -   
   -   
   -   
   -   
   -   
   -   
   -   
   -   
   -   
   -   
   -   
   -   
   -   
   -   
   -   
   -   
   -   
   -   
   -   
   -   
   -   
   -   
   -   
   -   
   -   
   -   
   -   
   -   
   -   
   -   
   -   
   -   
   -   
   -   
   -   
   -   
   -   
   C   
   A   
   A   
   T   
   G   
   G   
   C   
   A   
   A   
   G   
   C   
   C   
   A   
   A   
   A   
   C   
   A   
   A   
   G   
   A   
    A    
   T   
   T   
   A   
   G   
   C   
    C    
   G   
   A   
   A   
   G   
   C   
   G   
   A   
   A   
   G   
   A   
   A   
   A   
   A   
    C    
   A   
   G   
   C   
    T    
   A   
    C    
   T   
    A    
    C    
   A   
   T   
   T   
   T   
   T   
   T   
   A   
   A   
   A   
    C    
   G   
   T   
   A   
   T   
   T   
   G   
    T    
   C   
   A   
   A   
   A   
   A   
   C   
   A   
   G   
    G    
   A   
   A   
   T   
   T   
   T   
   G   
   A   
   T   
   A   
   A   
   G   
   T   
   T   
   A   
   C   
   C   
    G    
   T   
   C   
   C   
   G   
   T   
   T   
    G    
   T   
   T   
   C   
   A   
   A   
   G   
   A   
   A   
    G    
   C   
   T   
   A   
   G   
   C   
   T   
   T   
   A   
   A   
   A   
   G   
   A   
   A   
   A   
   A   
   A   
   T   
   G   
   G   
    C    
   T   
   A   
   T   
   G   
   A   
   T   
   A   
   C   
   T   
   A   
   A   
   A   
   T   
   C   
    T    
   G   
   T   
   T   
   G   
   T   
   T   
   G   
   A   
   A   
   A   
   A   
   A   
   T   
   A   
   C   
   C   
   A   
   A   
   G   
   C   
   A   
   A   
   T   
   T   
   T   
   A   
   T   
   T   
   C   
   A   
   G   
   G   
   G   
   A   
   T   
   T   
   C   
   A   
   A   
   G   
   C   
    A    
   G   
   A   
   A   
    G    
   G   
    A    
   G   
   T   
   C   
   A   
   A   
    A    
   G   
   C   
   T   
   A   
   G   
   T   
   G   
   A   
   T   
   G   
   T   
    T    
   C   
   A   
   A   
   G   
   T   
    C    
   A   
   A   
   A   
   A   
   A   
    G    
   G   
    C    
   G   
   A   
   A   
   A   
   G   
   A   
    C    
   A   
   A   
    T    
   C   
   A   
   A   
   T   
   A   
   C   
   A   
   C   
   A   
   T   
   T   
   T   
   A   
    C    
   C   
   T   
   A   
   T   
   A   
   A   
   A   
   T   
   T   
   A   
   T   
   C   
   G   
   A   
   T   
   G   
   A   
   G   
    C    
   A   
   C   
    G    
   C   
   C   
   T   
   T   
   T   
   A   
   G   
   G   
   C   
   G   
   A   
   A   
   A   
   T   
   G   
   A   
   A   
   A   
   G   
   A   
   T   
    T    
   T   
   G   
   T   
   C   
   T   
   T   
   A   
   T   
   C   
   A   
   A   
   T   
   C   
   A   
   A   
   G   
   T   
   A   
   T   
    C    
   G   
    C    
   C   
   A   
   A   
    A    
   A   
   A   
   A   
   G   
   G   
    C    
   G   
   A   
   T   
   A   
    C    
   C   
   T   
   A   
   C   
   C   
   A   
   A   
   A   
   T   
    C    
    G    
   C   
   T   
   T   
   G   
   G   
    A    
    A    
    G    
   C   
   C   
    A    
   T   
   C   
   T   
   T   
   T   
   A   
   A   
   T   
   T   
   T   
   T   
   T   
   C   
   C   
   A   
   G   
   A   
   T   
   A   
   T   
   G   
   T   
   C   
   A   
   G   
   G   
   A   
   A   
   A   
   T   
   G   
   A   
   T   
   A   
   A   
   A   
    A    
   T   
   T   
   T   
   C   
   G   
   A   
   T   
   T   
   C   
   A   
   A   
   G   
   T   
   A   
   G   
   A   
   T   
   A   
   A   
   T   
   G   
   C   
   C   
   A   
   A   
   A   
   C   
   G   
   T   
   G   
   G   
   A   
   G   
   A   
   A   
   A   
   T   
   T   
   G   
   T   
   C   
   G   
   A   
   T   
   C   
   G   
   T   
   A   
   A   
    T    
   G   
   G   
   T   
   A   
   G   
   T   
   G   
   G   
   G   
   C   
   T   
   A   
   G   
    C    
   A   
   A   
   T   
   T   
   A   
   A   
   C   
   A   
   A   
   A   
   G   
   T   
   G   
   T   
   T   
   T   
   G   
   A   
    C    
   G   
   A   
   A   
   G   
   T   
   G   
   G   
   G   
   C   
   G   
   T   
   A   
   G   
   T   
    G    
   C   
   C   
   T   
    G    
   G   
   C   
   A   
   A   
   A   
   C   
   T   
    C    
   G   
   G   
   T   
   T   
   C   
   T   
   G   
   G   
   C   
   G   
   C   
   A   
   G   
   A   
   A   
   A   
   A   
   A   
    A    
   C   
   A   
   G   
   C   
    C    
   A   
   A   
   T   
   A   
   T   
   C   
   A   
   A   
   A   
   G   
   C   
   T   
   T   
   T   
   T   
   A   
   G   
   T   
   G   
   A   
   T   
   A   
   A   
    A    
   T   
   T   
    C    
   G   
   G   
   C   
   G   
   T   
   T   
   T   
   C   
   T   
    G    
   T   
   T   
   G   
   A   
   T   
   G   
   A   
   A   
   A   
   T   
   C   
   A   
   A   
   T   
   C   
    A    
   A   
   A   
   A   
    G    
   T   
   T   
   A   
   A   
   G   
   C   
   C   
   A   
   A   
   G   
   G   
    A    
   T   
   G   
   G   
   G   
   T   
   C   
   C   
   A   
   A   
   G   
   C   
   A   
   G   
   A   
   C   
   T   
   C   
    C    
   T   
   T   
   T   
   G   
   T   
   A   
   C   
   C   
   A   
   A   
   T   
   C   
   A   
   C   
   A   
   G   
   T   
    C    
   G   
   C   
   T   
   T   
   C   
   T   
   G   
   A   
   A   
   C   
   C   
   A   
   G   
   T   
   G   
   A   
   C   
   A   
   G   
   A   
   A   
   T   
   T   
   A   
   C   
   C   
   A   
   A   
   C   
   A   
   G   
   G   
   G   
   G   
   C   
   T   
   G   
   C   
   G   
   A   
   C   
   A   
   A   
   A   
   A   
   G   
    A    
    T    
   A   
   C   
   A   
   G   
   A   
   G   
   T   
   C   
   A   
   C   
   G   
   T   
   T   
   A   
   T   
   T   
   A   
   T   
   C   
   C   
    G    
    C    
   T   
   G   
   G   
   G   
   G   
   G   
   A   
   A   
   G   
   C   
   A   
   G   
   C   
   T   
   G   
   C   
   G   
   C   
   A   
   A   
   T   
   T   
   A   
   A   
   T   
   C   
   G   
   G   
    G    
   T   
   A   
   T   
   A   
   C   
    G    
   G   
   G   
    C    
   A   
   C   
    C    
   A   
   T   
   T   
   A   
   C   
   T   
   G   
   C   
   A   
   G   
   A   
    A    
   G   
   A   
   T   
   A   
   T   
   T   
    G    
   A   
   A   
   A   
   A   
   A   
   A   
   A   
   T   
   C   
   C   
   A   
   G   
   A   
   G   
   T   
   T   
   A   
   A   
   G   
   T   
   A   
   G   
   T   
   A   
   C   
   G   
   G   
   G   
    C    
   G   
   T   
   A   
   A   
   T   
   T   
   G   
   G   
    T    
   A   
   A   
   A   
   A   
   C   
   T   
   G   
   G   
    C    
   T   
   T   
   A   
   G   
   A   
   A   
   C   
   G   
   A   
   G   
   C   
   G   
   T   
   T   
   T   
   G   
   A   
   T   
   A   
   A   
   A   
   G   
   A   
    A    
   T   
   T   
   A   
   A   
   G   
   A   
   G   
   G   
    G    
   C   
   A   
   A   
   G   
   A   
   T   
   G   
   G   
    T    
   G   
   G   
    C    
   T   
   C   
    A    
   C   
   T   
   A   
   G   
   T   
   T   
   A   
   T   
   T   
   T   
   T   
   A   
   G   
   A   
   T   
   G   
   A   
   C   
   A   
   A   
   A   
   G   
   A   
   G   
   A   
   A   
   T   
   G   
   T   
   C   
   A   
   A   
   A   
   A   
   A   
   A   
   G   
   C   
   T   
   T   
   T   
   A   
   C   
   A   
   A   
   A   
   C   
   C   
   A   
   A   
   A   
   G   
   A   
    G    
   A   
   A   
   A   
   A   
   A   
   A   
   G   
   A   
   T   
   G   
   G   
   T   
   C   
   A   
   A   
   A   
   C   
    G    
   A   
   T   
   T   
   A   
   A   
   A   
   T   
   T   
   A   
   A   
   C   
   G   
   A   
   T   
   T   
   G   
   A   
    C    
   A   
   G   
    T    
   G   
   G   
    C    
   G   
   T   
   A   
   C   
   A   
   A   
   C   
   A   
   A   
   C   
   A   
   A   
   G   
   C   
    T    
   T   
   T   
    T    
   G   
   C   
   T   
   A   
   T   
   A   
   T   
   T   
   T   
   G   
   A   
   C   
   A   
   A   
   A   
   C   
   G   
    C    
    C    
   C   
   T   
   G   
   G   
   T   
   A   
   G   
   T   
   G   
   C   
   G   
   G   
   T   
   C   
   A   
   T   
   T   
   A   
   C   
   C   
   G   
   A   
   T   
   C   
   C   
   T   
   C   
   A   
   A   
   A   
   A   
   A   
   G   
   G   
   C   
   G   
   A   
   T   
   T   
   T   
   A   
    T    
   T   
   A   
   G   
   C   
    A    
   A   
   C   
   G   
   G   
   T   
   T   
   A   
   G   
   T   
   T   
   C   
   A   
   C   
   C   
   T   
   T   
   C   
   G   
   T   
   A   
   T   
   G   
   A   
   T   
   C   
   C   
   T   
   A   
   A   
   T   
   A   
   A   
   A   
   A   
   T   
   G   
   G   
   C   
   C   
   A   
   A   
   T   
   G   
   G   
    C    
   A   
   T   
   T   
   T   
   C   
   T   
   C   
   A   
   A   
   A   
   A   
   A   
   G   
   A   
    A    
   T   
   A   
   T   
   G   
   A   
   T   
   G   
   C   
   T   
   T   
   A   
   C   
   A   
   A   
   T   
   A   
   A   
   C   
   A   
   A   
   C   
   A   
   A   
   A   
   G   
   A   
   T   
   T   
   T   
   A   
   C   
   C   
   A   
   T   
   T   
    C    
   A   
   C   
   A   
   G   
   C   
   A   
   C   
   G   
   T   
   T   
   T   
    T    
   G   
   C   
   G   
   A   
   C   
   A   
   G   
   G   
    C    
   T   
   A   
   T   
   G   
   C   
   A   
   C   
   C   
   A   
   G   
   G   
    C    
   T   
   C   
   T   
   A   
   C   
   C   
   T   
   T   
   T   
   A   
   A   
   A   
   A   
   C   
   A   
   A   
   T   
   T   
   A   
   C   
   C   
   G   
   G   
   T   
   G   
   C   
   C   
   A   
   T   
   T   
   G   
   G   
   T   
   T   
   T   
   A   
   G   
   A   
   T   
    G    
   C   
   A   
   G   
   G   
   G   
   A   
   C   
   A   
   T   
   T   
   G   
   A   
   A   
   G   
   C   
   C   
   A   
   G   
   A   
   T   
   G   
   A   
   A   
   G   
   A   
   G   
   C   
   T   
   A   
   G   
   A   
   A   
   A   
   T   
   T   
   A   
   A   
   T   
   G   
   G   
    T    
   T   
   T   
   G   
   A   
   A   
   A   
   T   
   G   
   G   
   C   
   A   
   A   
   A   
   A   
   A   
   G   
   A   
   T   
   A   
   A   
   A   
   T   
   C   
   T   
   T   
   G   
   G   
   G   
   G   
    C    
   G   
   G   
   C   
   T   
   A   
   T   
   T   
   T   
   T   
   G   
   C   
    G    
   A   
   C   
   A   
   C   
   G   
   T   
   G   
   T   
   A   
   A   
   A   
   A   
   G   
   A   
   A   
   G   
   C   
   A   
   A   
   G   
   T   
   C   
   C   
   A   
   G   
   T   
   C   
   A   
   A   
   T   
   C   
   T   
    G    
   C   
   G   
   T   
   A   
   C   
   C   
   G   
   C   
   T   
   T   
   T   
   A   
   G   
   T   
   T   
   A   
   A   
   T   
   T   
   C   
   A   
   G   
   A   
   T   
   A   
   A   
   T   
   A   
   T   
   T   
   T   
   A   
   T   
   T   
   T   
   T   
   G   
   C   
    C    
   C   
   A   
   A   
   C   
   A   
   A   
   A   
   C   
   A   
   T   
   T   
   A   
   C   
   G   
    G    
   A   
   T   
   G   
   G   
   G   
    A    
   G   
   A   
   A   
   G   
   A   
   C   
   A   
   A   
   A   
   T   
   T   
   T   
   A   
   G   
   A   
   G   
   C   
    G    
   G   
   G   
   T   
   C   
   T   
   T   
   A   
   A   
   T   
   A   
   A   
   A   
   T   
   T   
   C   
   A   
   T   
   T   
   T   
   T   
   C   
   G   
   G   
   T   
   G   
   A   
   G   
   G   
   A   
   A   
   C   
   T   
   A   
   G   
   A   
   T   
   T   
   T   
   A   
   C   
   C   
   A   
   A   
   T   
    C    
   G   
   C   
   C   
   A   
   T   
   G   
   A   
   C   
   G   
   C   
   C   
    T    
   G   
   C   
   T   
   C   
   A   
   A   
   A   
   T   
   T   
   T   
   C   
   A   
   A   
   A   
    T    
   G   
   A   
   A   
   G   
   A   
   T   
   A   
   A   
   A   
   T   
   T   
   T   
   A   
   A   
   T   
   T   
   C   
   T   
   G   
   A   
   A   
   A   
   T   
   T   
   C   
   T   
   T   
   T   
   T   
    A    
   G   
   C   
   A   
   G   
   A   
   T   
   A   
   C   
    G    
   G   
   G   
   C   
   T   
   A   
    C    
   G   
   G   
    T    
   C   
   A   
   A   
   G   
   G   
    T    
   C   
   A   
   A   
   T   
   T   
   G   
   T   
   T   
   A   
   A   
   T   
   T   
   T   
   C   
    G    
   C   
   C   
   A   
   A   
   T   
   C   
   C   
   A   
   G   
   C   
   A   
   A   
   G   
   C   
   T   
   A   
   C   
   C   
   A   
   T   
   G   
   T   
   A   
   T   
   A   
   G   
   T   
   G   
   T   
   T   
   T   
   T   
   C   
   C   
   A   
   A   
   A   
   A   
   T   
   A   
   A   
   T   
   G   
   G   
    A    
   A   
   C   
   A   
    T    
   T   
    A    
   G   
   T   
    C    
   T   
   A   
   T   
   C   
   C   
   A   
   A   
   A   
   A   
    C    
   T   
   A   
   G   
   T   
   T   
   T   
   T   
   A   
   G   
   A   
   C   
   A   
   A   
   A   
   G   
   A   
   A   
   A   
   C   
    G    
   A   
   A   
   G   
   A   
   A   
   A   
   A   
   A   
   A   
   G   
   A   
   C   
   A   
   A   
   T   
   G   
   T   
   A   
   A   
   T   
   C   
   A   
   G   
   C   
   G   
   C   
   C   
   A   
   A   
   C   
   G   
   C   
   A   
   G   
   C   
   G   
   A   
   A   
   T   
   A   
   C   
   A   
   A   
   T   
   T   
   G   
   C   
   C   
   A   
   C   
   A   
   G   
   A   
   T   
   C   
   T   
   T   
   T   
   T   
   A   
   G   
   G   
   T   
   A   
   G   
   T   
   G   
   T   
   G   
   G   
   A   
   A   
   G   
   A   
   T   
   C   
   C   
   T   
   T   
   C   
   A   
   G   
   G   
   T   
   T   
   A   
   T   
   G   
   T   
   T   
   T   
   A   
   T   
   A   
   A   
   T   
   A   
   T   
   G   
   T   
   A   
   C   
   A   
   A   
   T   
   C   
   C   
   G   
   A   
   A   
   C   
   T   
   T   
   C   
   T   
   C   
   A   
   T   
   T   
   G   
   G   
   C   
   T   
   G   
   C   
   T   
   A   
   A   
   A   
   A   
   C   
   A   
   G   
   G   
   A   
   A   
   C   
   A   
   G   
   C   
   T   
   G   
   A   
   A   
   A   
   T   
   A   
   A   
   A   
   A   
   G   
   A   
   T   
   A   
   A   
   A   
   C   
   A   
   A   
   G   
   A   
   T   
   A   
   C   
   A   
   G   
   A   
   T   
   G   
   G   
   C   
   A   
   A   
   A   
   G   
   A   
   A   
   A   
   A   
   C   
   A   
   G   
   T   
   T   
   T   
   C   
   C   
   T   
   T   
   C   
   T   
   A   
   A   
   C   
   G   
   T   
   T   
   A   
   G   
   A   
   T   
   C   
   G   
   T   
   A   
   G   
   T   
   A   
   A   
   C   
   A   
   A   
   T   
   A   
   A   
   A   
   T   
   T   
   C   
   T   
   T   
   A   
   A   
   C   
   A   
   A   
   T   
   G   
   A   
   T   
   T   
   A   
   T   
   G   
   G   
   T   
   T   
   G   
   A   
   A   
   A   
   A   
   T   
   T   
   C   
   A   
   G   
   G   
   A   
   G   
   A   
   A   
   A   
   A   
   T   
   G   
   G   
   T   
   T   
   C   
   T   
   G   
   C   
   A   
   A   
   C   
   A   
   G   
   A   
   T   
   A   
   T   
   C   
   A   
   G   
   T   
   A   
   A   
   A   
   C   
   C   
   A   
   T   
   T   
   A   
   A   
   T   
   T   
   G   
   A   
   T   
   T   
   A   
   C   
   T   
   T   
   A   
   G   
   A   
   A   
   G   
   C   
   A   
   A   
   C   
   C   
   A   
   T   
   T   
   A   
   A   
   A   
   -   
   -   
   -   
   -   
   -   
   -   
   -   
   -   
   -   
   -   
   -   
   -   
   -   
   -   
   -   
   -   
   -   
   -   
   -   
   -   
   -   
   -   
   -   
   -   
   -   
   -   
   -   
   -   
   -   
   -   
   -   
   -   
   -   
   -   
   -   
   -   
   -   
   -   
   -   
   -   
   -   
   -   
   -   
   -   
   -   
   -   
   -   
   -   
   -   
   -   
   -   
   -   
   -   
   -   
   -   
   -   
   -   
   -   
   -   
   -   
   -   
   -   
   -   
   -   
   -   
   -   
   -   
   -   
   -   
   -   
   -   
   -   
   -   
   -   
   -   
   -   
   -   
   -   
   -   
   -   
   -   
   -   
   -   
   -   
   -   
   -   
   -   
   -   
   -   
   -   
   -   
   -   
   -   
   -   
   -   
   -   
   -   
   -   
   -   
   -   
   -   
   -   
   -   
   -   
   -   
   -   
   -   
   -   
   -   
   -   
   -   
   -   
   -   
   -   
   -   
   -   
   -   
   -   
   -   
   -   
   -   
   -   
   -   
   -   
   -   
   -   
   -   
   -   
   -   
   -   
   -   
   -   
   -   
   -   
   -   
   -   
   -   
   -   
   -   
   -   
   -   
   -   
   -   
   -   
   -   
   -   
   -   
   -   
   -   
   -   
   -   
   -   
   -   
   -   
   -   
   -   
   -   
   -   
   -   
   -   
   -   
   -   
   -   
   -   
   -   
   -   
   -   
   -   
   -   
   C   
   A   
   A   
   T   
   G   
   G   
   C   
   A   
   A   
   G   
   C   
   C   
   A   
   A   
   A   
   C   
   A   
   A   
   G   
   A   
   A   
   T   
   T   
   A   
   G   
   C   
   C   
   G   
   A   
   A   
   G   
   C   
   G   
   A   
   A   
   G   
   A   
   A   
   A   
   A   
   C   
   A   
   G   
   C   
   T   
   A   
   C   
   T   
   A   
   C   
   A   
   T   
   T   
   T   
   T   
   T   
   A   
   A   
   A   
   C   
   G   
   T   
   A   
   T   
   T   
   G   
   T   
   C   
   A   
   A   
   A   
   A   
   C   
   A   
   G   
   G   
   A   
   A   
   T   
   T   
   T   
   G   
   A   
   T   
   A   
   A   
   G   
   T   
   T   
   A   
   C   
   C   
   G   
   T   
   C   
   C   
   G   
   T   
   T   
   G   
   T   
   T   
   C   
   A   
   A   
   G   
   A   
   A   
   G   
   C   
   T   
   A   
   G   
   C   
   T   
   T   
   A   
   A   
   A   
   G   
   A   
   A   
   A   
   A   
   A   
   T   
   G   
   G   
   C   
   T   
   A   
   T   
   G   
   A   
   T   
   A   
   C   
   T   
   A   
   A   
   A   
   T   
   C   
   T   
   G   
   T   
   T   
   G   
   T   
   T   
   G   
   A   
   A   
   A   
   A   
   A   
   T   
   A   
   C   
   C   
   A   
   A   
   G   
   C   
   A   
   A   
   T   
   T   
   T   
   A   
   T   
   T   
   C   
   A   
   G   
   G   
   G   
   A   
   T   
   T   
   C   
   A   
   A   
   G   
   C   
   A   
   G   
   A   
   A   
   G   
   G   
   A   
   G   
   T   
   C   
   A   
   A   
   A   
   G   
   C   
   T   
   A   
   G   
   T   
   G   
   A   
   T   
   G   
   T   
   T   
   C   
   A   
   A   
   G   
   T   
   C   
   A   
   A   
   A   
   A   
   A   
   G   
   G   
   C   
   G   
   A   
   A   
   A   
   G   
   A   
   C   
   A   
   A   
   T   
   C   
   A   
   A   
   T   
   A   
   C   
   A   
   C   
   A   
   T   
   T   
   T   
   A   
   C   
   C   
   T   
   A   
   T   
   A   
   A   
   A   
   T   
   T   
   A   
   T   
   C   
   G   
   A   
   T   
   G   
   A   
   G   
   C   
   A   
   C   
   G   
   C   
   C   
   T   
   T   
   T   
   A   
   G   
   G   
   C   
   G   
   A   
   A   
   A   
   T   
   G   
   A   
   A   
   A   
   G   
   A   
   T   
   T   
   T   
   G   
   T   
   C   
   T   
   T   
   A   
   T   
   C   
   A   
   A   
   T   
   C   
   A   
   A   
   G   
   T   
   A   
   T   
   C   
   G   
   C   
   C   
   A   
   A   
   A   
   A   
   A   
   A   
   G   
   G   
   C   
   G   
   A   
   T   
   A   
   C   
   C   
   T   
   A   
   C   
   C   
   A   
   A   
   A   
   T   
   C   
   G   
   C   
   T   
   T   
   G   
   G   
   A   
   A   
   G   
   C   
   C   
   A   
   T   
   C   
   T   
   T   
   T   
   A   
   A   
   T   
   T   
   T   
   T   
   T   
   C   
   C   
   A   
   G   
   A   
   T   
   A   
   T   
   G   
   T   
   C   
   A   
   G   
   G   
   A   
   A   
   A   
   T   
   G   
   A   
   T   
   A   
   A   
   A   
   A   
   T   
   T   
   T   
   C   
   G   
   A   
   T   
   T   
   C   
   A   
   A   
   G   
   T   
   A   
   G   
   A   
   T   
   A   
   A   
   T   
   G   
   C   
   C   
   A   
   A   
   A   
   C   
   G   
   T   
   G   
   G   
   A   
   G   
   A   
   A   
   A   
   T   
   T   
   G   
   T   
   C   
   G   
   A   
   T   
   C   
   G   
   T   
   A   
   A   
   T   
   G   
   G   
   T   
   A   
   G   
   T   
   G   
   G   
   G   
   C   
   T   
   A   
   G   
   C   
   A   
   A   
   T   
   T   
   A   
   A   
   C   
   A   
   A   
   A   
   G   
   T   
   G   
   T   
   T   
   T   
   G   
   A   
   C   
   G   
   A   
   A   
   G   
   T   
   G   
   G   
   G   
   C   
   G   
   T   
   A   
   G   
   T   
   G   
   C   
   C   
   T   
   G   
   G   
   C   
   A   
   A   
   A   
   C   
   T   
   C   
   G   
   G   
   T   
   T   
   C   
   T   
   G   
   G   
   C   
   G   
   C   
   A   
   G   
   A   
   A   
   A   
   A   
   A   
   A   
   C   
   A   
   G   
   C   
   C   
   A   
   A   
   T   
   A   
   T   
   C   
   A   
   A   
   A   
   G   
   C   
   T   
   T   
   T   
   T   
   A   
   G   
   T   
   G   
   A   
   T   
   A   
   A   
   A   
   T   
   T   
   C   
   G   
   G   
   C   
   G   
   T   
   T   
   T   
   C   
   T   
   G   
   T   
   T   
   G   
   A   
   T   
   G   
   A   
   A   
   A   
   T   
   C   
   A   
   A   
   T   
   C   
   A   
   A   
   A   
   A   
   G   
   T   
   T   
   A   
   A   
   G   
   C   
   C   
   A   
   A   
   G   
   G   
   A   
   T   
   G   
   G   
   G   
   T   
   C   
   C   
   A   
   A   
   G   
   C   
   A   
   G   
   A   
   C   
   T   
   C   
   C   
   T   
   T   
   T   
   G   
   T   
   A   
   C   
   C   
   A   
   A   
   T   
   C   
   A   
   C   
   A   
   G   
   T   
   C   
   G   
   C   
   T   
   T   
   C   
   T   
   G   
   A   
   A   
   C   
   C   
   A   
   G   
   T   
   G   
   A   
   C   
   A   
   G   
   A   
   A   
   T   
   T   
   A   
   C   
   C   
   A   
   A   
   C   
   A   
   G   
   G   
   G   
   G   
   C   
   T   
   G   
   C   
   G   
   A   
   C   
   A   
   A   
   A   
   A   
   G   
   A   
   T   
   A   
   C   
   A   
   G   
   A   
   G   
   T   
   C   
   A   
   C   
   G   
   T   
   T   
   A   
   T   
   T   
   A   
   T   
   C   
   C   
   G   
   C   
   T   
   G   
   G   
   G   
   G   
   G   
   A   
   A   
   G   
   C   
   A   
   G   
   C   
   T   
   G   
   C   
   G   
   C   
   A   
   A   
   T   
   T   
   A   
   A   
   T   
   C   
   G   
   G   
   G   
   T   
   A   
   T   
   A   
   C   
   G   
   G   
   G   
   C   
   A   
   C   
   C   
   A   
   T   
   T   
   A   
   C   
   T   
   G   
   C   
   A   
   G   
   A   
   A   
   G   
   A   
   T   
   A   
   T   
   T   
   G   
   A   
   A   
   A   
   A   
   A   
   A   
   A   
   T   
   C   
   C   
   A   
   G   
   A   
   G   
   T   
   T   
   A   
   A   
   G   
   T   
   A   
   G   
   T   
   A   
   C   
   G   
   G   
   G   
   C   
   G   
   T   
   A   
   A   
   T   
   T   
   G   
   G   
   T   
   A   
   A   
   A   
   A   
   C   
   T   
   G   
   G   
   C   
   T   
   T   
   A   
   G   
   A   
   A   
   C   
   G   
   A   
   G   
   C   
   G   
   T   
   T   
   T   
   G   
   A   
   T   
   A   
   A   
   A   
   G   
   A   
   A   
   T   
   T   
   A   
   A   
   G   
   A   
   G   
   G   
   G   
   C   
   A   
   A   
   G   
   A   
   T   
   G   
   G   
   T   
   G   
   G   
   C   
   T   
   C   
   A   
   C   
   T   
   A   
   G   
   T   
   T   
   A   
   T   
   T   
   T   
   T   
   A   
   G   
   A   
   T   
   G   
   A   
   C   
   A   
   A   
   A   
   G   
   A   
   G   
   A   
   A   
   T   
   G   
   T   
   C   
   A   
   A   
   A   
   A   
   A   
   A   
   G   
   C   
   T   
   T   
   T   
   A   
   C   
   A   
   A   
   A   
   C   
   C   
   A   
   A   
   A   
   G   
   A   
   G   
   A   
   A   
   A   
   A   
   A   
   A   
   G   
   A   
   T   
   G   
   G   
   T   
   C   
   A   
   A   
   A   
   C   
   G   
   A   
   T   
   T   
   A   
   A   
   A   
   T   
   T   
   A   
   A   
   C   
   G   
   A   
   T   
   T   
   G   
   A   
   C   
   A   
   G   
   T   
   G   
   G   
   C   
   G   
   T   
   A   
   C   
   A   
   A   
   C   
   A   
   A   
   C   
   A   
   A   
   G   
   C   
   T   
   T   
   T   
   T   
   G   
   C   
   T   
   A   
   T   
   A   
   T   
   T   
   T   
   G   
   A   
   C   
   A   
   A   
   A   
   C   
   G   
   C   
   C   
   C   
   T   
   G   
   G   
   T   
   A   
   G   
   T   
   G   
   C   
   G   
   G   
   T   
   C   
   A   
   T   
   T   
   A   
   C   
   C   
   G   
   A   
   T   
   C   
   C   
   T   
   C   
   A   
   A   
   A   
   A   
   A   
   G   
   G   
   C   
   G   
   A   
   T   
   T   
   T   
   A   
   T   
   T   
   A   
   G   
   C   
   A   
   A   
   C   
   G   
   G   
   T   
   T   
   A   
   G   
   T   
   T   
   C   
   A   
   C   
   C   
   T   
   T   
   C   
   G   
   T   
   A   
   T   
   G   
   A   
   T   
   C   
   C   
   T   
   A   
   A   
   T   
   A   
   A   
   A   
   A   
   T   
   G   
   G   
   C   
   C   
   A   
   A   
   T   
   G   
   G   
   C   
   A   
   T   
   T   
   T   
   C   
   T   
   C   
   A   
   A   
   A   
   A   
   A   
   G   
   A   
   A   
   T   
   A   
   T   
   G   
   A   
   T   
   G   
   C   
   T   
   T   
   A   
   C   
   A   
   A   
   T   
   A   
   A   
   C   
   A   
   A   
   C   
   A   
   A   
   A   
   G   
   A   
   T   
   T   
   T   
   A   
   C   
   C   
   A   
   T   
   T   
   C   
   A   
   C   
   A   
   G   
   C   
   A   
   C   
   G   
   T   
   T   
   T   
   T   
   G   
   C   
   G   
   A   
   C   
   A   
   G   
   G   
   C   
   T   
   A   
   T   
   G   
   C   
   A   
   C   
   C   
   A   
   G   
   G   
   C   
   T   
   C   
   T   
   A   
   C   
   C   
   T   
   T   
   T   
   A   
   A   
   A   
   A   
   C   
   A   
   A   
   T   
   T   
   A   
   C   
   C   
   G   
   G   
   T   
   G   
   C   
   C   
   A   
   T   
   T   
   G   
   G   
   T   
   T   
   T   
   A   
   G   
   A   
   T   
   G   
   C   
   A   
   G   
   G   
   G   
   A   
   C   
   A   
   T   
   T   
   G   
   A   
   A   
   G   
   C   
   C   
   A   
   G   
   A   
   T   
   G   
   A   
   A   
   G   
   A   
   G   
   C   
   T   
   A   
   G   
   A   
   A   
   A   
   T   
   T   
   A   
   A   
   T   
   G   
   G   
   T   
   T   
   T   
   G   
   A   
   A   
   A   
   T   
   G   
   G   
   C   
   A   
   A   
   A   
   A   
   A   
   G   
   A   
   T   
   A   
   A   
   A   
   T   
   C   
   T   
   T   
   G   
   G   
   G   
   G   
   C   
   G   
   G   
   C   
   T   
   A   
   T   
   T   
   T   
   T   
   G   
   C   
   G   
   A   
   C   
   A   
   C   
   G   
   T   
   G   
   T   
   A   
   A   
   A   
   A   
   G   
   A   
   A   
   G   
   C   
   A   
   A   
   G   
   T   
   C   
   C   
   A   
   G   
   T   
   C   
   A   
   A   
   T   
   C   
   T   
   G   
   C   
   G   
   T   
   A   
   C   
   C   
   G   
   C   
   T   
   T   
   T   
   A   
   G   
   T   
   T   
   A   
   A   
   T   
   T   
   C   
   A   
   G   
   A   
   T   
   A   
   A   
   T   
   A   
   T   
   T   
   T   
   A   
   T   
   T   
   T   
   T   
   G   
   C   
   C   
   C   
   A   
   A   
   C   
   A   
   A   
   A   
   C   
   A   
   T   
   T   
   A   
   C   
   G   
   G   
   A   
   T   
   G   
   G   
   G   
   A   
   G   
   A   
   A   
   G   
   A   
   C   
   A   
   A   
   A   
   T   
   T   
   T   
   A   
   G   
   A   
   G   
   C   
   G   
   G   
   G   
   T   
   C   
   T   
   T   
   A   
   A   
   T   
   A   
   A   
   A   
   T   
   T   
   C   
   A   
   T   
   T   
   T   
   T   
   C   
   G   
   G   
   T   
   G   
   A   
   G   
   G   
   A   
   A   
   C   
   T   
   A   
   G   
   A   
   T   
   T   
   T   
   A   
   C   
   C   
   A   
   A   
   T   
   C   
   G   
   C   
   C   
   A   
   T   
   G   
   A   
   C   
   G   
   C   
   C   
   T   
   G   
   C   
   T   
   C   
   A   
   A   
   A   
   T   
   T   
   T   
   C   
   A   
   A   
   A   
   T   
   G   
   A   
   A   
   G   
   A   
   T   
   A   
   A   
   A   
   T   
   T   
   T   
   A   
   A   
   T   
   T   
   C   
   T   
   G   
   A   
   A   
   A   
   T   
   T   
   C   
   T   
   T   
   T   
   T   
   A   
   G   
   C   
   A   
   G   
   A   
   T   
   A   
   C   
   G   
   G   
   G   
   C   
   T   
   A   
   C   
   G   
   G   
   T   
   C   
   A   
   A   
   G   
   G   
   T   
   C   
   A   
   A   
   T   
   T   
   G   
   T   
   T   
   A   
   A   
   T   
   T   
   T   
   C   
   G   
   C   
   C   
   A   
   A   
   T   
   C   
   C   
   A   
   G   
   C   
   A   
   A   
   G   
   C   
   T   
   A   
   C   
   C   
   A   
   T   
   G   
   T   
   A   
   T   
   A   
   G   
   T   
   G   
   T   
   T   
   T   
   T   
   C   
   C   
   A   
   A   
   A   
   A   
   T   
   A   
   A   
   T   
   G   
   G   
   A   
   A   
   C   
   A   
   T   
   T   
   A   
   G   
   T   
   C   
   T   
   A   
   T   
   C   
   C   
   A   
   A   
   A   
   A   
   C   
   T   
   A   
   G   
   T   
   T   
   T   
   T   
   A   
   G   
   A   
   C   
   A   
   A   
   A   
   G   
   A   
   A   
   A   
   C   
   G   
   A   
   A   
   G   
   A   
   A   
   A   
   A   
   A   
   A   
   G   
   A   
   C   
   A   
   A   
   T   
   G   
   T   
   A   
   A   
   T   
   C   
   A   
   G   
   C   
   G   
   C   
   C   
   A   
   A   
   C   
   G   
   C   
   A   
   G   
   C   
   G   
   A   
   A   
   T   
   A   
   C   
   A   
   A   
   T   
   T   
   G   
   C   
   C   
   A   
   C   
   A   
   G   
   A   
   T   
   C   
   T   
   T   
   T   
   T   
   A   
   G   
   G   
   T   
   A   
   G   
   T   
   G   
   T   
   G   
   G   
   A   
   A   
   G   
   A   
   T   
   C   
   C   
   T   
   T   
   C   
   A   
   G   
   G   
   T   
   T   
   A   
   T   
   G   
   T   
   T   
   T   
   A   
   T   
   A   
   A   
   T   
   A   
   T   
   G   
   T   
   A   
   C   
   A   
   A   
   T   
   C   
   C   
   G   
   A   
   A   
   C   
   T   
   T   
   C   
   T   
   C   
   A   
   T   
   T   
   G   
   G   
   C   
   T   
   G   
   C   
   T   
   A   
   A   
   A   
   A   
   C   
   A   
   G   
   G   
   A   
   A   
   C   
   A   
   G   
   C   
   T   
   G   
   A   
   A   
   A   
   T   
   A   
   A   
   A   
   A   
   G   
   A   
   T   
   A   
   A   
   A   
   C   
   A   
   A   
   G   
   A   
   T   
   A   
   C   
   A   
   G   
   A   
   T   
   G   
   G   
   C   
   A   
   A   
   A   
   G   
   A   
   A   
   A   
   A   
   C   
   A   
   G   
   T   
   T   
   T   
   C   
   C   
   T   
   T   
   C   
   T   
   A   
   A   
   C   
   G   
   T   
   T   
   A   
   G   
   A   
   T   
   C   
   G   
   T   
   A   
   G   
   T   
   A   
   A   
   C   
   A   
   A   
   T   
   A   
   A   
   A   
   T   
   T   
   C   
   T   
   T   
   A   
   A   
   C   
   A   
   A   
   T   
   G   
   A   
   T   
   T   
   A   
   T   
   G   
   G   
   T   
   T   
   G   
   A   
   A   
   A   
   A   
   T   
   T   
   C   
   A   
   G   
   G   
   A   
   G   
   A   
   A   
   A   
   A   
   T   
   G   
   G   
   T   
   T   
   C   
   T   
   G   
   C   
   A   
   A   
   C   
   A   
   G   
   A   
   T   
   A   
   T   
   C   
   A   
   G   
   T   
   A   
   A   
   A   
   C   
   C   
   A   
   T   
   T   
   A   
   A   
   T   
   T   
   G   
   A   
   T   
   T   
   A   
   C   
   T   
   T   
   A   
   G   
   A   
   A   
   G   
   C   
   A   
   A   
   C   
   C   
   A   
   T   
   T   
   A   
   A   
   A   
   -   
   -   
   -   
   -   
   -   
   -   
   -   
   -   
   -   
   -   
   -   
   -   
   -   
   -   
   -   
   -   
   -   
   -   
   -   
   -   
   -   
   -   
   -   
   -   
   -   
   -   
   -   
   -   
   -   
   -   
   -   
   -   
   -   
   -   
   -   
   -   
   -   
   -   
   -   
   -   
   -   
   -   
   -   
   -   
   -   
   -   
   -   
   -   
   -   
   -   
   -   
   -   
   -   
   -   
   -   
   -   
   -   
   -   
   -   
   -   
   -   
 

 
   N55706   
   -   
   -   
   -   
   -   
   -   
   -   
   -   
   -   
   -   
   -   
   -   
   -   
   -   
   -   
   -   
   -   
   -   
   -   
   -   
   -   
   -   
   -   
   -   
   -   
   -   
   -   
   -   
   -   
   -   
   -   
   -   
   -   
   -   
   -   
   -   
   -   
   -   
   -   
   -   
   -   
   -   
   -   
   -   
   -   
   -   
   -   
   -   
   -   
   -   
   -   
   -   
   -   
   -   
   -   
   -   
   -   
   -   
   -   
   -   
   -   
   -   
   -   
   -   
   -   
   -   
   -   
   -   
   -   
   -   
   -   
   -   
   -   
   -   
   -   
   -   
   -   
   -   
   -   
   -   
   -   
   -   
   -   
   -   
   -   
   -   
   -   
   -   
   -   
   -   
   -   
   -   
   -   
   -   
   -   
   -   
   -   
   -   
   -   
   -   
   -   
   -   
   -   
   -   
   -   
   -   
   -   
   -   
   -   
   C   
   A   
   A   
   T   
   G   
   G   
   C   
   A   
   A   
   G   
   C   
   C   
   A   
   A   
   A   
   C   
   A   
   A   
   G   
   A   
    A    
   T   
   T   
   A   
   G   
   C   
    C    
   G   
   A   
   A   
   G   
   C   
   G   
   A   
   A   
   G   
   A   
   A   
   A   
   A   
    C    
   A   
   G   
   C   
    T    
   A   
    C    
   T   
    A    
    A    
   A   
   T   
   T   
   T   
   T   
   T   
   A   
   A   
   A   
    C    
   G   
   T   
   A   
   T   
   T   
   G   
    T    
   C   
   A   
   A   
   A   
   A   
   C   
   A   
   G   
    G    
   A   
   A   
   T   
   T   
   T   
   G   
   A   
   T   
   A   
   A   
   G   
   T   
   T   
   A   
   C   
   C   
    G    
   T   
   C   
   C   
   G   
   T   
   T   
    G    
   T   
   T   
   C   
   A   
   A   
   G   
   A   
   A   
    G    
   C   
   T   
   A   
   G   
   C   
   T   
   T   
   A   
   A   
   A   
   G   
   A   
   A   
   A   
   A   
   A   
   T   
   G   
   G   
    C    
   T   
   A   
   T   
   G   
   A   
   T   
   A   
   C   
   T   
   A   
   A   
   A   
   T   
   C   
    T    
   G   
   T   
   T   
   G   
   T   
   T   
   G   
   A   
   A   
   A   
   A   
   A   
   T   
   A   
   C   
   C   
   A   
   A   
   G   
   C   
   A   
   A   
   T   
   T   
   T   
   A   
   T   
   T   
   C   
   A   
   G   
   G   
   G   
   A   
   T   
   T   
   C   
   A   
   A   
   G   
   C   
    A    
   G   
   A   
   A   
    G    
   G   
    A    
   G   
   T   
   C   
   A   
   A   
    A    
   G   
   C   
   T   
   A   
   G   
   T   
   G   
   A   
   T   
   G   
   T   
    C    
   C   
   A   
   A   
   G   
   T   
    C    
   A   
   A   
   A   
   A   
   A   
    G    
   G   
    C    
   G   
   A   
   A   
   A   
   G   
   A   
    C    
   A   
   A   
    T    
   C   
   A   
   A   
   T   
   A   
   C   
   A   
   C   
   A   
   T   
   T   
   T   
   A   
    C    
   C   
   T   
   A   
   T   
   A   
   A   
   A   
   T   
   T   
   A   
   T   
   C   
   G   
   A   
   T   
   G   
   A   
   G   
    C    
   A   
   C   
    G    
   C   
   C   
   T   
   T   
   T   
   A   
   G   
   G   
   C   
   G   
   A   
   A   
   A   
   T   
   G   
   A   
   A   
   A   
   G   
   A   
   T   
    T    
   T   
   G   
   T   
   C   
   T   
   T   
   A   
   T   
   C   
   A   
   A   
   T   
   C   
   A   
   A   
   G   
   T   
   A   
   T   
    C    
   G   
    C    
   C   
   A   
   A   
    A    
   A   
   A   
   A   
   G   
   G   
    C    
   G   
   A   
   T   
   A   
    C    
   C   
   T   
   A   
   C   
   C   
   A   
   A   
   A   
   T   
    C    
    G    
   C   
   T   
   T   
   G   
   G   
    A    
    A    
    G    
   C   
   C   
    G    
   T   
   C   
   T   
   T   
   T   
   A   
   A   
   T   
   T   
   T   
   T   
   T   
   C   
   C   
   A   
   G   
   A   
   T   
   A   
   T   
   G   
   T   
   C   
   A   
   G   
   G   
   A   
   A   
   A   
   T   
   G   
   A   
   T   
   A   
   A   
   A   
    A    
   T   
   T   
   T   
   C   
   G   
   A   
   T   
   T   
   C   
   A   
   A   
   G   
   T   
   A   
   G   
   A   
   T   
   A   
   A   
   T   
   G   
   C   
   C   
   A   
   A   
   A   
   C   
   G   
   T   
   G   
   G   
   A   
   G   
   A   
   A   
   A   
   T   
   T   
   G   
   T   
   C   
   G   
   A   
   T   
   C   
   G   
   T   
   A   
   A   
    T    
   G   
   G   
   T   
   A   
   G   
   T   
   G   
   G   
   G   
   C   
   T   
   A   
   G   
    C    
   A   
   A   
   T   
   T   
   A   
   A   
   C   
   A   
   A   
   A   
   G   
   T   
   G   
   T   
   T   
   T   
   G   
   A   
    C    
   G   
   A   
   A   
   G   
   T   
   G   
   G   
   G   
   C   
   G   
   T   
   A   
   G   
   T   
    G    
   C   
   C   
   T   
    G    
   G   
   C   
   A   
   A   
   A   
   C   
   T   
    C    
   G   
   G   
   T   
   T   
   C   
   T   
   G   
   G   
   C   
   G   
   C   
   A   
   G   
   A   
   A   
   A   
   A   
   A   
    A    
   C   
   A   
   G   
   C   
    C    
   A   
   A   
   T   
   A   
   T   
   C   
   A   
   A   
   A   
   G   
   C   
   T   
   T   
   T   
   T   
   A   
   G   
   T   
   G   
   A   
   T   
   A   
   A   
    G    
   T   
   T   
    C    
   G   
   G   
   C   
   G   
   T   
   T   
   T   
   C   
   T   
    G    
   T   
   T   
   G   
   A   
   T   
   G   
   A   
   A   
   A   
   T   
   C   
   A   
   A   
   T   
   C   
    A    
   A   
   A   
   A   
    G    
   T   
   T   
   A   
   A   
   G   
   C   
   C   
   A   
   A   
   G   
   G   
    G    
   T   
   G   
   G   
   G   
   T   
   C   
   C   
   A   
   A   
   G   
   C   
   A   
   G   
   A   
   C   
   T   
   C   
    C    
   T   
   T   
   T   
   G   
   T   
   A   
   C   
   C   
   A   
   A   
   T   
   C   
   A   
   C   
   A   
   G   
   T   
    C    
   G   
   C   
   T   
   T   
   C   
   T   
   G   
   A   
   A   
   C   
   C   
   A   
   G   
   T   
   G   
   A   
   C   
   A   
   G   
   A   
   A   
   T   
   T   
   A   
   C   
   C   
   A   
   A   
   C   
   A   
   G   
   G   
   G   
   G   
   C   
   T   
   G   
   C   
   G   
   A   
   C   
   A   
   A   
   A   
   A   
   G   
    A    
    T    
   A   
   C   
   A   
   G   
   A   
   G   
   T   
   C   
   A   
   C   
   G   
   T   
   T   
   A   
   T   
   T   
   A   
   T   
   C   
   C   
    G    
    C    
   T   
   G   
   G   
   G   
   G   
   G   
   A   
   A   
   G   
   C   
   A   
   G   
   C   
   T   
   G   
   C   
   G   
   C   
   A   
   A   
   T   
   T   
   A   
   A   
   T   
   C   
   G   
   G   
    G    
   T   
   A   
   T   
   A   
   C   
    G    
   G   
   G   
    C    
   A   
   C   
    C    
   A   
   T   
   T   
   A   
   C   
   T   
   G   
   C   
   A   
   G   
   A   
    A    
   G   
   A   
   T   
   A   
   T   
   T   
    A    
   A   
   A   
   A   
   A   
   A   
   A   
   A   
   T   
   C   
   C   
   A   
   G   
   A   
   G   
   T   
   T   
   A   
   A   
   G   
   T   
   A   
   G   
   T   
   A   
   C   
   G   
   G   
   G   
    C    
   G   
   T   
   A   
   A   
   T   
   T   
   G   
   G   
    T    
   A   
   A   
   A   
   A   
   C   
   T   
   G   
   G   
    C    
   T   
   T   
   A   
   G   
   A   
   A   
   C   
   G   
   A   
   G   
   C   
   G   
   T   
   T   
   T   
   G   
   A   
   T   
   A   
   A   
   A   
   G   
   A   
    A    
   T   
   T   
   A   
   A   
   G   
   A   
   G   
   G   
    G    
   C   
   A   
   A   
   G   
   A   
   T   
   G   
   G   
    T    
   G   
   G   
    C    
   T   
   C   
    A    
   C   
   T   
   A   
   G   
   T   
   T   
   A   
   T   
   T   
   T   
   T   
   A   
   G   
   A   
   T   
   G   
   A   
   C   
   A   
   A   
   A   
   G   
   A   
   G   
   A   
   A   
   T   
   G   
   T   
   C   
   A   
   A   
   A   
   A   
   A   
   A   
   G   
   C   
   T   
   T   
   T   
   A   
   C   
   A   
   A   
   A   
   C   
   C   
   A   
   A   
   A   
   G   
   A   
    G    
   A   
   A   
   A   
   A   
   A   
   A   
   G   
   A   
   T   
   G   
   G   
   T   
   C   
   A   
   A   
   A   
   C   
    G    
   A   
   T   
   T   
   A   
   A   
   A   
   T   
   T   
   A   
   A   
   C   
   G   
   A   
   T   
   T   
   G   
   A   
    C    
   A   
   G   
    T    
   G   
   G   
    C    
   G   
   T   
   A   
   C   
   A   
   A   
   C   
   A   
   A   
   C   
   A   
   A   
   G   
   C   
    C    
   T   
   T   
    C    
   G   
   C   
   T   
   A   
   T   
   A   
   T   
   T   
   T   
   G   
   A   
   C   
   A   
   A   
   A   
   C   
   G   
    C    
    C    
   C   
   T   
   G   
   G   
   T   
   A   
   G   
   T   
   G   
   C   
   G   
   G   
   T   
   C   
   A   
   T   
   T   
   A   
   C   
   C   
   G   
   A   
   T   
   C   
   C   
   T   
   C   
   A   
   A   
   A   
   A   
   A   
   G   
   G   
   C   
   G   
   A   
   T   
   T   
   T   
   A   
    T    
   T   
   A   
   G   
   C   
    A    
   A   
   C   
   G   
   G   
   T   
   T   
   A   
   G   
   T   
   T   
   C   
   A   
   C   
   C   
   T   
   T   
   C   
   G   
   T   
   A   
   T   
   G   
   A   
   T   
   C   
   C   
   T   
   A   
   A   
   T   
   A   
   A   
   A   
   A   
   T   
   G   
   G   
   C   
   C   
   A   
   A   
   T   
   G   
   G   
    C    
   A   
   T   
   T   
   T   
   C   
   T   
   C   
   A   
   A   
   A   
   A   
   A   
   G   
   A   
    A    
   T   
   A   
   T   
   G   
   A   
   T   
   G   
   C   
   T   
   T   
   A   
   C   
   A   
   A   
   T   
   A   
   A   
   C   
   A   
   A   
   C   
   A   
   A   
   A   
   G   
   A   
   T   
   T   
   T   
   A   
   C   
   C   
   A   
   T   
   T   
    C    
   A   
   C   
   A   
   G   
   C   
   A   
   C   
   G   
   T   
   T   
   T   
    T    
   G   
   C   
   G   
   A   
   C   
   A   
   G   
   G   
    C    
   T   
   A   
   T   
   G   
   C   
   A   
   C   
   C   
   A   
   G   
   G   
    C    
   T   
   C   
   T   
   A   
   C   
   C   
   T   
   T   
   T   
   A   
   A   
   A   
   A   
   C   
   A   
   A   
   T   
   T   
   A   
   C   
   C   
   G   
   G   
   T   
   G   
   C   
   C   
   A   
   T   
   T   
   G   
   G   
   T   
   T   
   T   
   A   
   G   
   A   
   T   
    G    
   C   
   A   
   G   
   G   
   G   
   A   
   C   
   A   
   T   
   T   
   G   
   A   
   A   
   G   
   C   
   C   
   A   
   G   
   A   
   T   
   G   
   A   
   A   
   G   
   A   
   G   
   C   
   T   
   A   
   G   
   A   
   A   
   A   
   T   
   T   
   A   
   A   
   T   
   G   
   G   
    T    
   T   
   T   
   G   
   A   
   A   
   A   
   T   
   G   
   G   
   C   
   A   
   A   
   A   
   A   
   A   
   G   
   A   
   T   
   A   
   A   
   A   
   T   
   C   
   T   
   T   
   G   
   G   
   G   
   G   
    C    
   G   
   G   
   C   
   T   
   A   
   T   
   T   
   T   
   T   
   G   
   C   
    G    
   A   
   C   
   A   
   C   
   G   
   T   
   G   
   T   
   A   
   A   
   A   
   A   
   G   
   A   
   A   
   G   
   C   
   A   
   A   
   G   
   T   
   C   
   C   
   A   
   G   
   T   
   C   
   A   
   A   
   T   
   C   
   T   
    G    
   C   
   G   
   T   
   A   
   C   
   C   
   G   
   C   
   T   
   T   
   T   
   A   
   G   
   T   
   T   
   A   
   A   
   T   
   T   
   C   
   A   
   G   
   A   
   T   
   A   
   A   
   T   
   A   
   T   
   T   
   T   
   A   
   T   
   T   
   T   
   T   
   G   
   C   
    C    
   C   
   A   
   A   
   C   
   A   
   A   
   A   
   C   
   A   
   T   
   T   
   A   
   C   
   G   
    G    
   A   
   T   
   G   
   G   
   G   
    A    
   G   
   A   
   A   
   G   
   A   
   C   
   A   
   A   
   A   
   T   
   T   
   T   
   A   
   G   
   A   
   G   
   C   
    G    
   G   
   G   
   T   
   C   
   T   
   T   
   A   
   A   
   T   
   A   
   A   
   A   
   T   
   T   
   C   
   A   
   T   
   T   
   T   
   T   
   C   
   G   
   G   
   T   
   G   
   A   
   G   
   G   
   A   
   A   
   C   
   T   
   A   
   G   
   A   
   T   
   T   
   T   
   A   
   C   
   C   
   A   
   A   
   T   
    T    
   G   
   C   
   C   
   A   
   T   
   G   
   A   
   C   
   G   
   C   
   C   
    T    
   G   
   C   
   T   
   C   
   A   
   A   
   A   
   T   
   T   
   T   
   C   
   A   
   A   
   A   
    T    
   G   
   A   
   A   
   G   
   A   
   T   
   A   
   A   
   A   
   T   
   T   
   T   
   A   
   A   
   T   
   T   
   C   
   T   
   G   
   A   
   A   
   A   
   T   
   T   
   C   
   T   
   T   
   T   
   T   
    A    
   G   
   C   
   A   
   G   
   A   
   T   
   A   
   C   
    G    
   G   
   G   
   C   
   T   
   A   
    C    
   G   
   G   
    T    
   C   
   A   
   A   
   G   
   G   
    C    
   C   
   A   
   A   
   T   
   T   
   G   
   T   
   T   
   A   
   A   
   T   
   T   
   T   
   C   
    G    
   C   
   C   
   A   
   A   
   T   
   C   
   C   
   A   
   G   
   C   
   A   
   A   
   G   
   C   
   T   
   A   
   C   
   C   
   A   
   T   
   G   
   T   
   A   
   T   
   A   
   G   
   T   
   G   
   T   
   T   
   T   
   T   
   C   
   C   
   A   
   A   
   A   
   A   
   T   
   A   
   A   
   T   
   G   
   G   
    G    
   A   
   C   
   A   
    T    
   T   
    A    
   G   
   T   
    C    
   T   
   A   
   T   
   C   
   C   
   A   
   A   
   A   
   A   
    T    
   T   
   A   
   G   
   T   
   T   
   T   
   T   
   A   
   G   
   A   
   C   
   A   
   A   
   A   
   G   
   A   
   A   
   A   
   C   
    G    
   A   
   A   
   G   
   A   
   A   
   A   
   A   
   A   
   A   
   G   
   A   
   C   
   A   
   A   
   T   
   G   
   T   
   A   
   A   
   T   
   C   
   A   
   G   
   C   
   G   
   C   
   C   
   A   
   A   
   C   
   G   
   C   
   A   
   G   
   C   
   G   
   A   
   A   
   T   
   A   
   C   
   A   
   A   
   T   
   T   
   G   
   C   
   C   
   A   
   C   
   A   
   G   
   A   
   T   
   C   
   T   
   T   
   T   
   T   
   A   
   G   
   G   
   T   
   A   
   G   
   T   
   G   
   T   
   G   
   G   
   A   
   A   
   G   
   A   
   T   
   C   
   C   
   T   
   T   
   C   
   A   
   G   
   G   
   T   
   T   
   A   
   T   
   G   
   T   
   T   
   T   
   A   
   T   
   A   
   A   
   T   
   A   
   T   
   G   
   T   
   A   
   C   
   A   
   A   
   T   
   C   
   C   
   G   
   A   
   A   
   C   
   T   
   T   
   C   
   T   
   C   
   A   
   T   
   T   
   G   
   G   
   C   
   T   
   G   
   C   
   T   
   A   
   A   
   A   
   A   
   C   
   A   
   G   
   G   
   A   
   A   
   C   
   A   
   G   
   C   
   T   
   G   
   A   
   A   
   A   
   T   
   A   
   A   
   A   
   A   
   G   
   A   
   T   
   A   
   A   
   A   
   C   
   A   
   A   
   G   
   A   
   T   
   A   
   C   
   A   
   G   
   A   
   T   
   G   
   G   
   C   
   A   
   A   
   A   
   G   
   A   
   A   
   A   
   A   
   C   
   A   
   G   
   T   
   T   
   T   
   C   
   C   
   T   
   T   
   C   
   T   
   A   
   A   
   C   
   G   
   T   
   T   
   A   
   G   
   A   
   T   
   C   
   G   
   T   
   A   
   G   
   T   
   A   
   A   
   C   
   A   
   A   
   T   
   A   
   A   
   A   
   T   
   T   
   C   
   T   
   T   
   A   
   A   
   C   
   A   
   A   
   T   
   G   
   A   
   T   
   T   
   A   
   T   
   G   
   G   
   T   
   T   
   G   
   A   
   A   
   A   
   A   
   T   
   T   
   C   
   A   
   G   
   G   
   A   
   G   
   A   
   A   
   A   
   A   
   T   
   G   
   G   
   T   
   T   
   C   
   T   
   G   
   C   
   A   
   A   
   C   
   A   
   G   
   A   
   T   
   A   
   T   
   C   
   A   
   G   
   T   
   A   
   A   
   A   
   C   
   C   
   A   
   T   
   T   
   A   
   A   
   T   
   T   
   G   
   A   
   T   
   T   
   A   
   C   
   T   
   T   
   A   
   G   
   A   
   A   
   G   
   C   
   A   
   A   
   C   
   C   
   A   
   T   
   T   
   A   
   A   
   A   
   -   
   -   
   -   
   -   
   -   
   -   
   -   
   -   
   -   
   -   
   -   
   -   
   -   
   -   
   -   
   -   
   -   
   -   
   -   
   -   
   -   
   -   
   -   
   -   
   -   
   -   
   -   
   -   
   -   
   -   
   -   
   -   
   -   
   -   
   -   
   -   
   -   
   -   
   -   
   -   
   -   
   -   
   -   
   -   
   -   
   -   
   -   
   -   
   -   
   -   
   -   
   -   
   -   
   -   
   -   
   -   
   -   
   -   
   -   
   -   
   -   
   -   
   -   
   -   
   -   
   -   
   -   
   -   
   -   
   -   
   -   
   -   
   -   
   -   
   -   
   -   
   -   
   -   
   -   
   -   
   -   
   -   
   -   
   -   
   -   
   -   
   -   
   -   
   -   
   -   
   -   
   -   
   -   
   -   
   -   
   -   
   -   
   -   
   -   
   -   
   -   
   -   
   -   
   -   
   -   
   -   
   -   
   -   
   -   
   -   
   -   
   -   
   -   
   -   
   -   
   -   
   -   
   -   
   -   
   -   
   -   
   -   
   -   
   -   
   -   
   -   
   -   
   -   
   -   
   -   
   -   
   -   
   -   
   -   
   -   
   -   
   -   
   -   
   -   
   -   
   -   
   -   
   -   
   -   
   -   
   -   
   -   
   -   
   -   
   -   
   -   
   -   
   -   
   -   
   -   
   -   
   -   
   -   
   -   
   -   
   -   
   -   
   -   
   -   
   -   
   -   
   -   
   -   
   -   
   C   
   A   
   A   
   T   
   G   
   G   
   C   
   A   
   A   
   G   
   C   
   C   
   A   
   A   
   A   
   C   
   A   
   A   
   G   
   A   
   A   
   T   
   T   
   A   
   G   
   C   
   C   
   G   
   A   
   A   
   G   
   C   
   G   
   A   
   A   
   G   
   A   
   A   
   A   
   A   
   C   
   A   
   G   
   C   
   T   
   A   
   C   
   T   
   A   
   A   
   A   
   T   
   T   
   T   
   T   
   T   
   A   
   A   
   A   
   C   
   G   
   T   
   A   
   T   
   T   
   G   
   T   
   C   
   A   
   A   
   A   
   A   
   C   
   A   
   G   
   G   
   A   
   A   
   T   
   T   
   T   
   G   
   A   
   T   
   A   
   A   
   G   
   T   
   T   
   A   
   C   
   C   
   G   
   T   
   C   
   C   
   G   
   T   
   T   
   G   
   T   
   T   
   C   
   A   
   A   
   G   
   A   
   A   
   G   
   C   
   T   
   A   
   G   
   C   
   T   
   T   
   A   
   A   
   A   
   G   
   A   
   A   
   A   
   A   
   A   
   T   
   G   
   G   
   C   
   T   
   A   
   T   
   G   
   A   
   T   
   A   
   C   
   T   
   A   
   A   
   A   
   T   
   C   
   T   
   G   
   T   
   T   
   G   
   T   
   T   
   G   
   A   
   A   
   A   
   A   
   A   
   T   
   A   
   C   
   C   
   A   
   A   
   G   
   C   
   A   
   A   
   T   
   T   
   T   
   A   
   T   
   T   
   C   
   A   
   G   
   G   
   G   
   A   
   T   
   T   
   C   
   A   
   A   
   G   
   C   
   A   
   G   
   A   
   A   
   G   
   G   
   A   
   G   
   T   
   C   
   A   
   A   
   A   
   G   
   C   
   T   
   A   
   G   
   T   
   G   
   A   
   T   
   G   
   T   
   C   
   C   
   A   
   A   
   G   
   T   
   C   
   A   
   A   
   A   
   A   
   A   
   G   
   G   
   C   
   G   
   A   
   A   
   A   
   G   
   A   
   C   
   A   
   A   
   T   
   C   
   A   
   A   
   T   
   A   
   C   
   A   
   C   
   A   
   T   
   T   
   T   
   A   
   C   
   C   
   T   
   A   
   T   
   A   
   A   
   A   
   T   
   T   
   A   
   T   
   C   
   G   
   A   
   T   
   G   
   A   
   G   
   C   
   A   
   C   
   G   
   C   
   C   
   T   
   T   
   T   
   A   
   G   
   G   
   C   
   G   
   A   
   A   
   A   
   T   
   G   
   A   
   A   
   A   
   G   
   A   
   T   
   T   
   T   
   G   
   T   
   C   
   T   
   T   
   A   
   T   
   C   
   A   
   A   
   T   
   C   
   A   
   A   
   G   
   T   
   A   
   T   
   C   
   G   
   C   
   C   
   A   
   A   
   A   
   A   
   A   
   A   
   G   
   G   
   C   
   G   
   A   
   T   
   A   
   C   
   C   
   T   
   A   
   C   
   C   
   A   
   A   
   A   
   T   
   C   
   G   
   C   
   T   
   T   
   G   
   G   
   A   
   A   
   G   
   C   
   C   
   G   
   T   
   C   
   T   
   T   
   T   
   A   
   A   
   T   
   T   
   T   
   T   
   T   
   C   
   C   
   A   
   G   
   A   
   T   
   A   
   T   
   G   
   T   
   C   
   A   
   G   
   G   
   A   
   A   
   A   
   T   
   G   
   A   
   T   
   A   
   A   
   A   
   A   
   T   
   T   
   T   
   C   
   G   
   A   
   T   
   T   
   C   
   A   
   A   
   G   
   T   
   A   
   G   
   A   
   T   
   A   
   A   
   T   
   G   
   C   
   C   
   A   
   A   
   A   
   C   
   G   
   T   
   G   
   G   
   A   
   G   
   A   
   A   
   A   
   T   
   T   
   G   
   T   
   C   
   G   
   A   
   T   
   C   
   G   
   T   
   A   
   A   
   T   
   G   
   G   
   T   
   A   
   G   
   T   
   G   
   G   
   G   
   C   
   T   
   A   
   G   
   C   
   A   
   A   
   T   
   T   
   A   
   A   
   C   
   A   
   A   
   A   
   G   
   T   
   G   
   T   
   T   
   T   
   G   
   A   
   C   
   G   
   A   
   A   
   G   
   T   
   G   
   G   
   G   
   C   
   G   
   T   
   A   
   G   
   T   
   G   
   C   
   C   
   T   
   G   
   G   
   C   
   A   
   A   
   A   
   C   
   T   
   C   
   G   
   G   
   T   
   T   
   C   
   T   
   G   
   G   
   C   
   G   
   C   
   A   
   G   
   A   
   A   
   A   
   A   
   A   
   A   
   C   
   A   
   G   
   C   
   C   
   A   
   A   
   T   
   A   
   T   
   C   
   A   
   A   
   A   
   G   
   C   
   T   
   T   
   T   
   T   
   A   
   G   
   T   
   G   
   A   
   T   
   A   
   A   
   G   
   T   
   T   
   C   
   G   
   G   
   C   
   G   
   T   
   T   
   T   
   C   
   T   
   G   
   T   
   T   
   G   
   A   
   T   
   G   
   A   
   A   
   A   
   T   
   C   
   A   
   A   
   T   
   C   
   A   
   A   
   A   
   A   
   G   
   T   
   T   
   A   
   A   
   G   
   C   
   C   
   A   
   A   
   G   
   G   
   G   
   T   
   G   
   G   
   G   
   T   
   C   
   C   
   A   
   A   
   G   
   C   
   A   
   G   
   A   
   C   
   T   
   C   
   C   
   T   
   T   
   T   
   G   
   T   
   A   
   C   
   C   
   A   
   A   
   T   
   C   
   A   
   C   
   A   
   G   
   T   
   C   
   G   
   C   
   T   
   T   
   C   
   T   
   G   
   A   
   A   
   C   
   C   
   A   
   G   
   T   
   G   
   A   
   C   
   A   
   G   
   A   
   A   
   T   
   T   
   A   
   C   
   C   
   A   
   A   
   C   
   A   
   G   
   G   
   G   
   G   
   C   
   T   
   G   
   C   
   G   
   A   
   C   
   A   
   A   
   A   
   A   
   G   
   A   
   T   
   A   
   C   
   A   
   G   
   A   
   G   
   T   
   C   
   A   
   C   
   G   
   T   
   T   
   A   
   T   
   T   
   A   
   T   
   C   
   C   
   G   
   C   
   T   
   G   
   G   
   G   
   G   
   G   
   A   
   A   
   G   
   C   
   A   
   G   
   C   
   T   
   G   
   C   
   G   
   C   
   A   
   A   
   T   
   T   
   A   
   A   
   T   
   C   
   G   
   G   
   G   
   T   
   A   
   T   
   A   
   C   
   G   
   G   
   G   
   C   
   A   
   C   
   C   
   A   
   T   
   T   
   A   
   C   
   T   
   G   
   C   
   A   
   G   
   A   
   A   
   G   
   A   
   T   
   A   
   T   
   T   
   A   
   A   
   A   
   A   
   A   
   A   
   A   
   A   
   T   
   C   
   C   
   A   
   G   
   A   
   G   
   T   
   T   
   A   
   A   
   G   
   T   
   A   
   G   
   T   
   A   
   C   
   G   
   G   
   G   
   C   
   G   
   T   
   A   
   A   
   T   
   T   
   G   
   G   
   T   
   A   
   A   
   A   
   A   
   C   
   T   
   G   
   G   
   C   
   T   
   T   
   A   
   G   
   A   
   A   
   C   
   G   
   A   
   G   
   C   
   G   
   T   
   T   
   T   
   G   
   A   
   T   
   A   
   A   
   A   
   G   
   A   
   A   
   T   
   T   
   A   
   A   
   G   
   A   
   G   
   G   
   G   
   C   
   A   
   A   
   G   
   A   
   T   
   G   
   G   
   T   
   G   
   G   
   C   
   T   
   C   
   A   
   C   
   T   
   A   
   G   
   T   
   T   
   A   
   T   
   T   
   T   
   T   
   A   
   G   
   A   
   T   
   G   
   A   
   C   
   A   
   A   
   A   
   G   
   A   
   G   
   A   
   A   
   T   
   G   
   T   
   C   
   A   
   A   
   A   
   A   
   A   
   A   
   G   
   C   
   T   
   T   
   T   
   A   
   C   
   A   
   A   
   A   
   C   
   C   
   A   
   A   
   A   
   G   
   A   
   G   
   A   
   A   
   A   
   A   
   A   
   A   
   G   
   A   
   T   
   G   
   G   
   T   
   C   
   A   
   A   
   A   
   C   
   G   
   A   
   T   
   T   
   A   
   A   
   A   
   T   
   T   
   A   
   A   
   C   
   G   
   A   
   T   
   T   
   G   
   A   
   C   
   A   
   G   
   T   
   G   
   G   
   C   
   G   
   T   
   A   
   C   
   A   
   A   
   C   
   A   
   A   
   C   
   A   
   A   
   G   
   C   
   C   
   T   
   T   
   C   
   G   
   C   
   T   
   A   
   T   
   A   
   T   
   T   
   T   
   G   
   A   
   C   
   A   
   A   
   A   
   C   
   G   
   C   
   C   
   C   
   T   
   G   
   G   
   T   
   A   
   G   
   T   
   G   
   C   
   G   
   G   
   T   
   C   
   A   
   T   
   T   
   A   
   C   
   C   
   G   
   A   
   T   
   C   
   C   
   T   
   C   
   A   
   A   
   A   
   A   
   A   
   G   
   G   
   C   
   G   
   A   
   T   
   T   
   T   
   A   
   T   
   T   
   A   
   G   
   C   
   A   
   A   
   C   
   G   
   G   
   T   
   T   
   A   
   G   
   T   
   T   
   C   
   A   
   C   
   C   
   T   
   T   
   C   
   G   
   T   
   A   
   T   
   G   
   A   
   T   
   C   
   C   
   T   
   A   
   A   
   T   
   A   
   A   
   A   
   A   
   T   
   G   
   G   
   C   
   C   
   A   
   A   
   T   
   G   
   G   
   C   
   A   
   T   
   T   
   T   
   C   
   T   
   C   
   A   
   A   
   A   
   A   
   A   
   G   
   A   
   A   
   T   
   A   
   T   
   G   
   A   
   T   
   G   
   C   
   T   
   T   
   A   
   C   
   A   
   A   
   T   
   A   
   A   
   C   
   A   
   A   
   C   
   A   
   A   
   A   
   G   
   A   
   T   
   T   
   T   
   A   
   C   
   C   
   A   
   T   
   T   
   C   
   A   
   C   
   A   
   G   
   C   
   A   
   C   
   G   
   T   
   T   
   T   
   T   
   G   
   C   
   G   
   A   
   C   
   A   
   G   
   G   
   C   
   T   
   A   
   T   
   G   
   C   
   A   
   C   
   C   
   A   
   G   
   G   
   C   
   T   
   C   
   T   
   A   
   C   
   C   
   T   
   T   
   T   
   A   
   A   
   A   
   A   
   C   
   A   
   A   
   T   
   T   
   A   
   C   
   C   
   G   
   G   
   T   
   G   
   C   
   C   
   A   
   T   
   T   
   G   
   G   
   T   
   T   
   T   
   A   
   G   
   A   
   T   
   G   
   C   
   A   
   G   
   G   
   G   
   A   
   C   
   A   
   T   
   T   
   G   
   A   
   A   
   G   
   C   
   C   
   A   
   G   
   A   
   T   
   G   
   A   
   A   
   G   
   A   
   G   
   C   
   T   
   A   
   G   
   A   
   A   
   A   
   T   
   T   
   A   
   A   
   T   
   G   
   G   
   T   
   T   
   T   
   G   
   A   
   A   
   A   
   T   
   G   
   G   
   C   
   A   
   A   
   A   
   A   
   A   
   G   
   A   
   T   
   A   
   A   
   A   
   T   
   C   
   T   
   T   
   G   
   G   
   G   
   G   
   C   
   G   
   G   
   C   
   T   
   A   
   T   
   T   
   T   
   T   
   G   
   C   
   G   
   A   
   C   
   A   
   C   
   G   
   T   
   G   
   T   
   A   
   A   
   A   
   A   
   G   
   A   
   A   
   G   
   C   
   A   
   A   
   G   
   T   
   C   
   C   
   A   
   G   
   T   
   C   
   A   
   A   
   T   
   C   
   T   
   G   
   C   
   G   
   T   
   A   
   C   
   C   
   G   
   C   
   T   
   T   
   T   
   A   
   G   
   T   
   T   
   A   
   A   
   T   
   T   
   C   
   A   
   G   
   A   
   T   
   A   
   A   
   T   
   A   
   T   
   T   
   T   
   A   
   T   
   T   
   T   
   T   
   G   
   C   
   C   
   C   
   A   
   A   
   C   
   A   
   A   
   A   
   C   
   A   
   T   
   T   
   A   
   C   
   G   
   G   
   A   
   T   
   G   
   G   
   G   
   A   
   G   
   A   
   A   
   G   
   A   
   C   
   A   
   A   
   A   
   T   
   T   
   T   
   A   
   G   
   A   
   G   
   C   
   G   
   G   
   G   
   T   
   C   
   T   
   T   
   A   
   A   
   T   
   A   
   A   
   A   
   T   
   T   
   C   
   A   
   T   
   T   
   T   
   T   
   C   
   G   
   G   
   T   
   G   
   A   
   G   
   G   
   A   
   A   
   C   
   T   
   A   
   G   
   A   
   T   
   T   
   T   
   A   
   C   
   C   
   A   
   A   
   T   
   T   
   G   
   C   
   C   
   A   
   T   
   G   
   A   
   C   
   G   
   C   
   C   
   T   
   G   
   C   
   T   
   C   
   A   
   A   
   A   
   T   
   T   
   T   
   C   
   A   
   A   
   A   
   T   
   G   
   A   
   A   
   G   
   A   
   T   
   A   
   A   
   A   
   T   
   T   
   T   
   A   
   A   
   T   
   T   
   C   
   T   
   G   
   A   
   A   
   A   
   T   
   T   
   C   
   T   
   T   
   T   
   T   
   A   
   G   
   C   
   A   
   G   
   A   
   T   
   A   
   C   
   G   
   G   
   G   
   C   
   T   
   A   
   C   
   G   
   G   
   T   
   C   
   A   
   A   
   G   
   G   
   C   
   C   
   A   
   A   
   T   
   T   
   G   
   T   
   T   
   A   
   A   
   T   
   T   
   T   
   C   
   G   
   C   
   C   
   A   
   A   
   T   
   C   
   C   
   A   
   G   
   C   
   A   
   A   
   G   
   C   
   T   
   A   
   C   
   C   
   A   
   T   
   G   
   T   
   A   
   T   
   A   
   G   
   T   
   G   
   T   
   T   
   T   
   T   
   C   
   C   
   A   
   A   
   A   
   A   
   T   
   A   
   A   
   T   
   G   
   G   
   G   
   A   
   C   
   A   
   T   
   T   
   A   
   G   
   T   
   C   
   T   
   A   
   T   
   C   
   C   
   A   
   A   
   A   
   A   
   T   
   T   
   A   
   G   
   T   
   T   
   T   
   T   
   A   
   G   
   A   
   C   
   A   
   A   
   A   
   G   
   A   
   A   
   A   
   C   
   G   
   A   
   A   
   G   
   A   
   A   
   A   
   A   
   A   
   A   
   G   
   A   
   C   
   A   
   A   
   T   
   G   
   T   
   A   
   A   
   T   
   C   
   A   
   G   
   C   
   G   
   C   
   C   
   A   
   A   
   C   
   G   
   C   
   A   
   G   
   C   
   G   
   A   
   A   
   T   
   A   
   C   
   A   
   A   
   T   
   T   
   G   
   C   
   C   
   A   
   C   
   A   
   G   
   A   
   T   
   C   
   T   
   T   
   T   
   T   
   A   
   G   
   G   
   T   
   A   
   G   
   T   
   G   
   T   
   G   
   G   
   A   
   A   
   G   
   A   
   T   
   C   
   C   
   T   
   T   
   C   
   A   
   G   
   G   
   T   
   T   
   A   
   T   
   G   
   T   
   T   
   T   
   A   
   T   
   A   
   A   
   T   
   A   
   T   
   G   
   T   
   A   
   C   
   A   
   A   
   T   
   C   
   C   
   G   
   A   
   A   
   C   
   T   
   T   
   C   
   T   
   C   
   A   
   T   
   T   
   G   
   G   
   C   
   T   
   G   
   C   
   T   
   A   
   A   
   A   
   A   
   C   
   A   
   G   
   G   
   A   
   A   
   C   
   A   
   G   
   C   
   T   
   G   
   A   
   A   
   A   
   T   
   A   
   A   
   A   
   A   
   G   
   A   
   T   
   A   
   A   
   A   
   C   
   A   
   A   
   G   
   A   
   T   
   A   
   C   
   A   
   G   
   A   
   T   
   G   
   G   
   C   
   A   
   A   
   A   
   G   
   A   
   A   
   A   
   A   
   C   
   A   
   G   
   T   
   T   
   T   
   C   
   C   
   T   
   T   
   C   
   T   
   A   
   A   
   C   
   G   
   T   
   T   
   A   
   G   
   A   
   T   
   C   
   G   
   T   
   A   
   G   
   T   
   A   
   A   
   C   
   A   
   A   
   T   
   A   
   A   
   A   
   T   
   T   
   C   
   T   
   T   
   A   
   A   
   C   
   A   
   A   
   T   
   G   
   A   
   T   
   T   
   A   
   T   
   G   
   G   
   T   
   T   
   G   
   A   
   A   
   A   
   A   
   T   
   T   
   C   
   A   
   G   
   G   
   A   
   G   
   A   
   A   
   A   
   A   
   T   
   G   
   G   
   T   
   T   
   C   
   T   
   G   
   C   
   A   
   A   
   C   
   A   
   G   
   A   
   T   
   A   
   T   
   C   
   A   
   G   
   T   
   A   
   A   
   A   
   C   
   C   
   A   
   T   
   T   
   A   
   A   
   T   
   T   
   G   
   A   
   T   
   T   
   A   
   C   
   T   
   T   
   A   
   G   
   A   
   A   
   G   
   C   
   A   
   A   
   C   
   C   
   A   
   T   
   T   
   A   
   A   
   A   
   -   
   -   
   -   
   -   
   -   
   -   
   -   
   -   
   -   
   -   
   -   
   -   
   -   
   -   
   -   
   -   
   -   
   -   
   -   
   -   
   -   
   -   
   -   
   -   
   -   
   -   
   -   
   -   
   -   
   -   
   -   
   -   
   -   
   -   
   -   
   -   
   -   
   -   
   -   
   -   
   -   
   -   
   -   
   -   
   -   
   -   
   -   
   -   
   -   
   -   
   -   
   -   
   -   
   -   
   -   
   -   
   -   
   -   
   -   
   -   
   -   
 

 
   N53766   
   -   
   -   
   -   
   -   
   -   
   -   
   -   
   -   
   -   
   -   
   -   
   -   
   -   
   -   
   -   
   -   
   -   
   -   
   -   
   -   
   -   
   -   
   -   
   -   
   -   
   -   
   -   
   -   
   -   
   -   
   -   
   -   
   -   
   -   
   -   
   -   
   -   
   -   
   -   
   -   
   -   
   -   
   -   
   -   
   -   
   -   
   -   
   -   
   -   
   -   
   -   
   -   
   -   
   -   
   -   
   -   
   -   
   -   
   -   
   -   
   -   
   -   
   -   
   -   
   -   
   -   
   -   
   -   
   -   
   -   
   -   
   -   
   -   
   -   
   -   
   -   
   -   
   -   
   -   
   -   
   -   
   -   
   -   
   -   
   -   
   -   
   -   
   -   
   -   
   -   
   -   
   -   
   -   
   -   
   -   
   -   
   -   
   -   
   -   
   -   
   -   
   -   
   -   
   -   
   -   
   -   
   -   
   -   
   C   
   A   
   A   
   T   
   G   
   G   
   C   
   A   
   A   
   G   
   C   
   C   
   A   
   A   
   A   
   C   
   A   
   A   
   G   
   A   
    A    
   T   
   T   
   A   
   G   
   C   
    C    
   G   
   A   
   A   
   G   
   C   
   G   
   A   
   A   
   G   
   A   
   A   
   A   
   A   
    C    
   A   
   G   
   C   
    T    
   A   
    C    
   T   
    A    
    C    
   A   
   T   
   T   
   T   
   T   
   T   
   A   
   A   
   A   
    C    
   G   
   T   
   A   
   T   
   T   
   G   
    T    
   C   
   A   
   A   
   A   
   A   
   C   
   A   
   G   
    G    
   A   
   A   
   T   
   T   
   T   
   G   
   A   
   T   
   A   
   A   
   G   
   T   
   T   
   A   
   C   
   C   
    G    
   T   
   C   
   C   
   G   
   T   
   T   
    G    
   T   
   T   
   C   
   A   
   A   
   G   
   A   
   A   
    G    
   C   
   T   
   A   
   G   
   C   
   T   
   T   
   A   
   A   
   A   
   G   
   A   
   A   
   A   
   A   
   A   
   T   
   G   
   G   
    C    
   T   
   A   
   T   
   G   
   A   
   T   
   A   
   C   
   T   
   A   
   A   
   A   
   T   
   C   
    T    
   G   
   T   
   T   
   G   
   T   
   T   
   G   
   A   
   A   
   A   
   A   
   A   
   T   
   A   
   C   
   C   
   A   
   A   
   G   
   C   
   A   
   A   
   T   
   T   
   T   
   A   
   T   
   T   
   C   
   A   
   G   
   G   
   G   
   A   
   T   
   T   
   C   
   A   
   A   
   G   
   C   
    A    
   G   
   A   
   A   
    G    
   G   
    A    
   G   
   T   
   C   
   A   
   A   
    A    
   G   
   C   
   T   
   A   
   G   
   T   
   G   
   A   
   T   
   G   
   T   
    T    
   C   
   A   
   A   
   G   
   T   
    C    
   A   
   A   
   A   
   A   
   A   
    G    
   G   
    C    
   G   
   A   
   A   
   A   
   G   
   A   
    C    
   A   
   A   
    T    
   C   
   A   
   A   
   T   
   A   
   C   
   A   
   C   
   A   
   T   
   T   
   T   
   A   
    C    
   C   
   T   
   A   
   T   
   A   
   A   
   A   
   T   
   T   
   A   
   T   
   C   
   G   
   A   
   T   
   G   
   A   
   G   
    C    
   A   
   C   
    G    
   C   
   C   
   T   
   T   
   T   
   A   
   G   
   G   
   C   
   G   
   A   
   A   
   A   
   T   
   G   
   A   
   A   
   A   
   G   
   A   
   T   
    T    
   T   
   G   
   T   
   C   
   T   
   T   
   A   
   T   
   C   
   A   
   A   
   T   
   C   
   A   
   A   
   G   
   T   
   A   
   T   
    C    
   G   
    C    
   C   
   A   
   A   
    A    
   A   
   A   
   A   
   G   
   G   
    C    
   G   
   A   
   T   
   A   
    C    
   C   
   T   
   A   
   C   
   C   
   A   
   A   
   A   
   T   
    C    
    G    
   C   
   T   
   T   
   G   
   G   
    A    
    A    
    G    
   C   
   C   
    A    
   T   
   C   
   T   
   T   
   T   
   A   
   A   
   T   
   T   
   T   
   T   
   T   
   C   
   C   
   A   
   G   
   A   
   T   
   A   
   T   
   G   
   T   
   C   
   A   
   G   
   G   
   A   
   A   
   A   
   T   
   G   
   A   
   T   
   A   
   A   
   A   
    A    
   T   
   T   
   T   
   C   
   G   
   A   
   T   
   T   
   C   
   A   
   A   
   G   
   T   
   A   
   G   
   A   
   T   
   A   
   A   
   T   
   G   
   C   
   C   
   A   
   A   
   A   
   C   
   G   
   T   
   G   
   G   
   A   
   G   
   A   
   A   
   A   
   T   
   T   
   G   
   T   
   C   
   G   
   A   
   T   
   C   
   G   
   T   
   A   
   A   
    T    
   G   
   G   
   T   
   A   
   G   
   T   
   G   
   G   
   G   
   C   
   T   
   A   
   G   
    C    
   A   
   A   
   T   
   T   
   A   
   A   
   C   
   A   
   A   
   A   
   G   
   T   
   G   
   T   
   T   
   T   
   G   
   A   
    C    
   G   
   A   
   A   
   G   
   T   
   G   
   G   
   G   
   C   
   G   
   T   
   A   
   G   
   T   
    G    
   C   
   C   
   T   
    G    
   G   
   C   
   A   
   A   
   A   
   C   
   T   
    C    
   G   
   G   
   T   
   T   
   C   
   T   
   G   
   G   
   C   
   G   
   C   
   A   
   G   
   A   
   A   
   A   
   A   
   A   
    A    
   C   
   A   
   G   
   C   
    C    
   A   
   A   
   T   
   A   
   T   
   C   
   A   
   A   
   A   
   G   
   C   
   T   
   T   
   T   
   T   
   A   
   G   
   T   
   G   
   A   
   T   
   A   
   A   
    A    
   T   
   T   
    C    
   G   
   G   
   C   
   G   
   T   
   T   
   T   
   C   
   T   
    G    
   T   
   T   
   G   
   A   
   T   
   G   
   A   
   A   
   A   
   T   
   C   
   A   
   A   
   T   
   C   
    A    
   A   
   A   
   A   
    G    
   T   
   T   
   A   
   A   
   G   
   C   
   C   
   A   
   A   
   G   
   G   
    A    
   T   
   G   
   G   
   G   
   T   
   C   
   C   
   A   
   A   
   G   
   C   
   A   
   G   
   A   
   C   
   T   
   C   
    C    
   T   
   T   
   T   
   G   
   T   
   A   
   C   
   C   
   A   
   A   
   T   
   C   
   A   
   C   
   A   
   G   
   T   
    C    
   G   
   C   
   T   
   T   
   C   
   T   
   G   
   A   
   A   
   C   
   C   
   A   
   G   
   T   
   G   
   A   
   C   
   A   
   G   
   A   
   A   
   T   
   T   
   A   
   C   
   C   
   A   
   A   
   C   
   A   
   G   
   G   
   G   
   G   
   C   
   T   
   G   
   C   
   G   
   A   
   C   
   A   
   A   
   A   
   A   
   G   
    A    
    T    
   A   
   C   
   A   
   G   
   A   
   G   
   T   
   C   
   A   
   C   
   G   
   T   
   T   
   A   
   T   
   T   
   A   
   T   
   C   
   C   
    G    
    C    
   T   
   G   
   G   
   G   
   G   
   G   
   A   
   A   
   G   
   C   
   A   
   G   
   C   
   T   
   G   
   C   
   G   
   C   
   A   
   A   
   T   
   T   
   A   
   A   
   T   
   C   
   G   
   G   
    G    
   T   
   A   
   T   
   A   
   C   
    G    
   G   
   G   
    C    
   A   
   C   
    C    
   A   
   T   
   T   
   A   
   C   
   T   
   G   
   C   
   A   
   G   
   A   
    A    
   G   
   A   
   T   
   A   
   T   
   T   
    G    
   A   
   A   
   A   
   A   
   A   
   A   
   A   
   T   
   C   
   C   
   A   
   G   
   A   
   G   
   T   
   T   
   A   
   A   
   G   
   T   
   A   
   G   
   T   
   A   
   C   
   G   
   G   
   G   
    C    
   G   
   T   
   A   
   A   
   T   
   T   
   G   
   G   
    T    
   A   
   A   
   A   
   A   
   C   
   T   
   G   
   G   
    C    
   T   
   T   
   A   
   G   
   A   
   A   
   C   
   G   
   A   
   G   
   C   
   G   
   T   
   T   
   T   
   G   
   A   
   T   
   A   
   A   
   A   
   G   
   A   
    A    
   T   
   T   
   A   
   A   
   G   
   A   
   G   
   G   
    G    
   C   
   A   
   A   
   G   
   A   
   T   
   G   
   G   
    T    
   G   
   G   
    C    
   T   
   C   
    A    
   C   
   T   
   A   
   G   
   T   
   T   
   A   
   T   
   T   
   T   
   T   
   A   
   G   
   A   
   T   
   G   
   A   
   C   
   A   
   A   
   A   
   G   
   A   
   G   
   A   
   A   
   T   
   G   
   T   
   C   
   A   
   A   
   A   
   A   
   A   
   A   
   G   
   C   
   T   
   T   
   T   
   A   
   C   
   A   
   A   
   A   
   C   
   C   
   A   
   A   
   A   
   G   
   A   
    G    
   A   
   A   
   A   
   A   
   A   
   A   
   G   
   A   
   T   
   G   
   G   
   T   
   C   
   A   
   A   
   A   
   C   
    G    
   A   
   T   
   T   
   A   
   A   
   A   
   T   
   T   
   A   
   A   
   C   
   G   
   A   
   T   
   T   
   G   
   A   
    C    
   A   
   G   
    T    
   G   
   G   
    C    
   G   
   T   
   A   
   C   
   A   
   A   
   C   
   A   
   A   
   C   
   A   
   A   
   G   
   C   
    T    
   T   
   T   
    T    
   G   
   C   
   T   
   A   
   T   
   A   
   T   
   T   
   T   
   G   
   A   
   C   
   A   
   A   
   A   
   C   
   G   
    C    
    C    
   C   
   T   
   G   
   G   
   T   
   A   
   G   
   T   
   G   
   C   
   G   
   G   
   T   
   C   
   A   
   T   
   T   
   A   
   C   
   C   
   G   
   A   
   T   
   C   
   C   
   T   
   C   
   A   
   A   
   A   
   A   
   A   
   G   
   G   
   C   
   G   
   A   
   T   
   T   
   T   
   A   
    T    
   T   
   A   
   G   
   C   
    A    
   A   
   C   
   G   
   G   
   T   
   T   
   A   
   G   
   T   
   T   
   C   
   A   
   C   
   C   
   T   
   T   
   C   
   G   
   T   
   A   
   T   
   G   
   A   
   T   
   C   
   C   
   T   
   A   
   A   
   T   
   A   
   A   
   A   
   A   
   T   
   G   
   G   
   C   
   C   
   A   
   A   
   T   
   G   
   G   
    C    
   A   
   T   
   T   
   T   
   C   
   T   
   C   
   A   
   A   
   A   
   A   
   A   
   G   
   A   
    A    
   T   
   A   
   T   
   G   
   A   
   T   
   G   
   C   
   T   
   T   
   A   
   C   
   A   
   A   
   T   
   A   
   A   
   C   
   A   
   A   
   C   
   A   
   A   
   A   
   G   
   A   
   T   
   T   
   T   
   A   
   C   
   C   
   A   
   T   
   T   
    C    
   A   
   C   
   A   
   G   
   C   
   A   
   C   
   G   
   T   
   T   
   T   
    T    
   G   
   C   
   G   
   A   
   C   
   A   
   G   
   G   
    C    
   T   
   A   
   T   
   G   
   C   
   A   
   C   
   C   
   A   
   G   
   G   
    C    
   T   
   C   
   T   
   A   
   C   
   C   
   T   
   T   
   T   
   A   
   A   
   A   
   A   
   C   
   A   
   A   
   T   
   T   
   A   
   C   
   C   
   G   
   G   
   T   
   G   
   C   
   C   
   A   
   T   
   T   
   G   
   G   
   T   
   T   
   T   
   A   
   G   
   A   
   T   
    G    
   C   
   A   
   G   
   G   
   G   
   A   
   C   
   A   
   T   
   T   
   G   
   A   
   A   
   G   
   C   
   C   
   A   
   G   
   A   
   T   
   G   
   A   
   A   
   G   
   A   
   G   
   C   
   T   
   A   
   G   
   A   
   A   
   A   
   T   
   T   
   A   
   A   
   T   
   G   
   G   
    T    
   T   
   T   
   G   
   A   
   A   
   A   
   T   
   G   
   G   
   C   
   A   
   A   
   A   
   A   
   A   
   G   
   A   
   T   
   A   
   A   
   A   
   T   
   C   
   T   
   T   
   G   
   G   
   G   
   G   
    C    
   G   
   G   
   C   
   T   
   A   
   T   
   T   
   T   
   T   
   G   
   C   
    G    
   A   
   C   
   A   
   C   
   G   
   T   
   G   
   T   
   A   
   A   
   A   
   A   
   G   
   A   
   A   
   G   
   C   
   A   
   A   
   G   
   T   
   C   
   C   
   A   
   G   
   T   
   C   
   A   
   A   
   T   
   C   
   T   
    G    
   C   
   G   
   T   
   A   
   C   
   C   
   G   
   C   
   T   
   T   
   T   
   A   
   G   
   T   
   T   
   A   
   A   
   T   
   T   
   C   
   A   
   G   
   A   
   T   
   A   
   A   
   T   
   A   
   T   
   T   
   T   
   A   
   T   
   T   
   T   
   T   
   G   
   C   
    C    
   C   
   A   
   A   
   C   
   A   
   A   
   A   
   C   
   A   
   T   
   T   
   A   
   C   
   G   
    G    
   A   
   T   
   G   
   G   
   G   
    A    
   G   
   A   
   A   
   G   
   A   
   C   
   A   
   A   
   A   
   T   
   T   
   T   
   A   
   G   
   A   
   G   
   C   
    G    
   G   
   G   
   T   
   C   
   T   
   T   
   A   
   A   
   T   
   A   
   A   
   A   
   T   
   T   
   C   
   A   
   T   
   T   
   T   
   T   
   C   
   G   
   G   
   T   
   G   
   A   
   G   
   G   
   A   
   A   
   C   
   T   
   A   
   G   
   A   
   T   
   T   
   T   
   A   
   C   
   C   
   A   
   A   
   T   
    C    
   G   
   C   
   C   
   A   
   T   
   G   
   A   
   C   
   G   
   C   
   C   
    T    
   G   
   C   
   T   
   C   
   A   
   A   
   A   
   T   
   T   
   T   
   C   
   A   
   A   
   A   
    T    
   G   
   A   
   A   
   G   
   A   
   T   
   A   
   A   
   A   
   T   
   T   
   T   
   A   
   A   
   T   
   T   
   C   
   T   
   G   
   A   
   A   
   A   
   T   
   T   
   C   
   T   
   T   
   T   
   T   
    A    
   G   
   C   
   A   
   G   
   A   
   T   
   A   
   C   
    G    
   G   
   G   
   C   
   T   
   A   
    C    
   G   
   G   
    T    
   C   
   A   
   A   
   G   
   G   
    T    
   C   
   A   
   A   
   T   
   T   
   G   
   T   
   T   
   A   
   A   
   T   
   T   
   T   
   C   
    G    
   C   
   C   
   A   
   A   
   T   
   C   
   C   
   A   
   G   
   C   
   A   
   A   
   G   
   C   
   T   
   A   
   C   
   C   
   A   
   T   
   G   
   T   
   A   
   T   
   A   
   G   
   T   
   G   
   T   
   T   
   T   
   T   
   C   
   C   
   A   
   A   
   A   
   A   
   T   
   A   
   A   
   T   
   G   
   G   
    A    
   A   
   C   
   A   
    T    
   T   
    A    
   G   
   T   
    C    
   T   
   A   
   T   
   C   
   C   
   A   
   A   
   A   
   A   
    C    
   T   
   A   
   G   
   T   
   T   
   T   
   T   
   A   
   G   
   A   
   C   
   A   
   A   
   A   
   G   
   A   
   A   
   A   
   C   
    G    
   A   
   A   
   G   
   A   
   A   
   A   
   A   
   A   
   A   
   G   
   A   
   C   
   A   
   A   
   T   
   G   
   T   
   A   
   A   
   T   
   C   
   A   
   G   
   C   
   G   
   C   
   C   
   A   
   A   
   C   
   G   
   C   
   A   
   G   
   C   
   G   
   A   
   A   
   T   
   A   
   C   
   A   
   A   
   T   
   T   
   G   
   C   
   C   
   A   
   C   
   A   
   G   
   A   
   T   
   C   
   T   
   T   
   T   
   T   
   A   
   G   
   G   
   T   
   A   
   G   
   T   
   G   
   T   
   G   
   G   
   A   
   A   
   G   
   A   
   T   
   C   
   C   
   T   
   T   
   C   
   A   
   G   
   G   
   T   
   T   
   A   
   T   
   G   
   T   
   T   
   T   
   A   
   T   
   A   
   A   
   T   
   A   
   T   
   G   
   T   
   A   
   C   
   A   
   A   
   T   
   C   
   C   
   G   
   A   
   A   
   C   
   T   
   T   
   C   
   T   
   C   
   A   
   T   
   T   
   G   
   G   
   C   
   T   
   G   
   C   
   T   
   A   
   A   
   A   
   A   
   C   
   A   
   G   
   G   
   A   
   A   
   C   
   A   
   G   
   C   
   T   
   G   
   A   
   A   
   A   
   T   
   A   
   A   
   A   
   A   
   G   
   A   
   T   
   A   
   A   
   A   
   C   
   A   
   A   
   G   
   A   
   T   
   A   
   C   
   A   
   G   
   A   
   T   
   G   
   G   
   C   
   A   
   A   
   A   
   G   
   A   
   A   
   A   
   A   
   C   
   A   
   G   
   T   
   T   
   T   
   C   
   C   
   T   
   T   
   C   
   T   
   A   
   A   
   C   
   G   
   T   
   T   
   A   
   G   
   A   
   T   
   C   
   G   
   T   
   A   
   G   
   T   
   A   
   A   
   C   
   A   
   A   
   T   
   A   
   A   
   A   
   T   
   T   
   C   
   T   
   T   
   A   
   A   
   C   
   A   
   A   
   T   
   G   
   A   
   T   
   T   
   A   
   T   
   G   
   G   
   T   
   T   
   G   
   A   
   A   
   A   
   A   
   T   
   T   
   C   
   A   
   G   
   G   
   A   
   G   
   A   
   A   
   A   
   A   
   T   
   G   
   G   
   T   
   T   
   C   
   T   
   G   
   C   
   A   
   A   
   C   
   A   
   G   
   A   
   T   
   A   
   T   
   C   
   A   
   G   
   T   
   A   
   A   
   A   
   C   
   C   
   A   
   T   
   T   
   A   
   A   
   T   
   T   
   G   
   A   
   T   
   T   
   A   
   C   
   T   
   T   
   A   
   G   
   A   
   A   
   G   
   C   
   A   
   A   
   C   
   C   
   A   
   T   
   T   
   A   
   A   
   A   
   -   
   -   
   -   
   -   
   -   
   -   
   -   
   -   
   -   
   -   
   -   
   -   
   -   
   -   
   -   
   -   
   -   
   -   
   -   
   -   
   -   
   -   
   -   
   -   
   -   
   -   
   -   
   -   
   -   
   -   
   -   
   -   
   -   
   -   
   -   
   -   
   -   
   -   
   -   
   -   
   -   
   -   
   -   
   -   
   -   
   -   
   -   
   -   
   -   
   -   
   -   
   -   
   -   
   -   
   -   
   -   
   -   
   -   
   -   
   -   
   -   
   -   
   -   
   -   
   -   
   -   
   -   
   -   
   -   
   -   
   -   
   -   
   -   
   -   
   -   
   -   
   -   
   -   
   -   
   -   
   -   
   -   
   -   
   -   
   -   
   -   
   -   
   -   
   -   
   -   
   -   
   -   
   -   
   -   
   -   
   -   
   -   
   -   
   -   
   -   
   -   
   -   
   -   
   -   
   -   
   -   
   -   
   -   
   -   
   -   
   -   
   -   
   -   
   -   
   -   
   -   
   -   
   -   
   -   
   -   
   -   
   -   
   -   
   -   
   -   
   -   
   -   
   -   
   -   
   -   
   -   
   -   
   -   
   -   
   -   
   -   
   -   
   -   
   -   
   -   
   -   
   -   
   -   
   -   
   -   
   -   
   -   
   -   
   -   
   -   
   -   
   -   
   -   
   -   
   -   
   -   
   -   
   -   
   -   
   -   
   -   
   -   
   -   
   -   
   -   
   -   
   -   
   -   
   -   
   C   
   A   
   A   
   T   
   G   
   G   
   C   
   A   
   A   
   G   
   C   
   C   
   A   
   A   
   A   
   C   
   A   
   A   
   G   
   A   
   A   
   T   
   T   
   A   
   G   
   C   
   C   
   G   
   A   
   A   
   G   
   C   
   G   
   A   
   A   
   G   
   A   
   A   
   A   
   A   
   C   
   A   
   G   
   C   
   T   
   A   
   C   
   T   
   A   
   C   
   A   
   T   
   T   
   T   
   T   
   T   
   A   
   A   
   A   
   C   
   G   
   T   
   A   
   T   
   T   
   G   
   T   
   C   
   A   
   A   
   A   
   A   
   C   
   A   
   G   
   G   
   A   
   A   
   T   
   T   
   T   
   G   
   A   
   T   
   A   
   A   
   G   
   T   
   T   
   A   
   C   
   C   
   G   
   T   
   C   
   C   
   G   
   T   
   T   
   G   
   T   
   T   
   C   
   A   
   A   
   G   
   A   
   A   
   G   
   C   
   T   
   A   
   G   
   C   
   T   
   T   
   A   
   A   
   A   
   G   
   A   
   A   
   A   
   A   
   A   
   T   
   G   
   G   
   C   
   T   
   A   
   T   
   G   
   A   
   T   
   A   
   C   
   T   
   A   
   A   
   A   
   T   
   C   
   T   
   G   
   T   
   T   
   G   
   T   
   T   
   G   
   A   
   A   
   A   
   A   
   A   
   T   
   A   
   C   
   C   
   A   
   A   
   G   
   C   
   A   
   A   
   T   
   T   
   T   
   A   
   T   
   T   
   C   
   A   
   G   
   G   
   G   
   A   
   T   
   T   
   C   
   A   
   A   
   G   
   C   
   A   
   G   
   A   
   A   
   G   
   G   
   A   
   G   
   T   
   C   
   A   
   A   
   A   
   G   
   C   
   T   
   A   
   G   
   T   
   G   
   A   
   T   
   G   
   T   
   T   
   C   
   A   
   A   
   G   
   T   
   C   
   A   
   A   
   A   
   A   
   A   
   G   
   G   
   C   
   G   
   A   
   A   
   A   
   G   
   A   
   C   
   A   
   A   
   T   
   C   
   A   
   A   
   T   
   A   
   C   
   A   
   C   
   A   
   T   
   T   
   T   
   A   
   C   
   C   
   T   
   A   
   T   
   A   
   A   
   A   
   T   
   T   
   A   
   T   
   C   
   G   
   A   
   T   
   G   
   A   
   G   
   C   
   A   
   C   
   G   
   C   
   C   
   T   
   T   
   T   
   A   
   G   
   G   
   C   
   G   
   A   
   A   
   A   
   T   
   G   
   A   
   A   
   A   
   G   
   A   
   T   
   T   
   T   
   G   
   T   
   C   
   T   
   T   
   A   
   T   
   C   
   A   
   A   
   T   
   C   
   A   
   A   
   G   
   T   
   A   
   T   
   C   
   G   
   C   
   C   
   A   
   A   
   A   
   A   
   A   
   A   
   G   
   G   
   C   
   G   
   A   
   T   
   A   
   C   
   C   
   T   
   A   
   C   
   C   
   A   
   A   
   A   
   T   
   C   
   G   
   C   
   T   
   T   
   G   
   G   
   A   
   A   
   G   
   C   
   C   
   A   
   T   
   C   
   T   
   T   
   T   
   A   
   A   
   T   
   T   
   T   
   T   
   T   
   C   
   C   
   A   
   G   
   A   
   T   
   A   
   T   
   G   
   T   
   C   
   A   
   G   
   G   
   A   
   A   
   A   
   T   
   G   
   A   
   T   
   A   
   A   
   A   
   A   
   T   
   T   
   T   
   C   
   G   
   A   
   T   
   T   
   C   
   A   
   A   
   G   
   T   
   A   
   G   
   A   
   T   
   A   
   A   
   T   
   G   
   C   
   C   
   A   
   A   
   A   
   C   
   G   
   T   
   G   
   G   
   A   
   G   
   A   
   A   
   A   
   T   
   T   
   G   
   T   
   C   
   G   
   A   
   T   
   C   
   G   
   T   
   A   
   A   
   T   
   G   
   G   
   T   
   A   
   G   
   T   
   G   
   G   
   G   
   C   
   T   
   A   
   G   
   C   
   A   
   A   
   T   
   T   
   A   
   A   
   C   
   A   
   A   
   A   
   G   
   T   
   G   
   T   
   T   
   T   
   G   
   A   
   C   
   G   
   A   
   A   
   G   
   T   
   G   
   G   
   G   
   C   
   G   
   T   
   A   
   G   
   T   
   G   
   C   
   C   
   T   
   G   
   G   
   C   
   A   
   A   
   A   
   C   
   T   
   C   
   G   
   G   
   T   
   T   
   C   
   T   
   G   
   G   
   C   
   G   
   C   
   A   
   G   
   A   
   A   
   A   
   A   
   A   
   A   
   C   
   A   
   G   
   C   
   C   
   A   
   A   
   T   
   A   
   T   
   C   
   A   
   A   
   A   
   G   
   C   
   T   
   T   
   T   
   T   
   A   
   G   
   T   
   G   
   A   
   T   
   A   
   A   
   A   
   T   
   T   
   C   
   G   
   G   
   C   
   G   
   T   
   T   
   T   
   C   
   T   
   G   
   T   
   T   
   G   
   A   
   T   
   G   
   A   
   A   
   A   
   T   
   C   
   A   
   A   
   T   
   C   
   A   
   A   
   A   
   A   
   G   
   T   
   T   
   A   
   A   
   G   
   C   
   C   
   A   
   A   
   G   
   G   
   A   
   T   
   G   
   G   
   G   
   T   
   C   
   C   
   A   
   A   
   G   
   C   
   A   
   G   
   A   
   C   
   T   
   C   
   C   
   T   
   T   
   T   
   G   
   T   
   A   
   C   
   C   
   A   
   A   
   T   
   C   
   A   
   C   
   A   
   G   
   T   
   C   
   G   
   C   
   T   
   T   
   C   
   T   
   G   
   A   
   A   
   C   
   C   
   A   
   G   
   T   
   G   
   A   
   C   
   A   
   G   
   A   
   A   
   T   
   T   
   A   
   C   
   C   
   A   
   A   
   C   
   A   
   G   
   G   
   G   
   G   
   C   
   T   
   G   
   C   
   G   
   A   
   C   
   A   
   A   
   A   
   A   
   G   
   A   
   T   
   A   
   C   
   A   
   G   
   A   
   G   
   T   
   C   
   A   
   C   
   G   
   T   
   T   
   A   
   T   
   T   
   A   
   T   
   C   
   C   
   G   
   C   
   T   
   G   
   G   
   G   
   G   
   G   
   A   
   A   
   G   
   C   
   A   
   G   
   C   
   T   
   G   
   C   
   G   
   C   
   A   
   A   
   T   
   T   
   A   
   A   
   T   
   C   
   G   
   G   
   G   
   T   
   A   
   T   
   A   
   C   
   G   
   G   
   G   
   C   
   A   
   C   
   C   
   A   
   T   
   T   
   A   
   C   
   T   
   G   
   C   
   A   
   G   
   A   
   A   
   G   
   A   
   T   
   A   
   T   
   T   
   G   
   A   
   A   
   A   
   A   
   A   
   A   
   A   
   T   
   C   
   C   
   A   
   G   
   A   
   G   
   T   
   T   
   A   
   A   
   G   
   T   
   A   
   G   
   T   
   A   
   C   
   G   
   G   
   G   
   C   
   G   
   T   
   A   
   A   
   T   
   T   
   G   
   G   
   T   
   A   
   A   
   A   
   A   
   C   
   T   
   G   
   G   
   C   
   T   
   T   
   A   
   G   
   A   
   A   
   C   
   G   
   A   
   G   
   C   
   G   
   T   
   T   
   T   
   G   
   A   
   T   
   A   
   A   
   A   
   G   
   A   
   A   
   T   
   T   
   A   
   A   
   G   
   A   
   G   
   G   
   G   
   C   
   A   
   A   
   G   
   A   
   T   
   G   
   G   
   T   
   G   
   G   
   C   
   T   
   C   
   A   
   C   
   T   
   A   
   G   
   T   
   T   
   A   
   T   
   T   
   T   
   T   
   A   
   G   
   A   
   T   
   G   
   A   
   C   
   A   
   A   
   A   
   G   
   A   
   G   
   A   
   A   
   T   
   G   
   T   
   C   
   A   
   A   
   A   
   A   
   A   
   A   
   G   
   C   
   T   
   T   
   T   
   A   
   C   
   A   
   A   
   A   
   C   
   C   
   A   
   A   
   A   
   G   
   A   
   G   
   A   
   A   
   A   
   A   
   A   
   A   
   G   
   A   
   T   
   G   
   G   
   T   
   C   
   A   
   A   
   A   
   C   
   G   
   A   
   T   
   T   
   A   
   A   
   A   
   T   
   T   
   A   
   A   
   C   
   G   
   A   
   T   
   T   
   G   
   A   
   C   
   A   
   G   
   T   
   G   
   G   
   C   
   G   
   T   
   A   
   C   
   A   
   A   
   C   
   A   
   A   
   C   
   A   
   A   
   G   
   C   
   T   
   T   
   T   
   T   
   G   
   C   
   T   
   A   
   T   
   A   
   T   
   T   
   T   
   G   
   A   
   C   
   A   
   A   
   A   
   C   
   G   
   C   
   C   
   C   
   T   
   G   
   G   
   T   
   A   
   G   
   T   
   G   
   C   
   G   
   G   
   T   
   C   
   A   
   T   
   T   
   A   
   C   
   C   
   G   
   A   
   T   
   C   
   C   
   T   
   C   
   A   
   A   
   A   
   A   
   A   
   G   
   G   
   C   
   G   
   A   
   T   
   T   
   T   
   A   
   T   
   T   
   A   
   G   
   C   
   A   
   A   
   C   
   G   
   G   
   T   
   T   
   A   
   G   
   T   
   T   
   C   
   A   
   C   
   C   
   T   
   T   
   C   
   G   
   T   
   A   
   T   
   G   
   A   
   T   
   C   
   C   
   T   
   A   
   A   
   T   
   A   
   A   
   A   
   A   
   T   
   G   
   G   
   C   
   C   
   A   
   A   
   T   
   G   
   G   
   C   
   A   
   T   
   T   
   T   
   C   
   T   
   C   
   A   
   A   
   A   
   A   
   A   
   G   
   A   
   A   
   T   
   A   
   T   
   G   
   A   
   T   
   G   
   C   
   T   
   T   
   A   
   C   
   A   
   A   
   T   
   A   
   A   
   C   
   A   
   A   
   C   
   A   
   A   
   A   
   G   
   A   
   T   
   T   
   T   
   A   
   C   
   C   
   A   
   T   
   T   
   C   
   A   
   C   
   A   
   G   
   C   
   A   
   C   
   G   
   T   
   T   
   T   
   T   
   G   
   C   
   G   
   A   
   C   
   A   
   G   
   G   
   C   
   T   
   A   
   T   
   G   
   C   
   A   
   C   
   C   
   A   
   G   
   G   
   C   
   T   
   C   
   T   
   A   
   C   
   C   
   T   
   T   
   T   
   A   
   A   
   A   
   A   
   C   
   A   
   A   
   T   
   T   
   A   
   C   
   C   
   G   
   G   
   T   
   G   
   C   
   C   
   A   
   T   
   T   
   G   
   G   
   T   
   T   
   T   
   A   
   G   
   A   
   T   
   G   
   C   
   A   
   G   
   G   
   G   
   A   
   C   
   A   
   T   
   T   
   G   
   A   
   A   
   G   
   C   
   C   
   A   
   G   
   A   
   T   
   G   
   A   
   A   
   G   
   A   
   G   
   C   
   T   
   A   
   G   
   A   
   A   
   A   
   T   
   T   
   A   
   A   
   T   
   G   
   G   
   T   
   T   
   T   
   G   
   A   
   A   
   A   
   T   
   G   
   G   
   C   
   A   
   A   
   A   
   A   
   A   
   G   
   A   
   T   
   A   
   A   
   A   
   T   
   C   
   T   
   T   
   G   
   G   
   G   
   G   
   C   
   G   
   G   
   C   
   T   
   A   
   T   
   T   
   T   
   T   
   G   
   C   
   G   
   A   
   C   
   A   
   C   
   G   
   T   
   G   
   T   
   A   
   A   
   A   
   A   
   G   
   A   
   A   
   G   
   C   
   A   
   A   
   G   
   T   
   C   
   C   
   A   
   G   
   T   
   C   
   A   
   A   
   T   
   C   
   T   
   G   
   C   
   G   
   T   
   A   
   C   
   C   
   G   
   C   
   T   
   T   
   T   
   A   
   G   
   T   
   T   
   A   
   A   
   T   
   T   
   C   
   A   
   G   
   A   
   T   
   A   
   A   
   T   
   A   
   T   
   T   
   T   
   A   
   T   
   T   
   T   
   T   
   G   
   C   
   C   
   C   
   A   
   A   
   C   
   A   
   A   
   A   
   C   
   A   
   T   
   T   
   A   
   C   
   G   
   G   
   A   
   T   
   G   
   G   
   G   
   A   
   G   
   A   
   A   
   G   
   A   
   C   
   A   
   A   
   A   
   T   
   T   
   T   
   A   
   G   
   A   
   G   
   C   
   G   
   G   
   G   
   T   
   C   
   T   
   T   
   A   
   A   
   T   
   A   
   A   
   A   
   T   
   T   
   C   
   A   
   T   
   T   
   T   
   T   
   C   
   G   
   G   
   T   
   G   
   A   
   G   
   G   
   A   
   A   
   C   
   T   
   A   
   G   
   A   
   T   
   T   
   T   
   A   
   C   
   C   
   A   
   A   
   T   
   C   
   G   
   C   
   C   
   A   
   T   
   G   
   A   
   C   
   G   
   C   
   C   
   T   
   G   
   C   
   T   
   C   
   A   
   A   
   A   
   T   
   T   
   T   
   C   
   A   
   A   
   A   
   T   
   G   
   A   
   A   
   G   
   A   
   T   
   A   
   A   
   A   
   T   
   T   
   T   
   A   
   A   
   T   
   T   
   C   
   T   
   G   
   A   
   A   
   A   
   T   
   T   
   C   
   T   
   T   
   T   
   T   
   A   
   G   
   C   
   A   
   G   
   A   
   T   
   A   
   C   
   G   
   G   
   G   
   C   
   T   
   A   
   C   
   G   
   G   
   T   
   C   
   A   
   A   
   G   
   G   
   T   
   C   
   A   
   A   
   T   
   T   
   G   
   T   
   T   
   A   
   A   
   T   
   T   
   T   
   C   
   G   
   C   
   C   
   A   
   A   
   T   
   C   
   C   
   A   
   G   
   C   
   A   
   A   
   G   
   C   
   T   
   A   
   C   
   C   
   A   
   T   
   G   
   T   
   A   
   T   
   A   
   G   
   T   
   G   
   T   
   T   
   T   
   T   
   C   
   C   
   A   
   A   
   A   
   A   
   T   
   A   
   A   
   T   
   G   
   G   
   A   
   A   
   C   
   A   
   T   
   T   
   A   
   G   
   T   
   C   
   T   
   A   
   T   
   C   
   C   
   A   
   A   
   A   
   A   
   C   
   T   
   A   
   G   
   T   
   T   
   T   
   T   
   A   
   G   
   A   
   C   
   A   
   A   
   A   
   G   
   A   
   A   
   A   
   C   
   G   
   A   
   A   
   G   
   A   
   A   
   A   
   A   
   A   
   A   
   G   
   A   
   C   
   A   
   A   
   T   
   G   
   T   
   A   
   A   
   T   
   C   
   A   
   G   
   C   
   G   
   C   
   C   
   A   
   A   
   C   
   G   
   C   
   A   
   G   
   C   
   G   
   A   
   A   
   T   
   A   
   C   
   A   
   A   
   T   
   T   
   G   
   C   
   C   
   A   
   C   
   A   
   G   
   A   
   T   
   C   
   T   
   T   
   T   
   T   
   A   
   G   
   G   
   T   
   A   
   G   
   T   
   G   
   T   
   G   
   G   
   A   
   A   
   G   
   A   
   T   
   C   
   C   
   T   
   T   
   C   
   A   
   G   
   G   
   T   
   T   
   A   
   T   
   G   
   T   
   T   
   T   
   A   
   T   
   A   
   A   
   T   
   A   
   T   
   G   
   T   
   A   
   C   
   A   
   A   
   T   
   C   
   C   
   G   
   A   
   A   
   C   
   T   
   T   
   C   
   T   
   C   
   A   
   T   
   T   
   G   
   G   
   C   
   T   
   G   
   C   
   T   
   A   
   A   
   A   
   A   
   C   
   A   
   G   
   G   
   A   
   A   
   C   
   A   
   G   
   C   
   T   
   G   
   A   
   A   
   A   
   T   
   A   
   A   
   A   
   A   
   G   
   A   
   T   
   A   
   A   
   A   
   C   
   A   
   A   
   G   
   A   
   T   
   A   
   C   
   A   
   G   
   A   
   T   
   G   
   G   
   C   
   A   
   A   
   A   
   G   
   A   
   A   
   A   
   A   
   C   
   A   
   G   
   T   
   T   
   T   
   C   
   C   
   T   
   T   
   C   
   T   
   A   
   A   
   C   
   G   
   T   
   T   
   A   
   G   
   A   
   T   
   C   
   G   
   T   
   A   
   G   
   T   
   A   
   A   
   C   
   A   
   A   
   T   
   A   
   A   
   A   
   T   
   T   
   C   
   T   
   T   
   A   
   A   
   C   
   A   
   A   
   T   
   G   
   A   
   T   
   T   
   A   
   T   
   G   
   G   
   T   
   T   
   G   
   A   
   A   
   A   
   A   
   T   
   T   
   C   
   A   
   G   
   G   
   A   
   G   
   A   
   A   
   A   
   A   
   T   
   G   
   G   
   T   
   T   
   C   
   T   
   G   
   C   
   A   
   A   
   C   
   A   
   G   
   A   
   T   
   A   
   T   
   C   
   A   
   G   
   T   
   A   
   A   
   A   
   C   
   C   
   A   
   T   
   T   
   A   
   A   
   T   
   T   
   G   
   A   
   T   
   T   
   A   
   C   
   T   
   T   
   A   
   G   
   A   
   A   
   G   
   C   
   A   
   A   
   C   
   C   
   A   
   T   
   T   
   A   
   A   
   A   
   -   
   -   
   -   
   -   
   -   
   -   
   -   
   -   
   -   
   -   
   -   
   -   
   -   
   -   
   -   
   -   
   -   
   -   
   -   
   -   
   -   
   -   
   -   
   -   
   -   
   -   
   -   
   -   
   -   
   -   
   -   
   -   
   -   
   -   
   -   
   -   
   -   
   -   
   -   
   -   
   -   
   -   
   -   
   -   
   -   
   -   
   -   
   -   
   -   
   -   
   -   
   -   
   -   
   -   
   -   
   -   
   -   
   -   
   -   
   -   
   -   
 

 
   N55181   
   -   
   -   
   -   
   -   
   -   
   -   
   -   
   -   
   -   
   -   
   -   
   -   
   -   
   -   
   -   
   -   
   -   
   -   
   -   
   -   
   -   
   -   
   -   
   -   
   -   
   -   
   -   
   -   
   -   
   -   
   -   
   -   
   -   
   -   
   -   
   -   
   -   
   -   
   -   
   -   
   -   
   -   
   -   
   -   
   -   
   -   
   -   
   -   
   -   
   -   
   -   
   -   
   -   
   -   
   -   
   -   
   -   
   -   
   -   
   -   
   -   
   -   
   -   
   -   
   -   
   -   
   -   
   -   
   -   
   -   
   -   
   -   
   -   
   -   
   -   
   -   
   -   
   -   
   -   
   -   
   -   
   -   
   -   
   -   
   -   
   -   
   -   
   -   
   -   
   -   
   -   
   -   
   -   
   -   
   -   
   -   
   -   
   -   
   -   
   -   
   -   
   -   
   -   
   -   
   -   
   -   
   -   
   -   
   C   
   A   
   A   
   T   
   G   
   G   
   C   
   A   
   A   
   G   
   C   
   C   
   A   
   A   
   A   
   C   
   A   
   A   
   G   
   A   
    A    
   T   
   T   
   A   
   G   
   C   
    C    
   G   
   A   
   A   
   G   
   C   
   G   
   A   
   A   
   G   
   A   
   A   
   A   
   A   
    C    
   A   
   G   
   C   
    T    
   A   
    A    
   T   
    A    
    C    
   A   
   T   
   T   
   T   
   T   
   T   
   A   
   A   
   A   
    T    
   G   
   T   
   A   
   T   
   T   
   G   
    T    
   C   
   A   
   A   
   A   
   A   
   C   
   A   
   G   
    G    
   A   
   A   
   T   
   T   
   T   
   G   
   A   
   T   
   A   
   A   
   G   
   T   
   T   
   A   
   C   
   C   
    G    
   T   
   C   
   C   
   G   
   T   
   T   
    G    
   T   
   T   
   C   
   A   
   A   
   G   
   A   
   A   
    T    
   C   
   T   
   A   
   G   
   C   
   T   
   T   
   A   
   A   
   A   
   G   
   A   
   A   
   A   
   A   
   A   
   T   
   G   
   G   
    C    
   T   
   A   
   T   
   G   
   A   
   T   
   A   
   C   
   T   
   A   
   A   
   A   
   T   
   C   
    T    
   G   
   T   
   T   
   G   
   T   
   T   
   G   
   A   
   A   
   A   
   A   
   A   
   T   
   A   
   C   
   C   
   A   
   A   
   G   
   C   
   A   
   A   
   T   
   T   
   T   
   A   
   T   
   T   
   C   
   A   
   G   
   G   
   G   
   A   
   T   
   T   
   C   
   A   
   A   
   G   
   C   
    A    
   G   
   A   
   A   
    G    
   G   
    A    
   G   
   T   
   C   
   A   
   A   
    A    
   G   
   C   
   T   
   A   
   G   
   T   
   G   
   A   
   T   
   G   
   T   
    C    
   C   
   A   
   A   
   G   
   T   
    C    
   A   
   A   
   A   
   A   
   A   
    G    
   G   
    C    
   G   
   A   
   A   
   A   
   G   
   A   
    C    
   A   
   A   
    T    
   C   
   A   
   A   
   T   
   A   
   C   
   A   
   C   
   A   
   T   
   T   
   T   
   A   
    C    
   C   
   T   
   A   
   T   
   A   
   A   
   A   
   T   
   T   
   A   
   T   
   C   
   G   
   A   
   T   
   G   
   A   
   G   
    C    
   A   
   C   
    T    
   C   
   C   
   T   
   T   
   T   
   A   
   G   
   G   
   C   
   G   
   A   
   A   
   A   
   T   
   G   
   A   
   A   
   A   
   G   
   A   
   T   
    C    
   T   
   G   
   T   
   C   
   T   
   T   
   A   
   T   
   C   
   A   
   A   
   T   
   C   
   A   
   A   
   G   
   T   
   A   
   T   
    C    
   G   
    C    
   C   
   A   
   A   
    A    
   A   
   A   
   A   
   G   
   G   
    C    
   G   
   A   
   T   
   A   
    C    
   C   
   T   
   A   
   C   
   C   
   A   
   A   
   A   
   T   
    C    
    T    
   C   
   T   
   T   
   G   
   G   
    A    
    A    
    G    
   C   
   C   
    G    
   T   
   C   
   T   
   T   
   T   
   A   
   A   
   T   
   T   
   T   
   T   
   T   
   C   
   C   
   A   
   G   
   A   
   T   
   A   
   T   
   G   
   T   
   C   
   A   
   G   
   G   
   A   
   A   
   A   
   T   
   G   
   A   
   T   
   A   
   A   
   A   
    A    
   T   
   T   
   T   
   C   
   G   
   A   
   T   
   T   
   C   
   A   
   A   
   G   
   T   
   A   
   G   
   A   
   T   
   A   
   A   
   T   
   G   
   C   
   C   
   A   
   A   
   A   
   C   
   G   
   T   
   G   
   G   
   A   
   G   
   A   
   A   
   A   
   T   
   T   
   G   
   T   
   C   
   G   
   A   
   T   
   C   
   G   
   T   
   A   
   A   
    T    
   G   
   G   
   T   
   A   
   G   
   T   
   G   
   G   
   G   
   C   
   T   
   A   
   G   
    C    
   A   
   A   
   T   
   T   
   A   
   A   
   C   
   A   
   A   
   A   
   G   
   T   
   G   
   T   
   T   
   T   
   G   
   A   
    C    
   G   
   A   
   A   
   G   
   T   
   G   
   G   
   G   
   C   
   G   
   T   
   A   
   G   
   T   
    G    
   C   
   C   
   T   
    G    
   G   
   C   
   A   
   A   
   A   
   C   
   T   
    C    
   G   
   G   
   T   
   T   
   C   
   T   
   G   
   G   
   C   
   G   
   C   
   A   
   G   
   A   
   A   
   A   
   A   
   A   
    A    
   C   
   A   
   G   
   C   
    C    
   A   
   A   
   T   
   A   
   T   
   C   
   A   
   A   
   A   
   G   
   C   
   T   
   T   
   T   
   T   
   A   
   G   
   T   
   G   
   A   
   T   
   A   
   A   
    G    
   T   
   T   
    C    
   G   
   G   
   C   
   G   
   T   
   T   
   T   
   C   
   T   
    G    
   T   
   T   
   G   
   A   
   T   
   G   
   A   
   A   
   A   
   T   
   C   
   A   
   A   
   T   
   C   
    A    
   A   
   A   
   A   
    G    
   T   
   T   
   A   
   A   
   G   
   C   
   C   
   A   
   A   
   G   
   G   
    G    
   T   
   G   
   G   
   G   
   T   
   C   
   C   
   A   
   A   
   G   
   C   
   A   
   G   
   A   
   C   
   T   
   C   
    C    
   T   
   T   
   T   
   G   
   T   
   A   
   C   
   C   
   A   
   A   
   T   
   C   
   A   
   C   
   A   
   G   
   T   
    T    
   G   
   C   
   T   
   T   
   C   
   T   
   G   
   A   
   A   
   C   
   C   
   A   
   G   
   T   
   G   
   A   
   C   
   A   
   G   
   A   
   A   
   T   
   T   
   A   
   C   
   C   
   A   
   A   
   C   
   A   
   G   
   G   
   G   
   G   
   C   
   T   
   G   
   C   
   G   
   A   
   C   
   A   
   A   
   A   
   A   
   G   
    A    
    T    
   A   
   C   
   A   
   G   
   A   
   G   
   T   
   C   
   A   
   C   
   G   
   T   
   T   
   A   
   T   
   T   
   A   
   T   
   C   
   C   
    A    
    C    
   T   
   G   
   G   
   G   
   G   
   G   
   A   
   A   
   G   
   C   
   A   
   G   
   C   
   T   
   G   
   C   
   G   
   C   
   A   
   A   
   T   
   T   
   A   
   A   
   T   
   C   
   G   
   G   
    G    
   T   
   A   
   T   
   A   
   C   
    G    
   G   
   G   
    C    
   A   
   C   
    C    
   A   
   T   
   T   
   A   
   C   
   T   
   G   
   C   
   A   
   G   
   A   
    A    
   G   
   A   
   T   
   A   
   T   
   T   
    G    
   A   
   A   
   A   
   A   
   A   
   A   
   A   
   T   
   C   
   C   
   A   
   G   
   A   
   G   
   T   
   T   
   A   
   A   
   G   
   T   
   A   
   G   
   T   
   A   
   C   
   G   
   G   
   G   
    T    
   G   
   T   
   A   
   A   
   T   
   T   
   G   
   G   
    T    
   A   
   A   
   A   
   A   
   C   
   T   
   G   
   G   
    C    
   T   
   T   
   A   
   G   
   A   
   A   
   C   
   G   
   A   
   G   
   C   
   G   
   T   
   T   
   T   
   G   
   A   
   T   
   A   
   A   
   A   
   G   
   A   
    G    
   T   
   T   
   A   
   A   
   G   
   A   
   G   
   G   
    G    
   C   
   A   
   A   
   G   
   A   
   T   
   G   
   G   
    T    
   G   
   G   
    C    
   T   
   C   
    A    
   C   
   T   
   A   
   G   
   T   
   T   
   A   
   T   
   T   
   T   
   T   
   A   
   G   
   A   
   T   
   G   
   A   
   C   
   A   
   A   
   A   
   G   
   A   
   G   
   A   
   A   
   T   
   G   
   T   
   C   
   A   
   A   
   A   
   A   
   A   
   A   
   G   
   C   
   T   
   T   
   T   
   A   
   C   
   A   
   A   
   A   
   C   
   C   
   A   
   A   
   A   
   G   
   A   
    G    
   A   
   A   
   A   
   A   
   A   
   A   
   G   
   A   
   T   
   G   
   G   
   T   
   C   
   A   
   A   
   A   
   C   
    G    
   A   
   T   
   T   
   A   
   A   
   A   
   T   
   T   
   A   
   A   
   C   
   G   
   A   
   T   
   T   
   G   
   A   
    C    
   A   
   G   
    T    
   G   
   G   
    C    
   G   
   T   
   A   
   C   
   A   
   A   
   C   
   A   
   A   
   C   
   A   
   A   
   G   
   C   
    C    
   T   
   T   
    C    
   G   
   C   
   T   
   A   
   T   
   A   
   T   
   T   
   T   
   G   
   A   
   C   
   A   
   A   
   A   
   C   
   G   
    T    
    C    
   C   
   T   
   G   
   G   
   T   
   A   
   G   
   T   
   G   
   C   
   G   
   G   
   T   
   C   
   A   
   T   
   T   
   A   
   C   
   C   
   G   
   A   
   T   
   C   
   C   
   T   
   C   
   A   
   A   
   A   
   A   
   A   
   G   
   G   
   C   
   G   
   A   
   T   
   T   
   T   
   A   
    T    
   T   
   A   
   G   
   C   
    A    
   A   
   C   
   G   
   G   
   T   
   T   
   A   
   G   
   T   
   T   
   C   
   A   
   C   
   C   
   T   
   T   
   C   
   G   
   T   
   A   
   T   
   G   
   A   
   T   
   C   
   C   
   T   
   A   
   A   
   T   
   A   
   A   
   A   
   A   
   T   
   G   
   G   
   C   
   C   
   A   
   A   
   T   
   G   
   G   
    T    
   A   
   T   
   T   
   T   
   C   
   T   
   C   
   A   
   A   
   A   
   A   
   A   
   G   
   A   
    A    
   T   
   A   
   T   
   G   
   A   
   T   
   G   
   C   
   T   
   T   
   A   
   C   
   A   
   A   
   T   
   A   
   A   
   C   
   A   
   A   
   C   
   A   
   A   
   A   
   G   
   A   
   T   
   T   
   T   
   A   
   C   
   C   
   A   
   T   
   T   
    C    
   A   
   C   
   A   
   G   
   C   
   A   
   C   
   G   
   T   
   T   
   T   
    T    
   G   
   C   
   G   
   A   
   C   
   A   
   G   
   G   
    C    
   T   
   A   
   T   
   G   
   C   
   A   
   C   
   C   
   A   
   G   
   G   
    C    
   T   
   C   
   T   
   A   
   C   
   C   
   T   
   T   
   T   
   A   
   A   
   A   
   A   
   C   
   A   
   A   
   T   
   T   
   A   
   C   
   C   
   G   
   G   
   T   
   G   
   C   
   C   
   A   
   T   
   T   
   G   
   G   
   T   
   T   
   T   
   A   
   G   
   A   
   T   
    G    
   C   
   A   
   G   
   G   
   G   
   A   
   C   
   A   
   T   
   T   
   G   
   A   
   A   
   G   
   C   
   C   
   A   
   G   
   A   
   T   
   G   
   A   
   A   
   G   
   A   
   G   
   C   
   T   
   A   
   G   
   A   
   A   
   A   
   T   
   T   
   A   
   A   
   T   
   G   
   G   
    C    
   T   
   T   
   G   
   A   
   A   
   A   
   T   
   G   
   G   
   C   
   A   
   A   
   A   
   A   
   A   
   G   
   A   
   T   
   A   
   A   
   A   
   T   
   C   
   T   
   T   
   G   
   G   
   G   
   G   
    C    
   G   
   G   
   C   
   T   
   A   
   T   
   T   
   T   
   T   
   G   
   C   
    A    
   A   
   C   
   A   
   C   
   G   
   T   
   G   
   T   
   A   
   A   
   A   
   A   
   G   
   A   
   A   
   G   
   C   
   A   
   A   
   G   
   T   
   C   
   C   
   A   
   G   
   T   
   C   
   A   
   A   
   T   
   C   
   T   
    G    
   C   
   G   
   T   
   A   
   C   
   C   
   G   
   C   
   T   
   T   
   T   
   A   
   G   
   T   
   T   
   A   
   A   
   T   
   T   
   C   
   A   
   G   
   A   
   T   
   A   
   A   
   T   
   A   
   T   
   T   
   T   
   A   
   T   
   T   
   T   
   T   
   G   
   C   
    C    
   C   
   A   
   A   
   C   
   A   
   A   
   A   
   C   
   A   
   T   
   T   
   A   
   C   
   G   
    G    
   A   
   T   
   G   
   G   
   G   
    G    
   G   
   A   
   A   
   G   
   A   
   C   
   A   
   A   
   A   
   T   
   T   
   T   
   A   
   G   
   A   
   G   
   C   
    A    
   G   
   G   
   T   
   C   
   T   
   T   
   A   
   A   
   T   
   A   
   A   
   A   
   T   
   T   
   C   
   A   
   T   
   T   
   T   
   T   
   C   
   G   
   G   
   T   
   G   
   A   
   G   
   G   
   A   
   A   
   C   
   T   
   A   
   G   
   A   
   T   
   T   
   T   
   A   
   C   
   C   
   A   
   A   
   T   
    T    
   G   
   C   
   C   
   A   
   T   
   G   
   A   
   C   
   G   
   C   
   C   
    T    
   G   
   C   
   T   
   C   
   A   
   A   
   A   
   T   
   T   
   T   
   C   
   A   
   A   
   A   
    T    
   G   
   A   
   A   
   G   
   A   
   T   
   A   
   A   
   A   
   T   
   T   
   T   
   A   
   A   
   T   
   T   
   C   
   T   
   G   
   A   
   A   
   A   
   T   
   T   
   C   
   T   
   T   
   T   
   T   
    A    
   G   
   C   
   A   
   G   
   A   
   T   
   A   
   C   
    G    
   G   
   G   
   C   
   T   
   A   
    C    
   G   
   G   
    C    
   C   
   A   
   A   
   G   
   G   
    C    
   C   
   A   
   A   
   T   
   T   
   G   
   T   
   T   
   A   
   A   
   T   
   T   
   T   
   C   
    G    
   C   
   C   
   A   
   A   
   T   
   C   
   C   
   A   
   G   
   C   
   A   
   A   
   G   
   C   
   T   
   A   
   C   
   C   
   A   
   T   
   G   
   T   
   A   
   T   
   A   
   G   
   T   
   G   
   T   
   T   
   T   
   T   
   C   
   C   
   A   
   A   
   A   
   A   
   T   
   A   
   A   
   T   
   G   
   G   
    A    
   A   
   C   
   A   
    T    
   T   
    A    
   G   
   T   
    C    
   T   
   A   
   T   
   C   
   C   
   A   
   A   
   A   
   A   
    T    
   T   
   A   
   G   
   T   
   T   
   T   
   T   
   A   
   G   
   A   
   C   
   A   
   A   
   A   
   G   
   A   
   A   
   A   
   C   
    G    
   A   
   A   
   G   
   A   
   A   
   A   
   A   
   A   
   A   
   G   
   A   
   C   
   A   
   A   
   T   
   G   
   T   
   A   
   A   
   T   
   C   
   A   
   G   
   C   
   G   
   C   
   C   
   A   
   A   
   C   
   G   
   C   
   A   
   G   
   C   
   G   
   A   
   A   
   T   
   A   
   C   
   A   
   A   
   T   
   T   
   G   
   C   
   C   
   A   
   C   
   A   
   G   
   A   
   T   
   C   
   T   
   T   
   T   
   T   
   A   
   G   
   G   
   T   
   A   
   G   
   T   
   G   
   T   
   G   
   G   
   A   
   A   
   G   
   A   
   T   
   C   
   C   
   T   
   T   
   C   
   A   
   G   
   G   
   T   
   T   
   A   
   T   
   G   
   T   
   T   
   T   
   A   
   T   
   A   
   A   
   T   
   A   
   T   
   G   
   T   
   A   
   C   
   A   
   A   
   T   
   C   
   C   
   G   
   A   
   A   
   C   
   T   
   T   
   C   
   T   
   C   
   A   
   T   
   T   
   G   
   G   
   C   
   T   
   G   
   C   
   T   
   A   
   A   
   A   
   A   
   C   
   A   
   G   
   G   
   A   
   A   
   C   
   A   
   G   
   C   
   T   
   G   
   A   
   A   
   A   
   T   
   A   
   A   
   A   
   A   
   G   
   A   
   T   
   A   
   A   
   A   
   C   
   A   
   A   
   G   
   A   
   T   
   A   
   C   
   A   
   G   
   A   
   T   
   G   
   G   
   C   
   A   
   A   
   A   
   G   
   A   
   A   
   A   
   A   
   C   
   A   
   G   
   T   
   T   
   T   
   C   
   C   
   T   
   C   
   C   
   T   
   A   
   A   
   C   
   G   
   T   
   T   
   A   
   G   
   A   
   T   
   C   
   G   
   T   
   A   
   G   
   T   
   A   
   A   
   C   
   A   
   A   
   T   
   A   
   A   
   A   
   T   
   T   
   C   
   T   
   T   
   A   
   A   
   C   
   A   
   A   
   T   
   G   
   A   
   T   
   T   
   A   
   T   
   G   
   G   
   T   
   T   
   G   
   A   
   A   
   A   
   A   
   T   
   T   
   C   
   A   
   G   
   G   
   A   
   G   
   A   
   A   
   A   
   A   
   T   
   G   
   G   
   T   
   T   
   C   
   T   
   G   
   C   
   A   
   A   
   C   
   A   
   G   
   A   
   T   
   A   
   T   
   C   
   A   
   G   
   T   
   A   
   A   
   A   
   C   
   C   
   A   
   T   
   T   
   A   
   A   
   T   
   T   
   G   
   A   
   T   
   T   
   A   
   C   
   T   
   T   
   A   
   G   
   A   
   A   
   G   
   C   
   A   
   A   
   C   
   C   
   A   
   T   
   T   
   A   
   A   
   A   
   -   
   -   
   -   
   -   
   -   
   -   
   -   
   -   
   -   
   -   
   -   
   -   
   -   
   -   
   -   
   -   
   -   
   -   
   -   
   -   
   -   
   -   
   -   
   -   
   -   
   -   
   -   
   -   
   -   
   -   
   -   
   -   
   -   
   -   
   -   
   -   
   -   
   -   
   -   
   -   
   -   
   -   
   -   
   -   
   -   
   -   
   -   
   -   
   -   
   -   
   -   
   -   
   -   
   -   
   -   
   -   
   -   
   -   
   -   
   -   
   -   
   -   
   -   
   -   
   -   
   -   
   -   
   -   
   -   
   -   
   -   
   -   
   -   
   -   
   -   
   -   
   -   
   -   
   -   
   -   
   -   
   -   
   -   
   -   
   -   
   -   
   -   
   -   
   -   
   -   
   -   
   -   
   -   
   -   
   -   
   -   
   -   
   -   
   -   
   -   
   -   
   -   
   -   
   -   
   -   
   -   
   -   
   -   
   -   
   -   
   -   
   -   
   -   
   -   
   -   
   -   
   -   
   -   
   -   
   -   
   -   
   -   
   -   
   -   
   -   
   -   
   -   
   -   
   -   
   -   
   -   
   -   
   -   
   -   
   -   
   -   
   -   
   -   
   -   
   -   
   -   
   -   
   -   
   -   
   -   
   -   
   -   
   -   
   -   
   -   
   -   
   -   
   -   
   -   
   -   
   -   
   -   
   -   
   -   
   -   
   -   
   -   
   -   
   -   
   -   
   -   
   -   
   -   
   -   
   C   
   A   
   A   
   T   
   G   
   G   
   C   
   A   
   A   
   G   
   C   
   C   
   A   
   A   
   A   
   C   
   A   
   A   
   G   
   A   
   A   
   T   
   T   
   A   
   G   
   C   
   C   
   G   
   A   
   A   
   G   
   C   
   G   
   A   
   A   
   G   
   A   
   A   
   A   
   A   
   C   
   A   
   G   
   C   
   T   
   A   
   A   
   T   
   A   
   C   
   A   
   T   
   T   
   T   
   T   
   T   
   A   
   A   
   A   
   T   
   G   
   T   
   A   
   T   
   T   
   G   
   T   
   C   
   A   
   A   
   A   
   A   
   C   
   A   
   G   
   G   
   A   
   A   
   T   
   T   
   T   
   G   
   A   
   T   
   A   
   A   
   G   
   T   
   T   
   A   
   C   
   C   
   G   
   T   
   C   
   C   
   G   
   T   
   T   
   G   
   T   
   T   
   C   
   A   
   A   
   G   
   A   
   A   
   T   
   C   
   T   
   A   
   G   
   C   
   T   
   T   
   A   
   A   
   A   
   G   
   A   
   A   
   A   
   A   
   A   
   T   
   G   
   G   
   C   
   T   
   A   
   T   
   G   
   A   
   T   
   A   
   C   
   T   
   A   
   A   
   A   
   T   
   C   
   T   
   G   
   T   
   T   
   G   
   T   
   T   
   G   
   A   
   A   
   A   
   A   
   A   
   T   
   A   
   C   
   C   
   A   
   A   
   G   
   C   
   A   
   A   
   T   
   T   
   T   
   A   
   T   
   T   
   C   
   A   
   G   
   G   
   G   
   A   
   T   
   T   
   C   
   A   
   A   
   G   
   C   
   A   
   G   
   A   
   A   
   G   
   G   
   A   
   G   
   T   
   C   
   A   
   A   
   A   
   G   
   C   
   T   
   A   
   G   
   T   
   G   
   A   
   T   
   G   
   T   
   C   
   C   
   A   
   A   
   G   
   T   
   C   
   A   
   A   
   A   
   A   
   A   
   G   
   G   
   C   
   G   
   A   
   A   
   A   
   G   
   A   
   C   
   A   
   A   
   T   
   C   
   A   
   A   
   T   
   A   
   C   
   A   
   C   
   A   
   T   
   T   
   T   
   A   
   C   
   C   
   T   
   A   
   T   
   A   
   A   
   A   
   T   
   T   
   A   
   T   
   C   
   G   
   A   
   T   
   G   
   A   
   G   
   C   
   A   
   C   
   T   
   C   
   C   
   T   
   T   
   T   
   A   
   G   
   G   
   C   
   G   
   A   
   A   
   A   
   T   
   G   
   A   
   A   
   A   
   G   
   A   
   T   
   C   
   T   
   G   
   T   
   C   
   T   
   T   
   A   
   T   
   C   
   A   
   A   
   T   
   C   
   A   
   A   
   G   
   T   
   A   
   T   
   C   
   G   
   C   
   C   
   A   
   A   
   A   
   A   
   A   
   A   
   G   
   G   
   C   
   G   
   A   
   T   
   A   
   C   
   C   
   T   
   A   
   C   
   C   
   A   
   A   
   A   
   T   
   C   
   T   
   C   
   T   
   T   
   G   
   G   
   A   
   A   
   G   
   C   
   C   
   G   
   T   
   C   
   T   
   T   
   T   
   A   
   A   
   T   
   T   
   T   
   T   
   T   
   C   
   C   
   A   
   G   
   A   
   T   
   A   
   T   
   G   
   T   
   C   
   A   
   G   
   G   
   A   
   A   
   A   
   T   
   G   
   A   
   T   
   A   
   A   
   A   
   A   
   T   
   T   
   T   
   C   
   G   
   A   
   T   
   T   
   C   
   A   
   A   
   G   
   T   
   A   
   G   
   A   
   T   
   A   
   A   
   T   
   G   
   C   
   C   
   A   
   A   
   A   
   C   
   G   
   T   
   G   
   G   
   A   
   G   
   A   
   A   
   A   
   T   
   T   
   G   
   T   
   C   
   G   
   A   
   T   
   C   
   G   
   T   
   A   
   A   
   T   
   G   
   G   
   T   
   A   
   G   
   T   
   G   
   G   
   G   
   C   
   T   
   A   
   G   
   C   
   A   
   A   
   T   
   T   
   A   
   A   
   C   
   A   
   A   
   A   
   G   
   T   
   G   
   T   
   T   
   T   
   G   
   A   
   C   
   G   
   A   
   A   
   G   
   T   
   G   
   G   
   G   
   C   
   G   
   T   
   A   
   G   
   T   
   G   
   C   
   C   
   T   
   G   
   G   
   C   
   A   
   A   
   A   
   C   
   T   
   C   
   G   
   G   
   T   
   T   
   C   
   T   
   G   
   G   
   C   
   G   
   C   
   A   
   G   
   A   
   A   
   A   
   A   
   A   
   A   
   C   
   A   
   G   
   C   
   C   
   A   
   A   
   T   
   A   
   T   
   C   
   A   
   A   
   A   
   G   
   C   
   T   
   T   
   T   
   T   
   A   
   G   
   T   
   G   
   A   
   T   
   A   
   A   
   G   
   T   
   T   
   C   
   G   
   G   
   C   
   G   
   T   
   T   
   T   
   C   
   T   
   G   
   T   
   T   
   G   
   A   
   T   
   G   
   A   
   A   
   A   
   T   
   C   
   A   
   A   
   T   
   C   
   A   
   A   
   A   
   A   
   G   
   T   
   T   
   A   
   A   
   G   
   C   
   C   
   A   
   A   
   G   
   G   
   G   
   T   
   G   
   G   
   G   
   T   
   C   
   C   
   A   
   A   
   G   
   C   
   A   
   G   
   A   
   C   
   T   
   C   
   C   
   T   
   T   
   T   
   G   
   T   
   A   
   C   
   C   
   A   
   A   
   T   
   C   
   A   
   C   
   A   
   G   
   T   
   T   
   G   
   C   
   T   
   T   
   C   
   T   
   G   
   A   
   A   
   C   
   C   
   A   
   G   
   T   
   G   
   A   
   C   
   A   
   G   
   A   
   A   
   T   
   T   
   A   
   C   
   C   
   A   
   A   
   C   
   A   
   G   
   G   
   G   
   G   
   C   
   T   
   G   
   C   
   G   
   A   
   C   
   A   
   A   
   A   
   A   
   G   
   A   
   T   
   A   
   C   
   A   
   G   
   A   
   G   
   T   
   C   
   A   
   C   
   G   
   T   
   T   
   A   
   T   
   T   
   A   
   T   
   C   
   C   
   A   
   C   
   T   
   G   
   G   
   G   
   G   
   G   
   A   
   A   
   G   
   C   
   A   
   G   
   C   
   T   
   G   
   C   
   G   
   C   
   A   
   A   
   T   
   T   
   A   
   A   
   T   
   C   
   G   
   G   
   G   
   T   
   A   
   T   
   A   
   C   
   G   
   G   
   G   
   C   
   A   
   C   
   C   
   A   
   T   
   T   
   A   
   C   
   T   
   G   
   C   
   A   
   G   
   A   
   A   
   G   
   A   
   T   
   A   
   T   
   T   
   G   
   A   
   A   
   A   
   A   
   A   
   A   
   A   
   T   
   C   
   C   
   A   
   G   
   A   
   G   
   T   
   T   
   A   
   A   
   G   
   T   
   A   
   G   
   T   
   A   
   C   
   G   
   G   
   G   
   T   
   G   
   T   
   A   
   A   
   T   
   T   
   G   
   G   
   T   
   A   
   A   
   A   
   A   
   C   
   T   
   G   
   G   
   C   
   T   
   T   
   A   
   G   
   A   
   A   
   C   
   G   
   A   
   G   
   C   
   G   
   T   
   T   
   T   
   G   
   A   
   T   
   A   
   A   
   A   
   G   
   A   
   G   
   T   
   T   
   A   
   A   
   G   
   A   
   G   
   G   
   G   
   C   
   A   
   A   
   G   
   A   
   T   
   G   
   G   
   T   
   G   
   G   
   C   
   T   
   C   
   A   
   C   
   T   
   A   
   G   
   T   
   T   
   A   
   T   
   T   
   T   
   T   
   A   
   G   
   A   
   T   
   G   
   A   
   C   
   A   
   A   
   A   
   G   
   A   
   G   
   A   
   A   
   T   
   G   
   T   
   C   
   A   
   A   
   A   
   A   
   A   
   A   
   G   
   C   
   T   
   T   
   T   
   A   
   C   
   A   
   A   
   A   
   C   
   C   
   A   
   A   
   A   
   G   
   A   
   G   
   A   
   A   
   A   
   A   
   A   
   A   
   G   
   A   
   T   
   G   
   G   
   T   
   C   
   A   
   A   
   A   
   C   
   G   
   A   
   T   
   T   
   A   
   A   
   A   
   T   
   T   
   A   
   A   
   C   
   G   
   A   
   T   
   T   
   G   
   A   
   C   
   A   
   G   
   T   
   G   
   G   
   C   
   G   
   T   
   A   
   C   
   A   
   A   
   C   
   A   
   A   
   C   
   A   
   A   
   G   
   C   
   C   
   T   
   T   
   C   
   G   
   C   
   T   
   A   
   T   
   A   
   T   
   T   
   T   
   G   
   A   
   C   
   A   
   A   
   A   
   C   
   G   
   T   
   C   
   C   
   T   
   G   
   G   
   T   
   A   
   G   
   T   
   G   
   C   
   G   
   G   
   T   
   C   
   A   
   T   
   T   
   A   
   C   
   C   
   G   
   A   
   T   
   C   
   C   
   T   
   C   
   A   
   A   
   A   
   A   
   A   
   G   
   G   
   C   
   G   
   A   
   T   
   T   
   T   
   A   
   T   
   T   
   A   
   G   
   C   
   A   
   A   
   C   
   G   
   G   
   T   
   T   
   A   
   G   
   T   
   T   
   C   
   A   
   C   
   C   
   T   
   T   
   C   
   G   
   T   
   A   
   T   
   G   
   A   
   T   
   C   
   C   
   T   
   A   
   A   
   T   
   A   
   A   
   A   
   A   
   T   
   G   
   G   
   C   
   C   
   A   
   A   
   T   
   G   
   G   
   T   
   A   
   T   
   T   
   T   
   C   
   T   
   C   
   A   
   A   
   A   
   A   
   A   
   G   
   A   
   A   
   T   
   A   
   T   
   G   
   A   
   T   
   G   
   C   
   T   
   T   
   A   
   C   
   A   
   A   
   T   
   A   
   A   
   C   
   A   
   A   
   C   
   A   
   A   
   A   
   G   
   A   
   T   
   T   
   T   
   A   
   C   
   C   
   A   
   T   
   T   
   C   
   A   
   C   
   A   
   G   
   C   
   A   
   C   
   G   
   T   
   T   
   T   
   T   
   G   
   C   
   G   
   A   
   C   
   A   
   G   
   G   
   C   
   T   
   A   
   T   
   G   
   C   
   A   
   C   
   C   
   A   
   G   
   G   
   C   
   T   
   C   
   T   
   A   
   C   
   C   
   T   
   T   
   T   
   A   
   A   
   A   
   A   
   C   
   A   
   A   
   T   
   T   
   A   
   C   
   C   
   G   
   G   
   T   
   G   
   C   
   C   
   A   
   T   
   T   
   G   
   G   
   T   
   T   
   T   
   A   
   G   
   A   
   T   
   G   
   C   
   A   
   G   
   G   
   G   
   A   
   C   
   A   
   T   
   T   
   G   
   A   
   A   
   G   
   C   
   C   
   A   
   G   
   A   
   T   
   G   
   A   
   A   
   G   
   A   
   G   
   C   
   T   
   A   
   G   
   A   
   A   
   A   
   T   
   T   
   A   
   A   
   T   
   G   
   G   
   C   
   T   
   T   
   G   
   A   
   A   
   A   
   T   
   G   
   G   
   C   
   A   
   A   
   A   
   A   
   A   
   G   
   A   
   T   
   A   
   A   
   A   
   T   
   C   
   T   
   T   
   G   
   G   
   G   
   G   
   C   
   G   
   G   
   C   
   T   
   A   
   T   
   T   
   T   
   T   
   G   
   C   
   A   
   A   
   C   
   A   
   C   
   G   
   T   
   G   
   T   
   A   
   A   
   A   
   A   
   G   
   A   
   A   
   G   
   C   
   A   
   A   
   G   
   T   
   C   
   C   
   A   
   G   
   T   
   C   
   A   
   A   
   T   
   C   
   T   
   G   
   C   
   G   
   T   
   A   
   C   
   C   
   G   
   C   
   T   
   T   
   T   
   A   
   G   
   T   
   T   
   A   
   A   
   T   
   T   
   C   
   A   
   G   
   A   
   T   
   A   
   A   
   T   
   A   
   T   
   T   
   T   
   A   
   T   
   T   
   T   
   T   
   G   
   C   
   C   
   C   
   A   
   A   
   C   
   A   
   A   
   A   
   C   
   A   
   T   
   T   
   A   
   C   
   G   
   G   
   A   
   T   
   G   
   G   
   G   
   G   
   G   
   A   
   A   
   G   
   A   
   C   
   A   
   A   
   A   
   T   
   T   
   T   
   A   
   G   
   A   
   G   
   C   
   A   
   G   
   G   
   T   
   C   
   T   
   T   
   A   
   A   
   T   
   A   
   A   
   A   
   T   
   T   
   C   
   A   
   T   
   T   
   T   
   T   
   C   
   G   
   G   
   T   
   G   
   A   
   G   
   G   
   A   
   A   
   C   
   T   
   A   
   G   
   A   
   T   
   T   
   T   
   A   
   C   
   C   
   A   
   A   
   T   
   T   
   G   
   C   
   C   
   A   
   T   
   G   
   A   
   C   
   G   
   C   
   C   
   T   
   G   
   C   
   T   
   C   
   A   
   A   
   A   
   T   
   T   
   T   
   C   
   A   
   A   
   A   
   T   
   G   
   A   
   A   
   G   
   A   
   T   
   A   
   A   
   A   
   T   
   T   
   T   
   A   
   A   
   T   
   T   
   C   
   T   
   G   
   A   
   A   
   A   
   T   
   T   
   C   
   T   
   T   
   T   
   T   
   A   
   G   
   C   
   A   
   G   
   A   
   T   
   A   
   C   
   G   
   G   
   G   
   C   
   T   
   A   
   C   
   G   
   G   
   C   
   C   
   A   
   A   
   G   
   G   
   C   
   C   
   A   
   A   
   T   
   T   
   G   
   T   
   T   
   A   
   A   
   T   
   T   
   T   
   C   
   G   
   C   
   C   
   A   
   A   
   T   
   C   
   C   
   A   
   G   
   C   
   A   
   A   
   G   
   C   
   T   
   A   
   C   
   C   
   A   
   T   
   G   
   T   
   A   
   T   
   A   
   G   
   T   
   G   
   T   
   T   
   T   
   T   
   C   
   C   
   A   
   A   
   A   
   A   
   T   
   A   
   A   
   T   
   G   
   G   
   A   
   A   
   C   
   A   
   T   
   T   
   A   
   G   
   T   
   C   
   T   
   A   
   T   
   C   
   C   
   A   
   A   
   A   
   A   
   T   
   T   
   A   
   G   
   T   
   T   
   T   
   T   
   A   
   G   
   A   
   C   
   A   
   A   
   A   
   G   
   A   
   A   
   A   
   C   
   G   
   A   
   A   
   G   
   A   
   A   
   A   
   A   
   A   
   A   
   G   
   A   
   C   
   A   
   A   
   T   
   G   
   T   
   A   
   A   
   T   
   C   
   A   
   G   
   C   
   G   
   C   
   C   
   A   
   A   
   C   
   G   
   C   
   A   
   G   
   C   
   G   
   A   
   A   
   T   
   A   
   C   
   A   
   A   
   T   
   T   
   G   
   C   
   C   
   A   
   C   
   A   
   G   
   A   
   T   
   C   
   T   
   T   
   T   
   T   
   A   
   G   
   G   
   T   
   A   
   G   
   T   
   G   
   T   
   G   
   G   
   A   
   A   
   G   
   A   
   T   
   C   
   C   
   T   
   T   
   C   
   A   
   G   
   G   
   T   
   T   
   A   
   T   
   G   
   T   
   T   
   T   
   A   
   T   
   A   
   A   
   T   
   A   
   T   
   G   
   T   
   A   
   C   
   A   
   A   
   T   
   C   
   C   
   G   
   A   
   A   
   C   
   T   
   T   
   C   
   T   
   C   
   A   
   T   
   T   
   G   
   G   
   C   
   T   
   G   
   C   
   T   
   A   
   A   
   A   
   A   
   C   
   A   
   G   
   G   
   A   
   A   
   C   
   A   
   G   
   C   
   T   
   G   
   A   
   A   
   A   
   T   
   A   
   A   
   A   
   A   
   G   
   A   
   T   
   A   
   A   
   A   
   C   
   A   
   A   
   G   
   A   
   T   
   A   
   C   
   A   
   G   
   A   
   T   
   G   
   G   
   C   
   A   
   A   
   A   
   G   
   A   
   A   
   A   
   A   
   C   
   A   
   G   
   T   
   T   
   T   
   C   
   C   
   T   
   C   
   C   
   T   
   A   
   A   
   C   
   G   
   T   
   T   
   A   
   G   
   A   
   T   
   C   
   G   
   T   
   A   
   G   
   T   
   A   
   A   
   C   
   A   
   A   
   T   
   A   
   A   
   A   
   T   
   T   
   C   
   T   
   T   
   A   
   A   
   C   
   A   
   A   
   T   
   G   
   A   
   T   
   T   
   A   
   T   
   G   
   G   
   T   
   T   
   G   
   A   
   A   
   A   
   A   
   T   
   T   
   C   
   A   
   G   
   G   
   A   
   G   
   A   
   A   
   A   
   A   
   T   
   G   
   G   
   T   
   T   
   C   
   T   
   G   
   C   
   A   
   A   
   C   
   A   
   G   
   A   
   T   
   A   
   T   
   C   
   A   
   G   
   T   
   A   
   A   
   A   
   C   
   C   
   A   
   T   
   T   
   A   
   A   
   T   
   T   
   G   
   A   
   T   
   T   
   A   
   C   
   T   
   T   
   A   
   G   
   A   
   A   
   G   
   C   
   A   
   A   
   C   
   C   
   A   
   T   
   T   
   A   
   A   
   A   
   -   
   -   
   -   
   -   
   -   
   -   
   -   
   -   
   -   
   -   
   -   
   -   
   -   
   -   
   -   
   -   
   -   
   -   
   -   
   -   
   -   
   -   
   -   
   -   
   -   
   -   
   -   
   -   
   -   
   -   
   -   
   -   
   -   
   -   
   -   
   -   
   -   
   -   
   -   
   -   
   -   
   -   
   -   
   -   
   -   
   -   
   -   
   -   
   -   
   -   
   -   
   -   
   -   
   -   
   -   
   -   
   -   
   -   
   -   
   -   
   -   
 

 
   N59841F   
   -   
   -   
   -   
   -   
   -   
   -   
   -   
   -   
   -   
   -   
   -   
   -   
   -   
   -   
   -   
   -   
   -   
   -   
   -   
   -   
   -   
   -   
   -   
   -   
   -   
   -   
   -   
   -   
   -   
   -   
   -   
   -   
   -   
   -   
   -   
   -   
   -   
   -   
   -   
   -   
   -   
   -   
   -   
   -   
   -   
   -   
   -   
   -   
   -   
   -   
   -   
   -   
   -   
   -   
   -   
   -   
   -   
   -   
   -   
   -   
   -   
   -   
   -   
   -   
   -   
   -   
   -   
   -   
   -   
   -   
   -   
   -   
   -   
   -   
   -   
   -   
   -   
   -   
   -   
   -   
   -   
   -   
   -   
   -   
   -   
   -   
   -   
   -   
   -   
   -   
   -   
   -   
   -   
   -   
   -   
   -   
   -   
   -   
   -   
   -   
   -   
   -   
   -   
   -   
   -   
   -   
   -   
   -   
   C   
   A   
   A   
   T   
   G   
   G   
   C   
   A   
   A   
   G   
   C   
   C   
   A   
   A   
   A   
   C   
   A   
   A   
   G   
   A   
    A    
   T   
   T   
   A   
   G   
   C   
    C    
   G   
   A   
   A   
   G   
   C   
   G   
   A   
   A   
   G   
   A   
   A   
   A   
   A   
    C    
   A   
   G   
   C   
    T    
   A   
    A    
   T   
    A    
    C    
   A   
   T   
   T   
   T   
   T   
   T   
   A   
   A   
   A   
    T    
   G   
   T   
   A   
   T   
   T   
   G   
    T    
   C   
   A   
   A   
   A   
   A   
   C   
   A   
   G   
    G    
   A   
   A   
   T   
   T   
   T   
   G   
   A   
   T   
   A   
   A   
   G   
   T   
   T   
   A   
   C   
   C   
    G    
   T   
   C   
   C   
   G   
   T   
   T   
    G    
   T   
   T   
   C   
   A   
   A   
   G   
   A   
   A   
    T    
   C   
   T   
   A   
   G   
   C   
   T   
   T   
   A   
   A   
   A   
   G   
   A   
   A   
   A   
   A   
   A   
   T   
   G   
   G   
    C    
   T   
   A   
   T   
   G   
   A   
   T   
   A   
   C   
   T   
   A   
   A   
   A   
   T   
   C   
    T    
   G   
   T   
   T   
   G   
   T   
   T   
   G   
   A   
   A   
   A   
   A   
   A   
   T   
   A   
   C   
   C   
   A   
   A   
   G   
   C   
   A   
   A   
   T   
   T   
   T   
   A   
   T   
   T   
   C   
   A   
   G   
   G   
   G   
   A   
   T   
   T   
   C   
   A   
   A   
   G   
   C   
    A    
   G   
   A   
   A   
    G    
   G   
    A    
   G   
   T   
   C   
   A   
   A   
    A    
   G   
   C   
   T   
   A   
   G   
   T   
   G   
   A   
   T   
   G   
   T   
    C    
   C   
   A   
   A   
   G   
   T   
    C    
   A   
   A   
   A   
   A   
   A   
    G    
   G   
    C    
   G   
   A   
   A   
   A   
   G   
   A   
    C    
   A   
   A   
    T    
   C   
   A   
   A   
   T   
   A   
   C   
   A   
   C   
   A   
   T   
   T   
   T   
   A   
    C    
   C   
   T   
   A   
   T   
   A   
   A   
   A   
   T   
   T   
   A   
   T   
   C   
   G   
   A   
   T   
   G   
   A   
   G   
    C    
   A   
   C   
    T    
   C   
   C   
   T   
   T   
   T   
   A   
   G   
   G   
   C   
   G   
   A   
   A   
   A   
   T   
   G   
   A   
   A   
   A   
   G   
   A   
   T   
    C    
   T   
   G   
   T   
   C   
   T   
   T   
   A   
   T   
   C   
   A   
   A   
   T   
   C   
   A   
   A   
   G   
   T   
   A   
   T   
    C    
   G   
    C    
   C   
   A   
   A   
    A    
   A   
   A   
   A   
   G   
   G   
    C    
   G   
   A   
   T   
   A   
    C    
   C   
   T   
   A   
   C   
   C   
   A   
   A   
   A   
   T   
    C    
    T    
   C   
   T   
   T   
   G   
   G   
    A    
    A    
    G    
   C   
   C   
    G    
   T   
   C   
   T   
   T   
   T   
   A   
   A   
   T   
   T   
   T   
   T   
   T   
   C   
   C   
   A   
   G   
   A   
   T   
   A   
   T   
   G   
   T   
   C   
   A   
   G   
   G   
   A   
   A   
   A   
   T   
   G   
   A   
   T   
   A   
   A   
   A   
    A    
   T   
   T   
   T   
   C   
   G   
   A   
   T   
   T   
   C   
   A   
   A   
   G   
   T   
   A   
   G   
   A   
   T   
   A   
   A   
   T   
   G   
   C   
   C   
   A   
   A   
   A   
   C   
   G   
   T   
   G   
   G   
   A   
   G   
   A   
   A   
   A   
   T   
   T   
   G   
   T   
   C   
   G   
   A   
   T   
   C   
   G   
   T   
   A   
   A   
    T    
   G   
   G   
   T   
   A   
   G   
   T   
   G   
   G   
   G   
   C   
   T   
   A   
   G   
    C    
   A   
   A   
   T   
   T   
   A   
   A   
   C   
   A   
   A   
   A   
   G   
   T   
   G   
   T   
   T   
   T   
   G   
   A   
    C    
   G   
   A   
   A   
   G   
   T   
   G   
   G   
   G   
   C   
   G   
   T   
   A   
   G   
   T   
    G    
   C   
   C   
   T   
    G    
   G   
   C   
   A   
   A   
   A   
   C   
   T   
    C    
   G   
   G   
   T   
   T   
   C   
   T   
   G   
   G   
   C   
   G   
   C   
   A   
   G   
   A   
   A   
   A   
   A   
   A   
    A    
   C   
   A   
   G   
   C   
    C    
   A   
   A   
   T   
   A   
   T   
   C   
   A   
   A   
   A   
   G   
   C   
   T   
   T   
   T   
   T   
   A   
   G   
   T   
   G   
   A   
   T   
   A   
   A   
    G    
   T   
   T   
    C    
   G   
   G   
   C   
   G   
   T   
   T   
   T   
   C   
   T   
    G    
   T   
   T   
   G   
   A   
   T   
   G   
   A   
   A   
   A   
   T   
   C   
   A   
   A   
   T   
   C   
    A    
   A   
   A   
   A   
    G    
   T   
   T   
   A   
   A   
   G   
   C   
   C   
   A   
   A   
   G   
   G   
    G    
   T   
   G   
   G   
   G   
   T   
   C   
   C   
   A   
   A   
   G   
   C   
   A   
   G   
   A   
   C   
   T   
   C   
    C    
   T   
   T   
   T   
   G   
   T   
   A   
   C   
   C   
   A   
   A   
   T   
   C   
   A   
   C   
   A   
   G   
   T   
    T    
   G   
   C   
   T   
   T   
   C   
   T   
   G   
   A   
   A   
   C   
   C   
   A   
   G   
   T   
   G   
   A   
   C   
   A   
   G   
   A   
   A   
   T   
   T   
   A   
   C   
   C   
   A   
   A   
   C   
   A   
   G   
   G   
   G   
   G   
   C   
   T   
   G   
   C   
   G   
   A   
   C   
   A   
   A   
   A   
   A   
   G   
    A    
    T    
   A   
   C   
   A   
   G   
   A   
   G   
   T   
   C   
   A   
   C   
   G   
   T   
   T   
   A   
   T   
   T   
   A   
   T   
   C   
   C   
    A    
    C    
   T   
   G   
   G   
   G   
   G   
   G   
   A   
   A   
   G   
   C   
   A   
   G   
   C   
   T   
   G   
   C   
   G   
   C   
   A   
   A   
   T   
   T   
   A   
   A   
   T   
   C   
   G   
   G   
    G    
   T   
   A   
   T   
   A   
   C   
    G    
   G   
   G   
    C    
   A   
   C   
    C    
   A   
   T   
   T   
   A   
   C   
   T   
   G   
   C   
   A   
   G   
   A   
    A    
   G   
   A   
   T   
   A   
   T   
   T   
    G    
   A   
   A   
   A   
   A   
   A   
   A   
   A   
   T   
   C   
   C   
   A   
   G   
   A   
   G   
   T   
   T   
   A   
   A   
   G   
   T   
   A   
   G   
   T   
   A   
   C   
   G   
   G   
   G   
    T    
   G   
   T   
   A   
   A   
   T   
   T   
   G   
   G   
    T    
   A   
   A   
   A   
   A   
   C   
   T   
   G   
   G   
    C    
   T   
   T   
   A   
   G   
   A   
   A   
   C   
   G   
   A   
   G   
   C   
   G   
   T   
   T   
   T   
   G   
   A   
   T   
   A   
   A   
   A   
   G   
   A   
    G    
   T   
   T   
   A   
   A   
   G   
   A   
   G   
   G   
    G    
   C   
   A   
   A   
   G   
   A   
   T   
   G   
   G   
    T    
   G   
   G   
    C    
   T   
   C   
    A    
   C   
   T   
   A   
   G   
   T   
   T   
   A   
   T   
   T   
   T   
   T   
   A   
   G   
   A   
   T   
   G   
   A   
   C   
   A   
   A   
   A   
   G   
   A   
   G   
   A   
   A   
   T   
   G   
   T   
   C   
   A   
   A   
   A   
   A   
   A   
   A   
   G   
   C   
   T   
   T   
   T   
   A   
   C   
   A   
   A   
   A   
   C   
   C   
   A   
   A   
   A   
   G   
   A   
    G    
   A   
   A   
   A   
   A   
   A   
   A   
   G   
   A   
   T   
   G   
   G   
   T   
   C   
   A   
   A   
   A   
   C   
    G    
   A   
   T   
   T   
   A   
   A   
   A   
   T   
   T   
   A   
   A   
   C   
   G   
   A   
   T   
   T   
   G   
   A   
    C    
   A   
   G   
    T    
   G   
   G   
    C    
   G   
   T   
   A   
   C   
   A   
   A   
   C   
   A   
   A   
   C   
   A   
   A   
   G   
   C   
    C    
   T   
   T   
    C    
   G   
   C   
   T   
   A   
   T   
   A   
   T   
   T   
   T   
   G   
   A   
   C   
   A   
   A   
   A   
   C   
   G   
    T    
    C    
   C   
   T   
   G   
   G   
   T   
   A   
   G   
   T   
   G   
   C   
   G   
   G   
   T   
   C   
   A   
   T   
   T   
   A   
   C   
   C   
   G   
   A   
   T   
   C   
   C   
   T   
   C   
   A   
   A   
   A   
   A   
   A   
   G   
   G   
   C   
   G   
   A   
   T   
   T   
   T   
   A   
    T    
   T   
   A   
   G   
   C   
    A    
   A   
   C   
   G   
   G   
   T   
   T   
   A   
   G   
   T   
   T   
   C   
   A   
   C   
   C   
   T   
   T   
   C   
   G   
   T   
   A   
   T   
   G   
   A   
   T   
   C   
   C   
   T   
   A   
   A   
   T   
   A   
   A   
   A   
   A   
   T   
   G   
   G   
   C   
   C   
   A   
   A   
   T   
   G   
   G   
    T    
   A   
   T   
   T   
   T   
   C   
   T   
   C   
   A   
   A   
   A   
   A   
   A   
   G   
   A   
    A    
   T   
   A   
   T   
   G   
   A   
   T   
   G   
   C   
   T   
   T   
   A   
   C   
   A   
   A   
   T   
   A   
   A   
   C   
   A   
   A   
   C   
   A   
   A   
   A   
   G   
   A   
   T   
   T   
   T   
   A   
   C   
   C   
   A   
   T   
   T   
    C    
   A   
   C   
   A   
   G   
   C   
   A   
   C   
   G   
   T   
   T   
   T   
    T    
   G   
   C   
   G   
   A   
   C   
   A   
   G   
   G   
    C    
   T   
   A   
   T   
   G   
   C   
   A   
   C   
   C   
   A   
   G   
   G   
    C    
   T   
   C   
   T   
   A   
   C   
   C   
   T   
   T   
   T   
   A   
   A   
   A   
   A   
   C   
   A   
   A   
   T   
   T   
   A   
   C   
   C   
   G   
   G   
   T   
   G   
   C   
   C   
   A   
   T   
   T   
   G   
   G   
   T   
   T   
   T   
   A   
   G   
   A   
   T   
    G    
   C   
   A   
   G   
   G   
   G   
   A   
   C   
   A   
   T   
   T   
   G   
   A   
   A   
   G   
   C   
   C   
   A   
   G   
   A   
   T   
   G   
   A   
   A   
   G   
   A   
   G   
   C   
   T   
   A   
   G   
   A   
   A   
   A   
   T   
   T   
   A   
   A   
   T   
   G   
   G   
    C    
   T   
   T   
   G   
   A   
   A   
   A   
   T   
   G   
   G   
   C   
   A   
   A   
   A   
   A   
   A   
   G   
   A   
   T   
   A   
   A   
   A   
   T   
   C   
   T   
   T   
   G   
   G   
   G   
   G   
    C    
   G   
   G   
   C   
   T   
   A   
   T   
   T   
   T   
   T   
   G   
   C   
    A    
   A   
   C   
   A   
   C   
   G   
   T   
   G   
   T   
   A   
   A   
   A   
   A   
   G   
   A   
   A   
   G   
   C   
   A   
   A   
   G   
   T   
   C   
   C   
   A   
   G   
   T   
   C   
   A   
   A   
   T   
   C   
   T   
    G    
   C   
   G   
   T   
   A   
   C   
   C   
   G   
   C   
   T   
   T   
   T   
   A   
   G   
   T   
   T   
   A   
   A   
   T   
   T   
   C   
   A   
   G   
   A   
   T   
   A   
   A   
   T   
   A   
   T   
   T   
   T   
   A   
   T   
   T   
   T   
   T   
   G   
   C   
    C    
   C   
   A   
   A   
   C   
   A   
   A   
   A   
   C   
   A   
   T   
   T   
   A   
   C   
   G   
    G    
   A   
   T   
   G   
   G   
   G   
    G    
   G   
   A   
   A   
   G   
   A   
   C   
   A   
   A   
   A   
   T   
   T   
   T   
   A   
   G   
   A   
   G   
   C   
    A    
   G   
   G   
   T   
   C   
   T   
   T   
   A   
   A   
   T   
   A   
   A   
   A   
   T   
   T   
   C   
   A   
   T   
   T   
   T   
   T   
   C   
   G   
   G   
   T   
   G   
   A   
   G   
   G   
   A   
   A   
   C   
   T   
   A   
   G   
   A   
   T   
   T   
   T   
   A   
   C   
   C   
   A   
   A   
   T   
    T    
   G   
   C   
   C   
   A   
   T   
   G   
   A   
   C   
   G   
   C   
   C   
    T    
   G   
   C   
   T   
   C   
   A   
   A   
   A   
   T   
   T   
   T   
   C   
   A   
   A   
   A   
    T    
   G   
   A   
   A   
   G   
   A   
   T   
   A   
   A   
   A   
   T   
   T   
   T   
   A   
   A   
   T   
   T   
   C   
   T   
   G   
   A   
   A   
   A   
   T   
   T   
   C   
   T   
   T   
   T   
   T   
    A    
   G   
   C   
   A   
   G   
   A   
   T   
   A   
   C   
    G    
   G   
   G   
   C   
   T   
   A   
    C    
   G   
   G   
    C    
   C   
   A   
   A   
   G   
   G   
    C    
   C   
   A   
   A   
   T   
   T   
   G   
   T   
   T   
   A   
   A   
   T   
   T   
   T   
   C   
    G    
   C   
   C   
   A   
   A   
   T   
   C   
   C   
   A   
   G   
   C   
   A   
   A   
   G   
   C   
   T   
   A   
   C   
   C   
   A   
   T   
   G   
   T   
   A   
   T   
   A   
   G   
   T   
   G   
   T   
   T   
   T   
   T   
   C   
   C   
   A   
   A   
   A   
   A   
   T   
   A   
   A   
   T   
   G   
   G   
    A    
   A   
   C   
   A   
    T    
   T   
    A    
   G   
   T   
    C    
   T   
   A   
   T   
   C   
   C   
   A   
   A   
   A   
   A   
    T    
   T   
   A   
   G   
   T   
   T   
   T   
   T   
   A   
   G   
   A   
   C   
   A   
   A   
   A   
   G   
   A   
   A   
   A   
   C   
    G    
   A   
   A   
   G   
   A   
   A   
   A   
   A   
   A   
   A   
   G   
   A   
   C   
   A   
   A   
   T   
   G   
   T   
   A   
   A   
   T   
   C   
   A   
   G   
   C   
   G   
   C   
   C   
   A   
   A   
   C   
   G   
   C   
   A   
   G   
   C   
   G   
   A   
   A   
   T   
   A   
   C   
   A   
   A   
   T   
   T   
   G   
   C   
   C   
   A   
   C   
   A   
   G   
   A   
   T   
   C   
   T   
   T   
   T   
   T   
   A   
   G   
   G   
   T   
   A   
   G   
   T   
   G   
   T   
   G   
   G   
   A   
   A   
   G   
   A   
   T   
   C   
   C   
   T   
   T   
   C   
   A   
   G   
   G   
   T   
   T   
   A   
   T   
   G   
   T   
   T   
   T   
   A   
   T   
   A   
   A   
   T   
   A   
   T   
   G   
   T   
   A   
   C   
   A   
   A   
   T   
   C   
   C   
   G   
   A   
   A   
   C   
   T   
   T   
   C   
   T   
   C   
   A   
   T   
   T   
   G   
   G   
   C   
   T   
   G   
   C   
   T   
   A   
   A   
   A   
   A   
   C   
   A   
   G   
   G   
   A   
   A   
   C   
   A   
   G   
   C   
   T   
   G   
   A   
   A   
   A   
   T   
   A   
   A   
   A   
   A   
   G   
   A   
   T   
   A   
   A   
   A   
   C   
   A   
   A   
   G   
   A   
   T   
   A   
   C   
   A   
   G   
   A   
   T   
   G   
   G   
   C   
   A   
   A   
   A   
   G   
   A   
   A   
   A   
   A   
   C   
   A   
   G   
   T   
   T   
   T   
   C   
   C   
   T   
   C   
   C   
   T   
   A   
   A   
   C   
   G   
   T   
   T   
   A   
   G   
   A   
   T   
   C   
   G   
   T   
   A   
   G   
   T   
   A   
   A   
   C   
   A   
   A   
   T   
   A   
   A   
   A   
   T   
   T   
   C   
   T   
   T   
   A   
   A   
   C   
   A   
   A   
   T   
   G   
   A   
   T   
   T   
   A   
   T   
   G   
   G   
   T   
   T   
   G   
   A   
   A   
   A   
   A   
   T   
   T   
   C   
   A   
   G   
   G   
   A   
   G   
   A   
   A   
   A   
   A   
   T   
   G   
   G   
   T   
   T   
   C   
   T   
   G   
   C   
   A   
   A   
   C   
   A   
   G   
   A   
   T   
   A   
   T   
   C   
   A   
   G   
   T   
   A   
   A   
   A   
   C   
   C   
   A   
   T   
   T   
   A   
   A   
   T   
   T   
   G   
   A   
   T   
   T   
   A   
   C   
   T   
   T   
   A   
   G   
   A   
   A   
   G   
   C   
   A   
   A   
   C   
   C   
   A   
   T   
   T   
   A   
   A   
   A   
   -   
   -   
   -   
   -   
   -   
   -   
   -   
   -   
   -   
   -   
   -   
   -   
   -   
   -   
   -   
   -   
   -   
   -   
   -   
   -   
   -   
   -   
   -   
   -   
   -   
   -   
   -   
   -   
   -   
   -   
   -   
   -   
   -   
   -   
   -   
   -   
   -   
   -   
   -   
   -   
   -   
   -   
   -   
   -   
   -   
   -   
   -   
   -   
   -   
   -   
   -   
   -   
   -   
   -   
   -   
   -   
   -   
   -   
   -   
   -   
   -   
   -   
   -   
   -   
   -   
   -   
   -   
   -   
   -   
   -   
   -   
   -   
   -   
   -   
   -   
   -   
   -   
   -   
   -   
   -   
   -   
   -   
   -   
   -   
   -   
   -   
   -   
   -   
   -   
   -   
   -   
   -   
   -   
   -   
   -   
   -   
   -   
   -   
   -   
   -   
   -   
   -   
   -   
   -   
   -   
   -   
   -   
   -   
   -   
   -   
   -   
   -   
   -   
   -   
   -   
   -   
   -   
   -   
   -   
   -   
   -   
   -   
   -   
   -   
   -   
   -   
   -   
   -   
   -   
   -   
   -   
   -   
   -   
   -   
   -   
   -   
   -   
   -   
   -   
   -   
   -   
   -   
   -   
   -   
   -   
   -   
   -   
   -   
   -   
   -   
   -   
   -   
   -   
   -   
   -   
   -   
   -   
   -   
   -   
   -   
   -   
   -   
   -   
   -   
   -   
   -   
   -   
   -   
   -   
   C   
   A   
   A   
   T   
   G   
   G   
   C   
   A   
   A   
   G   
   C   
   C   
   A   
   A   
   A   
   C   
   A   
   A   
   G   
   A   
   A   
   T   
   T   
   A   
   G   
   C   
   C   
   G   
   A   
   A   
   G   
   C   
   G   
   A   
   A   
   G   
   A   
   A   
   A   
   A   
   C   
   A   
   G   
   C   
   T   
   A   
   A   
   T   
   A   
   C   
   A   
   T   
   T   
   T   
   T   
   T   
   A   
   A   
   A   
   T   
   G   
   T   
   A   
   T   
   T   
   G   
   T   
   C   
   A   
   A   
   A   
   A   
   C   
   A   
   G   
   G   
   A   
   A   
   T   
   T   
   T   
   G   
   A   
   T   
   A   
   A   
   G   
   T   
   T   
   A   
   C   
   C   
   G   
   T   
   C   
   C   
   G   
   T   
   T   
   G   
   T   
   T   
   C   
   A   
   A   
   G   
   A   
   A   
   T   
   C   
   T   
   A   
   G   
   C   
   T   
   T   
   A   
   A   
   A   
   G   
   A   
   A   
   A   
   A   
   A   
   T   
   G   
   G   
   C   
   T   
   A   
   T   
   G   
   A   
   T   
   A   
   C   
   T   
   A   
   A   
   A   
   T   
   C   
   T   
   G   
   T   
   T   
   G   
   T   
   T   
   G   
   A   
   A   
   A   
   A   
   A   
   T   
   A   
   C   
   C   
   A   
   A   
   G   
   C   
   A   
   A   
   T   
   T   
   T   
   A   
   T   
   T   
   C   
   A   
   G   
   G   
   G   
   A   
   T   
   T   
   C   
   A   
   A   
   G   
   C   
   A   
   G   
   A   
   A   
   G   
   G   
   A   
   G   
   T   
   C   
   A   
   A   
   A   
   G   
   C   
   T   
   A   
   G   
   T   
   G   
   A   
   T   
   G   
   T   
   C   
   C   
   A   
   A   
   G   
   T   
   C   
   A   
   A   
   A   
   A   
   A   
   G   
   G   
   C   
   G   
   A   
   A   
   A   
   G   
   A   
   C   
   A   
   A   
   T   
   C   
   A   
   A   
   T   
   A   
   C   
   A   
   C   
   A   
   T   
   T   
   T   
   A   
   C   
   C   
   T   
   A   
   T   
   A   
   A   
   A   
   T   
   T   
   A   
   T   
   C   
   G   
   A   
   T   
   G   
   A   
   G   
   C   
   A   
   C   
   T   
   C   
   C   
   T   
   T   
   T   
   A   
   G   
   G   
   C   
   G   
   A   
   A   
   A   
   T   
   G   
   A   
   A   
   A   
   G   
   A   
   T   
   C   
   T   
   G   
   T   
   C   
   T   
   T   
   A   
   T   
   C   
   A   
   A   
   T   
   C   
   A   
   A   
   G   
   T   
   A   
   T   
   C   
   G   
   C   
   C   
   A   
   A   
   A   
   A   
   A   
   A   
   G   
   G   
   C   
   G   
   A   
   T   
   A   
   C   
   C   
   T   
   A   
   C   
   C   
   A   
   A   
   A   
   T   
   C   
   T   
   C   
   T   
   T   
   G   
   G   
   A   
   A   
   G   
   C   
   C   
   G   
   T   
   C   
   T   
   T   
   T   
   A   
   A   
   T   
   T   
   T   
   T   
   T   
   C   
   C   
   A   
   G   
   A   
   T   
   A   
   T   
   G   
   T   
   C   
   A   
   G   
   G   
   A   
   A   
   A   
   T   
   G   
   A   
   T   
   A   
   A   
   A   
   A   
   T   
   T   
   T   
   C   
   G   
   A   
   T   
   T   
   C   
   A   
   A   
   G   
   T   
   A   
   G   
   A   
   T   
   A   
   A   
   T   
   G   
   C   
   C   
   A   
   A   
   A   
   C   
   G   
   T   
   G   
   G   
   A   
   G   
   A   
   A   
   A   
   T   
   T   
   G   
   T   
   C   
   G   
   A   
   T   
   C   
   G   
   T   
   A   
   A   
   T   
   G   
   G   
   T   
   A   
   G   
   T   
   G   
   G   
   G   
   C   
   T   
   A   
   G   
   C   
   A   
   A   
   T   
   T   
   A   
   A   
   C   
   A   
   A   
   A   
   G   
   T   
   G   
   T   
   T   
   T   
   G   
   A   
   C   
   G   
   A   
   A   
   G   
   T   
   G   
   G   
   G   
   C   
   G   
   T   
   A   
   G   
   T   
   G   
   C   
   C   
   T   
   G   
   G   
   C   
   A   
   A   
   A   
   C   
   T   
   C   
   G   
   G   
   T   
   T   
   C   
   T   
   G   
   G   
   C   
   G   
   C   
   A   
   G   
   A   
   A   
   A   
   A   
   A   
   A   
   C   
   A   
   G   
   C   
   C   
   A   
   A   
   T   
   A   
   T   
   C   
   A   
   A   
   A   
   G   
   C   
   T   
   T   
   T   
   T   
   A   
   G   
   T   
   G   
   A   
   T   
   A   
   A   
   G   
   T   
   T   
   C   
   G   
   G   
   C   
   G   
   T   
   T   
   T   
   C   
   T   
   G   
   T   
   T   
   G   
   A   
   T   
   G   
   A   
   A   
   A   
   T   
   C   
   A   
   A   
   T   
   C   
   A   
   A   
   A   
   A   
   G   
   T   
   T   
   A   
   A   
   G   
   C   
   C   
   A   
   A   
   G   
   G   
   G   
   T   
   G   
   G   
   G   
   T   
   C   
   C   
   A   
   A   
   G   
   C   
   A   
   G   
   A   
   C   
   T   
   C   
   C   
   T   
   T   
   T   
   G   
   T   
   A   
   C   
   C   
   A   
   A   
   T   
   C   
   A   
   C   
   A   
   G   
   T   
   T   
   G   
   C   
   T   
   T   
   C   
   T   
   G   
   A   
   A   
   C   
   C   
   A   
   G   
   T   
   G   
   A   
   C   
   A   
   G   
   A   
   A   
   T   
   T   
   A   
   C   
   C   
   A   
   A   
   C   
   A   
   G   
   G   
   G   
   G   
   C   
   T   
   G   
   C   
   G   
   A   
   C   
   A   
   A   
   A   
   A   
   G   
   A   
   T   
   A   
   C   
   A   
   G   
   A   
   G   
   T   
   C   
   A   
   C   
   G   
   T   
   T   
   A   
   T   
   T   
   A   
   T   
   C   
   C   
   A   
   C   
   T   
   G   
   G   
   G   
   G   
   G   
   A   
   A   
   G   
   C   
   A   
   G   
   C   
   T   
   G   
   C   
   G   
   C   
   A   
   A   
   T   
   T   
   A   
   A   
   T   
   C   
   G   
   G   
   G   
   T   
   A   
   T   
   A   
   C   
   G   
   G   
   G   
   C   
   A   
   C   
   C   
   A   
   T   
   T   
   A   
   C   
   T   
   G   
   C   
   A   
   G   
   A   
   A   
   G   
   A   
   T   
   A   
   T   
   T   
   G   
   A   
   A   
   A   
   A   
   A   
   A   
   A   
   T   
   C   
   C   
   A   
   G   
   A   
   G   
   T   
   T   
   A   
   A   
   G   
   T   
   A   
   G   
   T   
   A   
   C   
   G   
   G   
   G   
   T   
   G   
   T   
   A   
   A   
   T   
   T   
   G   
   G   
   T   
   A   
   A   
   A   
   A   
   C   
   T   
   G   
   G   
   C   
   T   
   T   
   A   
   G   
   A   
   A   
   C   
   G   
   A   
   G   
   C   
   G   
   T   
   T   
   T   
   G   
   A   
   T   
   A   
   A   
   A   
   G   
   A   
   G   
   T   
   T   
   A   
   A   
   G   
   A   
   G   
   G   
   G   
   C   
   A   
   A   
   G   
   A   
   T   
   G   
   G   
   T   
   G   
   G   
   C   
   T   
   C   
   A   
   C   
   T   
   A   
   G   
   T   
   T   
   A   
   T   
   T   
   T   
   T   
   A   
   G   
   A   
   T   
   G   
   A   
   C   
   A   
   A   
   A   
   G   
   A   
   G   
   A   
   A   
   T   
   G   
   T   
   C   
   A   
   A   
   A   
   A   
   A   
   A   
   G   
   C   
   T   
   T   
   T   
   A   
   C   
   A   
   A   
   A   
   C   
   C   
   A   
   A   
   A   
   G   
   A   
   G   
   A   
   A   
   A   
   A   
   A   
   A   
   G   
   A   
   T   
   G   
   G   
   T   
   C   
   A   
   A   
   A   
   C   
   G   
   A   
   T   
   T   
   A   
   A   
   A   
   T   
   T   
   A   
   A   
   C   
   G   
   A   
   T   
   T   
   G   
   A   
   C   
   A   
   G   
   T   
   G   
   G   
   C   
   G   
   T   
   A   
   C   
   A   
   A   
   C   
   A   
   A   
   C   
   A   
   A   
   G   
   C   
   C   
   T   
   T   
   C   
   G   
   C   
   T   
   A   
   T   
   A   
   T   
   T   
   T   
   G   
   A   
   C   
   A   
   A   
   A   
   C   
   G   
   T   
   C   
   C   
   T   
   G   
   G   
   T   
   A   
   G   
   T   
   G   
   C   
   G   
   G   
   T   
   C   
   A   
   T   
   T   
   A   
   C   
   C   
   G   
   A   
   T   
   C   
   C   
   T   
   C   
   A   
   A   
   A   
   A   
   A   
   G   
   G   
   C   
   G   
   A   
   T   
   T   
   T   
   A   
   T   
   T   
   A   
   G   
   C   
   A   
   A   
   C   
   G   
   G   
   T   
   T   
   A   
   G   
   T   
   T   
   C   
   A   
   C   
   C   
   T   
   T   
   C   
   G   
   T   
   A   
   T   
   G   
   A   
   T   
   C   
   C   
   T   
   A   
   A   
   T   
   A   
   A   
   A   
   A   
   T   
   G   
   G   
   C   
   C   
   A   
   A   
   T   
   G   
   G   
   T   
   A   
   T   
   T   
   T   
   C   
   T   
   C   
   A   
   A   
   A   
   A   
   A   
   G   
   A   
   A   
   T   
   A   
   T   
   G   
   A   
   T   
   G   
   C   
   T   
   T   
   A   
   C   
   A   
   A   
   T   
   A   
   A   
   C   
   A   
   A   
   C   
   A   
   A   
   A   
   G   
   A   
   T   
   T   
   T   
   A   
   C   
   C   
   A   
   T   
   T   
   C   
   A   
   C   
   A   
   G   
   C   
   A   
   C   
   G   
   T   
   T   
   T   
   T   
   G   
   C   
   G   
   A   
   C   
   A   
   G   
   G   
   C   
   T   
   A   
   T   
   G   
   C   
   A   
   C   
   C   
   A   
   G   
   G   
   C   
   T   
   C   
   T   
   A   
   C   
   C   
   T   
   T   
   T   
   A   
   A   
   A   
   A   
   C   
   A   
   A   
   T   
   T   
   A   
   C   
   C   
   G   
   G   
   T   
   G   
   C   
   C   
   A   
   T   
   T   
   G   
   G   
   T   
   T   
   T   
   A   
   G   
   A   
   T   
   G   
   C   
   A   
   G   
   G   
   G   
   A   
   C   
   A   
   T   
   T   
   G   
   A   
   A   
   G   
   C   
   C   
   A   
   G   
   A   
   T   
   G   
   A   
   A   
   G   
   A   
   G   
   C   
   T   
   A   
   G   
   A   
   A   
   A   
   T   
   T   
   A   
   A   
   T   
   G   
   G   
   C   
   T   
   T   
   G   
   A   
   A   
   A   
   T   
   G   
   G   
   C   
   A   
   A   
   A   
   A   
   A   
   G   
   A   
   T   
   A   
   A   
   A   
   T   
   C   
   T   
   T   
   G   
   G   
   G   
   G   
   C   
   G   
   G   
   C   
   T   
   A   
   T   
   T   
   T   
   T   
   G   
   C   
   A   
   A   
   C   
   A   
   C   
   G   
   T   
   G   
   T   
   A   
   A   
   A   
   A   
   G   
   A   
   A   
   G   
   C   
   A   
   A   
   G   
   T   
   C   
   C   
   A   
   G   
   T   
   C   
   A   
   A   
   T   
   C   
   T   
   G   
   C   
   G   
   T   
   A   
   C   
   C   
   G   
   C   
   T   
   T   
   T   
   A   
   G   
   T   
   T   
   A   
   A   
   T   
   T   
   C   
   A   
   G   
   A   
   T   
   A   
   A   
   T   
   A   
   T   
   T   
   T   
   A   
   T   
   T   
   T   
   T   
   G   
   C   
   C   
   C   
   A   
   A   
   C   
   A   
   A   
   A   
   C   
   A   
   T   
   T   
   A   
   C   
   G   
   G   
   A   
   T   
   G   
   G   
   G   
   G   
   G   
   A   
   A   
   G   
   A   
   C   
   A   
   A   
   A   
   T   
   T   
   T   
   A   
   G   
   A   
   G   
   C   
   A   
   G   
   G   
   T   
   C   
   T   
   T   
   A   
   A   
   T   
   A   
   A   
   A   
   T   
   T   
   C   
   A   
   T   
   T   
   T   
   T   
   C   
   G   
   G   
   T   
   G   
   A   
   G   
   G   
   A   
   A   
   C   
   T   
   A   
   G   
   A   
   T   
   T   
   T   
   A   
   C   
   C   
   A   
   A   
   T   
   T   
   G   
   C   
   C   
   A   
   T   
   G   
   A   
   C   
   G   
   C   
   C   
   T   
   G   
   C   
   T   
   C   
   A   
   A   
   A   
   T   
   T   
   T   
   C   
   A   
   A   
   A   
   T   
   G   
   A   
   A   
   G   
   A   
   T   
   A   
   A   
   A   
   T   
   T   
   T   
   A   
   A   
   T   
   T   
   C   
   T   
   G   
   A   
   A   
   A   
   T   
   T   
   C   
   T   
   T   
   T   
   T   
   A   
   G   
   C   
   A   
   G   
   A   
   T   
   A   
   C   
   G   
   G   
   G   
   C   
   T   
   A   
   C   
   G   
   G   
   C   
   C   
   A   
   A   
   G   
   G   
   C   
   C   
   A   
   A   
   T   
   T   
   G   
   T   
   T   
   A   
   A   
   T   
   T   
   T   
   C   
   G   
   C   
   C   
   A   
   A   
   T   
   C   
   C   
   A   
   G   
   C   
   A   
   A   
   G   
   C   
   T   
   A   
   C   
   C   
   A   
   T   
   G   
   T   
   A   
   T   
   A   
   G   
   T   
   G   
   T   
   T   
   T   
   T   
   C   
   C   
   A   
   A   
   A   
   A   
   T   
   A   
   A   
   T   
   G   
   G   
   A   
   A   
   C   
   A   
   T   
   T   
   A   
   G   
   T   
   C   
   T   
   A   
   T   
   C   
   C   
   A   
   A   
   A   
   A   
   T   
   T   
   A   
   G   
   T   
   T   
   T   
   T   
   A   
   G   
   A   
   C   
   A   
   A   
   A   
   G   
   A   
   A   
   A   
   C   
   G   
   A   
   A   
   G   
   A   
   A   
   A   
   A   
   A   
   A   
   G   
   A   
   C   
   A   
   A   
   T   
   G   
   T   
   A   
   A   
   T   
   C   
   A   
   G   
   C   
   G   
   C   
   C   
   A   
   A   
   C   
   G   
   C   
   A   
   G   
   C   
   G   
   A   
   A   
   T   
   A   
   C   
   A   
   A   
   T   
   T   
   G   
   C   
   C   
   A   
   C   
   A   
   G   
   A   
   T   
   C   
   T   
   T   
   T   
   T   
   A   
   G   
   G   
   T   
   A   
   G   
   T   
   G   
   T   
   G   
   G   
   A   
   A   
   G   
   A   
   T   
   C   
   C   
   T   
   T   
   C   
   A   
   G   
   G   
   T   
   T   
   A   
   T   
   G   
   T   
   T   
   T   
   A   
   T   
   A   
   A   
   T   
   A   
   T   
   G   
   T   
   A   
   C   
   A   
   A   
   T   
   C   
   C   
   G   
   A   
   A   
   C   
   T   
   T   
   C   
   T   
   C   
   A   
   T   
   T   
   G   
   G   
   C   
   T   
   G   
   C   
   T   
   A   
   A   
   A   
   A   
   C   
   A   
   G   
   G   
   A   
   A   
   C   
   A   
   G   
   C   
   T   
   G   
   A   
   A   
   A   
   T   
   A   
   A   
   A   
   A   
   G   
   A   
   T   
   A   
   A   
   A   
   C   
   A   
   A   
   G   
   A   
   T   
   A   
   C   
   A   
   G   
   A   
   T   
   G   
   G   
   C   
   A   
   A   
   A   
   G   
   A   
   A   
   A   
   A   
   C   
   A   
   G   
   T   
   T   
   T   
   C   
   C   
   T   
   C   
   C   
   T   
   A   
   A   
   C   
   G   
   T   
   T   
   A   
   G   
   A   
   T   
   C   
   G   
   T   
   A   
   G   
   T   
   A   
   A   
   C   
   A   
   A   
   T   
   A   
   A   
   A   
   T   
   T   
   C   
   T   
   T   
   A   
   A   
   C   
   A   
   A   
   T   
   G   
   A   
   T   
   T   
   A   
   T   
   G   
   G   
   T   
   T   
   G   
   A   
   A   
   A   
   A   
   T   
   T   
   C   
   A   
   G   
   G   
   A   
   G   
   A   
   A   
   A   
   A   
   T   
   G   
   G   
   T   
   T   
   C   
   T   
   G   
   C   
   A   
   A   
   C   
   A   
   G   
   A   
   T   
   A   
   T   
   C   
   A   
   G   
   T   
   A   
   A   
   A   
   C   
   C   
   A   
   T   
   T   
   A   
   A   
   T   
   T   
   G   
   A   
   T   
   T   
   A   
   C   
   T   
   T   
   A   
   G   
   A   
   A   
   G   
   C   
   A   
   A   
   C   
   C   
   A   
   T   
   T   
   A   
   A   
   A   
   -   
   -   
   -   
   -   
   -   
   -   
   -   
   -   
   -   
   -   
   -   
   -   
   -   
   -   
   -   
   -   
   -   
   -   
   -   
   -   
   -   
   -   
   -   
   -   
   -   
   -   
   -   
   -   
   -   
   -   
   -   
   -   
   -   
   -   
   -   
   -   
   -   
   -   
   -   
   -   
   -   
   -   
   -   
   -   
   -   
   -   
   -   
   -   
   -   
   -   
   -   
   -   
   -   
   -   
   -   
   -   
   -   
   -   
   -   
   -   
   -   
 

 
   N60100F   
   -   
   -   
   -   
   -   
   -   
   -   
   -   
   -   
   -   
   -   
   -   
   -   
   -   
   -   
   -   
   -   
   -   
   -   
   -   
   -   
   -   
   -   
   -   
   -   
   -   
   -   
   -   
   -   
   -   
   -   
   -   
   -   
   -   
   -   
   -   
   -   
   -   
   -   
   -   
   -   
   -   
   -   
   -   
   -   
   -   
   -   
   -   
   -   
   -   
   -   
   -   
   -   
   -   
   -   
   -   
   -   
   -   
   -   
   -   
   -   
   -   
   -   
   -   
   -   
   -   
   -   
   -   
   -   
   -   
   -   
   -   
   -   
   -   
   -   
   -   
   -   
   -   
   -   
   -   
   -   
   -   
   -   
   -   
   -   
   -   
   -   
   -   
   -   
   -   
   -   
   -   
   -   
   -   
   -   
   -   
   -   
   -   
   -   
   -   
   -   
   -   
   -   
   -   
   -   
   -   
   -   
   -   
   -   
   C   
   A   
   A   
   T   
   G   
   G   
   C   
   A   
   A   
   G   
   C   
   C   
   A   
   A   
   A   
   C   
   A   
   A   
   G   
   A   
    A    
   T   
   T   
   A   
   G   
   C   
    C    
   G   
   A   
   A   
   G   
   C   
   G   
   A   
   A   
   G   
   A   
   A   
   A   
   A   
    C    
   A   
   G   
   C   
    T    
   A   
    A    
   T   
    A    
    C    
   A   
   T   
   T   
   T   
   T   
   T   
   A   
   A   
   A   
    T    
   G   
   T   
   A   
   T   
   T   
   G   
    T    
   C   
   A   
   A   
   A   
   A   
   C   
   A   
   G   
    G    
   A   
   A   
   T   
   T   
   T   
   G   
   A   
   T   
   A   
   A   
   G   
   T   
   T   
   A   
   C   
   C   
    G    
   T   
   C   
   C   
   G   
   T   
   T   
    G    
   T   
   T   
   C   
   A   
   A   
   G   
   A   
   A   
    G    
   C   
   T   
   A   
   G   
   C   
   T   
   T   
   A   
   A   
   A   
   G   
   A   
   A   
   A   
   A   
   A   
   T   
   G   
   G   
    C    
   T   
   A   
   T   
   G   
   A   
   T   
   A   
   C   
   T   
   A   
   A   
   A   
   T   
   C   
    T    
   G   
   T   
   T   
   G   
   T   
   T   
   G   
   A   
   A   
   A   
   A   
   A   
   T   
   A   
   C   
   C   
   A   
   A   
   G   
   C   
   A   
   A   
   T   
   T   
   T   
   A   
   T   
   T   
   C   
   A   
   G   
   G   
   G   
   A   
   T   
   T   
   C   
   A   
   A   
   G   
   C   
    A    
   G   
   A   
   A   
    G    
   G   
    A    
   G   
   T   
   C   
   A   
   A   
    A    
   G   
   C   
   T   
   A   
   G   
   T   
   G   
   A   
   T   
   G   
   T   
    C    
   C   
   A   
   A   
   G   
   T   
    C    
   A   
   A   
   A   
   A   
   A   
    G    
   G   
    C    
   G   
   A   
   A   
   A   
   G   
   A   
    C    
   A   
   A   
    T    
   C   
   A   
   A   
   T   
   A   
   C   
   A   
   C   
   A   
   T   
   T   
   T   
   A   
    C    
   C   
   T   
   A   
   T   
   A   
   A   
   A   
   T   
   T   
   A   
   T   
   C   
   G   
   A   
   T   
   G   
   A   
   G   
    C    
   A   
   C   
    T    
   C   
   C   
   T   
   T   
   T   
   A   
   G   
   G   
   C   
   G   
   A   
   A   
   A   
   T   
   G   
   A   
   A   
   A   
   G   
   A   
   T   
    C    
   T   
   G   
   T   
   C   
   T   
   T   
   A   
   T   
   C   
   A   
   A   
   T   
   C   
   A   
   A   
   G   
   T   
   A   
   T   
    C    
   G   
    C    
   C   
   A   
   A   
    A    
   A   
   A   
   A   
   G   
   G   
    C    
   G   
   A   
   T   
   A   
    C    
   C   
   T   
   A   
   C   
   C   
   A   
   A   
   A   
   T   
    C    
    G    
   C   
   T   
   T   
   G   
   G   
    A    
    A    
    G    
   C   
   C   
    G    
   T   
   C   
   T   
   T   
   T   
   A   
   A   
   T   
   T   
   T   
   T   
   T   
   C   
   C   
   A   
   G   
   A   
   T   
   A   
   T   
   G   
   T   
   C   
   A   
   G   
   G   
   A   
   A   
   A   
   T   
   G   
   A   
   T   
   A   
   A   
   A   
    A    
   T   
   T   
   T   
   C   
   G   
   A   
   T   
   T   
   C   
   A   
   A   
   G   
   T   
   A   
   G   
   A   
   T   
   A   
   A   
   T   
   G   
   C   
   C   
   A   
   A   
   A   
   C   
   G   
   T   
   G   
   G   
   A   
   G   
   A   
   A   
   A   
   T   
   T   
   G   
   T   
   C   
   G   
   A   
   T   
   C   
   G   
   T   
   A   
   A   
    T    
   G   
   G   
   T   
   A   
   G   
   T   
   G   
   G   
   G   
   C   
   T   
   A   
   G   
    C    
   A   
   A   
   T   
   T   
   A   
   A   
   C   
   A   
   A   
   A   
   G   
   T   
   G   
   T   
   T   
   T   
   G   
   A   
    C    
   G   
   A   
   A   
   G   
   T   
   G   
   G   
   G   
   C   
   G   
   T   
   A   
   G   
   T   
    G    
   C   
   C   
   T   
    G    
   G   
   C   
   A   
   A   
   A   
   C   
   T   
    C    
   G   
   G   
   T   
   T   
   C   
   T   
   G   
   G   
   C   
   G   
   C   
   A   
   G   
   A   
   A   
   A   
   A   
   A   
    A    
   C   
   A   
   G   
   C   
    C    
   A   
   A   
   T   
   A   
   T   
   C   
   A   
   A   
   A   
   G   
   C   
   T   
   T   
   T   
   T   
   A   
   G   
   T   
   G   
   A   
   T   
   A   
   A   
    G    
   T   
   T   
    C    
   G   
   G   
   C   
   G   
   T   
   T   
   T   
   C   
   T   
    G    
   T   
   T   
   G   
   A   
   T   
   G   
   A   
   A   
   A   
   T   
   C   
   A   
   A   
   T   
   C   
    A    
   A   
   A   
   A   
    G    
   T   
   T   
   A   
   A   
   G   
   C   
   C   
   A   
   A   
   G   
   G   
    G    
   T   
   G   
   G   
   G   
   T   
   C   
   C   
   A   
   A   
   G   
   C   
   A   
   G   
   A   
   C   
   T   
   C   
    C    
   T   
   T   
   T   
   G   
   T   
   A   
   C   
   C   
   A   
   A   
   T   
   C   
   A   
   C   
   A   
   G   
   T   
    T    
   G   
   C   
   T   
   T   
   C   
   T   
   G   
   A   
   A   
   C   
   C   
   A   
   G   
   T   
   G   
   A   
   C   
   A   
   G   
   A   
   A   
   T   
   T   
   A   
   C   
   C   
   A   
   A   
   C   
   A   
   G   
   G   
   G   
   G   
   C   
   T   
   G   
   C   
   G   
   A   
   C   
   A   
   A   
   A   
   A   
   G   
    A    
    T    
   A   
   C   
   A   
   G   
   A   
   G   
   T   
   C   
   A   
   C   
   G   
   T   
   T   
   A   
   T   
   T   
   A   
   T   
   C   
   C   
    A    
    C    
   T   
   G   
   G   
   G   
   G   
   G   
   A   
   A   
   G   
   C   
   A   
   G   
   C   
   T   
   G   
   C   
   G   
   C   
   A   
   A   
   T   
   T   
   A   
   A   
   T   
   C   
   G   
   G   
    G    
   T   
   A   
   T   
   A   
   C   
    G    
   G   
   G   
    C    
   A   
   C   
    C    
   A   
   T   
   T   
   A   
   C   
   T   
   G   
   C   
   A   
   G   
   A   
    A    
   G   
   A   
   T   
   A   
   T   
   T   
    G    
   A   
   A   
   A   
   A   
   A   
   A   
   A   
   T   
   C   
   C   
   A   
   G   
   A   
   G   
   T   
   T   
   A   
   A   
   G   
   T   
   A   
   G   
   T   
   A   
   C   
   G   
   G   
   G   
    C    
   G   
   T   
   A   
   A   
   T   
   T   
   G   
   G   
    T    
   A   
   A   
   A   
   A   
   C   
   T   
   G   
   G   
    C    
   T   
   T   
   A   
   G   
   A   
   A   
   C   
   G   
   A   
   G   
   C   
   G   
   T   
   T   
   T   
   G   
   A   
   T   
   A   
   A   
   A   
   G   
   A   
    A    
   T   
   T   
   A   
   A   
   G   
   A   
   G   
   G   
    G    
   C   
   A   
   A   
   G   
   A   
   T   
   G   
   G   
    T    
   G   
   G   
    C    
   T   
   C   
    A    
   C   
   T   
   A   
   G   
   T   
   T   
   A   
   T   
   T   
   T   
   T   
   A   
   G   
   A   
   T   
   G   
   A   
   C   
   A   
   A   
   A   
   G   
   A   
   G   
   A   
   A   
   T   
   G   
   T   
   C   
   A   
   A   
   A   
   A   
   A   
   A   
   G   
   C   
   T   
   T   
   T   
   A   
   C   
   A   
   A   
   A   
   C   
   C   
   A   
   A   
   A   
   G   
   A   
    G    
   A   
   A   
   A   
   A   
   A   
   A   
   G   
   A   
   T   
   G   
   G   
   T   
   C   
   A   
   A   
   A   
   C   
    G    
   A   
   T   
   T   
   A   
   A   
   A   
   T   
   T   
   A   
   A   
   C   
   G   
   A   
   T   
   T   
   G   
   A   
    C    
   A   
   G   
    C    
   G   
   G   
    G    
   G   
   T   
   A   
   C   
   A   
   A   
   C   
   A   
   A   
   C   
   A   
   A   
   G   
   C   
    T    
   T   
   T   
    T    
   G   
   C   
   T   
   A   
   T   
   A   
   T   
   T   
   T   
   G   
   A   
   C   
   A   
   A   
   A   
   C   
   G   
    T    
    C    
   C   
   T   
   G   
   G   
   T   
   A   
   G   
   T   
   G   
   C   
   G   
   G   
   T   
   C   
   A   
   T   
   T   
   A   
   C   
   C   
   G   
   A   
   T   
   C   
   C   
   T   
   C   
   A   
   A   
   A   
   A   
   A   
   G   
   G   
   C   
   G   
   A   
   T   
   T   
   T   
   A   
    T    
   T   
   A   
   G   
   C   
    A    
   A   
   C   
   G   
   G   
   T   
   T   
   A   
   G   
   T   
   T   
   C   
   A   
   C   
   C   
   T   
   T   
   C   
   G   
   T   
   A   
   T   
   G   
   A   
   T   
   C   
   C   
   T   
   A   
   A   
   T   
   A   
   A   
   A   
   A   
   T   
   G   
   G   
   C   
   C   
   A   
   A   
   T   
   G   
   G   
    T    
   A   
   T   
   T   
   T   
   C   
   T   
   C   
   A   
   A   
   A   
   A   
   A   
   G   
   A   
    C    
   T   
   A   
   T   
   G   
   A   
   T   
   G   
   C   
   T   
   T   
   A   
   C   
   A   
   A   
   T   
   A   
   A   
   C   
   A   
   A   
   C   
   A   
   A   
   A   
   G   
   A   
   T   
   T   
   T   
   A   
   C   
   C   
   A   
   T   
   T   
    C    
   A   
   C   
   A   
   G   
   C   
   A   
   C   
   G   
   T   
   T   
   T   
    T    
   G   
   C   
   G   
   A   
   C   
   A   
   G   
   G   
    C    
   T   
   A   
   T   
   G   
   C   
   A   
   C   
   C   
   A   
   G   
   G   
    C    
   T   
   C   
   T   
   A   
   C   
   C   
   T   
   T   
   T   
   A   
   A   
   A   
   A   
   C   
   A   
   A   
   T   
   T   
   A   
   C   
   C   
   G   
   G   
   T   
   G   
   C   
   C   
   A   
   T   
   T   
   G   
   G   
   T   
   T   
   T   
   A   
   G   
   A   
   T   
    G    
   C   
   A   
   G   
   G   
   G   
   A   
   C   
   A   
   T   
   T   
   G   
   A   
   A   
   G   
   C   
   C   
   A   
   G   
   A   
   T   
   G   
   A   
   A   
   G   
   A   
   G   
   C   
   T   
   A   
   G   
   A   
   A   
   A   
   T   
   T   
   A   
   A   
   T   
   G   
   G   
    C    
   T   
   T   
   G   
   A   
   A   
   A   
   T   
   G   
   G   
   C   
   A   
   A   
   A   
   A   
   A   
   G   
   A   
   T   
   A   
   A   
   A   
   T   
   C   
   T   
   T   
   G   
   G   
   G   
   G   
    C    
   G   
   G   
   C   
   T   
   A   
   T   
   T   
   T   
   T   
   G   
   C   
    G    
   A   
   C   
   A   
   C   
   G   
   T   
   G   
   T   
   A   
   A   
   A   
   A   
   G   
   A   
   A   
   G   
   C   
   A   
   A   
   G   
   T   
   C   
   C   
   A   
   G   
   T   
   C   
   A   
   A   
   T   
   C   
   T   
    G    
   C   
   G   
   T   
   A   
   C   
   C   
   G   
   C   
   T   
   T   
   T   
   A   
   G   
   T   
   T   
   A   
   A   
   T   
   T   
   C   
   A   
   G   
   A   
   T   
   A   
   A   
   T   
   A   
   T   
   T   
   T   
   A   
   T   
   T   
   T   
   T   
   G   
   C   
    C    
   C   
   A   
   A   
   C   
   A   
   A   
   A   
   C   
   A   
   T   
   T   
   A   
   C   
   G   
    G    
   A   
   T   
   G   
   G   
   G   
    G    
   G   
   A   
   A   
   G   
   A   
   C   
   A   
   A   
   A   
   T   
   T   
   T   
   A   
   G   
   A   
   G   
   C   
    A    
   G   
   G   
   T   
   C   
   T   
   T   
   A   
   A   
   T   
   A   
   A   
   A   
   T   
   T   
   C   
   A   
   T   
   T   
   T   
   T   
   C   
   G   
   G   
   T   
   G   
   A   
   G   
   G   
   A   
   A   
   C   
   T   
   A   
   G   
   A   
   T   
   T   
   T   
   A   
   C   
   C   
   A   
   A   
   T   
    T    
   G   
   C   
   C   
   A   
   T   
   G   
   A   
   C   
   G   
   C   
   C   
    T    
   G   
   C   
   T   
   C   
   A   
   A   
   A   
   T   
   T   
   T   
   C   
   A   
   A   
   A   
    T    
   G   
   A   
   A   
   G   
   A   
   T   
   A   
   A   
   A   
   T   
   T   
   T   
   A   
   A   
   T   
   T   
   C   
   T   
   G   
   A   
   A   
   A   
   T   
   T   
   C   
   T   
   T   
   T   
   T   
    A    
   G   
   C   
   A   
   G   
   A   
   T   
   A   
   C   
    G    
   G   
   G   
   C   
   T   
   A   
    T    
   G   
   G   
    T    
   C   
   A   
   A   
   G   
   G   
    T    
   C   
   A   
   A   
   T   
   T   
   G   
   T   
   T   
   A   
   A   
   T   
   T   
   T   
   C   
    G    
   C   
   C   
   A   
   A   
   T   
   C   
   C   
   A   
   G   
   C   
   A   
   A   
   G   
   C   
   T   
   A   
   C   
   C   
   A   
   T   
   G   
   T   
   A   
   T   
   A   
   G   
   T   
   G   
   T   
   T   
   T   
   T   
   C   
   C   
   A   
   A   
   A   
   A   
   T   
   A   
   A   
   T   
   G   
   G   
    A    
   A   
   C   
   A   
    T    
   T   
    A    
   G   
   T   
    C    
   T   
   A   
   T   
   C   
   C   
   A   
   A   
   A   
   A   
    C    
   T   
   A   
   G   
   T   
   T   
   T   
   T   
   A   
   G   
   A   
   C   
   A   
   A   
   A   
   G   
   A   
   A   
   A   
   C   
    G    
   A   
   A   
   G   
   A   
   A   
   A   
   A   
   A   
   A   
   G   
   A   
   C   
   A   
   A   
   T   
   G   
   T   
   A   
   A   
   T   
   C   
   A   
   G   
   C   
   G   
   C   
   C   
   A   
   A   
   C   
   G   
   C   
   A   
   G   
   C   
   G   
   A   
   A   
   T   
   A   
   C   
   A   
   A   
   T   
   T   
   G   
   C   
   C   
   A   
   C   
   A   
   G   
   A   
   T   
   C   
   T   
   T   
   T   
   T   
   A   
   G   
   G   
   T   
   A   
   G   
   T   
   G   
   T   
   G   
   G   
   A   
   A   
   G   
   A   
   T   
   C   
   C   
   T   
   T   
   C   
   A   
   G   
   G   
   T   
   T   
   A   
   T   
   G   
   T   
   T   
   T   
   A   
   T   
   A   
   A   
   T   
   A   
   T   
   G   
   T   
   A   
   C   
   A   
   A   
   T   
   C   
   C   
   G   
   A   
   A   
   C   
   T   
   T   
   C   
   T   
   C   
   A   
   T   
   T   
   G   
   G   
   C   
   T   
   G   
   C   
   T   
   A   
   A   
   A   
   A   
   C   
   A   
   G   
   G   
   A   
   A   
   C   
   A   
   G   
   C   
   T   
   G   
   A   
   A   
   A   
   T   
   A   
   A   
   A   
   A   
   G   
   A   
   T   
   A   
   A   
   A   
   C   
   A   
   A   
   G   
   A   
   T   
   A   
   C   
   A   
   G   
   A   
   T   
   G   
   G   
   C   
   A   
   A   
   A   
   G   
   A   
   A   
   A   
   A   
   C   
   A   
   G   
   T   
   T   
   T   
   C   
   C   
   T   
   T   
   C   
   T   
   A   
   A   
   C   
   G   
   T   
   T   
   A   
   G   
   A   
   T   
   C   
   G   
   T   
   A   
   G   
   T   
   A   
   A   
   C   
   A   
   A   
   T   
   A   
   A   
   A   
   T   
   T   
   C   
   T   
   T   
   A   
   A   
   C   
   A   
   A   
   T   
   G   
   A   
   T   
   T   
   A   
   T   
   G   
   G   
   T   
   T   
   G   
   A   
   A   
   A   
   A   
   T   
   T   
   C   
   A   
   G   
   G   
   A   
   G   
   A   
   A   
   A   
   A   
   T   
   G   
   G   
   T   
   T   
   C   
   T   
   G   
   C   
   A   
   A   
   C   
   A   
   G   
   A   
   T   
   A   
   T   
   C   
   A   
   G   
   T   
   A   
   A   
   A   
   C   
   C   
   A   
   T   
   T   
   A   
   A   
   T   
   T   
   G   
   A   
   T   
   T   
   A   
   C   
   T   
   T   
   A   
   G   
   A   
   A   
   G   
   C   
   A   
   A   
   C   
   C   
   A   
   T   
   T   
   A   
   A   
   A   
   -   
   -   
   -   
   -   
   -   
   -   
   -   
   -   
   -   
   -   
   -   
   -   
   -   
   -   
   -   
   -   
   -   
   -   
   -   
   -   
   -   
   -   
   -   
   -   
   -   
   -   
   -   
   -   
   -   
   -   
   -   
   -   
   -   
   -   
   -   
   -   
   -   
   -   
   -   
   -   
   -   
   -   
   -   
   -   
   -   
   -   
   -   
   -   
   -   
   -   
   -   
   -   
   -   
   -   
   -   
   -   
   -   
   -   
   -   
   -   
   -   
   -   
   -   
   -   
   -   
   -   
   -   
   -   
   -   
   -   
   -   
   -   
   -   
   -   
   -   
   -   
   -   
   -   
   -   
   -   
   -   
   -   
   -   
   -   
   -   
   -   
   -   
   -   
   -   
   -   
   -   
   -   
   -   
   -   
   -   
   -   
   -   
   -   
   -   
   -   
   -   
   -   
   -   
   -   
   -   
   -   
   -   
   -   
   -   
   -   
   -   
   -   
   -   
   -   
   -   
   -   
   -   
   -   
   -   
   -   
   -   
   -   
   -   
   -   
   -   
   -   
   -   
   -   
   -   
   -   
   -   
   -   
   -   
   -   
   -   
   -   
   -   
   -   
   -   
   -   
   -   
   -   
   -   
   -   
   -   
   -   
   -   
   -   
   -   
   -   
   -   
   -   
   -   
   -   
   -   
   -   
   -   
   -   
   -   
   -   
   -   
   -   
   -   
   -   
   -   
   -   
   -   
   -   
   -   
   C   
   A   
   A   
   T   
   G   
   G   
   C   
   A   
   A   
   G   
   C   
   C   
   A   
   A   
   A   
   C   
   A   
   A   
   G   
   A   
   A   
   T   
   T   
   A   
   G   
   C   
   C   
   G   
   A   
   A   
   G   
   C   
   G   
   A   
   A   
   G   
   A   
   A   
   A   
   A   
   C   
   A   
   G   
   C   
   T   
   A   
   A   
   T   
   A   
   C   
   A   
   T   
   T   
   T   
   T   
   T   
   A   
   A   
   A   
   T   
   G   
   T   
   A   
   T   
   T   
   G   
   T   
   C   
   A   
   A   
   A   
   A   
   C   
   A   
   G   
   G   
   A   
   A   
   T   
   T   
   T   
   G   
   A   
   T   
   A   
   A   
   G   
   T   
   T   
   A   
   C   
   C   
   G   
   T   
   C   
   C   
   G   
   T   
   T   
   G   
   T   
   T   
   C   
   A   
   A   
   G   
   A   
   A   
   G   
   C   
   T   
   A   
   G   
   C   
   T   
   T   
   A   
   A   
   A   
   G   
   A   
   A   
   A   
   A   
   A   
   T   
   G   
   G   
   C   
   T   
   A   
   T   
   G   
   A   
   T   
   A   
   C   
   T   
   A   
   A   
   A   
   T   
   C   
   T   
   G   
   T   
   T   
   G   
   T   
   T   
   G   
   A   
   A   
   A   
   A   
   A   
   T   
   A   
   C   
   C   
   A   
   A   
   G   
   C   
   A   
   A   
   T   
   T   
   T   
   A   
   T   
   T   
   C   
   A   
   G   
   G   
   G   
   A   
   T   
   T   
   C   
   A   
   A   
   G   
   C   
   A   
   G   
   A   
   A   
   G   
   G   
   A   
   G   
   T   
   C   
   A   
   A   
   A   
   G   
   C   
   T   
   A   
   G   
   T   
   G   
   A   
   T   
   G   
   T   
   C   
   C   
   A   
   A   
   G   
   T   
   C   
   A   
   A   
   A   
   A   
   A   
   G   
   G   
   C   
   G   
   A   
   A   
   A   
   G   
   A   
   C   
   A   
   A   
   T   
   C   
   A   
   A   
   T   
   A   
   C   
   A   
   C   
   A   
   T   
   T   
   T   
   A   
   C   
   C   
   T   
   A   
   T   
   A   
   A   
   A   
   T   
   T   
   A   
   T   
   C   
   G   
   A   
   T   
   G   
   A   
   G   
   C   
   A   
   C   
   T   
   C   
   C   
   T   
   T   
   T   
   A   
   G   
   G   
   C   
   G   
   A   
   A   
   A   
   T   
   G   
   A   
   A   
   A   
   G   
   A   
   T   
   C   
   T   
   G   
   T   
   C   
   T   
   T   
   A   
   T   
   C   
   A   
   A   
   T   
   C   
   A   
   A   
   G   
   T   
   A   
   T   
   C   
   G   
   C   
   C   
   A   
   A   
   A   
   A   
   A   
   A   
   G   
   G   
   C   
   G   
   A   
   T   
   A   
   C   
   C   
   T   
   A   
   C   
   C   
   A   
   A   
   A   
   T   
   C   
   G   
   C   
   T   
   T   
   G   
   G   
   A   
   A   
   G   
   C   
   C   
   G   
   T   
   C   
   T   
   T   
   T   
   A   
   A   
   T   
   T   
   T   
   T   
   T   
   C   
   C   
   A   
   G   
   A   
   T   
   A   
   T   
   G   
   T   
   C   
   A   
   G   
   G   
   A   
   A   
   A   
   T   
   G   
   A   
   T   
   A   
   A   
   A   
   A   
   T   
   T   
   T   
   C   
   G   
   A   
   T   
   T   
   C   
   A   
   A   
   G   
   T   
   A   
   G   
   A   
   T   
   A   
   A   
   T   
   G   
   C   
   C   
   A   
   A   
   A   
   C   
   G   
   T   
   G   
   G   
   A   
   G   
   A   
   A   
   A   
   T   
   T   
   G   
   T   
   C   
   G   
   A   
   T   
   C   
   G   
   T   
   A   
   A   
   T   
   G   
   G   
   T   
   A   
   G   
   T   
   G   
   G   
   G   
   C   
   T   
   A   
   G   
   C   
   A   
   A   
   T   
   T   
   A   
   A   
   C   
   A   
   A   
   A   
   G   
   T   
   G   
   T   
   T   
   T   
   G   
   A   
   C   
   G   
   A   
   A   
   G   
   T   
   G   
   G   
   G   
   C   
   G   
   T   
   A   
   G   
   T   
   G   
   C   
   C   
   T   
   G   
   G   
   C   
   A   
   A   
   A   
   C   
   T   
   C   
   G   
   G   
   T   
   T   
   C   
   T   
   G   
   G   
   C   
   G   
   C   
   A   
   G   
   A   
   A   
   A   
   A   
   A   
   A   
   C   
   A   
   G   
   C   
   C   
   A   
   A   
   T   
   A   
   T   
   C   
   A   
   A   
   A   
   G   
   C   
   T   
   T   
   T   
   T   
   A   
   G   
   T   
   G   
   A   
   T   
   A   
   A   
   G   
   T   
   T   
   C   
   G   
   G   
   C   
   G   
   T   
   T   
   T   
   C   
   T   
   G   
   T   
   T   
   G   
   A   
   T   
   G   
   A   
   A   
   A   
   T   
   C   
   A   
   A   
   T   
   C   
   A   
   A   
   A   
   A   
   G   
   T   
   T   
   A   
   A   
   G   
   C   
   C   
   A   
   A   
   G   
   G   
   G   
   T   
   G   
   G   
   G   
   T   
   C   
   C   
   A   
   A   
   G   
   C   
   A   
   G   
   A   
   C   
   T   
   C   
   C   
   T   
   T   
   T   
   G   
   T   
   A   
   C   
   C   
   A   
   A   
   T   
   C   
   A   
   C   
   A   
   G   
   T   
   T   
   G   
   C   
   T   
   T   
   C   
   T   
   G   
   A   
   A   
   C   
   C   
   A   
   G   
   T   
   G   
   A   
   C   
   A   
   G   
   A   
   A   
   T   
   T   
   A   
   C   
   C   
   A   
   A   
   C   
   A   
   G   
   G   
   G   
   G   
   C   
   T   
   G   
   C   
   G   
   A   
   C   
   A   
   A   
   A   
   A   
   G   
   A   
   T   
   A   
   C   
   A   
   G   
   A   
   G   
   T   
   C   
   A   
   C   
   G   
   T   
   T   
   A   
   T   
   T   
   A   
   T   
   C   
   C   
   A   
   C   
   T   
   G   
   G   
   G   
   G   
   G   
   A   
   A   
   G   
   C   
   A   
   G   
   C   
   T   
   G   
   C   
   G   
   C   
   A   
   A   
   T   
   T   
   A   
   A   
   T   
   C   
   G   
   G   
   G   
   T   
   A   
   T   
   A   
   C   
   G   
   G   
   G   
   C   
   A   
   C   
   C   
   A   
   T   
   T   
   A   
   C   
   T   
   G   
   C   
   A   
   G   
   A   
   A   
   G   
   A   
   T   
   A   
   T   
   T   
   G   
   A   
   A   
   A   
   A   
   A   
   A   
   A   
   T   
   C   
   C   
   A   
   G   
   A   
   G   
   T   
   T   
   A   
   A   
   G   
   T   
   A   
   G   
   T   
   A   
   C   
   G   
   G   
   G   
   C   
   G   
   T   
   A   
   A   
   T   
   T   
   G   
   G   
   T   
   A   
   A   
   A   
   A   
   C   
   T   
   G   
   G   
   C   
   T   
   T   
   A   
   G   
   A   
   A   
   C   
   G   
   A   
   G   
   C   
   G   
   T   
   T   
   T   
   G   
   A   
   T   
   A   
   A   
   A   
   G   
   A   
   A   
   T   
   T   
   A   
   A   
   G   
   A   
   G   
   G   
   G   
   C   
   A   
   A   
   G   
   A   
   T   
   G   
   G   
   T   
   G   
   G   
   C   
   T   
   C   
   A   
   C   
   T   
   A   
   G   
   T   
   T   
   A   
   T   
   T   
   T   
   T   
   A   
   G   
   A   
   T   
   G   
   A   
   C   
   A   
   A   
   A   
   G   
   A   
   G   
   A   
   A   
   T   
   G   
   T   
   C   
   A   
   A   
   A   
   A   
   A   
   A   
   G   
   C   
   T   
   T   
   T   
   A   
   C   
   A   
   A   
   A   
   C   
   C   
   A   
   A   
   A   
   G   
   A   
   G   
   A   
   A   
   A   
   A   
   A   
   A   
   G   
   A   
   T   
   G   
   G   
   T   
   C   
   A   
   A   
   A   
   C   
   G   
   A   
   T   
   T   
   A   
   A   
   A   
   T   
   T   
   A   
   A   
   C   
   G   
   A   
   T   
   T   
   G   
   A   
   C   
   A   
   G   
   C   
   G   
   G   
   G   
   G   
   T   
   A   
   C   
   A   
   A   
   C   
   A   
   A   
   C   
   A   
   A   
   G   
   C   
   T   
   T   
   T   
   T   
   G   
   C   
   T   
   A   
   T   
   A   
   T   
   T   
   T   
   G   
   A   
   C   
   A   
   A   
   A   
   C   
   G   
   T   
   C   
   C   
   T   
   G   
   G   
   T   
   A   
   G   
   T   
   G   
   C   
   G   
   G   
   T   
   C   
   A   
   T   
   T   
   A   
   C   
   C   
   G   
   A   
   T   
   C   
   C   
   T   
   C   
   A   
   A   
   A   
   A   
   A   
   G   
   G   
   C   
   G   
   A   
   T   
   T   
   T   
   A   
   T   
   T   
   A   
   G   
   C   
   A   
   A   
   C   
   G   
   G   
   T   
   T   
   A   
   G   
   T   
   T   
   C   
   A   
   C   
   C   
   T   
   T   
   C   
   G   
   T   
   A   
   T   
   G   
   A   
   T   
   C   
   C   
   T   
   A   
   A   
   T   
   A   
   A   
   A   
   A   
   T   
   G   
   G   
   C   
   C   
   A   
   A   
   T   
   G   
   G   
   T   
   A   
   T   
   T   
   T   
   C   
   T   
   C   
   A   
   A   
   A   
   A   
   A   
   G   
   A   
   C   
   T   
   A   
   T   
   G   
   A   
   T   
   G   
   C   
   T   
   T   
   A   
   C   
   A   
   A   
   T   
   A   
   A   
   C   
   A   
   A   
   C   
   A   
   A   
   A   
   G   
   A   
   T   
   T   
   T   
   A   
   C   
   C   
   A   
   T   
   T   
   C   
   A   
   C   
   A   
   G   
   C   
   A   
   C   
   G   
   T   
   T   
   T   
   T   
   G   
   C   
   G   
   A   
   C   
   A   
   G   
   G   
   C   
   T   
   A   
   T   
   G   
   C   
   A   
   C   
   C   
   A   
   G   
   G   
   C   
   T   
   C   
   T   
   A   
   C   
   C   
   T   
   T   
   T   
   A   
   A   
   A   
   A   
   C   
   A   
   A   
   T   
   T   
   A   
   C   
   C   
   G   
   G   
   T   
   G   
   C   
   C   
   A   
   T   
   T   
   G   
   G   
   T   
   T   
   T   
   A   
   G   
   A   
   T   
   G   
   C   
   A   
   G   
   G   
   G   
   A   
   C   
   A   
   T   
   T   
   G   
   A   
   A   
   G   
   C   
   C   
   A   
   G   
   A   
   T   
   G   
   A   
   A   
   G   
   A   
   G   
   C   
   T   
   A   
   G   
   A   
   A   
   A   
   T   
   T   
   A   
   A   
   T   
   G   
   G   
   C   
   T   
   T   
   G   
   A   
   A   
   A   
   T   
   G   
   G   
   C   
   A   
   A   
   A   
   A   
   A   
   G   
   A   
   T   
   A   
   A   
   A   
   T   
   C   
   T   
   T   
   G   
   G   
   G   
   G   
   C   
   G   
   G   
   C   
   T   
   A   
   T   
   T   
   T   
   T   
   G   
   C   
   G   
   A   
   C   
   A   
   C   
   G   
   T   
   G   
   T   
   A   
   A   
   A   
   A   
   G   
   A   
   A   
   G   
   C   
   A   
   A   
   G   
   T   
   C   
   C   
   A   
   G   
   T   
   C   
   A   
   A   
   T   
   C   
   T   
   G   
   C   
   G   
   T   
   A   
   C   
   C   
   G   
   C   
   T   
   T   
   T   
   A   
   G   
   T   
   T   
   A   
   A   
   T   
   T   
   C   
   A   
   G   
   A   
   T   
   A   
   A   
   T   
   A   
   T   
   T   
   T   
   A   
   T   
   T   
   T   
   T   
   G   
   C   
   C   
   C   
   A   
   A   
   C   
   A   
   A   
   A   
   C   
   A   
   T   
   T   
   A   
   C   
   G   
   G   
   A   
   T   
   G   
   G   
   G   
   G   
   G   
   A   
   A   
   G   
   A   
   C   
   A   
   A   
   A   
   T   
   T   
   T   
   A   
   G   
   A   
   G   
   C   
   A   
   G   
   G   
   T   
   C   
   T   
   T   
   A   
   A   
   T   
   A   
   A   
   A   
   T   
   T   
   C   
   A   
   T   
   T   
   T   
   T   
   C   
   G   
   G   
   T   
   G   
   A   
   G   
   G   
   A   
   A   
   C   
   T   
   A   
   G   
   A   
   T   
   T   
   T   
   A   
   C   
   C   
   A   
   A   
   T   
   T   
   G   
   C   
   C   
   A   
   T   
   G   
   A   
   C   
   G   
   C   
   C   
   T   
   G   
   C   
   T   
   C   
   A   
   A   
   A   
   T   
   T   
   T   
   C   
   A   
   A   
   A   
   T   
   G   
   A   
   A   
   G   
   A   
   T   
   A   
   A   
   A   
   T   
   T   
   T   
   A   
   A   
   T   
   T   
   C   
   T   
   G   
   A   
   A   
   A   
   T   
   T   
   C   
   T   
   T   
   T   
   T   
   A   
   G   
   C   
   A   
   G   
   A   
   T   
   A   
   C   
   G   
   G   
   G   
   C   
   T   
   A   
   T   
   G   
   G   
   T   
   C   
   A   
   A   
   G   
   G   
   T   
   C   
   A   
   A   
   T   
   T   
   G   
   T   
   T   
   A   
   A   
   T   
   T   
   T   
   C   
   G   
   C   
   C   
   A   
   A   
   T   
   C   
   C   
   A   
   G   
   C   
   A   
   A   
   G   
   C   
   T   
   A   
   C   
   C   
   A   
   T   
   G   
   T   
   A   
   T   
   A   
   G   
   T   
   G   
   T   
   T   
   T   
   T   
   C   
   C   
   A   
   A   
   A   
   A   
   T   
   A   
   A   
   T   
   G   
   G   
   A   
   A   
   C   
   A   
   T   
   T   
   A   
   G   
   T   
   C   
   T   
   A   
   T   
   C   
   C   
   A   
   A   
   A   
   A   
   C   
   T   
   A   
   G   
   T   
   T   
   T   
   T   
   A   
   G   
   A   
   C   
   A   
   A   
   A   
   G   
   A   
   A   
   A   
   C   
   G   
   A   
   A   
   G   
   A   
   A   
   A   
   A   
   A   
   A   
   G   
   A   
   C   
   A   
   A   
   T   
   G   
   T   
   A   
   A   
   T   
   C   
   A   
   G   
   C   
   G   
   C   
   C   
   A   
   A   
   C   
   G   
   C   
   A   
   G   
   C   
   G   
   A   
   A   
   T   
   A   
   C   
   A   
   A   
   T   
   T   
   G   
   C   
   C   
   A   
   C   
   A   
   G   
   A   
   T   
   C   
   T   
   T   
   T   
   T   
   A   
   G   
   G   
   T   
   A   
   G   
   T   
   G   
   T   
   G   
   G   
   A   
   A   
   G   
   A   
   T   
   C   
   C   
   T   
   T   
   C   
   A   
   G   
   G   
   T   
   T   
   A   
   T   
   G   
   T   
   T   
   T   
   A   
   T   
   A   
   A   
   T   
   A   
   T   
   G   
   T   
   A   
   C   
   A   
   A   
   T   
   C   
   C   
   G   
   A   
   A   
   C   
   T   
   T   
   C   
   T   
   C   
   A   
   T   
   T   
   G   
   G   
   C   
   T   
   G   
   C   
   T   
   A   
   A   
   A   
   A   
   C   
   A   
   G   
   G   
   A   
   A   
   C   
   A   
   G   
   C   
   T   
   G   
   A   
   A   
   A   
   T   
   A   
   A   
   A   
   A   
   G   
   A   
   T   
   A   
   A   
   A   
   C   
   A   
   A   
   G   
   A   
   T   
   A   
   C   
   A   
   G   
   A   
   T   
   G   
   G   
   C   
   A   
   A   
   A   
   G   
   A   
   A   
   A   
   A   
   C   
   A   
   G   
   T   
   T   
   T   
   C   
   C   
   T   
   T   
   C   
   T   
   A   
   A   
   C   
   G   
   T   
   T   
   A   
   G   
   A   
   T   
   C   
   G   
   T   
   A   
   G   
   T   
   A   
   A   
   C   
   A   
   A   
   T   
   A   
   A   
   A   
   T   
   T   
   C   
   T   
   T   
   A   
   A   
   C   
   A   
   A   
   T   
   G   
   A   
   T   
   T   
   A   
   T   
   G   
   G   
   T   
   T   
   G   
   A   
   A   
   A   
   A   
   T   
   T   
   C   
   A   
   G   
   G   
   A   
   G   
   A   
   A   
   A   
   A   
   T   
   G   
   G   
   T   
   T   
   C   
   T   
   G   
   C   
   A   
   A   
   C   
   A   
   G   
   A   
   T   
   A   
   T   
   C   
   A   
   G   
   T   
   A   
   A   
   A   
   C   
   C   
   A   
   T   
   T   
   A   
   A   
   T   
   T   
   G   
   A   
   T   
   T   
   A   
   C   
   T   
   T   
   A   
   G   
   A   
   A   
   G   
   C   
   A   
   A   
   C   
   C   
   A   
   T   
   T   
   A   
   A   
   A   
   -   
   -   
   -   
   -   
   -   
   -   
   -   
   -   
   -   
   -   
   -   
   -   
   -   
   -   
   -   
   -   
   -   
   -   
   -   
   -   
   -   
   -   
   -   
   -   
   -   
   -   
   -   
   -   
   -   
   -   
   -   
   -   
   -   
   -   
   -   
   -   
   -   
   -   
   -   
   -   
   -   
   -   
   -   
   -   
   -   
   -   
   -   
   -   
   -   
   -   
   -   
   -   
   -   
   -   
   -   
   -   
   -   
   -   
   -   
   -   
   -   
 

 
   N59455F   
   -   
   -   
   -   
   -   
   -   
   -   
   -   
   -   
   -   
   -   
   -   
   -   
   -   
   -   
   -   
   -   
   -   
   -   
   -   
   -   
   -   
   -   
   -   
   -   
   -   
   -   
   -   
   -   
   -   
   -   
   -   
   -   
   -   
   -   
   -   
   -   
   -   
   -   
   -   
   -   
   -   
   -   
   -   
   -   
   -   
   -   
   -   
   -   
   -   
   -   
   -   
   -   
   -   
   -   
   -   
   -   
   -   
   -   
   -   
   -   
   -   
   -   
   -   
   -   
   -   
   -   
   -   
   -   
   -   
   -   
   -   
   -   
   -   
   -   
   -   
   -   
   -   
   -   
   -   
   -   
   -   
   -   
   -   
   -   
   -   
   -   
   -   
   -   
   -   
   -   
   -   
   -   
   -   
   -   
   -   
   -   
   -   
   -   
   -   
   -   
   -   
   -   
   -   
   -   
   -   
   -   
   -   
   -   
   C   
   A   
   A   
   T   
   G   
   G   
   C   
   A   
   A   
   G   
   C   
   C   
   A   
   A   
   A   
   C   
   A   
   A   
   G   
   A   
    A    
   T   
   T   
   A   
   G   
   C   
    C    
   G   
   A   
   A   
   G   
   C   
   G   
   A   
   A   
   G   
   A   
   A   
   A   
   A   
    C    
   A   
   G   
   C   
    T    
   A   
    A    
   T   
    A    
    C    
   A   
   T   
   T   
   T   
   T   
   T   
   A   
   A   
   A   
    T    
   G   
   T   
   A   
   T   
   T   
   G   
    T    
   C   
   A   
   A   
   A   
   A   
   C   
   A   
   G   
    G    
   A   
   A   
   T   
   T   
   T   
   G   
   A   
   T   
   A   
   A   
   G   
   T   
   T   
   A   
   C   
   C   
    G    
   T   
   C   
   C   
   G   
   T   
   T   
    G    
   T   
   T   
   C   
   A   
   A   
   G   
   A   
   A   
    T    
   C   
   T   
   A   
   G   
   C   
   T   
   T   
   A   
   A   
   A   
   G   
   A   
   A   
   A   
   A   
   A   
   T   
   G   
   G   
    C    
   T   
   A   
   T   
   G   
   A   
   T   
   A   
   C   
   T   
   A   
   A   
   A   
   T   
   C   
    T    
   G   
   T   
   T   
   G   
   T   
   T   
   G   
   A   
   A   
   A   
   A   
   A   
   T   
   A   
   C   
   C   
   A   
   A   
   G   
   C   
   A   
   A   
   T   
   T   
   T   
   A   
   T   
   T   
   C   
   A   
   G   
   G   
   G   
   A   
   T   
   T   
   C   
   A   
   A   
   G   
   C   
    A    
   G   
   A   
   A   
    G    
   G   
    A    
   G   
   T   
   C   
   A   
   A   
    A    
   G   
   C   
   T   
   A   
   G   
   T   
   G   
   A   
   T   
   G   
   T   
    C    
   C   
   A   
   A   
   G   
   T   
    C    
   A   
   A   
   A   
   A   
   A   
    G    
   G   
    C    
   G   
   A   
   A   
   A   
   G   
   A   
    C    
   A   
   A   
    T    
   C   
   A   
   A   
   T   
   A   
   C   
   A   
   C   
   A   
   T   
   T   
   T   
   A   
    C    
   C   
   T   
   A   
   T   
   A   
   A   
   A   
   T   
   T   
   A   
   T   
   C   
   G   
   A   
   T   
   G   
   A   
   G   
    C    
   A   
   C   
    T    
   C   
   C   
   T   
   T   
   T   
   A   
   G   
   G   
   C   
   G   
   A   
   A   
   A   
   T   
   G   
   A   
   A   
   A   
   G   
   A   
   T   
    C    
   T   
   G   
   T   
   C   
   T   
   T   
   A   
   T   
   C   
   A   
   A   
   T   
   C   
   A   
   A   
   G   
   T   
   A   
   T   
    C    
   G   
    C    
   C   
   A   
   A   
    A    
   A   
   A   
   A   
   G   
   G   
    C    
   G   
   A   
   T   
   A   
    C    
   C   
   T   
   A   
   C   
   C   
   A   
   A   
   A   
   T   
    C    
    T    
   C   
   T   
   T   
   G   
   G   
    A    
    A    
    G    
   C   
   C   
    G    
   T   
   C   
   T   
   T   
   T   
   A   
   A   
   T   
   T   
   T   
   T   
   T   
   C   
   C   
   A   
   G   
   A   
   T   
   A   
   T   
   G   
   T   
   C   
   A   
   G   
   G   
   A   
   A   
   A   
   T   
   G   
   A   
   T   
   A   
   A   
   A   
    A    
   T   
   T   
   T   
   C   
   G   
   A   
   T   
   T   
   C   
   A   
   A   
   G   
   T   
   A   
   G   
   A   
   T   
   A   
   A   
   T   
   G   
   C   
   C   
   A   
   A   
   A   
   C   
   G   
   T   
   G   
   G   
   A   
   G   
   A   
   A   
   A   
   T   
   T   
   G   
   T   
   C   
   G   
   A   
   T   
   C   
   G   
   T   
   A   
   A   
    T    
   G   
   G   
   T   
   A   
   G   
   T   
   G   
   G   
   G   
   C   
   T   
   A   
   G   
    C    
   A   
   A   
   T   
   T   
   A   
   A   
   C   
   A   
   A   
   A   
   G   
   T   
   G   
   T   
   T   
   T   
   G   
   A   
    C    
   G   
   A   
   A   
   G   
   T   
   G   
   G   
   G   
   C   
   G   
   T   
   A   
   G   
   T   
    G    
   C   
   C   
   T   
    G    
   G   
   C   
   A   
   A   
   A   
   C   
   T   
    C    
   G   
   G   
   T   
   T   
   C   
   T   
   G   
   G   
   C   
   G   
   C   
   A   
   G   
   A   
   A   
   A   
   A   
   A   
    A    
   C   
   A   
   G   
   C   
    C    
   A   
   A   
   T   
   A   
   T   
   C   
   A   
   A   
   A   
   G   
   C   
   T   
   T   
   T   
   T   
   A   
   G   
   T   
   G   
   A   
   T   
   A   
   A   
    G    
   T   
   T   
    C    
   G   
   G   
   C   
   G   
   T   
   T   
   T   
   C   
   T   
    G    
   T   
   T   
   G   
   A   
   T   
   G   
   A   
   A   
   A   
   T   
   C   
   A   
   A   
   T   
   C   
    A    
   A   
   A   
   A   
    G    
   T   
   T   
   A   
   A   
   G   
   C   
   C   
   A   
   A   
   G   
   G   
    G    
   T   
   G   
   G   
   G   
   T   
   C   
   C   
   A   
   A   
   G   
   C   
   A   
   G   
   A   
   C   
   T   
   C   
    C    
   T   
   T   
   T   
   G   
   T   
   A   
   C   
   C   
   A   
   A   
   T   
   C   
   A   
   C   
   A   
   G   
   T   
    T    
   G   
   C   
   T   
   T   
   C   
   T   
   G   
   A   
   A   
   C   
   C   
   A   
   G   
   T   
   G   
   A   
   C   
   A   
   G   
   A   
   A   
   T   
   T   
   A   
   C   
   C   
   A   
   A   
   C   
   A   
   G   
   G   
   G   
   G   
   C   
   T   
   G   
   C   
   G   
   A   
   C   
   A   
   A   
   A   
   A   
   G   
    A    
    T    
   A   
   C   
   A   
   G   
   A   
   G   
   T   
   C   
   A   
   C   
   G   
   T   
   T   
   A   
   T   
   T   
   A   
   T   
   C   
   C   
    A    
    C    
   T   
   G   
   G   
   G   
   G   
   G   
   A   
   A   
   G   
   C   
   A   
   G   
   C   
   T   
   G   
   C   
   G   
   C   
   A   
   A   
   T   
   T   
   A   
   A   
   T   
   C   
   G   
   G   
    G    
   T   
   A   
   T   
   A   
   C   
    G    
   G   
   G   
    C    
   A   
   C   
    C    
   A   
   T   
   T   
   A   
   C   
   T   
   G   
   C   
   A   
   G   
   A   
    A    
   G   
   A   
   T   
   A   
   T   
   T   
    G    
   A   
   A   
   A   
   A   
   A   
   A   
   A   
   T   
   C   
   C   
   A   
   G   
   A   
   G   
   T   
   T   
   A   
   A   
   G   
   T   
   A   
   G   
   T   
   A   
   C   
   G   
   G   
   G   
    T    
   G   
   T   
   A   
   A   
   T   
   T   
   G   
   G   
    T    
   A   
   A   
   A   
   A   
   C   
   T   
   G   
   G   
    C    
   T   
   T   
   A   
   G   
   A   
   A   
   C   
   G   
   A   
   G   
   C   
   G   
   T   
   T   
   T   
   G   
   A   
   T   
   A   
   A   
   A   
   G   
   A   
    G    
   T   
   T   
   A   
   A   
   G   
   A   
   G   
   G   
    G    
   C   
   A   
   A   
   G   
   A   
   T   
   G   
   G   
    T    
   G   
   G   
    C    
   T   
   C   
    A    
   C   
   T   
   A   
   G   
   T   
   T   
   A   
   T   
   T   
   T   
   T   
   A   
   G   
   A   
   T   
   G   
   A   
   C   
   A   
   A   
   A   
   G   
   A   
   G   
   A   
   A   
   T   
   G   
   T   
   C   
   A   
   A   
   A   
   A   
   A   
   A   
   G   
   C   
   T   
   T   
   T   
   A   
   C   
   A   
   A   
   A   
   C   
   C   
   A   
   A   
   A   
   G   
   A   
    G    
   A   
   A   
   A   
   A   
   A   
   A   
   G   
   A   
   T   
   G   
   G   
   T   
   C   
   A   
   A   
   A   
   C   
    G    
   A   
   T   
   T   
   A   
   A   
   A   
   T   
   T   
   A   
   A   
   C   
   G   
   A   
   T   
   T   
   G   
   A   
    C    
   A   
   G   
    T    
   G   
   G   
    C    
   G   
   T   
   A   
   C   
   A   
   A   
   C   
   A   
   A   
   C   
   A   
   A   
   G   
   C   
    C    
   T   
   T   
    C    
   G   
   C   
   T   
   A   
   T   
   A   
   T   
   T   
   T   
   G   
   A   
   C   
   A   
   A   
   A   
   C   
   G   
    T    
    C    
   C   
   T   
   G   
   G   
   T   
   A   
   G   
   T   
   G   
   C   
   G   
   G   
   T   
   C   
   A   
   T   
   T   
   A   
   C   
   C   
   G   
   A   
   T   
   C   
   C   
   T   
   C   
   A   
   A   
   A   
   A   
   A   
   G   
   G   
   C   
   G   
   A   
   T   
   T   
   T   
   A   
    T    
   T   
   A   
   G   
   C   
    A    
   A   
   C   
   G   
   G   
   T   
   T   
   A   
   G   
   T   
   T   
   C   
   A   
   C   
   C   
   T   
   T   
   C   
   G   
   T   
   A   
   T   
   G   
   A   
   T   
   C   
   C   
   T   
   A   
   A   
   T   
   A   
   A   
   A   
   A   
   T   
   G   
   G   
   C   
   C   
   A   
   A   
   T   
   G   
   G   
    T    
   A   
   T   
   T   
   T   
   C   
   T   
   C   
   A   
   A   
   A   
   A   
   A   
   G   
   A   
    A    
   T   
   A   
   T   
   G   
   A   
   T   
   G   
   C   
   T   
   T   
   A   
   C   
   A   
   A   
   T   
   A   
   A   
   C   
   A   
   A   
   C   
   A   
   A   
   A   
   G   
   A   
   T   
   T   
   T   
   A   
   C   
   C   
   A   
   T   
   T   
    C    
   A   
   C   
   A   
   G   
   C   
   A   
   C   
   G   
   T   
   T   
   T   
    T    
   G   
   C   
   G   
   A   
   C   
   A   
   G   
   G   
    C    
   T   
   A   
   T   
   G   
   C   
   A   
   C   
   C   
   A   
   G   
   G   
    C    
   T   
   C   
   T   
   A   
   C   
   C   
   T   
   T   
   T   
   A   
   A   
   A   
   A   
   C   
   A   
   A   
   T   
   T   
   A   
   C   
   C   
   G   
   G   
   T   
   G   
   C   
   C   
   A   
   T   
   T   
   G   
   G   
   T   
   T   
   T   
   A   
   G   
   A   
   T   
    G    
   C   
   A   
   G   
   G   
   G   
   A   
   C   
   A   
   T   
   T   
   G   
   A   
   A   
   G   
   C   
   C   
   A   
   G   
   A   
   T   
   G   
   A   
   A   
   G   
   A   
   G   
   C   
   T   
   A   
   G   
   A   
   A   
   A   
   T   
   T   
   A   
   A   
   T   
   G   
   G   
    C    
   T   
   T   
   G   
   A   
   A   
   A   
   T   
   G   
   G   
   C   
   A   
   A   
   A   
   A   
   A   
   G   
   A   
   T   
   A   
   A   
   A   
   T   
   C   
   T   
   T   
   G   
   G   
   G   
   G   
    C    
   G   
   G   
   C   
   T   
   A   
   T   
   T   
   T   
   T   
   G   
   C   
    A    
   A   
   C   
   A   
   C   
   G   
   T   
   G   
   T   
   A   
   A   
   A   
   A   
   G   
   A   
   A   
   G   
   C   
   A   
   A   
   G   
   T   
   C   
   C   
   A   
   G   
   T   
   C   
   A   
   A   
   T   
   C   
   T   
    G    
   C   
   G   
   T   
   A   
   C   
   C   
   G   
   C   
   T   
   T   
   T   
   A   
   G   
   T   
   T   
   A   
   A   
   T   
   T   
   C   
   A   
   G   
   A   
   T   
   A   
   A   
   T   
   A   
   T   
   T   
   T   
   A   
   T   
   T   
   T   
   T   
   G   
   C   
    C    
   C   
   A   
   A   
   C   
   A   
   A   
   A   
   C   
   A   
   T   
   T   
   A   
   C   
   G   
    G    
   A   
   T   
   G   
   G   
   G   
    G    
   G   
   A   
   A   
   G   
   A   
   C   
   A   
   A   
   A   
   T   
   T   
   T   
   A   
   G   
   A   
   G   
   C   
    A    
   G   
   G   
   T   
   C   
   T   
   T   
   A   
   A   
   T   
   A   
   A   
   A   
   T   
   T   
   C   
   A   
   T   
   T   
   T   
   T   
   C   
   G   
   G   
   T   
   G   
   A   
   G   
   G   
   A   
   A   
   C   
   T   
   A   
   G   
   A   
   T   
   T   
   T   
   A   
   C   
   C   
   A   
   A   
   T   
    T    
   G   
   C   
   C   
   A   
   T   
   G   
   A   
   C   
   G   
   C   
   C   
    T    
   G   
   C   
   T   
   C   
   A   
   A   
   A   
   T   
   T   
   T   
   C   
   A   
   A   
   A   
    T    
   G   
   A   
   A   
   G   
   A   
   T   
   A   
   A   
   A   
   T   
   T   
   T   
   A   
   A   
   T   
   T   
   C   
   T   
   G   
   A   
   A   
   A   
   T   
   T   
   C   
   T   
   T   
   T   
   T   
    A    
   G   
   C   
   A   
   G   
   A   
   T   
   A   
   C   
    G    
   G   
   G   
   C   
   T   
   A   
    C    
   G   
   G   
    C    
   C   
   A   
   A   
   G   
   G   
    C    
   C   
   A   
   A   
   T   
   T   
   G   
   T   
   T   
   A   
   A   
   T   
   T   
   T   
   C   
    G    
   C   
   C   
   A   
   A   
   T   
   C   
   C   
   A   
   G   
   C   
   A   
   A   
   G   
   C   
   T   
   A   
   C   
   C   
   A   
   T   
   G   
   T   
   A   
   T   
   A   
   G   
   T   
   G   
   T   
   T   
   T   
   T   
   C   
   C   
   A   
   A   
   A   
   A   
   T   
   A   
   A   
   T   
   G   
   G   
    A    
   A   
   C   
   A   
    T    
   T   
    A    
   G   
   T   
    C    
   T   
   A   
   T   
   C   
   C   
   A   
   A   
   A   
   A   
    T    
   T   
   A   
   G   
   T   
   T   
   T   
   T   
   A   
   G   
   A   
   C   
   A   
   A   
   A   
   G   
   A   
   A   
   A   
   C   
    G    
   A   
   A   
   G   
   A   
   A   
   A   
   A   
   A   
   A   
   G   
   A   
   C   
   A   
   A   
   T   
   G   
   T   
   A   
   A   
   T   
   C   
   A   
   G   
   C   
   G   
   C   
   C   
   A   
   A   
   C   
   G   
   C   
   A   
   G   
   C   
   G   
   A   
   A   
   T   
   A   
   C   
   A   
   A   
   T   
   T   
   G   
   C   
   C   
   A   
   C   
   A   
   G   
   A   
   T   
   C   
   T   
   T   
   T   
   T   
   A   
   G   
   G   
   T   
   A   
   G   
   T   
   G   
   T   
   G   
   G   
   A   
   A   
   G   
   A   
   T   
   C   
   C   
   T   
   T   
   C   
   A   
   G   
   G   
   T   
   T   
   A   
   T   
   G   
   T   
   T   
   T   
   A   
   T   
   A   
   A   
   T   
   A   
   T   
   G   
   T   
   A   
   C   
   A   
   A   
   T   
   C   
   C   
   G   
   A   
   A   
   C   
   T   
   T   
   C   
   T   
   C   
   A   
   T   
   T   
   G   
   G   
   C   
   T   
   G   
   C   
   T   
   A   
   A   
   A   
   A   
   C   
   A   
   G   
   G   
   A   
   A   
   C   
   A   
   G   
   C   
   T   
   G   
   A   
   A   
   A   
   T   
   A   
   A   
   A   
   A   
   G   
   A   
   T   
   A   
   A   
   A   
   C   
   A   
   A   
   G   
   A   
   T   
   A   
   C   
   A   
   G   
   A   
   T   
   G   
   G   
   C   
   A   
   A   
   A   
   G   
   A   
   A   
   A   
   A   
   C   
   A   
   G   
   T   
   T   
   T   
   C   
   C   
   T   
   C   
   C   
   T   
   A   
   A   
   C   
   G   
   T   
   T   
   A   
   G   
   A   
   T   
   C   
   G   
   T   
   A   
   G   
   T   
   A   
   A   
   C   
   A   
   A   
   T   
   A   
   A   
   A   
   T   
   T   
   C   
   T   
   T   
   A   
   A   
   C   
   A   
   A   
   T   
   G   
   A   
   T   
   T   
   A   
   T   
   G   
   G   
   T   
   T   
   G   
   A   
   A   
   A   
   A   
   T   
   T   
   C   
   A   
   G   
   G   
   A   
   G   
   A   
   A   
   A   
   A   
   T   
   G   
   G   
   T   
   T   
   C   
   T   
   G   
   C   
   A   
   A   
   C   
   A   
   G   
   A   
   T   
   A   
   T   
   C   
   A   
   G   
   T   
   A   
   A   
   A   
   C   
   C   
   A   
   T   
   T   
   A   
   A   
   T   
   T   
   G   
   A   
   T   
   T   
   A   
   C   
   T   
   T   
   A   
   G   
   A   
   A   
   G   
   C   
   A   
   A   
   C   
   C   
   A   
   T   
   T   
   A   
   A   
   A   
   -   
   -   
   -   
   -   
   -   
   -   
   -   
   -   
   -   
   -   
   -   
   -   
   -   
   -   
   -   
   -   
   -   
   -   
   -   
   -   
   -   
   -   
   -   
   -   
   -   
   -   
   -   
   -   
   -   
   -   
   -   
   -   
   -   
   -   
   -   
   -   
   -   
   -   
   -   
   -   
   -   
   -   
   -   
   -   
   -   
   -   
   -   
   -   
   -   
   -   
   -   
   -   
   -   
   -   
   -   
   -   
   -   
   -   
   -   
   -   
   -   
   -   
   -   
   -   
   -   
   -   
   -   
   -   
   -   
   -   
   -   
   -   
   -   
   -   
   -   
   -   
   -   
   -   
   -   
   -   
   -   
   -   
   -   
   -   
   -   
   -   
   -   
   -   
   -   
   -   
   -   
   -   
   -   
   -   
   -   
   -   
   -   
   -   
   -   
   -   
   -   
   -   
   -   
   -   
   -   
   -   
   -   
   -   
   -   
   -   
   -   
   -   
   -   
   -   
   -   
   -   
   -   
   -   
   -   
   -   
   -   
   -   
   -   
   -   
   -   
   -   
   -   
   -   
   -   
   -   
   -   
   -   
   -   
   -   
   -   
   -   
   -   
   -   
   -   
   -   
   -   
   -   
   -   
   -   
   -   
   -   
   -   
   -   
   -   
   -   
   -   
   -   
   -   
   -   
   -   
   -   
   -   
   -   
   -   
   -   
   -   
   -   
   -   
   -   
   -   
   -   
   -   
   -   
   -   
   C   
   A   
   A   
   T   
   G   
   G   
   C   
   A   
   A   
   G   
   C   
   C   
   A   
   A   
   A   
   C   
   A   
   A   
   G   
   A   
   A   
   T   
   T   
   A   
   G   
   C   
   C   
   G   
   A   
   A   
   G   
   C   
   G   
   A   
   A   
   G   
   A   
   A   
   A   
   A   
   C   
   A   
   G   
   C   
   T   
   A   
   A   
   T   
   A   
   C   
   A   
   T   
   T   
   T   
   T   
   T   
   A   
   A   
   A   
   T   
   G   
   T   
   A   
   T   
   T   
   G   
   T   
   C   
   A   
   A   
   A   
   A   
   C   
   A   
   G   
   G   
   A   
   A   
   T   
   T   
   T   
   G   
   A   
   T   
   A   
   A   
   G   
   T   
   T   
   A   
   C   
   C   
   G   
   T   
   C   
   C   
   G   
   T   
   T   
   G   
   T   
   T   
   C   
   A   
   A   
   G   
   A   
   A   
   T   
   C   
   T   
   A   
   G   
   C   
   T   
   T   
   A   
   A   
   A   
   G   
   A   
   A   
   A   
   A   
   A   
   T   
   G   
   G   
   C   
   T   
   A   
   T   
   G   
   A   
   T   
   A   
   C   
   T   
   A   
   A   
   A   
   T   
   C   
   T   
   G   
   T   
   T   
   G   
   T   
   T   
   G   
   A   
   A   
   A   
   A   
   A   
   T   
   A   
   C   
   C   
   A   
   A   
   G   
   C   
   A   
   A   
   T   
   T   
   T   
   A   
   T   
   T   
   C   
   A   
   G   
   G   
   G   
   A   
   T   
   T   
   C   
   A   
   A   
   G   
   C   
   A   
   G   
   A   
   A   
   G   
   G   
   A   
   G   
   T   
   C   
   A   
   A   
   A   
   G   
   C   
   T   
   A   
   G   
   T   
   G   
   A   
   T   
   G   
   T   
   C   
   C   
   A   
   A   
   G   
   T   
   C   
   A   
   A   
   A   
   A   
   A   
   G   
   G   
   C   
   G   
   A   
   A   
   A   
   G   
   A   
   C   
   A   
   A   
   T   
   C   
   A   
   A   
   T   
   A   
   C   
   A   
   C   
   A   
   T   
   T   
   T   
   A   
   C   
   C   
   T   
   A   
   T   
   A   
   A   
   A   
   T   
   T   
   A   
   T   
   C   
   G   
   A   
   T   
   G   
   A   
   G   
   C   
   A   
   C   
   T   
   C   
   C   
   T   
   T   
   T   
   A   
   G   
   G   
   C   
   G   
   A   
   A   
   A   
   T   
   G   
   A   
   A   
   A   
   G   
   A   
   T   
   C   
   T   
   G   
   T   
   C   
   T   
   T   
   A   
   T   
   C   
   A   
   A   
   T   
   C   
   A   
   A   
   G   
   T   
   A   
   T   
   C   
   G   
   C   
   C   
   A   
   A   
   A   
   A   
   A   
   A   
   G   
   G   
   C   
   G   
   A   
   T   
   A   
   C   
   C   
   T   
   A   
   C   
   C   
   A   
   A   
   A   
   T   
   C   
   T   
   C   
   T   
   T   
   G   
   G   
   A   
   A   
   G   
   C   
   C   
   G   
   T   
   C   
   T   
   T   
   T   
   A   
   A   
   T   
   T   
   T   
   T   
   T   
   C   
   C   
   A   
   G   
   A   
   T   
   A   
   T   
   G   
   T   
   C   
   A   
   G   
   G   
   A   
   A   
   A   
   T   
   G   
   A   
   T   
   A   
   A   
   A   
   A   
   T   
   T   
   T   
   C   
   G   
   A   
   T   
   T   
   C   
   A   
   A   
   G   
   T   
   A   
   G   
   A   
   T   
   A   
   A   
   T   
   G   
   C   
   C   
   A   
   A   
   A   
   C   
   G   
   T   
   G   
   G   
   A   
   G   
   A   
   A   
   A   
   T   
   T   
   G   
   T   
   C   
   G   
   A   
   T   
   C   
   G   
   T   
   A   
   A   
   T   
   G   
   G   
   T   
   A   
   G   
   T   
   G   
   G   
   G   
   C   
   T   
   A   
   G   
   C   
   A   
   A   
   T   
   T   
   A   
   A   
   C   
   A   
   A   
   A   
   G   
   T   
   G   
   T   
   T   
   T   
   G   
   A   
   C   
   G   
   A   
   A   
   G   
   T   
   G   
   G   
   G   
   C   
   G   
   T   
   A   
   G   
   T   
   G   
   C   
   C   
   T   
   G   
   G   
   C   
   A   
   A   
   A   
   C   
   T   
   C   
   G   
   G   
   T   
   T   
   C   
   T   
   G   
   G   
   C   
   G   
   C   
   A   
   G   
   A   
   A   
   A   
   A   
   A   
   A   
   C   
   A   
   G   
   C   
   C   
   A   
   A   
   T   
   A   
   T   
   C   
   A   
   A   
   A   
   G   
   C   
   T   
   T   
   T   
   T   
   A   
   G   
   T   
   G   
   A   
   T   
   A   
   A   
   G   
   T   
   T   
   C   
   G   
   G   
   C   
   G   
   T   
   T   
   T   
   C   
   T   
   G   
   T   
   T   
   G   
   A   
   T   
   G   
   A   
   A   
   A   
   T   
   C   
   A   
   A   
   T   
   C   
   A   
   A   
   A   
   A   
   G   
   T   
   T   
   A   
   A   
   G   
   C   
   C   
   A   
   A   
   G   
   G   
   G   
   T   
   G   
   G   
   G   
   T   
   C   
   C   
   A   
   A   
   G   
   C   
   A   
   G   
   A   
   C   
   T   
   C   
   C   
   T   
   T   
   T   
   G   
   T   
   A   
   C   
   C   
   A   
   A   
   T   
   C   
   A   
   C   
   A   
   G   
   T   
   T   
   G   
   C   
   T   
   T   
   C   
   T   
   G   
   A   
   A   
   C   
   C   
   A   
   G   
   T   
   G   
   A   
   C   
   A   
   G   
   A   
   A   
   T   
   T   
   A   
   C   
   C   
   A   
   A   
   C   
   A   
   G   
   G   
   G   
   G   
   C   
   T   
   G   
   C   
   G   
   A   
   C   
   A   
   A   
   A   
   A   
   G   
   A   
   T   
   A   
   C   
   A   
   G   
   A   
   G   
   T   
   C   
   A   
   C   
   G   
   T   
   T   
   A   
   T   
   T   
   A   
   T   
   C   
   C   
   A   
   C   
   T   
   G   
   G   
   G   
   G   
   G   
   A   
   A   
   G   
   C   
   A   
   G   
   C   
   T   
   G   
   C   
   G   
   C   
   A   
   A   
   T   
   T   
   A   
   A   
   T   
   C   
   G   
   G   
   G   
   T   
   A   
   T   
   A   
   C   
   G   
   G   
   G   
   C   
   A   
   C   
   C   
   A   
   T   
   T   
   A   
   C   
   T   
   G   
   C   
   A   
   G   
   A   
   A   
   G   
   A   
   T   
   A   
   T   
   T   
   G   
   A   
   A   
   A   
   A   
   A   
   A   
   A   
   T   
   C   
   C   
   A   
   G   
   A   
   G   
   T   
   T   
   A   
   A   
   G   
   T   
   A   
   G   
   T   
   A   
   C   
   G   
   G   
   G   
   T   
   G   
   T   
   A   
   A   
   T   
   T   
   G   
   G   
   T   
   A   
   A   
   A   
   A   
   C   
   T   
   G   
   G   
   C   
   T   
   T   
   A   
   G   
   A   
   A   
   C   
   G   
   A   
   G   
   C   
   G   
   T   
   T   
   T   
   G   
   A   
   T   
   A   
   A   
   A   
   G   
   A   
   G   
   T   
   T   
   A   
   A   
   G   
   A   
   G   
   G   
   G   
   C   
   A   
   A   
   G   
   A   
   T   
   G   
   G   
   T   
   G   
   G   
   C   
   T   
   C   
   A   
   C   
   T   
   A   
   G   
   T   
   T   
   A   
   T   
   T   
   T   
   T   
   A   
   G   
   A   
   T   
   G   
   A   
   C   
   A   
   A   
   A   
   G   
   A   
   G   
   A   
   A   
   T   
   G   
   T   
   C   
   A   
   A   
   A   
   A   
   A   
   A   
   G   
   C   
   T   
   T   
   T   
   A   
   C   
   A   
   A   
   A   
   C   
   C   
   A   
   A   
   A   
   G   
   A   
   G   
   A   
   A   
   A   
   A   
   A   
   A   
   G   
   A   
   T   
   G   
   G   
   T   
   C   
   A   
   A   
   A   
   C   
   G   
   A   
   T   
   T   
   A   
   A   
   A   
   T   
   T   
   A   
   A   
   C   
   G   
   A   
   T   
   T   
   G   
   A   
   C   
   A   
   G   
   T   
   G   
   G   
   C   
   G   
   T   
   A   
   C   
   A   
   A   
   C   
   A   
   A   
   C   
   A   
   A   
   G   
   C   
   C   
   T   
   T   
   C   
   G   
   C   
   T   
   A   
   T   
   A   
   T   
   T   
   T   
   G   
   A   
   C   
   A   
   A   
   A   
   C   
   G   
   T   
   C   
   C   
   T   
   G   
   G   
   T   
   A   
   G   
   T   
   G   
   C   
   G   
   G   
   T   
   C   
   A   
   T   
   T   
   A   
   C   
   C   
   G   
   A   
   T   
   C   
   C   
   T   
   C   
   A   
   A   
   A   
   A   
   A   
   G   
   G   
   C   
   G   
   A   
   T   
   T   
   T   
   A   
   T   
   T   
   A   
   G   
   C   
   A   
   A   
   C   
   G   
   G   
   T   
   T   
   A   
   G   
   T   
   T   
   C   
   A   
   C   
   C   
   T   
   T   
   C   
   G   
   T   
   A   
   T   
   G   
   A   
   T   
   C   
   C   
   T   
   A   
   A   
   T   
   A   
   A   
   A   
   A   
   T   
   G   
   G   
   C   
   C   
   A   
   A   
   T   
   G   
   G   
   T   
   A   
   T   
   T   
   T   
   C   
   T   
   C   
   A   
   A   
   A   
   A   
   A   
   G   
   A   
   A   
   T   
   A   
   T   
   G   
   A   
   T   
   G   
   C   
   T   
   T   
   A   
   C   
   A   
   A   
   T   
   A   
   A   
   C   
   A   
   A   
   C   
   A   
   A   
   A   
   G   
   A   
   T   
   T   
   T   
   A   
   C   
   C   
   A   
   T   
   T   
   C   
   A   
   C   
   A   
   G   
   C   
   A   
   C   
   G   
   T   
   T   
   T   
   T   
   G   
   C   
   G   
   A   
   C   
   A   
   G   
   G   
   C   
   T   
   A   
   T   
   G   
   C   
   A   
   C   
   C   
   A   
   G   
   G   
   C   
   T   
   C   
   T   
   A   
   C   
   C   
   T   
   T   
   T   
   A   
   A   
   A   
   A   
   C   
   A   
   A   
   T   
   T   
   A   
   C   
   C   
   G   
   G   
   T   
   G   
   C   
   C   
   A   
   T   
   T   
   G   
   G   
   T   
   T   
   T   
   A   
   G   
   A   
   T   
   G   
   C   
   A   
   G   
   G   
   G   
   A   
   C   
   A   
   T   
   T   
   G   
   A   
   A   
   G   
   C   
   C   
   A   
   G   
   A   
   T   
   G   
   A   
   A   
   G   
   A   
   G   
   C   
   T   
   A   
   G   
   A   
   A   
   A   
   T   
   T   
   A   
   A   
   T   
   G   
   G   
   C   
   T   
   T   
   G   
   A   
   A   
   A   
   T   
   G   
   G   
   C   
   A   
   A   
   A   
   A   
   A   
   G   
   A   
   T   
   A   
   A   
   A   
   T   
   C   
   T   
   T   
   G   
   G   
   G   
   G   
   C   
   G   
   G   
   C   
   T   
   A   
   T   
   T   
   T   
   T   
   G   
   C   
   A   
   A   
   C   
   A   
   C   
   G   
   T   
   G   
   T   
   A   
   A   
   A   
   A   
   G   
   A   
   A   
   G   
   C   
   A   
   A   
   G   
   T   
   C   
   C   
   A   
   G   
   T   
   C   
   A   
   A   
   T   
   C   
   T   
   G   
   C   
   G   
   T   
   A   
   C   
   C   
   G   
   C   
   T   
   T   
   T   
   A   
   G   
   T   
   T   
   A   
   A   
   T   
   T   
   C   
   A   
   G   
   A   
   T   
   A   
   A   
   T   
   A   
   T   
   T   
   T   
   A   
   T   
   T   
   T   
   T   
   G   
   C   
   C   
   C   
   A   
   A   
   C   
   A   
   A   
   A   
   C   
   A   
   T   
   T   
   A   
   C   
   G   
   G   
   A   
   T   
   G   
   G   
   G   
   G   
   G   
   A   
   A   
   G   
   A   
   C   
   A   
   A   
   A   
   T   
   T   
   T   
   A   
   G   
   A   
   G   
   C   
   A   
   G   
   G   
   T   
   C   
   T   
   T   
   A   
   A   
   T   
   A   
   A   
   A   
   T   
   T   
   C   
   A   
   T   
   T   
   T   
   T   
   C   
   G   
   G   
   T   
   G   
   A   
   G   
   G   
   A   
   A   
   C   
   T   
   A   
   G   
   A   
   T   
   T   
   T   
   A   
   C   
   C   
   A   
   A   
   T   
   T   
   G   
   C   
   C   
   A   
   T   
   G   
   A   
   C   
   G   
   C   
   C   
   T   
   G   
   C   
   T   
   C   
   A   
   A   
   A   
   T   
   T   
   T   
   C   
   A   
   A   
   A   
   T   
   G   
   A   
   A   
   G   
   A   
   T   
   A   
   A   
   A   
   T   
   T   
   T   
   A   
   A   
   T   
   T   
   C   
   T   
   G   
   A   
   A   
   A   
   T   
   T   
   C   
   T   
   T   
   T   
   T   
   A   
   G   
   C   
   A   
   G   
   A   
   T   
   A   
   C   
   G   
   G   
   G   
   C   
   T   
   A   
   C   
   G   
   G   
   C   
   C   
   A   
   A   
   G   
   G   
   C   
   C   
   A   
   A   
   T   
   T   
   G   
   T   
   T   
   A   
   A   
   T   
   T   
   T   
   C   
   G   
   C   
   C   
   A   
   A   
   T   
   C   
   C   
   A   
   G   
   C   
   A   
   A   
   G   
   C   
   T   
   A   
   C   
   C   
   A   
   T   
   G   
   T   
   A   
   T   
   A   
   G   
   T   
   G   
   T   
   T   
   T   
   T   
   C   
   C   
   A   
   A   
   A   
   A   
   T   
   A   
   A   
   T   
   G   
   G   
   A   
   A   
   C   
   A   
   T   
   T   
   A   
   G   
   T   
   C   
   T   
   A   
   T   
   C   
   C   
   A   
   A   
   A   
   A   
   T   
   T   
   A   
   G   
   T   
   T   
   T   
   T   
   A   
   G   
   A   
   C   
   A   
   A   
   A   
   G   
   A   
   A   
   A   
   C   
   G   
   A   
   A   
   G   
   A   
   A   
   A   
   A   
   A   
   A   
   G   
   A   
   C   
   A   
   A   
   T   
   G   
   T   
   A   
   A   
   T   
   C   
   A   
   G   
   C   
   G   
   C   
   C   
   A   
   A   
   C   
   G   
   C   
   A   
   G   
   C   
   G   
   A   
   A   
   T   
   A   
   C   
   A   
   A   
   T   
   T   
   G   
   C   
   C   
   A   
   C   
   A   
   G   
   A   
   T   
   C   
   T   
   T   
   T   
   T   
   A   
   G   
   G   
   T   
   A   
   G   
   T   
   G   
   T   
   G   
   G   
   A   
   A   
   G   
   A   
   T   
   C   
   C   
   T   
   T   
   C   
   A   
   G   
   G   
   T   
   T   
   A   
   T   
   G   
   T   
   T   
   T   
   A   
   T   
   A   
   A   
   T   
   A   
   T   
   G   
   T   
   A   
   C   
   A   
   A   
   T   
   C   
   C   
   G   
   A   
   A   
   C   
   T   
   T   
   C   
   T   
   C   
   A   
   T   
   T   
   G   
   G   
   C   
   T   
   G   
   C   
   T   
   A   
   A   
   A   
   A   
   C   
   A   
   G   
   G   
   A   
   A   
   C   
   A   
   G   
   C   
   T   
   G   
   A   
   A   
   A   
   T   
   A   
   A   
   A   
   A   
   G   
   A   
   T   
   A   
   A   
   A   
   C   
   A   
   A   
   G   
   A   
   T   
   A   
   C   
   A   
   G   
   A   
   T   
   G   
   G   
   C   
   A   
   A   
   A   
   G   
   A   
   A   
   A   
   A   
   C   
   A   
   G   
   T   
   T   
   T   
   C   
   C   
   T   
   C   
   C   
   T   
   A   
   A   
   C   
   G   
   T   
   T   
   A   
   G   
   A   
   T   
   C   
   G   
   T   
   A   
   G   
   T   
   A   
   A   
   C   
   A   
   A   
   T   
   A   
   A   
   A   
   T   
   T   
   C   
   T   
   T   
   A   
   A   
   C   
   A   
   A   
   T   
   G   
   A   
   T   
   T   
   A   
   T   
   G   
   G   
   T   
   T   
   G   
   A   
   A   
   A   
   A   
   T   
   T   
   C   
   A   
   G   
   G   
   A   
   G   
   A   
   A   
   A   
   A   
   T   
   G   
   G   
   T   
   T   
   C   
   T   
   G   
   C   
   A   
   A   
   C   
   A   
   G   
   A   
   T   
   A   
   T   
   C   
   A   
   G   
   T   
   A   
   A   
   A   
   C   
   C   
   A   
   T   
   T   
   A   
   A   
   T   
   T   
   G   
   A   
   T   
   T   
   A   
   C   
   T   
   T   
   A   
   G   
   A   
   A   
   G   
   C   
   A   
   A   
   C   
   C   
   A   
   T   
   T   
   A   
   A   
   A   
   -   
   -   
   -   
   -   
   -   
   -   
   -   
   -   
   -   
   -   
   -   
   -   
   -   
   -   
   -   
   -   
   -   
   -   
   -   
   -   
   -   
   -   
   -   
   -   
   -   
   -   
   -   
   -   
   -   
   -   
   -   
   -   
   -   
   -   
   -   
   -   
   -   
   -   
   -   
   -   
   -   
   -   
   -   
   -   
   -   
   -   
   -   
   -   
   -   
   -   
   -   
   -   
   -   
   -   
   -   
   -   
   -   
   -   
   -   
   -   
   -   
 

 
   N59505F   
   -   
   -   
   -   
   -   
   -   
   -   
   -   
   -   
   -   
   -   
   -   
   -   
   -   
   -   
   -   
   -   
   -   
   -   
   -   
   -   
   -   
   -   
   -   
   -   
   -   
   -   
   -   
   -   
   -   
   -   
   -   
   -   
   -   
   -   
   -   
   -   
   -   
   -   
   -   
   -   
   -   
   -   
   -   
   -   
   -   
   -   
   -   
   -   
   -   
   -   
   -   
   -   
   -   
   -   
   -   
   -   
   -   
   -   
   -   
   -   
   -   
   -   
   -   
   -   
   -   
   -   
   -   
   -   
   -   
   -   
   -   
   -   
   -   
   -   
   -   
   -   
   -   
   -   
   -   
   -   
   -   
   -   
   -   
   -   
   -   
   -   
   -   
   -   
   -   
   -   
   -   
   -   
   -   
   -   
   -   
   -   
   -   
   -   
   -   
   -   
   -   
   -   
   -   
   -   
   -   
   -   
   -   
   -   
   C   
   A   
   A   
   T   
   G   
   G   
   C   
   A   
   A   
   G   
   C   
   C   
   A   
   A   
   A   
   C   
   A   
   A   
   G   
   A   
    A    
   T   
   T   
   A   
   G   
   C   
    C    
   G   
   A   
   A   
   G   
   C   
   G   
   A   
   A   
   G   
   A   
   A   
   A   
   A   
    C    
   A   
   G   
   C   
    T    
   A   
    A    
   T   
    A    
    C    
   A   
   T   
   T   
   T   
   T   
   T   
   A   
   A   
   A   
    T    
   G   
   T   
   A   
   T   
   T   
   G   
    T    
   C   
   A   
   A   
   A   
   A   
   C   
   A   
   G   
    G    
   A   
   A   
   T   
   T   
   T   
   G   
   A   
   T   
   A   
   A   
   G   
   T   
   T   
   A   
   C   
   C   
    G    
   T   
   C   
   C   
   G   
   T   
   T   
    G    
   T   
   T   
   C   
   A   
   A   
   G   
   A   
   A   
    T    
   C   
   T   
   A   
   G   
   C   
   T   
   T   
   A   
   A   
   A   
   G   
   A   
   A   
   A   
   A   
   A   
   T   
   G   
   G   
    C    
   T   
   A   
   T   
   G   
   A   
   T   
   A   
   C   
   T   
   A   
   A   
   A   
   T   
   C   
    T    
   G   
   T   
   T   
   G   
   T   
   T   
   G   
   A   
   A   
   A   
   A   
   A   
   T   
   A   
   C   
   C   
   A   
   A   
   G   
   C   
   A   
   A   
   T   
   T   
   T   
   A   
   T   
   T   
   C   
   A   
   G   
   G   
   G   
   A   
   T   
   T   
   C   
   A   
   A   
   G   
   C   
    A    
   G   
   A   
   A   
    G    
   G   
    A    
   G   
   T   
   C   
   A   
   A   
    A    
   G   
   C   
   T   
   A   
   G   
   T   
   G   
   A   
   T   
   G   
   T   
    C    
   C   
   A   
   A   
   G   
   T   
    C    
   A   
   A   
   A   
   A   
   A   
    G    
   G   
    C    
   G   
   A   
   A   
   A   
   G   
   A   
    C    
   A   
   A   
    T    
   C   
   A   
   A   
   T   
   A   
   C   
   A   
   C   
   A   
   T   
   T   
   T   
   A   
    C    
   C   
   T   
   A   
   T   
   A   
   A   
   A   
   T   
   T   
   A   
   T   
   C   
   G   
   A   
   T   
   G   
   A   
   G   
    C    
   A   
   C   
    T    
   C   
   C   
   T   
   T   
   T   
   A   
   G   
   G   
   C   
   G   
   A   
   A   
   A   
   T   
   G   
   A   
   A   
   A   
   G   
   A   
   T   
    C    
   T   
   G   
   T   
   C   
   T   
   T   
   A   
   T   
   C   
   A   
   A   
   T   
   C   
   A   
   A   
   G   
   T   
   A   
   T   
    C    
   G   
    C    
   C   
   A   
   A   
    A    
   A   
   A   
   A   
   G   
   G   
    C    
   G   
   A   
   T   
   A   
    C    
   C   
   T   
   A   
   C   
   C   
   A   
   A   
   A   
   T   
    C    
    T    
   C   
   T   
   T   
   G   
   G   
    A    
    A    
    G    
   C   
   C   
    G    
   T   
   C   
   T   
   T   
   T   
   A   
   A   
   T   
   T   
   T   
   T   
   T   
   C   
   C   
   A   
   G   
   A   
   T   
   A   
   T   
   G   
   T   
   C   
   A   
   G   
   G   
   A   
   A   
   A   
   T   
   G   
   A   
   T   
   A   
   A   
   A   
    A    
   T   
   T   
   T   
   C   
   G   
   A   
   T   
   T   
   C   
   A   
   A   
   G   
   T   
   A   
   G   
   A   
   T   
   A   
   A   
   T   
   G   
   C   
   C   
   A   
   A   
   A   
   C   
   G   
   T   
   G   
   G   
   A   
   G   
   A   
   A   
   A   
   T   
   T   
   G   
   T   
   C   
   G   
   A   
   T   
   C   
   G   
   T   
   A   
   A   
    T    
   G   
   G   
   T   
   A   
   G   
   T   
   G   
   G   
   G   
   C   
   T   
   A   
   G   
    C    
   A   
   A   
   T   
   T   
   A   
   A   
   C   
   A   
   A   
   A   
   G   
   T   
   G   
   T   
   T   
   T   
   G   
   A   
    C    
   G   
   A   
   A   
   G   
   T   
   G   
   G   
   G   
   C   
   G   
   T   
   A   
   G   
   T   
    G    
   C   
   C   
   T   
    G    
   G   
   C   
   A   
   A   
   A   
   C   
   T   
    C    
   G   
   G   
   T   
   T   
   C   
   T   
   G   
   G   
   C   
   G   
   C   
   A   
   G   
   A   
   A   
   A   
   A   
   A   
    A    
   C   
   A   
   G   
   C   
    C    
   A   
   A   
   T   
   A   
   T   
   C   
   A   
   A   
   A   
   G   
   C   
   T   
   T   
   T   
   T   
   A   
   G   
   T   
   G   
   A   
   T   
   A   
   A   
    G    
   T   
   T   
    C    
   G   
   G   
   C   
   G   
   T   
   T   
   T   
   C   
   T   
    G    
   T   
   T   
   G   
   A   
   T   
   G   
   A   
   A   
   A   
   T   
   C   
   A   
   A   
   T   
   C   
    A    
   A   
   A   
   A   
    G    
   T   
   T   
   A   
   A   
   G   
   C   
   C   
   A   
   A   
   G   
   G   
    G    
   T   
   G   
   G   
   G   
   T   
   C   
   C   
   A   
   A   
   G   
   C   
   A   
   G   
   A   
   C   
   T   
   C   
    C    
   T   
   T   
   T   
   G   
   T   
   A   
   C   
   C   
   A   
   A   
   T   
   C   
   A   
   C   
   A   
   G   
   T   
    T    
   G   
   C   
   T   
   T   
   C   
   T   
   G   
   A   
   A   
   C   
   C   
   A   
   G   
   T   
   G   
   A   
   C   
   A   
   G   
   A   
   A   
   T   
   T   
   A   
   C   
   C   
   A   
   A   
   C   
   A   
   G   
   G   
   G   
   G   
   C   
   T   
   G   
   C   
   G   
   A   
   C   
   A   
   A   
   A   
   A   
   G   
    A    
    T    
   A   
   C   
   A   
   G   
   A   
   G   
   T   
   C   
   A   
   C   
   G   
   T   
   T   
   A   
   T   
   T   
   A   
   T   
   C   
   C   
    A    
    C    
   T   
   G   
   G   
   G   
   G   
   G   
   A   
   A   
   G   
   C   
   A   
   G   
   C   
   T   
   G   
   C   
   G   
   C   
   A   
   A   
   T   
   T   
   A   
   A   
   T   
   C   
   G   
   G   
    G    
   T   
   A   
   T   
   A   
   C   
    G    
   G   
   G   
    C    
   A   
   C   
    C    
   A   
   T   
   T   
   A   
   C   
   T   
   G   
   C   
   A   
   G   
   A   
    A    
   G   
   A   
   T   
   A   
   T   
   T   
    G    
   A   
   A   
   A   
   A   
   A   
   A   
   A   
   T   
   C   
   C   
   A   
   G   
   A   
   G   
   T   
   T   
   A   
   A   
   G   
   T   
   A   
   G   
   T   
   A   
   C   
   G   
   G   
   G   
    T    
   G   
   T   
   A   
   A   
   T   
   T   
   G   
   G   
    T    
   A   
   A   
   A   
   A   
   C   
   T   
   G   
   G   
    C    
   T   
   T   
   A   
   G   
   A   
   A   
   C   
   G   
   A   
   G   
   C   
   G   
   T   
   T   
   T   
   G   
   A   
   T   
   A   
   A   
   A   
   G   
   A   
    G    
   T   
   T   
   A   
   A   
   G   
   A   
   G   
   G   
    G    
   C   
   A   
   A   
   G   
   A   
   T   
   G   
   G   
    T    
   G   
   G   
    C    
   T   
   C   
    A    
   C   
   T   
   A   
   G   
   T   
   T   
   A   
   T   
   T   
   T   
   T   
   A   
   G   
   A   
   T   
   G   
   A   
   C   
   A   
   A   
   A   
   G   
   A   
   G   
   A   
   A   
   T   
   G   
   T   
   C   
   A   
   A   
   A   
   A   
   A   
   A   
   G   
   C   
   T   
   T   
   T   
   A   
   C   
   A   
   A   
   A   
   C   
   C   
   A   
   A   
   A   
   G   
   A   
    G    
   A   
   A   
   A   
   A   
   A   
   A   
   G   
   A   
   T   
   G   
   G   
   T   
   C   
   A   
   A   
   A   
   C   
    G    
   A   
   T   
   T   
   A   
   A   
   A   
   T   
   T   
   A   
   A   
   C   
   G   
   A   
   T   
   T   
   G   
   A   
    C    
   A   
   G   
    T    
   G   
   G   
    C    
   G   
   T   
   A   
   C   
   A   
   A   
   C   
   A   
   A   
   C   
   A   
   A   
   G   
   C   
    C    
   T   
   T   
    C    
   G   
   C   
   T   
   A   
   T   
   A   
   T   
   T   
   T   
   G   
   A   
   C   
   A   
   A   
   A   
   C   
   G   
    T    
    C    
   C   
   T   
   G   
   G   
   T   
   A   
   G   
   T   
   G   
   C   
   G   
   G   
   T   
   C   
   A   
   T   
   T   
   A   
   C   
   C   
   G   
   A   
   T   
   C   
   C   
   T   
   C   
   A   
   A   
   A   
   A   
   A   
   G   
   G   
   C   
   G   
   A   
   T   
   T   
   T   
   A   
    T    
   T   
   A   
   G   
   C   
    A    
   A   
   C   
   G   
   G   
   T   
   T   
   A   
   G   
   T   
   T   
   C   
   A   
   C   
   C   
   T   
   T   
   C   
   G   
   T   
   A   
   T   
   G   
   A   
   T   
   C   
   C   
   T   
   A   
   A   
   T   
   A   
   A   
   A   
   A   
   T   
   G   
   G   
   C   
   C   
   A   
   A   
   T   
   G   
   G   
    T    
   A   
   T   
   T   
   T   
   C   
   T   
   C   
   A   
   A   
   A   
   A   
   A   
   G   
   A   
    A    
   T   
   A   
   T   
   G   
   A   
   T   
   G   
   C   
   T   
   T   
   A   
   C   
   A   
   A   
   T   
   A   
   A   
   C   
   A   
   A   
   C   
   A   
   A   
   A   
   G   
   A   
   T   
   T   
   T   
   A   
   C   
   C   
   A   
   T   
   T   
    C    
   A   
   C   
   A   
   G   
   C   
   A   
   C   
   G   
   T   
   T   
   T   
    T    
   G   
   C   
   G   
   A   
   C   
   A   
   G   
   G   
    C    
   T   
   A   
   T   
   G   
   C   
   A   
   C   
   C   
   A   
   G   
   G   
    C    
   T   
   C   
   T   
   A   
   C   
   C   
   T   
   T   
   T   
   A   
   A   
   A   
   A   
   C   
   A   
   A   
   T   
   T   
   A   
   C   
   C   
   G   
   G   
   T   
   G   
   C   
   C   
   A   
   T   
   T   
   G   
   G   
   T   
   T   
   T   
   A   
   G   
   A   
   T   
    G    
   C   
   A   
   G   
   G   
   G   
   A   
   C   
   A   
   T   
   T   
   G   
   A   
   A   
   G   
   C   
   C   
   A   
   G   
   A   
   T   
   G   
   A   
   A   
   G   
   A   
   G   
   C   
   T   
   A   
   G   
   A   
   A   
   A   
   T   
   T   
   A   
   A   
   T   
   G   
   G   
    C    
   T   
   T   
   G   
   A   
   A   
   A   
   T   
   G   
   G   
   C   
   A   
   A   
   A   
   A   
   A   
   G   
   A   
   T   
   A   
   A   
   A   
   T   
   C   
   T   
   T   
   G   
   G   
   G   
   G   
    C    
   G   
   G   
   C   
   T   
   A   
   T   
   T   
   T   
   T   
   G   
   C   
    A    
   A   
   C   
   A   
   C   
   G   
   T   
   G   
   T   
   A   
   A   
   A   
   A   
   G   
   A   
   A   
   G   
   C   
   A   
   A   
   G   
   T   
   C   
   C   
   A   
   G   
   T   
   C   
   A   
   A   
   T   
   C   
   T   
    G    
   C   
   G   
   T   
   A   
   C   
   C   
   G   
   C   
   T   
   T   
   T   
   A   
   G   
   T   
   T   
   A   
   A   
   T   
   T   
   C   
   A   
   G   
   A   
   T   
   A   
   A   
   T   
   A   
   T   
   T   
   T   
   A   
   T   
   T   
   T   
   T   
   G   
   C   
    C    
   C   
   A   
   A   
   C   
   A   
   A   
   A   
   C   
   A   
   T   
   T   
   A   
   C   
   G   
    G    
   A   
   T   
   G   
   G   
   G   
    G    
   G   
   A   
   A   
   G   
   A   
   C   
   A   
   A   
   A   
   T   
   T   
   T   
   A   
   G   
   A   
   G   
   C   
    A    
   G   
   G   
   T   
   C   
   T   
   T   
   A   
   A   
   T   
   A   
   A   
   A   
   T   
   T   
   C   
   A   
   T   
   T   
   T   
   T   
   C   
   G   
   G   
   T   
   G   
   A   
   G   
   G   
   A   
   A   
   C   
   T   
   A   
   G   
   A   
   T   
   T   
   T   
   A   
   C   
   C   
   A   
   A   
   T   
    T    
   G   
   C   
   C   
   A   
   T   
   G   
   A   
   C   
   G   
   C   
   C   
    T    
   G   
   C   
   T   
   C   
   A   
   A   
   A   
   T   
   T   
   T   
   C   
   A   
   A   
   A   
    T    
   G   
   A   
   A   
   G   
   A   
   T   
   A   
   A   
   A   
   T   
   T   
   T   
   A   
   A   
   T   
   T   
   C   
   T   
   G   
   A   
   A   
   A   
   T   
   T   
   C   
   T   
   T   
   T   
   T   
    A    
   G   
   C   
   A   
   G   
   A   
   T   
   A   
   C   
    G    
   G   
   G   
   C   
   T   
   A   
    C    
   G   
   G   
    C    
   C   
   A   
   A   
   G   
   G   
    C    
   C   
   A   
   A   
   T   
   T   
   G   
   T   
   T   
   A   
   A   
   T   
   T   
   T   
   C   
    G    
   C   
   C   
   A   
   A   
   T   
   C   
   C   
   A   
   G   
   C   
   A   
   A   
   G   
   C   
   T   
   A   
   C   
   C   
   A   
   T   
   G   
   T   
   A   
   T   
   A   
   G   
   T   
   G   
   T   
   T   
   T   
   T   
   C   
   C   
   A   
   A   
   A   
   A   
   T   
   A   
   A   
   T   
   G   
   G   
    A    
   A   
   C   
   A   
    T    
   T   
    A    
   G   
   T   
    C    
   T   
   A   
   T   
   C   
   C   
   A   
   A   
   A   
   A   
    T    
   T   
   A   
   G   
   T   
   T   
   T   
   T   
   A   
   G   
   A   
   C   
   A   
   A   
   A   
   G   
   A   
   A   
   A   
   C   
    G    
   A   
   A   
   G   
   A   
   A   
   A   
   A   
   A   
   A   
   G   
   A   
   C   
   A   
   A   
   T   
   G   
   T   
   A   
   A   
   T   
   C   
   A   
   G   
   C   
   G   
   C   
   C   
   A   
   A   
   C   
   G   
   C   
   A   
   G   
   C   
   G   
   A   
   A   
   T   
   A   
   C   
   A   
   A   
   T   
   T   
   G   
   C   
   C   
   A   
   C   
   A   
   G   
   A   
   T   
   C   
   T   
   T   
   T   
   T   
   A   
   G   
   G   
   T   
   A   
   G   
   T   
   G   
   T   
   G   
   G   
   A   
   A   
   G   
   A   
   T   
   C   
   C   
   T   
   T   
   C   
   A   
   G   
   G   
   T   
   T   
   A   
   T   
   G   
   T   
   T   
   T   
   A   
   T   
   A   
   A   
   T   
   A   
   T   
   G   
   T   
   A   
   C   
   A   
   A   
   T   
   C   
   C   
   G   
   A   
   A   
   C   
   T   
   T   
   C   
   T   
   C   
   A   
   T   
   T   
   G   
   G   
   C   
   T   
   G   
   C   
   T   
   A   
   A   
   A   
   A   
   C   
   A   
   G   
   G   
   A   
   A   
   C   
   A   
   G   
   C   
   T   
   G   
   A   
   A   
   A   
   T   
   A   
   A   
   A   
   A   
   G   
   A   
   T   
   A   
   A   
   A   
   C   
   A   
   A   
   G   
   A   
   T   
   A   
   C   
   A   
   G   
   A   
   T   
   G   
   G   
   C   
   A   
   A   
   A   
   G   
   A   
   A   
   A   
   A   
   C   
   A   
   G   
   T   
   T   
   T   
   C   
   C   
   T   
   C   
   C   
   T   
   A   
   A   
   C   
   G   
   T   
   T   
   A   
   G   
   A   
   T   
   C   
   G   
   T   
   A   
   G   
   T   
   A   
   A   
   C   
   A   
   A   
   T   
   A   
   A   
   A   
   T   
   T   
   C   
   T   
   T   
   A   
   A   
   C   
   A   
   A   
   T   
   G   
   A   
   T   
   T   
   A   
   T   
   G   
   G   
   T   
   T   
   G   
   A   
   A   
   A   
   A   
   T   
   T   
   C   
   A   
   G   
   G   
   A   
   G   
   A   
   A   
   A   
   A   
   T   
   G   
   G   
   T   
   T   
   C   
   T   
   G   
   C   
   A   
   A   
   C   
   A   
   G   
   A   
   T   
   A   
   T   
   C   
   A   
   G   
   T   
   A   
   A   
   A   
   C   
   C   
   A   
   T   
   T   
   A   
   A   
   T   
   T   
   G   
   A   
   T   
   T   
   A   
   C   
   T   
   T   
   A   
   G   
   A   
   A   
   G   
   C   
   A   
   A   
   C   
   C   
   A   
   T   
   T   
   A   
   A   
   A   
   -   
   -   
   -   
   -   
   -   
   -   
   -   
   -   
   -   
   -   
   -   
   -   
   -   
   -   
   -   
   -   
   -   
   -   
   -   
   -   
   -   
   -   
   -   
   -   
   -   
   -   
   -   
   -   
   -   
   -   
   -   
   -   
   -   
   -   
   -   
   -   
   -   
   -   
   -   
   -   
   -   
   -   
   -   
   -   
   -   
   -   
   -   
   -   
   -   
   -   
   -   
   -   
   -   
   -   
   -   
   -   
   -   
   -   
   -   
   -   
   -   
   -   
   -   
   -   
   -   
   -   
   -   
   -   
   -   
   -   
   -   
   -   
   -   
   -   
   -   
   -   
   -   
   -   
   -   
   -   
   -   
   -   
   -   
   -   
   -   
   -   
   -   
   -   
   -   
   -   
   -   
   -   
   -   
   -   
   -   
   -   
   -   
   -   
   -   
   -   
   -   
   -   
   -   
   -   
   -   
   -   
   -   
   -   
   -   
   -   
   -   
   -   
   -   
   -   
   -   
   -   
   -   
   -   
   -   
   -   
   -   
   -   
   -   
   -   
   -   
   -   
   -   
   -   
   -   
   -   
   -   
   -   
   -   
   -   
   -   
   -   
   -   
   -   
   -   
   -   
   -   
   -   
   -   
   -   
   -   
   -   
   -   
   -   
   -   
   -   
   -   
   -   
   -   
   -   
   -   
   -   
   -   
   -   
   -   
   -   
   -   
   -   
   -   
   -   
   -   
   -   
   -   
   -   
   -   
   C   
   A   
   A   
   T   
   G   
   G   
   C   
   A   
   A   
   G   
   C   
   C   
   A   
   A   
   A   
   C   
   A   
   A   
   G   
   A   
   A   
   T   
   T   
   A   
   G   
   C   
   C   
   G   
   A   
   A   
   G   
   C   
   G   
   A   
   A   
   G   
   A   
   A   
   A   
   A   
   C   
   A   
   G   
   C   
   T   
   A   
   A   
   T   
   A   
   C   
   A   
   T   
   T   
   T   
   T   
   T   
   A   
   A   
   A   
   T   
   G   
   T   
   A   
   T   
   T   
   G   
   T   
   C   
   A   
   A   
   A   
   A   
   C   
   A   
   G   
   G   
   A   
   A   
   T   
   T   
   T   
   G   
   A   
   T   
   A   
   A   
   G   
   T   
   T   
   A   
   C   
   C   
   G   
   T   
   C   
   C   
   G   
   T   
   T   
   G   
   T   
   T   
   C   
   A   
   A   
   G   
   A   
   A   
   T   
   C   
   T   
   A   
   G   
   C   
   T   
   T   
   A   
   A   
   A   
   G   
   A   
   A   
   A   
   A   
   A   
   T   
   G   
   G   
   C   
   T   
   A   
   T   
   G   
   A   
   T   
   A   
   C   
   T   
   A   
   A   
   A   
   T   
   C   
   T   
   G   
   T   
   T   
   G   
   T   
   T   
   G   
   A   
   A   
   A   
   A   
   A   
   T   
   A   
   C   
   C   
   A   
   A   
   G   
   C   
   A   
   A   
   T   
   T   
   T   
   A   
   T   
   T   
   C   
   A   
   G   
   G   
   G   
   A   
   T   
   T   
   C   
   A   
   A   
   G   
   C   
   A   
   G   
   A   
   A   
   G   
   G   
   A   
   G   
   T   
   C   
   A   
   A   
   A   
   G   
   C   
   T   
   A   
   G   
   T   
   G   
   A   
   T   
   G   
   T   
   C   
   C   
   A   
   A   
   G   
   T   
   C   
   A   
   A   
   A   
   A   
   A   
   G   
   G   
   C   
   G   
   A   
   A   
   A   
   G   
   A   
   C   
   A   
   A   
   T   
   C   
   A   
   A   
   T   
   A   
   C   
   A   
   C   
   A   
   T   
   T   
   T   
   A   
   C   
   C   
   T   
   A   
   T   
   A   
   A   
   A   
   T   
   T   
   A   
   T   
   C   
   G   
   A   
   T   
   G   
   A   
   G   
   C   
   A   
   C   
   T   
   C   
   C   
   T   
   T   
   T   
   A   
   G   
   G   
   C   
   G   
   A   
   A   
   A   
   T   
   G   
   A   
   A   
   A   
   G   
   A   
   T   
   C   
   T   
   G   
   T   
   C   
   T   
   T   
   A   
   T   
   C   
   A   
   A   
   T   
   C   
   A   
   A   
   G   
   T   
   A   
   T   
   C   
   G   
   C   
   C   
   A   
   A   
   A   
   A   
   A   
   A   
   G   
   G   
   C   
   G   
   A   
   T   
   A   
   C   
   C   
   T   
   A   
   C   
   C   
   A   
   A   
   A   
   T   
   C   
   T   
   C   
   T   
   T   
   G   
   G   
   A   
   A   
   G   
   C   
   C   
   G   
   T   
   C   
   T   
   T   
   T   
   A   
   A   
   T   
   T   
   T   
   T   
   T   
   C   
   C   
   A   
   G   
   A   
   T   
   A   
   T   
   G   
   T   
   C   
   A   
   G   
   G   
   A   
   A   
   A   
   T   
   G   
   A   
   T   
   A   
   A   
   A   
   A   
   T   
   T   
   T   
   C   
   G   
   A   
   T   
   T   
   C   
   A   
   A   
   G   
   T   
   A   
   G   
   A   
   T   
   A   
   A   
   T   
   G   
   C   
   C   
   A   
   A   
   A   
   C   
   G   
   T   
   G   
   G   
   A   
   G   
   A   
   A   
   A   
   T   
   T   
   G   
   T   
   C   
   G   
   A   
   T   
   C   
   G   
   T   
   A   
   A   
   T   
   G   
   G   
   T   
   A   
   G   
   T   
   G   
   G   
   G   
   C   
   T   
   A   
   G   
   C   
   A   
   A   
   T   
   T   
   A   
   A   
   C   
   A   
   A   
   A   
   G   
   T   
   G   
   T   
   T   
   T   
   G   
   A   
   C   
   G   
   A   
   A   
   G   
   T   
   G   
   G   
   G   
   C   
   G   
   T   
   A   
   G   
   T   
   G   
   C   
   C   
   T   
   G   
   G   
   C   
   A   
   A   
   A   
   C   
   T   
   C   
   G   
   G   
   T   
   T   
   C   
   T   
   G   
   G   
   C   
   G   
   C   
   A   
   G   
   A   
   A   
   A   
   A   
   A   
   A   
   C   
   A   
   G   
   C   
   C   
   A   
   A   
   T   
   A   
   T   
   C   
   A   
   A   
   A   
   G   
   C   
   T   
   T   
   T   
   T   
   A   
   G   
   T   
   G   
   A   
   T   
   A   
   A   
   G   
   T   
   T   
   C   
   G   
   G   
   C   
   G   
   T   
   T   
   T   
   C   
   T   
   G   
   T   
   T   
   G   
   A   
   T   
   G   
   A   
   A   
   A   
   T   
   C   
   A   
   A   
   T   
   C   
   A   
   A   
   A   
   A   
   G   
   T   
   T   
   A   
   A   
   G   
   C   
   C   
   A   
   A   
   G   
   G   
   G   
   T   
   G   
   G   
   G   
   T   
   C   
   C   
   A   
   A   
   G   
   C   
   A   
   G   
   A   
   C   
   T   
   C   
   C   
   T   
   T   
   T   
   G   
   T   
   A   
   C   
   C   
   A   
   A   
   T   
   C   
   A   
   C   
   A   
   G   
   T   
   T   
   G   
   C   
   T   
   T   
   C   
   T   
   G   
   A   
   A   
   C   
   C   
   A   
   G   
   T   
   G   
   A   
   C   
   A   
   G   
   A   
   A   
   T   
   T   
   A   
   C   
   C   
   A   
   A   
   C   
   A   
   G   
   G   
   G   
   G   
   C   
   T   
   G   
   C   
   G   
   A   
   C   
   A   
   A   
   A   
   A   
   G   
   A   
   T   
   A   
   C   
   A   
   G   
   A   
   G   
   T   
   C   
   A   
   C   
   G   
   T   
   T   
   A   
   T   
   T   
   A   
   T   
   C   
   C   
   A   
   C   
   T   
   G   
   G   
   G   
   G   
   G   
   A   
   A   
   G   
   C   
   A   
   G   
   C   
   T   
   G   
   C   
   G   
   C   
   A   
   A   
   T   
   T   
   A   
   A   
   T   
   C   
   G   
   G   
   G   
   T   
   A   
   T   
   A   
   C   
   G   
   G   
   G   
   C   
   A   
   C   
   C   
   A   
   T   
   T   
   A   
   C   
   T   
   G   
   C   
   A   
   G   
   A   
   A   
   G   
   A   
   T   
   A   
   T   
   T   
   G   
   A   
   A   
   A   
   A   
   A   
   A   
   A   
   T   
   C   
   C   
   A   
   G   
   A   
   G   
   T   
   T   
   A   
   A   
   G   
   T   
   A   
   G   
   T   
   A   
   C   
   G   
   G   
   G   
   T   
   G   
   T   
   A   
   A   
   T   
   T   
   G   
   G   
   T   
   A   
   A   
   A   
   A   
   C   
   T   
   G   
   G   
   C   
   T   
   T   
   A   
   G   
   A   
   A   
   C   
   G   
   A   
   G   
   C   
   G   
   T   
   T   
   T   
   G   
   A   
   T   
   A   
   A   
   A   
   G   
   A   
   G   
   T   
   T   
   A   
   A   
   G   
   A   
   G   
   G   
   G   
   C   
   A   
   A   
   G   
   A   
   T   
   G   
   G   
   T   
   G   
   G   
   C   
   T   
   C   
   A   
   C   
   T   
   A   
   G   
   T   
   T   
   A   
   T   
   T   
   T   
   T   
   A   
   G   
   A   
   T   
   G   
   A   
   C   
   A   
   A   
   A   
   G   
   A   
   G   
   A   
   A   
   T   
   G   
   T   
   C   
   A   
   A   
   A   
   A   
   A   
   A   
   G   
   C   
   T   
   T   
   T   
   A   
   C   
   A   
   A   
   A   
   C   
   C   
   A   
   A   
   A   
   G   
   A   
   G   
   A   
   A   
   A   
   A   
   A   
   A   
   G   
   A   
   T   
   G   
   G   
   T   
   C   
   A   
   A   
   A   
   C   
   G   
   A   
   T   
   T   
   A   
   A   
   A   
   T   
   T   
   A   
   A   
   C   
   G   
   A   
   T   
   T   
   G   
   A   
   C   
   A   
   G   
   T   
   G   
   G   
   C   
   G   
   T   
   A   
   C   
   A   
   A   
   C   
   A   
   A   
   C   
   A   
   A   
   G   
   C   
   C   
   T   
   T   
   C   
   G   
   C   
   T   
   A   
   T   
   A   
   T   
   T   
   T   
   G   
   A   
   C   
   A   
   A   
   A   
   C   
   G   
   T   
   C   
   C   
   T   
   G   
   G   
   T   
   A   
   G   
   T   
   G   
   C   
   G   
   G   
   T   
   C   
   A   
   T   
   T   
   A   
   C   
   C   
   G   
   A   
   T   
   C   
   C   
   T   
   C   
   A   
   A   
   A   
   A   
   A   
   G   
   G   
   C   
   G   
   A   
   T   
   T   
   T   
   A   
   T   
   T   
   A   
   G   
   C   
   A   
   A   
   C   
   G   
   G   
   T   
   T   
   A   
   G   
   T   
   T   
   C   
   A   
   C   
   C   
   T   
   T   
   C   
   G   
   T   
   A   
   T   
   G   
   A   
   T   
   C   
   C   
   T   
   A   
   A   
   T   
   A   
   A   
   A   
   A   
   T   
   G   
   G   
   C   
   C   
   A   
   A   
   T   
   G   
   G   
   T   
   A   
   T   
   T   
   T   
   C   
   T   
   C   
   A   
   A   
   A   
   A   
   A   
   G   
   A   
   A   
   T   
   A   
   T   
   G   
   A   
   T   
   G   
   C   
   T   
   T   
   A   
   C   
   A   
   A   
   T   
   A   
   A   
   C   
   A   
   A   
   C   
   A   
   A   
   A   
   G   
   A   
   T   
   T   
   T   
   A   
   C   
   C   
   A   
   T   
   T   
   C   
   A   
   C   
   A   
   G   
   C   
   A   
   C   
   G   
   T   
   T   
   T   
   T   
   G   
   C   
   G   
   A   
   C   
   A   
   G   
   G   
   C   
   T   
   A   
   T   
   G   
   C   
   A   
   C   
   C   
   A   
   G   
   G   
   C   
   T   
   C   
   T   
   A   
   C   
   C   
   T   
   T   
   T   
   A   
   A   
   A   
   A   
   C   
   A   
   A   
   T   
   T   
   A   
   C   
   C   
   G   
   G   
   T   
   G   
   C   
   C   
   A   
   T   
   T   
   G   
   G   
   T   
   T   
   T   
   A   
   G   
   A   
   T   
   G   
   C   
   A   
   G   
   G   
   G   
   A   
   C   
   A   
   T   
   T   
   G   
   A   
   A   
   G   
   C   
   C   
   A   
   G   
   A   
   T   
   G   
   A   
   A   
   G   
   A   
   G   
   C   
   T   
   A   
   G   
   A   
   A   
   A   
   T   
   T   
   A   
   A   
   T   
   G   
   G   
   C   
   T   
   T   
   G   
   A   
   A   
   A   
   T   
   G   
   G   
   C   
   A   
   A   
   A   
   A   
   A   
   G   
   A   
   T   
   A   
   A   
   A   
   T   
   C   
   T   
   T   
   G   
   G   
   G   
   G   
   C   
   G   
   G   
   C   
   T   
   A   
   T   
   T   
   T   
   T   
   G   
   C   
   A   
   A   
   C   
   A   
   C   
   G   
   T   
   G   
   T   
   A   
   A   
   A   
   A   
   G   
   A   
   A   
   G   
   C   
   A   
   A   
   G   
   T   
   C   
   C   
   A   
   G   
   T   
   C   
   A   
   A   
   T   
   C   
   T   
   G   
   C   
   G   
   T   
   A   
   C   
   C   
   G   
   C   
   T   
   T   
   T   
   A   
   G   
   T   
   T   
   A   
   A   
   T   
   T   
   C   
   A   
   G   
   A   
   T   
   A   
   A   
   T   
   A   
   T   
   T   
   T   
   A   
   T   
   T   
   T   
   T   
   G   
   C   
   C   
   C   
   A   
   A   
   C   
   A   
   A   
   A   
   C   
   A   
   T   
   T   
   A   
   C   
   G   
   G   
   A   
   T   
   G   
   G   
   G   
   G   
   G   
   A   
   A   
   G   
   A   
   C   
   A   
   A   
   A   
   T   
   T   
   T   
   A   
   G   
   A   
   G   
   C   
   A   
   G   
   G   
   T   
   C   
   T   
   T   
   A   
   A   
   T   
   A   
   A   
   A   
   T   
   T   
   C   
   A   
   T   
   T   
   T   
   T   
   C   
   G   
   G   
   T   
   G   
   A   
   G   
   G   
   A   
   A   
   C   
   T   
   A   
   G   
   A   
   T   
   T   
   T   
   A   
   C   
   C   
   A   
   A   
   T   
   T   
   G   
   C   
   C   
   A   
   T   
   G   
   A   
   C   
   G   
   C   
   C   
   T   
   G   
   C   
   T   
   C   
   A   
   A   
   A   
   T   
   T   
   T   
   C   
   A   
   A   
   A   
   T   
   G   
   A   
   A   
   G   
   A   
   T   
   A   
   A   
   A   
   T   
   T   
   T   
   A   
   A   
   T   
   T   
   C   
   T   
   G   
   A   
   A   
   A   
   T   
   T   
   C   
   T   
   T   
   T   
   T   
   A   
   G   
   C   
   A   
   G   
   A   
   T   
   A   
   C   
   G   
   G   
   G   
   C   
   T   
   A   
   C   
   G   
   G   
   C   
   C   
   A   
   A   
   G   
   G   
   C   
   C   
   A   
   A   
   T   
   T   
   G   
   T   
   T   
   A   
   A   
   T   
   T   
   T   
   C   
   G   
   C   
   C   
   A   
   A   
   T   
   C   
   C   
   A   
   G   
   C   
   A   
   A   
   G   
   C   
   T   
   A   
   C   
   C   
   A   
   T   
   G   
   T   
   A   
   T   
   A   
   G   
   T   
   G   
   T   
   T   
   T   
   T   
   C   
   C   
   A   
   A   
   A   
   A   
   T   
   A   
   A   
   T   
   G   
   G   
   A   
   A   
   C   
   A   
   T   
   T   
   A   
   G   
   T   
   C   
   T   
   A   
   T   
   C   
   C   
   A   
   A   
   A   
   A   
   T   
   T   
   A   
   G   
   T   
   T   
   T   
   T   
   A   
   G   
   A   
   C   
   A   
   A   
   A   
   G   
   A   
   A   
   A   
   C   
   G   
   A   
   A   
   G   
   A   
   A   
   A   
   A   
   A   
   A   
   G   
   A   
   C   
   A   
   A   
   T   
   G   
   T   
   A   
   A   
   T   
   C   
   A   
   G   
   C   
   G   
   C   
   C   
   A   
   A   
   C   
   G   
   C   
   A   
   G   
   C   
   G   
   A   
   A   
   T   
   A   
   C   
   A   
   A   
   T   
   T   
   G   
   C   
   C   
   A   
   C   
   A   
   G   
   A   
   T   
   C   
   T   
   T   
   T   
   T   
   A   
   G   
   G   
   T   
   A   
   G   
   T   
   G   
   T   
   G   
   G   
   A   
   A   
   G   
   A   
   T   
   C   
   C   
   T   
   T   
   C   
   A   
   G   
   G   
   T   
   T   
   A   
   T   
   G   
   T   
   T   
   T   
   A   
   T   
   A   
   A   
   T   
   A   
   T   
   G   
   T   
   A   
   C   
   A   
   A   
   T   
   C   
   C   
   G   
   A   
   A   
   C   
   T   
   T   
   C   
   T   
   C   
   A   
   T   
   T   
   G   
   G   
   C   
   T   
   G   
   C   
   T   
   A   
   A   
   A   
   A   
   C   
   A   
   G   
   G   
   A   
   A   
   C   
   A   
   G   
   C   
   T   
   G   
   A   
   A   
   A   
   T   
   A   
   A   
   A   
   A   
   G   
   A   
   T   
   A   
   A   
   A   
   C   
   A   
   A   
   G   
   A   
   T   
   A   
   C   
   A   
   G   
   A   
   T   
   G   
   G   
   C   
   A   
   A   
   A   
   G   
   A   
   A   
   A   
   A   
   C   
   A   
   G   
   T   
   T   
   T   
   C   
   C   
   T   
   C   
   C   
   T   
   A   
   A   
   C   
   G   
   T   
   T   
   A   
   G   
   A   
   T   
   C   
   G   
   T   
   A   
   G   
   T   
   A   
   A   
   C   
   A   
   A   
   T   
   A   
   A   
   A   
   T   
   T   
   C   
   T   
   T   
   A   
   A   
   C   
   A   
   A   
   T   
   G   
   A   
   T   
   T   
   A   
   T   
   G   
   G   
   T   
   T   
   G   
   A   
   A   
   A   
   A   
   T   
   T   
   C   
   A   
   G   
   G   
   A   
   G   
   A   
   A   
   A   
   A   
   T   
   G   
   G   
   T   
   T   
   C   
   T   
   G   
   C   
   A   
   A   
   C   
   A   
   G   
   A   
   T   
   A   
   T   
   C   
   A   
   G   
   T   
   A   
   A   
   A   
   C   
   C   
   A   
   T   
   T   
   A   
   A   
   T   
   T   
   G   
   A   
   T   
   T   
   A   
   C   
   T   
   T   
   A   
   G   
   A   
   A   
   G   
   C   
   A   
   A   
   C   
   C   
   A   
   T   
   T   
   A   
   A   
   A   
   -   
   -   
   -   
   -   
   -   
   -   
   -   
   -   
   -   
   -   
   -   
   -   
   -   
   -   
   -   
   -   
   -   
   -   
   -   
   -   
   -   
   -   
   -   
   -   
   -   
   -   
   -   
   -   
   -   
   -   
   -   
   -   
   -   
   -   
   -   
   -   
   -   
   -   
   -   
   -   
   -   
   -   
   -   
   -   
   -   
   -   
   -   
   -   
   -   
   -   
   -   
   -   
   -   
   -   
   -   
   -   
   -   
   -   
   -   
   -   
   -   
 

 
   N54553   
   -   
   -   
   -   
   -   
   -   
   -   
   -   
   -   
   -   
   -   
   -   
   -   
   -   
   -   
   -   
   -   
   -   
   -   
   -   
   -   
   -   
   -   
   -   
   -   
   -   
   -   
   -   
   -   
   -   
   -   
   -   
   -   
   -   
   -   
   -   
   -   
   -   
   -   
   -   
   -   
   -   
   -   
   -   
   -   
   -   
   -   
   -   
   -   
   -   
   -   
   -   
   -   
   -   
   -   
   -   
   -   
   -   
   -   
   -   
   -   
   -   
   -   
   -   
   -   
   -   
   -   
   -   
   -   
   -   
   -   
   -   
   -   
   -   
   -   
   -   
   -   
   -   
   -   
   -   
   -   
   -   
   -   
   -   
   -   
   -   
   -   
   -   
   -   
   -   
   -   
   -   
   -   
   -   
   -   
   -   
   -   
   -   
   -   
   -   
   -   
   -   
   -   
   -   
   -   
   -   
   -   
   -   
   -   
   C   
   A   
   A   
   T   
   G   
   G   
   C   
   A   
   A   
   G   
   C   
   C   
   A   
   A   
   A   
   C   
   A   
   A   
   G   
   A   
    A    
   T   
   T   
   A   
   G   
   C   
    C    
   G   
   A   
   A   
   G   
   C   
   G   
   A   
   A   
   G   
   A   
   A   
   A   
   A   
    C    
   A   
   G   
   C   
    T    
   A   
    C    
   T   
    A    
    C    
   A   
   T   
   T   
   T   
   T   
   T   
   A   
   A   
   A   
    C    
   G   
   T   
   A   
   T   
   T   
   G   
    A    
   C   
   A   
   A   
   A   
   A   
   C   
   A   
   G   
    A    
   A   
   A   
   T   
   T   
   T   
   G   
   A   
   T   
   A   
   A   
   G   
   T   
   T   
   A   
   C   
   C   
    G    
   T   
   C   
   C   
   G   
   T   
   T   
    G    
   T   
   T   
   C   
   A   
   A   
   G   
   A   
   A   
    G    
   C   
   T   
   A   
   G   
   C   
   T   
   T   
   A   
   A   
   A   
   G   
   A   
   A   
   A   
   A   
   A   
   T   
   G   
   G   
    C    
   T   
   A   
   T   
   G   
   A   
   T   
   A   
   C   
   T   
   A   
   A   
   A   
   T   
   C   
    T    
   G   
   T   
   T   
   G   
   T   
   T   
   G   
   A   
   A   
   A   
   A   
   A   
   T   
   A   
   C   
   C   
   A   
   A   
   G   
   C   
   A   
   A   
   T   
   T   
   T   
   A   
   T   
   T   
   C   
   A   
   G   
   G   
   G   
   A   
   T   
   T   
   C   
   A   
   A   
   G   
   C   
    A    
   G   
   A   
   A   
    G    
   G   
    A    
   G   
   T   
   C   
   A   
   A   
    A    
   G   
   C   
   T   
   A   
   G   
   T   
   G   
   A   
   T   
   G   
   T   
    T    
   C   
   A   
   A   
   G   
   T   
    C    
   A   
   A   
   A   
   A   
   A   
    A    
   G   
    C    
   G   
   A   
   A   
   A   
   G   
   A   
    C    
   A   
   A   
    T    
   C   
   A   
   A   
   T   
   A   
   C   
   A   
   C   
   A   
   T   
   T   
   T   
   A   
    C    
   C   
   T   
   A   
   T   
   A   
   A   
   A   
   T   
   T   
   A   
   T   
   C   
   G   
   A   
   T   
   G   
   A   
   G   
    T    
   A   
   C   
    C    
   C   
   C   
   T   
   T   
   T   
   A   
   G   
   G   
   C   
   G   
   A   
   A   
   A   
   T   
   G   
   A   
   A   
   A   
   G   
   A   
   T   
    T    
   T   
   G   
   T   
   C   
   T   
   T   
   A   
   T   
   C   
   A   
   A   
   T   
   C   
   A   
   A   
   G   
   T   
   A   
   T   
    C    
   G   
    C    
   C   
   A   
   A   
    A    
   A   
   A   
   A   
   G   
   G   
    C    
   G   
   A   
   T   
   A   
    C    
   C   
   T   
   A   
   C   
   C   
   A   
   A   
   A   
   T   
    C    
    G    
   C   
   T   
   T   
   G   
   G   
    A    
    A    
    G    
   C   
   C   
    G    
   T   
   C   
   T   
   T   
   T   
   A   
   A   
   T   
   T   
   T   
   T   
   T   
   C   
   C   
   A   
   G   
   A   
   T   
   A   
   T   
   G   
   T   
   C   
   A   
   G   
   G   
   A   
   A   
   A   
   T   
   G   
   A   
   T   
   A   
   A   
   A   
    A    
   T   
   T   
   T   
   C   
   G   
   A   
   T   
   T   
   C   
   A   
   A   
   G   
   T   
   A   
   G   
   A   
   T   
   A   
   A   
   T   
   G   
   C   
   C   
   A   
   A   
   A   
   C   
   G   
   T   
   G   
   G   
   A   
   G   
   A   
   A   
   A   
   T   
   T   
   G   
   T   
   C   
   G   
   A   
   T   
   C   
   G   
   T   
   A   
   A   
    T    
   G   
   G   
   T   
   A   
   G   
   T   
   G   
   G   
   G   
   C   
   T   
   A   
   G   
    C    
   A   
   A   
   T   
   T   
   A   
   A   
   C   
   A   
   A   
   A   
   G   
   T   
   G   
   T   
   T   
   T   
   G   
   A   
    C    
   G   
   A   
   A   
   G   
   T   
   G   
   G   
   G   
   C   
   G   
   T   
   A   
   G   
   T   
    G    
   C   
   C   
   T   
    G    
   G   
   C   
   A   
   A   
   A   
   C   
   T   
    C    
   G   
   G   
   T   
   T   
   C   
   T   
   G   
   G   
   C   
   G   
   C   
   A   
   G   
   A   
   A   
   A   
   A   
   A   
    A    
   C   
   A   
   G   
   C   
    C    
   A   
   A   
   T   
   A   
   T   
   C   
   A   
   A   
   A   
   G   
   C   
   T   
   T   
   T   
   T   
   A   
   G   
   T   
   G   
   A   
   T   
   A   
   A   
    G    
   T   
   T   
    C    
   G   
   G   
   C   
   G   
   T   
   T   
   T   
   C   
   T   
    G    
   T   
   T   
   G   
   A   
   T   
   G   
   A   
   A   
   A   
   T   
   C   
   A   
   A   
   T   
   C   
    A    
   A   
   A   
   A   
    G    
   T   
   T   
   A   
   A   
   G   
   C   
   C   
   A   
   A   
   G   
   G   
    G    
   T   
   G   
   G   
   G   
   T   
   C   
   C   
   A   
   A   
   G   
   C   
   A   
   G   
   A   
   C   
   T   
   C   
    C    
   T   
   T   
   T   
   G   
   T   
   A   
   C   
   C   
   A   
   A   
   T   
   C   
   A   
   C   
   A   
   G   
   T   
    C    
   G   
   C   
   T   
   T   
   C   
   T   
   G   
   A   
   A   
   C   
   C   
   A   
   G   
   T   
   G   
   A   
   C   
   A   
   G   
   A   
   A   
   T   
   T   
   A   
   C   
   C   
   A   
   A   
   C   
   A   
   G   
   G   
   G   
   G   
   C   
   T   
   G   
   C   
   G   
   A   
   C   
   A   
   A   
   A   
   A   
   G   
    A    
    T    
   A   
   C   
   A   
   G   
   A   
   G   
   T   
   C   
   A   
   C   
   G   
   T   
   T   
   A   
   T   
   T   
   A   
   T   
   C   
   C   
    G    
    C    
   T   
   G   
   G   
   G   
   G   
   G   
   A   
   A   
   G   
   C   
   A   
   G   
   C   
   T   
   G   
   C   
   G   
   C   
   A   
   A   
   T   
   T   
   A   
   A   
   T   
   C   
   G   
   G   
    G    
   T   
   A   
   T   
   A   
   C   
    A    
   G   
   G   
    C    
   A   
   C   
    C    
   A   
   T   
   T   
   A   
   C   
   T   
   G   
   C   
   A   
   G   
   A   
    A    
   G   
   A   
   T   
   A   
   T   
   T   
    A    
   A   
   A   
   A   
   A   
   A   
   A   
   A   
   T   
   C   
   C   
   A   
   G   
   A   
   G   
   T   
   T   
   A   
   A   
   G   
   T   
   A   
   G   
   T   
   A   
   C   
   G   
   G   
   G   
    C    
   G   
   T   
   A   
   A   
   T   
   T   
   G   
   G   
    T    
   A   
   A   
   A   
   A   
   C   
   T   
   G   
   G   
    C    
   T   
   T   
   A   
   G   
   A   
   A   
   C   
   G   
   A   
   G   
   C   
   G   
   T   
   T   
   T   
   G   
   A   
   T   
   A   
   A   
   A   
   G   
   A   
    A    
   T   
   T   
   A   
   A   
   G   
   A   
   G   
   G   
    G    
   C   
   A   
   A   
   G   
   A   
   T   
   G   
   G   
    T    
   G   
   G   
    C    
   T   
   C   
    A    
   C   
   T   
   A   
   G   
   T   
   T   
   A   
   T   
   T   
   T   
   T   
   A   
   G   
   A   
   T   
   G   
   A   
   C   
   A   
   A   
   A   
   G   
   A   
   G   
   A   
   A   
   T   
   G   
   T   
   C   
   A   
   A   
   A   
   A   
   A   
   A   
   G   
   C   
   T   
   T   
   T   
   A   
   C   
   A   
   A   
   A   
   C   
   C   
   A   
   A   
   A   
   G   
   A   
    G    
   A   
   A   
   A   
   A   
   A   
   A   
   G   
   A   
   T   
   G   
   G   
   T   
   C   
   A   
   A   
   A   
   C   
    G    
   A   
   T   
   T   
   A   
   A   
   A   
   T   
   T   
   A   
   A   
   C   
   G   
   A   
   T   
   T   
   G   
   A   
    C    
   A   
   G   
    T    
   G   
   G   
    C    
   G   
   T   
   A   
   C   
   A   
   A   
   C   
   A   
   A   
   C   
   A   
   A   
   G   
   C   
    C    
   T   
   T   
    C    
   G   
   C   
   T   
   A   
   T   
   A   
   T   
   T   
   T   
   G   
   A   
   C   
   A   
   A   
   A   
   C   
   G   
    C    
    C    
   C   
   T   
   G   
   G   
   T   
   A   
   G   
   T   
   G   
   C   
   G   
   G   
   T   
   C   
   A   
   T   
   T   
   A   
   C   
   C   
   G   
   A   
   T   
   C   
   C   
   T   
   C   
   A   
   A   
   A   
   A   
   A   
   G   
   G   
   C   
   G   
   A   
   T   
   T   
   T   
   A   
    T    
   T   
   A   
   G   
   C   
    A    
   A   
   C   
   G   
   G   
   T   
   T   
   A   
   G   
   T   
   T   
   C   
   A   
   C   
   C   
   T   
   T   
   C   
   G   
   T   
   A   
   T   
   G   
   A   
   T   
   C   
   C   
   T   
   A   
   A   
   T   
   A   
   A   
   A   
   A   
   T   
   G   
   G   
   C   
   C   
   A   
   A   
   T   
   G   
   G   
    T    
   A   
   T   
   T   
   T   
   C   
   T   
   C   
   A   
   A   
   A   
   A   
   A   
   G   
   A   
    A    
   T   
   A   
   T   
   G   
   A   
   T   
   G   
   C   
   T   
   T   
   A   
   C   
   A   
   A   
   T   
   A   
   A   
   C   
   A   
   A   
   C   
   A   
   A   
   A   
   G   
   A   
   T   
   T   
   T   
   A   
   C   
   C   
   A   
   T   
   T   
    C    
   A   
   C   
   A   
   G   
   C   
   A   
   C   
   G   
   T   
   T   
   T   
    T    
   G   
   C   
   G   
   A   
   C   
   A   
   G   
   G   
    C    
   T   
   A   
   T   
   G   
   C   
   A   
   C   
   C   
   A   
   G   
   G   
    C    
   T   
   C   
   T   
   A   
   C   
   C   
   T   
   T   
   T   
   A   
   A   
   A   
   A   
   C   
   A   
   A   
   T   
   T   
   A   
   C   
   C   
   G   
   G   
   T   
   G   
   C   
   C   
   A   
   T   
   T   
   G   
   G   
   T   
   T   
   T   
   A   
   G   
   A   
   T   
    G    
   C   
   A   
   G   
   G   
   G   
   A   
   C   
   A   
   T   
   T   
   G   
   A   
   A   
   G   
   C   
   C   
   A   
   G   
   A   
   T   
   G   
   A   
   A   
   G   
   A   
   G   
   C   
   T   
   A   
   G   
   A   
   A   
   A   
   T   
   T   
   A   
   A   
   T   
   G   
   G   
    C    
   T   
   T   
   G   
   A   
   A   
   A   
   T   
   G   
   G   
   C   
   A   
   A   
   A   
   A   
   A   
   G   
   A   
   T   
   A   
   A   
   A   
   T   
   C   
   T   
   T   
   G   
   G   
   G   
   G   
    C    
   G   
   G   
   C   
   T   
   A   
   T   
   T   
   T   
   T   
   G   
   C   
    A    
   A   
   C   
   A   
   C   
   G   
   T   
   G   
   T   
   A   
   A   
   A   
   A   
   G   
   A   
   A   
   G   
   C   
   A   
   A   
   G   
   T   
   C   
   C   
   A   
   G   
   T   
   C   
   A   
   A   
   T   
   C   
   T   
    G    
   C   
   G   
   T   
   A   
   C   
   C   
   G   
   C   
   T   
   T   
   T   
   A   
   G   
   T   
   T   
   A   
   A   
   T   
   T   
   C   
   A   
   G   
   A   
   T   
   A   
   A   
   T   
   A   
   T   
   T   
   T   
   A   
   T   
   T   
   T   
   T   
   G   
   C   
    C    
   C   
   A   
   A   
   C   
   A   
   A   
   A   
   C   
   A   
   T   
   T   
   A   
   C   
   G   
    T    
   A   
   T   
   G   
   G   
   G   
    G    
   G   
   A   
   A   
   G   
   A   
   C   
   A   
   A   
   A   
   T   
   T   
   T   
   A   
   G   
   A   
   G   
   C   
    G    
   G   
   G   
   T   
   C   
   T   
   T   
   A   
   A   
   T   
   A   
   A   
   A   
   T   
   T   
   C   
   A   
   T   
   T   
   T   
   T   
   C   
   G   
   G   
   T   
   G   
   A   
   G   
   G   
   A   
   A   
   C   
   T   
   A   
   G   
   A   
   T   
   T   
   T   
   A   
   C   
   C   
   A   
   A   
   T   
    T    
   G   
   C   
   C   
   A   
   T   
   G   
   A   
   C   
   G   
   C   
   C   
    T    
   G   
   C   
   T   
   C   
   A   
   A   
   A   
   T   
   T   
   T   
   C   
   A   
   A   
   A   
    T    
   G   
   A   
   A   
   G   
   A   
   T   
   A   
   A   
   A   
   T   
   T   
   T   
   A   
   A   
   T   
   T   
   C   
   T   
   G   
   A   
   A   
   A   
   T   
   T   
   C   
   T   
   T   
   T   
   T   
    G    
   G   
   C   
   A   
   G   
   A   
   T   
   A   
   C   
    G    
   G   
   G   
   C   
   T   
   A   
    C    
   G   
   G   
    C    
   C   
   A   
   A   
   G   
   G   
    C    
   C   
   A   
   A   
   T   
   T   
   G   
   T   
   T   
   A   
   A   
   T   
   T   
   T   
   C   
    G    
   C   
   C   
   A   
   A   
   T   
   C   
   C   
   A   
   G   
   C   
   A   
   A   
   G   
   C   
   T   
   A   
   C   
   C   
   A   
   T   
   G   
   T   
   A   
   T   
   A   
   G   
   T   
   G   
   T   
   T   
   T   
   T   
   C   
   C   
   A   
   A   
   A   
   A   
   T   
   A   
   A   
   T   
   G   
   G   
    G    
   A   
   C   
   A   
    T    
   T   
    A    
   G   
   T   
    C    
   T   
   A   
   T   
   C   
   C   
   A   
   A   
   A   
   A   
    C    
   T   
   A   
   G   
   T   
   T   
   T   
   T   
   A   
   G   
   A   
   C   
   A   
   A   
   A   
   G   
   A   
   A   
   A   
   C   
    G    
   A   
   A   
   G   
   A   
   A   
   A   
   A   
   A   
   A   
   G   
   A   
   C   
   A   
   A   
   T   
   G   
   T   
   A   
   A   
   T   
   C   
   A   
   G   
   C   
   G   
   C   
   C   
   A   
   A   
   C   
   G   
   C   
   A   
   G   
   C   
   G   
   A   
   A   
   T   
   A   
   C   
   A   
   A   
   T   
   T   
   G   
   C   
   C   
   A   
   C   
   A   
   G   
   A   
   T   
   C   
   T   
   T   
   T   
   T   
   A   
   G   
   G   
   T   
   A   
   G   
   T   
   G   
   T   
   G   
   G   
   A   
   A   
   G   
   A   
   T   
   C   
   C   
   T   
   T   
   C   
   A   
   G   
   G   
   T   
   T   
   A   
   T   
   G   
   T   
   T   
   T   
   A   
   T   
   A   
   A   
   T   
   A   
   T   
   G   
   T   
   A   
   C   
   A   
   A   
   T   
   C   
   C   
   G   
   A   
   A   
   C   
   T   
   T   
   C   
   T   
   C   
   A   
   T   
   T   
   G   
   G   
   C   
   T   
   G   
   C   
   C   
   A   
   A   
   A   
   A   
   C   
   A   
   G   
   G   
   A   
   A   
   C   
   A   
   G   
   C   
   T   
   G   
   A   
   A   
   A   
   T   
   A   
   A   
   A   
   A   
   G   
   A   
   T   
   A   
   A   
   A   
   C   
   A   
   A   
   G   
   A   
   T   
   A   
   C   
   A   
   G   
   A   
   T   
   G   
   G   
   C   
   A   
   A   
   A   
   G   
   A   
   A   
   A   
   A   
   C   
   A   
   G   
   T   
   T   
   T   
   C   
   C   
   T   
   T   
   C   
   T   
   A   
   A   
   C   
   G   
   T   
   T   
   A   
   G   
   A   
   T   
   C   
   G   
   T   
   A   
   G   
   T   
   A   
   A   
   C   
   A   
   A   
   T   
   A   
   A   
   A   
   T   
   T   
   C   
   T   
   T   
   A   
   A   
   C   
   A   
   A   
   T   
   G   
   A   
   T   
   T   
   A   
   T   
   G   
   G   
   T   
   T   
   G   
   A   
   A   
   A   
   A   
   T   
   T   
   C   
   A   
   G   
   G   
   A   
   G   
   A   
   A   
   A   
   A   
   T   
   G   
   G   
   T   
   T   
   C   
   T   
   G   
   C   
   A   
   A   
   C   
   A   
   G   
   A   
   T   
   A   
   T   
   C   
   A   
   G   
   T   
   A   
   A   
   A   
   C   
   C   
   A   
   T   
   T   
   A   
   A   
   T   
   T   
   G   
   A   
   T   
   T   
   A   
   C   
   T   
   T   
   A   
   G   
   A   
   A   
   G   
   C   
   A   
   A   
   C   
   C   
   A   
   T   
   T   
   A   
   A   
   A   
   -   
   -   
   -   
   -   
   -   
   -   
   -   
   -   
   -   
   -   
   -   
   -   
   -   
   -   
   -   
   -   
   -   
   -   
   -   
   -   
   -   
   -   
   -   
   -   
   -   
   -   
   -   
   -   
   -   
   -   
   -   
   -   
   -   
   -   
   -   
   -   
   -   
   -   
   -   
   -   
   -   
   -   
   -   
   -   
   -   
   -   
   -   
   -   
   -   
   -   
   -   
   -   
   -   
   -   
   -   
   -   
   -   
   -   
   -   
   -   
   -   
   -   
   -   
   -   
   -   
   -   
   -   
   -   
   -   
   -   
   -   
   -   
   -   
   -   
   -   
   -   
   -   
   -   
   -   
   -   
   -   
   -   
   -   
   -   
   -   
   -   
   -   
   -   
   -   
   -   
   -   
   -   
   -   
   -   
   -   
   -   
   -   
   -   
   -   
   -   
   -   
   -   
   -   
   -   
   -   
   -   
   -   
   -   
   -   
   -   
   -   
   -   
   -   
   -   
   -   
   -   
   -   
   -   
   -   
   -   
   -   
   -   
   -   
   -   
   -   
   -   
   -   
   -   
   -   
   -   
   -   
   -   
   -   
   -   
   -   
   -   
   -   
   -   
   -   
   -   
   -   
   -   
   -   
   -   
   -   
   -   
   -   
   -   
   -   
   -   
   -   
   -   
   -   
   -   
   -   
   -   
   -   
   -   
   -   
   -   
   -   
   -   
   -   
   -   
   -   
   -   
   -   
   -   
   -   
   C   
   A   
   A   
   T   
   G   
   G   
   C   
   A   
   A   
   G   
   C   
   C   
   A   
   A   
   A   
   C   
   A   
   A   
   G   
   A   
   A   
   T   
   T   
   A   
   G   
   C   
   C   
   G   
   A   
   A   
   G   
   C   
   G   
   A   
   A   
   G   
   A   
   A   
   A   
   A   
   C   
   A   
   G   
   C   
   T   
   A   
   C   
   T   
   A   
   C   
   A   
   T   
   T   
   T   
   T   
   T   
   A   
   A   
   A   
   C   
   G   
   T   
   A   
   T   
   T   
   G   
   A   
   C   
   A   
   A   
   A   
   A   
   C   
   A   
   G   
   A   
   A   
   A   
   T   
   T   
   T   
   G   
   A   
   T   
   A   
   A   
   G   
   T   
   T   
   A   
   C   
   C   
   G   
   T   
   C   
   C   
   G   
   T   
   T   
   G   
   T   
   T   
   C   
   A   
   A   
   G   
   A   
   A   
   G   
   C   
   T   
   A   
   G   
   C   
   T   
   T   
   A   
   A   
   A   
   G   
   A   
   A   
   A   
   A   
   A   
   T   
   G   
   G   
   C   
   T   
   A   
   T   
   G   
   A   
   T   
   A   
   C   
   T   
   A   
   A   
   A   
   T   
   C   
   T   
   G   
   T   
   T   
   G   
   T   
   T   
   G   
   A   
   A   
   A   
   A   
   A   
   T   
   A   
   C   
   C   
   A   
   A   
   G   
   C   
   A   
   A   
   T   
   T   
   T   
   A   
   T   
   T   
   C   
   A   
   G   
   G   
   G   
   A   
   T   
   T   
   C   
   A   
   A   
   G   
   C   
   A   
   G   
   A   
   A   
   G   
   G   
   A   
   G   
   T   
   C   
   A   
   A   
   A   
   G   
   C   
   T   
   A   
   G   
   T   
   G   
   A   
   T   
   G   
   T   
   T   
   C   
   A   
   A   
   G   
   T   
   C   
   A   
   A   
   A   
   A   
   A   
   A   
   G   
   C   
   G   
   A   
   A   
   A   
   G   
   A   
   C   
   A   
   A   
   T   
   C   
   A   
   A   
   T   
   A   
   C   
   A   
   C   
   A   
   T   
   T   
   T   
   A   
   C   
   C   
   T   
   A   
   T   
   A   
   A   
   A   
   T   
   T   
   A   
   T   
   C   
   G   
   A   
   T   
   G   
   A   
   G   
   T   
   A   
   C   
   C   
   C   
   C   
   T   
   T   
   T   
   A   
   G   
   G   
   C   
   G   
   A   
   A   
   A   
   T   
   G   
   A   
   A   
   A   
   G   
   A   
   T   
   T   
   T   
   G   
   T   
   C   
   T   
   T   
   A   
   T   
   C   
   A   
   A   
   T   
   C   
   A   
   A   
   G   
   T   
   A   
   T   
   C   
   G   
   C   
   C   
   A   
   A   
   A   
   A   
   A   
   A   
   G   
   G   
   C   
   G   
   A   
   T   
   A   
   C   
   C   
   T   
   A   
   C   
   C   
   A   
   A   
   A   
   T   
   C   
   G   
   C   
   T   
   T   
   G   
   G   
   A   
   A   
   G   
   C   
   C   
   G   
   T   
   C   
   T   
   T   
   T   
   A   
   A   
   T   
   T   
   T   
   T   
   T   
   C   
   C   
   A   
   G   
   A   
   T   
   A   
   T   
   G   
   T   
   C   
   A   
   G   
   G   
   A   
   A   
   A   
   T   
   G   
   A   
   T   
   A   
   A   
   A   
   A   
   T   
   T   
   T   
   C   
   G   
   A   
   T   
   T   
   C   
   A   
   A   
   G   
   T   
   A   
   G   
   A   
   T   
   A   
   A   
   T   
   G   
   C   
   C   
   A   
   A   
   A   
   C   
   G   
   T   
   G   
   G   
   A   
   G   
   A   
   A   
   A   
   T   
   T   
   G   
   T   
   C   
   G   
   A   
   T   
   C   
   G   
   T   
   A   
   A   
   T   
   G   
   G   
   T   
   A   
   G   
   T   
   G   
   G   
   G   
   C   
   T   
   A   
   G   
   C   
   A   
   A   
   T   
   T   
   A   
   A   
   C   
   A   
   A   
   A   
   G   
   T   
   G   
   T   
   T   
   T   
   G   
   A   
   C   
   G   
   A   
   A   
   G   
   T   
   G   
   G   
   G   
   C   
   G   
   T   
   A   
   G   
   T   
   G   
   C   
   C   
   T   
   G   
   G   
   C   
   A   
   A   
   A   
   C   
   T   
   C   
   G   
   G   
   T   
   T   
   C   
   T   
   G   
   G   
   C   
   G   
   C   
   A   
   G   
   A   
   A   
   A   
   A   
   A   
   A   
   C   
   A   
   G   
   C   
   C   
   A   
   A   
   T   
   A   
   T   
   C   
   A   
   A   
   A   
   G   
   C   
   T   
   T   
   T   
   T   
   A   
   G   
   T   
   G   
   A   
   T   
   A   
   A   
   G   
   T   
   T   
   C   
   G   
   G   
   C   
   G   
   T   
   T   
   T   
   C   
   T   
   G   
   T   
   T   
   G   
   A   
   T   
   G   
   A   
   A   
   A   
   T   
   C   
   A   
   A   
   T   
   C   
   A   
   A   
   A   
   A   
   G   
   T   
   T   
   A   
   A   
   G   
   C   
   C   
   A   
   A   
   G   
   G   
   G   
   T   
   G   
   G   
   G   
   T   
   C   
   C   
   A   
   A   
   G   
   C   
   A   
   G   
   A   
   C   
   T   
   C   
   C   
   T   
   T   
   T   
   G   
   T   
   A   
   C   
   C   
   A   
   A   
   T   
   C   
   A   
   C   
   A   
   G   
   T   
   C   
   G   
   C   
   T   
   T   
   C   
   T   
   G   
   A   
   A   
   C   
   C   
   A   
   G   
   T   
   G   
   A   
   C   
   A   
   G   
   A   
   A   
   T   
   T   
   A   
   C   
   C   
   A   
   A   
   C   
   A   
   G   
   G   
   G   
   G   
   C   
   T   
   G   
   C   
   G   
   A   
   C   
   A   
   A   
   A   
   A   
   G   
   A   
   T   
   A   
   C   
   A   
   G   
   A   
   G   
   T   
   C   
   A   
   C   
   G   
   T   
   T   
   A   
   T   
   T   
   A   
   T   
   C   
   C   
   G   
   C   
   T   
   G   
   G   
   G   
   G   
   G   
   A   
   A   
   G   
   C   
   A   
   G   
   C   
   T   
   G   
   C   
   G   
   C   
   A   
   A   
   T   
   T   
   A   
   A   
   T   
   C   
   G   
   G   
   G   
   T   
   A   
   T   
   A   
   C   
   A   
   G   
   G   
   C   
   A   
   C   
   C   
   A   
   T   
   T   
   A   
   C   
   T   
   G   
   C   
   A   
   G   
   A   
   A   
   G   
   A   
   T   
   A   
   T   
   T   
   A   
   A   
   A   
   A   
   A   
   A   
   A   
   A   
   T   
   C   
   C   
   A   
   G   
   A   
   G   
   T   
   T   
   A   
   A   
   G   
   T   
   A   
   G   
   T   
   A   
   C   
   G   
   G   
   G   
   C   
   G   
   T   
   A   
   A   
   T   
   T   
   G   
   G   
   T   
   A   
   A   
   A   
   A   
   C   
   T   
   G   
   G   
   C   
   T   
   T   
   A   
   G   
   A   
   A   
   C   
   G   
   A   
   G   
   C   
   G   
   T   
   T   
   T   
   G   
   A   
   T   
   A   
   A   
   A   
   G   
   A   
   A   
   T   
   T   
   A   
   A   
   G   
   A   
   G   
   G   
   G   
   C   
   A   
   A   
   G   
   A   
   T   
   G   
   G   
   T   
   G   
   G   
   C   
   T   
   C   
   A   
   C   
   T   
   A   
   G   
   T   
   T   
   A   
   T   
   T   
   T   
   T   
   A   
   G   
   A   
   T   
   G   
   A   
   C   
   A   
   A   
   A   
   G   
   A   
   G   
   A   
   A   
   T   
   G   
   T   
   C   
   A   
   A   
   A   
   A   
   A   
   A   
   G   
   C   
   T   
   T   
   T   
   A   
   C   
   A   
   A   
   A   
   C   
   C   
   A   
   A   
   A   
   G   
   A   
   G   
   A   
   A   
   A   
   A   
   A   
   A   
   G   
   A   
   T   
   G   
   G   
   T   
   C   
   A   
   A   
   A   
   C   
   G   
   A   
   T   
   T   
   A   
   A   
   A   
   T   
   T   
   A   
   A   
   C   
   G   
   A   
   T   
   T   
   G   
   A   
   C   
   A   
   G   
   T   
   G   
   G   
   C   
   G   
   T   
   A   
   C   
   A   
   A   
   C   
   A   
   A   
   C   
   A   
   A   
   G   
   C   
   C   
   T   
   T   
   C   
   G   
   C   
   T   
   A   
   T   
   A   
   T   
   T   
   T   
   G   
   A   
   C   
   A   
   A   
   A   
   C   
   G   
   C   
   C   
   C   
   T   
   G   
   G   
   T   
   A   
   G   
   T   
   G   
   C   
   G   
   G   
   T   
   C   
   A   
   T   
   T   
   A   
   C   
   C   
   G   
   A   
   T   
   C   
   C   
   T   
   C   
   A   
   A   
   A   
   A   
   A   
   G   
   G   
   C   
   G   
   A   
   T   
   T   
   T   
   A   
   T   
   T   
   A   
   G   
   C   
   A   
   A   
   C   
   G   
   G   
   T   
   T   
   A   
   G   
   T   
   T   
   C   
   A   
   C   
   C   
   T   
   T   
   C   
   G   
   T   
   A   
   T   
   G   
   A   
   T   
   C   
   C   
   T   
   A   
   A   
   T   
   A   
   A   
   A   
   A   
   T   
   G   
   G   
   C   
   C   
   A   
   A   
   T   
   G   
   G   
   T   
   A   
   T   
   T   
   T   
   C   
   T   
   C   
   A   
   A   
   A   
   A   
   A   
   G   
   A   
   A   
   T   
   A   
   T   
   G   
   A   
   T   
   G   
   C   
   T   
   T   
   A   
   C   
   A   
   A   
   T   
   A   
   A   
   C   
   A   
   A   
   C   
   A   
   A   
   A   
   G   
   A   
   T   
   T   
   T   
   A   
   C   
   C   
   A   
   T   
   T   
   C   
   A   
   C   
   A   
   G   
   C   
   A   
   C   
   G   
   T   
   T   
   T   
   T   
   G   
   C   
   G   
   A   
   C   
   A   
   G   
   G   
   C   
   T   
   A   
   T   
   G   
   C   
   A   
   C   
   C   
   A   
   G   
   G   
   C   
   T   
   C   
   T   
   A   
   C   
   C   
   T   
   T   
   T   
   A   
   A   
   A   
   A   
   C   
   A   
   A   
   T   
   T   
   A   
   C   
   C   
   G   
   G   
   T   
   G   
   C   
   C   
   A   
   T   
   T   
   G   
   G   
   T   
   T   
   T   
   A   
   G   
   A   
   T   
   G   
   C   
   A   
   G   
   G   
   G   
   A   
   C   
   A   
   T   
   T   
   G   
   A   
   A   
   G   
   C   
   C   
   A   
   G   
   A   
   T   
   G   
   A   
   A   
   G   
   A   
   G   
   C   
   T   
   A   
   G   
   A   
   A   
   A   
   T   
   T   
   A   
   A   
   T   
   G   
   G   
   C   
   T   
   T   
   G   
   A   
   A   
   A   
   T   
   G   
   G   
   C   
   A   
   A   
   A   
   A   
   A   
   G   
   A   
   T   
   A   
   A   
   A   
   T   
   C   
   T   
   T   
   G   
   G   
   G   
   G   
   C   
   G   
   G   
   C   
   T   
   A   
   T   
   T   
   T   
   T   
   G   
   C   
   A   
   A   
   C   
   A   
   C   
   G   
   T   
   G   
   T   
   A   
   A   
   A   
   A   
   G   
   A   
   A   
   G   
   C   
   A   
   A   
   G   
   T   
   C   
   C   
   A   
   G   
   T   
   C   
   A   
   A   
   T   
   C   
   T   
   G   
   C   
   G   
   T   
   A   
   C   
   C   
   G   
   C   
   T   
   T   
   T   
   A   
   G   
   T   
   T   
   A   
   A   
   T   
   T   
   C   
   A   
   G   
   A   
   T   
   A   
   A   
   T   
   A   
   T   
   T   
   T   
   A   
   T   
   T   
   T   
   T   
   G   
   C   
   C   
   C   
   A   
   A   
   C   
   A   
   A   
   A   
   C   
   A   
   T   
   T   
   A   
   C   
   G   
   T   
   A   
   T   
   G   
   G   
   G   
   G   
   G   
   A   
   A   
   G   
   A   
   C   
   A   
   A   
   A   
   T   
   T   
   T   
   A   
   G   
   A   
   G   
   C   
   G   
   G   
   G   
   T   
   C   
   T   
   T   
   A   
   A   
   T   
   A   
   A   
   A   
   T   
   T   
   C   
   A   
   T   
   T   
   T   
   T   
   C   
   G   
   G   
   T   
   G   
   A   
   G   
   G   
   A   
   A   
   C   
   T   
   A   
   G   
   A   
   T   
   T   
   T   
   A   
   C   
   C   
   A   
   A   
   T   
   T   
   G   
   C   
   C   
   A   
   T   
   G   
   A   
   C   
   G   
   C   
   C   
   T   
   G   
   C   
   T   
   C   
   A   
   A   
   A   
   T   
   T   
   T   
   C   
   A   
   A   
   A   
   T   
   G   
   A   
   A   
   G   
   A   
   T   
   A   
   A   
   A   
   T   
   T   
   T   
   A   
   A   
   T   
   T   
   C   
   T   
   G   
   A   
   A   
   A   
   T   
   T   
   C   
   T   
   T   
   T   
   T   
   G   
   G   
   C   
   A   
   G   
   A   
   T   
   A   
   C   
   G   
   G   
   G   
   C   
   T   
   A   
   C   
   G   
   G   
   C   
   C   
   A   
   A   
   G   
   G   
   C   
   C   
   A   
   A   
   T   
   T   
   G   
   T   
   T   
   A   
   A   
   T   
   T   
   T   
   C   
   G   
   C   
   C   
   A   
   A   
   T   
   C   
   C   
   A   
   G   
   C   
   A   
   A   
   G   
   C   
   T   
   A   
   C   
   C   
   A   
   T   
   G   
   T   
   A   
   T   
   A   
   G   
   T   
   G   
   T   
   T   
   T   
   T   
   C   
   C   
   A   
   A   
   A   
   A   
   T   
   A   
   A   
   T   
   G   
   G   
   G   
   A   
   C   
   A   
   T   
   T   
   A   
   G   
   T   
   C   
   T   
   A   
   T   
   C   
   C   
   A   
   A   
   A   
   A   
   C   
   T   
   A   
   G   
   T   
   T   
   T   
   T   
   A   
   G   
   A   
   C   
   A   
   A   
   A   
   G   
   A   
   A   
   A   
   C   
   G   
   A   
   A   
   G   
   A   
   A   
   A   
   A   
   A   
   A   
   G   
   A   
   C   
   A   
   A   
   T   
   G   
   T   
   A   
   A   
   T   
   C   
   A   
   G   
   C   
   G   
   C   
   C   
   A   
   A   
   C   
   G   
   C   
   A   
   G   
   C   
   G   
   A   
   A   
   T   
   A   
   C   
   A   
   A   
   T   
   T   
   G   
   C   
   C   
   A   
   C   
   A   
   G   
   A   
   T   
   C   
   T   
   T   
   T   
   T   
   A   
   G   
   G   
   T   
   A   
   G   
   T   
   G   
   T   
   G   
   G   
   A   
   A   
   G   
   A   
   T   
   C   
   C   
   T   
   T   
   C   
   A   
   G   
   G   
   T   
   T   
   A   
   T   
   G   
   T   
   T   
   T   
   A   
   T   
   A   
   A   
   T   
   A   
   T   
   G   
   T   
   A   
   C   
   A   
   A   
   T   
   C   
   C   
   G   
   A   
   A   
   C   
   T   
   T   
   C   
   T   
   C   
   A   
   T   
   T   
   G   
   G   
   C   
   T   
   G   
   C   
   C   
   A   
   A   
   A   
   A   
   C   
   A   
   G   
   G   
   A   
   A   
   C   
   A   
   G   
   C   
   T   
   G   
   A   
   A   
   A   
   T   
   A   
   A   
   A   
   A   
   G   
   A   
   T   
   A   
   A   
   A   
   C   
   A   
   A   
   G   
   A   
   T   
   A   
   C   
   A   
   G   
   A   
   T   
   G   
   G   
   C   
   A   
   A   
   A   
   G   
   A   
   A   
   A   
   A   
   C   
   A   
   G   
   T   
   T   
   T   
   C   
   C   
   T   
   T   
   C   
   T   
   A   
   A   
   C   
   G   
   T   
   T   
   A   
   G   
   A   
   T   
   C   
   G   
   T   
   A   
   G   
   T   
   A   
   A   
   C   
   A   
   A   
   T   
   A   
   A   
   A   
   T   
   T   
   C   
   T   
   T   
   A   
   A   
   C   
   A   
   A   
   T   
   G   
   A   
   T   
   T   
   A   
   T   
   G   
   G   
   T   
   T   
   G   
   A   
   A   
   A   
   A   
   T   
   T   
   C   
   A   
   G   
   G   
   A   
   G   
   A   
   A   
   A   
   A   
   T   
   G   
   G   
   T   
   T   
   C   
   T   
   G   
   C   
   A   
   A   
   C   
   A   
   G   
   A   
   T   
   A   
   T   
   C   
   A   
   G   
   T   
   A   
   A   
   A   
   C   
   C   
   A   
   T   
   T   
   A   
   A   
   T   
   T   
   G   
   A   
   T   
   T   
   A   
   C   
   T   
   T   
   A   
   G   
   A   
   A   
   G   
   C   
   A   
   A   
   C   
   C   
   A   
   T   
   T   
   A   
   A   
   A   
   -   
   -   
   -   
   -   
   -   
   -   
   -   
   -   
   -   
   -   
   -   
   -   
   -   
   -   
   -   
   -   
   -   
   -   
   -   
   -   
   -   
   -   
   -   
   -   
   -   
   -   
   -   
   -   
   -   
   -   
   -   
   -   
   -   
   -   
   -   
   -   
   -   
   -   
   -   
   -   
   -   
   -   
   -   
   -   
   -   
   -   
   -   
   -   
   -   
   -   
   -   
   -   
   -   
   -   
   -   
   -   
   -   
   -   
   -   
   -   
   -   
 

 
   N60063F   
   -   
   -   
   -   
   -   
   -   
   -   
   -   
   -   
   -   
   -   
   -   
   -   
   -   
   -   
   -   
   -   
   -   
   -   
   -   
   -   
   -   
   -   
   -   
   -   
   -   
   -   
   -   
   -   
   -   
   -   
   -   
   -   
   -   
   -   
   -   
   -   
   -   
   -   
   -   
   -   
   -   
   -   
   -   
   -   
   -   
   -   
   -   
   -   
   -   
   -   
   -   
   -   
   -   
   -   
   -   
   -   
   -   
   -   
   -   
   -   
   -   
   -   
   -   
   -   
   -   
   -   
   -   
   -   
   -   
   -   
   -   
   -   
   -   
   -   
   -   
   -   
   -   
   -   
   -   
   -   
   -   
   -   
   -   
   -   
   -   
   -   
   -   
   -   
   -   
   -   
   -   
   -   
   -   
   -   
   -   
   -   
   -   
   -   
   -   
   -   
   -   
   -   
   -   
   -   
   -   
   -   
   -   
   -   
   C   
   A   
   A   
   T   
   G   
   G   
   C   
   A   
   A   
   G   
   C   
   C   
   A   
   A   
   A   
   C   
   A   
   A   
   G   
   A   
    A    
   T   
   T   
   A   
   G   
   C   
    C    
   G   
   A   
   A   
   G   
   C   
   G   
   A   
   A   
   G   
   A   
   A   
   A   
   A   
    C    
   A   
   G   
   C   
    T    
   A   
    C    
   T   
    A    
    C    
   A   
   T   
   T   
   T   
   T   
   T   
   A   
   A   
   A   
    C    
   G   
   T   
   A   
   T   
   T   
   G   
    T    
   C   
   A   
   A   
   A   
   A   
   C   
   A   
   G   
    G    
   A   
   A   
   T   
   T   
   T   
   G   
   A   
   T   
   A   
   A   
   G   
   T   
   T   
   A   
   C   
   C   
    G    
   T   
   C   
   C   
   G   
   T   
   T   
    G    
   T   
   T   
   C   
   A   
   A   
   G   
   A   
   A   
    G    
   C   
   T   
   A   
   G   
   C   
   T   
   T   
   A   
   A   
   A   
   G   
   A   
   A   
   A   
   A   
   A   
   T   
   G   
   G   
    C    
   T   
   A   
   T   
   G   
   A   
   T   
   A   
   C   
   T   
   A   
   A   
   A   
   T   
   C   
    T    
   G   
   T   
   T   
   G   
   T   
   T   
   G   
   A   
   A   
   A   
   A   
   A   
   T   
   A   
   C   
   C   
   A   
   A   
   G   
   C   
   A   
   A   
   T   
   T   
   T   
   A   
   T   
   T   
   C   
   A   
   G   
   G   
   G   
   A   
   T   
   T   
   C   
   A   
   A   
   G   
   C   
    A    
   G   
   A   
   A   
    G    
   G   
    A    
   G   
   T   
   C   
   A   
   A   
    A    
   G   
   C   
   T   
   A   
   G   
   T   
   G   
   A   
   T   
   G   
   T   
    T    
   C   
   A   
   A   
   G   
   T   
    C    
   A   
   A   
   A   
   A   
   A   
    G    
   G   
    C    
   G   
   A   
   A   
   A   
   G   
   A   
    C    
   A   
   A   
    T    
   C   
   A   
   A   
   T   
   A   
   C   
   A   
   C   
   A   
   T   
   T   
   T   
   A   
    C    
   C   
   T   
   A   
   T   
   A   
   A   
   A   
   T   
   T   
   A   
   T   
   C   
   G   
   A   
   T   
   G   
   A   
   G   
    T    
   A   
   C   
    C    
   C   
   C   
   T   
   T   
   T   
   A   
   G   
   G   
   C   
   G   
   A   
   A   
   A   
   T   
   G   
   A   
   A   
   A   
   G   
   A   
   T   
    T    
   T   
   G   
   T   
   C   
   T   
   T   
   A   
   T   
   C   
   A   
   A   
   T   
   C   
   A   
   A   
   G   
   T   
   A   
   T   
    C    
   G   
    C    
   C   
   A   
   A   
    A    
   A   
   A   
   A   
   G   
   G   
    C    
   G   
   A   
   T   
   A   
    C    
   C   
   T   
   A   
   C   
   C   
   A   
   A   
   A   
   T   
    C    
    G    
   C   
   T   
   T   
   G   
   G   
    A    
    A    
    G    
   C   
   C   
    G    
   T   
   C   
   T   
   T   
   T   
   A   
   A   
   T   
   T   
   T   
   T   
   T   
   C   
   C   
   A   
   G   
   A   
   T   
   A   
   T   
   G   
   T   
   C   
   A   
   G   
   G   
   A   
   A   
   A   
   T   
   G   
   A   
   T   
   A   
   A   
   A   
    A    
   T   
   T   
   T   
   C   
   G   
   A   
   T   
   T   
   C   
   A   
   A   
   G   
   T   
   A   
   G   
   A   
   T   
   A   
   A   
   T   
   G   
   C   
   C   
   A   
   A   
   A   
   C   
   G   
   T   
   G   
   G   
   A   
   G   
   A   
   A   
   A   
   T   
   T   
   G   
   T   
   C   
   G   
   A   
   T   
   C   
   G   
   T   
   A   
   A   
    T    
   G   
   G   
   T   
   A   
   G   
   T   
   G   
   G   
   G   
   C   
   T   
   A   
   G   
    C    
   A   
   A   
   T   
   T   
   A   
   A   
   C   
   A   
   A   
   A   
   G   
   T   
   G   
   T   
   T   
   T   
   G   
   A   
    C    
   G   
   A   
   A   
   G   
   T   
   G   
   G   
   G   
   C   
   G   
   T   
   A   
   G   
   T   
    G    
   C   
   C   
   T   
    G    
   G   
   C   
   A   
   A   
   A   
   C   
   T   
    C    
   G   
   G   
   T   
   T   
   C   
   T   
   G   
   G   
   C   
   G   
   C   
   A   
   G   
   A   
   A   
   A   
   A   
   A   
    A    
   C   
   A   
   G   
   C   
    C    
   A   
   A   
   T   
   A   
   T   
   C   
   A   
   A   
   A   
   G   
   C   
   T   
   T   
   T   
   T   
   A   
   G   
   T   
   G   
   A   
   T   
   A   
   A   
    G    
   T   
   T   
    C    
   G   
   G   
   C   
   G   
   T   
   T   
   T   
   C   
   T   
    A    
   T   
   T   
   G   
   A   
   T   
   G   
   A   
   A   
   A   
   T   
   C   
   A   
   A   
   T   
   C   
    A    
   A   
   A   
   A   
    G    
   T   
   T   
   A   
   A   
   G   
   C   
   C   
   A   
   A   
   G   
   G   
    A    
   T   
   G   
   G   
   G   
   T   
   C   
   C   
   A   
   A   
   G   
   C   
   A   
   G   
   A   
   C   
   T   
   C   
    C    
   T   
   T   
   T   
   G   
   T   
   A   
   C   
   C   
   A   
   A   
   T   
   C   
   A   
   C   
   A   
   G   
   T   
    C    
   G   
   C   
   T   
   T   
   C   
   T   
   G   
   A   
   A   
   C   
   C   
   A   
   G   
   T   
   G   
   A   
   C   
   A   
   G   
   A   
   A   
   T   
   T   
   A   
   C   
   C   
   A   
   A   
   C   
   A   
   G   
   G   
   G   
   G   
   C   
   T   
   G   
   C   
   G   
   A   
   C   
   A   
   A   
   A   
   A   
   G   
    A    
    T    
   A   
   C   
   A   
   G   
   A   
   G   
   T   
   C   
   A   
   C   
   G   
   T   
   T   
   A   
   T   
   T   
   A   
   T   
   C   
   C   
    G    
    C    
   T   
   G   
   G   
   G   
   G   
   G   
   A   
   A   
   G   
   C   
   A   
   G   
   C   
   T   
   G   
   C   
   G   
   C   
   A   
   A   
   T   
   T   
   A   
   A   
   T   
   C   
   G   
   G   
    G    
   T   
   A   
   T   
   A   
   C   
    G    
   G   
   G   
    C    
   A   
   C   
    C    
   A   
   T   
   T   
   A   
   C   
   T   
   G   
   C   
   A   
   G   
   A   
    A    
   G   
   A   
   T   
   A   
   T   
   T   
    G    
   A   
   A   
   A   
   A   
   A   
   A   
   A   
   T   
   C   
   C   
   A   
   G   
   A   
   G   
   T   
   T   
   A   
   A   
   G   
   T   
   A   
   G   
   T   
   A   
   C   
   G   
   G   
   G   
    C    
   G   
   T   
   A   
   A   
   T   
   T   
   G   
   G   
    T    
   A   
   A   
   A   
   A   
   C   
   T   
   G   
   G   
    C    
   T   
   T   
   A   
   G   
   A   
   A   
   C   
   G   
   A   
   G   
   C   
   G   
   T   
   T   
   T   
   G   
   A   
   T   
   A   
   A   
   A   
   G   
   A   
    A    
   T   
   T   
   A   
   A   
   G   
   A   
   G   
   G   
    G    
   C   
   A   
   A   
   G   
   A   
   T   
   G   
   G   
    T    
   G   
   G   
    C    
   T   
   C   
    A    
   C   
   T   
   A   
   G   
   T   
   T   
   A   
   T   
   T   
   T   
   T   
   A   
   G   
   A   
   T   
   G   
   A   
   C   
   A   
   A   
   A   
   G   
   A   
   G   
   A   
   A   
   T   
   G   
   T   
   C   
   A   
   A   
   A   
   A   
   A   
   A   
   G   
   C   
   T   
   T   
   T   
   A   
   C   
   A   
   A   
   A   
   C   
   C   
   A   
   A   
   A   
   G   
   A   
    G    
   A   
   A   
   A   
   A   
   A   
   A   
   G   
   A   
   T   
   G   
   G   
   T   
   C   
   A   
   A   
   A   
   C   
    G    
   A   
   T   
   T   
   A   
   A   
   A   
   T   
   T   
   A   
   A   
   C   
   G   
   A   
   T   
   T   
   G   
   A   
    C    
   A   
   G   
    T    
   G   
   G   
    C    
   G   
   T   
   A   
   C   
   A   
   A   
   C   
   A   
   A   
   C   
   A   
   A   
   G   
   C   
    C    
   T   
   T   
    C    
   G   
   C   
   T   
   A   
   T   
   A   
   T   
   T   
   T   
   G   
   A   
   C   
   A   
   A   
   A   
   C   
   G   
    T    
    C    
   C   
   T   
   G   
   G   
   T   
   A   
   G   
   T   
   G   
   C   
   G   
   G   
   T   
   C   
   A   
   T   
   T   
   A   
   C   
   C   
   G   
   A   
   T   
   C   
   C   
   T   
   C   
   A   
   A   
   A   
   A   
   A   
   G   
   G   
   C   
   G   
   A   
   T   
   T   
   T   
   A   
    T    
   T   
   A   
   G   
   C   
    A    
   A   
   C   
   G   
   G   
   T   
   T   
   A   
   G   
   T   
   T   
   C   
   A   
   C   
   C   
   T   
   T   
   C   
   G   
   T   
   A   
   T   
   G   
   A   
   T   
   C   
   C   
   T   
   A   
   A   
   T   
   A   
   A   
   A   
   A   
   T   
   G   
   G   
   C   
   C   
   A   
   A   
   T   
   G   
   G   
    T    
   A   
   T   
   T   
   T   
   C   
   T   
   C   
   A   
   A   
   A   
   A   
   A   
   G   
   A   
    A    
   T   
   A   
   T   
   G   
   A   
   T   
   G   
   C   
   T   
   T   
   A   
   C   
   A   
   A   
   T   
   A   
   A   
   C   
   A   
   A   
   C   
   A   
   A   
   A   
   G   
   A   
   T   
   T   
   T   
   A   
   C   
   C   
   A   
   T   
   T   
    C    
   A   
   C   
   A   
   G   
   C   
   A   
   C   
   G   
   T   
   T   
   T   
    T    
   G   
   C   
   G   
   A   
   C   
   A   
   G   
   G   
    C    
   T   
   A   
   T   
   G   
   C   
   A   
   C   
   C   
   A   
   G   
   G   
    C    
   T   
   C   
   T   
   A   
   C   
   C   
   T   
   T   
   T   
   A   
   A   
   A   
   A   
   C   
   A   
   A   
   T   
   T   
   A   
   C   
   C   
   G   
   G   
   T   
   G   
   C   
   C   
   A   
   T   
   T   
   G   
   G   
   T   
   T   
   T   
   A   
   G   
   A   
   T   
    G    
   C   
   A   
   G   
   G   
   G   
   A   
   C   
   A   
   T   
   T   
   G   
   A   
   A   
   G   
   C   
   C   
   A   
   G   
   A   
   T   
   G   
   A   
   A   
   G   
   A   
   G   
   C   
   T   
   A   
   G   
   A   
   A   
   A   
   T   
   T   
   A   
   A   
   T   
   G   
   G   
    C    
   T   
   T   
   G   
   A   
   A   
   A   
   T   
   G   
   G   
   C   
   A   
   A   
   A   
   A   
   A   
   G   
   A   
   T   
   A   
   A   
   A   
   T   
   C   
   T   
   T   
   G   
   G   
   G   
   G   
    C    
   G   
   G   
   C   
   T   
   A   
   T   
   T   
   T   
   T   
   G   
   C   
    G    
   A   
   C   
   A   
   C   
   G   
   T   
   G   
   T   
   A   
   A   
   A   
   A   
   G   
   A   
   A   
   G   
   C   
   A   
   A   
   G   
   T   
   C   
   C   
   A   
   G   
   T   
   C   
   A   
   A   
   T   
   C   
   T   
    G    
   C   
   G   
   T   
   A   
   C   
   C   
   G   
   C   
   T   
   T   
   T   
   A   
   G   
   T   
   T   
   A   
   A   
   T   
   T   
   C   
   A   
   G   
   A   
   T   
   A   
   A   
   T   
   A   
   T   
   T   
   T   
   A   
   T   
   T   
   T   
   T   
   G   
   C   
    T    
   C   
   A   
   A   
   C   
   A   
   A   
   A   
   C   
   A   
   T   
   T   
   A   
   C   
   G   
    G    
   A   
   T   
   G   
   G   
   G   
    A    
   G   
   A   
   A   
   G   
   A   
   C   
   A   
   A   
   A   
   T   
   T   
   T   
   A   
   G   
   A   
   G   
   C   
    G    
   G   
   G   
   T   
   C   
   T   
   T   
   A   
   A   
   T   
   A   
   A   
   A   
   T   
   T   
   C   
   A   
   T   
   T   
   T   
   T   
   C   
   G   
   G   
   T   
   G   
   A   
   G   
   G   
   A   
   A   
   C   
   T   
   A   
   G   
   A   
   T   
   T   
   T   
   A   
   C   
   C   
   A   
   A   
   T   
    T    
   G   
   C   
   C   
   A   
   T   
   G   
   A   
   C   
   G   
   C   
   C   
    T    
   G   
   C   
   T   
   C   
   A   
   A   
   A   
   T   
   T   
   T   
   C   
   A   
   A   
   A   
    T    
   G   
   A   
   A   
   G   
   A   
   T   
   A   
   A   
   A   
   T   
   T   
   T   
   A   
   A   
   T   
   T   
   C   
   T   
   G   
   A   
   A   
   A   
   T   
   T   
   C   
   T   
   T   
   T   
   T   
    A    
   G   
   C   
   A   
   G   
   A   
   T   
   A   
   C   
    G    
   G   
   G   
   C   
   T   
   A   
    C    
   G   
   G   
    T    
   C   
   A   
   A   
   G   
   G   
    C    
   C   
   A   
   A   
   T   
   T   
   G   
   T   
   T   
   A   
   A   
   T   
   T   
   T   
   C   
    G    
   C   
   C   
   A   
   A   
   T   
   C   
   C   
   A   
   G   
   C   
   A   
   A   
   G   
   C   
   T   
   A   
   C   
   C   
   A   
   T   
   G   
   T   
   A   
   T   
   A   
   G   
   T   
   G   
   T   
   T   
   T   
   T   
   C   
   C   
   A   
   A   
   A   
   A   
   T   
   A   
   A   
   T   
   G   
   G   
    A    
   A   
   C   
   A   
    T    
   T   
    G    
   G   
   T   
    C    
   T   
   A   
   T   
   C   
   C   
   A   
   A   
   A   
   A   
    A    
   T   
   A   
   G   
   T   
   T   
   T   
   T   
   A   
   G   
   A   
   C   
   A   
   A   
   A   
   G   
   A   
   A   
   A   
   C   
    G    
   A   
   A   
   G   
   A   
   A   
   A   
   A   
   A   
   A   
   G   
   A   
   C   
   A   
   A   
   T   
   G   
   T   
   A   
   A   
   T   
   C   
   A   
   G   
   C   
   G   
   C   
   C   
   A   
   A   
   C   
   G   
   C   
   A   
   G   
   C   
   G   
   A   
   A   
   T   
   A   
   C   
   A   
   A   
   T   
   T   
   G   
   C   
   C   
   A   
   C   
   A   
   G   
   A   
   T   
   C   
   T   
   T   
   T   
   T   
   A   
   G   
   G   
   T   
   A   
   G   
   T   
   G   
   T   
   G   
   G   
   A   
   A   
   G   
   A   
   C   
   C   
   C   
   T   
   T   
   C   
   A   
   G   
   G   
   T   
   T   
   A   
   T   
   G   
   T   
   T   
   T   
   A   
   T   
   A   
   A   
   T   
   A   
   T   
   G   
   T   
   A   
   C   
   A   
   A   
   T   
   C   
   C   
   G   
   A   
   A   
   C   
   T   
   T   
   C   
   T   
   C   
   A   
   T   
   T   
   G   
   G   
   C   
   T   
   G   
   C   
   T   
   A   
   A   
   A   
   A   
   C   
   A   
   G   
   G   
   A   
   A   
   C   
   A   
   G   
   C   
   T   
   G   
   A   
   A   
   A   
   T   
   A   
   A   
   A   
   A   
   G   
   A   
   T   
   A   
   A   
   A   
   C   
   A   
   A   
   G   
   A   
   T   
   A   
   C   
   A   
   G   
   A   
   T   
   G   
   G   
   C   
   A   
   A   
   A   
   G   
   A   
   A   
   A   
   A   
   C   
   A   
   G   
   T   
   T   
   T   
   C   
   C   
   T   
   T   
   C   
   T   
   A   
   A   
   C   
   G   
   T   
   T   
   A   
   G   
   A   
   T   
   C   
   G   
   T   
   A   
   G   
   T   
   A   
   A   
   C   
   A   
   A   
   T   
   A   
   A   
   A   
   T   
   T   
   C   
   T   
   T   
   A   
   A   
   C   
   A   
   A   
   T   
   G   
   A   
   T   
   T   
   A   
   T   
   G   
   G   
   T   
   T   
   G   
   A   
   A   
   A   
   A   
   T   
   T   
   C   
   A   
   G   
   G   
   A   
   G   
   A   
   A   
   A   
   A   
   T   
   G   
   G   
   T   
   T   
   C   
   T   
   G   
   C   
   A   
   A   
   C   
   A   
   G   
   A   
   T   
   A   
   T   
   C   
   A   
   G   
   T   
   A   
   A   
   A   
   C   
   C   
   A   
   T   
   T   
   A   
   A   
   T   
   T   
   G   
   A   
   T   
   T   
   A   
   C   
   T   
   T   
   A   
   G   
   A   
   A   
   G   
   C   
   A   
   A   
   C   
   C   
   A   
   T   
   T   
   A   
   A   
   A   
   -   
   -   
   -   
   -   
   -   
   -   
   -   
   -   
   -   
   -   
   -   
   -   
   -   
   -   
   -   
   -   
   -   
   -   
   -   
   -   
   -   
   -   
   -   
   -   
   -   
   -   
   -   
   -   
   -   
   -   
   -   
   -   
   -   
   -   
   -   
   -   
   -   
   -   
   -   
   -   
   -   
   -   
   -   
   -   
   -   
   -   
   -   
   -   
   -   
   -   
   -   
   -   
   -   
   -   
   -   
   -   
   -   
   -   
   -   
   -   
   -   
   -   
   -   
   -   
   -   
   -   
   -   
   -   
   -   
   -   
   -   
   -   
   -   
   -   
   -   
   -   
   -   
   -   
   -   
   -   
   -   
   -   
   -   
   -   
   -   
   -   
   -   
   -   
   -   
   -   
   -   
   -   
   -   
   -   
   -   
   -   
   -   
   -   
   -   
   -   
   -   
   -   
   -   
   -   
   -   
   -   
   -   
   -   
   -   
   -   
   -   
   -   
   -   
   -   
   -   
   -   
   -   
   -   
   -   
   -   
   -   
   -   
   -   
   -   
   -   
   -   
   -   
   -   
   -   
   -   
   -   
   -   
   -   
   -   
   -   
   -   
   -   
   -   
   -   
   -   
   -   
   -   
   -   
   -   
   -   
   -   
   -   
   -   
   -   
   -   
   -   
   -   
   -   
   -   
   -   
   -   
   -   
   -   
   -   
   -   
   -   
   -   
   -   
   -   
   -   
   -   
   -   
   -   
   -   
   C   
   A   
   A   
   T   
   G   
   G   
   C   
   A   
   A   
   G   
   C   
   C   
   A   
   A   
   A   
   C   
   A   
   A   
   G   
   A   
   A   
   T   
   T   
   A   
   G   
   C   
   C   
   G   
   A   
   A   
   G   
   C   
   G   
   A   
   A   
   G   
   A   
   A   
   A   
   A   
   C   
   A   
   G   
   C   
   T   
   A   
   C   
   T   
   A   
   C   
   A   
   T   
   T   
   T   
   T   
   T   
   A   
   A   
   A   
   C   
   G   
   T   
   A   
   T   
   T   
   G   
   T   
   C   
   A   
   A   
   A   
   A   
   C   
   A   
   G   
   G   
   A   
   A   
   T   
   T   
   T   
   G   
   A   
   T   
   A   
   A   
   G   
   T   
   T   
   A   
   C   
   C   
   G   
   T   
   C   
   C   
   G   
   T   
   T   
   G   
   T   
   T   
   C   
   A   
   A   
   G   
   A   
   A   
   G   
   C   
   T   
   A   
   G   
   C   
   T   
   T   
   A   
   A   
   A   
   G   
   A   
   A   
   A   
   A   
   A   
   T   
   G   
   G   
   C   
   T   
   A   
   T   
   G   
   A   
   T   
   A   
   C   
   T   
   A   
   A   
   A   
   T   
   C   
   T   
   G   
   T   
   T   
   G   
   T   
   T   
   G   
   A   
   A   
   A   
   A   
   A   
   T   
   A   
   C   
   C   
   A   
   A   
   G   
   C   
   A   
   A   
   T   
   T   
   T   
   A   
   T   
   T   
   C   
   A   
   G   
   G   
   G   
   A   
   T   
   T   
   C   
   A   
   A   
   G   
   C   
   A   
   G   
   A   
   A   
   G   
   G   
   A   
   G   
   T   
   C   
   A   
   A   
   A   
   G   
   C   
   T   
   A   
   G   
   T   
   G   
   A   
   T   
   G   
   T   
   T   
   C   
   A   
   A   
   G   
   T   
   C   
   A   
   A   
   A   
   A   
   A   
   G   
   G   
   C   
   G   
   A   
   A   
   A   
   G   
   A   
   C   
   A   
   A   
   T   
   C   
   A   
   A   
   T   
   A   
   C   
   A   
   C   
   A   
   T   
   T   
   T   
   A   
   C   
   C   
   T   
   A   
   T   
   A   
   A   
   A   
   T   
   T   
   A   
   T   
   C   
   G   
   A   
   T   
   G   
   A   
   G   
   T   
   A   
   C   
   C   
   C   
   C   
   T   
   T   
   T   
   A   
   G   
   G   
   C   
   G   
   A   
   A   
   A   
   T   
   G   
   A   
   A   
   A   
   G   
   A   
   T   
   T   
   T   
   G   
   T   
   C   
   T   
   T   
   A   
   T   
   C   
   A   
   A   
   T   
   C   
   A   
   A   
   G   
   T   
   A   
   T   
   C   
   G   
   C   
   C   
   A   
   A   
   A   
   A   
   A   
   A   
   G   
   G   
   C   
   G   
   A   
   T   
   A   
   C   
   C   
   T   
   A   
   C   
   C   
   A   
   A   
   A   
   T   
   C   
   G   
   C   
   T   
   T   
   G   
   G   
   A   
   A   
   G   
   C   
   C   
   G   
   T   
   C   
   T   
   T   
   T   
   A   
   A   
   T   
   T   
   T   
   T   
   T   
   C   
   C   
   A   
   G   
   A   
   T   
   A   
   T   
   G   
   T   
   C   
   A   
   G   
   G   
   A   
   A   
   A   
   T   
   G   
   A   
   T   
   A   
   A   
   A   
   A   
   T   
   T   
   T   
   C   
   G   
   A   
   T   
   T   
   C   
   A   
   A   
   G   
   T   
   A   
   G   
   A   
   T   
   A   
   A   
   T   
   G   
   C   
   C   
   A   
   A   
   A   
   C   
   G   
   T   
   G   
   G   
   A   
   G   
   A   
   A   
   A   
   T   
   T   
   G   
   T   
   C   
   G   
   A   
   T   
   C   
   G   
   T   
   A   
   A   
   T   
   G   
   G   
   T   
   A   
   G   
   T   
   G   
   G   
   G   
   C   
   T   
   A   
   G   
   C   
   A   
   A   
   T   
   T   
   A   
   A   
   C   
   A   
   A   
   A   
   G   
   T   
   G   
   T   
   T   
   T   
   G   
   A   
   C   
   G   
   A   
   A   
   G   
   T   
   G   
   G   
   G   
   C   
   G   
   T   
   A   
   G   
   T   
   G   
   C   
   C   
   T   
   G   
   G   
   C   
   A   
   A   
   A   
   C   
   T   
   C   
   G   
   G   
   T   
   T   
   C   
   T   
   G   
   G   
   C   
   G   
   C   
   A   
   G   
   A   
   A   
   A   
   A   
   A   
   A   
   C   
   A   
   G   
   C   
   C   
   A   
   A   
   T   
   A   
   T   
   C   
   A   
   A   
   A   
   G   
   C   
   T   
   T   
   T   
   T   
   A   
   G   
   T   
   G   
   A   
   T   
   A   
   A   
   G   
   T   
   T   
   C   
   G   
   G   
   C   
   G   
   T   
   T   
   T   
   C   
   T   
   A   
   T   
   T   
   G   
   A   
   T   
   G   
   A   
   A   
   A   
   T   
   C   
   A   
   A   
   T   
   C   
   A   
   A   
   A   
   A   
   G   
   T   
   T   
   A   
   A   
   G   
   C   
   C   
   A   
   A   
   G   
   G   
   A   
   T   
   G   
   G   
   G   
   T   
   C   
   C   
   A   
   A   
   G   
   C   
   A   
   G   
   A   
   C   
   T   
   C   
   C   
   T   
   T   
   T   
   G   
   T   
   A   
   C   
   C   
   A   
   A   
   T   
   C   
   A   
   C   
   A   
   G   
   T   
   C   
   G   
   C   
   T   
   T   
   C   
   T   
   G   
   A   
   A   
   C   
   C   
   A   
   G   
   T   
   G   
   A   
   C   
   A   
   G   
   A   
   A   
   T   
   T   
   A   
   C   
   C   
   A   
   A   
   C   
   A   
   G   
   G   
   G   
   G   
   C   
   T   
   G   
   C   
   G   
   A   
   C   
   A   
   A   
   A   
   A   
   G   
   A   
   T   
   A   
   C   
   A   
   G   
   A   
   G   
   T   
   C   
   A   
   C   
   G   
   T   
   T   
   A   
   T   
   T   
   A   
   T   
   C   
   C   
   G   
   C   
   T   
   G   
   G   
   G   
   G   
   G   
   A   
   A   
   G   
   C   
   A   
   G   
   C   
   T   
   G   
   C   
   G   
   C   
   A   
   A   
   T   
   T   
   A   
   A   
   T   
   C   
   G   
   G   
   G   
   T   
   A   
   T   
   A   
   C   
   G   
   G   
   G   
   C   
   A   
   C   
   C   
   A   
   T   
   T   
   A   
   C   
   T   
   G   
   C   
   A   
   G   
   A   
   A   
   G   
   A   
   T   
   A   
   T   
   T   
   G   
   A   
   A   
   A   
   A   
   A   
   A   
   A   
   T   
   C   
   C   
   A   
   G   
   A   
   G   
   T   
   T   
   A   
   A   
   G   
   T   
   A   
   G   
   T   
   A   
   C   
   G   
   G   
   G   
   C   
   G   
   T   
   A   
   A   
   T   
   T   
   G   
   G   
   T   
   A   
   A   
   A   
   A   
   C   
   T   
   G   
   G   
   C   
   T   
   T   
   A   
   G   
   A   
   A   
   C   
   G   
   A   
   G   
   C   
   G   
   T   
   T   
   T   
   G   
   A   
   T   
   A   
   A   
   A   
   G   
   A   
   A   
   T   
   T   
   A   
   A   
   G   
   A   
   G   
   G   
   G   
   C   
   A   
   A   
   G   
   A   
   T   
   G   
   G   
   T   
   G   
   G   
   C   
   T   
   C   
   A   
   C   
   T   
   A   
   G   
   T   
   T   
   A   
   T   
   T   
   T   
   T   
   A   
   G   
   A   
   T   
   G   
   A   
   C   
   A   
   A   
   A   
   G   
   A   
   G   
   A   
   A   
   T   
   G   
   T   
   C   
   A   
   A   
   A   
   A   
   A   
   A   
   G   
   C   
   T   
   T   
   T   
   A   
   C   
   A   
   A   
   A   
   C   
   C   
   A   
   A   
   A   
   G   
   A   
   G   
   A   
   A   
   A   
   A   
   A   
   A   
   G   
   A   
   T   
   G   
   G   
   T   
   C   
   A   
   A   
   A   
   C   
   G   
   A   
   T   
   T   
   A   
   A   
   A   
   T   
   T   
   A   
   A   
   C   
   G   
   A   
   T   
   T   
   G   
   A   
   C   
   A   
   G   
   T   
   G   
   G   
   C   
   G   
   T   
   A   
   C   
   A   
   A   
   C   
   A   
   A   
   C   
   A   
   A   
   G   
   C   
   C   
   T   
   T   
   C   
   G   
   C   
   T   
   A   
   T   
   A   
   T   
   T   
   T   
   G   
   A   
   C   
   A   
   A   
   A   
   C   
   G   
   T   
   C   
   C   
   T   
   G   
   G   
   T   
   A   
   G   
   T   
   G   
   C   
   G   
   G   
   T   
   C   
   A   
   T   
   T   
   A   
   C   
   C   
   G   
   A   
   T   
   C   
   C   
   T   
   C   
   A   
   A   
   A   
   A   
   A   
   G   
   G   
   C   
   G   
   A   
   T   
   T   
   T   
   A   
   T   
   T   
   A   
   G   
   C   
   A   
   A   
   C   
   G   
   G   
   T   
   T   
   A   
   G   
   T   
   T   
   C   
   A   
   C   
   C   
   T   
   T   
   C   
   G   
   T   
   A   
   T   
   G   
   A   
   T   
   C   
   C   
   T   
   A   
   A   
   T   
   A   
   A   
   A   
   A   
   T   
   G   
   G   
   C   
   C   
   A   
   A   
   T   
   G   
   G   
   T   
   A   
   T   
   T   
   T   
   C   
   T   
   C   
   A   
   A   
   A   
   A   
   A   
   G   
   A   
   A   
   T   
   A   
   T   
   G   
   A   
   T   
   G   
   C   
   T   
   T   
   A   
   C   
   A   
   A   
   T   
   A   
   A   
   C   
   A   
   A   
   C   
   A   
   A   
   A   
   G   
   A   
   T   
   T   
   T   
   A   
   C   
   C   
   A   
   T   
   T   
   C   
   A   
   C   
   A   
   G   
   C   
   A   
   C   
   G   
   T   
   T   
   T   
   T   
   G   
   C   
   G   
   A   
   C   
   A   
   G   
   G   
   C   
   T   
   A   
   T   
   G   
   C   
   A   
   C   
   C   
   A   
   G   
   G   
   C   
   T   
   C   
   T   
   A   
   C   
   C   
   T   
   T   
   T   
   A   
   A   
   A   
   A   
   C   
   A   
   A   
   T   
   T   
   A   
   C   
   C   
   G   
   G   
   T   
   G   
   C   
   C   
   A   
   T   
   T   
   G   
   G   
   T   
   T   
   T   
   A   
   G   
   A   
   T   
   G   
   C   
   A   
   G   
   G   
   G   
   A   
   C   
   A   
   T   
   T   
   G   
   A   
   A   
   G   
   C   
   C   
   A   
   G   
   A   
   T   
   G   
   A   
   A   
   G   
   A   
   G   
   C   
   T   
   A   
   G   
   A   
   A   
   A   
   T   
   T   
   A   
   A   
   T   
   G   
   G   
   C   
   T   
   T   
   G   
   A   
   A   
   A   
   T   
   G   
   G   
   C   
   A   
   A   
   A   
   A   
   A   
   G   
   A   
   T   
   A   
   A   
   A   
   T   
   C   
   T   
   T   
   G   
   G   
   G   
   G   
   C   
   G   
   G   
   C   
   T   
   A   
   T   
   T   
   T   
   T   
   G   
   C   
   G   
   A   
   C   
   A   
   C   
   G   
   T   
   G   
   T   
   A   
   A   
   A   
   A   
   G   
   A   
   A   
   G   
   C   
   A   
   A   
   G   
   T   
   C   
   C   
   A   
   G   
   T   
   C   
   A   
   A   
   T   
   C   
   T   
   G   
   C   
   G   
   T   
   A   
   C   
   C   
   G   
   C   
   T   
   T   
   T   
   A   
   G   
   T   
   T   
   A   
   A   
   T   
   T   
   C   
   A   
   G   
   A   
   T   
   A   
   A   
   T   
   A   
   T   
   T   
   T   
   A   
   T   
   T   
   T   
   T   
   G   
   C   
   T   
   C   
   A   
   A   
   C   
   A   
   A   
   A   
   C   
   A   
   T   
   T   
   A   
   C   
   G   
   G   
   A   
   T   
   G   
   G   
   G   
   A   
   G   
   A   
   A   
   G   
   A   
   C   
   A   
   A   
   A   
   T   
   T   
   T   
   A   
   G   
   A   
   G   
   C   
   G   
   G   
   G   
   T   
   C   
   T   
   T   
   A   
   A   
   T   
   A   
   A   
   A   
   T   
   T   
   C   
   A   
   T   
   T   
   T   
   T   
   C   
   G   
   G   
   T   
   G   
   A   
   G   
   G   
   A   
   A   
   C   
   T   
   A   
   G   
   A   
   T   
   T   
   T   
   A   
   C   
   C   
   A   
   A   
   T   
   T   
   G   
   C   
   C   
   A   
   T   
   G   
   A   
   C   
   G   
   C   
   C   
   T   
   G   
   C   
   T   
   C   
   A   
   A   
   A   
   T   
   T   
   T   
   C   
   A   
   A   
   A   
   T   
   G   
   A   
   A   
   G   
   A   
   T   
   A   
   A   
   A   
   T   
   T   
   T   
   A   
   A   
   T   
   T   
   C   
   T   
   G   
   A   
   A   
   A   
   T   
   T   
   C   
   T   
   T   
   T   
   T   
   A   
   G   
   C   
   A   
   G   
   A   
   T   
   A   
   C   
   G   
   G   
   G   
   C   
   T   
   A   
   C   
   G   
   G   
   T   
   C   
   A   
   A   
   G   
   G   
   C   
   C   
   A   
   A   
   T   
   T   
   G   
   T   
   T   
   A   
   A   
   T   
   T   
   T   
   C   
   G   
   C   
   C   
   A   
   A   
   T   
   C   
   C   
   A   
   G   
   C   
   A   
   A   
   G   
   C   
   T   
   A   
   C   
   C   
   A   
   T   
   G   
   T   
   A   
   T   
   A   
   G   
   T   
   G   
   T   
   T   
   T   
   T   
   C   
   C   
   A   
   A   
   A   
   A   
   T   
   A   
   A   
   T   
   G   
   G   
   A   
   A   
   C   
   A   
   T   
   T   
   G   
   G   
   T   
   C   
   T   
   A   
   T   
   C   
   C   
   A   
   A   
   A   
   A   
   A   
   T   
   A   
   G   
   T   
   T   
   T   
   T   
   A   
   G   
   A   
   C   
   A   
   A   
   A   
   G   
   A   
   A   
   A   
   C   
   G   
   A   
   A   
   G   
   A   
   A   
   A   
   A   
   A   
   A   
   G   
   A   
   C   
   A   
   A   
   T   
   G   
   T   
   A   
   A   
   T   
   C   
   A   
   G   
   C   
   G   
   C   
   C   
   A   
   A   
   C   
   G   
   C   
   A   
   G   
   C   
   G   
   A   
   A   
   T   
   A   
   C   
   A   
   A   
   T   
   T   
   G   
   C   
   C   
   A   
   C   
   A   
   G   
   A   
   T   
   C   
   T   
   T   
   T   
   T   
   A   
   G   
   G   
   T   
   A   
   G   
   T   
   G   
   T   
   G   
   G   
   A   
   A   
   G   
   A   
   C   
   C   
   C   
   T   
   T   
   C   
   A   
   G   
   G   
   T   
   T   
   A   
   T   
   G   
   T   
   T   
   T   
   A   
   T   
   A   
   A   
   T   
   A   
   T   
   G   
   T   
   A   
   C   
   A   
   A   
   T   
   C   
   C   
   G   
   A   
   A   
   C   
   T   
   T   
   C   
   T   
   C   
   A   
   T   
   T   
   G   
   G   
   C   
   T   
   G   
   C   
   T   
   A   
   A   
   A   
   A   
   C   
   A   
   G   
   G   
   A   
   A   
   C   
   A   
   G   
   C   
   T   
   G   
   A   
   A   
   A   
   T   
   A   
   A   
   A   
   A   
   G   
   A   
   T   
   A   
   A   
   A   
   C   
   A   
   A   
   G   
   A   
   T   
   A   
   C   
   A   
   G   
   A   
   T   
   G   
   G   
   C   
   A   
   A   
   A   
   G   
   A   
   A   
   A   
   A   
   C   
   A   
   G   
   T   
   T   
   T   
   C   
   C   
   T   
   T   
   C   
   T   
   A   
   A   
   C   
   G   
   T   
   T   
   A   
   G   
   A   
   T   
   C   
   G   
   T   
   A   
   G   
   T   
   A   
   A   
   C   
   A   
   A   
   T   
   A   
   A   
   A   
   T   
   T   
   C   
   T   
   T   
   A   
   A   
   C   
   A   
   A   
   T   
   G   
   A   
   T   
   T   
   A   
   T   
   G   
   G   
   T   
   T   
   G   
   A   
   A   
   A   
   A   
   T   
   T   
   C   
   A   
   G   
   G   
   A   
   G   
   A   
   A   
   A   
   A   
   T   
   G   
   G   
   T   
   T   
   C   
   T   
   G   
   C   
   A   
   A   
   C   
   A   
   G   
   A   
   T   
   A   
   T   
   C   
   A   
   G   
   T   
   A   
   A   
   A   
   C   
   C   
   A   
   T   
   T   
   A   
   A   
   T   
   T   
   G   
   A   
   T   
   T   
   A   
   C   
   T   
   T   
   A   
   G   
   A   
   A   
   G   
   C   
   A   
   A   
   C   
   C   
   A   
   T   
   T   
   A   
   A   
   A   
   -   
   -   
   -   
   -   
   -   
   -   
   -   
   -   
   -   
   -   
   -   
   -   
   -   
   -   
   -   
   -   
   -   
   -   
   -   
   -   
   -   
   -   
   -   
   -   
   -   
   -   
   -   
   -   
   -   
   -   
   -   
   -   
   -   
   -   
   -   
   -   
   -   
   -   
   -   
   -   
   -   
   -   
   -   
   -   
   -   
   -   
   -   
   -   
   -   
   -   
   -   
   -   
   -   
   -   
   -   
   -   
   -   
   -   
   -   
   -   
   -   
 

 
   N54676   
   -   
   -   
   -   
   -   
   -   
   -   
   -   
   -   
   -   
   -   
   -   
   -   
   -   
   -   
   -   
   -   
   -   
   -   
   -   
   -   
   -   
   -   
   -   
   -   
   -   
   -   
   -   
   -   
   -   
   -   
   -   
   -   
   -   
   -   
   -   
   -   
   -   
   -   
   -   
   -   
   -   
   -   
   -   
   -   
   -   
   -   
   -   
   -   
   -   
   -   
   -   
   -   
   -   
   -   
   -   
   -   
   -   
   -   
   -   
   -   
   -   
   -   
   -   
   -   
   -   
   -   
   -   
   -   
   -   
   -   
   -   
   -   
   -   
   -   
   -   
   -   
   -   
   -   
   -   
   -   
   -   
   -   
   -   
   -   
   -   
   -   
   -   
   -   
   -   
   -   
   -   
   -   
   -   
   -   
   -   
   -   
   -   
   -   
   -   
   -   
   -   
   -   
   -   
   -   
   -   
   -   
   -   
   -   
   C   
   A   
   A   
   T   
   G   
   G   
   C   
   A   
   A   
   G   
   C   
   C   
   A   
   A   
   A   
   C   
   A   
   A   
   G   
   A   
    A    
   T   
   T   
   A   
   G   
   C   
    C    
   G   
   A   
   A   
   G   
   C   
   G   
   A   
   A   
   G   
   A   
   A   
   A   
   A   
    C    
   A   
   G   
   C   
    T    
   A   
    A    
   T   
    A    
    C    
   A   
   T   
   T   
   T   
   T   
   T   
   A   
   A   
   A   
    T    
   G   
   T   
   A   
   T   
   T   
   G   
    T    
   C   
   A   
   A   
   A   
   A   
   C   
   A   
   G   
    G    
   A   
   A   
   T   
   T   
   T   
   G   
   A   
   T   
   A   
   A   
   G   
   T   
   T   
   A   
   C   
   C   
    G    
   T   
   C   
   C   
   G   
   T   
   T   
    G    
   T   
   T   
   C   
   A   
   A   
   G   
   A   
   A   
    T    
   C   
   T   
   A   
   G   
   C   
   T   
   T   
   A   
   A   
   A   
   G   
   A   
   A   
   A   
   A   
   A   
   T   
   G   
   G   
    C    
   T   
   A   
   T   
   G   
   A   
   T   
   A   
   C   
   T   
   A   
   A   
   A   
   T   
   C   
    T    
   G   
   T   
   T   
   G   
   T   
   T   
   G   
   A   
   A   
   A   
   A   
   A   
   T   
   A   
   C   
   C   
   A   
   A   
   G   
   C   
   A   
   A   
   T   
   T   
   T   
   A   
   T   
   T   
   C   
   A   
   G   
   G   
   G   
   A   
   T   
   T   
   C   
   A   
   A   
   G   
   C   
    A    
   G   
   A   
   A   
    G    
   G   
    A    
   G   
   T   
   C   
   A   
   A   
    A    
   G   
   C   
   T   
   A   
   G   
   T   
   G   
   A   
   T   
   G   
   T   
    C    
   C   
   A   
   A   
   G   
   T   
    C    
   A   
   A   
   A   
   A   
   A   
    G    
   G   
    C    
   G   
   A   
   A   
   A   
   G   
   A   
    C    
   A   
   A   
    T    
   C   
   A   
   A   
   T   
   A   
   C   
   A   
   C   
   A   
   T   
   T   
   T   
   A   
    C    
   C   
   T   
   A   
   T   
   A   
   A   
   A   
   T   
   T   
   A   
   T   
   C   
   G   
   A   
   T   
   G   
   A   
   G   
    C    
   A   
   C   
    T    
   C   
   C   
   T   
   T   
   T   
   A   
   G   
   G   
   C   
   G   
   A   
   A   
   A   
   T   
   G   
   A   
   A   
   A   
   G   
   A   
   T   
    C    
   T   
   G   
   T   
   C   
   T   
   T   
   A   
   T   
   C   
   A   
   A   
   T   
   C   
   A   
   A   
   G   
   T   
   A   
   T   
    C    
   G   
    C    
   C   
   A   
   A   
    A    
   A   
   A   
   A   
   G   
   G   
    C    
   G   
   A   
   T   
   A   
    C    
   C   
   T   
   A   
   C   
   C   
   A   
   A   
   A   
   T   
    C    
    T    
   C   
   T   
   T   
   G   
   G   
    A    
    A    
    G    
   C   
   C   
    G    
   T   
   C   
   T   
   T   
   T   
   A   
   A   
   T   
   T   
   T   
   T   
   T   
   C   
   C   
   A   
   G   
   A   
   T   
   A   
   T   
   G   
   T   
   C   
   A   
   G   
   G   
   A   
   A   
   A   
   T   
   G   
   A   
   T   
   A   
   A   
   A   
    A    
   T   
   T   
   T   
   C   
   G   
   A   
   T   
   T   
   C   
   A   
   A   
   G   
   T   
   A   
   G   
   A   
   T   
   A   
   A   
   T   
   G   
   C   
   C   
   A   
   A   
   A   
   C   
   G   
   T   
   G   
   G   
   A   
   G   
   A   
   A   
   A   
   T   
   T   
   G   
   T   
   C   
   G   
   A   
   T   
   C   
   G   
   T   
   A   
   A   
    T    
   G   
   G   
   T   
   A   
   G   
   T   
   G   
   G   
   G   
   C   
   T   
   A   
   G   
    C    
   A   
   A   
   T   
   T   
   A   
   A   
   C   
   A   
   A   
   A   
   G   
   T   
   G   
   T   
   T   
   T   
   G   
   A   
    C    
   G   
   A   
   A   
   G   
   T   
   G   
   G   
   G   
   C   
   G   
   T   
   A   
   G   
   T   
    G    
   C   
   C   
   T   
    G    
   G   
   C   
   A   
   A   
   A   
   C   
   T   
    C    
   G   
   G   
   T   
   T   
   C   
   T   
   G   
   G   
   C   
   G   
   C   
   A   
   G   
   A   
   A   
   A   
   A   
   A   
    A    
   C   
   A   
   G   
   C   
    C    
   A   
   A   
   T   
   A   
   T   
   C   
   A   
   A   
   A   
   G   
   C   
   T   
   T   
   T   
   T   
   A   
   G   
   T   
   G   
   A   
   T   
   A   
   A   
    G    
   T   
   T   
    C    
   G   
   G   
   C   
   G   
   T   
   T   
   T   
   C   
   T   
    G    
   T   
   T   
   G   
   A   
   T   
   G   
   A   
   A   
   A   
   T   
   C   
   A   
   A   
   T   
   C   
    A    
   A   
   A   
   A   
    G    
   T   
   T   
   A   
   A   
   G   
   C   
   C   
   A   
   A   
   G   
   G   
    G    
   T   
   G   
   G   
   G   
   T   
   C   
   C   
   A   
   A   
   G   
   C   
   A   
   G   
   A   
   C   
   T   
   C   
    C    
   T   
   T   
   T   
   G   
   T   
   A   
   C   
   C   
   A   
   A   
   T   
   C   
   A   
   C   
   A   
   G   
   T   
    T    
   G   
   C   
   T   
   T   
   C   
   T   
   G   
   A   
   A   
   C   
   C   
   A   
   G   
   T   
   G   
   A   
   C   
   A   
   G   
   A   
   A   
   T   
   T   
   A   
   C   
   C   
   A   
   A   
   C   
   A   
   G   
   G   
   G   
   G   
   C   
   T   
   G   
   C   
   G   
   A   
   C   
   A   
   A   
   A   
   A   
   G   
    A    
    T    
   A   
   C   
   A   
   G   
   A   
   G   
   T   
   C   
   A   
   C   
   G   
   T   
   T   
   A   
   T   
   T   
   A   
   T   
   C   
   C   
    A    
    C    
   T   
   G   
   G   
   G   
   G   
   G   
   A   
   A   
   G   
   C   
   A   
   G   
   C   
   T   
   G   
   C   
   G   
   C   
   A   
   A   
   T   
   T   
   A   
   A   
   T   
   C   
   G   
   G   
    G    
   T   
   A   
   T   
   A   
   C   
    G    
   G   
   G   
    C    
   A   
   C   
    C    
   A   
   T   
   T   
   A   
   C   
   T   
   G   
   C   
   A   
   G   
   A   
    A    
   G   
   A   
   T   
   A   
   T   
   T   
    G    
   A   
   A   
   A   
   A   
   A   
   A   
   A   
   T   
   C   
   C   
   A   
   G   
   A   
   G   
   T   
   T   
   A   
   A   
   G   
   T   
   A   
   G   
   T   
   A   
   C   
   G   
   G   
   G   
    T    
   G   
   T   
   A   
   A   
   T   
   T   
   G   
   G   
    T    
   A   
   A   
   A   
   A   
   C   
   T   
   G   
   G   
    C    
   T   
   T   
   A   
   G   
   A   
   A   
   C   
   G   
   A   
   G   
   C   
   G   
   T   
   T   
   T   
   G   
   A   
   T   
   A   
   A   
   A   
   G   
   A   
    G    
   T   
   T   
   A   
   A   
   G   
   A   
   G   
   G   
    G    
   C   
   A   
   A   
   G   
   A   
   T   
   G   
   G   
    T    
   G   
   G   
    C    
   T   
   C   
    A    
   C   
   T   
   A   
   G   
   T   
   T   
   A   
   T   
   T   
   T   
   T   
   A   
   G   
   A   
   T   
   G   
   A   
   C   
   A   
   A   
   A   
   G   
   A   
   G   
   A   
   A   
   T   
   G   
   T   
   C   
   A   
   A   
   A   
   A   
   A   
   A   
   G   
   C   
   T   
   T   
   T   
   A   
   C   
   A   
   A   
   A   
   C   
   C   
   A   
   A   
   A   
   G   
   A   
    G    
   A   
   A   
   A   
   A   
   A   
   A   
   G   
   A   
   T   
   G   
   G   
   T   
   C   
   A   
   A   
   A   
   C   
    G    
   A   
   T   
   T   
   A   
   A   
   A   
   T   
   T   
   A   
   A   
   C   
   G   
   A   
   T   
   T   
   G   
   A   
    C    
   A   
   G   
    T    
   G   
   G   
    C    
   G   
   T   
   A   
   C   
   A   
   A   
   C   
   A   
   A   
   C   
   A   
   A   
   G   
   C   
    C    
   T   
   T   
    C    
   G   
   C   
   T   
   A   
   T   
   A   
   T   
   T   
   T   
   G   
   A   
   C   
   A   
   A   
   A   
   C   
   G   
    T    
    C    
   C   
   T   
   G   
   G   
   T   
   A   
   G   
   T   
   G   
   C   
   G   
   G   
   T   
   C   
   A   
   T   
   T   
   A   
   C   
   C   
   G   
   A   
   T   
   C   
   C   
   T   
   C   
   A   
   A   
   A   
   A   
   A   
   G   
   G   
   C   
   G   
   A   
   T   
   T   
   T   
   A   
    T    
   T   
   A   
   G   
   C   
    A    
   A   
   C   
   G   
   G   
   T   
   T   
   A   
   G   
   T   
   T   
   C   
   A   
   C   
   C   
   T   
   T   
   C   
   G   
   T   
   A   
   T   
   G   
   A   
   T   
   C   
   C   
   T   
   A   
   A   
   T   
   A   
   A   
   A   
   A   
   T   
   G   
   G   
   C   
   C   
   A   
   A   
   T   
   G   
   G   
    T    
   A   
   T   
   T   
   T   
   C   
   T   
   C   
   A   
   A   
   A   
   A   
   A   
   G   
   A   
    A    
   T   
   A   
   T   
   G   
   A   
   T   
   G   
   C   
   T   
   T   
   A   
   C   
   A   
   A   
   T   
   A   
   A   
   C   
   A   
   A   
   C   
   A   
   A   
   A   
   G   
   A   
   T   
   T   
   T   
   A   
   C   
   C   
   A   
   T   
   T   
    C    
   A   
   C   
   A   
   G   
   C   
   A   
   C   
   G   
   T   
   T   
   T   
    T    
   G   
   C   
   G   
   A   
   C   
   A   
   G   
   G   
    C    
   T   
   A   
   T   
   G   
   C   
   A   
   C   
   C   
   A   
   G   
   G   
    C    
   T   
   C   
   T   
   A   
   C   
   C   
   T   
   T   
   T   
   A   
   A   
   A   
   A   
   C   
   A   
   A   
   T   
   T   
   A   
   C   
   C   
   G   
   G   
   T   
   G   
   C   
   C   
   A   
   T   
   T   
   G   
   G   
   T   
   T   
   T   
   A   
   G   
   A   
   T   
    G    
   C   
   A   
   G   
   G   
   G   
   A   
   C   
   A   
   T   
   T   
   G   
   A   
   A   
   G   
   C   
   C   
   A   
   G   
   A   
   T   
   G   
   A   
   A   
   G   
   A   
   G   
   C   
   T   
   A   
   G   
   A   
   A   
   A   
   T   
   T   
   A   
   A   
   T   
   G   
   G   
    C    
   T   
   T   
   G   
   A   
   A   
   A   
   T   
   G   
   G   
   C   
   A   
   A   
   A   
   A   
   A   
   G   
   A   
   T   
   A   
   A   
   A   
   T   
   C   
   T   
   T   
   G   
   G   
   G   
   G   
    C    
   G   
   G   
   C   
   T   
   A   
   T   
   T   
   T   
   T   
   G   
   C   
    A    
   A   
   C   
   A   
   C   
   G   
   T   
   G   
   T   
   A   
   A   
   A   
   A   
   G   
   A   
   A   
   G   
   C   
   A   
   A   
   G   
   T   
   C   
   C   
   A   
   G   
   T   
   C   
   A   
   A   
   T   
   C   
   T   
    G    
   C   
   G   
   T   
   A   
   C   
   C   
   G   
   C   
   T   
   T   
   T   
   A   
   G   
   T   
   T   
   A   
   A   
   T   
   T   
   C   
   A   
   G   
   A   
   T   
   A   
   A   
   T   
   A   
   T   
   T   
   T   
   A   
   T   
   T   
   T   
   T   
   G   
   C   
    C    
   C   
   A   
   A   
   C   
   A   
   A   
   A   
   C   
   A   
   T   
   T   
   A   
   C   
   G   
    G    
   A   
   T   
   G   
   G   
   G   
    G    
   G   
   A   
   A   
   G   
   A   
   C   
   A   
   A   
   A   
   T   
   T   
   T   
   A   
   G   
   A   
   G   
   C   
    A    
   G   
   G   
   T   
   C   
   T   
   T   
   A   
   A   
   T   
   A   
   A   
   A   
   T   
   T   
   C   
   A   
   T   
   T   
   T   
   T   
   C   
   G   
   G   
   T   
   G   
   A   
   G   
   G   
   A   
   A   
   C   
   T   
   A   
   G   
   A   
   T   
   T   
   T   
   A   
   C   
   C   
   A   
   A   
   T   
    T    
   G   
   C   
   C   
   A   
   T   
   G   
   A   
   C   
   G   
   C   
   C   
    T    
   G   
   C   
   T   
   C   
   A   
   A   
   A   
   T   
   T   
   T   
   C   
   A   
   A   
   A   
    T    
   G   
   A   
   A   
   G   
   A   
   T   
   A   
   A   
   A   
   T   
   T   
   T   
   A   
   A   
   T   
   T   
   C   
   T   
   G   
   A   
   A   
   A   
   T   
   T   
   C   
   T   
   T   
   T   
   T   
    A    
   G   
   C   
   A   
   G   
   A   
   T   
   A   
   C   
    G    
   G   
   G   
   C   
   T   
   A   
    C    
   G   
   G   
    C    
   C   
   A   
   A   
   G   
   G   
    C    
   C   
   A   
   A   
   T   
   T   
   G   
   T   
   T   
   A   
   A   
   T   
   T   
   T   
   C   
    G    
   C   
   C   
   A   
   A   
   T   
   C   
   C   
   A   
   G   
   C   
   A   
   A   
   G   
   C   
   T   
   A   
   C   
   C   
   A   
   T   
   G   
   T   
   A   
   T   
   A   
   G   
   T   
   G   
   T   
   T   
   T   
   T   
   C   
   C   
   A   
   A   
   A   
   A   
   T   
   A   
   A   
   T   
   G   
   G   
    A    
   A   
   C   
   A   
    T    
   T   
    A    
   G   
   T   
    C    
   T   
   A   
   T   
   C   
   C   
   A   
   A   
   A   
   A   
    T    
   T   
   A   
   G   
   T   
   T   
   T   
   T   
   A   
   G   
   A   
   C   
   A   
   A   
   A   
   G   
   A   
   A   
   A   
   C   
    G    
   A   
   A   
   G   
   A   
   A   
   A   
   A   
   A   
   A   
   G   
   A   
   C   
   A   
   A   
   T   
   G   
   T   
   A   
   A   
   T   
   C   
   A   
   G   
   C   
   G   
   C   
   C   
   A   
   A   
   C   
   G   
   C   
   A   
   G   
   C   
   G   
   A   
   A   
   T   
   A   
   C   
   A   
   A   
   T   
   T   
   G   
   C   
   C   
   A   
   C   
   A   
   G   
   A   
   T   
   C   
   T   
   T   
   T   
   T   
   A   
   G   
   G   
   T   
   A   
   G   
   T   
   G   
   T   
   G   
   G   
   A   
   A   
   G   
   A   
   T   
   C   
   C   
   T   
   T   
   C   
   A   
   G   
   G   
   T   
   T   
   A   
   T   
   G   
   T   
   T   
   T   
   A   
   T   
   A   
   A   
   T   
   A   
   T   
   G   
   T   
   A   
   C   
   A   
   A   
   T   
   C   
   C   
   G   
   A   
   A   
   C   
   T   
   T   
   C   
   T   
   C   
   A   
   T   
   T   
   G   
   G   
   C   
   T   
   G   
   C   
   T   
   A   
   A   
   A   
   A   
   C   
   A   
   G   
   G   
   A   
   A   
   C   
   A   
   G   
   C   
   T   
   G   
   A   
   A   
   A   
   T   
   A   
   A   
   A   
   A   
   G   
   A   
   T   
   A   
   A   
   A   
   C   
   A   
   A   
   G   
   A   
   T   
   A   
   C   
   A   
   G   
   A   
   T   
   G   
   G   
   C   
   A   
   A   
   A   
   G   
   A   
   A   
   A   
   A   
   C   
   A   
   G   
   T   
   T   
   T   
   C   
   C   
   T   
   C   
   C   
   T   
   A   
   A   
   C   
   G   
   T   
   T   
   A   
   G   
   A   
   T   
   C   
   G   
   T   
   A   
   G   
   T   
   A   
   A   
   C   
   A   
   A   
   T   
   A   
   A   
   A   
   T   
   T   
   C   
   T   
   T   
   A   
   A   
   C   
   A   
   A   
   T   
   G   
   A   
   T   
   T   
   A   
   T   
   G   
   G   
   T   
   T   
   G   
   A   
   A   
   A   
   A   
   T   
   T   
   C   
   A   
   G   
   G   
   A   
   G   
   A   
   A   
   A   
   A   
   T   
   G   
   G   
   T   
   T   
   C   
   T   
   G   
   C   
   A   
   A   
   C   
   A   
   G   
   A   
   T   
   A   
   T   
   C   
   A   
   G   
   T   
   A   
   A   
   A   
   C   
   C   
   A   
   T   
   T   
   A   
   A   
   T   
   T   
   G   
   A   
   T   
   T   
   A   
   C   
   T   
   T   
   A   
   G   
   A   
   A   
   G   
   C   
   A   
   A   
   C   
   C   
   A   
   T   
   T   
   A   
   A   
   A   
   -   
   -   
   -   
   -   
   -   
   -   
   -   
   -   
   -   
   -   
   -   
   -   
   -   
   -   
   -   
   -   
   -   
   -   
   -   
   -   
   -   
   -   
   -   
   -   
   -   
   -   
   -   
   -   
   -   
   -   
   -   
   -   
   -   
   -   
   -   
   -   
   -   
   -   
   -   
   -   
   -   
   -   
   -   
   -   
   -   
   -   
   -   
   -   
   -   
   -   
   -   
   -   
   -   
   -   
   -   
   -   
   -   
   -   
   -   
   -   
   -   
   -   
   -   
   -   
   -   
   -   
   -   
   -   
   -   
   -   
   -   
   -   
   -   
   -   
   -   
   -   
   -   
   -   
   -   
   -   
   -   
   -   
   -   
   -   
   -   
   -   
   -   
   -   
   -   
   -   
   -   
   -   
   -   
   -   
   -   
   -   
   -   
   -   
   -   
   -   
   -   
   -   
   -   
   -   
   -   
   -   
   -   
   -   
   -   
   -   
   -   
   -   
   -   
   -   
   -   
   -   
   -   
   -   
   -   
   -   
   -   
   -   
   -   
   -   
   -   
   -   
   -   
   -   
   -   
   -   
   -   
   -   
   -   
   -   
   -   
   -   
   -   
   -   
   -   
   -   
   -   
   -   
   -   
   -   
   -   
   -   
   -   
   -   
   -   
   -   
   -   
   -   
   -   
   -   
   -   
   -   
   -   
   -   
   -   
   -   
   -   
   -   
   -   
   -   
   -   
   -   
   -   
   -   
   -   
   C   
   A   
   A   
   T   
   G   
   G   
   C   
   A   
   A   
   G   
   C   
   C   
   A   
   A   
   A   
   C   
   A   
   A   
   G   
   A   
   A   
   T   
   T   
   A   
   G   
   C   
   C   
   G   
   A   
   A   
   G   
   C   
   G   
   A   
   A   
   G   
   A   
   A   
   A   
   A   
   C   
   A   
   G   
   C   
   T   
   A   
   A   
   T   
   A   
   C   
   A   
   T   
   T   
   T   
   T   
   T   
   A   
   A   
   A   
   T   
   G   
   T   
   A   
   T   
   T   
   G   
   T   
   C   
   A   
   A   
   A   
   A   
   C   
   A   
   G   
   G   
   A   
   A   
   T   
   T   
   T   
   G   
   A   
   T   
   A   
   A   
   G   
   T   
   T   
   A   
   C   
   C   
   G   
   T   
   C   
   C   
   G   
   T   
   T   
   G   
   T   
   T   
   C   
   A   
   A   
   G   
   A   
   A   
   T   
   C   
   T   
   A   
   G   
   C   
   T   
   T   
   A   
   A   
   A   
   G   
   A   
   A   
   A   
   A   
   A   
   T   
   G   
   G   
   C   
   T   
   A   
   T   
   G   
   A   
   T   
   A   
   C   
   T   
   A   
   A   
   A   
   T   
   C   
   T   
   G   
   T   
   T   
   G   
   T   
   T   
   G   
   A   
   A   
   A   
   A   
   A   
   T   
   A   
   C   
   C   
   A   
   A   
   G   
   C   
   A   
   A   
   T   
   T   
   T   
   A   
   T   
   T   
   C   
   A   
   G   
   G   
   G   
   A   
   T   
   T   
   C   
   A   
   A   
   G   
   C   
   A   
   G   
   A   
   A   
   G   
   G   
   A   
   G   
   T   
   C   
   A   
   A   
   A   
   G   
   C   
   T   
   A   
   G   
   T   
   G   
   A   
   T   
   G   
   T   
   C   
   C   
   A   
   A   
   G   
   T   
   C   
   A   
   A   
   A   
   A   
   A   
   G   
   G   
   C   
   G   
   A   
   A   
   A   
   G   
   A   
   C   
   A   
   A   
   T   
   C   
   A   
   A   
   T   
   A   
   C   
   A   
   C   
   A   
   T   
   T   
   T   
   A   
   C   
   C   
   T   
   A   
   T   
   A   
   A   
   A   
   T   
   T   
   A   
   T   
   C   
   G   
   A   
   T   
   G   
   A   
   G   
   C   
   A   
   C   
   T   
   C   
   C   
   T   
   T   
   T   
   A   
   G   
   G   
   C   
   G   
   A   
   A   
   A   
   T   
   G   
   A   
   A   
   A   
   G   
   A   
   T   
   C   
   T   
   G   
   T   
   C   
   T   
   T   
   A   
   T   
   C   
   A   
   A   
   T   
   C   
   A   
   A   
   G   
   T   
   A   
   T   
   C   
   G   
   C   
   C   
   A   
   A   
   A   
   A   
   A   
   A   
   G   
   G   
   C   
   G   
   A   
   T   
   A   
   C   
   C   
   T   
   A   
   C   
   C   
   A   
   A   
   A   
   T   
   C   
   T   
   C   
   T   
   T   
   G   
   G   
   A   
   A   
   G   
   C   
   C   
   G   
   T   
   C   
   T   
   T   
   T   
   A   
   A   
   T   
   T   
   T   
   T   
   T   
   C   
   C   
   A   
   G   
   A   
   T   
   A   
   T   
   G   
   T   
   C   
   A   
   G   
   G   
   A   
   A   
   A   
   T   
   G   
   A   
   T   
   A   
   A   
   A   
   A   
   T   
   T   
   T   
   C   
   G   
   A   
   T   
   T   
   C   
   A   
   A   
   G   
   T   
   A   
   G   
   A   
   T   
   A   
   A   
   T   
   G   
   C   
   C   
   A   
   A   
   A   
   C   
   G   
   T   
   G   
   G   
   A   
   G   
   A   
   A   
   A   
   T   
   T   
   G   
   T   
   C   
   G   
   A   
   T   
   C   
   G   
   T   
   A   
   A   
   T   
   G   
   G   
   T   
   A   
   G   
   T   
   G   
   G   
   G   
   C   
   T   
   A   
   G   
   C   
   A   
   A   
   T   
   T   
   A   
   A   
   C   
   A   
   A   
   A   
   G   
   T   
   G   
   T   
   T   
   T   
   G   
   A   
   C   
   G   
   A   
   A   
   G   
   T   
   G   
   G   
   G   
   C   
   G   
   T   
   A   
   G   
   T   
   G   
   C   
   C   
   T   
   G   
   G   
   C   
   A   
   A   
   A   
   C   
   T   
   C   
   G   
   G   
   T   
   T   
   C   
   T   
   G   
   G   
   C   
   G   
   C   
   A   
   G   
   A   
   A   
   A   
   A   
   A   
   A   
   C   
   A   
   G   
   C   
   C   
   A   
   A   
   T   
   A   
   T   
   C   
   A   
   A   
   A   
   G   
   C   
   T   
   T   
   T   
   T   
   A   
   G   
   T   
   G   
   A   
   T   
   A   
   A   
   G   
   T   
   T   
   C   
   G   
   G   
   C   
   G   
   T   
   T   
   T   
   C   
   T   
   G   
   T   
   T   
   G   
   A   
   T   
   G   
   A   
   A   
   A   
   T   
   C   
   A   
   A   
   T   
   C   
   A   
   A   
   A   
   A   
   G   
   T   
   T   
   A   
   A   
   G   
   C   
   C   
   A   
   A   
   G   
   G   
   G   
   T   
   G   
   G   
   G   
   T   
   C   
   C   
   A   
   A   
   G   
   C   
   A   
   G   
   A   
   C   
   T   
   C   
   C   
   T   
   T   
   T   
   G   
   T   
   A   
   C   
   C   
   A   
   A   
   T   
   C   
   A   
   C   
   A   
   G   
   T   
   T   
   G   
   C   
   T   
   T   
   C   
   T   
   G   
   A   
   A   
   C   
   C   
   A   
   G   
   T   
   G   
   A   
   C   
   A   
   G   
   A   
   A   
   T   
   T   
   A   
   C   
   C   
   A   
   A   
   C   
   A   
   G   
   G   
   G   
   G   
   C   
   T   
   G   
   C   
   G   
   A   
   C   
   A   
   A   
   A   
   A   
   G   
   A   
   T   
   A   
   C   
   A   
   G   
   A   
   G   
   T   
   C   
   A   
   C   
   G   
   T   
   T   
   A   
   T   
   T   
   A   
   T   
   C   
   C   
   A   
   C   
   T   
   G   
   G   
   G   
   G   
   G   
   A   
   A   
   G   
   C   
   A   
   G   
   C   
   T   
   G   
   C   
   G   
   C   
   A   
   A   
   T   
   T   
   A   
   A   
   T   
   C   
   G   
   G   
   G   
   T   
   A   
   T   
   A   
   C   
   G   
   G   
   G   
   C   
   A   
   C   
   C   
   A   
   T   
   T   
   A   
   C   
   T   
   G   
   C   
   A   
   G   
   A   
   A   
   G   
   A   
   T   
   A   
   T   
   T   
   G   
   A   
   A   
   A   
   A   
   A   
   A   
   A   
   T   
   C   
   C   
   A   
   G   
   A   
   G   
   T   
   T   
   A   
   A   
   G   
   T   
   A   
   G   
   T   
   A   
   C   
   G   
   G   
   G   
   T   
   G   
   T   
   A   
   A   
   T   
   T   
   G   
   G   
   T   
   A   
   A   
   A   
   A   
   C   
   T   
   G   
   G   
   C   
   T   
   T   
   A   
   G   
   A   
   A   
   C   
   G   
   A   
   G   
   C   
   G   
   T   
   T   
   T   
   G   
   A   
   T   
   A   
   A   
   A   
   G   
   A   
   G   
   T   
   T   
   A   
   A   
   G   
   A   
   G   
   G   
   G   
   C   
   A   
   A   
   G   
   A   
   T   
   G   
   G   
   T   
   G   
   G   
   C   
   T   
   C   
   A   
   C   
   T   
   A   
   G   
   T   
   T   
   A   
   T   
   T   
   T   
   T   
   A   
   G   
   A   
   T   
   G   
   A   
   C   
   A   
   A   
   A   
   G   
   A   
   G   
   A   
   A   
   T   
   G   
   T   
   C   
   A   
   A   
   A   
   A   
   A   
   A   
   G   
   C   
   T   
   T   
   T   
   A   
   C   
   A   
   A   
   A   
   C   
   C   
   A   
   A   
   A   
   G   
   A   
   G   
   A   
   A   
   A   
   A   
   A   
   A   
   G   
   A   
   T   
   G   
   G   
   T   
   C   
   A   
   A   
   A   
   C   
   G   
   A   
   T   
   T   
   A   
   A   
   A   
   T   
   T   
   A   
   A   
   C   
   G   
   A   
   T   
   T   
   G   
   A   
   C   
   A   
   G   
   T   
   G   
   G   
   C   
   G   
   T   
   A   
   C   
   A   
   A   
   C   
   A   
   A   
   C   
   A   
   A   
   G   
   C   
   C   
   T   
   T   
   C   
   G   
   C   
   T   
   A   
   T   
   A   
   T   
   T   
   T   
   G   
   A   
   C   
   A   
   A   
   A   
   C   
   G   
   T   
   C   
   C   
   T   
   G   
   G   
   T   
   A   
   G   
   T   
   G   
   C   
   G   
   G   
   T   
   C   
   A   
   T   
   T   
   A   
   C   
   C   
   G   
   A   
   T   
   C   
   C   
   T   
   C   
   A   
   A   
   A   
   A   
   A   
   G   
   G   
   C   
   G   
   A   
   T   
   T   
   T   
   A   
   T   
   T   
   A   
   G   
   C   
   A   
   A   
   C   
   G   
   G   
   T   
   T   
   A   
   G   
   T   
   T   
   C   
   A   
   C   
   C   
   T   
   T   
   C   
   G   
   T   
   A   
   T   
   G   
   A   
   T   
   C   
   C   
   T   
   A   
   A   
   T   
   A   
   A   
   A   
   A   
   T   
   G   
   G   
   C   
   C   
   A   
   A   
   T   
   G   
   G   
   T   
   A   
   T   
   T   
   T   
   C   
   T   
   C   
   A   
   A   
   A   
   A   
   A   
   G   
   A   
   A   
   T   
   A   
   T   
   G   
   A   
   T   
   G   
   C   
   T   
   T   
   A   
   C   
   A   
   A   
   T   
   A   
   A   
   C   
   A   
   A   
   C   
   A   
   A   
   A   
   G   
   A   
   T   
   T   
   T   
   A   
   C   
   C   
   A   
   T   
   T   
   C   
   A   
   C   
   A   
   G   
   C   
   A   
   C   
   G   
   T   
   T   
   T   
   T   
   G   
   C   
   G   
   A   
   C   
   A   
   G   
   G   
   C   
   T   
   A   
   T   
   G   
   C   
   A   
   C   
   C   
   A   
   G   
   G   
   C   
   T   
   C   
   T   
   A   
   C   
   C   
   T   
   T   
   T   
   A   
   A   
   A   
   A   
   C   
   A   
   A   
   T   
   T   
   A   
   C   
   C   
   G   
   G   
   T   
   G   
   C   
   C   
   A   
   T   
   T   
   G   
   G   
   T   
   T   
   T   
   A   
   G   
   A   
   T   
   G   
   C   
   A   
   G   
   G   
   G   
   A   
   C   
   A   
   T   
   T   
   G   
   A   
   A   
   G   
   C   
   C   
   A   
   G   
   A   
   T   
   G   
   A   
   A   
   G   
   A   
   G   
   C   
   T   
   A   
   G   
   A   
   A   
   A   
   T   
   T   
   A   
   A   
   T   
   G   
   G   
   C   
   T   
   T   
   G   
   A   
   A   
   A   
   T   
   G   
   G   
   C   
   A   
   A   
   A   
   A   
   A   
   G   
   A   
   T   
   A   
   A   
   A   
   T   
   C   
   T   
   T   
   G   
   G   
   G   
   G   
   C   
   G   
   G   
   C   
   T   
   A   
   T   
   T   
   T   
   T   
   G   
   C   
   A   
   A   
   C   
   A   
   C   
   G   
   T   
   G   
   T   
   A   
   A   
   A   
   A   
   G   
   A   
   A   
   G   
   C   
   A   
   A   
   G   
   T   
   C   
   C   
   A   
   G   
   T   
   C   
   A   
   A   
   T   
   C   
   T   
   G   
   C   
   G   
   T   
   A   
   C   
   C   
   G   
   C   
   T   
   T   
   T   
   A   
   G   
   T   
   T   
   A   
   A   
   T   
   T   
   C   
   A   
   G   
   A   
   T   
   A   
   A   
   T   
   A   
   T   
   T   
   T   
   A   
   T   
   T   
   T   
   T   
   G   
   C   
   C   
   C   
   A   
   A   
   C   
   A   
   A   
   A   
   C   
   A   
   T   
   T   
   A   
   C   
   G   
   G   
   A   
   T   
   G   
   G   
   G   
   G   
   G   
   A   
   A   
   G   
   A   
   C   
   A   
   A   
   A   
   T   
   T   
   T   
   A   
   G   
   A   
   G   
   C   
   A   
   G   
   G   
   T   
   C   
   T   
   T   
   A   
   A   
   T   
   A   
   A   
   A   
   T   
   T   
   C   
   A   
   T   
   T   
   T   
   T   
   C   
   G   
   G   
   T   
   G   
   A   
   G   
   G   
   A   
   A   
   C   
   T   
   A   
   G   
   A   
   T   
   T   
   T   
   A   
   C   
   C   
   A   
   A   
   T   
   T   
   G   
   C   
   C   
   A   
   T   
   G   
   A   
   C   
   G   
   C   
   C   
   T   
   G   
   C   
   T   
   C   
   A   
   A   
   A   
   T   
   T   
   T   
   C   
   A   
   A   
   A   
   T   
   G   
   A   
   A   
   G   
   A   
   T   
   A   
   A   
   A   
   T   
   T   
   T   
   A   
   A   
   T   
   T   
   C   
   T   
   G   
   A   
   A   
   A   
   T   
   T   
   C   
   T   
   T   
   T   
   T   
   A   
   G   
   C   
   A   
   G   
   A   
   T   
   A   
   C   
   G   
   G   
   G   
   C   
   T   
   A   
   C   
   G   
   G   
   C   
   C   
   A   
   A   
   G   
   G   
   C   
   C   
   A   
   A   
   T   
   T   
   G   
   T   
   T   
   A   
   A   
   T   
   T   
   T   
   C   
   G   
   C   
   C   
   A   
   A   
   T   
   C   
   C   
   A   
   G   
   C   
   A   
   A   
   G   
   C   
   T   
   A   
   C   
   C   
   A   
   T   
   G   
   T   
   A   
   T   
   A   
   G   
   T   
   G   
   T   
   T   
   T   
   T   
   C   
   C   
   A   
   A   
   A   
   A   
   T   
   A   
   A   
   T   
   G   
   G   
   A   
   A   
   C   
   A   
   T   
   T   
   A   
   G   
   T   
   C   
   T   
   A   
   T   
   C   
   C   
   A   
   A   
   A   
   A   
   T   
   T   
   A   
   G   
   T   
   T   
   T   
   T   
   A   
   G   
   A   
   C   
   A   
   A   
   A   
   G   
   A   
   A   
   A   
   C   
   G   
   A   
   A   
   G   
   A   
   A   
   A   
   A   
   A   
   A   
   G   
   A   
   C   
   A   
   A   
   T   
   G   
   T   
   A   
   A   
   T   
   C   
   A   
   G   
   C   
   G   
   C   
   C   
   A   
   A   
   C   
   G   
   C   
   A   
   G   
   C   
   G   
   A   
   A   
   T   
   A   
   C   
   A   
   A   
   T   
   T   
   G   
   C   
   C   
   A   
   C   
   A   
   G   
   A   
   T   
   C   
   T   
   T   
   T   
   T   
   A   
   G   
   G   
   T   
   A   
   G   
   T   
   G   
   T   
   G   
   G   
   A   
   A   
   G   
   A   
   T   
   C   
   C   
   T   
   T   
   C   
   A   
   G   
   G   
   T   
   T   
   A   
   T   
   G   
   T   
   T   
   T   
   A   
   T   
   A   
   A   
   T   
   A   
   T   
   G   
   T   
   A   
   C   
   A   
   A   
   T   
   C   
   C   
   G   
   A   
   A   
   C   
   T   
   T   
   C   
   T   
   C   
   A   
   T   
   T   
   G   
   G   
   C   
   T   
   G   
   C   
   T   
   A   
   A   
   A   
   A   
   C   
   A   
   G   
   G   
   A   
   A   
   C   
   A   
   G   
   C   
   T   
   G   
   A   
   A   
   A   
   T   
   A   
   A   
   A   
   A   
   G   
   A   
   T   
   A   
   A   
   A   
   C   
   A   
   A   
   G   
   A   
   T   
   A   
   C   
   A   
   G   
   A   
   T   
   G   
   G   
   C   
   A   
   A   
   A   
   G   
   A   
   A   
   A   
   A   
   C   
   A   
   G   
   T   
   T   
   T   
   C   
   C   
   T   
   C   
   C   
   T   
   A   
   A   
   C   
   G   
   T   
   T   
   A   
   G   
   A   
   T   
   C   
   G   
   T   
   A   
   G   
   T   
   A   
   A   
   C   
   A   
   A   
   T   
   A   
   A   
   A   
   T   
   T   
   C   
   T   
   T   
   A   
   A   
   C   
   A   
   A   
   T   
   G   
   A   
   T   
   T   
   A   
   T   
   G   
   G   
   T   
   T   
   G   
   A   
   A   
   A   
   A   
   T   
   T   
   C   
   A   
   G   
   G   
   A   
   G   
   A   
   A   
   A   
   A   
   T   
   G   
   G   
   T   
   T   
   C   
   T   
   G   
   C   
   A   
   A   
   C   
   A   
   G   
   A   
   T   
   A   
   T   
   C   
   A   
   G   
   T   
   A   
   A   
   A   
   C   
   C   
   A   
   T   
   T   
   A   
   A   
   T   
   T   
   G   
   A   
   T   
   T   
   A   
   C   
   T   
   T   
   A   
   G   
   A   
   A   
   G   
   C   
   A   
   A   
   C   
   C   
   A   
   T   
   T   
   A   
   A   
   A   
   -   
   -   
   -   
   -   
   -   
   -   
   -   
   -   
   -   
   -   
   -   
   -   
   -   
   -   
   -   
   -   
   -   
   -   
   -   
   -   
   -   
   -   
   -   
   -   
   -   
   -   
   -   
   -   
   -   
   -   
   -   
   -   
   -   
   -   
   -   
   -   
   -   
   -   
   -   
   -   
   -   
   -   
   -   
   -   
   -   
   -   
   -   
   -   
   -   
   -   
   -   
   -   
   -   
   -   
   -   
   -   
   -   
   -   
   -   
   -   
   -   
 

 
   N59554F   
   -   
   -   
   -   
   -   
   -   
   -   
   -   
   -   
   -   
   -   
   -   
   -   
   -   
   -   
   -   
   -   
   -   
   -   
   -   
   -   
   -   
   -   
   -   
   -   
   -   
   -   
   -   
   -   
   -   
   -   
   -   
   -   
   -   
   -   
   -   
   -   
   -   
   -   
   -   
   -   
   -   
   -   
   -   
   -   
   -   
   -   
   -   
   -   
   -   
   -   
   -   
   -   
   -   
   -   
   -   
   -   
   -   
   -   
   -   
   -   
   -   
   -   
   -   
   -   
   -   
   -   
   -   
   -   
   -   
   -   
   -   
   -   
   -   
   -   
   -   
   -   
   -   
   -   
   -   
   -   
   -   
   -   
   -   
   -   
   -   
   -   
   -   
   -   
   -   
   -   
   -   
   -   
   -   
   -   
   -   
   -   
   -   
   -   
   -   
   -   
   -   
   -   
   -   
   -   
   -   
   -   
   -   
   -   
   C   
   A   
   A   
   T   
   G   
   G   
   C   
   A   
   A   
   G   
   C   
   C   
   A   
   A   
   A   
   C   
   A   
   A   
   G   
   A   
    A    
   T   
   T   
   A   
   G   
   C   
    C    
   G   
   A   
   A   
   G   
   C   
   G   
   A   
   A   
   G   
   A   
   A   
   A   
   A   
    C    
   A   
   G   
   C   
    T    
   A   
    C    
   T   
    A    
    C    
   A   
   T   
   T   
   T   
   T   
   T   
   A   
   A   
   A   
    C    
   G   
   T   
   A   
   T   
   T   
   G   
    T    
   C   
   A   
   A   
   A   
   A   
   C   
   A   
   G   
    G    
   A   
   A   
   T   
   T   
   T   
   G   
   A   
   T   
   A   
   A   
   G   
   T   
   T   
   A   
   C   
   C   
    G    
   T   
   C   
   C   
   G   
   T   
   T   
    G    
   T   
   T   
   C   
   A   
   A   
   G   
   A   
   A   
    G    
   C   
   T   
   A   
   G   
   C   
   T   
   T   
   A   
   A   
   A   
   G   
   A   
   A   
   A   
   A   
   A   
   T   
   G   
   G   
    C    
   T   
   A   
   T   
   G   
   A   
   T   
   A   
   C   
   T   
   A   
   A   
   A   
   T   
   C   
    T    
   G   
   T   
   T   
   G   
   T   
   T   
   G   
   A   
   A   
   A   
   A   
   A   
   T   
   A   
   C   
   C   
   A   
   A   
   G   
   C   
   A   
   A   
   T   
   T   
   T   
   A   
   T   
   T   
   C   
   A   
   G   
   G   
   G   
   A   
   T   
   T   
   C   
   A   
   A   
   G   
   C   
    A    
   G   
   A   
   A   
    G    
   G   
    A    
   G   
   T   
   C   
   A   
   A   
    A    
   G   
   C   
   T   
   A   
   G   
   T   
   G   
   A   
   T   
   G   
   T   
    T    
   C   
   A   
   A   
   G   
   T   
    C    
   A   
   A   
   A   
   A   
   A   
    G    
   G   
    C    
   G   
   A   
   A   
   A   
   G   
   A   
    C    
   A   
   A   
    T    
   C   
   A   
   A   
   T   
   A   
   C   
   A   
   C   
   A   
   T   
   T   
   T   
   A   
    C    
   C   
   T   
   A   
   T   
   A   
   A   
   A   
   T   
   T   
   A   
   T   
   C   
   G   
   A   
   T   
   G   
   A   
   G   
    C    
   A   
   C   
    G    
   C   
   C   
   T   
   T   
   T   
   A   
   G   
   G   
   C   
   G   
   A   
   A   
   A   
   T   
   G   
   A   
   A   
   A   
   G   
   A   
   T   
    T    
   T   
   G   
   T   
   C   
   T   
   T   
   A   
   T   
   C   
   A   
   A   
   T   
   C   
   A   
   A   
   G   
   T   
   A   
   T   
    C    
   G   
    C    
   C   
   A   
   A   
    A    
   A   
   A   
   A   
   G   
   G   
    C    
   G   
   A   
   T   
   A   
    C    
   C   
   T   
   A   
   C   
   C   
   A   
   A   
   A   
   T   
    C    
    G    
   C   
   T   
   T   
   G   
   G   
    A    
    A    
    G    
   C   
   C   
    A    
   T   
   C   
   T   
   T   
   T   
   A   
   A   
   T   
   T   
   T   
   T   
   T   
   C   
   C   
   A   
   G   
   A   
   T   
   A   
   T   
   G   
   T   
   C   
   A   
   G   
   G   
   A   
   A   
   A   
   T   
   G   
   A   
   T   
   A   
   A   
   A   
    A    
   T   
   T   
   T   
   C   
   G   
   A   
   T   
   T   
   C   
   A   
   A   
   G   
   T   
   A   
   G   
   A   
   T   
   A   
   A   
   T   
   G   
   C   
   C   
   A   
   A   
   A   
   C   
   G   
   T   
   G   
   G   
   A   
   G   
   A   
   A   
   A   
   T   
   T   
   G   
   T   
   C   
   G   
   A   
   T   
   C   
   G   
   T   
   A   
   A   
    T    
   G   
   G   
   T   
   A   
   G   
   T   
   G   
   G   
   G   
   C   
   T   
   A   
   G   
    C    
   A   
   A   
   T   
   T   
   A   
   A   
   C   
   A   
   A   
   A   
   G   
   T   
   G   
   T   
   T   
   T   
   G   
   A   
    C    
   G   
   A   
   A   
   G   
   T   
   G   
   G   
   G   
   C   
   G   
   T   
   A   
   G   
   T   
    G    
   C   
   C   
   T   
    G    
   G   
   C   
   A   
   A   
   A   
   C   
   T   
    C    
   G   
   G   
   T   
   T   
   C   
   T   
   G   
   G   
   C   
   G   
   C   
   A   
   G   
   A   
   A   
   A   
   A   
   A   
    A    
   C   
   A   
   G   
   C   
    C    
   A   
   A   
   T   
   A   
   T   
   C   
   A   
   A   
   A   
   G   
   C   
   T   
   T   
   T   
   T   
   A   
   G   
   T   
   G   
   A   
   T   
   A   
   A   
    A    
   T   
   T   
    C    
   G   
   G   
   C   
   G   
   T   
   T   
   T   
   C   
   T   
    G    
   T   
   T   
   G   
   A   
   T   
   G   
   A   
   A   
   A   
   T   
   C   
   A   
   A   
   T   
   C   
    A    
   A   
   A   
   A   
    G    
   T   
   T   
   A   
   A   
   G   
   C   
   C   
   A   
   A   
   G   
   G   
    A    
   T   
   G   
   G   
   G   
   T   
   C   
   C   
   A   
   A   
   G   
   C   
   A   
   G   
   A   
   C   
   T   
   C   
    C    
   T   
   T   
   T   
   G   
   T   
   A   
   C   
   C   
   A   
   A   
   T   
   C   
   A   
   C   
   A   
   G   
   T   
    C    
   G   
   C   
   T   
   T   
   C   
   T   
   G   
   A   
   A   
   C   
   C   
   A   
   G   
   T   
   G   
   A   
   C   
   A   
   G   
   A   
   A   
   T   
   T   
   A   
   C   
   C   
   A   
   A   
   C   
   A   
   G   
   G   
   G   
   G   
   C   
   T   
   G   
   C   
   G   
   A   
   C   
   A   
   A   
   A   
   A   
   G   
    A    
    T    
   A   
   C   
   A   
   G   
   A   
   G   
   T   
   C   
   A   
   C   
   G   
   T   
   T   
   A   
   T   
   T   
   A   
   T   
   C   
   C   
    G    
    C    
   T   
   G   
   G   
   G   
   G   
   G   
   A   
   A   
   G   
   C   
   A   
   G   
   C   
   T   
   G   
   C   
   G   
   C   
   A   
   A   
   T   
   T   
   A   
   A   
   T   
   C   
   G   
   G   
    G    
   T   
   A   
   T   
   A   
   C   
    G    
   G   
   G   
    C    
   A   
   C   
    C    
   A   
   T   
   T   
   A   
   C   
   T   
   G   
   C   
   A   
   G   
   A   
    A    
   G   
   A   
   T   
   A   
   T   
   T   
    G    
   A   
   A   
   A   
   A   
   A   
   A   
   A   
   T   
   C   
   C   
   A   
   G   
   A   
   G   
   T   
   T   
   A   
   A   
   G   
   T   
   A   
   G   
   T   
   A   
   C   
   G   
   G   
   G   
    C    
   G   
   T   
   A   
   A   
   T   
   T   
   G   
   G   
    T    
   A   
   A   
   A   
   A   
   C   
   T   
   G   
   G   
    C    
   T   
   T   
   A   
   G   
   A   
   A   
   C   
   G   
   A   
   G   
   C   
   G   
   T   
   T   
   T   
   G   
   A   
   T   
   A   
   A   
   A   
   G   
   A   
    A    
   T   
   T   
   A   
   A   
   G   
   A   
   G   
   G   
    G    
   C   
   A   
   A   
   G   
   A   
   T   
   G   
   G   
    T    
   G   
   G   
    C    
   T   
   C   
    A    
   C   
   T   
   A   
   G   
   T   
   T   
   A   
   T   
   T   
   T   
   T   
   A   
   G   
   A   
   T   
   G   
   A   
   C   
   A   
   A   
   A   
   G   
   A   
   G   
   A   
   A   
   T   
   G   
   T   
   C   
   A   
   A   
   A   
   A   
   A   
   A   
   G   
   C   
   T   
   T   
   T   
   A   
   C   
   A   
   A   
   A   
   C   
   C   
   A   
   A   
   A   
   G   
   A   
    G    
   A   
   A   
   A   
   A   
   A   
   A   
   G   
   A   
   T   
   G   
   G   
   T   
   C   
   A   
   A   
   A   
   C   
    G    
   A   
   T   
   T   
   A   
   A   
   A   
   T   
   T   
   A   
   A   
   C   
   G   
   A   
   T   
   T   
   G   
   A   
    C    
   A   
   G   
    T    
   G   
   G   
    C    
   G   
   T   
   A   
   C   
   A   
   A   
   C   
   A   
   A   
   C   
   A   
   A   
   G   
   C   
    T    
   T   
   T   
    T    
   G   
   C   
   T   
   A   
   T   
   A   
   T   
   T   
   T   
   G   
   A   
   C   
   A   
   A   
   A   
   C   
   G   
    C    
    C    
   C   
   T   
   G   
   G   
   T   
   A   
   G   
   T   
   G   
   C   
   G   
   G   
   T   
   C   
   A   
   T   
   T   
   A   
   C   
   C   
   G   
   A   
   T   
   C   
   C   
   T   
   C   
   A   
   A   
   A   
   A   
   A   
   G   
   G   
   C   
   G   
   A   
   T   
   T   
   T   
   A   
    T    
   T   
   A   
   G   
   C   
    A    
   A   
   C   
   G   
   G   
   T   
   T   
   A   
   G   
   T   
   T   
   C   
   A   
   C   
   C   
   T   
   T   
   C   
   G   
   T   
   A   
   T   
   G   
   A   
   T   
   C   
   C   
   T   
   A   
   A   
   T   
   A   
   A   
   A   
   A   
   T   
   G   
   G   
   C   
   C   
   A   
   A   
   T   
   G   
   G   
    C    
   A   
   T   
   T   
   T   
   C   
   T   
   C   
   A   
   A   
   A   
   A   
   A   
   G   
   A   
    A    
   T   
   A   
   T   
   G   
   A   
   T   
   G   
   C   
   T   
   T   
   A   
   C   
   A   
   A   
   T   
   A   
   A   
   C   
   A   
   A   
   C   
   A   
   A   
   A   
   G   
   A   
   T   
   T   
   T   
   A   
   C   
   C   
   A   
   T   
   T   
    C    
   A   
   C   
   A   
   G   
   C   
   A   
   C   
   G   
   T   
   T   
   T   
    T    
   G   
   C   
   G   
   A   
   C   
   A   
   G   
   G   
    C    
   T   
   A   
   T   
   G   
   C   
   A   
   C   
   C   
   A   
   G   
   G   
    C    
   T   
   C   
   T   
   A   
   C   
   C   
   T   
   T   
   T   
   A   
   A   
   A   
   A   
   C   
   A   
   A   
   T   
   T   
   A   
   C   
   C   
   G   
   G   
   T   
   G   
   C   
   C   
   A   
   T   
   T   
   G   
   G   
   T   
   T   
   T   
   A   
   G   
   A   
   T   
    G    
   C   
   A   
   G   
   G   
   G   
   A   
   C   
   A   
   T   
   T   
   G   
   A   
   A   
   G   
   C   
   C   
   A   
   G   
   A   
   T   
   G   
   A   
   A   
   G   
   A   
   G   
   C   
   T   
   A   
   G   
   A   
   A   
   A   
   T   
   T   
   A   
   A   
   T   
   G   
   G   
    T    
   T   
   T   
   G   
   A   
   A   
   A   
   T   
   G   
   G   
   C   
   A   
   A   
   A   
   A   
   A   
   G   
   A   
   T   
   A   
   A   
   A   
   T   
   C   
   T   
   T   
   G   
   G   
   G   
   G   
    C    
   G   
   G   
   C   
   T   
   A   
   T   
   T   
   T   
   T   
   G   
   C   
    G    
   A   
   C   
   A   
   C   
   G   
   T   
   G   
   T   
   A   
   A   
   A   
   A   
   G   
   A   
   A   
   G   
   C   
   A   
   A   
   G   
   T   
   C   
   C   
   A   
   G   
   T   
   C   
   A   
   A   
   T   
   C   
   T   
    G    
   C   
   G   
   T   
   A   
   C   
   C   
   G   
   C   
   T   
   T   
   T   
   A   
   G   
   T   
   T   
   A   
   A   
   T   
   T   
   C   
   A   
   G   
   A   
   T   
   A   
   A   
   T   
   A   
   T   
   T   
   T   
   A   
   T   
   T   
   T   
   T   
   G   
   C   
    C    
   C   
   A   
   A   
   C   
   A   
   A   
   A   
   C   
   A   
   T   
   T   
   A   
   C   
   G   
    G    
   A   
   T   
   G   
   G   
   G   
    A    
   G   
   A   
   A   
   G   
   A   
   C   
   A   
   A   
   A   
   T   
   T   
   T   
   A   
   G   
   A   
   G   
   C   
    G    
   G   
   G   
   T   
   C   
   T   
   T   
   A   
   A   
   T   
   A   
   A   
   A   
   T   
   T   
   C   
   A   
   T   
   T   
   T   
   T   
   C   
   G   
   G   
   T   
   G   
   A   
   G   
   G   
   A   
   A   
   C   
   T   
   A   
   G   
   A   
   T   
   T   
   T   
   A   
   C   
   C   
   A   
   A   
   T   
    T    
   G   
   C   
   C   
   A   
   T   
   G   
   A   
   C   
   G   
   C   
   C   
    T    
   G   
   C   
   T   
   C   
   A   
   A   
   A   
   T   
   T   
   T   
   C   
   A   
   A   
   A   
    T    
   G   
   A   
   A   
   G   
   A   
   T   
   A   
   A   
   A   
   T   
   T   
   T   
   A   
   A   
   T   
   T   
   C   
   T   
   G   
   A   
   A   
   A   
   T   
   T   
   C   
   T   
   T   
   T   
   T   
    A    
   G   
   C   
   A   
   G   
   A   
   T   
   A   
   C   
    G    
   G   
   G   
   C   
   T   
   A   
    C    
   G   
   G   
    T    
   C   
   A   
   A   
   G   
   G   
    T    
   C   
   A   
   A   
   T   
   T   
   G   
   T   
   T   
   A   
   A   
   T   
   T   
   T   
   C   
    G    
   C   
   C   
   A   
   A   
   T   
   C   
   C   
   A   
   G   
   C   
   A   
   A   
   G   
   C   
   T   
   A   
   C   
   C   
   A   
   T   
   G   
   T   
   A   
   T   
   A   
   G   
   T   
   G   
   T   
   T   
   T   
   T   
   C   
   C   
   A   
   A   
   A   
   A   
   T   
   A   
   A   
   T   
   G   
   G   
    A    
   A   
   C   
   A   
    T    
   T   
    A    
   G   
   T   
    C    
   T   
   A   
   T   
   C   
   C   
   A   
   A   
   A   
   A   
    C    
   T   
   A   
   G   
   T   
   T   
   T   
   T   
   A   
   G   
   A   
   C   
   A   
   A   
   A   
   G   
   A   
   A   
   A   
   C   
    G    
   A   
   A   
   G   
   A   
   A   
   A   
   A   
   A   
   A   
   G   
   A   
   C   
   A   
   A   
   T   
   G   
   T   
   A   
   A   
   T   
   C   
   A   
   G   
   C   
   G   
   C   
   C   
   A   
   A   
   C   
   G   
   C   
   A   
   G   
   C   
   G   
   A   
   A   
   T   
   A   
   C   
   A   
   A   
   T   
   T   
   G   
   C   
   C   
   A   
   C   
   A   
   G   
   A   
   T   
   C   
   T   
   T   
   T   
   T   
   A   
   G   
   G   
   T   
   A   
   G   
   T   
   G   
   T   
   G   
   G   
   A   
   A   
   G   
   A   
   T   
   C   
   C   
   T   
   T   
   C   
   A   
   G   
   G   
   T   
   T   
   A   
   T   
   G   
   T   
   T   
   T   
   A   
   T   
   A   
   A   
   T   
   A   
   T   
   G   
   T   
   A   
   C   
   A   
   A   
   T   
   C   
   C   
   G   
   A   
   A   
   C   
   T   
   T   
   C   
   T   
   C   
   A   
   T   
   T   
   G   
   G   
   C   
   T   
   G   
   C   
   T   
   A   
   A   
   A   
   A   
   C   
   A   
   G   
   G   
   A   
   A   
   C   
   A   
   G   
   C   
   T   
   G   
   A   
   A   
   A   
   T   
   A   
   A   
   A   
   A   
   G   
   A   
   T   
   A   
   A   
   A   
   C   
   A   
   A   
   G   
   A   
   T   
   A   
   C   
   A   
   G   
   A   
   T   
   G   
   G   
   C   
   A   
   A   
   A   
   G   
   A   
   A   
   A   
   A   
   C   
   A   
   G   
   T   
   T   
   T   
   C   
   C   
   T   
   T   
   C   
   T   
   A   
   A   
   C   
   G   
   T   
   T   
   A   
   G   
   A   
   T   
   C   
   G   
   T   
   A   
   G   
   T   
   A   
   A   
   C   
   A   
   A   
   T   
   A   
   A   
   A   
   T   
   T   
   C   
   T   
   T   
   A   
   A   
   C   
   A   
   A   
   T   
   G   
   A   
   T   
   T   
   A   
   T   
   G   
   G   
   T   
   T   
   G   
   A   
   A   
   A   
   A   
   T   
   T   
   C   
   A   
   G   
   G   
   A   
   G   
   A   
   A   
   A   
   A   
   T   
   G   
   G   
   T   
   T   
   C   
   T   
   G   
   C   
   A   
   A   
   C   
   A   
   G   
   A   
   T   
   A   
   T   
   C   
   A   
   G   
   T   
   A   
   A   
   A   
   C   
   C   
   A   
   T   
   T   
   A   
   A   
   T   
   T   
   G   
   A   
   T   
   T   
   A   
   C   
   T   
   T   
   A   
   G   
   A   
   A   
   G   
   C   
   A   
   A   
   C   
   C   
   A   
   T   
   T   
   A   
   A   
   A   
   -   
   -   
   -   
   -   
   -   
   -   
   -   
   -   
   -   
   -   
   -   
   -   
   -   
   -   
   -   
   -   
   -   
   -   
   -   
   -   
   -   
   -   
   -   
   -   
   -   
   -   
   -   
   -   
   -   
   -   
   -   
   -   
   -   
   -   
   -   
   -   
   -   
   -   
   -   
   -   
   -   
   -   
   -   
   -   
   -   
   -   
   -   
   -   
   -   
   -   
   -   
   -   
   -   
   -   
   -   
   -   
   -   
   -   
   -   
   -   
   -   
   -   
   -   
   -   
   -   
   -   
   -   
   -   
   -   
   -   
   -   
   -   
   -   
   -   
   -   
   -   
   -   
   -   
   -   
   -   
   -   
   -   
   -   
   -   
   -   
   -   
   -   
   -   
   -   
   -   
   -   
   -   
   -   
   -   
   -   
   -   
   -   
   -   
   -   
   -   
   -   
   -   
   -   
   -   
   -   
   -   
   -   
   -   
   -   
   -   
   -   
   -   
   -   
   -   
   -   
   -   
   -   
   -   
   -   
   -   
   -   
   -   
   -   
   -   
   -   
   -   
   -   
   -   
   -   
   -   
   -   
   -   
   -   
   -   
   -   
   -   
   -   
   -   
   -   
   -   
   -   
   -   
   -   
   -   
   -   
   -   
   -   
   -   
   -   
   -   
   -   
   -   
   -   
   -   
   -   
   -   
   -   
   -   
   -   
   -   
   -   
   -   
   -   
   -   
   -   
   -   
   -   
   -   
   -   
   C   
   A   
   A   
   T   
   G   
   G   
   C   
   A   
   A   
   G   
   C   
   C   
   A   
   A   
   A   
   C   
   A   
   A   
   G   
   A   
   A   
   T   
   T   
   A   
   G   
   C   
   C   
   G   
   A   
   A   
   G   
   C   
   G   
   A   
   A   
   G   
   A   
   A   
   A   
   A   
   C   
   A   
   G   
   C   
   T   
   A   
   C   
   T   
   A   
   C   
   A   
   T   
   T   
   T   
   T   
   T   
   A   
   A   
   A   
   C   
   G   
   T   
   A   
   T   
   T   
   G   
   T   
   C   
   A   
   A   
   A   
   A   
   C   
   A   
   G   
   G   
   A   
   A   
   T   
   T   
   T   
   G   
   A   
   T   
   A   
   A   
   G   
   T   
   T   
   A   
   C   
   C   
   G   
   T   
   C   
   C   
   G   
   T   
   T   
   G   
   T   
   T   
   C   
   A   
   A   
   G   
   A   
   A   
   G   
   C   
   T   
   A   
   G   
   C   
   T   
   T   
   A   
   A   
   A   
   G   
   A   
   A   
   A   
   A   
   A   
   T   
   G   
   G   
   C   
   T   
   A   
   T   
   G   
   A   
   T   
   A   
   C   
   T   
   A   
   A   
   A   
   T   
   C   
   T   
   G   
   T   
   T   
   G   
   T   
   T   
   G   
   A   
   A   
   A   
   A   
   A   
   T   
   A   
   C   
   C   
   A   
   A   
   G   
   C   
   A   
   A   
   T   
   T   
   T   
   A   
   T   
   T   
   C   
   A   
   G   
   G   
   G   
   A   
   T   
   T   
   C   
   A   
   A   
   G   
   C   
   A   
   G   
   A   
   A   
   G   
   G   
   A   
   G   
   T   
   C   
   A   
   A   
   A   
   G   
   C   
   T   
   A   
   G   
   T   
   G   
   A   
   T   
   G   
   T   
   T   
   C   
   A   
   A   
   G   
   T   
   C   
   A   
   A   
   A   
   A   
   A   
   G   
   G   
   C   
   G   
   A   
   A   
   A   
   G   
   A   
   C   
   A   
   A   
   T   
   C   
   A   
   A   
   T   
   A   
   C   
   A   
   C   
   A   
   T   
   T   
   T   
   A   
   C   
   C   
   T   
   A   
   T   
   A   
   A   
   A   
   T   
   T   
   A   
   T   
   C   
   G   
   A   
   T   
   G   
   A   
   G   
   C   
   A   
   C   
   G   
   C   
   C   
   T   
   T   
   T   
   A   
   G   
   G   
   C   
   G   
   A   
   A   
   A   
   T   
   G   
   A   
   A   
   A   
   G   
   A   
   T   
   T   
   T   
   G   
   T   
   C   
   T   
   T   
   A   
   T   
   C   
   A   
   A   
   T   
   C   
   A   
   A   
   G   
   T   
   A   
   T   
   C   
   G   
   C   
   C   
   A   
   A   
   A   
   A   
   A   
   A   
   G   
   G   
   C   
   G   
   A   
   T   
   A   
   C   
   C   
   T   
   A   
   C   
   C   
   A   
   A   
   A   
   T   
   C   
   G   
   C   
   T   
   T   
   G   
   G   
   A   
   A   
   G   
   C   
   C   
   A   
   T   
   C   
   T   
   T   
   T   
   A   
   A   
   T   
   T   
   T   
   T   
   T   
   C   
   C   
   A   
   G   
   A   
   T   
   A   
   T   
   G   
   T   
   C   
   A   
   G   
   G   
   A   
   A   
   A   
   T   
   G   
   A   
   T   
   A   
   A   
   A   
   A   
   T   
   T   
   T   
   C   
   G   
   A   
   T   
   T   
   C   
   A   
   A   
   G   
   T   
   A   
   G   
   A   
   T   
   A   
   A   
   T   
   G   
   C   
   C   
   A   
   A   
   A   
   C   
   G   
   T   
   G   
   G   
   A   
   G   
   A   
   A   
   A   
   T   
   T   
   G   
   T   
   C   
   G   
   A   
   T   
   C   
   G   
   T   
   A   
   A   
   T   
   G   
   G   
   T   
   A   
   G   
   T   
   G   
   G   
   G   
   C   
   T   
   A   
   G   
   C   
   A   
   A   
   T   
   T   
   A   
   A   
   C   
   A   
   A   
   A   
   G   
   T   
   G   
   T   
   T   
   T   
   G   
   A   
   C   
   G   
   A   
   A   
   G   
   T   
   G   
   G   
   G   
   C   
   G   
   T   
   A   
   G   
   T   
   G   
   C   
   C   
   T   
   G   
   G   
   C   
   A   
   A   
   A   
   C   
   T   
   C   
   G   
   G   
   T   
   T   
   C   
   T   
   G   
   G   
   C   
   G   
   C   
   A   
   G   
   A   
   A   
   A   
   A   
   A   
   A   
   C   
   A   
   G   
   C   
   C   
   A   
   A   
   T   
   A   
   T   
   C   
   A   
   A   
   A   
   G   
   C   
   T   
   T   
   T   
   T   
   A   
   G   
   T   
   G   
   A   
   T   
   A   
   A   
   A   
   T   
   T   
   C   
   G   
   G   
   C   
   G   
   T   
   T   
   T   
   C   
   T   
   G   
   T   
   T   
   G   
   A   
   T   
   G   
   A   
   A   
   A   
   T   
   C   
   A   
   A   
   T   
   C   
   A   
   A   
   A   
   A   
   G   
   T   
   T   
   A   
   A   
   G   
   C   
   C   
   A   
   A   
   G   
   G   
   A   
   T   
   G   
   G   
   G   
   T   
   C   
   C   
   A   
   A   
   G   
   C   
   A   
   G   
   A   
   C   
   T   
   C   
   C   
   T   
   T   
   T   
   G   
   T   
   A   
   C   
   C   
   A   
   A   
   T   
   C   
   A   
   C   
   A   
   G   
   T   
   C   
   G   
   C   
   T   
   T   
   C   
   T   
   G   
   A   
   A   
   C   
   C   
   A   
   G   
   T   
   G   
   A   
   C   
   A   
   G   
   A   
   A   
   T   
   T   
   A   
   C   
   C   
   A   
   A   
   C   
   A   
   G   
   G   
   G   
   G   
   C   
   T   
   G   
   C   
   G   
   A   
   C   
   A   
   A   
   A   
   A   
   G   
   A   
   T   
   A   
   C   
   A   
   G   
   A   
   G   
   T   
   C   
   A   
   C   
   G   
   T   
   T   
   A   
   T   
   T   
   A   
   T   
   C   
   C   
   G   
   C   
   T   
   G   
   G   
   G   
   G   
   G   
   A   
   A   
   G   
   C   
   A   
   G   
   C   
   T   
   G   
   C   
   G   
   C   
   A   
   A   
   T   
   T   
   A   
   A   
   T   
   C   
   G   
   G   
   G   
   T   
   A   
   T   
   A   
   C   
   G   
   G   
   G   
   C   
   A   
   C   
   C   
   A   
   T   
   T   
   A   
   C   
   T   
   G   
   C   
   A   
   G   
   A   
   A   
   G   
   A   
   T   
   A   
   T   
   T   
   G   
   A   
   A   
   A   
   A   
   A   
   A   
   A   
   T   
   C   
   C   
   A   
   G   
   A   
   G   
   T   
   T   
   A   
   A   
   G   
   T   
   A   
   G   
   T   
   A   
   C   
   G   
   G   
   G   
   C   
   G   
   T   
   A   
   A   
   T   
   T   
   G   
   G   
   T   
   A   
   A   
   A   
   A   
   C   
   T   
   G   
   G   
   C   
   T   
   T   
   A   
   G   
   A   
   A   
   C   
   G   
   A   
   G   
   C   
   G   
   T   
   T   
   T   
   G   
   A   
   T   
   A   
   A   
   A   
   G   
   A   
   A   
   T   
   T   
   A   
   A   
   G   
   A   
   G   
   G   
   G   
   C   
   A   
   A   
   G   
   A   
   T   
   G   
   G   
   T   
   G   
   G   
   C   
   T   
   C   
   A   
   C   
   T   
   A   
   G   
   T   
   T   
   A   
   T   
   T   
   T   
   T   
   A   
   G   
   A   
   T   
   G   
   A   
   C   
   A   
   A   
   A   
   G   
   A   
   G   
   A   
   A   
   T   
   G   
   T   
   C   
   A   
   A   
   A   
   A   
   A   
   A   
   G   
   C   
   T   
   T   
   T   
   A   
   C   
   A   
   A   
   A   
   C   
   C   
   A   
   A   
   A   
   G   
   A   
   G   
   A   
   A   
   A   
   A   
   A   
   A   
   G   
   A   
   T   
   G   
   G   
   T   
   C   
   A   
   A   
   A   
   C   
   G   
   A   
   T   
   T   
   A   
   A   
   A   
   T   
   T   
   A   
   A   
   C   
   G   
   A   
   T   
   T   
   G   
   A   
   C   
   A   
   G   
   T   
   G   
   G   
   C   
   G   
   T   
   A   
   C   
   A   
   A   
   C   
   A   
   A   
   C   
   A   
   A   
   G   
   C   
   T   
   T   
   T   
   T   
   G   
   C   
   T   
   A   
   T   
   A   
   T   
   T   
   T   
   G   
   A   
   C   
   A   
   A   
   A   
   C   
   G   
   C   
   C   
   C   
   T   
   G   
   G   
   T   
   A   
   G   
   T   
   G   
   C   
   G   
   G   
   T   
   C   
   A   
   T   
   T   
   A   
   C   
   C   
   G   
   A   
   T   
   C   
   C   
   T   
   C   
   A   
   A   
   A   
   A   
   A   
   G   
   G   
   C   
   G   
   A   
   T   
   T   
   T   
   A   
   T   
   T   
   A   
   G   
   C   
   A   
   A   
   C   
   G   
   G   
   T   
   T   
   A   
   G   
   T   
   T   
   C   
   A   
   C   
   C   
   T   
   T   
   C   
   G   
   T   
   A   
   T   
   G   
   A   
   T   
   C   
   C   
   T   
   A   
   A   
   T   
   A   
   A   
   A   
   A   
   T   
   G   
   G   
   C   
   C   
   A   
   A   
   T   
   G   
   G   
   C   
   A   
   T   
   T   
   T   
   C   
   T   
   C   
   A   
   A   
   A   
   A   
   A   
   G   
   A   
   A   
   T   
   A   
   T   
   G   
   A   
   T   
   G   
   C   
   T   
   T   
   A   
   C   
   A   
   A   
   T   
   A   
   A   
   C   
   A   
   A   
   C   
   A   
   A   
   A   
   G   
   A   
   T   
   T   
   T   
   A   
   C   
   C   
   A   
   T   
   T   
   C   
   A   
   C   
   A   
   G   
   C   
   A   
   C   
   G   
   T   
   T   
   T   
   T   
   G   
   C   
   G   
   A   
   C   
   A   
   G   
   G   
   C   
   T   
   A   
   T   
   G   
   C   
   A   
   C   
   C   
   A   
   G   
   G   
   C   
   T   
   C   
   T   
   A   
   C   
   C   
   T   
   T   
   T   
   A   
   A   
   A   
   A   
   C   
   A   
   A   
   T   
   T   
   A   
   C   
   C   
   G   
   G   
   T   
   G   
   C   
   C   
   A   
   T   
   T   
   G   
   G   
   T   
   T   
   T   
   A   
   G   
   A   
   T   
   G   
   C   
   A   
   G   
   G   
   G   
   A   
   C   
   A   
   T   
   T   
   G   
   A   
   A   
   G   
   C   
   C   
   A   
   G   
   A   
   T   
   G   
   A   
   A   
   G   
   A   
   G   
   C   
   T   
   A   
   G   
   A   
   A   
   A   
   T   
   T   
   A   
   A   
   T   
   G   
   G   
   T   
   T   
   T   
   G   
   A   
   A   
   A   
   T   
   G   
   G   
   C   
   A   
   A   
   A   
   A   
   A   
   G   
   A   
   T   
   A   
   A   
   A   
   T   
   C   
   T   
   T   
   G   
   G   
   G   
   G   
   C   
   G   
   G   
   C   
   T   
   A   
   T   
   T   
   T   
   T   
   G   
   C   
   G   
   A   
   C   
   A   
   C   
   G   
   T   
   G   
   T   
   A   
   A   
   A   
   A   
   G   
   A   
   A   
   G   
   C   
   A   
   A   
   G   
   T   
   C   
   C   
   A   
   G   
   T   
   C   
   A   
   A   
   T   
   C   
   T   
   G   
   C   
   G   
   T   
   A   
   C   
   C   
   G   
   C   
   T   
   T   
   T   
   A   
   G   
   T   
   T   
   A   
   A   
   T   
   T   
   C   
   A   
   G   
   A   
   T   
   A   
   A   
   T   
   A   
   T   
   T   
   T   
   A   
   T   
   T   
   T   
   T   
   G   
   C   
   C   
   C   
   A   
   A   
   C   
   A   
   A   
   A   
   C   
   A   
   T   
   T   
   A   
   C   
   G   
   G   
   A   
   T   
   G   
   G   
   G   
   A   
   G   
   A   
   A   
   G   
   A   
   C   
   A   
   A   
   A   
   T   
   T   
   T   
   A   
   G   
   A   
   G   
   C   
   G   
   G   
   G   
   T   
   C   
   T   
   T   
   A   
   A   
   T   
   A   
   A   
   A   
   T   
   T   
   C   
   A   
   T   
   T   
   T   
   T   
   C   
   G   
   G   
   T   
   G   
   A   
   G   
   G   
   A   
   A   
   C   
   T   
   A   
   G   
   A   
   T   
   T   
   T   
   A   
   C   
   C   
   A   
   A   
   T   
   T   
   G   
   C   
   C   
   A   
   T   
   G   
   A   
   C   
   G   
   C   
   C   
   T   
   G   
   C   
   T   
   C   
   A   
   A   
   A   
   T   
   T   
   T   
   C   
   A   
   A   
   A   
   T   
   G   
   A   
   A   
   G   
   A   
   T   
   A   
   A   
   A   
   T   
   T   
   T   
   A   
   A   
   T   
   T   
   C   
   T   
   G   
   A   
   A   
   A   
   T   
   T   
   C   
   T   
   T   
   T   
   T   
   A   
   G   
   C   
   A   
   G   
   A   
   T   
   A   
   C   
   G   
   G   
   G   
   C   
   T   
   A   
   C   
   G   
   G   
   T   
   C   
   A   
   A   
   G   
   G   
   T   
   C   
   A   
   A   
   T   
   T   
   G   
   T   
   T   
   A   
   A   
   T   
   T   
   T   
   C   
   G   
   C   
   C   
   A   
   A   
   T   
   C   
   C   
   A   
   G   
   C   
   A   
   A   
   G   
   C   
   T   
   A   
   C   
   C   
   A   
   T   
   G   
   T   
   A   
   T   
   A   
   G   
   T   
   G   
   T   
   T   
   T   
   T   
   C   
   C   
   A   
   A   
   A   
   A   
   T   
   A   
   A   
   T   
   G   
   G   
   A   
   A   
   C   
   A   
   T   
   T   
   A   
   G   
   T   
   C   
   T   
   A   
   T   
   C   
   C   
   A   
   A   
   A   
   A   
   C   
   T   
   A   
   G   
   T   
   T   
   T   
   T   
   A   
   G   
   A   
   C   
   A   
   A   
   A   
   G   
   A   
   A   
   A   
   C   
   G   
   A   
   A   
   G   
   A   
   A   
   A   
   A   
   A   
   A   
   G   
   A   
   C   
   A   
   A   
   T   
   G   
   T   
   A   
   A   
   T   
   C   
   A   
   G   
   C   
   G   
   C   
   C   
   A   
   A   
   C   
   G   
   C   
   A   
   G   
   C   
   G   
   A   
   A   
   T   
   A   
   C   
   A   
   A   
   T   
   T   
   G   
   C   
   C   
   A   
   C   
   A   
   G   
   A   
   T   
   C   
   T   
   T   
   T   
   T   
   A   
   G   
   G   
   T   
   A   
   G   
   T   
   G   
   T   
   G   
   G   
   A   
   A   
   G   
   A   
   T   
   C   
   C   
   T   
   T   
   C   
   A   
   G   
   G   
   T   
   T   
   A   
   T   
   G   
   T   
   T   
   T   
   A   
   T   
   A   
   A   
   T   
   A   
   T   
   G   
   T   
   A   
   C   
   A   
   A   
   T   
   C   
   C   
   G   
   A   
   A   
   C   
   T   
   T   
   C   
   T   
   C   
   A   
   T   
   T   
   G   
   G   
   C   
   T   
   G   
   C   
   T   
   A   
   A   
   A   
   A   
   C   
   A   
   G   
   G   
   A   
   A   
   C   
   A   
   G   
   C   
   T   
   G   
   A   
   A   
   A   
   T   
   A   
   A   
   A   
   A   
   G   
   A   
   T   
   A   
   A   
   A   
   C   
   A   
   A   
   G   
   A   
   T   
   A   
   C   
   A   
   G   
   A   
   T   
   G   
   G   
   C   
   A   
   A   
   A   
   G   
   A   
   A   
   A   
   A   
   C   
   A   
   G   
   T   
   T   
   T   
   C   
   C   
   T   
   T   
   C   
   T   
   A   
   A   
   C   
   G   
   T   
   T   
   A   
   G   
   A   
   T   
   C   
   G   
   T   
   A   
   G   
   T   
   A   
   A   
   C   
   A   
   A   
   T   
   A   
   A   
   A   
   T   
   T   
   C   
   T   
   T   
   A   
   A   
   C   
   A   
   A   
   T   
   G   
   A   
   T   
   T   
   A   
   T   
   G   
   G   
   T   
   T   
   G   
   A   
   A   
   A   
   A   
   T   
   T   
   C   
   A   
   G   
   G   
   A   
   G   
   A   
   A   
   A   
   A   
   T   
   G   
   G   
   T   
   T   
   C   
   T   
   G   
   C   
   A   
   A   
   C   
   A   
   G   
   A   
   T   
   A   
   T   
   C   
   A   
   G   
   T   
   A   
   A   
   A   
   C   
   C   
   A   
   T   
   T   
   A   
   A   
   T   
   T   
   G   
   A   
   T   
   T   
   A   
   C   
   T   
   T   
   A   
   G   
   A   
   A   
   G   
   C   
   A   
   A   
   C   
   C   
   A   
   T   
   T   
   A   
   A   
   A   
   -   
   -   
   -   
   -   
   -   
   -   
   -   
   -   
   -   
   -   
   -   
   -   
   -   
   -   
   -   
   -   
   -   
   -   
   -   
   -   
   -   
   -   
   -   
   -   
   -   
   -   
   -   
   -   
   -   
   -   
   -   
   -   
   -   
   -   
   -   
   -   
   -   
   -   
   -   
   -   
   -   
   -   
   -   
   -   
   -   
   -   
   -   
   -   
   -   
   -   
   -   
   -   
   -   
   -   
   -   
   -   
   -   
   -   
   -   
   -   
   -   
 

 
   N60268F   
   -   
   -   
   -   
   -   
   -   
   -   
   -   
   -   
   -   
   -   
   -   
   -   
   -   
   -   
   -   
   -   
   -   
   -   
   -   
   -   
   -   
   -   
   -   
   -   
   -   
   -   
   -   
   -   
   -   
   -   
   -   
   -   
   -   
   -   
   -   
   -   
   -   
   -   
   -   
   -   
   -   
   -   
   -   
   -   
   -   
   -   
   -   
   -   
   -   
   -   
   -   
   -   
   -   
   -   
   -   
   -   
   -   
   -   
   -   
   -   
   -   
   -   
   -   
   -   
   -   
   -   
   -   
   -   
   -   
   -   
   -   
   -   
   -   
   -   
   -   
   -   
   -   
   -   
   -   
   -   
   -   
   -   
   -   
   -   
   -   
   -   
   -   
   -   
   -   
   -   
   -   
   -   
   -   
   -   
   -   
   -   
   -   
   -   
   -   
   -   
   -   
   -   
   -   
   -   
   -   
   -   
   -   
   -   
   C   
   A   
   A   
   T   
   G   
   G   
   C   
   A   
   A   
   G   
   C   
   C   
   A   
   A   
   A   
   C   
   A   
   A   
   G   
   A   
    A    
   T   
   T   
   A   
   G   
   C   
    C    
   G   
   A   
   A   
   G   
   C   
   G   
   A   
   A   
   G   
   A   
   A   
   A   
   A   
    C    
   A   
   G   
   C   
    T    
   A   
    C    
   T   
    A    
    C    
   A   
   T   
   T   
   T   
   T   
   T   
   A   
   A   
   A   
    C    
   G   
   T   
   A   
   T   
   T   
   G   
    A    
   C   
   A   
   A   
   A   
   A   
   C   
   A   
   G   
    A    
   A   
   A   
   T   
   T   
   T   
   G   
   A   
   T   
   A   
   A   
   G   
   T   
   T   
   A   
   C   
   C   
    G    
   T   
   C   
   C   
   G   
   T   
   T   
    G    
   T   
   T   
   C   
   A   
   A   
   G   
   A   
   A   
    G    
   C   
   T   
   A   
   G   
   C   
   T   
   T   
   A   
   A   
   A   
   G   
   A   
   A   
   A   
   A   
   A   
   T   
   G   
   G   
    C    
   T   
   A   
   T   
   G   
   A   
   T   
   A   
   C   
   T   
   A   
   A   
   A   
   T   
   C   
    T    
   G   
   T   
   T   
   G   
   T   
   T   
   G   
   A   
   A   
   A   
   A   
   A   
   T   
   A   
   C   
   C   
   A   
   A   
   G   
   C   
   A   
   A   
   T   
   T   
   T   
   A   
   T   
   T   
   C   
   A   
   G   
   G   
   G   
   A   
   T   
   T   
   C   
   A   
   A   
   G   
   C   
    A    
   G   
   A   
   A   
    G    
   G   
    A    
   G   
   T   
   C   
   A   
   A   
    A    
   G   
   C   
   T   
   A   
   G   
   T   
   G   
   A   
   T   
   G   
   T   
    T    
   C   
   A   
   A   
   G   
   T   
    C    
   A   
   A   
   A   
   A   
   A   
    G    
   G   
    C    
   G   
   A   
   A   
   A   
   G   
   A   
    T    
   A   
   A   
    T    
   C   
   A   
   A   
   T   
   A   
   C   
   A   
   C   
   A   
   T   
   T   
   T   
   A   
    C    
   C   
   T   
   A   
   T   
   A   
   A   
   A   
   T   
   T   
   A   
   T   
   C   
   G   
   A   
   T   
   G   
   A   
   G   
    C    
   A   
   C   
    T    
   C   
   C   
   T   
   T   
   T   
   A   
   G   
   G   
   C   
   G   
   A   
   A   
   A   
   T   
   G   
   A   
   A   
   A   
   G   
   A   
   T   
    T    
   T   
   G   
   T   
   C   
   T   
   T   
   A   
   T   
   C   
   A   
   A   
   T   
   C   
   A   
   A   
   G   
   T   
   A   
   T   
    C    
   G   
    C    
   C   
   A   
   A   
    A    
   A   
   A   
   A   
   G   
   G   
    C    
   G   
   A   
   T   
   A   
    C    
   C   
   T   
   A   
   C   
   C   
   A   
   A   
   A   
   T   
    C    
    G    
   C   
   T   
   T   
   G   
   G   
    A    
    A    
    G    
   C   
   C   
    G    
   T   
   C   
   T   
   T   
   T   
   A   
   A   
   T   
   T   
   T   
   T   
   T   
   C   
   C   
   A   
   G   
   A   
   T   
   A   
   T   
   G   
   T   
   C   
   A   
   G   
   G   
   A   
   A   
   A   
   T   
   G   
   A   
   T   
   A   
   A   
   A   
    A    
   T   
   T   
   T   
   C   
   G   
   A   
   T   
   T   
   C   
   A   
   A   
   G   
   T   
   A   
   G   
   A   
   T   
   A   
   A   
   T   
   G   
   C   
   C   
   A   
   A   
   A   
   C   
   G   
   T   
   G   
   G   
   A   
   G   
   A   
   A   
   A   
   T   
   T   
   G   
   T   
   C   
   G   
   A   
   T   
   C   
   G   
   T   
   A   
   A   
    T    
   G   
   G   
   T   
   A   
   G   
   T   
   G   
   G   
   G   
   C   
   T   
   A   
   G   
    C    
   A   
   A   
   T   
   T   
   A   
   A   
   C   
   A   
   A   
   A   
   G   
   T   
   G   
   T   
   T   
   T   
   G   
   A   
    C    
   G   
   A   
   A   
   G   
   T   
   G   
   G   
   G   
   C   
   G   
   T   
   A   
   G   
   T   
    G    
   C   
   C   
   T   
    G    
   G   
   C   
   A   
   A   
   A   
   C   
   T   
    C    
   G   
   G   
   T   
   T   
   C   
   T   
   G   
   G   
   C   
   G   
   C   
   A   
   G   
   A   
   A   
   A   
   A   
   A   
    A    
   C   
   A   
   G   
   C   
    C    
   A   
   A   
   T   
   A   
   T   
   C   
   A   
   A   
   A   
   G   
   C   
   T   
   T   
   T   
   T   
   A   
   G   
   T   
   G   
   A   
   T   
   A   
   A   
    G    
   T   
   T   
    C    
   G   
   G   
   C   
   G   
   T   
   T   
   T   
   C   
   T   
    G    
   T   
   T   
   G   
   A   
   T   
   G   
   A   
   A   
   A   
   T   
   C   
   A   
   A   
   T   
   C   
    A    
   A   
   A   
   A   
    G    
   T   
   T   
   A   
   A   
   G   
   C   
   C   
   A   
   A   
   G   
   G   
    G    
   T   
   G   
   G   
   G   
   T   
   C   
   C   
   A   
   A   
   G   
   C   
   A   
   G   
   A   
   C   
   T   
   C   
    C    
   T   
   T   
   T   
   G   
   T   
   A   
   C   
   C   
   A   
   A   
   T   
   C   
   A   
   C   
   A   
   G   
   T   
    C    
   G   
   C   
   T   
   T   
   C   
   T   
   G   
   A   
   A   
   C   
   C   
   A   
   G   
   T   
   G   
   A   
   C   
   A   
   G   
   A   
   A   
   T   
   T   
   A   
   C   
   C   
   A   
   A   
   C   
   A   
   G   
   G   
   G   
   G   
   C   
   T   
   G   
   C   
   G   
   A   
   C   
   A   
   A   
   A   
   A   
   G   
    A    
    T    
   A   
   C   
   A   
   G   
   A   
   G   
   T   
   C   
   A   
   C   
   G   
   T   
   T   
   A   
   T   
   T   
   A   
   T   
   C   
   C   
    G    
    C    
   T   
   G   
   G   
   G   
   G   
   G   
   A   
   A   
   G   
   C   
   A   
   G   
   C   
   T   
   G   
   C   
   G   
   C   
   A   
   A   
   T   
   T   
   A   
   A   
   T   
   C   
   G   
   G   
    G    
   T   
   A   
   T   
   A   
   C   
    G    
   G   
   G   
    C    
   A   
   C   
    C    
   A   
   T   
   T   
   A   
   C   
   T   
   G   
   C   
   A   
   G   
   A   
    A    
   G   
   A   
   T   
   A   
   T   
   T   
    A    
   A   
   A   
   A   
   A   
   A   
   A   
   A   
   T   
   C   
   C   
   A   
   G   
   A   
   G   
   T   
   T   
   A   
   A   
   G   
   T   
   A   
   G   
   T   
   A   
   C   
   G   
   G   
   G   
    C    
   G   
   T   
   A   
   A   
   T   
   T   
   G   
   G   
    T    
   A   
   A   
   A   
   A   
   C   
   T   
   G   
   G   
    C    
   T   
   T   
   A   
   G   
   A   
   A   
   C   
   G   
   A   
   G   
   C   
   G   
   T   
   T   
   T   
   G   
   A   
   T   
   A   
   A   
   A   
   G   
   A   
    A    
   T   
   T   
   A   
   A   
   G   
   A   
   G   
   G   
    G    
   C   
   A   
   A   
   G   
   A   
   T   
   G   
   G   
    T    
   G   
   G   
    C    
   T   
   C   
    A    
   C   
   T   
   A   
   G   
   T   
   T   
   A   
   T   
   T   
   T   
   T   
   A   
   G   
   A   
   T   
   G   
   A   
   C   
   A   
   A   
   A   
   G   
   A   
   G   
   A   
   A   
   T   
   G   
   T   
   C   
   A   
   A   
   A   
   A   
   A   
   A   
   G   
   C   
   T   
   T   
   T   
   A   
   C   
   A   
   A   
   A   
   C   
   C   
   A   
   A   
   A   
   G   
   A   
    G    
   A   
   A   
   A   
   A   
   A   
   A   
   G   
   A   
   T   
   G   
   G   
   T   
   C   
   A   
   A   
   A   
   C   
    G    
   A   
   T   
   T   
   A   
   A   
   A   
   T   
   T   
   A   
   A   
   C   
   G   
   A   
   T   
   T   
   G   
   A   
    C    
   A   
   G   
    T    
   G   
   G   
    C    
   G   
   T   
   A   
   C   
   A   
   A   
   C   
   A   
   A   
   C   
   A   
   A   
   G   
   C   
    C    
   T   
   T   
    C    
   G   
   C   
   T   
   A   
   T   
   A   
   T   
   T   
   T   
   G   
   A   
   C   
   A   
   A   
   A   
   C   
   G   
    C    
    C    
   C   
   T   
   G   
   G   
   T   
   A   
   G   
   T   
   G   
   C   
   G   
   G   
   T   
   C   
   A   
   T   
   T   
   A   
   C   
   C   
   G   
   A   
   T   
   C   
   C   
   T   
   C   
   A   
   A   
   A   
   A   
   A   
   G   
   G   
   C   
   G   
   A   
   T   
   T   
   T   
   A   
    T    
   T   
   A   
   G   
   C   
    A    
   A   
   C   
   G   
   G   
   T   
   T   
   A   
   G   
   T   
   T   
   C   
   A   
   C   
   C   
   T   
   T   
   C   
   G   
   T   
   A   
   T   
   G   
   A   
   T   
   C   
   C   
   T   
   A   
   A   
   T   
   A   
   A   
   A   
   A   
   T   
   G   
   G   
   C   
   C   
   A   
   A   
   T   
   G   
   G   
    C    
   A   
   T   
   T   
   T   
   C   
   T   
   C   
   A   
   A   
   A   
   A   
   A   
   G   
   A   
    A    
   T   
   A   
   T   
   G   
   A   
   T   
   G   
   C   
   T   
   T   
   A   
   C   
   A   
   A   
   T   
   A   
   A   
   C   
   A   
   A   
   C   
   A   
   A   
   A   
   G   
   A   
   T   
   T   
   T   
   A   
   C   
   C   
   A   
   T   
   T   
    T    
   A   
   C   
   A   
   G   
   C   
   A   
   C   
   G   
   T   
   T   
   T   
    T    
   G   
   C   
   G   
   A   
   C   
   A   
   G   
   G   
    C    
   T   
   A   
   T   
   G   
   C   
   A   
   C   
   C   
   A   
   G   
   G   
    C    
   T   
   C   
   T   
   A   
   C   
   C   
   T   
   T   
   T   
   A   
   A   
   A   
   A   
   C   
   A   
   A   
   T   
   T   
   A   
   C   
   C   
   G   
   G   
   T   
   G   
   C   
   C   
   A   
   T   
   T   
   G   
   G   
   T   
   T   
   T   
   A   
   G   
   A   
   T   
    G    
   C   
   A   
   G   
   G   
   G   
   A   
   C   
   A   
   T   
   T   
   G   
   A   
   A   
   G   
   C   
   C   
   A   
   G   
   A   
   T   
   G   
   A   
   A   
   G   
   A   
   G   
   C   
   T   
   A   
   G   
   A   
   A   
   A   
   T   
   T   
   A   
   A   
   T   
   G   
   G   
    T    
   T   
   T   
   G   
   A   
   A   
   A   
   T   
   G   
   G   
   C   
   A   
   A   
   A   
   A   
   A   
   G   
   A   
   T   
   A   
   A   
   A   
   T   
   C   
   T   
   T   
   G   
   G   
   G   
   G   
    C    
   G   
   G   
   C   
   T   
   A   
   T   
   T   
   T   
   T   
   G   
   C   
    G    
   A   
   C   
   A   
   C   
   G   
   T   
   G   
   T   
   A   
   A   
   A   
   A   
   G   
   A   
   A   
   G   
   C   
   A   
   A   
   G   
   T   
   C   
   C   
   A   
   G   
   T   
   C   
   A   
   A   
   T   
   C   
   T   
    G    
   C   
   G   
   T   
   A   
   C   
   C   
   G   
   C   
   T   
   T   
   T   
   A   
   G   
   T   
   T   
   A   
   A   
   T   
   T   
   C   
   A   
   G   
   A   
   T   
   A   
   A   
   T   
   A   
   T   
   T   
   T   
   A   
   T   
   T   
   T   
   T   
   G   
   C   
    C    
   C   
   A   
   A   
   C   
   A   
   A   
   A   
   C   
   A   
   T   
   T   
   A   
   C   
   G   
    G    
   A   
   T   
   G   
   G   
   G   
    A    
   G   
   A   
   A   
   G   
   A   
   C   
   A   
   A   
   A   
   T   
   T   
   T   
   A   
   G   
   A   
   G   
   C   
    G    
   G   
   G   
   T   
   C   
   T   
   T   
   A   
   A   
   T   
   A   
   A   
   A   
   T   
   T   
   C   
   A   
   T   
   T   
   T   
   T   
   C   
   G   
   G   
   T   
   G   
   A   
   G   
   G   
   A   
   A   
   C   
   T   
   A   
   G   
   A   
   T   
   T   
   T   
   A   
   C   
   C   
   A   
   A   
   T   
    T    
   G   
   C   
   C   
   A   
   T   
   G   
   A   
   C   
   G   
   C   
   C   
    T    
   G   
   C   
   T   
   C   
   A   
   A   
   A   
   T   
   T   
   T   
   C   
   A   
   A   
   A   
    T    
   G   
   A   
   A   
   G   
   A   
   T   
   A   
   A   
   A   
   T   
   T   
   T   
   A   
   A   
   T   
   T   
   C   
   T   
   G   
   A   
   A   
   A   
   T   
   T   
   C   
   T   
   T   
   T   
   T   
    A    
   G   
   C   
   A   
   G   
   A   
   T   
   A   
   C   
    G    
   G   
   G   
   C   
   T   
   A   
    C    
   G   
   G   
    T    
   C   
   A   
   A   
   G   
   G   
    T    
   C   
   A   
   A   
   T   
   T   
   G   
   T   
   T   
   A   
   A   
   T   
   T   
   T   
   C   
    G    
   C   
   C   
   A   
   A   
   T   
   C   
   C   
   A   
   G   
   C   
   A   
   A   
   G   
   C   
   T   
   A   
   C   
   C   
   A   
   T   
   G   
   T   
   A   
   T   
   A   
   G   
   T   
   G   
   T   
   T   
   T   
   T   
   C   
   C   
   A   
   A   
   A   
   A   
   T   
   A   
   A   
   T   
   G   
   G   
    A    
   A   
   C   
   A   
    T    
   T   
    A    
   G   
   T   
    C    
   T   
   A   
   T   
   C   
   C   
   A   
   A   
   A   
   A   
    C    
   T   
   A   
   G   
   T   
   T   
   T   
   T   
   A   
   G   
   A   
   C   
   A   
   A   
   A   
   G   
   A   
   A   
   A   
   C   
    G    
   A   
   A   
   G   
   A   
   A   
   A   
   A   
   A   
   A   
   G   
   A   
   C   
   A   
   A   
   T   
   G   
   T   
   A   
   A   
   T   
   C   
   A   
   G   
   C   
   G   
   C   
   C   
   A   
   A   
   C   
   G   
   C   
   A   
   G   
   C   
   G   
   A   
   A   
   T   
   A   
   C   
   A   
   A   
   T   
   T   
   G   
   C   
   C   
   A   
   C   
   A   
   G   
   A   
   T   
   C   
   T   
   T   
   T   
   T   
   A   
   G   
   G   
   T   
   A   
   G   
   T   
   G   
   T   
   G   
   G   
   A   
   A   
   G   
   A   
   T   
   C   
   C   
   T   
   T   
   C   
   A   
   G   
   G   
   T   
   T   
   A   
   T   
   G   
   T   
   T   
   T   
   A   
   T   
   A   
   A   
   T   
   A   
   T   
   G   
   T   
   A   
   C   
   A   
   A   
   T   
   C   
   C   
   G   
   A   
   A   
   C   
   T   
   T   
   C   
   T   
   C   
   A   
   T   
   T   
   G   
   G   
   C   
   T   
   G   
   C   
   T   
   A   
   A   
   A   
   A   
   C   
   A   
   G   
   G   
   A   
   A   
   C   
   A   
   G   
   C   
   T   
   G   
   A   
   A   
   A   
   T   
   A   
   A   
   A   
   A   
   G   
   A   
   T   
   A   
   A   
   A   
   C   
   A   
   A   
   G   
   A   
   T   
   A   
   C   
   A   
   G   
   A   
   T   
   G   
   G   
   C   
   A   
   A   
   A   
   G   
   A   
   A   
   A   
   A   
   C   
   A   
   G   
   T   
   T   
   T   
   C   
   C   
   T   
   T   
   C   
   T   
   A   
   A   
   C   
   G   
   T   
   T   
   A   
   G   
   A   
   T   
   C   
   G   
   T   
   A   
   G   
   T   
   A   
   A   
   C   
   A   
   A   
   T   
   A   
   A   
   A   
   T   
   T   
   C   
   T   
   T   
   A   
   A   
   C   
   A   
   A   
   T   
   G   
   A   
   T   
   T   
   A   
   T   
   G   
   G   
   T   
   T   
   G   
   A   
   A   
   A   
   A   
   T   
   T   
   C   
   A   
   G   
   G   
   A   
   G   
   A   
   A   
   A   
   A   
   T   
   G   
   G   
   T   
   T   
   C   
   T   
   G   
   C   
   A   
   A   
   C   
   A   
   G   
   A   
   T   
   A   
   T   
   C   
   A   
   G   
   T   
   A   
   A   
   A   
   C   
   C   
   A   
   T   
   T   
   A   
   A   
   T   
   T   
   G   
   A   
   T   
   T   
   A   
   C   
   T   
   T   
   A   
   G   
   A   
   A   
   G   
   C   
   A   
   A   
   C   
   C   
   A   
   T   
   T   
   A   
   A   
   A   
   -   
   -   
   -   
   -   
   -   
   -   
   -   
   -   
   -   
   -   
   -   
   -   
   -   
   -   
   -   
   -   
   -   
   -   
   -   
   -   
   -   
   -   
   -   
   -   
   -   
   -   
   -   
   -   
   -   
   -   
   -   
   -   
   -   
   -   
   -   
   -   
   -   
   -   
   -   
   -   
   -   
   -   
   -   
   -   
   -   
   -   
   -   
   -   
   -   
   -   
   -   
   -   
   -   
   -   
   -   
   -   
   -   
   -   
   -   
   -   
   -   
   -   
   -   
   -   
   -   
   -   
   -   
   -   
   -   
   -   
   -   
   -   
   -   
   -   
   -   
   -   
   -   
   -   
   -   
   -   
   -   
   -   
   -   
   -   
   -   
   -   
   -   
   -   
   -   
   -   
   -   
   -   
   -   
   -   
   -   
   -   
   -   
   -   
   -   
   -   
   -   
   -   
   -   
   -   
   -   
   -   
   -   
   -   
   -   
   -   
   -   
   -   
   -   
   -   
   -   
   -   
   -   
   -   
   -   
   -   
   -   
   -   
   -   
   -   
   -   
   -   
   -   
   -   
   -   
   -   
   -   
   -   
   -   
   -   
   -   
   -   
   -   
   -   
   -   
   -   
   -   
   -   
   -   
   -   
   -   
   -   
   -   
   -   
   -   
   -   
   -   
   -   
   -   
   -   
   -   
   -   
   -   
   -   
   -   
   -   
   -   
   -   
   -   
   -   
   -   
   -   
   -   
   -   
   -   
   C   
   A   
   A   
   T   
   G   
   G   
   C   
   A   
   A   
   G   
   C   
   C   
   A   
   A   
   A   
   C   
   A   
   A   
   G   
   A   
   A   
   T   
   T   
   A   
   G   
   C   
   C   
   G   
   A   
   A   
   G   
   C   
   G   
   A   
   A   
   G   
   A   
   A   
   A   
   A   
   C   
   A   
   G   
   C   
   T   
   A   
   C   
   T   
   A   
   C   
   A   
   T   
   T   
   T   
   T   
   T   
   A   
   A   
   A   
   C   
   G   
   T   
   A   
   T   
   T   
   G   
   A   
   C   
   A   
   A   
   A   
   A   
   C   
   A   
   G   
   A   
   A   
   A   
   T   
   T   
   T   
   G   
   A   
   T   
   A   
   A   
   G   
   T   
   T   
   A   
   C   
   C   
   G   
   T   
   C   
   C   
   G   
   T   
   T   
   G   
   T   
   T   
   C   
   A   
   A   
   G   
   A   
   A   
   G   
   C   
   T   
   A   
   G   
   C   
   T   
   T   
   A   
   A   
   A   
   G   
   A   
   A   
   A   
   A   
   A   
   T   
   G   
   G   
   C   
   T   
   A   
   T   
   G   
   A   
   T   
   A   
   C   
   T   
   A   
   A   
   A   
   T   
   C   
   T   
   G   
   T   
   T   
   G   
   T   
   T   
   G   
   A   
   A   
   A   
   A   
   A   
   T   
   A   
   C   
   C   
   A   
   A   
   G   
   C   
   A   
   A   
   T   
   T   
   T   
   A   
   T   
   T   
   C   
   A   
   G   
   G   
   G   
   A   
   T   
   T   
   C   
   A   
   A   
   G   
   C   
   A   
   G   
   A   
   A   
   G   
   G   
   A   
   G   
   T   
   C   
   A   
   A   
   A   
   G   
   C   
   T   
   A   
   G   
   T   
   G   
   A   
   T   
   G   
   T   
   T   
   C   
   A   
   A   
   G   
   T   
   C   
   A   
   A   
   A   
   A   
   A   
   G   
   G   
   C   
   G   
   A   
   A   
   A   
   G   
   A   
   T   
   A   
   A   
   T   
   C   
   A   
   A   
   T   
   A   
   C   
   A   
   C   
   A   
   T   
   T   
   T   
   A   
   C   
   C   
   T   
   A   
   T   
   A   
   A   
   A   
   T   
   T   
   A   
   T   
   C   
   G   
   A   
   T   
   G   
   A   
   G   
   C   
   A   
   C   
   T   
   C   
   C   
   T   
   T   
   T   
   A   
   G   
   G   
   C   
   G   
   A   
   A   
   A   
   T   
   G   
   A   
   A   
   A   
   G   
   A   
   T   
   T   
   T   
   G   
   T   
   C   
   T   
   T   
   A   
   T   
   C   
   A   
   A   
   T   
   C   
   A   
   A   
   G   
   T   
   A   
   T   
   C   
   G   
   C   
   C   
   A   
   A   
   A   
   A   
   A   
   A   
   G   
   G   
   C   
   G   
   A   
   T   
   A   
   C   
   C   
   T   
   A   
   C   
   C   
   A   
   A   
   A   
   T   
   C   
   G   
   C   
   T   
   T   
   G   
   G   
   A   
   A   
   G   
   C   
   C   
   G   
   T   
   C   
   T   
   T   
   T   
   A   
   A   
   T   
   T   
   T   
   T   
   T   
   C   
   C   
   A   
   G   
   A   
   T   
   A   
   T   
   G   
   T   
   C   
   A   
   G   
   G   
   A   
   A   
   A   
   T   
   G   
   A   
   T   
   A   
   A   
   A   
   A   
   T   
   T   
   T   
   C   
   G   
   A   
   T   
   T   
   C   
   A   
   A   
   G   
   T   
   A   
   G   
   A   
   T   
   A   
   A   
   T   
   G   
   C   
   C   
   A   
   A   
   A   
   C   
   G   
   T   
   G   
   G   
   A   
   G   
   A   
   A   
   A   
   T   
   T   
   G   
   T   
   C   
   G   
   A   
   T   
   C   
   G   
   T   
   A   
   A   
   T   
   G   
   G   
   T   
   A   
   G   
   T   
   G   
   G   
   G   
   C   
   T   
   A   
   G   
   C   
   A   
   A   
   T   
   T   
   A   
   A   
   C   
   A   
   A   
   A   
   G   
   T   
   G   
   T   
   T   
   T   
   G   
   A   
   C   
   G   
   A   
   A   
   G   
   T   
   G   
   G   
   G   
   C   
   G   
   T   
   A   
   G   
   T   
   G   
   C   
   C   
   T   
   G   
   G   
   C   
   A   
   A   
   A   
   C   
   T   
   C   
   G   
   G   
   T   
   T   
   C   
   T   
   G   
   G   
   C   
   G   
   C   
   A   
   G   
   A   
   A   
   A   
   A   
   A   
   A   
   C   
   A   
   G   
   C   
   C   
   A   
   A   
   T   
   A   
   T   
   C   
   A   
   A   
   A   
   G   
   C   
   T   
   T   
   T   
   T   
   A   
   G   
   T   
   G   
   A   
   T   
   A   
   A   
   G   
   T   
   T   
   C   
   G   
   G   
   C   
   G   
   T   
   T   
   T   
   C   
   T   
   G   
   T   
   T   
   G   
   A   
   T   
   G   
   A   
   A   
   A   
   T   
   C   
   A   
   A   
   T   
   C   
   A   
   A   
   A   
   A   
   G   
   T   
   T   
   A   
   A   
   G   
   C   
   C   
   A   
   A   
   G   
   G   
   G   
   T   
   G   
   G   
   G   
   T   
   C   
   C   
   A   
   A   
   G   
   C   
   A   
   G   
   A   
   C   
   T   
   C   
   C   
   T   
   T   
   T   
   G   
   T   
   A   
   C   
   C   
   A   
   A   
   T   
   C   
   A   
   C   
   A   
   G   
   T   
   C   
   G   
   C   
   T   
   T   
   C   
   T   
   G   
   A   
   A   
   C   
   C   
   A   
   G   
   T   
   G   
   A   
   C   
   A   
   G   
   A   
   A   
   T   
   T   
   A   
   C   
   C   
   A   
   A   
   C   
   A   
   G   
   G   
   G   
   G   
   C   
   T   
   G   
   C   
   G   
   A   
   C   
   A   
   A   
   A   
   A   
   G   
   A   
   T   
   A   
   C   
   A   
   G   
   A   
   G   
   T   
   C   
   A   
   C   
   G   
   T   
   T   
   A   
   T   
   T   
   A   
   T   
   C   
   C   
   G   
   C   
   T   
   G   
   G   
   G   
   G   
   G   
   A   
   A   
   G   
   C   
   A   
   G   
   C   
   T   
   G   
   C   
   G   
   C   
   A   
   A   
   T   
   T   
   A   
   A   
   T   
   C   
   G   
   G   
   G   
   T   
   A   
   T   
   A   
   C   
   G   
   G   
   G   
   C   
   A   
   C   
   C   
   A   
   T   
   T   
   A   
   C   
   T   
   G   
   C   
   A   
   G   
   A   
   A   
   G   
   A   
   T   
   A   
   T   
   T   
   A   
   A   
   A   
   A   
   A   
   A   
   A   
   A   
   T   
   C   
   C   
   A   
   G   
   A   
   G   
   T   
   T   
   A   
   A   
   G   
   T   
   A   
   G   
   T   
   A   
   C   
   G   
   G   
   G   
   C   
   G   
   T   
   A   
   A   
   T   
   T   
   G   
   G   
   T   
   A   
   A   
   A   
   A   
   C   
   T   
   G   
   G   
   C   
   T   
   T   
   A   
   G   
   A   
   A   
   C   
   G   
   A   
   G   
   C   
   G   
   T   
   T   
   T   
   G   
   A   
   T   
   A   
   A   
   A   
   G   
   A   
   A   
   T   
   T   
   A   
   A   
   G   
   A   
   G   
   G   
   G   
   C   
   A   
   A   
   G   
   A   
   T   
   G   
   G   
   T   
   G   
   G   
   C   
   T   
   C   
   A   
   C   
   T   
   A   
   G   
   T   
   T   
   A   
   T   
   T   
   T   
   T   
   A   
   G   
   A   
   T   
   G   
   A   
   C   
   A   
   A   
   A   
   G   
   A   
   G   
   A   
   A   
   T   
   G   
   T   
   C   
   A   
   A   
   A   
   A   
   A   
   A   
   G   
   C   
   T   
   T   
   T   
   A   
   C   
   A   
   A   
   A   
   C   
   C   
   A   
   A   
   A   
   G   
   A   
   G   
   A   
   A   
   A   
   A   
   A   
   A   
   G   
   A   
   T   
   G   
   G   
   T   
   C   
   A   
   A   
   A   
   C   
   G   
   A   
   T   
   T   
   A   
   A   
   A   
   T   
   T   
   A   
   A   
   C   
   G   
   A   
   T   
   T   
   G   
   A   
   C   
   A   
   G   
   T   
   G   
   G   
   C   
   G   
   T   
   A   
   C   
   A   
   A   
   C   
   A   
   A   
   C   
   A   
   A   
   G   
   C   
   C   
   T   
   T   
   C   
   G   
   C   
   T   
   A   
   T   
   A   
   T   
   T   
   T   
   G   
   A   
   C   
   A   
   A   
   A   
   C   
   G   
   C   
   C   
   C   
   T   
   G   
   G   
   T   
   A   
   G   
   T   
   G   
   C   
   G   
   G   
   T   
   C   
   A   
   T   
   T   
   A   
   C   
   C   
   G   
   A   
   T   
   C   
   C   
   T   
   C   
   A   
   A   
   A   
   A   
   A   
   G   
   G   
   C   
   G   
   A   
   T   
   T   
   T   
   A   
   T   
   T   
   A   
   G   
   C   
   A   
   A   
   C   
   G   
   G   
   T   
   T   
   A   
   G   
   T   
   T   
   C   
   A   
   C   
   C   
   T   
   T   
   C   
   G   
   T   
   A   
   T   
   G   
   A   
   T   
   C   
   C   
   T   
   A   
   A   
   T   
   A   
   A   
   A   
   A   
   T   
   G   
   G   
   C   
   C   
   A   
   A   
   T   
   G   
   G   
   C   
   A   
   T   
   T   
   T   
   C   
   T   
   C   
   A   
   A   
   A   
   A   
   A   
   G   
   A   
   A   
   T   
   A   
   T   
   G   
   A   
   T   
   G   
   C   
   T   
   T   
   A   
   C   
   A   
   A   
   T   
   A   
   A   
   C   
   A   
   A   
   C   
   A   
   A   
   A   
   G   
   A   
   T   
   T   
   T   
   A   
   C   
   C   
   A   
   T   
   T   
   T   
   A   
   C   
   A   
   G   
   C   
   A   
   C   
   G   
   T   
   T   
   T   
   T   
   G   
   C   
   G   
   A   
   C   
   A   
   G   
   G   
   C   
   T   
   A   
   T   
   G   
   C   
   A   
   C   
   C   
   A   
   G   
   G   
   C   
   T   
   C   
   T   
   A   
   C   
   C   
   T   
   T   
   T   
   A   
   A   
   A   
   A   
   C   
   A   
   A   
   T   
   T   
   A   
   C   
   C   
   G   
   G   
   T   
   G   
   C   
   C   
   A   
   T   
   T   
   G   
   G   
   T   
   T   
   T   
   A   
   G   
   A   
   T   
   G   
   C   
   A   
   G   
   G   
   G   
   A   
   C   
   A   
   T   
   T   
   G   
   A   
   A   
   G   
   C   
   C   
   A   
   G   
   A   
   T   
   G   
   A   
   A   
   G   
   A   
   G   
   C   
   T   
   A   
   G   
   A   
   A   
   A   
   T   
   T   
   A   
   A   
   T   
   G   
   G   
   T   
   T   
   T   
   G   
   A   
   A   
   A   
   T   
   G   
   G   
   C   
   A   
   A   
   A   
   A   
   A   
   G   
   A   
   T   
   A   
   A   
   A   
   T   
   C   
   T   
   T   
   G   
   G   
   G   
   G   
   C   
   G   
   G   
   C   
   T   
   A   
   T   
   T   
   T   
   T   
   G   
   C   
   G   
   A   
   C   
   A   
   C   
   G   
   T   
   G   
   T   
   A   
   A   
   A   
   A   
   G   
   A   
   A   
   G   
   C   
   A   
   A   
   G   
   T   
   C   
   C   
   A   
   G   
   T   
   C   
   A   
   A   
   T   
   C   
   T   
   G   
   C   
   G   
   T   
   A   
   C   
   C   
   G   
   C   
   T   
   T   
   T   
   A   
   G   
   T   
   T   
   A   
   A   
   T   
   T   
   C   
   A   
   G   
   A   
   T   
   A   
   A   
   T   
   A   
   T   
   T   
   T   
   A   
   T   
   T   
   T   
   T   
   G   
   C   
   C   
   C   
   A   
   A   
   C   
   A   
   A   
   A   
   C   
   A   
   T   
   T   
   A   
   C   
   G   
   G   
   A   
   T   
   G   
   G   
   G   
   A   
   G   
   A   
   A   
   G   
   A   
   C   
   A   
   A   
   A   
   T   
   T   
   T   
   A   
   G   
   A   
   G   
   C   
   G   
   G   
   G   
   T   
   C   
   T   
   T   
   A   
   A   
   T   
   A   
   A   
   A   
   T   
   T   
   C   
   A   
   T   
   T   
   T   
   T   
   C   
   G   
   G   
   T   
   G   
   A   
   G   
   G   
   A   
   A   
   C   
   T   
   A   
   G   
   A   
   T   
   T   
   T   
   A   
   C   
   C   
   A   
   A   
   T   
   T   
   G   
   C   
   C   
   A   
   T   
   G   
   A   
   C   
   G   
   C   
   C   
   T   
   G   
   C   
   T   
   C   
   A   
   A   
   A   
   T   
   T   
   T   
   C   
   A   
   A   
   A   
   T   
   G   
   A   
   A   
   G   
   A   
   T   
   A   
   A   
   A   
   T   
   T   
   T   
   A   
   A   
   T   
   T   
   C   
   T   
   G   
   A   
   A   
   A   
   T   
   T   
   C   
   T   
   T   
   T   
   T   
   A   
   G   
   C   
   A   
   G   
   A   
   T   
   A   
   C   
   G   
   G   
   G   
   C   
   T   
   A   
   C   
   G   
   G   
   T   
   C   
   A   
   A   
   G   
   G   
   T   
   C   
   A   
   A   
   T   
   T   
   G   
   T   
   T   
   A   
   A   
   T   
   T   
   T   
   C   
   G   
   C   
   C   
   A   
   A   
   T   
   C   
   C   
   A   
   G   
   C   
   A   
   A   
   G   
   C   
   T   
   A   
   C   
   C   
   A   
   T   
   G   
   T   
   A   
   T   
   A   
   G   
   T   
   G   
   T   
   T   
   T   
   T   
   C   
   C   
   A   
   A   
   A   
   A   
   T   
   A   
   A   
   T   
   G   
   G   
   A   
   A   
   C   
   A   
   T   
   T   
   A   
   G   
   T   
   C   
   T   
   A   
   T   
   C   
   C   
   A   
   A   
   A   
   A   
   C   
   T   
   A   
   G   
   T   
   T   
   T   
   T   
   A   
   G   
   A   
   C   
   A   
   A   
   A   
   G   
   A   
   A   
   A   
   C   
   G   
   A   
   A   
   G   
   A   
   A   
   A   
   A   
   A   
   A   
   G   
   A   
   C   
   A   
   A   
   T   
   G   
   T   
   A   
   A   
   T   
   C   
   A   
   G   
   C   
   G   
   C   
   C   
   A   
   A   
   C   
   G   
   C   
   A   
   G   
   C   
   G   
   A   
   A   
   T   
   A   
   C   
   A   
   A   
   T   
   T   
   G   
   C   
   C   
   A   
   C   
   A   
   G   
   A   
   T   
   C   
   T   
   T   
   T   
   T   
   A   
   G   
   G   
   T   
   A   
   G   
   T   
   G   
   T   
   G   
   G   
   A   
   A   
   G   
   A   
   T   
   C   
   C   
   T   
   T   
   C   
   A   
   G   
   G   
   T   
   T   
   A   
   T   
   G   
   T   
   T   
   T   
   A   
   T   
   A   
   A   
   T   
   A   
   T   
   G   
   T   
   A   
   C   
   A   
   A   
   T   
   C   
   C   
   G   
   A   
   A   
   C   
   T   
   T   
   C   
   T   
   C   
   A   
   T   
   T   
   G   
   G   
   C   
   T   
   G   
   C   
   T   
   A   
   A   
   A   
   A   
   C   
   A   
   G   
   G   
   A   
   A   
   C   
   A   
   G   
   C   
   T   
   G   
   A   
   A   
   A   
   T   
   A   
   A   
   A   
   A   
   G   
   A   
   T   
   A   
   A   
   A   
   C   
   A   
   A   
   G   
   A   
   T   
   A   
   C   
   A   
   G   
   A   
   T   
   G   
   G   
   C   
   A   
   A   
   A   
   G   
   A   
   A   
   A   
   A   
   C   
   A   
   G   
   T   
   T   
   T   
   C   
   C   
   T   
   T   
   C   
   T   
   A   
   A   
   C   
   G   
   T   
   T   
   A   
   G   
   A   
   T   
   C   
   G   
   T   
   A   
   G   
   T   
   A   
   A   
   C   
   A   
   A   
   T   
   A   
   A   
   A   
   T   
   T   
   C   
   T   
   T   
   A   
   A   
   C   
   A   
   A   
   T   
   G   
   A   
   T   
   T   
   A   
   T   
   G   
   G   
   T   
   T   
   G   
   A   
   A   
   A   
   A   
   T   
   T   
   C   
   A   
   G   
   G   
   A   
   G   
   A   
   A   
   A   
   A   
   T   
   G   
   G   
   T   
   T   
   C   
   T   
   G   
   C   
   A   
   A   
   C   
   A   
   G   
   A   
   T   
   A   
   T   
   C   
   A   
   G   
   T   
   A   
   A   
   A   
   C   
   C   
   A   
   T   
   T   
   A   
   A   
   T   
   T   
   G   
   A   
   T   
   T   
   A   
   C   
   T   
   T   
   A   
   G   
   A   
   A   
   G   
   C   
   A   
   A   
   C   
   C   
   A   
   T   
   T   
   A   
   A   
   A   
   -   
   -   
   -   
   -   
   -   
   -   
   -   
   -   
   -   
   -   
   -   
   -   
   -   
   -   
   -   
   -   
   -   
   -   
   -   
   -   
   -   
   -   
   -   
   -   
   -   
   -   
   -   
   -   
   -   
   -   
   -   
   -   
   -   
   -   
   -   
   -   
   -   
   -   
   -   
   -   
   -   
   -   
   -   
   -   
   -   
   -   
   -   
   -   
   -   
   -   
   -   
   -   
   -   
   -   
   -   
   -   
   -   
   -   
   -   
   -   
   -   
 

 
   N52648   
   -   
   -   
   -   
   -   
   -   
   -   
   -   
   -   
   -   
   -   
   -   
   -   
   -   
   -   
   -   
   -   
   -   
   -   
   -   
   -   
   -   
   -   
   -   
   -   
   -   
   -   
   -   
   -   
   -   
   -   
   -   
   -   
   -   
   -   
   -   
   -   
   -   
   -   
   -   
   -   
   -   
   -   
   -   
   -   
   -   
   -   
   -   
   -   
   -   
   -   
   -   
   -   
   -   
   -   
   -   
   -   
   -   
   -   
   -   
   -   
   -   
   -   
   -   
   -   
   -   
   -   
   -   
   -   
   -   
   -   
   -   
   -   
   -   
   -   
   -   
   -   
   -   
   -   
   -   
   -   
   -   
   -   
   -   
   -   
   -   
   -   
   -   
   -   
   -   
   -   
   -   
   -   
   -   
   -   
   -   
   -   
   -   
   -   
   -   
   -   
   -   
   -   
   -   
   -   
   -   
   -   
   -   
   -   
   C   
   A   
   A   
   T   
   G   
   G   
   C   
   A   
   A   
   G   
   C   
   C   
   A   
   A   
   A   
   C   
   A   
   A   
   G   
   A   
    A    
   T   
   T   
   A   
   G   
   C   
    C    
   G   
   A   
   A   
   G   
   C   
   G   
   A   
   A   
   G   
   A   
   A   
   A   
   A   
    C    
   A   
   G   
   C   
    T    
   A   
    A    
   T   
    A    
    C    
   A   
   T   
   T   
   T   
   T   
   T   
   A   
   A   
   A   
    T    
   G   
   T   
   A   
   T   
   T   
   G   
    T    
   C   
   A   
   A   
   A   
   A   
   C   
   A   
   G   
    G    
   A   
   A   
   T   
   T   
   T   
   G   
   A   
   T   
   A   
   A   
   G   
   T   
   T   
   A   
   C   
   C   
    G    
   T   
   C   
   C   
   G   
   T   
   T   
    G    
   T   
   T   
   C   
   A   
   A   
   G   
   A   
   A   
    T    
   C   
   T   
   A   
   G   
   C   
   T   
   T   
   A   
   A   
   A   
   G   
   A   
   A   
   A   
   A   
   A   
   T   
   G   
   G   
    C    
   T   
   A   
   T   
   G   
   A   
   T   
   A   
   C   
   T   
   A   
   A   
   A   
   T   
   C   
    T    
   G   
   T   
   T   
   G   
   T   
   T   
   G   
   A   
   A   
   A   
   A   
   A   
   T   
   A   
   C   
   C   
   A   
   A   
   G   
   C   
   A   
   A   
   T   
   T   
   T   
   A   
   T   
   T   
   C   
   A   
   G   
   G   
   G   
   A   
   T   
   T   
   C   
   A   
   A   
   G   
   C   
    A    
   G   
   A   
   A   
    G    
   G   
    A    
   G   
   T   
   C   
   A   
   A   
    A    
   G   
   C   
   T   
   A   
   G   
   T   
   G   
   A   
   T   
   G   
   T   
    C    
   C   
   A   
   A   
   G   
   T   
    C    
   A   
   A   
   A   
   A   
   A   
    G    
   G   
    C    
   G   
   A   
   A   
   A   
   G   
   A   
    C    
   A   
   A   
    T    
   C   
   A   
   A   
   T   
   A   
   C   
   A   
   C   
   A   
   T   
   T   
   T   
   A   
    C    
   C   
   T   
   A   
   T   
   A   
   A   
   A   
   T   
   T   
   A   
   T   
   C   
   G   
   A   
   T   
   G   
   A   
   G   
    C    
   A   
   C   
    T    
   C   
   C   
   T   
   T   
   T   
   A   
   G   
   G   
   C   
   G   
   A   
   A   
   A   
   T   
   G   
   A   
   A   
   A   
   G   
   A   
   T   
    C    
   T   
   G   
   T   
   C   
   T   
   T   
   A   
   T   
   C   
   A   
   A   
   T   
   C   
   A   
   A   
   G   
   T   
   A   
   T   
    C    
   G   
    C    
   C   
   A   
   A   
    A    
   A   
   A   
   A   
   G   
   G   
    C    
   G   
   A   
   T   
   A   
    C    
   C   
   T   
   A   
   C   
   C   
   A   
   A   
   A   
   T   
    C    
    T    
   C   
   T   
   T   
   G   
   G   
    A    
    A    
    G    
   C   
   C   
    G    
   T   
   C   
   T   
   T   
   T   
   A   
   A   
   T   
   T   
   T   
   T   
   T   
   C   
   C   
   A   
   G   
   A   
   T   
   A   
   T   
   G   
   T   
   C   
   A   
   G   
   G   
   A   
   A   
   A   
   T   
   G   
   A   
   T   
   A   
   A   
   A   
    A    
   T   
   T   
   T   
   C   
   G   
   A   
   T   
   T   
   C   
   A   
   A   
   G   
   T   
   A   
   G   
   A   
   T   
   A   
   A   
   T   
   G   
   C   
   C   
   A   
   A   
   A   
   C   
   G   
   T   
   G   
   G   
   A   
   G   
   A   
   A   
   A   
   T   
   T   
   G   
   T   
   C   
   G   
   A   
   T   
   C   
   G   
   T   
   A   
   A   
    T    
   G   
   G   
   T   
   A   
   G   
   T   
   G   
   G   
   G   
   C   
   T   
   A   
   G   
    C    
   A   
   A   
   T   
   T   
   A   
   A   
   C   
   A   
   A   
   A   
   G   
   T   
   G   
   T   
   T   
   T   
   G   
   A   
    C    
   G   
   A   
   A   
   G   
   T   
   G   
   G   
   G   
   C   
   G   
   T   
   A   
   G   
   T   
    G    
   C   
   C   
   T   
    G    
   G   
   C   
   A   
   A   
   A   
   C   
   T   
    C    
   G   
   G   
   T   
   T   
   C   
   T   
   G   
   G   
   C   
   G   
   C   
   A   
   G   
   A   
   A   
   A   
   A   
   A   
    A    
   C   
   A   
   G   
   C   
    C    
   A   
   A   
   T   
   A   
   T   
   C   
   A   
   A   
   A   
   G   
   C   
   T   
   T   
   T   
   T   
   A   
   G   
   T   
   G   
   A   
   T   
   A   
   A   
    G    
   T   
   T   
    C    
   G   
   G   
   C   
   G   
   T   
   T   
   T   
   C   
   T   
    G    
   T   
   T   
   G   
   A   
   T   
   G   
   A   
   A   
   A   
   T   
   C   
   A   
   A   
   T   
   C   
    A    
   A   
   A   
   A   
    G    
   T   
   T   
   A   
   A   
   G   
   C   
   C   
   A   
   A   
   G   
   G   
    G    
   T   
   G   
   G   
   G   
   T   
   C   
   C   
   A   
   A   
   G   
   C   
   A   
   G   
   A   
   C   
   T   
   C   
    C    
   T   
   T   
   T   
   G   
   T   
   A   
   C   
   C   
   A   
   A   
   T   
   C   
   A   
   C   
   A   
   G   
   T   
    T    
   G   
   C   
   T   
   T   
   C   
   T   
   G   
   A   
   A   
   C   
   C   
   A   
   G   
   T   
   G   
   A   
   C   
   A   
   G   
   A   
   A   
   T   
   T   
   A   
   C   
   C   
   A   
   A   
   C   
   A   
   G   
   G   
   G   
   G   
   C   
   T   
   G   
   C   
   G   
   A   
   C   
   A   
   A   
   A   
   A   
   G   
    A    
    T    
   A   
   C   
   A   
   G   
   A   
   G   
   T   
   C   
   A   
   C   
   G   
   T   
   T   
   A   
   T   
   T   
   A   
   T   
   C   
   C   
    A    
    C    
   T   
   G   
   G   
   G   
   G   
   G   
   A   
   A   
   G   
   C   
   A   
   G   
   C   
   T   
   G   
   C   
   G   
   C   
   A   
   A   
   T   
   T   
   A   
   A   
   T   
   C   
   G   
   G   
    G    
   T   
   A   
   T   
   A   
   C   
    G    
   G   
   G   
    C    
   A   
   C   
    C    
   A   
   T   
   T   
   A   
   C   
   T   
   G   
   C   
   A   
   G   
   A   
    A    
   G   
   A   
   T   
   A   
   T   
   T   
    G    
   A   
   A   
   A   
   A   
   A   
   A   
   A   
   T   
   C   
   C   
   A   
   G   
   A   
   G   
   T   
   T   
   A   
   A   
   G   
   T   
   A   
   G   
   T   
   A   
   C   
   G   
   G   
   G   
    T    
   G   
   T   
   A   
   A   
   T   
   T   
   G   
   G   
    T    
   A   
   A   
   A   
   A   
   C   
   T   
   G   
   G   
    C    
   T   
   T   
   A   
   G   
   A   
   A   
   C   
   G   
   A   
   G   
   C   
   G   
   T   
   T   
   T   
   G   
   A   
   T   
   A   
   A   
   A   
   G   
   A   
    G    
   T   
   T   
   A   
   A   
   G   
   A   
   G   
   G   
    G    
   C   
   A   
   A   
   G   
   A   
   T   
   G   
   G   
    T    
   G   
   G   
    C    
   T   
   C   
    A    
   C   
   T   
   A   
   G   
   T   
   T   
   A   
   T   
   T   
   T   
   T   
   A   
   G   
   A   
   T   
   G   
   A   
   C   
   A   
   A   
   A   
   G   
   A   
   G   
   A   
   A   
   T   
   G   
   T   
   C   
   A   
   A   
   A   
   A   
   A   
   A   
   G   
   C   
   T   
   T   
   T   
   A   
   C   
   A   
   A   
   A   
   C   
   C   
   A   
   A   
   A   
   G   
   A   
    G    
   A   
   A   
   A   
   A   
   A   
   A   
   G   
   A   
   T   
   G   
   G   
   T   
   C   
   A   
   A   
   A   
   C   
    G    
   A   
   T   
   T   
   A   
   A   
   A   
   T   
   T   
   A   
   A   
   C   
   G   
   A   
   T   
   T   
   G   
   A   
    C    
   A   
   G   
    T    
   G   
   G   
    C    
   G   
   T   
   A   
   C   
   A   
   A   
   C   
   A   
   A   
   C   
   A   
   A   
   G   
   C   
    C    
   T   
   T   
    C    
   G   
   C   
   T   
   A   
   T   
   A   
   T   
   T   
   T   
   G   
   A   
   C   
   A   
   A   
   A   
   C   
   G   
    T    
    C    
   C   
   T   
   G   
   G   
   T   
   A   
   G   
   T   
   G   
   C   
   G   
   G   
   T   
   C   
   A   
   T   
   T   
   A   
   C   
   C   
   G   
   A   
   T   
   C   
   C   
   T   
   C   
   A   
   A   
   A   
   A   
   A   
   G   
   G   
   C   
   G   
   A   
   T   
   T   
   T   
   A   
    T    
   T   
   A   
   G   
   C   
    A    
   A   
   C   
   G   
   G   
   T   
   T   
   A   
   G   
   T   
   T   
   C   
   A   
   C   
   C   
   T   
   T   
   C   
   G   
   T   
   A   
   T   
   G   
   A   
   T   
   C   
   C   
   T   
   A   
   A   
   T   
   A   
   A   
   A   
   A   
   T   
   G   
   G   
   C   
   C   
   A   
   A   
   T   
   G   
   G   
    T    
   A   
   T   
   T   
   T   
   C   
   T   
   C   
   A   
   A   
   A   
   A   
   A   
   G   
   A   
    A    
   T   
   A   
   T   
   G   
   A   
   T   
   G   
   C   
   T   
   T   
   A   
   C   
   A   
   A   
   T   
   A   
   A   
   C   
   A   
   A   
   C   
   A   
   A   
   A   
   G   
   A   
   T   
   T   
   T   
   A   
   C   
   C   
   A   
   T   
   T   
    C    
   A   
   C   
   A   
   G   
   C   
   A   
   C   
   G   
   T   
   T   
   T   
    T    
   G   
   C   
   G   
   A   
   C   
   A   
   G   
   G   
    C    
   T   
   A   
   T   
   G   
   C   
   A   
   C   
   C   
   A   
   G   
   G   
    C    
   T   
   C   
   T   
   A   
   C   
   C   
   T   
   T   
   T   
   A   
   A   
   A   
   A   
   C   
   A   
   A   
   T   
   T   
   A   
   C   
   C   
   G   
   G   
   T   
   G   
   C   
   C   
   A   
   T   
   T   
   G   
   G   
   T   
   T   
   T   
   A   
   G   
   A   
   T   
    G    
   C   
   A   
   G   
   G   
   G   
   A   
   C   
   A   
   T   
   T   
   G   
   A   
   A   
   G   
   C   
   C   
   A   
   G   
   A   
   T   
   G   
   A   
   A   
   G   
   A   
   G   
   C   
   T   
   A   
   G   
   A   
   A   
   A   
   T   
   T   
   A   
   A   
   T   
   G   
   G   
    C    
   T   
   T   
   G   
   A   
   A   
   A   
   T   
   G   
   G   
   C   
   A   
   A   
   A   
   A   
   A   
   G   
   A   
   T   
   A   
   A   
   A   
   T   
   C   
   T   
   T   
   G   
   G   
   G   
   G   
    C    
   G   
   G   
   C   
   T   
   A   
   T   
   T   
   T   
   T   
   G   
   C   
    A    
   A   
   C   
   A   
   C   
   G   
   T   
   G   
   T   
   A   
   A   
   A   
   A   
   G   
   A   
   A   
   G   
   C   
   A   
   A   
   G   
   T   
   C   
   C   
   A   
   G   
   T   
   C   
   A   
   A   
   T   
   C   
   T   
    G    
   C   
   G   
   T   
   A   
   C   
   C   
   G   
   C   
   T   
   T   
   T   
   A   
   G   
   T   
   T   
   A   
   A   
   T   
   T   
   C   
   A   
   G   
   A   
   T   
   A   
   A   
   T   
   A   
   T   
   T   
   T   
   A   
   T   
   T   
   T   
   T   
   G   
   C   
    C    
   C   
   A   
   A   
   C   
   A   
   A   
   A   
   C   
   A   
   T   
   T   
   A   
   C   
   G   
    G    
   A   
   T   
   G   
   G   
   G   
    G    
   G   
   A   
   A   
   G   
   A   
   C   
   A   
   A   
   A   
   T   
   T   
   T   
   A   
   G   
   A   
   G   
   C   
    A    
   G   
   G   
   T   
   C   
   T   
   T   
   A   
   A   
   T   
   A   
   A   
   A   
   T   
   T   
   C   
   A   
   T   
   T   
   T   
   T   
   C   
   G   
   G   
   T   
   G   
   A   
   G   
   G   
   A   
   A   
   C   
   T   
   A   
   G   
   A   
   T   
   T   
   T   
   A   
   C   
   C   
   A   
   A   
   T   
    T    
   G   
   C   
   C   
   A   
   T   
   G   
   A   
   C   
   G   
   C   
   C   
    T    
   G   
   C   
   T   
   C   
   A   
   A   
   A   
   T   
   T   
   T   
   C   
   A   
   A   
   A   
    T    
   G   
   A   
   A   
   G   
   A   
   T   
   A   
   A   
   A   
   T   
   T   
   T   
   A   
   A   
   T   
   T   
   C   
   T   
   G   
   A   
   A   
   A   
   T   
   T   
   C   
   T   
   T   
   T   
   T   
    A    
   G   
   C   
   A   
   G   
   A   
   T   
   A   
   C   
    G    
   G   
   G   
   C   
   T   
   A   
    C    
   G   
   G   
    C    
   C   
   A   
   A   
   G   
   G   
    C    
   C   
   A   
   A   
   T   
   T   
   G   
   T   
   T   
   A   
   A   
   T   
   T   
   T   
   C   
    G    
   C   
   C   
   A   
   A   
   T   
   C   
   C   
   A   
   G   
   C   
   A   
   A   
   G   
   C   
   T   
   A   
   C   
   C   
   A   
   T   
   G   
   T   
   A   
   T   
   A   
   G   
   T   
   G   
   T   
   T   
   T   
   T   
   C   
   C   
   A   
   A   
   A   
   A   
   T   
   A   
   A   
   T   
   G   
   G   
    A    
   A   
   C   
   A   
    T    
   T   
    A    
   G   
   T   
    C    
   T   
   A   
   T   
   C   
   C   
   A   
   A   
   A   
   A   
    T    
   T   
   A   
   G   
   T   
   T   
   T   
   T   
   A   
   G   
   A   
   C   
   A   
   A   
   A   
   G   
   A   
   A   
   A   
   C   
    G    
   A   
   A   
   G   
   A   
   A   
   A   
   A   
   A   
   A   
   G   
   A   
   C   
   A   
   A   
   T   
   G   
   T   
   A   
   A   
   T   
   C   
   A   
   G   
   C   
   G   
   C   
   C   
   A   
   A   
   C   
   G   
   C   
   A   
   G   
   C   
   G   
   A   
   A   
   T   
   A   
   C   
   A   
   A   
   T   
   T   
   G   
   C   
   C   
   A   
   C   
   A   
   G   
   A   
   T   
   C   
   T   
   T   
   T   
   T   
   A   
   G   
   G   
   T   
   A   
   G   
   T   
   G   
   T   
   G   
   G   
   A   
   A   
   G   
   A   
   T   
   C   
   C   
   T   
   T   
   C   
   A   
   G   
   G   
   T   
   T   
   A   
   T   
   G   
   T   
   T   
   T   
   A   
   T   
   A   
   A   
   T   
   A   
   T   
   G   
   T   
   A   
   C   
   A   
   A   
   T   
   C   
   C   
   G   
   A   
   A   
   C   
   T   
   T   
   C   
   T   
   C   
   A   
   T   
   T   
   G   
   G   
   C   
   T   
   G   
   C   
   T   
   A   
   A   
   A   
   A   
   C   
   A   
   G   
   G   
   A   
   A   
   C   
   A   
   G   
   C   
   T   
   G   
   A   
   A   
   A   
   T   
   A   
   A   
   A   
   A   
   G   
   A   
   T   
   A   
   A   
   A   
   C   
   A   
   A   
   G   
   A   
   T   
   A   
   C   
   A   
   G   
   A   
   T   
   G   
   G   
   C   
   A   
   A   
   A   
   G   
   A   
   A   
   A   
   A   
   C   
   A   
   G   
   T   
   T   
   T   
   C   
   C   
   T   
   C   
   C   
   T   
   A   
   A   
   C   
   G   
   T   
   T   
   A   
   G   
   A   
   T   
   C   
   G   
   T   
   A   
   G   
   T   
   A   
   A   
   C   
   A   
   A   
   T   
   A   
   A   
   A   
   T   
   T   
   C   
   T   
   T   
   A   
   A   
   C   
   A   
   A   
   T   
   G   
   A   
   T   
   T   
   A   
   T   
   G   
   G   
   T   
   T   
   G   
   A   
   A   
   A   
   A   
   T   
   T   
   C   
   A   
   G   
   G   
   A   
   G   
   A   
   A   
   A   
   A   
   T   
   G   
   G   
   T   
   T   
   C   
   T   
   G   
   C   
   A   
   A   
   C   
   A   
   G   
   A   
   T   
   A   
   T   
   C   
   A   
   G   
   T   
   A   
   A   
   A   
   C   
   C   
   A   
   T   
   T   
   A   
   A   
   T   
   T   
   G   
   A   
   T   
   T   
   A   
   C   
   T   
   T   
   A   
   G   
   A   
   A   
   G   
   C   
   A   
   A   
   C   
   C   
   A   
   T   
   T   
   A   
   A   
   A   
   -   
   -   
   -   
   -   
   -   
   -   
   -   
   -   
   -   
   -   
   -   
   -   
   -   
   -   
   -   
   -   
   -   
   -   
   -   
   -   
   -   
   -   
   -   
   -   
   -   
   -   
   -   
   -   
   -   
   -   
   -   
   -   
   -   
   -   
   -   
   -   
   -   
   -   
   -   
   -   
   -   
   -   
   -   
   -   
   -   
   -   
   -   
   -   
   -   
   -   
   -   
   -   
   -   
   -   
   -   
   -   
   -   
   -   
   -   
   -   
   -   
   -   
   -   
   -   
   -   
   -   
   -   
   -   
   -   
   -   
   -   
   -   
   -   
   -   
   -   
   -   
   -   
   -   
   -   
   -   
   -   
   -   
   -   
   -   
   -   
   -   
   -   
   -   
   -   
   -   
   -   
   -   
   -   
   -   
   -   
   -   
   -   
   -   
   -   
   -   
   -   
   -   
   -   
   -   
   -   
   -   
   -   
   -   
   -   
   -   
   -   
   -   
   -   
   -   
   -   
   -   
   -   
   -   
   -   
   -   
   -   
   -   
   -   
   -   
   -   
   -   
   -   
   -   
   -   
   -   
   -   
   -   
   -   
   -   
   -   
   -   
   -   
   -   
   -   
   -   
   -   
   -   
   -   
   -   
   -   
   -   
   -   
   -   
   -   
   -   
   -   
   -   
   -   
   -   
   -   
   -   
   -   
   -   
   -   
   -   
   -   
   -   
   -   
   -   
   -   
   -   
   -   
   -   
   -   
   C   
   A   
   A   
   T   
   G   
   G   
   C   
   A   
   A   
   G   
   C   
   C   
   A   
   A   
   A   
   C   
   A   
   A   
   G   
   A   
   A   
   T   
   T   
   A   
   G   
   C   
   C   
   G   
   A   
   A   
   G   
   C   
   G   
   A   
   A   
   G   
   A   
   A   
   A   
   A   
   C   
   A   
   G   
   C   
   T   
   A   
   A   
   T   
   A   
   C   
   A   
   T   
   T   
   T   
   T   
   T   
   A   
   A   
   A   
   T   
   G   
   T   
   A   
   T   
   T   
   G   
   T   
   C   
   A   
   A   
   A   
   A   
   C   
   A   
   G   
   G   
   A   
   A   
   T   
   T   
   T   
   G   
   A   
   T   
   A   
   A   
   G   
   T   
   T   
   A   
   C   
   C   
   G   
   T   
   C   
   C   
   G   
   T   
   T   
   G   
   T   
   T   
   C   
   A   
   A   
   G   
   A   
   A   
   T   
   C   
   T   
   A   
   G   
   C   
   T   
   T   
   A   
   A   
   A   
   G   
   A   
   A   
   A   
   A   
   A   
   T   
   G   
   G   
   C   
   T   
   A   
   T   
   G   
   A   
   T   
   A   
   C   
   T   
   A   
   A   
   A   
   T   
   C   
   T   
   G   
   T   
   T   
   G   
   T   
   T   
   G   
   A   
   A   
   A   
   A   
   A   
   T   
   A   
   C   
   C   
   A   
   A   
   G   
   C   
   A   
   A   
   T   
   T   
   T   
   A   
   T   
   T   
   C   
   A   
   G   
   G   
   G   
   A   
   T   
   T   
   C   
   A   
   A   
   G   
   C   
   A   
   G   
   A   
   A   
   G   
   G   
   A   
   G   
   T   
   C   
   A   
   A   
   A   
   G   
   C   
   T   
   A   
   G   
   T   
   G   
   A   
   T   
   G   
   T   
   C   
   C   
   A   
   A   
   G   
   T   
   C   
   A   
   A   
   A   
   A   
   A   
   G   
   G   
   C   
   G   
   A   
   A   
   A   
   G   
   A   
   C   
   A   
   A   
   T   
   C   
   A   
   A   
   T   
   A   
   C   
   A   
   C   
   A   
   T   
   T   
   T   
   A   
   C   
   C   
   T   
   A   
   T   
   A   
   A   
   A   
   T   
   T   
   A   
   T   
   C   
   G   
   A   
   T   
   G   
   A   
   G   
   C   
   A   
   C   
   T   
   C   
   C   
   T   
   T   
   T   
   A   
   G   
   G   
   C   
   G   
   A   
   A   
   A   
   T   
   G   
   A   
   A   
   A   
   G   
   A   
   T   
   C   
   T   
   G   
   T   
   C   
   T   
   T   
   A   
   T   
   C   
   A   
   A   
   T   
   C   
   A   
   A   
   G   
   T   
   A   
   T   
   C   
   G   
   C   
   C   
   A   
   A   
   A   
   A   
   A   
   A   
   G   
   G   
   C   
   G   
   A   
   T   
   A   
   C   
   C   
   T   
   A   
   C   
   C   
   A   
   A   
   A   
   T   
   C   
   T   
   C   
   T   
   T   
   G   
   G   
   A   
   A   
   G   
   C   
   C   
   G   
   T   
   C   
   T   
   T   
   T   
   A   
   A   
   T   
   T   
   T   
   T   
   T   
   C   
   C   
   A   
   G   
   A   
   T   
   A   
   T   
   G   
   T   
   C   
   A   
   G   
   G   
   A   
   A   
   A   
   T   
   G   
   A   
   T   
   A   
   A   
   A   
   A   
   T   
   T   
   T   
   C   
   G   
   A   
   T   
   T   
   C   
   A   
   A   
   G   
   T   
   A   
   G   
   A   
   T   
   A   
   A   
   T   
   G   
   C   
   C   
   A   
   A   
   A   
   C   
   G   
   T   
   G   
   G   
   A   
   G   
   A   
   A   
   A   
   T   
   T   
   G   
   T   
   C   
   G   
   A   
   T   
   C   
   G   
   T   
   A   
   A   
   T   
   G   
   G   
   T   
   A   
   G   
   T   
   G   
   G   
   G   
   C   
   T   
   A   
   G   
   C   
   A   
   A   
   T   
   T   
   A   
   A   
   C   
   A   
   A   
   A   
   G   
   T   
   G   
   T   
   T   
   T   
   G   
   A   
   C   
   G   
   A   
   A   
   G   
   T   
   G   
   G   
   G   
   C   
   G   
   T   
   A   
   G   
   T   
   G   
   C   
   C   
   T   
   G   
   G   
   C   
   A   
   A   
   A   
   C   
   T   
   C   
   G   
   G   
   T   
   T   
   C   
   T   
   G   
   G   
   C   
   G   
   C   
   A   
   G   
   A   
   A   
   A   
   A   
   A   
   A   
   C   
   A   
   G   
   C   
   C   
   A   
   A   
   T   
   A   
   T   
   C   
   A   
   A   
   A   
   G   
   C   
   T   
   T   
   T   
   T   
   A   
   G   
   T   
   G   
   A   
   T   
   A   
   A   
   G   
   T   
   T   
   C   
   G   
   G   
   C   
   G   
   T   
   T   
   T   
   C   
   T   
   G   
   T   
   T   
   G   
   A   
   T   
   G   
   A   
   A   
   A   
   T   
   C   
   A   
   A   
   T   
   C   
   A   
   A   
   A   
   A   
   G   
   T   
   T   
   A   
   A   
   G   
   C   
   C   
   A   
   A   
   G   
   G   
   G   
   T   
   G   
   G   
   G   
   T   
   C   
   C   
   A   
   A   
   G   
   C   
   A   
   G   
   A   
   C   
   T   
   C   
   C   
   T   
   T   
   T   
   G   
   T   
   A   
   C   
   C   
   A   
   A   
   T   
   C   
   A   
   C   
   A   
   G   
   T   
   T   
   G   
   C   
   T   
   T   
   C   
   T   
   G   
   A   
   A   
   C   
   C   
   A   
   G   
   T   
   G   
   A   
   C   
   A   
   G   
   A   
   A   
   T   
   T   
   A   
   C   
   C   
   A   
   A   
   C   
   A   
   G   
   G   
   G   
   G   
   C   
   T   
   G   
   C   
   G   
   A   
   C   
   A   
   A   
   A   
   A   
   G   
   A   
   T   
   A   
   C   
   A   
   G   
   A   
   G   
   T   
   C   
   A   
   C   
   G   
   T   
   T   
   A   
   T   
   T   
   A   
   T   
   C   
   C   
   A   
   C   
   T   
   G   
   G   
   G   
   G   
   G   
   A   
   A   
   G   
   C   
   A   
   G   
   C   
   T   
   G   
   C   
   G   
   C   
   A   
   A   
   T   
   T   
   A   
   A   
   T   
   C   
   G   
   G   
   G   
   T   
   A   
   T   
   A   
   C   
   G   
   G   
   G   
   C   
   A   
   C   
   C   
   A   
   T   
   T   
   A   
   C   
   T   
   G   
   C   
   A   
   G   
   A   
   A   
   G   
   A   
   T   
   A   
   T   
   T   
   G   
   A   
   A   
   A   
   A   
   A   
   A   
   A   
   T   
   C   
   C   
   A   
   G   
   A   
   G   
   T   
   T   
   A   
   A   
   G   
   T   
   A   
   G   
   T   
   A   
   C   
   G   
   G   
   G   
   T   
   G   
   T   
   A   
   A   
   T   
   T   
   G   
   G   
   T   
   A   
   A   
   A   
   A   
   C   
   T   
   G   
   G   
   C   
   T   
   T   
   A   
   G   
   A   
   A   
   C   
   G   
   A   
   G   
   C   
   G   
   T   
   T   
   T   
   G   
   A   
   T   
   A   
   A   
   A   
   G   
   A   
   G   
   T   
   T   
   A   
   A   
   G   
   A   
   G   
   G   
   G   
   C   
   A   
   A   
   G   
   A   
   T   
   G   
   G   
   T   
   G   
   G   
   C   
   T   
   C   
   A   
   C   
   T   
   A   
   G   
   T   
   T   
   A   
   T   
   T   
   T   
   T   
   A   
   G   
   A   
   T   
   G   
   A   
   C   
   A   
   A   
   A   
   G   
   A   
   G   
   A   
   A   
   T   
   G   
   T   
   C   
   A   
   A   
   A   
   A   
   A   
   A   
   G   
   C   
   T   
   T   
   T   
   A   
   C   
   A   
   A   
   A   
   C   
   C   
   A   
   A   
   A   
   G   
   A   
   G   
   A   
   A   
   A   
   A   
   A   
   A   
   G   
   A   
   T   
   G   
   G   
   T   
   C   
   A   
   A   
   A   
   C   
   G   
   A   
   T   
   T   
   A   
   A   
   A   
   T   
   T   
   A   
   A   
   C   
   G   
   A   
   T   
   T   
   G   
   A   
   C   
   A   
   G   
   T   
   G   
   G   
   C   
   G   
   T   
   A   
   C   
   A   
   A   
   C   
   A   
   A   
   C   
   A   
   A   
   G   
   C   
   C   
   T   
   T   
   C   
   G   
   C   
   T   
   A   
   T   
   A   
   T   
   T   
   T   
   G   
   A   
   C   
   A   
   A   
   A   
   C   
   G   
   T   
   C   
   C   
   T   
   G   
   G   
   T   
   A   
   G   
   T   
   G   
   C   
   G   
   G   
   T   
   C   
   A   
   T   
   T   
   A   
   C   
   C   
   G   
   A   
   T   
   C   
   C   
   T   
   C   
   A   
   A   
   A   
   A   
   A   
   G   
   G   
   C   
   G   
   A   
   T   
   T   
   T   
   A   
   T   
   T   
   A   
   G   
   C   
   A   
   A   
   C   
   G   
   G   
   T   
   T   
   A   
   G   
   T   
   T   
   C   
   A   
   C   
   C   
   T   
   T   
   C   
   G   
   T   
   A   
   T   
   G   
   A   
   T   
   C   
   C   
   T   
   A   
   A   
   T   
   A   
   A   
   A   
   A   
   T   
   G   
   G   
   C   
   C   
   A   
   A   
   T   
   G   
   G   
   T   
   A   
   T   
   T   
   T   
   C   
   T   
   C   
   A   
   A   
   A   
   A   
   A   
   G   
   A   
   A   
   T   
   A   
   T   
   G   
   A   
   T   
   G   
   C   
   T   
   T   
   A   
   C   
   A   
   A   
   T   
   A   
   A   
   C   
   A   
   A   
   C   
   A   
   A   
   A   
   G   
   A   
   T   
   T   
   T   
   A   
   C   
   C   
   A   
   T   
   T   
   C   
   A   
   C   
   A   
   G   
   C   
   A   
   C   
   G   
   T   
   T   
   T   
   T   
   G   
   C   
   G   
   A   
   C   
   A   
   G   
   G   
   C   
   T   
   A   
   T   
   G   
   C   
   A   
   C   
   C   
   A   
   G   
   G   
   C   
   T   
   C   
   T   
   A   
   C   
   C   
   T   
   T   
   T   
   A   
   A   
   A   
   A   
   C   
   A   
   A   
   T   
   T   
   A   
   C   
   C   
   G   
   G   
   T   
   G   
   C   
   C   
   A   
   T   
   T   
   G   
   G   
   T   
   T   
   T   
   A   
   G   
   A   
   T   
   G   
   C   
   A   
   G   
   G   
   G   
   A   
   C   
   A   
   T   
   T   
   G   
   A   
   A   
   G   
   C   
   C   
   A   
   G   
   A   
   T   
   G   
   A   
   A   
   G   
   A   
   G   
   C   
   T   
   A   
   G   
   A   
   A   
   A   
   T   
   T   
   A   
   A   
   T   
   G   
   G   
   C   
   T   
   T   
   G   
   A   
   A   
   A   
   T   
   G   
   G   
   C   
   A   
   A   
   A   
   A   
   A   
   G   
   A   
   T   
   A   
   A   
   A   
   T   
   C   
   T   
   T   
   G   
   G   
   G   
   G   
   C   
   G   
   G   
   C   
   T   
   A   
   T   
   T   
   T   
   T   
   G   
   C   
   A   
   A   
   C   
   A   
   C   
   G   
   T   
   G   
   T   
   A   
   A   
   A   
   A   
   G   
   A   
   A   
   G   
   C   
   A   
   A   
   G   
   T   
   C   
   C   
   A   
   G   
   T   
   C   
   A   
   A   
   T   
   C   
   T   
   G   
   C   
   G   
   T   
   A   
   C   
   C   
   G   
   C   
   T   
   T   
   T   
   A   
   G   
   T   
   T   
   A   
   A   
   T   
   T   
   C   
   A   
   G   
   A   
   T   
   A   
   A   
   T   
   A   
   T   
   T   
   T   
   A   
   T   
   T   
   T   
   T   
   G   
   C   
   C   
   C   
   A   
   A   
   C   
   A   
   A   
   A   
   C   
   A   
   T   
   T   
   A   
   C   
   G   
   G   
   A   
   T   
   G   
   G   
   G   
   G   
   G   
   A   
   A   
   G   
   A   
   C   
   A   
   A   
   A   
   T   
   T   
   T   
   A   
   G   
   A   
   G   
   C   
   A   
   G   
   G   
   T   
   C   
   T   
   T   
   A   
   A   
   T   
   A   
   A   
   A   
   T   
   T   
   C   
   A   
   T   
   T   
   T   
   T   
   C   
   G   
   G   
   T   
   G   
   A   
   G   
   G   
   A   
   A   
   C   
   T   
   A   
   G   
   A   
   T   
   T   
   T   
   A   
   C   
   C   
   A   
   A   
   T   
   T   
   G   
   C   
   C   
   A   
   T   
   G   
   A   
   C   
   G   
   C   
   C   
   T   
   G   
   C   
   T   
   C   
   A   
   A   
   A   
   T   
   T   
   T   
   C   
   A   
   A   
   A   
   T   
   G   
   A   
   A   
   G   
   A   
   T   
   A   
   A   
   A   
   T   
   T   
   T   
   A   
   A   
   T   
   T   
   C   
   T   
   G   
   A   
   A   
   A   
   T   
   T   
   C   
   T   
   T   
   T   
   T   
   A   
   G   
   C   
   A   
   G   
   A   
   T   
   A   
   C   
   G   
   G   
   G   
   C   
   T   
   A   
   C   
   G   
   G   
   C   
   C   
   A   
   A   
   G   
   G   
   C   
   C   
   A   
   A   
   T   
   T   
   G   
   T   
   T   
   A   
   A   
   T   
   T   
   T   
   C   
   G   
   C   
   C   
   A   
   A   
   T   
   C   
   C   
   A   
   G   
   C   
   A   
   A   
   G   
   C   
   T   
   A   
   C   
   C   
   A   
   T   
   G   
   T   
   A   
   T   
   A   
   G   
   T   
   G   
   T   
   T   
   T   
   T   
   C   
   C   
   A   
   A   
   A   
   A   
   T   
   A   
   A   
   T   
   G   
   G   
   A   
   A   
   C   
   A   
   T   
   T   
   A   
   G   
   T   
   C   
   T   
   A   
   T   
   C   
   C   
   A   
   A   
   A   
   A   
   T   
   T   
   A   
   G   
   T   
   T   
   T   
   T   
   A   
   G   
   A   
   C   
   A   
   A   
   A   
   G   
   A   
   A   
   A   
   C   
   G   
   A   
   A   
   G   
   A   
   A   
   A   
   A   
   A   
   A   
   G   
   A   
   C   
   A   
   A   
   T   
   G   
   T   
   A   
   A   
   T   
   C   
   A   
   G   
   C   
   G   
   C   
   C   
   A   
   A   
   C   
   G   
   C   
   A   
   G   
   C   
   G   
   A   
   A   
   T   
   A   
   C   
   A   
   A   
   T   
   T   
   G   
   C   
   C   
   A   
   C   
   A   
   G   
   A   
   T   
   C   
   T   
   T   
   T   
   T   
   A   
   G   
   G   
   T   
   A   
   G   
   T   
   G   
   T   
   G   
   G   
   A   
   A   
   G   
   A   
   T   
   C   
   C   
   T   
   T   
   C   
   A   
   G   
   G   
   T   
   T   
   A   
   T   
   G   
   T   
   T   
   T   
   A   
   T   
   A   
   A   
   T   
   A   
   T   
   G   
   T   
   A   
   C   
   A   
   A   
   T   
   C   
   C   
   G   
   A   
   A   
   C   
   T   
   T   
   C   
   T   
   C   
   A   
   T   
   T   
   G   
   G   
   C   
   T   
   G   
   C   
   T   
   A   
   A   
   A   
   A   
   C   
   A   
   G   
   G   
   A   
   A   
   C   
   A   
   G   
   C   
   T   
   G   
   A   
   A   
   A   
   T   
   A   
   A   
   A   
   A   
   G   
   A   
   T   
   A   
   A   
   A   
   C   
   A   
   A   
   G   
   A   
   T   
   A   
   C   
   A   
   G   
   A   
   T   
   G   
   G   
   C   
   A   
   A   
   A   
   G   
   A   
   A   
   A   
   A   
   C   
   A   
   G   
   T   
   T   
   T   
   C   
   C   
   T   
   C   
   C   
   T   
   A   
   A   
   C   
   G   
   T   
   T   
   A   
   G   
   A   
   T   
   C   
   G   
   T   
   A   
   G   
   T   
   A   
   A   
   C   
   A   
   A   
   T   
   A   
   A   
   A   
   T   
   T   
   C   
   T   
   T   
   A   
   A   
   C   
   A   
   A   
   T   
   G   
   A   
   T   
   T   
   A   
   T   
   G   
   G   
   T   
   T   
   G   
   A   
   A   
   A   
   A   
   T   
   T   
   C   
   A   
   G   
   G   
   A   
   G   
   A   
   A   
   A   
   A   
   T   
   G   
   G   
   T   
   T   
   C   
   T   
   G   
   C   
   A   
   A   
   C   
   A   
   G   
   A   
   T   
   A   
   T   
   C   
   A   
   G   
   T   
   A   
   A   
   A   
   C   
   C   
   A   
   T   
   T   
   A   
   A   
   T   
   T   
   G   
   A   
   T   
   T   
   A   
   C   
   T   
   T   
   A   
   G   
   A   
   A   
   G   
   C   
   A   
   A   
   C   
   C   
   A   
   T   
   T   
   A   
   A   
   A   
   -   
   -   
   -   
   -   
   -   
   -   
   -   
   -   
   -   
   -   
   -   
   -   
   -   
   -   
   -   
   -   
   -   
   -   
   -   
   -   
   -   
   -   
   -   
   -   
   -   
   -   
   -   
   -   
   -   
   -   
   -   
   -   
   -   
   -   
   -   
   -   
   -   
   -   
   -   
   -   
   -   
   -   
   -   
   -   
   -   
   -   
   -   
   -   
   -   
   -   
   -   
   -   
   -   
   -   
   -   
   -   
   -   
   -   
   -   
   -   
   -   
 

 
   N59544F   
   -   
   -   
   -   
   -   
   -   
   -   
   -   
   -   
   -   
   -   
   -   
   -   
   -   
   -   
   -   
   -   
   -   
   -   
   -   
   -   
   -   
   -   
   -   
   -   
   -   
   -   
   -   
   -   
   -   
   -   
   -   
   -   
   -   
   -   
   -   
   -   
   -   
   -   
   -   
   -   
   -   
   -   
   -   
   -   
   -   
   -   
   -   
   -   
   -   
   -   
   -   
   -   
   -   
   -   
   -   
   -   
   -   
   -   
   -   
   -   
   -   
   -   
   -   
   -   
   -   
   -   
   -   
   -   
   -   
   -   
   -   
   -   
   -   
   -   
   -   
   -   
   -   
   -   
   -   
   -   
   -   
   -   
   -   
   -   
   -   
   -   
   -   
   -   
   -   
   -   
   -   
   -   
   -   
   -   
   -   
   -   
   -   
   -   
   -   
   -   
   -   
   -   
   -   
   -   
   -   
   -   
   -   
   -   
   C   
   A   
   A   
   T   
   G   
   G   
   C   
   A   
   A   
   G   
   C   
   C   
   A   
   A   
   A   
   C   
   A   
   A   
   G   
   A   
    A    
   T   
   T   
   A   
   G   
   C   
    C    
   G   
   A   
   A   
   G   
   C   
   G   
   A   
   A   
   G   
   A   
   A   
   A   
   A   
    C    
   A   
   G   
   C   
    T    
   A   
    A    
   T   
    A    
    C    
   A   
   T   
   T   
   T   
   T   
   T   
   A   
   A   
   A   
    T    
   G   
   T   
   A   
   T   
   T   
   G   
    T    
   C   
   A   
   A   
   A   
   A   
   C   
   A   
   G   
    G    
   A   
   A   
   T   
   T   
   T   
   G   
   A   
   T   
   A   
   A   
   G   
   T   
   T   
   A   
   C   
   C   
    G    
   T   
   C   
   C   
   G   
   T   
   T   
    G    
   T   
   T   
   C   
   A   
   A   
   G   
   A   
   A   
    T    
   C   
   T   
   A   
   G   
   C   
   T   
   T   
   A   
   A   
   A   
   G   
   A   
   A   
   A   
   A   
   A   
   T   
   G   
   G   
    C    
   T   
   A   
   T   
   G   
   A   
   T   
   A   
   C   
   T   
   A   
   A   
   A   
   T   
   C   
    T    
   G   
   T   
   T   
   G   
   T   
   T   
   G   
   A   
   A   
   A   
   A   
   A   
   T   
   A   
   C   
   C   
   A   
   A   
   G   
   C   
   A   
   A   
   T   
   T   
   T   
   A   
   T   
   T   
   C   
   A   
   G   
   G   
   G   
   A   
   T   
   T   
   C   
   A   
   A   
   G   
   C   
    A    
   G   
   A   
   A   
    G    
   G   
    A    
   G   
   T   
   C   
   A   
   A   
    A    
   G   
   C   
   T   
   A   
   G   
   T   
   G   
   A   
   T   
   G   
   T   
    C    
   C   
   A   
   A   
   G   
   T   
    C    
   A   
   A   
   A   
   A   
   A   
    G    
   G   
    C    
   G   
   A   
   A   
   A   
   G   
   A   
    C    
   A   
   A   
    T    
   C   
   A   
   A   
   T   
   A   
   C   
   A   
   C   
   A   
   T   
   T   
   T   
   A   
    C    
   C   
   T   
   A   
   T   
   A   
   A   
   A   
   T   
   T   
   A   
   T   
   C   
   G   
   A   
   T   
   G   
   A   
   G   
    C    
   A   
   C   
    T    
   C   
   C   
   T   
   T   
   T   
   A   
   G   
   G   
   C   
   G   
   A   
   A   
   A   
   T   
   G   
   A   
   A   
   A   
   G   
   A   
   T   
    C    
   T   
   G   
   T   
   C   
   T   
   T   
   A   
   T   
   C   
   A   
   A   
   T   
   C   
   A   
   A   
   G   
   T   
   A   
   T   
    C    
   G   
    C    
   C   
   A   
   A   
    A    
   A   
   A   
   A   
   G   
   G   
    C    
   G   
   A   
   T   
   A   
    C    
   C   
   T   
   A   
   C   
   C   
   A   
   A   
   A   
   T   
    C    
    T    
   C   
   T   
   T   
   G   
   G   
    A    
    A    
    G    
   C   
   C   
    G    
   T   
   C   
   T   
   T   
   T   
   A   
   A   
   T   
   T   
   T   
   T   
   T   
   C   
   C   
   A   
   G   
   A   
   T   
   A   
   T   
   G   
   T   
   C   
   A   
   G   
   G   
   A   
   A   
   A   
   T   
   G   
   A   
   T   
   A   
   A   
   A   
    A    
   T   
   T   
   T   
   C   
   G   
   A   
   T   
   T   
   C   
   A   
   A   
   G   
   T   
   A   
   G   
   A   
   T   
   A   
   A   
   T   
   G   
   C   
   C   
   A   
   A   
   A   
   C   
   G   
   T   
   G   
   G   
   A   
   G   
   A   
   A   
   A   
   T   
   T   
   G   
   T   
   C   
   G   
   A   
   T   
   C   
   G   
   T   
   A   
   A   
    T    
   G   
   G   
   T   
   A   
   G   
   T   
   G   
   G   
   G   
   C   
   T   
   A   
   G   
    C    
   A   
   A   
   T   
   T   
   A   
   A   
   C   
   A   
   A   
   A   
   G   
   T   
   G   
   T   
   T   
   T   
   G   
   A   
    C    
   G   
   A   
   A   
   G   
   T   
   G   
   G   
   G   
   C   
   G   
   T   
   A   
   G   
   T   
    G    
   C   
   C   
   T   
    G    
   G   
   C   
   A   
   A   
   A   
   C   
   T   
    C    
   G   
   G   
   T   
   T   
   C   
   T   
   G   
   G   
   C   
   G   
   C   
   A   
   G   
   A   
   A   
   A   
   A   
   A   
    A    
   C   
   A   
   G   
   C   
    C    
   A   
   A   
   T   
   A   
   T   
   C   
   A   
   A   
   A   
   G   
   C   
   T   
   T   
   T   
   T   
   A   
   G   
   T   
   G   
   A   
   T   
   A   
   A   
    G    
   T   
   T   
    C    
   G   
   G   
   C   
   G   
   T   
   T   
   T   
   C   
   T   
    G    
   T   
   T   
   G   
   A   
   T   
   G   
   A   
   A   
   A   
   T   
   C   
   A   
   A   
   T   
   C   
    A    
   A   
   A   
   A   
    G    
   T   
   T   
   A   
   A   
   G   
   C   
   C   
   A   
   A   
   G   
   G   
    G    
   T   
   G   
   G   
   G   
   T   
   C   
   C   
   A   
   A   
   G   
   C   
   A   
   G   
   A   
   C   
   T   
   C   
    C    
   T   
   T   
   T   
   G   
   T   
   A   
   C   
   C   
   A   
   A   
   T   
   C   
   A   
   C   
   A   
   G   
   T   
    T    
   G   
   C   
   T   
   T   
   C   
   T   
   G   
   A   
   A   
   C   
   C   
   A   
   G   
   T   
   G   
   A   
   C   
   A   
   G   
   A   
   A   
   T   
   T   
   A   
   C   
   C   
   A   
   A   
   C   
   A   
   G   
   G   
   G   
   G   
   C   
   T   
   G   
   C   
   G   
   A   
   C   
   A   
   A   
   A   
   A   
   G   
    A    
    T    
   A   
   C   
   A   
   G   
   A   
   G   
   T   
   C   
   A   
   C   
   G   
   T   
   T   
   A   
   T   
   T   
   A   
   T   
   C   
   C   
    A    
    C    
   T   
   G   
   G   
   G   
   G   
   G   
   A   
   A   
   G   
   C   
   A   
   G   
   C   
   T   
   G   
   C   
   G   
   C   
   A   
   A   
   T   
   T   
   A   
   A   
   T   
   C   
   G   
   G   
    G    
   T   
   A   
   T   
   A   
   C   
    G    
   G   
   G   
    C    
   A   
   C   
    C    
   A   
   T   
   T   
   A   
   C   
   T   
   G   
   C   
   A   
   G   
   A   
    A    
   G   
   A   
   T   
   A   
   T   
   T   
    G    
   A   
   A   
   A   
   A   
   A   
   A   
   A   
   T   
   C   
   C   
   A   
   G   
   A   
   G   
   T   
   T   
   A   
   A   
   G   
   T   
   A   
   G   
   T   
   A   
   C   
   G   
   G   
   G   
    T    
   G   
   T   
   A   
   A   
   T   
   T   
   G   
   G   
    T    
   A   
   A   
   A   
   A   
   C   
   T   
   G   
   G   
    C    
   T   
   T   
   A   
   G   
   A   
   A   
   C   
   G   
   A   
   G   
   C   
   G   
   T   
   T   
   T   
   G   
   A   
   T   
   A   
   A   
   A   
   G   
   A   
    G    
   T   
   T   
   A   
   A   
   G   
   A   
   G   
   G   
    G    
   C   
   A   
   A   
   G   
   A   
   T   
   G   
   G   
    T    
   G   
   G   
    C    
   T   
   C   
    A    
   C   
   T   
   A   
   G   
   T   
   T   
   A   
   T   
   T   
   T   
   T   
   A   
   G   
   A   
   T   
   G   
   A   
   C   
   A   
   A   
   A   
   G   
   A   
   G   
   A   
   A   
   T   
   G   
   T   
   C   
   A   
   A   
   A   
   A   
   A   
   A   
   G   
   C   
   T   
   T   
   T   
   A   
   C   
   A   
   A   
   A   
   C   
   C   
   A   
   A   
   A   
   G   
   A   
    G    
   A   
   A   
   A   
   A   
   A   
   A   
   G   
   A   
   T   
   G   
   G   
   T   
   C   
   A   
   A   
   A   
   C   
    G    
   A   
   T   
   T   
   A   
   A   
   A   
   T   
   T   
   A   
   A   
   C   
   G   
   A   
   T   
   T   
   G   
   A   
    C    
   A   
   G   
    T    
   G   
   G   
    C    
   G   
   T   
   A   
   C   
   A   
   A   
   C   
   A   
   A   
   C   
   A   
   A   
   G   
   C   
    C    
   T   
   T   
    C    
   G   
   C   
   T   
   A   
   T   
   A   
   T   
   T   
   T   
   G   
   A   
   C   
   A   
   A   
   A   
   C   
   G   
    T    
    C    
   C   
   T   
   G   
   G   
   T   
   A   
   G   
   T   
   G   
   C   
   G   
   G   
   T   
   C   
   A   
   T   
   T   
   A   
   C   
   C   
   G   
   A   
   T   
   C   
   C   
   T   
   C   
   A   
   A   
   A   
   A   
   A   
   G   
   G   
   C   
   G   
   A   
   T   
   T   
   T   
   A   
    T    
   T   
   A   
   G   
   C   
    A    
   A   
   C   
   G   
   G   
   T   
   T   
   A   
   G   
   T   
   T   
   C   
   A   
   C   
   C   
   T   
   T   
   C   
   G   
   T   
   A   
   T   
   G   
   A   
   T   
   C   
   C   
   T   
   A   
   A   
   T   
   A   
   A   
   A   
   A   
   T   
   G   
   G   
   C   
   C   
   A   
   A   
   T   
   G   
   G   
    T    
   A   
   T   
   T   
   T   
   C   
   T   
   C   
   A   
   A   
   A   
   A   
   A   
   G   
   A   
    A    
   T   
   A   
   T   
   G   
   A   
   T   
   G   
   C   
   T   
   T   
   A   
   C   
   A   
   A   
   T   
   A   
   A   
   C   
   A   
   A   
   C   
   A   
   A   
   A   
   G   
   A   
   T   
   T   
   T   
   A   
   C   
   C   
   A   
   T   
   T   
    C    
   A   
   C   
   A   
   G   
   C   
   A   
   C   
   G   
   T   
   T   
   T   
    T    
   G   
   C   
   G   
   A   
   C   
   A   
   G   
   G   
    C    
   T   
   A   
   T   
   G   
   C   
   A   
   C   
   C   
   A   
   G   
   G   
    C    
   T   
   C   
   T   
   A   
   C   
   C   
   T   
   T   
   T   
   A   
   A   
   A   
   A   
   C   
   A   
   A   
   T   
   T   
   A   
   C   
   C   
   G   
   G   
   T   
   G   
   C   
   C   
   A   
   T   
   T   
   G   
   G   
   T   
   T   
   T   
   A   
   G   
   A   
   T   
    G    
   C   
   A   
   G   
   G   
   G   
   A   
   C   
   A   
   T   
   T   
   G   
   A   
   A   
   G   
   C   
   C   
   A   
   G   
   A   
   T   
   G   
   A   
   A   
   G   
   A   
   G   
   C   
   T   
   A   
   G   
   A   
   A   
   A   
   T   
   T   
   A   
   A   
   T   
   G   
   G   
    C    
   T   
   T   
   G   
   A   
   A   
   A   
   T   
   G   
   G   
   C   
   A   
   A   
   A   
   A   
   A   
   G   
   A   
   T   
   A   
   A   
   A   
   T   
   C   
   T   
   T   
   G   
   G   
   G   
   G   
    C    
   G   
   G   
   C   
   T   
   A   
   T   
   T   
   T   
   T   
   G   
   C   
    A    
   A   
   C   
   A   
   C   
   G   
   T   
   G   
   T   
   A   
   A   
   A   
   A   
   G   
   A   
   A   
   G   
   C   
   A   
   A   
   G   
   T   
   C   
   C   
   A   
   G   
   T   
   C   
   A   
   A   
   T   
   C   
   T   
    G    
   C   
   G   
   T   
   A   
   C   
   C   
   G   
   C   
   T   
   T   
   T   
   A   
   G   
   T   
   T   
   A   
   A   
   T   
   T   
   C   
   A   
   G   
   A   
   T   
   A   
   A   
   T   
   A   
   T   
   T   
   T   
   A   
   T   
   T   
   T   
   T   
   G   
   C   
    C    
   C   
   A   
   A   
   C   
   A   
   A   
   A   
   C   
   A   
   T   
   T   
   A   
   C   
   G   
    G    
   A   
   T   
   G   
   G   
   G   
    G    
   G   
   A   
   A   
   G   
   A   
   C   
   A   
   A   
   A   
   T   
   T   
   T   
   A   
   G   
   A   
   G   
   C   
    A    
   G   
   G   
   T   
   C   
   T   
   T   
   A   
   A   
   T   
   A   
   A   
   A   
   T   
   T   
   C   
   A   
   T   
   T   
   T   
   T   
   C   
   G   
   G   
   T   
   G   
   A   
   G   
   G   
   A   
   A   
   C   
   T   
   A   
   G   
   A   
   T   
   T   
   T   
   A   
   C   
   C   
   A   
   A   
   T   
    T    
   G   
   C   
   C   
   A   
   T   
   G   
   A   
   C   
   G   
   C   
   C   
    T    
   G   
   C   
   T   
   C   
   A   
   A   
   A   
   T   
   T   
   T   
   C   
   A   
   A   
   A   
    T    
   G   
   A   
   A   
   G   
   A   
   T   
   A   
   A   
   A   
   T   
   T   
   T   
   A   
   A   
   T   
   T   
   C   
   T   
   G   
   A   
   A   
   A   
   T   
   T   
   C   
   T   
   T   
   T   
   T   
    A    
   G   
   C   
   A   
   G   
   A   
   T   
   A   
   C   
    G    
   G   
   G   
   C   
   T   
   A   
    C    
   G   
   G   
    C    
   C   
   A   
   A   
   G   
   G   
    C    
   C   
   A   
   A   
   T   
   T   
   G   
   T   
   T   
   A   
   A   
   T   
   T   
   T   
   C   
    G    
   C   
   C   
   A   
   A   
   T   
   C   
   C   
   A   
   G   
   C   
   A   
   A   
   G   
   C   
   T   
   A   
   C   
   C   
   A   
   T   
   G   
   T   
   A   
   T   
   A   
   G   
   T   
   G   
   T   
   T   
   T   
   T   
   C   
   C   
   A   
   A   
   A   
   A   
   T   
   A   
   A   
   T   
   G   
   G   
    A    
   A   
   C   
   A   
    T    
   T   
    A    
   G   
   T   
    C    
   T   
   A   
   T   
   C   
   C   
   A   
   A   
   A   
   A   
    T    
   T   
   A   
   G   
   T   
   T   
   T   
   T   
   A   
   G   
   A   
   C   
   A   
   A   
   A   
   G   
   A   
   A   
   A   
   C   
    G    
   A   
   A   
   G   
   A   
   A   
   A   
   A   
   A   
   A   
   G   
   A   
   C   
   A   
   A   
   T   
   G   
   T   
   A   
   A   
   T   
   C   
   A   
   G   
   C   
   G   
   C   
   C   
   A   
   A   
   C   
   G   
   C   
   A   
   G   
   C   
   G   
   A   
   A   
   T   
   A   
   C   
   A   
   A   
   T   
   T   
   G   
   C   
   C   
   A   
   C   
   A   
   G   
   A   
   T   
   C   
   T   
   T   
   T   
   T   
   A   
   G   
   G   
   T   
   A   
   G   
   T   
   G   
   T   
   G   
   G   
   A   
   A   
   G   
   A   
   T   
   C   
   C   
   T   
   T   
   C   
   A   
   G   
   G   
   T   
   T   
   A   
   T   
   G   
   T   
   T   
   T   
   A   
   T   
   A   
   A   
   T   
   A   
   T   
   G   
   T   
   A   
   C   
   A   
   A   
   T   
   C   
   C   
   G   
   A   
   A   
   C   
   T   
   T   
   C   
   T   
   C   
   A   
   T   
   T   
   G   
   G   
   C   
   T   
   G   
   C   
   T   
   A   
   A   
   A   
   A   
   C   
   A   
   G   
   G   
   A   
   A   
   C   
   A   
   G   
   C   
   T   
   G   
   A   
   A   
   A   
   T   
   A   
   A   
   A   
   A   
   G   
   A   
   T   
   A   
   A   
   A   
   C   
   A   
   A   
   G   
   A   
   T   
   A   
   C   
   A   
   G   
   A   
   T   
   G   
   G   
   C   
   A   
   A   
   A   
   G   
   A   
   A   
   A   
   A   
   C   
   A   
   G   
   T   
   T   
   T   
   C   
   C   
   T   
   C   
   C   
   T   
   A   
   A   
   C   
   G   
   T   
   T   
   A   
   G   
   A   
   T   
   C   
   G   
   T   
   A   
   G   
   T   
   A   
   A   
   C   
   A   
   A   
   T   
   A   
   A   
   A   
   T   
   T   
   C   
   T   
   T   
   A   
   A   
   C   
   A   
   A   
   T   
   G   
   A   
   T   
   T   
   A   
   T   
   G   
   G   
   T   
   T   
   G   
   A   
   A   
   A   
   A   
   T   
   T   
   C   
   A   
   G   
   G   
   A   
   G   
   A   
   A   
   A   
   A   
   T   
   G   
   G   
   T   
   T   
   C   
   T   
   G   
   C   
   A   
   A   
   C   
   A   
   G   
   A   
   T   
   A   
   T   
   C   
   A   
   G   
   T   
   A   
   A   
   A   
   C   
   C   
   A   
   T   
   T   
   A   
   A   
   T   
   T   
   G   
   A   
   T   
   T   
   A   
   C   
   T   
   T   
   A   
   G   
   A   
   A   
   G   
   C   
   A   
   A   
   C   
   C   
   A   
   T   
   T   
   A   
   A   
   A   
   -   
   -   
   -   
   -   
   -   
   -   
   -   
   -   
   -   
   -   
   -   
   -   
   -   
   -   
   -   
   -   
   -   
   -   
   -   
   -   
   -   
   -   
   -   
   -   
   -   
   -   
   -   
   -   
   -   
   -   
   -   
   -   
   -   
   -   
   -   
   -   
   -   
   -   
   -   
   -   
   -   
   -   
   -   
   -   
   -   
   -   
   -   
   -   
   -   
   -   
   -   
   -   
   -   
   -   
   -   
   -   
   -   
   -   
   -   
   -   
   -   
   -   
   -   
   -   
   -   
   -   
   -   
   -   
   -   
   -   
   -   
   -   
   -   
   -   
   -   
   -   
   -   
   -   
   -   
   -   
   -   
   -   
   -   
   -   
   -   
   -   
   -   
   -   
   -   
   -   
   -   
   -   
   -   
   -   
   -   
   -   
   -   
   -   
   -   
   -   
   -   
   -   
   -   
   -   
   -   
   -   
   -   
   -   
   -   
   -   
   -   
   -   
   -   
   -   
   -   
   -   
   -   
   -   
   -   
   -   
   -   
   -   
   -   
   -   
   -   
   -   
   -   
   -   
   -   
   -   
   -   
   -   
   -   
   -   
   -   
   -   
   -   
   -   
   -   
   -   
   -   
   -   
   -   
   -   
   -   
   -   
   -   
   -   
   -   
   -   
   -   
   -   
   -   
   -   
   -   
   -   
   -   
   -   
   -   
   -   
   -   
   -   
   -   
   -   
   -   
   -   
   -   
   -   
   -   
   C   
   A   
   A   
   T   
   G   
   G   
   C   
   A   
   A   
   G   
   C   
   C   
   A   
   A   
   A   
   C   
   A   
   A   
   G   
   A   
   A   
   T   
   T   
   A   
   G   
   C   
   C   
   G   
   A   
   A   
   G   
   C   
   G   
   A   
   A   
   G   
   A   
   A   
   A   
   A   
   C   
   A   
   G   
   C   
   T   
   A   
   A   
   T   
   A   
   C   
   A   
   T   
   T   
   T   
   T   
   T   
   A   
   A   
   A   
   T   
   G   
   T   
   A   
   T   
   T   
   G   
   T   
   C   
   A   
   A   
   A   
   A   
   C   
   A   
   G   
   G   
   A   
   A   
   T   
   T   
   T   
   G   
   A   
   T   
   A   
   A   
   G   
   T   
   T   
   A   
   C   
   C   
   G   
   T   
   C   
   C   
   G   
   T   
   T   
   G   
   T   
   T   
   C   
   A   
   A   
   G   
   A   
   A   
   T   
   C   
   T   
   A   
   G   
   C   
   T   
   T   
   A   
   A   
   A   
   G   
   A   
   A   
   A   
   A   
   A   
   T   
   G   
   G   
   C   
   T   
   A   
   T   
   G   
   A   
   T   
   A   
   C   
   T   
   A   
   A   
   A   
   T   
   C   
   T   
   G   
   T   
   T   
   G   
   T   
   T   
   G   
   A   
   A   
   A   
   A   
   A   
   T   
   A   
   C   
   C   
   A   
   A   
   G   
   C   
   A   
   A   
   T   
   T   
   T   
   A   
   T   
   T   
   C   
   A   
   G   
   G   
   G   
   A   
   T   
   T   
   C   
   A   
   A   
   G   
   C   
   A   
   G   
   A   
   A   
   G   
   G   
   A   
   G   
   T   
   C   
   A   
   A   
   A   
   G   
   C   
   T   
   A   
   G   
   T   
   G   
   A   
   T   
   G   
   T   
   C   
   C   
   A   
   A   
   G   
   T   
   C   
   A   
   A   
   A   
   A   
   A   
   G   
   G   
   C   
   G   
   A   
   A   
   A   
   G   
   A   
   C   
   A   
   A   
   T   
   C   
   A   
   A   
   T   
   A   
   C   
   A   
   C   
   A   
   T   
   T   
   T   
   A   
   C   
   C   
   T   
   A   
   T   
   A   
   A   
   A   
   T   
   T   
   A   
   T   
   C   
   G   
   A   
   T   
   G   
   A   
   G   
   C   
   A   
   C   
   T   
   C   
   C   
   T   
   T   
   T   
   A   
   G   
   G   
   C   
   G   
   A   
   A   
   A   
   T   
   G   
   A   
   A   
   A   
   G   
   A   
   T   
   C   
   T   
   G   
   T   
   C   
   T   
   T   
   A   
   T   
   C   
   A   
   A   
   T   
   C   
   A   
   A   
   G   
   T   
   A   
   T   
   C   
   G   
   C   
   C   
   A   
   A   
   A   
   A   
   A   
   A   
   G   
   G   
   C   
   G   
   A   
   T   
   A   
   C   
   C   
   T   
   A   
   C   
   C   
   A   
   A   
   A   
   T   
   C   
   T   
   C   
   T   
   T   
   G   
   G   
   A   
   A   
   G   
   C   
   C   
   G   
   T   
   C   
   T   
   T   
   T   
   A   
   A   
   T   
   T   
   T   
   T   
   T   
   C   
   C   
   A   
   G   
   A   
   T   
   A   
   T   
   G   
   T   
   C   
   A   
   G   
   G   
   A   
   A   
   A   
   T   
   G   
   A   
   T   
   A   
   A   
   A   
   A   
   T   
   T   
   T   
   C   
   G   
   A   
   T   
   T   
   C   
   A   
   A   
   G   
   T   
   A   
   G   
   A   
   T   
   A   
   A   
   T   
   G   
   C   
   C   
   A   
   A   
   A   
   C   
   G   
   T   
   G   
   G   
   A   
   G   
   A   
   A   
   A   
   T   
   T   
   G   
   T   
   C   
   G   
   A   
   T   
   C   
   G   
   T   
   A   
   A   
   T   
   G   
   G   
   T   
   A   
   G   
   T   
   G   
   G   
   G   
   C   
   T   
   A   
   G   
   C   
   A   
   A   
   T   
   T   
   A   
   A   
   C   
   A   
   A   
   A   
   G   
   T   
   G   
   T   
   T   
   T   
   G   
   A   
   C   
   G   
   A   
   A   
   G   
   T   
   G   
   G   
   G   
   C   
   G   
   T   
   A   
   G   
   T   
   G   
   C   
   C   
   T   
   G   
   G   
   C   
   A   
   A   
   A   
   C   
   T   
   C   
   G   
   G   
   T   
   T   
   C   
   T   
   G   
   G   
   C   
   G   
   C   
   A   
   G   
   A   
   A   
   A   
   A   
   A   
   A   
   C   
   A   
   G   
   C   
   C   
   A   
   A   
   T   
   A   
   T   
   C   
   A   
   A   
   A   
   G   
   C   
   T   
   T   
   T   
   T   
   A   
   G   
   T   
   G   
   A   
   T   
   A   
   A   
   G   
   T   
   T   
   C   
   G   
   G   
   C   
   G   
   T   
   T   
   T   
   C   
   T   
   G   
   T   
   T   
   G   
   A   
   T   
   G   
   A   
   A   
   A   
   T   
   C   
   A   
   A   
   T   
   C   
   A   
   A   
   A   
   A   
   G   
   T   
   T   
   A   
   A   
   G   
   C   
   C   
   A   
   A   
   G   
   G   
   G   
   T   
   G   
   G   
   G   
   T   
   C   
   C   
   A   
   A   
   G   
   C   
   A   
   G   
   A   
   C   
   T   
   C   
   C   
   T   
   T   
   T   
   G   
   T   
   A   
   C   
   C   
   A   
   A   
   T   
   C   
   A   
   C   
   A   
   G   
   T   
   T   
   G   
   C   
   T   
   T   
   C   
   T   
   G   
   A   
   A   
   C   
   C   
   A   
   G   
   T   
   G   
   A   
   C   
   A   
   G   
   A   
   A   
   T   
   T   
   A   
   C   
   C   
   A   
   A   
   C   
   A   
   G   
   G   
   G   
   G   
   C   
   T   
   G   
   C   
   G   
   A   
   C   
   A   
   A   
   A   
   A   
   G   
   A   
   T   
   A   
   C   
   A   
   G   
   A   
   G   
   T   
   C   
   A   
   C   
   G   
   T   
   T   
   A   
   T   
   T   
   A   
   T   
   C   
   C   
   A   
   C   
   T   
   G   
   G   
   G   
   G   
   G   
   A   
   A   
   G   
   C   
   A   
   G   
   C   
   T   
   G   
   C   
   G   
   C   
   A   
   A   
   T   
   T   
   A   
   A   
   T   
   C   
   G   
   G   
   G   
   T   
   A   
   T   
   A   
   C   
   G   
   G   
   G   
   C   
   A   
   C   
   C   
   A   
   T   
   T   
   A   
   C   
   T   
   G   
   C   
   A   
   G   
   A   
   A   
   G   
   A   
   T   
   A   
   T   
   T   
   G   
   A   
   A   
   A   
   A   
   A   
   A   
   A   
   T   
   C   
   C   
   A   
   G   
   A   
   G   
   T   
   T   
   A   
   A   
   G   
   T   
   A   
   G   
   T   
   A   
   C   
   G   
   G   
   G   
   T   
   G   
   T   
   A   
   A   
   T   
   T   
   G   
   G   
   T   
   A   
   A   
   A   
   A   
   C   
   T   
   G   
   G   
   C   
   T   
   T   
   A   
   G   
   A   
   A   
   C   
   G   
   A   
   G   
   C   
   G   
   T   
   T   
   T   
   G   
   A   
   T   
   A   
   A   
   A   
   G   
   A   
   G   
   T   
   T   
   A   
   A   
   G   
   A   
   G   
   G   
   G   
   C   
   A   
   A   
   G   
   A   
   T   
   G   
   G   
   T   
   G   
   G   
   C   
   T   
   C   
   A   
   C   
   T   
   A   
   G   
   T   
   T   
   A   
   T   
   T   
   T   
   T   
   A   
   G   
   A   
   T   
   G   
   A   
   C   
   A   
   A   
   A   
   G   
   A   
   G   
   A   
   A   
   T   
   G   
   T   
   C   
   A   
   A   
   A   
   A   
   A   
   A   
   G   
   C   
   T   
   T   
   T   
   A   
   C   
   A   
   A   
   A   
   C   
   C   
   A   
   A   
   A   
   G   
   A   
   G   
   A   
   A   
   A   
   A   
   A   
   A   
   G   
   A   
   T   
   G   
   G   
   T   
   C   
   A   
   A   
   A   
   C   
   G   
   A   
   T   
   T   
   A   
   A   
   A   
   T   
   T   
   A   
   A   
   C   
   G   
   A   
   T   
   T   
   G   
   A   
   C   
   A   
   G   
   T   
   G   
   G   
   C   
   G   
   T   
   A   
   C   
   A   
   A   
   C   
   A   
   A   
   C   
   A   
   A   
   G   
   C   
   C   
   T   
   T   
   C   
   G   
   C   
   T   
   A   
   T   
   A   
   T   
   T   
   T   
   G   
   A   
   C   
   A   
   A   
   A   
   C   
   G   
   T   
   C   
   C   
   T   
   G   
   G   
   T   
   A   
   G   
   T   
   G   
   C   
   G   
   G   
   T   
   C   
   A   
   T   
   T   
   A   
   C   
   C   
   G   
   A   
   T   
   C   
   C   
   T   
   C   
   A   
   A   
   A   
   A   
   A   
   G   
   G   
   C   
   G   
   A   
   T   
   T   
   T   
   A   
   T   
   T   
   A   
   G   
   C   
   A   
   A   
   C   
   G   
   G   
   T   
   T   
   A   
   G   
   T   
   T   
   C   
   A   
   C   
   C   
   T   
   T   
   C   
   G   
   T   
   A   
   T   
   G   
   A   
   T   
   C   
   C   
   T   
   A   
   A   
   T   
   A   
   A   
   A   
   A   
   T   
   G   
   G   
   C   
   C   
   A   
   A   
   T   
   G   
   G   
   T   
   A   
   T   
   T   
   T   
   C   
   T   
   C   
   A   
   A   
   A   
   A   
   A   
   G   
   A   
   A   
   T   
   A   
   T   
   G   
   A   
   T   
   G   
   C   
   T   
   T   
   A   
   C   
   A   
   A   
   T   
   A   
   A   
   C   
   A   
   A   
   C   
   A   
   A   
   A   
   G   
   A   
   T   
   T   
   T   
   A   
   C   
   C   
   A   
   T   
   T   
   C   
   A   
   C   
   A   
   G   
   C   
   A   
   C   
   G   
   T   
   T   
   T   
   T   
   G   
   C   
   G   
   A   
   C   
   A   
   G   
   G   
   C   
   T   
   A   
   T   
   G   
   C   
   A   
   C   
   C   
   A   
   G   
   G   
   C   
   T   
   C   
   T   
   A   
   C   
   C   
   T   
   T   
   T   
   A   
   A   
   A   
   A   
   C   
   A   
   A   
   T   
   T   
   A   
   C   
   C   
   G   
   G   
   T   
   G   
   C   
   C   
   A   
   T   
   T   
   G   
   G   
   T   
   T   
   T   
   A   
   G   
   A   
   T   
   G   
   C   
   A   
   G   
   G   
   G   
   A   
   C   
   A   
   T   
   T   
   G   
   A   
   A   
   G   
   C   
   C   
   A   
   G   
   A   
   T   
   G   
   A   
   A   
   G   
   A   
   G   
   C   
   T   
   A   
   G   
   A   
   A   
   A   
   T   
   T   
   A   
   A   
   T   
   G   
   G   
   C   
   T   
   T   
   G   
   A   
   A   
   A   
   T   
   G   
   G   
   C   
   A   
   A   
   A   
   A   
   A   
   G   
   A   
   T   
   A   
   A   
   A   
   T   
   C   
   T   
   T   
   G   
   G   
   G   
   G   
   C   
   G   
   G   
   C   
   T   
   A   
   T   
   T   
   T   
   T   
   G   
   C   
   A   
   A   
   C   
   A   
   C   
   G   
   T   
   G   
   T   
   A   
   A   
   A   
   A   
   G   
   A   
   A   
   G   
   C   
   A   
   A   
   G   
   T   
   C   
   C   
   A   
   G   
   T   
   C   
   A   
   A   
   T   
   C   
   T   
   G   
   C   
   G   
   T   
   A   
   C   
   C   
   G   
   C   
   T   
   T   
   T   
   A   
   G   
   T   
   T   
   A   
   A   
   T   
   T   
   C   
   A   
   G   
   A   
   T   
   A   
   A   
   T   
   A   
   T   
   T   
   T   
   A   
   T   
   T   
   T   
   T   
   G   
   C   
   C   
   C   
   A   
   A   
   C   
   A   
   A   
   A   
   C   
   A   
   T   
   T   
   A   
   C   
   G   
   G   
   A   
   T   
   G   
   G   
   G   
   G   
   G   
   A   
   A   
   G   
   A   
   C   
   A   
   A   
   A   
   T   
   T   
   T   
   A   
   G   
   A   
   G   
   C   
   A   
   G   
   G   
   T   
   C   
   T   
   T   
   A   
   A   
   T   
   A   
   A   
   A   
   T   
   T   
   C   
   A   
   T   
   T   
   T   
   T   
   C   
   G   
   G   
   T   
   G   
   A   
   G   
   G   
   A   
   A   
   C   
   T   
   A   
   G   
   A   
   T   
   T   
   T   
   A   
   C   
   C   
   A   
   A   
   T   
   T   
   G   
   C   
   C   
   A   
   T   
   G   
   A   
   C   
   G   
   C   
   C   
   T   
   G   
   C   
   T   
   C   
   A   
   A   
   A   
   T   
   T   
   T   
   C   
   A   
   A   
   A   
   T   
   G   
   A   
   A   
   G   
   A   
   T   
   A   
   A   
   A   
   T   
   T   
   T   
   A   
   A   
   T   
   T   
   C   
   T   
   G   
   A   
   A   
   A   
   T   
   T   
   C   
   T   
   T   
   T   
   T   
   A   
   G   
   C   
   A   
   G   
   A   
   T   
   A   
   C   
   G   
   G   
   G   
   C   
   T   
   A   
   C   
   G   
   G   
   C   
   C   
   A   
   A   
   G   
   G   
   C   
   C   
   A   
   A   
   T   
   T   
   G   
   T   
   T   
   A   
   A   
   T   
   T   
   T   
   C   
   G   
   C   
   C   
   A   
   A   
   T   
   C   
   C   
   A   
   G   
   C   
   A   
   A   
   G   
   C   
   T   
   A   
   C   
   C   
   A   
   T   
   G   
   T   
   A   
   T   
   A   
   G   
   T   
   G   
   T   
   T   
   T   
   T   
   C   
   C   
   A   
   A   
   A   
   A   
   T   
   A   
   A   
   T   
   G   
   G   
   A   
   A   
   C   
   A   
   T   
   T   
   A   
   G   
   T   
   C   
   T   
   A   
   T   
   C   
   C   
   A   
   A   
   A   
   A   
   T   
   T   
   A   
   G   
   T   
   T   
   T   
   T   
   A   
   G   
   A   
   C   
   A   
   A   
   A   
   G   
   A   
   A   
   A   
   C   
   G   
   A   
   A   
   G   
   A   
   A   
   A   
   A   
   A   
   A   
   G   
   A   
   C   
   A   
   A   
   T   
   G   
   T   
   A   
   A   
   T   
   C   
   A   
   G   
   C   
   G   
   C   
   C   
   A   
   A   
   C   
   G   
   C   
   A   
   G   
   C   
   G   
   A   
   A   
   T   
   A   
   C   
   A   
   A   
   T   
   T   
   G   
   C   
   C   
   A   
   C   
   A   
   G   
   A   
   T   
   C   
   T   
   T   
   T   
   T   
   A   
   G   
   G   
   T   
   A   
   G   
   T   
   G   
   T   
   G   
   G   
   A   
   A   
   G   
   A   
   T   
   C   
   C   
   T   
   T   
   C   
   A   
   G   
   G   
   T   
   T   
   A   
   T   
   G   
   T   
   T   
   T   
   A   
   T   
   A   
   A   
   T   
   A   
   T   
   G   
   T   
   A   
   C   
   A   
   A   
   T   
   C   
   C   
   G   
   A   
   A   
   C   
   T   
   T   
   C   
   T   
   C   
   A   
   T   
   T   
   G   
   G   
   C   
   T   
   G   
   C   
   T   
   A   
   A   
   A   
   A   
   C   
   A   
   G   
   G   
   A   
   A   
   C   
   A   
   G   
   C   
   T   
   G   
   A   
   A   
   A   
   T   
   A   
   A   
   A   
   A   
   G   
   A   
   T   
   A   
   A   
   A   
   C   
   A   
   A   
   G   
   A   
   T   
   A   
   C   
   A   
   G   
   A   
   T   
   G   
   G   
   C   
   A   
   A   
   A   
   G   
   A   
   A   
   A   
   A   
   C   
   A   
   G   
   T   
   T   
   T   
   C   
   C   
   T   
   C   
   C   
   T   
   A   
   A   
   C   
   G   
   T   
   T   
   A   
   G   
   A   
   T   
   C   
   G   
   T   
   A   
   G   
   T   
   A   
   A   
   C   
   A   
   A   
   T   
   A   
   A   
   A   
   T   
   T   
   C   
   T   
   T   
   A   
   A   
   C   
   A   
   A   
   T   
   G   
   A   
   T   
   T   
   A   
   T   
   G   
   G   
   T   
   T   
   G   
   A   
   A   
   A   
   A   
   T   
   T   
   C   
   A   
   G   
   G   
   A   
   G   
   A   
   A   
   A   
   A   
   T   
   G   
   G   
   T   
   T   
   C   
   T   
   G   
   C   
   A   
   A   
   C   
   A   
   G   
   A   
   T   
   A   
   T   
   C   
   A   
   G   
   T   
   A   
   A   
   A   
   C   
   C   
   A   
   T   
   T   
   A   
   A   
   T   
   T   
   G   
   A   
   T   
   T   
   A   
   C   
   T   
   T   
   A   
   G   
   A   
   A   
   G   
   C   
   A   
   A   
   C   
   C   
   A   
   T   
   T   
   A   
   A   
   A   
   -   
   -   
   -   
   -   
   -   
   -   
   -   
   -   
   -   
   -   
   -   
   -   
   -   
   -   
   -   
   -   
   -   
   -   
   -   
   -   
   -   
   -   
   -   
   -   
   -   
   -   
   -   
   -   
   -   
   -   
   -   
   -   
   -   
   -   
   -   
   -   
   -   
   -   
   -   
   -   
   -   
   -   
   -   
   -   
   -   
   -   
   -   
   -   
   -   
   -   
   -   
   -   
   -   
   -   
   -   
   -   
   -   
   -   
   -   
   -   
   -   
 

 
   N59611F   
   -   
   -   
   -   
   -   
   -   
   -   
   -   
   -   
   -   
   -   
   -   
   -   
   -   
   -   
   -   
   -   
   -   
   -   
   -   
   -   
   -   
   -   
   -   
   -   
   -   
   -   
   -   
   -   
   -   
   -   
   -   
   -   
   -   
   -   
   -   
   -   
   -   
   -   
   -   
   -   
   -   
   -   
   -   
   -   
   -   
   -   
   -   
   -   
   -   
   -   
   -   
   -   
   -   
   -   
   -   
   -   
   -   
   -   
   -   
   -   
   -   
   -   
   -   
   -   
   -   
   -   
   -   
   -   
   -   
   -   
   -   
   -   
   -   
   -   
   -   
   -   
   -   
   -   
   -   
   -   
   -   
   -   
   -   
   -   
   -   
   -   
   -   
   -   
   -   
   -   
   -   
   -   
   -   
   -   
   -   
   -   
   -   
   -   
   -   
   -   
   -   
   -   
   -   
   -   
   -   
   -   
   -   
   -   
   C   
   A   
   A   
   T   
   G   
   G   
   C   
   A   
   A   
   G   
   C   
   C   
   A   
   A   
   A   
   C   
   A   
   A   
   G   
   A   
    A    
   T   
   T   
   A   
   G   
   C   
    C    
   G   
   A   
   A   
   G   
   C   
   G   
   A   
   A   
   G   
   A   
   A   
   A   
   A   
    C    
   A   
   G   
   C   
    T    
   A   
    C    
   T   
    G    
    A    
   A   
   T   
   T   
   T   
   T   
   T   
   A   
   A   
   A   
    C    
   G   
   T   
   A   
   T   
   T   
   G   
    T    
   C   
   A   
   A   
   A   
   A   
   C   
   A   
   G   
    G    
   A   
   A   
   T   
   T   
   T   
   G   
   A   
   T   
   A   
   A   
   G   
   T   
   T   
   A   
   C   
   C   
    G    
   T   
   C   
   C   
   G   
   T   
   T   
    G    
   T   
   T   
   C   
   A   
   A   
   G   
   A   
   A   
    G    
   C   
   T   
   A   
   G   
   C   
   T   
   T   
   A   
   A   
   A   
   G   
   A   
   A   
   A   
   A   
   A   
   T   
   G   
   G   
    C    
   T   
   A   
   T   
   G   
   A   
   T   
   A   
   C   
   T   
   A   
   A   
   A   
   T   
   C   
    T    
   G   
   T   
   T   
   G   
   T   
   T   
   G   
   A   
   A   
   A   
   A   
   A   
   T   
   A   
   C   
   C   
   A   
   A   
   G   
   C   
   A   
   A   
   T   
   T   
   T   
   A   
   T   
   T   
   C   
   A   
   G   
   G   
   G   
   A   
   T   
   T   
   C   
   A   
   A   
   G   
   C   
    A    
   G   
   A   
   A   
    G    
   G   
    A    
   G   
   T   
   C   
   A   
   A   
    A    
   G   
   C   
   T   
   A   
   G   
   T   
   G   
   A   
   T   
   G   
   T   
    C    
   C   
   A   
   A   
   G   
   T   
    C    
   A   
   A   
   A   
   A   
   A   
    G    
   G   
    C    
   G   
   A   
   A   
   A   
   G   
   A   
    C    
   A   
   A   
    T    
   C   
   A   
   A   
   T   
   A   
   C   
   A   
   C   
   A   
   T   
   T   
   T   
   A   
    C    
   C   
   T   
   A   
   T   
   A   
   A   
   A   
   T   
   T   
   A   
   T   
   C   
   G   
   A   
   T   
   G   
   A   
   G   
    C    
   A   
   C   
    T    
   C   
   C   
   T   
   T   
   T   
   A   
   G   
   G   
   C   
   G   
   A   
   A   
   A   
   T   
   G   
   A   
   A   
   A   
   G   
   A   
   T   
    T    
   T   
   G   
   T   
   C   
   T   
   T   
   A   
   T   
   C   
   A   
   A   
   T   
   C   
   A   
   A   
   G   
   T   
   A   
   T   
    C    
   G   
    C    
   C   
   A   
   A   
    A    
   A   
   A   
   A   
   G   
   G   
    C    
   G   
   A   
   T   
   A   
    C    
   C   
   T   
   A   
   C   
   C   
   A   
   A   
   A   
   T   
    C    
    G    
   C   
   T   
   T   
   G   
   G   
    A    
    A    
    G    
   C   
   C   
    G    
   T   
   C   
   T   
   T   
   T   
   A   
   A   
   T   
   T   
   T   
   T   
   T   
   C   
   C   
   A   
   G   
   A   
   T   
   A   
   T   
   G   
   T   
   C   
   A   
   G   
   G   
   A   
   A   
   A   
   T   
   G   
   A   
   T   
   A   
   A   
   A   
    A    
   T   
   T   
   T   
   C   
   G   
   A   
   T   
   T   
   C   
   A   
   A   
   G   
   T   
   A   
   G   
   A   
   T   
   A   
   A   
   T   
   G   
   C   
   C   
   A   
   A   
   A   
   C   
   G   
   T   
   G   
   G   
   A   
   G   
   A   
   A   
   A   
   T   
   T   
   G   
   T   
   C   
   G   
   A   
   T   
   C   
   G   
   T   
   A   
   A   
    T    
   G   
   G   
   T   
   A   
   G   
   T   
   G   
   G   
   G   
   C   
   T   
   A   
   G   
    C    
   A   
   A   
   T   
   T   
   A   
   A   
   C   
   A   
   A   
   A   
   G   
   T   
   G   
   T   
   T   
   T   
   G   
   A   
    C    
   G   
   A   
   A   
   G   
   T   
   G   
   G   
   G   
   C   
   G   
   T   
   A   
   G   
   T   
    G    
   C   
   C   
   T   
    G    
   G   
   C   
   A   
   A   
   A   
   C   
   T   
    C    
   G   
   G   
   T   
   T   
   C   
   T   
   G   
   G   
   C   
   G   
   C   
   A   
   G   
   A   
   A   
   A   
   A   
   A   
    A    
   C   
   A   
   G   
   C   
    C    
   A   
   A   
   T   
   A   
   T   
   C   
   A   
   A   
   A   
   G   
   C   
   T   
   T   
   T   
   T   
   A   
   G   
   T   
   G   
   A   
   T   
   A   
   A   
    A    
   T   
   T   
    C    
   G   
   G   
   C   
   G   
   T   
   T   
   T   
   C   
   T   
    G    
   T   
   T   
   G   
   A   
   T   
   G   
   A   
   A   
   A   
   T   
   C   
   A   
   A   
   T   
   C   
    A    
   A   
   A   
   A   
    A    
   T   
   T   
   A   
   A   
   G   
   C   
   C   
   A   
   A   
   G   
   G   
    A    
   T   
   G   
   G   
   G   
   T   
   C   
   C   
   A   
   A   
   G   
   C   
   A   
   G   
   A   
   C   
   T   
   C   
    C    
   T   
   T   
   T   
   G   
   T   
   A   
   C   
   C   
   A   
   A   
   T   
   C   
   A   
   C   
   A   
   G   
   T   
    C    
   G   
   C   
   T   
   T   
   C   
   T   
   G   
   A   
   A   
   C   
   C   
   A   
   G   
   T   
   G   
   A   
   C   
   A   
   G   
   A   
   A   
   T   
   T   
   A   
   C   
   C   
   A   
   A   
   C   
   A   
   G   
   G   
   G   
   G   
   C   
   T   
   G   
   C   
   G   
   A   
   C   
   A   
   A   
   A   
   A   
   G   
    A    
    T    
   A   
   C   
   A   
   G   
   A   
   G   
   T   
   C   
   A   
   C   
   G   
   T   
   T   
   A   
   T   
   T   
   A   
   T   
   C   
   C   
    G    
    C    
   T   
   G   
   G   
   G   
   G   
   G   
   A   
   A   
   G   
   C   
   A   
   G   
   C   
   T   
   G   
   C   
   G   
   C   
   A   
   A   
   T   
   T   
   A   
   A   
   T   
   C   
   G   
   G   
    G    
   T   
   A   
   T   
   A   
   C   
    G    
   G   
   G   
    C    
   A   
   C   
    C    
   A   
   T   
   T   
   A   
   C   
   T   
   G   
   C   
   A   
   G   
   A   
    A    
   G   
   A   
   T   
   A   
   T   
   T   
    G    
   A   
   A   
   A   
   A   
   A   
   A   
   A   
   T   
   C   
   C   
   A   
   G   
   A   
   G   
   T   
   T   
   A   
   A   
   G   
   T   
   A   
   G   
   T   
   A   
   C   
   G   
   G   
   G   
    C    
   G   
   T   
   A   
   A   
   T   
   T   
   G   
   G   
    T    
   A   
   A   
   A   
   A   
   C   
   T   
   G   
   G   
    C    
   T   
   T   
   A   
   G   
   A   
   A   
   C   
   G   
   A   
   G   
   C   
   G   
   T   
   T   
   T   
   G   
   A   
   T   
   A   
   A   
   A   
   G   
   A   
    A    
   T   
   T   
   A   
   A   
   G   
   A   
   G   
   G   
    G    
   C   
   A   
   A   
   G   
   A   
   T   
   G   
   G   
    T    
   G   
   G   
    C    
   T   
   C   
    A    
   C   
   T   
   A   
   G   
   T   
   T   
   A   
   T   
   T   
   T   
   T   
   A   
   G   
   A   
   T   
   G   
   A   
   C   
   A   
   A   
   A   
   G   
   A   
   G   
   A   
   A   
   T   
   G   
   T   
   C   
   A   
   A   
   A   
   A   
   A   
   A   
   G   
   C   
   T   
   T   
   T   
   A   
   C   
   A   
   A   
   A   
   C   
   C   
   A   
   A   
   A   
   G   
   A   
    G    
   A   
   A   
   A   
   A   
   A   
   A   
   G   
   A   
   T   
   G   
   G   
   T   
   C   
   A   
   A   
   A   
   C   
    G    
   A   
   T   
   T   
   A   
   A   
   A   
   T   
   T   
   A   
   A   
   C   
   G   
   A   
   T   
   T   
   G   
   A   
    C    
   A   
   G   
    T    
   G   
   G   
    C    
   G   
   T   
   A   
   C   
   A   
   A   
   C   
   A   
   A   
   C   
   A   
   A   
   G   
   C   
    C    
   T   
   T   
    T    
   G   
   C   
   T   
   A   
   T   
   A   
   T   
   T   
   T   
   G   
   A   
   C   
   A   
   A   
   A   
   C   
   G   
    C    
    C    
   C   
   T   
   G   
   G   
   T   
   A   
   G   
   T   
   G   
   C   
   G   
   G   
   T   
   C   
   A   
   T   
   T   
   A   
   C   
   C   
   G   
   A   
   T   
   C   
   C   
   T   
   C   
   A   
   A   
   A   
   A   
   A   
   G   
   G   
   C   
   G   
   A   
   T   
   T   
   T   
   A   
    T    
   T   
   A   
   G   
   C   
    A    
   A   
   C   
   G   
   G   
   T   
   T   
   A   
   G   
   T   
   T   
   C   
   A   
   C   
   C   
   T   
   T   
   C   
   G   
   T   
   A   
   T   
   G   
   A   
   T   
   C   
   C   
   T   
   A   
   A   
   T   
   A   
   A   
   A   
   A   
   T   
   G   
   G   
   C   
   C   
   A   
   A   
   T   
   G   
   G   
    C    
   A   
   T   
   T   
   T   
   C   
   T   
   C   
   A   
   A   
   A   
   A   
   A   
   G   
   A   
    A    
   T   
   A   
   T   
   G   
   A   
   T   
   G   
   C   
   T   
   T   
   A   
   C   
   A   
   A   
   T   
   A   
   A   
   C   
   A   
   A   
   C   
   A   
   A   
   A   
   G   
   A   
   T   
   T   
   T   
   A   
   C   
   C   
   A   
   T   
   T   
    C    
   A   
   C   
   A   
   G   
   C   
   A   
   C   
   G   
   T   
   T   
   T   
    T    
   G   
   C   
   G   
   A   
   C   
   A   
   G   
   G   
    C    
   T   
   A   
   T   
   G   
   C   
   A   
   C   
   C   
   A   
   G   
   G   
    C    
   T   
   C   
   T   
   A   
   C   
   C   
   T   
   T   
   T   
   A   
   A   
   A   
   A   
   C   
   A   
   A   
   T   
   T   
   A   
   C   
   C   
   G   
   G   
   T   
   G   
   C   
   C   
   A   
   T   
   T   
   G   
   G   
   T   
   T   
   T   
   A   
   G   
   A   
   T   
    G    
   C   
   A   
   G   
   G   
   G   
   A   
   C   
   A   
   T   
   T   
   G   
   A   
   A   
   G   
   C   
   C   
   A   
   G   
   A   
   T   
   G   
   A   
   A   
   G   
   A   
   G   
   C   
   T   
   A   
   G   
   A   
   A   
   A   
   T   
   T   
   A   
   A   
   T   
   G   
   G   
    T    
   T   
   T   
   G   
   A   
   A   
   A   
   T   
   G   
   G   
   C   
   A   
   A   
   A   
   A   
   A   
   G   
   A   
   T   
   A   
   A   
   A   
   T   
   C   
   T   
   T   
   G   
   G   
   G   
   G   
    C    
   G   
   G   
   C   
   T   
   A   
   T   
   T   
   T   
   T   
   G   
   C   
    G    
   A   
   C   
   A   
   C   
   G   
   T   
   G   
   T   
   A   
   A   
   A   
   A   
   G   
   A   
   A   
   G   
   C   
   A   
   A   
   G   
   T   
   C   
   C   
   A   
   G   
   T   
   C   
   A   
   A   
   T   
   C   
   T   
    G    
   C   
   G   
   T   
   A   
   C   
   C   
   G   
   C   
   T   
   T   
   T   
   A   
   G   
   T   
   T   
   A   
   A   
   T   
   T   
   C   
   A   
   G   
   A   
   T   
   A   
   A   
   T   
   A   
   T   
   T   
   T   
   A   
   T   
   T   
   T   
   T   
   G   
   C   
    C    
   C   
   A   
   A   
   C   
   A   
   A   
   A   
   C   
   A   
   T   
   T   
   A   
   C   
   G   
    T    
   A   
   T   
   G   
   G   
   G   
    G    
   G   
   A   
   A   
   G   
   A   
   C   
   A   
   A   
   A   
   T   
   T   
   T   
   A   
   G   
   A   
   G   
   C   
    G    
   G   
   G   
   T   
   C   
   T   
   T   
   A   
   A   
   T   
   A   
   A   
   A   
   T   
   T   
   C   
   A   
   T   
   T   
   T   
   T   
   C   
   G   
   G   
   T   
   G   
   A   
   G   
   G   
   A   
   A   
   C   
   T   
   A   
   G   
   A   
   T   
   T   
   T   
   A   
   C   
   C   
   A   
   A   
   T   
    T    
   G   
   C   
   C   
   A   
   T   
   G   
   A   
   C   
   G   
   C   
   C   
    T    
   G   
   C   
   T   
   C   
   A   
   A   
   A   
   T   
   T   
   T   
   C   
   A   
   A   
   A   
    T    
   G   
   A   
   A   
   G   
   A   
   T   
   A   
   A   
   A   
   T   
   T   
   T   
   A   
   A   
   T   
   T   
   C   
   T   
   G   
   A   
   A   
   A   
   T   
   T   
   C   
   T   
   T   
   T   
   T   
    A    
   G   
   C   
   A   
   G   
   A   
   T   
   A   
   C   
    G    
   G   
   G   
   C   
   T   
   A   
    C    
   G   
   G   
    T    
   C   
   A   
   A   
   G   
   G   
    C    
   C   
   A   
   A   
   T   
   T   
   G   
   T   
   T   
   A   
   A   
   T   
   T   
   T   
   C   
    G    
   C   
   C   
   A   
   A   
   T   
   C   
   C   
   A   
   G   
   C   
   A   
   A   
   G   
   C   
   T   
   A   
   C   
   C   
   A   
   T   
   G   
   T   
   A   
   T   
   A   
   G   
   T   
   G   
   T   
   T   
   T   
   T   
   C   
   C   
   A   
   A   
   A   
   A   
   T   
   A   
   A   
   T   
   G   
   G   
    A    
   A   
   C   
   A   
    T    
   T   
    G    
   G   
   T   
    C    
   T   
   A   
   T   
   C   
   C   
   A   
   A   
   A   
   A   
    A    
   T   
   A   
   G   
   T   
   T   
   T   
   T   
   A   
   G   
   A   
   C   
   A   
   A   
   A   
   G   
   A   
   A   
   A   
   C   
    G    
   A   
   A   
   G   
   A   
   A   
   A   
   A   
   A   
   A   
   G   
   A   
   C   
   A   
   A   
   T   
   G   
   T   
   A   
   A   
   T   
   C   
   A   
   G   
   C   
   G   
   C   
   C   
   A   
   A   
   C   
   G   
   C   
   A   
   G   
   C   
   G   
   A   
   A   
   T   
   A   
   C   
   A   
   A   
   T   
   T   
   G   
   C   
   C   
   A   
   C   
   A   
   G   
   A   
   T   
   C   
   T   
   T   
   T   
   T   
   A   
   G   
   G   
   T   
   A   
   G   
   T   
   G   
   T   
   G   
   G   
   A   
   A   
   G   
   A   
   C   
   C   
   C   
   T   
   T   
   C   
   A   
   G   
   G   
   T   
   T   
   A   
   T   
   G   
   T   
   T   
   T   
   A   
   T   
   A   
   A   
   T   
   A   
   T   
   G   
   T   
   A   
   C   
   A   
   A   
   T   
   C   
   C   
   G   
   A   
   A   
   C   
   T   
   T   
   C   
   T   
   C   
   A   
   T   
   T   
   G   
   G   
   C   
   T   
   G   
   C   
   T   
   A   
   A   
   A   
   A   
   C   
   A   
   G   
   G   
   A   
   A   
   C   
   A   
   G   
   C   
   T   
   G   
   A   
   A   
   A   
   T   
   A   
   A   
   A   
   A   
   G   
   A   
   T   
   A   
   A   
   A   
   C   
   A   
   A   
   G   
   A   
   T   
   A   
   C   
   A   
   G   
   A   
   T   
   G   
   G   
   C   
   A   
   A   
   A   
   G   
   A   
   A   
   A   
   A   
   C   
   A   
   G   
   T   
   T   
   T   
   C   
   C   
   T   
   T   
   C   
   T   
   A   
   A   
   C   
   G   
   T   
   T   
   A   
   G   
   A   
   T   
   C   
   G   
   T   
   A   
   G   
   T   
   A   
   A   
   C   
   A   
   A   
   T   
   A   
   A   
   A   
   T   
   T   
   C   
   T   
   T   
   A   
   A   
   C   
   A   
   A   
   T   
   G   
   A   
   T   
   T   
   A   
   T   
   G   
   G   
   T   
   T   
   G   
   A   
   A   
   A   
   A   
   T   
   T   
   C   
   A   
   G   
   G   
   A   
   G   
   A   
   A   
   A   
   A   
   T   
   G   
   G   
   T   
   T   
   C   
   T   
   G   
   C   
   A   
   A   
   C   
   A   
   G   
   A   
   T   
   A   
   T   
   C   
   A   
   G   
   T   
   A   
   A   
   A   
   C   
   C   
   A   
   T   
   T   
   A   
   A   
   T   
   T   
   G   
   A   
   T   
   T   
   A   
   C   
   T   
   T   
   A   
   G   
   A   
   A   
   G   
   C   
   A   
   A   
   C   
   C   
   A   
   T   
   T   
   A   
   A   
   A   
   -   
   -   
   -   
   -   
   -   
   -   
   -   
   -   
   -   
   -   
   -   
   -   
   -   
   -   
   -   
   -   
   -   
   -   
   -   
   -   
   -   
   -   
   -   
   -   
   -   
   -   
   -   
   -   
   -   
   -   
   -   
   -   
   -   
   -   
   -   
   -   
   -   
   -   
   -   
   -   
   -   
   -   
   -   
   -   
   -   
   -   
   -   
   -   
   -   
   -   
   -   
   -   
   -   
   -   
   -   
   -   
   -   
   -   
   -   
   -   
   -   
   -   
   -   
   -   
   -   
   -   
   -   
   -   
   -   
   -   
   -   
   -   
   -   
   -   
   -   
   -   
   -   
   -   
   -   
   -   
   -   
   -   
   -   
   -   
   -   
   -   
   -   
   -   
   -   
   -   
   -   
   -   
   -   
   -   
   -   
   -   
   -   
   -   
   -   
   -   
   -   
   -   
   -   
   -   
   -   
   -   
   -   
   -   
   -   
   -   
   -   
   -   
   -   
   -   
   -   
   -   
   -   
   -   
   -   
   -   
   -   
   -   
   -   
   -   
   -   
   -   
   -   
   -   
   -   
   -   
   -   
   -   
   -   
   -   
   -   
   -   
   -   
   -   
   -   
   -   
   -   
   -   
   -   
   -   
   -   
   -   
   -   
   -   
   -   
   -   
   -   
   -   
   -   
   -   
   -   
   -   
   -   
   -   
   -   
   -   
   -   
   -   
   -   
   -   
   -   
   -   
   -   
   -   
   -   
   C   
   A   
   A   
   T   
   G   
   G   
   C   
   A   
   A   
   G   
   C   
   C   
   A   
   A   
   A   
   C   
   A   
   A   
   G   
   A   
   A   
   T   
   T   
   A   
   G   
   C   
   C   
   G   
   A   
   A   
   G   
   C   
   G   
   A   
   A   
   G   
   A   
   A   
   A   
   A   
   C   
   A   
   G   
   C   
   T   
   A   
   C   
   T   
   G   
   A   
   A   
   T   
   T   
   T   
   T   
   T   
   A   
   A   
   A   
   C   
   G   
   T   
   A   
   T   
   T   
   G   
   T   
   C   
   A   
   A   
   A   
   A   
   C   
   A   
   G   
   G   
   A   
   A   
   T   
   T   
   T   
   G   
   A   
   T   
   A   
   A   
   G   
   T   
   T   
   A   
   C   
   C   
   G   
   T   
   C   
   C   
   G   
   T   
   T   
   G   
   T   
   T   
   C   
   A   
   A   
   G   
   A   
   A   
   G   
   C   
   T   
   A   
   G   
   C   
   T   
   T   
   A   
   A   
   A   
   G   
   A   
   A   
   A   
   A   
   A   
   T   
   G   
   G   
   C   
   T   
   A   
   T   
   G   
   A   
   T   
   A   
   C   
   T   
   A   
   A   
   A   
   T   
   C   
   T   
   G   
   T   
   T   
   G   
   T   
   T   
   G   
   A   
   A   
   A   
   A   
   A   
   T   
   A   
   C   
   C   
   A   
   A   
   G   
   C   
   A   
   A   
   T   
   T   
   T   
   A   
   T   
   T   
   C   
   A   
   G   
   G   
   G   
   A   
   T   
   T   
   C   
   A   
   A   
   G   
   C   
   A   
   G   
   A   
   A   
   G   
   G   
   A   
   G   
   T   
   C   
   A   
   A   
   A   
   G   
   C   
   T   
   A   
   G   
   T   
   G   
   A   
   T   
   G   
   T   
   C   
   C   
   A   
   A   
   G   
   T   
   C   
   A   
   A   
   A   
   A   
   A   
   G   
   G   
   C   
   G   
   A   
   A   
   A   
   G   
   A   
   C   
   A   
   A   
   T   
   C   
   A   
   A   
   T   
   A   
   C   
   A   
   C   
   A   
   T   
   T   
   T   
   A   
   C   
   C   
   T   
   A   
   T   
   A   
   A   
   A   
   T   
   T   
   A   
   T   
   C   
   G   
   A   
   T   
   G   
   A   
   G   
   C   
   A   
   C   
   T   
   C   
   C   
   T   
   T   
   T   
   A   
   G   
   G   
   C   
   G   
   A   
   A   
   A   
   T   
   G   
   A   
   A   
   A   
   G   
   A   
   T   
   T   
   T   
   G   
   T   
   C   
   T   
   T   
   A   
   T   
   C   
   A   
   A   
   T   
   C   
   A   
   A   
   G   
   T   
   A   
   T   
   C   
   G   
   C   
   C   
   A   
   A   
   A   
   A   
   A   
   A   
   G   
   G   
   C   
   G   
   A   
   T   
   A   
   C   
   C   
   T   
   A   
   C   
   C   
   A   
   A   
   A   
   T   
   C   
   G   
   C   
   T   
   T   
   G   
   G   
   A   
   A   
   G   
   C   
   C   
   G   
   T   
   C   
   T   
   T   
   T   
   A   
   A   
   T   
   T   
   T   
   T   
   T   
   C   
   C   
   A   
   G   
   A   
   T   
   A   
   T   
   G   
   T   
   C   
   A   
   G   
   G   
   A   
   A   
   A   
   T   
   G   
   A   
   T   
   A   
   A   
   A   
   A   
   T   
   T   
   T   
   C   
   G   
   A   
   T   
   T   
   C   
   A   
   A   
   G   
   T   
   A   
   G   
   A   
   T   
   A   
   A   
   T   
   G   
   C   
   C   
   A   
   A   
   A   
   C   
   G   
   T   
   G   
   G   
   A   
   G   
   A   
   A   
   A   
   T   
   T   
   G   
   T   
   C   
   G   
   A   
   T   
   C   
   G   
   T   
   A   
   A   
   T   
   G   
   G   
   T   
   A   
   G   
   T   
   G   
   G   
   G   
   C   
   T   
   A   
   G   
   C   
   A   
   A   
   T   
   T   
   A   
   A   
   C   
   A   
   A   
   A   
   G   
   T   
   G   
   T   
   T   
   T   
   G   
   A   
   C   
   G   
   A   
   A   
   G   
   T   
   G   
   G   
   G   
   C   
   G   
   T   
   A   
   G   
   T   
   G   
   C   
   C   
   T   
   G   
   G   
   C   
   A   
   A   
   A   
   C   
   T   
   C   
   G   
   G   
   T   
   T   
   C   
   T   
   G   
   G   
   C   
   G   
   C   
   A   
   G   
   A   
   A   
   A   
   A   
   A   
   A   
   C   
   A   
   G   
   C   
   C   
   A   
   A   
   T   
   A   
   T   
   C   
   A   
   A   
   A   
   G   
   C   
   T   
   T   
   T   
   T   
   A   
   G   
   T   
   G   
   A   
   T   
   A   
   A   
   A   
   T   
   T   
   C   
   G   
   G   
   C   
   G   
   T   
   T   
   T   
   C   
   T   
   G   
   T   
   T   
   G   
   A   
   T   
   G   
   A   
   A   
   A   
   T   
   C   
   A   
   A   
   T   
   C   
   A   
   A   
   A   
   A   
   A   
   T   
   T   
   A   
   A   
   G   
   C   
   C   
   A   
   A   
   G   
   G   
   A   
   T   
   G   
   G   
   G   
   T   
   C   
   C   
   A   
   A   
   G   
   C   
   A   
   G   
   A   
   C   
   T   
   C   
   C   
   T   
   T   
   T   
   G   
   T   
   A   
   C   
   C   
   A   
   A   
   T   
   C   
   A   
   C   
   A   
   G   
   T   
   C   
   G   
   C   
   T   
   T   
   C   
   T   
   G   
   A   
   A   
   C   
   C   
   A   
   G   
   T   
   G   
   A   
   C   
   A   
   G   
   A   
   A   
   T   
   T   
   A   
   C   
   C   
   A   
   A   
   C   
   A   
   G   
   G   
   G   
   G   
   C   
   T   
   G   
   C   
   G   
   A   
   C   
   A   
   A   
   A   
   A   
   G   
   A   
   T   
   A   
   C   
   A   
   G   
   A   
   G   
   T   
   C   
   A   
   C   
   G   
   T   
   T   
   A   
   T   
   T   
   A   
   T   
   C   
   C   
   G   
   C   
   T   
   G   
   G   
   G   
   G   
   G   
   A   
   A   
   G   
   C   
   A   
   G   
   C   
   T   
   G   
   C   
   G   
   C   
   A   
   A   
   T   
   T   
   A   
   A   
   T   
   C   
   G   
   G   
   G   
   T   
   A   
   T   
   A   
   C   
   G   
   G   
   G   
   C   
   A   
   C   
   C   
   A   
   T   
   T   
   A   
   C   
   T   
   G   
   C   
   A   
   G   
   A   
   A   
   G   
   A   
   T   
   A   
   T   
   T   
   G   
   A   
   A   
   A   
   A   
   A   
   A   
   A   
   T   
   C   
   C   
   A   
   G   
   A   
   G   
   T   
   T   
   A   
   A   
   G   
   T   
   A   
   G   
   T   
   A   
   C   
   G   
   G   
   G   
   C   
   G   
   T   
   A   
   A   
   T   
   T   
   G   
   G   
   T   
   A   
   A   
   A   
   A   
   C   
   T   
   G   
   G   
   C   
   T   
   T   
   A   
   G   
   A   
   A   
   C   
   G   
   A   
   G   
   C   
   G   
   T   
   T   
   T   
   G   
   A   
   T   
   A   
   A   
   A   
   G   
   A   
   A   
   T   
   T   
   A   
   A   
   G   
   A   
   G   
   G   
   G   
   C   
   A   
   A   
   G   
   A   
   T   
   G   
   G   
   T   
   G   
   G   
   C   
   T   
   C   
   A   
   C   
   T   
   A   
   G   
   T   
   T   
   A   
   T   
   T   
   T   
   T   
   A   
   G   
   A   
   T   
   G   
   A   
   C   
   A   
   A   
   A   
   G   
   A   
   G   
   A   
   A   
   T   
   G   
   T   
   C   
   A   
   A   
   A   
   A   
   A   
   A   
   G   
   C   
   T   
   T   
   T   
   A   
   C   
   A   
   A   
   A   
   C   
   C   
   A   
   A   
   A   
   G   
   A   
   G   
   A   
   A   
   A   
   A   
   A   
   A   
   G   
   A   
   T   
   G   
   G   
   T   
   C   
   A   
   A   
   A   
   C   
   G   
   A   
   T   
   T   
   A   
   A   
   A   
   T   
   T   
   A   
   A   
   C   
   G   
   A   
   T   
   T   
   G   
   A   
   C   
   A   
   G   
   T   
   G   
   G   
   C   
   G   
   T   
   A   
   C   
   A   
   A   
   C   
   A   
   A   
   C   
   A   
   A   
   G   
   C   
   C   
   T   
   T   
   T   
   G   
   C   
   T   
   A   
   T   
   A   
   T   
   T   
   T   
   G   
   A   
   C   
   A   
   A   
   A   
   C   
   G   
   C   
   C   
   C   
   T   
   G   
   G   
   T   
   A   
   G   
   T   
   G   
   C   
   G   
   G   
   T   
   C   
   A   
   T   
   T   
   A   
   C   
   C   
   G   
   A   
   T   
   C   
   C   
   T   
   C   
   A   
   A   
   A   
   A   
   A   
   G   
   G   
   C   
   G   
   A   
   T   
   T   
   T   
   A   
   T   
   T   
   A   
   G   
   C   
   A   
   A   
   C   
   G   
   G   
   T   
   T   
   A   
   G   
   T   
   T   
   C   
   A   
   C   
   C   
   T   
   T   
   C   
   G   
   T   
   A   
   T   
   G   
   A   
   T   
   C   
   C   
   T   
   A   
   A   
   T   
   A   
   A   
   A   
   A   
   T   
   G   
   G   
   C   
   C   
   A   
   A   
   T   
   G   
   G   
   C   
   A   
   T   
   T   
   T   
   C   
   T   
   C   
   A   
   A   
   A   
   A   
   A   
   G   
   A   
   A   
   T   
   A   
   T   
   G   
   A   
   T   
   G   
   C   
   T   
   T   
   A   
   C   
   A   
   A   
   T   
   A   
   A   
   C   
   A   
   A   
   C   
   A   
   A   
   A   
   G   
   A   
   T   
   T   
   T   
   A   
   C   
   C   
   A   
   T   
   T   
   C   
   A   
   C   
   A   
   G   
   C   
   A   
   C   
   G   
   T   
   T   
   T   
   T   
   G   
   C   
   G   
   A   
   C   
   A   
   G   
   G   
   C   
   T   
   A   
   T   
   G   
   C   
   A   
   C   
   C   
   A   
   G   
   G   
   C   
   T   
   C   
   T   
   A   
   C   
   C   
   T   
   T   
   T   
   A   
   A   
   A   
   A   
   C   
   A   
   A   
   T   
   T   
   A   
   C   
   C   
   G   
   G   
   T   
   G   
   C   
   C   
   A   
   T   
   T   
   G   
   G   
   T   
   T   
   T   
   A   
   G   
   A   
   T   
   G   
   C   
   A   
   G   
   G   
   G   
   A   
   C   
   A   
   T   
   T   
   G   
   A   
   A   
   G   
   C   
   C   
   A   
   G   
   A   
   T   
   G   
   A   
   A   
   G   
   A   
   G   
   C   
   T   
   A   
   G   
   A   
   A   
   A   
   T   
   T   
   A   
   A   
   T   
   G   
   G   
   T   
   T   
   T   
   G   
   A   
   A   
   A   
   T   
   G   
   G   
   C   
   A   
   A   
   A   
   A   
   A   
   G   
   A   
   T   
   A   
   A   
   A   
   T   
   C   
   T   
   T   
   G   
   G   
   G   
   G   
   C   
   G   
   G   
   C   
   T   
   A   
   T   
   T   
   T   
   T   
   G   
   C   
   G   
   A   
   C   
   A   
   C   
   G   
   T   
   G   
   T   
   A   
   A   
   A   
   A   
   G   
   A   
   A   
   G   
   C   
   A   
   A   
   G   
   T   
   C   
   C   
   A   
   G   
   T   
   C   
   A   
   A   
   T   
   C   
   T   
   G   
   C   
   G   
   T   
   A   
   C   
   C   
   G   
   C   
   T   
   T   
   T   
   A   
   G   
   T   
   T   
   A   
   A   
   T   
   T   
   C   
   A   
   G   
   A   
   T   
   A   
   A   
   T   
   A   
   T   
   T   
   T   
   A   
   T   
   T   
   T   
   T   
   G   
   C   
   C   
   C   
   A   
   A   
   C   
   A   
   A   
   A   
   C   
   A   
   T   
   T   
   A   
   C   
   G   
   T   
   A   
   T   
   G   
   G   
   G   
   G   
   G   
   A   
   A   
   G   
   A   
   C   
   A   
   A   
   A   
   T   
   T   
   T   
   A   
   G   
   A   
   G   
   C   
   G   
   G   
   G   
   T   
   C   
   T   
   T   
   A   
   A   
   T   
   A   
   A   
   A   
   T   
   T   
   C   
   A   
   T   
   T   
   T   
   T   
   C   
   G   
   G   
   T   
   G   
   A   
   G   
   G   
   A   
   A   
   C   
   T   
   A   
   G   
   A   
   T   
   T   
   T   
   A   
   C   
   C   
   A   
   A   
   T   
   T   
   G   
   C   
   C   
   A   
   T   
   G   
   A   
   C   
   G   
   C   
   C   
   T   
   G   
   C   
   T   
   C   
   A   
   A   
   A   
   T   
   T   
   T   
   C   
   A   
   A   
   A   
   T   
   G   
   A   
   A   
   G   
   A   
   T   
   A   
   A   
   A   
   T   
   T   
   T   
   A   
   A   
   T   
   T   
   C   
   T   
   G   
   A   
   A   
   A   
   T   
   T   
   C   
   T   
   T   
   T   
   T   
   A   
   G   
   C   
   A   
   G   
   A   
   T   
   A   
   C   
   G   
   G   
   G   
   C   
   T   
   A   
   C   
   G   
   G   
   T   
   C   
   A   
   A   
   G   
   G   
   C   
   C   
   A   
   A   
   T   
   T   
   G   
   T   
   T   
   A   
   A   
   T   
   T   
   T   
   C   
   G   
   C   
   C   
   A   
   A   
   T   
   C   
   C   
   A   
   G   
   C   
   A   
   A   
   G   
   C   
   T   
   A   
   C   
   C   
   A   
   T   
   G   
   T   
   A   
   T   
   A   
   G   
   T   
   G   
   T   
   T   
   T   
   T   
   C   
   C   
   A   
   A   
   A   
   A   
   T   
   A   
   A   
   T   
   G   
   G   
   A   
   A   
   C   
   A   
   T   
   T   
   G   
   G   
   T   
   C   
   T   
   A   
   T   
   C   
   C   
   A   
   A   
   A   
   A   
   A   
   T   
   A   
   G   
   T   
   T   
   T   
   T   
   A   
   G   
   A   
   C   
   A   
   A   
   A   
   G   
   A   
   A   
   A   
   C   
   G   
   A   
   A   
   G   
   A   
   A   
   A   
   A   
   A   
   A   
   G   
   A   
   C   
   A   
   A   
   T   
   G   
   T   
   A   
   A   
   T   
   C   
   A   
   G   
   C   
   G   
   C   
   C   
   A   
   A   
   C   
   G   
   C   
   A   
   G   
   C   
   G   
   A   
   A   
   T   
   A   
   C   
   A   
   A   
   T   
   T   
   G   
   C   
   C   
   A   
   C   
   A   
   G   
   A   
   T   
   C   
   T   
   T   
   T   
   T   
   A   
   G   
   G   
   T   
   A   
   G   
   T   
   G   
   T   
   G   
   G   
   A   
   A   
   G   
   A   
   C   
   C   
   C   
   T   
   T   
   C   
   A   
   G   
   G   
   T   
   T   
   A   
   T   
   G   
   T   
   T   
   T   
   A   
   T   
   A   
   A   
   T   
   A   
   T   
   G   
   T   
   A   
   C   
   A   
   A   
   T   
   C   
   C   
   G   
   A   
   A   
   C   
   T   
   T   
   C   
   T   
   C   
   A   
   T   
   T   
   G   
   G   
   C   
   T   
   G   
   C   
   T   
   A   
   A   
   A   
   A   
   C   
   A   
   G   
   G   
   A   
   A   
   C   
   A   
   G   
   C   
   T   
   G   
   A   
   A   
   A   
   T   
   A   
   A   
   A   
   A   
   G   
   A   
   T   
   A   
   A   
   A   
   C   
   A   
   A   
   G   
   A   
   T   
   A   
   C   
   A   
   G   
   A   
   T   
   G   
   G   
   C   
   A   
   A   
   A   
   G   
   A   
   A   
   A   
   A   
   C   
   A   
   G   
   T   
   T   
   T   
   C   
   C   
   T   
   T   
   C   
   T   
   A   
   A   
   C   
   G   
   T   
   T   
   A   
   G   
   A   
   T   
   C   
   G   
   T   
   A   
   G   
   T   
   A   
   A   
   C   
   A   
   A   
   T   
   A   
   A   
   A   
   T   
   T   
   C   
   T   
   T   
   A   
   A   
   C   
   A   
   A   
   T   
   G   
   A   
   T   
   T   
   A   
   T   
   G   
   G   
   T   
   T   
   G   
   A   
   A   
   A   
   A   
   T   
   T   
   C   
   A   
   G   
   G   
   A   
   G   
   A   
   A   
   A   
   A   
   T   
   G   
   G   
   T   
   T   
   C   
   T   
   G   
   C   
   A   
   A   
   C   
   A   
   G   
   A   
   T   
   A   
   T   
   C   
   A   
   G   
   T   
   A   
   A   
   A   
   C   
   C   
   A   
   T   
   T   
   A   
   A   
   T   
   T   
   G   
   A   
   T   
   T   
   A   
   C   
   T   
   T   
   A   
   G   
   A   
   A   
   G   
   C   
   A   
   A   
   C   
   C   
   A   
   T   
   T   
   A   
   A   
   A   
   -   
   -   
   -   
   -   
   -   
   -   
   -   
   -   
   -   
   -   
   -   
   -   
   -   
   -   
   -   
   -   
   -   
   -   
   -   
   -   
   -   
   -   
   -   
   -   
   -   
   -   
   -   
   -   
   -   
   -   
   -   
   -   
   -   
   -   
   -   
   -   
   -   
   -   
   -   
   -   
   -   
   -   
   -   
   -   
   -   
   -   
   -   
   -   
   -   
   -   
   -   
   -   
   -   
   -   
   -   
   -   
   -   
   -   
   -   
   -   
   -   
 

 
   N59584F   
   -   
   -   
   -   
   -   
   -   
   -   
   -   
   -   
   -   
   -   
   -   
   -   
   -   
   -   
   -   
   -   
   -   
   -   
   -   
   -   
   -   
   -   
   -   
   -   
   -   
   -   
   -   
   -   
   -   
   -   
   -   
   -   
   -   
   -   
   -   
   -   
   -   
   -   
   -   
   -   
   -   
   -   
   -   
   -   
   -   
   -   
   -   
   -   
   -   
   -   
   -   
   -   
   -   
   -   
   -   
   -   
   -   
   -   
   -   
   -   
   -   
   -   
   -   
   -   
   -   
   -   
   -   
   -   
   -   
   -   
   -   
   -   
   -   
   -   
   -   
   -   
   -   
   -   
   -   
   -   
   -   
   -   
   -   
   -   
   -   
   -   
   -   
   -   
   -   
   -   
   -   
   -   
   -   
   -   
   -   
   -   
   -   
   -   
   -   
   -   
   -   
   -   
   -   
   -   
   -   
   -   
   -   
   -   
   C   
   A   
   A   
   T   
   G   
   G   
   C   
   A   
   A   
   G   
   C   
   C   
   A   
   A   
   A   
   C   
   A   
   A   
   G   
   A   
    A    
   T   
   T   
   A   
   G   
   C   
    C    
   G   
   A   
   A   
   G   
   C   
   G   
   A   
   A   
   G   
   A   
   A   
   A   
   A   
    C    
   A   
   G   
   C   
    T    
   A   
    C    
   T   
    A    
    C    
   A   
   T   
   T   
   T   
   T   
   T   
   A   
   A   
   A   
    C    
   G   
   T   
   A   
   T   
   T   
   G   
    T    
   C   
   A   
   A   
   A   
   A   
   C   
   A   
   G   
    G    
   A   
   A   
   T   
   T   
   T   
   G   
   A   
   T   
   A   
   A   
   G   
   T   
   T   
   A   
   C   
   C   
    G    
   T   
   C   
   C   
   G   
   T   
   T   
    G    
   T   
   T   
   C   
   A   
   A   
   G   
   A   
   A   
    G    
   C   
   T   
   A   
   G   
   C   
   T   
   T   
   A   
   A   
   A   
   G   
   A   
   A   
   A   
   A   
   A   
   T   
   G   
   G   
    C    
   T   
   A   
   T   
   G   
   A   
   T   
   A   
   C   
   T   
   A   
   A   
   A   
   T   
   C   
    T    
   G   
   T   
   T   
   G   
   T   
   T   
   G   
   A   
   A   
   A   
   A   
   A   
   T   
   A   
   C   
   C   
   A   
   A   
   G   
   C   
   A   
   A   
   T   
   T   
   T   
   A   
   T   
   T   
   C   
   A   
   G   
   G   
   G   
   A   
   T   
   T   
   C   
   A   
   A   
   G   
   C   
    A    
   G   
   A   
   A   
    G    
   G   
    A    
   G   
   T   
   C   
   A   
   A   
    A    
   G   
   C   
   T   
   A   
   G   
   T   
   G   
   A   
   T   
   G   
   T   
    T    
   C   
   A   
   A   
   G   
   T   
    C    
   A   
   A   
   A   
   A   
   A   
    G    
   G   
    C    
   G   
   A   
   A   
   A   
   G   
   A   
    C    
   A   
   A   
    T    
   C   
   A   
   A   
   T   
   A   
   C   
   A   
   C   
   A   
   T   
   T   
   T   
   A   
    C    
   C   
   T   
   A   
   T   
   A   
   A   
   A   
   T   
   T   
   A   
   T   
   C   
   G   
   A   
   T   
   G   
   A   
   G   
    T    
   A   
   C   
    C    
   C   
   C   
   T   
   T   
   T   
   A   
   G   
   G   
   C   
   G   
   A   
   A   
   A   
   T   
   G   
   A   
   A   
   A   
   G   
   A   
   T   
    T    
   T   
   G   
   T   
   C   
   T   
   T   
   A   
   T   
   C   
   A   
   A   
   T   
   C   
   A   
   A   
   G   
   T   
   A   
   T   
    C    
   G   
    C    
   C   
   A   
   A   
    G    
   A   
   A   
   A   
   G   
   G   
    C    
   G   
   A   
   T   
   A   
    C    
   C   
   T   
   A   
   C   
   C   
   A   
   A   
   A   
   T   
    C    
    G    
   C   
   T   
   T   
   G   
   G   
    A    
    A    
    G    
   C   
   C   
    G    
   T   
   C   
   T   
   T   
   T   
   A   
   A   
   T   
   T   
   T   
   T   
   T   
   C   
   C   
   A   
   G   
   A   
   T   
   A   
   T   
   G   
   T   
   C   
   A   
   G   
   G   
   A   
   A   
   A   
   T   
   G   
   A   
   T   
   A   
   A   
   A   
    A    
   T   
   T   
   T   
   C   
   G   
   A   
   T   
   T   
   C   
   A   
   A   
   G   
   T   
   A   
   G   
   A   
   T   
   A   
   A   
   T   
   G   
   C   
   C   
   A   
   A   
   A   
   C   
   G   
   T   
   G   
   G   
   A   
   G   
   A   
   A   
   A   
   T   
   T   
   G   
   T   
   C   
   G   
   A   
   T   
   C   
   G   
   T   
   A   
   A   
    T    
   G   
   G   
   T   
   A   
   G   
   T   
   G   
   G   
   G   
   C   
   T   
   A   
   G   
    C    
   A   
   A   
   T   
   T   
   A   
   A   
   C   
   A   
   A   
   A   
   G   
   T   
   G   
   T   
   T   
   T   
   G   
   A   
    C    
   G   
   A   
   A   
   G   
   T   
   G   
   G   
   G   
   C   
   G   
   T   
   A   
   G   
   T   
    G    
   C   
   C   
   T   
    G    
   G   
   C   
   A   
   A   
   A   
   C   
   T   
    C    
   G   
   G   
   T   
   T   
   C   
   T   
   G   
   G   
   C   
   G   
   C   
   A   
   G   
   A   
   A   
   A   
   A   
   A   
    A    
   C   
   A   
   G   
   C   
    C    
   A   
   A   
   T   
   A   
   T   
   C   
   A   
   A   
   A   
   G   
   C   
   T   
   T   
   T   
   T   
   A   
   G   
   T   
   G   
   A   
   T   
   A   
   A   
    G    
   T   
   T   
    C    
   G   
   G   
   C   
   G   
   T   
   T   
   T   
   C   
   T   
    G    
   T   
   T   
   G   
   A   
   T   
   G   
   A   
   A   
   A   
   T   
   C   
   A   
   A   
   T   
   C   
    A    
   A   
   A   
   A   
    G    
   T   
   T   
   A   
   A   
   G   
   C   
   C   
   A   
   A   
   G   
   G   
    A    
   T   
   G   
   G   
   G   
   T   
   C   
   C   
   A   
   A   
   G   
   C   
   A   
   G   
   A   
   C   
   T   
   C   
    C    
   T   
   T   
   T   
   G   
   T   
   A   
   C   
   C   
   A   
   A   
   T   
   C   
   A   
   C   
   A   
   G   
   T   
    T    
   G   
   C   
   T   
   T   
   C   
   T   
   G   
   A   
   A   
   C   
   C   
   A   
   G   
   T   
   G   
   A   
   C   
   A   
   G   
   A   
   A   
   T   
   T   
   A   
   C   
   C   
   A   
   A   
   C   
   A   
   G   
   G   
   G   
   G   
   C   
   T   
   G   
   C   
   G   
   A   
   C   
   A   
   A   
   A   
   A   
   G   
    A    
    C    
   A   
   C   
   A   
   G   
   A   
   G   
   T   
   C   
   A   
   C   
   G   
   T   
   T   
   A   
   T   
   T   
   A   
   T   
   C   
   C   
    G    
    T    
   T   
   G   
   G   
   G   
   G   
   G   
   A   
   A   
   G   
   C   
   A   
   G   
   C   
   T   
   G   
   C   
   G   
   C   
   A   
   A   
   T   
   T   
   A   
   A   
   T   
   C   
   G   
   G   
    G    
   T   
   A   
   T   
   A   
   C   
    G    
   G   
   G   
    C    
   A   
   C   
    C    
   A   
   T   
   T   
   A   
   C   
   T   
   G   
   C   
   A   
   G   
   A   
    A    
   G   
   A   
   T   
   A   
   T   
   T   
    G    
   A   
   A   
   A   
   A   
   A   
   A   
   A   
   T   
   C   
   C   
   A   
   G   
   A   
   G   
   T   
   T   
   A   
   A   
   G   
   T   
   A   
   G   
   T   
   A   
   C   
   G   
   G   
   G   
    C    
   G   
   T   
   A   
   A   
   T   
   T   
   G   
   G   
    T    
   A   
   A   
   A   
   A   
   C   
   T   
   G   
   G   
    C    
   T   
   T   
   A   
   G   
   A   
   A   
   C   
   G   
   A   
   G   
   C   
   G   
   T   
   T   
   T   
   G   
   A   
   T   
   A   
   A   
   A   
   G   
   A   
    A    
   T   
   T   
   A   
   A   
   G   
   A   
   G   
   G   
    G    
   C   
   A   
   A   
   G   
   A   
   T   
   G   
   G   
    T    
   G   
   G   
    C    
   T   
   C   
    A    
   C   
   T   
   A   
   G   
   T   
   T   
   A   
   T   
   T   
   T   
   T   
   A   
   G   
   A   
   T   
   G   
   A   
   C   
   A   
   A   
   A   
   G   
   A   
   G   
   A   
   A   
   T   
   G   
   T   
   C   
   A   
   A   
   A   
   A   
   A   
   A   
   G   
   C   
   T   
   T   
   T   
   A   
   C   
   A   
   A   
   A   
   C   
   C   
   A   
   A   
   A   
   G   
   A   
    G    
   A   
   A   
   A   
   A   
   A   
   A   
   G   
   A   
   T   
   G   
   G   
   T   
   C   
   A   
   A   
   A   
   C   
    G    
   A   
   T   
   T   
   A   
   A   
   A   
   T   
   T   
   A   
   A   
   C   
   G   
   A   
   T   
   T   
   G   
   A   
    C    
   A   
   G   
    T    
   G   
   G   
    C    
   G   
   T   
   A   
   C   
   A   
   A   
   C   
   A   
   A   
   C   
   A   
   A   
   G   
   C   
    C    
   T   
   T   
    C    
   G   
   C   
   T   
   A   
   T   
   A   
   T   
   T   
   T   
   G   
   A   
   C   
   A   
   A   
   A   
   C   
   G   
    T    
    C    
   C   
   T   
   G   
   G   
   T   
   A   
   G   
   T   
   G   
   C   
   G   
   G   
   T   
   C   
   A   
   T   
   T   
   A   
   C   
   C   
   G   
   A   
   T   
   C   
   C   
   T   
   C   
   A   
   A   
   A   
   A   
   A   
   G   
   G   
   C   
   G   
   A   
   T   
   T   
   T   
   A   
    T    
   T   
   A   
   G   
   C   
    A    
   A   
   C   
   G   
   G   
   T   
   T   
   A   
   G   
   T   
   T   
   C   
   A   
   C   
   C   
   T   
   T   
   C   
   G   
   T   
   A   
   T   
   G   
   A   
   T   
   C   
   C   
   T   
   A   
   A   
   T   
   A   
   A   
   A   
   A   
   T   
   G   
   G   
   C   
   C   
   A   
   A   
   T   
   G   
   G   
    T    
   A   
   T   
   T   
   T   
   C   
   T   
   C   
   A   
   A   
   A   
   A   
   A   
   G   
   A   
    A    
   T   
   A   
   T   
   G   
   A   
   T   
   G   
   C   
   T   
   T   
   A   
   C   
   A   
   A   
   T   
   A   
   A   
   C   
   A   
   A   
   C   
   A   
   A   
   A   
   G   
   A   
   T   
   T   
   T   
   A   
   C   
   C   
   A   
   T   
   T   
    C    
   A   
   C   
   A   
   G   
   C   
   A   
   C   
   G   
   T   
   T   
   T   
    T    
   G   
   C   
   G   
   A   
   C   
   A   
   G   
   G   
    C    
   T   
   A   
   T   
   G   
   C   
   A   
   C   
   C   
   A   
   G   
   G   
    C    
   T   
   C   
   T   
   A   
   C   
   C   
   T   
   T   
   T   
   A   
   A   
   A   
   A   
   C   
   A   
   A   
   T   
   T   
   A   
   C   
   C   
   G   
   G   
   T   
   G   
   C   
   C   
   A   
   T   
   T   
   G   
   G   
   T   
   T   
   T   
   A   
   G   
   A   
   T   
    G    
   C   
   A   
   G   
   G   
   G   
   A   
   C   
   A   
   T   
   T   
   G   
   A   
   A   
   G   
   C   
   C   
   A   
   G   
   A   
   T   
   G   
   A   
   A   
   G   
   A   
   G   
   C   
   T   
   A   
   G   
   A   
   A   
   A   
   T   
   T   
   A   
   A   
   T   
   G   
   G   
    C    
   T   
   T   
   G   
   A   
   A   
   A   
   T   
   G   
   G   
   C   
   A   
   A   
   A   
   A   
   A   
   G   
   A   
   T   
   A   
   A   
   A   
   T   
   C   
   T   
   T   
   G   
   G   
   G   
   G   
    C    
   G   
   G   
   C   
   T   
   A   
   T   
   T   
   T   
   T   
   G   
   C   
    G    
   A   
   C   
   A   
   C   
   G   
   T   
   G   
   T   
   A   
   A   
   A   
   A   
   G   
   A   
   A   
   G   
   C   
   A   
   A   
   G   
   T   
   C   
   C   
   A   
   G   
   T   
   C   
   A   
   A   
   T   
   C   
   T   
    G    
   C   
   G   
   T   
   A   
   C   
   C   
   G   
   C   
   T   
   T   
   T   
   A   
   G   
   T   
   T   
   A   
   A   
   T   
   T   
   C   
   A   
   G   
   A   
   T   
   A   
   A   
   T   
   A   
   T   
   T   
   T   
   A   
   T   
   T   
   T   
   T   
   G   
   C   
    C    
   C   
   A   
   A   
   C   
   A   
   A   
   A   
   C   
   A   
   T   
   T   
   A   
   C   
   G   
    G    
   A   
   T   
   G   
   G   
   G   
    A    
   G   
   A   
   A   
   G   
   A   
   C   
   A   
   A   
   A   
   T   
   T   
   T   
   A   
   G   
   A   
   G   
   C   
    G    
   G   
   G   
   T   
   C   
   T   
   T   
   A   
   A   
   T   
   A   
   A   
   A   
   T   
   T   
   C   
   A   
   T   
   T   
   T   
   T   
   C   
   G   
   G   
   T   
   G   
   A   
   G   
   G   
   A   
   A   
   C   
   T   
   A   
   G   
   A   
   T   
   T   
   T   
   A   
   C   
   C   
   A   
   A   
   T   
    T    
   G   
   C   
   C   
   A   
   T   
   G   
   A   
   C   
   G   
   C   
   C   
    T    
   G   
   C   
   T   
   C   
   A   
   A   
   A   
   T   
   T   
   T   
   C   
   A   
   A   
   A   
    T    
   G   
   A   
   A   
   G   
   A   
   T   
   A   
   A   
   A   
   T   
   T   
   T   
   A   
   A   
   T   
   T   
   C   
   T   
   G   
   A   
   A   
   A   
   T   
   T   
   C   
   T   
   T   
   T   
   T   
    A    
   G   
   C   
   A   
   G   
   A   
   T   
   A   
   C   
    G    
   G   
   G   
   C   
   T   
   A   
    C    
   G   
   G   
    T    
   C   
   A   
   A   
   G   
   G   
    C    
   C   
   A   
   A   
   T   
   T   
   G   
   T   
   T   
   A   
   A   
   T   
   T   
   T   
   C   
    G    
   C   
   C   
   A   
   A   
   T   
   C   
   C   
   A   
   G   
   C   
   A   
   A   
   G   
   C   
   T   
   A   
   C   
   C   
   A   
   T   
   G   
   T   
   A   
   T   
   A   
   G   
   T   
   G   
   T   
   T   
   T   
   T   
   C   
   C   
   A   
   A   
   A   
   A   
   T   
   A   
   A   
   T   
   G   
   G   
    A    
   A   
   C   
   A   
    T    
   T   
    G    
   G   
   T   
    C    
   T   
   A   
   T   
   C   
   C   
   A   
   A   
   A   
   A   
    A    
   T   
   A   
   G   
   T   
   T   
   T   
   T   
   A   
   G   
   A   
   C   
   A   
   A   
   A   
   G   
   A   
   A   
   A   
   C   
    G    
   A   
   A   
   G   
   A   
   A   
   A   
   A   
   A   
   A   
   G   
   A   
   C   
   A   
   A   
   T   
   G   
   T   
   A   
   A   
   T   
   C   
   A   
   G   
   C   
   G   
   C   
   C   
   A   
   A   
   C   
   G   
   C   
   A   
   G   
   C   
   G   
   A   
   A   
   T   
   A   
   C   
   A   
   A   
   T   
   T   
   G   
   C   
   C   
   A   
   C   
   A   
   G   
   A   
   T   
   C   
   T   
   T   
   T   
   T   
   A   
   G   
   G   
   T   
   A   
   G   
   T   
   G   
   T   
   G   
   G   
   A   
   A   
   G   
   A   
   C   
   C   
   C   
   T   
   T   
   C   
   A   
   G   
   G   
   T   
   T   
   A   
   T   
   G   
   T   
   T   
   T   
   A   
   T   
   A   
   A   
   T   
   A   
   T   
   G   
   T   
   A   
   C   
   A   
   A   
   T   
   C   
   C   
   G   
   A   
   A   
   C   
   T   
   T   
   C   
   T   
   C   
   A   
   T   
   T   
   G   
   G   
   C   
   T   
   G   
   C   
   T   
   A   
   A   
   A   
   A   
   C   
   A   
   G   
   G   
   A   
   A   
   C   
   A   
   G   
   C   
   T   
   G   
   A   
   A   
   A   
   T   
   A   
   A   
   A   
   A   
   G   
   A   
   T   
   A   
   A   
   A   
   C   
   A   
   A   
   G   
   A   
   T   
   A   
   C   
   A   
   G   
   A   
   T   
   G   
   G   
   C   
   A   
   A   
   A   
   G   
   A   
   A   
   A   
   A   
   C   
   A   
   G   
   T   
   T   
   T   
   C   
   C   
   T   
   T   
   C   
   T   
   A   
   A   
   C   
   G   
   T   
   T   
   A   
   G   
   A   
   T   
   C   
   G   
   T   
   A   
   G   
   T   
   A   
   A   
   C   
   A   
   A   
   T   
   A   
   A   
   A   
   T   
   T   
   C   
   T   
   T   
   A   
   A   
   C   
   A   
   A   
   T   
   G   
   A   
   T   
   T   
   A   
   T   
   G   
   G   
   T   
   T   
   G   
   A   
   A   
   A   
   A   
   T   
   T   
   C   
   A   
   G   
   G   
   A   
   G   
   A   
   A   
   A   
   A   
   T   
   G   
   G   
   T   
   T   
   C   
   T   
   G   
   C   
   A   
   A   
   C   
   A   
   G   
   A   
   T   
   A   
   T   
   C   
   A   
   G   
   T   
   A   
   A   
   A   
   C   
   C   
   A   
   T   
   T   
   A   
   A   
   T   
   T   
   G   
   A   
   T   
   T   
   A   
   C   
   T   
   T   
   A   
   G   
   A   
   A   
   G   
   C   
   A   
   A   
   C   
   C   
   A   
   T   
   T   
   A   
   A   
   A   
   -   
   -   
   -   
   -   
   -   
   -   
   -   
   -   
   -   
   -   
   -   
   -   
   -   
   -   
   -   
   -   
   -   
   -   
   -   
   -   
   -   
   -   
   -   
   -   
   -   
   -   
   -   
   -   
   -   
   -   
   -   
   -   
   -   
   -   
   -   
   -   
   -   
   -   
   -   
   -   
   -   
   -   
   -   
   -   
   -   
   -   
   -   
   -   
   -   
   -   
   -   
   -   
   -   
   -   
   -   
   -   
   -   
   -   
   -   
   -   
   -   
   -   
   -   
   -   
   -   
   -   
   -   
   -   
   -   
   -   
   -   
   -   
   -   
   -   
   -   
   -   
   -   
   -   
   -   
   -   
   -   
   -   
   -   
   -   
   -   
   -   
   -   
   -   
   -   
   -   
   -   
   -   
   -   
   -   
   -   
   -   
   -   
   -   
   -   
   -   
   -   
   -   
   -   
   -   
   -   
   -   
   -   
   -   
   -   
   -   
   -   
   -   
   -   
   -   
   -   
   -   
   -   
   -   
   -   
   -   
   -   
   -   
   -   
   -   
   -   
   -   
   -   
   -   
   -   
   -   
   -   
   -   
   -   
   -   
   -   
   -   
   -   
   -   
   -   
   -   
   -   
   -   
   -   
   -   
   -   
   -   
   -   
   -   
   -   
   -   
   -   
   -   
   -   
   -   
   -   
   -   
   -   
   -   
   -   
   -   
   -   
   -   
   -   
   -   
   -   
   -   
   -   
   -   
   -   
   C   
   A   
   A   
   T   
   G   
   G   
   C   
   A   
   A   
   G   
   C   
   C   
   A   
   A   
   A   
   C   
   A   
   A   
   G   
   A   
   A   
   T   
   T   
   A   
   G   
   C   
   C   
   G   
   A   
   A   
   G   
   C   
   G   
   A   
   A   
   G   
   A   
   A   
   A   
   A   
   C   
   A   
   G   
   C   
   T   
   A   
   C   
   T   
   A   
   C   
   A   
   T   
   T   
   T   
   T   
   T   
   A   
   A   
   A   
   C   
   G   
   T   
   A   
   T   
   T   
   G   
   T   
   C   
   A   
   A   
   A   
   A   
   C   
   A   
   G   
   G   
   A   
   A   
   T   
   T   
   T   
   G   
   A   
   T   
   A   
   A   
   G   
   T   
   T   
   A   
   C   
   C   
   G   
   T   
   C   
   C   
   G   
   T   
   T   
   G   
   T   
   T   
   C   
   A   
   A   
   G   
   A   
   A   
   G   
   C   
   T   
   A   
   G   
   C   
   T   
   T   
   A   
   A   
   A   
   G   
   A   
   A   
   A   
   A   
   A   
   T   
   G   
   G   
   C   
   T   
   A   
   T   
   G   
   A   
   T   
   A   
   C   
   T   
   A   
   A   
   A   
   T   
   C   
   T   
   G   
   T   
   T   
   G   
   T   
   T   
   G   
   A   
   A   
   A   
   A   
   A   
   T   
   A   
   C   
   C   
   A   
   A   
   G   
   C   
   A   
   A   
   T   
   T   
   T   
   A   
   T   
   T   
   C   
   A   
   G   
   G   
   G   
   A   
   T   
   T   
   C   
   A   
   A   
   G   
   C   
   A   
   G   
   A   
   A   
   G   
   G   
   A   
   G   
   T   
   C   
   A   
   A   
   A   
   G   
   C   
   T   
   A   
   G   
   T   
   G   
   A   
   T   
   G   
   T   
   T   
   C   
   A   
   A   
   G   
   T   
   C   
   A   
   A   
   A   
   A   
   A   
   G   
   G   
   C   
   G   
   A   
   A   
   A   
   G   
   A   
   C   
   A   
   A   
   T   
   C   
   A   
   A   
   T   
   A   
   C   
   A   
   C   
   A   
   T   
   T   
   T   
   A   
   C   
   C   
   T   
   A   
   T   
   A   
   A   
   A   
   T   
   T   
   A   
   T   
   C   
   G   
   A   
   T   
   G   
   A   
   G   
   T   
   A   
   C   
   C   
   C   
   C   
   T   
   T   
   T   
   A   
   G   
   G   
   C   
   G   
   A   
   A   
   A   
   T   
   G   
   A   
   A   
   A   
   G   
   A   
   T   
   T   
   T   
   G   
   T   
   C   
   T   
   T   
   A   
   T   
   C   
   A   
   A   
   T   
   C   
   A   
   A   
   G   
   T   
   A   
   T   
   C   
   G   
   C   
   C   
   A   
   A   
   G   
   A   
   A   
   A   
   G   
   G   
   C   
   G   
   A   
   T   
   A   
   C   
   C   
   T   
   A   
   C   
   C   
   A   
   A   
   A   
   T   
   C   
   G   
   C   
   T   
   T   
   G   
   G   
   A   
   A   
   G   
   C   
   C   
   G   
   T   
   C   
   T   
   T   
   T   
   A   
   A   
   T   
   T   
   T   
   T   
   T   
   C   
   C   
   A   
   G   
   A   
   T   
   A   
   T   
   G   
   T   
   C   
   A   
   G   
   G   
   A   
   A   
   A   
   T   
   G   
   A   
   T   
   A   
   A   
   A   
   A   
   T   
   T   
   T   
   C   
   G   
   A   
   T   
   T   
   C   
   A   
   A   
   G   
   T   
   A   
   G   
   A   
   T   
   A   
   A   
   T   
   G   
   C   
   C   
   A   
   A   
   A   
   C   
   G   
   T   
   G   
   G   
   A   
   G   
   A   
   A   
   A   
   T   
   T   
   G   
   T   
   C   
   G   
   A   
   T   
   C   
   G   
   T   
   A   
   A   
   T   
   G   
   G   
   T   
   A   
   G   
   T   
   G   
   G   
   G   
   C   
   T   
   A   
   G   
   C   
   A   
   A   
   T   
   T   
   A   
   A   
   C   
   A   
   A   
   A   
   G   
   T   
   G   
   T   
   T   
   T   
   G   
   A   
   C   
   G   
   A   
   A   
   G   
   T   
   G   
   G   
   G   
   C   
   G   
   T   
   A   
   G   
   T   
   G   
   C   
   C   
   T   
   G   
   G   
   C   
   A   
   A   
   A   
   C   
   T   
   C   
   G   
   G   
   T   
   T   
   C   
   T   
   G   
   G   
   C   
   G   
   C   
   A   
   G   
   A   
   A   
   A   
   A   
   A   
   A   
   C   
   A   
   G   
   C   
   C   
   A   
   A   
   T   
   A   
   T   
   C   
   A   
   A   
   A   
   G   
   C   
   T   
   T   
   T   
   T   
   A   
   G   
   T   
   G   
   A   
   T   
   A   
   A   
   G   
   T   
   T   
   C   
   G   
   G   
   C   
   G   
   T   
   T   
   T   
   C   
   T   
   G   
   T   
   T   
   G   
   A   
   T   
   G   
   A   
   A   
   A   
   T   
   C   
   A   
   A   
   T   
   C   
   A   
   A   
   A   
   A   
   G   
   T   
   T   
   A   
   A   
   G   
   C   
   C   
   A   
   A   
   G   
   G   
   A   
   T   
   G   
   G   
   G   
   T   
   C   
   C   
   A   
   A   
   G   
   C   
   A   
   G   
   A   
   C   
   T   
   C   
   C   
   T   
   T   
   T   
   G   
   T   
   A   
   C   
   C   
   A   
   A   
   T   
   C   
   A   
   C   
   A   
   G   
   T   
   T   
   G   
   C   
   T   
   T   
   C   
   T   
   G   
   A   
   A   
   C   
   C   
   A   
   G   
   T   
   G   
   A   
   C   
   A   
   G   
   A   
   A   
   T   
   T   
   A   
   C   
   C   
   A   
   A   
   C   
   A   
   G   
   G   
   G   
   G   
   C   
   T   
   G   
   C   
   G   
   A   
   C   
   A   
   A   
   A   
   A   
   G   
   A   
   C   
   A   
   C   
   A   
   G   
   A   
   G   
   T   
   C   
   A   
   C   
   G   
   T   
   T   
   A   
   T   
   T   
   A   
   T   
   C   
   C   
   G   
   T   
   T   
   G   
   G   
   G   
   G   
   G   
   A   
   A   
   G   
   C   
   A   
   G   
   C   
   T   
   G   
   C   
   G   
   C   
   A   
   A   
   T   
   T   
   A   
   A   
   T   
   C   
   G   
   G   
   G   
   T   
   A   
   T   
   A   
   C   
   G   
   G   
   G   
   C   
   A   
   C   
   C   
   A   
   T   
   T   
   A   
   C   
   T   
   G   
   C   
   A   
   G   
   A   
   A   
   G   
   A   
   T   
   A   
   T   
   T   
   G   
   A   
   A   
   A   
   A   
   A   
   A   
   A   
   T   
   C   
   C   
   A   
   G   
   A   
   G   
   T   
   T   
   A   
   A   
   G   
   T   
   A   
   G   
   T   
   A   
   C   
   G   
   G   
   G   
   C   
   G   
   T   
   A   
   A   
   T   
   T   
   G   
   G   
   T   
   A   
   A   
   A   
   A   
   C   
   T   
   G   
   G   
   C   
   T   
   T   
   A   
   G   
   A   
   A   
   C   
   G   
   A   
   G   
   C   
   G   
   T   
   T   
   T   
   G   
   A   
   T   
   A   
   A   
   A   
   G   
   A   
   A   
   T   
   T   
   A   
   A   
   G   
   A   
   G   
   G   
   G   
   C   
   A   
   A   
   G   
   A   
   T   
   G   
   G   
   T   
   G   
   G   
   C   
   T   
   C   
   A   
   C   
   T   
   A   
   G   
   T   
   T   
   A   
   T   
   T   
   T   
   T   
   A   
   G   
   A   
   T   
   G   
   A   
   C   
   A   
   A   
   A   
   G   
   A   
   G   
   A   
   A   
   T   
   G   
   T   
   C   
   A   
   A   
   A   
   A   
   A   
   A   
   G   
   C   
   T   
   T   
   T   
   A   
   C   
   A   
   A   
   A   
   C   
   C   
   A   
   A   
   A   
   G   
   A   
   G   
   A   
   A   
   A   
   A   
   A   
   A   
   G   
   A   
   T   
   G   
   G   
   T   
   C   
   A   
   A   
   A   
   C   
   G   
   A   
   T   
   T   
   A   
   A   
   A   
   T   
   T   
   A   
   A   
   C   
   G   
   A   
   T   
   T   
   G   
   A   
   C   
   A   
   G   
   T   
   G   
   G   
   C   
   G   
   T   
   A   
   C   
   A   
   A   
   C   
   A   
   A   
   C   
   A   
   A   
   G   
   C   
   C   
   T   
   T   
   C   
   G   
   C   
   T   
   A   
   T   
   A   
   T   
   T   
   T   
   G   
   A   
   C   
   A   
   A   
   A   
   C   
   G   
   T   
   C   
   C   
   T   
   G   
   G   
   T   
   A   
   G   
   T   
   G   
   C   
   G   
   G   
   T   
   C   
   A   
   T   
   T   
   A   
   C   
   C   
   G   
   A   
   T   
   C   
   C   
   T   
   C   
   A   
   A   
   A   
   A   
   A   
   G   
   G   
   C   
   G   
   A   
   T   
   T   
   T   
   A   
   T   
   T   
   A   
   G   
   C   
   A   
   A   
   C   
   G   
   G   
   T   
   T   
   A   
   G   
   T   
   T   
   C   
   A   
   C   
   C   
   T   
   T   
   C   
   G   
   T   
   A   
   T   
   G   
   A   
   T   
   C   
   C   
   T   
   A   
   A   
   T   
   A   
   A   
   A   
   A   
   T   
   G   
   G   
   C   
   C   
   A   
   A   
   T   
   G   
   G   
   T   
   A   
   T   
   T   
   T   
   C   
   T   
   C   
   A   
   A   
   A   
   A   
   A   
   G   
   A   
   A   
   T   
   A   
   T   
   G   
   A   
   T   
   G   
   C   
   T   
   T   
   A   
   C   
   A   
   A   
   T   
   A   
   A   
   C   
   A   
   A   
   C   
   A   
   A   
   A   
   G   
   A   
   T   
   T   
   T   
   A   
   C   
   C   
   A   
   T   
   T   
   C   
   A   
   C   
   A   
   G   
   C   
   A   
   C   
   G   
   T   
   T   
   T   
   T   
   G   
   C   
   G   
   A   
   C   
   A   
   G   
   G   
   C   
   T   
   A   
   T   
   G   
   C   
   A   
   C   
   C   
   A   
   G   
   G   
   C   
   T   
   C   
   T   
   A   
   C   
   C   
   T   
   T   
   T   
   A   
   A   
   A   
   A   
   C   
   A   
   A   
   T   
   T   
   A   
   C   
   C   
   G   
   G   
   T   
   G   
   C   
   C   
   A   
   T   
   T   
   G   
   G   
   T   
   T   
   T   
   A   
   G   
   A   
   T   
   G   
   C   
   A   
   G   
   G   
   G   
   A   
   C   
   A   
   T   
   T   
   G   
   A   
   A   
   G   
   C   
   C   
   A   
   G   
   A   
   T   
   G   
   A   
   A   
   G   
   A   
   G   
   C   
   T   
   A   
   G   
   A   
   A   
   A   
   T   
   T   
   A   
   A   
   T   
   G   
   G   
   C   
   T   
   T   
   G   
   A   
   A   
   A   
   T   
   G   
   G   
   C   
   A   
   A   
   A   
   A   
   A   
   G   
   A   
   T   
   A   
   A   
   A   
   T   
   C   
   T   
   T   
   G   
   G   
   G   
   G   
   C   
   G   
   G   
   C   
   T   
   A   
   T   
   T   
   T   
   T   
   G   
   C   
   G   
   A   
   C   
   A   
   C   
   G   
   T   
   G   
   T   
   A   
   A   
   A   
   A   
   G   
   A   
   A   
   G   
   C   
   A   
   A   
   G   
   T   
   C   
   C   
   A   
   G   
   T   
   C   
   A   
   A   
   T   
   C   
   T   
   G   
   C   
   G   
   T   
   A   
   C   
   C   
   G   
   C   
   T   
   T   
   T   
   A   
   G   
   T   
   T   
   A   
   A   
   T   
   T   
   C   
   A   
   G   
   A   
   T   
   A   
   A   
   T   
   A   
   T   
   T   
   T   
   A   
   T   
   T   
   T   
   T   
   G   
   C   
   C   
   C   
   A   
   A   
   C   
   A   
   A   
   A   
   C   
   A   
   T   
   T   
   A   
   C   
   G   
   G   
   A   
   T   
   G   
   G   
   G   
   A   
   G   
   A   
   A   
   G   
   A   
   C   
   A   
   A   
   A   
   T   
   T   
   T   
   A   
   G   
   A   
   G   
   C   
   G   
   G   
   G   
   T   
   C   
   T   
   T   
   A   
   A   
   T   
   A   
   A   
   A   
   T   
   T   
   C   
   A   
   T   
   T   
   T   
   T   
   C   
   G   
   G   
   T   
   G   
   A   
   G   
   G   
   A   
   A   
   C   
   T   
   A   
   G   
   A   
   T   
   T   
   T   
   A   
   C   
   C   
   A   
   A   
   T   
   T   
   G   
   C   
   C   
   A   
   T   
   G   
   A   
   C   
   G   
   C   
   C   
   T   
   G   
   C   
   T   
   C   
   A   
   A   
   A   
   T   
   T   
   T   
   C   
   A   
   A   
   A   
   T   
   G   
   A   
   A   
   G   
   A   
   T   
   A   
   A   
   A   
   T   
   T   
   T   
   A   
   A   
   T   
   T   
   C   
   T   
   G   
   A   
   A   
   A   
   T   
   T   
   C   
   T   
   T   
   T   
   T   
   A   
   G   
   C   
   A   
   G   
   A   
   T   
   A   
   C   
   G   
   G   
   G   
   C   
   T   
   A   
   C   
   G   
   G   
   T   
   C   
   A   
   A   
   G   
   G   
   C   
   C   
   A   
   A   
   T   
   T   
   G   
   T   
   T   
   A   
   A   
   T   
   T   
   T   
   C   
   G   
   C   
   C   
   A   
   A   
   T   
   C   
   C   
   A   
   G   
   C   
   A   
   A   
   G   
   C   
   T   
   A   
   C   
   C   
   A   
   T   
   G   
   T   
   A   
   T   
   A   
   G   
   T   
   G   
   T   
   T   
   T   
   T   
   C   
   C   
   A   
   A   
   A   
   A   
   T   
   A   
   A   
   T   
   G   
   G   
   A   
   A   
   C   
   A   
   T   
   T   
   G   
   G   
   T   
   C   
   T   
   A   
   T   
   C   
   C   
   A   
   A   
   A   
   A   
   A   
   T   
   A   
   G   
   T   
   T   
   T   
   T   
   A   
   G   
   A   
   C   
   A   
   A   
   A   
   G   
   A   
   A   
   A   
   C   
   G   
   A   
   A   
   G   
   A   
   A   
   A   
   A   
   A   
   A   
   G   
   A   
   C   
   A   
   A   
   T   
   G   
   T   
   A   
   A   
   T   
   C   
   A   
   G   
   C   
   G   
   C   
   C   
   A   
   A   
   C   
   G   
   C   
   A   
   G   
   C   
   G   
   A   
   A   
   T   
   A   
   C   
   A   
   A   
   T   
   T   
   G   
   C   
   C   
   A   
   C   
   A   
   G   
   A   
   T   
   C   
   T   
   T   
   T   
   T   
   A   
   G   
   G   
   T   
   A   
   G   
   T   
   G   
   T   
   G   
   G   
   A   
   A   
   G   
   A   
   C   
   C   
   C   
   T   
   T   
   C   
   A   
   G   
   G   
   T   
   T   
   A   
   T   
   G   
   T   
   T   
   T   
   A   
   T   
   A   
   A   
   T   
   A   
   T   
   G   
   T   
   A   
   C   
   A   
   A   
   T   
   C   
   C   
   G   
   A   
   A   
   C   
   T   
   T   
   C   
   T   
   C   
   A   
   T   
   T   
   G   
   G   
   C   
   T   
   G   
   C   
   T   
   A   
   A   
   A   
   A   
   C   
   A   
   G   
   G   
   A   
   A   
   C   
   A   
   G   
   C   
   T   
   G   
   A   
   A   
   A   
   T   
   A   
   A   
   A   
   A   
   G   
   A   
   T   
   A   
   A   
   A   
   C   
   A   
   A   
   G   
   A   
   T   
   A   
   C   
   A   
   G   
   A   
   T   
   G   
   G   
   C   
   A   
   A   
   A   
   G   
   A   
   A   
   A   
   A   
   C   
   A   
   G   
   T   
   T   
   T   
   C   
   C   
   T   
   T   
   C   
   T   
   A   
   A   
   C   
   G   
   T   
   T   
   A   
   G   
   A   
   T   
   C   
   G   
   T   
   A   
   G   
   T   
   A   
   A   
   C   
   A   
   A   
   T   
   A   
   A   
   A   
   T   
   T   
   C   
   T   
   T   
   A   
   A   
   C   
   A   
   A   
   T   
   G   
   A   
   T   
   T   
   A   
   T   
   G   
   G   
   T   
   T   
   G   
   A   
   A   
   A   
   A   
   T   
   T   
   C   
   A   
   G   
   G   
   A   
   G   
   A   
   A   
   A   
   A   
   T   
   G   
   G   
   T   
   T   
   C   
   T   
   G   
   C   
   A   
   A   
   C   
   A   
   G   
   A   
   T   
   A   
   T   
   C   
   A   
   G   
   T   
   A   
   A   
   A   
   C   
   C   
   A   
   T   
   T   
   A   
   A   
   T   
   T   
   G   
   A   
   T   
   T   
   A   
   C   
   T   
   T   
   A   
   G   
   A   
   A   
   G   
   C   
   A   
   A   
   C   
   C   
   A   
   T   
   T   
   A   
   A   
   A   
   -   
   -   
   -   
   -   
   -   
   -   
   -   
   -   
   -   
   -   
   -   
   -   
   -   
   -   
   -   
   -   
   -   
   -   
   -   
   -   
   -   
   -   
   -   
   -   
   -   
   -   
   -   
   -   
   -   
   -   
   -   
   -   
   -   
   -   
   -   
   -   
   -   
   -   
   -   
   -   
   -   
   -   
   -   
   -   
   -   
   -   
   -   
   -   
   -   
   -   
   -   
   -   
   -   
   -   
   -   
   -   
   -   
   -   
   -   
   -   
   -   
 

 
   N55724   
   -   
   -   
   -   
   -   
   -   
   -   
   -   
   -   
   -   
   -   
   -   
   -   
   -   
   -   
   -   
   -   
   -   
   -   
   -   
   -   
   -   
   -   
   -   
   -   
   -   
   -   
   -   
   -   
   -   
   -   
   -   
   -   
   -   
   -   
   -   
   -   
   -   
   -   
   -   
   -   
   -   
   -   
   -   
   -   
   -   
   -   
   -   
   -   
   -   
   -   
   -   
   -   
   -   
   -   
   -   
   -   
   -   
   -   
   -   
   -   
   -   
   -   
   -   
   -   
   -   
   -   
   -   
   -   
   -   
   -   
   -   
   -   
   -   
   -   
   -   
   -   
   -   
   -   
   -   
   -   
   -   
   -   
   -   
   -   
   -   
   -   
   -   
   -   
   -   
   -   
   -   
   -   
   -   
   -   
   -   
   -   
   -   
   -   
   -   
   -   
   -   
   -   
   -   
   -   
   -   
   -   
   -   
   -   
   C   
   A   
   A   
   T   
   G   
   G   
   C   
   A   
   A   
   G   
   C   
   C   
   A   
   A   
   A   
   C   
   A   
   A   
   G   
   A   
    A    
   T   
   T   
   A   
   G   
   C   
    C    
   G   
   A   
   A   
   G   
   C   
   G   
   A   
   A   
   G   
   A   
   A   
   A   
   A   
    C    
   A   
   G   
   C   
    T    
   A   
    C    
   T   
    A    
    C    
   A   
   T   
   T   
   T   
   T   
   T   
   A   
   A   
   A   
    C    
   G   
   T   
   A   
   T   
   T   
   G   
    T    
   C   
   A   
   A   
   A   
   A   
   C   
   A   
   G   
    G    
   A   
   A   
   T   
   T   
   T   
   G   
   A   
   T   
   A   
   A   
   G   
   T   
   T   
   A   
   C   
   C   
    G    
   T   
   C   
   C   
   G   
   T   
   T   
    G    
   T   
   T   
   C   
   A   
   A   
   G   
   A   
   A   
    G    
   C   
   T   
   A   
   G   
   C   
   T   
   T   
   A   
   A   
   A   
   G   
   A   
   A   
   A   
   A   
   A   
   T   
   G   
   G   
    C    
   T   
   A   
   T   
   G   
   A   
   T   
   A   
   C   
   T   
   A   
   A   
   A   
   T   
   C   
    T    
   G   
   T   
   T   
   G   
   T   
   T   
   G   
   A   
   A   
   A   
   A   
   A   
   T   
   A   
   C   
   C   
   A   
   A   
   G   
   C   
   A   
   A   
   T   
   T   
   T   
   A   
   T   
   T   
   C   
   A   
   G   
   G   
   G   
   A   
   T   
   T   
   C   
   A   
   A   
   G   
   C   
    A    
   G   
   A   
   A   
    G    
   G   
    A    
   G   
   T   
   C   
   A   
   A   
    A    
   G   
   C   
   T   
   A   
   G   
   T   
   G   
   A   
   T   
   G   
   T   
    T    
   C   
   A   
   A   
   G   
   T   
    C    
   A   
   A   
   A   
   A   
   A   
    G    
   G   
    C    
   G   
   A   
   A   
   A   
   G   
   A   
    C    
   A   
   A   
    T    
   C   
   A   
   A   
   T   
   A   
   C   
   A   
   C   
   A   
   T   
   T   
   T   
   A   
    C    
   C   
   T   
   A   
   T   
   A   
   A   
   A   
   T   
   T   
   A   
   T   
   C   
   G   
   A   
   T   
   G   
   A   
   G   
    C    
   A   
   C   
    T    
   C   
   C   
   T   
   T   
   T   
   A   
   G   
   G   
   C   
   G   
   A   
   A   
   A   
   T   
   G   
   A   
   A   
   A   
   G   
   A   
   T   
    T    
   T   
   G   
   T   
   C   
   T   
   T   
   A   
   T   
   C   
   A   
   A   
   T   
   C   
   A   
   A   
   G   
   T   
   A   
   T   
    C    
   G   
    C    
   C   
   A   
   A   
    G    
   A   
   A   
   A   
   G   
   G   
    C    
   G   
   A   
   T   
   A   
    C    
   C   
   T   
   A   
   C   
   C   
   A   
   A   
   A   
   T   
    C    
    G    
   C   
   T   
   T   
   G   
   G   
    A    
    A    
    G    
   C   
   C   
    G    
   T   
   C   
   T   
   T   
   T   
   A   
   A   
   T   
   T   
   T   
   T   
   T   
   C   
   C   
   A   
   G   
   A   
   T   
   A   
   T   
   G   
   T   
   C   
   A   
   G   
   G   
   A   
   A   
   A   
   T   
   G   
   A   
   T   
   A   
   A   
   A   
    A    
   T   
   T   
   T   
   C   
   G   
   A   
   T   
   T   
   C   
   A   
   A   
   G   
   T   
   A   
   G   
   A   
   T   
   A   
   A   
   T   
   G   
   C   
   C   
   A   
   A   
   A   
   C   
   G   
   T   
   G   
   G   
   A   
   G   
   A   
   A   
   A   
   T   
   T   
   G   
   T   
   C   
   G   
   A   
   T   
   C   
   G   
   T   
   A   
   A   
    T    
   G   
   G   
   T   
   A   
   G   
   T   
   G   
   G   
   G   
   C   
   T   
   A   
   G   
    C    
   A   
   A   
   T   
   T   
   A   
   A   
   C   
   A   
   A   
   A   
   G   
   T   
   G   
   T   
   T   
   T   
   G   
   A   
    C    
   G   
   A   
   A   
   G   
   T   
   G   
   G   
   G   
   C   
   G   
   T   
   A   
   G   
   T   
    G    
   C   
   C   
   T   
    G    
   G   
   C   
   A   
   A   
   A   
   C   
   T   
    C    
   G   
   G   
   T   
   T   
   C   
   T   
   G   
   G   
   C   
   G   
   C   
   A   
   G   
   A   
   A   
   A   
   A   
   A   
    A    
   C   
   A   
   G   
   C   
    C    
   A   
   A   
   T   
   A   
   T   
   C   
   A   
   A   
   A   
   G   
   C   
   T   
   T   
   T   
   T   
   A   
   G   
   T   
   G   
   A   
   T   
   A   
   A   
    A    
   T   
   T   
    C    
   G   
   G   
   C   
   G   
   T   
   T   
   T   
   C   
   T   
    G    
   T   
   T   
   G   
   A   
   T   
   G   
   A   
   A   
   A   
   T   
   C   
   A   
   A   
   T   
   C   
    A    
   A   
   A   
   A   
    G    
   T   
   T   
   A   
   A   
   G   
   C   
   C   
   A   
   A   
   G   
   G   
    A    
   T   
   G   
   G   
   G   
   T   
   C   
   C   
   A   
   A   
   G   
   C   
   A   
   G   
   A   
   C   
   T   
   C   
    C    
   T   
   T   
   T   
   G   
   T   
   A   
   C   
   C   
   A   
   A   
   T   
   C   
   A   
   C   
   A   
   G   
   T   
    C    
   G   
   C   
   T   
   T   
   C   
   T   
   G   
   A   
   A   
   C   
   C   
   A   
   G   
   T   
   G   
   A   
   C   
   A   
   G   
   A   
   A   
   T   
   T   
   A   
   C   
   C   
   A   
   A   
   C   
   A   
   G   
   G   
   G   
   G   
   C   
   T   
   G   
   C   
   G   
   A   
   C   
   A   
   A   
   A   
   A   
   G   
    A    
    T    
   A   
   C   
   A   
   G   
   A   
   G   
   T   
   C   
   A   
   C   
   G   
   T   
   T   
   A   
   T   
   T   
   A   
   T   
   C   
   C   
    G    
    C    
   T   
   G   
   G   
   G   
   G   
   G   
   A   
   A   
   G   
   C   
   A   
   G   
   C   
   T   
   G   
   C   
   G   
   C   
   A   
   A   
   T   
   T   
   A   
   A   
   T   
   C   
   G   
   G   
    G    
   T   
   A   
   T   
   A   
   C   
    G    
   G   
   G   
    C    
   A   
   C   
    C    
   A   
   T   
   T   
   A   
   C   
   T   
   G   
   C   
   A   
   G   
   A   
    A    
   G   
   A   
   T   
   A   
   T   
   T   
    G    
   A   
   A   
   A   
   A   
   A   
   A   
   A   
   T   
   C   
   C   
   A   
   G   
   A   
   G   
   T   
   T   
   A   
   A   
   G   
   T   
   A   
   G   
   T   
   A   
   C   
   G   
   G   
   G   
    C    
   G   
   T   
   A   
   A   
   T   
   T   
   G   
   G   
    T    
   A   
   A   
   A   
   A   
   C   
   T   
   G   
   G   
    T    
   T   
   T   
   A   
   G   
   A   
   A   
   C   
   G   
   A   
   G   
   C   
   G   
   T   
   T   
   T   
   G   
   A   
   T   
   A   
   A   
   A   
   G   
   A   
    A    
   T   
   T   
   A   
   A   
   G   
   A   
   G   
   G   
    G    
   C   
   A   
   A   
   G   
   A   
   T   
   G   
   G   
    T    
   G   
   G   
    C    
   T   
   C   
    A    
   C   
   T   
   A   
   G   
   T   
   T   
   A   
   T   
   T   
   T   
   T   
   A   
   G   
   A   
   T   
   G   
   A   
   C   
   A   
   A   
   A   
   G   
   A   
   G   
   A   
   A   
   T   
   G   
   T   
   C   
   A   
   A   
   A   
   A   
   A   
   A   
   G   
   C   
   T   
   T   
   T   
   A   
   C   
   A   
   A   
   A   
   C   
   C   
   A   
   A   
   A   
   G   
   A   
    G    
   A   
   A   
   A   
   A   
   A   
   A   
   G   
   A   
   T   
   G   
   G   
   T   
   C   
   A   
   A   
   A   
   C   
    G    
   A   
   T   
   T   
   A   
   A   
   A   
   T   
   T   
   A   
   A   
   C   
   G   
   A   
   T   
   T   
   G   
   A   
    C    
   A   
   G   
    T    
   G   
   G   
    C    
   G   
   T   
   A   
   C   
   A   
   A   
   C   
   A   
   A   
   C   
   A   
   A   
   G   
   C   
    C    
   T   
   T   
    C    
   G   
   C   
   T   
   A   
   T   
   A   
   T   
   T   
   T   
   G   
   A   
   C   
   A   
   A   
   A   
   C   
   G   
    T    
    C    
   C   
   T   
   G   
   G   
   T   
   A   
   G   
   T   
   G   
   C   
   G   
   G   
   T   
   C   
   A   
   T   
   T   
   A   
   C   
   C   
   G   
   A   
   T   
   C   
   C   
   T   
   C   
   A   
   A   
   A   
   A   
   A   
   G   
   G   
   C   
   G   
   A   
   T   
   T   
   T   
   A   
    T    
   T   
   A   
   G   
   C   
    A    
   A   
   C   
   G   
   G   
   T   
   T   
   A   
   G   
   T   
   T   
   C   
   A   
   C   
   C   
   T   
   T   
   C   
   G   
   T   
   A   
   T   
   G   
   A   
   T   
   C   
   C   
   T   
   A   
   A   
   T   
   A   
   A   
   A   
   A   
   T   
   G   
   G   
   C   
   C   
   A   
   A   
   T   
   G   
   G   
    T    
   A   
   T   
   T   
   T   
   C   
   T   
   C   
   A   
   A   
   A   
   A   
   A   
   G   
   A   
    A    
   T   
   A   
   T   
   G   
   A   
   T   
   G   
   C   
   T   
   T   
   A   
   C   
   A   
   A   
   T   
   A   
   A   
   C   
   A   
   A   
   C   
   A   
   A   
   A   
   G   
   A   
   T   
   T   
   T   
   A   
   C   
   C   
   A   
   T   
   T   
    C    
   A   
   C   
   A   
   G   
   C   
   A   
   C   
   G   
   T   
   T   
   T   
    T    
   G   
   C   
   G   
   A   
   C   
   A   
   G   
   G   
    C    
   T   
   A   
   T   
   G   
   C   
   A   
   C   
   C   
   A   
   G   
   G   
    C    
   T   
   C   
   T   
   A   
   C   
   C   
   T   
   T   
   T   
   A   
   A   
   A   
   A   
   C   
   A   
   A   
   T   
   T   
   A   
   C   
   C   
   G   
   G   
   T   
   G   
   C   
   C   
   A   
   T   
   T   
   G   
   G   
   T   
   T   
   T   
   A   
   G   
   A   
   T   
    G    
   C   
   A   
   G   
   G   
   G   
   A   
   C   
   A   
   T   
   T   
   G   
   A   
   A   
   G   
   C   
   C   
   A   
   G   
   A   
   T   
   G   
   A   
   A   
   G   
   A   
   G   
   C   
   T   
   A   
   G   
   A   
   A   
   A   
   T   
   T   
   A   
   A   
   T   
   G   
   G   
    T    
   T   
   T   
   G   
   A   
   A   
   A   
   T   
   G   
   G   
   C   
   A   
   A   
   A   
   A   
   A   
   G   
   A   
   T   
   A   
   A   
   A   
   T   
   C   
   T   
   T   
   G   
   G   
   G   
   G   
    C    
   G   
   G   
   C   
   T   
   A   
   T   
   T   
   T   
   T   
   G   
   C   
    G    
   A   
   C   
   A   
   C   
   G   
   T   
   G   
   T   
   A   
   A   
   A   
   A   
   G   
   A   
   A   
   G   
   C   
   A   
   A   
   G   
   T   
   C   
   C   
   A   
   G   
   T   
   C   
   A   
   A   
   T   
   C   
   T   
    G    
   C   
   G   
   T   
   A   
   C   
   C   
   G   
   C   
   T   
   T   
   T   
   A   
   G   
   T   
   T   
   A   
   A   
   T   
   T   
   C   
   A   
   G   
   A   
   T   
   A   
   A   
   T   
   A   
   T   
   T   
   T   
   A   
   T   
   T   
   T   
   T   
   G   
   C   
    C    
   C   
   A   
   A   
   C   
   A   
   A   
   A   
   C   
   A   
   T   
   T   
   A   
   C   
   G   
    G    
   A   
   T   
   G   
   G   
   G   
    A    
   G   
   A   
   A   
   G   
   A   
   C   
   A   
   A   
   A   
   T   
   T   
   T   
   A   
   G   
   A   
   G   
   C   
    G    
   G   
   G   
   T   
   C   
   T   
   T   
   A   
   A   
   T   
   A   
   A   
   A   
   T   
   T   
   C   
   A   
   T   
   T   
   T   
   T   
   C   
   G   
   G   
   T   
   G   
   A   
   G   
   G   
   A   
   A   
   C   
   T   
   A   
   G   
   A   
   T   
   T   
   T   
   A   
   C   
   C   
   A   
   A   
   T   
    T    
   G   
   C   
   C   
   A   
   T   
   G   
   A   
   C   
   G   
   C   
   C   
    T    
   G   
   C   
   T   
   C   
   A   
   A   
   A   
   T   
   T   
   T   
   C   
   A   
   A   
   A   
    T    
   G   
   A   
   A   
   G   
   A   
   T   
   A   
   A   
   A   
   T   
   T   
   T   
   A   
   A   
   T   
   T   
   C   
   T   
   G   
   A   
   A   
   A   
   T   
   T   
   C   
   T   
   T   
   T   
   T   
    A    
   G   
   C   
   A   
   G   
   A   
   T   
   A   
   C   
    G    
   G   
   G   
   C   
   T   
   A   
    C    
   G   
   G   
    T    
   C   
   A   
   A   
   G   
   G   
    T    
   C   
   A   
   A   
   T   
   T   
   G   
   T   
   T   
   A   
   A   
   T   
   T   
   T   
   C   
    G    
   C   
   C   
   A   
   A   
   T   
   C   
   C   
   A   
   G   
   C   
   A   
   A   
   G   
   C   
   T   
   A   
   C   
   C   
   A   
   T   
   G   
   T   
   A   
   T   
   A   
   G   
   T   
   G   
   T   
   T   
   T   
   T   
   C   
   C   
   A   
   A   
   A   
   A   
   T   
   A   
   A   
   T   
   G   
   G   
    A    
   A   
   C   
   A   
    T    
   T   
    A    
   G   
   T   
    C    
   T   
   A   
   T   
   C   
   C   
   A   
   A   
   A   
   A   
    C    
   T   
   A   
   G   
   T   
   T   
   T   
   T   
   A   
   G   
   A   
   C   
   A   
   A   
   A   
   G   
   A   
   A   
   A   
   C   
    G    
   A   
   A   
   G   
   A   
   A   
   A   
   A   
   A   
   A   
   G   
   A   
   C   
   A   
   A   
   T   
   G   
   T   
   A   
   A   
   T   
   C   
   A   
   G   
   C   
   G   
   C   
   C   
   A   
   A   
   C   
   G   
   C   
   A   
   G   
   C   
   G   
   A   
   A   
   T   
   A   
   C   
   A   
   A   
   T   
   T   
   G   
   C   
   C   
   A   
   C   
   A   
   G   
   A   
   T   
   C   
   T   
   T   
   T   
   T   
   A   
   G   
   G   
   T   
   A   
   G   
   T   
   G   
   T   
   G   
   G   
   A   
   A   
   G   
   A   
   T   
   C   
   C   
   T   
   T   
   C   
   A   
   G   
   G   
   T   
   T   
   A   
   T   
   G   
   T   
   T   
   T   
   A   
   T   
   A   
   A   
   T   
   A   
   T   
   G   
   T   
   A   
   C   
   A   
   A   
   T   
   C   
   C   
   G   
   A   
   A   
   C   
   T   
   T   
   C   
   T   
   C   
   A   
   T   
   T   
   G   
   G   
   C   
   T   
   G   
   C   
   T   
   A   
   A   
   A   
   A   
   C   
   A   
   G   
   G   
   A   
   A   
   C   
   A   
   G   
   C   
   T   
   G   
   A   
   A   
   A   
   T   
   A   
   A   
   A   
   A   
   G   
   A   
   T   
   A   
   A   
   A   
   C   
   A   
   A   
   G   
   A   
   T   
   A   
   C   
   A   
   G   
   A   
   T   
   G   
   G   
   C   
   A   
   A   
   A   
   G   
   A   
   A   
   A   
   A   
   C   
   A   
   G   
   T   
   T   
   T   
   C   
   C   
   T   
   T   
   C   
   T   
   A   
   A   
   C   
   G   
   T   
   T   
   A   
   G   
   A   
   T   
   C   
   G   
   T   
   A   
   G   
   T   
   A   
   A   
   C   
   A   
   A   
   T   
   A   
   A   
   A   
   T   
   T   
   C   
   T   
   T   
   A   
   A   
   C   
   A   
   A   
   T   
   G   
   A   
   T   
   T   
   A   
   T   
   G   
   G   
   T   
   T   
   G   
   A   
   A   
   A   
   A   
   T   
   T   
   C   
   A   
   G   
   G   
   A   
   G   
   A   
   A   
   A   
   A   
   T   
   G   
   G   
   T   
   T   
   C   
   T   
   G   
   C   
   A   
   A   
   C   
   A   
   G   
   A   
   T   
   A   
   T   
   C   
   A   
   G   
   T   
   A   
   A   
   A   
   C   
   C   
   A   
   T   
   T   
   A   
   A   
   T   
   T   
   G   
   A   
   T   
   T   
   A   
   C   
   T   
   T   
   A   
   G   
   A   
   A   
   G   
   C   
   A   
   A   
   C   
   C   
   A   
   T   
   T   
   A   
   A   
   A   
   -   
   -   
   -   
   -   
   -   
   -   
   -   
   -   
   -   
   -   
   -   
   -   
   -   
   -   
   -   
   -   
   -   
   -   
   -   
   -   
   -   
   -   
   -   
   -   
   -   
   -   
   -   
   -   
   -   
   -   
   -   
   -   
   -   
   -   
   -   
   -   
   -   
   -   
   -   
   -   
   -   
   -   
   -   
   -   
   -   
   -   
   -   
   -   
   -   
   -   
   -   
   -   
   -   
   -   
   -   
   -   
   -   
   -   
   -   
   -   
   -   
   -   
   -   
   -   
   -   
   -   
   -   
   -   
   -   
   -   
   -   
   -   
   -   
   -   
   -   
   -   
   -   
   -   
   -   
   -   
   -   
   -   
   -   
   -   
   -   
   -   
   -   
   -   
   -   
   -   
   -   
   -   
   -   
   -   
   -   
   -   
   -   
   -   
   -   
   -   
   -   
   -   
   -   
   -   
   -   
   -   
   -   
   -   
   -   
   -   
   -   
   -   
   -   
   -   
   -   
   -   
   -   
   -   
   -   
   -   
   -   
   -   
   -   
   -   
   -   
   -   
   -   
   -   
   -   
   -   
   -   
   -   
   -   
   -   
   -   
   -   
   -   
   -   
   -   
   -   
   -   
   -   
   -   
   -   
   -   
   -   
   -   
   -   
   -   
   -   
   -   
   -   
   -   
   -   
   -   
   -   
   -   
   -   
   -   
   -   
   -   
   -   
   -   
   -   
   -   
   -   
   -   
   -   
   -   
   C   
   A   
   A   
   T   
   G   
   G   
   C   
   A   
   A   
   G   
   C   
   C   
   A   
   A   
   A   
   C   
   A   
   A   
   G   
   A   
   A   
   T   
   T   
   A   
   G   
   C   
   C   
   G   
   A   
   A   
   G   
   C   
   G   
   A   
   A   
   G   
   A   
   A   
   A   
   A   
   C   
   A   
   G   
   C   
   T   
   A   
   C   
   T   
   A   
   C   
   A   
   T   
   T   
   T   
   T   
   T   
   A   
   A   
   A   
   C   
   G   
   T   
   A   
   T   
   T   
   G   
   T   
   C   
   A   
   A   
   A   
   A   
   C   
   A   
   G   
   G   
   A   
   A   
   T   
   T   
   T   
   G   
   A   
   T   
   A   
   A   
   G   
   T   
   T   
   A   
   C   
   C   
   G   
   T   
   C   
   C   
   G   
   T   
   T   
   G   
   T   
   T   
   C   
   A   
   A   
   G   
   A   
   A   
   G   
   C   
   T   
   A   
   G   
   C   
   T   
   T   
   A   
   A   
   A   
   G   
   A   
   A   
   A   
   A   
   A   
   T   
   G   
   G   
   C   
   T   
   A   
   T   
   G   
   A   
   T   
   A   
   C   
   T   
   A   
   A   
   A   
   T   
   C   
   T   
   G   
   T   
   T   
   G   
   T   
   T   
   G   
   A   
   A   
   A   
   A   
   A   
   T   
   A   
   C   
   C   
   A   
   A   
   G   
   C   
   A   
   A   
   T   
   T   
   T   
   A   
   T   
   T   
   C   
   A   
   G   
   G   
   G   
   A   
   T   
   T   
   C   
   A   
   A   
   G   
   C   
   A   
   G   
   A   
   A   
   G   
   G   
   A   
   G   
   T   
   C   
   A   
   A   
   A   
   G   
   C   
   T   
   A   
   G   
   T   
   G   
   A   
   T   
   G   
   T   
   T   
   C   
   A   
   A   
   G   
   T   
   C   
   A   
   A   
   A   
   A   
   A   
   G   
   G   
   C   
   G   
   A   
   A   
   A   
   G   
   A   
   C   
   A   
   A   
   T   
   C   
   A   
   A   
   T   
   A   
   C   
   A   
   C   
   A   
   T   
   T   
   T   
   A   
   C   
   C   
   T   
   A   
   T   
   A   
   A   
   A   
   T   
   T   
   A   
   T   
   C   
   G   
   A   
   T   
   G   
   A   
   G   
   C   
   A   
   C   
   T   
   C   
   C   
   T   
   T   
   T   
   A   
   G   
   G   
   C   
   G   
   A   
   A   
   A   
   T   
   G   
   A   
   A   
   A   
   G   
   A   
   T   
   T   
   T   
   G   
   T   
   C   
   T   
   T   
   A   
   T   
   C   
   A   
   A   
   T   
   C   
   A   
   A   
   G   
   T   
   A   
   T   
   C   
   G   
   C   
   C   
   A   
   A   
   G   
   A   
   A   
   A   
   G   
   G   
   C   
   G   
   A   
   T   
   A   
   C   
   C   
   T   
   A   
   C   
   C   
   A   
   A   
   A   
   T   
   C   
   G   
   C   
   T   
   T   
   G   
   G   
   A   
   A   
   G   
   C   
   C   
   G   
   T   
   C   
   T   
   T   
   T   
   A   
   A   
   T   
   T   
   T   
   T   
   T   
   C   
   C   
   A   
   G   
   A   
   T   
   A   
   T   
   G   
   T   
   C   
   A   
   G   
   G   
   A   
   A   
   A   
   T   
   G   
   A   
   T   
   A   
   A   
   A   
   A   
   T   
   T   
   T   
   C   
   G   
   A   
   T   
   T   
   C   
   A   
   A   
   G   
   T   
   A   
   G   
   A   
   T   
   A   
   A   
   T   
   G   
   C   
   C   
   A   
   A   
   A   
   C   
   G   
   T   
   G   
   G   
   A   
   G   
   A   
   A   
   A   
   T   
   T   
   G   
   T   
   C   
   G   
   A   
   T   
   C   
   G   
   T   
   A   
   A   
   T   
   G   
   G   
   T   
   A   
   G   
   T   
   G   
   G   
   G   
   C   
   T   
   A   
   G   
   C   
   A   
   A   
   T   
   T   
   A   
   A   
   C   
   A   
   A   
   A   
   G   
   T   
   G   
   T   
   T   
   T   
   G   
   A   
   C   
   G   
   A   
   A   
   G   
   T   
   G   
   G   
   G   
   C   
   G   
   T   
   A   
   G   
   T   
   G   
   C   
   C   
   T   
   G   
   G   
   C   
   A   
   A   
   A   
   C   
   T   
   C   
   G   
   G   
   T   
   T   
   C   
   T   
   G   
   G   
   C   
   G   
   C   
   A   
   G   
   A   
   A   
   A   
   A   
   A   
   A   
   C   
   A   
   G   
   C   
   C   
   A   
   A   
   T   
   A   
   T   
   C   
   A   
   A   
   A   
   G   
   C   
   T   
   T   
   T   
   T   
   A   
   G   
   T   
   G   
   A   
   T   
   A   
   A   
   A   
   T   
   T   
   C   
   G   
   G   
   C   
   G   
   T   
   T   
   T   
   C   
   T   
   G   
   T   
   T   
   G   
   A   
   T   
   G   
   A   
   A   
   A   
   T   
   C   
   A   
   A   
   T   
   C   
   A   
   A   
   A   
   A   
   G   
   T   
   T   
   A   
   A   
   G   
   C   
   C   
   A   
   A   
   G   
   G   
   A   
   T   
   G   
   G   
   G   
   T   
   C   
   C   
   A   
   A   
   G   
   C   
   A   
   G   
   A   
   C   
   T   
   C   
   C   
   T   
   T   
   T   
   G   
   T   
   A   
   C   
   C   
   A   
   A   
   T   
   C   
   A   
   C   
   A   
   G   
   T   
   C   
   G   
   C   
   T   
   T   
   C   
   T   
   G   
   A   
   A   
   C   
   C   
   A   
   G   
   T   
   G   
   A   
   C   
   A   
   G   
   A   
   A   
   T   
   T   
   A   
   C   
   C   
   A   
   A   
   C   
   A   
   G   
   G   
   G   
   G   
   C   
   T   
   G   
   C   
   G   
   A   
   C   
   A   
   A   
   A   
   A   
   G   
   A   
   T   
   A   
   C   
   A   
   G   
   A   
   G   
   T   
   C   
   A   
   C   
   G   
   T   
   T   
   A   
   T   
   T   
   A   
   T   
   C   
   C   
   G   
   C   
   T   
   G   
   G   
   G   
   G   
   G   
   A   
   A   
   G   
   C   
   A   
   G   
   C   
   T   
   G   
   C   
   G   
   C   
   A   
   A   
   T   
   T   
   A   
   A   
   T   
   C   
   G   
   G   
   G   
   T   
   A   
   T   
   A   
   C   
   G   
   G   
   G   
   C   
   A   
   C   
   C   
   A   
   T   
   T   
   A   
   C   
   T   
   G   
   C   
   A   
   G   
   A   
   A   
   G   
   A   
   T   
   A   
   T   
   T   
   G   
   A   
   A   
   A   
   A   
   A   
   A   
   A   
   T   
   C   
   C   
   A   
   G   
   A   
   G   
   T   
   T   
   A   
   A   
   G   
   T   
   A   
   G   
   T   
   A   
   C   
   G   
   G   
   G   
   C   
   G   
   T   
   A   
   A   
   T   
   T   
   G   
   G   
   T   
   A   
   A   
   A   
   A   
   C   
   T   
   G   
   G   
   T   
   T   
   T   
   A   
   G   
   A   
   A   
   C   
   G   
   A   
   G   
   C   
   G   
   T   
   T   
   T   
   G   
   A   
   T   
   A   
   A   
   A   
   G   
   A   
   A   
   T   
   T   
   A   
   A   
   G   
   A   
   G   
   G   
   G   
   C   
   A   
   A   
   G   
   A   
   T   
   G   
   G   
   T   
   G   
   G   
   C   
   T   
   C   
   A   
   C   
   T   
   A   
   G   
   T   
   T   
   A   
   T   
   T   
   T   
   T   
   A   
   G   
   A   
   T   
   G   
   A   
   C   
   A   
   A   
   A   
   G   
   A   
   G   
   A   
   A   
   T   
   G   
   T   
   C   
   A   
   A   
   A   
   A   
   A   
   A   
   G   
   C   
   T   
   T   
   T   
   A   
   C   
   A   
   A   
   A   
   C   
   C   
   A   
   A   
   A   
   G   
   A   
   G   
   A   
   A   
   A   
   A   
   A   
   A   
   G   
   A   
   T   
   G   
   G   
   T   
   C   
   A   
   A   
   A   
   C   
   G   
   A   
   T   
   T   
   A   
   A   
   A   
   T   
   T   
   A   
   A   
   C   
   G   
   A   
   T   
   T   
   G   
   A   
   C   
   A   
   G   
   T   
   G   
   G   
   C   
   G   
   T   
   A   
   C   
   A   
   A   
   C   
   A   
   A   
   C   
   A   
   A   
   G   
   C   
   C   
   T   
   T   
   C   
   G   
   C   
   T   
   A   
   T   
   A   
   T   
   T   
   T   
   G   
   A   
   C   
   A   
   A   
   A   
   C   
   G   
   T   
   C   
   C   
   T   
   G   
   G   
   T   
   A   
   G   
   T   
   G   
   C   
   G   
   G   
   T   
   C   
   A   
   T   
   T   
   A   
   C   
   C   
   G   
   A   
   T   
   C   
   C   
   T   
   C   
   A   
   A   
   A   
   A   
   A   
   G   
   G   
   C   
   G   
   A   
   T   
   T   
   T   
   A   
   T   
   T   
   A   
   G   
   C   
   A   
   A   
   C   
   G   
   G   
   T   
   T   
   A   
   G   
   T   
   T   
   C   
   A   
   C   
   C   
   T   
   T   
   C   
   G   
   T   
   A   
   T   
   G   
   A   
   T   
   C   
   C   
   T   
   A   
   A   
   T   
   A   
   A   
   A   
   A   
   T   
   G   
   G   
   C   
   C   
   A   
   A   
   T   
   G   
   G   
   T   
   A   
   T   
   T   
   T   
   C   
   T   
   C   
   A   
   A   
   A   
   A   
   A   
   G   
   A   
   A   
   T   
   A   
   T   
   G   
   A   
   T   
   G   
   C   
   T   
   T   
   A   
   C   
   A   
   A   
   T   
   A   
   A   
   C   
   A   
   A   
   C   
   A   
   A   
   A   
   G   
   A   
   T   
   T   
   T   
   A   
   C   
   C   
   A   
   T   
   T   
   C   
   A   
   C   
   A   
   G   
   C   
   A   
   C   
   G   
   T   
   T   
   T   
   T   
   G   
   C   
   G   
   A   
   C   
   A   
   G   
   G   
   C   
   T   
   A   
   T   
   G   
   C   
   A   
   C   
   C   
   A   
   G   
   G   
   C   
   T   
   C   
   T   
   A   
   C   
   C   
   T   
   T   
   T   
   A   
   A   
   A   
   A   
   C   
   A   
   A   
   T   
   T   
   A   
   C   
   C   
   G   
   G   
   T   
   G   
   C   
   C   
   A   
   T   
   T   
   G   
   G   
   T   
   T   
   T   
   A   
   G   
   A   
   T   
   G   
   C   
   A   
   G   
   G   
   G   
   A   
   C   
   A   
   T   
   T   
   G   
   A   
   A   
   G   
   C   
   C   
   A   
   G   
   A   
   T   
   G   
   A   
   A   
   G   
   A   
   G   
   C   
   T   
   A   
   G   
   A   
   A   
   A   
   T   
   T   
   A   
   A   
   T   
   G   
   G   
   T   
   T   
   T   
   G   
   A   
   A   
   A   
   T   
   G   
   G   
   C   
   A   
   A   
   A   
   A   
   A   
   G   
   A   
   T   
   A   
   A   
   A   
   T   
   C   
   T   
   T   
   G   
   G   
   G   
   G   
   C   
   G   
   G   
   C   
   T   
   A   
   T   
   T   
   T   
   T   
   G   
   C   
   G   
   A   
   C   
   A   
   C   
   G   
   T   
   G   
   T   
   A   
   A   
   A   
   A   
   G   
   A   
   A   
   G   
   C   
   A   
   A   
   G   
   T   
   C   
   C   
   A   
   G   
   T   
   C   
   A   
   A   
   T   
   C   
   T   
   G   
   C   
   G   
   T   
   A   
   C   
   C   
   G   
   C   
   T   
   T   
   T   
   A   
   G   
   T   
   T   
   A   
   A   
   T   
   T   
   C   
   A   
   G   
   A   
   T   
   A   
   A   
   T   
   A   
   T   
   T   
   T   
   A   
   T   
   T   
   T   
   T   
   G   
   C   
   C   
   C   
   A   
   A   
   C   
   A   
   A   
   A   
   C   
   A   
   T   
   T   
   A   
   C   
   G   
   G   
   A   
   T   
   G   
   G   
   G   
   A   
   G   
   A   
   A   
   G   
   A   
   C   
   A   
   A   
   A   
   T   
   T   
   T   
   A   
   G   
   A   
   G   
   C   
   G   
   G   
   G   
   T   
   C   
   T   
   T   
   A   
   A   
   T   
   A   
   A   
   A   
   T   
   T   
   C   
   A   
   T   
   T   
   T   
   T   
   C   
   G   
   G   
   T   
   G   
   A   
   G   
   G   
   A   
   A   
   C   
   T   
   A   
   G   
   A   
   T   
   T   
   T   
   A   
   C   
   C   
   A   
   A   
   T   
   T   
   G   
   C   
   C   
   A   
   T   
   G   
   A   
   C   
   G   
   C   
   C   
   T   
   G   
   C   
   T   
   C   
   A   
   A   
   A   
   T   
   T   
   T   
   C   
   A   
   A   
   A   
   T   
   G   
   A   
   A   
   G   
   A   
   T   
   A   
   A   
   A   
   T   
   T   
   T   
   A   
   A   
   T   
   T   
   C   
   T   
   G   
   A   
   A   
   A   
   T   
   T   
   C   
   T   
   T   
   T   
   T   
   A   
   G   
   C   
   A   
   G   
   A   
   T   
   A   
   C   
   G   
   G   
   G   
   C   
   T   
   A   
   C   
   G   
   G   
   T   
   C   
   A   
   A   
   G   
   G   
   T   
   C   
   A   
   A   
   T   
   T   
   G   
   T   
   T   
   A   
   A   
   T   
   T   
   T   
   C   
   G   
   C   
   C   
   A   
   A   
   T   
   C   
   C   
   A   
   G   
   C   
   A   
   A   
   G   
   C   
   T   
   A   
   C   
   C   
   A   
   T   
   G   
   T   
   A   
   T   
   A   
   G   
   T   
   G   
   T   
   T   
   T   
   T   
   C   
   C   
   A   
   A   
   A   
   A   
   T   
   A   
   A   
   T   
   G   
   G   
   A   
   A   
   C   
   A   
   T   
   T   
   A   
   G   
   T   
   C   
   T   
   A   
   T   
   C   
   C   
   A   
   A   
   A   
   A   
   C   
   T   
   A   
   G   
   T   
   T   
   T   
   T   
   A   
   G   
   A   
   C   
   A   
   A   
   A   
   G   
   A   
   A   
   A   
   C   
   G   
   A   
   A   
   G   
   A   
   A   
   A   
   A   
   A   
   A   
   G   
   A   
   C   
   A   
   A   
   T   
   G   
   T   
   A   
   A   
   T   
   C   
   A   
   G   
   C   
   G   
   C   
   C   
   A   
   A   
   C   
   G   
   C   
   A   
   G   
   C   
   G   
   A   
   A   
   T   
   A   
   C   
   A   
   A   
   T   
   T   
   G   
   C   
   C   
   A   
   C   
   A   
   G   
   A   
   T   
   C   
   T   
   T   
   T   
   T   
   A   
   G   
   G   
   T   
   A   
   G   
   T   
   G   
   T   
   G   
   G   
   A   
   A   
   G   
   A   
   T   
   C   
   C   
   T   
   T   
   C   
   A   
   G   
   G   
   T   
   T   
   A   
   T   
   G   
   T   
   T   
   T   
   A   
   T   
   A   
   A   
   T   
   A   
   T   
   G   
   T   
   A   
   C   
   A   
   A   
   T   
   C   
   C   
   G   
   A   
   A   
   C   
   T   
   T   
   C   
   T   
   C   
   A   
   T   
   T   
   G   
   G   
   C   
   T   
   G   
   C   
   T   
   A   
   A   
   A   
   A   
   C   
   A   
   G   
   G   
   A   
   A   
   C   
   A   
   G   
   C   
   T   
   G   
   A   
   A   
   A   
   T   
   A   
   A   
   A   
   A   
   G   
   A   
   T   
   A   
   A   
   A   
   C   
   A   
   A   
   G   
   A   
   T   
   A   
   C   
   A   
   G   
   A   
   T   
   G   
   G   
   C   
   A   
   A   
   A   
   G   
   A   
   A   
   A   
   A   
   C   
   A   
   G   
   T   
   T   
   T   
   C   
   C   
   T   
   T   
   C   
   T   
   A   
   A   
   C   
   G   
   T   
   T   
   A   
   G   
   A   
   T   
   C   
   G   
   T   
   A   
   G   
   T   
   A   
   A   
   C   
   A   
   A   
   T   
   A   
   A   
   A   
   T   
   T   
   C   
   T   
   T   
   A   
   A   
   C   
   A   
   A   
   T   
   G   
   A   
   T   
   T   
   A   
   T   
   G   
   G   
   T   
   T   
   G   
   A   
   A   
   A   
   A   
   T   
   T   
   C   
   A   
   G   
   G   
   A   
   G   
   A   
   A   
   A   
   A   
   T   
   G   
   G   
   T   
   T   
   C   
   T   
   G   
   C   
   A   
   A   
   C   
   A   
   G   
   A   
   T   
   A   
   T   
   C   
   A   
   G   
   T   
   A   
   A   
   A   
   C   
   C   
   A   
   T   
   T   
   A   
   A   
   T   
   T   
   G   
   A   
   T   
   T   
   A   
   C   
   T   
   T   
   A   
   G   
   A   
   A   
   G   
   C   
   A   
   A   
   C   
   C   
   A   
   T   
   T   
   A   
   A   
   A   
   -   
   -   
   -   
   -   
   -   
   -   
   -   
   -   
   -   
   -   
   -   
   -   
   -   
   -   
   -   
   -   
   -   
   -   
   -   
   -   
   -   
   -   
   -   
   -   
   -   
   -   
   -   
   -   
   -   
   -   
   -   
   -   
   -   
   -   
   -   
   -   
   -   
   -   
   -   
   -   
   -   
   -   
   -   
   -   
   -   
   -   
   -   
   -   
   -   
   -   
   -   
   -   
   -   
   -   
   -   
   -   
   -   
   -   
   -   
   -   
   -   
 

 
   N54156   
   -   
   -   
   -   
   -   
   -   
   -   
   -   
   -   
   -   
   -   
   -   
   -   
   -   
   -   
   -   
   -   
   -   
   -   
   -   
   -   
   -   
   -   
   -   
   -   
   -   
   -   
   -   
   -   
   -   
   -   
   -   
   -   
   -   
   -   
   -   
   -   
   -   
   -   
   -   
   -   
   -   
   -   
   -   
   -   
   -   
   -   
   -   
   -   
   -   
   -   
   -   
   -   
   -   
   -   
   -   
   -   
   -   
   -   
   -   
   -   
   -   
   -   
   -   
   -   
   -   
   -   
   -   
   -   
   -   
   -   
   -   
   -   
   -   
   -   
   -   
   -   
   -   
   -   
   -   
   -   
   -   
   -   
   -   
   -   
   -   
   -   
   -   
   -   
   -   
   -   
   -   
   -   
   -   
   -   
   -   
   -   
   -   
   -   
   -   
   -   
   -   
   -   
   -   
   -   
   -   
   -   
   -   
   -   
   C   
   A   
   A   
   T   
   G   
   G   
   C   
   A   
   A   
   G   
   C   
   C   
   A   
   A   
   A   
   C   
   A   
   A   
   G   
   A   
    A    
   T   
   T   
   A   
   G   
   C   
    C    
   G   
   A   
   A   
   G   
   C   
   G   
   A   
   A   
   G   
   A   
   A   
   A   
   A   
    C    
   A   
   G   
   C   
    T    
   A   
    C    
   T   
    A    
    C    
   A   
   T   
   T   
   T   
   T   
   T   
   A   
   A   
   A   
    C    
   G   
   T   
   A   
   T   
   T   
   G   
    T    
   C   
   A   
   A   
   A   
   A   
   C   
   A   
   G   
    G    
   A   
   A   
   T   
   T   
   T   
   G   
   A   
   T   
   A   
   A   
   G   
   T   
   T   
   A   
   C   
   C   
    G    
   T   
   C   
   C   
   G   
   T   
   T   
    G    
   T   
   T   
   C   
   A   
   A   
   G   
   A   
   A   
    G    
   C   
   T   
   A   
   G   
   C   
   T   
   T   
   A   
   A   
   A   
   G   
   A   
   A   
   A   
   A   
   A   
   T   
   G   
   G   
    C    
   T   
   A   
   T   
   G   
   A   
   T   
   A   
   C   
   T   
   A   
   A   
   A   
   T   
   C   
    T    
   G   
   T   
   T   
   G   
   T   
   T   
   G   
   A   
   A   
   A   
   A   
   A   
   T   
   A   
   C   
   C   
   A   
   A   
   G   
   C   
   A   
   A   
   T   
   T   
   T   
   A   
   T   
   T   
   C   
   A   
   G   
   G   
   G   
   A   
   T   
   T   
   C   
   A   
   A   
   G   
   C   
    A    
   G   
   A   
   A   
    G    
   G   
    A    
   G   
   T   
   C   
   A   
   A   
    A    
   G   
   C   
   T   
   A   
   G   
   T   
   G   
   A   
   T   
   G   
   T   
    T    
   C   
   A   
   A   
   G   
   T   
    A    
   A   
   A   
   A   
   A   
   A   
    G    
   G   
    C    
   G   
   A   
   A   
   A   
   G   
   A   
    C    
   A   
   A   
    T    
   C   
   A   
   A   
   T   
   A   
   C   
   A   
   C   
   A   
   T   
   T   
   T   
   A   
    C    
   C   
   T   
   A   
   T   
   A   
   A   
   A   
   T   
   T   
   A   
   T   
   C   
   G   
   A   
   T   
   G   
   A   
   G   
    T    
   A   
   C   
    C    
   C   
   C   
   T   
   T   
   T   
   A   
   G   
   G   
   C   
   G   
   A   
   A   
   A   
   T   
   G   
   A   
   A   
   A   
   G   
   A   
   T   
    T    
   T   
   G   
   T   
   C   
   T   
   T   
   A   
   T   
   C   
   A   
   A   
   T   
   C   
   A   
   A   
   G   
   T   
   A   
   T   
    C    
   G   
    C    
   C   
   A   
   A   
    A    
   A   
   A   
   A   
   G   
   G   
    C    
   G   
   A   
   T   
   A   
    C    
   C   
   T   
   A   
   C   
   C   
   A   
   A   
   A   
   T   
    C    
    G    
   C   
   T   
   T   
   G   
   G   
    A    
    A    
    G    
   C   
   C   
    G    
   T   
   C   
   T   
   T   
   T   
   A   
   A   
   T   
   T   
   T   
   T   
   T   
   C   
   C   
   A   
   G   
   A   
   T   
   A   
   T   
   G   
   T   
   C   
   A   
   G   
   G   
   A   
   A   
   A   
   T   
   G   
   A   
   T   
   A   
   A   
   A   
    A    
   T   
   T   
   T   
   C   
   G   
   A   
   T   
   T   
   C   
   A   
   A   
   G   
   T   
   A   
   G   
   A   
   T   
   A   
   A   
   T   
   G   
   C   
   C   
   A   
   A   
   A   
   C   
   G   
   T   
   G   
   G   
   A   
   G   
   A   
   A   
   A   
   T   
   T   
   G   
   T   
   C   
   G   
   A   
   T   
   C   
   G   
   T   
   A   
   A   
    T    
   G   
   G   
   T   
   A   
   G   
   T   
   G   
   G   
   G   
   C   
   T   
   A   
   G   
    C    
   A   
   A   
   T   
   T   
   A   
   A   
   C   
   A   
   A   
   A   
   G   
   T   
   G   
   T   
   T   
   T   
   G   
   A   
    T    
   G   
   A   
   A   
   G   
   T   
   G   
   G   
   G   
   C   
   G   
   T   
   A   
   G   
   T   
    G    
   C   
   C   
   T   
    G    
   G   
   C   
   A   
   A   
   A   
   C   
   T   
    C    
   G   
   G   
   T   
   T   
   C   
   T   
   G   
   G   
   C   
   G   
   C   
   A   
   G   
   A   
   A   
   A   
   A   
   A   
    A    
   C   
   A   
   G   
   C   
    C    
   A   
   A   
   T   
   A   
   T   
   C   
   A   
   A   
   A   
   G   
   C   
   T   
   T   
   T   
   T   
   A   
   G   
   T   
   G   
   A   
   T   
   A   
   A   
    A    
   T   
   T   
    C    
   G   
   G   
   C   
   G   
   T   
   T   
   T   
   C   
   T   
    G    
   T   
   T   
   G   
   A   
   T   
   G   
   A   
   A   
   A   
   T   
   C   
   A   
   A   
   T   
   C   
    A    
   A   
   A   
   A   
    G    
   T   
   T   
   A   
   A   
   G   
   C   
   C   
   A   
   A   
   G   
   G   
    A    
   T   
   G   
   G   
   G   
   T   
   C   
   C   
   A   
   A   
   G   
   C   
   A   
   G   
   A   
   C   
   T   
   C   
    C    
   T   
   T   
   T   
   G   
   T   
   A   
   C   
   C   
   A   
   A   
   T   
   C   
   A   
   C   
   A   
   G   
   T   
    C    
   G   
   C   
   T   
   T   
   C   
   T   
   G   
   A   
   A   
   C   
   C   
   A   
   G   
   T   
   G   
   A   
   C   
   A   
   G   
   A   
   A   
   T   
   T   
   A   
   C   
   C   
   A   
   A   
   C   
   A   
   G   
   G   
   G   
   G   
   C   
   T   
   G   
   C   
   G   
   A   
   C   
   A   
   A   
   A   
   A   
   G   
    A    
    T    
   A   
   C   
   A   
   G   
   A   
   G   
   T   
   C   
   A   
   C   
   G   
   T   
   T   
   A   
   T   
   T   
   A   
   T   
   C   
   C   
    G    
    C    
   T   
   G   
   G   
   G   
   G   
   G   
   A   
   A   
   G   
   C   
   A   
   G   
   C   
   T   
   G   
   C   
   G   
   C   
   A   
   A   
   T   
   T   
   A   
   A   
   T   
   C   
   G   
   G   
    G    
   T   
   A   
   T   
   A   
   C   
    G    
   G   
   G   
    C    
   A   
   C   
    C    
   A   
   T   
   T   
   A   
   C   
   T   
   G   
   C   
   A   
   G   
   A   
    A    
   G   
   A   
   T   
   A   
   T   
   T   
    G    
   A   
   A   
   A   
   A   
   A   
   A   
   A   
   T   
   C   
   C   
   A   
   G   
   A   
   G   
   T   
   T   
   A   
   A   
   G   
   T   
   A   
   G   
   T   
   A   
   C   
   G   
   G   
   G   
    C    
   G   
   T   
   A   
   A   
   T   
   T   
   G   
   G   
    T    
   A   
   A   
   A   
   A   
   C   
   T   
   G   
   G   
    T    
   T   
   T   
   A   
   G   
   A   
   A   
   C   
   G   
   A   
   G   
   C   
   G   
   T   
   T   
   T   
   G   
   A   
   T   
   A   
   A   
   A   
   G   
   A   
    A    
   T   
   T   
   A   
   A   
   G   
   A   
   G   
   G   
    G    
   C   
   A   
   A   
   G   
   A   
   T   
   G   
   G   
    T    
   G   
   G   
    C    
   T   
   C   
    A    
   C   
   T   
   A   
   G   
   T   
   T   
   A   
   T   
   T   
   T   
   T   
   A   
   G   
   A   
   T   
   G   
   A   
   C   
   A   
   A   
   A   
   G   
   A   
   G   
   A   
   A   
   T   
   G   
   T   
   C   
   A   
   A   
   A   
   A   
   A   
   A   
   G   
   C   
   T   
   T   
   T   
   A   
   C   
   A   
   A   
   A   
   C   
   C   
   A   
   A   
   A   
   G   
   A   
    G    
   A   
   A   
   A   
   A   
   A   
   A   
   G   
   A   
   T   
   G   
   G   
   T   
   C   
   A   
   A   
   A   
   C   
    G    
   A   
   T   
   T   
   A   
   A   
   A   
   T   
   T   
   A   
   A   
   C   
   G   
   A   
   T   
   T   
   G   
   A   
    C    
   A   
   G   
    T    
   G   
   G   
    C    
   G   
   T   
   A   
   C   
   A   
   A   
   C   
   A   
   A   
   C   
   A   
   A   
   G   
   C   
    C    
   T   
   T   
    C    
   G   
   C   
   T   
   A   
   T   
   A   
   T   
   T   
   T   
   G   
   A   
   C   
   A   
   A   
   A   
   C   
   G   
    T    
    C    
   C   
   T   
   G   
   G   
   T   
   A   
   G   
   T   
   G   
   C   
   G   
   G   
   T   
   C   
   A   
   T   
   T   
   A   
   C   
   C   
   G   
   A   
   T   
   C   
   C   
   T   
   C   
   A   
   A   
   A   
   A   
   A   
   G   
   G   
   C   
   G   
   A   
   T   
   T   
   T   
   A   
    T    
   T   
   A   
   G   
   C   
    A    
   A   
   C   
   G   
   G   
   T   
   T   
   A   
   G   
   T   
   T   
   C   
   A   
   C   
   C   
   T   
   T   
   C   
   G   
   T   
   A   
   T   
   G   
   A   
   T   
   C   
   C   
   T   
   A   
   A   
   T   
   A   
   A   
   A   
   A   
   T   
   G   
   G   
   C   
   C   
   A   
   A   
   T   
   G   
   G   
    T    
   A   
   T   
   T   
   T   
   C   
   T   
   C   
   A   
   A   
   A   
   A   
   A   
   G   
   A   
    A    
   T   
   A   
   T   
   G   
   A   
   T   
   G   
   C   
   T   
   T   
   A   
   C   
   A   
   A   
   T   
   A   
   A   
   C   
   A   
   A   
   C   
   A   
   A   
   A   
   G   
   A   
   T   
   T   
   T   
   A   
   C   
   C   
   A   
   T   
   T   
    C    
   A   
   C   
   A   
   G   
   C   
   A   
   C   
   G   
   T   
   T   
   T   
    T    
   G   
   C   
   G   
   A   
   C   
   A   
   G   
   G   
    C    
   T   
   A   
   T   
   G   
   C   
   A   
   C   
   C   
   A   
   G   
   G   
    C    
   T   
   C   
   T   
   A   
   C   
   C   
   T   
   T   
   T   
   A   
   A   
   A   
   A   
   C   
   A   
   A   
   T   
   T   
   A   
   C   
   C   
   G   
   G   
   T   
   G   
   C   
   C   
   A   
   T   
   T   
   G   
   G   
   T   
   T   
   T   
   A   
   G   
   A   
   T   
    G    
   C   
   A   
   G   
   G   
   G   
   A   
   C   
   A   
   T   
   T   
   G   
   A   
   A   
   G   
   C   
   C   
   A   
   G   
   A   
   T   
   G   
   A   
   A   
   G   
   A   
   G   
   C   
   T   
   A   
   G   
   A   
   A   
   A   
   T   
   T   
   A   
   A   
   T   
   G   
   G   
    T    
   T   
   T   
   G   
   A   
   A   
   A   
   T   
   G   
   G   
   C   
   A   
   A   
   A   
   A   
   A   
   G   
   A   
   T   
   A   
   A   
   A   
   T   
   C   
   T   
   T   
   G   
   G   
   G   
   G   
    C    
   G   
   G   
   C   
   T   
   A   
   T   
   T   
   T   
   T   
   G   
   C   
    G    
   A   
   C   
   A   
   C   
   G   
   T   
   G   
   T   
   A   
   A   
   A   
   A   
   G   
   A   
   A   
   G   
   C   
   A   
   A   
   G   
   T   
   C   
   C   
   A   
   G   
   T   
   C   
   A   
   A   
   T   
   C   
   T   
    G    
   C   
   G   
   T   
   A   
   C   
   C   
   G   
   C   
   T   
   T   
   T   
   A   
   G   
   T   
   T   
   A   
   A   
   T   
   T   
   C   
   A   
   G   
   A   
   T   
   A   
   A   
   T   
   A   
   T   
   T   
   T   
   A   
   T   
   T   
   T   
   T   
   G   
   C   
    C    
   C   
   A   
   A   
   C   
   A   
   A   
   A   
   C   
   A   
   T   
   T   
   A   
   C   
   G   
    G    
   A   
   T   
   G   
   G   
   G   
    A    
   G   
   A   
   A   
   G   
   A   
   C   
   A   
   A   
   A   
   T   
   T   
   T   
   A   
   G   
   A   
   G   
   C   
    G    
   G   
   G   
   T   
   C   
   T   
   T   
   A   
   A   
   T   
   A   
   A   
   A   
   T   
   T   
   C   
   A   
   T   
   T   
   T   
   T   
   C   
   G   
   G   
   T   
   G   
   A   
   G   
   G   
   A   
   A   
   C   
   T   
   A   
   G   
   A   
   T   
   T   
   T   
   A   
   C   
   C   
   A   
   A   
   T   
    T    
   G   
   C   
   C   
   A   
   T   
   G   
   A   
   C   
   G   
   C   
   C   
    T    
   G   
   C   
   T   
   C   
   A   
   A   
   A   
   T   
   T   
   T   
   C   
   A   
   A   
   A   
    T    
   G   
   A   
   A   
   G   
   A   
   T   
   A   
   A   
   A   
   T   
   T   
   T   
   A   
   A   
   T   
   T   
   C   
   T   
   G   
   A   
   A   
   A   
   T   
   T   
   C   
   T   
   T   
   T   
   T   
    A    
   G   
   C   
   A   
   G   
   A   
   T   
   A   
   C   
    G    
   G   
   G   
   C   
   T   
   A   
    C    
   G   
   G   
    T    
   C   
   A   
   A   
   G   
   G   
    T    
   C   
   A   
   A   
   T   
   T   
   G   
   T   
   T   
   A   
   A   
   T   
   T   
   T   
   C   
    G    
   C   
   C   
   A   
   A   
   T   
   C   
   C   
   A   
   G   
   C   
   A   
   A   
   G   
   C   
   T   
   A   
   C   
   C   
   A   
   T   
   G   
   T   
   A   
   T   
   A   
   G   
   T   
   G   
   T   
   T   
   T   
   T   
   C   
   C   
   A   
   A   
   A   
   A   
   T   
   A   
   A   
   T   
   G   
   G   
    A    
   A   
   C   
   A   
    T    
   T   
    A    
   G   
   T   
    C    
   T   
   A   
   T   
   C   
   C   
   A   
   A   
   A   
   A   
    C    
   T   
   A   
   G   
   T   
   T   
   T   
   T   
   A   
   G   
   A   
   C   
   A   
   A   
   A   
   G   
   A   
   A   
   A   
   C   
    G    
   A   
   A   
   G   
   A   
   A   
   A   
   A   
   A   
   A   
   G   
   A   
   C   
   A   
   A   
   T   
   G   
   T   
   A   
   A   
   T   
   C   
   A   
   G   
   C   
   G   
   C   
   C   
   A   
   A   
   C   
   G   
   C   
   A   
   G   
   C   
   G   
   A   
   A   
   T   
   A   
   C   
   A   
   A   
   T   
   T   
   G   
   C   
   C   
   A   
   C   
   A   
   G   
   A   
   T   
   C   
   T   
   T   
   T   
   T   
   A   
   G   
   G   
   T   
   A   
   G   
   T   
   G   
   T   
   G   
   G   
   A   
   A   
   G   
   A   
   T   
   C   
   C   
   T   
   T   
   C   
   A   
   G   
   G   
   T   
   T   
   A   
   T   
   G   
   T   
   T   
   T   
   A   
   T   
   A   
   A   
   T   
   A   
   T   
   G   
   T   
   A   
   C   
   A   
   A   
   T   
   C   
   C   
   G   
   A   
   A   
   C   
   T   
   T   
   C   
   T   
   C   
   A   
   T   
   T   
   G   
   G   
   C   
   T   
   G   
   C   
   T   
   A   
   A   
   A   
   A   
   C   
   A   
   G   
   G   
   A   
   A   
   C   
   A   
   G   
   C   
   T   
   G   
   A   
   A   
   A   
   T   
   A   
   A   
   A   
   A   
   G   
   A   
   T   
   A   
   A   
   A   
   C   
   A   
   A   
   G   
   A   
   T   
   A   
   C   
   A   
   G   
   A   
   T   
   G   
   G   
   C   
   A   
   A   
   A   
   G   
   A   
   A   
   A   
   A   
   C   
   A   
   G   
   T   
   T   
   T   
   C   
   C   
   T   
   T   
   C   
   T   
   A   
   A   
   C   
   G   
   T   
   T   
   A   
   G   
   A   
   T   
   C   
   G   
   T   
   A   
   G   
   T   
   A   
   A   
   C   
   A   
   A   
   T   
   A   
   A   
   A   
   T   
   T   
   C   
   T   
   T   
   A   
   A   
   C   
   A   
   A   
   T   
   G   
   A   
   T   
   T   
   A   
   T   
   G   
   G   
   T   
   T   
   G   
   A   
   A   
   A   
   A   
   T   
   T   
   C   
   A   
   G   
   G   
   A   
   G   
   A   
   A   
   A   
   A   
   T   
   G   
   G   
   T   
   T   
   C   
   T   
   G   
   C   
   A   
   A   
   C   
   A   
   G   
   A   
   T   
   A   
   T   
   C   
   A   
   G   
   T   
   A   
   A   
   A   
   C   
   C   
   A   
   T   
   T   
   A   
   A   
   T   
   T   
   G   
   A   
   T   
   T   
   A   
   C   
   T   
   T   
   A   
   G   
   A   
   A   
   G   
   C   
   A   
   A   
   C   
   C   
   A   
   T   
   T   
   A   
   A   
   A   
   -   
   -   
   -   
   -   
   -   
   -   
   -   
   -   
   -   
   -   
   -   
   -   
   -   
   -   
   -   
   -   
   -   
   -   
   -   
   -   
   -   
   -   
   -   
   -   
   -   
   -   
   -   
   -   
   -   
   -   
   -   
   -   
   -   
   -   
   -   
   -   
   -   
   -   
   -   
   -   
   -   
   -   
   -   
   -   
   -   
   -   
   -   
   -   
   -   
   -   
   -   
   -   
   -   
   -   
   -   
   -   
   -   
   -   
   -   
   -   
   -   
   -   
   -   
   -   
   -   
   -   
   -   
   -   
   -   
   -   
   -   
   -   
   -   
   -   
   -   
   -   
   -   
   -   
   -   
   -   
   -   
   -   
   -   
   -   
   -   
   -   
   -   
   -   
   -   
   -   
   -   
   -   
   -   
   -   
   -   
   -   
   -   
   -   
   -   
   -   
   -   
   -   
   -   
   -   
   -   
   -   
   -   
   -   
   -   
   -   
   -   
   -   
   -   
   -   
   -   
   -   
   -   
   -   
   -   
   -   
   -   
   -   
   -   
   -   
   -   
   -   
   -   
   -   
   -   
   -   
   -   
   -   
   -   
   -   
   -   
   -   
   -   
   -   
   -   
   -   
   -   
   -   
   -   
   -   
   -   
   -   
   -   
   -   
   -   
   -   
   -   
   -   
   -   
   -   
   -   
   -   
   -   
   -   
   -   
   -   
   -   
   -   
   -   
   -   
   -   
   -   
   -   
   -   
   -   
   C   
   A   
   A   
   T   
   G   
   G   
   C   
   A   
   A   
   G   
   C   
   C   
   A   
   A   
   A   
   C   
   A   
   A   
   G   
   A   
   A   
   T   
   T   
   A   
   G   
   C   
   C   
   G   
   A   
   A   
   G   
   C   
   G   
   A   
   A   
   G   
   A   
   A   
   A   
   A   
   C   
   A   
   G   
   C   
   T   
   A   
   C   
   T   
   A   
   C   
   A   
   T   
   T   
   T   
   T   
   T   
   A   
   A   
   A   
   C   
   G   
   T   
   A   
   T   
   T   
   G   
   T   
   C   
   A   
   A   
   A   
   A   
   C   
   A   
   G   
   G   
   A   
   A   
   T   
   T   
   T   
   G   
   A   
   T   
   A   
   A   
   G   
   T   
   T   
   A   
   C   
   C   
   G   
   T   
   C   
   C   
   G   
   T   
   T   
   G   
   T   
   T   
   C   
   A   
   A   
   G   
   A   
   A   
   G   
   C   
   T   
   A   
   G   
   C   
   T   
   T   
   A   
   A   
   A   
   G   
   A   
   A   
   A   
   A   
   A   
   T   
   G   
   G   
   C   
   T   
   A   
   T   
   G   
   A   
   T   
   A   
   C   
   T   
   A   
   A   
   A   
   T   
   C   
   T   
   G   
   T   
   T   
   G   
   T   
   T   
   G   
   A   
   A   
   A   
   A   
   A   
   T   
   A   
   C   
   C   
   A   
   A   
   G   
   C   
   A   
   A   
   T   
   T   
   T   
   A   
   T   
   T   
   C   
   A   
   G   
   G   
   G   
   A   
   T   
   T   
   C   
   A   
   A   
   G   
   C   
   A   
   G   
   A   
   A   
   G   
   G   
   A   
   G   
   T   
   C   
   A   
   A   
   A   
   G   
   C   
   T   
   A   
   G   
   T   
   G   
   A   
   T   
   G   
   T   
   T   
   C   
   A   
   A   
   G   
   T   
   A   
   A   
   A   
   A   
   A   
   A   
   G   
   G   
   C   
   G   
   A   
   A   
   A   
   G   
   A   
   C   
   A   
   A   
   T   
   C   
   A   
   A   
   T   
   A   
   C   
   A   
   C   
   A   
   T   
   T   
   T   
   A   
   C   
   C   
   T   
   A   
   T   
   A   
   A   
   A   
   T   
   T   
   A   
   T   
   C   
   G   
   A   
   T   
   G   
   A   
   G   
   T   
   A   
   C   
   C   
   C   
   C   
   T   
   T   
   T   
   A   
   G   
   G   
   C   
   G   
   A   
   A   
   A   
   T   
   G   
   A   
   A   
   A   
   G   
   A   
   T   
   T   
   T   
   G   
   T   
   C   
   T   
   T   
   A   
   T   
   C   
   A   
   A   
   T   
   C   
   A   
   A   
   G   
   T   
   A   
   T   
   C   
   G   
   C   
   C   
   A   
   A   
   A   
   A   
   A   
   A   
   G   
   G   
   C   
   G   
   A   
   T   
   A   
   C   
   C   
   T   
   A   
   C   
   C   
   A   
   A   
   A   
   T   
   C   
   G   
   C   
   T   
   T   
   G   
   G   
   A   
   A   
   G   
   C   
   C   
   G   
   T   
   C   
   T   
   T   
   T   
   A   
   A   
   T   
   T   
   T   
   T   
   T   
   C   
   C   
   A   
   G   
   A   
   T   
   A   
   T   
   G   
   T   
   C   
   A   
   G   
   G   
   A   
   A   
   A   
   T   
   G   
   A   
   T   
   A   
   A   
   A   
   A   
   T   
   T   
   T   
   C   
   G   
   A   
   T   
   T   
   C   
   A   
   A   
   G   
   T   
   A   
   G   
   A   
   T   
   A   
   A   
   T   
   G   
   C   
   C   
   A   
   A   
   A   
   C   
   G   
   T   
   G   
   G   
   A   
   G   
   A   
   A   
   A   
   T   
   T   
   G   
   T   
   C   
   G   
   A   
   T   
   C   
   G   
   T   
   A   
   A   
   T   
   G   
   G   
   T   
   A   
   G   
   T   
   G   
   G   
   G   
   C   
   T   
   A   
   G   
   C   
   A   
   A   
   T   
   T   
   A   
   A   
   C   
   A   
   A   
   A   
   G   
   T   
   G   
   T   
   T   
   T   
   G   
   A   
   T   
   G   
   A   
   A   
   G   
   T   
   G   
   G   
   G   
   C   
   G   
   T   
   A   
   G   
   T   
   G   
   C   
   C   
   T   
   G   
   G   
   C   
   A   
   A   
   A   
   C   
   T   
   C   
   G   
   G   
   T   
   T   
   C   
   T   
   G   
   G   
   C   
   G   
   C   
   A   
   G   
   A   
   A   
   A   
   A   
   A   
   A   
   C   
   A   
   G   
   C   
   C   
   A   
   A   
   T   
   A   
   T   
   C   
   A   
   A   
   A   
   G   
   C   
   T   
   T   
   T   
   T   
   A   
   G   
   T   
   G   
   A   
   T   
   A   
   A   
   A   
   T   
   T   
   C   
   G   
   G   
   C   
   G   
   T   
   T   
   T   
   C   
   T   
   G   
   T   
   T   
   G   
   A   
   T   
   G   
   A   
   A   
   A   
   T   
   C   
   A   
   A   
   T   
   C   
   A   
   A   
   A   
   A   
   G   
   T   
   T   
   A   
   A   
   G   
   C   
   C   
   A   
   A   
   G   
   G   
   A   
   T   
   G   
   G   
   G   
   T   
   C   
   C   
   A   
   A   
   G   
   C   
   A   
   G   
   A   
   C   
   T   
   C   
   C   
   T   
   T   
   T   
   G   
   T   
   A   
   C   
   C   
   A   
   A   
   T   
   C   
   A   
   C   
   A   
   G   
   T   
   C   
   G   
   C   
   T   
   T   
   C   
   T   
   G   
   A   
   A   
   C   
   C   
   A   
   G   
   T   
   G   
   A   
   C   
   A   
   G   
   A   
   A   
   T   
   T   
   A   
   C   
   C   
   A   
   A   
   C   
   A   
   G   
   G   
   G   
   G   
   C   
   T   
   G   
   C   
   G   
   A   
   C   
   A   
   A   
   A   
   A   
   G   
   A   
   T   
   A   
   C   
   A   
   G   
   A   
   G   
   T   
   C   
   A   
   C   
   G   
   T   
   T   
   A   
   T   
   T   
   A   
   T   
   C   
   C   
   G   
   C   
   T   
   G   
   G   
   G   
   G   
   G   
   A   
   A   
   G   
   C   
   A   
   G   
   C   
   T   
   G   
   C   
   G   
   C   
   A   
   A   
   T   
   T   
   A   
   A   
   T   
   C   
   G   
   G   
   G   
   T   
   A   
   T   
   A   
   C   
   G   
   G   
   G   
   C   
   A   
   C   
   C   
   A   
   T   
   T   
   A   
   C   
   T   
   G   
   C   
   A   
   G   
   A   
   A   
   G   
   A   
   T   
   A   
   T   
   T   
   G   
   A   
   A   
   A   
   A   
   A   
   A   
   A   
   T   
   C   
   C   
   A   
   G   
   A   
   G   
   T   
   T   
   A   
   A   
   G   
   T   
   A   
   G   
   T   
   A   
   C   
   G   
   G   
   G   
   C   
   G   
   T   
   A   
   A   
   T   
   T   
   G   
   G   
   T   
   A   
   A   
   A   
   A   
   C   
   T   
   G   
   G   
   T   
   T   
   T   
   A   
   G   
   A   
   A   
   C   
   G   
   A   
   G   
   C   
   G   
   T   
   T   
   T   
   G   
   A   
   T   
   A   
   A   
   A   
   G   
   A   
   A   
   T   
   T   
   A   
   A   
   G   
   A   
   G   
   G   
   G   
   C   
   A   
   A   
   G   
   A   
   T   
   G   
   G   
   T   
   G   
   G   
   C   
   T   
   C   
   A   
   C   
   T   
   A   
   G   
   T   
   T   
   A   
   T   
   T   
   T   
   T   
   A   
   G   
   A   
   T   
   G   
   A   
   C   
   A   
   A   
   A   
   G   
   A   
   G   
   A   
   A   
   T   
   G   
   T   
   C   
   A   
   A   
   A   
   A   
   A   
   A   
   G   
   C   
   T   
   T   
   T   
   A   
   C   
   A   
   A   
   A   
   C   
   C   
   A   
   A   
   A   
   G   
   A   
   G   
   A   
   A   
   A   
   A   
   A   
   A   
   G   
   A   
   T   
   G   
   G   
   T   
   C   
   A   
   A   
   A   
   C   
   G   
   A   
   T   
   T   
   A   
   A   
   A   
   T   
   T   
   A   
   A   
   C   
   G   
   A   
   T   
   T   
   G   
   A   
   C   
   A   
   G   
   T   
   G   
   G   
   C   
   G   
   T   
   A   
   C   
   A   
   A   
   C   
   A   
   A   
   C   
   A   
   A   
   G   
   C   
   C   
   T   
   T   
   C   
   G   
   C   
   T   
   A   
   T   
   A   
   T   
   T   
   T   
   G   
   A   
   C   
   A   
   A   
   A   
   C   
   G   
   T   
   C   
   C   
   T   
   G   
   G   
   T   
   A   
   G   
   T   
   G   
   C   
   G   
   G   
   T   
   C   
   A   
   T   
   T   
   A   
   C   
   C   
   G   
   A   
   T   
   C   
   C   
   T   
   C   
   A   
   A   
   A   
   A   
   A   
   G   
   G   
   C   
   G   
   A   
   T   
   T   
   T   
   A   
   T   
   T   
   A   
   G   
   C   
   A   
   A   
   C   
   G   
   G   
   T   
   T   
   A   
   G   
   T   
   T   
   C   
   A   
   C   
   C   
   T   
   T   
   C   
   G   
   T   
   A   
   T   
   G   
   A   
   T   
   C   
   C   
   T   
   A   
   A   
   T   
   A   
   A   
   A   
   A   
   T   
   G   
   G   
   C   
   C   
   A   
   A   
   T   
   G   
   G   
   T   
   A   
   T   
   T   
   T   
   C   
   T   
   C   
   A   
   A   
   A   
   A   
   A   
   G   
   A   
   A   
   T   
   A   
   T   
   G   
   A   
   T   
   G   
   C   
   T   
   T   
   A   
   C   
   A   
   A   
   T   
   A   
   A   
   C   
   A   
   A   
   C   
   A   
   A   
   A   
   G   
   A   
   T   
   T   
   T   
   A   
   C   
   C   
   A   
   T   
   T   
   C   
   A   
   C   
   A   
   G   
   C   
   A   
   C   
   G   
   T   
   T   
   T   
   T   
   G   
   C   
   G   
   A   
   C   
   A   
   G   
   G   
   C   
   T   
   A   
   T   
   G   
   C   
   A   
   C   
   C   
   A   
   G   
   G   
   C   
   T   
   C   
   T   
   A   
   C   
   C   
   T   
   T   
   T   
   A   
   A   
   A   
   A   
   C   
   A   
   A   
   T   
   T   
   A   
   C   
   C   
   G   
   G   
   T   
   G   
   C   
   C   
   A   
   T   
   T   
   G   
   G   
   T   
   T   
   T   
   A   
   G   
   A   
   T   
   G   
   C   
   A   
   G   
   G   
   G   
   A   
   C   
   A   
   T   
   T   
   G   
   A   
   A   
   G   
   C   
   C   
   A   
   G   
   A   
   T   
   G   
   A   
   A   
   G   
   A   
   G   
   C   
   T   
   A   
   G   
   A   
   A   
   A   
   T   
   T   
   A   
   A   
   T   
   G   
   G   
   T   
   T   
   T   
   G   
   A   
   A   
   A   
   T   
   G   
   G   
   C   
   A   
   A   
   A   
   A   
   A   
   G   
   A   
   T   
   A   
   A   
   A   
   T   
   C   
   T   
   T   
   G   
   G   
   G   
   G   
   C   
   G   
   G   
   C   
   T   
   A   
   T   
   T   
   T   
   T   
   G   
   C   
   G   
   A   
   C   
   A   
   C   
   G   
   T   
   G   
   T   
   A   
   A   
   A   
   A   
   G   
   A   
   A   
   G   
   C   
   A   
   A   
   G   
   T   
   C   
   C   
   A   
   G   
   T   
   C   
   A   
   A   
   T   
   C   
   T   
   G   
   C   
   G   
   T   
   A   
   C   
   C   
   G   
   C   
   T   
   T   
   T   
   A   
   G   
   T   
   T   
   A   
   A   
   T   
   T   
   C   
   A   
   G   
   A   
   T   
   A   
   A   
   T   
   A   
   T   
   T   
   T   
   A   
   T   
   T   
   T   
   T   
   G   
   C   
   C   
   C   
   A   
   A   
   C   
   A   
   A   
   A   
   C   
   A   
   T   
   T   
   A   
   C   
   G   
   G   
   A   
   T   
   G   
   G   
   G   
   A   
   G   
   A   
   A   
   G   
   A   
   C   
   A   
   A   
   A   
   T   
   T   
   T   
   A   
   G   
   A   
   G   
   C   
   G   
   G   
   G   
   T   
   C   
   T   
   T   
   A   
   A   
   T   
   A   
   A   
   A   
   T   
   T   
   C   
   A   
   T   
   T   
   T   
   T   
   C   
   G   
   G   
   T   
   G   
   A   
   G   
   G   
   A   
   A   
   C   
   T   
   A   
   G   
   A   
   T   
   T   
   T   
   A   
   C   
   C   
   A   
   A   
   T   
   T   
   G   
   C   
   C   
   A   
   T   
   G   
   A   
   C   
   G   
   C   
   C   
   T   
   G   
   C   
   T   
   C   
   A   
   A   
   A   
   T   
   T   
   T   
   C   
   A   
   A   
   A   
   T   
   G   
   A   
   A   
   G   
   A   
   T   
   A   
   A   
   A   
   T   
   T   
   T   
   A   
   A   
   T   
   T   
   C   
   T   
   G   
   A   
   A   
   A   
   T   
   T   
   C   
   T   
   T   
   T   
   T   
   A   
   G   
   C   
   A   
   G   
   A   
   T   
   A   
   C   
   G   
   G   
   G   
   C   
   T   
   A   
   C   
   G   
   G   
   T   
   C   
   A   
   A   
   G   
   G   
   T   
   C   
   A   
   A   
   T   
   T   
   G   
   T   
   T   
   A   
   A   
   T   
   T   
   T   
   C   
   G   
   C   
   C   
   A   
   A   
   T   
   C   
   C   
   A   
   G   
   C   
   A   
   A   
   G   
   C   
   T   
   A   
   C   
   C   
   A   
   T   
   G   
   T   
   A   
   T   
   A   
   G   
   T   
   G   
   T   
   T   
   T   
   T   
   C   
   C   
   A   
   A   
   A   
   A   
   T   
   A   
   A   
   T   
   G   
   G   
   A   
   A   
   C   
   A   
   T   
   T   
   A   
   G   
   T   
   C   
   T   
   A   
   T   
   C   
   C   
   A   
   A   
   A   
   A   
   C   
   T   
   A   
   G   
   T   
   T   
   T   
   T   
   A   
   G   
   A   
   C   
   A   
   A   
   A   
   G   
   A   
   A   
   A   
   C   
   G   
   A   
   A   
   G   
   A   
   A   
   A   
   A   
   A   
   A   
   G   
   A   
   C   
   A   
   A   
   T   
   G   
   T   
   A   
   A   
   T   
   C   
   A   
   G   
   C   
   G   
   C   
   C   
   A   
   A   
   C   
   G   
   C   
   A   
   G   
   C   
   G   
   A   
   A   
   T   
   A   
   C   
   A   
   A   
   T   
   T   
   G   
   C   
   C   
   A   
   C   
   A   
   G   
   A   
   T   
   C   
   T   
   T   
   T   
   T   
   A   
   G   
   G   
   T   
   A   
   G   
   T   
   G   
   T   
   G   
   G   
   A   
   A   
   G   
   A   
   T   
   C   
   C   
   T   
   T   
   C   
   A   
   G   
   G   
   T   
   T   
   A   
   T   
   G   
   T   
   T   
   T   
   A   
   T   
   A   
   A   
   T   
   A   
   T   
   G   
   T   
   A   
   C   
   A   
   A   
   T   
   C   
   C   
   G   
   A   
   A   
   C   
   T   
   T   
   C   
   T   
   C   
   A   
   T   
   T   
   G   
   G   
   C   
   T   
   G   
   C   
   T   
   A   
   A   
   A   
   A   
   C   
   A   
   G   
   G   
   A   
   A   
   C   
   A   
   G   
   C   
   T   
   G   
   A   
   A   
   A   
   T   
   A   
   A   
   A   
   A   
   G   
   A   
   T   
   A   
   A   
   A   
   C   
   A   
   A   
   G   
   A   
   T   
   A   
   C   
   A   
   G   
   A   
   T   
   G   
   G   
   C   
   A   
   A   
   A   
   G   
   A   
   A   
   A   
   A   
   C   
   A   
   G   
   T   
   T   
   T   
   C   
   C   
   T   
   T   
   C   
   T   
   A   
   A   
   C   
   G   
   T   
   T   
   A   
   G   
   A   
   T   
   C   
   G   
   T   
   A   
   G   
   T   
   A   
   A   
   C   
   A   
   A   
   T   
   A   
   A   
   A   
   T   
   T   
   C   
   T   
   T   
   A   
   A   
   C   
   A   
   A   
   T   
   G   
   A   
   T   
   T   
   A   
   T   
   G   
   G   
   T   
   T   
   G   
   A   
   A   
   A   
   A   
   T   
   T   
   C   
   A   
   G   
   G   
   A   
   G   
   A   
   A   
   A   
   A   
   T   
   G   
   G   
   T   
   T   
   C   
   T   
   G   
   C   
   A   
   A   
   C   
   A   
   G   
   A   
   T   
   A   
   T   
   C   
   A   
   G   
   T   
   A   
   A   
   A   
   C   
   C   
   A   
   T   
   T   
   A   
   A   
   T   
   T   
   G   
   A   
   T   
   T   
   A   
   C   
   T   
   T   
   A   
   G   
   A   
   A   
   G   
   C   
   A   
   A   
   C   
   C   
   A   
   T   
   T   
   A   
   A   
   A   
   -   
   -   
   -   
   -   
   -   
   -   
   -   
   -   
   -   
   -   
   -   
   -   
   -   
   -   
   -   
   -   
   -   
   -   
   -   
   -   
   -   
   -   
   -   
   -   
   -   
   -   
   -   
   -   
   -   
   -   
   -   
   -   
   -   
   -   
   -   
   -   
   -   
   -   
   -   
   -   
   -   
   -   
   -   
   -   
   -   
   -   
   -   
   -   
   -   
   -   
   -   
   -   
   -   
   -   
   -   
   -   
   -   
   -   
   -   
   -   
   -   
 

 
   N53519   
   -   
   -   
   -   
   -   
   -   
   -   
   -   
   -   
   -   
   -   
   -   
   -   
   -   
   -   
   -   
   -   
   -   
   -   
   -   
   -   
   -   
   -   
   -   
   -   
   -   
   -   
   -   
   -   
   -   
   -   
   -   
   -   
   -   
   -   
   -   
   -   
   -   
   -   
   -   
   -   
   -   
   -   
   -   
   -   
   -   
   -   
   -   
   -   
   -   
   -   
   -   
   -   
   -   
   -   
   -   
   -   
   -   
   -   
   -   
   -   
   -   
   -   
   -   
   -   
   -   
   -   
   -   
   -   
   -   
   -   
   -   
   -   
   -   
   -   
   -   
   -   
   -   
   -   
   -   
   -   
   -   
   -   
   -   
   -   
   -   
   -   
   -   
   -   
   -   
   -   
   -   
   -   
   -   
   -   
   -   
   -   
   -   
   -   
   -   
   -   
   -   
   -   
   -   
   -   
   -   
   -   
   -   
   -   
   C   
   A   
   A   
   T   
   G   
   G   
   C   
   A   
   A   
   G   
   C   
   C   
   A   
   A   
   A   
   C   
   A   
   A   
   G   
   A   
    A    
   T   
   T   
   A   
   G   
   C   
    C    
   G   
   A   
   A   
   G   
   C   
   G   
   A   
   A   
   G   
   A   
   A   
   A   
   A   
    C    
   A   
   G   
   C   
    T    
   A   
    C    
   T   
    A    
    C    
   A   
   T   
   T   
   T   
   T   
   T   
   A   
   A   
   A   
    C    
   G   
   T   
   A   
   T   
   T   
   G   
    T    
   C   
   A   
   A   
   A   
   A   
   C   
   A   
   G   
    G    
   A   
   A   
   T   
   T   
   T   
   G   
   A   
   T   
   A   
   A   
   G   
   T   
   T   
   A   
   C   
   C   
    G    
   T   
   C   
   C   
   G   
   T   
   T   
    G    
   T   
   T   
   C   
   A   
   A   
   G   
   A   
   A   
    G    
   C   
   T   
   A   
   G   
   C   
   T   
   T   
   A   
   A   
   A   
   G   
   A   
   A   
   A   
   A   
   A   
   T   
   G   
   G   
    C    
   T   
   A   
   T   
   G   
   A   
   T   
   A   
   C   
   T   
   A   
   A   
   A   
   T   
   C   
    T    
   G   
   T   
   T   
   G   
   T   
   T   
   G   
   A   
   A   
   A   
   A   
   A   
   T   
   A   
   C   
   C   
   A   
   A   
   G   
   C   
   A   
   A   
   T   
   T   
   T   
   A   
   T   
   T   
   C   
   A   
   G   
   G   
   G   
   A   
   T   
   T   
   C   
   A   
   A   
   G   
   C   
    A    
   G   
   A   
   A   
    G    
   G   
    A    
   G   
   T   
   C   
   A   
   A   
    A    
   G   
   C   
   T   
   A   
   G   
   T   
   G   
   A   
   T   
   G   
   T   
    T    
   C   
   A   
   A   
   G   
   T   
    C    
   A   
   A   
   A   
   A   
   A   
    G    
   G   
    C    
   G   
   A   
   A   
   A   
   G   
   A   
    C    
   A   
   A   
    T    
   C   
   A   
   A   
   T   
   A   
   C   
   A   
   C   
   A   
   T   
   T   
   T   
   A   
    C    
   C   
   T   
   A   
   T   
   A   
   A   
   A   
   T   
   T   
   A   
   T   
   C   
   G   
   A   
   T   
   G   
   A   
   G   
    T    
   A   
   C   
    C    
   C   
   C   
   T   
   T   
   T   
   A   
   G   
   G   
   C   
   G   
   A   
   A   
   A   
   T   
   G   
   A   
   A   
   A   
   G   
   A   
   T   
    T    
   T   
   G   
   T   
   C   
   T   
   T   
   A   
   T   
   C   
   A   
   A   
   T   
   C   
   A   
   A   
   G   
   T   
   A   
   T   
    C    
   G   
    C    
   C   
   A   
   A   
    A    
   A   
   A   
   A   
   G   
   G   
    C    
   G   
   A   
   T   
   A   
    C    
   C   
   T   
   A   
   C   
   C   
   A   
   A   
   A   
   T   
    C    
    G    
   C   
   T   
   T   
   G   
   G   
    A    
    A    
    G    
   C   
   C   
    G    
   T   
   C   
   T   
   T   
   T   
   A   
   A   
   T   
   T   
   T   
   T   
   T   
   C   
   C   
   A   
   G   
   A   
   T   
   A   
   T   
   G   
   T   
   C   
   A   
   G   
   G   
   A   
   A   
   A   
   T   
   G   
   A   
   T   
   A   
   A   
   A   
    A    
   T   
   T   
   T   
   C   
   G   
   A   
   T   
   T   
   C   
   A   
   A   
   G   
   T   
   A   
   G   
   A   
   T   
   A   
   A   
   T   
   G   
   C   
   C   
   A   
   A   
   A   
   C   
   G   
   T   
   G   
   G   
   A   
   G   
   A   
   A   
   A   
   T   
   T   
   G   
   T   
   C   
   G   
   A   
   T   
   C   
   G   
   T   
   A   
   A   
    T    
   G   
   G   
   T   
   A   
   G   
   T   
   G   
   G   
   G   
   C   
   T   
   A   
   G   
    C    
   A   
   A   
   T   
   T   
   A   
   A   
   C   
   A   
   A   
   A   
   G   
   T   
   G   
   T   
   T   
   T   
   G   
   A   
    C    
   G   
   A   
   A   
   G   
   T   
   G   
   G   
   G   
   C   
   G   
   T   
   A   
   G   
   T   
    G    
   C   
   C   
   T   
    G    
   G   
   C   
   A   
   A   
   A   
   C   
   T   
    C    
   G   
   G   
   T   
   T   
   C   
   T   
   G   
   G   
   C   
   G   
   C   
   A   
   G   
   A   
   A   
   A   
   A   
   A   
    A    
   C   
   A   
   G   
   C   
    C    
   A   
   A   
   T   
   A   
   T   
   C   
   A   
   A   
   A   
   G   
   C   
   T   
   T   
   T   
   T   
   A   
   G   
   T   
   G   
   A   
   T   
   A   
   A   
    G    
   T   
   T   
    C    
   G   
   G   
   C   
   G   
   T   
   T   
   T   
   C   
   T   
    A    
   T   
   T   
   G   
   A   
   T   
   G   
   A   
   A   
   A   
   T   
   C   
   A   
   A   
   T   
   C   
    A    
   A   
   A   
   A   
    G    
   T   
   T   
   A   
   A   
   G   
   C   
   C   
   A   
   A   
   G   
   G   
    A    
   T   
   G   
   G   
   G   
   T   
   C   
   C   
   A   
   A   
   G   
   C   
   A   
   G   
   A   
   C   
   T   
   C   
    C    
   T   
   T   
   T   
   G   
   T   
   A   
   C   
   C   
   A   
   A   
   T   
   C   
   A   
   C   
   A   
   G   
   T   
    C    
   G   
   C   
   T   
   T   
   C   
   T   
   G   
   A   
   A   
   C   
   C   
   A   
   G   
   T   
   G   
   A   
   C   
   A   
   G   
   A   
   A   
   T   
   T   
   A   
   C   
   C   
   A   
   A   
   C   
   A   
   G   
   G   
   G   
   G   
   C   
   T   
   G   
   C   
   G   
   A   
   C   
   A   
   A   
   A   
   A   
   G   
    A    
    T    
   A   
   C   
   A   
   G   
   A   
   G   
   T   
   C   
   A   
   C   
   G   
   T   
   T   
   A   
   T   
   T   
   A   
   T   
   C   
   C   
    G    
    C    
   T   
   G   
   G   
   G   
   G   
   G   
   A   
   A   
   G   
   C   
   A   
   G   
   C   
   T   
   G   
   C   
   G   
   C   
   A   
   A   
   T   
   T   
   A   
   A   
   T   
   C   
   G   
   G   
    G    
   T   
   A   
   T   
   A   
   C   
    G    
   G   
   G   
    C    
   A   
   C   
    C    
   A   
   T   
   T   
   A   
   C   
   T   
   G   
   C   
   A   
   G   
   A   
    A    
   G   
   A   
   T   
   A   
   T   
   T   
    G    
   A   
   A   
   A   
   A   
   A   
   A   
   A   
   T   
   C   
   C   
   A   
   G   
   A   
   G   
   T   
   T   
   A   
   A   
   G   
   T   
   A   
   G   
   T   
   A   
   C   
   G   
   G   
   G   
    C    
   G   
   T   
   A   
   A   
   T   
   T   
   G   
   G   
    T    
   A   
   A   
   A   
   A   
   C   
   T   
   G   
   G   
    C    
   T   
   T   
   A   
   G   
   A   
   A   
   C   
   G   
   A   
   G   
   C   
   G   
   T   
   T   
   T   
   G   
   A   
   T   
   A   
   A   
   A   
   G   
   A   
    A    
   T   
   T   
   A   
   A   
   G   
   A   
   G   
   G   
    G    
   C   
   A   
   A   
   G   
   A   
   T   
   G   
   G   
    T    
   G   
   G   
    C    
   T   
   C   
    A    
   C   
   T   
   A   
   G   
   T   
   T   
   A   
   T   
   T   
   T   
   T   
   A   
   G   
   A   
   T   
   G   
   A   
   C   
   A   
   A   
   A   
   G   
   A   
   G   
   A   
   A   
   T   
   G   
   T   
   C   
   A   
   A   
   A   
   A   
   A   
   A   
   G   
   C   
   T   
   T   
   T   
   A   
   C   
   A   
   A   
   A   
   C   
   C   
   A   
   A   
   A   
   G   
   A   
    G    
   A   
   A   
   A   
   A   
   A   
   A   
   G   
   A   
   T   
   G   
   G   
   T   
   C   
   A   
   A   
   A   
   C   
    G    
   A   
   T   
   T   
   A   
   A   
   A   
   T   
   T   
   A   
   A   
   C   
   G   
   A   
   T   
   T   
   G   
   A   
    C    
   A   
   G   
    T    
   G   
   G   
    C    
   G   
   T   
   A   
   C   
   A   
   A   
   C   
   A   
   A   
   C   
   A   
   A   
   G   
   C   
    C    
   T   
   T   
    C    
   G   
   C   
   T   
   A   
   T   
   A   
   T   
   T   
   T   
   G   
   A   
   C   
   A   
   A   
   A   
   C   
   G   
    T    
    C    
   C   
   T   
   G   
   G   
   T   
   A   
   G   
   T   
   G   
   C   
   G   
   G   
   T   
   C   
   A   
   T   
   T   
   A   
   C   
   C   
   G   
   A   
   T   
   C   
   C   
   T   
   C   
   A   
   A   
   A   
   A   
   A   
   G   
   G   
   C   
   G   
   A   
   T   
   T   
   T   
   A   
    T    
   T   
   A   
   G   
   C   
    A    
   A   
   C   
   G   
   G   
   T   
   T   
   A   
   G   
   T   
   T   
   C   
   A   
   C   
   C   
   T   
   T   
   C   
   G   
   T   
   A   
   T   
   G   
   A   
   T   
   C   
   C   
   T   
   A   
   A   
   T   
   A   
   A   
   A   
   A   
   T   
   G   
   G   
   C   
   C   
   A   
   A   
   T   
   G   
   G   
    T    
   A   
   T   
   T   
   T   
   C   
   T   
   C   
   A   
   A   
   A   
   A   
   A   
   G   
   A   
    A    
   T   
   A   
   T   
   G   
   A   
   T   
   G   
   C   
   T   
   T   
   A   
   C   
   A   
   A   
   T   
   A   
   A   
   C   
   A   
   A   
   C   
   A   
   A   
   A   
   G   
   A   
   T   
   T   
   T   
   A   
   C   
   C   
   A   
   T   
   T   
    C    
   A   
   C   
   A   
   G   
   C   
   A   
   C   
   G   
   T   
   T   
   T   
    T    
   G   
   C   
   G   
   A   
   C   
   A   
   G   
   G   
    C    
   T   
   A   
   T   
   G   
   C   
   A   
   C   
   C   
   A   
   G   
   G   
    C    
   T   
   C   
   T   
   A   
   C   
   C   
   T   
   T   
   T   
   A   
   A   
   A   
   A   
   C   
   A   
   A   
   T   
   T   
   A   
   C   
   C   
   G   
   G   
   T   
   G   
   C   
   C   
   A   
   T   
   T   
   G   
   G   
   T   
   T   
   T   
   A   
   G   
   A   
   T   
    G    
   C   
   A   
   G   
   G   
   G   
   A   
   C   
   A   
   T   
   T   
   G   
   A   
   A   
   G   
   C   
   C   
   A   
   G   
   A   
   T   
   G   
   A   
   A   
   G   
   A   
   G   
   C   
   T   
   A   
   G   
   A   
   A   
   A   
   T   
   T   
   A   
   A   
   T   
   G   
   G   
    C    
   T   
   T   
   G   
   A   
   A   
   A   
   T   
   G   
   G   
   C   
   A   
   A   
   A   
   A   
   A   
   G   
   A   
   T   
   A   
   A   
   A   
   T   
   C   
   T   
   T   
   G   
   G   
   G   
   G   
    C    
   G   
   G   
   C   
   T   
   A   
   T   
   T   
   T   
   T   
   G   
   C   
    G    
   A   
   C   
   A   
   C   
   G   
   T   
   G   
   T   
   A   
   A   
   A   
   A   
   G   
   A   
   A   
   G   
   C   
   A   
   A   
   G   
   T   
   C   
   C   
   A   
   G   
   T   
   C   
   A   
   A   
   T   
   C   
   T   
    G    
   C   
   G   
   T   
   A   
   C   
   C   
   G   
   C   
   T   
   T   
   T   
   A   
   G   
   T   
   T   
   A   
   A   
   T   
   T   
   C   
   A   
   G   
   A   
   T   
   A   
   A   
   T   
   A   
   T   
   T   
   T   
   A   
   T   
   T   
   T   
   T   
   G   
   C   
    C    
   C   
   A   
   A   
   C   
   A   
   A   
   A   
   C   
   A   
   T   
   T   
   A   
   C   
   G   
    G    
   A   
   T   
   G   
   G   
   G   
    A    
   G   
   A   
   A   
   G   
   A   
   C   
   A   
   A   
   A   
   T   
   T   
   T   
   A   
   G   
   A   
   G   
   C   
    G    
   G   
   G   
   T   
   C   
   T   
   T   
   A   
   A   
   T   
   A   
   A   
   A   
   T   
   T   
   C   
   A   
   T   
   T   
   T   
   T   
   C   
   G   
   G   
   T   
   G   
   A   
   G   
   G   
   A   
   A   
   C   
   T   
   A   
   G   
   A   
   T   
   T   
   T   
   A   
   C   
   C   
   A   
   A   
   T   
    T    
   G   
   C   
   C   
   A   
   T   
   G   
   A   
   C   
   G   
   C   
   C   
    T    
   G   
   C   
   T   
   C   
   A   
   A   
   A   
   T   
   T   
   T   
   C   
   A   
   A   
   A   
    T    
   G   
   A   
   A   
   G   
   A   
   T   
   A   
   A   
   A   
   T   
   T   
   T   
   A   
   A   
   T   
   T   
   C   
   T   
   G   
   A   
   A   
   A   
   T   
   T   
   C   
   T   
   T   
   T   
   T   
    A    
   G   
   C   
   A   
   G   
   A   
   T   
   A   
   C   
    G    
   G   
   G   
   C   
   T   
   A   
    C    
   G   
   G   
    T    
   C   
   A   
   A   
   G   
   G   
    C    
   C   
   A   
   A   
   T   
   T   
   G   
   T   
   T   
   A   
   A   
   T   
   T   
   T   
   C   
    G    
   C   
   C   
   A   
   A   
   T   
   C   
   C   
   A   
   G   
   C   
   A   
   A   
   G   
   C   
   T   
   A   
   C   
   C   
   A   
   T   
   G   
   T   
   A   
   T   
   A   
   G   
   T   
   G   
   T   
   T   
   T   
   T   
   C   
   C   
   A   
   A   
   A   
   A   
   T   
   A   
   A   
   T   
   G   
   G   
    A    
   A   
   C   
   A   
    T    
   T   
    G    
   G   
   T   
    C    
   T   
   A   
   T   
   C   
   C   
   A   
   A   
   A   
   A   
    A    
   T   
   A   
   G   
   T   
   T   
   T   
   T   
   A   
   G   
   A   
   C   
   A   
   A   
   A   
   G   
   A   
   A   
   A   
   C   
    G    
   A   
   A   
   G   
   A   
   A   
   A   
   A   
   A   
   A   
   G   
   A   
   C   
   A   
   A   
   T   
   G   
   T   
   A   
   A   
   T   
   C   
   A   
   G   
   C   
   G   
   C   
   C   
   A   
   A   
   C   
   G   
   C   
   A   
   G   
   C   
   G   
   A   
   A   
   T   
   A   
   C   
   A   
   A   
   T   
   T   
   G   
   C   
   C   
   A   
   C   
   A   
   G   
   A   
   T   
   C   
   T   
   T   
   T   
   T   
   A   
   G   
   G   
   T   
   A   
   G   
   T   
   G   
   T   
   G   
   G   
   A   
   A   
   G   
   A   
   C   
   C   
   C   
   T   
   T   
   C   
   A   
   G   
   G   
   T   
   T   
   A   
   T   
   G   
   T   
   T   
   T   
   A   
   T   
   A   
   A   
   T   
   A   
   T   
   G   
   T   
   A   
   C   
   A   
   A   
   T   
   C   
   C   
   G   
   A   
   A   
   C   
   T   
   T   
   C   
   T   
   C   
   A   
   T   
   T   
   G   
   G   
   C   
   T   
   G   
   C   
   T   
   A   
   A   
   A   
   A   
   C   
   A   
   G   
   G   
   A   
   A   
   C   
   A   
   G   
   C   
   T   
   G   
   A   
   A   
   A   
   T   
   A   
   A   
   A   
   A   
   G   
   A   
   T   
   A   
   A   
   A   
   C   
   A   
   A   
   G   
   A   
   T   
   A   
   C   
   A   
   G   
   A   
   T   
   G   
   G   
   C   
   A   
   A   
   A   
   G   
   A   
   A   
   A   
   A   
   C   
   A   
   G   
   T   
   T   
   T   
   C   
   C   
   T   
   T   
   C   
   T   
   A   
   A   
   C   
   G   
   T   
   T   
   A   
   G   
   A   
   T   
   C   
   G   
   T   
   A   
   G   
   T   
   A   
   A   
   C   
   A   
   A   
   T   
   A   
   A   
   A   
   T   
   T   
   C   
   T   
   T   
   A   
   A   
   C   
   A   
   A   
   T   
   G   
   A   
   T   
   T   
   A   
   T   
   G   
   G   
   T   
   T   
   G   
   A   
   A   
   A   
   A   
   T   
   T   
   C   
   A   
   G   
   G   
   A   
   G   
   A   
   A   
   A   
   A   
   T   
   G   
   G   
   T   
   T   
   C   
   T   
   G   
   C   
   A   
   A   
   C   
   A   
   G   
   A   
   T   
   A   
   T   
   C   
   A   
   G   
   T   
   A   
   A   
   A   
   C   
   C   
   A   
   T   
   T   
   A   
   A   
   T   
   T   
   G   
   A   
   T   
   T   
   A   
   C   
   T   
   T   
   A   
   G   
   A   
   A   
   G   
   C   
   A   
   A   
   C   
   C   
   A   
   T   
   T   
   A   
   A   
   A   
   -   
   -   
   -   
   -   
   -   
   -   
   -   
   -   
   -   
   -   
   -   
   -   
   -   
   -   
   -   
   -   
   -   
   -   
   -   
   -   
   -   
   -   
   -   
   -   
   -   
   -   
   -   
   -   
   -   
   -   
   -   
   -   
   -   
   -   
   -   
   -   
   -   
   -   
   -   
   -   
   -   
   -   
   -   
   -   
   -   
   -   
   -   
   -   
   -   
   -   
   -   
   -   
   -   
   -   
   -   
   -   
   -   
   -   
   -   
   -   
   -   
   -   
   -   
   -   
   -   
   -   
   -   
   -   
   -   
   -   
   -   
   -   
   -   
   -   
   -   
   -   
   -   
   -   
   -   
   -   
   -   
   -   
   -   
   -   
   -   
   -   
   -   
   -   
   -   
   -   
   -   
   -   
   -   
   -   
   -   
   -   
   -   
   -   
   -   
   -   
   -   
   -   
   -   
   -   
   -   
   -   
   -   
   -   
   -   
   -   
   -   
   -   
   -   
   -   
   -   
   -   
   -   
   -   
   -   
   -   
   -   
   -   
   -   
   -   
   -   
   -   
   -   
   -   
   -   
   -   
   -   
   -   
   -   
   -   
   -   
   -   
   -   
   -   
   -   
   -   
   -   
   -   
   -   
   -   
   -   
   -   
   -   
   -   
   -   
   -   
   -   
   -   
   -   
   -   
   -   
   -   
   -   
   -   
   -   
   -   
   -   
   -   
   -   
   -   
   -   
   -   
   -   
   -   
   -   
   C   
   A   
   A   
   T   
   G   
   G   
   C   
   A   
   A   
   G   
   C   
   C   
   A   
   A   
   A   
   C   
   A   
   A   
   G   
   A   
   A   
   T   
   T   
   A   
   G   
   C   
   C   
   G   
   A   
   A   
   G   
   C   
   G   
   A   
   A   
   G   
   A   
   A   
   A   
   A   
   C   
   A   
   G   
   C   
   T   
   A   
   C   
   T   
   A   
   C   
   A   
   T   
   T   
   T   
   T   
   T   
   A   
   A   
   A   
   C   
   G   
   T   
   A   
   T   
   T   
   G   
   T   
   C   
   A   
   A   
   A   
   A   
   C   
   A   
   G   
   G   
   A   
   A   
   T   
   T   
   T   
   G   
   A   
   T   
   A   
   A   
   G   
   T   
   T   
   A   
   C   
   C   
   G   
   T   
   C   
   C   
   G   
   T   
   T   
   G   
   T   
   T   
   C   
   A   
   A   
   G   
   A   
   A   
   G   
   C   
   T   
   A   
   G   
   C   
   T   
   T   
   A   
   A   
   A   
   G   
   A   
   A   
   A   
   A   
   A   
   T   
   G   
   G   
   C   
   T   
   A   
   T   
   G   
   A   
   T   
   A   
   C   
   T   
   A   
   A   
   A   
   T   
   C   
   T   
   G   
   T   
   T   
   G   
   T   
   T   
   G   
   A   
   A   
   A   
   A   
   A   
   T   
   A   
   C   
   C   
   A   
   A   
   G   
   C   
   A   
   A   
   T   
   T   
   T   
   A   
   T   
   T   
   C   
   A   
   G   
   G   
   G   
   A   
   T   
   T   
   C   
   A   
   A   
   G   
   C   
   A   
   G   
   A   
   A   
   G   
   G   
   A   
   G   
   T   
   C   
   A   
   A   
   A   
   G   
   C   
   T   
   A   
   G   
   T   
   G   
   A   
   T   
   G   
   T   
   T   
   C   
   A   
   A   
   G   
   T   
   C   
   A   
   A   
   A   
   A   
   A   
   G   
   G   
   C   
   G   
   A   
   A   
   A   
   G   
   A   
   C   
   A   
   A   
   T   
   C   
   A   
   A   
   T   
   A   
   C   
   A   
   C   
   A   
   T   
   T   
   T   
   A   
   C   
   C   
   T   
   A   
   T   
   A   
   A   
   A   
   T   
   T   
   A   
   T   
   C   
   G   
   A   
   T   
   G   
   A   
   G   
   T   
   A   
   C   
   C   
   C   
   C   
   T   
   T   
   T   
   A   
   G   
   G   
   C   
   G   
   A   
   A   
   A   
   T   
   G   
   A   
   A   
   A   
   G   
   A   
   T   
   T   
   T   
   G   
   T   
   C   
   T   
   T   
   A   
   T   
   C   
   A   
   A   
   T   
   C   
   A   
   A   
   G   
   T   
   A   
   T   
   C   
   G   
   C   
   C   
   A   
   A   
   A   
   A   
   A   
   A   
   G   
   G   
   C   
   G   
   A   
   T   
   A   
   C   
   C   
   T   
   A   
   C   
   C   
   A   
   A   
   A   
   T   
   C   
   G   
   C   
   T   
   T   
   G   
   G   
   A   
   A   
   G   
   C   
   C   
   G   
   T   
   C   
   T   
   T   
   T   
   A   
   A   
   T   
   T   
   T   
   T   
   T   
   C   
   C   
   A   
   G   
   A   
   T   
   A   
   T   
   G   
   T   
   C   
   A   
   G   
   G   
   A   
   A   
   A   
   T   
   G   
   A   
   T   
   A   
   A   
   A   
   A   
   T   
   T   
   T   
   C   
   G   
   A   
   T   
   T   
   C   
   A   
   A   
   G   
   T   
   A   
   G   
   A   
   T   
   A   
   A   
   T   
   G   
   C   
   C   
   A   
   A   
   A   
   C   
   G   
   T   
   G   
   G   
   A   
   G   
   A   
   A   
   A   
   T   
   T   
   G   
   T   
   C   
   G   
   A   
   T   
   C   
   G   
   T   
   A   
   A   
   T   
   G   
   G   
   T   
   A   
   G   
   T   
   G   
   G   
   G   
   C   
   T   
   A   
   G   
   C   
   A   
   A   
   T   
   T   
   A   
   A   
   C   
   A   
   A   
   A   
   G   
   T   
   G   
   T   
   T   
   T   
   G   
   A   
   C   
   G   
   A   
   A   
   G   
   T   
   G   
   G   
   G   
   C   
   G   
   T   
   A   
   G   
   T   
   G   
   C   
   C   
   T   
   G   
   G   
   C   
   A   
   A   
   A   
   C   
   T   
   C   
   G   
   G   
   T   
   T   
   C   
   T   
   G   
   G   
   C   
   G   
   C   
   A   
   G   
   A   
   A   
   A   
   A   
   A   
   A   
   C   
   A   
   G   
   C   
   C   
   A   
   A   
   T   
   A   
   T   
   C   
   A   
   A   
   A   
   G   
   C   
   T   
   T   
   T   
   T   
   A   
   G   
   T   
   G   
   A   
   T   
   A   
   A   
   G   
   T   
   T   
   C   
   G   
   G   
   C   
   G   
   T   
   T   
   T   
   C   
   T   
   A   
   T   
   T   
   G   
   A   
   T   
   G   
   A   
   A   
   A   
   T   
   C   
   A   
   A   
   T   
   C   
   A   
   A   
   A   
   A   
   G   
   T   
   T   
   A   
   A   
   G   
   C   
   C   
   A   
   A   
   G   
   G   
   A   
   T   
   G   
   G   
   G   
   T   
   C   
   C   
   A   
   A   
   G   
   C   
   A   
   G   
   A   
   C   
   T   
   C   
   C   
   T   
   T   
   T   
   G   
   T   
   A   
   C   
   C   
   A   
   A   
   T   
   C   
   A   
   C   
   A   
   G   
   T   
   C   
   G   
   C   
   T   
   T   
   C   
   T   
   G   
   A   
   A   
   C   
   C   
   A   
   G   
   T   
   G   
   A   
   C   
   A   
   G   
   A   
   A   
   T   
   T   
   A   
   C   
   C   
   A   
   A   
   C   
   A   
   G   
   G   
   G   
   G   
   C   
   T   
   G   
   C   
   G   
   A   
   C   
   A   
   A   
   A   
   A   
   G   
   A   
   T   
   A   
   C   
   A   
   G   
   A   
   G   
   T   
   C   
   A   
   C   
   G   
   T   
   T   
   A   
   T   
   T   
   A   
   T   
   C   
   C   
   G   
   C   
   T   
   G   
   G   
   G   
   G   
   G   
   A   
   A   
   G   
   C   
   A   
   G   
   C   
   T   
   G   
   C   
   G   
   C   
   A   
   A   
   T   
   T   
   A   
   A   
   T   
   C   
   G   
   G   
   G   
   T   
   A   
   T   
   A   
   C   
   G   
   G   
   G   
   C   
   A   
   C   
   C   
   A   
   T   
   T   
   A   
   C   
   T   
   G   
   C   
   A   
   G   
   A   
   A   
   G   
   A   
   T   
   A   
   T   
   T   
   G   
   A   
   A   
   A   
   A   
   A   
   A   
   A   
   T   
   C   
   C   
   A   
   G   
   A   
   G   
   T   
   T   
   A   
   A   
   G   
   T   
   A   
   G   
   T   
   A   
   C   
   G   
   G   
   G   
   C   
   G   
   T   
   A   
   A   
   T   
   T   
   G   
   G   
   T   
   A   
   A   
   A   
   A   
   C   
   T   
   G   
   G   
   C   
   T   
   T   
   A   
   G   
   A   
   A   
   C   
   G   
   A   
   G   
   C   
   G   
   T   
   T   
   T   
   G   
   A   
   T   
   A   
   A   
   A   
   G   
   A   
   A   
   T   
   T   
   A   
   A   
   G   
   A   
   G   
   G   
   G   
   C   
   A   
   A   
   G   
   A   
   T   
   G   
   G   
   T   
   G   
   G   
   C   
   T   
   C   
   A   
   C   
   T   
   A   
   G   
   T   
   T   
   A   
   T   
   T   
   T   
   T   
   A   
   G   
   A   
   T   
   G   
   A   
   C   
   A   
   A   
   A   
   G   
   A   
   G   
   A   
   A   
   T   
   G   
   T   
   C   
   A   
   A   
   A   
   A   
   A   
   A   
   G   
   C   
   T   
   T   
   T   
   A   
   C   
   A   
   A   
   A   
   C   
   C   
   A   
   A   
   A   
   G   
   A   
   G   
   A   
   A   
   A   
   A   
   A   
   A   
   G   
   A   
   T   
   G   
   G   
   T   
   C   
   A   
   A   
   A   
   C   
   G   
   A   
   T   
   T   
   A   
   A   
   A   
   T   
   T   
   A   
   A   
   C   
   G   
   A   
   T   
   T   
   G   
   A   
   C   
   A   
   G   
   T   
   G   
   G   
   C   
   G   
   T   
   A   
   C   
   A   
   A   
   C   
   A   
   A   
   C   
   A   
   A   
   G   
   C   
   C   
   T   
   T   
   C   
   G   
   C   
   T   
   A   
   T   
   A   
   T   
   T   
   T   
   G   
   A   
   C   
   A   
   A   
   A   
   C   
   G   
   T   
   C   
   C   
   T   
   G   
   G   
   T   
   A   
   G   
   T   
   G   
   C   
   G   
   G   
   T   
   C   
   A   
   T   
   T   
   A   
   C   
   C   
   G   
   A   
   T   
   C   
   C   
   T   
   C   
   A   
   A   
   A   
   A   
   A   
   G   
   G   
   C   
   G   
   A   
   T   
   T   
   T   
   A   
   T   
   T   
   A   
   G   
   C   
   A   
   A   
   C   
   G   
   G   
   T   
   T   
   A   
   G   
   T   
   T   
   C   
   A   
   C   
   C   
   T   
   T   
   C   
   G   
   T   
   A   
   T   
   G   
   A   
   T   
   C   
   C   
   T   
   A   
   A   
   T   
   A   
   A   
   A   
   A   
   T   
   G   
   G   
   C   
   C   
   A   
   A   
   T   
   G   
   G   
   T   
   A   
   T   
   T   
   T   
   C   
   T   
   C   
   A   
   A   
   A   
   A   
   A   
   G   
   A   
   A   
   T   
   A   
   T   
   G   
   A   
   T   
   G   
   C   
   T   
   T   
   A   
   C   
   A   
   A   
   T   
   A   
   A   
   C   
   A   
   A   
   C   
   A   
   A   
   A   
   G   
   A   
   T   
   T   
   T   
   A   
   C   
   C   
   A   
   T   
   T   
   C   
   A   
   C   
   A   
   G   
   C   
   A   
   C   
   G   
   T   
   T   
   T   
   T   
   G   
   C   
   G   
   A   
   C   
   A   
   G   
   G   
   C   
   T   
   A   
   T   
   G   
   C   
   A   
   C   
   C   
   A   
   G   
   G   
   C   
   T   
   C   
   T   
   A   
   C   
   C   
   T   
   T   
   T   
   A   
   A   
   A   
   A   
   C   
   A   
   A   
   T   
   T   
   A   
   C   
   C   
   G   
   G   
   T   
   G   
   C   
   C   
   A   
   T   
   T   
   G   
   G   
   T   
   T   
   T   
   A   
   G   
   A   
   T   
   G   
   C   
   A   
   G   
   G   
   G   
   A   
   C   
   A   
   T   
   T   
   G   
   A   
   A   
   G   
   C   
   C   
   A   
   G   
   A   
   T   
   G   
   A   
   A   
   G   
   A   
   G   
   C   
   T   
   A   
   G   
   A   
   A   
   A   
   T   
   T   
   A   
   A   
   T   
   G   
   G   
   C   
   T   
   T   
   G   
   A   
   A   
   A   
   T   
   G   
   G   
   C   
   A   
   A   
   A   
   A   
   A   
   G   
   A   
   T   
   A   
   A   
   A   
   T   
   C   
   T   
   T   
   G   
   G   
   G   
   G   
   C   
   G   
   G   
   C   
   T   
   A   
   T   
   T   
   T   
   T   
   G   
   C   
   G   
   A   
   C   
   A   
   C   
   G   
   T   
   G   
   T   
   A   
   A   
   A   
   A   
   G   
   A   
   A   
   G   
   C   
   A   
   A   
   G   
   T   
   C   
   C   
   A   
   G   
   T   
   C   
   A   
   A   
   T   
   C   
   T   
   G   
   C   
   G   
   T   
   A   
   C   
   C   
   G   
   C   
   T   
   T   
   T   
   A   
   G   
   T   
   T   
   A   
   A   
   T   
   T   
   C   
   A   
   G   
   A   
   T   
   A   
   A   
   T   
   A   
   T   
   T   
   T   
   A   
   T   
   T   
   T   
   T   
   G   
   C   
   C   
   C   
   A   
   A   
   C   
   A   
   A   
   A   
   C   
   A   
   T   
   T   
   A   
   C   
   G   
   G   
   A   
   T   
   G   
   G   
   G   
   A   
   G   
   A   
   A   
   G   
   A   
   C   
   A   
   A   
   A   
   T   
   T   
   T   
   A   
   G   
   A   
   G   
   C   
   G   
   G   
   G   
   T   
   C   
   T   
   T   
   A   
   A   
   T   
   A   
   A   
   A   
   T   
   T   
   C   
   A   
   T   
   T   
   T   
   T   
   C   
   G   
   G   
   T   
   G   
   A   
   G   
   G   
   A   
   A   
   C   
   T   
   A   
   G   
   A   
   T   
   T   
   T   
   A   
   C   
   C   
   A   
   A   
   T   
   T   
   G   
   C   
   C   
   A   
   T   
   G   
   A   
   C   
   G   
   C   
   C   
   T   
   G   
   C   
   T   
   C   
   A   
   A   
   A   
   T   
   T   
   T   
   C   
   A   
   A   
   A   
   T   
   G   
   A   
   A   
   G   
   A   
   T   
   A   
   A   
   A   
   T   
   T   
   T   
   A   
   A   
   T   
   T   
   C   
   T   
   G   
   A   
   A   
   A   
   T   
   T   
   C   
   T   
   T   
   T   
   T   
   A   
   G   
   C   
   A   
   G   
   A   
   T   
   A   
   C   
   G   
   G   
   G   
   C   
   T   
   A   
   C   
   G   
   G   
   T   
   C   
   A   
   A   
   G   
   G   
   C   
   C   
   A   
   A   
   T   
   T   
   G   
   T   
   T   
   A   
   A   
   T   
   T   
   T   
   C   
   G   
   C   
   C   
   A   
   A   
   T   
   C   
   C   
   A   
   G   
   C   
   A   
   A   
   G   
   C   
   T   
   A   
   C   
   C   
   A   
   T   
   G   
   T   
   A   
   T   
   A   
   G   
   T   
   G   
   T   
   T   
   T   
   T   
   C   
   C   
   A   
   A   
   A   
   A   
   T   
   A   
   A   
   T   
   G   
   G   
   A   
   A   
   C   
   A   
   T   
   T   
   G   
   G   
   T   
   C   
   T   
   A   
   T   
   C   
   C   
   A   
   A   
   A   
   A   
   A   
   T   
   A   
   G   
   T   
   T   
   T   
   T   
   A   
   G   
   A   
   C   
   A   
   A   
   A   
   G   
   A   
   A   
   A   
   C   
   G   
   A   
   A   
   G   
   A   
   A   
   A   
   A   
   A   
   A   
   G   
   A   
   C   
   A   
   A   
   T   
   G   
   T   
   A   
   A   
   T   
   C   
   A   
   G   
   C   
   G   
   C   
   C   
   A   
   A   
   C   
   G   
   C   
   A   
   G   
   C   
   G   
   A   
   A   
   T   
   A   
   C   
   A   
   A   
   T   
   T   
   G   
   C   
   C   
   A   
   C   
   A   
   G   
   A   
   T   
   C   
   T   
   T   
   T   
   T   
   A   
   G   
   G   
   T   
   A   
   G   
   T   
   G   
   T   
   G   
   G   
   A   
   A   
   G   
   A   
   C   
   C   
   C   
   T   
   T   
   C   
   A   
   G   
   G   
   T   
   T   
   A   
   T   
   G   
   T   
   T   
   T   
   A   
   T   
   A   
   A   
   T   
   A   
   T   
   G   
   T   
   A   
   C   
   A   
   A   
   T   
   C   
   C   
   G   
   A   
   A   
   C   
   T   
   T   
   C   
   T   
   C   
   A   
   T   
   T   
   G   
   G   
   C   
   T   
   G   
   C   
   T   
   A   
   A   
   A   
   A   
   C   
   A   
   G   
   G   
   A   
   A   
   C   
   A   
   G   
   C   
   T   
   G   
   A   
   A   
   A   
   T   
   A   
   A   
   A   
   A   
   G   
   A   
   T   
   A   
   A   
   A   
   C   
   A   
   A   
   G   
   A   
   T   
   A   
   C   
   A   
   G   
   A   
   T   
   G   
   G   
   C   
   A   
   A   
   A   
   G   
   A   
   A   
   A   
   A   
   C   
   A   
   G   
   T   
   T   
   T   
   C   
   C   
   T   
   T   
   C   
   T   
   A   
   A   
   C   
   G   
   T   
   T   
   A   
   G   
   A   
   T   
   C   
   G   
   T   
   A   
   G   
   T   
   A   
   A   
   C   
   A   
   A   
   T   
   A   
   A   
   A   
   T   
   T   
   C   
   T   
   T   
   A   
   A   
   C   
   A   
   A   
   T   
   G   
   A   
   T   
   T   
   A   
   T   
   G   
   G   
   T   
   T   
   G   
   A   
   A   
   A   
   A   
   T   
   T   
   C   
   A   
   G   
   G   
   A   
   G   
   A   
   A   
   A   
   A   
   T   
   G   
   G   
   T   
   T   
   C   
   T   
   G   
   C   
   A   
   A   
   C   
   A   
   G   
   A   
   T   
   A   
   T   
   C   
   A   
   G   
   T   
   A   
   A   
   A   
   C   
   C   
   A   
   T   
   T   
   A   
   A   
   T   
   T   
   G   
   A   
   T   
   T   
   A   
   C   
   T   
   T   
   A   
   G   
   A   
   A   
   G   
   C   
   A   
   A   
   C   
   C   
   A   
   T   
   T   
   A   
   A   
   A   
   -   
   -   
   -   
   -   
   -   
   -   
   -   
   -   
   -   
   -   
   -   
   -   
   -   
   -   
   -   
   -   
   -   
   -   
   -   
   -   
   -   
   -   
   -   
   -   
   -   
   -   
   -   
   -   
   -   
   -   
   -   
   -   
   -   
   -   
   -   
   -   
   -   
   -   
   -   
   -   
   -   
   -   
   -   
   -   
   -   
   -   
   -   
   -   
   -   
   -   
   -   
   -   
   -   
   -   
   -   
   -   
   -   
   -   
   -   
   -   
   -   
 

 
   N54574   
   -   
   -   
   -   
   -   
   -   
   -   
   -   
   -   
   -   
   -   
   -   
   -   
   -   
   -   
   -   
   -   
   -   
   -   
   -   
   -   
   -   
   -   
   -   
   -   
   -   
   -   
   -   
   -   
   -   
   -   
   -   
   -   
   -   
   -   
   -   
   -   
   -   
   -   
   -   
   -   
   -   
   -   
   -   
   -   
   -   
   -   
   -   
   -   
   -   
   -   
   -   
   -   
   -   
   -   
   -   
   -   
   -   
   -   
   -   
   -   
   -   
   -   
   -   
   -   
   -   
   -   
   -   
   -   
   -   
   -   
   -   
   -   
   -   
   -   
   -   
   -   
   -   
   -   
   -   
   -   
   -   
   -   
   -   
   -   
   -   
   -   
   -   
   -   
   -   
   -   
   -   
   -   
   -   
   -   
   -   
   -   
   -   
   -   
   -   
   -   
   -   
   -   
   -   
   -   
   -   
   -   
   -   
   -   
   C   
   A   
   A   
   T   
   G   
   G   
   C   
   A   
   A   
   G   
   C   
   C   
   A   
   A   
   A   
   C   
   A   
   A   
   G   
   A   
    A    
   T   
   T   
   A   
   G   
   C   
    C    
   G   
   A   
   A   
   G   
   C   
   G   
   A   
   A   
   G   
   A   
   A   
   A   
   A   
    C    
   A   
   G   
   C   
    T    
   A   
    C    
   T   
    A    
    C    
   A   
   T   
   T   
   T   
   T   
   T   
   A   
   A   
   A   
    C    
   G   
   T   
   A   
   T   
   T   
   G   
    T    
   C   
   A   
   A   
   A   
   A   
   C   
   A   
   G   
    G    
   A   
   A   
   T   
   T   
   T   
   G   
   A   
   T   
   A   
   A   
   G   
   T   
   T   
   A   
   C   
   C   
    G    
   T   
   C   
   C   
   G   
   T   
   T   
    G    
   T   
   T   
   C   
   A   
   A   
   G   
   A   
   A   
    G    
   C   
   T   
   A   
   G   
   C   
   T   
   T   
   A   
   A   
   A   
   G   
   A   
   A   
   A   
   A   
   A   
   T   
   G   
   G   
    C    
   T   
   A   
   T   
   G   
   A   
   T   
   A   
   C   
   T   
   A   
   A   
   A   
   T   
   C   
    T    
   G   
   T   
   T   
   G   
   T   
   T   
   G   
   A   
   A   
   A   
   A   
   A   
   T   
   A   
   C   
   C   
   A   
   A   
   G   
   C   
   A   
   A   
   T   
   T   
   T   
   A   
   T   
   T   
   C   
   A   
   G   
   G   
   G   
   A   
   T   
   T   
   C   
   A   
   A   
   G   
   C   
    A    
   G   
   A   
   A   
    G    
   G   
    A    
   G   
   T   
   C   
   A   
   A   
    A    
   G   
   C   
   T   
   A   
   G   
   T   
   G   
   A   
   T   
   G   
   T   
    T    
   C   
   A   
   A   
   G   
   T   
    C    
   A   
   A   
   A   
   A   
   A   
    G    
   G   
    C    
   G   
   A   
   A   
   A   
   G   
   A   
    C    
   A   
   A   
    T    
   C   
   A   
   A   
   T   
   A   
   C   
   A   
   C   
   A   
   T   
   T   
   T   
   A   
    C    
   C   
   T   
   A   
   T   
   A   
   A   
   A   
   T   
   T   
   A   
   T   
   C   
   G   
   A   
   T   
   G   
   A   
   G   
    T    
   A   
   C   
    C    
   C   
   C   
   T   
   T   
   T   
   A   
   G   
   G   
   C   
   G   
   A   
   A   
   A   
   T   
   G   
   A   
   A   
   A   
   G   
   A   
   T   
    T    
   T   
   G   
   T   
   C   
   T   
   T   
   A   
   T   
   C   
   A   
   A   
   T   
   C   
   A   
   A   
   G   
   T   
   A   
   T   
    C    
   G   
    C    
   C   
   A   
   A   
    A    
   A   
   A   
   A   
   G   
   G   
    C    
   G   
   A   
   T   
   A   
    C    
   C   
   T   
   A   
   C   
   C   
   A   
   A   
   A   
   T   
    C    
    G    
   C   
   T   
   T   
   G   
   G   
    A    
    A    
    G    
   C   
   C   
    G    
   T   
   C   
   T   
   T   
   T   
   A   
   A   
   T   
   T   
   T   
   T   
   T   
   C   
   C   
   A   
   G   
   A   
   T   
   A   
   T   
   G   
   T   
   C   
   A   
   G   
   G   
   A   
   A   
   A   
   T   
   G   
   A   
   T   
   A   
   A   
   A   
    A    
   T   
   T   
   T   
   C   
   G   
   A   
   T   
   T   
   C   
   A   
   A   
   G   
   T   
   A   
   G   
   A   
   T   
   A   
   A   
   T   
   G   
   C   
   C   
   A   
   A   
   A   
   C   
   G   
   T   
   G   
   G   
   A   
   G   
   A   
   A   
   A   
   T   
   T   
   G   
   T   
   C   
   G   
   A   
   T   
   C   
   G   
   T   
   A   
   A   
    T    
   G   
   G   
   T   
   A   
   G   
   T   
   G   
   G   
   G   
   C   
   T   
   A   
   G   
    C    
   A   
   A   
   T   
   T   
   A   
   A   
   C   
   A   
   A   
   A   
   G   
   T   
   G   
   T   
   T   
   T   
   G   
   A   
    C    
   G   
   A   
   A   
   G   
   T   
   G   
   G   
   G   
   C   
   G   
   T   
   A   
   G   
   T   
    G    
   C   
   C   
   T   
    G    
   G   
   C   
   A   
   A   
   A   
   C   
   T   
    C    
   G   
   G   
   T   
   T   
   C   
   T   
   G   
   G   
   C   
   G   
   C   
   A   
   G   
   A   
   A   
   A   
   A   
   A   
    A    
   C   
   A   
   G   
   C   
    C    
   A   
   A   
   T   
   A   
   T   
   C   
   A   
   A   
   A   
   G   
   C   
   T   
   T   
   T   
   T   
   A   
   G   
   T   
   G   
   A   
   T   
   A   
   A   
    A    
   T   
   T   
    C    
   G   
   G   
   C   
   G   
   T   
   T   
   T   
   C   
   T   
    G    
   T   
   T   
   G   
   A   
   T   
   G   
   A   
   A   
   A   
   T   
   C   
   A   
   A   
   T   
   C   
    A    
   A   
   A   
   A   
    G    
   T   
   T   
   A   
   A   
   G   
   C   
   C   
   A   
   A   
   G   
   G   
    A    
   T   
   G   
   G   
   G   
   T   
   C   
   C   
   A   
   A   
   G   
   C   
   A   
   G   
   A   
   C   
   T   
   C   
    C    
   T   
   T   
   T   
   G   
   T   
   A   
   C   
   C   
   A   
   A   
   T   
   C   
   A   
   C   
   A   
   G   
   T   
    C    
   G   
   C   
   T   
   T   
   C   
   T   
   G   
   A   
   A   
   C   
   C   
   A   
   G   
   T   
   G   
   A   
   C   
   A   
   G   
   A   
   A   
   T   
   T   
   A   
   C   
   C   
   A   
   A   
   C   
   A   
   G   
   G   
   G   
   G   
   C   
   T   
   G   
   C   
   G   
   A   
   C   
   A   
   A   
   A   
   A   
   G   
    A    
    T    
   A   
   C   
   A   
   G   
   A   
   G   
   T   
   C   
   A   
   C   
   G   
   T   
   T   
   A   
   T   
   T   
   A   
   T   
   C   
   C   
    G    
    C    
   T   
   G   
   G   
   G   
   G   
   G   
   A   
   A   
   G   
   C   
   A   
   G   
   C   
   T   
   G   
   C   
   G   
   C   
   A   
   A   
   T   
   T   
   A   
   A   
   T   
   C   
   G   
   G   
    G    
   T   
   A   
   T   
   A   
   C   
    G    
   G   
   G   
    C    
   A   
   C   
    C    
   A   
   T   
   T   
   A   
   C   
   T   
   G   
   C   
   A   
   G   
   A   
    A    
   G   
   A   
   T   
   A   
   T   
   T   
    G    
   A   
   A   
   A   
   A   
   A   
   A   
   A   
   T   
   C   
   C   
   A   
   G   
   A   
   G   
   T   
   T   
   A   
   A   
   G   
   T   
   A   
   G   
   T   
   A   
   C   
   G   
   G   
   G   
    C    
   G   
   T   
   A   
   A   
   T   
   T   
   G   
   G   
    T    
   A   
   A   
   A   
   A   
   C   
   T   
   G   
   G   
    T    
   T   
   T   
   A   
   G   
   A   
   A   
   C   
   G   
   A   
   G   
   C   
   G   
   T   
   T   
   T   
   G   
   A   
   T   
   A   
   A   
   A   
   G   
   A   
    A    
   T   
   T   
   A   
   A   
   G   
   A   
   G   
   G   
    G    
   C   
   A   
   A   
   G   
   A   
   T   
   G   
   G   
    T    
   G   
   G   
    C    
   T   
   C   
    A    
   C   
   T   
   A   
   G   
   T   
   T   
   A   
   T   
   T   
   T   
   T   
   A   
   G   
   A   
   T   
   G   
   A   
   C   
   A   
   A   
   A   
   G   
   A   
   G   
   A   
   A   
   T   
   G   
   T   
   C   
   A   
   A   
   A   
   A   
   A   
   A   
   G   
   C   
   T   
   T   
   T   
   A   
   C   
   A   
   A   
   A   
   C   
   C   
   A   
   A   
   A   
   G   
   A   
    G    
   A   
   A   
   A   
   A   
   A   
   A   
   G   
   A   
   T   
   G   
   G   
   T   
   C   
   A   
   A   
   A   
   C   
    G    
   A   
   T   
   T   
   A   
   A   
   A   
   T   
   T   
   A   
   A   
   C   
   G   
   A   
   T   
   T   
   G   
   A   
    C    
   A   
   G   
    T    
   G   
   G   
    C    
   G   
   T   
   A   
   C   
   A   
   A   
   C   
   A   
   A   
   C   
   A   
   A   
   G   
   C   
    T    
   T   
   T   
    T    
   G   
   C   
   T   
   A   
   T   
   A   
   T   
   T   
   T   
   G   
   A   
   C   
   A   
   A   
   A   
   C   
   G   
    C    
    C    
   C   
   T   
   G   
   G   
   T   
   A   
   G   
   T   
   G   
   C   
   G   
   G   
   T   
   C   
   A   
   T   
   T   
   A   
   C   
   C   
   G   
   A   
   T   
   C   
   C   
   T   
   C   
   A   
   A   
   A   
   A   
   A   
   G   
   G   
   C   
   G   
   A   
   T   
   T   
   T   
   A   
    T    
   T   
   A   
   G   
   C   
    A    
   A   
   C   
   G   
   G   
   T   
   T   
   A   
   G   
   T   
   T   
   C   
   A   
   C   
   C   
   T   
   T   
   C   
   G   
   T   
   A   
   T   
   G   
   A   
   T   
   C   
   C   
   T   
   A   
   A   
   T   
   A   
   A   
   A   
   A   
   T   
   G   
   G   
   C   
   C   
   A   
   A   
   T   
   G   
   G   
    C    
   A   
   T   
   T   
   T   
   C   
   T   
   C   
   A   
   A   
   A   
   A   
   A   
   G   
   A   
    A    
   T   
   A   
   T   
   G   
   A   
   T   
   G   
   C   
   T   
   T   
   A   
   C   
   A   
   A   
   T   
   A   
   A   
   C   
   A   
   A   
   C   
   A   
   A   
   A   
   G   
   A   
   T   
   T   
   T   
   A   
   C   
   C   
   A   
   T   
   T   
    C    
   A   
   C   
   A   
   G   
   C   
   A   
   C   
   G   
   T   
   T   
   T   
    T    
   G   
   C   
   G   
   A   
   C   
   A   
   G   
   G   
    C    
   T   
   A   
   T   
   G   
   C   
   A   
   C   
   C   
   A   
   G   
   G   
    C    
   T   
   C   
   T   
   A   
   C   
   C   
   T   
   T   
   T   
   A   
   A   
   A   
   A   
   C   
   A   
   A   
   T   
   T   
   A   
   C   
   C   
   G   
   G   
   T   
   G   
   C   
   C   
   A   
   T   
   T   
   G   
   G   
   T   
   T   
   T   
   A   
   G   
   A   
   T   
    G    
   C   
   A   
   G   
   G   
   G   
   A   
   C   
   A   
   T   
   T   
   G   
   A   
   A   
   G   
   C   
   C   
   A   
   G   
   A   
   T   
   G   
   A   
   A   
   G   
   A   
   G   
   C   
   T   
   A   
   G   
   A   
   A   
   A   
   T   
   T   
   A   
   A   
   T   
   G   
   G   
    T    
   T   
   T   
   G   
   A   
   A   
   A   
   T   
   G   
   G   
   C   
   A   
   A   
   A   
   A   
   A   
   G   
   A   
   T   
   A   
   A   
   A   
   T   
   C   
   T   
   T   
   G   
   G   
   G   
   G   
    C    
   G   
   G   
   C   
   T   
   A   
   T   
   T   
   T   
   T   
   G   
   C   
    G    
   A   
   C   
   A   
   C   
   G   
   T   
   G   
   T   
   A   
   A   
   A   
   A   
   G   
   A   
   A   
   G   
   C   
   A   
   A   
   G   
   T   
   C   
   C   
   A   
   G   
   T   
   C   
   A   
   A   
   T   
   C   
   T   
    G    
   C   
   G   
   T   
   A   
   C   
   C   
   G   
   C   
   T   
   T   
   T   
   A   
   G   
   T   
   T   
   A   
   A   
   T   
   T   
   C   
   A   
   G   
   A   
   T   
   A   
   A   
   T   
   A   
   T   
   T   
   T   
   A   
   T   
   T   
   T   
   T   
   G   
   C   
    C    
   C   
   A   
   A   
   C   
   A   
   A   
   A   
   C   
   A   
   T   
   T   
   A   
   C   
   G   
    G    
   A   
   T   
   G   
   G   
   G   
    A    
   G   
   A   
   A   
   G   
   A   
   C   
   A   
   A   
   A   
   T   
   T   
   T   
   A   
   G   
   A   
   G   
   C   
    G    
   G   
   G   
   T   
   C   
   T   
   T   
   A   
   A   
   T   
   A   
   A   
   A   
   T   
   T   
   C   
   A   
   T   
   T   
   T   
   T   
   C   
   G   
   G   
   T   
   G   
   A   
   G   
   G   
   A   
   A   
   C   
   T   
   A   
   G   
   A   
   T   
   T   
   T   
   A   
   C   
   C   
   A   
   A   
   T   
    T    
   G   
   C   
   C   
   A   
   T   
   G   
   A   
   C   
   G   
   C   
   C   
    T    
   G   
   C   
   T   
   C   
   A   
   A   
   A   
   T   
   T   
   T   
   C   
   A   
   A   
   A   
    T    
   G   
   A   
   A   
   G   
   A   
   T   
   A   
   A   
   A   
   T   
   T   
   T   
   A   
   A   
   T   
   T   
   C   
   T   
   G   
   A   
   A   
   A   
   T   
   T   
   C   
   T   
   T   
   T   
   T   
    A    
   G   
   C   
   A   
   G   
   A   
   T   
   A   
   C   
    G    
   G   
   G   
   C   
   T   
   A   
    C    
   G   
   G   
    T    
   C   
   A   
   A   
   G   
   G   
    T    
   C   
   A   
   A   
   T   
   T   
   G   
   T   
   T   
   A   
   A   
   T   
   T   
   T   
   C   
    G    
   C   
   C   
   A   
   A   
   T   
   C   
   C   
   A   
   G   
   C   
   A   
   A   
   G   
   C   
   T   
   A   
   C   
   C   
   A   
   T   
   G   
   T   
   A   
   T   
   A   
   G   
   T   
   G   
   T   
   T   
   T   
   T   
   C   
   C   
   A   
   A   
   A   
   A   
   T   
   A   
   A   
   T   
   G   
   G   
    A    
   A   
   C   
   A   
    T    
   T   
    A    
   G   
   T   
    C    
   T   
   A   
   T   
   C   
   C   
   A   
   A   
   A   
   A   
    C    
   T   
   A   
   G   
   T   
   T   
   T   
   T   
   A   
   G   
   A   
   A   
   A   
   A   
   A   
   G   
   A   
   A   
   A   
   C   
    G    
   A   
   A   
   G   
   A   
   A   
   A   
   A   
   A   
   A   
   G   
   A   
   C   
   A   
   A   
   T   
   G   
   T   
   A   
   A   
   T   
   C   
   A   
   G   
   C   
   G   
   C   
   C   
   A   
   A   
   C   
   G   
   C   
   A   
   G   
   C   
   G   
   A   
   A   
   T   
   A   
   C   
   A   
   A   
   T   
   T   
   G   
   C   
   C   
   A   
   C   
   A   
   G   
   A   
   T   
   C   
   T   
   T   
   T   
   T   
   A   
   G   
   G   
   T   
   A   
   G   
   T   
   G   
   T   
   G   
   G   
   A   
   A   
   G   
   A   
   T   
   C   
   C   
   T   
   T   
   C   
   A   
   G   
   G   
   T   
   T   
   A   
   T   
   G   
   T   
   T   
   T   
   A   
   T   
   A   
   A   
   T   
   A   
   T   
   G   
   T   
   A   
   C   
   A   
   A   
   T   
   C   
   C   
   G   
   A   
   A   
   C   
   T   
   T   
   C   
   T   
   C   
   A   
   T   
   T   
   G   
   G   
   C   
   T   
   G   
   C   
   T   
   A   
   A   
   A   
   A   
   C   
   A   
   G   
   G   
   A   
   A   
   C   
   A   
   G   
   C   
   T   
   G   
   A   
   A   
   A   
   T   
   A   
   A   
   A   
   A   
   G   
   A   
   T   
   A   
   A   
   A   
   C   
   A   
   A   
   G   
   A   
   T   
   A   
   C   
   A   
   G   
   A   
   T   
   G   
   G   
   C   
   A   
   A   
   A   
   G   
   A   
   A   
   A   
   A   
   C   
   A   
   G   
   T   
   T   
   T   
   C   
   C   
   T   
   T   
   C   
   T   
   A   
   A   
   C   
   G   
   T   
   T   
   A   
   G   
   A   
   T   
   C   
   G   
   T   
   A   
   G   
   T   
   A   
   A   
   C   
   A   
   A   
   T   
   A   
   A   
   A   
   T   
   T   
   C   
   T   
   T   
   A   
   A   
   C   
   A   
   A   
   T   
   G   
   A   
   T   
   T   
   A   
   T   
   G   
   G   
   T   
   T   
   G   
   A   
   A   
   A   
   A   
   T   
   T   
   C   
   A   
   G   
   G   
   A   
   G   
   A   
   A   
   A   
   A   
   T   
   G   
   G   
   T   
   T   
   C   
   T   
   G   
   C   
   A   
   A   
   C   
   A   
   G   
   A   
   T   
   A   
   T   
   C   
   A   
   G   
   T   
   A   
   A   
   A   
   C   
   C   
   A   
   T   
   T   
   A   
   A   
   T   
   T   
   G   
   A   
   T   
   T   
   A   
   C   
   T   
   T   
   A   
   G   
   A   
   A   
   G   
   C   
   A   
   A   
   C   
   C   
   A   
   T   
   T   
   A   
   A   
   A   
   -   
   -   
   -   
   -   
   -   
   -   
   -   
   -   
   -   
   -   
   -   
   -   
   -   
   -   
   -   
   -   
   -   
   -   
   -   
   -   
   -   
   -   
   -   
   -   
   -   
   -   
   -   
   -   
   -   
   -   
   -   
   -   
   -   
   -   
   -   
   -   
   -   
   -   
   -   
   -   
   -   
   -   
   -   
   -   
   -   
   -   
   -   
   -   
   -   
   -   
   -   
   -   
   -   
   -   
   -   
   -   
   -   
   -   
   -   
   -   
   -   
   -   
   -   
   -   
   -   
   -   
   -   
   -   
   -   
   -   
   -   
   -   
   -   
   -   
   -   
   -   
   -   
   -   
   -   
   -   
   -   
   -   
   -   
   -   
   -   
   -   
   -   
   -   
   -   
   -   
   -   
   -   
   -   
   -   
   -   
   -   
   -   
   -   
   -   
   -   
   -   
   -   
   -   
   -   
   -   
   -   
   -   
   -   
   -   
   -   
   -   
   -   
   -   
   -   
   -   
   -   
   -   
   -   
   -   
   -   
   -   
   -   
   -   
   -   
   -   
   -   
   -   
   -   
   -   
   -   
   -   
   -   
   -   
   -   
   -   
   -   
   -   
   -   
   -   
   -   
   -   
   -   
   -   
   -   
   -   
   -   
   -   
   -   
   -   
   -   
   -   
   -   
   -   
   -   
   -   
   -   
   -   
   -   
   -   
   -   
   -   
   -   
   -   
   -   
   -   
   -   
   -   
   -   
   -   
   C   
   A   
   A   
   T   
   G   
   G   
   C   
   A   
   A   
   G   
   C   
   C   
   A   
   A   
   A   
   C   
   A   
   A   
   G   
   A   
   A   
   T   
   T   
   A   
   G   
   C   
   C   
   G   
   A   
   A   
   G   
   C   
   G   
   A   
   A   
   G   
   A   
   A   
   A   
   A   
   C   
   A   
   G   
   C   
   T   
   A   
   C   
   T   
   A   
   C   
   A   
   T   
   T   
   T   
   T   
   T   
   A   
   A   
   A   
   C   
   G   
   T   
   A   
   T   
   T   
   G   
   T   
   C   
   A   
   A   
   A   
   A   
   C   
   A   
   G   
   G   
   A   
   A   
   T   
   T   
   T   
   G   
   A   
   T   
   A   
   A   
   G   
   T   
   T   
   A   
   C   
   C   
   G   
   T   
   C   
   C   
   G   
   T   
   T   
   G   
   T   
   T   
   C   
   A   
   A   
   G   
   A   
   A   
   G   
   C   
   T   
   A   
   G   
   C   
   T   
   T   
   A   
   A   
   A   
   G   
   A   
   A   
   A   
   A   
   A   
   T   
   G   
   G   
   C   
   T   
   A   
   T   
   G   
   A   
   T   
   A   
   C   
   T   
   A   
   A   
   A   
   T   
   C   
   T   
   G   
   T   
   T   
   G   
   T   
   T   
   G   
   A   
   A   
   A   
   A   
   A   
   T   
   A   
   C   
   C   
   A   
   A   
   G   
   C   
   A   
   A   
   T   
   T   
   T   
   A   
   T   
   T   
   C   
   A   
   G   
   G   
   G   
   A   
   T   
   T   
   C   
   A   
   A   
   G   
   C   
   A   
   G   
   A   
   A   
   G   
   G   
   A   
   G   
   T   
   C   
   A   
   A   
   A   
   G   
   C   
   T   
   A   
   G   
   T   
   G   
   A   
   T   
   G   
   T   
   T   
   C   
   A   
   A   
   G   
   T   
   C   
   A   
   A   
   A   
   A   
   A   
   G   
   G   
   C   
   G   
   A   
   A   
   A   
   G   
   A   
   C   
   A   
   A   
   T   
   C   
   A   
   A   
   T   
   A   
   C   
   A   
   C   
   A   
   T   
   T   
   T   
   A   
   C   
   C   
   T   
   A   
   T   
   A   
   A   
   A   
   T   
   T   
   A   
   T   
   C   
   G   
   A   
   T   
   G   
   A   
   G   
   T   
   A   
   C   
   C   
   C   
   C   
   T   
   T   
   T   
   A   
   G   
   G   
   C   
   G   
   A   
   A   
   A   
   T   
   G   
   A   
   A   
   A   
   G   
   A   
   T   
   T   
   T   
   G   
   T   
   C   
   T   
   T   
   A   
   T   
   C   
   A   
   A   
   T   
   C   
   A   
   A   
   G   
   T   
   A   
   T   
   C   
   G   
   C   
   C   
   A   
   A   
   A   
   A   
   A   
   A   
   G   
   G   
   C   
   G   
   A   
   T   
   A   
   C   
   C   
   T   
   A   
   C   
   C   
   A   
   A   
   A   
   T   
   C   
   G   
   C   
   T   
   T   
   G   
   G   
   A   
   A   
   G   
   C   
   C   
   G   
   T   
   C   
   T   
   T   
   T   
   A   
   A   
   T   
   T   
   T   
   T   
   T   
   C   
   C   
   A   
   G   
   A   
   T   
   A   
   T   
   G   
   T   
   C   
   A   
   G   
   G   
   A   
   A   
   A   
   T   
   G   
   A   
   T   
   A   
   A   
   A   
   A   
   T   
   T   
   T   
   C   
   G   
   A   
   T   
   T   
   C   
   A   
   A   
   G   
   T   
   A   
   G   
   A   
   T   
   A   
   A   
   T   
   G   
   C   
   C   
   A   
   A   
   A   
   C   
   G   
   T   
   G   
   G   
   A   
   G   
   A   
   A   
   A   
   T   
   T   
   G   
   T   
   C   
   G   
   A   
   T   
   C   
   G   
   T   
   A   
   A   
   T   
   G   
   G   
   T   
   A   
   G   
   T   
   G   
   G   
   G   
   C   
   T   
   A   
   G   
   C   
   A   
   A   
   T   
   T   
   A   
   A   
   C   
   A   
   A   
   A   
   G   
   T   
   G   
   T   
   T   
   T   
   G   
   A   
   C   
   G   
   A   
   A   
   G   
   T   
   G   
   G   
   G   
   C   
   G   
   T   
   A   
   G   
   T   
   G   
   C   
   C   
   T   
   G   
   G   
   C   
   A   
   A   
   A   
   C   
   T   
   C   
   G   
   G   
   T   
   T   
   C   
   T   
   G   
   G   
   C   
   G   
   C   
   A   
   G   
   A   
   A   
   A   
   A   
   A   
   A   
   C   
   A   
   G   
   C   
   C   
   A   
   A   
   T   
   A   
   T   
   C   
   A   
   A   
   A   
   G   
   C   
   T   
   T   
   T   
   T   
   A   
   G   
   T   
   G   
   A   
   T   
   A   
   A   
   A   
   T   
   T   
   C   
   G   
   G   
   C   
   G   
   T   
   T   
   T   
   C   
   T   
   G   
   T   
   T   
   G   
   A   
   T   
   G   
   A   
   A   
   A   
   T   
   C   
   A   
   A   
   T   
   C   
   A   
   A   
   A   
   A   
   G   
   T   
   T   
   A   
   A   
   G   
   C   
   C   
   A   
   A   
   G   
   G   
   A   
   T   
   G   
   G   
   G   
   T   
   C   
   C   
   A   
   A   
   G   
   C   
   A   
   G   
   A   
   C   
   T   
   C   
   C   
   T   
   T   
   T   
   G   
   T   
   A   
   C   
   C   
   A   
   A   
   T   
   C   
   A   
   C   
   A   
   G   
   T   
   C   
   G   
   C   
   T   
   T   
   C   
   T   
   G   
   A   
   A   
   C   
   C   
   A   
   G   
   T   
   G   
   A   
   C   
   A   
   G   
   A   
   A   
   T   
   T   
   A   
   C   
   C   
   A   
   A   
   C   
   A   
   G   
   G   
   G   
   G   
   C   
   T   
   G   
   C   
   G   
   A   
   C   
   A   
   A   
   A   
   A   
   G   
   A   
   T   
   A   
   C   
   A   
   G   
   A   
   G   
   T   
   C   
   A   
   C   
   G   
   T   
   T   
   A   
   T   
   T   
   A   
   T   
   C   
   C   
   G   
   C   
   T   
   G   
   G   
   G   
   G   
   G   
   A   
   A   
   G   
   C   
   A   
   G   
   C   
   T   
   G   
   C   
   G   
   C   
   A   
   A   
   T   
   T   
   A   
   A   
   T   
   C   
   G   
   G   
   G   
   T   
   A   
   T   
   A   
   C   
   G   
   G   
   G   
   C   
   A   
   C   
   C   
   A   
   T   
   T   
   A   
   C   
   T   
   G   
   C   
   A   
   G   
   A   
   A   
   G   
   A   
   T   
   A   
   T   
   T   
   G   
   A   
   A   
   A   
   A   
   A   
   A   
   A   
   T   
   C   
   C   
   A   
   G   
   A   
   G   
   T   
   T   
   A   
   A   
   G   
   T   
   A   
   G   
   T   
   A   
   C   
   G   
   G   
   G   
   C   
   G   
   T   
   A   
   A   
   T   
   T   
   G   
   G   
   T   
   A   
   A   
   A   
   A   
   C   
   T   
   G   
   G   
   T   
   T   
   T   
   A   
   G   
   A   
   A   
   C   
   G   
   A   
   G   
   C   
   G   
   T   
   T   
   T   
   G   
   A   
   T   
   A   
   A   
   A   
   G   
   A   
   A   
   T   
   T   
   A   
   A   
   G   
   A   
   G   
   G   
   G   
   C   
   A   
   A   
   G   
   A   
   T   
   G   
   G   
   T   
   G   
   G   
   C   
   T   
   C   
   A   
   C   
   T   
   A   
   G   
   T   
   T   
   A   
   T   
   T   
   T   
   T   
   A   
   G   
   A   
   T   
   G   
   A   
   C   
   A   
   A   
   A   
   G   
   A   
   G   
   A   
   A   
   T   
   G   
   T   
   C   
   A   
   A   
   A   
   A   
   A   
   A   
   G   
   C   
   T   
   T   
   T   
   A   
   C   
   A   
   A   
   A   
   C   
   C   
   A   
   A   
   A   
   G   
   A   
   G   
   A   
   A   
   A   
   A   
   A   
   A   
   G   
   A   
   T   
   G   
   G   
   T   
   C   
   A   
   A   
   A   
   C   
   G   
   A   
   T   
   T   
   A   
   A   
   A   
   T   
   T   
   A   
   A   
   C   
   G   
   A   
   T   
   T   
   G   
   A   
   C   
   A   
   G   
   T   
   G   
   G   
   C   
   G   
   T   
   A   
   C   
   A   
   A   
   C   
   A   
   A   
   C   
   A   
   A   
   G   
   C   
   T   
   T   
   T   
   T   
   G   
   C   
   T   
   A   
   T   
   A   
   T   
   T   
   T   
   G   
   A   
   C   
   A   
   A   
   A   
   C   
   G   
   C   
   C   
   C   
   T   
   G   
   G   
   T   
   A   
   G   
   T   
   G   
   C   
   G   
   G   
   T   
   C   
   A   
   T   
   T   
   A   
   C   
   C   
   G   
   A   
   T   
   C   
   C   
   T   
   C   
   A   
   A   
   A   
   A   
   A   
   G   
   G   
   C   
   G   
   A   
   T   
   T   
   T   
   A   
   T   
   T   
   A   
   G   
   C   
   A   
   A   
   C   
   G   
   G   
   T   
   T   
   A   
   G   
   T   
   T   
   C   
   A   
   C   
   C   
   T   
   T   
   C   
   G   
   T   
   A   
   T   
   G   
   A   
   T   
   C   
   C   
   T   
   A   
   A   
   T   
   A   
   A   
   A   
   A   
   T   
   G   
   G   
   C   
   C   
   A   
   A   
   T   
   G   
   G   
   C   
   A   
   T   
   T   
   T   
   C   
   T   
   C   
   A   
   A   
   A   
   A   
   A   
   G   
   A   
   A   
   T   
   A   
   T   
   G   
   A   
   T   
   G   
   C   
   T   
   T   
   A   
   C   
   A   
   A   
   T   
   A   
   A   
   C   
   A   
   A   
   C   
   A   
   A   
   A   
   G   
   A   
   T   
   T   
   T   
   A   
   C   
   C   
   A   
   T   
   T   
   C   
   A   
   C   
   A   
   G   
   C   
   A   
   C   
   G   
   T   
   T   
   T   
   T   
   G   
   C   
   G   
   A   
   C   
   A   
   G   
   G   
   C   
   T   
   A   
   T   
   G   
   C   
   A   
   C   
   C   
   A   
   G   
   G   
   C   
   T   
   C   
   T   
   A   
   C   
   C   
   T   
   T   
   T   
   A   
   A   
   A   
   A   
   C   
   A   
   A   
   T   
   T   
   A   
   C   
   C   
   G   
   G   
   T   
   G   
   C   
   C   
   A   
   T   
   T   
   G   
   G   
   T   
   T   
   T   
   A   
   G   
   A   
   T   
   G   
   C   
   A   
   G   
   G   
   G   
   A   
   C   
   A   
   T   
   T   
   G   
   A   
   A   
   G   
   C   
   C   
   A   
   G   
   A   
   T   
   G   
   A   
   A   
   G   
   A   
   G   
   C   
   T   
   A   
   G   
   A   
   A   
   A   
   T   
   T   
   A   
   A   
   T   
   G   
   G   
   T   
   T   
   T   
   G   
   A   
   A   
   A   
   T   
   G   
   G   
   C   
   A   
   A   
   A   
   A   
   A   
   G   
   A   
   T   
   A   
   A   
   A   
   T   
   C   
   T   
   T   
   G   
   G   
   G   
   G   
   C   
   G   
   G   
   C   
   T   
   A   
   T   
   T   
   T   
   T   
   G   
   C   
   G   
   A   
   C   
   A   
   C   
   G   
   T   
   G   
   T   
   A   
   A   
   A   
   A   
   G   
   A   
   A   
   G   
   C   
   A   
   A   
   G   
   T   
   C   
   C   
   A   
   G   
   T   
   C   
   A   
   A   
   T   
   C   
   T   
   G   
   C   
   G   
   T   
   A   
   C   
   C   
   G   
   C   
   T   
   T   
   T   
   A   
   G   
   T   
   T   
   A   
   A   
   T   
   T   
   C   
   A   
   G   
   A   
   T   
   A   
   A   
   T   
   A   
   T   
   T   
   T   
   A   
   T   
   T   
   T   
   T   
   G   
   C   
   C   
   C   
   A   
   A   
   C   
   A   
   A   
   A   
   C   
   A   
   T   
   T   
   A   
   C   
   G   
   G   
   A   
   T   
   G   
   G   
   G   
   A   
   G   
   A   
   A   
   G   
   A   
   C   
   A   
   A   
   A   
   T   
   T   
   T   
   A   
   G   
   A   
   G   
   C   
   G   
   G   
   G   
   T   
   C   
   T   
   T   
   A   
   A   
   T   
   A   
   A   
   A   
   T   
   T   
   C   
   A   
   T   
   T   
   T   
   T   
   C   
   G   
   G   
   T   
   G   
   A   
   G   
   G   
   A   
   A   
   C   
   T   
   A   
   G   
   A   
   T   
   T   
   T   
   A   
   C   
   C   
   A   
   A   
   T   
   T   
   G   
   C   
   C   
   A   
   T   
   G   
   A   
   C   
   G   
   C   
   C   
   T   
   G   
   C   
   T   
   C   
   A   
   A   
   A   
   T   
   T   
   T   
   C   
   A   
   A   
   A   
   T   
   G   
   A   
   A   
   G   
   A   
   T   
   A   
   A   
   A   
   T   
   T   
   T   
   A   
   A   
   T   
   T   
   C   
   T   
   G   
   A   
   A   
   A   
   T   
   T   
   C   
   T   
   T   
   T   
   T   
   A   
   G   
   C   
   A   
   G   
   A   
   T   
   A   
   C   
   G   
   G   
   G   
   C   
   T   
   A   
   C   
   G   
   G   
   T   
   C   
   A   
   A   
   G   
   G   
   T   
   C   
   A   
   A   
   T   
   T   
   G   
   T   
   T   
   A   
   A   
   T   
   T   
   T   
   C   
   G   
   C   
   C   
   A   
   A   
   T   
   C   
   C   
   A   
   G   
   C   
   A   
   A   
   G   
   C   
   T   
   A   
   C   
   C   
   A   
   T   
   G   
   T   
   A   
   T   
   A   
   G   
   T   
   G   
   T   
   T   
   T   
   T   
   C   
   C   
   A   
   A   
   A   
   A   
   T   
   A   
   A   
   T   
   G   
   G   
   A   
   A   
   C   
   A   
   T   
   T   
   A   
   G   
   T   
   C   
   T   
   A   
   T   
   C   
   C   
   A   
   A   
   A   
   A   
   C   
   T   
   A   
   G   
   T   
   T   
   T   
   T   
   A   
   G   
   A   
   A   
   A   
   A   
   A   
   G   
   A   
   A   
   A   
   C   
   G   
   A   
   A   
   G   
   A   
   A   
   A   
   A   
   A   
   A   
   G   
   A   
   C   
   A   
   A   
   T   
   G   
   T   
   A   
   A   
   T   
   C   
   A   
   G   
   C   
   G   
   C   
   C   
   A   
   A   
   C   
   G   
   C   
   A   
   G   
   C   
   G   
   A   
   A   
   T   
   A   
   C   
   A   
   A   
   T   
   T   
   G   
   C   
   C   
   A   
   C   
   A   
   G   
   A   
   T   
   C   
   T   
   T   
   T   
   T   
   A   
   G   
   G   
   T   
   A   
   G   
   T   
   G   
   T   
   G   
   G   
   A   
   A   
   G   
   A   
   T   
   C   
   C   
   T   
   T   
   C   
   A   
   G   
   G   
   T   
   T   
   A   
   T   
   G   
   T   
   T   
   T   
   A   
   T   
   A   
   A   
   T   
   A   
   T   
   G   
   T   
   A   
   C   
   A   
   A   
   T   
   C   
   C   
   G   
   A   
   A   
   C   
   T   
   T   
   C   
   T   
   C   
   A   
   T   
   T   
   G   
   G   
   C   
   T   
   G   
   C   
   T   
   A   
   A   
   A   
   A   
   C   
   A   
   G   
   G   
   A   
   A   
   C   
   A   
   G   
   C   
   T   
   G   
   A   
   A   
   A   
   T   
   A   
   A   
   A   
   A   
   G   
   A   
   T   
   A   
   A   
   A   
   C   
   A   
   A   
   G   
   A   
   T   
   A   
   C   
   A   
   G   
   A   
   T   
   G   
   G   
   C   
   A   
   A   
   A   
   G   
   A   
   A   
   A   
   A   
   C   
   A   
   G   
   T   
   T   
   T   
   C   
   C   
   T   
   T   
   C   
   T   
   A   
   A   
   C   
   G   
   T   
   T   
   A   
   G   
   A   
   T   
   C   
   G   
   T   
   A   
   G   
   T   
   A   
   A   
   C   
   A   
   A   
   T   
   A   
   A   
   A   
   T   
   T   
   C   
   T   
   T   
   A   
   A   
   C   
   A   
   A   
   T   
   G   
   A   
   T   
   T   
   A   
   T   
   G   
   G   
   T   
   T   
   G   
   A   
   A   
   A   
   A   
   T   
   T   
   C   
   A   
   G   
   G   
   A   
   G   
   A   
   A   
   A   
   A   
   T   
   G   
   G   
   T   
   T   
   C   
   T   
   G   
   C   
   A   
   A   
   C   
   A   
   G   
   A   
   T   
   A   
   T   
   C   
   A   
   G   
   T   
   A   
   A   
   A   
   C   
   C   
   A   
   T   
   T   
   A   
   A   
   T   
   T   
   G   
   A   
   T   
   T   
   A   
   C   
   T   
   T   
   A   
   G   
   A   
   A   
   G   
   C   
   A   
   A   
   C   
   C   
   A   
   T   
   T   
   A   
   A   
   A   
   -   
   -   
   -   
   -   
   -   
   -   
   -   
   -   
   -   
   -   
   -   
   -   
   -   
   -   
   -   
   -   
   -   
   -   
   -   
   -   
   -   
   -   
   -   
   -   
   -   
   -   
   -   
   -   
   -   
   -   
   -   
   -   
   -   
   -   
   -   
   -   
   -   
   -   
   -   
   -   
   -   
   -   
   -   
   -   
   -   
   -   
   -   
   -   
   -   
   -   
   -   
   -   
   -   
   -   
   -   
   -   
   -   
   -   
   -   
   -   
   -   
 

 
   N59845F   
   -   
   -   
   -   
   -   
   -   
   -   
   -   
   -   
   -   
   -   
   -   
   -   
   -   
   -   
   -   
   -   
   -   
   -   
   -   
   -   
   -   
   -   
   -   
   -   
   -   
   -   
   -   
   -   
   -   
   -   
   -   
   -   
   -   
   -   
   -   
   -   
   -   
   -   
   -   
   -   
   -   
   -   
   -   
   -   
   -   
   -   
   -   
   -   
   -   
   -   
   -   
   -   
   -   
   -   
   -   
   -   
   -   
   -   
   -   
   -   
   -   
   -   
   -   
   -   
   -   
   -   
   -   
   -   
   -   
   -   
   -   
   -   
   -   
   -   
   -   
   -   
   -   
   -   
   -   
   -   
   -   
   -   
   -   
   -   
   -   
   -   
   -   
   -   
   -   
   -   
   -   
   -   
   -   
   -   
   -   
   -   
   -   
   -   
   -   
   -   
   -   
   -   
   -   
   -   
   -   
   -   
   -   
   -   
   C   
   A   
   A   
   T   
   G   
   G   
   C   
   A   
   A   
   G   
   C   
   C   
   A   
   A   
   A   
   C   
   A   
   A   
   G   
   A   
    A    
   T   
   T   
   A   
   G   
   C   
    C    
   G   
   A   
   A   
   G   
   C   
   G   
   A   
   A   
   G   
   A   
   A   
   A   
   A   
    C    
   A   
   G   
   C   
    T    
   A   
    C    
   T   
    A    
    C    
   A   
   T   
   T   
   T   
   T   
   T   
   A   
   A   
   A   
    C    
   G   
   T   
   A   
   T   
   T   
   G   
    T    
   C   
   A   
   A   
   A   
   A   
   C   
   A   
   G   
    G    
   A   
   A   
   T   
   T   
   T   
   G   
   A   
   T   
   A   
   A   
   G   
   T   
   T   
   A   
   C   
   C   
    G    
   T   
   C   
   C   
   G   
   T   
   T   
    G    
   T   
   T   
   C   
   A   
   A   
   G   
   A   
   A   
    G    
   C   
   T   
   A   
   G   
   C   
   T   
   T   
   A   
   A   
   A   
   G   
   A   
   A   
   A   
   A   
   A   
   T   
   G   
   G   
    C    
   T   
   A   
   T   
   G   
   A   
   T   
   A   
   C   
   T   
   A   
   A   
   A   
   T   
   C   
    T    
   G   
   T   
   T   
   G   
   T   
   T   
   G   
   A   
   A   
   A   
   A   
   A   
   T   
   A   
   C   
   C   
   A   
   A   
   G   
   C   
   A   
   A   
   T   
   T   
   T   
   A   
   T   
   T   
   C   
   A   
   G   
   G   
   G   
   A   
   T   
   T   
   C   
   A   
   A   
   G   
   C   
    A    
   G   
   A   
   A   
    G    
   G   
    A    
   G   
   T   
   C   
   A   
   A   
    A    
   G   
   C   
   T   
   A   
   G   
   T   
   G   
   A   
   T   
   G   
   T   
    T    
   C   
   A   
   A   
   G   
   T   
    C    
   A   
   A   
   A   
   A   
   A   
    G    
   G   
    C    
   G   
   A   
   A   
   A   
   G   
   A   
    C    
   A   
   A   
    T    
   C   
   A   
   A   
   T   
   A   
   C   
   A   
   C   
   A   
   T   
   T   
   T   
   A   
    C    
   C   
   T   
   A   
   T   
   A   
   A   
   A   
   T   
   T   
   A   
   T   
   C   
   G   
   A   
   T   
   G   
   A   
   G   
    T    
   A   
   C   
    C    
   C   
   C   
   T   
   T   
   T   
   A   
   G   
   G   
   C   
   G   
   A   
   A   
   A   
   T   
   G   
   A   
   A   
   A   
   G   
   A   
   T   
    T    
   T   
   G   
   T   
   C   
   T   
   T   
   A   
   T   
   C   
   A   
   A   
   T   
   C   
   A   
   A   
   G   
   T   
   A   
   T   
    C    
   G   
    C    
   C   
   A   
   A   
    G    
   A   
   A   
   A   
   G   
   G   
    C    
   G   
   A   
   T   
   A   
    C    
   C   
   T   
   A   
   C   
   C   
   A   
   A   
   A   
   T   
    C    
    G    
   C   
   T   
   T   
   G   
   G   
    A    
    A    
    G    
   C   
   C   
    G    
   T   
   C   
   T   
   T   
   T   
   A   
   A   
   T   
   T   
   T   
   T   
   T   
   C   
   C   
   A   
   G   
   A   
   T   
   A   
   T   
   G   
   T   
   C   
   A   
   G   
   G   
   A   
   A   
   A   
   T   
   G   
   A   
   T   
   A   
   A   
   A   
    A    
   T   
   T   
   T   
   C   
   G   
   A   
   T   
   T   
   C   
   A   
   A   
   G   
   T   
   A   
   G   
   A   
   T   
   A   
   A   
   T   
   G   
   C   
   C   
   A   
   A   
   A   
   C   
   G   
   T   
   G   
   G   
   A   
   G   
   A   
   A   
   A   
   T   
   T   
   G   
   T   
   C   
   G   
   A   
   T   
   C   
   G   
   T   
   A   
   A   
    C    
   G   
   G   
   T   
   A   
   G   
   T   
   G   
   G   
   G   
   C   
   T   
   A   
   G   
    C    
   A   
   A   
   T   
   T   
   A   
   A   
   C   
   A   
   A   
   A   
   G   
   T   
   G   
   T   
   T   
   T   
   G   
   A   
    C    
   G   
   A   
   A   
   G   
   T   
   G   
   G   
   G   
   C   
   G   
   T   
   A   
   G   
   T   
    G    
   C   
   C   
   T   
    G    
   G   
   C   
   A   
   A   
   A   
   C   
   T   
    C    
   G   
   G   
   T   
   T   
   C   
   T   
   G   
   G   
   C   
   G   
   C   
   A   
   G   
   A   
   A   
   A   
   A   
   A   
    A    
   C   
   A   
   G   
   C   
    C    
   A   
   A   
   T   
   A   
   T   
   C   
   A   
   A   
   A   
   G   
   C   
   T   
   T   
   T   
   T   
   A   
   G   
   T   
   G   
   A   
   T   
   A   
   A   
    A    
   T   
   T   
    C    
   G   
   G   
   C   
   G   
   T   
   T   
   T   
   C   
   T   
    G    
   T   
   T   
   G   
   A   
   T   
   G   
   A   
   A   
   A   
   T   
   C   
   A   
   A   
   T   
   C   
    A    
   A   
   A   
   A   
    G    
   T   
   T   
   A   
   A   
   G   
   C   
   C   
   A   
   A   
   G   
   G   
    A    
   T   
   G   
   G   
   G   
   T   
   C   
   C   
   A   
   A   
   G   
   C   
   A   
   G   
   A   
   C   
   T   
   C   
    C    
   T   
   T   
   T   
   G   
   T   
   A   
   C   
   C   
   A   
   A   
   T   
   C   
   A   
   C   
   A   
   G   
   T   
    C    
   G   
   C   
   T   
   T   
   C   
   T   
   G   
   A   
   A   
   C   
   C   
   A   
   G   
   T   
   G   
   A   
   C   
   A   
   G   
   A   
   A   
   T   
   T   
   A   
   C   
   C   
   A   
   A   
   C   
   A   
   G   
   G   
   G   
   G   
   C   
   T   
   G   
   C   
   G   
   A   
   C   
   A   
   A   
   A   
   A   
   G   
    A    
    T    
   A   
   C   
   A   
   G   
   A   
   G   
   T   
   C   
   A   
   C   
   G   
   T   
   T   
   A   
   T   
   T   
   A   
   T   
   C   
   C   
    G    
    C    
   T   
   G   
   G   
   G   
   G   
   G   
   A   
   A   
   G   
   C   
   A   
   G   
   C   
   T   
   G   
   C   
   G   
   C   
   A   
   A   
   T   
   T   
   A   
   A   
   T   
   C   
   G   
   G   
    G    
   T   
   A   
   T   
   A   
   C   
    G    
   G   
   G   
    C    
   A   
   C   
    C    
   A   
   T   
   T   
   A   
   C   
   T   
   G   
   C   
   A   
   G   
   A   
    A    
   G   
   A   
   T   
   A   
   T   
   T   
    G    
   A   
   A   
   A   
   A   
   A   
   A   
   A   
   T   
   C   
   C   
   A   
   G   
   A   
   G   
   T   
   T   
   A   
   A   
   G   
   T   
   A   
   G   
   T   
   A   
   C   
   G   
   G   
   G   
    C    
   G   
   T   
   A   
   A   
   T   
   T   
   G   
   G   
    T    
   A   
   A   
   A   
   A   
   C   
   T   
   G   
   G   
    T    
   T   
   T   
   A   
   G   
   A   
   A   
   C   
   G   
   A   
   G   
   C   
   G   
   T   
   T   
   T   
   G   
   A   
   T   
   A   
   A   
   A   
   G   
   A   
    A    
   T   
   T   
   A   
   A   
   G   
   A   
   G   
   G   
    G    
   C   
   A   
   A   
   G   
   A   
   T   
   G   
   G   
    T    
   G   
   G   
    C    
   T   
   C   
    A    
   C   
   T   
   A   
   G   
   T   
   T   
   A   
   T   
   T   
   T   
   T   
   A   
   G   
   A   
   T   
   G   
   A   
   C   
   A   
   A   
   A   
   G   
   A   
   G   
   A   
   A   
   T   
   G   
   T   
   C   
   A   
   A   
   A   
   A   
   A   
   A   
   G   
   C   
   T   
   T   
   T   
   A   
   C   
   A   
   A   
   A   
   C   
   C   
   A   
   A   
   A   
   G   
   A   
    G    
   A   
   A   
   A   
   A   
   A   
   A   
   G   
   A   
   T   
   G   
   G   
   T   
   C   
   A   
   A   
   A   
   C   
    G    
   A   
   T   
   T   
   A   
   A   
   A   
   T   
   T   
   A   
   A   
   C   
   G   
   A   
   T   
   T   
   G   
   A   
    C    
   A   
   G   
    T    
   G   
   G   
    C    
   G   
   T   
   A   
   C   
   A   
   A   
   C   
   A   
   A   
   C   
   A   
   A   
   G   
   C   
    C    
   T   
   T   
    C    
   G   
   C   
   T   
   A   
   T   
   A   
   T   
   T   
   T   
   G   
   A   
   C   
   A   
   A   
   A   
   C   
   G   
    T    
    C    
   C   
   T   
   G   
   G   
   T   
   A   
   G   
   T   
   G   
   C   
   G   
   G   
   T   
   C   
   A   
   T   
   T   
   A   
   C   
   C   
   G   
   A   
   T   
   C   
   C   
   T   
   C   
   A   
   A   
   A   
   A   
   A   
   G   
   G   
   C   
   G   
   A   
   T   
   T   
   T   
   A   
    T    
   T   
   A   
   G   
   C   
    A    
   A   
   C   
   G   
   G   
   T   
   T   
   A   
   G   
   T   
   T   
   C   
   A   
   C   
   C   
   T   
   T   
   C   
   G   
   T   
   A   
   T   
   G   
   A   
   T   
   C   
   C   
   T   
   A   
   A   
   T   
   A   
   A   
   A   
   A   
   T   
   G   
   G   
   C   
   C   
   A   
   A   
   T   
   G   
   G   
    T    
   A   
   T   
   T   
   T   
   C   
   T   
   C   
   A   
   A   
   A   
   A   
   A   
   G   
   A   
    A    
   T   
   A   
   T   
   G   
   A   
   T   
   G   
   C   
   T   
   T   
   A   
   C   
   A   
   A   
   T   
   A   
   A   
   C   
   A   
   A   
   C   
   A   
   A   
   A   
   G   
   A   
   T   
   T   
   T   
   A   
   C   
   C   
   A   
   T   
   T   
    C    
   A   
   C   
   A   
   G   
   C   
   A   
   C   
   G   
   T   
   T   
   T   
    T    
   G   
   C   
   G   
   A   
   C   
   A   
   G   
   G   
    C    
   T   
   A   
   T   
   G   
   C   
   A   
   C   
   C   
   A   
   G   
   G   
    C    
   T   
   C   
   T   
   A   
   C   
   C   
   T   
   T   
   T   
   A   
   A   
   A   
   A   
   C   
   A   
   A   
   T   
   T   
   A   
   C   
   C   
   G   
   G   
   T   
   G   
   C   
   C   
   A   
   T   
   T   
   G   
   G   
   T   
   T   
   T   
   A   
   G   
   A   
   T   
    G    
   C   
   A   
   G   
   G   
   G   
   A   
   C   
   A   
   T   
   T   
   G   
   A   
   A   
   G   
   C   
   C   
   A   
   G   
   A   
   T   
   G   
   A   
   A   
   G   
   A   
   G   
   C   
   T   
   A   
   G   
   A   
   A   
   A   
   T   
   T   
   A   
   A   
   T   
   G   
   G   
    T    
   T   
   T   
   G   
   A   
   A   
   A   
   T   
   G   
   G   
   C   
   A   
   A   
   A   
   A   
   A   
   G   
   A   
   T   
   A   
   A   
   A   
   T   
   C   
   T   
   T   
   G   
   G   
   G   
   G   
    C    
   G   
   G   
   C   
   T   
   A   
   T   
   T   
   T   
   T   
   G   
   C   
    G    
   A   
   C   
   A   
   C   
   G   
   T   
   G   
   T   
   A   
   A   
   A   
   A   
   G   
   A   
   A   
   G   
   C   
   A   
   A   
   G   
   T   
   C   
   C   
   A   
   G   
   T   
   C   
   A   
   A   
   T   
   C   
   T   
    G    
   C   
   G   
   T   
   A   
   C   
   C   
   G   
   C   
   T   
   T   
   T   
   A   
   G   
   T   
   T   
   A   
   A   
   T   
   T   
   C   
   A   
   G   
   A   
   T   
   A   
   A   
   T   
   A   
   T   
   T   
   T   
   A   
   T   
   T   
   T   
   T   
   G   
   C   
    C    
   C   
   A   
   A   
   C   
   A   
   A   
   A   
   C   
   A   
   T   
   T   
   A   
   C   
   G   
    G    
   A   
   T   
   G   
   G   
   G   
    A    
   G   
   A   
   A   
   G   
   A   
   C   
   A   
   A   
   A   
   T   
   T   
   T   
   A   
   G   
   A   
   G   
   C   
    G    
   G   
   G   
   T   
   C   
   T   
   T   
   A   
   A   
   T   
   A   
   A   
   A   
   T   
   T   
   C   
   A   
   T   
   T   
   T   
   T   
   C   
   G   
   G   
   T   
   G   
   A   
   G   
   G   
   A   
   A   
   C   
   T   
   A   
   G   
   A   
   T   
   T   
   T   
   A   
   C   
   C   
   A   
   A   
   T   
    T    
   G   
   C   
   C   
   A   
   T   
   G   
   A   
   C   
   G   
   C   
   C   
    T    
   G   
   C   
   T   
   C   
   A   
   A   
   A   
   T   
   T   
   T   
   C   
   A   
   A   
   A   
    T    
   G   
   A   
   A   
   G   
   A   
   T   
   A   
   A   
   A   
   T   
   T   
   T   
   A   
   A   
   T   
   T   
   C   
   T   
   G   
   A   
   A   
   A   
   T   
   T   
   C   
   T   
   T   
   T   
   T   
    A    
   G   
   C   
   A   
   G   
   A   
   T   
   A   
   C   
    G    
   G   
   G   
   C   
   T   
   A   
    C    
   G   
   G   
    T    
   C   
   A   
   A   
   G   
   G   
    T    
   C   
   A   
   A   
   T   
   T   
   G   
   T   
   T   
   A   
   A   
   T   
   T   
   T   
   C   
    G    
   C   
   C   
   A   
   A   
   T   
   C   
   C   
   A   
   G   
   C   
   A   
   A   
   G   
   C   
   T   
   A   
   C   
   C   
   A   
   T   
   G   
   T   
   A   
   T   
   A   
   G   
   T   
   G   
   T   
   T   
   T   
   T   
   C   
   C   
   A   
   A   
   A   
   A   
   T   
   A   
   A   
   T   
   G   
   G   
    A    
   A   
   C   
   A   
    T    
   T   
    A    
   G   
   T   
    C    
   T   
   A   
   T   
   C   
   C   
   A   
   A   
   A   
   A   
    C    
   T   
   A   
   G   
   T   
   T   
   T   
   T   
   A   
   G   
   A   
   C   
   A   
   A   
   A   
   G   
   A   
   A   
   A   
   C   
    G    
   A   
   A   
   G   
   A   
   A   
   A   
   A   
   A   
   A   
   G   
   A   
   C   
   A   
   A   
   T   
   G   
   T   
   A   
   A   
   T   
   C   
   A   
   G   
   C   
   G   
   C   
   C   
   A   
   A   
   C   
   G   
   C   
   A   
   G   
   C   
   G   
   A   
   A   
   T   
   A   
   C   
   A   
   A   
   T   
   T   
   G   
   C   
   C   
   A   
   C   
   A   
   G   
   A   
   T   
   C   
   T   
   T   
   T   
   T   
   A   
   G   
   G   
   T   
   A   
   G   
   T   
   G   
   T   
   G   
   G   
   A   
   A   
   G   
   A   
   T   
   C   
   C   
   T   
   T   
   C   
   A   
   G   
   G   
   T   
   T   
   A   
   T   
   G   
   T   
   T   
   T   
   A   
   T   
   A   
   A   
   T   
   A   
   T   
   G   
   T   
   A   
   C   
   A   
   A   
   T   
   C   
   C   
   G   
   A   
   A   
   C   
   T   
   T   
   C   
   T   
   C   
   A   
   T   
   T   
   G   
   G   
   C   
   T   
   G   
   C   
   T   
   A   
   A   
   A   
   A   
   C   
   A   
   G   
   G   
   A   
   A   
   C   
   A   
   G   
   C   
   T   
   G   
   A   
   A   
   A   
   T   
   A   
   A   
   A   
   A   
   G   
   A   
   T   
   A   
   A   
   A   
   C   
   A   
   A   
   G   
   A   
   T   
   A   
   C   
   A   
   G   
   A   
   T   
   G   
   G   
   C   
   A   
   A   
   A   
   G   
   A   
   A   
   A   
   A   
   C   
   A   
   G   
   T   
   T   
   T   
   C   
   C   
   T   
   T   
   C   
   T   
   A   
   A   
   C   
   G   
   T   
   T   
   A   
   G   
   A   
   T   
   C   
   G   
   T   
   A   
   G   
   T   
   A   
   A   
   C   
   A   
   A   
   T   
   A   
   A   
   A   
   T   
   T   
   C   
   T   
   T   
   A   
   A   
   C   
   A   
   A   
   T   
   G   
   A   
   T   
   T   
   A   
   T   
   G   
   G   
   T   
   T   
   G   
   A   
   A   
   A   
   A   
   T   
   T   
   C   
   A   
   G   
   G   
   A   
   G   
   A   
   A   
   A   
   A   
   T   
   G   
   G   
   T   
   T   
   C   
   T   
   G   
   C   
   A   
   A   
   C   
   A   
   G   
   A   
   T   
   A   
   T   
   C   
   A   
   G   
   T   
   A   
   A   
   A   
   C   
   C   
   A   
   T   
   T   
   A   
   A   
   T   
   T   
   G   
   A   
   T   
   T   
   A   
   C   
   T   
   T   
   A   
   G   
   A   
   A   
   G   
   C   
   A   
   A   
   C   
   C   
   A   
   T   
   T   
   A   
   A   
   A   
   -   
   -   
   -   
   -   
   -   
   -   
   -   
   -   
   -   
   -   
   -   
   -   
   -   
   -   
   -   
   -   
   -   
   -   
   -   
   -   
   -   
   -   
   -   
   -   
   -   
   -   
   -   
   -   
   -   
   -   
   -   
   -   
   -   
   -   
   -   
   -   
   -   
   -   
   -   
   -   
   -   
   -   
   -   
   -   
   -   
   -   
   -   
   -   
   -   
   -   
   -   
   -   
   -   
   -   
   -   
   -   
   -   
   -   
   -   
   -   
   -   
   -   
   -   
   -   
   -   
   -   
   -   
   -   
   -   
   -   
   -   
   -   
   -   
   -   
   -   
   -   
   -   
   -   
   -   
   -   
   -   
   -   
   -   
   -   
   -   
   -   
   -   
   -   
   -   
   -   
   -   
   -   
   -   
   -   
   -   
   -   
   -   
   -   
   -   
   -   
   -   
   -   
   -   
   -   
   -   
   -   
   -   
   -   
   -   
   -   
   -   
   -   
   -   
   -   
   -   
   -   
   -   
   -   
   -   
   -   
   -   
   -   
   -   
   -   
   -   
   -   
   -   
   -   
   -   
   -   
   -   
   -   
   -   
   -   
   -   
   -   
   -   
   -   
   -   
   -   
   -   
   -   
   -   
   -   
   -   
   -   
   -   
   -   
   -   
   -   
   -   
   -   
   -   
   -   
   -   
   -   
   -   
   -   
   -   
   -   
   -   
   -   
   -   
   -   
   -   
   -   
   -   
   -   
   -   
   C   
   A   
   A   
   T   
   G   
   G   
   C   
   A   
   A   
   G   
   C   
   C   
   A   
   A   
   A   
   C   
   A   
   A   
   G   
   A   
   A   
   T   
   T   
   A   
   G   
   C   
   C   
   G   
   A   
   A   
   G   
   C   
   G   
   A   
   A   
   G   
   A   
   A   
   A   
   A   
   C   
   A   
   G   
   C   
   T   
   A   
   C   
   T   
   A   
   C   
   A   
   T   
   T   
   T   
   T   
   T   
   A   
   A   
   A   
   C   
   G   
   T   
   A   
   T   
   T   
   G   
   T   
   C   
   A   
   A   
   A   
   A   
   C   
   A   
   G   
   G   
   A   
   A   
   T   
   T   
   T   
   G   
   A   
   T   
   A   
   A   
   G   
   T   
   T   
   A   
   C   
   C   
   G   
   T   
   C   
   C   
   G   
   T   
   T   
   G   
   T   
   T   
   C   
   A   
   A   
   G   
   A   
   A   
   G   
   C   
   T   
   A   
   G   
   C   
   T   
   T   
   A   
   A   
   A   
   G   
   A   
   A   
   A   
   A   
   A   
   T   
   G   
   G   
   C   
   T   
   A   
   T   
   G   
   A   
   T   
   A   
   C   
   T   
   A   
   A   
   A   
   T   
   C   
   T   
   G   
   T   
   T   
   G   
   T   
   T   
   G   
   A   
   A   
   A   
   A   
   A   
   T   
   A   
   C   
   C   
   A   
   A   
   G   
   C   
   A   
   A   
   T   
   T   
   T   
   A   
   T   
   T   
   C   
   A   
   G   
   G   
   G   
   A   
   T   
   T   
   C   
   A   
   A   
   G   
   C   
   A   
   G   
   A   
   A   
   G   
   G   
   A   
   G   
   T   
   C   
   A   
   A   
   A   
   G   
   C   
   T   
   A   
   G   
   T   
   G   
   A   
   T   
   G   
   T   
   T   
   C   
   A   
   A   
   G   
   T   
   C   
   A   
   A   
   A   
   A   
   A   
   G   
   G   
   C   
   G   
   A   
   A   
   A   
   G   
   A   
   C   
   A   
   A   
   T   
   C   
   A   
   A   
   T   
   A   
   C   
   A   
   C   
   A   
   T   
   T   
   T   
   A   
   C   
   C   
   T   
   A   
   T   
   A   
   A   
   A   
   T   
   T   
   A   
   T   
   C   
   G   
   A   
   T   
   G   
   A   
   G   
   T   
   A   
   C   
   C   
   C   
   C   
   T   
   T   
   T   
   A   
   G   
   G   
   C   
   G   
   A   
   A   
   A   
   T   
   G   
   A   
   A   
   A   
   G   
   A   
   T   
   T   
   T   
   G   
   T   
   C   
   T   
   T   
   A   
   T   
   C   
   A   
   A   
   T   
   C   
   A   
   A   
   G   
   T   
   A   
   T   
   C   
   G   
   C   
   C   
   A   
   A   
   G   
   A   
   A   
   A   
   G   
   G   
   C   
   G   
   A   
   T   
   A   
   C   
   C   
   T   
   A   
   C   
   C   
   A   
   A   
   A   
   T   
   C   
   G   
   C   
   T   
   T   
   G   
   G   
   A   
   A   
   G   
   C   
   C   
   G   
   T   
   C   
   T   
   T   
   T   
   A   
   A   
   T   
   T   
   T   
   T   
   T   
   C   
   C   
   A   
   G   
   A   
   T   
   A   
   T   
   G   
   T   
   C   
   A   
   G   
   G   
   A   
   A   
   A   
   T   
   G   
   A   
   T   
   A   
   A   
   A   
   A   
   T   
   T   
   T   
   C   
   G   
   A   
   T   
   T   
   C   
   A   
   A   
   G   
   T   
   A   
   G   
   A   
   T   
   A   
   A   
   T   
   G   
   C   
   C   
   A   
   A   
   A   
   C   
   G   
   T   
   G   
   G   
   A   
   G   
   A   
   A   
   A   
   T   
   T   
   G   
   T   
   C   
   G   
   A   
   T   
   C   
   G   
   T   
   A   
   A   
   C   
   G   
   G   
   T   
   A   
   G   
   T   
   G   
   G   
   G   
   C   
   T   
   A   
   G   
   C   
   A   
   A   
   T   
   T   
   A   
   A   
   C   
   A   
   A   
   A   
   G   
   T   
   G   
   T   
   T   
   T   
   G   
   A   
   C   
   G   
   A   
   A   
   G   
   T   
   G   
   G   
   G   
   C   
   G   
   T   
   A   
   G   
   T   
   G   
   C   
   C   
   T   
   G   
   G   
   C   
   A   
   A   
   A   
   C   
   T   
   C   
   G   
   G   
   T   
   T   
   C   
   T   
   G   
   G   
   C   
   G   
   C   
   A   
   G   
   A   
   A   
   A   
   A   
   A   
   A   
   C   
   A   
   G   
   C   
   C   
   A   
   A   
   T   
   A   
   T   
   C   
   A   
   A   
   A   
   G   
   C   
   T   
   T   
   T   
   T   
   A   
   G   
   T   
   G   
   A   
   T   
   A   
   A   
   A   
   T   
   T   
   C   
   G   
   G   
   C   
   G   
   T   
   T   
   T   
   C   
   T   
   G   
   T   
   T   
   G   
   A   
   T   
   G   
   A   
   A   
   A   
   T   
   C   
   A   
   A   
   T   
   C   
   A   
   A   
   A   
   A   
   G   
   T   
   T   
   A   
   A   
   G   
   C   
   C   
   A   
   A   
   G   
   G   
   A   
   T   
   G   
   G   
   G   
   T   
   C   
   C   
   A   
   A   
   G   
   C   
   A   
   G   
   A   
   C   
   T   
   C   
   C   
   T   
   T   
   T   
   G   
   T   
   A   
   C   
   C   
   A   
   A   
   T   
   C   
   A   
   C   
   A   
   G   
   T   
   C   
   G   
   C   
   T   
   T   
   C   
   T   
   G   
   A   
   A   
   C   
   C   
   A   
   G   
   T   
   G   
   A   
   C   
   A   
   G   
   A   
   A   
   T   
   T   
   A   
   C   
   C   
   A   
   A   
   C   
   A   
   G   
   G   
   G   
   G   
   C   
   T   
   G   
   C   
   G   
   A   
   C   
   A   
   A   
   A   
   A   
   G   
   A   
   T   
   A   
   C   
   A   
   G   
   A   
   G   
   T   
   C   
   A   
   C   
   G   
   T   
   T   
   A   
   T   
   T   
   A   
   T   
   C   
   C   
   G   
   C   
   T   
   G   
   G   
   G   
   G   
   G   
   A   
   A   
   G   
   C   
   A   
   G   
   C   
   T   
   G   
   C   
   G   
   C   
   A   
   A   
   T   
   T   
   A   
   A   
   T   
   C   
   G   
   G   
   G   
   T   
   A   
   T   
   A   
   C   
   G   
   G   
   G   
   C   
   A   
   C   
   C   
   A   
   T   
   T   
   A   
   C   
   T   
   G   
   C   
   A   
   G   
   A   
   A   
   G   
   A   
   T   
   A   
   T   
   T   
   G   
   A   
   A   
   A   
   A   
   A   
   A   
   A   
   T   
   C   
   C   
   A   
   G   
   A   
   G   
   T   
   T   
   A   
   A   
   G   
   T   
   A   
   G   
   T   
   A   
   C   
   G   
   G   
   G   
   C   
   G   
   T   
   A   
   A   
   T   
   T   
   G   
   G   
   T   
   A   
   A   
   A   
   A   
   C   
   T   
   G   
   G   
   T   
   T   
   T   
   A   
   G   
   A   
   A   
   C   
   G   
   A   
   G   
   C   
   G   
   T   
   T   
   T   
   G   
   A   
   T   
   A   
   A   
   A   
   G   
   A   
   A   
   T   
   T   
   A   
   A   
   G   
   A   
   G   
   G   
   G   
   C   
   A   
   A   
   G   
   A   
   T   
   G   
   G   
   T   
   G   
   G   
   C   
   T   
   C   
   A   
   C   
   T   
   A   
   G   
   T   
   T   
   A   
   T   
   T   
   T   
   T   
   A   
   G   
   A   
   T   
   G   
   A   
   C   
   A   
   A   
   A   
   G   
   A   
   G   
   A   
   A   
   T   
   G   
   T   
   C   
   A   
   A   
   A   
   A   
   A   
   A   
   G   
   C   
   T   
   T   
   T   
   A   
   C   
   A   
   A   
   A   
   C   
   C   
   A   
   A   
   A   
   G   
   A   
   G   
   A   
   A   
   A   
   A   
   A   
   A   
   G   
   A   
   T   
   G   
   G   
   T   
   C   
   A   
   A   
   A   
   C   
   G   
   A   
   T   
   T   
   A   
   A   
   A   
   T   
   T   
   A   
   A   
   C   
   G   
   A   
   T   
   T   
   G   
   A   
   C   
   A   
   G   
   T   
   G   
   G   
   C   
   G   
   T   
   A   
   C   
   A   
   A   
   C   
   A   
   A   
   C   
   A   
   A   
   G   
   C   
   C   
   T   
   T   
   C   
   G   
   C   
   T   
   A   
   T   
   A   
   T   
   T   
   T   
   G   
   A   
   C   
   A   
   A   
   A   
   C   
   G   
   T   
   C   
   C   
   T   
   G   
   G   
   T   
   A   
   G   
   T   
   G   
   C   
   G   
   G   
   T   
   C   
   A   
   T   
   T   
   A   
   C   
   C   
   G   
   A   
   T   
   C   
   C   
   T   
   C   
   A   
   A   
   A   
   A   
   A   
   G   
   G   
   C   
   G   
   A   
   T   
   T   
   T   
   A   
   T   
   T   
   A   
   G   
   C   
   A   
   A   
   C   
   G   
   G   
   T   
   T   
   A   
   G   
   T   
   T   
   C   
   A   
   C   
   C   
   T   
   T   
   C   
   G   
   T   
   A   
   T   
   G   
   A   
   T   
   C   
   C   
   T   
   A   
   A   
   T   
   A   
   A   
   A   
   A   
   T   
   G   
   G   
   C   
   C   
   A   
   A   
   T   
   G   
   G   
   T   
   A   
   T   
   T   
   T   
   C   
   T   
   C   
   A   
   A   
   A   
   A   
   A   
   G   
   A   
   A   
   T   
   A   
   T   
   G   
   A   
   T   
   G   
   C   
   T   
   T   
   A   
   C   
   A   
   A   
   T   
   A   
   A   
   C   
   A   
   A   
   C   
   A   
   A   
   A   
   G   
   A   
   T   
   T   
   T   
   A   
   C   
   C   
   A   
   T   
   T   
   C   
   A   
   C   
   A   
   G   
   C   
   A   
   C   
   G   
   T   
   T   
   T   
   T   
   G   
   C   
   G   
   A   
   C   
   A   
   G   
   G   
   C   
   T   
   A   
   T   
   G   
   C   
   A   
   C   
   C   
   A   
   G   
   G   
   C   
   T   
   C   
   T   
   A   
   C   
   C   
   T   
   T   
   T   
   A   
   A   
   A   
   A   
   C   
   A   
   A   
   T   
   T   
   A   
   C   
   C   
   G   
   G   
   T   
   G   
   C   
   C   
   A   
   T   
   T   
   G   
   G   
   T   
   T   
   T   
   A   
   G   
   A   
   T   
   G   
   C   
   A   
   G   
   G   
   G   
   A   
   C   
   A   
   T   
   T   
   G   
   A   
   A   
   G   
   C   
   C   
   A   
   G   
   A   
   T   
   G   
   A   
   A   
   G   
   A   
   G   
   C   
   T   
   A   
   G   
   A   
   A   
   A   
   T   
   T   
   A   
   A   
   T   
   G   
   G   
   T   
   T   
   T   
   G   
   A   
   A   
   A   
   T   
   G   
   G   
   C   
   A   
   A   
   A   
   A   
   A   
   G   
   A   
   T   
   A   
   A   
   A   
   T   
   C   
   T   
   T   
   G   
   G   
   G   
   G   
   C   
   G   
   G   
   C   
   T   
   A   
   T   
   T   
   T   
   T   
   G   
   C   
   G   
   A   
   C   
   A   
   C   
   G   
   T   
   G   
   T   
   A   
   A   
   A   
   A   
   G   
   A   
   A   
   G   
   C   
   A   
   A   
   G   
   T   
   C   
   C   
   A   
   G   
   T   
   C   
   A   
   A   
   T   
   C   
   T   
   G   
   C   
   G   
   T   
   A   
   C   
   C   
   G   
   C   
   T   
   T   
   T   
   A   
   G   
   T   
   T   
   A   
   A   
   T   
   T   
   C   
   A   
   G   
   A   
   T   
   A   
   A   
   T   
   A   
   T   
   T   
   T   
   A   
   T   
   T   
   T   
   T   
   G   
   C   
   C   
   C   
   A   
   A   
   C   
   A   
   A   
   A   
   C   
   A   
   T   
   T   
   A   
   C   
   G   
   G   
   A   
   T   
   G   
   G   
   G   
   A   
   G   
   A   
   A   
   G   
   A   
   C   
   A   
   A   
   A   
   T   
   T   
   T   
   A   
   G   
   A   
   G   
   C   
   G   
   G   
   G   
   T   
   C   
   T   
   T   
   A   
   A   
   T   
   A   
   A   
   A   
   T   
   T   
   C   
   A   
   T   
   T   
   T   
   T   
   C   
   G   
   G   
   T   
   G   
   A   
   G   
   G   
   A   
   A   
   C   
   T   
   A   
   G   
   A   
   T   
   T   
   T   
   A   
   C   
   C   
   A   
   A   
   T   
   T   
   G   
   C   
   C   
   A   
   T   
   G   
   A   
   C   
   G   
   C   
   C   
   T   
   G   
   C   
   T   
   C   
   A   
   A   
   A   
   T   
   T   
   T   
   C   
   A   
   A   
   A   
   T   
   G   
   A   
   A   
   G   
   A   
   T   
   A   
   A   
   A   
   T   
   T   
   T   
   A   
   A   
   T   
   T   
   C   
   T   
   G   
   A   
   A   
   A   
   T   
   T   
   C   
   T   
   T   
   T   
   T   
   A   
   G   
   C   
   A   
   G   
   A   
   T   
   A   
   C   
   G   
   G   
   G   
   C   
   T   
   A   
   C   
   G   
   G   
   T   
   C   
   A   
   A   
   G   
   G   
   T   
   C   
   A   
   A   
   T   
   T   
   G   
   T   
   T   
   A   
   A   
   T   
   T   
   T   
   C   
   G   
   C   
   C   
   A   
   A   
   T   
   C   
   C   
   A   
   G   
   C   
   A   
   A   
   G   
   C   
   T   
   A   
   C   
   C   
   A   
   T   
   G   
   T   
   A   
   T   
   A   
   G   
   T   
   G   
   T   
   T   
   T   
   T   
   C   
   C   
   A   
   A   
   A   
   A   
   T   
   A   
   A   
   T   
   G   
   G   
   A   
   A   
   C   
   A   
   T   
   T   
   A   
   G   
   T   
   C   
   T   
   A   
   T   
   C   
   C   
   A   
   A   
   A   
   A   
   C   
   T   
   A   
   G   
   T   
   T   
   T   
   T   
   A   
   G   
   A   
   C   
   A   
   A   
   A   
   G   
   A   
   A   
   A   
   C   
   G   
   A   
   A   
   G   
   A   
   A   
   A   
   A   
   A   
   A   
   G   
   A   
   C   
   A   
   A   
   T   
   G   
   T   
   A   
   A   
   T   
   C   
   A   
   G   
   C   
   G   
   C   
   C   
   A   
   A   
   C   
   G   
   C   
   A   
   G   
   C   
   G   
   A   
   A   
   T   
   A   
   C   
   A   
   A   
   T   
   T   
   G   
   C   
   C   
   A   
   C   
   A   
   G   
   A   
   T   
   C   
   T   
   T   
   T   
   T   
   A   
   G   
   G   
   T   
   A   
   G   
   T   
   G   
   T   
   G   
   G   
   A   
   A   
   G   
   A   
   T   
   C   
   C   
   T   
   T   
   C   
   A   
   G   
   G   
   T   
   T   
   A   
   T   
   G   
   T   
   T   
   T   
   A   
   T   
   A   
   A   
   T   
   A   
   T   
   G   
   T   
   A   
   C   
   A   
   A   
   T   
   C   
   C   
   G   
   A   
   A   
   C   
   T   
   T   
   C   
   T   
   C   
   A   
   T   
   T   
   G   
   G   
   C   
   T   
   G   
   C   
   T   
   A   
   A   
   A   
   A   
   C   
   A   
   G   
   G   
   A   
   A   
   C   
   A   
   G   
   C   
   T   
   G   
   A   
   A   
   A   
   T   
   A   
   A   
   A   
   A   
   G   
   A   
   T   
   A   
   A   
   A   
   C   
   A   
   A   
   G   
   A   
   T   
   A   
   C   
   A   
   G   
   A   
   T   
   G   
   G   
   C   
   A   
   A   
   A   
   G   
   A   
   A   
   A   
   A   
   C   
   A   
   G   
   T   
   T   
   T   
   C   
   C   
   T   
   T   
   C   
   T   
   A   
   A   
   C   
   G   
   T   
   T   
   A   
   G   
   A   
   T   
   C   
   G   
   T   
   A   
   G   
   T   
   A   
   A   
   C   
   A   
   A   
   T   
   A   
   A   
   A   
   T   
   T   
   C   
   T   
   T   
   A   
   A   
   C   
   A   
   A   
   T   
   G   
   A   
   T   
   T   
   A   
   T   
   G   
   G   
   T   
   T   
   G   
   A   
   A   
   A   
   A   
   T   
   T   
   C   
   A   
   G   
   G   
   A   
   G   
   A   
   A   
   A   
   A   
   T   
   G   
   G   
   T   
   T   
   C   
   T   
   G   
   C   
   A   
   A   
   C   
   A   
   G   
   A   
   T   
   A   
   T   
   C   
   A   
   G   
   T   
   A   
   A   
   A   
   C   
   C   
   A   
   T   
   T   
   A   
   A   
   T   
   T   
   G   
   A   
   T   
   T   
   A   
   C   
   T   
   T   
   A   
   G   
   A   
   A   
   G   
   C   
   A   
   A   
   C   
   C   
   A   
   T   
   T   
   A   
   A   
   A   
   -   
   -   
   -   
   -   
   -   
   -   
   -   
   -   
   -   
   -   
   -   
   -   
   -   
   -   
   -   
   -   
   -   
   -   
   -   
   -   
   -   
   -   
   -   
   -   
   -   
   -   
   -   
   -   
   -   
   -   
   -   
   -   
   -   
   -   
   -   
   -   
   -   
   -   
   -   
   -   
   -   
   -   
   -   
   -   
   -   
   -   
   -   
   -   
   -   
   -   
   -   
   -   
   -   
   -   
   -   
   -   
   -   
   -   
   -   
   -   
   -   
 

 
   N52724   
   -   
   -   
   -   
   -   
   -   
   -   
   -   
   -   
   -   
   -   
   -   
   -   
   -   
   -   
   -   
   -   
   -   
   -   
   -   
   -   
   -   
   -   
   -   
   -   
   -   
   -   
   -   
   -   
   -   
   -   
   -   
   -   
   -   
   -   
   -   
   -   
   -   
   -   
   -   
   -   
   -   
   -   
   -   
   -   
   -   
   -   
   -   
   -   
   -   
   -   
   -   
   -   
   -   
   -   
   -   
   -   
   -   
   -   
   -   
   -   
   -   
   -   
   -   
   -   
   -   
   -   
   -   
   -   
   -   
   -   
   -   
   -   
   -   
   -   
   -   
   -   
   -   
   -   
   -   
   -   
   -   
   -   
   -   
   -   
   -   
   -   
   -   
   -   
   -   
   -   
   -   
   -   
   -   
   -   
   -   
   -   
   -   
   -   
   -   
   -   
   -   
   -   
   -   
   -   
   -   
   -   
   -   
   -   
   C   
   A   
   A   
   T   
   G   
   G   
   C   
   A   
   A   
   G   
   C   
   C   
   A   
   A   
   A   
   C   
   A   
   A   
   G   
   A   
    A    
   T   
   T   
   A   
   G   
   C   
    C    
   G   
   A   
   A   
   G   
   C   
   G   
   A   
   A   
   G   
   A   
   A   
   A   
   A   
    C    
   A   
   G   
   C   
    T    
   A   
    C    
   T   
    A    
    C    
   A   
   T   
   T   
   T   
   T   
   T   
   A   
   A   
   A   
    C    
   G   
   T   
   A   
   T   
   T   
   G   
    T    
   C   
   A   
   A   
   A   
   A   
   C   
   A   
   G   
    G    
   A   
   A   
   T   
   T   
   T   
   G   
   A   
   T   
   A   
   A   
   G   
   T   
   T   
   A   
   C   
   C   
    G    
   T   
   C   
   C   
   G   
   T   
   T   
    A    
   T   
   T   
   C   
   A   
   A   
   G   
   A   
   A   
    G    
   C   
   T   
   A   
   G   
   C   
   T   
   T   
   A   
   A   
   A   
   G   
   A   
   A   
   A   
   A   
   A   
   T   
   G   
   G   
    C    
   T   
   A   
   T   
   G   
   A   
   T   
   A   
   C   
   T   
   A   
   A   
   A   
   T   
   C   
    T    
   G   
   T   
   T   
   G   
   T   
   T   
   G   
   A   
   A   
   A   
   A   
   A   
   T   
   A   
   C   
   C   
   A   
   A   
   G   
   C   
   A   
   A   
   T   
   T   
   T   
   A   
   T   
   T   
   C   
   A   
   G   
   G   
   G   
   A   
   T   
   T   
   C   
   A   
   A   
   G   
   C   
    A    
   G   
   A   
   A   
    G    
   G   
    A    
   G   
   T   
   C   
   A   
   A   
    A    
   G   
   C   
   T   
   A   
   G   
   T   
   G   
   A   
   T   
   G   
   T   
    T    
   C   
   A   
   A   
   G   
   T   
    C    
   A   
   A   
   A   
   A   
   A   
    G    
   G   
    C    
   G   
   A   
   A   
   A   
   G   
   A   
    C    
   A   
   A   
    T    
   C   
   A   
   A   
   T   
   A   
   C   
   A   
   C   
   A   
   T   
   T   
   T   
   A   
    C    
   C   
   T   
   A   
   T   
   A   
   A   
   A   
   T   
   T   
   A   
   T   
   C   
   G   
   A   
   T   
   G   
   A   
   G   
    T    
   A   
   C   
    C    
   C   
   C   
   T   
   T   
   T   
   A   
   G   
   G   
   C   
   G   
   A   
   A   
   A   
   T   
   G   
   A   
   A   
   A   
   G   
   A   
   T   
    T    
   T   
   G   
   T   
   C   
   T   
   T   
   A   
   T   
   C   
   A   
   A   
   T   
   C   
   A   
   A   
   G   
   T   
   A   
   T   
    C    
   G   
    C    
   C   
   A   
   A   
    A    
   A   
   A   
   A   
   G   
   G   
    C    
   G   
   A   
   T   
   A   
    C    
   C   
   T   
   A   
   C   
   C   
   A   
   A   
   A   
   T   
    C    
    G    
   C   
   T   
   T   
   G   
   G   
    A    
    A    
    G    
   C   
   C   
    G    
   T   
   C   
   T   
   T   
   T   
   A   
   A   
   T   
   T   
   T   
   T   
   T   
   C   
   C   
   A   
   G   
   A   
   T   
   A   
   T   
   G   
   T   
   C   
   A   
   G   
   G   
   A   
   A   
   A   
   T   
   G   
   A   
   T   
   A   
   A   
   A   
    A    
   T   
   T   
   T   
   C   
   G   
   A   
   T   
   T   
   C   
   A   
   A   
   G   
   T   
   A   
   G   
   A   
   T   
   A   
   A   
   T   
   G   
   C   
   C   
   A   
   A   
   A   
   C   
   G   
   T   
   G   
   G   
   A   
   G   
   A   
   A   
   A   
   T   
   T   
   G   
   T   
   C   
   G   
   A   
   T   
   C   
   G   
   T   
   A   
   A   
    T    
   G   
   G   
   T   
   A   
   G   
   T   
   G   
   G   
   G   
   C   
   T   
   A   
   G   
    C    
   A   
   A   
   T   
   T   
   A   
   A   
   C   
   A   
   A   
   A   
   G   
   T   
   G   
   T   
   T   
   T   
   G   
   A   
    C    
   G   
   A   
   A   
   G   
   T   
   G   
   G   
   G   
   C   
   G   
   T   
   A   
   G   
   T   
    G    
   C   
   C   
   T   
    G    
   G   
   C   
   A   
   A   
   A   
   C   
   T   
    C    
   G   
   G   
   T   
   T   
   C   
   T   
   G   
   G   
   C   
   G   
   C   
   A   
   G   
   A   
   A   
   A   
   A   
   A   
    A    
   C   
   A   
   G   
   C   
    A    
   A   
   A   
   T   
   A   
   T   
   C   
   A   
   A   
   A   
   G   
   C   
   T   
   T   
   T   
   T   
   A   
   G   
   T   
   G   
   A   
   T   
   A   
   A   
    A    
   T   
   T   
    C    
   G   
   G   
   C   
   G   
   T   
   T   
   T   
   C   
   T   
    G    
   T   
   T   
   G   
   A   
   T   
   G   
   A   
   A   
   A   
   T   
   C   
   A   
   A   
   T   
   C   
    A    
   A   
   A   
   A   
    G    
   T   
   T   
   A   
   A   
   G   
   C   
   C   
   A   
   A   
   G   
   G   
    A    
   T   
   G   
   G   
   G   
   T   
   C   
   C   
   A   
   A   
   G   
   C   
   A   
   G   
   A   
   C   
   T   
   C   
    C    
   T   
   T   
   T   
   G   
   T   
   A   
   C   
   C   
   A   
   A   
   T   
   C   
   A   
   C   
   A   
   G   
   T   
    C    
   G   
   C   
   T   
   T   
   C   
   T   
   G   
   A   
   A   
   C   
   C   
   A   
   G   
   T   
   G   
   A   
   C   
   A   
   G   
   A   
   A   
   T   
   T   
   A   
   C   
   C   
   A   
   A   
   C   
   A   
   G   
   G   
   G   
   G   
   C   
   T   
   G   
   C   
   G   
   A   
   C   
   A   
   A   
   A   
   A   
   G   
    A    
    T    
   A   
   C   
   A   
   G   
   A   
   G   
   T   
   C   
   A   
   C   
   G   
   T   
   T   
   A   
   T   
   T   
   A   
   T   
   C   
   C   
    G    
    C    
   T   
   G   
   G   
   G   
   G   
   G   
   A   
   A   
   G   
   C   
   A   
   G   
   C   
   T   
   G   
   C   
   G   
   C   
   A   
   A   
   T   
   T   
   A   
   A   
   T   
   C   
   G   
   G   
    G    
   T   
   A   
   T   
   A   
   C   
    G    
   G   
   G   
    C    
   A   
   C   
    C    
   A   
   T   
   T   
   A   
   C   
   T   
   G   
   C   
   A   
   G   
   A   
    A    
   G   
   A   
   T   
   A   
   T   
   T   
    G    
   A   
   A   
   A   
   A   
   A   
   A   
   A   
   T   
   C   
   C   
   A   
   G   
   A   
   G   
   T   
   T   
   A   
   A   
   G   
   T   
   A   
   G   
   T   
   A   
   C   
   G   
   G   
   G   
    C    
   G   
   T   
   A   
   A   
   T   
   T   
   G   
   G   
    T    
   A   
   A   
   A   
   A   
   C   
   T   
   G   
   G   
    C    
   T   
   T   
   A   
   G   
   A   
   A   
   C   
   G   
   A   
   G   
   C   
   G   
   T   
   T   
   T   
   G   
   A   
   T   
   A   
   A   
   A   
   G   
   A   
    A    
   T   
   T   
   A   
   A   
   G   
   A   
   G   
   G   
    G    
   C   
   A   
   A   
   G   
   A   
   T   
   G   
   G   
    T    
   G   
   G   
    C    
   T   
   C   
    A    
   C   
   T   
   A   
   G   
   T   
   T   
   A   
   T   
   T   
   T   
   T   
   A   
   G   
   A   
   T   
   G   
   A   
   C   
   A   
   A   
   A   
   G   
   A   
   G   
   A   
   A   
   T   
   G   
   T   
   C   
   A   
   A   
   A   
   A   
   A   
   A   
   G   
   C   
   T   
   T   
   T   
   A   
   C   
   A   
   A   
   A   
   C   
   C   
   A   
   A   
   A   
   G   
   A   
    G    
   A   
   A   
   A   
   A   
   A   
   A   
   G   
   A   
   T   
   G   
   G   
   T   
   C   
   A   
   A   
   A   
   C   
    G    
   A   
   T   
   T   
   A   
   A   
   A   
   T   
   T   
   A   
   A   
   C   
   G   
   A   
   T   
   T   
   G   
   A   
    C    
   A   
   G   
    T    
   G   
   G   
    C    
   G   
   T   
   A   
   C   
   A   
   A   
   C   
   A   
   A   
   C   
   A   
   A   
   G   
   C   
    C    
   T   
   T   
    C    
   G   
   C   
   T   
   A   
   T   
   A   
   T   
   T   
   T   
   G   
   A   
   C   
   A   
   A   
   A   
   C   
   G   
    C    
    C    
   C   
   T   
   G   
   G   
   T   
   A   
   G   
   T   
   G   
   C   
   G   
   G   
   T   
   C   
   A   
   T   
   T   
   A   
   C   
   C   
   G   
   A   
   T   
   C   
   C   
   T   
   C   
   A   
   A   
   A   
   A   
   A   
   G   
   G   
   C   
   G   
   A   
   T   
   T   
   T   
   A   
    T    
   T   
   A   
   G   
   C   
    A    
   A   
   C   
   G   
   G   
   T   
   T   
   A   
   G   
   T   
   T   
   C   
   A   
   C   
   C   
   T   
   T   
   C   
   G   
   T   
   A   
   T   
   G   
   A   
   T   
   C   
   C   
   T   
   A   
   A   
   T   
   A   
   A   
   A   
   A   
   T   
   G   
   G   
   C   
   C   
   A   
   A   
   T   
   G   
   G   
    C    
   A   
   T   
   T   
   T   
   C   
   T   
   C   
   A   
   A   
   A   
   A   
   A   
   G   
   A   
    A    
   T   
   A   
   T   
   G   
   A   
   T   
   G   
   C   
   T   
   T   
   A   
   C   
   A   
   A   
   T   
   A   
   A   
   C   
   A   
   A   
   C   
   A   
   A   
   A   
   G   
   A   
   T   
   T   
   T   
   A   
   C   
   C   
   A   
   T   
   T   
    C    
   A   
   C   
   A   
   G   
   C   
   A   
   C   
   G   
   T   
   T   
   T   
    T    
   G   
   C   
   G   
   A   
   C   
   A   
   G   
   G   
    C    
   T   
   A   
   T   
   G   
   C   
   A   
   C   
   C   
   A   
   G   
   G   
    C    
   T   
   C   
   T   
   A   
   C   
   C   
   T   
   T   
   T   
   A   
   A   
   A   
   A   
   C   
   A   
   A   
   T   
   T   
   A   
   C   
   C   
   G   
   G   
   T   
   G   
   C   
   C   
   A   
   T   
   T   
   G   
   G   
   T   
   T   
   T   
   A   
   G   
   A   
   T   
    G    
   C   
   A   
   G   
   G   
   G   
   A   
   C   
   A   
   T   
   T   
   G   
   A   
   A   
   G   
   C   
   C   
   A   
   G   
   A   
   T   
   G   
   A   
   A   
   G   
   A   
   G   
   C   
   T   
   A   
   G   
   A   
   A   
   A   
   T   
   T   
   A   
   A   
   T   
   G   
   G   
    T    
   T   
   T   
   G   
   A   
   A   
   A   
   T   
   G   
   G   
   C   
   A   
   A   
   A   
   A   
   A   
   G   
   A   
   T   
   A   
   A   
   A   
   T   
   C   
   T   
   T   
   G   
   G   
   G   
   G   
    C    
   G   
   G   
   C   
   T   
   A   
   T   
   T   
   T   
   T   
   G   
   C   
    G    
   A   
   C   
   A   
   C   
   G   
   T   
   G   
   T   
   A   
   A   
   A   
   A   
   G   
   A   
   A   
   G   
   C   
   A   
   A   
   G   
   T   
   C   
   C   
   A   
   G   
   T   
   C   
   A   
   A   
   T   
   C   
   T   
    G    
   C   
   G   
   T   
   A   
   C   
   C   
   G   
   C   
   T   
   T   
   T   
   A   
   G   
   T   
   T   
   A   
   A   
   T   
   T   
   C   
   A   
   G   
   A   
   T   
   A   
   A   
   T   
   A   
   T   
   T   
   T   
   A   
   T   
   T   
   T   
   T   
   G   
   C   
    C    
   C   
   A   
   A   
   C   
   A   
   A   
   A   
   C   
   A   
   T   
   T   
   A   
   C   
   G   
    G    
   A   
   T   
   G   
   G   
   G   
    G    
   G   
   A   
   A   
   G   
   A   
   C   
   A   
   A   
   A   
   T   
   T   
   T   
   A   
   G   
   A   
   G   
   C   
    G    
   G   
   G   
   T   
   C   
   T   
   T   
   A   
   A   
   T   
   A   
   A   
   A   
   T   
   T   
   C   
   A   
   T   
   T   
   T   
   T   
   C   
   G   
   G   
   T   
   G   
   A   
   G   
   G   
   A   
   A   
   C   
   T   
   A   
   G   
   A   
   T   
   T   
   T   
   A   
   C   
   C   
   A   
   A   
   T   
    T    
   G   
   C   
   C   
   A   
   T   
   G   
   A   
   C   
   G   
   C   
   C   
    T    
   G   
   C   
   T   
   C   
   A   
   A   
   A   
   T   
   T   
   T   
   C   
   A   
   A   
   A   
    T    
   G   
   A   
   A   
   G   
   A   
   T   
   A   
   A   
   A   
   T   
   T   
   T   
   A   
   A   
   T   
   T   
   C   
   T   
   G   
   A   
   A   
   A   
   T   
   T   
   C   
   T   
   T   
   T   
   T   
    A    
   G   
   C   
   A   
   G   
   A   
   T   
   A   
   C   
    G    
   G   
   G   
   C   
   T   
   A   
    C    
   G   
   G   
    T    
   C   
   A   
   A   
   G   
   G   
    C    
   C   
   A   
   A   
   T   
   T   
   G   
   T   
   T   
   A   
   A   
   T   
   T   
   T   
   C   
    G    
   C   
   C   
   A   
   A   
   T   
   C   
   C   
   A   
   G   
   C   
   A   
   A   
   G   
   C   
   T   
   A   
   C   
   C   
   A   
   T   
   G   
   T   
   A   
   T   
   A   
   G   
   T   
   G   
   T   
   T   
   T   
   T   
   C   
   C   
   A   
   A   
   A   
   A   
   T   
   A   
   A   
   T   
   G   
   G   
    A    
   A   
   C   
   A   
    T    
   T   
    G    
   G   
   T   
    C    
   T   
   A   
   T   
   C   
   C   
   A   
   A   
   A   
   A   
    T    
   T   
   A   
   G   
   T   
   T   
   T   
   T   
   A   
   G   
   A   
   C   
   A   
   A   
   A   
   G   
   A   
   A   
   A   
   C   
    G    
   A   
   A   
   G   
   A   
   A   
   A   
   A   
   A   
   A   
   G   
   A   
   C   
   A   
   A   
   T   
   G   
   T   
   A   
   A   
   T   
   C   
   A   
   G   
   C   
   G   
   C   
   C   
   A   
   A   
   C   
   G   
   C   
   A   
   G   
   C   
   G   
   A   
   A   
   T   
   A   
   C   
   A   
   A   
   T   
   T   
   G   
   C   
   C   
   A   
   C   
   A   
   G   
   A   
   T   
   C   
   T   
   T   
   T   
   T   
   A   
   G   
   G   
   T   
   A   
   G   
   T   
   G   
   T   
   G   
   G   
   A   
   A   
   G   
   A   
   T   
   C   
   C   
   T   
   T   
   C   
   A   
   G   
   G   
   T   
   T   
   A   
   T   
   G   
   T   
   T   
   T   
   A   
   T   
   A   
   A   
   T   
   A   
   T   
   G   
   T   
   A   
   C   
   A   
   A   
   T   
   C   
   C   
   G   
   A   
   A   
   C   
   T   
   T   
   C   
   T   
   C   
   A   
   T   
   T   
   G   
   G   
   C   
   T   
   G   
   C   
   T   
   A   
   A   
   A   
   A   
   C   
   A   
   G   
   G   
   A   
   A   
   C   
   A   
   G   
   C   
   T   
   G   
   A   
   A   
   A   
   T   
   A   
   A   
   A   
   A   
   G   
   A   
   T   
   A   
   A   
   A   
   C   
   A   
   A   
   G   
   A   
   T   
   A   
   C   
   A   
   G   
   A   
   T   
   G   
   G   
   C   
   A   
   A   
   A   
   G   
   A   
   A   
   A   
   A   
   C   
   A   
   G   
   T   
   T   
   T   
   C   
   C   
   T   
   T   
   C   
   T   
   A   
   A   
   C   
   G   
   T   
   T   
   A   
   G   
   A   
   T   
   C   
   G   
   T   
   A   
   G   
   T   
   A   
   A   
   C   
   A   
   A   
   T   
   A   
   A   
   A   
   T   
   T   
   C   
   T   
   T   
   A   
   A   
   C   
   A   
   A   
   T   
   G   
   A   
   T   
   T   
   A   
   T   
   G   
   G   
   T   
   T   
   G   
   A   
   A   
   A   
   A   
   T   
   T   
   C   
   A   
   G   
   G   
   A   
   G   
   A   
   A   
   A   
   A   
   T   
   G   
   G   
   T   
   T   
   C   
   T   
   G   
   C   
   A   
   A   
   C   
   A   
   G   
   A   
   T   
   A   
   T   
   C   
   A   
   G   
   T   
   A   
   A   
   A   
   C   
   C   
   A   
   T   
   T   
   A   
   A   
   T   
   T   
   G   
   A   
   T   
   T   
   A   
   C   
   T   
   T   
   A   
   G   
   A   
   A   
   G   
   C   
   A   
   A   
   C   
   C   
   A   
   T   
   T   
   A   
   A   
   A   
   -   
   -   
   -   
   -   
   -   
   -   
   -   
   -   
   -   
   -   
   -   
   -   
   -   
   -   
   -   
   -   
   -   
   -   
   -   
   -   
   -   
   -   
   -   
   -   
   -   
   -   
   -   
   -   
   -   
   -   
   -   
   -   
   -   
   -   
   -   
   -   
   -   
   -   
   -   
   -   
   -   
   -   
   -   
   -   
   -   
   -   
   -   
   -   
   -   
   -   
   -   
   -   
   -   
   -   
   -   
   -   
   -   
   -   
   -   
   -   
   -   
   -   
   -   
   -   
   -   
   -   
   -   
   -   
   -   
   -   
   -   
   -   
   -   
   -   
   -   
   -   
   -   
   -   
   -   
   -   
   -   
   -   
   -   
   -   
   -   
   -   
   -   
   -   
   -   
   -   
   -   
   -   
   -   
   -   
   -   
   -   
   -   
   -   
   -   
   -   
   -   
   -   
   -   
   -   
   -   
   -   
   -   
   -   
   -   
   -   
   -   
   -   
   -   
   -   
   -   
   -   
   -   
   -   
   -   
   -   
   -   
   -   
   -   
   -   
   -   
   -   
   -   
   -   
   -   
   -   
   -   
   -   
   -   
   -   
   -   
   -   
   -   
   -   
   -   
   -   
   -   
   -   
   -   
   -   
   -   
   -   
   -   
   -   
   -   
   -   
   -   
   -   
   -   
   -   
   -   
   -   
   -   
   -   
   -   
   -   
   -   
   -   
   -   
   -   
   -   
   -   
   -   
   -   
   -   
   C   
   A   
   A   
   T   
   G   
   G   
   C   
   A   
   A   
   G   
   C   
   C   
   A   
   A   
   A   
   C   
   A   
   A   
   G   
   A   
   A   
   T   
   T   
   A   
   G   
   C   
   C   
   G   
   A   
   A   
   G   
   C   
   G   
   A   
   A   
   G   
   A   
   A   
   A   
   A   
   C   
   A   
   G   
   C   
   T   
   A   
   C   
   T   
   A   
   C   
   A   
   T   
   T   
   T   
   T   
   T   
   A   
   A   
   A   
   C   
   G   
   T   
   A   
   T   
   T   
   G   
   T   
   C   
   A   
   A   
   A   
   A   
   C   
   A   
   G   
   G   
   A   
   A   
   T   
   T   
   T   
   G   
   A   
   T   
   A   
   A   
   G   
   T   
   T   
   A   
   C   
   C   
   G   
   T   
   C   
   C   
   G   
   T   
   T   
   A   
   T   
   T   
   C   
   A   
   A   
   G   
   A   
   A   
   G   
   C   
   T   
   A   
   G   
   C   
   T   
   T   
   A   
   A   
   A   
   G   
   A   
   A   
   A   
   A   
   A   
   T   
   G   
   G   
   C   
   T   
   A   
   T   
   G   
   A   
   T   
   A   
   C   
   T   
   A   
   A   
   A   
   T   
   C   
   T   
   G   
   T   
   T   
   G   
   T   
   T   
   G   
   A   
   A   
   A   
   A   
   A   
   T   
   A   
   C   
   C   
   A   
   A   
   G   
   C   
   A   
   A   
   T   
   T   
   T   
   A   
   T   
   T   
   C   
   A   
   G   
   G   
   G   
   A   
   T   
   T   
   C   
   A   
   A   
   G   
   C   
   A   
   G   
   A   
   A   
   G   
   G   
   A   
   G   
   T   
   C   
   A   
   A   
   A   
   G   
   C   
   T   
   A   
   G   
   T   
   G   
   A   
   T   
   G   
   T   
   T   
   C   
   A   
   A   
   G   
   T   
   C   
   A   
   A   
   A   
   A   
   A   
   G   
   G   
   C   
   G   
   A   
   A   
   A   
   G   
   A   
   C   
   A   
   A   
   T   
   C   
   A   
   A   
   T   
   A   
   C   
   A   
   C   
   A   
   T   
   T   
   T   
   A   
   C   
   C   
   T   
   A   
   T   
   A   
   A   
   A   
   T   
   T   
   A   
   T   
   C   
   G   
   A   
   T   
   G   
   A   
   G   
   T   
   A   
   C   
   C   
   C   
   C   
   T   
   T   
   T   
   A   
   G   
   G   
   C   
   G   
   A   
   A   
   A   
   T   
   G   
   A   
   A   
   A   
   G   
   A   
   T   
   T   
   T   
   G   
   T   
   C   
   T   
   T   
   A   
   T   
   C   
   A   
   A   
   T   
   C   
   A   
   A   
   G   
   T   
   A   
   T   
   C   
   G   
   C   
   C   
   A   
   A   
   A   
   A   
   A   
   A   
   G   
   G   
   C   
   G   
   A   
   T   
   A   
   C   
   C   
   T   
   A   
   C   
   C   
   A   
   A   
   A   
   T   
   C   
   G   
   C   
   T   
   T   
   G   
   G   
   A   
   A   
   G   
   C   
   C   
   G   
   T   
   C   
   T   
   T   
   T   
   A   
   A   
   T   
   T   
   T   
   T   
   T   
   C   
   C   
   A   
   G   
   A   
   T   
   A   
   T   
   G   
   T   
   C   
   A   
   G   
   G   
   A   
   A   
   A   
   T   
   G   
   A   
   T   
   A   
   A   
   A   
   A   
   T   
   T   
   T   
   C   
   G   
   A   
   T   
   T   
   C   
   A   
   A   
   G   
   T   
   A   
   G   
   A   
   T   
   A   
   A   
   T   
   G   
   C   
   C   
   A   
   A   
   A   
   C   
   G   
   T   
   G   
   G   
   A   
   G   
   A   
   A   
   A   
   T   
   T   
   G   
   T   
   C   
   G   
   A   
   T   
   C   
   G   
   T   
   A   
   A   
   T   
   G   
   G   
   T   
   A   
   G   
   T   
   G   
   G   
   G   
   C   
   T   
   A   
   G   
   C   
   A   
   A   
   T   
   T   
   A   
   A   
   C   
   A   
   A   
   A   
   G   
   T   
   G   
   T   
   T   
   T   
   G   
   A   
   C   
   G   
   A   
   A   
   G   
   T   
   G   
   G   
   G   
   C   
   G   
   T   
   A   
   G   
   T   
   G   
   C   
   C   
   T   
   G   
   G   
   C   
   A   
   A   
   A   
   C   
   T   
   C   
   G   
   G   
   T   
   T   
   C   
   T   
   G   
   G   
   C   
   G   
   C   
   A   
   G   
   A   
   A   
   A   
   A   
   A   
   A   
   C   
   A   
   G   
   C   
   A   
   A   
   A   
   T   
   A   
   T   
   C   
   A   
   A   
   A   
   G   
   C   
   T   
   T   
   T   
   T   
   A   
   G   
   T   
   G   
   A   
   T   
   A   
   A   
   A   
   T   
   T   
   C   
   G   
   G   
   C   
   G   
   T   
   T   
   T   
   C   
   T   
   G   
   T   
   T   
   G   
   A   
   T   
   G   
   A   
   A   
   A   
   T   
   C   
   A   
   A   
   T   
   C   
   A   
   A   
   A   
   A   
   G   
   T   
   T   
   A   
   A   
   G   
   C   
   C   
   A   
   A   
   G   
   G   
   A   
   T   
   G   
   G   
   G   
   T   
   C   
   C   
   A   
   A   
   G   
   C   
   A   
   G   
   A   
   C   
   T   
   C   
   C   
   T   
   T   
   T   
   G   
   T   
   A   
   C   
   C   
   A   
   A   
   T   
   C   
   A   
   C   
   A   
   G   
   T   
   C   
   G   
   C   
   T   
   T   
   C   
   T   
   G   
   A   
   A   
   C   
   C   
   A   
   G   
   T   
   G   
   A   
   C   
   A   
   G   
   A   
   A   
   T   
   T   
   A   
   C   
   C   
   A   
   A   
   C   
   A   
   G   
   G   
   G   
   G   
   C   
   T   
   G   
   C   
   G   
   A   
   C   
   A   
   A   
   A   
   A   
   G   
   A   
   T   
   A   
   C   
   A   
   G   
   A   
   G   
   T   
   C   
   A   
   C   
   G   
   T   
   T   
   A   
   T   
   T   
   A   
   T   
   C   
   C   
   G   
   C   
   T   
   G   
   G   
   G   
   G   
   G   
   A   
   A   
   G   
   C   
   A   
   G   
   C   
   T   
   G   
   C   
   G   
   C   
   A   
   A   
   T   
   T   
   A   
   A   
   T   
   C   
   G   
   G   
   G   
   T   
   A   
   T   
   A   
   C   
   G   
   G   
   G   
   C   
   A   
   C   
   C   
   A   
   T   
   T   
   A   
   C   
   T   
   G   
   C   
   A   
   G   
   A   
   A   
   G   
   A   
   T   
   A   
   T   
   T   
   G   
   A   
   A   
   A   
   A   
   A   
   A   
   A   
   T   
   C   
   C   
   A   
   G   
   A   
   G   
   T   
   T   
   A   
   A   
   G   
   T   
   A   
   G   
   T   
   A   
   C   
   G   
   G   
   G   
   C   
   G   
   T   
   A   
   A   
   T   
   T   
   G   
   G   
   T   
   A   
   A   
   A   
   A   
   C   
   T   
   G   
   G   
   C   
   T   
   T   
   A   
   G   
   A   
   A   
   C   
   G   
   A   
   G   
   C   
   G   
   T   
   T   
   T   
   G   
   A   
   T   
   A   
   A   
   A   
   G   
   A   
   A   
   T   
   T   
   A   
   A   
   G   
   A   
   G   
   G   
   G   
   C   
   A   
   A   
   G   
   A   
   T   
   G   
   G   
   T   
   G   
   G   
   C   
   T   
   C   
   A   
   C   
   T   
   A   
   G   
   T   
   T   
   A   
   T   
   T   
   T   
   T   
   A   
   G   
   A   
   T   
   G   
   A   
   C   
   A   
   A   
   A   
   G   
   A   
   G   
   A   
   A   
   T   
   G   
   T   
   C   
   A   
   A   
   A   
   A   
   A   
   A   
   G   
   C   
   T   
   T   
   T   
   A   
   C   
   A   
   A   
   A   
   C   
   C   
   A   
   A   
   A   
   G   
   A   
   G   
   A   
   A   
   A   
   A   
   A   
   A   
   G   
   A   
   T   
   G   
   G   
   T   
   C   
   A   
   A   
   A   
   C   
   G   
   A   
   T   
   T   
   A   
   A   
   A   
   T   
   T   
   A   
   A   
   C   
   G   
   A   
   T   
   T   
   G   
   A   
   C   
   A   
   G   
   T   
   G   
   G   
   C   
   G   
   T   
   A   
   C   
   A   
   A   
   C   
   A   
   A   
   C   
   A   
   A   
   G   
   C   
   C   
   T   
   T   
   C   
   G   
   C   
   T   
   A   
   T   
   A   
   T   
   T   
   T   
   G   
   A   
   C   
   A   
   A   
   A   
   C   
   G   
   C   
   C   
   C   
   T   
   G   
   G   
   T   
   A   
   G   
   T   
   G   
   C   
   G   
   G   
   T   
   C   
   A   
   T   
   T   
   A   
   C   
   C   
   G   
   A   
   T   
   C   
   C   
   T   
   C   
   A   
   A   
   A   
   A   
   A   
   G   
   G   
   C   
   G   
   A   
   T   
   T   
   T   
   A   
   T   
   T   
   A   
   G   
   C   
   A   
   A   
   C   
   G   
   G   
   T   
   T   
   A   
   G   
   T   
   T   
   C   
   A   
   C   
   C   
   T   
   T   
   C   
   G   
   T   
   A   
   T   
   G   
   A   
   T   
   C   
   C   
   T   
   A   
   A   
   T   
   A   
   A   
   A   
   A   
   T   
   G   
   G   
   C   
   C   
   A   
   A   
   T   
   G   
   G   
   C   
   A   
   T   
   T   
   T   
   C   
   T   
   C   
   A   
   A   
   A   
   A   
   A   
   G   
   A   
   A   
   T   
   A   
   T   
   G   
   A   
   T   
   G   
   C   
   T   
   T   
   A   
   C   
   A   
   A   
   T   
   A   
   A   
   C   
   A   
   A   
   C   
   A   
   A   
   A   
   G   
   A   
   T   
   T   
   T   
   A   
   C   
   C   
   A   
   T   
   T   
   C   
   A   
   C   
   A   
   G   
   C   
   A   
   C   
   G   
   T   
   T   
   T   
   T   
   G   
   C   
   G   
   A   
   C   
   A   
   G   
   G   
   C   
   T   
   A   
   T   
   G   
   C   
   A   
   C   
   C   
   A   
   G   
   G   
   C   
   T   
   C   
   T   
   A   
   C   
   C   
   T   
   T   
   T   
   A   
   A   
   A   
   A   
   C   
   A   
   A   
   T   
   T   
   A   
   C   
   C   
   G   
   G   
   T   
   G   
   C   
   C   
   A   
   T   
   T   
   G   
   G   
   T   
   T   
   T   
   A   
   G   
   A   
   T   
   G   
   C   
   A   
   G   
   G   
   G   
   A   
   C   
   A   
   T   
   T   
   G   
   A   
   A   
   G   
   C   
   C   
   A   
   G   
   A   
   T   
   G   
   A   
   A   
   G   
   A   
   G   
   C   
   T   
   A   
   G   
   A   
   A   
   A   
   T   
   T   
   A   
   A   
   T   
   G   
   G   
   T   
   T   
   T   
   G   
   A   
   A   
   A   
   T   
   G   
   G   
   C   
   A   
   A   
   A   
   A   
   A   
   G   
   A   
   T   
   A   
   A   
   A   
   T   
   C   
   T   
   T   
   G   
   G   
   G   
   G   
   C   
   G   
   G   
   C   
   T   
   A   
   T   
   T   
   T   
   T   
   G   
   C   
   G   
   A   
   C   
   A   
   C   
   G   
   T   
   G   
   T   
   A   
   A   
   A   
   A   
   G   
   A   
   A   
   G   
   C   
   A   
   A   
   G   
   T   
   C   
   C   
   A   
   G   
   T   
   C   
   A   
   A   
   T   
   C   
   T   
   G   
   C   
   G   
   T   
   A   
   C   
   C   
   G   
   C   
   T   
   T   
   T   
   A   
   G   
   T   
   T   
   A   
   A   
   T   
   T   
   C   
   A   
   G   
   A   
   T   
   A   
   A   
   T   
   A   
   T   
   T   
   T   
   A   
   T   
   T   
   T   
   T   
   G   
   C   
   C   
   C   
   A   
   A   
   C   
   A   
   A   
   A   
   C   
   A   
   T   
   T   
   A   
   C   
   G   
   G   
   A   
   T   
   G   
   G   
   G   
   G   
   G   
   A   
   A   
   G   
   A   
   C   
   A   
   A   
   A   
   T   
   T   
   T   
   A   
   G   
   A   
   G   
   C   
   G   
   G   
   G   
   T   
   C   
   T   
   T   
   A   
   A   
   T   
   A   
   A   
   A   
   T   
   T   
   C   
   A   
   T   
   T   
   T   
   T   
   C   
   G   
   G   
   T   
   G   
   A   
   G   
   G   
   A   
   A   
   C   
   T   
   A   
   G   
   A   
   T   
   T   
   T   
   A   
   C   
   C   
   A   
   A   
   T   
   T   
   G   
   C   
   C   
   A   
   T   
   G   
   A   
   C   
   G   
   C   
   C   
   T   
   G   
   C   
   T   
   C   
   A   
   A   
   A   
   T   
   T   
   T   
   C   
   A   
   A   
   A   
   T   
   G   
   A   
   A   
   G   
   A   
   T   
   A   
   A   
   A   
   T   
   T   
   T   
   A   
   A   
   T   
   T   
   C   
   T   
   G   
   A   
   A   
   A   
   T   
   T   
   C   
   T   
   T   
   T   
   T   
   A   
   G   
   C   
   A   
   G   
   A   
   T   
   A   
   C   
   G   
   G   
   G   
   C   
   T   
   A   
   C   
   G   
   G   
   T   
   C   
   A   
   A   
   G   
   G   
   C   
   C   
   A   
   A   
   T   
   T   
   G   
   T   
   T   
   A   
   A   
   T   
   T   
   T   
   C   
   G   
   C   
   C   
   A   
   A   
   T   
   C   
   C   
   A   
   G   
   C   
   A   
   A   
   G   
   C   
   T   
   A   
   C   
   C   
   A   
   T   
   G   
   T   
   A   
   T   
   A   
   G   
   T   
   G   
   T   
   T   
   T   
   T   
   C   
   C   
   A   
   A   
   A   
   A   
   T   
   A   
   A   
   T   
   G   
   G   
   A   
   A   
   C   
   A   
   T   
   T   
   G   
   G   
   T   
   C   
   T   
   A   
   T   
   C   
   C   
   A   
   A   
   A   
   A   
   T   
   T   
   A   
   G   
   T   
   T   
   T   
   T   
   A   
   G   
   A   
   C   
   A   
   A   
   A   
   G   
   A   
   A   
   A   
   C   
   G   
   A   
   A   
   G   
   A   
   A   
   A   
   A   
   A   
   A   
   G   
   A   
   C   
   A   
   A   
   T   
   G   
   T   
   A   
   A   
   T   
   C   
   A   
   G   
   C   
   G   
   C   
   C   
   A   
   A   
   C   
   G   
   C   
   A   
   G   
   C   
   G   
   A   
   A   
   T   
   A   
   C   
   A   
   A   
   T   
   T   
   G   
   C   
   C   
   A   
   C   
   A   
   G   
   A   
   T   
   C   
   T   
   T   
   T   
   T   
   A   
   G   
   G   
   T   
   A   
   G   
   T   
   G   
   T   
   G   
   G   
   A   
   A   
   G   
   A   
   T   
   C   
   C   
   T   
   T   
   C   
   A   
   G   
   G   
   T   
   T   
   A   
   T   
   G   
   T   
   T   
   T   
   A   
   T   
   A   
   A   
   T   
   A   
   T   
   G   
   T   
   A   
   C   
   A   
   A   
   T   
   C   
   C   
   G   
   A   
   A   
   C   
   T   
   T   
   C   
   T   
   C   
   A   
   T   
   T   
   G   
   G   
   C   
   T   
   G   
   C   
   T   
   A   
   A   
   A   
   A   
   C   
   A   
   G   
   G   
   A   
   A   
   C   
   A   
   G   
   C   
   T   
   G   
   A   
   A   
   A   
   T   
   A   
   A   
   A   
   A   
   G   
   A   
   T   
   A   
   A   
   A   
   C   
   A   
   A   
   G   
   A   
   T   
   A   
   C   
   A   
   G   
   A   
   T   
   G   
   G   
   C   
   A   
   A   
   A   
   G   
   A   
   A   
   A   
   A   
   C   
   A   
   G   
   T   
   T   
   T   
   C   
   C   
   T   
   T   
   C   
   T   
   A   
   A   
   C   
   G   
   T   
   T   
   A   
   G   
   A   
   T   
   C   
   G   
   T   
   A   
   G   
   T   
   A   
   A   
   C   
   A   
   A   
   T   
   A   
   A   
   A   
   T   
   T   
   C   
   T   
   T   
   A   
   A   
   C   
   A   
   A   
   T   
   G   
   A   
   T   
   T   
   A   
   T   
   G   
   G   
   T   
   T   
   G   
   A   
   A   
   A   
   A   
   T   
   T   
   C   
   A   
   G   
   G   
   A   
   G   
   A   
   A   
   A   
   A   
   T   
   G   
   G   
   T   
   T   
   C   
   T   
   G   
   C   
   A   
   A   
   C   
   A   
   G   
   A   
   T   
   A   
   T   
   C   
   A   
   G   
   T   
   A   
   A   
   A   
   C   
   C   
   A   
   T   
   T   
   A   
   A   
   T   
   T   
   G   
   A   
   T   
   T   
   A   
   C   
   T   
   T   
   A   
   G   
   A   
   A   
   G   
   C   
   A   
   A   
   C   
   C   
   A   
   T   
   T   
   A   
   A   
   A   
   -   
   -   
   -   
   -   
   -   
   -   
   -   
   -   
   -   
   -   
   -   
   -   
   -   
   -   
   -   
   -   
   -   
   -   
   -   
   -   
   -   
   -   
   -   
   -   
   -   
   -   
   -   
   -   
   -   
   -   
   -   
   -   
   -   
   -   
   -   
   -   
   -   
   -   
   -   
   -   
   -   
   -   
   -   
   -   
   -   
   -   
   -   
   -   
   -   
   -   
   -   
   -   
   -   
   -   
   -   
   -   
   -   
   -   
   -   
   -   
   -   
 

 
   N52729   
   -   
   -   
   -   
   -   
   -   
   -   
   -   
   -   
   -   
   -   
   -   
   -   
   -   
   -   
   -   
   -   
   -   
   -   
   -   
   -   
   -   
   -   
   -   
   -   
   -   
   -   
   -   
   -   
   -   
   -   
   -   
   -   
   -   
   -   
   -   
   -   
   -   
   -   
   -   
   -   
   -   
   -   
   -   
   -   
   -   
   -   
   -   
   -   
   -   
   -   
   -   
   -   
   -   
   -   
   -   
   -   
   -   
   -   
   -   
   -   
   -   
   -   
   -   
   -   
   -   
   -   
   -   
   -   
   -   
   -   
   -   
   -   
   -   
   -   
   -   
   -   
   -   
   -   
   -   
   -   
   -   
   -   
   -   
   -   
   -   
   -   
   -   
   -   
   -   
   -   
   -   
   -   
   -   
   -   
   -   
   -   
   -   
   -   
   -   
   -   
   -   
   -   
   -   
   -   
   -   
   -   
   -   
   -   
   C   
   A   
   A   
   T   
   G   
   G   
   C   
   A   
   A   
   G   
   C   
   C   
   A   
   A   
   A   
   C   
   A   
   A   
   G   
   A   
    A    
   T   
   T   
   A   
   G   
   C   
    C    
   G   
   A   
   A   
   G   
   C   
   G   
   A   
   A   
   G   
   A   
   A   
   A   
   A   
    C    
   A   
   G   
   C   
    T    
   A   
    C    
   T   
    A    
    C    
   A   
   T   
   T   
   T   
   T   
   T   
   A   
   A   
   A   
    C    
   G   
   T   
   A   
   T   
   T   
   G   
    A    
   C   
   A   
   A   
   A   
   A   
   C   
   A   
   G   
    A    
   A   
   A   
   T   
   T   
   T   
   G   
   A   
   T   
   A   
   A   
   G   
   T   
   T   
   A   
   C   
   C   
    G    
   T   
   C   
   C   
   G   
   T   
   T   
    G    
   T   
   T   
   C   
   A   
   A   
   G   
   A   
   A   
    G    
   C   
   T   
   A   
   G   
   C   
   T   
   T   
   A   
   A   
   A   
   G   
   A   
   A   
   A   
   A   
   A   
   T   
   G   
   G   
    C    
   T   
   A   
   T   
   G   
   A   
   T   
   A   
   C   
   T   
   A   
   A   
   A   
   T   
   C   
    T    
   G   
   T   
   T   
   G   
   T   
   T   
   G   
   A   
   A   
   A   
   A   
   A   
   T   
   A   
   C   
   C   
   A   
   A   
   G   
   C   
   A   
   A   
   T   
   T   
   T   
   A   
   T   
   T   
   C   
   A   
   G   
   G   
   G   
   A   
   T   
   T   
   C   
   A   
   A   
   G   
   C   
    A    
   G   
   A   
   A   
    G    
   G   
    A    
   G   
   T   
   C   
   A   
   A   
    A    
   G   
   C   
   T   
   A   
   G   
   T   
   G   
   A   
   T   
   G   
   T   
    T    
   C   
   A   
   A   
   G   
   T   
    C    
   A   
   A   
   A   
   A   
   A   
    A    
   G   
    C    
   G   
   A   
   A   
   A   
   G   
   A   
    C    
   A   
   A   
    T    
   C   
   A   
   A   
   T   
   A   
   C   
   A   
   C   
   A   
   T   
   T   
   T   
   A   
    C    
   C   
   T   
   A   
   T   
   A   
   A   
   A   
   T   
   T   
   A   
   T   
   C   
   G   
   A   
   T   
   G   
   A   
   G   
    T    
   A   
   C   
    C    
   C   
   C   
   T   
   T   
   T   
   A   
   G   
   G   
   C   
   G   
   A   
   A   
   A   
   T   
   G   
   A   
   A   
   A   
   G   
   A   
   T   
    T    
   T   
   G   
   T   
   C   
   T   
   T   
   A   
   T   
   C   
   A   
   A   
   T   
   C   
   A   
   A   
   G   
   T   
   A   
   T   
    C    
   G   
    C    
   C   
   A   
   A   
    A    
   A   
   A   
   A   
   G   
   G   
    C    
   G   
   A   
   T   
   A   
    C    
   C   
   T   
   A   
   C   
   C   
   A   
   A   
   A   
   T   
    C    
    G    
   C   
   T   
   T   
   G   
   G   
    A    
    A    
    G    
   C   
   C   
    G    
   T   
   C   
   T   
   T   
   T   
   A   
   A   
   T   
   T   
   T   
   T   
   T   
   C   
   C   
   A   
   G   
   A   
   T   
   A   
   T   
   G   
   T   
   C   
   A   
   G   
   G   
   A   
   A   
   A   
   T   
   G   
   A   
   T   
   A   
   A   
   A   
    A    
   T   
   T   
   T   
   C   
   G   
   A   
   T   
   T   
   C   
   A   
   A   
   G   
   T   
   A   
   G   
   A   
   T   
   A   
   A   
   T   
   G   
   C   
   C   
   A   
   A   
   A   
   C   
   G   
   T   
   G   
   G   
   A   
   G   
   A   
   A   
   A   
   T   
   T   
   G   
   T   
   C   
   G   
   A   
   T   
   C   
   G   
   T   
   A   
   A   
    T    
   G   
   G   
   T   
   A   
   G   
   T   
   G   
   G   
   G   
   C   
   T   
   A   
   G   
    C    
   A   
   A   
   T   
   T   
   A   
   A   
   C   
   A   
   A   
   A   
   G   
   T   
   G   
   T   
   T   
   T   
   G   
   A   
    C    
   G   
   A   
   A   
   G   
   T   
   G   
   G   
   G   
   C   
   G   
   T   
   A   
   G   
   T   
    G    
   C   
   C   
   T   
    G    
   G   
   C   
   A   
   A   
   A   
   C   
   T   
    C    
   G   
   G   
   T   
   T   
   C   
   T   
   G   
   G   
   C   
   G   
   C   
   A   
   G   
   A   
   A   
   A   
   A   
   A   
    A    
   C   
   A   
   G   
   C   
    C    
   A   
   A   
   T   
   A   
   T   
   C   
   A   
   A   
   A   
   G   
   C   
   T   
   T   
   T   
   T   
   A   
   G   
   T   
   G   
   A   
   T   
   A   
   A   
    G    
   T   
   T   
    C    
   G   
   G   
   C   
   G   
   T   
   T   
   T   
   C   
   T   
    G    
   T   
   T   
   G   
   A   
   T   
   G   
   A   
   A   
   A   
   T   
   C   
   A   
   A   
   T   
   C   
    A    
   A   
   A   
   A   
    G    
   T   
   T   
   A   
   A   
   G   
   C   
   C   
   A   
   A   
   G   
   G   
    G    
   T   
   G   
   G   
   G   
   T   
   C   
   C   
   A   
   A   
   G   
   C   
   A   
   G   
   A   
   C   
   T   
   C   
    C    
   T   
   T   
   T   
   G   
   T   
   A   
   C   
   C   
   A   
   A   
   T   
   C   
   A   
   C   
   A   
   G   
   T   
    C    
   G   
   C   
   T   
   T   
   C   
   T   
   G   
   A   
   A   
   C   
   C   
   A   
   G   
   T   
   G   
   A   
   C   
   A   
   G   
   A   
   A   
   T   
   T   
   A   
   C   
   C   
   A   
   A   
   C   
   A   
   G   
   G   
   G   
   G   
   C   
   T   
   G   
   C   
   G   
   A   
   C   
   A   
   A   
   A   
   A   
   G   
    A    
    T    
   A   
   C   
   A   
   G   
   A   
   G   
   T   
   C   
   A   
   C   
   G   
   T   
   T   
   A   
   T   
   T   
   A   
   T   
   C   
   C   
    G    
    C    
   T   
   G   
   G   
   G   
   G   
   G   
   A   
   A   
   G   
   C   
   A   
   G   
   C   
   T   
   G   
   C   
   G   
   C   
   A   
   A   
   T   
   T   
   A   
   A   
   T   
   C   
   G   
   G   
    G    
   T   
   A   
   T   
   A   
   C   
    A    
   G   
   G   
    C    
   A   
   C   
    C    
   A   
   T   
   T   
   A   
   C   
   T   
   G   
   C   
   A   
   G   
   A   
    A    
   G   
   A   
   T   
   A   
   T   
   T   
    A    
   A   
   A   
   A   
   A   
   A   
   A   
   A   
   T   
   C   
   C   
   A   
   G   
   A   
   G   
   T   
   T   
   A   
   A   
   G   
   T   
   A   
   G   
   T   
   A   
   C   
   G   
   G   
   G   
    C    
   G   
   T   
   A   
   A   
   T   
   T   
   G   
   G   
    T    
   A   
   A   
   A   
   A   
   C   
   T   
   G   
   G   
    C    
   T   
   T   
   A   
   G   
   A   
   A   
   C   
   G   
   A   
   G   
   C   
   G   
   T   
   T   
   T   
   G   
   A   
   T   
   A   
   A   
   A   
   G   
   A   
    A    
   T   
   T   
   A   
   A   
   G   
   A   
   G   
   G   
    G    
   C   
   A   
   A   
   G   
   A   
   T   
   G   
   G   
    T    
   G   
   G   
    C    
   T   
   C   
    A    
   C   
   T   
   A   
   G   
   T   
   T   
   A   
   T   
   T   
   T   
   T   
   A   
   G   
   A   
   T   
   G   
   A   
   C   
   A   
   A   
   A   
   G   
   A   
   G   
   A   
   A   
   T   
   G   
   T   
   C   
   A   
   A   
   A   
   A   
   A   
   A   
   G   
   C   
   T   
   T   
   T   
   A   
   C   
   A   
   A   
   A   
   C   
   C   
   A   
   A   
   A   
   G   
   A   
    G    
   A   
   A   
   A   
   A   
   A   
   A   
   G   
   A   
   T   
   G   
   G   
   T   
   C   
   A   
   A   
   A   
   C   
    G    
   A   
   T   
   T   
   A   
   A   
   A   
   T   
   T   
   A   
   A   
   C   
   G   
   A   
   T   
   T   
   G   
   A   
    C    
   A   
   G   
    T    
   G   
   G   
    C    
   G   
   T   
   A   
   C   
   A   
   A   
   C   
   A   
   A   
   C   
   A   
   A   
   G   
   C   
    C    
   T   
   T   
    C    
   G   
   C   
   T   
   A   
   T   
   A   
   T   
   T   
   T   
   G   
   A   
   C   
   A   
   A   
   A   
   C   
   G   
    C    
    C    
   C   
   T   
   G   
   G   
   T   
   A   
   G   
   T   
   G   
   C   
   G   
   G   
   T   
   C   
   A   
   T   
   T   
   A   
   C   
   C   
   G   
   A   
   T   
   C   
   C   
   T   
   C   
   A   
   A   
   A   
   A   
   A   
   G   
   G   
   C   
   G   
   A   
   T   
   T   
   T   
   A   
    T    
   T   
   A   
   G   
   C   
    A    
   A   
   C   
   G   
   G   
   T   
   T   
   A   
   G   
   T   
   T   
   C   
   A   
   C   
   C   
   T   
   T   
   C   
   G   
   T   
   A   
   T   
   G   
   A   
   T   
   C   
   C   
   T   
   A   
   A   
   T   
   A   
   A   
   A   
   A   
   T   
   G   
   G   
   C   
   C   
   A   
   A   
   T   
   G   
   G   
    C    
   A   
   T   
   T   
   T   
   C   
   T   
   C   
   A   
   A   
   A   
   A   
   A   
   G   
   A   
    A    
   T   
   A   
   T   
   G   
   A   
   T   
   G   
   C   
   T   
   T   
   A   
   C   
   A   
   A   
   T   
   A   
   A   
   C   
   A   
   A   
   C   
   A   
   A   
   A   
   G   
   A   
   T   
   T   
   T   
   A   
   C   
   C   
   A   
   T   
   T   
    C    
   A   
   C   
   A   
   G   
   C   
   A   
   C   
   G   
   T   
   T   
   T   
    T    
   G   
   C   
   G   
   A   
   C   
   A   
   G   
   G   
    C    
   T   
   A   
   T   
   G   
   C   
   A   
   C   
   C   
   A   
   G   
   G   
    C    
   T   
   C   
   T   
   A   
   C   
   C   
   T   
   T   
   T   
   A   
   A   
   A   
   A   
   C   
   A   
   A   
   T   
   T   
   A   
   C   
   C   
   G   
   G   
   T   
   G   
   C   
   C   
   A   
   T   
   T   
   G   
   G   
   T   
   T   
   T   
   A   
   G   
   A   
   T   
    A    
   C   
   A   
   G   
   G   
   G   
   A   
   C   
   A   
   T   
   T   
   G   
   A   
   A   
   G   
   C   
   C   
   A   
   G   
   A   
   T   
   G   
   A   
   A   
   G   
   A   
   G   
   C   
   T   
   A   
   G   
   A   
   A   
   A   
   T   
   T   
   A   
   A   
   T   
   G   
   G   
    T    
   T   
   T   
   G   
   A   
   A   
   A   
   T   
   G   
   G   
   C   
   A   
   A   
   A   
   A   
   A   
   G   
   A   
   T   
   A   
   A   
   A   
   T   
   C   
   T   
   T   
   G   
   G   
   G   
   G   
    C    
   G   
   G   
   C   
   T   
   A   
   T   
   T   
   T   
   T   
   G   
   C   
    G    
   A   
   C   
   A   
   C   
   G   
   T   
   G   
   T   
   A   
   A   
   A   
   A   
   G   
   A   
   A   
   G   
   C   
   A   
   A   
   G   
   T   
   C   
   C   
   A   
   G   
   T   
   C   
   A   
   A   
   T   
   C   
   T   
    G    
   C   
   G   
   T   
   A   
   C   
   C   
   G   
   C   
   T   
   T   
   T   
   A   
   G   
   T   
   T   
   A   
   A   
   T   
   T   
   C   
   A   
   G   
   A   
   T   
   A   
   A   
   T   
   A   
   T   
   T   
   T   
   A   
   T   
   T   
   T   
   T   
   G   
   C   
    C    
   C   
   A   
   A   
   C   
   A   
   A   
   A   
   C   
   A   
   T   
   T   
   A   
   C   
   G   
    G    
   A   
   T   
   G   
   G   
   G   
    A    
   G   
   A   
   A   
   G   
   A   
   C   
   A   
   A   
   A   
   T   
   T   
   T   
   A   
   G   
   A   
   G   
   C   
    G    
   G   
   G   
   T   
   C   
   T   
   T   
   A   
   A   
   T   
   A   
   A   
   A   
   T   
   T   
   C   
   A   
   T   
   T   
   T   
   T   
   C   
   G   
   G   
   T   
   G   
   A   
   G   
   G   
   A   
   A   
   C   
   T   
   A   
   G   
   A   
   T   
   T   
   T   
   A   
   C   
   C   
   A   
   A   
   T   
    T    
   G   
   C   
   C   
   A   
   T   
   G   
   A   
   C   
   G   
   C   
   C   
    T    
   G   
   C   
   T   
   C   
   A   
   A   
   A   
   T   
   T   
   T   
   C   
   A   
   A   
   A   
    T    
   G   
   A   
   A   
   G   
   A   
   T   
   A   
   A   
   A   
   T   
   T   
   T   
   A   
   A   
   T   
   T   
   C   
   T   
   G   
   A   
   A   
   A   
   T   
   T   
   C   
   T   
   T   
   T   
   T   
    G    
   G   
   C   
   A   
   G   
   A   
   T   
   A   
   C   
    G    
   G   
   G   
   C   
   T   
   A   
    C    
   G   
   G   
    C    
   C   
   A   
   A   
   G   
   G   
    C    
   C   
   A   
   A   
   T   
   T   
   G   
   T   
   T   
   A   
   A   
   T   
   T   
   T   
   C   
    G    
   C   
   C   
   A   
   A   
   T   
   C   
   C   
   A   
   G   
   C   
   A   
   A   
   G   
   C   
   T   
   A   
   C   
   C   
   A   
   T   
   G   
   T   
   A   
   T   
   A   
   G   
   T   
   G   
   T   
   T   
   T   
   T   
   C   
   C   
   A   
   A   
   A   
   A   
   T   
   A   
   A   
   T   
   G   
   G   
    G    
   A   
   C   
   A   
    T    
   T   
    A    
   G   
   T   
    C    
   T   
   A   
   T   
   C   
   C   
   A   
   A   
   A   
   A   
    T    
   T   
   A   
   G   
   T   
   T   
   T   
   T   
   A   
   G   
   A   
   C   
   A   
   A   
   A   
   G   
   A   
   A   
   A   
   C   
    G    
   A   
   A   
   G   
   A   
   A   
   A   
   A   
   A   
   A   
   G   
   A   
   C   
   A   
   A   
   T   
   G   
   T   
   A   
   A   
   T   
   C   
   A   
   G   
   C   
   G   
   C   
   C   
   A   
   A   
   C   
   G   
   C   
   A   
   G   
   C   
   G   
   A   
   A   
   T   
   A   
   C   
   A   
   A   
   T   
   T   
   G   
   C   
   C   
   A   
   C   
   A   
   G   
   A   
   T   
   C   
   T   
   T   
   T   
   T   
   A   
   G   
   G   
   T   
   A   
   G   
   T   
   G   
   T   
   G   
   G   
   A   
   A   
   G   
   A   
   T   
   C   
   C   
   T   
   T   
   C   
   A   
   G   
   G   
   T   
   T   
   A   
   T   
   G   
   T   
   T   
   T   
   A   
   T   
   A   
   A   
   T   
   A   
   T   
   G   
   T   
   A   
   C   
   A   
   A   
   T   
   C   
   C   
   G   
   A   
   A   
   C   
   T   
   T   
   C   
   T   
   C   
   A   
   T   
   T   
   G   
   G   
   C   
   T   
   G   
   C   
   C   
   A   
   A   
   A   
   A   
   C   
   A   
   G   
   G   
   A   
   A   
   C   
   A   
   G   
   C   
   T   
   G   
   A   
   A   
   A   
   T   
   A   
   A   
   A   
   A   
   G   
   A   
   T   
   A   
   A   
   A   
   C   
   A   
   A   
   G   
   A   
   T   
   A   
   C   
   A   
   G   
   A   
   T   
   G   
   G   
   C   
   A   
   A   
   A   
   G   
   A   
   A   
   A   
   A   
   C   
   A   
   G   
   T   
   T   
   T   
   C   
   C   
   T   
   T   
   C   
   T   
   A   
   A   
   C   
   G   
   T   
   T   
   A   
   G   
   A   
   T   
   C   
   G   
   T   
   A   
   G   
   T   
   A   
   A   
   C   
   A   
   A   
   T   
   A   
   A   
   A   
   T   
   T   
   C   
   T   
   T   
   A   
   A   
   C   
   A   
   A   
   T   
   G   
   A   
   T   
   T   
   A   
   T   
   G   
   G   
   T   
   T   
   G   
   A   
   A   
   A   
   A   
   T   
   T   
   C   
   A   
   G   
   G   
   A   
   G   
   A   
   A   
   A   
   A   
   T   
   G   
   G   
   T   
   T   
   C   
   T   
   G   
   C   
   A   
   A   
   C   
   A   
   G   
   A   
   T   
   A   
   T   
   C   
   A   
   G   
   T   
   A   
   A   
   A   
   C   
   C   
   A   
   T   
   T   
   A   
   A   
   T   
   T   
   G   
   A   
   T   
   T   
   A   
   C   
   T   
   T   
   A   
   G   
   A   
   A   
   G   
   C   
   A   
   A   
   C   
   C   
   A   
   T   
   T   
   A   
   A   
   A   
   -   
   -   
   -   
   -   
   -   
   -   
   -   
   -   
   -   
   -   
   -   
   -   
   -   
   -   
   -   
   -   
   -   
   -   
   -   
   -   
   -   
   -   
   -   
   -   
   -   
   -   
   -   
   -   
   -   
   -   
   -   
   -   
   -   
   -   
   -   
   -   
   -   
   -   
   -   
   -   
   -   
   -   
   -   
   -   
   -   
   -   
   -   
   -   
   -   
   -   
   -   
   -   
   -   
   -   
   -   
   -   
   -   
   -   
   -   
   -   
   -   
   -   
   -   
   -   
   -   
   -   
   -   
   -   
   -   
   -   
   -   
   -   
   -   
   -   
   -   
   -   
   -   
   -   
   -   
   -   
   -   
   -   
   -   
   -   
   -   
   -   
   -   
   -   
   -   
   -   
   -   
   -   
   -   
   -   
   -   
   -   
   -   
   -   
   -   
   -   
   -   
   -   
   -   
   -   
   -   
   -   
   -   
   -   
   -   
   -   
   -   
   -   
   -   
   -   
   -   
   -   
   -   
   -   
   -   
   -   
   -   
   -   
   -   
   -   
   -   
   -   
   -   
   -   
   -   
   -   
   -   
   -   
   -   
   -   
   -   
   -   
   -   
   -   
   -   
   -   
   -   
   -   
   -   
   -   
   -   
   -   
   -   
   -   
   -   
   -   
   -   
   -   
   -   
   -   
   -   
   -   
   -   
   -   
   -   
   -   
   -   
   -   
   -   
   -   
   -   
   -   
   -   
   -   
   -   
   C   
   A   
   A   
   T   
   G   
   G   
   C   
   A   
   A   
   G   
   C   
   C   
   A   
   A   
   A   
   C   
   A   
   A   
   G   
   A   
   A   
   T   
   T   
   A   
   G   
   C   
   C   
   G   
   A   
   A   
   G   
   C   
   G   
   A   
   A   
   G   
   A   
   A   
   A   
   A   
   C   
   A   
   G   
   C   
   T   
   A   
   C   
   T   
   A   
   C   
   A   
   T   
   T   
   T   
   T   
   T   
   A   
   A   
   A   
   C   
   G   
   T   
   A   
   T   
   T   
   G   
   A   
   C   
   A   
   A   
   A   
   A   
   C   
   A   
   G   
   A   
   A   
   A   
   T   
   T   
   T   
   G   
   A   
   T   
   A   
   A   
   G   
   T   
   T   
   A   
   C   
   C   
   G   
   T   
   C   
   C   
   G   
   T   
   T   
   G   
   T   
   T   
   C   
   A   
   A   
   G   
   A   
   A   
   G   
   C   
   T   
   A   
   G   
   C   
   T   
   T   
   A   
   A   
   A   
   G   
   A   
   A   
   A   
   A   
   A   
   T   
   G   
   G   
   C   
   T   
   A   
   T   
   G   
   A   
   T   
   A   
   C   
   T   
   A   
   A   
   A   
   T   
   C   
   T   
   G   
   T   
   T   
   G   
   T   
   T   
   G   
   A   
   A   
   A   
   A   
   A   
   T   
   A   
   C   
   C   
   A   
   A   
   G   
   C   
   A   
   A   
   T   
   T   
   T   
   A   
   T   
   T   
   C   
   A   
   G   
   G   
   G   
   A   
   T   
   T   
   C   
   A   
   A   
   G   
   C   
   A   
   G   
   A   
   A   
   G   
   G   
   A   
   G   
   T   
   C   
   A   
   A   
   A   
   G   
   C   
   T   
   A   
   G   
   T   
   G   
   A   
   T   
   G   
   T   
   T   
   C   
   A   
   A   
   G   
   T   
   C   
   A   
   A   
   A   
   A   
   A   
   A   
   G   
   C   
   G   
   A   
   A   
   A   
   G   
   A   
   C   
   A   
   A   
   T   
   C   
   A   
   A   
   T   
   A   
   C   
   A   
   C   
   A   
   T   
   T   
   T   
   A   
   C   
   C   
   T   
   A   
   T   
   A   
   A   
   A   
   T   
   T   
   A   
   T   
   C   
   G   
   A   
   T   
   G   
   A   
   G   
   T   
   A   
   C   
   C   
   C   
   C   
   T   
   T   
   T   
   A   
   G   
   G   
   C   
   G   
   A   
   A   
   A   
   T   
   G   
   A   
   A   
   A   
   G   
   A   
   T   
   T   
   T   
   G   
   T   
   C   
   T   
   T   
   A   
   T   
   C   
   A   
   A   
   T   
   C   
   A   
   A   
   G   
   T   
   A   
   T   
   C   
   G   
   C   
   C   
   A   
   A   
   A   
   A   
   A   
   A   
   G   
   G   
   C   
   G   
   A   
   T   
   A   
   C   
   C   
   T   
   A   
   C   
   C   
   A   
   A   
   A   
   T   
   C   
   G   
   C   
   T   
   T   
   G   
   G   
   A   
   A   
   G   
   C   
   C   
   G   
   T   
   C   
   T   
   T   
   T   
   A   
   A   
   T   
   T   
   T   
   T   
   T   
   C   
   C   
   A   
   G   
   A   
   T   
   A   
   T   
   G   
   T   
   C   
   A   
   G   
   G   
   A   
   A   
   A   
   T   
   G   
   A   
   T   
   A   
   A   
   A   
   A   
   T   
   T   
   T   
   C   
   G   
   A   
   T   
   T   
   C   
   A   
   A   
   G   
   T   
   A   
   G   
   A   
   T   
   A   
   A   
   T   
   G   
   C   
   C   
   A   
   A   
   A   
   C   
   G   
   T   
   G   
   G   
   A   
   G   
   A   
   A   
   A   
   T   
   T   
   G   
   T   
   C   
   G   
   A   
   T   
   C   
   G   
   T   
   A   
   A   
   T   
   G   
   G   
   T   
   A   
   G   
   T   
   G   
   G   
   G   
   C   
   T   
   A   
   G   
   C   
   A   
   A   
   T   
   T   
   A   
   A   
   C   
   A   
   A   
   A   
   G   
   T   
   G   
   T   
   T   
   T   
   G   
   A   
   C   
   G   
   A   
   A   
   G   
   T   
   G   
   G   
   G   
   C   
   G   
   T   
   A   
   G   
   T   
   G   
   C   
   C   
   T   
   G   
   G   
   C   
   A   
   A   
   A   
   C   
   T   
   C   
   G   
   G   
   T   
   T   
   C   
   T   
   G   
   G   
   C   
   G   
   C   
   A   
   G   
   A   
   A   
   A   
   A   
   A   
   A   
   C   
   A   
   G   
   C   
   C   
   A   
   A   
   T   
   A   
   T   
   C   
   A   
   A   
   A   
   G   
   C   
   T   
   T   
   T   
   T   
   A   
   G   
   T   
   G   
   A   
   T   
   A   
   A   
   G   
   T   
   T   
   C   
   G   
   G   
   C   
   G   
   T   
   T   
   T   
   C   
   T   
   G   
   T   
   T   
   G   
   A   
   T   
   G   
   A   
   A   
   A   
   T   
   C   
   A   
   A   
   T   
   C   
   A   
   A   
   A   
   A   
   G   
   T   
   T   
   A   
   A   
   G   
   C   
   C   
   A   
   A   
   G   
   G   
   G   
   T   
   G   
   G   
   G   
   T   
   C   
   C   
   A   
   A   
   G   
   C   
   A   
   G   
   A   
   C   
   T   
   C   
   C   
   T   
   T   
   T   
   G   
   T   
   A   
   C   
   C   
   A   
   A   
   T   
   C   
   A   
   C   
   A   
   G   
   T   
   C   
   G   
   C   
   T   
   T   
   C   
   T   
   G   
   A   
   A   
   C   
   C   
   A   
   G   
   T   
   G   
   A   
   C   
   A   
   G   
   A   
   A   
   T   
   T   
   A   
   C   
   C   
   A   
   A   
   C   
   A   
   G   
   G   
   G   
   G   
   C   
   T   
   G   
   C   
   G   
   A   
   C   
   A   
   A   
   A   
   A   
   G   
   A   
   T   
   A   
   C   
   A   
   G   
   A   
   G   
   T   
   C   
   A   
   C   
   G   
   T   
   T   
   A   
   T   
   T   
   A   
   T   
   C   
   C   
   G   
   C   
   T   
   G   
   G   
   G   
   G   
   G   
   A   
   A   
   G   
   C   
   A   
   G   
   C   
   T   
   G   
   C   
   G   
   C   
   A   
   A   
   T   
   T   
   A   
   A   
   T   
   C   
   G   
   G   
   G   
   T   
   A   
   T   
   A   
   C   
   A   
   G   
   G   
   C   
   A   
   C   
   C   
   A   
   T   
   T   
   A   
   C   
   T   
   G   
   C   
   A   
   G   
   A   
   A   
   G   
   A   
   T   
   A   
   T   
   T   
   A   
   A   
   A   
   A   
   A   
   A   
   A   
   A   
   T   
   C   
   C   
   A   
   G   
   A   
   G   
   T   
   T   
   A   
   A   
   G   
   T   
   A   
   G   
   T   
   A   
   C   
   G   
   G   
   G   
   C   
   G   
   T   
   A   
   A   
   T   
   T   
   G   
   G   
   T   
   A   
   A   
   A   
   A   
   C   
   T   
   G   
   G   
   C   
   T   
   T   
   A   
   G   
   A   
   A   
   C   
   G   
   A   
   G   
   C   
   G   
   T   
   T   
   T   
   G   
   A   
   T   
   A   
   A   
   A   
   G   
   A   
   A   
   T   
   T   
   A   
   A   
   G   
   A   
   G   
   G   
   G   
   C   
   A   
   A   
   G   
   A   
   T   
   G   
   G   
   T   
   G   
   G   
   C   
   T   
   C   
   A   
   C   
   T   
   A   
   G   
   T   
   T   
   A   
   T   
   T   
   T   
   T   
   A   
   G   
   A   
   T   
   G   
   A   
   C   
   A   
   A   
   A   
   G   
   A   
   G   
   A   
   A   
   T   
   G   
   T   
   C   
   A   
   A   
   A   
   A   
   A   
   A   
   G   
   C   
   T   
   T   
   T   
   A   
   C   
   A   
   A   
   A   
   C   
   C   
   A   
   A   
   A   
   G   
   A   
   G   
   A   
   A   
   A   
   A   
   A   
   A   
   G   
   A   
   T   
   G   
   G   
   T   
   C   
   A   
   A   
   A   
   C   
   G   
   A   
   T   
   T   
   A   
   A   
   A   
   T   
   T   
   A   
   A   
   C   
   G   
   A   
   T   
   T   
   G   
   A   
   C   
   A   
   G   
   T   
   G   
   G   
   C   
   G   
   T   
   A   
   C   
   A   
   A   
   C   
   A   
   A   
   C   
   A   
   A   
   G   
   C   
   C   
   T   
   T   
   C   
   G   
   C   
   T   
   A   
   T   
   A   
   T   
   T   
   T   
   G   
   A   
   C   
   A   
   A   
   A   
   C   
   G   
   C   
   C   
   C   
   T   
   G   
   G   
   T   
   A   
   G   
   T   
   G   
   C   
   G   
   G   
   T   
   C   
   A   
   T   
   T   
   A   
   C   
   C   
   G   
   A   
   T   
   C   
   C   
   T   
   C   
   A   
   A   
   A   
   A   
   A   
   G   
   G   
   C   
   G   
   A   
   T   
   T   
   T   
   A   
   T   
   T   
   A   
   G   
   C   
   A   
   A   
   C   
   G   
   G   
   T   
   T   
   A   
   G   
   T   
   T   
   C   
   A   
   C   
   C   
   T   
   T   
   C   
   G   
   T   
   A   
   T   
   G   
   A   
   T   
   C   
   C   
   T   
   A   
   A   
   T   
   A   
   A   
   A   
   A   
   T   
   G   
   G   
   C   
   C   
   A   
   A   
   T   
   G   
   G   
   C   
   A   
   T   
   T   
   T   
   C   
   T   
   C   
   A   
   A   
   A   
   A   
   A   
   G   
   A   
   A   
   T   
   A   
   T   
   G   
   A   
   T   
   G   
   C   
   T   
   T   
   A   
   C   
   A   
   A   
   T   
   A   
   A   
   C   
   A   
   A   
   C   
   A   
   A   
   A   
   G   
   A   
   T   
   T   
   T   
   A   
   C   
   C   
   A   
   T   
   T   
   C   
   A   
   C   
   A   
   G   
   C   
   A   
   C   
   G   
   T   
   T   
   T   
   T   
   G   
   C   
   G   
   A   
   C   
   A   
   G   
   G   
   C   
   T   
   A   
   T   
   G   
   C   
   A   
   C   
   C   
   A   
   G   
   G   
   C   
   T   
   C   
   T   
   A   
   C   
   C   
   T   
   T   
   T   
   A   
   A   
   A   
   A   
   C   
   A   
   A   
   T   
   T   
   A   
   C   
   C   
   G   
   G   
   T   
   G   
   C   
   C   
   A   
   T   
   T   
   G   
   G   
   T   
   T   
   T   
   A   
   G   
   A   
   T   
   A   
   C   
   A   
   G   
   G   
   G   
   A   
   C   
   A   
   T   
   T   
   G   
   A   
   A   
   G   
   C   
   C   
   A   
   G   
   A   
   T   
   G   
   A   
   A   
   G   
   A   
   G   
   C   
   T   
   A   
   G   
   A   
   A   
   A   
   T   
   T   
   A   
   A   
   T   
   G   
   G   
   T   
   T   
   T   
   G   
   A   
   A   
   A   
   T   
   G   
   G   
   C   
   A   
   A   
   A   
   A   
   A   
   G   
   A   
   T   
   A   
   A   
   A   
   T   
   C   
   T   
   T   
   G   
   G   
   G   
   G   
   C   
   G   
   G   
   C   
   T   
   A   
   T   
   T   
   T   
   T   
   G   
   C   
   G   
   A   
   C   
   A   
   C   
   G   
   T   
   G   
   T   
   A   
   A   
   A   
   A   
   G   
   A   
   A   
   G   
   C   
   A   
   A   
   G   
   T   
   C   
   C   
   A   
   G   
   T   
   C   
   A   
   A   
   T   
   C   
   T   
   G   
   C   
   G   
   T   
   A   
   C   
   C   
   G   
   C   
   T   
   T   
   T   
   A   
   G   
   T   
   T   
   A   
   A   
   T   
   T   
   C   
   A   
   G   
   A   
   T   
   A   
   A   
   T   
   A   
   T   
   T   
   T   
   A   
   T   
   T   
   T   
   T   
   G   
   C   
   C   
   C   
   A   
   A   
   C   
   A   
   A   
   A   
   C   
   A   
   T   
   T   
   A   
   C   
   G   
   G   
   A   
   T   
   G   
   G   
   G   
   A   
   G   
   A   
   A   
   G   
   A   
   C   
   A   
   A   
   A   
   T   
   T   
   T   
   A   
   G   
   A   
   G   
   C   
   G   
   G   
   G   
   T   
   C   
   T   
   T   
   A   
   A   
   T   
   A   
   A   
   A   
   T   
   T   
   C   
   A   
   T   
   T   
   T   
   T   
   C   
   G   
   G   
   T   
   G   
   A   
   G   
   G   
   A   
   A   
   C   
   T   
   A   
   G   
   A   
   T   
   T   
   T   
   A   
   C   
   C   
   A   
   A   
   T   
   T   
   G   
   C   
   C   
   A   
   T   
   G   
   A   
   C   
   G   
   C   
   C   
   T   
   G   
   C   
   T   
   C   
   A   
   A   
   A   
   T   
   T   
   T   
   C   
   A   
   A   
   A   
   T   
   G   
   A   
   A   
   G   
   A   
   T   
   A   
   A   
   A   
   T   
   T   
   T   
   A   
   A   
   T   
   T   
   C   
   T   
   G   
   A   
   A   
   A   
   T   
   T   
   C   
   T   
   T   
   T   
   T   
   G   
   G   
   C   
   A   
   G   
   A   
   T   
   A   
   C   
   G   
   G   
   G   
   C   
   T   
   A   
   C   
   G   
   G   
   C   
   C   
   A   
   A   
   G   
   G   
   C   
   C   
   A   
   A   
   T   
   T   
   G   
   T   
   T   
   A   
   A   
   T   
   T   
   T   
   C   
   G   
   C   
   C   
   A   
   A   
   T   
   C   
   C   
   A   
   G   
   C   
   A   
   A   
   G   
   C   
   T   
   A   
   C   
   C   
   A   
   T   
   G   
   T   
   A   
   T   
   A   
   G   
   T   
   G   
   T   
   T   
   T   
   T   
   C   
   C   
   A   
   A   
   A   
   A   
   T   
   A   
   A   
   T   
   G   
   G   
   G   
   A   
   C   
   A   
   T   
   T   
   A   
   G   
   T   
   C   
   T   
   A   
   T   
   C   
   C   
   A   
   A   
   A   
   A   
   T   
   T   
   A   
   G   
   T   
   T   
   T   
   T   
   A   
   G   
   A   
   C   
   A   
   A   
   A   
   G   
   A   
   A   
   A   
   C   
   G   
   A   
   A   
   G   
   A   
   A   
   A   
   A   
   A   
   A   
   G   
   A   
   C   
   A   
   A   
   T   
   G   
   T   
   A   
   A   
   T   
   C   
   A   
   G   
   C   
   G   
   C   
   C   
   A   
   A   
   C   
   G   
   C   
   A   
   G   
   C   
   G   
   A   
   A   
   T   
   A   
   C   
   A   
   A   
   T   
   T   
   G   
   C   
   C   
   A   
   C   
   A   
   G   
   A   
   T   
   C   
   T   
   T   
   T   
   T   
   A   
   G   
   G   
   T   
   A   
   G   
   T   
   G   
   T   
   G   
   G   
   A   
   A   
   G   
   A   
   T   
   C   
   C   
   T   
   T   
   C   
   A   
   G   
   G   
   T   
   T   
   A   
   T   
   G   
   T   
   T   
   T   
   A   
   T   
   A   
   A   
   T   
   A   
   T   
   G   
   T   
   A   
   C   
   A   
   A   
   T   
   C   
   C   
   G   
   A   
   A   
   C   
   T   
   T   
   C   
   T   
   C   
   A   
   T   
   T   
   G   
   G   
   C   
   T   
   G   
   C   
   C   
   A   
   A   
   A   
   A   
   C   
   A   
   G   
   G   
   A   
   A   
   C   
   A   
   G   
   C   
   T   
   G   
   A   
   A   
   A   
   T   
   A   
   A   
   A   
   A   
   G   
   A   
   T   
   A   
   A   
   A   
   C   
   A   
   A   
   G   
   A   
   T   
   A   
   C   
   A   
   G   
   A   
   T   
   G   
   G   
   C   
   A   
   A   
   A   
   G   
   A   
   A   
   A   
   A   
   C   
   A   
   G   
   T   
   T   
   T   
   C   
   C   
   T   
   T   
   C   
   T   
   A   
   A   
   C   
   G   
   T   
   T   
   A   
   G   
   A   
   T   
   C   
   G   
   T   
   A   
   G   
   T   
   A   
   A   
   C   
   A   
   A   
   T   
   A   
   A   
   A   
   T   
   T   
   C   
   T   
   T   
   A   
   A   
   C   
   A   
   A   
   T   
   G   
   A   
   T   
   T   
   A   
   T   
   G   
   G   
   T   
   T   
   G   
   A   
   A   
   A   
   A   
   T   
   T   
   C   
   A   
   G   
   G   
   A   
   G   
   A   
   A   
   A   
   A   
   T   
   G   
   G   
   T   
   T   
   C   
   T   
   G   
   C   
   A   
   A   
   C   
   A   
   G   
   A   
   T   
   A   
   T   
   C   
   A   
   G   
   T   
   A   
   A   
   A   
   C   
   C   
   A   
   T   
   T   
   A   
   A   
   T   
   T   
   G   
   A   
   T   
   T   
   A   
   C   
   T   
   T   
   A   
   G   
   A   
   A   
   G   
   C   
   A   
   A   
   C   
   C   
   A   
   T   
   T   
   A   
   A   
   A   
   -   
   -   
   -   
   -   
   -   
   -   
   -   
   -   
   -   
   -   
   -   
   -   
   -   
   -   
   -   
   -   
   -   
   -   
   -   
   -   
   -   
   -   
   -   
   -   
   -   
   -   
   -   
   -   
   -   
   -   
   -   
   -   
   -   
   -   
   -   
   -   
   -   
   -   
   -   
   -   
   -   
   -   
   -   
   -   
   -   
   -   
   -   
   -   
   -   
   -   
   -   
   -   
   -   
   -   
   -   
   -   
   -   
   -   
   -   
   -   
   -   
 

 
   N59847F   
   -   
   -   
   -   
   -   
   -   
   -   
   -   
   -   
   -   
   -   
   -   
   -   
   -   
   -   
   -   
   -   
   -   
   -   
   -   
   -   
   -   
   -   
   -   
   -   
   -   
   -   
   -   
   -   
   -   
   -   
   -   
   -   
   -   
   -   
   -   
   -   
   -   
   -   
   -   
   -   
   -   
   -   
   -   
   -   
   -   
   -   
   -   
   -   
   -   
   -   
   -   
   -   
   -   
   -   
   -   
   -   
   -   
   -   
   -   
   -   
   -   
   -   
   -   
   -   
   -   
   -   
   -   
   -   
   -   
   -   
   -   
   -   
   -   
   -   
   -   
   -   
   -   
   -   
   -   
   -   
   -   
   -   
   -   
   -   
   -   
   -   
   -   
   -   
   -   
   -   
   -   
   -   
   -   
   -   
   -   
   -   
   -   
   -   
   -   
   -   
   -   
   -   
   -   
   -   
   -   
   -   
   -   
   -   
   C   
   A   
   A   
   T   
   G   
   G   
   C   
   A   
   A   
   G   
   C   
   C   
   A   
   A   
   A   
   C   
   A   
   A   
   G   
   A   
    A    
   T   
   T   
   A   
   G   
   C   
    C    
   G   
   A   
   A   
   G   
   C   
   G   
   A   
   A   
   G   
   A   
   A   
   A   
   A   
    C    
   A   
   G   
   C   
    T    
   A   
    A    
   T   
    A    
    C    
   A   
   T   
   T   
   T   
   T   
   T   
   A   
   A   
   A   
    T    
   G   
   T   
   A   
   T   
   T   
   G   
    T    
   C   
   A   
   A   
   A   
   A   
   C   
   A   
   G   
    G    
   A   
   A   
   T   
   T   
   T   
   G   
   A   
   T   
   A   
   A   
   G   
   T   
   T   
   A   
   C   
   C   
    G    
   T   
   C   
   C   
   G   
   T   
   T   
    G    
   T   
   T   
   C   
   A   
   A   
   G   
   A   
   A   
    T    
   C   
   T   
   A   
   G   
   C   
   T   
   T   
   A   
   A   
   A   
   G   
   A   
   A   
   A   
   A   
   A   
   T   
   G   
   G   
    C    
   T   
   A   
   T   
   G   
   A   
   T   
   A   
   C   
   T   
   A   
   A   
   A   
   T   
   C   
    T    
   G   
   T   
   T   
   G   
   T   
   T   
   G   
   A   
   A   
   A   
   A   
   A   
   T   
   A   
   C   
   C   
   A   
   A   
   G   
   C   
   A   
   A   
   T   
   T   
   T   
   A   
   T   
   T   
   C   
   A   
   G   
   G   
   G   
   A   
   T   
   T   
   C   
   A   
   A   
   G   
   C   
    A    
   G   
   A   
   A   
    G    
   G   
    A    
   G   
   T   
   C   
   A   
   A   
    A    
   G   
   C   
   T   
   A   
   G   
   T   
   G   
   A   
   T   
   G   
   T   
    C    
   C   
   A   
   A   
   G   
   T   
    C    
   A   
   A   
   A   
   A   
   A   
    G    
   G   
    C    
   G   
   A   
   A   
   A   
   G   
   A   
    C    
   A   
   A   
    T    
   C   
   A   
   A   
   T   
   A   
   C   
   A   
   C   
   A   
   T   
   T   
   T   
   A   
    C    
   C   
   T   
   A   
   T   
   A   
   A   
   A   
   T   
   T   
   A   
   T   
   C   
   G   
   A   
   T   
   G   
   A   
   G   
    C    
   A   
   C   
    T    
   C   
   C   
   T   
   T   
   T   
   A   
   G   
   G   
   C   
   G   
   A   
   A   
   A   
   T   
   G   
   A   
   A   
   A   
   G   
   A   
   T   
    C    
   T   
   G   
   T   
   C   
   T   
   T   
   A   
   T   
   C   
   A   
   A   
   T   
   C   
   A   
   A   
   G   
   T   
   A   
   T   
    C    
   G   
    C    
   C   
   A   
   A   
    A    
   A   
   A   
   A   
   G   
   G   
    C    
   G   
   A   
   T   
   A   
    C    
   C   
   T   
   A   
   C   
   C   
   A   
   A   
   A   
   T   
    C    
    T    
   C   
   T   
   T   
   G   
   G   
    A    
    A    
    G    
   C   
   C   
    G    
   T   
   C   
   T   
   T   
   T   
   A   
   A   
   T   
   T   
   T   
   T   
   T   
   C   
   C   
   A   
   G   
   A   
   T   
   A   
   T   
   G   
   T   
   C   
   A   
   G   
   G   
   A   
   A   
   A   
   T   
   G   
   A   
   T   
   A   
   A   
   A   
    A    
   T   
   T   
   T   
   C   
   G   
   A   
   T   
   T   
   C   
   A   
   A   
   G   
   T   
   A   
   G   
   A   
   T   
   A   
   A   
   T   
   G   
   C   
   C   
   A   
   A   
   A   
   C   
   G   
   T   
   G   
   G   
   A   
   G   
   A   
   A   
   A   
   T   
   T   
   G   
   T   
   C   
   G   
   A   
   T   
   C   
   G   
   T   
   A   
   A   
    T    
   G   
   G   
   T   
   A   
   G   
   T   
   G   
   G   
   G   
   C   
   T   
   A   
   G   
    C    
   A   
   A   
   T   
   T   
   A   
   A   
   C   
   A   
   A   
   A   
   G   
   T   
   G   
   T   
   T   
   T   
   G   
   A   
    C    
   G   
   A   
   A   
   G   
   T   
   G   
   G   
   G   
   C   
   G   
   T   
   A   
   G   
   T   
    G    
   C   
   C   
   T   
    G    
   G   
   C   
   A   
   A   
   A   
   C   
   T   
    C    
   G   
   G   
   T   
   T   
   C   
   T   
   G   
   G   
   C   
   G   
   C   
   A   
   G   
   A   
   A   
   A   
   A   
   A   
    A    
   C   
   A   
   G   
   C   
    C    
   A   
   A   
   T   
   A   
   T   
   C   
   A   
   A   
   A   
   G   
   C   
   T   
   T   
   T   
   T   
   A   
   G   
   T   
   G   
   A   
   T   
   A   
   A   
    G    
   T   
   T   
    C    
   G   
   G   
   C   
   G   
   T   
   T   
   T   
   C   
   T   
    G    
   T   
   T   
   G   
   A   
   T   
   G   
   A   
   A   
   A   
   T   
   C   
   A   
   A   
   T   
   C   
    A    
   A   
   A   
   A   
    G    
   T   
   T   
   A   
   A   
   G   
   C   
   C   
   A   
   A   
   G   
   G   
    G    
   T   
   G   
   G   
   G   
   T   
   C   
   C   
   A   
   A   
   G   
   C   
   A   
   G   
   A   
   C   
   T   
   C   
    C    
   T   
   T   
   T   
   G   
   T   
   A   
   C   
   C   
   A   
   A   
   T   
   C   
   A   
   C   
   A   
   G   
   T   
    T    
   G   
   C   
   T   
   T   
   C   
   T   
   G   
   A   
   A   
   C   
   C   
   A   
   G   
   T   
   G   
   A   
   C   
   A   
   G   
   A   
   A   
   T   
   T   
   A   
   C   
   C   
   A   
   A   
   C   
   A   
   G   
   G   
   G   
   G   
   C   
   T   
   G   
   C   
   G   
   A   
   C   
   A   
   A   
   A   
   A   
   G   
    A    
    T    
   A   
   C   
   A   
   G   
   A   
   G   
   T   
   C   
   A   
   C   
   G   
   T   
   T   
   A   
   T   
   T   
   A   
   T   
   C   
   C   
    A    
    C    
   T   
   G   
   G   
   G   
   G   
   G   
   A   
   A   
   G   
   C   
   A   
   G   
   C   
   T   
   G   
   C   
   G   
   C   
   A   
   A   
   T   
   T   
   A   
   A   
   T   
   C   
   G   
   G   
    G    
   T   
   A   
   T   
   A   
   C   
    G    
   G   
   G   
    C    
   A   
   C   
    C    
   A   
   T   
   T   
   A   
   C   
   T   
   G   
   C   
   A   
   G   
   A   
    A    
   G   
   A   
   T   
   A   
   T   
   T   
    G    
   A   
   A   
   A   
   A   
   A   
   A   
   A   
   T   
   C   
   C   
   A   
   G   
   A   
   G   
   T   
   T   
   A   
   A   
   G   
   T   
   A   
   G   
   T   
   A   
   C   
   G   
   G   
   G   
    T    
   G   
   T   
   A   
   A   
   T   
   T   
   G   
   G   
    T    
   A   
   A   
   A   
   A   
   C   
   T   
   G   
   G   
    C    
   T   
   T   
   A   
   G   
   A   
   A   
   C   
   G   
   A   
   G   
   C   
   G   
   T   
   T   
   T   
   G   
   A   
   T   
   A   
   A   
   A   
   G   
   A   
    G    
   T   
   T   
   A   
   A   
   G   
   A   
   G   
   G   
    G    
   C   
   A   
   A   
   G   
   A   
   T   
   G   
   G   
    T    
   G   
   G   
    C    
   T   
   C   
    A    
   C   
   T   
   A   
   G   
   T   
   T   
   A   
   T   
   T   
   T   
   T   
   A   
   G   
   A   
   T   
   G   
   A   
   C   
   A   
   A   
   A   
   G   
   A   
   G   
   A   
   A   
   T   
   G   
   T   
   C   
   A   
   A   
   A   
   A   
   A   
   A   
   G   
   C   
   T   
   T   
   T   
   A   
   C   
   A   
   A   
   A   
   C   
   C   
   A   
   A   
   A   
   G   
   A   
    G    
   A   
   A   
   A   
   A   
   A   
   A   
   G   
   A   
   T   
   G   
   G   
   T   
   C   
   A   
   A   
   A   
   C   
    G    
   A   
   T   
   T   
   A   
   A   
   A   
   T   
   T   
   A   
   A   
   C   
   G   
   A   
   T   
   T   
   G   
   A   
    C    
   A   
   G   
    T    
   G   
   G   
    C    
   G   
   T   
   A   
   C   
   A   
   A   
   C   
   A   
   A   
   C   
   A   
   A   
   G   
   C   
    C    
   T   
   T   
    C    
   G   
   C   
   T   
   A   
   T   
   A   
   T   
   T   
   T   
   G   
   A   
   C   
   A   
   A   
   A   
   C   
   G   
    T    
    C    
   C   
   T   
   G   
   G   
   T   
   A   
   G   
   T   
   G   
   C   
   G   
   G   
   T   
   C   
   A   
   T   
   T   
   A   
   C   
   C   
   G   
   A   
   T   
   C   
   C   
   T   
   C   
   A   
   A   
   A   
   A   
   A   
   G   
   G   
   C   
   G   
   A   
   T   
   T   
   T   
   A   
    T    
   T   
   A   
   G   
   C   
    A    
   A   
   C   
   G   
   G   
   T   
   T   
   A   
   G   
   T   
   T   
   C   
   A   
   C   
   C   
   T   
   T   
   C   
   G   
   T   
   A   
   T   
   G   
   A   
   T   
   C   
   C   
   T   
   A   
   A   
   T   
   A   
   A   
   A   
   A   
   T   
   G   
   G   
   C   
   C   
   A   
   A   
   T   
   G   
   G   
    T    
   A   
   T   
   T   
   T   
   C   
   T   
   C   
   A   
   A   
   A   
   A   
   A   
   G   
   A   
    A    
   T   
   A   
   T   
   G   
   A   
   T   
   G   
   C   
   T   
   T   
   A   
   C   
   A   
   A   
   T   
   A   
   A   
   C   
   A   
   A   
   C   
   A   
   A   
   A   
   G   
   A   
   T   
   T   
   T   
   A   
   C   
   C   
   A   
   T   
   T   
    C    
   A   
   C   
   A   
   G   
   C   
   A   
   C   
   G   
   T   
   T   
   T   
    T    
   G   
   C   
   G   
   A   
   C   
   A   
   G   
   G   
    C    
   T   
   A   
   T   
   G   
   C   
   A   
   C   
   C   
   A   
   G   
   G   
    C    
   T   
   C   
   T   
   A   
   C   
   C   
   T   
   T   
   T   
   A   
   A   
   A   
   A   
   C   
   A   
   A   
   T   
   T   
   A   
   C   
   C   
   G   
   G   
   T   
   G   
   C   
   C   
   A   
   T   
   T   
   G   
   G   
   T   
   T   
   T   
   A   
   G   
   A   
   T   
    G    
   C   
   A   
   G   
   G   
   G   
   A   
   C   
   A   
   T   
   T   
   G   
   A   
   A   
   G   
   C   
   C   
   A   
   G   
   A   
   T   
   G   
   A   
   A   
   G   
   A   
   G   
   C   
   T   
   A   
   G   
   A   
   A   
   A   
   T   
   T   
   A   
   A   
   T   
   G   
   G   
    C    
   T   
   T   
   G   
   A   
   A   
   A   
   T   
   G   
   G   
   C   
   A   
   A   
   A   
   A   
   A   
   G   
   A   
   T   
   A   
   A   
   A   
   T   
   C   
   T   
   T   
   G   
   G   
   G   
   G   
    C    
   G   
   G   
   C   
   T   
   A   
   T   
   T   
   T   
   T   
   G   
   C   
    A    
   A   
   C   
   A   
   C   
   G   
   T   
   G   
   T   
   A   
   A   
   A   
   A   
   G   
   A   
   A   
   G   
   C   
   A   
   A   
   G   
   T   
   C   
   C   
   A   
   G   
   T   
   C   
   A   
   A   
   T   
   C   
   T   
    G    
   C   
   G   
   T   
   A   
   C   
   C   
   G   
   C   
   T   
   T   
   T   
   A   
   G   
   T   
   T   
   A   
   A   
   T   
   T   
   C   
   A   
   G   
   A   
   T   
   A   
   A   
   T   
   A   
   T   
   T   
   T   
   A   
   T   
   T   
   T   
   T   
   G   
   C   
    C    
   C   
   A   
   A   
   C   
   A   
   A   
   A   
   C   
   A   
   T   
   T   
   A   
   C   
   G   
    G    
   A   
   T   
   G   
   G   
   G   
    G    
   G   
   A   
   A   
   G   
   A   
   C   
   A   
   A   
   A   
   T   
   T   
   T   
   A   
   G   
   A   
   G   
   C   
    A    
   G   
   G   
   T   
   C   
   T   
   T   
   A   
   A   
   T   
   A   
   A   
   A   
   T   
   T   
   C   
   A   
   T   
   T   
   T   
   T   
   C   
   G   
   G   
   T   
   G   
   A   
   G   
   G   
   A   
   A   
   C   
   T   
   A   
   G   
   A   
   T   
   T   
   T   
   A   
   C   
   C   
   A   
   A   
   T   
    T    
   G   
   C   
   C   
   A   
   T   
   G   
   A   
   C   
   G   
   C   
   C   
    T    
   G   
   C   
   T   
   C   
   A   
   A   
   A   
   T   
   T   
   T   
   C   
   A   
   A   
   A   
    T    
   G   
   A   
   A   
   G   
   A   
   T   
   A   
   A   
   A   
   T   
   T   
   T   
   A   
   A   
   T   
   T   
   C   
   T   
   G   
   A   
   A   
   A   
   T   
   T   
   C   
   T   
   T   
   T   
   T   
    A    
   G   
   C   
   A   
   G   
   A   
   T   
   A   
   C   
    G    
   G   
   G   
   C   
   T   
   A   
    C    
   G   
   G   
    C    
   C   
   A   
   A   
   G   
   G   
    C    
   C   
   A   
   A   
   T   
   T   
   G   
   T   
   T   
   A   
   A   
   T   
   T   
   T   
   C   
    G    
   C   
   C   
   A   
   A   
   T   
   C   
   C   
   A   
   G   
   C   
   A   
   A   
   G   
   C   
   T   
   A   
   C   
   C   
   A   
   T   
   G   
   T   
   A   
   T   
   A   
   G   
   T   
   G   
   T   
   T   
   T   
   T   
   C   
   C   
   A   
   A   
   A   
   A   
   T   
   A   
   A   
   T   
   G   
   G   
    A    
   A   
   C   
   A   
    T    
   T   
    A    
   G   
   T   
    C    
   T   
   A   
   T   
   C   
   C   
   A   
   A   
   A   
   A   
    T    
   T   
   A   
   G   
   T   
   T   
   T   
   T   
   A   
   G   
   A   
   C   
   A   
   A   
   A   
   G   
   A   
   A   
   A   
   C   
    G    
   A   
   A   
   G   
   A   
   A   
   A   
   A   
   A   
   A   
   G   
   A   
   C   
   A   
   A   
   T   
   G   
   T   
   A   
   A   
   T   
   C   
   A   
   G   
   C   
   G   
   C   
   C   
   A   
   A   
   C   
   G   
   C   
   A   
   G   
   C   
   G   
   A   
   A   
   T   
   A   
   C   
   A   
   A   
   T   
   T   
   G   
   C   
   C   
   A   
   C   
   A   
   G   
   A   
   T   
   C   
   T   
   T   
   T   
   T   
   A   
   G   
   G   
   T   
   A   
   G   
   T   
   G   
   T   
   G   
   G   
   A   
   A   
   G   
   A   
   T   
   C   
   C   
   T   
   T   
   C   
   A   
   G   
   G   
   T   
   T   
   A   
   T   
   G   
   T   
   T   
   T   
   A   
   T   
   A   
   A   
   T   
   A   
   T   
   G   
   T   
   A   
   C   
   A   
   A   
   T   
   C   
   C   
   G   
   A   
   A   
   C   
   T   
   T   
   C   
   T   
   C   
   A   
   T   
   T   
   G   
   G   
   C   
   T   
   G   
   C   
   T   
   A   
   A   
   A   
   A   
   C   
   A   
   G   
   G   
   A   
   A   
   C   
   A   
   G   
   C   
   T   
   G   
   A   
   A   
   A   
   T   
   A   
   A   
   A   
   A   
   G   
   A   
   T   
   A   
   A   
   A   
   C   
   A   
   A   
   G   
   A   
   T   
   A   
   C   
   A   
   G   
   A   
   T   
   G   
   G   
   C   
   A   
   A   
   A   
   G   
   A   
   A   
   A   
   A   
   C   
   A   
   G   
   T   
   T   
   T   
   C   
   C   
   T   
   C   
   C   
   T   
   A   
   A   
   C   
   G   
   T   
   T   
   A   
   G   
   A   
   T   
   C   
   G   
   T   
   A   
   G   
   T   
   A   
   A   
   C   
   A   
   A   
   T   
   A   
   A   
   A   
   T   
   T   
   C   
   T   
   T   
   A   
   A   
   C   
   A   
   A   
   T   
   G   
   A   
   T   
   T   
   A   
   T   
   G   
   G   
   T   
   T   
   G   
   A   
   A   
   A   
   A   
   T   
   T   
   C   
   A   
   G   
   G   
   A   
   G   
   A   
   A   
   A   
   A   
   T   
   G   
   G   
   T   
   T   
   C   
   T   
   G   
   C   
   A   
   A   
   C   
   A   
   G   
   A   
   T   
   A   
   T   
   C   
   A   
   G   
   T   
   A   
   A   
   A   
   C   
   C   
   A   
   T   
   T   
   A   
   A   
   T   
   T   
   G   
   A   
   T   
   T   
   A   
   C   
   T   
   T   
   A   
   G   
   A   
   A   
   G   
   C   
   A   
   A   
   C   
   C   
   A   
   T   
   T   
   A   
   A   
   A   
   -   
   -   
   -   
   -   
   -   
   -   
   -   
   -   
   -   
   -   
   -   
   -   
   -   
   -   
   -   
   -   
   -   
   -   
   -   
   -   
   -   
   -   
   -   
   -   
   -   
   -   
   -   
   -   
   -   
   -   
   -   
   -   
   -   
   -   
   -   
   -   
   -   
   -   
   -   
   -   
   -   
   -   
   -   
   -   
   -   
   -   
   -   
   -   
   -   
   -   
   -   
   -   
   -   
   -   
   -   
   -   
   -   
   -   
   -   
   -   
   -   
   -   
   -   
   -   
   -   
   -   
   -   
   -   
   -   
   -   
   -   
   -   
   -   
   -   
   -   
   -   
   -   
   -   
   -   
   -   
   -   
   -   
   -   
   -   
   -   
   -   
   -   
   -   
   -   
   -   
   -   
   -   
   -   
   -   
   -   
   -   
   -   
   -   
   -   
   -   
   -   
   -   
   -   
   -   
   -   
   -   
   -   
   -   
   -   
   -   
   -   
   -   
   -   
   -   
   -   
   -   
   -   
   -   
   -   
   -   
   -   
   -   
   -   
   -   
   -   
   -   
   -   
   -   
   -   
   -   
   -   
   -   
   -   
   -   
   -   
   -   
   -   
   -   
   -   
   -   
   -   
   -   
   -   
   -   
   -   
   -   
   -   
   -   
   -   
   -   
   -   
   -   
   -   
   -   
   -   
   -   
   -   
   -   
   -   
   -   
   -   
   -   
   -   
   -   
   -   
   -   
   -   
   -   
   -   
   C   
   A   
   A   
   T   
   G   
   G   
   C   
   A   
   A   
   G   
   C   
   C   
   A   
   A   
   A   
   C   
   A   
   A   
   G   
   A   
   A   
   T   
   T   
   A   
   G   
   C   
   C   
   G   
   A   
   A   
   G   
   C   
   G   
   A   
   A   
   G   
   A   
   A   
   A   
   A   
   C   
   A   
   G   
   C   
   T   
   A   
   A   
   T   
   A   
   C   
   A   
   T   
   T   
   T   
   T   
   T   
   A   
   A   
   A   
   T   
   G   
   T   
   A   
   T   
   T   
   G   
   T   
   C   
   A   
   A   
   A   
   A   
   C   
   A   
   G   
   G   
   A   
   A   
   T   
   T   
   T   
   G   
   A   
   T   
   A   
   A   
   G   
   T   
   T   
   A   
   C   
   C   
   G   
   T   
   C   
   C   
   G   
   T   
   T   
   G   
   T   
   T   
   C   
   A   
   A   
   G   
   A   
   A   
   T   
   C   
   T   
   A   
   G   
   C   
   T   
   T   
   A   
   A   
   A   
   G   
   A   
   A   
   A   
   A   
   A   
   T   
   G   
   G   
   C   
   T   
   A   
   T   
   G   
   A   
   T   
   A   
   C   
   T   
   A   
   A   
   A   
   T   
   C   
   T   
   G   
   T   
   T   
   G   
   T   
   T   
   G   
   A   
   A   
   A   
   A   
   A   
   T   
   A   
   C   
   C   
   A   
   A   
   G   
   C   
   A   
   A   
   T   
   T   
   T   
   A   
   T   
   T   
   C   
   A   
   G   
   G   
   G   
   A   
   T   
   T   
   C   
   A   
   A   
   G   
   C   
   A   
   G   
   A   
   A   
   G   
   G   
   A   
   G   
   T   
   C   
   A   
   A   
   A   
   G   
   C   
   T   
   A   
   G   
   T   
   G   
   A   
   T   
   G   
   T   
   C   
   C   
   A   
   A   
   G   
   T   
   C   
   A   
   A   
   A   
   A   
   A   
   G   
   G   
   C   
   G   
   A   
   A   
   A   
   G   
   A   
   C   
   A   
   A   
   T   
   C   
   A   
   A   
   T   
   A   
   C   
   A   
   C   
   A   
   T   
   T   
   T   
   A   
   C   
   C   
   T   
   A   
   T   
   A   
   A   
   A   
   T   
   T   
   A   
   T   
   C   
   G   
   A   
   T   
   G   
   A   
   G   
   C   
   A   
   C   
   T   
   C   
   C   
   T   
   T   
   T   
   A   
   G   
   G   
   C   
   G   
   A   
   A   
   A   
   T   
   G   
   A   
   A   
   A   
   G   
   A   
   T   
   C   
   T   
   G   
   T   
   C   
   T   
   T   
   A   
   T   
   C   
   A   
   A   
   T   
   C   
   A   
   A   
   G   
   T   
   A   
   T   
   C   
   G   
   C   
   C   
   A   
   A   
   A   
   A   
   A   
   A   
   G   
   G   
   C   
   G   
   A   
   T   
   A   
   C   
   C   
   T   
   A   
   C   
   C   
   A   
   A   
   A   
   T   
   C   
   T   
   C   
   T   
   T   
   G   
   G   
   A   
   A   
   G   
   C   
   C   
   G   
   T   
   C   
   T   
   T   
   T   
   A   
   A   
   T   
   T   
   T   
   T   
   T   
   C   
   C   
   A   
   G   
   A   
   T   
   A   
   T   
   G   
   T   
   C   
   A   
   G   
   G   
   A   
   A   
   A   
   T   
   G   
   A   
   T   
   A   
   A   
   A   
   A   
   T   
   T   
   T   
   C   
   G   
   A   
   T   
   T   
   C   
   A   
   A   
   G   
   T   
   A   
   G   
   A   
   T   
   A   
   A   
   T   
   G   
   C   
   C   
   A   
   A   
   A   
   C   
   G   
   T   
   G   
   G   
   A   
   G   
   A   
   A   
   A   
   T   
   T   
   G   
   T   
   C   
   G   
   A   
   T   
   C   
   G   
   T   
   A   
   A   
   T   
   G   
   G   
   T   
   A   
   G   
   T   
   G   
   G   
   G   
   C   
   T   
   A   
   G   
   C   
   A   
   A   
   T   
   T   
   A   
   A   
   C   
   A   
   A   
   A   
   G   
   T   
   G   
   T   
   T   
   T   
   G   
   A   
   C   
   G   
   A   
   A   
   G   
   T   
   G   
   G   
   G   
   C   
   G   
   T   
   A   
   G   
   T   
   G   
   C   
   C   
   T   
   G   
   G   
   C   
   A   
   A   
   A   
   C   
   T   
   C   
   G   
   G   
   T   
   T   
   C   
   T   
   G   
   G   
   C   
   G   
   C   
   A   
   G   
   A   
   A   
   A   
   A   
   A   
   A   
   C   
   A   
   G   
   C   
   C   
   A   
   A   
   T   
   A   
   T   
   C   
   A   
   A   
   A   
   G   
   C   
   T   
   T   
   T   
   T   
   A   
   G   
   T   
   G   
   A   
   T   
   A   
   A   
   G   
   T   
   T   
   C   
   G   
   G   
   C   
   G   
   T   
   T   
   T   
   C   
   T   
   G   
   T   
   T   
   G   
   A   
   T   
   G   
   A   
   A   
   A   
   T   
   C   
   A   
   A   
   T   
   C   
   A   
   A   
   A   
   A   
   G   
   T   
   T   
   A   
   A   
   G   
   C   
   C   
   A   
   A   
   G   
   G   
   G   
   T   
   G   
   G   
   G   
   T   
   C   
   C   
   A   
   A   
   G   
   C   
   A   
   G   
   A   
   C   
   T   
   C   
   C   
   T   
   T   
   T   
   G   
   T   
   A   
   C   
   C   
   A   
   A   
   T   
   C   
   A   
   C   
   A   
   G   
   T   
   T   
   G   
   C   
   T   
   T   
   C   
   T   
   G   
   A   
   A   
   C   
   C   
   A   
   G   
   T   
   G   
   A   
   C   
   A   
   G   
   A   
   A   
   T   
   T   
   A   
   C   
   C   
   A   
   A   
   C   
   A   
   G   
   G   
   G   
   G   
   C   
   T   
   G   
   C   
   G   
   A   
   C   
   A   
   A   
   A   
   A   
   G   
   A   
   T   
   A   
   C   
   A   
   G   
   A   
   G   
   T   
   C   
   A   
   C   
   G   
   T   
   T   
   A   
   T   
   T   
   A   
   T   
   C   
   C   
   A   
   C   
   T   
   G   
   G   
   G   
   G   
   G   
   A   
   A   
   G   
   C   
   A   
   G   
   C   
   T   
   G   
   C   
   G   
   C   
   A   
   A   
   T   
   T   
   A   
   A   
   T   
   C   
   G   
   G   
   G   
   T   
   A   
   T   
   A   
   C   
   G   
   G   
   G   
   C   
   A   
   C   
   C   
   A   
   T   
   T   
   A   
   C   
   T   
   G   
   C   
   A   
   G   
   A   
   A   
   G   
   A   
   T   
   A   
   T   
   T   
   G   
   A   
   A   
   A   
   A   
   A   
   A   
   A   
   T   
   C   
   C   
   A   
   G   
   A   
   G   
   T   
   T   
   A   
   A   
   G   
   T   
   A   
   G   
   T   
   A   
   C   
   G   
   G   
   G   
   T   
   G   
   T   
   A   
   A   
   T   
   T   
   G   
   G   
   T   
   A   
   A   
   A   
   A   
   C   
   T   
   G   
   G   
   C   
   T   
   T   
   A   
   G   
   A   
   A   
   C   
   G   
   A   
   G   
   C   
   G   
   T   
   T   
   T   
   G   
   A   
   T   
   A   
   A   
   A   
   G   
   A   
   G   
   T   
   T   
   A   
   A   
   G   
   A   
   G   
   G   
   G   
   C   
   A   
   A   
   G   
   A   
   T   
   G   
   G   
   T   
   G   
   G   
   C   
   T   
   C   
   A   
   C   
   T   
   A   
   G   
   T   
   T   
   A   
   T   
   T   
   T   
   T   
   A   
   G   
   A   
   T   
   G   
   A   
   C   
   A   
   A   
   A   
   G   
   A   
   G   
   A   
   A   
   T   
   G   
   T   
   C   
   A   
   A   
   A   
   A   
   A   
   A   
   G   
   C   
   T   
   T   
   T   
   A   
   C   
   A   
   A   
   A   
   C   
   C   
   A   
   A   
   A   
   G   
   A   
   G   
   A   
   A   
   A   
   A   
   A   
   A   
   G   
   A   
   T   
   G   
   G   
   T   
   C   
   A   
   A   
   A   
   C   
   G   
   A   
   T   
   T   
   A   
   A   
   A   
   T   
   T   
   A   
   A   
   C   
   G   
   A   
   T   
   T   
   G   
   A   
   C   
   A   
   G   
   T   
   G   
   G   
   C   
   G   
   T   
   A   
   C   
   A   
   A   
   C   
   A   
   A   
   C   
   A   
   A   
   G   
   C   
   C   
   T   
   T   
   C   
   G   
   C   
   T   
   A   
   T   
   A   
   T   
   T   
   T   
   G   
   A   
   C   
   A   
   A   
   A   
   C   
   G   
   T   
   C   
   C   
   T   
   G   
   G   
   T   
   A   
   G   
   T   
   G   
   C   
   G   
   G   
   T   
   C   
   A   
   T   
   T   
   A   
   C   
   C   
   G   
   A   
   T   
   C   
   C   
   T   
   C   
   A   
   A   
   A   
   A   
   A   
   G   
   G   
   C   
   G   
   A   
   T   
   T   
   T   
   A   
   T   
   T   
   A   
   G   
   C   
   A   
   A   
   C   
   G   
   G   
   T   
   T   
   A   
   G   
   T   
   T   
   C   
   A   
   C   
   C   
   T   
   T   
   C   
   G   
   T   
   A   
   T   
   G   
   A   
   T   
   C   
   C   
   T   
   A   
   A   
   T   
   A   
   A   
   A   
   A   
   T   
   G   
   G   
   C   
   C   
   A   
   A   
   T   
   G   
   G   
   T   
   A   
   T   
   T   
   T   
   C   
   T   
   C   
   A   
   A   
   A   
   A   
   A   
   G   
   A   
   A   
   T   
   A   
   T   
   G   
   A   
   T   
   G   
   C   
   T   
   T   
   A   
   C   
   A   
   A   
   T   
   A   
   A   
   C   
   A   
   A   
   C   
   A   
   A   
   A   
   G   
   A   
   T   
   T   
   T   
   A   
   C   
   C   
   A   
   T   
   T   
   C   
   A   
   C   
   A   
   G   
   C   
   A   
   C   
   G   
   T   
   T   
   T   
   T   
   G   
   C   
   G   
   A   
   C   
   A   
   G   
   G   
   C   
   T   
   A   
   T   
   G   
   C   
   A   
   C   
   C   
   A   
   G   
   G   
   C   
   T   
   C   
   T   
   A   
   C   
   C   
   T   
   T   
   T   
   A   
   A   
   A   
   A   
   C   
   A   
   A   
   T   
   T   
   A   
   C   
   C   
   G   
   G   
   T   
   G   
   C   
   C   
   A   
   T   
   T   
   G   
   G   
   T   
   T   
   T   
   A   
   G   
   A   
   T   
   G   
   C   
   A   
   G   
   G   
   G   
   A   
   C   
   A   
   T   
   T   
   G   
   A   
   A   
   G   
   C   
   C   
   A   
   G   
   A   
   T   
   G   
   A   
   A   
   G   
   A   
   G   
   C   
   T   
   A   
   G   
   A   
   A   
   A   
   T   
   T   
   A   
   A   
   T   
   G   
   G   
   C   
   T   
   T   
   G   
   A   
   A   
   A   
   T   
   G   
   G   
   C   
   A   
   A   
   A   
   A   
   A   
   G   
   A   
   T   
   A   
   A   
   A   
   T   
   C   
   T   
   T   
   G   
   G   
   G   
   G   
   C   
   G   
   G   
   C   
   T   
   A   
   T   
   T   
   T   
   T   
   G   
   C   
   A   
   A   
   C   
   A   
   C   
   G   
   T   
   G   
   T   
   A   
   A   
   A   
   A   
   G   
   A   
   A   
   G   
   C   
   A   
   A   
   G   
   T   
   C   
   C   
   A   
   G   
   T   
   C   
   A   
   A   
   T   
   C   
   T   
   G   
   C   
   G   
   T   
   A   
   C   
   C   
   G   
   C   
   T   
   T   
   T   
   A   
   G   
   T   
   T   
   A   
   A   
   T   
   T   
   C   
   A   
   G   
   A   
   T   
   A   
   A   
   T   
   A   
   T   
   T   
   T   
   A   
   T   
   T   
   T   
   T   
   G   
   C   
   C   
   C   
   A   
   A   
   C   
   A   
   A   
   A   
   C   
   A   
   T   
   T   
   A   
   C   
   G   
   G   
   A   
   T   
   G   
   G   
   G   
   G   
   G   
   A   
   A   
   G   
   A   
   C   
   A   
   A   
   A   
   T   
   T   
   T   
   A   
   G   
   A   
   G   
   C   
   A   
   G   
   G   
   T   
   C   
   T   
   T   
   A   
   A   
   T   
   A   
   A   
   A   
   T   
   T   
   C   
   A   
   T   
   T   
   T   
   T   
   C   
   G   
   G   
   T   
   G   
   A   
   G   
   G   
   A   
   A   
   C   
   T   
   A   
   G   
   A   
   T   
   T   
   T   
   A   
   C   
   C   
   A   
   A   
   T   
   T   
   G   
   C   
   C   
   A   
   T   
   G   
   A   
   C   
   G   
   C   
   C   
   T   
   G   
   C   
   T   
   C   
   A   
   A   
   A   
   T   
   T   
   T   
   C   
   A   
   A   
   A   
   T   
   G   
   A   
   A   
   G   
   A   
   T   
   A   
   A   
   A   
   T   
   T   
   T   
   A   
   A   
   T   
   T   
   C   
   T   
   G   
   A   
   A   
   A   
   T   
   T   
   C   
   T   
   T   
   T   
   T   
   A   
   G   
   C   
   A   
   G   
   A   
   T   
   A   
   C   
   G   
   G   
   G   
   C   
   T   
   A   
   C   
   G   
   G   
   C   
   C   
   A   
   A   
   G   
   G   
   C   
   C   
   A   
   A   
   T   
   T   
   G   
   T   
   T   
   A   
   A   
   T   
   T   
   T   
   C   
   G   
   C   
   C   
   A   
   A   
   T   
   C   
   C   
   A   
   G   
   C   
   A   
   A   
   G   
   C   
   T   
   A   
   C   
   C   
   A   
   T   
   G   
   T   
   A   
   T   
   A   
   G   
   T   
   G   
   T   
   T   
   T   
   T   
   C   
   C   
   A   
   A   
   A   
   A   
   T   
   A   
   A   
   T   
   G   
   G   
   A   
   A   
   C   
   A   
   T   
   T   
   A   
   G   
   T   
   C   
   T   
   A   
   T   
   C   
   C   
   A   
   A   
   A   
   A   
   T   
   T   
   A   
   G   
   T   
   T   
   T   
   T   
   A   
   G   
   A   
   C   
   A   
   A   
   A   
   G   
   A   
   A   
   A   
   C   
   G   
   A   
   A   
   G   
   A   
   A   
   A   
   A   
   A   
   A   
   G   
   A   
   C   
   A   
   A   
   T   
   G   
   T   
   A   
   A   
   T   
   C   
   A   
   G   
   C   
   G   
   C   
   C   
   A   
   A   
   C   
   G   
   C   
   A   
   G   
   C   
   G   
   A   
   A   
   T   
   A   
   C   
   A   
   A   
   T   
   T   
   G   
   C   
   C   
   A   
   C   
   A   
   G   
   A   
   T   
   C   
   T   
   T   
   T   
   T   
   A   
   G   
   G   
   T   
   A   
   G   
   T   
   G   
   T   
   G   
   G   
   A   
   A   
   G   
   A   
   T   
   C   
   C   
   T   
   T   
   C   
   A   
   G   
   G   
   T   
   T   
   A   
   T   
   G   
   T   
   T   
   T   
   A   
   T   
   A   
   A   
   T   
   A   
   T   
   G   
   T   
   A   
   C   
   A   
   A   
   T   
   C   
   C   
   G   
   A   
   A   
   C   
   T   
   T   
   C   
   T   
   C   
   A   
   T   
   T   
   G   
   G   
   C   
   T   
   G   
   C   
   T   
   A   
   A   
   A   
   A   
   C   
   A   
   G   
   G   
   A   
   A   
   C   
   A   
   G   
   C   
   T   
   G   
   A   
   A   
   A   
   T   
   A   
   A   
   A   
   A   
   G   
   A   
   T   
   A   
   A   
   A   
   C   
   A   
   A   
   G   
   A   
   T   
   A   
   C   
   A   
   G   
   A   
   T   
   G   
   G   
   C   
   A   
   A   
   A   
   G   
   A   
   A   
   A   
   A   
   C   
   A   
   G   
   T   
   T   
   T   
   C   
   C   
   T   
   C   
   C   
   T   
   A   
   A   
   C   
   G   
   T   
   T   
   A   
   G   
   A   
   T   
   C   
   G   
   T   
   A   
   G   
   T   
   A   
   A   
   C   
   A   
   A   
   T   
   A   
   A   
   A   
   T   
   T   
   C   
   T   
   T   
   A   
   A   
   C   
   A   
   A   
   T   
   G   
   A   
   T   
   T   
   A   
   T   
   G   
   G   
   T   
   T   
   G   
   A   
   A   
   A   
   A   
   T   
   T   
   C   
   A   
   G   
   G   
   A   
   G   
   A   
   A   
   A   
   A   
   T   
   G   
   G   
   T   
   T   
   C   
   T   
   G   
   C   
   A   
   A   
   C   
   A   
   G   
   A   
   T   
   A   
   T   
   C   
   A   
   G   
   T   
   A   
   A   
   A   
   C   
   C   
   A   
   T   
   T   
   A   
   A   
   T   
   T   
   G   
   A   
   T   
   T   
   A   
   C   
   T   
   T   
   A   
   G   
   A   
   A   
   G   
   C   
   A   
   A   
   C   
   C   
   A   
   T   
   T   
   A   
   A   
   A   
   -   
   -   
   -   
   -   
   -   
   -   
   -   
   -   
   -   
   -   
   -   
   -   
   -   
   -   
   -   
   -   
   -   
   -   
   -   
   -   
   -   
   -   
   -   
   -   
   -   
   -   
   -   
   -   
   -   
   -   
   -   
   -   
   -   
   -   
   -   
   -   
   -   
   -   
   -   
   -   
   -   
   -   
   -   
   -   
   -   
   -   
   -   
   -   
   -   
   -   
   -   
   -   
   -   
   -   
   -   
   -   
   -   
   -   
   -   
   -   
   -   
 

 
   N53420   
   A   
   T   
   G   
   G   
   A   
   A   
   C   
   G   
   A   
   A   
   G   
   C   
   A   
   A   
   T   
   C   
   G   
   T   
   A   
   A   
   T   
   A   
   A   
   A   
   A   
   A   
   A   
   T   
   C   
   T   
   T   
   C   
   A   
   A   
   A   
   A   
   A   
   A   
   A   
   C   
   C   
   A   
   C   
   T   
   T   
   A   
   T   
   T   
   C   
   T   
   T   
   G   
   G   
   C   
   G   
   T   
   T   
   T   
   C   
   T   
   G   
   C   
   C   
   T   
   T   
   G   
   G   
   T   
   T   
   C   
   T   
   G   
   A   
   T   
   C   
   G   
   C   
   T   
   G   
   C   
   T   
   G   
   T   
   C   
   G   
   G   
   T   
   G   
   G   
   T   
   G   
   G   
   G   
   T   
   A   
   T   
   T   
   A   
   T   
   G   
   C   
   T   
   T   
   A   
   T   
   A   
   G   
   C   
   C   
   A   
   A   
   T   
   G   
   G   
   C   
   A   
   A   
   G   
   C   
   C   
   A   
   A   
   A   
   C   
   A   
   A   
   G   
   A   
    A    
   T   
   T   
   A   
   G   
   C   
    T    
   G   
   A   
   A   
   G   
   C   
   G   
   A   
   A   
   G   
   A   
   A   
   A   
   A   
    C    
   A   
   G   
   C   
    C    
   A   
    A    
   T   
    A    
    C    
   A   
   T   
   T   
   T   
   T   
   T   
   A   
   A   
   A   
    C    
   G   
   T   
   A   
   T   
   T   
   G   
    T    
   C   
   A   
   A   
   A   
   A   
   C   
   A   
   G   
    G    
   A   
   A   
   T   
   T   
   T   
   G   
   A   
   T   
   A   
   A   
   G   
   T   
   T   
   A   
   C   
   C   
    A    
   T   
   C   
   C   
   G   
   T   
   T   
    G    
   T   
   T   
   C   
   A   
   A   
   G   
   A   
   A   
    A    
   C   
   T   
   A   
   G   
   C   
   T   
   T   
   A   
   A   
   A   
   G   
   A   
   A   
   A   
   A   
   A   
   T   
   G   
   G   
    C    
   T   
   A   
   T   
   G   
   A   
   T   
   A   
   C   
   T   
   A   
   A   
   A   
   T   
   C   
    T    
   G   
   T   
   T   
   G   
   T   
   T   
   G   
   A   
   A   
   A   
   A   
   A   
   T   
   A   
   C   
   C   
   A   
   A   
   G   
   C   
   A   
   A   
   T   
   T   
   T   
   A   
   T   
   T   
   C   
   A   
   G   
   G   
   G   
   A   
   T   
   T   
   C   
   A   
   A   
   G   
   C   
    A    
   G   
   A   
   A   
    G    
   G   
    G    
   G   
   T   
   C   
   A   
   A   
    A    
   G   
   C   
   T   
   A   
   G   
   T   
   G   
   A   
   T   
   G   
   T   
    T    
   C   
   A   
   A   
   G   
   T   
    C    
   A   
   A   
   A   
   A   
   A   
    A    
   G   
    G    
   G   
   A   
   A   
   A   
   G   
   A   
    C    
   A   
   A   
    C    
   C   
   A   
   A   
   T   
   A   
   C   
   A   
   C   
   A   
   T   
   T   
   T   
   A   
    C    
   C   
   T   
   A   
   T   
   A   
   A   
   A   
   T   
   T   
   A   
   T   
   C   
   G   
   A   
   T   
   G   
   A   
   G   
    C    
   A   
   C   
    T    
   C   
   C   
   T   
   T   
   T   
   A   
   G   
   G   
   C   
   G   
   A   
   A   
   A   
   T   
   G   
   A   
   A   
   A   
   G   
   A   
   T   
    T    
   T   
   G   
   T   
   C   
   T   
   T   
   A   
   T   
   C   
   A   
   A   
   T   
   C   
   A   
   A   
   G   
   T   
   A   
   T   
    C    
   G   
    C    
   C   
   A   
   A   
    A    
   A   
   A   
   A   
   G   
   G   
    C    
   G   
   A   
   T   
   A   
    A    
   C   
   T   
   A   
   C   
   C   
   A   
   A   
   A   
   T   
    C    
    G    
   C   
   T   
   T   
   G   
   G   
    G    
    C    
    A    
   C   
   C   
    G    
   T   
   C   
   T   
   T   
   T   
   A   
   A   
   T   
   T   
   T   
   T   
   T   
   C   
   C   
   A   
   G   
   A   
   T   
   A   
   T   
   G   
   T   
   C   
   A   
   G   
   G   
   A   
   A   
   A   
   T   
   G   
   A   
   T   
   A   
   A   
   A   
    A    
   T   
   T   
   T   
   C   
   G   
   A   
   T   
   T   
   C   
   A   
   A   
   G   
   T   
   A   
   G   
   A   
   T   
   A   
   A   
   T   
   G   
   C   
   C   
   A   
   A   
   A   
   C   
   G   
   T   
   G   
   G   
   A   
   G   
   A   
   A   
   A   
   T   
   T   
   G   
   T   
   C   
   G   
   A   
   T   
   C   
   G   
   T   
   A   
   A   
    T    
   G   
   G   
   T   
   A   
   G   
   T   
   G   
   G   
   G   
   C   
   T   
   A   
   G   
    C    
   A   
   A   
   T   
   T   
   A   
   A   
   C   
   A   
   A   
   A   
   G   
   T   
   G   
   T   
   T   
   T   
   G   
   A   
    T    
   G   
   A   
   A   
   G   
   T   
   G   
   G   
   G   
   C   
   G   
   T   
   A   
   G   
   T   
    G    
   C   
   C   
   T   
    G    
   G   
   C   
   A   
   A   
   A   
   C   
   T   
    C    
   G   
   G   
   T   
   T   
   C   
   T   
   G   
   G   
   C   
   G   
   C   
   A   
   G   
   A   
   A   
   A   
   A   
   A   
    A    
   C   
   A   
   G   
   C   
    C    
   A   
   A   
   T   
   A   
   T   
   C   
   A   
   A   
   A   
   G   
   C   
   T   
   T   
   T   
   T   
   A   
   G   
   T   
   G   
   A   
   T   
   A   
   A   
    G    
   T   
   T   
    T    
   G   
   G   
   C   
   G   
   T   
   T   
   T   
   C   
   T   
    G    
   T   
   T   
   G   
   A   
   T   
   G   
   A   
   A   
   A   
   T   
   C   
   A   
   A   
   T   
   C   
    A    
   A   
   A   
   A   
    G    
   T   
   T   
   A   
   A   
   G   
   C   
   C   
   A   
   A   
   G   
   G   
    A    
   T   
   G   
   G   
   G   
   T   
   C   
   C   
   A   
   A   
   G   
   C   
   A   
   G   
   A   
   C   
   T   
   C   
    G    
   T   
   T   
   T   
   G   
   T   
   A   
   C   
   C   
   A   
   A   
   T   
   C   
   A   
   C   
   A   
   G   
   T   
    T    
   G   
   C   
   T   
   T   
   C   
   T   
   G   
   A   
   A   
   C   
   C   
   A   
   G   
   T   
   G   
   A   
   C   
   A   
   G   
   A   
   A   
   T   
   T   
   A   
   C   
   C   
   A   
   A   
   C   
   A   
   G   
   G   
   G   
   G   
   C   
   T   
   G   
   C   
   G   
   A   
   C   
   A   
   A   
   A   
   A   
   G   
    A    
    T    
   A   
   C   
   A   
   G   
   A   
   G   
   T   
   C   
   A   
   C   
   G   
   T   
   T   
   A   
   T   
   T   
   A   
   T   
   C   
   C   
    A    
    T    
   T   
   G   
   G   
   G   
   G   
   G   
   A   
   A   
   G   
   C   
   A   
   G   
   C   
   T   
   G   
   C   
   G   
   C   
   A   
   A   
   T   
   T   
   A   
   A   
   T   
   C   
   G   
   G   
    G    
   T   
   A   
   T   
   A   
   C   
    G    
   G   
   G   
    C    
   A   
   C   
    T    
   A   
   T   
   T   
   A   
   C   
   T   
   G   
   C   
   A   
   G   
   A   
    A    
   G   
   A   
   T   
   A   
   T   
   T   
    G    
   A   
   A   
   A   
   A   
   A   
   A   
   A   
   T   
   C   
   C   
   A   
   G   
   A   
   G   
   T   
   T   
   A   
   A   
   G   
   T   
   A   
   G   
   T   
   A   
   C   
   G   
   G   
   G   
    C    
   G   
   T   
   A   
   A   
   T   
   T   
   G   
   G   
    C    
   A   
   A   
   A   
   A   
   C   
   T   
   G   
   G   
    C    
   T   
   T   
   A   
   G   
   A   
   A   
   C   
   G   
   A   
   G   
   C   
   G   
   T   
   T   
   T   
   G   
   A   
   T   
   A   
   A   
   A   
   G   
   A   
    A    
   T   
   T   
   A   
   A   
   G   
   A   
   G   
   G   
    A    
   C   
   A   
   A   
   G   
   A   
   T   
   G   
   G   
    T    
   G   
   G   
    T    
   T   
   C   
    A    
   C   
   T   
   A   
   G   
   T   
   T   
   A   
   T   
   T   
   T   
   T   
   A   
   G   
   A   
   T   
   G   
   A   
   C   
   A   
   A   
   A   
   G   
   A   
   G   
   A   
   A   
   T   
   G   
   T   
   C   
   A   
   A   
   A   
   A   
   A   
   A   
   G   
   C   
   T   
   T   
   T   
   A   
   C   
   A   
   A   
   A   
   C   
   C   
   A   
   A   
   A   
   G   
   A   
    G    
   A   
   A   
   A   
   A   
   A   
   A   
   G   
   A   
   T   
   G   
   G   
   T   
   C   
   A   
   A   
   A   
   C   
    A    
   A   
   T   
   T   
   A   
   A   
   A   
   T   
   T   
   A   
   A   
   C   
   G   
   A   
   T   
   T   
   G   
   A   
    T    
   A   
   G   
    T    
   G   
   G   
    C    
   G   
   T   
   A   
   C   
   A   
   A   
   C   
   A   
   A   
   C   
   A   
   A   
   G   
   C   
    C    
   T   
   T   
    C    
   G   
   C   
   T   
   A   
   T   
   A   
   T   
   T   
   T   
   G   
   A   
   C   
   A   
   A   
   A   
   C   
   G   
    T    
    C    
   C   
   T   
   G   
   G   
   T   
   A   
   G   
   T   
   G   
   C   
   G   
   G   
   T   
   C   
   A   
   T   
   T   
   A   
   C   
   C   
   G   
   A   
   T   
   C   
   C   
   T   
   C   
   A   
   A   
   A   
   A   
   A   
   G   
   G   
   C   
   G   
   A   
   T   
   T   
   T   
   A   
    C    
   T   
   A   
   G   
   C   
    G    
   A   
   C   
   G   
   G   
   T   
   T   
   A   
   G   
   T   
   T   
   C   
   A   
   C   
   C   
   T   
   T   
   C   
   G   
   T   
   A   
   T   
   G   
   A   
   T   
   C   
   C   
   T   
   A   
   A   
   T   
   A   
   A   
   A   
   A   
   T   
   G   
   G   
   C   
   C   
   A   
   A   
   T   
   G   
   G   
    C    
   A   
   T   
   T   
   T   
   C   
   T   
   C   
   A   
   A   
   A   
   A   
   A   
   G   
   A   
    A    
   T   
   A   
   T   
   G   
   A   
   T   
   G   
   C   
   T   
   T   
   A   
   C   
   A   
   A   
   T   
   A   
   A   
   C   
   A   
   A   
   C   
   A   
   A   
   A   
   G   
   A   
   T   
   T   
   T   
   A   
   C   
   C   
   A   
   T   
   T   
    C    
   A   
   C   
   A   
   G   
   C   
   A   
   C   
   G   
   T   
   T   
   T   
    T    
   G   
   C   
   G   
   A   
   C   
   A   
   G   
   G   
    C    
   T   
   A   
   T   
   G   
   C   
   A   
   C   
   C   
   A   
   G   
   G   
    C    
   T   
   C   
   T   
   A   
   C   
   C   
   T   
   T   
   T   
   A   
   A   
   A   
   A   
   C   
   A   
   A   
   T   
   T   
   A   
   C   
   C   
   G   
   G   
   T   
   G   
   C   
   C   
   A   
   T   
   T   
   G   
   G   
   T   
   T   
   T   
   A   
   G   
   A   
   T   
    G    
   C   
   A   
   G   
   G   
   G   
   A   
   C   
   A   
   T   
   T   
   G   
   A   
   A   
   G   
   C   
   C   
   A   
   G   
   A   
   T   
   G   
   A   
   A   
   G   
   A   
   G   
   C   
   T   
   A   
   G   
   A   
   A   
   A   
   T   
   T   
   A   
   A   
   T   
   G   
   G   
    C    
   T   
   T   
   G   
   A   
   A   
   A   
   T   
   G   
   G   
   C   
   A   
   A   
   A   
   A   
   A   
   G   
   A   
   T   
   A   
   A   
   A   
   T   
   C   
   T   
   T   
   G   
   G   
   G   
   G   
    C    
   G   
   G   
   C   
   T   
   A   
   T   
   T   
   T   
   T   
   G   
   C   
    G    
   A   
   C   
   A   
   C   
   G   
   T   
   G   
   T   
   A   
   A   
   A   
   A   
   G   
   A   
   A   
   G   
   C   
   A   
   A   
   G   
   T   
   C   
   C   
   A   
   G   
   T   
   C   
   A   
   A   
   T   
   C   
   T   
    T    
   C   
   G   
   T   
   A   
   C   
   C   
   G   
   C   
   T   
   T   
   T   
   A   
   G   
   T   
   T   
   A   
   A   
   T   
   T   
   C   
   A   
   G   
   A   
   T   
   A   
   A   
   T   
   A   
   T   
   T   
   T   
   A   
   T   
   T   
   T   
   T   
   G   
   C   
    C    
   C   
   A   
   A   
   C   
   A   
   A   
   A   
   C   
   A   
   T   
   T   
   A   
   C   
   G   
    G    
   A   
   T   
   G   
   G   
   G   
    G    
   G   
   A   
   A   
   G   
   A   
   C   
   A   
   A   
   A   
   T   
   T   
   T   
   A   
   G   
   A   
   G   
   C   
    G    
   G   
   G   
   T   
   C   
   T   
   T   
   A   
   A   
   T   
   A   
   A   
   A   
   T   
   T   
   C   
   A   
   T   
   T   
   T   
   T   
   C   
   G   
   G   
   T   
   G   
   A   
   G   
   G   
   A   
   A   
   C   
   T   
   A   
   G   
   A   
   T   
   T   
   T   
   A   
   C   
   C   
   A   
   A   
   T   
    T    
   G   
   C   
   C   
   A   
   T   
   G   
   A   
   C   
   G   
   C   
   C   
    T    
   G   
   C   
   T   
   C   
   A   
   A   
   A   
   T   
   T   
   T   
   C   
   A   
   A   
   A   
    C    
   G   
   A   
   A   
   G   
   A   
   T   
   A   
   A   
   A   
   T   
   T   
   T   
   A   
   A   
   T   
   T   
   C   
   T   
   G   
   A   
   A   
   A   
   T   
   T   
   C   
   T   
   T   
   T   
   T   
    A    
   G   
   C   
   A   
   G   
   A   
   T   
   A   
   C   
    A    
   G   
   G   
   C   
   T   
   A   
    T    
   G   
   G   
    C    
   C   
   A   
   A   
   G   
   G   
    C    
   C   
   A   
   A   
   T   
   T   
   G   
   T   
   T   
   A   
   A   
   T   
   T   
   T   
   C   
    G    
   C   
   C   
   A   
   A   
   T   
   C   
   C   
   A   
   G   
   C   
   A   
   A   
   G   
   C   
   T   
   A   
   C   
   C   
   A   
   T   
   G   
   T   
   A   
   T   
   A   
   G   
   T   
   G   
   T   
   T   
   T   
   T   
   C   
   C   
   A   
   A   
   A   
   A   
   T   
   A   
   A   
   T   
   G   
   G   
    A    
   A   
   C   
   A   
    T    
   T   
    A    
   G   
   T   
    T    
   T   
   A   
   T   
   C   
   C   
   A   
   A   
   A   
   A   
    T    
   T   
   A   
   G   
   T   
   T   
   T   
   T   
   A   
   G   
   A   
   G   
   A   
   A   
   A   
   G   
   A   
   A   
   A   
   C   
    G    
   A   
   A   
   G   
   A   
   A   
   A   
   A   
   A   
   A   
   G   
   A   
   C   
   A   
   A   
   T   
   G   
   T   
   A   
   A   
   T   
   C   
   A   
   G   
   C   
   G   
   C   
   C   
   A   
   A   
   C   
   G   
   C   
   A   
   G   
   C   
   G   
   A   
   A   
   T   
   A   
   C   
   A   
   A   
   T   
   A   
   G   
   C   
   C   
   A   
   C   
   A   
   G   
   A   
   T   
   C   
   T   
   T   
   T   
   T   
   A   
   G   
   G   
   T   
   A   
   G   
   T   
   G   
   T   
   G   
   G   
   A   
   A   
   G   
   A   
   T   
   C   
   C   
   T   
   T   
   C   
   A   
   G   
   G   
   T   
   T   
   A   
   T   
   G   
   T   
   T   
   T   
   A   
   T   
   A   
   A   
   T   
   A   
   T   
   G   
   T   
   A   
   C   
   A   
   A   
   T   
   C   
   C   
   G   
   A   
   A   
   C   
   T   
   T   
   C   
   T   
   C   
   A   
   T   
   T   
   G   
   G   
   C   
   T   
   G   
   C   
   C   
   A   
   A   
   A   
   A   
   C   
   A   
   G   
   G   
   A   
   A   
   C   
   A   
   G   
   C   
   T   
   G   
   A   
   A   
   A   
   T   
   A   
   A   
   A   
   A   
   G   
   A   
   T   
   A   
   A   
   A   
   C   
   A   
   A   
   G   
   A   
   T   
   A   
   C   
   A   
   G   
   A   
   T   
   G   
   G   
   C   
   A   
   A   
   A   
   G   
   A   
   A   
   A   
   A   
   C   
   A   
   G   
   T   
   T   
   T   
   C   
   C   
   T   
   T   
   C   
   T   
   A   
   A   
   C   
   G   
   T   
   T   
   A   
   G   
   A   
   T   
   C   
   G   
   T   
   A   
   G   
   T   
   A   
   A   
   C   
   A   
   A   
   T   
   A   
   A   
   A   
   T   
   T   
   T   
   T   
   T   
   A   
   A   
   C   
   A   
   A   
   T   
   G   
   A   
   T   
   T   
   A   
   T   
   G   
   G   
   T   
   T   
   G   
   A   
   A   
   A   
   A   
   T   
   T   
   C   
   A   
   G   
   G   
   A   
   G   
   A   
   A   
   A   
   A   
   T   
   G   
   G   
   T   
   T   
   C   
   T   
   G   
   C   
   A   
   A   
   C   
   A   
   G   
   A   
   T   
   A   
   T   
   C   
   A   
   G   
   T   
   A   
   A   
   A   
   C   
   C   
   A   
   T   
   T   
   A   
   A   
   T   
   T   
   G   
   A   
   T   
   T   
   A   
   C   
   T   
   T   
   A   
   G   
   A   
   A   
   G   
   C   
   A   
   A   
   C   
   C   
   A   
   T   
   T   
   A   
   A   
   A   
   -   
   -   
   -   
   -   
   -   
   -   
   -   
   -   
   -   
   -   
   -   
   -   
   -   
   -   
   -   
   -   
   -   
   -   
   -   
   -   
   -   
   -   
   -   
   -   
   -   
   -   
   -   
   -   
   -   
   -   
   -   
   -   
   -   
   -   
   -   
   -   
   -   
   -   
   -   
   -   
   -   
   -   
   -   
   -   
   -   
   -   
   -   
   -   
   -   
   -   
   -   
   -   
   -   
   -   
   -   
   -   
   -   
   -   
   -   
   -   
   -   
   A   
   T   
   G   
   G   
   A   
   A   
   C   
   G   
   A   
   A   
   G   
   C   
   A   
   A   
   T   
   C   
   G   
   T   
   A   
   A   
   T   
   A   
   A   
   A   
   A   
   A   
   A   
   T   
   C   
   T   
   T   
   C   
   A   
   A   
   A   
   A   
   A   
   A   
   A   
   C   
   C   
   A   
   C   
   T   
   T   
   A   
   T   
   T   
   C   
   T   
   T   
   G   
   G   
   C   
   G   
   T   
   T   
   T   
   C   
   T   
   G   
   C   
   C   
   T   
   T   
   G   
   G   
   T   
   T   
   C   
   T   
   G   
   A   
   T   
   C   
   G   
   C   
   T   
   G   
   C   
   T   
   G   
   T   
   C   
   G   
   G   
   T   
   G   
   G   
   T   
   G   
   G   
   G   
   T   
   A   
   T   
   T   
   A   
   T   
   G   
   C   
   T   
   T   
   A   
   T   
   A   
   G   
   C   
   C   
   A   
   A   
   T   
   G   
   G   
   C   
   A   
   A   
   G   
   C   
   C   
   A   
   A   
   A   
   C   
   A   
   A   
   G   
   A   
   A   
   T   
   T   
   A   
   G   
   C   
   T   
   G   
   A   
   A   
   G   
   C   
   G   
   A   
   A   
   G   
   A   
   A   
   A   
   A   
   C   
   A   
   G   
   C   
   C   
   A   
   A   
   T   
   A   
   C   
   A   
   T   
   T   
   T   
   T   
   T   
   A   
   A   
   A   
   C   
   G   
   T   
   A   
   T   
   T   
   G   
   T   
   C   
   A   
   A   
   A   
   A   
   C   
   A   
   G   
   G   
   A   
   A   
   T   
   T   
   T   
   G   
   A   
   T   
   A   
   A   
   G   
   T   
   T   
   A   
   C   
   C   
   A   
   T   
   C   
   C   
   G   
   T   
   T   
   G   
   T   
   T   
   C   
   A   
   A   
   G   
   A   
   A   
   A   
   C   
   T   
   A   
   G   
   C   
   T   
   T   
   A   
   A   
   A   
   G   
   A   
   A   
   A   
   A   
   A   
   T   
   G   
   G   
   C   
   T   
   A   
   T   
   G   
   A   
   T   
   A   
   C   
   T   
   A   
   A   
   A   
   T   
   C   
   T   
   G   
   T   
   T   
   G   
   T   
   T   
   G   
   A   
   A   
   A   
   A   
   A   
   T   
   A   
   C   
   C   
   A   
   A   
   G   
   C   
   A   
   A   
   T   
   T   
   T   
   A   
   T   
   T   
   C   
   A   
   G   
   G   
   G   
   A   
   T   
   T   
   C   
   A   
   A   
   G   
   C   
   A   
   G   
   A   
   A   
   G   
   G   
   G   
   G   
   T   
   C   
   A   
   A   
   A   
   G   
   C   
   T   
   A   
   G   
   T   
   G   
   A   
   T   
   G   
   T   
   T   
   C   
   A   
   A   
   G   
   T   
   C   
   A   
   A   
   A   
   A   
   A   
   A   
   G   
   G   
   G   
   A   
   A   
   A   
   G   
   A   
   C   
   A   
   A   
   C   
   C   
   A   
   A   
   T   
   A   
   C   
   A   
   C   
   A   
   T   
   T   
   T   
   A   
   C   
   C   
   T   
   A   
   T   
   A   
   A   
   A   
   T   
   T   
   A   
   T   
   C   
   G   
   A   
   T   
   G   
   A   
   G   
   C   
   A   
   C   
   T   
   C   
   C   
   T   
   T   
   T   
   A   
   G   
   G   
   C   
   G   
   A   
   A   
   A   
   T   
   G   
   A   
   A   
   A   
   G   
   A   
   T   
   T   
   T   
   G   
   T   
   C   
   T   
   T   
   A   
   T   
   C   
   A   
   A   
   T   
   C   
   A   
   A   
   G   
   T   
   A   
   T   
   C   
   G   
   C   
   C   
   A   
   A   
   A   
   A   
   A   
   A   
   G   
   G   
   C   
   G   
   A   
   T   
   A   
   A   
   C   
   T   
   A   
   C   
   C   
   A   
   A   
   A   
   T   
   C   
   G   
   C   
   T   
   T   
   G   
   G   
   G   
   C   
   A   
   C   
   C   
   G   
   T   
   C   
   T   
   T   
   T   
   A   
   A   
   T   
   T   
   T   
   T   
   T   
   C   
   C   
   A   
   G   
   A   
   T   
   A   
   T   
   G   
   T   
   C   
   A   
   G   
   G   
   A   
   A   
   A   
   T   
   G   
   A   
   T   
   A   
   A   
   A   
   A   
   T   
   T   
   T   
   C   
   G   
   A   
   T   
   T   
   C   
   A   
   A   
   G   
   T   
   A   
   G   
   A   
   T   
   A   
   A   
   T   
   G   
   C   
   C   
   A   
   A   
   A   
   C   
   G   
   T   
   G   
   G   
   A   
   G   
   A   
   A   
   A   
   T   
   T   
   G   
   T   
   C   
   G   
   A   
   T   
   C   
   G   
   T   
   A   
   A   
   T   
   G   
   G   
   T   
   A   
   G   
   T   
   G   
   G   
   G   
   C   
   T   
   A   
   G   
   C   
   A   
   A   
   T   
   T   
   A   
   A   
   C   
   A   
   A   
   A   
   G   
   T   
   G   
   T   
   T   
   T   
   G   
   A   
   T   
   G   
   A   
   A   
   G   
   T   
   G   
   G   
   G   
   C   
   G   
   T   
   A   
   G   
   T   
   G   
   C   
   C   
   T   
   G   
   G   
   C   
   A   
   A   
   A   
   C   
   T   
   C   
   G   
   G   
   T   
   T   
   C   
   T   
   G   
   G   
   C   
   G   
   C   
   A   
   G   
   A   
   A   
   A   
   A   
   A   
   A   
   C   
   A   
   G   
   C   
   C   
   A   
   A   
   T   
   A   
   T   
   C   
   A   
   A   
   A   
   G   
   C   
   T   
   T   
   T   
   T   
   A   
   G   
   T   
   G   
   A   
   T   
   A   
   A   
   G   
   T   
   T   
   T   
   G   
   G   
   C   
   G   
   T   
   T   
   T   
   C   
   T   
   G   
   T   
   T   
   G   
   A   
   T   
   G   
   A   
   A   
   A   
   T   
   C   
   A   
   A   
   T   
   C   
   A   
   A   
   A   
   A   
   G   
   T   
   T   
   A   
   A   
   G   
   C   
   C   
   A   
   A   
   G   
   G   
   A   
   T   
   G   
   G   
   G   
   T   
   C   
   C   
   A   
   A   
   G   
   C   
   A   
   G   
   A   
   C   
   T   
   C   
   G   
   T   
   T   
   T   
   G   
   T   
   A   
   C   
   C   
   A   
   A   
   T   
   C   
   A   
   C   
   A   
   G   
   T   
   T   
   G   
   C   
   T   
   T   
   C   
   T   
   G   
   A   
   A   
   C   
   C   
   A   
   G   
   T   
   G   
   A   
   C   
   A   
   G   
   A   
   A   
   T   
   T   
   A   
   C   
   C   
   A   
   A   
   C   
   A   
   G   
   G   
   G   
   G   
   C   
   T   
   G   
   C   
   G   
   A   
   C   
   A   
   A   
   A   
   A   
   G   
   A   
   T   
   A   
   C   
   A   
   G   
   A   
   G   
   T   
   C   
   A   
   C   
   G   
   T   
   T   
   A   
   T   
   T   
   A   
   T   
   C   
   C   
   A   
   T   
   T   
   G   
   G   
   G   
   G   
   G   
   A   
   A   
   G   
   C   
   A   
   G   
   C   
   T   
   G   
   C   
   G   
   C   
   A   
   A   
   T   
   T   
   A   
   A   
   T   
   C   
   G   
   G   
   G   
   T   
   A   
   T   
   A   
   C   
   G   
   G   
   G   
   C   
   A   
   C   
   T   
   A   
   T   
   T   
   A   
   C   
   T   
   G   
   C   
   A   
   G   
   A   
   A   
   G   
   A   
   T   
   A   
   T   
   T   
   G   
   A   
   A   
   A   
   A   
   A   
   A   
   A   
   T   
   C   
   C   
   A   
   G   
   A   
   G   
   T   
   T   
   A   
   A   
   G   
   T   
   A   
   G   
   T   
   A   
   C   
   G   
   G   
   G   
   C   
   G   
   T   
   A   
   A   
   T   
   T   
   G   
   G   
   C   
   A   
   A   
   A   
   A   
   C   
   T   
   G   
   G   
   C   
   T   
   T   
   A   
   G   
   A   
   A   
   C   
   G   
   A   
   G   
   C   
   G   
   T   
   T   
   T   
   G   
   A   
   T   
   A   
   A   
   A   
   G   
   A   
   A   
   T   
   T   
   A   
   A   
   G   
   A   
   G   
   G   
   A   
   C   
   A   
   A   
   G   
   A   
   T   
   G   
   G   
   T   
   G   
   G   
   T   
   T   
   C   
   A   
   C   
   T   
   A   
   G   
   T   
   T   
   A   
   T   
   T   
   T   
   T   
   A   
   G   
   A   
   T   
   G   
   A   
   C   
   A   
   A   
   A   
   G   
   A   
   G   
   A   
   A   
   T   
   G   
   T   
   C   
   A   
   A   
   A   
   A   
   A   
   A   
   G   
   C   
   T   
   T   
   T   
   A   
   C   
   A   
   A   
   A   
   C   
   C   
   A   
   A   
   A   
   G   
   A   
   G   
   A   
   A   
   A   
   A   
   A   
   A   
   G   
   A   
   T   
   G   
   G   
   T   
   C   
   A   
   A   
   A   
   C   
   A   
   A   
   T   
   T   
   A   
   A   
   A   
   T   
   T   
   A   
   A   
   C   
   G   
   A   
   T   
   T   
   G   
   A   
   T   
   A   
   G   
   T   
   G   
   G   
   C   
   G   
   T   
   A   
   C   
   A   
   A   
   C   
   A   
   A   
   C   
   A   
   A   
   G   
   C   
   C   
   T   
   T   
   C   
   G   
   C   
   T   
   A   
   T   
   A   
   T   
   T   
   T   
   G   
   A   
   C   
   A   
   A   
   A   
   C   
   G   
   T   
   C   
   C   
   T   
   G   
   G   
   T   
   A   
   G   
   T   
   G   
   C   
   G   
   G   
   T   
   C   
   A   
   T   
   T   
   A   
   C   
   C   
   G   
   A   
   T   
   C   
   C   
   T   
   C   
   A   
   A   
   A   
   A   
   A   
   G   
   G   
   C   
   G   
   A   
   T   
   T   
   T   
   A   
   C   
   T   
   A   
   G   
   C   
   G   
   A   
   C   
   G   
   G   
   T   
   T   
   A   
   G   
   T   
   T   
   C   
   A   
   C   
   C   
   T   
   T   
   C   
   G   
   T   
   A   
   T   
   G   
   A   
   T   
   C   
   C   
   T   
   A   
   A   
   T   
   A   
   A   
   A   
   A   
   T   
   G   
   G   
   C   
   C   
   A   
   A   
   T   
   G   
   G   
   C   
   A   
   T   
   T   
   T   
   C   
   T   
   C   
   A   
   A   
   A   
   A   
   A   
   G   
   A   
   A   
   T   
   A   
   T   
   G   
   A   
   T   
   G   
   C   
   T   
   T   
   A   
   C   
   A   
   A   
   T   
   A   
   A   
   C   
   A   
   A   
   C   
   A   
   A   
   A   
   G   
   A   
   T   
   T   
   T   
   A   
   C   
   C   
   A   
   T   
   T   
   C   
   A   
   C   
   A   
   G   
   C   
   A   
   C   
   G   
   T   
   T   
   T   
   T   
   G   
   C   
   G   
   A   
   C   
   A   
   G   
   G   
   C   
   T   
   A   
   T   
   G   
   C   
   A   
   C   
   C   
   A   
   G   
   G   
   C   
   T   
   C   
   T   
   A   
   C   
   C   
   T   
   T   
   T   
   A   
   A   
   A   
   A   
   C   
   A   
   A   
   T   
   T   
   A   
   C   
   C   
   G   
   G   
   T   
   G   
   C   
   C   
   A   
   T   
   T   
   G   
   G   
   T   
   T   
   T   
   A   
   G   
   A   
   T   
   G   
   C   
   A   
   G   
   G   
   G   
   A   
   C   
   A   
   T   
   T   
   G   
   A   
   A   
   G   
   C   
   C   
   A   
   G   
   A   
   T   
   G   
   A   
   A   
   G   
   A   
   G   
   C   
   T   
   A   
   G   
   A   
   A   
   A   
   T   
   T   
   A   
   A   
   T   
   G   
   G   
   C   
   T   
   T   
   G   
   A   
   A   
   A   
   T   
   G   
   G   
   C   
   A   
   A   
   A   
   A   
   A   
   G   
   A   
   T   
   A   
   A   
   A   
   T   
   C   
   T   
   T   
   G   
   G   
   G   
   G   
   C   
   G   
   G   
   C   
   T   
   A   
   T   
   T   
   T   
   T   
   G   
   C   
   G   
   A   
   C   
   A   
   C   
   G   
   T   
   G   
   T   
   A   
   A   
   A   
   A   
   G   
   A   
   A   
   G   
   C   
   A   
   A   
   G   
   T   
   C   
   C   
   A   
   G   
   T   
   C   
   A   
   A   
   T   
   C   
   T   
   T   
   C   
   G   
   T   
   A   
   C   
   C   
   G   
   C   
   T   
   T   
   T   
   A   
   G   
   T   
   T   
   A   
   A   
   T   
   T   
   C   
   A   
   G   
   A   
   T   
   A   
   A   
   T   
   A   
   T   
   T   
   T   
   A   
   T   
   T   
   T   
   T   
   G   
   C   
   C   
   C   
   A   
   A   
   C   
   A   
   A   
   A   
   C   
   A   
   T   
   T   
   A   
   C   
   G   
   G   
   A   
   T   
   G   
   G   
   G   
   G   
   G   
   A   
   A   
   G   
   A   
   C   
   A   
   A   
   A   
   T   
   T   
   T   
   A   
   G   
   A   
   G   
   C   
   G   
   G   
   G   
   T   
   C   
   T   
   T   
   A   
   A   
   T   
   A   
   A   
   A   
   T   
   T   
   C   
   A   
   T   
   T   
   T   
   T   
   C   
   G   
   G   
   T   
   G   
   A   
   G   
   G   
   A   
   A   
   C   
   T   
   A   
   G   
   A   
   T   
   T   
   T   
   A   
   C   
   C   
   A   
   A   
   T   
   T   
   G   
   C   
   C   
   A   
   T   
   G   
   A   
   C   
   G   
   C   
   C   
   T   
   G   
   C   
   T   
   C   
   A   
   A   
   A   
   T   
   T   
   T   
   C   
   A   
   A   
   A   
   C   
   G   
   A   
   A   
   G   
   A   
   T   
   A   
   A   
   A   
   T   
   T   
   T   
   A   
   A   
   T   
   T   
   C   
   T   
   G   
   A   
   A   
   A   
   T   
   T   
   C   
   T   
   T   
   T   
   T   
   A   
   G   
   C   
   A   
   G   
   A   
   T   
   A   
   C   
   A   
   G   
   G   
   C   
   T   
   A   
   T   
   G   
   G   
   C   
   C   
   A   
   A   
   G   
   G   
   C   
   C   
   A   
   A   
   T   
   T   
   G   
   T   
   T   
   A   
   A   
   T   
   T   
   T   
   C   
   G   
   C   
   C   
   A   
   A   
   T   
   C   
   C   
   A   
   G   
   C   
   A   
   A   
   G   
   C   
   T   
   A   
   C   
   C   
   A   
   T   
   G   
   T   
   A   
   T   
   A   
   G   
   T   
   G   
   T   
   T   
   T   
   T   
   C   
   C   
   A   
   A   
   A   
   A   
   T   
   A   
   A   
   T   
   G   
   G   
   A   
   A   
   C   
   A   
   T   
   T   
   A   
   G   
   T   
   T   
   T   
   A   
   T   
   C   
   C   
   A   
   A   
   A   
   A   
   T   
   T   
   A   
   G   
   T   
   T   
   T   
   T   
   A   
   G   
   A   
   G   
   A   
   A   
   A   
   G   
   A   
   A   
   A   
   C   
   G   
   A   
   A   
   G   
   A   
   A   
   A   
   A   
   A   
   A   
   G   
   A   
   C   
   A   
   A   
   T   
   G   
   T   
   A   
   A   
   T   
   C   
   A   
   G   
   C   
   G   
   C   
   C   
   A   
   A   
   C   
   G   
   C   
   A   
   G   
   C   
   G   
   A   
   A   
   T   
   A   
   C   
   A   
   A   
   T   
   A   
   G   
   C   
   C   
   A   
   C   
   A   
   G   
   A   
   T   
   C   
   T   
   T   
   T   
   T   
   A   
   G   
   G   
   T   
   A   
   G   
   T   
   G   
   T   
   G   
   G   
   A   
   A   
   G   
   A   
   T   
   C   
   C   
   T   
   T   
   C   
   A   
   G   
   G   
   T   
   T   
   A   
   T   
   G   
   T   
   T   
   T   
   A   
   T   
   A   
   A   
   T   
   A   
   T   
   G   
   T   
   A   
   C   
   A   
   A   
   T   
   C   
   C   
   G   
   A   
   A   
   C   
   T   
   T   
   C   
   T   
   C   
   A   
   T   
   T   
   G   
   G   
   C   
   T   
   G   
   C   
   C   
   A   
   A   
   A   
   A   
   C   
   A   
   G   
   G   
   A   
   A   
   C   
   A   
   G   
   C   
   T   
   G   
   A   
   A   
   A   
   T   
   A   
   A   
   A   
   A   
   G   
   A   
   T   
   A   
   A   
   A   
   C   
   A   
   A   
   G   
   A   
   T   
   A   
   C   
   A   
   G   
   A   
   T   
   G   
   G   
   C   
   A   
   A   
   A   
   G   
   A   
   A   
   A   
   A   
   C   
   A   
   G   
   T   
   T   
   T   
   C   
   C   
   T   
   T   
   C   
   T   
   A   
   A   
   C   
   G   
   T   
   T   
   A   
   G   
   A   
   T   
   C   
   G   
   T   
   A   
   G   
   T   
   A   
   A   
   C   
   A   
   A   
   T   
   A   
   A   
   A   
   T   
   T   
   T   
   T   
   T   
   A   
   A   
   C   
   A   
   A   
   T   
   G   
   A   
   T   
   T   
   A   
   T   
   G   
   G   
   T   
   T   
   G   
   A   
   A   
   A   
   A   
   T   
   T   
   C   
   A   
   G   
   G   
   A   
   G   
   A   
   A   
   A   
   A   
   T   
   G   
   G   
   T   
   T   
   C   
   T   
   G   
   C   
   A   
   A   
   C   
   A   
   G   
   A   
   T   
   A   
   T   
   C   
   A   
   G   
   T   
   A   
   A   
   A   
   C   
   C   
   A   
   T   
   T   
   A   
   A   
   T   
   T   
   G   
   A   
   T   
   T   
   A   
   C   
   T   
   T   
   A   
   G   
   A   
   A   
   G   
   C   
   A   
   A   
   C   
   C   
   A   
   T   
   T   
   A   
   A   
   A   
   -   
   -   
   -   
   -   
   -   
   -   
   -   
   -   
   -   
   -   
   -   
   -   
   -   
   -   
   -   
   -   
   -   
   -   
   -   
   -   
   -   
   -   
   -   
   -   
   -   
   -   
   -   
   -   
   -   
   -   
   -   
   -   
   -   
   -   
   -   
   -   
   -   
   -   
   -   
   -   
   -   
   -   
   -   
   -   
   -   
   -   
   -   
   -   
   -   
   -   
   -   
   -   
   -   
   -   
   -   
   -   
   -   
   -   
   -   
   -   
   -   
 

 
   N55725   
   -   
   -   
   -   
   -   
   -   
   -   
   -   
   -   
   -   
   -   
   -   
   -   
   -   
   -   
   -   
   -   
   -   
   -   
   -   
   -   
   -   
   -   
   -   
   -   
   -   
   -   
   -   
   -   
   -   
   -   
   -   
   -   
   -   
   -   
   -   
   -   
   -   
   -   
   -   
   -   
   -   
   -   
   -   
   -   
   -   
   -   
   -   
   -   
   -   
   -   
   -   
   -   
   -   
   -   
   -   
   -   
   -   
   -   
   -   
   -   
   -   
   -   
   -   
   -   
   -   
   -   
   -   
   -   
   -   
   -   
   -   
   -   
   -   
   -   
   -   
   -   
   -   
   -   
   -   
   -   
   -   
   -   
   -   
   -   
   -   
   -   
   -   
   -   
   -   
   -   
   -   
   -   
   -   
   -   
   -   
   -   
   -   
   -   
   -   
   -   
   -   
   -   
   -   
   -   
   -   
   -   
   -   
   -   
   C   
   A   
   A   
   T   
   G   
   G   
   C   
   A   
   A   
   G   
   C   
   C   
   A   
   A   
   A   
   C   
   A   
   A   
   G   
   A   
    A    
   T   
   T   
   A   
   G   
   C   
    C    
   G   
   A   
   A   
   G   
   C   
   G   
   A   
   A   
   G   
   A   
   A   
   A   
   A   
    C    
   A   
   G   
   C   
    T    
   A   
    C    
   T   
    A    
    C    
   A   
   T   
   T   
   T   
   T   
   T   
   A   
   A   
   A   
    C    
   G   
   T   
   A   
   T   
   T   
   G   
    T    
   C   
   A   
   A   
   A   
   A   
   C   
   A   
   G   
    G    
   A   
   A   
   T   
   T   
   T   
   G   
   A   
   T   
   A   
   A   
   G   
   T   
   T   
   A   
   C   
   C   
    G    
   T   
   C   
   C   
   G   
   T   
   T   
    G    
   T   
   T   
   C   
   A   
   A   
   G   
   A   
   A   
    G    
   C   
   T   
   A   
   G   
   C   
   T   
   T   
   A   
   A   
   A   
   G   
   A   
   A   
   A   
   A   
   A   
   T   
   G   
   G   
    C    
   T   
   A   
   T   
   G   
   A   
   T   
   A   
   C   
   T   
   A   
   A   
   A   
   T   
   C   
    T    
   G   
   T   
   T   
   G   
   T   
   T   
   G   
   A   
   A   
   A   
   A   
   A   
   T   
   A   
   C   
   C   
   A   
   A   
   G   
   C   
   A   
   A   
   T   
   T   
   T   
   A   
   T   
   T   
   C   
   A   
   G   
   G   
   G   
   A   
   T   
   T   
   C   
   A   
   A   
   G   
   C   
    T    
   G   
   A   
   A   
    G    
   G   
    A    
   G   
   T   
   C   
   A   
   A   
    A    
   G   
   C   
   T   
   A   
   G   
   T   
   G   
   A   
   T   
   G   
   T   
    T    
   C   
   A   
   A   
   G   
   T   
    C    
   A   
   A   
   A   
   A   
   A   
    A    
   G   
    C    
   G   
   A   
   A   
   A   
   G   
   A   
    C    
   A   
   A   
    T    
   C   
   A   
   A   
   T   
   A   
   C   
   A   
   C   
   A   
   T   
   T   
   T   
   A   
    C    
   C   
   T   
   A   
   T   
   A   
   A   
   A   
   T   
   T   
   A   
   T   
   C   
   G   
   A   
   T   
   G   
   A   
   G   
    C    
   A   
   C   
    G    
   C   
   C   
   T   
   T   
   T   
   A   
   G   
   G   
   C   
   G   
   A   
   A   
   A   
   T   
   G   
   A   
   A   
   A   
   G   
   A   
   T   
    T    
   T   
   G   
   T   
   C   
   T   
   T   
   A   
   T   
   C   
   A   
   A   
   T   
   C   
   A   
   A   
   G   
   T   
   A   
   T   
    C    
   G   
    A    
   C   
   A   
   A   
    A    
   A   
   A   
   A   
   G   
   G   
    C    
   G   
   A   
   T   
   A   
    C    
   C   
   T   
   A   
   C   
   C   
   A   
   A   
   A   
   T   
    C    
    G    
   C   
   T   
   T   
   G   
   G   
    A    
    A    
    G    
   C   
   C   
    A    
   T   
   C   
   T   
   T   
   T   
   A   
   A   
   T   
   T   
   T   
   T   
   T   
   C   
   C   
   A   
   G   
   A   
   T   
   A   
   T   
   G   
   T   
   C   
   A   
   G   
   G   
   A   
   A   
   A   
   T   
   G   
   A   
   T   
   A   
   A   
   A   
    A    
   T   
   T   
   T   
   C   
   G   
   A   
   T   
   T   
   C   
   A   
   A   
   G   
   T   
   A   
   G   
   A   
   T   
   A   
   A   
   T   
   G   
   C   
   C   
   A   
   A   
   A   
   C   
   G   
   T   
   G   
   G   
   A   
   G   
   A   
   A   
   A   
   T   
   T   
   G   
   T   
   C   
   G   
   A   
   T   
   C   
   G   
   T   
   A   
   A   
    T    
   G   
   G   
   T   
   A   
   G   
   T   
   G   
   G   
   G   
   C   
   T   
   A   
   G   
    C    
   A   
   A   
   T   
   T   
   A   
   A   
   C   
   A   
   A   
   A   
   G   
   T   
   G   
   T   
   T   
   T   
   G   
   A   
    C    
   G   
   A   
   A   
   G   
   T   
   G   
   G   
   G   
   C   
   G   
   T   
   A   
   G   
   T   
    G    
   C   
   C   
   T   
    A    
   G   
   C   
   A   
   A   
   A   
   C   
   T   
    C    
   G   
   G   
   T   
   T   
   C   
   T   
   G   
   G   
   C   
   G   
   C   
   A   
   G   
   A   
   A   
   A   
   A   
   A   
    G    
   C   
   A   
   G   
   C   
    C    
   A   
   A   
   T   
   A   
   T   
   C   
   A   
   A   
   A   
   G   
   C   
   T   
   T   
   T   
   T   
   A   
   G   
   T   
   G   
   A   
   T   
   A   
   A   
    G    
   T   
   T   
    C    
   G   
   G   
   C   
   G   
   T   
   T   
   T   
   C   
   T   
    G    
   T   
   T   
   G   
   A   
   T   
   G   
   A   
   A   
   A   
   T   
   C   
   A   
   A   
   T   
   C   
    A    
   A   
   A   
   A   
    G    
   T   
   T   
   A   
   A   
   G   
   C   
   C   
   A   
   A   
   G   
   G   
    A    
   T   
   G   
   G   
   G   
   T   
   C   
   C   
   A   
   A   
   G   
   C   
   A   
   G   
   A   
   C   
   T   
   C   
    C    
   T   
   T   
   T   
   G   
   T   
   A   
   C   
   C   
   A   
   A   
   T   
   C   
   A   
   C   
   A   
   G   
   T   
    T    
   G   
   C   
   T   
   T   
   C   
   T   
   G   
   A   
   A   
   C   
   C   
   A   
   G   
   T   
   G   
   A   
   C   
   A   
   G   
   A   
   A   
   T   
   T   
   A   
   C   
   C   
   A   
   A   
   C   
   A   
   G   
   G   
   G   
   G   
   C   
   T   
   G   
   C   
   G   
   A   
   C   
   A   
   A   
   A   
   A   
   G   
    A    
    C    
   A   
   C   
   A   
   G   
   A   
   G   
   T   
   C   
   A   
   C   
   G   
   T   
   T   
   A   
   T   
   T   
   A   
   T   
   C   
   C   
    G    
    T    
   T   
   G   
   G   
   G   
   G   
   G   
   A   
   A   
   G   
   C   
   A   
   G   
   C   
   T   
   G   
   C   
   G   
   C   
   A   
   A   
   T   
   T   
   A   
   A   
   T   
   C   
   G   
   G   
    G    
   T   
   A   
   T   
   A   
   C   
    G    
   G   
   G   
    C    
   A   
   C   
    C    
   A   
   T   
   T   
   A   
   C   
   T   
   G   
   C   
   A   
   G   
   A   
    A    
   G   
   A   
   T   
   A   
   T   
   T   
    A    
   A   
   A   
   A   
   A   
   A   
   A   
   A   
   T   
   C   
   C   
   A   
   G   
   A   
   G   
   T   
   T   
   A   
   A   
   G   
   T   
   A   
   G   
   T   
   A   
   C   
   G   
   G   
   G   
    C    
   G   
   T   
   A   
   A   
   T   
   T   
   G   
   G   
    T    
   A   
   A   
   A   
   A   
   C   
   T   
   G   
   G   
    C    
   T   
   T   
   A   
   G   
   A   
   A   
   C   
   G   
   A   
   G   
   C   
   G   
   T   
   T   
   T   
   G   
   A   
   T   
   A   
   A   
   A   
   G   
   A   
    A    
   T   
   T   
   A   
   A   
   G   
   A   
   G   
   G   
    G    
   C   
   A   
   A   
   G   
   A   
   T   
   G   
   G   
    T    
   G   
   G   
    C    
   T   
   C   
    A    
   C   
   T   
   A   
   G   
   T   
   T   
   A   
   T   
   T   
   T   
   T   
   A   
   G   
   A   
   T   
   G   
   A   
   C   
   A   
   A   
   A   
   G   
   A   
   G   
   A   
   A   
   T   
   G   
   T   
   C   
   A   
   A   
   A   
   A   
   A   
   A   
   G   
   C   
   T   
   T   
   T   
   A   
   C   
   A   
   A   
   A   
   C   
   C   
   A   
   A   
   A   
   G   
   A   
    G    
   A   
   A   
   A   
   A   
   A   
   A   
   G   
   A   
   T   
   G   
   G   
   T   
   C   
   A   
   A   
   A   
   C   
    G    
   A   
   T   
   T   
   A   
   A   
   A   
   T   
   T   
   A   
   A   
   C   
   G   
   A   
   T   
   T   
   G   
   A   
    C    
   A   
   G   
    C    
   G   
   G   
    G    
   G   
   T   
   A   
   C   
   A   
   A   
   C   
   A   
   A   
   C   
   A   
   A   
   G   
   C   
    T    
   T   
   T   
    T    
   G   
   C   
   T   
   A   
   T   
   A   
   T   
   T   
   T   
   G   
   A   
   C   
   A   
   A   
   A   
   C   
   G   
    T    
    C    
   C   
   T   
   G   
   G   
   T   
   A   
   G   
   T   
   G   
   C   
   G   
   G   
   T   
   C   
   A   
   T   
   T   
   A   
   C   
   C   
   G   
   A   
   T   
   C   
   C   
   T   
   C   
   A   
   A   
   A   
   A   
   A   
   G   
   G   
   C   
   G   
   A   
   T   
   T   
   T   
   A   
    T    
   T   
   A   
   G   
   C   
    A    
   A   
   C   
   G   
   G   
   T   
   T   
   A   
   G   
   T   
   T   
   C   
   A   
   C   
   C   
   T   
   T   
   C   
   G   
   T   
   A   
   T   
   G   
   A   
   T   
   C   
   C   
   T   
   A   
   A   
   T   
   A   
   A   
   A   
   A   
   T   
   G   
   G   
   C   
   C   
   A   
   A   
   T   
   G   
   G   
    C    
   A   
   T   
   T   
   T   
   C   
   T   
   C   
   A   
   A   
   A   
   A   
   A   
   G   
   A   
    A    
   T   
   A   
   T   
   G   
   A   
   T   
   G   
   C   
   T   
   T   
   A   
   C   
   A   
   A   
   T   
   A   
   A   
   C   
   A   
   A   
   C   
   A   
   A   
   A   
   G   
   A   
   T   
   T   
   T   
   A   
   C   
   C   
   A   
   T   
   T   
    C    
   A   
   C   
   A   
   G   
   C   
   A   
   C   
   G   
   T   
   T   
   T   
    C    
   G   
   C   
   G   
   A   
   C   
   A   
   G   
   G   
    C    
   T   
   A   
   T   
   G   
   C   
   A   
   C   
   C   
   A   
   G   
   G   
    C    
   T   
   C   
   T   
   A   
   C   
   C   
   T   
   T   
   T   
   A   
   A   
   A   
   A   
   C   
   A   
   A   
   T   
   T   
   A   
   C   
   C   
   G   
   G   
   T   
   G   
   C   
   C   
   A   
   T   
   T   
   G   
   G   
   T   
   T   
   T   
   A   
   G   
   A   
   T   
    G    
   C   
   A   
   G   
   G   
   G   
   A   
   C   
   A   
   T   
   T   
   G   
   A   
   A   
   G   
   C   
   C   
   A   
   G   
   A   
   T   
   G   
   A   
   A   
   G   
   A   
   G   
   C   
   T   
   A   
   G   
   A   
   A   
   A   
   T   
   T   
   A   
   A   
   T   
   G   
   G   
    C    
   T   
   T   
   G   
   A   
   A   
   A   
   T   
   G   
   G   
   C   
   A   
   A   
   A   
   A   
   A   
   G   
   A   
   T   
   A   
   A   
   A   
   T   
   C   
   T   
   T   
   G   
   G   
   G   
   G   
    C    
   G   
   G   
   C   
   T   
   A   
   T   
   T   
   T   
   T   
   G   
   C   
    G    
   A   
   C   
   A   
   C   
   G   
   T   
   G   
   T   
   A   
   A   
   A   
   A   
   G   
   A   
   A   
   G   
   C   
   A   
   A   
   G   
   T   
   C   
   C   
   A   
   G   
   T   
   C   
   A   
   A   
   T   
   C   
   T   
    G    
   C   
   G   
   T   
   A   
   C   
   C   
   G   
   C   
   T   
   T   
   T   
   A   
   G   
   T   
   T   
   A   
   A   
   T   
   T   
   C   
   A   
   G   
   A   
   T   
   A   
   A   
   T   
   A   
   T   
   T   
   T   
   A   
   T   
   T   
   T   
   T   
   G   
   C   
    C    
   C   
   A   
   A   
   C   
   A   
   A   
   A   
   C   
   A   
   T   
   T   
   A   
   C   
   G   
    G    
   A   
   T   
   G   
   G   
   G   
    G    
   G   
   A   
   A   
   G   
   A   
   C   
   A   
   A   
   A   
   T   
   T   
   T   
   A   
   G   
   A   
   G   
   C   
    G    
   G   
   G   
   T   
   C   
   T   
   T   
   A   
   A   
   T   
   A   
   A   
   A   
   T   
   T   
   C   
   A   
   T   
   T   
   T   
   T   
   C   
   G   
   G   
   T   
   G   
   A   
   G   
   G   
   A   
   A   
   C   
   T   
   A   
   G   
   A   
   T   
   T   
   T   
   A   
   C   
   C   
   A   
   A   
   T   
    T    
   G   
   C   
   C   
   A   
   T   
   G   
   A   
   C   
   G   
   C   
   C   
    T    
   G   
   C   
   T   
   C   
   A   
   A   
   A   
   T   
   T   
   T   
   C   
   A   
   A   
   A   
    T    
   G   
   A   
   A   
   G   
   A   
   T   
   A   
   A   
   A   
   T   
   T   
   T   
   A   
   A   
   T   
   T   
   C   
   T   
   G   
   A   
   A   
   A   
   T   
   T   
   C   
   T   
   T   
   T   
   T   
    A    
   G   
   C   
   A   
   G   
   A   
   T   
   A   
   C   
    G    
   G   
   G   
   C   
   T   
   A   
    T    
   G   
   G   
    T    
   C   
   A   
   A   
   G   
   G   
    T    
   C   
   A   
   A   
   T   
   T   
   G   
   T   
   T   
   A   
   A   
   T   
   T   
   T   
   C   
    G    
   C   
   C   
   A   
   A   
   T   
   C   
   C   
   A   
   G   
   C   
   A   
   A   
   G   
   C   
   T   
   A   
   C   
   C   
   A   
   T   
   G   
   T   
   A   
   T   
   A   
   G   
   T   
   G   
   T   
   T   
   T   
   T   
   C   
   C   
   A   
   A   
   A   
   A   
   T   
   A   
   A   
   T   
   G   
   G   
    A    
   A   
   C   
   A   
    T    
   T   
    A    
   G   
   T   
    C    
   T   
   A   
   T   
   C   
   C   
   A   
   A   
   A   
   A   
    C    
   T   
   A   
   G   
   T   
   T   
   T   
   T   
   A   
   G   
   A   
   C   
   A   
   A   
   A   
   G   
   A   
   A   
   A   
   C   
    G    
   A   
   A   
   G   
   A   
   A   
   A   
   A   
   A   
   A   
   G   
   A   
   C   
   A   
   A   
   T   
   G   
   T   
   A   
   A   
   T   
   C   
   A   
   G   
   C   
   G   
   C   
   C   
   A   
   A   
   T   
   G   
   C   
   A   
   G   
   C   
   G   
   A   
   A   
   T   
   A   
   C   
   A   
   A   
   T   
   T   
   G   
   C   
   C   
   A   
   C   
   A   
   G   
   A   
   T   
   C   
   T   
   T   
   T   
   T   
   A   
   G   
   G   
   T   
   A   
   G   
   T   
   G   
   T   
   G   
   G   
   A   
   A   
   G   
   A   
   T   
   C   
   C   
   T   
   T   
   C   
   A   
   G   
   G   
   T   
   T   
   A   
   T   
   G   
   T   
   T   
   T   
   A   
   T   
   A   
   A   
   T   
   A   
   T   
   G   
   T   
   A   
   C   
   A   
   A   
   T   
   C   
   C   
   G   
   A   
   A   
   C   
   T   
   T   
   C   
   T   
   C   
   A   
   T   
   T   
   G   
   G   
   C   
   T   
   G   
   C   
   T   
   A   
   A   
   A   
   A   
   C   
   A   
   G   
   G   
   A   
   A   
   C   
   A   
   G   
   C   
   T   
   G   
   A   
   A   
   A   
   T   
   A   
   A   
   A   
   A   
   G   
   A   
   T   
   A   
   A   
   A   
   C   
   A   
   A   
   G   
   A   
   T   
   A   
   C   
   A   
   G   
   A   
   T   
   G   
   G   
   C   
   A   
   A   
   A   
   G   
   A   
   A   
   A   
   A   
   C   
   A   
   G   
   T   
   T   
   T   
   C   
   C   
   T   
   T   
   C   
   T   
   A   
   A   
   C   
   G   
   T   
   T   
   A   
   G   
   A   
   T   
   C   
   G   
   T   
   A   
   G   
   T   
   A   
   A   
   C   
   A   
   A   
   T   
   A   
   A   
   A   
   T   
   T   
   C   
   T   
   T   
   A   
   A   
   C   
   A   
   A   
   T   
   G   
   A   
   T   
   T   
   A   
   T   
   G   
   G   
   T   
   T   
   G   
   A   
   A   
   A   
   A   
   T   
   T   
   C   
   A   
   G   
   G   
   A   
   G   
   A   
   A   
   A   
   A   
   T   
   G   
   G   
   T   
   T   
   C   
   T   
   G   
   C   
   A   
   A   
   C   
   A   
   G   
   A   
   T   
   A   
   T   
   C   
   A   
   G   
   T   
   A   
   A   
   A   
   C   
   C   
   A   
   T   
   T   
   A   
   A   
   T   
   T   
   G   
   A   
   T   
   T   
   A   
   C   
   T   
   T   
   A   
   G   
   A   
   A   
   G   
   C   
   A   
   A   
   C   
   C   
   A   
   T   
   T   
   A   
   A   
   A   
   -   
   -   
   -   
   -   
   -   
   -   
   -   
   -   
   -   
   -   
   -   
   -   
   -   
   -   
   -   
   -   
   -   
   -   
   -   
   -   
   -   
   -   
   -   
   -   
   -   
   -   
   -   
   -   
   -   
   -   
   -   
   -   
   -   
   -   
   -   
   -   
   -   
   -   
   -   
   -   
   -   
   -   
   -   
   -   
   -   
   -   
   -   
   -   
   -   
   -   
   -   
   -   
   -   
   -   
   -   
   -   
   -   
   -   
   -   
   -   
   -   
   -   
   -   
   -   
   -   
   -   
   -   
   -   
   -   
   -   
   -   
   -   
   -   
   -   
   -   
   -   
   -   
   -   
   -   
   -   
   -   
   -   
   -   
   -   
   -   
   -   
   -   
   -   
   -   
   -   
   -   
   -   
   -   
   -   
   -   
   -   
   -   
   -   
   -   
   -   
   -   
   -   
   -   
   -   
   -   
   -   
   -   
   -   
   -   
   -   
   -   
   -   
   -   
   -   
   -   
   -   
   -   
   -   
   -   
   -   
   -   
   -   
   -   
   -   
   -   
   -   
   -   
   -   
   -   
   -   
   -   
   -   
   -   
   -   
   -   
   -   
   -   
   -   
   -   
   -   
   -   
   -   
   -   
   -   
   -   
   -   
   -   
   -   
   -   
   -   
   -   
   -   
   -   
   -   
   -   
   -   
   -   
   -   
   -   
   -   
   -   
   -   
   -   
   -   
   -   
   -   
   -   
   -   
   -   
   C   
   A   
   A   
   T   
   G   
   G   
   C   
   A   
   A   
   G   
   C   
   C   
   A   
   A   
   A   
   C   
   A   
   A   
   G   
   A   
   A   
   T   
   T   
   A   
   G   
   C   
   C   
   G   
   A   
   A   
   G   
   C   
   G   
   A   
   A   
   G   
   A   
   A   
   A   
   A   
   C   
   A   
   G   
   C   
   T   
   A   
   C   
   T   
   A   
   C   
   A   
   T   
   T   
   T   
   T   
   T   
   A   
   A   
   A   
   C   
   G   
   T   
   A   
   T   
   T   
   G   
   T   
   C   
   A   
   A   
   A   
   A   
   C   
   A   
   G   
   G   
   A   
   A   
   T   
   T   
   T   
   G   
   A   
   T   
   A   
   A   
   G   
   T   
   T   
   A   
   C   
   C   
   G   
   T   
   C   
   C   
   G   
   T   
   T   
   G   
   T   
   T   
   C   
   A   
   A   
   G   
   A   
   A   
   G   
   C   
   T   
   A   
   G   
   C   
   T   
   T   
   A   
   A   
   A   
   G   
   A   
   A   
   A   
   A   
   A   
   T   
   G   
   G   
   C   
   T   
   A   
   T   
   G   
   A   
   T   
   A   
   C   
   T   
   A   
   A   
   A   
   T   
   C   
   T   
   G   
   T   
   T   
   G   
   T   
   T   
   G   
   A   
   A   
   A   
   A   
   A   
   T   
   A   
   C   
   C   
   A   
   A   
   G   
   C   
   A   
   A   
   T   
   T   
   T   
   A   
   T   
   T   
   C   
   A   
   G   
   G   
   G   
   A   
   T   
   T   
   C   
   A   
   A   
   G   
   C   
   T   
   G   
   A   
   A   
   G   
   G   
   A   
   G   
   T   
   C   
   A   
   A   
   A   
   G   
   C   
   T   
   A   
   G   
   T   
   G   
   A   
   T   
   G   
   T   
   T   
   C   
   A   
   A   
   G   
   T   
   C   
   A   
   A   
   A   
   A   
   A   
   A   
   G   
   C   
   G   
   A   
   A   
   A   
   G   
   A   
   C   
   A   
   A   
   T   
   C   
   A   
   A   
   T   
   A   
   C   
   A   
   C   
   A   
   T   
   T   
   T   
   A   
   C   
   C   
   T   
   A   
   T   
   A   
   A   
   A   
   T   
   T   
   A   
   T   
   C   
   G   
   A   
   T   
   G   
   A   
   G   
   C   
   A   
   C   
   G   
   C   
   C   
   T   
   T   
   T   
   A   
   G   
   G   
   C   
   G   
   A   
   A   
   A   
   T   
   G   
   A   
   A   
   A   
   G   
   A   
   T   
   T   
   T   
   G   
   T   
   C   
   T   
   T   
   A   
   T   
   C   
   A   
   A   
   T   
   C   
   A   
   A   
   G   
   T   
   A   
   T   
   C   
   G   
   A   
   C   
   A   
   A   
   A   
   A   
   A   
   A   
   G   
   G   
   C   
   G   
   A   
   T   
   A   
   C   
   C   
   T   
   A   
   C   
   C   
   A   
   A   
   A   
   T   
   C   
   G   
   C   
   T   
   T   
   G   
   G   
   A   
   A   
   G   
   C   
   C   
   A   
   T   
   C   
   T   
   T   
   T   
   A   
   A   
   T   
   T   
   T   
   T   
   T   
   C   
   C   
   A   
   G   
   A   
   T   
   A   
   T   
   G   
   T   
   C   
   A   
   G   
   G   
   A   
   A   
   A   
   T   
   G   
   A   
   T   
   A   
   A   
   A   
   A   
   T   
   T   
   T   
   C   
   G   
   A   
   T   
   T   
   C   
   A   
   A   
   G   
   T   
   A   
   G   
   A   
   T   
   A   
   A   
   T   
   G   
   C   
   C   
   A   
   A   
   A   
   C   
   G   
   T   
   G   
   G   
   A   
   G   
   A   
   A   
   A   
   T   
   T   
   G   
   T   
   C   
   G   
   A   
   T   
   C   
   G   
   T   
   A   
   A   
   T   
   G   
   G   
   T   
   A   
   G   
   T   
   G   
   G   
   G   
   C   
   T   
   A   
   G   
   C   
   A   
   A   
   T   
   T   
   A   
   A   
   C   
   A   
   A   
   A   
   G   
   T   
   G   
   T   
   T   
   T   
   G   
   A   
   C   
   G   
   A   
   A   
   G   
   T   
   G   
   G   
   G   
   C   
   G   
   T   
   A   
   G   
   T   
   G   
   C   
   C   
   T   
   A   
   G   
   C   
   A   
   A   
   A   
   C   
   T   
   C   
   G   
   G   
   T   
   T   
   C   
   T   
   G   
   G   
   C   
   G   
   C   
   A   
   G   
   A   
   A   
   A   
   A   
   A   
   G   
   C   
   A   
   G   
   C   
   C   
   A   
   A   
   T   
   A   
   T   
   C   
   A   
   A   
   A   
   G   
   C   
   T   
   T   
   T   
   T   
   A   
   G   
   T   
   G   
   A   
   T   
   A   
   A   
   G   
   T   
   T   
   C   
   G   
   G   
   C   
   G   
   T   
   T   
   T   
   C   
   T   
   G   
   T   
   T   
   G   
   A   
   T   
   G   
   A   
   A   
   A   
   T   
   C   
   A   
   A   
   T   
   C   
   A   
   A   
   A   
   A   
   G   
   T   
   T   
   A   
   A   
   G   
   C   
   C   
   A   
   A   
   G   
   G   
   A   
   T   
   G   
   G   
   G   
   T   
   C   
   C   
   A   
   A   
   G   
   C   
   A   
   G   
   A   
   C   
   T   
   C   
   C   
   T   
   T   
   T   
   G   
   T   
   A   
   C   
   C   
   A   
   A   
   T   
   C   
   A   
   C   
   A   
   G   
   T   
   T   
   G   
   C   
   T   
   T   
   C   
   T   
   G   
   A   
   A   
   C   
   C   
   A   
   G   
   T   
   G   
   A   
   C   
   A   
   G   
   A   
   A   
   T   
   T   
   A   
   C   
   C   
   A   
   A   
   C   
   A   
   G   
   G   
   G   
   G   
   C   
   T   
   G   
   C   
   G   
   A   
   C   
   A   
   A   
   A   
   A   
   G   
   A   
   C   
   A   
   C   
   A   
   G   
   A   
   G   
   T   
   C   
   A   
   C   
   G   
   T   
   T   
   A   
   T   
   T   
   A   
   T   
   C   
   C   
   G   
   T   
   T   
   G   
   G   
   G   
   G   
   G   
   A   
   A   
   G   
   C   
   A   
   G   
   C   
   T   
   G   
   C   
   G   
   C   
   A   
   A   
   T   
   T   
   A   
   A   
   T   
   C   
   G   
   G   
   G   
   T   
   A   
   T   
   A   
   C   
   G   
   G   
   G   
   C   
   A   
   C   
   C   
   A   
   T   
   T   
   A   
   C   
   T   
   G   
   C   
   A   
   G   
   A   
   A   
   G   
   A   
   T   
   A   
   T   
   T   
   A   
   A   
   A   
   A   
   A   
   A   
   A   
   A   
   T   
   C   
   C   
   A   
   G   
   A   
   G   
   T   
   T   
   A   
   A   
   G   
   T   
   A   
   G   
   T   
   A   
   C   
   G   
   G   
   G   
   C   
   G   
   T   
   A   
   A   
   T   
   T   
   G   
   G   
   T   
   A   
   A   
   A   
   A   
   C   
   T   
   G   
   G   
   C   
   T   
   T   
   A   
   G   
   A   
   A   
   C   
   G   
   A   
   G   
   C   
   G   
   T   
   T   
   T   
   G   
   A   
   T   
   A   
   A   
   A   
   G   
   A   
   A   
   T   
   T   
   A   
   A   
   G   
   A   
   G   
   G   
   G   
   C   
   A   
   A   
   G   
   A   
   T   
   G   
   G   
   T   
   G   
   G   
   C   
   T   
   C   
   A   
   C   
   T   
   A   
   G   
   T   
   T   
   A   
   T   
   T   
   T   
   T   
   A   
   G   
   A   
   T   
   G   
   A   
   C   
   A   
   A   
   A   
   G   
   A   
   G   
   A   
   A   
   T   
   G   
   T   
   C   
   A   
   A   
   A   
   A   
   A   
   A   
   G   
   C   
   T   
   T   
   T   
   A   
   C   
   A   
   A   
   A   
   C   
   C   
   A   
   A   
   A   
   G   
   A   
   G   
   A   
   A   
   A   
   A   
   A   
   A   
   G   
   A   
   T   
   G   
   G   
   T   
   C   
   A   
   A   
   A   
   C   
   G   
   A   
   T   
   T   
   A   
   A   
   A   
   T   
   T   
   A   
   A   
   C   
   G   
   A   
   T   
   T   
   G   
   A   
   C   
   A   
   G   
   C   
   G   
   G   
   G   
   G   
   T   
   A   
   C   
   A   
   A   
   C   
   A   
   A   
   C   
   A   
   A   
   G   
   C   
   T   
   T   
   T   
   T   
   G   
   C   
   T   
   A   
   T   
   A   
   T   
   T   
   T   
   G   
   A   
   C   
   A   
   A   
   A   
   C   
   G   
   T   
   C   
   C   
   T   
   G   
   G   
   T   
   A   
   G   
   T   
   G   
   C   
   G   
   G   
   T   
   C   
   A   
   T   
   T   
   A   
   C   
   C   
   G   
   A   
   T   
   C   
   C   
   T   
   C   
   A   
   A   
   A   
   A   
   A   
   G   
   G   
   C   
   G   
   A   
   T   
   T   
   T   
   A   
   T   
   T   
   A   
   G   
   C   
   A   
   A   
   C   
   G   
   G   
   T   
   T   
   A   
   G   
   T   
   T   
   C   
   A   
   C   
   C   
   T   
   T   
   C   
   G   
   T   
   A   
   T   
   G   
   A   
   T   
   C   
   C   
   T   
   A   
   A   
   T   
   A   
   A   
   A   
   A   
   T   
   G   
   G   
   C   
   C   
   A   
   A   
   T   
   G   
   G   
   C   
   A   
   T   
   T   
   T   
   C   
   T   
   C   
   A   
   A   
   A   
   A   
   A   
   G   
   A   
   A   
   T   
   A   
   T   
   G   
   A   
   T   
   G   
   C   
   T   
   T   
   A   
   C   
   A   
   A   
   T   
   A   
   A   
   C   
   A   
   A   
   C   
   A   
   A   
   A   
   G   
   A   
   T   
   T   
   T   
   A   
   C   
   C   
   A   
   T   
   T   
   C   
   A   
   C   
   A   
   G   
   C   
   A   
   C   
   G   
   T   
   T   
   T   
   C   
   G   
   C   
   G   
   A   
   C   
   A   
   G   
   G   
   C   
   T   
   A   
   T   
   G   
   C   
   A   
   C   
   C   
   A   
   G   
   G   
   C   
   T   
   C   
   T   
   A   
   C   
   C   
   T   
   T   
   T   
   A   
   A   
   A   
   A   
   C   
   A   
   A   
   T   
   T   
   A   
   C   
   C   
   G   
   G   
   T   
   G   
   C   
   C   
   A   
   T   
   T   
   G   
   G   
   T   
   T   
   T   
   A   
   G   
   A   
   T   
   G   
   C   
   A   
   G   
   G   
   G   
   A   
   C   
   A   
   T   
   T   
   G   
   A   
   A   
   G   
   C   
   C   
   A   
   G   
   A   
   T   
   G   
   A   
   A   
   G   
   A   
   G   
   C   
   T   
   A   
   G   
   A   
   A   
   A   
   T   
   T   
   A   
   A   
   T   
   G   
   G   
   C   
   T   
   T   
   G   
   A   
   A   
   A   
   T   
   G   
   G   
   C   
   A   
   A   
   A   
   A   
   A   
   G   
   A   
   T   
   A   
   A   
   A   
   T   
   C   
   T   
   T   
   G   
   G   
   G   
   G   
   C   
   G   
   G   
   C   
   T   
   A   
   T   
   T   
   T   
   T   
   G   
   C   
   G   
   A   
   C   
   A   
   C   
   G   
   T   
   G   
   T   
   A   
   A   
   A   
   A   
   G   
   A   
   A   
   G   
   C   
   A   
   A   
   G   
   T   
   C   
   C   
   A   
   G   
   T   
   C   
   A   
   A   
   T   
   C   
   T   
   G   
   C   
   G   
   T   
   A   
   C   
   C   
   G   
   C   
   T   
   T   
   T   
   A   
   G   
   T   
   T   
   A   
   A   
   T   
   T   
   C   
   A   
   G   
   A   
   T   
   A   
   A   
   T   
   A   
   T   
   T   
   T   
   A   
   T   
   T   
   T   
   T   
   G   
   C   
   C   
   C   
   A   
   A   
   C   
   A   
   A   
   A   
   C   
   A   
   T   
   T   
   A   
   C   
   G   
   G   
   A   
   T   
   G   
   G   
   G   
   G   
   G   
   A   
   A   
   G   
   A   
   C   
   A   
   A   
   A   
   T   
   T   
   T   
   A   
   G   
   A   
   G   
   C   
   G   
   G   
   G   
   T   
   C   
   T   
   T   
   A   
   A   
   T   
   A   
   A   
   A   
   T   
   T   
   C   
   A   
   T   
   T   
   T   
   T   
   C   
   G   
   G   
   T   
   G   
   A   
   G   
   G   
   A   
   A   
   C   
   T   
   A   
   G   
   A   
   T   
   T   
   T   
   A   
   C   
   C   
   A   
   A   
   T   
   T   
   G   
   C   
   C   
   A   
   T   
   G   
   A   
   C   
   G   
   C   
   C   
   T   
   G   
   C   
   T   
   C   
   A   
   A   
   A   
   T   
   T   
   T   
   C   
   A   
   A   
   A   
   T   
   G   
   A   
   A   
   G   
   A   
   T   
   A   
   A   
   A   
   T   
   T   
   T   
   A   
   A   
   T   
   T   
   C   
   T   
   G   
   A   
   A   
   A   
   T   
   T   
   C   
   T   
   T   
   T   
   T   
   A   
   G   
   C   
   A   
   G   
   A   
   T   
   A   
   C   
   G   
   G   
   G   
   C   
   T   
   A   
   T   
   G   
   G   
   T   
   C   
   A   
   A   
   G   
   G   
   T   
   C   
   A   
   A   
   T   
   T   
   G   
   T   
   T   
   A   
   A   
   T   
   T   
   T   
   C   
   G   
   C   
   C   
   A   
   A   
   T   
   C   
   C   
   A   
   G   
   C   
   A   
   A   
   G   
   C   
   T   
   A   
   C   
   C   
   A   
   T   
   G   
   T   
   A   
   T   
   A   
   G   
   T   
   G   
   T   
   T   
   T   
   T   
   C   
   C   
   A   
   A   
   A   
   A   
   T   
   A   
   A   
   T   
   G   
   G   
   A   
   A   
   C   
   A   
   T   
   T   
   A   
   G   
   T   
   C   
   T   
   A   
   T   
   C   
   C   
   A   
   A   
   A   
   A   
   C   
   T   
   A   
   G   
   T   
   T   
   T   
   T   
   A   
   G   
   A   
   C   
   A   
   A   
   A   
   G   
   A   
   A   
   A   
   C   
   G   
   A   
   A   
   G   
   A   
   A   
   A   
   A   
   A   
   A   
   G   
   A   
   C   
   A   
   A   
   T   
   G   
   T   
   A   
   A   
   T   
   C   
   A   
   G   
   C   
   G   
   C   
   C   
   A   
   A   
   T   
   G   
   C   
   A   
   G   
   C   
   G   
   A   
   A   
   T   
   A   
   C   
   A   
   A   
   T   
   T   
   G   
   C   
   C   
   A   
   C   
   A   
   G   
   A   
   T   
   C   
   T   
   T   
   T   
   T   
   A   
   G   
   G   
   T   
   A   
   G   
   T   
   G   
   T   
   G   
   G   
   A   
   A   
   G   
   A   
   T   
   C   
   C   
   T   
   T   
   C   
   A   
   G   
   G   
   T   
   T   
   A   
   T   
   G   
   T   
   T   
   T   
   A   
   T   
   A   
   A   
   T   
   A   
   T   
   G   
   T   
   A   
   C   
   A   
   A   
   T   
   C   
   C   
   G   
   A   
   A   
   C   
   T   
   T   
   C   
   T   
   C   
   A   
   T   
   T   
   G   
   G   
   C   
   T   
   G   
   C   
   T   
   A   
   A   
   A   
   A   
   C   
   A   
   G   
   G   
   A   
   A   
   C   
   A   
   G   
   C   
   T   
   G   
   A   
   A   
   A   
   T   
   A   
   A   
   A   
   A   
   G   
   A   
   T   
   A   
   A   
   A   
   C   
   A   
   A   
   G   
   A   
   T   
   A   
   C   
   A   
   G   
   A   
   T   
   G   
   G   
   C   
   A   
   A   
   A   
   G   
   A   
   A   
   A   
   A   
   C   
   A   
   G   
   T   
   T   
   T   
   C   
   C   
   T   
   T   
   C   
   T   
   A   
   A   
   C   
   G   
   T   
   T   
   A   
   G   
   A   
   T   
   C   
   G   
   T   
   A   
   G   
   T   
   A   
   A   
   C   
   A   
   A   
   T   
   A   
   A   
   A   
   T   
   T   
   C   
   T   
   T   
   A   
   A   
   C   
   A   
   A   
   T   
   G   
   A   
   T   
   T   
   A   
   T   
   G   
   G   
   T   
   T   
   G   
   A   
   A   
   A   
   A   
   T   
   T   
   C   
   A   
   G   
   G   
   A   
   G   
   A   
   A   
   A   
   A   
   T   
   G   
   G   
   T   
   T   
   C   
   T   
   G   
   C   
   A   
   A   
   C   
   A   
   G   
   A   
   T   
   A   
   T   
   C   
   A   
   G   
   T   
   A   
   A   
   A   
   C   
   C   
   A   
   T   
   T   
   A   
   A   
   T   
   T   
   G   
   A   
   T   
   T   
   A   
   C   
   T   
   T   
   A   
   G   
   A   
   A   
   G   
   C   
   A   
   A   
   C   
   C   
   A   
   T   
   T   
   A   
   A   
   A   
   -   
   -   
   -   
   -   
   -   
   -   
   -   
   -   
   -   
   -   
   -   
   -   
   -   
   -   
   -   
   -   
   -   
   -   
   -   
   -   
   -   
   -   
   -   
   -   
   -   
   -   
   -   
   -   
   -   
   -   
   -   
   -   
   -   
   -   
   -   
   -   
   -   
   -   
   -   
   -   
   -   
   -   
   -   
   -   
   -   
   -   
   -   
   -   
   -   
   -   
   -   
   -   
   -   
   -   
   -   
   -   
   -   
   -   
   -   
   -   
   -   
 

 
   N59606F   
   -   
   -   
   -   
   -   
   -   
   -   
   -   
   -   
   -   
   -   
   -   
   -   
   -   
   -   
   -   
   -   
   -   
   -   
   -   
   -   
   -   
   -   
   -   
   -   
   -   
   -   
   -   
   -   
   -   
   -   
   -   
   -   
   -   
   -   
   -   
   -   
   -   
   -   
   -   
   -   
   -   
   -   
   -   
   -   
   -   
   -   
   -   
   -   
   -   
   -   
   -   
   -   
   -   
   -   
   -   
   -   
   -   
   -   
   -   
   -   
   -   
   -   
   -   
   -   
   -   
   -   
   -   
   -   
   -   
   -   
   -   
   -   
   -   
   -   
   -   
   -   
   -   
   -   
   -   
   -   
   -   
   -   
   -   
   -   
   -   
   -   
   -   
   -   
   -   
   -   
   -   
   -   
   -   
   -   
   -   
   -   
   -   
   -   
   -   
   -   
   -   
   -   
   -   
   -   
   -   
   -   
   -   
   -   
   C   
   A   
   A   
   T   
   G   
   G   
   C   
   A   
   A   
   G   
   C   
   C   
   A   
   A   
   A   
   C   
   A   
   A   
   G   
   A   
    A    
   T   
   T   
   A   
   G   
   C   
    C    
   G   
   A   
   A   
   G   
   C   
   G   
   A   
   A   
   G   
   A   
   A   
   A   
   A   
    C    
   A   
   G   
   C   
    T    
   A   
    C    
   T   
    A    
    C    
   A   
   T   
   T   
   T   
   T   
   T   
   A   
   A   
   A   
    C    
   G   
   T   
   A   
   T   
   T   
   G   
    A    
   C   
   A   
   A   
   A   
   A   
   C   
   A   
   G   
    A    
   A   
   A   
   T   
   T   
   T   
   G   
   A   
   T   
   A   
   A   
   G   
   T   
   T   
   A   
   C   
   C   
    G    
   T   
   C   
   C   
   G   
   T   
   T   
    G    
   T   
   T   
   C   
   A   
   A   
   G   
   A   
   A   
    G    
   C   
   T   
   A   
   G   
   C   
   T   
   T   
   A   
   A   
   A   
   G   
   A   
   A   
   A   
   A   
   A   
   T   
   G   
   G   
    C    
   T   
   A   
   T   
   G   
   A   
   T   
   A   
   C   
   T   
   A   
   A   
   A   
   T   
   C   
    T    
   G   
   T   
   T   
   G   
   T   
   T   
   G   
   A   
   A   
   A   
   A   
   A   
   T   
   A   
   C   
   C   
   A   
   A   
   G   
   C   
   A   
   A   
   T   
   T   
   T   
   A   
   T   
   T   
   C   
   A   
   G   
   G   
   G   
   A   
   T   
   T   
   C   
   A   
   A   
   G   
   C   
    A    
   G   
   A   
   A   
    G    
   G   
    A    
   G   
   T   
   C   
   A   
   A   
    A    
   G   
   C   
   T   
   A   
   G   
   T   
   G   
   A   
   T   
   G   
   T   
    T    
   C   
   A   
   A   
   G   
   T   
    C    
   A   
   A   
   A   
   A   
   A   
    A    
   G   
    C    
   G   
   A   
   A   
   A   
   G   
   A   
    C    
   A   
   A   
    T    
   C   
   A   
   A   
   T   
   A   
   C   
   A   
   C   
   A   
   T   
   T   
   T   
   A   
    A    
   C   
   T   
   A   
   T   
   A   
   A   
   A   
   T   
   T   
   A   
   T   
   C   
   G   
   A   
   T   
   G   
   A   
   G   
    T    
   A   
   C   
    C    
   C   
   C   
   T   
   T   
   T   
   A   
   G   
   G   
   C   
   G   
   A   
   A   
   A   
   T   
   G   
   A   
   A   
   A   
   G   
   A   
   T   
    T    
   T   
   G   
   T   
   C   
   T   
   T   
   A   
   T   
   C   
   A   
   A   
   T   
   C   
   A   
   A   
   G   
   T   
   A   
   T   
    C    
   G   
    C    
   C   
   A   
   A   
    A    
   A   
   A   
   A   
   G   
   G   
    C    
   G   
   A   
   T   
   A   
    C    
   C   
   T   
   A   
   C   
   C   
   A   
   A   
   A   
   T   
    C    
    G    
   C   
   T   
   T   
   G   
   G   
    A    
    A    
    G    
   C   
   C   
    G    
   T   
   C   
   T   
   T   
   T   
   A   
   A   
   T   
   T   
   T   
   T   
   T   
   C   
   C   
   A   
   G   
   A   
   T   
   A   
   T   
   G   
   T   
   C   
   A   
   G   
   G   
   A   
   A   
   A   
   T   
   G   
   A   
   T   
   A   
   A   
   A   
    A    
   T   
   T   
   T   
   C   
   G   
   A   
   T   
   T   
   C   
   A   
   A   
   G   
   T   
   A   
   G   
   A   
   T   
   A   
   A   
   T   
   G   
   C   
   C   
   A   
   A   
   A   
   C   
   G   
   T   
   G   
   G   
   A   
   G   
   A   
   A   
   A   
   T   
   T   
   G   
   T   
   C   
   G   
   A   
   T   
   C   
   G   
   T   
   A   
   A   
    T    
   G   
   G   
   T   
   A   
   G   
   T   
   G   
   G   
   G   
   C   
   T   
   A   
   G   
    C    
   A   
   A   
   T   
   T   
   A   
   A   
   C   
   A   
   A   
   A   
   G   
   T   
   G   
   T   
   T   
   T   
   G   
   A   
    C    
   G   
   A   
   A   
   G   
   T   
   G   
   G   
   G   
   C   
   G   
   T   
   A   
   G   
   T   
    G    
   C   
   C   
   T   
    G    
   G   
   C   
   A   
   A   
   A   
   C   
   T   
    C    
   G   
   G   
   T   
   T   
   C   
   T   
   G   
   G   
   C   
   G   
   C   
   A   
   G   
   A   
   A   
   A   
   A   
   A   
    A    
   C   
   A   
   G   
   C   
    C    
   A   
   A   
   T   
   A   
   T   
   C   
   A   
   A   
   A   
   G   
   C   
   T   
   T   
   T   
   T   
   A   
   G   
   T   
   G   
   A   
   T   
   A   
   A   
    G    
   T   
   T   
    C    
   G   
   G   
   C   
   G   
   T   
   T   
   T   
   C   
   T   
    G    
   T   
   T   
   G   
   A   
   T   
   G   
   A   
   A   
   A   
   T   
   C   
   A   
   A   
   T   
   C   
    A    
   A   
   A   
   A   
    G    
   T   
   T   
   A   
   A   
   G   
   C   
   C   
   A   
   A   
   G   
   G   
    G    
   T   
   G   
   G   
   G   
   T   
   C   
   C   
   A   
   A   
   G   
   C   
   A   
   G   
   A   
   C   
   T   
   C   
    C    
   T   
   T   
   T   
   G   
   T   
   A   
   C   
   C   
   A   
   A   
   T   
   C   
   A   
   C   
   A   
   G   
   T   
    C    
   G   
   C   
   T   
   T   
   C   
   T   
   G   
   A   
   A   
   C   
   C   
   A   
   G   
   T   
   G   
   A   
   C   
   A   
   G   
   A   
   A   
   T   
   T   
   A   
   C   
   C   
   A   
   A   
   C   
   A   
   G   
   G   
   G   
   G   
   C   
   T   
   G   
   C   
   G   
   A   
   C   
   A   
   A   
   A   
   A   
   G   
    A    
    T    
   A   
   C   
   A   
   G   
   A   
   G   
   T   
   C   
   A   
   C   
   G   
   T   
   T   
   A   
   T   
   T   
   A   
   T   
   C   
   C   
    G    
    C    
   T   
   G   
   G   
   G   
   G   
   G   
   A   
   A   
   G   
   C   
   A   
   G   
   C   
   T   
   G   
   C   
   G   
   C   
   A   
   A   
   T   
   T   
   A   
   A   
   T   
   C   
   G   
   G   
    G    
   T   
   A   
   T   
   A   
   C   
    A    
   G   
   G   
    C    
   A   
   C   
    C    
   A   
   T   
   T   
   A   
   C   
   T   
   G   
   C   
   A   
   G   
   A   
    A    
   G   
   A   
   T   
   A   
   T   
   T   
    A    
   A   
   A   
   A   
   A   
   A   
   A   
   A   
   T   
   C   
   C   
   A   
   G   
   A   
   G   
   T   
   T   
   A   
   A   
   G   
   T   
   A   
   G   
   T   
   A   
   C   
   G   
   G   
   G   
    C    
   G   
   T   
   A   
   A   
   T   
   T   
   G   
   G   
    T    
   A   
   A   
   A   
   A   
   C   
   T   
   G   
   G   
    C    
   T   
   T   
   A   
   G   
   A   
   A   
   C   
   G   
   A   
   G   
   C   
   G   
   T   
   T   
   T   
   G   
   A   
   T   
   A   
   A   
   A   
   G   
   A   
    A    
   T   
   T   
   A   
   A   
   G   
   A   
   G   
   G   
    G    
   C   
   A   
   A   
   G   
   A   
   T   
   G   
   G   
    T    
   G   
   G   
    C    
   T   
   C   
    A    
   C   
   T   
   A   
   G   
   T   
   T   
   A   
   T   
   T   
   T   
   T   
   A   
   G   
   A   
   T   
   G   
   A   
   C   
   A   
   A   
   A   
   G   
   A   
   G   
   A   
   A   
   T   
   G   
   T   
   C   
   A   
   A   
   A   
   A   
   A   
   A   
   G   
   C   
   T   
   T   
   T   
   A   
   C   
   A   
   A   
   A   
   C   
   C   
   A   
   A   
   A   
   G   
   A   
    G    
   A   
   A   
   A   
   A   
   A   
   A   
   G   
   A   
   T   
   G   
   G   
   T   
   C   
   A   
   A   
   A   
   C   
    G    
   A   
   T   
   T   
   A   
   A   
   A   
   T   
   T   
   A   
   A   
   C   
   G   
   A   
   T   
   T   
   G   
   A   
    C    
   A   
   G   
    T    
   G   
   G   
    C    
   G   
   T   
   A   
   C   
   A   
   A   
   C   
   A   
   A   
   C   
   A   
   A   
   G   
   C   
    C    
   T   
   T   
    C    
   G   
   C   
   T   
   A   
   T   
   A   
   T   
   T   
   T   
   G   
   A   
   C   
   A   
   A   
   A   
   C   
   G   
    C    
    C    
   C   
   T   
   G   
   G   
   T   
   A   
   G   
   T   
   G   
   C   
   G   
   G   
   T   
   C   
   A   
   T   
   T   
   A   
   C   
   C   
   G   
   A   
   T   
   C   
   C   
   T   
   C   
   A   
   A   
   A   
   A   
   A   
   G   
   G   
   C   
   G   
   A   
   T   
   T   
   T   
   A   
    T    
   T   
   A   
   G   
   C   
    A    
   A   
   C   
   G   
   G   
   T   
   T   
   A   
   G   
   T   
   T   
   C   
   A   
   C   
   C   
   T   
   T   
   C   
   G   
   T   
   A   
   T   
   G   
   A   
   T   
   C   
   C   
   T   
   A   
   A   
   T   
   A   
   A   
   A   
   A   
   T   
   G   
   G   
   C   
   C   
   A   
   A   
   T   
   G   
   G   
    T    
   A   
   T   
   T   
   T   
   C   
   T   
   C   
   A   
   A   
   A   
   A   
   A   
   G   
   A   
    A    
   T   
   A   
   T   
   G   
   A   
   T   
   G   
   C   
   T   
   T   
   A   
   C   
   A   
   A   
   T   
   A   
   A   
   C   
   A   
   A   
   C   
   A   
   A   
   A   
   G   
   A   
   T   
   T   
   T   
   A   
   C   
   C   
   A   
   T   
   T   
    C    
   A   
   C   
   A   
   G   
   C   
   A   
   C   
   G   
   T   
   T   
   T   
    T    
   G   
   C   
   G   
   A   
   C   
   A   
   G   
   G   
    C    
   T   
   A   
   T   
   G   
   C   
   A   
   C   
   C   
   A   
   G   
   G   
    T    
   T   
   C   
   T   
   A   
   C   
   C   
   T   
   T   
   T   
   A   
   A   
   A   
   A   
   C   
   A   
   A   
   T   
   T   
   A   
   C   
   C   
   G   
   G   
   T   
   G   
   C   
   C   
   A   
   T   
   T   
   G   
   G   
   T   
   T   
   T   
   A   
   G   
   A   
   T   
    G    
   C   
   A   
   G   
   G   
   G   
   A   
   C   
   A   
   T   
   T   
   G   
   A   
   A   
   G   
   C   
   C   
   A   
   G   
   A   
   T   
   G   
   A   
   A   
   G   
   A   
   G   
   C   
   T   
   A   
   G   
   A   
   A   
   A   
   T   
   T   
   A   
   A   
   T   
   G   
   G   
    C    
   T   
   T   
   G   
   A   
   A   
   A   
   T   
   G   
   G   
   C   
   A   
   A   
   A   
   A   
   A   
   G   
   A   
   T   
   A   
   A   
   A   
   T   
   C   
   T   
   T   
   G   
   G   
   G   
   G   
    C    
   G   
   G   
   C   
   T   
   A   
   T   
   T   
   T   
   T   
   G   
   C   
    A    
   A   
   C   
   A   
   C   
   G   
   T   
   G   
   T   
   A   
   A   
   A   
   A   
   G   
   A   
   A   
   G   
   C   
   A   
   A   
   G   
   T   
   C   
   C   
   A   
   G   
   T   
   C   
   A   
   A   
   T   
   C   
   T   
    G    
   C   
   G   
   T   
   A   
   C   
   C   
   G   
   C   
   T   
   T   
   T   
   A   
   G   
   T   
   T   
   A   
   A   
   T   
   T   
   C   
   A   
   G   
   A   
   T   
   A   
   A   
   T   
   A   
   T   
   T   
   T   
   A   
   T   
   T   
   T   
   T   
   G   
   C   
    C    
   C   
   A   
   A   
   C   
   A   
   A   
   A   
   C   
   A   
   T   
   T   
   A   
   C   
   G   
    G    
   A   
   T   
   G   
   G   
   G   
    A    
   G   
   A   
   A   
   G   
   A   
   C   
   A   
   A   
   A   
   T   
   T   
   T   
   A   
   G   
   A   
   G   
   C   
    G    
   G   
   G   
   T   
   C   
   T   
   T   
   A   
   A   
   T   
   A   
   A   
   A   
   T   
   T   
   C   
   A   
   T   
   T   
   T   
   T   
   C   
   G   
   G   
   T   
   G   
   A   
   G   
   G   
   A   
   A   
   C   
   T   
   A   
   G   
   A   
   T   
   T   
   T   
   A   
   C   
   C   
   A   
   A   
   T   
    T    
   G   
   C   
   C   
   A   
   T   
   G   
   A   
   C   
   G   
   C   
   C   
    T    
   G   
   C   
   T   
   C   
   A   
   A   
   A   
   T   
   T   
   T   
   C   
   A   
   A   
   A   
    T    
   G   
   A   
   A   
   G   
   A   
   T   
   A   
   A   
   A   
   T   
   T   
   T   
   A   
   A   
   T   
   T   
   C   
   T   
   G   
   A   
   A   
   A   
   T   
   T   
   C   
   T   
   T   
   T   
   T   
    G    
   G   
   C   
   A   
   G   
   A   
   T   
   A   
   C   
    G    
   G   
   G   
   C   
   T   
   A   
    C    
   G   
   G   
    C    
   C   
   A   
   A   
   G   
   G   
    C    
   C   
   A   
   A   
   T   
   T   
   G   
   T   
   T   
   A   
   A   
   T   
   T   
   T   
   C   
    G    
   C   
   C   
   A   
   A   
   T   
   C   
   C   
   A   
   G   
   C   
   A   
   A   
   G   
   C   
   T   
   A   
   C   
   C   
   A   
   T   
   G   
   T   
   A   
   T   
   A   
   G   
   T   
   G   
   T   
   T   
   T   
   T   
   C   
   C   
   A   
   A   
   A   
   A   
   T   
   A   
   A   
   T   
   G   
   G   
    G    
   A   
   C   
   A   
    T    
   T   
    A    
   G   
   T   
    C    
   T   
   A   
   T   
   C   
   C   
   A   
   A   
   A   
   A   
    T    
   T   
   A   
   G   
   T   
   T   
   T   
   T   
   A   
   G   
   A   
   C   
   A   
   A   
   A   
   G   
   A   
   A   
   A   
   C   
    G    
   A   
   A   
   G   
   A   
   A   
   A   
   A   
   A   
   A   
   G   
   A   
   C   
   A   
   A   
   T   
   G   
   T   
   A   
   A   
   T   
   C   
   A   
   G   
   C   
   G   
   C   
   C   
   A   
   A   
   C   
   G   
   C   
   A   
   G   
   C   
   G   
   A   
   A   
   T   
   A   
   C   
   A   
   A   
   T   
   T   
   G   
   C   
   C   
   A   
   C   
   A   
   G   
   A   
   T   
   C   
   T   
   T   
   T   
   T   
   A   
   G   
   G   
   T   
   A   
   G   
   T   
   G   
   T   
   G   
   G   
   A   
   A   
   G   
   A   
   T   
   C   
   C   
   T   
   T   
   C   
   A   
   G   
   G   
   T   
   T   
   A   
   T   
   G   
   T   
   T   
   T   
   A   
   T   
   A   
   A   
   T   
   A   
   T   
   G   
   T   
   A   
   C   
   A   
   A   
   T   
   C   
   C   
   G   
   A   
   A   
   C   
   T   
   T   
   C   
   T   
   C   
   A   
   T   
   T   
   G   
   G   
   C   
   T   
   G   
   C   
   C   
   A   
   A   
   A   
   A   
   C   
   A   
   G   
   G   
   A   
   A   
   C   
   A   
   G   
   C   
   T   
   G   
   A   
   A   
   A   
   T   
   A   
   A   
   A   
   A   
   G   
   A   
   T   
   A   
   A   
   A   
   C   
   A   
   A   
   G   
   A   
   T   
   A   
   C   
   A   
   G   
   A   
   T   
   G   
   G   
   C   
   A   
   A   
   A   
   G   
   A   
   A   
   A   
   A   
   C   
   A   
   G   
   T   
   T   
   T   
   C   
   C   
   T   
   T   
   C   
   T   
   A   
   A   
   C   
   G   
   T   
   T   
   A   
   G   
   A   
   T   
   C   
   G   
   T   
   A   
   G   
   T   
   A   
   A   
   C   
   A   
   A   
   T   
   A   
   A   
   A   
   T   
   T   
   C   
   T   
   T   
   A   
   A   
   C   
   A   
   A   
   T   
   G   
   A   
   T   
   T   
   A   
   T   
   G   
   G   
   T   
   T   
   G   
   A   
   A   
   A   
   A   
   T   
   T   
   C   
   A   
   G   
   G   
   A   
   G   
   A   
   A   
   A   
   A   
   T   
   G   
   G   
   T   
   T   
   C   
   T   
   G   
   C   
   A   
   A   
   C   
   A   
   G   
   A   
   T   
   A   
   T   
   C   
   A   
   G   
   T   
   A   
   A   
   A   
   C   
   C   
   A   
   T   
   T   
   A   
   A   
   T   
   T   
   G   
   A   
   T   
   T   
   A   
   C   
   T   
   T   
   A   
   G   
   A   
   A   
   G   
   C   
   A   
   A   
   C   
   C   
   A   
   T   
   T   
   A   
   A   
   A   
   -   
   -   
   -   
   -   
   -   
   -   
   -   
   -   
   -   
   -   
   -   
   -   
   -   
   -   
   -   
   -   
   -   
   -   
   -   
   -   
   -   
   -   
   -   
   -   
   -   
   -   
   -   
   -   
   -   
   -   
   -   
   -   
   -   
   -   
   -   
   -   
   -   
   -   
   -   
   -   
   -   
   -   
   -   
   -   
   -   
   -   
   -   
   -   
   -   
   -   
   -   
   -   
   -   
   -   
   -   
   -   
   -   
   -   
   -   
   -   
   -   
   -   
   -   
   -   
   -   
   -   
   -   
   -   
   -   
   -   
   -   
   -   
   -   
   -   
   -   
   -   
   -   
   -   
   -   
   -   
   -   
   -   
   -   
   -   
   -   
   -   
   -   
   -   
   -   
   -   
   -   
   -   
   -   
   -   
   -   
   -   
   -   
   -   
   -   
   -   
   -   
   -   
   -   
   -   
   -   
   -   
   -   
   -   
   -   
   -   
   -   
   -   
   -   
   -   
   -   
   -   
   -   
   -   
   -   
   -   
   -   
   -   
   -   
   -   
   -   
   -   
   -   
   -   
   -   
   -   
   -   
   -   
   -   
   -   
   -   
   -   
   -   
   -   
   -   
   -   
   -   
   -   
   -   
   -   
   -   
   -   
   -   
   -   
   -   
   -   
   -   
   -   
   -   
   -   
   -   
   -   
   -   
   -   
   -   
   -   
   -   
   -   
   -   
   -   
   -   
   -   
   -   
   -   
   -   
   C   
   A   
   A   
   T   
   G   
   G   
   C   
   A   
   A   
   G   
   C   
   C   
   A   
   A   
   A   
   C   
   A   
   A   
   G   
   A   
   A   
   T   
   T   
   A   
   G   
   C   
   C   
   G   
   A   
   A   
   G   
   C   
   G   
   A   
   A   
   G   
   A   
   A   
   A   
   A   
   C   
   A   
   G   
   C   
   T   
   A   
   C   
   T   
   A   
   C   
   A   
   T   
   T   
   T   
   T   
   T   
   A   
   A   
   A   
   C   
   G   
   T   
   A   
   T   
   T   
   G   
   A   
   C   
   A   
   A   
   A   
   A   
   C   
   A   
   G   
   A   
   A   
   A   
   T   
   T   
   T   
   G   
   A   
   T   
   A   
   A   
   G   
   T   
   T   
   A   
   C   
   C   
   G   
   T   
   C   
   C   
   G   
   T   
   T   
   G   
   T   
   T   
   C   
   A   
   A   
   G   
   A   
   A   
   G   
   C   
   T   
   A   
   G   
   C   
   T   
   T   
   A   
   A   
   A   
   G   
   A   
   A   
   A   
   A   
   A   
   T   
   G   
   G   
   C   
   T   
   A   
   T   
   G   
   A   
   T   
   A   
   C   
   T   
   A   
   A   
   A   
   T   
   C   
   T   
   G   
   T   
   T   
   G   
   T   
   T   
   G   
   A   
   A   
   A   
   A   
   A   
   T   
   A   
   C   
   C   
   A   
   A   
   G   
   C   
   A   
   A   
   T   
   T   
   T   
   A   
   T   
   T   
   C   
   A   
   G   
   G   
   G   
   A   
   T   
   T   
   C   
   A   
   A   
   G   
   C   
   A   
   G   
   A   
   A   
   G   
   G   
   A   
   G   
   T   
   C   
   A   
   A   
   A   
   G   
   C   
   T   
   A   
   G   
   T   
   G   
   A   
   T   
   G   
   T   
   T   
   C   
   A   
   A   
   G   
   T   
   C   
   A   
   A   
   A   
   A   
   A   
   A   
   G   
   C   
   G   
   A   
   A   
   A   
   G   
   A   
   C   
   A   
   A   
   T   
   C   
   A   
   A   
   T   
   A   
   C   
   A   
   C   
   A   
   T   
   T   
   T   
   A   
   A   
   C   
   T   
   A   
   T   
   A   
   A   
   A   
   T   
   T   
   A   
   T   
   C   
   G   
   A   
   T   
   G   
   A   
   G   
   T   
   A   
   C   
   C   
   C   
   C   
   T   
   T   
   T   
   A   
   G   
   G   
   C   
   G   
   A   
   A   
   A   
   T   
   G   
   A   
   A   
   A   
   G   
   A   
   T   
   T   
   T   
   G   
   T   
   C   
   T   
   T   
   A   
   T   
   C   
   A   
   A   
   T   
   C   
   A   
   A   
   G   
   T   
   A   
   T   
   C   
   G   
   C   
   C   
   A   
   A   
   A   
   A   
   A   
   A   
   G   
   G   
   C   
   G   
   A   
   T   
   A   
   C   
   C   
   T   
   A   
   C   
   C   
   A   
   A   
   A   
   T   
   C   
   G   
   C   
   T   
   T   
   G   
   G   
   A   
   A   
   G   
   C   
   C   
   G   
   T   
   C   
   T   
   T   
   T   
   A   
   A   
   T   
   T   
   T   
   T   
   T   
   C   
   C   
   A   
   G   
   A   
   T   
   A   
   T   
   G   
   T   
   C   
   A   
   G   
   G   
   A   
   A   
   A   
   T   
   G   
   A   
   T   
   A   
   A   
   A   
   A   
   T   
   T   
   T   
   C   
   G   
   A   
   T   
   T   
   C   
   A   
   A   
   G   
   T   
   A   
   G   
   A   
   T   
   A   
   A   
   T   
   G   
   C   
   C   
   A   
   A   
   A   
   C   
   G   
   T   
   G   
   G   
   A   
   G   
   A   
   A   
   A   
   T   
   T   
   G   
   T   
   C   
   G   
   A   
   T   
   C   
   G   
   T   
   A   
   A   
   T   
   G   
   G   
   T   
   A   
   G   
   T   
   G   
   G   
   G   
   C   
   T   
   A   
   G   
   C   
   A   
   A   
   T   
   T   
   A   
   A   
   C   
   A   
   A   
   A   
   G   
   T   
   G   
   T   
   T   
   T   
   G   
   A   
   C   
   G   
   A   
   A   
   G   
   T   
   G   
   G   
   G   
   C   
   G   
   T   
   A   
   G   
   T   
   G   
   C   
   C   
   T   
   G   
   G   
   C   
   A   
   A   
   A   
   C   
   T   
   C   
   G   
   G   
   T   
   T   
   C   
   T   
   G   
   G   
   C   
   G   
   C   
   A   
   G   
   A   
   A   
   A   
   A   
   A   
   A   
   C   
   A   
   G   
   C   
   C   
   A   
   A   
   T   
   A   
   T   
   C   
   A   
   A   
   A   
   G   
   C   
   T   
   T   
   T   
   T   
   A   
   G   
   T   
   G   
   A   
   T   
   A   
   A   
   G   
   T   
   T   
   C   
   G   
   G   
   C   
   G   
   T   
   T   
   T   
   C   
   T   
   G   
   T   
   T   
   G   
   A   
   T   
   G   
   A   
   A   
   A   
   T   
   C   
   A   
   A   
   T   
   C   
   A   
   A   
   A   
   A   
   G   
   T   
   T   
   A   
   A   
   G   
   C   
   C   
   A   
   A   
   G   
   G   
   G   
   T   
   G   
   G   
   G   
   T   
   C   
   C   
   A   
   A   
   G   
   C   
   A   
   G   
   A   
   C   
   T   
   C   
   C   
   T   
   T   
   T   
   G   
   T   
   A   
   C   
   C   
   A   
   A   
   T   
   C   
   A   
   C   
   A   
   G   
   T   
   C   
   G   
   C   
   T   
   T   
   C   
   T   
   G   
   A   
   A   
   C   
   C   
   A   
   G   
   T   
   G   
   A   
   C   
   A   
   G   
   A   
   A   
   T   
   T   
   A   
   C   
   C   
   A   
   A   
   C   
   A   
   G   
   G   
   G   
   G   
   C   
   T   
   G   
   C   
   G   
   A   
   C   
   A   
   A   
   A   
   A   
   G   
   A   
   T   
   A   
   C   
   A   
   G   
   A   
   G   
   T   
   C   
   A   
   C   
   G   
   T   
   T   
   A   
   T   
   T   
   A   
   T   
   C   
   C   
   G   
   C   
   T   
   G   
   G   
   G   
   G   
   G   
   A   
   A   
   G   
   C   
   A   
   G   
   C   
   T   
   G   
   C   
   G   
   C   
   A   
   A   
   T   
   T   
   A   
   A   
   T   
   C   
   G   
   G   
   G   
   T   
   A   
   T   
   A   
   C   
   A   
   G   
   G   
   C   
   A   
   C   
   C   
   A   
   T   
   T   
   A   
   C   
   T   
   G   
   C   
   A   
   G   
   A   
   A   
   G   
   A   
   T   
   A   
   T   
   T   
   A   
   A   
   A   
   A   
   A   
   A   
   A   
   A   
   T   
   C   
   C   
   A   
   G   
   A   
   G   
   T   
   T   
   A   
   A   
   G   
   T   
   A   
   G   
   T   
   A   
   C   
   G   
   G   
   G   
   C   
   G   
   T   
   A   
   A   
   T   
   T   
   G   
   G   
   T   
   A   
   A   
   A   
   A   
   C   
   T   
   G   
   G   
   C   
   T   
   T   
   A   
   G   
   A   
   A   
   C   
   G   
   A   
   G   
   C   
   G   
   T   
   T   
   T   
   G   
   A   
   T   
   A   
   A   
   A   
   G   
   A   
   A   
   T   
   T   
   A   
   A   
   G   
   A   
   G   
   G   
   G   
   C   
   A   
   A   
   G   
   A   
   T   
   G   
   G   
   T   
   G   
   G   
   C   
   T   
   C   
   A   
   C   
   T   
   A   
   G   
   T   
   T   
   A   
   T   
   T   
   T   
   T   
   A   
   G   
   A   
   T   
   G   
   A   
   C   
   A   
   A   
   A   
   G   
   A   
   G   
   A   
   A   
   T   
   G   
   T   
   C   
   A   
   A   
   A   
   A   
   A   
   A   
   G   
   C   
   T   
   T   
   T   
   A   
   C   
   A   
   A   
   A   
   C   
   C   
   A   
   A   
   A   
   G   
   A   
   G   
   A   
   A   
   A   
   A   
   A   
   A   
   G   
   A   
   T   
   G   
   G   
   T   
   C   
   A   
   A   
   A   
   C   
   G   
   A   
   T   
   T   
   A   
   A   
   A   
   T   
   T   
   A   
   A   
   C   
   G   
   A   
   T   
   T   
   G   
   A   
   C   
   A   
   G   
   T   
   G   
   G   
   C   
   G   
   T   
   A   
   C   
   A   
   A   
   C   
   A   
   A   
   C   
   A   
   A   
   G   
   C   
   C   
   T   
   T   
   C   
   G   
   C   
   T   
   A   
   T   
   A   
   T   
   T   
   T   
   G   
   A   
   C   
   A   
   A   
   A   
   C   
   G   
   C   
   C   
   C   
   T   
   G   
   G   
   T   
   A   
   G   
   T   
   G   
   C   
   G   
   G   
   T   
   C   
   A   
   T   
   T   
   A   
   C   
   C   
   G   
   A   
   T   
   C   
   C   
   T   
   C   
   A   
   A   
   A   
   A   
   A   
   G   
   G   
   C   
   G   
   A   
   T   
   T   
   T   
   A   
   T   
   T   
   A   
   G   
   C   
   A   
   A   
   C   
   G   
   G   
   T   
   T   
   A   
   G   
   T   
   T   
   C   
   A   
   C   
   C   
   T   
   T   
   C   
   G   
   T   
   A   
   T   
   G   
   A   
   T   
   C   
   C   
   T   
   A   
   A   
   T   
   A   
   A   
   A   
   A   
   T   
   G   
   G   
   C   
   C   
   A   
   A   
   T   
   G   
   G   
   T   
   A   
   T   
   T   
   T   
   C   
   T   
   C   
   A   
   A   
   A   
   A   
   A   
   G   
   A   
   A   
   T   
   A   
   T   
   G   
   A   
   T   
   G   
   C   
   T   
   T   
   A   
   C   
   A   
   A   
   T   
   A   
   A   
   C   
   A   
   A   
   C   
   A   
   A   
   A   
   G   
   A   
   T   
   T   
   T   
   A   
   C   
   C   
   A   
   T   
   T   
   C   
   A   
   C   
   A   
   G   
   C   
   A   
   C   
   G   
   T   
   T   
   T   
   T   
   G   
   C   
   G   
   A   
   C   
   A   
   G   
   G   
   C   
   T   
   A   
   T   
   G   
   C   
   A   
   C   
   C   
   A   
   G   
   G   
   T   
   T   
   C   
   T   
   A   
   C   
   C   
   T   
   T   
   T   
   A   
   A   
   A   
   A   
   C   
   A   
   A   
   T   
   T   
   A   
   C   
   C   
   G   
   G   
   T   
   G   
   C   
   C   
   A   
   T   
   T   
   G   
   G   
   T   
   T   
   T   
   A   
   G   
   A   
   T   
   G   
   C   
   A   
   G   
   G   
   G   
   A   
   C   
   A   
   T   
   T   
   G   
   A   
   A   
   G   
   C   
   C   
   A   
   G   
   A   
   T   
   G   
   A   
   A   
   G   
   A   
   G   
   C   
   T   
   A   
   G   
   A   
   A   
   A   
   T   
   T   
   A   
   A   
   T   
   G   
   G   
   C   
   T   
   T   
   G   
   A   
   A   
   A   
   T   
   G   
   G   
   C   
   A   
   A   
   A   
   A   
   A   
   G   
   A   
   T   
   A   
   A   
   A   
   T   
   C   
   T   
   T   
   G   
   G   
   G   
   G   
   C   
   G   
   G   
   C   
   T   
   A   
   T   
   T   
   T   
   T   
   G   
   C   
   A   
   A   
   C   
   A   
   C   
   G   
   T   
   G   
   T   
   A   
   A   
   A   
   A   
   G   
   A   
   A   
   G   
   C   
   A   
   A   
   G   
   T   
   C   
   C   
   A   
   G   
   T   
   C   
   A   
   A   
   T   
   C   
   T   
   G   
   C   
   G   
   T   
   A   
   C   
   C   
   G   
   C   
   T   
   T   
   T   
   A   
   G   
   T   
   T   
   A   
   A   
   T   
   T   
   C   
   A   
   G   
   A   
   T   
   A   
   A   
   T   
   A   
   T   
   T   
   T   
   A   
   T   
   T   
   T   
   T   
   G   
   C   
   C   
   C   
   A   
   A   
   C   
   A   
   A   
   A   
   C   
   A   
   T   
   T   
   A   
   C   
   G   
   G   
   A   
   T   
   G   
   G   
   G   
   A   
   G   
   A   
   A   
   G   
   A   
   C   
   A   
   A   
   A   
   T   
   T   
   T   
   A   
   G   
   A   
   G   
   C   
   G   
   G   
   G   
   T   
   C   
   T   
   T   
   A   
   A   
   T   
   A   
   A   
   A   
   T   
   T   
   C   
   A   
   T   
   T   
   T   
   T   
   C   
   G   
   G   
   T   
   G   
   A   
   G   
   G   
   A   
   A   
   C   
   T   
   A   
   G   
   A   
   T   
   T   
   T   
   A   
   C   
   C   
   A   
   A   
   T   
   T   
   G   
   C   
   C   
   A   
   T   
   G   
   A   
   C   
   G   
   C   
   C   
   T   
   G   
   C   
   T   
   C   
   A   
   A   
   A   
   T   
   T   
   T   
   C   
   A   
   A   
   A   
   T   
   G   
   A   
   A   
   G   
   A   
   T   
   A   
   A   
   A   
   T   
   T   
   T   
   A   
   A   
   T   
   T   
   C   
   T   
   G   
   A   
   A   
   A   
   T   
   T   
   C   
   T   
   T   
   T   
   T   
   G   
   G   
   C   
   A   
   G   
   A   
   T   
   A   
   C   
   G   
   G   
   G   
   C   
   T   
   A   
   C   
   G   
   G   
   C   
   C   
   A   
   A   
   G   
   G   
   C   
   C   
   A   
   A   
   T   
   T   
   G   
   T   
   T   
   A   
   A   
   T   
   T   
   T   
   C   
   G   
   C   
   C   
   A   
   A   
   T   
   C   
   C   
   A   
   G   
   C   
   A   
   A   
   G   
   C   
   T   
   A   
   C   
   C   
   A   
   T   
   G   
   T   
   A   
   T   
   A   
   G   
   T   
   G   
   T   
   T   
   T   
   T   
   C   
   C   
   A   
   A   
   A   
   A   
   T   
   A   
   A   
   T   
   G   
   G   
   G   
   A   
   C   
   A   
   T   
   T   
   A   
   G   
   T   
   C   
   T   
   A   
   T   
   C   
   C   
   A   
   A   
   A   
   A   
   T   
   T   
   A   
   G   
   T   
   T   
   T   
   T   
   A   
   G   
   A   
   C   
   A   
   A   
   A   
   G   
   A   
   A   
   A   
   C   
   G   
   A   
   A   
   G   
   A   
   A   
   A   
   A   
   A   
   A   
   G   
   A   
   C   
   A   
   A   
   T   
   G   
   T   
   A   
   A   
   T   
   C   
   A   
   G   
   C   
   G   
   C   
   C   
   A   
   A   
   C   
   G   
   C   
   A   
   G   
   C   
   G   
   A   
   A   
   T   
   A   
   C   
   A   
   A   
   T   
   T   
   G   
   C   
   C   
   A   
   C   
   A   
   G   
   A   
   T   
   C   
   T   
   T   
   T   
   T   
   A   
   G   
   G   
   T   
   A   
   G   
   T   
   G   
   T   
   G   
   G   
   A   
   A   
   G   
   A   
   T   
   C   
   C   
   T   
   T   
   C   
   A   
   G   
   G   
   T   
   T   
   A   
   T   
   G   
   T   
   T   
   T   
   A   
   T   
   A   
   A   
   T   
   A   
   T   
   G   
   T   
   A   
   C   
   A   
   A   
   T   
   C   
   C   
   G   
   A   
   A   
   C   
   T   
   T   
   C   
   T   
   C   
   A   
   T   
   T   
   G   
   G   
   C   
   T   
   G   
   C   
   C   
   A   
   A   
   A   
   A   
   C   
   A   
   G   
   G   
   A   
   A   
   C   
   A   
   G   
   C   
   T   
   G   
   A   
   A   
   A   
   T   
   A   
   A   
   A   
   A   
   G   
   A   
   T   
   A   
   A   
   A   
   C   
   A   
   A   
   G   
   A   
   T   
   A   
   C   
   A   
   G   
   A   
   T   
   G   
   G   
   C   
   A   
   A   
   A   
   G   
   A   
   A   
   A   
   A   
   C   
   A   
   G   
   T   
   T   
   T   
   C   
   C   
   T   
   T   
   C   
   T   
   A   
   A   
   C   
   G   
   T   
   T   
   A   
   G   
   A   
   T   
   C   
   G   
   T   
   A   
   G   
   T   
   A   
   A   
   C   
   A   
   A   
   T   
   A   
   A   
   A   
   T   
   T   
   C   
   T   
   T   
   A   
   A   
   C   
   A   
   A   
   T   
   G   
   A   
   T   
   T   
   A   
   T   
   G   
   G   
   T   
   T   
   G   
   A   
   A   
   A   
   A   
   T   
   T   
   C   
   A   
   G   
   G   
   A   
   G   
   A   
   A   
   A   
   A   
   T   
   G   
   G   
   T   
   T   
   C   
   T   
   G   
   C   
   A   
   A   
   C   
   A   
   G   
   A   
   T   
   A   
   T   
   C   
   A   
   G   
   T   
   A   
   A   
   A   
   C   
   C   
   A   
   T   
   T   
   A   
   A   
   T   
   T   
   G   
   A   
   T   
   T   
   A   
   C   
   T   
   T   
   A   
   G   
   A   
   A   
   G   
   C   
   A   
   A   
   C   
   C   
   A   
   T   
   T   
   A   
   A   
   A   
   -   
   -   
   -   
   -   
   -   
   -   
   -   
   -   
   -   
   -   
   -   
   -   
   -   
   -   
   -   
   -   
   -   
   -   
   -   
   -   
   -   
   -   
   -   
   -   
   -   
   -   
   -   
   -   
   -   
   -   
   -   
   -   
   -   
   -   
   -   
   -   
   -   
   -   
   -   
   -   
   -   
   -   
   -   
   -   
   -   
   -   
   -   
   -   
   -   
   -   
   -   
   -   
   -   
   -   
   -   
   -   
   -   
   -   
   -   
   -   
   -   
 

 
   N59599F   
   -   
   -   
   -   
   -   
   -   
   -   
   -   
   -   
   -   
   -   
   -   
   -   
   -   
   -   
   -   
   -   
   -   
   -   
   -   
   -   
   -   
   -   
   -   
   -   
   -   
   -   
   -   
   -   
   -   
   -   
   -   
   -   
   -   
   -   
   -   
   -   
   -   
   -   
   -   
   -   
   -   
   -   
   -   
   -   
   -   
   -   
   -   
   -   
   -   
   -   
   -   
   -   
   -   
   -   
   -   
   -   
   -   
   -   
   -   
   -   
   -   
   -   
   -   
   -   
   -   
   -   
   -   
   -   
   -   
   -   
   -   
   -   
   -   
   -   
   -   
   -   
   -   
   -   
   -   
   -   
   -   
   -   
   -   
   -   
   -   
   -   
   -   
   -   
   -   
   -   
   -   
   -   
   -   
   -   
   -   
   -   
   -   
   -   
   -   
   -   
   -   
   -   
   -   
   -   
   -   
   -   
   -   
   -   
   C   
   A   
   A   
   T   
   G   
   G   
   C   
   A   
   A   
   G   
   C   
   C   
   A   
   A   
   A   
   C   
   A   
   A   
   G   
   A   
    A    
   T   
   T   
   A   
   G   
   C   
    C    
   G   
   A   
   A   
   G   
   C   
   G   
   A   
   A   
   G   
   A   
   A   
   A   
   A   
    C    
   A   
   G   
   C   
    T    
   A   
    C    
   T   
    A    
    C    
   A   
   T   
   T   
   T   
   T   
   T   
   A   
   A   
   A   
    C    
   G   
   T   
   A   
   T   
   T   
   G   
    T    
   C   
   A   
   A   
   A   
   A   
   C   
   A   
   G   
    G    
   A   
   A   
   T   
   T   
   T   
   G   
   A   
   T   
   A   
   A   
   G   
   T   
   T   
   A   
   C   
   C   
    G    
   T   
   C   
   C   
   G   
   T   
   T   
    G    
   T   
   T   
   C   
   A   
   A   
   G   
   A   
   A   
    G    
   C   
   T   
   A   
   G   
   C   
   T   
   T   
   A   
   A   
   A   
   G   
   A   
   A   
   A   
   A   
   A   
   T   
   G   
   G   
    C    
   T   
   A   
   T   
   G   
   A   
   T   
   A   
   C   
   T   
   A   
   A   
   A   
   T   
   C   
    T    
   G   
   T   
   T   
   G   
   T   
   T   
   G   
   A   
   A   
   A   
   A   
   A   
   T   
   A   
   C   
   C   
   A   
   A   
   G   
   C   
   A   
   A   
   T   
   T   
   T   
   A   
   T   
   T   
   C   
   A   
   G   
   G   
   G   
   A   
   T   
   T   
   C   
   A   
   A   
   G   
   C   
    A    
   G   
   A   
   A   
    G    
   G   
    A    
   G   
   T   
   C   
   A   
   A   
    A    
   G   
   C   
   T   
   A   
   G   
   T   
   G   
   A   
   T   
   G   
   T   
    T    
   C   
   A   
   A   
   G   
   T   
    C    
   A   
   A   
   A   
   A   
   A   
    G    
   G   
    C    
   G   
   A   
   A   
   A   
   G   
   A   
    T    
   A   
   A   
    T    
   C   
   A   
   A   
   T   
   A   
   C   
   A   
   C   
   A   
   T   
   T   
   T   
   A   
    C    
   C   
   T   
   A   
   T   
   A   
   A   
   A   
   T   
   T   
   A   
   T   
   C   
   G   
   A   
   T   
   G   
   A   
   G   
    C    
   A   
   C   
    T    
   C   
   C   
   T   
   T   
   T   
   A   
   G   
   G   
   C   
   G   
   A   
   A   
   A   
   T   
   G   
   A   
   A   
   A   
   G   
   A   
   T   
    T    
   T   
   G   
   T   
   C   
   T   
   T   
   A   
   T   
   C   
   A   
   A   
   T   
   C   
   A   
   A   
   G   
   T   
   A   
   T   
    T    
   G   
    C    
   C   
   A   
   A   
    G    
   A   
   A   
   A   
   G   
   G   
    C    
   G   
   A   
   T   
   A   
    C    
   C   
   T   
   A   
   C   
   C   
   A   
   A   
   A   
   T   
    C    
    G    
   C   
   T   
   T   
   G   
   G   
    A    
    A    
    T    
   C   
   C   
    G    
   T   
   C   
   T   
   T   
   T   
   A   
   A   
   T   
   T   
   T   
   T   
   T   
   C   
   C   
   A   
   G   
   A   
   T   
   A   
   T   
   G   
   T   
   C   
   A   
   G   
   G   
   A   
   A   
   A   
   T   
   G   
   A   
   T   
   A   
   A   
   A   
    G    
   T   
   T   
   T   
   C   
   G   
   A   
   T   
   T   
   C   
   A   
   A   
   G   
   T   
   A   
   G   
   A   
   T   
   A   
   A   
   T   
   G   
   C   
   C   
   A   
   A   
   A   
   C   
   G   
   T   
   G   
   G   
   A   
   G   
   A   
   A   
   A   
   T   
   T   
   G   
   T   
   C   
   G   
   A   
   T   
   C   
   G   
   T   
   A   
   A   
    T    
   G   
   G   
   T   
   A   
   G   
   T   
   G   
   G   
   G   
   C   
   T   
   A   
   G   
    C    
   A   
   A   
   T   
   T   
   A   
   A   
   C   
   A   
   A   
   A   
   G   
   T   
   G   
   T   
   T   
   T   
   G   
   A   
    T    
   G   
   A   
   A   
   G   
   T   
   G   
   G   
   G   
   C   
   G   
   T   
   A   
   G   
   T   
    G    
   C   
   C   
   T   
    G    
   G   
   C   
   A   
   A   
   A   
   C   
   T   
    C    
   G   
   G   
   T   
   T   
   C   
   T   
   G   
   G   
   C   
   G   
   C   
   A   
   G   
   A   
   A   
   A   
   A   
   A   
    A    
   C   
   A   
   G   
   C   
    C    
   A   
   A   
   T   
   A   
   T   
   C   
   A   
   A   
   A   
   G   
   C   
   T   
   T   
   T   
   T   
   A   
   G   
   T   
   G   
   A   
   T   
   A   
   A   
    G    
   T   
   T   
    C    
   G   
   G   
   C   
   G   
   T   
   T   
   T   
   C   
   T   
    G    
   T   
   T   
   G   
   A   
   T   
   G   
   A   
   A   
   A   
   T   
   C   
   A   
   A   
   T   
   C   
    G    
   A   
   A   
   A   
    G    
   T   
   T   
   A   
   A   
   G   
   C   
   C   
   A   
   A   
   G   
   G   
    G    
   T   
   G   
   G   
   G   
   T   
   C   
   C   
   A   
   A   
   G   
   C   
   A   
   G   
   A   
   C   
   T   
   C   
    C    
   T   
   T   
   T   
   G   
   T   
   A   
   C   
   C   
   A   
   A   
   T   
   C   
   A   
   C   
   A   
   G   
   T   
    C    
   G   
   C   
   T   
   T   
   C   
   T   
   G   
   A   
   A   
   C   
   C   
   A   
   G   
   T   
   G   
   A   
   C   
   A   
   G   
   A   
   A   
   T   
   T   
   A   
   C   
   C   
   A   
   A   
   C   
   A   
   G   
   G   
   G   
   G   
   C   
   T   
   G   
   C   
   G   
   A   
   C   
   A   
   A   
   A   
   A   
   G   
    A    
    C    
   A   
   C   
   A   
   G   
   A   
   G   
   T   
   C   
   A   
   C   
   G   
   T   
   T   
   A   
   T   
   T   
   A   
   T   
   C   
   C   
    G    
    T    
   T   
   G   
   G   
   G   
   G   
   G   
   A   
   A   
   G   
   C   
   A   
   G   
   C   
   T   
   G   
   C   
   G   
   C   
   A   
   A   
   T   
   T   
   A   
   A   
   T   
   C   
   G   
   G   
    G    
   T   
   A   
   T   
   A   
   C   
    G    
   G   
   G   
    C    
   A   
   C   
    C    
   A   
   T   
   T   
   A   
   C   
   T   
   G   
   C   
   A   
   G   
   A   
    T    
   G   
   A   
   T   
   A   
   T   
   T   
    A    
   A   
   A   
   A   
   A   
   A   
   A   
   A   
   T   
   C   
   C   
   A   
   G   
   A   
   G   
   T   
   T   
   A   
   A   
   G   
   T   
   A   
   G   
   T   
   A   
   C   
   G   
   G   
   G   
    C    
   G   
   T   
   A   
   A   
   T   
   T   
   G   
   G   
    T    
   A   
   A   
   A   
   A   
   C   
   T   
   G   
   G   
    C    
   T   
   T   
   A   
   G   
   A   
   A   
   C   
   G   
   A   
   G   
   C   
   G   
   T   
   T   
   T   
   G   
   A   
   T   
   A   
   A   
   A   
   G   
   A   
    A    
   T   
   T   
   A   
   A   
   G   
   A   
   G   
   G   
    G    
   C   
   A   
   A   
   G   
   A   
   T   
   G   
   G   
    T    
   G   
   G   
    C    
   T   
   C   
    A    
   C   
   T   
   A   
   G   
   T   
   T   
   A   
   T   
   T   
   T   
   T   
   A   
   G   
   A   
   T   
   G   
   A   
   C   
   A   
   A   
   A   
   G   
   A   
   G   
   A   
   A   
   T   
   G   
   T   
   C   
   A   
   A   
   A   
   A   
   A   
   A   
   G   
   C   
   T   
   T   
   T   
   A   
   C   
   A   
   A   
   A   
   C   
   C   
   A   
   A   
   A   
   G   
   A   
    G    
   A   
   A   
   A   
   A   
   A   
   A   
   G   
   A   
   T   
   G   
   G   
   T   
   C   
   A   
   A   
   A   
   C   
    G    
   A   
   T   
   T   
   A   
   A   
   A   
   T   
   T   
   A   
   A   
   C   
   G   
   A   
   T   
   T   
   G   
   A   
    C    
   A   
   G   
    T    
   G   
   G   
    C    
   G   
   T   
   A   
   C   
   A   
   A   
   C   
   A   
   A   
   C   
   A   
   A   
   G   
   C   
    C    
   T   
   T   
    C    
   G   
   C   
   T   
   A   
   T   
   A   
   T   
   T   
   T   
   G   
   A   
   C   
   A   
   A   
   A   
   C   
   G   
    C    
    C    
   C   
   T   
   G   
   G   
   T   
   A   
   G   
   T   
   G   
   C   
   G   
   G   
   T   
   C   
   A   
   T   
   T   
   A   
   C   
   C   
   G   
   A   
   T   
   C   
   C   
   T   
   C   
   A   
   A   
   A   
   A   
   A   
   G   
   G   
   C   
   G   
   A   
   T   
   T   
   T   
   A   
    T    
   T   
   A   
   G   
   C   
    A    
   A   
   C   
   G   
   G   
   T   
   T   
   A   
   G   
   T   
   T   
   C   
   A   
   C   
   C   
   T   
   T   
   C   
   G   
   T   
   A   
   T   
   G   
   A   
   T   
   C   
   C   
   T   
   A   
   A   
   T   
   A   
   A   
   A   
   A   
   T   
   G   
   G   
   C   
   C   
   A   
   A   
   T   
   G   
   G   
    C    
   A   
   T   
   T   
   T   
   C   
   T   
   C   
   A   
   A   
   A   
   A   
   A   
   G   
   A   
    A    
   T   
   A   
   T   
   G   
   A   
   T   
   G   
   C   
   T   
   T   
   A   
   C   
   A   
   A   
   T   
   A   
   A   
   C   
   A   
   A   
   C   
   A   
   A   
   A   
   G   
   A   
   T   
   T   
   T   
   A   
   C   
   C   
   A   
   T   
   T   
    C    
   A   
   C   
   A   
   G   
   C   
   A   
   C   
   G   
   T   
   T   
   T   
    T    
   G   
   C   
   G   
   A   
   C   
   A   
   G   
   G   
    C    
   T   
   A   
   T   
   G   
   C   
   A   
   C   
   C   
   A   
   G   
   G   
    C    
   T   
   C   
   T   
   A   
   C   
   C   
   T   
   T   
   T   
   A   
   A   
   A   
   A   
   C   
   A   
   A   
   T   
   T   
   A   
   C   
   C   
   G   
   G   
   T   
   G   
   C   
   C   
   A   
   T   
   T   
   G   
   G   
   T   
   T   
   T   
   A   
   G   
   A   
   T   
    G    
   C   
   A   
   G   
   G   
   G   
   A   
   C   
   A   
   T   
   T   
   G   
   A   
   A   
   G   
   C   
   C   
   A   
   G   
   A   
   T   
   G   
   A   
   A   
   G   
   A   
   G   
   C   
   T   
   A   
   G   
   A   
   A   
   A   
   T   
   T   
   A   
   A   
   T   
   G   
   G   
    T    
   T   
   T   
   G   
   A   
   A   
   A   
   T   
   G   
   G   
   C   
   A   
   A   
   A   
   A   
   A   
   G   
   A   
   T   
   A   
   A   
   A   
   T   
   C   
   T   
   T   
   G   
   G   
   G   
   G   
    C    
   G   
   G   
   C   
   T   
   A   
   T   
   T   
   T   
   T   
   G   
   C   
    G    
   A   
   C   
   A   
   C   
   G   
   T   
   G   
   T   
   A   
   A   
   A   
   A   
   G   
   A   
   A   
   G   
   C   
   A   
   A   
   G   
   T   
   C   
   C   
   A   
   G   
   T   
   C   
   A   
   A   
   T   
   C   
   T   
    G    
   C   
   G   
   T   
   A   
   C   
   C   
   G   
   C   
   T   
   T   
   T   
   A   
   G   
   T   
   T   
   A   
   A   
   T   
   T   
   C   
   A   
   G   
   A   
   T   
   A   
   A   
   T   
   A   
   T   
   T   
   T   
   A   
   T   
   T   
   T   
   T   
   G   
   C   
    C    
   C   
   A   
   A   
   C   
   A   
   A   
   A   
   C   
   A   
   T   
   T   
   A   
   C   
   G   
    G    
   A   
   T   
   G   
   G   
   G   
    A    
   G   
   A   
   A   
   G   
   A   
   C   
   A   
   A   
   A   
   T   
   T   
   T   
   A   
   G   
   A   
   G   
   C   
    G    
   G   
   G   
   T   
   C   
   T   
   T   
   A   
   A   
   T   
   A   
   A   
   A   
   T   
   T   
   C   
   A   
   T   
   T   
   T   
   T   
   C   
   G   
   G   
   T   
   G   
   A   
   G   
   G   
   A   
   A   
   C   
   T   
   A   
   G   
   A   
   T   
   T   
   T   
   A   
   C   
   C   
   A   
   A   
   T   
    T    
   G   
   C   
   C   
   A   
   T   
   G   
   A   
   C   
   G   
   C   
   C   
    T    
   G   
   C   
   T   
   C   
   A   
   A   
   A   
   T   
   T   
   T   
   C   
   A   
   A   
   A   
    T    
   G   
   A   
   A   
   G   
   A   
   T   
   A   
   A   
   A   
   T   
   T   
   T   
   A   
   A   
   T   
   T   
   C   
   T   
   G   
   A   
   A   
   A   
   T   
   T   
   C   
   T   
   T   
   T   
   T   
    A    
   G   
   C   
   A   
   G   
   A   
   T   
   A   
   C   
    G    
   G   
   G   
   C   
   T   
   A   
    C    
   G   
   G   
    T    
   C   
   A   
   A   
   G   
   G   
    T    
   C   
   A   
   A   
   T   
   T   
   G   
   T   
   T   
   A   
   A   
   T   
   T   
   T   
   C   
    G    
   C   
   C   
   A   
   A   
   T   
   C   
   C   
   A   
   G   
   C   
   A   
   A   
   G   
   C   
   T   
   A   
   C   
   C   
   A   
   T   
   G   
   T   
   A   
   T   
   A   
   G   
   T   
   G   
   T   
   T   
   T   
   T   
   C   
   C   
   A   
   A   
   A   
   A   
   T   
   A   
   A   
   T   
   G   
   G   
    A    
   A   
   C   
   A   
    T    
   T   
    A    
   G   
   T   
    C    
   T   
   A   
   T   
   C   
   C   
   A   
   A   
   A   
   A   
    C    
   T   
   A   
   G   
   T   
   T   
   T   
   T   
   A   
   G   
   A   
   C   
   A   
   A   
   A   
   G   
   A   
   A   
   A   
   C   
    G    
   A   
   A   
   G   
   A   
   A   
   A   
   A   
   A   
   A   
   G   
   A   
   C   
   A   
   A   
   T   
   G   
   T   
   A   
   A   
   T   
   C   
   A   
   G   
   C   
   G   
   C   
   C   
   A   
   A   
   C   
   G   
   C   
   A   
   G   
   C   
   G   
   A   
   A   
   T   
   A   
   C   
   A   
   A   
   T   
   T   
   G   
   C   
   C   
   A   
   C   
   A   
   G   
   A   
   T   
   C   
   T   
   T   
   T   
   T   
   A   
   G   
   G   
   T   
   A   
   G   
   T   
   G   
   T   
   G   
   G   
   A   
   A   
   G   
   A   
   T   
   C   
   C   
   T   
   T   
   C   
   A   
   G   
   G   
   T   
   T   
   A   
   T   
   G   
   T   
   T   
   T   
   A   
   T   
   A   
   A   
   T   
   A   
   T   
   G   
   T   
   A   
   C   
   A   
   A   
   T   
   C   
   C   
   G   
   A   
   A   
   C   
   T   
   T   
   C   
   T   
   C   
   A   
   T   
   T   
   G   
   G   
   C   
   T   
   G   
   C   
   T   
   A   
   A   
   A   
   A   
   C   
   A   
   G   
   G   
   A   
   A   
   C   
   A   
   G   
   C   
   T   
   G   
   A   
   A   
   A   
   T   
   A   
   A   
   A   
   A   
   G   
   A   
   T   
   A   
   A   
   A   
   C   
   A   
   A   
   G   
   A   
   T   
   A   
   C   
   A   
   G   
   A   
   T   
   G   
   G   
   C   
   A   
   A   
   A   
   G   
   A   
   A   
   A   
   A   
   C   
   A   
   G   
   T   
   T   
   T   
   C   
   C   
   T   
   T   
   C   
   T   
   A   
   A   
   C   
   G   
   T   
   T   
   A   
   G   
   A   
   T   
   C   
   G   
   T   
   A   
   G   
   T   
   A   
   A   
   C   
   A   
   A   
   T   
   A   
   A   
   A   
   T   
   T   
   C   
   T   
   T   
   A   
   A   
   C   
   A   
   A   
   T   
   G   
   A   
   T   
   T   
   A   
   T   
   G   
   G   
   T   
   T   
   G   
   A   
   A   
   A   
   A   
   T   
   T   
   C   
   A   
   G   
   G   
   A   
   G   
   A   
   A   
   A   
   A   
   T   
   G   
   G   
   T   
   T   
   C   
   T   
   G   
   C   
   A   
   A   
   C   
   A   
   G   
   A   
   T   
   A   
   T   
   C   
   A   
   G   
   T   
   A   
   A   
   A   
   C   
   C   
   A   
   T   
   T   
   A   
   A   
   T   
   T   
   G   
   A   
   T   
   T   
   A   
   C   
   T   
   T   
   A   
   G   
   A   
   A   
   G   
   C   
   A   
   A   
   C   
   C   
   A   
   T   
   T   
   A   
   A   
   A   
   -   
   -   
   -   
   -   
   -   
   -   
   -   
   -   
   -   
   -   
   -   
   -   
   -   
   -   
   -   
   -   
   -   
   -   
   -   
   -   
   -   
   -   
   -   
   -   
   -   
   -   
   -   
   -   
   -   
   -   
   -   
   -   
   -   
   -   
   -   
   -   
   -   
   -   
   -   
   -   
   -   
   -   
   -   
   -   
   -   
   -   
   -   
   -   
   -   
   -   
   -   
   -   
   -   
   -   
   -   
   -   
   -   
   -   
   -   
   -   
   -   
   -   
   -   
   -   
   -   
   -   
   -   
   -   
   -   
   -   
   -   
   -   
   -   
   -   
   -   
   -   
   -   
   -   
   -   
   -   
   -   
   -   
   -   
   -   
   -   
   -   
   -   
   -   
   -   
   -   
   -   
   -   
   -   
   -   
   -   
   -   
   -   
   -   
   -   
   -   
   -   
   -   
   -   
   -   
   -   
   -   
   -   
   -   
   -   
   -   
   -   
   -   
   -   
   -   
   -   
   -   
   -   
   -   
   -   
   -   
   -   
   -   
   -   
   -   
   -   
   -   
   -   
   -   
   -   
   -   
   -   
   -   
   -   
   -   
   -   
   -   
   -   
   -   
   -   
   -   
   -   
   -   
   -   
   -   
   -   
   -   
   -   
   -   
   -   
   -   
   -   
   -   
   -   
   -   
   -   
   -   
   -   
   -   
   -   
   -   
   -   
   -   
   -   
   -   
   -   
   -   
   -   
   -   
   -   
   C   
   A   
   A   
   T   
   G   
   G   
   C   
   A   
   A   
   G   
   C   
   C   
   A   
   A   
   A   
   C   
   A   
   A   
   G   
   A   
   A   
   T   
   T   
   A   
   G   
   C   
   C   
   G   
   A   
   A   
   G   
   C   
   G   
   A   
   A   
   G   
   A   
   A   
   A   
   A   
   C   
   A   
   G   
   C   
   T   
   A   
   C   
   T   
   A   
   C   
   A   
   T   
   T   
   T   
   T   
   T   
   A   
   A   
   A   
   C   
   G   
   T   
   A   
   T   
   T   
   G   
   T   
   C   
   A   
   A   
   A   
   A   
   C   
   A   
   G   
   G   
   A   
   A   
   T   
   T   
   T   
   G   
   A   
   T   
   A   
   A   
   G   
   T   
   T   
   A   
   C   
   C   
   G   
   T   
   C   
   C   
   G   
   T   
   T   
   G   
   T   
   T   
   C   
   A   
   A   
   G   
   A   
   A   
   G   
   C   
   T   
   A   
   G   
   C   
   T   
   T   
   A   
   A   
   A   
   G   
   A   
   A   
   A   
   A   
   A   
   T   
   G   
   G   
   C   
   T   
   A   
   T   
   G   
   A   
   T   
   A   
   C   
   T   
   A   
   A   
   A   
   T   
   C   
   T   
   G   
   T   
   T   
   G   
   T   
   T   
   G   
   A   
   A   
   A   
   A   
   A   
   T   
   A   
   C   
   C   
   A   
   A   
   G   
   C   
   A   
   A   
   T   
   T   
   T   
   A   
   T   
   T   
   C   
   A   
   G   
   G   
   G   
   A   
   T   
   T   
   C   
   A   
   A   
   G   
   C   
   A   
   G   
   A   
   A   
   G   
   G   
   A   
   G   
   T   
   C   
   A   
   A   
   A   
   G   
   C   
   T   
   A   
   G   
   T   
   G   
   A   
   T   
   G   
   T   
   T   
   C   
   A   
   A   
   G   
   T   
   C   
   A   
   A   
   A   
   A   
   A   
   G   
   G   
   C   
   G   
   A   
   A   
   A   
   G   
   A   
   T   
   A   
   A   
   T   
   C   
   A   
   A   
   T   
   A   
   C   
   A   
   C   
   A   
   T   
   T   
   T   
   A   
   C   
   C   
   T   
   A   
   T   
   A   
   A   
   A   
   T   
   T   
   A   
   T   
   C   
   G   
   A   
   T   
   G   
   A   
   G   
   C   
   A   
   C   
   T   
   C   
   C   
   T   
   T   
   T   
   A   
   G   
   G   
   C   
   G   
   A   
   A   
   A   
   T   
   G   
   A   
   A   
   A   
   G   
   A   
   T   
   T   
   T   
   G   
   T   
   C   
   T   
   T   
   A   
   T   
   C   
   A   
   A   
   T   
   C   
   A   
   A   
   G   
   T   
   A   
   T   
   T   
   G   
   C   
   C   
   A   
   A   
   G   
   A   
   A   
   A   
   G   
   G   
   C   
   G   
   A   
   T   
   A   
   C   
   C   
   T   
   A   
   C   
   C   
   A   
   A   
   A   
   T   
   C   
   G   
   C   
   T   
   T   
   G   
   G   
   A   
   A   
   T   
   C   
   C   
   G   
   T   
   C   
   T   
   T   
   T   
   A   
   A   
   T   
   T   
   T   
   T   
   T   
   C   
   C   
   A   
   G   
   A   
   T   
   A   
   T   
   G   
   T   
   C   
   A   
   G   
   G   
   A   
   A   
   A   
   T   
   G   
   A   
   T   
   A   
   A   
   A   
   G   
   T   
   T   
   T   
   C   
   G   
   A   
   T   
   T   
   C   
   A   
   A   
   G   
   T   
   A   
   G   
   A   
   T   
   A   
   A   
   T   
   G   
   C   
   C   
   A   
   A   
   A   
   C   
   G   
   T   
   G   
   G   
   A   
   G   
   A   
   A   
   A   
   T   
   T   
   G   
   T   
   C   
   G   
   A   
   T   
   C   
   G   
   T   
   A   
   A   
   T   
   G   
   G   
   T   
   A   
   G   
   T   
   G   
   G   
   G   
   C   
   T   
   A   
   G   
   C   
   A   
   A   
   T   
   T   
   A   
   A   
   C   
   A   
   A   
   A   
   G   
   T   
   G   
   T   
   T   
   T   
   G   
   A   
   T   
   G   
   A   
   A   
   G   
   T   
   G   
   G   
   G   
   C   
   G   
   T   
   A   
   G   
   T   
   G   
   C   
   C   
   T   
   G   
   G   
   C   
   A   
   A   
   A   
   C   
   T   
   C   
   G   
   G   
   T   
   T   
   C   
   T   
   G   
   G   
   C   
   G   
   C   
   A   
   G   
   A   
   A   
   A   
   A   
   A   
   A   
   C   
   A   
   G   
   C   
   C   
   A   
   A   
   T   
   A   
   T   
   C   
   A   
   A   
   A   
   G   
   C   
   T   
   T   
   T   
   T   
   A   
   G   
   T   
   G   
   A   
   T   
   A   
   A   
   G   
   T   
   T   
   C   
   G   
   G   
   C   
   G   
   T   
   T   
   T   
   C   
   T   
   G   
   T   
   T   
   G   
   A   
   T   
   G   
   A   
   A   
   A   
   T   
   C   
   A   
   A   
   T   
   C   
   G   
   A   
   A   
   A   
   G   
   T   
   T   
   A   
   A   
   G   
   C   
   C   
   A   
   A   
   G   
   G   
   G   
   T   
   G   
   G   
   G   
   T   
   C   
   C   
   A   
   A   
   G   
   C   
   A   
   G   
   A   
   C   
   T   
   C   
   C   
   T   
   T   
   T   
   G   
   T   
   A   
   C   
   C   
   A   
   A   
   T   
   C   
   A   
   C   
   A   
   G   
   T   
   C   
   G   
   C   
   T   
   T   
   C   
   T   
   G   
   A   
   A   
   C   
   C   
   A   
   G   
   T   
   G   
   A   
   C   
   A   
   G   
   A   
   A   
   T   
   T   
   A   
   C   
   C   
   A   
   A   
   C   
   A   
   G   
   G   
   G   
   G   
   C   
   T   
   G   
   C   
   G   
   A   
   C   
   A   
   A   
   A   
   A   
   G   
   A   
   C   
   A   
   C   
   A   
   G   
   A   
   G   
   T   
   C   
   A   
   C   
   G   
   T   
   T   
   A   
   T   
   T   
   A   
   T   
   C   
   C   
   G   
   T   
   T   
   G   
   G   
   G   
   G   
   G   
   A   
   A   
   G   
   C   
   A   
   G   
   C   
   T   
   G   
   C   
   G   
   C   
   A   
   A   
   T   
   T   
   A   
   A   
   T   
   C   
   G   
   G   
   G   
   T   
   A   
   T   
   A   
   C   
   G   
   G   
   G   
   C   
   A   
   C   
   C   
   A   
   T   
   T   
   A   
   C   
   T   
   G   
   C   
   A   
   G   
   A   
   T   
   G   
   A   
   T   
   A   
   T   
   T   
   A   
   A   
   A   
   A   
   A   
   A   
   A   
   A   
   T   
   C   
   C   
   A   
   G   
   A   
   G   
   T   
   T   
   A   
   A   
   G   
   T   
   A   
   G   
   T   
   A   
   C   
   G   
   G   
   G   
   C   
   G   
   T   
   A   
   A   
   T   
   T   
   G   
   G   
   T   
   A   
   A   
   A   
   A   
   C   
   T   
   G   
   G   
   C   
   T   
   T   
   A   
   G   
   A   
   A   
   C   
   G   
   A   
   G   
   C   
   G   
   T   
   T   
   T   
   G   
   A   
   T   
   A   
   A   
   A   
   G   
   A   
   A   
   T   
   T   
   A   
   A   
   G   
   A   
   G   
   G   
   G   
   C   
   A   
   A   
   G   
   A   
   T   
   G   
   G   
   T   
   G   
   G   
   C   
   T   
   C   
   A   
   C   
   T   
   A   
   G   
   T   
   T   
   A   
   T   
   T   
   T   
   T   
   A   
   G   
   A   
   T   
   G   
   A   
   C   
   A   
   A   
   A   
   G   
   A   
   G   
   A   
   A   
   T   
   G   
   T   
   C   
   A   
   A   
   A   
   A   
   A   
   A   
   G   
   C   
   T   
   T   
   T   
   A   
   C   
   A   
   A   
   A   
   C   
   C   
   A   
   A   
   A   
   G   
   A   
   G   
   A   
   A   
   A   
   A   
   A   
   A   
   G   
   A   
   T   
   G   
   G   
   T   
   C   
   A   
   A   
   A   
   C   
   G   
   A   
   T   
   T   
   A   
   A   
   A   
   T   
   T   
   A   
   A   
   C   
   G   
   A   
   T   
   T   
   G   
   A   
   C   
   A   
   G   
   T   
   G   
   G   
   C   
   G   
   T   
   A   
   C   
   A   
   A   
   C   
   A   
   A   
   C   
   A   
   A   
   G   
   C   
   C   
   T   
   T   
   C   
   G   
   C   
   T   
   A   
   T   
   A   
   T   
   T   
   T   
   G   
   A   
   C   
   A   
   A   
   A   
   C   
   G   
   C   
   C   
   C   
   T   
   G   
   G   
   T   
   A   
   G   
   T   
   G   
   C   
   G   
   G   
   T   
   C   
   A   
   T   
   T   
   A   
   C   
   C   
   G   
   A   
   T   
   C   
   C   
   T   
   C   
   A   
   A   
   A   
   A   
   A   
   G   
   G   
   C   
   G   
   A   
   T   
   T   
   T   
   A   
   T   
   T   
   A   
   G   
   C   
   A   
   A   
   C   
   G   
   G   
   T   
   T   
   A   
   G   
   T   
   T   
   C   
   A   
   C   
   C   
   T   
   T   
   C   
   G   
   T   
   A   
   T   
   G   
   A   
   T   
   C   
   C   
   T   
   A   
   A   
   T   
   A   
   A   
   A   
   A   
   T   
   G   
   G   
   C   
   C   
   A   
   A   
   T   
   G   
   G   
   C   
   A   
   T   
   T   
   T   
   C   
   T   
   C   
   A   
   A   
   A   
   A   
   A   
   G   
   A   
   A   
   T   
   A   
   T   
   G   
   A   
   T   
   G   
   C   
   T   
   T   
   A   
   C   
   A   
   A   
   T   
   A   
   A   
   C   
   A   
   A   
   C   
   A   
   A   
   A   
   G   
   A   
   T   
   T   
   T   
   A   
   C   
   C   
   A   
   T   
   T   
   C   
   A   
   C   
   A   
   G   
   C   
   A   
   C   
   G   
   T   
   T   
   T   
   T   
   G   
   C   
   G   
   A   
   C   
   A   
   G   
   G   
   C   
   T   
   A   
   T   
   G   
   C   
   A   
   C   
   C   
   A   
   G   
   G   
   C   
   T   
   C   
   T   
   A   
   C   
   C   
   T   
   T   
   T   
   A   
   A   
   A   
   A   
   C   
   A   
   A   
   T   
   T   
   A   
   C   
   C   
   G   
   G   
   T   
   G   
   C   
   C   
   A   
   T   
   T   
   G   
   G   
   T   
   T   
   T   
   A   
   G   
   A   
   T   
   G   
   C   
   A   
   G   
   G   
   G   
   A   
   C   
   A   
   T   
   T   
   G   
   A   
   A   
   G   
   C   
   C   
   A   
   G   
   A   
   T   
   G   
   A   
   A   
   G   
   A   
   G   
   C   
   T   
   A   
   G   
   A   
   A   
   A   
   T   
   T   
   A   
   A   
   T   
   G   
   G   
   T   
   T   
   T   
   G   
   A   
   A   
   A   
   T   
   G   
   G   
   C   
   A   
   A   
   A   
   A   
   A   
   G   
   A   
   T   
   A   
   A   
   A   
   T   
   C   
   T   
   T   
   G   
   G   
   G   
   G   
   C   
   G   
   G   
   C   
   T   
   A   
   T   
   T   
   T   
   T   
   G   
   C   
   G   
   A   
   C   
   A   
   C   
   G   
   T   
   G   
   T   
   A   
   A   
   A   
   A   
   G   
   A   
   A   
   G   
   C   
   A   
   A   
   G   
   T   
   C   
   C   
   A   
   G   
   T   
   C   
   A   
   A   
   T   
   C   
   T   
   G   
   C   
   G   
   T   
   A   
   C   
   C   
   G   
   C   
   T   
   T   
   T   
   A   
   G   
   T   
   T   
   A   
   A   
   T   
   T   
   C   
   A   
   G   
   A   
   T   
   A   
   A   
   T   
   A   
   T   
   T   
   T   
   A   
   T   
   T   
   T   
   T   
   G   
   C   
   C   
   C   
   A   
   A   
   C   
   A   
   A   
   A   
   C   
   A   
   T   
   T   
   A   
   C   
   G   
   G   
   A   
   T   
   G   
   G   
   G   
   A   
   G   
   A   
   A   
   G   
   A   
   C   
   A   
   A   
   A   
   T   
   T   
   T   
   A   
   G   
   A   
   G   
   C   
   G   
   G   
   G   
   T   
   C   
   T   
   T   
   A   
   A   
   T   
   A   
   A   
   A   
   T   
   T   
   C   
   A   
   T   
   T   
   T   
   T   
   C   
   G   
   G   
   T   
   G   
   A   
   G   
   G   
   A   
   A   
   C   
   T   
   A   
   G   
   A   
   T   
   T   
   T   
   A   
   C   
   C   
   A   
   A   
   T   
   T   
   G   
   C   
   C   
   A   
   T   
   G   
   A   
   C   
   G   
   C   
   C   
   T   
   G   
   C   
   T   
   C   
   A   
   A   
   A   
   T   
   T   
   T   
   C   
   A   
   A   
   A   
   T   
   G   
   A   
   A   
   G   
   A   
   T   
   A   
   A   
   A   
   T   
   T   
   T   
   A   
   A   
   T   
   T   
   C   
   T   
   G   
   A   
   A   
   A   
   T   
   T   
   C   
   T   
   T   
   T   
   T   
   A   
   G   
   C   
   A   
   G   
   A   
   T   
   A   
   C   
   G   
   G   
   G   
   C   
   T   
   A   
   C   
   G   
   G   
   T   
   C   
   A   
   A   
   G   
   G   
   T   
   C   
   A   
   A   
   T   
   T   
   G   
   T   
   T   
   A   
   A   
   T   
   T   
   T   
   C   
   G   
   C   
   C   
   A   
   A   
   T   
   C   
   C   
   A   
   G   
   C   
   A   
   A   
   G   
   C   
   T   
   A   
   C   
   C   
   A   
   T   
   G   
   T   
   A   
   T   
   A   
   G   
   T   
   G   
   T   
   T   
   T   
   T   
   C   
   C   
   A   
   A   
   A   
   A   
   T   
   A   
   A   
   T   
   G   
   G   
   A   
   A   
   C   
   A   
   T   
   T   
   A   
   G   
   T   
   C   
   T   
   A   
   T   
   C   
   C   
   A   
   A   
   A   
   A   
   C   
   T   
   A   
   G   
   T   
   T   
   T   
   T   
   A   
   G   
   A   
   C   
   A   
   A   
   A   
   G   
   A   
   A   
   A   
   C   
   G   
   A   
   A   
   G   
   A   
   A   
   A   
   A   
   A   
   A   
   G   
   A   
   C   
   A   
   A   
   T   
   G   
   T   
   A   
   A   
   T   
   C   
   A   
   G   
   C   
   G   
   C   
   C   
   A   
   A   
   C   
   G   
   C   
   A   
   G   
   C   
   G   
   A   
   A   
   T   
   A   
   C   
   A   
   A   
   T   
   T   
   G   
   C   
   C   
   A   
   C   
   A   
   G   
   A   
   T   
   C   
   T   
   T   
   T   
   T   
   A   
   G   
   G   
   T   
   A   
   G   
   T   
   G   
   T   
   G   
   G   
   A   
   A   
   G   
   A   
   T   
   C   
   C   
   T   
   T   
   C   
   A   
   G   
   G   
   T   
   T   
   A   
   T   
   G   
   T   
   T   
   T   
   A   
   T   
   A   
   A   
   T   
   A   
   T   
   G   
   T   
   A   
   C   
   A   
   A   
   T   
   C   
   C   
   G   
   A   
   A   
   C   
   T   
   T   
   C   
   T   
   C   
   A   
   T   
   T   
   G   
   G   
   C   
   T   
   G   
   C   
   T   
   A   
   A   
   A   
   A   
   C   
   A   
   G   
   G   
   A   
   A   
   C   
   A   
   G   
   C   
   T   
   G   
   A   
   A   
   A   
   T   
   A   
   A   
   A   
   A   
   G   
   A   
   T   
   A   
   A   
   A   
   C   
   A   
   A   
   G   
   A   
   T   
   A   
   C   
   A   
   G   
   A   
   T   
   G   
   G   
   C   
   A   
   A   
   A   
   G   
   A   
   A   
   A   
   A   
   C   
   A   
   G   
   T   
   T   
   T   
   C   
   C   
   T   
   T   
   C   
   T   
   A   
   A   
   C   
   G   
   T   
   T   
   A   
   G   
   A   
   T   
   C   
   G   
   T   
   A   
   G   
   T   
   A   
   A   
   C   
   A   
   A   
   T   
   A   
   A   
   A   
   T   
   T   
   C   
   T   
   T   
   A   
   A   
   C   
   A   
   A   
   T   
   G   
   A   
   T   
   T   
   A   
   T   
   G   
   G   
   T   
   T   
   G   
   A   
   A   
   A   
   A   
   T   
   T   
   C   
   A   
   G   
   G   
   A   
   G   
   A   
   A   
   A   
   A   
   T   
   G   
   G   
   T   
   T   
   C   
   T   
   G   
   C   
   A   
   A   
   C   
   A   
   G   
   A   
   T   
   A   
   T   
   C   
   A   
   G   
   T   
   A   
   A   
   A   
   C   
   C   
   A   
   T   
   T   
   A   
   A   
   T   
   T   
   G   
   A   
   T   
   T   
   A   
   C   
   T   
   T   
   A   
   G   
   A   
   A   
   G   
   C   
   A   
   A   
   C   
   C   
   A   
   T   
   T   
   A   
   A   
   A   
   -   
   -   
   -   
   -   
   -   
   -   
   -   
   -   
   -   
   -   
   -   
   -   
   -   
   -   
   -   
   -   
   -   
   -   
   -   
   -   
   -   
   -   
   -   
   -   
   -   
   -   
   -   
   -   
   -   
   -   
   -   
   -   
   -   
   -   
   -   
   -   
   -   
   -   
   -   
   -   
   -   
   -   
   -   
   -   
   -   
   -   
   -   
   -   
   -   
   -   
   -   
   -   
   -   
   -   
   -   
   -   
   -   
   -   
   -   
   -   
   -   
 

 
   N59910F   
   -   
   -   
   -   
   -   
   -   
   -   
   -   
   -   
   -   
   -   
   -   
   -   
   -   
   -   
   -   
   -   
   -   
   -   
   -   
   -   
   -   
   -   
   -   
   -   
   -   
   -   
   -   
   -   
   -   
   -   
   -   
   -   
   -   
   -   
   -   
   -   
   -   
   -   
   -   
   -   
   -   
   -   
   -   
   -   
   -   
   -   
   -   
   -   
   -   
   -   
   -   
   -   
   -   
   -   
   -   
   -   
   -   
   -   
   -   
   -   
   -   
   -   
   -   
   -   
   -   
   -   
   -   
   -   
   -   
   -   
   -   
   -   
   -   
   -   
   -   
   -   
   -   
   -   
   -   
   -   
   -   
   -   
   -   
   -   
   -   
   -   
   -   
   -   
   -   
   -   
   -   
   -   
   -   
   -   
   -   
   -   
   -   
   -   
   -   
   -   
   -   
   -   
   -   
   -   
   -   
   -   
   -   
   -   
   C   
   A   
   A   
   T   
   G   
   G   
   C   
   A   
   A   
   G   
   C   
   C   
   A   
   A   
   A   
   C   
   A   
   A   
   G   
   A   
    A    
   T   
   T   
   A   
   G   
   C   
    C    
   G   
   A   
   A   
   G   
   C   
   G   
   A   
   A   
   G   
   A   
   A   
   A   
   A   
    C    
   A   
   G   
   C   
    T    
   A   
    C    
   T   
    A    
    C    
   A   
   T   
   T   
   T   
   T   
   T   
   A   
   A   
   A   
    C    
   G   
   T   
   A   
   T   
   T   
   G   
    A    
   C   
   A   
   A   
   A   
   A   
   C   
   A   
   G   
    A    
   A   
   A   
   T   
   T   
   T   
   G   
   A   
   T   
   A   
   A   
   G   
   T   
   T   
   A   
   C   
   C   
    G    
   T   
   C   
   C   
   G   
   T   
   T   
    G    
   T   
   T   
   C   
   A   
   A   
   G   
   A   
   A   
    G    
   C   
   T   
   A   
   G   
   C   
   T   
   T   
   A   
   A   
   A   
   G   
   A   
   A   
   A   
   A   
   A   
   T   
   G   
   G   
    C    
   T   
   A   
   T   
   G   
   A   
   T   
   A   
   C   
   T   
   A   
   A   
   A   
   T   
   C   
    T    
   G   
   T   
   T   
   G   
   T   
   T   
   G   
   A   
   A   
   A   
   A   
   A   
   T   
   A   
   C   
   C   
   A   
   A   
   G   
   C   
   A   
   A   
   T   
   T   
   T   
   A   
   T   
   T   
   C   
   A   
   G   
   G   
   G   
   A   
   T   
   T   
   C   
   A   
   A   
   G   
   C   
    A    
   G   
   A   
   A   
    G    
   G   
    A    
   G   
   T   
   C   
   A   
   A   
    A    
   G   
   C   
   T   
   A   
   G   
   T   
   G   
   A   
   T   
   G   
   T   
    T    
   C   
   A   
   A   
   G   
   T   
    C    
   A   
   A   
   A   
   A   
   A   
    G    
   G   
    C    
   G   
   A   
   A   
   A   
   G   
   A   
    T    
   A   
   A   
    T    
   C   
   A   
   A   
   T   
   A   
   C   
   A   
   C   
   A   
   T   
   T   
   T   
   A   
    C    
   C   
   T   
   A   
   T   
   A   
   A   
   A   
   T   
   T   
   A   
   T   
   C   
   G   
   A   
   T   
   G   
   A   
   G   
    C    
   A   
   C   
    T    
   C   
   C   
   T   
   T   
   T   
   A   
   G   
   G   
   C   
   G   
   A   
   A   
   A   
   T   
   G   
   A   
   A   
   A   
   G   
   A   
   T   
    T    
   T   
   G   
   T   
   C   
   T   
   T   
   A   
   T   
   C   
   A   
   A   
   T   
   C   
   A   
   A   
   G   
   T   
   A   
   T   
    C    
   G   
    C    
   C   
   A   
   A   
    A    
   A   
   A   
   A   
   G   
   G   
    C    
   G   
   A   
   T   
   A   
    C    
   C   
   T   
   A   
   C   
   C   
   A   
   A   
   A   
   T   
    C    
    G    
   C   
   T   
   T   
   G   
   G   
    A    
    A    
    G    
   C   
   C   
    G    
   T   
   C   
   T   
   T   
   T   
   A   
   A   
   T   
   T   
   T   
   T   
   T   
   C   
   C   
   A   
   G   
   A   
   T   
   A   
   T   
   G   
   T   
   C   
   A   
   G   
   G   
   A   
   A   
   A   
   T   
   G   
   A   
   T   
   A   
   A   
   A   
    A    
   T   
   T   
   T   
   C   
   G   
   A   
   T   
   T   
   C   
   A   
   A   
   G   
   T   
   A   
   G   
   A   
   T   
   A   
   A   
   T   
   G   
   C   
   C   
   A   
   A   
   A   
   C   
   G   
   T   
   G   
   G   
   A   
   G   
   A   
   A   
   A   
   T   
   T   
   G   
   T   
   C   
   G   
   A   
   T   
   C   
   G   
   T   
   A   
   A   
    T    
   G   
   G   
   T   
   A   
   G   
   T   
   G   
   G   
   G   
   C   
   T   
   A   
   G   
    C    
   A   
   A   
   T   
   T   
   A   
   A   
   C   
   A   
   A   
   A   
   G   
   T   
   G   
   T   
   T   
   T   
   G   
   A   
    C    
   G   
   A   
   A   
   G   
   T   
   G   
   G   
   G   
   C   
   G   
   T   
   A   
   G   
   T   
    G    
   C   
   C   
   T   
    G    
   G   
   C   
   A   
   A   
   A   
   C   
   T   
    C    
   G   
   G   
   T   
   T   
   C   
   T   
   G   
   G   
   C   
   G   
   C   
   A   
   G   
   A   
   A   
   A   
   A   
   A   
    A    
   C   
   A   
   G   
   C   
    C    
   A   
   A   
   T   
   A   
   T   
   C   
   A   
   A   
   A   
   G   
   C   
   T   
   T   
   T   
   T   
   A   
   G   
   T   
   G   
   A   
   T   
   A   
   A   
    G    
   T   
   T   
    C    
   G   
   G   
   C   
   G   
   T   
   T   
   T   
   C   
   T   
    G    
   T   
   T   
   G   
   A   
   T   
   G   
   A   
   A   
   A   
   T   
   C   
   A   
   A   
   T   
   C   
    A    
   A   
   A   
   A   
    G    
   T   
   T   
   A   
   A   
   G   
   C   
   C   
   A   
   A   
   G   
   G   
    G    
   T   
   G   
   G   
   G   
   T   
   C   
   C   
   A   
   A   
   G   
   C   
   A   
   G   
   A   
   C   
   T   
   C   
    C    
   T   
   T   
   T   
   G   
   T   
   A   
   C   
   C   
   A   
   A   
   T   
   C   
   A   
   C   
   A   
   G   
   T   
    C    
   G   
   C   
   T   
   T   
   C   
   T   
   G   
   A   
   A   
   C   
   C   
   A   
   G   
   T   
   G   
   A   
   C   
   A   
   G   
   A   
   A   
   T   
   T   
   A   
   C   
   C   
   A   
   A   
   C   
   A   
   G   
   G   
   G   
   G   
   C   
   T   
   G   
   C   
   G   
   A   
   C   
   A   
   A   
   A   
   A   
   G   
    A    
    T    
   A   
   C   
   A   
   G   
   A   
   G   
   T   
   C   
   A   
   C   
   G   
   T   
   T   
   A   
   T   
   T   
   A   
   T   
   C   
   C   
    G    
    C    
   T   
   G   
   G   
   G   
   G   
   G   
   A   
   A   
   G   
   C   
   A   
   G   
   C   
   T   
   G   
   C   
   G   
   C   
   A   
   A   
   T   
   T   
   A   
   A   
   T   
   C   
   G   
   G   
    G    
   T   
   A   
   T   
   A   
   C   
    G    
   G   
   G   
    C    
   A   
   C   
    C    
   A   
   T   
   T   
   A   
   C   
   T   
   G   
   C   
   A   
   G   
   A   
    A    
   G   
   A   
   T   
   A   
   T   
   T   
    A    
   A   
   A   
   A   
   A   
   A   
   A   
   A   
   T   
   C   
   C   
   A   
   G   
   A   
   G   
   T   
   T   
   A   
   A   
   G   
   T   
   A   
   G   
   T   
   A   
   C   
   G   
   G   
   G   
    C    
   G   
   T   
   A   
   A   
   T   
   T   
   G   
   G   
    T    
   A   
   A   
   A   
   A   
   C   
   T   
   G   
   G   
    C    
   T   
   T   
   A   
   G   
   A   
   A   
   C   
   G   
   A   
   G   
   C   
   G   
   T   
   T   
   T   
   G   
   A   
   T   
   A   
   A   
   A   
   G   
   A   
    A    
   T   
   T   
   A   
   A   
   G   
   A   
   G   
   G   
    G    
   C   
   A   
   A   
   G   
   A   
   T   
   G   
   G   
    T    
   G   
   G   
    C    
   T   
   C   
    A    
   C   
   T   
   A   
   G   
   T   
   T   
   A   
   T   
   T   
   T   
   T   
   A   
   G   
   A   
   T   
   G   
   A   
   C   
   A   
   A   
   A   
   G   
   A   
   G   
   A   
   A   
   T   
   G   
   T   
   C   
   A   
   A   
   A   
   A   
   A   
   A   
   G   
   C   
   T   
   T   
   T   
   A   
   C   
   A   
   A   
   A   
   C   
   C   
   A   
   A   
   A   
   G   
   A   
    G    
   A   
   A   
   A   
   A   
   A   
   A   
   G   
   A   
   T   
   G   
   G   
   T   
   C   
   A   
   A   
   A   
   C   
    G    
   A   
   T   
   T   
   A   
   A   
   A   
   T   
   T   
   A   
   A   
   C   
   G   
   A   
   T   
   T   
   G   
   A   
    C    
   A   
   G   
    T    
   G   
   G   
    C    
   G   
   T   
   A   
   C   
   A   
   A   
   C   
   A   
   A   
   C   
   A   
   A   
   G   
   C   
    C    
   T   
   T   
    C    
   G   
   C   
   T   
   A   
   T   
   A   
   T   
   T   
   T   
   G   
   A   
   C   
   A   
   A   
   A   
   C   
   G   
    C    
    C    
   C   
   T   
   G   
   G   
   T   
   A   
   G   
   T   
   G   
   C   
   G   
   G   
   T   
   C   
   A   
   T   
   T   
   A   
   C   
   C   
   G   
   A   
   T   
   C   
   C   
   T   
   C   
   A   
   A   
   A   
   A   
   A   
   G   
   G   
   C   
   G   
   A   
   T   
   T   
   T   
   A   
    T    
   T   
   A   
   G   
   C   
    A    
   A   
   C   
   G   
   G   
   T   
   T   
   A   
   G   
   T   
   T   
   C   
   A   
   C   
   C   
   T   
   T   
   C   
   G   
   T   
   A   
   T   
   G   
   A   
   T   
   C   
   C   
   T   
   A   
   A   
   T   
   A   
   A   
   A   
   A   
   T   
   G   
   G   
   C   
   C   
   A   
   A   
   T   
   G   
   G   
    C    
   A   
   T   
   T   
   T   
   C   
   T   
   C   
   A   
   A   
   A   
   A   
   A   
   G   
   A   
    A    
   T   
   A   
   T   
   G   
   A   
   T   
   G   
   C   
   T   
   T   
   A   
   C   
   A   
   A   
   T   
   A   
   A   
   C   
   A   
   A   
   C   
   A   
   A   
   A   
   G   
   A   
   T   
   T   
   T   
   A   
   C   
   C   
   A   
   T   
   T   
    T    
   A   
   C   
   A   
   G   
   C   
   A   
   C   
   G   
   T   
   T   
   T   
    T    
   G   
   C   
   G   
   A   
   C   
   A   
   G   
   G   
    C    
   T   
   A   
   T   
   G   
   C   
   A   
   C   
   C   
   A   
   G   
   G   
    C    
   T   
   C   
   T   
   A   
   C   
   C   
   T   
   T   
   T   
   A   
   A   
   A   
   A   
   C   
   A   
   A   
   T   
   T   
   A   
   C   
   C   
   G   
   G   
   T   
   G   
   C   
   C   
   A   
   T   
   T   
   G   
   G   
   T   
   T   
   T   
   A   
   G   
   A   
   T   
    G    
   C   
   A   
   G   
   G   
   G   
   A   
   C   
   A   
   T   
   T   
   G   
   A   
   A   
   G   
   C   
   C   
   A   
   G   
   A   
   T   
   G   
   A   
   A   
   G   
   A   
   G   
   C   
   T   
   A   
   G   
   A   
   A   
   A   
   T   
   T   
   A   
   A   
   T   
   G   
   G   
    T    
   T   
   T   
   G   
   A   
   A   
   A   
   T   
   G   
   G   
   C   
   A   
   A   
   A   
   A   
   A   
   G   
   A   
   T   
   A   
   A   
   A   
   T   
   C   
   T   
   T   
   G   
   G   
   G   
   G   
    C    
   G   
   G   
   C   
   T   
   A   
   T   
   T   
   T   
   T   
   G   
   C   
    G    
   A   
   C   
   A   
   C   
   G   
   T   
   G   
   T   
   A   
   A   
   A   
   A   
   G   
   A   
   A   
   G   
   C   
   A   
   A   
   G   
   T   
   C   
   C   
   A   
   G   
   T   
   C   
   A   
   A   
   T   
   C   
   T   
    G    
   C   
   G   
   T   
   A   
   C   
   C   
   G   
   C   
   T   
   T   
   T   
   A   
   G   
   T   
   T   
   A   
   A   
   T   
   T   
   C   
   A   
   G   
   A   
   T   
   A   
   A   
   T   
   A   
   T   
   T   
   T   
   A   
   T   
   T   
   T   
   T   
   G   
   C   
    C    
   C   
   A   
   A   
   C   
   A   
   A   
   A   
   C   
   A   
   T   
   T   
   A   
   C   
   G   
    G    
   A   
   T   
   G   
   G   
   G   
    A    
   G   
   A   
   A   
   G   
   A   
   C   
   A   
   A   
   A   
   T   
   T   
   T   
   A   
   G   
   A   
   G   
   C   
    G    
   G   
   G   
   T   
   C   
   T   
   T   
   A   
   A   
   T   
   A   
   A   
   A   
   T   
   T   
   C   
   A   
   T   
   T   
   T   
   T   
   C   
   G   
   G   
   T   
   G   
   A   
   G   
   G   
   A   
   A   
   C   
   T   
   A   
   G   
   A   
   T   
   T   
   T   
   A   
   C   
   C   
   A   
   A   
   T   
    T    
   G   
   C   
   C   
   A   
   T   
   G   
   A   
   C   
   G   
   C   
   C   
    T    
   G   
   C   
   T   
   C   
   A   
   A   
   A   
   T   
   T   
   T   
   C   
   A   
   A   
   A   
    T    
   G   
   A   
   A   
   G   
   A   
   T   
   A   
   A   
   A   
   T   
   T   
   T   
   A   
   A   
   T   
   T   
   C   
   T   
   G   
   A   
   A   
   A   
   T   
   T   
   C   
   T   
   T   
   T   
   T   
    A    
   G   
   C   
   A   
   G   
   A   
   T   
   A   
   C   
    G    
   G   
   G   
   C   
   T   
   A   
    C    
   G   
   G   
    T    
   C   
   A   
   A   
   G   
   G   
    T    
   C   
   A   
   A   
   T   
   T   
   G   
   T   
   T   
   A   
   A   
   T   
   T   
   T   
   C   
    G    
   C   
   C   
   A   
   A   
   T   
   C   
   C   
   A   
   G   
   C   
   A   
   A   
   G   
   C   
   T   
   A   
   C   
   C   
   A   
   T   
   G   
   T   
   A   
   T   
   A   
   G   
   T   
   G   
   T   
   T   
   T   
   T   
   C   
   C   
   A   
   A   
   A   
   A   
   T   
   A   
   A   
   T   
   G   
   G   
    A    
   A   
   C   
   A   
    T    
   T   
    A    
   G   
   T   
    C    
   T   
   A   
   T   
   C   
   C   
   A   
   A   
   A   
   A   
    C    
   T   
   A   
   G   
   T   
   T   
   T   
   T   
   A   
   G   
   A   
   C   
   A   
   A   
   A   
   G   
   A   
   A   
   A   
   C   
    G    
   A   
   A   
   G   
   A   
   A   
   A   
   A   
   A   
   A   
   G   
   A   
   C   
   A   
   A   
   T   
   G   
   T   
   A   
   A   
   T   
   C   
   A   
   G   
   C   
   G   
   C   
   C   
   A   
   A   
   C   
   G   
   C   
   A   
   G   
   C   
   G   
   A   
   A   
   T   
   A   
   C   
   A   
   A   
   T   
   T   
   G   
   C   
   C   
   A   
   C   
   A   
   G   
   A   
   T   
   C   
   T   
   T   
   T   
   T   
   A   
   G   
   G   
   T   
   A   
   G   
   T   
   G   
   T   
   G   
   G   
   A   
   A   
   G   
   A   
   T   
   C   
   C   
   T   
   T   
   C   
   A   
   G   
   G   
   T   
   T   
   A   
   T   
   G   
   T   
   T   
   T   
   A   
   T   
   A   
   A   
   T   
   A   
   T   
   G   
   T   
   A   
   C   
   A   
   A   
   T   
   C   
   C   
   G   
   A   
   A   
   C   
   T   
   T   
   C   
   T   
   C   
   A   
   T   
   T   
   G   
   G   
   C   
   T   
   G   
   C   
   T   
   A   
   A   
   A   
   A   
   C   
   A   
   G   
   G   
   A   
   A   
   C   
   A   
   G   
   C   
   T   
   G   
   A   
   A   
   A   
   T   
   A   
   A   
   A   
   A   
   G   
   A   
   T   
   A   
   A   
   A   
   C   
   A   
   A   
   G   
   A   
   T   
   A   
   C   
   A   
   G   
   A   
   T   
   G   
   G   
   C   
   A   
   A   
   A   
   G   
   A   
   A   
   A   
   A   
   C   
   A   
   G   
   T   
   T   
   T   
   C   
   C   
   T   
   T   
   C   
   T   
   A   
   A   
   C   
   G   
   T   
   T   
   A   
   G   
   A   
   T   
   C   
   G   
   T   
   A   
   G   
   T   
   A   
   A   
   C   
   A   
   A   
   T   
   A   
   A   
   A   
   T   
   T   
   C   
   T   
   T   
   A   
   A   
   C   
   A   
   A   
   T   
   G   
   A   
   T   
   T   
   A   
   T   
   G   
   G   
   T   
   T   
   G   
   A   
   A   
   A   
   A   
   T   
   T   
   C   
   A   
   G   
   G   
   A   
   G   
   A   
   A   
   A   
   A   
   T   
   G   
   G   
   T   
   T   
   C   
   T   
   G   
   C   
   A   
   A   
   C   
   A   
   G   
   A   
   T   
   A   
   T   
   C   
   A   
   G   
   T   
   A   
   A   
   A   
   C   
   C   
   A   
   T   
   T   
   A   
   A   
   T   
   T   
   G   
   A   
   T   
   T   
   A   
   C   
   T   
   T   
   A   
   G   
   A   
   A   
   G   
   C   
   A   
   A   
   C   
   C   
   A   
   T   
   T   
   A   
   A   
   A   
   -   
   -   
   -   
   -   
   -   
   -   
   -   
   -   
   -   
   -   
   -   
   -   
   -   
   -   
   -   
   -   
   -   
   -   
   -   
   -   
   -   
   -   
   -   
   -   
   -   
   -   
   -   
   -   
   -   
   -   
   -   
   -   
   -   
   -   
   -   
   -   
   -   
   -   
   -   
   -   
   -   
   -   
   -   
   -   
   -   
   -   
   -   
   -   
   -   
   -   
   -   
   -   
   -   
   -   
   -   
   -   
   -   
   -   
   -   
   -   
   -   
   -   
   -   
   -   
   -   
   -   
   -   
   -   
   -   
   -   
   -   
   -   
   -   
   -   
   -   
   -   
   -   
   -   
   -   
   -   
   -   
   -   
   -   
   -   
   -   
   -   
   -   
   -   
   -   
   -   
   -   
   -   
   -   
   -   
   -   
   -   
   -   
   -   
   -   
   -   
   -   
   -   
   -   
   -   
   -   
   -   
   -   
   -   
   -   
   -   
   -   
   -   
   -   
   -   
   -   
   -   
   -   
   -   
   -   
   -   
   -   
   -   
   -   
   -   
   -   
   -   
   -   
   -   
   -   
   -   
   -   
   -   
   -   
   -   
   -   
   -   
   -   
   -   
   -   
   -   
   -   
   -   
   -   
   -   
   -   
   -   
   -   
   -   
   -   
   -   
   -   
   -   
   -   
   -   
   -   
   -   
   -   
   -   
   -   
   -   
   -   
   -   
   -   
   -   
   -   
   -   
   -   
   -   
   -   
   C   
   A   
   A   
   T   
   G   
   G   
   C   
   A   
   A   
   G   
   C   
   C   
   A   
   A   
   A   
   C   
   A   
   A   
   G   
   A   
   A   
   T   
   T   
   A   
   G   
   C   
   C   
   G   
   A   
   A   
   G   
   C   
   G   
   A   
   A   
   G   
   A   
   A   
   A   
   A   
   C   
   A   
   G   
   C   
   T   
   A   
   C   
   T   
   A   
   C   
   A   
   T   
   T   
   T   
   T   
   T   
   A   
   A   
   A   
   C   
   G   
   T   
   A   
   T   
   T   
   G   
   A   
   C   
   A   
   A   
   A   
   A   
   C   
   A   
   G   
   A   
   A   
   A   
   T   
   T   
   T   
   G   
   A   
   T   
   A   
   A   
   G   
   T   
   T   
   A   
   C   
   C   
   G   
   T   
   C   
   C   
   G   
   T   
   T   
   G   
   T   
   T   
   C   
   A   
   A   
   G   
   A   
   A   
   G   
   C   
   T   
   A   
   G   
   C   
   T   
   T   
   A   
   A   
   A   
   G   
   A   
   A   
   A   
   A   
   A   
   T   
   G   
   G   
   C   
   T   
   A   
   T   
   G   
   A   
   T   
   A   
   C   
   T   
   A   
   A   
   A   
   T   
   C   
   T   
   G   
   T   
   T   
   G   
   T   
   T   
   G   
   A   
   A   
   A   
   A   
   A   
   T   
   A   
   C   
   C   
   A   
   A   
   G   
   C   
   A   
   A   
   T   
   T   
   T   
   A   
   T   
   T   
   C   
   A   
   G   
   G   
   G   
   A   
   T   
   T   
   C   
   A   
   A   
   G   
   C   
   A   
   G   
   A   
   A   
   G   
   G   
   A   
   G   
   T   
   C   
   A   
   A   
   A   
   G   
   C   
   T   
   A   
   G   
   T   
   G   
   A   
   T   
   G   
   T   
   T   
   C   
   A   
   A   
   G   
   T   
   C   
   A   
   A   
   A   
   A   
   A   
   G   
   G   
   C   
   G   
   A   
   A   
   A   
   G   
   A   
   T   
   A   
   A   
   T   
   C   
   A   
   A   
   T   
   A   
   C   
   A   
   C   
   A   
   T   
   T   
   T   
   A   
   C   
   C   
   T   
   A   
   T   
   A   
   A   
   A   
   T   
   T   
   A   
   T   
   C   
   G   
   A   
   T   
   G   
   A   
   G   
   C   
   A   
   C   
   T   
   C   
   C   
   T   
   T   
   T   
   A   
   G   
   G   
   C   
   G   
   A   
   A   
   A   
   T   
   G   
   A   
   A   
   A   
   G   
   A   
   T   
   T   
   T   
   G   
   T   
   C   
   T   
   T   
   A   
   T   
   C   
   A   
   A   
   T   
   C   
   A   
   A   
   G   
   T   
   A   
   T   
   C   
   G   
   C   
   C   
   A   
   A   
   A   
   A   
   A   
   A   
   G   
   G   
   C   
   G   
   A   
   T   
   A   
   C   
   C   
   T   
   A   
   C   
   C   
   A   
   A   
   A   
   T   
   C   
   G   
   C   
   T   
   T   
   G   
   G   
   A   
   A   
   G   
   C   
   C   
   G   
   T   
   C   
   T   
   T   
   T   
   A   
   A   
   T   
   T   
   T   
   T   
   T   
   C   
   C   
   A   
   G   
   A   
   T   
   A   
   T   
   G   
   T   
   C   
   A   
   G   
   G   
   A   
   A   
   A   
   T   
   G   
   A   
   T   
   A   
   A   
   A   
   A   
   T   
   T   
   T   
   C   
   G   
   A   
   T   
   T   
   C   
   A   
   A   
   G   
   T   
   A   
   G   
   A   
   T   
   A   
   A   
   T   
   G   
   C   
   C   
   A   
   A   
   A   
   C   
   G   
   T   
   G   
   G   
   A   
   G   
   A   
   A   
   A   
   T   
   T   
   G   
   T   
   C   
   G   
   A   
   T   
   C   
   G   
   T   
   A   
   A   
   T   
   G   
   G   
   T   
   A   
   G   
   T   
   G   
   G   
   G   
   C   
   T   
   A   
   G   
   C   
   A   
   A   
   T   
   T   
   A   
   A   
   C   
   A   
   A   
   A   
   G   
   T   
   G   
   T   
   T   
   T   
   G   
   A   
   C   
   G   
   A   
   A   
   G   
   T   
   G   
   G   
   G   
   C   
   G   
   T   
   A   
   G   
   T   
   G   
   C   
   C   
   T   
   G   
   G   
   C   
   A   
   A   
   A   
   C   
   T   
   C   
   G   
   G   
   T   
   T   
   C   
   T   
   G   
   G   
   C   
   G   
   C   
   A   
   G   
   A   
   A   
   A   
   A   
   A   
   A   
   C   
   A   
   G   
   C   
   C   
   A   
   A   
   T   
   A   
   T   
   C   
   A   
   A   
   A   
   G   
   C   
   T   
   T   
   T   
   T   
   A   
   G   
   T   
   G   
   A   
   T   
   A   
   A   
   G   
   T   
   T   
   C   
   G   
   G   
   C   
   G   
   T   
   T   
   T   
   C   
   T   
   G   
   T   
   T   
   G   
   A   
   T   
   G   
   A   
   A   
   A   
   T   
   C   
   A   
   A   
   T   
   C   
   A   
   A   
   A   
   A   
   G   
   T   
   T   
   A   
   A   
   G   
   C   
   C   
   A   
   A   
   G   
   G   
   G   
   T   
   G   
   G   
   G   
   T   
   C   
   C   
   A   
   A   
   G   
   C   
   A   
   G   
   A   
   C   
   T   
   C   
   C   
   T   
   T   
   T   
   G   
   T   
   A   
   C   
   C   
   A   
   A   
   T   
   C   
   A   
   C   
   A   
   G   
   T   
   C   
   G   
   C   
   T   
   T   
   C   
   T   
   G   
   A   
   A   
   C   
   C   
   A   
   G   
   T   
   G   
   A   
   C   
   A   
   G   
   A   
   A   
   T   
   T   
   A   
   C   
   C   
   A   
   A   
   C   
   A   
   G   
   G   
   G   
   G   
   C   
   T   
   G   
   C   
   G   
   A   
   C   
   A   
   A   
   A   
   A   
   G   
   A   
   T   
   A   
   C   
   A   
   G   
   A   
   G   
   T   
   C   
   A   
   C   
   G   
   T   
   T   
   A   
   T   
   T   
   A   
   T   
   C   
   C   
   G   
   C   
   T   
   G   
   G   
   G   
   G   
   G   
   A   
   A   
   G   
   C   
   A   
   G   
   C   
   T   
   G   
   C   
   G   
   C   
   A   
   A   
   T   
   T   
   A   
   A   
   T   
   C   
   G   
   G   
   G   
   T   
   A   
   T   
   A   
   C   
   G   
   G   
   G   
   C   
   A   
   C   
   C   
   A   
   T   
   T   
   A   
   C   
   T   
   G   
   C   
   A   
   G   
   A   
   A   
   G   
   A   
   T   
   A   
   T   
   T   
   A   
   A   
   A   
   A   
   A   
   A   
   A   
   A   
   T   
   C   
   C   
   A   
   G   
   A   
   G   
   T   
   T   
   A   
   A   
   G   
   T   
   A   
   G   
   T   
   A   
   C   
   G   
   G   
   G   
   C   
   G   
   T   
   A   
   A   
   T   
   T   
   G   
   G   
   T   
   A   
   A   
   A   
   A   
   C   
   T   
   G   
   G   
   C   
   T   
   T   
   A   
   G   
   A   
   A   
   C   
   G   
   A   
   G   
   C   
   G   
   T   
   T   
   T   
   G   
   A   
   T   
   A   
   A   
   A   
   G   
   A   
   A   
   T   
   T   
   A   
   A   
   G   
   A   
   G   
   G   
   G   
   C   
   A   
   A   
   G   
   A   
   T   
   G   
   G   
   T   
   G   
   G   
   C   
   T   
   C   
   A   
   C   
   T   
   A   
   G   
   T   
   T   
   A   
   T   
   T   
   T   
   T   
   A   
   G   
   A   
   T   
   G   
   A   
   C   
   A   
   A   
   A   
   G   
   A   
   G   
   A   
   A   
   T   
   G   
   T   
   C   
   A   
   A   
   A   
   A   
   A   
   A   
   G   
   C   
   T   
   T   
   T   
   A   
   C   
   A   
   A   
   A   
   C   
   C   
   A   
   A   
   A   
   G   
   A   
   G   
   A   
   A   
   A   
   A   
   A   
   A   
   G   
   A   
   T   
   G   
   G   
   T   
   C   
   A   
   A   
   A   
   C   
   G   
   A   
   T   
   T   
   A   
   A   
   A   
   T   
   T   
   A   
   A   
   C   
   G   
   A   
   T   
   T   
   G   
   A   
   C   
   A   
   G   
   T   
   G   
   G   
   C   
   G   
   T   
   A   
   C   
   A   
   A   
   C   
   A   
   A   
   C   
   A   
   A   
   G   
   C   
   C   
   T   
   T   
   C   
   G   
   C   
   T   
   A   
   T   
   A   
   T   
   T   
   T   
   G   
   A   
   C   
   A   
   A   
   A   
   C   
   G   
   C   
   C   
   C   
   T   
   G   
   G   
   T   
   A   
   G   
   T   
   G   
   C   
   G   
   G   
   T   
   C   
   A   
   T   
   T   
   A   
   C   
   C   
   G   
   A   
   T   
   C   
   C   
   T   
   C   
   A   
   A   
   A   
   A   
   A   
   G   
   G   
   C   
   G   
   A   
   T   
   T   
   T   
   A   
   T   
   T   
   A   
   G   
   C   
   A   
   A   
   C   
   G   
   G   
   T   
   T   
   A   
   G   
   T   
   T   
   C   
   A   
   C   
   C   
   T   
   T   
   C   
   G   
   T   
   A   
   T   
   G   
   A   
   T   
   C   
   C   
   T   
   A   
   A   
   T   
   A   
   A   
   A   
   A   
   T   
   G   
   G   
   C   
   C   
   A   
   A   
   T   
   G   
   G   
   C   
   A   
   T   
   T   
   T   
   C   
   T   
   C   
   A   
   A   
   A   
   A   
   A   
   G   
   A   
   A   
   T   
   A   
   T   
   G   
   A   
   T   
   G   
   C   
   T   
   T   
   A   
   C   
   A   
   A   
   T   
   A   
   A   
   C   
   A   
   A   
   C   
   A   
   A   
   A   
   G   
   A   
   T   
   T   
   T   
   A   
   C   
   C   
   A   
   T   
   T   
   T   
   A   
   C   
   A   
   G   
   C   
   A   
   C   
   G   
   T   
   T   
   T   
   T   
   G   
   C   
   G   
   A   
   C   
   A   
   G   
   G   
   C   
   T   
   A   
   T   
   G   
   C   
   A   
   C   
   C   
   A   
   G   
   G   
   C   
   T   
   C   
   T   
   A   
   C   
   C   
   T   
   T   
   T   
   A   
   A   
   A   
   A   
   C   
   A   
   A   
   T   
   T   
   A   
   C   
   C   
   G   
   G   
   T   
   G   
   C   
   C   
   A   
   T   
   T   
   G   
   G   
   T   
   T   
   T   
   A   
   G   
   A   
   T   
   G   
   C   
   A   
   G   
   G   
   G   
   A   
   C   
   A   
   T   
   T   
   G   
   A   
   A   
   G   
   C   
   C   
   A   
   G   
   A   
   T   
   G   
   A   
   A   
   G   
   A   
   G   
   C   
   T   
   A   
   G   
   A   
   A   
   A   
   T   
   T   
   A   
   A   
   T   
   G   
   G   
   T   
   T   
   T   
   G   
   A   
   A   
   A   
   T   
   G   
   G   
   C   
   A   
   A   
   A   
   A   
   A   
   G   
   A   
   T   
   A   
   A   
   A   
   T   
   C   
   T   
   T   
   G   
   G   
   G   
   G   
   C   
   G   
   G   
   C   
   T   
   A   
   T   
   T   
   T   
   T   
   G   
   C   
   G   
   A   
   C   
   A   
   C   
   G   
   T   
   G   
   T   
   A   
   A   
   A   
   A   
   G   
   A   
   A   
   G   
   C   
   A   
   A   
   G   
   T   
   C   
   C   
   A   
   G   
   T   
   C   
   A   
   A   
   T   
   C   
   T   
   G   
   C   
   G   
   T   
   A   
   C   
   C   
   G   
   C   
   T   
   T   
   T   
   A   
   G   
   T   
   T   
   A   
   A   
   T   
   T   
   C   
   A   
   G   
   A   
   T   
   A   
   A   
   T   
   A   
   T   
   T   
   T   
   A   
   T   
   T   
   T   
   T   
   G   
   C   
   C   
   C   
   A   
   A   
   C   
   A   
   A   
   A   
   C   
   A   
   T   
   T   
   A   
   C   
   G   
   G   
   A   
   T   
   G   
   G   
   G   
   A   
   G   
   A   
   A   
   G   
   A   
   C   
   A   
   A   
   A   
   T   
   T   
   T   
   A   
   G   
   A   
   G   
   C   
   G   
   G   
   G   
   T   
   C   
   T   
   T   
   A   
   A   
   T   
   A   
   A   
   A   
   T   
   T   
   C   
   A   
   T   
   T   
   T   
   T   
   C   
   G   
   G   
   T   
   G   
   A   
   G   
   G   
   A   
   A   
   C   
   T   
   A   
   G   
   A   
   T   
   T   
   T   
   A   
   C   
   C   
   A   
   A   
   T   
   T   
   G   
   C   
   C   
   A   
   T   
   G   
   A   
   C   
   G   
   C   
   C   
   T   
   G   
   C   
   T   
   C   
   A   
   A   
   A   
   T   
   T   
   T   
   C   
   A   
   A   
   A   
   T   
   G   
   A   
   A   
   G   
   A   
   T   
   A   
   A   
   A   
   T   
   T   
   T   
   A   
   A   
   T   
   T   
   C   
   T   
   G   
   A   
   A   
   A   
   T   
   T   
   C   
   T   
   T   
   T   
   T   
   A   
   G   
   C   
   A   
   G   
   A   
   T   
   A   
   C   
   G   
   G   
   G   
   C   
   T   
   A   
   C   
   G   
   G   
   T   
   C   
   A   
   A   
   G   
   G   
   T   
   C   
   A   
   A   
   T   
   T   
   G   
   T   
   T   
   A   
   A   
   T   
   T   
   T   
   C   
   G   
   C   
   C   
   A   
   A   
   T   
   C   
   C   
   A   
   G   
   C   
   A   
   A   
   G   
   C   
   T   
   A   
   C   
   C   
   A   
   T   
   G   
   T   
   A   
   T   
   A   
   G   
   T   
   G   
   T   
   T   
   T   
   T   
   C   
   C   
   A   
   A   
   A   
   A   
   T   
   A   
   A   
   T   
   G   
   G   
   A   
   A   
   C   
   A   
   T   
   T   
   A   
   G   
   T   
   C   
   T   
   A   
   T   
   C   
   C   
   A   
   A   
   A   
   A   
   C   
   T   
   A   
   G   
   T   
   T   
   T   
   T   
   A   
   G   
   A   
   C   
   A   
   A   
   A   
   G   
   A   
   A   
   A   
   C   
   G   
   A   
   A   
   G   
   A   
   A   
   A   
   A   
   A   
   A   
   G   
   A   
   C   
   A   
   A   
   T   
   G   
   T   
   A   
   A   
   T   
   C   
   A   
   G   
   C   
   G   
   C   
   C   
   A   
   A   
   C   
   G   
   C   
   A   
   G   
   C   
   G   
   A   
   A   
   T   
   A   
   C   
   A   
   A   
   T   
   T   
   G   
   C   
   C   
   A   
   C   
   A   
   G   
   A   
   T   
   C   
   T   
   T   
   T   
   T   
   A   
   G   
   G   
   T   
   A   
   G   
   T   
   G   
   T   
   G   
   G   
   A   
   A   
   G   
   A   
   T   
   C   
   C   
   T   
   T   
   C   
   A   
   G   
   G   
   T   
   T   
   A   
   T   
   G   
   T   
   T   
   T   
   A   
   T   
   A   
   A   
   T   
   A   
   T   
   G   
   T   
   A   
   C   
   A   
   A   
   T   
   C   
   C   
   G   
   A   
   A   
   C   
   T   
   T   
   C   
   T   
   C   
   A   
   T   
   T   
   G   
   G   
   C   
   T   
   G   
   C   
   T   
   A   
   A   
   A   
   A   
   C   
   A   
   G   
   G   
   A   
   A   
   C   
   A   
   G   
   C   
   T   
   G   
   A   
   A   
   A   
   T   
   A   
   A   
   A   
   A   
   G   
   A   
   T   
   A   
   A   
   A   
   C   
   A   
   A   
   G   
   A   
   T   
   A   
   C   
   A   
   G   
   A   
   T   
   G   
   G   
   C   
   A   
   A   
   A   
   G   
   A   
   A   
   A   
   A   
   C   
   A   
   G   
   T   
   T   
   T   
   C   
   C   
   T   
   T   
   C   
   T   
   A   
   A   
   C   
   G   
   T   
   T   
   A   
   G   
   A   
   T   
   C   
   G   
   T   
   A   
   G   
   T   
   A   
   A   
   C   
   A   
   A   
   T   
   A   
   A   
   A   
   T   
   T   
   C   
   T   
   T   
   A   
   A   
   C   
   A   
   A   
   T   
   G   
   A   
   T   
   T   
   A   
   T   
   G   
   G   
   T   
   T   
   G   
   A   
   A   
   A   
   A   
   T   
   T   
   C   
   A   
   G   
   G   
   A   
   G   
   A   
   A   
   A   
   A   
   T   
   G   
   G   
   T   
   T   
   C   
   T   
   G   
   C   
   A   
   A   
   C   
   A   
   G   
   A   
   T   
   A   
   T   
   C   
   A   
   G   
   T   
   A   
   A   
   A   
   C   
   C   
   A   
   T   
   T   
   A   
   A   
   T   
   T   
   G   
   A   
   T   
   T   
   A   
   C   
   T   
   T   
   A   
   G   
   A   
   A   
   G   
   C   
   A   
   A   
   C   
   C   
   A   
   T   
   T   
   A   
   A   
   A   
   -   
   -   
   -   
   -   
   -   
   -   
   -   
   -   
   -   
   -   
   -   
   -   
   -   
   -   
   -   
   -   
   -   
   -   
   -   
   -   
   -   
   -   
   -   
   -   
   -   
   -   
   -   
   -   
   -   
   -   
   -   
   -   
   -   
   -   
   -   
   -   
   -   
   -   
   -   
   -   
   -   
   -   
   -   
   -   
   -   
   -   
   -   
   -   
   -   
   -   
   -   
   -   
   -   
   -   
   -   
   -   
   -   
   -   
   -   
   -   
   -   
 

 
   N59896F   
   -   
   -   
   -   
   -   
   -   
   -   
   -   
   -   
   -   
   -   
   -   
   -   
   -   
   -   
   -   
   -   
   -   
   -   
   -   
   -   
   -   
   -   
   -   
   -   
   -   
   -   
   -   
   -   
   -   
   -   
   -   
   -   
   -   
   -   
   -   
   -   
   -   
   -   
   -   
   -   
   -   
   -   
   -   
   -   
   -   
   -   
   -   
   -   
   -   
   -   
   -   
   -   
   -   
   -   
   -   
   -   
   -   
   -   
   -   
   -   
   -   
   -   
   -   
   -   
   -   
   -   
   -   
   -   
   -   
   -   
   -   
   -   
   -   
   -   
   -   
   -   
   -   
   -   
   -   
   -   
   -   
   -   
   -   
   -   
   -   
   -   
   -   
   -   
   -   
   -   
   -   
   -   
   -   
   -   
   -   
   -   
   -   
   -   
   -   
   -   
   -   
   -   
   -   
   -   
   -   
   -   
   -   
   -   
   C   
   A   
   A   
   T   
   G   
   G   
   C   
   A   
   A   
   G   
   C   
   C   
   A   
   A   
   A   
   C   
   A   
   A   
   G   
   A   
    A    
   T   
   T   
   A   
   G   
   C   
    C    
   G   
   A   
   A   
   G   
   C   
   G   
   A   
   A   
   G   
   A   
   A   
   A   
   A   
    C    
   A   
   G   
   C   
    T    
   A   
    C    
   T   
    A    
    A    
   A   
   T   
   T   
   T   
   T   
   T   
   A   
   A   
   A   
    C    
   G   
   T   
   A   
   T   
   T   
   G   
    T    
   C   
   A   
   A   
   A   
   A   
   C   
   A   
   G   
    G    
   A   
   A   
   T   
   T   
   T   
   G   
   A   
   T   
   A   
   A   
   G   
   T   
   T   
   A   
   C   
   C   
    G    
   T   
   C   
   C   
   G   
   T   
   T   
    G    
   T   
   T   
   C   
   A   
   A   
   G   
   A   
   A   
    G    
   C   
   T   
   A   
   G   
   C   
   T   
   T   
   A   
   A   
   A   
   G   
   A   
   A   
   A   
   A   
   A   
   T   
   G   
   G   
    C    
   T   
   A   
   T   
   G   
   A   
   T   
   A   
   C   
   T   
   A   
   A   
   A   
   T   
   C   
    T    
   G   
   T   
   T   
   G   
   T   
   T   
   G   
   A   
   A   
   A   
   A   
   A   
   T   
   A   
   C   
   C   
   A   
   A   
   G   
   C   
   A   
   A   
   T   
   T   
   T   
   A   
   T   
   T   
   C   
   A   
   G   
   G   
   G   
   A   
   T   
   T   
   C   
   A   
   A   
   G   
   C   
    A    
   G   
   A   
   A   
    G    
   G   
    A    
   G   
   T   
   C   
   A   
   A   
    A    
   G   
   C   
   T   
   A   
   G   
   T   
   G   
   A   
   T   
   G   
   T   
    C    
   C   
   A   
   A   
   G   
   T   
    C    
   A   
   A   
   A   
   A   
   A   
    G    
   G   
    C    
   G   
   A   
   A   
   A   
   G   
   A   
    C    
   A   
   A   
    T    
   C   
   A   
   A   
   T   
   A   
   C   
   A   
   C   
   A   
   T   
   T   
   T   
   A   
    C    
   C   
   T   
   A   
   T   
   A   
   A   
   A   
   T   
   T   
   A   
   T   
   C   
   G   
   A   
   T   
   G   
   A   
   G   
    C    
   A   
   C   
    G    
   C   
   C   
   T   
   T   
   T   
   A   
   G   
   G   
   C   
   G   
   A   
   A   
   A   
   T   
   G   
   A   
   A   
   A   
   G   
   A   
   T   
    T    
   T   
   G   
   T   
   C   
   T   
   T   
   A   
   T   
   C   
   A   
   A   
   T   
   C   
   A   
   A   
   G   
   T   
   A   
   T   
    C    
   G   
    C    
   C   
   A   
   A   
    A    
   A   
   A   
   A   
   G   
   G   
    C    
   G   
   A   
   T   
   A   
    C    
   C   
   T   
   A   
   C   
   C   
   A   
   A   
   A   
   T   
    C    
    G    
   C   
   T   
   T   
   G   
   G   
    A    
    A    
    G    
   C   
   C   
    G    
   T   
   C   
   T   
   T   
   T   
   A   
   A   
   T   
   T   
   T   
   T   
   T   
   C   
   C   
   A   
   G   
   A   
   T   
   A   
   T   
   G   
   T   
   C   
   A   
   G   
   G   
   A   
   A   
   A   
   T   
   G   
   A   
   T   
   A   
   A   
   A   
    A    
   T   
   T   
   T   
   C   
   G   
   A   
   T   
   T   
   C   
   A   
   A   
   G   
   T   
   A   
   G   
   A   
   T   
   A   
   A   
   T   
   G   
   C   
   C   
   A   
   A   
   A   
   C   
   G   
   T   
   G   
   G   
   A   
   G   
   A   
   A   
   A   
   T   
   T   
   G   
   T   
   C   
   G   
   A   
   T   
   C   
   G   
   T   
   A   
   A   
    T    
   G   
   G   
   T   
   A   
   G   
   T   
   G   
   G   
   G   
   C   
   T   
   A   
   G   
    C    
   A   
   A   
   T   
   T   
   A   
   A   
   C   
   A   
   A   
   A   
   G   
   T   
   G   
   T   
   T   
   T   
   G   
   A   
    C    
   G   
   A   
   A   
   G   
   T   
   G   
   G   
   G   
   C   
   G   
   T   
   A   
   G   
   T   
    G    
   C   
   C   
   T   
    G    
   G   
   C   
   A   
   A   
   A   
   C   
   T   
    C    
   G   
   G   
   T   
   T   
   C   
   T   
   G   
   G   
   C   
   G   
   C   
   A   
   G   
   A   
   A   
   A   
   A   
   A   
    A    
   C   
   A   
   G   
   C   
    C    
   A   
   A   
   T   
   A   
   T   
   C   
   A   
   A   
   A   
   G   
   C   
   T   
   T   
   T   
   T   
   A   
   G   
   T   
   G   
   A   
   T   
   A   
   A   
    G    
   T   
   T   
    C    
   G   
   G   
   C   
   G   
   T   
   T   
   T   
   C   
   T   
    G    
   T   
   T   
   G   
   A   
   T   
   G   
   A   
   A   
   A   
   T   
   C   
   A   
   A   
   T   
   C   
    A    
   A   
   A   
   A   
    G    
   T   
   T   
   A   
   A   
   G   
   C   
   C   
   A   
   A   
   G   
   G   
    G    
   T   
   G   
   G   
   G   
   T   
   C   
   C   
   A   
   A   
   G   
   C   
   A   
   G   
   A   
   C   
   T   
   C   
    C    
   T   
   T   
   T   
   G   
   T   
   A   
   C   
   C   
   A   
   A   
   T   
   C   
   A   
   C   
   A   
   G   
   T   
    C    
   G   
   C   
   T   
   T   
   C   
   T   
   G   
   A   
   A   
   C   
   C   
   A   
   G   
   T   
   G   
   A   
   C   
   A   
   G   
   A   
   A   
   T   
   T   
   A   
   C   
   C   
   A   
   A   
   C   
   A   
   G   
   G   
   G   
   G   
   C   
   T   
   G   
   C   
   G   
   A   
   C   
   A   
   A   
   A   
   A   
   G   
    A    
    T    
   A   
   C   
   A   
   G   
   A   
   G   
   T   
   C   
   A   
   C   
   G   
   T   
   T   
   A   
   T   
   T   
   A   
   T   
   C   
   C   
    G    
    C    
   T   
   G   
   G   
   G   
   G   
   G   
   A   
   A   
   G   
   C   
   A   
   G   
   C   
   T   
   G   
   C   
   G   
   C   
   A   
   A   
   T   
   T   
   A   
   A   
   T   
   C   
   G   
   G   
    G    
   T   
   A   
   T   
   A   
   C   
    G    
   G   
   G   
    C    
   A   
   C   
    C    
   A   
   T   
   T   
   A   
   C   
   T   
   G   
   C   
   A   
   G   
   A   
    A    
   G   
   A   
   T   
   A   
   T   
   T   
    A    
   A   
   A   
   A   
   A   
   A   
   A   
   A   
   T   
   C   
   C   
   A   
   G   
   A   
   G   
   T   
   T   
   A   
   A   
   G   
   T   
   A   
   G   
   T   
   A   
   C   
   G   
   G   
   G   
    C    
   G   
   T   
   A   
   A   
   T   
   T   
   G   
   G   
    T    
   A   
   A   
   A   
   A   
   C   
   T   
   G   
   G   
    C    
   T   
   T   
   A   
   G   
   A   
   A   
   C   
   G   
   A   
   G   
   C   
   G   
   T   
   T   
   T   
   G   
   A   
   T   
   A   
   A   
   A   
   G   
   A   
    A    
   T   
   T   
   A   
   A   
   G   
   A   
   G   
   G   
    G    
   C   
   A   
   A   
   G   
   A   
   T   
   G   
   G   
    T    
   G   
   G   
    C    
   T   
   C   
    A    
   C   
   T   
   A   
   G   
   T   
   T   
   A   
   T   
   T   
   T   
   T   
   A   
   G   
   A   
   T   
   G   
   A   
   C   
   A   
   A   
   A   
   G   
   A   
   G   
   A   
   A   
   T   
   G   
   T   
   C   
   A   
   A   
   A   
   A   
   A   
   A   
   G   
   C   
   T   
   T   
   T   
   A   
   C   
   A   
   A   
   A   
   C   
   C   
   A   
   A   
   A   
   G   
   A   
    G    
   A   
   A   
   A   
   A   
   A   
   A   
   G   
   A   
   T   
   G   
   G   
   T   
   C   
   A   
   A   
   A   
   C   
    G    
   A   
   T   
   T   
   A   
   A   
   A   
   T   
   T   
   A   
   A   
   C   
   G   
   A   
   T   
   T   
   G   
   A   
    C    
   A   
   G   
    T    
   G   
   G   
    C    
   G   
   T   
   A   
   C   
   A   
   A   
   C   
   A   
   A   
   C   
   A   
   A   
   G   
   C   
    C    
   T   
   T   
    C    
   G   
   C   
   T   
   A   
   T   
   A   
   T   
   T   
   T   
   G   
   A   
   C   
   A   
   A   
   A   
   C   
   G   
    C    
    C    
   C   
   T   
   G   
   G   
   T   
   A   
   G   
   T   
   G   
   C   
   G   
   G   
   T   
   C   
   A   
   T   
   T   
   A   
   C   
   C   
   G   
   A   
   T   
   C   
   C   
   T   
   C   
   A   
   A   
   A   
   A   
   A   
   G   
   G   
   C   
   G   
   A   
   T   
   T   
   T   
   A   
    T    
   T   
   A   
   G   
   C   
    A    
   A   
   C   
   G   
   G   
   T   
   T   
   A   
   G   
   T   
   T   
   C   
   A   
   C   
   C   
   T   
   T   
   C   
   G   
   T   
   A   
   T   
   G   
   A   
   T   
   C   
   C   
   T   
   A   
   A   
   T   
   A   
   A   
   A   
   A   
   T   
   G   
   G   
   C   
   C   
   A   
   A   
   T   
   G   
   G   
    C    
   A   
   T   
   T   
   T   
   C   
   T   
   C   
   A   
   A   
   A   
   A   
   A   
   G   
   A   
    A    
   T   
   A   
   T   
   G   
   A   
   T   
   G   
   C   
   T   
   T   
   A   
   C   
   A   
   A   
   T   
   A   
   A   
   C   
   A   
   A   
   C   
   A   
   A   
   A   
   G   
   A   
   T   
   T   
   T   
   A   
   C   
   C   
   A   
   T   
   T   
    C    
   A   
   C   
   A   
   G   
   C   
   A   
   C   
   G   
   T   
   T   
   T   
    T    
   G   
   C   
   G   
   A   
   C   
   A   
   G   
   G   
    C    
   T   
   A   
   T   
   G   
   C   
   A   
   C   
   C   
   A   
   G   
   G   
    C    
   T   
   C   
   T   
   A   
   C   
   C   
   T   
   T   
   T   
   A   
   A   
   A   
   A   
   C   
   A   
   A   
   T   
   T   
   A   
   C   
   C   
   G   
   G   
   T   
   G   
   C   
   C   
   A   
   T   
   T   
   G   
   G   
   T   
   T   
   T   
   A   
   G   
   A   
   T   
    G    
   C   
   A   
   G   
   G   
   G   
   A   
   C   
   A   
   T   
   T   
   G   
   A   
   A   
   G   
   C   
   C   
   A   
   G   
   A   
   T   
   G   
   A   
   A   
   G   
   A   
   G   
   C   
   T   
   A   
   G   
   A   
   A   
   A   
   T   
   T   
   A   
   A   
   T   
   G   
   G   
    T    
   T   
   T   
   G   
   A   
   A   
   A   
   T   
   G   
   G   
   C   
   A   
   A   
   A   
   A   
   A   
   G   
   A   
   T   
   A   
   A   
   A   
   T   
   C   
   T   
   T   
   G   
   G   
   G   
   G   
    C    
   G   
   G   
   C   
   T   
   A   
   T   
   T   
   T   
   T   
   G   
   C   
    G    
   A   
   C   
   A   
   C   
   G   
   T   
   G   
   T   
   A   
   A   
   A   
   A   
   G   
   A   
   A   
   G   
   C   
   A   
   A   
   G   
   T   
   C   
   C   
   A   
   G   
   T   
   C   
   A   
   A   
   T   
   C   
   T   
    G    
   C   
   G   
   T   
   A   
   C   
   C   
   G   
   C   
   T   
   T   
   T   
   A   
   G   
   T   
   T   
   A   
   A   
   T   
   T   
   C   
   A   
   G   
   A   
   T   
   A   
   A   
   T   
   A   
   T   
   T   
   T   
   A   
   T   
   T   
   T   
   T   
   G   
   C   
    C    
   C   
   A   
   A   
   C   
   A   
   A   
   A   
   C   
   A   
   T   
   T   
   A   
   C   
   G   
    G    
   A   
   T   
   G   
   G   
   G   
    A    
   G   
   A   
   A   
   G   
   A   
   C   
   A   
   A   
   A   
   T   
   T   
   T   
   A   
   G   
   A   
   G   
   C   
    G    
   G   
   G   
   T   
   C   
   T   
   T   
   A   
   A   
   T   
   A   
   A   
   A   
   T   
   T   
   C   
   A   
   T   
   T   
   T   
   T   
   C   
   G   
   G   
   T   
   G   
   A   
   G   
   G   
   A   
   A   
   C   
   T   
   A   
   G   
   A   
   T   
   T   
   T   
   A   
   C   
   C   
   A   
   A   
   T   
    T    
   G   
   C   
   C   
   A   
   T   
   G   
   A   
   C   
   G   
   C   
   C   
    T    
   G   
   C   
   T   
   C   
   A   
   A   
   A   
   T   
   T   
   T   
   C   
   A   
   A   
   A   
    T    
   G   
   A   
   A   
   G   
   A   
   T   
   A   
   A   
   A   
   T   
   T   
   T   
   A   
   A   
   T   
   T   
   C   
   T   
   G   
   A   
   A   
   A   
   T   
   T   
   C   
   T   
   T   
   T   
   T   
    A    
   G   
   C   
   A   
   G   
   A   
   T   
   A   
   C   
    G    
   G   
   G   
   C   
   T   
   A   
    C    
   G   
   G   
    T    
   C   
   A   
   A   
   G   
   G   
    C    
   C   
   A   
   A   
   T   
   T   
   G   
   T   
   T   
   A   
   A   
   T   
   T   
   T   
   C   
    G    
   C   
   C   
   A   
   A   
   T   
   C   
   C   
   A   
   G   
   C   
   A   
   A   
   G   
   C   
   T   
   A   
   C   
   C   
   A   
   T   
   G   
   T   
   A   
   T   
   A   
   G   
   T   
   G   
   T   
   T   
   T   
   T   
   C   
   C   
   A   
   A   
   A   
   A   
   T   
   A   
   A   
   T   
   G   
   G   
    G    
   A   
   C   
   A   
    T    
   T   
    A    
   G   
   T   
    C    
   T   
   A   
   T   
   C   
   C   
   A   
   A   
   A   
   A   
    T    
   T   
   A   
   G   
   T   
   T   
   T   
   T   
   A   
   G   
   A   
   C   
   A   
   A   
   A   
   G   
   A   
   A   
   A   
   C   
    G    
   A   
   A   
   G   
   A   
   A   
   A   
   A   
   A   
   A   
   G   
   A   
   C   
   A   
   A   
   T   
   G   
   T   
   A   
   A   
   T   
   C   
   A   
   G   
   C   
   G   
   C   
   C   
   A   
   A   
   C   
   G   
   C   
   A   
   G   
   C   
   G   
   A   
   A   
   T   
   A   
   C   
   A   
   A   
   T   
   T   
   G   
   C   
   C   
   A   
   C   
   A   
   G   
   A   
   T   
   C   
   T   
   T   
   T   
   T   
   A   
   G   
   G   
   T   
   A   
   G   
   T   
   G   
   T   
   G   
   G   
   A   
   A   
   G   
   A   
   T   
   C   
   C   
   T   
   T   
   C   
   A   
   G   
   G   
   T   
   T   
   A   
   T   
   G   
   T   
   T   
   T   
   A   
   T   
   A   
   A   
   T   
   A   
   T   
   G   
   T   
   A   
   C   
   A   
   A   
   T   
   C   
   C   
   G   
   A   
   A   
   C   
   T   
   T   
   C   
   T   
   C   
   A   
   T   
   T   
   G   
   G   
   C   
   T   
   G   
   C   
   T   
   A   
   A   
   A   
   A   
   C   
   A   
   G   
   G   
   A   
   A   
   C   
   A   
   G   
   C   
   T   
   G   
   A   
   A   
   A   
   T   
   A   
   A   
   A   
   A   
   G   
   A   
   T   
   A   
   A   
   A   
   C   
   A   
   A   
   G   
   A   
   T   
   A   
   C   
   A   
   G   
   A   
   T   
   G   
   G   
   C   
   A   
   A   
   A   
   G   
   A   
   A   
   A   
   A   
   C   
   A   
   G   
   T   
   T   
   T   
   C   
   C   
   T   
   T   
   C   
   T   
   A   
   A   
   C   
   G   
   T   
   T   
   A   
   G   
   A   
   T   
   C   
   G   
   T   
   A   
   G   
   T   
   A   
   A   
   C   
   A   
   A   
   T   
   A   
   A   
   A   
   T   
   T   
   C   
   T   
   T   
   A   
   A   
   C   
   A   
   A   
   T   
   G   
   A   
   T   
   T   
   A   
   T   
   G   
   G   
   T   
   T   
   G   
   A   
   A   
   A   
   A   
   T   
   T   
   C   
   A   
   G   
   G   
   A   
   G   
   A   
   A   
   A   
   A   
   T   
   G   
   G   
   T   
   T   
   C   
   T   
   G   
   C   
   A   
   A   
   C   
   A   
   G   
   A   
   T   
   A   
   T   
   C   
   A   
   G   
   T   
   A   
   A   
   A   
   C   
   C   
   A   
   T   
   T   
   A   
   A   
   T   
   T   
   G   
   A   
   T   
   T   
   A   
   C   
   T   
   T   
   A   
   G   
   A   
   A   
   G   
   C   
   A   
   A   
   C   
   C   
   A   
   T   
   T   
   A   
   A   
   A   
   -   
   -   
   -   
   -   
   -   
   -   
   -   
   -   
   -   
   -   
   -   
   -   
   -   
   -   
   -   
   -   
   -   
   -   
   -   
   -   
   -   
   -   
   -   
   -   
   -   
   -   
   -   
   -   
   -   
   -   
   -   
   -   
   -   
   -   
   -   
   -   
   -   
   -   
   -   
   -   
   -   
   -   
   -   
   -   
   -   
   -   
   -   
   -   
   -   
   -   
   -   
   -   
   -   
   -   
   -   
   -   
   -   
   -   
   -   
   -   
   -   
   -   
   -   
   -   
   -   
   -   
   -   
   -   
   -   
   -   
   -   
   -   
   -   
   -   
   -   
   -   
   -   
   -   
   -   
   -   
   -   
   -   
   -   
   -   
   -   
   -   
   -   
   -   
   -   
   -   
   -   
   -   
   -   
   -   
   -   
   -   
   -   
   -   
   -   
   -   
   -   
   -   
   -   
   -   
   -   
   -   
   -   
   -   
   -   
   -   
   -   
   -   
   -   
   -   
   -   
   -   
   -   
   -   
   -   
   -   
   -   
   -   
   -   
   -   
   -   
   -   
   -   
   -   
   -   
   -   
   -   
   -   
   -   
   -   
   -   
   -   
   -   
   -   
   -   
   -   
   -   
   -   
   -   
   -   
   -   
   -   
   -   
   -   
   -   
   -   
   -   
   -   
   -   
   -   
   -   
   -   
   -   
   -   
   -   
   -   
   -   
   -   
   -   
   -   
   -   
   -   
   -   
   -   
   -   
   C   
   A   
   A   
   T   
   G   
   G   
   C   
   A   
   A   
   G   
   C   
   C   
   A   
   A   
   A   
   C   
   A   
   A   
   G   
   A   
   A   
   T   
   T   
   A   
   G   
   C   
   C   
   G   
   A   
   A   
   G   
   C   
   G   
   A   
   A   
   G   
   A   
   A   
   A   
   A   
   C   
   A   
   G   
   C   
   T   
   A   
   C   
   T   
   A   
   A   
   A   
   T   
   T   
   T   
   T   
   T   
   A   
   A   
   A   
   C   
   G   
   T   
   A   
   T   
   T   
   G   
   T   
   C   
   A   
   A   
   A   
   A   
   C   
   A   
   G   
   G   
   A   
   A   
   T   
   T   
   T   
   G   
   A   
   T   
   A   
   A   
   G   
   T   
   T   
   A   
   C   
   C   
   G   
   T   
   C   
   C   
   G   
   T   
   T   
   G   
   T   
   T   
   C   
   A   
   A   
   G   
   A   
   A   
   G   
   C   
   T   
   A   
   G   
   C   
   T   
   T   
   A   
   A   
   A   
   G   
   A   
   A   
   A   
   A   
   A   
   T   
   G   
   G   
   C   
   T   
   A   
   T   
   G   
   A   
   T   
   A   
   C   
   T   
   A   
   A   
   A   
   T   
   C   
   T   
   G   
   T   
   T   
   G   
   T   
   T   
   G   
   A   
   A   
   A   
   A   
   A   
   T   
   A   
   C   
   C   
   A   
   A   
   G   
   C   
   A   
   A   
   T   
   T   
   T   
   A   
   T   
   T   
   C   
   A   
   G   
   G   
   G   
   A   
   T   
   T   
   C   
   A   
   A   
   G   
   C   
   A   
   G   
   A   
   A   
   G   
   G   
   A   
   G   
   T   
   C   
   A   
   A   
   A   
   G   
   C   
   T   
   A   
   G   
   T   
   G   
   A   
   T   
   G   
   T   
   C   
   C   
   A   
   A   
   G   
   T   
   C   
   A   
   A   
   A   
   A   
   A   
   G   
   G   
   C   
   G   
   A   
   A   
   A   
   G   
   A   
   C   
   A   
   A   
   T   
   C   
   A   
   A   
   T   
   A   
   C   
   A   
   C   
   A   
   T   
   T   
   T   
   A   
   C   
   C   
   T   
   A   
   T   
   A   
   A   
   A   
   T   
   T   
   A   
   T   
   C   
   G   
   A   
   T   
   G   
   A   
   G   
   C   
   A   
   C   
   G   
   C   
   C   
   T   
   T   
   T   
   A   
   G   
   G   
   C   
   G   
   A   
   A   
   A   
   T   
   G   
   A   
   A   
   A   
   G   
   A   
   T   
   T   
   T   
   G   
   T   
   C   
   T   
   T   
   A   
   T   
   C   
   A   
   A   
   T   
   C   
   A   
   A   
   G   
   T   
   A   
   T   
   C   
   G   
   C   
   C   
   A   
   A   
   A   
   A   
   A   
   A   
   G   
   G   
   C   
   G   
   A   
   T   
   A   
   C   
   C   
   T   
   A   
   C   
   C   
   A   
   A   
   A   
   T   
   C   
   G   
   C   
   T   
   T   
   G   
   G   
   A   
   A   
   G   
   C   
   C   
   G   
   T   
   C   
   T   
   T   
   T   
   A   
   A   
   T   
   T   
   T   
   T   
   T   
   C   
   C   
   A   
   G   
   A   
   T   
   A   
   T   
   G   
   T   
   C   
   A   
   G   
   G   
   A   
   A   
   A   
   T   
   G   
   A   
   T   
   A   
   A   
   A   
   A   
   T   
   T   
   T   
   C   
   G   
   A   
   T   
   T   
   C   
   A   
   A   
   G   
   T   
   A   
   G   
   A   
   T   
   A   
   A   
   T   
   G   
   C   
   C   
   A   
   A   
   A   
   C   
   G   
   T   
   G   
   G   
   A   
   G   
   A   
   A   
   A   
   T   
   T   
   G   
   T   
   C   
   G   
   A   
   T   
   C   
   G   
   T   
   A   
   A   
   T   
   G   
   G   
   T   
   A   
   G   
   T   
   G   
   G   
   G   
   C   
   T   
   A   
   G   
   C   
   A   
   A   
   T   
   T   
   A   
   A   
   C   
   A   
   A   
   A   
   G   
   T   
   G   
   T   
   T   
   T   
   G   
   A   
   C   
   G   
   A   
   A   
   G   
   T   
   G   
   G   
   G   
   C   
   G   
   T   
   A   
   G   
   T   
   G   
   C   
   C   
   T   
   G   
   G   
   C   
   A   
   A   
   A   
   C   
   T   
   C   
   G   
   G   
   T   
   T   
   C   
   T   
   G   
   G   
   C   
   G   
   C   
   A   
   G   
   A   
   A   
   A   
   A   
   A   
   A   
   C   
   A   
   G   
   C   
   C   
   A   
   A   
   T   
   A   
   T   
   C   
   A   
   A   
   A   
   G   
   C   
   T   
   T   
   T   
   T   
   A   
   G   
   T   
   G   
   A   
   T   
   A   
   A   
   G   
   T   
   T   
   C   
   G   
   G   
   C   
   G   
   T   
   T   
   T   
   C   
   T   
   G   
   T   
   T   
   G   
   A   
   T   
   G   
   A   
   A   
   A   
   T   
   C   
   A   
   A   
   T   
   C   
   A   
   A   
   A   
   A   
   G   
   T   
   T   
   A   
   A   
   G   
   C   
   C   
   A   
   A   
   G   
   G   
   G   
   T   
   G   
   G   
   G   
   T   
   C   
   C   
   A   
   A   
   G   
   C   
   A   
   G   
   A   
   C   
   T   
   C   
   C   
   T   
   T   
   T   
   G   
   T   
   A   
   C   
   C   
   A   
   A   
   T   
   C   
   A   
   C   
   A   
   G   
   T   
   C   
   G   
   C   
   T   
   T   
   C   
   T   
   G   
   A   
   A   
   C   
   C   
   A   
   G   
   T   
   G   
   A   
   C   
   A   
   G   
   A   
   A   
   T   
   T   
   A   
   C   
   C   
   A   
   A   
   C   
   A   
   G   
   G   
   G   
   G   
   C   
   T   
   G   
   C   
   G   
   A   
   C   
   A   
   A   
   A   
   A   
   G   
   A   
   T   
   A   
   C   
   A   
   G   
   A   
   G   
   T   
   C   
   A   
   C   
   G   
   T   
   T   
   A   
   T   
   T   
   A   
   T   
   C   
   C   
   G   
   C   
   T   
   G   
   G   
   G   
   G   
   G   
   A   
   A   
   G   
   C   
   A   
   G   
   C   
   T   
   G   
   C   
   G   
   C   
   A   
   A   
   T   
   T   
   A   
   A   
   T   
   C   
   G   
   G   
   G   
   T   
   A   
   T   
   A   
   C   
   G   
   G   
   G   
   C   
   A   
   C   
   C   
   A   
   T   
   T   
   A   
   C   
   T   
   G   
   C   
   A   
   G   
   A   
   A   
   G   
   A   
   T   
   A   
   T   
   T   
   A   
   A   
   A   
   A   
   A   
   A   
   A   
   A   
   T   
   C   
   C   
   A   
   G   
   A   
   G   
   T   
   T   
   A   
   A   
   G   
   T   
   A   
   G   
   T   
   A   
   C   
   G   
   G   
   G   
   C   
   G   
   T   
   A   
   A   
   T   
   T   
   G   
   G   
   T   
   A   
   A   
   A   
   A   
   C   
   T   
   G   
   G   
   C   
   T   
   T   
   A   
   G   
   A   
   A   
   C   
   G   
   A   
   G   
   C   
   G   
   T   
   T   
   T   
   G   
   A   
   T   
   A   
   A   
   A   
   G   
   A   
   A   
   T   
   T   
   A   
   A   
   G   
   A   
   G   
   G   
   G   
   C   
   A   
   A   
   G   
   A   
   T   
   G   
   G   
   T   
   G   
   G   
   C   
   T   
   C   
   A   
   C   
   T   
   A   
   G   
   T   
   T   
   A   
   T   
   T   
   T   
   T   
   A   
   G   
   A   
   T   
   G   
   A   
   C   
   A   
   A   
   A   
   G   
   A   
   G   
   A   
   A   
   T   
   G   
   T   
   C   
   A   
   A   
   A   
   A   
   A   
   A   
   G   
   C   
   T   
   T   
   T   
   A   
   C   
   A   
   A   
   A   
   C   
   C   
   A   
   A   
   A   
   G   
   A   
   G   
   A   
   A   
   A   
   A   
   A   
   A   
   G   
   A   
   T   
   G   
   G   
   T   
   C   
   A   
   A   
   A   
   C   
   G   
   A   
   T   
   T   
   A   
   A   
   A   
   T   
   T   
   A   
   A   
   C   
   G   
   A   
   T   
   T   
   G   
   A   
   C   
   A   
   G   
   T   
   G   
   G   
   C   
   G   
   T   
   A   
   C   
   A   
   A   
   C   
   A   
   A   
   C   
   A   
   A   
   G   
   C   
   C   
   T   
   T   
   C   
   G   
   C   
   T   
   A   
   T   
   A   
   T   
   T   
   T   
   G   
   A   
   C   
   A   
   A   
   A   
   C   
   G   
   C   
   C   
   C   
   T   
   G   
   G   
   T   
   A   
   G   
   T   
   G   
   C   
   G   
   G   
   T   
   C   
   A   
   T   
   T   
   A   
   C   
   C   
   G   
   A   
   T   
   C   
   C   
   T   
   C   
   A   
   A   
   A   
   A   
   A   
   G   
   G   
   C   
   G   
   A   
   T   
   T   
   T   
   A   
   T   
   T   
   A   
   G   
   C   
   A   
   A   
   C   
   G   
   G   
   T   
   T   
   A   
   G   
   T   
   T   
   C   
   A   
   C   
   C   
   T   
   T   
   C   
   G   
   T   
   A   
   T   
   G   
   A   
   T   
   C   
   C   
   T   
   A   
   A   
   T   
   A   
   A   
   A   
   A   
   T   
   G   
   G   
   C   
   C   
   A   
   A   
   T   
   G   
   G   
   C   
   A   
   T   
   T   
   T   
   C   
   T   
   C   
   A   
   A   
   A   
   A   
   A   
   G   
   A   
   A   
   T   
   A   
   T   
   G   
   A   
   T   
   G   
   C   
   T   
   T   
   A   
   C   
   A   
   A   
   T   
   A   
   A   
   C   
   A   
   A   
   C   
   A   
   A   
   A   
   G   
   A   
   T   
   T   
   T   
   A   
   C   
   C   
   A   
   T   
   T   
   C   
   A   
   C   
   A   
   G   
   C   
   A   
   C   
   G   
   T   
   T   
   T   
   T   
   G   
   C   
   G   
   A   
   C   
   A   
   G   
   G   
   C   
   T   
   A   
   T   
   G   
   C   
   A   
   C   
   C   
   A   
   G   
   G   
   C   
   T   
   C   
   T   
   A   
   C   
   C   
   T   
   T   
   T   
   A   
   A   
   A   
   A   
   C   
   A   
   A   
   T   
   T   
   A   
   C   
   C   
   G   
   G   
   T   
   G   
   C   
   C   
   A   
   T   
   T   
   G   
   G   
   T   
   T   
   T   
   A   
   G   
   A   
   T   
   G   
   C   
   A   
   G   
   G   
   G   
   A   
   C   
   A   
   T   
   T   
   G   
   A   
   A   
   G   
   C   
   C   
   A   
   G   
   A   
   T   
   G   
   A   
   A   
   G   
   A   
   G   
   C   
   T   
   A   
   G   
   A   
   A   
   A   
   T   
   T   
   A   
   A   
   T   
   G   
   G   
   T   
   T   
   T   
   G   
   A   
   A   
   A   
   T   
   G   
   G   
   C   
   A   
   A   
   A   
   A   
   A   
   G   
   A   
   T   
   A   
   A   
   A   
   T   
   C   
   T   
   T   
   G   
   G   
   G   
   G   
   C   
   G   
   G   
   C   
   T   
   A   
   T   
   T   
   T   
   T   
   G   
   C   
   G   
   A   
   C   
   A   
   C   
   G   
   T   
   G   
   T   
   A   
   A   
   A   
   A   
   G   
   A   
   A   
   G   
   C   
   A   
   A   
   G   
   T   
   C   
   C   
   A   
   G   
   T   
   C   
   A   
   A   
   T   
   C   
   T   
   G   
   C   
   G   
   T   
   A   
   C   
   C   
   G   
   C   
   T   
   T   
   T   
   A   
   G   
   T   
   T   
   A   
   A   
   T   
   T   
   C   
   A   
   G   
   A   
   T   
   A   
   A   
   T   
   A   
   T   
   T   
   T   
   A   
   T   
   T   
   T   
   T   
   G   
   C   
   C   
   C   
   A   
   A   
   C   
   A   
   A   
   A   
   C   
   A   
   T   
   T   
   A   
   C   
   G   
   G   
   A   
   T   
   G   
   G   
   G   
   A   
   G   
   A   
   A   
   G   
   A   
   C   
   A   
   A   
   A   
   T   
   T   
   T   
   A   
   G   
   A   
   G   
   C   
   G   
   G   
   G   
   T   
   C   
   T   
   T   
   A   
   A   
   T   
   A   
   A   
   A   
   T   
   T   
   C   
   A   
   T   
   T   
   T   
   T   
   C   
   G   
   G   
   T   
   G   
   A   
   G   
   G   
   A   
   A   
   C   
   T   
   A   
   G   
   A   
   T   
   T   
   T   
   A   
   C   
   C   
   A   
   A   
   T   
   T   
   G   
   C   
   C   
   A   
   T   
   G   
   A   
   C   
   G   
   C   
   C   
   T   
   G   
   C   
   T   
   C   
   A   
   A   
   A   
   T   
   T   
   T   
   C   
   A   
   A   
   A   
   T   
   G   
   A   
   A   
   G   
   A   
   T   
   A   
   A   
   A   
   T   
   T   
   T   
   A   
   A   
   T   
   T   
   C   
   T   
   G   
   A   
   A   
   A   
   T   
   T   
   C   
   T   
   T   
   T   
   T   
   A   
   G   
   C   
   A   
   G   
   A   
   T   
   A   
   C   
   G   
   G   
   G   
   C   
   T   
   A   
   C   
   G   
   G   
   T   
   C   
   A   
   A   
   G   
   G   
   C   
   C   
   A   
   A   
   T   
   T   
   G   
   T   
   T   
   A   
   A   
   T   
   T   
   T   
   C   
   G   
   C   
   C   
   A   
   A   
   T   
   C   
   C   
   A   
   G   
   C   
   A   
   A   
   G   
   C   
   T   
   A   
   C   
   C   
   A   
   T   
   G   
   T   
   A   
   T   
   A   
   G   
   T   
   G   
   T   
   T   
   T   
   T   
   C   
   C   
   A   
   A   
   A   
   A   
   T   
   A   
   A   
   T   
   G   
   G   
   G   
   A   
   C   
   A   
   T   
   T   
   A   
   G   
   T   
   C   
   T   
   A   
   T   
   C   
   C   
   A   
   A   
   A   
   A   
   T   
   T   
   A   
   G   
   T   
   T   
   T   
   T   
   A   
   G   
   A   
   C   
   A   
   A   
   A   
   G   
   A   
   A   
   A   
   C   
   G   
   A   
   A   
   G   
   A   
   A   
   A   
   A   
   A   
   A   
   G   
   A   
   C   
   A   
   A   
   T   
   G   
   T   
   A   
   A   
   T   
   C   
   A   
   G   
   C   
   G   
   C   
   C   
   A   
   A   
   C   
   G   
   C   
   A   
   G   
   C   
   G   
   A   
   A   
   T   
   A   
   C   
   A   
   A   
   T   
   T   
   G   
   C   
   C   
   A   
   C   
   A   
   G   
   A   
   T   
   C   
   T   
   T   
   T   
   T   
   A   
   G   
   G   
   T   
   A   
   G   
   T   
   G   
   T   
   G   
   G   
   A   
   A   
   G   
   A   
   T   
   C   
   C   
   T   
   T   
   C   
   A   
   G   
   G   
   T   
   T   
   A   
   T   
   G   
   T   
   T   
   T   
   A   
   T   
   A   
   A   
   T   
   A   
   T   
   G   
   T   
   A   
   C   
   A   
   A   
   T   
   C   
   C   
   G   
   A   
   A   
   C   
   T   
   T   
   C   
   T   
   C   
   A   
   T   
   T   
   G   
   G   
   C   
   T   
   G   
   C   
   T   
   A   
   A   
   A   
   A   
   C   
   A   
   G   
   G   
   A   
   A   
   C   
   A   
   G   
   C   
   T   
   G   
   A   
   A   
   A   
   T   
   A   
   A   
   A   
   A   
   G   
   A   
   T   
   A   
   A   
   A   
   C   
   A   
   A   
   G   
   A   
   T   
   A   
   C   
   A   
   G   
   A   
   T   
   G   
   G   
   C   
   A   
   A   
   A   
   G   
   A   
   A   
   A   
   A   
   C   
   A   
   G   
   T   
   T   
   T   
   C   
   C   
   T   
   T   
   C   
   T   
   A   
   A   
   C   
   G   
   T   
   T   
   A   
   G   
   A   
   T   
   C   
   G   
   T   
   A   
   G   
   T   
   A   
   A   
   C   
   A   
   A   
   T   
   A   
   A   
   A   
   T   
   T   
   C   
   T   
   T   
   A   
   A   
   C   
   A   
   A   
   T   
   G   
   A   
   T   
   T   
   A   
   T   
   G   
   G   
   T   
   T   
   G   
   A   
   A   
   A   
   A   
   T   
   T   
   C   
   A   
   G   
   G   
   A   
   G   
   A   
   A   
   A   
   A   
   T   
   G   
   G   
   T   
   T   
   C   
   T   
   G   
   C   
   A   
   A   
   C   
   A   
   G   
   A   
   T   
   A   
   T   
   C   
   A   
   G   
   T   
   A   
   A   
   A   
   C   
   C   
   A   
   T   
   T   
   A   
   A   
   T   
   T   
   G   
   A   
   T   
   T   
   A   
   C   
   T   
   T   
   A   
   G   
   A   
   A   
   G   
   C   
   A   
   A   
   C   
   C   
   A   
   T   
   T   
   A   
   A   
   A   
   -   
   -   
   -   
   -   
   -   
   -   
   -   
   -   
   -   
   -   
   -   
   -   
   -   
   -   
   -   
   -   
   -   
   -   
   -   
   -   
   -   
   -   
   -   
   -   
   -   
   -   
   -   
   -   
   -   
   -   
   -   
   -   
   -   
   -   
   -   
   -   
   -   
   -   
   -   
   -   
   -   
   -   
   -   
   -   
   -   
   -   
   -   
   -   
   -   
   -   
   -   
   -   
   -   
   -   
   -   
   -   
   -   
   -   
   -   
   -   
   -   
 

 
   N52467   
   A   
   T   
   G   
   G   
   A   
   A   
   C   
   G   
   A   
   A   
   G   
   C   
   A   
   A   
   T   
   C   
   G   
   T   
   A   
   A   
   T   
   A   
   A   
   A   
   A   
   A   
   A   
   T   
   C   
   T   
   T   
   C   
   A   
   A   
   A   
   A   
   A   
   A   
   A   
   C   
   C   
   A   
   C   
   T   
   T   
   A   
   T   
   T   
   C   
   T   
   T   
   G   
   G   
   C   
   G   
   T   
   T   
   T   
   C   
   T   
   G   
   C   
   C   
   T   
   T   
   G   
   G   
   T   
   T   
   C   
   T   
   G   
   A   
   T   
   C   
   G   
   C   
   T   
   G   
   C   
   T   
   G   
   T   
   C   
   G   
   G   
   T   
   G   
   G   
   T   
   G   
   G   
   G   
   T   
   A   
   T   
   T   
   A   
   T   
   G   
   C   
   T   
   T   
   A   
   T   
   A   
   G   
   C   
   C   
   A   
   A   
   T   
   G   
   G   
   C   
   A   
   A   
   G   
   C   
   C   
   A   
   A   
   A   
   C   
   A   
   A   
   G   
   A   
    A    
   T   
   T   
   A   
   G   
   C   
    T    
   G   
   A   
   A   
   G   
   C   
   G   
   A   
   A   
   G   
   A   
   A   
   A   
   A   
    C    
   A   
   G   
   C   
    C    
   A   
    A    
   T   
    A    
    C    
   A   
   T   
   T   
   T   
   T   
   T   
   A   
   A   
   A   
    C    
   G   
   T   
   A   
   T   
   T   
   G   
    T    
   C   
   A   
   A   
   A   
   A   
   C   
   A   
   G   
    G    
   A   
   A   
   T   
   T   
   T   
   G   
   A   
   T   
   A   
   A   
   G   
   T   
   T   
   A   
   C   
   C   
    A    
   T   
   C   
   C   
   G   
   T   
   T   
    G    
   T   
   T   
   C   
   A   
   A   
   G   
   A   
   A   
    A    
   C   
   T   
   A   
   G   
   C   
   T   
   T   
   A   
   A   
   A   
   G   
   A   
   A   
   A   
   A   
   A   
   T   
   G   
   G   
    C    
   T   
   A   
   T   
   G   
   A   
   T   
   A   
   C   
   T   
   A   
   A   
   A   
   T   
   C   
    T    
   G   
   T   
   T   
   G   
   T   
   T   
   G   
   A   
   A   
   A   
   A   
   A   
   T   
   A   
   C   
   C   
   A   
   A   
   G   
   C   
   A   
   A   
   T   
   T   
   T   
   A   
   T   
   T   
   C   
   A   
   G   
   G   
   G   
   A   
   T   
   T   
   C   
   A   
   A   
   G   
   C   
    A    
   G   
   A   
   A   
    G    
   G   
    G    
   G   
   T   
   C   
   A   
   A   
    A    
   G   
   C   
   T   
   A   
   G   
   T   
   G   
   A   
   T   
   G   
   T   
    T    
   C   
   A   
   A   
   G   
   T   
    C    
   A   
   A   
   A   
   A   
   A   
    A    
   G   
    G    
   G   
   A   
   A   
   A   
   G   
   A   
    C    
   A   
   A   
    C    
   C   
   A   
   A   
   T   
   A   
   C   
   A   
   C   
   A   
   T   
   T   
   T   
   A   
    C    
   C   
   T   
   A   
   T   
   A   
   A   
   A   
   T   
   T   
   A   
   T   
   C   
   G   
   A   
   T   
   G   
   A   
   G   
    C    
   A   
   C   
    T    
   C   
   C   
   T   
   T   
   T   
   A   
   G   
   G   
   C   
   G   
   A   
   A   
   A   
   T   
   G   
   A   
   A   
   A   
   G   
   A   
   T   
    T    
   T   
   G   
   T   
   C   
   T   
   T   
   A   
   T   
   C   
   A   
   A   
   T   
   C   
   A   
   A   
   G   
   T   
   A   
   T   
    C    
   G   
    C    
   C   
   A   
   A   
    A    
   A   
   A   
   A   
   G   
   G   
    C    
   G   
   A   
   T   
   A   
    A    
   C   
   T   
   A   
   C   
   C   
   A   
   A   
   A   
   T   
    C    
    G    
   C   
   T   
   T   
   G   
   G   
    G    
    C    
    A    
   C   
   C   
    G    
   T   
   C   
   T   
   T   
   T   
   A   
   A   
   T   
   T   
   T   
   T   
   T   
   C   
   C   
   A   
   G   
   A   
   T   
   A   
   T   
   G   
   T   
   C   
   A   
   G   
   G   
   A   
   A   
   A   
   T   
   G   
   A   
   T   
   A   
   A   
   A   
    A    
   T   
   T   
   T   
   C   
   G   
   A   
   T   
   T   
   C   
   A   
   A   
   G   
   T   
   A   
   G   
   A   
   T   
   A   
   A   
   T   
   G   
   C   
   C   
   A   
   A   
   A   
   C   
   G   
   T   
   G   
   G   
   A   
   G   
   A   
   A   
   A   
   T   
   T   
   G   
   T   
   C   
   G   
   A   
   T   
   C   
   G   
   T   
   A   
   A   
    T    
   G   
   G   
   T   
   A   
   G   
   T   
   G   
   G   
   G   
   C   
   T   
   A   
   G   
    C    
   A   
   A   
   T   
   T   
   A   
   A   
   C   
   A   
   A   
   A   
   G   
   T   
   G   
   T   
   T   
   T   
   G   
   A   
    T    
   G   
   A   
   A   
   G   
   T   
   G   
   G   
   G   
   C   
   G   
   T   
   A   
   G   
   T   
    G    
   C   
   C   
   T   
    G    
   G   
   C   
   A   
   A   
   A   
   C   
   T   
    C    
   G   
   G   
   T   
   T   
   C   
   T   
   G   
   G   
   C   
   G   
   C   
   A   
   G   
   A   
   A   
   A   
   A   
   A   
    A    
   C   
   A   
   G   
   C   
    C    
   A   
   A   
   T   
   A   
   T   
   C   
   A   
   A   
   A   
   G   
   C   
   T   
   T   
   T   
   T   
   A   
   G   
   T   
   G   
   A   
   T   
   A   
   A   
    G    
   T   
   T   
    T    
   G   
   G   
   C   
   G   
   T   
   T   
   T   
   C   
   T   
    G    
   T   
   T   
   G   
   A   
   T   
   G   
   A   
   A   
   A   
   T   
   C   
   A   
   A   
   T   
   C   
    A    
   A   
   A   
   A   
    G    
   T   
   T   
   A   
   A   
   G   
   C   
   C   
   A   
   A   
   G   
   G   
    A    
   T   
   G   
   G   
   G   
   T   
   C   
   C   
   A   
   A   
   G   
   C   
   A   
   G   
   A   
   C   
   T   
   C   
    G    
   T   
   T   
   T   
   G   
   T   
   A   
   C   
   C   
   A   
   A   
   T   
   C   
   A   
   C   
   A   
   G   
   T   
    T    
   G   
   C   
   T   
   T   
   C   
   T   
   G   
   A   
   A   
   C   
   C   
   A   
   G   
   T   
   G   
   A   
   C   
   A   
   G   
   A   
   A   
   T   
   T   
   A   
   C   
   C   
   A   
   A   
   C   
   A   
   G   
   G   
   G   
   G   
   C   
   T   
   G   
   C   
   G   
   A   
   C   
   A   
   A   
   A   
   A   
   G   
    A    
    T    
   A   
   C   
   A   
   G   
   A   
   G   
   T   
   C   
   A   
   C   
   G   
   T   
   T   
   A   
   T   
   T   
   A   
   T   
   C   
   C   
    A    
    T    
   T   
   G   
   G   
   G   
   G   
   G   
   A   
   A   
   G   
   C   
   A   
   G   
   C   
   T   
   G   
   C   
   G   
   C   
   A   
   A   
   T   
   T   
   A   
   A   
   T   
   C   
   G   
   G   
    G    
   T   
   A   
   T   
   A   
   C   
    G    
   G   
   G   
    C    
   A   
   C   
    T    
   A   
   T   
   T   
   A   
   C   
   T   
   G   
   C   
   A   
   G   
   A   
    A    
   G   
   A   
   T   
   A   
   T   
   T   
    G    
   A   
   A   
   A   
   A   
   A   
   A   
   A   
   T   
   C   
   C   
   A   
   G   
   A   
   G   
   T   
   T   
   A   
   A   
   G   
   T   
   A   
   G   
   T   
   A   
   C   
   G   
   G   
   G   
    C    
   G   
   T   
   A   
   A   
   T   
   T   
   G   
   G   
    C    
   A   
   A   
   A   
   A   
   C   
   T   
   G   
   G   
    C    
   T   
   T   
   A   
   G   
   A   
   A   
   C   
   G   
   A   
   G   
   C   
   G   
   T   
   T   
   T   
   G   
   A   
   T   
   A   
   A   
   A   
   G   
   A   
    A    
   T   
   T   
   A   
   A   
   G   
   A   
   G   
   G   
    A    
   C   
   A   
   A   
   G   
   A   
   T   
   G   
   G   
    T    
   G   
   G   
    T    
   T   
   C   
    A    
   C   
   T   
   A   
   G   
   T   
   T   
   A   
   T   
   T   
   T   
   T   
   A   
   G   
   A   
   T   
   G   
   A   
   C   
   A   
   A   
   A   
   G   
   A   
   G   
   A   
   A   
   T   
   G   
   T   
   C   
   A   
   A   
   A   
   A   
   A   
   A   
   G   
   C   
   T   
   T   
   T   
   A   
   C   
   A   
   A   
   A   
   C   
   C   
   A   
   A   
   A   
   G   
   A   
    G    
   A   
   A   
   A   
   A   
   A   
   A   
   G   
   A   
   T   
   G   
   G   
   T   
   C   
   A   
   A   
   A   
   C   
    A    
   A   
   T   
   T   
   A   
   A   
   A   
   T   
   T   
   A   
   A   
   C   
   G   
   A   
   T   
   T   
   G   
   A   
    T    
   A   
   G   
    T    
   G   
   G   
    C    
   G   
   T   
   A   
   C   
   A   
   A   
   C   
   A   
   A   
   C   
   A   
   A   
   G   
   C   
    C    
   T   
   T   
    C    
   G   
   C   
   T   
   A   
   T   
   A   
   T   
   T   
   T   
   G   
   A   
   C   
   A   
   A   
   A   
   C   
   G   
    T    
    C    
   C   
   T   
   G   
   G   
   T   
   A   
   G   
   T   
   G   
   C   
   G   
   G   
   T   
   C   
   A   
   T   
   T   
   A   
   C   
   C   
   G   
   A   
   T   
   C   
   C   
   T   
   C   
   A   
   A   
   A   
   A   
   A   
   G   
   G   
   C   
   G   
   A   
   T   
   T   
   T   
   A   
    C    
   T   
   A   
   G   
   C   
    G    
   A   
   C   
   G   
   G   
   T   
   T   
   A   
   G   
   T   
   T   
   C   
   A   
   C   
   C   
   T   
   T   
   C   
   G   
   T   
   A   
   T   
   G   
   A   
   T   
   C   
   C   
   T   
   A   
   A   
   T   
   A   
   A   
   A   
   A   
   T   
   G   
   G   
   C   
   C   
   A   
   A   
   T   
   G   
   G   
    C    
   A   
   T   
   T   
   T   
   C   
   T   
   C   
   A   
   A   
   A   
   A   
   A   
   G   
   A   
    A    
   T   
   A   
   T   
   G   
   A   
   T   
   G   
   C   
   T   
   T   
   A   
   C   
   A   
   A   
   T   
   A   
   A   
   C   
   A   
   A   
   C   
   A   
   A   
   A   
   G   
   A   
   T   
   T   
   T   
   A   
   C   
   C   
   A   
   T   
   T   
    C    
   A   
   C   
   A   
   G   
   C   
   A   
   C   
   G   
   T   
   T   
   T   
    T    
   G   
   C   
   G   
   A   
   C   
   A   
   G   
   G   
    C    
   T   
   A   
   T   
   G   
   C   
   A   
   C   
   C   
   A   
   G   
   G   
    C    
   T   
   C   
   T   
   A   
   C   
   C   
   T   
   T   
   T   
   A   
   A   
   A   
   A   
   C   
   A   
   A   
   T   
   T   
   A   
   C   
   C   
   G   
   G   
   T   
   G   
   C   
   C   
   A   
   T   
   T   
   G   
   G   
   T   
   T   
   T   
   A   
   G   
   A   
   T   
    G    
   C   
   A   
   G   
   G   
   G   
   A   
   C   
   A   
   T   
   T   
   G   
   A   
   A   
   G   
   C   
   C   
   A   
   G   
   A   
   T   
   G   
   A   
   A   
   G   
   A   
   G   
   C   
   T   
   A   
   G   
   A   
   A   
   A   
   T   
   T   
   A   
   A   
   T   
   G   
   G   
    C    
   T   
   T   
   G   
   A   
   A   
   A   
   T   
   G   
   G   
   C   
   A   
   A   
   A   
   A   
   A   
   G   
   A   
   T   
   A   
   A   
   A   
   T   
   C   
   T   
   T   
   G   
   G   
   G   
   G   
    C    
   G   
   G   
   C   
   T   
   A   
   T   
   T   
   T   
   T   
   G   
   C   
    G    
   A   
   C   
   A   
   C   
   G   
   T   
   G   
   T   
   A   
   A   
   A   
   A   
   G   
   A   
   A   
   G   
   C   
   A   
   A   
   G   
   T   
   C   
   C   
   A   
   G   
   T   
   C   
   A   
   A   
   T   
   C   
   T   
    T    
   C   
   G   
   T   
   A   
   C   
   C   
   G   
   C   
   T   
   T   
   T   
   A   
   G   
   T   
   T   
   A   
   A   
   T   
   T   
   C   
   A   
   G   
   A   
   T   
   A   
   A   
   T   
   A   
   T   
   T   
   T   
   A   
   T   
   T   
   T   
   T   
   G   
   C   
    C    
   C   
   A   
   A   
   C   
   A   
   A   
   A   
   C   
   A   
   T   
   T   
   A   
   C   
   G   
    G    
   A   
   T   
   G   
   G   
   G   
    G    
   G   
   A   
   A   
   G   
   A   
   C   
   A   
   A   
   A   
   T   
   T   
   T   
   A   
   G   
   A   
   G   
   C   
    G    
   G   
   G   
   T   
   C   
   T   
   T   
   A   
   A   
   T   
   A   
   A   
   A   
   T   
   T   
   C   
   A   
   T   
   T   
   T   
   T   
   C   
   G   
   G   
   T   
   G   
   A   
   G   
   G   
   A   
   A   
   C   
   T   
   A   
   G   
   A   
   T   
   T   
   T   
   A   
   C   
   C   
   A   
   A   
   T   
    T    
   G   
   C   
   C   
   A   
   T   
   G   
   A   
   C   
   G   
   C   
   C   
    T    
   G   
   C   
   T   
   C   
   A   
   A   
   A   
   T   
   T   
   T   
   C   
   A   
   A   
   A   
    C    
   G   
   A   
   A   
   G   
   A   
   T   
   A   
   A   
   A   
   T   
   T   
   T   
   A   
   A   
   T   
   T   
   C   
   T   
   G   
   A   
   A   
   A   
   T   
   T   
   C   
   T   
   T   
   T   
   T   
    A    
   G   
   C   
   A   
   G   
   A   
   T   
   A   
   C   
    A    
   G   
   G   
   C   
   T   
   A   
    T    
   G   
   G   
    C    
   C   
   A   
   A   
   G   
   G   
    C    
   C   
   A   
   A   
   T   
   T   
   G   
   T   
   T   
   A   
   A   
   T   
   T   
   T   
   C   
    G    
   C   
   C   
   A   
   A   
   T   
   C   
   C   
   A   
   G   
   C   
   A   
   A   
   G   
   C   
   T   
   A   
   C   
   C   
   A   
   T   
   G   
   T   
   A   
   T   
   A   
   G   
   T   
   G   
   T   
   T   
   T   
   T   
   C   
   C   
   A   
   A   
   A   
   A   
   T   
   A   
   A   
   T   
   G   
   G   
    A    
   A   
   C   
   A   
    T    
   T   
    A    
   G   
   T   
    T    
   T   
   A   
   T   
   C   
   C   
   A   
   A   
   A   
   A   
    T    
   T   
   A   
   G   
   T   
   T   
   T   
   T   
   A   
   G   
   A   
   G   
   A   
   A   
   A   
   G   
   A   
   A   
   A   
   C   
    G    
   A   
   A   
   G   
   A   
   A   
   A   
   A   
   A   
   A   
   G   
   A   
   C   
   A   
   A   
   T   
   G   
   T   
   A   
   A   
   T   
   C   
   A   
   G   
   C   
   G   
   C   
   C   
   A   
   A   
   C   
   G   
   C   
   A   
   G   
   C   
   G   
   A   
   A   
   T   
   A   
   C   
   A   
   A   
   T   
   A   
   G   
   C   
   C   
   A   
   C   
   A   
   G   
   A   
   T   
   C   
   T   
   T   
   T   
   T   
   A   
   G   
   G   
   T   
   A   
   G   
   T   
   G   
   T   
   G   
   G   
   A   
   A   
   G   
   A   
   T   
   C   
   C   
   T   
   T   
   C   
   A   
   G   
   G   
   T   
   T   
   A   
   T   
   G   
   T   
   T   
   T   
   A   
   T   
   A   
   A   
   T   
   A   
   T   
   G   
   T   
   A   
   C   
   A   
   A   
   T   
   C   
   C   
   G   
   A   
   A   
   C   
   T   
   T   
   C   
   T   
   C   
   A   
   T   
   T   
   G   
   G   
   C   
   T   
   G   
   C   
   C   
   A   
   A   
   A   
   A   
   C   
   A   
   G   
   G   
   A   
   A   
   C   
   A   
   G   
   C   
   T   
   G   
   A   
   A   
   A   
   T   
   A   
   A   
   A   
   A   
   G   
   A   
   T   
   A   
   A   
   A   
   C   
   A   
   A   
   G   
   A   
   T   
   A   
   C   
   A   
   G   
   A   
   T   
   G   
   G   
   C   
   A   
   A   
   A   
   G   
   A   
   A   
   A   
   A   
   C   
   A   
   G   
   T   
   T   
   T   
   C   
   C   
   T   
   T   
   C   
   T   
   A   
   A   
   C   
   G   
   T   
   T   
   A   
   G   
   A   
   T   
   C   
   G   
   T   
   A   
   G   
   T   
   A   
   A   
   C   
   A   
   A   
   T   
   A   
   A   
   A   
   T   
   T   
   T   
   T   
   T   
   A   
   A   
   C   
   A   
   A   
   T   
   G   
   A   
   T   
   T   
   A   
   T   
   G   
   G   
   T   
   T   
   G   
   A   
   A   
   A   
   A   
   T   
   T   
   C   
   A   
   G   
   G   
   A   
   G   
   A   
   A   
   A   
   A   
   T   
   G   
   G   
   T   
   T   
   C   
   T   
   G   
   C   
   A   
   A   
   C   
   A   
   G   
   A   
   T   
   A   
   T   
   C   
   A   
   G   
   T   
   A   
   A   
   A   
   C   
   C   
   A   
   T   
   T   
   A   
   A   
   T   
   T   
   G   
   A   
   T   
   T   
   A   
   C   
   T   
   T   
   A   
   G   
   A   
   A   
   G   
   C   
   A   
   A   
   C   
   C   
   A   
   T   
   T   
   A   
   A   
   A   
   -   
   -   
   -   
   -   
   -   
   -   
   -   
   -   
   -   
   -   
   -   
   -   
   -   
   -   
   -   
   -   
   -   
   -   
   -   
   -   
   -   
   -   
   -   
   -   
   -   
   -   
   -   
   -   
   -   
   -   
   -   
   -   
   -   
   -   
   -   
   -   
   -   
   -   
   -   
   -   
   -   
   -   
   -   
   -   
   -   
   -   
   -   
   -   
   -   
   -   
   -   
   -   
   -   
   -   
   -   
   -   
   -   
   -   
   -   
   -   
   -   
   A   
   T   
   G   
   G   
   A   
   A   
   C   
   G   
   A   
   A   
   G   
   C   
   A   
   A   
   T   
   C   
   G   
   T   
   A   
   A   
   T   
   A   
   A   
   A   
   A   
   A   
   A   
   T   
   C   
   T   
   T   
   C   
   A   
   A   
   A   
   A   
   A   
   A   
   A   
   C   
   C   
   A   
   C   
   T   
   T   
   A   
   T   
   T   
   C   
   T   
   T   
   G   
   G   
   C   
   G   
   T   
   T   
   T   
   C   
   T   
   G   
   C   
   C   
   T   
   T   
   G   
   G   
   T   
   T   
   C   
   T   
   G   
   A   
   T   
   C   
   G   
   C   
   T   
   G   
   C   
   T   
   G   
   T   
   C   
   G   
   G   
   T   
   G   
   G   
   T   
   G   
   G   
   G   
   T   
   A   
   T   
   T   
   A   
   T   
   G   
   C   
   T   
   T   
   A   
   T   
   A   
   G   
   C   
   C   
   A   
   A   
   T   
   G   
   G   
   C   
   A   
   A   
   G   
   C   
   C   
   A   
   A   
   A   
   C   
   A   
   A   
   G   
   A   
   A   
   T   
   T   
   A   
   G   
   C   
   T   
   G   
   A   
   A   
   G   
   C   
   G   
   A   
   A   
   G   
   A   
   A   
   A   
   A   
   C   
   A   
   G   
   C   
   C   
   A   
   A   
   T   
   A   
   C   
   A   
   T   
   T   
   T   
   T   
   T   
   A   
   A   
   A   
   C   
   G   
   T   
   A   
   T   
   T   
   G   
   T   
   C   
   A   
   A   
   A   
   A   
   C   
   A   
   G   
   G   
   A   
   A   
   T   
   T   
   T   
   G   
   A   
   T   
   A   
   A   
   G   
   T   
   T   
   A   
   C   
   C   
   A   
   T   
   C   
   C   
   G   
   T   
   T   
   G   
   T   
   T   
   C   
   A   
   A   
   G   
   A   
   A   
   A   
   C   
   T   
   A   
   G   
   C   
   T   
   T   
   A   
   A   
   A   
   G   
   A   
   A   
   A   
   A   
   A   
   T   
   G   
   G   
   C   
   T   
   A   
   T   
   G   
   A   
   T   
   A   
   C   
   T   
   A   
   A   
   A   
   T   
   C   
   T   
   G   
   T   
   T   
   G   
   T   
   T   
   G   
   A   
   A   
   A   
   A   
   A   
   T   
   A   
   C   
   C   
   A   
   A   
   G   
   C   
   A   
   A   
   T   
   T   
   T   
   A   
   T   
   T   
   C   
   A   
   G   
   G   
   G   
   A   
   T   
   T   
   C   
   A   
   A   
   G   
   C   
   A   
   G   
   A   
   A   
   G   
   G   
   G   
   G   
   T   
   C   
   A   
   A   
   A   
   G   
   C   
   T   
   A   
   G   
   T   
   G   
   A   
   T   
   G   
   T   
   T   
   C   
   A   
   A   
   G   
   T   
   C   
   A   
   A   
   A   
   A   
   A   
   A   
   G   
   G   
   G   
   A   
   A   
   A   
   G   
   A   
   C   
   A   
   A   
   C   
   C   
   A   
   A   
   T   
   A   
   C   
   A   
   C   
   A   
   T   
   T   
   T   
   A   
   C   
   C   
   T   
   A   
   T   
   A   
   A   
   A   
   T   
   T   
   A   
   T   
   C   
   G   
   A   
   T   
   G   
   A   
   G   
   C   
   A   
   C   
   T   
   C   
   C   
   T   
   T   
   T   
   A   
   G   
   G   
   C   
   G   
   A   
   A   
   A   
   T   
   G   
   A   
   A   
   A   
   G   
   A   
   T   
   T   
   T   
   G   
   T   
   C   
   T   
   T   
   A   
   T   
   C   
   A   
   A   
   T   
   C   
   A   
   A   
   G   
   T   
   A   
   T   
   C   
   G   
   C   
   C   
   A   
   A   
   A   
   A   
   A   
   A   
   G   
   G   
   C   
   G   
   A   
   T   
   A   
   A   
   C   
   T   
   A   
   C   
   C   
   A   
   A   
   A   
   T   
   C   
   G   
   C   
   T   
   T   
   G   
   G   
   G   
   C   
   A   
   C   
   C   
   G   
   T   
   C   
   T   
   T   
   T   
   A   
   A   
   T   
   T   
   T   
   T   
   T   
   C   
   C   
   A   
   G   
   A   
   T   
   A   
   T   
   G   
   T   
   C   
   A   
   G   
   G   
   A   
   A   
   A   
   T   
   G   
   A   
   T   
   A   
   A   
   A   
   A   
   T   
   T   
   T   
   C   
   G   
   A   
   T   
   T   
   C   
   A   
   A   
   G   
   T   
   A   
   G   
   A   
   T   
   A   
   A   
   T   
   G   
   C   
   C   
   A   
   A   
   A   
   C   
   G   
   T   
   G   
   G   
   A   
   G   
   A   
   A   
   A   
   T   
   T   
   G   
   T   
   C   
   G   
   A   
   T   
   C   
   G   
   T   
   A   
   A   
   T   
   G   
   G   
   T   
   A   
   G   
   T   
   G   
   G   
   G   
   C   
   T   
   A   
   G   
   C   
   A   
   A   
   T   
   T   
   A   
   A   
   C   
   A   
   A   
   A   
   G   
   T   
   G   
   T   
   T   
   T   
   G   
   A   
   T   
   G   
   A   
   A   
   G   
   T   
   G   
   G   
   G   
   C   
   G   
   T   
   A   
   G   
   T   
   G   
   C   
   C   
   T   
   G   
   G   
   C   
   A   
   A   
   A   
   C   
   T   
   C   
   G   
   G   
   T   
   T   
   C   
   T   
   G   
   G   
   C   
   G   
   C   
   A   
   G   
   A   
   A   
   A   
   A   
   A   
   A   
   C   
   A   
   G   
   C   
   C   
   A   
   A   
   T   
   A   
   T   
   C   
   A   
   A   
   A   
   G   
   C   
   T   
   T   
   T   
   T   
   A   
   G   
   T   
   G   
   A   
   T   
   A   
   A   
   G   
   T   
   T   
   T   
   G   
   G   
   C   
   G   
   T   
   T   
   T   
   C   
   T   
   G   
   T   
   T   
   G   
   A   
   T   
   G   
   A   
   A   
   A   
   T   
   C   
   A   
   A   
   T   
   C   
   A   
   A   
   A   
   A   
   G   
   T   
   T   
   A   
   A   
   G   
   C   
   C   
   A   
   A   
   G   
   G   
   A   
   T   
   G   
   G   
   G   
   T   
   C   
   C   
   A   
   A   
   G   
   C   
   A   
   G   
   A   
   C   
   T   
   C   
   G   
   T   
   T   
   T   
   G   
   T   
   A   
   C   
   C   
   A   
   A   
   T   
   C   
   A   
   C   
   A   
   G   
   T   
   T   
   G   
   C   
   T   
   T   
   C   
   T   
   G   
   A   
   A   
   C   
   C   
   A   
   G   
   T   
   G   
   A   
   C   
   A   
   G   
   A   
   A   
   T   
   T   
   A   
   C   
   C   
   A   
   A   
   C   
   A   
   G   
   G   
   G   
   G   
   C   
   T   
   G   
   C   
   G   
   A   
   C   
   A   
   A   
   A   
   A   
   G   
   A   
   T   
   A   
   C   
   A   
   G   
   A   
   G   
   T   
   C   
   A   
   C   
   G   
   T   
   T   
   A   
   T   
   T   
   A   
   T   
   C   
   C   
   A   
   T   
   T   
   G   
   G   
   G   
   G   
   G   
   A   
   A   
   G   
   C   
   A   
   G   
   C   
   T   
   G   
   C   
   G   
   C   
   A   
   A   
   T   
   T   
   A   
   A   
   T   
   C   
   G   
   G   
   G   
   T   
   A   
   T   
   A   
   C   
   G   
   G   
   G   
   C   
   A   
   C   
   T   
   A   
   T   
   T   
   A   
   C   
   T   
   G   
   C   
   A   
   G   
   A   
   A   
   G   
   A   
   T   
   A   
   T   
   T   
   G   
   A   
   A   
   A   
   A   
   A   
   A   
   A   
   T   
   C   
   C   
   A   
   G   
   A   
   G   
   T   
   T   
   A   
   A   
   G   
   T   
   A   
   G   
   T   
   A   
   C   
   G   
   G   
   G   
   C   
   G   
   T   
   A   
   A   
   T   
   T   
   G   
   G   
   C   
   A   
   A   
   A   
   A   
   C   
   T   
   G   
   G   
   C   
   T   
   T   
   A   
   G   
   A   
   A   
   C   
   G   
   A   
   G   
   C   
   G   
   T   
   T   
   T   
   G   
   A   
   T   
   A   
   A   
   A   
   G   
   A   
   A   
   T   
   T   
   A   
   A   
   G   
   A   
   G   
   G   
   A   
   C   
   A   
   A   
   G   
   A   
   T   
   G   
   G   
   T   
   G   
   G   
   T   
   T   
   C   
   A   
   C   
   T   
   A   
   G   
   T   
   T   
   A   
   T   
   T   
   T   
   T   
   A   
   G   
   A   
   T   
   G   
   A   
   C   
   A   
   A   
   A   
   G   
   A   
   G   
   A   
   A   
   T   
   G   
   T   
   C   
   A   
   A   
   A   
   A   
   A   
   A   
   G   
   C   
   T   
   T   
   T   
   A   
   C   
   A   
   A   
   A   
   C   
   C   
   A   
   A   
   A   
   G   
   A   
   G   
   A   
   A   
   A   
   A   
   A   
   A   
   G   
   A   
   T   
   G   
   G   
   T   
   C   
   A   
   A   
   A   
   C   
   A   
   A   
   T   
   T   
   A   
   A   
   A   
   T   
   T   
   A   
   A   
   C   
   G   
   A   
   T   
   T   
   G   
   A   
   T   
   A   
   G   
   T   
   G   
   G   
   C   
   G   
   T   
   A   
   C   
   A   
   A   
   C   
   A   
   A   
   C   
   A   
   A   
   G   
   C   
   C   
   T   
   T   
   C   
   G   
   C   
   T   
   A   
   T   
   A   
   T   
   T   
   T   
   G   
   A   
   C   
   A   
   A   
   A   
   C   
   G   
   T   
   C   
   C   
   T   
   G   
   G   
   T   
   A   
   G   
   T   
   G   
   C   
   G   
   G   
   T   
   C   
   A   
   T   
   T   
   A   
   C   
   C   
   G   
   A   
   T   
   C   
   C   
   T   
   C   
   A   
   A   
   A   
   A   
   A   
   G   
   G   
   C   
   G   
   A   
   T   
   T   
   T   
   A   
   C   
   T   
   A   
   G   
   C   
   G   
   A   
   C   
   G   
   G   
   T   
   T   
   A   
   G   
   T   
   T   
   C   
   A   
   C   
   C   
   T   
   T   
   C   
   G   
   T   
   A   
   T   
   G   
   A   
   T   
   C   
   C   
   T   
   A   
   A   
   T   
   A   
   A   
   A   
   A   
   T   
   G   
   G   
   C   
   C   
   A   
   A   
   T   
   G   
   G   
   C   
   A   
   T   
   T   
   T   
   C   
   T   
   C   
   A   
   A   
   A   
   A   
   A   
   G   
   A   
   A   
   T   
   A   
   T   
   G   
   A   
   T   
   G   
   C   
   T   
   T   
   A   
   C   
   A   
   A   
   T   
   A   
   A   
   C   
   A   
   A   
   C   
   A   
   A   
   A   
   G   
   A   
   T   
   T   
   T   
   A   
   C   
   C   
   A   
   T   
   T   
   C   
   A   
   C   
   A   
   G   
   C   
   A   
   C   
   G   
   T   
   T   
   T   
   T   
   G   
   C   
   G   
   A   
   C   
   A   
   G   
   G   
   C   
   T   
   A   
   T   
   G   
   C   
   A   
   C   
   C   
   A   
   G   
   G   
   C   
   T   
   C   
   T   
   A   
   C   
   C   
   T   
   T   
   T   
   A   
   A   
   A   
   A   
   C   
   A   
   A   
   T   
   T   
   A   
   C   
   C   
   G   
   G   
   T   
   G   
   C   
   C   
   A   
   T   
   T   
   G   
   G   
   T   
   T   
   T   
   A   
   G   
   A   
   T   
   G   
   C   
   A   
   G   
   G   
   G   
   A   
   C   
   A   
   T   
   T   
   G   
   A   
   A   
   G   
   C   
   C   
   A   
   G   
   A   
   T   
   G   
   A   
   A   
   G   
   A   
   G   
   C   
   T   
   A   
   G   
   A   
   A   
   A   
   T   
   T   
   A   
   A   
   T   
   G   
   G   
   C   
   T   
   T   
   G   
   A   
   A   
   A   
   T   
   G   
   G   
   C   
   A   
   A   
   A   
   A   
   A   
   G   
   A   
   T   
   A   
   A   
   A   
   T   
   C   
   T   
   T   
   G   
   G   
   G   
   G   
   C   
   G   
   G   
   C   
   T   
   A   
   T   
   T   
   T   
   T   
   G   
   C   
   G   
   A   
   C   
   A   
   C   
   G   
   T   
   G   
   T   
   A   
   A   
   A   
   A   
   G   
   A   
   A   
   G   
   C   
   A   
   A   
   G   
   T   
   C   
   C   
   A   
   G   
   T   
   C   
   A   
   A   
   T   
   C   
   T   
   T   
   C   
   G   
   T   
   A   
   C   
   C   
   G   
   C   
   T   
   T   
   T   
   A   
   G   
   T   
   T   
   A   
   A   
   T   
   T   
   C   
   A   
   G   
   A   
   T   
   A   
   A   
   T   
   A   
   T   
   T   
   T   
   A   
   T   
   T   
   T   
   T   
   G   
   C   
   C   
   C   
   A   
   A   
   C   
   A   
   A   
   A   
   C   
   A   
   T   
   T   
   A   
   C   
   G   
   G   
   A   
   T   
   G   
   G   
   G   
   G   
   G   
   A   
   A   
   G   
   A   
   C   
   A   
   A   
   A   
   T   
   T   
   T   
   A   
   G   
   A   
   G   
   C   
   G   
   G   
   G   
   T   
   C   
   T   
   T   
   A   
   A   
   T   
   A   
   A   
   A   
   T   
   T   
   C   
   A   
   T   
   T   
   T   
   T   
   C   
   G   
   G   
   T   
   G   
   A   
   G   
   G   
   A   
   A   
   C   
   T   
   A   
   G   
   A   
   T   
   T   
   T   
   A   
   C   
   C   
   A   
   A   
   T   
   T   
   G   
   C   
   C   
   A   
   T   
   G   
   A   
   C   
   G   
   C   
   C   
   T   
   G   
   C   
   T   
   C   
   A   
   A   
   A   
   T   
   T   
   T   
   C   
   A   
   A   
   A   
   C   
   G   
   A   
   A   
   G   
   A   
   T   
   A   
   A   
   A   
   T   
   T   
   T   
   A   
   A   
   T   
   T   
   C   
   T   
   G   
   A   
   A   
   A   
   T   
   T   
   C   
   T   
   T   
   T   
   T   
   A   
   G   
   C   
   A   
   G   
   A   
   T   
   A   
   C   
   A   
   G   
   G   
   C   
   T   
   A   
   T   
   G   
   G   
   C   
   C   
   A   
   A   
   G   
   G   
   C   
   C   
   A   
   A   
   T   
   T   
   G   
   T   
   T   
   A   
   A   
   T   
   T   
   T   
   C   
   G   
   C   
   C   
   A   
   A   
   T   
   C   
   C   
   A   
   G   
   C   
   A   
   A   
   G   
   C   
   T   
   A   
   C   
   C   
   A   
   T   
   G   
   T   
   A   
   T   
   A   
   G   
   T   
   G   
   T   
   T   
   T   
   T   
   C   
   C   
   A   
   A   
   A   
   A   
   T   
   A   
   A   
   T   
   G   
   G   
   A   
   A   
   C   
   A   
   T   
   T   
   A   
   G   
   T   
   T   
   T   
   A   
   T   
   C   
   C   
   A   
   A   
   A   
   A   
   T   
   T   
   A   
   G   
   T   
   T   
   T   
   T   
   A   
   G   
   A   
   G   
   A   
   A   
   A   
   G   
   A   
   A   
   A   
   C   
   G   
   A   
   A   
   G   
   A   
   A   
   A   
   A   
   A   
   A   
   G   
   A   
   C   
   A   
   A   
   T   
   G   
   T   
   A   
   A   
   T   
   C   
   A   
   G   
   C   
   G   
   C   
   C   
   A   
   A   
   C   
   G   
   C   
   A   
   G   
   C   
   G   
   A   
   A   
   T   
   A   
   C   
   A   
   A   
   T   
   A   
   G   
   C   
   C   
   A   
   C   
   A   
   G   
   A   
   T   
   C   
   T   
   T   
   T   
   T   
   A   
   G   
   G   
   T   
   A   
   G   
   T   
   G   
   T   
   G   
   G   
   A   
   A   
   G   
   A   
   T   
   C   
   C   
   T   
   T   
   C   
   A   
   G   
   G   
   T   
   T   
   A   
   T   
   G   
   T   
   T   
   T   
   A   
   T   
   A   
   A   
   T   
   A   
   T   
   G   
   T   
   A   
   C   
   A   
   A   
   T   
   C   
   C   
   G   
   A   
   A   
   C   
   T   
   T   
   C   
   T   
   C   
   A   
   T   
   T   
   G   
   G   
   C   
   T   
   G   
   C   
   C   
   A   
   A   
   A   
   A   
   C   
   A   
   G   
   G   
   A   
   A   
   C   
   A   
   G   
   C   
   T   
   G   
   A   
   A   
   A   
   T   
   A   
   A   
   A   
   A   
   G   
   A   
   T   
   A   
   A   
   A   
   C   
   A   
   A   
   G   
   A   
   T   
   A   
   C   
   A   
   G   
   A   
   T   
   G   
   G   
   C   
   A   
   A   
   A   
   G   
   A   
   A   
   A   
   A   
   C   
   A   
   G   
   T   
   T   
   T   
   C   
   C   
   T   
   T   
   C   
   T   
   A   
   A   
   C   
   G   
   T   
   T   
   A   
   G   
   A   
   T   
   C   
   G   
   T   
   A   
   G   
   T   
   A   
   A   
   C   
   A   
   A   
   T   
   A   
   A   
   A   
   T   
   T   
   T   
   T   
   T   
   A   
   A   
   C   
   A   
   A   
   T   
   G   
   A   
   T   
   T   
   A   
   T   
   G   
   G   
   T   
   T   
   G   
   A   
   A   
   A   
   A   
   T   
   T   
   C   
   A   
   G   
   G   
   A   
   G   
   A   
   A   
   A   
   A   
   T   
   G   
   G   
   T   
   T   
   C   
   T   
   G   
   C   
   A   
   A   
   C   
   A   
   G   
   A   
   T   
   A   
   T   
   C   
   A   
   G   
   T   
   A   
   A   
   A   
   C   
   C   
   A   
   T   
   T   
   A   
   A   
   T   
   T   
   G   
   A   
   T   
   T   
   A   
   C   
   T   
   T   
   A   
   G   
   A   
   A   
   G   
   C   
   A   
   A   
   C   
   C   
   A   
   T   
   T   
   A   
   A   
   A   
   -   
   -   
   -   
   -   
   -   
   -   
   -   
   -   
   -   
   -   
   -   
   -   
   -   
   -   
   -   
   -   
   -   
   -   
   -   
   -   
   -   
   -   
   -   
   -   
   -   
   -   
   -   
   -   
   -   
   -   
   -   
   -   
   -   
   -   
   -   
   -   
   -   
   -   
   -   
   -   
   -   
   -   
   -   
   -   
   -   
   -   
   -   
   -   
   -   
   -   
   -   
   -   
   -   
   -   
   -   
   -   
   -   
   -   
   -   
   -   
   -   
 

 
   N59689F   
   -   
   -   
   -   
   -   
   -   
   -   
   -   
   -   
   -   
   -   
   -   
   -   
   -   
   -   
   -   
   -   
   -   
   -   
   -   
   -   
   -   
   -   
   -   
   -   
   -   
   -   
   -   
   -   
   -   
   -   
   -   
   -   
   -   
   -   
   -   
   -   
   -   
   -   
   -   
   -   
   -   
   -   
   -   
   -   
   -   
   -   
   -   
   -   
   -   
   -   
   -   
   -   
   -   
   -   
   -   
   -   
   -   
   -   
   -   
   -   
   -   
   -   
   -   
   -   
   -   
   -   
   -   
   -   
   -   
   -   
   -   
   -   
   -   
   -   
   -   
   -   
   -   
   -   
   -   
   -   
   -   
   -   
   -   
   -   
   -   
   -   
   -   
   -   
   -   
   -   
   -   
   -   
   -   
   -   
   -   
   -   
   -   
   -   
   -   
   -   
   -   
   -   
   -   
   -   
   -   
   -   
   -   
   -   
   C   
   A   
   A   
   T   
   G   
   G   
   C   
   A   
   A   
   G   
   C   
   C   
   A   
   A   
   A   
   C   
   A   
   A   
   G   
   A   
    A    
   T   
   T   
   A   
   G   
   C   
    C    
   G   
   A   
   A   
   G   
   C   
   G   
   A   
   A   
   G   
   A   
   A   
   A   
   A   
    C    
   A   
   G   
   C   
    T    
   A   
    C    
   T   
    A    
    C    
   A   
   T   
   T   
   T   
   T   
   T   
   A   
   A   
   A   
    C    
   G   
   T   
   A   
   T   
   T   
   G   
    T    
   C   
   A   
   A   
   A   
   A   
   C   
   A   
   G   
    G    
   A   
   A   
   T   
   T   
   T   
   G   
   A   
   T   
   A   
   A   
   G   
   T   
   T   
   A   
   C   
   C   
    G    
   T   
   C   
   C   
   G   
   T   
   T   
    G    
   T   
   T   
   C   
   A   
   A   
   G   
   A   
   A   
    G    
   C   
   T   
   A   
   G   
   C   
   T   
   T   
   A   
   A   
   A   
   G   
   A   
   A   
   A   
   A   
   A   
   T   
   G   
   G   
    C    
   T   
   A   
   T   
   G   
   A   
   T   
   A   
   C   
   T   
   A   
   A   
   A   
   T   
   C   
    T    
   G   
   T   
   T   
   G   
   T   
   T   
   G   
   A   
   A   
   A   
   A   
   A   
   T   
   A   
   C   
   C   
   A   
   A   
   G   
   C   
   A   
   A   
   T   
   T   
   T   
   A   
   T   
   T   
   C   
   A   
   G   
   G   
   G   
   A   
   T   
   T   
   C   
   A   
   A   
   G   
   C   
    A    
   G   
   A   
   A   
    G    
   G   
    A    
   G   
   T   
   C   
   A   
   A   
    A    
   G   
   C   
   T   
   A   
   G   
   T   
   G   
   A   
   T   
   G   
   T   
    T    
   C   
   A   
   A   
   G   
   T   
    A    
   A   
   A   
   A   
   A   
   A   
    G    
   G   
    C    
   G   
   A   
   A   
   A   
   G   
   A   
    C    
   A   
   A   
    T    
   C   
   A   
   A   
   T   
   A   
   C   
   A   
   C   
   A   
   T   
   T   
   T   
   A   
    C    
   C   
   T   
   A   
   T   
   A   
   A   
   A   
   T   
   T   
   A   
   T   
   C   
   G   
   A   
   T   
   G   
   A   
   G   
    T    
   A   
   C   
    C    
   C   
   C   
   T   
   T   
   T   
   A   
   G   
   G   
   C   
   G   
   A   
   A   
   A   
   T   
   G   
   A   
   A   
   A   
   G   
   A   
   T   
    T    
   T   
   G   
   T   
   C   
   T   
   T   
   A   
   T   
   C   
   A   
   A   
   T   
   C   
   A   
   A   
   G   
   T   
   A   
   T   
    C    
   G   
    C    
   C   
   A   
   A   
    A    
   A   
   A   
   A   
   G   
   G   
    C    
   G   
   A   
   T   
   A   
    C    
   C   
   T   
   A   
   C   
   C   
   A   
   A   
   A   
   T   
    C    
    G    
   C   
   T   
   T   
   G   
   G   
    A    
    A    
    G    
   C   
   C   
    G    
   T   
   C   
   T   
   T   
   T   
   A   
   A   
   T   
   T   
   T   
   T   
   T   
   C   
   C   
   A   
   G   
   A   
   T   
   A   
   T   
   G   
   T   
   C   
   A   
   G   
   G   
   A   
   A   
   A   
   T   
   G   
   A   
   T   
   A   
   A   
   A   
    A    
   T   
   T   
   T   
   C   
   G   
   A   
   T   
   T   
   C   
   A   
   A   
   G   
   T   
   A   
   G   
   A   
   T   
   A   
   A   
   T   
   G   
   C   
   C   
   A   
   A   
   A   
   C   
   G   
   T   
   G   
   G   
   A   
   G   
   A   
   A   
   A   
   T   
   T   
   G   
   T   
   C   
   G   
   A   
   T   
   C   
   G   
   T   
   A   
   A   
    T    
   G   
   G   
   T   
   A   
   G   
   T   
   G   
   G   
   G   
   C   
   T   
   A   
   G   
    C    
   A   
   A   
   T   
   T   
   A   
   A   
   C   
   A   
   A   
   A   
   G   
   T   
   G   
   T   
   T   
   T   
   G   
   A   
    T    
   G   
   A   
   A   
   G   
   T   
   G   
   G   
   G   
   C   
   G   
   T   
   A   
   G   
   T   
    G    
   C   
   C   
   T   
    G    
   G   
   C   
   A   
   A   
   A   
   C   
   T   
    C    
   G   
   G   
   T   
   T   
   C   
   T   
   G   
   G   
   C   
   G   
   C   
   A   
   G   
   A   
   A   
   A   
   A   
   A   
    A    
   C   
   A   
   G   
   C   
    C    
   A   
   A   
   T   
   A   
   T   
   C   
   A   
   A   
   A   
   G   
   C   
   T   
   T   
   T   
   T   
   A   
   G   
   T   
   G   
   A   
   T   
   A   
   A   
    A    
   T   
   T   
    C    
   G   
   G   
   C   
   G   
   T   
   T   
   T   
   C   
   T   
    G    
   T   
   T   
   G   
   A   
   T   
   G   
   A   
   A   
   A   
   T   
   C   
   A   
   A   
   T   
   C   
    A    
   A   
   A   
   A   
    G    
   T   
   T   
   A   
   A   
   G   
   C   
   C   
   A   
   A   
   G   
   G   
    A    
   T   
   G   
   G   
   G   
   T   
   C   
   C   
   A   
   A   
   G   
   C   
   A   
   G   
   A   
   C   
   T   
   C   
    C    
   T   
   T   
   T   
   G   
   T   
   A   
   C   
   C   
   A   
   A   
   T   
   C   
   A   
   C   
   A   
   G   
   T   
    C    
   G   
   C   
   T   
   T   
   C   
   T   
   G   
   A   
   A   
   C   
   C   
   A   
   G   
   T   
   G   
   A   
   C   
   A   
   G   
   A   
   A   
   T   
   T   
   A   
   C   
   C   
   A   
   A   
   C   
   A   
   G   
   G   
   G   
   G   
   C   
   T   
   G   
   C   
   G   
   A   
   C   
   A   
   A   
   A   
   A   
   G   
    A    
    T    
   A   
   C   
   A   
   G   
   A   
   G   
   T   
   C   
   A   
   C   
   G   
   T   
   T   
   A   
   T   
   T   
   A   
   T   
   C   
   C   
    G    
    C    
   T   
   G   
   G   
   G   
   G   
   G   
   A   
   A   
   G   
   C   
   A   
   G   
   C   
   T   
   G   
   C   
   G   
   C   
   A   
   A   
   T   
   T   
   A   
   A   
   T   
   C   
   G   
   G   
    G    
   T   
   A   
   T   
   A   
   C   
    G    
   G   
   G   
    C    
   A   
   C   
    C    
   A   
   T   
   T   
   A   
   C   
   T   
   G   
   C   
   A   
   G   
   A   
    A    
   G   
   A   
   T   
   A   
   T   
   T   
    G    
   A   
   A   
   A   
   A   
   A   
   A   
   A   
   T   
   C   
   C   
   A   
   G   
   A   
   G   
   T   
   T   
   A   
   A   
   G   
   T   
   A   
   G   
   T   
   A   
   C   
   G   
   G   
   G   
    C    
   G   
   T   
   A   
   A   
   T   
   T   
   G   
   G   
    T    
   A   
   A   
   A   
   A   
   C   
   T   
   G   
   G   
    T    
   T   
   T   
   A   
   G   
   A   
   A   
   C   
   G   
   A   
   G   
   C   
   G   
   T   
   T   
   T   
   G   
   A   
   T   
   A   
   A   
   A   
   G   
   A   
    A    
   T   
   T   
   A   
   A   
   G   
   A   
   G   
   G   
    G    
   C   
   A   
   A   
   G   
   A   
   T   
   G   
   G   
    T    
   G   
   G   
    C    
   T   
   C   
    A    
   C   
   T   
   A   
   G   
   T   
   T   
   A   
   T   
   T   
   T   
   T   
   A   
   G   
   A   
   T   
   G   
   A   
   C   
   A   
   A   
   A   
   G   
   A   
   G   
   A   
   A   
   T   
   G   
   T   
   C   
   A   
   A   
   A   
   A   
   A   
   A   
   G   
   C   
   T   
   T   
   T   
   A   
   C   
   A   
   A   
   A   
   C   
   C   
   A   
   A   
   A   
   G   
   A   
    G    
   A   
   A   
   A   
   A   
   A   
   A   
   G   
   A   
   T   
   G   
   G   
   T   
   C   
   A   
   A   
   A   
   C   
    G    
   A   
   T   
   T   
   A   
   A   
   A   
   T   
   T   
   A   
   A   
   C   
   G   
   A   
   T   
   T   
   G   
   A   
    C    
   A   
   G   
    T    
   G   
   G   
    C    
   G   
   T   
   A   
   C   
   A   
   A   
   C   
   A   
   A   
   C   
   A   
   A   
   G   
   C   
    C    
   T   
   T   
    C    
   G   
   C   
   T   
   A   
   T   
   A   
   T   
   T   
   T   
   G   
   A   
   C   
   A   
   A   
   A   
   C   
   G   
    T    
    C    
   C   
   T   
   G   
   G   
   T   
   A   
   G   
   T   
   G   
   C   
   G   
   G   
   T   
   C   
   A   
   T   
   T   
   A   
   C   
   C   
   G   
   A   
   T   
   C   
   C   
   T   
   C   
   A   
   A   
   A   
   A   
   A   
   G   
   G   
   C   
   G   
   A   
   T   
   T   
   T   
   A   
    T    
   T   
   A   
   G   
   C   
    A    
   A   
   C   
   G   
   G   
   T   
   T   
   A   
   G   
   T   
   T   
   C   
   A   
   C   
   C   
   T   
   T   
   C   
   G   
   T   
   A   
   T   
   G   
   A   
   T   
   C   
   C   
   T   
   A   
   A   
   T   
   A   
   A   
   A   
   A   
   T   
   G   
   G   
   C   
   C   
   A   
   A   
   T   
   G   
   G   
    T    
   A   
   T   
   T   
   T   
   C   
   T   
   C   
   A   
   A   
   A   
   A   
   A   
   G   
   A   
    A    
   T   
   A   
   T   
   G   
   A   
   T   
   G   
   C   
   T   
   T   
   A   
   C   
   A   
   A   
   T   
   A   
   A   
   C   
   A   
   A   
   C   
   A   
   A   
   A   
   G   
   A   
   T   
   T   
   T   
   A   
   C   
   C   
   A   
   T   
   T   
    C    
   A   
   C   
   A   
   G   
   C   
   A   
   C   
   G   
   T   
   T   
   T   
    T    
   G   
   C   
   G   
   A   
   C   
   A   
   G   
   G   
    C    
   T   
   A   
   T   
   G   
   C   
   A   
   C   
   C   
   A   
   G   
   G   
    C    
   T   
   C   
   T   
   A   
   C   
   C   
   T   
   T   
   T   
   A   
   A   
   A   
   A   
   C   
   A   
   A   
   T   
   T   
   A   
   C   
   C   
   G   
   G   
   T   
   G   
   C   
   C   
   A   
   T   
   T   
   G   
   G   
   T   
   T   
   T   
   A   
   G   
   A   
   T   
    G    
   C   
   A   
   G   
   G   
   G   
   A   
   C   
   A   
   T   
   T   
   G   
   A   
   A   
   G   
   C   
   C   
   A   
   G   
   A   
   T   
   G   
   A   
   A   
   G   
   A   
   G   
   C   
   T   
   A   
   G   
   A   
   A   
   A   
   T   
   T   
   A   
   A   
   T   
   G   
   G   
    T    
   T   
   T   
   G   
   A   
   A   
   A   
   T   
   G   
   G   
   C   
   A   
   A   
   A   
   A   
   A   
   G   
   A   
   T   
   A   
   A   
   A   
   T   
   C   
   T   
   T   
   G   
   G   
   G   
   G   
    C    
   G   
   G   
   C   
   T   
   A   
   T   
   T   
   T   
   T   
   G   
   C   
    G    
   A   
   C   
   A   
   C   
   G   
   T   
   G   
   T   
   A   
   A   
   A   
   A   
   G   
   A   
   A   
   G   
   C   
   A   
   A   
   G   
   T   
   C   
   C   
   A   
   G   
   T   
   C   
   A   
   A   
   T   
   C   
   T   
    G    
   C   
   G   
   T   
   A   
   C   
   C   
   G   
   C   
   T   
   T   
   T   
   A   
   G   
   T   
   T   
   A   
   A   
   T   
   T   
   C   
   A   
   G   
   A   
   T   
   A   
   A   
   T   
   A   
   T   
   T   
   T   
   A   
   T   
   T   
   T   
   T   
   G   
   C   
    C    
   C   
   A   
   A   
   C   
   A   
   A   
   A   
   C   
   A   
   T   
   T   
   A   
   C   
   G   
    G    
   A   
   T   
   G   
   G   
   G   
    A    
   G   
   A   
   A   
   G   
   A   
   C   
   A   
   A   
   A   
   T   
   T   
   T   
   A   
   G   
   A   
   G   
   C   
    G    
   G   
   G   
   T   
   C   
   T   
   T   
   A   
   A   
   T   
   A   
   A   
   A   
   T   
   T   
   C   
   A   
   T   
   T   
   T   
   T   
   C   
   G   
   G   
   T   
   G   
   A   
   G   
   G   
   A   
   A   
   C   
   T   
   A   
   G   
   A   
   T   
   T   
   T   
   A   
   C   
   C   
   A   
   A   
   T   
    T    
   G   
   C   
   C   
   A   
   T   
   G   
   A   
   C   
   G   
   C   
   C   
    T    
   G   
   C   
   T   
   C   
   A   
   A   
   A   
   T   
   T   
   T   
   C   
   A   
   A   
   A   
    T    
   G   
   A   
   A   
   G   
   A   
   T   
   A   
   A   
   A   
   T   
   T   
   T   
   A   
   A   
   T   
   T   
   C   
   T   
   G   
   A   
   A   
   A   
   T   
   T   
   C   
   T   
   T   
   T   
   T   
    A    
   G   
   C   
   A   
   G   
   A   
   T   
   A   
   C   
    G    
   G   
   G   
   C   
   T   
   A   
    C    
   G   
   G   
    T    
   C   
   A   
   A   
   G   
   G   
    T    
   C   
   A   
   A   
   T   
   T   
   G   
   T   
   T   
   A   
   A   
   T   
   T   
   T   
   C   
    G    
   C   
   C   
   A   
   A   
   T   
   C   
   C   
   A   
   G   
   C   
   A   
   A   
   G   
   C   
   T   
   A   
   C   
   C   
   A   
   T   
   G   
   T   
   A   
   T   
   A   
   G   
   T   
   G   
   T   
   T   
   T   
   T   
   C   
   C   
   A   
   A   
   A   
   A   
   T   
   A   
   A   
   T   
   G   
   G   
    A    
   A   
   C   
   A   
    T    
   T   
    A    
   G   
   T   
    C    
   T   
   A   
   T   
   C   
   C   
   A   
   A   
   A   
   A   
    C    
   T   
   A   
   G   
   T   
   T   
   T   
   T   
   A   
   G   
   A   
   C   
   A   
   A   
   A   
   G   
   A   
   A   
   A   
   C   
    G    
   A   
   A   
   G   
   A   
   A   
   A   
   A   
   A   
   A   
   G   
   A   
   C   
   A   
   A   
   T   
   G   
   T   
   A   
   A   
   T   
   C   
   A   
   G   
   C   
   G   
   C   
   C   
   A   
   A   
   C   
   G   
   C   
   A   
   G   
   C   
   G   
   A   
   A   
   T   
   A   
   C   
   A   
   A   
   T   
   T   
   G   
   C   
   C   
   A   
   C   
   A   
   G   
   A   
   T   
   C   
   T   
   T   
   T   
   T   
   A   
   G   
   G   
   T   
   A   
   G   
   T   
   G   
   T   
   G   
   G   
   A   
   A   
   G   
   A   
   T   
   C   
   C   
   T   
   T   
   C   
   A   
   G   
   G   
   T   
   T   
   A   
   T   
   G   
   T   
   T   
   T   
   A   
   T   
   A   
   A   
   T   
   A   
   T   
   G   
   T   
   A   
   C   
   A   
   A   
   T   
   C   
   C   
   G   
   A   
   A   
   C   
   T   
   T   
   C   
   T   
   C   
   A   
   T   
   T   
   G   
   G   
   C   
   T   
   G   
   C   
   T   
   A   
   A   
   A   
   A   
   C   
   A   
   G   
   G   
   A   
   A   
   C   
   A   
   G   
   C   
   T   
   G   
   A   
   A   
   A   
   T   
   A   
   A   
   A   
   A   
   G   
   A   
   T   
   A   
   A   
   A   
   C   
   A   
   A   
   G   
   A   
   T   
   A   
   C   
   A   
   G   
   A   
   T   
   G   
   G   
   C   
   A   
   A   
   A   
   G   
   A   
   A   
   A   
   A   
   C   
   A   
   G   
   T   
   T   
   T   
   C   
   C   
   T   
   T   
   C   
   T   
   A   
   A   
   C   
   G   
   T   
   T   
   A   
   G   
   A   
   T   
   C   
   G   
   T   
   A   
   G   
   T   
   A   
   A   
   C   
   A   
   A   
   T   
   A   
   A   
   A   
   T   
   T   
   C   
   T   
   T   
   A   
   A   
   C   
   A   
   A   
   T   
   G   
   A   
   T   
   T   
   A   
   T   
   G   
   G   
   T   
   T   
   G   
   A   
   A   
   A   
   A   
   T   
   T   
   C   
   A   
   G   
   G   
   A   
   G   
   A   
   A   
   A   
   A   
   T   
   G   
   G   
   T   
   T   
   C   
   T   
   G   
   C   
   A   
   A   
   C   
   A   
   G   
   A   
   T   
   A   
   T   
   C   
   A   
   G   
   T   
   A   
   A   
   A   
   C   
   C   
   A   
   T   
   T   
   A   
   A   
   T   
   T   
   G   
   A   
   T   
   T   
   A   
   C   
   T   
   T   
   A   
   G   
   A   
   A   
   G   
   C   
   A   
   A   
   C   
   C   
   A   
   T   
   T   
   A   
   A   
   A   
   -   
   -   
   -   
   -   
   -   
   -   
   -   
   -   
   -   
   -   
   -   
   -   
   -   
   -   
   -   
   -   
   -   
   -   
   -   
   -   
   -   
   -   
   -   
   -   
   -   
   -   
   -   
   -   
   -   
   -   
   -   
   -   
   -   
   -   
   -   
   -   
   -   
   -   
   -   
   -   
   -   
   -   
   -   
   -   
   -   
   -   
   -   
   -   
   -   
   -   
   -   
   -   
   -   
   -   
   -   
   -   
   -   
   -   
   -   
   -   
   -   
   -   
   -   
   -   
   -   
   -   
   -   
   -   
   -   
   -   
   -   
   -   
   -   
   -   
   -   
   -   
   -   
   -   
   -   
   -   
   -   
   -   
   -   
   -   
   -   
   -   
   -   
   -   
   -   
   -   
   -   
   -   
   -   
   -   
   -   
   -   
   -   
   -   
   -   
   -   
   -   
   -   
   -   
   -   
   -   
   -   
   -   
   -   
   -   
   -   
   -   
   -   
   -   
   -   
   -   
   -   
   -   
   -   
   -   
   -   
   -   
   -   
   -   
   -   
   -   
   -   
   -   
   -   
   -   
   -   
   -   
   -   
   -   
   -   
   -   
   -   
   -   
   -   
   -   
   -   
   -   
   -   
   -   
   -   
   -   
   -   
   -   
   -   
   -   
   -   
   -   
   -   
   -   
   -   
   -   
   -   
   -   
   -   
   -   
   -   
   -   
   -   
   -   
   -   
   -   
   -   
   -   
   -   
   -   
   C   
   A   
   A   
   T   
   G   
   G   
   C   
   A   
   A   
   G   
   C   
   C   
   A   
   A   
   A   
   C   
   A   
   A   
   G   
   A   
   A   
   T   
   T   
   A   
   G   
   C   
   C   
   G   
   A   
   A   
   G   
   C   
   G   
   A   
   A   
   G   
   A   
   A   
   A   
   A   
   C   
   A   
   G   
   C   
   T   
   A   
   C   
   T   
   A   
   C   
   A   
   T   
   T   
   T   
   T   
   T   
   A   
   A   
   A   
   C   
   G   
   T   
   A   
   T   
   T   
   G   
   T   
   C   
   A   
   A   
   A   
   A   
   C   
   A   
   G   
   G   
   A   
   A   
   T   
   T   
   T   
   G   
   A   
   T   
   A   
   A   
   G   
   T   
   T   
   A   
   C   
   C   
   G   
   T   
   C   
   C   
   G   
   T   
   T   
   G   
   T   
   T   
   C   
   A   
   A   
   G   
   A   
   A   
   G   
   C   
   T   
   A   
   G   
   C   
   T   
   T   
   A   
   A   
   A   
   G   
   A   
   A   
   A   
   A   
   A   
   T   
   G   
   G   
   C   
   T   
   A   
   T   
   G   
   A   
   T   
   A   
   C   
   T   
   A   
   A   
   A   
   T   
   C   
   T   
   G   
   T   
   T   
   G   
   T   
   T   
   G   
   A   
   A   
   A   
   A   
   A   
   T   
   A   
   C   
   C   
   A   
   A   
   G   
   C   
   A   
   A   
   T   
   T   
   T   
   A   
   T   
   T   
   C   
   A   
   G   
   G   
   G   
   A   
   T   
   T   
   C   
   A   
   A   
   G   
   C   
   A   
   G   
   A   
   A   
   G   
   G   
   A   
   G   
   T   
   C   
   A   
   A   
   A   
   G   
   C   
   T   
   A   
   G   
   T   
   G   
   A   
   T   
   G   
   T   
   T   
   C   
   A   
   A   
   G   
   T   
   A   
   A   
   A   
   A   
   A   
   A   
   G   
   G   
   C   
   G   
   A   
   A   
   A   
   G   
   A   
   C   
   A   
   A   
   T   
   C   
   A   
   A   
   T   
   A   
   C   
   A   
   C   
   A   
   T   
   T   
   T   
   A   
   C   
   C   
   T   
   A   
   T   
   A   
   A   
   A   
   T   
   T   
   A   
   T   
   C   
   G   
   A   
   T   
   G   
   A   
   G   
   T   
   A   
   C   
   C   
   C   
   C   
   T   
   T   
   T   
   A   
   G   
   G   
   C   
   G   
   A   
   A   
   A   
   T   
   G   
   A   
   A   
   A   
   G   
   A   
   T   
   T   
   T   
   G   
   T   
   C   
   T   
   T   
   A   
   T   
   C   
   A   
   A   
   T   
   C   
   A   
   A   
   G   
   T   
   A   
   T   
   C   
   G   
   C   
   C   
   A   
   A   
   A   
   A   
   A   
   A   
   G   
   G   
   C   
   G   
   A   
   T   
   A   
   C   
   C   
   T   
   A   
   C   
   C   
   A   
   A   
   A   
   T   
   C   
   G   
   C   
   T   
   T   
   G   
   G   
   A   
   A   
   G   
   C   
   C   
   G   
   T   
   C   
   T   
   T   
   T   
   A   
   A   
   T   
   T   
   T   
   T   
   T   
   C   
   C   
   A   
   G   
   A   
   T   
   A   
   T   
   G   
   T   
   C   
   A   
   G   
   G   
   A   
   A   
   A   
   T   
   G   
   A   
   T   
   A   
   A   
   A   
   A   
   T   
   T   
   T   
   C   
   G   
   A   
   T   
   T   
   C   
   A   
   A   
   G   
   T   
   A   
   G   
   A   
   T   
   A   
   A   
   T   
   G   
   C   
   C   
   A   
   A   
   A   
   C   
   G   
   T   
   G   
   G   
   A   
   G   
   A   
   A   
   A   
   T   
   T   
   G   
   T   
   C   
   G   
   A   
   T   
   C   
   G   
   T   
   A   
   A   
   T   
   G   
   G   
   T   
   A   
   G   
   T   
   G   
   G   
   G   
   C   
   T   
   A   
   G   
   C   
   A   
   A   
   T   
   T   
   A   
   A   
   C   
   A   
   A   
   A   
   G   
   T   
   G   
   T   
   T   
   T   
   G   
   A   
   T   
   G   
   A   
   A   
   G   
   T   
   G   
   G   
   G   
   C   
   G   
   T   
   A   
   G   
   T   
   G   
   C   
   C   
   T   
   G   
   G   
   C   
   A   
   A   
   A   
   C   
   T   
   C   
   G   
   G   
   T   
   T   
   C   
   T   
   G   
   G   
   C   
   G   
   C   
   A   
   G   
   A   
   A   
   A   
   A   
   A   
   A   
   C   
   A   
   G   
   C   
   C   
   A   
   A   
   T   
   A   
   T   
   C   
   A   
   A   
   A   
   G   
   C   
   T   
   T   
   T   
   T   
   A   
   G   
   T   
   G   
   A   
   T   
   A   
   A   
   A   
   T   
   T   
   C   
   G   
   G   
   C   
   G   
   T   
   T   
   T   
   C   
   T   
   G   
   T   
   T   
   G   
   A   
   T   
   G   
   A   
   A   
   A   
   T   
   C   
   A   
   A   
   T   
   C   
   A   
   A   
   A   
   A   
   G   
   T   
   T   
   A   
   A   
   G   
   C   
   C   
   A   
   A   
   G   
   G   
   A   
   T   
   G   
   G   
   G   
   T   
   C   
   C   
   A   
   A   
   G   
   C   
   A   
   G   
   A   
   C   
   T   
   C   
   C   
   T   
   T   
   T   
   G   
   T   
   A   
   C   
   C   
   A   
   A   
   T   
   C   
   A   
   C   
   A   
   G   
   T   
   C   
   G   
   C   
   T   
   T   
   C   
   T   
   G   
   A   
   A   
   C   
   C   
   A   
   G   
   T   
   G   
   A   
   C   
   A   
   G   
   A   
   A   
   T   
   T   
   A   
   C   
   C   
   A   
   A   
   C   
   A   
   G   
   G   
   G   
   G   
   C   
   T   
   G   
   C   
   G   
   A   
   C   
   A   
   A   
   A   
   A   
   G   
   A   
   T   
   A   
   C   
   A   
   G   
   A   
   G   
   T   
   C   
   A   
   C   
   G   
   T   
   T   
   A   
   T   
   T   
   A   
   T   
   C   
   C   
   G   
   C   
   T   
   G   
   G   
   G   
   G   
   G   
   A   
   A   
   G   
   C   
   A   
   G   
   C   
   T   
   G   
   C   
   G   
   C   
   A   
   A   
   T   
   T   
   A   
   A   
   T   
   C   
   G   
   G   
   G   
   T   
   A   
   T   
   A   
   C   
   G   
   G   
   G   
   C   
   A   
   C   
   C   
   A   
   T   
   T   
   A   
   C   
   T   
   G   
   C   
   A   
   G   
   A   
   A   
   G   
   A   
   T   
   A   
   T   
   T   
   G   
   A   
   A   
   A   
   A   
   A   
   A   
   A   
   T   
   C   
   C   
   A   
   G   
   A   
   G   
   T   
   T   
   A   
   A   
   G   
   T   
   A   
   G   
   T   
   A   
   C   
   G   
   G   
   G   
   C   
   G   
   T   
   A   
   A   
   T   
   T   
   G   
   G   
   T   
   A   
   A   
   A   
   A   
   C   
   T   
   G   
   G   
   T   
   T   
   T   
   A   
   G   
   A   
   A   
   C   
   G   
   A   
   G   
   C   
   G   
   T   
   T   
   T   
   G   
   A   
   T   
   A   
   A   
   A   
   G   
   A   
   A   
   T   
   T   
   A   
   A   
   G   
   A   
   G   
   G   
   G   
   C   
   A   
   A   
   G   
   A   
   T   
   G   
   G   
   T   
   G   
   G   
   C   
   T   
   C   
   A   
   C   
   T   
   A   
   G   
   T   
   T   
   A   
   T   
   T   
   T   
   T   
   A   
   G   
   A   
   T   
   G   
   A   
   C   
   A   
   A   
   A   
   G   
   A   
   G   
   A   
   A   
   T   
   G   
   T   
   C   
   A   
   A   
   A   
   A   
   A   
   A   
   G   
   C   
   T   
   T   
   T   
   A   
   C   
   A   
   A   
   A   
   C   
   C   
   A   
   A   
   A   
   G   
   A   
   G   
   A   
   A   
   A   
   A   
   A   
   A   
   G   
   A   
   T   
   G   
   G   
   T   
   C   
   A   
   A   
   A   
   C   
   G   
   A   
   T   
   T   
   A   
   A   
   A   
   T   
   T   
   A   
   A   
   C   
   G   
   A   
   T   
   T   
   G   
   A   
   C   
   A   
   G   
   T   
   G   
   G   
   C   
   G   
   T   
   A   
   C   
   A   
   A   
   C   
   A   
   A   
   C   
   A   
   A   
   G   
   C   
   C   
   T   
   T   
   C   
   G   
   C   
   T   
   A   
   T   
   A   
   T   
   T   
   T   
   G   
   A   
   C   
   A   
   A   
   A   
   C   
   G   
   T   
   C   
   C   
   T   
   G   
   G   
   T   
   A   
   G   
   T   
   G   
   C   
   G   
   G   
   T   
   C   
   A   
   T   
   T   
   A   
   C   
   C   
   G   
   A   
   T   
   C   
   C   
   T   
   C   
   A   
   A   
   A   
   A   
   A   
   G   
   G   
   C   
   G   
   A   
   T   
   T   
   T   
   A   
   T   
   T   
   A   
   G   
   C   
   A   
   A   
   C   
   G   
   G   
   T   
   T   
   A   
   G   
   T   
   T   
   C   
   A   
   C   
   C   
   T   
   T   
   C   
   G   
   T   
   A   
   T   
   G   
   A   
   T   
   C   
   C   
   T   
   A   
   A   
   T   
   A   
   A   
   A   
   A   
   T   
   G   
   G   
   C   
   C   
   A   
   A   
   T   
   G   
   G   
   T   
   A   
   T   
   T   
   T   
   C   
   T   
   C   
   A   
   A   
   A   
   A   
   A   
   G   
   A   
   A   
   T   
   A   
   T   
   G   
   A   
   T   
   G   
   C   
   T   
   T   
   A   
   C   
   A   
   A   
   T   
   A   
   A   
   C   
   A   
   A   
   C   
   A   
   A   
   A   
   G   
   A   
   T   
   T   
   T   
   A   
   C   
   C   
   A   
   T   
   T   
   C   
   A   
   C   
   A   
   G   
   C   
   A   
   C   
   G   
   T   
   T   
   T   
   T   
   G   
   C   
   G   
   A   
   C   
   A   
   G   
   G   
   C   
   T   
   A   
   T   
   G   
   C   
   A   
   C   
   C   
   A   
   G   
   G   
   C   
   T   
   C   
   T   
   A   
   C   
   C   
   T   
   T   
   T   
   A   
   A   
   A   
   A   
   C   
   A   
   A   
   T   
   T   
   A   
   C   
   C   
   G   
   G   
   T   
   G   
   C   
   C   
   A   
   T   
   T   
   G   
   G   
   T   
   T   
   T   
   A   
   G   
   A   
   T   
   G   
   C   
   A   
   G   
   G   
   G   
   A   
   C   
   A   
   T   
   T   
   G   
   A   
   A   
   G   
   C   
   C   
   A   
   G   
   A   
   T   
   G   
   A   
   A   
   G   
   A   
   G   
   C   
   T   
   A   
   G   
   A   
   A   
   A   
   T   
   T   
   A   
   A   
   T   
   G   
   G   
   T   
   T   
   T   
   G   
   A   
   A   
   A   
   T   
   G   
   G   
   C   
   A   
   A   
   A   
   A   
   A   
   G   
   A   
   T   
   A   
   A   
   A   
   T   
   C   
   T   
   T   
   G   
   G   
   G   
   G   
   C   
   G   
   G   
   C   
   T   
   A   
   T   
   T   
   T   
   T   
   G   
   C   
   G   
   A   
   C   
   A   
   C   
   G   
   T   
   G   
   T   
   A   
   A   
   A   
   A   
   G   
   A   
   A   
   G   
   C   
   A   
   A   
   G   
   T   
   C   
   C   
   A   
   G   
   T   
   C   
   A   
   A   
   T   
   C   
   T   
   G   
   C   
   G   
   T   
   A   
   C   
   C   
   G   
   C   
   T   
   T   
   T   
   A   
   G   
   T   
   T   
   A   
   A   
   T   
   T   
   C   
   A   
   G   
   A   
   T   
   A   
   A   
   T   
   A   
   T   
   T   
   T   
   A   
   T   
   T   
   T   
   T   
   G   
   C   
   C   
   C   
   A   
   A   
   C   
   A   
   A   
   A   
   C   
   A   
   T   
   T   
   A   
   C   
   G   
   G   
   A   
   T   
   G   
   G   
   G   
   A   
   G   
   A   
   A   
   G   
   A   
   C   
   A   
   A   
   A   
   T   
   T   
   T   
   A   
   G   
   A   
   G   
   C   
   G   
   G   
   G   
   T   
   C   
   T   
   T   
   A   
   A   
   T   
   A   
   A   
   A   
   T   
   T   
   C   
   A   
   T   
   T   
   T   
   T   
   C   
   G   
   G   
   T   
   G   
   A   
   G   
   G   
   A   
   A   
   C   
   T   
   A   
   G   
   A   
   T   
   T   
   T   
   A   
   C   
   C   
   A   
   A   
   T   
   T   
   G   
   C   
   C   
   A   
   T   
   G   
   A   
   C   
   G   
   C   
   C   
   T   
   G   
   C   
   T   
   C   
   A   
   A   
   A   
   T   
   T   
   T   
   C   
   A   
   A   
   A   
   T   
   G   
   A   
   A   
   G   
   A   
   T   
   A   
   A   
   A   
   T   
   T   
   T   
   A   
   A   
   T   
   T   
   C   
   T   
   G   
   A   
   A   
   A   
   T   
   T   
   C   
   T   
   T   
   T   
   T   
   A   
   G   
   C   
   A   
   G   
   A   
   T   
   A   
   C   
   G   
   G   
   G   
   C   
   T   
   A   
   C   
   G   
   G   
   T   
   C   
   A   
   A   
   G   
   G   
   T   
   C   
   A   
   A   
   T   
   T   
   G   
   T   
   T   
   A   
   A   
   T   
   T   
   T   
   C   
   G   
   C   
   C   
   A   
   A   
   T   
   C   
   C   
   A   
   G   
   C   
   A   
   A   
   G   
   C   
   T   
   A   
   C   
   C   
   A   
   T   
   G   
   T   
   A   
   T   
   A   
   G   
   T   
   G   
   T   
   T   
   T   
   T   
   C   
   C   
   A   
   A   
   A   
   A   
   T   
   A   
   A   
   T   
   G   
   G   
   A   
   A   
   C   
   A   
   T   
   T   
   A   
   G   
   T   
   C   
   T   
   A   
   T   
   C   
   C   
   A   
   A   
   A   
   A   
   C   
   T   
   A   
   G   
   T   
   T   
   T   
   T   
   A   
   G   
   A   
   C   
   A   
   A   
   A   
   G   
   A   
   A   
   A   
   C   
   G   
   A   
   A   
   G   
   A   
   A   
   A   
   A   
   A   
   A   
   G   
   A   
   C   
   A   
   A   
   T   
   G   
   T   
   A   
   A   
   T   
   C   
   A   
   G   
   C   
   G   
   C   
   C   
   A   
   A   
   C   
   G   
   C   
   A   
   G   
   C   
   G   
   A   
   A   
   T   
   A   
   C   
   A   
   A   
   T   
   T   
   G   
   C   
   C   
   A   
   C   
   A   
   G   
   A   
   T   
   C   
   T   
   T   
   T   
   T   
   A   
   G   
   G   
   T   
   A   
   G   
   T   
   G   
   T   
   G   
   G   
   A   
   A   
   G   
   A   
   T   
   C   
   C   
   T   
   T   
   C   
   A   
   G   
   G   
   T   
   T   
   A   
   T   
   G   
   T   
   T   
   T   
   A   
   T   
   A   
   A   
   T   
   A   
   T   
   G   
   T   
   A   
   C   
   A   
   A   
   T   
   C   
   C   
   G   
   A   
   A   
   C   
   T   
   T   
   C   
   T   
   C   
   A   
   T   
   T   
   G   
   G   
   C   
   T   
   G   
   C   
   T   
   A   
   A   
   A   
   A   
   C   
   A   
   G   
   G   
   A   
   A   
   C   
   A   
   G   
   C   
   T   
   G   
   A   
   A   
   A   
   T   
   A   
   A   
   A   
   A   
   G   
   A   
   T   
   A   
   A   
   A   
   C   
   A   
   A   
   G   
   A   
   T   
   A   
   C   
   A   
   G   
   A   
   T   
   G   
   G   
   C   
   A   
   A   
   A   
   G   
   A   
   A   
   A   
   A   
   C   
   A   
   G   
   T   
   T   
   T   
   C   
   C   
   T   
   T   
   C   
   T   
   A   
   A   
   C   
   G   
   T   
   T   
   A   
   G   
   A   
   T   
   C   
   G   
   T   
   A   
   G   
   T   
   A   
   A   
   C   
   A   
   A   
   T   
   A   
   A   
   A   
   T   
   T   
   C   
   T   
   T   
   A   
   A   
   C   
   A   
   A   
   T   
   G   
   A   
   T   
   T   
   A   
   T   
   G   
   G   
   T   
   T   
   G   
   A   
   A   
   A   
   A   
   T   
   T   
   C   
   A   
   G   
   G   
   A   
   G   
   A   
   A   
   A   
   A   
   T   
   G   
   G   
   T   
   T   
   C   
   T   
   G   
   C   
   A   
   A   
   C   
   A   
   G   
   A   
   T   
   A   
   T   
   C   
   A   
   G   
   T   
   A   
   A   
   A   
   C   
   C   
   A   
   T   
   T   
   A   
   A   
   T   
   T   
   G   
   A   
   T   
   T   
   A   
   C   
   T   
   T   
   A   
   G   
   A   
   A   
   G   
   C   
   A   
   A   
   C   
   C   
   A   
   T   
   T   
   A   
   A   
   A   
   -   
   -   
   -   
   -   
   -   
   -   
   -   
   -   
   -   
   -   
   -   
   -   
   -   
   -   
   -   
   -   
   -   
   -   
   -   
   -   
   -   
   -   
   -   
   -   
   -   
   -   
   -   
   -   
   -   
   -   
   -   
   -   
   -   
   -   
   -   
   -   
   -   
   -   
   -   
   -   
   -   
   -   
   -   
   -   
   -   
   -   
   -   
   -   
   -   
   -   
   -   
   -   
   -   
   -   
   -   
   -   
   -   
   -   
   -   
   -   
   -   
 

 
   N52587   
   A   
   T   
   G   
   G   
   A   
   A   
   C   
   G   
   A   
   A   
   G   
   C   
   A   
   A   
   T   
   C   
   G   
   T   
   A   
   A   
   T   
   A   
   A   
   A   
   A   
   A   
   A   
   T   
   C   
   T   
   T   
   C   
   A   
   A   
   A   
   A   
   A   
   A   
   A   
   C   
   C   
   A   
   C   
   T   
   T   
   A   
   T   
   T   
   C   
   T   
   T   
   G   
   G   
   C   
   G   
   T   
   T   
   T   
   C   
   T   
   G   
   C   
   C   
   T   
   T   
   G   
   G   
   T   
   T   
   C   
   T   
   G   
   A   
   T   
   C   
   G   
   C   
   T   
   G   
   C   
   T   
   G   
   T   
   C   
   G   
   G   
   T   
   G   
   G   
   T   
   G   
   G   
   G   
   T   
   A   
   T   
   T   
   A   
   T   
   G   
   C   
   T   
   T   
   A   
   T   
   A   
   G   
   C   
   C   
   A   
   A   
   T   
   G   
   G   
   C   
   A   
   A   
   G   
   C   
   C   
   A   
   A   
   A   
   C   
   A   
   A   
   G   
   A   
    A    
   T   
   T   
   A   
   G   
   C   
    T    
   G   
   A   
   A   
   G   
   C   
   G   
   A   
   A   
   G   
   A   
   A   
   A   
   A   
    C    
   A   
   G   
   C   
    C    
   A   
    A    
   T   
    A    
    C    
   A   
   T   
   T   
   T   
   T   
   T   
   A   
   A   
   A   
    C    
   G   
   T   
   A   
   T   
   T   
   G   
    T    
   C   
   A   
   A   
   A   
   A   
   C   
   A   
   G   
    G    
   A   
   A   
   T   
   T   
   T   
   G   
   A   
   T   
   A   
   A   
   G   
   T   
   T   
   A   
   C   
   C   
    A    
   T   
   C   
   C   
   G   
   T   
   T   
    G    
   T   
   T   
   C   
   A   
   A   
   G   
   A   
   A   
    A    
   C   
   T   
   A   
   G   
   C   
   T   
   T   
   A   
   A   
   A   
   G   
   A   
   A   
   A   
   A   
   A   
   T   
   G   
   G   
    C    
   T   
   A   
   T   
   G   
   A   
   T   
   A   
   C   
   T   
   A   
   A   
   A   
   T   
   C   
    T    
   G   
   T   
   T   
   G   
   T   
   T   
   G   
   A   
   A   
   A   
   A   
   A   
   T   
   A   
   C   
   C   
   A   
   A   
   G   
   C   
   A   
   A   
   T   
   T   
   T   
   A   
   T   
   T   
   C   
   A   
   G   
   G   
   G   
   A   
   T   
   T   
   C   
   A   
   A   
   G   
   C   
    A    
   G   
   A   
   A   
    G    
   G   
    G    
   G   
   T   
   C   
   A   
   A   
    A    
   G   
   C   
   T   
   A   
   G   
   T   
   G   
   A   
   T   
   G   
   T   
    T    
   C   
   A   
   A   
   G   
   T   
    C    
   A   
   A   
   A   
   A   
   A   
    A    
   G   
    G    
   G   
   A   
   A   
   A   
   G   
   A   
    C    
   A   
   A   
    C    
   C   
   A   
   A   
   T   
   A   
   C   
   A   
   C   
   A   
   T   
   T   
   T   
   A   
    C    
   C   
   T   
   A   
   T   
   A   
   A   
   A   
   T   
   T   
   A   
   T   
   C   
   G   
   A   
   T   
   G   
   A   
   G   
    C    
   A   
   C   
    T    
   C   
   C   
   T   
   T   
   T   
   A   
   G   
   G   
   C   
   G   
   A   
   A   
   A   
   T   
   G   
   A   
   A   
   A   
   G   
   A   
   T   
    T    
   T   
   G   
   T   
   C   
   T   
   T   
   A   
   T   
   C   
   A   
   A   
   T   
   C   
   A   
   A   
   G   
   T   
   A   
   T   
    C    
   G   
    C    
   C   
   A   
   A   
    A    
   A   
   A   
   A   
   G   
   G   
    C    
   G   
   A   
   T   
   A   
    A    
   C   
   T   
   A   
   C   
   C   
   A   
   A   
   A   
   T   
    C    
    G    
   C   
   T   
   T   
   G   
   G   
    G    
    C    
    A    
   C   
   C   
    G    
   T   
   C   
   T   
   T   
   T   
   A   
   A   
   T   
   T   
   T   
   T   
   T   
   C   
   C   
   A   
   G   
   A   
   T   
   A   
   T   
   G   
   T   
   C   
   A   
   G   
   G   
   A   
   A   
   A   
   T   
   G   
   A   
   T   
   A   
   A   
   A   
    A    
   T   
   T   
   T   
   C   
   G   
   A   
   T   
   T   
   C   
   A   
   A   
   G   
   T   
   A   
   G   
   A   
   T   
   A   
   A   
   T   
   G   
   C   
   C   
   A   
   A   
   A   
   C   
   G   
   T   
   G   
   G   
   A   
   G   
   A   
   A   
   A   
   T   
   T   
   G   
   T   
   C   
   G   
   A   
   T   
   C   
   G   
   T   
   A   
   A   
    T    
   G   
   G   
   T   
   A   
   G   
   T   
   G   
   G   
   G   
   C   
   T   
   A   
   G   
    C    
   A   
   A   
   T   
   T   
   A   
   A   
   C   
   A   
   A   
   A   
   G   
   T   
   G   
   T   
   T   
   T   
   G   
   A   
    T    
   G   
   A   
   A   
   G   
   T   
   G   
   G   
   G   
   C   
   G   
   T   
   A   
   G   
   T   
    G    
   C   
   C   
   T   
    G    
   G   
   C   
   A   
   A   
   A   
   C   
   T   
    C    
   G   
   G   
   T   
   T   
   C   
   T   
   G   
   G   
   C   
   G   
   C   
   A   
   G   
   A   
   A   
   A   
   A   
   A   
    A    
   C   
   A   
   G   
   C   
    C    
   A   
   A   
   T   
   A   
   T   
   C   
   A   
   A   
   A   
   G   
   C   
   T   
   T   
   T   
   T   
   A   
   G   
   T   
   G   
   A   
   T   
   A   
   A   
    G    
   T   
   T   
    T    
   G   
   G   
   C   
   G   
   T   
   T   
   T   
   C   
   T   
    G    
   T   
   T   
   G   
   A   
   T   
   G   
   A   
   A   
   A   
   T   
   C   
   A   
   A   
   T   
   C   
    A    
   A   
   A   
   A   
    G    
   T   
   T   
   A   
   A   
   G   
   C   
   C   
   A   
   A   
   G   
   G   
    A    
   T   
   G   
   G   
   G   
   T   
   C   
   C   
   A   
   A   
   G   
   C   
   A   
   G   
   A   
   C   
   T   
   C   
    G    
   T   
   T   
   T   
   G   
   T   
   A   
   C   
   C   
   A   
   A   
   T   
   C   
   A   
   C   
   A   
   G   
   T   
    T    
   G   
   C   
   T   
   T   
   C   
   T   
   G   
   A   
   A   
   C   
   C   
   A   
   G   
   T   
   G   
   A   
   C   
   A   
   G   
   A   
   A   
   T   
   T   
   A   
   C   
   C   
   A   
   A   
   C   
   A   
   G   
   G   
   G   
   G   
   C   
   T   
   G   
   C   
   G   
   A   
   C   
   A   
   A   
   A   
   A   
   G   
    A    
    T    
   A   
   C   
   A   
   G   
   A   
   G   
   T   
   C   
   A   
   C   
   G   
   T   
   T   
   A   
   T   
   T   
   A   
   T   
   C   
   C   
    A    
    T    
   T   
   G   
   G   
   G   
   G   
   G   
   A   
   A   
   G   
   C   
   A   
   G   
   C   
   T   
   G   
   C   
   G   
   C   
   A   
   A   
   T   
   T   
   A   
   A   
   T   
   C   
   G   
   G   
    G    
   T   
   A   
   T   
   A   
   C   
    G    
   G   
   G   
    C    
   A   
   C   
    T    
   A   
   T   
   T   
   A   
   C   
   T   
   G   
   C   
   A   
   G   
   A   
    A    
   G   
   A   
   T   
   A   
   T   
   T   
    G    
   A   
   A   
   A   
   A   
   A   
   A   
   A   
   T   
   C   
   C   
   A   
   G   
   A   
   G   
   T   
   T   
   A   
   A   
   G   
   T   
   A   
   G   
   T   
   A   
   C   
   G   
   G   
   G   
    C    
   G   
   T   
   A   
   A   
   T   
   T   
   G   
   G   
    C    
   A   
   A   
   A   
   A   
   C   
   T   
   G   
   G   
    C    
   T   
   T   
   A   
   G   
   A   
   A   
   C   
   G   
   A   
   G   
   C   
   G   
   T   
   T   
   T   
   G   
   A   
   T   
   A   
   A   
   A   
   G   
   A   
    A    
   T   
   T   
   A   
   A   
   G   
   A   
   G   
   G   
    A    
   C   
   A   
   A   
   G   
   A   
   T   
   G   
   G   
    T    
   G   
   G   
    T    
   T   
   C   
    A    
   C   
   T   
   A   
   G   
   T   
   T   
   A   
   T   
   T   
   T   
   T   
   A   
   G   
   A   
   T   
   G   
   A   
   C   
   A   
   A   
   A   
   G   
   A   
   G   
   A   
   A   
   T   
   G   
   T   
   C   
   A   
   A   
   A   
   A   
   A   
   A   
   G   
   C   
   T   
   T   
   T   
   A   
   C   
   A   
   A   
   A   
   C   
   C   
   A   
   A   
   A   
   G   
   A   
    G    
   A   
   A   
   A   
   A   
   A   
   A   
   G   
   A   
   T   
   G   
   G   
   T   
   C   
   A   
   A   
   A   
   C   
    A    
   A   
   T   
   T   
   A   
   A   
   A   
   T   
   T   
   A   
   A   
   C   
   G   
   A   
   T   
   T   
   G   
   A   
    T    
   A   
   G   
    T    
   G   
   G   
    C    
   G   
   T   
   A   
   C   
   A   
   A   
   C   
   A   
   A   
   C   
   A   
   A   
   G   
   C   
    C    
   T   
   T   
    C    
   G   
   C   
   T   
   A   
   T   
   A   
   T   
   T   
   T   
   G   
   A   
   C   
   A   
   A   
   A   
   C   
   G   
    T    
    C    
   C   
   T   
   G   
   G   
   T   
   A   
   G   
   T   
   G   
   C   
   G   
   G   
   T   
   C   
   A   
   T   
   T   
   A   
   C   
   C   
   G   
   A   
   T   
   C   
   C   
   T   
   C   
   A   
   A   
   A   
   A   
   A   
   G   
   G   
   C   
   G   
   A   
   T   
   T   
   T   
   A   
    C    
   T   
   A   
   G   
   C   
    G    
   A   
   C   
   G   
   G   
   T   
   T   
   A   
   G   
   T   
   T   
   C   
   A   
   C   
   C   
   T   
   T   
   C   
   G   
   T   
   A   
   T   
   G   
   A   
   T   
   C   
   C   
   T   
   A   
   A   
   T   
   A   
   A   
   A   
   A   
   T   
   G   
   G   
   C   
   C   
   A   
   A   
   T   
   G   
   G   
    C    
   A   
   T   
   T   
   T   
   C   
   T   
   C   
   A   
   A   
   A   
   A   
   A   
   G   
   A   
    A    
   T   
   A   
   T   
   G   
   A   
   T   
   G   
   C   
   T   
   T   
   A   
   C   
   A   
   A   
   T   
   A   
   A   
   C   
   A   
   A   
   C   
   A   
   A   
   A   
   G   
   A   
   T   
   T   
   T   
   A   
   C   
   C   
   A   
   T   
   T   
    C    
   A   
   C   
   A   
   G   
   C   
   A   
   C   
   G   
   T   
   T   
   T   
    T    
   G   
   C   
   G   
   A   
   C   
   A   
   G   
   G   
    C    
   T   
   A   
   T   
   G   
   C   
   A   
   C   
   C   
   A   
   G   
   G   
    C    
   T   
   C   
   T   
   A   
   C   
   C   
   T   
   T   
   T   
   A   
   A   
   A   
   A   
   C   
   A   
   A   
   T   
   T   
   A   
   C   
   C   
   G   
   G   
   T   
   G   
   C   
   C   
   A   
   T   
   T   
   G   
   G   
   T   
   T   
   T   
   A   
   G   
   A   
   T   
    G    
   C   
   A   
   G   
   G   
   G   
   A   
   C   
   A   
   T   
   T   
   G   
   A   
   A   
   G   
   C   
   C   
   A   
   G   
   A   
   T   
   G   
   A   
   A   
   G   
   A   
   G   
   C   
   T   
   A   
   G   
   A   
   A   
   A   
   T   
   T   
   A   
   A   
   T   
   G   
   G   
    C    
   T   
   T   
   G   
   A   
   A   
   A   
   T   
   G   
   G   
   C   
   A   
   A   
   A   
   A   
   A   
   G   
   A   
   T   
   A   
   A   
   A   
   T   
   C   
   T   
   T   
   G   
   G   
   G   
   G   
    C    
   G   
   G   
   C   
   T   
   A   
   T   
   T   
   T   
   T   
   G   
   C   
    G    
   A   
   C   
   A   
   C   
   G   
   T   
   G   
   T   
   A   
   A   
   A   
   A   
   G   
   A   
   A   
   G   
   C   
   A   
   A   
   G   
   T   
   C   
   C   
   A   
   G   
   T   
   C   
   A   
   A   
   T   
   C   
   T   
    T    
   C   
   G   
   T   
   A   
   C   
   C   
   G   
   C   
   T   
   T   
   T   
   A   
   G   
   T   
   T   
   A   
   A   
   T   
   T   
   C   
   A   
   G   
   A   
   T   
   A   
   A   
   T   
   A   
   T   
   T   
   T   
   A   
   T   
   T   
   T   
   T   
   G   
   C   
    C    
   C   
   A   
   A   
   C   
   A   
   A   
   A   
   C   
   A   
   T   
   T   
   A   
   C   
   G   
    G    
   A   
   T   
   G   
   G   
   G   
    G    
   G   
   A   
   A   
   G   
   A   
   C   
   A   
   A   
   A   
   T   
   T   
   T   
   A   
   G   
   A   
   G   
   C   
    G    
   G   
   G   
   T   
   C   
   T   
   T   
   A   
   A   
   T   
   A   
   A   
   A   
   T   
   T   
   C   
   A   
   T   
   T   
   T   
   T   
   C   
   G   
   G   
   T   
   G   
   A   
   G   
   G   
   A   
   A   
   C   
   T   
   A   
   G   
   A   
   T   
   T   
   T   
   A   
   C   
   C   
   A   
   A   
   T   
    T    
   G   
   C   
   C   
   A   
   T   
   G   
   A   
   C   
   G   
   C   
   C   
    T    
   G   
   C   
   T   
   C   
   A   
   A   
   A   
   T   
   T   
   T   
   C   
   A   
   A   
   A   
    C    
   G   
   A   
   A   
   G   
   A   
   T   
   A   
   A   
   A   
   T   
   T   
   T   
   A   
   A   
   T   
   T   
   C   
   T   
   G   
   A   
   A   
   A   
   T   
   T   
   C   
   T   
   T   
   T   
   T   
    A    
   G   
   C   
   A   
   G   
   A   
   T   
   A   
   C   
    A    
   G   
   G   
   C   
   T   
   A   
    T    
   G   
   G   
    C    
   C   
   A   
   A   
   G   
   G   
    C    
   C   
   A   
   A   
   T   
   T   
   G   
   T   
   T   
   A   
   A   
   T   
   T   
   T   
   C   
    G    
   C   
   C   
   A   
   A   
   T   
   C   
   C   
   A   
   G   
   C   
   A   
   A   
   G   
   C   
   T   
   A   
   C   
   C   
   A   
   T   
   G   
   T   
   A   
   T   
   A   
   G   
   T   
   G   
   T   
   T   
   T   
   T   
   C   
   C   
   A   
   A   
   A   
   A   
   T   
   A   
   A   
   T   
   G   
   G   
    A    
   A   
   C   
   A   
    T    
   T   
    A    
   G   
   T   
    T    
   T   
   A   
   T   
   C   
   C   
   A   
   A   
   A   
   A   
    T    
   T   
   A   
   G   
   T   
   T   
   T   
   T   
   A   
   G   
   A   
   G   
   A   
   A   
   A   
   G   
   A   
   A   
   A   
   C   
    G    
   A   
   A   
   G   
   A   
   A   
   A   
   A   
   A   
   A   
   G   
   A   
   C   
   A   
   A   
   T   
   G   
   T   
   A   
   A   
   T   
   C   
   A   
   G   
   C   
   G   
   C   
   C   
   A   
   A   
   C   
   G   
   C   
   A   
   G   
   C   
   G   
   A   
   A   
   T   
   A   
   C   
   A   
   A   
   T   
   A   
   G   
   C   
   C   
   A   
   C   
   A   
   G   
   A   
   T   
   C   
   T   
   T   
   T   
   T   
   A   
   G   
   G   
   T   
   A   
   G   
   T   
   G   
   T   
   G   
   G   
   A   
   A   
   G   
   A   
   T   
   C   
   C   
   T   
   T   
   C   
   A   
   G   
   G   
   T   
   T   
   A   
   T   
   G   
   T   
   T   
   T   
   A   
   T   
   A   
   A   
   T   
   A   
   T   
   G   
   T   
   A   
   C   
   A   
   A   
   T   
   C   
   C   
   G   
   A   
   A   
   C   
   T   
   T   
   C   
   T   
   C   
   A   
   T   
   T   
   G   
   G   
   C   
   T   
   G   
   C   
   C   
   A   
   A   
   A   
   A   
   C   
   A   
   G   
   G   
   A   
   A   
   C   
   A   
   G   
   C   
   T   
   G   
   A   
   A   
   A   
   T   
   A   
   A   
   A   
   A   
   G   
   A   
   T   
   A   
   A   
   A   
   C   
   A   
   A   
   G   
   A   
   T   
   A   
   C   
   A   
   G   
   A   
   T   
   G   
   G   
   C   
   A   
   A   
   A   
   G   
   A   
   A   
   A   
   A   
   C   
   A   
   G   
   T   
   T   
   T   
   C   
   C   
   T   
   T   
   C   
   T   
   A   
   A   
   C   
   G   
   T   
   T   
   A   
   G   
   A   
   T   
   C   
   G   
   T   
   A   
   G   
   T   
   A   
   A   
   C   
   A   
   A   
   T   
   A   
   A   
   A   
   T   
   T   
   T   
   T   
   T   
   A   
   A   
   C   
   A   
   A   
   T   
   G   
   A   
   T   
   T   
   A   
   T   
   G   
   G   
   T   
   T   
   G   
   A   
   A   
   A   
   A   
   T   
   T   
   C   
   A   
   G   
   G   
   A   
   G   
   A   
   A   
   A   
   A   
   T   
   G   
   G   
   T   
   T   
   C   
   T   
   G   
   C   
   A   
   A   
   C   
   A   
   G   
   A   
   T   
   A   
   T   
   C   
   A   
   G   
   T   
   A   
   A   
   A   
   C   
   C   
   A   
   T   
   T   
   A   
   A   
   T   
   T   
   G   
   A   
   T   
   T   
   A   
   C   
   T   
   T   
   A   
   G   
   A   
   A   
   G   
   C   
   A   
   A   
   C   
   C   
   A   
   T   
   T   
   A   
   A   
   A   
   -   
   -   
   -   
   -   
   -   
   -   
   -   
   -   
   -   
   -   
   -   
   -   
   -   
   -   
   -   
   -   
   -   
   -   
   -   
   -   
   -   
   -   
   -   
   -   
   -   
   -   
   -   
   -   
   -   
   -   
   -   
   -   
   -   
   -   
   -   
   -   
   -   
   -   
   -   
   -   
   -   
   -   
   -   
   -   
   -   
   -   
   -   
   -   
   -   
   -   
   -   
   -   
   -   
   -   
   -   
   -   
   -   
   -   
   -   
   -   
   -   
   A   
   T   
   G   
   G   
   A   
   A   
   C   
   G   
   A   
   A   
   G   
   C   
   A   
   A   
   T   
   C   
   G   
   T   
   A   
   A   
   T   
   A   
   A   
   A   
   A   
   A   
   A   
   T   
   C   
   T   
   T   
   C   
   A   
   A   
   A   
   A   
   A   
   A   
   A   
   C   
   C   
   A   
   C   
   T   
   T   
   A   
   T   
   T   
   C   
   T   
   T   
   G   
   G   
   C   
   G   
   T   
   T   
   T   
   C   
   T   
   G   
   C   
   C   
   T   
   T   
   G   
   G   
   T   
   T   
   C   
   T   
   G   
   A   
   T   
   C   
   G   
   C   
   T   
   G   
   C   
   T   
   G   
   T   
   C   
   G   
   G   
   T   
   G   
   G   
   T   
   G   
   G   
   G   
   T   
   A   
   T   
   T   
   A   
   T   
   G   
   C   
   T   
   T   
   A   
   T   
   A   
   G   
   C   
   C   
   A   
   A   
   T   
   G   
   G   
   C   
   A   
   A   
   G   
   C   
   C   
   A   
   A   
   A   
   C   
   A   
   A   
   G   
   A   
   A   
   T   
   T   
   A   
   G   
   C   
   T   
   G   
   A   
   A   
   G   
   C   
   G   
   A   
   A   
   G   
   A   
   A   
   A   
   A   
   C   
   A   
   G   
   C   
   C   
   A   
   A   
   T   
   A   
   C   
   A   
   T   
   T   
   T   
   T   
   T   
   A   
   A   
   A   
   C   
   G   
   T   
   A   
   T   
   T   
   G   
   T   
   C   
   A   
   A   
   A   
   A   
   C   
   A   
   G   
   G   
   A   
   A   
   T   
   T   
   T   
   G   
   A   
   T   
   A   
   A   
   G   
   T   
   T   
   A   
   C   
   C   
   A   
   T   
   C   
   C   
   G   
   T   
   T   
   G   
   T   
   T   
   C   
   A   
   A   
   G   
   A   
   A   
   A   
   C   
   T   
   A   
   G   
   C   
   T   
   T   
   A   
   A   
   A   
   G   
   A   
   A   
   A   
   A   
   A   
   T   
   G   
   G   
   C   
   T   
   A   
   T   
   G   
   A   
   T   
   A   
   C   
   T   
   A   
   A   
   A   
   T   
   C   
   T   
   G   
   T   
   T   
   G   
   T   
   T   
   G   
   A   
   A   
   A   
   A   
   A   
   T   
   A   
   C   
   C   
   A   
   A   
   G   
   C   
   A   
   A   
   T   
   T   
   T   
   A   
   T   
   T   
   C   
   A   
   G   
   G   
   G   
   A   
   T   
   T   
   C   
   A   
   A   
   G   
   C   
   A   
   G   
   A   
   A   
   G   
   G   
   G   
   G   
   T   
   C   
   A   
   A   
   A   
   G   
   C   
   T   
   A   
   G   
   T   
   G   
   A   
   T   
   G   
   T   
   T   
   C   
   A   
   A   
   G   
   T   
   C   
   A   
   A   
   A   
   A   
   A   
   A   
   G   
   G   
   G   
   A   
   A   
   A   
   G   
   A   
   C   
   A   
   A   
   C   
   C   
   A   
   A   
   T   
   A   
   C   
   A   
   C   
   A   
   T   
   T   
   T   
   A   
   C   
   C   
   T   
   A   
   T   
   A   
   A   
   A   
   T   
   T   
   A   
   T   
   C   
   G   
   A   
   T   
   G   
   A   
   G   
   C   
   A   
   C   
   T   
   C   
   C   
   T   
   T   
   T   
   A   
   G   
   G   
   C   
   G   
   A   
   A   
   A   
   T   
   G   
   A   
   A   
   A   
   G   
   A   
   T   
   T   
   T   
   G   
   T   
   C   
   T   
   T   
   A   
   T   
   C   
   A   
   A   
   T   
   C   
   A   
   A   
   G   
   T   
   A   
   T   
   C   
   G   
   C   
   C   
   A   
   A   
   A   
   A   
   A   
   A   
   G   
   G   
   C   
   G   
   A   
   T   
   A   
   A   
   C   
   T   
   A   
   C   
   C   
   A   
   A   
   A   
   T   
   C   
   G   
   C   
   T   
   T   
   G   
   G   
   G   
   C   
   A   
   C   
   C   
   G   
   T   
   C   
   T   
   T   
   T   
   A   
   A   
   T   
   T   
   T   
   T   
   T   
   C   
   C   
   A   
   G   
   A   
   T   
   A   
   T   
   G   
   T   
   C   
   A   
   G   
   G   
   A   
   A   
   A   
   T   
   G   
   A   
   T   
   A   
   A   
   A   
   A   
   T   
   T   
   T   
   C   
   G   
   A   
   T   
   T   
   C   
   A   
   A   
   G   
   T   
   A   
   G   
   A   
   T   
   A   
   A   
   T   
   G   
   C   
   C   
   A   
   A   
   A   
   C   
   G   
   T   
   G   
   G   
   A   
   G   
   A   
   A   
   A   
   T   
   T   
   G   
   T   
   C   
   G   
   A   
   T   
   C   
   G   
   T   
   A   
   A   
   T   
   G   
   G   
   T   
   A   
   G   
   T   
   G   
   G   
   G   
   C   
   T   
   A   
   G   
   C   
   A   
   A   
   T   
   T   
   A   
   A   
   C   
   A   
   A   
   A   
   G   
   T   
   G   
   T   
   T   
   T   
   G   
   A   
   T   
   G   
   A   
   A   
   G   
   T   
   G   
   G   
   G   
   C   
   G   
   T   
   A   
   G   
   T   
   G   
   C   
   C   
   T   
   G   
   G   
   C   
   A   
   A   
   A   
   C   
   T   
   C   
   G   
   G   
   T   
   T   
   C   
   T   
   G   
   G   
   C   
   G   
   C   
   A   
   G   
   A   
   A   
   A   
   A   
   A   
   A   
   C   
   A   
   G   
   C   
   C   
   A   
   A   
   T   
   A   
   T   
   C   
   A   
   A   
   A   
   G   
   C   
   T   
   T   
   T   
   T   
   A   
   G   
   T   
   G   
   A   
   T   
   A   
   A   
   G   
   T   
   T   
   T   
   G   
   G   
   C   
   G   
   T   
   T   
   T   
   C   
   T   
   G   
   T   
   T   
   G   
   A   
   T   
   G   
   A   
   A   
   A   
   T   
   C   
   A   
   A   
   T   
   C   
   A   
   A   
   A   
   A   
   G   
   T   
   T   
   A   
   A   
   G   
   C   
   C   
   A   
   A   
   G   
   G   
   A   
   T   
   G   
   G   
   G   
   T   
   C   
   C   
   A   
   A   
   G   
   C   
   A   
   G   
   A   
   C   
   T   
   C   
   G   
   T   
   T   
   T   
   G   
   T   
   A   
   C   
   C   
   A   
   A   
   T   
   C   
   A   
   C   
   A   
   G   
   T   
   T   
   G   
   C   
   T   
   T   
   C   
   T   
   G   
   A   
   A   
   C   
   C   
   A   
   G   
   T   
   G   
   A   
   C   
   A   
   G   
   A   
   A   
   T   
   T   
   A   
   C   
   C   
   A   
   A   
   C   
   A   
   G   
   G   
   G   
   G   
   C   
   T   
   G   
   C   
   G   
   A   
   C   
   A   
   A   
   A   
   A   
   G   
   A   
   T   
   A   
   C   
   A   
   G   
   A   
   G   
   T   
   C   
   A   
   C   
   G   
   T   
   T   
   A   
   T   
   T   
   A   
   T   
   C   
   C   
   A   
   T   
   T   
   G   
   G   
   G   
   G   
   G   
   A   
   A   
   G   
   C   
   A   
   G   
   C   
   T   
   G   
   C   
   G   
   C   
   A   
   A   
   T   
   T   
   A   
   A   
   T   
   C   
   G   
   G   
   G   
   T   
   A   
   T   
   A   
   C   
   G   
   G   
   G   
   C   
   A   
   C   
   T   
   A   
   T   
   T   
   A   
   C   
   T   
   G   
   C   
   A   
   G   
   A   
   A   
   G   
   A   
   T   
   A   
   T   
   T   
   G   
   A   
   A   
   A   
   A   
   A   
   A   
   A   
   T   
   C   
   C   
   A   
   G   
   A   
   G   
   T   
   T   
   A   
   A   
   G   
   T   
   A   
   G   
   T   
   A   
   C   
   G   
   G   
   G   
   C   
   G   
   T   
   A   
   A   
   T   
   T   
   G   
   G   
   C   
   A   
   A   
   A   
   A   
   C   
   T   
   G   
   G   
   C   
   T   
   T   
   A   
   G   
   A   
   A   
   C   
   G   
   A   
   G   
   C   
   G   
   T   
   T   
   T   
   G   
   A   
   T   
   A   
   A   
   A   
   G   
   A   
   A   
   T   
   T   
   A   
   A   
   G   
   A   
   G   
   G   
   A   
   C   
   A   
   A   
   G   
   A   
   T   
   G   
   G   
   T   
   G   
   G   
   T   
   T   
   C   
   A   
   C   
   T   
   A   
   G   
   T   
   T   
   A   
   T   
   T   
   T   
   T   
   A   
   G   
   A   
   T   
   G   
   A   
   C   
   A   
   A   
   A   
   G   
   A   
   G   
   A   
   A   
   T   
   G   
   T   
   C   
   A   
   A   
   A   
   A   
   A   
   A   
   G   
   C   
   T   
   T   
   T   
   A   
   C   
   A   
   A   
   A   
   C   
   C   
   A   
   A   
   A   
   G   
   A   
   G   
   A   
   A   
   A   
   A   
   A   
   A   
   G   
   A   
   T   
   G   
   G   
   T   
   C   
   A   
   A   
   A   
   C   
   A   
   A   
   T   
   T   
   A   
   A   
   A   
   T   
   T   
   A   
   A   
   C   
   G   
   A   
   T   
   T   
   G   
   A   
   T   
   A   
   G   
   T   
   G   
   G   
   C   
   G   
   T   
   A   
   C   
   A   
   A   
   C   
   A   
   A   
   C   
   A   
   A   
   G   
   C   
   C   
   T   
   T   
   C   
   G   
   C   
   T   
   A   
   T   
   A   
   T   
   T   
   T   
   G   
   A   
   C   
   A   
   A   
   A   
   C   
   G   
   T   
   C   
   C   
   T   
   G   
   G   
   T   
   A   
   G   
   T   
   G   
   C   
   G   
   G   
   T   
   C   
   A   
   T   
   T   
   A   
   C   
   C   
   G   
   A   
   T   
   C   
   C   
   T   
   C   
   A   
   A   
   A   
   A   
   A   
   G   
   G   
   C   
   G   
   A   
   T   
   T   
   T   
   A   
   C   
   T   
   A   
   G   
   C   
   G   
   A   
   C   
   G   
   G   
   T   
   T   
   A   
   G   
   T   
   T   
   C   
   A   
   C   
   C   
   T   
   T   
   C   
   G   
   T   
   A   
   T   
   G   
   A   
   T   
   C   
   C   
   T   
   A   
   A   
   T   
   A   
   A   
   A   
   A   
   T   
   G   
   G   
   C   
   C   
   A   
   A   
   T   
   G   
   G   
   C   
   A   
   T   
   T   
   T   
   C   
   T   
   C   
   A   
   A   
   A   
   A   
   A   
   G   
   A   
   A   
   T   
   A   
   T   
   G   
   A   
   T   
   G   
   C   
   T   
   T   
   A   
   C   
   A   
   A   
   T   
   A   
   A   
   C   
   A   
   A   
   C   
   A   
   A   
   A   
   G   
   A   
   T   
   T   
   T   
   A   
   C   
   C   
   A   
   T   
   T   
   C   
   A   
   C   
   A   
   G   
   C   
   A   
   C   
   G   
   T   
   T   
   T   
   T   
   G   
   C   
   G   
   A   
   C   
   A   
   G   
   G   
   C   
   T   
   A   
   T   
   G   
   C   
   A   
   C   
   C   
   A   
   G   
   G   
   C   
   T   
   C   
   T   
   A   
   C   
   C   
   T   
   T   
   T   
   A   
   A   
   A   
   A   
   C   
   A   
   A   
   T   
   T   
   A   
   C   
   C   
   G   
   G   
   T   
   G   
   C   
   C   
   A   
   T   
   T   
   G   
   G   
   T   
   T   
   T   
   A   
   G   
   A   
   T   
   G   
   C   
   A   
   G   
   G   
   G   
   A   
   C   
   A   
   T   
   T   
   G   
   A   
   A   
   G   
   C   
   C   
   A   
   G   
   A   
   T   
   G   
   A   
   A   
   G   
   A   
   G   
   C   
   T   
   A   
   G   
   A   
   A   
   A   
   T   
   T   
   A   
   A   
   T   
   G   
   G   
   C   
   T   
   T   
   G   
   A   
   A   
   A   
   T   
   G   
   G   
   C   
   A   
   A   
   A   
   A   
   A   
   G   
   A   
   T   
   A   
   A   
   A   
   T   
   C   
   T   
   T   
   G   
   G   
   G   
   G   
   C   
   G   
   G   
   C   
   T   
   A   
   T   
   T   
   T   
   T   
   G   
   C   
   G   
   A   
   C   
   A   
   C   
   G   
   T   
   G   
   T   
   A   
   A   
   A   
   A   
   G   
   A   
   A   
   G   
   C   
   A   
   A   
   G   
   T   
   C   
   C   
   A   
   G   
   T   
   C   
   A   
   A   
   T   
   C   
   T   
   T   
   C   
   G   
   T   
   A   
   C   
   C   
   G   
   C   
   T   
   T   
   T   
   A   
   G   
   T   
   T   
   A   
   A   
   T   
   T   
   C   
   A   
   G   
   A   
   T   
   A   
   A   
   T   
   A   
   T   
   T   
   T   
   A   
   T   
   T   
   T   
   T   
   G   
   C   
   C   
   C   
   A   
   A   
   C   
   A   
   A   
   A   
   C   
   A   
   T   
   T   
   A   
   C   
   G   
   G   
   A   
   T   
   G   
   G   
   G   
   G   
   G   
   A   
   A   
   G   
   A   
   C   
   A   
   A   
   A   
   T   
   T   
   T   
   A   
   G   
   A   
   G   
   C   
   G   
   G   
   G   
   T   
   C   
   T   
   T   
   A   
   A   
   T   
   A   
   A   
   A   
   T   
   T   
   C   
   A   
   T   
   T   
   T   
   T   
   C   
   G   
   G   
   T   
   G   
   A   
   G   
   G   
   A   
   A   
   C   
   T   
   A   
   G   
   A   
   T   
   T   
   T   
   A   
   C   
   C   
   A   
   A   
   T   
   T   
   G   
   C   
   C   
   A   
   T   
   G   
   A   
   C   
   G   
   C   
   C   
   T   
   G   
   C   
   T   
   C   
   A   
   A   
   A   
   T   
   T   
   T   
   C   
   A   
   A   
   A   
   C   
   G   
   A   
   A   
   G   
   A   
   T   
   A   
   A   
   A   
   T   
   T   
   T   
   A   
   A   
   T   
   T   
   C   
   T   
   G   
   A   
   A   
   A   
   T   
   T   
   C   
   T   
   T   
   T   
   T   
   A   
   G   
   C   
   A   
   G   
   A   
   T   
   A   
   C   
   A   
   G   
   G   
   C   
   T   
   A   
   T   
   G   
   G   
   C   
   C   
   A   
   A   
   G   
   G   
   C   
   C   
   A   
   A   
   T   
   T   
   G   
   T   
   T   
   A   
   A   
   T   
   T   
   T   
   C   
   G   
   C   
   C   
   A   
   A   
   T   
   C   
   C   
   A   
   G   
   C   
   A   
   A   
   G   
   C   
   T   
   A   
   C   
   C   
   A   
   T   
   G   
   T   
   A   
   T   
   A   
   G   
   T   
   G   
   T   
   T   
   T   
   T   
   C   
   C   
   A   
   A   
   A   
   A   
   T   
   A   
   A   
   T   
   G   
   G   
   A   
   A   
   C   
   A   
   T   
   T   
   A   
   G   
   T   
   T   
   T   
   A   
   T   
   C   
   C   
   A   
   A   
   A   
   A   
   T   
   T   
   A   
   G   
   T   
   T   
   T   
   T   
   A   
   G   
   A   
   G   
   A   
   A   
   A   
   G   
   A   
   A   
   A   
   C   
   G   
   A   
   A   
   G   
   A   
   A   
   A   
   A   
   A   
   A   
   G   
   A   
   C   
   A   
   A   
   T   
   G   
   T   
   A   
   A   
   T   
   C   
   A   
   G   
   C   
   G   
   C   
   C   
   A   
   A   
   C   
   G   
   C   
   A   
   G   
   C   
   G   
   A   
   A   
   T   
   A   
   C   
   A   
   A   
   T   
   A   
   G   
   C   
   C   
   A   
   C   
   A   
   G   
   A   
   T   
   C   
   T   
   T   
   T   
   T   
   A   
   G   
   G   
   T   
   A   
   G   
   T   
   G   
   T   
   G   
   G   
   A   
   A   
   G   
   A   
   T   
   C   
   C   
   T   
   T   
   C   
   A   
   G   
   G   
   T   
   T   
   A   
   T   
   G   
   T   
   T   
   T   
   A   
   T   
   A   
   A   
   T   
   A   
   T   
   G   
   T   
   A   
   C   
   A   
   A   
   T   
   C   
   C   
   G   
   A   
   A   
   C   
   T   
   T   
   C   
   T   
   C   
   A   
   T   
   T   
   G   
   G   
   C   
   T   
   G   
   C   
   C   
   A   
   A   
   A   
   A   
   C   
   A   
   G   
   G   
   A   
   A   
   C   
   A   
   G   
   C   
   T   
   G   
   A   
   A   
   A   
   T   
   A   
   A   
   A   
   A   
   G   
   A   
   T   
   A   
   A   
   A   
   C   
   A   
   A   
   G   
   A   
   T   
   A   
   C   
   A   
   G   
   A   
   T   
   G   
   G   
   C   
   A   
   A   
   A   
   G   
   A   
   A   
   A   
   A   
   C   
   A   
   G   
   T   
   T   
   T   
   C   
   C   
   T   
   T   
   C   
   T   
   A   
   A   
   C   
   G   
   T   
   T   
   A   
   G   
   A   
   T   
   C   
   G   
   T   
   A   
   G   
   T   
   A   
   A   
   C   
   A   
   A   
   T   
   A   
   A   
   A   
   T   
   T   
   T   
   T   
   T   
   A   
   A   
   C   
   A   
   A   
   T   
   G   
   A   
   T   
   T   
   A   
   T   
   G   
   G   
   T   
   T   
   G   
   A   
   A   
   A   
   A   
   T   
   T   
   C   
   A   
   G   
   G   
   A   
   G   
   A   
   A   
   A   
   A   
   T   
   G   
   G   
   T   
   T   
   C   
   T   
   G   
   C   
   A   
   A   
   C   
   A   
   G   
   A   
   T   
   A   
   T   
   C   
   A   
   G   
   T   
   A   
   A   
   A   
   C   
   C   
   A   
   T   
   T   
   A   
   A   
   T   
   T   
   G   
   A   
   T   
   T   
   A   
   C   
   T   
   T   
   A   
   G   
   A   
   A   
   G   
   C   
   A   
   A   
   C   
   C   
   A   
   T   
   T   
   A   
   A   
   A   
   -   
   -   
   -   
   -   
   -   
   -   
   -   
   -   
   -   
   -   
   -   
   -   
   -   
   -   
   -   
   -   
   -   
   -   
   -   
   -   
   -   
   -   
   -   
   -   
   -   
   -   
   -   
   -   
   -   
   -   
   -   
   -   
   -   
   -   
   -   
   -   
   -   
   -   
   -   
   -   
   -   
   -   
   -   
   -   
   -   
   -   
   -   
   -   
   -   
   -   
   -   
   -   
   -   
   -   
   -   
   -   
   -   
   -   
   -   
   -   
   -   
 

 
   N55240   
   -   
   -   
   -   
   -   
   -   
   -   
   -   
   -   
   -   
   -   
   -   
   -   
   -   
   -   
   -   
   -   
   -   
   -   
   -   
   -   
   -   
   -   
   -   
   -   
   -   
   -   
   -   
   -   
   -   
   -   
   -   
   -   
   -   
   -   
   -   
   -   
   -   
   -   
   -   
   -   
   -   
   -   
   -   
   -   
   -   
   -   
   -   
   -   
   -   
   -   
   -   
   -   
   -   
   -   
   -   
   -   
   -   
   -   
   -   
   -   
   -   
   -   
   -   
   -   
   -   
   -   
   -   
   -   
   -   
   -   
   -   
   -   
   -   
   -   
   -   
   -   
   -   
   -   
   -   
   -   
   -   
   -   
   -   
   -   
   -   
   -   
   -   
   -   
   -   
   -   
   -   
   -   
   -   
   -   
   -   
   -   
   -   
   -   
   -   
   -   
   -   
   -   
   -   
   -   
   -   
   -   
   -   
   -   
   C   
   A   
   A   
   T   
   G   
   G   
   C   
   A   
   A   
   G   
   C   
   C   
   A   
   A   
   A   
   C   
   A   
   A   
   G   
   A   
    A    
   T   
   T   
   A   
   G   
   C   
    C    
   G   
   A   
   A   
   G   
   C   
   G   
   A   
   A   
   G   
   A   
   A   
   A   
   A   
    C    
   A   
   G   
   C   
    T    
   A   
    C    
   T   
    A    
    C    
   A   
   T   
   T   
   T   
   T   
   T   
   A   
   A   
   A   
    C    
   G   
   T   
   A   
   T   
   T   
   G   
    T    
   C   
   A   
   A   
   A   
   A   
   C   
   A   
   G   
    G    
   A   
   A   
   T   
   T   
   T   
   G   
   A   
   T   
   A   
   A   
   G   
   T   
   T   
   A   
   C   
   C   
    G    
   T   
   C   
   C   
   G   
   T   
   T   
    G    
   T   
   T   
   C   
   A   
   A   
   G   
   A   
   A   
    G    
   C   
   T   
   A   
   G   
   C   
   T   
   T   
   A   
   A   
   A   
   G   
   A   
   A   
   A   
   A   
   A   
   T   
   G   
   G   
    C    
   T   
   A   
   T   
   G   
   A   
   T   
   A   
   C   
   T   
   A   
   A   
   A   
   T   
   C   
    T    
   G   
   T   
   T   
   G   
   T   
   T   
   G   
   A   
   A   
   A   
   A   
   A   
   T   
   A   
   C   
   C   
   A   
   A   
   G   
   C   
   A   
   A   
   T   
   T   
   T   
   A   
   T   
   T   
   C   
   A   
   G   
   G   
   G   
   A   
   T   
   T   
   C   
   A   
   A   
   G   
   C   
    A    
   G   
   A   
   A   
    G    
   G   
    A    
   G   
   T   
   C   
   A   
   A   
    A    
   G   
   C   
   T   
   A   
   G   
   T   
   G   
   A   
   T   
   G   
   T   
    T    
   C   
   A   
   A   
   G   
   T   
    A    
   A   
   A   
   A   
   A   
   A   
    G    
   G   
    C    
   G   
   A   
   A   
   A   
   G   
   A   
    C    
   A   
   A   
    T    
   C   
   A   
   A   
   T   
   A   
   C   
   A   
   C   
   A   
   T   
   T   
   T   
   A   
    C    
   C   
   T   
   A   
   T   
   A   
   A   
   A   
   T   
   T   
   A   
   T   
   C   
   G   
   A   
   T   
   G   
   A   
   G   
    T    
   A   
   C   
    C    
   C   
   C   
   T   
   T   
   T   
   A   
   G   
   G   
   C   
   G   
   A   
   A   
   A   
   T   
   G   
   A   
   A   
   A   
   G   
   A   
   T   
    T    
   T   
   G   
   T   
   C   
   T   
   T   
   A   
   T   
   C   
   A   
   A   
   T   
   C   
   A   
   A   
   G   
   T   
   A   
   T   
    C    
   G   
    C    
   C   
   A   
   A   
    A    
   A   
   A   
   A   
   G   
   G   
    C    
   G   
   A   
   T   
   A   
    C    
   C   
   T   
   A   
   C   
   C   
   A   
   A   
   A   
   T   
    C    
    G    
   C   
   T   
   T   
   G   
   G   
    A    
    A    
    G    
   C   
   C   
    G    
   T   
   C   
   T   
   T   
   T   
   A   
   A   
   T   
   T   
   T   
   T   
   T   
   C   
   C   
   A   
   G   
   A   
   T   
   A   
   T   
   G   
   T   
   C   
   A   
   G   
   G   
   A   
   A   
   A   
   T   
   G   
   A   
   T   
   A   
   A   
   A   
    A    
   T   
   T   
   T   
   C   
   G   
   A   
   T   
   T   
   C   
   A   
   A   
   G   
   T   
   A   
   G   
   A   
   T   
   A   
   A   
   T   
   G   
   C   
   C   
   A   
   A   
   A   
   C   
   G   
   T   
   G   
   G   
   A   
   G   
   A   
   A   
   A   
   T   
   T   
   G   
   T   
   C   
   G   
   A   
   T   
   C   
   G   
   T   
   A   
   A   
    T    
   G   
   G   
   T   
   A   
   G   
   T   
   G   
   G   
   G   
   C   
   T   
   A   
   G   
    C    
   A   
   A   
   T   
   T   
   A   
   A   
   C   
   A   
   A   
   A   
   G   
   T   
   G   
   T   
   T   
   T   
   G   
   A   
    T    
   G   
   A   
   A   
   G   
   T   
   G   
   G   
   G   
   C   
   G   
   T   
   A   
   G   
   T   
    G    
   C   
   C   
   T   
    G    
   G   
   C   
   A   
   A   
   A   
   C   
   T   
    C    
   G   
   G   
   T   
   T   
   C   
   T   
   G   
   G   
   C   
   G   
   C   
   A   
   G   
   A   
   A   
   A   
   A   
   A   
    A    
   C   
   A   
   G   
   C   
    C    
   A   
   A   
   T   
   A   
   T   
   C   
   A   
   A   
   A   
   G   
   C   
   T   
   T   
   T   
   T   
   A   
   G   
   T   
   G   
   A   
   T   
   A   
   A   
    A    
   T   
   T   
    C    
   G   
   G   
   C   
   G   
   T   
   T   
   T   
   C   
   T   
    G    
   T   
   T   
   G   
   A   
   T   
   G   
   A   
   A   
   A   
   T   
   C   
   A   
   A   
   T   
   C   
    A    
   A   
   A   
   A   
    G    
   T   
   T   
   A   
   A   
   G   
   C   
   C   
   A   
   A   
   G   
   G   
    A    
   T   
   G   
   G   
   G   
   T   
   C   
   C   
   A   
   A   
   G   
   C   
   A   
   G   
   A   
   C   
   T   
   C   
    C    
   T   
   T   
   T   
   G   
   T   
   A   
   C   
   C   
   A   
   A   
   T   
   C   
   A   
   C   
   A   
   G   
   T   
    C    
   G   
   C   
   T   
   T   
   C   
   T   
   G   
   A   
   A   
   C   
   C   
   A   
   G   
   T   
   G   
   A   
   C   
   A   
   G   
   A   
   A   
   T   
   T   
   A   
   C   
   C   
   A   
   A   
   C   
   A   
   G   
   G   
   G   
   G   
   C   
   T   
   G   
   C   
   G   
   A   
   C   
   A   
   A   
   A   
   A   
   G   
    A    
    T    
   A   
   C   
   A   
   G   
   A   
   G   
   T   
   C   
   A   
   C   
   G   
   T   
   T   
   A   
   T   
   T   
   A   
   T   
   C   
   C   
    G    
    C    
   T   
   G   
   G   
   G   
   G   
   G   
   A   
   A   
   G   
   C   
   A   
   G   
   C   
   T   
   G   
   C   
   G   
   C   
   A   
   A   
   T   
   T   
   A   
   A   
   T   
   C   
   G   
   G   
    G    
   T   
   A   
   T   
   A   
   C   
    G    
   G   
   G   
    C    
   A   
   C   
    C    
   A   
   T   
   T   
   A   
   C   
   T   
   G   
   C   
   A   
   G   
   A   
    A    
   G   
   A   
   T   
   A   
   T   
   T   
    G    
   A   
   A   
   A   
   A   
   A   
   A   
   A   
   T   
   C   
   C   
   A   
   G   
   A   
   G   
   T   
   T   
   A   
   A   
   G   
   T   
   A   
   G   
   T   
   A   
   C   
   G   
   G   
   G   
    C    
   G   
   T   
   A   
   A   
   T   
   T   
   G   
   G   
    T    
   A   
   A   
   A   
   A   
   C   
   T   
   G   
   G   
    T    
   T   
   T   
   A   
   G   
   A   
   A   
   C   
   G   
   A   
   G   
   C   
   G   
   T   
   T   
   T   
   G   
   A   
   T   
   A   
   A   
   A   
   G   
   A   
    A    
   T   
   T   
   A   
   A   
   G   
   A   
   G   
   G   
    G    
   C   
   A   
   A   
   G   
   A   
   T   
   G   
   G   
    T    
   G   
   G   
    C    
   T   
   C   
    A    
   C   
   T   
   A   
   G   
   T   
   T   
   A   
   T   
   T   
   T   
   T   
   A   
   G   
   A   
   T   
   G   
   A   
   C   
   A   
   A   
   A   
   G   
   A   
   G   
   A   
   A   
   T   
   G   
   T   
   C   
   A   
   A   
   A   
   A   
   A   
   A   
   G   
   C   
   T   
   T   
   T   
   A   
   C   
   A   
   A   
   A   
   C   
   C   
   A   
   A   
   A   
   G   
   A   
    G    
   A   
   A   
   A   
   A   
   A   
   A   
   G   
   A   
   T   
   G   
   G   
   T   
   C   
   A   
   A   
   A   
   C   
    G    
   A   
   T   
   T   
   A   
   A   
   A   
   T   
   T   
   A   
   A   
   C   
   G   
   A   
   T   
   T   
   G   
   A   
    C    
   A   
   G   
    T    
   G   
   G   
    C    
   G   
   T   
   A   
   C   
   A   
   A   
   C   
   A   
   A   
   C   
   A   
   A   
   G   
   C   
    C    
   T   
   T   
    C    
   G   
   C   
   T   
   A   
   T   
   A   
   T   
   T   
   T   
   G   
   A   
   C   
   A   
   A   
   A   
   C   
   G   
    T    
    C    
   C   
   T   
   G   
   G   
   T   
   A   
   G   
   T   
   G   
   C   
   G   
   G   
   T   
   C   
   A   
   T   
   T   
   A   
   C   
   C   
   G   
   A   
   T   
   C   
   C   
   T   
   C   
   A   
   A   
   A   
   A   
   A   
   G   
   G   
   C   
   G   
   A   
   T   
   T   
   T   
   A   
    T    
   T   
   A   
   G   
   C   
    A    
   A   
   C   
   G   
   G   
   T   
   T   
   A   
   G   
   T   
   T   
   C   
   A   
   C   
   C   
   T   
   T   
   C   
   G   
   T   
   A   
   T   
   G   
   A   
   T   
   C   
   C   
   T   
   A   
   A   
   T   
   A   
   A   
   A   
   A   
   T   
   G   
   G   
   C   
   C   
   A   
   A   
   T   
   G   
   G   
    T    
   A   
   T   
   T   
   T   
   C   
   T   
   C   
   A   
   A   
   A   
   A   
   A   
   G   
   A   
    A    
   T   
   A   
   T   
   G   
   A   
   T   
   G   
   C   
   T   
   T   
   A   
   C   
   A   
   A   
   T   
   A   
   A   
   C   
   A   
   A   
   C   
   A   
   A   
   A   
   G   
   A   
   T   
   T   
   T   
   A   
   C   
   C   
   A   
   T   
   T   
    C    
   A   
   C   
   A   
   G   
   C   
   A   
   C   
   G   
   T   
   T   
   T   
    T    
   G   
   C   
   G   
   A   
   C   
   A   
   G   
   G   
    C    
   T   
   A   
   T   
   G   
   C   
   A   
   C   
   C   
   A   
   G   
   G   
    C    
   T   
   C   
   T   
   A   
   C   
   C   
   T   
   T   
   T   
   A   
   A   
   A   
   A   
   C   
   A   
   A   
   T   
   T   
   A   
   C   
   C   
   G   
   G   
   T   
   G   
   C   
   C   
   A   
   T   
   T   
   G   
   G   
   T   
   T   
   T   
   A   
   G   
   A   
   T   
    G    
   C   
   A   
   G   
   G   
   G   
   A   
   C   
   A   
   T   
   T   
   G   
   A   
   A   
   G   
   C   
   C   
   A   
   G   
   A   
   T   
   G   
   A   
   A   
   G   
   A   
   G   
   C   
   T   
   A   
   G   
   A   
   A   
   A   
   T   
   T   
   A   
   A   
   T   
   G   
   G   
    T    
   T   
   T   
   G   
   A   
   A   
   A   
   T   
   G   
   G   
   C   
   A   
   A   
   A   
   A   
   A   
   G   
   A   
   T   
   A   
   A   
   A   
   T   
   C   
   T   
   T   
   G   
   G   
   G   
   G   
    T    
   G   
   G   
   C   
   T   
   A   
   T   
   T   
   T   
   T   
   G   
   C   
    G    
   A   
   C   
   A   
   C   
   G   
   T   
   G   
   T   
   A   
   A   
   A   
   A   
   G   
   A   
   A   
   G   
   C   
   A   
   A   
   G   
   T   
   C   
   C   
   A   
   G   
   T   
   C   
   A   
   A   
   T   
   C   
   T   
    G    
   C   
   G   
   T   
   A   
   C   
   C   
   G   
   C   
   T   
   T   
   T   
   A   
   G   
   T   
   T   
   A   
   A   
   T   
   T   
   C   
   A   
   G   
   A   
   T   
   A   
   A   
   T   
   A   
   T   
   T   
   T   
   A   
   T   
   T   
   T   
   T   
   G   
   C   
    C    
   C   
   A   
   A   
   C   
   A   
   A   
   A   
   C   
   A   
   T   
   T   
   A   
   C   
   G   
    G    
   A   
   T   
   G   
   G   
   G   
    A    
   G   
   A   
   A   
   G   
   A   
   C   
   A   
   A   
   A   
   T   
   T   
   T   
   A   
   G   
   A   
   G   
   C   
    G    
   G   
   G   
   T   
   C   
   T   
   T   
   A   
   A   
   T   
   A   
   A   
   A   
   T   
   T   
   C   
   A   
   T   
   T   
   T   
   T   
   C   
   G   
   G   
   T   
   G   
   A   
   G   
   G   
   A   
   A   
   C   
   T   
   A   
   G   
   A   
   T   
   T   
   T   
   A   
   C   
   C   
   A   
   A   
   T   
    T    
   G   
   C   
   C   
   A   
   T   
   G   
   A   
   C   
   G   
   C   
   C   
    T    
   G   
   C   
   T   
   C   
   A   
   A   
   A   
   T   
   T   
   T   
   C   
   A   
   A   
   A   
    T    
   G   
   A   
   A   
   G   
   A   
   T   
   A   
   A   
   A   
   T   
   T   
   T   
   A   
   A   
   T   
   T   
   C   
   T   
   G   
   A   
   A   
   A   
   T   
   T   
   C   
   T   
   T   
   T   
   T   
    A    
   G   
   C   
   A   
   G   
   A   
   T   
   A   
   C   
    G    
   G   
   G   
   C   
   T   
   A   
    C    
   G   
   G   
    T    
   C   
   A   
   A   
   G   
   G   
    T    
   C   
   A   
   A   
   T   
   T   
   G   
   T   
   T   
   A   
   A   
   T   
   T   
   T   
   C   
    G    
   C   
   C   
   A   
   A   
   T   
   C   
   C   
   A   
   G   
   C   
   A   
   A   
   G   
   C   
   T   
   A   
   C   
   C   
   A   
   T   
   G   
   T   
   A   
   T   
   A   
   G   
   T   
   G   
   T   
   T   
   T   
   T   
   C   
   C   
   A   
   A   
   A   
   A   
   T   
   A   
   A   
   T   
   G   
   G   
    A    
   A   
   C   
   A   
    T    
   T   
    A    
   G   
   T   
    C    
   T   
   A   
   T   
   C   
   C   
   A   
   A   
   A   
   A   
    C    
   T   
   A   
   G   
   T   
   T   
   T   
   T   
   A   
   G   
   A   
   C   
   A   
   A   
   A   
   G   
   A   
   A   
   A   
   C   
    G    
   A   
   A   
   G   
   A   
   A   
   A   
   A   
   A   
   A   
   G   
   A   
   C   
   A   
   A   
   T   
   G   
   T   
   A   
   A   
   T   
   C   
   A   
   G   
   C   
   G   
   C   
   C   
   A   
   A   
   C   
   G   
   C   
   A   
   G   
   C   
   G   
   A   
   A   
   T   
   A   
   C   
   A   
   A   
   T   
   T   
   G   
   C   
   C   
   A   
   C   
   A   
   G   
   A   
   T   
   C   
   T   
   T   
   T   
   T   
   A   
   G   
   G   
   T   
   A   
   G   
   T   
   G   
   T   
   G   
   G   
   A   
   A   
   G   
   A   
   T   
   C   
   C   
   T   
   T   
   C   
   A   
   G   
   G   
   T   
   T   
   A   
   T   
   G   
   T   
   T   
   T   
   A   
   T   
   A   
   A   
   T   
   A   
   T   
   G   
   T   
   A   
   C   
   A   
   A   
   T   
   C   
   C   
   G   
   A   
   A   
   C   
   T   
   T   
   C   
   T   
   C   
   A   
   T   
   T   
   G   
   G   
   C   
   T   
   G   
   C   
   T   
   A   
   A   
   A   
   A   
   C   
   A   
   G   
   G   
   A   
   A   
   C   
   A   
   G   
   C   
   T   
   G   
   A   
   A   
   A   
   T   
   A   
   A   
   A   
   A   
   G   
   A   
   T   
   A   
   A   
   A   
   C   
   A   
   A   
   G   
   A   
   T   
   A   
   C   
   A   
   G   
   A   
   T   
   G   
   G   
   C   
   A   
   A   
   A   
   G   
   A   
   A   
   A   
   A   
   C   
   A   
   G   
   T   
   T   
   T   
   C   
   C   
   T   
   T   
   C   
   T   
   A   
   A   
   C   
   G   
   T   
   T   
   A   
   G   
   A   
   T   
   C   
   G   
   T   
   A   
   G   
   T   
   A   
   A   
   C   
   A   
   A   
   T   
   A   
   A   
   A   
   T   
   T   
   C   
   T   
   T   
   A   
   A   
   C   
   A   
   A   
   T   
   G   
   A   
   T   
   T   
   A   
   T   
   G   
   G   
   T   
   T   
   G   
   A   
   A   
   A   
   A   
   T   
   T   
   C   
   A   
   G   
   G   
   A   
   G   
   A   
   A   
   A   
   A   
   T   
   G   
   G   
   T   
   T   
   C   
   T   
   G   
   C   
   A   
   A   
   C   
   A   
   G   
   A   
   T   
   A   
   T   
   C   
   A   
   G   
   T   
   A   
   A   
   A   
   C   
   C   
   A   
   T   
   T   
   A   
   A   
   T   
   T   
   G   
   A   
   T   
   T   
   A   
   C   
   T   
   T   
   A   
   G   
   A   
   A   
   G   
   C   
   A   
   A   
   C   
   C   
   A   
   T   
   T   
   A   
   A   
   A   
   -   
   -   
   -   
   -   
   -   
   -   
   -   
   -   
   -   
   -   
   -   
   -   
   -   
   -   
   -   
   -   
   -   
   -   
   -   
   -   
   -   
   -   
   -   
   -   
   -   
   -   
   -   
   -   
   -   
   -   
   -   
   -   
   -   
   -   
   -   
   -   
   -   
   -   
   -   
   -   
   -   
   -   
   -   
   -   
   -   
   -   
   -   
   -   
   -   
   -   
   -   
   -   
   -   
   -   
   -   
   -   
   -   
   -   
   -   
   -   
   -   
   -   
   -   
   -   
   -   
   -   
   -   
   -   
   -   
   -   
   -   
   -   
   -   
   -   
   -   
   -   
   -   
   -   
   -   
   -   
   -   
   -   
   -   
   -   
   -   
   -   
   -   
   -   
   -   
   -   
   -   
   -   
   -   
   -   
   -   
   -   
   -   
   -   
   -   
   -   
   -   
   -   
   -   
   -   
   -   
   -   
   -   
   -   
   -   
   -   
   -   
   -   
   -   
   -   
   -   
   -   
   -   
   -   
   -   
   -   
   -   
   -   
   -   
   -   
   -   
   -   
   -   
   -   
   -   
   -   
   -   
   -   
   -   
   -   
   -   
   -   
   -   
   -   
   -   
   -   
   -   
   -   
   -   
   -   
   -   
   -   
   -   
   -   
   -   
   -   
   -   
   -   
   -   
   -   
   -   
   -   
   -   
   -   
   -   
   -   
   -   
   -   
   -   
   -   
   -   
   -   
   -   
   -   
   -   
   C   
   A   
   A   
   T   
   G   
   G   
   C   
   A   
   A   
   G   
   C   
   C   
   A   
   A   
   A   
   C   
   A   
   A   
   G   
   A   
   A   
   T   
   T   
   A   
   G   
   C   
   C   
   G   
   A   
   A   
   G   
   C   
   G   
   A   
   A   
   G   
   A   
   A   
   A   
   A   
   C   
   A   
   G   
   C   
   T   
   A   
   C   
   T   
   A   
   C   
   A   
   T   
   T   
   T   
   T   
   T   
   A   
   A   
   A   
   C   
   G   
   T   
   A   
   T   
   T   
   G   
   T   
   C   
   A   
   A   
   A   
   A   
   C   
   A   
   G   
   G   
   A   
   A   
   T   
   T   
   T   
   G   
   A   
   T   
   A   
   A   
   G   
   T   
   T   
   A   
   C   
   C   
   G   
   T   
   C   
   C   
   G   
   T   
   T   
   G   
   T   
   T   
   C   
   A   
   A   
   G   
   A   
   A   
   G   
   C   
   T   
   A   
   G   
   C   
   T   
   T   
   A   
   A   
   A   
   G   
   A   
   A   
   A   
   A   
   A   
   T   
   G   
   G   
   C   
   T   
   A   
   T   
   G   
   A   
   T   
   A   
   C   
   T   
   A   
   A   
   A   
   T   
   C   
   T   
   G   
   T   
   T   
   G   
   T   
   T   
   G   
   A   
   A   
   A   
   A   
   A   
   T   
   A   
   C   
   C   
   A   
   A   
   G   
   C   
   A   
   A   
   T   
   T   
   T   
   A   
   T   
   T   
   C   
   A   
   G   
   G   
   G   
   A   
   T   
   T   
   C   
   A   
   A   
   G   
   C   
   A   
   G   
   A   
   A   
   G   
   G   
   A   
   G   
   T   
   C   
   A   
   A   
   A   
   G   
   C   
   T   
   A   
   G   
   T   
   G   
   A   
   T   
   G   
   T   
   T   
   C   
   A   
   A   
   G   
   T   
   A   
   A   
   A   
   A   
   A   
   A   
   G   
   G   
   C   
   G   
   A   
   A   
   A   
   G   
   A   
   C   
   A   
   A   
   T   
   C   
   A   
   A   
   T   
   A   
   C   
   A   
   C   
   A   
   T   
   T   
   T   
   A   
   C   
   C   
   T   
   A   
   T   
   A   
   A   
   A   
   T   
   T   
   A   
   T   
   C   
   G   
   A   
   T   
   G   
   A   
   G   
   T   
   A   
   C   
   C   
   C   
   C   
   T   
   T   
   T   
   A   
   G   
   G   
   C   
   G   
   A   
   A   
   A   
   T   
   G   
   A   
   A   
   A   
   G   
   A   
   T   
   T   
   T   
   G   
   T   
   C   
   T   
   T   
   A   
   T   
   C   
   A   
   A   
   T   
   C   
   A   
   A   
   G   
   T   
   A   
   T   
   C   
   G   
   C   
   C   
   A   
   A   
   A   
   A   
   A   
   A   
   G   
   G   
   C   
   G   
   A   
   T   
   A   
   C   
   C   
   T   
   A   
   C   
   C   
   A   
   A   
   A   
   T   
   C   
   G   
   C   
   T   
   T   
   G   
   G   
   A   
   A   
   G   
   C   
   C   
   G   
   T   
   C   
   T   
   T   
   T   
   A   
   A   
   T   
   T   
   T   
   T   
   T   
   C   
   C   
   A   
   G   
   A   
   T   
   A   
   T   
   G   
   T   
   C   
   A   
   G   
   G   
   A   
   A   
   A   
   T   
   G   
   A   
   T   
   A   
   A   
   A   
   A   
   T   
   T   
   T   
   C   
   G   
   A   
   T   
   T   
   C   
   A   
   A   
   G   
   T   
   A   
   G   
   A   
   T   
   A   
   A   
   T   
   G   
   C   
   C   
   A   
   A   
   A   
   C   
   G   
   T   
   G   
   G   
   A   
   G   
   A   
   A   
   A   
   T   
   T   
   G   
   T   
   C   
   G   
   A   
   T   
   C   
   G   
   T   
   A   
   A   
   T   
   G   
   G   
   T   
   A   
   G   
   T   
   G   
   G   
   G   
   C   
   T   
   A   
   G   
   C   
   A   
   A   
   T   
   T   
   A   
   A   
   C   
   A   
   A   
   A   
   G   
   T   
   G   
   T   
   T   
   T   
   G   
   A   
   T   
   G   
   A   
   A   
   G   
   T   
   G   
   G   
   G   
   C   
   G   
   T   
   A   
   G   
   T   
   G   
   C   
   C   
   T   
   G   
   G   
   C   
   A   
   A   
   A   
   C   
   T   
   C   
   G   
   G   
   T   
   T   
   C   
   T   
   G   
   G   
   C   
   G   
   C   
   A   
   G   
   A   
   A   
   A   
   A   
   A   
   A   
   C   
   A   
   G   
   C   
   C   
   A   
   A   
   T   
   A   
   T   
   C   
   A   
   A   
   A   
   G   
   C   
   T   
   T   
   T   
   T   
   A   
   G   
   T   
   G   
   A   
   T   
   A   
   A   
   A   
   T   
   T   
   C   
   G   
   G   
   C   
   G   
   T   
   T   
   T   
   C   
   T   
   G   
   T   
   T   
   G   
   A   
   T   
   G   
   A   
   A   
   A   
   T   
   C   
   A   
   A   
   T   
   C   
   A   
   A   
   A   
   A   
   G   
   T   
   T   
   A   
   A   
   G   
   C   
   C   
   A   
   A   
   G   
   G   
   A   
   T   
   G   
   G   
   G   
   T   
   C   
   C   
   A   
   A   
   G   
   C   
   A   
   G   
   A   
   C   
   T   
   C   
   C   
   T   
   T   
   T   
   G   
   T   
   A   
   C   
   C   
   A   
   A   
   T   
   C   
   A   
   C   
   A   
   G   
   T   
   C   
   G   
   C   
   T   
   T   
   C   
   T   
   G   
   A   
   A   
   C   
   C   
   A   
   G   
   T   
   G   
   A   
   C   
   A   
   G   
   A   
   A   
   T   
   T   
   A   
   C   
   C   
   A   
   A   
   C   
   A   
   G   
   G   
   G   
   G   
   C   
   T   
   G   
   C   
   G   
   A   
   C   
   A   
   A   
   A   
   A   
   G   
   A   
   T   
   A   
   C   
   A   
   G   
   A   
   G   
   T   
   C   
   A   
   C   
   G   
   T   
   T   
   A   
   T   
   T   
   A   
   T   
   C   
   C   
   G   
   C   
   T   
   G   
   G   
   G   
   G   
   G   
   A   
   A   
   G   
   C   
   A   
   G   
   C   
   T   
   G   
   C   
   G   
   C   
   A   
   A   
   T   
   T   
   A   
   A   
   T   
   C   
   G   
   G   
   G   
   T   
   A   
   T   
   A   
   C   
   G   
   G   
   G   
   C   
   A   
   C   
   C   
   A   
   T   
   T   
   A   
   C   
   T   
   G   
   C   
   A   
   G   
   A   
   A   
   G   
   A   
   T   
   A   
   T   
   T   
   G   
   A   
   A   
   A   
   A   
   A   
   A   
   A   
   T   
   C   
   C   
   A   
   G   
   A   
   G   
   T   
   T   
   A   
   A   
   G   
   T   
   A   
   G   
   T   
   A   
   C   
   G   
   G   
   G   
   C   
   G   
   T   
   A   
   A   
   T   
   T   
   G   
   G   
   T   
   A   
   A   
   A   
   A   
   C   
   T   
   G   
   G   
   T   
   T   
   T   
   A   
   G   
   A   
   A   
   C   
   G   
   A   
   G   
   C   
   G   
   T   
   T   
   T   
   G   
   A   
   T   
   A   
   A   
   A   
   G   
   A   
   A   
   T   
   T   
   A   
   A   
   G   
   A   
   G   
   G   
   G   
   C   
   A   
   A   
   G   
   A   
   T   
   G   
   G   
   T   
   G   
   G   
   C   
   T   
   C   
   A   
   C   
   T   
   A   
   G   
   T   
   T   
   A   
   T   
   T   
   T   
   T   
   A   
   G   
   A   
   T   
   G   
   A   
   C   
   A   
   A   
   A   
   G   
   A   
   G   
   A   
   A   
   T   
   G   
   T   
   C   
   A   
   A   
   A   
   A   
   A   
   A   
   G   
   C   
   T   
   T   
   T   
   A   
   C   
   A   
   A   
   A   
   C   
   C   
   A   
   A   
   A   
   G   
   A   
   G   
   A   
   A   
   A   
   A   
   A   
   A   
   G   
   A   
   T   
   G   
   G   
   T   
   C   
   A   
   A   
   A   
   C   
   G   
   A   
   T   
   T   
   A   
   A   
   A   
   T   
   T   
   A   
   A   
   C   
   G   
   A   
   T   
   T   
   G   
   A   
   C   
   A   
   G   
   T   
   G   
   G   
   C   
   G   
   T   
   A   
   C   
   A   
   A   
   C   
   A   
   A   
   C   
   A   
   A   
   G   
   C   
   C   
   T   
   T   
   C   
   G   
   C   
   T   
   A   
   T   
   A   
   T   
   T   
   T   
   G   
   A   
   C   
   A   
   A   
   A   
   C   
   G   
   T   
   C   
   C   
   T   
   G   
   G   
   T   
   A   
   G   
   T   
   G   
   C   
   G   
   G   
   T   
   C   
   A   
   T   
   T   
   A   
   C   
   C   
   G   
   A   
   T   
   C   
   C   
   T   
   C   
   A   
   A   
   A   
   A   
   A   
   G   
   G   
   C   
   G   
   A   
   T   
   T   
   T   
   A   
   T   
   T   
   A   
   G   
   C   
   A   
   A   
   C   
   G   
   G   
   T   
   T   
   A   
   G   
   T   
   T   
   C   
   A   
   C   
   C   
   T   
   T   
   C   
   G   
   T   
   A   
   T   
   G   
   A   
   T   
   C   
   C   
   T   
   A   
   A   
   T   
   A   
   A   
   A   
   A   
   T   
   G   
   G   
   C   
   C   
   A   
   A   
   T   
   G   
   G   
   T   
   A   
   T   
   T   
   T   
   C   
   T   
   C   
   A   
   A   
   A   
   A   
   A   
   G   
   A   
   A   
   T   
   A   
   T   
   G   
   A   
   T   
   G   
   C   
   T   
   T   
   A   
   C   
   A   
   A   
   T   
   A   
   A   
   C   
   A   
   A   
   C   
   A   
   A   
   A   
   G   
   A   
   T   
   T   
   T   
   A   
   C   
   C   
   A   
   T   
   T   
   C   
   A   
   C   
   A   
   G   
   C   
   A   
   C   
   G   
   T   
   T   
   T   
   T   
   G   
   C   
   G   
   A   
   C   
   A   
   G   
   G   
   C   
   T   
   A   
   T   
   G   
   C   
   A   
   C   
   C   
   A   
   G   
   G   
   C   
   T   
   C   
   T   
   A   
   C   
   C   
   T   
   T   
   T   
   A   
   A   
   A   
   A   
   C   
   A   
   A   
   T   
   T   
   A   
   C   
   C   
   G   
   G   
   T   
   G   
   C   
   C   
   A   
   T   
   T   
   G   
   G   
   T   
   T   
   T   
   A   
   G   
   A   
   T   
   G   
   C   
   A   
   G   
   G   
   G   
   A   
   C   
   A   
   T   
   T   
   G   
   A   
   A   
   G   
   C   
   C   
   A   
   G   
   A   
   T   
   G   
   A   
   A   
   G   
   A   
   G   
   C   
   T   
   A   
   G   
   A   
   A   
   A   
   T   
   T   
   A   
   A   
   T   
   G   
   G   
   T   
   T   
   T   
   G   
   A   
   A   
   A   
   T   
   G   
   G   
   C   
   A   
   A   
   A   
   A   
   A   
   G   
   A   
   T   
   A   
   A   
   A   
   T   
   C   
   T   
   T   
   G   
   G   
   G   
   G   
   T   
   G   
   G   
   C   
   T   
   A   
   T   
   T   
   T   
   T   
   G   
   C   
   G   
   A   
   C   
   A   
   C   
   G   
   T   
   G   
   T   
   A   
   A   
   A   
   A   
   G   
   A   
   A   
   G   
   C   
   A   
   A   
   G   
   T   
   C   
   C   
   A   
   G   
   T   
   C   
   A   
   A   
   T   
   C   
   T   
   G   
   C   
   G   
   T   
   A   
   C   
   C   
   G   
   C   
   T   
   T   
   T   
   A   
   G   
   T   
   T   
   A   
   A   
   T   
   T   
   C   
   A   
   G   
   A   
   T   
   A   
   A   
   T   
   A   
   T   
   T   
   T   
   A   
   T   
   T   
   T   
   T   
   G   
   C   
   C   
   C   
   A   
   A   
   C   
   A   
   A   
   A   
   C   
   A   
   T   
   T   
   A   
   C   
   G   
   G   
   A   
   T   
   G   
   G   
   G   
   A   
   G   
   A   
   A   
   G   
   A   
   C   
   A   
   A   
   A   
   T   
   T   
   T   
   A   
   G   
   A   
   G   
   C   
   G   
   G   
   G   
   T   
   C   
   T   
   T   
   A   
   A   
   T   
   A   
   A   
   A   
   T   
   T   
   C   
   A   
   T   
   T   
   T   
   T   
   C   
   G   
   G   
   T   
   G   
   A   
   G   
   G   
   A   
   A   
   C   
   T   
   A   
   G   
   A   
   T   
   T   
   T   
   A   
   C   
   C   
   A   
   A   
   T   
   T   
   G   
   C   
   C   
   A   
   T   
   G   
   A   
   C   
   G   
   C   
   C   
   T   
   G   
   C   
   T   
   C   
   A   
   A   
   A   
   T   
   T   
   T   
   C   
   A   
   A   
   A   
   T   
   G   
   A   
   A   
   G   
   A   
   T   
   A   
   A   
   A   
   T   
   T   
   T   
   A   
   A   
   T   
   T   
   C   
   T   
   G   
   A   
   A   
   A   
   T   
   T   
   C   
   T   
   T   
   T   
   T   
   A   
   G   
   C   
   A   
   G   
   A   
   T   
   A   
   C   
   G   
   G   
   G   
   C   
   T   
   A   
   C   
   G   
   G   
   T   
   C   
   A   
   A   
   G   
   G   
   T   
   C   
   A   
   A   
   T   
   T   
   G   
   T   
   T   
   A   
   A   
   T   
   T   
   T   
   C   
   G   
   C   
   C   
   A   
   A   
   T   
   C   
   C   
   A   
   G   
   C   
   A   
   A   
   G   
   C   
   T   
   A   
   C   
   C   
   A   
   T   
   G   
   T   
   A   
   T   
   A   
   G   
   T   
   G   
   T   
   T   
   T   
   T   
   C   
   C   
   A   
   A   
   A   
   A   
   T   
   A   
   A   
   T   
   G   
   G   
   A   
   A   
   C   
   A   
   T   
   T   
   A   
   G   
   T   
   C   
   T   
   A   
   T   
   C   
   C   
   A   
   A   
   A   
   A   
   C   
   T   
   A   
   G   
   T   
   T   
   T   
   T   
   A   
   G   
   A   
   C   
   A   
   A   
   A   
   G   
   A   
   A   
   A   
   C   
   G   
   A   
   A   
   G   
   A   
   A   
   A   
   A   
   A   
   A   
   G   
   A   
   C   
   A   
   A   
   T   
   G   
   T   
   A   
   A   
   T   
   C   
   A   
   G   
   C   
   G   
   C   
   C   
   A   
   A   
   C   
   G   
   C   
   A   
   G   
   C   
   G   
   A   
   A   
   T   
   A   
   C   
   A   
   A   
   T   
   T   
   G   
   C   
   C   
   A   
   C   
   A   
   G   
   A   
   T   
   C   
   T   
   T   
   T   
   T   
   A   
   G   
   G   
   T   
   A   
   G   
   T   
   G   
   T   
   G   
   G   
   A   
   A   
   G   
   A   
   T   
   C   
   C   
   T   
   T   
   C   
   A   
   G   
   G   
   T   
   T   
   A   
   T   
   G   
   T   
   T   
   T   
   A   
   T   
   A   
   A   
   T   
   A   
   T   
   G   
   T   
   A   
   C   
   A   
   A   
   T   
   C   
   C   
   G   
   A   
   A   
   C   
   T   
   T   
   C   
   T   
   C   
   A   
   T   
   T   
   G   
   G   
   C   
   T   
   G   
   C   
   T   
   A   
   A   
   A   
   A   
   C   
   A   
   G   
   G   
   A   
   A   
   C   
   A   
   G   
   C   
   T   
   G   
   A   
   A   
   A   
   T   
   A   
   A   
   A   
   A   
   G   
   A   
   T   
   A   
   A   
   A   
   C   
   A   
   A   
   G   
   A   
   T   
   A   
   C   
   A   
   G   
   A   
   T   
   G   
   G   
   C   
   A   
   A   
   A   
   G   
   A   
   A   
   A   
   A   
   C   
   A   
   G   
   T   
   T   
   T   
   C   
   C   
   T   
   T   
   C   
   T   
   A   
   A   
   C   
   G   
   T   
   T   
   A   
   G   
   A   
   T   
   C   
   G   
   T   
   A   
   G   
   T   
   A   
   A   
   C   
   A   
   A   
   T   
   A   
   A   
   A   
   T   
   T   
   C   
   T   
   T   
   A   
   A   
   C   
   A   
   A   
   T   
   G   
   A   
   T   
   T   
   A   
   T   
   G   
   G   
   T   
   T   
   G   
   A   
   A   
   A   
   A   
   T   
   T   
   C   
   A   
   G   
   G   
   A   
   G   
   A   
   A   
   A   
   A   
   T   
   G   
   G   
   T   
   T   
   C   
   T   
   G   
   C   
   A   
   A   
   C   
   A   
   G   
   A   
   T   
   A   
   T   
   C   
   A   
   G   
   T   
   A   
   A   
   A   
   C   
   C   
   A   
   T   
   T   
   A   
   A   
   T   
   T   
   G   
   A   
   T   
   T   
   A   
   C   
   T   
   T   
   A   
   G   
   A   
   A   
   G   
   C   
   A   
   A   
   C   
   C   
   A   
   T   
   T   
   A   
   A   
   A   
   -   
   -   
   -   
   -   
   -   
   -   
   -   
   -   
   -   
   -   
   -   
   -   
   -   
   -   
   -   
   -   
   -   
   -   
   -   
   -   
   -   
   -   
   -   
   -   
   -   
   -   
   -   
   -   
   -   
   -   
   -   
   -   
   -   
   -   
   -   
   -   
   -   
   -   
   -   
   -   
   -   
   -   
   -   
   -   
   -   
   -   
   -   
   -   
   -   
   -   
   -   
   -   
   -   
   -   
   -   
   -   
   -   
   -   
   -   
   -   
   -   
 

 
   N54595   
   -   
   -   
   -   
   -   
   -   
   -   
   -   
   -   
   -   
   -   
   -   
   -   
   -   
   -   
   -   
   -   
   -   
   -   
   -   
   -   
   -   
   -   
   -   
   -   
   -   
   -   
   -   
   -   
   -   
   -   
   -   
   -   
   -   
   -   
   -   
   -   
   -   
   -   
   -   
   -   
   -   
   -   
   -   
   -   
   -   
   -   
   -   
   -   
   -   
   -   
   -   
   -   
   -   
   -   
   -   
   -   
   -   
   -   
   -   
   -   
   -   
   -   
   -   
   -   
   -   
   -   
   -   
   -   
   -   
   -   
   -   
   -   
   -   
   -   
   -   
   -   
   -   
   -   
   -   
   -   
   -   
   -   
   -   
   -   
   -   
   -   
   -   
   -   
   -   
   -   
   -   
   -   
   -   
   -   
   -   
   -   
   -   
   -   
   -   
   -   
   -   
   -   
   -   
   -   
   -   
   -   
   -   
   -   
   C   
   A   
   A   
   T   
   G   
   G   
   C   
   A   
   A   
   G   
   C   
   C   
   A   
   A   
   A   
   C   
   A   
   A   
   G   
   A   
    A    
   T   
   T   
   A   
   G   
   C   
    C    
   G   
   A   
   A   
   G   
   C   
   G   
   A   
   A   
   G   
   A   
   A   
   A   
   A   
    C    
   A   
   G   
   C   
    T    
   A   
    C    
   T   
    A    
    C    
   A   
   T   
   T   
   T   
   T   
   T   
   A   
   A   
   A   
    C    
   G   
   T   
   A   
   T   
   T   
   G   
    T    
   C   
   A   
   A   
   A   
   A   
   C   
   A   
   G   
    G    
   A   
   A   
   T   
   T   
   T   
   G   
   A   
   T   
   A   
   A   
   G   
   T   
   T   
   A   
   C   
   C   
    G    
   T   
   C   
   C   
   G   
   T   
   T   
    G    
   T   
   T   
   C   
   A   
   A   
   G   
   A   
   A   
    G    
   C   
   T   
   A   
   G   
   C   
   T   
   T   
   A   
   A   
   A   
   G   
   A   
   A   
   A   
   A   
   A   
   T   
   G   
   G   
    C    
   T   
   A   
   T   
   G   
   A   
   T   
   A   
   C   
   T   
   A   
   A   
   A   
   T   
   C   
    T    
   G   
   T   
   T   
   G   
   T   
   T   
   G   
   A   
   A   
   A   
   A   
   A   
   T   
   A   
   C   
   C   
   A   
   A   
   G   
   C   
   A   
   A   
   T   
   T   
   T   
   A   
   T   
   T   
   C   
   A   
   G   
   G   
   G   
   A   
   T   
   T   
   C   
   A   
   A   
   G   
   C   
    A    
   G   
   A   
   A   
    G    
   G   
    A    
   G   
   T   
   C   
   A   
   A   
    A    
   G   
   C   
   T   
   A   
   G   
   T   
   G   
   A   
   T   
   G   
   T   
    T    
   C   
   A   
   A   
   G   
   T   
    C    
   A   
   A   
   A   
   A   
   A   
    G    
   G   
    C    
   G   
   A   
   A   
   A   
   G   
   A   
    C    
   A   
   A   
    T    
   C   
   A   
   A   
   T   
   A   
   C   
   A   
   C   
   A   
   T   
   T   
   T   
   A   
    C    
   C   
   T   
   A   
   T   
   A   
   A   
   A   
   T   
   T   
   A   
   T   
   C   
   G   
   A   
   T   
   G   
   A   
   G   
    T    
   A   
   C   
    C    
   C   
   C   
   T   
   T   
   T   
   A   
   G   
   G   
   C   
   G   
   A   
   A   
   A   
   T   
   G   
   A   
   A   
   A   
   G   
   A   
   T   
    T    
   T   
   G   
   T   
   C   
   T   
   T   
   A   
   T   
   C   
   A   
   A   
   T   
   C   
   A   
   A   
   G   
   T   
   A   
   T   
    C    
   G   
    C    
   C   
   A   
   A   
    A    
   A   
   A   
   A   
   G   
   G   
    C    
   G   
   A   
   T   
   A   
    C    
   C   
   T   
   A   
   C   
   C   
   A   
   A   
   A   
   T   
    C    
    G    
   C   
   T   
   T   
   G   
   G   
    A    
    A    
    G    
   C   
   C   
    G    
   T   
   C   
   T   
   T   
   T   
   A   
   A   
   T   
   T   
   T   
   T   
   T   
   C   
   C   
   A   
   G   
   A   
   T   
   A   
   T   
   G   
   T   
   C   
   A   
   G   
   G   
   A   
   A   
   A   
   T   
   G   
   A   
   T   
   A   
   A   
   A   
    A    
   T   
   T   
   T   
   C   
   G   
   A   
   T   
   T   
   C   
   A   
   A   
   G   
   T   
   A   
   G   
   A   
   T   
   A   
   A   
   T   
   G   
   C   
   C   
   A   
   A   
   A   
   C   
   G   
   T   
   G   
   G   
   A   
   G   
   A   
   A   
   A   
   T   
   T   
   G   
   T   
   C   
   G   
   A   
   T   
   C   
   G   
   T   
   A   
   A   
    T    
   G   
   G   
   T   
   A   
   G   
   T   
   G   
   G   
   G   
   C   
   T   
   A   
   G   
    C    
   A   
   A   
   T   
   T   
   A   
   A   
   C   
   A   
   A   
   A   
   G   
   T   
   G   
   T   
   T   
   T   
   G   
   A   
    C    
   G   
   A   
   A   
   G   
   T   
   G   
   G   
   G   
   C   
   G   
   T   
   A   
   G   
   T   
    G    
   C   
   C   
   T   
    G    
   G   
   C   
   A   
   A   
   A   
   C   
   T   
    C    
   G   
   G   
   T   
   T   
   C   
   T   
   G   
   G   
   C   
   G   
   C   
   A   
   G   
   A   
   A   
   A   
   A   
   A   
    A    
   C   
   A   
   G   
   C   
    C    
   A   
   A   
   T   
   A   
   T   
   C   
   A   
   A   
   A   
   G   
   C   
   T   
   T   
   T   
   T   
   A   
   G   
   T   
   G   
   A   
   T   
   A   
   A   
    A    
   T   
   T   
    C    
   G   
   G   
   C   
   G   
   T   
   T   
   T   
   C   
   T   
    G    
   T   
   T   
   G   
   A   
   T   
   G   
   A   
   A   
   A   
   T   
   C   
   A   
   A   
   T   
   C   
    A    
   A   
   A   
   A   
    G    
   T   
   T   
   A   
   A   
   G   
   C   
   C   
   A   
   A   
   G   
   G   
    A    
   T   
   G   
   G   
   G   
   T   
   C   
   C   
   A   
   A   
   G   
   C   
   A   
   G   
   A   
   C   
   T   
   C   
    C    
   T   
   T   
   T   
   G   
   T   
   A   
   C   
   C   
   A   
   A   
   T   
   C   
   A   
   C   
   A   
   G   
   T   
    C    
   G   
   C   
   T   
   T   
   C   
   T   
   G   
   A   
   A   
   C   
   C   
   A   
   G   
   T   
   G   
   A   
   C   
   A   
   G   
   A   
   A   
   T   
   T   
   A   
   C   
   C   
   A   
   A   
   C   
   A   
   G   
   G   
   G   
   G   
   C   
   T   
   G   
   C   
   G   
   A   
   C   
   A   
   A   
   A   
   A   
   G   
    A    
    T    
   A   
   C   
   A   
   G   
   A   
   G   
   T   
   C   
   A   
   C   
   G   
   T   
   T   
   A   
   T   
   T   
   A   
   T   
   C   
   C   
    G    
    C    
   T   
   G   
   G   
   G   
   G   
   G   
   A   
   A   
   G   
   C   
   A   
   G   
   C   
   T   
   G   
   C   
   G   
   C   
   A   
   A   
   T   
   T   
   A   
   A   
   T   
   C   
   G   
   G   
    G    
   T   
   A   
   T   
   A   
   C   
    G    
   G   
   G   
    C    
   A   
   C   
    C    
   A   
   T   
   T   
   A   
   C   
   T   
   G   
   C   
   A   
   G   
   A   
    A    
   G   
   A   
   T   
   A   
   T   
   T   
    G    
   A   
   A   
   A   
   A   
   A   
   A   
   A   
   T   
   C   
   C   
   A   
   G   
   A   
   G   
   T   
   T   
   A   
   A   
   G   
   T   
   A   
   G   
   T   
   A   
   C   
   G   
   G   
   G   
    C    
   G   
   T   
   A   
   A   
   T   
   T   
   G   
   G   
    T    
   A   
   A   
   A   
   A   
   C   
   T   
   G   
   G   
    T    
   T   
   T   
   A   
   G   
   A   
   A   
   C   
   G   
   A   
   G   
   C   
   G   
   T   
   T   
   T   
   G   
   A   
   T   
   A   
   A   
   A   
   G   
   A   
    A    
   T   
   T   
   A   
   A   
   G   
   A   
   G   
   G   
    G    
   C   
   A   
   A   
   G   
   A   
   T   
   G   
   G   
    T    
   G   
   G   
    C    
   T   
   C   
    A    
   C   
   T   
   A   
   G   
   T   
   T   
   A   
   T   
   T   
   T   
   T   
   A   
   G   
   A   
   T   
   G   
   A   
   C   
   A   
   A   
   A   
   G   
   A   
   G   
   A   
   A   
   T   
   G   
   T   
   C   
   A   
   A   
   A   
   A   
   A   
   A   
   G   
   C   
   T   
   T   
   T   
   A   
   C   
   A   
   A   
   A   
   C   
   C   
   A   
   A   
   A   
   G   
   A   
    G    
   A   
   A   
   A   
   A   
   A   
   A   
   G   
   A   
   T   
   G   
   G   
   T   
   C   
   A   
   A   
   A   
   C   
    G    
   A   
   T   
   T   
   A   
   A   
   A   
   T   
   T   
   A   
   A   
   C   
   G   
   A   
   T   
   T   
   G   
   A   
    C    
   A   
   G   
    T    
   G   
   G   
    C    
   G   
   T   
   A   
   C   
   A   
   A   
   C   
   A   
   A   
   C   
   A   
   A   
   G   
   C   
    T    
   T   
   T   
    T    
   G   
   C   
   T   
   A   
   T   
   A   
   T   
   T   
   T   
   G   
   A   
   C   
   A   
   A   
   A   
   C   
   G   
    C    
    C    
   C   
   T   
   G   
   G   
   T   
   A   
   G   
   T   
   G   
   C   
   G   
   G   
   T   
   C   
   A   
   T   
   T   
   A   
   C   
   C   
   G   
   A   
   T   
   C   
   C   
   T   
   C   
   A   
   A   
   A   
   A   
   A   
   G   
   G   
   C   
   G   
   A   
   T   
   T   
   T   
   A   
    T    
   T   
   A   
   G   
   C   
    A    
   A   
   C   
   G   
   G   
   T   
   T   
   A   
   G   
   T   
   T   
   C   
   A   
   C   
   C   
   T   
   T   
   C   
   G   
   T   
   A   
   T   
   G   
   A   
   T   
   C   
   C   
   T   
   A   
   A   
   T   
   A   
   A   
   A   
   A   
   T   
   G   
   G   
   C   
   C   
   A   
   A   
   T   
   G   
   G   
    C    
   A   
   T   
   T   
   T   
   C   
   T   
   C   
   A   
   A   
   A   
   A   
   A   
   G   
   A   
    A    
   T   
   A   
   T   
   G   
   A   
   T   
   G   
   C   
   T   
   T   
   A   
   C   
   A   
   A   
   T   
   A   
   A   
   C   
   A   
   A   
   C   
   A   
   A   
   A   
   G   
   A   
   T   
   T   
   T   
   A   
   C   
   C   
   A   
   T   
   T   
    C    
   A   
   C   
   A   
   G   
   C   
   A   
   C   
   G   
   T   
   T   
   T   
    T    
   G   
   C   
   G   
   A   
   C   
   A   
   G   
   G   
    C    
   T   
   A   
   T   
   G   
   C   
   A   
   C   
   C   
   A   
   G   
   G   
    C    
   T   
   C   
   T   
   A   
   C   
   C   
   T   
   T   
   T   
   A   
   A   
   A   
   A   
   C   
   A   
   A   
   T   
   T   
   A   
   C   
   C   
   G   
   G   
   T   
   G   
   C   
   C   
   A   
   T   
   T   
   G   
   G   
   T   
   T   
   T   
   A   
   G   
   A   
   T   
    G    
   C   
   A   
   G   
   G   
   G   
   A   
   C   
   A   
   T   
   T   
   G   
   A   
   A   
   G   
   C   
   C   
   A   
   G   
   A   
   T   
   G   
   A   
   A   
   G   
   A   
   G   
   C   
   T   
   A   
   G   
   A   
   A   
   A   
   T   
   T   
   A   
   A   
   T   
   G   
   G   
    T    
   T   
   T   
   G   
   A   
   A   
   A   
   T   
   G   
   G   
   C   
   A   
   A   
   A   
   A   
   A   
   G   
   A   
   T   
   A   
   A   
   A   
   T   
   C   
   T   
   T   
   G   
   G   
   G   
   G   
    C    
   G   
   G   
   C   
   T   
   A   
   T   
   T   
   T   
   T   
   G   
   C   
    G    
   A   
   C   
   A   
   C   
   G   
   T   
   G   
   T   
   A   
   A   
   A   
   A   
   G   
   A   
   A   
   G   
   C   
   A   
   A   
   G   
   T   
   C   
   C   
   A   
   G   
   T   
   C   
   A   
   A   
   T   
   C   
   T   
    G    
   C   
   G   
   T   
   A   
   C   
   C   
   G   
   C   
   T   
   T   
   T   
   A   
   G   
   T   
   T   
   A   
   A   
   T   
   T   
   C   
   A   
   G   
   A   
   T   
   A   
   A   
   T   
   A   
   T   
   T   
   T   
   A   
   T   
   T   
   T   
   T   
   G   
   C   
    C    
   C   
   A   
   A   
   C   
   A   
   A   
   A   
   C   
   A   
   T   
   T   
   A   
   C   
   G   
    G    
   A   
   T   
   G   
   G   
   G   
    A    
   G   
   A   
   A   
   G   
   A   
   C   
   A   
   A   
   A   
   T   
   T   
   T   
   A   
   G   
   A   
   G   
   C   
    G    
   G   
   G   
   T   
   C   
   T   
   T   
   A   
   A   
   T   
   A   
   A   
   A   
   T   
   T   
   C   
   A   
   T   
   T   
   T   
   T   
   C   
   G   
   G   
   T   
   G   
   A   
   G   
   G   
   A   
   A   
   C   
   T   
   A   
   G   
   A   
   T   
   T   
   T   
   A   
   C   
   C   
   A   
   A   
   T   
    T    
   G   
   C   
   C   
   A   
   T   
   G   
   A   
   C   
   G   
   C   
   C   
    T    
   G   
   C   
   T   
   C   
   A   
   A   
   A   
   T   
   T   
   T   
   C   
   A   
   A   
   A   
    T    
   G   
   A   
   A   
   G   
   A   
   T   
   A   
   A   
   A   
   T   
   T   
   T   
   A   
   A   
   T   
   T   
   C   
   T   
   G   
   A   
   A   
   A   
   T   
   T   
   C   
   T   
   T   
   T   
   T   
    A    
   G   
   C   
   A   
   G   
   A   
   T   
   A   
   C   
    G    
   G   
   G   
   C   
   T   
   A   
    C    
   G   
   G   
    T    
   C   
   A   
   A   
   G   
   G   
    T    
   C   
   A   
   A   
   T   
   T   
   G   
   T   
   T   
   A   
   A   
   T   
   T   
   T   
   C   
    G    
   C   
   C   
   A   
   A   
   T   
   C   
   C   
   A   
   G   
   C   
   A   
   A   
   G   
   C   
   T   
   A   
   C   
   C   
   A   
   T   
   G   
   T   
   A   
   T   
   A   
   G   
   T   
   G   
   T   
   T   
   T   
   T   
   C   
   C   
   A   
   A   
   A   
   A   
   T   
   A   
   A   
   T   
   G   
   G   
    A    
   A   
   C   
   A   
    T    
   T   
    A    
   G   
   T   
    C    
   T   
   A   
   T   
   C   
   C   
   A   
   A   
   A   
   A   
    C    
   T   
   A   
   G   
   T   
   T   
   T   
   T   
   A   
   G   
   A   
   A   
   A   
   A   
   A   
   G   
   A   
   A   
   A   
   C   
    G    
   A   
   A   
   G   
   A   
   A   
   A   
   A   
   A   
   A   
   G   
   A   
   C   
   A   
   A   
   T   
   G   
   T   
   A   
   A   
   T   
   C   
   A   
   G   
   C   
   G   
   C   
   C   
   A   
   A   
   C   
   G   
   C   
   A   
   G   
   C   
   G   
   A   
   A   
   T   
   A   
   C   
   A   
   A   
   T   
   T   
   G   
   C   
   C   
   A   
   C   
   A   
   G   
   A   
   T   
   C   
   T   
   T   
   T   
   T   
   A   
   G   
   G   
   T   
   A   
   G   
   T   
   G   
   T   
   G   
   G   
   A   
   A   
   G   
   A   
   T   
   C   
   C   
   T   
   T   
   C   
   A   
   G   
   G   
   T   
   T   
   A   
   T   
   G   
   T   
   T   
   T   
   A   
   T   
   A   
   A   
   T   
   A   
   T   
   G   
   T   
   A   
   C   
   A   
   A   
   T   
   C   
   C   
   G   
   A   
   A   
   C   
   T   
   T   
   C   
   T   
   C   
   A   
   T   
   T   
   G   
   G   
   C   
   T   
   G   
   C   
   T   
   A   
   A   
   A   
   A   
   C   
   A   
   G   
   G   
   A   
   A   
   C   
   A   
   G   
   C   
   T   
   G   
   A   
   A   
   A   
   T   
   A   
   A   
   A   
   A   
   G   
   A   
   T   
   A   
   A   
   A   
   C   
   A   
   A   
   G   
   A   
   T   
   A   
   C   
   A   
   G   
   A   
   T   
   G   
   G   
   C   
   A   
   A   
   A   
   G   
   A   
   A   
   A   
   A   
   C   
   A   
   G   
   T   
   T   
   T   
   C   
   C   
   T   
   T   
   C   
   T   
   A   
   A   
   C   
   G   
   T   
   T   
   A   
   G   
   A   
   T   
   C   
   G   
   T   
   A   
   G   
   T   
   A   
   A   
   C   
   A   
   A   
   T   
   A   
   A   
   A   
   T   
   T   
   C   
   T   
   T   
   A   
   A   
   C   
   A   
   A   
   T   
   G   
   A   
   T   
   T   
   A   
   T   
   G   
   G   
   T   
   T   
   G   
   A   
   A   
   A   
   A   
   T   
   T   
   C   
   A   
   G   
   G   
   A   
   G   
   A   
   A   
   A   
   A   
   T   
   G   
   G   
   T   
   T   
   C   
   T   
   G   
   C   
   A   
   A   
   C   
   A   
   G   
   A   
   T   
   A   
   T   
   C   
   A   
   G   
   T   
   A   
   A   
   A   
   C   
   C   
   A   
   T   
   T   
   A   
   A   
   T   
   T   
   G   
   A   
   T   
   T   
   A   
   C   
   T   
   T   
   A   
   G   
   A   
   A   
   G   
   C   
   A   
   A   
   C   
   C   
   A   
   T   
   T   
   A   
   A   
   A   
   -   
   -   
   -   
   -   
   -   
   -   
   -   
   -   
   -   
   -   
   -   
   -   
   -   
   -   
   -   
   -   
   -   
   -   
   -   
   -   
   -   
   -   
   -   
   -   
   -   
   -   
   -   
   -   
   -   
   -   
   -   
   -   
   -   
   -   
   -   
   -   
   -   
   -   
   -   
   -   
   -   
   -   
   -   
   -   
   -   
   -   
   -   
   -   
   -   
   -   
   -   
   -   
   -   
   -   
   -   
   -   
   -   
   -   
   -   
   -   
   -   
   -   
   -   
   -   
   -   
   -   
   -   
   -   
   -   
   -   
   -   
   -   
   -   
   -   
   -   
   -   
   -   
   -   
   -   
   -   
   -   
   -   
   -   
   -   
   -   
   -   
   -   
   -   
   -   
   -   
   -   
   -   
   -   
   -   
   -   
   -   
   -   
   -   
   -   
   -   
   -   
   -   
   -   
   -   
   -   
   -   
   -   
   -   
   -   
   -   
   -   
   -   
   -   
   -   
   -   
   -   
   -   
   -   
   -   
   -   
   -   
   -   
   -   
   -   
   -   
   -   
   -   
   -   
   -   
   -   
   -   
   -   
   -   
   -   
   -   
   -   
   -   
   -   
   -   
   -   
   -   
   -   
   -   
   -   
   -   
   -   
   -   
   -   
   -   
   -   
   -   
   -   
   -   
   -   
   -   
   -   
   -   
   -   
   -   
   -   
   -   
   -   
   -   
   -   
   -   
   -   
   -   
   -   
   -   
   C   
   A   
   A   
   T   
   G   
   G   
   C   
   A   
   A   
   G   
   C   
   C   
   A   
   A   
   A   
   C   
   A   
   A   
   G   
   A   
   A   
   T   
   T   
   A   
   G   
   C   
   C   
   G   
   A   
   A   
   G   
   C   
   G   
   A   
   A   
   G   
   A   
   A   
   A   
   A   
   C   
   A   
   G   
   C   
   T   
   A   
   C   
   T   
   A   
   C   
   A   
   T   
   T   
   T   
   T   
   T   
   A   
   A   
   A   
   C   
   G   
   T   
   A   
   T   
   T   
   G   
   T   
   C   
   A   
   A   
   A   
   A   
   C   
   A   
   G   
   G   
   A   
   A   
   T   
   T   
   T   
   G   
   A   
   T   
   A   
   A   
   G   
   T   
   T   
   A   
   C   
   C   
   G   
   T   
   C   
   C   
   G   
   T   
   T   
   G   
   T   
   T   
   C   
   A   
   A   
   G   
   A   
   A   
   G   
   C   
   T   
   A   
   G   
   C   
   T   
   T   
   A   
   A   
   A   
   G   
   A   
   A   
   A   
   A   
   A   
   T   
   G   
   G   
   C   
   T   
   A   
   T   
   G   
   A   
   T   
   A   
   C   
   T   
   A   
   A   
   A   
   T   
   C   
   T   
   G   
   T   
   T   
   G   
   T   
   T   
   G   
   A   
   A   
   A   
   A   
   A   
   T   
   A   
   C   
   C   
   A   
   A   
   G   
   C   
   A   
   A   
   T   
   T   
   T   
   A   
   T   
   T   
   C   
   A   
   G   
   G   
   G   
   A   
   T   
   T   
   C   
   A   
   A   
   G   
   C   
   A   
   G   
   A   
   A   
   G   
   G   
   A   
   G   
   T   
   C   
   A   
   A   
   A   
   G   
   C   
   T   
   A   
   G   
   T   
   G   
   A   
   T   
   G   
   T   
   T   
   C   
   A   
   A   
   G   
   T   
   C   
   A   
   A   
   A   
   A   
   A   
   G   
   G   
   C   
   G   
   A   
   A   
   A   
   G   
   A   
   C   
   A   
   A   
   T   
   C   
   A   
   A   
   T   
   A   
   C   
   A   
   C   
   A   
   T   
   T   
   T   
   A   
   C   
   C   
   T   
   A   
   T   
   A   
   A   
   A   
   T   
   T   
   A   
   T   
   C   
   G   
   A   
   T   
   G   
   A   
   G   
   T   
   A   
   C   
   C   
   C   
   C   
   T   
   T   
   T   
   A   
   G   
   G   
   C   
   G   
   A   
   A   
   A   
   T   
   G   
   A   
   A   
   A   
   G   
   A   
   T   
   T   
   T   
   G   
   T   
   C   
   T   
   T   
   A   
   T   
   C   
   A   
   A   
   T   
   C   
   A   
   A   
   G   
   T   
   A   
   T   
   C   
   G   
   C   
   C   
   A   
   A   
   A   
   A   
   A   
   A   
   G   
   G   
   C   
   G   
   A   
   T   
   A   
   C   
   C   
   T   
   A   
   C   
   C   
   A   
   A   
   A   
   T   
   C   
   G   
   C   
   T   
   T   
   G   
   G   
   A   
   A   
   G   
   C   
   C   
   G   
   T   
   C   
   T   
   T   
   T   
   A   
   A   
   T   
   T   
   T   
   T   
   T   
   C   
   C   
   A   
   G   
   A   
   T   
   A   
   T   
   G   
   T   
   C   
   A   
   G   
   G   
   A   
   A   
   A   
   T   
   G   
   A   
   T   
   A   
   A   
   A   
   A   
   T   
   T   
   T   
   C   
   G   
   A   
   T   
   T   
   C   
   A   
   A   
   G   
   T   
   A   
   G   
   A   
   T   
   A   
   A   
   T   
   G   
   C   
   C   
   A   
   A   
   A   
   C   
   G   
   T   
   G   
   G   
   A   
   G   
   A   
   A   
   A   
   T   
   T   
   G   
   T   
   C   
   G   
   A   
   T   
   C   
   G   
   T   
   A   
   A   
   T   
   G   
   G   
   T   
   A   
   G   
   T   
   G   
   G   
   G   
   C   
   T   
   A   
   G   
   C   
   A   
   A   
   T   
   T   
   A   
   A   
   C   
   A   
   A   
   A   
   G   
   T   
   G   
   T   
   T   
   T   
   G   
   A   
   C   
   G   
   A   
   A   
   G   
   T   
   G   
   G   
   G   
   C   
   G   
   T   
   A   
   G   
   T   
   G   
   C   
   C   
   T   
   G   
   G   
   C   
   A   
   A   
   A   
   C   
   T   
   C   
   G   
   G   
   T   
   T   
   C   
   T   
   G   
   G   
   C   
   G   
   C   
   A   
   G   
   A   
   A   
   A   
   A   
   A   
   A   
   C   
   A   
   G   
   C   
   C   
   A   
   A   
   T   
   A   
   T   
   C   
   A   
   A   
   A   
   G   
   C   
   T   
   T   
   T   
   T   
   A   
   G   
   T   
   G   
   A   
   T   
   A   
   A   
   A   
   T   
   T   
   C   
   G   
   G   
   C   
   G   
   T   
   T   
   T   
   C   
   T   
   G   
   T   
   T   
   G   
   A   
   T   
   G   
   A   
   A   
   A   
   T   
   C   
   A   
   A   
   T   
   C   
   A   
   A   
   A   
   A   
   G   
   T   
   T   
   A   
   A   
   G   
   C   
   C   
   A   
   A   
   G   
   G   
   A   
   T   
   G   
   G   
   G   
   T   
   C   
   C   
   A   
   A   
   G   
   C   
   A   
   G   
   A   
   C   
   T   
   C   
   C   
   T   
   T   
   T   
   G   
   T   
   A   
   C   
   C   
   A   
   A   
   T   
   C   
   A   
   C   
   A   
   G   
   T   
   C   
   G   
   C   
   T   
   T   
   C   
   T   
   G   
   A   
   A   
   C   
   C   
   A   
   G   
   T   
   G   
   A   
   C   
   A   
   G   
   A   
   A   
   T   
   T   
   A   
   C   
   C   
   A   
   A   
   C   
   A   
   G   
   G   
   G   
   G   
   C   
   T   
   G   
   C   
   G   
   A   
   C   
   A   
   A   
   A   
   A   
   G   
   A   
   T   
   A   
   C   
   A   
   G   
   A   
   G   
   T   
   C   
   A   
   C   
   G   
   T   
   T   
   A   
   T   
   T   
   A   
   T   
   C   
   C   
   G   
   C   
   T   
   G   
   G   
   G   
   G   
   G   
   A   
   A   
   G   
   C   
   A   
   G   
   C   
   T   
   G   
   C   
   G   
   C   
   A   
   A   
   T   
   T   
   A   
   A   
   T   
   C   
   G   
   G   
   G   
   T   
   A   
   T   
   A   
   C   
   G   
   G   
   G   
   C   
   A   
   C   
   C   
   A   
   T   
   T   
   A   
   C   
   T   
   G   
   C   
   A   
   G   
   A   
   A   
   G   
   A   
   T   
   A   
   T   
   T   
   G   
   A   
   A   
   A   
   A   
   A   
   A   
   A   
   T   
   C   
   C   
   A   
   G   
   A   
   G   
   T   
   T   
   A   
   A   
   G   
   T   
   A   
   G   
   T   
   A   
   C   
   G   
   G   
   G   
   C   
   G   
   T   
   A   
   A   
   T   
   T   
   G   
   G   
   T   
   A   
   A   
   A   
   A   
   C   
   T   
   G   
   G   
   T   
   T   
   T   
   A   
   G   
   A   
   A   
   C   
   G   
   A   
   G   
   C   
   G   
   T   
   T   
   T   
   G   
   A   
   T   
   A   
   A   
   A   
   G   
   A   
   A   
   T   
   T   
   A   
   A   
   G   
   A   
   G   
   G   
   G   
   C   
   A   
   A   
   G   
   A   
   T   
   G   
   G   
   T   
   G   
   G   
   C   
   T   
   C   
   A   
   C   
   T   
   A   
   G   
   T   
   T   
   A   
   T   
   T   
   T   
   T   
   A   
   G   
   A   
   T   
   G   
   A   
   C   
   A   
   A   
   A   
   G   
   A   
   G   
   A   
   A   
   T   
   G   
   T   
   C   
   A   
   A   
   A   
   A   
   A   
   A   
   G   
   C   
   T   
   T   
   T   
   A   
   C   
   A   
   A   
   A   
   C   
   C   
   A   
   A   
   A   
   G   
   A   
   G   
   A   
   A   
   A   
   A   
   A   
   A   
   G   
   A   
   T   
   G   
   G   
   T   
   C   
   A   
   A   
   A   
   C   
   G   
   A   
   T   
   T   
   A   
   A   
   A   
   T   
   T   
   A   
   A   
   C   
   G   
   A   
   T   
   T   
   G   
   A   
   C   
   A   
   G   
   T   
   G   
   G   
   C   
   G   
   T   
   A   
   C   
   A   
   A   
   C   
   A   
   A   
   C   
   A   
   A   
   G   
   C   
   T   
   T   
   T   
   T   
   G   
   C   
   T   
   A   
   T   
   A   
   T   
   T   
   T   
   G   
   A   
   C   
   A   
   A   
   A   
   C   
   G   
   C   
   C   
   C   
   T   
   G   
   G   
   T   
   A   
   G   
   T   
   G   
   C   
   G   
   G   
   T   
   C   
   A   
   T   
   T   
   A   
   C   
   C   
   G   
   A   
   T   
   C   
   C   
   T   
   C   
   A   
   A   
   A   
   A   
   A   
   G   
   G   
   C   
   G   
   A   
   T   
   T   
   T   
   A   
   T   
   T   
   A   
   G   
   C   
   A   
   A   
   C   
   G   
   G   
   T   
   T   
   A   
   G   
   T   
   T   
   C   
   A   
   C   
   C   
   T   
   T   
   C   
   G   
   T   
   A   
   T   
   G   
   A   
   T   
   C   
   C   
   T   
   A   
   A   
   T   
   A   
   A   
   A   
   A   
   T   
   G   
   G   
   C   
   C   
   A   
   A   
   T   
   G   
   G   
   C   
   A   
   T   
   T   
   T   
   C   
   T   
   C   
   A   
   A   
   A   
   A   
   A   
   G   
   A   
   A   
   T   
   A   
   T   
   G   
   A   
   T   
   G   
   C   
   T   
   T   
   A   
   C   
   A   
   A   
   T   
   A   
   A   
   C   
   A   
   A   
   C   
   A   
   A   
   A   
   G   
   A   
   T   
   T   
   T   
   A   
   C   
   C   
   A   
   T   
   T   
   C   
   A   
   C   
   A   
   G   
   C   
   A   
   C   
   G   
   T   
   T   
   T   
   T   
   G   
   C   
   G   
   A   
   C   
   A   
   G   
   G   
   C   
   T   
   A   
   T   
   G   
   C   
   A   
   C   
   C   
   A   
   G   
   G   
   C   
   T   
   C   
   T   
   A   
   C   
   C   
   T   
   T   
   T   
   A   
   A   
   A   
   A   
   C   
   A   
   A   
   T   
   T   
   A   
   C   
   C   
   G   
   G   
   T   
   G   
   C   
   C   
   A   
   T   
   T   
   G   
   G   
   T   
   T   
   T   
   A   
   G   
   A   
   T   
   G   
   C   
   A   
   G   
   G   
   G   
   A   
   C   
   A   
   T   
   T   
   G   
   A   
   A   
   G   
   C   
   C   
   A   
   G   
   A   
   T   
   G   
   A   
   A   
   G   
   A   
   G   
   C   
   T   
   A   
   G   
   A   
   A   
   A   
   T   
   T   
   A   
   A   
   T   
   G   
   G   
   T   
   T   
   T   
   G   
   A   
   A   
   A   
   T   
   G   
   G   
   C   
   A   
   A   
   A   
   A   
   A   
   G   
   A   
   T   
   A   
   A   
   A   
   T   
   C   
   T   
   T   
   G   
   G   
   G   
   G   
   C   
   G   
   G   
   C   
   T   
   A   
   T   
   T   
   T   
   T   
   G   
   C   
   G   
   A   
   C   
   A   
   C   
   G   
   T   
   G   
   T   
   A   
   A   
   A   
   A   
   G   
   A   
   A   
   G   
   C   
   A   
   A   
   G   
   T   
   C   
   C   
   A   
   G   
   T   
   C   
   A   
   A   
   T   
   C   
   T   
   G   
   C   
   G   
   T   
   A   
   C   
   C   
   G   
   C   
   T   
   T   
   T   
   A   
   G   
   T   
   T   
   A   
   A   
   T   
   T   
   C   
   A   
   G   
   A   
   T   
   A   
   A   
   T   
   A   
   T   
   T   
   T   
   A   
   T   
   T   
   T   
   T   
   G   
   C   
   C   
   C   
   A   
   A   
   C   
   A   
   A   
   A   
   C   
   A   
   T   
   T   
   A   
   C   
   G   
   G   
   A   
   T   
   G   
   G   
   G   
   A   
   G   
   A   
   A   
   G   
   A   
   C   
   A   
   A   
   A   
   T   
   T   
   T   
   A   
   G   
   A   
   G   
   C   
   G   
   G   
   G   
   T   
   C   
   T   
   T   
   A   
   A   
   T   
   A   
   A   
   A   
   T   
   T   
   C   
   A   
   T   
   T   
   T   
   T   
   C   
   G   
   G   
   T   
   G   
   A   
   G   
   G   
   A   
   A   
   C   
   T   
   A   
   G   
   A   
   T   
   T   
   T   
   A   
   C   
   C   
   A   
   A   
   T   
   T   
   G   
   C   
   C   
   A   
   T   
   G   
   A   
   C   
   G   
   C   
   C   
   T   
   G   
   C   
   T   
   C   
   A   
   A   
   A   
   T   
   T   
   T   
   C   
   A   
   A   
   A   
   T   
   G   
   A   
   A   
   G   
   A   
   T   
   A   
   A   
   A   
   T   
   T   
   T   
   A   
   A   
   T   
   T   
   C   
   T   
   G   
   A   
   A   
   A   
   T   
   T   
   C   
   T   
   T   
   T   
   T   
   A   
   G   
   C   
   A   
   G   
   A   
   T   
   A   
   C   
   G   
   G   
   G   
   C   
   T   
   A   
   C   
   G   
   G   
   T   
   C   
   A   
   A   
   G   
   G   
   T   
   C   
   A   
   A   
   T   
   T   
   G   
   T   
   T   
   A   
   A   
   T   
   T   
   T   
   C   
   G   
   C   
   C   
   A   
   A   
   T   
   C   
   C   
   A   
   G   
   C   
   A   
   A   
   G   
   C   
   T   
   A   
   C   
   C   
   A   
   T   
   G   
   T   
   A   
   T   
   A   
   G   
   T   
   G   
   T   
   T   
   T   
   T   
   C   
   C   
   A   
   A   
   A   
   A   
   T   
   A   
   A   
   T   
   G   
   G   
   A   
   A   
   C   
   A   
   T   
   T   
   A   
   G   
   T   
   C   
   T   
   A   
   T   
   C   
   C   
   A   
   A   
   A   
   A   
   C   
   T   
   A   
   G   
   T   
   T   
   T   
   T   
   A   
   G   
   A   
   A   
   A   
   A   
   A   
   G   
   A   
   A   
   A   
   C   
   G   
   A   
   A   
   G   
   A   
   A   
   A   
   A   
   A   
   A   
   G   
   A   
   C   
   A   
   A   
   T   
   G   
   T   
   A   
   A   
   T   
   C   
   A   
   G   
   C   
   G   
   C   
   C   
   A   
   A   
   C   
   G   
   C   
   A   
   G   
   C   
   G   
   A   
   A   
   T   
   A   
   C   
   A   
   A   
   T   
   T   
   G   
   C   
   C   
   A   
   C   
   A   
   G   
   A   
   T   
   C   
   T   
   T   
   T   
   T   
   A   
   G   
   G   
   T   
   A   
   G   
   T   
   G   
   T   
   G   
   G   
   A   
   A   
   G   
   A   
   T   
   C   
   C   
   T   
   T   
   C   
   A   
   G   
   G   
   T   
   T   
   A   
   T   
   G   
   T   
   T   
   T   
   A   
   T   
   A   
   A   
   T   
   A   
   T   
   G   
   T   
   A   
   C   
   A   
   A   
   T   
   C   
   C   
   G   
   A   
   A   
   C   
   T   
   T   
   C   
   T   
   C   
   A   
   T   
   T   
   G   
   G   
   C   
   T   
   G   
   C   
   T   
   A   
   A   
   A   
   A   
   C   
   A   
   G   
   G   
   A   
   A   
   C   
   A   
   G   
   C   
   T   
   G   
   A   
   A   
   A   
   T   
   A   
   A   
   A   
   A   
   G   
   A   
   T   
   A   
   A   
   A   
   C   
   A   
   A   
   G   
   A   
   T   
   A   
   C   
   A   
   G   
   A   
   T   
   G   
   G   
   C   
   A   
   A   
   A   
   G   
   A   
   A   
   A   
   A   
   C   
   A   
   G   
   T   
   T   
   T   
   C   
   C   
   T   
   T   
   C   
   T   
   A   
   A   
   C   
   G   
   T   
   T   
   A   
   G   
   A   
   T   
   C   
   G   
   T   
   A   
   G   
   T   
   A   
   A   
   C   
   A   
   A   
   T   
   A   
   A   
   A   
   T   
   T   
   C   
   T   
   T   
   A   
   A   
   C   
   A   
   A   
   T   
   G   
   A   
   T   
   T   
   A   
   T   
   G   
   G   
   T   
   T   
   G   
   A   
   A   
   A   
   A   
   T   
   T   
   C   
   A   
   G   
   G   
   A   
   G   
   A   
   A   
   A   
   A   
   T   
   G   
   G   
   T   
   T   
   C   
   T   
   G   
   C   
   A   
   A   
   C   
   A   
   G   
   A   
   T   
   A   
   T   
   C   
   A   
   G   
   T   
   A   
   A   
   A   
   C   
   C   
   A   
   T   
   T   
   A   
   A   
   T   
   T   
   G   
   A   
   T   
   T   
   A   
   C   
   T   
   T   
   A   
   G   
   A   
   A   
   G   
   C   
   A   
   A   
   C   
   C   
   A   
   T   
   T   
   A   
   A   
   A   
   -   
   -   
   -   
   -   
   -   
   -   
   -   
   -   
   -   
   -   
   -   
   -   
   -   
   -   
   -   
   -   
   -   
   -   
   -   
   -   
   -   
   -   
   -   
   -   
   -   
   -   
   -   
   -   
   -   
   -   
   -   
   -   
   -   
   -   
   -   
   -   
   -   
   -   
   -   
   -   
   -   
   -   
   -   
   -   
   -   
   -   
   -   
   -   
   -   
   -   
   -   
   -   
   -   
   -   
   -   
   -   
   -   
   -   
   -   
   -   
   -   
 

 
   N54576   
   -   
   -   
   -   
   -   
   -   
   -   
   -   
   -   
   -   
   -   
   -   
   -   
   -   
   -   
   -   
   -   
   -   
   -   
   -   
   -   
   -   
   -   
   -   
   -   
   -   
   -   
   -   
   -   
   -   
   -   
   -   
   -   
   -   
   -   
   -   
   -   
   -   
   -   
   -   
   -   
   -   
   -   
   -   
   -   
   -   
   -   
   -   
   -   
   -   
   -   
   -   
   -   
   -   
   -   
   -   
   -   
   -   
   -   
   -   
   -   
   -   
   -   
   -   
   -   
   -   
   -   
   -   
   -   
   -   
   -   
   -   
   -   
   -   
   -   
   -   
   -   
   -   
   -   
   -   
   -   
   -   
   -   
   -   
   -   
   -   
   -   
   -   
   -   
   -   
   -   
   -   
   -   
   -   
   -   
   -   
   -   
   -   
   -   
   -   
   -   
   -   
   -   
   -   
   -   
   -   
   -   
   -   
   -   
   C   
   A   
   A   
   T   
   G   
   G   
   C   
   A   
   A   
   G   
   C   
   C   
   A   
   A   
   A   
   C   
   A   
   A   
   G   
   A   
    A    
   T   
   T   
   A   
   G   
   C   
    C    
   G   
   A   
   A   
   G   
   C   
   G   
   A   
   A   
   G   
   A   
   A   
   A   
   A   
    C    
   A   
   G   
   C   
    T    
   A   
    C    
   T   
    A    
    C    
   A   
   T   
   T   
   T   
   T   
   T   
   A   
   A   
   A   
    C    
   G   
   T   
   A   
   T   
   T   
   G   
    A    
   C   
   A   
   A   
   A   
   A   
   C   
   A   
   G   
    A    
   A   
   A   
   T   
   T   
   T   
   G   
   A   
   T   
   A   
   A   
   G   
   T   
   T   
   A   
   C   
   C   
    G    
   T   
   C   
   C   
   G   
   T   
   T   
    G    
   T   
   T   
   C   
   A   
   A   
   G   
   A   
   A   
    G    
   C   
   T   
   A   
   G   
   C   
   T   
   T   
   A   
   A   
   A   
   G   
   A   
   A   
   A   
   A   
   A   
   T   
   G   
   G   
    C    
   T   
   A   
   T   
   G   
   A   
   T   
   A   
   C   
   T   
   A   
   A   
   A   
   T   
   C   
    C    
   G   
   T   
   T   
   G   
   T   
   T   
   G   
   A   
   A   
   A   
   A   
   A   
   T   
   A   
   C   
   C   
   A   
   A   
   G   
   C   
   A   
   A   
   T   
   T   
   T   
   A   
   T   
   T   
   C   
   A   
   G   
   G   
   G   
   A   
   T   
   T   
   C   
   A   
   A   
   G   
   C   
    A    
   G   
   A   
   A   
    G    
   G   
    A    
   G   
   T   
   C   
   A   
   A   
    A    
   G   
   C   
   T   
   A   
   G   
   T   
   G   
   A   
   T   
   G   
   T   
    T    
   C   
   A   
   A   
   G   
   T   
    C    
   A   
   A   
   A   
   A   
   A   
    A    
   G   
    C    
   G   
   A   
   A   
   A   
   G   
   A   
    C    
   A   
   A   
    T    
   C   
   A   
   A   
   T   
   A   
   C   
   A   
   C   
   A   
   T   
   T   
   T   
   A   
    A    
   C   
   T   
   A   
   T   
   A   
   A   
   A   
   T   
   T   
   A   
   T   
   C   
   G   
   A   
   T   
   G   
   A   
   G   
    T    
   A   
   C   
    C    
   C   
   C   
   T   
   T   
   T   
   A   
   G   
   G   
   C   
   G   
   A   
   A   
   A   
   T   
   G   
   A   
   A   
   A   
   G   
   A   
   T   
    T    
   T   
   G   
   T   
   C   
   T   
   T   
   A   
   T   
   C   
   A   
   A   
   T   
   C   
   A   
   A   
   G   
   T   
   A   
   T   
    C    
   G   
    C    
   C   
   A   
   A   
    A    
   A   
   A   
   A   
   G   
   G   
    C    
   G   
   A   
   T   
   A   
    C    
   C   
   T   
   A   
   C   
   C   
   A   
   A   
   A   
   T   
    C    
    G    
   C   
   T   
   T   
   G   
   G   
    A    
    A    
    G    
   C   
   C   
    G    
   T   
   C   
   T   
   T   
   T   
   A   
   A   
   T   
   T   
   T   
   T   
   T   
   C   
   C   
   A   
   G   
   A   
   T   
   A   
   T   
   G   
   T   
   C   
   A   
   G   
   G   
   A   
   A   
   A   
   T   
   G   
   A   
   T   
   A   
   A   
   A   
    A    
   T   
   T   
   T   
   C   
   G   
   A   
   T   
   T   
   C   
   A   
   A   
   G   
   T   
   A   
   G   
   A   
   T   
   A   
   A   
   T   
   G   
   C   
   C   
   A   
   A   
   A   
   C   
   G   
   T   
   G   
   G   
   A   
   G   
   A   
   A   
   A   
   T   
   T   
   G   
   T   
   C   
   G   
   A   
   T   
   C   
   G   
   T   
   A   
   A   
    T    
   G   
   G   
   T   
   A   
   G   
   T   
   G   
   G   
   G   
   C   
   T   
   A   
   G   
    C    
   A   
   A   
   T   
   T   
   A   
   A   
   C   
   A   
   A   
   A   
   G   
   T   
   G   
   T   
   T   
   T   
   G   
   A   
    C    
   G   
   A   
   A   
   G   
   T   
   G   
   G   
   G   
   C   
   G   
   T   
   A   
   G   
   T   
    G    
   C   
   C   
   T   
    G    
   G   
   C   
   A   
   A   
   A   
   C   
   T   
    C    
   G   
   G   
   T   
   T   
   C   
   T   
   G   
   G   
   C   
   G   
   C   
   A   
   G   
   A   
   A   
   A   
   A   
   A   
    A    
   C   
   A   
   G   
   C   
    C    
   A   
   A   
   T   
   A   
   T   
   C   
   A   
   A   
   A   
   G   
   C   
   T   
   T   
   T   
   T   
   A   
   G   
   T   
   G   
   A   
   T   
   A   
   A   
    G    
   T   
   T   
    C    
   G   
   G   
   C   
   G   
   T   
   T   
   T   
   C   
   T   
    G    
   T   
   T   
   G   
   A   
   T   
   G   
   A   
   A   
   A   
   T   
   C   
   A   
   A   
   T   
   C   
    A    
   A   
   A   
   A   
    G    
   T   
   T   
   A   
   A   
   G   
   C   
   C   
   A   
   A   
   G   
   G   
    G    
   T   
   G   
   G   
   G   
   T   
   C   
   C   
   A   
   A   
   G   
   C   
   A   
   G   
   A   
   C   
   T   
   C   
    C    
   T   
   T   
   T   
   G   
   T   
   A   
   C   
   C   
   A   
   A   
   T   
   C   
   A   
   C   
   A   
   G   
   T   
    C    
   G   
   C   
   T   
   T   
   C   
   T   
   G   
   A   
   A   
   C   
   C   
   A   
   G   
   T   
   G   
   A   
   C   
   A   
   G   
   A   
   A   
   T   
   T   
   A   
   C   
   C   
   A   
   A   
   C   
   A   
   G   
   G   
   G   
   G   
   C   
   T   
   G   
   C   
   G   
   A   
   C   
   A   
   A   
   A   
   A   
   G   
    A    
    T    
   A   
   C   
   A   
   G   
   A   
   G   
   T   
   C   
   A   
   C   
   G   
   T   
   T   
   A   
   T   
   T   
   A   
   T   
   C   
   C   
    G    
    C    
   T   
   G   
   G   
   G   
   G   
   G   
   A   
   A   
   G   
   C   
   A   
   G   
   C   
   T   
   G   
   C   
   G   
   C   
   A   
   A   
   T   
   T   
   A   
   A   
   T   
   C   
   G   
   G   
    G    
   T   
   A   
   T   
   A   
   C   
    A    
   G   
   G   
    C    
   A   
   C   
    C    
   A   
   T   
   T   
   A   
   C   
   T   
   G   
   C   
   A   
   G   
   A   
    A    
   G   
   A   
   T   
   A   
   T   
   T   
    A    
   A   
   A   
   A   
   A   
   A   
   A   
   A   
   T   
   C   
   C   
   A   
   G   
   A   
   G   
   T   
   T   
   A   
   A   
   G   
   T   
   A   
   G   
   T   
   A   
   C   
   G   
   G   
   G   
    C    
   G   
   T   
   A   
   A   
   T   
   T   
   G   
   G   
    T    
   A   
   A   
   A   
   A   
   C   
   T   
   G   
   G   
    C    
   T   
   T   
   A   
   G   
   A   
   A   
   C   
   G   
   A   
   G   
   C   
   G   
   T   
   T   
   T   
   G   
   A   
   T   
   A   
   A   
   A   
   G   
   A   
    A    
   T   
   T   
   A   
   A   
   G   
   A   
   G   
   G   
    G    
   C   
   A   
   A   
   G   
   A   
   T   
   G   
   G   
    T    
   G   
   G   
    C    
   T   
   C   
    A    
   C   
   T   
   A   
   G   
   T   
   T   
   A   
   T   
   T   
   T   
   T   
   A   
   G   
   A   
   T   
   G   
   A   
   C   
   A   
   A   
   A   
   G   
   A   
   G   
   A   
   A   
   T   
   G   
   T   
   C   
   A   
   A   
   A   
   A   
   A   
   A   
   G   
   C   
   T   
   T   
   T   
   A   
   C   
   A   
   A   
   A   
   C   
   C   
   A   
   A   
   A   
   G   
   A   
    G    
   A   
   A   
   A   
   A   
   A   
   A   
   G   
   A   
   T   
   G   
   G   
   T   
   C   
   A   
   A   
   A   
   C   
    G    
   A   
   T   
   T   
   A   
   A   
   A   
   T   
   T   
   A   
   A   
   C   
   G   
   A   
   T   
   T   
   G   
   A   
    C    
   A   
   G   
    T    
   G   
   G   
    C    
   G   
   T   
   A   
   C   
   A   
   A   
   C   
   A   
   A   
   C   
   A   
   A   
   G   
   C   
    C    
   T   
   T   
    C    
   G   
   C   
   T   
   A   
   T   
   A   
   T   
   T   
   T   
   G   
   A   
   C   
   A   
   A   
   A   
   C   
   G   
    C    
    C    
   C   
   T   
   G   
   G   
   T   
   A   
   G   
   T   
   G   
   C   
   G   
   G   
   T   
   C   
   A   
   T   
   T   
   A   
   C   
   C   
   G   
   A   
   T   
   C   
   C   
   T   
   C   
   A   
   A   
   A   
   A   
   A   
   G   
   G   
   C   
   G   
   A   
   T   
   T   
   T   
   A   
    T    
   T   
   A   
   G   
   C   
    A    
   A   
   C   
   G   
   G   
   T   
   T   
   A   
   G   
   T   
   T   
   C   
   A   
   C   
   C   
   T   
   T   
   C   
   G   
   T   
   A   
   T   
   G   
   A   
   T   
   C   
   C   
   T   
   A   
   A   
   T   
   A   
   A   
   A   
   A   
   T   
   G   
   G   
   C   
   C   
   A   
   A   
   T   
   G   
   G   
    T    
   A   
   T   
   T   
   T   
   C   
   T   
   C   
   A   
   A   
   A   
   A   
   A   
   G   
   A   
    A    
   T   
   A   
   T   
   G   
   A   
   T   
   G   
   C   
   T   
   T   
   A   
   C   
   A   
   A   
   T   
   A   
   A   
   C   
   A   
   A   
   C   
   A   
   A   
   A   
   G   
   A   
   T   
   T   
   T   
   A   
   C   
   C   
   A   
   T   
   T   
    C    
   A   
   C   
   A   
   G   
   C   
   A   
   C   
   G   
   T   
   T   
   T   
    T    
   G   
   C   
   G   
   A   
   C   
   A   
   G   
   G   
    C    
   T   
   A   
   T   
   G   
   C   
   A   
   C   
   C   
   A   
   G   
   G   
    T    
   T   
   C   
   T   
   A   
   C   
   C   
   T   
   T   
   T   
   A   
   A   
   A   
   A   
   C   
   A   
   A   
   T   
   T   
   A   
   C   
   C   
   G   
   G   
   T   
   G   
   C   
   C   
   A   
   T   
   T   
   G   
   G   
   T   
   T   
   T   
   A   
   G   
   A   
   T   
    G    
   C   
   A   
   G   
   G   
   G   
   A   
   C   
   A   
   T   
   T   
   G   
   A   
   A   
   G   
   C   
   C   
   A   
   G   
   A   
   T   
   G   
   A   
   A   
   G   
   A   
   G   
   C   
   T   
   A   
   G   
   A   
   A   
   A   
   T   
   T   
   A   
   A   
   T   
   G   
   G   
    C    
   T   
   T   
   G   
   A   
   A   
   A   
   T   
   G   
   G   
   C   
   A   
   A   
   A   
   A   
   A   
   G   
   A   
   T   
   A   
   A   
   A   
   T   
   C   
   T   
   T   
   G   
   G   
   G   
   G   
    C    
   G   
   G   
   C   
   T   
   A   
   T   
   T   
   T   
   T   
   G   
   C   
    A    
   A   
   C   
   A   
   C   
   G   
   T   
   G   
   T   
   A   
   A   
   A   
   A   
   G   
   A   
   A   
   G   
   C   
   A   
   A   
   G   
   T   
   C   
   C   
   A   
   G   
   T   
   C   
   A   
   A   
   T   
   C   
   T   
    G    
   C   
   G   
   T   
   A   
   C   
   C   
   G   
   C   
   T   
   T   
   T   
   A   
   G   
   T   
   T   
   A   
   A   
   T   
   T   
   C   
   A   
   G   
   A   
   T   
   A   
   A   
   T   
   A   
   T   
   T   
   T   
   A   
   T   
   T   
   T   
   T   
   G   
   C   
    C    
   C   
   A   
   A   
   C   
   A   
   A   
   A   
   C   
   A   
   T   
   T   
   A   
   C   
   G   
    G    
   A   
   T   
   G   
   G   
   G   
    A    
   G   
   A   
   A   
   G   
   A   
   C   
   A   
   A   
   A   
   T   
   T   
   T   
   A   
   G   
   A   
   G   
   C   
    G    
   G   
   G   
   T   
   C   
   T   
   T   
   A   
   A   
   T   
   A   
   A   
   A   
   T   
   T   
   C   
   A   
   T   
   T   
   T   
   T   
   C   
   G   
   G   
   T   
   G   
   A   
   G   
   G   
   A   
   A   
   C   
   T   
   A   
   G   
   A   
   T   
   T   
   T   
   A   
   C   
   C   
   A   
   A   
   T   
    T    
   G   
   C   
   C   
   A   
   T   
   G   
   A   
   C   
   G   
   C   
   C   
    T    
   G   
   C   
   T   
   C   
   A   
   A   
   A   
   T   
   T   
   T   
   C   
   A   
   A   
   A   
    T    
   G   
   A   
   A   
   G   
   A   
   T   
   A   
   A   
   A   
   T   
   T   
   T   
   A   
   A   
   T   
   T   
   C   
   T   
   G   
   A   
   A   
   A   
   T   
   T   
   C   
   T   
   T   
   T   
   T   
    G    
   G   
   C   
   A   
   G   
   A   
   T   
   A   
   C   
    G    
   G   
   G   
   C   
   T   
   A   
    C    
   G   
   G   
    C    
   C   
   A   
   A   
   G   
   G   
    C    
   C   
   A   
   A   
   T   
   T   
   G   
   T   
   T   
   A   
   A   
   T   
   T   
   T   
   C   
    G    
   C   
   C   
   A   
   A   
   T   
   C   
   C   
   A   
   G   
   C   
   A   
   A   
   G   
   C   
   T   
   A   
   C   
   C   
   A   
   T   
   G   
   T   
   A   
   T   
   A   
   G   
   T   
   G   
   T   
   T   
   T   
   T   
   C   
   C   
   A   
   A   
   A   
   A   
   T   
   A   
   A   
   T   
   G   
   G   
    G    
   A   
   C   
   A   
    T    
   T   
    A    
   G   
   T   
    C    
   T   
   A   
   T   
   C   
   C   
   A   
   A   
   A   
   A   
    T    
   T   
   A   
   G   
   T   
   T   
   T   
   T   
   A   
   G   
   A   
   C   
   A   
   A   
   A   
   G   
   A   
   A   
   A   
   C   
    G    
   A   
   A   
   G   
   A   
   A   
   A   
   A   
   A   
   A   
   G   
   A   
   C   
   A   
   A   
   T   
   G   
   T   
   A   
   A   
   T   
   C   
   A   
   G   
   C   
   G   
   C   
   C   
   A   
   A   
   C   
   G   
   C   
   A   
   G   
   C   
   G   
   A   
   A   
   T   
   A   
   C   
   A   
   A   
   T   
   T   
   G   
   C   
   C   
   A   
   C   
   A   
   G   
   A   
   T   
   C   
   T   
   T   
   T   
   T   
   A   
   G   
   G   
   T   
   A   
   G   
   T   
   G   
   T   
   G   
   G   
   A   
   A   
   G   
   A   
   T   
   C   
   C   
   T   
   T   
   C   
   A   
   G   
   G   
   T   
   T   
   A   
   T   
   G   
   T   
   T   
   T   
   A   
   T   
   A   
   A   
   T   
   A   
   T   
   G   
   T   
   A   
   C   
   A   
   A   
   T   
   C   
   C   
   G   
   A   
   A   
   C   
   T   
   T   
   C   
   T   
   C   
   A   
   T   
   T   
   G   
   G   
   C   
   T   
   G   
   C   
   C   
   A   
   A   
   A   
   A   
   C   
   A   
   G   
   G   
   A   
   A   
   C   
   A   
   G   
   C   
   T   
   G   
   A   
   A   
   A   
   T   
   A   
   A   
   A   
   A   
   G   
   A   
   T   
   A   
   A   
   A   
   C   
   A   
   A   
   G   
   A   
   T   
   A   
   C   
   A   
   G   
   A   
   T   
   G   
   G   
   C   
   A   
   A   
   A   
   G   
   A   
   A   
   A   
   A   
   C   
   A   
   G   
   T   
   T   
   T   
   C   
   C   
   T   
   T   
   C   
   T   
   A   
   A   
   C   
   G   
   T   
   T   
   A   
   G   
   A   
   T   
   C   
   G   
   T   
   A   
   G   
   T   
   A   
   A   
   C   
   A   
   A   
   T   
   A   
   A   
   A   
   T   
   T   
   C   
   T   
   T   
   A   
   A   
   C   
   A   
   A   
   T   
   G   
   A   
   T   
   T   
   A   
   T   
   G   
   G   
   T   
   T   
   G   
   A   
   A   
   A   
   A   
   T   
   T   
   C   
   A   
   G   
   G   
   A   
   G   
   A   
   A   
   A   
   A   
   T   
   G   
   G   
   T   
   T   
   C   
   T   
   G   
   C   
   A   
   A   
   C   
   A   
   G   
   A   
   T   
   A   
   T   
   C   
   A   
   G   
   T   
   A   
   A   
   A   
   C   
   C   
   A   
   T   
   T   
   A   
   A   
   T   
   T   
   G   
   A   
   T   
   T   
   A   
   C   
   T   
   T   
   A   
   G   
   A   
   A   
   G   
   C   
   A   
   A   
   C   
   C   
   A   
   T   
   T   
   A   
   A   
   A   
   -   
   -   
   -   
   -   
   -   
   -   
   -   
   -   
   -   
   -   
   -   
   -   
   -   
   -   
   -   
   -   
   -   
   -   
   -   
   -   
   -   
   -   
   -   
   -   
   -   
   -   
   -   
   -   
   -   
   -   
   -   
   -   
   -   
   -   
   -   
   -   
   -   
   -   
   -   
   -   
   -   
   -   
   -   
   -   
   -   
   -   
   -   
   -   
   -   
   -   
   -   
   -   
   -   
   -   
   -   
   -   
   -   
   -   
   -   
   -   
   -   
   -   
   -   
   -   
   -   
   -   
   -   
   -   
   -   
   -   
   -   
   -   
   -   
   -   
   -   
   -   
   -   
   -   
   -   
   -   
   -   
   -   
   -   
   -   
   -   
   -   
   -   
   -   
   -   
   -   
   -   
   -   
   -   
   -   
   -   
   -   
   -   
   -   
   -   
   -   
   -   
   -   
   -   
   -   
   -   
   -   
   -   
   -   
   -   
   -   
   -   
   -   
   -   
   -   
   -   
   -   
   -   
   -   
   -   
   -   
   -   
   -   
   -   
   -   
   -   
   -   
   -   
   -   
   -   
   -   
   -   
   -   
   -   
   -   
   -   
   -   
   -   
   -   
   -   
   -   
   -   
   -   
   -   
   -   
   -   
   -   
   -   
   -   
   -   
   -   
   -   
   -   
   -   
   -   
   -   
   -   
   -   
   -   
   -   
   -   
   -   
   -   
   -   
   -   
   -   
   -   
   -   
   -   
   -   
   C   
   A   
   A   
   T   
   G   
   G   
   C   
   A   
   A   
   G   
   C   
   C   
   A   
   A   
   A   
   C   
   A   
   A   
   G   
   A   
   A   
   T   
   T   
   A   
   G   
   C   
   C   
   G   
   A   
   A   
   G   
   C   
   G   
   A   
   A   
   G   
   A   
   A   
   A   
   A   
   C   
   A   
   G   
   C   
   T   
   A   
   C   
   T   
   A   
   C   
   A   
   T   
   T   
   T   
   T   
   T   
   A   
   A   
   A   
   C   
   G   
   T   
   A   
   T   
   T   
   G   
   A   
   C   
   A   
   A   
   A   
   A   
   C   
   A   
   G   
   A   
   A   
   A   
   T   
   T   
   T   
   G   
   A   
   T   
   A   
   A   
   G   
   T   
   T   
   A   
   C   
   C   
   G   
   T   
   C   
   C   
   G   
   T   
   T   
   G   
   T   
   T   
   C   
   A   
   A   
   G   
   A   
   A   
   G   
   C   
   T   
   A   
   G   
   C   
   T   
   T   
   A   
   A   
   A   
   G   
   A   
   A   
   A   
   A   
   A   
   T   
   G   
   G   
   C   
   T   
   A   
   T   
   G   
   A   
   T   
   A   
   C   
   T   
   A   
   A   
   A   
   T   
   C   
   C   
   G   
   T   
   T   
   G   
   T   
   T   
   G   
   A   
   A   
   A   
   A   
   A   
   T   
   A   
   C   
   C   
   A   
   A   
   G   
   C   
   A   
   A   
   T   
   T   
   T   
   A   
   T   
   T   
   C   
   A   
   G   
   G   
   G   
   A   
   T   
   T   
   C   
   A   
   A   
   G   
   C   
   A   
   G   
   A   
   A   
   G   
   G   
   A   
   G   
   T   
   C   
   A   
   A   
   A   
   G   
   C   
   T   
   A   
   G   
   T   
   G   
   A   
   T   
   G   
   T   
   T   
   C   
   A   
   A   
   G   
   T   
   C   
   A   
   A   
   A   
   A   
   A   
   A   
   G   
   C   
   G   
   A   
   A   
   A   
   G   
   A   
   C   
   A   
   A   
   T   
   C   
   A   
   A   
   T   
   A   
   C   
   A   
   C   
   A   
   T   
   T   
   T   
   A   
   A   
   C   
   T   
   A   
   T   
   A   
   A   
   A   
   T   
   T   
   A   
   T   
   C   
   G   
   A   
   T   
   G   
   A   
   G   
   T   
   A   
   C   
   C   
   C   
   C   
   T   
   T   
   T   
   A   
   G   
   G   
   C   
   G   
   A   
   A   
   A   
   T   
   G   
   A   
   A   
   A   
   G   
   A   
   T   
   T   
   T   
   G   
   T   
   C   
   T   
   T   
   A   
   T   
   C   
   A   
   A   
   T   
   C   
   A   
   A   
   G   
   T   
   A   
   T   
   C   
   G   
   C   
   C   
   A   
   A   
   A   
   A   
   A   
   A   
   G   
   G   
   C   
   G   
   A   
   T   
   A   
   C   
   C   
   T   
   A   
   C   
   C   
   A   
   A   
   A   
   T   
   C   
   G   
   C   
   T   
   T   
   G   
   G   
   A   
   A   
   G   
   C   
   C   
   G   
   T   
   C   
   T   
   T   
   T   
   A   
   A   
   T   
   T   
   T   
   T   
   T   
   C   
   C   
   A   
   G   
   A   
   T   
   A   
   T   
   G   
   T   
   C   
   A   
   G   
   G   
   A   
   A   
   A   
   T   
   G   
   A   
   T   
   A   
   A   
   A   
   A   
   T   
   T   
   T   
   C   
   G   
   A   
   T   
   T   
   C   
   A   
   A   
   G   
   T   
   A   
   G   
   A   
   T   
   A   
   A   
   T   
   G   
   C   
   C   
   A   
   A   
   A   
   C   
   G   
   T   
   G   
   G   
   A   
   G   
   A   
   A   
   A   
   T   
   T   
   G   
   T   
   C   
   G   
   A   
   T   
   C   
   G   
   T   
   A   
   A   
   T   
   G   
   G   
   T   
   A   
   G   
   T   
   G   
   G   
   G   
   C   
   T   
   A   
   G   
   C   
   A   
   A   
   T   
   T   
   A   
   A   
   C   
   A   
   A   
   A   
   G   
   T   
   G   
   T   
   T   
   T   
   G   
   A   
   C   
   G   
   A   
   A   
   G   
   T   
   G   
   G   
   G   
   C   
   G   
   T   
   A   
   G   
   T   
   G   
   C   
   C   
   T   
   G   
   G   
   C   
   A   
   A   
   A   
   C   
   T   
   C   
   G   
   G   
   T   
   T   
   C   
   T   
   G   
   G   
   C   
   G   
   C   
   A   
   G   
   A   
   A   
   A   
   A   
   A   
   A   
   C   
   A   
   G   
   C   
   C   
   A   
   A   
   T   
   A   
   T   
   C   
   A   
   A   
   A   
   G   
   C   
   T   
   T   
   T   
   T   
   A   
   G   
   T   
   G   
   A   
   T   
   A   
   A   
   G   
   T   
   T   
   C   
   G   
   G   
   C   
   G   
   T   
   T   
   T   
   C   
   T   
   G   
   T   
   T   
   G   
   A   
   T   
   G   
   A   
   A   
   A   
   T   
   C   
   A   
   A   
   T   
   C   
   A   
   A   
   A   
   A   
   G   
   T   
   T   
   A   
   A   
   G   
   C   
   C   
   A   
   A   
   G   
   G   
   G   
   T   
   G   
   G   
   G   
   T   
   C   
   C   
   A   
   A   
   G   
   C   
   A   
   G   
   A   
   C   
   T   
   C   
   C   
   T   
   T   
   T   
   G   
   T   
   A   
   C   
   C   
   A   
   A   
   T   
   C   
   A   
   C   
   A   
   G   
   T   
   C   
   G   
   C   
   T   
   T   
   C   
   T   
   G   
   A   
   A   
   C   
   C   
   A   
   G   
   T   
   G   
   A   
   C   
   A   
   G   
   A   
   A   
   T   
   T   
   A   
   C   
   C   
   A   
   A   
   C   
   A   
   G   
   G   
   G   
   G   
   C   
   T   
   G   
   C   
   G   
   A   
   C   
   A   
   A   
   A   
   A   
   G   
   A   
   T   
   A   
   C   
   A   
   G   
   A   
   G   
   T   
   C   
   A   
   C   
   G   
   T   
   T   
   A   
   T   
   T   
   A   
   T   
   C   
   C   
   G   
   C   
   T   
   G   
   G   
   G   
   G   
   G   
   A   
   A   
   G   
   C   
   A   
   G   
   C   
   T   
   G   
   C   
   G   
   C   
   A   
   A   
   T   
   T   
   A   
   A   
   T   
   C   
   G   
   G   
   G   
   T   
   A   
   T   
   A   
   C   
   A   
   G   
   G   
   C   
   A   
   C   
   C   
   A   
   T   
   T   
   A   
   C   
   T   
   G   
   C   
   A   
   G   
   A   
   A   
   G   
   A   
   T   
   A   
   T   
   T   
   A   
   A   
   A   
   A   
   A   
   A   
   A   
   A   
   T   
   C   
   C   
   A   
   G   
   A   
   G   
   T   
   T   
   A   
   A   
   G   
   T   
   A   
   G   
   T   
   A   
   C   
   G   
   G   
   G   
   C   
   G   
   T   
   A   
   A   
   T   
   T   
   G   
   G   
   T   
   A   
   A   
   A   
   A   
   C   
   T   
   G   
   G   
   C   
   T   
   T   
   A   
   G   
   A   
   A   
   C   
   G   
   A   
   G   
   C   
   G   
   T   
   T   
   T   
   G   
   A   
   T   
   A   
   A   
   A   
   G   
   A   
   A   
   T   
   T   
   A   
   A   
   G   
   A   
   G   
   G   
   G   
   C   
   A   
   A   
   G   
   A   
   T   
   G   
   G   
   T   
   G   
   G   
   C   
   T   
   C   
   A   
   C   
   T   
   A   
   G   
   T   
   T   
   A   
   T   
   T   
   T   
   T   
   A   
   G   
   A   
   T   
   G   
   A   
   C   
   A   
   A   
   A   
   G   
   A   
   G   
   A   
   A   
   T   
   G   
   T   
   C   
   A   
   A   
   A   
   A   
   A   
   A   
   G   
   C   
   T   
   T   
   T   
   A   
   C   
   A   
   A   
   A   
   C   
   C   
   A   
   A   
   A   
   G   
   A   
   G   
   A   
   A   
   A   
   A   
   A   
   A   
   G   
   A   
   T   
   G   
   G   
   T   
   C   
   A   
   A   
   A   
   C   
   G   
   A   
   T   
   T   
   A   
   A   
   A   
   T   
   T   
   A   
   A   
   C   
   G   
   A   
   T   
   T   
   G   
   A   
   C   
   A   
   G   
   T   
   G   
   G   
   C   
   G   
   T   
   A   
   C   
   A   
   A   
   C   
   A   
   A   
   C   
   A   
   A   
   G   
   C   
   C   
   T   
   T   
   C   
   G   
   C   
   T   
   A   
   T   
   A   
   T   
   T   
   T   
   G   
   A   
   C   
   A   
   A   
   A   
   C   
   G   
   C   
   C   
   C   
   T   
   G   
   G   
   T   
   A   
   G   
   T   
   G   
   C   
   G   
   G   
   T   
   C   
   A   
   T   
   T   
   A   
   C   
   C   
   G   
   A   
   T   
   C   
   C   
   T   
   C   
   A   
   A   
   A   
   A   
   A   
   G   
   G   
   C   
   G   
   A   
   T   
   T   
   T   
   A   
   T   
   T   
   A   
   G   
   C   
   A   
   A   
   C   
   G   
   G   
   T   
   T   
   A   
   G   
   T   
   T   
   C   
   A   
   C   
   C   
   T   
   T   
   C   
   G   
   T   
   A   
   T   
   G   
   A   
   T   
   C   
   C   
   T   
   A   
   A   
   T   
   A   
   A   
   A   
   A   
   T   
   G   
   G   
   C   
   C   
   A   
   A   
   T   
   G   
   G   
   T   
   A   
   T   
   T   
   T   
   C   
   T   
   C   
   A   
   A   
   A   
   A   
   A   
   G   
   A   
   A   
   T   
   A   
   T   
   G   
   A   
   T   
   G   
   C   
   T   
   T   
   A   
   C   
   A   
   A   
   T   
   A   
   A   
   C   
   A   
   A   
   C   
   A   
   A   
   A   
   G   
   A   
   T   
   T   
   T   
   A   
   C   
   C   
   A   
   T   
   T   
   C   
   A   
   C   
   A   
   G   
   C   
   A   
   C   
   G   
   T   
   T   
   T   
   T   
   G   
   C   
   G   
   A   
   C   
   A   
   G   
   G   
   C   
   T   
   A   
   T   
   G   
   C   
   A   
   C   
   C   
   A   
   G   
   G   
   T   
   T   
   C   
   T   
   A   
   C   
   C   
   T   
   T   
   T   
   A   
   A   
   A   
   A   
   C   
   A   
   A   
   T   
   T   
   A   
   C   
   C   
   G   
   G   
   T   
   G   
   C   
   C   
   A   
   T   
   T   
   G   
   G   
   T   
   T   
   T   
   A   
   G   
   A   
   T   
   G   
   C   
   A   
   G   
   G   
   G   
   A   
   C   
   A   
   T   
   T   
   G   
   A   
   A   
   G   
   C   
   C   
   A   
   G   
   A   
   T   
   G   
   A   
   A   
   G   
   A   
   G   
   C   
   T   
   A   
   G   
   A   
   A   
   A   
   T   
   T   
   A   
   A   
   T   
   G   
   G   
   C   
   T   
   T   
   G   
   A   
   A   
   A   
   T   
   G   
   G   
   C   
   A   
   A   
   A   
   A   
   A   
   G   
   A   
   T   
   A   
   A   
   A   
   T   
   C   
   T   
   T   
   G   
   G   
   G   
   G   
   C   
   G   
   G   
   C   
   T   
   A   
   T   
   T   
   T   
   T   
   G   
   C   
   A   
   A   
   C   
   A   
   C   
   G   
   T   
   G   
   T   
   A   
   A   
   A   
   A   
   G   
   A   
   A   
   G   
   C   
   A   
   A   
   G   
   T   
   C   
   C   
   A   
   G   
   T   
   C   
   A   
   A   
   T   
   C   
   T   
   G   
   C   
   G   
   T   
   A   
   C   
   C   
   G   
   C   
   T   
   T   
   T   
   A   
   G   
   T   
   T   
   A   
   A   
   T   
   T   
   C   
   A   
   G   
   A   
   T   
   A   
   A   
   T   
   A   
   T   
   T   
   T   
   A   
   T   
   T   
   T   
   T   
   G   
   C   
   C   
   C   
   A   
   A   
   C   
   A   
   A   
   A   
   C   
   A   
   T   
   T   
   A   
   C   
   G   
   G   
   A   
   T   
   G   
   G   
   G   
   A   
   G   
   A   
   A   
   G   
   A   
   C   
   A   
   A   
   A   
   T   
   T   
   T   
   A   
   G   
   A   
   G   
   C   
   G   
   G   
   G   
   T   
   C   
   T   
   T   
   A   
   A   
   T   
   A   
   A   
   A   
   T   
   T   
   C   
   A   
   T   
   T   
   T   
   T   
   C   
   G   
   G   
   T   
   G   
   A   
   G   
   G   
   A   
   A   
   C   
   T   
   A   
   G   
   A   
   T   
   T   
   T   
   A   
   C   
   C   
   A   
   A   
   T   
   T   
   G   
   C   
   C   
   A   
   T   
   G   
   A   
   C   
   G   
   C   
   C   
   T   
   G   
   C   
   T   
   C   
   A   
   A   
   A   
   T   
   T   
   T   
   C   
   A   
   A   
   A   
   T   
   G   
   A   
   A   
   G   
   A   
   T   
   A   
   A   
   A   
   T   
   T   
   T   
   A   
   A   
   T   
   T   
   C   
   T   
   G   
   A   
   A   
   A   
   T   
   T   
   C   
   T   
   T   
   T   
   T   
   G   
   G   
   C   
   A   
   G   
   A   
   T   
   A   
   C   
   G   
   G   
   G   
   C   
   T   
   A   
   C   
   G   
   G   
   C   
   C   
   A   
   A   
   G   
   G   
   C   
   C   
   A   
   A   
   T   
   T   
   G   
   T   
   T   
   A   
   A   
   T   
   T   
   T   
   C   
   G   
   C   
   C   
   A   
   A   
   T   
   C   
   C   
   A   
   G   
   C   
   A   
   A   
   G   
   C   
   T   
   A   
   C   
   C   
   A   
   T   
   G   
   T   
   A   
   T   
   A   
   G   
   T   
   G   
   T   
   T   
   T   
   T   
   C   
   C   
   A   
   A   
   A   
   A   
   T   
   A   
   A   
   T   
   G   
   G   
   G   
   A   
   C   
   A   
   T   
   T   
   A   
   G   
   T   
   C   
   T   
   A   
   T   
   C   
   C   
   A   
   A   
   A   
   A   
   T   
   T   
   A   
   G   
   T   
   T   
   T   
   T   
   A   
   G   
   A   
   C   
   A   
   A   
   A   
   G   
   A   
   A   
   A   
   C   
   G   
   A   
   A   
   G   
   A   
   A   
   A   
   A   
   A   
   A   
   G   
   A   
   C   
   A   
   A   
   T   
   G   
   T   
   A   
   A   
   T   
   C   
   A   
   G   
   C   
   G   
   C   
   C   
   A   
   A   
   C   
   G   
   C   
   A   
   G   
   C   
   G   
   A   
   A   
   T   
   A   
   C   
   A   
   A   
   T   
   T   
   G   
   C   
   C   
   A   
   C   
   A   
   G   
   A   
   T   
   C   
   T   
   T   
   T   
   T   
   A   
   G   
   G   
   T   
   A   
   G   
   T   
   G   
   T   
   G   
   G   
   A   
   A   
   G   
   A   
   T   
   C   
   C   
   T   
   T   
   C   
   A   
   G   
   G   
   T   
   T   
   A   
   T   
   G   
   T   
   T   
   T   
   A   
   T   
   A   
   A   
   T   
   A   
   T   
   G   
   T   
   A   
   C   
   A   
   A   
   T   
   C   
   C   
   G   
   A   
   A   
   C   
   T   
   T   
   C   
   T   
   C   
   A   
   T   
   T   
   G   
   G   
   C   
   T   
   G   
   C   
   C   
   A   
   A   
   A   
   A   
   C   
   A   
   G   
   G   
   A   
   A   
   C   
   A   
   G   
   C   
   T   
   G   
   A   
   A   
   A   
   T   
   A   
   A   
   A   
   A   
   G   
   A   
   T   
   A   
   A   
   A   
   C   
   A   
   A   
   G   
   A   
   T   
   A   
   C   
   A   
   G   
   A   
   T   
   G   
   G   
   C   
   A   
   A   
   A   
   G   
   A   
   A   
   A   
   A   
   C   
   A   
   G   
   T   
   T   
   T   
   C   
   C   
   T   
   T   
   C   
   T   
   A   
   A   
   C   
   G   
   T   
   T   
   A   
   G   
   A   
   T   
   C   
   G   
   T   
   A   
   G   
   T   
   A   
   A   
   C   
   A   
   A   
   T   
   A   
   A   
   A   
   T   
   T   
   C   
   T   
   T   
   A   
   A   
   C   
   A   
   A   
   T   
   G   
   A   
   T   
   T   
   A   
   T   
   G   
   G   
   T   
   T   
   G   
   A   
   A   
   A   
   A   
   T   
   T   
   C   
   A   
   G   
   G   
   A   
   G   
   A   
   A   
   A   
   A   
   T   
   G   
   G   
   T   
   T   
   C   
   T   
   G   
   C   
   A   
   A   
   C   
   A   
   G   
   A   
   T   
   A   
   T   
   C   
   A   
   G   
   T   
   A   
   A   
   A   
   C   
   C   
   A   
   T   
   T   
   A   
   A   
   T   
   T   
   G   
   A   
   T   
   T   
   A   
   C   
   T   
   T   
   A   
   G   
   A   
   A   
   G   
   C   
   A   
   A   
   C   
   C   
   A   
   T   
   T   
   A   
   A   
   A   
   -   
   -   
   -   
   -   
   -   
   -   
   -   
   -   
   -   
   -   
   -   
   -   
   -   
   -   
   -   
   -   
   -   
   -   
   -   
   -   
   -   
   -   
   -   
   -   
   -   
   -   
   -   
   -   
   -   
   -   
   -   
   -   
   -   
   -   
   -   
   -   
   -   
   -   
   -   
   -   
   -   
   -   
   -   
   -   
   -   
   -   
   -   
   -   
   -   
   -   
   -   
   -   
   -   
   -   
   -   
   -   
   -   
   -   
   -   
   -   
   -   
 

 
   N59409F   
   -   
   -   
   -   
   -   
   -   
   -   
   -   
   -   
   -   
   -   
   -   
   -   
   -   
   -   
   -   
   -   
   -   
   -   
   -   
   -   
   -   
   -   
   -   
   -   
   -   
   -   
   -   
   -   
   -   
   -   
   -   
   -   
   -   
   -   
   -   
   -   
   -   
   -   
   -   
   -   
   -   
   -   
   -   
   -   
   -   
   -   
   -   
   -   
   -   
   -   
   -   
   -   
   -   
   -   
   -   
   -   
   -   
   -   
   -   
   -   
   -   
   -   
   -   
   -   
   -   
   -   
   -   
   -   
   -   
   -   
   -   
   -   
   -   
   -   
   -   
   -   
   -   
   -   
   -   
   -   
   -   
   -   
   -   
   -   
   -   
   -   
   -   
   -   
   -   
   -   
   -   
   -   
   -   
   -   
   -   
   -   
   -   
   -   
   -   
   -   
   -   
   -   
   -   
   -   
   -   
   -   
   -   
   -   
   C   
   A   
   A   
   T   
   G   
   G   
   C   
   A   
   A   
   G   
   C   
   C   
   A   
   A   
   A   
   C   
   A   
   A   
   G   
   A   
    G    
   T   
   T   
   A   
   G   
   C   
    C    
   G   
   A   
   A   
   G   
   C   
   G   
   A   
   A   
   G   
   A   
   A   
   A   
   A   
    C    
   A   
   G   
   C   
    T    
   A   
    C    
   T   
    A    
    C    
   A   
   T   
   T   
   T   
   T   
   T   
   A   
   A   
   A   
    C    
   G   
   T   
   A   
   T   
   T   
   G   
    T    
   C   
   A   
   A   
   A   
   A   
   C   
   A   
   G   
    G    
   A   
   A   
   T   
   T   
   T   
   G   
   A   
   T   
   A   
   A   
   G   
   T   
   T   
   A   
   C   
   C   
    G    
   T   
   C   
   C   
   G   
   T   
   T   
    G    
   T   
   T   
   C   
   A   
   A   
   G   
   A   
   A   
    G    
   C   
   T   
   A   
   G   
   C   
   T   
   T   
   A   
   A   
   A   
   G   
   A   
   A   
   A   
   A   
   A   
   T   
   G   
   G   
    C    
   T   
   A   
   T   
   G   
   A   
   T   
   A   
   C   
   T   
   A   
   A   
   A   
   T   
   C   
    T    
   G   
   T   
   T   
   G   
   T   
   T   
   G   
   A   
   A   
   A   
   A   
   A   
   T   
   A   
   C   
   C   
   A   
   A   
   G   
   C   
   A   
   A   
   T   
   T   
   T   
   A   
   T   
   T   
   C   
   A   
   G   
   G   
   G   
   A   
   T   
   T   
   C   
   A   
   A   
   G   
   C   
    A    
   G   
   A   
   A   
    G    
   G   
    A    
   G   
   T   
   C   
   A   
   A   
    A    
   G   
   C   
   T   
   A   
   G   
   T   
   G   
   A   
   T   
   G   
   T   
    T    
   C   
   A   
   A   
   G   
   T   
    C    
   A   
   A   
   A   
   A   
   A   
    G    
   G   
    C    
   G   
   A   
   A   
   A   
   G   
   A   
    C    
   A   
   A   
    T    
   C   
   A   
   A   
   T   
   A   
   C   
   A   
   C   
   A   
   T   
   T   
   T   
   A   
    C    
   C   
   T   
   A   
   T   
   A   
   A   
   A   
   T   
   T   
   A   
   T   
   C   
   G   
   A   
   T   
   G   
   A   
   G   
    C    
   A   
   C   
    T    
   C   
   C   
   T   
   T   
   T   
   A   
   G   
   G   
   C   
   G   
   A   
   A   
   A   
   T   
   G   
   A   
   A   
   A   
   G   
   A   
   T   
    T    
   T   
   G   
   T   
   C   
   T   
   T   
   A   
   T   
   C   
   A   
   A   
   T   
   C   
   A   
   A   
   G   
   T   
   A   
   T   
    C    
   G   
    C    
   C   
   A   
   A   
    G    
   A   
   A   
   A   
   G   
   G   
    C    
   G   
   A   
   T   
   A   
    T    
   C   
   T   
   A   
   C   
   C   
   A   
   A   
   A   
   T   
    C    
    G    
   C   
   T   
   T   
   G   
   G   
    A    
    A    
    G    
   C   
   C   
    G    
   T   
   C   
   T   
   T   
   T   
   A   
   A   
   T   
   T   
   T   
   T   
   T   
   C   
   C   
   A   
   G   
   A   
   T   
   A   
   T   
   G   
   T   
   C   
   A   
   G   
   G   
   A   
   A   
   A   
   T   
   G   
   A   
   T   
   A   
   A   
   A   
    A    
   T   
   T   
   T   
   C   
   G   
   A   
   T   
   T   
   C   
   A   
   A   
   G   
   T   
   A   
   G   
   A   
   T   
   A   
   A   
   T   
   G   
   C   
   C   
   A   
   A   
   A   
   C   
   G   
   T   
   G   
   G   
   A   
   G   
   A   
   A   
   A   
   T   
   T   
   G   
   T   
   C   
   G   
   A   
   T   
   C   
   G   
   T   
   A   
   A   
    T    
   G   
   G   
   T   
   A   
   G   
   T   
   G   
   G   
   G   
   C   
   T   
   A   
   G   
    C    
   A   
   A   
   T   
   T   
   A   
   A   
   C   
   A   
   A   
   A   
   G   
   T   
   G   
   T   
   T   
   T   
   G   
   A   
    C    
   G   
   A   
   A   
   G   
   T   
   G   
   G   
   G   
   C   
   G   
   T   
   A   
   G   
   T   
    G    
   C   
   C   
   T   
    G    
   G   
   C   
   A   
   A   
   A   
   C   
   T   
    T    
   G   
   G   
   T   
   T   
   C   
   T   
   G   
   G   
   C   
   G   
   C   
   A   
   G   
   A   
   A   
   A   
   A   
   A   
    A    
   C   
   A   
   G   
   C   
    C    
   A   
   A   
   T   
   A   
   T   
   C   
   A   
   A   
   A   
   G   
   C   
   T   
   T   
   T   
   T   
   A   
   G   
   T   
   G   
   A   
   T   
   A   
   A   
    G    
   T   
   T   
    C    
   G   
   G   
   C   
   G   
   T   
   T   
   T   
   C   
   T   
    G    
   T   
   T   
   G   
   A   
   T   
   G   
   A   
   A   
   A   
   T   
   C   
   A   
   A   
   T   
   C   
    A    
   A   
   A   
   A   
    G    
   T   
   T   
   A   
   A   
   G   
   C   
   C   
   A   
   A   
   G   
   G   
    A    
   T   
   G   
   G   
   G   
   T   
   C   
   C   
   A   
   A   
   G   
   C   
   A   
   G   
   A   
   C   
   T   
   C   
    C    
   T   
   T   
   T   
   G   
   T   
   A   
   C   
   C   
   A   
   A   
   T   
   C   
   A   
   C   
   A   
   G   
   T   
    C    
   G   
   C   
   T   
   T   
   C   
   T   
   G   
   A   
   A   
   C   
   C   
   A   
   G   
   T   
   G   
   A   
   C   
   A   
   G   
   A   
   A   
   T   
   T   
   A   
   C   
   C   
   A   
   A   
   C   
   A   
   G   
   G   
   G   
   G   
   C   
   T   
   G   
   C   
   G   
   A   
   C   
   A   
   A   
   A   
   A   
   G   
    A    
    T    
   A   
   C   
   A   
   G   
   A   
   G   
   T   
   C   
   A   
   C   
   G   
   T   
   T   
   A   
   T   
   T   
   A   
   T   
   C   
   C   
    G    
    C    
   T   
   G   
   G   
   G   
   G   
   G   
   A   
   A   
   G   
   C   
   A   
   G   
   C   
   T   
   G   
   C   
   G   
   C   
   A   
   A   
   T   
   T   
   A   
   A   
   T   
   C   
   G   
   G   
    G    
   T   
   A   
   T   
   A   
   C   
    G    
   G   
   G   
    C    
   A   
   C   
    C    
   A   
   T   
   T   
   A   
   C   
   T   
   G   
   C   
   A   
   G   
   A   
    A    
   G   
   A   
   T   
   A   
   T   
   T   
    G    
   A   
   A   
   A   
   A   
   A   
   A   
   A   
   T   
   C   
   C   
   A   
   G   
   A   
   G   
   T   
   T   
   A   
   A   
   G   
   T   
   A   
   G   
   T   
   A   
   C   
   G   
   G   
   G   
    C    
   G   
   T   
   A   
   A   
   T   
   T   
   G   
   G   
    T    
   A   
   A   
   A   
   A   
   C   
   T   
   G   
   G   
    C    
   T   
   T   
   A   
   G   
   A   
   A   
   C   
   G   
   A   
   G   
   C   
   G   
   T   
   T   
   T   
   G   
   A   
   T   
   A   
   A   
   A   
   G   
   A   
    A    
   T   
   T   
   A   
   A   
   G   
   A   
   G   
   G   
    G    
   C   
   A   
   A   
   G   
   A   
   T   
   G   
   G   
    T    
   G   
   G   
    C    
   T   
   C   
    A    
   C   
   T   
   A   
   G   
   T   
   T   
   A   
   T   
   T   
   T   
   T   
   A   
   G   
   A   
   T   
   G   
   A   
   C   
   A   
   A   
   A   
   G   
   A   
   G   
   A   
   A   
   T   
   G   
   T   
   C   
   A   
   A   
   A   
   A   
   A   
   A   
   G   
   C   
   T   
   T   
   T   
   A   
   C   
   A   
   A   
   A   
   C   
   C   
   A   
   A   
   A   
   G   
   A   
    G    
   A   
   A   
   A   
   A   
   A   
   A   
   G   
   A   
   T   
   G   
   G   
   T   
   C   
   A   
   A   
   A   
   C   
    G    
   A   
   T   
   T   
   A   
   A   
   A   
   T   
   T   
   A   
   A   
   C   
   G   
   A   
   T   
   T   
   G   
   A   
    C    
   A   
   G   
    T    
   G   
   G   
    C    
   G   
   T   
   A   
   C   
   A   
   A   
   C   
   A   
   A   
   C   
   A   
   A   
   G   
   C   
    C    
   T   
   T   
    C    
   G   
   C   
   T   
   A   
   T   
   A   
   T   
   T   
   T   
   G   
   A   
   C   
   A   
   A   
   A   
   C   
   G   
    T    
    C    
   C   
   T   
   G   
   G   
   T   
   A   
   G   
   T   
   G   
   C   
   G   
   G   
   T   
   C   
   A   
   T   
   T   
   A   
   C   
   C   
   G   
   A   
   T   
   C   
   C   
   T   
   C   
   A   
   A   
   A   
   A   
   A   
   G   
   G   
   C   
   G   
   A   
   T   
   T   
   T   
   A   
    T    
   T   
   A   
   G   
   C   
    A    
   A   
   C   
   G   
   G   
   T   
   T   
   A   
   G   
   T   
   T   
   C   
   A   
   C   
   C   
   T   
   T   
   C   
   G   
   T   
   A   
   T   
   G   
   A   
   T   
   C   
   C   
   T   
   A   
   A   
   T   
   A   
   A   
   A   
   A   
   T   
   G   
   G   
   C   
   C   
   A   
   A   
   T   
   G   
   G   
    T    
   A   
   T   
   T   
   T   
   C   
   T   
   C   
   A   
   A   
   A   
   A   
   A   
   G   
   A   
    A    
   T   
   A   
   T   
   G   
   A   
   T   
   G   
   C   
   T   
   T   
   A   
   C   
   A   
   A   
   T   
   A   
   A   
   C   
   A   
   A   
   C   
   A   
   A   
   A   
   G   
   A   
   T   
   T   
   T   
   A   
   C   
   C   
   A   
   T   
   T   
    C    
   A   
   C   
   A   
   G   
   C   
   A   
   C   
   G   
   T   
   T   
   T   
    T    
   G   
   C   
   G   
   A   
   C   
   A   
   G   
   G   
    C    
   T   
   A   
   T   
   G   
   C   
   A   
   C   
   C   
   A   
   G   
   G   
    C    
   T   
   C   
   T   
   A   
   C   
   C   
   T   
   T   
   T   
   A   
   A   
   A   
   A   
   C   
   A   
   A   
   T   
   T   
   A   
   C   
   C   
   G   
   G   
   T   
   G   
   C   
   C   
   A   
   T   
   T   
   G   
   G   
   T   
   T   
   T   
   A   
   G   
   A   
   T   
    G    
   C   
   A   
   G   
   G   
   G   
   A   
   C   
   A   
   T   
   T   
   G   
   A   
   A   
   G   
   C   
   C   
   A   
   G   
   A   
   T   
   G   
   A   
   A   
   G   
   A   
   G   
   C   
   T   
   A   
   G   
   A   
   A   
   A   
   T   
   T   
   A   
   A   
   T   
   G   
   G   
    C    
   T   
   T   
   G   
   A   
   A   
   A   
   T   
   G   
   G   
   C   
   A   
   A   
   A   
   A   
   A   
   G   
   A   
   T   
   A   
   A   
   A   
   T   
   C   
   T   
   T   
   G   
   G   
   G   
   G   
    C    
   G   
   G   
   C   
   T   
   A   
   T   
   T   
   T   
   T   
   G   
   C   
    G    
   A   
   C   
   A   
   C   
   G   
   T   
   G   
   T   
   A   
   A   
   A   
   A   
   G   
   A   
   A   
   G   
   C   
   A   
   A   
   G   
   T   
   C   
   C   
   A   
   G   
   T   
   C   
   A   
   A   
   T   
   C   
   T   
    G    
   C   
   G   
   T   
   A   
   C   
   C   
   G   
   C   
   T   
   T   
   T   
   A   
   G   
   T   
   T   
   A   
   A   
   T   
   T   
   C   
   A   
   G   
   A   
   T   
   A   
   A   
   T   
   A   
   T   
   T   
   T   
   A   
   T   
   T   
   T   
   T   
   G   
   C   
    C    
   C   
   A   
   A   
   C   
   A   
   A   
   A   
   C   
   A   
   T   
   T   
   A   
   C   
   G   
    G    
   A   
   T   
   G   
   G   
   G   
    A    
   G   
   A   
   A   
   G   
   A   
   C   
   A   
   A   
   A   
   T   
   T   
   T   
   A   
   G   
   A   
   G   
   C   
    G    
   G   
   G   
   T   
   C   
   T   
   T   
   A   
   A   
   T   
   A   
   A   
   A   
   T   
   T   
   C   
   A   
   T   
   T   
   T   
   T   
   C   
   G   
   G   
   T   
   G   
   A   
   G   
   G   
   A   
   A   
   C   
   T   
   A   
   G   
   A   
   T   
   T   
   T   
   A   
   C   
   C   
   A   
   A   
   T   
    T    
   G   
   C   
   C   
   A   
   T   
   G   
   A   
   C   
   G   
   C   
   C   
    T    
   G   
   C   
   T   
   C   
   A   
   A   
   A   
   T   
   T   
   T   
   C   
   A   
   A   
   A   
    T    
   G   
   A   
   A   
   G   
   A   
   T   
   A   
   A   
   A   
   T   
   T   
   T   
   A   
   A   
   T   
   T   
   C   
   T   
   G   
   A   
   A   
   A   
   T   
   T   
   C   
   T   
   T   
   T   
   T   
    A    
   G   
   C   
   A   
   G   
   A   
   T   
   A   
   C   
    G    
   G   
   G   
   C   
   T   
   A   
    C    
   G   
   G   
    T    
   C   
   A   
   A   
   G   
   G   
    C    
   C   
   A   
   A   
   T   
   T   
   G   
   T   
   T   
   A   
   A   
   T   
   T   
   T   
   C   
    A    
   C   
   C   
   A   
   A   
   T   
   C   
   C   
   A   
   G   
   C   
   A   
   A   
   G   
   C   
   T   
   A   
   C   
   C   
   A   
   T   
   G   
   T   
   A   
   T   
   A   
   G   
   T   
   G   
   T   
   T   
   T   
   T   
   C   
   C   
   A   
   A   
   A   
   A   
   T   
   A   
   A   
   T   
   G   
   G   
    A    
   A   
   C   
   A   
    T    
   T   
    A    
   G   
   T   
    C    
   T   
   A   
   T   
   C   
   C   
   A   
   A   
   A   
   A   
    T    
   T   
   A   
   G   
   T   
   T   
   T   
   T   
   A   
   G   
   A   
   C   
   A   
   A   
   A   
   G   
   A   
   A   
   A   
   C   
    G    
   A   
   A   
   G   
   A   
   A   
   A   
   A   
   A   
   A   
   G   
   A   
   C   
   A   
   A   
   T   
   G   
   T   
   A   
   A   
   T   
   C   
   A   
   G   
   C   
   G   
   C   
   C   
   A   
   A   
   C   
   G   
   C   
   A   
   G   
   C   
   G   
   A   
   A   
   T   
   A   
   C   
   A   
   A   
   T   
   T   
   G   
   C   
   C   
   A   
   C   
   A   
   G   
   A   
   T   
   C   
   T   
   T   
   T   
   T   
   A   
   G   
   G   
   T   
   A   
   G   
   T   
   G   
   T   
   G   
   G   
   A   
   A   
   G   
   A   
   T   
   C   
   C   
   T   
   T   
   C   
   A   
   G   
   G   
   T   
   T   
   A   
   T   
   G   
   T   
   T   
   T   
   A   
   T   
   A   
   A   
   T   
   A   
   T   
   G   
   T   
   A   
   C   
   A   
   A   
   T   
   C   
   C   
   G   
   A   
   A   
   C   
   T   
   T   
   C   
   T   
   C   
   A   
   T   
   T   
   G   
   G   
   C   
   T   
   G   
   C   
   T   
   A   
   A   
   A   
   A   
   C   
   A   
   G   
   G   
   A   
   A   
   C   
   A   
   G   
   C   
   T   
   G   
   A   
   A   
   A   
   T   
   A   
   A   
   A   
   A   
   G   
   A   
   T   
   A   
   A   
   A   
   C   
   A   
   A   
   G   
   A   
   T   
   A   
   C   
   A   
   G   
   A   
   T   
   G   
   G   
   C   
   A   
   A   
   A   
   G   
   A   
   A   
   A   
   A   
   C   
   A   
   G   
   T   
   T   
   T   
   C   
   C   
   T   
   T   
   C   
   T   
   A   
   A   
   C   
   G   
   T   
   T   
   A   
   G   
   A   
   T   
   C   
   G   
   T   
   A   
   G   
   T   
   A   
   A   
   C   
   A   
   A   
   T   
   A   
   A   
   A   
   T   
   T   
   C   
   T   
   T   
   A   
   A   
   C   
   A   
   A   
   T   
   G   
   A   
   T   
   T   
   A   
   T   
   G   
   G   
   T   
   T   
   G   
   A   
   A   
   A   
   A   
   T   
   T   
   C   
   A   
   G   
   G   
   A   
   G   
   A   
   A   
   A   
   A   
   T   
   G   
   G   
   T   
   T   
   C   
   T   
   G   
   C   
   A   
   A   
   C   
   A   
   G   
   A   
   T   
   A   
   T   
   C   
   A   
   G   
   T   
   A   
   A   
   A   
   C   
   C   
   A   
   T   
   T   
   A   
   A   
   T   
   T   
   G   
   A   
   T   
   T   
   A   
   C   
   T   
   T   
   A   
   G   
   A   
   A   
   G   
   C   
   A   
   A   
   C   
   C   
   A   
   T   
   T   
   A   
   A   
   A   
   -   
   -   
   -   
   -   
   -   
   -   
   -   
   -   
   -   
   -   
   -   
   -   
   -   
   -   
   -   
   -   
   -   
   -   
   -   
   -   
   -   
   -   
   -   
   -   
   -   
   -   
   -   
   -   
   -   
   -   
   -   
   -   
   -   
   -   
   -   
   -   
   -   
   -   
   -   
   -   
   -   
   -   
   -   
   -   
   -   
   -   
   -   
   -   
   -   
   -   
   -   
   -   
   -   
   -   
   -   
   -   
   -   
   -   
   -   
   -   
   -   
   -   
   -   
   -   
   -   
   -   
   -   
   -   
   -   
   -   
   -   
   -   
   -   
   -   
   -   
   -   
   -   
   -   
   -   
   -   
   -   
   -   
   -   
   -   
   -   
   -   
   -   
   -   
   -   
   -   
   -   
   -   
   -   
   -   
   -   
   -   
   -   
   -   
   -   
   -   
   -   
   -   
   -   
   -   
   -   
   -   
   -   
   -   
   -   
   -   
   -   
   -   
   -   
   -   
   -   
   -   
   -   
   -   
   -   
   -   
   -   
   -   
   -   
   -   
   -   
   -   
   -   
   -   
   -   
   -   
   -   
   -   
   -   
   -   
   -   
   -   
   -   
   -   
   -   
   -   
   -   
   -   
   -   
   -   
   -   
   -   
   -   
   -   
   -   
   -   
   -   
   -   
   -   
   -   
   -   
   -   
   -   
   -   
   -   
   -   
   -   
   -   
   -   
   -   
   -   
   -   
   -   
   -   
   -   
   C   
   A   
   A   
   T   
   G   
   G   
   C   
   A   
   A   
   G   
   C   
   C   
   A   
   A   
   A   
   C   
   A   
   A   
   G   
   A   
   G   
   T   
   T   
   A   
   G   
   C   
   C   
   G   
   A   
   A   
   G   
   C   
   G   
   A   
   A   
   G   
   A   
   A   
   A   
   A   
   C   
   A   
   G   
   C   
   T   
   A   
   C   
   T   
   A   
   C   
   A   
   T   
   T   
   T   
   T   
   T   
   A   
   A   
   A   
   C   
   G   
   T   
   A   
   T   
   T   
   G   
   T   
   C   
   A   
   A   
   A   
   A   
   C   
   A   
   G   
   G   
   A   
   A   
   T   
   T   
   T   
   G   
   A   
   T   
   A   
   A   
   G   
   T   
   T   
   A   
   C   
   C   
   G   
   T   
   C   
   C   
   G   
   T   
   T   
   G   
   T   
   T   
   C   
   A   
   A   
   G   
   A   
   A   
   G   
   C   
   T   
   A   
   G   
   C   
   T   
   T   
   A   
   A   
   A   
   G   
   A   
   A   
   A   
   A   
   A   
   T   
   G   
   G   
   C   
   T   
   A   
   T   
   G   
   A   
   T   
   A   
   C   
   T   
   A   
   A   
   A   
   T   
   C   
   T   
   G   
   T   
   T   
   G   
   T   
   T   
   G   
   A   
   A   
   A   
   A   
   A   
   T   
   A   
   C   
   C   
   A   
   A   
   G   
   C   
   A   
   A   
   T   
   T   
   T   
   A   
   T   
   T   
   C   
   A   
   G   
   G   
   G   
   A   
   T   
   T   
   C   
   A   
   A   
   G   
   C   
   A   
   G   
   A   
   A   
   G   
   G   
   A   
   G   
   T   
   C   
   A   
   A   
   A   
   G   
   C   
   T   
   A   
   G   
   T   
   G   
   A   
   T   
   G   
   T   
   T   
   C   
   A   
   A   
   G   
   T   
   C   
   A   
   A   
   A   
   A   
   A   
   G   
   G   
   C   
   G   
   A   
   A   
   A   
   G   
   A   
   C   
   A   
   A   
   T   
   C   
   A   
   A   
   T   
   A   
   C   
   A   
   C   
   A   
   T   
   T   
   T   
   A   
   C   
   C   
   T   
   A   
   T   
   A   
   A   
   A   
   T   
   T   
   A   
   T   
   C   
   G   
   A   
   T   
   G   
   A   
   G   
   C   
   A   
   C   
   T   
   C   
   C   
   T   
   T   
   T   
   A   
   G   
   G   
   C   
   G   
   A   
   A   
   A   
   T   
   G   
   A   
   A   
   A   
   G   
   A   
   T   
   T   
   T   
   G   
   T   
   C   
   T   
   T   
   A   
   T   
   C   
   A   
   A   
   T   
   C   
   A   
   A   
   G   
   T   
   A   
   T   
   C   
   G   
   C   
   C   
   A   
   A   
   G   
   A   
   A   
   A   
   G   
   G   
   C   
   G   
   A   
   T   
   A   
   T   
   C   
   T   
   A   
   C   
   C   
   A   
   A   
   A   
   T   
   C   
   G   
   C   
   T   
   T   
   G   
   G   
   A   
   A   
   G   
   C   
   C   
   G   
   T   
   C   
   T   
   T   
   T   
   A   
   A   
   T   
   T   
   T   
   T   
   T   
   C   
   C   
   A   
   G   
   A   
   T   
   A   
   T   
   G   
   T   
   C   
   A   
   G   
   G   
   A   
   A   
   A   
   T   
   G   
   A   
   T   
   A   
   A   
   A   
   A   
   T   
   T   
   T   
   C   
   G   
   A   
   T   
   T   
   C   
   A   
   A   
   G   
   T   
   A   
   G   
   A   
   T   
   A   
   A   
   T   
   G   
   C   
   C   
   A   
   A   
   A   
   C   
   G   
   T   
   G   
   G   
   A   
   G   
   A   
   A   
   A   
   T   
   T   
   G   
   T   
   C   
   G   
   A   
   T   
   C   
   G   
   T   
   A   
   A   
   T   
   G   
   G   
   T   
   A   
   G   
   T   
   G   
   G   
   G   
   C   
   T   
   A   
   G   
   C   
   A   
   A   
   T   
   T   
   A   
   A   
   C   
   A   
   A   
   A   
   G   
   T   
   G   
   T   
   T   
   T   
   G   
   A   
   C   
   G   
   A   
   A   
   G   
   T   
   G   
   G   
   G   
   C   
   G   
   T   
   A   
   G   
   T   
   G   
   C   
   C   
   T   
   G   
   G   
   C   
   A   
   A   
   A   
   C   
   T   
   T   
   G   
   G   
   T   
   T   
   C   
   T   
   G   
   G   
   C   
   G   
   C   
   A   
   G   
   A   
   A   
   A   
   A   
   A   
   A   
   C   
   A   
   G   
   C   
   C   
   A   
   A   
   T   
   A   
   T   
   C   
   A   
   A   
   A   
   G   
   C   
   T   
   T   
   T   
   T   
   A   
   G   
   T   
   G   
   A   
   T   
   A   
   A   
   G   
   T   
   T   
   C   
   G   
   G   
   C   
   G   
   T   
   T   
   T   
   C   
   T   
   G   
   T   
   T   
   G   
   A   
   T   
   G   
   A   
   A   
   A   
   T   
   C   
   A   
   A   
   T   
   C   
   A   
   A   
   A   
   A   
   G   
   T   
   T   
   A   
   A   
   G   
   C   
   C   
   A   
   A   
   G   
   G   
   A   
   T   
   G   
   G   
   G   
   T   
   C   
   C   
   A   
   A   
   G   
   C   
   A   
   G   
   A   
   C   
   T   
   C   
   C   
   T   
   T   
   T   
   G   
   T   
   A   
   C   
   C   
   A   
   A   
   T   
   C   
   A   
   C   
   A   
   G   
   T   
   C   
   G   
   C   
   T   
   T   
   C   
   T   
   G   
   A   
   A   
   C   
   C   
   A   
   G   
   T   
   G   
   A   
   C   
   A   
   G   
   A   
   A   
   T   
   T   
   A   
   C   
   C   
   A   
   A   
   C   
   A   
   G   
   G   
   G   
   G   
   C   
   T   
   G   
   C   
   G   
   A   
   C   
   A   
   A   
   A   
   A   
   G   
   A   
   T   
   A   
   C   
   A   
   G   
   A   
   G   
   T   
   C   
   A   
   C   
   G   
   T   
   T   
   A   
   T   
   T   
   A   
   T   
   C   
   C   
   G   
   C   
   T   
   G   
   G   
   G   
   G   
   G   
   A   
   A   
   G   
   C   
   A   
   G   
   C   
   T   
   G   
   C   
   G   
   C   
   A   
   A   
   T   
   T   
   A   
   A   
   T   
   C   
   G   
   G   
   G   
   T   
   A   
   T   
   A   
   C   
   G   
   G   
   G   
   C   
   A   
   C   
   C   
   A   
   T   
   T   
   A   
   C   
   T   
   G   
   C   
   A   
   G   
   A   
   A   
   G   
   A   
   T   
   A   
   T   
   T   
   G   
   A   
   A   
   A   
   A   
   A   
   A   
   A   
   T   
   C   
   C   
   A   
   G   
   A   
   G   
   T   
   T   
   A   
   A   
   G   
   T   
   A   
   G   
   T   
   A   
   C   
   G   
   G   
   G   
   C   
   G   
   T   
   A   
   A   
   T   
   T   
   G   
   G   
   T   
   A   
   A   
   A   
   A   
   C   
   T   
   G   
   G   
   C   
   T   
   T   
   A   
   G   
   A   
   A   
   C   
   G   
   A   
   G   
   C   
   G   
   T   
   T   
   T   
   G   
   A   
   T   
   A   
   A   
   A   
   G   
   A   
   A   
   T   
   T   
   A   
   A   
   G   
   A   
   G   
   G   
   G   
   C   
   A   
   A   
   G   
   A   
   T   
   G   
   G   
   T   
   G   
   G   
   C   
   T   
   C   
   A   
   C   
   T   
   A   
   G   
   T   
   T   
   A   
   T   
   T   
   T   
   T   
   A   
   G   
   A   
   T   
   G   
   A   
   C   
   A   
   A   
   A   
   G   
   A   
   G   
   A   
   A   
   T   
   G   
   T   
   C   
   A   
   A   
   A   
   A   
   A   
   A   
   G   
   C   
   T   
   T   
   T   
   A   
   C   
   A   
   A   
   A   
   C   
   C   
   A   
   A   
   A   
   G   
   A   
   G   
   A   
   A   
   A   
   A   
   A   
   A   
   G   
   A   
   T   
   G   
   G   
   T   
   C   
   A   
   A   
   A   
   C   
   G   
   A   
   T   
   T   
   A   
   A   
   A   
   T   
   T   
   A   
   A   
   C   
   G   
   A   
   T   
   T   
   G   
   A   
   C   
   A   
   G   
   T   
   G   
   G   
   C   
   G   
   T   
   A   
   C   
   A   
   A   
   C   
   A   
   A   
   C   
   A   
   A   
   G   
   C   
   C   
   T   
   T   
   C   
   G   
   C   
   T   
   A   
   T   
   A   
   T   
   T   
   T   
   G   
   A   
   C   
   A   
   A   
   A   
   C   
   G   
   T   
   C   
   C   
   T   
   G   
   G   
   T   
   A   
   G   
   T   
   G   
   C   
   G   
   G   
   T   
   C   
   A   
   T   
   T   
   A   
   C   
   C   
   G   
   A   
   T   
   C   
   C   
   T   
   C   
   A   
   A   
   A   
   A   
   A   
   G   
   G   
   C   
   G   
   A   
   T   
   T   
   T   
   A   
   T   
   T   
   A   
   G   
   C   
   A   
   A   
   C   
   G   
   G   
   T   
   T   
   A   
   G   
   T   
   T   
   C   
   A   
   C   
   C   
   T   
   T   
   C   
   G   
   T   
   A   
   T   
   G   
   A   
   T   
   C   
   C   
   T   
   A   
   A   
   T   
   A   
   A   
   A   
   A   
   T   
   G   
   G   
   C   
   C   
   A   
   A   
   T   
   G   
   G   
   T   
   A   
   T   
   T   
   T   
   C   
   T   
   C   
   A   
   A   
   A   
   A   
   A   
   G   
   A   
   A   
   T   
   A   
   T   
   G   
   A   
   T   
   G   
   C   
   T   
   T   
   A   
   C   
   A   
   A   
   T   
   A   
   A   
   C   
   A   
   A   
   C   
   A   
   A   
   A   
   G   
   A   
   T   
   T   
   T   
   A   
   C   
   C   
   A   
   T   
   T   
   C   
   A   
   C   
   A   
   G   
   C   
   A   
   C   
   G   
   T   
   T   
   T   
   T   
   G   
   C   
   G   
   A   
   C   
   A   
   G   
   G   
   C   
   T   
   A   
   T   
   G   
   C   
   A   
   C   
   C   
   A   
   G   
   G   
   C   
   T   
   C   
   T   
   A   
   C   
   C   
   T   
   T   
   T   
   A   
   A   
   A   
   A   
   C   
   A   
   A   
   T   
   T   
   A   
   C   
   C   
   G   
   G   
   T   
   G   
   C   
   C   
   A   
   T   
   T   
   G   
   G   
   T   
   T   
   T   
   A   
   G   
   A   
   T   
   G   
   C   
   A   
   G   
   G   
   G   
   A   
   C   
   A   
   T   
   T   
   G   
   A   
   A   
   G   
   C   
   C   
   A   
   G   
   A   
   T   
   G   
   A   
   A   
   G   
   A   
   G   
   C   
   T   
   A   
   G   
   A   
   A   
   A   
   T   
   T   
   A   
   A   
   T   
   G   
   G   
   C   
   T   
   T   
   G   
   A   
   A   
   A   
   T   
   G   
   G   
   C   
   A   
   A   
   A   
   A   
   A   
   G   
   A   
   T   
   A   
   A   
   A   
   T   
   C   
   T   
   T   
   G   
   G   
   G   
   G   
   C   
   G   
   G   
   C   
   T   
   A   
   T   
   T   
   T   
   T   
   G   
   C   
   G   
   A   
   C   
   A   
   C   
   G   
   T   
   G   
   T   
   A   
   A   
   A   
   A   
   G   
   A   
   A   
   G   
   C   
   A   
   A   
   G   
   T   
   C   
   C   
   A   
   G   
   T   
   C   
   A   
   A   
   T   
   C   
   T   
   G   
   C   
   G   
   T   
   A   
   C   
   C   
   G   
   C   
   T   
   T   
   T   
   A   
   G   
   T   
   T   
   A   
   A   
   T   
   T   
   C   
   A   
   G   
   A   
   T   
   A   
   A   
   T   
   A   
   T   
   T   
   T   
   A   
   T   
   T   
   T   
   T   
   G   
   C   
   C   
   C   
   A   
   A   
   C   
   A   
   A   
   A   
   C   
   A   
   T   
   T   
   A   
   C   
   G   
   G   
   A   
   T   
   G   
   G   
   G   
   A   
   G   
   A   
   A   
   G   
   A   
   C   
   A   
   A   
   A   
   T   
   T   
   T   
   A   
   G   
   A   
   G   
   C   
   G   
   G   
   G   
   T   
   C   
   T   
   T   
   A   
   A   
   T   
   A   
   A   
   A   
   T   
   T   
   C   
   A   
   T   
   T   
   T   
   T   
   C   
   G   
   G   
   T   
   G   
   A   
   G   
   G   
   A   
   A   
   C   
   T   
   A   
   G   
   A   
   T   
   T   
   T   
   A   
   C   
   C   
   A   
   A   
   T   
   T   
   G   
   C   
   C   
   A   
   T   
   G   
   A   
   C   
   G   
   C   
   C   
   T   
   G   
   C   
   T   
   C   
   A   
   A   
   A   
   T   
   T   
   T   
   C   
   A   
   A   
   A   
   T   
   G   
   A   
   A   
   G   
   A   
   T   
   A   
   A   
   A   
   T   
   T   
   T   
   A   
   A   
   T   
   T   
   C   
   T   
   G   
   A   
   A   
   A   
   T   
   T   
   C   
   T   
   T   
   T   
   T   
   A   
   G   
   C   
   A   
   G   
   A   
   T   
   A   
   C   
   G   
   G   
   G   
   C   
   T   
   A   
   C   
   G   
   G   
   T   
   C   
   A   
   A   
   G   
   G   
   C   
   C   
   A   
   A   
   T   
   T   
   G   
   T   
   T   
   A   
   A   
   T   
   T   
   T   
   C   
   A   
   C   
   C   
   A   
   A   
   T   
   C   
   C   
   A   
   G   
   C   
   A   
   A   
   G   
   C   
   T   
   A   
   C   
   C   
   A   
   T   
   G   
   T   
   A   
   T   
   A   
   G   
   T   
   G   
   T   
   T   
   T   
   T   
   C   
   C   
   A   
   A   
   A   
   A   
   T   
   A   
   A   
   T   
   G   
   G   
   A   
   A   
   C   
   A   
   T   
   T   
   A   
   G   
   T   
   C   
   T   
   A   
   T   
   C   
   C   
   A   
   A   
   A   
   A   
   T   
   T   
   A   
   G   
   T   
   T   
   T   
   T   
   A   
   G   
   A   
   C   
   A   
   A   
   A   
   G   
   A   
   A   
   A   
   C   
   G   
   A   
   A   
   G   
   A   
   A   
   A   
   A   
   A   
   A   
   G   
   A   
   C   
   A   
   A   
   T   
   G   
   T   
   A   
   A   
   T   
   C   
   A   
   G   
   C   
   G   
   C   
   C   
   A   
   A   
   C   
   G   
   C   
   A   
   G   
   C   
   G   
   A   
   A   
   T   
   A   
   C   
   A   
   A   
   T   
   T   
   G   
   C   
   C   
   A   
   C   
   A   
   G   
   A   
   T   
   C   
   T   
   T   
   T   
   T   
   A   
   G   
   G   
   T   
   A   
   G   
   T   
   G   
   T   
   G   
   G   
   A   
   A   
   G   
   A   
   T   
   C   
   C   
   T   
   T   
   C   
   A   
   G   
   G   
   T   
   T   
   A   
   T   
   G   
   T   
   T   
   T   
   A   
   T   
   A   
   A   
   T   
   A   
   T   
   G   
   T   
   A   
   C   
   A   
   A   
   T   
   C   
   C   
   G   
   A   
   A   
   C   
   T   
   T   
   C   
   T   
   C   
   A   
   T   
   T   
   G   
   G   
   C   
   T   
   G   
   C   
   T   
   A   
   A   
   A   
   A   
   C   
   A   
   G   
   G   
   A   
   A   
   C   
   A   
   G   
   C   
   T   
   G   
   A   
   A   
   A   
   T   
   A   
   A   
   A   
   A   
   G   
   A   
   T   
   A   
   A   
   A   
   C   
   A   
   A   
   G   
   A   
   T   
   A   
   C   
   A   
   G   
   A   
   T   
   G   
   G   
   C   
   A   
   A   
   A   
   G   
   A   
   A   
   A   
   A   
   C   
   A   
   G   
   T   
   T   
   T   
   C   
   C   
   T   
   T   
   C   
   T   
   A   
   A   
   C   
   G   
   T   
   T   
   A   
   G   
   A   
   T   
   C   
   G   
   T   
   A   
   G   
   T   
   A   
   A   
   C   
   A   
   A   
   T   
   A   
   A   
   A   
   T   
   T   
   C   
   T   
   T   
   A   
   A   
   C   
   A   
   A   
   T   
   G   
   A   
   T   
   T   
   A   
   T   
   G   
   G   
   T   
   T   
   G   
   A   
   A   
   A   
   A   
   T   
   T   
   C   
   A   
   G   
   G   
   A   
   G   
   A   
   A   
   A   
   A   
   T   
   G   
   G   
   T   
   T   
   C   
   T   
   G   
   C   
   A   
   A   
   C   
   A   
   G   
   A   
   T   
   A   
   T   
   C   
   A   
   G   
   T   
   A   
   A   
   A   
   C   
   C   
   A   
   T   
   T   
   A   
   A   
   T   
   T   
   G   
   A   
   T   
   T   
   A   
   C   
   T   
   T   
   A   
   G   
   A   
   A   
   G   
   C   
   A   
   A   
   C   
   C   
   A   
   T   
   T   
   A   
   A   
   A   
   -   
   -   
   -   
   -   
   -   
   -   
   -   
   -   
   -   
   -   
   -   
   -   
   -   
   -   
   -   
   -   
   -   
   -   
   -   
   -   
   -   
   -   
   -   
   -   
   -   
   -   
   -   
   -   
   -   
   -   
   -   
   -   
   -   
   -   
   -   
   -   
   -   
   -   
   -   
   -   
   -   
   -   
   -   
   -   
   -   
   -   
   -   
   -   
   -   
   -   
   -   
   -   
   -   
   -   
   -   
   -   
   -   
   -   
   -   
   -   
   -   
 

 
   N59457F   
   -   
   -   
   -   
   -   
   -   
   -   
   -   
   -   
   -   
   -   
   -   
   -   
   -   
   -   
   -   
   -   
   -   
   -   
   -   
   -   
   -   
   -   
   -   
   -   
   -   
   -   
   -   
   -   
   -   
   -   
   -   
   -   
   -   
   -   
   -   
   -   
   -   
   -   
   -   
   -   
   -   
   -   
   -   
   -   
   -   
   -   
   -   
   -   
   -   
   -   
   -   
   -   
   -   
   -   
   -   
   -   
   -   
   -   
   -   
   -   
   -   
   -   
   -   
   -   
   -   
   -   
   -   
   -   
   -   
   -   
   -   
   -   
   -   
   -   
   -   
   -   
   -   
   -   
   -   
   -   
   -   
   -   
   -   
   -   
   -   
   -   
   -   
   -   
   -   
   -   
   -   
   -   
   -   
   -   
   -   
   -   
   -   
   -   
   -   
   -   
   -   
   -   
   -   
   -   
   -   
   -   
   -   
   -   
   C   
   A   
   A   
   T   
   G   
   G   
   C   
   A   
   A   
   G   
   C   
   C   
   A   
   A   
   A   
   C   
   A   
   A   
   G   
   A   
    A    
   T   
   T   
   A   
   G   
   C   
    C    
   G   
   A   
   A   
   G   
   C   
   G   
   A   
   A   
   G   
   A   
   A   
   A   
   A   
    C    
   A   
   G   
   C   
    T    
   A   
    C    
   T   
    A    
    C    
   A   
   T   
   T   
   T   
   T   
   T   
   A   
   A   
   A   
    C    
   G   
   T   
   A   
   T   
   T   
   G   
    A    
   C   
   A   
   A   
   A   
   A   
   C   
   A   
   G   
    A    
   A   
   A   
   T   
   T   
   T   
   G   
   A   
   T   
   A   
   A   
   G   
   T   
   T   
   A   
   C   
   C   
    G    
   T   
   C   
   C   
   G   
   T   
   T   
    G    
   T   
   T   
   C   
   A   
   A   
   G   
   A   
   A   
    G    
   C   
   T   
   A   
   G   
   C   
   T   
   T   
   A   
   A   
   A   
   G   
   A   
   A   
   A   
   A   
   A   
   T   
   G   
   G   
    C    
   T   
   A   
   T   
   G   
   A   
   T   
   A   
   C   
   T   
   A   
   A   
   A   
   T   
   C   
    T    
   G   
   T   
   T   
   G   
   T   
   T   
   G   
   A   
   A   
   A   
   A   
   A   
   T   
   A   
   C   
   C   
   A   
   A   
   G   
   C   
   A   
   A   
   T   
   T   
   T   
   A   
   T   
   T   
   C   
   A   
   G   
   G   
   G   
   A   
   T   
   T   
   C   
   A   
   A   
   G   
   C   
    A    
   G   
   A   
   A   
    G    
   G   
    A    
   G   
   T   
   C   
   A   
   A   
    A    
   G   
   C   
   T   
   A   
   G   
   T   
   G   
   A   
   T   
   G   
   T   
    T    
   C   
   A   
   A   
   G   
   T   
    C    
   A   
   A   
   A   
   A   
   A   
    A    
   G   
    C    
   G   
   A   
   A   
   A   
   G   
   A   
    C    
   A   
   A   
    T    
   C   
   A   
   A   
   T   
   A   
   C   
   A   
   C   
   A   
   T   
   T   
   T   
   A   
    C    
   C   
   T   
   A   
   T   
   A   
   A   
   A   
   T   
   T   
   A   
   T   
   C   
   G   
   A   
   T   
   G   
   A   
   G   
    T    
   A   
   C   
    C    
   C   
   C   
   T   
   T   
   T   
   A   
   G   
   G   
   C   
   G   
   A   
   A   
   A   
   T   
   G   
   A   
   A   
   A   
   G   
   A   
   T   
    T    
   T   
   G   
   T   
   C   
   T   
   T   
   A   
   T   
   C   
   A   
   A   
   T   
   C   
   A   
   A   
   G   
   T   
   A   
   T   
    C    
   G   
    C    
   C   
   A   
   A   
    A    
   A   
   A   
   A   
   G   
   G   
    C    
   G   
   A   
   T   
   A   
    C    
   C   
   T   
   A   
   C   
   C   
   A   
   A   
   A   
   T   
    C    
    G    
   C   
   T   
   T   
   G   
   G   
    A    
    A    
    G    
   C   
   C   
    G    
   T   
   C   
   T   
   T   
   T   
   A   
   A   
   T   
   T   
   T   
   T   
   T   
   C   
   C   
   A   
   G   
   A   
   T   
   A   
   T   
   G   
   T   
   C   
   A   
   G   
   G   
   A   
   A   
   A   
   T   
   G   
   A   
   T   
   A   
   A   
   A   
    A    
   T   
   T   
   T   
   C   
   G   
   A   
   T   
   T   
   C   
   A   
   A   
   G   
   T   
   A   
   G   
   A   
   T   
   A   
   A   
   T   
   G   
   C   
   C   
   A   
   A   
   A   
   C   
   G   
   T   
   G   
   G   
   A   
   G   
   A   
   A   
   A   
   T   
   T   
   G   
   T   
   C   
   G   
   A   
   T   
   C   
   G   
   T   
   A   
   A   
    T    
   G   
   G   
   T   
   A   
   G   
   T   
   G   
   G   
   G   
   C   
   T   
   A   
   G   
    C    
   A   
   A   
   T   
   T   
   A   
   A   
   C   
   A   
   A   
   A   
   G   
   T   
   G   
   T   
   T   
   T   
   G   
   A   
    C    
   G   
   A   
   A   
   G   
   T   
   G   
   G   
   G   
   C   
   G   
   T   
   A   
   G   
   T   
    G    
   C   
   C   
   T   
    G    
   G   
   C   
   A   
   A   
   A   
   C   
   T   
    A    
   G   
   G   
   T   
   T   
   C   
   T   
   G   
   G   
   C   
   G   
   C   
   A   
   G   
   A   
   A   
   A   
   A   
   A   
    A    
   C   
   A   
   G   
   C   
    C    
   A   
   A   
   T   
   A   
   T   
   C   
   A   
   A   
   A   
   G   
   C   
   T   
   T   
   T   
   T   
   A   
   G   
   T   
   G   
   A   
   T   
   A   
   A   
    G    
   T   
   T   
    C    
   G   
   G   
   C   
   G   
   T   
   T   
   T   
   C   
   T   
    G    
   T   
   T   
   G   
   A   
   T   
   G   
   A   
   A   
   A   
   T   
   C   
   A   
   A   
   T   
   C   
    A    
   A   
   A   
   A   
    G    
   T   
   T   
   A   
   A   
   G   
   C   
   C   
   A   
   A   
   G   
   G   
    G    
   T   
   G   
   G   
   G   
   T   
   C   
   C   
   A   
   A   
   G   
   C   
   A   
   G   
   A   
   C   
   T   
   C   
    C    
   T   
   T   
   T   
   G   
   T   
   A   
   C   
   C   
   A   
   A   
   T   
   C   
   A   
   C   
   A   
   G   
   T   
    C    
   G   
   C   
   T   
   T   
   C   
   T   
   G   
   A   
   A   
   C   
   C   
   A   
   G   
   T   
   G   
   A   
   C   
   A   
   G   
   A   
   A   
   T   
   T   
   A   
   C   
   C   
   A   
   A   
   C   
   A   
   G   
   G   
   G   
   G   
   C   
   T   
   G   
   C   
   G   
   A   
   C   
   A   
   A   
   A   
   A   
   G   
    A    
    T    
   A   
   C   
   A   
   G   
   A   
   G   
   T   
   C   
   A   
   C   
   G   
   T   
   T   
   A   
   T   
   T   
   A   
   T   
   C   
   C   
    G    
    C    
   T   
   G   
   G   
   G   
   G   
   G   
   A   
   A   
   G   
   C   
   A   
   G   
   C   
   T   
   G   
   C   
   G   
   C   
   A   
   A   
   T   
   T   
   A   
   A   
   T   
   C   
   G   
   G   
    G    
   T   
   A   
   T   
   A   
   C   
    A    
   G   
   G   
    C    
   A   
   C   
    C    
   A   
   T   
   T   
   A   
   C   
   T   
   G   
   C   
   A   
   G   
   A   
    A    
   G   
   A   
   T   
   A   
   T   
   T   
    A    
   A   
   A   
   A   
   A   
   A   
   A   
   A   
   T   
   C   
   C   
   A   
   G   
   A   
   G   
   T   
   T   
   A   
   A   
   G   
   T   
   A   
   G   
   T   
   A   
   C   
   G   
   G   
   G   
    C    
   G   
   T   
   A   
   A   
   T   
   T   
   G   
   G   
    T    
   A   
   A   
   A   
   A   
   C   
   T   
   G   
   G   
    C    
   T   
   T   
   A   
   G   
   A   
   A   
   C   
   G   
   A   
   G   
   C   
   G   
   T   
   T   
   T   
   G   
   A   
   T   
   A   
   A   
   A   
   G   
   A   
    A    
   T   
   T   
   A   
   A   
   G   
   A   
   G   
   G   
    G    
   C   
   A   
   A   
   G   
   A   
   T   
   G   
   G   
    T    
   G   
   G   
    C    
   T   
   C   
    A    
   C   
   T   
   A   
   G   
   T   
   T   
   A   
   T   
   T   
   T   
   T   
   A   
   G   
   A   
   T   
   G   
   A   
   C   
   A   
   A   
   A   
   G   
   A   
   G   
   A   
   A   
   T   
   G   
   T   
   C   
   A   
   A   
   A   
   A   
   A   
   A   
   G   
   C   
   T   
   T   
   T   
   A   
   C   
   A   
   A   
   A   
   C   
   C   
   A   
   A   
   A   
   G   
   A   
    G    
   A   
   A   
   A   
   A   
   A   
   A   
   G   
   A   
   T   
   G   
   G   
   T   
   C   
   A   
   A   
   A   
   C   
    G    
   A   
   T   
   T   
   A   
   A   
   A   
   T   
   T   
   A   
   A   
   C   
   G   
   A   
   T   
   T   
   G   
   A   
    C    
   A   
   G   
    T    
   G   
   G   
    C    
   G   
   T   
   A   
   C   
   A   
   A   
   C   
   A   
   A   
   C   
   A   
   A   
   G   
   C   
    C    
   T   
   T   
    C    
   G   
   C   
   T   
   A   
   T   
   A   
   T   
   T   
   T   
   G   
   A   
   C   
   A   
   A   
   A   
   C   
   G   
    C    
    C    
   C   
   T   
   G   
   G   
   T   
   A   
   G   
   T   
   G   
   C   
   G   
   G   
   T   
   C   
   A   
   T   
   T   
   A   
   C   
   C   
   G   
   A   
   T   
   C   
   C   
   T   
   C   
   A   
   A   
   A   
   A   
   A   
   G   
   G   
   C   
   G   
   A   
   T   
   T   
   T   
   A   
    T    
   T   
   A   
   G   
   C   
    A    
   A   
   C   
   G   
   G   
   T   
   T   
   A   
   G   
   T   
   T   
   C   
   A   
   C   
   C   
   T   
   T   
   C   
   G   
   T   
   A   
   T   
   G   
   A   
   T   
   C   
   C   
   T   
   A   
   A   
   T   
   A   
   A   
   A   
   A   
   T   
   G   
   G   
   C   
   C   
   A   
   A   
   T   
   G   
   G   
    C    
   A   
   T   
   T   
   T   
   C   
   T   
   C   
   A   
   A   
   A   
   A   
   A   
   G   
   A   
    A    
   T   
   A   
   T   
   G   
   A   
   T   
   G   
   C   
   T   
   T   
   A   
   C   
   A   
   A   
   T   
   A   
   A   
   C   
   A   
   A   
   C   
   A   
   A   
   A   
   G   
   A   
   T   
   T   
   T   
   A   
   C   
   C   
   A   
   T   
   T   
    C    
   A   
   C   
   A   
   G   
   C   
   A   
   C   
   G   
   T   
   T   
   T   
    T    
   G   
   C   
   G   
   A   
   C   
   A   
   G   
   G   
    C    
   T   
   A   
   T   
   G   
   C   
   A   
   C   
   C   
   A   
   G   
   G   
    C    
   T   
   C   
   T   
   A   
   C   
   C   
   T   
   T   
   T   
   A   
   A   
   A   
   A   
   C   
   A   
   A   
   T   
   T   
   A   
   C   
   C   
   G   
   G   
   T   
   G   
   C   
   C   
   A   
   T   
   T   
   G   
   G   
   T   
   T   
   T   
   A   
   G   
   A   
   T   
    A    
   C   
   A   
   G   
   G   
   G   
   A   
   C   
   A   
   T   
   T   
   G   
   A   
   A   
   G   
   C   
   C   
   A   
   G   
   A   
   T   
   G   
   A   
   A   
   G   
   A   
   G   
   C   
   T   
   A   
   G   
   A   
   A   
   A   
   T   
   T   
   A   
   A   
   T   
   G   
   G   
    T    
   T   
   T   
   G   
   A   
   A   
   A   
   T   
   G   
   G   
   C   
   A   
   A   
   A   
   A   
   A   
   G   
   A   
   T   
   A   
   A   
   A   
   T   
   C   
   T   
   T   
   G   
   G   
   G   
   G   
    C    
   G   
   G   
   C   
   T   
   A   
   T   
   T   
   T   
   T   
   G   
   C   
    G    
   A   
   C   
   A   
   C   
   G   
   T   
   G   
   T   
   A   
   A   
   A   
   A   
   G   
   A   
   A   
   G   
   C   
   A   
   A   
   G   
   T   
   C   
   C   
   A   
   G   
   T   
   C   
   A   
   A   
   T   
   C   
   T   
    G    
   C   
   G   
   T   
   A   
   C   
   C   
   G   
   C   
   T   
   T   
   T   
   A   
   G   
   T   
   T   
   A   
   A   
   T   
   T   
   C   
   A   
   G   
   A   
   T   
   A   
   A   
   T   
   A   
   T   
   T   
   T   
   A   
   T   
   T   
   T   
   T   
   G   
   C   
    C    
   C   
   A   
   A   
   C   
   A   
   A   
   A   
   C   
   A   
   T   
   T   
   A   
   C   
   G   
    G    
   A   
   T   
   G   
   G   
   G   
    A    
   G   
   A   
   A   
   G   
   A   
   C   
   A   
   A   
   A   
   T   
   T   
   T   
   A   
   G   
   A   
   G   
   C   
    G    
   G   
   G   
   T   
   C   
   T   
   T   
   A   
   A   
   T   
   A   
   A   
   A   
   T   
   T   
   C   
   A   
   T   
   T   
   T   
   T   
   C   
   G   
   G   
   T   
   G   
   A   
   G   
   G   
   A   
   A   
   C   
   T   
   A   
   G   
   A   
   T   
   T   
   T   
   A   
   C   
   C   
   A   
   A   
   T   
    T    
   G   
   C   
   C   
   A   
   T   
   G   
   A   
   C   
   G   
   C   
   C   
    T    
   G   
   C   
   T   
   C   
   A   
   A   
   A   
   T   
   T   
   T   
   C   
   A   
   A   
   A   
    T    
   G   
   A   
   A   
   G   
   A   
   T   
   A   
   A   
   A   
   T   
   T   
   T   
   A   
   A   
   T   
   T   
   C   
   T   
   G   
   A   
   A   
   A   
   T   
   T   
   C   
   T   
   T   
   T   
   T   
    G    
   G   
   C   
   A   
   G   
   A   
   T   
   A   
   C   
    G    
   G   
   G   
   C   
   T   
   A   
    C    
   G   
   G   
    C    
   C   
   A   
   A   
   G   
   G   
    C    
   C   
   A   
   A   
   T   
   T   
   G   
   T   
   T   
   A   
   A   
   T   
   T   
   T   
   C   
    G    
   C   
   C   
   A   
   A   
   T   
   C   
   C   
   A   
   G   
   C   
   A   
   A   
   G   
   C   
   T   
   A   
   C   
   C   
   A   
   T   
   G   
   T   
   A   
   T   
   A   
   G   
   T   
   G   
   T   
   T   
   T   
   T   
   C   
   C   
   A   
   A   
   A   
   A   
   T   
   A   
   A   
   T   
   G   
   G   
    G    
   A   
   C   
   A   
    T    
   T   
    A    
   G   
   T   
    C    
   T   
   A   
   T   
   C   
   C   
   A   
   A   
   A   
   A   
    T    
   T   
   A   
   G   
   T   
   T   
   T   
   T   
   A   
   G   
   A   
   C   
   A   
   A   
   A   
   G   
   A   
   A   
   A   
   C   
    G    
   A   
   A   
   G   
   A   
   A   
   A   
   A   
   A   
   A   
   G   
   A   
   C   
   A   
   A   
   T   
   G   
   T   
   A   
   A   
   T   
   C   
   A   
   G   
   C   
   G   
   C   
   C   
   A   
   A   
   C   
   G   
   C   
   A   
   G   
   C   
   G   
   A   
   A   
   T   
   A   
   C   
   A   
   A   
   T   
   T   
   G   
   C   
   C   
   A   
   C   
   A   
   G   
   A   
   T   
   C   
   T   
   T   
   T   
   T   
   A   
   G   
   G   
   T   
   A   
   G   
   T   
   G   
   T   
   G   
   G   
   A   
   A   
   G   
   A   
   T   
   C   
   C   
   T   
   T   
   C   
   A   
   G   
   G   
   T   
   T   
   A   
   T   
   G   
   T   
   T   
   T   
   A   
   T   
   A   
   A   
   T   
   A   
   T   
   G   
   T   
   A   
   C   
   A   
   A   
   T   
   C   
   C   
   G   
   A   
   A   
   C   
   T   
   T   
   C   
   T   
   C   
   A   
   T   
   T   
   G   
   G   
   C   
   T   
   G   
   C   
   C   
   A   
   A   
   A   
   A   
   C   
   A   
   G   
   G   
   A   
   A   
   C   
   A   
   G   
   C   
   T   
   G   
   A   
   A   
   A   
   T   
   A   
   A   
   A   
   A   
   G   
   A   
   T   
   A   
   A   
   A   
   C   
   A   
   A   
   G   
   A   
   T   
   A   
   C   
   A   
   G   
   A   
   T   
   G   
   G   
   C   
   A   
   A   
   A   
   G   
   A   
   A   
   A   
   A   
   C   
   A   
   G   
   T   
   T   
   T   
   C   
   C   
   T   
   T   
   C   
   T   
   A   
   A   
   C   
   G   
   T   
   T   
   A   
   G   
   A   
   T   
   C   
   G   
   T   
   A   
   G   
   T   
   A   
   A   
   C   
   A   
   A   
   T   
   A   
   A   
   A   
   T   
   T   
   C   
   T   
   T   
   A   
   A   
   C   
   A   
   A   
   T   
   G   
   A   
   T   
   T   
   A   
   T   
   G   
   G   
   T   
   T   
   G   
   A   
   A   
   A   
   A   
   T   
   T   
   C   
   A   
   G   
   G   
   A   
   G   
   A   
   A   
   A   
   A   
   T   
   G   
   G   
   T   
   T   
   C   
   T   
   G   
   C   
   A   
   A   
   C   
   A   
   G   
   A   
   T   
   A   
   T   
   C   
   A   
   G   
   T   
   A   
   A   
   A   
   C   
   C   
   A   
   T   
   T   
   A   
   A   
   T   
   T   
   G   
   A   
   T   
   T   
   A   
   C   
   T   
   T   
   A   
   G   
   A   
   A   
   G   
   C   
   A   
   A   
   C   
   C   
   A   
   T   
   T   
   A   
   A   
   A   
   -   
   -   
   -   
   -   
   -   
   -   
   -   
   -   
   -   
   -   
   -   
   -   
   -   
   -   
   -   
   -   
   -   
   -   
   -   
   -   
   -   
   -   
   -   
   -   
   -   
   -   
   -   
   -   
   -   
   -   
   -   
   -   
   -   
   -   
   -   
   -   
   -   
   -   
   -   
   -   
   -   
   -   
   -   
   -   
   -   
   -   
   -   
   -   
   -   
   -   
   -   
   -   
   -   
   -   
   -   
   -   
   -   
   -   
   -   
   -   
   -   
   -   
   -   
   -   
   -   
   -   
   -   
   -   
   -   
   -   
   -   
   -   
   -   
   -   
   -   
   -   
   -   
   -   
   -   
   -   
   -   
   -   
   -   
   -   
   -   
   -   
   -   
   -   
   -   
   -   
   -   
   -   
   -   
   -   
   -   
   -   
   -   
   -   
   -   
   -   
   -   
   -   
   -   
   -   
   -   
   -   
   -   
   -   
   -   
   -   
   -   
   -   
   -   
   -   
   -   
   -   
   -   
   -   
   -   
   -   
   -   
   -   
   -   
   -   
   -   
   -   
   -   
   -   
   -   
   -   
   -   
   -   
   -   
   -   
   -   
   -   
   -   
   -   
   -   
   -   
   -   
   -   
   -   
   -   
   -   
   -   
   -   
   -   
   -   
   -   
   -   
   -   
   -   
   -   
   -   
   -   
   -   
   -   
   -   
   -   
   -   
   -   
   -   
   -   
   -   
   -   
   -   
   -   
   -   
   C   
   A   
   A   
   T   
   G   
   G   
   C   
   A   
   A   
   G   
   C   
   C   
   A   
   A   
   A   
   C   
   A   
   A   
   G   
   A   
   A   
   T   
   T   
   A   
   G   
   C   
   C   
   G   
   A   
   A   
   G   
   C   
   G   
   A   
   A   
   G   
   A   
   A   
   A   
   A   
   C   
   A   
   G   
   C   
   T   
   A   
   C   
   T   
   A   
   C   
   A   
   T   
   T   
   T   
   T   
   T   
   A   
   A   
   A   
   C   
   G   
   T   
   A   
   T   
   T   
   G   
   A   
   C   
   A   
   A   
   A   
   A   
   C   
   A   
   G   
   A   
   A   
   A   
   T   
   T   
   T   
   G   
   A   
   T   
   A   
   A   
   G   
   T   
   T   
   A   
   C   
   C   
   G   
   T   
   C   
   C   
   G   
   T   
   T   
   G   
   T   
   T   
   C   
   A   
   A   
   G   
   A   
   A   
   G   
   C   
   T   
   A   
   G   
   C   
   T   
   T   
   A   
   A   
   A   
   G   
   A   
   A   
   A   
   A   
   A   
   T   
   G   
   G   
   C   
   T   
   A   
   T   
   G   
   A   
   T   
   A   
   C   
   T   
   A   
   A   
   A   
   T   
   C   
   T   
   G   
   T   
   T   
   G   
   T   
   T   
   G   
   A   
   A   
   A   
   A   
   A   
   T   
   A   
   C   
   C   
   A   
   A   
   G   
   C   
   A   
   A   
   T   
   T   
   T   
   A   
   T   
   T   
   C   
   A   
   G   
   G   
   G   
   A   
   T   
   T   
   C   
   A   
   A   
   G   
   C   
   A   
   G   
   A   
   A   
   G   
   G   
   A   
   G   
   T   
   C   
   A   
   A   
   A   
   G   
   C   
   T   
   A   
   G   
   T   
   G   
   A   
   T   
   G   
   T   
   T   
   C   
   A   
   A   
   G   
   T   
   C   
   A   
   A   
   A   
   A   
   A   
   A   
   G   
   C   
   G   
   A   
   A   
   A   
   G   
   A   
   C   
   A   
   A   
   T   
   C   
   A   
   A   
   T   
   A   
   C   
   A   
   C   
   A   
   T   
   T   
   T   
   A   
   C   
   C   
   T   
   A   
   T   
   A   
   A   
   A   
   T   
   T   
   A   
   T   
   C   
   G   
   A   
   T   
   G   
   A   
   G   
   T   
   A   
   C   
   C   
   C   
   C   
   T   
   T   
   T   
   A   
   G   
   G   
   C   
   G   
   A   
   A   
   A   
   T   
   G   
   A   
   A   
   A   
   G   
   A   
   T   
   T   
   T   
   G   
   T   
   C   
   T   
   T   
   A   
   T   
   C   
   A   
   A   
   T   
   C   
   A   
   A   
   G   
   T   
   A   
   T   
   C   
   G   
   C   
   C   
   A   
   A   
   A   
   A   
   A   
   A   
   G   
   G   
   C   
   G   
   A   
   T   
   A   
   C   
   C   
   T   
   A   
   C   
   C   
   A   
   A   
   A   
   T   
   C   
   G   
   C   
   T   
   T   
   G   
   G   
   A   
   A   
   G   
   C   
   C   
   G   
   T   
   C   
   T   
   T   
   T   
   A   
   A   
   T   
   T   
   T   
   T   
   T   
   C   
   C   
   A   
   G   
   A   
   T   
   A   
   T   
   G   
   T   
   C   
   A   
   G   
   G   
   A   
   A   
   A   
   T   
   G   
   A   
   T   
   A   
   A   
   A   
   A   
   T   
   T   
   T   
   C   
   G   
   A   
   T   
   T   
   C   
   A   
   A   
   G   
   T   
   A   
   G   
   A   
   T   
   A   
   A   
   T   
   G   
   C   
   C   
   A   
   A   
   A   
   C   
   G   
   T   
   G   
   G   
   A   
   G   
   A   
   A   
   A   
   T   
   T   
   G   
   T   
   C   
   G   
   A   
   T   
   C   
   G   
   T   
   A   
   A   
   T   
   G   
   G   
   T   
   A   
   G   
   T   
   G   
   G   
   G   
   C   
   T   
   A   
   G   
   C   
   A   
   A   
   T   
   T   
   A   
   A   
   C   
   A   
   A   
   A   
   G   
   T   
   G   
   T   
   T   
   T   
   G   
   A   
   C   
   G   
   A   
   A   
   G   
   T   
   G   
   G   
   G   
   C   
   G   
   T   
   A   
   G   
   T   
   G   
   C   
   C   
   T   
   G   
   G   
   C   
   A   
   A   
   A   
   C   
   T   
   A   
   G   
   G   
   T   
   T   
   C   
   T   
   G   
   G   
   C   
   G   
   C   
   A   
   G   
   A   
   A   
   A   
   A   
   A   
   A   
   C   
   A   
   G   
   C   
   C   
   A   
   A   
   T   
   A   
   T   
   C   
   A   
   A   
   A   
   G   
   C   
   T   
   T   
   T   
   T   
   A   
   G   
   T   
   G   
   A   
   T   
   A   
   A   
   G   
   T   
   T   
   C   
   G   
   G   
   C   
   G   
   T   
   T   
   T   
   C   
   T   
   G   
   T   
   T   
   G   
   A   
   T   
   G   
   A   
   A   
   A   
   T   
   C   
   A   
   A   
   T   
   C   
   A   
   A   
   A   
   A   
   G   
   T   
   T   
   A   
   A   
   G   
   C   
   C   
   A   
   A   
   G   
   G   
   G   
   T   
   G   
   G   
   G   
   T   
   C   
   C   
   A   
   A   
   G   
   C   
   A   
   G   
   A   
   C   
   T   
   C   
   C   
   T   
   T   
   T   
   G   
   T   
   A   
   C   
   C   
   A   
   A   
   T   
   C   
   A   
   C   
   A   
   G   
   T   
   C   
   G   
   C   
   T   
   T   
   C   
   T   
   G   
   A   
   A   
   C   
   C   
   A   
   G   
   T   
   G   
   A   
   C   
   A   
   G   
   A   
   A   
   T   
   T   
   A   
   C   
   C   
   A   
   A   
   C   
   A   
   G   
   G   
   G   
   G   
   C   
   T   
   G   
   C   
   G   
   A   
   C   
   A   
   A   
   A   
   A   
   G   
   A   
   T   
   A   
   C   
   A   
   G   
   A   
   G   
   T   
   C   
   A   
   C   
   G   
   T   
   T   
   A   
   T   
   T   
   A   
   T   
   C   
   C   
   G   
   C   
   T   
   G   
   G   
   G   
   G   
   G   
   A   
   A   
   G   
   C   
   A   
   G   
   C   
   T   
   G   
   C   
   G   
   C   
   A   
   A   
   T   
   T   
   A   
   A   
   T   
   C   
   G   
   G   
   G   
   T   
   A   
   T   
   A   
   C   
   A   
   G   
   G   
   C   
   A   
   C   
   C   
   A   
   T   
   T   
   A   
   C   
   T   
   G   
   C   
   A   
   G   
   A   
   A   
   G   
   A   
   T   
   A   
   T   
   T   
   A   
   A   
   A   
   A   
   A   
   A   
   A   
   A   
   T   
   C   
   C   
   A   
   G   
   A   
   G   
   T   
   T   
   A   
   A   
   G   
   T   
   A   
   G   
   T   
   A   
   C   
   G   
   G   
   G   
   C   
   G   
   T   
   A   
   A   
   T   
   T   
   G   
   G   
   T   
   A   
   A   
   A   
   A   
   C   
   T   
   G   
   G   
   C   
   T   
   T   
   A   
   G   
   A   
   A   
   C   
   G   
   A   
   G   
   C   
   G   
   T   
   T   
   T   
   G   
   A   
   T   
   A   
   A   
   A   
   G   
   A   
   A   
   T   
   T   
   A   
   A   
   G   
   A   
   G   
   G   
   G   
   C   
   A   
   A   
   G   
   A   
   T   
   G   
   G   
   T   
   G   
   G   
   C   
   T   
   C   
   A   
   C   
   T   
   A   
   G   
   T   
   T   
   A   
   T   
   T   
   T   
   T   
   A   
   G   
   A   
   T   
   G   
   A   
   C   
   A   
   A   
   A   
   G   
   A   
   G   
   A   
   A   
   T   
   G   
   T   
   C   
   A   
   A   
   A   
   A   
   A   
   A   
   G   
   C   
   T   
   T   
   T   
   A   
   C   
   A   
   A   
   A   
   C   
   C   
   A   
   A   
   A   
   G   
   A   
   G   
   A   
   A   
   A   
   A   
   A   
   A   
   G   
   A   
   T   
   G   
   G   
   T   
   C   
   A   
   A   
   A   
   C   
   G   
   A   
   T   
   T   
   A   
   A   
   A   
   T   
   T   
   A   
   A   
   C   
   G   
   A   
   T   
   T   
   G   
   A   
   C   
   A   
   G   
   T   
   G   
   G   
   C   
   G   
   T   
   A   
   C   
   A   
   A   
   C   
   A   
   A   
   C   
   A   
   A   
   G   
   C   
   C   
   T   
   T   
   C   
   G   
   C   
   T   
   A   
   T   
   A   
   T   
   T   
   T   
   G   
   A   
   C   
   A   
   A   
   A   
   C   
   G   
   C   
   C   
   C   
   T   
   G   
   G   
   T   
   A   
   G   
   T   
   G   
   C   
   G   
   G   
   T   
   C   
   A   
   T   
   T   
   A   
   C   
   C   
   G   
   A   
   T   
   C   
   C   
   T   
   C   
   A   
   A   
   A   
   A   
   A   
   G   
   G   
   C   
   G   
   A   
   T   
   T   
   T   
   A   
   T   
   T   
   A   
   G   
   C   
   A   
   A   
   C   
   G   
   G   
   T   
   T   
   A   
   G   
   T   
   T   
   C   
   A   
   C   
   C   
   T   
   T   
   C   
   G   
   T   
   A   
   T   
   G   
   A   
   T   
   C   
   C   
   T   
   A   
   A   
   T   
   A   
   A   
   A   
   A   
   T   
   G   
   G   
   C   
   C   
   A   
   A   
   T   
   G   
   G   
   C   
   A   
   T   
   T   
   T   
   C   
   T   
   C   
   A   
   A   
   A   
   A   
   A   
   G   
   A   
   A   
   T   
   A   
   T   
   G   
   A   
   T   
   G   
   C   
   T   
   T   
   A   
   C   
   A   
   A   
   T   
   A   
   A   
   C   
   A   
   A   
   C   
   A   
   A   
   A   
   G   
   A   
   T   
   T   
   T   
   A   
   C   
   C   
   A   
   T   
   T   
   C   
   A   
   C   
   A   
   G   
   C   
   A   
   C   
   G   
   T   
   T   
   T   
   T   
   G   
   C   
   G   
   A   
   C   
   A   
   G   
   G   
   C   
   T   
   A   
   T   
   G   
   C   
   A   
   C   
   C   
   A   
   G   
   G   
   C   
   T   
   C   
   T   
   A   
   C   
   C   
   T   
   T   
   T   
   A   
   A   
   A   
   A   
   C   
   A   
   A   
   T   
   T   
   A   
   C   
   C   
   G   
   G   
   T   
   G   
   C   
   C   
   A   
   T   
   T   
   G   
   G   
   T   
   T   
   T   
   A   
   G   
   A   
   T   
   A   
   C   
   A   
   G   
   G   
   G   
   A   
   C   
   A   
   T   
   T   
   G   
   A   
   A   
   G   
   C   
   C   
   A   
   G   
   A   
   T   
   G   
   A   
   A   
   G   
   A   
   G   
   C   
   T   
   A   
   G   
   A   
   A   
   A   
   T   
   T   
   A   
   A   
   T   
   G   
   G   
   T   
   T   
   T   
   G   
   A   
   A   
   A   
   T   
   G   
   G   
   C   
   A   
   A   
   A   
   A   
   A   
   G   
   A   
   T   
   A   
   A   
   A   
   T   
   C   
   T   
   T   
   G   
   G   
   G   
   G   
   C   
   G   
   G   
   C   
   T   
   A   
   T   
   T   
   T   
   T   
   G   
   C   
   G   
   A   
   C   
   A   
   C   
   G   
   T   
   G   
   T   
   A   
   A   
   A   
   A   
   G   
   A   
   A   
   G   
   C   
   A   
   A   
   G   
   T   
   C   
   C   
   A   
   G   
   T   
   C   
   A   
   A   
   T   
   C   
   T   
   G   
   C   
   G   
   T   
   A   
   C   
   C   
   G   
   C   
   T   
   T   
   T   
   A   
   G   
   T   
   T   
   A   
   A   
   T   
   T   
   C   
   A   
   G   
   A   
   T   
   A   
   A   
   T   
   A   
   T   
   T   
   T   
   A   
   T   
   T   
   T   
   T   
   G   
   C   
   C   
   C   
   A   
   A   
   C   
   A   
   A   
   A   
   C   
   A   
   T   
   T   
   A   
   C   
   G   
   G   
   A   
   T   
   G   
   G   
   G   
   A   
   G   
   A   
   A   
   G   
   A   
   C   
   A   
   A   
   A   
   T   
   T   
   T   
   A   
   G   
   A   
   G   
   C   
   G   
   G   
   G   
   T   
   C   
   T   
   T   
   A   
   A   
   T   
   A   
   A   
   A   
   T   
   T   
   C   
   A   
   T   
   T   
   T   
   T   
   C   
   G   
   G   
   T   
   G   
   A   
   G   
   G   
   A   
   A   
   C   
   T   
   A   
   G   
   A   
   T   
   T   
   T   
   A   
   C   
   C   
   A   
   A   
   T   
   T   
   G   
   C   
   C   
   A   
   T   
   G   
   A   
   C   
   G   
   C   
   C   
   T   
   G   
   C   
   T   
   C   
   A   
   A   
   A   
   T   
   T   
   T   
   C   
   A   
   A   
   A   
   T   
   G   
   A   
   A   
   G   
   A   
   T   
   A   
   A   
   A   
   T   
   T   
   T   
   A   
   A   
   T   
   T   
   C   
   T   
   G   
   A   
   A   
   A   
   T   
   T   
   C   
   T   
   T   
   T   
   T   
   G   
   G   
   C   
   A   
   G   
   A   
   T   
   A   
   C   
   G   
   G   
   G   
   C   
   T   
   A   
   C   
   G   
   G   
   C   
   C   
   A   
   A   
   G   
   G   
   C   
   C   
   A   
   A   
   T   
   T   
   G   
   T   
   T   
   A   
   A   
   T   
   T   
   T   
   C   
   G   
   C   
   C   
   A   
   A   
   T   
   C   
   C   
   A   
   G   
   C   
   A   
   A   
   G   
   C   
   T   
   A   
   C   
   C   
   A   
   T   
   G   
   T   
   A   
   T   
   A   
   G   
   T   
   G   
   T   
   T   
   T   
   T   
   C   
   C   
   A   
   A   
   A   
   A   
   T   
   A   
   A   
   T   
   G   
   G   
   G   
   A   
   C   
   A   
   T   
   T   
   A   
   G   
   T   
   C   
   T   
   A   
   T   
   C   
   C   
   A   
   A   
   A   
   A   
   T   
   T   
   A   
   G   
   T   
   T   
   T   
   T   
   A   
   G   
   A   
   C   
   A   
   A   
   A   
   G   
   A   
   A   
   A   
   C   
   G   
   A   
   A   
   G   
   A   
   A   
   A   
   A   
   A   
   A   
   G   
   A   
   C   
   A   
   A   
   T   
   G   
   T   
   A   
   A   
   T   
   C   
   A   
   G   
   C   
   G   
   C   
   C   
   A   
   A   
   C   
   G   
   C   
   A   
   G   
   C   
   G   
   A   
   A   
   T   
   A   
   C   
   A   
   A   
   T   
   T   
   G   
   C   
   C   
   A   
   C   
   A   
   G   
   A   
   T   
   C   
   T   
   T   
   T   
   T   
   A   
   G   
   G   
   T   
   A   
   G   
   T   
   G   
   T   
   G   
   G   
   A   
   A   
   G   
   A   
   T   
   C   
   C   
   T   
   T   
   C   
   A   
   G   
   G   
   T   
   T   
   A   
   T   
   G   
   T   
   T   
   T   
   A   
   T   
   A   
   A   
   T   
   A   
   T   
   G   
   T   
   A   
   C   
   A   
   A   
   T   
   C   
   C   
   G   
   A   
   A   
   C   
   T   
   T   
   C   
   T   
   C   
   A   
   T   
   T   
   G   
   G   
   C   
   T   
   G   
   C   
   C   
   A   
   A   
   A   
   A   
   C   
   A   
   G   
   G   
   A   
   A   
   C   
   A   
   G   
   C   
   T   
   G   
   A   
   A   
   A   
   T   
   A   
   A   
   A   
   A   
   G   
   A   
   T   
   A   
   A   
   A   
   C   
   A   
   A   
   G   
   A   
   T   
   A   
   C   
   A   
   G   
   A   
   T   
   G   
   G   
   C   
   A   
   A   
   A   
   G   
   A   
   A   
   A   
   A   
   C   
   A   
   G   
   T   
   T   
   T   
   C   
   C   
   T   
   T   
   C   
   T   
   A   
   A   
   C   
   G   
   T   
   T   
   A   
   G   
   A   
   T   
   C   
   G   
   T   
   A   
   G   
   T   
   A   
   A   
   C   
   A   
   A   
   T   
   A   
   A   
   A   
   T   
   T   
   C   
   T   
   T   
   A   
   A   
   C   
   A   
   A   
   T   
   G   
   A   
   T   
   T   
   A   
   T   
   G   
   G   
   T   
   T   
   G   
   A   
   A   
   A   
   A   
   T   
   T   
   C   
   A   
   G   
   G   
   A   
   G   
   A   
   A   
   A   
   A   
   T   
   G   
   G   
   T   
   T   
   C   
   T   
   G   
   C   
   A   
   A   
   C   
   A   
   G   
   A   
   T   
   A   
   T   
   C   
   A   
   G   
   T   
   A   
   A   
   A   
   C   
   C   
   A   
   T   
   T   
   A   
   A   
   T   
   T   
   G   
   A   
   T   
   T   
   A   
   C   
   T   
   T   
   A   
   G   
   A   
   A   
   G   
   C   
   A   
   A   
   C   
   C   
   A   
   T   
   T   
   A   
   A   
   A   
   -   
   -   
   -   
   -   
   -   
   -   
   -   
   -   
   -   
   -   
   -   
   -   
   -   
   -   
   -   
   -   
   -   
   -   
   -   
   -   
   -   
   -   
   -   
   -   
   -   
   -   
   -   
   -   
   -   
   -   
   -   
   -   
   -   
   -   
   -   
   -   
   -   
   -   
   -   
   -   
   -   
   -   
   -   
   -   
   -   
   -   
   -   
   -   
   -   
   -   
   -   
   -   
   -   
   -   
   -   
   -   
   -   
   -   
   -   
   -   
   -   
 

 
   N59836F   
   -   
   -   
   -   
   -   
   -   
   -   
   -   
   -   
   -   
   -   
   -   
   -   
   -   
   -   
   -   
   -   
   -   
   -   
   -   
   -   
   -   
   -   
   -   
   -   
   -   
   -   
   -   
   -   
   -   
   -   
   -   
   -   
   -   
   -   
   -   
   -   
   -   
   -   
   -   
   -   
   -   
   -   
   -   
   -   
   -   
   -   
   -   
   -   
   -   
   -   
   -   
   -   
   -   
   -   
   -   
   -   
   -   
   -   
   -   
   -   
   -   
   -   
   -   
   -   
   -   
   -   
   -   
   -   
   -   
   -   
   -   
   -   
   -   
   -   
   -   
   -   
   -   
   -   
   -   
   -   
   -   
   -   
   -   
   -   
   -   
   -   
   -   
   -   
   -   
   -   
   -   
   -   
   -   
   -   
   -   
   -   
   -   
   -   
   -   
   -   
   -   
   -   
   -   
   -   
   -   
   -   
   -   
   -   
   C   
   A   
   A   
   T   
   G   
   G   
   C   
   A   
   A   
   G   
   C   
   C   
   A   
   A   
   A   
   C   
   A   
   A   
   G   
   A   
    A    
   T   
   T   
   A   
   G   
   C   
    C    
   G   
   A   
   A   
   G   
   C   
   G   
   A   
   A   
   G   
   A   
   A   
   A   
   A   
    C    
   A   
   G   
   C   
    T    
   A   
    C    
   T   
    A    
    C    
   A   
   T   
   T   
   T   
   T   
   T   
   A   
   A   
   A   
    C    
   G   
   T   
   A   
   T   
   T   
   G   
    T    
   C   
   A   
   A   
   A   
   A   
   C   
   A   
   G   
    G    
   A   
   A   
   T   
   T   
   T   
   G   
   A   
   T   
   A   
   A   
   G   
   T   
   T   
   A   
   C   
   C   
    G    
   T   
   C   
   C   
   G   
   T   
   T   
    G    
   T   
   T   
   C   
   A   
   A   
   G   
   A   
   A   
    G    
   C   
   T   
   A   
   G   
   C   
   T   
   T   
   A   
   A   
   A   
   G   
   A   
   A   
   A   
   A   
   A   
   T   
   G   
   G   
    C    
   T   
   A   
   T   
   G   
   A   
   T   
   A   
   C   
   T   
   A   
   A   
   A   
   T   
   C   
    T    
   G   
   T   
   T   
   G   
   T   
   T   
   G   
   A   
   A   
   A   
   A   
   A   
   T   
   A   
   C   
   C   
   A   
   A   
   G   
   C   
   A   
   A   
   T   
   T   
   T   
   A   
   T   
   T   
   C   
   A   
   G   
   G   
   G   
   A   
   T   
   T   
   C   
   A   
   A   
   G   
   C   
    A    
   G   
   A   
   A   
    G    
   G   
    A    
   G   
   T   
   C   
   A   
   A   
    A    
   G   
   C   
   T   
   A   
   G   
   T   
   G   
   A   
   T   
   G   
   T   
    T    
   C   
   A   
   A   
   G   
   T   
    A    
   A   
   A   
   A   
   A   
   A   
    G    
   G   
    C    
   G   
   A   
   A   
   A   
   G   
   A   
    C    
   A   
   A   
    T    
   C   
   A   
   A   
   T   
   A   
   C   
   A   
   C   
   A   
   T   
   T   
   T   
   A   
    C    
   C   
   T   
   A   
   T   
   A   
   A   
   A   
   T   
   T   
   A   
   T   
   C   
   G   
   A   
   T   
   G   
   A   
   G   
    T    
   A   
   C   
    C    
   C   
   C   
   T   
   T   
   T   
   A   
   G   
   G   
   C   
   G   
   A   
   A   
   A   
   T   
   G   
   A   
   A   
   A   
   G   
   A   
   T   
    T    
   T   
   G   
   T   
   C   
   T   
   T   
   A   
   T   
   C   
   A   
   A   
   T   
   C   
   A   
   A   
   G   
   T   
   A   
   T   
    C    
   G   
    C    
   C   
   A   
   A   
    A    
   A   
   A   
   A   
   G   
   G   
    C    
   G   
   A   
   T   
   A   
    C    
   C   
   T   
   A   
   C   
   C   
   A   
   A   
   A   
   T   
    C    
    G    
   C   
   T   
   T   
   G   
   G   
    A    
    A    
    G    
   C   
   C   
    G    
   T   
   C   
   T   
   T   
   T   
   A   
   A   
   T   
   T   
   T   
   T   
   T   
   C   
   C   
   A   
   G   
   A   
   T   
   A   
   T   
   G   
   T   
   C   
   A   
   G   
   G   
   A   
   A   
   A   
   T   
   G   
   A   
   T   
   A   
   A   
   A   
    A    
   T   
   T   
   T   
   C   
   G   
   A   
   T   
   T   
   C   
   A   
   A   
   G   
   T   
   A   
   G   
   A   
   T   
   A   
   A   
   T   
   G   
   C   
   C   
   A   
   A   
   A   
   C   
   G   
   T   
   G   
   G   
   A   
   G   
   A   
   A   
   A   
   T   
   T   
   G   
   T   
   C   
   G   
   A   
   T   
   C   
   G   
   T   
   A   
   A   
    T    
   G   
   G   
   T   
   A   
   G   
   T   
   G   
   G   
   G   
   C   
   T   
   A   
   G   
    C    
   A   
   A   
   T   
   T   
   A   
   A   
   C   
   A   
   A   
   A   
   G   
   T   
   G   
   T   
   T   
   T   
   G   
   A   
    T    
   G   
   A   
   A   
   G   
   T   
   G   
   G   
   G   
   C   
   G   
   T   
   A   
   G   
   T   
    G    
   C   
   C   
   T   
    G    
   G   
   C   
   A   
   A   
   A   
   C   
   T   
    C    
   G   
   G   
   T   
   T   
   C   
   T   
   G   
   G   
   C   
   G   
   C   
   A   
   G   
   A   
   A   
   A   
   A   
   A   
    A    
   C   
   A   
   G   
   C   
    C    
   A   
   A   
   T   
   A   
   T   
   C   
   A   
   A   
   A   
   G   
   C   
   T   
   T   
   T   
   T   
   A   
   G   
   T   
   G   
   A   
   T   
   A   
   A   
    A    
   T   
   T   
    C    
   G   
   G   
   C   
   G   
   T   
   T   
   T   
   C   
   T   
    G    
   T   
   T   
   G   
   A   
   T   
   G   
   A   
   A   
   A   
   T   
   C   
   A   
   A   
   T   
   C   
    A    
   A   
   A   
   A   
    G    
   T   
   T   
   A   
   A   
   G   
   C   
   C   
   A   
   A   
   G   
   G   
    A    
   T   
   G   
   G   
   G   
   T   
   C   
   C   
   A   
   A   
   G   
   C   
   A   
   G   
   A   
   C   
   T   
   C   
    C    
   T   
   T   
   T   
   G   
   T   
   A   
   C   
   C   
   A   
   A   
   T   
   C   
   A   
   C   
   A   
   G   
   T   
    C    
   G   
   C   
   T   
   T   
   C   
   T   
   G   
   A   
   A   
   C   
   C   
   A   
   G   
   T   
   G   
   A   
   C   
   A   
   G   
   A   
   A   
   T   
   T   
   A   
   C   
   C   
   A   
   A   
   C   
   A   
   G   
   G   
   G   
   G   
   C   
   T   
   G   
   C   
   G   
   A   
   C   
   A   
   A   
   A   
   A   
   G   
    A    
    T    
   A   
   C   
   A   
   G   
   A   
   G   
   T   
   C   
   A   
   C   
   G   
   T   
   T   
   A   
   T   
   T   
   A   
   T   
   C   
   C   
    G    
    C    
   T   
   G   
   G   
   G   
   G   
   G   
   A   
   A   
   G   
   C   
   A   
   G   
   C   
   T   
   G   
   C   
   G   
   C   
   A   
   A   
   T   
   T   
   A   
   A   
   T   
   C   
   G   
   G   
    G    
   T   
   A   
   T   
   A   
   C   
    G    
   G   
   G   
    C    
   A   
   C   
    C    
   A   
   T   
   T   
   A   
   C   
   T   
   G   
   C   
   A   
   G   
   A   
    A    
   G   
   A   
   T   
   A   
   T   
   T   
    G    
   A   
   A   
   A   
   A   
   A   
   A   
   A   
   T   
   C   
   C   
   A   
   G   
   A   
   G   
   T   
   T   
   A   
   A   
   G   
   T   
   A   
   G   
   T   
   A   
   C   
   G   
   G   
   G   
    C    
   G   
   T   
   A   
   A   
   T   
   T   
   G   
   G   
    T    
   A   
   A   
   A   
   A   
   C   
   T   
   G   
   G   
    T    
   T   
   T   
   A   
   G   
   A   
   A   
   C   
   G   
   A   
   G   
   C   
   G   
   T   
   T   
   T   
   G   
   A   
   T   
   A   
   A   
   A   
   G   
   A   
    A    
   T   
   T   
   A   
   A   
   G   
   A   
   G   
   G   
    G    
   C   
   A   
   A   
   G   
   A   
   T   
   G   
   G   
    T    
   G   
   G   
    C    
   T   
   C   
    A    
   C   
   T   
   A   
   G   
   T   
   T   
   A   
   T   
   T   
   T   
   T   
   A   
   G   
   A   
   T   
   G   
   A   
   C   
   A   
   A   
   A   
   G   
   A   
   G   
   A   
   A   
   T   
   G   
   T   
   C   
   A   
   A   
   A   
   A   
   A   
   A   
   G   
   C   
   T   
   T   
   T   
   A   
   C   
   A   
   A   
   A   
   C   
   C   
   A   
   A   
   A   
   G   
   A   
    G    
   A   
   A   
   A   
   A   
   A   
   A   
   G   
   A   
   T   
   G   
   G   
   T   
   C   
   A   
   A   
   A   
   C   
    G    
   A   
   T   
   T   
   A   
   A   
   A   
   T   
   T   
   A   
   A   
   C   
   G   
   A   
   T   
   T   
   G   
   A   
    C    
   A   
   G   
    T    
   G   
   G   
    C    
   G   
   T   
   A   
   C   
   A   
   A   
   C   
   A   
   A   
   C   
   A   
   A   
   G   
   C   
    C    
   T   
   T   
    C    
   G   
   C   
   T   
   A   
   T   
   A   
   T   
   T   
   T   
   G   
   A   
   C   
   A   
   A   
   A   
   C   
   G   
    T    
    C    
   C   
   T   
   G   
   G   
   T   
   A   
   G   
   T   
   G   
   C   
   G   
   G   
   T   
   C   
   A   
   T   
   T   
   A   
   C   
   C   
   G   
   A   
   T   
   C   
   C   
   T   
   C   
   A   
   A   
   A   
   A   
   A   
   G   
   G   
   C   
   G   
   A   
   T   
   T   
   T   
   A   
    T    
   T   
   A   
   G   
   C   
    A    
   A   
   C   
   G   
   G   
   T   
   T   
   A   
   G   
   T   
   T   
   C   
   A   
   C   
   C   
   T   
   T   
   C   
   G   
   T   
   A   
   T   
   G   
   A   
   T   
   C   
   C   
   T   
   A   
   A   
   T   
   A   
   A   
   A   
   A   
   T   
   G   
   G   
   C   
   C   
   A   
   A   
   T   
   G   
   G   
    T    
   A   
   T   
   T   
   T   
   C   
   T   
   C   
   A   
   A   
   A   
   A   
   A   
   G   
   A   
    A    
   T   
   A   
   T   
   G   
   A   
   T   
   G   
   C   
   T   
   T   
   A   
   C   
   A   
   A   
   T   
   A   
   A   
   C   
   A   
   A   
   C   
   A   
   A   
   A   
   G   
   A   
   T   
   T   
   T   
   A   
   C   
   C   
   A   
   T   
   T   
    C    
   A   
   C   
   A   
   G   
   C   
   A   
   C   
   G   
   T   
   T   
   T   
    T    
   G   
   C   
   G   
   A   
   C   
   A   
   G   
   G   
    C    
   T   
   A   
   T   
   G   
   C   
   A   
   C   
   C   
   A   
   G   
   G   
    C    
   T   
   C   
   T   
   A   
   C   
   C   
   T   
   T   
   T   
   A   
   A   
   A   
   A   
   C   
   A   
   A   
   T   
   T   
   A   
   C   
   C   
   G   
   G   
   T   
   G   
   C   
   C   
   A   
   T   
   T   
   G   
   G   
   T   
   T   
   T   
   A   
   G   
   A   
   T   
    G    
   C   
   A   
   G   
   G   
   G   
   A   
   C   
   A   
   T   
   T   
   G   
   A   
   A   
   G   
   C   
   C   
   A   
   G   
   A   
   T   
   G   
   A   
   A   
   G   
   A   
   G   
   C   
   T   
   A   
   G   
   A   
   A   
   A   
   T   
   T   
   A   
   A   
   T   
   G   
   G   
    T    
   T   
   T   
   G   
   A   
   A   
   A   
   T   
   G   
   G   
   C   
   A   
   A   
   A   
   A   
   A   
   G   
   A   
   T   
   A   
   A   
   A   
   T   
   C   
   T   
   T   
   G   
   G   
   G   
   G   
    C    
   G   
   G   
   C   
   T   
   A   
   T   
   T   
   T   
   T   
   G   
   C   
    G    
   A   
   C   
   A   
   C   
   G   
   T   
   G   
   T   
   A   
   A   
   A   
   A   
   G   
   A   
   A   
   G   
   C   
   A   
   A   
   G   
   T   
   C   
   C   
   A   
   G   
   T   
   C   
   A   
   A   
   T   
   C   
   T   
    G    
   C   
   G   
   T   
   A   
   C   
   C   
   G   
   C   
   T   
   T   
   T   
   A   
   G   
   T   
   T   
   A   
   A   
   T   
   T   
   C   
   A   
   G   
   A   
   T   
   A   
   A   
   T   
   A   
   T   
   T   
   T   
   A   
   T   
   T   
   T   
   T   
   G   
   C   
    C    
   C   
   A   
   A   
   C   
   A   
   A   
   A   
   C   
   A   
   T   
   T   
   A   
   C   
   G   
    G    
   A   
   T   
   G   
   G   
   G   
    A    
   G   
   A   
   A   
   G   
   A   
   C   
   A   
   A   
   A   
   T   
   T   
   T   
   A   
   G   
   A   
   G   
   C   
    G    
   G   
   G   
   T   
   C   
   T   
   T   
   A   
   A   
   T   
   A   
   A   
   A   
   T   
   T   
   C   
   A   
   T   
   T   
   T   
   T   
   C   
   G   
   G   
   T   
   G   
   A   
   G   
   G   
   A   
   A   
   C   
   T   
   A   
   G   
   A   
   T   
   T   
   T   
   A   
   C   
   C   
   A   
   A   
   T   
    T    
   G   
   C   
   C   
   A   
   T   
   G   
   A   
   C   
   G   
   C   
   C   
    T    
   G   
   C   
   T   
   C   
   A   
   A   
   A   
   T   
   T   
   T   
   C   
   A   
   A   
   A   
    T    
   G   
   A   
   A   
   G   
   A   
   T   
   A   
   A   
   A   
   T   
   T   
   T   
   A   
   A   
   T   
   T   
   C   
   T   
   G   
   A   
   A   
   A   
   T   
   T   
   C   
   T   
   T   
   T   
   T   
    A    
   G   
   C   
   A   
   G   
   A   
   T   
   A   
   C   
    G    
   G   
   G   
   C   
   T   
   A   
    C    
   G   
   G   
    T    
   C   
   A   
   A   
   G   
   G   
    T    
   C   
   A   
   A   
   T   
   T   
   G   
   T   
   T   
   A   
   A   
   T   
   T   
   T   
   C   
    G    
   C   
   C   
   A   
   A   
   T   
   C   
   C   
   A   
   G   
   C   
   A   
   A   
   G   
   C   
   T   
   A   
   C   
   C   
   A   
   T   
   G   
   T   
   A   
   T   
   A   
   G   
   T   
   G   
   T   
   T   
   T   
   T   
   C   
   C   
   A   
   A   
   A   
   A   
   T   
   A   
   A   
   T   
   G   
   G   
    A    
   A   
   C   
   A   
    T    
   T   
    A    
   G   
   T   
    C    
   T   
   A   
   T   
   C   
   C   
   A   
   A   
   A   
   A   
    C    
   T   
   A   
   G   
   T   
   T   
   T   
   T   
   A   
   G   
   A   
   C   
   A   
   A   
   A   
   G   
   A   
   A   
   A   
   C   
    G    
   A   
   A   
   G   
   A   
   A   
   A   
   A   
   A   
   A   
   G   
   A   
   C   
   A   
   A   
   T   
   G   
   T   
   A   
   A   
   T   
   C   
   A   
   G   
   C   
   G   
   C   
   C   
   A   
   A   
   C   
   G   
   C   
   A   
   G   
   C   
   G   
   A   
   A   
   T   
   A   
   C   
   A   
   A   
   T   
   T   
   G   
   C   
   C   
   A   
   C   
   A   
   G   
   A   
   T   
   C   
   T   
   T   
   T   
   T   
   A   
   G   
   G   
   T   
   A   
   G   
   T   
   G   
   T   
   G   
   G   
   A   
   A   
   G   
   A   
   T   
   C   
   C   
   T   
   T   
   C   
   A   
   G   
   G   
   T   
   T   
   A   
   T   
   G   
   T   
   T   
   T   
   A   
   T   
   A   
   A   
   T   
   A   
   T   
   G   
   T   
   A   
   C   
   A   
   A   
   T   
   C   
   C   
   G   
   A   
   A   
   C   
   T   
   T   
   C   
   T   
   C   
   A   
   T   
   T   
   G   
   G   
   C   
   T   
   G   
   C   
   T   
   A   
   A   
   A   
   A   
   C   
   A   
   G   
   G   
   A   
   A   
   C   
   A   
   G   
   C   
   T   
   G   
   A   
   A   
   A   
   T   
   A   
   A   
   A   
   A   
   G   
   A   
   T   
   A   
   A   
   A   
   C   
   A   
   A   
   G   
   A   
   T   
   A   
   C   
   A   
   G   
   A   
   T   
   G   
   G   
   C   
   A   
   A   
   A   
   G   
   A   
   A   
   A   
   A   
   C   
   A   
   G   
   T   
   T   
   T   
   C   
   C   
   T   
   T   
   C   
   T   
   A   
   A   
   C   
   G   
   T   
   T   
   A   
   G   
   A   
   T   
   C   
   G   
   T   
   A   
   G   
   T   
   A   
   A   
   C   
   A   
   A   
   T   
   A   
   A   
   A   
   T   
   T   
   C   
   T   
   T   
   A   
   A   
   C   
   A   
   A   
   T   
   G   
   A   
   T   
   T   
   A   
   T   
   G   
   G   
   T   
   T   
   G   
   A   
   A   
   A   
   A   
   T   
   T   
   C   
   A   
   G   
   G   
   A   
   G   
   A   
   A   
   A   
   A   
   T   
   G   
   G   
   T   
   T   
   C   
   T   
   G   
   C   
   A   
   A   
   C   
   A   
   G   
   A   
   T   
   A   
   T   
   C   
   A   
   G   
   T   
   A   
   A   
   A   
   C   
   C   
   A   
   T   
   T   
   A   
   A   
   T   
   T   
   G   
   A   
   T   
   T   
   A   
   C   
   T   
   T   
   A   
   G   
   A   
   A   
   G   
   C   
   A   
   A   
   C   
   C   
   A   
   T   
   T   
   A   
   A   
   A   
   -   
   -   
   -   
   -   
   -   
   -   
   -   
   -   
   -   
   -   
   -   
   -   
   -   
   -   
   -   
   -   
   -   
   -   
   -   
   -   
   -   
   -   
   -   
   -   
   -   
   -   
   -   
   -   
   -   
   -   
   -   
   -   
   -   
   -   
   -   
   -   
   -   
   -   
   -   
   -   
   -   
   -   
   -   
   -   
   -   
   -   
   -   
   -   
   -   
   -   
   -   
   -   
   -   
   -   
   -   
   -   
   -   
   -   
   -   
   -   
   -   
   -   
   -   
   -   
   -   
   -   
   -   
   -   
   -   
   -   
   -   
   -   
   -   
   -   
   -   
   -   
   -   
   -   
   -   
   -   
   -   
   -   
   -   
   -   
   -   
   -   
   -   
   -   
   -   
   -   
   -   
   -   
   -   
   -   
   -   
   -   
   -   
   -   
   -   
   -   
   -   
   -   
   -   
   -   
   -   
   -   
   -   
   -   
   -   
   -   
   -   
   -   
   -   
   -   
   -   
   -   
   -   
   -   
   -   
   -   
   -   
   -   
   -   
   -   
   -   
   -   
   -   
   -   
   -   
   -   
   -   
   -   
   -   
   -   
   -   
   -   
   -   
   -   
   -   
   -   
   -   
   -   
   -   
   -   
   -   
   -   
   -   
   -   
   -   
   -   
   -   
   -   
   -   
   -   
   -   
   -   
   -   
   -   
   -   
   -   
   -   
   -   
   -   
   -   
   -   
   -   
   -   
   -   
   -   
   C   
   A   
   A   
   T   
   G   
   G   
   C   
   A   
   A   
   G   
   C   
   C   
   A   
   A   
   A   
   C   
   A   
   A   
   G   
   A   
   A   
   T   
   T   
   A   
   G   
   C   
   C   
   G   
   A   
   A   
   G   
   C   
   G   
   A   
   A   
   G   
   A   
   A   
   A   
   A   
   C   
   A   
   G   
   C   
   T   
   A   
   C   
   T   
   A   
   C   
   A   
   T
[truncated: 1,315,441 more chars]
