# Supplementary material for: sraX: A Novel Comprehensive Resistome Analysis Tool
Source: Front Microbiol. 2020 Feb 5;11:52. doi: 10.3389/fmicb.2020.00052 (PMC7025521; doi:10.3389/fmicb.2020.00052)
Supplement: Supplementary file 4 [file Data_Sheet_3.ZIP › pbp4/pbp4_aa.html]

# pbp4

|  |
| --- |
|  |

|  |
| --- |
|  |

|  |  |
| --- | --- |
| **Definition:** | Not indicated |

|  |
| --- |
|  |

|  |
| --- |
|  |

|  |  |
| --- | --- |
| **Reference organism:** | Not indicated |

|  |
| --- |
|  |

|  |
| --- |
|  |

|  |  |
| --- | --- |
| **Summary Data** | || **Parameter** | **Value_(n)_** || Total number of genomes | 97 |
| MSA length | 680 |
| Total number of SNPs | 38 |
| SNP density (# SNPs/MSA length) | 0.056 |

|  |
| --- |
|  |

|  |
| --- |
|  |

|  |
| --- |
|  |

|  |
| --- |
|  |

|  |  |  |  |  |
| --- | --- | --- | --- | --- |
| **SNPs conferring antibiotic resistance** | | | | || **Reference gene** | **Seq. pos.** | **Ref\_base** | **Alt\_base** | **Confirmation SNP?** |||  |  |  |  |  |
| --- | --- | --- | --- | --- |
| pbp4 50 I Y **SNP variant (I50T)** | | | | |
||  |  |  |  |  |
| --- | --- | --- | --- | --- |
| pbp4 218 K N **No** | | | | |
||  |  |  |  |  |
| --- | --- | --- | --- | --- |
| pbp4 243 I F **No** | | | | |
||  |  |  |  |  |
| --- | --- | --- | --- | --- |
| pbp4 369 V A **No** | | | | |
||  |  |  |  |  |
| --- | --- | --- | --- | --- |
| pbp4 475 L M **No** | | | | |
||  |  |  |  |  |
| --- | --- | --- | --- | --- |
| pbp4 520 P S **No** | | | | |
||  |  |  |  |  |
| --- | --- | --- | --- | --- |
| pbp4 573 D E **Yes** | | | | |
||  |  |  |  |  |
| --- | --- | --- | --- | --- |
| pbp4 582 V I **No** | | | | |
||  |  |  |  |  |
| --- | --- | --- | --- | --- |
| pbp4 605 Y H **No** | | | | |
||  |  |  |  |  |
| --- | --- | --- | --- | --- |
| pbp4 623 A P **No** | | | | |
||  |  |  |  |  |
| --- | --- | --- | --- | --- |
| pbp4 632 D E **No** | | | | |

|  |
| --- |
|  |

|  |
| --- |
|  |

|  |
| --- |
|  |

|  |  |  |  |  |  |  |  |  |  |  |  |  |  |  |  |  |  |  |  |  |  |  |  |  |  |  |  |  |  |  |  |  |  |  |  |  |  |  |  |  |  |  |  |  |  |  |  |  |  |  |  |  |  |  |  |  |  |  |  |  |  |  |  |  |  |  |  |  |  |  |  |  |  |  |  |  |  |  |  |  |  |  |  |  |  |  |  |  |  |  |  |  |  |  |  |  |  |  |  |  |  |  |  |  |  |  |  |  |  |  |  |  |  |  |  |  |  |  |  |  |  |  |  |  |  |  |  |  |  |  |  |  |  |  |  |  |  |  |  |  |  |  |  |  |  |  |  |  |  |  |  |  |  |  |  |  |  |  |  |  |  |  |  |  |  |  |  |  |  |  |  |  |  |  |  |  |  |  |  |  |  |  |  |  |  |  |  |  |  |  |  |  |  |  |  |  |  |  |  |  |  |  |  |  |  |  |  |  |  |  |  |  |  |  |  |  |  |  |  |  |  |  |  |  |  |  |  |  |  |  |  |  |  |  |  |  |  |  |  |  |  |  |  |  |  |  |  |  |  |  |  |  |  |  |  |  |  |  |  |  |  |  |  |  |  |  |  |  |  |  |  |  |  |  |  |  |  |  |  |  |  |  |  |  |  |  |  |  |  |  |  |  |  |  |  |  |  |  |  |  |  |  |  |  |  |  |  |  |  |  |  |  |  |  |  |  |  |  |  |  |  |  |  |  |  |  |  |  |  |  |  |  |  |  |  |  |  |  |  |  |  |  |  |  |  |  |  |  |  |  |  |  |  |  |  |  |  |  |  |  |  |  |  |  |  |  |  |  |  |  |  |  |  |  |  |  |  |  |  |  |  |  |  |  |  |  |  |  |  |  |  |  |  |  |  |  |  |  |  |  |  |  |  |  |  |  |  |  |  |  |  |  |  |  |  |  |  |  |  |  |  |  |  |  |  |  |  |  |  |  |  |  |  |  |  |  |  |  |  |  |  |  |  |  |  |  |  |  |  |  |  |  |  |  |  |  |  |  |  |  |  |  |  |  |  |  |  |  |  |  |  |  |  |  |  |  |  |  |  |  |  |  |  |  |  |  |  |  |  |  |  |  |  |  |  |  |  |  |  |  |  |  |  |  |  |  |  |  |  |  |  |  |  |  |  |  |  |  |  |  |  |  |  |  |  |  |  |  |  |  |  |  |  |  |  |  |  |  |  |  |  |  |  |  |  |  |  |  |  |  |  |  |  |  |  |  |  |  |  |  |  |  |  |  |  |  |  |  |  |  |  |  |  |  |  |  |  |  |  |  |  |  |  |  |  |  |  |  |  |  |  |  |  |  |  |  |  |  |  |  |  |  |  |  |  |  |  |  |  |  |  |  |  |  |  |  |  |  |  |  |  |  |  |  |  |  |  |  |  |  |  |  |  |  |  |  |  |  |  |  |  |  |  |  |  |  |  |  |  |  |  |  |  |  |  |  |  |  |  |  |  |  |  |  |  |  |  |  |  |  |  |  |  |  |  |  |  |  |  |  |  |
| --- | --- | --- | --- | --- | --- | --- | --- | --- | --- | --- | --- | --- | --- | --- | --- | --- | --- | --- | --- | --- | --- | --- | --- | --- | --- | --- | --- | --- | --- | --- | --- | --- | --- | --- | --- | --- | --- | --- | --- | --- | --- | --- | --- | --- | --- | --- | --- | --- | --- | --- | --- | --- | --- | --- | --- | --- | --- | --- | --- | --- | --- | --- | --- | --- | --- | --- | --- | --- | --- | --- | --- | --- | --- | --- | --- | --- | --- | --- | --- | --- | --- | --- | --- | --- | --- | --- | --- | --- | --- | --- | --- | --- | --- | --- | --- | --- | --- | --- | --- | --- | --- | --- | --- | --- | --- | --- | --- | --- | --- | --- | --- | --- | --- | --- | --- | --- | --- | --- | --- | --- | --- | --- | --- | --- | --- | --- | --- | --- | --- | --- | --- | --- | --- | --- | --- | --- | --- | --- | --- | --- | --- | --- | --- | --- | --- | --- | --- | --- | --- | --- | --- | --- | --- | --- | --- | --- | --- | --- | --- | --- | --- | --- | --- | --- | --- | --- | --- | --- | --- | --- | --- | --- | --- | --- | --- | --- | --- | --- | --- | --- | --- | --- | --- | --- | --- | --- | --- | --- | --- | --- | --- | --- | --- | --- | --- | --- | --- | --- | --- | --- | --- | --- | --- | --- | --- | --- | --- | --- | --- | --- | --- | --- | --- | --- | --- | --- | --- | --- | --- | --- | --- | --- | --- | --- | --- | --- | --- | --- | --- | --- | --- | --- | --- | --- | --- | --- | --- | --- | --- | --- | --- | --- | --- | --- | --- | --- | --- | --- | --- | --- | --- | --- | --- | --- | --- | --- | --- | --- | --- | --- | --- | --- | --- | --- | --- | --- | --- | --- | --- | --- | --- | --- | --- | --- | --- | --- | --- | --- | --- | --- | --- | --- | --- | --- | --- | --- | --- | --- | --- | --- | --- | --- | --- | --- | --- | --- | --- | --- | --- | --- | --- | --- | --- | --- | --- | --- | --- | --- | --- | --- | --- | --- | --- | --- | --- | --- | --- | --- | --- | --- | --- | --- | --- | --- | --- | --- | --- | --- | --- | --- | --- | --- | --- | --- | --- | --- | --- | --- | --- | --- | --- | --- | --- | --- | --- | --- | --- | --- | --- | --- | --- | --- | --- | --- | --- | --- | --- | --- | --- | --- | --- | --- | --- | --- | --- | --- | --- | --- | --- | --- | --- | --- | --- | --- | --- | --- | --- | --- | --- | --- | --- | --- | --- | --- | --- | --- | --- | --- | --- | --- | --- | --- | --- | --- | --- | --- | --- | --- | --- | --- | --- | --- | --- | --- | --- | --- | --- | --- | --- | --- | --- | --- | --- | --- | --- | --- | --- | --- | --- | --- | --- | --- | --- | --- | --- | --- | --- | --- | --- | --- | --- | --- | --- | --- | --- | --- | --- | --- | --- | --- | --- | --- | --- | --- | --- | --- | --- | --- | --- | --- | --- | --- | --- | --- | --- | --- | --- | --- | --- | --- | --- | --- | --- | --- | --- | --- | --- | --- | --- | --- | --- | --- | --- | --- | --- | --- | --- | --- | --- | --- | --- | --- | --- | --- | --- | --- | --- | --- | --- | --- | --- | --- | --- | --- | --- | --- | --- | --- | --- | --- | --- | --- | --- | --- | --- | --- | --- | --- | --- | --- | --- | --- | --- | --- | --- | --- | --- | --- | --- | --- | --- | --- | --- | --- | --- | --- | --- | --- | --- | --- | --- | --- | --- | --- | --- | --- | --- | --- | --- | --- | --- | --- | --- | --- | --- | --- | --- | --- | --- | --- | --- | --- | --- | --- | --- | --- | --- | --- | --- | --- | --- | --- | --- | --- | --- | --- | --- | --- | --- | --- | --- | --- | --- | --- | --- | --- | --- | --- | --- | --- | --- | --- | --- | --- | --- | --- | --- | --- | --- | --- | --- | --- | --- | --- | --- | --- | --- | --- | --- | --- | --- | --- | --- | --- | --- | --- | --- | --- | --- | --- | --- | --- | --- | --- | --- | --- | --- | --- | --- | --- | --- | --- | --- | --- | --- | --- | --- | --- | --- | --- | --- | --- | --- | --- | --- | --- | --- | --- | --- | --- | --- | --- | --- | --- | --- | --- | --- | --- | --- | --- | --- | --- | --- | --- | --- | --- | --- | --- | --- | --- | --- | --- | --- | --- | --- | --- | --- | --- | --- | --- | --- | --- | --- | --- | --- | --- | --- | --- | --- | --- | --- |
|  || **pbp4** | **M** | **E** | **R** | **S** | **N** | **R** | **N** | **K** | **K** | **S** | **S** | **K** | **K** | **P** | **L** | **I** | **L** | **G** | **V** | **S** | **A** | **L** | **V** | **L** | **I** | **A** | **A** | **A** | **G** | **G** | **G** | **Y** | **Y** | **A** | **Y** | **S** | **Q** | **W** | **Q** | **A** | **K** | **Q** | **E** | **L** | **A** | **E** | **A** | **K** | **K** | **I** | **A** | **T** | **T** | **F** | **L** | **N** | **V** | **L** | **S** | **K** | **Q** | **E** | **F** | **D** | **K** | **L** | **P** | **S** | **V** | **V** | **Q** | **E** | **A** | **S** | **L** | **K** | **K** | **N** | **G** | **Y** | **D** | **T** | **K** | **S** | **V** | **V** | **E** | **K** | **Y** | **Q** | **A** | **I** | **Y** | **S** | **G** | **I** | **Q** | **A** | **E** | **G** | **V** | **K** | **A** | **S** | **D** | **V** | **Q** | **V** | **K** | **K** | **A** | **K** | **D** | **N** | **Q** | **Y** | **T** | **F** | **T** | **Y** | **K** | **L** | **S** | **M** | **S** | **T** | **P** | **L** | **G** | **E** | **M** | **K** | **D** | **L** | **S** | **Y** | **Q** | **S** | **S** | **I** | **A** | **K** | **K** | **G** | **D** | **T** | **Y** | **Q** | **I** | **A** | **W** | **K** | **P** | **S** | **L** | **I** | **F** | **P** | **D** | **M** | **S** | **G** | **N** | **D** | **K** | **I** | **S** | **I** | **Q** | **V** | **D** | **N** | **A** | **K** | **R** | **G** | **E** | **I** | **V** | **D** | **R** | **N** | **G** | **S** | **G** | **L** | **A** | **I** | **N** | **K** | **V** | **F** | **D** | **E** | **V** | **G** | **V** | **V** | **P** | **G** | **K** | **L** | **G** | **S** | **G** | **A** | **E** | **K** | **T** | **A** | **N** | **I** | **K** | **A** | **F** | **S** | **D** | **K** | **F** | **G** | **V** | **S** | **V** | **D** | **E** | **I** | **N** | **Q** | **K** | **L** | **S** | **Q** | **G** | **W** | **V** | **Q** | **A** | **D** | **S** | **F** | **V** | **P** | **I** | **T** | **V** | **A** | **S** | **E** | **P** | **V** | **T** | **E** | **L** | **P** | **T** | **G** | **A** | **A** | **T** | **K** | **D** | **T** | **E** | **S** | **R** | **Y** | **Y** | **P** | **L** | **G** | **E** | **A** | **A** | **A** | **Q** | **L** | **I** | **G** | **Y** | **T** | **G** | **T** | **I** | **T** | **A** | **E** | **D** | **I** | **E** | **K** | **N** | **P** | **E** | **L** | **S** | **S** | **T** | **G** | **V** | **I** | **G** | **K** | **T** | **G** | **L** | **E** | **R** | **A** | **F** | **D** | **K** | **E** | **L** | **R** | **G** | **Q** | **D** | **G** | **G** | **S** | **L** | **V** | **I** | **L** | **D** | **D** | **K** | **E** | **N** | **V** | **K** | **K** | **A** | **L** | **Q** | **T** | **K** | **E** | **K** | **K** | **D** | **G** | **Q** | **T** | **I** | **K** | **L** | **T** | **I** | **D** | **S** | **G** | **V** | **Q** | **Q** | **Q** | **A** | **F** | **A** | **I** | **F** | **D** | **K** | **R** | **P** | **G** | **S** | **A** | **V** | **I** | **T** | **D** | **P** | **Q** | **K** | **G** | **D** | **L** | **L** | **A** | **T** | **V** | **S** | **S** | **P** | **S** | **Y** | **D** | **P** | **N** | **K** | **M** | **A** | **N** | **G** | **I** | **S** | **Q** | **K** | **E** | **Y** | **D** | **A** | **Y** | **N** | **N** | **N** | **K** | **D** | **L** | **P** | **F** | **T** | **A** | **R** | **F** | **A** | **T** | **G** | **Y** | **A** | **P** | **G** | **S** | **T** | **F** | **K** | **T** | **I** | **T** | **G** | **A** | **I** | **G** | **L** | **D** | **A** | **G** | **T** | **L** | **K** | **P** | **D** | **E** | **E** | **L** | **E** | **I** | **N** | **G** | **L** | **K** | **W** | **Q** | **K** | **D** | **K** | **S** | **W** | **G** | **G** | **Y** | **F** | **A** | **T** | **R** | **V** | **K** | **E** | **A** | **S** | **P** | **V** | **N** | **L** | **R** | **T** | **A** | **L** | **V** | **N** | **S** | **D** | **N** | **I** | **Y** | **F** | **A** | **Q** | **Q** | **T** | **L** | **R** | **M** | **G** | **E** | **D** | **K** | **F** | **R** | **A** | **G** | **L** | **N** | **K** | **F** | **I** | **F** | **G** | **E** | **E** | **L** | **D** | **L** | **P** | **I** | **A** | **M** | **T** | **P** | **A** | **Q** | **I** | **S** | **N** | **E** | **D** | **K** | **F** | **N** | **S** | **E** | **I** | **L** | **L** | **A** | **D** | **T** | **G** | **Y** | **G** | **Q** | **G** | **Q** | **L** | **L** | **I** | **S** | **P** | **I** | **Q** | **Q** | **A** | **T** | **M** | **Y** | **S** | **V** | **F** | **Q** | **N** | **N** | **G** | **T** | **L** | **V** | **Y** | **P** | **K** | **L** | **V** | **L** | **D** | **K** | **E** | **T** | **K** | **K** | **K** | **D** | **N** | **V** | **I** | **S** | **A** | **N** | **A** | **A** | **N** | **T** | **I** | **A** | **T** | **D** | **L** | **L** | **G** | **S** | **V** | **E** | **D** | **P** | **S** | **G** | **Y** | **V** | **Y** | **N** | **M** | **Y** | **N** | **P** | **N** | **F** | **S** | **L** | **A** | **A** | **K** | **T** | **G** | **T** | **A** | **E** | **I** | **K** | **D** | **K** | **Q** | **D** | **T** | **D** | **G** | **K** | **E** | **N** | **S** | **F** | **L** | **L** | **T** | **L** | **D** | **R** | **S** | **N** | **N** | **K** | **F** | **L** | **T** | **M** | **I** | **M** | **V** | **E** | **N** | **S** | **G** | **E** | **N** | **G** | **S** | **A** | **T** | **D** | **I** | **S** | **K** | **P** | **L** | **I** | **D** | **Y** | **L** | **E** | **A** | **T** | **I** | **K** |
| **N52755** | **-** | **-** | **-** | **-** | **-** | **-** | **-** | **-** | **-** | **-** | **-** | **-** | **-** | **-** | **-** | **-** | **-** | **-** | **-** | **-** | **-** | **-** | **-** | **-** | **-** | **-** | **-** | **-** | **-** | **-** | **-** | **-** | **-** | **-** | **-** | **-** | **Q** | **W** | **Q** | **A** | **K** | **Q** | **E** | **L** | **A** | **E** | **A** | **K** | **K** | **T** | **A** | **T** | **T** | **F** | **L** | **N** | **V** | **L** | **S** | **K** | **Q** | **E** | **F** | **D** | **K** | **L** | **P** | **S** | **V** | **V** | **Q** | **E** | **A** | **S** | **L** | **K** | **K** | **N** | **G** | **Y** | **D** | **T** | **K** | **S** | **V** | **V** | **E** | **K** | **Y** | **Q** | **A** | **I** | **Y** | **S** | **G** | **I** | **Q** | **A** | **E** | **G** | **V** | **K** | **A** | **S** | **D** | **V** | **Q** | **V** | **K** | **K** | **A** | **K** | **D** | **N** | **Q** | **Y** | **T** | **F** | **T** | **Y** | **K** | **L** | **S** | **M** | **S** | **T** | **P** | **L** | **G** | **E** | **M** | **K** | **D** | **L** | **S** | **Y** | **Q** | **S** | **S** | **I** | **A** | **K** | **K** | **G** | **D** | **T** | **Y** | **Q** | **I** | **A** | **W** | **K** | **P** | **S** | **L** | **I** | **F** | **P** | **D** | **M** | **S** | **G** | **N** | **D** | **K** | **I** | **S** | **I** | **Q** | **V** | **D** | **N** | **A** | **K** | **R** | **G** | **E** | **I** | **V** | **D** | **R** | **N** | **G** | **S** | **G** | **L** | **A** | **I** | **N** | **K** | **V** | **F** | **D** | **E** | **V** | **G** | **V** | **V** | **P** | **G** | **K** | **L** | **G** | **S** | **G** | **A** | **E** | **K** | **T** | **A** | **N** | **I** | **K** | **A** | **F** | **S** | **D** | **K** | **F** | **G** | **V** | **S** | **I** | **D** | **E** | **I** | **N** | **Q** | **K** | **L** | **S** | **Q** | **G** | **W** | **V** | **Q** | **A** | **D** | **S** | **F** | **V** | **P** | **I** | **T** | **V** | **A** | **S** | **E** | **P** | **V** | **T** | **E** | **L** | **P** | **T** | **G** | **A** | **A** | **T** | **K** | **D** | **T** | **E** | **S** | **R** | **Y** | **Y** | **P** | **L** | **G** | **E** | **A** | **A** | **A** | **Q** | **L** | **I** | **G** | **Y** | **T** | **G** | **T** | **I** | **T** | **A** | **E** | **D** | **I** | **E** | **K** | **N** | **P** | **E** | **L** | **S** | **S** | **T** | **G** | **V** | **I** | **G** | **K** | **T** | **G** | **L** | **E** | **R** | **A** | **F** | **D** | **K** | **E** | **L** | **R** | **G** | **Q** | **D** | **G** | **G** | **S** | **L** | **V** | **I** | **L** | **D** | **D** | **K** | **E** | **N** | **V** | **K** | **K** | **A** | **L** | **Q** | **T** | **K** | **E** | **K** | **K** | **D** | **G** | **Q** | **T** | **I** | **K** | **L** | **T** | **I** | **D** | **S** | **G** | **V** | **Q** | **Q** | **Q** | **A** | **F** | **A** | **I** | **F** | **D** | **K** | **R** | **P** | **G** | **S** | **A** | **V** | **I** | **T** | **D** | **P** | **Q** | **K** | **G** | **D** | **L** | **L** | **A** | **T** | **V** | **S** | **S** | **P** | **S** | **Y** | **D** | **P** | **N** | **K** | **M** | **A** | **N** | **G** | **I** | **S** | **Q** | **K** | **E** | **Y** | **D** | **A** | **Y** | **N** | **N** | **N** | **K** | **D** | **L** | **P** | **F** | **T** | **A** | **R** | **F** | **A** | **T** | **G** | **Y** | **A** | **P** | **G** | **S** | **T** | **F** | **K** | **T** | **I** | **T** | **G** | **A** | **I** | **G** | **L** | **D** | **A** | **G** | **T** | **L** | **K** | **P** | **D** | **E** | **E** | **L** | **E** | **I** | **N** | **G** | **L** | **K** | **W** | **Q** | **K** | **D** | **K** | **S** | **W** | **G** | **G** | **Y** | **F** | **A** | **T** | **R** | **V** | **K** | **E** | **A** | **S** | **P** | **V** | **N** | **L** | **R** | **T** | **A** | **L** | **V** | **N** | **S** | **D** | **N** | **I** | **Y** | **F** | **A** | **Q** | **Q** | **T** | **L** | **R** | **M** | **G** | **E** | **D** | **K** | **F** | **R** | **A** | **G** | **L** | **N** | **K** | **F** | **I** | **F** | **G** | **E** | **E** | **L** | **D** | **L** | **P** | **I** | **A** | **M** | **T** | **P** | **A** | **Q** | **I** | **S** | **N** | **E** | **D** | **K** | **F** | **N** | **S** | **E** | **I** | **L** | **L** | **A** | **D** | **T** | **G** | **Y** | **G** | **Q** | **G** | **Q** | **L** | **L** | **I** | **S** | **P** | **I** | **Q** | **Q** | **A** | **T** | **M** | **Y** | **S** | **V** | **F** | **Q** | **N** | **N** | **G** | **T** | **L** | **V** | **Y** | **P** | **K** | **L** | **V** | **L** | **D** | **K** | **E** | **T** | **K** | **K** | **K** | **D** | **N** | **V** | **I** | **S** | **A** | **N** | **A** | **A** | **N** | **T** | **I** | **A** | **T** | **D** | **L** | **L** | **G** | **S** | **V** | **E** | **D** | **P** | **S** | **G** | **Y** | **V** | **Y** | **N** | **M** | **Y** | **N** | **P** | **N** | **F** | **S** | **L** | **A** | **A** | **K** | **T** | **G** | **T** | **A** | **E** | **I** | **K** | **D** | **K** | **Q** | **D** | **T** | **D** | **G** | **K** | **E** | **N** | **S** | **F** | **L** | **L** | **T** | **L** | **D** | **R** | **S** | **N** | **N** | **K** | **F** | **L** | **T** | **M** | **I** | **M** | **V** | **E** | **N** | **S** | **G** | **E** | **N** | **G** | **S** | **A** | **T** | **D** | **I** | **S** | **K** | **P** | **L** | **I** | **D** | **Y** | **L** | **E** | **A** | **T** | **I** | **K** | **-** | **-** | **-** | **-** | **-** | **-** | **-** | **-** | **-** | **-** | **-** | **-** | **-** | **-** | **-** | **-** | **-** | **-** | **-** | **-** | **-** | **-** | **-** | **-** | **-** | **-** | **-** | **-** | **-** | **-** | **-** | **-** | **-** | **-** | **-** | **-** | **Q** | **W** | **Q** | **A** | **K** | **Q** | **E** | **L** | **A** | **E** | **A** | **K** | **K** | **T** | **A** | **T** | **T** | **F** | **L** | **N** | **V** | **L** | **S** | **K** | **Q** | **E** | **F** | **D** | **K** | **L** | **P** | **S** | **V** | **V** | **Q** | **E** | **A** | **S** | **L** | **K** | **K** | **N** | **G** | **Y** | **D** | **T** | **K** | **S** | **V** | **V** | **E** | **K** | **Y** | **Q** | **A** | **I** | **Y** | **S** | **G** | **I** | **Q** | **A** | **E** | **G** | **V** | **K** | **A** | **S** | **D** | **V** | **Q** | **V** | **K** | **K** | **A** | **K** | **D** | **N** | **Q** | **Y** | **T** | **F** | **T** | **Y** | **K** | **L** | **S** | **M** | **S** | **T** | **P** | **L** | **G** | **E** | **M** | **K** | **D** | **L** | **S** | **Y** | **Q** | **S** | **S** | **I** | **A** | **K** | **K** | **G** | **D** | **T** | **Y** | **Q** | **I** | **A** | **W** | **K** | **P** | **S** | **L** | **I** | **F** | **P** | **D** | **M** | **S** | **G** | **N** | **D** | **K** | **I** | **S** | **I** | **Q** | **V** | **D** | **N** | **A** | **K** | **R** | **G** | **E** | **I** | **V** | **D** | **R** | **N** | **G** | **S** | **G** | **L** | **A** | **I** | **N** | **K** | **V** | **F** | **D** | **E** | **V** | **G** | **V** | **V** | **P** | **G** | **K** | **L** | **G** | **S** | **G** | **A** | **E** | **K** | **T** | **A** | **N** | **I** | **K** | **A** | **F** | **S** | **D** | **K** | **F** | **G** | **V** | **S** | **I** | **D** | **E** | **I** | **N** | **Q** | **K** | **L** | **S** | **Q** | **G** | **W** | **V** | **Q** | **A** | **D** | **S** | **F** | **V** | **P** | **I** | **T** | **V** | **A** | **S** | **E** | **P** | **V** | **T** | **E** | **L** | **P** | **T** | **G** | **A** | **A** | **T** | **K** | **D** | **T** | **E** | **S** | **R** | **Y** | **Y** | **P** | **L** | **G** | **E** | **A** | **A** | **A** | **Q** | **L** | **I** | **G** | **Y** | **T** | **G** | **T** | **I** | **T** | **A** | **E** | **D** | **I** | **E** | **K** | **N** | **P** | **E** | **L** | **S** | **S** | **T** | **G** | **V** | **I** | **G** | **K** | **T** | **G** | **L** | **E** | **R** | **A** | **F** | **D** | **K** | **E** | **L** | **R** | **G** | **Q** | **D** | **G** | **G** | **S** | **L** | **V** | **I** | **L** | **D** | **D** | **K** | **E** | **N** | **V** | **K** | **K** | **A** | **L** | **Q** | **T** | **K** | **E** | **K** | **K** | **D** | **G** | **Q** | **T** | **I** | **K** | **L** | **T** | **I** | **D** | **S** | **G** | **V** | **Q** | **Q** | **Q** | **A** | **F** | **A** | **I** | **F** | **D** | **K** | **R** | **P** | **G** | **S** | **A** | **V** | **I** | **T** | **D** | **P** | **Q** | **K** | **G** | **D** | **L** | **L** | **A** | **T** | **V** | **S** | **S** | **P** | **S** | **Y** | **D** | **P** | **N** | **K** | **M** | **A** | **N** | **G** | **I** | **S** | **Q** | **K** | **E** | **Y** | **D** | **A** | **Y** | **N** | **N** | **N** | **K** | **D** | **L** | **P** | **F** | **T** | **A** | **R** | **F** | **A** | **T** | **G** | **Y** | **A** | **P** | **G** | **S** | **T** | **F** | **K** | **T** | **I** | **T** | **G** | **A** | **I** | **G** | **L** | **D** | **A** | **G** | **T** | **L** | **K** | **P** | **D** | **E** | **E** | **L** | **E** | **I** | **N** | **G** | **L** | **K** | **W** | **Q** | **K** | **D** | **K** | **S** | **W** | **G** | **G** | **Y** | **F** | **A** | **T** | **R** | **V** | **K** | **E** | **A** | **S** | **P** | **V** | **N** | **L** | **R** | **T** | **A** | **L** | **V** | **N** | **S** | **D** | **N** | **I** | **Y** | **F** | **A** | **Q** | **Q** | **T** | **L** | **R** | **M** | **G** | **E** | **D** | **K** | **F** | **R** | **A** | **G** | **L** | **N** | **K** | **F** | **I** | **F** | **G** | **E** | **E** | **L** | **D** | **L** | **P** | **I** | **A** | **M** | **T** | **P** | **A** | **Q** | **I** | **S** | **N** | **E** | **D** | **K** | **F** | **N** | **S** | **E** | **I** | **L** | **L** | **A** | **D** | **T** | **G** | **Y** | **G** | **Q** | **G** | **Q** | **L** | **L** | **I** | **S** | **P** | **I** | **Q** | **Q** | **A** | **T** | **M** | **Y** | **S** | **V** | **F** | **Q** | **N** | **N** | **G** | **T** | **L** | **V** | **Y** | **P** | **K** | **L** | **V** | **L** | **D** | **K** | **E** | **T** | **K** | **K** | **K** | **D** | **N** | **V** | **I** | **S** | **A** | **N** | **A** | **A** | **N** | **T** | **I** | **A** | **T** | **D** | **L** | **L** | **G** | **S** | **V** | **E** | **D** | **P** | **S** | **G** | **Y** | **V** | **Y** | **N** | **M** | **Y** | **N** | **P** | **N** | **F** | **S** | **L** | **A** | **A** | **K** | **T** | **G** | **T** | **A** | **E** | **I** | **K** | **D** | **K** | **Q** | **D** | **T** | **D** | **G** | **K** | **E** | **N** | **S** | **F** | **L** | **L** | **T** | **L** | **D** | **R** | **S** | **N** | **N** | **K** | **F** | **L** | **T** | **M** | **I** | **M** | **V** | **E** | **N** | **S** | **G** | **E** | **N** | **G** | **S** | **A** | **T** | **D** | **I** | **S** | **K** | **P** | **L** | **I** | **D** | **Y** | **L** | **E** | **A** | **T** | **I** | **K** |
| **N59845F** | **-** | **-** | **-** | **-** | **-** | **-** | **-** | **-** | **-** | **-** | **-** | **-** | **-** | **-** | **-** | **-** | **-** | **-** | **-** | **-** | **-** | **-** | **-** | **-** | **-** | **-** | **-** | **-** | **-** | **-** | **-** | **-** | **-** | **-** | **-** | **-** | **Q** | **W** | **Q** | **A** | **K** | **Q** | **E** | **L** | **A** | **E** | **A** | **K** | **K** | **T** | **A** | **T** | **T** | **F** | **L** | **N** | **V** | **L** | **S** | **K** | **Q** | **E** | **F** | **D** | **K** | **L** | **P** | **S** | **V** | **V** | **Q** | **E** | **A** | **S** | **L** | **K** | **K** | **N** | **G** | **Y** | **D** | **T** | **K** | **S** | **V** | **V** | **E** | **K** | **Y** | **Q** | **A** | **I** | **Y** | **S** | **G** | **I** | **Q** | **A** | **E** | **G** | **V** | **K** | **A** | **S** | **D** | **V** | **Q** | **V** | **K** | **K** | **A** | **K** | **D** | **N** | **Q** | **Y** | **T** | **F** | **T** | **Y** | **K** | **L** | **S** | **M** | **S** | **T** | **P** | **L** | **G** | **E** | **M** | **K** | **D** | **L** | **S** | **Y** | **Q** | **S** | **S** | **I** | **A** | **K** | **K** | **G** | **D** | **T** | **Y** | **Q** | **I** | **A** | **W** | **K** | **P** | **S** | **L** | **I** | **F** | **P** | **D** | **M** | **S** | **G** | **N** | **D** | **K** | **I** | **S** | **I** | **Q** | **V** | **D** | **N** | **A** | **K** | **R** | **G** | **E** | **I** | **V** | **D** | **R** | **N** | **G** | **S** | **G** | **L** | **A** | **I** | **N** | **K** | **V** | **F** | **D** | **E** | **V** | **G** | **V** | **V** | **P** | **G** | **K** | **L** | **G** | **S** | **G** | **A** | **E** | **K** | **T** | **A** | **N** | **I** | **K** | **A** | **F** | **S** | **D** | **K** | **F** | **G** | **V** | **S** | **V** | **D** | **E** | **I** | **N** | **Q** | **K** | **L** | **S** | **Q** | **G** | **W** | **V** | **Q** | **A** | **D** | **S** | **F** | **V** | **P** | **I** | **T** | **V** | **A** | **S** | **E** | **P** | **V** | **T** | **E** | **L** | **P** | **T** | **G** | **A** | **A** | **T** | **K** | **D** | **T** | **E** | **S** | **R** | **Y** | **Y** | **P** | **L** | **G** | **E** | **A** | **A** | **A** | **Q** | **L** | **I** | **G** | **Y** | **T** | **G** | **T** | **I** | **T** | **A** | **E** | **D** | **I** | **E** | **K** | **N** | **P** | **E** | **L** | **S** | **S** | **T** | **G** | **V** | **I** | **G** | **K** | **T** | **G** | **L** | **E** | **R** | **A** | **F** | **D** | **K** | **E** | **L** | **R** | **G** | **Q** | **D** | **G** | **G** | **S** | **L** | **V** | **I** | **L** | **D** | **D** | **K** | **E** | **N** | **V** | **K** | **K** | **A** | **L** | **Q** | **T** | **K** | **E** | **K** | **K** | **D** | **G** | **Q** | **T** | **I** | **K** | **L** | **T** | **I** | **D** | **S** | **G** | **V** | **Q** | **Q** | **Q** | **A** | **F** | **A** | **I** | **F** | **D** | **K** | **R** | **P** | **G** | **S** | **A** | **V** | **I** | **T** | **D** | **P** | **Q** | **K** | **G** | **D** | **L** | **L** | **A** | **T** | **V** | **S** | **S** | **P** | **S** | **Y** | **D** | **P** | **N** | **K** | **M** | **A** | **N** | **G** | **I** | **S** | **Q** | **K** | **E** | **Y** | **D** | **A** | **Y** | **N** | **N** | **N** | **K** | **D** | **L** | **P** | **F** | **T** | **A** | **R** | **F** | **A** | **T** | **G** | **Y** | **A** | **P** | **G** | **S** | **T** | **F** | **K** | **T** | **I** | **T** | **G** | **A** | **I** | **G** | **L** | **D** | **A** | **G** | **T** | **L** | **K** | **P** | **D** | **E** | **E** | **L** | **E** | **I** | **N** | **G** | **L** | **K** | **W** | **Q** | **K** | **D** | **K** | **S** | **W** | **G** | **G** | **Y** | **F** | **A** | **T** | **R** | **V** | **K** | **E** | **A** | **S** | **P** | **V** | **N** | **L** | **R** | **T** | **A** | **L** | **V** | **N** | **S** | **D** | **N** | **I** | **Y** | **F** | **A** | **Q** | **Q** | **T** | **L** | **R** | **M** | **G** | **E** | **D** | **K** | **F** | **R** | **A** | **G** | **L** | **N** | **K** | **F** | **I** | **F** | **G** | **E** | **E** | **L** | **D** | **L** | **P** | **I** | **A** | **M** | **T** | **P** | **A** | **Q** | **I** | **S** | **N** | **E** | **D** | **K** | **F** | **N** | **S** | **E** | **I** | **L** | **L** | **A** | **D** | **T** | **G** | **Y** | **G** | **Q** | **G** | **Q** | **L** | **L** | **I** | **S** | **P** | **I** | **Q** | **Q** | **A** | **T** | **M** | **Y** | **S** | **V** | **F** | **Q** | **N** | **N** | **G** | **T** | **L** | **V** | **Y** | **P** | **K** | **L** | **V** | **L** | **D** | **K** | **E** | **T** | **K** | **K** | **K** | **D** | **N** | **V** | **I** | **S** | **A** | **N** | **A** | **A** | **N** | **T** | **I** | **A** | **T** | **D** | **L** | **L** | **G** | **S** | **V** | **E** | **D** | **P** | **S** | **G** | **Y** | **V** | **Y** | **N** | **M** | **Y** | **N** | **P** | **N** | **F** | **S** | **L** | **A** | **A** | **K** | **T** | **G** | **T** | **A** | **E** | **I** | **K** | **D** | **K** | **Q** | **D** | **T** | **D** | **G** | **K** | **E** | **N** | **S** | **F** | **L** | **L** | **T** | **L** | **D** | **R** | **S** | **N** | **N** | **K** | **F** | **L** | **T** | **M** | **I** | **M** | **V** | **E** | **N** | **S** | **G** | **E** | **N** | **G** | **S** | **A** | **T** | **D** | **I** | **S** | **K** | **P** | **L** | **I** | **D** | **Y** | **L** | **E** | **A** | **T** | **I** | **K** | **-** | **-** | **-** | **-** | **-** | **-** | **-** | **-** | **-** | **-** | **-** | **-** | **-** | **-** | **-** | **-** | **-** | **-** | **-** | **-** | **-** | **-** | **-** | **-** | **-** | **-** | **-** | **-** | **-** | **-** | **-** | **-** | **-** | **-** | **-** | **-** | **Q** | **W** | **Q** | **A** | **K** | **Q** | **E** | **L** | **A** | **E** | **A** | **K** | **K** | **T** | **A** | **T** | **T** | **F** | **L** | **N** | **V** | **L** | **S** | **K** | **Q** | **E** | **F** | **D** | **K** | **L** | **P** | **S** | **V** | **V** | **Q** | **E** | **A** | **S** | **L** | **K** | **K** | **N** | **G** | **Y** | **D** | **T** | **K** | **S** | **V** | **V** | **E** | **K** | **Y** | **Q** | **A** | **I** | **Y** | **S** | **G** | **I** | **Q** | **A** | **E** | **G** | **V** | **K** | **A** | **S** | **D** | **V** | **Q** | **V** | **K** | **K** | **A** | **K** | **D** | **N** | **Q** | **Y** | **T** | **F** | **T** | **Y** | **K** | **L** | **S** | **M** | **S** | **T** | **P** | **L** | **G** | **E** | **M** | **K** | **D** | **L** | **S** | **Y** | **Q** | **S** | **S** | **I** | **A** | **K** | **K** | **G** | **D** | **T** | **Y** | **Q** | **I** | **A** | **W** | **K** | **P** | **S** | **L** | **I** | **F** | **P** | **D** | **M** | **S** | **G** | **N** | **D** | **K** | **I** | **S** | **I** | **Q** | **V** | **D** | **N** | **A** | **K** | **R** | **G** | **E** | **I** | **V** | **D** | **R** | **N** | **G** | **S** | **G** | **L** | **A** | **I** | **N** | **K** | **V** | **F** | **D** | **E** | **V** | **G** | **V** | **V** | **P** | **G** | **K** | **L** | **G** | **S** | **G** | **A** | **E** | **K** | **T** | **A** | **N** | **I** | **K** | **A** | **F** | **S** | **D** | **K** | **F** | **G** | **V** | **S** | **V** | **D** | **E** | **I** | **N** | **Q** | **K** | **L** | **S** | **Q** | **G** | **W** | **V** | **Q** | **A** | **D** | **S** | **F** | **V** | **P** | **I** | **T** | **V** | **A** | **S** | **E** | **P** | **V** | **T** | **E** | **L** | **P** | **T** | **G** | **A** | **A** | **T** | **K** | **D** | **T** | **E** | **S** | **R** | **Y** | **Y** | **P** | **L** | **G** | **E** | **A** | **A** | **A** | **Q** | **L** | **I** | **G** | **Y** | **T** | **G** | **T** | **I** | **T** | **A** | **E** | **D** | **I** | **E** | **K** | **N** | **P** | **E** | **L** | **S** | **S** | **T** | **G** | **V** | **I** | **G** | **K** | **T** | **G** | **L** | **E** | **R** | **A** | **F** | **D** | **K** | **E** | **L** | **R** | **G** | **Q** | **D** | **G** | **G** | **S** | **L** | **V** | **I** | **L** | **D** | **D** | **K** | **E** | **N** | **V** | **K** | **K** | **A** | **L** | **Q** | **T** | **K** | **E** | **K** | **K** | **D** | **G** | **Q** | **T** | **I** | **K** | **L** | **T** | **I** | **D** | **S** | **G** | **V** | **Q** | **Q** | **Q** | **A** | **F** | **A** | **I** | **F** | **D** | **K** | **R** | **P** | **G** | **S** | **A** | **V** | **I** | **T** | **D** | **P** | **Q** | **K** | **G** | **D** | **L** | **L** | **A** | **T** | **V** | **S** | **S** | **P** | **S** | **Y** | **D** | **P** | **N** | **K** | **M** | **A** | **N** | **G** | **I** | **S** | **Q** | **K** | **E** | **Y** | **D** | **A** | **Y** | **N** | **N** | **N** | **K** | **D** | **L** | **P** | **F** | **T** | **A** | **R** | **F** | **A** | **T** | **G** | **Y** | **A** | **P** | **G** | **S** | **T** | **F** | **K** | **T** | **I** | **T** | **G** | **A** | **I** | **G** | **L** | **D** | **A** | **G** | **T** | **L** | **K** | **P** | **D** | **E** | **E** | **L** | **E** | **I** | **N** | **G** | **L** | **K** | **W** | **Q** | **K** | **D** | **K** | **S** | **W** | **G** | **G** | **Y** | **F** | **A** | **T** | **R** | **V** | **K** | **E** | **A** | **S** | **P** | **V** | **N** | **L** | **R** | **T** | **A** | **L** | **V** | **N** | **S** | **D** | **N** | **I** | **Y** | **F** | **A** | **Q** | **Q** | **T** | **L** | **R** | **M** | **G** | **E** | **D** | **K** | **F** | **R** | **A** | **G** | **L** | **N** | **K** | **F** | **I** | **F** | **G** | **E** | **E** | **L** | **D** | **L** | **P** | **I** | **A** | **M** | **T** | **P** | **A** | **Q** | **I** | **S** | **N** | **E** | **D** | **K** | **F** | **N** | **S** | **E** | **I** | **L** | **L** | **A** | **D** | **T** | **G** | **Y** | **G** | **Q** | **G** | **Q** | **L** | **L** | **I** | **S** | **P** | **I** | **Q** | **Q** | **A** | **T** | **M** | **Y** | **S** | **V** | **F** | **Q** | **N** | **N** | **G** | **T** | **L** | **V** | **Y** | **P** | **K** | **L** | **V** | **L** | **D** | **K** | **E** | **T** | **K** | **K** | **K** | **D** | **N** | **V** | **I** | **S** | **A** | **N** | **A** | **A** | **N** | **T** | **I** | **A** | **T** | **D** | **L** | **L** | **G** | **S** | **V** | **E** | **D** | **P** | **S** | **G** | **Y** | **V** | **Y** | **N** | **M** | **Y** | **N** | **P** | **N** | **F** | **S** | **L** | **A** | **A** | **K** | **T** | **G** | **T** | **A** | **E** | **I** | **K** | **D** | **K** | **Q** | **D** | **T** | **D** | **G** | **K** | **E** | **N** | **S** | **F** | **L** | **L** | **T** | **L** | **D** | **R** | **S** | **N** | **N** | **K** | **F** | **L** | **T** | **M** | **I** | **M** | **V** | **E** | **N** | **S** | **G** | **E** | **N** | **G** | **S** | **A** | **T** | **D** | **I** | **S** | **K** | **P** | **L** | **I** | **D** | **Y** | **L** | **E** | **A** | **T** | **I** | **K** |
| **N53661** | **-** | **-** | **-** | **-** | **-** | **-** | **-** | **-** | **-** | **-** | **-** | **-** | **-** | **-** | **-** | **-** | **-** | **-** | **-** | **-** | **-** | **-** | **-** | **-** | **-** | **-** | **-** | **-** | **-** | **-** | **-** | **-** | **-** | **-** | **-** | **-** | **Q** | **W** | **Q** | **A** | **K** | **Q** | **E** | **L** | **A** | **E** | **A** | **K** | **K** | **T** | **A** | **T** | **T** | **F** | **L** | **N** | **V** | **L** | **T** | **K** | **Q** | **K** | **F** | **D** | **K** | **L** | **P** | **S** | **V** | **V** | **Q** | **E** | **A** | **S** | **L** | **K** | **K** | **N** | **G** | **Y** | **D** | **T** | **K** | **S** | **V** | **V** | **E** | **K** | **Y** | **Q** | **A** | **I** | **Y** | **S** | **G** | **I** | **Q** | **A** | **E** | **G** | **V** | **K** | **A** | **S** | **D** | **V** | **Q** | **V** | **K** | **K** | **A** | **K** | **D** | **N** | **Q** | **Y** | **T** | **F** | **N** | **Y** | **K** | **L** | **S** | **M** | **S** | **T** | **P** | **L** | **G** | **E** | **M** | **K** | **D** | **L** | **S** | **Y** | **Q** | **S** | **S** | **I** | **A** | **K** | **K** | **G** | **D** | **T** | **Y** | **Q** | **I** | **A** | **W** | **K** | **P** | **S** | **L** | **I** | **F** | **P** | **D** | **M** | **S** | **G** | **N** | **D** | **K** | **I** | **S** | **I** | **Q** | **V** | **D** | **N** | **A** | **K** | **R** | **G** | **E** | **I** | **V** | **D** | **R** | **N** | **G** | **S** | **G** | **L** | **A** | **I** | **N** | **K** | **V** | **F** | **D** | **E** | **V** | **G** | **V** | **V** | **P** | **G** | **K** | **L** | **G** | **S** | **G** | **A** | **E** | **K** | **T** | **A** | **N** | **I** | **K** | **A** | **F** | **S** | **D** | **K** | **F** | **G** | **V** | **S** | **V** | **D** | **E** | **I** | **N** | **Q** | **K** | **L** | **S** | **Q** | **G** | **W** | **V** | **Q** | **A** | **D** | **S** | **F** | **V** | **P** | **I** | **T** | **V** | **A** | **S** | **E** | **P** | **V** | **T** | **E** | **L** | **P** | **T** | **G** | **A** | **A** | **T** | **K** | **D** | **T** | **E** | **S** | **R** | **Y** | **Y** | **P** | **L** | **G** | **E** | **A** | **A** | **A** | **Q** | **L** | **I** | **G** | **Y** | **T** | **G** | **T** | **I** | **T** | **A** | **E** | **D** | **I** | **K** | **K** | **N** | **P** | **E** | **L** | **S** | **S** | **T** | **G** | **V** | **I** | **G** | **K** | **T** | **G** | **L** | **E** | **R** | **A** | **F** | **D** | **K** | **E** | **L** | **R** | **G** | **Q** | **D** | **G** | **G** | **S** | **L** | **V** | **I** | **L** | **D** | **D** | **K** | **E** | **N** | **V** | **K** | **K** | **A** | **L** | **Q** | **T** | **K** | **E** | **K** | **K** | **D** | **G** | **Q** | **T** | **I** | **K** | **L** | **T** | **I** | **D** | **S** | **G** | **V** | **Q** | **Q** | **Q** | **A** | **F** | **A** | **I** | **F** | **D** | **K** | **R** | **P** | **G** | **S** | **A** | **V** | **I** | **T** | **D** | **P** | **Q** | **K** | **G** | **D** | **L** | **L** | **A** | **T** | **V** | **S** | **S** | **P** | **S** | **Y** | **D** | **P** | **N** | **K** | **M** | **A** | **N** | **G** | **I** | **S** | **Q** | **K** | **E** | **Y** | **D** | **A** | **Y** | **N** | **N** | **N** | **K** | **D** | **L** | **P** | **F** | **T** | **A** | **R** | **F** | **A** | **T** | **G** | **Y** | **A** | **P** | **G** | **S** | **T** | **F** | **K** | **T** | **I** | **T** | **G** | **A** | **I** | **G** | **L** | **D** | **A** | **G** | **T** | **L** | **K** | **P** | **D** | **E** | **E** | **L** | **E** | **I** | **N** | **G** | **L** | **K** | **W** | **Q** | **K** | **D** | **K** | **S** | **W** | **G** | **G** | **Y** | **F** | **A** | **T** | **R** | **V** | **K** | **E** | **A** | **S** | **P** | **V** | **N** | **L** | **R** | **T** | **A** | **L** | **V** | **N** | **S** | **D** | **N** | **I** | **Y** | **F** | **A** | **Q** | **Q** | **T** | **L** | **R** | **M** | **G** | **E** | **D** | **K** | **F** | **R** | **A** | **G** | **L** | **N** | **K** | **F** | **I** | **F** | **G** | **E** | **E** | **L** | **D** | **L** | **P** | **I** | **A** | **M** | **T** | **P** | **A** | **Q** | **I** | **S** | **N** | **E** | **D** | **K** | **F** | **N** | **S** | **E** | **I** | **L** | **L** | **A** | **D** | **T** | **G** | **Y** | **G** | **Q** | **G** | **Q** | **L** | **L** | **I** | **S** | **P** | **I** | **Q** | **Q** | **A** | **T** | **M** | **Y** | **S** | **V** | **F** | **Q** | **N** | **N** | **G** | **T** | **L** | **V** | **Y** | **P** | **K** | **L** | **V** | **L** | **D** | **K** | **E** | **T** | **K** | **K** | **K** | **D** | **N** | **V** | **I** | **S** | **A** | **N** | **A** | **A** | **N** | **T** | **I** | **A** | **T** | **D** | **L** | **L** | **G** | **S** | **V** | **E** | **D** | **P** | **S** | **G** | **Y** | **V** | **Y** | **N** | **M** | **Y** | **N** | **P** | **N** | **F** | **S** | **L** | **A** | **A** | **K** | **T** | **G** | **T** | **A** | **E** | **I** | **K** | **D** | **K** | **Q** | **D** | **T** | **D** | **G** | **K** | **E** | **N** | **S** | **F** | **L** | **L** | **T** | **L** | **D** | **R** | **S** | **N** | **N** | **K** | **F** | **L** | **T** | **M** | **I** | **M** | **V** | **E** | **N** | **S** | **G** | **E** | **N** | **G** | **S** | **A** | **T** | **D** | **I** | **S** | **K** | **P** | **L** | **I** | **D** | **Y** | **L** | **E** | **A** | **T** | **I** | **K** | **-** | **-** | **-** | **-** | **-** | **-** | **-** | **-** | **-** | **-** | **-** | **-** | **-** | **-** | **-** | **-** | **-** | **-** | **-** | **-** | **-** | **-** | **-** | **-** | **-** | **-** | **-** | **-** | **-** | **-** | **-** | **-** | **-** | **-** | **-** | **-** | **Q** | **W** | **Q** | **A** | **K** | **Q** | **E** | **L** | **A** | **E** | **A** | **K** | **K** | **T** | **A** | **T** | **T** | **F** | **L** | **N** | **V** | **L** | **T** | **K** | **Q** | **K** | **F** | **D** | **K** | **L** | **P** | **S** | **V** | **V** | **Q** | **E** | **A** | **S** | **L** | **K** | **K** | **N** | **G** | **Y** | **D** | **T** | **K** | **S** | **V** | **V** | **E** | **K** | **Y** | **Q** | **A** | **I** | **Y** | **S** | **G** | **I** | **Q** | **A** | **E** | **G** | **V** | **K** | **A** | **S** | **D** | **V** | **Q** | **V** | **K** | **K** | **A** | **K** | **D** | **N** | **Q** | **Y** | **T** | **F** | **N** | **Y** | **K** | **L** | **S** | **M** | **S** | **T** | **P** | **L** | **G** | **E** | **M** | **K** | **D** | **L** | **S** | **Y** | **Q** | **S** | **S** | **I** | **A** | **K** | **K** | **G** | **D** | **T** | **Y** | **Q** | **I** | **A** | **W** | **K** | **P** | **S** | **L** | **I** | **F** | **P** | **D** | **M** | **S** | **G** | **N** | **D** | **K** | **I** | **S** | **I** | **Q** | **V** | **D** | **N** | **A** | **K** | **R** | **G** | **E** | **I** | **V** | **D** | **R** | **N** | **G** | **S** | **G** | **L** | **A** | **I** | **N** | **K** | **V** | **F** | **D** | **E** | **V** | **G** | **V** | **V** | **P** | **G** | **K** | **L** | **G** | **S** | **G** | **A** | **E** | **K** | **T** | **A** | **N** | **I** | **K** | **A** | **F** | **S** | **D** | **K** | **F** | **G** | **V** | **S** | **V** | **D** | **E** | **I** | **N** | **Q** | **K** | **L** | **S** | **Q** | **G** | **W** | **V** | **Q** | **A** | **D** | **S** | **F** | **V** | **P** | **I** | **T** | **V** | **A** | **S** | **E** | **P** | **V** | **T** | **E** | **L** | **P** | **T** | **G** | **A** | **A** | **T** | **K** | **D** | **T** | **E** | **S** | **R** | **Y** | **Y** | **P** | **L** | **G** | **E** | **A** | **A** | **A** | **Q** | **L** | **I** | **G** | **Y** | **T** | **G** | **T** | **I** | **T** | **A** | **E** | **D** | **I** | **K** | **K** | **N** | **P** | **E** | **L** | **S** | **S** | **T** | **G** | **V** | **I** | **G** | **K** | **T** | **G** | **L** | **E** | **R** | **A** | **F** | **D** | **K** | **E** | **L** | **R** | **G** | **Q** | **D** | **G** | **G** | **S** | **L** | **V** | **I** | **L** | **D** | **D** | **K** | **E** | **N** | **V** | **K** | **K** | **A** | **L** | **Q** | **T** | **K** | **E** | **K** | **K** | **D** | **G** | **Q** | **T** | **I** | **K** | **L** | **T** | **I** | **D** | **S** | **G** | **V** | **Q** | **Q** | **Q** | **A** | **F** | **A** | **I** | **F** | **D** | **K** | **R** | **P** | **G** | **S** | **A** | **V** | **I** | **T** | **D** | **P** | **Q** | **K** | **G** | **D** | **L** | **L** | **A** | **T** | **V** | **S** | **S** | **P** | **S** | **Y** | **D** | **P** | **N** | **K** | **M** | **A** | **N** | **G** | **I** | **S** | **Q** | **K** | **E** | **Y** | **D** | **A** | **Y** | **N** | **N** | **N** | **K** | **D** | **L** | **P** | **F** | **T** | **A** | **R** | **F** | **A** | **T** | **G** | **Y** | **A** | **P** | **G** | **S** | **T** | **F** | **K** | **T** | **I** | **T** | **G** | **A** | **I** | **G** | **L** | **D** | **A** | **G** | **T** | **L** | **K** | **P** | **D** | **E** | **E** | **L** | **E** | **I** | **N** | **G** | **L** | **K** | **W** | **Q** | **K** | **D** | **K** | **S** | **W** | **G** | **G** | **Y** | **F** | **A** | **T** | **R** | **V** | **K** | **E** | **A** | **S** | **P** | **V** | **N** | **L** | **R** | **T** | **A** | **L** | **V** | **N** | **S** | **D** | **N** | **I** | **Y** | **F** | **A** | **Q** | **Q** | **T** | **L** | **R** | **M** | **G** | **E** | **D** | **K** | **F** | **R** | **A** | **G** | **L** | **N** | **K** | **F** | **I** | **F** | **G** | **E** | **E** | **L** | **D** | **L** | **P** | **I** | **A** | **M** | **T** | **P** | **A** | **Q** | **I** | **S** | **N** | **E** | **D** | **K** | **F** | **N** | **S** | **E** | **I** | **L** | **L** | **A** | **D** | **T** | **G** | **Y** | **G** | **Q** | **G** | **Q** | **L** | **L** | **I** | **S** | **P** | **I** | **Q** | **Q** | **A** | **T** | **M** | **Y** | **S** | **V** | **F** | **Q** | **N** | **N** | **G** | **T** | **L** | **V** | **Y** | **P** | **K** | **L** | **V** | **L** | **D** | **K** | **E** | **T** | **K** | **K** | **K** | **D** | **N** | **V** | **I** | **S** | **A** | **N** | **A** | **A** | **N** | **T** | **I** | **A** | **T** | **D** | **L** | **L** | **G** | **S** | **V** | **E** | **D** | **P** | **S** | **G** | **Y** | **V** | **Y** | **N** | **M** | **Y** | **N** | **P** | **N** | **F** | **S** | **L** | **A** | **A** | **K** | **T** | **G** | **T** | **A** | **E** | **I** | **K** | **D** | **K** | **Q** | **D** | **T** | **D** | **G** | **K** | **E** | **N** | **S** | **F** | **L** | **L** | **T** | **L** | **D** | **R** | **S** | **N** | **N** | **K** | **F** | **L** | **T** | **M** | **I** | **M** | **V** | **E** | **N** | **S** | **G** | **E** | **N** | **G** | **S** | **A** | **T** | **D** | **I** | **S** | **K** | **P** | **L** | **I** | **D** | **Y** | **L** | **E** | **A** | **T** | **I** | **K** |
| **N59841F** | **-** | **-** | **-** | **-** | **-** | **-** | **-** | **-** | **-** | **-** | **-** | **-** | **-** | **-** | **-** | **-** | **-** | **-** | **-** | **-** | **-** | **-** | **-** | **-** | **-** | **-** | **-** | **-** | **-** | **-** | **-** | **-** | **-** | **-** | **-** | **-** | **Q** | **W** | **Q** | **A** | **K** | **Q** | **E** | **L** | **A** | **E** | **A** | **K** | **K** | **T** | **A** | **N** | **T** | **F** | **L** | **N** | **V** | **L** | **S** | **K** | **Q** | **E** | **F** | **D** | **K** | **L** | **P** | **S** | **V** | **V** | **Q** | **E** | **S** | **S** | **L** | **K** | **K** | **N** | **G** | **Y** | **D** | **T** | **K** | **S** | **V** | **V** | **E** | **K** | **Y** | **Q** | **A** | **I** | **Y** | **S** | **G** | **I** | **Q** | **A** | **E** | **G** | **V** | **K** | **A** | **S** | **D** | **V** | **Q** | **V** | **K** | **K** | **A** | **K** | **D** | **N** | **Q** | **Y** | **T** | **F** | **T** | **Y** | **K** | **L** | **S** | **M** | **S** | **T** | **P** | **L** | **G** | **E** | **M** | **K** | **D** | **L** | **S** | **Y** | **Q** | **S** | **S** | **I** | **A** | **K** | **K** | **G** | **D** | **T** | **Y** | **Q** | **I** | **S** | **W** | **K** | **P** | **S** | **L** | **I** | **F** | **P** | **D** | **M** | **S** | **G** | **N** | **D** | **K** | **I** | **S** | **I** | **Q** | **V** | **D** | **N** | **A** | **K** | **R** | **G** | **E** | **I** | **V** | **D** | **R** | **N** | **G** | **S** | **G** | **L** | **A** | **I** | **N** | **K** | **V** | **F** | **D** | **E** | **V** | **G** | **V** | **V** | **P** | **G** | **K** | **L** | **G** | **S** | **G** | **A** | **E** | **K** | **T** | **A** | **N** | **I** | **K** | **A** | **F** | **S** | **D** | **K** | **F** | **G** | **V** | **S** | **V** | **D** | **E** | **I** | **N** | **Q** | **K** | **L** | **S** | **Q** | **G** | **W** | **V** | **Q** | **A** | **D** | **S** | **F** | **V** | **P** | **I** | **T** | **V** | **A** | **S** | **E** | **P** | **V** | **T** | **E** | **L** | **P** | **T** | **G** | **A** | **A** | **T** | **K** | **D** | **T** | **E** | **S** | **R** | **Y** | **Y** | **P** | **L** | **G** | **E** | **A** | **A** | **A** | **Q** | **L** | **I** | **G** | **Y** | **T** | **G** | **T** | **I** | **T** | **A** | **E** | **D** | **I** | **E** | **K** | **N** | **P** | **E** | **L** | **S** | **S** | **T** | **G** | **V** | **I** | **G** | **K** | **T** | **G** | **L** | **E** | **R** | **A** | **F** | **D** | **K** | **E** | **L** | **R** | **G** | **Q** | **D** | **G** | **G** | **S** | **L** | **V** | **I** | **L** | **D** | **D** | **K** | **E** | **N** | **V** | **K** | **K** | **A** | **L** | **Q** | **T** | **K** | **E** | **K** | **K** | **D** | **G** | **Q** | **T** | **I** | **K** | **L** | **T** | **I** | **D** | **S** | **G** | **V** | **Q** | **Q** | **Q** | **A** | **F** | **A** | **I** | **F** | **D** | **K** | **R** | **P** | **G** | **S** | **A** | **V** | **I** | **T** | **D** | **P** | **Q** | **K** | **G** | **D** | **L** | **L** | **A** | **T** | **V** | **S** | **S** | **P** | **S** | **Y** | **D** | **P** | **N** | **K** | **M** | **A** | **N** | **G** | **I** | **S** | **Q** | **K** | **E** | **Y** | **D** | **A** | **Y** | **N** | **N** | **N** | **K** | **D** | **L** | **P** | **F** | **T** | **A** | **R** | **F** | **A** | **T** | **G** | **Y** | **A** | **P** | **G** | **S** | **T** | **F** | **K** | **T** | **I** | **T** | **G** | **A** | **I** | **G** | **L** | **D** | **A** | **G** | **T** | **L** | **K** | **P** | **D** | **E** | **E** | **L** | **E** | **I** | **N** | **G** | **L** | **K** | **W** | **Q** | **K** | **D** | **K** | **S** | **W** | **G** | **G** | **Y** | **F** | **A** | **T** | **R** | **V** | **K** | **E** | **A** | **S** | **P** | **V** | **N** | **L** | **R** | **T** | **A** | **L** | **V** | **N** | **S** | **D** | **N** | **I** | **Y** | **F** | **A** | **Q** | **Q** | **T** | **L** | **R** | **M** | **G** | **E** | **D** | **K** | **F** | **R** | **A** | **G** | **L** | **N** | **K** | **F** | **I** | **F** | **G** | **E** | **E** | **L** | **D** | **L** | **P** | **I** | **A** | **M** | **T** | **P** | **A** | **Q** | **I** | **S** | **N** | **E** | **D** | **K** | **F** | **N** | **S** | **E** | **I** | **L** | **L** | **A** | **D** | **T** | **G** | **Y** | **G** | **Q** | **G** | **Q** | **L** | **L** | **I** | **S** | **P** | **I** | **Q** | **Q** | **A** | **T** | **M** | **Y** | **S** | **V** | **F** | **Q** | **N** | **N** | **G** | **T** | **L** | **V** | **Y** | **P** | **K** | **L** | **V** | **L** | **D** | **K** | **E** | **T** | **K** | **K** | **K** | **D** | **N** | **V** | **I** | **S** | **A** | **N** | **A** | **A** | **N** | **T** | **I** | **A** | **T** | **D** | **L** | **L** | **G** | **S** | **V** | **E** | **D** | **P** | **S** | **G** | **Y** | **V** | **Y** | **N** | **M** | **Y** | **N** | **P** | **N** | **F** | **S** | **L** | **A** | **A** | **K** | **T** | **G** | **T** | **A** | **E** | **I** | **K** | **D** | **K** | **Q** | **D** | **T** | **D** | **G** | **K** | **E** | **N** | **S** | **F** | **L** | **L** | **T** | **L** | **D** | **R** | **S** | **N** | **N** | **K** | **F** | **L** | **T** | **M** | **I** | **M** | **V** | **E** | **N** | **S** | **G** | **E** | **N** | **G** | **S** | **A** | **T** | **D** | **I** | **S** | **K** | **P** | **L** | **I** | **D** | **Y** | **L** | **E** | **A** | **T** | **I** | **K** | **-** | **-** | **-** | **-** | **-** | **-** | **-** | **-** | **-** | **-** | **-** | **-** | **-** | **-** | **-** | **-** | **-** | **-** | **-** | **-** | **-** | **-** | **-** | **-** | **-** | **-** | **-** | **-** | **-** | **-** | **-** | **-** | **-** | **-** | **-** | **-** | **Q** | **W** | **Q** | **A** | **K** | **Q** | **E** | **L** | **A** | **E** | **A** | **K** | **K** | **T** | **A** | **N** | **T** | **F** | **L** | **N** | **V** | **L** | **S** | **K** | **Q** | **E** | **F** | **D** | **K** | **L** | **P** | **S** | **V** | **V** | **Q** | **E** | **S** | **S** | **L** | **K** | **K** | **N** | **G** | **Y** | **D** | **T** | **K** | **S** | **V** | **V** | **E** | **K** | **Y** | **Q** | **A** | **I** | **Y** | **S** | **G** | **I** | **Q** | **A** | **E** | **G** | **V** | **K** | **A** | **S** | **D** | **V** | **Q** | **V** | **K** | **K** | **A** | **K** | **D** | **N** | **Q** | **Y** | **T** | **F** | **T** | **Y** | **K** | **L** | **S** | **M** | **S** | **T** | **P** | **L** | **G** | **E** | **M** | **K** | **D** | **L** | **S** | **Y** | **Q** | **S** | **S** | **I** | **A** | **K** | **K** | **G** | **D** | **T** | **Y** | **Q** | **I** | **S** | **W** | **K** | **P** | **S** | **L** | **I** | **F** | **P** | **D** | **M** | **S** | **G** | **N** | **D** | **K** | **I** | **S** | **I** | **Q** | **V** | **D** | **N** | **A** | **K** | **R** | **G** | **E** | **I** | **V** | **D** | **R** | **N** | **G** | **S** | **G** | **L** | **A** | **I** | **N** | **K** | **V** | **F** | **D** | **E** | **V** | **G** | **V** | **V** | **P** | **G** | **K** | **L** | **G** | **S** | **G** | **A** | **E** | **K** | **T** | **A** | **N** | **I** | **K** | **A** | **F** | **S** | **D** | **K** | **F** | **G** | **V** | **S** | **V** | **D** | **E** | **I** | **N** | **Q** | **K** | **L** | **S** | **Q** | **G** | **W** | **V** | **Q** | **A** | **D** | **S** | **F** | **V** | **P** | **I** | **T** | **V** | **A** | **S** | **E** | **P** | **V** | **T** | **E** | **L** | **P** | **T** | **G** | **A** | **A** | **T** | **K** | **D** | **T** | **E** | **S** | **R** | **Y** | **Y** | **P** | **L** | **G** | **E** | **A** | **A** | **A** | **Q** | **L** | **I** | **G** | **Y** | **T** | **G** | **T** | **I** | **T** | **A** | **E** | **D** | **I** | **E** | **K** | **N** | **P** | **E** | **L** | **S** | **S** | **T** | **G** | **V** | **I** | **G** | **K** | **T** | **G** | **L** | **E** | **R** | **A** | **F** | **D** | **K** | **E** | **L** | **R** | **G** | **Q** | **D** | **G** | **G** | **S** | **L** | **V** | **I** | **L** | **D** | **D** | **K** | **E** | **N** | **V** | **K** | **K** | **A** | **L** | **Q** | **T** | **K** | **E** | **K** | **K** | **D** | **G** | **Q** | **T** | **I** | **K** | **L** | **T** | **I** | **D** | **S** | **G** | **V** | **Q** | **Q** | **Q** | **A** | **F** | **A** | **I** | **F** | **D** | **K** | **R** | **P** | **G** | **S** | **A** | **V** | **I** | **T** | **D** | **P** | **Q** | **K** | **G** | **D** | **L** | **L** | **A** | **T** | **V** | **S** | **S** | **P** | **S** | **Y** | **D** | **P** | **N** | **K** | **M** | **A** | **N** | **G** | **I** | **S** | **Q** | **K** | **E** | **Y** | **D** | **A** | **Y** | **N** | **N** | **N** | **K** | **D** | **L** | **P** | **F** | **T** | **A** | **R** | **F** | **A** | **T** | **G** | **Y** | **A** | **P** | **G** | **S** | **T** | **F** | **K** | **T** | **I** | **T** | **G** | **A** | **I** | **G** | **L** | **D** | **A** | **G** | **T** | **L** | **K** | **P** | **D** | **E** | **E** | **L** | **E** | **I** | **N** | **G** | **L** | **K** | **W** | **Q** | **K** | **D** | **K** | **S** | **W** | **G** | **G** | **Y** | **F** | **A** | **T** | **R** | **V** | **K** | **E** | **A** | **S** | **P** | **V** | **N** | **L** | **R** | **T** | **A** | **L** | **V** | **N** | **S** | **D** | **N** | **I** | **Y** | **F** | **A** | **Q** | **Q** | **T** | **L** | **R** | **M** | **G** | **E** | **D** | **K** | **F** | **R** | **A** | **G** | **L** | **N** | **K** | **F** | **I** | **F** | **G** | **E** | **E** | **L** | **D** | **L** | **P** | **I** | **A** | **M** | **T** | **P** | **A** | **Q** | **I** | **S** | **N** | **E** | **D** | **K** | **F** | **N** | **S** | **E** | **I** | **L** | **L** | **A** | **D** | **T** | **G** | **Y** | **G** | **Q** | **G** | **Q** | **L** | **L** | **I** | **S** | **P** | **I** | **Q** | **Q** | **A** | **T** | **M** | **Y** | **S** | **V** | **F** | **Q** | **N** | **N** | **G** | **T** | **L** | **V** | **Y** | **P** | **K** | **L** | **V** | **L** | **D** | **K** | **E** | **T** | **K** | **K** | **K** | **D** | **N** | **V** | **I** | **S** | **A** | **N** | **A** | **A** | **N** | **T** | **I** | **A** | **T** | **D** | **L** | **L** | **G** | **S** | **V** | **E** | **D** | **P** | **S** | **G** | **Y** | **V** | **Y** | **N** | **M** | **Y** | **N** | **P** | **N** | **F** | **S** | **L** | **A** | **A** | **K** | **T** | **G** | **T** | **A** | **E** | **I** | **K** | **D** | **K** | **Q** | **D** | **T** | **D** | **G** | **K** | **E** | **N** | **S** | **F** | **L** | **L** | **T** | **L** | **D** | **R** | **S** | **N** | **N** | **K** | **F** | **L** | **T** | **M** | **I** | **M** | **V** | **E** | **N** | **S** | **G** | **E** | **N** | **G** | **S** | **A** | **T** | **D** | **I** | **S** | **K** | **P** | **L** | **I** | **D** | **Y** | **L** | **E** | **A** | **T** | **I** | **K** |
| **N59491F** | **-** | **-** | **-** | **-** | **-** | **-** | **-** | **-** | **-** | **-** | **-** | **-** | **-** | **-** | **-** | **-** | **-** | **-** | **-** | **-** | **-** | **-** | **-** | **-** | **-** | **-** | **-** | **-** | **-** | **-** | **-** | **-** | **-** | **-** | **-** | **-** | **Q** | **W** | **Q** | **A** | **K** | **Q** | **E** | **L** | **A** | **E** | **A** | **K** | **K** | **T** | **A** | **N** | **T** | **F** | **L** | **N** | **V** | **L** | **S** | **K** | **Q** | **E** | **F** | **D** | **K** | **L** | **P** | **S** | **V** | **V** | **Q** | **E** | **S** | **S** | **L** | **K** | **K** | **N** | **G** | **Y** | **D** | **T** | **K** | **S** | **V** | **V** | **E** | **K** | **Y** | **Q** | **A** | **I** | **Y** | **S** | **G** | **I** | **Q** | **A** | **E** | **G** | **V** | **K** | **A** | **S** | **D** | **V** | **Q** | **V** | **K** | **K** | **A** | **K** | **D** | **N** | **Q** | **Y** | **T** | **F** | **T** | **Y** | **K** | **L** | **S** | **M** | **S** | **T** | **P** | **L** | **G** | **E** | **M** | **K** | **D** | **L** | **S** | **Y** | **Q** | **S** | **S** | **I** | **A** | **K** | **K** | **G** | **D** | **T** | **Y** | **Q** | **I** | **S** | **W** | **K** | **P** | **S** | **L** | **I** | **F** | **P** | **D** | **M** | **S** | **G** | **N** | **D** | **K** | **I** | **S** | **I** | **Q** | **V** | **D** | **N** | **A** | **K** | **R** | **G** | **E** | **I** | **V** | **D** | **R** | **N** | **G** | **S** | **G** | **L** | **A** | **I** | **N** | **K** | **V** | **F** | **D** | **E** | **V** | **G** | **V** | **V** | **P** | **G** | **K** | **L** | **G** | **S** | **G** | **A** | **E** | **K** | **T** | **A** | **N** | **I** | **K** | **A** | **F** | **S** | **D** | **K** | **F** | **G** | **V** | **S** | **V** | **D** | **E** | **I** | **N** | **Q** | **K** | **L** | **S** | **Q** | **G** | **W** | **V** | **Q** | **A** | **D** | **S** | **F** | **V** | **P** | **I** | **T** | **V** | **A** | **S** | **E** | **P** | **V** | **T** | **E** | **L** | **P** | **T** | **G** | **A** | **A** | **T** | **K** | **D** | **T** | **E** | **S** | **R** | **Y** | **Y** | **P** | **L** | **G** | **E** | **A** | **A** | **A** | **Q** | **L** | **I** | **G** | **Y** | **T** | **G** | **T** | **I** | **T** | **A** | **E** | **D** | **I** | **E** | **K** | **N** | **P** | **E** | **L** | **S** | **S** | **T** | **G** | **V** | **I** | **G** | **K** | **T** | **G** | **L** | **E** | **R** | **A** | **F** | **D** | **K** | **E** | **L** | **R** | **G** | **Q** | **D** | **G** | **G** | **S** | **L** | **V** | **I** | **L** | **D** | **D** | **K** | **E** | **N** | **V** | **K** | **K** | **A** | **L** | **Q** | **T** | **K** | **E** | **K** | **K** | **D** | **G** | **Q** | **T** | **I** | **K** | **L** | **T** | **I** | **D** | **S** | **G** | **V** | **Q** | **Q** | **Q** | **A** | **F** | **A** | **I** | **F** | **D** | **K** | **R** | **P** | **G** | **S** | **A** | **V** | **I** | **T** | **D** | **P** | **Q** | **K** | **G** | **D** | **L** | **L** | **A** | **T** | **V** | **S** | **S** | **P** | **S** | **Y** | **D** | **P** | **N** | **K** | **M** | **A** | **N** | **G** | **I** | **S** | **Q** | **K** | **E** | **Y** | **D** | **A** | **Y** | **N** | **N** | **N** | **K** | **D** | **L** | **P** | **F** | **T** | **A** | **R** | **F** | **A** | **T** | **G** | **Y** | **A** | **P** | **G** | **S** | **T** | **F** | **K** | **T** | **I** | **T** | **G** | **A** | **I** | **G** | **L** | **D** | **A** | **G** | **T** | **L** | **K** | **P** | **D** | **E** | **E** | **L** | **E** | **I** | **N** | **G** | **L** | **K** | **W** | **Q** | **K** | **D** | **K** | **S** | **W** | **G** | **G** | **Y** | **F** | **A** | **T** | **R** | **V** | **K** | **E** | **A** | **S** | **P** | **V** | **N** | **L** | **R** | **T** | **A** | **L** | **V** | **N** | **S** | **D** | **N** | **I** | **Y** | **F** | **A** | **Q** | **Q** | **T** | **L** | **R** | **M** | **G** | **E** | **D** | **K** | **F** | **R** | **A** | **G** | **L** | **N** | **K** | **F** | **I** | **F** | **G** | **E** | **E** | **L** | **D** | **L** | **P** | **I** | **A** | **M** | **T** | **P** | **A** | **Q** | **I** | **S** | **N** | **E** | **D** | **K** | **F** | **N** | **S** | **E** | **I** | **L** | **L** | **A** | **D** | **T** | **G** | **Y** | **G** | **Q** | **G** | **Q** | **L** | **L** | **I** | **S** | **P** | **I** | **Q** | **Q** | **A** | **T** | **M** | **Y** | **S** | **V** | **F** | **Q** | **N** | **N** | **G** | **T** | **L** | **V** | **Y** | **P** | **K** | **L** | **V** | **L** | **D** | **K** | **E** | **T** | **K** | **K** | **K** | **D** | **N** | **V** | **I** | **S** | **A** | **N** | **A** | **A** | **N** | **T** | **I** | **A** | **T** | **D** | **L** | **L** | **G** | **S** | **V** | **E** | **D** | **P** | **S** | **G** | **Y** | **V** | **Y** | **N** | **M** | **Y** | **N** | **P** | **N** | **F** | **S** | **L** | **A** | **A** | **K** | **T** | **G** | **T** | **A** | **E** | **I** | **K** | **D** | **K** | **Q** | **D** | **T** | **D** | **G** | **K** | **E** | **N** | **S** | **F** | **L** | **L** | **T** | **L** | **D** | **R** | **S** | **N** | **N** | **K** | **F** | **L** | **T** | **M** | **I** | **M** | **V** | **E** | **N** | **S** | **G** | **E** | **N** | **G** | **S** | **A** | **T** | **D** | **I** | **S** | **K** | **P** | **L** | **I** | **D** | **Y** | **L** | **E** | **A** | **T** | **I** | **K** | **-** | **-** | **-** | **-** | **-** | **-** | **-** | **-** | **-** | **-** | **-** | **-** | **-** | **-** | **-** | **-** | **-** | **-** | **-** | **-** | **-** | **-** | **-** | **-** | **-** | **-** | **-** | **-** | **-** | **-** | **-** | **-** | **-** | **-** | **-** | **-** | **Q** | **W** | **Q** | **A** | **K** | **Q** | **E** | **L** | **A** | **E** | **A** | **K** | **K** | **T** | **A** | **N** | **T** | **F** | **L** | **N** | **V** | **L** | **S** | **K** | **Q** | **E** | **F** | **D** | **K** | **L** | **P** | **S** | **V** | **V** | **Q** | **E** | **S** | **S** | **L** | **K** | **K** | **N** | **G** | **Y** | **D** | **T** | **K** | **S** | **V** | **V** | **E** | **K** | **Y** | **Q** | **A** | **I** | **Y** | **S** | **G** | **I** | **Q** | **A** | **E** | **G** | **V** | **K** | **A** | **S** | **D** | **V** | **Q** | **V** | **K** | **K** | **A** | **K** | **D** | **N** | **Q** | **Y** | **T** | **F** | **T** | **Y** | **K** | **L** | **S** | **M** | **S** | **T** | **P** | **L** | **G** | **E** | **M** | **K** | **D** | **L** | **S** | **Y** | **Q** | **S** | **S** | **I** | **A** | **K** | **K** | **G** | **D** | **T** | **Y** | **Q** | **I** | **S** | **W** | **K** | **P** | **S** | **L** | **I** | **F** | **P** | **D** | **M** | **S** | **G** | **N** | **D** | **K** | **I** | **S** | **I** | **Q** | **V** | **D** | **N** | **A** | **K** | **R** | **G** | **E** | **I** | **V** | **D** | **R** | **N** | **G** | **S** | **G** | **L** | **A** | **I** | **N** | **K** | **V** | **F** | **D** | **E** | **V** | **G** | **V** | **V** | **P** | **G** | **K** | **L** | **G** | **S** | **G** | **A** | **E** | **K** | **T** | **A** | **N** | **I** | **K** | **A** | **F** | **S** | **D** | **K** | **F** | **G** | **V** | **S** | **V** | **D** | **E** | **I** | **N** | **Q** | **K** | **L** | **S** | **Q** | **G** | **W** | **V** | **Q** | **A** | **D** | **S** | **F** | **V** | **P** | **I** | **T** | **V** | **A** | **S** | **E** | **P** | **V** | **T** | **E** | **L** | **P** | **T** | **G** | **A** | **A** | **T** | **K** | **D** | **T** | **E** | **S** | **R** | **Y** | **Y** | **P** | **L** | **G** | **E** | **A** | **A** | **A** | **Q** | **L** | **I** | **G** | **Y** | **T** | **G** | **T** | **I** | **T** | **A** | **E** | **D** | **I** | **E** | **K** | **N** | **P** | **E** | **L** | **S** | **S** | **T** | **G** | **V** | **I** | **G** | **K** | **T** | **G** | **L** | **E** | **R** | **A** | **F** | **D** | **K** | **E** | **L** | **R** | **G** | **Q** | **D** | **G** | **G** | **S** | **L** | **V** | **I** | **L** | **D** | **D** | **K** | **E** | **N** | **V** | **K** | **K** | **A** | **L** | **Q** | **T** | **K** | **E** | **K** | **K** | **D** | **G** | **Q** | **T** | **I** | **K** | **L** | **T** | **I** | **D** | **S** | **G** | **V** | **Q** | **Q** | **Q** | **A** | **F** | **A** | **I** | **F** | **D** | **K** | **R** | **P** | **G** | **S** | **A** | **V** | **I** | **T** | **D** | **P** | **Q** | **K** | **G** | **D** | **L** | **L** | **A** | **T** | **V** | **S** | **S** | **P** | **S** | **Y** | **D** | **P** | **N** | **K** | **M** | **A** | **N** | **G** | **I** | **S** | **Q** | **K** | **E** | **Y** | **D** | **A** | **Y** | **N** | **N** | **N** | **K** | **D** | **L** | **P** | **F** | **T** | **A** | **R** | **F** | **A** | **T** | **G** | **Y** | **A** | **P** | **G** | **S** | **T** | **F** | **K** | **T** | **I** | **T** | **G** | **A** | **I** | **G** | **L** | **D** | **A** | **G** | **T** | **L** | **K** | **P** | **D** | **E** | **E** | **L** | **E** | **I** | **N** | **G** | **L** | **K** | **W** | **Q** | **K** | **D** | **K** | **S** | **W** | **G** | **G** | **Y** | **F** | **A** | **T** | **R** | **V** | **K** | **E** | **A** | **S** | **P** | **V** | **N** | **L** | **R** | **T** | **A** | **L** | **V** | **N** | **S** | **D** | **N** | **I** | **Y** | **F** | **A** | **Q** | **Q** | **T** | **L** | **R** | **M** | **G** | **E** | **D** | **K** | **F** | **R** | **A** | **G** | **L** | **N** | **K** | **F** | **I** | **F** | **G** | **E** | **E** | **L** | **D** | **L** | **P** | **I** | **A** | **M** | **T** | **P** | **A** | **Q** | **I** | **S** | **N** | **E** | **D** | **K** | **F** | **N** | **S** | **E** | **I** | **L** | **L** | **A** | **D** | **T** | **G** | **Y** | **G** | **Q** | **G** | **Q** | **L** | **L** | **I** | **S** | **P** | **I** | **Q** | **Q** | **A** | **T** | **M** | **Y** | **S** | **V** | **F** | **Q** | **N** | **N** | **G** | **T** | **L** | **V** | **Y** | **P** | **K** | **L** | **V** | **L** | **D** | **K** | **E** | **T** | **K** | **K** | **K** | **D** | **N** | **V** | **I** | **S** | **A** | **N** | **A** | **A** | **N** | **T** | **I** | **A** | **T** | **D** | **L** | **L** | **G** | **S** | **V** | **E** | **D** | **P** | **S** | **G** | **Y** | **V** | **Y** | **N** | **M** | **Y** | **N** | **P** | **N** | **F** | **S** | **L** | **A** | **A** | **K** | **T** | **G** | **T** | **A** | **E** | **I** | **K** | **D** | **K** | **Q** | **D** | **T** | **D** | **G** | **K** | **E** | **N** | **S** | **F** | **L** | **L** | **T** | **L** | **D** | **R** | **S** | **N** | **N** | **K** | **F** | **L** | **T** | **M** | **I** | **M** | **V** | **E** | **N** | **S** | **G** | **E** | **N** | **G** | **S** | **A** | **T** | **D** | **I** | **S** | **K** | **P** | **L** | **I** | **D** | **Y** | **L** | **E** | **A** | **T** | **I** | **K** |
| **N53476** | **-** | **-** | **-** | **-** | **-** | **-** | **-** | **-** | **-** | **-** | **-** | **-** | **-** | **-** | **-** | **-** | **-** | **-** | **-** | **-** | **-** | **-** | **-** | **-** | **-** | **-** | **-** | **-** | **-** | **-** | **-** | **-** | **-** | **-** | **-** | **-** | **Q** | **W** | **Q** | **A** | **K** | **Q** | **E** | **L** | **A** | **E** | **A** | **K** | **K** | **T** | **A** | **T** | **T** | **F** | **L** | **N** | **V** | **L** | **S** | **K** | **Q** | **E** | **F** | **D** | **K** | **L** | **P** | **S** | **V** | **V** | **Q** | **E** | **A** | **S** | **L** | **K** | **K** | **N** | **G** | **Y** | **D** | **T** | **K** | **S** | **V** | **V** | **E** | **K** | **Y** | **Q** | **A** | **I** | **Y** | **S** | **G** | **I** | **Q** | **A** | **E** | **G** | **V** | **K** | **A** | **S** | **D** | **V** | **Q** | **V** | **K** | **K** | **A** | **K** | **D** | **N** | **Q** | **Y** | **T** | **F** | **T** | **Y** | **K** | **L** | **S** | **M** | **S** | **T** | **P** | **L** | **G** | **E** | **M** | **K** | **D** | **L** | **S** | **Y** | **Q** | **S** | **S** | **I** | **A** | **K** | **K** | **G** | **D** | **T** | **Y** | **Q** | **I** | **A** | **W** | **K** | **P** | **S** | **L** | **I** | **F** | **P** | **D** | **M** | **S** | **G** | **N** | **D** | **K** | **I** | **S** | **I** | **Q** | **V** | **D** | **N** | **A** | **K** | **R** | **G** | **E** | **I** | **V** | **D** | **R** | **N** | **G** | **S** | **G** | **L** | **A** | **I** | **N** | **K** | **V** | **F** | **D** | **E** | **V** | **G** | **V** | **V** | **P** | **G** | **K** | **L** | **G** | **S** | **G** | **A** | **E** | **K** | **T** | **A** | **N** | **I** | **K** | **A** | **F** | **S** | **D** | **K** | **F** | **G** | **V** | **S** | **V** | **D** | **E** | **I** | **N** | **Q** | **K** | **L** | **S** | **Q** | **G** | **W** | **V** | **Q** | **A** | **D** | **S** | **F** | **V** | **P** | **I** | **T** | **V** | **A** | **S** | **E** | **P** | **V** | **T** | **E** | **L** | **P** | **T** | **G** | **A** | **A** | **T** | **K** | **D** | **T** | **E** | **S** | **R** | **Y** | **Y** | **P** | **L** | **G** | **E** | **A** | **A** | **A** | **Q** | **L** | **I** | **G** | **Y** | **T** | **G** | **T** | **I** | **T** | **A** | **E** | **D** | **I** | **E** | **K** | **N** | **P** | **E** | **L** | **S** | **S** | **T** | **G** | **V** | **I** | **G** | **K** | **T** | **G** | **L** | **E** | **R** | **A** | **F** | **D** | **K** | **E** | **L** | **R** | **G** | **Q** | **D** | **G** | **G** | **S** | **L** | **V** | **I** | **L** | **D** | **D** | **K** | **E** | **N** | **V** | **K** | **K** | **A** | **L** | **Q** | **T** | **K** | **E** | **K** | **K** | **D** | **G** | **Q** | **T** | **I** | **K** | **L** | **T** | **I** | **D** | **S** | **G** | **V** | **Q** | **Q** | **Q** | **A** | **F** | **A** | **I** | **F** | **D** | **K** | **R** | **P** | **G** | **S** | **A** | **V** | **I** | **T** | **D** | **P** | **Q** | **K** | **G** | **D** | **L** | **L** | **A** | **T** | **V** | **S** | **S** | **P** | **S** | **Y** | **D** | **P** | **N** | **K** | **M** | **A** | **N** | **G** | **I** | **S** | **Q** | **K** | **E** | **Y** | **D** | **A** | **Y** | **N** | **N** | **N** | **K** | **D** | **L** | **P** | **F** | **T** | **A** | **R** | **F** | **A** | **T** | **G** | **Y** | **A** | **P** | **G** | **S** | **T** | **F** | **K** | **T** | **I** | **T** | **G** | **A** | **I** | **G** | **L** | **D** | **A** | **G** | **T** | **L** | **K** | **P** | **D** | **E** | **E** | **L** | **E** | **I** | **N** | **G** | **L** | **K** | **W** | **Q** | **K** | **D** | **K** | **S** | **W** | **G** | **G** | **Y** | **F** | **A** | **T** | **R** | **V** | **K** | **E** | **A** | **S** | **P** | **V** | **N** | **L** | **R** | **T** | **A** | **L** | **V** | **N** | **S** | **D** | **N** | **I** | **Y** | **F** | **A** | **Q** | **Q** | **T** | **L** | **R** | **M** | **G** | **E** | **D** | **K** | **F** | **R** | **A** | **G** | **L** | **N** | **K** | **F** | **I** | **F** | **G** | **E** | **E** | **L** | **D** | **L** | **P** | **I** | **A** | **M** | **T** | **P** | **A** | **Q** | **I** | **S** | **N** | **E** | **D** | **K** | **F** | **N** | **S** | **E** | **I** | **L** | **L** | **A** | **D** | **T** | **G** | **Y** | **G** | **Q** | **G** | **Q** | **L** | **L** | **I** | **S** | **P** | **I** | **Q** | **Q** | **A** | **T** | **M** | **Y** | **S** | **V** | **F** | **Q** | **N** | **N** | **G** | **T** | **L** | **V** | **Y** | **P** | **K** | **L** | **V** | **L** | **D** | **K** | **E** | **T** | **K** | **K** | **K** | **D** | **N** | **V** | **I** | **S** | **A** | **N** | **A** | **A** | **N** | **T** | **I** | **A** | **T** | **D** | **L** | **L** | **G** | **S** | **V** | **E** | **D** | **P** | **S** | **G** | **Y** | **V** | **Y** | **N** | **M** | **Y** | **N** | **P** | **N** | **F** | **S** | **L** | **A** | **A** | **K** | **T** | **G** | **T** | **A** | **E** | **I** | **K** | **D** | **K** | **Q** | **D** | **T** | **D** | **G** | **K** | **E** | **N** | **S** | **F** | **L** | **L** | **T** | **L** | **D** | **R** | **S** | **N** | **N** | **K** | **F** | **L** | **T** | **M** | **I** | **M** | **V** | **E** | **N** | **S** | **G** | **E** | **N** | **G** | **S** | **A** | **T** | **D** | **I** | **S** | **K** | **P** | **L** | **I** | **D** | **Y** | **L** | **E** | **A** | **T** | **I** | **K** | **-** | **-** | **-** | **-** | **-** | **-** | **-** | **-** | **-** | **-** | **-** | **-** | **-** | **-** | **-** | **-** | **-** | **-** | **-** | **-** | **-** | **-** | **-** | **-** | **-** | **-** | **-** | **-** | **-** | **-** | **-** | **-** | **-** | **-** | **-** | **-** | **Q** | **W** | **Q** | **A** | **K** | **Q** | **E** | **L** | **A** | **E** | **A** | **K** | **K** | **T** | **A** | **T** | **T** | **F** | **L** | **N** | **V** | **L** | **S** | **K** | **Q** | **E** | **F** | **D** | **K** | **L** | **P** | **S** | **V** | **V** | **Q** | **E** | **A** | **S** | **L** | **K** | **K** | **N** | **G** | **Y** | **D** | **T** | **K** | **S** | **V** | **V** | **E** | **K** | **Y** | **Q** | **A** | **I** | **Y** | **S** | **G** | **I** | **Q** | **A** | **E** | **G** | **V** | **K** | **A** | **S** | **D** | **V** | **Q** | **V** | **K** | **K** | **A** | **K** | **D** | **N** | **Q** | **Y** | **T** | **F** | **T** | **Y** | **K** | **L** | **S** | **M** | **S** | **T** | **P** | **L** | **G** | **E** | **M** | **K** | **D** | **L** | **S** | **Y** | **Q** | **S** | **S** | **I** | **A** | **K** | **K** | **G** | **D** | **T** | **Y** | **Q** | **I** | **A** | **W** | **K** | **P** | **S** | **L** | **I** | **F** | **P** | **D** | **M** | **S** | **G** | **N** | **D** | **K** | **I** | **S** | **I** | **Q** | **V** | **D** | **N** | **A** | **K** | **R** | **G** | **E** | **I** | **V** | **D** | **R** | **N** | **G** | **S** | **G** | **L** | **A** | **I** | **N** | **K** | **V** | **F** | **D** | **E** | **V** | **G** | **V** | **V** | **P** | **G** | **K** | **L** | **G** | **S** | **G** | **A** | **E** | **K** | **T** | **A** | **N** | **I** | **K** | **A** | **F** | **S** | **D** | **K** | **F** | **G** | **V** | **S** | **V** | **D** | **E** | **I** | **N** | **Q** | **K** | **L** | **S** | **Q** | **G** | **W** | **V** | **Q** | **A** | **D** | **S** | **F** | **V** | **P** | **I** | **T** | **V** | **A** | **S** | **E** | **P** | **V** | **T** | **E** | **L** | **P** | **T** | **G** | **A** | **A** | **T** | **K** | **D** | **T** | **E** | **S** | **R** | **Y** | **Y** | **P** | **L** | **G** | **E** | **A** | **A** | **A** | **Q** | **L** | **I** | **G** | **Y** | **T** | **G** | **T** | **I** | **T** | **A** | **E** | **D** | **I** | **E** | **K** | **N** | **P** | **E** | **L** | **S** | **S** | **T** | **G** | **V** | **I** | **G** | **K** | **T** | **G** | **L** | **E** | **R** | **A** | **F** | **D** | **K** | **E** | **L** | **R** | **G** | **Q** | **D** | **G** | **G** | **S** | **L** | **V** | **I** | **L** | **D** | **D** | **K** | **E** | **N** | **V** | **K** | **K** | **A** | **L** | **Q** | **T** | **K** | **E** | **K** | **K** | **D** | **G** | **Q** | **T** | **I** | **K** | **L** | **T** | **I** | **D** | **S** | **G** | **V** | **Q** | **Q** | **Q** | **A** | **F** | **A** | **I** | **F** | **D** | **K** | **R** | **P** | **G** | **S** | **A** | **V** | **I** | **T** | **D** | **P** | **Q** | **K** | **G** | **D** | **L** | **L** | **A** | **T** | **V** | **S** | **S** | **P** | **S** | **Y** | **D** | **P** | **N** | **K** | **M** | **A** | **N** | **G** | **I** | **S** | **Q** | **K** | **E** | **Y** | **D** | **A** | **Y** | **N** | **N** | **N** | **K** | **D** | **L** | **P** | **F** | **T** | **A** | **R** | **F** | **A** | **T** | **G** | **Y** | **A** | **P** | **G** | **S** | **T** | **F** | **K** | **T** | **I** | **T** | **G** | **A** | **I** | **G** | **L** | **D** | **A** | **G** | **T** | **L** | **K** | **P** | **D** | **E** | **E** | **L** | **E** | **I** | **N** | **G** | **L** | **K** | **W** | **Q** | **K** | **D** | **K** | **S** | **W** | **G** | **G** | **Y** | **F** | **A** | **T** | **R** | **V** | **K** | **E** | **A** | **S** | **P** | **V** | **N** | **L** | **R** | **T** | **A** | **L** | **V** | **N** | **S** | **D** | **N** | **I** | **Y** | **F** | **A** | **Q** | **Q** | **T** | **L** | **R** | **M** | **G** | **E** | **D** | **K** | **F** | **R** | **A** | **G** | **L** | **N** | **K** | **F** | **I** | **F** | **G** | **E** | **E** | **L** | **D** | **L** | **P** | **I** | **A** | **M** | **T** | **P** | **A** | **Q** | **I** | **S** | **N** | **E** | **D** | **K** | **F** | **N** | **S** | **E** | **I** | **L** | **L** | **A** | **D** | **T** | **G** | **Y** | **G** | **Q** | **G** | **Q** | **L** | **L** | **I** | **S** | **P** | **I** | **Q** | **Q** | **A** | **T** | **M** | **Y** | **S** | **V** | **F** | **Q** | **N** | **N** | **G** | **T** | **L** | **V** | **Y** | **P** | **K** | **L** | **V** | **L** | **D** | **K** | **E** | **T** | **K** | **K** | **K** | **D** | **N** | **V** | **I** | **S** | **A** | **N** | **A** | **A** | **N** | **T** | **I** | **A** | **T** | **D** | **L** | **L** | **G** | **S** | **V** | **E** | **D** | **P** | **S** | **G** | **Y** | **V** | **Y** | **N** | **M** | **Y** | **N** | **P** | **N** | **F** | **S** | **L** | **A** | **A** | **K** | **T** | **G** | **T** | **A** | **E** | **I** | **K** | **D** | **K** | **Q** | **D** | **T** | **D** | **G** | **K** | **E** | **N** | **S** | **F** | **L** | **L** | **T** | **L** | **D** | **R** | **S** | **N** | **N** | **K** | **F** | **L** | **T** | **M** | **I** | **M** | **V** | **E** | **N** | **S** | **G** | **E** | **N** | **G** | **S** | **A** | **T** | **D** | **I** | **S** | **K** | **P** | **L** | **I** | **D** | **Y** | **L** | **E** | **A** | **T** | **I** | **K** |
| **N60392F** | **-** | **-** | **-** | **-** | **-** | **-** | **-** | **-** | **-** | **-** | **-** | **-** | **-** | **-** | **-** | **-** | **-** | **-** | **-** | **-** | **-** | **-** | **-** | **-** | **-** | **-** | **-** | **-** | **-** | **-** | **-** | **-** | **-** | **-** | **-** | **-** | **Q** | **W** | **Q** | **A** | **K** | **Q** | **E** | **L** | **A** | **E** | **A** | **K** | **K** | **T** | **A** | **T** | **T** | **F** | **L** | **N** | **V** | **L** | **S** | **K** | **Q** | **E** | **F** | **D** | **K** | **L** | **P** | **S** | **V** | **V** | **Q** | **E** | **A** | **S** | **L** | **K** | **K** | **N** | **G** | **Y** | **D** | **T** | **K** | **S** | **V** | **V** | **E** | **K** | **Y** | **Q** | **A** | **I** | **Y** | **S** | **G** | **I** | **Q** | **A** | **E** | **G** | **V** | **K** | **A** | **S** | **D** | **V** | **Q** | **V** | **K** | **K** | **A** | **K** | **D** | **N** | **Q** | **Y** | **T** | **F** | **T** | **Y** | **K** | **L** | **S** | **M** | **S** | **T** | **P** | **L** | **G** | **E** | **M** | **K** | **D** | **L** | **S** | **Y** | **Q** | **S** | **S** | **I** | **A** | **K** | **K** | **G** | **D** | **T** | **Y** | **Q** | **I** | **A** | **W** | **K** | **P** | **S** | **L** | **I** | **F** | **P** | **D** | **M** | **S** | **G** | **N** | **D** | **K** | **I** | **S** | **I** | **Q** | **V** | **D** | **N** | **A** | **K** | **R** | **G** | **E** | **I** | **V** | **D** | **R** | **N** | **G** | **S** | **G** | **L** | **A** | **I** | **N** | **K** | **V** | **F** | **D** | **E** | **V** | **G** | **V** | **V** | **P** | **G** | **K** | **L** | **G** | **S** | **G** | **A** | **E** | **K** | **T** | **A** | **N** | **I** | **K** | **A** | **F** | **S** | **D** | **K** | **F** | **G** | **V** | **S** | **V** | **D** | **E** | **I** | **N** | **Q** | **K** | **L** | **S** | **Q** | **G** | **W** | **V** | **Q** | **A** | **D** | **S** | **F** | **V** | **P** | **I** | **T** | **V** | **A** | **S** | **E** | **P** | **V** | **T** | **E** | **L** | **P** | **T** | **G** | **A** | **A** | **T** | **K** | **G** | **T** | **E** | **S** | **R** | **Y** | **Y** | **P** | **L** | **G** | **E** | **A** | **A** | **A** | **Q** | **L** | **I** | **G** | **Y** | **T** | **G** | **T** | **I** | **T** | **A** | **E** | **D** | **I** | **E** | **K** | **N** | **P** | **E** | **L** | **S** | **S** | **T** | **G** | **V** | **I** | **G** | **K** | **T** | **G** | **L** | **E** | **R** | **A** | **F** | **D** | **K** | **E** | **L** | **R** | **G** | **Q** | **D** | **G** | **G** | **S** | **L** | **V** | **I** | **L** | **D** | **D** | **K** | **E** | **N** | **V** | **K** | **K** | **A** | **L** | **Q** | **T** | **K** | **E** | **K** | **K** | **D** | **G** | **Q** | **T** | **I** | **K** | **L** | **T** | **I** | **D** | **S** | **G** | **V** | **Q** | **Q** | **Q** | **A** | **F** | **A** | **I** | **F** | **D** | **K** | **R** | **P** | **G** | **S** | **A** | **V** | **I** | **T** | **D** | **P** | **Q** | **K** | **G** | **D** | **L** | **L** | **A** | **T** | **V** | **S** | **S** | **P** | **S** | **Y** | **D** | **P** | **N** | **K** | **M** | **A** | **N** | **G** | **I** | **S** | **Q** | **K** | **E** | **Y** | **D** | **A** | **Y** | **N** | **N** | **N** | **K** | **D** | **L** | **P** | **F** | **T** | **A** | **R** | **F** | **A** | **T** | **G** | **Y** | **A** | **P** | **G** | **S** | **T** | **F** | **K** | **T** | **I** | **T** | **G** | **A** | **I** | **G** | **L** | **D** | **A** | **G** | **T** | **L** | **K** | **P** | **D** | **E** | **E** | **L** | **E** | **I** | **N** | **G** | **L** | **K** | **W** | **Q** | **K** | **D** | **K** | **S** | **W** | **G** | **G** | **Y** | **F** | **A** | **T** | **R** | **V** | **K** | **E** | **A** | **S** | **P** | **V** | **N** | **L** | **R** | **T** | **A** | **L** | **V** | **N** | **S** | **D** | **N** | **I** | **Y** | **F** | **A** | **Q** | **Q** | **T** | **L** | **R** | **M** | **G** | **E** | **D** | **K** | **F** | **R** | **A** | **G** | **L** | **N** | **K** | **F** | **I** | **F** | **G** | **E** | **E** | **L** | **D** | **L** | **P** | **I** | **A** | **M** | **T** | **P** | **A** | **Q** | **I** | **S** | **N** | **E** | **D** | **K** | **F** | **N** | **S** | **E** | **I** | **L** | **L** | **A** | **D** | **T** | **G** | **Y** | **G** | **Q** | **G** | **Q** | **L** | **L** | **I** | **S** | **P** | **I** | **Q** | **Q** | **A** | **T** | **M** | **Y** | **S** | **V** | **F** | **Q** | **N** | **N** | **G** | **T** | **L** | **V** | **Y** | **P** | **K** | **L** | **V** | **L** | **D** | **K** | **E** | **T** | **K** | **K** | **K** | **D** | **N** | **V** | **I** | **S** | **A** | **N** | **A** | **A** | **N** | **T** | **I** | **A** | **T** | **D** | **L** | **L** | **G** | **S** | **V** | **E** | **D** | **P** | **S** | **G** | **Y** | **V** | **Y** | **N** | **M** | **Y** | **N** | **P** | **N** | **F** | **S** | **L** | **A** | **A** | **K** | **T** | **G** | **T** | **A** | **E** | **I** | **K** | **D** | **K** | **Q** | **D** | **T** | **D** | **G** | **K** | **E** | **N** | **S** | **F** | **L** | **L** | **T** | **L** | **D** | **R** | **S** | **N** | **N** | **K** | **F** | **L** | **T** | **M** | **I** | **M** | **V** | **E** | **N** | **S** | **G** | **E** | **N** | **G** | **S** | **A** | **T** | **D** | **I** | **S** | **K** | **P** | **L** | **I** | **D** | **Y** | **L** | **E** | **A** | **T** | **I** | **K** | **-** | **-** | **-** | **-** | **-** | **-** | **-** | **-** | **-** | **-** | **-** | **-** | **-** | **-** | **-** | **-** | **-** | **-** | **-** | **-** | **-** | **-** | **-** | **-** | **-** | **-** | **-** | **-** | **-** | **-** | **-** | **-** | **-** | **-** | **-** | **-** | **Q** | **W** | **Q** | **A** | **K** | **Q** | **E** | **L** | **A** | **E** | **A** | **K** | **K** | **T** | **A** | **T** | **T** | **F** | **L** | **N** | **V** | **L** | **S** | **K** | **Q** | **E** | **F** | **D** | **K** | **L** | **P** | **S** | **V** | **V** | **Q** | **E** | **A** | **S** | **L** | **K** | **K** | **N** | **G** | **Y** | **D** | **T** | **K** | **S** | **V** | **V** | **E** | **K** | **Y** | **Q** | **A** | **I** | **Y** | **S** | **G** | **I** | **Q** | **A** | **E** | **G** | **V** | **K** | **A** | **S** | **D** | **V** | **Q** | **V** | **K** | **K** | **A** | **K** | **D** | **N** | **Q** | **Y** | **T** | **F** | **T** | **Y** | **K** | **L** | **S** | **M** | **S** | **T** | **P** | **L** | **G** | **E** | **M** | **K** | **D** | **L** | **S** | **Y** | **Q** | **S** | **S** | **I** | **A** | **K** | **K** | **G** | **D** | **T** | **Y** | **Q** | **I** | **A** | **W** | **K** | **P** | **S** | **L** | **I** | **F** | **P** | **D** | **M** | **S** | **G** | **N** | **D** | **K** | **I** | **S** | **I** | **Q** | **V** | **D** | **N** | **A** | **K** | **R** | **G** | **E** | **I** | **V** | **D** | **R** | **N** | **G** | **S** | **G** | **L** | **A** | **I** | **N** | **K** | **V** | **F** | **D** | **E** | **V** | **G** | **V** | **V** | **P** | **G** | **K** | **L** | **G** | **S** | **G** | **A** | **E** | **K** | **T** | **A** | **N** | **I** | **K** | **A** | **F** | **S** | **D** | **K** | **F** | **G** | **V** | **S** | **V** | **D** | **E** | **I** | **N** | **Q** | **K** | **L** | **S** | **Q** | **G** | **W** | **V** | **Q** | **A** | **D** | **S** | **F** | **V** | **P** | **I** | **T** | **V** | **A** | **S** | **E** | **P** | **V** | **T** | **E** | **L** | **P** | **T** | **G** | **A** | **A** | **T** | **K** | **G** | **T** | **E** | **S** | **R** | **Y** | **Y** | **P** | **L** | **G** | **E** | **A** | **A** | **A** | **Q** | **L** | **I** | **G** | **Y** | **T** | **G** | **T** | **I** | **T** | **A** | **E** | **D** | **I** | **E** | **K** | **N** | **P** | **E** | **L** | **S** | **S** | **T** | **G** | **V** | **I** | **G** | **K** | **T** | **G** | **L** | **E** | **R** | **A** | **F** | **D** | **K** | **E** | **L** | **R** | **G** | **Q** | **D** | **G** | **G** | **S** | **L** | **V** | **I** | **L** | **D** | **D** | **K** | **E** | **N** | **V** | **K** | **K** | **A** | **L** | **Q** | **T** | **K** | **E** | **K** | **K** | **D** | **G** | **Q** | **T** | **I** | **K** | **L** | **T** | **I** | **D** | **S** | **G** | **V** | **Q** | **Q** | **Q** | **A** | **F** | **A** | **I** | **F** | **D** | **K** | **R** | **P** | **G** | **S** | **A** | **V** | **I** | **T** | **D** | **P** | **Q** | **K** | **G** | **D** | **L** | **L** | **A** | **T** | **V** | **S** | **S** | **P** | **S** | **Y** | **D** | **P** | **N** | **K** | **M** | **A** | **N** | **G** | **I** | **S** | **Q** | **K** | **E** | **Y** | **D** | **A** | **Y** | **N** | **N** | **N** | **K** | **D** | **L** | **P** | **F** | **T** | **A** | **R** | **F** | **A** | **T** | **G** | **Y** | **A** | **P** | **G** | **S** | **T** | **F** | **K** | **T** | **I** | **T** | **G** | **A** | **I** | **G** | **L** | **D** | **A** | **G** | **T** | **L** | **K** | **P** | **D** | **E** | **E** | **L** | **E** | **I** | **N** | **G** | **L** | **K** | **W** | **Q** | **K** | **D** | **K** | **S** | **W** | **G** | **G** | **Y** | **F** | **A** | **T** | **R** | **V** | **K** | **E** | **A** | **S** | **P** | **V** | **N** | **L** | **R** | **T** | **A** | **L** | **V** | **N** | **S** | **D** | **N** | **I** | **Y** | **F** | **A** | **Q** | **Q** | **T** | **L** | **R** | **M** | **G** | **E** | **D** | **K** | **F** | **R** | **A** | **G** | **L** | **N** | **K** | **F** | **I** | **F** | **G** | **E** | **E** | **L** | **D** | **L** | **P** | **I** | **A** | **M** | **T** | **P** | **A** | **Q** | **I** | **S** | **N** | **E** | **D** | **K** | **F** | **N** | **S** | **E** | **I** | **L** | **L** | **A** | **D** | **T** | **G** | **Y** | **G** | **Q** | **G** | **Q** | **L** | **L** | **I** | **S** | **P** | **I** | **Q** | **Q** | **A** | **T** | **M** | **Y** | **S** | **V** | **F** | **Q** | **N** | **N** | **G** | **T** | **L** | **V** | **Y** | **P** | **K** | **L** | **V** | **L** | **D** | **K** | **E** | **T** | **K** | **K** | **K** | **D** | **N** | **V** | **I** | **S** | **A** | **N** | **A** | **A** | **N** | **T** | **I** | **A** | **T** | **D** | **L** | **L** | **G** | **S** | **V** | **E** | **D** | **P** | **S** | **G** | **Y** | **V** | **Y** | **N** | **M** | **Y** | **N** | **P** | **N** | **F** | **S** | **L** | **A** | **A** | **K** | **T** | **G** | **T** | **A** | **E** | **I** | **K** | **D** | **K** | **Q** | **D** | **T** | **D** | **G** | **K** | **E** | **N** | **S** | **F** | **L** | **L** | **T** | **L** | **D** | **R** | **S** | **N** | **N** | **K** | **F** | **L** | **T** | **M** | **I** | **M** | **V** | **E** | **N** | **S** | **G** | **E** | **N** | **G** | **S** | **A** | **T** | **D** | **I** | **S** | **K** | **P** | **L** | **I** | **D** | **Y** | **L** | **E** | **A** | **T** | **I** | **K** |
| **N52773** | **-** | **-** | **-** | **-** | **-** | **-** | **-** | **-** | **-** | **-** | **-** | **-** | **-** | **-** | **-** | **-** | **-** | **-** | **-** | **-** | **-** | **-** | **-** | **-** | **-** | **-** | **-** | **-** | **-** | **-** | **-** | **-** | **-** | **-** | **-** | **-** | **Q** | **W** | **Q** | **A** | **K** | **Q** | **E** | **L** | **A** | **E** | **A** | **K** | **K** | **T** | **A** | **T** | **T** | **F** | **L** | **N** | **V** | **L** | **S** | **K** | **Q** | **E** | **F** | **D** | **K** | **L** | **P** | **S** | **V** | **V** | **Q** | **E** | **A** | **S** | **L** | **K** | **K** | **N** | **G** | **Y** | **D** | **T** | **K** | **S** | **V** | **V** | **E** | **K** | **Y** | **Q** | **A** | **I** | **Y** | **S** | **G** | **I** | **Q** | **A** | **E** | **G** | **V** | **K** | **A** | **S** | **D** | **V** | **Q** | **V** | **K** | **K** | **A** | **K** | **D** | **N** | **Q** | **Y** | **T** | **F** | **T** | **Y** | **K** | **L** | **S** | **M** | **S** | **T** | **P** | **L** | **G** | **E** | **M** | **K** | **D** | **L** | **S** | **Y** | **Q** | **S** | **S** | **I** | **A** | **K** | **K** | **G** | **D** | **T** | **Y** | **Q** | **I** | **A** | **W** | **K** | **P** | **S** | **L** | **I** | **F** | **P** | **D** | **M** | **S** | **G** | **N** | **D** | **K** | **I** | **S** | **I** | **Q** | **V** | **D** | **N** | **A** | **K** | **R** | **G** | **E** | **I** | **V** | **D** | **R** | **N** | **G** | **S** | **G** | **L** | **V** | **I** | **N** | **K** | **V** | **F** | **D** | **E** | **V** | **G** | **V** | **V** | **P** | **G** | **K** | **L** | **G** | **S** | **G** | **A** | **E** | **K** | **T** | **A** | **N** | **I** | **K** | **A** | **F** | **S** | **D** | **K** | **F** | **G** | **V** | **S** | **V** | **D** | **E** | **I** | **N** | **Q** | **K** | **L** | **S** | **Q** | **G** | **W** | **V** | **Q** | **A** | **D** | **S** | **F** | **V** | **P** | **I** | **T** | **V** | **A** | **S** | **E** | **P** | **V** | **T** | **E** | **L** | **P** | **T** | **G** | **A** | **A** | **T** | **K** | **D** | **T** | **E** | **S** | **R** | **Y** | **Y** | **P** | **L** | **G** | **E** | **A** | **A** | **A** | **Q** | **L** | **I** | **G** | **Y** | **T** | **G** | **T** | **I** | **T** | **A** | **E** | **D** | **I** | **E** | **K** | **N** | **P** | **E** | **L** | **S** | **S** | **T** | **G** | **V** | **I** | **G** | **K** | **T** | **G** | **L** | **E** | **R** | **A** | **F** | **D** | **K** | **E** | **L** | **R** | **G** | **Q** | **D** | **G** | **G** | **S** | **L** | **V** | **I** | **L** | **D** | **D** | **K** | **E** | **N** | **V** | **K** | **K** | **A** | **L** | **Q** | **T** | **K** | **E** | **K** | **K** | **D** | **G** | **Q** | **T** | **I** | **K** | **L** | **T** | **I** | **D** | **S** | **G** | **V** | **Q** | **Q** | **Q** | **A** | **F** | **A** | **I** | **F** | **D** | **K** | **R** | **T** | **G** | **S** | **A** | **V** | **I** | **T** | **D** | **P** | **Q** | **K** | **G** | **D** | **L** | **L** | **A** | **T** | **V** | **S** | **S** | **P** | **S** | **Y** | **D** | **P** | **N** | **K** | **M** | **A** | **N** | **G** | **I** | **S** | **Q** | **K** | **E** | **Y** | **D** | **A** | **Y** | **N** | **N** | **N** | **K** | **D** | **L** | **P** | **F** | **T** | **A** | **R** | **F** | **A** | **T** | **G** | **Y** | **A** | **P** | **G** | **S** | **T** | **F** | **K** | **T** | **I** | **T** | **G** | **A** | **I** | **G** | **L** | **D** | **A** | **G** | **T** | **L** | **K** | **P** | **D** | **E** | **E** | **L** | **E** | **I** | **N** | **G** | **L** | **K** | **W** | **Q** | **K** | **D** | **K** | **S** | **W** | **G** | **G** | **Y** | **F** | **A** | **T** | **R** | **V** | **K** | **E** | **A** | **S** | **P** | **V** | **N** | **L** | **R** | **T** | **A** | **L** | **V** | **N** | **S** | **D** | **N** | **I** | **Y** | **F** | **A** | **Q** | **Q** | **T** | **L** | **R** | **M** | **G** | **E** | **D** | **K** | **F** | **R** | **A** | **G** | **L** | **N** | **K** | **F** | **I** | **F** | **G** | **E** | **E** | **L** | **D** | **L** | **P** | **I** | **A** | **M** | **T** | **P** | **A** | **Q** | **I** | **S** | **N** | **E** | **D** | **K** | **F** | **N** | **S** | **E** | **I** | **L** | **L** | **A** | **D** | **T** | **G** | **Y** | **G** | **Q** | **G** | **Q** | **L** | **L** | **I** | **S** | **P** | **I** | **Q** | **Q** | **A** | **T** | **M** | **Y** | **S** | **V** | **F** | **Q** | **N** | **N** | **G** | **T** | **L** | **V** | **Y** | **P** | **K** | **I** | **V** | **L** | **D** | **K** | **E** | **T** | **K** | **K** | **K** | **D** | **N** | **V** | **I** | **S** | **A** | **N** | **A** | **A** | **N** | **T** | **I** | **A** | **T** | **D** | **L** | **L** | **G** | **S** | **V** | **E** | **D** | **P** | **S** | **G** | **Y** | **V** | **Y** | **N** | **M** | **Y** | **N** | **P** | **N** | **F** | **S** | **L** | **A** | **A** | **K** | **T** | **G** | **T** | **A** | **E** | **I** | **K** | **D** | **K** | **Q** | **D** | **T** | **D** | **G** | **K** | **E** | **N** | **S** | **F** | **L** | **L** | **T** | **L** | **D** | **R** | **S** | **N** | **N** | **K** | **F** | **L** | **T** | **M** | **I** | **M** | **V** | **E** | **N** | **S** | **G** | **E** | **N** | **G** | **S** | **A** | **T** | **D** | **I** | **S** | **K** | **P** | **L** | **I** | **D** | **Y** | **L** | **E** | **A** | **T** | **I** | **K** | **-** | **-** | **-** | **-** | **-** | **-** | **-** | **-** | **-** | **-** | **-** | **-** | **-** | **-** | **-** | **-** | **-** | **-** | **-** | **-** | **-** | **-** | **-** | **-** | **-** | **-** | **-** | **-** | **-** | **-** | **-** | **-** | **-** | **-** | **-** | **-** | **Q** | **W** | **Q** | **A** | **K** | **Q** | **E** | **L** | **A** | **E** | **A** | **K** | **K** | **T** | **A** | **T** | **T** | **F** | **L** | **N** | **V** | **L** | **S** | **K** | **Q** | **E** | **F** | **D** | **K** | **L** | **P** | **S** | **V** | **V** | **Q** | **E** | **A** | **S** | **L** | **K** | **K** | **N** | **G** | **Y** | **D** | **T** | **K** | **S** | **V** | **V** | **E** | **K** | **Y** | **Q** | **A** | **I** | **Y** | **S** | **G** | **I** | **Q** | **A** | **E** | **G** | **V** | **K** | **A** | **S** | **D** | **V** | **Q** | **V** | **K** | **K** | **A** | **K** | **D** | **N** | **Q** | **Y** | **T** | **F** | **T** | **Y** | **K** | **L** | **S** | **M** | **S** | **T** | **P** | **L** | **G** | **E** | **M** | **K** | **D** | **L** | **S** | **Y** | **Q** | **S** | **S** | **I** | **A** | **K** | **K** | **G** | **D** | **T** | **Y** | **Q** | **I** | **A** | **W** | **K** | **P** | **S** | **L** | **I** | **F** | **P** | **D** | **M** | **S** | **G** | **N** | **D** | **K** | **I** | **S** | **I** | **Q** | **V** | **D** | **N** | **A** | **K** | **R** | **G** | **E** | **I** | **V** | **D** | **R** | **N** | **G** | **S** | **G** | **L** | **V** | **I** | **N** | **K** | **V** | **F** | **D** | **E** | **V** | **G** | **V** | **V** | **P** | **G** | **K** | **L** | **G** | **S** | **G** | **A** | **E** | **K** | **T** | **A** | **N** | **I** | **K** | **A** | **F** | **S** | **D** | **K** | **F** | **G** | **V** | **S** | **V** | **D** | **E** | **I** | **N** | **Q** | **K** | **L** | **S** | **Q** | **G** | **W** | **V** | **Q** | **A** | **D** | **S** | **F** | **V** | **P** | **I** | **T** | **V** | **A** | **S** | **E** | **P** | **V** | **T** | **E** | **L** | **P** | **T** | **G** | **A** | **A** | **T** | **K** | **D** | **T** | **E** | **S** | **R** | **Y** | **Y** | **P** | **L** | **G** | **E** | **A** | **A** | **A** | **Q** | **L** | **I** | **G** | **Y** | **T** | **G** | **T** | **I** | **T** | **A** | **E** | **D** | **I** | **E** | **K** | **N** | **P** | **E** | **L** | **S** | **S** | **T** | **G** | **V** | **I** | **G** | **K** | **T** | **G** | **L** | **E** | **R** | **A** | **F** | **D** | **K** | **E** | **L** | **R** | **G** | **Q** | **D** | **G** | **G** | **S** | **L** | **V** | **I** | **L** | **D** | **D** | **K** | **E** | **N** | **V** | **K** | **K** | **A** | **L** | **Q** | **T** | **K** | **E** | **K** | **K** | **D** | **G** | **Q** | **T** | **I** | **K** | **L** | **T** | **I** | **D** | **S** | **G** | **V** | **Q** | **Q** | **Q** | **A** | **F** | **A** | **I** | **F** | **D** | **K** | **R** | **T** | **G** | **S** | **A** | **V** | **I** | **T** | **D** | **P** | **Q** | **K** | **G** | **D** | **L** | **L** | **A** | **T** | **V** | **S** | **S** | **P** | **S** | **Y** | **D** | **P** | **N** | **K** | **M** | **A** | **N** | **G** | **I** | **S** | **Q** | **K** | **E** | **Y** | **D** | **A** | **Y** | **N** | **N** | **N** | **K** | **D** | **L** | **P** | **F** | **T** | **A** | **R** | **F** | **A** | **T** | **G** | **Y** | **A** | **P** | **G** | **S** | **T** | **F** | **K** | **T** | **I** | **T** | **G** | **A** | **I** | **G** | **L** | **D** | **A** | **G** | **T** | **L** | **K** | **P** | **D** | **E** | **E** | **L** | **E** | **I** | **N** | **G** | **L** | **K** | **W** | **Q** | **K** | **D** | **K** | **S** | **W** | **G** | **G** | **Y** | **F** | **A** | **T** | **R** | **V** | **K** | **E** | **A** | **S** | **P** | **V** | **N** | **L** | **R** | **T** | **A** | **L** | **V** | **N** | **S** | **D** | **N** | **I** | **Y** | **F** | **A** | **Q** | **Q** | **T** | **L** | **R** | **M** | **G** | **E** | **D** | **K** | **F** | **R** | **A** | **G** | **L** | **N** | **K** | **F** | **I** | **F** | **G** | **E** | **E** | **L** | **D** | **L** | **P** | **I** | **A** | **M** | **T** | **P** | **A** | **Q** | **I** | **S** | **N** | **E** | **D** | **K** | **F** | **N** | **S** | **E** | **I** | **L** | **L** | **A** | **D** | **T** | **G** | **Y** | **G** | **Q** | **G** | **Q** | **L** | **L** | **I** | **S** | **P** | **I** | **Q** | **Q** | **A** | **T** | **M** | **Y** | **S** | **V** | **F** | **Q** | **N** | **N** | **G** | **T** | **L** | **V** | **Y** | **P** | **K** | **I** | **V** | **L** | **D** | **K** | **E** | **T** | **K** | **K** | **K** | **D** | **N** | **V** | **I** | **S** | **A** | **N** | **A** | **A** | **N** | **T** | **I** | **A** | **T** | **D** | **L** | **L** | **G** | **S** | **V** | **E** | **D** | **P** | **S** | **G** | **Y** | **V** | **Y** | **N** | **M** | **Y** | **N** | **P** | **N** | **F** | **S** | **L** | **A** | **A** | **K** | **T** | **G** | **T** | **A** | **E** | **I** | **K** | **D** | **K** | **Q** | **D** | **T** | **D** | **G** | **K** | **E** | **N** | **S** | **F** | **L** | **L** | **T** | **L** | **D** | **R** | **S** | **N** | **N** | **K** | **F** | **L** | **T** | **M** | **I** | **M** | **V** | **E** | **N** | **S** | **G** | **E** | **N** | **G** | **S** | **A** | **T** | **D** | **I** | **S** | **K** | **P** | **L** | **I** | **D** | **Y** | **L** | **E** | **A** | **T** | **I** | **K** |
| **N59409F** | **-** | **-** | **-** | **-** | **-** | **-** | **-** | **-** | **-** | **-** | **-** | **-** | **-** | **-** | **-** | **-** | **-** | **-** | **-** | **-** | **-** | **-** | **-** | **-** | **-** | **-** | **-** | **-** | **-** | **-** | **-** | **-** | **-** | **-** | **-** | **-** | **Q** | **W** | **Q** | **A** | **K** | **Q** | **E** | **L** | **A** | **E** | **A** | **K** | **K** | **T** | **A** | **T** | **T** | **F** | **L** | **N** | **V** | **L** | **S** | **K** | **Q** | **E** | **F** | **D** | **K** | **L** | **P** | **S** | **V** | **V** | **Q** | **E** | **A** | **S** | **L** | **K** | **K** | **N** | **G** | **Y** | **D** | **T** | **K** | **S** | **V** | **V** | **E** | **K** | **Y** | **Q** | **A** | **I** | **Y** | **S** | **G** | **I** | **Q** | **A** | **E** | **G** | **V** | **K** | **A** | **S** | **D** | **V** | **Q** | **V** | **K** | **K** | **A** | **K** | **D** | **N** | **Q** | **Y** | **T** | **F** | **T** | **Y** | **K** | **L** | **S** | **M** | **S** | **T** | **P** | **L** | **G** | **E** | **M** | **K** | **D** | **L** | **S** | **Y** | **Q** | **S** | **S** | **I** | **A** | **K** | **K** | **G** | **D** | **I** | **Y** | **Q** | **I** | **A** | **W** | **K** | **P** | **S** | **L** | **I** | **F** | **P** | **D** | **M** | **S** | **G** | **N** | **D** | **K** | **I** | **S** | **I** | **Q** | **V** | **D** | **N** | **A** | **K** | **R** | **G** | **E** | **I** | **V** | **D** | **R** | **N** | **G** | **S** | **G** | **L** | **A** | **I** | **N** | **K** | **V** | **F** | **D** | **E** | **V** | **G** | **V** | **V** | **P** | **G** | **K** | **L** | **G** | **S** | **G** | **A** | **E** | **K** | **T** | **A** | **N** | **I** | **K** | **A** | **F** | **S** | **D** | **K** | **F** | **G** | **V** | **S** | **V** | **D** | **E** | **I** | **N** | **Q** | **K** | **L** | **S** | **Q** | **G** | **W** | **V** | **Q** | **A** | **D** | **S** | **F** | **V** | **P** | **I** | **T** | **V** | **A** | **S** | **E** | **P** | **V** | **T** | **E** | **L** | **P** | **T** | **G** | **A** | **A** | **T** | **K** | **D** | **T** | **E** | **S** | **R** | **Y** | **Y** | **P** | **L** | **G** | **E** | **A** | **A** | **A** | **Q** | **L** | **I** | **G** | **Y** | **T** | **G** | **T** | **I** | **T** | **A** | **E** | **D** | **I** | **E** | **K** | **N** | **P** | **E** | **L** | **S** | **S** | **T** | **G** | **V** | **I** | **G** | **K** | **T** | **G** | **L** | **E** | **R** | **A** | **F** | **D** | **K** | **E** | **L** | **R** | **G** | **Q** | **D** | **G** | **G** | **S** | **L** | **V** | **I** | **L** | **D** | **D** | **K** | **E** | **N** | **V** | **K** | **K** | **A** | **L** | **Q** | **T** | **K** | **E** | **K** | **K** | **D** | **G** | **Q** | **T** | **I** | **K** | **L** | **T** | **I** | **D** | **S** | **G** | **V** | **Q** | **Q** | **Q** | **A** | **F** | **A** | **I** | **F** | **D** | **K** | **R** | **P** | **G** | **S** | **A** | **V** | **I** | **T** | **D** | **P** | **Q** | **K** | **G** | **D** | **L** | **L** | **A** | **T** | **V** | **S** | **S** | **P** | **S** | **Y** | **D** | **P** | **N** | **K** | **M** | **A** | **N** | **G** | **I** | **S** | **Q** | **K** | **E** | **Y** | **D** | **A** | **Y** | **N** | **N** | **N** | **K** | **D** | **L** | **P** | **F** | **T** | **A** | **R** | **F** | **A** | **T** | **G** | **Y** | **A** | **P** | **G** | **S** | **T** | **F** | **K** | **T** | **I** | **T** | **G** | **A** | **I** | **G** | **L** | **D** | **A** | **G** | **T** | **L** | **K** | **P** | **D** | **E** | **E** | **L** | **E** | **I** | **N** | **G** | **L** | **K** | **W** | **Q** | **K** | **D** | **K** | **S** | **W** | **G** | **G** | **Y** | **F** | **A** | **T** | **R** | **V** | **K** | **E** | **A** | **S** | **P** | **V** | **N** | **L** | **R** | **T** | **A** | **L** | **V** | **N** | **S** | **D** | **N** | **I** | **Y** | **F** | **A** | **Q** | **Q** | **T** | **L** | **R** | **M** | **G** | **E** | **D** | **K** | **F** | **R** | **A** | **G** | **L** | **N** | **K** | **F** | **I** | **F** | **G** | **E** | **E** | **L** | **D** | **L** | **P** | **I** | **A** | **M** | **T** | **P** | **A** | **Q** | **I** | **S** | **N** | **E** | **D** | **K** | **F** | **N** | **S** | **E** | **I** | **L** | **L** | **A** | **D** | **T** | **G** | **Y** | **G** | **Q** | **G** | **Q** | **L** | **L** | **I** | **S** | **P** | **I** | **Q** | **Q** | **A** | **T** | **M** | **Y** | **S** | **V** | **F** | **Q** | **N** | **N** | **G** | **T** | **L** | **V** | **Y** | **P** | **K** | **L** | **V** | **L** | **D** | **K** | **E** | **T** | **K** | **K** | **K** | **D** | **N** | **V** | **I** | **S** | **A** | **N** | **A** | **A** | **N** | **T** | **I** | **A** | **T** | **D** | **L** | **L** | **G** | **S** | **V** | **E** | **D** | **P** | **S** | **G** | **Y** | **V** | **Y** | **N** | **M** | **Y** | **N** | **P** | **N** | **F** | **S** | **L** | **A** | **A** | **K** | **T** | **G** | **T** | **A** | **E** | **I** | **K** | **D** | **K** | **Q** | **D** | **T** | **D** | **G** | **K** | **E** | **N** | **S** | **F** | **L** | **L** | **T** | **L** | **D** | **R** | **S** | **N** | **N** | **K** | **F** | **L** | **T** | **M** | **I** | **M** | **V** | **E** | **N** | **S** | **G** | **E** | **N** | **G** | **S** | **A** | **T** | **D** | **I** | **S** | **K** | **P** | **L** | **I** | **D** | **Y** | **L** | **E** | **A** | **T** | **I** | **K** | **-** | **-** | **-** | **-** | **-** | **-** | **-** | **-** | **-** | **-** | **-** | **-** | **-** | **-** | **-** | **-** | **-** | **-** | **-** | **-** | **-** | **-** | **-** | **-** | **-** | **-** | **-** | **-** | **-** | **-** | **-** | **-** | **-** | **-** | **-** | **-** | **Q** | **W** | **Q** | **A** | **K** | **Q** | **E** | **L** | **A** | **E** | **A** | **K** | **K** | **T** | **A** | **T** | **T** | **F** | **L** | **N** | **V** | **L** | **S** | **K** | **Q** | **E** | **F** | **D** | **K** | **L** | **P** | **S** | **V** | **V** | **Q** | **E** | **A** | **S** | **L** | **K** | **K** | **N** | **G** | **Y** | **D** | **T** | **K** | **S** | **V** | **V** | **E** | **K** | **Y** | **Q** | **A** | **I** | **Y** | **S** | **G** | **I** | **Q** | **A** | **E** | **G** | **V** | **K** | **A** | **S** | **D** | **V** | **Q** | **V** | **K** | **K** | **A** | **K** | **D** | **N** | **Q** | **Y** | **T** | **F** | **T** | **Y** | **K** | **L** | **S** | **M** | **S** | **T** | **P** | **L** | **G** | **E** | **M** | **K** | **D** | **L** | **S** | **Y** | **Q** | **S** | **S** | **I** | **A** | **K** | **K** | **G** | **D** | **I** | **Y** | **Q** | **I** | **A** | **W** | **K** | **P** | **S** | **L** | **I** | **F** | **P** | **D** | **M** | **S** | **G** | **N** | **D** | **K** | **I** | **S** | **I** | **Q** | **V** | **D** | **N** | **A** | **K** | **R** | **G** | **E** | **I** | **V** | **D** | **R** | **N** | **G** | **S** | **G** | **L** | **A** | **I** | **N** | **K** | **V** | **F** | **D** | **E** | **V** | **G** | **V** | **V** | **P** | **G** | **K** | **L** | **G** | **S** | **G** | **A** | **E** | **K** | **T** | **A** | **N** | **I** | **K** | **A** | **F** | **S** | **D** | **K** | **F** | **G** | **V** | **S** | **V** | **D** | **E** | **I** | **N** | **Q** | **K** | **L** | **S** | **Q** | **G** | **W** | **V** | **Q** | **A** | **D** | **S** | **F** | **V** | **P** | **I** | **T** | **V** | **A** | **S** | **E** | **P** | **V** | **T** | **E** | **L** | **P** | **T** | **G** | **A** | **A** | **T** | **K** | **D** | **T** | **E** | **S** | **R** | **Y** | **Y** | **P** | **L** | **G** | **E** | **A** | **A** | **A** | **Q** | **L** | **I** | **G** | **Y** | **T** | **G** | **T** | **I** | **T** | **A** | **E** | **D** | **I** | **E** | **K** | **N** | **P** | **E** | **L** | **S** | **S** | **T** | **G** | **V** | **I** | **G** | **K** | **T** | **G** | **L** | **E** | **R** | **A** | **F** | **D** | **K** | **E** | **L** | **R** | **G** | **Q** | **D** | **G** | **G** | **S** | **L** | **V** | **I** | **L** | **D** | **D** | **K** | **E** | **N** | **V** | **K** | **K** | **A** | **L** | **Q** | **T** | **K** | **E** | **K** | **K** | **D** | **G** | **Q** | **T** | **I** | **K** | **L** | **T** | **I** | **D** | **S** | **G** | **V** | **Q** | **Q** | **Q** | **A** | **F** | **A** | **I** | **F** | **D** | **K** | **R** | **P** | **G** | **S** | **A** | **V** | **I** | **T** | **D** | **P** | **Q** | **K** | **G** | **D** | **L** | **L** | **A** | **T** | **V** | **S** | **S** | **P** | **S** | **Y** | **D** | **P** | **N** | **K** | **M** | **A** | **N** | **G** | **I** | **S** | **Q** | **K** | **E** | **Y** | **D** | **A** | **Y** | **N** | **N** | **N** | **K** | **D** | **L** | **P** | **F** | **T** | **A** | **R** | **F** | **A** | **T** | **G** | **Y** | **A** | **P** | **G** | **S** | **T** | **F** | **K** | **T** | **I** | **T** | **G** | **A** | **I** | **G** | **L** | **D** | **A** | **G** | **T** | **L** | **K** | **P** | **D** | **E** | **E** | **L** | **E** | **I** | **N** | **G** | **L** | **K** | **W** | **Q** | **K** | **D** | **K** | **S** | **W** | **G** | **G** | **Y** | **F** | **A** | **T** | **R** | **V** | **K** | **E** | **A** | **S** | **P** | **V** | **N** | **L** | **R** | **T** | **A** | **L** | **V** | **N** | **S** | **D** | **N** | **I** | **Y** | **F** | **A** | **Q** | **Q** | **T** | **L** | **R** | **M** | **G** | **E** | **D** | **K** | **F** | **R** | **A** | **G** | **L** | **N** | **K** | **F** | **I** | **F** | **G** | **E** | **E** | **L** | **D** | **L** | **P** | **I** | **A** | **M** | **T** | **P** | **A** | **Q** | **I** | **S** | **N** | **E** | **D** | **K** | **F** | **N** | **S** | **E** | **I** | **L** | **L** | **A** | **D** | **T** | **G** | **Y** | **G** | **Q** | **G** | **Q** | **L** | **L** | **I** | **S** | **P** | **I** | **Q** | **Q** | **A** | **T** | **M** | **Y** | **S** | **V** | **F** | **Q** | **N** | **N** | **G** | **T** | **L** | **V** | **Y** | **P** | **K** | **L** | **V** | **L** | **D** | **K** | **E** | **T** | **K** | **K** | **K** | **D** | **N** | **V** | **I** | **S** | **A** | **N** | **A** | **A** | **N** | **T** | **I** | **A** | **T** | **D** | **L** | **L** | **G** | **S** | **V** | **E** | **D** | **P** | **S** | **G** | **Y** | **V** | **Y** | **N** | **M** | **Y** | **N** | **P** | **N** | **F** | **S** | **L** | **A** | **A** | **K** | **T** | **G** | **T** | **A** | **E** | **I** | **K** | **D** | **K** | **Q** | **D** | **T** | **D** | **G** | **K** | **E** | **N** | **S** | **F** | **L** | **L** | **T** | **L** | **D** | **R** | **S** | **N** | **N** | **K** | **F** | **L** | **T** | **M** | **I** | **M** | **V** | **E** | **N** | **S** | **G** | **E** | **N** | **G** | **S** | **A** | **T** | **D** | **I** | **S** | **K** | **P** | **L** | **I** | **D** | **Y** | **L** | **E** | **A** | **T** | **I** | **K** |
| **N59458F** | **-** | **-** | **-** | **-** | **-** | **-** | **-** | **-** | **-** | **-** | **-** | **-** | **-** | **-** | **-** | **-** | **-** | **-** | **-** | **-** | **-** | **-** | **-** | **-** | **-** | **-** | **-** | **-** | **-** | **-** | **-** | **-** | **-** | **-** | **-** | **-** | **Q** | **W** | **Q** | **A** | **K** | **Q** | **E** | **L** | **A** | **E** | **A** | **K** | **K** | **T** | **A** | **T** | **E** | **F** | **L** | **N** | **V** | **L** | **S** | **K** | **Q** | **E** | **F** | **D** | **K** | **L** | **P** | **S** | **V** | **V** | **Q** | **E** | **A** | **S** | **L** | **K** | **K** | **N** | **G** | **Y** | **D** | **T** | **K** | **S** | **V** | **V** | **E** | **K** | **Y** | **Q** | **A** | **I** | **Y** | **S** | **G** | **I** | **Q** | **A** | **E** | **G** | **V** | **K** | **A** | **S** | **D** | **V** | **Q** | **V** | **K** | **K** | **A** | **K** | **D** | **N** | **Q** | **Y** | **T** | **F** | **T** | **Y** | **K** | **L** | **S** | **M** | **S** | **T** | **P** | **L** | **G** | **E** | **M** | **K** | **D** | **L** | **S** | **Y** | **Q** | **S** | **S** | **I** | **A** | **K** | **K** | **G** | **D** | **T** | **Y** | **Q** | **I** | **A** | **W** | **K** | **P** | **S** | **L** | **I** | **F** | **P** | **D** | **M** | **S** | **G** | **N** | **D** | **K** | **I** | **S** | **I** | **Q** | **V** | **D** | **N** | **A** | **K** | **R** | **G** | **E** | **I** | **V** | **D** | **R** | **N** | **G** | **S** | **G** | **L** | **A** | **I** | **N** | **K** | **V** | **F** | **D** | **E** | **V** | **G** | **V** | **V** | **P** | **G** | **K** | **L** | **G** | **S** | **G** | **A** | **E** | **K** | **T** | **A** | **N** | **I** | **K** | **A** | **F** | **S** | **D** | **K** | **F** | **G** | **V** | **S** | **V** | **D** | **E** | **I** | **N** | **Q** | **K** | **L** | **S** | **Q** | **G** | **W** | **V** | **Q** | **A** | **D** | **S** | **F** | **V** | **P** | **I** | **T** | **V** | **A** | **S** | **E** | **P** | **V** | **T** | **E** | **L** | **P** | **T** | **G** | **A** | **A** | **T** | **K** | **D** | **T** | **E** | **S** | **R** | **Y** | **Y** | **P** | **L** | **G** | **E** | **A** | **A** | **A** | **Q** | **L** | **I** | **G** | **Y** | **T** | **G** | **T** | **I** | **T** | **A** | **E** | **D** | **I** | **E** | **K** | **N** | **P** | **E** | **L** | **S** | **S** | **T** | **G** | **V** | **I** | **G** | **K** | **T** | **G** | **L** | **E** | **R** | **A** | **F** | **D** | **K** | **E** | **L** | **R** | **G** | **Q** | **D** | **G** | **G** | **S** | **L** | **V** | **I** | **L** | **D** | **D** | **K** | **E** | **N** | **V** | **K** | **K** | **A** | **L** | **Q** | **T** | **K** | **E** | **K** | **K** | **D** | **G** | **Q** | **T** | **I** | **K** | **L** | **T** | **I** | **D** | **S** | **G** | **V** | **Q** | **Q** | **Q** | **A** | **F** | **A** | **I** | **F** | **D** | **K** | **R** | **P** | **G** | **S** | **A** | **V** | **I** | **T** | **D** | **P** | **Q** | **K** | **G** | **D** | **L** | **L** | **A** | **T** | **V** | **S** | **S** | **P** | **S** | **Y** | **D** | **P** | **N** | **K** | **M** | **A** | **N** | **G** | **I** | **S** | **Q** | **K** | **E** | **Y** | **D** | **A** | **Y** | **N** | **N** | **N** | **K** | **D** | **L** | **P** | **F** | **T** | **A** | **R** | **F** | **A** | **T** | **G** | **Y** | **A** | **P** | **G** | **S** | **T** | **F** | **K** | **T** | **I** | **T** | **G** | **A** | **I** | **G** | **L** | **D** | **A** | **G** | **T** | **L** | **K** | **P** | **D** | **E** | **E** | **L** | **E** | **I** | **N** | **G** | **L** | **K** | **W** | **Q** | **K** | **D** | **K** | **S** | **W** | **G** | **G** | **Y** | **F** | **A** | **T** | **R** | **V** | **K** | **E** | **A** | **S** | **P** | **V** | **N** | **L** | **R** | **T** | **A** | **L** | **V** | **N** | **S** | **D** | **N** | **I** | **Y** | **F** | **A** | **Q** | **Q** | **T** | **L** | **R** | **M** | **G** | **E** | **D** | **K** | **F** | **R** | **A** | **G** | **L** | **N** | **K** | **F** | **I** | **F** | **G** | **E** | **E** | **L** | **D** | **L** | **P** | **I** | **A** | **M** | **T** | **P** | **A** | **Q** | **I** | **S** | **N** | **E** | **D** | **K** | **F** | **N** | **S** | **E** | **I** | **L** | **L** | **A** | **D** | **T** | **G** | **Y** | **G** | **Q** | **G** | **Q** | **L** | **L** | **I** | **S** | **P** | **I** | **Q** | **Q** | **A** | **T** | **M** | **Y** | **S** | **V** | **F** | **Q** | **N** | **N** | **G** | **T** | **L** | **V** | **Y** | **P** | **K** | **I** | **V** | **L** | **D** | **K** | **E** | **T** | **K** | **K** | **K** | **D** | **N** | **V** | **I** | **S** | **A** | **N** | **A** | **A** | **N** | **T** | **I** | **A** | **T** | **D** | **L** | **L** | **G** | **S** | **V** | **E** | **D** | **P** | **S** | **G** | **Y** | **V** | **Y** | **N** | **M** | **Y** | **N** | **P** | **N** | **F** | **S** | **L** | **A** | **A** | **K** | **T** | **G** | **T** | **A** | **E** | **I** | **K** | **D** | **K** | **Q** | **D** | **T** | **D** | **G** | **K** | **E** | **N** | **S** | **F** | **L** | **L** | **T** | **L** | **D** | **R** | **S** | **N** | **N** | **K** | **F** | **L** | **T** | **M** | **I** | **M** | **V** | **E** | **N** | **S** | **G** | **E** | **N** | **G** | **S** | **A** | **T** | **D** | **I** | **S** | **K** | **P** | **L** | **I** | **D** | **Y** | **L** | **E** | **A** | **T** | **I** | **K** | **-** | **-** | **-** | **-** | **-** | **-** | **-** | **-** | **-** | **-** | **-** | **-** | **-** | **-** | **-** | **-** | **-** | **-** | **-** | **-** | **-** | **-** | **-** | **-** | **-** | **-** | **-** | **-** | **-** | **-** | **-** | **-** | **-** | **-** | **-** | **-** | **Q** | **W** | **Q** | **A** | **K** | **Q** | **E** | **L** | **A** | **E** | **A** | **K** | **K** | **T** | **A** | **T** | **E** | **F** | **L** | **N** | **V** | **L** | **S** | **K** | **Q** | **E** | **F** | **D** | **K** | **L** | **P** | **S** | **V** | **V** | **Q** | **E** | **A** | **S** | **L** | **K** | **K** | **N** | **G** | **Y** | **D** | **T** | **K** | **S** | **V** | **V** | **E** | **K** | **Y** | **Q** | **A** | **I** | **Y** | **S** | **G** | **I** | **Q** | **A** | **E** | **G** | **V** | **K** | **A** | **S** | **D** | **V** | **Q** | **V** | **K** | **K** | **A** | **K** | **D** | **N** | **Q** | **Y** | **T** | **F** | **T** | **Y** | **K** | **L** | **S** | **M** | **S** | **T** | **P** | **L** | **G** | **E** | **M** | **K** | **D** | **L** | **S** | **Y** | **Q** | **S** | **S** | **I** | **A** | **K** | **K** | **G** | **D** | **T** | **Y** | **Q** | **I** | **A** | **W** | **K** | **P** | **S** | **L** | **I** | **F** | **P** | **D** | **M** | **S** | **G** | **N** | **D** | **K** | **I** | **S** | **I** | **Q** | **V** | **D** | **N** | **A** | **K** | **R** | **G** | **E** | **I** | **V** | **D** | **R** | **N** | **G** | **S** | **G** | **L** | **A** | **I** | **N** | **K** | **V** | **F** | **D** | **E** | **V** | **G** | **V** | **V** | **P** | **G** | **K** | **L** | **G** | **S** | **G** | **A** | **E** | **K** | **T** | **A** | **N** | **I** | **K** | **A** | **F** | **S** | **D** | **K** | **F** | **G** | **V** | **S** | **V** | **D** | **E** | **I** | **N** | **Q** | **K** | **L** | **S** | **Q** | **G** | **W** | **V** | **Q** | **A** | **D** | **S** | **F** | **V** | **P** | **I** | **T** | **V** | **A** | **S** | **E** | **P** | **V** | **T** | **E** | **L** | **P** | **T** | **G** | **A** | **A** | **T** | **K** | **D** | **T** | **E** | **S** | **R** | **Y** | **Y** | **P** | **L** | **G** | **E** | **A** | **A** | **A** | **Q** | **L** | **I** | **G** | **Y** | **T** | **G** | **T** | **I** | **T** | **A** | **E** | **D** | **I** | **E** | **K** | **N** | **P** | **E** | **L** | **S** | **S** | **T** | **G** | **V** | **I** | **G** | **K** | **T** | **G** | **L** | **E** | **R** | **A** | **F** | **D** | **K** | **E** | **L** | **R** | **G** | **Q** | **D** | **G** | **G** | **S** | **L** | **V** | **I** | **L** | **D** | **D** | **K** | **E** | **N** | **V** | **K** | **K** | **A** | **L** | **Q** | **T** | **K** | **E** | **K** | **K** | **D** | **G** | **Q** | **T** | **I** | **K** | **L** | **T** | **I** | **D** | **S** | **G** | **V** | **Q** | **Q** | **Q** | **A** | **F** | **A** | **I** | **F** | **D** | **K** | **R** | **P** | **G** | **S** | **A** | **V** | **I** | **T** | **D** | **P** | **Q** | **K** | **G** | **D** | **L** | **L** | **A** | **T** | **V** | **S** | **S** | **P** | **S** | **Y** | **D** | **P** | **N** | **K** | **M** | **A** | **N** | **G** | **I** | **S** | **Q** | **K** | **E** | **Y** | **D** | **A** | **Y** | **N** | **N** | **N** | **K** | **D** | **L** | **P** | **F** | **T** | **A** | **R** | **F** | **A** | **T** | **G** | **Y** | **A** | **P** | **G** | **S** | **T** | **F** | **K** | **T** | **I** | **T** | **G** | **A** | **I** | **G** | **L** | **D** | **A** | **G** | **T** | **L** | **K** | **P** | **D** | **E** | **E** | **L** | **E** | **I** | **N** | **G** | **L** | **K** | **W** | **Q** | **K** | **D** | **K** | **S** | **W** | **G** | **G** | **Y** | **F** | **A** | **T** | **R** | **V** | **K** | **E** | **A** | **S** | **P** | **V** | **N** | **L** | **R** | **T** | **A** | **L** | **V** | **N** | **S** | **D** | **N** | **I** | **Y** | **F** | **A** | **Q** | **Q** | **T** | **L** | **R** | **M** | **G** | **E** | **D** | **K** | **F** | **R** | **A** | **G** | **L** | **N** | **K** | **F** | **I** | **F** | **G** | **E** | **E** | **L** | **D** | **L** | **P** | **I** | **A** | **M** | **T** | **P** | **A** | **Q** | **I** | **S** | **N** | **E** | **D** | **K** | **F** | **N** | **S** | **E** | **I** | **L** | **L** | **A** | **D** | **T** | **G** | **Y** | **G** | **Q** | **G** | **Q** | **L** | **L** | **I** | **S** | **P** | **I** | **Q** | **Q** | **A** | **T** | **M** | **Y** | **S** | **V** | **F** | **Q** | **N** | **N** | **G** | **T** | **L** | **V** | **Y** | **P** | **K** | **I** | **V** | **L** | **D** | **K** | **E** | **T** | **K** | **K** | **K** | **D** | **N** | **V** | **I** | **S** | **A** | **N** | **A** | **A** | **N** | **T** | **I** | **A** | **T** | **D** | **L** | **L** | **G** | **S** | **V** | **E** | **D** | **P** | **S** | **G** | **Y** | **V** | **Y** | **N** | **M** | **Y** | **N** | **P** | **N** | **F** | **S** | **L** | **A** | **A** | **K** | **T** | **G** | **T** | **A** | **E** | **I** | **K** | **D** | **K** | **Q** | **D** | **T** | **D** | **G** | **K** | **E** | **N** | **S** | **F** | **L** | **L** | **T** | **L** | **D** | **R** | **S** | **N** | **N** | **K** | **F** | **L** | **T** | **M** | **I** | **M** | **V** | **E** | **N** | **S** | **G** | **E** | **N** | **G** | **S** | **A** | **T** | **D** | **I** | **S** | **K** | **P** | **L** | **I** | **D** | **Y** | **L** | **E** | **A** | **T** | **I** | **K** |
| **N53766** | **-** | **-** | **-** | **-** | **-** | **-** | **-** | **-** | **-** | **-** | **-** | **-** | **-** | **-** | **-** | **-** | **-** | **-** | **-** | **-** | **-** | **-** | **-** | **-** | **-** | **-** | **-** | **-** | **-** | **-** | **-** | **-** | **-** | **-** | **-** | **-** | **Q** | **W** | **Q** | **A** | **K** | **Q** | **E** | **L** | **A** | **E** | **A** | **K** | **K** | **T** | **A** | **T** | **T** | **F** | **L** | **N** | **V** | **L** | **S** | **K** | **Q** | **E** | **F** | **D** | **K** | **L** | **P** | **S** | **V** | **V** | **Q** | **E** | **A** | **S** | **L** | **K** | **K** | **N** | **G** | **Y** | **D** | **T** | **K** | **S** | **V** | **V** | **E** | **K** | **Y** | **Q** | **A** | **I** | **Y** | **S** | **G** | **I** | **Q** | **A** | **E** | **G** | **V** | **K** | **A** | **S** | **D** | **V** | **Q** | **V** | **K** | **K** | **A** | **K** | **D** | **N** | **Q** | **Y** | **T** | **F** | **T** | **Y** | **K** | **L** | **S** | **M** | **S** | **T** | **P** | **L** | **G** | **E** | **M** | **K** | **D** | **L** | **S** | **Y** | **Q** | **S** | **S** | **I** | **A** | **K** | **K** | **G** | **D** | **T** | **Y** | **Q** | **I** | **A** | **W** | **K** | **P** | **S** | **L** | **I** | **F** | **P** | **D** | **M** | **S** | **G** | **N** | **D** | **K** | **I** | **S** | **I** | **Q** | **V** | **D** | **N** | **A** | **K** | **R** | **G** | **E** | **I** | **V** | **D** | **R** | **N** | **G** | **S** | **G** | **L** | **A** | **I** | **N** | **K** | **V** | **F** | **D** | **E** | **V** | **G** | **V** | **V** | **P** | **G** | **K** | **L** | **G** | **S** | **G** | **A** | **E** | **K** | **T** | **A** | **N** | **I** | **K** | **A** | **F** | **S** | **D** | **K** | **F** | **G** | **V** | **S** | **V** | **D** | **E** | **I** | **N** | **Q** | **K** | **L** | **S** | **Q** | **G** | **W** | **V** | **Q** | **A** | **D** | **S** | **F** | **V** | **P** | **I** | **T** | **V** | **A** | **S** | **E** | **P** | **V** | **T** | **E** | **L** | **P** | **T** | **G** | **A** | **A** | **T** | **K** | **D** | **T** | **E** | **S** | **R** | **Y** | **Y** | **P** | **L** | **G** | **E** | **A** | **A** | **A** | **Q** | **L** | **I** | **G** | **Y** | **T** | **G** | **T** | **I** | **T** | **A** | **E** | **D** | **I** | **E** | **K** | **N** | **P** | **E** | **L** | **S** | **S** | **T** | **G** | **V** | **I** | **G** | **K** | **T** | **G** | **L** | **E** | **R** | **A** | **F** | **D** | **K** | **E** | **L** | **R** | **G** | **Q** | **D** | **G** | **G** | **S** | **L** | **V** | **I** | **L** | **D** | **D** | **K** | **E** | **N** | **V** | **K** | **K** | **A** | **L** | **Q** | **T** | **K** | **E** | **K** | **K** | **D** | **G** | **Q** | **T** | **I** | **K** | **L** | **T** | **I** | **D** | **S** | **G** | **V** | **Q** | **Q** | **Q** | **A** | **F** | **A** | **I** | **F** | **D** | **K** | **R** | **P** | **G** | **S** | **A** | **V** | **I** | **T** | **D** | **P** | **Q** | **K** | **G** | **D** | **L** | **L** | **A** | **T** | **V** | **S** | **S** | **P** | **S** | **Y** | **D** | **P** | **N** | **K** | **M** | **A** | **N** | **G** | **I** | **S** | **Q** | **K** | **E** | **Y** | **D** | **A** | **Y** | **N** | **N** | **N** | **K** | **D** | **L** | **P** | **F** | **T** | **A** | **R** | **F** | **A** | **T** | **G** | **Y** | **A** | **P** | **G** | **S** | **T** | **F** | **K** | **T** | **I** | **T** | **G** | **A** | **I** | **G** | **L** | **D** | **A** | **G** | **T** | **L** | **K** | **P** | **D** | **E** | **E** | **L** | **E** | **I** | **N** | **G** | **L** | **K** | **W** | **Q** | **K** | **D** | **K** | **S** | **W** | **G** | **G** | **Y** | **F** | **A** | **T** | **R** | **V** | **K** | **E** | **A** | **S** | **P** | **V** | **N** | **L** | **R** | **T** | **A** | **L** | **V** | **N** | **S** | **D** | **N** | **I** | **Y** | **F** | **A** | **Q** | **Q** | **T** | **L** | **R** | **M** | **G** | **E** | **D** | **K** | **F** | **R** | **A** | **G** | **L** | **N** | **K** | **F** | **I** | **F** | **G** | **E** | **E** | **L** | **D** | **L** | **P** | **I** | **A** | **M** | **T** | **P** | **A** | **Q** | **I** | **S** | **N** | **E** | **D** | **K** | **F** | **N** | **S** | **E** | **I** | **L** | **L** | **A** | **D** | **T** | **G** | **Y** | **G** | **Q** | **G** | **Q** | **L** | **L** | **I** | **S** | **P** | **I** | **Q** | **Q** | **A** | **T** | **M** | **Y** | **S** | **V** | **F** | **Q** | **N** | **N** | **G** | **T** | **L** | **V** | **Y** | **P** | **K** | **L** | **V** | **L** | **D** | **K** | **E** | **T** | **K** | **K** | **K** | **D** | **N** | **V** | **I** | **S** | **A** | **N** | **A** | **A** | **N** | **T** | **I** | **A** | **T** | **D** | **L** | **L** | **G** | **S** | **V** | **E** | **D** | **P** | **S** | **G** | **Y** | **V** | **Y** | **N** | **M** | **Y** | **N** | **P** | **N** | **F** | **S** | **L** | **A** | **A** | **K** | **T** | **G** | **T** | **A** | **E** | **I** | **K** | **D** | **K** | **Q** | **D** | **T** | **D** | **G** | **K** | **E** | **N** | **S** | **F** | **L** | **L** | **T** | **L** | **D** | **R** | **S** | **N** | **N** | **K** | **F** | **L** | **T** | **M** | **I** | **M** | **V** | **E** | **N** | **S** | **G** | **E** | **N** | **G** | **S** | **A** | **T** | **D** | **I** | **S** | **K** | **P** | **L** | **I** | **D** | **Y** | **L** | **E** | **A** | **T** | **I** | **K** | **-** | **-** | **-** | **-** | **-** | **-** | **-** | **-** | **-** | **-** | **-** | **-** | **-** | **-** | **-** | **-** | **-** | **-** | **-** | **-** | **-** | **-** | **-** | **-** | **-** | **-** | **-** | **-** | **-** | **-** | **-** | **-** | **-** | **-** | **-** | **-** | **Q** | **W** | **Q** | **A** | **K** | **Q** | **E** | **L** | **A** | **E** | **A** | **K** | **K** | **T** | **A** | **T** | **T** | **F** | **L** | **N** | **V** | **L** | **S** | **K** | **Q** | **E** | **F** | **D** | **K** | **L** | **P** | **S** | **V** | **V** | **Q** | **E** | **A** | **S** | **L** | **K** | **K** | **N** | **G** | **Y** | **D** | **T** | **K** | **S** | **V** | **V** | **E** | **K** | **Y** | **Q** | **A** | **I** | **Y** | **S** | **G** | **I** | **Q** | **A** | **E** | **G** | **V** | **K** | **A** | **S** | **D** | **V** | **Q** | **V** | **K** | **K** | **A** | **K** | **D** | **N** | **Q** | **Y** | **T** | **F** | **T** | **Y** | **K** | **L** | **S** | **M** | **S** | **T** | **P** | **L** | **G** | **E** | **M** | **K** | **D** | **L** | **S** | **Y** | **Q** | **S** | **S** | **I** | **A** | **K** | **K** | **G** | **D** | **T** | **Y** | **Q** | **I** | **A** | **W** | **K** | **P** | **S** | **L** | **I** | **F** | **P** | **D** | **M** | **S** | **G** | **N** | **D** | **K** | **I** | **S** | **I** | **Q** | **V** | **D** | **N** | **A** | **K** | **R** | **G** | **E** | **I** | **V** | **D** | **R** | **N** | **G** | **S** | **G** | **L** | **A** | **I** | **N** | **K** | **V** | **F** | **D** | **E** | **V** | **G** | **V** | **V** | **P** | **G** | **K** | **L** | **G** | **S** | **G** | **A** | **E** | **K** | **T** | **A** | **N** | **I** | **K** | **A** | **F** | **S** | **D** | **K** | **F** | **G** | **V** | **S** | **V** | **D** | **E** | **I** | **N** | **Q** | **K** | **L** | **S** | **Q** | **G** | **W** | **V** | **Q** | **A** | **D** | **S** | **F** | **V** | **P** | **I** | **T** | **V** | **A** | **S** | **E** | **P** | **V** | **T** | **E** | **L** | **P** | **T** | **G** | **A** | **A** | **T** | **K** | **D** | **T** | **E** | **S** | **R** | **Y** | **Y** | **P** | **L** | **G** | **E** | **A** | **A** | **A** | **Q** | **L** | **I** | **G** | **Y** | **T** | **G** | **T** | **I** | **T** | **A** | **E** | **D** | **I** | **E** | **K** | **N** | **P** | **E** | **L** | **S** | **S** | **T** | **G** | **V** | **I** | **G** | **K** | **T** | **G** | **L** | **E** | **R** | **A** | **F** | **D** | **K** | **E** | **L** | **R** | **G** | **Q** | **D** | **G** | **G** | **S** | **L** | **V** | **I** | **L** | **D** | **D** | **K** | **E** | **N** | **V** | **K** | **K** | **A** | **L** | **Q** | **T** | **K** | **E** | **K** | **K** | **D** | **G** | **Q** | **T** | **I** | **K** | **L** | **T** | **I** | **D** | **S** | **G** | **V** | **Q** | **Q** | **Q** | **A** | **F** | **A** | **I** | **F** | **D** | **K** | **R** | **P** | **G** | **S** | **A** | **V** | **I** | **T** | **D** | **P** | **Q** | **K** | **G** | **D** | **L** | **L** | **A** | **T** | **V** | **S** | **S** | **P** | **S** | **Y** | **D** | **P** | **N** | **K** | **M** | **A** | **N** | **G** | **I** | **S** | **Q** | **K** | **E** | **Y** | **D** | **A** | **Y** | **N** | **N** | **N** | **K** | **D** | **L** | **P** | **F** | **T** | **A** | **R** | **F** | **A** | **T** | **G** | **Y** | **A** | **P** | **G** | **S** | **T** | **F** | **K** | **T** | **I** | **T** | **G** | **A** | **I** | **G** | **L** | **D** | **A** | **G** | **T** | **L** | **K** | **P** | **D** | **E** | **E** | **L** | **E** | **I** | **N** | **G** | **L** | **K** | **W** | **Q** | **K** | **D** | **K** | **S** | **W** | **G** | **G** | **Y** | **F** | **A** | **T** | **R** | **V** | **K** | **E** | **A** | **S** | **P** | **V** | **N** | **L** | **R** | **T** | **A** | **L** | **V** | **N** | **S** | **D** | **N** | **I** | **Y** | **F** | **A** | **Q** | **Q** | **T** | **L** | **R** | **M** | **G** | **E** | **D** | **K** | **F** | **R** | **A** | **G** | **L** | **N** | **K** | **F** | **I** | **F** | **G** | **E** | **E** | **L** | **D** | **L** | **P** | **I** | **A** | **M** | **T** | **P** | **A** | **Q** | **I** | **S** | **N** | **E** | **D** | **K** | **F** | **N** | **S** | **E** | **I** | **L** | **L** | **A** | **D** | **T** | **G** | **Y** | **G** | **Q** | **G** | **Q** | **L** | **L** | **I** | **S** | **P** | **I** | **Q** | **Q** | **A** | **T** | **M** | **Y** | **S** | **V** | **F** | **Q** | **N** | **N** | **G** | **T** | **L** | **V** | **Y** | **P** | **K** | **L** | **V** | **L** | **D** | **K** | **E** | **T** | **K** | **K** | **K** | **D** | **N** | **V** | **I** | **S** | **A** | **N** | **A** | **A** | **N** | **T** | **I** | **A** | **T** | **D** | **L** | **L** | **G** | **S** | **V** | **E** | **D** | **P** | **S** | **G** | **Y** | **V** | **Y** | **N** | **M** | **Y** | **N** | **P** | **N** | **F** | **S** | **L** | **A** | **A** | **K** | **T** | **G** | **T** | **A** | **E** | **I** | **K** | **D** | **K** | **Q** | **D** | **T** | **D** | **G** | **K** | **E** | **N** | **S** | **F** | **L** | **L** | **T** | **L** | **D** | **R** | **S** | **N** | **N** | **K** | **F** | **L** | **T** | **M** | **I** | **M** | **V** | **E** | **N** | **S** | **G** | **E** | **N** | **G** | **S** | **A** | **T** | **D** | **I** | **S** | **K** | **P** | **L** | **I** | **D** | **Y** | **L** | **E** | **A** | **T** | **I** | **K** |
| **N59508F** | **-** | **-** | **-** | **-** | **-** | **-** | **-** | **-** | **-** | **-** | **-** | **-** | **-** | **-** | **-** | **-** | **-** | **-** | **-** | **-** | **-** | **-** | **-** | **-** | **-** | **-** | **-** | **-** | **-** | **-** | **-** | **-** | **-** | **-** | **-** | **-** | **Q** | **W** | **Q** | **A** | **K** | **Q** | **E** | **L** | **A** | **E** | **A** | **K** | **K** | **T** | **A** | **T** | **T** | **F** | **L** | **N** | **V** | **L** | **T** | **K** | **Q** | **K** | **F** | **D** | **K** | **L** | **P** | **S** | **V** | **V** | **Q** | **E** | **A** | **S** | **L** | **K** | **K** | **N** | **G** | **Y** | **D** | **T** | **K** | **S** | **V** | **V** | **E** | **K** | **Y** | **Q** | **A** | **I** | **Y** | **S** | **G** | **I** | **Q** | **A** | **E** | **G** | **V** | **K** | **A** | **S** | **D** | **V** | **Q** | **V** | **K** | **K** | **A** | **K** | **D** | **N** | **Q** | **Y** | **T** | **F** | **T** | **Y** | **K** | **L** | **S** | **M** | **S** | **T** | **P** | **L** | **G** | **E** | **M** | **K** | **D** | **L** | **S** | **Y** | **Q** | **S** | **S** | **I** | **A** | **K** | **K** | **G** | **D** | **T** | **Y** | **Q** | **I** | **A** | **W** | **K** | **P** | **S** | **L** | **I** | **F** | **P** | **D** | **M** | **S** | **G** | **N** | **D** | **K** | **I** | **S** | **I** | **Q** | **V** | **D** | **N** | **A** | **K** | **R** | **G** | **E** | **I** | **V** | **D** | **R** | **N** | **G** | **S** | **G** | **L** | **A** | **I** | **N** | **K** | **V** | **F** | **D** | **E** | **V** | **G** | **V** | **V** | **P** | **G** | **K** | **L** | **G** | **S** | **G** | **A** | **E** | **K** | **T** | **A** | **N** | **I** | **K** | **A** | **F** | **S** | **D** | **K** | **F** | **G** | **V** | **S** | **V** | **D** | **E** | **I** | **N** | **Q** | **K** | **L** | **S** | **Q** | **G** | **W** | **V** | **Q** | **A** | **D** | **S** | **F** | **V** | **P** | **I** | **T** | **V** | **A** | **S** | **E** | **P** | **V** | **T** | **E** | **L** | **P** | **T** | **G** | **A** | **A** | **T** | **K** | **D** | **T** | **E** | **S** | **R** | **Y** | **Y** | **P** | **L** | **G** | **E** | **A** | **A** | **A** | **Q** | **L** | **I** | **G** | **Y** | **T** | **G** | **T** | **I** | **T** | **A** | **E** | **D** | **I** | **K** | **K** | **N** | **P** | **E** | **L** | **S** | **S** | **T** | **G** | **V** | **I** | **G** | **K** | **T** | **G** | **L** | **E** | **R** | **A** | **F** | **D** | **K** | **E** | **L** | **R** | **G** | **Q** | **D** | **G** | **G** | **S** | **L** | **V** | **I** | **L** | **D** | **D** | **K** | **E** | **N** | **V** | **K** | **K** | **A** | **L** | **Q** | **T** | **K** | **E** | **K** | **K** | **D** | **G** | **Q** | **T** | **I** | **K** | **L** | **T** | **I** | **D** | **S** | **G** | **V** | **Q** | **Q** | **Q** | **A** | **F** | **A** | **I** | **F** | **D** | **K** | **R** | **P** | **G** | **S** | **A** | **V** | **I** | **T** | **D** | **P** | **Q** | **K** | **G** | **D** | **L** | **L** | **A** | **T** | **V** | **S** | **S** | **P** | **S** | **Y** | **D** | **P** | **N** | **K** | **M** | **A** | **N** | **G** | **I** | **S** | **Q** | **K** | **E** | **Y** | **D** | **A** | **Y** | **N** | **N** | **N** | **K** | **D** | **L** | **P** | **F** | **T** | **A** | **R** | **F** | **A** | **T** | **G** | **Y** | **A** | **P** | **G** | **S** | **T** | **F** | **K** | **T** | **I** | **T** | **G** | **A** | **I** | **G** | **L** | **D** | **T** | **G** | **T** | **L** | **K** | **P** | **D** | **E** | **E** | **L** | **E** | **I** | **N** | **G** | **L** | **K** | **W** | **Q** | **K** | **D** | **K** | **S** | **W** | **G** | **G** | **Y** | **F** | **A** | **T** | **R** | **V** | **K** | **E** | **A** | **S** | **P** | **V** | **N** | **L** | **R** | **T** | **A** | **L** | **V** | **N** | **S** | **D** | **N** | **I** | **Y** | **F** | **A** | **Q** | **Q** | **T** | **L** | **R** | **M** | **G** | **E** | **D** | **K** | **F** | **R** | **A** | **G** | **L** | **N** | **K** | **F** | **I** | **F** | **G** | **E** | **E** | **L** | **D** | **L** | **P** | **I** | **A** | **M** | **T** | **P** | **A** | **Q** | **I** | **S** | **N** | **E** | **D** | **K** | **F** | **N** | **S** | **E** | **I** | **L** | **L** | **A** | **D** | **T** | **G** | **Y** | **G** | **Q** | **G** | **Q** | **L** | **L** | **I** | **S** | **P** | **I** | **Q** | **Q** | **A** | **T** | **M** | **Y** | **S** | **V** | **F** | **Q** | **N** | **N** | **G** | **T** | **L** | **V** | **Y** | **P** | **K** | **L** | **V** | **L** | **D** | **K** | **E** | **T** | **K** | **K** | **K** | **D** | **N** | **V** | **I** | **S** | **A** | **N** | **A** | **A** | **N** | **T** | **I** | **A** | **T** | **D** | **L** | **L** | **G** | **S** | **V** | **E** | **D** | **P** | **S** | **G** | **Y** | **V** | **Y** | **N** | **M** | **Y** | **N** | **P** | **N** | **F** | **S** | **L** | **A** | **A** | **K** | **T** | **G** | **T** | **A** | **E** | **I** | **K** | **D** | **K** | **Q** | **D** | **T** | **D** | **G** | **K** | **E** | **N** | **S** | **F** | **L** | **L** | **T** | **L** | **D** | **R** | **S** | **N** | **N** | **K** | **F** | **L** | **T** | **M** | **I** | **M** | **V** | **E** | **N** | **S** | **G** | **E** | **N** | **G** | **S** | **A** | **T** | **D** | **I** | **S** | **K** | **P** | **L** | **I** | **D** | **Y** | **L** | **E** | **A** | **T** | **I** | **K** | **-** | **-** | **-** | **-** | **-** | **-** | **-** | **-** | **-** | **-** | **-** | **-** | **-** | **-** | **-** | **-** | **-** | **-** | **-** | **-** | **-** | **-** | **-** | **-** | **-** | **-** | **-** | **-** | **-** | **-** | **-** | **-** | **-** | **-** | **-** | **-** | **Q** | **W** | **Q** | **A** | **K** | **Q** | **E** | **L** | **A** | **E** | **A** | **K** | **K** | **T** | **A** | **T** | **T** | **F** | **L** | **N** | **V** | **L** | **T** | **K** | **Q** | **K** | **F** | **D** | **K** | **L** | **P** | **S** | **V** | **V** | **Q** | **E** | **A** | **S** | **L** | **K** | **K** | **N** | **G** | **Y** | **D** | **T** | **K** | **S** | **V** | **V** | **E** | **K** | **Y** | **Q** | **A** | **I** | **Y** | **S** | **G** | **I** | **Q** | **A** | **E** | **G** | **V** | **K** | **A** | **S** | **D** | **V** | **Q** | **V** | **K** | **K** | **A** | **K** | **D** | **N** | **Q** | **Y** | **T** | **F** | **T** | **Y** | **K** | **L** | **S** | **M** | **S** | **T** | **P** | **L** | **G** | **E** | **M** | **K** | **D** | **L** | **S** | **Y** | **Q** | **S** | **S** | **I** | **A** | **K** | **K** | **G** | **D** | **T** | **Y** | **Q** | **I** | **A** | **W** | **K** | **P** | **S** | **L** | **I** | **F** | **P** | **D** | **M** | **S** | **G** | **N** | **D** | **K** | **I** | **S** | **I** | **Q** | **V** | **D** | **N** | **A** | **K** | **R** | **G** | **E** | **I** | **V** | **D** | **R** | **N** | **G** | **S** | **G** | **L** | **A** | **I** | **N** | **K** | **V** | **F** | **D** | **E** | **V** | **G** | **V** | **V** | **P** | **G** | **K** | **L** | **G** | **S** | **G** | **A** | **E** | **K** | **T** | **A** | **N** | **I** | **K** | **A** | **F** | **S** | **D** | **K** | **F** | **G** | **V** | **S** | **V** | **D** | **E** | **I** | **N** | **Q** | **K** | **L** | **S** | **Q** | **G** | **W** | **V** | **Q** | **A** | **D** | **S** | **F** | **V** | **P** | **I** | **T** | **V** | **A** | **S** | **E** | **P** | **V** | **T** | **E** | **L** | **P** | **T** | **G** | **A** | **A** | **T** | **K** | **D** | **T** | **E** | **S** | **R** | **Y** | **Y** | **P** | **L** | **G** | **E** | **A** | **A** | **A** | **Q** | **L** | **I** | **G** | **Y** | **T** | **G** | **T** | **I** | **T** | **A** | **E** | **D** | **I** | **K** | **K** | **N** | **P** | **E** | **L** | **S** | **S** | **T** | **G** | **V** | **I** | **G** | **K** | **T** | **G** | **L** | **E** | **R** | **A** | **F** | **D** | **K** | **E** | **L** | **R** | **G** | **Q** | **D** | **G** | **G** | **S** | **L** | **V** | **I** | **L** | **D** | **D** | **K** | **E** | **N** | **V** | **K** | **K** | **A** | **L** | **Q** | **T** | **K** | **E** | **K** | **K** | **D** | **G** | **Q** | **T** | **I** | **K** | **L** | **T** | **I** | **D** | **S** | **G** | **V** | **Q** | **Q** | **Q** | **A** | **F** | **A** | **I** | **F** | **D** | **K** | **R** | **P** | **G** | **S** | **A** | **V** | **I** | **T** | **D** | **P** | **Q** | **K** | **G** | **D** | **L** | **L** | **A** | **T** | **V** | **S** | **S** | **P** | **S** | **Y** | **D** | **P** | **N** | **K** | **M** | **A** | **N** | **G** | **I** | **S** | **Q** | **K** | **E** | **Y** | **D** | **A** | **Y** | **N** | **N** | **N** | **K** | **D** | **L** | **P** | **F** | **T** | **A** | **R** | **F** | **A** | **T** | **G** | **Y** | **A** | **P** | **G** | **S** | **T** | **F** | **K** | **T** | **I** | **T** | **G** | **A** | **I** | **G** | **L** | **D** | **T** | **G** | **T** | **L** | **K** | **P** | **D** | **E** | **E** | **L** | **E** | **I** | **N** | **G** | **L** | **K** | **W** | **Q** | **K** | **D** | **K** | **S** | **W** | **G** | **G** | **Y** | **F** | **A** | **T** | **R** | **V** | **K** | **E** | **A** | **S** | **P** | **V** | **N** | **L** | **R** | **T** | **A** | **L** | **V** | **N** | **S** | **D** | **N** | **I** | **Y** | **F** | **A** | **Q** | **Q** | **T** | **L** | **R** | **M** | **G** | **E** | **D** | **K** | **F** | **R** | **A** | **G** | **L** | **N** | **K** | **F** | **I** | **F** | **G** | **E** | **E** | **L** | **D** | **L** | **P** | **I** | **A** | **M** | **T** | **P** | **A** | **Q** | **I** | **S** | **N** | **E** | **D** | **K** | **F** | **N** | **S** | **E** | **I** | **L** | **L** | **A** | **D** | **T** | **G** | **Y** | **G** | **Q** | **G** | **Q** | **L** | **L** | **I** | **S** | **P** | **I** | **Q** | **Q** | **A** | **T** | **M** | **Y** | **S** | **V** | **F** | **Q** | **N** | **N** | **G** | **T** | **L** | **V** | **Y** | **P** | **K** | **L** | **V** | **L** | **D** | **K** | **E** | **T** | **K** | **K** | **K** | **D** | **N** | **V** | **I** | **S** | **A** | **N** | **A** | **A** | **N** | **T** | **I** | **A** | **T** | **D** | **L** | **L** | **G** | **S** | **V** | **E** | **D** | **P** | **S** | **G** | **Y** | **V** | **Y** | **N** | **M** | **Y** | **N** | **P** | **N** | **F** | **S** | **L** | **A** | **A** | **K** | **T** | **G** | **T** | **A** | **E** | **I** | **K** | **D** | **K** | **Q** | **D** | **T** | **D** | **G** | **K** | **E** | **N** | **S** | **F** | **L** | **L** | **T** | **L** | **D** | **R** | **S** | **N** | **N** | **K** | **F** | **L** | **T** | **M** | **I** | **M** | **V** | **E** | **N** | **S** | **G** | **E** | **N** | **G** | **S** | **A** | **T** | **D** | **I** | **S** | **K** | **P** | **L** | **I** | **D** | **Y** | **L** | **E** | **A** | **T** | **I** | **K** |
| **N55724** | **-** | **-** | **-** | **-** | **-** | **-** | **-** | **-** | **-** | **-** | **-** | **-** | **-** | **-** | **-** | **-** | **-** | **-** | **-** | **-** | **-** | **-** | **-** | **-** | **-** | **-** | **-** | **-** | **-** | **-** | **-** | **-** | **-** | **-** | **-** | **-** | **Q** | **W** | **Q** | **A** | **K** | **Q** | **E** | **L** | **A** | **E** | **A** | **K** | **K** | **T** | **A** | **T** | **T** | **F** | **L** | **N** | **V** | **L** | **S** | **K** | **Q** | **E** | **F** | **D** | **K** | **L** | **P** | **S** | **V** | **V** | **Q** | **E** | **A** | **S** | **L** | **K** | **K** | **N** | **G** | **Y** | **D** | **T** | **K** | **S** | **V** | **V** | **E** | **K** | **Y** | **Q** | **A** | **I** | **Y** | **S** | **G** | **I** | **Q** | **A** | **E** | **G** | **V** | **K** | **A** | **S** | **D** | **V** | **Q** | **V** | **K** | **K** | **A** | **K** | **D** | **N** | **Q** | **Y** | **T** | **F** | **T** | **Y** | **K** | **L** | **S** | **M** | **S** | **T** | **P** | **L** | **G** | **E** | **M** | **K** | **D** | **L** | **S** | **Y** | **Q** | **S** | **S** | **I** | **A** | **K** | **K** | **G** | **D** | **T** | **Y** | **Q** | **I** | **A** | **W** | **K** | **P** | **S** | **L** | **I** | **F** | **P** | **D** | **M** | **S** | **G** | **N** | **D** | **K** | **I** | **S** | **I** | **Q** | **V** | **D** | **N** | **A** | **K** | **R** | **G** | **E** | **I** | **V** | **D** | **R** | **N** | **G** | **S** | **G** | **L** | **A** | **I** | **N** | **K** | **V** | **F** | **D** | **E** | **V** | **G** | **V** | **V** | **P** | **G** | **K** | **L** | **G** | **S** | **G** | **A** | **E** | **K** | **T** | **A** | **N** | **I** | **K** | **A** | **F** | **S** | **D** | **K** | **F** | **G** | **V** | **S** | **V** | **D** | **E** | **I** | **N** | **Q** | **K** | **L** | **S** | **Q** | **G** | **W** | **V** | **Q** | **A** | **D** | **S** | **F** | **V** | **P** | **I** | **T** | **V** | **A** | **S** | **E** | **P** | **V** | **T** | **E** | **L** | **P** | **T** | **G** | **A** | **A** | **T** | **K** | **D** | **T** | **E** | **S** | **R** | **Y** | **Y** | **P** | **L** | **G** | **E** | **A** | **A** | **A** | **Q** | **L** | **I** | **G** | **Y** | **T** | **G** | **T** | **I** | **T** | **A** | **E** | **D** | **I** | **E** | **K** | **N** | **P** | **E** | **L** | **S** | **S** | **T** | **G** | **V** | **I** | **G** | **K** | **T** | **G** | **L** | **E** | **R** | **A** | **F** | **D** | **K** | **E** | **L** | **R** | **G** | **Q** | **D** | **G** | **G** | **S** | **L** | **V** | **I** | **L** | **D** | **D** | **K** | **E** | **N** | **V** | **K** | **K** | **A** | **L** | **Q** | **T** | **K** | **E** | **K** | **K** | **D** | **G** | **Q** | **T** | **I** | **K** | **L** | **T** | **I** | **D** | **S** | **G** | **V** | **Q** | **Q** | **Q** | **A** | **F** | **A** | **I** | **F** | **D** | **K** | **R** | **P** | **G** | **S** | **A** | **V** | **I** | **T** | **D** | **P** | **Q** | **K** | **G** | **D** | **L** | **L** | **A** | **T** | **V** | **S** | **S** | **P** | **S** | **Y** | **D** | **P** | **N** | **K** | **M** | **A** | **N** | **G** | **I** | **S** | **Q** | **K** | **E** | **Y** | **D** | **A** | **Y** | **N** | **N** | **N** | **K** | **D** | **L** | **P** | **F** | **T** | **A** | **R** | **F** | **A** | **T** | **G** | **Y** | **A** | **P** | **G** | **S** | **T** | **F** | **K** | **T** | **I** | **T** | **G** | **A** | **I** | **G** | **L** | **D** | **A** | **G** | **T** | **L** | **K** | **P** | **D** | **E** | **E** | **L** | **E** | **I** | **N** | **G** | **L** | **K** | **W** | **Q** | **K** | **D** | **K** | **S** | **W** | **G** | **G** | **Y** | **F** | **A** | **T** | **R** | **V** | **K** | **E** | **A** | **S** | **P** | **V** | **N** | **L** | **R** | **T** | **A** | **L** | **V** | **N** | **S** | **D** | **N** | **I** | **Y** | **F** | **A** | **Q** | **Q** | **T** | **L** | **R** | **M** | **G** | **E** | **D** | **K** | **F** | **R** | **A** | **G** | **L** | **N** | **K** | **F** | **I** | **F** | **G** | **E** | **E** | **L** | **D** | **L** | **P** | **I** | **A** | **M** | **T** | **P** | **A** | **Q** | **I** | **S** | **N** | **E** | **D** | **K** | **F** | **N** | **S** | **E** | **I** | **L** | **L** | **A** | **D** | **T** | **G** | **Y** | **G** | **Q** | **G** | **Q** | **L** | **L** | **I** | **S** | **P** | **I** | **Q** | **Q** | **A** | **T** | **M** | **Y** | **S** | **V** | **F** | **Q** | **N** | **N** | **G** | **T** | **L** | **V** | **Y** | **P** | **K** | **L** | **V** | **L** | **D** | **K** | **E** | **T** | **K** | **K** | **K** | **D** | **N** | **V** | **I** | **S** | **A** | **N** | **A** | **A** | **N** | **T** | **I** | **A** | **T** | **D** | **L** | **L** | **G** | **S** | **V** | **E** | **D** | **P** | **S** | **G** | **Y** | **V** | **Y** | **N** | **M** | **Y** | **N** | **P** | **N** | **F** | **S** | **L** | **A** | **A** | **K** | **T** | **G** | **T** | **A** | **E** | **I** | **K** | **D** | **K** | **Q** | **D** | **T** | **D** | **G** | **K** | **E** | **N** | **S** | **F** | **L** | **L** | **T** | **L** | **D** | **R** | **S** | **N** | **N** | **K** | **F** | **L** | **T** | **M** | **I** | **M** | **V** | **E** | **N** | **S** | **G** | **E** | **N** | **G** | **S** | **A** | **T** | **D** | **I** | **S** | **K** | **P** | **L** | **I** | **D** | **Y** | **L** | **E** | **A** | **T** | **I** | **K** | **-** | **-** | **-** | **-** | **-** | **-** | **-** | **-** | **-** | **-** | **-** | **-** | **-** | **-** | **-** | **-** | **-** | **-** | **-** | **-** | **-** | **-** | **-** | **-** | **-** | **-** | **-** | **-** | **-** | **-** | **-** | **-** | **-** | **-** | **-** | **-** | **Q** | **W** | **Q** | **A** | **K** | **Q** | **E** | **L** | **A** | **E** | **A** | **K** | **K** | **T** | **A** | **T** | **T** | **F** | **L** | **N** | **V** | **L** | **S** | **K** | **Q** | **E** | **F** | **D** | **K** | **L** | **P** | **S** | **V** | **V** | **Q** | **E** | **A** | **S** | **L** | **K** | **K** | **N** | **G** | **Y** | **D** | **T** | **K** | **S** | **V** | **V** | **E** | **K** | **Y** | **Q** | **A** | **I** | **Y** | **S** | **G** | **I** | **Q** | **A** | **E** | **G** | **V** | **K** | **A** | **S** | **D** | **V** | **Q** | **V** | **K** | **K** | **A** | **K** | **D** | **N** | **Q** | **Y** | **T** | **F** | **T** | **Y** | **K** | **L** | **S** | **M** | **S** | **T** | **P** | **L** | **G** | **E** | **M** | **K** | **D** | **L** | **S** | **Y** | **Q** | **S** | **S** | **I** | **A** | **K** | **K** | **G** | **D** | **T** | **Y** | **Q** | **I** | **A** | **W** | **K** | **P** | **S** | **L** | **I** | **F** | **P** | **D** | **M** | **S** | **G** | **N** | **D** | **K** | **I** | **S** | **I** | **Q** | **V** | **D** | **N** | **A** | **K** | **R** | **G** | **E** | **I** | **V** | **D** | **R** | **N** | **G** | **S** | **G** | **L** | **A** | **I** | **N** | **K** | **V** | **F** | **D** | **E** | **V** | **G** | **V** | **V** | **P** | **G** | **K** | **L** | **G** | **S** | **G** | **A** | **E** | **K** | **T** | **A** | **N** | **I** | **K** | **A** | **F** | **S** | **D** | **K** | **F** | **G** | **V** | **S** | **V** | **D** | **E** | **I** | **N** | **Q** | **K** | **L** | **S** | **Q** | **G** | **W** | **V** | **Q** | **A** | **D** | **S** | **F** | **V** | **P** | **I** | **T** | **V** | **A** | **S** | **E** | **P** | **V** | **T** | **E** | **L** | **P** | **T** | **G** | **A** | **A** | **T** | **K** | **D** | **T** | **E** | **S** | **R** | **Y** | **Y** | **P** | **L** | **G** | **E** | **A** | **A** | **A** | **Q** | **L** | **I** | **G** | **Y** | **T** | **G** | **T** | **I** | **T** | **A** | **E** | **D** | **I** | **E** | **K** | **N** | **P** | **E** | **L** | **S** | **S** | **T** | **G** | **V** | **I** | **G** | **K** | **T** | **G** | **L** | **E** | **R** | **A** | **F** | **D** | **K** | **E** | **L** | **R** | **G** | **Q** | **D** | **G** | **G** | **S** | **L** | **V** | **I** | **L** | **D** | **D** | **K** | **E** | **N** | **V** | **K** | **K** | **A** | **L** | **Q** | **T** | **K** | **E** | **K** | **K** | **D** | **G** | **Q** | **T** | **I** | **K** | **L** | **T** | **I** | **D** | **S** | **G** | **V** | **Q** | **Q** | **Q** | **A** | **F** | **A** | **I** | **F** | **D** | **K** | **R** | **P** | **G** | **S** | **A** | **V** | **I** | **T** | **D** | **P** | **Q** | **K** | **G** | **D** | **L** | **L** | **A** | **T** | **V** | **S** | **S** | **P** | **S** | **Y** | **D** | **P** | **N** | **K** | **M** | **A** | **N** | **G** | **I** | **S** | **Q** | **K** | **E** | **Y** | **D** | **A** | **Y** | **N** | **N** | **N** | **K** | **D** | **L** | **P** | **F** | **T** | **A** | **R** | **F** | **A** | **T** | **G** | **Y** | **A** | **P** | **G** | **S** | **T** | **F** | **K** | **T** | **I** | **T** | **G** | **A** | **I** | **G** | **L** | **D** | **A** | **G** | **T** | **L** | **K** | **P** | **D** | **E** | **E** | **L** | **E** | **I** | **N** | **G** | **L** | **K** | **W** | **Q** | **K** | **D** | **K** | **S** | **W** | **G** | **G** | **Y** | **F** | **A** | **T** | **R** | **V** | **K** | **E** | **A** | **S** | **P** | **V** | **N** | **L** | **R** | **T** | **A** | **L** | **V** | **N** | **S** | **D** | **N** | **I** | **Y** | **F** | **A** | **Q** | **Q** | **T** | **L** | **R** | **M** | **G** | **E** | **D** | **K** | **F** | **R** | **A** | **G** | **L** | **N** | **K** | **F** | **I** | **F** | **G** | **E** | **E** | **L** | **D** | **L** | **P** | **I** | **A** | **M** | **T** | **P** | **A** | **Q** | **I** | **S** | **N** | **E** | **D** | **K** | **F** | **N** | **S** | **E** | **I** | **L** | **L** | **A** | **D** | **T** | **G** | **Y** | **G** | **Q** | **G** | **Q** | **L** | **L** | **I** | **S** | **P** | **I** | **Q** | **Q** | **A** | **T** | **M** | **Y** | **S** | **V** | **F** | **Q** | **N** | **N** | **G** | **T** | **L** | **V** | **Y** | **P** | **K** | **L** | **V** | **L** | **D** | **K** | **E** | **T** | **K** | **K** | **K** | **D** | **N** | **V** | **I** | **S** | **A** | **N** | **A** | **A** | **N** | **T** | **I** | **A** | **T** | **D** | **L** | **L** | **G** | **S** | **V** | **E** | **D** | **P** | **S** | **G** | **Y** | **V** | **Y** | **N** | **M** | **Y** | **N** | **P** | **N** | **F** | **S** | **L** | **A** | **A** | **K** | **T** | **G** | **T** | **A** | **E** | **I** | **K** | **D** | **K** | **Q** | **D** | **T** | **D** | **G** | **K** | **E** | **N** | **S** | **F** | **L** | **L** | **T** | **L** | **D** | **R** | **S** | **N** | **N** | **K** | **F** | **L** | **T** | **M** | **I** | **M** | **V** | **E** | **N** | **S** | **G** | **E** | **N** | **G** | **S** | **A** | **T** | **D** | **I** | **S** | **K** | **P** | **L** | **I** | **D** | **Y** | **L** | **E** | **A** | **T** | **I** | **K** |
| **N52724** | **-** | **-** | **-** | **-** | **-** | **-** | **-** | **-** | **-** | **-** | **-** | **-** | **-** | **-** | **-** | **-** | **-** | **-** | **-** | **-** | **-** | **-** | **-** | **-** | **-** | **-** | **-** | **-** | **-** | **-** | **-** | **-** | **-** | **-** | **-** | **-** | **Q** | **W** | **Q** | **A** | **K** | **Q** | **E** | **L** | **A** | **E** | **A** | **K** | **K** | **T** | **A** | **T** | **T** | **F** | **L** | **N** | **V** | **L** | **S** | **K** | **Q** | **E** | **F** | **D** | **K** | **L** | **P** | **S** | **V** | **I** | **Q** | **E** | **A** | **S** | **L** | **K** | **K** | **N** | **G** | **Y** | **D** | **T** | **K** | **S** | **V** | **V** | **E** | **K** | **Y** | **Q** | **A** | **I** | **Y** | **S** | **G** | **I** | **Q** | **A** | **E** | **G** | **V** | **K** | **A** | **S** | **D** | **V** | **Q** | **V** | **K** | **K** | **A** | **K** | **D** | **N** | **Q** | **Y** | **T** | **F** | **T** | **Y** | **K** | **L** | **S** | **M** | **S** | **T** | **P** | **L** | **G** | **E** | **M** | **K** | **D** | **L** | **S** | **Y** | **Q** | **S** | **S** | **I** | **A** | **K** | **K** | **G** | **D** | **T** | **Y** | **Q** | **I** | **A** | **W** | **K** | **P** | **S** | **L** | **I** | **F** | **P** | **D** | **M** | **S** | **G** | **N** | **D** | **K** | **I** | **S** | **I** | **Q** | **V** | **D** | **N** | **A** | **K** | **R** | **G** | **E** | **I** | **V** | **D** | **R** | **N** | **G** | **S** | **G** | **L** | **A** | **I** | **N** | **K** | **V** | **F** | **D** | **E** | **V** | **G** | **V** | **V** | **P** | **G** | **K** | **L** | **G** | **S** | **G** | **A** | **E** | **K** | **T** | **A** | **N** | **I** | **K** | **A** | **F** | **S** | **D** | **K** | **F** | **G** | **V** | **S** | **V** | **D** | **E** | **I** | **N** | **Q** | **K** | **L** | **S** | **Q** | **G** | **W** | **V** | **Q** | **A** | **D** | **S** | **F** | **V** | **P** | **I** | **T** | **V** | **A** | **S** | **E** | **P** | **V** | **T** | **E** | **L** | **P** | **T** | **G** | **A** | **A** | **T** | **K** | **D** | **T** | **E** | **S** | **R** | **Y** | **Y** | **P** | **L** | **G** | **E** | **A** | **A** | **A** | **Q** | **L** | **I** | **G** | **Y** | **T** | **G** | **T** | **I** | **T** | **A** | **E** | **D** | **I** | **E** | **K** | **N** | **P** | **E** | **L** | **S** | **S** | **T** | **G** | **V** | **I** | **G** | **K** | **T** | **G** | **L** | **E** | **R** | **A** | **F** | **D** | **K** | **E** | **L** | **R** | **G** | **Q** | **D** | **G** | **G** | **S** | **L** | **V** | **I** | **L** | **D** | **D** | **K** | **E** | **N** | **V** | **K** | **K** | **A** | **L** | **Q** | **T** | **K** | **E** | **K** | **K** | **D** | **G** | **Q** | **T** | **I** | **K** | **L** | **T** | **I** | **D** | **S** | **G** | **V** | **Q** | **Q** | **Q** | **A** | **F** | **A** | **I** | **F** | **D** | **K** | **R** | **P** | **G** | **S** | **A** | **V** | **I** | **T** | **D** | **P** | **Q** | **K** | **G** | **D** | **L** | **L** | **A** | **T** | **V** | **S** | **S** | **P** | **S** | **Y** | **D** | **P** | **N** | **K** | **M** | **A** | **N** | **G** | **I** | **S** | **Q** | **K** | **E** | **Y** | **D** | **A** | **Y** | **N** | **N** | **N** | **K** | **D** | **L** | **P** | **F** | **T** | **A** | **R** | **F** | **A** | **T** | **G** | **Y** | **A** | **P** | **G** | **S** | **T** | **F** | **K** | **T** | **I** | **T** | **G** | **A** | **I** | **G** | **L** | **D** | **A** | **G** | **T** | **L** | **K** | **P** | **D** | **E** | **E** | **L** | **E** | **I** | **N** | **G** | **L** | **K** | **W** | **Q** | **K** | **D** | **K** | **S** | **W** | **G** | **G** | **Y** | **F** | **A** | **T** | **R** | **V** | **K** | **E** | **A** | **S** | **P** | **V** | **N** | **L** | **R** | **T** | **A** | **L** | **V** | **N** | **S** | **D** | **N** | **I** | **Y** | **F** | **A** | **Q** | **Q** | **T** | **L** | **R** | **M** | **G** | **E** | **D** | **K** | **F** | **R** | **A** | **G** | **L** | **N** | **K** | **F** | **I** | **F** | **G** | **E** | **E** | **L** | **D** | **L** | **P** | **I** | **A** | **M** | **T** | **P** | **A** | **Q** | **I** | **S** | **N** | **E** | **D** | **K** | **F** | **N** | **S** | **E** | **I** | **L** | **L** | **A** | **D** | **T** | **G** | **Y** | **G** | **Q** | **G** | **Q** | **L** | **L** | **I** | **S** | **P** | **I** | **Q** | **Q** | **A** | **T** | **M** | **Y** | **S** | **V** | **F** | **Q** | **N** | **N** | **G** | **T** | **L** | **V** | **Y** | **P** | **K** | **L** | **V** | **L** | **D** | **K** | **E** | **T** | **K** | **K** | **K** | **D** | **N** | **V** | **I** | **S** | **A** | **N** | **A** | **A** | **N** | **T** | **I** | **A** | **T** | **D** | **L** | **L** | **G** | **S** | **V** | **E** | **D** | **P** | **S** | **G** | **Y** | **V** | **Y** | **N** | **M** | **Y** | **N** | **P** | **N** | **F** | **S** | **L** | **A** | **A** | **K** | **T** | **G** | **T** | **A** | **E** | **I** | **K** | **D** | **K** | **Q** | **D** | **T** | **D** | **G** | **K** | **E** | **N** | **S** | **F** | **L** | **L** | **T** | **L** | **D** | **R** | **S** | **N** | **N** | **K** | **F** | **L** | **T** | **M** | **I** | **M** | **V** | **E** | **N** | **S** | **G** | **E** | **N** | **G** | **S** | **A** | **T** | **D** | **I** | **S** | **K** | **P** | **L** | **I** | **D** | **Y** | **L** | **E** | **A** | **T** | **I** | **K** | **-** | **-** | **-** | **-** | **-** | **-** | **-** | **-** | **-** | **-** | **-** | **-** | **-** | **-** | **-** | **-** | **-** | **-** | **-** | **-** | **-** | **-** | **-** | **-** | **-** | **-** | **-** | **-** | **-** | **-** | **-** | **-** | **-** | **-** | **-** | **-** | **Q** | **W** | **Q** | **A** | **K** | **Q** | **E** | **L** | **A** | **E** | **A** | **K** | **K** | **T** | **A** | **T** | **T** | **F** | **L** | **N** | **V** | **L** | **S** | **K** | **Q** | **E** | **F** | **D** | **K** | **L** | **P** | **S** | **V** | **I** | **Q** | **E** | **A** | **S** | **L** | **K** | **K** | **N** | **G** | **Y** | **D** | **T** | **K** | **S** | **V** | **V** | **E** | **K** | **Y** | **Q** | **A** | **I** | **Y** | **S** | **G** | **I** | **Q** | **A** | **E** | **G** | **V** | **K** | **A** | **S** | **D** | **V** | **Q** | **V** | **K** | **K** | **A** | **K** | **D** | **N** | **Q** | **Y** | **T** | **F** | **T** | **Y** | **K** | **L** | **S** | **M** | **S** | **T** | **P** | **L** | **G** | **E** | **M** | **K** | **D** | **L** | **S** | **Y** | **Q** | **S** | **S** | **I** | **A** | **K** | **K** | **G** | **D** | **T** | **Y** | **Q** | **I** | **A** | **W** | **K** | **P** | **S** | **L** | **I** | **F** | **P** | **D** | **M** | **S** | **G** | **N** | **D** | **K** | **I** | **S** | **I** | **Q** | **V** | **D** | **N** | **A** | **K** | **R** | **G** | **E** | **I** | **V** | **D** | **R** | **N** | **G** | **S** | **G** | **L** | **A** | **I** | **N** | **K** | **V** | **F** | **D** | **E** | **V** | **G** | **V** | **V** | **P** | **G** | **K** | **L** | **G** | **S** | **G** | **A** | **E** | **K** | **T** | **A** | **N** | **I** | **K** | **A** | **F** | **S** | **D** | **K** | **F** | **G** | **V** | **S** | **V** | **D** | **E** | **I** | **N** | **Q** | **K** | **L** | **S** | **Q** | **G** | **W** | **V** | **Q** | **A** | **D** | **S** | **F** | **V** | **P** | **I** | **T** | **V** | **A** | **S** | **E** | **P** | **V** | **T** | **E** | **L** | **P** | **T** | **G** | **A** | **A** | **T** | **K** | **D** | **T** | **E** | **S** | **R** | **Y** | **Y** | **P** | **L** | **G** | **E** | **A** | **A** | **A** | **Q** | **L** | **I** | **G** | **Y** | **T** | **G** | **T** | **I** | **T** | **A** | **E** | **D** | **I** | **E** | **K** | **N** | **P** | **E** | **L** | **S** | **S** | **T** | **G** | **V** | **I** | **G** | **K** | **T** | **G** | **L** | **E** | **R** | **A** | **F** | **D** | **K** | **E** | **L** | **R** | **G** | **Q** | **D** | **G** | **G** | **S** | **L** | **V** | **I** | **L** | **D** | **D** | **K** | **E** | **N** | **V** | **K** | **K** | **A** | **L** | **Q** | **T** | **K** | **E** | **K** | **K** | **D** | **G** | **Q** | **T** | **I** | **K** | **L** | **T** | **I** | **D** | **S** | **G** | **V** | **Q** | **Q** | **Q** | **A** | **F** | **A** | **I** | **F** | **D** | **K** | **R** | **P** | **G** | **S** | **A** | **V** | **I** | **T** | **D** | **P** | **Q** | **K** | **G** | **D** | **L** | **L** | **A** | **T** | **V** | **S** | **S** | **P** | **S** | **Y** | **D** | **P** | **N** | **K** | **M** | **A** | **N** | **G** | **I** | **S** | **Q** | **K** | **E** | **Y** | **D** | **A** | **Y** | **N** | **N** | **N** | **K** | **D** | **L** | **P** | **F** | **T** | **A** | **R** | **F** | **A** | **T** | **G** | **Y** | **A** | **P** | **G** | **S** | **T** | **F** | **K** | **T** | **I** | **T** | **G** | **A** | **I** | **G** | **L** | **D** | **A** | **G** | **T** | **L** | **K** | **P** | **D** | **E** | **E** | **L** | **E** | **I** | **N** | **G** | **L** | **K** | **W** | **Q** | **K** | **D** | **K** | **S** | **W** | **G** | **G** | **Y** | **F** | **A** | **T** | **R** | **V** | **K** | **E** | **A** | **S** | **P** | **V** | **N** | **L** | **R** | **T** | **A** | **L** | **V** | **N** | **S** | **D** | **N** | **I** | **Y** | **F** | **A** | **Q** | **Q** | **T** | **L** | **R** | **M** | **G** | **E** | **D** | **K** | **F** | **R** | **A** | **G** | **L** | **N** | **K** | **F** | **I** | **F** | **G** | **E** | **E** | **L** | **D** | **L** | **P** | **I** | **A** | **M** | **T** | **P** | **A** | **Q** | **I** | **S** | **N** | **E** | **D** | **K** | **F** | **N** | **S** | **E** | **I** | **L** | **L** | **A** | **D** | **T** | **G** | **Y** | **G** | **Q** | **G** | **Q** | **L** | **L** | **I** | **S** | **P** | **I** | **Q** | **Q** | **A** | **T** | **M** | **Y** | **S** | **V** | **F** | **Q** | **N** | **N** | **G** | **T** | **L** | **V** | **Y** | **P** | **K** | **L** | **V** | **L** | **D** | **K** | **E** | **T** | **K** | **K** | **K** | **D** | **N** | **V** | **I** | **S** | **A** | **N** | **A** | **A** | **N** | **T** | **I** | **A** | **T** | **D** | **L** | **L** | **G** | **S** | **V** | **E** | **D** | **P** | **S** | **G** | **Y** | **V** | **Y** | **N** | **M** | **Y** | **N** | **P** | **N** | **F** | **S** | **L** | **A** | **A** | **K** | **T** | **G** | **T** | **A** | **E** | **I** | **K** | **D** | **K** | **Q** | **D** | **T** | **D** | **G** | **K** | **E** | **N** | **S** | **F** | **L** | **L** | **T** | **L** | **D** | **R** | **S** | **N** | **N** | **K** | **F** | **L** | **T** | **M** | **I** | **M** | **V** | **E** | **N** | **S** | **G** | **E** | **N** | **G** | **S** | **A** | **T** | **D** | **I** | **S** | **K** | **P** | **L** | **I** | **D** | **Y** | **L** | **E** | **A** | **T** | **I** | **K** |
| **N52467** | **M** | **E** | **R** | **S** | **N** | **R** | **N** | **K** | **K** | **S** | **S** | **K** | **K** | **P** | **L** | **I** | **L** | **G** | **V** | **S** | **A** | **L** | **V** | **L** | **I** | **A** | **A** | **V** | **G** | **G** | **G** | **Y** | **Y** | **A** | **Y** | **S** | **Q** | **W** | **Q** | **A** | **K** | **Q** | **E** | **L** | **A** | **E** | **A** | **K** | **K** | **T** | **A** | **N** | **T** | **F** | **L** | **N** | **V** | **L** | **S** | **K** | **Q** | **E** | **F** | **D** | **K** | **L** | **P** | **S** | **V** | **V** | **Q** | **E** | **T** | **S** | **L** | **K** | **K** | **N** | **G** | **Y** | **D** | **T** | **K** | **S** | **V** | **V** | **E** | **K** | **Y** | **Q** | **A** | **I** | **Y** | **S** | **G** | **I** | **Q** | **A** | **E** | **G** | **V** | **K** | **A** | **S** | **D** | **V** | **Q** | **V** | **K** | **K** | **G** | **K** | **D** | **N** | **Q** | **Y** | **T** | **F** | **T** | **Y** | **K** | **L** | **S** | **M** | **S** | **T** | **P** | **L** | **G** | **E** | **M** | **K** | **D** | **L** | **S** | **Y** | **Q** | **S** | **S** | **I** | **A** | **K** | **K** | **G** | **D** | **N** | **Y** | **Q** | **I** | **A** | **W** | **A** | **P** | **S** | **L** | **I** | **F** | **P** | **D** | **M** | **S** | **G** | **N** | **D** | **K** | **I** | **S** | **I** | **Q** | **V** | **D** | **N** | **A** | **K** | **R** | **G** | **E** | **I** | **V** | **D** | **R** | **N** | **G** | **S** | **G** | **L** | **A** | **I** | **N** | **K** | **V** | **F** | **D** | **E** | **V** | **G** | **V** | **V** | **P** | **G** | **K** | **L** | **G** | **S** | **G** | **A** | **E** | **K** | **T** | **A** | **N** | **I** | **K** | **A** | **F** | **S** | **D** | **K** | **F** | **G** | **V** | **S** | **V** | **D** | **E** | **I** | **N** | **Q** | **K** | **L** | **S** | **Q** | **G** | **W** | **V** | **Q** | **A** | **D** | **S** | **F** | **V** | **P** | **I** | **T** | **V** | **A** | **S** | **E** | **P** | **V** | **T** | **E** | **L** | **P** | **T** | **G** | **A** | **A** | **T** | **K** | **D** | **T** | **E** | **S** | **R** | **Y** | **Y** | **P** | **L** | **G** | **E** | **A** | **A** | **A** | **Q** | **L** | **I** | **G** | **Y** | **T** | **G** | **T** | **I** | **T** | **A** | **E** | **D** | **I** | **E** | **K** | **N** | **P** | **E** | **L** | **S** | **S** | **T** | **G** | **V** | **I** | **G** | **K** | **T** | **G** | **L** | **E** | **R** | **A** | **F** | **D** | **K** | **E** | **L** | **R** | **G** | **Q** | **D** | **G** | **G** | **S** | **L** | **V** | **I** | **L** | **D** | **D** | **K** | **E** | **N** | **V** | **K** | **K** | **A** | **L** | **Q** | **T** | **K** | **E** | **K** | **K** | **D** | **G** | **Q** | **T** | **I** | **K** | **L** | **T** | **I** | **D** | **S** | **G** | **V** | **Q** | **Q** | **Q** | **A** | **F** | **A** | **I** | **F** | **D** | **K** | **R** | **P** | **G** | **S** | **A** | **V** | **I** | **T** | **D** | **P** | **Q** | **K** | **G** | **D** | **L** | **L** | **A** | **T** | **V** | **S** | **S** | **P** | **S** | **Y** | **D** | **P** | **N** | **K** | **M** | **A** | **N** | **G** | **I** | **S** | **Q** | **K** | **E** | **Y** | **D** | **A** | **Y** | **N** | **N** | **N** | **K** | **D** | **L** | **P** | **F** | **T** | **A** | **R** | **F** | **A** | **T** | **G** | **Y** | **A** | **P** | **G** | **S** | **T** | **F** | **K** | **T** | **I** | **T** | **G** | **A** | **I** | **G** | **L** | **D** | **A** | **G** | **T** | **L** | **K** | **P** | **D** | **E** | **E** | **L** | **E** | **I** | **N** | **G** | **L** | **K** | **W** | **Q** | **K** | **D** | **K** | **S** | **W** | **G** | **G** | **Y** | **F** | **A** | **T** | **R** | **V** | **K** | **E** | **A** | **S** | **P** | **V** | **N** | **L** | **R** | **T** | **A** | **L** | **V** | **N** | **S** | **D** | **N** | **I** | **Y** | **F** | **A** | **Q** | **Q** | **T** | **L** | **R** | **M** | **G** | **E** | **D** | **K** | **F** | **R** | **A** | **G** | **L** | **N** | **K** | **F** | **I** | **F** | **G** | **E** | **E** | **L** | **D** | **L** | **P** | **I** | **A** | **M** | **T** | **P** | **A** | **Q** | **I** | **S** | **N** | **E** | **D** | **K** | **F** | **N** | **S** | **E** | **I** | **L** | **L** | **A** | **D** | **T** | **G** | **Y** | **G** | **Q** | **G** | **Q** | **L** | **L** | **I** | **S** | **P** | **I** | **Q** | **Q** | **A** | **T** | **M** | **Y** | **S** | **V** | **F** | **Q** | **N** | **N** | **G** | **T** | **L** | **V** | **Y** | **P** | **K** | **L** | **V** | **L** | **E** | **K** | **E** | **T** | **K** | **K** | **K** | **D** | **N** | **V** | **I** | **S** | **A** | **N** | **A** | **A** | **N** | **T** | **I** | **A** | **T** | **D** | **L** | **L** | **G** | **S** | **V** | **E** | **D** | **P** | **S** | **G** | **Y** | **V** | **Y** | **N** | **M** | **Y** | **N** | **P** | **N** | **F** | **S** | **L** | **A** | **A** | **K** | **T** | **G** | **T** | **A** | **E** | **I** | **K** | **D** | **K** | **Q** | **D** | **T** | **D** | **G** | **K** | **E** | **N** | **S** | **F** | **L** | **L** | **T** | **L** | **D** | **R** | **S** | **N** | **N** | **K** | **F** | **L** | **T** | **M** | **I** | **M** | **V** | **E** | **N** | **S** | **G** | **E** | **N** | **G** | **S** | **A** | **T** | **D** | **I** | **S** | **K** | **P** | **L** | **I** | **D** | **Y** | **L** | **E** | **A** | **T** | **I** | **K** | **M** | **E** | **R** | **S** | **N** | **R** | **N** | **K** | **K** | **S** | **S** | **K** | **K** | **P** | **L** | **I** | **L** | **G** | **V** | **S** | **A** | **L** | **V** | **L** | **I** | **A** | **A** | **V** | **G** | **G** | **G** | **Y** | **Y** | **A** | **Y** | **S** | **Q** | **W** | **Q** | **A** | **K** | **Q** | **E** | **L** | **A** | **E** | **A** | **K** | **K** | **T** | **A** | **N** | **T** | **F** | **L** | **N** | **V** | **L** | **S** | **K** | **Q** | **E** | **F** | **D** | **K** | **L** | **P** | **S** | **V** | **V** | **Q** | **E** | **T** | **S** | **L** | **K** | **K** | **N** | **G** | **Y** | **D** | **T** | **K** | **S** | **V** | **V** | **E** | **K** | **Y** | **Q** | **A** | **I** | **Y** | **S** | **G** | **I** | **Q** | **A** | **E** | **G** | **V** | **K** | **A** | **S** | **D** | **V** | **Q** | **V** | **K** | **K** | **G** | **K** | **D** | **N** | **Q** | **Y** | **T** | **F** | **T** | **Y** | **K** | **L** | **S** | **M** | **S** | **T** | **P** | **L** | **G** | **E** | **M** | **K** | **D** | **L** | **S** | **Y** | **Q** | **S** | **S** | **I** | **A** | **K** | **K** | **G** | **D** | **N** | **Y** | **Q** | **I** | **A** | **W** | **A** | **P** | **S** | **L** | **I** | **F** | **P** | **D** | **M** | **S** | **G** | **N** | **D** | **K** | **I** | **S** | **I** | **Q** | **V** | **D** | **N** | **A** | **K** | **R** | **G** | **E** | **I** | **V** | **D** | **R** | **N** | **G** | **S** | **G** | **L** | **A** | **I** | **N** | **K** | **V** | **F** | **D** | **E** | **V** | **G** | **V** | **V** | **P** | **G** | **K** | **L** | **G** | **S** | **G** | **A** | **E** | **K** | **T** | **A** | **N** | **I** | **K** | **A** | **F** | **S** | **D** | **K** | **F** | **G** | **V** | **S** | **V** | **D** | **E** | **I** | **N** | **Q** | **K** | **L** | **S** | **Q** | **G** | **W** | **V** | **Q** | **A** | **D** | **S** | **F** | **V** | **P** | **I** | **T** | **V** | **A** | **S** | **E** | **P** | **V** | **T** | **E** | **L** | **P** | **T** | **G** | **A** | **A** | **T** | **K** | **D** | **T** | **E** | **S** | **R** | **Y** | **Y** | **P** | **L** | **G** | **E** | **A** | **A** | **A** | **Q** | **L** | **I** | **G** | **Y** | **T** | **G** | **T** | **I** | **T** | **A** | **E** | **D** | **I** | **E** | **K** | **N** | **P** | **E** | **L** | **S** | **S** | **T** | **G** | **V** | **I** | **G** | **K** | **T** | **G** | **L** | **E** | **R** | **A** | **F** | **D** | **K** | **E** | **L** | **R** | **G** | **Q** | **D** | **G** | **G** | **S** | **L** | **V** | **I** | **L** | **D** | **D** | **K** | **E** | **N** | **V** | **K** | **K** | **A** | **L** | **Q** | **T** | **K** | **E** | **K** | **K** | **D** | **G** | **Q** | **T** | **I** | **K** | **L** | **T** | **I** | **D** | **S** | **G** | **V** | **Q** | **Q** | **Q** | **A** | **F** | **A** | **I** | **F** | **D** | **K** | **R** | **P** | **G** | **S** | **A** | **V** | **I** | **T** | **D** | **P** | **Q** | **K** | **G** | **D** | **L** | **L** | **A** | **T** | **V** | **S** | **S** | **P** | **S** | **Y** | **D** | **P** | **N** | **K** | **M** | **A** | **N** | **G** | **I** | **S** | **Q** | **K** | **E** | **Y** | **D** | **A** | **Y** | **N** | **N** | **N** | **K** | **D** | **L** | **P** | **F** | **T** | **A** | **R** | **F** | **A** | **T** | **G** | **Y** | **A** | **P** | **G** | **S** | **T** | **F** | **K** | **T** | **I** | **T** | **G** | **A** | **I** | **G** | **L** | **D** | **A** | **G** | **T** | **L** | **K** | **P** | **D** | **E** | **E** | **L** | **E** | **I** | **N** | **G** | **L** | **K** | **W** | **Q** | **K** | **D** | **K** | **S** | **W** | **G** | **G** | **Y** | **F** | **A** | **T** | **R** | **V** | **K** | **E** | **A** | **S** | **P** | **V** | **N** | **L** | **R** | **T** | **A** | **L** | **V** | **N** | **S** | **D** | **N** | **I** | **Y** | **F** | **A** | **Q** | **Q** | **T** | **L** | **R** | **M** | **G** | **E** | **D** | **K** | **F** | **R** | **A** | **G** | **L** | **N** | **K** | **F** | **I** | **F** | **G** | **E** | **E** | **L** | **D** | **L** | **P** | **I** | **A** | **M** | **T** | **P** | **A** | **Q** | **I** | **S** | **N** | **E** | **D** | **K** | **F** | **N** | **S** | **E** | **I** | **L** | **L** | **A** | **D** | **T** | **G** | **Y** | **G** | **Q** | **G** | **Q** | **L** | **L** | **I** | **S** | **P** | **I** | **Q** | **Q** | **A** | **T** | **M** | **Y** | **S** | **V** | **F** | **Q** | **N** | **N** | **G** | **T** | **L** | **V** | **Y** | **P** | **K** | **L** | **V** | **L** | **E** | **K** | **E** | **T** | **K** | **K** | **K** | **D** | **N** | **V** | **I** | **S** | **A** | **N** | **A** | **A** | **N** | **T** | **I** | **A** | **T** | **D** | **L** | **L** | **G** | **S** | **V** | **E** | **D** | **P** | **S** | **G** | **Y** | **V** | **Y** | **N** | **M** | **Y** | **N** | **P** | **N** | **F** | **S** | **L** | **A** | **A** | **K** | **T** | **G** | **T** | **A** | **E** | **I** | **K** | **D** | **K** | **Q** | **D** | **T** | **D** | **G** | **K** | **E** | **N** | **S** | **F** | **L** | **L** | **T** | **L** | **D** | **R** | **S** | **N** | **N** | **K** | **F** | **L** | **T** | **M** | **I** | **M** | **V** | **E** | **N** | **S** | **G** | **E** | **N** | **G** | **S** | **A** | **T** | **D** | **I** | **S** | **K** | **P** | **L** | **I** | **D** | **Y** | **L** | **E** | **A** | **T** | **I** | **K** |
| **N60268F** | **-** | **-** | **-** | **-** | **-** | **-** | **-** | **-** | **-** | **-** | **-** | **-** | **-** | **-** | **-** | **-** | **-** | **-** | **-** | **-** | **-** | **-** | **-** | **-** | **-** | **-** | **-** | **-** | **-** | **-** | **-** | **-** | **-** | **-** | **-** | **-** | **Q** | **W** | **Q** | **A** | **K** | **Q** | **E** | **L** | **A** | **E** | **A** | **K** | **K** | **T** | **A** | **T** | **T** | **F** | **L** | **N** | **V** | **L** | **T** | **K** | **Q** | **K** | **F** | **D** | **K** | **L** | **P** | **S** | **V** | **V** | **Q** | **E** | **A** | **S** | **L** | **K** | **K** | **N** | **G** | **Y** | **D** | **T** | **K** | **S** | **V** | **V** | **E** | **K** | **Y** | **Q** | **A** | **I** | **Y** | **S** | **G** | **I** | **Q** | **A** | **E** | **G** | **V** | **K** | **A** | **S** | **D** | **V** | **Q** | **V** | **K** | **K** | **A** | **K** | **D** | **N** | **Q** | **Y** | **T** | **F** | **T** | **Y** | **K** | **L** | **S** | **M** | **S** | **T** | **P** | **L** | **G** | **E** | **M** | **K** | **D** | **L** | **S** | **Y** | **Q** | **S** | **S** | **I** | **A** | **K** | **K** | **G** | **D** | **T** | **Y** | **Q** | **I** | **A** | **W** | **K** | **P** | **S** | **L** | **I** | **F** | **P** | **D** | **M** | **S** | **G** | **N** | **D** | **K** | **I** | **S** | **I** | **Q** | **V** | **D** | **N** | **A** | **K** | **R** | **G** | **E** | **I** | **V** | **D** | **R** | **N** | **G** | **S** | **G** | **L** | **A** | **I** | **N** | **K** | **V** | **F** | **D** | **E** | **V** | **G** | **V** | **V** | **P** | **G** | **K** | **L** | **G** | **S** | **G** | **A** | **E** | **K** | **T** | **A** | **N** | **I** | **K** | **A** | **F** | **S** | **D** | **K** | **F** | **G** | **V** | **S** | **V** | **D** | **E** | **I** | **N** | **Q** | **K** | **L** | **S** | **Q** | **G** | **W** | **V** | **Q** | **A** | **D** | **S** | **F** | **V** | **P** | **I** | **T** | **V** | **A** | **S** | **E** | **P** | **V** | **T** | **E** | **L** | **P** | **T** | **G** | **A** | **A** | **T** | **K** | **D** | **T** | **E** | **S** | **R** | **Y** | **Y** | **P** | **L** | **G** | **E** | **A** | **A** | **A** | **Q** | **L** | **I** | **G** | **Y** | **T** | **G** | **T** | **I** | **T** | **A** | **E** | **D** | **I** | **K** | **K** | **N** | **P** | **E** | **L** | **S** | **S** | **T** | **G** | **V** | **I** | **G** | **K** | **T** | **G** | **L** | **E** | **R** | **A** | **F** | **D** | **K** | **E** | **L** | **R** | **G** | **Q** | **D** | **G** | **G** | **S** | **L** | **V** | **I** | **L** | **D** | **D** | **K** | **E** | **N** | **V** | **K** | **K** | **A** | **L** | **Q** | **T** | **K** | **E** | **K** | **K** | **D** | **G** | **Q** | **T** | **I** | **K** | **L** | **T** | **I** | **D** | **S** | **G** | **V** | **Q** | **Q** | **Q** | **A** | **F** | **A** | **I** | **F** | **D** | **K** | **R** | **P** | **G** | **S** | **A** | **V** | **I** | **T** | **D** | **P** | **Q** | **K** | **G** | **D** | **L** | **L** | **A** | **T** | **V** | **S** | **S** | **P** | **S** | **Y** | **D** | **P** | **N** | **K** | **M** | **A** | **N** | **G** | **I** | **S** | **Q** | **K** | **E** | **Y** | **D** | **A** | **Y** | **N** | **N** | **N** | **K** | **D** | **L** | **P** | **F** | **T** | **A** | **R** | **F** | **A** | **T** | **G** | **Y** | **A** | **P** | **G** | **S** | **T** | **F** | **K** | **T** | **I** | **T** | **G** | **A** | **I** | **G** | **L** | **D** | **A** | **G** | **T** | **L** | **K** | **P** | **D** | **E** | **E** | **L** | **E** | **I** | **N** | **G** | **L** | **K** | **W** | **Q** | **K** | **D** | **K** | **S** | **W** | **G** | **G** | **Y** | **F** | **A** | **T** | **R** | **V** | **K** | **E** | **A** | **S** | **P** | **V** | **N** | **L** | **R** | **T** | **A** | **L** | **V** | **N** | **S** | **D** | **N** | **I** | **Y** | **F** | **A** | **Q** | **Q** | **T** | **L** | **R** | **M** | **G** | **E** | **D** | **K** | **F** | **R** | **A** | **G** | **L** | **N** | **K** | **F** | **I** | **F** | **G** | **E** | **E** | **L** | **D** | **L** | **P** | **I** | **A** | **M** | **T** | **P** | **A** | **Q** | **I** | **S** | **N** | **E** | **D** | **K** | **F** | **N** | **S** | **E** | **I** | **L** | **L** | **A** | **D** | **T** | **G** | **Y** | **G** | **Q** | **G** | **Q** | **L** | **L** | **I** | **S** | **P** | **I** | **Q** | **Q** | **A** | **T** | **M** | **Y** | **S** | **V** | **F** | **Q** | **N** | **N** | **G** | **T** | **L** | **V** | **Y** | **P** | **K** | **L** | **V** | **L** | **D** | **K** | **E** | **T** | **K** | **K** | **K** | **D** | **N** | **V** | **I** | **S** | **A** | **N** | **A** | **A** | **N** | **T** | **I** | **A** | **T** | **D** | **L** | **L** | **G** | **S** | **V** | **E** | **D** | **P** | **S** | **G** | **Y** | **V** | **Y** | **N** | **M** | **Y** | **N** | **P** | **N** | **F** | **S** | **L** | **A** | **A** | **K** | **T** | **G** | **T** | **A** | **E** | **I** | **K** | **D** | **K** | **Q** | **D** | **T** | **D** | **G** | **K** | **E** | **N** | **S** | **F** | **L** | **L** | **T** | **L** | **D** | **R** | **S** | **N** | **N** | **K** | **F** | **L** | **T** | **M** | **I** | **M** | **V** | **E** | **N** | **S** | **G** | **E** | **N** | **G** | **S** | **A** | **T** | **D** | **I** | **S** | **K** | **P** | **L** | **I** | **D** | **Y** | **L** | **E** | **A** | **T** | **I** | **K** | **-** | **-** | **-** | **-** | **-** | **-** | **-** | **-** | **-** | **-** | **-** | **-** | **-** | **-** | **-** | **-** | **-** | **-** | **-** | **-** | **-** | **-** | **-** | **-** | **-** | **-** | **-** | **-** | **-** | **-** | **-** | **-** | **-** | **-** | **-** | **-** | **Q** | **W** | **Q** | **A** | **K** | **Q** | **E** | **L** | **A** | **E** | **A** | **K** | **K** | **T** | **A** | **T** | **T** | **F** | **L** | **N** | **V** | **L** | **T** | **K** | **Q** | **K** | **F** | **D** | **K** | **L** | **P** | **S** | **V** | **V** | **Q** | **E** | **A** | **S** | **L** | **K** | **K** | **N** | **G** | **Y** | **D** | **T** | **K** | **S** | **V** | **V** | **E** | **K** | **Y** | **Q** | **A** | **I** | **Y** | **S** | **G** | **I** | **Q** | **A** | **E** | **G** | **V** | **K** | **A** | **S** | **D** | **V** | **Q** | **V** | **K** | **K** | **A** | **K** | **D** | **N** | **Q** | **Y** | **T** | **F** | **T** | **Y** | **K** | **L** | **S** | **M** | **S** | **T** | **P** | **L** | **G** | **E** | **M** | **K** | **D** | **L** | **S** | **Y** | **Q** | **S** | **S** | **I** | **A** | **K** | **K** | **G** | **D** | **T** | **Y** | **Q** | **I** | **A** | **W** | **K** | **P** | **S** | **L** | **I** | **F** | **P** | **D** | **M** | **S** | **G** | **N** | **D** | **K** | **I** | **S** | **I** | **Q** | **V** | **D** | **N** | **A** | **K** | **R** | **G** | **E** | **I** | **V** | **D** | **R** | **N** | **G** | **S** | **G** | **L** | **A** | **I** | **N** | **K** | **V** | **F** | **D** | **E** | **V** | **G** | **V** | **V** | **P** | **G** | **K** | **L** | **G** | **S** | **G** | **A** | **E** | **K** | **T** | **A** | **N** | **I** | **K** | **A** | **F** | **S** | **D** | **K** | **F** | **G** | **V** | **S** | **V** | **D** | **E** | **I** | **N** | **Q** | **K** | **L** | **S** | **Q** | **G** | **W** | **V** | **Q** | **A** | **D** | **S** | **F** | **V** | **P** | **I** | **T** | **V** | **A** | **S** | **E** | **P** | **V** | **T** | **E** | **L** | **P** | **T** | **G** | **A** | **A** | **T** | **K** | **D** | **T** | **E** | **S** | **R** | **Y** | **Y** | **P** | **L** | **G** | **E** | **A** | **A** | **A** | **Q** | **L** | **I** | **G** | **Y** | **T** | **G** | **T** | **I** | **T** | **A** | **E** | **D** | **I** | **K** | **K** | **N** | **P** | **E** | **L** | **S** | **S** | **T** | **G** | **V** | **I** | **G** | **K** | **T** | **G** | **L** | **E** | **R** | **A** | **F** | **D** | **K** | **E** | **L** | **R** | **G** | **Q** | **D** | **G** | **G** | **S** | **L** | **V** | **I** | **L** | **D** | **D** | **K** | **E** | **N** | **V** | **K** | **K** | **A** | **L** | **Q** | **T** | **K** | **E** | **K** | **K** | **D** | **G** | **Q** | **T** | **I** | **K** | **L** | **T** | **I** | **D** | **S** | **G** | **V** | **Q** | **Q** | **Q** | **A** | **F** | **A** | **I** | **F** | **D** | **K** | **R** | **P** | **G** | **S** | **A** | **V** | **I** | **T** | **D** | **P** | **Q** | **K** | **G** | **D** | **L** | **L** | **A** | **T** | **V** | **S** | **S** | **P** | **S** | **Y** | **D** | **P** | **N** | **K** | **M** | **A** | **N** | **G** | **I** | **S** | **Q** | **K** | **E** | **Y** | **D** | **A** | **Y** | **N** | **N** | **N** | **K** | **D** | **L** | **P** | **F** | **T** | **A** | **R** | **F** | **A** | **T** | **G** | **Y** | **A** | **P** | **G** | **S** | **T** | **F** | **K** | **T** | **I** | **T** | **G** | **A** | **I** | **G** | **L** | **D** | **A** | **G** | **T** | **L** | **K** | **P** | **D** | **E** | **E** | **L** | **E** | **I** | **N** | **G** | **L** | **K** | **W** | **Q** | **K** | **D** | **K** | **S** | **W** | **G** | **G** | **Y** | **F** | **A** | **T** | **R** | **V** | **K** | **E** | **A** | **S** | **P** | **V** | **N** | **L** | **R** | **T** | **A** | **L** | **V** | **N** | **S** | **D** | **N** | **I** | **Y** | **F** | **A** | **Q** | **Q** | **T** | **L** | **R** | **M** | **G** | **E** | **D** | **K** | **F** | **R** | **A** | **G** | **L** | **N** | **K** | **F** | **I** | **F** | **G** | **E** | **E** | **L** | **D** | **L** | **P** | **I** | **A** | **M** | **T** | **P** | **A** | **Q** | **I** | **S** | **N** | **E** | **D** | **K** | **F** | **N** | **S** | **E** | **I** | **L** | **L** | **A** | **D** | **T** | **G** | **Y** | **G** | **Q** | **G** | **Q** | **L** | **L** | **I** | **S** | **P** | **I** | **Q** | **Q** | **A** | **T** | **M** | **Y** | **S** | **V** | **F** | **Q** | **N** | **N** | **G** | **T** | **L** | **V** | **Y** | **P** | **K** | **L** | **V** | **L** | **D** | **K** | **E** | **T** | **K** | **K** | **K** | **D** | **N** | **V** | **I** | **S** | **A** | **N** | **A** | **A** | **N** | **T** | **I** | **A** | **T** | **D** | **L** | **L** | **G** | **S** | **V** | **E** | **D** | **P** | **S** | **G** | **Y** | **V** | **Y** | **N** | **M** | **Y** | **N** | **P** | **N** | **F** | **S** | **L** | **A** | **A** | **K** | **T** | **G** | **T** | **A** | **E** | **I** | **K** | **D** | **K** | **Q** | **D** | **T** | **D** | **G** | **K** | **E** | **N** | **S** | **F** | **L** | **L** | **T** | **L** | **D** | **R** | **S** | **N** | **N** | **K** | **F** | **L** | **T** | **M** | **I** | **M** | **V** | **E** | **N** | **S** | **G** | **E** | **N** | **G** | **S** | **A** | **T** | **D** | **I** | **S** | **K** | **P** | **L** | **I** | **D** | **Y** | **L** | **E** | **A** | **T** | **I** | **K** |
| **N59584F** | **-** | **-** | **-** | **-** | **-** | **-** | **-** | **-** | **-** | **-** | **-** | **-** | **-** | **-** | **-** | **-** | **-** | **-** | **-** | **-** | **-** | **-** | **-** | **-** | **-** | **-** | **-** | **-** | **-** | **-** | **-** | **-** | **-** | **-** | **-** | **-** | **Q** | **W** | **Q** | **A** | **K** | **Q** | **E** | **L** | **A** | **E** | **A** | **K** | **K** | **T** | **A** | **T** | **T** | **F** | **L** | **N** | **V** | **L** | **S** | **K** | **Q** | **E** | **F** | **D** | **K** | **L** | **P** | **S** | **V** | **V** | **Q** | **E** | **A** | **S** | **L** | **K** | **K** | **N** | **G** | **Y** | **D** | **T** | **K** | **S** | **V** | **V** | **E** | **K** | **Y** | **Q** | **A** | **I** | **Y** | **S** | **G** | **I** | **Q** | **A** | **E** | **G** | **V** | **K** | **A** | **S** | **D** | **V** | **Q** | **V** | **K** | **K** | **A** | **K** | **D** | **N** | **Q** | **Y** | **T** | **F** | **T** | **Y** | **K** | **L** | **S** | **M** | **S** | **T** | **P** | **L** | **G** | **E** | **M** | **K** | **D** | **L** | **S** | **Y** | **Q** | **S** | **S** | **I** | **A** | **K** | **K** | **G** | **D** | **T** | **Y** | **Q** | **I** | **A** | **W** | **K** | **P** | **S** | **L** | **I** | **F** | **P** | **D** | **M** | **S** | **G** | **N** | **D** | **K** | **I** | **S** | **I** | **Q** | **V** | **D** | **N** | **A** | **K** | **R** | **G** | **E** | **I** | **V** | **D** | **R** | **N** | **G** | **S** | **G** | **L** | **A** | **I** | **N** | **K** | **V** | **F** | **D** | **E** | **V** | **G** | **V** | **V** | **P** | **G** | **K** | **L** | **G** | **S** | **G** | **A** | **E** | **K** | **T** | **A** | **N** | **I** | **K** | **A** | **F** | **S** | **D** | **K** | **F** | **G** | **V** | **S** | **V** | **D** | **E** | **I** | **N** | **Q** | **K** | **L** | **S** | **Q** | **G** | **W** | **V** | **Q** | **A** | **D** | **S** | **F** | **V** | **P** | **I** | **T** | **V** | **A** | **S** | **E** | **P** | **V** | **T** | **E** | **L** | **P** | **T** | **G** | **A** | **A** | **T** | **K** | **D** | **T** | **E** | **S** | **R** | **Y** | **Y** | **P** | **L** | **G** | **E** | **A** | **A** | **A** | **Q** | **L** | **I** | **G** | **Y** | **T** | **G** | **T** | **I** | **T** | **A** | **E** | **D** | **I** | **E** | **K** | **N** | **P** | **E** | **L** | **S** | **S** | **T** | **G** | **V** | **I** | **G** | **K** | **T** | **G** | **L** | **E** | **R** | **A** | **F** | **D** | **K** | **E** | **L** | **R** | **G** | **Q** | **D** | **G** | **G** | **S** | **L** | **V** | **I** | **L** | **D** | **D** | **K** | **E** | **N** | **V** | **K** | **K** | **A** | **L** | **Q** | **T** | **K** | **E** | **K** | **K** | **D** | **G** | **Q** | **T** | **I** | **K** | **L** | **T** | **I** | **D** | **S** | **G** | **V** | **Q** | **Q** | **Q** | **A** | **F** | **A** | **I** | **F** | **D** | **K** | **R** | **P** | **G** | **S** | **A** | **V** | **I** | **T** | **D** | **P** | **Q** | **K** | **G** | **D** | **L** | **L** | **A** | **T** | **V** | **S** | **S** | **P** | **S** | **Y** | **D** | **P** | **N** | **K** | **M** | **A** | **N** | **G** | **I** | **S** | **Q** | **K** | **E** | **Y** | **D** | **A** | **Y** | **N** | **N** | **N** | **K** | **D** | **L** | **P** | **F** | **T** | **A** | **R** | **F** | **A** | **T** | **G** | **Y** | **A** | **P** | **G** | **S** | **T** | **F** | **K** | **T** | **I** | **T** | **G** | **A** | **I** | **G** | **L** | **D** | **A** | **G** | **T** | **L** | **K** | **P** | **D** | **E** | **E** | **L** | **E** | **I** | **N** | **G** | **L** | **K** | **W** | **Q** | **K** | **D** | **K** | **S** | **W** | **G** | **G** | **Y** | **F** | **A** | **T** | **R** | **V** | **K** | **E** | **A** | **S** | **P** | **V** | **N** | **L** | **R** | **T** | **A** | **L** | **V** | **N** | **S** | **D** | **N** | **I** | **Y** | **F** | **A** | **Q** | **Q** | **T** | **L** | **R** | **M** | **G** | **E** | **D** | **K** | **F** | **R** | **A** | **G** | **L** | **N** | **K** | **F** | **I** | **F** | **G** | **E** | **E** | **L** | **D** | **L** | **P** | **I** | **A** | **M** | **T** | **P** | **A** | **Q** | **I** | **S** | **N** | **E** | **D** | **K** | **F** | **N** | **S** | **E** | **I** | **L** | **L** | **A** | **D** | **T** | **G** | **Y** | **G** | **Q** | **G** | **Q** | **L** | **L** | **I** | **S** | **P** | **I** | **Q** | **Q** | **A** | **T** | **M** | **Y** | **S** | **V** | **F** | **Q** | **N** | **N** | **G** | **T** | **L** | **V** | **Y** | **P** | **K** | **I** | **V** | **L** | **D** | **K** | **E** | **T** | **K** | **K** | **K** | **D** | **N** | **V** | **I** | **S** | **A** | **N** | **A** | **A** | **N** | **T** | **I** | **A** | **T** | **D** | **L** | **L** | **G** | **S** | **V** | **E** | **D** | **P** | **S** | **G** | **Y** | **V** | **Y** | **N** | **M** | **Y** | **N** | **P** | **N** | **F** | **S** | **L** | **A** | **A** | **K** | **T** | **G** | **T** | **A** | **E** | **I** | **K** | **D** | **K** | **Q** | **D** | **T** | **D** | **G** | **K** | **E** | **N** | **S** | **F** | **L** | **L** | **T** | **L** | **D** | **R** | **S** | **N** | **N** | **K** | **F** | **L** | **T** | **M** | **I** | **M** | **V** | **E** | **N** | **S** | **G** | **E** | **N** | **G** | **S** | **A** | **T** | **D** | **I** | **S** | **K** | **P** | **L** | **I** | **D** | **Y** | **L** | **E** | **A** | **T** | **I** | **K** | **-** | **-** | **-** | **-** | **-** | **-** | **-** | **-** | **-** | **-** | **-** | **-** | **-** | **-** | **-** | **-** | **-** | **-** | **-** | **-** | **-** | **-** | **-** | **-** | **-** | **-** | **-** | **-** | **-** | **-** | **-** | **-** | **-** | **-** | **-** | **-** | **Q** | **W** | **Q** | **A** | **K** | **Q** | **E** | **L** | **A** | **E** | **A** | **K** | **K** | **T** | **A** | **T** | **T** | **F** | **L** | **N** | **V** | **L** | **S** | **K** | **Q** | **E** | **F** | **D** | **K** | **L** | **P** | **S** | **V** | **V** | **Q** | **E** | **A** | **S** | **L** | **K** | **K** | **N** | **G** | **Y** | **D** | **T** | **K** | **S** | **V** | **V** | **E** | **K** | **Y** | **Q** | **A** | **I** | **Y** | **S** | **G** | **I** | **Q** | **A** | **E** | **G** | **V** | **K** | **A** | **S** | **D** | **V** | **Q** | **V** | **K** | **K** | **A** | **K** | **D** | **N** | **Q** | **Y** | **T** | **F** | **T** | **Y** | **K** | **L** | **S** | **M** | **S** | **T** | **P** | **L** | **G** | **E** | **M** | **K** | **D** | **L** | **S** | **Y** | **Q** | **S** | **S** | **I** | **A** | **K** | **K** | **G** | **D** | **T** | **Y** | **Q** | **I** | **A** | **W** | **K** | **P** | **S** | **L** | **I** | **F** | **P** | **D** | **M** | **S** | **G** | **N** | **D** | **K** | **I** | **S** | **I** | **Q** | **V** | **D** | **N** | **A** | **K** | **R** | **G** | **E** | **I** | **V** | **D** | **R** | **N** | **G** | **S** | **G** | **L** | **A** | **I** | **N** | **K** | **V** | **F** | **D** | **E** | **V** | **G** | **V** | **V** | **P** | **G** | **K** | **L** | **G** | **S** | **G** | **A** | **E** | **K** | **T** | **A** | **N** | **I** | **K** | **A** | **F** | **S** | **D** | **K** | **F** | **G** | **V** | **S** | **V** | **D** | **E** | **I** | **N** | **Q** | **K** | **L** | **S** | **Q** | **G** | **W** | **V** | **Q** | **A** | **D** | **S** | **F** | **V** | **P** | **I** | **T** | **V** | **A** | **S** | **E** | **P** | **V** | **T** | **E** | **L** | **P** | **T** | **G** | **A** | **A** | **T** | **K** | **D** | **T** | **E** | **S** | **R** | **Y** | **Y** | **P** | **L** | **G** | **E** | **A** | **A** | **A** | **Q** | **L** | **I** | **G** | **Y** | **T** | **G** | **T** | **I** | **T** | **A** | **E** | **D** | **I** | **E** | **K** | **N** | **P** | **E** | **L** | **S** | **S** | **T** | **G** | **V** | **I** | **G** | **K** | **T** | **G** | **L** | **E** | **R** | **A** | **F** | **D** | **K** | **E** | **L** | **R** | **G** | **Q** | **D** | **G** | **G** | **S** | **L** | **V** | **I** | **L** | **D** | **D** | **K** | **E** | **N** | **V** | **K** | **K** | **A** | **L** | **Q** | **T** | **K** | **E** | **K** | **K** | **D** | **G** | **Q** | **T** | **I** | **K** | **L** | **T** | **I** | **D** | **S** | **G** | **V** | **Q** | **Q** | **Q** | **A** | **F** | **A** | **I** | **F** | **D** | **K** | **R** | **P** | **G** | **S** | **A** | **V** | **I** | **T** | **D** | **P** | **Q** | **K** | **G** | **D** | **L** | **L** | **A** | **T** | **V** | **S** | **S** | **P** | **S** | **Y** | **D** | **P** | **N** | **K** | **M** | **A** | **N** | **G** | **I** | **S** | **Q** | **K** | **E** | **Y** | **D** | **A** | **Y** | **N** | **N** | **N** | **K** | **D** | **L** | **P** | **F** | **T** | **A** | **R** | **F** | **A** | **T** | **G** | **Y** | **A** | **P** | **G** | **S** | **T** | **F** | **K** | **T** | **I** | **T** | **G** | **A** | **I** | **G** | **L** | **D** | **A** | **G** | **T** | **L** | **K** | **P** | **D** | **E** | **E** | **L** | **E** | **I** | **N** | **G** | **L** | **K** | **W** | **Q** | **K** | **D** | **K** | **S** | **W** | **G** | **G** | **Y** | **F** | **A** | **T** | **R** | **V** | **K** | **E** | **A** | **S** | **P** | **V** | **N** | **L** | **R** | **T** | **A** | **L** | **V** | **N** | **S** | **D** | **N** | **I** | **Y** | **F** | **A** | **Q** | **Q** | **T** | **L** | **R** | **M** | **G** | **E** | **D** | **K** | **F** | **R** | **A** | **G** | **L** | **N** | **K** | **F** | **I** | **F** | **G** | **E** | **E** | **L** | **D** | **L** | **P** | **I** | **A** | **M** | **T** | **P** | **A** | **Q** | **I** | **S** | **N** | **E** | **D** | **K** | **F** | **N** | **S** | **E** | **I** | **L** | **L** | **A** | **D** | **T** | **G** | **Y** | **G** | **Q** | **G** | **Q** | **L** | **L** | **I** | **S** | **P** | **I** | **Q** | **Q** | **A** | **T** | **M** | **Y** | **S** | **V** | **F** | **Q** | **N** | **N** | **G** | **T** | **L** | **V** | **Y** | **P** | **K** | **I** | **V** | **L** | **D** | **K** | **E** | **T** | **K** | **K** | **K** | **D** | **N** | **V** | **I** | **S** | **A** | **N** | **A** | **A** | **N** | **T** | **I** | **A** | **T** | **D** | **L** | **L** | **G** | **S** | **V** | **E** | **D** | **P** | **S** | **G** | **Y** | **V** | **Y** | **N** | **M** | **Y** | **N** | **P** | **N** | **F** | **S** | **L** | **A** | **A** | **K** | **T** | **G** | **T** | **A** | **E** | **I** | **K** | **D** | **K** | **Q** | **D** | **T** | **D** | **G** | **K** | **E** | **N** | **S** | **F** | **L** | **L** | **T** | **L** | **D** | **R** | **S** | **N** | **N** | **K** | **F** | **L** | **T** | **M** | **I** | **M** | **V** | **E** | **N** | **S** | **G** | **E** | **N** | **G** | **S** | **A** | **T** | **D** | **I** | **S** | **K** | **P** | **L** | **I** | **D** | **Y** | **L** | **E** | **A** | **T** | **I** | **K** |
| **N60034F** | **-** | **-** | **-** | **-** | **-** | **-** | **-** | **-** | **-** | **-** | **-** | **-** | **-** | **-** | **-** | **-** | **-** | **-** | **-** | **-** | **-** | **-** | **-** | **-** | **-** | **-** | **-** | **-** | **-** | **-** | **-** | **-** | **-** | **-** | **-** | **-** | **Q** | **W** | **Q** | **A** | **K** | **Q** | **E** | **L** | **A** | **E** | **A** | **K** | **K** | **T** | **A** | **T** | **T** | **F** | **L** | **N** | **V** | **L** | **S** | **K** | **Q** | **E** | **F** | **D** | **K** | **L** | **P** | **S** | **V** | **V** | **Q** | **E** | **A** | **S** | **L** | **K** | **K** | **N** | **G** | **Y** | **D** | **T** | **K** | **S** | **V** | **V** | **E** | **K** | **Y** | **Q** | **A** | **I** | **Y** | **S** | **G** | **I** | **Q** | **A** | **E** | **G** | **V** | **K** | **A** | **S** | **D** | **V** | **Q** | **V** | **K** | **K** | **A** | **K** | **D** | **N** | **Q** | **Y** | **T** | **F** | **T** | **Y** | **K** | **L** | **S** | **M** | **S** | **T** | **P** | **L** | **G** | **E** | **M** | **K** | **D** | **L** | **S** | **Y** | **Q** | **S** | **S** | **I** | **A** | **K** | **K** | **G** | **D** | **T** | **Y** | **Q** | **I** | **A** | **W** | **K** | **P** | **S** | **L** | **I** | **F** | **P** | **D** | **M** | **S** | **G** | **N** | **D** | **K** | **I** | **S** | **I** | **Q** | **V** | **D** | **N** | **A** | **K** | **R** | **G** | **E** | **I** | **V** | **D** | **R** | **N** | **G** | **S** | **G** | **L** | **A** | **I** | **N** | **K** | **V** | **F** | **D** | **E** | **V** | **G** | **V** | **V** | **P** | **G** | **K** | **L** | **G** | **S** | **G** | **A** | **E** | **K** | **T** | **A** | **N** | **I** | **K** | **A** | **F** | **S** | **D** | **K** | **F** | **G** | **V** | **S** | **V** | **D** | **E** | **I** | **N** | **Q** | **K** | **L** | **S** | **Q** | **G** | **W** | **V** | **Q** | **A** | **D** | **S** | **F** | **V** | **P** | **I** | **T** | **V** | **A** | **S** | **E** | **P** | **V** | **T** | **E** | **L** | **P** | **T** | **G** | **A** | **A** | **T** | **K** | **D** | **T** | **E** | **S** | **R** | **Y** | **Y** | **P** | **L** | **G** | **E** | **A** | **A** | **A** | **Q** | **L** | **I** | **G** | **Y** | **T** | **G** | **T** | **I** | **T** | **A** | **E** | **D** | **I** | **E** | **K** | **N** | **P** | **E** | **L** | **S** | **S** | **T** | **G** | **V** | **I** | **G** | **K** | **T** | **G** | **L** | **E** | **R** | **A** | **F** | **D** | **K** | **E** | **L** | **R** | **G** | **Q** | **D** | **G** | **G** | **S** | **L** | **V** | **I** | **L** | **D** | **D** | **K** | **E** | **N** | **V** | **K** | **K** | **A** | **L** | **Q** | **T** | **K** | **E** | **K** | **K** | **D** | **G** | **Q** | **T** | **I** | **K** | **L** | **T** | **I** | **D** | **S** | **G** | **V** | **Q** | **Q** | **Q** | **A** | **F** | **A** | **I** | **F** | **D** | **K** | **R** | **P** | **G** | **S** | **A** | **V** | **I** | **T** | **D** | **P** | **Q** | **K** | **G** | **D** | **L** | **L** | **A** | **T** | **V** | **S** | **S** | **P** | **S** | **Y** | **D** | **P** | **N** | **K** | **M** | **A** | **N** | **G** | **I** | **S** | **Q** | **K** | **E** | **Y** | **D** | **A** | **Y** | **N** | **N** | **N** | **K** | **D** | **L** | **P** | **F** | **T** | **A** | **R** | **F** | **A** | **T** | **G** | **Y** | **A** | **P** | **G** | **S** | **T** | **F** | **K** | **T** | **I** | **T** | **G** | **A** | **I** | **G** | **L** | **D** | **A** | **G** | **T** | **L** | **K** | **P** | **D** | **E** | **E** | **L** | **E** | **I** | **N** | **G** | **L** | **K** | **W** | **Q** | **K** | **D** | **K** | **S** | **W** | **G** | **G** | **Y** | **F** | **A** | **T** | **R** | **V** | **K** | **E** | **A** | **S** | **P** | **V** | **N** | **L** | **R** | **T** | **A** | **L** | **V** | **N** | **S** | **D** | **N** | **I** | **Y** | **F** | **A** | **Q** | **Q** | **T** | **L** | **R** | **M** | **G** | **E** | **D** | **K** | **F** | **R** | **A** | **G** | **L** | **N** | **K** | **F** | **I** | **F** | **G** | **E** | **E** | **L** | **D** | **L** | **P** | **I** | **A** | **M** | **T** | **P** | **A** | **Q** | **I** | **S** | **N** | **E** | **D** | **K** | **F** | **N** | **S** | **E** | **I** | **L** | **L** | **A** | **D** | **T** | **G** | **Y** | **G** | **Q** | **G** | **Q** | **L** | **L** | **I** | **S** | **P** | **I** | **Q** | **Q** | **A** | **T** | **M** | **Y** | **S** | **V** | **F** | **Q** | **N** | **N** | **G** | **T** | **L** | **V** | **Y** | **P** | **K** | **L** | **V** | **L** | **D** | **K** | **E** | **T** | **K** | **K** | **K** | **D** | **N** | **V** | **I** | **S** | **A** | **N** | **A** | **A** | **N** | **T** | **I** | **A** | **T** | **D** | **L** | **L** | **G** | **S** | **V** | **E** | **D** | **P** | **S** | **G** | **Y** | **V** | **Y** | **N** | **M** | **Y** | **N** | **P** | **N** | **F** | **S** | **L** | **A** | **A** | **K** | **T** | **G** | **T** | **A** | **E** | **I** | **K** | **D** | **K** | **Q** | **D** | **T** | **D** | **G** | **K** | **E** | **N** | **S** | **F** | **L** | **L** | **T** | **L** | **D** | **R** | **S** | **N** | **N** | **K** | **F** | **L** | **T** | **M** | **I** | **M** | **V** | **E** | **N** | **S** | **G** | **E** | **N** | **G** | **S** | **A** | **T** | **D** | **I** | **S** | **K** | **P** | **L** | **I** | **D** | **Y** | **L** | **E** | **A** | **T** | **I** | **K** | **-** | **-** | **-** | **-** | **-** | **-** | **-** | **-** | **-** | **-** | **-** | **-** | **-** | **-** | **-** | **-** | **-** | **-** | **-** | **-** | **-** | **-** | **-** | **-** | **-** | **-** | **-** | **-** | **-** | **-** | **-** | **-** | **-** | **-** | **-** | **-** | **Q** | **W** | **Q** | **A** | **K** | **Q** | **E** | **L** | **A** | **E** | **A** | **K** | **K** | **T** | **A** | **T** | **T** | **F** | **L** | **N** | **V** | **L** | **S** | **K** | **Q** | **E** | **F** | **D** | **K** | **L** | **P** | **S** | **V** | **V** | **Q** | **E** | **A** | **S** | **L** | **K** | **K** | **N** | **G** | **Y** | **D** | **T** | **K** | **S** | **V** | **V** | **E** | **K** | **Y** | **Q** | **A** | **I** | **Y** | **S** | **G** | **I** | **Q** | **A** | **E** | **G** | **V** | **K** | **A** | **S** | **D** | **V** | **Q** | **V** | **K** | **K** | **A** | **K** | **D** | **N** | **Q** | **Y** | **T** | **F** | **T** | **Y** | **K** | **L** | **S** | **M** | **S** | **T** | **P** | **L** | **G** | **E** | **M** | **K** | **D** | **L** | **S** | **Y** | **Q** | **S** | **S** | **I** | **A** | **K** | **K** | **G** | **D** | **T** | **Y** | **Q** | **I** | **A** | **W** | **K** | **P** | **S** | **L** | **I** | **F** | **P** | **D** | **M** | **S** | **G** | **N** | **D** | **K** | **I** | **S** | **I** | **Q** | **V** | **D** | **N** | **A** | **K** | **R** | **G** | **E** | **I** | **V** | **D** | **R** | **N** | **G** | **S** | **G** | **L** | **A** | **I** | **N** | **K** | **V** | **F** | **D** | **E** | **V** | **G** | **V** | **V** | **P** | **G** | **K** | **L** | **G** | **S** | **G** | **A** | **E** | **K** | **T** | **A** | **N** | **I** | **K** | **A** | **F** | **S** | **D** | **K** | **F** | **G** | **V** | **S** | **V** | **D** | **E** | **I** | **N** | **Q** | **K** | **L** | **S** | **Q** | **G** | **W** | **V** | **Q** | **A** | **D** | **S** | **F** | **V** | **P** | **I** | **T** | **V** | **A** | **S** | **E** | **P** | **V** | **T** | **E** | **L** | **P** | **T** | **G** | **A** | **A** | **T** | **K** | **D** | **T** | **E** | **S** | **R** | **Y** | **Y** | **P** | **L** | **G** | **E** | **A** | **A** | **A** | **Q** | **L** | **I** | **G** | **Y** | **T** | **G** | **T** | **I** | **T** | **A** | **E** | **D** | **I** | **E** | **K** | **N** | **P** | **E** | **L** | **S** | **S** | **T** | **G** | **V** | **I** | **G** | **K** | **T** | **G** | **L** | **E** | **R** | **A** | **F** | **D** | **K** | **E** | **L** | **R** | **G** | **Q** | **D** | **G** | **G** | **S** | **L** | **V** | **I** | **L** | **D** | **D** | **K** | **E** | **N** | **V** | **K** | **K** | **A** | **L** | **Q** | **T** | **K** | **E** | **K** | **K** | **D** | **G** | **Q** | **T** | **I** | **K** | **L** | **T** | **I** | **D** | **S** | **G** | **V** | **Q** | **Q** | **Q** | **A** | **F** | **A** | **I** | **F** | **D** | **K** | **R** | **P** | **G** | **S** | **A** | **V** | **I** | **T** | **D** | **P** | **Q** | **K** | **G** | **D** | **L** | **L** | **A** | **T** | **V** | **S** | **S** | **P** | **S** | **Y** | **D** | **P** | **N** | **K** | **M** | **A** | **N** | **G** | **I** | **S** | **Q** | **K** | **E** | **Y** | **D** | **A** | **Y** | **N** | **N** | **N** | **K** | **D** | **L** | **P** | **F** | **T** | **A** | **R** | **F** | **A** | **T** | **G** | **Y** | **A** | **P** | **G** | **S** | **T** | **F** | **K** | **T** | **I** | **T** | **G** | **A** | **I** | **G** | **L** | **D** | **A** | **G** | **T** | **L** | **K** | **P** | **D** | **E** | **E** | **L** | **E** | **I** | **N** | **G** | **L** | **K** | **W** | **Q** | **K** | **D** | **K** | **S** | **W** | **G** | **G** | **Y** | **F** | **A** | **T** | **R** | **V** | **K** | **E** | **A** | **S** | **P** | **V** | **N** | **L** | **R** | **T** | **A** | **L** | **V** | **N** | **S** | **D** | **N** | **I** | **Y** | **F** | **A** | **Q** | **Q** | **T** | **L** | **R** | **M** | **G** | **E** | **D** | **K** | **F** | **R** | **A** | **G** | **L** | **N** | **K** | **F** | **I** | **F** | **G** | **E** | **E** | **L** | **D** | **L** | **P** | **I** | **A** | **M** | **T** | **P** | **A** | **Q** | **I** | **S** | **N** | **E** | **D** | **K** | **F** | **N** | **S** | **E** | **I** | **L** | **L** | **A** | **D** | **T** | **G** | **Y** | **G** | **Q** | **G** | **Q** | **L** | **L** | **I** | **S** | **P** | **I** | **Q** | **Q** | **A** | **T** | **M** | **Y** | **S** | **V** | **F** | **Q** | **N** | **N** | **G** | **T** | **L** | **V** | **Y** | **P** | **K** | **L** | **V** | **L** | **D** | **K** | **E** | **T** | **K** | **K** | **K** | **D** | **N** | **V** | **I** | **S** | **A** | **N** | **A** | **A** | **N** | **T** | **I** | **A** | **T** | **D** | **L** | **L** | **G** | **S** | **V** | **E** | **D** | **P** | **S** | **G** | **Y** | **V** | **Y** | **N** | **M** | **Y** | **N** | **P** | **N** | **F** | **S** | **L** | **A** | **A** | **K** | **T** | **G** | **T** | **A** | **E** | **I** | **K** | **D** | **K** | **Q** | **D** | **T** | **D** | **G** | **K** | **E** | **N** | **S** | **F** | **L** | **L** | **T** | **L** | **D** | **R** | **S** | **N** | **N** | **K** | **F** | **L** | **T** | **M** | **I** | **M** | **V** | **E** | **N** | **S** | **G** | **E** | **N** | **G** | **S** | **A** | **T** | **D** | **I** | **S** | **K** | **P** | **L** | **I** | **D** | **Y** | **L** | **E** | **A** | **T** | **I** | **K** |
| **N55185** | **-** | **-** | **-** | **-** | **-** | **-** | **-** | **-** | **-** | **-** | **-** | **-** | **-** | **-** | **-** | **-** | **-** | **-** | **-** | **-** | **-** | **-** | **-** | **-** | **-** | **-** | **-** | **-** | **-** | **-** | **-** | **-** | **-** | **-** | **-** | **-** | **Q** | **W** | **Q** | **A** | **K** | **Q** | **E** | **L** | **A** | **E** | **A** | **K** | **K** | **T** | **A** | **T** | **T** | **F** | **L** | **N** | **V** | **L** | **S** | **K** | **Q** | **E** | **F** | **D** | **K** | **L** | **P** | **S** | **V** | **I** | **Q** | **E** | **A** | **S** | **L** | **K** | **K** | **N** | **G** | **Y** | **D** | **T** | **K** | **S** | **V** | **V** | **E** | **K** | **Y** | **Q** | **A** | **I** | **Y** | **S** | **G** | **I** | **Q** | **A** | **E** | **G** | **V** | **K** | **A** | **S** | **D** | **V** | **Q** | **V** | **K** | **K** | **A** | **K** | **D** | **N** | **Q** | **Y** | **T** | **F** | **T** | **Y** | **K** | **L** | **S** | **M** | **S** | **T** | **P** | **L** | **G** | **E** | **M** | **K** | **D** | **L** | **S** | **Y** | **Q** | **S** | **S** | **I** | **A** | **K** | **K** | **G** | **D** | **T** | **Y** | **Q** | **I** | **A** | **W** | **K** | **P** | **S** | **L** | **I** | **F** | **P** | **D** | **M** | **S** | **G** | **N** | **D** | **K** | **I** | **S** | **I** | **Q** | **V** | **D** | **N** | **A** | **K** | **R** | **G** | **E** | **I** | **V** | **D** | **R** | **N** | **G** | **S** | **G** | **L** | **A** | **I** | **N** | **K** | **V** | **F** | **D** | **E** | **V** | **G** | **V** | **V** | **P** | **G** | **K** | **L** | **G** | **S** | **G** | **A** | **E** | **K** | **T** | **A** | **N** | **I** | **K** | **A** | **F** | **S** | **D** | **K** | **F** | **G** | **V** | **S** | **V** | **D** | **E** | **I** | **N** | **Q** | **K** | **L** | **S** | **Q** | **G** | **W** | **V** | **Q** | **A** | **D** | **S** | **F** | **V** | **P** | **I** | **T** | **V** | **A** | **S** | **E** | **P** | **V** | **T** | **E** | **L** | **P** | **T** | **G** | **A** | **A** | **T** | **K** | **D** | **T** | **E** | **S** | **R** | **Y** | **Y** | **P** | **L** | **G** | **E** | **A** | **A** | **A** | **Q** | **L** | **I** | **G** | **Y** | **T** | **G** | **T** | **I** | **T** | **A** | **E** | **D** | **I** | **E** | **K** | **N** | **P** | **E** | **L** | **S** | **S** | **T** | **G** | **V** | **I** | **G** | **K** | **T** | **G** | **L** | **E** | **R** | **A** | **F** | **D** | **K** | **E** | **L** | **R** | **G** | **Q** | **D** | **G** | **G** | **S** | **L** | **V** | **I** | **L** | **D** | **D** | **K** | **E** | **N** | **V** | **K** | **K** | **A** | **L** | **Q** | **T** | **K** | **E** | **K** | **K** | **D** | **G** | **Q** | **T** | **I** | **K** | **L** | **T** | **I** | **D** | **S** | **G** | **V** | **Q** | **Q** | **Q** | **A** | **F** | **A** | **I** | **F** | **D** | **K** | **R** | **P** | **G** | **S** | **A** | **V** | **I** | **T** | **D** | **P** | **Q** | **K** | **G** | **D** | **L** | **L** | **A** | **T** | **V** | **S** | **S** | **P** | **S** | **Y** | **D** | **P** | **N** | **K** | **M** | **A** | **N** | **G** | **I** | **S** | **Q** | **K** | **E** | **Y** | **D** | **A** | **Y** | **N** | **N** | **N** | **K** | **D** | **L** | **P** | **F** | **T** | **A** | **R** | **F** | **A** | **T** | **G** | **Y** | **A** | **P** | **G** | **S** | **T** | **F** | **K** | **T** | **I** | **T** | **G** | **A** | **I** | **G** | **L** | **D** | **A** | **G** | **T** | **L** | **K** | **P** | **D** | **E** | **E** | **L** | **E** | **I** | **N** | **G** | **L** | **K** | **W** | **Q** | **K** | **D** | **K** | **S** | **W** | **G** | **G** | **Y** | **F** | **A** | **T** | **R** | **V** | **K** | **E** | **A** | **S** | **P** | **V** | **N** | **L** | **R** | **T** | **A** | **L** | **V** | **N** | **S** | **D** | **N** | **I** | **Y** | **F** | **A** | **Q** | **Q** | **T** | **L** | **R** | **M** | **G** | **E** | **D** | **K** | **F** | **R** | **A** | **G** | **L** | **N** | **K** | **F** | **I** | **F** | **G** | **E** | **E** | **L** | **D** | **L** | **P** | **I** | **A** | **M** | **T** | **P** | **A** | **Q** | **I** | **S** | **N** | **E** | **D** | **K** | **F** | **N** | **S** | **E** | **I** | **L** | **L** | **A** | **D** | **T** | **G** | **Y** | **G** | **Q** | **G** | **Q** | **L** | **L** | **I** | **S** | **P** | **I** | **Q** | **Q** | **A** | **T** | **M** | **Y** | **S** | **V** | **F** | **Q** | **N** | **N** | **G** | **T** | **L** | **V** | **Y** | **P** | **K** | **L** | **V** | **L** | **D** | **K** | **E** | **T** | **K** | **K** | **K** | **D** | **N** | **V** | **I** | **S** | **A** | **N** | **A** | **A** | **N** | **T** | **I** | **A** | **T** | **D** | **L** | **L** | **G** | **S** | **V** | **E** | **D** | **P** | **S** | **G** | **Y** | **V** | **Y** | **N** | **M** | **Y** | **N** | **P** | **N** | **F** | **S** | **L** | **A** | **A** | **K** | **T** | **G** | **T** | **A** | **E** | **I** | **K** | **D** | **K** | **Q** | **D** | **T** | **D** | **G** | **K** | **E** | **N** | **S** | **F** | **L** | **L** | **T** | **L** | **D** | **R** | **S** | **N** | **N** | **K** | **F** | **L** | **T** | **M** | **I** | **M** | **V** | **E** | **N** | **S** | **G** | **E** | **N** | **G** | **S** | **A** | **T** | **D** | **I** | **S** | **K** | **P** | **L** | **I** | **D** | **Y** | **L** | **E** | **A** | **T** | **I** | **K** | **-** | **-** | **-** | **-** | **-** | **-** | **-** | **-** | **-** | **-** | **-** | **-** | **-** | **-** | **-** | **-** | **-** | **-** | **-** | **-** | **-** | **-** | **-** | **-** | **-** | **-** | **-** | **-** | **-** | **-** | **-** | **-** | **-** | **-** | **-** | **-** | **Q** | **W** | **Q** | **A** | **K** | **Q** | **E** | **L** | **A** | **E** | **A** | **K** | **K** | **T** | **A** | **T** | **T** | **F** | **L** | **N** | **V** | **L** | **S** | **K** | **Q** | **E** | **F** | **D** | **K** | **L** | **P** | **S** | **V** | **I** | **Q** | **E** | **A** | **S** | **L** | **K** | **K** | **N** | **G** | **Y** | **D** | **T** | **K** | **S** | **V** | **V** | **E** | **K** | **Y** | **Q** | **A** | **I** | **Y** | **S** | **G** | **I** | **Q** | **A** | **E** | **G** | **V** | **K** | **A** | **S** | **D** | **V** | **Q** | **V** | **K** | **K** | **A** | **K** | **D** | **N** | **Q** | **Y** | **T** | **F** | **T** | **Y** | **K** | **L** | **S** | **M** | **S** | **T** | **P** | **L** | **G** | **E** | **M** | **K** | **D** | **L** | **S** | **Y** | **Q** | **S** | **S** | **I** | **A** | **K** | **K** | **G** | **D** | **T** | **Y** | **Q** | **I** | **A** | **W** | **K** | **P** | **S** | **L** | **I** | **F** | **P** | **D** | **M** | **S** | **G** | **N** | **D** | **K** | **I** | **S** | **I** | **Q** | **V** | **D** | **N** | **A** | **K** | **R** | **G** | **E** | **I** | **V** | **D** | **R** | **N** | **G** | **S** | **G** | **L** | **A** | **I** | **N** | **K** | **V** | **F** | **D** | **E** | **V** | **G** | **V** | **V** | **P** | **G** | **K** | **L** | **G** | **S** | **G** | **A** | **E** | **K** | **T** | **A** | **N** | **I** | **K** | **A** | **F** | **S** | **D** | **K** | **F** | **G** | **V** | **S** | **V** | **D** | **E** | **I** | **N** | **Q** | **K** | **L** | **S** | **Q** | **G** | **W** | **V** | **Q** | **A** | **D** | **S** | **F** | **V** | **P** | **I** | **T** | **V** | **A** | **S** | **E** | **P** | **V** | **T** | **E** | **L** | **P** | **T** | **G** | **A** | **A** | **T** | **K** | **D** | **T** | **E** | **S** | **R** | **Y** | **Y** | **P** | **L** | **G** | **E** | **A** | **A** | **A** | **Q** | **L** | **I** | **G** | **Y** | **T** | **G** | **T** | **I** | **T** | **A** | **E** | **D** | **I** | **E** | **K** | **N** | **P** | **E** | **L** | **S** | **S** | **T** | **G** | **V** | **I** | **G** | **K** | **T** | **G** | **L** | **E** | **R** | **A** | **F** | **D** | **K** | **E** | **L** | **R** | **G** | **Q** | **D** | **G** | **G** | **S** | **L** | **V** | **I** | **L** | **D** | **D** | **K** | **E** | **N** | **V** | **K** | **K** | **A** | **L** | **Q** | **T** | **K** | **E** | **K** | **K** | **D** | **G** | **Q** | **T** | **I** | **K** | **L** | **T** | **I** | **D** | **S** | **G** | **V** | **Q** | **Q** | **Q** | **A** | **F** | **A** | **I** | **F** | **D** | **K** | **R** | **P** | **G** | **S** | **A** | **V** | **I** | **T** | **D** | **P** | **Q** | **K** | **G** | **D** | **L** | **L** | **A** | **T** | **V** | **S** | **S** | **P** | **S** | **Y** | **D** | **P** | **N** | **K** | **M** | **A** | **N** | **G** | **I** | **S** | **Q** | **K** | **E** | **Y** | **D** | **A** | **Y** | **N** | **N** | **N** | **K** | **D** | **L** | **P** | **F** | **T** | **A** | **R** | **F** | **A** | **T** | **G** | **Y** | **A** | **P** | **G** | **S** | **T** | **F** | **K** | **T** | **I** | **T** | **G** | **A** | **I** | **G** | **L** | **D** | **A** | **G** | **T** | **L** | **K** | **P** | **D** | **E** | **E** | **L** | **E** | **I** | **N** | **G** | **L** | **K** | **W** | **Q** | **K** | **D** | **K** | **S** | **W** | **G** | **G** | **Y** | **F** | **A** | **T** | **R** | **V** | **K** | **E** | **A** | **S** | **P** | **V** | **N** | **L** | **R** | **T** | **A** | **L** | **V** | **N** | **S** | **D** | **N** | **I** | **Y** | **F** | **A** | **Q** | **Q** | **T** | **L** | **R** | **M** | **G** | **E** | **D** | **K** | **F** | **R** | **A** | **G** | **L** | **N** | **K** | **F** | **I** | **F** | **G** | **E** | **E** | **L** | **D** | **L** | **P** | **I** | **A** | **M** | **T** | **P** | **A** | **Q** | **I** | **S** | **N** | **E** | **D** | **K** | **F** | **N** | **S** | **E** | **I** | **L** | **L** | **A** | **D** | **T** | **G** | **Y** | **G** | **Q** | **G** | **Q** | **L** | **L** | **I** | **S** | **P** | **I** | **Q** | **Q** | **A** | **T** | **M** | **Y** | **S** | **V** | **F** | **Q** | **N** | **N** | **G** | **T** | **L** | **V** | **Y** | **P** | **K** | **L** | **V** | **L** | **D** | **K** | **E** | **T** | **K** | **K** | **K** | **D** | **N** | **V** | **I** | **S** | **A** | **N** | **A** | **A** | **N** | **T** | **I** | **A** | **T** | **D** | **L** | **L** | **G** | **S** | **V** | **E** | **D** | **P** | **S** | **G** | **Y** | **V** | **Y** | **N** | **M** | **Y** | **N** | **P** | **N** | **F** | **S** | **L** | **A** | **A** | **K** | **T** | **G** | **T** | **A** | **E** | **I** | **K** | **D** | **K** | **Q** | **D** | **T** | **D** | **G** | **K** | **E** | **N** | **S** | **F** | **L** | **L** | **T** | **L** | **D** | **R** | **S** | **N** | **N** | **K** | **F** | **L** | **T** | **M** | **I** | **M** | **V** | **E** | **N** | **S** | **G** | **E** | **N** | **G** | **S** | **A** | **T** | **D** | **I** | **S** | **K** | **P** | **L** | **I** | **D** | **Y** | **L** | **E** | **A** | **T** | **I** | **K** |
| **N60280F** | **-** | **-** | **-** | **-** | **-** | **-** | **-** | **-** | **-** | **-** | **-** | **-** | **-** | **-** | **-** | **-** | **-** | **-** | **-** | **-** | **-** | **-** | **-** | **-** | **-** | **-** | **-** | **-** | **-** | **-** | **-** | **-** | **-** | **-** | **-** | **-** | **Q** | **W** | **Q** | **A** | **K** | **Q** | **E** | **L** | **A** | **E** | **A** | **K** | **K** | **T** | **A** | **T** | **T** | **F** | **L** | **N** | **V** | **L** | **S** | **K** | **Q** | **E** | **F** | **D** | **K** | **L** | **P** | **S** | **V** | **V** | **Q** | **E** | **A** | **S** | **L** | **K** | **K** | **N** | **G** | **Y** | **D** | **T** | **K** | **S** | **V** | **V** | **E** | **K** | **Y** | **Q** | **A** | **I** | **Y** | **S** | **G** | **I** | **Q** | **A** | **E** | **G** | **V** | **K** | **A** | **S** | **D** | **V** | **Q** | **V** | **K** | **K** | **A** | **K** | **D** | **N** | **Q** | **Y** | **T** | **F** | **T** | **Y** | **K** | **L** | **S** | **M** | **S** | **T** | **P** | **L** | **G** | **E** | **M** | **K** | **D** | **L** | **S** | **Y** | **Q** | **S** | **S** | **I** | **A** | **K** | **K** | **G** | **D** | **T** | **Y** | **Q** | **I** | **A** | **W** | **K** | **P** | **S** | **L** | **I** | **F** | **P** | **D** | **M** | **S** | **G** | **N** | **D** | **K** | **I** | **S** | **I** | **Q** | **V** | **D** | **N** | **A** | **K** | **R** | **G** | **E** | **I** | **V** | **D** | **R** | **N** | **G** | **S** | **G** | **L** | **A** | **I** | **N** | **K** | **V** | **F** | **D** | **E** | **V** | **G** | **V** | **V** | **P** | **G** | **K** | **L** | **G** | **S** | **G** | **A** | **E** | **K** | **T** | **A** | **N** | **I** | **K** | **A** | **F** | **S** | **D** | **K** | **F** | **G** | **V** | **S** | **V** | **D** | **E** | **I** | **N** | **Q** | **K** | **L** | **S** | **Q** | **G** | **W** | **V** | **Q** | **A** | **D** | **S** | **F** | **V** | **P** | **I** | **T** | **V** | **A** | **S** | **E** | **P** | **V** | **T** | **E** | **L** | **P** | **T** | **G** | **A** | **A** | **T** | **K** | **D** | **T** | **E** | **S** | **R** | **Y** | **Y** | **P** | **L** | **G** | **E** | **A** | **A** | **A** | **Q** | **L** | **I** | **G** | **Y** | **T** | **G** | **T** | **I** | **T** | **A** | **E** | **D** | **I** | **E** | **K** | **N** | **P** | **E** | **L** | **S** | **S** | **T** | **G** | **V** | **I** | **G** | **K** | **T** | **G** | **L** | **E** | **R** | **A** | **F** | **D** | **K** | **E** | **L** | **R** | **G** | **Q** | **D** | **G** | **G** | **S** | **L** | **V** | **I** | **L** | **D** | **D** | **K** | **E** | **N** | **V** | **K** | **K** | **A** | **L** | **Q** | **T** | **K** | **E** | **K** | **K** | **D** | **G** | **Q** | **T** | **I** | **K** | **L** | **T** | **I** | **D** | **S** | **G** | **V** | **Q** | **Q** | **Q** | **A** | **F** | **A** | **I** | **F** | **D** | **K** | **R** | **P** | **G** | **S** | **A** | **V** | **I** | **T** | **D** | **P** | **Q** | **K** | **G** | **D** | **L** | **L** | **A** | **T** | **V** | **S** | **S** | **P** | **S** | **Y** | **D** | **P** | **N** | **K** | **M** | **A** | **N** | **G** | **I** | **S** | **Q** | **K** | **E** | **Y** | **D** | **A** | **Y** | **N** | **N** | **N** | **K** | **D** | **L** | **P** | **F** | **T** | **A** | **R** | **F** | **A** | **T** | **G** | **Y** | **A** | **P** | **G** | **S** | **T** | **F** | **K** | **T** | **I** | **T** | **G** | **A** | **I** | **G** | **L** | **D** | **A** | **G** | **T** | **L** | **K** | **P** | **D** | **E** | **E** | **L** | **E** | **I** | **N** | **G** | **L** | **K** | **W** | **Q** | **K** | **D** | **K** | **S** | **W** | **G** | **G** | **Y** | **F** | **A** | **T** | **R** | **V** | **K** | **E** | **A** | **S** | **P** | **V** | **N** | **L** | **R** | **T** | **A** | **L** | **V** | **N** | **S** | **D** | **N** | **I** | **Y** | **F** | **A** | **Q** | **Q** | **T** | **L** | **R** | **M** | **G** | **E** | **D** | **K** | **F** | **R** | **A** | **G** | **L** | **N** | **K** | **F** | **I** | **F** | **G** | **E** | **E** | **L** | **D** | **L** | **P** | **I** | **A** | **M** | **T** | **P** | **A** | **Q** | **I** | **S** | **N** | **E** | **D** | **K** | **F** | **N** | **S** | **E** | **I** | **L** | **L** | **A** | **D** | **T** | **G** | **Y** | **G** | **Q** | **G** | **Q** | **L** | **L** | **I** | **S** | **P** | **I** | **Q** | **Q** | **A** | **T** | **M** | **Y** | **S** | **V** | **F** | **Q** | **N** | **N** | **G** | **T** | **L** | **V** | **Y** | **P** | **K** | **L** | **V** | **L** | **D** | **K** | **E** | **T** | **K** | **K** | **K** | **D** | **N** | **V** | **I** | **S** | **A** | **N** | **A** | **A** | **N** | **T** | **I** | **A** | **T** | **D** | **L** | **L** | **G** | **S** | **V** | **E** | **D** | **P** | **S** | **G** | **Y** | **V** | **Y** | **N** | **M** | **Y** | **N** | **P** | **N** | **F** | **S** | **L** | **A** | **A** | **K** | **T** | **G** | **T** | **A** | **E** | **I** | **K** | **D** | **K** | **Q** | **D** | **T** | **D** | **G** | **K** | **E** | **N** | **S** | **F** | **L** | **L** | **T** | **L** | **D** | **R** | **S** | **N** | **N** | **K** | **F** | **L** | **T** | **M** | **I** | **M** | **V** | **E** | **N** | **S** | **G** | **E** | **N** | **G** | **S** | **A** | **T** | **D** | **I** | **S** | **K** | **P** | **L** | **I** | **D** | **Y** | **L** | **E** | **A** | **T** | **I** | **K** | **-** | **-** | **-** | **-** | **-** | **-** | **-** | **-** | **-** | **-** | **-** | **-** | **-** | **-** | **-** | **-** | **-** | **-** | **-** | **-** | **-** | **-** | **-** | **-** | **-** | **-** | **-** | **-** | **-** | **-** | **-** | **-** | **-** | **-** | **-** | **-** | **Q** | **W** | **Q** | **A** | **K** | **Q** | **E** | **L** | **A** | **E** | **A** | **K** | **K** | **T** | **A** | **T** | **T** | **F** | **L** | **N** | **V** | **L** | **S** | **K** | **Q** | **E** | **F** | **D** | **K** | **L** | **P** | **S** | **V** | **V** | **Q** | **E** | **A** | **S** | **L** | **K** | **K** | **N** | **G** | **Y** | **D** | **T** | **K** | **S** | **V** | **V** | **E** | **K** | **Y** | **Q** | **A** | **I** | **Y** | **S** | **G** | **I** | **Q** | **A** | **E** | **G** | **V** | **K** | **A** | **S** | **D** | **V** | **Q** | **V** | **K** | **K** | **A** | **K** | **D** | **N** | **Q** | **Y** | **T** | **F** | **T** | **Y** | **K** | **L** | **S** | **M** | **S** | **T** | **P** | **L** | **G** | **E** | **M** | **K** | **D** | **L** | **S** | **Y** | **Q** | **S** | **S** | **I** | **A** | **K** | **K** | **G** | **D** | **T** | **Y** | **Q** | **I** | **A** | **W** | **K** | **P** | **S** | **L** | **I** | **F** | **P** | **D** | **M** | **S** | **G** | **N** | **D** | **K** | **I** | **S** | **I** | **Q** | **V** | **D** | **N** | **A** | **K** | **R** | **G** | **E** | **I** | **V** | **D** | **R** | **N** | **G** | **S** | **G** | **L** | **A** | **I** | **N** | **K** | **V** | **F** | **D** | **E** | **V** | **G** | **V** | **V** | **P** | **G** | **K** | **L** | **G** | **S** | **G** | **A** | **E** | **K** | **T** | **A** | **N** | **I** | **K** | **A** | **F** | **S** | **D** | **K** | **F** | **G** | **V** | **S** | **V** | **D** | **E** | **I** | **N** | **Q** | **K** | **L** | **S** | **Q** | **G** | **W** | **V** | **Q** | **A** | **D** | **S** | **F** | **V** | **P** | **I** | **T** | **V** | **A** | **S** | **E** | **P** | **V** | **T** | **E** | **L** | **P** | **T** | **G** | **A** | **A** | **T** | **K** | **D** | **T** | **E** | **S** | **R** | **Y** | **Y** | **P** | **L** | **G** | **E** | **A** | **A** | **A** | **Q** | **L** | **I** | **G** | **Y** | **T** | **G** | **T** | **I** | **T** | **A** | **E** | **D** | **I** | **E** | **K** | **N** | **P** | **E** | **L** | **S** | **S** | **T** | **G** | **V** | **I** | **G** | **K** | **T** | **G** | **L** | **E** | **R** | **A** | **F** | **D** | **K** | **E** | **L** | **R** | **G** | **Q** | **D** | **G** | **G** | **S** | **L** | **V** | **I** | **L** | **D** | **D** | **K** | **E** | **N** | **V** | **K** | **K** | **A** | **L** | **Q** | **T** | **K** | **E** | **K** | **K** | **D** | **G** | **Q** | **T** | **I** | **K** | **L** | **T** | **I** | **D** | **S** | **G** | **V** | **Q** | **Q** | **Q** | **A** | **F** | **A** | **I** | **F** | **D** | **K** | **R** | **P** | **G** | **S** | **A** | **V** | **I** | **T** | **D** | **P** | **Q** | **K** | **G** | **D** | **L** | **L** | **A** | **T** | **V** | **S** | **S** | **P** | **S** | **Y** | **D** | **P** | **N** | **K** | **M** | **A** | **N** | **G** | **I** | **S** | **Q** | **K** | **E** | **Y** | **D** | **A** | **Y** | **N** | **N** | **N** | **K** | **D** | **L** | **P** | **F** | **T** | **A** | **R** | **F** | **A** | **T** | **G** | **Y** | **A** | **P** | **G** | **S** | **T** | **F** | **K** | **T** | **I** | **T** | **G** | **A** | **I** | **G** | **L** | **D** | **A** | **G** | **T** | **L** | **K** | **P** | **D** | **E** | **E** | **L** | **E** | **I** | **N** | **G** | **L** | **K** | **W** | **Q** | **K** | **D** | **K** | **S** | **W** | **G** | **G** | **Y** | **F** | **A** | **T** | **R** | **V** | **K** | **E** | **A** | **S** | **P** | **V** | **N** | **L** | **R** | **T** | **A** | **L** | **V** | **N** | **S** | **D** | **N** | **I** | **Y** | **F** | **A** | **Q** | **Q** | **T** | **L** | **R** | **M** | **G** | **E** | **D** | **K** | **F** | **R** | **A** | **G** | **L** | **N** | **K** | **F** | **I** | **F** | **G** | **E** | **E** | **L** | **D** | **L** | **P** | **I** | **A** | **M** | **T** | **P** | **A** | **Q** | **I** | **S** | **N** | **E** | **D** | **K** | **F** | **N** | **S** | **E** | **I** | **L** | **L** | **A** | **D** | **T** | **G** | **Y** | **G** | **Q** | **G** | **Q** | **L** | **L** | **I** | **S** | **P** | **I** | **Q** | **Q** | **A** | **T** | **M** | **Y** | **S** | **V** | **F** | **Q** | **N** | **N** | **G** | **T** | **L** | **V** | **Y** | **P** | **K** | **L** | **V** | **L** | **D** | **K** | **E** | **T** | **K** | **K** | **K** | **D** | **N** | **V** | **I** | **S** | **A** | **N** | **A** | **A** | **N** | **T** | **I** | **A** | **T** | **D** | **L** | **L** | **G** | **S** | **V** | **E** | **D** | **P** | **S** | **G** | **Y** | **V** | **Y** | **N** | **M** | **Y** | **N** | **P** | **N** | **F** | **S** | **L** | **A** | **A** | **K** | **T** | **G** | **T** | **A** | **E** | **I** | **K** | **D** | **K** | **Q** | **D** | **T** | **D** | **G** | **K** | **E** | **N** | **S** | **F** | **L** | **L** | **T** | **L** | **D** | **R** | **S** | **N** | **N** | **K** | **F** | **L** | **T** | **M** | **I** | **M** | **V** | **E** | **N** | **S** | **G** | **E** | **N** | **G** | **S** | **A** | **T** | **D** | **I** | **S** | **K** | **P** | **L** | **I** | **D** | **Y** | **L** | **E** | **A** | **T** | **I** | **K** |
| **N54574** | **-** | **-** | **-** | **-** | **-** | **-** | **-** | **-** | **-** | **-** | **-** | **-** | **-** | **-** | **-** | **-** | **-** | **-** | **-** | **-** | **-** | **-** | **-** | **-** | **-** | **-** | **-** | **-** | **-** | **-** | **-** | **-** | **-** | **-** | **-** | **-** | **Q** | **W** | **Q** | **A** | **K** | **Q** | **E** | **L** | **A** | **E** | **A** | **K** | **K** | **T** | **A** | **T** | **T** | **F** | **L** | **N** | **V** | **L** | **S** | **K** | **Q** | **E** | **F** | **D** | **K** | **L** | **P** | **S** | **V** | **V** | **Q** | **E** | **A** | **S** | **L** | **K** | **K** | **N** | **G** | **Y** | **D** | **T** | **K** | **S** | **V** | **V** | **E** | **K** | **Y** | **Q** | **A** | **I** | **Y** | **S** | **G** | **I** | **Q** | **A** | **E** | **G** | **V** | **K** | **A** | **S** | **D** | **V** | **Q** | **V** | **K** | **K** | **A** | **K** | **D** | **N** | **Q** | **Y** | **T** | **F** | **T** | **Y** | **K** | **L** | **S** | **M** | **S** | **T** | **P** | **L** | **G** | **E** | **M** | **K** | **D** | **L** | **S** | **Y** | **Q** | **S** | **S** | **I** | **A** | **K** | **K** | **G** | **D** | **T** | **Y** | **Q** | **I** | **A** | **W** | **K** | **P** | **S** | **L** | **I** | **F** | **P** | **D** | **M** | **S** | **G** | **N** | **D** | **K** | **I** | **S** | **I** | **Q** | **V** | **D** | **N** | **A** | **K** | **R** | **G** | **E** | **I** | **V** | **D** | **R** | **N** | **G** | **S** | **G** | **L** | **A** | **I** | **N** | **K** | **V** | **F** | **D** | **E** | **V** | **G** | **V** | **V** | **P** | **G** | **K** | **L** | **G** | **S** | **G** | **A** | **E** | **K** | **T** | **A** | **N** | **I** | **K** | **A** | **F** | **S** | **D** | **K** | **F** | **G** | **V** | **S** | **V** | **D** | **E** | **I** | **N** | **Q** | **K** | **L** | **S** | **Q** | **G** | **W** | **V** | **Q** | **A** | **D** | **S** | **F** | **V** | **P** | **I** | **T** | **V** | **A** | **S** | **E** | **P** | **V** | **T** | **E** | **L** | **P** | **T** | **G** | **A** | **A** | **T** | **K** | **D** | **T** | **E** | **S** | **R** | **Y** | **Y** | **P** | **L** | **G** | **E** | **A** | **A** | **A** | **Q** | **L** | **I** | **G** | **Y** | **T** | **G** | **T** | **I** | **T** | **A** | **E** | **D** | **I** | **E** | **K** | **N** | **P** | **E** | **L** | **S** | **S** | **T** | **G** | **V** | **I** | **G** | **K** | **T** | **G** | **L** | **E** | **R** | **A** | **F** | **D** | **K** | **E** | **L** | **R** | **G** | **Q** | **D** | **G** | **G** | **S** | **L** | **V** | **I** | **L** | **D** | **D** | **K** | **E** | **N** | **V** | **K** | **K** | **A** | **L** | **Q** | **T** | **K** | **E** | **K** | **K** | **D** | **G** | **Q** | **T** | **I** | **K** | **L** | **T** | **I** | **D** | **S** | **G** | **V** | **Q** | **Q** | **Q** | **A** | **F** | **A** | **I** | **F** | **D** | **K** | **R** | **P** | **G** | **S** | **A** | **V** | **I** | **T** | **D** | **P** | **Q** | **K** | **G** | **D** | **L** | **L** | **A** | **T** | **V** | **S** | **S** | **P** | **S** | **Y** | **D** | **P** | **N** | **K** | **M** | **A** | **N** | **G** | **I** | **S** | **Q** | **K** | **E** | **Y** | **D** | **A** | **Y** | **N** | **N** | **N** | **K** | **D** | **L** | **P** | **F** | **T** | **A** | **R** | **F** | **A** | **T** | **G** | **Y** | **A** | **P** | **G** | **S** | **T** | **F** | **K** | **T** | **I** | **T** | **G** | **A** | **I** | **G** | **L** | **D** | **A** | **G** | **T** | **L** | **K** | **P** | **D** | **E** | **E** | **L** | **E** | **I** | **N** | **G** | **L** | **K** | **W** | **Q** | **K** | **D** | **K** | **S** | **W** | **G** | **G** | **Y** | **F** | **A** | **T** | **R** | **V** | **K** | **E** | **A** | **S** | **P** | **V** | **N** | **L** | **R** | **T** | **A** | **L** | **V** | **N** | **S** | **D** | **N** | **I** | **Y** | **F** | **A** | **Q** | **Q** | **T** | **L** | **R** | **M** | **G** | **E** | **D** | **K** | **F** | **R** | **A** | **G** | **L** | **N** | **K** | **F** | **I** | **F** | **G** | **E** | **E** | **L** | **D** | **L** | **P** | **I** | **A** | **M** | **T** | **P** | **A** | **Q** | **I** | **S** | **N** | **E** | **D** | **K** | **F** | **N** | **S** | **E** | **I** | **L** | **L** | **A** | **D** | **T** | **G** | **Y** | **G** | **Q** | **G** | **Q** | **L** | **L** | **I** | **S** | **P** | **I** | **Q** | **Q** | **A** | **T** | **M** | **Y** | **S** | **V** | **F** | **Q** | **N** | **N** | **G** | **T** | **L** | **V** | **Y** | **P** | **K** | **L** | **V** | **L** | **E** | **K** | **E** | **T** | **K** | **K** | **K** | **D** | **N** | **V** | **I** | **S** | **A** | **N** | **A** | **A** | **N** | **T** | **I** | **A** | **T** | **D** | **L** | **L** | **G** | **S** | **V** | **E** | **D** | **P** | **S** | **G** | **Y** | **V** | **Y** | **N** | **M** | **Y** | **N** | **P** | **N** | **F** | **S** | **L** | **A** | **A** | **K** | **T** | **G** | **T** | **A** | **E** | **I** | **K** | **D** | **K** | **Q** | **D** | **T** | **D** | **G** | **K** | **E** | **N** | **S** | **F** | **L** | **L** | **T** | **L** | **D** | **R** | **S** | **N** | **N** | **K** | **F** | **L** | **T** | **M** | **I** | **M** | **V** | **E** | **N** | **S** | **G** | **E** | **N** | **G** | **S** | **A** | **T** | **D** | **I** | **S** | **K** | **P** | **L** | **I** | **D** | **Y** | **L** | **E** | **A** | **T** | **I** | **K** | **-** | **-** | **-** | **-** | **-** | **-** | **-** | **-** | **-** | **-** | **-** | **-** | **-** | **-** | **-** | **-** | **-** | **-** | **-** | **-** | **-** | **-** | **-** | **-** | **-** | **-** | **-** | **-** | **-** | **-** | **-** | **-** | **-** | **-** | **-** | **-** | **Q** | **W** | **Q** | **A** | **K** | **Q** | **E** | **L** | **A** | **E** | **A** | **K** | **K** | **T** | **A** | **T** | **T** | **F** | **L** | **N** | **V** | **L** | **S** | **K** | **Q** | **E** | **F** | **D** | **K** | **L** | **P** | **S** | **V** | **V** | **Q** | **E** | **A** | **S** | **L** | **K** | **K** | **N** | **G** | **Y** | **D** | **T** | **K** | **S** | **V** | **V** | **E** | **K** | **Y** | **Q** | **A** | **I** | **Y** | **S** | **G** | **I** | **Q** | **A** | **E** | **G** | **V** | **K** | **A** | **S** | **D** | **V** | **Q** | **V** | **K** | **K** | **A** | **K** | **D** | **N** | **Q** | **Y** | **T** | **F** | **T** | **Y** | **K** | **L** | **S** | **M** | **S** | **T** | **P** | **L** | **G** | **E** | **M** | **K** | **D** | **L** | **S** | **Y** | **Q** | **S** | **S** | **I** | **A** | **K** | **K** | **G** | **D** | **T** | **Y** | **Q** | **I** | **A** | **W** | **K** | **P** | **S** | **L** | **I** | **F** | **P** | **D** | **M** | **S** | **G** | **N** | **D** | **K** | **I** | **S** | **I** | **Q** | **V** | **D** | **N** | **A** | **K** | **R** | **G** | **E** | **I** | **V** | **D** | **R** | **N** | **G** | **S** | **G** | **L** | **A** | **I** | **N** | **K** | **V** | **F** | **D** | **E** | **V** | **G** | **V** | **V** | **P** | **G** | **K** | **L** | **G** | **S** | **G** | **A** | **E** | **K** | **T** | **A** | **N** | **I** | **K** | **A** | **F** | **S** | **D** | **K** | **F** | **G** | **V** | **S** | **V** | **D** | **E** | **I** | **N** | **Q** | **K** | **L** | **S** | **Q** | **G** | **W** | **V** | **Q** | **A** | **D** | **S** | **F** | **V** | **P** | **I** | **T** | **V** | **A** | **S** | **E** | **P** | **V** | **T** | **E** | **L** | **P** | **T** | **G** | **A** | **A** | **T** | **K** | **D** | **T** | **E** | **S** | **R** | **Y** | **Y** | **P** | **L** | **G** | **E** | **A** | **A** | **A** | **Q** | **L** | **I** | **G** | **Y** | **T** | **G** | **T** | **I** | **T** | **A** | **E** | **D** | **I** | **E** | **K** | **N** | **P** | **E** | **L** | **S** | **S** | **T** | **G** | **V** | **I** | **G** | **K** | **T** | **G** | **L** | **E** | **R** | **A** | **F** | **D** | **K** | **E** | **L** | **R** | **G** | **Q** | **D** | **G** | **G** | **S** | **L** | **V** | **I** | **L** | **D** | **D** | **K** | **E** | **N** | **V** | **K** | **K** | **A** | **L** | **Q** | **T** | **K** | **E** | **K** | **K** | **D** | **G** | **Q** | **T** | **I** | **K** | **L** | **T** | **I** | **D** | **S** | **G** | **V** | **Q** | **Q** | **Q** | **A** | **F** | **A** | **I** | **F** | **D** | **K** | **R** | **P** | **G** | **S** | **A** | **V** | **I** | **T** | **D** | **P** | **Q** | **K** | **G** | **D** | **L** | **L** | **A** | **T** | **V** | **S** | **S** | **P** | **S** | **Y** | **D** | **P** | **N** | **K** | **M** | **A** | **N** | **G** | **I** | **S** | **Q** | **K** | **E** | **Y** | **D** | **A** | **Y** | **N** | **N** | **N** | **K** | **D** | **L** | **P** | **F** | **T** | **A** | **R** | **F** | **A** | **T** | **G** | **Y** | **A** | **P** | **G** | **S** | **T** | **F** | **K** | **T** | **I** | **T** | **G** | **A** | **I** | **G** | **L** | **D** | **A** | **G** | **T** | **L** | **K** | **P** | **D** | **E** | **E** | **L** | **E** | **I** | **N** | **G** | **L** | **K** | **W** | **Q** | **K** | **D** | **K** | **S** | **W** | **G** | **G** | **Y** | **F** | **A** | **T** | **R** | **V** | **K** | **E** | **A** | **S** | **P** | **V** | **N** | **L** | **R** | **T** | **A** | **L** | **V** | **N** | **S** | **D** | **N** | **I** | **Y** | **F** | **A** | **Q** | **Q** | **T** | **L** | **R** | **M** | **G** | **E** | **D** | **K** | **F** | **R** | **A** | **G** | **L** | **N** | **K** | **F** | **I** | **F** | **G** | **E** | **E** | **L** | **D** | **L** | **P** | **I** | **A** | **M** | **T** | **P** | **A** | **Q** | **I** | **S** | **N** | **E** | **D** | **K** | **F** | **N** | **S** | **E** | **I** | **L** | **L** | **A** | **D** | **T** | **G** | **Y** | **G** | **Q** | **G** | **Q** | **L** | **L** | **I** | **S** | **P** | **I** | **Q** | **Q** | **A** | **T** | **M** | **Y** | **S** | **V** | **F** | **Q** | **N** | **N** | **G** | **T** | **L** | **V** | **Y** | **P** | **K** | **L** | **V** | **L** | **E** | **K** | **E** | **T** | **K** | **K** | **K** | **D** | **N** | **V** | **I** | **S** | **A** | **N** | **A** | **A** | **N** | **T** | **I** | **A** | **T** | **D** | **L** | **L** | **G** | **S** | **V** | **E** | **D** | **P** | **S** | **G** | **Y** | **V** | **Y** | **N** | **M** | **Y** | **N** | **P** | **N** | **F** | **S** | **L** | **A** | **A** | **K** | **T** | **G** | **T** | **A** | **E** | **I** | **K** | **D** | **K** | **Q** | **D** | **T** | **D** | **G** | **K** | **E** | **N** | **S** | **F** | **L** | **L** | **T** | **L** | **D** | **R** | **S** | **N** | **N** | **K** | **F** | **L** | **T** | **M** | **I** | **M** | **V** | **E** | **N** | **S** | **G** | **E** | **N** | **G** | **S** | **A** | **T** | **D** | **I** | **S** | **K** | **P** | **L** | **I** | **D** | **Y** | **L** | **E** | **A** | **T** | **I** | **K** |
| **N60063F** | **-** | **-** | **-** | **-** | **-** | **-** | **-** | **-** | **-** | **-** | **-** | **-** | **-** | **-** | **-** | **-** | **-** | **-** | **-** | **-** | **-** | **-** | **-** | **-** | **-** | **-** | **-** | **-** | **-** | **-** | **-** | **-** | **-** | **-** | **-** | **-** | **Q** | **W** | **Q** | **A** | **K** | **Q** | **E** | **L** | **A** | **E** | **A** | **K** | **K** | **T** | **A** | **T** | **T** | **F** | **L** | **N** | **V** | **L** | **S** | **K** | **Q** | **E** | **F** | **D** | **K** | **L** | **P** | **S** | **V** | **V** | **Q** | **E** | **A** | **S** | **L** | **K** | **K** | **N** | **G** | **Y** | **D** | **T** | **K** | **S** | **V** | **V** | **E** | **K** | **Y** | **Q** | **A** | **I** | **Y** | **S** | **G** | **I** | **Q** | **A** | **E** | **G** | **V** | **K** | **A** | **S** | **D** | **V** | **Q** | **V** | **K** | **K** | **A** | **K** | **D** | **N** | **Q** | **Y** | **T** | **F** | **T** | **Y** | **K** | **L** | **S** | **M** | **S** | **T** | **P** | **L** | **G** | **E** | **M** | **K** | **D** | **L** | **S** | **Y** | **Q** | **S** | **S** | **I** | **A** | **K** | **K** | **G** | **D** | **T** | **Y** | **Q** | **I** | **A** | **W** | **K** | **P** | **S** | **L** | **I** | **F** | **P** | **D** | **M** | **S** | **G** | **N** | **D** | **K** | **I** | **S** | **I** | **Q** | **V** | **D** | **N** | **A** | **K** | **R** | **G** | **E** | **I** | **V** | **D** | **R** | **N** | **G** | **S** | **G** | **L** | **A** | **I** | **N** | **K** | **V** | **F** | **D** | **E** | **V** | **G** | **V** | **V** | **P** | **G** | **K** | **L** | **G** | **S** | **G** | **A** | **E** | **K** | **T** | **A** | **N** | **I** | **K** | **A** | **F** | **S** | **D** | **K** | **F** | **G** | **V** | **S** | **I** | **D** | **E** | **I** | **N** | **Q** | **K** | **L** | **S** | **Q** | **G** | **W** | **V** | **Q** | **A** | **D** | **S** | **F** | **V** | **P** | **I** | **T** | **V** | **A** | **S** | **E** | **P** | **V** | **T** | **E** | **L** | **P** | **T** | **G** | **A** | **A** | **T** | **K** | **D** | **T** | **E** | **S** | **R** | **Y** | **Y** | **P** | **L** | **G** | **E** | **A** | **A** | **A** | **Q** | **L** | **I** | **G** | **Y** | **T** | **G** | **T** | **I** | **T** | **A** | **E** | **D** | **I** | **E** | **K** | **N** | **P** | **E** | **L** | **S** | **S** | **T** | **G** | **V** | **I** | **G** | **K** | **T** | **G** | **L** | **E** | **R** | **A** | **F** | **D** | **K** | **E** | **L** | **R** | **G** | **Q** | **D** | **G** | **G** | **S** | **L** | **V** | **I** | **L** | **D** | **D** | **K** | **E** | **N** | **V** | **K** | **K** | **A** | **L** | **Q** | **T** | **K** | **E** | **K** | **K** | **D** | **G** | **Q** | **T** | **I** | **K** | **L** | **T** | **I** | **D** | **S** | **G** | **V** | **Q** | **Q** | **Q** | **A** | **F** | **A** | **I** | **F** | **D** | **K** | **R** | **P** | **G** | **S** | **A** | **V** | **I** | **T** | **D** | **P** | **Q** | **K** | **G** | **D** | **L** | **L** | **A** | **T** | **V** | **S** | **S** | **P** | **S** | **Y** | **D** | **P** | **N** | **K** | **M** | **A** | **N** | **G** | **I** | **S** | **Q** | **K** | **E** | **Y** | **D** | **A** | **Y** | **N** | **N** | **N** | **K** | **D** | **L** | **P** | **F** | **T** | **A** | **R** | **F** | **A** | **T** | **G** | **Y** | **A** | **P** | **G** | **S** | **T** | **F** | **K** | **T** | **I** | **T** | **G** | **A** | **I** | **G** | **L** | **D** | **A** | **G** | **T** | **L** | **K** | **P** | **D** | **E** | **E** | **L** | **E** | **I** | **N** | **G** | **L** | **K** | **W** | **Q** | **K** | **D** | **K** | **S** | **W** | **G** | **G** | **Y** | **F** | **A** | **T** | **R** | **V** | **K** | **E** | **A** | **S** | **P** | **V** | **N** | **L** | **R** | **T** | **A** | **L** | **V** | **N** | **S** | **D** | **N** | **I** | **Y** | **F** | **A** | **Q** | **Q** | **T** | **L** | **R** | **M** | **G** | **E** | **D** | **K** | **F** | **R** | **A** | **G** | **L** | **N** | **K** | **F** | **I** | **F** | **G** | **E** | **E** | **L** | **D** | **L** | **P** | **I** | **A** | **M** | **T** | **P** | **A** | **Q** | **I** | **S** | **N** | **E** | **D** | **K** | **F** | **N** | **S** | **E** | **I** | **L** | **L** | **A** | **D** | **T** | **G** | **Y** | **G** | **Q** | **G** | **Q** | **L** | **L** | **I** | **S** | **P** | **I** | **Q** | **Q** | **A** | **T** | **M** | **Y** | **S** | **V** | **F** | **Q** | **N** | **N** | **G** | **T** | **L** | **V** | **Y** | **P** | **K** | **I** | **V** | **L** | **D** | **K** | **E** | **T** | **K** | **K** | **K** | **D** | **N** | **V** | **I** | **S** | **A** | **N** | **A** | **A** | **N** | **T** | **I** | **A** | **T** | **D** | **L** | **L** | **G** | **S** | **V** | **E** | **D** | **P** | **S** | **G** | **Y** | **V** | **Y** | **N** | **M** | **Y** | **N** | **P** | **N** | **F** | **S** | **L** | **A** | **A** | **K** | **T** | **G** | **T** | **A** | **E** | **I** | **K** | **D** | **K** | **Q** | **D** | **T** | **D** | **G** | **K** | **E** | **N** | **S** | **F** | **L** | **L** | **T** | **L** | **D** | **R** | **S** | **N** | **N** | **K** | **F** | **L** | **T** | **M** | **I** | **M** | **V** | **E** | **N** | **S** | **G** | **E** | **N** | **G** | **S** | **A** | **T** | **D** | **I** | **S** | **K** | **P** | **L** | **I** | **D** | **Y** | **L** | **E** | **A** | **T** | **I** | **K** | **-** | **-** | **-** | **-** | **-** | **-** | **-** | **-** | **-** | **-** | **-** | **-** | **-** | **-** | **-** | **-** | **-** | **-** | **-** | **-** | **-** | **-** | **-** | **-** | **-** | **-** | **-** | **-** | **-** | **-** | **-** | **-** | **-** | **-** | **-** | **-** | **Q** | **W** | **Q** | **A** | **K** | **Q** | **E** | **L** | **A** | **E** | **A** | **K** | **K** | **T** | **A** | **T** | **T** | **F** | **L** | **N** | **V** | **L** | **S** | **K** | **Q** | **E** | **F** | **D** | **K** | **L** | **P** | **S** | **V** | **V** | **Q** | **E** | **A** | **S** | **L** | **K** | **K** | **N** | **G** | **Y** | **D** | **T** | **K** | **S** | **V** | **V** | **E** | **K** | **Y** | **Q** | **A** | **I** | **Y** | **S** | **G** | **I** | **Q** | **A** | **E** | **G** | **V** | **K** | **A** | **S** | **D** | **V** | **Q** | **V** | **K** | **K** | **A** | **K** | **D** | **N** | **Q** | **Y** | **T** | **F** | **T** | **Y** | **K** | **L** | **S** | **M** | **S** | **T** | **P** | **L** | **G** | **E** | **M** | **K** | **D** | **L** | **S** | **Y** | **Q** | **S** | **S** | **I** | **A** | **K** | **K** | **G** | **D** | **T** | **Y** | **Q** | **I** | **A** | **W** | **K** | **P** | **S** | **L** | **I** | **F** | **P** | **D** | **M** | **S** | **G** | **N** | **D** | **K** | **I** | **S** | **I** | **Q** | **V** | **D** | **N** | **A** | **K** | **R** | **G** | **E** | **I** | **V** | **D** | **R** | **N** | **G** | **S** | **G** | **L** | **A** | **I** | **N** | **K** | **V** | **F** | **D** | **E** | **V** | **G** | **V** | **V** | **P** | **G** | **K** | **L** | **G** | **S** | **G** | **A** | **E** | **K** | **T** | **A** | **N** | **I** | **K** | **A** | **F** | **S** | **D** | **K** | **F** | **G** | **V** | **S** | **I** | **D** | **E** | **I** | **N** | **Q** | **K** | **L** | **S** | **Q** | **G** | **W** | **V** | **Q** | **A** | **D** | **S** | **F** | **V** | **P** | **I** | **T** | **V** | **A** | **S** | **E** | **P** | **V** | **T** | **E** | **L** | **P** | **T** | **G** | **A** | **A** | **T** | **K** | **D** | **T** | **E** | **S** | **R** | **Y** | **Y** | **P** | **L** | **G** | **E** | **A** | **A** | **A** | **Q** | **L** | **I** | **G** | **Y** | **T** | **G** | **T** | **I** | **T** | **A** | **E** | **D** | **I** | **E** | **K** | **N** | **P** | **E** | **L** | **S** | **S** | **T** | **G** | **V** | **I** | **G** | **K** | **T** | **G** | **L** | **E** | **R** | **A** | **F** | **D** | **K** | **E** | **L** | **R** | **G** | **Q** | **D** | **G** | **G** | **S** | **L** | **V** | **I** | **L** | **D** | **D** | **K** | **E** | **N** | **V** | **K** | **K** | **A** | **L** | **Q** | **T** | **K** | **E** | **K** | **K** | **D** | **G** | **Q** | **T** | **I** | **K** | **L** | **T** | **I** | **D** | **S** | **G** | **V** | **Q** | **Q** | **Q** | **A** | **F** | **A** | **I** | **F** | **D** | **K** | **R** | **P** | **G** | **S** | **A** | **V** | **I** | **T** | **D** | **P** | **Q** | **K** | **G** | **D** | **L** | **L** | **A** | **T** | **V** | **S** | **S** | **P** | **S** | **Y** | **D** | **P** | **N** | **K** | **M** | **A** | **N** | **G** | **I** | **S** | **Q** | **K** | **E** | **Y** | **D** | **A** | **Y** | **N** | **N** | **N** | **K** | **D** | **L** | **P** | **F** | **T** | **A** | **R** | **F** | **A** | **T** | **G** | **Y** | **A** | **P** | **G** | **S** | **T** | **F** | **K** | **T** | **I** | **T** | **G** | **A** | **I** | **G** | **L** | **D** | **A** | **G** | **T** | **L** | **K** | **P** | **D** | **E** | **E** | **L** | **E** | **I** | **N** | **G** | **L** | **K** | **W** | **Q** | **K** | **D** | **K** | **S** | **W** | **G** | **G** | **Y** | **F** | **A** | **T** | **R** | **V** | **K** | **E** | **A** | **S** | **P** | **V** | **N** | **L** | **R** | **T** | **A** | **L** | **V** | **N** | **S** | **D** | **N** | **I** | **Y** | **F** | **A** | **Q** | **Q** | **T** | **L** | **R** | **M** | **G** | **E** | **D** | **K** | **F** | **R** | **A** | **G** | **L** | **N** | **K** | **F** | **I** | **F** | **G** | **E** | **E** | **L** | **D** | **L** | **P** | **I** | **A** | **M** | **T** | **P** | **A** | **Q** | **I** | **S** | **N** | **E** | **D** | **K** | **F** | **N** | **S** | **E** | **I** | **L** | **L** | **A** | **D** | **T** | **G** | **Y** | **G** | **Q** | **G** | **Q** | **L** | **L** | **I** | **S** | **P** | **I** | **Q** | **Q** | **A** | **T** | **M** | **Y** | **S** | **V** | **F** | **Q** | **N** | **N** | **G** | **T** | **L** | **V** | **Y** | **P** | **K** | **I** | **V** | **L** | **D** | **K** | **E** | **T** | **K** | **K** | **K** | **D** | **N** | **V** | **I** | **S** | **A** | **N** | **A** | **A** | **N** | **T** | **I** | **A** | **T** | **D** | **L** | **L** | **G** | **S** | **V** | **E** | **D** | **P** | **S** | **G** | **Y** | **V** | **Y** | **N** | **M** | **Y** | **N** | **P** | **N** | **F** | **S** | **L** | **A** | **A** | **K** | **T** | **G** | **T** | **A** | **E** | **I** | **K** | **D** | **K** | **Q** | **D** | **T** | **D** | **G** | **K** | **E** | **N** | **S** | **F** | **L** | **L** | **T** | **L** | **D** | **R** | **S** | **N** | **N** | **K** | **F** | **L** | **T** | **M** | **I** | **M** | **V** | **E** | **N** | **S** | **G** | **E** | **N** | **G** | **S** | **A** | **T** | **D** | **I** | **S** | **K** | **P** | **L** | **I** | **D** | **Y** | **L** | **E** | **A** | **T** | **I** | **K** |
| **N53457** | **-** | **-** | **-** | **-** | **-** | **-** | **-** | **-** | **-** | **-** | **-** | **-** | **-** | **-** | **-** | **-** | **-** | **-** | **-** | **-** | **-** | **-** | **-** | **-** | **-** | **-** | **-** | **-** | **-** | **-** | **-** | **-** | **-** | **-** | **-** | **-** | **Q** | **W** | **Q** | **A** | **K** | **Q** | **E** | **L** | **A** | **E** | **A** | **K** | **K** | **T** | **A** | **T** | **T** | **F** | **L** | **N** | **V** | **L** | **S** | **K** | **Q** | **E** | **F** | **D** | **K** | **L** | **P** | **S** | **V** | **V** | **Q** | **E** | **A** | **S** | **L** | **K** | **K** | **N** | **G** | **Y** | **D** | **T** | **K** | **S** | **V** | **V** | **E** | **K** | **Y** | **Q** | **A** | **I** | **Y** | **S** | **G** | **I** | **Q** | **A** | **E** | **G** | **V** | **K** | **A** | **S** | **D** | **V** | **Q** | **V** | **K** | **K** | **A** | **K** | **D** | **N** | **Q** | **Y** | **T** | **F** | **T** | **Y** | **K** | **L** | **S** | **M** | **S** | **T** | **P** | **L** | **G** | **E** | **M** | **K** | **D** | **L** | **S** | **Y** | **Q** | **S** | **S** | **I** | **A** | **K** | **K** | **G** | **D** | **T** | **Y** | **Q** | **I** | **A** | **W** | **K** | **P** | **S** | **L** | **I** | **F** | **P** | **D** | **M** | **S** | **G** | **N** | **D** | **K** | **I** | **S** | **I** | **Q** | **V** | **D** | **N** | **A** | **K** | **R** | **G** | **E** | **I** | **V** | **D** | **R** | **N** | **G** | **S** | **G** | **L** | **A** | **I** | **N** | **K** | **V** | **F** | **D** | **E** | **V** | **G** | **V** | **V** | **P** | **G** | **K** | **L** | **G** | **S** | **G** | **A** | **E** | **K** | **T** | **A** | **N** | **I** | **K** | **A** | **F** | **S** | **D** | **K** | **F** | **G** | **V** | **S** | **V** | **D** | **E** | **I** | **N** | **Q** | **K** | **L** | **S** | **Q** | **G** | **W** | **V** | **Q** | **A** | **D** | **S** | **F** | **V** | **P** | **I** | **T** | **V** | **A** | **S** | **E** | **P** | **V** | **T** | **E** | **L** | **P** | **T** | **G** | **A** | **A** | **T** | **K** | **D** | **T** | **E** | **S** | **R** | **Y** | **Y** | **P** | **L** | **G** | **E** | **A** | **A** | **A** | **Q** | **L** | **I** | **G** | **Y** | **T** | **G** | **T** | **I** | **T** | **A** | **E** | **D** | **I** | **K** | **K** | **N** | **P** | **E** | **L** | **S** | **S** | **T** | **G** | **V** | **I** | **G** | **K** | **T** | **G** | **L** | **E** | **R** | **A** | **F** | **D** | **K** | **E** | **L** | **R** | **G** | **Q** | **D** | **G** | **G** | **S** | **L** | **V** | **I** | **L** | **D** | **D** | **K** | **E** | **N** | **V** | **K** | **K** | **A** | **L** | **Q** | **T** | **K** | **E** | **K** | **K** | **D** | **G** | **Q** | **T** | **I** | **K** | **L** | **T** | **I** | **D** | **S** | **G** | **V** | **Q** | **Q** | **Q** | **A** | **F** | **A** | **I** | **F** | **D** | **K** | **R** | **P** | **G** | **S** | **A** | **V** | **I** | **T** | **D** | **P** | **Q** | **K** | **G** | **D** | **L** | **L** | **A** | **T** | **V** | **S** | **S** | **P** | **S** | **Y** | **D** | **P** | **N** | **K** | **M** | **A** | **N** | **G** | **I** | **S** | **Q** | **K** | **E** | **Y** | **D** | **A** | **Y** | **N** | **N** | **N** | **K** | **D** | **L** | **P** | **F** | **T** | **A** | **R** | **F** | **A** | **T** | **G** | **Y** | **A** | **P** | **G** | **S** | **T** | **F** | **K** | **T** | **I** | **T** | **G** | **A** | **I** | **G** | **L** | **D** | **A** | **G** | **T** | **L** | **K** | **P** | **D** | **E** | **E** | **L** | **E** | **I** | **N** | **G** | **L** | **K** | **W** | **Q** | **K** | **D** | **K** | **S** | **W** | **G** | **G** | **Y** | **F** | **A** | **T** | **R** | **V** | **K** | **E** | **A** | **S** | **P** | **V** | **N** | **L** | **R** | **T** | **A** | **L** | **V** | **N** | **S** | **D** | **N** | **I** | **Y** | **F** | **A** | **Q** | **Q** | **T** | **L** | **R** | **M** | **G** | **E** | **D** | **K** | **F** | **R** | **A** | **G** | **L** | **N** | **K** | **F** | **I** | **F** | **G** | **E** | **E** | **L** | **D** | **L** | **P** | **I** | **A** | **M** | **T** | **P** | **A** | **Q** | **I** | **S** | **N** | **E** | **D** | **K** | **F** | **N** | **S** | **E** | **I** | **L** | **L** | **A** | **D** | **T** | **G** | **Y** | **G** | **Q** | **G** | **Q** | **L** | **L** | **I** | **S** | **P** | **I** | **Q** | **Q** | **A** | **T** | **M** | **Y** | **S** | **V** | **F** | **Q** | **N** | **N** | **G** | **T** | **L** | **V** | **Y** | **P** | **K** | **L** | **V** | **L** | **D** | **K** | **E** | **T** | **K** | **K** | **K** | **D** | **N** | **V** | **I** | **S** | **A** | **N** | **A** | **A** | **N** | **T** | **I** | **A** | **T** | **D** | **L** | **L** | **G** | **S** | **V** | **E** | **D** | **P** | **S** | **G** | **Y** | **V** | **Y** | **N** | **M** | **Y** | **N** | **P** | **N** | **F** | **S** | **L** | **A** | **A** | **K** | **T** | **G** | **T** | **A** | **E** | **I** | **K** | **D** | **K** | **Q** | **D** | **T** | **D** | **G** | **K** | **E** | **N** | **S** | **F** | **L** | **L** | **T** | **L** | **D** | **R** | **S** | **N** | **N** | **K** | **F** | **L** | **T** | **M** | **I** | **M** | **V** | **E** | **N** | **S** | **G** | **E** | **N** | **G** | **S** | **A** | **T** | **D** | **I** | **S** | **K** | **P** | **L** | **I** | **D** | **Y** | **L** | **E** | **A** | **T** | **I** | **K** | **-** | **-** | **-** | **-** | **-** | **-** | **-** | **-** | **-** | **-** | **-** | **-** | **-** | **-** | **-** | **-** | **-** | **-** | **-** | **-** | **-** | **-** | **-** | **-** | **-** | **-** | **-** | **-** | **-** | **-** | **-** | **-** | **-** | **-** | **-** | **-** | **Q** | **W** | **Q** | **A** | **K** | **Q** | **E** | **L** | **A** | **E** | **A** | **K** | **K** | **T** | **A** | **T** | **T** | **F** | **L** | **N** | **V** | **L** | **S** | **K** | **Q** | **E** | **F** | **D** | **K** | **L** | **P** | **S** | **V** | **V** | **Q** | **E** | **A** | **S** | **L** | **K** | **K** | **N** | **G** | **Y** | **D** | **T** | **K** | **S** | **V** | **V** | **E** | **K** | **Y** | **Q** | **A** | **I** | **Y** | **S** | **G** | **I** | **Q** | **A** | **E** | **G** | **V** | **K** | **A** | **S** | **D** | **V** | **Q** | **V** | **K** | **K** | **A** | **K** | **D** | **N** | **Q** | **Y** | **T** | **F** | **T** | **Y** | **K** | **L** | **S** | **M** | **S** | **T** | **P** | **L** | **G** | **E** | **M** | **K** | **D** | **L** | **S** | **Y** | **Q** | **S** | **S** | **I** | **A** | **K** | **K** | **G** | **D** | **T** | **Y** | **Q** | **I** | **A** | **W** | **K** | **P** | **S** | **L** | **I** | **F** | **P** | **D** | **M** | **S** | **G** | **N** | **D** | **K** | **I** | **S** | **I** | **Q** | **V** | **D** | **N** | **A** | **K** | **R** | **G** | **E** | **I** | **V** | **D** | **R** | **N** | **G** | **S** | **G** | **L** | **A** | **I** | **N** | **K** | **V** | **F** | **D** | **E** | **V** | **G** | **V** | **V** | **P** | **G** | **K** | **L** | **G** | **S** | **G** | **A** | **E** | **K** | **T** | **A** | **N** | **I** | **K** | **A** | **F** | **S** | **D** | **K** | **F** | **G** | **V** | **S** | **V** | **D** | **E** | **I** | **N** | **Q** | **K** | **L** | **S** | **Q** | **G** | **W** | **V** | **Q** | **A** | **D** | **S** | **F** | **V** | **P** | **I** | **T** | **V** | **A** | **S** | **E** | **P** | **V** | **T** | **E** | **L** | **P** | **T** | **G** | **A** | **A** | **T** | **K** | **D** | **T** | **E** | **S** | **R** | **Y** | **Y** | **P** | **L** | **G** | **E** | **A** | **A** | **A** | **Q** | **L** | **I** | **G** | **Y** | **T** | **G** | **T** | **I** | **T** | **A** | **E** | **D** | **I** | **K** | **K** | **N** | **P** | **E** | **L** | **S** | **S** | **T** | **G** | **V** | **I** | **G** | **K** | **T** | **G** | **L** | **E** | **R** | **A** | **F** | **D** | **K** | **E** | **L** | **R** | **G** | **Q** | **D** | **G** | **G** | **S** | **L** | **V** | **I** | **L** | **D** | **D** | **K** | **E** | **N** | **V** | **K** | **K** | **A** | **L** | **Q** | **T** | **K** | **E** | **K** | **K** | **D** | **G** | **Q** | **T** | **I** | **K** | **L** | **T** | **I** | **D** | **S** | **G** | **V** | **Q** | **Q** | **Q** | **A** | **F** | **A** | **I** | **F** | **D** | **K** | **R** | **P** | **G** | **S** | **A** | **V** | **I** | **T** | **D** | **P** | **Q** | **K** | **G** | **D** | **L** | **L** | **A** | **T** | **V** | **S** | **S** | **P** | **S** | **Y** | **D** | **P** | **N** | **K** | **M** | **A** | **N** | **G** | **I** | **S** | **Q** | **K** | **E** | **Y** | **D** | **A** | **Y** | **N** | **N** | **N** | **K** | **D** | **L** | **P** | **F** | **T** | **A** | **R** | **F** | **A** | **T** | **G** | **Y** | **A** | **P** | **G** | **S** | **T** | **F** | **K** | **T** | **I** | **T** | **G** | **A** | **I** | **G** | **L** | **D** | **A** | **G** | **T** | **L** | **K** | **P** | **D** | **E** | **E** | **L** | **E** | **I** | **N** | **G** | **L** | **K** | **W** | **Q** | **K** | **D** | **K** | **S** | **W** | **G** | **G** | **Y** | **F** | **A** | **T** | **R** | **V** | **K** | **E** | **A** | **S** | **P** | **V** | **N** | **L** | **R** | **T** | **A** | **L** | **V** | **N** | **S** | **D** | **N** | **I** | **Y** | **F** | **A** | **Q** | **Q** | **T** | **L** | **R** | **M** | **G** | **E** | **D** | **K** | **F** | **R** | **A** | **G** | **L** | **N** | **K** | **F** | **I** | **F** | **G** | **E** | **E** | **L** | **D** | **L** | **P** | **I** | **A** | **M** | **T** | **P** | **A** | **Q** | **I** | **S** | **N** | **E** | **D** | **K** | **F** | **N** | **S** | **E** | **I** | **L** | **L** | **A** | **D** | **T** | **G** | **Y** | **G** | **Q** | **G** | **Q** | **L** | **L** | **I** | **S** | **P** | **I** | **Q** | **Q** | **A** | **T** | **M** | **Y** | **S** | **V** | **F** | **Q** | **N** | **N** | **G** | **T** | **L** | **V** | **Y** | **P** | **K** | **L** | **V** | **L** | **D** | **K** | **E** | **T** | **K** | **K** | **K** | **D** | **N** | **V** | **I** | **S** | **A** | **N** | **A** | **A** | **N** | **T** | **I** | **A** | **T** | **D** | **L** | **L** | **G** | **S** | **V** | **E** | **D** | **P** | **S** | **G** | **Y** | **V** | **Y** | **N** | **M** | **Y** | **N** | **P** | **N** | **F** | **S** | **L** | **A** | **A** | **K** | **T** | **G** | **T** | **A** | **E** | **I** | **K** | **D** | **K** | **Q** | **D** | **T** | **D** | **G** | **K** | **E** | **N** | **S** | **F** | **L** | **L** | **T** | **L** | **D** | **R** | **S** | **N** | **N** | **K** | **F** | **L** | **T** | **M** | **I** | **M** | **V** | **E** | **N** | **S** | **G** | **E** | **N** | **G** | **S** | **A** | **T** | **D** | **I** | **S** | **K** | **P** | **L** | **I** | **D** | **Y** | **L** | **E** | **A** | **T** | **I** | **K** |
| **N55113** | **-** | **-** | **-** | **-** | **-** | **-** | **-** | **-** | **-** | **-** | **-** | **-** | **-** | **-** | **-** | **-** | **-** | **-** | **-** | **-** | **-** | **-** | **-** | **-** | **-** | **-** | **-** | **-** | **-** | **-** | **-** | **-** | **-** | **-** | **-** | **-** | **Q** | **W** | **Q** | **A** | **K** | **Q** | **E** | **L** | **A** | **E** | **A** | **K** | **K** | **T** | **A** | **T** | **K** | **F** | **L** | **N** | **V** | **L** | **S** | **K** | **Q** | **E** | **F** | **D** | **K** | **L** | **P** | **S** | **V** | **V** | **Q** | **E** | **A** | **S** | **L** | **K** | **K** | **N** | **G** | **Y** | **D** | **T** | **K** | **S** | **V** | **V** | **E** | **K** | **Y** | **Q** | **A** | **I** | **Y** | **S** | **G** | **I** | **Q** | **A** | **E** | **G** | **V** | **K** | **A** | **S** | **D** | **V** | **Q** | **V** | **K** | **K** | **A** | **K** | **D** | **N** | **Q** | **Y** | **T** | **F** | **T** | **Y** | **K** | **L** | **S** | **M** | **S** | **T** | **P** | **L** | **G** | **E** | **M** | **K** | **D** | **L** | **S** | **Y** | **Q** | **S** | **S** | **I** | **A** | **K** | **K** | **G** | **D** | **T** | **Y** | **Q** | **I** | **A** | **W** | **K** | **P** | **S** | **L** | **I** | **F** | **P** | **D** | **M** | **S** | **G** | **N** | **D** | **K** | **I** | **S** | **I** | **Q** | **V** | **D** | **N** | **A** | **K** | **R** | **G** | **E** | **I** | **V** | **D** | **R** | **N** | **G** | **S** | **G** | **L** | **A** | **I** | **N** | **K** | **V** | **F** | **D** | **E** | **V** | **G** | **V** | **V** | **P** | **G** | **K** | **L** | **G** | **S** | **G** | **A** | **E** | **K** | **T** | **A** | **N** | **I** | **K** | **A** | **F** | **S** | **D** | **K** | **F** | **G** | **V** | **S** | **V** | **D** | **E** | **I** | **N** | **Q** | **K** | **L** | **S** | **Q** | **G** | **W** | **V** | **Q** | **A** | **D** | **S** | **F** | **V** | **P** | **I** | **T** | **V** | **A** | **S** | **E** | **P** | **V** | **T** | **E** | **L** | **P** | **T** | **G** | **A** | **A** | **T** | **K** | **D** | **T** | **E** | **S** | **R** | **Y** | **Y** | **P** | **L** | **G** | **E** | **A** | **A** | **A** | **Q** | **L** | **I** | **G** | **Y** | **T** | **G** | **T** | **I** | **T** | **A** | **E** | **D** | **I** | **E** | **K** | **N** | **P** | **E** | **L** | **S** | **S** | **T** | **G** | **V** | **I** | **G** | **K** | **T** | **G** | **L** | **E** | **R** | **A** | **F** | **D** | **K** | **E** | **L** | **R** | **G** | **Q** | **D** | **G** | **G** | **S** | **L** | **V** | **I** | **L** | **D** | **D** | **K** | **E** | **N** | **V** | **K** | **K** | **A** | **L** | **Q** | **T** | **K** | **E** | **K** | **K** | **D** | **G** | **Q** | **T** | **I** | **K** | **L** | **T** | **I** | **D** | **S** | **G** | **V** | **Q** | **Q** | **Q** | **A** | **F** | **A** | **I** | **F** | **D** | **K** | **R** | **P** | **G** | **S** | **A** | **V** | **I** | **T** | **D** | **P** | **Q** | **K** | **G** | **D** | **L** | **L** | **A** | **T** | **V** | **S** | **S** | **P** | **S** | **Y** | **D** | **P** | **N** | **K** | **M** | **A** | **N** | **G** | **I** | **S** | **Q** | **K** | **E** | **Y** | **D** | **A** | **Y** | **N** | **N** | **N** | **K** | **D** | **L** | **P** | **F** | **T** | **A** | **R** | **F** | **A** | **T** | **G** | **Y** | **A** | **P** | **G** | **S** | **T** | **F** | **K** | **T** | **I** | **T** | **G** | **A** | **I** | **G** | **L** | **D** | **A** | **G** | **T** | **L** | **K** | **P** | **D** | **E** | **E** | **L** | **E** | **I** | **N** | **G** | **L** | **K** | **W** | **Q** | **K** | **D** | **K** | **S** | **W** | **G** | **G** | **Y** | **F** | **A** | **T** | **R** | **V** | **K** | **E** | **A** | **S** | **P** | **V** | **N** | **L** | **R** | **T** | **A** | **L** | **V** | **N** | **S** | **D** | **N** | **I** | **Y** | **F** | **A** | **Q** | **Q** | **T** | **L** | **R** | **M** | **G** | **E** | **D** | **K** | **F** | **R** | **A** | **G** | **L** | **N** | **K** | **F** | **I** | **F** | **G** | **E** | **E** | **L** | **D** | **L** | **P** | **I** | **A** | **M** | **T** | **P** | **A** | **Q** | **I** | **S** | **N** | **E** | **D** | **K** | **F** | **N** | **S** | **E** | **I** | **L** | **L** | **A** | **D** | **T** | **G** | **Y** | **G** | **Q** | **G** | **Q** | **L** | **L** | **I** | **S** | **P** | **I** | **Q** | **Q** | **A** | **T** | **M** | **Y** | **S** | **V** | **F** | **Q** | **N** | **N** | **G** | **T** | **L** | **V** | **Y** | **P** | **K** | **L** | **V** | **L** | **D** | **K** | **E** | **T** | **K** | **K** | **K** | **D** | **N** | **V** | **I** | **S** | **A** | **N** | **A** | **A** | **N** | **T** | **I** | **A** | **T** | **D** | **L** | **L** | **G** | **S** | **V** | **E** | **D** | **P** | **S** | **G** | **Y** | **V** | **Y** | **N** | **M** | **Y** | **N** | **P** | **N** | **F** | **S** | **L** | **A** | **A** | **K** | **T** | **G** | **T** | **A** | **E** | **I** | **K** | **D** | **K** | **Q** | **D** | **T** | **D** | **G** | **K** | **E** | **N** | **S** | **F** | **L** | **L** | **T** | **L** | **D** | **R** | **S** | **N** | **N** | **K** | **F** | **L** | **T** | **M** | **I** | **M** | **V** | **E** | **N** | **S** | **G** | **E** | **N** | **G** | **S** | **A** | **T** | **D** | **I** | **S** | **K** | **P** | **L** | **I** | **D** | **Y** | **L** | **E** | **A** | **T** | **I** | **K** | **-** | **-** | **-** | **-** | **-** | **-** | **-** | **-** | **-** | **-** | **-** | **-** | **-** | **-** | **-** | **-** | **-** | **-** | **-** | **-** | **-** | **-** | **-** | **-** | **-** | **-** | **-** | **-** | **-** | **-** | **-** | **-** | **-** | **-** | **-** | **-** | **Q** | **W** | **Q** | **A** | **K** | **Q** | **E** | **L** | **A** | **E** | **A** | **K** | **K** | **T** | **A** | **T** | **K** | **F** | **L** | **N** | **V** | **L** | **S** | **K** | **Q** | **E** | **F** | **D** | **K** | **L** | **P** | **S** | **V** | **V** | **Q** | **E** | **A** | **S** | **L** | **K** | **K** | **N** | **G** | **Y** | **D** | **T** | **K** | **S** | **V** | **V** | **E** | **K** | **Y** | **Q** | **A** | **I** | **Y** | **S** | **G** | **I** | **Q** | **A** | **E** | **G** | **V** | **K** | **A** | **S** | **D** | **V** | **Q** | **V** | **K** | **K** | **A** | **K** | **D** | **N** | **Q** | **Y** | **T** | **F** | **T** | **Y** | **K** | **L** | **S** | **M** | **S** | **T** | **P** | **L** | **G** | **E** | **M** | **K** | **D** | **L** | **S** | **Y** | **Q** | **S** | **S** | **I** | **A** | **K** | **K** | **G** | **D** | **T** | **Y** | **Q** | **I** | **A** | **W** | **K** | **P** | **S** | **L** | **I** | **F** | **P** | **D** | **M** | **S** | **G** | **N** | **D** | **K** | **I** | **S** | **I** | **Q** | **V** | **D** | **N** | **A** | **K** | **R** | **G** | **E** | **I** | **V** | **D** | **R** | **N** | **G** | **S** | **G** | **L** | **A** | **I** | **N** | **K** | **V** | **F** | **D** | **E** | **V** | **G** | **V** | **V** | **P** | **G** | **K** | **L** | **G** | **S** | **G** | **A** | **E** | **K** | **T** | **A** | **N** | **I** | **K** | **A** | **F** | **S** | **D** | **K** | **F** | **G** | **V** | **S** | **V** | **D** | **E** | **I** | **N** | **Q** | **K** | **L** | **S** | **Q** | **G** | **W** | **V** | **Q** | **A** | **D** | **S** | **F** | **V** | **P** | **I** | **T** | **V** | **A** | **S** | **E** | **P** | **V** | **T** | **E** | **L** | **P** | **T** | **G** | **A** | **A** | **T** | **K** | **D** | **T** | **E** | **S** | **R** | **Y** | **Y** | **P** | **L** | **G** | **E** | **A** | **A** | **A** | **Q** | **L** | **I** | **G** | **Y** | **T** | **G** | **T** | **I** | **T** | **A** | **E** | **D** | **I** | **E** | **K** | **N** | **P** | **E** | **L** | **S** | **S** | **T** | **G** | **V** | **I** | **G** | **K** | **T** | **G** | **L** | **E** | **R** | **A** | **F** | **D** | **K** | **E** | **L** | **R** | **G** | **Q** | **D** | **G** | **G** | **S** | **L** | **V** | **I** | **L** | **D** | **D** | **K** | **E** | **N** | **V** | **K** | **K** | **A** | **L** | **Q** | **T** | **K** | **E** | **K** | **K** | **D** | **G** | **Q** | **T** | **I** | **K** | **L** | **T** | **I** | **D** | **S** | **G** | **V** | **Q** | **Q** | **Q** | **A** | **F** | **A** | **I** | **F** | **D** | **K** | **R** | **P** | **G** | **S** | **A** | **V** | **I** | **T** | **D** | **P** | **Q** | **K** | **G** | **D** | **L** | **L** | **A** | **T** | **V** | **S** | **S** | **P** | **S** | **Y** | **D** | **P** | **N** | **K** | **M** | **A** | **N** | **G** | **I** | **S** | **Q** | **K** | **E** | **Y** | **D** | **A** | **Y** | **N** | **N** | **N** | **K** | **D** | **L** | **P** | **F** | **T** | **A** | **R** | **F** | **A** | **T** | **G** | **Y** | **A** | **P** | **G** | **S** | **T** | **F** | **K** | **T** | **I** | **T** | **G** | **A** | **I** | **G** | **L** | **D** | **A** | **G** | **T** | **L** | **K** | **P** | **D** | **E** | **E** | **L** | **E** | **I** | **N** | **G** | **L** | **K** | **W** | **Q** | **K** | **D** | **K** | **S** | **W** | **G** | **G** | **Y** | **F** | **A** | **T** | **R** | **V** | **K** | **E** | **A** | **S** | **P** | **V** | **N** | **L** | **R** | **T** | **A** | **L** | **V** | **N** | **S** | **D** | **N** | **I** | **Y** | **F** | **A** | **Q** | **Q** | **T** | **L** | **R** | **M** | **G** | **E** | **D** | **K** | **F** | **R** | **A** | **G** | **L** | **N** | **K** | **F** | **I** | **F** | **G** | **E** | **E** | **L** | **D** | **L** | **P** | **I** | **A** | **M** | **T** | **P** | **A** | **Q** | **I** | **S** | **N** | **E** | **D** | **K** | **F** | **N** | **S** | **E** | **I** | **L** | **L** | **A** | **D** | **T** | **G** | **Y** | **G** | **Q** | **G** | **Q** | **L** | **L** | **I** | **S** | **P** | **I** | **Q** | **Q** | **A** | **T** | **M** | **Y** | **S** | **V** | **F** | **Q** | **N** | **N** | **G** | **T** | **L** | **V** | **Y** | **P** | **K** | **L** | **V** | **L** | **D** | **K** | **E** | **T** | **K** | **K** | **K** | **D** | **N** | **V** | **I** | **S** | **A** | **N** | **A** | **A** | **N** | **T** | **I** | **A** | **T** | **D** | **L** | **L** | **G** | **S** | **V** | **E** | **D** | **P** | **S** | **G** | **Y** | **V** | **Y** | **N** | **M** | **Y** | **N** | **P** | **N** | **F** | **S** | **L** | **A** | **A** | **K** | **T** | **G** | **T** | **A** | **E** | **I** | **K** | **D** | **K** | **Q** | **D** | **T** | **D** | **G** | **K** | **E** | **N** | **S** | **F** | **L** | **L** | **T** | **L** | **D** | **R** | **S** | **N** | **N** | **K** | **F** | **L** | **T** | **M** | **I** | **M** | **V** | **E** | **N** | **S** | **G** | **E** | **N** | **G** | **S** | **A** | **T** | **D** | **I** | **S** | **K** | **P** | **L** | **I** | **D** | **Y** | **L** | **E** | **A** | **T** | **I** | **K** |
| **N52662** | **M** | **E** | **R** | **S** | **N** | **R** | **N** | **K** | **K** | **S** | **S** | **K** | **K** | **P** | **L** | **I** | **L** | **G** | **V** | **S** | **A** | **L** | **V** | **L** | **I** | **A** | **A** | **V** | **G** | **G** | **G** | **Y** | **Y** | **A** | **Y** | **S** | **Q** | **W** | **Q** | **A** | **K** | **Q** | **E** | **L** | **A** | **E** | **A** | **K** | **K** | **T** | **A** | **N** | **T** | **F** | **L** | **N** | **V** | **L** | **S** | **K** | **Q** | **E** | **F** | **D** | **K** | **L** | **P** | **S** | **V** | **V** | **Q** | **E** | **T** | **S** | **L** | **K** | **K** | **N** | **G** | **Y** | **D** | **T** | **K** | **S** | **V** | **V** | **E** | **K** | **Y** | **Q** | **A** | **I** | **Y** | **S** | **G** | **I** | **Q** | **A** | **E** | **G** | **V** | **K** | **A** | **S** | **D** | **V** | **Q** | **V** | **K** | **K** | **G** | **K** | **D** | **N** | **Q** | **Y** | **T** | **F** | **T** | **Y** | **K** | **L** | **S** | **M** | **S** | **T** | **P** | **L** | **G** | **E** | **M** | **K** | **D** | **L** | **S** | **Y** | **Q** | **S** | **S** | **I** | **A** | **K** | **K** | **G** | **D** | **N** | **Y** | **Q** | **I** | **A** | **W** | **A** | **P** | **S** | **L** | **I** | **F** | **P** | **D** | **M** | **S** | **G** | **N** | **D** | **K** | **I** | **S** | **I** | **Q** | **V** | **D** | **N** | **A** | **K** | **R** | **G** | **E** | **I** | **V** | **D** | **R** | **N** | **G** | **S** | **G** | **L** | **A** | **I** | **N** | **K** | **V** | **F** | **D** | **E** | **V** | **G** | **V** | **V** | **P** | **G** | **K** | **L** | **G** | **S** | **G** | **A** | **E** | **K** | **T** | **A** | **N** | **I** | **K** | **A** | **F** | **S** | **D** | **K** | **F** | **G** | **V** | **S** | **V** | **D** | **E** | **I** | **N** | **Q** | **K** | **L** | **S** | **Q** | **G** | **W** | **V** | **Q** | **A** | **D** | **S** | **F** | **V** | **P** | **I** | **T** | **V** | **A** | **S** | **E** | **P** | **V** | **T** | **E** | **L** | **P** | **T** | **G** | **A** | **A** | **T** | **K** | **D** | **T** | **E** | **S** | **R** | **Y** | **Y** | **P** | **L** | **G** | **E** | **A** | **A** | **A** | **Q** | **L** | **I** | **G** | **Y** | **T** | **G** | **T** | **I** | **T** | **A** | **E** | **D** | **I** | **E** | **K** | **N** | **P** | **E** | **L** | **S** | **S** | **T** | **G** | **V** | **I** | **G** | **K** | **T** | **G** | **L** | **E** | **R** | **A** | **F** | **D** | **K** | **E** | **L** | **R** | **G** | **Q** | **D** | **G** | **G** | **S** | **L** | **V** | **I** | **L** | **D** | **D** | **K** | **E** | **N** | **V** | **K** | **K** | **A** | **L** | **Q** | **T** | **K** | **E** | **K** | **K** | **D** | **G** | **Q** | **T** | **I** | **K** | **L** | **T** | **I** | **D** | **S** | **G** | **V** | **Q** | **Q** | **Q** | **A** | **F** | **A** | **I** | **F** | **D** | **K** | **R** | **P** | **G** | **S** | **A** | **V** | **I** | **T** | **D** | **P** | **Q** | **K** | **G** | **D** | **L** | **L** | **A** | **T** | **V** | **S** | **S** | **P** | **S** | **Y** | **D** | **P** | **N** | **K** | **M** | **A** | **N** | **G** | **I** | **S** | **Q** | **K** | **E** | **Y** | **D** | **A** | **Y** | **N** | **N** | **N** | **K** | **D** | **L** | **P** | **F** | **T** | **A** | **R** | **F** | **A** | **T** | **G** | **Y** | **A** | **P** | **G** | **S** | **T** | **F** | **K** | **T** | **I** | **T** | **G** | **A** | **I** | **G** | **L** | **D** | **A** | **G** | **T** | **L** | **K** | **P** | **D** | **E** | **E** | **L** | **E** | **I** | **N** | **G** | **L** | **K** | **W** | **Q** | **K** | **D** | **K** | **S** | **W** | **G** | **G** | **Y** | **F** | **A** | **T** | **R** | **V** | **K** | **E** | **A** | **S** | **P** | **V** | **N** | **L** | **R** | **T** | **A** | **L** | **V** | **N** | **S** | **D** | **N** | **I** | **Y** | **F** | **A** | **Q** | **Q** | **T** | **L** | **R** | **M** | **G** | **E** | **D** | **K** | **F** | **R** | **A** | **G** | **L** | **N** | **K** | **F** | **I** | **F** | **G** | **E** | **E** | **L** | **D** | **L** | **P** | **I** | **A** | **M** | **T** | **P** | **A** | **Q** | **I** | **S** | **N** | **E** | **D** | **K** | **F** | **N** | **S** | **E** | **I** | **L** | **L** | **A** | **D** | **T** | **G** | **Y** | **G** | **Q** | **G** | **Q** | **L** | **L** | **I** | **S** | **P** | **I** | **Q** | **Q** | **A** | **T** | **M** | **Y** | **S** | **V** | **F** | **Q** | **N** | **N** | **G** | **T** | **L** | **V** | **Y** | **P** | **K** | **L** | **V** | **L** | **E** | **K** | **E** | **T** | **K** | **K** | **K** | **D** | **N** | **V** | **I** | **S** | **A** | **N** | **A** | **A** | **N** | **T** | **I** | **A** | **T** | **D** | **L** | **L** | **G** | **S** | **V** | **E** | **D** | **P** | **S** | **G** | **Y** | **V** | **Y** | **N** | **M** | **Y** | **N** | **P** | **N** | **F** | **S** | **L** | **A** | **A** | **K** | **T** | **G** | **T** | **A** | **E** | **I** | **K** | **D** | **K** | **Q** | **D** | **T** | **D** | **G** | **K** | **E** | **N** | **S** | **F** | **L** | **L** | **T** | **L** | **D** | **R** | **S** | **N** | **N** | **K** | **F** | **L** | **T** | **M** | **I** | **M** | **V** | **E** | **N** | **S** | **G** | **E** | **N** | **G** | **S** | **A** | **T** | **D** | **I** | **S** | **K** | **P** | **L** | **I** | **D** | **Y** | **L** | **E** | **A** | **T** | **I** | **K** | **M** | **E** | **R** | **S** | **N** | **R** | **N** | **K** | **K** | **S** | **S** | **K** | **K** | **P** | **L** | **I** | **L** | **G** | **V** | **S** | **A** | **L** | **V** | **L** | **I** | **A** | **A** | **V** | **G** | **G** | **G** | **Y** | **Y** | **A** | **Y** | **S** | **Q** | **W** | **Q** | **A** | **K** | **Q** | **E** | **L** | **A** | **E** | **A** | **K** | **K** | **T** | **A** | **N** | **T** | **F** | **L** | **N** | **V** | **L** | **S** | **K** | **Q** | **E** | **F** | **D** | **K** | **L** | **P** | **S** | **V** | **V** | **Q** | **E** | **T** | **S** | **L** | **K** | **K** | **N** | **G** | **Y** | **D** | **T** | **K** | **S** | **V** | **V** | **E** | **K** | **Y** | **Q** | **A** | **I** | **Y** | **S** | **G** | **I** | **Q** | **A** | **E** | **G** | **V** | **K** | **A** | **S** | **D** | **V** | **Q** | **V** | **K** | **K** | **G** | **K** | **D** | **N** | **Q** | **Y** | **T** | **F** | **T** | **Y** | **K** | **L** | **S** | **M** | **S** | **T** | **P** | **L** | **G** | **E** | **M** | **K** | **D** | **L** | **S** | **Y** | **Q** | **S** | **S** | **I** | **A** | **K** | **K** | **G** | **D** | **N** | **Y** | **Q** | **I** | **A** | **W** | **A** | **P** | **S** | **L** | **I** | **F** | **P** | **D** | **M** | **S** | **G** | **N** | **D** | **K** | **I** | **S** | **I** | **Q** | **V** | **D** | **N** | **A** | **K** | **R** | **G** | **E** | **I** | **V** | **D** | **R** | **N** | **G** | **S** | **G** | **L** | **A** | **I** | **N** | **K** | **V** | **F** | **D** | **E** | **V** | **G** | **V** | **V** | **P** | **G** | **K** | **L** | **G** | **S** | **G** | **A** | **E** | **K** | **T** | **A** | **N** | **I** | **K** | **A** | **F** | **S** | **D** | **K** | **F** | **G** | **V** | **S** | **V** | **D** | **E** | **I** | **N** | **Q** | **K** | **L** | **S** | **Q** | **G** | **W** | **V** | **Q** | **A** | **D** | **S** | **F** | **V** | **P** | **I** | **T** | **V** | **A** | **S** | **E** | **P** | **V** | **T** | **E** | **L** | **P** | **T** | **G** | **A** | **A** | **T** | **K** | **D** | **T** | **E** | **S** | **R** | **Y** | **Y** | **P** | **L** | **G** | **E** | **A** | **A** | **A** | **Q** | **L** | **I** | **G** | **Y** | **T** | **G** | **T** | **I** | **T** | **A** | **E** | **D** | **I** | **E** | **K** | **N** | **P** | **E** | **L** | **S** | **S** | **T** | **G** | **V** | **I** | **G** | **K** | **T** | **G** | **L** | **E** | **R** | **A** | **F** | **D** | **K** | **E** | **L** | **R** | **G** | **Q** | **D** | **G** | **G** | **S** | **L** | **V** | **I** | **L** | **D** | **D** | **K** | **E** | **N** | **V** | **K** | **K** | **A** | **L** | **Q** | **T** | **K** | **E** | **K** | **K** | **D** | **G** | **Q** | **T** | **I** | **K** | **L** | **T** | **I** | **D** | **S** | **G** | **V** | **Q** | **Q** | **Q** | **A** | **F** | **A** | **I** | **F** | **D** | **K** | **R** | **P** | **G** | **S** | **A** | **V** | **I** | **T** | **D** | **P** | **Q** | **K** | **G** | **D** | **L** | **L** | **A** | **T** | **V** | **S** | **S** | **P** | **S** | **Y** | **D** | **P** | **N** | **K** | **M** | **A** | **N** | **G** | **I** | **S** | **Q** | **K** | **E** | **Y** | **D** | **A** | **Y** | **N** | **N** | **N** | **K** | **D** | **L** | **P** | **F** | **T** | **A** | **R** | **F** | **A** | **T** | **G** | **Y** | **A** | **P** | **G** | **S** | **T** | **F** | **K** | **T** | **I** | **T** | **G** | **A** | **I** | **G** | **L** | **D** | **A** | **G** | **T** | **L** | **K** | **P** | **D** | **E** | **E** | **L** | **E** | **I** | **N** | **G** | **L** | **K** | **W** | **Q** | **K** | **D** | **K** | **S** | **W** | **G** | **G** | **Y** | **F** | **A** | **T** | **R** | **V** | **K** | **E** | **A** | **S** | **P** | **V** | **N** | **L** | **R** | **T** | **A** | **L** | **V** | **N** | **S** | **D** | **N** | **I** | **Y** | **F** | **A** | **Q** | **Q** | **T** | **L** | **R** | **M** | **G** | **E** | **D** | **K** | **F** | **R** | **A** | **G** | **L** | **N** | **K** | **F** | **I** | **F** | **G** | **E** | **E** | **L** | **D** | **L** | **P** | **I** | **A** | **M** | **T** | **P** | **A** | **Q** | **I** | **S** | **N** | **E** | **D** | **K** | **F** | **N** | **S** | **E** | **I** | **L** | **L** | **A** | **D** | **T** | **G** | **Y** | **G** | **Q** | **G** | **Q** | **L** | **L** | **I** | **S** | **P** | **I** | **Q** | **Q** | **A** | **T** | **M** | **Y** | **S** | **V** | **F** | **Q** | **N** | **N** | **G** | **T** | **L** | **V** | **Y** | **P** | **K** | **L** | **V** | **L** | **E** | **K** | **E** | **T** | **K** | **K** | **K** | **D** | **N** | **V** | **I** | **S** | **A** | **N** | **A** | **A** | **N** | **T** | **I** | **A** | **T** | **D** | **L** | **L** | **G** | **S** | **V** | **E** | **D** | **P** | **S** | **G** | **Y** | **V** | **Y** | **N** | **M** | **Y** | **N** | **P** | **N** | **F** | **S** | **L** | **A** | **A** | **K** | **T** | **G** | **T** | **A** | **E** | **I** | **K** | **D** | **K** | **Q** | **D** | **T** | **D** | **G** | **K** | **E** | **N** | **S** | **F** | **L** | **L** | **T** | **L** | **D** | **R** | **S** | **N** | **N** | **K** | **F** | **L** | **T** | **M** | **I** | **M** | **V** | **E** | **N** | **S** | **G** | **E** | **N** | **G** | **S** | **A** | **T** | **D** | **I** | **S** | **K** | **P** | **L** | **I** | **D** | **Y** | **L** | **E** | **A** | **T** | **I** | **K** |
| **N59550F** | **-** | **-** | **-** | **-** | **-** | **-** | **-** | **-** | **-** | **-** | **-** | **-** | **-** | **-** | **-** | **-** | **-** | **-** | **-** | **-** | **-** | **-** | **-** | **-** | **-** | **-** | **-** | **-** | **-** | **-** | **-** | **-** | **-** | **-** | **-** | **-** | **Q** | **W** | **Q** | **A** | **K** | **Q** | **E** | **L** | **A** | **E** | **A** | **K** | **K** | **T** | **A** | **T** | **T** | **F** | **L** | **N** | **V** | **L** | **T** | **K** | **Q** | **K** | **F** | **D** | **K** | **L** | **P** | **S** | **V** | **V** | **Q** | **E** | **A** | **S** | **L** | **K** | **K** | **N** | **G** | **Y** | **D** | **T** | **K** | **S** | **V** | **V** | **E** | **K** | **Y** | **Q** | **A** | **I** | **Y** | **S** | **G** | **I** | **Q** | **A** | **E** | **G** | **V** | **K** | **A** | **S** | **D** | **V** | **Q** | **V** | **K** | **K** | **A** | **K** | **D** | **N** | **Q** | **Y** | **T** | **F** | **T** | **Y** | **K** | **L** | **S** | **M** | **S** | **T** | **P** | **L** | **G** | **E** | **M** | **K** | **D** | **L** | **S** | **Y** | **Q** | **S** | **S** | **I** | **A** | **K** | **K** | **G** | **D** | **T** | **Y** | **Q** | **I** | **A** | **W** | **K** | **P** | **S** | **L** | **I** | **F** | **P** | **D** | **M** | **S** | **G** | **N** | **D** | **K** | **I** | **S** | **I** | **Q** | **V** | **D** | **N** | **A** | **K** | **R** | **G** | **E** | **I** | **V** | **D** | **R** | **N** | **G** | **S** | **G** | **L** | **A** | **I** | **N** | **K** | **V** | **F** | **D** | **E** | **V** | **G** | **V** | **V** | **P** | **G** | **K** | **L** | **G** | **S** | **G** | **A** | **E** | **K** | **T** | **A** | **N** | **I** | **K** | **A** | **F** | **S** | **D** | **K** | **F** | **G** | **V** | **S** | **V** | **D** | **E** | **I** | **N** | **Q** | **K** | **L** | **S** | **Q** | **G** | **W** | **V** | **Q** | **A** | **D** | **S** | **F** | **V** | **P** | **I** | **T** | **V** | **A** | **S** | **E** | **P** | **V** | **T** | **E** | **L** | **P** | **T** | **G** | **A** | **A** | **T** | **K** | **D** | **T** | **E** | **S** | **R** | **Y** | **Y** | **P** | **L** | **G** | **E** | **A** | **A** | **A** | **Q** | **L** | **I** | **G** | **Y** | **T** | **G** | **T** | **I** | **T** | **A** | **E** | **D** | **I** | **K** | **K** | **N** | **P** | **E** | **L** | **S** | **S** | **T** | **G** | **V** | **I** | **G** | **K** | **T** | **G** | **L** | **E** | **R** | **A** | **F** | **D** | **K** | **E** | **L** | **R** | **G** | **Q** | **D** | **G** | **G** | **S** | **L** | **V** | **I** | **L** | **D** | **D** | **K** | **E** | **N** | **V** | **K** | **K** | **A** | **L** | **Q** | **T** | **K** | **E** | **K** | **K** | **D** | **G** | **Q** | **T** | **I** | **K** | **L** | **T** | **I** | **D** | **S** | **G** | **V** | **Q** | **Q** | **Q** | **A** | **F** | **A** | **I** | **F** | **D** | **K** | **R** | **P** | **G** | **S** | **A** | **V** | **I** | **T** | **D** | **P** | **Q** | **K** | **G** | **D** | **L** | **L** | **A** | **T** | **V** | **S** | **S** | **P** | **S** | **Y** | **D** | **P** | **N** | **K** | **M** | **A** | **N** | **G** | **I** | **S** | **Q** | **K** | **E** | **Y** | **D** | **A** | **Y** | **N** | **N** | **N** | **K** | **D** | **L** | **P** | **F** | **T** | **A** | **R** | **F** | **A** | **T** | **G** | **Y** | **A** | **P** | **G** | **S** | **T** | **F** | **K** | **T** | **I** | **T** | **G** | **A** | **I** | **G** | **L** | **D** | **A** | **G** | **T** | **L** | **K** | **P** | **D** | **E** | **E** | **L** | **E** | **I** | **N** | **G** | **L** | **K** | **W** | **Q** | **K** | **D** | **K** | **S** | **W** | **G** | **G** | **Y** | **F** | **A** | **T** | **R** | **V** | **K** | **E** | **A** | **S** | **P** | **V** | **N** | **L** | **R** | **T** | **A** | **L** | **V** | **N** | **S** | **D** | **N** | **I** | **Y** | **F** | **A** | **Q** | **Q** | **T** | **L** | **R** | **M** | **G** | **E** | **D** | **K** | **F** | **R** | **A** | **G** | **L** | **N** | **K** | **F** | **I** | **F** | **G** | **E** | **E** | **L** | **D** | **L** | **P** | **I** | **A** | **M** | **T** | **P** | **A** | **Q** | **I** | **S** | **N** | **E** | **D** | **K** | **F** | **N** | **S** | **E** | **I** | **L** | **L** | **A** | **D** | **T** | **G** | **Y** | **G** | **Q** | **G** | **Q** | **L** | **L** | **I** | **S** | **P** | **I** | **Q** | **Q** | **A** | **T** | **M** | **Y** | **S** | **V** | **F** | **Q** | **N** | **N** | **G** | **T** | **L** | **V** | **Y** | **P** | **K** | **L** | **V** | **L** | **D** | **K** | **E** | **T** | **K** | **K** | **K** | **D** | **N** | **V** | **I** | **S** | **A** | **N** | **A** | **A** | **N** | **T** | **I** | **A** | **T** | **D** | **L** | **L** | **G** | **S** | **V** | **E** | **D** | **P** | **S** | **G** | **Y** | **V** | **Y** | **N** | **M** | **Y** | **N** | **P** | **N** | **F** | **S** | **L** | **A** | **A** | **K** | **T** | **G** | **T** | **A** | **E** | **I** | **K** | **D** | **K** | **Q** | **D** | **T** | **D** | **G** | **K** | **E** | **N** | **S** | **F** | **L** | **L** | **T** | **L** | **D** | **R** | **S** | **N** | **N** | **K** | **F** | **L** | **T** | **M** | **I** | **M** | **V** | **E** | **N** | **S** | **G** | **E** | **N** | **G** | **S** | **A** | **T** | **D** | **I** | **S** | **K** | **P** | **L** | **I** | **D** | **Y** | **L** | **E** | **A** | **T** | **I** | **K** | **-** | **-** | **-** | **-** | **-** | **-** | **-** | **-** | **-** | **-** | **-** | **-** | **-** | **-** | **-** | **-** | **-** | **-** | **-** | **-** | **-** | **-** | **-** | **-** | **-** | **-** | **-** | **-** | **-** | **-** | **-** | **-** | **-** | **-** | **-** | **-** | **Q** | **W** | **Q** | **A** | **K** | **Q** | **E** | **L** | **A** | **E** | **A** | **K** | **K** | **T** | **A** | **T** | **T** | **F** | **L** | **N** | **V** | **L** | **T** | **K** | **Q** | **K** | **F** | **D** | **K** | **L** | **P** | **S** | **V** | **V** | **Q** | **E** | **A** | **S** | **L** | **K** | **K** | **N** | **G** | **Y** | **D** | **T** | **K** | **S** | **V** | **V** | **E** | **K** | **Y** | **Q** | **A** | **I** | **Y** | **S** | **G** | **I** | **Q** | **A** | **E** | **G** | **V** | **K** | **A** | **S** | **D** | **V** | **Q** | **V** | **K** | **K** | **A** | **K** | **D** | **N** | **Q** | **Y** | **T** | **F** | **T** | **Y** | **K** | **L** | **S** | **M** | **S** | **T** | **P** | **L** | **G** | **E** | **M** | **K** | **D** | **L** | **S** | **Y** | **Q** | **S** | **S** | **I** | **A** | **K** | **K** | **G** | **D** | **T** | **Y** | **Q** | **I** | **A** | **W** | **K** | **P** | **S** | **L** | **I** | **F** | **P** | **D** | **M** | **S** | **G** | **N** | **D** | **K** | **I** | **S** | **I** | **Q** | **V** | **D** | **N** | **A** | **K** | **R** | **G** | **E** | **I** | **V** | **D** | **R** | **N** | **G** | **S** | **G** | **L** | **A** | **I** | **N** | **K** | **V** | **F** | **D** | **E** | **V** | **G** | **V** | **V** | **P** | **G** | **K** | **L** | **G** | **S** | **G** | **A** | **E** | **K** | **T** | **A** | **N** | **I** | **K** | **A** | **F** | **S** | **D** | **K** | **F** | **G** | **V** | **S** | **V** | **D** | **E** | **I** | **N** | **Q** | **K** | **L** | **S** | **Q** | **G** | **W** | **V** | **Q** | **A** | **D** | **S** | **F** | **V** | **P** | **I** | **T** | **V** | **A** | **S** | **E** | **P** | **V** | **T** | **E** | **L** | **P** | **T** | **G** | **A** | **A** | **T** | **K** | **D** | **T** | **E** | **S** | **R** | **Y** | **Y** | **P** | **L** | **G** | **E** | **A** | **A** | **A** | **Q** | **L** | **I** | **G** | **Y** | **T** | **G** | **T** | **I** | **T** | **A** | **E** | **D** | **I** | **K** | **K** | **N** | **P** | **E** | **L** | **S** | **S** | **T** | **G** | **V** | **I** | **G** | **K** | **T** | **G** | **L** | **E** | **R** | **A** | **F** | **D** | **K** | **E** | **L** | **R** | **G** | **Q** | **D** | **G** | **G** | **S** | **L** | **V** | **I** | **L** | **D** | **D** | **K** | **E** | **N** | **V** | **K** | **K** | **A** | **L** | **Q** | **T** | **K** | **E** | **K** | **K** | **D** | **G** | **Q** | **T** | **I** | **K** | **L** | **T** | **I** | **D** | **S** | **G** | **V** | **Q** | **Q** | **Q** | **A** | **F** | **A** | **I** | **F** | **D** | **K** | **R** | **P** | **G** | **S** | **A** | **V** | **I** | **T** | **D** | **P** | **Q** | **K** | **G** | **D** | **L** | **L** | **A** | **T** | **V** | **S** | **S** | **P** | **S** | **Y** | **D** | **P** | **N** | **K** | **M** | **A** | **N** | **G** | **I** | **S** | **Q** | **K** | **E** | **Y** | **D** | **A** | **Y** | **N** | **N** | **N** | **K** | **D** | **L** | **P** | **F** | **T** | **A** | **R** | **F** | **A** | **T** | **G** | **Y** | **A** | **P** | **G** | **S** | **T** | **F** | **K** | **T** | **I** | **T** | **G** | **A** | **I** | **G** | **L** | **D** | **A** | **G** | **T** | **L** | **K** | **P** | **D** | **E** | **E** | **L** | **E** | **I** | **N** | **G** | **L** | **K** | **W** | **Q** | **K** | **D** | **K** | **S** | **W** | **G** | **G** | **Y** | **F** | **A** | **T** | **R** | **V** | **K** | **E** | **A** | **S** | **P** | **V** | **N** | **L** | **R** | **T** | **A** | **L** | **V** | **N** | **S** | **D** | **N** | **I** | **Y** | **F** | **A** | **Q** | **Q** | **T** | **L** | **R** | **M** | **G** | **E** | **D** | **K** | **F** | **R** | **A** | **G** | **L** | **N** | **K** | **F** | **I** | **F** | **G** | **E** | **E** | **L** | **D** | **L** | **P** | **I** | **A** | **M** | **T** | **P** | **A** | **Q** | **I** | **S** | **N** | **E** | **D** | **K** | **F** | **N** | **S** | **E** | **I** | **L** | **L** | **A** | **D** | **T** | **G** | **Y** | **G** | **Q** | **G** | **Q** | **L** | **L** | **I** | **S** | **P** | **I** | **Q** | **Q** | **A** | **T** | **M** | **Y** | **S** | **V** | **F** | **Q** | **N** | **N** | **G** | **T** | **L** | **V** | **Y** | **P** | **K** | **L** | **V** | **L** | **D** | **K** | **E** | **T** | **K** | **K** | **K** | **D** | **N** | **V** | **I** | **S** | **A** | **N** | **A** | **A** | **N** | **T** | **I** | **A** | **T** | **D** | **L** | **L** | **G** | **S** | **V** | **E** | **D** | **P** | **S** | **G** | **Y** | **V** | **Y** | **N** | **M** | **Y** | **N** | **P** | **N** | **F** | **S** | **L** | **A** | **A** | **K** | **T** | **G** | **T** | **A** | **E** | **I** | **K** | **D** | **K** | **Q** | **D** | **T** | **D** | **G** | **K** | **E** | **N** | **S** | **F** | **L** | **L** | **T** | **L** | **D** | **R** | **S** | **N** | **N** | **K** | **F** | **L** | **T** | **M** | **I** | **M** | **V** | **E** | **N** | **S** | **G** | **E** | **N** | **G** | **S** | **A** | **T** | **D** | **I** | **S** | **K** | **P** | **L** | **I** | **D** | **Y** | **L** | **E** | **A** | **T** | **I** | **K** |
| **N54553** | **-** | **-** | **-** | **-** | **-** | **-** | **-** | **-** | **-** | **-** | **-** | **-** | **-** | **-** | **-** | **-** | **-** | **-** | **-** | **-** | **-** | **-** | **-** | **-** | **-** | **-** | **-** | **-** | **-** | **-** | **-** | **-** | **-** | **-** | **-** | **-** | **Q** | **W** | **Q** | **A** | **K** | **Q** | **E** | **L** | **A** | **E** | **A** | **K** | **K** | **T** | **A** | **T** | **T** | **F** | **L** | **N** | **V** | **L** | **T** | **K** | **Q** | **K** | **F** | **D** | **K** | **L** | **P** | **S** | **V** | **V** | **Q** | **E** | **A** | **S** | **L** | **K** | **K** | **N** | **G** | **Y** | **D** | **T** | **K** | **S** | **V** | **V** | **E** | **K** | **Y** | **Q** | **A** | **I** | **Y** | **S** | **G** | **I** | **Q** | **A** | **E** | **G** | **V** | **K** | **A** | **S** | **D** | **V** | **Q** | **V** | **K** | **K** | **A** | **K** | **D** | **N** | **Q** | **Y** | **T** | **F** | **T** | **Y** | **K** | **L** | **S** | **M** | **S** | **T** | **P** | **L** | **G** | **E** | **M** | **K** | **D** | **L** | **S** | **Y** | **Q** | **S** | **S** | **I** | **A** | **K** | **K** | **G** | **D** | **T** | **Y** | **Q** | **I** | **A** | **W** | **K** | **P** | **S** | **L** | **I** | **F** | **P** | **D** | **M** | **S** | **G** | **N** | **D** | **K** | **I** | **S** | **I** | **Q** | **V** | **D** | **N** | **A** | **K** | **R** | **G** | **E** | **I** | **V** | **D** | **R** | **N** | **G** | **S** | **G** | **L** | **A** | **I** | **N** | **K** | **V** | **F** | **D** | **E** | **V** | **G** | **V** | **V** | **P** | **G** | **K** | **L** | **G** | **S** | **G** | **A** | **E** | **K** | **T** | **A** | **N** | **I** | **K** | **A** | **F** | **S** | **D** | **K** | **F** | **G** | **V** | **S** | **V** | **D** | **E** | **I** | **N** | **Q** | **K** | **L** | **S** | **Q** | **G** | **W** | **V** | **Q** | **A** | **D** | **S** | **F** | **V** | **P** | **I** | **T** | **V** | **A** | **S** | **E** | **P** | **V** | **T** | **E** | **L** | **P** | **T** | **G** | **A** | **A** | **T** | **K** | **D** | **T** | **E** | **S** | **R** | **Y** | **Y** | **P** | **L** | **G** | **E** | **A** | **A** | **A** | **Q** | **L** | **I** | **G** | **Y** | **T** | **G** | **T** | **I** | **T** | **A** | **E** | **D** | **I** | **K** | **K** | **N** | **P** | **E** | **L** | **S** | **S** | **T** | **G** | **V** | **I** | **G** | **K** | **T** | **G** | **L** | **E** | **R** | **A** | **F** | **D** | **K** | **E** | **L** | **R** | **G** | **Q** | **D** | **G** | **G** | **S** | **L** | **V** | **I** | **L** | **D** | **D** | **K** | **E** | **N** | **V** | **K** | **K** | **A** | **L** | **Q** | **T** | **K** | **E** | **K** | **K** | **D** | **G** | **Q** | **T** | **I** | **K** | **L** | **T** | **I** | **D** | **S** | **G** | **V** | **Q** | **Q** | **Q** | **A** | **F** | **A** | **I** | **F** | **D** | **K** | **R** | **P** | **G** | **S** | **A** | **V** | **I** | **T** | **D** | **P** | **Q** | **K** | **G** | **D** | **L** | **L** | **A** | **T** | **V** | **S** | **S** | **P** | **S** | **Y** | **D** | **P** | **N** | **K** | **M** | **A** | **N** | **G** | **I** | **S** | **Q** | **K** | **E** | **Y** | **D** | **A** | **Y** | **N** | **N** | **N** | **K** | **D** | **L** | **P** | **F** | **T** | **A** | **R** | **F** | **A** | **T** | **G** | **Y** | **A** | **P** | **G** | **S** | **T** | **F** | **K** | **T** | **I** | **T** | **G** | **A** | **I** | **G** | **L** | **D** | **A** | **G** | **T** | **L** | **K** | **P** | **D** | **E** | **E** | **L** | **E** | **I** | **N** | **G** | **L** | **K** | **W** | **Q** | **K** | **D** | **K** | **S** | **W** | **G** | **G** | **Y** | **F** | **A** | **T** | **R** | **V** | **K** | **E** | **A** | **S** | **P** | **V** | **N** | **L** | **R** | **T** | **A** | **L** | **V** | **N** | **S** | **D** | **N** | **I** | **Y** | **F** | **A** | **Q** | **Q** | **T** | **L** | **R** | **M** | **G** | **E** | **D** | **K** | **F** | **R** | **A** | **G** | **L** | **N** | **K** | **F** | **I** | **F** | **G** | **E** | **E** | **L** | **D** | **L** | **P** | **I** | **A** | **M** | **T** | **P** | **A** | **Q** | **I** | **S** | **N** | **E** | **D** | **K** | **F** | **N** | **S** | **E** | **I** | **L** | **L** | **A** | **D** | **T** | **G** | **Y** | **G** | **Q** | **G** | **Q** | **L** | **L** | **I** | **S** | **P** | **I** | **Q** | **Q** | **A** | **T** | **M** | **Y** | **S** | **V** | **F** | **Q** | **N** | **N** | **G** | **T** | **L** | **V** | **Y** | **P** | **K** | **L** | **V** | **L** | **D** | **K** | **E** | **T** | **K** | **K** | **K** | **D** | **N** | **V** | **I** | **S** | **A** | **N** | **A** | **A** | **N** | **T** | **I** | **A** | **T** | **D** | **L** | **L** | **G** | **S** | **V** | **E** | **D** | **P** | **S** | **G** | **Y** | **V** | **Y** | **N** | **M** | **Y** | **N** | **P** | **N** | **F** | **S** | **L** | **A** | **A** | **K** | **T** | **G** | **T** | **A** | **E** | **I** | **K** | **D** | **K** | **Q** | **D** | **T** | **D** | **G** | **K** | **E** | **N** | **S** | **F** | **L** | **L** | **T** | **L** | **D** | **R** | **S** | **N** | **N** | **K** | **F** | **L** | **T** | **M** | **I** | **M** | **V** | **E** | **N** | **S** | **G** | **E** | **N** | **G** | **S** | **A** | **T** | **D** | **I** | **S** | **K** | **P** | **L** | **I** | **D** | **Y** | **L** | **E** | **A** | **T** | **I** | **K** | **-** | **-** | **-** | **-** | **-** | **-** | **-** | **-** | **-** | **-** | **-** | **-** | **-** | **-** | **-** | **-** | **-** | **-** | **-** | **-** | **-** | **-** | **-** | **-** | **-** | **-** | **-** | **-** | **-** | **-** | **-** | **-** | **-** | **-** | **-** | **-** | **Q** | **W** | **Q** | **A** | **K** | **Q** | **E** | **L** | **A** | **E** | **A** | **K** | **K** | **T** | **A** | **T** | **T** | **F** | **L** | **N** | **V** | **L** | **T** | **K** | **Q** | **K** | **F** | **D** | **K** | **L** | **P** | **S** | **V** | **V** | **Q** | **E** | **A** | **S** | **L** | **K** | **K** | **N** | **G** | **Y** | **D** | **T** | **K** | **S** | **V** | **V** | **E** | **K** | **Y** | **Q** | **A** | **I** | **Y** | **S** | **G** | **I** | **Q** | **A** | **E** | **G** | **V** | **K** | **A** | **S** | **D** | **V** | **Q** | **V** | **K** | **K** | **A** | **K** | **D** | **N** | **Q** | **Y** | **T** | **F** | **T** | **Y** | **K** | **L** | **S** | **M** | **S** | **T** | **P** | **L** | **G** | **E** | **M** | **K** | **D** | **L** | **S** | **Y** | **Q** | **S** | **S** | **I** | **A** | **K** | **K** | **G** | **D** | **T** | **Y** | **Q** | **I** | **A** | **W** | **K** | **P** | **S** | **L** | **I** | **F** | **P** | **D** | **M** | **S** | **G** | **N** | **D** | **K** | **I** | **S** | **I** | **Q** | **V** | **D** | **N** | **A** | **K** | **R** | **G** | **E** | **I** | **V** | **D** | **R** | **N** | **G** | **S** | **G** | **L** | **A** | **I** | **N** | **K** | **V** | **F** | **D** | **E** | **V** | **G** | **V** | **V** | **P** | **G** | **K** | **L** | **G** | **S** | **G** | **A** | **E** | **K** | **T** | **A** | **N** | **I** | **K** | **A** | **F** | **S** | **D** | **K** | **F** | **G** | **V** | **S** | **V** | **D** | **E** | **I** | **N** | **Q** | **K** | **L** | **S** | **Q** | **G** | **W** | **V** | **Q** | **A** | **D** | **S** | **F** | **V** | **P** | **I** | **T** | **V** | **A** | **S** | **E** | **P** | **V** | **T** | **E** | **L** | **P** | **T** | **G** | **A** | **A** | **T** | **K** | **D** | **T** | **E** | **S** | **R** | **Y** | **Y** | **P** | **L** | **G** | **E** | **A** | **A** | **A** | **Q** | **L** | **I** | **G** | **Y** | **T** | **G** | **T** | **I** | **T** | **A** | **E** | **D** | **I** | **K** | **K** | **N** | **P** | **E** | **L** | **S** | **S** | **T** | **G** | **V** | **I** | **G** | **K** | **T** | **G** | **L** | **E** | **R** | **A** | **F** | **D** | **K** | **E** | **L** | **R** | **G** | **Q** | **D** | **G** | **G** | **S** | **L** | **V** | **I** | **L** | **D** | **D** | **K** | **E** | **N** | **V** | **K** | **K** | **A** | **L** | **Q** | **T** | **K** | **E** | **K** | **K** | **D** | **G** | **Q** | **T** | **I** | **K** | **L** | **T** | **I** | **D** | **S** | **G** | **V** | **Q** | **Q** | **Q** | **A** | **F** | **A** | **I** | **F** | **D** | **K** | **R** | **P** | **G** | **S** | **A** | **V** | **I** | **T** | **D** | **P** | **Q** | **K** | **G** | **D** | **L** | **L** | **A** | **T** | **V** | **S** | **S** | **P** | **S** | **Y** | **D** | **P** | **N** | **K** | **M** | **A** | **N** | **G** | **I** | **S** | **Q** | **K** | **E** | **Y** | **D** | **A** | **Y** | **N** | **N** | **N** | **K** | **D** | **L** | **P** | **F** | **T** | **A** | **R** | **F** | **A** | **T** | **G** | **Y** | **A** | **P** | **G** | **S** | **T** | **F** | **K** | **T** | **I** | **T** | **G** | **A** | **I** | **G** | **L** | **D** | **A** | **G** | **T** | **L** | **K** | **P** | **D** | **E** | **E** | **L** | **E** | **I** | **N** | **G** | **L** | **K** | **W** | **Q** | **K** | **D** | **K** | **S** | **W** | **G** | **G** | **Y** | **F** | **A** | **T** | **R** | **V** | **K** | **E** | **A** | **S** | **P** | **V** | **N** | **L** | **R** | **T** | **A** | **L** | **V** | **N** | **S** | **D** | **N** | **I** | **Y** | **F** | **A** | **Q** | **Q** | **T** | **L** | **R** | **M** | **G** | **E** | **D** | **K** | **F** | **R** | **A** | **G** | **L** | **N** | **K** | **F** | **I** | **F** | **G** | **E** | **E** | **L** | **D** | **L** | **P** | **I** | **A** | **M** | **T** | **P** | **A** | **Q** | **I** | **S** | **N** | **E** | **D** | **K** | **F** | **N** | **S** | **E** | **I** | **L** | **L** | **A** | **D** | **T** | **G** | **Y** | **G** | **Q** | **G** | **Q** | **L** | **L** | **I** | **S** | **P** | **I** | **Q** | **Q** | **A** | **T** | **M** | **Y** | **S** | **V** | **F** | **Q** | **N** | **N** | **G** | **T** | **L** | **V** | **Y** | **P** | **K** | **L** | **V** | **L** | **D** | **K** | **E** | **T** | **K** | **K** | **K** | **D** | **N** | **V** | **I** | **S** | **A** | **N** | **A** | **A** | **N** | **T** | **I** | **A** | **T** | **D** | **L** | **L** | **G** | **S** | **V** | **E** | **D** | **P** | **S** | **G** | **Y** | **V** | **Y** | **N** | **M** | **Y** | **N** | **P** | **N** | **F** | **S** | **L** | **A** | **A** | **K** | **T** | **G** | **T** | **A** | **E** | **I** | **K** | **D** | **K** | **Q** | **D** | **T** | **D** | **G** | **K** | **E** | **N** | **S** | **F** | **L** | **L** | **T** | **L** | **D** | **R** | **S** | **N** | **N** | **K** | **F** | **L** | **T** | **M** | **I** | **M** | **V** | **E** | **N** | **S** | **G** | **E** | **N** | **G** | **S** | **A** | **T** | **D** | **I** | **S** | **K** | **P** | **L** | **I** | **D** | **Y** | **L** | **E** | **A** | **T** | **I** | **K** |
| **N54595** | **-** | **-** | **-** | **-** | **-** | **-** | **-** | **-** | **-** | **-** | **-** | **-** | **-** | **-** | **-** | **-** | **-** | **-** | **-** | **-** | **-** | **-** | **-** | **-** | **-** | **-** | **-** | **-** | **-** | **-** | **-** | **-** | **-** | **-** | **-** | **-** | **Q** | **W** | **Q** | **A** | **K** | **Q** | **E** | **L** | **A** | **E** | **A** | **K** | **K** | **T** | **A** | **T** | **T** | **F** | **L** | **N** | **V** | **L** | **S** | **K** | **Q** | **E** | **F** | **D** | **K** | **L** | **P** | **S** | **V** | **V** | **Q** | **E** | **A** | **S** | **L** | **K** | **K** | **N** | **G** | **Y** | **D** | **T** | **K** | **S** | **V** | **V** | **E** | **K** | **Y** | **Q** | **A** | **I** | **Y** | **S** | **G** | **I** | **Q** | **A** | **E** | **G** | **V** | **K** | **A** | **S** | **D** | **V** | **Q** | **V** | **K** | **K** | **A** | **K** | **D** | **N** | **Q** | **Y** | **T** | **F** | **T** | **Y** | **K** | **L** | **S** | **M** | **S** | **T** | **P** | **L** | **G** | **E** | **M** | **K** | **D** | **L** | **S** | **Y** | **Q** | **S** | **S** | **I** | **A** | **K** | **K** | **G** | **D** | **T** | **Y** | **Q** | **I** | **A** | **W** | **K** | **P** | **S** | **L** | **I** | **F** | **P** | **D** | **M** | **S** | **G** | **N** | **D** | **K** | **I** | **S** | **I** | **Q** | **V** | **D** | **N** | **A** | **K** | **R** | **G** | **E** | **I** | **V** | **D** | **R** | **N** | **G** | **S** | **G** | **L** | **A** | **I** | **N** | **K** | **V** | **F** | **D** | **E** | **V** | **G** | **V** | **V** | **P** | **G** | **K** | **L** | **G** | **S** | **G** | **A** | **E** | **K** | **T** | **A** | **N** | **I** | **K** | **A** | **F** | **S** | **D** | **K** | **F** | **G** | **V** | **S** | **V** | **D** | **E** | **I** | **N** | **Q** | **K** | **L** | **S** | **Q** | **G** | **W** | **V** | **Q** | **A** | **D** | **S** | **F** | **V** | **P** | **I** | **T** | **V** | **A** | **S** | **E** | **P** | **V** | **T** | **E** | **L** | **P** | **T** | **G** | **A** | **A** | **T** | **K** | **D** | **T** | **E** | **S** | **R** | **Y** | **Y** | **P** | **L** | **G** | **E** | **A** | **A** | **A** | **Q** | **L** | **I** | **G** | **Y** | **T** | **G** | **T** | **I** | **T** | **A** | **E** | **D** | **I** | **E** | **K** | **N** | **P** | **E** | **L** | **S** | **S** | **T** | **G** | **V** | **I** | **G** | **K** | **T** | **G** | **L** | **E** | **R** | **A** | **F** | **D** | **K** | **E** | **L** | **R** | **G** | **Q** | **D** | **G** | **G** | **S** | **L** | **V** | **I** | **L** | **D** | **D** | **K** | **E** | **N** | **V** | **K** | **K** | **A** | **L** | **Q** | **T** | **K** | **E** | **K** | **K** | **D** | **G** | **Q** | **T** | **I** | **K** | **L** | **T** | **I** | **D** | **S** | **G** | **V** | **Q** | **Q** | **Q** | **A** | **F** | **A** | **I** | **F** | **D** | **K** | **R** | **P** | **G** | **S** | **A** | **V** | **I** | **T** | **D** | **P** | **Q** | **K** | **G** | **D** | **L** | **L** | **A** | **T** | **V** | **S** | **S** | **P** | **S** | **Y** | **D** | **P** | **N** | **K** | **M** | **A** | **N** | **G** | **I** | **S** | **Q** | **K** | **E** | **Y** | **D** | **A** | **Y** | **N** | **N** | **N** | **K** | **D** | **L** | **P** | **F** | **T** | **A** | **R** | **F** | **A** | **T** | **G** | **Y** | **A** | **P** | **G** | **S** | **T** | **F** | **K** | **T** | **I** | **T** | **G** | **A** | **I** | **G** | **L** | **D** | **A** | **G** | **T** | **L** | **K** | **P** | **D** | **E** | **E** | **L** | **E** | **I** | **N** | **G** | **L** | **K** | **W** | **Q** | **K** | **D** | **K** | **S** | **W** | **G** | **G** | **Y** | **F** | **A** | **T** | **R** | **V** | **K** | **E** | **A** | **S** | **P** | **V** | **N** | **L** | **R** | **T** | **A** | **L** | **V** | **N** | **S** | **D** | **N** | **I** | **Y** | **F** | **A** | **Q** | **Q** | **T** | **L** | **R** | **M** | **G** | **E** | **D** | **K** | **F** | **R** | **A** | **G** | **L** | **N** | **K** | **F** | **I** | **F** | **G** | **E** | **E** | **L** | **D** | **L** | **P** | **I** | **A** | **M** | **T** | **P** | **A** | **Q** | **I** | **S** | **N** | **E** | **D** | **K** | **F** | **N** | **S** | **E** | **I** | **L** | **L** | **A** | **D** | **T** | **G** | **Y** | **G** | **Q** | **G** | **Q** | **L** | **L** | **I** | **S** | **P** | **I** | **Q** | **Q** | **A** | **T** | **M** | **Y** | **S** | **V** | **F** | **Q** | **N** | **N** | **G** | **T** | **L** | **V** | **Y** | **P** | **K** | **L** | **V** | **L** | **E** | **K** | **E** | **T** | **K** | **K** | **K** | **D** | **N** | **V** | **I** | **S** | **A** | **N** | **A** | **A** | **N** | **T** | **I** | **A** | **T** | **D** | **L** | **L** | **G** | **S** | **V** | **E** | **D** | **P** | **S** | **G** | **Y** | **V** | **Y** | **N** | **M** | **Y** | **N** | **P** | **N** | **F** | **S** | **L** | **A** | **A** | **K** | **T** | **G** | **T** | **A** | **E** | **I** | **K** | **D** | **K** | **Q** | **D** | **T** | **D** | **G** | **K** | **E** | **N** | **S** | **F** | **L** | **L** | **T** | **L** | **D** | **R** | **S** | **N** | **N** | **K** | **F** | **L** | **T** | **M** | **I** | **M** | **V** | **E** | **N** | **S** | **G** | **E** | **N** | **G** | **S** | **A** | **T** | **D** | **I** | **S** | **K** | **P** | **L** | **I** | **D** | **Y** | **L** | **E** | **A** | **T** | **I** | **K** | **-** | **-** | **-** | **-** | **-** | **-** | **-** | **-** | **-** | **-** | **-** | **-** | **-** | **-** | **-** | **-** | **-** | **-** | **-** | **-** | **-** | **-** | **-** | **-** | **-** | **-** | **-** | **-** | **-** | **-** | **-** | **-** | **-** | **-** | **-** | **-** | **Q** | **W** | **Q** | **A** | **K** | **Q** | **E** | **L** | **A** | **E** | **A** | **K** | **K** | **T** | **A** | **T** | **T** | **F** | **L** | **N** | **V** | **L** | **S** | **K** | **Q** | **E** | **F** | **D** | **K** | **L** | **P** | **S** | **V** | **V** | **Q** | **E** | **A** | **S** | **L** | **K** | **K** | **N** | **G** | **Y** | **D** | **T** | **K** | **S** | **V** | **V** | **E** | **K** | **Y** | **Q** | **A** | **I** | **Y** | **S** | **G** | **I** | **Q** | **A** | **E** | **G** | **V** | **K** | **A** | **S** | **D** | **V** | **Q** | **V** | **K** | **K** | **A** | **K** | **D** | **N** | **Q** | **Y** | **T** | **F** | **T** | **Y** | **K** | **L** | **S** | **M** | **S** | **T** | **P** | **L** | **G** | **E** | **M** | **K** | **D** | **L** | **S** | **Y** | **Q** | **S** | **S** | **I** | **A** | **K** | **K** | **G** | **D** | **T** | **Y** | **Q** | **I** | **A** | **W** | **K** | **P** | **S** | **L** | **I** | **F** | **P** | **D** | **M** | **S** | **G** | **N** | **D** | **K** | **I** | **S** | **I** | **Q** | **V** | **D** | **N** | **A** | **K** | **R** | **G** | **E** | **I** | **V** | **D** | **R** | **N** | **G** | **S** | **G** | **L** | **A** | **I** | **N** | **K** | **V** | **F** | **D** | **E** | **V** | **G** | **V** | **V** | **P** | **G** | **K** | **L** | **G** | **S** | **G** | **A** | **E** | **K** | **T** | **A** | **N** | **I** | **K** | **A** | **F** | **S** | **D** | **K** | **F** | **G** | **V** | **S** | **V** | **D** | **E** | **I** | **N** | **Q** | **K** | **L** | **S** | **Q** | **G** | **W** | **V** | **Q** | **A** | **D** | **S** | **F** | **V** | **P** | **I** | **T** | **V** | **A** | **S** | **E** | **P** | **V** | **T** | **E** | **L** | **P** | **T** | **G** | **A** | **A** | **T** | **K** | **D** | **T** | **E** | **S** | **R** | **Y** | **Y** | **P** | **L** | **G** | **E** | **A** | **A** | **A** | **Q** | **L** | **I** | **G** | **Y** | **T** | **G** | **T** | **I** | **T** | **A** | **E** | **D** | **I** | **E** | **K** | **N** | **P** | **E** | **L** | **S** | **S** | **T** | **G** | **V** | **I** | **G** | **K** | **T** | **G** | **L** | **E** | **R** | **A** | **F** | **D** | **K** | **E** | **L** | **R** | **G** | **Q** | **D** | **G** | **G** | **S** | **L** | **V** | **I** | **L** | **D** | **D** | **K** | **E** | **N** | **V** | **K** | **K** | **A** | **L** | **Q** | **T** | **K** | **E** | **K** | **K** | **D** | **G** | **Q** | **T** | **I** | **K** | **L** | **T** | **I** | **D** | **S** | **G** | **V** | **Q** | **Q** | **Q** | **A** | **F** | **A** | **I** | **F** | **D** | **K** | **R** | **P** | **G** | **S** | **A** | **V** | **I** | **T** | **D** | **P** | **Q** | **K** | **G** | **D** | **L** | **L** | **A** | **T** | **V** | **S** | **S** | **P** | **S** | **Y** | **D** | **P** | **N** | **K** | **M** | **A** | **N** | **G** | **I** | **S** | **Q** | **K** | **E** | **Y** | **D** | **A** | **Y** | **N** | **N** | **N** | **K** | **D** | **L** | **P** | **F** | **T** | **A** | **R** | **F** | **A** | **T** | **G** | **Y** | **A** | **P** | **G** | **S** | **T** | **F** | **K** | **T** | **I** | **T** | **G** | **A** | **I** | **G** | **L** | **D** | **A** | **G** | **T** | **L** | **K** | **P** | **D** | **E** | **E** | **L** | **E** | **I** | **N** | **G** | **L** | **K** | **W** | **Q** | **K** | **D** | **K** | **S** | **W** | **G** | **G** | **Y** | **F** | **A** | **T** | **R** | **V** | **K** | **E** | **A** | **S** | **P** | **V** | **N** | **L** | **R** | **T** | **A** | **L** | **V** | **N** | **S** | **D** | **N** | **I** | **Y** | **F** | **A** | **Q** | **Q** | **T** | **L** | **R** | **M** | **G** | **E** | **D** | **K** | **F** | **R** | **A** | **G** | **L** | **N** | **K** | **F** | **I** | **F** | **G** | **E** | **E** | **L** | **D** | **L** | **P** | **I** | **A** | **M** | **T** | **P** | **A** | **Q** | **I** | **S** | **N** | **E** | **D** | **K** | **F** | **N** | **S** | **E** | **I** | **L** | **L** | **A** | **D** | **T** | **G** | **Y** | **G** | **Q** | **G** | **Q** | **L** | **L** | **I** | **S** | **P** | **I** | **Q** | **Q** | **A** | **T** | **M** | **Y** | **S** | **V** | **F** | **Q** | **N** | **N** | **G** | **T** | **L** | **V** | **Y** | **P** | **K** | **L** | **V** | **L** | **E** | **K** | **E** | **T** | **K** | **K** | **K** | **D** | **N** | **V** | **I** | **S** | **A** | **N** | **A** | **A** | **N** | **T** | **I** | **A** | **T** | **D** | **L** | **L** | **G** | **S** | **V** | **E** | **D** | **P** | **S** | **G** | **Y** | **V** | **Y** | **N** | **M** | **Y** | **N** | **P** | **N** | **F** | **S** | **L** | **A** | **A** | **K** | **T** | **G** | **T** | **A** | **E** | **I** | **K** | **D** | **K** | **Q** | **D** | **T** | **D** | **G** | **K** | **E** | **N** | **S** | **F** | **L** | **L** | **T** | **L** | **D** | **R** | **S** | **N** | **N** | **K** | **F** | **L** | **T** | **M** | **I** | **M** | **V** | **E** | **N** | **S** | **G** | **E** | **N** | **G** | **S** | **A** | **T** | **D** | **I** | **S** | **K** | **P** | **L** | **I** | **D** | **Y** | **L** | **E** | **A** | **T** | **I** | **K** |
| **N59862F** | **-** | **-** | **-** | **-** | **-** | **-** | **-** | **-** | **-** | **-** | **-** | **-** | **-** | **-** | **-** | **-** | **-** | **-** | **-** | **-** | **-** | **-** | **-** | **-** | **-** | **-** | **-** | **-** | **-** | **-** | **-** | **-** | **-** | **-** | **-** | **-** | **Q** | **W** | **Q** | **A** | **K** | **Q** | **E** | **L** | **A** | **E** | **A** | **K** | **K** | **T** | **A** | **T** | **T** | **F** | **L** | **N** | **V** | **L** | **S** | **K** | **Q** | **E** | **F** | **D** | **K** | **L** | **P** | **S** | **V** | **V** | **Q** | **E** | **A** | **S** | **L** | **K** | **K** | **N** | **G** | **Y** | **D** | **T** | **K** | **S** | **V** | **V** | **E** | **K** | **Y** | **Q** | **A** | **I** | **Y** | **S** | **G** | **I** | **Q** | **A** | **E** | **G** | **V** | **K** | **A** | **S** | **D** | **V** | **Q** | **V** | **K** | **K** | **A** | **K** | **D** | **N** | **Q** | **Y** | **T** | **F** | **T** | **Y** | **K** | **L** | **S** | **M** | **S** | **T** | **P** | **L** | **G** | **E** | **M** | **K** | **D** | **L** | **S** | **Y** | **Q** | **S** | **S** | **I** | **A** | **K** | **K** | **G** | **D** | **T** | **Y** | **Q** | **I** | **A** | **W** | **K** | **P** | **S** | **L** | **I** | **F** | **P** | **D** | **M** | **S** | **G** | **N** | **D** | **K** | **I** | **S** | **I** | **Q** | **V** | **D** | **N** | **A** | **K** | **R** | **G** | **E** | **I** | **V** | **D** | **R** | **N** | **G** | **S** | **G** | **L** | **A** | **I** | **N** | **K** | **V** | **F** | **D** | **E** | **V** | **G** | **V** | **V** | **P** | **G** | **K** | **L** | **G** | **S** | **G** | **A** | **E** | **K** | **T** | **A** | **N** | **I** | **K** | **A** | **F** | **S** | **D** | **K** | **F** | **G** | **V** | **S** | **V** | **D** | **E** | **I** | **N** | **Q** | **K** | **L** | **S** | **Q** | **G** | **W** | **V** | **Q** | **A** | **D** | **S** | **F** | **V** | **P** | **I** | **T** | **V** | **A** | **S** | **E** | **P** | **V** | **T** | **E** | **L** | **P** | **T** | **G** | **A** | **A** | **T** | **K** | **D** | **T** | **E** | **S** | **R** | **Y** | **Y** | **P** | **L** | **G** | **E** | **A** | **A** | **A** | **Q** | **L** | **I** | **G** | **Y** | **T** | **G** | **T** | **I** | **T** | **A** | **E** | **D** | **I** | **E** | **K** | **N** | **P** | **E** | **L** | **S** | **S** | **T** | **G** | **V** | **I** | **G** | **K** | **T** | **G** | **L** | **E** | **R** | **A** | **F** | **D** | **K** | **E** | **L** | **R** | **G** | **Q** | **D** | **G** | **G** | **S** | **L** | **V** | **I** | **L** | **D** | **D** | **K** | **E** | **N** | **V** | **K** | **K** | **A** | **L** | **Q** | **T** | **K** | **E** | **K** | **K** | **D** | **G** | **Q** | **T** | **I** | **K** | **L** | **T** | **I** | **D** | **S** | **G** | **V** | **Q** | **Q** | **Q** | **A** | **F** | **A** | **I** | **F** | **D** | **K** | **R** | **P** | **G** | **S** | **A** | **V** | **I** | **T** | **D** | **P** | **Q** | **K** | **G** | **D** | **L** | **L** | **A** | **T** | **V** | **S** | **S** | **P** | **S** | **Y** | **D** | **P** | **N** | **K** | **M** | **A** | **N** | **G** | **I** | **S** | **Q** | **K** | **E** | **Y** | **D** | **A** | **Y** | **N** | **N** | **N** | **K** | **D** | **L** | **P** | **F** | **T** | **A** | **R** | **F** | **A** | **T** | **G** | **Y** | **A** | **P** | **G** | **S** | **T** | **F** | **K** | **T** | **I** | **T** | **G** | **A** | **I** | **G** | **L** | **D** | **A** | **G** | **T** | **L** | **K** | **P** | **D** | **E** | **E** | **L** | **E** | **I** | **N** | **G** | **L** | **K** | **W** | **Q** | **K** | **D** | **K** | **S** | **W** | **G** | **G** | **Y** | **F** | **A** | **T** | **R** | **V** | **K** | **E** | **A** | **S** | **P** | **V** | **N** | **L** | **R** | **T** | **A** | **L** | **V** | **N** | **S** | **D** | **N** | **I** | **Y** | **F** | **A** | **Q** | **Q** | **T** | **L** | **R** | **M** | **G** | **E** | **D** | **K** | **F** | **R** | **A** | **G** | **L** | **N** | **K** | **F** | **I** | **F** | **G** | **E** | **E** | **L** | **D** | **L** | **P** | **I** | **A** | **M** | **T** | **P** | **A** | **Q** | **I** | **S** | **N** | **E** | **D** | **K** | **F** | **N** | **S** | **E** | **I** | **L** | **L** | **A** | **D** | **T** | **G** | **Y** | **G** | **Q** | **G** | **Q** | **L** | **L** | **I** | **S** | **P** | **I** | **Q** | **Q** | **A** | **T** | **M** | **Y** | **S** | **V** | **F** | **Q** | **N** | **N** | **G** | **T** | **L** | **V** | **Y** | **P** | **K** | **L** | **V** | **L** | **D** | **K** | **E** | **T** | **K** | **K** | **K** | **D** | **N** | **V** | **I** | **S** | **A** | **N** | **A** | **A** | **N** | **T** | **I** | **A** | **T** | **D** | **L** | **L** | **G** | **S** | **V** | **E** | **D** | **P** | **S** | **G** | **Y** | **V** | **Y** | **N** | **M** | **Y** | **N** | **P** | **N** | **F** | **S** | **L** | **A** | **A** | **K** | **T** | **G** | **T** | **A** | **E** | **I** | **K** | **D** | **K** | **Q** | **D** | **T** | **D** | **G** | **K** | **E** | **N** | **S** | **F** | **L** | **L** | **T** | **L** | **D** | **R** | **S** | **N** | **N** | **K** | **F** | **L** | **T** | **M** | **I** | **M** | **V** | **E** | **N** | **S** | **G** | **E** | **N** | **G** | **S** | **A** | **T** | **D** | **I** | **S** | **K** | **P** | **L** | **I** | **D** | **Y** | **L** | **E** | **A** | **T** | **I** | **K** | **-** | **-** | **-** | **-** | **-** | **-** | **-** | **-** | **-** | **-** | **-** | **-** | **-** | **-** | **-** | **-** | **-** | **-** | **-** | **-** | **-** | **-** | **-** | **-** | **-** | **-** | **-** | **-** | **-** | **-** | **-** | **-** | **-** | **-** | **-** | **-** | **Q** | **W** | **Q** | **A** | **K** | **Q** | **E** | **L** | **A** | **E** | **A** | **K** | **K** | **T** | **A** | **T** | **T** | **F** | **L** | **N** | **V** | **L** | **S** | **K** | **Q** | **E** | **F** | **D** | **K** | **L** | **P** | **S** | **V** | **V** | **Q** | **E** | **A** | **S** | **L** | **K** | **K** | **N** | **G** | **Y** | **D** | **T** | **K** | **S** | **V** | **V** | **E** | **K** | **Y** | **Q** | **A** | **I** | **Y** | **S** | **G** | **I** | **Q** | **A** | **E** | **G** | **V** | **K** | **A** | **S** | **D** | **V** | **Q** | **V** | **K** | **K** | **A** | **K** | **D** | **N** | **Q** | **Y** | **T** | **F** | **T** | **Y** | **K** | **L** | **S** | **M** | **S** | **T** | **P** | **L** | **G** | **E** | **M** | **K** | **D** | **L** | **S** | **Y** | **Q** | **S** | **S** | **I** | **A** | **K** | **K** | **G** | **D** | **T** | **Y** | **Q** | **I** | **A** | **W** | **K** | **P** | **S** | **L** | **I** | **F** | **P** | **D** | **M** | **S** | **G** | **N** | **D** | **K** | **I** | **S** | **I** | **Q** | **V** | **D** | **N** | **A** | **K** | **R** | **G** | **E** | **I** | **V** | **D** | **R** | **N** | **G** | **S** | **G** | **L** | **A** | **I** | **N** | **K** | **V** | **F** | **D** | **E** | **V** | **G** | **V** | **V** | **P** | **G** | **K** | **L** | **G** | **S** | **G** | **A** | **E** | **K** | **T** | **A** | **N** | **I** | **K** | **A** | **F** | **S** | **D** | **K** | **F** | **G** | **V** | **S** | **V** | **D** | **E** | **I** | **N** | **Q** | **K** | **L** | **S** | **Q** | **G** | **W** | **V** | **Q** | **A** | **D** | **S** | **F** | **V** | **P** | **I** | **T** | **V** | **A** | **S** | **E** | **P** | **V** | **T** | **E** | **L** | **P** | **T** | **G** | **A** | **A** | **T** | **K** | **D** | **T** | **E** | **S** | **R** | **Y** | **Y** | **P** | **L** | **G** | **E** | **A** | **A** | **A** | **Q** | **L** | **I** | **G** | **Y** | **T** | **G** | **T** | **I** | **T** | **A** | **E** | **D** | **I** | **E** | **K** | **N** | **P** | **E** | **L** | **S** | **S** | **T** | **G** | **V** | **I** | **G** | **K** | **T** | **G** | **L** | **E** | **R** | **A** | **F** | **D** | **K** | **E** | **L** | **R** | **G** | **Q** | **D** | **G** | **G** | **S** | **L** | **V** | **I** | **L** | **D** | **D** | **K** | **E** | **N** | **V** | **K** | **K** | **A** | **L** | **Q** | **T** | **K** | **E** | **K** | **K** | **D** | **G** | **Q** | **T** | **I** | **K** | **L** | **T** | **I** | **D** | **S** | **G** | **V** | **Q** | **Q** | **Q** | **A** | **F** | **A** | **I** | **F** | **D** | **K** | **R** | **P** | **G** | **S** | **A** | **V** | **I** | **T** | **D** | **P** | **Q** | **K** | **G** | **D** | **L** | **L** | **A** | **T** | **V** | **S** | **S** | **P** | **S** | **Y** | **D** | **P** | **N** | **K** | **M** | **A** | **N** | **G** | **I** | **S** | **Q** | **K** | **E** | **Y** | **D** | **A** | **Y** | **N** | **N** | **N** | **K** | **D** | **L** | **P** | **F** | **T** | **A** | **R** | **F** | **A** | **T** | **G** | **Y** | **A** | **P** | **G** | **S** | **T** | **F** | **K** | **T** | **I** | **T** | **G** | **A** | **I** | **G** | **L** | **D** | **A** | **G** | **T** | **L** | **K** | **P** | **D** | **E** | **E** | **L** | **E** | **I** | **N** | **G** | **L** | **K** | **W** | **Q** | **K** | **D** | **K** | **S** | **W** | **G** | **G** | **Y** | **F** | **A** | **T** | **R** | **V** | **K** | **E** | **A** | **S** | **P** | **V** | **N** | **L** | **R** | **T** | **A** | **L** | **V** | **N** | **S** | **D** | **N** | **I** | **Y** | **F** | **A** | **Q** | **Q** | **T** | **L** | **R** | **M** | **G** | **E** | **D** | **K** | **F** | **R** | **A** | **G** | **L** | **N** | **K** | **F** | **I** | **F** | **G** | **E** | **E** | **L** | **D** | **L** | **P** | **I** | **A** | **M** | **T** | **P** | **A** | **Q** | **I** | **S** | **N** | **E** | **D** | **K** | **F** | **N** | **S** | **E** | **I** | **L** | **L** | **A** | **D** | **T** | **G** | **Y** | **G** | **Q** | **G** | **Q** | **L** | **L** | **I** | **S** | **P** | **I** | **Q** | **Q** | **A** | **T** | **M** | **Y** | **S** | **V** | **F** | **Q** | **N** | **N** | **G** | **T** | **L** | **V** | **Y** | **P** | **K** | **L** | **V** | **L** | **D** | **K** | **E** | **T** | **K** | **K** | **K** | **D** | **N** | **V** | **I** | **S** | **A** | **N** | **A** | **A** | **N** | **T** | **I** | **A** | **T** | **D** | **L** | **L** | **G** | **S** | **V** | **E** | **D** | **P** | **S** | **G** | **Y** | **V** | **Y** | **N** | **M** | **Y** | **N** | **P** | **N** | **F** | **S** | **L** | **A** | **A** | **K** | **T** | **G** | **T** | **A** | **E** | **I** | **K** | **D** | **K** | **Q** | **D** | **T** | **D** | **G** | **K** | **E** | **N** | **S** | **F** | **L** | **L** | **T** | **L** | **D** | **R** | **S** | **N** | **N** | **K** | **F** | **L** | **T** | **M** | **I** | **M** | **V** | **E** | **N** | **S** | **G** | **E** | **N** | **G** | **S** | **A** | **T** | **D** | **I** | **S** | **K** | **P** | **L** | **I** | **D** | **Y** | **L** | **E** | **A** | **T** | **I** | **K** |
| **N54097** | **-** | **-** | **-** | **-** | **-** | **-** | **-** | **-** | **-** | **-** | **-** | **-** | **-** | **-** | **-** | **-** | **-** | **-** | **-** | **-** | **-** | **-** | **-** | **-** | **-** | **-** | **-** | **-** | **-** | **-** | **-** | **-** | **-** | **-** | **-** | **-** | **Q** | **W** | **Q** | **A** | **K** | **Q** | **E** | **L** | **A** | **E** | **A** | **K** | **K** | **T** | **A** | **T** | **T** | **F** | **L** | **N** | **V** | **L** | **S** | **K** | **Q** | **E** | **F** | **D** | **K** | **L** | **P** | **S** | **V** | **V** | **Q** | **E** | **A** | **S** | **L** | **K** | **K** | **N** | **G** | **Y** | **D** | **T** | **K** | **S** | **V** | **V** | **E** | **K** | **Y** | **Q** | **A** | **I** | **Y** | **S** | **G** | **I** | **Q** | **A** | **E** | **G** | **V** | **K** | **A** | **S** | **D** | **V** | **Q** | **V** | **K** | **K** | **A** | **K** | **D** | **N** | **Q** | **Y** | **T** | **F** | **T** | **Y** | **K** | **L** | **S** | **M** | **S** | **T** | **P** | **L** | **G** | **E** | **M** | **K** | **D** | **L** | **S** | **Y** | **Q** | **S** | **S** | **I** | **A** | **K** | **K** | **G** | **D** | **T** | **Y** | **Q** | **I** | **A** | **W** | **K** | **P** | **S** | **L** | **I** | **F** | **P** | **D** | **M** | **S** | **G** | **N** | **D** | **K** | **I** | **S** | **I** | **Q** | **V** | **D** | **N** | **A** | **K** | **R** | **G** | **E** | **I** | **V** | **D** | **R** | **N** | **G** | **S** | **G** | **L** | **A** | **I** | **N** | **K** | **V** | **F** | **D** | **E** | **V** | **G** | **V** | **V** | **P** | **G** | **K** | **L** | **G** | **S** | **G** | **A** | **E** | **K** | **T** | **A** | **N** | **I** | **K** | **A** | **F** | **S** | **D** | **K** | **F** | **G** | **V** | **S** | **V** | **D** | **E** | **I** | **N** | **Q** | **K** | **L** | **S** | **Q** | **G** | **W** | **V** | **Q** | **A** | **D** | **S** | **F** | **V** | **P** | **I** | **T** | **V** | **A** | **S** | **E** | **P** | **V** | **T** | **E** | **L** | **P** | **T** | **G** | **A** | **A** | **T** | **K** | **D** | **T** | **E** | **S** | **R** | **Y** | **Y** | **P** | **L** | **G** | **E** | **A** | **A** | **A** | **Q** | **L** | **I** | **G** | **Y** | **T** | **G** | **T** | **I** | **T** | **A** | **E** | **D** | **I** | **E** | **K** | **N** | **P** | **E** | **L** | **S** | **S** | **T** | **G** | **V** | **I** | **G** | **K** | **T** | **G** | **L** | **E** | **R** | **A** | **F** | **D** | **K** | **E** | **L** | **R** | **G** | **Q** | **D** | **G** | **G** | **S** | **L** | **V** | **I** | **L** | **D** | **D** | **K** | **E** | **N** | **V** | **K** | **K** | **A** | **L** | **Q** | **T** | **K** | **E** | **K** | **K** | **D** | **G** | **Q** | **T** | **I** | **K** | **L** | **T** | **I** | **D** | **S** | **G** | **V** | **Q** | **Q** | **Q** | **A** | **F** | **A** | **I** | **F** | **D** | **K** | **R** | **P** | **G** | **S** | **A** | **V** | **I** | **T** | **D** | **P** | **Q** | **K** | **G** | **D** | **L** | **L** | **A** | **T** | **V** | **S** | **S** | **P** | **S** | **Y** | **D** | **P** | **N** | **K** | **M** | **A** | **N** | **G** | **I** | **S** | **Q** | **K** | **E** | **Y** | **D** | **A** | **Y** | **N** | **N** | **N** | **K** | **D** | **L** | **P** | **F** | **T** | **A** | **R** | **F** | **A** | **T** | **G** | **Y** | **A** | **P** | **G** | **S** | **T** | **F** | **K** | **T** | **I** | **T** | **G** | **A** | **I** | **G** | **L** | **D** | **A** | **G** | **T** | **L** | **K** | **P** | **D** | **E** | **E** | **L** | **E** | **I** | **N** | **G** | **L** | **K** | **W** | **Q** | **K** | **D** | **K** | **S** | **W** | **G** | **G** | **Y** | **F** | **A** | **T** | **R** | **V** | **K** | **E** | **A** | **S** | **P** | **V** | **N** | **L** | **R** | **T** | **A** | **L** | **V** | **N** | **S** | **D** | **N** | **I** | **Y** | **F** | **A** | **Q** | **Q** | **T** | **L** | **R** | **M** | **G** | **E** | **D** | **K** | **F** | **R** | **A** | **G** | **L** | **N** | **K** | **F** | **I** | **F** | **G** | **E** | **E** | **L** | **D** | **L** | **P** | **I** | **A** | **M** | **T** | **P** | **A** | **Q** | **I** | **S** | **N** | **E** | **D** | **K** | **F** | **N** | **S** | **E** | **I** | **L** | **L** | **A** | **D** | **T** | **G** | **Y** | **G** | **Q** | **G** | **Q** | **L** | **L** | **I** | **S** | **P** | **I** | **Q** | **Q** | **A** | **T** | **M** | **Y** | **S** | **V** | **F** | **Q** | **N** | **N** | **G** | **T** | **L** | **V** | **Y** | **P** | **K** | **L** | **V** | **L** | **D** | **K** | **E** | **T** | **K** | **K** | **K** | **D** | **N** | **V** | **I** | **S** | **A** | **N** | **A** | **A** | **N** | **T** | **I** | **A** | **T** | **D** | **L** | **L** | **G** | **S** | **V** | **E** | **D** | **P** | **S** | **G** | **Y** | **V** | **Y** | **N** | **M** | **Y** | **N** | **P** | **N** | **F** | **S** | **L** | **A** | **A** | **K** | **T** | **G** | **T** | **A** | **E** | **I** | **K** | **D** | **K** | **Q** | **D** | **T** | **D** | **G** | **K** | **E** | **N** | **S** | **F** | **L** | **L** | **T** | **L** | **D** | **R** | **S** | **N** | **N** | **K** | **F** | **L** | **T** | **M** | **I** | **M** | **V** | **E** | **N** | **S** | **G** | **E** | **N** | **G** | **S** | **A** | **T** | **D** | **I** | **S** | **K** | **P** | **L** | **I** | **D** | **Y** | **L** | **E** | **A** | **T** | **I** | **K** | **-** | **-** | **-** | **-** | **-** | **-** | **-** | **-** | **-** | **-** | **-** | **-** | **-** | **-** | **-** | **-** | **-** | **-** | **-** | **-** | **-** | **-** | **-** | **-** | **-** | **-** | **-** | **-** | **-** | **-** | **-** | **-** | **-** | **-** | **-** | **-** | **Q** | **W** | **Q** | **A** | **K** | **Q** | **E** | **L** | **A** | **E** | **A** | **K** | **K** | **T** | **A** | **T** | **T** | **F** | **L** | **N** | **V** | **L** | **S** | **K** | **Q** | **E** | **F** | **D** | **K** | **L** | **P** | **S** | **V** | **V** | **Q** | **E** | **A** | **S** | **L** | **K** | **K** | **N** | **G** | **Y** | **D** | **T** | **K** | **S** | **V** | **V** | **E** | **K** | **Y** | **Q** | **A** | **I** | **Y** | **S** | **G** | **I** | **Q** | **A** | **E** | **G** | **V** | **K** | **A** | **S** | **D** | **V** | **Q** | **V** | **K** | **K** | **A** | **K** | **D** | **N** | **Q** | **Y** | **T** | **F** | **T** | **Y** | **K** | **L** | **S** | **M** | **S** | **T** | **P** | **L** | **G** | **E** | **M** | **K** | **D** | **L** | **S** | **Y** | **Q** | **S** | **S** | **I** | **A** | **K** | **K** | **G** | **D** | **T** | **Y** | **Q** | **I** | **A** | **W** | **K** | **P** | **S** | **L** | **I** | **F** | **P** | **D** | **M** | **S** | **G** | **N** | **D** | **K** | **I** | **S** | **I** | **Q** | **V** | **D** | **N** | **A** | **K** | **R** | **G** | **E** | **I** | **V** | **D** | **R** | **N** | **G** | **S** | **G** | **L** | **A** | **I** | **N** | **K** | **V** | **F** | **D** | **E** | **V** | **G** | **V** | **V** | **P** | **G** | **K** | **L** | **G** | **S** | **G** | **A** | **E** | **K** | **T** | **A** | **N** | **I** | **K** | **A** | **F** | **S** | **D** | **K** | **F** | **G** | **V** | **S** | **V** | **D** | **E** | **I** | **N** | **Q** | **K** | **L** | **S** | **Q** | **G** | **W** | **V** | **Q** | **A** | **D** | **S** | **F** | **V** | **P** | **I** | **T** | **V** | **A** | **S** | **E** | **P** | **V** | **T** | **E** | **L** | **P** | **T** | **G** | **A** | **A** | **T** | **K** | **D** | **T** | **E** | **S** | **R** | **Y** | **Y** | **P** | **L** | **G** | **E** | **A** | **A** | **A** | **Q** | **L** | **I** | **G** | **Y** | **T** | **G** | **T** | **I** | **T** | **A** | **E** | **D** | **I** | **E** | **K** | **N** | **P** | **E** | **L** | **S** | **S** | **T** | **G** | **V** | **I** | **G** | **K** | **T** | **G** | **L** | **E** | **R** | **A** | **F** | **D** | **K** | **E** | **L** | **R** | **G** | **Q** | **D** | **G** | **G** | **S** | **L** | **V** | **I** | **L** | **D** | **D** | **K** | **E** | **N** | **V** | **K** | **K** | **A** | **L** | **Q** | **T** | **K** | **E** | **K** | **K** | **D** | **G** | **Q** | **T** | **I** | **K** | **L** | **T** | **I** | **D** | **S** | **G** | **V** | **Q** | **Q** | **Q** | **A** | **F** | **A** | **I** | **F** | **D** | **K** | **R** | **P** | **G** | **S** | **A** | **V** | **I** | **T** | **D** | **P** | **Q** | **K** | **G** | **D** | **L** | **L** | **A** | **T** | **V** | **S** | **S** | **P** | **S** | **Y** | **D** | **P** | **N** | **K** | **M** | **A** | **N** | **G** | **I** | **S** | **Q** | **K** | **E** | **Y** | **D** | **A** | **Y** | **N** | **N** | **N** | **K** | **D** | **L** | **P** | **F** | **T** | **A** | **R** | **F** | **A** | **T** | **G** | **Y** | **A** | **P** | **G** | **S** | **T** | **F** | **K** | **T** | **I** | **T** | **G** | **A** | **I** | **G** | **L** | **D** | **A** | **G** | **T** | **L** | **K** | **P** | **D** | **E** | **E** | **L** | **E** | **I** | **N** | **G** | **L** | **K** | **W** | **Q** | **K** | **D** | **K** | **S** | **W** | **G** | **G** | **Y** | **F** | **A** | **T** | **R** | **V** | **K** | **E** | **A** | **S** | **P** | **V** | **N** | **L** | **R** | **T** | **A** | **L** | **V** | **N** | **S** | **D** | **N** | **I** | **Y** | **F** | **A** | **Q** | **Q** | **T** | **L** | **R** | **M** | **G** | **E** | **D** | **K** | **F** | **R** | **A** | **G** | **L** | **N** | **K** | **F** | **I** | **F** | **G** | **E** | **E** | **L** | **D** | **L** | **P** | **I** | **A** | **M** | **T** | **P** | **A** | **Q** | **I** | **S** | **N** | **E** | **D** | **K** | **F** | **N** | **S** | **E** | **I** | **L** | **L** | **A** | **D** | **T** | **G** | **Y** | **G** | **Q** | **G** | **Q** | **L** | **L** | **I** | **S** | **P** | **I** | **Q** | **Q** | **A** | **T** | **M** | **Y** | **S** | **V** | **F** | **Q** | **N** | **N** | **G** | **T** | **L** | **V** | **Y** | **P** | **K** | **L** | **V** | **L** | **D** | **K** | **E** | **T** | **K** | **K** | **K** | **D** | **N** | **V** | **I** | **S** | **A** | **N** | **A** | **A** | **N** | **T** | **I** | **A** | **T** | **D** | **L** | **L** | **G** | **S** | **V** | **E** | **D** | **P** | **S** | **G** | **Y** | **V** | **Y** | **N** | **M** | **Y** | **N** | **P** | **N** | **F** | **S** | **L** | **A** | **A** | **K** | **T** | **G** | **T** | **A** | **E** | **I** | **K** | **D** | **K** | **Q** | **D** | **T** | **D** | **G** | **K** | **E** | **N** | **S** | **F** | **L** | **L** | **T** | **L** | **D** | **R** | **S** | **N** | **N** | **K** | **F** | **L** | **T** | **M** | **I** | **M** | **V** | **E** | **N** | **S** | **G** | **E** | **N** | **G** | **S** | **A** | **T** | **D** | **I** | **S** | **K** | **P** | **L** | **I** | **D** | **Y** | **L** | **E** | **A** | **T** | **I** | **K** |
| **N59606F** | **-** | **-** | **-** | **-** | **-** | **-** | **-** | **-** | **-** | **-** | **-** | **-** | **-** | **-** | **-** | **-** | **-** | **-** | **-** | **-** | **-** | **-** | **-** | **-** | **-** | **-** | **-** | **-** | **-** | **-** | **-** | **-** | **-** | **-** | **-** | **-** | **Q** | **W** | **Q** | **A** | **K** | **Q** | **E** | **L** | **A** | **E** | **A** | **K** | **K** | **T** | **A** | **T** | **T** | **F** | **L** | **N** | **V** | **L** | **T** | **K** | **Q** | **K** | **F** | **D** | **K** | **L** | **P** | **S** | **V** | **V** | **Q** | **E** | **A** | **S** | **L** | **K** | **K** | **N** | **G** | **Y** | **D** | **T** | **K** | **S** | **V** | **V** | **E** | **K** | **Y** | **Q** | **A** | **I** | **Y** | **S** | **G** | **I** | **Q** | **A** | **E** | **G** | **V** | **K** | **A** | **S** | **D** | **V** | **Q** | **V** | **K** | **K** | **A** | **K** | **D** | **N** | **Q** | **Y** | **T** | **F** | **N** | **Y** | **K** | **L** | **S** | **M** | **S** | **T** | **P** | **L** | **G** | **E** | **M** | **K** | **D** | **L** | **S** | **Y** | **Q** | **S** | **S** | **I** | **A** | **K** | **K** | **G** | **D** | **T** | **Y** | **Q** | **I** | **A** | **W** | **K** | **P** | **S** | **L** | **I** | **F** | **P** | **D** | **M** | **S** | **G** | **N** | **D** | **K** | **I** | **S** | **I** | **Q** | **V** | **D** | **N** | **A** | **K** | **R** | **G** | **E** | **I** | **V** | **D** | **R** | **N** | **G** | **S** | **G** | **L** | **A** | **I** | **N** | **K** | **V** | **F** | **D** | **E** | **V** | **G** | **V** | **V** | **P** | **G** | **K** | **L** | **G** | **S** | **G** | **A** | **E** | **K** | **T** | **A** | **N** | **I** | **K** | **A** | **F** | **S** | **D** | **K** | **F** | **G** | **V** | **S** | **V** | **D** | **E** | **I** | **N** | **Q** | **K** | **L** | **S** | **Q** | **G** | **W** | **V** | **Q** | **A** | **D** | **S** | **F** | **V** | **P** | **I** | **T** | **V** | **A** | **S** | **E** | **P** | **V** | **T** | **E** | **L** | **P** | **T** | **G** | **A** | **A** | **T** | **K** | **D** | **T** | **E** | **S** | **R** | **Y** | **Y** | **P** | **L** | **G** | **E** | **A** | **A** | **A** | **Q** | **L** | **I** | **G** | **Y** | **T** | **G** | **T** | **I** | **T** | **A** | **E** | **D** | **I** | **K** | **K** | **N** | **P** | **E** | **L** | **S** | **S** | **T** | **G** | **V** | **I** | **G** | **K** | **T** | **G** | **L** | **E** | **R** | **A** | **F** | **D** | **K** | **E** | **L** | **R** | **G** | **Q** | **D** | **G** | **G** | **S** | **L** | **V** | **I** | **L** | **D** | **D** | **K** | **E** | **N** | **V** | **K** | **K** | **A** | **L** | **Q** | **T** | **K** | **E** | **K** | **K** | **D** | **G** | **Q** | **T** | **I** | **K** | **L** | **T** | **I** | **D** | **S** | **G** | **V** | **Q** | **Q** | **Q** | **A** | **F** | **A** | **I** | **F** | **D** | **K** | **R** | **P** | **G** | **S** | **A** | **V** | **I** | **T** | **D** | **P** | **Q** | **K** | **G** | **D** | **L** | **L** | **A** | **T** | **V** | **S** | **S** | **P** | **S** | **Y** | **D** | **P** | **N** | **K** | **M** | **A** | **N** | **G** | **I** | **S** | **Q** | **K** | **E** | **Y** | **D** | **A** | **Y** | **N** | **N** | **N** | **K** | **D** | **L** | **P** | **F** | **T** | **A** | **R** | **F** | **A** | **T** | **G** | **Y** | **A** | **P** | **G** | **S** | **T** | **F** | **K** | **T** | **I** | **T** | **G** | **A** | **I** | **G** | **L** | **D** | **A** | **G** | **T** | **L** | **K** | **P** | **D** | **E** | **E** | **L** | **E** | **I** | **N** | **G** | **L** | **K** | **W** | **Q** | **K** | **D** | **K** | **S** | **W** | **G** | **G** | **Y** | **F** | **A** | **T** | **R** | **V** | **K** | **E** | **A** | **S** | **P** | **V** | **N** | **L** | **R** | **T** | **A** | **L** | **V** | **N** | **S** | **D** | **N** | **I** | **Y** | **F** | **A** | **Q** | **Q** | **T** | **L** | **R** | **M** | **G** | **E** | **D** | **K** | **F** | **R** | **A** | **G** | **L** | **N** | **K** | **F** | **I** | **F** | **G** | **E** | **E** | **L** | **D** | **L** | **P** | **I** | **A** | **M** | **T** | **P** | **A** | **Q** | **I** | **S** | **N** | **E** | **D** | **K** | **F** | **N** | **S** | **E** | **I** | **L** | **L** | **A** | **D** | **T** | **G** | **Y** | **G** | **Q** | **G** | **Q** | **L** | **L** | **I** | **S** | **P** | **I** | **Q** | **Q** | **A** | **T** | **M** | **Y** | **S** | **V** | **F** | **Q** | **N** | **N** | **G** | **T** | **L** | **V** | **Y** | **P** | **K** | **L** | **V** | **L** | **D** | **K** | **E** | **T** | **K** | **K** | **K** | **D** | **N** | **V** | **I** | **S** | **A** | **N** | **A** | **A** | **N** | **T** | **I** | **A** | **T** | **D** | **L** | **L** | **G** | **S** | **V** | **E** | **D** | **P** | **S** | **G** | **Y** | **V** | **Y** | **N** | **M** | **Y** | **N** | **P** | **N** | **F** | **S** | **L** | **A** | **A** | **K** | **T** | **G** | **T** | **A** | **E** | **I** | **K** | **D** | **K** | **Q** | **D** | **T** | **D** | **G** | **K** | **E** | **N** | **S** | **F** | **L** | **L** | **T** | **L** | **D** | **R** | **S** | **N** | **N** | **K** | **F** | **L** | **T** | **M** | **I** | **M** | **V** | **E** | **N** | **S** | **G** | **E** | **N** | **G** | **S** | **A** | **T** | **D** | **I** | **S** | **K** | **P** | **L** | **I** | **D** | **Y** | **L** | **E** | **A** | **T** | **I** | **K** | **-** | **-** | **-** | **-** | **-** | **-** | **-** | **-** | **-** | **-** | **-** | **-** | **-** | **-** | **-** | **-** | **-** | **-** | **-** | **-** | **-** | **-** | **-** | **-** | **-** | **-** | **-** | **-** | **-** | **-** | **-** | **-** | **-** | **-** | **-** | **-** | **Q** | **W** | **Q** | **A** | **K** | **Q** | **E** | **L** | **A** | **E** | **A** | **K** | **K** | **T** | **A** | **T** | **T** | **F** | **L** | **N** | **V** | **L** | **T** | **K** | **Q** | **K** | **F** | **D** | **K** | **L** | **P** | **S** | **V** | **V** | **Q** | **E** | **A** | **S** | **L** | **K** | **K** | **N** | **G** | **Y** | **D** | **T** | **K** | **S** | **V** | **V** | **E** | **K** | **Y** | **Q** | **A** | **I** | **Y** | **S** | **G** | **I** | **Q** | **A** | **E** | **G** | **V** | **K** | **A** | **S** | **D** | **V** | **Q** | **V** | **K** | **K** | **A** | **K** | **D** | **N** | **Q** | **Y** | **T** | **F** | **N** | **Y** | **K** | **L** | **S** | **M** | **S** | **T** | **P** | **L** | **G** | **E** | **M** | **K** | **D** | **L** | **S** | **Y** | **Q** | **S** | **S** | **I** | **A** | **K** | **K** | **G** | **D** | **T** | **Y** | **Q** | **I** | **A** | **W** | **K** | **P** | **S** | **L** | **I** | **F** | **P** | **D** | **M** | **S** | **G** | **N** | **D** | **K** | **I** | **S** | **I** | **Q** | **V** | **D** | **N** | **A** | **K** | **R** | **G** | **E** | **I** | **V** | **D** | **R** | **N** | **G** | **S** | **G** | **L** | **A** | **I** | **N** | **K** | **V** | **F** | **D** | **E** | **V** | **G** | **V** | **V** | **P** | **G** | **K** | **L** | **G** | **S** | **G** | **A** | **E** | **K** | **T** | **A** | **N** | **I** | **K** | **A** | **F** | **S** | **D** | **K** | **F** | **G** | **V** | **S** | **V** | **D** | **E** | **I** | **N** | **Q** | **K** | **L** | **S** | **Q** | **G** | **W** | **V** | **Q** | **A** | **D** | **S** | **F** | **V** | **P** | **I** | **T** | **V** | **A** | **S** | **E** | **P** | **V** | **T** | **E** | **L** | **P** | **T** | **G** | **A** | **A** | **T** | **K** | **D** | **T** | **E** | **S** | **R** | **Y** | **Y** | **P** | **L** | **G** | **E** | **A** | **A** | **A** | **Q** | **L** | **I** | **G** | **Y** | **T** | **G** | **T** | **I** | **T** | **A** | **E** | **D** | **I** | **K** | **K** | **N** | **P** | **E** | **L** | **S** | **S** | **T** | **G** | **V** | **I** | **G** | **K** | **T** | **G** | **L** | **E** | **R** | **A** | **F** | **D** | **K** | **E** | **L** | **R** | **G** | **Q** | **D** | **G** | **G** | **S** | **L** | **V** | **I** | **L** | **D** | **D** | **K** | **E** | **N** | **V** | **K** | **K** | **A** | **L** | **Q** | **T** | **K** | **E** | **K** | **K** | **D** | **G** | **Q** | **T** | **I** | **K** | **L** | **T** | **I** | **D** | **S** | **G** | **V** | **Q** | **Q** | **Q** | **A** | **F** | **A** | **I** | **F** | **D** | **K** | **R** | **P** | **G** | **S** | **A** | **V** | **I** | **T** | **D** | **P** | **Q** | **K** | **G** | **D** | **L** | **L** | **A** | **T** | **V** | **S** | **S** | **P** | **S** | **Y** | **D** | **P** | **N** | **K** | **M** | **A** | **N** | **G** | **I** | **S** | **Q** | **K** | **E** | **Y** | **D** | **A** | **Y** | **N** | **N** | **N** | **K** | **D** | **L** | **P** | **F** | **T** | **A** | **R** | **F** | **A** | **T** | **G** | **Y** | **A** | **P** | **G** | **S** | **T** | **F** | **K** | **T** | **I** | **T** | **G** | **A** | **I** | **G** | **L** | **D** | **A** | **G** | **T** | **L** | **K** | **P** | **D** | **E** | **E** | **L** | **E** | **I** | **N** | **G** | **L** | **K** | **W** | **Q** | **K** | **D** | **K** | **S** | **W** | **G** | **G** | **Y** | **F** | **A** | **T** | **R** | **V** | **K** | **E** | **A** | **S** | **P** | **V** | **N** | **L** | **R** | **T** | **A** | **L** | **V** | **N** | **S** | **D** | **N** | **I** | **Y** | **F** | **A** | **Q** | **Q** | **T** | **L** | **R** | **M** | **G** | **E** | **D** | **K** | **F** | **R** | **A** | **G** | **L** | **N** | **K** | **F** | **I** | **F** | **G** | **E** | **E** | **L** | **D** | **L** | **P** | **I** | **A** | **M** | **T** | **P** | **A** | **Q** | **I** | **S** | **N** | **E** | **D** | **K** | **F** | **N** | **S** | **E** | **I** | **L** | **L** | **A** | **D** | **T** | **G** | **Y** | **G** | **Q** | **G** | **Q** | **L** | **L** | **I** | **S** | **P** | **I** | **Q** | **Q** | **A** | **T** | **M** | **Y** | **S** | **V** | **F** | **Q** | **N** | **N** | **G** | **T** | **L** | **V** | **Y** | **P** | **K** | **L** | **V** | **L** | **D** | **K** | **E** | **T** | **K** | **K** | **K** | **D** | **N** | **V** | **I** | **S** | **A** | **N** | **A** | **A** | **N** | **T** | **I** | **A** | **T** | **D** | **L** | **L** | **G** | **S** | **V** | **E** | **D** | **P** | **S** | **G** | **Y** | **V** | **Y** | **N** | **M** | **Y** | **N** | **P** | **N** | **F** | **S** | **L** | **A** | **A** | **K** | **T** | **G** | **T** | **A** | **E** | **I** | **K** | **D** | **K** | **Q** | **D** | **T** | **D** | **G** | **K** | **E** | **N** | **S** | **F** | **L** | **L** | **T** | **L** | **D** | **R** | **S** | **N** | **N** | **K** | **F** | **L** | **T** | **M** | **I** | **M** | **V** | **E** | **N** | **S** | **G** | **E** | **N** | **G** | **S** | **A** | **T** | **D** | **I** | **S** | **K** | **P** | **L** | **I** | **D** | **Y** | **L** | **E** | **A** | **T** | **I** | **K** |
| **N55265** | **M** | **E** | **R** | **S** | **N** | **R** | **N** | **K** | **K** | **S** | **S** | **K** | **K** | **P** | **L** | **I** | **L** | **G** | **V** | **S** | **A** | **L** | **V** | **L** | **I** | **A** | **A** | **V** | **G** | **G** | **G** | **Y** | **Y** | **A** | **Y** | **S** | **Q** | **W** | **Q** | **A** | **K** | **Q** | **E** | **L** | **A** | **E** | **A** | **K** | **K** | **T** | **A** | **N** | **T** | **F** | **L** | **N** | **V** | **L** | **S** | **K** | **Q** | **E** | **F** | **D** | **K** | **L** | **P** | **S** | **V** | **V** | **Q** | **E** | **T** | **S** | **L** | **K** | **K** | **N** | **G** | **Y** | **D** | **T** | **K** | **S** | **V** | **V** | **E** | **K** | **Y** | **Q** | **A** | **I** | **Y** | **S** | **G** | **I** | **Q** | **A** | **E** | **G** | **V** | **K** | **A** | **S** | **D** | **V** | **Q** | **V** | **K** | **K** | **G** | **K** | **D** | **N** | **Q** | **Y** | **T** | **F** | **T** | **Y** | **K** | **L** | **S** | **M** | **S** | **T** | **P** | **L** | **G** | **E** | **M** | **K** | **D** | **L** | **S** | **Y** | **Q** | **S** | **S** | **I** | **A** | **K** | **K** | **G** | **D** | **N** | **Y** | **Q** | **I** | **A** | **W** | **A** | **P** | **S** | **L** | **I** | **F** | **P** | **D** | **M** | **S** | **G** | **N** | **D** | **K** | **I** | **S** | **I** | **Q** | **V** | **D** | **N** | **A** | **K** | **R** | **G** | **E** | **I** | **V** | **D** | **R** | **N** | **G** | **S** | **G** | **L** | **A** | **I** | **N** | **K** | **V** | **F** | **D** | **E** | **V** | **G** | **V** | **V** | **P** | **G** | **K** | **L** | **G** | **S** | **G** | **A** | **E** | **K** | **T** | **A** | **N** | **I** | **K** | **A** | **F** | **S** | **D** | **K** | **F** | **G** | **V** | **S** | **V** | **D** | **E** | **I** | **N** | **Q** | **K** | **L** | **S** | **Q** | **G** | **W** | **V** | **Q** | **A** | **D** | **S** | **F** | **V** | **P** | **I** | **T** | **V** | **A** | **S** | **E** | **P** | **V** | **T** | **E** | **L** | **P** | **T** | **G** | **A** | **A** | **T** | **K** | **D** | **T** | **E** | **S** | **R** | **Y** | **Y** | **P** | **L** | **G** | **E** | **A** | **A** | **A** | **Q** | **L** | **I** | **G** | **Y** | **T** | **G** | **T** | **I** | **T** | **A** | **E** | **D** | **I** | **E** | **K** | **N** | **P** | **E** | **L** | **S** | **S** | **T** | **G** | **V** | **I** | **G** | **K** | **T** | **G** | **L** | **E** | **R** | **A** | **F** | **D** | **K** | **E** | **L** | **R** | **G** | **Q** | **D** | **G** | **G** | **S** | **L** | **V** | **I** | **L** | **D** | **D** | **K** | **E** | **N** | **V** | **K** | **K** | **A** | **L** | **Q** | **T** | **K** | **E** | **K** | **K** | **D** | **G** | **Q** | **T** | **I** | **K** | **L** | **T** | **I** | **D** | **S** | **G** | **V** | **Q** | **Q** | **Q** | **A** | **F** | **A** | **I** | **F** | **D** | **K** | **R** | **P** | **G** | **S** | **A** | **V** | **I** | **T** | **D** | **P** | **Q** | **K** | **G** | **D** | **L** | **L** | **A** | **T** | **V** | **S** | **S** | **P** | **S** | **Y** | **D** | **P** | **N** | **K** | **M** | **A** | **N** | **G** | **I** | **S** | **Q** | **K** | **E** | **Y** | **D** | **A** | **Y** | **N** | **N** | **N** | **K** | **D** | **L** | **P** | **F** | **T** | **A** | **R** | **F** | **A** | **T** | **G** | **Y** | **A** | **P** | **G** | **S** | **T** | **F** | **K** | **T** | **I** | **T** | **G** | **A** | **I** | **G** | **L** | **D** | **A** | **G** | **T** | **L** | **K** | **P** | **D** | **E** | **E** | **L** | **E** | **I** | **N** | **G** | **L** | **K** | **W** | **Q** | **K** | **D** | **K** | **S** | **W** | **G** | **G** | **Y** | **F** | **A** | **T** | **R** | **V** | **K** | **E** | **A** | **S** | **P** | **V** | **N** | **L** | **R** | **T** | **A** | **L** | **V** | **N** | **S** | **D** | **N** | **I** | **Y** | **F** | **A** | **Q** | **Q** | **T** | **L** | **R** | **M** | **G** | **E** | **D** | **K** | **F** | **R** | **A** | **G** | **L** | **N** | **K** | **F** | **I** | **F** | **G** | **E** | **E** | **L** | **D** | **L** | **P** | **I** | **A** | **M** | **T** | **P** | **A** | **Q** | **I** | **S** | **N** | **E** | **D** | **K** | **F** | **N** | **S** | **E** | **I** | **L** | **L** | **A** | **D** | **T** | **G** | **Y** | **G** | **Q** | **G** | **Q** | **L** | **L** | **I** | **S** | **P** | **I** | **Q** | **Q** | **A** | **T** | **M** | **Y** | **S** | **V** | **F** | **Q** | **N** | **N** | **G** | **T** | **L** | **V** | **Y** | **P** | **K** | **L** | **V** | **L** | **E** | **K** | **E** | **T** | **K** | **K** | **K** | **D** | **N** | **V** | **I** | **S** | **A** | **N** | **A** | **A** | **N** | **T** | **I** | **A** | **T** | **D** | **L** | **L** | **G** | **S** | **V** | **E** | **D** | **P** | **S** | **G** | **Y** | **V** | **Y** | **N** | **M** | **Y** | **N** | **P** | **N** | **F** | **S** | **L** | **A** | **A** | **K** | **T** | **G** | **T** | **A** | **E** | **I** | **K** | **D** | **K** | **Q** | **D** | **T** | **D** | **G** | **K** | **E** | **N** | **S** | **F** | **L** | **L** | **T** | **L** | **D** | **R** | **S** | **N** | **N** | **K** | **F** | **L** | **T** | **M** | **I** | **M** | **V** | **E** | **N** | **S** | **G** | **E** | **N** | **G** | **S** | **A** | **T** | **D** | **I** | **S** | **K** | **P** | **L** | **I** | **D** | **Y** | **L** | **E** | **A** | **T** | **I** | **K** | **M** | **E** | **R** | **S** | **N** | **R** | **N** | **K** | **K** | **S** | **S** | **K** | **K** | **P** | **L** | **I** | **L** | **G** | **V** | **S** | **A** | **L** | **V** | **L** | **I** | **A** | **A** | **V** | **G** | **G** | **G** | **Y** | **Y** | **A** | **Y** | **S** | **Q** | **W** | **Q** | **A** | **K** | **Q** | **E** | **L** | **A** | **E** | **A** | **K** | **K** | **T** | **A** | **N** | **T** | **F** | **L** | **N** | **V** | **L** | **S** | **K** | **Q** | **E** | **F** | **D** | **K** | **L** | **P** | **S** | **V** | **V** | **Q** | **E** | **T** | **S** | **L** | **K** | **K** | **N** | **G** | **Y** | **D** | **T** | **K** | **S** | **V** | **V** | **E** | **K** | **Y** | **Q** | **A** | **I** | **Y** | **S** | **G** | **I** | **Q** | **A** | **E** | **G** | **V** | **K** | **A** | **S** | **D** | **V** | **Q** | **V** | **K** | **K** | **G** | **K** | **D** | **N** | **Q** | **Y** | **T** | **F** | **T** | **Y** | **K** | **L** | **S** | **M** | **S** | **T** | **P** | **L** | **G** | **E** | **M** | **K** | **D** | **L** | **S** | **Y** | **Q** | **S** | **S** | **I** | **A** | **K** | **K** | **G** | **D** | **N** | **Y** | **Q** | **I** | **A** | **W** | **A** | **P** | **S** | **L** | **I** | **F** | **P** | **D** | **M** | **S** | **G** | **N** | **D** | **K** | **I** | **S** | **I** | **Q** | **V** | **D** | **N** | **A** | **K** | **R** | **G** | **E** | **I** | **V** | **D** | **R** | **N** | **G** | **S** | **G** | **L** | **A** | **I** | **N** | **K** | **V** | **F** | **D** | **E** | **V** | **G** | **V** | **V** | **P** | **G** | **K** | **L** | **G** | **S** | **G** | **A** | **E** | **K** | **T** | **A** | **N** | **I** | **K** | **A** | **F** | **S** | **D** | **K** | **F** | **G** | **V** | **S** | **V** | **D** | **E** | **I** | **N** | **Q** | **K** | **L** | **S** | **Q** | **G** | **W** | **V** | **Q** | **A** | **D** | **S** | **F** | **V** | **P** | **I** | **T** | **V** | **A** | **S** | **E** | **P** | **V** | **T** | **E** | **L** | **P** | **T** | **G** | **A** | **A** | **T** | **K** | **D** | **T** | **E** | **S** | **R** | **Y** | **Y** | **P** | **L** | **G** | **E** | **A** | **A** | **A** | **Q** | **L** | **I** | **G** | **Y** | **T** | **G** | **T** | **I** | **T** | **A** | **E** | **D** | **I** | **E** | **K** | **N** | **P** | **E** | **L** | **S** | **S** | **T** | **G** | **V** | **I** | **G** | **K** | **T** | **G** | **L** | **E** | **R** | **A** | **F** | **D** | **K** | **E** | **L** | **R** | **G** | **Q** | **D** | **G** | **G** | **S** | **L** | **V** | **I** | **L** | **D** | **D** | **K** | **E** | **N** | **V** | **K** | **K** | **A** | **L** | **Q** | **T** | **K** | **E** | **K** | **K** | **D** | **G** | **Q** | **T** | **I** | **K** | **L** | **T** | **I** | **D** | **S** | **G** | **V** | **Q** | **Q** | **Q** | **A** | **F** | **A** | **I** | **F** | **D** | **K** | **R** | **P** | **G** | **S** | **A** | **V** | **I** | **T** | **D** | **P** | **Q** | **K** | **G** | **D** | **L** | **L** | **A** | **T** | **V** | **S** | **S** | **P** | **S** | **Y** | **D** | **P** | **N** | **K** | **M** | **A** | **N** | **G** | **I** | **S** | **Q** | **K** | **E** | **Y** | **D** | **A** | **Y** | **N** | **N** | **N** | **K** | **D** | **L** | **P** | **F** | **T** | **A** | **R** | **F** | **A** | **T** | **G** | **Y** | **A** | **P** | **G** | **S** | **T** | **F** | **K** | **T** | **I** | **T** | **G** | **A** | **I** | **G** | **L** | **D** | **A** | **G** | **T** | **L** | **K** | **P** | **D** | **E** | **E** | **L** | **E** | **I** | **N** | **G** | **L** | **K** | **W** | **Q** | **K** | **D** | **K** | **S** | **W** | **G** | **G** | **Y** | **F** | **A** | **T** | **R** | **V** | **K** | **E** | **A** | **S** | **P** | **V** | **N** | **L** | **R** | **T** | **A** | **L** | **V** | **N** | **S** | **D** | **N** | **I** | **Y** | **F** | **A** | **Q** | **Q** | **T** | **L** | **R** | **M** | **G** | **E** | **D** | **K** | **F** | **R** | **A** | **G** | **L** | **N** | **K** | **F** | **I** | **F** | **G** | **E** | **E** | **L** | **D** | **L** | **P** | **I** | **A** | **M** | **T** | **P** | **A** | **Q** | **I** | **S** | **N** | **E** | **D** | **K** | **F** | **N** | **S** | **E** | **I** | **L** | **L** | **A** | **D** | **T** | **G** | **Y** | **G** | **Q** | **G** | **Q** | **L** | **L** | **I** | **S** | **P** | **I** | **Q** | **Q** | **A** | **T** | **M** | **Y** | **S** | **V** | **F** | **Q** | **N** | **N** | **G** | **T** | **L** | **V** | **Y** | **P** | **K** | **L** | **V** | **L** | **E** | **K** | **E** | **T** | **K** | **K** | **K** | **D** | **N** | **V** | **I** | **S** | **A** | **N** | **A** | **A** | **N** | **T** | **I** | **A** | **T** | **D** | **L** | **L** | **G** | **S** | **V** | **E** | **D** | **P** | **S** | **G** | **Y** | **V** | **Y** | **N** | **M** | **Y** | **N** | **P** | **N** | **F** | **S** | **L** | **A** | **A** | **K** | **T** | **G** | **T** | **A** | **E** | **I** | **K** | **D** | **K** | **Q** | **D** | **T** | **D** | **G** | **K** | **E** | **N** | **S** | **F** | **L** | **L** | **T** | **L** | **D** | **R** | **S** | **N** | **N** | **K** | **F** | **L** | **T** | **M** | **I** | **M** | **V** | **E** | **N** | **S** | **G** | **E** | **N** | **G** | **S** | **A** | **T** | **D** | **I** | **S** | **K** | **P** | **L** | **I** | **D** | **Y** | **L** | **E** | **A** | **T** | **I** | **K** |
| **N59910F** | **-** | **-** | **-** | **-** | **-** | **-** | **-** | **-** | **-** | **-** | **-** | **-** | **-** | **-** | **-** | **-** | **-** | **-** | **-** | **-** | **-** | **-** | **-** | **-** | **-** | **-** | **-** | **-** | **-** | **-** | **-** | **-** | **-** | **-** | **-** | **-** | **Q** | **W** | **Q** | **A** | **K** | **Q** | **E** | **L** | **A** | **E** | **A** | **K** | **K** | **T** | **A** | **T** | **T** | **F** | **L** | **N** | **V** | **L** | **T** | **K** | **Q** | **K** | **F** | **D** | **K** | **L** | **P** | **S** | **V** | **V** | **Q** | **E** | **A** | **S** | **L** | **K** | **K** | **N** | **G** | **Y** | **D** | **T** | **K** | **S** | **V** | **V** | **E** | **K** | **Y** | **Q** | **A** | **I** | **Y** | **S** | **G** | **I** | **Q** | **A** | **E** | **G** | **V** | **K** | **A** | **S** | **D** | **V** | **Q** | **V** | **K** | **K** | **A** | **K** | **D** | **N** | **Q** | **Y** | **T** | **F** | **T** | **Y** | **K** | **L** | **S** | **M** | **S** | **T** | **P** | **L** | **G** | **E** | **M** | **K** | **D** | **L** | **S** | **Y** | **Q** | **S** | **S** | **I** | **A** | **K** | **K** | **G** | **D** | **T** | **Y** | **Q** | **I** | **A** | **W** | **K** | **P** | **S** | **L** | **I** | **F** | **P** | **D** | **M** | **S** | **G** | **N** | **D** | **K** | **I** | **S** | **I** | **Q** | **V** | **D** | **N** | **A** | **K** | **R** | **G** | **E** | **I** | **V** | **D** | **R** | **N** | **G** | **S** | **G** | **L** | **A** | **I** | **N** | **K** | **V** | **F** | **D** | **E** | **V** | **G** | **V** | **V** | **P** | **G** | **K** | **L** | **G** | **S** | **G** | **A** | **E** | **K** | **T** | **A** | **N** | **I** | **K** | **A** | **F** | **S** | **D** | **K** | **F** | **G** | **V** | **S** | **V** | **D** | **E** | **I** | **N** | **Q** | **K** | **L** | **S** | **Q** | **G** | **W** | **V** | **Q** | **A** | **D** | **S** | **F** | **V** | **P** | **I** | **T** | **V** | **A** | **S** | **E** | **P** | **V** | **T** | **E** | **L** | **P** | **T** | **G** | **A** | **A** | **T** | **K** | **D** | **T** | **E** | **S** | **R** | **Y** | **Y** | **P** | **L** | **G** | **E** | **A** | **A** | **A** | **Q** | **L** | **I** | **G** | **Y** | **T** | **G** | **T** | **I** | **T** | **A** | **E** | **D** | **I** | **K** | **K** | **N** | **P** | **E** | **L** | **S** | **S** | **T** | **G** | **V** | **I** | **G** | **K** | **T** | **G** | **L** | **E** | **R** | **A** | **F** | **D** | **K** | **E** | **L** | **R** | **G** | **Q** | **D** | **G** | **G** | **S** | **L** | **V** | **I** | **L** | **D** | **D** | **K** | **E** | **N** | **V** | **K** | **K** | **A** | **L** | **Q** | **T** | **K** | **E** | **K** | **K** | **D** | **G** | **Q** | **T** | **I** | **K** | **L** | **T** | **I** | **D** | **S** | **G** | **V** | **Q** | **Q** | **Q** | **A** | **F** | **A** | **I** | **F** | **D** | **K** | **R** | **P** | **G** | **S** | **A** | **V** | **I** | **T** | **D** | **P** | **Q** | **K** | **G** | **D** | **L** | **L** | **A** | **T** | **V** | **S** | **S** | **P** | **S** | **Y** | **D** | **P** | **N** | **K** | **M** | **A** | **N** | **G** | **I** | **S** | **Q** | **K** | **E** | **Y** | **D** | **A** | **Y** | **N** | **N** | **N** | **K** | **D** | **L** | **P** | **F** | **T** | **A** | **R** | **F** | **A** | **T** | **G** | **Y** | **A** | **P** | **G** | **S** | **T** | **F** | **K** | **T** | **I** | **T** | **G** | **A** | **I** | **G** | **L** | **D** | **A** | **G** | **T** | **L** | **K** | **P** | **D** | **E** | **E** | **L** | **E** | **I** | **N** | **G** | **L** | **K** | **W** | **Q** | **K** | **D** | **K** | **S** | **W** | **G** | **G** | **Y** | **F** | **A** | **T** | **R** | **V** | **K** | **E** | **A** | **S** | **P** | **V** | **N** | **L** | **R** | **T** | **A** | **L** | **V** | **N** | **S** | **D** | **N** | **I** | **Y** | **F** | **A** | **Q** | **Q** | **T** | **L** | **R** | **M** | **G** | **E** | **D** | **K** | **F** | **R** | **A** | **G** | **L** | **N** | **K** | **F** | **I** | **F** | **G** | **E** | **E** | **L** | **D** | **L** | **P** | **I** | **A** | **M** | **T** | **P** | **A** | **Q** | **I** | **S** | **N** | **E** | **D** | **K** | **F** | **N** | **S** | **E** | **I** | **L** | **L** | **A** | **D** | **T** | **G** | **Y** | **G** | **Q** | **G** | **Q** | **L** | **L** | **I** | **S** | **P** | **I** | **Q** | **Q** | **A** | **T** | **M** | **Y** | **S** | **V** | **F** | **Q** | **N** | **N** | **G** | **T** | **L** | **V** | **Y** | **P** | **K** | **L** | **V** | **L** | **D** | **K** | **E** | **T** | **K** | **K** | **K** | **D** | **N** | **V** | **I** | **S** | **A** | **N** | **A** | **A** | **N** | **T** | **I** | **A** | **T** | **D** | **L** | **L** | **G** | **S** | **V** | **E** | **D** | **P** | **S** | **G** | **Y** | **V** | **Y** | **N** | **M** | **Y** | **N** | **P** | **N** | **F** | **S** | **L** | **A** | **A** | **K** | **T** | **G** | **T** | **A** | **E** | **I** | **K** | **D** | **K** | **Q** | **D** | **T** | **D** | **G** | **K** | **E** | **N** | **S** | **F** | **L** | **L** | **T** | **L** | **D** | **R** | **S** | **N** | **N** | **K** | **F** | **L** | **T** | **M** | **I** | **M** | **V** | **E** | **N** | **S** | **G** | **E** | **N** | **G** | **S** | **A** | **T** | **D** | **I** | **S** | **K** | **P** | **L** | **I** | **D** | **Y** | **L** | **E** | **A** | **T** | **I** | **K** | **-** | **-** | **-** | **-** | **-** | **-** | **-** | **-** | **-** | **-** | **-** | **-** | **-** | **-** | **-** | **-** | **-** | **-** | **-** | **-** | **-** | **-** | **-** | **-** | **-** | **-** | **-** | **-** | **-** | **-** | **-** | **-** | **-** | **-** | **-** | **-** | **Q** | **W** | **Q** | **A** | **K** | **Q** | **E** | **L** | **A** | **E** | **A** | **K** | **K** | **T** | **A** | **T** | **T** | **F** | **L** | **N** | **V** | **L** | **T** | **K** | **Q** | **K** | **F** | **D** | **K** | **L** | **P** | **S** | **V** | **V** | **Q** | **E** | **A** | **S** | **L** | **K** | **K** | **N** | **G** | **Y** | **D** | **T** | **K** | **S** | **V** | **V** | **E** | **K** | **Y** | **Q** | **A** | **I** | **Y** | **S** | **G** | **I** | **Q** | **A** | **E** | **G** | **V** | **K** | **A** | **S** | **D** | **V** | **Q** | **V** | **K** | **K** | **A** | **K** | **D** | **N** | **Q** | **Y** | **T** | **F** | **T** | **Y** | **K** | **L** | **S** | **M** | **S** | **T** | **P** | **L** | **G** | **E** | **M** | **K** | **D** | **L** | **S** | **Y** | **Q** | **S** | **S** | **I** | **A** | **K** | **K** | **G** | **D** | **T** | **Y** | **Q** | **I** | **A** | **W** | **K** | **P** | **S** | **L** | **I** | **F** | **P** | **D** | **M** | **S** | **G** | **N** | **D** | **K** | **I** | **S** | **I** | **Q** | **V** | **D** | **N** | **A** | **K** | **R** | **G** | **E** | **I** | **V** | **D** | **R** | **N** | **G** | **S** | **G** | **L** | **A** | **I** | **N** | **K** | **V** | **F** | **D** | **E** | **V** | **G** | **V** | **V** | **P** | **G** | **K** | **L** | **G** | **S** | **G** | **A** | **E** | **K** | **T** | **A** | **N** | **I** | **K** | **A** | **F** | **S** | **D** | **K** | **F** | **G** | **V** | **S** | **V** | **D** | **E** | **I** | **N** | **Q** | **K** | **L** | **S** | **Q** | **G** | **W** | **V** | **Q** | **A** | **D** | **S** | **F** | **V** | **P** | **I** | **T** | **V** | **A** | **S** | **E** | **P** | **V** | **T** | **E** | **L** | **P** | **T** | **G** | **A** | **A** | **T** | **K** | **D** | **T** | **E** | **S** | **R** | **Y** | **Y** | **P** | **L** | **G** | **E** | **A** | **A** | **A** | **Q** | **L** | **I** | **G** | **Y** | **T** | **G** | **T** | **I** | **T** | **A** | **E** | **D** | **I** | **K** | **K** | **N** | **P** | **E** | **L** | **S** | **S** | **T** | **G** | **V** | **I** | **G** | **K** | **T** | **G** | **L** | **E** | **R** | **A** | **F** | **D** | **K** | **E** | **L** | **R** | **G** | **Q** | **D** | **G** | **G** | **S** | **L** | **V** | **I** | **L** | **D** | **D** | **K** | **E** | **N** | **V** | **K** | **K** | **A** | **L** | **Q** | **T** | **K** | **E** | **K** | **K** | **D** | **G** | **Q** | **T** | **I** | **K** | **L** | **T** | **I** | **D** | **S** | **G** | **V** | **Q** | **Q** | **Q** | **A** | **F** | **A** | **I** | **F** | **D** | **K** | **R** | **P** | **G** | **S** | **A** | **V** | **I** | **T** | **D** | **P** | **Q** | **K** | **G** | **D** | **L** | **L** | **A** | **T** | **V** | **S** | **S** | **P** | **S** | **Y** | **D** | **P** | **N** | **K** | **M** | **A** | **N** | **G** | **I** | **S** | **Q** | **K** | **E** | **Y** | **D** | **A** | **Y** | **N** | **N** | **N** | **K** | **D** | **L** | **P** | **F** | **T** | **A** | **R** | **F** | **A** | **T** | **G** | **Y** | **A** | **P** | **G** | **S** | **T** | **F** | **K** | **T** | **I** | **T** | **G** | **A** | **I** | **G** | **L** | **D** | **A** | **G** | **T** | **L** | **K** | **P** | **D** | **E** | **E** | **L** | **E** | **I** | **N** | **G** | **L** | **K** | **W** | **Q** | **K** | **D** | **K** | **S** | **W** | **G** | **G** | **Y** | **F** | **A** | **T** | **R** | **V** | **K** | **E** | **A** | **S** | **P** | **V** | **N** | **L** | **R** | **T** | **A** | **L** | **V** | **N** | **S** | **D** | **N** | **I** | **Y** | **F** | **A** | **Q** | **Q** | **T** | **L** | **R** | **M** | **G** | **E** | **D** | **K** | **F** | **R** | **A** | **G** | **L** | **N** | **K** | **F** | **I** | **F** | **G** | **E** | **E** | **L** | **D** | **L** | **P** | **I** | **A** | **M** | **T** | **P** | **A** | **Q** | **I** | **S** | **N** | **E** | **D** | **K** | **F** | **N** | **S** | **E** | **I** | **L** | **L** | **A** | **D** | **T** | **G** | **Y** | **G** | **Q** | **G** | **Q** | **L** | **L** | **I** | **S** | **P** | **I** | **Q** | **Q** | **A** | **T** | **M** | **Y** | **S** | **V** | **F** | **Q** | **N** | **N** | **G** | **T** | **L** | **V** | **Y** | **P** | **K** | **L** | **V** | **L** | **D** | **K** | **E** | **T** | **K** | **K** | **K** | **D** | **N** | **V** | **I** | **S** | **A** | **N** | **A** | **A** | **N** | **T** | **I** | **A** | **T** | **D** | **L** | **L** | **G** | **S** | **V** | **E** | **D** | **P** | **S** | **G** | **Y** | **V** | **Y** | **N** | **M** | **Y** | **N** | **P** | **N** | **F** | **S** | **L** | **A** | **A** | **K** | **T** | **G** | **T** | **A** | **E** | **I** | **K** | **D** | **K** | **Q** | **D** | **T** | **D** | **G** | **K** | **E** | **N** | **S** | **F** | **L** | **L** | **T** | **L** | **D** | **R** | **S** | **N** | **N** | **K** | **F** | **L** | **T** | **M** | **I** | **M** | **V** | **E** | **N** | **S** | **G** | **E** | **N** | **G** | **S** | **A** | **T** | **D** | **I** | **S** | **K** | **P** | **L** | **I** | **D** | **Y** | **L** | **E** | **A** | **T** | **I** | **K** |
| **N59554F** | **-** | **-** | **-** | **-** | **-** | **-** | **-** | **-** | **-** | **-** | **-** | **-** | **-** | **-** | **-** | **-** | **-** | **-** | **-** | **-** | **-** | **-** | **-** | **-** | **-** | **-** | **-** | **-** | **-** | **-** | **-** | **-** | **-** | **-** | **-** | **-** | **Q** | **W** | **Q** | **A** | **K** | **Q** | **E** | **L** | **A** | **E** | **A** | **K** | **K** | **T** | **A** | **T** | **T** | **F** | **L** | **N** | **V** | **L** | **S** | **K** | **Q** | **E** | **F** | **D** | **K** | **L** | **P** | **S** | **V** | **V** | **Q** | **E** | **A** | **S** | **L** | **K** | **K** | **N** | **G** | **Y** | **D** | **T** | **K** | **S** | **V** | **V** | **E** | **K** | **Y** | **Q** | **A** | **I** | **Y** | **S** | **G** | **I** | **Q** | **A** | **E** | **G** | **V** | **K** | **A** | **S** | **D** | **V** | **Q** | **V** | **K** | **K** | **A** | **K** | **D** | **N** | **Q** | **Y** | **T** | **F** | **T** | **Y** | **K** | **L** | **S** | **M** | **S** | **T** | **P** | **L** | **G** | **E** | **M** | **K** | **D** | **L** | **S** | **Y** | **Q** | **S** | **S** | **I** | **A** | **K** | **K** | **G** | **D** | **T** | **Y** | **Q** | **I** | **A** | **W** | **K** | **P** | **S** | **L** | **I** | **F** | **P** | **D** | **M** | **S** | **G** | **N** | **D** | **K** | **I** | **S** | **I** | **Q** | **V** | **D** | **N** | **A** | **K** | **R** | **G** | **E** | **I** | **V** | **D** | **R** | **N** | **G** | **S** | **G** | **L** | **A** | **I** | **N** | **K** | **V** | **F** | **D** | **E** | **V** | **G** | **V** | **V** | **P** | **G** | **K** | **L** | **G** | **S** | **G** | **A** | **E** | **K** | **T** | **A** | **N** | **I** | **K** | **A** | **F** | **S** | **D** | **K** | **F** | **G** | **V** | **S** | **V** | **D** | **E** | **I** | **N** | **Q** | **K** | **L** | **S** | **Q** | **G** | **W** | **V** | **Q** | **A** | **D** | **S** | **F** | **V** | **P** | **I** | **T** | **V** | **A** | **S** | **E** | **P** | **V** | **T** | **E** | **L** | **P** | **T** | **G** | **A** | **A** | **T** | **K** | **D** | **T** | **E** | **S** | **R** | **Y** | **Y** | **P** | **L** | **G** | **E** | **A** | **A** | **A** | **Q** | **L** | **I** | **G** | **Y** | **T** | **G** | **T** | **I** | **T** | **A** | **E** | **D** | **I** | **E** | **K** | **N** | **P** | **E** | **L** | **S** | **S** | **T** | **G** | **V** | **I** | **G** | **K** | **T** | **G** | **L** | **E** | **R** | **A** | **F** | **D** | **K** | **E** | **L** | **R** | **G** | **Q** | **D** | **G** | **G** | **S** | **L** | **V** | **I** | **L** | **D** | **D** | **K** | **E** | **N** | **V** | **K** | **K** | **A** | **L** | **Q** | **T** | **K** | **E** | **K** | **K** | **D** | **G** | **Q** | **T** | **I** | **K** | **L** | **T** | **I** | **D** | **S** | **G** | **V** | **Q** | **Q** | **Q** | **A** | **F** | **A** | **I** | **F** | **D** | **K** | **R** | **P** | **G** | **S** | **A** | **V** | **I** | **T** | **D** | **P** | **Q** | **K** | **G** | **D** | **L** | **L** | **A** | **T** | **V** | **S** | **S** | **P** | **S** | **Y** | **D** | **P** | **N** | **K** | **M** | **A** | **N** | **G** | **I** | **S** | **Q** | **K** | **E** | **Y** | **D** | **A** | **Y** | **N** | **N** | **N** | **K** | **D** | **L** | **P** | **F** | **T** | **A** | **R** | **F** | **A** | **T** | **G** | **Y** | **A** | **P** | **G** | **S** | **T** | **F** | **K** | **T** | **I** | **T** | **G** | **A** | **I** | **G** | **L** | **D** | **A** | **G** | **T** | **L** | **K** | **P** | **D** | **E** | **E** | **L** | **E** | **I** | **N** | **G** | **L** | **K** | **W** | **Q** | **K** | **D** | **K** | **S** | **W** | **G** | **G** | **Y** | **F** | **A** | **T** | **R** | **V** | **K** | **E** | **A** | **S** | **P** | **V** | **N** | **L** | **R** | **T** | **A** | **L** | **V** | **N** | **S** | **D** | **N** | **I** | **Y** | **F** | **A** | **Q** | **Q** | **T** | **L** | **R** | **M** | **G** | **E** | **D** | **K** | **F** | **R** | **A** | **G** | **L** | **N** | **K** | **F** | **I** | **F** | **G** | **E** | **E** | **L** | **D** | **L** | **P** | **I** | **A** | **M** | **T** | **P** | **A** | **Q** | **I** | **S** | **N** | **E** | **D** | **K** | **F** | **N** | **S** | **E** | **I** | **L** | **L** | **A** | **D** | **T** | **G** | **Y** | **G** | **Q** | **G** | **Q** | **L** | **L** | **I** | **S** | **P** | **I** | **Q** | **Q** | **A** | **T** | **M** | **Y** | **S** | **V** | **F** | **Q** | **N** | **N** | **G** | **T** | **L** | **V** | **Y** | **P** | **K** | **L** | **V** | **L** | **D** | **K** | **E** | **T** | **K** | **K** | **K** | **D** | **N** | **V** | **I** | **S** | **A** | **N** | **A** | **A** | **N** | **T** | **I** | **A** | **T** | **D** | **L** | **L** | **G** | **S** | **V** | **E** | **D** | **P** | **S** | **G** | **Y** | **V** | **Y** | **N** | **M** | **Y** | **N** | **P** | **N** | **F** | **S** | **L** | **A** | **A** | **K** | **T** | **G** | **T** | **A** | **E** | **I** | **K** | **D** | **K** | **Q** | **D** | **T** | **D** | **G** | **K** | **E** | **N** | **S** | **F** | **L** | **L** | **T** | **L** | **D** | **R** | **S** | **N** | **N** | **K** | **F** | **L** | **T** | **M** | **I** | **M** | **V** | **E** | **N** | **S** | **G** | **E** | **N** | **G** | **S** | **A** | **T** | **D** | **I** | **S** | **K** | **P** | **L** | **I** | **D** | **Y** | **L** | **E** | **A** | **T** | **I** | **K** | **-** | **-** | **-** | **-** | **-** | **-** | **-** | **-** | **-** | **-** | **-** | **-** | **-** | **-** | **-** | **-** | **-** | **-** | **-** | **-** | **-** | **-** | **-** | **-** | **-** | **-** | **-** | **-** | **-** | **-** | **-** | **-** | **-** | **-** | **-** | **-** | **Q** | **W** | **Q** | **A** | **K** | **Q** | **E** | **L** | **A** | **E** | **A** | **K** | **K** | **T** | **A** | **T** | **T** | **F** | **L** | **N** | **V** | **L** | **S** | **K** | **Q** | **E** | **F** | **D** | **K** | **L** | **P** | **S** | **V** | **V** | **Q** | **E** | **A** | **S** | **L** | **K** | **K** | **N** | **G** | **Y** | **D** | **T** | **K** | **S** | **V** | **V** | **E** | **K** | **Y** | **Q** | **A** | **I** | **Y** | **S** | **G** | **I** | **Q** | **A** | **E** | **G** | **V** | **K** | **A** | **S** | **D** | **V** | **Q** | **V** | **K** | **K** | **A** | **K** | **D** | **N** | **Q** | **Y** | **T** | **F** | **T** | **Y** | **K** | **L** | **S** | **M** | **S** | **T** | **P** | **L** | **G** | **E** | **M** | **K** | **D** | **L** | **S** | **Y** | **Q** | **S** | **S** | **I** | **A** | **K** | **K** | **G** | **D** | **T** | **Y** | **Q** | **I** | **A** | **W** | **K** | **P** | **S** | **L** | **I** | **F** | **P** | **D** | **M** | **S** | **G** | **N** | **D** | **K** | **I** | **S** | **I** | **Q** | **V** | **D** | **N** | **A** | **K** | **R** | **G** | **E** | **I** | **V** | **D** | **R** | **N** | **G** | **S** | **G** | **L** | **A** | **I** | **N** | **K** | **V** | **F** | **D** | **E** | **V** | **G** | **V** | **V** | **P** | **G** | **K** | **L** | **G** | **S** | **G** | **A** | **E** | **K** | **T** | **A** | **N** | **I** | **K** | **A** | **F** | **S** | **D** | **K** | **F** | **G** | **V** | **S** | **V** | **D** | **E** | **I** | **N** | **Q** | **K** | **L** | **S** | **Q** | **G** | **W** | **V** | **Q** | **A** | **D** | **S** | **F** | **V** | **P** | **I** | **T** | **V** | **A** | **S** | **E** | **P** | **V** | **T** | **E** | **L** | **P** | **T** | **G** | **A** | **A** | **T** | **K** | **D** | **T** | **E** | **S** | **R** | **Y** | **Y** | **P** | **L** | **G** | **E** | **A** | **A** | **A** | **Q** | **L** | **I** | **G** | **Y** | **T** | **G** | **T** | **I** | **T** | **A** | **E** | **D** | **I** | **E** | **K** | **N** | **P** | **E** | **L** | **S** | **S** | **T** | **G** | **V** | **I** | **G** | **K** | **T** | **G** | **L** | **E** | **R** | **A** | **F** | **D** | **K** | **E** | **L** | **R** | **G** | **Q** | **D** | **G** | **G** | **S** | **L** | **V** | **I** | **L** | **D** | **D** | **K** | **E** | **N** | **V** | **K** | **K** | **A** | **L** | **Q** | **T** | **K** | **E** | **K** | **K** | **D** | **G** | **Q** | **T** | **I** | **K** | **L** | **T** | **I** | **D** | **S** | **G** | **V** | **Q** | **Q** | **Q** | **A** | **F** | **A** | **I** | **F** | **D** | **K** | **R** | **P** | **G** | **S** | **A** | **V** | **I** | **T** | **D** | **P** | **Q** | **K** | **G** | **D** | **L** | **L** | **A** | **T** | **V** | **S** | **S** | **P** | **S** | **Y** | **D** | **P** | **N** | **K** | **M** | **A** | **N** | **G** | **I** | **S** | **Q** | **K** | **E** | **Y** | **D** | **A** | **Y** | **N** | **N** | **N** | **K** | **D** | **L** | **P** | **F** | **T** | **A** | **R** | **F** | **A** | **T** | **G** | **Y** | **A** | **P** | **G** | **S** | **T** | **F** | **K** | **T** | **I** | **T** | **G** | **A** | **I** | **G** | **L** | **D** | **A** | **G** | **T** | **L** | **K** | **P** | **D** | **E** | **E** | **L** | **E** | **I** | **N** | **G** | **L** | **K** | **W** | **Q** | **K** | **D** | **K** | **S** | **W** | **G** | **G** | **Y** | **F** | **A** | **T** | **R** | **V** | **K** | **E** | **A** | **S** | **P** | **V** | **N** | **L** | **R** | **T** | **A** | **L** | **V** | **N** | **S** | **D** | **N** | **I** | **Y** | **F** | **A** | **Q** | **Q** | **T** | **L** | **R** | **M** | **G** | **E** | **D** | **K** | **F** | **R** | **A** | **G** | **L** | **N** | **K** | **F** | **I** | **F** | **G** | **E** | **E** | **L** | **D** | **L** | **P** | **I** | **A** | **M** | **T** | **P** | **A** | **Q** | **I** | **S** | **N** | **E** | **D** | **K** | **F** | **N** | **S** | **E** | **I** | **L** | **L** | **A** | **D** | **T** | **G** | **Y** | **G** | **Q** | **G** | **Q** | **L** | **L** | **I** | **S** | **P** | **I** | **Q** | **Q** | **A** | **T** | **M** | **Y** | **S** | **V** | **F** | **Q** | **N** | **N** | **G** | **T** | **L** | **V** | **Y** | **P** | **K** | **L** | **V** | **L** | **D** | **K** | **E** | **T** | **K** | **K** | **K** | **D** | **N** | **V** | **I** | **S** | **A** | **N** | **A** | **A** | **N** | **T** | **I** | **A** | **T** | **D** | **L** | **L** | **G** | **S** | **V** | **E** | **D** | **P** | **S** | **G** | **Y** | **V** | **Y** | **N** | **M** | **Y** | **N** | **P** | **N** | **F** | **S** | **L** | **A** | **A** | **K** | **T** | **G** | **T** | **A** | **E** | **I** | **K** | **D** | **K** | **Q** | **D** | **T** | **D** | **G** | **K** | **E** | **N** | **S** | **F** | **L** | **L** | **T** | **L** | **D** | **R** | **S** | **N** | **N** | **K** | **F** | **L** | **T** | **M** | **I** | **M** | **V** | **E** | **N** | **S** | **G** | **E** | **N** | **G** | **S** | **A** | **T** | **D** | **I** | **S** | **K** | **P** | **L** | **I** | **D** | **Y** | **L** | **E** | **A** | **T** | **I** | **K** |
| **N52454** | **-** | **-** | **-** | **-** | **-** | **-** | **-** | **-** | **-** | **-** | **-** | **-** | **-** | **-** | **-** | **-** | **-** | **-** | **-** | **-** | **-** | **-** | **-** | **-** | **-** | **-** | **-** | **-** | **-** | **-** | **-** | **-** | **-** | **-** | **-** | **-** | **Q** | **W** | **Q** | **A** | **K** | **Q** | **E** | **L** | **A** | **E** | **A** | **K** | **K** | **T** | **A** | **T** | **T** | **F** | **L** | **N** | **V** | **L** | **S** | **K** | **Q** | **E** | **F** | **D** | **K** | **L** | **P** | **S** | **V** | **V** | **Q** | **E** | **A** | **S** | **L** | **K** | **K** | **N** | **G** | **Y** | **D** | **T** | **K** | **S** | **V** | **V** | **E** | **K** | **Y** | **Q** | **A** | **I** | **Y** | **S** | **G** | **I** | **Q** | **A** | **E** | **G** | **V** | **K** | **A** | **S** | **D** | **V** | **Q** | **V** | **K** | **K** | **A** | **K** | **D** | **N** | **Q** | **Y** | **T** | **F** | **T** | **Y** | **K** | **L** | **S** | **M** | **S** | **T** | **P** | **L** | **G** | **E** | **M** | **K** | **D** | **L** | **S** | **Y** | **Q** | **S** | **S** | **I** | **A** | **K** | **K** | **G** | **D** | **T** | **Y** | **Q** | **I** | **A** | **W** | **K** | **P** | **S** | **L** | **I** | **F** | **P** | **D** | **M** | **S** | **G** | **N** | **D** | **K** | **I** | **S** | **I** | **Q** | **V** | **D** | **N** | **A** | **K** | **R** | **G** | **E** | **I** | **V** | **D** | **R** | **N** | **G** | **S** | **G** | **L** | **A** | **I** | **N** | **K** | **V** | **F** | **D** | **E** | **V** | **G** | **V** | **V** | **P** | **G** | **K** | **L** | **G** | **S** | **G** | **A** | **E** | **K** | **T** | **A** | **N** | **I** | **K** | **A** | **F** | **S** | **D** | **K** | **F** | **G** | **V** | **S** | **V** | **D** | **E** | **I** | **N** | **Q** | **K** | **L** | **S** | **Q** | **G** | **W** | **V** | **Q** | **A** | **D** | **S** | **F** | **V** | **P** | **I** | **T** | **V** | **A** | **S** | **E** | **P** | **V** | **T** | **E** | **L** | **P** | **T** | **G** | **A** | **A** | **T** | **K** | **D** | **T** | **E** | **S** | **R** | **Y** | **Y** | **P** | **L** | **G** | **E** | **A** | **A** | **A** | **Q** | **L** | **I** | **G** | **Y** | **T** | **G** | **T** | **I** | **T** | **A** | **E** | **D** | **I** | **E** | **K** | **N** | **P** | **E** | **L** | **S** | **S** | **T** | **G** | **V** | **I** | **G** | **K** | **T** | **G** | **L** | **E** | **R** | **A** | **F** | **D** | **K** | **E** | **L** | **R** | **G** | **Q** | **D** | **G** | **G** | **S** | **L** | **V** | **I** | **L** | **D** | **D** | **K** | **E** | **N** | **V** | **K** | **K** | **A** | **L** | **Q** | **T** | **K** | **E** | **K** | **K** | **D** | **G** | **Q** | **T** | **I** | **K** | **L** | **T** | **I** | **D** | **S** | **G** | **V** | **Q** | **Q** | **Q** | **A** | **F** | **A** | **I** | **F** | **D** | **K** | **R** | **P** | **G** | **S** | **A** | **V** | **I** | **T** | **D** | **P** | **Q** | **K** | **G** | **D** | **L** | **L** | **A** | **T** | **V** | **S** | **S** | **P** | **S** | **Y** | **D** | **P** | **N** | **K** | **M** | **A** | **N** | **G** | **I** | **S** | **Q** | **K** | **E** | **Y** | **D** | **A** | **Y** | **N** | **N** | **N** | **K** | **D** | **L** | **P** | **F** | **T** | **A** | **R** | **F** | **A** | **T** | **G** | **Y** | **A** | **P** | **G** | **S** | **T** | **F** | **K** | **T** | **I** | **T** | **G** | **A** | **I** | **G** | **L** | **D** | **A** | **G** | **T** | **L** | **K** | **P** | **D** | **E** | **E** | **L** | **E** | **I** | **N** | **G** | **L** | **K** | **W** | **Q** | **K** | **D** | **K** | **S** | **W** | **G** | **G** | **Y** | **F** | **A** | **T** | **R** | **V** | **K** | **E** | **A** | **S** | **P** | **V** | **N** | **L** | **R** | **T** | **A** | **L** | **V** | **N** | **S** | **D** | **N** | **I** | **Y** | **F** | **A** | **Q** | **Q** | **T** | **L** | **R** | **M** | **G** | **E** | **D** | **K** | **F** | **R** | **A** | **G** | **L** | **N** | **K** | **F** | **I** | **F** | **G** | **E** | **E** | **L** | **D** | **L** | **P** | **I** | **A** | **M** | **T** | **P** | **A** | **Q** | **I** | **S** | **N** | **E** | **D** | **K** | **F** | **N** | **S** | **E** | **I** | **L** | **L** | **A** | **D** | **T** | **G** | **Y** | **G** | **Q** | **G** | **Q** | **L** | **L** | **I** | **S** | **P** | **I** | **Q** | **Q** | **A** | **T** | **M** | **Y** | **S** | **V** | **F** | **Q** | **N** | **N** | **G** | **T** | **L** | **V** | **Y** | **P** | **K** | **L** | **V** | **L** | **D** | **K** | **E** | **T** | **K** | **K** | **K** | **D** | **N** | **V** | **I** | **S** | **A** | **N** | **A** | **A** | **N** | **T** | **I** | **A** | **T** | **D** | **L** | **L** | **G** | **S** | **V** | **E** | **D** | **P** | **S** | **G** | **Y** | **V** | **Y** | **N** | **M** | **Y** | **N** | **P** | **N** | **F** | **S** | **L** | **A** | **A** | **K** | **T** | **G** | **T** | **A** | **E** | **I** | **K** | **D** | **K** | **Q** | **D** | **T** | **D** | **G** | **K** | **E** | **N** | **S** | **F** | **L** | **L** | **T** | **L** | **D** | **R** | **S** | **N** | **N** | **K** | **F** | **L** | **T** | **M** | **I** | **M** | **V** | **E** | **N** | **S** | **G** | **E** | **N** | **G** | **S** | **A** | **T** | **D** | **I** | **S** | **K** | **P** | **L** | **I** | **D** | **Y** | **L** | **E** | **A** | **T** | **I** | **K** | **-** | **-** | **-** | **-** | **-** | **-** | **-** | **-** | **-** | **-** | **-** | **-** | **-** | **-** | **-** | **-** | **-** | **-** | **-** | **-** | **-** | **-** | **-** | **-** | **-** | **-** | **-** | **-** | **-** | **-** | **-** | **-** | **-** | **-** | **-** | **-** | **Q** | **W** | **Q** | **A** | **K** | **Q** | **E** | **L** | **A** | **E** | **A** | **K** | **K** | **T** | **A** | **T** | **T** | **F** | **L** | **N** | **V** | **L** | **S** | **K** | **Q** | **E** | **F** | **D** | **K** | **L** | **P** | **S** | **V** | **V** | **Q** | **E** | **A** | **S** | **L** | **K** | **K** | **N** | **G** | **Y** | **D** | **T** | **K** | **S** | **V** | **V** | **E** | **K** | **Y** | **Q** | **A** | **I** | **Y** | **S** | **G** | **I** | **Q** | **A** | **E** | **G** | **V** | **K** | **A** | **S** | **D** | **V** | **Q** | **V** | **K** | **K** | **A** | **K** | **D** | **N** | **Q** | **Y** | **T** | **F** | **T** | **Y** | **K** | **L** | **S** | **M** | **S** | **T** | **P** | **L** | **G** | **E** | **M** | **K** | **D** | **L** | **S** | **Y** | **Q** | **S** | **S** | **I** | **A** | **K** | **K** | **G** | **D** | **T** | **Y** | **Q** | **I** | **A** | **W** | **K** | **P** | **S** | **L** | **I** | **F** | **P** | **D** | **M** | **S** | **G** | **N** | **D** | **K** | **I** | **S** | **I** | **Q** | **V** | **D** | **N** | **A** | **K** | **R** | **G** | **E** | **I** | **V** | **D** | **R** | **N** | **G** | **S** | **G** | **L** | **A** | **I** | **N** | **K** | **V** | **F** | **D** | **E** | **V** | **G** | **V** | **V** | **P** | **G** | **K** | **L** | **G** | **S** | **G** | **A** | **E** | **K** | **T** | **A** | **N** | **I** | **K** | **A** | **F** | **S** | **D** | **K** | **F** | **G** | **V** | **S** | **V** | **D** | **E** | **I** | **N** | **Q** | **K** | **L** | **S** | **Q** | **G** | **W** | **V** | **Q** | **A** | **D** | **S** | **F** | **V** | **P** | **I** | **T** | **V** | **A** | **S** | **E** | **P** | **V** | **T** | **E** | **L** | **P** | **T** | **G** | **A** | **A** | **T** | **K** | **D** | **T** | **E** | **S** | **R** | **Y** | **Y** | **P** | **L** | **G** | **E** | **A** | **A** | **A** | **Q** | **L** | **I** | **G** | **Y** | **T** | **G** | **T** | **I** | **T** | **A** | **E** | **D** | **I** | **E** | **K** | **N** | **P** | **E** | **L** | **S** | **S** | **T** | **G** | **V** | **I** | **G** | **K** | **T** | **G** | **L** | **E** | **R** | **A** | **F** | **D** | **K** | **E** | **L** | **R** | **G** | **Q** | **D** | **G** | **G** | **S** | **L** | **V** | **I** | **L** | **D** | **D** | **K** | **E** | **N** | **V** | **K** | **K** | **A** | **L** | **Q** | **T** | **K** | **E** | **K** | **K** | **D** | **G** | **Q** | **T** | **I** | **K** | **L** | **T** | **I** | **D** | **S** | **G** | **V** | **Q** | **Q** | **Q** | **A** | **F** | **A** | **I** | **F** | **D** | **K** | **R** | **P** | **G** | **S** | **A** | **V** | **I** | **T** | **D** | **P** | **Q** | **K** | **G** | **D** | **L** | **L** | **A** | **T** | **V** | **S** | **S** | **P** | **S** | **Y** | **D** | **P** | **N** | **K** | **M** | **A** | **N** | **G** | **I** | **S** | **Q** | **K** | **E** | **Y** | **D** | **A** | **Y** | **N** | **N** | **N** | **K** | **D** | **L** | **P** | **F** | **T** | **A** | **R** | **F** | **A** | **T** | **G** | **Y** | **A** | **P** | **G** | **S** | **T** | **F** | **K** | **T** | **I** | **T** | **G** | **A** | **I** | **G** | **L** | **D** | **A** | **G** | **T** | **L** | **K** | **P** | **D** | **E** | **E** | **L** | **E** | **I** | **N** | **G** | **L** | **K** | **W** | **Q** | **K** | **D** | **K** | **S** | **W** | **G** | **G** | **Y** | **F** | **A** | **T** | **R** | **V** | **K** | **E** | **A** | **S** | **P** | **V** | **N** | **L** | **R** | **T** | **A** | **L** | **V** | **N** | **S** | **D** | **N** | **I** | **Y** | **F** | **A** | **Q** | **Q** | **T** | **L** | **R** | **M** | **G** | **E** | **D** | **K** | **F** | **R** | **A** | **G** | **L** | **N** | **K** | **F** | **I** | **F** | **G** | **E** | **E** | **L** | **D** | **L** | **P** | **I** | **A** | **M** | **T** | **P** | **A** | **Q** | **I** | **S** | **N** | **E** | **D** | **K** | **F** | **N** | **S** | **E** | **I** | **L** | **L** | **A** | **D** | **T** | **G** | **Y** | **G** | **Q** | **G** | **Q** | **L** | **L** | **I** | **S** | **P** | **I** | **Q** | **Q** | **A** | **T** | **M** | **Y** | **S** | **V** | **F** | **Q** | **N** | **N** | **G** | **T** | **L** | **V** | **Y** | **P** | **K** | **L** | **V** | **L** | **D** | **K** | **E** | **T** | **K** | **K** | **K** | **D** | **N** | **V** | **I** | **S** | **A** | **N** | **A** | **A** | **N** | **T** | **I** | **A** | **T** | **D** | **L** | **L** | **G** | **S** | **V** | **E** | **D** | **P** | **S** | **G** | **Y** | **V** | **Y** | **N** | **M** | **Y** | **N** | **P** | **N** | **F** | **S** | **L** | **A** | **A** | **K** | **T** | **G** | **T** | **A** | **E** | **I** | **K** | **D** | **K** | **Q** | **D** | **T** | **D** | **G** | **K** | **E** | **N** | **S** | **F** | **L** | **L** | **T** | **L** | **D** | **R** | **S** | **N** | **N** | **K** | **F** | **L** | **T** | **M** | **I** | **M** | **V** | **E** | **N** | **S** | **G** | **E** | **N** | **G** | **S** | **A** | **T** | **D** | **I** | **S** | **K** | **P** | **L** | **I** | **D** | **Y** | **L** | **E** | **A** | **T** | **I** | **K** |
| **N53761** | **-** | **-** | **-** | **-** | **-** | **-** | **-** | **-** | **-** | **-** | **-** | **-** | **-** | **-** | **-** | **-** | **-** | **-** | **-** | **-** | **-** | **-** | **-** | **-** | **-** | **-** | **-** | **-** | **-** | **-** | **-** | **-** | **-** | **-** | **-** | **-** | **Q** | **W** | **Q** | **A** | **K** | **Q** | **E** | **L** | **A** | **E** | **A** | **K** | **K** | **T** | **A** | **T** | **T** | **F** | **L** | **N** | **V** | **L** | **S** | **K** | **Q** | **E** | **F** | **D** | **K** | **L** | **P** | **S** | **V** | **V** | **Q** | **E** | **A** | **S** | **L** | **K** | **K** | **N** | **G** | **Y** | **D** | **T** | **K** | **S** | **V** | **V** | **E** | **K** | **Y** | **Q** | **A** | **I** | **Y** | **S** | **G** | **I** | **Q** | **A** | **E** | **G** | **V** | **K** | **A** | **S** | **D** | **V** | **Q** | **V** | **K** | **K** | **A** | **K** | **D** | **N** | **Q** | **Y** | **T** | **F** | **T** | **Y** | **K** | **L** | **S** | **M** | **S** | **T** | **P** | **L** | **G** | **E** | **M** | **K** | **D** | **L** | **S** | **Y** | **Q** | **S** | **S** | **I** | **A** | **K** | **K** | **G** | **D** | **T** | **Y** | **Q** | **I** | **A** | **W** | **K** | **P** | **S** | **L** | **I** | **F** | **P** | **D** | **M** | **S** | **G** | **N** | **D** | **K** | **I** | **S** | **I** | **Q** | **V** | **D** | **N** | **A** | **K** | **R** | **G** | **E** | **I** | **V** | **D** | **R** | **N** | **G** | **S** | **G** | **L** | **A** | **I** | **N** | **K** | **V** | **F** | **D** | **E** | **V** | **G** | **V** | **V** | **P** | **G** | **K** | **L** | **G** | **S** | **G** | **A** | **E** | **K** | **T** | **A** | **N** | **I** | **K** | **A** | **F** | **S** | **D** | **K** | **F** | **G** | **V** | **S** | **V** | **D** | **E** | **I** | **N** | **Q** | **K** | **L** | **S** | **Q** | **G** | **W** | **V** | **Q** | **A** | **D** | **S** | **F** | **V** | **P** | **I** | **T** | **V** | **A** | **S** | **E** | **P** | **V** | **T** | **E** | **L** | **P** | **T** | **G** | **A** | **A** | **T** | **K** | **D** | **T** | **E** | **S** | **R** | **Y** | **Y** | **P** | **L** | **G** | **E** | **A** | **A** | **A** | **Q** | **L** | **I** | **G** | **Y** | **T** | **G** | **T** | **I** | **T** | **A** | **E** | **D** | **I** | **E** | **K** | **N** | **P** | **E** | **L** | **S** | **S** | **T** | **G** | **V** | **I** | **G** | **K** | **T** | **G** | **L** | **E** | **R** | **A** | **F** | **D** | **K** | **E** | **L** | **R** | **G** | **Q** | **D** | **G** | **G** | **S** | **L** | **V** | **I** | **L** | **D** | **D** | **K** | **E** | **N** | **V** | **K** | **K** | **A** | **L** | **Q** | **T** | **K** | **E** | **K** | **K** | **D** | **G** | **Q** | **T** | **I** | **K** | **L** | **T** | **I** | **D** | **S** | **G** | **V** | **Q** | **Q** | **Q** | **A** | **F** | **A** | **I** | **F** | **D** | **K** | **R** | **P** | **G** | **S** | **A** | **V** | **I** | **T** | **D** | **P** | **Q** | **K** | **G** | **D** | **L** | **L** | **A** | **T** | **V** | **S** | **S** | **P** | **S** | **Y** | **D** | **P** | **N** | **K** | **M** | **A** | **N** | **G** | **I** | **S** | **Q** | **K** | **E** | **Y** | **D** | **A** | **Y** | **N** | **N** | **N** | **K** | **D** | **L** | **P** | **F** | **T** | **A** | **R** | **F** | **A** | **T** | **G** | **Y** | **A** | **P** | **G** | **S** | **T** | **F** | **K** | **T** | **I** | **T** | **G** | **A** | **I** | **G** | **L** | **D** | **A** | **G** | **T** | **L** | **K** | **P** | **D** | **E** | **E** | **L** | **E** | **I** | **N** | **G** | **L** | **K** | **W** | **Q** | **K** | **D** | **K** | **S** | **W** | **G** | **G** | **Y** | **F** | **A** | **T** | **R** | **V** | **K** | **E** | **A** | **S** | **P** | **V** | **N** | **L** | **R** | **T** | **A** | **L** | **V** | **N** | **S** | **D** | **N** | **I** | **Y** | **F** | **A** | **Q** | **Q** | **T** | **L** | **R** | **M** | **G** | **E** | **D** | **K** | **F** | **R** | **A** | **G** | **L** | **N** | **K** | **F** | **I** | **F** | **G** | **E** | **E** | **L** | **D** | **L** | **P** | **I** | **A** | **M** | **T** | **P** | **A** | **Q** | **I** | **S** | **N** | **E** | **D** | **K** | **F** | **N** | **S** | **E** | **I** | **L** | **L** | **A** | **D** | **T** | **G** | **Y** | **G** | **Q** | **G** | **Q** | **L** | **L** | **I** | **S** | **P** | **I** | **Q** | **Q** | **A** | **T** | **M** | **Y** | **S** | **V** | **F** | **Q** | **N** | **N** | **G** | **T** | **L** | **V** | **Y** | **P** | **K** | **L** | **V** | **L** | **D** | **K** | **E** | **T** | **K** | **K** | **K** | **D** | **N** | **V** | **I** | **S** | **A** | **N** | **A** | **A** | **N** | **T** | **I** | **A** | **T** | **D** | **L** | **L** | **G** | **S** | **V** | **E** | **D** | **P** | **S** | **G** | **Y** | **V** | **Y** | **N** | **M** | **Y** | **N** | **P** | **N** | **F** | **S** | **L** | **A** | **A** | **K** | **T** | **G** | **T** | **A** | **E** | **I** | **K** | **D** | **K** | **Q** | **D** | **T** | **D** | **G** | **K** | **E** | **N** | **S** | **F** | **L** | **L** | **T** | **L** | **D** | **R** | **S** | **N** | **N** | **K** | **F** | **L** | **T** | **M** | **I** | **M** | **V** | **E** | **N** | **S** | **G** | **E** | **N** | **G** | **S** | **A** | **T** | **D** | **I** | **S** | **K** | **P** | **L** | **I** | **D** | **Y** | **L** | **E** | **A** | **T** | **I** | **K** | **-** | **-** | **-** | **-** | **-** | **-** | **-** | **-** | **-** | **-** | **-** | **-** | **-** | **-** | **-** | **-** | **-** | **-** | **-** | **-** | **-** | **-** | **-** | **-** | **-** | **-** | **-** | **-** | **-** | **-** | **-** | **-** | **-** | **-** | **-** | **-** | **Q** | **W** | **Q** | **A** | **K** | **Q** | **E** | **L** | **A** | **E** | **A** | **K** | **K** | **T** | **A** | **T** | **T** | **F** | **L** | **N** | **V** | **L** | **S** | **K** | **Q** | **E** | **F** | **D** | **K** | **L** | **P** | **S** | **V** | **V** | **Q** | **E** | **A** | **S** | **L** | **K** | **K** | **N** | **G** | **Y** | **D** | **T** | **K** | **S** | **V** | **V** | **E** | **K** | **Y** | **Q** | **A** | **I** | **Y** | **S** | **G** | **I** | **Q** | **A** | **E** | **G** | **V** | **K** | **A** | **S** | **D** | **V** | **Q** | **V** | **K** | **K** | **A** | **K** | **D** | **N** | **Q** | **Y** | **T** | **F** | **T** | **Y** | **K** | **L** | **S** | **M** | **S** | **T** | **P** | **L** | **G** | **E** | **M** | **K** | **D** | **L** | **S** | **Y** | **Q** | **S** | **S** | **I** | **A** | **K** | **K** | **G** | **D** | **T** | **Y** | **Q** | **I** | **A** | **W** | **K** | **P** | **S** | **L** | **I** | **F** | **P** | **D** | **M** | **S** | **G** | **N** | **D** | **K** | **I** | **S** | **I** | **Q** | **V** | **D** | **N** | **A** | **K** | **R** | **G** | **E** | **I** | **V** | **D** | **R** | **N** | **G** | **S** | **G** | **L** | **A** | **I** | **N** | **K** | **V** | **F** | **D** | **E** | **V** | **G** | **V** | **V** | **P** | **G** | **K** | **L** | **G** | **S** | **G** | **A** | **E** | **K** | **T** | **A** | **N** | **I** | **K** | **A** | **F** | **S** | **D** | **K** | **F** | **G** | **V** | **S** | **V** | **D** | **E** | **I** | **N** | **Q** | **K** | **L** | **S** | **Q** | **G** | **W** | **V** | **Q** | **A** | **D** | **S** | **F** | **V** | **P** | **I** | **T** | **V** | **A** | **S** | **E** | **P** | **V** | **T** | **E** | **L** | **P** | **T** | **G** | **A** | **A** | **T** | **K** | **D** | **T** | **E** | **S** | **R** | **Y** | **Y** | **P** | **L** | **G** | **E** | **A** | **A** | **A** | **Q** | **L** | **I** | **G** | **Y** | **T** | **G** | **T** | **I** | **T** | **A** | **E** | **D** | **I** | **E** | **K** | **N** | **P** | **E** | **L** | **S** | **S** | **T** | **G** | **V** | **I** | **G** | **K** | **T** | **G** | **L** | **E** | **R** | **A** | **F** | **D** | **K** | **E** | **L** | **R** | **G** | **Q** | **D** | **G** | **G** | **S** | **L** | **V** | **I** | **L** | **D** | **D** | **K** | **E** | **N** | **V** | **K** | **K** | **A** | **L** | **Q** | **T** | **K** | **E** | **K** | **K** | **D** | **G** | **Q** | **T** | **I** | **K** | **L** | **T** | **I** | **D** | **S** | **G** | **V** | **Q** | **Q** | **Q** | **A** | **F** | **A** | **I** | **F** | **D** | **K** | **R** | **P** | **G** | **S** | **A** | **V** | **I** | **T** | **D** | **P** | **Q** | **K** | **G** | **D** | **L** | **L** | **A** | **T** | **V** | **S** | **S** | **P** | **S** | **Y** | **D** | **P** | **N** | **K** | **M** | **A** | **N** | **G** | **I** | **S** | **Q** | **K** | **E** | **Y** | **D** | **A** | **Y** | **N** | **N** | **N** | **K** | **D** | **L** | **P** | **F** | **T** | **A** | **R** | **F** | **A** | **T** | **G** | **Y** | **A** | **P** | **G** | **S** | **T** | **F** | **K** | **T** | **I** | **T** | **G** | **A** | **I** | **G** | **L** | **D** | **A** | **G** | **T** | **L** | **K** | **P** | **D** | **E** | **E** | **L** | **E** | **I** | **N** | **G** | **L** | **K** | **W** | **Q** | **K** | **D** | **K** | **S** | **W** | **G** | **G** | **Y** | **F** | **A** | **T** | **R** | **V** | **K** | **E** | **A** | **S** | **P** | **V** | **N** | **L** | **R** | **T** | **A** | **L** | **V** | **N** | **S** | **D** | **N** | **I** | **Y** | **F** | **A** | **Q** | **Q** | **T** | **L** | **R** | **M** | **G** | **E** | **D** | **K** | **F** | **R** | **A** | **G** | **L** | **N** | **K** | **F** | **I** | **F** | **G** | **E** | **E** | **L** | **D** | **L** | **P** | **I** | **A** | **M** | **T** | **P** | **A** | **Q** | **I** | **S** | **N** | **E** | **D** | **K** | **F** | **N** | **S** | **E** | **I** | **L** | **L** | **A** | **D** | **T** | **G** | **Y** | **G** | **Q** | **G** | **Q** | **L** | **L** | **I** | **S** | **P** | **I** | **Q** | **Q** | **A** | **T** | **M** | **Y** | **S** | **V** | **F** | **Q** | **N** | **N** | **G** | **T** | **L** | **V** | **Y** | **P** | **K** | **L** | **V** | **L** | **D** | **K** | **E** | **T** | **K** | **K** | **K** | **D** | **N** | **V** | **I** | **S** | **A** | **N** | **A** | **A** | **N** | **T** | **I** | **A** | **T** | **D** | **L** | **L** | **G** | **S** | **V** | **E** | **D** | **P** | **S** | **G** | **Y** | **V** | **Y** | **N** | **M** | **Y** | **N** | **P** | **N** | **F** | **S** | **L** | **A** | **A** | **K** | **T** | **G** | **T** | **A** | **E** | **I** | **K** | **D** | **K** | **Q** | **D** | **T** | **D** | **G** | **K** | **E** | **N** | **S** | **F** | **L** | **L** | **T** | **L** | **D** | **R** | **S** | **N** | **N** | **K** | **F** | **L** | **T** | **M** | **I** | **M** | **V** | **E** | **N** | **S** | **G** | **E** | **N** | **G** | **S** | **A** | **T** | **D** | **I** | **S** | **K** | **P** | **L** | **I** | **D** | **Y** | **L** | **E** | **A** | **T** | **I** | **K** |
| **N52729** | **-** | **-** | **-** | **-** | **-** | **-** | **-** | **-** | **-** | **-** | **-** | **-** | **-** | **-** | **-** | **-** | **-** | **-** | **-** | **-** | **-** | **-** | **-** | **-** | **-** | **-** | **-** | **-** | **-** | **-** | **-** | **-** | **-** | **-** | **-** | **-** | **Q** | **W** | **Q** | **A** | **K** | **Q** | **E** | **L** | **A** | **E** | **A** | **K** | **K** | **T** | **A** | **T** | **T** | **F** | **L** | **N** | **V** | **L** | **T** | **K** | **Q** | **K** | **F** | **D** | **K** | **L** | **P** | **S** | **V** | **V** | **Q** | **E** | **A** | **S** | **L** | **K** | **K** | **N** | **G** | **Y** | **D** | **T** | **K** | **S** | **V** | **V** | **E** | **K** | **Y** | **Q** | **A** | **I** | **Y** | **S** | **G** | **I** | **Q** | **A** | **E** | **G** | **V** | **K** | **A** | **S** | **D** | **V** | **Q** | **V** | **K** | **K** | **A** | **K** | **D** | **N** | **Q** | **Y** | **T** | **F** | **T** | **Y** | **K** | **L** | **S** | **M** | **S** | **T** | **P** | **L** | **G** | **E** | **M** | **K** | **D** | **L** | **S** | **Y** | **Q** | **S** | **S** | **I** | **A** | **K** | **K** | **G** | **D** | **T** | **Y** | **Q** | **I** | **A** | **W** | **K** | **P** | **S** | **L** | **I** | **F** | **P** | **D** | **M** | **S** | **G** | **N** | **D** | **K** | **I** | **S** | **I** | **Q** | **V** | **D** | **N** | **A** | **K** | **R** | **G** | **E** | **I** | **V** | **D** | **R** | **N** | **G** | **S** | **G** | **L** | **A** | **I** | **N** | **K** | **V** | **F** | **D** | **E** | **V** | **G** | **V** | **V** | **P** | **G** | **K** | **L** | **G** | **S** | **G** | **A** | **E** | **K** | **T** | **A** | **N** | **I** | **K** | **A** | **F** | **S** | **D** | **K** | **F** | **G** | **V** | **S** | **V** | **D** | **E** | **I** | **N** | **Q** | **K** | **L** | **S** | **Q** | **G** | **W** | **V** | **Q** | **A** | **D** | **S** | **F** | **V** | **P** | **I** | **T** | **V** | **A** | **S** | **E** | **P** | **V** | **T** | **E** | **L** | **P** | **T** | **G** | **A** | **A** | **T** | **K** | **D** | **T** | **E** | **S** | **R** | **Y** | **Y** | **P** | **L** | **G** | **E** | **A** | **A** | **A** | **Q** | **L** | **I** | **G** | **Y** | **T** | **G** | **T** | **I** | **T** | **A** | **E** | **D** | **I** | **K** | **K** | **N** | **P** | **E** | **L** | **S** | **S** | **T** | **G** | **V** | **I** | **G** | **K** | **T** | **G** | **L** | **E** | **R** | **A** | **F** | **D** | **K** | **E** | **L** | **R** | **G** | **Q** | **D** | **G** | **G** | **S** | **L** | **V** | **I** | **L** | **D** | **D** | **K** | **E** | **N** | **V** | **K** | **K** | **A** | **L** | **Q** | **T** | **K** | **E** | **K** | **K** | **D** | **G** | **Q** | **T** | **I** | **K** | **L** | **T** | **I** | **D** | **S** | **G** | **V** | **Q** | **Q** | **Q** | **A** | **F** | **A** | **I** | **F** | **D** | **K** | **R** | **P** | **G** | **S** | **A** | **V** | **I** | **T** | **D** | **P** | **Q** | **K** | **G** | **D** | **L** | **L** | **A** | **T** | **V** | **S** | **S** | **P** | **S** | **Y** | **D** | **P** | **N** | **K** | **M** | **A** | **N** | **G** | **I** | **S** | **Q** | **K** | **E** | **Y** | **D** | **A** | **Y** | **N** | **N** | **N** | **K** | **D** | **L** | **P** | **F** | **T** | **A** | **R** | **F** | **A** | **T** | **G** | **Y** | **A** | **P** | **G** | **S** | **T** | **F** | **K** | **T** | **I** | **T** | **G** | **A** | **I** | **G** | **L** | **D** | **T** | **G** | **T** | **L** | **K** | **P** | **D** | **E** | **E** | **L** | **E** | **I** | **N** | **G** | **L** | **K** | **W** | **Q** | **K** | **D** | **K** | **S** | **W** | **G** | **G** | **Y** | **F** | **A** | **T** | **R** | **V** | **K** | **E** | **A** | **S** | **P** | **V** | **N** | **L** | **R** | **T** | **A** | **L** | **V** | **N** | **S** | **D** | **N** | **I** | **Y** | **F** | **A** | **Q** | **Q** | **T** | **L** | **R** | **M** | **G** | **E** | **D** | **K** | **F** | **R** | **A** | **G** | **L** | **N** | **K** | **F** | **I** | **F** | **G** | **E** | **E** | **L** | **D** | **L** | **P** | **I** | **A** | **M** | **T** | **P** | **A** | **Q** | **I** | **S** | **N** | **E** | **D** | **K** | **F** | **N** | **S** | **E** | **I** | **L** | **L** | **A** | **D** | **T** | **G** | **Y** | **G** | **Q** | **G** | **Q** | **L** | **L** | **I** | **S** | **P** | **I** | **Q** | **Q** | **A** | **T** | **M** | **Y** | **S** | **V** | **F** | **Q** | **N** | **N** | **G** | **T** | **L** | **V** | **Y** | **P** | **K** | **L** | **V** | **L** | **D** | **K** | **E** | **T** | **K** | **K** | **K** | **D** | **N** | **V** | **I** | **S** | **A** | **N** | **A** | **A** | **N** | **T** | **I** | **A** | **T** | **D** | **L** | **L** | **G** | **S** | **V** | **E** | **D** | **P** | **S** | **G** | **Y** | **V** | **Y** | **N** | **M** | **Y** | **N** | **P** | **N** | **F** | **S** | **L** | **A** | **A** | **K** | **T** | **G** | **T** | **A** | **E** | **I** | **K** | **D** | **K** | **Q** | **D** | **T** | **D** | **G** | **K** | **E** | **N** | **S** | **F** | **L** | **L** | **T** | **L** | **D** | **R** | **S** | **N** | **N** | **K** | **F** | **L** | **T** | **M** | **I** | **M** | **V** | **E** | **N** | **S** | **G** | **E** | **N** | **G** | **S** | **A** | **T** | **D** | **I** | **S** | **K** | **P** | **L** | **I** | **D** | **Y** | **L** | **E** | **A** | **T** | **I** | **K** | **-** | **-** | **-** | **-** | **-** | **-** | **-** | **-** | **-** | **-** | **-** | **-** | **-** | **-** | **-** | **-** | **-** | **-** | **-** | **-** | **-** | **-** | **-** | **-** | **-** | **-** | **-** | **-** | **-** | **-** | **-** | **-** | **-** | **-** | **-** | **-** | **Q** | **W** | **Q** | **A** | **K** | **Q** | **E** | **L** | **A** | **E** | **A** | **K** | **K** | **T** | **A** | **T** | **T** | **F** | **L** | **N** | **V** | **L** | **T** | **K** | **Q** | **K** | **F** | **D** | **K** | **L** | **P** | **S** | **V** | **V** | **Q** | **E** | **A** | **S** | **L** | **K** | **K** | **N** | **G** | **Y** | **D** | **T** | **K** | **S** | **V** | **V** | **E** | **K** | **Y** | **Q** | **A** | **I** | **Y** | **S** | **G** | **I** | **Q** | **A** | **E** | **G** | **V** | **K** | **A** | **S** | **D** | **V** | **Q** | **V** | **K** | **K** | **A** | **K** | **D** | **N** | **Q** | **Y** | **T** | **F** | **T** | **Y** | **K** | **L** | **S** | **M** | **S** | **T** | **P** | **L** | **G** | **E** | **M** | **K** | **D** | **L** | **S** | **Y** | **Q** | **S** | **S** | **I** | **A** | **K** | **K** | **G** | **D** | **T** | **Y** | **Q** | **I** | **A** | **W** | **K** | **P** | **S** | **L** | **I** | **F** | **P** | **D** | **M** | **S** | **G** | **N** | **D** | **K** | **I** | **S** | **I** | **Q** | **V** | **D** | **N** | **A** | **K** | **R** | **G** | **E** | **I** | **V** | **D** | **R** | **N** | **G** | **S** | **G** | **L** | **A** | **I** | **N** | **K** | **V** | **F** | **D** | **E** | **V** | **G** | **V** | **V** | **P** | **G** | **K** | **L** | **G** | **S** | **G** | **A** | **E** | **K** | **T** | **A** | **N** | **I** | **K** | **A** | **F** | **S** | **D** | **K** | **F** | **G** | **V** | **S** | **V** | **D** | **E** | **I** | **N** | **Q** | **K** | **L** | **S** | **Q** | **G** | **W** | **V** | **Q** | **A** | **D** | **S** | **F** | **V** | **P** | **I** | **T** | **V** | **A** | **S** | **E** | **P** | **V** | **T** | **E** | **L** | **P** | **T** | **G** | **A** | **A** | **T** | **K** | **D** | **T** | **E** | **S** | **R** | **Y** | **Y** | **P** | **L** | **G** | **E** | **A** | **A** | **A** | **Q** | **L** | **I** | **G** | **Y** | **T** | **G** | **T** | **I** | **T** | **A** | **E** | **D** | **I** | **K** | **K** | **N** | **P** | **E** | **L** | **S** | **S** | **T** | **G** | **V** | **I** | **G** | **K** | **T** | **G** | **L** | **E** | **R** | **A** | **F** | **D** | **K** | **E** | **L** | **R** | **G** | **Q** | **D** | **G** | **G** | **S** | **L** | **V** | **I** | **L** | **D** | **D** | **K** | **E** | **N** | **V** | **K** | **K** | **A** | **L** | **Q** | **T** | **K** | **E** | **K** | **K** | **D** | **G** | **Q** | **T** | **I** | **K** | **L** | **T** | **I** | **D** | **S** | **G** | **V** | **Q** | **Q** | **Q** | **A** | **F** | **A** | **I** | **F** | **D** | **K** | **R** | **P** | **G** | **S** | **A** | **V** | **I** | **T** | **D** | **P** | **Q** | **K** | **G** | **D** | **L** | **L** | **A** | **T** | **V** | **S** | **S** | **P** | **S** | **Y** | **D** | **P** | **N** | **K** | **M** | **A** | **N** | **G** | **I** | **S** | **Q** | **K** | **E** | **Y** | **D** | **A** | **Y** | **N** | **N** | **N** | **K** | **D** | **L** | **P** | **F** | **T** | **A** | **R** | **F** | **A** | **T** | **G** | **Y** | **A** | **P** | **G** | **S** | **T** | **F** | **K** | **T** | **I** | **T** | **G** | **A** | **I** | **G** | **L** | **D** | **T** | **G** | **T** | **L** | **K** | **P** | **D** | **E** | **E** | **L** | **E** | **I** | **N** | **G** | **L** | **K** | **W** | **Q** | **K** | **D** | **K** | **S** | **W** | **G** | **G** | **Y** | **F** | **A** | **T** | **R** | **V** | **K** | **E** | **A** | **S** | **P** | **V** | **N** | **L** | **R** | **T** | **A** | **L** | **V** | **N** | **S** | **D** | **N** | **I** | **Y** | **F** | **A** | **Q** | **Q** | **T** | **L** | **R** | **M** | **G** | **E** | **D** | **K** | **F** | **R** | **A** | **G** | **L** | **N** | **K** | **F** | **I** | **F** | **G** | **E** | **E** | **L** | **D** | **L** | **P** | **I** | **A** | **M** | **T** | **P** | **A** | **Q** | **I** | **S** | **N** | **E** | **D** | **K** | **F** | **N** | **S** | **E** | **I** | **L** | **L** | **A** | **D** | **T** | **G** | **Y** | **G** | **Q** | **G** | **Q** | **L** | **L** | **I** | **S** | **P** | **I** | **Q** | **Q** | **A** | **T** | **M** | **Y** | **S** | **V** | **F** | **Q** | **N** | **N** | **G** | **T** | **L** | **V** | **Y** | **P** | **K** | **L** | **V** | **L** | **D** | **K** | **E** | **T** | **K** | **K** | **K** | **D** | **N** | **V** | **I** | **S** | **A** | **N** | **A** | **A** | **N** | **T** | **I** | **A** | **T** | **D** | **L** | **L** | **G** | **S** | **V** | **E** | **D** | **P** | **S** | **G** | **Y** | **V** | **Y** | **N** | **M** | **Y** | **N** | **P** | **N** | **F** | **S** | **L** | **A** | **A** | **K** | **T** | **G** | **T** | **A** | **E** | **I** | **K** | **D** | **K** | **Q** | **D** | **T** | **D** | **G** | **K** | **E** | **N** | **S** | **F** | **L** | **L** | **T** | **L** | **D** | **R** | **S** | **N** | **N** | **K** | **F** | **L** | **T** | **M** | **I** | **M** | **V** | **E** | **N** | **S** | **G** | **E** | **N** | **G** | **S** | **A** | **T** | **D** | **I** | **S** | **K** | **P** | **L** | **I** | **D** | **Y** | **L** | **E** | **A** | **T** | **I** | **K** |
| **N54676** | **-** | **-** | **-** | **-** | **-** | **-** | **-** | **-** | **-** | **-** | **-** | **-** | **-** | **-** | **-** | **-** | **-** | **-** | **-** | **-** | **-** | **-** | **-** | **-** | **-** | **-** | **-** | **-** | **-** | **-** | **-** | **-** | **-** | **-** | **-** | **-** | **Q** | **W** | **Q** | **A** | **K** | **Q** | **E** | **L** | **A** | **E** | **A** | **K** | **K** | **T** | **A** | **N** | **T** | **F** | **L** | **N** | **V** | **L** | **S** | **K** | **Q** | **E** | **F** | **D** | **K** | **L** | **P** | **S** | **V** | **V** | **Q** | **E** | **S** | **S** | **L** | **K** | **K** | **N** | **G** | **Y** | **D** | **T** | **K** | **S** | **V** | **V** | **E** | **K** | **Y** | **Q** | **A** | **I** | **Y** | **S** | **G** | **I** | **Q** | **A** | **E** | **G** | **V** | **K** | **A** | **S** | **D** | **V** | **Q** | **V** | **K** | **K** | **A** | **K** | **D** | **N** | **Q** | **Y** | **T** | **F** | **T** | **Y** | **K** | **L** | **S** | **M** | **S** | **T** | **P** | **L** | **G** | **E** | **M** | **K** | **D** | **L** | **S** | **Y** | **Q** | **S** | **S** | **I** | **A** | **K** | **K** | **G** | **D** | **T** | **Y** | **Q** | **I** | **S** | **W** | **K** | **P** | **S** | **L** | **I** | **F** | **P** | **D** | **M** | **S** | **G** | **N** | **D** | **K** | **I** | **S** | **I** | **Q** | **V** | **D** | **N** | **A** | **K** | **R** | **G** | **E** | **I** | **V** | **D** | **R** | **N** | **G** | **S** | **G** | **L** | **A** | **I** | **N** | **K** | **V** | **F** | **D** | **E** | **V** | **G** | **V** | **V** | **P** | **G** | **K** | **L** | **G** | **S** | **G** | **A** | **E** | **K** | **T** | **A** | **N** | **I** | **K** | **A** | **F** | **S** | **D** | **K** | **F** | **G** | **V** | **S** | **V** | **D** | **E** | **I** | **N** | **Q** | **K** | **L** | **S** | **Q** | **G** | **W** | **V** | **Q** | **A** | **D** | **S** | **F** | **V** | **P** | **I** | **T** | **V** | **A** | **S** | **E** | **P** | **V** | **T** | **E** | **L** | **P** | **T** | **G** | **A** | **A** | **T** | **K** | **D** | **T** | **E** | **S** | **R** | **Y** | **Y** | **P** | **L** | **G** | **E** | **A** | **A** | **A** | **Q** | **L** | **I** | **G** | **Y** | **T** | **G** | **T** | **I** | **T** | **A** | **E** | **D** | **I** | **E** | **K** | **N** | **P** | **E** | **L** | **S** | **S** | **T** | **G** | **V** | **I** | **G** | **K** | **T** | **G** | **L** | **E** | **R** | **A** | **F** | **D** | **K** | **E** | **L** | **R** | **G** | **Q** | **D** | **G** | **G** | **S** | **L** | **V** | **I** | **L** | **D** | **D** | **K** | **E** | **N** | **V** | **K** | **K** | **A** | **L** | **Q** | **T** | **K** | **E** | **K** | **K** | **D** | **G** | **Q** | **T** | **I** | **K** | **L** | **T** | **I** | **D** | **S** | **G** | **V** | **Q** | **Q** | **Q** | **A** | **F** | **A** | **I** | **F** | **D** | **K** | **R** | **P** | **G** | **S** | **A** | **V** | **I** | **T** | **D** | **P** | **Q** | **K** | **G** | **D** | **L** | **L** | **A** | **T** | **V** | **S** | **S** | **P** | **S** | **Y** | **D** | **P** | **N** | **K** | **M** | **A** | **N** | **G** | **I** | **S** | **Q** | **K** | **E** | **Y** | **D** | **A** | **Y** | **N** | **N** | **N** | **K** | **D** | **L** | **P** | **F** | **T** | **A** | **R** | **F** | **A** | **T** | **G** | **Y** | **A** | **P** | **G** | **S** | **T** | **F** | **K** | **T** | **I** | **T** | **G** | **A** | **I** | **G** | **L** | **D** | **A** | **G** | **T** | **L** | **K** | **P** | **D** | **E** | **E** | **L** | **E** | **I** | **N** | **G** | **L** | **K** | **W** | **Q** | **K** | **D** | **K** | **S** | **W** | **G** | **G** | **Y** | **F** | **A** | **T** | **R** | **V** | **K** | **E** | **A** | **S** | **P** | **V** | **N** | **L** | **R** | **T** | **A** | **L** | **V** | **N** | **S** | **D** | **N** | **I** | **Y** | **F** | **A** | **Q** | **Q** | **T** | **L** | **R** | **M** | **G** | **E** | **D** | **K** | **F** | **R** | **A** | **G** | **L** | **N** | **K** | **F** | **I** | **F** | **G** | **E** | **E** | **L** | **D** | **L** | **P** | **I** | **A** | **M** | **T** | **P** | **A** | **Q** | **I** | **S** | **N** | **E** | **D** | **K** | **F** | **N** | **S** | **E** | **I** | **L** | **L** | **A** | **D** | **T** | **G** | **Y** | **G** | **Q** | **G** | **Q** | **L** | **L** | **I** | **S** | **P** | **I** | **Q** | **Q** | **A** | **T** | **M** | **Y** | **S** | **V** | **F** | **Q** | **N** | **N** | **G** | **T** | **L** | **V** | **Y** | **P** | **K** | **L** | **V** | **L** | **D** | **K** | **E** | **T** | **K** | **K** | **K** | **D** | **N** | **V** | **I** | **S** | **A** | **N** | **A** | **A** | **N** | **T** | **I** | **A** | **T** | **D** | **L** | **L** | **G** | **S** | **V** | **E** | **D** | **P** | **S** | **G** | **Y** | **V** | **Y** | **N** | **M** | **Y** | **N** | **P** | **N** | **F** | **S** | **L** | **A** | **A** | **K** | **T** | **G** | **T** | **A** | **E** | **I** | **K** | **D** | **K** | **Q** | **D** | **T** | **D** | **G** | **K** | **E** | **N** | **S** | **F** | **L** | **L** | **T** | **L** | **D** | **R** | **S** | **N** | **N** | **K** | **F** | **L** | **T** | **M** | **I** | **M** | **V** | **E** | **N** | **S** | **G** | **E** | **N** | **G** | **S** | **A** | **T** | **D** | **I** | **S** | **K** | **P** | **L** | **I** | **D** | **Y** | **L** | **E** | **A** | **T** | **I** | **K** | **-** | **-** | **-** | **-** | **-** | **-** | **-** | **-** | **-** | **-** | **-** | **-** | **-** | **-** | **-** | **-** | **-** | **-** | **-** | **-** | **-** | **-** | **-** | **-** | **-** | **-** | **-** | **-** | **-** | **-** | **-** | **-** | **-** | **-** | **-** | **-** | **Q** | **W** | **Q** | **A** | **K** | **Q** | **E** | **L** | **A** | **E** | **A** | **K** | **K** | **T** | **A** | **N** | **T** | **F** | **L** | **N** | **V** | **L** | **S** | **K** | **Q** | **E** | **F** | **D** | **K** | **L** | **P** | **S** | **V** | **V** | **Q** | **E** | **S** | **S** | **L** | **K** | **K** | **N** | **G** | **Y** | **D** | **T** | **K** | **S** | **V** | **V** | **E** | **K** | **Y** | **Q** | **A** | **I** | **Y** | **S** | **G** | **I** | **Q** | **A** | **E** | **G** | **V** | **K** | **A** | **S** | **D** | **V** | **Q** | **V** | **K** | **K** | **A** | **K** | **D** | **N** | **Q** | **Y** | **T** | **F** | **T** | **Y** | **K** | **L** | **S** | **M** | **S** | **T** | **P** | **L** | **G** | **E** | **M** | **K** | **D** | **L** | **S** | **Y** | **Q** | **S** | **S** | **I** | **A** | **K** | **K** | **G** | **D** | **T** | **Y** | **Q** | **I** | **S** | **W** | **K** | **P** | **S** | **L** | **I** | **F** | **P** | **D** | **M** | **S** | **G** | **N** | **D** | **K** | **I** | **S** | **I** | **Q** | **V** | **D** | **N** | **A** | **K** | **R** | **G** | **E** | **I** | **V** | **D** | **R** | **N** | **G** | **S** | **G** | **L** | **A** | **I** | **N** | **K** | **V** | **F** | **D** | **E** | **V** | **G** | **V** | **V** | **P** | **G** | **K** | **L** | **G** | **S** | **G** | **A** | **E** | **K** | **T** | **A** | **N** | **I** | **K** | **A** | **F** | **S** | **D** | **K** | **F** | **G** | **V** | **S** | **V** | **D** | **E** | **I** | **N** | **Q** | **K** | **L** | **S** | **Q** | **G** | **W** | **V** | **Q** | **A** | **D** | **S** | **F** | **V** | **P** | **I** | **T** | **V** | **A** | **S** | **E** | **P** | **V** | **T** | **E** | **L** | **P** | **T** | **G** | **A** | **A** | **T** | **K** | **D** | **T** | **E** | **S** | **R** | **Y** | **Y** | **P** | **L** | **G** | **E** | **A** | **A** | **A** | **Q** | **L** | **I** | **G** | **Y** | **T** | **G** | **T** | **I** | **T** | **A** | **E** | **D** | **I** | **E** | **K** | **N** | **P** | **E** | **L** | **S** | **S** | **T** | **G** | **V** | **I** | **G** | **K** | **T** | **G** | **L** | **E** | **R** | **A** | **F** | **D** | **K** | **E** | **L** | **R** | **G** | **Q** | **D** | **G** | **G** | **S** | **L** | **V** | **I** | **L** | **D** | **D** | **K** | **E** | **N** | **V** | **K** | **K** | **A** | **L** | **Q** | **T** | **K** | **E** | **K** | **K** | **D** | **G** | **Q** | **T** | **I** | **K** | **L** | **T** | **I** | **D** | **S** | **G** | **V** | **Q** | **Q** | **Q** | **A** | **F** | **A** | **I** | **F** | **D** | **K** | **R** | **P** | **G** | **S** | **A** | **V** | **I** | **T** | **D** | **P** | **Q** | **K** | **G** | **D** | **L** | **L** | **A** | **T** | **V** | **S** | **S** | **P** | **S** | **Y** | **D** | **P** | **N** | **K** | **M** | **A** | **N** | **G** | **I** | **S** | **Q** | **K** | **E** | **Y** | **D** | **A** | **Y** | **N** | **N** | **N** | **K** | **D** | **L** | **P** | **F** | **T** | **A** | **R** | **F** | **A** | **T** | **G** | **Y** | **A** | **P** | **G** | **S** | **T** | **F** | **K** | **T** | **I** | **T** | **G** | **A** | **I** | **G** | **L** | **D** | **A** | **G** | **T** | **L** | **K** | **P** | **D** | **E** | **E** | **L** | **E** | **I** | **N** | **G** | **L** | **K** | **W** | **Q** | **K** | **D** | **K** | **S** | **W** | **G** | **G** | **Y** | **F** | **A** | **T** | **R** | **V** | **K** | **E** | **A** | **S** | **P** | **V** | **N** | **L** | **R** | **T** | **A** | **L** | **V** | **N** | **S** | **D** | **N** | **I** | **Y** | **F** | **A** | **Q** | **Q** | **T** | **L** | **R** | **M** | **G** | **E** | **D** | **K** | **F** | **R** | **A** | **G** | **L** | **N** | **K** | **F** | **I** | **F** | **G** | **E** | **E** | **L** | **D** | **L** | **P** | **I** | **A** | **M** | **T** | **P** | **A** | **Q** | **I** | **S** | **N** | **E** | **D** | **K** | **F** | **N** | **S** | **E** | **I** | **L** | **L** | **A** | **D** | **T** | **G** | **Y** | **G** | **Q** | **G** | **Q** | **L** | **L** | **I** | **S** | **P** | **I** | **Q** | **Q** | **A** | **T** | **M** | **Y** | **S** | **V** | **F** | **Q** | **N** | **N** | **G** | **T** | **L** | **V** | **Y** | **P** | **K** | **L** | **V** | **L** | **D** | **K** | **E** | **T** | **K** | **K** | **K** | **D** | **N** | **V** | **I** | **S** | **A** | **N** | **A** | **A** | **N** | **T** | **I** | **A** | **T** | **D** | **L** | **L** | **G** | **S** | **V** | **E** | **D** | **P** | **S** | **G** | **Y** | **V** | **Y** | **N** | **M** | **Y** | **N** | **P** | **N** | **F** | **S** | **L** | **A** | **A** | **K** | **T** | **G** | **T** | **A** | **E** | **I** | **K** | **D** | **K** | **Q** | **D** | **T** | **D** | **G** | **K** | **E** | **N** | **S** | **F** | **L** | **L** | **T** | **L** | **D** | **R** | **S** | **N** | **N** | **K** | **F** | **L** | **T** | **M** | **I** | **M** | **V** | **E** | **N** | **S** | **G** | **E** | **N** | **G** | **S** | **A** | **T** | **D** | **I** | **S** | **K** | **P** | **L** | **I** | **D** | **Y** | **L** | **E** | **A** | **T** | **I** | **K** |
| **N59457F** | **-** | **-** | **-** | **-** | **-** | **-** | **-** | **-** | **-** | **-** | **-** | **-** | **-** | **-** | **-** | **-** | **-** | **-** | **-** | **-** | **-** | **-** | **-** | **-** | **-** | **-** | **-** | **-** | **-** | **-** | **-** | **-** | **-** | **-** | **-** | **-** | **Q** | **W** | **Q** | **A** | **K** | **Q** | **E** | **L** | **A** | **E** | **A** | **K** | **K** | **T** | **A** | **T** | **T** | **F** | **L** | **N** | **V** | **L** | **T** | **K** | **Q** | **K** | **F** | **D** | **K** | **L** | **P** | **S** | **V** | **V** | **Q** | **E** | **A** | **S** | **L** | **K** | **K** | **N** | **G** | **Y** | **D** | **T** | **K** | **S** | **V** | **V** | **E** | **K** | **Y** | **Q** | **A** | **I** | **Y** | **S** | **G** | **I** | **Q** | **A** | **E** | **G** | **V** | **K** | **A** | **S** | **D** | **V** | **Q** | **V** | **K** | **K** | **A** | **K** | **D** | **N** | **Q** | **Y** | **T** | **F** | **T** | **Y** | **K** | **L** | **S** | **M** | **S** | **T** | **P** | **L** | **G** | **E** | **M** | **K** | **D** | **L** | **S** | **Y** | **Q** | **S** | **S** | **I** | **A** | **K** | **K** | **G** | **D** | **T** | **Y** | **Q** | **I** | **A** | **W** | **K** | **P** | **S** | **L** | **I** | **F** | **P** | **D** | **M** | **S** | **G** | **N** | **D** | **K** | **I** | **S** | **I** | **Q** | **V** | **D** | **N** | **A** | **K** | **R** | **G** | **E** | **I** | **V** | **D** | **R** | **N** | **G** | **S** | **G** | **L** | **A** | **I** | **N** | **K** | **V** | **F** | **D** | **E** | **V** | **G** | **V** | **V** | **P** | **G** | **K** | **L** | **G** | **S** | **G** | **A** | **E** | **K** | **T** | **A** | **N** | **I** | **K** | **A** | **F** | **S** | **D** | **K** | **F** | **G** | **V** | **S** | **V** | **D** | **E** | **I** | **N** | **Q** | **K** | **L** | **S** | **Q** | **G** | **W** | **V** | **Q** | **A** | **D** | **S** | **F** | **V** | **P** | **I** | **T** | **V** | **A** | **S** | **E** | **P** | **V** | **T** | **E** | **L** | **P** | **T** | **G** | **A** | **A** | **T** | **K** | **D** | **T** | **E** | **S** | **R** | **Y** | **Y** | **P** | **L** | **G** | **E** | **A** | **A** | **A** | **Q** | **L** | **I** | **G** | **Y** | **T** | **G** | **T** | **I** | **T** | **A** | **E** | **D** | **I** | **K** | **K** | **N** | **P** | **E** | **L** | **S** | **S** | **T** | **G** | **V** | **I** | **G** | **K** | **T** | **G** | **L** | **E** | **R** | **A** | **F** | **D** | **K** | **E** | **L** | **R** | **G** | **Q** | **D** | **G** | **G** | **S** | **L** | **V** | **I** | **L** | **D** | **D** | **K** | **E** | **N** | **V** | **K** | **K** | **A** | **L** | **Q** | **T** | **K** | **E** | **K** | **K** | **D** | **G** | **Q** | **T** | **I** | **K** | **L** | **T** | **I** | **D** | **S** | **G** | **V** | **Q** | **Q** | **Q** | **A** | **F** | **A** | **I** | **F** | **D** | **K** | **R** | **P** | **G** | **S** | **A** | **V** | **I** | **T** | **D** | **P** | **Q** | **K** | **G** | **D** | **L** | **L** | **A** | **T** | **V** | **S** | **S** | **P** | **S** | **Y** | **D** | **P** | **N** | **K** | **M** | **A** | **N** | **G** | **I** | **S** | **Q** | **K** | **E** | **Y** | **D** | **A** | **Y** | **N** | **N** | **N** | **K** | **D** | **L** | **P** | **F** | **T** | **A** | **R** | **F** | **A** | **T** | **G** | **Y** | **A** | **P** | **G** | **S** | **T** | **F** | **K** | **T** | **I** | **T** | **G** | **A** | **I** | **G** | **L** | **D** | **T** | **G** | **T** | **L** | **K** | **P** | **D** | **E** | **E** | **L** | **E** | **I** | **N** | **G** | **L** | **K** | **W** | **Q** | **K** | **D** | **K** | **S** | **W** | **G** | **G** | **Y** | **F** | **A** | **T** | **R** | **V** | **K** | **E** | **A** | **S** | **P** | **V** | **N** | **L** | **R** | **T** | **A** | **L** | **V** | **N** | **S** | **D** | **N** | **I** | **Y** | **F** | **A** | **Q** | **Q** | **T** | **L** | **R** | **M** | **G** | **E** | **D** | **K** | **F** | **R** | **A** | **G** | **L** | **N** | **K** | **F** | **I** | **F** | **G** | **E** | **E** | **L** | **D** | **L** | **P** | **I** | **A** | **M** | **T** | **P** | **A** | **Q** | **I** | **S** | **N** | **E** | **D** | **K** | **F** | **N** | **S** | **E** | **I** | **L** | **L** | **A** | **D** | **T** | **G** | **Y** | **G** | **Q** | **G** | **Q** | **L** | **L** | **I** | **S** | **P** | **I** | **Q** | **Q** | **A** | **T** | **M** | **Y** | **S** | **V** | **F** | **Q** | **N** | **N** | **G** | **T** | **L** | **V** | **Y** | **P** | **K** | **L** | **V** | **L** | **D** | **K** | **E** | **T** | **K** | **K** | **K** | **D** | **N** | **V** | **I** | **S** | **A** | **N** | **A** | **A** | **N** | **T** | **I** | **A** | **T** | **D** | **L** | **L** | **G** | **S** | **V** | **E** | **D** | **P** | **S** | **G** | **Y** | **V** | **Y** | **N** | **M** | **Y** | **N** | **P** | **N** | **F** | **S** | **L** | **A** | **A** | **K** | **T** | **G** | **T** | **A** | **E** | **I** | **K** | **D** | **K** | **Q** | **D** | **T** | **D** | **G** | **K** | **E** | **N** | **S** | **F** | **L** | **L** | **T** | **L** | **D** | **R** | **S** | **N** | **N** | **K** | **F** | **L** | **T** | **M** | **I** | **M** | **V** | **E** | **N** | **S** | **G** | **E** | **N** | **G** | **S** | **A** | **T** | **D** | **I** | **S** | **K** | **P** | **L** | **I** | **D** | **Y** | **L** | **E** | **A** | **T** | **I** | **K** | **-** | **-** | **-** | **-** | **-** | **-** | **-** | **-** | **-** | **-** | **-** | **-** | **-** | **-** | **-** | **-** | **-** | **-** | **-** | **-** | **-** | **-** | **-** | **-** | **-** | **-** | **-** | **-** | **-** | **-** | **-** | **-** | **-** | **-** | **-** | **-** | **Q** | **W** | **Q** | **A** | **K** | **Q** | **E** | **L** | **A** | **E** | **A** | **K** | **K** | **T** | **A** | **T** | **T** | **F** | **L** | **N** | **V** | **L** | **T** | **K** | **Q** | **K** | **F** | **D** | **K** | **L** | **P** | **S** | **V** | **V** | **Q** | **E** | **A** | **S** | **L** | **K** | **K** | **N** | **G** | **Y** | **D** | **T** | **K** | **S** | **V** | **V** | **E** | **K** | **Y** | **Q** | **A** | **I** | **Y** | **S** | **G** | **I** | **Q** | **A** | **E** | **G** | **V** | **K** | **A** | **S** | **D** | **V** | **Q** | **V** | **K** | **K** | **A** | **K** | **D** | **N** | **Q** | **Y** | **T** | **F** | **T** | **Y** | **K** | **L** | **S** | **M** | **S** | **T** | **P** | **L** | **G** | **E** | **M** | **K** | **D** | **L** | **S** | **Y** | **Q** | **S** | **S** | **I** | **A** | **K** | **K** | **G** | **D** | **T** | **Y** | **Q** | **I** | **A** | **W** | **K** | **P** | **S** | **L** | **I** | **F** | **P** | **D** | **M** | **S** | **G** | **N** | **D** | **K** | **I** | **S** | **I** | **Q** | **V** | **D** | **N** | **A** | **K** | **R** | **G** | **E** | **I** | **V** | **D** | **R** | **N** | **G** | **S** | **G** | **L** | **A** | **I** | **N** | **K** | **V** | **F** | **D** | **E** | **V** | **G** | **V** | **V** | **P** | **G** | **K** | **L** | **G** | **S** | **G** | **A** | **E** | **K** | **T** | **A** | **N** | **I** | **K** | **A** | **F** | **S** | **D** | **K** | **F** | **G** | **V** | **S** | **V** | **D** | **E** | **I** | **N** | **Q** | **K** | **L** | **S** | **Q** | **G** | **W** | **V** | **Q** | **A** | **D** | **S** | **F** | **V** | **P** | **I** | **T** | **V** | **A** | **S** | **E** | **P** | **V** | **T** | **E** | **L** | **P** | **T** | **G** | **A** | **A** | **T** | **K** | **D** | **T** | **E** | **S** | **R** | **Y** | **Y** | **P** | **L** | **G** | **E** | **A** | **A** | **A** | **Q** | **L** | **I** | **G** | **Y** | **T** | **G** | **T** | **I** | **T** | **A** | **E** | **D** | **I** | **K** | **K** | **N** | **P** | **E** | **L** | **S** | **S** | **T** | **G** | **V** | **I** | **G** | **K** | **T** | **G** | **L** | **E** | **R** | **A** | **F** | **D** | **K** | **E** | **L** | **R** | **G** | **Q** | **D** | **G** | **G** | **S** | **L** | **V** | **I** | **L** | **D** | **D** | **K** | **E** | **N** | **V** | **K** | **K** | **A** | **L** | **Q** | **T** | **K** | **E** | **K** | **K** | **D** | **G** | **Q** | **T** | **I** | **K** | **L** | **T** | **I** | **D** | **S** | **G** | **V** | **Q** | **Q** | **Q** | **A** | **F** | **A** | **I** | **F** | **D** | **K** | **R** | **P** | **G** | **S** | **A** | **V** | **I** | **T** | **D** | **P** | **Q** | **K** | **G** | **D** | **L** | **L** | **A** | **T** | **V** | **S** | **S** | **P** | **S** | **Y** | **D** | **P** | **N** | **K** | **M** | **A** | **N** | **G** | **I** | **S** | **Q** | **K** | **E** | **Y** | **D** | **A** | **Y** | **N** | **N** | **N** | **K** | **D** | **L** | **P** | **F** | **T** | **A** | **R** | **F** | **A** | **T** | **G** | **Y** | **A** | **P** | **G** | **S** | **T** | **F** | **K** | **T** | **I** | **T** | **G** | **A** | **I** | **G** | **L** | **D** | **T** | **G** | **T** | **L** | **K** | **P** | **D** | **E** | **E** | **L** | **E** | **I** | **N** | **G** | **L** | **K** | **W** | **Q** | **K** | **D** | **K** | **S** | **W** | **G** | **G** | **Y** | **F** | **A** | **T** | **R** | **V** | **K** | **E** | **A** | **S** | **P** | **V** | **N** | **L** | **R** | **T** | **A** | **L** | **V** | **N** | **S** | **D** | **N** | **I** | **Y** | **F** | **A** | **Q** | **Q** | **T** | **L** | **R** | **M** | **G** | **E** | **D** | **K** | **F** | **R** | **A** | **G** | **L** | **N** | **K** | **F** | **I** | **F** | **G** | **E** | **E** | **L** | **D** | **L** | **P** | **I** | **A** | **M** | **T** | **P** | **A** | **Q** | **I** | **S** | **N** | **E** | **D** | **K** | **F** | **N** | **S** | **E** | **I** | **L** | **L** | **A** | **D** | **T** | **G** | **Y** | **G** | **Q** | **G** | **Q** | **L** | **L** | **I** | **S** | **P** | **I** | **Q** | **Q** | **A** | **T** | **M** | **Y** | **S** | **V** | **F** | **Q** | **N** | **N** | **G** | **T** | **L** | **V** | **Y** | **P** | **K** | **L** | **V** | **L** | **D** | **K** | **E** | **T** | **K** | **K** | **K** | **D** | **N** | **V** | **I** | **S** | **A** | **N** | **A** | **A** | **N** | **T** | **I** | **A** | **T** | **D** | **L** | **L** | **G** | **S** | **V** | **E** | **D** | **P** | **S** | **G** | **Y** | **V** | **Y** | **N** | **M** | **Y** | **N** | **P** | **N** | **F** | **S** | **L** | **A** | **A** | **K** | **T** | **G** | **T** | **A** | **E** | **I** | **K** | **D** | **K** | **Q** | **D** | **T** | **D** | **G** | **K** | **E** | **N** | **S** | **F** | **L** | **L** | **T** | **L** | **D** | **R** | **S** | **N** | **N** | **K** | **F** | **L** | **T** | **M** | **I** | **M** | **V** | **E** | **N** | **S** | **G** | **E** | **N** | **G** | **S** | **A** | **T** | **D** | **I** | **S** | **K** | **P** | **L** | **I** | **D** | **Y** | **L** | **E** | **A** | **T** | **I** | **K** |
| **N55725** | **-** | **-** | **-** | **-** | **-** | **-** | **-** | **-** | **-** | **-** | **-** | **-** | **-** | **-** | **-** | **-** | **-** | **-** | **-** | **-** | **-** | **-** | **-** | **-** | **-** | **-** | **-** | **-** | **-** | **-** | **-** | **-** | **-** | **-** | **-** | **-** | **Q** | **W** | **Q** | **A** | **K** | **Q** | **E** | **L** | **A** | **E** | **A** | **K** | **K** | **T** | **A** | **T** | **T** | **F** | **L** | **N** | **V** | **L** | **S** | **K** | **Q** | **E** | **F** | **D** | **K** | **L** | **P** | **S** | **V** | **V** | **Q** | **E** | **A** | **S** | **L** | **K** | **K** | **N** | **G** | **Y** | **D** | **T** | **K** | **S** | **V** | **V** | **E** | **K** | **Y** | **Q** | **A** | **I** | **Y** | **S** | **G** | **I** | **Q** | **A** | **E** | **G** | **V** | **K** | **A** | **S** | **D** | **V** | **Q** | **V** | **K** | **K** | **A** | **K** | **D** | **N** | **Q** | **Y** | **T** | **F** | **T** | **Y** | **K** | **L** | **S** | **M** | **S** | **T** | **P** | **L** | **G** | **E** | **M** | **K** | **D** | **L** | **S** | **Y** | **Q** | **S** | **S** | **I** | **D** | **K** | **K** | **G** | **D** | **T** | **Y** | **Q** | **I** | **A** | **W** | **K** | **P** | **S** | **L** | **I** | **F** | **P** | **D** | **M** | **S** | **G** | **N** | **D** | **K** | **I** | **S** | **I** | **Q** | **V** | **D** | **N** | **A** | **K** | **R** | **G** | **E** | **I** | **V** | **D** | **R** | **N** | **G** | **S** | **G** | **L** | **A** | **I** | **N** | **K** | **V** | **F** | **D** | **E** | **V** | **G** | **V** | **V** | **P** | **S** | **K** | **L** | **G** | **S** | **G** | **A** | **E** | **K** | **A** | **A** | **N** | **I** | **K** | **A** | **F** | **S** | **D** | **K** | **F** | **G** | **V** | **S** | **V** | **D** | **E** | **I** | **N** | **Q** | **K** | **L** | **S** | **Q** | **G** | **W** | **V** | **Q** | **A** | **D** | **S** | **F** | **V** | **P** | **I** | **T** | **V** | **A** | **S** | **E** | **P** | **V** | **T** | **E** | **L** | **P** | **T** | **G** | **A** | **A** | **T** | **K** | **D** | **T** | **E** | **S** | **R** | **Y** | **Y** | **P** | **L** | **G** | **E** | **A** | **A** | **A** | **Q** | **L** | **I** | **G** | **Y** | **T** | **G** | **T** | **I** | **T** | **A** | **E** | **D** | **I** | **K** | **K** | **N** | **P** | **E** | **L** | **S** | **S** | **T** | **G** | **V** | **I** | **G** | **K** | **T** | **G** | **L** | **E** | **R** | **A** | **F** | **D** | **K** | **E** | **L** | **R** | **G** | **Q** | **D** | **G** | **G** | **S** | **L** | **V** | **I** | **L** | **D** | **D** | **K** | **E** | **N** | **V** | **K** | **K** | **A** | **L** | **Q** | **T** | **K** | **E** | **K** | **K** | **D** | **G** | **Q** | **T** | **I** | **K** | **L** | **T** | **I** | **D** | **S** | **G** | **V** | **Q** | **Q** | **Q** | **A** | **F** | **A** | **I** | **F** | **D** | **K** | **R** | **P** | **G** | **S** | **A** | **V** | **I** | **T** | **D** | **P** | **Q** | **K** | **G** | **D** | **L** | **L** | **A** | **T** | **V** | **S** | **S** | **P** | **S** | **Y** | **D** | **P** | **N** | **K** | **M** | **A** | **N** | **G** | **I** | **S** | **Q** | **K** | **E** | **Y** | **D** | **A** | **Y** | **N** | **N** | **N** | **K** | **D** | **L** | **P** | **F** | **T** | **A** | **R** | **F** | **A** | **T** | **G** | **Y** | **A** | **P** | **G** | **S** | **T** | **F** | **K** | **T** | **I** | **T** | **G** | **A** | **I** | **G** | **L** | **D** | **A** | **G** | **T** | **L** | **K** | **P** | **D** | **E** | **E** | **L** | **E** | **I** | **N** | **G** | **L** | **K** | **W** | **Q** | **K** | **D** | **K** | **S** | **W** | **G** | **G** | **Y** | **F** | **A** | **T** | **R** | **V** | **K** | **E** | **A** | **S** | **P** | **V** | **N** | **L** | **R** | **T** | **A** | **L** | **V** | **N** | **S** | **D** | **N** | **I** | **Y** | **F** | **A** | **Q** | **Q** | **T** | **L** | **R** | **M** | **G** | **E** | **D** | **K** | **F** | **R** | **A** | **G** | **L** | **N** | **K** | **F** | **I** | **F** | **G** | **E** | **E** | **L** | **D** | **L** | **P** | **I** | **A** | **M** | **T** | **P** | **A** | **Q** | **I** | **S** | **N** | **E** | **D** | **K** | **F** | **N** | **S** | **E** | **I** | **L** | **L** | **A** | **D** | **T** | **G** | **Y** | **G** | **Q** | **G** | **Q** | **L** | **L** | **I** | **S** | **P** | **I** | **Q** | **Q** | **A** | **T** | **M** | **Y** | **S** | **V** | **F** | **Q** | **N** | **N** | **G** | **T** | **L** | **V** | **Y** | **P** | **K** | **L** | **V** | **L** | **D** | **K** | **E** | **T** | **K** | **K** | **K** | **D** | **N** | **V** | **I** | **S** | **A** | **N** | **A** | **A** | **N** | **T** | **I** | **A** | **T** | **D** | **L** | **L** | **G** | **S** | **V** | **E** | **D** | **P** | **S** | **G** | **Y** | **V** | **Y** | **N** | **M** | **Y** | **N** | **P** | **N** | **F** | **S** | **L** | **A** | **A** | **K** | **T** | **G** | **T** | **A** | **E** | **I** | **K** | **D** | **K** | **Q** | **D** | **T** | **D** | **G** | **K** | **E** | **N** | **S** | **F** | **L** | **L** | **T** | **L** | **D** | **R** | **S** | **N** | **N** | **K** | **F** | **L** | **T** | **M** | **I** | **M** | **V** | **E** | **N** | **S** | **G** | **E** | **N** | **G** | **S** | **A** | **T** | **D** | **I** | **S** | **K** | **P** | **L** | **I** | **D** | **Y** | **L** | **E** | **A** | **T** | **I** | **K** | **-** | **-** | **-** | **-** | **-** | **-** | **-** | **-** | **-** | **-** | **-** | **-** | **-** | **-** | **-** | **-** | **-** | **-** | **-** | **-** | **-** | **-** | **-** | **-** | **-** | **-** | **-** | **-** | **-** | **-** | **-** | **-** | **-** | **-** | **-** | **-** | **Q** | **W** | **Q** | **A** | **K** | **Q** | **E** | **L** | **A** | **E** | **A** | **K** | **K** | **T** | **A** | **T** | **T** | **F** | **L** | **N** | **V** | **L** | **S** | **K** | **Q** | **E** | **F** | **D** | **K** | **L** | **P** | **S** | **V** | **V** | **Q** | **E** | **A** | **S** | **L** | **K** | **K** | **N** | **G** | **Y** | **D** | **T** | **K** | **S** | **V** | **V** | **E** | **K** | **Y** | **Q** | **A** | **I** | **Y** | **S** | **G** | **I** | **Q** | **A** | **E** | **G** | **V** | **K** | **A** | **S** | **D** | **V** | **Q** | **V** | **K** | **K** | **A** | **K** | **D** | **N** | **Q** | **Y** | **T** | **F** | **T** | **Y** | **K** | **L** | **S** | **M** | **S** | **T** | **P** | **L** | **G** | **E** | **M** | **K** | **D** | **L** | **S** | **Y** | **Q** | **S** | **S** | **I** | **D** | **K** | **K** | **G** | **D** | **T** | **Y** | **Q** | **I** | **A** | **W** | **K** | **P** | **S** | **L** | **I** | **F** | **P** | **D** | **M** | **S** | **G** | **N** | **D** | **K** | **I** | **S** | **I** | **Q** | **V** | **D** | **N** | **A** | **K** | **R** | **G** | **E** | **I** | **V** | **D** | **R** | **N** | **G** | **S** | **G** | **L** | **A** | **I** | **N** | **K** | **V** | **F** | **D** | **E** | **V** | **G** | **V** | **V** | **P** | **S** | **K** | **L** | **G** | **S** | **G** | **A** | **E** | **K** | **A** | **A** | **N** | **I** | **K** | **A** | **F** | **S** | **D** | **K** | **F** | **G** | **V** | **S** | **V** | **D** | **E** | **I** | **N** | **Q** | **K** | **L** | **S** | **Q** | **G** | **W** | **V** | **Q** | **A** | **D** | **S** | **F** | **V** | **P** | **I** | **T** | **V** | **A** | **S** | **E** | **P** | **V** | **T** | **E** | **L** | **P** | **T** | **G** | **A** | **A** | **T** | **K** | **D** | **T** | **E** | **S** | **R** | **Y** | **Y** | **P** | **L** | **G** | **E** | **A** | **A** | **A** | **Q** | **L** | **I** | **G** | **Y** | **T** | **G** | **T** | **I** | **T** | **A** | **E** | **D** | **I** | **K** | **K** | **N** | **P** | **E** | **L** | **S** | **S** | **T** | **G** | **V** | **I** | **G** | **K** | **T** | **G** | **L** | **E** | **R** | **A** | **F** | **D** | **K** | **E** | **L** | **R** | **G** | **Q** | **D** | **G** | **G** | **S** | **L** | **V** | **I** | **L** | **D** | **D** | **K** | **E** | **N** | **V** | **K** | **K** | **A** | **L** | **Q** | **T** | **K** | **E** | **K** | **K** | **D** | **G** | **Q** | **T** | **I** | **K** | **L** | **T** | **I** | **D** | **S** | **G** | **V** | **Q** | **Q** | **Q** | **A** | **F** | **A** | **I** | **F** | **D** | **K** | **R** | **P** | **G** | **S** | **A** | **V** | **I** | **T** | **D** | **P** | **Q** | **K** | **G** | **D** | **L** | **L** | **A** | **T** | **V** | **S** | **S** | **P** | **S** | **Y** | **D** | **P** | **N** | **K** | **M** | **A** | **N** | **G** | **I** | **S** | **Q** | **K** | **E** | **Y** | **D** | **A** | **Y** | **N** | **N** | **N** | **K** | **D** | **L** | **P** | **F** | **T** | **A** | **R** | **F** | **A** | **T** | **G** | **Y** | **A** | **P** | **G** | **S** | **T** | **F** | **K** | **T** | **I** | **T** | **G** | **A** | **I** | **G** | **L** | **D** | **A** | **G** | **T** | **L** | **K** | **P** | **D** | **E** | **E** | **L** | **E** | **I** | **N** | **G** | **L** | **K** | **W** | **Q** | **K** | **D** | **K** | **S** | **W** | **G** | **G** | **Y** | **F** | **A** | **T** | **R** | **V** | **K** | **E** | **A** | **S** | **P** | **V** | **N** | **L** | **R** | **T** | **A** | **L** | **V** | **N** | **S** | **D** | **N** | **I** | **Y** | **F** | **A** | **Q** | **Q** | **T** | **L** | **R** | **M** | **G** | **E** | **D** | **K** | **F** | **R** | **A** | **G** | **L** | **N** | **K** | **F** | **I** | **F** | **G** | **E** | **E** | **L** | **D** | **L** | **P** | **I** | **A** | **M** | **T** | **P** | **A** | **Q** | **I** | **S** | **N** | **E** | **D** | **K** | **F** | **N** | **S** | **E** | **I** | **L** | **L** | **A** | **D** | **T** | **G** | **Y** | **G** | **Q** | **G** | **Q** | **L** | **L** | **I** | **S** | **P** | **I** | **Q** | **Q** | **A** | **T** | **M** | **Y** | **S** | **V** | **F** | **Q** | **N** | **N** | **G** | **T** | **L** | **V** | **Y** | **P** | **K** | **L** | **V** | **L** | **D** | **K** | **E** | **T** | **K** | **K** | **K** | **D** | **N** | **V** | **I** | **S** | **A** | **N** | **A** | **A** | **N** | **T** | **I** | **A** | **T** | **D** | **L** | **L** | **G** | **S** | **V** | **E** | **D** | **P** | **S** | **G** | **Y** | **V** | **Y** | **N** | **M** | **Y** | **N** | **P** | **N** | **F** | **S** | **L** | **A** | **A** | **K** | **T** | **G** | **T** | **A** | **E** | **I** | **K** | **D** | **K** | **Q** | **D** | **T** | **D** | **G** | **K** | **E** | **N** | **S** | **F** | **L** | **L** | **T** | **L** | **D** | **R** | **S** | **N** | **N** | **K** | **F** | **L** | **T** | **M** | **I** | **M** | **V** | **E** | **N** | **S** | **G** | **E** | **N** | **G** | **S** | **A** | **T** | **D** | **I** | **S** | **K** | **P** | **L** | **I** | **D** | **Y** | **L** | **E** | **A** | **T** | **I** | **K** |
| **N59599F** | **-** | **-** | **-** | **-** | **-** | **-** | **-** | **-** | **-** | **-** | **-** | **-** | **-** | **-** | **-** | **-** | **-** | **-** | **-** | **-** | **-** | **-** | **-** | **-** | **-** | **-** | **-** | **-** | **-** | **-** | **-** | **-** | **-** | **-** | **-** | **-** | **Q** | **W** | **Q** | **A** | **K** | **Q** | **E** | **L** | **A** | **E** | **A** | **K** | **K** | **T** | **A** | **T** | **T** | **F** | **L** | **N** | **V** | **L** | **S** | **K** | **Q** | **E** | **F** | **D** | **K** | **L** | **P** | **S** | **V** | **V** | **Q** | **E** | **A** | **S** | **L** | **K** | **K** | **N** | **G** | **Y** | **D** | **T** | **K** | **S** | **V** | **V** | **E** | **K** | **Y** | **Q** | **A** | **I** | **Y** | **S** | **G** | **I** | **Q** | **A** | **E** | **G** | **V** | **K** | **A** | **S** | **D** | **V** | **Q** | **V** | **K** | **K** | **A** | **K** | **D** | **N** | **Q** | **Y** | **T** | **F** | **T** | **Y** | **K** | **L** | **S** | **M** | **S** | **T** | **P** | **L** | **G** | **E** | **M** | **K** | **D** | **L** | **S** | **Y** | **Q** | **S** | **S** | **I** | **A** | **K** | **K** | **G** | **D** | **T** | **Y** | **Q** | **I** | **A** | **W** | **N** | **P** | **S** | **L** | **I** | **F** | **P** | **D** | **M** | **S** | **G** | **N** | **D** | **K** | **V** | **S** | **I** | **Q** | **V** | **D** | **N** | **A** | **K** | **R** | **G** | **E** | **I** | **V** | **D** | **R** | **N** | **G** | **S** | **G** | **L** | **A** | **I** | **N** | **K** | **V** | **F** | **D** | **E** | **V** | **G** | **V** | **V** | **P** | **G** | **K** | **L** | **G** | **S** | **G** | **A** | **E** | **K** | **T** | **A** | **N** | **I** | **K** | **A** | **F** | **S** | **D** | **K** | **F** | **G** | **V** | **S** | **V** | **D** | **E** | **I** | **N** | **R** | **K** | **L** | **S** | **Q** | **G** | **W** | **V** | **Q** | **A** | **D** | **S** | **F** | **V** | **P** | **I** | **T** | **V** | **A** | **S** | **E** | **P** | **V** | **T** | **E** | **L** | **P** | **T** | **G** | **A** | **A** | **T** | **K** | **D** | **T** | **E** | **S** | **R** | **Y** | **Y** | **P** | **L** | **G** | **E** | **A** | **A** | **A** | **Q** | **L** | **I** | **G** | **Y** | **T** | **G** | **T** | **I** | **T** | **A** | **D** | **D** | **I** | **K** | **K** | **N** | **P** | **E** | **L** | **S** | **S** | **T** | **G** | **V** | **I** | **G** | **K** | **T** | **G** | **L** | **E** | **R** | **A** | **F** | **D** | **K** | **E** | **L** | **R** | **G** | **Q** | **D** | **G** | **G** | **S** | **L** | **V** | **I** | **L** | **D** | **D** | **K** | **E** | **N** | **V** | **K** | **K** | **A** | **L** | **Q** | **T** | **K** | **E** | **K** | **K** | **D** | **G** | **Q** | **T** | **I** | **K** | **L** | **T** | **I** | **D** | **S** | **G** | **V** | **Q** | **Q** | **Q** | **A** | **F** | **A** | **I** | **F** | **D** | **K** | **R** | **P** | **G** | **S** | **A** | **V** | **I** | **T** | **D** | **P** | **Q** | **K** | **G** | **D** | **L** | **L** | **A** | **T** | **V** | **S** | **S** | **P** | **S** | **Y** | **D** | **P** | **N** | **K** | **M** | **A** | **N** | **G** | **I** | **S** | **Q** | **K** | **E** | **Y** | **D** | **A** | **Y** | **N** | **N** | **N** | **K** | **D** | **L** | **P** | **F** | **T** | **A** | **R** | **F** | **A** | **T** | **G** | **Y** | **A** | **P** | **G** | **S** | **T** | **F** | **K** | **T** | **I** | **T** | **G** | **A** | **I** | **G** | **L** | **D** | **A** | **G** | **T** | **L** | **K** | **P** | **D** | **E** | **E** | **L** | **E** | **I** | **N** | **G** | **L** | **K** | **W** | **Q** | **K** | **D** | **K** | **S** | **W** | **G** | **G** | **Y** | **F** | **A** | **T** | **R** | **V** | **K** | **E** | **A** | **S** | **P** | **V** | **N** | **L** | **R** | **T** | **A** | **L** | **V** | **N** | **S** | **D** | **N** | **I** | **Y** | **F** | **A** | **Q** | **Q** | **T** | **L** | **R** | **M** | **G** | **E** | **D** | **K** | **F** | **R** | **A** | **G** | **L** | **N** | **K** | **F** | **I** | **F** | **G** | **E** | **E** | **L** | **D** | **L** | **P** | **I** | **A** | **M** | **T** | **P** | **A** | **Q** | **I** | **S** | **N** | **E** | **D** | **K** | **F** | **N** | **S** | **E** | **I** | **L** | **L** | **A** | **D** | **T** | **G** | **Y** | **G** | **Q** | **G** | **Q** | **L** | **L** | **I** | **S** | **P** | **I** | **Q** | **Q** | **A** | **T** | **M** | **Y** | **S** | **V** | **F** | **Q** | **N** | **N** | **G** | **T** | **L** | **V** | **Y** | **P** | **K** | **L** | **V** | **L** | **D** | **K** | **E** | **T** | **K** | **K** | **K** | **D** | **N** | **V** | **I** | **S** | **A** | **N** | **A** | **A** | **N** | **T** | **I** | **A** | **T** | **D** | **L** | **L** | **G** | **S** | **V** | **E** | **D** | **P** | **S** | **G** | **Y** | **V** | **Y** | **N** | **M** | **Y** | **N** | **P** | **N** | **F** | **S** | **L** | **A** | **A** | **K** | **T** | **G** | **T** | **A** | **E** | **I** | **K** | **D** | **K** | **Q** | **D** | **T** | **D** | **G** | **K** | **E** | **N** | **S** | **F** | **L** | **L** | **T** | **L** | **D** | **R** | **S** | **N** | **N** | **K** | **F** | **L** | **T** | **M** | **I** | **M** | **V** | **E** | **N** | **S** | **G** | **E** | **N** | **G** | **S** | **A** | **T** | **D** | **I** | **S** | **K** | **P** | **L** | **I** | **D** | **Y** | **L** | **E** | **A** | **T** | **I** | **K** | **-** | **-** | **-** | **-** | **-** | **-** | **-** | **-** | **-** | **-** | **-** | **-** | **-** | **-** | **-** | **-** | **-** | **-** | **-** | **-** | **-** | **-** | **-** | **-** | **-** | **-** | **-** | **-** | **-** | **-** | **-** | **-** | **-** | **-** | **-** | **-** | **Q** | **W** | **Q** | **A** | **K** | **Q** | **E** | **L** | **A** | **E** | **A** | **K** | **K** | **T** | **A** | **T** | **T** | **F** | **L** | **N** | **V** | **L** | **S** | **K** | **Q** | **E** | **F** | **D** | **K** | **L** | **P** | **S** | **V** | **V** | **Q** | **E** | **A** | **S** | **L** | **K** | **K** | **N** | **G** | **Y** | **D** | **T** | **K** | **S** | **V** | **V** | **E** | **K** | **Y** | **Q** | **A** | **I** | **Y** | **S** | **G** | **I** | **Q** | **A** | **E** | **G** | **V** | **K** | **A** | **S** | **D** | **V** | **Q** | **V** | **K** | **K** | **A** | **K** | **D** | **N** | **Q** | **Y** | **T** | **F** | **T** | **Y** | **K** | **L** | **S** | **M** | **S** | **T** | **P** | **L** | **G** | **E** | **M** | **K** | **D** | **L** | **S** | **Y** | **Q** | **S** | **S** | **I** | **A** | **K** | **K** | **G** | **D** | **T** | **Y** | **Q** | **I** | **A** | **W** | **N** | **P** | **S** | **L** | **I** | **F** | **P** | **D** | **M** | **S** | **G** | **N** | **D** | **K** | **V** | **S** | **I** | **Q** | **V** | **D** | **N** | **A** | **K** | **R** | **G** | **E** | **I** | **V** | **D** | **R** | **N** | **G** | **S** | **G** | **L** | **A** | **I** | **N** | **K** | **V** | **F** | **D** | **E** | **V** | **G** | **V** | **V** | **P** | **G** | **K** | **L** | **G** | **S** | **G** | **A** | **E** | **K** | **T** | **A** | **N** | **I** | **K** | **A** | **F** | **S** | **D** | **K** | **F** | **G** | **V** | **S** | **V** | **D** | **E** | **I** | **N** | **R** | **K** | **L** | **S** | **Q** | **G** | **W** | **V** | **Q** | **A** | **D** | **S** | **F** | **V** | **P** | **I** | **T** | **V** | **A** | **S** | **E** | **P** | **V** | **T** | **E** | **L** | **P** | **T** | **G** | **A** | **A** | **T** | **K** | **D** | **T** | **E** | **S** | **R** | **Y** | **Y** | **P** | **L** | **G** | **E** | **A** | **A** | **A** | **Q** | **L** | **I** | **G** | **Y** | **T** | **G** | **T** | **I** | **T** | **A** | **D** | **D** | **I** | **K** | **K** | **N** | **P** | **E** | **L** | **S** | **S** | **T** | **G** | **V** | **I** | **G** | **K** | **T** | **G** | **L** | **E** | **R** | **A** | **F** | **D** | **K** | **E** | **L** | **R** | **G** | **Q** | **D** | **G** | **G** | **S** | **L** | **V** | **I** | **L** | **D** | **D** | **K** | **E** | **N** | **V** | **K** | **K** | **A** | **L** | **Q** | **T** | **K** | **E** | **K** | **K** | **D** | **G** | **Q** | **T** | **I** | **K** | **L** | **T** | **I** | **D** | **S** | **G** | **V** | **Q** | **Q** | **Q** | **A** | **F** | **A** | **I** | **F** | **D** | **K** | **R** | **P** | **G** | **S** | **A** | **V** | **I** | **T** | **D** | **P** | **Q** | **K** | **G** | **D** | **L** | **L** | **A** | **T** | **V** | **S** | **S** | **P** | **S** | **Y** | **D** | **P** | **N** | **K** | **M** | **A** | **N** | **G** | **I** | **S** | **Q** | **K** | **E** | **Y** | **D** | **A** | **Y** | **N** | **N** | **N** | **K** | **D** | **L** | **P** | **F** | **T** | **A** | **R** | **F** | **A** | **T** | **G** | **Y** | **A** | **P** | **G** | **S** | **T** | **F** | **K** | **T** | **I** | **T** | **G** | **A** | **I** | **G** | **L** | **D** | **A** | **G** | **T** | **L** | **K** | **P** | **D** | **E** | **E** | **L** | **E** | **I** | **N** | **G** | **L** | **K** | **W** | **Q** | **K** | **D** | **K** | **S** | **W** | **G** | **G** | **Y** | **F** | **A** | **T** | **R** | **V** | **K** | **E** | **A** | **S** | **P** | **V** | **N** | **L** | **R** | **T** | **A** | **L** | **V** | **N** | **S** | **D** | **N** | **I** | **Y** | **F** | **A** | **Q** | **Q** | **T** | **L** | **R** | **M** | **G** | **E** | **D** | **K** | **F** | **R** | **A** | **G** | **L** | **N** | **K** | **F** | **I** | **F** | **G** | **E** | **E** | **L** | **D** | **L** | **P** | **I** | **A** | **M** | **T** | **P** | **A** | **Q** | **I** | **S** | **N** | **E** | **D** | **K** | **F** | **N** | **S** | **E** | **I** | **L** | **L** | **A** | **D** | **T** | **G** | **Y** | **G** | **Q** | **G** | **Q** | **L** | **L** | **I** | **S** | **P** | **I** | **Q** | **Q** | **A** | **T** | **M** | **Y** | **S** | **V** | **F** | **Q** | **N** | **N** | **G** | **T** | **L** | **V** | **Y** | **P** | **K** | **L** | **V** | **L** | **D** | **K** | **E** | **T** | **K** | **K** | **K** | **D** | **N** | **V** | **I** | **S** | **A** | **N** | **A** | **A** | **N** | **T** | **I** | **A** | **T** | **D** | **L** | **L** | **G** | **S** | **V** | **E** | **D** | **P** | **S** | **G** | **Y** | **V** | **Y** | **N** | **M** | **Y** | **N** | **P** | **N** | **F** | **S** | **L** | **A** | **A** | **K** | **T** | **G** | **T** | **A** | **E** | **I** | **K** | **D** | **K** | **Q** | **D** | **T** | **D** | **G** | **K** | **E** | **N** | **S** | **F** | **L** | **L** | **T** | **L** | **D** | **R** | **S** | **N** | **N** | **K** | **F** | **L** | **T** | **M** | **I** | **M** | **V** | **E** | **N** | **S** | **G** | **E** | **N** | **G** | **S** | **A** | **T** | **D** | **I** | **S** | **K** | **P** | **L** | **I** | **D** | **Y** | **L** | **E** | **A** | **T** | **I** | **K** |
| **N59466F** | **-** | **-** | **-** | **-** | **-** | **-** | **-** | **-** | **-** | **-** | **-** | **-** | **-** | **-** | **-** | **-** | **-** | **-** | **-** | **-** | **-** | **-** | **-** | **-** | **-** | **-** | **-** | **-** | **-** | **-** | **-** | **-** | **-** | **-** | **-** | **-** | **Q** | **W** | **Q** | **A** | **K** | **Q** | **E** | **L** | **A** | **E** | **A** | **K** | **K** | **T** | **A** | **T** | **T** | **F** | **L** | **N** | **V** | **L** | **S** | **K** | **Q** | **E** | **F** | **D** | **K** | **L** | **P** | **S** | **V** | **V** | **Q** | **E** | **A** | **S** | **L** | **K** | **K** | **N** | **G** | **Y** | **D** | **T** | **K** | **S** | **V** | **V** | **E** | **K** | **Y** | **Q** | **A** | **I** | **Y** | **S** | **G** | **I** | **Q** | **A** | **E** | **G** | **V** | **K** | **A** | **S** | **D** | **V** | **Q** | **V** | **K** | **K** | **A** | **K** | **D** | **N** | **Q** | **Y** | **T** | **F** | **T** | **Y** | **K** | **L** | **S** | **M** | **S** | **T** | **P** | **L** | **G** | **E** | **M** | **K** | **D** | **L** | **S** | **Y** | **Q** | **S** | **S** | **I** | **A** | **K** | **K** | **G** | **D** | **T** | **Y** | **Q** | **I** | **A** | **W** | **K** | **P** | **S** | **L** | **I** | **F** | **P** | **D** | **M** | **S** | **G** | **N** | **D** | **K** | **I** | **S** | **I** | **Q** | **V** | **D** | **N** | **A** | **K** | **R** | **G** | **E** | **I** | **V** | **D** | **R** | **N** | **G** | **S** | **G** | **L** | **A** | **I** | **N** | **K** | **V** | **F** | **D** | **E** | **V** | **G** | **V** | **V** | **P** | **G** | **K** | **L** | **G** | **S** | **G** | **A** | **E** | **K** | **T** | **A** | **N** | **I** | **K** | **A** | **F** | **S** | **D** | **K** | **F** | **G** | **V** | **S** | **I** | **D** | **E** | **I** | **N** | **Q** | **K** | **L** | **S** | **Q** | **G** | **W** | **V** | **Q** | **A** | **D** | **S** | **F** | **V** | **P** | **I** | **T** | **V** | **A** | **S** | **E** | **P** | **V** | **T** | **E** | **L** | **P** | **T** | **G** | **A** | **A** | **T** | **K** | **D** | **T** | **E** | **S** | **R** | **Y** | **Y** | **P** | **L** | **G** | **E** | **A** | **A** | **A** | **Q** | **L** | **I** | **G** | **Y** | **T** | **G** | **T** | **I** | **T** | **A** | **E** | **D** | **I** | **E** | **K** | **N** | **P** | **E** | **L** | **S** | **S** | **T** | **G** | **V** | **I** | **G** | **K** | **T** | **G** | **L** | **E** | **R** | **A** | **F** | **D** | **K** | **E** | **L** | **R** | **G** | **Q** | **D** | **G** | **G** | **S** | **L** | **V** | **I** | **L** | **D** | **D** | **K** | **E** | **N** | **V** | **K** | **K** | **A** | **L** | **Q** | **T** | **K** | **E** | **K** | **K** | **D** | **G** | **Q** | **T** | **I** | **K** | **L** | **T** | **I** | **D** | **S** | **G** | **V** | **Q** | **Q** | **Q** | **A** | **F** | **A** | **I** | **F** | **D** | **K** | **R** | **P** | **G** | **S** | **A** | **V** | **I** | **T** | **D** | **P** | **Q** | **K** | **G** | **D** | **L** | **L** | **A** | **T** | **V** | **S** | **S** | **P** | **S** | **Y** | **D** | **P** | **N** | **K** | **M** | **A** | **N** | **G** | **I** | **S** | **Q** | **K** | **E** | **Y** | **D** | **A** | **Y** | **N** | **N** | **N** | **K** | **D** | **L** | **P** | **F** | **T** | **A** | **R** | **F** | **A** | **T** | **G** | **Y** | **A** | **P** | **G** | **S** | **T** | **F** | **K** | **T** | **I** | **T** | **G** | **A** | **I** | **G** | **L** | **D** | **A** | **G** | **T** | **L** | **K** | **P** | **D** | **E** | **E** | **L** | **E** | **I** | **N** | **G** | **L** | **K** | **W** | **Q** | **K** | **D** | **K** | **S** | **W** | **G** | **G** | **Y** | **F** | **A** | **T** | **R** | **V** | **K** | **E** | **A** | **S** | **P** | **V** | **N** | **L** | **R** | **T** | **A** | **L** | **V** | **N** | **S** | **D** | **N** | **I** | **Y** | **F** | **A** | **Q** | **Q** | **T** | **L** | **R** | **M** | **G** | **E** | **D** | **K** | **F** | **R** | **A** | **G** | **L** | **N** | **K** | **F** | **I** | **F** | **G** | **E** | **E** | **L** | **D** | **L** | **P** | **I** | **A** | **M** | **T** | **P** | **A** | **Q** | **I** | **S** | **N** | **E** | **D** | **K** | **F** | **N** | **S** | **E** | **I** | **L** | **L** | **A** | **D** | **T** | **G** | **Y** | **G** | **Q** | **G** | **Q** | **L** | **L** | **I** | **S** | **P** | **I** | **Q** | **Q** | **A** | **T** | **M** | **Y** | **S** | **V** | **F** | **Q** | **N** | **N** | **G** | **T** | **L** | **V** | **Y** | **P** | **K** | **L** | **V** | **L** | **D** | **K** | **E** | **T** | **K** | **K** | **K** | **D** | **N** | **V** | **I** | **S** | **A** | **N** | **A** | **A** | **N** | **T** | **I** | **A** | **T** | **D** | **L** | **L** | **G** | **S** | **V** | **E** | **D** | **P** | **S** | **G** | **Y** | **V** | **Y** | **N** | **M** | **Y** | **N** | **P** | **N** | **F** | **S** | **L** | **A** | **A** | **K** | **T** | **G** | **T** | **A** | **E** | **I** | **K** | **D** | **K** | **Q** | **D** | **T** | **D** | **G** | **K** | **E** | **N** | **S** | **F** | **L** | **L** | **T** | **L** | **D** | **R** | **S** | **N** | **N** | **K** | **F** | **L** | **T** | **M** | **I** | **M** | **V** | **E** | **N** | **S** | **G** | **E** | **N** | **G** | **S** | **A** | **T** | **D** | **I** | **S** | **K** | **P** | **L** | **I** | **D** | **Y** | **L** | **E** | **A** | **T** | **I** | **K** | **-** | **-** | **-** | **-** | **-** | **-** | **-** | **-** | **-** | **-** | **-** | **-** | **-** | **-** | **-** | **-** | **-** | **-** | **-** | **-** | **-** | **-** | **-** | **-** | **-** | **-** | **-** | **-** | **-** | **-** | **-** | **-** | **-** | **-** | **-** | **-** | **Q** | **W** | **Q** | **A** | **K** | **Q** | **E** | **L** | **A** | **E** | **A** | **K** | **K** | **T** | **A** | **T** | **T** | **F** | **L** | **N** | **V** | **L** | **S** | **K** | **Q** | **E** | **F** | **D** | **K** | **L** | **P** | **S** | **V** | **V** | **Q** | **E** | **A** | **S** | **L** | **K** | **K** | **N** | **G** | **Y** | **D** | **T** | **K** | **S** | **V** | **V** | **E** | **K** | **Y** | **Q** | **A** | **I** | **Y** | **S** | **G** | **I** | **Q** | **A** | **E** | **G** | **V** | **K** | **A** | **S** | **D** | **V** | **Q** | **V** | **K** | **K** | **A** | **K** | **D** | **N** | **Q** | **Y** | **T** | **F** | **T** | **Y** | **K** | **L** | **S** | **M** | **S** | **T** | **P** | **L** | **G** | **E** | **M** | **K** | **D** | **L** | **S** | **Y** | **Q** | **S** | **S** | **I** | **A** | **K** | **K** | **G** | **D** | **T** | **Y** | **Q** | **I** | **A** | **W** | **K** | **P** | **S** | **L** | **I** | **F** | **P** | **D** | **M** | **S** | **G** | **N** | **D** | **K** | **I** | **S** | **I** | **Q** | **V** | **D** | **N** | **A** | **K** | **R** | **G** | **E** | **I** | **V** | **D** | **R** | **N** | **G** | **S** | **G** | **L** | **A** | **I** | **N** | **K** | **V** | **F** | **D** | **E** | **V** | **G** | **V** | **V** | **P** | **G** | **K** | **L** | **G** | **S** | **G** | **A** | **E** | **K** | **T** | **A** | **N** | **I** | **K** | **A** | **F** | **S** | **D** | **K** | **F** | **G** | **V** | **S** | **I** | **D** | **E** | **I** | **N** | **Q** | **K** | **L** | **S** | **Q** | **G** | **W** | **V** | **Q** | **A** | **D** | **S** | **F** | **V** | **P** | **I** | **T** | **V** | **A** | **S** | **E** | **P** | **V** | **T** | **E** | **L** | **P** | **T** | **G** | **A** | **A** | **T** | **K** | **D** | **T** | **E** | **S** | **R** | **Y** | **Y** | **P** | **L** | **G** | **E** | **A** | **A** | **A** | **Q** | **L** | **I** | **G** | **Y** | **T** | **G** | **T** | **I** | **T** | **A** | **E** | **D** | **I** | **E** | **K** | **N** | **P** | **E** | **L** | **S** | **S** | **T** | **G** | **V** | **I** | **G** | **K** | **T** | **G** | **L** | **E** | **R** | **A** | **F** | **D** | **K** | **E** | **L** | **R** | **G** | **Q** | **D** | **G** | **G** | **S** | **L** | **V** | **I** | **L** | **D** | **D** | **K** | **E** | **N** | **V** | **K** | **K** | **A** | **L** | **Q** | **T** | **K** | **E** | **K** | **K** | **D** | **G** | **Q** | **T** | **I** | **K** | **L** | **T** | **I** | **D** | **S** | **G** | **V** | **Q** | **Q** | **Q** | **A** | **F** | **A** | **I** | **F** | **D** | **K** | **R** | **P** | **G** | **S** | **A** | **V** | **I** | **T** | **D** | **P** | **Q** | **K** | **G** | **D** | **L** | **L** | **A** | **T** | **V** | **S** | **S** | **P** | **S** | **Y** | **D** | **P** | **N** | **K** | **M** | **A** | **N** | **G** | **I** | **S** | **Q** | **K** | **E** | **Y** | **D** | **A** | **Y** | **N** | **N** | **N** | **K** | **D** | **L** | **P** | **F** | **T** | **A** | **R** | **F** | **A** | **T** | **G** | **Y** | **A** | **P** | **G** | **S** | **T** | **F** | **K** | **T** | **I** | **T** | **G** | **A** | **I** | **G** | **L** | **D** | **A** | **G** | **T** | **L** | **K** | **P** | **D** | **E** | **E** | **L** | **E** | **I** | **N** | **G** | **L** | **K** | **W** | **Q** | **K** | **D** | **K** | **S** | **W** | **G** | **G** | **Y** | **F** | **A** | **T** | **R** | **V** | **K** | **E** | **A** | **S** | **P** | **V** | **N** | **L** | **R** | **T** | **A** | **L** | **V** | **N** | **S** | **D** | **N** | **I** | **Y** | **F** | **A** | **Q** | **Q** | **T** | **L** | **R** | **M** | **G** | **E** | **D** | **K** | **F** | **R** | **A** | **G** | **L** | **N** | **K** | **F** | **I** | **F** | **G** | **E** | **E** | **L** | **D** | **L** | **P** | **I** | **A** | **M** | **T** | **P** | **A** | **Q** | **I** | **S** | **N** | **E** | **D** | **K** | **F** | **N** | **S** | **E** | **I** | **L** | **L** | **A** | **D** | **T** | **G** | **Y** | **G** | **Q** | **G** | **Q** | **L** | **L** | **I** | **S** | **P** | **I** | **Q** | **Q** | **A** | **T** | **M** | **Y** | **S** | **V** | **F** | **Q** | **N** | **N** | **G** | **T** | **L** | **V** | **Y** | **P** | **K** | **L** | **V** | **L** | **D** | **K** | **E** | **T** | **K** | **K** | **K** | **D** | **N** | **V** | **I** | **S** | **A** | **N** | **A** | **A** | **N** | **T** | **I** | **A** | **T** | **D** | **L** | **L** | **G** | **S** | **V** | **E** | **D** | **P** | **S** | **G** | **Y** | **V** | **Y** | **N** | **M** | **Y** | **N** | **P** | **N** | **F** | **S** | **L** | **A** | **A** | **K** | **T** | **G** | **T** | **A** | **E** | **I** | **K** | **D** | **K** | **Q** | **D** | **T** | **D** | **G** | **K** | **E** | **N** | **S** | **F** | **L** | **L** | **T** | **L** | **D** | **R** | **S** | **N** | **N** | **K** | **F** | **L** | **T** | **M** | **I** | **M** | **V** | **E** | **N** | **S** | **G** | **E** | **N** | **G** | **S** | **A** | **T** | **D** | **I** | **S** | **K** | **P** | **L** | **I** | **D** | **Y** | **L** | **E** | **A** | **T** | **I** | **K** |
| **N55181** | **-** | **-** | **-** | **-** | **-** | **-** | **-** | **-** | **-** | **-** | **-** | **-** | **-** | **-** | **-** | **-** | **-** | **-** | **-** | **-** | **-** | **-** | **-** | **-** | **-** | **-** | **-** | **-** | **-** | **-** | **-** | **-** | **-** | **-** | **-** | **-** | **Q** | **W** | **Q** | **A** | **K** | **Q** | **E** | **L** | **A** | **E** | **A** | **K** | **K** | **T** | **A** | **N** | **T** | **F** | **L** | **N** | **V** | **L** | **S** | **K** | **Q** | **E** | **F** | **D** | **K** | **L** | **P** | **S** | **V** | **V** | **Q** | **E** | **S** | **S** | **L** | **K** | **K** | **N** | **G** | **Y** | **D** | **T** | **K** | **S** | **V** | **V** | **E** | **K** | **Y** | **Q** | **A** | **I** | **Y** | **S** | **G** | **I** | **Q** | **A** | **E** | **G** | **V** | **K** | **A** | **S** | **D** | **V** | **Q** | **V** | **K** | **K** | **A** | **K** | **D** | **N** | **Q** | **Y** | **T** | **F** | **T** | **Y** | **K** | **L** | **S** | **M** | **S** | **T** | **P** | **L** | **G** | **E** | **M** | **K** | **D** | **L** | **S** | **Y** | **Q** | **S** | **S** | **I** | **A** | **K** | **K** | **G** | **D** | **T** | **Y** | **Q** | **I** | **S** | **W** | **K** | **P** | **S** | **L** | **I** | **F** | **P** | **D** | **M** | **S** | **G** | **N** | **D** | **K** | **I** | **S** | **I** | **Q** | **V** | **D** | **N** | **A** | **K** | **R** | **G** | **E** | **I** | **V** | **D** | **R** | **N** | **G** | **S** | **G** | **L** | **A** | **I** | **N** | **K** | **V** | **F** | **D** | **E** | **V** | **G** | **V** | **V** | **P** | **G** | **K** | **L** | **G** | **S** | **G** | **A** | **E** | **K** | **T** | **A** | **N** | **I** | **K** | **A** | **F** | **S** | **D** | **K** | **F** | **G** | **V** | **S** | **V** | **D** | **E** | **I** | **N** | **Q** | **K** | **L** | **S** | **Q** | **G** | **W** | **V** | **Q** | **A** | **D** | **S** | **F** | **V** | **P** | **I** | **T** | **V** | **A** | **S** | **E** | **P** | **V** | **T** | **E** | **L** | **P** | **T** | **G** | **A** | **A** | **T** | **K** | **D** | **T** | **E** | **S** | **R** | **Y** | **Y** | **P** | **L** | **G** | **E** | **A** | **A** | **A** | **Q** | **L** | **I** | **G** | **Y** | **T** | **G** | **T** | **I** | **T** | **A** | **E** | **D** | **I** | **E** | **K** | **N** | **P** | **E** | **L** | **S** | **S** | **T** | **G** | **V** | **I** | **G** | **K** | **T** | **G** | **L** | **E** | **R** | **A** | **F** | **D** | **K** | **E** | **L** | **R** | **G** | **Q** | **D** | **G** | **G** | **S** | **L** | **V** | **I** | **L** | **D** | **D** | **K** | **E** | **N** | **V** | **K** | **K** | **A** | **L** | **Q** | **T** | **K** | **E** | **K** | **K** | **D** | **G** | **Q** | **T** | **I** | **K** | **L** | **T** | **I** | **D** | **S** | **G** | **V** | **Q** | **Q** | **Q** | **A** | **F** | **A** | **I** | **F** | **D** | **K** | **R** | **P** | **G** | **S** | **A** | **V** | **I** | **T** | **D** | **P** | **Q** | **K** | **G** | **D** | **L** | **L** | **A** | **T** | **V** | **S** | **S** | **P** | **S** | **Y** | **D** | **P** | **N** | **K** | **M** | **A** | **N** | **G** | **I** | **S** | **Q** | **K** | **E** | **Y** | **D** | **A** | **Y** | **N** | **N** | **N** | **K** | **D** | **L** | **P** | **F** | **T** | **A** | **R** | **F** | **A** | **T** | **G** | **Y** | **A** | **P** | **G** | **S** | **T** | **F** | **K** | **T** | **I** | **T** | **G** | **A** | **I** | **G** | **L** | **D** | **A** | **G** | **T** | **L** | **K** | **P** | **D** | **E** | **E** | **L** | **E** | **I** | **N** | **G** | **L** | **K** | **W** | **Q** | **K** | **D** | **K** | **S** | **W** | **G** | **G** | **Y** | **F** | **A** | **T** | **R** | **V** | **K** | **E** | **A** | **S** | **P** | **V** | **N** | **L** | **R** | **T** | **A** | **L** | **V** | **N** | **S** | **D** | **N** | **I** | **Y** | **F** | **A** | **Q** | **Q** | **T** | **L** | **R** | **M** | **G** | **E** | **D** | **K** | **F** | **R** | **A** | **G** | **L** | **N** | **K** | **F** | **I** | **F** | **G** | **E** | **E** | **L** | **D** | **L** | **P** | **I** | **A** | **M** | **T** | **P** | **A** | **Q** | **I** | **S** | **N** | **E** | **D** | **K** | **F** | **N** | **S** | **E** | **I** | **L** | **L** | **A** | **D** | **T** | **G** | **Y** | **G** | **Q** | **G** | **Q** | **L** | **L** | **I** | **S** | **P** | **I** | **Q** | **Q** | **A** | **T** | **M** | **Y** | **S** | **V** | **F** | **Q** | **N** | **N** | **G** | **T** | **L** | **V** | **Y** | **P** | **K** | **L** | **V** | **L** | **D** | **K** | **E** | **T** | **K** | **K** | **K** | **D** | **N** | **V** | **I** | **S** | **A** | **N** | **A** | **A** | **N** | **T** | **I** | **A** | **T** | **D** | **L** | **L** | **G** | **S** | **V** | **E** | **D** | **P** | **S** | **G** | **Y** | **V** | **Y** | **N** | **M** | **Y** | **N** | **P** | **N** | **F** | **S** | **L** | **A** | **A** | **K** | **T** | **G** | **T** | **A** | **E** | **I** | **K** | **D** | **K** | **Q** | **D** | **T** | **D** | **G** | **K** | **E** | **N** | **S** | **F** | **L** | **L** | **T** | **L** | **D** | **R** | **S** | **N** | **N** | **K** | **F** | **L** | **T** | **M** | **I** | **M** | **V** | **E** | **N** | **S** | **G** | **E** | **N** | **G** | **S** | **A** | **T** | **D** | **I** | **S** | **K** | **P** | **L** | **I** | **D** | **Y** | **L** | **E** | **A** | **T** | **I** | **K** | **-** | **-** | **-** | **-** | **-** | **-** | **-** | **-** | **-** | **-** | **-** | **-** | **-** | **-** | **-** | **-** | **-** | **-** | **-** | **-** | **-** | **-** | **-** | **-** | **-** | **-** | **-** | **-** | **-** | **-** | **-** | **-** | **-** | **-** | **-** | **-** | **Q** | **W** | **Q** | **A** | **K** | **Q** | **E** | **L** | **A** | **E** | **A** | **K** | **K** | **T** | **A** | **N** | **T** | **F** | **L** | **N** | **V** | **L** | **S** | **K** | **Q** | **E** | **F** | **D** | **K** | **L** | **P** | **S** | **V** | **V** | **Q** | **E** | **S** | **S** | **L** | **K** | **K** | **N** | **G** | **Y** | **D** | **T** | **K** | **S** | **V** | **V** | **E** | **K** | **Y** | **Q** | **A** | **I** | **Y** | **S** | **G** | **I** | **Q** | **A** | **E** | **G** | **V** | **K** | **A** | **S** | **D** | **V** | **Q** | **V** | **K** | **K** | **A** | **K** | **D** | **N** | **Q** | **Y** | **T** | **F** | **T** | **Y** | **K** | **L** | **S** | **M** | **S** | **T** | **P** | **L** | **G** | **E** | **M** | **K** | **D** | **L** | **S** | **Y** | **Q** | **S** | **S** | **I** | **A** | **K** | **K** | **G** | **D** | **T** | **Y** | **Q** | **I** | **S** | **W** | **K** | **P** | **S** | **L** | **I** | **F** | **P** | **D** | **M** | **S** | **G** | **N** | **D** | **K** | **I** | **S** | **I** | **Q** | **V** | **D** | **N** | **A** | **K** | **R** | **G** | **E** | **I** | **V** | **D** | **R** | **N** | **G** | **S** | **G** | **L** | **A** | **I** | **N** | **K** | **V** | **F** | **D** | **E** | **V** | **G** | **V** | **V** | **P** | **G** | **K** | **L** | **G** | **S** | **G** | **A** | **E** | **K** | **T** | **A** | **N** | **I** | **K** | **A** | **F** | **S** | **D** | **K** | **F** | **G** | **V** | **S** | **V** | **D** | **E** | **I** | **N** | **Q** | **K** | **L** | **S** | **Q** | **G** | **W** | **V** | **Q** | **A** | **D** | **S** | **F** | **V** | **P** | **I** | **T** | **V** | **A** | **S** | **E** | **P** | **V** | **T** | **E** | **L** | **P** | **T** | **G** | **A** | **A** | **T** | **K** | **D** | **T** | **E** | **S** | **R** | **Y** | **Y** | **P** | **L** | **G** | **E** | **A** | **A** | **A** | **Q** | **L** | **I** | **G** | **Y** | **T** | **G** | **T** | **I** | **T** | **A** | **E** | **D** | **I** | **E** | **K** | **N** | **P** | **E** | **L** | **S** | **S** | **T** | **G** | **V** | **I** | **G** | **K** | **T** | **G** | **L** | **E** | **R** | **A** | **F** | **D** | **K** | **E** | **L** | **R** | **G** | **Q** | **D** | **G** | **G** | **S** | **L** | **V** | **I** | **L** | **D** | **D** | **K** | **E** | **N** | **V** | **K** | **K** | **A** | **L** | **Q** | **T** | **K** | **E** | **K** | **K** | **D** | **G** | **Q** | **T** | **I** | **K** | **L** | **T** | **I** | **D** | **S** | **G** | **V** | **Q** | **Q** | **Q** | **A** | **F** | **A** | **I** | **F** | **D** | **K** | **R** | **P** | **G** | **S** | **A** | **V** | **I** | **T** | **D** | **P** | **Q** | **K** | **G** | **D** | **L** | **L** | **A** | **T** | **V** | **S** | **S** | **P** | **S** | **Y** | **D** | **P** | **N** | **K** | **M** | **A** | **N** | **G** | **I** | **S** | **Q** | **K** | **E** | **Y** | **D** | **A** | **Y** | **N** | **N** | **N** | **K** | **D** | **L** | **P** | **F** | **T** | **A** | **R** | **F** | **A** | **T** | **G** | **Y** | **A** | **P** | **G** | **S** | **T** | **F** | **K** | **T** | **I** | **T** | **G** | **A** | **I** | **G** | **L** | **D** | **A** | **G** | **T** | **L** | **K** | **P** | **D** | **E** | **E** | **L** | **E** | **I** | **N** | **G** | **L** | **K** | **W** | **Q** | **K** | **D** | **K** | **S** | **W** | **G** | **G** | **Y** | **F** | **A** | **T** | **R** | **V** | **K** | **E** | **A** | **S** | **P** | **V** | **N** | **L** | **R** | **T** | **A** | **L** | **V** | **N** | **S** | **D** | **N** | **I** | **Y** | **F** | **A** | **Q** | **Q** | **T** | **L** | **R** | **M** | **G** | **E** | **D** | **K** | **F** | **R** | **A** | **G** | **L** | **N** | **K** | **F** | **I** | **F** | **G** | **E** | **E** | **L** | **D** | **L** | **P** | **I** | **A** | **M** | **T** | **P** | **A** | **Q** | **I** | **S** | **N** | **E** | **D** | **K** | **F** | **N** | **S** | **E** | **I** | **L** | **L** | **A** | **D** | **T** | **G** | **Y** | **G** | **Q** | **G** | **Q** | **L** | **L** | **I** | **S** | **P** | **I** | **Q** | **Q** | **A** | **T** | **M** | **Y** | **S** | **V** | **F** | **Q** | **N** | **N** | **G** | **T** | **L** | **V** | **Y** | **P** | **K** | **L** | **V** | **L** | **D** | **K** | **E** | **T** | **K** | **K** | **K** | **D** | **N** | **V** | **I** | **S** | **A** | **N** | **A** | **A** | **N** | **T** | **I** | **A** | **T** | **D** | **L** | **L** | **G** | **S** | **V** | **E** | **D** | **P** | **S** | **G** | **Y** | **V** | **Y** | **N** | **M** | **Y** | **N** | **P** | **N** | **F** | **S** | **L** | **A** | **A** | **K** | **T** | **G** | **T** | **A** | **E** | **I** | **K** | **D** | **K** | **Q** | **D** | **T** | **D** | **G** | **K** | **E** | **N** | **S** | **F** | **L** | **L** | **T** | **L** | **D** | **R** | **S** | **N** | **N** | **K** | **F** | **L** | **T** | **M** | **I** | **M** | **V** | **E** | **N** | **S** | **G** | **E** | **N** | **G** | **S** | **A** | **T** | **D** | **I** | **S** | **K** | **P** | **L** | **I** | **D** | **Y** | **L** | **E** | **A** | **T** | **I** | **K** |
| **N59847F** | **-** | **-** | **-** | **-** | **-** | **-** | **-** | **-** | **-** | **-** | **-** | **-** | **-** | **-** | **-** | **-** | **-** | **-** | **-** | **-** | **-** | **-** | **-** | **-** | **-** | **-** | **-** | **-** | **-** | **-** | **-** | **-** | **-** | **-** | **-** | **-** | **Q** | **W** | **Q** | **A** | **K** | **Q** | **E** | **L** | **A** | **E** | **A** | **K** | **K** | **T** | **A** | **N** | **T** | **F** | **L** | **N** | **V** | **L** | **S** | **K** | **Q** | **E** | **F** | **D** | **K** | **L** | **P** | **S** | **V** | **V** | **Q** | **E** | **S** | **S** | **L** | **K** | **K** | **N** | **G** | **Y** | **D** | **T** | **K** | **S** | **V** | **V** | **E** | **K** | **Y** | **Q** | **A** | **I** | **Y** | **S** | **G** | **I** | **Q** | **A** | **E** | **G** | **V** | **K** | **A** | **S** | **D** | **V** | **Q** | **V** | **K** | **K** | **A** | **K** | **D** | **N** | **Q** | **Y** | **T** | **F** | **T** | **Y** | **K** | **L** | **S** | **M** | **S** | **T** | **P** | **L** | **G** | **E** | **M** | **K** | **D** | **L** | **S** | **Y** | **Q** | **S** | **S** | **I** | **A** | **K** | **K** | **G** | **D** | **T** | **Y** | **Q** | **I** | **S** | **W** | **K** | **P** | **S** | **L** | **I** | **F** | **P** | **D** | **M** | **S** | **G** | **N** | **D** | **K** | **I** | **S** | **I** | **Q** | **V** | **D** | **N** | **A** | **K** | **R** | **G** | **E** | **I** | **V** | **D** | **R** | **N** | **G** | **S** | **G** | **L** | **A** | **I** | **N** | **K** | **V** | **F** | **D** | **E** | **V** | **G** | **V** | **V** | **P** | **G** | **K** | **L** | **G** | **S** | **G** | **A** | **E** | **K** | **T** | **A** | **N** | **I** | **K** | **A** | **F** | **S** | **D** | **K** | **F** | **G** | **V** | **S** | **V** | **D** | **E** | **I** | **N** | **Q** | **K** | **L** | **S** | **Q** | **G** | **W** | **V** | **Q** | **A** | **D** | **S** | **F** | **V** | **P** | **I** | **T** | **V** | **A** | **S** | **E** | **P** | **V** | **T** | **E** | **L** | **P** | **T** | **G** | **A** | **A** | **T** | **K** | **D** | **T** | **E** | **S** | **R** | **Y** | **Y** | **P** | **L** | **G** | **E** | **A** | **A** | **A** | **Q** | **L** | **I** | **G** | **Y** | **T** | **G** | **T** | **I** | **T** | **A** | **E** | **D** | **I** | **E** | **K** | **N** | **P** | **E** | **L** | **S** | **S** | **T** | **G** | **V** | **I** | **G** | **K** | **T** | **G** | **L** | **E** | **R** | **A** | **F** | **D** | **K** | **E** | **L** | **R** | **G** | **Q** | **D** | **G** | **G** | **S** | **L** | **V** | **I** | **L** | **D** | **D** | **K** | **E** | **N** | **V** | **K** | **K** | **A** | **L** | **Q** | **T** | **K** | **E** | **K** | **K** | **D** | **G** | **Q** | **T** | **I** | **K** | **L** | **T** | **I** | **D** | **S** | **G** | **V** | **Q** | **Q** | **Q** | **A** | **F** | **A** | **I** | **F** | **D** | **K** | **R** | **P** | **G** | **S** | **A** | **V** | **I** | **T** | **D** | **P** | **Q** | **K** | **G** | **D** | **L** | **L** | **A** | **T** | **V** | **S** | **S** | **P** | **S** | **Y** | **D** | **P** | **N** | **K** | **M** | **A** | **N** | **G** | **I** | **S** | **Q** | **K** | **E** | **Y** | **D** | **A** | **Y** | **N** | **N** | **N** | **K** | **D** | **L** | **P** | **F** | **T** | **A** | **R** | **F** | **A** | **T** | **G** | **Y** | **A** | **P** | **G** | **S** | **T** | **F** | **K** | **T** | **I** | **T** | **G** | **A** | **I** | **G** | **L** | **D** | **A** | **G** | **T** | **L** | **K** | **P** | **D** | **E** | **E** | **L** | **E** | **I** | **N** | **G** | **L** | **K** | **W** | **Q** | **K** | **D** | **K** | **S** | **W** | **G** | **G** | **Y** | **F** | **A** | **T** | **R** | **V** | **K** | **E** | **A** | **S** | **P** | **V** | **N** | **L** | **R** | **T** | **A** | **L** | **V** | **N** | **S** | **D** | **N** | **I** | **Y** | **F** | **A** | **Q** | **Q** | **T** | **L** | **R** | **M** | **G** | **E** | **D** | **K** | **F** | **R** | **A** | **G** | **L** | **N** | **K** | **F** | **I** | **F** | **G** | **E** | **E** | **L** | **D** | **L** | **P** | **I** | **A** | **M** | **T** | **P** | **A** | **Q** | **I** | **S** | **N** | **E** | **D** | **K** | **F** | **N** | **S** | **E** | **I** | **L** | **L** | **A** | **D** | **T** | **G** | **Y** | **G** | **Q** | **G** | **Q** | **L** | **L** | **I** | **S** | **P** | **I** | **Q** | **Q** | **A** | **T** | **M** | **Y** | **S** | **V** | **F** | **Q** | **N** | **N** | **G** | **T** | **L** | **V** | **Y** | **P** | **K** | **L** | **V** | **L** | **D** | **K** | **E** | **T** | **K** | **K** | **K** | **D** | **N** | **V** | **I** | **S** | **A** | **N** | **A** | **A** | **N** | **T** | **I** | **A** | **T** | **D** | **L** | **L** | **G** | **S** | **V** | **E** | **D** | **P** | **S** | **G** | **Y** | **V** | **Y** | **N** | **M** | **Y** | **N** | **P** | **N** | **F** | **S** | **L** | **A** | **A** | **K** | **T** | **G** | **T** | **A** | **E** | **I** | **K** | **D** | **K** | **Q** | **D** | **T** | **D** | **G** | **K** | **E** | **N** | **S** | **F** | **L** | **L** | **T** | **L** | **D** | **R** | **S** | **N** | **N** | **K** | **F** | **L** | **T** | **M** | **I** | **M** | **V** | **E** | **N** | **S** | **G** | **E** | **N** | **G** | **S** | **A** | **T** | **D** | **I** | **S** | **K** | **P** | **L** | **I** | **D** | **Y** | **L** | **E** | **A** | **T** | **I** | **K** | **-** | **-** | **-** | **-** | **-** | **-** | **-** | **-** | **-** | **-** | **-** | **-** | **-** | **-** | **-** | **-** | **-** | **-** | **-** | **-** | **-** | **-** | **-** | **-** | **-** | **-** | **-** | **-** | **-** | **-** | **-** | **-** | **-** | **-** | **-** | **-** | **Q** | **W** | **Q** | **A** | **K** | **Q** | **E** | **L** | **A** | **E** | **A** | **K** | **K** | **T** | **A** | **N** | **T** | **F** | **L** | **N** | **V** | **L** | **S** | **K** | **Q** | **E** | **F** | **D** | **K** | **L** | **P** | **S** | **V** | **V** | **Q** | **E** | **S** | **S** | **L** | **K** | **K** | **N** | **G** | **Y** | **D** | **T** | **K** | **S** | **V** | **V** | **E** | **K** | **Y** | **Q** | **A** | **I** | **Y** | **S** | **G** | **I** | **Q** | **A** | **E** | **G** | **V** | **K** | **A** | **S** | **D** | **V** | **Q** | **V** | **K** | **K** | **A** | **K** | **D** | **N** | **Q** | **Y** | **T** | **F** | **T** | **Y** | **K** | **L** | **S** | **M** | **S** | **T** | **P** | **L** | **G** | **E** | **M** | **K** | **D** | **L** | **S** | **Y** | **Q** | **S** | **S** | **I** | **A** | **K** | **K** | **G** | **D** | **T** | **Y** | **Q** | **I** | **S** | **W** | **K** | **P** | **S** | **L** | **I** | **F** | **P** | **D** | **M** | **S** | **G** | **N** | **D** | **K** | **I** | **S** | **I** | **Q** | **V** | **D** | **N** | **A** | **K** | **R** | **G** | **E** | **I** | **V** | **D** | **R** | **N** | **G** | **S** | **G** | **L** | **A** | **I** | **N** | **K** | **V** | **F** | **D** | **E** | **V** | **G** | **V** | **V** | **P** | **G** | **K** | **L** | **G** | **S** | **G** | **A** | **E** | **K** | **T** | **A** | **N** | **I** | **K** | **A** | **F** | **S** | **D** | **K** | **F** | **G** | **V** | **S** | **V** | **D** | **E** | **I** | **N** | **Q** | **K** | **L** | **S** | **Q** | **G** | **W** | **V** | **Q** | **A** | **D** | **S** | **F** | **V** | **P** | **I** | **T** | **V** | **A** | **S** | **E** | **P** | **V** | **T** | **E** | **L** | **P** | **T** | **G** | **A** | **A** | **T** | **K** | **D** | **T** | **E** | **S** | **R** | **Y** | **Y** | **P** | **L** | **G** | **E** | **A** | **A** | **A** | **Q** | **L** | **I** | **G** | **Y** | **T** | **G** | **T** | **I** | **T** | **A** | **E** | **D** | **I** | **E** | **K** | **N** | **P** | **E** | **L** | **S** | **S** | **T** | **G** | **V** | **I** | **G** | **K** | **T** | **G** | **L** | **E** | **R** | **A** | **F** | **D** | **K** | **E** | **L** | **R** | **G** | **Q** | **D** | **G** | **G** | **S** | **L** | **V** | **I** | **L** | **D** | **D** | **K** | **E** | **N** | **V** | **K** | **K** | **A** | **L** | **Q** | **T** | **K** | **E** | **K** | **K** | **D** | **G** | **Q** | **T** | **I** | **K** | **L** | **T** | **I** | **D** | **S** | **G** | **V** | **Q** | **Q** | **Q** | **A** | **F** | **A** | **I** | **F** | **D** | **K** | **R** | **P** | **G** | **S** | **A** | **V** | **I** | **T** | **D** | **P** | **Q** | **K** | **G** | **D** | **L** | **L** | **A** | **T** | **V** | **S** | **S** | **P** | **S** | **Y** | **D** | **P** | **N** | **K** | **M** | **A** | **N** | **G** | **I** | **S** | **Q** | **K** | **E** | **Y** | **D** | **A** | **Y** | **N** | **N** | **N** | **K** | **D** | **L** | **P** | **F** | **T** | **A** | **R** | **F** | **A** | **T** | **G** | **Y** | **A** | **P** | **G** | **S** | **T** | **F** | **K** | **T** | **I** | **T** | **G** | **A** | **I** | **G** | **L** | **D** | **A** | **G** | **T** | **L** | **K** | **P** | **D** | **E** | **E** | **L** | **E** | **I** | **N** | **G** | **L** | **K** | **W** | **Q** | **K** | **D** | **K** | **S** | **W** | **G** | **G** | **Y** | **F** | **A** | **T** | **R** | **V** | **K** | **E** | **A** | **S** | **P** | **V** | **N** | **L** | **R** | **T** | **A** | **L** | **V** | **N** | **S** | **D** | **N** | **I** | **Y** | **F** | **A** | **Q** | **Q** | **T** | **L** | **R** | **M** | **G** | **E** | **D** | **K** | **F** | **R** | **A** | **G** | **L** | **N** | **K** | **F** | **I** | **F** | **G** | **E** | **E** | **L** | **D** | **L** | **P** | **I** | **A** | **M** | **T** | **P** | **A** | **Q** | **I** | **S** | **N** | **E** | **D** | **K** | **F** | **N** | **S** | **E** | **I** | **L** | **L** | **A** | **D** | **T** | **G** | **Y** | **G** | **Q** | **G** | **Q** | **L** | **L** | **I** | **S** | **P** | **I** | **Q** | **Q** | **A** | **T** | **M** | **Y** | **S** | **V** | **F** | **Q** | **N** | **N** | **G** | **T** | **L** | **V** | **Y** | **P** | **K** | **L** | **V** | **L** | **D** | **K** | **E** | **T** | **K** | **K** | **K** | **D** | **N** | **V** | **I** | **S** | **A** | **N** | **A** | **A** | **N** | **T** | **I** | **A** | **T** | **D** | **L** | **L** | **G** | **S** | **V** | **E** | **D** | **P** | **S** | **G** | **Y** | **V** | **Y** | **N** | **M** | **Y** | **N** | **P** | **N** | **F** | **S** | **L** | **A** | **A** | **K** | **T** | **G** | **T** | **A** | **E** | **I** | **K** | **D** | **K** | **Q** | **D** | **T** | **D** | **G** | **K** | **E** | **N** | **S** | **F** | **L** | **L** | **T** | **L** | **D** | **R** | **S** | **N** | **N** | **K** | **F** | **L** | **T** | **M** | **I** | **M** | **V** | **E** | **N** | **S** | **G** | **E** | **N** | **G** | **S** | **A** | **T** | **D** | **I** | **S** | **K** | **P** | **L** | **I** | **D** | **Y** | **L** | **E** | **A** | **T** | **I** | **K** |
| **N59611F** | **-** | **-** | **-** | **-** | **-** | **-** | **-** | **-** | **-** | **-** | **-** | **-** | **-** | **-** | **-** | **-** | **-** | **-** | **-** | **-** | **-** | **-** | **-** | **-** | **-** | **-** | **-** | **-** | **-** | **-** | **-** | **-** | **-** | **-** | **-** | **-** | **Q** | **W** | **Q** | **A** | **K** | **Q** | **E** | **L** | **A** | **E** | **A** | **K** | **K** | **T** | **A** | **T** | **E** | **F** | **L** | **N** | **V** | **L** | **S** | **K** | **Q** | **E** | **F** | **D** | **K** | **L** | **P** | **S** | **V** | **V** | **Q** | **E** | **A** | **S** | **L** | **K** | **K** | **N** | **G** | **Y** | **D** | **T** | **K** | **S** | **V** | **V** | **E** | **K** | **Y** | **Q** | **A** | **I** | **Y** | **S** | **G** | **I** | **Q** | **A** | **E** | **G** | **V** | **K** | **A** | **S** | **D** | **V** | **Q** | **V** | **K** | **K** | **A** | **K** | **D** | **N** | **Q** | **Y** | **T** | **F** | **T** | **Y** | **K** | **L** | **S** | **M** | **S** | **T** | **P** | **L** | **G** | **E** | **M** | **K** | **D** | **L** | **S** | **Y** | **Q** | **S** | **S** | **I** | **A** | **K** | **K** | **G** | **D** | **T** | **Y** | **Q** | **I** | **A** | **W** | **K** | **P** | **S** | **L** | **I** | **F** | **P** | **D** | **M** | **S** | **G** | **N** | **D** | **K** | **I** | **S** | **I** | **Q** | **V** | **D** | **N** | **A** | **K** | **R** | **G** | **E** | **I** | **V** | **D** | **R** | **N** | **G** | **S** | **G** | **L** | **A** | **I** | **N** | **K** | **V** | **F** | **D** | **E** | **V** | **G** | **V** | **V** | **P** | **G** | **K** | **L** | **G** | **S** | **G** | **A** | **E** | **K** | **T** | **A** | **N** | **I** | **K** | **A** | **F** | **S** | **D** | **K** | **F** | **G** | **V** | **S** | **V** | **D** | **E** | **I** | **N** | **Q** | **K** | **L** | **S** | **Q** | **G** | **W** | **V** | **Q** | **A** | **D** | **S** | **F** | **V** | **P** | **I** | **T** | **V** | **A** | **S** | **E** | **P** | **V** | **T** | **E** | **L** | **P** | **T** | **G** | **A** | **A** | **T** | **K** | **D** | **T** | **E** | **S** | **R** | **Y** | **Y** | **P** | **L** | **G** | **E** | **A** | **A** | **A** | **Q** | **L** | **I** | **G** | **Y** | **T** | **G** | **T** | **I** | **T** | **A** | **E** | **D** | **I** | **E** | **K** | **N** | **P** | **E** | **L** | **S** | **S** | **T** | **G** | **V** | **I** | **G** | **K** | **T** | **G** | **L** | **E** | **R** | **A** | **F** | **D** | **K** | **E** | **L** | **R** | **G** | **Q** | **D** | **G** | **G** | **S** | **L** | **V** | **I** | **L** | **D** | **D** | **K** | **E** | **N** | **V** | **K** | **K** | **A** | **L** | **Q** | **T** | **K** | **E** | **K** | **K** | **D** | **G** | **Q** | **T** | **I** | **K** | **L** | **T** | **I** | **D** | **S** | **G** | **V** | **Q** | **Q** | **Q** | **A** | **F** | **A** | **I** | **F** | **D** | **K** | **R** | **P** | **G** | **S** | **A** | **V** | **I** | **T** | **D** | **P** | **Q** | **K** | **G** | **D** | **L** | **L** | **A** | **T** | **V** | **S** | **S** | **P** | **S** | **Y** | **D** | **P** | **N** | **K** | **M** | **A** | **N** | **G** | **I** | **S** | **Q** | **K** | **E** | **Y** | **D** | **A** | **Y** | **N** | **N** | **N** | **K** | **D** | **L** | **P** | **F** | **T** | **A** | **R** | **F** | **A** | **T** | **G** | **Y** | **A** | **P** | **G** | **S** | **T** | **F** | **K** | **T** | **I** | **T** | **G** | **A** | **I** | **G** | **L** | **D** | **A** | **G** | **T** | **L** | **K** | **P** | **D** | **E** | **E** | **L** | **E** | **I** | **N** | **G** | **L** | **K** | **W** | **Q** | **K** | **D** | **K** | **S** | **W** | **G** | **G** | **Y** | **F** | **A** | **T** | **R** | **V** | **K** | **E** | **A** | **S** | **P** | **V** | **N** | **L** | **R** | **T** | **A** | **L** | **V** | **N** | **S** | **D** | **N** | **I** | **Y** | **F** | **A** | **Q** | **Q** | **T** | **L** | **R** | **M** | **G** | **E** | **D** | **K** | **F** | **R** | **A** | **G** | **L** | **N** | **K** | **F** | **I** | **F** | **G** | **E** | **E** | **L** | **D** | **L** | **P** | **I** | **A** | **M** | **T** | **P** | **A** | **Q** | **I** | **S** | **N** | **E** | **D** | **K** | **F** | **N** | **S** | **E** | **I** | **L** | **L** | **A** | **D** | **T** | **G** | **Y** | **G** | **Q** | **G** | **Q** | **L** | **L** | **I** | **S** | **P** | **I** | **Q** | **Q** | **A** | **T** | **M** | **Y** | **S** | **V** | **F** | **Q** | **N** | **N** | **G** | **T** | **L** | **V** | **Y** | **P** | **K** | **I** | **V** | **L** | **D** | **K** | **E** | **T** | **K** | **K** | **K** | **D** | **N** | **V** | **I** | **S** | **A** | **N** | **A** | **A** | **N** | **T** | **I** | **A** | **T** | **D** | **L** | **L** | **G** | **S** | **V** | **E** | **D** | **P** | **S** | **G** | **Y** | **V** | **Y** | **N** | **M** | **Y** | **N** | **P** | **N** | **F** | **S** | **L** | **A** | **A** | **K** | **T** | **G** | **T** | **A** | **E** | **I** | **K** | **D** | **K** | **Q** | **D** | **T** | **D** | **G** | **K** | **E** | **N** | **S** | **F** | **L** | **L** | **T** | **L** | **D** | **R** | **S** | **N** | **N** | **K** | **F** | **L** | **T** | **M** | **I** | **M** | **V** | **E** | **N** | **S** | **G** | **E** | **N** | **G** | **S** | **A** | **T** | **D** | **I** | **S** | **K** | **P** | **L** | **I** | **D** | **Y** | **L** | **E** | **A** | **T** | **I** | **K** | **-** | **-** | **-** | **-** | **-** | **-** | **-** | **-** | **-** | **-** | **-** | **-** | **-** | **-** | **-** | **-** | **-** | **-** | **-** | **-** | **-** | **-** | **-** | **-** | **-** | **-** | **-** | **-** | **-** | **-** | **-** | **-** | **-** | **-** | **-** | **-** | **Q** | **W** | **Q** | **A** | **K** | **Q** | **E** | **L** | **A** | **E** | **A** | **K** | **K** | **T** | **A** | **T** | **E** | **F** | **L** | **N** | **V** | **L** | **S** | **K** | **Q** | **E** | **F** | **D** | **K** | **L** | **P** | **S** | **V** | **V** | **Q** | **E** | **A** | **S** | **L** | **K** | **K** | **N** | **G** | **Y** | **D** | **T** | **K** | **S** | **V** | **V** | **E** | **K** | **Y** | **Q** | **A** | **I** | **Y** | **S** | **G** | **I** | **Q** | **A** | **E** | **G** | **V** | **K** | **A** | **S** | **D** | **V** | **Q** | **V** | **K** | **K** | **A** | **K** | **D** | **N** | **Q** | **Y** | **T** | **F** | **T** | **Y** | **K** | **L** | **S** | **M** | **S** | **T** | **P** | **L** | **G** | **E** | **M** | **K** | **D** | **L** | **S** | **Y** | **Q** | **S** | **S** | **I** | **A** | **K** | **K** | **G** | **D** | **T** | **Y** | **Q** | **I** | **A** | **W** | **K** | **P** | **S** | **L** | **I** | **F** | **P** | **D** | **M** | **S** | **G** | **N** | **D** | **K** | **I** | **S** | **I** | **Q** | **V** | **D** | **N** | **A** | **K** | **R** | **G** | **E** | **I** | **V** | **D** | **R** | **N** | **G** | **S** | **G** | **L** | **A** | **I** | **N** | **K** | **V** | **F** | **D** | **E** | **V** | **G** | **V** | **V** | **P** | **G** | **K** | **L** | **G** | **S** | **G** | **A** | **E** | **K** | **T** | **A** | **N** | **I** | **K** | **A** | **F** | **S** | **D** | **K** | **F** | **G** | **V** | **S** | **V** | **D** | **E** | **I** | **N** | **Q** | **K** | **L** | **S** | **Q** | **G** | **W** | **V** | **Q** | **A** | **D** | **S** | **F** | **V** | **P** | **I** | **T** | **V** | **A** | **S** | **E** | **P** | **V** | **T** | **E** | **L** | **P** | **T** | **G** | **A** | **A** | **T** | **K** | **D** | **T** | **E** | **S** | **R** | **Y** | **Y** | **P** | **L** | **G** | **E** | **A** | **A** | **A** | **Q** | **L** | **I** | **G** | **Y** | **T** | **G** | **T** | **I** | **T** | **A** | **E** | **D** | **I** | **E** | **K** | **N** | **P** | **E** | **L** | **S** | **S** | **T** | **G** | **V** | **I** | **G** | **K** | **T** | **G** | **L** | **E** | **R** | **A** | **F** | **D** | **K** | **E** | **L** | **R** | **G** | **Q** | **D** | **G** | **G** | **S** | **L** | **V** | **I** | **L** | **D** | **D** | **K** | **E** | **N** | **V** | **K** | **K** | **A** | **L** | **Q** | **T** | **K** | **E** | **K** | **K** | **D** | **G** | **Q** | **T** | **I** | **K** | **L** | **T** | **I** | **D** | **S** | **G** | **V** | **Q** | **Q** | **Q** | **A** | **F** | **A** | **I** | **F** | **D** | **K** | **R** | **P** | **G** | **S** | **A** | **V** | **I** | **T** | **D** | **P** | **Q** | **K** | **G** | **D** | **L** | **L** | **A** | **T** | **V** | **S** | **S** | **P** | **S** | **Y** | **D** | **P** | **N** | **K** | **M** | **A** | **N** | **G** | **I** | **S** | **Q** | **K** | **E** | **Y** | **D** | **A** | **Y** | **N** | **N** | **N** | **K** | **D** | **L** | **P** | **F** | **T** | **A** | **R** | **F** | **A** | **T** | **G** | **Y** | **A** | **P** | **G** | **S** | **T** | **F** | **K** | **T** | **I** | **T** | **G** | **A** | **I** | **G** | **L** | **D** | **A** | **G** | **T** | **L** | **K** | **P** | **D** | **E** | **E** | **L** | **E** | **I** | **N** | **G** | **L** | **K** | **W** | **Q** | **K** | **D** | **K** | **S** | **W** | **G** | **G** | **Y** | **F** | **A** | **T** | **R** | **V** | **K** | **E** | **A** | **S** | **P** | **V** | **N** | **L** | **R** | **T** | **A** | **L** | **V** | **N** | **S** | **D** | **N** | **I** | **Y** | **F** | **A** | **Q** | **Q** | **T** | **L** | **R** | **M** | **G** | **E** | **D** | **K** | **F** | **R** | **A** | **G** | **L** | **N** | **K** | **F** | **I** | **F** | **G** | **E** | **E** | **L** | **D** | **L** | **P** | **I** | **A** | **M** | **T** | **P** | **A** | **Q** | **I** | **S** | **N** | **E** | **D** | **K** | **F** | **N** | **S** | **E** | **I** | **L** | **L** | **A** | **D** | **T** | **G** | **Y** | **G** | **Q** | **G** | **Q** | **L** | **L** | **I** | **S** | **P** | **I** | **Q** | **Q** | **A** | **T** | **M** | **Y** | **S** | **V** | **F** | **Q** | **N** | **N** | **G** | **T** | **L** | **V** | **Y** | **P** | **K** | **I** | **V** | **L** | **D** | **K** | **E** | **T** | **K** | **K** | **K** | **D** | **N** | **V** | **I** | **S** | **A** | **N** | **A** | **A** | **N** | **T** | **I** | **A** | **T** | **D** | **L** | **L** | **G** | **S** | **V** | **E** | **D** | **P** | **S** | **G** | **Y** | **V** | **Y** | **N** | **M** | **Y** | **N** | **P** | **N** | **F** | **S** | **L** | **A** | **A** | **K** | **T** | **G** | **T** | **A** | **E** | **I** | **K** | **D** | **K** | **Q** | **D** | **T** | **D** | **G** | **K** | **E** | **N** | **S** | **F** | **L** | **L** | **T** | **L** | **D** | **R** | **S** | **N** | **N** | **K** | **F** | **L** | **T** | **M** | **I** | **M** | **V** | **E** | **N** | **S** | **G** | **E** | **N** | **G** | **S** | **A** | **T** | **D** | **I** | **S** | **K** | **P** | **L** | **I** | **D** | **Y** | **L** | **E** | **A** | **T** | **I** | **K** |
| **N52648** | **-** | **-** | **-** | **-** | **-** | **-** | **-** | **-** | **-** | **-** | **-** | **-** | **-** | **-** | **-** | **-** | **-** | **-** | **-** | **-** | **-** | **-** | **-** | **-** | **-** | **-** | **-** | **-** | **-** | **-** | **-** | **-** | **-** | **-** | **-** | **-** | **Q** | **W** | **Q** | **A** | **K** | **Q** | **E** | **L** | **A** | **E** | **A** | **K** | **K** | **T** | **A** | **N** | **T** | **F** | **L** | **N** | **V** | **L** | **S** | **K** | **Q** | **E** | **F** | **D** | **K** | **L** | **P** | **S** | **V** | **V** | **Q** | **E** | **S** | **S** | **L** | **K** | **K** | **N** | **G** | **Y** | **D** | **T** | **K** | **S** | **V** | **V** | **E** | **K** | **Y** | **Q** | **A** | **I** | **Y** | **S** | **G** | **I** | **Q** | **A** | **E** | **G** | **V** | **K** | **A** | **S** | **D** | **V** | **Q** | **V** | **K** | **K** | **A** | **K** | **D** | **N** | **Q** | **Y** | **T** | **F** | **T** | **Y** | **K** | **L** | **S** | **M** | **S** | **T** | **P** | **L** | **G** | **E** | **M** | **K** | **D** | **L** | **S** | **Y** | **Q** | **S** | **S** | **I** | **A** | **K** | **K** | **G** | **D** | **T** | **Y** | **Q** | **I** | **S** | **W** | **K** | **P** | **S** | **L** | **I** | **F** | **P** | **D** | **M** | **S** | **G** | **N** | **D** | **K** | **I** | **S** | **I** | **Q** | **V** | **D** | **N** | **A** | **K** | **R** | **G** | **E** | **I** | **V** | **D** | **R** | **N** | **G** | **S** | **G** | **L** | **A** | **I** | **N** | **K** | **V** | **F** | **D** | **E** | **V** | **G** | **V** | **V** | **P** | **G** | **K** | **L** | **G** | **S** | **G** | **A** | **E** | **K** | **T** | **A** | **N** | **I** | **K** | **A** | **F** | **S** | **D** | **K** | **F** | **G** | **V** | **S** | **V** | **D** | **E** | **I** | **N** | **Q** | **K** | **L** | **S** | **Q** | **G** | **W** | **V** | **Q** | **A** | **D** | **S** | **F** | **V** | **P** | **I** | **T** | **V** | **A** | **S** | **E** | **P** | **V** | **T** | **E** | **L** | **P** | **T** | **G** | **A** | **A** | **T** | **K** | **D** | **T** | **E** | **S** | **R** | **Y** | **Y** | **P** | **L** | **G** | **E** | **A** | **A** | **A** | **Q** | **L** | **I** | **G** | **Y** | **T** | **G** | **T** | **I** | **T** | **A** | **E** | **D** | **I** | **E** | **K** | **N** | **P** | **E** | **L** | **S** | **S** | **T** | **G** | **V** | **I** | **G** | **K** | **T** | **G** | **L** | **E** | **R** | **A** | **F** | **D** | **K** | **E** | **L** | **R** | **G** | **Q** | **D** | **G** | **G** | **S** | **L** | **V** | **I** | **L** | **D** | **D** | **K** | **E** | **N** | **V** | **K** | **K** | **A** | **L** | **Q** | **T** | **K** | **E** | **K** | **K** | **D** | **G** | **Q** | **T** | **I** | **K** | **L** | **T** | **I** | **D** | **S** | **G** | **V** | **Q** | **Q** | **Q** | **A** | **F** | **A** | **I** | **F** | **D** | **K** | **R** | **P** | **G** | **S** | **A** | **V** | **I** | **T** | **D** | **P** | **Q** | **K** | **G** | **D** | **L** | **L** | **A** | **T** | **V** | **S** | **S** | **P** | **S** | **Y** | **D** | **P** | **N** | **K** | **M** | **A** | **N** | **G** | **I** | **S** | **Q** | **K** | **E** | **Y** | **D** | **A** | **Y** | **N** | **N** | **N** | **K** | **D** | **L** | **P** | **F** | **T** | **A** | **R** | **F** | **A** | **T** | **G** | **Y** | **A** | **P** | **G** | **S** | **T** | **F** | **K** | **T** | **I** | **T** | **G** | **A** | **I** | **G** | **L** | **D** | **A** | **G** | **T** | **L** | **K** | **P** | **D** | **E** | **E** | **L** | **E** | **I** | **N** | **G** | **L** | **K** | **W** | **Q** | **K** | **D** | **K** | **S** | **W** | **G** | **G** | **Y** | **F** | **A** | **T** | **R** | **V** | **K** | **E** | **A** | **S** | **P** | **V** | **N** | **L** | **R** | **T** | **A** | **L** | **V** | **N** | **S** | **D** | **N** | **I** | **Y** | **F** | **A** | **Q** | **Q** | **T** | **L** | **R** | **M** | **G** | **E** | **D** | **K** | **F** | **R** | **A** | **G** | **L** | **N** | **K** | **F** | **I** | **F** | **G** | **E** | **E** | **L** | **D** | **L** | **P** | **I** | **A** | **M** | **T** | **P** | **A** | **Q** | **I** | **S** | **N** | **E** | **D** | **K** | **F** | **N** | **S** | **E** | **I** | **L** | **L** | **A** | **D** | **T** | **G** | **Y** | **G** | **Q** | **G** | **Q** | **L** | **L** | **I** | **S** | **P** | **I** | **Q** | **Q** | **A** | **T** | **M** | **Y** | **S** | **V** | **F** | **Q** | **N** | **N** | **G** | **T** | **L** | **V** | **Y** | **P** | **K** | **L** | **V** | **L** | **D** | **K** | **E** | **T** | **K** | **K** | **K** | **D** | **N** | **V** | **I** | **S** | **A** | **N** | **A** | **A** | **N** | **T** | **I** | **A** | **T** | **D** | **L** | **L** | **G** | **S** | **V** | **E** | **D** | **P** | **S** | **G** | **Y** | **V** | **Y** | **N** | **M** | **Y** | **N** | **P** | **N** | **F** | **S** | **L** | **A** | **A** | **K** | **T** | **G** | **T** | **A** | **E** | **I** | **K** | **D** | **K** | **Q** | **D** | **T** | **D** | **G** | **K** | **E** | **N** | **S** | **F** | **L** | **L** | **T** | **L** | **D** | **R** | **S** | **N** | **N** | **K** | **F** | **L** | **T** | **M** | **I** | **M** | **V** | **E** | **N** | **S** | **G** | **E** | **N** | **G** | **S** | **A** | **T** | **D** | **I** | **S** | **K** | **P** | **L** | **I** | **D** | **Y** | **L** | **E** | **A** | **T** | **I** | **K** | **-** | **-** | **-** | **-** | **-** | **-** | **-** | **-** | **-** | **-** | **-** | **-** | **-** | **-** | **-** | **-** | **-** | **-** | **-** | **-** | **-** | **-** | **-** | **-** | **-** | **-** | **-** | **-** | **-** | **-** | **-** | **-** | **-** | **-** | **-** | **-** | **Q** | **W** | **Q** | **A** | **K** | **Q** | **E** | **L** | **A** | **E** | **A** | **K** | **K** | **T** | **A** | **N** | **T** | **F** | **L** | **N** | **V** | **L** | **S** | **K** | **Q** | **E** | **F** | **D** | **K** | **L** | **P** | **S** | **V** | **V** | **Q** | **E** | **S** | **S** | **L** | **K** | **K** | **N** | **G** | **Y** | **D** | **T** | **K** | **S** | **V** | **V** | **E** | **K** | **Y** | **Q** | **A** | **I** | **Y** | **S** | **G** | **I** | **Q** | **A** | **E** | **G** | **V** | **K** | **A** | **S** | **D** | **V** | **Q** | **V** | **K** | **K** | **A** | **K** | **D** | **N** | **Q** | **Y** | **T** | **F** | **T** | **Y** | **K** | **L** | **S** | **M** | **S** | **T** | **P** | **L** | **G** | **E** | **M** | **K** | **D** | **L** | **S** | **Y** | **Q** | **S** | **S** | **I** | **A** | **K** | **K** | **G** | **D** | **T** | **Y** | **Q** | **I** | **S** | **W** | **K** | **P** | **S** | **L** | **I** | **F** | **P** | **D** | **M** | **S** | **G** | **N** | **D** | **K** | **I** | **S** | **I** | **Q** | **V** | **D** | **N** | **A** | **K** | **R** | **G** | **E** | **I** | **V** | **D** | **R** | **N** | **G** | **S** | **G** | **L** | **A** | **I** | **N** | **K** | **V** | **F** | **D** | **E** | **V** | **G** | **V** | **V** | **P** | **G** | **K** | **L** | **G** | **S** | **G** | **A** | **E** | **K** | **T** | **A** | **N** | **I** | **K** | **A** | **F** | **S** | **D** | **K** | **F** | **G** | **V** | **S** | **V** | **D** | **E** | **I** | **N** | **Q** | **K** | **L** | **S** | **Q** | **G** | **W** | **V** | **Q** | **A** | **D** | **S** | **F** | **V** | **P** | **I** | **T** | **V** | **A** | **S** | **E** | **P** | **V** | **T** | **E** | **L** | **P** | **T** | **G** | **A** | **A** | **T** | **K** | **D** | **T** | **E** | **S** | **R** | **Y** | **Y** | **P** | **L** | **G** | **E** | **A** | **A** | **A** | **Q** | **L** | **I** | **G** | **Y** | **T** | **G** | **T** | **I** | **T** | **A** | **E** | **D** | **I** | **E** | **K** | **N** | **P** | **E** | **L** | **S** | **S** | **T** | **G** | **V** | **I** | **G** | **K** | **T** | **G** | **L** | **E** | **R** | **A** | **F** | **D** | **K** | **E** | **L** | **R** | **G** | **Q** | **D** | **G** | **G** | **S** | **L** | **V** | **I** | **L** | **D** | **D** | **K** | **E** | **N** | **V** | **K** | **K** | **A** | **L** | **Q** | **T** | **K** | **E** | **K** | **K** | **D** | **G** | **Q** | **T** | **I** | **K** | **L** | **T** | **I** | **D** | **S** | **G** | **V** | **Q** | **Q** | **Q** | **A** | **F** | **A** | **I** | **F** | **D** | **K** | **R** | **P** | **G** | **S** | **A** | **V** | **I** | **T** | **D** | **P** | **Q** | **K** | **G** | **D** | **L** | **L** | **A** | **T** | **V** | **S** | **S** | **P** | **S** | **Y** | **D** | **P** | **N** | **K** | **M** | **A** | **N** | **G** | **I** | **S** | **Q** | **K** | **E** | **Y** | **D** | **A** | **Y** | **N** | **N** | **N** | **K** | **D** | **L** | **P** | **F** | **T** | **A** | **R** | **F** | **A** | **T** | **G** | **Y** | **A** | **P** | **G** | **S** | **T** | **F** | **K** | **T** | **I** | **T** | **G** | **A** | **I** | **G** | **L** | **D** | **A** | **G** | **T** | **L** | **K** | **P** | **D** | **E** | **E** | **L** | **E** | **I** | **N** | **G** | **L** | **K** | **W** | **Q** | **K** | **D** | **K** | **S** | **W** | **G** | **G** | **Y** | **F** | **A** | **T** | **R** | **V** | **K** | **E** | **A** | **S** | **P** | **V** | **N** | **L** | **R** | **T** | **A** | **L** | **V** | **N** | **S** | **D** | **N** | **I** | **Y** | **F** | **A** | **Q** | **Q** | **T** | **L** | **R** | **M** | **G** | **E** | **D** | **K** | **F** | **R** | **A** | **G** | **L** | **N** | **K** | **F** | **I** | **F** | **G** | **E** | **E** | **L** | **D** | **L** | **P** | **I** | **A** | **M** | **T** | **P** | **A** | **Q** | **I** | **S** | **N** | **E** | **D** | **K** | **F** | **N** | **S** | **E** | **I** | **L** | **L** | **A** | **D** | **T** | **G** | **Y** | **G** | **Q** | **G** | **Q** | **L** | **L** | **I** | **S** | **P** | **I** | **Q** | **Q** | **A** | **T** | **M** | **Y** | **S** | **V** | **F** | **Q** | **N** | **N** | **G** | **T** | **L** | **V** | **Y** | **P** | **K** | **L** | **V** | **L** | **D** | **K** | **E** | **T** | **K** | **K** | **K** | **D** | **N** | **V** | **I** | **S** | **A** | **N** | **A** | **A** | **N** | **T** | **I** | **A** | **T** | **D** | **L** | **L** | **G** | **S** | **V** | **E** | **D** | **P** | **S** | **G** | **Y** | **V** | **Y** | **N** | **M** | **Y** | **N** | **P** | **N** | **F** | **S** | **L** | **A** | **A** | **K** | **T** | **G** | **T** | **A** | **E** | **I** | **K** | **D** | **K** | **Q** | **D** | **T** | **D** | **G** | **K** | **E** | **N** | **S** | **F** | **L** | **L** | **T** | **L** | **D** | **R** | **S** | **N** | **N** | **K** | **F** | **L** | **T** | **M** | **I** | **M** | **V** | **E** | **N** | **S** | **G** | **E** | **N** | **G** | **S** | **A** | **T** | **D** | **I** | **S** | **K** | **P** | **L** | **I** | **D** | **Y** | **L** | **E** | **A** | **T** | **I** | **K** |
| **N59609F** | **-** | **-** | **-** | **-** | **-** | **-** | **-** | **-** | **-** | **-** | **-** | **-** | **-** | **-** | **-** | **-** | **-** | **-** | **-** | **-** | **-** | **-** | **-** | **-** | **-** | **-** | **-** | **-** | **-** | **-** | **-** | **-** | **-** | **-** | **-** | **-** | **Q** | **W** | **Q** | **A** | **K** | **Q** | **E** | **L** | **A** | **E** | **A** | **K** | **K** | **T** | **A** | **T** | **T** | **F** | **L** | **N** | **V** | **L** | **S** | **K** | **Q** | **E** | **F** | **D** | **K** | **L** | **P** | **S** | **V** | **V** | **Q** | **E** | **A** | **S** | **L** | **K** | **K** | **N** | **G** | **Y** | **D** | **T** | **K** | **S** | **V** | **V** | **E** | **K** | **Y** | **Q** | **A** | **I** | **Y** | **S** | **G** | **I** | **Q** | **A** | **E** | **G** | **V** | **K** | **A** | **S** | **D** | **V** | **Q** | **V** | **K** | **K** | **A** | **K** | **D** | **N** | **Q** | **Y** | **T** | **F** | **T** | **Y** | **K** | **L** | **S** | **M** | **S** | **T** | **P** | **L** | **G** | **E** | **M** | **K** | **D** | **L** | **S** | **Y** | **Q** | **S** | **S** | **I** | **A** | **K** | **K** | **G** | **D** | **T** | **Y** | **Q** | **I** | **A** | **W** | **N** | **P** | **S** | **L** | **I** | **F** | **P** | **D** | **M** | **S** | **G** | **N** | **D** | **K** | **V** | **S** | **I** | **Q** | **V** | **D** | **N** | **A** | **K** | **R** | **G** | **E** | **I** | **V** | **D** | **R** | **N** | **G** | **S** | **G** | **L** | **A** | **I** | **N** | **K** | **V** | **F** | **D** | **E** | **V** | **G** | **V** | **V** | **P** | **G** | **K** | **L** | **G** | **S** | **G** | **A** | **E** | **K** | **T** | **A** | **N** | **I** | **K** | **A** | **F** | **S** | **D** | **K** | **F** | **G** | **V** | **S** | **V** | **D** | **E** | **I** | **N** | **R** | **K** | **L** | **S** | **Q** | **G** | **W** | **V** | **Q** | **A** | **D** | **S** | **F** | **V** | **P** | **I** | **T** | **V** | **A** | **S** | **E** | **P** | **V** | **T** | **E** | **L** | **P** | **T** | **G** | **A** | **A** | **T** | **K** | **D** | **T** | **E** | **S** | **R** | **Y** | **Y** | **P** | **L** | **G** | **E** | **A** | **A** | **A** | **Q** | **L** | **I** | **G** | **Y** | **T** | **G** | **T** | **I** | **T** | **A** | **D** | **D** | **I** | **K** | **K** | **N** | **P** | **E** | **L** | **S** | **S** | **T** | **G** | **V** | **I** | **G** | **K** | **T** | **G** | **L** | **E** | **R** | **A** | **F** | **D** | **K** | **E** | **L** | **R** | **G** | **Q** | **D** | **G** | **G** | **S** | **L** | **V** | **I** | **L** | **D** | **D** | **K** | **E** | **N** | **V** | **K** | **K** | **A** | **L** | **Q** | **T** | **K** | **E** | **K** | **K** | **D** | **G** | **Q** | **T** | **I** | **K** | **L** | **T** | **I** | **D** | **S** | **G** | **V** | **Q** | **Q** | **Q** | **A** | **F** | **A** | **I** | **F** | **D** | **K** | **R** | **P** | **G** | **S** | **A** | **V** | **I** | **T** | **D** | **P** | **Q** | **K** | **G** | **D** | **L** | **L** | **A** | **T** | **V** | **S** | **S** | **P** | **S** | **Y** | **D** | **P** | **N** | **K** | **M** | **A** | **N** | **G** | **I** | **S** | **Q** | **K** | **E** | **Y** | **D** | **A** | **Y** | **N** | **N** | **N** | **K** | **D** | **L** | **P** | **F** | **T** | **A** | **R** | **F** | **A** | **T** | **G** | **Y** | **A** | **P** | **G** | **S** | **T** | **F** | **K** | **T** | **I** | **T** | **G** | **A** | **I** | **G** | **L** | **D** | **A** | **G** | **T** | **L** | **K** | **P** | **D** | **E** | **E** | **L** | **E** | **I** | **N** | **G** | **L** | **K** | **W** | **Q** | **K** | **D** | **K** | **S** | **W** | **G** | **G** | **Y** | **F** | **A** | **T** | **R** | **V** | **K** | **E** | **A** | **S** | **P** | **V** | **N** | **L** | **R** | **T** | **A** | **L** | **V** | **N** | **S** | **D** | **N** | **I** | **Y** | **F** | **A** | **Q** | **Q** | **T** | **L** | **R** | **M** | **G** | **E** | **D** | **K** | **F** | **R** | **A** | **G** | **L** | **N** | **K** | **F** | **I** | **F** | **G** | **E** | **E** | **L** | **D** | **L** | **P** | **I** | **A** | **M** | **T** | **P** | **A** | **Q** | **I** | **S** | **N** | **E** | **D** | **K** | **F** | **N** | **S** | **E** | **I** | **L** | **L** | **A** | **D** | **T** | **G** | **Y** | **G** | **Q** | **G** | **Q** | **L** | **L** | **I** | **S** | **P** | **I** | **Q** | **Q** | **A** | **T** | **M** | **Y** | **S** | **V** | **F** | **Q** | **N** | **N** | **G** | **T** | **L** | **V** | **Y** | **P** | **K** | **L** | **V** | **L** | **D** | **K** | **E** | **T** | **K** | **K** | **K** | **D** | **N** | **V** | **I** | **S** | **A** | **N** | **A** | **A** | **N** | **T** | **I** | **A** | **T** | **D** | **L** | **L** | **G** | **S** | **V** | **E** | **D** | **P** | **S** | **G** | **Y** | **V** | **Y** | **N** | **M** | **Y** | **N** | **P** | **N** | **F** | **S** | **L** | **A** | **A** | **K** | **T** | **G** | **T** | **A** | **E** | **I** | **K** | **D** | **K** | **Q** | **D** | **T** | **D** | **G** | **K** | **E** | **N** | **S** | **F** | **L** | **L** | **T** | **L** | **D** | **R** | **S** | **N** | **N** | **K** | **F** | **L** | **T** | **M** | **I** | **M** | **V** | **E** | **N** | **S** | **G** | **E** | **N** | **G** | **S** | **A** | **T** | **D** | **I** | **S** | **K** | **P** | **L** | **I** | **D** | **Y** | **L** | **E** | **A** | **T** | **I** | **K** | **-** | **-** | **-** | **-** | **-** | **-** | **-** | **-** | **-** | **-** | **-** | **-** | **-** | **-** | **-** | **-** | **-** | **-** | **-** | **-** | **-** | **-** | **-** | **-** | **-** | **-** | **-** | **-** | **-** | **-** | **-** | **-** | **-** | **-** | **-** | **-** | **Q** | **W** | **Q** | **A** | **K** | **Q** | **E** | **L** | **A** | **E** | **A** | **K** | **K** | **T** | **A** | **T** | **T** | **F** | **L** | **N** | **V** | **L** | **S** | **K** | **Q** | **E** | **F** | **D** | **K** | **L** | **P** | **S** | **V** | **V** | **Q** | **E** | **A** | **S** | **L** | **K** | **K** | **N** | **G** | **Y** | **D** | **T** | **K** | **S** | **V** | **V** | **E** | **K** | **Y** | **Q** | **A** | **I** | **Y** | **S** | **G** | **I** | **Q** | **A** | **E** | **G** | **V** | **K** | **A** | **S** | **D** | **V** | **Q** | **V** | **K** | **K** | **A** | **K** | **D** | **N** | **Q** | **Y** | **T** | **F** | **T** | **Y** | **K** | **L** | **S** | **M** | **S** | **T** | **P** | **L** | **G** | **E** | **M** | **K** | **D** | **L** | **S** | **Y** | **Q** | **S** | **S** | **I** | **A** | **K** | **K** | **G** | **D** | **T** | **Y** | **Q** | **I** | **A** | **W** | **N** | **P** | **S** | **L** | **I** | **F** | **P** | **D** | **M** | **S** | **G** | **N** | **D** | **K** | **V** | **S** | **I** | **Q** | **V** | **D** | **N** | **A** | **K** | **R** | **G** | **E** | **I** | **V** | **D** | **R** | **N** | **G** | **S** | **G** | **L** | **A** | **I** | **N** | **K** | **V** | **F** | **D** | **E** | **V** | **G** | **V** | **V** | **P** | **G** | **K** | **L** | **G** | **S** | **G** | **A** | **E** | **K** | **T** | **A** | **N** | **I** | **K** | **A** | **F** | **S** | **D** | **K** | **F** | **G** | **V** | **S** | **V** | **D** | **E** | **I** | **N** | **R** | **K** | **L** | **S** | **Q** | **G** | **W** | **V** | **Q** | **A** | **D** | **S** | **F** | **V** | **P** | **I** | **T** | **V** | **A** | **S** | **E** | **P** | **V** | **T** | **E** | **L** | **P** | **T** | **G** | **A** | **A** | **T** | **K** | **D** | **T** | **E** | **S** | **R** | **Y** | **Y** | **P** | **L** | **G** | **E** | **A** | **A** | **A** | **Q** | **L** | **I** | **G** | **Y** | **T** | **G** | **T** | **I** | **T** | **A** | **D** | **D** | **I** | **K** | **K** | **N** | **P** | **E** | **L** | **S** | **S** | **T** | **G** | **V** | **I** | **G** | **K** | **T** | **G** | **L** | **E** | **R** | **A** | **F** | **D** | **K** | **E** | **L** | **R** | **G** | **Q** | **D** | **G** | **G** | **S** | **L** | **V** | **I** | **L** | **D** | **D** | **K** | **E** | **N** | **V** | **K** | **K** | **A** | **L** | **Q** | **T** | **K** | **E** | **K** | **K** | **D** | **G** | **Q** | **T** | **I** | **K** | **L** | **T** | **I** | **D** | **S** | **G** | **V** | **Q** | **Q** | **Q** | **A** | **F** | **A** | **I** | **F** | **D** | **K** | **R** | **P** | **G** | **S** | **A** | **V** | **I** | **T** | **D** | **P** | **Q** | **K** | **G** | **D** | **L** | **L** | **A** | **T** | **V** | **S** | **S** | **P** | **S** | **Y** | **D** | **P** | **N** | **K** | **M** | **A** | **N** | **G** | **I** | **S** | **Q** | **K** | **E** | **Y** | **D** | **A** | **Y** | **N** | **N** | **N** | **K** | **D** | **L** | **P** | **F** | **T** | **A** | **R** | **F** | **A** | **T** | **G** | **Y** | **A** | **P** | **G** | **S** | **T** | **F** | **K** | **T** | **I** | **T** | **G** | **A** | **I** | **G** | **L** | **D** | **A** | **G** | **T** | **L** | **K** | **P** | **D** | **E** | **E** | **L** | **E** | **I** | **N** | **G** | **L** | **K** | **W** | **Q** | **K** | **D** | **K** | **S** | **W** | **G** | **G** | **Y** | **F** | **A** | **T** | **R** | **V** | **K** | **E** | **A** | **S** | **P** | **V** | **N** | **L** | **R** | **T** | **A** | **L** | **V** | **N** | **S** | **D** | **N** | **I** | **Y** | **F** | **A** | **Q** | **Q** | **T** | **L** | **R** | **M** | **G** | **E** | **D** | **K** | **F** | **R** | **A** | **G** | **L** | **N** | **K** | **F** | **I** | **F** | **G** | **E** | **E** | **L** | **D** | **L** | **P** | **I** | **A** | **M** | **T** | **P** | **A** | **Q** | **I** | **S** | **N** | **E** | **D** | **K** | **F** | **N** | **S** | **E** | **I** | **L** | **L** | **A** | **D** | **T** | **G** | **Y** | **G** | **Q** | **G** | **Q** | **L** | **L** | **I** | **S** | **P** | **I** | **Q** | **Q** | **A** | **T** | **M** | **Y** | **S** | **V** | **F** | **Q** | **N** | **N** | **G** | **T** | **L** | **V** | **Y** | **P** | **K** | **L** | **V** | **L** | **D** | **K** | **E** | **T** | **K** | **K** | **K** | **D** | **N** | **V** | **I** | **S** | **A** | **N** | **A** | **A** | **N** | **T** | **I** | **A** | **T** | **D** | **L** | **L** | **G** | **S** | **V** | **E** | **D** | **P** | **S** | **G** | **Y** | **V** | **Y** | **N** | **M** | **Y** | **N** | **P** | **N** | **F** | **S** | **L** | **A** | **A** | **K** | **T** | **G** | **T** | **A** | **E** | **I** | **K** | **D** | **K** | **Q** | **D** | **T** | **D** | **G** | **K** | **E** | **N** | **S** | **F** | **L** | **L** | **T** | **L** | **D** | **R** | **S** | **N** | **N** | **K** | **F** | **L** | **T** | **M** | **I** | **M** | **V** | **E** | **N** | **S** | **G** | **E** | **N** | **G** | **S** | **A** | **T** | **D** | **I** | **S** | **K** | **P** | **L** | **I** | **D** | **Y** | **L** | **E** | **A** | **T** | **I** | **K** |
| **N60440F** | **-** | **-** | **-** | **-** | **-** | **-** | **-** | **-** | **-** | **-** | **-** | **-** | **-** | **-** | **-** | **-** | **-** | **-** | **-** | **-** | **-** | **-** | **-** | **-** | **-** | **-** | **-** | **-** | **-** | **-** | **-** | **-** | **-** | **-** | **-** | **-** | **Q** | **W** | **Q** | **A** | **K** | **Q** | **E** | **L** | **A** | **E** | **A** | **K** | **K** | **T** | **A** | **T** | **T** | **F** | **L** | **N** | **V** | **L** | **S** | **K** | **Q** | **E** | **F** | **D** | **K** | **L** | **P** | **S** | **V** | **V** | **Q** | **E** | **A** | **S** | **L** | **K** | **K** | **N** | **G** | **Y** | **D** | **T** | **K** | **S** | **V** | **V** | **E** | **K** | **Y** | **Q** | **A** | **I** | **Y** | **S** | **G** | **I** | **Q** | **A** | **E** | **G** | **V** | **K** | **A** | **S** | **D** | **V** | **Q** | **V** | **K** | **K** | **A** | **K** | **D** | **N** | **Q** | **Y** | **T** | **F** | **T** | **Y** | **K** | **L** | **S** | **M** | **S** | **T** | **P** | **L** | **G** | **E** | **M** | **K** | **D** | **L** | **S** | **Y** | **Q** | **S** | **S** | **I** | **A** | **K** | **K** | **G** | **D** | **T** | **Y** | **Q** | **I** | **A** | **W** | **K** | **P** | **S** | **L** | **I** | **F** | **P** | **D** | **M** | **S** | **G** | **N** | **D** | **K** | **I** | **S** | **I** | **Q** | **V** | **D** | **N** | **A** | **K** | **R** | **G** | **E** | **I** | **V** | **D** | **R** | **N** | **G** | **S** | **G** | **L** | **A** | **I** | **N** | **K** | **V** | **F** | **D** | **E** | **V** | **G** | **V** | **V** | **P** | **G** | **K** | **L** | **G** | **S** | **G** | **A** | **E** | **K** | **T** | **A** | **N** | **I** | **K** | **A** | **F** | **S** | **D** | **K** | **F** | **G** | **V** | **S** | **V** | **D** | **E** | **I** | **N** | **Q** | **K** | **L** | **S** | **Q** | **G** | **W** | **V** | **Q** | **A** | **D** | **S** | **F** | **V** | **P** | **I** | **T** | **V** | **A** | **S** | **E** | **P** | **V** | **T** | **E** | **L** | **P** | **T** | **G** | **A** | **A** | **T** | **K** | **D** | **T** | **E** | **S** | **R** | **Y** | **Y** | **P** | **L** | **G** | **E** | **A** | **A** | **A** | **Q** | **L** | **I** | **G** | **Y** | **T** | **G** | **T** | **I** | **T** | **A** | **E** | **D** | **I** | **K** | **K** | **N** | **P** | **E** | **L** | **S** | **S** | **T** | **G** | **V** | **I** | **G** | **K** | **T** | **G** | **L** | **E** | **R** | **A** | **F** | **D** | **K** | **E** | **L** | **R** | **G** | **Q** | **D** | **G** | **G** | **S** | **L** | **V** | **I** | **L** | **D** | **D** | **K** | **E** | **N** | **V** | **K** | **K** | **A** | **L** | **Q** | **T** | **K** | **E** | **K** | **K** | **D** | **G** | **Q** | **T** | **I** | **K** | **L** | **T** | **I** | **D** | **S** | **G** | **V** | **Q** | **Q** | **Q** | **A** | **F** | **A** | **I** | **F** | **D** | **K** | **R** | **P** | **G** | **S** | **A** | **V** | **I** | **T** | **D** | **P** | **Q** | **K** | **G** | **D** | **L** | **L** | **A** | **T** | **V** | **S** | **S** | **P** | **S** | **Y** | **D** | **P** | **N** | **K** | **M** | **A** | **N** | **G** | **I** | **S** | **Q** | **K** | **E** | **Y** | **D** | **A** | **Y** | **N** | **N** | **N** | **K** | **D** | **L** | **P** | **F** | **T** | **A** | **R** | **F** | **A** | **T** | **G** | **Y** | **A** | **P** | **G** | **S** | **T** | **F** | **K** | **T** | **I** | **T** | **G** | **A** | **I** | **G** | **L** | **D** | **A** | **G** | **T** | **L** | **K** | **P** | **D** | **E** | **E** | **L** | **E** | **I** | **N** | **G** | **L** | **K** | **W** | **Q** | **K** | **D** | **K** | **S** | **W** | **G** | **G** | **Y** | **F** | **A** | **T** | **R** | **V** | **K** | **E** | **A** | **S** | **P** | **V** | **N** | **L** | **R** | **T** | **A** | **L** | **V** | **N** | **S** | **D** | **N** | **I** | **Y** | **F** | **A** | **Q** | **Q** | **T** | **L** | **R** | **M** | **G** | **E** | **D** | **K** | **F** | **R** | **A** | **G** | **L** | **N** | **K** | **F** | **I** | **F** | **G** | **E** | **E** | **L** | **D** | **L** | **P** | **I** | **A** | **M** | **T** | **P** | **A** | **Q** | **I** | **S** | **N** | **E** | **D** | **K** | **F** | **N** | **S** | **E** | **I** | **L** | **L** | **A** | **D** | **T** | **G** | **Y** | **G** | **Q** | **G** | **Q** | **L** | **L** | **I** | **S** | **P** | **I** | **Q** | **Q** | **A** | **T** | **M** | **Y** | **S** | **V** | **F** | **Q** | **N** | **N** | **G** | **T** | **L** | **V** | **Y** | **P** | **K** | **L** | **V** | **L** | **D** | **K** | **E** | **T** | **K** | **K** | **K** | **D** | **N** | **V** | **I** | **S** | **A** | **N** | **A** | **A** | **N** | **T** | **I** | **A** | **T** | **D** | **L** | **L** | **G** | **S** | **V** | **E** | **D** | **P** | **S** | **G** | **Y** | **V** | **Y** | **N** | **M** | **Y** | **N** | **P** | **N** | **F** | **S** | **L** | **A** | **A** | **K** | **T** | **G** | **T** | **A** | **E** | **I** | **K** | **D** | **K** | **Q** | **D** | **T** | **D** | **G** | **K** | **E** | **N** | **S** | **F** | **L** | **L** | **T** | **L** | **D** | **R** | **S** | **N** | **N** | **K** | **F** | **L** | **T** | **M** | **I** | **M** | **V** | **E** | **N** | **S** | **G** | **E** | **N** | **G** | **S** | **A** | **T** | **D** | **I** | **S** | **K** | **P** | **L** | **I** | **D** | **Y** | **L** | **E** | **A** | **T** | **I** | **K** | **-** | **-** | **-** | **-** | **-** | **-** | **-** | **-** | **-** | **-** | **-** | **-** | **-** | **-** | **-** | **-** | **-** | **-** | **-** | **-** | **-** | **-** | **-** | **-** | **-** | **-** | **-** | **-** | **-** | **-** | **-** | **-** | **-** | **-** | **-** | **-** | **Q** | **W** | **Q** | **A** | **K** | **Q** | **E** | **L** | **A** | **E** | **A** | **K** | **K** | **T** | **A** | **T** | **T** | **F** | **L** | **N** | **V** | **L** | **S** | **K** | **Q** | **E** | **F** | **D** | **K** | **L** | **P** | **S** | **V** | **V** | **Q** | **E** | **A** | **S** | **L** | **K** | **K** | **N** | **G** | **Y** | **D** | **T** | **K** | **S** | **V** | **V** | **E** | **K** | **Y** | **Q** | **A** | **I** | **Y** | **S** | **G** | **I** | **Q** | **A** | **E** | **G** | **V** | **K** | **A** | **S** | **D** | **V** | **Q** | **V** | **K** | **K** | **A** | **K** | **D** | **N** | **Q** | **Y** | **T** | **F** | **T** | **Y** | **K** | **L** | **S** | **M** | **S** | **T** | **P** | **L** | **G** | **E** | **M** | **K** | **D** | **L** | **S** | **Y** | **Q** | **S** | **S** | **I** | **A** | **K** | **K** | **G** | **D** | **T** | **Y** | **Q** | **I** | **A** | **W** | **K** | **P** | **S** | **L** | **I** | **F** | **P** | **D** | **M** | **S** | **G** | **N** | **D** | **K** | **I** | **S** | **I** | **Q** | **V** | **D** | **N** | **A** | **K** | **R** | **G** | **E** | **I** | **V** | **D** | **R** | **N** | **G** | **S** | **G** | **L** | **A** | **I** | **N** | **K** | **V** | **F** | **D** | **E** | **V** | **G** | **V** | **V** | **P** | **G** | **K** | **L** | **G** | **S** | **G** | **A** | **E** | **K** | **T** | **A** | **N** | **I** | **K** | **A** | **F** | **S** | **D** | **K** | **F** | **G** | **V** | **S** | **V** | **D** | **E** | **I** | **N** | **Q** | **K** | **L** | **S** | **Q** | **G** | **W** | **V** | **Q** | **A** | **D** | **S** | **F** | **V** | **P** | **I** | **T** | **V** | **A** | **S** | **E** | **P** | **V** | **T** | **E** | **L** | **P** | **T** | **G** | **A** | **A** | **T** | **K** | **D** | **T** | **E** | **S** | **R** | **Y** | **Y** | **P** | **L** | **G** | **E** | **A** | **A** | **A** | **Q** | **L** | **I** | **G** | **Y** | **T** | **G** | **T** | **I** | **T** | **A** | **E** | **D** | **I** | **K** | **K** | **N** | **P** | **E** | **L** | **S** | **S** | **T** | **G** | **V** | **I** | **G** | **K** | **T** | **G** | **L** | **E** | **R** | **A** | **F** | **D** | **K** | **E** | **L** | **R** | **G** | **Q** | **D** | **G** | **G** | **S** | **L** | **V** | **I** | **L** | **D** | **D** | **K** | **E** | **N** | **V** | **K** | **K** | **A** | **L** | **Q** | **T** | **K** | **E** | **K** | **K** | **D** | **G** | **Q** | **T** | **I** | **K** | **L** | **T** | **I** | **D** | **S** | **G** | **V** | **Q** | **Q** | **Q** | **A** | **F** | **A** | **I** | **F** | **D** | **K** | **R** | **P** | **G** | **S** | **A** | **V** | **I** | **T** | **D** | **P** | **Q** | **K** | **G** | **D** | **L** | **L** | **A** | **T** | **V** | **S** | **S** | **P** | **S** | **Y** | **D** | **P** | **N** | **K** | **M** | **A** | **N** | **G** | **I** | **S** | **Q** | **K** | **E** | **Y** | **D** | **A** | **Y** | **N** | **N** | **N** | **K** | **D** | **L** | **P** | **F** | **T** | **A** | **R** | **F** | **A** | **T** | **G** | **Y** | **A** | **P** | **G** | **S** | **T** | **F** | **K** | **T** | **I** | **T** | **G** | **A** | **I** | **G** | **L** | **D** | **A** | **G** | **T** | **L** | **K** | **P** | **D** | **E** | **E** | **L** | **E** | **I** | **N** | **G** | **L** | **K** | **W** | **Q** | **K** | **D** | **K** | **S** | **W** | **G** | **G** | **Y** | **F** | **A** | **T** | **R** | **V** | **K** | **E** | **A** | **S** | **P** | **V** | **N** | **L** | **R** | **T** | **A** | **L** | **V** | **N** | **S** | **D** | **N** | **I** | **Y** | **F** | **A** | **Q** | **Q** | **T** | **L** | **R** | **M** | **G** | **E** | **D** | **K** | **F** | **R** | **A** | **G** | **L** | **N** | **K** | **F** | **I** | **F** | **G** | **E** | **E** | **L** | **D** | **L** | **P** | **I** | **A** | **M** | **T** | **P** | **A** | **Q** | **I** | **S** | **N** | **E** | **D** | **K** | **F** | **N** | **S** | **E** | **I** | **L** | **L** | **A** | **D** | **T** | **G** | **Y** | **G** | **Q** | **G** | **Q** | **L** | **L** | **I** | **S** | **P** | **I** | **Q** | **Q** | **A** | **T** | **M** | **Y** | **S** | **V** | **F** | **Q** | **N** | **N** | **G** | **T** | **L** | **V** | **Y** | **P** | **K** | **L** | **V** | **L** | **D** | **K** | **E** | **T** | **K** | **K** | **K** | **D** | **N** | **V** | **I** | **S** | **A** | **N** | **A** | **A** | **N** | **T** | **I** | **A** | **T** | **D** | **L** | **L** | **G** | **S** | **V** | **E** | **D** | **P** | **S** | **G** | **Y** | **V** | **Y** | **N** | **M** | **Y** | **N** | **P** | **N** | **F** | **S** | **L** | **A** | **A** | **K** | **T** | **G** | **T** | **A** | **E** | **I** | **K** | **D** | **K** | **Q** | **D** | **T** | **D** | **G** | **K** | **E** | **N** | **S** | **F** | **L** | **L** | **T** | **L** | **D** | **R** | **S** | **N** | **N** | **K** | **F** | **L** | **T** | **M** | **I** | **M** | **V** | **E** | **N** | **S** | **G** | **E** | **N** | **G** | **S** | **A** | **T** | **D** | **I** | **S** | **K** | **P** | **L** | **I** | **D** | **Y** | **L** | **E** | **A** | **T** | **I** | **K** |
| **N55240** | **-** | **-** | **-** | **-** | **-** | **-** | **-** | **-** | **-** | **-** | **-** | **-** | **-** | **-** | **-** | **-** | **-** | **-** | **-** | **-** | **-** | **-** | **-** | **-** | **-** | **-** | **-** | **-** | **-** | **-** | **-** | **-** | **-** | **-** | **-** | **-** | **Q** | **W** | **Q** | **A** | **K** | **Q** | **E** | **L** | **A** | **E** | **A** | **K** | **K** | **T** | **A** | **T** | **T** | **F** | **L** | **N** | **V** | **L** | **S** | **K** | **Q** | **E** | **F** | **D** | **K** | **L** | **P** | **S** | **V** | **V** | **Q** | **E** | **A** | **S** | **L** | **K** | **K** | **N** | **G** | **Y** | **D** | **T** | **K** | **S** | **V** | **V** | **E** | **K** | **Y** | **Q** | **A** | **I** | **Y** | **S** | **G** | **I** | **Q** | **A** | **E** | **G** | **V** | **K** | **A** | **S** | **D** | **V** | **Q** | **V** | **K** | **K** | **A** | **K** | **D** | **N** | **Q** | **Y** | **T** | **F** | **T** | **Y** | **K** | **L** | **S** | **M** | **S** | **T** | **P** | **L** | **G** | **E** | **M** | **K** | **D** | **L** | **S** | **Y** | **Q** | **S** | **S** | **I** | **A** | **K** | **K** | **G** | **D** | **T** | **Y** | **Q** | **I** | **A** | **W** | **K** | **P** | **S** | **L** | **I** | **F** | **P** | **D** | **M** | **S** | **G** | **N** | **D** | **K** | **I** | **S** | **I** | **Q** | **V** | **D** | **N** | **A** | **K** | **R** | **G** | **E** | **I** | **V** | **D** | **R** | **N** | **G** | **S** | **G** | **L** | **A** | **I** | **N** | **K** | **V** | **F** | **D** | **E** | **V** | **G** | **V** | **V** | **P** | **G** | **K** | **L** | **G** | **S** | **G** | **A** | **E** | **K** | **T** | **A** | **N** | **I** | **K** | **A** | **F** | **S** | **D** | **K** | **F** | **G** | **V** | **S** | **V** | **D** | **E** | **I** | **N** | **Q** | **K** | **L** | **S** | **Q** | **G** | **W** | **V** | **Q** | **A** | **D** | **S** | **F** | **V** | **P** | **I** | **T** | **V** | **A** | **S** | **E** | **P** | **V** | **T** | **E** | **L** | **P** | **T** | **G** | **A** | **A** | **T** | **K** | **D** | **T** | **E** | **S** | **R** | **Y** | **Y** | **P** | **L** | **G** | **E** | **A** | **A** | **A** | **Q** | **L** | **I** | **G** | **Y** | **T** | **G** | **T** | **I** | **T** | **A** | **E** | **D** | **I** | **E** | **K** | **N** | **P** | **E** | **L** | **S** | **S** | **T** | **G** | **V** | **I** | **G** | **K** | **T** | **G** | **L** | **E** | **R** | **A** | **F** | **D** | **K** | **E** | **L** | **R** | **G** | **Q** | **D** | **G** | **G** | **S** | **L** | **V** | **I** | **L** | **D** | **D** | **K** | **E** | **N** | **V** | **K** | **K** | **A** | **L** | **Q** | **T** | **K** | **E** | **K** | **K** | **D** | **G** | **Q** | **T** | **I** | **K** | **L** | **T** | **I** | **D** | **S** | **G** | **V** | **Q** | **Q** | **Q** | **A** | **F** | **A** | **I** | **F** | **D** | **K** | **R** | **P** | **G** | **S** | **A** | **V** | **I** | **T** | **D** | **P** | **Q** | **K** | **G** | **D** | **L** | **L** | **A** | **T** | **V** | **S** | **S** | **P** | **S** | **Y** | **D** | **P** | **N** | **K** | **M** | **A** | **N** | **G** | **I** | **S** | **Q** | **K** | **E** | **Y** | **D** | **A** | **Y** | **N** | **N** | **N** | **K** | **D** | **L** | **P** | **F** | **T** | **A** | **R** | **F** | **A** | **T** | **G** | **Y** | **A** | **P** | **G** | **S** | **T** | **F** | **K** | **T** | **I** | **T** | **G** | **A** | **I** | **G** | **L** | **D** | **A** | **G** | **T** | **L** | **K** | **P** | **D** | **E** | **E** | **L** | **E** | **I** | **N** | **G** | **L** | **K** | **W** | **Q** | **K** | **D** | **K** | **S** | **W** | **G** | **G** | **Y** | **F** | **A** | **T** | **R** | **V** | **K** | **E** | **A** | **S** | **P** | **V** | **N** | **L** | **R** | **T** | **A** | **L** | **V** | **N** | **S** | **D** | **N** | **I** | **Y** | **F** | **A** | **Q** | **Q** | **T** | **L** | **R** | **M** | **G** | **E** | **D** | **K** | **F** | **R** | **A** | **G** | **L** | **N** | **K** | **F** | **I** | **F** | **G** | **E** | **E** | **L** | **D** | **L** | **P** | **I** | **A** | **M** | **T** | **P** | **A** | **Q** | **I** | **S** | **N** | **E** | **D** | **K** | **F** | **N** | **S** | **E** | **I** | **L** | **L** | **A** | **D** | **T** | **G** | **Y** | **G** | **Q** | **G** | **Q** | **L** | **L** | **I** | **S** | **P** | **I** | **Q** | **Q** | **A** | **T** | **M** | **Y** | **S** | **V** | **F** | **Q** | **N** | **N** | **G** | **T** | **L** | **V** | **Y** | **P** | **K** | **L** | **V** | **L** | **D** | **K** | **E** | **T** | **K** | **K** | **K** | **D** | **N** | **V** | **I** | **S** | **A** | **N** | **A** | **A** | **N** | **T** | **I** | **A** | **T** | **D** | **L** | **L** | **G** | **S** | **V** | **E** | **D** | **P** | **S** | **G** | **Y** | **V** | **Y** | **N** | **M** | **Y** | **N** | **P** | **N** | **F** | **S** | **L** | **A** | **A** | **K** | **T** | **G** | **T** | **A** | **E** | **I** | **K** | **D** | **K** | **Q** | **D** | **T** | **D** | **G** | **K** | **E** | **N** | **S** | **F** | **L** | **L** | **T** | **L** | **D** | **R** | **S** | **N** | **N** | **K** | **F** | **L** | **T** | **M** | **I** | **M** | **V** | **E** | **N** | **S** | **G** | **E** | **N** | **G** | **S** | **A** | **T** | **D** | **I** | **S** | **K** | **P** | **L** | **I** | **D** | **Y** | **L** | **E** | **A** | **T** | **I** | **K** | **-** | **-** | **-** | **-** | **-** | **-** | **-** | **-** | **-** | **-** | **-** | **-** | **-** | **-** | **-** | **-** | **-** | **-** | **-** | **-** | **-** | **-** | **-** | **-** | **-** | **-** | **-** | **-** | **-** | **-** | **-** | **-** | **-** | **-** | **-** | **-** | **Q** | **W** | **Q** | **A** | **K** | **Q** | **E** | **L** | **A** | **E** | **A** | **K** | **K** | **T** | **A** | **T** | **T** | **F** | **L** | **N** | **V** | **L** | **S** | **K** | **Q** | **E** | **F** | **D** | **K** | **L** | **P** | **S** | **V** | **V** | **Q** | **E** | **A** | **S** | **L** | **K** | **K** | **N** | **G** | **Y** | **D** | **T** | **K** | **S** | **V** | **V** | **E** | **K** | **Y** | **Q** | **A** | **I** | **Y** | **S** | **G** | **I** | **Q** | **A** | **E** | **G** | **V** | **K** | **A** | **S** | **D** | **V** | **Q** | **V** | **K** | **K** | **A** | **K** | **D** | **N** | **Q** | **Y** | **T** | **F** | **T** | **Y** | **K** | **L** | **S** | **M** | **S** | **T** | **P** | **L** | **G** | **E** | **M** | **K** | **D** | **L** | **S** | **Y** | **Q** | **S** | **S** | **I** | **A** | **K** | **K** | **G** | **D** | **T** | **Y** | **Q** | **I** | **A** | **W** | **K** | **P** | **S** | **L** | **I** | **F** | **P** | **D** | **M** | **S** | **G** | **N** | **D** | **K** | **I** | **S** | **I** | **Q** | **V** | **D** | **N** | **A** | **K** | **R** | **G** | **E** | **I** | **V** | **D** | **R** | **N** | **G** | **S** | **G** | **L** | **A** | **I** | **N** | **K** | **V** | **F** | **D** | **E** | **V** | **G** | **V** | **V** | **P** | **G** | **K** | **L** | **G** | **S** | **G** | **A** | **E** | **K** | **T** | **A** | **N** | **I** | **K** | **A** | **F** | **S** | **D** | **K** | **F** | **G** | **V** | **S** | **V** | **D** | **E** | **I** | **N** | **Q** | **K** | **L** | **S** | **Q** | **G** | **W** | **V** | **Q** | **A** | **D** | **S** | **F** | **V** | **P** | **I** | **T** | **V** | **A** | **S** | **E** | **P** | **V** | **T** | **E** | **L** | **P** | **T** | **G** | **A** | **A** | **T** | **K** | **D** | **T** | **E** | **S** | **R** | **Y** | **Y** | **P** | **L** | **G** | **E** | **A** | **A** | **A** | **Q** | **L** | **I** | **G** | **Y** | **T** | **G** | **T** | **I** | **T** | **A** | **E** | **D** | **I** | **E** | **K** | **N** | **P** | **E** | **L** | **S** | **S** | **T** | **G** | **V** | **I** | **G** | **K** | **T** | **G** | **L** | **E** | **R** | **A** | **F** | **D** | **K** | **E** | **L** | **R** | **G** | **Q** | **D** | **G** | **G** | **S** | **L** | **V** | **I** | **L** | **D** | **D** | **K** | **E** | **N** | **V** | **K** | **K** | **A** | **L** | **Q** | **T** | **K** | **E** | **K** | **K** | **D** | **G** | **Q** | **T** | **I** | **K** | **L** | **T** | **I** | **D** | **S** | **G** | **V** | **Q** | **Q** | **Q** | **A** | **F** | **A** | **I** | **F** | **D** | **K** | **R** | **P** | **G** | **S** | **A** | **V** | **I** | **T** | **D** | **P** | **Q** | **K** | **G** | **D** | **L** | **L** | **A** | **T** | **V** | **S** | **S** | **P** | **S** | **Y** | **D** | **P** | **N** | **K** | **M** | **A** | **N** | **G** | **I** | **S** | **Q** | **K** | **E** | **Y** | **D** | **A** | **Y** | **N** | **N** | **N** | **K** | **D** | **L** | **P** | **F** | **T** | **A** | **R** | **F** | **A** | **T** | **G** | **Y** | **A** | **P** | **G** | **S** | **T** | **F** | **K** | **T** | **I** | **T** | **G** | **A** | **I** | **G** | **L** | **D** | **A** | **G** | **T** | **L** | **K** | **P** | **D** | **E** | **E** | **L** | **E** | **I** | **N** | **G** | **L** | **K** | **W** | **Q** | **K** | **D** | **K** | **S** | **W** | **G** | **G** | **Y** | **F** | **A** | **T** | **R** | **V** | **K** | **E** | **A** | **S** | **P** | **V** | **N** | **L** | **R** | **T** | **A** | **L** | **V** | **N** | **S** | **D** | **N** | **I** | **Y** | **F** | **A** | **Q** | **Q** | **T** | **L** | **R** | **M** | **G** | **E** | **D** | **K** | **F** | **R** | **A** | **G** | **L** | **N** | **K** | **F** | **I** | **F** | **G** | **E** | **E** | **L** | **D** | **L** | **P** | **I** | **A** | **M** | **T** | **P** | **A** | **Q** | **I** | **S** | **N** | **E** | **D** | **K** | **F** | **N** | **S** | **E** | **I** | **L** | **L** | **A** | **D** | **T** | **G** | **Y** | **G** | **Q** | **G** | **Q** | **L** | **L** | **I** | **S** | **P** | **I** | **Q** | **Q** | **A** | **T** | **M** | **Y** | **S** | **V** | **F** | **Q** | **N** | **N** | **G** | **T** | **L** | **V** | **Y** | **P** | **K** | **L** | **V** | **L** | **D** | **K** | **E** | **T** | **K** | **K** | **K** | **D** | **N** | **V** | **I** | **S** | **A** | **N** | **A** | **A** | **N** | **T** | **I** | **A** | **T** | **D** | **L** | **L** | **G** | **S** | **V** | **E** | **D** | **P** | **S** | **G** | **Y** | **V** | **Y** | **N** | **M** | **Y** | **N** | **P** | **N** | **F** | **S** | **L** | **A** | **A** | **K** | **T** | **G** | **T** | **A** | **E** | **I** | **K** | **D** | **K** | **Q** | **D** | **T** | **D** | **G** | **K** | **E** | **N** | **S** | **F** | **L** | **L** | **T** | **L** | **D** | **R** | **S** | **N** | **N** | **K** | **F** | **L** | **T** | **M** | **I** | **M** | **V** | **E** | **N** | **S** | **G** | **E** | **N** | **G** | **S** | **A** | **T** | **D** | **I** | **S** | **K** | **P** | **L** | **I** | **D** | **Y** | **L** | **E** | **A** | **T** | **I** | **K** |
| **N55119** | **-** | **-** | **-** | **-** | **-** | **-** | **-** | **-** | **-** | **-** | **-** | **-** | **-** | **-** | **-** | **-** | **-** | **-** | **-** | **-** | **-** | **-** | **-** | **-** | **-** | **-** | **-** | **-** | **-** | **-** | **-** | **-** | **-** | **-** | **-** | **-** | **Q** | **W** | **Q** | **A** | **K** | **Q** | **E** | **L** | **A** | **E** | **A** | **K** | **K** | **T** | **A** | **T** | **T** | **F** | **L** | **N** | **V** | **L** | **T** | **K** | **Q** | **K** | **F** | **D** | **K** | **L** | **P** | **S** | **V** | **V** | **Q** | **E** | **A** | **S** | **L** | **K** | **K** | **N** | **G** | **Y** | **D** | **T** | **K** | **S** | **V** | **V** | **E** | **K** | **Y** | **Q** | **A** | **I** | **Y** | **S** | **G** | **I** | **Q** | **A** | **E** | **G** | **V** | **K** | **A** | **S** | **D** | **V** | **Q** | **V** | **K** | **K** | **A** | **K** | **D** | **N** | **Q** | **Y** | **T** | **F** | **T** | **Y** | **K** | **L** | **S** | **M** | **S** | **T** | **P** | **L** | **G** | **E** | **M** | **K** | **D** | **L** | **S** | **Y** | **Q** | **S** | **S** | **I** | **A** | **K** | **K** | **G** | **D** | **T** | **Y** | **Q** | **I** | **A** | **W** | **K** | **P** | **S** | **L** | **I** | **F** | **P** | **D** | **M** | **S** | **G** | **N** | **D** | **K** | **I** | **S** | **I** | **Q** | **V** | **D** | **N** | **A** | **K** | **R** | **G** | **E** | **I** | **V** | **D** | **R** | **N** | **G** | **S** | **G** | **L** | **A** | **I** | **N** | **K** | **V** | **F** | **D** | **E** | **V** | **G** | **V** | **V** | **P** | **G** | **K** | **L** | **G** | **S** | **G** | **A** | **E** | **K** | **T** | **A** | **N** | **I** | **K** | **A** | **F** | **S** | **D** | **K** | **F** | **G** | **V** | **S** | **V** | **D** | **E** | **I** | **N** | **Q** | **K** | **L** | **S** | **Q** | **G** | **W** | **V** | **Q** | **A** | **D** | **S** | **F** | **V** | **P** | **I** | **T** | **V** | **A** | **S** | **E** | **P** | **V** | **T** | **E** | **L** | **P** | **T** | **G** | **A** | **A** | **T** | **K** | **D** | **T** | **E** | **S** | **R** | **Y** | **Y** | **P** | **L** | **G** | **E** | **A** | **A** | **A** | **Q** | **L** | **I** | **G** | **Y** | **T** | **G** | **T** | **I** | **T** | **A** | **E** | **D** | **I** | **K** | **K** | **N** | **P** | **E** | **L** | **S** | **S** | **T** | **G** | **V** | **I** | **G** | **K** | **T** | **G** | **L** | **E** | **R** | **A** | **F** | **D** | **K** | **E** | **L** | **R** | **G** | **Q** | **D** | **G** | **G** | **S** | **L** | **V** | **I** | **L** | **D** | **D** | **K** | **E** | **N** | **V** | **K** | **K** | **A** | **L** | **Q** | **T** | **K** | **E** | **K** | **K** | **D** | **G** | **Q** | **T** | **I** | **K** | **L** | **T** | **I** | **D** | **S** | **G** | **V** | **Q** | **Q** | **Q** | **A** | **F** | **A** | **I** | **F** | **D** | **K** | **R** | **P** | **G** | **S** | **A** | **V** | **I** | **T** | **D** | **P** | **Q** | **K** | **G** | **D** | **L** | **L** | **A** | **T** | **V** | **S** | **S** | **P** | **S** | **Y** | **D** | **P** | **N** | **K** | **M** | **A** | **N** | **G** | **I** | **S** | **Q** | **K** | **E** | **Y** | **D** | **A** | **Y** | **N** | **N** | **N** | **K** | **D** | **L** | **P** | **F** | **T** | **A** | **R** | **F** | **A** | **T** | **G** | **Y** | **A** | **P** | **G** | **S** | **T** | **F** | **K** | **T** | **I** | **T** | **G** | **A** | **I** | **G** | **L** | **D** | **T** | **G** | **T** | **L** | **K** | **P** | **D** | **E** | **E** | **L** | **E** | **I** | **N** | **G** | **L** | **K** | **W** | **Q** | **K** | **D** | **K** | **S** | **W** | **G** | **G** | **Y** | **F** | **A** | **T** | **R** | **V** | **K** | **E** | **A** | **S** | **P** | **V** | **N** | **L** | **R** | **T** | **A** | **L** | **V** | **N** | **S** | **D** | **N** | **I** | **Y** | **F** | **A** | **Q** | **Q** | **T** | **L** | **R** | **M** | **G** | **E** | **D** | **K** | **F** | **R** | **A** | **G** | **L** | **N** | **K** | **F** | **I** | **F** | **G** | **E** | **E** | **L** | **D** | **L** | **P** | **I** | **A** | **M** | **T** | **P** | **A** | **Q** | **I** | **S** | **N** | **E** | **D** | **K** | **F** | **N** | **S** | **E** | **I** | **L** | **L** | **A** | **D** | **T** | **G** | **Y** | **G** | **Q** | **G** | **Q** | **L** | **L** | **I** | **S** | **P** | **I** | **Q** | **Q** | **A** | **T** | **M** | **Y** | **S** | **V** | **F** | **Q** | **N** | **N** | **G** | **T** | **L** | **V** | **Y** | **P** | **K** | **L** | **V** | **L** | **D** | **K** | **E** | **T** | **K** | **K** | **K** | **D** | **N** | **V** | **I** | **S** | **A** | **N** | **A** | **A** | **N** | **T** | **I** | **A** | **T** | **D** | **L** | **L** | **G** | **S** | **V** | **E** | **D** | **P** | **S** | **G** | **Y** | **V** | **Y** | **N** | **M** | **Y** | **N** | **P** | **N** | **F** | **S** | **L** | **A** | **A** | **K** | **T** | **G** | **T** | **A** | **E** | **I** | **K** | **D** | **K** | **Q** | **D** | **T** | **D** | **G** | **K** | **E** | **N** | **S** | **F** | **L** | **L** | **T** | **L** | **D** | **R** | **S** | **N** | **N** | **K** | **F** | **L** | **T** | **M** | **I** | **M** | **V** | **E** | **N** | **S** | **G** | **E** | **N** | **G** | **S** | **A** | **T** | **D** | **I** | **S** | **K** | **P** | **L** | **I** | **D** | **Y** | **L** | **E** | **A** | **T** | **I** | **K** | **-** | **-** | **-** | **-** | **-** | **-** | **-** | **-** | **-** | **-** | **-** | **-** | **-** | **-** | **-** | **-** | **-** | **-** | **-** | **-** | **-** | **-** | **-** | **-** | **-** | **-** | **-** | **-** | **-** | **-** | **-** | **-** | **-** | **-** | **-** | **-** | **Q** | **W** | **Q** | **A** | **K** | **Q** | **E** | **L** | **A** | **E** | **A** | **K** | **K** | **T** | **A** | **T** | **T** | **F** | **L** | **N** | **V** | **L** | **T** | **K** | **Q** | **K** | **F** | **D** | **K** | **L** | **P** | **S** | **V** | **V** | **Q** | **E** | **A** | **S** | **L** | **K** | **K** | **N** | **G** | **Y** | **D** | **T** | **K** | **S** | **V** | **V** | **E** | **K** | **Y** | **Q** | **A** | **I** | **Y** | **S** | **G** | **I** | **Q** | **A** | **E** | **G** | **V** | **K** | **A** | **S** | **D** | **V** | **Q** | **V** | **K** | **K** | **A** | **K** | **D** | **N** | **Q** | **Y** | **T** | **F** | **T** | **Y** | **K** | **L** | **S** | **M** | **S** | **T** | **P** | **L** | **G** | **E** | **M** | **K** | **D** | **L** | **S** | **Y** | **Q** | **S** | **S** | **I** | **A** | **K** | **K** | **G** | **D** | **T** | **Y** | **Q** | **I** | **A** | **W** | **K** | **P** | **S** | **L** | **I** | **F** | **P** | **D** | **M** | **S** | **G** | **N** | **D** | **K** | **I** | **S** | **I** | **Q** | **V** | **D** | **N** | **A** | **K** | **R** | **G** | **E** | **I** | **V** | **D** | **R** | **N** | **G** | **S** | **G** | **L** | **A** | **I** | **N** | **K** | **V** | **F** | **D** | **E** | **V** | **G** | **V** | **V** | **P** | **G** | **K** | **L** | **G** | **S** | **G** | **A** | **E** | **K** | **T** | **A** | **N** | **I** | **K** | **A** | **F** | **S** | **D** | **K** | **F** | **G** | **V** | **S** | **V** | **D** | **E** | **I** | **N** | **Q** | **K** | **L** | **S** | **Q** | **G** | **W** | **V** | **Q** | **A** | **D** | **S** | **F** | **V** | **P** | **I** | **T** | **V** | **A** | **S** | **E** | **P** | **V** | **T** | **E** | **L** | **P** | **T** | **G** | **A** | **A** | **T** | **K** | **D** | **T** | **E** | **S** | **R** | **Y** | **Y** | **P** | **L** | **G** | **E** | **A** | **A** | **A** | **Q** | **L** | **I** | **G** | **Y** | **T** | **G** | **T** | **I** | **T** | **A** | **E** | **D** | **I** | **K** | **K** | **N** | **P** | **E** | **L** | **S** | **S** | **T** | **G** | **V** | **I** | **G** | **K** | **T** | **G** | **L** | **E** | **R** | **A** | **F** | **D** | **K** | **E** | **L** | **R** | **G** | **Q** | **D** | **G** | **G** | **S** | **L** | **V** | **I** | **L** | **D** | **D** | **K** | **E** | **N** | **V** | **K** | **K** | **A** | **L** | **Q** | **T** | **K** | **E** | **K** | **K** | **D** | **G** | **Q** | **T** | **I** | **K** | **L** | **T** | **I** | **D** | **S** | **G** | **V** | **Q** | **Q** | **Q** | **A** | **F** | **A** | **I** | **F** | **D** | **K** | **R** | **P** | **G** | **S** | **A** | **V** | **I** | **T** | **D** | **P** | **Q** | **K** | **G** | **D** | **L** | **L** | **A** | **T** | **V** | **S** | **S** | **P** | **S** | **Y** | **D** | **P** | **N** | **K** | **M** | **A** | **N** | **G** | **I** | **S** | **Q** | **K** | **E** | **Y** | **D** | **A** | **Y** | **N** | **N** | **N** | **K** | **D** | **L** | **P** | **F** | **T** | **A** | **R** | **F** | **A** | **T** | **G** | **Y** | **A** | **P** | **G** | **S** | **T** | **F** | **K** | **T** | **I** | **T** | **G** | **A** | **I** | **G** | **L** | **D** | **T** | **G** | **T** | **L** | **K** | **P** | **D** | **E** | **E** | **L** | **E** | **I** | **N** | **G** | **L** | **K** | **W** | **Q** | **K** | **D** | **K** | **S** | **W** | **G** | **G** | **Y** | **F** | **A** | **T** | **R** | **V** | **K** | **E** | **A** | **S** | **P** | **V** | **N** | **L** | **R** | **T** | **A** | **L** | **V** | **N** | **S** | **D** | **N** | **I** | **Y** | **F** | **A** | **Q** | **Q** | **T** | **L** | **R** | **M** | **G** | **E** | **D** | **K** | **F** | **R** | **A** | **G** | **L** | **N** | **K** | **F** | **I** | **F** | **G** | **E** | **E** | **L** | **D** | **L** | **P** | **I** | **A** | **M** | **T** | **P** | **A** | **Q** | **I** | **S** | **N** | **E** | **D** | **K** | **F** | **N** | **S** | **E** | **I** | **L** | **L** | **A** | **D** | **T** | **G** | **Y** | **G** | **Q** | **G** | **Q** | **L** | **L** | **I** | **S** | **P** | **I** | **Q** | **Q** | **A** | **T** | **M** | **Y** | **S** | **V** | **F** | **Q** | **N** | **N** | **G** | **T** | **L** | **V** | **Y** | **P** | **K** | **L** | **V** | **L** | **D** | **K** | **E** | **T** | **K** | **K** | **K** | **D** | **N** | **V** | **I** | **S** | **A** | **N** | **A** | **A** | **N** | **T** | **I** | **A** | **T** | **D** | **L** | **L** | **G** | **S** | **V** | **E** | **D** | **P** | **S** | **G** | **Y** | **V** | **Y** | **N** | **M** | **Y** | **N** | **P** | **N** | **F** | **S** | **L** | **A** | **A** | **K** | **T** | **G** | **T** | **A** | **E** | **I** | **K** | **D** | **K** | **Q** | **D** | **T** | **D** | **G** | **K** | **E** | **N** | **S** | **F** | **L** | **L** | **T** | **L** | **D** | **R** | **S** | **N** | **N** | **K** | **F** | **L** | **T** | **M** | **I** | **M** | **V** | **E** | **N** | **S** | **G** | **E** | **N** | **G** | **S** | **A** | **T** | **D** | **I** | **S** | **K** | **P** | **L** | **I** | **D** | **Y** | **L** | **E** | **A** | **T** | **I** | **K** |
| **N60114F** | **-** | **-** | **-** | **-** | **-** | **-** | **-** | **-** | **-** | **-** | **-** | **-** | **-** | **-** | **-** | **-** | **-** | **-** | **-** | **-** | **-** | **-** | **-** | **-** | **-** | **-** | **-** | **-** | **-** | **-** | **-** | **-** | **-** | **-** | **-** | **-** | **Q** | **W** | **Q** | **A** | **K** | **Q** | **E** | **L** | **A** | **E** | **A** | **K** | **K** | **T** | **A** | **T** | **T** | **F** | **L** | **N** | **V** | **L** | **S** | **K** | **Q** | **E** | **F** | **D** | **K** | **L** | **P** | **S** | **V** | **V** | **Q** | **E** | **A** | **S** | **L** | **K** | **K** | **N** | **G** | **Y** | **D** | **T** | **K** | **S** | **V** | **V** | **E** | **K** | **Y** | **Q** | **A** | **I** | **Y** | **S** | **G** | **I** | **Q** | **A** | **E** | **G** | **V** | **K** | **A** | **S** | **D** | **V** | **Q** | **V** | **K** | **K** | **A** | **K** | **D** | **N** | **Q** | **Y** | **T** | **F** | **T** | **Y** | **K** | **L** | **S** | **M** | **S** | **T** | **P** | **L** | **G** | **E** | **M** | **K** | **D** | **L** | **S** | **Y** | **Q** | **S** | **S** | **I** | **A** | **K** | **K** | **G** | **D** | **T** | **Y** | **Q** | **I** | **A** | **W** | **K** | **P** | **S** | **L** | **I** | **F** | **P** | **D** | **M** | **S** | **G** | **N** | **D** | **K** | **I** | **S** | **I** | **Q** | **V** | **D** | **N** | **A** | **K** | **R** | **G** | **E** | **I** | **V** | **D** | **R** | **N** | **G** | **S** | **G** | **L** | **A** | **I** | **N** | **K** | **V** | **F** | **D** | **E** | **V** | **G** | **V** | **V** | **P** | **G** | **K** | **L** | **G** | **S** | **G** | **A** | **E** | **K** | **T** | **A** | **N** | **I** | **K** | **A** | **F** | **S** | **D** | **K** | **F** | **G** | **V** | **S** | **V** | **D** | **E** | **I** | **N** | **Q** | **K** | **L** | **S** | **Q** | **G** | **W** | **V** | **Q** | **A** | **D** | **S** | **F** | **V** | **P** | **I** | **T** | **V** | **A** | **S** | **E** | **P** | **V** | **T** | **E** | **L** | **P** | **T** | **G** | **A** | **A** | **T** | **K** | **D** | **T** | **E** | **S** | **R** | **Y** | **Y** | **P** | **L** | **G** | **E** | **A** | **A** | **A** | **Q** | **L** | **I** | **G** | **Y** | **T** | **G** | **T** | **I** | **T** | **A** | **E** | **D** | **I** | **E** | **K** | **N** | **P** | **E** | **L** | **S** | **S** | **T** | **G** | **V** | **I** | **G** | **K** | **T** | **G** | **L** | **E** | **R** | **A** | **F** | **D** | **K** | **E** | **L** | **R** | **G** | **Q** | **D** | **G** | **G** | **S** | **L** | **V** | **I** | **L** | **D** | **D** | **K** | **E** | **N** | **V** | **K** | **K** | **A** | **L** | **Q** | **T** | **K** | **E** | **K** | **K** | **D** | **G** | **Q** | **T** | **I** | **K** | **L** | **T** | **I** | **D** | **S** | **G** | **V** | **Q** | **Q** | **Q** | **A** | **F** | **A** | **I** | **F** | **D** | **K** | **R** | **P** | **G** | **S** | **A** | **V** | **I** | **T** | **D** | **P** | **Q** | **K** | **G** | **D** | **L** | **L** | **A** | **T** | **V** | **S** | **S** | **P** | **S** | **Y** | **D** | **P** | **N** | **K** | **M** | **A** | **N** | **G** | **I** | **S** | **Q** | **K** | **E** | **Y** | **D** | **A** | **Y** | **N** | **N** | **N** | **K** | **D** | **L** | **P** | **F** | **T** | **A** | **R** | **F** | **A** | **T** | **G** | **Y** | **A** | **P** | **G** | **S** | **T** | **F** | **K** | **T** | **I** | **T** | **G** | **A** | **I** | **G** | **L** | **D** | **A** | **G** | **T** | **L** | **K** | **P** | **D** | **E** | **E** | **L** | **E** | **I** | **N** | **G** | **L** | **K** | **W** | **Q** | **K** | **D** | **K** | **S** | **W** | **G** | **G** | **Y** | **F** | **A** | **T** | **R** | **V** | **K** | **E** | **A** | **S** | **P** | **V** | **N** | **L** | **R** | **T** | **A** | **L** | **V** | **N** | **S** | **D** | **N** | **I** | **Y** | **F** | **A** | **Q** | **Q** | **T** | **L** | **R** | **M** | **G** | **E** | **D** | **K** | **F** | **R** | **A** | **G** | **L** | **N** | **K** | **F** | **I** | **F** | **G** | **E** | **E** | **L** | **D** | **L** | **P** | **I** | **A** | **M** | **T** | **P** | **A** | **Q** | **I** | **S** | **N** | **E** | **D** | **K** | **F** | **N** | **S** | **E** | **I** | **L** | **L** | **A** | **D** | **T** | **G** | **Y** | **G** | **Q** | **G** | **Q** | **L** | **L** | **I** | **S** | **P** | **I** | **Q** | **Q** | **A** | **T** | **M** | **Y** | **S** | **V** | **F** | **Q** | **N** | **N** | **G** | **T** | **L** | **V** | **Y** | **P** | **K** | **L** | **V** | **L** | **D** | **K** | **E** | **T** | **K** | **K** | **K** | **D** | **N** | **V** | **I** | **S** | **A** | **N** | **A** | **A** | **N** | **T** | **I** | **A** | **T** | **D** | **L** | **L** | **G** | **S** | **V** | **E** | **D** | **P** | **S** | **G** | **Y** | **V** | **Y** | **N** | **M** | **Y** | **N** | **P** | **N** | **F** | **S** | **L** | **A** | **A** | **K** | **T** | **G** | **T** | **A** | **E** | **I** | **K** | **D** | **K** | **Q** | **D** | **T** | **D** | **G** | **K** | **E** | **N** | **S** | **F** | **L** | **L** | **T** | **L** | **D** | **R** | **S** | **N** | **N** | **K** | **F** | **L** | **T** | **M** | **I** | **M** | **V** | **E** | **N** | **S** | **G** | **E** | **N** | **G** | **S** | **A** | **T** | **D** | **I** | **S** | **K** | **P** | **L** | **I** | **D** | **Y** | **L** | **E** | **A** | **T** | **I** | **K** | **-** | **-** | **-** | **-** | **-** | **-** | **-** | **-** | **-** | **-** | **-** | **-** | **-** | **-** | **-** | **-** | **-** | **-** | **-** | **-** | **-** | **-** | **-** | **-** | **-** | **-** | **-** | **-** | **-** | **-** | **-** | **-** | **-** | **-** | **-** | **-** | **Q** | **W** | **Q** | **A** | **K** | **Q** | **E** | **L** | **A** | **E** | **A** | **K** | **K** | **T** | **A** | **T** | **T** | **F** | **L** | **N** | **V** | **L** | **S** | **K** | **Q** | **E** | **F** | **D** | **K** | **L** | **P** | **S** | **V** | **V** | **Q** | **E** | **A** | **S** | **L** | **K** | **K** | **N** | **G** | **Y** | **D** | **T** | **K** | **S** | **V** | **V** | **E** | **K** | **Y** | **Q** | **A** | **I** | **Y** | **S** | **G** | **I** | **Q** | **A** | **E** | **G** | **V** | **K** | **A** | **S** | **D** | **V** | **Q** | **V** | **K** | **K** | **A** | **K** | **D** | **N** | **Q** | **Y** | **T** | **F** | **T** | **Y** | **K** | **L** | **S** | **M** | **S** | **T** | **P** | **L** | **G** | **E** | **M** | **K** | **D** | **L** | **S** | **Y** | **Q** | **S** | **S** | **I** | **A** | **K** | **K** | **G** | **D** | **T** | **Y** | **Q** | **I** | **A** | **W** | **K** | **P** | **S** | **L** | **I** | **F** | **P** | **D** | **M** | **S** | **G** | **N** | **D** | **K** | **I** | **S** | **I** | **Q** | **V** | **D** | **N** | **A** | **K** | **R** | **G** | **E** | **I** | **V** | **D** | **R** | **N** | **G** | **S** | **G** | **L** | **A** | **I** | **N** | **K** | **V** | **F** | **D** | **E** | **V** | **G** | **V** | **V** | **P** | **G** | **K** | **L** | **G** | **S** | **G** | **A** | **E** | **K** | **T** | **A** | **N** | **I** | **K** | **A** | **F** | **S** | **D** | **K** | **F** | **G** | **V** | **S** | **V** | **D** | **E** | **I** | **N** | **Q** | **K** | **L** | **S** | **Q** | **G** | **W** | **V** | **Q** | **A** | **D** | **S** | **F** | **V** | **P** | **I** | **T** | **V** | **A** | **S** | **E** | **P** | **V** | **T** | **E** | **L** | **P** | **T** | **G** | **A** | **A** | **T** | **K** | **D** | **T** | **E** | **S** | **R** | **Y** | **Y** | **P** | **L** | **G** | **E** | **A** | **A** | **A** | **Q** | **L** | **I** | **G** | **Y** | **T** | **G** | **T** | **I** | **T** | **A** | **E** | **D** | **I** | **E** | **K** | **N** | **P** | **E** | **L** | **S** | **S** | **T** | **G** | **V** | **I** | **G** | **K** | **T** | **G** | **L** | **E** | **R** | **A** | **F** | **D** | **K** | **E** | **L** | **R** | **G** | **Q** | **D** | **G** | **G** | **S** | **L** | **V** | **I** | **L** | **D** | **D** | **K** | **E** | **N** | **V** | **K** | **K** | **A** | **L** | **Q** | **T** | **K** | **E** | **K** | **K** | **D** | **G** | **Q** | **T** | **I** | **K** | **L** | **T** | **I** | **D** | **S** | **G** | **V** | **Q** | **Q** | **Q** | **A** | **F** | **A** | **I** | **F** | **D** | **K** | **R** | **P** | **G** | **S** | **A** | **V** | **I** | **T** | **D** | **P** | **Q** | **K** | **G** | **D** | **L** | **L** | **A** | **T** | **V** | **S** | **S** | **P** | **S** | **Y** | **D** | **P** | **N** | **K** | **M** | **A** | **N** | **G** | **I** | **S** | **Q** | **K** | **E** | **Y** | **D** | **A** | **Y** | **N** | **N** | **N** | **K** | **D** | **L** | **P** | **F** | **T** | **A** | **R** | **F** | **A** | **T** | **G** | **Y** | **A** | **P** | **G** | **S** | **T** | **F** | **K** | **T** | **I** | **T** | **G** | **A** | **I** | **G** | **L** | **D** | **A** | **G** | **T** | **L** | **K** | **P** | **D** | **E** | **E** | **L** | **E** | **I** | **N** | **G** | **L** | **K** | **W** | **Q** | **K** | **D** | **K** | **S** | **W** | **G** | **G** | **Y** | **F** | **A** | **T** | **R** | **V** | **K** | **E** | **A** | **S** | **P** | **V** | **N** | **L** | **R** | **T** | **A** | **L** | **V** | **N** | **S** | **D** | **N** | **I** | **Y** | **F** | **A** | **Q** | **Q** | **T** | **L** | **R** | **M** | **G** | **E** | **D** | **K** | **F** | **R** | **A** | **G** | **L** | **N** | **K** | **F** | **I** | **F** | **G** | **E** | **E** | **L** | **D** | **L** | **P** | **I** | **A** | **M** | **T** | **P** | **A** | **Q** | **I** | **S** | **N** | **E** | **D** | **K** | **F** | **N** | **S** | **E** | **I** | **L** | **L** | **A** | **D** | **T** | **G** | **Y** | **G** | **Q** | **G** | **Q** | **L** | **L** | **I** | **S** | **P** | **I** | **Q** | **Q** | **A** | **T** | **M** | **Y** | **S** | **V** | **F** | **Q** | **N** | **N** | **G** | **T** | **L** | **V** | **Y** | **P** | **K** | **L** | **V** | **L** | **D** | **K** | **E** | **T** | **K** | **K** | **K** | **D** | **N** | **V** | **I** | **S** | **A** | **N** | **A** | **A** | **N** | **T** | **I** | **A** | **T** | **D** | **L** | **L** | **G** | **S** | **V** | **E** | **D** | **P** | **S** | **G** | **Y** | **V** | **Y** | **N** | **M** | **Y** | **N** | **P** | **N** | **F** | **S** | **L** | **A** | **A** | **K** | **T** | **G** | **T** | **A** | **E** | **I** | **K** | **D** | **K** | **Q** | **D** | **T** | **D** | **G** | **K** | **E** | **N** | **S** | **F** | **L** | **L** | **T** | **L** | **D** | **R** | **S** | **N** | **N** | **K** | **F** | **L** | **T** | **M** | **I** | **M** | **V** | **E** | **N** | **S** | **G** | **E** | **N** | **G** | **S** | **A** | **T** | **D** | **I** | **S** | **K** | **P** | **L** | **I** | **D** | **Y** | **L** | **E** | **A** | **T** | **I** | **K** |
| **N54118** | **-** | **-** | **-** | **-** | **-** | **-** | **-** | **-** | **-** | **-** | **-** | **-** | **-** | **-** | **-** | **-** | **-** | **-** | **-** | **-** | **-** | **-** | **-** | **-** | **-** | **-** | **-** | **-** | **-** | **-** | **-** | **-** | **-** | **-** | **-** | **-** | **Q** | **W** | **Q** | **A** | **K** | **Q** | **E** | **L** | **A** | **E** | **A** | **K** | **K** | **T** | **A** | **T** | **T** | **F** | **L** | **N** | **V** | **L** | **T** | **K** | **Q** | **K** | **F** | **D** | **K** | **L** | **P** | **S** | **V** | **V** | **Q** | **E** | **A** | **S** | **L** | **K** | **K** | **N** | **G** | **Y** | **D** | **T** | **K** | **S** | **V** | **V** | **E** | **K** | **Y** | **Q** | **A** | **I** | **Y** | **S** | **G** | **I** | **Q** | **A** | **E** | **G** | **V** | **K** | **A** | **S** | **D** | **V** | **Q** | **V** | **K** | **K** | **A** | **K** | **D** | **N** | **Q** | **Y** | **T** | **F** | **T** | **Y** | **K** | **L** | **S** | **M** | **S** | **T** | **P** | **L** | **G** | **E** | **M** | **K** | **D** | **L** | **S** | **Y** | **Q** | **S** | **S** | **I** | **A** | **K** | **K** | **G** | **D** | **T** | **Y** | **Q** | **I** | **A** | **W** | **K** | **P** | **S** | **L** | **I** | **F** | **P** | **D** | **M** | **S** | **G** | **N** | **D** | **K** | **I** | **S** | **I** | **Q** | **V** | **D** | **N** | **A** | **K** | **R** | **G** | **E** | **I** | **V** | **D** | **R** | **N** | **G** | **S** | **G** | **L** | **A** | **I** | **N** | **K** | **V** | **F** | **D** | **E** | **V** | **G** | **V** | **V** | **P** | **G** | **K** | **L** | **G** | **S** | **G** | **A** | **E** | **K** | **T** | **A** | **N** | **I** | **K** | **A** | **F** | **S** | **D** | **K** | **F** | **G** | **V** | **S** | **V** | **D** | **E** | **I** | **N** | **Q** | **K** | **L** | **S** | **Q** | **G** | **W** | **V** | **Q** | **A** | **D** | **S** | **F** | **V** | **P** | **I** | **T** | **V** | **A** | **S** | **E** | **P** | **V** | **T** | **E** | **L** | **P** | **T** | **G** | **A** | **A** | **T** | **K** | **D** | **T** | **E** | **S** | **R** | **Y** | **Y** | **P** | **L** | **G** | **E** | **A** | **A** | **A** | **Q** | **L** | **I** | **G** | **Y** | **T** | **G** | **T** | **I** | **T** | **A** | **E** | **D** | **I** | **K** | **K** | **N** | **P** | **E** | **L** | **S** | **S** | **T** | **G** | **V** | **I** | **G** | **K** | **T** | **G** | **L** | **E** | **R** | **A** | **F** | **D** | **K** | **E** | **L** | **R** | **G** | **Q** | **D** | **G** | **G** | **S** | **L** | **V** | **I** | **L** | **D** | **D** | **K** | **E** | **N** | **V** | **K** | **K** | **A** | **L** | **Q** | **T** | **K** | **E** | **K** | **K** | **D** | **G** | **Q** | **T** | **I** | **K** | **L** | **T** | **I** | **D** | **S** | **G** | **V** | **Q** | **Q** | **Q** | **A** | **F** | **A** | **I** | **F** | **D** | **K** | **R** | **P** | **G** | **S** | **A** | **V** | **I** | **T** | **D** | **P** | **Q** | **K** | **G** | **D** | **L** | **L** | **A** | **T** | **V** | **S** | **S** | **P** | **S** | **Y** | **D** | **P** | **N** | **K** | **M** | **A** | **N** | **G** | **I** | **S** | **Q** | **K** | **E** | **Y** | **D** | **A** | **Y** | **N** | **N** | **N** | **K** | **D** | **L** | **P** | **F** | **T** | **A** | **R** | **F** | **A** | **T** | **G** | **Y** | **A** | **P** | **G** | **S** | **T** | **F** | **K** | **T** | **I** | **T** | **G** | **A** | **I** | **G** | **L** | **D** | **T** | **G** | **T** | **L** | **K** | **P** | **D** | **E** | **E** | **L** | **E** | **I** | **N** | **G** | **L** | **K** | **W** | **Q** | **K** | **D** | **K** | **S** | **W** | **G** | **G** | **Y** | **F** | **A** | **T** | **R** | **V** | **K** | **E** | **A** | **S** | **P** | **V** | **N** | **L** | **R** | **T** | **A** | **L** | **V** | **N** | **S** | **D** | **N** | **I** | **Y** | **F** | **A** | **Q** | **Q** | **T** | **L** | **R** | **M** | **G** | **E** | **D** | **K** | **F** | **R** | **A** | **G** | **L** | **N** | **K** | **F** | **I** | **F** | **G** | **E** | **E** | **L** | **D** | **L** | **P** | **I** | **A** | **M** | **T** | **P** | **A** | **Q** | **I** | **S** | **N** | **E** | **D** | **K** | **F** | **N** | **S** | **E** | **I** | **L** | **L** | **A** | **D** | **T** | **G** | **Y** | **G** | **Q** | **G** | **Q** | **L** | **L** | **I** | **S** | **P** | **I** | **Q** | **Q** | **A** | **T** | **M** | **Y** | **S** | **V** | **F** | **Q** | **N** | **N** | **G** | **T** | **L** | **V** | **Y** | **P** | **K** | **L** | **V** | **L** | **D** | **K** | **E** | **T** | **K** | **K** | **K** | **D** | **N** | **V** | **I** | **S** | **A** | **N** | **A** | **A** | **N** | **T** | **I** | **A** | **T** | **D** | **L** | **L** | **G** | **S** | **V** | **E** | **D** | **P** | **S** | **G** | **Y** | **V** | **Y** | **N** | **M** | **Y** | **N** | **P** | **N** | **F** | **S** | **L** | **A** | **A** | **K** | **T** | **G** | **T** | **A** | **E** | **I** | **K** | **D** | **K** | **Q** | **D** | **T** | **D** | **G** | **K** | **E** | **N** | **S** | **F** | **L** | **L** | **T** | **L** | **D** | **R** | **S** | **N** | **N** | **K** | **F** | **L** | **T** | **M** | **I** | **M** | **V** | **E** | **N** | **S** | **G** | **E** | **N** | **G** | **S** | **A** | **T** | **D** | **I** | **S** | **K** | **P** | **L** | **I** | **D** | **Y** | **L** | **E** | **A** | **T** | **I** | **K** | **-** | **-** | **-** | **-** | **-** | **-** | **-** | **-** | **-** | **-** | **-** | **-** | **-** | **-** | **-** | **-** | **-** | **-** | **-** | **-** | **-** | **-** | **-** | **-** | **-** | **-** | **-** | **-** | **-** | **-** | **-** | **-** | **-** | **-** | **-** | **-** | **Q** | **W** | **Q** | **A** | **K** | **Q** | **E** | **L** | **A** | **E** | **A** | **K** | **K** | **T** | **A** | **T** | **T** | **F** | **L** | **N** | **V** | **L** | **T** | **K** | **Q** | **K** | **F** | **D** | **K** | **L** | **P** | **S** | **V** | **V** | **Q** | **E** | **A** | **S** | **L** | **K** | **K** | **N** | **G** | **Y** | **D** | **T** | **K** | **S** | **V** | **V** | **E** | **K** | **Y** | **Q** | **A** | **I** | **Y** | **S** | **G** | **I** | **Q** | **A** | **E** | **G** | **V** | **K** | **A** | **S** | **D** | **V** | **Q** | **V** | **K** | **K** | **A** | **K** | **D** | **N** | **Q** | **Y** | **T** | **F** | **T** | **Y** | **K** | **L** | **S** | **M** | **S** | **T** | **P** | **L** | **G** | **E** | **M** | **K** | **D** | **L** | **S** | **Y** | **Q** | **S** | **S** | **I** | **A** | **K** | **K** | **G** | **D** | **T** | **Y** | **Q** | **I** | **A** | **W** | **K** | **P** | **S** | **L** | **I** | **F** | **P** | **D** | **M** | **S** | **G** | **N** | **D** | **K** | **I** | **S** | **I** | **Q** | **V** | **D** | **N** | **A** | **K** | **R** | **G** | **E** | **I** | **V** | **D** | **R** | **N** | **G** | **S** | **G** | **L** | **A** | **I** | **N** | **K** | **V** | **F** | **D** | **E** | **V** | **G** | **V** | **V** | **P** | **G** | **K** | **L** | **G** | **S** | **G** | **A** | **E** | **K** | **T** | **A** | **N** | **I** | **K** | **A** | **F** | **S** | **D** | **K** | **F** | **G** | **V** | **S** | **V** | **D** | **E** | **I** | **N** | **Q** | **K** | **L** | **S** | **Q** | **G** | **W** | **V** | **Q** | **A** | **D** | **S** | **F** | **V** | **P** | **I** | **T** | **V** | **A** | **S** | **E** | **P** | **V** | **T** | **E** | **L** | **P** | **T** | **G** | **A** | **A** | **T** | **K** | **D** | **T** | **E** | **S** | **R** | **Y** | **Y** | **P** | **L** | **G** | **E** | **A** | **A** | **A** | **Q** | **L** | **I** | **G** | **Y** | **T** | **G** | **T** | **I** | **T** | **A** | **E** | **D** | **I** | **K** | **K** | **N** | **P** | **E** | **L** | **S** | **S** | **T** | **G** | **V** | **I** | **G** | **K** | **T** | **G** | **L** | **E** | **R** | **A** | **F** | **D** | **K** | **E** | **L** | **R** | **G** | **Q** | **D** | **G** | **G** | **S** | **L** | **V** | **I** | **L** | **D** | **D** | **K** | **E** | **N** | **V** | **K** | **K** | **A** | **L** | **Q** | **T** | **K** | **E** | **K** | **K** | **D** | **G** | **Q** | **T** | **I** | **K** | **L** | **T** | **I** | **D** | **S** | **G** | **V** | **Q** | **Q** | **Q** | **A** | **F** | **A** | **I** | **F** | **D** | **K** | **R** | **P** | **G** | **S** | **A** | **V** | **I** | **T** | **D** | **P** | **Q** | **K** | **G** | **D** | **L** | **L** | **A** | **T** | **V** | **S** | **S** | **P** | **S** | **Y** | **D** | **P** | **N** | **K** | **M** | **A** | **N** | **G** | **I** | **S** | **Q** | **K** | **E** | **Y** | **D** | **A** | **Y** | **N** | **N** | **N** | **K** | **D** | **L** | **P** | **F** | **T** | **A** | **R** | **F** | **A** | **T** | **G** | **Y** | **A** | **P** | **G** | **S** | **T** | **F** | **K** | **T** | **I** | **T** | **G** | **A** | **I** | **G** | **L** | **D** | **T** | **G** | **T** | **L** | **K** | **P** | **D** | **E** | **E** | **L** | **E** | **I** | **N** | **G** | **L** | **K** | **W** | **Q** | **K** | **D** | **K** | **S** | **W** | **G** | **G** | **Y** | **F** | **A** | **T** | **R** | **V** | **K** | **E** | **A** | **S** | **P** | **V** | **N** | **L** | **R** | **T** | **A** | **L** | **V** | **N** | **S** | **D** | **N** | **I** | **Y** | **F** | **A** | **Q** | **Q** | **T** | **L** | **R** | **M** | **G** | **E** | **D** | **K** | **F** | **R** | **A** | **G** | **L** | **N** | **K** | **F** | **I** | **F** | **G** | **E** | **E** | **L** | **D** | **L** | **P** | **I** | **A** | **M** | **T** | **P** | **A** | **Q** | **I** | **S** | **N** | **E** | **D** | **K** | **F** | **N** | **S** | **E** | **I** | **L** | **L** | **A** | **D** | **T** | **G** | **Y** | **G** | **Q** | **G** | **Q** | **L** | **L** | **I** | **S** | **P** | **I** | **Q** | **Q** | **A** | **T** | **M** | **Y** | **S** | **V** | **F** | **Q** | **N** | **N** | **G** | **T** | **L** | **V** | **Y** | **P** | **K** | **L** | **V** | **L** | **D** | **K** | **E** | **T** | **K** | **K** | **K** | **D** | **N** | **V** | **I** | **S** | **A** | **N** | **A** | **A** | **N** | **T** | **I** | **A** | **T** | **D** | **L** | **L** | **G** | **S** | **V** | **E** | **D** | **P** | **S** | **G** | **Y** | **V** | **Y** | **N** | **M** | **Y** | **N** | **P** | **N** | **F** | **S** | **L** | **A** | **A** | **K** | **T** | **G** | **T** | **A** | **E** | **I** | **K** | **D** | **K** | **Q** | **D** | **T** | **D** | **G** | **K** | **E** | **N** | **S** | **F** | **L** | **L** | **T** | **L** | **D** | **R** | **S** | **N** | **N** | **K** | **F** | **L** | **T** | **M** | **I** | **M** | **V** | **E** | **N** | **S** | **G** | **E** | **N** | **G** | **S** | **A** | **T** | **D** | **I** | **S** | **K** | **P** | **L** | **I** | **D** | **Y** | **L** | **E** | **A** | **T** | **I** | **K** |
| **N54548** | **M** | **E** | **R** | **S** | **N** | **R** | **N** | **K** | **K** | **S** | **S** | **K** | **K** | **P** | **L** | **I** | **L** | **G** | **V** | **S** | **A** | **L** | **V** | **L** | **I** | **A** | **A** | **V** | **G** | **G** | **G** | **Y** | **Y** | **A** | **Y** | **S** | **Q** | **W** | **Q** | **A** | **K** | **Q** | **E** | **L** | **A** | **E** | **A** | **K** | **K** | **T** | **A** | **N** | **T** | **F** | **L** | **N** | **V** | **L** | **S** | **K** | **Q** | **E** | **F** | **D** | **K** | **L** | **P** | **S** | **V** | **V** | **Q** | **E** | **T** | **S** | **L** | **K** | **K** | **N** | **G** | **Y** | **D** | **T** | **K** | **S** | **V** | **V** | **E** | **K** | **Y** | **Q** | **A** | **I** | **Y** | **S** | **G** | **I** | **Q** | **A** | **E** | **G** | **V** | **K** | **A** | **S** | **D** | **V** | **Q** | **V** | **K** | **K** | **G** | **K** | **D** | **N** | **Q** | **Y** | **T** | **F** | **T** | **Y** | **K** | **L** | **S** | **M** | **S** | **T** | **P** | **L** | **G** | **E** | **M** | **K** | **D** | **L** | **S** | **Y** | **Q** | **S** | **S** | **I** | **A** | **K** | **K** | **G** | **D** | **N** | **Y** | **Q** | **I** | **A** | **W** | **A** | **P** | **S** | **L** | **I** | **F** | **P** | **D** | **M** | **S** | **G** | **N** | **D** | **K** | **I** | **S** | **I** | **Q** | **V** | **D** | **N** | **A** | **K** | **R** | **G** | **E** | **I** | **V** | **D** | **R** | **N** | **G** | **S** | **G** | **L** | **A** | **I** | **N** | **K** | **V** | **F** | **D** | **E** | **V** | **G** | **V** | **V** | **P** | **G** | **K** | **L** | **G** | **S** | **G** | **A** | **E** | **K** | **T** | **A** | **N** | **I** | **K** | **A** | **F** | **S** | **D** | **K** | **F** | **G** | **V** | **S** | **V** | **D** | **E** | **I** | **N** | **Q** | **K** | **L** | **S** | **Q** | **G** | **W** | **V** | **Q** | **A** | **D** | **S** | **F** | **V** | **P** | **I** | **T** | **V** | **A** | **S** | **E** | **P** | **V** | **T** | **E** | **L** | **P** | **T** | **G** | **A** | **A** | **T** | **K** | **D** | **T** | **E** | **S** | **R** | **Y** | **Y** | **P** | **L** | **G** | **E** | **A** | **A** | **A** | **Q** | **L** | **I** | **G** | **Y** | **T** | **G** | **T** | **I** | **T** | **A** | **E** | **D** | **I** | **E** | **K** | **N** | **P** | **E** | **L** | **S** | **S** | **T** | **G** | **V** | **I** | **G** | **K** | **T** | **G** | **L** | **E** | **R** | **A** | **F** | **D** | **K** | **E** | **L** | **R** | **G** | **Q** | **D** | **G** | **G** | **S** | **L** | **V** | **I** | **L** | **D** | **D** | **K** | **E** | **N** | **V** | **K** | **K** | **A** | **L** | **Q** | **T** | **K** | **E** | **K** | **K** | **D** | **G** | **Q** | **T** | **I** | **K** | **L** | **T** | **I** | **D** | **S** | **G** | **V** | **Q** | **Q** | **Q** | **A** | **F** | **A** | **I** | **F** | **D** | **K** | **R** | **P** | **G** | **S** | **A** | **V** | **I** | **T** | **D** | **P** | **Q** | **K** | **G** | **D** | **L** | **L** | **A** | **T** | **V** | **S** | **S** | **P** | **S** | **Y** | **D** | **P** | **N** | **K** | **M** | **A** | **N** | **G** | **I** | **S** | **Q** | **K** | **E** | **Y** | **D** | **A** | **Y** | **N** | **N** | **N** | **K** | **D** | **L** | **P** | **F** | **T** | **A** | **R** | **F** | **A** | **T** | **G** | **Y** | **A** | **P** | **G** | **S** | **T** | **F** | **K** | **T** | **I** | **T** | **G** | **A** | **I** | **G** | **L** | **D** | **A** | **G** | **T** | **L** | **K** | **P** | **D** | **E** | **E** | **L** | **E** | **I** | **N** | **G** | **L** | **K** | **W** | **Q** | **K** | **D** | **K** | **S** | **W** | **G** | **G** | **Y** | **F** | **A** | **T** | **R** | **V** | **K** | **E** | **A** | **S** | **P** | **V** | **N** | **L** | **R** | **T** | **A** | **L** | **V** | **N** | **S** | **D** | **N** | **I** | **Y** | **F** | **A** | **Q** | **Q** | **T** | **L** | **R** | **M** | **G** | **E** | **D** | **K** | **F** | **R** | **A** | **G** | **L** | **N** | **K** | **F** | **I** | **F** | **G** | **E** | **E** | **L** | **D** | **L** | **P** | **I** | **A** | **M** | **T** | **P** | **A** | **Q** | **I** | **S** | **N** | **E** | **D** | **K** | **F** | **N** | **S** | **E** | **I** | **L** | **L** | **A** | **D** | **T** | **G** | **Y** | **G** | **Q** | **G** | **Q** | **L** | **L** | **I** | **S** | **P** | **I** | **Q** | **Q** | **A** | **T** | **M** | **Y** | **S** | **V** | **F** | **Q** | **N** | **N** | **G** | **T** | **L** | **V** | **Y** | **P** | **K** | **L** | **V** | **L** | **E** | **K** | **E** | **T** | **K** | **K** | **K** | **D** | **N** | **V** | **I** | **S** | **A** | **N** | **A** | **A** | **N** | **T** | **I** | **A** | **T** | **D** | **L** | **L** | **G** | **S** | **V** | **E** | **D** | **P** | **S** | **G** | **Y** | **V** | **Y** | **N** | **M** | **Y** | **N** | **P** | **N** | **F** | **S** | **L** | **A** | **A** | **K** | **T** | **G** | **T** | **A** | **E** | **I** | **K** | **D** | **K** | **Q** | **D** | **T** | **D** | **G** | **K** | **E** | **N** | **S** | **F** | **L** | **L** | **T** | **L** | **D** | **R** | **S** | **N** | **N** | **K** | **F** | **L** | **T** | **M** | **I** | **M** | **V** | **E** | **N** | **S** | **G** | **E** | **N** | **G** | **S** | **A** | **T** | **D** | **I** | **S** | **K** | **P** | **L** | **I** | **D** | **Y** | **L** | **E** | **A** | **T** | **I** | **K** | **M** | **E** | **R** | **S** | **N** | **R** | **N** | **K** | **K** | **S** | **S** | **K** | **K** | **P** | **L** | **I** | **L** | **G** | **V** | **S** | **A** | **L** | **V** | **L** | **I** | **A** | **A** | **V** | **G** | **G** | **G** | **Y** | **Y** | **A** | **Y** | **S** | **Q** | **W** | **Q** | **A** | **K** | **Q** | **E** | **L** | **A** | **E** | **A** | **K** | **K** | **T** | **A** | **N** | **T** | **F** | **L** | **N** | **V** | **L** | **S** | **K** | **Q** | **E** | **F** | **D** | **K** | **L** | **P** | **S** | **V** | **V** | **Q** | **E** | **T** | **S** | **L** | **K** | **K** | **N** | **G** | **Y** | **D** | **T** | **K** | **S** | **V** | **V** | **E** | **K** | **Y** | **Q** | **A** | **I** | **Y** | **S** | **G** | **I** | **Q** | **A** | **E** | **G** | **V** | **K** | **A** | **S** | **D** | **V** | **Q** | **V** | **K** | **K** | **G** | **K** | **D** | **N** | **Q** | **Y** | **T** | **F** | **T** | **Y** | **K** | **L** | **S** | **M** | **S** | **T** | **P** | **L** | **G** | **E** | **M** | **K** | **D** | **L** | **S** | **Y** | **Q** | **S** | **S** | **I** | **A** | **K** | **K** | **G** | **D** | **N** | **Y** | **Q** | **I** | **A** | **W** | **A** | **P** | **S** | **L** | **I** | **F** | **P** | **D** | **M** | **S** | **G** | **N** | **D** | **K** | **I** | **S** | **I** | **Q** | **V** | **D** | **N** | **A** | **K** | **R** | **G** | **E** | **I** | **V** | **D** | **R** | **N** | **G** | **S** | **G** | **L** | **A** | **I** | **N** | **K** | **V** | **F** | **D** | **E** | **V** | **G** | **V** | **V** | **P** | **G** | **K** | **L** | **G** | **S** | **G** | **A** | **E** | **K** | **T** | **A** | **N** | **I** | **K** | **A** | **F** | **S** | **D** | **K** | **F** | **G** | **V** | **S** | **V** | **D** | **E** | **I** | **N** | **Q** | **K** | **L** | **S** | **Q** | **G** | **W** | **V** | **Q** | **A** | **D** | **S** | **F** | **V** | **P** | **I** | **T** | **V** | **A** | **S** | **E** | **P** | **V** | **T** | **E** | **L** | **P** | **T** | **G** | **A** | **A** | **T** | **K** | **D** | **T** | **E** | **S** | **R** | **Y** | **Y** | **P** | **L** | **G** | **E** | **A** | **A** | **A** | **Q** | **L** | **I** | **G** | **Y** | **T** | **G** | **T** | **I** | **T** | **A** | **E** | **D** | **I** | **E** | **K** | **N** | **P** | **E** | **L** | **S** | **S** | **T** | **G** | **V** | **I** | **G** | **K** | **T** | **G** | **L** | **E** | **R** | **A** | **F** | **D** | **K** | **E** | **L** | **R** | **G** | **Q** | **D** | **G** | **G** | **S** | **L** | **V** | **I** | **L** | **D** | **D** | **K** | **E** | **N** | **V** | **K** | **K** | **A** | **L** | **Q** | **T** | **K** | **E** | **K** | **K** | **D** | **G** | **Q** | **T** | **I** | **K** | **L** | **T** | **I** | **D** | **S** | **G** | **V** | **Q** | **Q** | **Q** | **A** | **F** | **A** | **I** | **F** | **D** | **K** | **R** | **P** | **G** | **S** | **A** | **V** | **I** | **T** | **D** | **P** | **Q** | **K** | **G** | **D** | **L** | **L** | **A** | **T** | **V** | **S** | **S** | **P** | **S** | **Y** | **D** | **P** | **N** | **K** | **M** | **A** | **N** | **G** | **I** | **S** | **Q** | **K** | **E** | **Y** | **D** | **A** | **Y** | **N** | **N** | **N** | **K** | **D** | **L** | **P** | **F** | **T** | **A** | **R** | **F** | **A** | **T** | **G** | **Y** | **A** | **P** | **G** | **S** | **T** | **F** | **K** | **T** | **I** | **T** | **G** | **A** | **I** | **G** | **L** | **D** | **A** | **G** | **T** | **L** | **K** | **P** | **D** | **E** | **E** | **L** | **E** | **I** | **N** | **G** | **L** | **K** | **W** | **Q** | **K** | **D** | **K** | **S** | **W** | **G** | **G** | **Y** | **F** | **A** | **T** | **R** | **V** | **K** | **E** | **A** | **S** | **P** | **V** | **N** | **L** | **R** | **T** | **A** | **L** | **V** | **N** | **S** | **D** | **N** | **I** | **Y** | **F** | **A** | **Q** | **Q** | **T** | **L** | **R** | **M** | **G** | **E** | **D** | **K** | **F** | **R** | **A** | **G** | **L** | **N** | **K** | **F** | **I** | **F** | **G** | **E** | **E** | **L** | **D** | **L** | **P** | **I** | **A** | **M** | **T** | **P** | **A** | **Q** | **I** | **S** | **N** | **E** | **D** | **K** | **F** | **N** | **S** | **E** | **I** | **L** | **L** | **A** | **D** | **T** | **G** | **Y** | **G** | **Q** | **G** | **Q** | **L** | **L** | **I** | **S** | **P** | **I** | **Q** | **Q** | **A** | **T** | **M** | **Y** | **S** | **V** | **F** | **Q** | **N** | **N** | **G** | **T** | **L** | **V** | **Y** | **P** | **K** | **L** | **V** | **L** | **E** | **K** | **E** | **T** | **K** | **K** | **K** | **D** | **N** | **V** | **I** | **S** | **A** | **N** | **A** | **A** | **N** | **T** | **I** | **A** | **T** | **D** | **L** | **L** | **G** | **S** | **V** | **E** | **D** | **P** | **S** | **G** | **Y** | **V** | **Y** | **N** | **M** | **Y** | **N** | **P** | **N** | **F** | **S** | **L** | **A** | **A** | **K** | **T** | **G** | **T** | **A** | **E** | **I** | **K** | **D** | **K** | **Q** | **D** | **T** | **D** | **G** | **K** | **E** | **N** | **S** | **F** | **L** | **L** | **T** | **L** | **D** | **R** | **S** | **N** | **N** | **K** | **F** | **L** | **T** | **M** | **I** | **M** | **V** | **E** | **N** | **S** | **G** | **E** | **N** | **G** | **S** | **A** | **T** | **D** | **I** | **S** | **K** | **P** | **L** | **I** | **D** | **Y** | **L** | **E** | **A** | **T** | **I** | **K** |
| **N54588** | **-** | **-** | **-** | **-** | **-** | **-** | **-** | **-** | **-** | **-** | **-** | **-** | **-** | **-** | **-** | **-** | **-** | **-** | **-** | **-** | **-** | **-** | **-** | **-** | **-** | **-** | **-** | **-** | **-** | **-** | **-** | **-** | **-** | **-** | **-** | **-** | **Q** | **W** | **Q** | **A** | **K** | **Q** | **E** | **L** | **A** | **E** | **A** | **K** | **K** | **T** | **A** | **T** | **T** | **F** | **L** | **N** | **V** | **L** | **S** | **K** | **Q** | **E** | **F** | **D** | **K** | **L** | **P** | **S** | **V** | **V** | **Q** | **E** | **A** | **S** | **L** | **K** | **K** | **N** | **G** | **Y** | **D** | **T** | **K** | **S** | **V** | **V** | **E** | **K** | **Y** | **Q** | **A** | **I** | **Y** | **S** | **G** | **I** | **Q** | **A** | **E** | **G** | **V** | **K** | **A** | **S** | **D** | **V** | **Q** | **V** | **K** | **K** | **A** | **K** | **D** | **N** | **Q** | **Y** | **T** | **F** | **T** | **Y** | **K** | **L** | **S** | **M** | **S** | **T** | **P** | **L** | **G** | **E** | **M** | **K** | **D** | **L** | **S** | **Y** | **Q** | **S** | **S** | **I** | **A** | **K** | **K** | **G** | **D** | **T** | **Y** | **Q** | **I** | **A** | **W** | **K** | **P** | **S** | **L** | **I** | **F** | **P** | **D** | **M** | **S** | **G** | **N** | **D** | **K** | **I** | **S** | **I** | **Q** | **V** | **D** | **N** | **A** | **K** | **R** | **G** | **E** | **I** | **V** | **D** | **R** | **N** | **G** | **S** | **G** | **L** | **A** | **I** | **N** | **K** | **V** | **F** | **D** | **E** | **V** | **G** | **V** | **V** | **P** | **G** | **K** | **L** | **G** | **S** | **G** | **A** | **E** | **K** | **T** | **A** | **N** | **I** | **K** | **A** | **F** | **S** | **D** | **K** | **F** | **G** | **V** | **S** | **V** | **D** | **E** | **I** | **N** | **Q** | **K** | **L** | **S** | **Q** | **G** | **W** | **V** | **Q** | **A** | **D** | **S** | **F** | **V** | **P** | **I** | **T** | **V** | **A** | **S** | **E** | **P** | **V** | **T** | **E** | **L** | **P** | **T** | **G** | **A** | **A** | **T** | **K** | **D** | **T** | **E** | **S** | **R** | **Y** | **Y** | **P** | **L** | **G** | **E** | **A** | **A** | **A** | **Q** | **L** | **I** | **G** | **Y** | **T** | **G** | **T** | **I** | **T** | **A** | **E** | **D** | **I** | **E** | **K** | **N** | **P** | **E** | **L** | **S** | **S** | **T** | **G** | **V** | **I** | **G** | **K** | **T** | **G** | **L** | **E** | **R** | **A** | **F** | **D** | **K** | **E** | **L** | **R** | **G** | **Q** | **D** | **G** | **G** | **S** | **L** | **V** | **I** | **L** | **D** | **D** | **K** | **E** | **N** | **V** | **K** | **K** | **A** | **L** | **Q** | **T** | **K** | **E** | **K** | **K** | **D** | **G** | **Q** | **T** | **I** | **K** | **L** | **T** | **I** | **D** | **S** | **G** | **V** | **Q** | **Q** | **Q** | **A** | **F** | **A** | **I** | **F** | **D** | **K** | **R** | **P** | **G** | **S** | **A** | **V** | **I** | **T** | **D** | **P** | **Q** | **K** | **G** | **D** | **L** | **L** | **A** | **T** | **V** | **S** | **S** | **P** | **S** | **Y** | **D** | **P** | **N** | **K** | **M** | **A** | **N** | **G** | **I** | **S** | **Q** | **K** | **E** | **Y** | **D** | **A** | **Y** | **N** | **N** | **N** | **K** | **D** | **L** | **P** | **F** | **T** | **A** | **R** | **F** | **A** | **T** | **G** | **Y** | **A** | **P** | **G** | **S** | **T** | **F** | **K** | **T** | **I** | **T** | **G** | **A** | **I** | **G** | **L** | **D** | **A** | **G** | **T** | **L** | **K** | **P** | **D** | **E** | **E** | **L** | **E** | **I** | **N** | **G** | **L** | **K** | **W** | **Q** | **K** | **D** | **K** | **S** | **W** | **G** | **G** | **Y** | **F** | **A** | **T** | **R** | **V** | **K** | **E** | **A** | **S** | **P** | **V** | **N** | **L** | **R** | **T** | **A** | **L** | **V** | **N** | **S** | **D** | **N** | **I** | **Y** | **F** | **A** | **Q** | **Q** | **T** | **L** | **R** | **M** | **G** | **E** | **D** | **K** | **F** | **R** | **A** | **G** | **L** | **N** | **K** | **F** | **I** | **F** | **G** | **E** | **E** | **L** | **D** | **L** | **P** | **I** | **A** | **M** | **T** | **P** | **A** | **Q** | **I** | **S** | **N** | **E** | **D** | **K** | **F** | **N** | **S** | **E** | **I** | **L** | **L** | **A** | **D** | **T** | **G** | **Y** | **G** | **Q** | **G** | **Q** | **L** | **L** | **I** | **S** | **P** | **I** | **Q** | **Q** | **A** | **T** | **M** | **Y** | **S** | **V** | **F** | **Q** | **N** | **N** | **G** | **T** | **L** | **V** | **Y** | **P** | **K** | **L** | **V** | **L** | **D** | **K** | **E** | **T** | **K** | **K** | **K** | **D** | **N** | **V** | **I** | **S** | **A** | **N** | **A** | **A** | **N** | **T** | **I** | **A** | **T** | **D** | **L** | **L** | **G** | **S** | **V** | **E** | **D** | **P** | **S** | **G** | **Y** | **V** | **Y** | **N** | **M** | **Y** | **N** | **P** | **N** | **F** | **S** | **L** | **A** | **A** | **K** | **T** | **G** | **T** | **A** | **E** | **I** | **K** | **D** | **K** | **Q** | **D** | **T** | **D** | **G** | **K** | **E** | **N** | **S** | **F** | **L** | **L** | **T** | **L** | **D** | **R** | **S** | **N** | **N** | **K** | **F** | **L** | **T** | **M** | **I** | **M** | **V** | **E** | **N** | **S** | **G** | **E** | **N** | **G** | **S** | **A** | **T** | **D** | **I** | **S** | **K** | **P** | **L** | **I** | **D** | **Y** | **L** | **E** | **A** | **T** | **I** | **K** | **-** | **-** | **-** | **-** | **-** | **-** | **-** | **-** | **-** | **-** | **-** | **-** | **-** | **-** | **-** | **-** | **-** | **-** | **-** | **-** | **-** | **-** | **-** | **-** | **-** | **-** | **-** | **-** | **-** | **-** | **-** | **-** | **-** | **-** | **-** | **-** | **Q** | **W** | **Q** | **A** | **K** | **Q** | **E** | **L** | **A** | **E** | **A** | **K** | **K** | **T** | **A** | **T** | **T** | **F** | **L** | **N** | **V** | **L** | **S** | **K** | **Q** | **E** | **F** | **D** | **K** | **L** | **P** | **S** | **V** | **V** | **Q** | **E** | **A** | **S** | **L** | **K** | **K** | **N** | **G** | **Y** | **D** | **T** | **K** | **S** | **V** | **V** | **E** | **K** | **Y** | **Q** | **A** | **I** | **Y** | **S** | **G** | **I** | **Q** | **A** | **E** | **G** | **V** | **K** | **A** | **S** | **D** | **V** | **Q** | **V** | **K** | **K** | **A** | **K** | **D** | **N** | **Q** | **Y** | **T** | **F** | **T** | **Y** | **K** | **L** | **S** | **M** | **S** | **T** | **P** | **L** | **G** | **E** | **M** | **K** | **D** | **L** | **S** | **Y** | **Q** | **S** | **S** | **I** | **A** | **K** | **K** | **G** | **D** | **T** | **Y** | **Q** | **I** | **A** | **W** | **K** | **P** | **S** | **L** | **I** | **F** | **P** | **D** | **M** | **S** | **G** | **N** | **D** | **K** | **I** | **S** | **I** | **Q** | **V** | **D** | **N** | **A** | **K** | **R** | **G** | **E** | **I** | **V** | **D** | **R** | **N** | **G** | **S** | **G** | **L** | **A** | **I** | **N** | **K** | **V** | **F** | **D** | **E** | **V** | **G** | **V** | **V** | **P** | **G** | **K** | **L** | **G** | **S** | **G** | **A** | **E** | **K** | **T** | **A** | **N** | **I** | **K** | **A** | **F** | **S** | **D** | **K** | **F** | **G** | **V** | **S** | **V** | **D** | **E** | **I** | **N** | **Q** | **K** | **L** | **S** | **Q** | **G** | **W** | **V** | **Q** | **A** | **D** | **S** | **F** | **V** | **P** | **I** | **T** | **V** | **A** | **S** | **E** | **P** | **V** | **T** | **E** | **L** | **P** | **T** | **G** | **A** | **A** | **T** | **K** | **D** | **T** | **E** | **S** | **R** | **Y** | **Y** | **P** | **L** | **G** | **E** | **A** | **A** | **A** | **Q** | **L** | **I** | **G** | **Y** | **T** | **G** | **T** | **I** | **T** | **A** | **E** | **D** | **I** | **E** | **K** | **N** | **P** | **E** | **L** | **S** | **S** | **T** | **G** | **V** | **I** | **G** | **K** | **T** | **G** | **L** | **E** | **R** | **A** | **F** | **D** | **K** | **E** | **L** | **R** | **G** | **Q** | **D** | **G** | **G** | **S** | **L** | **V** | **I** | **L** | **D** | **D** | **K** | **E** | **N** | **V** | **K** | **K** | **A** | **L** | **Q** | **T** | **K** | **E** | **K** | **K** | **D** | **G** | **Q** | **T** | **I** | **K** | **L** | **T** | **I** | **D** | **S** | **G** | **V** | **Q** | **Q** | **Q** | **A** | **F** | **A** | **I** | **F** | **D** | **K** | **R** | **P** | **G** | **S** | **A** | **V** | **I** | **T** | **D** | **P** | **Q** | **K** | **G** | **D** | **L** | **L** | **A** | **T** | **V** | **S** | **S** | **P** | **S** | **Y** | **D** | **P** | **N** | **K** | **M** | **A** | **N** | **G** | **I** | **S** | **Q** | **K** | **E** | **Y** | **D** | **A** | **Y** | **N** | **N** | **N** | **K** | **D** | **L** | **P** | **F** | **T** | **A** | **R** | **F** | **A** | **T** | **G** | **Y** | **A** | **P** | **G** | **S** | **T** | **F** | **K** | **T** | **I** | **T** | **G** | **A** | **I** | **G** | **L** | **D** | **A** | **G** | **T** | **L** | **K** | **P** | **D** | **E** | **E** | **L** | **E** | **I** | **N** | **G** | **L** | **K** | **W** | **Q** | **K** | **D** | **K** | **S** | **W** | **G** | **G** | **Y** | **F** | **A** | **T** | **R** | **V** | **K** | **E** | **A** | **S** | **P** | **V** | **N** | **L** | **R** | **T** | **A** | **L** | **V** | **N** | **S** | **D** | **N** | **I** | **Y** | **F** | **A** | **Q** | **Q** | **T** | **L** | **R** | **M** | **G** | **E** | **D** | **K** | **F** | **R** | **A** | **G** | **L** | **N** | **K** | **F** | **I** | **F** | **G** | **E** | **E** | **L** | **D** | **L** | **P** | **I** | **A** | **M** | **T** | **P** | **A** | **Q** | **I** | **S** | **N** | **E** | **D** | **K** | **F** | **N** | **S** | **E** | **I** | **L** | **L** | **A** | **D** | **T** | **G** | **Y** | **G** | **Q** | **G** | **Q** | **L** | **L** | **I** | **S** | **P** | **I** | **Q** | **Q** | **A** | **T** | **M** | **Y** | **S** | **V** | **F** | **Q** | **N** | **N** | **G** | **T** | **L** | **V** | **Y** | **P** | **K** | **L** | **V** | **L** | **D** | **K** | **E** | **T** | **K** | **K** | **K** | **D** | **N** | **V** | **I** | **S** | **A** | **N** | **A** | **A** | **N** | **T** | **I** | **A** | **T** | **D** | **L** | **L** | **G** | **S** | **V** | **E** | **D** | **P** | **S** | **G** | **Y** | **V** | **Y** | **N** | **M** | **Y** | **N** | **P** | **N** | **F** | **S** | **L** | **A** | **A** | **K** | **T** | **G** | **T** | **A** | **E** | **I** | **K** | **D** | **K** | **Q** | **D** | **T** | **D** | **G** | **K** | **E** | **N** | **S** | **F** | **L** | **L** | **T** | **L** | **D** | **R** | **S** | **N** | **N** | **K** | **F** | **L** | **T** | **M** | **I** | **M** | **V** | **E** | **N** | **S** | **G** | **E** | **N** | **G** | **S** | **A** | **T** | **D** | **I** | **S** | **K** | **P** | **L** | **I** | **D** | **Y** | **L** | **E** | **A** | **T** | **I** | **K** |
| **N59420F** | **-** | **-** | **-** | **-** | **-** | **-** | **-** | **-** | **-** | **-** | **-** | **-** | **-** | **-** | **-** | **-** | **-** | **-** | **-** | **-** | **-** | **-** | **-** | **-** | **-** | **-** | **-** | **-** | **-** | **-** | **-** | **-** | **-** | **-** | **-** | **-** | **Q** | **W** | **Q** | **A** | **K** | **Q** | **E** | **L** | **A** | **E** | **A** | **K** | **K** | **T** | **A** | **T** | **T** | **F** | **L** | **N** | **V** | **L** | **S** | **K** | **Q** | **E** | **F** | **D** | **K** | **L** | **P** | **S** | **V** | **V** | **Q** | **E** | **A** | **S** | **L** | **K** | **K** | **N** | **G** | **Y** | **D** | **T** | **K** | **S** | **V** | **V** | **E** | **K** | **Y** | **Q** | **A** | **I** | **Y** | **S** | **G** | **I** | **Q** | **A** | **E** | **G** | **V** | **K** | **A** | **S** | **D** | **V** | **Q** | **V** | **K** | **K** | **A** | **K** | **D** | **N** | **Q** | **Y** | **T** | **F** | **T** | **Y** | **K** | **L** | **S** | **M** | **S** | **T** | **P** | **L** | **G** | **E** | **M** | **K** | **D** | **L** | **S** | **Y** | **Q** | **S** | **S** | **I** | **A** | **K** | **K** | **G** | **D** | **T** | **Y** | **Q** | **I** | **A** | **W** | **K** | **P** | **S** | **L** | **I** | **F** | **P** | **D** | **M** | **S** | **G** | **N** | **D** | **K** | **I** | **S** | **I** | **Q** | **V** | **D** | **N** | **A** | **K** | **R** | **G** | **E** | **I** | **V** | **D** | **R** | **N** | **G** | **S** | **G** | **L** | **A** | **I** | **N** | **K** | **V** | **F** | **D** | **E** | **V** | **G** | **V** | **V** | **P** | **G** | **K** | **L** | **G** | **S** | **G** | **A** | **E** | **K** | **T** | **A** | **N** | **I** | **K** | **A** | **F** | **S** | **D** | **K** | **F** | **G** | **V** | **S** | **V** | **D** | **E** | **I** | **N** | **Q** | **K** | **L** | **S** | **Q** | **G** | **W** | **V** | **Q** | **A** | **D** | **S** | **F** | **V** | **P** | **I** | **T** | **V** | **A** | **S** | **E** | **P** | **V** | **T** | **E** | **L** | **P** | **T** | **G** | **A** | **A** | **T** | **K** | **D** | **T** | **E** | **S** | **R** | **Y** | **Y** | **P** | **L** | **G** | **E** | **A** | **A** | **A** | **Q** | **L** | **I** | **G** | **Y** | **T** | **G** | **T** | **I** | **T** | **A** | **E** | **D** | **I** | **E** | **K** | **N** | **P** | **E** | **L** | **S** | **S** | **T** | **G** | **V** | **I** | **G** | **K** | **T** | **G** | **L** | **E** | **R** | **A** | **F** | **D** | **K** | **E** | **L** | **R** | **G** | **Q** | **D** | **G** | **G** | **S** | **L** | **V** | **I** | **L** | **D** | **D** | **K** | **E** | **N** | **V** | **K** | **K** | **A** | **L** | **Q** | **T** | **K** | **E** | **K** | **K** | **D** | **G** | **Q** | **T** | **I** | **K** | **L** | **T** | **I** | **D** | **S** | **G** | **V** | **Q** | **Q** | **Q** | **A** | **F** | **A** | **I** | **F** | **D** | **K** | **R** | **P** | **G** | **S** | **A** | **V** | **I** | **T** | **D** | **P** | **Q** | **K** | **G** | **D** | **L** | **L** | **A** | **T** | **V** | **S** | **S** | **P** | **S** | **Y** | **D** | **P** | **N** | **K** | **M** | **A** | **N** | **G** | **I** | **S** | **Q** | **K** | **E** | **Y** | **D** | **A** | **Y** | **N** | **N** | **N** | **K** | **D** | **L** | **P** | **F** | **T** | **A** | **R** | **F** | **A** | **T** | **G** | **Y** | **A** | **P** | **G** | **S** | **T** | **F** | **K** | **T** | **I** | **T** | **G** | **A** | **I** | **G** | **L** | **D** | **A** | **G** | **T** | **L** | **K** | **P** | **D** | **E** | **E** | **L** | **E** | **I** | **N** | **G** | **L** | **K** | **W** | **Q** | **K** | **D** | **K** | **S** | **W** | **G** | **G** | **Y** | **F** | **A** | **T** | **R** | **V** | **K** | **E** | **A** | **S** | **P** | **V** | **N** | **L** | **R** | **T** | **A** | **L** | **V** | **N** | **S** | **D** | **N** | **I** | **Y** | **F** | **A** | **Q** | **Q** | **T** | **L** | **R** | **M** | **G** | **E** | **D** | **K** | **F** | **R** | **A** | **G** | **L** | **N** | **K** | **F** | **I** | **F** | **G** | **E** | **E** | **L** | **D** | **L** | **P** | **I** | **A** | **M** | **T** | **P** | **A** | **Q** | **I** | **S** | **N** | **E** | **D** | **K** | **F** | **N** | **S** | **E** | **I** | **L** | **L** | **A** | **D** | **T** | **G** | **Y** | **G** | **Q** | **G** | **Q** | **L** | **L** | **I** | **S** | **P** | **I** | **Q** | **Q** | **A** | **T** | **M** | **Y** | **S** | **V** | **F** | **Q** | **N** | **N** | **G** | **T** | **L** | **V** | **Y** | **P** | **K** | **L** | **V** | **L** | **D** | **K** | **E** | **T** | **K** | **K** | **K** | **D** | **N** | **V** | **I** | **S** | **A** | **N** | **A** | **A** | **N** | **T** | **I** | **A** | **T** | **D** | **L** | **L** | **G** | **S** | **V** | **E** | **D** | **P** | **S** | **G** | **Y** | **V** | **Y** | **N** | **M** | **Y** | **N** | **P** | **N** | **F** | **S** | **L** | **A** | **A** | **K** | **T** | **G** | **T** | **A** | **E** | **I** | **K** | **D** | **K** | **Q** | **D** | **T** | **D** | **G** | **K** | **E** | **N** | **S** | **F** | **L** | **L** | **T** | **L** | **D** | **R** | **S** | **N** | **N** | **K** | **F** | **L** | **T** | **M** | **I** | **M** | **V** | **E** | **N** | **S** | **G** | **E** | **N** | **G** | **S** | **A** | **T** | **D** | **I** | **S** | **K** | **P** | **L** | **I** | **D** | **Y** | **L** | **E** | **A** | **T** | **I** | **K** | **-** | **-** | **-** | **-** | **-** | **-** | **-** | **-** | **-** | **-** | **-** | **-** | **-** | **-** | **-** | **-** | **-** | **-** | **-** | **-** | **-** | **-** | **-** | **-** | **-** | **-** | **-** | **-** | **-** | **-** | **-** | **-** | **-** | **-** | **-** | **-** | **Q** | **W** | **Q** | **A** | **K** | **Q** | **E** | **L** | **A** | **E** | **A** | **K** | **K** | **T** | **A** | **T** | **T** | **F** | **L** | **N** | **V** | **L** | **S** | **K** | **Q** | **E** | **F** | **D** | **K** | **L** | **P** | **S** | **V** | **V** | **Q** | **E** | **A** | **S** | **L** | **K** | **K** | **N** | **G** | **Y** | **D** | **T** | **K** | **S** | **V** | **V** | **E** | **K** | **Y** | **Q** | **A** | **I** | **Y** | **S** | **G** | **I** | **Q** | **A** | **E** | **G** | **V** | **K** | **A** | **S** | **D** | **V** | **Q** | **V** | **K** | **K** | **A** | **K** | **D** | **N** | **Q** | **Y** | **T** | **F** | **T** | **Y** | **K** | **L** | **S** | **M** | **S** | **T** | **P** | **L** | **G** | **E** | **M** | **K** | **D** | **L** | **S** | **Y** | **Q** | **S** | **S** | **I** | **A** | **K** | **K** | **G** | **D** | **T** | **Y** | **Q** | **I** | **A** | **W** | **K** | **P** | **S** | **L** | **I** | **F** | **P** | **D** | **M** | **S** | **G** | **N** | **D** | **K** | **I** | **S** | **I** | **Q** | **V** | **D** | **N** | **A** | **K** | **R** | **G** | **E** | **I** | **V** | **D** | **R** | **N** | **G** | **S** | **G** | **L** | **A** | **I** | **N** | **K** | **V** | **F** | **D** | **E** | **V** | **G** | **V** | **V** | **P** | **G** | **K** | **L** | **G** | **S** | **G** | **A** | **E** | **K** | **T** | **A** | **N** | **I** | **K** | **A** | **F** | **S** | **D** | **K** | **F** | **G** | **V** | **S** | **V** | **D** | **E** | **I** | **N** | **Q** | **K** | **L** | **S** | **Q** | **G** | **W** | **V** | **Q** | **A** | **D** | **S** | **F** | **V** | **P** | **I** | **T** | **V** | **A** | **S** | **E** | **P** | **V** | **T** | **E** | **L** | **P** | **T** | **G** | **A** | **A** | **T** | **K** | **D** | **T** | **E** | **S** | **R** | **Y** | **Y** | **P** | **L** | **G** | **E** | **A** | **A** | **A** | **Q** | **L** | **I** | **G** | **Y** | **T** | **G** | **T** | **I** | **T** | **A** | **E** | **D** | **I** | **E** | **K** | **N** | **P** | **E** | **L** | **S** | **S** | **T** | **G** | **V** | **I** | **G** | **K** | **T** | **G** | **L** | **E** | **R** | **A** | **F** | **D** | **K** | **E** | **L** | **R** | **G** | **Q** | **D** | **G** | **G** | **S** | **L** | **V** | **I** | **L** | **D** | **D** | **K** | **E** | **N** | **V** | **K** | **K** | **A** | **L** | **Q** | **T** | **K** | **E** | **K** | **K** | **D** | **G** | **Q** | **T** | **I** | **K** | **L** | **T** | **I** | **D** | **S** | **G** | **V** | **Q** | **Q** | **Q** | **A** | **F** | **A** | **I** | **F** | **D** | **K** | **R** | **P** | **G** | **S** | **A** | **V** | **I** | **T** | **D** | **P** | **Q** | **K** | **G** | **D** | **L** | **L** | **A** | **T** | **V** | **S** | **S** | **P** | **S** | **Y** | **D** | **P** | **N** | **K** | **M** | **A** | **N** | **G** | **I** | **S** | **Q** | **K** | **E** | **Y** | **D** | **A** | **Y** | **N** | **N** | **N** | **K** | **D** | **L** | **P** | **F** | **T** | **A** | **R** | **F** | **A** | **T** | **G** | **Y** | **A** | **P** | **G** | **S** | **T** | **F** | **K** | **T** | **I** | **T** | **G** | **A** | **I** | **G** | **L** | **D** | **A** | **G** | **T** | **L** | **K** | **P** | **D** | **E** | **E** | **L** | **E** | **I** | **N** | **G** | **L** | **K** | **W** | **Q** | **K** | **D** | **K** | **S** | **W** | **G** | **G** | **Y** | **F** | **A** | **T** | **R** | **V** | **K** | **E** | **A** | **S** | **P** | **V** | **N** | **L** | **R** | **T** | **A** | **L** | **V** | **N** | **S** | **D** | **N** | **I** | **Y** | **F** | **A** | **Q** | **Q** | **T** | **L** | **R** | **M** | **G** | **E** | **D** | **K** | **F** | **R** | **A** | **G** | **L** | **N** | **K** | **F** | **I** | **F** | **G** | **E** | **E** | **L** | **D** | **L** | **P** | **I** | **A** | **M** | **T** | **P** | **A** | **Q** | **I** | **S** | **N** | **E** | **D** | **K** | **F** | **N** | **S** | **E** | **I** | **L** | **L** | **A** | **D** | **T** | **G** | **Y** | **G** | **Q** | **G** | **Q** | **L** | **L** | **I** | **S** | **P** | **I** | **Q** | **Q** | **A** | **T** | **M** | **Y** | **S** | **V** | **F** | **Q** | **N** | **N** | **G** | **T** | **L** | **V** | **Y** | **P** | **K** | **L** | **V** | **L** | **D** | **K** | **E** | **T** | **K** | **K** | **K** | **D** | **N** | **V** | **I** | **S** | **A** | **N** | **A** | **A** | **N** | **T** | **I** | **A** | **T** | **D** | **L** | **L** | **G** | **S** | **V** | **E** | **D** | **P** | **S** | **G** | **Y** | **V** | **Y** | **N** | **M** | **Y** | **N** | **P** | **N** | **F** | **S** | **L** | **A** | **A** | **K** | **T** | **G** | **T** | **A** | **E** | **I** | **K** | **D** | **K** | **Q** | **D** | **T** | **D** | **G** | **K** | **E** | **N** | **S** | **F** | **L** | **L** | **T** | **L** | **D** | **R** | **S** | **N** | **N** | **K** | **F** | **L** | **T** | **M** | **I** | **M** | **V** | **E** | **N** | **S** | **G** | **E** | **N** | **G** | **S** | **A** | **T** | **D** | **I** | **S** | **K** | **P** | **L** | **I** | **D** | **Y** | **L** | **E** | **A** | **T** | **I** | **K** |
| **N59959F** | **-** | **-** | **-** | **-** | **-** | **-** | **-** | **-** | **-** | **-** | **-** | **-** | **-** | **-** | **-** | **-** | **-** | **-** | **-** | **-** | **-** | **-** | **-** | **-** | **-** | **-** | **-** | **-** | **-** | **-** | **-** | **-** | **-** | **-** | **-** | **-** | **Q** | **W** | **Q** | **A** | **K** | **Q** | **E** | **L** | **A** | **E** | **A** | **K** | **K** | **T** | **A** | **T** | **T** | **F** | **L** | **N** | **V** | **L** | **S** | **K** | **Q** | **E** | **F** | **D** | **K** | **L** | **P** | **S** | **V** | **V** | **Q** | **E** | **A** | **S** | **L** | **K** | **K** | **N** | **G** | **Y** | **D** | **T** | **K** | **S** | **V** | **V** | **E** | **K** | **Y** | **Q** | **A** | **I** | **Y** | **S** | **G** | **I** | **Q** | **A** | **E** | **G** | **V** | **K** | **A** | **S** | **D** | **V** | **Q** | **V** | **K** | **K** | **A** | **K** | **D** | **N** | **Q** | **Y** | **T** | **F** | **T** | **Y** | **K** | **L** | **S** | **M** | **S** | **T** | **P** | **L** | **G** | **E** | **M** | **K** | **D** | **L** | **S** | **Y** | **Q** | **S** | **S** | **I** | **A** | **K** | **K** | **G** | **D** | **T** | **Y** | **Q** | **I** | **A** | **W** | **K** | **P** | **S** | **L** | **I** | **F** | **P** | **D** | **M** | **S** | **G** | **N** | **D** | **K** | **I** | **S** | **I** | **Q** | **V** | **D** | **N** | **A** | **K** | **R** | **G** | **E** | **I** | **V** | **D** | **R** | **N** | **G** | **S** | **G** | **L** | **A** | **I** | **N** | **K** | **V** | **F** | **D** | **E** | **V** | **G** | **V** | **V** | **P** | **G** | **K** | **L** | **G** | **S** | **G** | **A** | **E** | **K** | **T** | **A** | **N** | **I** | **K** | **A** | **F** | **S** | **D** | **K** | **F** | **G** | **V** | **S** | **V** | **D** | **E** | **I** | **N** | **Q** | **K** | **L** | **S** | **Q** | **G** | **W** | **V** | **Q** | **A** | **D** | **S** | **F** | **V** | **P** | **I** | **T** | **V** | **A** | **S** | **E** | **P** | **V** | **T** | **E** | **L** | **P** | **T** | **G** | **A** | **A** | **T** | **K** | **D** | **T** | **E** | **S** | **R** | **Y** | **Y** | **P** | **L** | **G** | **E** | **A** | **A** | **A** | **Q** | **L** | **I** | **G** | **Y** | **T** | **G** | **T** | **I** | **T** | **A** | **E** | **D** | **I** | **K** | **K** | **N** | **P** | **E** | **L** | **S** | **S** | **T** | **G** | **V** | **I** | **G** | **K** | **T** | **G** | **L** | **E** | **R** | **A** | **F** | **D** | **K** | **E** | **L** | **R** | **G** | **Q** | **D** | **G** | **G** | **S** | **L** | **V** | **I** | **L** | **D** | **D** | **K** | **E** | **N** | **V** | **K** | **K** | **A** | **L** | **Q** | **T** | **K** | **E** | **K** | **K** | **D** | **G** | **Q** | **T** | **I** | **K** | **L** | **T** | **I** | **D** | **S** | **G** | **V** | **Q** | **Q** | **Q** | **A** | **F** | **A** | **I** | **F** | **D** | **K** | **R** | **P** | **G** | **S** | **A** | **V** | **I** | **T** | **D** | **P** | **Q** | **K** | **G** | **D** | **L** | **L** | **A** | **T** | **V** | **S** | **S** | **P** | **S** | **Y** | **D** | **P** | **N** | **K** | **M** | **A** | **N** | **G** | **I** | **S** | **Q** | **K** | **E** | **Y** | **D** | **A** | **Y** | **N** | **N** | **N** | **K** | **D** | **L** | **P** | **F** | **T** | **A** | **R** | **F** | **A** | **T** | **G** | **Y** | **A** | **P** | **G** | **S** | **T** | **F** | **K** | **T** | **I** | **T** | **G** | **A** | **I** | **G** | **L** | **D** | **A** | **G** | **T** | **L** | **K** | **P** | **D** | **E** | **E** | **L** | **E** | **I** | **N** | **G** | **L** | **K** | **W** | **Q** | **K** | **D** | **K** | **S** | **W** | **G** | **G** | **Y** | **F** | **A** | **T** | **R** | **V** | **K** | **E** | **A** | **S** | **P** | **V** | **N** | **L** | **R** | **T** | **A** | **L** | **V** | **N** | **S** | **D** | **N** | **I** | **Y** | **F** | **A** | **Q** | **Q** | **T** | **L** | **R** | **M** | **G** | **E** | **D** | **K** | **F** | **R** | **A** | **G** | **L** | **N** | **K** | **F** | **I** | **F** | **G** | **E** | **E** | **L** | **D** | **L** | **P** | **I** | **A** | **M** | **T** | **P** | **A** | **Q** | **I** | **S** | **N** | **E** | **D** | **K** | **F** | **N** | **S** | **E** | **I** | **L** | **L** | **A** | **D** | **T** | **G** | **Y** | **G** | **Q** | **G** | **Q** | **L** | **L** | **I** | **S** | **P** | **I** | **Q** | **Q** | **A** | **T** | **M** | **Y** | **S** | **V** | **F** | **Q** | **N** | **N** | **G** | **T** | **L** | **V** | **Y** | **P** | **K** | **L** | **V** | **L** | **D** | **K** | **E** | **T** | **K** | **K** | **K** | **D** | **N** | **V** | **I** | **S** | **A** | **N** | **A** | **A** | **N** | **T** | **I** | **A** | **T** | **D** | **L** | **L** | **G** | **S** | **V** | **E** | **D** | **P** | **S** | **G** | **Y** | **V** | **Y** | **N** | **M** | **Y** | **N** | **P** | **N** | **F** | **S** | **L** | **A** | **A** | **K** | **T** | **G** | **T** | **A** | **E** | **I** | **K** | **D** | **K** | **Q** | **D** | **T** | **D** | **G** | **K** | **E** | **N** | **S** | **F** | **L** | **L** | **T** | **L** | **D** | **R** | **S** | **N** | **N** | **K** | **F** | **L** | **T** | **M** | **I** | **M** | **V** | **E** | **N** | **S** | **G** | **E** | **N** | **G** | **S** | **A** | **T** | **D** | **I** | **S** | **K** | **P** | **L** | **I** | **D** | **Y** | **L** | **E** | **A** | **T** | **I** | **K** | **-** | **-** | **-** | **-** | **-** | **-** | **-** | **-** | **-** | **-** | **-** | **-** | **-** | **-** | **-** | **-** | **-** | **-** | **-** | **-** | **-** | **-** | **-** | **-** | **-** | **-** | **-** | **-** | **-** | **-** | **-** | **-** | **-** | **-** | **-** | **-** | **Q** | **W** | **Q** | **A** | **K** | **Q** | **E** | **L** | **A** | **E** | **A** | **K** | **K** | **T** | **A** | **T** | **T** | **F** | **L** | **N** | **V** | **L** | **S** | **K** | **Q** | **E** | **F** | **D** | **K** | **L** | **P** | **S** | **V** | **V** | **Q** | **E** | **A** | **S** | **L** | **K** | **K** | **N** | **G** | **Y** | **D** | **T** | **K** | **S** | **V** | **V** | **E** | **K** | **Y** | **Q** | **A** | **I** | **Y** | **S** | **G** | **I** | **Q** | **A** | **E** | **G** | **V** | **K** | **A** | **S** | **D** | **V** | **Q** | **V** | **K** | **K** | **A** | **K** | **D** | **N** | **Q** | **Y** | **T** | **F** | **T** | **Y** | **K** | **L** | **S** | **M** | **S** | **T** | **P** | **L** | **G** | **E** | **M** | **K** | **D** | **L** | **S** | **Y** | **Q** | **S** | **S** | **I** | **A** | **K** | **K** | **G** | **D** | **T** | **Y** | **Q** | **I** | **A** | **W** | **K** | **P** | **S** | **L** | **I** | **F** | **P** | **D** | **M** | **S** | **G** | **N** | **D** | **K** | **I** | **S** | **I** | **Q** | **V** | **D** | **N** | **A** | **K** | **R** | **G** | **E** | **I** | **V** | **D** | **R** | **N** | **G** | **S** | **G** | **L** | **A** | **I** | **N** | **K** | **V** | **F** | **D** | **E** | **V** | **G** | **V** | **V** | **P** | **G** | **K** | **L** | **G** | **S** | **G** | **A** | **E** | **K** | **T** | **A** | **N** | **I** | **K** | **A** | **F** | **S** | **D** | **K** | **F** | **G** | **V** | **S** | **V** | **D** | **E** | **I** | **N** | **Q** | **K** | **L** | **S** | **Q** | **G** | **W** | **V** | **Q** | **A** | **D** | **S** | **F** | **V** | **P** | **I** | **T** | **V** | **A** | **S** | **E** | **P** | **V** | **T** | **E** | **L** | **P** | **T** | **G** | **A** | **A** | **T** | **K** | **D** | **T** | **E** | **S** | **R** | **Y** | **Y** | **P** | **L** | **G** | **E** | **A** | **A** | **A** | **Q** | **L** | **I** | **G** | **Y** | **T** | **G** | **T** | **I** | **T** | **A** | **E** | **D** | **I** | **K** | **K** | **N** | **P** | **E** | **L** | **S** | **S** | **T** | **G** | **V** | **I** | **G** | **K** | **T** | **G** | **L** | **E** | **R** | **A** | **F** | **D** | **K** | **E** | **L** | **R** | **G** | **Q** | **D** | **G** | **G** | **S** | **L** | **V** | **I** | **L** | **D** | **D** | **K** | **E** | **N** | **V** | **K** | **K** | **A** | **L** | **Q** | **T** | **K** | **E** | **K** | **K** | **D** | **G** | **Q** | **T** | **I** | **K** | **L** | **T** | **I** | **D** | **S** | **G** | **V** | **Q** | **Q** | **Q** | **A** | **F** | **A** | **I** | **F** | **D** | **K** | **R** | **P** | **G** | **S** | **A** | **V** | **I** | **T** | **D** | **P** | **Q** | **K** | **G** | **D** | **L** | **L** | **A** | **T** | **V** | **S** | **S** | **P** | **S** | **Y** | **D** | **P** | **N** | **K** | **M** | **A** | **N** | **G** | **I** | **S** | **Q** | **K** | **E** | **Y** | **D** | **A** | **Y** | **N** | **N** | **N** | **K** | **D** | **L** | **P** | **F** | **T** | **A** | **R** | **F** | **A** | **T** | **G** | **Y** | **A** | **P** | **G** | **S** | **T** | **F** | **K** | **T** | **I** | **T** | **G** | **A** | **I** | **G** | **L** | **D** | **A** | **G** | **T** | **L** | **K** | **P** | **D** | **E** | **E** | **L** | **E** | **I** | **N** | **G** | **L** | **K** | **W** | **Q** | **K** | **D** | **K** | **S** | **W** | **G** | **G** | **Y** | **F** | **A** | **T** | **R** | **V** | **K** | **E** | **A** | **S** | **P** | **V** | **N** | **L** | **R** | **T** | **A** | **L** | **V** | **N** | **S** | **D** | **N** | **I** | **Y** | **F** | **A** | **Q** | **Q** | **T** | **L** | **R** | **M** | **G** | **E** | **D** | **K** | **F** | **R** | **A** | **G** | **L** | **N** | **K** | **F** | **I** | **F** | **G** | **E** | **E** | **L** | **D** | **L** | **P** | **I** | **A** | **M** | **T** | **P** | **A** | **Q** | **I** | **S** | **N** | **E** | **D** | **K** | **F** | **N** | **S** | **E** | **I** | **L** | **L** | **A** | **D** | **T** | **G** | **Y** | **G** | **Q** | **G** | **Q** | **L** | **L** | **I** | **S** | **P** | **I** | **Q** | **Q** | **A** | **T** | **M** | **Y** | **S** | **V** | **F** | **Q** | **N** | **N** | **G** | **T** | **L** | **V** | **Y** | **P** | **K** | **L** | **V** | **L** | **D** | **K** | **E** | **T** | **K** | **K** | **K** | **D** | **N** | **V** | **I** | **S** | **A** | **N** | **A** | **A** | **N** | **T** | **I** | **A** | **T** | **D** | **L** | **L** | **G** | **S** | **V** | **E** | **D** | **P** | **S** | **G** | **Y** | **V** | **Y** | **N** | **M** | **Y** | **N** | **P** | **N** | **F** | **S** | **L** | **A** | **A** | **K** | **T** | **G** | **T** | **A** | **E** | **I** | **K** | **D** | **K** | **Q** | **D** | **T** | **D** | **G** | **K** | **E** | **N** | **S** | **F** | **L** | **L** | **T** | **L** | **D** | **R** | **S** | **N** | **N** | **K** | **F** | **L** | **T** | **M** | **I** | **M** | **V** | **E** | **N** | **S** | **G** | **E** | **N** | **G** | **S** | **A** | **T** | **D** | **I** | **S** | **K** | **P** | **L** | **I** | **D** | **Y** | **L** | **E** | **A** | **T** | **I** | **K** |
| **N52623** | **-** | **-** | **-** | **-** | **-** | **-** | **-** | **-** | **-** | **-** | **-** | **-** | **-** | **-** | **-** | **-** | **-** | **-** | **-** | **-** | **-** | **-** | **-** | **-** | **-** | **-** | **-** | **-** | **-** | **-** | **-** | **-** | **-** | **-** | **-** | **-** | **Q** | **W** | **Q** | **A** | **K** | **Q** | **E** | **L** | **A** | **E** | **A** | **K** | **K** | **T** | **A** | **T** | **E** | **F** | **L** | **N** | **V** | **L** | **S** | **K** | **Q** | **E** | **F** | **D** | **K** | **L** | **P** | **S** | **V** | **V** | **Q** | **E** | **A** | **S** | **L** | **K** | **K** | **N** | **G** | **Y** | **D** | **T** | **K** | **S** | **V** | **V** | **E** | **K** | **Y** | **Q** | **A** | **I** | **Y** | **S** | **G** | **I** | **Q** | **A** | **E** | **G** | **V** | **K** | **A** | **S** | **D** | **V** | **Q** | **V** | **K** | **K** | **A** | **K** | **D** | **N** | **Q** | **Y** | **T** | **F** | **T** | **Y** | **K** | **L** | **S** | **M** | **S** | **T** | **P** | **L** | **G** | **E** | **M** | **K** | **D** | **L** | **S** | **Y** | **Q** | **S** | **S** | **I** | **A** | **K** | **K** | **G** | **D** | **T** | **Y** | **Q** | **I** | **A** | **W** | **K** | **P** | **S** | **L** | **I** | **F** | **P** | **D** | **M** | **S** | **G** | **N** | **D** | **K** | **I** | **S** | **I** | **Q** | **V** | **D** | **N** | **A** | **K** | **R** | **G** | **E** | **I** | **V** | **D** | **R** | **N** | **G** | **S** | **G** | **L** | **A** | **I** | **N** | **K** | **V** | **F** | **D** | **E** | **V** | **G** | **V** | **V** | **P** | **G** | **K** | **L** | **G** | **S** | **G** | **A** | **E** | **K** | **T** | **A** | **N** | **I** | **K** | **A** | **F** | **S** | **D** | **K** | **F** | **G** | **V** | **S** | **V** | **D** | **E** | **I** | **N** | **Q** | **K** | **L** | **S** | **Q** | **G** | **W** | **V** | **Q** | **A** | **D** | **S** | **F** | **V** | **P** | **I** | **T** | **V** | **A** | **S** | **E** | **P** | **V** | **T** | **E** | **L** | **P** | **T** | **G** | **A** | **A** | **T** | **K** | **D** | **T** | **E** | **S** | **R** | **Y** | **Y** | **P** | **L** | **G** | **E** | **A** | **A** | **A** | **Q** | **L** | **I** | **G** | **Y** | **T** | **G** | **T** | **I** | **T** | **A** | **E** | **D** | **I** | **E** | **K** | **N** | **P** | **E** | **L** | **S** | **S** | **T** | **G** | **V** | **I** | **G** | **K** | **T** | **G** | **L** | **E** | **R** | **A** | **F** | **D** | **K** | **E** | **L** | **R** | **G** | **Q** | **D** | **G** | **G** | **S** | **L** | **V** | **I** | **L** | **D** | **D** | **K** | **E** | **N** | **V** | **K** | **K** | **A** | **L** | **Q** | **T** | **K** | **E** | **K** | **K** | **D** | **G** | **Q** | **T** | **I** | **K** | **L** | **T** | **I** | **D** | **S** | **G** | **V** | **Q** | **Q** | **Q** | **A** | **F** | **A** | **I** | **F** | **D** | **K** | **R** | **P** | **G** | **S** | **A** | **V** | **I** | **T** | **D** | **P** | **Q** | **K** | **G** | **D** | **L** | **L** | **A** | **T** | **V** | **S** | **S** | **P** | **S** | **Y** | **D** | **P** | **N** | **K** | **M** | **A** | **N** | **G** | **I** | **S** | **Q** | **K** | **E** | **Y** | **D** | **A** | **Y** | **N** | **N** | **N** | **K** | **D** | **L** | **P** | **F** | **T** | **A** | **R** | **F** | **A** | **T** | **G** | **Y** | **A** | **P** | **G** | **S** | **T** | **F** | **K** | **T** | **I** | **T** | **G** | **A** | **I** | **G** | **L** | **D** | **A** | **G** | **T** | **L** | **K** | **P** | **D** | **E** | **E** | **L** | **E** | **I** | **N** | **G** | **L** | **K** | **W** | **Q** | **K** | **D** | **K** | **S** | **W** | **G** | **G** | **Y** | **F** | **A** | **T** | **R** | **V** | **K** | **E** | **A** | **S** | **P** | **V** | **N** | **L** | **R** | **T** | **A** | **L** | **V** | **N** | **S** | **D** | **N** | **I** | **Y** | **F** | **A** | **Q** | **Q** | **T** | **L** | **R** | **M** | **G** | **E** | **D** | **K** | **F** | **R** | **A** | **G** | **L** | **N** | **K** | **F** | **I** | **F** | **G** | **E** | **E** | **L** | **D** | **L** | **P** | **I** | **A** | **M** | **T** | **P** | **A** | **Q** | **I** | **S** | **N** | **E** | **D** | **K** | **F** | **N** | **S** | **E** | **I** | **L** | **L** | **A** | **D** | **T** | **G** | **Y** | **G** | **Q** | **G** | **Q** | **L** | **L** | **I** | **S** | **P** | **I** | **Q** | **Q** | **A** | **T** | **M** | **Y** | **S** | **V** | **F** | **Q** | **N** | **N** | **G** | **T** | **L** | **V** | **Y** | **P** | **K** | **I** | **V** | **L** | **D** | **K** | **E** | **T** | **K** | **K** | **K** | **D** | **N** | **V** | **I** | **S** | **A** | **N** | **A** | **A** | **N** | **T** | **I** | **A** | **T** | **D** | **L** | **L** | **G** | **S** | **V** | **E** | **D** | **P** | **S** | **G** | **Y** | **V** | **Y** | **N** | **M** | **Y** | **N** | **P** | **N** | **F** | **S** | **L** | **A** | **A** | **K** | **T** | **G** | **T** | **A** | **E** | **I** | **K** | **D** | **K** | **Q** | **D** | **T** | **D** | **G** | **K** | **E** | **N** | **S** | **F** | **L** | **L** | **T** | **L** | **D** | **R** | **S** | **N** | **N** | **K** | **F** | **L** | **T** | **M** | **I** | **M** | **V** | **E** | **N** | **S** | **G** | **E** | **N** | **G** | **S** | **A** | **T** | **D** | **I** | **S** | **K** | **P** | **L** | **I** | **D** | **Y** | **L** | **E** | **A** | **T** | **I** | **K** | **-** | **-** | **-** | **-** | **-** | **-** | **-** | **-** | **-** | **-** | **-** | **-** | **-** | **-** | **-** | **-** | **-** | **-** | **-** | **-** | **-** | **-** | **-** | **-** | **-** | **-** | **-** | **-** | **-** | **-** | **-** | **-** | **-** | **-** | **-** | **-** | **Q** | **W** | **Q** | **A** | **K** | **Q** | **E** | **L** | **A** | **E** | **A** | **K** | **K** | **T** | **A** | **T** | **E** | **F** | **L** | **N** | **V** | **L** | **S** | **K** | **Q** | **E** | **F** | **D** | **K** | **L** | **P** | **S** | **V** | **V** | **Q** | **E** | **A** | **S** | **L** | **K** | **K** | **N** | **G** | **Y** | **D** | **T** | **K** | **S** | **V** | **V** | **E** | **K** | **Y** | **Q** | **A** | **I** | **Y** | **S** | **G** | **I** | **Q** | **A** | **E** | **G** | **V** | **K** | **A** | **S** | **D** | **V** | **Q** | **V** | **K** | **K** | **A** | **K** | **D** | **N** | **Q** | **Y** | **T** | **F** | **T** | **Y** | **K** | **L** | **S** | **M** | **S** | **T** | **P** | **L** | **G** | **E** | **M** | **K** | **D** | **L** | **S** | **Y** | **Q** | **S** | **S** | **I** | **A** | **K** | **K** | **G** | **D** | **T** | **Y** | **Q** | **I** | **A** | **W** | **K** | **P** | **S** | **L** | **I** | **F** | **P** | **D** | **M** | **S** | **G** | **N** | **D** | **K** | **I** | **S** | **I** | **Q** | **V** | **D** | **N** | **A** | **K** | **R** | **G** | **E** | **I** | **V** | **D** | **R** | **N** | **G** | **S** | **G** | **L** | **A** | **I** | **N** | **K** | **V** | **F** | **D** | **E** | **V** | **G** | **V** | **V** | **P** | **G** | **K** | **L** | **G** | **S** | **G** | **A** | **E** | **K** | **T** | **A** | **N** | **I** | **K** | **A** | **F** | **S** | **D** | **K** | **F** | **G** | **V** | **S** | **V** | **D** | **E** | **I** | **N** | **Q** | **K** | **L** | **S** | **Q** | **G** | **W** | **V** | **Q** | **A** | **D** | **S** | **F** | **V** | **P** | **I** | **T** | **V** | **A** | **S** | **E** | **P** | **V** | **T** | **E** | **L** | **P** | **T** | **G** | **A** | **A** | **T** | **K** | **D** | **T** | **E** | **S** | **R** | **Y** | **Y** | **P** | **L** | **G** | **E** | **A** | **A** | **A** | **Q** | **L** | **I** | **G** | **Y** | **T** | **G** | **T** | **I** | **T** | **A** | **E** | **D** | **I** | **E** | **K** | **N** | **P** | **E** | **L** | **S** | **S** | **T** | **G** | **V** | **I** | **G** | **K** | **T** | **G** | **L** | **E** | **R** | **A** | **F** | **D** | **K** | **E** | **L** | **R** | **G** | **Q** | **D** | **G** | **G** | **S** | **L** | **V** | **I** | **L** | **D** | **D** | **K** | **E** | **N** | **V** | **K** | **K** | **A** | **L** | **Q** | **T** | **K** | **E** | **K** | **K** | **D** | **G** | **Q** | **T** | **I** | **K** | **L** | **T** | **I** | **D** | **S** | **G** | **V** | **Q** | **Q** | **Q** | **A** | **F** | **A** | **I** | **F** | **D** | **K** | **R** | **P** | **G** | **S** | **A** | **V** | **I** | **T** | **D** | **P** | **Q** | **K** | **G** | **D** | **L** | **L** | **A** | **T** | **V** | **S** | **S** | **P** | **S** | **Y** | **D** | **P** | **N** | **K** | **M** | **A** | **N** | **G** | **I** | **S** | **Q** | **K** | **E** | **Y** | **D** | **A** | **Y** | **N** | **N** | **N** | **K** | **D** | **L** | **P** | **F** | **T** | **A** | **R** | **F** | **A** | **T** | **G** | **Y** | **A** | **P** | **G** | **S** | **T** | **F** | **K** | **T** | **I** | **T** | **G** | **A** | **I** | **G** | **L** | **D** | **A** | **G** | **T** | **L** | **K** | **P** | **D** | **E** | **E** | **L** | **E** | **I** | **N** | **G** | **L** | **K** | **W** | **Q** | **K** | **D** | **K** | **S** | **W** | **G** | **G** | **Y** | **F** | **A** | **T** | **R** | **V** | **K** | **E** | **A** | **S** | **P** | **V** | **N** | **L** | **R** | **T** | **A** | **L** | **V** | **N** | **S** | **D** | **N** | **I** | **Y** | **F** | **A** | **Q** | **Q** | **T** | **L** | **R** | **M** | **G** | **E** | **D** | **K** | **F** | **R** | **A** | **G** | **L** | **N** | **K** | **F** | **I** | **F** | **G** | **E** | **E** | **L** | **D** | **L** | **P** | **I** | **A** | **M** | **T** | **P** | **A** | **Q** | **I** | **S** | **N** | **E** | **D** | **K** | **F** | **N** | **S** | **E** | **I** | **L** | **L** | **A** | **D** | **T** | **G** | **Y** | **G** | **Q** | **G** | **Q** | **L** | **L** | **I** | **S** | **P** | **I** | **Q** | **Q** | **A** | **T** | **M** | **Y** | **S** | **V** | **F** | **Q** | **N** | **N** | **G** | **T** | **L** | **V** | **Y** | **P** | **K** | **I** | **V** | **L** | **D** | **K** | **E** | **T** | **K** | **K** | **K** | **D** | **N** | **V** | **I** | **S** | **A** | **N** | **A** | **A** | **N** | **T** | **I** | **A** | **T** | **D** | **L** | **L** | **G** | **S** | **V** | **E** | **D** | **P** | **S** | **G** | **Y** | **V** | **Y** | **N** | **M** | **Y** | **N** | **P** | **N** | **F** | **S** | **L** | **A** | **A** | **K** | **T** | **G** | **T** | **A** | **E** | **I** | **K** | **D** | **K** | **Q** | **D** | **T** | **D** | **G** | **K** | **E** | **N** | **S** | **F** | **L** | **L** | **T** | **L** | **D** | **R** | **S** | **N** | **N** | **K** | **F** | **L** | **T** | **M** | **I** | **M** | **V** | **E** | **N** | **S** | **G** | **E** | **N** | **G** | **S** | **A** | **T** | **D** | **I** | **S** | **K** | **P** | **L** | **I** | **D** | **Y** | **L** | **E** | **A** | **T** | **I** | **K** |
| **N59455F** | **-** | **-** | **-** | **-** | **-** | **-** | **-** | **-** | **-** | **-** | **-** | **-** | **-** | **-** | **-** | **-** | **-** | **-** | **-** | **-** | **-** | **-** | **-** | **-** | **-** | **-** | **-** | **-** | **-** | **-** | **-** | **-** | **-** | **-** | **-** | **-** | **Q** | **W** | **Q** | **A** | **K** | **Q** | **E** | **L** | **A** | **E** | **A** | **K** | **K** | **T** | **A** | **N** | **T** | **F** | **L** | **N** | **V** | **L** | **S** | **K** | **Q** | **E** | **F** | **D** | **K** | **L** | **P** | **S** | **V** | **V** | **Q** | **E** | **S** | **S** | **L** | **K** | **K** | **N** | **G** | **Y** | **D** | **T** | **K** | **S** | **V** | **V** | **E** | **K** | **Y** | **Q** | **A** | **I** | **Y** | **S** | **G** | **I** | **Q** | **A** | **E** | **G** | **V** | **K** | **A** | **S** | **D** | **V** | **Q** | **V** | **K** | **K** | **A** | **K** | **D** | **N** | **Q** | **Y** | **T** | **F** | **T** | **Y** | **K** | **L** | **S** | **M** | **S** | **T** | **P** | **L** | **G** | **E** | **M** | **K** | **D** | **L** | **S** | **Y** | **Q** | **S** | **S** | **I** | **A** | **K** | **K** | **G** | **D** | **T** | **Y** | **Q** | **I** | **S** | **W** | **K** | **P** | **S** | **L** | **I** | **F** | **P** | **D** | **M** | **S** | **G** | **N** | **D** | **K** | **I** | **S** | **I** | **Q** | **V** | **D** | **N** | **A** | **K** | **R** | **G** | **E** | **I** | **V** | **D** | **R** | **N** | **G** | **S** | **G** | **L** | **A** | **I** | **N** | **K** | **V** | **F** | **D** | **E** | **V** | **G** | **V** | **V** | **P** | **G** | **K** | **L** | **G** | **S** | **G** | **A** | **E** | **K** | **T** | **A** | **N** | **I** | **K** | **A** | **F** | **S** | **D** | **K** | **F** | **G** | **V** | **S** | **V** | **D** | **E** | **I** | **N** | **Q** | **K** | **L** | **S** | **Q** | **G** | **W** | **V** | **Q** | **A** | **D** | **S** | **F** | **V** | **P** | **I** | **T** | **V** | **A** | **S** | **E** | **P** | **V** | **T** | **E** | **L** | **P** | **T** | **G** | **A** | **A** | **T** | **K** | **D** | **T** | **E** | **S** | **R** | **Y** | **Y** | **P** | **L** | **G** | **E** | **A** | **A** | **A** | **Q** | **L** | **I** | **G** | **Y** | **T** | **G** | **T** | **I** | **T** | **A** | **E** | **D** | **I** | **E** | **K** | **N** | **P** | **E** | **L** | **S** | **S** | **T** | **G** | **V** | **I** | **G** | **K** | **T** | **G** | **L** | **E** | **R** | **A** | **F** | **D** | **K** | **E** | **L** | **R** | **G** | **Q** | **D** | **G** | **G** | **S** | **L** | **V** | **I** | **L** | **D** | **D** | **K** | **E** | **N** | **V** | **K** | **K** | **A** | **L** | **Q** | **T** | **K** | **E** | **K** | **K** | **D** | **G** | **Q** | **T** | **I** | **K** | **L** | **T** | **I** | **D** | **S** | **G** | **V** | **Q** | **Q** | **Q** | **A** | **F** | **A** | **I** | **F** | **D** | **K** | **R** | **P** | **G** | **S** | **A** | **V** | **I** | **T** | **D** | **P** | **Q** | **K** | **G** | **D** | **L** | **L** | **A** | **T** | **V** | **S** | **S** | **P** | **S** | **Y** | **D** | **P** | **N** | **K** | **M** | **A** | **N** | **G** | **I** | **S** | **Q** | **K** | **E** | **Y** | **D** | **A** | **Y** | **N** | **N** | **N** | **K** | **D** | **L** | **P** | **F** | **T** | **A** | **R** | **F** | **A** | **T** | **G** | **Y** | **A** | **P** | **G** | **S** | **T** | **F** | **K** | **T** | **I** | **T** | **G** | **A** | **I** | **G** | **L** | **D** | **A** | **G** | **T** | **L** | **K** | **P** | **D** | **E** | **E** | **L** | **E** | **I** | **N** | **G** | **L** | **K** | **W** | **Q** | **K** | **D** | **K** | **S** | **W** | **G** | **G** | **Y** | **F** | **A** | **T** | **R** | **V** | **K** | **E** | **A** | **S** | **P** | **V** | **N** | **L** | **R** | **T** | **A** | **L** | **V** | **N** | **S** | **D** | **N** | **I** | **Y** | **F** | **A** | **Q** | **Q** | **T** | **L** | **R** | **M** | **G** | **E** | **D** | **K** | **F** | **R** | **A** | **G** | **L** | **N** | **K** | **F** | **I** | **F** | **G** | **E** | **E** | **L** | **D** | **L** | **P** | **I** | **A** | **M** | **T** | **P** | **A** | **Q** | **I** | **S** | **N** | **E** | **D** | **K** | **F** | **N** | **S** | **E** | **I** | **L** | **L** | **A** | **D** | **T** | **G** | **Y** | **G** | **Q** | **G** | **Q** | **L** | **L** | **I** | **S** | **P** | **I** | **Q** | **Q** | **A** | **T** | **M** | **Y** | **S** | **V** | **F** | **Q** | **N** | **N** | **G** | **T** | **L** | **V** | **Y** | **P** | **K** | **L** | **V** | **L** | **D** | **K** | **E** | **T** | **K** | **K** | **K** | **D** | **N** | **V** | **I** | **S** | **A** | **N** | **A** | **A** | **N** | **T** | **I** | **A** | **T** | **D** | **L** | **L** | **G** | **S** | **V** | **E** | **D** | **P** | **S** | **G** | **Y** | **V** | **Y** | **N** | **M** | **Y** | **N** | **P** | **N** | **F** | **S** | **L** | **A** | **A** | **K** | **T** | **G** | **T** | **A** | **E** | **I** | **K** | **D** | **K** | **Q** | **D** | **T** | **D** | **G** | **K** | **E** | **N** | **S** | **F** | **L** | **L** | **T** | **L** | **D** | **R** | **S** | **N** | **N** | **K** | **F** | **L** | **T** | **M** | **I** | **M** | **V** | **E** | **N** | **S** | **G** | **E** | **N** | **G** | **S** | **A** | **T** | **D** | **I** | **S** | **K** | **P** | **L** | **I** | **D** | **Y** | **L** | **E** | **A** | **T** | **I** | **K** | **-** | **-** | **-** | **-** | **-** | **-** | **-** | **-** | **-** | **-** | **-** | **-** | **-** | **-** | **-** | **-** | **-** | **-** | **-** | **-** | **-** | **-** | **-** | **-** | **-** | **-** | **-** | **-** | **-** | **-** | **-** | **-** | **-** | **-** | **-** | **-** | **Q** | **W** | **Q** | **A** | **K** | **Q** | **E** | **L** | **A** | **E** | **A** | **K** | **K** | **T** | **A** | **N** | **T** | **F** | **L** | **N** | **V** | **L** | **S** | **K** | **Q** | **E** | **F** | **D** | **K** | **L** | **P** | **S** | **V** | **V** | **Q** | **E** | **S** | **S** | **L** | **K** | **K** | **N** | **G** | **Y** | **D** | **T** | **K** | **S** | **V** | **V** | **E** | **K** | **Y** | **Q** | **A** | **I** | **Y** | **S** | **G** | **I** | **Q** | **A** | **E** | **G** | **V** | **K** | **A** | **S** | **D** | **V** | **Q** | **V** | **K** | **K** | **A** | **K** | **D** | **N** | **Q** | **Y** | **T** | **F** | **T** | **Y** | **K** | **L** | **S** | **M** | **S** | **T** | **P** | **L** | **G** | **E** | **M** | **K** | **D** | **L** | **S** | **Y** | **Q** | **S** | **S** | **I** | **A** | **K** | **K** | **G** | **D** | **T** | **Y** | **Q** | **I** | **S** | **W** | **K** | **P** | **S** | **L** | **I** | **F** | **P** | **D** | **M** | **S** | **G** | **N** | **D** | **K** | **I** | **S** | **I** | **Q** | **V** | **D** | **N** | **A** | **K** | **R** | **G** | **E** | **I** | **V** | **D** | **R** | **N** | **G** | **S** | **G** | **L** | **A** | **I** | **N** | **K** | **V** | **F** | **D** | **E** | **V** | **G** | **V** | **V** | **P** | **G** | **K** | **L** | **G** | **S** | **G** | **A** | **E** | **K** | **T** | **A** | **N** | **I** | **K** | **A** | **F** | **S** | **D** | **K** | **F** | **G** | **V** | **S** | **V** | **D** | **E** | **I** | **N** | **Q** | **K** | **L** | **S** | **Q** | **G** | **W** | **V** | **Q** | **A** | **D** | **S** | **F** | **V** | **P** | **I** | **T** | **V** | **A** | **S** | **E** | **P** | **V** | **T** | **E** | **L** | **P** | **T** | **G** | **A** | **A** | **T** | **K** | **D** | **T** | **E** | **S** | **R** | **Y** | **Y** | **P** | **L** | **G** | **E** | **A** | **A** | **A** | **Q** | **L** | **I** | **G** | **Y** | **T** | **G** | **T** | **I** | **T** | **A** | **E** | **D** | **I** | **E** | **K** | **N** | **P** | **E** | **L** | **S** | **S** | **T** | **G** | **V** | **I** | **G** | **K** | **T** | **G** | **L** | **E** | **R** | **A** | **F** | **D** | **K** | **E** | **L** | **R** | **G** | **Q** | **D** | **G** | **G** | **S** | **L** | **V** | **I** | **L** | **D** | **D** | **K** | **E** | **N** | **V** | **K** | **K** | **A** | **L** | **Q** | **T** | **K** | **E** | **K** | **K** | **D** | **G** | **Q** | **T** | **I** | **K** | **L** | **T** | **I** | **D** | **S** | **G** | **V** | **Q** | **Q** | **Q** | **A** | **F** | **A** | **I** | **F** | **D** | **K** | **R** | **P** | **G** | **S** | **A** | **V** | **I** | **T** | **D** | **P** | **Q** | **K** | **G** | **D** | **L** | **L** | **A** | **T** | **V** | **S** | **S** | **P** | **S** | **Y** | **D** | **P** | **N** | **K** | **M** | **A** | **N** | **G** | **I** | **S** | **Q** | **K** | **E** | **Y** | **D** | **A** | **Y** | **N** | **N** | **N** | **K** | **D** | **L** | **P** | **F** | **T** | **A** | **R** | **F** | **A** | **T** | **G** | **Y** | **A** | **P** | **G** | **S** | **T** | **F** | **K** | **T** | **I** | **T** | **G** | **A** | **I** | **G** | **L** | **D** | **A** | **G** | **T** | **L** | **K** | **P** | **D** | **E** | **E** | **L** | **E** | **I** | **N** | **G** | **L** | **K** | **W** | **Q** | **K** | **D** | **K** | **S** | **W** | **G** | **G** | **Y** | **F** | **A** | **T** | **R** | **V** | **K** | **E** | **A** | **S** | **P** | **V** | **N** | **L** | **R** | **T** | **A** | **L** | **V** | **N** | **S** | **D** | **N** | **I** | **Y** | **F** | **A** | **Q** | **Q** | **T** | **L** | **R** | **M** | **G** | **E** | **D** | **K** | **F** | **R** | **A** | **G** | **L** | **N** | **K** | **F** | **I** | **F** | **G** | **E** | **E** | **L** | **D** | **L** | **P** | **I** | **A** | **M** | **T** | **P** | **A** | **Q** | **I** | **S** | **N** | **E** | **D** | **K** | **F** | **N** | **S** | **E** | **I** | **L** | **L** | **A** | **D** | **T** | **G** | **Y** | **G** | **Q** | **G** | **Q** | **L** | **L** | **I** | **S** | **P** | **I** | **Q** | **Q** | **A** | **T** | **M** | **Y** | **S** | **V** | **F** | **Q** | **N** | **N** | **G** | **T** | **L** | **V** | **Y** | **P** | **K** | **L** | **V** | **L** | **D** | **K** | **E** | **T** | **K** | **K** | **K** | **D** | **N** | **V** | **I** | **S** | **A** | **N** | **A** | **A** | **N** | **T** | **I** | **A** | **T** | **D** | **L** | **L** | **G** | **S** | **V** | **E** | **D** | **P** | **S** | **G** | **Y** | **V** | **Y** | **N** | **M** | **Y** | **N** | **P** | **N** | **F** | **S** | **L** | **A** | **A** | **K** | **T** | **G** | **T** | **A** | **E** | **I** | **K** | **D** | **K** | **Q** | **D** | **T** | **D** | **G** | **K** | **E** | **N** | **S** | **F** | **L** | **L** | **T** | **L** | **D** | **R** | **S** | **N** | **N** | **K** | **F** | **L** | **T** | **M** | **I** | **M** | **V** | **E** | **N** | **S** | **G** | **E** | **N** | **G** | **S** | **A** | **T** | **D** | **I** | **S** | **K** | **P** | **L** | **I** | **D** | **Y** | **L** | **E** | **A** | **T** | **I** | **K** |
| **N60027F** | **-** | **-** | **-** | **-** | **-** | **-** | **-** | **-** | **-** | **-** | **-** | **-** | **-** | **-** | **-** | **-** | **-** | **-** | **-** | **-** | **-** | **-** | **-** | **-** | **-** | **-** | **-** | **-** | **-** | **-** | **-** | **-** | **-** | **-** | **-** | **-** | **Q** | **W** | **Q** | **A** | **K** | **Q** | **E** | **L** | **A** | **E** | **A** | **K** | **K** | **T** | **A** | **T** | **T** | **F** | **L** | **N** | **V** | **L** | **S** | **K** | **Q** | **E** | **F** | **D** | **K** | **L** | **P** | **S** | **V** | **V** | **Q** | **E** | **A** | **S** | **L** | **K** | **K** | **N** | **G** | **Y** | **D** | **T** | **K** | **S** | **V** | **V** | **E** | **K** | **Y** | **Q** | **A** | **I** | **Y** | **S** | **G** | **I** | **Q** | **A** | **E** | **G** | **V** | **K** | **A** | **S** | **D** | **V** | **Q** | **V** | **K** | **K** | **A** | **K** | **D** | **N** | **Q** | **Y** | **T** | **F** | **T** | **Y** | **K** | **L** | **S** | **M** | **S** | **T** | **P** | **L** | **G** | **E** | **M** | **K** | **D** | **L** | **S** | **Y** | **Q** | **S** | **S** | **I** | **A** | **K** | **K** | **G** | **D** | **T** | **Y** | **Q** | **I** | **A** | **W** | **K** | **P** | **S** | **L** | **I** | **F** | **P** | **D** | **M** | **S** | **G** | **N** | **D** | **K** | **I** | **S** | **I** | **Q** | **V** | **D** | **N** | **A** | **K** | **R** | **G** | **E** | **I** | **V** | **D** | **R** | **N** | **G** | **S** | **G** | **L** | **A** | **I** | **N** | **K** | **V** | **F** | **D** | **E** | **V** | **G** | **V** | **V** | **P** | **G** | **K** | **L** | **G** | **S** | **G** | **A** | **E** | **K** | **T** | **A** | **N** | **I** | **K** | **A** | **F** | **S** | **D** | **K** | **F** | **G** | **V** | **S** | **V** | **D** | **E** | **I** | **N** | **Q** | **K** | **L** | **S** | **Q** | **G** | **W** | **V** | **Q** | **A** | **D** | **S** | **F** | **V** | **P** | **I** | **T** | **V** | **A** | **S** | **E** | **P** | **V** | **T** | **E** | **L** | **P** | **T** | **G** | **A** | **A** | **T** | **K** | **D** | **T** | **E** | **S** | **R** | **Y** | **Y** | **P** | **L** | **G** | **E** | **A** | **A** | **A** | **Q** | **L** | **I** | **G** | **Y** | **T** | **G** | **T** | **I** | **T** | **A** | **E** | **D** | **I** | **E** | **K** | **N** | **P** | **E** | **L** | **S** | **S** | **T** | **G** | **V** | **I** | **G** | **K** | **T** | **G** | **L** | **E** | **R** | **A** | **F** | **D** | **K** | **E** | **L** | **R** | **G** | **Q** | **D** | **G** | **G** | **S** | **L** | **V** | **I** | **L** | **D** | **D** | **K** | **E** | **N** | **V** | **K** | **K** | **A** | **L** | **Q** | **T** | **K** | **D** | **K** | **K** | **D** | **G** | **Q** | **T** | **I** | **K** | **L** | **T** | **I** | **D** | **S** | **G** | **V** | **Q** | **Q** | **Q** | **A** | **F** | **A** | **I** | **F** | **D** | **K** | **R** | **P** | **G** | **S** | **A** | **V** | **I** | **T** | **D** | **P** | **Q** | **K** | **G** | **D** | **L** | **L** | **A** | **T** | **V** | **S** | **S** | **P** | **S** | **Y** | **D** | **P** | **N** | **K** | **M** | **A** | **N** | **G** | **I** | **S** | **Q** | **K** | **E** | **Y** | **D** | **A** | **Y** | **N** | **N** | **N** | **K** | **D** | **L** | **P** | **F** | **T** | **A** | **R** | **F** | **A** | **T** | **G** | **Y** | **A** | **P** | **G** | **S** | **T** | **F** | **K** | **T** | **I** | **T** | **G** | **A** | **I** | **G** | **L** | **D** | **A** | **G** | **T** | **L** | **K** | **P** | **D** | **E** | **E** | **L** | **E** | **I** | **N** | **G** | **L** | **K** | **W** | **Q** | **K** | **D** | **K** | **S** | **W** | **G** | **G** | **Y** | **F** | **A** | **T** | **R** | **V** | **K** | **E** | **A** | **S** | **P** | **V** | **N** | **L** | **R** | **T** | **A** | **L** | **V** | **N** | **S** | **D** | **N** | **I** | **Y** | **F** | **A** | **Q** | **Q** | **T** | **L** | **R** | **M** | **G** | **E** | **D** | **K** | **F** | **R** | **A** | **G** | **L** | **N** | **K** | **F** | **I** | **F** | **G** | **E** | **E** | **L** | **D** | **L** | **P** | **I** | **A** | **M** | **T** | **P** | **A** | **Q** | **I** | **S** | **N** | **E** | **D** | **K** | **F** | **N** | **S** | **E** | **I** | **L** | **L** | **A** | **D** | **T** | **G** | **Y** | **G** | **Q** | **G** | **Q** | **L** | **L** | **I** | **S** | **P** | **I** | **Q** | **Q** | **A** | **T** | **M** | **Y** | **S** | **V** | **F** | **Q** | **N** | **N** | **G** | **T** | **L** | **V** | **Y** | **P** | **K** | **L** | **V** | **L** | **D** | **K** | **E** | **T** | **K** | **K** | **K** | **D** | **N** | **V** | **I** | **S** | **A** | **N** | **A** | **A** | **N** | **T** | **I** | **A** | **T** | **D** | **L** | **L** | **G** | **S** | **V** | **E** | **D** | **P** | **S** | **G** | **Y** | **V** | **Y** | **N** | **M** | **Y** | **N** | **P** | **N** | **F** | **S** | **L** | **A** | **A** | **K** | **T** | **G** | **T** | **A** | **E** | **I** | **K** | **D** | **K** | **Q** | **D** | **T** | **D** | **G** | **K** | **E** | **N** | **S** | **F** | **L** | **L** | **T** | **L** | **D** | **R** | **S** | **N** | **N** | **K** | **F** | **L** | **T** | **M** | **I** | **M** | **V** | **E** | **N** | **S** | **G** | **E** | **N** | **G** | **S** | **A** | **T** | **D** | **I** | **S** | **K** | **P** | **L** | **I** | **D** | **Y** | **L** | **E** | **A** | **T** | **I** | **K** | **-** | **-** | **-** | **-** | **-** | **-** | **-** | **-** | **-** | **-** | **-** | **-** | **-** | **-** | **-** | **-** | **-** | **-** | **-** | **-** | **-** | **-** | **-** | **-** | **-** | **-** | **-** | **-** | **-** | **-** | **-** | **-** | **-** | **-** | **-** | **-** | **Q** | **W** | **Q** | **A** | **K** | **Q** | **E** | **L** | **A** | **E** | **A** | **K** | **K** | **T** | **A** | **T** | **T** | **F** | **L** | **N** | **V** | **L** | **S** | **K** | **Q** | **E** | **F** | **D** | **K** | **L** | **P** | **S** | **V** | **V** | **Q** | **E** | **A** | **S** | **L** | **K** | **K** | **N** | **G** | **Y** | **D** | **T** | **K** | **S** | **V** | **V** | **E** | **K** | **Y** | **Q** | **A** | **I** | **Y** | **S** | **G** | **I** | **Q** | **A** | **E** | **G** | **V** | **K** | **A** | **S** | **D** | **V** | **Q** | **V** | **K** | **K** | **A** | **K** | **D** | **N** | **Q** | **Y** | **T** | **F** | **T** | **Y** | **K** | **L** | **S** | **M** | **S** | **T** | **P** | **L** | **G** | **E** | **M** | **K** | **D** | **L** | **S** | **Y** | **Q** | **S** | **S** | **I** | **A** | **K** | **K** | **G** | **D** | **T** | **Y** | **Q** | **I** | **A** | **W** | **K** | **P** | **S** | **L** | **I** | **F** | **P** | **D** | **M** | **S** | **G** | **N** | **D** | **K** | **I** | **S** | **I** | **Q** | **V** | **D** | **N** | **A** | **K** | **R** | **G** | **E** | **I** | **V** | **D** | **R** | **N** | **G** | **S** | **G** | **L** | **A** | **I** | **N** | **K** | **V** | **F** | **D** | **E** | **V** | **G** | **V** | **V** | **P** | **G** | **K** | **L** | **G** | **S** | **G** | **A** | **E** | **K** | **T** | **A** | **N** | **I** | **K** | **A** | **F** | **S** | **D** | **K** | **F** | **G** | **V** | **S** | **V** | **D** | **E** | **I** | **N** | **Q** | **K** | **L** | **S** | **Q** | **G** | **W** | **V** | **Q** | **A** | **D** | **S** | **F** | **V** | **P** | **I** | **T** | **V** | **A** | **S** | **E** | **P** | **V** | **T** | **E** | **L** | **P** | **T** | **G** | **A** | **A** | **T** | **K** | **D** | **T** | **E** | **S** | **R** | **Y** | **Y** | **P** | **L** | **G** | **E** | **A** | **A** | **A** | **Q** | **L** | **I** | **G** | **Y** | **T** | **G** | **T** | **I** | **T** | **A** | **E** | **D** | **I** | **E** | **K** | **N** | **P** | **E** | **L** | **S** | **S** | **T** | **G** | **V** | **I** | **G** | **K** | **T** | **G** | **L** | **E** | **R** | **A** | **F** | **D** | **K** | **E** | **L** | **R** | **G** | **Q** | **D** | **G** | **G** | **S** | **L** | **V** | **I** | **L** | **D** | **D** | **K** | **E** | **N** | **V** | **K** | **K** | **A** | **L** | **Q** | **T** | **K** | **D** | **K** | **K** | **D** | **G** | **Q** | **T** | **I** | **K** | **L** | **T** | **I** | **D** | **S** | **G** | **V** | **Q** | **Q** | **Q** | **A** | **F** | **A** | **I** | **F** | **D** | **K** | **R** | **P** | **G** | **S** | **A** | **V** | **I** | **T** | **D** | **P** | **Q** | **K** | **G** | **D** | **L** | **L** | **A** | **T** | **V** | **S** | **S** | **P** | **S** | **Y** | **D** | **P** | **N** | **K** | **M** | **A** | **N** | **G** | **I** | **S** | **Q** | **K** | **E** | **Y** | **D** | **A** | **Y** | **N** | **N** | **N** | **K** | **D** | **L** | **P** | **F** | **T** | **A** | **R** | **F** | **A** | **T** | **G** | **Y** | **A** | **P** | **G** | **S** | **T** | **F** | **K** | **T** | **I** | **T** | **G** | **A** | **I** | **G** | **L** | **D** | **A** | **G** | **T** | **L** | **K** | **P** | **D** | **E** | **E** | **L** | **E** | **I** | **N** | **G** | **L** | **K** | **W** | **Q** | **K** | **D** | **K** | **S** | **W** | **G** | **G** | **Y** | **F** | **A** | **T** | **R** | **V** | **K** | **E** | **A** | **S** | **P** | **V** | **N** | **L** | **R** | **T** | **A** | **L** | **V** | **N** | **S** | **D** | **N** | **I** | **Y** | **F** | **A** | **Q** | **Q** | **T** | **L** | **R** | **M** | **G** | **E** | **D** | **K** | **F** | **R** | **A** | **G** | **L** | **N** | **K** | **F** | **I** | **F** | **G** | **E** | **E** | **L** | **D** | **L** | **P** | **I** | **A** | **M** | **T** | **P** | **A** | **Q** | **I** | **S** | **N** | **E** | **D** | **K** | **F** | **N** | **S** | **E** | **I** | **L** | **L** | **A** | **D** | **T** | **G** | **Y** | **G** | **Q** | **G** | **Q** | **L** | **L** | **I** | **S** | **P** | **I** | **Q** | **Q** | **A** | **T** | **M** | **Y** | **S** | **V** | **F** | **Q** | **N** | **N** | **G** | **T** | **L** | **V** | **Y** | **P** | **K** | **L** | **V** | **L** | **D** | **K** | **E** | **T** | **K** | **K** | **K** | **D** | **N** | **V** | **I** | **S** | **A** | **N** | **A** | **A** | **N** | **T** | **I** | **A** | **T** | **D** | **L** | **L** | **G** | **S** | **V** | **E** | **D** | **P** | **S** | **G** | **Y** | **V** | **Y** | **N** | **M** | **Y** | **N** | **P** | **N** | **F** | **S** | **L** | **A** | **A** | **K** | **T** | **G** | **T** | **A** | **E** | **I** | **K** | **D** | **K** | **Q** | **D** | **T** | **D** | **G** | **K** | **E** | **N** | **S** | **F** | **L** | **L** | **T** | **L** | **D** | **R** | **S** | **N** | **N** | **K** | **F** | **L** | **T** | **M** | **I** | **M** | **V** | **E** | **N** | **S** | **G** | **E** | **N** | **G** | **S** | **A** | **T** | **D** | **I** | **S** | **K** | **P** | **L** | **I** | **D** | **Y** | **L** | **E** | **A** | **T** | **I** | **K** |
| **N55706** | **-** | **-** | **-** | **-** | **-** | **-** | **-** | **-** | **-** | **-** | **-** | **-** | **-** | **-** | **-** | **-** | **-** | **-** | **-** | **-** | **-** | **-** | **-** | **-** | **-** | **-** | **-** | **-** | **-** | **-** | **-** | **-** | **-** | **-** | **-** | **-** | **Q** | **W** | **Q** | **A** | **K** | **Q** | **E** | **L** | **A** | **E** | **A** | **K** | **K** | **T** | **A** | **T** | **K** | **F** | **L** | **N** | **V** | **L** | **S** | **K** | **Q** | **E** | **F** | **D** | **K** | **L** | **P** | **S** | **V** | **V** | **Q** | **E** | **A** | **S** | **L** | **K** | **K** | **N** | **G** | **Y** | **D** | **T** | **K** | **S** | **V** | **V** | **E** | **K** | **Y** | **Q** | **A** | **I** | **Y** | **S** | **G** | **I** | **Q** | **A** | **E** | **G** | **V** | **K** | **A** | **S** | **D** | **V** | **Q** | **V** | **K** | **K** | **A** | **K** | **D** | **N** | **Q** | **Y** | **T** | **F** | **T** | **Y** | **K** | **L** | **S** | **M** | **S** | **T** | **P** | **L** | **G** | **E** | **M** | **K** | **D** | **L** | **S** | **Y** | **Q** | **S** | **S** | **I** | **A** | **K** | **K** | **G** | **D** | **T** | **Y** | **Q** | **I** | **A** | **W** | **K** | **P** | **S** | **L** | **I** | **F** | **P** | **D** | **M** | **S** | **G** | **N** | **D** | **K** | **I** | **S** | **I** | **Q** | **V** | **D** | **N** | **A** | **K** | **R** | **G** | **E** | **I** | **V** | **D** | **R** | **N** | **G** | **S** | **G** | **L** | **A** | **I** | **N** | **K** | **V** | **F** | **D** | **E** | **V** | **G** | **V** | **V** | **P** | **G** | **K** | **L** | **G** | **S** | **G** | **A** | **E** | **K** | **T** | **A** | **N** | **I** | **K** | **A** | **F** | **S** | **D** | **K** | **F** | **G** | **V** | **S** | **V** | **D** | **E** | **I** | **N** | **Q** | **K** | **L** | **S** | **Q** | **G** | **W** | **V** | **Q** | **A** | **D** | **S** | **F** | **V** | **P** | **I** | **T** | **V** | **A** | **S** | **E** | **P** | **V** | **T** | **E** | **L** | **P** | **T** | **G** | **A** | **A** | **T** | **K** | **D** | **T** | **E** | **S** | **R** | **Y** | **Y** | **P** | **L** | **G** | **E** | **A** | **A** | **A** | **Q** | **L** | **I** | **G** | **Y** | **T** | **G** | **T** | **I** | **T** | **A** | **E** | **D** | **I** | **K** | **K** | **N** | **P** | **E** | **L** | **S** | **S** | **T** | **G** | **V** | **I** | **G** | **K** | **T** | **G** | **L** | **E** | **R** | **A** | **F** | **D** | **K** | **E** | **L** | **R** | **G** | **Q** | **D** | **G** | **G** | **S** | **L** | **V** | **I** | **L** | **D** | **D** | **K** | **E** | **N** | **V** | **K** | **K** | **A** | **L** | **Q** | **T** | **K** | **E** | **K** | **K** | **D** | **G** | **Q** | **T** | **I** | **K** | **L** | **T** | **I** | **D** | **S** | **G** | **V** | **Q** | **Q** | **Q** | **A** | **F** | **A** | **I** | **F** | **D** | **K** | **R** | **P** | **G** | **S** | **A** | **V** | **I** | **T** | **D** | **P** | **Q** | **K** | **G** | **D** | **L** | **L** | **A** | **T** | **V** | **S** | **S** | **P** | **S** | **Y** | **D** | **P** | **N** | **K** | **M** | **A** | **N** | **G** | **I** | **S** | **Q** | **K** | **E** | **Y** | **D** | **A** | **Y** | **N** | **N** | **N** | **K** | **D** | **L** | **P** | **F** | **T** | **A** | **R** | **F** | **A** | **T** | **G** | **Y** | **A** | **P** | **G** | **S** | **T** | **F** | **K** | **T** | **I** | **T** | **G** | **A** | **I** | **G** | **L** | **D** | **A** | **G** | **T** | **L** | **K** | **P** | **D** | **E** | **E** | **L** | **E** | **I** | **N** | **G** | **L** | **K** | **W** | **Q** | **K** | **D** | **K** | **S** | **W** | **G** | **G** | **Y** | **F** | **A** | **T** | **R** | **V** | **K** | **E** | **A** | **S** | **P** | **V** | **N** | **L** | **R** | **T** | **A** | **L** | **V** | **N** | **S** | **D** | **N** | **I** | **Y** | **F** | **A** | **Q** | **Q** | **T** | **L** | **R** | **M** | **G** | **E** | **D** | **K** | **F** | **R** | **A** | **G** | **L** | **N** | **K** | **F** | **I** | **F** | **G** | **E** | **E** | **L** | **D** | **L** | **P** | **I** | **A** | **M** | **T** | **P** | **A** | **Q** | **I** | **S** | **N** | **E** | **D** | **K** | **F** | **N** | **S** | **E** | **I** | **L** | **L** | **A** | **D** | **T** | **G** | **Y** | **G** | **Q** | **G** | **Q** | **L** | **L** | **I** | **S** | **P** | **I** | **Q** | **Q** | **A** | **T** | **M** | **Y** | **S** | **V** | **F** | **Q** | **N** | **N** | **G** | **T** | **L** | **V** | **Y** | **P** | **K** | **L** | **V** | **L** | **D** | **K** | **E** | **T** | **K** | **K** | **K** | **D** | **N** | **V** | **I** | **S** | **A** | **N** | **A** | **A** | **N** | **T** | **I** | **A** | **T** | **D** | **L** | **L** | **G** | **S** | **V** | **E** | **D** | **P** | **S** | **G** | **Y** | **V** | **Y** | **N** | **M** | **Y** | **N** | **P** | **N** | **F** | **S** | **L** | **A** | **A** | **K** | **T** | **G** | **T** | **A** | **E** | **I** | **K** | **D** | **K** | **Q** | **D** | **T** | **D** | **G** | **K** | **E** | **N** | **S** | **F** | **L** | **L** | **T** | **L** | **D** | **R** | **S** | **N** | **N** | **K** | **F** | **L** | **T** | **M** | **I** | **M** | **V** | **E** | **N** | **S** | **G** | **E** | **N** | **G** | **S** | **A** | **T** | **D** | **I** | **S** | **K** | **P** | **L** | **I** | **D** | **Y** | **L** | **E** | **A** | **T** | **I** | **K** | **-** | **-** | **-** | **-** | **-** | **-** | **-** | **-** | **-** | **-** | **-** | **-** | **-** | **-** | **-** | **-** | **-** | **-** | **-** | **-** | **-** | **-** | **-** | **-** | **-** | **-** | **-** | **-** | **-** | **-** | **-** | **-** | **-** | **-** | **-** | **-** | **Q** | **W** | **Q** | **A** | **K** | **Q** | **E** | **L** | **A** | **E** | **A** | **K** | **K** | **T** | **A** | **T** | **K** | **F** | **L** | **N** | **V** | **L** | **S** | **K** | **Q** | **E** | **F** | **D** | **K** | **L** | **P** | **S** | **V** | **V** | **Q** | **E** | **A** | **S** | **L** | **K** | **K** | **N** | **G** | **Y** | **D** | **T** | **K** | **S** | **V** | **V** | **E** | **K** | **Y** | **Q** | **A** | **I** | **Y** | **S** | **G** | **I** | **Q** | **A** | **E** | **G** | **V** | **K** | **A** | **S** | **D** | **V** | **Q** | **V** | **K** | **K** | **A** | **K** | **D** | **N** | **Q** | **Y** | **T** | **F** | **T** | **Y** | **K** | **L** | **S** | **M** | **S** | **T** | **P** | **L** | **G** | **E** | **M** | **K** | **D** | **L** | **S** | **Y** | **Q** | **S** | **S** | **I** | **A** | **K** | **K** | **G** | **D** | **T** | **Y** | **Q** | **I** | **A** | **W** | **K** | **P** | **S** | **L** | **I** | **F** | **P** | **D** | **M** | **S** | **G** | **N** | **D** | **K** | **I** | **S** | **I** | **Q** | **V** | **D** | **N** | **A** | **K** | **R** | **G** | **E** | **I** | **V** | **D** | **R** | **N** | **G** | **S** | **G** | **L** | **A** | **I** | **N** | **K** | **V** | **F** | **D** | **E** | **V** | **G** | **V** | **V** | **P** | **G** | **K** | **L** | **G** | **S** | **G** | **A** | **E** | **K** | **T** | **A** | **N** | **I** | **K** | **A** | **F** | **S** | **D** | **K** | **F** | **G** | **V** | **S** | **V** | **D** | **E** | **I** | **N** | **Q** | **K** | **L** | **S** | **Q** | **G** | **W** | **V** | **Q** | **A** | **D** | **S** | **F** | **V** | **P** | **I** | **T** | **V** | **A** | **S** | **E** | **P** | **V** | **T** | **E** | **L** | **P** | **T** | **G** | **A** | **A** | **T** | **K** | **D** | **T** | **E** | **S** | **R** | **Y** | **Y** | **P** | **L** | **G** | **E** | **A** | **A** | **A** | **Q** | **L** | **I** | **G** | **Y** | **T** | **G** | **T** | **I** | **T** | **A** | **E** | **D** | **I** | **K** | **K** | **N** | **P** | **E** | **L** | **S** | **S** | **T** | **G** | **V** | **I** | **G** | **K** | **T** | **G** | **L** | **E** | **R** | **A** | **F** | **D** | **K** | **E** | **L** | **R** | **G** | **Q** | **D** | **G** | **G** | **S** | **L** | **V** | **I** | **L** | **D** | **D** | **K** | **E** | **N** | **V** | **K** | **K** | **A** | **L** | **Q** | **T** | **K** | **E** | **K** | **K** | **D** | **G** | **Q** | **T** | **I** | **K** | **L** | **T** | **I** | **D** | **S** | **G** | **V** | **Q** | **Q** | **Q** | **A** | **F** | **A** | **I** | **F** | **D** | **K** | **R** | **P** | **G** | **S** | **A** | **V** | **I** | **T** | **D** | **P** | **Q** | **K** | **G** | **D** | **L** | **L** | **A** | **T** | **V** | **S** | **S** | **P** | **S** | **Y** | **D** | **P** | **N** | **K** | **M** | **A** | **N** | **G** | **I** | **S** | **Q** | **K** | **E** | **Y** | **D** | **A** | **Y** | **N** | **N** | **N** | **K** | **D** | **L** | **P** | **F** | **T** | **A** | **R** | **F** | **A** | **T** | **G** | **Y** | **A** | **P** | **G** | **S** | **T** | **F** | **K** | **T** | **I** | **T** | **G** | **A** | **I** | **G** | **L** | **D** | **A** | **G** | **T** | **L** | **K** | **P** | **D** | **E** | **E** | **L** | **E** | **I** | **N** | **G** | **L** | **K** | **W** | **Q** | **K** | **D** | **K** | **S** | **W** | **G** | **G** | **Y** | **F** | **A** | **T** | **R** | **V** | **K** | **E** | **A** | **S** | **P** | **V** | **N** | **L** | **R** | **T** | **A** | **L** | **V** | **N** | **S** | **D** | **N** | **I** | **Y** | **F** | **A** | **Q** | **Q** | **T** | **L** | **R** | **M** | **G** | **E** | **D** | **K** | **F** | **R** | **A** | **G** | **L** | **N** | **K** | **F** | **I** | **F** | **G** | **E** | **E** | **L** | **D** | **L** | **P** | **I** | **A** | **M** | **T** | **P** | **A** | **Q** | **I** | **S** | **N** | **E** | **D** | **K** | **F** | **N** | **S** | **E** | **I** | **L** | **L** | **A** | **D** | **T** | **G** | **Y** | **G** | **Q** | **G** | **Q** | **L** | **L** | **I** | **S** | **P** | **I** | **Q** | **Q** | **A** | **T** | **M** | **Y** | **S** | **V** | **F** | **Q** | **N** | **N** | **G** | **T** | **L** | **V** | **Y** | **P** | **K** | **L** | **V** | **L** | **D** | **K** | **E** | **T** | **K** | **K** | **K** | **D** | **N** | **V** | **I** | **S** | **A** | **N** | **A** | **A** | **N** | **T** | **I** | **A** | **T** | **D** | **L** | **L** | **G** | **S** | **V** | **E** | **D** | **P** | **S** | **G** | **Y** | **V** | **Y** | **N** | **M** | **Y** | **N** | **P** | **N** | **F** | **S** | **L** | **A** | **A** | **K** | **T** | **G** | **T** | **A** | **E** | **I** | **K** | **D** | **K** | **Q** | **D** | **T** | **D** | **G** | **K** | **E** | **N** | **S** | **F** | **L** | **L** | **T** | **L** | **D** | **R** | **S** | **N** | **N** | **K** | **F** | **L** | **T** | **M** | **I** | **M** | **V** | **E** | **N** | **S** | **G** | **E** | **N** | **G** | **S** | **A** | **T** | **D** | **I** | **S** | **K** | **P** | **L** | **I** | **D** | **Y** | **L** | **E** | **A** | **T** | **I** | **K** |
| **N53519** | **-** | **-** | **-** | **-** | **-** | **-** | **-** | **-** | **-** | **-** | **-** | **-** | **-** | **-** | **-** | **-** | **-** | **-** | **-** | **-** | **-** | **-** | **-** | **-** | **-** | **-** | **-** | **-** | **-** | **-** | **-** | **-** | **-** | **-** | **-** | **-** | **Q** | **W** | **Q** | **A** | **K** | **Q** | **E** | **L** | **A** | **E** | **A** | **K** | **K** | **T** | **A** | **T** | **T** | **F** | **L** | **N** | **V** | **L** | **S** | **K** | **Q** | **E** | **F** | **D** | **K** | **L** | **P** | **S** | **V** | **V** | **Q** | **E** | **A** | **S** | **L** | **K** | **K** | **N** | **G** | **Y** | **D** | **T** | **K** | **S** | **V** | **V** | **E** | **K** | **Y** | **Q** | **A** | **I** | **Y** | **S** | **G** | **I** | **Q** | **A** | **E** | **G** | **V** | **K** | **A** | **S** | **D** | **V** | **Q** | **V** | **K** | **K** | **A** | **K** | **D** | **N** | **Q** | **Y** | **T** | **F** | **T** | **Y** | **K** | **L** | **S** | **M** | **S** | **T** | **P** | **L** | **G** | **E** | **M** | **K** | **D** | **L** | **S** | **Y** | **Q** | **S** | **S** | **I** | **A** | **K** | **K** | **G** | **D** | **T** | **Y** | **Q** | **I** | **A** | **W** | **K** | **P** | **S** | **L** | **I** | **F** | **P** | **D** | **M** | **S** | **G** | **N** | **D** | **K** | **I** | **S** | **I** | **Q** | **V** | **D** | **N** | **A** | **K** | **R** | **G** | **E** | **I** | **V** | **D** | **R** | **N** | **G** | **S** | **G** | **L** | **A** | **I** | **N** | **K** | **V** | **F** | **D** | **E** | **V** | **G** | **V** | **V** | **P** | **G** | **K** | **L** | **G** | **S** | **G** | **A** | **E** | **K** | **T** | **A** | **N** | **I** | **K** | **A** | **F** | **S** | **D** | **K** | **F** | **G** | **V** | **S** | **I** | **D** | **E** | **I** | **N** | **Q** | **K** | **L** | **S** | **Q** | **G** | **W** | **V** | **Q** | **A** | **D** | **S** | **F** | **V** | **P** | **I** | **T** | **V** | **A** | **S** | **E** | **P** | **V** | **T** | **E** | **L** | **P** | **T** | **G** | **A** | **A** | **T** | **K** | **D** | **T** | **E** | **S** | **R** | **Y** | **Y** | **P** | **L** | **G** | **E** | **A** | **A** | **A** | **Q** | **L** | **I** | **G** | **Y** | **T** | **G** | **T** | **I** | **T** | **A** | **E** | **D** | **I** | **E** | **K** | **N** | **P** | **E** | **L** | **S** | **S** | **T** | **G** | **V** | **I** | **G** | **K** | **T** | **G** | **L** | **E** | **R** | **A** | **F** | **D** | **K** | **E** | **L** | **R** | **G** | **Q** | **D** | **G** | **G** | **S** | **L** | **V** | **I** | **L** | **D** | **D** | **K** | **E** | **N** | **V** | **K** | **K** | **A** | **L** | **Q** | **T** | **K** | **E** | **K** | **K** | **D** | **G** | **Q** | **T** | **I** | **K** | **L** | **T** | **I** | **D** | **S** | **G** | **V** | **Q** | **Q** | **Q** | **A** | **F** | **A** | **I** | **F** | **D** | **K** | **R** | **P** | **G** | **S** | **A** | **V** | **I** | **T** | **D** | **P** | **Q** | **K** | **G** | **D** | **L** | **L** | **A** | **T** | **V** | **S** | **S** | **P** | **S** | **Y** | **D** | **P** | **N** | **K** | **M** | **A** | **N** | **G** | **I** | **S** | **Q** | **K** | **E** | **Y** | **D** | **A** | **Y** | **N** | **N** | **N** | **K** | **D** | **L** | **P** | **F** | **T** | **A** | **R** | **F** | **A** | **T** | **G** | **Y** | **A** | **P** | **G** | **S** | **T** | **F** | **K** | **T** | **I** | **T** | **G** | **A** | **I** | **G** | **L** | **D** | **A** | **G** | **T** | **L** | **K** | **P** | **D** | **E** | **E** | **L** | **E** | **I** | **N** | **G** | **L** | **K** | **W** | **Q** | **K** | **D** | **K** | **S** | **W** | **G** | **G** | **Y** | **F** | **A** | **T** | **R** | **V** | **K** | **E** | **A** | **S** | **P** | **V** | **N** | **L** | **R** | **T** | **A** | **L** | **V** | **N** | **S** | **D** | **N** | **I** | **Y** | **F** | **A** | **Q** | **Q** | **T** | **L** | **R** | **M** | **G** | **E** | **D** | **K** | **F** | **R** | **A** | **G** | **L** | **N** | **K** | **F** | **I** | **F** | **G** | **E** | **E** | **L** | **D** | **L** | **P** | **I** | **A** | **M** | **T** | **P** | **A** | **Q** | **I** | **S** | **N** | **E** | **D** | **K** | **F** | **N** | **S** | **E** | **I** | **L** | **L** | **A** | **D** | **T** | **G** | **Y** | **G** | **Q** | **G** | **Q** | **L** | **L** | **I** | **S** | **P** | **I** | **Q** | **Q** | **A** | **T** | **M** | **Y** | **S** | **V** | **F** | **Q** | **N** | **N** | **G** | **T** | **L** | **V** | **Y** | **P** | **K** | **I** | **V** | **L** | **D** | **K** | **E** | **T** | **K** | **K** | **K** | **D** | **N** | **V** | **I** | **S** | **A** | **N** | **A** | **A** | **N** | **T** | **I** | **A** | **T** | **D** | **L** | **L** | **G** | **S** | **V** | **E** | **D** | **P** | **S** | **G** | **Y** | **V** | **Y** | **N** | **M** | **Y** | **N** | **P** | **N** | **F** | **S** | **L** | **A** | **A** | **K** | **T** | **G** | **T** | **A** | **E** | **I** | **K** | **D** | **K** | **Q** | **D** | **T** | **D** | **G** | **K** | **E** | **N** | **S** | **F** | **L** | **L** | **T** | **L** | **D** | **R** | **S** | **N** | **N** | **K** | **F** | **L** | **T** | **M** | **I** | **M** | **V** | **E** | **N** | **S** | **G** | **E** | **N** | **G** | **S** | **A** | **T** | **D** | **I** | **S** | **K** | **P** | **L** | **I** | **D** | **Y** | **L** | **E** | **A** | **T** | **I** | **K** | **-** | **-** | **-** | **-** | **-** | **-** | **-** | **-** | **-** | **-** | **-** | **-** | **-** | **-** | **-** | **-** | **-** | **-** | **-** | **-** | **-** | **-** | **-** | **-** | **-** | **-** | **-** | **-** | **-** | **-** | **-** | **-** | **-** | **-** | **-** | **-** | **Q** | **W** | **Q** | **A** | **K** | **Q** | **E** | **L** | **A** | **E** | **A** | **K** | **K** | **T** | **A** | **T** | **T** | **F** | **L** | **N** | **V** | **L** | **S** | **K** | **Q** | **E** | **F** | **D** | **K** | **L** | **P** | **S** | **V** | **V** | **Q** | **E** | **A** | **S** | **L** | **K** | **K** | **N** | **G** | **Y** | **D** | **T** | **K** | **S** | **V** | **V** | **E** | **K** | **Y** | **Q** | **A** | **I** | **Y** | **S** | **G** | **I** | **Q** | **A** | **E** | **G** | **V** | **K** | **A** | **S** | **D** | **V** | **Q** | **V** | **K** | **K** | **A** | **K** | **D** | **N** | **Q** | **Y** | **T** | **F** | **T** | **Y** | **K** | **L** | **S** | **M** | **S** | **T** | **P** | **L** | **G** | **E** | **M** | **K** | **D** | **L** | **S** | **Y** | **Q** | **S** | **S** | **I** | **A** | **K** | **K** | **G** | **D** | **T** | **Y** | **Q** | **I** | **A** | **W** | **K** | **P** | **S** | **L** | **I** | **F** | **P** | **D** | **M** | **S** | **G** | **N** | **D** | **K** | **I** | **S** | **I** | **Q** | **V** | **D** | **N** | **A** | **K** | **R** | **G** | **E** | **I** | **V** | **D** | **R** | **N** | **G** | **S** | **G** | **L** | **A** | **I** | **N** | **K** | **V** | **F** | **D** | **E** | **V** | **G** | **V** | **V** | **P** | **G** | **K** | **L** | **G** | **S** | **G** | **A** | **E** | **K** | **T** | **A** | **N** | **I** | **K** | **A** | **F** | **S** | **D** | **K** | **F** | **G** | **V** | **S** | **I** | **D** | **E** | **I** | **N** | **Q** | **K** | **L** | **S** | **Q** | **G** | **W** | **V** | **Q** | **A** | **D** | **S** | **F** | **V** | **P** | **I** | **T** | **V** | **A** | **S** | **E** | **P** | **V** | **T** | **E** | **L** | **P** | **T** | **G** | **A** | **A** | **T** | **K** | **D** | **T** | **E** | **S** | **R** | **Y** | **Y** | **P** | **L** | **G** | **E** | **A** | **A** | **A** | **Q** | **L** | **I** | **G** | **Y** | **T** | **G** | **T** | **I** | **T** | **A** | **E** | **D** | **I** | **E** | **K** | **N** | **P** | **E** | **L** | **S** | **S** | **T** | **G** | **V** | **I** | **G** | **K** | **T** | **G** | **L** | **E** | **R** | **A** | **F** | **D** | **K** | **E** | **L** | **R** | **G** | **Q** | **D** | **G** | **G** | **S** | **L** | **V** | **I** | **L** | **D** | **D** | **K** | **E** | **N** | **V** | **K** | **K** | **A** | **L** | **Q** | **T** | **K** | **E** | **K** | **K** | **D** | **G** | **Q** | **T** | **I** | **K** | **L** | **T** | **I** | **D** | **S** | **G** | **V** | **Q** | **Q** | **Q** | **A** | **F** | **A** | **I** | **F** | **D** | **K** | **R** | **P** | **G** | **S** | **A** | **V** | **I** | **T** | **D** | **P** | **Q** | **K** | **G** | **D** | **L** | **L** | **A** | **T** | **V** | **S** | **S** | **P** | **S** | **Y** | **D** | **P** | **N** | **K** | **M** | **A** | **N** | **G** | **I** | **S** | **Q** | **K** | **E** | **Y** | **D** | **A** | **Y** | **N** | **N** | **N** | **K** | **D** | **L** | **P** | **F** | **T** | **A** | **R** | **F** | **A** | **T** | **G** | **Y** | **A** | **P** | **G** | **S** | **T** | **F** | **K** | **T** | **I** | **T** | **G** | **A** | **I** | **G** | **L** | **D** | **A** | **G** | **T** | **L** | **K** | **P** | **D** | **E** | **E** | **L** | **E** | **I** | **N** | **G** | **L** | **K** | **W** | **Q** | **K** | **D** | **K** | **S** | **W** | **G** | **G** | **Y** | **F** | **A** | **T** | **R** | **V** | **K** | **E** | **A** | **S** | **P** | **V** | **N** | **L** | **R** | **T** | **A** | **L** | **V** | **N** | **S** | **D** | **N** | **I** | **Y** | **F** | **A** | **Q** | **Q** | **T** | **L** | **R** | **M** | **G** | **E** | **D** | **K** | **F** | **R** | **A** | **G** | **L** | **N** | **K** | **F** | **I** | **F** | **G** | **E** | **E** | **L** | **D** | **L** | **P** | **I** | **A** | **M** | **T** | **P** | **A** | **Q** | **I** | **S** | **N** | **E** | **D** | **K** | **F** | **N** | **S** | **E** | **I** | **L** | **L** | **A** | **D** | **T** | **G** | **Y** | **G** | **Q** | **G** | **Q** | **L** | **L** | **I** | **S** | **P** | **I** | **Q** | **Q** | **A** | **T** | **M** | **Y** | **S** | **V** | **F** | **Q** | **N** | **N** | **G** | **T** | **L** | **V** | **Y** | **P** | **K** | **I** | **V** | **L** | **D** | **K** | **E** | **T** | **K** | **K** | **K** | **D** | **N** | **V** | **I** | **S** | **A** | **N** | **A** | **A** | **N** | **T** | **I** | **A** | **T** | **D** | **L** | **L** | **G** | **S** | **V** | **E** | **D** | **P** | **S** | **G** | **Y** | **V** | **Y** | **N** | **M** | **Y** | **N** | **P** | **N** | **F** | **S** | **L** | **A** | **A** | **K** | **T** | **G** | **T** | **A** | **E** | **I** | **K** | **D** | **K** | **Q** | **D** | **T** | **D** | **G** | **K** | **E** | **N** | **S** | **F** | **L** | **L** | **T** | **L** | **D** | **R** | **S** | **N** | **N** | **K** | **F** | **L** | **T** | **M** | **I** | **M** | **V** | **E** | **N** | **S** | **G** | **E** | **N** | **G** | **S** | **A** | **T** | **D** | **I** | **S** | **K** | **P** | **L** | **I** | **D** | **Y** | **L** | **E** | **A** | **T** | **I** | **K** |
| **N59860F** | **-** | **-** | **-** | **-** | **-** | **-** | **-** | **-** | **-** | **-** | **-** | **-** | **-** | **-** | **-** | **-** | **-** | **-** | **-** | **-** | **-** | **-** | **-** | **-** | **-** | **-** | **-** | **-** | **-** | **-** | **-** | **-** | **-** | **-** | **-** | **-** | **Q** | **W** | **Q** | **A** | **K** | **Q** | **E** | **L** | **A** | **E** | **A** | **K** | **K** | **T** | **A** | **T** | **T** | **F** | **L** | **N** | **V** | **L** | **S** | **K** | **Q** | **E** | **F** | **D** | **K** | **L** | **P** | **S** | **V** | **V** | **Q** | **E** | **A** | **S** | **L** | **K** | **K** | **N** | **G** | **Y** | **D** | **T** | **K** | **S** | **V** | **V** | **E** | **K** | **Y** | **Q** | **A** | **I** | **Y** | **S** | **G** | **I** | **Q** | **A** | **E** | **G** | **V** | **K** | **A** | **S** | **D** | **V** | **Q** | **V** | **K** | **K** | **A** | **K** | **D** | **N** | **Q** | **Y** | **T** | **F** | **T** | **Y** | **K** | **L** | **S** | **M** | **S** | **T** | **P** | **L** | **G** | **E** | **M** | **K** | **D** | **L** | **S** | **Y** | **Q** | **S** | **S** | **I** | **A** | **K** | **K** | **G** | **D** | **T** | **Y** | **Q** | **I** | **A** | **W** | **K** | **P** | **S** | **L** | **I** | **F** | **P** | **D** | **M** | **S** | **G** | **N** | **D** | **K** | **I** | **S** | **I** | **Q** | **V** | **D** | **N** | **A** | **K** | **R** | **G** | **E** | **I** | **V** | **D** | **R** | **N** | **G** | **S** | **G** | **L** | **A** | **I** | **N** | **K** | **V** | **F** | **D** | **E** | **V** | **G** | **V** | **V** | **P** | **G** | **K** | **L** | **G** | **S** | **G** | **A** | **E** | **K** | **T** | **A** | **N** | **I** | **K** | **A** | **F** | **S** | **D** | **K** | **F** | **G** | **V** | **S** | **V** | **D** | **E** | **I** | **N** | **Q** | **K** | **L** | **S** | **Q** | **G** | **W** | **V** | **Q** | **A** | **D** | **S** | **F** | **V** | **P** | **I** | **T** | **V** | **A** | **S** | **E** | **P** | **V** | **T** | **E** | **L** | **P** | **T** | **G** | **A** | **A** | **T** | **K** | **D** | **T** | **E** | **S** | **R** | **Y** | **Y** | **P** | **L** | **G** | **E** | **A** | **A** | **A** | **Q** | **L** | **I** | **G** | **Y** | **T** | **G** | **T** | **I** | **T** | **A** | **E** | **D** | **I** | **E** | **K** | **N** | **P** | **E** | **L** | **S** | **S** | **T** | **G** | **V** | **I** | **G** | **K** | **T** | **G** | **L** | **E** | **R** | **A** | **F** | **D** | **K** | **E** | **L** | **R** | **G** | **Q** | **D** | **G** | **G** | **S** | **L** | **V** | **I** | **L** | **D** | **D** | **K** | **E** | **N** | **V** | **K** | **K** | **A** | **L** | **Q** | **T** | **K** | **E** | **K** | **K** | **D** | **G** | **Q** | **T** | **I** | **K** | **L** | **T** | **I** | **D** | **S** | **G** | **V** | **Q** | **Q** | **Q** | **A** | **F** | **A** | **I** | **F** | **D** | **K** | **R** | **P** | **G** | **S** | **A** | **V** | **I** | **T** | **D** | **P** | **Q** | **K** | **G** | **D** | **L** | **L** | **A** | **T** | **V** | **S** | **S** | **P** | **S** | **Y** | **D** | **P** | **N** | **K** | **M** | **A** | **N** | **G** | **I** | **S** | **Q** | **K** | **E** | **Y** | **D** | **A** | **Y** | **N** | **N** | **N** | **K** | **D** | **L** | **P** | **F** | **T** | **A** | **R** | **F** | **A** | **T** | **G** | **Y** | **A** | **P** | **G** | **S** | **T** | **F** | **K** | **T** | **I** | **T** | **G** | **A** | **I** | **G** | **L** | **D** | **A** | **G** | **T** | **L** | **K** | **P** | **D** | **E** | **E** | **L** | **E** | **I** | **N** | **G** | **L** | **K** | **W** | **Q** | **K** | **D** | **K** | **S** | **W** | **G** | **G** | **Y** | **F** | **A** | **T** | **R** | **V** | **K** | **E** | **A** | **S** | **P** | **V** | **N** | **L** | **R** | **T** | **A** | **L** | **V** | **N** | **S** | **D** | **N** | **I** | **Y** | **F** | **A** | **Q** | **Q** | **T** | **L** | **R** | **M** | **G** | **E** | **D** | **K** | **F** | **R** | **A** | **G** | **L** | **N** | **K** | **F** | **I** | **F** | **G** | **E** | **E** | **L** | **D** | **L** | **P** | **I** | **A** | **M** | **T** | **P** | **A** | **Q** | **I** | **S** | **N** | **E** | **D** | **K** | **F** | **N** | **S** | **E** | **I** | **L** | **L** | **A** | **D** | **T** | **G** | **Y** | **G** | **Q** | **G** | **Q** | **L** | **L** | **I** | **S** | **P** | **I** | **Q** | **Q** | **A** | **T** | **M** | **Y** | **S** | **V** | **F** | **Q** | **N** | **N** | **G** | **T** | **L** | **V** | **Y** | **P** | **K** | **L** | **V** | **L** | **D** | **K** | **E** | **T** | **K** | **K** | **K** | **D** | **N** | **V** | **I** | **S** | **A** | **N** | **A** | **A** | **N** | **T** | **I** | **A** | **T** | **D** | **L** | **L** | **G** | **S** | **V** | **E** | **D** | **P** | **S** | **G** | **Y** | **V** | **Y** | **N** | **M** | **Y** | **N** | **P** | **N** | **F** | **S** | **L** | **A** | **A** | **K** | **T** | **G** | **T** | **A** | **E** | **I** | **K** | **D** | **K** | **Q** | **D** | **T** | **D** | **G** | **K** | **E** | **N** | **S** | **F** | **L** | **L** | **T** | **L** | **D** | **R** | **S** | **N** | **N** | **K** | **F** | **L** | **T** | **M** | **I** | **M** | **V** | **E** | **N** | **S** | **G** | **E** | **N** | **G** | **S** | **A** | **T** | **D** | **I** | **S** | **K** | **P** | **L** | **I** | **D** | **Y** | **L** | **E** | **A** | **T** | **I** | **K** | **-** | **-** | **-** | **-** | **-** | **-** | **-** | **-** | **-** | **-** | **-** | **-** | **-** | **-** | **-** | **-** | **-** | **-** | **-** | **-** | **-** | **-** | **-** | **-** | **-** | **-** | **-** | **-** | **-** | **-** | **-** | **-** | **-** | **-** | **-** | **-** | **Q** | **W** | **Q** | **A** | **K** | **Q** | **E** | **L** | **A** | **E** | **A** | **K** | **K** | **T** | **A** | **T** | **T** | **F** | **L** | **N** | **V** | **L** | **S** | **K** | **Q** | **E** | **F** | **D** | **K** | **L** | **P** | **S** | **V** | **V** | **Q** | **E** | **A** | **S** | **L** | **K** | **K** | **N** | **G** | **Y** | **D** | **T** | **K** | **S** | **V** | **V** | **E** | **K** | **Y** | **Q** | **A** | **I** | **Y** | **S** | **G** | **I** | **Q** | **A** | **E** | **G** | **V** | **K** | **A** | **S** | **D** | **V** | **Q** | **V** | **K** | **K** | **A** | **K** | **D** | **N** | **Q** | **Y** | **T** | **F** | **T** | **Y** | **K** | **L** | **S** | **M** | **S** | **T** | **P** | **L** | **G** | **E** | **M** | **K** | **D** | **L** | **S** | **Y** | **Q** | **S** | **S** | **I** | **A** | **K** | **K** | **G** | **D** | **T** | **Y** | **Q** | **I** | **A** | **W** | **K** | **P** | **S** | **L** | **I** | **F** | **P** | **D** | **M** | **S** | **G** | **N** | **D** | **K** | **I** | **S** | **I** | **Q** | **V** | **D** | **N** | **A** | **K** | **R** | **G** | **E** | **I** | **V** | **D** | **R** | **N** | **G** | **S** | **G** | **L** | **A** | **I** | **N** | **K** | **V** | **F** | **D** | **E** | **V** | **G** | **V** | **V** | **P** | **G** | **K** | **L** | **G** | **S** | **G** | **A** | **E** | **K** | **T** | **A** | **N** | **I** | **K** | **A** | **F** | **S** | **D** | **K** | **F** | **G** | **V** | **S** | **V** | **D** | **E** | **I** | **N** | **Q** | **K** | **L** | **S** | **Q** | **G** | **W** | **V** | **Q** | **A** | **D** | **S** | **F** | **V** | **P** | **I** | **T** | **V** | **A** | **S** | **E** | **P** | **V** | **T** | **E** | **L** | **P** | **T** | **G** | **A** | **A** | **T** | **K** | **D** | **T** | **E** | **S** | **R** | **Y** | **Y** | **P** | **L** | **G** | **E** | **A** | **A** | **A** | **Q** | **L** | **I** | **G** | **Y** | **T** | **G** | **T** | **I** | **T** | **A** | **E** | **D** | **I** | **E** | **K** | **N** | **P** | **E** | **L** | **S** | **S** | **T** | **G** | **V** | **I** | **G** | **K** | **T** | **G** | **L** | **E** | **R** | **A** | **F** | **D** | **K** | **E** | **L** | **R** | **G** | **Q** | **D** | **G** | **G** | **S** | **L** | **V** | **I** | **L** | **D** | **D** | **K** | **E** | **N** | **V** | **K** | **K** | **A** | **L** | **Q** | **T** | **K** | **E** | **K** | **K** | **D** | **G** | **Q** | **T** | **I** | **K** | **L** | **T** | **I** | **D** | **S** | **G** | **V** | **Q** | **Q** | **Q** | **A** | **F** | **A** | **I** | **F** | **D** | **K** | **R** | **P** | **G** | **S** | **A** | **V** | **I** | **T** | **D** | **P** | **Q** | **K** | **G** | **D** | **L** | **L** | **A** | **T** | **V** | **S** | **S** | **P** | **S** | **Y** | **D** | **P** | **N** | **K** | **M** | **A** | **N** | **G** | **I** | **S** | **Q** | **K** | **E** | **Y** | **D** | **A** | **Y** | **N** | **N** | **N** | **K** | **D** | **L** | **P** | **F** | **T** | **A** | **R** | **F** | **A** | **T** | **G** | **Y** | **A** | **P** | **G** | **S** | **T** | **F** | **K** | **T** | **I** | **T** | **G** | **A** | **I** | **G** | **L** | **D** | **A** | **G** | **T** | **L** | **K** | **P** | **D** | **E** | **E** | **L** | **E** | **I** | **N** | **G** | **L** | **K** | **W** | **Q** | **K** | **D** | **K** | **S** | **W** | **G** | **G** | **Y** | **F** | **A** | **T** | **R** | **V** | **K** | **E** | **A** | **S** | **P** | **V** | **N** | **L** | **R** | **T** | **A** | **L** | **V** | **N** | **S** | **D** | **N** | **I** | **Y** | **F** | **A** | **Q** | **Q** | **T** | **L** | **R** | **M** | **G** | **E** | **D** | **K** | **F** | **R** | **A** | **G** | **L** | **N** | **K** | **F** | **I** | **F** | **G** | **E** | **E** | **L** | **D** | **L** | **P** | **I** | **A** | **M** | **T** | **P** | **A** | **Q** | **I** | **S** | **N** | **E** | **D** | **K** | **F** | **N** | **S** | **E** | **I** | **L** | **L** | **A** | **D** | **T** | **G** | **Y** | **G** | **Q** | **G** | **Q** | **L** | **L** | **I** | **S** | **P** | **I** | **Q** | **Q** | **A** | **T** | **M** | **Y** | **S** | **V** | **F** | **Q** | **N** | **N** | **G** | **T** | **L** | **V** | **Y** | **P** | **K** | **L** | **V** | **L** | **D** | **K** | **E** | **T** | **K** | **K** | **K** | **D** | **N** | **V** | **I** | **S** | **A** | **N** | **A** | **A** | **N** | **T** | **I** | **A** | **T** | **D** | **L** | **L** | **G** | **S** | **V** | **E** | **D** | **P** | **S** | **G** | **Y** | **V** | **Y** | **N** | **M** | **Y** | **N** | **P** | **N** | **F** | **S** | **L** | **A** | **A** | **K** | **T** | **G** | **T** | **A** | **E** | **I** | **K** | **D** | **K** | **Q** | **D** | **T** | **D** | **G** | **K** | **E** | **N** | **S** | **F** | **L** | **L** | **T** | **L** | **D** | **R** | **S** | **N** | **N** | **K** | **F** | **L** | **T** | **M** | **I** | **M** | **V** | **E** | **N** | **S** | **G** | **E** | **N** | **G** | **S** | **A** | **T** | **D** | **I** | **S** | **K** | **P** | **L** | **I** | **D** | **Y** | **L** | **E** | **A** | **T** | **I** | **K** |
| **N55106** | **-** | **-** | **-** | **-** | **-** | **-** | **-** | **-** | **-** | **-** | **-** | **-** | **-** | **-** | **-** | **-** | **-** | **-** | **-** | **-** | **-** | **-** | **-** | **-** | **-** | **-** | **-** | **-** | **-** | **-** | **-** | **-** | **-** | **-** | **-** | **-** | **Q** | **W** | **Q** | **A** | **K** | **Q** | **E** | **L** | **A** | **E** | **A** | **K** | **K** | **T** | **A** | **T** | **T** | **F** | **L** | **N** | **V** | **L** | **S** | **K** | **Q** | **E** | **F** | **D** | **K** | **L** | **P** | **S** | **V** | **V** | **Q** | **E** | **A** | **S** | **L** | **K** | **K** | **N** | **G** | **Y** | **D** | **T** | **K** | **S** | **V** | **V** | **E** | **K** | **Y** | **Q** | **A** | **I** | **Y** | **S** | **G** | **I** | **Q** | **A** | **E** | **G** | **V** | **K** | **A** | **S** | **D** | **V** | **Q** | **V** | **K** | **K** | **A** | **K** | **D** | **N** | **Q** | **Y** | **T** | **F** | **T** | **Y** | **K** | **L** | **S** | **M** | **S** | **T** | **P** | **L** | **G** | **E** | **M** | **K** | **D** | **L** | **S** | **Y** | **Q** | **S** | **S** | **I** | **A** | **K** | **K** | **G** | **D** | **T** | **Y** | **Q** | **I** | **A** | **W** | **K** | **P** | **S** | **L** | **I** | **F** | **P** | **D** | **M** | **S** | **G** | **N** | **D** | **K** | **I** | **S** | **I** | **Q** | **V** | **D** | **N** | **A** | **K** | **R** | **G** | **E** | **I** | **V** | **D** | **R** | **N** | **G** | **S** | **G** | **L** | **A** | **I** | **N** | **K** | **V** | **F** | **D** | **E** | **V** | **G** | **V** | **V** | **P** | **G** | **K** | **L** | **G** | **S** | **G** | **A** | **E** | **K** | **T** | **A** | **N** | **I** | **K** | **A** | **F** | **S** | **D** | **K** | **F** | **G** | **V** | **S** | **V** | **D** | **E** | **I** | **N** | **Q** | **K** | **L** | **S** | **Q** | **G** | **W** | **V** | **Q** | **A** | **D** | **S** | **F** | **V** | **P** | **I** | **T** | **V** | **A** | **S** | **E** | **P** | **V** | **T** | **E** | **L** | **P** | **T** | **G** | **A** | **A** | **T** | **K** | **D** | **T** | **E** | **S** | **R** | **Y** | **Y** | **P** | **L** | **G** | **E** | **A** | **A** | **A** | **Q** | **L** | **I** | **G** | **Y** | **T** | **G** | **T** | **I** | **T** | **A** | **E** | **D** | **I** | **E** | **K** | **N** | **P** | **E** | **L** | **S** | **S** | **T** | **G** | **V** | **I** | **G** | **K** | **T** | **G** | **L** | **E** | **R** | **A** | **F** | **D** | **K** | **E** | **L** | **R** | **G** | **Q** | **D** | **G** | **G** | **S** | **L** | **V** | **I** | **L** | **D** | **D** | **K** | **E** | **N** | **V** | **K** | **K** | **A** | **L** | **Q** | **T** | **K** | **E** | **K** | **K** | **D** | **G** | **Q** | **T** | **I** | **K** | **L** | **T** | **I** | **D** | **S** | **G** | **V** | **Q** | **Q** | **Q** | **A** | **F** | **A** | **I** | **F** | **D** | **K** | **R** | **P** | **G** | **S** | **A** | **V** | **I** | **T** | **D** | **P** | **Q** | **K** | **G** | **D** | **L** | **L** | **A** | **T** | **V** | **S** | **S** | **P** | **S** | **Y** | **D** | **P** | **N** | **K** | **M** | **A** | **N** | **G** | **I** | **S** | **Q** | **K** | **E** | **Y** | **D** | **A** | **Y** | **N** | **N** | **N** | **K** | **D** | **L** | **P** | **F** | **T** | **A** | **R** | **F** | **A** | **T** | **G** | **Y** | **A** | **P** | **G** | **S** | **T** | **F** | **K** | **T** | **I** | **T** | **G** | **A** | **I** | **G** | **L** | **D** | **A** | **G** | **T** | **L** | **K** | **P** | **D** | **E** | **E** | **L** | **E** | **I** | **N** | **G** | **L** | **K** | **W** | **Q** | **K** | **D** | **K** | **S** | **W** | **G** | **G** | **Y** | **F** | **A** | **T** | **R** | **V** | **K** | **E** | **A** | **S** | **P** | **V** | **N** | **L** | **R** | **T** | **A** | **L** | **V** | **N** | **S** | **D** | **N** | **I** | **Y** | **F** | **A** | **Q** | **Q** | **T** | **L** | **R** | **M** | **G** | **E** | **D** | **K** | **F** | **R** | **A** | **G** | **L** | **N** | **K** | **F** | **I** | **F** | **G** | **E** | **E** | **L** | **D** | **L** | **P** | **I** | **A** | **M** | **T** | **P** | **A** | **Q** | **I** | **S** | **N** | **E** | **D** | **K** | **F** | **N** | **S** | **E** | **I** | **L** | **L** | **A** | **D** | **T** | **G** | **Y** | **G** | **Q** | **G** | **Q** | **L** | **L** | **I** | **S** | **P** | **I** | **Q** | **Q** | **A** | **T** | **M** | **Y** | **S** | **V** | **F** | **Q** | **N** | **N** | **G** | **T** | **L** | **V** | **Y** | **P** | **K** | **L** | **V** | **L** | **D** | **K** | **E** | **T** | **K** | **K** | **K** | **D** | **N** | **V** | **I** | **S** | **A** | **N** | **A** | **A** | **N** | **T** | **I** | **A** | **T** | **D** | **L** | **L** | **G** | **S** | **V** | **E** | **D** | **P** | **S** | **G** | **Y** | **V** | **Y** | **N** | **M** | **Y** | **N** | **P** | **N** | **F** | **S** | **L** | **A** | **A** | **K** | **T** | **G** | **T** | **A** | **E** | **I** | **K** | **D** | **K** | **Q** | **D** | **T** | **D** | **G** | **K** | **E** | **N** | **S** | **F** | **L** | **L** | **T** | **L** | **D** | **R** | **S** | **N** | **N** | **K** | **F** | **L** | **T** | **M** | **I** | **M** | **V** | **E** | **N** | **S** | **G** | **E** | **N** | **G** | **S** | **A** | **T** | **D** | **I** | **S** | **K** | **P** | **L** | **I** | **D** | **Y** | **L** | **E** | **A** | **T** | **I** | **K** | **-** | **-** | **-** | **-** | **-** | **-** | **-** | **-** | **-** | **-** | **-** | **-** | **-** | **-** | **-** | **-** | **-** | **-** | **-** | **-** | **-** | **-** | **-** | **-** | **-** | **-** | **-** | **-** | **-** | **-** | **-** | **-** | **-** | **-** | **-** | **-** | **Q** | **W** | **Q** | **A** | **K** | **Q** | **E** | **L** | **A** | **E** | **A** | **K** | **K** | **T** | **A** | **T** | **T** | **F** | **L** | **N** | **V** | **L** | **S** | **K** | **Q** | **E** | **F** | **D** | **K** | **L** | **P** | **S** | **V** | **V** | **Q** | **E** | **A** | **S** | **L** | **K** | **K** | **N** | **G** | **Y** | **D** | **T** | **K** | **S** | **V** | **V** | **E** | **K** | **Y** | **Q** | **A** | **I** | **Y** | **S** | **G** | **I** | **Q** | **A** | **E** | **G** | **V** | **K** | **A** | **S** | **D** | **V** | **Q** | **V** | **K** | **K** | **A** | **K** | **D** | **N** | **Q** | **Y** | **T** | **F** | **T** | **Y** | **K** | **L** | **S** | **M** | **S** | **T** | **P** | **L** | **G** | **E** | **M** | **K** | **D** | **L** | **S** | **Y** | **Q** | **S** | **S** | **I** | **A** | **K** | **K** | **G** | **D** | **T** | **Y** | **Q** | **I** | **A** | **W** | **K** | **P** | **S** | **L** | **I** | **F** | **P** | **D** | **M** | **S** | **G** | **N** | **D** | **K** | **I** | **S** | **I** | **Q** | **V** | **D** | **N** | **A** | **K** | **R** | **G** | **E** | **I** | **V** | **D** | **R** | **N** | **G** | **S** | **G** | **L** | **A** | **I** | **N** | **K** | **V** | **F** | **D** | **E** | **V** | **G** | **V** | **V** | **P** | **G** | **K** | **L** | **G** | **S** | **G** | **A** | **E** | **K** | **T** | **A** | **N** | **I** | **K** | **A** | **F** | **S** | **D** | **K** | **F** | **G** | **V** | **S** | **V** | **D** | **E** | **I** | **N** | **Q** | **K** | **L** | **S** | **Q** | **G** | **W** | **V** | **Q** | **A** | **D** | **S** | **F** | **V** | **P** | **I** | **T** | **V** | **A** | **S** | **E** | **P** | **V** | **T** | **E** | **L** | **P** | **T** | **G** | **A** | **A** | **T** | **K** | **D** | **T** | **E** | **S** | **R** | **Y** | **Y** | **P** | **L** | **G** | **E** | **A** | **A** | **A** | **Q** | **L** | **I** | **G** | **Y** | **T** | **G** | **T** | **I** | **T** | **A** | **E** | **D** | **I** | **E** | **K** | **N** | **P** | **E** | **L** | **S** | **S** | **T** | **G** | **V** | **I** | **G** | **K** | **T** | **G** | **L** | **E** | **R** | **A** | **F** | **D** | **K** | **E** | **L** | **R** | **G** | **Q** | **D** | **G** | **G** | **S** | **L** | **V** | **I** | **L** | **D** | **D** | **K** | **E** | **N** | **V** | **K** | **K** | **A** | **L** | **Q** | **T** | **K** | **E** | **K** | **K** | **D** | **G** | **Q** | **T** | **I** | **K** | **L** | **T** | **I** | **D** | **S** | **G** | **V** | **Q** | **Q** | **Q** | **A** | **F** | **A** | **I** | **F** | **D** | **K** | **R** | **P** | **G** | **S** | **A** | **V** | **I** | **T** | **D** | **P** | **Q** | **K** | **G** | **D** | **L** | **L** | **A** | **T** | **V** | **S** | **S** | **P** | **S** | **Y** | **D** | **P** | **N** | **K** | **M** | **A** | **N** | **G** | **I** | **S** | **Q** | **K** | **E** | **Y** | **D** | **A** | **Y** | **N** | **N** | **N** | **K** | **D** | **L** | **P** | **F** | **T** | **A** | **R** | **F** | **A** | **T** | **G** | **Y** | **A** | **P** | **G** | **S** | **T** | **F** | **K** | **T** | **I** | **T** | **G** | **A** | **I** | **G** | **L** | **D** | **A** | **G** | **T** | **L** | **K** | **P** | **D** | **E** | **E** | **L** | **E** | **I** | **N** | **G** | **L** | **K** | **W** | **Q** | **K** | **D** | **K** | **S** | **W** | **G** | **G** | **Y** | **F** | **A** | **T** | **R** | **V** | **K** | **E** | **A** | **S** | **P** | **V** | **N** | **L** | **R** | **T** | **A** | **L** | **V** | **N** | **S** | **D** | **N** | **I** | **Y** | **F** | **A** | **Q** | **Q** | **T** | **L** | **R** | **M** | **G** | **E** | **D** | **K** | **F** | **R** | **A** | **G** | **L** | **N** | **K** | **F** | **I** | **F** | **G** | **E** | **E** | **L** | **D** | **L** | **P** | **I** | **A** | **M** | **T** | **P** | **A** | **Q** | **I** | **S** | **N** | **E** | **D** | **K** | **F** | **N** | **S** | **E** | **I** | **L** | **L** | **A** | **D** | **T** | **G** | **Y** | **G** | **Q** | **G** | **Q** | **L** | **L** | **I** | **S** | **P** | **I** | **Q** | **Q** | **A** | **T** | **M** | **Y** | **S** | **V** | **F** | **Q** | **N** | **N** | **G** | **T** | **L** | **V** | **Y** | **P** | **K** | **L** | **V** | **L** | **D** | **K** | **E** | **T** | **K** | **K** | **K** | **D** | **N** | **V** | **I** | **S** | **A** | **N** | **A** | **A** | **N** | **T** | **I** | **A** | **T** | **D** | **L** | **L** | **G** | **S** | **V** | **E** | **D** | **P** | **S** | **G** | **Y** | **V** | **Y** | **N** | **M** | **Y** | **N** | **P** | **N** | **F** | **S** | **L** | **A** | **A** | **K** | **T** | **G** | **T** | **A** | **E** | **I** | **K** | **D** | **K** | **Q** | **D** | **T** | **D** | **G** | **K** | **E** | **N** | **S** | **F** | **L** | **L** | **T** | **L** | **D** | **R** | **S** | **N** | **N** | **K** | **F** | **L** | **T** | **M** | **I** | **M** | **V** | **E** | **N** | **S** | **G** | **E** | **N** | **G** | **S** | **A** | **T** | **D** | **I** | **S** | **K** | **P** | **L** | **I** | **D** | **Y** | **L** | **E** | **A** | **T** | **I** | **K** |
| **N52469** | **-** | **-** | **-** | **-** | **-** | **-** | **-** | **-** | **-** | **-** | **-** | **-** | **-** | **-** | **-** | **-** | **-** | **-** | **-** | **-** | **-** | **-** | **-** | **-** | **-** | **-** | **-** | **-** | **-** | **-** | **-** | **-** | **-** | **-** | **-** | **-** | **Q** | **W** | **Q** | **A** | **K** | **Q** | **E** | **L** | **A** | **E** | **A** | **K** | **K** | **T** | **A** | **T** | **T** | **F** | **L** | **N** | **V** | **L** | **S** | **K** | **Q** | **E** | **F** | **D** | **K** | **L** | **P** | **S** | **V** | **V** | **Q** | **E** | **A** | **S** | **L** | **K** | **K** | **N** | **G** | **Y** | **D** | **T** | **K** | **S** | **V** | **V** | **E** | **K** | **Y** | **Q** | **A** | **I** | **Y** | **S** | **G** | **I** | **Q** | **A** | **E** | **G** | **V** | **K** | **A** | **S** | **D** | **V** | **Q** | **V** | **K** | **K** | **A** | **K** | **D** | **N** | **Q** | **Y** | **T** | **F** | **T** | **Y** | **K** | **L** | **S** | **M** | **S** | **T** | **P** | **L** | **G** | **E** | **M** | **K** | **D** | **L** | **S** | **Y** | **Q** | **S** | **S** | **I** | **A** | **K** | **K** | **G** | **D** | **T** | **Y** | **Q** | **I** | **A** | **W** | **K** | **P** | **S** | **L** | **I** | **F** | **P** | **D** | **M** | **S** | **G** | **N** | **D** | **K** | **I** | **S** | **I** | **Q** | **V** | **D** | **N** | **A** | **K** | **R** | **G** | **E** | **I** | **V** | **D** | **R** | **N** | **G** | **S** | **G** | **L** | **A** | **I** | **N** | **K** | **V** | **F** | **D** | **E** | **V** | **G** | **V** | **V** | **P** | **G** | **K** | **L** | **G** | **S** | **G** | **A** | **E** | **K** | **T** | **A** | **N** | **I** | **K** | **A** | **F** | **S** | **D** | **K** | **F** | **G** | **V** | **S** | **V** | **D** | **E** | **I** | **N** | **Q** | **K** | **L** | **S** | **Q** | **G** | **W** | **V** | **Q** | **A** | **D** | **S** | **F** | **V** | **P** | **I** | **T** | **V** | **A** | **S** | **E** | **P** | **V** | **T** | **E** | **L** | **P** | **T** | **G** | **A** | **A** | **T** | **K** | **D** | **T** | **E** | **S** | **R** | **Y** | **Y** | **P** | **L** | **G** | **E** | **A** | **A** | **A** | **Q** | **L** | **I** | **G** | **Y** | **T** | **G** | **T** | **I** | **T** | **A** | **E** | **D** | **I** | **E** | **K** | **N** | **P** | **E** | **L** | **S** | **S** | **T** | **G** | **V** | **I** | **G** | **K** | **T** | **G** | **L** | **E** | **R** | **A** | **F** | **D** | **K** | **E** | **L** | **R** | **G** | **Q** | **D** | **G** | **G** | **S** | **L** | **V** | **I** | **L** | **D** | **D** | **K** | **E** | **N** | **V** | **K** | **K** | **A** | **L** | **Q** | **T** | **K** | **E** | **K** | **K** | **D** | **G** | **Q** | **T** | **I** | **K** | **L** | **T** | **I** | **D** | **S** | **G** | **V** | **Q** | **Q** | **Q** | **A** | **F** | **A** | **I** | **F** | **D** | **K** | **R** | **P** | **G** | **S** | **A** | **V** | **I** | **T** | **D** | **P** | **Q** | **K** | **G** | **D** | **L** | **L** | **A** | **T** | **V** | **S** | **S** | **P** | **S** | **Y** | **D** | **P** | **N** | **K** | **M** | **A** | **N** | **G** | **I** | **S** | **Q** | **K** | **E** | **Y** | **D** | **A** | **Y** | **N** | **N** | **N** | **K** | **D** | **L** | **P** | **F** | **T** | **A** | **R** | **F** | **A** | **T** | **G** | **Y** | **A** | **P** | **G** | **S** | **T** | **F** | **K** | **T** | **I** | **T** | **G** | **A** | **I** | **G** | **L** | **D** | **A** | **G** | **T** | **L** | **K** | **P** | **D** | **E** | **E** | **L** | **E** | **I** | **N** | **G** | **L** | **K** | **W** | **Q** | **K** | **D** | **K** | **S** | **W** | **G** | **G** | **Y** | **F** | **A** | **T** | **R** | **V** | **K** | **E** | **A** | **S** | **P** | **V** | **N** | **L** | **R** | **T** | **A** | **L** | **V** | **N** | **S** | **D** | **N** | **I** | **Y** | **F** | **A** | **Q** | **Q** | **T** | **L** | **R** | **M** | **G** | **E** | **D** | **K** | **F** | **R** | **A** | **G** | **L** | **N** | **K** | **F** | **I** | **F** | **G** | **E** | **E** | **L** | **D** | **L** | **P** | **I** | **A** | **M** | **T** | **P** | **A** | **Q** | **I** | **S** | **N** | **E** | **D** | **K** | **F** | **N** | **S** | **E** | **I** | **L** | **L** | **A** | **D** | **T** | **G** | **Y** | **G** | **Q** | **G** | **Q** | **L** | **L** | **I** | **S** | **P** | **I** | **Q** | **Q** | **A** | **T** | **M** | **Y** | **S** | **V** | **F** | **Q** | **N** | **N** | **G** | **T** | **L** | **V** | **Y** | **P** | **K** | **L** | **V** | **L** | **E** | **K** | **E** | **T** | **K** | **K** | **K** | **D** | **N** | **V** | **I** | **S** | **A** | **N** | **A** | **A** | **N** | **T** | **I** | **A** | **T** | **D** | **L** | **L** | **G** | **S** | **V** | **E** | **D** | **P** | **S** | **G** | **Y** | **V** | **Y** | **N** | **M** | **Y** | **N** | **P** | **N** | **F** | **S** | **L** | **A** | **A** | **K** | **T** | **G** | **T** | **A** | **E** | **I** | **K** | **D** | **K** | **Q** | **D** | **T** | **D** | **G** | **K** | **E** | **N** | **S** | **F** | **L** | **L** | **T** | **L** | **D** | **R** | **S** | **N** | **N** | **K** | **F** | **L** | **T** | **M** | **I** | **M** | **V** | **E** | **N** | **S** | **G** | **E** | **N** | **G** | **S** | **A** | **T** | **D** | **I** | **S** | **K** | **P** | **L** | **I** | **D** | **Y** | **L** | **E** | **A** | **T** | **I** | **K** | **-** | **-** | **-** | **-** | **-** | **-** | **-** | **-** | **-** | **-** | **-** | **-** | **-** | **-** | **-** | **-** | **-** | **-** | **-** | **-** | **-** | **-** | **-** | **-** | **-** | **-** | **-** | **-** | **-** | **-** | **-** | **-** | **-** | **-** | **-** | **-** | **Q** | **W** | **Q** | **A** | **K** | **Q** | **E** | **L** | **A** | **E** | **A** | **K** | **K** | **T** | **A** | **T** | **T** | **F** | **L** | **N** | **V** | **L** | **S** | **K** | **Q** | **E** | **F** | **D** | **K** | **L** | **P** | **S** | **V** | **V** | **Q** | **E** | **A** | **S** | **L** | **K** | **K** | **N** | **G** | **Y** | **D** | **T** | **K** | **S** | **V** | **V** | **E** | **K** | **Y** | **Q** | **A** | **I** | **Y** | **S** | **G** | **I** | **Q** | **A** | **E** | **G** | **V** | **K** | **A** | **S** | **D** | **V** | **Q** | **V** | **K** | **K** | **A** | **K** | **D** | **N** | **Q** | **Y** | **T** | **F** | **T** | **Y** | **K** | **L** | **S** | **M** | **S** | **T** | **P** | **L** | **G** | **E** | **M** | **K** | **D** | **L** | **S** | **Y** | **Q** | **S** | **S** | **I** | **A** | **K** | **K** | **G** | **D** | **T** | **Y** | **Q** | **I** | **A** | **W** | **K** | **P** | **S** | **L** | **I** | **F** | **P** | **D** | **M** | **S** | **G** | **N** | **D** | **K** | **I** | **S** | **I** | **Q** | **V** | **D** | **N** | **A** | **K** | **R** | **G** | **E** | **I** | **V** | **D** | **R** | **N** | **G** | **S** | **G** | **L** | **A** | **I** | **N** | **K** | **V** | **F** | **D** | **E** | **V** | **G** | **V** | **V** | **P** | **G** | **K** | **L** | **G** | **S** | **G** | **A** | **E** | **K** | **T** | **A** | **N** | **I** | **K** | **A** | **F** | **S** | **D** | **K** | **F** | **G** | **V** | **S** | **V** | **D** | **E** | **I** | **N** | **Q** | **K** | **L** | **S** | **Q** | **G** | **W** | **V** | **Q** | **A** | **D** | **S** | **F** | **V** | **P** | **I** | **T** | **V** | **A** | **S** | **E** | **P** | **V** | **T** | **E** | **L** | **P** | **T** | **G** | **A** | **A** | **T** | **K** | **D** | **T** | **E** | **S** | **R** | **Y** | **Y** | **P** | **L** | **G** | **E** | **A** | **A** | **A** | **Q** | **L** | **I** | **G** | **Y** | **T** | **G** | **T** | **I** | **T** | **A** | **E** | **D** | **I** | **E** | **K** | **N** | **P** | **E** | **L** | **S** | **S** | **T** | **G** | **V** | **I** | **G** | **K** | **T** | **G** | **L** | **E** | **R** | **A** | **F** | **D** | **K** | **E** | **L** | **R** | **G** | **Q** | **D** | **G** | **G** | **S** | **L** | **V** | **I** | **L** | **D** | **D** | **K** | **E** | **N** | **V** | **K** | **K** | **A** | **L** | **Q** | **T** | **K** | **E** | **K** | **K** | **D** | **G** | **Q** | **T** | **I** | **K** | **L** | **T** | **I** | **D** | **S** | **G** | **V** | **Q** | **Q** | **Q** | **A** | **F** | **A** | **I** | **F** | **D** | **K** | **R** | **P** | **G** | **S** | **A** | **V** | **I** | **T** | **D** | **P** | **Q** | **K** | **G** | **D** | **L** | **L** | **A** | **T** | **V** | **S** | **S** | **P** | **S** | **Y** | **D** | **P** | **N** | **K** | **M** | **A** | **N** | **G** | **I** | **S** | **Q** | **K** | **E** | **Y** | **D** | **A** | **Y** | **N** | **N** | **N** | **K** | **D** | **L** | **P** | **F** | **T** | **A** | **R** | **F** | **A** | **T** | **G** | **Y** | **A** | **P** | **G** | **S** | **T** | **F** | **K** | **T** | **I** | **T** | **G** | **A** | **I** | **G** | **L** | **D** | **A** | **G** | **T** | **L** | **K** | **P** | **D** | **E** | **E** | **L** | **E** | **I** | **N** | **G** | **L** | **K** | **W** | **Q** | **K** | **D** | **K** | **S** | **W** | **G** | **G** | **Y** | **F** | **A** | **T** | **R** | **V** | **K** | **E** | **A** | **S** | **P** | **V** | **N** | **L** | **R** | **T** | **A** | **L** | **V** | **N** | **S** | **D** | **N** | **I** | **Y** | **F** | **A** | **Q** | **Q** | **T** | **L** | **R** | **M** | **G** | **E** | **D** | **K** | **F** | **R** | **A** | **G** | **L** | **N** | **K** | **F** | **I** | **F** | **G** | **E** | **E** | **L** | **D** | **L** | **P** | **I** | **A** | **M** | **T** | **P** | **A** | **Q** | **I** | **S** | **N** | **E** | **D** | **K** | **F** | **N** | **S** | **E** | **I** | **L** | **L** | **A** | **D** | **T** | **G** | **Y** | **G** | **Q** | **G** | **Q** | **L** | **L** | **I** | **S** | **P** | **I** | **Q** | **Q** | **A** | **T** | **M** | **Y** | **S** | **V** | **F** | **Q** | **N** | **N** | **G** | **T** | **L** | **V** | **Y** | **P** | **K** | **L** | **V** | **L** | **E** | **K** | **E** | **T** | **K** | **K** | **K** | **D** | **N** | **V** | **I** | **S** | **A** | **N** | **A** | **A** | **N** | **T** | **I** | **A** | **T** | **D** | **L** | **L** | **G** | **S** | **V** | **E** | **D** | **P** | **S** | **G** | **Y** | **V** | **Y** | **N** | **M** | **Y** | **N** | **P** | **N** | **F** | **S** | **L** | **A** | **A** | **K** | **T** | **G** | **T** | **A** | **E** | **I** | **K** | **D** | **K** | **Q** | **D** | **T** | **D** | **G** | **K** | **E** | **N** | **S** | **F** | **L** | **L** | **T** | **L** | **D** | **R** | **S** | **N** | **N** | **K** | **F** | **L** | **T** | **M** | **I** | **M** | **V** | **E** | **N** | **S** | **G** | **E** | **N** | **G** | **S** | **A** | **T** | **D** | **I** | **S** | **K** | **P** | **L** | **I** | **D** | **Y** | **L** | **E** | **A** | **T** | **I** | **K** |
| **N59592F** | **-** | **-** | **-** | **-** | **-** | **-** | **-** | **-** | **-** | **-** | **-** | **-** | **-** | **-** | **-** | **-** | **-** | **-** | **-** | **-** | **-** | **-** | **-** | **-** | **-** | **-** | **-** | **-** | **-** | **-** | **-** | **-** | **-** | **-** | **-** | **-** | **Q** | **W** | **Q** | **A** | **K** | **Q** | **E** | **L** | **A** | **E** | **A** | **K** | **K** | **T** | **A** | **T** | **T** | **F** | **L** | **N** | **V** | **L** | **S** | **K** | **Q** | **E** | **F** | **D** | **K** | **L** | **P** | **S** | **V** | **V** | **Q** | **E** | **A** | **S** | **L** | **K** | **K** | **N** | **G** | **Y** | **D** | **T** | **K** | **S** | **V** | **V** | **E** | **K** | **Y** | **Q** | **A** | **I** | **Y** | **S** | **G** | **I** | **Q** | **A** | **E** | **G** | **V** | **K** | **A** | **S** | **D** | **V** | **Q** | **V** | **K** | **K** | **A** | **K** | **D** | **N** | **Q** | **Y** | **T** | **F** | **T** | **Y** | **K** | **L** | **S** | **M** | **S** | **T** | **P** | **L** | **G** | **E** | **M** | **K** | **D** | **L** | **S** | **Y** | **Q** | **S** | **S** | **I** | **A** | **K** | **K** | **G** | **D** | **T** | **Y** | **Q** | **I** | **A** | **W** | **K** | **P** | **S** | **L** | **I** | **F** | **P** | **D** | **M** | **S** | **G** | **N** | **D** | **K** | **I** | **S** | **I** | **Q** | **V** | **D** | **N** | **A** | **K** | **R** | **G** | **E** | **I** | **V** | **D** | **R** | **N** | **G** | **S** | **G** | **L** | **A** | **I** | **N** | **K** | **V** | **F** | **D** | **E** | **V** | **G** | **V** | **V** | **P** | **G** | **K** | **L** | **G** | **S** | **G** | **A** | **E** | **K** | **T** | **A** | **N** | **I** | **K** | **A** | **F** | **S** | **D** | **K** | **F** | **G** | **V** | **S** | **V** | **D** | **E** | **I** | **N** | **Q** | **K** | **L** | **S** | **Q** | **G** | **W** | **V** | **Q** | **A** | **D** | **S** | **F** | **V** | **P** | **I** | **T** | **V** | **A** | **S** | **E** | **P** | **V** | **T** | **E** | **L** | **P** | **T** | **G** | **A** | **A** | **T** | **K** | **D** | **T** | **E** | **S** | **R** | **Y** | **Y** | **P** | **L** | **G** | **E** | **A** | **A** | **A** | **Q** | **L** | **I** | **G** | **Y** | **T** | **G** | **T** | **I** | **T** | **A** | **E** | **D** | **I** | **E** | **K** | **N** | **P** | **E** | **L** | **S** | **S** | **T** | **G** | **V** | **I** | **G** | **K** | **T** | **G** | **L** | **E** | **R** | **A** | **F** | **D** | **K** | **E** | **L** | **R** | **G** | **Q** | **D** | **G** | **G** | **S** | **L** | **V** | **I** | **L** | **D** | **D** | **K** | **E** | **N** | **V** | **K** | **K** | **A** | **L** | **Q** | **T** | **K** | **E** | **K** | **K** | **D** | **G** | **Q** | **T** | **I** | **K** | **L** | **T** | **I** | **D** | **S** | **G** | **V** | **Q** | **Q** | **Q** | **A** | **F** | **A** | **I** | **F** | **D** | **K** | **R** | **P** | **G** | **S** | **A** | **V** | **I** | **T** | **D** | **P** | **Q** | **K** | **G** | **D** | **L** | **L** | **A** | **T** | **V** | **S** | **S** | **P** | **S** | **Y** | **D** | **P** | **N** | **K** | **M** | **A** | **N** | **G** | **I** | **S** | **Q** | **K** | **E** | **Y** | **D** | **A** | **Y** | **N** | **N** | **N** | **K** | **D** | **L** | **P** | **F** | **T** | **A** | **R** | **F** | **A** | **T** | **G** | **Y** | **A** | **P** | **G** | **S** | **T** | **F** | **K** | **T** | **I** | **T** | **G** | **A** | **I** | **G** | **L** | **D** | **A** | **G** | **T** | **L** | **K** | **P** | **D** | **E** | **E** | **L** | **E** | **I** | **N** | **G** | **L** | **K** | **W** | **Q** | **K** | **D** | **K** | **S** | **W** | **G** | **G** | **Y** | **F** | **A** | **T** | **R** | **V** | **K** | **E** | **A** | **S** | **P** | **V** | **N** | **L** | **R** | **T** | **A** | **L** | **V** | **N** | **S** | **D** | **N** | **I** | **Y** | **F** | **A** | **Q** | **Q** | **T** | **L** | **R** | **M** | **G** | **E** | **D** | **K** | **F** | **R** | **A** | **G** | **L** | **N** | **K** | **F** | **I** | **F** | **G** | **E** | **E** | **L** | **D** | **L** | **P** | **I** | **A** | **M** | **T** | **P** | **A** | **Q** | **I** | **S** | **N** | **E** | **D** | **K** | **F** | **N** | **S** | **E** | **I** | **L** | **L** | **A** | **D** | **T** | **G** | **Y** | **G** | **Q** | **G** | **Q** | **L** | **L** | **I** | **S** | **P** | **I** | **Q** | **Q** | **A** | **T** | **M** | **Y** | **S** | **V** | **F** | **Q** | **N** | **N** | **G** | **T** | **L** | **V** | **Y** | **P** | **K** | **L** | **V** | **L** | **D** | **K** | **E** | **T** | **K** | **K** | **K** | **D** | **N** | **V** | **I** | **S** | **A** | **N** | **A** | **A** | **N** | **T** | **I** | **A** | **T** | **D** | **L** | **L** | **G** | **S** | **V** | **E** | **D** | **P** | **S** | **G** | **Y** | **V** | **Y** | **N** | **M** | **Y** | **N** | **P** | **N** | **F** | **S** | **L** | **A** | **A** | **K** | **T** | **G** | **T** | **A** | **E** | **I** | **K** | **D** | **K** | **Q** | **D** | **T** | **D** | **G** | **K** | **E** | **N** | **S** | **F** | **L** | **L** | **T** | **L** | **D** | **R** | **S** | **N** | **N** | **K** | **F** | **L** | **T** | **M** | **I** | **M** | **V** | **E** | **N** | **S** | **G** | **E** | **N** | **G** | **S** | **A** | **T** | **D** | **I** | **S** | **K** | **P** | **L** | **I** | **D** | **Y** | **L** | **E** | **A** | **T** | **I** | **K** | **-** | **-** | **-** | **-** | **-** | **-** | **-** | **-** | **-** | **-** | **-** | **-** | **-** | **-** | **-** | **-** | **-** | **-** | **-** | **-** | **-** | **-** | **-** | **-** | **-** | **-** | **-** | **-** | **-** | **-** | **-** | **-** | **-** | **-** | **-** | **-** | **Q** | **W** | **Q** | **A** | **K** | **Q** | **E** | **L** | **A** | **E** | **A** | **K** | **K** | **T** | **A** | **T** | **T** | **F** | **L** | **N** | **V** | **L** | **S** | **K** | **Q** | **E** | **F** | **D** | **K** | **L** | **P** | **S** | **V** | **V** | **Q** | **E** | **A** | **S** | **L** | **K** | **K** | **N** | **G** | **Y** | **D** | **T** | **K** | **S** | **V** | **V** | **E** | **K** | **Y** | **Q** | **A** | **I** | **Y** | **S** | **G** | **I** | **Q** | **A** | **E** | **G** | **V** | **K** | **A** | **S** | **D** | **V** | **Q** | **V** | **K** | **K** | **A** | **K** | **D** | **N** | **Q** | **Y** | **T** | **F** | **T** | **Y** | **K** | **L** | **S** | **M** | **S** | **T** | **P** | **L** | **G** | **E** | **M** | **K** | **D** | **L** | **S** | **Y** | **Q** | **S** | **S** | **I** | **A** | **K** | **K** | **G** | **D** | **T** | **Y** | **Q** | **I** | **A** | **W** | **K** | **P** | **S** | **L** | **I** | **F** | **P** | **D** | **M** | **S** | **G** | **N** | **D** | **K** | **I** | **S** | **I** | **Q** | **V** | **D** | **N** | **A** | **K** | **R** | **G** | **E** | **I** | **V** | **D** | **R** | **N** | **G** | **S** | **G** | **L** | **A** | **I** | **N** | **K** | **V** | **F** | **D** | **E** | **V** | **G** | **V** | **V** | **P** | **G** | **K** | **L** | **G** | **S** | **G** | **A** | **E** | **K** | **T** | **A** | **N** | **I** | **K** | **A** | **F** | **S** | **D** | **K** | **F** | **G** | **V** | **S** | **V** | **D** | **E** | **I** | **N** | **Q** | **K** | **L** | **S** | **Q** | **G** | **W** | **V** | **Q** | **A** | **D** | **S** | **F** | **V** | **P** | **I** | **T** | **V** | **A** | **S** | **E** | **P** | **V** | **T** | **E** | **L** | **P** | **T** | **G** | **A** | **A** | **T** | **K** | **D** | **T** | **E** | **S** | **R** | **Y** | **Y** | **P** | **L** | **G** | **E** | **A** | **A** | **A** | **Q** | **L** | **I** | **G** | **Y** | **T** | **G** | **T** | **I** | **T** | **A** | **E** | **D** | **I** | **E** | **K** | **N** | **P** | **E** | **L** | **S** | **S** | **T** | **G** | **V** | **I** | **G** | **K** | **T** | **G** | **L** | **E** | **R** | **A** | **F** | **D** | **K** | **E** | **L** | **R** | **G** | **Q** | **D** | **G** | **G** | **S** | **L** | **V** | **I** | **L** | **D** | **D** | **K** | **E** | **N** | **V** | **K** | **K** | **A** | **L** | **Q** | **T** | **K** | **E** | **K** | **K** | **D** | **G** | **Q** | **T** | **I** | **K** | **L** | **T** | **I** | **D** | **S** | **G** | **V** | **Q** | **Q** | **Q** | **A** | **F** | **A** | **I** | **F** | **D** | **K** | **R** | **P** | **G** | **S** | **A** | **V** | **I** | **T** | **D** | **P** | **Q** | **K** | **G** | **D** | **L** | **L** | **A** | **T** | **V** | **S** | **S** | **P** | **S** | **Y** | **D** | **P** | **N** | **K** | **M** | **A** | **N** | **G** | **I** | **S** | **Q** | **K** | **E** | **Y** | **D** | **A** | **Y** | **N** | **N** | **N** | **K** | **D** | **L** | **P** | **F** | **T** | **A** | **R** | **F** | **A** | **T** | **G** | **Y** | **A** | **P** | **G** | **S** | **T** | **F** | **K** | **T** | **I** | **T** | **G** | **A** | **I** | **G** | **L** | **D** | **A** | **G** | **T** | **L** | **K** | **P** | **D** | **E** | **E** | **L** | **E** | **I** | **N** | **G** | **L** | **K** | **W** | **Q** | **K** | **D** | **K** | **S** | **W** | **G** | **G** | **Y** | **F** | **A** | **T** | **R** | **V** | **K** | **E** | **A** | **S** | **P** | **V** | **N** | **L** | **R** | **T** | **A** | **L** | **V** | **N** | **S** | **D** | **N** | **I** | **Y** | **F** | **A** | **Q** | **Q** | **T** | **L** | **R** | **M** | **G** | **E** | **D** | **K** | **F** | **R** | **A** | **G** | **L** | **N** | **K** | **F** | **I** | **F** | **G** | **E** | **E** | **L** | **D** | **L** | **P** | **I** | **A** | **M** | **T** | **P** | **A** | **Q** | **I** | **S** | **N** | **E** | **D** | **K** | **F** | **N** | **S** | **E** | **I** | **L** | **L** | **A** | **D** | **T** | **G** | **Y** | **G** | **Q** | **G** | **Q** | **L** | **L** | **I** | **S** | **P** | **I** | **Q** | **Q** | **A** | **T** | **M** | **Y** | **S** | **V** | **F** | **Q** | **N** | **N** | **G** | **T** | **L** | **V** | **Y** | **P** | **K** | **L** | **V** | **L** | **D** | **K** | **E** | **T** | **K** | **K** | **K** | **D** | **N** | **V** | **I** | **S** | **A** | **N** | **A** | **A** | **N** | **T** | **I** | **A** | **T** | **D** | **L** | **L** | **G** | **S** | **V** | **E** | **D** | **P** | **S** | **G** | **Y** | **V** | **Y** | **N** | **M** | **Y** | **N** | **P** | **N** | **F** | **S** | **L** | **A** | **A** | **K** | **T** | **G** | **T** | **A** | **E** | **I** | **K** | **D** | **K** | **Q** | **D** | **T** | **D** | **G** | **K** | **E** | **N** | **S** | **F** | **L** | **L** | **T** | **L** | **D** | **R** | **S** | **N** | **N** | **K** | **F** | **L** | **T** | **M** | **I** | **M** | **V** | **E** | **N** | **S** | **G** | **E** | **N** | **G** | **S** | **A** | **T** | **D** | **I** | **S** | **K** | **P** | **L** | **I** | **D** | **Y** | **L** | **E** | **A** | **T** | **I** | **K** |
| **N59462F** | **-** | **-** | **-** | **-** | **-** | **-** | **-** | **-** | **-** | **-** | **-** | **-** | **-** | **-** | **-** | **-** | **-** | **-** | **-** | **-** | **-** | **-** | **-** | **-** | **-** | **-** | **-** | **-** | **-** | **-** | **-** | **-** | **-** | **-** | **-** | **-** | **Q** | **W** | **Q** | **A** | **K** | **Q** | **E** | **L** | **A** | **E** | **A** | **K** | **K** | **T** | **A** | **T** | **T** | **F** | **L** | **N** | **V** | **L** | **S** | **K** | **Q** | **E** | **F** | **D** | **K** | **L** | **P** | **S** | **V** | **V** | **Q** | **E** | **A** | **S** | **L** | **K** | **K** | **N** | **G** | **Y** | **D** | **T** | **K** | **S** | **V** | **V** | **E** | **K** | **Y** | **Q** | **A** | **I** | **Y** | **S** | **G** | **I** | **Q** | **A** | **E** | **G** | **V** | **K** | **A** | **S** | **D** | **V** | **Q** | **V** | **K** | **K** | **A** | **K** | **D** | **N** | **Q** | **Y** | **T** | **F** | **T** | **Y** | **K** | **L** | **S** | **M** | **S** | **T** | **P** | **L** | **G** | **E** | **M** | **K** | **D** | **L** | **S** | **Y** | **Q** | **S** | **S** | **I** | **A** | **K** | **K** | **G** | **D** | **T** | **Y** | **Q** | **I** | **A** | **W** | **K** | **P** | **S** | **L** | **I** | **F** | **P** | **D** | **M** | **S** | **G** | **N** | **D** | **K** | **I** | **S** | **I** | **Q** | **V** | **D** | **N** | **A** | **K** | **R** | **G** | **E** | **I** | **V** | **D** | **R** | **N** | **G** | **S** | **G** | **L** | **A** | **I** | **N** | **K** | **V** | **F** | **D** | **E** | **V** | **G** | **V** | **V** | **P** | **G** | **K** | **L** | **G** | **S** | **G** | **A** | **E** | **K** | **T** | **A** | **N** | **I** | **K** | **A** | **F** | **S** | **D** | **K** | **F** | **G** | **V** | **S** | **V** | **D** | **E** | **I** | **N** | **Q** | **K** | **L** | **S** | **Q** | **G** | **W** | **V** | **Q** | **A** | **D** | **S** | **F** | **V** | **P** | **I** | **T** | **V** | **A** | **S** | **E** | **P** | **V** | **T** | **E** | **L** | **P** | **T** | **G** | **A** | **A** | **T** | **K** | **D** | **T** | **E** | **S** | **R** | **Y** | **Y** | **P** | **L** | **G** | **E** | **A** | **A** | **A** | **Q** | **L** | **I** | **G** | **Y** | **T** | **G** | **T** | **I** | **T** | **A** | **E** | **D** | **I** | **E** | **K** | **N** | **P** | **E** | **L** | **S** | **S** | **T** | **G** | **V** | **I** | **G** | **K** | **T** | **G** | **L** | **E** | **R** | **A** | **F** | **D** | **K** | **E** | **L** | **R** | **G** | **Q** | **D** | **G** | **G** | **S** | **L** | **V** | **I** | **L** | **D** | **D** | **K** | **E** | **N** | **V** | **K** | **K** | **A** | **L** | **Q** | **T** | **K** | **E** | **K** | **K** | **D** | **G** | **Q** | **T** | **I** | **K** | **L** | **T** | **I** | **D** | **S** | **G** | **V** | **Q** | **Q** | **Q** | **A** | **F** | **A** | **I** | **F** | **D** | **K** | **R** | **P** | **G** | **S** | **A** | **V** | **I** | **T** | **D** | **P** | **Q** | **K** | **G** | **D** | **L** | **L** | **A** | **T** | **V** | **S** | **S** | **P** | **S** | **Y** | **D** | **P** | **N** | **K** | **M** | **A** | **N** | **G** | **I** | **S** | **Q** | **K** | **E** | **Y** | **D** | **A** | **Y** | **N** | **N** | **N** | **K** | **D** | **L** | **P** | **F** | **T** | **A** | **R** | **F** | **A** | **T** | **G** | **Y** | **A** | **P** | **G** | **S** | **T** | **F** | **K** | **T** | **I** | **T** | **G** | **A** | **I** | **G** | **L** | **D** | **A** | **G** | **T** | **L** | **K** | **P** | **D** | **E** | **E** | **L** | **E** | **I** | **N** | **G** | **L** | **K** | **W** | **Q** | **K** | **D** | **K** | **S** | **W** | **G** | **G** | **Y** | **F** | **A** | **T** | **R** | **V** | **K** | **E** | **A** | **S** | **P** | **V** | **N** | **L** | **R** | **T** | **A** | **L** | **V** | **N** | **S** | **D** | **N** | **I** | **Y** | **F** | **A** | **Q** | **Q** | **T** | **L** | **R** | **M** | **G** | **E** | **D** | **K** | **F** | **R** | **A** | **G** | **L** | **N** | **K** | **F** | **I** | **F** | **G** | **E** | **E** | **L** | **D** | **L** | **P** | **I** | **A** | **M** | **T** | **P** | **A** | **Q** | **I** | **S** | **N** | **E** | **D** | **K** | **F** | **N** | **S** | **E** | **I** | **L** | **L** | **A** | **D** | **T** | **G** | **Y** | **G** | **Q** | **G** | **Q** | **L** | **L** | **I** | **S** | **P** | **I** | **Q** | **Q** | **A** | **T** | **M** | **Y** | **S** | **V** | **F** | **Q** | **N** | **N** | **G** | **T** | **L** | **V** | **Y** | **P** | **K** | **L** | **V** | **L** | **D** | **K** | **E** | **T** | **K** | **K** | **K** | **D** | **N** | **V** | **I** | **S** | **A** | **N** | **A** | **A** | **N** | **T** | **I** | **A** | **T** | **D** | **L** | **L** | **G** | **S** | **V** | **E** | **D** | **P** | **S** | **G** | **Y** | **V** | **Y** | **N** | **M** | **Y** | **N** | **P** | **N** | **F** | **S** | **L** | **A** | **A** | **K** | **T** | **G** | **T** | **A** | **E** | **I** | **K** | **D** | **K** | **Q** | **D** | **T** | **D** | **G** | **K** | **E** | **N** | **S** | **F** | **L** | **L** | **T** | **L** | **D** | **R** | **S** | **N** | **N** | **K** | **F** | **L** | **T** | **M** | **I** | **M** | **V** | **E** | **N** | **S** | **G** | **E** | **N** | **G** | **S** | **A** | **T** | **D** | **I** | **S** | **K** | **P** | **L** | **I** | **D** | **Y** | **L** | **E** | **A** | **T** | **I** | **K** | **-** | **-** | **-** | **-** | **-** | **-** | **-** | **-** | **-** | **-** | **-** | **-** | **-** | **-** | **-** | **-** | **-** | **-** | **-** | **-** | **-** | **-** | **-** | **-** | **-** | **-** | **-** | **-** | **-** | **-** | **-** | **-** | **-** | **-** | **-** | **-** | **Q** | **W** | **Q** | **A** | **K** | **Q** | **E** | **L** | **A** | **E** | **A** | **K** | **K** | **T** | **A** | **T** | **T** | **F** | **L** | **N** | **V** | **L** | **S** | **K** | **Q** | **E** | **F** | **D** | **K** | **L** | **P** | **S** | **V** | **V** | **Q** | **E** | **A** | **S** | **L** | **K** | **K** | **N** | **G** | **Y** | **D** | **T** | **K** | **S** | **V** | **V** | **E** | **K** | **Y** | **Q** | **A** | **I** | **Y** | **S** | **G** | **I** | **Q** | **A** | **E** | **G** | **V** | **K** | **A** | **S** | **D** | **V** | **Q** | **V** | **K** | **K** | **A** | **K** | **D** | **N** | **Q** | **Y** | **T** | **F** | **T** | **Y** | **K** | **L** | **S** | **M** | **S** | **T** | **P** | **L** | **G** | **E** | **M** | **K** | **D** | **L** | **S** | **Y** | **Q** | **S** | **S** | **I** | **A** | **K** | **K** | **G** | **D** | **T** | **Y** | **Q** | **I** | **A** | **W** | **K** | **P** | **S** | **L** | **I** | **F** | **P** | **D** | **M** | **S** | **G** | **N** | **D** | **K** | **I** | **S** | **I** | **Q** | **V** | **D** | **N** | **A** | **K** | **R** | **G** | **E** | **I** | **V** | **D** | **R** | **N** | **G** | **S** | **G** | **L** | **A** | **I** | **N** | **K** | **V** | **F** | **D** | **E** | **V** | **G** | **V** | **V** | **P** | **G** | **K** | **L** | **G** | **S** | **G** | **A** | **E** | **K** | **T** | **A** | **N** | **I** | **K** | **A** | **F** | **S** | **D** | **K** | **F** | **G** | **V** | **S** | **V** | **D** | **E** | **I** | **N** | **Q** | **K** | **L** | **S** | **Q** | **G** | **W** | **V** | **Q** | **A** | **D** | **S** | **F** | **V** | **P** | **I** | **T** | **V** | **A** | **S** | **E** | **P** | **V** | **T** | **E** | **L** | **P** | **T** | **G** | **A** | **A** | **T** | **K** | **D** | **T** | **E** | **S** | **R** | **Y** | **Y** | **P** | **L** | **G** | **E** | **A** | **A** | **A** | **Q** | **L** | **I** | **G** | **Y** | **T** | **G** | **T** | **I** | **T** | **A** | **E** | **D** | **I** | **E** | **K** | **N** | **P** | **E** | **L** | **S** | **S** | **T** | **G** | **V** | **I** | **G** | **K** | **T** | **G** | **L** | **E** | **R** | **A** | **F** | **D** | **K** | **E** | **L** | **R** | **G** | **Q** | **D** | **G** | **G** | **S** | **L** | **V** | **I** | **L** | **D** | **D** | **K** | **E** | **N** | **V** | **K** | **K** | **A** | **L** | **Q** | **T** | **K** | **E** | **K** | **K** | **D** | **G** | **Q** | **T** | **I** | **K** | **L** | **T** | **I** | **D** | **S** | **G** | **V** | **Q** | **Q** | **Q** | **A** | **F** | **A** | **I** | **F** | **D** | **K** | **R** | **P** | **G** | **S** | **A** | **V** | **I** | **T** | **D** | **P** | **Q** | **K** | **G** | **D** | **L** | **L** | **A** | **T** | **V** | **S** | **S** | **P** | **S** | **Y** | **D** | **P** | **N** | **K** | **M** | **A** | **N** | **G** | **I** | **S** | **Q** | **K** | **E** | **Y** | **D** | **A** | **Y** | **N** | **N** | **N** | **K** | **D** | **L** | **P** | **F** | **T** | **A** | **R** | **F** | **A** | **T** | **G** | **Y** | **A** | **P** | **G** | **S** | **T** | **F** | **K** | **T** | **I** | **T** | **G** | **A** | **I** | **G** | **L** | **D** | **A** | **G** | **T** | **L** | **K** | **P** | **D** | **E** | **E** | **L** | **E** | **I** | **N** | **G** | **L** | **K** | **W** | **Q** | **K** | **D** | **K** | **S** | **W** | **G** | **G** | **Y** | **F** | **A** | **T** | **R** | **V** | **K** | **E** | **A** | **S** | **P** | **V** | **N** | **L** | **R** | **T** | **A** | **L** | **V** | **N** | **S** | **D** | **N** | **I** | **Y** | **F** | **A** | **Q** | **Q** | **T** | **L** | **R** | **M** | **G** | **E** | **D** | **K** | **F** | **R** | **A** | **G** | **L** | **N** | **K** | **F** | **I** | **F** | **G** | **E** | **E** | **L** | **D** | **L** | **P** | **I** | **A** | **M** | **T** | **P** | **A** | **Q** | **I** | **S** | **N** | **E** | **D** | **K** | **F** | **N** | **S** | **E** | **I** | **L** | **L** | **A** | **D** | **T** | **G** | **Y** | **G** | **Q** | **G** | **Q** | **L** | **L** | **I** | **S** | **P** | **I** | **Q** | **Q** | **A** | **T** | **M** | **Y** | **S** | **V** | **F** | **Q** | **N** | **N** | **G** | **T** | **L** | **V** | **Y** | **P** | **K** | **L** | **V** | **L** | **D** | **K** | **E** | **T** | **K** | **K** | **K** | **D** | **N** | **V** | **I** | **S** | **A** | **N** | **A** | **A** | **N** | **T** | **I** | **A** | **T** | **D** | **L** | **L** | **G** | **S** | **V** | **E** | **D** | **P** | **S** | **G** | **Y** | **V** | **Y** | **N** | **M** | **Y** | **N** | **P** | **N** | **F** | **S** | **L** | **A** | **A** | **K** | **T** | **G** | **T** | **A** | **E** | **I** | **K** | **D** | **K** | **Q** | **D** | **T** | **D** | **G** | **K** | **E** | **N** | **S** | **F** | **L** | **L** | **T** | **L** | **D** | **R** | **S** | **N** | **N** | **K** | **F** | **L** | **T** | **M** | **I** | **M** | **V** | **E** | **N** | **S** | **G** | **E** | **N** | **G** | **S** | **A** | **T** | **D** | **I** | **S** | **K** | **P** | **L** | **I** | **D** | **Y** | **L** | **E** | **A** | **T** | **I** | **K** |
| **N60271F** | **-** | **-** | **-** | **-** | **-** | **-** | **-** | **-** | **-** | **-** | **-** | **-** | **-** | **-** | **-** | **-** | **-** | **-** | **-** | **-** | **-** | **-** | **-** | **-** | **-** | **-** | **-** | **-** | **-** | **-** | **-** | **-** | **-** | **-** | **-** | **-** | **Q** | **W** | **Q** | **A** | **K** | **Q** | **E** | **L** | **A** | **E** | **A** | **K** | **K** | **T** | **A** | **T** | **T** | **F** | **L** | **N** | **V** | **L** | **S** | **K** | **Q** | **E** | **F** | **D** | **K** | **L** | **P** | **S** | **V** | **V** | **Q** | **E** | **A** | **S** | **L** | **K** | **K** | **N** | **G** | **Y** | **D** | **T** | **K** | **S** | **V** | **V** | **E** | **K** | **Y** | **Q** | **A** | **I** | **Y** | **S** | **G** | **I** | **Q** | **A** | **E** | **G** | **V** | **K** | **A** | **S** | **D** | **V** | **Q** | **V** | **K** | **K** | **A** | **K** | **D** | **N** | **Q** | **Y** | **T** | **F** | **T** | **Y** | **K** | **L** | **S** | **M** | **S** | **T** | **P** | **L** | **G** | **E** | **M** | **K** | **D** | **L** | **S** | **Y** | **Q** | **S** | **S** | **I** | **A** | **K** | **K** | **G** | **D** | **T** | **Y** | **Q** | **I** | **A** | **W** | **K** | **P** | **S** | **L** | **I** | **F** | **P** | **D** | **M** | **S** | **G** | **N** | **D** | **K** | **I** | **S** | **I** | **Q** | **V** | **D** | **N** | **A** | **K** | **R** | **G** | **E** | **I** | **V** | **D** | **R** | **N** | **G** | **S** | **G** | **L** | **A** | **I** | **N** | **K** | **V** | **F** | **D** | **E** | **V** | **G** | **V** | **V** | **P** | **G** | **K** | **L** | **G** | **S** | **G** | **A** | **E** | **K** | **T** | **A** | **N** | **I** | **K** | **A** | **F** | **S** | **D** | **K** | **F** | **G** | **V** | **S** | **V** | **D** | **E** | **I** | **N** | **Q** | **K** | **L** | **S** | **Q** | **G** | **W** | **V** | **Q** | **A** | **D** | **S** | **F** | **V** | **P** | **I** | **T** | **V** | **A** | **S** | **E** | **P** | **V** | **T** | **E** | **L** | **P** | **T** | **G** | **A** | **A** | **T** | **K** | **D** | **T** | **E** | **S** | **R** | **Y** | **Y** | **P** | **L** | **G** | **E** | **A** | **A** | **A** | **Q** | **L** | **I** | **G** | **Y** | **T** | **G** | **T** | **I** | **T** | **A** | **E** | **D** | **I** | **E** | **K** | **N** | **P** | **E** | **L** | **S** | **S** | **T** | **G** | **V** | **I** | **G** | **K** | **T** | **G** | **L** | **E** | **R** | **A** | **F** | **D** | **K** | **E** | **L** | **R** | **G** | **Q** | **D** | **G** | **G** | **S** | **L** | **V** | **I** | **L** | **D** | **D** | **K** | **E** | **N** | **V** | **K** | **K** | **A** | **L** | **Q** | **T** | **K** | **D** | **K** | **K** | **D** | **G** | **Q** | **T** | **I** | **K** | **L** | **T** | **I** | **D** | **S** | **G** | **V** | **Q** | **Q** | **Q** | **A** | **F** | **A** | **I** | **F** | **D** | **K** | **R** | **P** | **G** | **S** | **A** | **V** | **I** | **T** | **D** | **P** | **Q** | **K** | **G** | **D** | **L** | **L** | **A** | **T** | **V** | **S** | **S** | **P** | **S** | **Y** | **D** | **P** | **N** | **K** | **M** | **A** | **N** | **G** | **I** | **S** | **Q** | **K** | **E** | **Y** | **D** | **A** | **Y** | **N** | **N** | **N** | **K** | **D** | **L** | **P** | **F** | **T** | **A** | **R** | **F** | **A** | **T** | **G** | **Y** | **A** | **P** | **G** | **S** | **T** | **F** | **K** | **T** | **I** | **T** | **G** | **A** | **I** | **G** | **L** | **D** | **A** | **G** | **T** | **L** | **K** | **P** | **D** | **E** | **E** | **L** | **E** | **I** | **N** | **G** | **L** | **K** | **W** | **Q** | **K** | **D** | **K** | **S** | **W** | **G** | **G** | **Y** | **F** | **A** | **T** | **R** | **V** | **K** | **E** | **A** | **S** | **P** | **V** | **N** | **L** | **R** | **T** | **A** | **L** | **V** | **N** | **S** | **D** | **N** | **I** | **Y** | **F** | **A** | **Q** | **Q** | **T** | **L** | **R** | **M** | **G** | **E** | **D** | **K** | **F** | **R** | **A** | **G** | **L** | **N** | **K** | **F** | **I** | **F** | **G** | **E** | **E** | **L** | **D** | **L** | **P** | **I** | **A** | **M** | **T** | **P** | **A** | **Q** | **I** | **S** | **N** | **E** | **D** | **K** | **F** | **N** | **S** | **E** | **I** | **L** | **L** | **A** | **D** | **T** | **G** | **Y** | **G** | **Q** | **G** | **Q** | **L** | **L** | **I** | **S** | **P** | **I** | **Q** | **Q** | **A** | **T** | **M** | **Y** | **S** | **V** | **F** | **Q** | **N** | **N** | **G** | **T** | **L** | **V** | **Y** | **P** | **K** | **L** | **V** | **L** | **D** | **K** | **E** | **T** | **K** | **K** | **K** | **D** | **N** | **V** | **I** | **S** | **A** | **N** | **A** | **A** | **N** | **T** | **I** | **A** | **T** | **D** | **L** | **L** | **G** | **S** | **V** | **E** | **D** | **P** | **S** | **G** | **Y** | **V** | **Y** | **N** | **M** | **Y** | **N** | **P** | **N** | **F** | **S** | **L** | **A** | **A** | **K** | **T** | **G** | **T** | **A** | **E** | **I** | **K** | **D** | **K** | **Q** | **D** | **T** | **D** | **G** | **K** | **E** | **N** | **S** | **F** | **L** | **L** | **T** | **L** | **D** | **R** | **S** | **N** | **N** | **K** | **F** | **L** | **T** | **M** | **I** | **M** | **V** | **E** | **N** | **S** | **G** | **E** | **N** | **G** | **S** | **A** | **T** | **D** | **I** | **S** | **K** | **P** | **L** | **I** | **D** | **Y** | **L** | **E** | **A** | **T** | **I** | **K** | **-** | **-** | **-** | **-** | **-** | **-** | **-** | **-** | **-** | **-** | **-** | **-** | **-** | **-** | **-** | **-** | **-** | **-** | **-** | **-** | **-** | **-** | **-** | **-** | **-** | **-** | **-** | **-** | **-** | **-** | **-** | **-** | **-** | **-** | **-** | **-** | **Q** | **W** | **Q** | **A** | **K** | **Q** | **E** | **L** | **A** | **E** | **A** | **K** | **K** | **T** | **A** | **T** | **T** | **F** | **L** | **N** | **V** | **L** | **S** | **K** | **Q** | **E** | **F** | **D** | **K** | **L** | **P** | **S** | **V** | **V** | **Q** | **E** | **A** | **S** | **L** | **K** | **K** | **N** | **G** | **Y** | **D** | **T** | **K** | **S** | **V** | **V** | **E** | **K** | **Y** | **Q** | **A** | **I** | **Y** | **S** | **G** | **I** | **Q** | **A** | **E** | **G** | **V** | **K** | **A** | **S** | **D** | **V** | **Q** | **V** | **K** | **K** | **A** | **K** | **D** | **N** | **Q** | **Y** | **T** | **F** | **T** | **Y** | **K** | **L** | **S** | **M** | **S** | **T** | **P** | **L** | **G** | **E** | **M** | **K** | **D** | **L** | **S** | **Y** | **Q** | **S** | **S** | **I** | **A** | **K** | **K** | **G** | **D** | **T** | **Y** | **Q** | **I** | **A** | **W** | **K** | **P** | **S** | **L** | **I** | **F** | **P** | **D** | **M** | **S** | **G** | **N** | **D** | **K** | **I** | **S** | **I** | **Q** | **V** | **D** | **N** | **A** | **K** | **R** | **G** | **E** | **I** | **V** | **D** | **R** | **N** | **G** | **S** | **G** | **L** | **A** | **I** | **N** | **K** | **V** | **F** | **D** | **E** | **V** | **G** | **V** | **V** | **P** | **G** | **K** | **L** | **G** | **S** | **G** | **A** | **E** | **K** | **T** | **A** | **N** | **I** | **K** | **A** | **F** | **S** | **D** | **K** | **F** | **G** | **V** | **S** | **V** | **D** | **E** | **I** | **N** | **Q** | **K** | **L** | **S** | **Q** | **G** | **W** | **V** | **Q** | **A** | **D** | **S** | **F** | **V** | **P** | **I** | **T** | **V** | **A** | **S** | **E** | **P** | **V** | **T** | **E** | **L** | **P** | **T** | **G** | **A** | **A** | **T** | **K** | **D** | **T** | **E** | **S** | **R** | **Y** | **Y** | **P** | **L** | **G** | **E** | **A** | **A** | **A** | **Q** | **L** | **I** | **G** | **Y** | **T** | **G** | **T** | **I** | **T** | **A** | **E** | **D** | **I** | **E** | **K** | **N** | **P** | **E** | **L** | **S** | **S** | **T** | **G** | **V** | **I** | **G** | **K** | **T** | **G** | **L** | **E** | **R** | **A** | **F** | **D** | **K** | **E** | **L** | **R** | **G** | **Q** | **D** | **G** | **G** | **S** | **L** | **V** | **I** | **L** | **D** | **D** | **K** | **E** | **N** | **V** | **K** | **K** | **A** | **L** | **Q** | **T** | **K** | **D** | **K** | **K** | **D** | **G** | **Q** | **T** | **I** | **K** | **L** | **T** | **I** | **D** | **S** | **G** | **V** | **Q** | **Q** | **Q** | **A** | **F** | **A** | **I** | **F** | **D** | **K** | **R** | **P** | **G** | **S** | **A** | **V** | **I** | **T** | **D** | **P** | **Q** | **K** | **G** | **D** | **L** | **L** | **A** | **T** | **V** | **S** | **S** | **P** | **S** | **Y** | **D** | **P** | **N** | **K** | **M** | **A** | **N** | **G** | **I** | **S** | **Q** | **K** | **E** | **Y** | **D** | **A** | **Y** | **N** | **N** | **N** | **K** | **D** | **L** | **P** | **F** | **T** | **A** | **R** | **F** | **A** | **T** | **G** | **Y** | **A** | **P** | **G** | **S** | **T** | **F** | **K** | **T** | **I** | **T** | **G** | **A** | **I** | **G** | **L** | **D** | **A** | **G** | **T** | **L** | **K** | **P** | **D** | **E** | **E** | **L** | **E** | **I** | **N** | **G** | **L** | **K** | **W** | **Q** | **K** | **D** | **K** | **S** | **W** | **G** | **G** | **Y** | **F** | **A** | **T** | **R** | **V** | **K** | **E** | **A** | **S** | **P** | **V** | **N** | **L** | **R** | **T** | **A** | **L** | **V** | **N** | **S** | **D** | **N** | **I** | **Y** | **F** | **A** | **Q** | **Q** | **T** | **L** | **R** | **M** | **G** | **E** | **D** | **K** | **F** | **R** | **A** | **G** | **L** | **N** | **K** | **F** | **I** | **F** | **G** | **E** | **E** | **L** | **D** | **L** | **P** | **I** | **A** | **M** | **T** | **P** | **A** | **Q** | **I** | **S** | **N** | **E** | **D** | **K** | **F** | **N** | **S** | **E** | **I** | **L** | **L** | **A** | **D** | **T** | **G** | **Y** | **G** | **Q** | **G** | **Q** | **L** | **L** | **I** | **S** | **P** | **I** | **Q** | **Q** | **A** | **T** | **M** | **Y** | **S** | **V** | **F** | **Q** | **N** | **N** | **G** | **T** | **L** | **V** | **Y** | **P** | **K** | **L** | **V** | **L** | **D** | **K** | **E** | **T** | **K** | **K** | **K** | **D** | **N** | **V** | **I** | **S** | **A** | **N** | **A** | **A** | **N** | **T** | **I** | **A** | **T** | **D** | **L** | **L** | **G** | **S** | **V** | **E** | **D** | **P** | **S** | **G** | **Y** | **V** | **Y** | **N** | **M** | **Y** | **N** | **P** | **N** | **F** | **S** | **L** | **A** | **A** | **K** | **T** | **G** | **T** | **A** | **E** | **I** | **K** | **D** | **K** | **Q** | **D** | **T** | **D** | **G** | **K** | **E** | **N** | **S** | **F** | **L** | **L** | **T** | **L** | **D** | **R** | **S** | **N** | **N** | **K** | **F** | **L** | **T** | **M** | **I** | **M** | **V** | **E** | **N** | **S** | **G** | **E** | **N** | **G** | **S** | **A** | **T** | **D** | **I** | **S** | **K** | **P** | **L** | **I** | **D** | **Y** | **L** | **E** | **A** | **T** | **I** | **K** |
| **N59600F** | **-** | **-** | **-** | **-** | **-** | **-** | **-** | **-** | **-** | **-** | **-** | **-** | **-** | **-** | **-** | **-** | **-** | **-** | **-** | **-** | **-** | **-** | **-** | **-** | **-** | **-** | **-** | **-** | **-** | **-** | **-** | **-** | **-** | **-** | **-** | **-** | **Q** | **W** | **Q** | **A** | **K** | **Q** | **E** | **L** | **A** | **E** | **A** | **K** | **K** | **T** | **A** | **T** | **T** | **F** | **L** | **N** | **V** | **L** | **T** | **K** | **Q** | **K** | **F** | **D** | **K** | **L** | **P** | **S** | **V** | **V** | **Q** | **E** | **A** | **S** | **L** | **K** | **K** | **N** | **G** | **Y** | **D** | **T** | **K** | **S** | **V** | **V** | **E** | **K** | **Y** | **Q** | **A** | **I** | **Y** | **S** | **G** | **I** | **Q** | **A** | **E** | **G** | **V** | **K** | **A** | **S** | **D** | **V** | **Q** | **V** | **K** | **K** | **A** | **K** | **D** | **N** | **Q** | **Y** | **T** | **F** | **T** | **Y** | **K** | **L** | **S** | **M** | **S** | **T** | **P** | **L** | **G** | **E** | **M** | **K** | **D** | **L** | **S** | **Y** | **Q** | **S** | **S** | **I** | **A** | **K** | **K** | **G** | **D** | **T** | **Y** | **Q** | **I** | **A** | **W** | **K** | **P** | **S** | **L** | **I** | **F** | **P** | **D** | **M** | **S** | **G** | **N** | **D** | **K** | **I** | **S** | **I** | **Q** | **V** | **D** | **N** | **A** | **K** | **R** | **G** | **E** | **I** | **V** | **D** | **R** | **N** | **G** | **S** | **G** | **L** | **A** | **I** | **N** | **K** | **V** | **F** | **D** | **E** | **V** | **G** | **V** | **V** | **P** | **G** | **K** | **L** | **G** | **S** | **G** | **A** | **E** | **K** | **T** | **A** | **N** | **I** | **K** | **A** | **F** | **S** | **D** | **K** | **F** | **G** | **V** | **S** | **V** | **D** | **E** | **I** | **N** | **Q** | **K** | **L** | **S** | **Q** | **G** | **W** | **V** | **Q** | **A** | **D** | **S** | **F** | **V** | **P** | **I** | **T** | **V** | **A** | **S** | **E** | **P** | **V** | **T** | **E** | **L** | **P** | **T** | **G** | **A** | **A** | **T** | **K** | **D** | **T** | **E** | **S** | **R** | **Y** | **Y** | **P** | **L** | **G** | **E** | **A** | **A** | **A** | **Q** | **L** | **I** | **G** | **Y** | **T** | **G** | **T** | **I** | **T** | **A** | **E** | **D** | **I** | **K** | **K** | **N** | **P** | **E** | **L** | **S** | **S** | **T** | **G** | **V** | **I** | **G** | **K** | **T** | **G** | **L** | **E** | **R** | **A** | **F** | **D** | **K** | **E** | **L** | **R** | **G** | **Q** | **D** | **G** | **G** | **S** | **L** | **V** | **I** | **L** | **D** | **D** | **K** | **E** | **N** | **V** | **K** | **K** | **A** | **L** | **Q** | **T** | **K** | **E** | **K** | **K** | **D** | **G** | **Q** | **T** | **I** | **K** | **L** | **T** | **I** | **D** | **S** | **G** | **V** | **Q** | **Q** | **Q** | **A** | **F** | **A** | **I** | **F** | **D** | **K** | **R** | **P** | **G** | **S** | **A** | **V** | **I** | **T** | **D** | **P** | **Q** | **K** | **G** | **D** | **L** | **L** | **A** | **T** | **V** | **S** | **S** | **P** | **S** | **Y** | **D** | **P** | **N** | **K** | **M** | **A** | **N** | **G** | **I** | **S** | **Q** | **K** | **E** | **Y** | **D** | **A** | **Y** | **N** | **N** | **N** | **K** | **D** | **L** | **P** | **F** | **T** | **A** | **R** | **F** | **A** | **T** | **G** | **Y** | **A** | **P** | **G** | **S** | **T** | **F** | **K** | **T** | **I** | **T** | **G** | **A** | **I** | **G** | **L** | **D** | **T** | **G** | **T** | **L** | **K** | **P** | **D** | **E** | **E** | **L** | **E** | **I** | **N** | **G** | **L** | **K** | **W** | **Q** | **K** | **D** | **K** | **S** | **W** | **G** | **G** | **Y** | **F** | **A** | **T** | **R** | **V** | **K** | **E** | **A** | **S** | **P** | **V** | **N** | **L** | **R** | **T** | **A** | **L** | **V** | **N** | **S** | **D** | **N** | **I** | **Y** | **F** | **A** | **Q** | **Q** | **T** | **L** | **R** | **M** | **G** | **E** | **D** | **K** | **F** | **R** | **A** | **G** | **L** | **N** | **K** | **F** | **I** | **F** | **G** | **E** | **E** | **L** | **D** | **L** | **P** | **I** | **A** | **M** | **T** | **P** | **A** | **Q** | **I** | **S** | **N** | **E** | **D** | **K** | **F** | **N** | **S** | **E** | **I** | **L** | **L** | **A** | **D** | **T** | **G** | **Y** | **G** | **Q** | **G** | **Q** | **L** | **L** | **I** | **S** | **P** | **I** | **Q** | **Q** | **A** | **T** | **M** | **Y** | **S** | **V** | **F** | **Q** | **N** | **N** | **G** | **T** | **L** | **V** | **Y** | **P** | **K** | **L** | **V** | **L** | **D** | **K** | **E** | **T** | **K** | **K** | **K** | **D** | **N** | **V** | **I** | **S** | **A** | **N** | **A** | **A** | **N** | **T** | **I** | **A** | **T** | **D** | **L** | **L** | **G** | **S** | **V** | **E** | **D** | **P** | **S** | **G** | **Y** | **V** | **Y** | **N** | **M** | **Y** | **N** | **P** | **N** | **F** | **S** | **L** | **A** | **A** | **K** | **T** | **G** | **T** | **A** | **E** | **I** | **K** | **D** | **K** | **Q** | **D** | **T** | **D** | **G** | **K** | **E** | **N** | **S** | **F** | **L** | **L** | **T** | **L** | **D** | **R** | **S** | **N** | **N** | **K** | **F** | **L** | **T** | **M** | **I** | **M** | **V** | **E** | **N** | **S** | **G** | **E** | **N** | **G** | **S** | **A** | **T** | **D** | **I** | **S** | **K** | **P** | **L** | **I** | **D** | **Y** | **L** | **E** | **A** | **T** | **I** | **K** | **-** | **-** | **-** | **-** | **-** | **-** | **-** | **-** | **-** | **-** | **-** | **-** | **-** | **-** | **-** | **-** | **-** | **-** | **-** | **-** | **-** | **-** | **-** | **-** | **-** | **-** | **-** | **-** | **-** | **-** | **-** | **-** | **-** | **-** | **-** | **-** | **Q** | **W** | **Q** | **A** | **K** | **Q** | **E** | **L** | **A** | **E** | **A** | **K** | **K** | **T** | **A** | **T** | **T** | **F** | **L** | **N** | **V** | **L** | **T** | **K** | **Q** | **K** | **F** | **D** | **K** | **L** | **P** | **S** | **V** | **V** | **Q** | **E** | **A** | **S** | **L** | **K** | **K** | **N** | **G** | **Y** | **D** | **T** | **K** | **S** | **V** | **V** | **E** | **K** | **Y** | **Q** | **A** | **I** | **Y** | **S** | **G** | **I** | **Q** | **A** | **E** | **G** | **V** | **K** | **A** | **S** | **D** | **V** | **Q** | **V** | **K** | **K** | **A** | **K** | **D** | **N** | **Q** | **Y** | **T** | **F** | **T** | **Y** | **K** | **L** | **S** | **M** | **S** | **T** | **P** | **L** | **G** | **E** | **M** | **K** | **D** | **L** | **S** | **Y** | **Q** | **S** | **S** | **I** | **A** | **K** | **K** | **G** | **D** | **T** | **Y** | **Q** | **I** | **A** | **W** | **K** | **P** | **S** | **L** | **I** | **F** | **P** | **D** | **M** | **S** | **G** | **N** | **D** | **K** | **I** | **S** | **I** | **Q** | **V** | **D** | **N** | **A** | **K** | **R** | **G** | **E** | **I** | **V** | **D** | **R** | **N** | **G** | **S** | **G** | **L** | **A** | **I** | **N** | **K** | **V** | **F** | **D** | **E** | **V** | **G** | **V** | **V** | **P** | **G** | **K** | **L** | **G** | **S** | **G** | **A** | **E** | **K** | **T** | **A** | **N** | **I** | **K** | **A** | **F** | **S** | **D** | **K** | **F** | **G** | **V** | **S** | **V** | **D** | **E** | **I** | **N** | **Q** | **K** | **L** | **S** | **Q** | **G** | **W** | **V** | **Q** | **A** | **D** | **S** | **F** | **V** | **P** | **I** | **T** | **V** | **A** | **S** | **E** | **P** | **V** | **T** | **E** | **L** | **P** | **T** | **G** | **A** | **A** | **T** | **K** | **D** | **T** | **E** | **S** | **R** | **Y** | **Y** | **P** | **L** | **G** | **E** | **A** | **A** | **A** | **Q** | **L** | **I** | **G** | **Y** | **T** | **G** | **T** | **I** | **T** | **A** | **E** | **D** | **I** | **K** | **K** | **N** | **P** | **E** | **L** | **S** | **S** | **T** | **G** | **V** | **I** | **G** | **K** | **T** | **G** | **L** | **E** | **R** | **A** | **F** | **D** | **K** | **E** | **L** | **R** | **G** | **Q** | **D** | **G** | **G** | **S** | **L** | **V** | **I** | **L** | **D** | **D** | **K** | **E** | **N** | **V** | **K** | **K** | **A** | **L** | **Q** | **T** | **K** | **E** | **K** | **K** | **D** | **G** | **Q** | **T** | **I** | **K** | **L** | **T** | **I** | **D** | **S** | **G** | **V** | **Q** | **Q** | **Q** | **A** | **F** | **A** | **I** | **F** | **D** | **K** | **R** | **P** | **G** | **S** | **A** | **V** | **I** | **T** | **D** | **P** | **Q** | **K** | **G** | **D** | **L** | **L** | **A** | **T** | **V** | **S** | **S** | **P** | **S** | **Y** | **D** | **P** | **N** | **K** | **M** | **A** | **N** | **G** | **I** | **S** | **Q** | **K** | **E** | **Y** | **D** | **A** | **Y** | **N** | **N** | **N** | **K** | **D** | **L** | **P** | **F** | **T** | **A** | **R** | **F** | **A** | **T** | **G** | **Y** | **A** | **P** | **G** | **S** | **T** | **F** | **K** | **T** | **I** | **T** | **G** | **A** | **I** | **G** | **L** | **D** | **T** | **G** | **T** | **L** | **K** | **P** | **D** | **E** | **E** | **L** | **E** | **I** | **N** | **G** | **L** | **K** | **W** | **Q** | **K** | **D** | **K** | **S** | **W** | **G** | **G** | **Y** | **F** | **A** | **T** | **R** | **V** | **K** | **E** | **A** | **S** | **P** | **V** | **N** | **L** | **R** | **T** | **A** | **L** | **V** | **N** | **S** | **D** | **N** | **I** | **Y** | **F** | **A** | **Q** | **Q** | **T** | **L** | **R** | **M** | **G** | **E** | **D** | **K** | **F** | **R** | **A** | **G** | **L** | **N** | **K** | **F** | **I** | **F** | **G** | **E** | **E** | **L** | **D** | **L** | **P** | **I** | **A** | **M** | **T** | **P** | **A** | **Q** | **I** | **S** | **N** | **E** | **D** | **K** | **F** | **N** | **S** | **E** | **I** | **L** | **L** | **A** | **D** | **T** | **G** | **Y** | **G** | **Q** | **G** | **Q** | **L** | **L** | **I** | **S** | **P** | **I** | **Q** | **Q** | **A** | **T** | **M** | **Y** | **S** | **V** | **F** | **Q** | **N** | **N** | **G** | **T** | **L** | **V** | **Y** | **P** | **K** | **L** | **V** | **L** | **D** | **K** | **E** | **T** | **K** | **K** | **K** | **D** | **N** | **V** | **I** | **S** | **A** | **N** | **A** | **A** | **N** | **T** | **I** | **A** | **T** | **D** | **L** | **L** | **G** | **S** | **V** | **E** | **D** | **P** | **S** | **G** | **Y** | **V** | **Y** | **N** | **M** | **Y** | **N** | **P** | **N** | **F** | **S** | **L** | **A** | **A** | **K** | **T** | **G** | **T** | **A** | **E** | **I** | **K** | **D** | **K** | **Q** | **D** | **T** | **D** | **G** | **K** | **E** | **N** | **S** | **F** | **L** | **L** | **T** | **L** | **D** | **R** | **S** | **N** | **N** | **K** | **F** | **L** | **T** | **M** | **I** | **M** | **V** | **E** | **N** | **S** | **G** | **E** | **N** | **G** | **S** | **A** | **T** | **D** | **I** | **S** | **K** | **P** | **L** | **I** | **D** | **Y** | **L** | **E** | **A** | **T** | **I** | **K** |
| **N59555F** | **-** | **-** | **-** | **-** | **-** | **-** | **-** | **-** | **-** | **-** | **-** | **-** | **-** | **-** | **-** | **-** | **-** | **-** | **-** | **-** | **-** | **-** | **-** | **-** | **-** | **-** | **-** | **-** | **-** | **-** | **-** | **-** | **-** | **-** | **-** | **-** | **Q** | **W** | **Q** | **A** | **K** | **Q** | **E** | **L** | **A** | **E** | **A** | **K** | **K** | **T** | **A** | **N** | **T** | **F** | **L** | **N** | **V** | **L** | **S** | **K** | **Q** | **E** | **F** | **D** | **K** | **L** | **P** | **S** | **V** | **V** | **Q** | **E** | **S** | **S** | **L** | **K** | **K** | **N** | **G** | **Y** | **D** | **T** | **K** | **S** | **V** | **V** | **E** | **K** | **Y** | **Q** | **A** | **I** | **Y** | **S** | **G** | **I** | **Q** | **A** | **E** | **G** | **V** | **K** | **A** | **S** | **D** | **V** | **Q** | **V** | **K** | **K** | **A** | **K** | **D** | **N** | **Q** | **Y** | **T** | **F** | **T** | **Y** | **K** | **L** | **S** | **M** | **S** | **T** | **P** | **L** | **G** | **E** | **M** | **K** | **D** | **L** | **S** | **Y** | **Q** | **S** | **S** | **I** | **A** | **K** | **K** | **G** | **D** | **T** | **Y** | **Q** | **I** | **S** | **W** | **K** | **P** | **S** | **L** | **I** | **F** | **P** | **D** | **M** | **S** | **G** | **N** | **D** | **K** | **I** | **S** | **I** | **Q** | **V** | **D** | **N** | **A** | **K** | **R** | **G** | **E** | **I** | **V** | **D** | **R** | **N** | **G** | **S** | **G** | **L** | **A** | **I** | **N** | **K** | **V** | **F** | **D** | **E** | **V** | **G** | **V** | **V** | **P** | **G** | **K** | **L** | **G** | **S** | **G** | **A** | **E** | **K** | **T** | **A** | **N** | **I** | **K** | **A** | **F** | **S** | **D** | **K** | **F** | **G** | **V** | **S** | **V** | **D** | **E** | **I** | **N** | **Q** | **K** | **L** | **S** | **Q** | **G** | **W** | **V** | **Q** | **A** | **D** | **S** | **F** | **V** | **P** | **I** | **T** | **V** | **A** | **S** | **E** | **P** | **V** | **T** | **E** | **L** | **P** | **T** | **G** | **A** | **A** | **T** | **K** | **D** | **T** | **E** | **S** | **R** | **Y** | **Y** | **P** | **L** | **G** | **E** | **A** | **A** | **A** | **Q** | **L** | **I** | **G** | **Y** | **T** | **G** | **T** | **I** | **T** | **A** | **E** | **D** | **I** | **E** | **K** | **N** | **P** | **E** | **L** | **S** | **S** | **T** | **G** | **V** | **I** | **G** | **K** | **T** | **G** | **L** | **E** | **R** | **A** | **F** | **D** | **K** | **E** | **L** | **R** | **G** | **Q** | **D** | **G** | **G** | **S** | **L** | **V** | **I** | **L** | **D** | **D** | **K** | **E** | **N** | **V** | **K** | **K** | **A** | **L** | **Q** | **T** | **K** | **E** | **K** | **K** | **D** | **G** | **Q** | **T** | **I** | **K** | **L** | **T** | **I** | **D** | **S** | **G** | **V** | **Q** | **Q** | **Q** | **A** | **F** | **A** | **I** | **F** | **D** | **K** | **R** | **P** | **G** | **S** | **A** | **V** | **I** | **T** | **D** | **P** | **Q** | **K** | **G** | **D** | **L** | **L** | **A** | **T** | **V** | **S** | **S** | **P** | **S** | **Y** | **D** | **P** | **N** | **K** | **M** | **A** | **N** | **G** | **I** | **S** | **Q** | **K** | **E** | **Y** | **D** | **A** | **Y** | **N** | **N** | **N** | **K** | **D** | **L** | **P** | **F** | **T** | **A** | **R** | **F** | **A** | **T** | **G** | **Y** | **A** | **P** | **G** | **S** | **T** | **F** | **K** | **T** | **I** | **T** | **G** | **A** | **I** | **G** | **L** | **D** | **A** | **G** | **T** | **L** | **K** | **P** | **D** | **E** | **E** | **L** | **E** | **I** | **N** | **G** | **L** | **K** | **W** | **Q** | **K** | **D** | **K** | **S** | **W** | **G** | **G** | **Y** | **F** | **A** | **T** | **R** | **V** | **K** | **E** | **A** | **S** | **P** | **V** | **N** | **L** | **R** | **T** | **A** | **L** | **V** | **N** | **S** | **D** | **N** | **I** | **Y** | **F** | **A** | **Q** | **Q** | **T** | **L** | **R** | **M** | **G** | **E** | **D** | **K** | **F** | **R** | **A** | **G** | **L** | **N** | **K** | **F** | **I** | **F** | **G** | **E** | **E** | **L** | **D** | **L** | **P** | **I** | **A** | **M** | **T** | **P** | **A** | **Q** | **I** | **S** | **N** | **E** | **D** | **K** | **F** | **N** | **S** | **E** | **I** | **L** | **L** | **A** | **D** | **T** | **G** | **Y** | **G** | **Q** | **G** | **Q** | **L** | **L** | **I** | **S** | **P** | **I** | **Q** | **Q** | **A** | **T** | **M** | **Y** | **S** | **V** | **F** | **Q** | **N** | **N** | **G** | **T** | **L** | **V** | **Y** | **P** | **K** | **L** | **V** | **L** | **D** | **K** | **E** | **T** | **K** | **K** | **K** | **D** | **N** | **V** | **I** | **S** | **A** | **N** | **A** | **A** | **N** | **T** | **I** | **A** | **T** | **D** | **L** | **L** | **G** | **S** | **V** | **E** | **D** | **P** | **S** | **G** | **Y** | **V** | **Y** | **N** | **M** | **Y** | **N** | **P** | **N** | **F** | **S** | **L** | **A** | **A** | **K** | **T** | **G** | **T** | **A** | **E** | **I** | **K** | **D** | **K** | **Q** | **D** | **T** | **D** | **G** | **K** | **E** | **N** | **S** | **F** | **L** | **L** | **T** | **L** | **D** | **R** | **S** | **N** | **N** | **K** | **F** | **L** | **T** | **M** | **I** | **M** | **V** | **E** | **N** | **S** | **G** | **E** | **N** | **G** | **S** | **A** | **T** | **D** | **I** | **S** | **K** | **P** | **L** | **I** | **D** | **Y** | **L** | **E** | **A** | **T** | **I** | **K** | **-** | **-** | **-** | **-** | **-** | **-** | **-** | **-** | **-** | **-** | **-** | **-** | **-** | **-** | **-** | **-** | **-** | **-** | **-** | **-** | **-** | **-** | **-** | **-** | **-** | **-** | **-** | **-** | **-** | **-** | **-** | **-** | **-** | **-** | **-** | **-** | **Q** | **W** | **Q** | **A** | **K** | **Q** | **E** | **L** | **A** | **E** | **A** | **K** | **K** | **T** | **A** | **N** | **T** | **F** | **L** | **N** | **V** | **L** | **S** | **K** | **Q** | **E** | **F** | **D** | **K** | **L** | **P** | **S** | **V** | **V** | **Q** | **E** | **S** | **S** | **L** | **K** | **K** | **N** | **G** | **Y** | **D** | **T** | **K** | **S** | **V** | **V** | **E** | **K** | **Y** | **Q** | **A** | **I** | **Y** | **S** | **G** | **I** | **Q** | **A** | **E** | **G** | **V** | **K** | **A** | **S** | **D** | **V** | **Q** | **V** | **K** | **K** | **A** | **K** | **D** | **N** | **Q** | **Y** | **T** | **F** | **T** | **Y** | **K** | **L** | **S** | **M** | **S** | **T** | **P** | **L** | **G** | **E** | **M** | **K** | **D** | **L** | **S** | **Y** | **Q** | **S** | **S** | **I** | **A** | **K** | **K** | **G** | **D** | **T** | **Y** | **Q** | **I** | **S** | **W** | **K** | **P** | **S** | **L** | **I** | **F** | **P** | **D** | **M** | **S** | **G** | **N** | **D** | **K** | **I** | **S** | **I** | **Q** | **V** | **D** | **N** | **A** | **K** | **R** | **G** | **E** | **I** | **V** | **D** | **R** | **N** | **G** | **S** | **G** | **L** | **A** | **I** | **N** | **K** | **V** | **F** | **D** | **E** | **V** | **G** | **V** | **V** | **P** | **G** | **K** | **L** | **G** | **S** | **G** | **A** | **E** | **K** | **T** | **A** | **N** | **I** | **K** | **A** | **F** | **S** | **D** | **K** | **F** | **G** | **V** | **S** | **V** | **D** | **E** | **I** | **N** | **Q** | **K** | **L** | **S** | **Q** | **G** | **W** | **V** | **Q** | **A** | **D** | **S** | **F** | **V** | **P** | **I** | **T** | **V** | **A** | **S** | **E** | **P** | **V** | **T** | **E** | **L** | **P** | **T** | **G** | **A** | **A** | **T** | **K** | **D** | **T** | **E** | **S** | **R** | **Y** | **Y** | **P** | **L** | **G** | **E** | **A** | **A** | **A** | **Q** | **L** | **I** | **G** | **Y** | **T** | **G** | **T** | **I** | **T** | **A** | **E** | **D** | **I** | **E** | **K** | **N** | **P** | **E** | **L** | **S** | **S** | **T** | **G** | **V** | **I** | **G** | **K** | **T** | **G** | **L** | **E** | **R** | **A** | **F** | **D** | **K** | **E** | **L** | **R** | **G** | **Q** | **D** | **G** | **G** | **S** | **L** | **V** | **I** | **L** | **D** | **D** | **K** | **E** | **N** | **V** | **K** | **K** | **A** | **L** | **Q** | **T** | **K** | **E** | **K** | **K** | **D** | **G** | **Q** | **T** | **I** | **K** | **L** | **T** | **I** | **D** | **S** | **G** | **V** | **Q** | **Q** | **Q** | **A** | **F** | **A** | **I** | **F** | **D** | **K** | **R** | **P** | **G** | **S** | **A** | **V** | **I** | **T** | **D** | **P** | **Q** | **K** | **G** | **D** | **L** | **L** | **A** | **T** | **V** | **S** | **S** | **P** | **S** | **Y** | **D** | **P** | **N** | **K** | **M** | **A** | **N** | **G** | **I** | **S** | **Q** | **K** | **E** | **Y** | **D** | **A** | **Y** | **N** | **N** | **N** | **K** | **D** | **L** | **P** | **F** | **T** | **A** | **R** | **F** | **A** | **T** | **G** | **Y** | **A** | **P** | **G** | **S** | **T** | **F** | **K** | **T** | **I** | **T** | **G** | **A** | **I** | **G** | **L** | **D** | **A** | **G** | **T** | **L** | **K** | **P** | **D** | **E** | **E** | **L** | **E** | **I** | **N** | **G** | **L** | **K** | **W** | **Q** | **K** | **D** | **K** | **S** | **W** | **G** | **G** | **Y** | **F** | **A** | **T** | **R** | **V** | **K** | **E** | **A** | **S** | **P** | **V** | **N** | **L** | **R** | **T** | **A** | **L** | **V** | **N** | **S** | **D** | **N** | **I** | **Y** | **F** | **A** | **Q** | **Q** | **T** | **L** | **R** | **M** | **G** | **E** | **D** | **K** | **F** | **R** | **A** | **G** | **L** | **N** | **K** | **F** | **I** | **F** | **G** | **E** | **E** | **L** | **D** | **L** | **P** | **I** | **A** | **M** | **T** | **P** | **A** | **Q** | **I** | **S** | **N** | **E** | **D** | **K** | **F** | **N** | **S** | **E** | **I** | **L** | **L** | **A** | **D** | **T** | **G** | **Y** | **G** | **Q** | **G** | **Q** | **L** | **L** | **I** | **S** | **P** | **I** | **Q** | **Q** | **A** | **T** | **M** | **Y** | **S** | **V** | **F** | **Q** | **N** | **N** | **G** | **T** | **L** | **V** | **Y** | **P** | **K** | **L** | **V** | **L** | **D** | **K** | **E** | **T** | **K** | **K** | **K** | **D** | **N** | **V** | **I** | **S** | **A** | **N** | **A** | **A** | **N** | **T** | **I** | **A** | **T** | **D** | **L** | **L** | **G** | **S** | **V** | **E** | **D** | **P** | **S** | **G** | **Y** | **V** | **Y** | **N** | **M** | **Y** | **N** | **P** | **N** | **F** | **S** | **L** | **A** | **A** | **K** | **T** | **G** | **T** | **A** | **E** | **I** | **K** | **D** | **K** | **Q** | **D** | **T** | **D** | **G** | **K** | **E** | **N** | **S** | **F** | **L** | **L** | **T** | **L** | **D** | **R** | **S** | **N** | **N** | **K** | **F** | **L** | **T** | **M** | **I** | **M** | **V** | **E** | **N** | **S** | **G** | **E** | **N** | **G** | **S** | **A** | **T** | **D** | **I** | **S** | **K** | **P** | **L** | **I** | **D** | **Y** | **L** | **E** | **A** | **T** | **I** | **K** |
| **N53453** | **-** | **-** | **-** | **-** | **-** | **-** | **-** | **-** | **-** | **-** | **-** | **-** | **-** | **-** | **-** | **-** | **-** | **-** | **-** | **-** | **-** | **-** | **-** | **-** | **-** | **-** | **-** | **-** | **-** | **-** | **-** | **-** | **-** | **-** | **-** | **-** | **Q** | **W** | **Q** | **A** | **K** | **Q** | **E** | **L** | **A** | **E** | **A** | **K** | **K** | **T** | **A** | **T** | **T** | **F** | **L** | **N** | **V** | **L** | **S** | **K** | **Q** | **E** | **F** | **D** | **K** | **L** | **P** | **S** | **V** | **V** | **Q** | **E** | **A** | **S** | **L** | **K** | **K** | **N** | **G** | **Y** | **D** | **T** | **K** | **S** | **V** | **V** | **E** | **K** | **Y** | **Q** | **A** | **I** | **Y** | **S** | **G** | **I** | **Q** | **A** | **E** | **G** | **V** | **K** | **A** | **S** | **D** | **V** | **Q** | **V** | **K** | **K** | **A** | **K** | **D** | **N** | **Q** | **Y** | **T** | **F** | **T** | **Y** | **K** | **L** | **S** | **M** | **S** | **T** | **P** | **L** | **G** | **E** | **M** | **K** | **D** | **L** | **S** | **Y** | **Q** | **S** | **S** | **I** | **A** | **K** | **K** | **G** | **D** | **T** | **Y** | **Q** | **I** | **A** | **W** | **K** | **P** | **S** | **L** | **I** | **F** | **P** | **D** | **M** | **S** | **G** | **N** | **D** | **K** | **I** | **S** | **I** | **Q** | **V** | **D** | **N** | **A** | **K** | **R** | **G** | **E** | **I** | **V** | **D** | **R** | **N** | **G** | **S** | **G** | **L** | **A** | **I** | **N** | **K** | **V** | **F** | **D** | **E** | **V** | **G** | **V** | **V** | **P** | **G** | **K** | **L** | **G** | **S** | **G** | **A** | **E** | **K** | **T** | **A** | **N** | **I** | **K** | **A** | **F** | **S** | **D** | **K** | **F** | **G** | **V** | **S** | **V** | **D** | **E** | **I** | **N** | **Q** | **K** | **L** | **S** | **Q** | **G** | **W** | **V** | **Q** | **A** | **D** | **S** | **F** | **V** | **P** | **I** | **T** | **V** | **A** | **S** | **E** | **P** | **V** | **T** | **E** | **L** | **P** | **T** | **G** | **A** | **A** | **T** | **K** | **D** | **T** | **E** | **S** | **R** | **Y** | **Y** | **P** | **L** | **G** | **E** | **A** | **A** | **A** | **Q** | **L** | **I** | **G** | **Y** | **T** | **G** | **T** | **I** | **T** | **A** | **E** | **D** | **I** | **E** | **K** | **N** | **P** | **E** | **L** | **S** | **S** | **T** | **G** | **V** | **I** | **G** | **K** | **T** | **G** | **L** | **E** | **R** | **A** | **F** | **D** | **K** | **E** | **L** | **R** | **G** | **Q** | **D** | **G** | **G** | **S** | **L** | **V** | **I** | **L** | **D** | **D** | **K** | **E** | **N** | **V** | **K** | **K** | **A** | **L** | **Q** | **T** | **K** | **E** | **K** | **K** | **D** | **G** | **Q** | **T** | **I** | **K** | **L** | **T** | **I** | **D** | **S** | **G** | **V** | **Q** | **Q** | **Q** | **A** | **F** | **A** | **I** | **F** | **D** | **K** | **R** | **P** | **G** | **S** | **A** | **V** | **I** | **T** | **D** | **P** | **Q** | **K** | **G** | **D** | **L** | **L** | **A** | **T** | **V** | **S** | **S** | **P** | **S** | **Y** | **D** | **P** | **N** | **K** | **M** | **A** | **N** | **G** | **I** | **S** | **Q** | **K** | **E** | **Y** | **D** | **A** | **Y** | **N** | **N** | **N** | **K** | **D** | **L** | **P** | **F** | **T** | **A** | **R** | **F** | **A** | **T** | **G** | **Y** | **A** | **P** | **G** | **S** | **T** | **F** | **K** | **T** | **I** | **T** | **G** | **A** | **I** | **G** | **L** | **D** | **A** | **G** | **T** | **L** | **K** | **P** | **D** | **E** | **E** | **L** | **E** | **I** | **N** | **G** | **L** | **K** | **W** | **Q** | **K** | **D** | **K** | **S** | **W** | **G** | **G** | **Y** | **F** | **A** | **T** | **R** | **V** | **K** | **E** | **A** | **S** | **P** | **V** | **N** | **L** | **R** | **T** | **A** | **L** | **V** | **N** | **S** | **D** | **N** | **I** | **Y** | **F** | **A** | **Q** | **Q** | **T** | **L** | **R** | **M** | **G** | **E** | **D** | **K** | **F** | **R** | **A** | **G** | **L** | **N** | **K** | **F** | **I** | **F** | **G** | **E** | **E** | **L** | **D** | **L** | **P** | **I** | **A** | **M** | **T** | **P** | **A** | **Q** | **I** | **S** | **N** | **E** | **D** | **K** | **F** | **N** | **S** | **E** | **I** | **L** | **L** | **A** | **D** | **T** | **G** | **Y** | **G** | **Q** | **G** | **Q** | **L** | **L** | **I** | **S** | **P** | **I** | **Q** | **Q** | **A** | **T** | **M** | **Y** | **S** | **V** | **F** | **Q** | **N** | **N** | **G** | **T** | **L** | **V** | **Y** | **P** | **K** | **L** | **V** | **L** | **E** | **K** | **E** | **T** | **K** | **K** | **K** | **D** | **N** | **V** | **I** | **S** | **A** | **N** | **A** | **A** | **N** | **T** | **I** | **A** | **T** | **D** | **L** | **L** | **G** | **S** | **V** | **E** | **D** | **P** | **S** | **G** | **Y** | **V** | **Y** | **N** | **M** | **Y** | **N** | **P** | **N** | **F** | **S** | **L** | **A** | **A** | **K** | **T** | **G** | **T** | **A** | **E** | **I** | **K** | **D** | **K** | **Q** | **D** | **T** | **D** | **G** | **K** | **E** | **N** | **S** | **F** | **L** | **L** | **T** | **L** | **D** | **R** | **S** | **N** | **N** | **K** | **F** | **L** | **T** | **M** | **I** | **M** | **V** | **E** | **N** | **S** | **G** | **E** | **N** | **G** | **S** | **A** | **T** | **D** | **I** | **S** | **K** | **P** | **L** | **I** | **D** | **Y** | **L** | **E** | **A** | **T** | **I** | **K** | **-** | **-** | **-** | **-** | **-** | **-** | **-** | **-** | **-** | **-** | **-** | **-** | **-** | **-** | **-** | **-** | **-** | **-** | **-** | **-** | **-** | **-** | **-** | **-** | **-** | **-** | **-** | **-** | **-** | **-** | **-** | **-** | **-** | **-** | **-** | **-** | **Q** | **W** | **Q** | **A** | **K** | **Q** | **E** | **L** | **A** | **E** | **A** | **K** | **K** | **T** | **A** | **T** | **T** | **F** | **L** | **N** | **V** | **L** | **S** | **K** | **Q** | **E** | **F** | **D** | **K** | **L** | **P** | **S** | **V** | **V** | **Q** | **E** | **A** | **S** | **L** | **K** | **K** | **N** | **G** | **Y** | **D** | **T** | **K** | **S** | **V** | **V** | **E** | **K** | **Y** | **Q** | **A** | **I** | **Y** | **S** | **G** | **I** | **Q** | **A** | **E** | **G** | **V** | **K** | **A** | **S** | **D** | **V** | **Q** | **V** | **K** | **K** | **A** | **K** | **D** | **N** | **Q** | **Y** | **T** | **F** | **T** | **Y** | **K** | **L** | **S** | **M** | **S** | **T** | **P** | **L** | **G** | **E** | **M** | **K** | **D** | **L** | **S** | **Y** | **Q** | **S** | **S** | **I** | **A** | **K** | **K** | **G** | **D** | **T** | **Y** | **Q** | **I** | **A** | **W** | **K** | **P** | **S** | **L** | **I** | **F** | **P** | **D** | **M** | **S** | **G** | **N** | **D** | **K** | **I** | **S** | **I** | **Q** | **V** | **D** | **N** | **A** | **K** | **R** | **G** | **E** | **I** | **V** | **D** | **R** | **N** | **G** | **S** | **G** | **L** | **A** | **I** | **N** | **K** | **V** | **F** | **D** | **E** | **V** | **G** | **V** | **V** | **P** | **G** | **K** | **L** | **G** | **S** | **G** | **A** | **E** | **K** | **T** | **A** | **N** | **I** | **K** | **A** | **F** | **S** | **D** | **K** | **F** | **G** | **V** | **S** | **V** | **D** | **E** | **I** | **N** | **Q** | **K** | **L** | **S** | **Q** | **G** | **W** | **V** | **Q** | **A** | **D** | **S** | **F** | **V** | **P** | **I** | **T** | **V** | **A** | **S** | **E** | **P** | **V** | **T** | **E** | **L** | **P** | **T** | **G** | **A** | **A** | **T** | **K** | **D** | **T** | **E** | **S** | **R** | **Y** | **Y** | **P** | **L** | **G** | **E** | **A** | **A** | **A** | **Q** | **L** | **I** | **G** | **Y** | **T** | **G** | **T** | **I** | **T** | **A** | **E** | **D** | **I** | **E** | **K** | **N** | **P** | **E** | **L** | **S** | **S** | **T** | **G** | **V** | **I** | **G** | **K** | **T** | **G** | **L** | **E** | **R** | **A** | **F** | **D** | **K** | **E** | **L** | **R** | **G** | **Q** | **D** | **G** | **G** | **S** | **L** | **V** | **I** | **L** | **D** | **D** | **K** | **E** | **N** | **V** | **K** | **K** | **A** | **L** | **Q** | **T** | **K** | **E** | **K** | **K** | **D** | **G** | **Q** | **T** | **I** | **K** | **L** | **T** | **I** | **D** | **S** | **G** | **V** | **Q** | **Q** | **Q** | **A** | **F** | **A** | **I** | **F** | **D** | **K** | **R** | **P** | **G** | **S** | **A** | **V** | **I** | **T** | **D** | **P** | **Q** | **K** | **G** | **D** | **L** | **L** | **A** | **T** | **V** | **S** | **S** | **P** | **S** | **Y** | **D** | **P** | **N** | **K** | **M** | **A** | **N** | **G** | **I** | **S** | **Q** | **K** | **E** | **Y** | **D** | **A** | **Y** | **N** | **N** | **N** | **K** | **D** | **L** | **P** | **F** | **T** | **A** | **R** | **F** | **A** | **T** | **G** | **Y** | **A** | **P** | **G** | **S** | **T** | **F** | **K** | **T** | **I** | **T** | **G** | **A** | **I** | **G** | **L** | **D** | **A** | **G** | **T** | **L** | **K** | **P** | **D** | **E** | **E** | **L** | **E** | **I** | **N** | **G** | **L** | **K** | **W** | **Q** | **K** | **D** | **K** | **S** | **W** | **G** | **G** | **Y** | **F** | **A** | **T** | **R** | **V** | **K** | **E** | **A** | **S** | **P** | **V** | **N** | **L** | **R** | **T** | **A** | **L** | **V** | **N** | **S** | **D** | **N** | **I** | **Y** | **F** | **A** | **Q** | **Q** | **T** | **L** | **R** | **M** | **G** | **E** | **D** | **K** | **F** | **R** | **A** | **G** | **L** | **N** | **K** | **F** | **I** | **F** | **G** | **E** | **E** | **L** | **D** | **L** | **P** | **I** | **A** | **M** | **T** | **P** | **A** | **Q** | **I** | **S** | **N** | **E** | **D** | **K** | **F** | **N** | **S** | **E** | **I** | **L** | **L** | **A** | **D** | **T** | **G** | **Y** | **G** | **Q** | **G** | **Q** | **L** | **L** | **I** | **S** | **P** | **I** | **Q** | **Q** | **A** | **T** | **M** | **Y** | **S** | **V** | **F** | **Q** | **N** | **N** | **G** | **T** | **L** | **V** | **Y** | **P** | **K** | **L** | **V** | **L** | **E** | **K** | **E** | **T** | **K** | **K** | **K** | **D** | **N** | **V** | **I** | **S** | **A** | **N** | **A** | **A** | **N** | **T** | **I** | **A** | **T** | **D** | **L** | **L** | **G** | **S** | **V** | **E** | **D** | **P** | **S** | **G** | **Y** | **V** | **Y** | **N** | **M** | **Y** | **N** | **P** | **N** | **F** | **S** | **L** | **A** | **A** | **K** | **T** | **G** | **T** | **A** | **E** | **I** | **K** | **D** | **K** | **Q** | **D** | **T** | **D** | **G** | **K** | **E** | **N** | **S** | **F** | **L** | **L** | **T** | **L** | **D** | **R** | **S** | **N** | **N** | **K** | **F** | **L** | **T** | **M** | **I** | **M** | **V** | **E** | **N** | **S** | **G** | **E** | **N** | **G** | **S** | **A** | **T** | **D** | **I** | **S** | **K** | **P** | **L** | **I** | **D** | **Y** | **L** | **E** | **A** | **T** | **I** | **K** |
| **N59505F** | **-** | **-** | **-** | **-** | **-** | **-** | **-** | **-** | **-** | **-** | **-** | **-** | **-** | **-** | **-** | **-** | **-** | **-** | **-** | **-** | **-** | **-** | **-** | **-** | **-** | **-** | **-** | **-** | **-** | **-** | **-** | **-** | **-** | **-** | **-** | **-** | **Q** | **W** | **Q** | **A** | **K** | **Q** | **E** | **L** | **A** | **E** | **A** | **K** | **K** | **T** | **A** | **N** | **T** | **F** | **L** | **N** | **V** | **L** | **S** | **K** | **Q** | **E** | **F** | **D** | **K** | **L** | **P** | **S** | **V** | **V** | **Q** | **E** | **S** | **S** | **L** | **K** | **K** | **N** | **G** | **Y** | **D** | **T** | **K** | **S** | **V** | **V** | **E** | **K** | **Y** | **Q** | **A** | **I** | **Y** | **S** | **G** | **I** | **Q** | **A** | **E** | **G** | **V** | **K** | **A** | **S** | **D** | **V** | **Q** | **V** | **K** | **K** | **A** | **K** | **D** | **N** | **Q** | **Y** | **T** | **F** | **T** | **Y** | **K** | **L** | **S** | **M** | **S** | **T** | **P** | **L** | **G** | **E** | **M** | **K** | **D** | **L** | **S** | **Y** | **Q** | **S** | **S** | **I** | **A** | **K** | **K** | **G** | **D** | **T** | **Y** | **Q** | **I** | **S** | **W** | **K** | **P** | **S** | **L** | **I** | **F** | **P** | **D** | **M** | **S** | **G** | **N** | **D** | **K** | **I** | **S** | **I** | **Q** | **V** | **D** | **N** | **A** | **K** | **R** | **G** | **E** | **I** | **V** | **D** | **R** | **N** | **G** | **S** | **G** | **L** | **A** | **I** | **N** | **K** | **V** | **F** | **D** | **E** | **V** | **G** | **V** | **V** | **P** | **G** | **K** | **L** | **G** | **S** | **G** | **A** | **E** | **K** | **T** | **A** | **N** | **I** | **K** | **A** | **F** | **S** | **D** | **K** | **F** | **G** | **V** | **S** | **V** | **D** | **E** | **I** | **N** | **Q** | **K** | **L** | **S** | **Q** | **G** | **W** | **V** | **Q** | **A** | **D** | **S** | **F** | **V** | **P** | **I** | **T** | **V** | **A** | **S** | **E** | **P** | **V** | **T** | **E** | **L** | **P** | **T** | **G** | **A** | **A** | **T** | **K** | **D** | **T** | **E** | **S** | **R** | **Y** | **Y** | **P** | **L** | **G** | **E** | **A** | **A** | **A** | **Q** | **L** | **I** | **G** | **Y** | **T** | **G** | **T** | **I** | **T** | **A** | **E** | **D** | **I** | **E** | **K** | **N** | **P** | **E** | **L** | **S** | **S** | **T** | **G** | **V** | **I** | **G** | **K** | **T** | **G** | **L** | **E** | **R** | **A** | **F** | **D** | **K** | **E** | **L** | **R** | **G** | **Q** | **D** | **G** | **G** | **S** | **L** | **V** | **I** | **L** | **D** | **D** | **K** | **E** | **N** | **V** | **K** | **K** | **A** | **L** | **Q** | **T** | **K** | **E** | **K** | **K** | **D** | **G** | **Q** | **T** | **I** | **K** | **L** | **T** | **I** | **D** | **S** | **G** | **V** | **Q** | **Q** | **Q** | **A** | **F** | **A** | **I** | **F** | **D** | **K** | **R** | **P** | **G** | **S** | **A** | **V** | **I** | **T** | **D** | **P** | **Q** | **K** | **G** | **D** | **L** | **L** | **A** | **T** | **V** | **S** | **S** | **P** | **S** | **Y** | **D** | **P** | **N** | **K** | **M** | **A** | **N** | **G** | **I** | **S** | **Q** | **K** | **E** | **Y** | **D** | **A** | **Y** | **N** | **N** | **N** | **K** | **D** | **L** | **P** | **F** | **T** | **A** | **R** | **F** | **A** | **T** | **G** | **Y** | **A** | **P** | **G** | **S** | **T** | **F** | **K** | **T** | **I** | **T** | **G** | **A** | **I** | **G** | **L** | **D** | **A** | **G** | **T** | **L** | **K** | **P** | **D** | **E** | **E** | **L** | **E** | **I** | **N** | **G** | **L** | **K** | **W** | **Q** | **K** | **D** | **K** | **S** | **W** | **G** | **G** | **Y** | **F** | **A** | **T** | **R** | **V** | **K** | **E** | **A** | **S** | **P** | **V** | **N** | **L** | **R** | **T** | **A** | **L** | **V** | **N** | **S** | **D** | **N** | **I** | **Y** | **F** | **A** | **Q** | **Q** | **T** | **L** | **R** | **M** | **G** | **E** | **D** | **K** | **F** | **R** | **A** | **G** | **L** | **N** | **K** | **F** | **I** | **F** | **G** | **E** | **E** | **L** | **D** | **L** | **P** | **I** | **A** | **M** | **T** | **P** | **A** | **Q** | **I** | **S** | **N** | **E** | **D** | **K** | **F** | **N** | **S** | **E** | **I** | **L** | **L** | **A** | **D** | **T** | **G** | **Y** | **G** | **Q** | **G** | **Q** | **L** | **L** | **I** | **S** | **P** | **I** | **Q** | **Q** | **A** | **T** | **M** | **Y** | **S** | **V** | **F** | **Q** | **N** | **N** | **G** | **T** | **L** | **V** | **Y** | **P** | **K** | **L** | **V** | **L** | **D** | **K** | **E** | **T** | **K** | **K** | **K** | **D** | **N** | **V** | **I** | **S** | **A** | **N** | **A** | **A** | **N** | **T** | **I** | **A** | **T** | **D** | **L** | **L** | **G** | **S** | **V** | **E** | **D** | **P** | **S** | **G** | **Y** | **V** | **Y** | **N** | **M** | **Y** | **N** | **P** | **N** | **F** | **S** | **L** | **A** | **A** | **K** | **T** | **G** | **T** | **A** | **E** | **I** | **K** | **D** | **K** | **Q** | **D** | **T** | **D** | **G** | **K** | **E** | **N** | **S** | **F** | **L** | **L** | **T** | **L** | **D** | **R** | **S** | **N** | **N** | **K** | **F** | **L** | **T** | **M** | **I** | **M** | **V** | **E** | **N** | **S** | **G** | **E** | **N** | **G** | **S** | **A** | **T** | **D** | **I** | **S** | **K** | **P** | **L** | **I** | **D** | **Y** | **L** | **E** | **A** | **T** | **I** | **K** | **-** | **-** | **-** | **-** | **-** | **-** | **-** | **-** | **-** | **-** | **-** | **-** | **-** | **-** | **-** | **-** | **-** | **-** | **-** | **-** | **-** | **-** | **-** | **-** | **-** | **-** | **-** | **-** | **-** | **-** | **-** | **-** | **-** | **-** | **-** | **-** | **Q** | **W** | **Q** | **A** | **K** | **Q** | **E** | **L** | **A** | **E** | **A** | **K** | **K** | **T** | **A** | **N** | **T** | **F** | **L** | **N** | **V** | **L** | **S** | **K** | **Q** | **E** | **F** | **D** | **K** | **L** | **P** | **S** | **V** | **V** | **Q** | **E** | **S** | **S** | **L** | **K** | **K** | **N** | **G** | **Y** | **D** | **T** | **K** | **S** | **V** | **V** | **E** | **K** | **Y** | **Q** | **A** | **I** | **Y** | **S** | **G** | **I** | **Q** | **A** | **E** | **G** | **V** | **K** | **A** | **S** | **D** | **V** | **Q** | **V** | **K** | **K** | **A** | **K** | **D** | **N** | **Q** | **Y** | **T** | **F** | **T** | **Y** | **K** | **L** | **S** | **M** | **S** | **T** | **P** | **L** | **G** | **E** | **M** | **K** | **D** | **L** | **S** | **Y** | **Q** | **S** | **S** | **I** | **A** | **K** | **K** | **G** | **D** | **T** | **Y** | **Q** | **I** | **S** | **W** | **K** | **P** | **S** | **L** | **I** | **F** | **P** | **D** | **M** | **S** | **G** | **N** | **D** | **K** | **I** | **S** | **I** | **Q** | **V** | **D** | **N** | **A** | **K** | **R** | **G** | **E** | **I** | **V** | **D** | **R** | **N** | **G** | **S** | **G** | **L** | **A** | **I** | **N** | **K** | **V** | **F** | **D** | **E** | **V** | **G** | **V** | **V** | **P** | **G** | **K** | **L** | **G** | **S** | **G** | **A** | **E** | **K** | **T** | **A** | **N** | **I** | **K** | **A** | **F** | **S** | **D** | **K** | **F** | **G** | **V** | **S** | **V** | **D** | **E** | **I** | **N** | **Q** | **K** | **L** | **S** | **Q** | **G** | **W** | **V** | **Q** | **A** | **D** | **S** | **F** | **V** | **P** | **I** | **T** | **V** | **A** | **S** | **E** | **P** | **V** | **T** | **E** | **L** | **P** | **T** | **G** | **A** | **A** | **T** | **K** | **D** | **T** | **E** | **S** | **R** | **Y** | **Y** | **P** | **L** | **G** | **E** | **A** | **A** | **A** | **Q** | **L** | **I** | **G** | **Y** | **T** | **G** | **T** | **I** | **T** | **A** | **E** | **D** | **I** | **E** | **K** | **N** | **P** | **E** | **L** | **S** | **S** | **T** | **G** | **V** | **I** | **G** | **K** | **T** | **G** | **L** | **E** | **R** | **A** | **F** | **D** | **K** | **E** | **L** | **R** | **G** | **Q** | **D** | **G** | **G** | **S** | **L** | **V** | **I** | **L** | **D** | **D** | **K** | **E** | **N** | **V** | **K** | **K** | **A** | **L** | **Q** | **T** | **K** | **E** | **K** | **K** | **D** | **G** | **Q** | **T** | **I** | **K** | **L** | **T** | **I** | **D** | **S** | **G** | **V** | **Q** | **Q** | **Q** | **A** | **F** | **A** | **I** | **F** | **D** | **K** | **R** | **P** | **G** | **S** | **A** | **V** | **I** | **T** | **D** | **P** | **Q** | **K** | **G** | **D** | **L** | **L** | **A** | **T** | **V** | **S** | **S** | **P** | **S** | **Y** | **D** | **P** | **N** | **K** | **M** | **A** | **N** | **G** | **I** | **S** | **Q** | **K** | **E** | **Y** | **D** | **A** | **Y** | **N** | **N** | **N** | **K** | **D** | **L** | **P** | **F** | **T** | **A** | **R** | **F** | **A** | **T** | **G** | **Y** | **A** | **P** | **G** | **S** | **T** | **F** | **K** | **T** | **I** | **T** | **G** | **A** | **I** | **G** | **L** | **D** | **A** | **G** | **T** | **L** | **K** | **P** | **D** | **E** | **E** | **L** | **E** | **I** | **N** | **G** | **L** | **K** | **W** | **Q** | **K** | **D** | **K** | **S** | **W** | **G** | **G** | **Y** | **F** | **A** | **T** | **R** | **V** | **K** | **E** | **A** | **S** | **P** | **V** | **N** | **L** | **R** | **T** | **A** | **L** | **V** | **N** | **S** | **D** | **N** | **I** | **Y** | **F** | **A** | **Q** | **Q** | **T** | **L** | **R** | **M** | **G** | **E** | **D** | **K** | **F** | **R** | **A** | **G** | **L** | **N** | **K** | **F** | **I** | **F** | **G** | **E** | **E** | **L** | **D** | **L** | **P** | **I** | **A** | **M** | **T** | **P** | **A** | **Q** | **I** | **S** | **N** | **E** | **D** | **K** | **F** | **N** | **S** | **E** | **I** | **L** | **L** | **A** | **D** | **T** | **G** | **Y** | **G** | **Q** | **G** | **Q** | **L** | **L** | **I** | **S** | **P** | **I** | **Q** | **Q** | **A** | **T** | **M** | **Y** | **S** | **V** | **F** | **Q** | **N** | **N** | **G** | **T** | **L** | **V** | **Y** | **P** | **K** | **L** | **V** | **L** | **D** | **K** | **E** | **T** | **K** | **K** | **K** | **D** | **N** | **V** | **I** | **S** | **A** | **N** | **A** | **A** | **N** | **T** | **I** | **A** | **T** | **D** | **L** | **L** | **G** | **S** | **V** | **E** | **D** | **P** | **S** | **G** | **Y** | **V** | **Y** | **N** | **M** | **Y** | **N** | **P** | **N** | **F** | **S** | **L** | **A** | **A** | **K** | **T** | **G** | **T** | **A** | **E** | **I** | **K** | **D** | **K** | **Q** | **D** | **T** | **D** | **G** | **K** | **E** | **N** | **S** | **F** | **L** | **L** | **T** | **L** | **D** | **R** | **S** | **N** | **N** | **K** | **F** | **L** | **T** | **M** | **I** | **M** | **V** | **E** | **N** | **S** | **G** | **E** | **N** | **G** | **S** | **A** | **T** | **D** | **I** | **S** | **K** | **P** | **L** | **I** | **D** | **Y** | **L** | **E** | **A** | **T** | **I** | **K** |
| **N59544F** | **-** | **-** | **-** | **-** | **-** | **-** | **-** | **-** | **-** | **-** | **-** | **-** | **-** | **-** | **-** | **-** | **-** | **-** | **-** | **-** | **-** | **-** | **-** | **-** | **-** | **-** | **-** | **-** | **-** | **-** | **-** | **-** | **-** | **-** | **-** | **-** | **Q** | **W** | **Q** | **A** | **K** | **Q** | **E** | **L** | **A** | **E** | **A** | **K** | **K** | **T** | **A** | **N** | **T** | **F** | **L** | **N** | **V** | **L** | **S** | **K** | **Q** | **E** | **F** | **D** | **K** | **L** | **P** | **S** | **V** | **V** | **Q** | **E** | **S** | **S** | **L** | **K** | **K** | **N** | **G** | **Y** | **D** | **T** | **K** | **S** | **V** | **V** | **E** | **K** | **Y** | **Q** | **A** | **I** | **Y** | **S** | **G** | **I** | **Q** | **A** | **E** | **G** | **V** | **K** | **A** | **S** | **D** | **V** | **Q** | **V** | **K** | **K** | **A** | **K** | **D** | **N** | **Q** | **Y** | **T** | **F** | **T** | **Y** | **K** | **L** | **S** | **M** | **S** | **T** | **P** | **L** | **G** | **E** | **M** | **K** | **D** | **L** | **S** | **Y** | **Q** | **S** | **S** | **I** | **A** | **K** | **K** | **G** | **D** | **T** | **Y** | **Q** | **I** | **S** | **W** | **K** | **P** | **S** | **L** | **I** | **F** | **P** | **D** | **M** | **S** | **G** | **N** | **D** | **K** | **I** | **S** | **I** | **Q** | **V** | **D** | **N** | **A** | **K** | **R** | **G** | **E** | **I** | **V** | **D** | **R** | **N** | **G** | **S** | **G** | **L** | **A** | **I** | **N** | **K** | **V** | **F** | **D** | **E** | **V** | **G** | **V** | **V** | **P** | **G** | **K** | **L** | **G** | **S** | **G** | **A** | **E** | **K** | **T** | **A** | **N** | **I** | **K** | **A** | **F** | **S** | **D** | **K** | **F** | **G** | **V** | **S** | **V** | **D** | **E** | **I** | **N** | **Q** | **K** | **L** | **S** | **Q** | **G** | **W** | **V** | **Q** | **A** | **D** | **S** | **F** | **V** | **P** | **I** | **T** | **V** | **A** | **S** | **E** | **P** | **V** | **T** | **E** | **L** | **P** | **T** | **G** | **A** | **A** | **T** | **K** | **D** | **T** | **E** | **S** | **R** | **Y** | **Y** | **P** | **L** | **G** | **E** | **A** | **A** | **A** | **Q** | **L** | **I** | **G** | **Y** | **T** | **G** | **T** | **I** | **T** | **A** | **E** | **D** | **I** | **E** | **K** | **N** | **P** | **E** | **L** | **S** | **S** | **T** | **G** | **V** | **I** | **G** | **K** | **T** | **G** | **L** | **E** | **R** | **A** | **F** | **D** | **K** | **E** | **L** | **R** | **G** | **Q** | **D** | **G** | **G** | **S** | **L** | **V** | **I** | **L** | **D** | **D** | **K** | **E** | **N** | **V** | **K** | **K** | **A** | **L** | **Q** | **T** | **K** | **E** | **K** | **K** | **D** | **G** | **Q** | **T** | **I** | **K** | **L** | **T** | **I** | **D** | **S** | **G** | **V** | **Q** | **Q** | **Q** | **A** | **F** | **A** | **I** | **F** | **D** | **K** | **R** | **P** | **G** | **S** | **A** | **V** | **I** | **T** | **D** | **P** | **Q** | **K** | **G** | **D** | **L** | **L** | **A** | **T** | **V** | **S** | **S** | **P** | **S** | **Y** | **D** | **P** | **N** | **K** | **M** | **A** | **N** | **G** | **I** | **S** | **Q** | **K** | **E** | **Y** | **D** | **A** | **Y** | **N** | **N** | **N** | **K** | **D** | **L** | **P** | **F** | **T** | **A** | **R** | **F** | **A** | **T** | **G** | **Y** | **A** | **P** | **G** | **S** | **T** | **F** | **K** | **T** | **I** | **T** | **G** | **A** | **I** | **G** | **L** | **D** | **A** | **G** | **T** | **L** | **K** | **P** | **D** | **E** | **E** | **L** | **E** | **I** | **N** | **G** | **L** | **K** | **W** | **Q** | **K** | **D** | **K** | **S** | **W** | **G** | **G** | **Y** | **F** | **A** | **T** | **R** | **V** | **K** | **E** | **A** | **S** | **P** | **V** | **N** | **L** | **R** | **T** | **A** | **L** | **V** | **N** | **S** | **D** | **N** | **I** | **Y** | **F** | **A** | **Q** | **Q** | **T** | **L** | **R** | **M** | **G** | **E** | **D** | **K** | **F** | **R** | **A** | **G** | **L** | **N** | **K** | **F** | **I** | **F** | **G** | **E** | **E** | **L** | **D** | **L** | **P** | **I** | **A** | **M** | **T** | **P** | **A** | **Q** | **I** | **S** | **N** | **E** | **D** | **K** | **F** | **N** | **S** | **E** | **I** | **L** | **L** | **A** | **D** | **T** | **G** | **Y** | **G** | **Q** | **G** | **Q** | **L** | **L** | **I** | **S** | **P** | **I** | **Q** | **Q** | **A** | **T** | **M** | **Y** | **S** | **V** | **F** | **Q** | **N** | **N** | **G** | **T** | **L** | **V** | **Y** | **P** | **K** | **L** | **V** | **L** | **D** | **K** | **E** | **T** | **K** | **K** | **K** | **D** | **N** | **V** | **I** | **S** | **A** | **N** | **A** | **A** | **N** | **T** | **I** | **A** | **T** | **D** | **L** | **L** | **G** | **S** | **V** | **E** | **D** | **P** | **S** | **G** | **Y** | **V** | **Y** | **N** | **M** | **Y** | **N** | **P** | **N** | **F** | **S** | **L** | **A** | **A** | **K** | **T** | **G** | **T** | **A** | **E** | **I** | **K** | **D** | **K** | **Q** | **D** | **T** | **D** | **G** | **K** | **E** | **N** | **S** | **F** | **L** | **L** | **T** | **L** | **D** | **R** | **S** | **N** | **N** | **K** | **F** | **L** | **T** | **M** | **I** | **M** | **V** | **E** | **N** | **S** | **G** | **E** | **N** | **G** | **S** | **A** | **T** | **D** | **I** | **S** | **K** | **P** | **L** | **I** | **D** | **Y** | **L** | **E** | **A** | **T** | **I** | **K** | **-** | **-** | **-** | **-** | **-** | **-** | **-** | **-** | **-** | **-** | **-** | **-** | **-** | **-** | **-** | **-** | **-** | **-** | **-** | **-** | **-** | **-** | **-** | **-** | **-** | **-** | **-** | **-** | **-** | **-** | **-** | **-** | **-** | **-** | **-** | **-** | **Q** | **W** | **Q** | **A** | **K** | **Q** | **E** | **L** | **A** | **E** | **A** | **K** | **K** | **T** | **A** | **N** | **T** | **F** | **L** | **N** | **V** | **L** | **S** | **K** | **Q** | **E** | **F** | **D** | **K** | **L** | **P** | **S** | **V** | **V** | **Q** | **E** | **S** | **S** | **L** | **K** | **K** | **N** | **G** | **Y** | **D** | **T** | **K** | **S** | **V** | **V** | **E** | **K** | **Y** | **Q** | **A** | **I** | **Y** | **S** | **G** | **I** | **Q** | **A** | **E** | **G** | **V** | **K** | **A** | **S** | **D** | **V** | **Q** | **V** | **K** | **K** | **A** | **K** | **D** | **N** | **Q** | **Y** | **T** | **F** | **T** | **Y** | **K** | **L** | **S** | **M** | **S** | **T** | **P** | **L** | **G** | **E** | **M** | **K** | **D** | **L** | **S** | **Y** | **Q** | **S** | **S** | **I** | **A** | **K** | **K** | **G** | **D** | **T** | **Y** | **Q** | **I** | **S** | **W** | **K** | **P** | **S** | **L** | **I** | **F** | **P** | **D** | **M** | **S** | **G** | **N** | **D** | **K** | **I** | **S** | **I** | **Q** | **V** | **D** | **N** | **A** | **K** | **R** | **G** | **E** | **I** | **V** | **D** | **R** | **N** | **G** | **S** | **G** | **L** | **A** | **I** | **N** | **K** | **V** | **F** | **D** | **E** | **V** | **G** | **V** | **V** | **P** | **G** | **K** | **L** | **G** | **S** | **G** | **A** | **E** | **K** | **T** | **A** | **N** | **I** | **K** | **A** | **F** | **S** | **D** | **K** | **F** | **G** | **V** | **S** | **V** | **D** | **E** | **I** | **N** | **Q** | **K** | **L** | **S** | **Q** | **G** | **W** | **V** | **Q** | **A** | **D** | **S** | **F** | **V** | **P** | **I** | **T** | **V** | **A** | **S** | **E** | **P** | **V** | **T** | **E** | **L** | **P** | **T** | **G** | **A** | **A** | **T** | **K** | **D** | **T** | **E** | **S** | **R** | **Y** | **Y** | **P** | **L** | **G** | **E** | **A** | **A** | **A** | **Q** | **L** | **I** | **G** | **Y** | **T** | **G** | **T** | **I** | **T** | **A** | **E** | **D** | **I** | **E** | **K** | **N** | **P** | **E** | **L** | **S** | **S** | **T** | **G** | **V** | **I** | **G** | **K** | **T** | **G** | **L** | **E** | **R** | **A** | **F** | **D** | **K** | **E** | **L** | **R** | **G** | **Q** | **D** | **G** | **G** | **S** | **L** | **V** | **I** | **L** | **D** | **D** | **K** | **E** | **N** | **V** | **K** | **K** | **A** | **L** | **Q** | **T** | **K** | **E** | **K** | **K** | **D** | **G** | **Q** | **T** | **I** | **K** | **L** | **T** | **I** | **D** | **S** | **G** | **V** | **Q** | **Q** | **Q** | **A** | **F** | **A** | **I** | **F** | **D** | **K** | **R** | **P** | **G** | **S** | **A** | **V** | **I** | **T** | **D** | **P** | **Q** | **K** | **G** | **D** | **L** | **L** | **A** | **T** | **V** | **S** | **S** | **P** | **S** | **Y** | **D** | **P** | **N** | **K** | **M** | **A** | **N** | **G** | **I** | **S** | **Q** | **K** | **E** | **Y** | **D** | **A** | **Y** | **N** | **N** | **N** | **K** | **D** | **L** | **P** | **F** | **T** | **A** | **R** | **F** | **A** | **T** | **G** | **Y** | **A** | **P** | **G** | **S** | **T** | **F** | **K** | **T** | **I** | **T** | **G** | **A** | **I** | **G** | **L** | **D** | **A** | **G** | **T** | **L** | **K** | **P** | **D** | **E** | **E** | **L** | **E** | **I** | **N** | **G** | **L** | **K** | **W** | **Q** | **K** | **D** | **K** | **S** | **W** | **G** | **G** | **Y** | **F** | **A** | **T** | **R** | **V** | **K** | **E** | **A** | **S** | **P** | **V** | **N** | **L** | **R** | **T** | **A** | **L** | **V** | **N** | **S** | **D** | **N** | **I** | **Y** | **F** | **A** | **Q** | **Q** | **T** | **L** | **R** | **M** | **G** | **E** | **D** | **K** | **F** | **R** | **A** | **G** | **L** | **N** | **K** | **F** | **I** | **F** | **G** | **E** | **E** | **L** | **D** | **L** | **P** | **I** | **A** | **M** | **T** | **P** | **A** | **Q** | **I** | **S** | **N** | **E** | **D** | **K** | **F** | **N** | **S** | **E** | **I** | **L** | **L** | **A** | **D** | **T** | **G** | **Y** | **G** | **Q** | **G** | **Q** | **L** | **L** | **I** | **S** | **P** | **I** | **Q** | **Q** | **A** | **T** | **M** | **Y** | **S** | **V** | **F** | **Q** | **N** | **N** | **G** | **T** | **L** | **V** | **Y** | **P** | **K** | **L** | **V** | **L** | **D** | **K** | **E** | **T** | **K** | **K** | **K** | **D** | **N** | **V** | **I** | **S** | **A** | **N** | **A** | **A** | **N** | **T** | **I** | **A** | **T** | **D** | **L** | **L** | **G** | **S** | **V** | **E** | **D** | **P** | **S** | **G** | **Y** | **V** | **Y** | **N** | **M** | **Y** | **N** | **P** | **N** | **F** | **S** | **L** | **A** | **A** | **K** | **T** | **G** | **T** | **A** | **E** | **I** | **K** | **D** | **K** | **Q** | **D** | **T** | **D** | **G** | **K** | **E** | **N** | **S** | **F** | **L** | **L** | **T** | **L** | **D** | **R** | **S** | **N** | **N** | **K** | **F** | **L** | **T** | **M** | **I** | **M** | **V** | **E** | **N** | **S** | **G** | **E** | **N** | **G** | **S** | **A** | **T** | **D** | **I** | **S** | **K** | **P** | **L** | **I** | **D** | **Y** | **L** | **E** | **A** | **T** | **I** | **K** |
| **N52734** | **-** | **-** | **-** | **-** | **-** | **-** | **-** | **-** | **-** | **-** | **-** | **-** | **-** | **-** | **-** | **-** | **-** | **-** | **-** | **-** | **-** | **-** | **-** | **-** | **-** | **-** | **-** | **-** | **-** | **-** | **-** | **-** | **-** | **-** | **-** | **-** | **Q** | **W** | **Q** | **A** | **K** | **Q** | **E** | **L** | **A** | **E** | **A** | **K** | **K** | **T** | **A** | **T** | **T** | **F** | **L** | **N** | **V** | **L** | **S** | **K** | **Q** | **E** | **F** | **D** | **K** | **L** | **P** | **S** | **V** | **V** | **Q** | **E** | **A** | **S** | **L** | **K** | **K** | **N** | **G** | **Y** | **D** | **T** | **K** | **S** | **V** | **V** | **E** | **K** | **Y** | **Q** | **A** | **I** | **Y** | **S** | **G** | **I** | **Q** | **A** | **E** | **G** | **V** | **K** | **A** | **S** | **D** | **V** | **Q** | **V** | **K** | **K** | **A** | **K** | **D** | **N** | **Q** | **Y** | **T** | **F** | **T** | **Y** | **K** | **L** | **S** | **M** | **S** | **T** | **P** | **L** | **G** | **E** | **M** | **K** | **D** | **L** | **S** | **Y** | **Q** | **S** | **S** | **I** | **A** | **K** | **K** | **G** | **D** | **T** | **Y** | **Q** | **I** | **A** | **W** | **K** | **P** | **S** | **L** | **I** | **F** | **P** | **D** | **M** | **S** | **G** | **N** | **D** | **K** | **I** | **S** | **I** | **Q** | **V** | **D** | **N** | **A** | **K** | **R** | **G** | **E** | **I** | **V** | **D** | **R** | **N** | **G** | **S** | **G** | **L** | **A** | **I** | **N** | **K** | **V** | **F** | **D** | **E** | **V** | **G** | **V** | **V** | **P** | **G** | **K** | **L** | **G** | **S** | **G** | **A** | **E** | **K** | **T** | **A** | **N** | **I** | **K** | **A** | **F** | **S** | **D** | **K** | **F** | **G** | **V** | **S** | **I** | **D** | **E** | **I** | **N** | **Q** | **K** | **L** | **S** | **Q** | **G** | **W** | **V** | **Q** | **A** | **D** | **S** | **F** | **V** | **P** | **I** | **T** | **V** | **A** | **S** | **E** | **P** | **V** | **T** | **E** | **L** | **P** | **T** | **G** | **A** | **A** | **T** | **K** | **D** | **T** | **E** | **S** | **R** | **Y** | **Y** | **P** | **L** | **G** | **E** | **A** | **A** | **A** | **Q** | **L** | **I** | **G** | **Y** | **T** | **G** | **T** | **I** | **T** | **A** | **E** | **D** | **I** | **E** | **K** | **N** | **P** | **E** | **L** | **S** | **S** | **T** | **G** | **V** | **I** | **G** | **K** | **T** | **G** | **L** | **E** | **R** | **A** | **F** | **D** | **K** | **E** | **L** | **R** | **G** | **Q** | **D** | **G** | **G** | **S** | **L** | **V** | **I** | **L** | **D** | **D** | **K** | **E** | **N** | **V** | **K** | **K** | **A** | **L** | **Q** | **T** | **K** | **E** | **K** | **K** | **D** | **G** | **Q** | **T** | **I** | **K** | **L** | **T** | **I** | **D** | **S** | **G** | **V** | **Q** | **Q** | **Q** | **A** | **F** | **A** | **I** | **F** | **D** | **K** | **R** | **P** | **G** | **S** | **A** | **V** | **I** | **T** | **D** | **P** | **Q** | **K** | **G** | **D** | **L** | **L** | **A** | **T** | **V** | **S** | **S** | **P** | **S** | **Y** | **D** | **P** | **N** | **K** | **M** | **A** | **N** | **G** | **I** | **S** | **Q** | **K** | **E** | **Y** | **D** | **A** | **Y** | **N** | **N** | **N** | **K** | **D** | **L** | **P** | **F** | **T** | **A** | **R** | **F** | **A** | **T** | **G** | **Y** | **A** | **P** | **G** | **S** | **T** | **F** | **K** | **T** | **I** | **T** | **G** | **A** | **I** | **G** | **L** | **D** | **A** | **G** | **T** | **L** | **K** | **P** | **D** | **E** | **E** | **L** | **E** | **I** | **N** | **G** | **L** | **K** | **W** | **Q** | **K** | **D** | **K** | **S** | **W** | **G** | **G** | **Y** | **F** | **A** | **T** | **R** | **V** | **K** | **E** | **A** | **S** | **P** | **V** | **N** | **L** | **R** | **T** | **A** | **L** | **V** | **N** | **S** | **D** | **N** | **I** | **Y** | **F** | **A** | **Q** | **Q** | **T** | **L** | **R** | **M** | **G** | **E** | **D** | **K** | **F** | **R** | **A** | **G** | **L** | **N** | **K** | **F** | **I** | **F** | **G** | **E** | **E** | **L** | **D** | **L** | **P** | **I** | **A** | **M** | **T** | **P** | **A** | **Q** | **I** | **S** | **N** | **E** | **D** | **K** | **F** | **N** | **S** | **E** | **I** | **L** | **L** | **A** | **D** | **T** | **G** | **Y** | **G** | **Q** | **G** | **Q** | **L** | **L** | **I** | **S** | **P** | **I** | **Q** | **Q** | **A** | **T** | **M** | **Y** | **S** | **V** | **F** | **Q** | **N** | **N** | **G** | **T** | **L** | **V** | **Y** | **P** | **K** | **I** | **V** | **L** | **E** | **K** | **K** | **R** | **R** | **K** | **K** | **-** | **-** | **-** | **-** | **-** | **-** | **-** | **-** | **-** | **-** | **-** | **-** | **-** | **-** | **-** | **-** | **-** | **-** | **-** | **-** | **-** | **-** | **-** | **-** | **-** | **-** | **-** | **-** | **-** | **-** | **-** | **-** | **-** | **-** | **-** | **-** | **-** | **-** | **-** | **-** | **-** | **-** | **-** | **-** | **-** | **-** | **-** | **-** | **-** | **-** | **-** | **-** | **-** | **-** | **-** | **-** | **-** | **-** | **-** | **-** | **-** | **-** | **-** | **-** | **-** | **-** | **-** | **-** | **-** | **-** | **-** | **-** | **-** | **-** | **-** | **-** | **-** | **-** | **-** | **-** | **-** | **-** | **-** | **-** | **-** | **-** | **-** | **-** | **-** | **-** | **-** | **-** | **-** | **-** | **-** | **-** | **-** | **-** | **-** | **-** | **-** | **-** | **-** | **-** | **-** | **-** | **-** | **-** | **-** | **-** | **-** | **-** | **-** | **-** | **-** | **-** | **-** | **-** | **-** | **-** | **-** | **-** | **-** | **-** | **-** | **-** | **-** | **-** | **-** | **-** | **-** | **-** | **-** | **-** | **-** | **-** | **-** | **Q** | **W** | **Q** | **A** | **K** | **Q** | **E** | **L** | **A** | **E** | **A** | **K** | **K** | **T** | **A** | **T** | **T** | **F** | **L** | **N** | **V** | **L** | **S** | **K** | **Q** | **E** | **F** | **D** | **K** | **L** | **P** | **S** | **V** | **V** | **Q** | **E** | **A** | **S** | **L** | **K** | **K** | **N** | **G** | **Y** | **D** | **T** | **K** | **S** | **V** | **V** | **E** | **K** | **Y** | **Q** | **A** | **I** | **Y** | **S** | **G** | **I** | **Q** | **A** | **E** | **G** | **V** | **K** | **A** | **S** | **D** | **V** | **Q** | **V** | **K** | **K** | **A** | **K** | **D** | **N** | **Q** | **Y** | **T** | **F** | **T** | **Y** | **K** | **L** | **S** | **M** | **S** | **T** | **P** | **L** | **G** | **E** | **M** | **K** | **D** | **L** | **S** | **Y** | **Q** | **S** | **S** | **I** | **A** | **K** | **K** | **G** | **D** | **T** | **Y** | **Q** | **I** | **A** | **W** | **K** | **P** | **S** | **L** | **I** | **F** | **P** | **D** | **M** | **S** | **G** | **N** | **D** | **K** | **I** | **S** | **I** | **Q** | **V** | **D** | **N** | **A** | **K** | **R** | **G** | **E** | **I** | **V** | **D** | **R** | **N** | **G** | **S** | **G** | **L** | **A** | **I** | **N** | **K** | **V** | **F** | **D** | **E** | **V** | **G** | **V** | **V** | **P** | **G** | **K** | **L** | **G** | **S** | **G** | **A** | **E** | **K** | **T** | **A** | **N** | **I** | **K** | **A** | **F** | **S** | **D** | **K** | **F** | **G** | **V** | **S** | **I** | **D** | **E** | **I** | **N** | **Q** | **K** | **L** | **S** | **Q** | **G** | **W** | **V** | **Q** | **A** | **D** | **S** | **F** | **V** | **P** | **I** | **T** | **V** | **A** | **S** | **E** | **P** | **V** | **T** | **E** | **L** | **P** | **T** | **G** | **A** | **A** | **T** | **K** | **D** | **T** | **E** | **S** | **R** | **Y** | **Y** | **P** | **L** | **G** | **E** | **A** | **A** | **A** | **Q** | **L** | **I** | **G** | **Y** | **T** | **G** | **T** | **I** | **T** | **A** | **E** | **D** | **I** | **E** | **K** | **N** | **P** | **E** | **L** | **S** | **S** | **T** | **G** | **V** | **I** | **G** | **K** | **T** | **G** | **L** | **E** | **R** | **A** | **F** | **D** | **K** | **E** | **L** | **R** | **G** | **Q** | **D** | **G** | **G** | **S** | **L** | **V** | **I** | **L** | **D** | **D** | **K** | **E** | **N** | **V** | **K** | **K** | **A** | **L** | **Q** | **T** | **K** | **E** | **K** | **K** | **D** | **G** | **Q** | **T** | **I** | **K** | **L** | **T** | **I** | **D** | **S** | **G** | **V** | **Q** | **Q** | **Q** | **A** | **F** | **A** | **I** | **F** | **D** | **K** | **R** | **P** | **G** | **S** | **A** | **V** | **I** | **T** | **D** | **P** | **Q** | **K** | **G** | **D** | **L** | **L** | **A** | **T** | **V** | **S** | **S** | **P** | **S** | **Y** | **D** | **P** | **N** | **K** | **M** | **A** | **N** | **G** | **I** | **S** | **Q** | **K** | **E** | **Y** | **D** | **A** | **Y** | **N** | **N** | **N** | **K** | **D** | **L** | **P** | **F** | **T** | **A** | **R** | **F** | **A** | **T** | **G** | **Y** | **A** | **P** | **G** | **S** | **T** | **F** | **K** | **T** | **I** | **T** | **G** | **A** | **I** | **G** | **L** | **D** | **A** | **G** | **T** | **L** | **K** | **P** | **D** | **E** | **E** | **L** | **E** | **I** | **N** | **G** | **L** | **K** | **W** | **Q** | **K** | **D** | **K** | **S** | **W** | **G** | **G** | **Y** | **F** | **A** | **T** | **R** | **V** | **K** | **E** | **A** | **S** | **P** | **V** | **N** | **L** | **R** | **T** | **A** | **L** | **V** | **N** | **S** | **D** | **N** | **I** | **Y** | **F** | **A** | **Q** | **Q** | **T** | **L** | **R** | **M** | **G** | **E** | **D** | **K** | **F** | **R** | **A** | **G** | **L** | **N** | **K** | **F** | **I** | **F** | **G** | **E** | **E** | **L** | **D** | **L** | **P** | **I** | **A** | **M** | **T** | **P** | **A** | **Q** | **I** | **S** | **N** | **E** | **D** | **K** | **F** | **N** | **S** | **E** | **I** | **L** | **L** | **A** | **D** | **T** | **G** | **Y** | **G** | **Q** | **G** | **Q** | **L** | **L** | **I** | **S** | **P** | **I** | **Q** | **Q** | **A** | **T** | **M** | **Y** | **S** | **V** | **F** | **Q** | **N** | **N** | **G** | **T** | **L** | **V** | **Y** | **P** | **K** | **I** | **V** | **L** | **E** | **K** | **K** | **R** | **R** | **K** | **K** | **-** | **-** | **-** | **-** | **-** | **-** | **-** | **-** | **-** | **-** | **-** | **-** | **-** | **-** | **-** | **-** | **-** | **-** | **-** | **-** | **-** | **-** | **-** | **-** | **-** | **-** | **-** | **-** | **-** | **-** | **-** | **-** | **-** | **-** | **-** | **-** | **-** | **-** | **-** | **-** | **-** | **-** | **-** | **-** | **-** | **-** | **-** | **-** | **-** | **-** | **-** | **-** | **-** | **-** | **-** | **-** | **-** | **-** | **-** | **-** | **-** | **-** | **-** | **-** | **-** | **-** | **-** | **-** | **-** | **-** | **-** | **-** | **-** | **-** | **-** | **-** | **-** | **-** | **-** | **-** | **-** | **-** | **-** | **-** | **-** | **-** | **-** | **-** | **-** | **-** | **-** | **-** | **-** | **-** | **-** | **-** | **-** | **-** | **-** | **-** | **-** |
| **N59646F** | **-** | **-** | **-** | **-** | **-** | **-** | **-** | **-** | **-** | **-** | **-** | **-** | **-** | **-** | **-** | **-** | **-** | **-** | **-** | **-** | **-** | **-** | **-** | **-** | **-** | **-** | **-** | **-** | **-** | **-** | **-** | **-** | **-** | **-** | **-** | **-** | **Q** | **W** | **Q** | **A** | **K** | **Q** | **E** | **L** | **A** | **E** | **A** | **K** | **K** | **T** | **A** | **T** | **T** | **F** | **L** | **N** | **V** | **L** | **S** | **K** | **Q** | **E** | **F** | **D** | **K** | **L** | **P** | **S** | **V** | **V** | **Q** | **E** | **A** | **S** | **L** | **K** | **K** | **N** | **G** | **Y** | **D** | **T** | **K** | **S** | **V** | **V** | **E** | **K** | **Y** | **Q** | **A** | **I** | **Y** | **S** | **G** | **I** | **Q** | **A** | **E** | **G** | **V** | **K** | **A** | **S** | **D** | **V** | **Q** | **V** | **K** | **K** | **A** | **K** | **D** | **N** | **Q** | **Y** | **T** | **F** | **T** | **Y** | **K** | **L** | **S** | **M** | **S** | **T** | **P** | **L** | **G** | **E** | **M** | **K** | **D** | **L** | **S** | **Y** | **Q** | **S** | **S** | **I** | **A** | **K** | **K** | **G** | **D** | **T** | **Y** | **Q** | **I** | **A** | **W** | **K** | **P** | **S** | **L** | **I** | **F** | **P** | **D** | **M** | **S** | **G** | **N** | **D** | **K** | **I** | **S** | **I** | **Q** | **V** | **D** | **N** | **A** | **K** | **R** | **G** | **E** | **I** | **V** | **D** | **R** | **N** | **G** | **S** | **G** | **L** | **A** | **I** | **N** | **K** | **V** | **F** | **D** | **E** | **V** | **G** | **V** | **V** | **P** | **G** | **K** | **L** | **G** | **S** | **G** | **A** | **E** | **K** | **T** | **A** | **N** | **I** | **K** | **A** | **F** | **S** | **D** | **K** | **F** | **G** | **V** | **S** | **V** | **D** | **E** | **I** | **N** | **Q** | **K** | **L** | **S** | **Q** | **G** | **W** | **V** | **Q** | **A** | **D** | **S** | **F** | **V** | **P** | **I** | **T** | **V** | **A** | **S** | **E** | **P** | **V** | **T** | **E** | **L** | **P** | **T** | **G** | **A** | **A** | **T** | **K** | **D** | **T** | **E** | **S** | **R** | **Y** | **Y** | **P** | **L** | **G** | **E** | **A** | **A** | **A** | **Q** | **L** | **I** | **G** | **Y** | **T** | **G** | **T** | **I** | **T** | **A** | **E** | **D** | **I** | **E** | **K** | **N** | **P** | **E** | **L** | **S** | **S** | **T** | **G** | **V** | **I** | **G** | **K** | **T** | **G** | **L** | **E** | **R** | **A** | **F** | **D** | **K** | **E** | **L** | **R** | **G** | **Q** | **D** | **G** | **G** | **S** | **L** | **V** | **I** | **L** | **D** | **D** | **K** | **E** | **N** | **V** | **K** | **K** | **A** | **L** | **Q** | **T** | **K** | **E** | **K** | **K** | **D** | **G** | **Q** | **T** | **I** | **K** | **L** | **T** | **I** | **D** | **S** | **G** | **V** | **Q** | **Q** | **Q** | **A** | **F** | **A** | **I** | **F** | **D** | **K** | **R** | **P** | **G** | **S** | **A** | **V** | **I** | **T** | **D** | **P** | **Q** | **K** | **G** | **D** | **L** | **L** | **A** | **T** | **V** | **S** | **S** | **P** | **S** | **Y** | **D** | **P** | **N** | **K** | **M** | **A** | **N** | **G** | **I** | **S** | **Q** | **K** | **E** | **Y** | **D** | **A** | **Y** | **N** | **N** | **N** | **K** | **D** | **L** | **P** | **F** | **T** | **A** | **R** | **F** | **A** | **T** | **G** | **Y** | **A** | **P** | **G** | **S** | **T** | **F** | **K** | **T** | **I** | **T** | **G** | **A** | **I** | **G** | **L** | **D** | **A** | **G** | **T** | **L** | **K** | **P** | **D** | **E** | **E** | **L** | **E** | **I** | **N** | **G** | **L** | **K** | **W** | **Q** | **K** | **D** | **K** | **S** | **W** | **G** | **G** | **Y** | **F** | **A** | **T** | **R** | **V** | **K** | **E** | **A** | **S** | **P** | **V** | **N** | **L** | **R** | **T** | **A** | **L** | **V** | **N** | **S** | **D** | **N** | **I** | **Y** | **F** | **A** | **Q** | **Q** | **T** | **L** | **R** | **M** | **G** | **E** | **D** | **K** | **F** | **R** | **A** | **G** | **L** | **N** | **K** | **F** | **I** | **F** | **G** | **E** | **E** | **L** | **D** | **L** | **P** | **I** | **A** | **M** | **T** | **P** | **A** | **Q** | **I** | **S** | **N** | **E** | **D** | **K** | **F** | **N** | **S** | **E** | **I** | **L** | **L** | **A** | **D** | **T** | **G** | **Y** | **G** | **Q** | **G** | **Q** | **L** | **L** | **I** | **S** | **P** | **I** | **Q** | **Q** | **A** | **T** | **M** | **Y** | **S** | **V** | **F** | **Q** | **N** | **N** | **G** | **T** | **L** | **V** | **Y** | **P** | **K** | **L** | **V** | **L** | **D** | **K** | **E** | **T** | **K** | **K** | **K** | **D** | **N** | **V** | **I** | **S** | **A** | **N** | **A** | **A** | **N** | **T** | **I** | **A** | **T** | **D** | **L** | **L** | **G** | **S** | **V** | **E** | **D** | **P** | **S** | **G** | **Y** | **V** | **Y** | **N** | **M** | **Y** | **N** | **P** | **N** | **F** | **S** | **L** | **A** | **A** | **K** | **T** | **G** | **T** | **A** | **E** | **I** | **K** | **D** | **K** | **Q** | **D** | **T** | **D** | **G** | **K** | **E** | **N** | **S** | **F** | **L** | **L** | **T** | **L** | **D** | **R** | **S** | **N** | **N** | **K** | **F** | **L** | **T** | **M** | **I** | **M** | **V** | **E** | **N** | **S** | **G** | **E** | **N** | **G** | **S** | **A** | **T** | **D** | **I** | **S** | **K** | **P** | **L** | **I** | **D** | **Y** | **L** | **E** | **A** | **T** | **I** | **K** | **-** | **-** | **-** | **-** | **-** | **-** | **-** | **-** | **-** | **-** | **-** | **-** | **-** | **-** | **-** | **-** | **-** | **-** | **-** | **-** | **-** | **-** | **-** | **-** | **-** | **-** | **-** | **-** | **-** | **-** | **-** | **-** | **-** | **-** | **-** | **-** | **Q** | **W** | **Q** | **A** | **K** | **Q** | **E** | **L** | **A** | **E** | **A** | **K** | **K** | **T** | **A** | **T** | **T** | **F** | **L** | **N** | **V** | **L** | **S** | **K** | **Q** | **E** | **F** | **D** | **K** | **L** | **P** | **S** | **V** | **V** | **Q** | **E** | **A** | **S** | **L** | **K** | **K** | **N** | **G** | **Y** | **D** | **T** | **K** | **S** | **V** | **V** | **E** | **K** | **Y** | **Q** | **A** | **I** | **Y** | **S** | **G** | **I** | **Q** | **A** | **E** | **G** | **V** | **K** | **A** | **S** | **D** | **V** | **Q** | **V** | **K** | **K** | **A** | **K** | **D** | **N** | **Q** | **Y** | **T** | **F** | **T** | **Y** | **K** | **L** | **S** | **M** | **S** | **T** | **P** | **L** | **G** | **E** | **M** | **K** | **D** | **L** | **S** | **Y** | **Q** | **S** | **S** | **I** | **A** | **K** | **K** | **G** | **D** | **T** | **Y** | **Q** | **I** | **A** | **W** | **K** | **P** | **S** | **L** | **I** | **F** | **P** | **D** | **M** | **S** | **G** | **N** | **D** | **K** | **I** | **S** | **I** | **Q** | **V** | **D** | **N** | **A** | **K** | **R** | **G** | **E** | **I** | **V** | **D** | **R** | **N** | **G** | **S** | **G** | **L** | **A** | **I** | **N** | **K** | **V** | **F** | **D** | **E** | **V** | **G** | **V** | **V** | **P** | **G** | **K** | **L** | **G** | **S** | **G** | **A** | **E** | **K** | **T** | **A** | **N** | **I** | **K** | **A** | **F** | **S** | **D** | **K** | **F** | **G** | **V** | **S** | **V** | **D** | **E** | **I** | **N** | **Q** | **K** | **L** | **S** | **Q** | **G** | **W** | **V** | **Q** | **A** | **D** | **S** | **F** | **V** | **P** | **I** | **T** | **V** | **A** | **S** | **E** | **P** | **V** | **T** | **E** | **L** | **P** | **T** | **G** | **A** | **A** | **T** | **K** | **D** | **T** | **E** | **S** | **R** | **Y** | **Y** | **P** | **L** | **G** | **E** | **A** | **A** | **A** | **Q** | **L** | **I** | **G** | **Y** | **T** | **G** | **T** | **I** | **T** | **A** | **E** | **D** | **I** | **E** | **K** | **N** | **P** | **E** | **L** | **S** | **S** | **T** | **G** | **V** | **I** | **G** | **K** | **T** | **G** | **L** | **E** | **R** | **A** | **F** | **D** | **K** | **E** | **L** | **R** | **G** | **Q** | **D** | **G** | **G** | **S** | **L** | **V** | **I** | **L** | **D** | **D** | **K** | **E** | **N** | **V** | **K** | **K** | **A** | **L** | **Q** | **T** | **K** | **E** | **K** | **K** | **D** | **G** | **Q** | **T** | **I** | **K** | **L** | **T** | **I** | **D** | **S** | **G** | **V** | **Q** | **Q** | **Q** | **A** | **F** | **A** | **I** | **F** | **D** | **K** | **R** | **P** | **G** | **S** | **A** | **V** | **I** | **T** | **D** | **P** | **Q** | **K** | **G** | **D** | **L** | **L** | **A** | **T** | **V** | **S** | **S** | **P** | **S** | **Y** | **D** | **P** | **N** | **K** | **M** | **A** | **N** | **G** | **I** | **S** | **Q** | **K** | **E** | **Y** | **D** | **A** | **Y** | **N** | **N** | **N** | **K** | **D** | **L** | **P** | **F** | **T** | **A** | **R** | **F** | **A** | **T** | **G** | **Y** | **A** | **P** | **G** | **S** | **T** | **F** | **K** | **T** | **I** | **T** | **G** | **A** | **I** | **G** | **L** | **D** | **A** | **G** | **T** | **L** | **K** | **P** | **D** | **E** | **E** | **L** | **E** | **I** | **N** | **G** | **L** | **K** | **W** | **Q** | **K** | **D** | **K** | **S** | **W** | **G** | **G** | **Y** | **F** | **A** | **T** | **R** | **V** | **K** | **E** | **A** | **S** | **P** | **V** | **N** | **L** | **R** | **T** | **A** | **L** | **V** | **N** | **S** | **D** | **N** | **I** | **Y** | **F** | **A** | **Q** | **Q** | **T** | **L** | **R** | **M** | **G** | **E** | **D** | **K** | **F** | **R** | **A** | **G** | **L** | **N** | **K** | **F** | **I** | **F** | **G** | **E** | **E** | **L** | **D** | **L** | **P** | **I** | **A** | **M** | **T** | **P** | **A** | **Q** | **I** | **S** | **N** | **E** | **D** | **K** | **F** | **N** | **S** | **E** | **I** | **L** | **L** | **A** | **D** | **T** | **G** | **Y** | **G** | **Q** | **G** | **Q** | **L** | **L** | **I** | **S** | **P** | **I** | **Q** | **Q** | **A** | **T** | **M** | **Y** | **S** | **V** | **F** | **Q** | **N** | **N** | **G** | **T** | **L** | **V** | **Y** | **P** | **K** | **L** | **V** | **L** | **D** | **K** | **E** | **T** | **K** | **K** | **K** | **D** | **N** | **V** | **I** | **S** | **A** | **N** | **A** | **A** | **N** | **T** | **I** | **A** | **T** | **D** | **L** | **L** | **G** | **S** | **V** | **E** | **D** | **P** | **S** | **G** | **Y** | **V** | **Y** | **N** | **M** | **Y** | **N** | **P** | **N** | **F** | **S** | **L** | **A** | **A** | **K** | **T** | **G** | **T** | **A** | **E** | **I** | **K** | **D** | **K** | **Q** | **D** | **T** | **D** | **G** | **K** | **E** | **N** | **S** | **F** | **L** | **L** | **T** | **L** | **D** | **R** | **S** | **N** | **N** | **K** | **F** | **L** | **T** | **M** | **I** | **M** | **V** | **E** | **N** | **S** | **G** | **E** | **N** | **G** | **S** | **A** | **T** | **D** | **I** | **S** | **K** | **P** | **L** | **I** | **D** | **Y** | **L** | **E** | **A** | **T** | **I** | **K** |
| **N53405** | **-** | **-** | **-** | **-** | **-** | **-** | **-** | **-** | **-** | **-** | **-** | **-** | **-** | **-** | **-** | **-** | **-** | **-** | **-** | **-** | **-** | **-** | **-** | **-** | **-** | **-** | **-** | **-** | **-** | **-** | **-** | **-** | **-** | **-** | **-** | **-** | **Q** | **W** | **Q** | **A** | **K** | **Q** | **E** | **L** | **A** | **E** | **A** | **K** | **K** | **T** | **A** | **T** | **K** | **F** | **L** | **N** | **V** | **L** | **S** | **K** | **Q** | **E** | **F** | **D** | **K** | **L** | **P** | **S** | **V** | **V** | **Q** | **E** | **A** | **S** | **L** | **K** | **K** | **N** | **G** | **Y** | **D** | **T** | **K** | **S** | **V** | **V** | **E** | **K** | **Y** | **Q** | **A** | **I** | **Y** | **S** | **G** | **I** | **Q** | **A** | **E** | **G** | **V** | **K** | **A** | **S** | **D** | **V** | **Q** | **V** | **K** | **K** | **A** | **K** | **D** | **N** | **Q** | **Y** | **T** | **F** | **T** | **Y** | **K** | **L** | **S** | **M** | **S** | **T** | **P** | **L** | **G** | **E** | **M** | **K** | **D** | **L** | **S** | **Y** | **Q** | **S** | **S** | **I** | **A** | **K** | **K** | **G** | **D** | **T** | **Y** | **Q** | **I** | **A** | **W** | **K** | **P** | **S** | **L** | **I** | **F** | **P** | **D** | **M** | **S** | **G** | **N** | **D** | **K** | **I** | **S** | **I** | **Q** | **V** | **D** | **N** | **A** | **K** | **R** | **G** | **E** | **I** | **V** | **D** | **R** | **N** | **G** | **S** | **G** | **L** | **A** | **I** | **N** | **K** | **V** | **F** | **D** | **E** | **V** | **G** | **V** | **V** | **P** | **G** | **K** | **L** | **G** | **S** | **G** | **A** | **E** | **K** | **T** | **A** | **N** | **I** | **K** | **A** | **F** | **S** | **D** | **K** | **F** | **G** | **V** | **S** | **V** | **D** | **E** | **I** | **N** | **Q** | **K** | **L** | **S** | **Q** | **G** | **W** | **V** | **Q** | **A** | **D** | **S** | **F** | **V** | **P** | **I** | **T** | **V** | **A** | **S** | **E** | **P** | **V** | **T** | **E** | **L** | **P** | **T** | **G** | **A** | **A** | **T** | **K** | **D** | **T** | **E** | **S** | **R** | **Y** | **Y** | **P** | **L** | **G** | **E** | **A** | **A** | **A** | **Q** | **L** | **I** | **G** | **Y** | **T** | **G** | **T** | **I** | **T** | **A** | **E** | **D** | **I** | **K** | **K** | **N** | **P** | **E** | **L** | **S** | **S** | **T** | **G** | **V** | **I** | **G** | **K** | **T** | **G** | **L** | **E** | **R** | **A** | **F** | **D** | **K** | **E** | **L** | **R** | **G** | **Q** | **D** | **G** | **G** | **S** | **L** | **V** | **I** | **L** | **D** | **D** | **K** | **E** | **N** | **V** | **K** | **K** | **A** | **L** | **Q** | **T** | **K** | **E** | **K** | **K** | **D** | **G** | **Q** | **T** | **I** | **K** | **L** | **T** | **I** | **D** | **S** | **G** | **V** | **Q** | **Q** | **Q** | **A** | **F** | **A** | **I** | **F** | **D** | **K** | **R** | **P** | **G** | **S** | **A** | **V** | **I** | **T** | **D** | **P** | **Q** | **K** | **G** | **D** | **L** | **L** | **A** | **T** | **V** | **S** | **S** | **P** | **S** | **Y** | **D** | **P** | **N** | **K** | **M** | **A** | **N** | **G** | **I** | **S** | **Q** | **K** | **E** | **Y** | **D** | **A** | **Y** | **N** | **N** | **N** | **K** | **D** | **L** | **P** | **F** | **T** | **A** | **R** | **F** | **A** | **T** | **G** | **Y** | **A** | **P** | **G** | **S** | **T** | **F** | **K** | **T** | **I** | **T** | **G** | **A** | **I** | **G** | **L** | **D** | **A** | **G** | **T** | **L** | **K** | **P** | **D** | **E** | **E** | **L** | **E** | **I** | **N** | **G** | **L** | **K** | **W** | **Q** | **K** | **D** | **K** | **S** | **W** | **G** | **G** | **Y** | **F** | **A** | **T** | **R** | **V** | **K** | **E** | **A** | **S** | **P** | **V** | **N** | **L** | **R** | **T** | **A** | **L** | **V** | **N** | **S** | **D** | **N** | **I** | **Y** | **F** | **A** | **Q** | **Q** | **T** | **L** | **R** | **M** | **G** | **E** | **D** | **K** | **F** | **R** | **A** | **G** | **L** | **N** | **K** | **F** | **I** | **F** | **G** | **E** | **E** | **L** | **D** | **L** | **P** | **I** | **A** | **M** | **T** | **P** | **A** | **Q** | **I** | **S** | **N** | **E** | **D** | **K** | **F** | **N** | **S** | **E** | **I** | **L** | **L** | **A** | **D** | **T** | **G** | **Y** | **G** | **Q** | **G** | **Q** | **L** | **L** | **I** | **S** | **P** | **I** | **Q** | **Q** | **A** | **T** | **M** | **Y** | **S** | **V** | **F** | **Q** | **N** | **N** | **G** | **T** | **L** | **V** | **Y** | **P** | **K** | **L** | **V** | **L** | **D** | **K** | **E** | **T** | **K** | **K** | **K** | **D** | **N** | **V** | **I** | **S** | **A** | **N** | **A** | **A** | **N** | **T** | **I** | **A** | **T** | **D** | **L** | **L** | **G** | **S** | **V** | **E** | **D** | **P** | **S** | **G** | **Y** | **V** | **Y** | **N** | **M** | **Y** | **N** | **P** | **N** | **F** | **S** | **L** | **A** | **A** | **K** | **T** | **G** | **T** | **A** | **E** | **I** | **K** | **D** | **K** | **Q** | **D** | **T** | **D** | **G** | **K** | **E** | **N** | **S** | **F** | **L** | **L** | **T** | **L** | **D** | **R** | **S** | **N** | **N** | **K** | **F** | **L** | **T** | **M** | **I** | **M** | **V** | **E** | **N** | **S** | **G** | **E** | **N** | **G** | **S** | **A** | **T** | **D** | **I** | **S** | **K** | **P** | **L** | **I** | **D** | **Y** | **L** | **E** | **A** | **T** | **I** | **K** | **-** | **-** | **-** | **-** | **-** | **-** | **-** | **-** | **-** | **-** | **-** | **-** | **-** | **-** | **-** | **-** | **-** | **-** | **-** | **-** | **-** | **-** | **-** | **-** | **-** | **-** | **-** | **-** | **-** | **-** | **-** | **-** | **-** | **-** | **-** | **-** | **Q** | **W** | **Q** | **A** | **K** | **Q** | **E** | **L** | **A** | **E** | **A** | **K** | **K** | **T** | **A** | **T** | **K** | **F** | **L** | **N** | **V** | **L** | **S** | **K** | **Q** | **E** | **F** | **D** | **K** | **L** | **P** | **S** | **V** | **V** | **Q** | **E** | **A** | **S** | **L** | **K** | **K** | **N** | **G** | **Y** | **D** | **T** | **K** | **S** | **V** | **V** | **E** | **K** | **Y** | **Q** | **A** | **I** | **Y** | **S** | **G** | **I** | **Q** | **A** | **E** | **G** | **V** | **K** | **A** | **S** | **D** | **V** | **Q** | **V** | **K** | **K** | **A** | **K** | **D** | **N** | **Q** | **Y** | **T** | **F** | **T** | **Y** | **K** | **L** | **S** | **M** | **S** | **T** | **P** | **L** | **G** | **E** | **M** | **K** | **D** | **L** | **S** | **Y** | **Q** | **S** | **S** | **I** | **A** | **K** | **K** | **G** | **D** | **T** | **Y** | **Q** | **I** | **A** | **W** | **K** | **P** | **S** | **L** | **I** | **F** | **P** | **D** | **M** | **S** | **G** | **N** | **D** | **K** | **I** | **S** | **I** | **Q** | **V** | **D** | **N** | **A** | **K** | **R** | **G** | **E** | **I** | **V** | **D** | **R** | **N** | **G** | **S** | **G** | **L** | **A** | **I** | **N** | **K** | **V** | **F** | **D** | **E** | **V** | **G** | **V** | **V** | **P** | **G** | **K** | **L** | **G** | **S** | **G** | **A** | **E** | **K** | **T** | **A** | **N** | **I** | **K** | **A** | **F** | **S** | **D** | **K** | **F** | **G** | **V** | **S** | **V** | **D** | **E** | **I** | **N** | **Q** | **K** | **L** | **S** | **Q** | **G** | **W** | **V** | **Q** | **A** | **D** | **S** | **F** | **V** | **P** | **I** | **T** | **V** | **A** | **S** | **E** | **P** | **V** | **T** | **E** | **L** | **P** | **T** | **G** | **A** | **A** | **T** | **K** | **D** | **T** | **E** | **S** | **R** | **Y** | **Y** | **P** | **L** | **G** | **E** | **A** | **A** | **A** | **Q** | **L** | **I** | **G** | **Y** | **T** | **G** | **T** | **I** | **T** | **A** | **E** | **D** | **I** | **K** | **K** | **N** | **P** | **E** | **L** | **S** | **S** | **T** | **G** | **V** | **I** | **G** | **K** | **T** | **G** | **L** | **E** | **R** | **A** | **F** | **D** | **K** | **E** | **L** | **R** | **G** | **Q** | **D** | **G** | **G** | **S** | **L** | **V** | **I** | **L** | **D** | **D** | **K** | **E** | **N** | **V** | **K** | **K** | **A** | **L** | **Q** | **T** | **K** | **E** | **K** | **K** | **D** | **G** | **Q** | **T** | **I** | **K** | **L** | **T** | **I** | **D** | **S** | **G** | **V** | **Q** | **Q** | **Q** | **A** | **F** | **A** | **I** | **F** | **D** | **K** | **R** | **P** | **G** | **S** | **A** | **V** | **I** | **T** | **D** | **P** | **Q** | **K** | **G** | **D** | **L** | **L** | **A** | **T** | **V** | **S** | **S** | **P** | **S** | **Y** | **D** | **P** | **N** | **K** | **M** | **A** | **N** | **G** | **I** | **S** | **Q** | **K** | **E** | **Y** | **D** | **A** | **Y** | **N** | **N** | **N** | **K** | **D** | **L** | **P** | **F** | **T** | **A** | **R** | **F** | **A** | **T** | **G** | **Y** | **A** | **P** | **G** | **S** | **T** | **F** | **K** | **T** | **I** | **T** | **G** | **A** | **I** | **G** | **L** | **D** | **A** | **G** | **T** | **L** | **K** | **P** | **D** | **E** | **E** | **L** | **E** | **I** | **N** | **G** | **L** | **K** | **W** | **Q** | **K** | **D** | **K** | **S** | **W** | **G** | **G** | **Y** | **F** | **A** | **T** | **R** | **V** | **K** | **E** | **A** | **S** | **P** | **V** | **N** | **L** | **R** | **T** | **A** | **L** | **V** | **N** | **S** | **D** | **N** | **I** | **Y** | **F** | **A** | **Q** | **Q** | **T** | **L** | **R** | **M** | **G** | **E** | **D** | **K** | **F** | **R** | **A** | **G** | **L** | **N** | **K** | **F** | **I** | **F** | **G** | **E** | **E** | **L** | **D** | **L** | **P** | **I** | **A** | **M** | **T** | **P** | **A** | **Q** | **I** | **S** | **N** | **E** | **D** | **K** | **F** | **N** | **S** | **E** | **I** | **L** | **L** | **A** | **D** | **T** | **G** | **Y** | **G** | **Q** | **G** | **Q** | **L** | **L** | **I** | **S** | **P** | **I** | **Q** | **Q** | **A** | **T** | **M** | **Y** | **S** | **V** | **F** | **Q** | **N** | **N** | **G** | **T** | **L** | **V** | **Y** | **P** | **K** | **L** | **V** | **L** | **D** | **K** | **E** | **T** | **K** | **K** | **K** | **D** | **N** | **V** | **I** | **S** | **A** | **N** | **A** | **A** | **N** | **T** | **I** | **A** | **T** | **D** | **L** | **L** | **G** | **S** | **V** | **E** | **D** | **P** | **S** | **G** | **Y** | **V** | **Y** | **N** | **M** | **Y** | **N** | **P** | **N** | **F** | **S** | **L** | **A** | **A** | **K** | **T** | **G** | **T** | **A** | **E** | **I** | **K** | **D** | **K** | **Q** | **D** | **T** | **D** | **G** | **K** | **E** | **N** | **S** | **F** | **L** | **L** | **T** | **L** | **D** | **R** | **S** | **N** | **N** | **K** | **F** | **L** | **T** | **M** | **I** | **M** | **V** | **E** | **N** | **S** | **G** | **E** | **N** | **G** | **S** | **A** | **T** | **D** | **I** | **S** | **K** | **P** | **L** | **I** | **D** | **Y** | **L** | **E** | **A** | **T** | **I** | **K** |
| **N55268** | **-** | **-** | **-** | **-** | **-** | **-** | **-** | **-** | **-** | **-** | **-** | **-** | **-** | **-** | **-** | **-** | **-** | **-** | **-** | **-** | **-** | **-** | **-** | **-** | **-** | **-** | **-** | **-** | **-** | **-** | **-** | **-** | **-** | **-** | **-** | **-** | **Q** | **W** | **Q** | **A** | **K** | **Q** | **E** | **L** | **A** | **E** | **A** | **K** | **K** | **T** | **A** | **T** | **K** | **F** | **L** | **N** | **V** | **L** | **S** | **K** | **Q** | **E** | **F** | **D** | **K** | **L** | **P** | **S** | **V** | **V** | **Q** | **E** | **A** | **S** | **L** | **K** | **K** | **N** | **G** | **Y** | **D** | **T** | **K** | **S** | **V** | **V** | **E** | **K** | **Y** | **Q** | **A** | **I** | **Y** | **S** | **G** | **I** | **Q** | **A** | **E** | **G** | **V** | **K** | **A** | **S** | **D** | **V** | **Q** | **V** | **K** | **K** | **A** | **K** | **D** | **N** | **Q** | **Y** | **T** | **F** | **T** | **Y** | **K** | **L** | **S** | **M** | **S** | **T** | **P** | **L** | **G** | **E** | **M** | **K** | **D** | **L** | **S** | **Y** | **Q** | **S** | **S** | **I** | **A** | **K** | **K** | **G** | **D** | **T** | **Y** | **Q** | **I** | **A** | **W** | **K** | **P** | **S** | **L** | **I** | **F** | **P** | **D** | **M** | **S** | **G** | **N** | **D** | **K** | **I** | **S** | **I** | **Q** | **V** | **D** | **N** | **A** | **K** | **R** | **G** | **E** | **I** | **V** | **D** | **R** | **N** | **G** | **S** | **G** | **L** | **A** | **I** | **N** | **K** | **V** | **F** | **D** | **E** | **V** | **G** | **V** | **V** | **P** | **G** | **K** | **L** | **G** | **S** | **G** | **A** | **E** | **K** | **T** | **A** | **N** | **I** | **K** | **A** | **F** | **S** | **D** | **K** | **F** | **G** | **V** | **S** | **V** | **D** | **E** | **I** | **N** | **Q** | **K** | **L** | **S** | **Q** | **G** | **W** | **V** | **Q** | **A** | **D** | **S** | **F** | **V** | **P** | **I** | **T** | **V** | **A** | **S** | **E** | **P** | **V** | **T** | **E** | **L** | **P** | **T** | **G** | **A** | **A** | **T** | **K** | **D** | **T** | **E** | **S** | **R** | **Y** | **Y** | **P** | **L** | **G** | **E** | **A** | **A** | **A** | **Q** | **L** | **I** | **G** | **Y** | **T** | **G** | **T** | **I** | **T** | **A** | **E** | **D** | **I** | **E** | **K** | **N** | **P** | **E** | **L** | **S** | **S** | **T** | **G** | **V** | **I** | **G** | **K** | **T** | **G** | **L** | **E** | **R** | **A** | **F** | **D** | **K** | **E** | **L** | **R** | **G** | **Q** | **D** | **G** | **G** | **S** | **L** | **V** | **I** | **L** | **D** | **D** | **K** | **E** | **N** | **V** | **K** | **K** | **A** | **L** | **Q** | **T** | **K** | **E** | **K** | **K** | **D** | **G** | **Q** | **T** | **I** | **K** | **L** | **T** | **I** | **D** | **S** | **G** | **V** | **Q** | **Q** | **Q** | **A** | **F** | **A** | **I** | **F** | **D** | **K** | **R** | **P** | **G** | **S** | **A** | **V** | **I** | **T** | **D** | **P** | **Q** | **K** | **G** | **D** | **L** | **L** | **A** | **T** | **V** | **S** | **S** | **P** | **S** | **Y** | **D** | **P** | **N** | **K** | **M** | **A** | **N** | **G** | **I** | **S** | **Q** | **K** | **E** | **Y** | **D** | **A** | **Y** | **N** | **N** | **N** | **K** | **D** | **L** | **P** | **F** | **T** | **A** | **R** | **F** | **A** | **T** | **G** | **Y** | **A** | **P** | **G** | **S** | **T** | **F** | **K** | **T** | **I** | **T** | **G** | **A** | **I** | **G** | **L** | **D** | **A** | **G** | **T** | **L** | **K** | **P** | **D** | **E** | **E** | **L** | **E** | **I** | **N** | **G** | **L** | **K** | **W** | **Q** | **K** | **D** | **K** | **S** | **W** | **G** | **G** | **Y** | **F** | **A** | **T** | **R** | **V** | **K** | **E** | **A** | **S** | **P** | **V** | **N** | **L** | **R** | **T** | **A** | **L** | **V** | **N** | **S** | **D** | **N** | **I** | **Y** | **F** | **A** | **Q** | **Q** | **T** | **L** | **R** | **M** | **G** | **E** | **D** | **K** | **F** | **R** | **A** | **G** | **L** | **N** | **K** | **F** | **I** | **F** | **G** | **E** | **E** | **L** | **D** | **L** | **P** | **I** | **A** | **M** | **T** | **P** | **A** | **Q** | **I** | **S** | **N** | **E** | **D** | **K** | **F** | **N** | **S** | **E** | **I** | **L** | **L** | **A** | **D** | **T** | **G** | **Y** | **G** | **Q** | **G** | **Q** | **L** | **L** | **I** | **S** | **P** | **I** | **Q** | **Q** | **A** | **T** | **M** | **Y** | **S** | **V** | **F** | **Q** | **N** | **N** | **G** | **T** | **L** | **V** | **Y** | **P** | **K** | **L** | **V** | **L** | **D** | **K** | **E** | **T** | **K** | **K** | **K** | **D** | **N** | **V** | **I** | **S** | **A** | **N** | **A** | **A** | **N** | **T** | **I** | **A** | **T** | **D** | **L** | **L** | **G** | **S** | **V** | **E** | **D** | **P** | **S** | **G** | **Y** | **V** | **Y** | **N** | **M** | **Y** | **N** | **P** | **N** | **F** | **S** | **L** | **A** | **A** | **K** | **T** | **G** | **T** | **A** | **E** | **I** | **K** | **D** | **K** | **Q** | **D** | **T** | **D** | **G** | **K** | **E** | **N** | **S** | **F** | **L** | **L** | **T** | **L** | **D** | **R** | **S** | **N** | **N** | **K** | **F** | **L** | **T** | **M** | **I** | **M** | **V** | **E** | **N** | **S** | **G** | **E** | **N** | **G** | **S** | **A** | **T** | **D** | **I** | **S** | **K** | **P** | **L** | **I** | **D** | **Y** | **L** | **E** | **A** | **T** | **I** | **K** | **-** | **-** | **-** | **-** | **-** | **-** | **-** | **-** | **-** | **-** | **-** | **-** | **-** | **-** | **-** | **-** | **-** | **-** | **-** | **-** | **-** | **-** | **-** | **-** | **-** | **-** | **-** | **-** | **-** | **-** | **-** | **-** | **-** | **-** | **-** | **-** | **Q** | **W** | **Q** | **A** | **K** | **Q** | **E** | **L** | **A** | **E** | **A** | **K** | **K** | **T** | **A** | **T** | **K** | **F** | **L** | **N** | **V** | **L** | **S** | **K** | **Q** | **E** | **F** | **D** | **K** | **L** | **P** | **S** | **V** | **V** | **Q** | **E** | **A** | **S** | **L** | **K** | **K** | **N** | **G** | **Y** | **D** | **T** | **K** | **S** | **V** | **V** | **E** | **K** | **Y** | **Q** | **A** | **I** | **Y** | **S** | **G** | **I** | **Q** | **A** | **E** | **G** | **V** | **K** | **A** | **S** | **D** | **V** | **Q** | **V** | **K** | **K** | **A** | **K** | **D** | **N** | **Q** | **Y** | **T** | **F** | **T** | **Y** | **K** | **L** | **S** | **M** | **S** | **T** | **P** | **L** | **G** | **E** | **M** | **K** | **D** | **L** | **S** | **Y** | **Q** | **S** | **S** | **I** | **A** | **K** | **K** | **G** | **D** | **T** | **Y** | **Q** | **I** | **A** | **W** | **K** | **P** | **S** | **L** | **I** | **F** | **P** | **D** | **M** | **S** | **G** | **N** | **D** | **K** | **I** | **S** | **I** | **Q** | **V** | **D** | **N** | **A** | **K** | **R** | **G** | **E** | **I** | **V** | **D** | **R** | **N** | **G** | **S** | **G** | **L** | **A** | **I** | **N** | **K** | **V** | **F** | **D** | **E** | **V** | **G** | **V** | **V** | **P** | **G** | **K** | **L** | **G** | **S** | **G** | **A** | **E** | **K** | **T** | **A** | **N** | **I** | **K** | **A** | **F** | **S** | **D** | **K** | **F** | **G** | **V** | **S** | **V** | **D** | **E** | **I** | **N** | **Q** | **K** | **L** | **S** | **Q** | **G** | **W** | **V** | **Q** | **A** | **D** | **S** | **F** | **V** | **P** | **I** | **T** | **V** | **A** | **S** | **E** | **P** | **V** | **T** | **E** | **L** | **P** | **T** | **G** | **A** | **A** | **T** | **K** | **D** | **T** | **E** | **S** | **R** | **Y** | **Y** | **P** | **L** | **G** | **E** | **A** | **A** | **A** | **Q** | **L** | **I** | **G** | **Y** | **T** | **G** | **T** | **I** | **T** | **A** | **E** | **D** | **I** | **E** | **K** | **N** | **P** | **E** | **L** | **S** | **S** | **T** | **G** | **V** | **I** | **G** | **K** | **T** | **G** | **L** | **E** | **R** | **A** | **F** | **D** | **K** | **E** | **L** | **R** | **G** | **Q** | **D** | **G** | **G** | **S** | **L** | **V** | **I** | **L** | **D** | **D** | **K** | **E** | **N** | **V** | **K** | **K** | **A** | **L** | **Q** | **T** | **K** | **E** | **K** | **K** | **D** | **G** | **Q** | **T** | **I** | **K** | **L** | **T** | **I** | **D** | **S** | **G** | **V** | **Q** | **Q** | **Q** | **A** | **F** | **A** | **I** | **F** | **D** | **K** | **R** | **P** | **G** | **S** | **A** | **V** | **I** | **T** | **D** | **P** | **Q** | **K** | **G** | **D** | **L** | **L** | **A** | **T** | **V** | **S** | **S** | **P** | **S** | **Y** | **D** | **P** | **N** | **K** | **M** | **A** | **N** | **G** | **I** | **S** | **Q** | **K** | **E** | **Y** | **D** | **A** | **Y** | **N** | **N** | **N** | **K** | **D** | **L** | **P** | **F** | **T** | **A** | **R** | **F** | **A** | **T** | **G** | **Y** | **A** | **P** | **G** | **S** | **T** | **F** | **K** | **T** | **I** | **T** | **G** | **A** | **I** | **G** | **L** | **D** | **A** | **G** | **T** | **L** | **K** | **P** | **D** | **E** | **E** | **L** | **E** | **I** | **N** | **G** | **L** | **K** | **W** | **Q** | **K** | **D** | **K** | **S** | **W** | **G** | **G** | **Y** | **F** | **A** | **T** | **R** | **V** | **K** | **E** | **A** | **S** | **P** | **V** | **N** | **L** | **R** | **T** | **A** | **L** | **V** | **N** | **S** | **D** | **N** | **I** | **Y** | **F** | **A** | **Q** | **Q** | **T** | **L** | **R** | **M** | **G** | **E** | **D** | **K** | **F** | **R** | **A** | **G** | **L** | **N** | **K** | **F** | **I** | **F** | **G** | **E** | **E** | **L** | **D** | **L** | **P** | **I** | **A** | **M** | **T** | **P** | **A** | **Q** | **I** | **S** | **N** | **E** | **D** | **K** | **F** | **N** | **S** | **E** | **I** | **L** | **L** | **A** | **D** | **T** | **G** | **Y** | **G** | **Q** | **G** | **Q** | **L** | **L** | **I** | **S** | **P** | **I** | **Q** | **Q** | **A** | **T** | **M** | **Y** | **S** | **V** | **F** | **Q** | **N** | **N** | **G** | **T** | **L** | **V** | **Y** | **P** | **K** | **L** | **V** | **L** | **D** | **K** | **E** | **T** | **K** | **K** | **K** | **D** | **N** | **V** | **I** | **S** | **A** | **N** | **A** | **A** | **N** | **T** | **I** | **A** | **T** | **D** | **L** | **L** | **G** | **S** | **V** | **E** | **D** | **P** | **S** | **G** | **Y** | **V** | **Y** | **N** | **M** | **Y** | **N** | **P** | **N** | **F** | **S** | **L** | **A** | **A** | **K** | **T** | **G** | **T** | **A** | **E** | **I** | **K** | **D** | **K** | **Q** | **D** | **T** | **D** | **G** | **K** | **E** | **N** | **S** | **F** | **L** | **L** | **T** | **L** | **D** | **R** | **S** | **N** | **N** | **K** | **F** | **L** | **T** | **M** | **I** | **M** | **V** | **E** | **N** | **S** | **G** | **E** | **N** | **G** | **S** | **A** | **T** | **D** | **I** | **S** | **K** | **P** | **L** | **I** | **D** | **Y** | **L** | **E** | **A** | **T** | **I** | **K** |
| **N59509F** | **-** | **-** | **-** | **-** | **-** | **-** | **-** | **-** | **-** | **-** | **-** | **-** | **-** | **-** | **-** | **-** | **-** | **-** | **-** | **-** | **-** | **-** | **-** | **-** | **-** | **-** | **-** | **-** | **-** | **-** | **-** | **-** | **-** | **-** | **-** | **-** | **Q** | **W** | **Q** | **A** | **K** | **Q** | **E** | **L** | **A** | **E** | **A** | **K** | **K** | **T** | **A** | **T** | **T** | **F** | **L** | **N** | **V** | **L** | **T** | **K** | **Q** | **K** | **F** | **D** | **K** | **L** | **P** | **S** | **V** | **V** | **Q** | **E** | **A** | **S** | **L** | **K** | **K** | **N** | **G** | **Y** | **D** | **T** | **K** | **S** | **V** | **V** | **E** | **K** | **Y** | **Q** | **A** | **I** | **Y** | **S** | **G** | **I** | **Q** | **A** | **E** | **G** | **V** | **K** | **A** | **S** | **D** | **V** | **Q** | **V** | **K** | **K** | **A** | **K** | **D** | **N** | **Q** | **Y** | **T** | **F** | **T** | **Y** | **K** | **L** | **S** | **M** | **S** | **T** | **P** | **L** | **G** | **E** | **M** | **K** | **D** | **L** | **S** | **Y** | **Q** | **S** | **S** | **I** | **A** | **K** | **K** | **G** | **D** | **T** | **Y** | **Q** | **I** | **A** | **W** | **K** | **P** | **S** | **L** | **I** | **F** | **P** | **D** | **M** | **S** | **G** | **N** | **D** | **K** | **I** | **S** | **I** | **Q** | **V** | **D** | **N** | **A** | **K** | **R** | **G** | **E** | **I** | **V** | **D** | **R** | **N** | **G** | **S** | **G** | **L** | **A** | **I** | **N** | **K** | **V** | **F** | **D** | **E** | **V** | **G** | **V** | **V** | **P** | **G** | **K** | **L** | **G** | **S** | **G** | **A** | **E** | **K** | **T** | **A** | **N** | **I** | **K** | **A** | **F** | **S** | **D** | **K** | **F** | **G** | **V** | **S** | **V** | **D** | **E** | **I** | **N** | **Q** | **K** | **L** | **S** | **Q** | **G** | **W** | **V** | **Q** | **A** | **D** | **S** | **F** | **V** | **P** | **I** | **T** | **V** | **A** | **S** | **E** | **P** | **V** | **T** | **E** | **L** | **P** | **T** | **G** | **A** | **A** | **T** | **K** | **D** | **T** | **E** | **S** | **R** | **Y** | **Y** | **P** | **L** | **G** | **E** | **A** | **A** | **A** | **Q** | **L** | **I** | **G** | **Y** | **T** | **G** | **T** | **I** | **T** | **A** | **E** | **D** | **I** | **K** | **K** | **N** | **P** | **E** | **L** | **S** | **S** | **T** | **G** | **V** | **I** | **G** | **K** | **T** | **G** | **L** | **E** | **R** | **A** | **F** | **D** | **K** | **E** | **L** | **R** | **G** | **Q** | **D** | **G** | **G** | **S** | **L** | **V** | **I** | **L** | **D** | **D** | **K** | **E** | **N** | **V** | **K** | **K** | **A** | **L** | **Q** | **T** | **K** | **E** | **K** | **K** | **D** | **G** | **Q** | **T** | **I** | **K** | **L** | **T** | **I** | **D** | **S** | **G** | **V** | **Q** | **Q** | **Q** | **A** | **F** | **A** | **I** | **F** | **D** | **K** | **R** | **P** | **G** | **S** | **A** | **V** | **I** | **T** | **D** | **P** | **Q** | **K** | **G** | **D** | **L** | **L** | **A** | **T** | **V** | **S** | **S** | **P** | **S** | **Y** | **D** | **P** | **N** | **K** | **M** | **A** | **N** | **G** | **I** | **S** | **Q** | **K** | **E** | **Y** | **D** | **A** | **Y** | **N** | **N** | **N** | **K** | **D** | **L** | **P** | **F** | **T** | **A** | **R** | **F** | **A** | **T** | **G** | **Y** | **A** | **P** | **G** | **S** | **T** | **F** | **K** | **T** | **I** | **T** | **G** | **A** | **I** | **G** | **L** | **D** | **A** | **G** | **T** | **L** | **K** | **P** | **D** | **E** | **E** | **L** | **E** | **I** | **N** | **G** | **L** | **K** | **W** | **Q** | **K** | **D** | **K** | **S** | **W** | **G** | **G** | **Y** | **F** | **A** | **T** | **R** | **V** | **K** | **E** | **A** | **S** | **P** | **V** | **N** | **L** | **R** | **T** | **A** | **L** | **V** | **N** | **S** | **D** | **N** | **I** | **Y** | **F** | **A** | **Q** | **Q** | **T** | **L** | **R** | **M** | **G** | **E** | **D** | **K** | **F** | **R** | **A** | **G** | **L** | **N** | **K** | **F** | **I** | **F** | **G** | **E** | **E** | **L** | **D** | **L** | **P** | **I** | **A** | **M** | **T** | **P** | **A** | **Q** | **I** | **S** | **N** | **E** | **D** | **K** | **F** | **N** | **S** | **E** | **I** | **L** | **L** | **A** | **D** | **T** | **G** | **Y** | **G** | **Q** | **G** | **Q** | **L** | **L** | **I** | **S** | **P** | **I** | **Q** | **Q** | **A** | **T** | **M** | **Y** | **S** | **V** | **F** | **Q** | **N** | **N** | **G** | **T** | **L** | **V** | **Y** | **P** | **K** | **L** | **V** | **L** | **D** | **K** | **E** | **T** | **K** | **K** | **K** | **D** | **N** | **V** | **I** | **S** | **A** | **N** | **A** | **A** | **N** | **T** | **I** | **A** | **T** | **D** | **L** | **L** | **G** | **S** | **V** | **E** | **D** | **P** | **S** | **G** | **Y** | **V** | **Y** | **N** | **M** | **Y** | **N** | **P** | **N** | **F** | **S** | **L** | **A** | **A** | **K** | **T** | **G** | **T** | **A** | **E** | **I** | **K** | **D** | **K** | **Q** | **D** | **T** | **D** | **G** | **K** | **E** | **N** | **S** | **F** | **L** | **L** | **T** | **L** | **D** | **R** | **S** | **N** | **N** | **K** | **F** | **L** | **T** | **M** | **I** | **M** | **V** | **E** | **N** | **S** | **G** | **E** | **N** | **G** | **S** | **A** | **T** | **D** | **I** | **S** | **K** | **P** | **L** | **I** | **D** | **Y** | **L** | **E** | **A** | **T** | **I** | **K** | **-** | **-** | **-** | **-** | **-** | **-** | **-** | **-** | **-** | **-** | **-** | **-** | **-** | **-** | **-** | **-** | **-** | **-** | **-** | **-** | **-** | **-** | **-** | **-** | **-** | **-** | **-** | **-** | **-** | **-** | **-** | **-** | **-** | **-** | **-** | **-** | **Q** | **W** | **Q** | **A** | **K** | **Q** | **E** | **L** | **A** | **E** | **A** | **K** | **K** | **T** | **A** | **T** | **T** | **F** | **L** | **N** | **V** | **L** | **T** | **K** | **Q** | **K** | **F** | **D** | **K** | **L** | **P** | **S** | **V** | **V** | **Q** | **E** | **A** | **S** | **L** | **K** | **K** | **N** | **G** | **Y** | **D** | **T** | **K** | **S** | **V** | **V** | **E** | **K** | **Y** | **Q** | **A** | **I** | **Y** | **S** | **G** | **I** | **Q** | **A** | **E** | **G** | **V** | **K** | **A** | **S** | **D** | **V** | **Q** | **V** | **K** | **K** | **A** | **K** | **D** | **N** | **Q** | **Y** | **T** | **F** | **T** | **Y** | **K** | **L** | **S** | **M** | **S** | **T** | **P** | **L** | **G** | **E** | **M** | **K** | **D** | **L** | **S** | **Y** | **Q** | **S** | **S** | **I** | **A** | **K** | **K** | **G** | **D** | **T** | **Y** | **Q** | **I** | **A** | **W** | **K** | **P** | **S** | **L** | **I** | **F** | **P** | **D** | **M** | **S** | **G** | **N** | **D** | **K** | **I** | **S** | **I** | **Q** | **V** | **D** | **N** | **A** | **K** | **R** | **G** | **E** | **I** | **V** | **D** | **R** | **N** | **G** | **S** | **G** | **L** | **A** | **I** | **N** | **K** | **V** | **F** | **D** | **E** | **V** | **G** | **V** | **V** | **P** | **G** | **K** | **L** | **G** | **S** | **G** | **A** | **E** | **K** | **T** | **A** | **N** | **I** | **K** | **A** | **F** | **S** | **D** | **K** | **F** | **G** | **V** | **S** | **V** | **D** | **E** | **I** | **N** | **Q** | **K** | **L** | **S** | **Q** | **G** | **W** | **V** | **Q** | **A** | **D** | **S** | **F** | **V** | **P** | **I** | **T** | **V** | **A** | **S** | **E** | **P** | **V** | **T** | **E** | **L** | **P** | **T** | **G** | **A** | **A** | **T** | **K** | **D** | **T** | **E** | **S** | **R** | **Y** | **Y** | **P** | **L** | **G** | **E** | **A** | **A** | **A** | **Q** | **L** | **I** | **G** | **Y** | **T** | **G** | **T** | **I** | **T** | **A** | **E** | **D** | **I** | **K** | **K** | **N** | **P** | **E** | **L** | **S** | **S** | **T** | **G** | **V** | **I** | **G** | **K** | **T** | **G** | **L** | **E** | **R** | **A** | **F** | **D** | **K** | **E** | **L** | **R** | **G** | **Q** | **D** | **G** | **G** | **S** | **L** | **V** | **I** | **L** | **D** | **D** | **K** | **E** | **N** | **V** | **K** | **K** | **A** | **L** | **Q** | **T** | **K** | **E** | **K** | **K** | **D** | **G** | **Q** | **T** | **I** | **K** | **L** | **T** | **I** | **D** | **S** | **G** | **V** | **Q** | **Q** | **Q** | **A** | **F** | **A** | **I** | **F** | **D** | **K** | **R** | **P** | **G** | **S** | **A** | **V** | **I** | **T** | **D** | **P** | **Q** | **K** | **G** | **D** | **L** | **L** | **A** | **T** | **V** | **S** | **S** | **P** | **S** | **Y** | **D** | **P** | **N** | **K** | **M** | **A** | **N** | **G** | **I** | **S** | **Q** | **K** | **E** | **Y** | **D** | **A** | **Y** | **N** | **N** | **N** | **K** | **D** | **L** | **P** | **F** | **T** | **A** | **R** | **F** | **A** | **T** | **G** | **Y** | **A** | **P** | **G** | **S** | **T** | **F** | **K** | **T** | **I** | **T** | **G** | **A** | **I** | **G** | **L** | **D** | **A** | **G** | **T** | **L** | **K** | **P** | **D** | **E** | **E** | **L** | **E** | **I** | **N** | **G** | **L** | **K** | **W** | **Q** | **K** | **D** | **K** | **S** | **W** | **G** | **G** | **Y** | **F** | **A** | **T** | **R** | **V** | **K** | **E** | **A** | **S** | **P** | **V** | **N** | **L** | **R** | **T** | **A** | **L** | **V** | **N** | **S** | **D** | **N** | **I** | **Y** | **F** | **A** | **Q** | **Q** | **T** | **L** | **R** | **M** | **G** | **E** | **D** | **K** | **F** | **R** | **A** | **G** | **L** | **N** | **K** | **F** | **I** | **F** | **G** | **E** | **E** | **L** | **D** | **L** | **P** | **I** | **A** | **M** | **T** | **P** | **A** | **Q** | **I** | **S** | **N** | **E** | **D** | **K** | **F** | **N** | **S** | **E** | **I** | **L** | **L** | **A** | **D** | **T** | **G** | **Y** | **G** | **Q** | **G** | **Q** | **L** | **L** | **I** | **S** | **P** | **I** | **Q** | **Q** | **A** | **T** | **M** | **Y** | **S** | **V** | **F** | **Q** | **N** | **N** | **G** | **T** | **L** | **V** | **Y** | **P** | **K** | **L** | **V** | **L** | **D** | **K** | **E** | **T** | **K** | **K** | **K** | **D** | **N** | **V** | **I** | **S** | **A** | **N** | **A** | **A** | **N** | **T** | **I** | **A** | **T** | **D** | **L** | **L** | **G** | **S** | **V** | **E** | **D** | **P** | **S** | **G** | **Y** | **V** | **Y** | **N** | **M** | **Y** | **N** | **P** | **N** | **F** | **S** | **L** | **A** | **A** | **K** | **T** | **G** | **T** | **A** | **E** | **I** | **K** | **D** | **K** | **Q** | **D** | **T** | **D** | **G** | **K** | **E** | **N** | **S** | **F** | **L** | **L** | **T** | **L** | **D** | **R** | **S** | **N** | **N** | **K** | **F** | **L** | **T** | **M** | **I** | **M** | **V** | **E** | **N** | **S** | **G** | **E** | **N** | **G** | **S** | **A** | **T** | **D** | **I** | **S** | **K** | **P** | **L** | **I** | **D** | **Y** | **L** | **E** | **A** | **T** | **I** | **K** |
| **N54576** | **-** | **-** | **-** | **-** | **-** | **-** | **-** | **-** | **-** | **-** | **-** | **-** | **-** | **-** | **-** | **-** | **-** | **-** | **-** | **-** | **-** | **-** | **-** | **-** | **-** | **-** | **-** | **-** | **-** | **-** | **-** | **-** | **-** | **-** | **-** | **-** | **Q** | **W** | **Q** | **A** | **K** | **Q** | **E** | **L** | **A** | **E** | **A** | **K** | **K** | **T** | **A** | **T** | **T** | **F** | **L** | **N** | **V** | **L** | **T** | **K** | **Q** | **K** | **F** | **D** | **K** | **L** | **P** | **S** | **V** | **V** | **Q** | **E** | **A** | **S** | **L** | **K** | **K** | **N** | **G** | **Y** | **D** | **T** | **K** | **S** | **V** | **V** | **E** | **K** | **Y** | **Q** | **A** | **I** | **Y** | **S** | **G** | **I** | **Q** | **A** | **E** | **G** | **V** | **K** | **A** | **S** | **D** | **V** | **Q** | **V** | **K** | **K** | **A** | **K** | **D** | **N** | **Q** | **Y** | **T** | **F** | **N** | **Y** | **K** | **L** | **S** | **M** | **S** | **T** | **P** | **L** | **G** | **E** | **M** | **K** | **D** | **L** | **S** | **Y** | **Q** | **S** | **S** | **I** | **A** | **K** | **K** | **G** | **D** | **T** | **Y** | **Q** | **I** | **A** | **W** | **K** | **P** | **S** | **L** | **I** | **F** | **P** | **D** | **M** | **S** | **G** | **N** | **D** | **K** | **I** | **S** | **I** | **Q** | **V** | **D** | **N** | **A** | **K** | **R** | **G** | **E** | **I** | **V** | **D** | **R** | **N** | **G** | **S** | **G** | **L** | **A** | **I** | **N** | **K** | **V** | **F** | **D** | **E** | **V** | **G** | **V** | **V** | **P** | **G** | **K** | **L** | **G** | **S** | **G** | **A** | **E** | **K** | **T** | **A** | **N** | **I** | **K** | **A** | **F** | **S** | **D** | **K** | **F** | **G** | **V** | **S** | **V** | **D** | **E** | **I** | **N** | **Q** | **K** | **L** | **S** | **Q** | **G** | **W** | **V** | **Q** | **A** | **D** | **S** | **F** | **V** | **P** | **I** | **T** | **V** | **A** | **S** | **E** | **P** | **V** | **T** | **E** | **L** | **P** | **T** | **G** | **A** | **A** | **T** | **K** | **D** | **T** | **E** | **S** | **R** | **Y** | **Y** | **P** | **L** | **G** | **E** | **A** | **A** | **A** | **Q** | **L** | **I** | **G** | **Y** | **T** | **G** | **T** | **I** | **T** | **A** | **E** | **D** | **I** | **K** | **K** | **N** | **P** | **E** | **L** | **S** | **S** | **T** | **G** | **V** | **I** | **G** | **K** | **T** | **G** | **L** | **E** | **R** | **A** | **F** | **D** | **K** | **E** | **L** | **R** | **G** | **Q** | **D** | **G** | **G** | **S** | **L** | **V** | **I** | **L** | **D** | **D** | **K** | **E** | **N** | **V** | **K** | **K** | **A** | **L** | **Q** | **T** | **K** | **E** | **K** | **K** | **D** | **G** | **Q** | **T** | **I** | **K** | **L** | **T** | **I** | **D** | **S** | **G** | **V** | **Q** | **Q** | **Q** | **A** | **F** | **A** | **I** | **F** | **D** | **K** | **R** | **P** | **G** | **S** | **A** | **V** | **I** | **T** | **D** | **P** | **Q** | **K** | **G** | **D** | **L** | **L** | **A** | **T** | **V** | **S** | **S** | **P** | **S** | **Y** | **D** | **P** | **N** | **K** | **M** | **A** | **N** | **G** | **I** | **S** | **Q** | **K** | **E** | **Y** | **D** | **A** | **Y** | **N** | **N** | **N** | **K** | **D** | **L** | **P** | **F** | **T** | **A** | **R** | **F** | **A** | **T** | **G** | **Y** | **A** | **P** | **G** | **S** | **T** | **F** | **K** | **T** | **I** | **T** | **G** | **A** | **I** | **G** | **L** | **D** | **A** | **G** | **T** | **L** | **K** | **P** | **D** | **E** | **E** | **L** | **E** | **I** | **N** | **G** | **L** | **K** | **W** | **Q** | **K** | **D** | **K** | **S** | **W** | **G** | **G** | **Y** | **F** | **A** | **T** | **R** | **V** | **K** | **E** | **A** | **S** | **P** | **V** | **N** | **L** | **R** | **T** | **A** | **L** | **V** | **N** | **S** | **D** | **N** | **I** | **Y** | **F** | **A** | **Q** | **Q** | **T** | **L** | **R** | **M** | **G** | **E** | **D** | **K** | **F** | **R** | **A** | **G** | **L** | **N** | **K** | **F** | **I** | **F** | **G** | **E** | **E** | **L** | **D** | **L** | **P** | **I** | **A** | **M** | **T** | **P** | **A** | **Q** | **I** | **S** | **N** | **E** | **D** | **K** | **F** | **N** | **S** | **E** | **I** | **L** | **L** | **A** | **D** | **T** | **G** | **Y** | **G** | **Q** | **G** | **Q** | **L** | **L** | **I** | **S** | **P** | **I** | **Q** | **Q** | **A** | **T** | **M** | **Y** | **S** | **V** | **F** | **Q** | **N** | **N** | **G** | **T** | **L** | **V** | **Y** | **P** | **K** | **L** | **V** | **L** | **D** | **K** | **E** | **T** | **K** | **K** | **K** | **D** | **N** | **V** | **I** | **S** | **A** | **N** | **A** | **A** | **N** | **T** | **I** | **A** | **T** | **D** | **L** | **L** | **G** | **S** | **V** | **E** | **D** | **P** | **S** | **G** | **Y** | **V** | **Y** | **N** | **M** | **Y** | **N** | **P** | **N** | **F** | **S** | **L** | **A** | **A** | **K** | **T** | **G** | **T** | **A** | **E** | **I** | **K** | **D** | **K** | **Q** | **D** | **T** | **D** | **G** | **K** | **E** | **N** | **S** | **F** | **L** | **L** | **T** | **L** | **D** | **R** | **S** | **N** | **N** | **K** | **F** | **L** | **T** | **M** | **I** | **M** | **V** | **E** | **N** | **S** | **G** | **E** | **N** | **G** | **S** | **A** | **T** | **D** | **I** | **S** | **K** | **P** | **L** | **I** | **D** | **Y** | **L** | **E** | **A** | **T** | **I** | **K** | **-** | **-** | **-** | **-** | **-** | **-** | **-** | **-** | **-** | **-** | **-** | **-** | **-** | **-** | **-** | **-** | **-** | **-** | **-** | **-** | **-** | **-** | **-** | **-** | **-** | **-** | **-** | **-** | **-** | **-** | **-** | **-** | **-** | **-** | **-** | **-** | **Q** | **W** | **Q** | **A** | **K** | **Q** | **E** | **L** | **A** | **E** | **A** | **K** | **K** | **T** | **A** | **T** | **T** | **F** | **L** | **N** | **V** | **L** | **T** | **K** | **Q** | **K** | **F** | **D** | **K** | **L** | **P** | **S** | **V** | **V** | **Q** | **E** | **A** | **S** | **L** | **K** | **K** | **N** | **G** | **Y** | **D** | **T** | **K** | **S** | **V** | **V** | **E** | **K** | **Y** | **Q** | **A** | **I** | **Y** | **S** | **G** | **I** | **Q** | **A** | **E** | **G** | **V** | **K** | **A** | **S** | **D** | **V** | **Q** | **V** | **K** | **K** | **A** | **K** | **D** | **N** | **Q** | **Y** | **T** | **F** | **N** | **Y** | **K** | **L** | **S** | **M** | **S** | **T** | **P** | **L** | **G** | **E** | **M** | **K** | **D** | **L** | **S** | **Y** | **Q** | **S** | **S** | **I** | **A** | **K** | **K** | **G** | **D** | **T** | **Y** | **Q** | **I** | **A** | **W** | **K** | **P** | **S** | **L** | **I** | **F** | **P** | **D** | **M** | **S** | **G** | **N** | **D** | **K** | **I** | **S** | **I** | **Q** | **V** | **D** | **N** | **A** | **K** | **R** | **G** | **E** | **I** | **V** | **D** | **R** | **N** | **G** | **S** | **G** | **L** | **A** | **I** | **N** | **K** | **V** | **F** | **D** | **E** | **V** | **G** | **V** | **V** | **P** | **G** | **K** | **L** | **G** | **S** | **G** | **A** | **E** | **K** | **T** | **A** | **N** | **I** | **K** | **A** | **F** | **S** | **D** | **K** | **F** | **G** | **V** | **S** | **V** | **D** | **E** | **I** | **N** | **Q** | **K** | **L** | **S** | **Q** | **G** | **W** | **V** | **Q** | **A** | **D** | **S** | **F** | **V** | **P** | **I** | **T** | **V** | **A** | **S** | **E** | **P** | **V** | **T** | **E** | **L** | **P** | **T** | **G** | **A** | **A** | **T** | **K** | **D** | **T** | **E** | **S** | **R** | **Y** | **Y** | **P** | **L** | **G** | **E** | **A** | **A** | **A** | **Q** | **L** | **I** | **G** | **Y** | **T** | **G** | **T** | **I** | **T** | **A** | **E** | **D** | **I** | **K** | **K** | **N** | **P** | **E** | **L** | **S** | **S** | **T** | **G** | **V** | **I** | **G** | **K** | **T** | **G** | **L** | **E** | **R** | **A** | **F** | **D** | **K** | **E** | **L** | **R** | **G** | **Q** | **D** | **G** | **G** | **S** | **L** | **V** | **I** | **L** | **D** | **D** | **K** | **E** | **N** | **V** | **K** | **K** | **A** | **L** | **Q** | **T** | **K** | **E** | **K** | **K** | **D** | **G** | **Q** | **T** | **I** | **K** | **L** | **T** | **I** | **D** | **S** | **G** | **V** | **Q** | **Q** | **Q** | **A** | **F** | **A** | **I** | **F** | **D** | **K** | **R** | **P** | **G** | **S** | **A** | **V** | **I** | **T** | **D** | **P** | **Q** | **K** | **G** | **D** | **L** | **L** | **A** | **T** | **V** | **S** | **S** | **P** | **S** | **Y** | **D** | **P** | **N** | **K** | **M** | **A** | **N** | **G** | **I** | **S** | **Q** | **K** | **E** | **Y** | **D** | **A** | **Y** | **N** | **N** | **N** | **K** | **D** | **L** | **P** | **F** | **T** | **A** | **R** | **F** | **A** | **T** | **G** | **Y** | **A** | **P** | **G** | **S** | **T** | **F** | **K** | **T** | **I** | **T** | **G** | **A** | **I** | **G** | **L** | **D** | **A** | **G** | **T** | **L** | **K** | **P** | **D** | **E** | **E** | **L** | **E** | **I** | **N** | **G** | **L** | **K** | **W** | **Q** | **K** | **D** | **K** | **S** | **W** | **G** | **G** | **Y** | **F** | **A** | **T** | **R** | **V** | **K** | **E** | **A** | **S** | **P** | **V** | **N** | **L** | **R** | **T** | **A** | **L** | **V** | **N** | **S** | **D** | **N** | **I** | **Y** | **F** | **A** | **Q** | **Q** | **T** | **L** | **R** | **M** | **G** | **E** | **D** | **K** | **F** | **R** | **A** | **G** | **L** | **N** | **K** | **F** | **I** | **F** | **G** | **E** | **E** | **L** | **D** | **L** | **P** | **I** | **A** | **M** | **T** | **P** | **A** | **Q** | **I** | **S** | **N** | **E** | **D** | **K** | **F** | **N** | **S** | **E** | **I** | **L** | **L** | **A** | **D** | **T** | **G** | **Y** | **G** | **Q** | **G** | **Q** | **L** | **L** | **I** | **S** | **P** | **I** | **Q** | **Q** | **A** | **T** | **M** | **Y** | **S** | **V** | **F** | **Q** | **N** | **N** | **G** | **T** | **L** | **V** | **Y** | **P** | **K** | **L** | **V** | **L** | **D** | **K** | **E** | **T** | **K** | **K** | **K** | **D** | **N** | **V** | **I** | **S** | **A** | **N** | **A** | **A** | **N** | **T** | **I** | **A** | **T** | **D** | **L** | **L** | **G** | **S** | **V** | **E** | **D** | **P** | **S** | **G** | **Y** | **V** | **Y** | **N** | **M** | **Y** | **N** | **P** | **N** | **F** | **S** | **L** | **A** | **A** | **K** | **T** | **G** | **T** | **A** | **E** | **I** | **K** | **D** | **K** | **Q** | **D** | **T** | **D** | **G** | **K** | **E** | **N** | **S** | **F** | **L** | **L** | **T** | **L** | **D** | **R** | **S** | **N** | **N** | **K** | **F** | **L** | **T** | **M** | **I** | **M** | **V** | **E** | **N** | **S** | **G** | **E** | **N** | **G** | **S** | **A** | **T** | **D** | **I** | **S** | **K** | **P** | **L** | **I** | **D** | **Y** | **L** | **E** | **A** | **T** | **I** | **K** |
| **N59591F** | **-** | **-** | **-** | **-** | **-** | **-** | **-** | **-** | **-** | **-** | **-** | **-** | **-** | **-** | **-** | **-** | **-** | **-** | **-** | **-** | **-** | **-** | **-** | **-** | **-** | **-** | **-** | **-** | **-** | **-** | **-** | **-** | **-** | **-** | **-** | **-** | **Q** | **W** | **Q** | **A** | **K** | **Q** | **E** | **L** | **A** | **E** | **A** | **K** | **K** | **T** | **A** | **T** | **T** | **F** | **L** | **N** | **V** | **L** | **S** | **K** | **Q** | **E** | **F** | **D** | **K** | **L** | **P** | **S** | **V** | **V** | **Q** | **E** | **A** | **S** | **L** | **K** | **K** | **N** | **G** | **Y** | **D** | **T** | **K** | **S** | **V** | **V** | **E** | **K** | **Y** | **Q** | **A** | **I** | **Y** | **S** | **G** | **I** | **Q** | **A** | **E** | **R** | **V** | **K** | **A** | **S** | **D** | **V** | **Q** | **V** | **K** | **K** | **A** | **K** | **D** | **N** | **Q** | **Y** | **T** | **F** | **T** | **Y** | **K** | **L** | **S** | **M** | **S** | **T** | **P** | **L** | **G** | **E** | **M** | **K** | **D** | **L** | **S** | **Y** | **Q** | **S** | **S** | **I** | **A** | **K** | **K** | **G** | **D** | **T** | **Y** | **Q** | **I** | **A** | **W** | **K** | **P** | **S** | **L** | **I** | **F** | **P** | **D** | **M** | **S** | **G** | **N** | **D** | **K** | **I** | **S** | **I** | **Q** | **V** | **D** | **N** | **A** | **K** | **R** | **G** | **E** | **I** | **V** | **D** | **R** | **N** | **G** | **S** | **G** | **L** | **A** | **I** | **N** | **K** | **V** | **F** | **D** | **E** | **V** | **G** | **V** | **V** | **P** | **G** | **K** | **L** | **G** | **S** | **G** | **A** | **E** | **K** | **T** | **A** | **N** | **I** | **K** | **A** | **F** | **S** | **D** | **K** | **F** | **G** | **V** | **S** | **V** | **D** | **E** | **I** | **N** | **Q** | **K** | **L** | **S** | **Q** | **G** | **W** | **V** | **Q** | **A** | **D** | **S** | **F** | **V** | **P** | **I** | **T** | **V** | **A** | **S** | **E** | **P** | **V** | **T** | **E** | **L** | **P** | **T** | **G** | **A** | **A** | **T** | **K** | **D** | **T** | **E** | **S** | **R** | **Y** | **Y** | **P** | **L** | **G** | **E** | **A** | **A** | **A** | **Q** | **L** | **I** | **G** | **Y** | **T** | **G** | **T** | **I** | **T** | **A** | **E** | **D** | **I** | **K** | **K** | **N** | **P** | **E** | **L** | **S** | **S** | **T** | **G** | **V** | **I** | **G** | **K** | **T** | **G** | **L** | **E** | **R** | **A** | **F** | **D** | **K** | **E** | **L** | **R** | **G** | **Q** | **D** | **G** | **G** | **S** | **L** | **V** | **I** | **L** | **D** | **D** | **K** | **E** | **N** | **V** | **K** | **K** | **A** | **L** | **Q** | **T** | **K** | **E** | **K** | **K** | **D** | **G** | **Q** | **T** | **I** | **K** | **L** | **T** | **I** | **D** | **S** | **G** | **V** | **Q** | **Q** | **Q** | **A** | **F** | **A** | **I** | **F** | **D** | **K** | **R** | **P** | **G** | **S** | **A** | **V** | **I** | **T** | **D** | **P** | **Q** | **K** | **G** | **D** | **L** | **L** | **A** | **T** | **V** | **S** | **S** | **P** | **S** | **Y** | **D** | **P** | **N** | **K** | **M** | **A** | **N** | **G** | **I** | **S** | **Q** | **K** | **E** | **Y** | **D** | **A** | **Y** | **N** | **N** | **N** | **K** | **D** | **L** | **P** | **F** | **T** | **A** | **R** | **F** | **A** | **T** | **G** | **Y** | **A** | **P** | **G** | **S** | **T** | **F** | **K** | **T** | **I** | **T** | **G** | **A** | **I** | **G** | **L** | **D** | **A** | **G** | **T** | **L** | **K** | **P** | **D** | **E** | **E** | **L** | **E** | **I** | **N** | **G** | **L** | **K** | **W** | **Q** | **K** | **D** | **K** | **S** | **W** | **G** | **G** | **Y** | **F** | **A** | **T** | **R** | **V** | **K** | **E** | **A** | **S** | **P** | **V** | **N** | **L** | **R** | **T** | **A** | **L** | **V** | **N** | **S** | **D** | **N** | **I** | **Y** | **F** | **A** | **Q** | **Q** | **T** | **L** | **R** | **M** | **G** | **E** | **D** | **K** | **F** | **R** | **A** | **G** | **L** | **N** | **K** | **F** | **I** | **F** | **G** | **E** | **E** | **L** | **D** | **L** | **P** | **I** | **A** | **M** | **T** | **P** | **A** | **Q** | **I** | **S** | **N** | **E** | **D** | **K** | **F** | **N** | **S** | **E** | **I** | **L** | **L** | **A** | **D** | **T** | **G** | **Y** | **G** | **Q** | **G** | **Q** | **L** | **L** | **I** | **S** | **P** | **I** | **Q** | **Q** | **A** | **T** | **M** | **Y** | **S** | **V** | **F** | **Q** | **N** | **N** | **G** | **T** | **L** | **V** | **Y** | **P** | **K** | **L** | **V** | **L** | **D** | **K** | **E** | **T** | **K** | **K** | **K** | **D** | **N** | **V** | **I** | **S** | **A** | **N** | **A** | **A** | **N** | **T** | **I** | **A** | **T** | **D** | **L** | **L** | **G** | **S** | **V** | **E** | **D** | **P** | **S** | **G** | **Y** | **V** | **Y** | **N** | **M** | **Y** | **N** | **P** | **N** | **F** | **S** | **L** | **A** | **A** | **K** | **T** | **G** | **T** | **A** | **E** | **I** | **K** | **D** | **K** | **Q** | **D** | **T** | **D** | **G** | **K** | **E** | **N** | **S** | **F** | **L** | **L** | **T** | **L** | **D** | **R** | **S** | **N** | **N** | **K** | **F** | **L** | **T** | **M** | **I** | **M** | **V** | **E** | **N** | **S** | **G** | **E** | **N** | **G** | **S** | **A** | **T** | **D** | **I** | **S** | **K** | **P** | **L** | **I** | **D** | **Y** | **L** | **E** | **A** | **T** | **I** | **K** | **-** | **-** | **-** | **-** | **-** | **-** | **-** | **-** | **-** | **-** | **-** | **-** | **-** | **-** | **-** | **-** | **-** | **-** | **-** | **-** | **-** | **-** | **-** | **-** | **-** | **-** | **-** | **-** | **-** | **-** | **-** | **-** | **-** | **-** | **-** | **-** | **Q** | **W** | **Q** | **A** | **K** | **Q** | **E** | **L** | **A** | **E** | **A** | **K** | **K** | **T** | **A** | **T** | **T** | **F** | **L** | **N** | **V** | **L** | **S** | **K** | **Q** | **E** | **F** | **D** | **K** | **L** | **P** | **S** | **V** | **V** | **Q** | **E** | **A** | **S** | **L** | **K** | **K** | **N** | **G** | **Y** | **D** | **T** | **K** | **S** | **V** | **V** | **E** | **K** | **Y** | **Q** | **A** | **I** | **Y** | **S** | **G** | **I** | **Q** | **A** | **E** | **R** | **V** | **K** | **A** | **S** | **D** | **V** | **Q** | **V** | **K** | **K** | **A** | **K** | **D** | **N** | **Q** | **Y** | **T** | **F** | **T** | **Y** | **K** | **L** | **S** | **M** | **S** | **T** | **P** | **L** | **G** | **E** | **M** | **K** | **D** | **L** | **S** | **Y** | **Q** | **S** | **S** | **I** | **A** | **K** | **K** | **G** | **D** | **T** | **Y** | **Q** | **I** | **A** | **W** | **K** | **P** | **S** | **L** | **I** | **F** | **P** | **D** | **M** | **S** | **G** | **N** | **D** | **K** | **I** | **S** | **I** | **Q** | **V** | **D** | **N** | **A** | **K** | **R** | **G** | **E** | **I** | **V** | **D** | **R** | **N** | **G** | **S** | **G** | **L** | **A** | **I** | **N** | **K** | **V** | **F** | **D** | **E** | **V** | **G** | **V** | **V** | **P** | **G** | **K** | **L** | **G** | **S** | **G** | **A** | **E** | **K** | **T** | **A** | **N** | **I** | **K** | **A** | **F** | **S** | **D** | **K** | **F** | **G** | **V** | **S** | **V** | **D** | **E** | **I** | **N** | **Q** | **K** | **L** | **S** | **Q** | **G** | **W** | **V** | **Q** | **A** | **D** | **S** | **F** | **V** | **P** | **I** | **T** | **V** | **A** | **S** | **E** | **P** | **V** | **T** | **E** | **L** | **P** | **T** | **G** | **A** | **A** | **T** | **K** | **D** | **T** | **E** | **S** | **R** | **Y** | **Y** | **P** | **L** | **G** | **E** | **A** | **A** | **A** | **Q** | **L** | **I** | **G** | **Y** | **T** | **G** | **T** | **I** | **T** | **A** | **E** | **D** | **I** | **K** | **K** | **N** | **P** | **E** | **L** | **S** | **S** | **T** | **G** | **V** | **I** | **G** | **K** | **T** | **G** | **L** | **E** | **R** | **A** | **F** | **D** | **K** | **E** | **L** | **R** | **G** | **Q** | **D** | **G** | **G** | **S** | **L** | **V** | **I** | **L** | **D** | **D** | **K** | **E** | **N** | **V** | **K** | **K** | **A** | **L** | **Q** | **T** | **K** | **E** | **K** | **K** | **D** | **G** | **Q** | **T** | **I** | **K** | **L** | **T** | **I** | **D** | **S** | **G** | **V** | **Q** | **Q** | **Q** | **A** | **F** | **A** | **I** | **F** | **D** | **K** | **R** | **P** | **G** | **S** | **A** | **V** | **I** | **T** | **D** | **P** | **Q** | **K** | **G** | **D** | **L** | **L** | **A** | **T** | **V** | **S** | **S** | **P** | **S** | **Y** | **D** | **P** | **N** | **K** | **M** | **A** | **N** | **G** | **I** | **S** | **Q** | **K** | **E** | **Y** | **D** | **A** | **Y** | **N** | **N** | **N** | **K** | **D** | **L** | **P** | **F** | **T** | **A** | **R** | **F** | **A** | **T** | **G** | **Y** | **A** | **P** | **G** | **S** | **T** | **F** | **K** | **T** | **I** | **T** | **G** | **A** | **I** | **G** | **L** | **D** | **A** | **G** | **T** | **L** | **K** | **P** | **D** | **E** | **E** | **L** | **E** | **I** | **N** | **G** | **L** | **K** | **W** | **Q** | **K** | **D** | **K** | **S** | **W** | **G** | **G** | **Y** | **F** | **A** | **T** | **R** | **V** | **K** | **E** | **A** | **S** | **P** | **V** | **N** | **L** | **R** | **T** | **A** | **L** | **V** | **N** | **S** | **D** | **N** | **I** | **Y** | **F** | **A** | **Q** | **Q** | **T** | **L** | **R** | **M** | **G** | **E** | **D** | **K** | **F** | **R** | **A** | **G** | **L** | **N** | **K** | **F** | **I** | **F** | **G** | **E** | **E** | **L** | **D** | **L** | **P** | **I** | **A** | **M** | **T** | **P** | **A** | **Q** | **I** | **S** | **N** | **E** | **D** | **K** | **F** | **N** | **S** | **E** | **I** | **L** | **L** | **A** | **D** | **T** | **G** | **Y** | **G** | **Q** | **G** | **Q** | **L** | **L** | **I** | **S** | **P** | **I** | **Q** | **Q** | **A** | **T** | **M** | **Y** | **S** | **V** | **F** | **Q** | **N** | **N** | **G** | **T** | **L** | **V** | **Y** | **P** | **K** | **L** | **V** | **L** | **D** | **K** | **E** | **T** | **K** | **K** | **K** | **D** | **N** | **V** | **I** | **S** | **A** | **N** | **A** | **A** | **N** | **T** | **I** | **A** | **T** | **D** | **L** | **L** | **G** | **S** | **V** | **E** | **D** | **P** | **S** | **G** | **Y** | **V** | **Y** | **N** | **M** | **Y** | **N** | **P** | **N** | **F** | **S** | **L** | **A** | **A** | **K** | **T** | **G** | **T** | **A** | **E** | **I** | **K** | **D** | **K** | **Q** | **D** | **T** | **D** | **G** | **K** | **E** | **N** | **S** | **F** | **L** | **L** | **T** | **L** | **D** | **R** | **S** | **N** | **N** | **K** | **F** | **L** | **T** | **M** | **I** | **M** | **V** | **E** | **N** | **S** | **G** | **E** | **N** | **G** | **S** | **A** | **T** | **D** | **I** | **S** | **K** | **P** | **L** | **I** | **D** | **Y** | **L** | **E** | **A** | **T** | **I** | **K** |
| **N59896F** | **-** | **-** | **-** | **-** | **-** | **-** | **-** | **-** | **-** | **-** | **-** | **-** | **-** | **-** | **-** | **-** | **-** | **-** | **-** | **-** | **-** | **-** | **-** | **-** | **-** | **-** | **-** | **-** | **-** | **-** | **-** | **-** | **-** | **-** | **-** | **-** | **Q** | **W** | **Q** | **A** | **K** | **Q** | **E** | **L** | **A** | **E** | **A** | **K** | **K** | **T** | **A** | **T** | **K** | **F** | **L** | **N** | **V** | **L** | **S** | **K** | **Q** | **E** | **F** | **D** | **K** | **L** | **P** | **S** | **V** | **V** | **Q** | **E** | **A** | **S** | **L** | **K** | **K** | **N** | **G** | **Y** | **D** | **T** | **K** | **S** | **V** | **V** | **E** | **K** | **Y** | **Q** | **A** | **I** | **Y** | **S** | **G** | **I** | **Q** | **A** | **E** | **G** | **V** | **K** | **A** | **S** | **D** | **V** | **Q** | **V** | **K** | **K** | **A** | **K** | **D** | **N** | **Q** | **Y** | **T** | **F** | **T** | **Y** | **K** | **L** | **S** | **M** | **S** | **T** | **P** | **L** | **G** | **E** | **M** | **K** | **D** | **L** | **S** | **Y** | **Q** | **S** | **S** | **I** | **A** | **K** | **K** | **G** | **D** | **T** | **Y** | **Q** | **I** | **A** | **W** | **K** | **P** | **S** | **L** | **I** | **F** | **P** | **D** | **M** | **S** | **G** | **N** | **D** | **K** | **I** | **S** | **I** | **Q** | **V** | **D** | **N** | **A** | **K** | **R** | **G** | **E** | **I** | **V** | **D** | **R** | **N** | **G** | **S** | **G** | **L** | **A** | **I** | **N** | **K** | **V** | **F** | **D** | **E** | **V** | **G** | **V** | **V** | **P** | **G** | **K** | **L** | **G** | **S** | **G** | **A** | **E** | **K** | **T** | **A** | **N** | **I** | **K** | **A** | **F** | **S** | **D** | **K** | **F** | **G** | **V** | **S** | **V** | **D** | **E** | **I** | **N** | **Q** | **K** | **L** | **S** | **Q** | **G** | **W** | **V** | **Q** | **A** | **D** | **S** | **F** | **V** | **P** | **I** | **T** | **V** | **A** | **S** | **E** | **P** | **V** | **T** | **E** | **L** | **P** | **T** | **G** | **A** | **A** | **T** | **K** | **D** | **T** | **E** | **S** | **R** | **Y** | **Y** | **P** | **L** | **G** | **E** | **A** | **A** | **A** | **Q** | **L** | **I** | **G** | **Y** | **T** | **G** | **T** | **I** | **T** | **A** | **E** | **D** | **I** | **K** | **K** | **N** | **P** | **E** | **L** | **S** | **S** | **T** | **G** | **V** | **I** | **G** | **K** | **T** | **G** | **L** | **E** | **R** | **A** | **F** | **D** | **K** | **E** | **L** | **R** | **G** | **Q** | **D** | **G** | **G** | **S** | **L** | **V** | **I** | **L** | **D** | **D** | **K** | **E** | **N** | **V** | **K** | **K** | **A** | **L** | **Q** | **T** | **K** | **E** | **K** | **K** | **D** | **G** | **Q** | **T** | **I** | **K** | **L** | **T** | **I** | **D** | **S** | **G** | **V** | **Q** | **Q** | **Q** | **A** | **F** | **A** | **I** | **F** | **D** | **K** | **R** | **P** | **G** | **S** | **A** | **V** | **I** | **T** | **D** | **P** | **Q** | **K** | **G** | **D** | **L** | **L** | **A** | **T** | **V** | **S** | **S** | **P** | **S** | **Y** | **D** | **P** | **N** | **K** | **M** | **A** | **N** | **G** | **I** | **S** | **Q** | **K** | **E** | **Y** | **D** | **A** | **Y** | **N** | **N** | **N** | **K** | **D** | **L** | **P** | **F** | **T** | **A** | **R** | **F** | **A** | **T** | **G** | **Y** | **A** | **P** | **G** | **S** | **T** | **F** | **K** | **T** | **I** | **T** | **G** | **A** | **I** | **G** | **L** | **D** | **A** | **G** | **T** | **L** | **K** | **P** | **D** | **E** | **E** | **L** | **E** | **I** | **N** | **G** | **L** | **K** | **W** | **Q** | **K** | **D** | **K** | **S** | **W** | **G** | **G** | **Y** | **F** | **A** | **T** | **R** | **V** | **K** | **E** | **A** | **S** | **P** | **V** | **N** | **L** | **R** | **T** | **A** | **L** | **V** | **N** | **S** | **D** | **N** | **I** | **Y** | **F** | **A** | **Q** | **Q** | **T** | **L** | **R** | **M** | **G** | **E** | **D** | **K** | **F** | **R** | **A** | **G** | **L** | **N** | **K** | **F** | **I** | **F** | **G** | **E** | **E** | **L** | **D** | **L** | **P** | **I** | **A** | **M** | **T** | **P** | **A** | **Q** | **I** | **S** | **N** | **E** | **D** | **K** | **F** | **N** | **S** | **E** | **I** | **L** | **L** | **A** | **D** | **T** | **G** | **Y** | **G** | **Q** | **G** | **Q** | **L** | **L** | **I** | **S** | **P** | **I** | **Q** | **Q** | **A** | **T** | **M** | **Y** | **S** | **V** | **F** | **Q** | **N** | **N** | **G** | **T** | **L** | **V** | **Y** | **P** | **K** | **L** | **V** | **L** | **D** | **K** | **E** | **T** | **K** | **K** | **K** | **D** | **N** | **V** | **I** | **S** | **A** | **N** | **A** | **A** | **N** | **T** | **I** | **A** | **T** | **D** | **L** | **L** | **G** | **S** | **V** | **E** | **D** | **P** | **S** | **G** | **Y** | **V** | **Y** | **N** | **M** | **Y** | **N** | **P** | **N** | **F** | **S** | **L** | **A** | **A** | **K** | **T** | **G** | **T** | **A** | **E** | **I** | **K** | **D** | **K** | **Q** | **D** | **T** | **D** | **G** | **K** | **E** | **N** | **S** | **F** | **L** | **L** | **T** | **L** | **D** | **R** | **S** | **N** | **N** | **K** | **F** | **L** | **T** | **M** | **I** | **M** | **V** | **E** | **N** | **S** | **G** | **E** | **N** | **G** | **S** | **A** | **T** | **D** | **I** | **S** | **K** | **P** | **L** | **I** | **D** | **Y** | **L** | **E** | **A** | **T** | **I** | **K** | **-** | **-** | **-** | **-** | **-** | **-** | **-** | **-** | **-** | **-** | **-** | **-** | **-** | **-** | **-** | **-** | **-** | **-** | **-** | **-** | **-** | **-** | **-** | **-** | **-** | **-** | **-** | **-** | **-** | **-** | **-** | **-** | **-** | **-** | **-** | **-** | **Q** | **W** | **Q** | **A** | **K** | **Q** | **E** | **L** | **A** | **E** | **A** | **K** | **K** | **T** | **A** | **T** | **K** | **F** | **L** | **N** | **V** | **L** | **S** | **K** | **Q** | **E** | **F** | **D** | **K** | **L** | **P** | **S** | **V** | **V** | **Q** | **E** | **A** | **S** | **L** | **K** | **K** | **N** | **G** | **Y** | **D** | **T** | **K** | **S** | **V** | **V** | **E** | **K** | **Y** | **Q** | **A** | **I** | **Y** | **S** | **G** | **I** | **Q** | **A** | **E** | **G** | **V** | **K** | **A** | **S** | **D** | **V** | **Q** | **V** | **K** | **K** | **A** | **K** | **D** | **N** | **Q** | **Y** | **T** | **F** | **T** | **Y** | **K** | **L** | **S** | **M** | **S** | **T** | **P** | **L** | **G** | **E** | **M** | **K** | **D** | **L** | **S** | **Y** | **Q** | **S** | **S** | **I** | **A** | **K** | **K** | **G** | **D** | **T** | **Y** | **Q** | **I** | **A** | **W** | **K** | **P** | **S** | **L** | **I** | **F** | **P** | **D** | **M** | **S** | **G** | **N** | **D** | **K** | **I** | **S** | **I** | **Q** | **V** | **D** | **N** | **A** | **K** | **R** | **G** | **E** | **I** | **V** | **D** | **R** | **N** | **G** | **S** | **G** | **L** | **A** | **I** | **N** | **K** | **V** | **F** | **D** | **E** | **V** | **G** | **V** | **V** | **P** | **G** | **K** | **L** | **G** | **S** | **G** | **A** | **E** | **K** | **T** | **A** | **N** | **I** | **K** | **A** | **F** | **S** | **D** | **K** | **F** | **G** | **V** | **S** | **V** | **D** | **E** | **I** | **N** | **Q** | **K** | **L** | **S** | **Q** | **G** | **W** | **V** | **Q** | **A** | **D** | **S** | **F** | **V** | **P** | **I** | **T** | **V** | **A** | **S** | **E** | **P** | **V** | **T** | **E** | **L** | **P** | **T** | **G** | **A** | **A** | **T** | **K** | **D** | **T** | **E** | **S** | **R** | **Y** | **Y** | **P** | **L** | **G** | **E** | **A** | **A** | **A** | **Q** | **L** | **I** | **G** | **Y** | **T** | **G** | **T** | **I** | **T** | **A** | **E** | **D** | **I** | **K** | **K** | **N** | **P** | **E** | **L** | **S** | **S** | **T** | **G** | **V** | **I** | **G** | **K** | **T** | **G** | **L** | **E** | **R** | **A** | **F** | **D** | **K** | **E** | **L** | **R** | **G** | **Q** | **D** | **G** | **G** | **S** | **L** | **V** | **I** | **L** | **D** | **D** | **K** | **E** | **N** | **V** | **K** | **K** | **A** | **L** | **Q** | **T** | **K** | **E** | **K** | **K** | **D** | **G** | **Q** | **T** | **I** | **K** | **L** | **T** | **I** | **D** | **S** | **G** | **V** | **Q** | **Q** | **Q** | **A** | **F** | **A** | **I** | **F** | **D** | **K** | **R** | **P** | **G** | **S** | **A** | **V** | **I** | **T** | **D** | **P** | **Q** | **K** | **G** | **D** | **L** | **L** | **A** | **T** | **V** | **S** | **S** | **P** | **S** | **Y** | **D** | **P** | **N** | **K** | **M** | **A** | **N** | **G** | **I** | **S** | **Q** | **K** | **E** | **Y** | **D** | **A** | **Y** | **N** | **N** | **N** | **K** | **D** | **L** | **P** | **F** | **T** | **A** | **R** | **F** | **A** | **T** | **G** | **Y** | **A** | **P** | **G** | **S** | **T** | **F** | **K** | **T** | **I** | **T** | **G** | **A** | **I** | **G** | **L** | **D** | **A** | **G** | **T** | **L** | **K** | **P** | **D** | **E** | **E** | **L** | **E** | **I** | **N** | **G** | **L** | **K** | **W** | **Q** | **K** | **D** | **K** | **S** | **W** | **G** | **G** | **Y** | **F** | **A** | **T** | **R** | **V** | **K** | **E** | **A** | **S** | **P** | **V** | **N** | **L** | **R** | **T** | **A** | **L** | **V** | **N** | **S** | **D** | **N** | **I** | **Y** | **F** | **A** | **Q** | **Q** | **T** | **L** | **R** | **M** | **G** | **E** | **D** | **K** | **F** | **R** | **A** | **G** | **L** | **N** | **K** | **F** | **I** | **F** | **G** | **E** | **E** | **L** | **D** | **L** | **P** | **I** | **A** | **M** | **T** | **P** | **A** | **Q** | **I** | **S** | **N** | **E** | **D** | **K** | **F** | **N** | **S** | **E** | **I** | **L** | **L** | **A** | **D** | **T** | **G** | **Y** | **G** | **Q** | **G** | **Q** | **L** | **L** | **I** | **S** | **P** | **I** | **Q** | **Q** | **A** | **T** | **M** | **Y** | **S** | **V** | **F** | **Q** | **N** | **N** | **G** | **T** | **L** | **V** | **Y** | **P** | **K** | **L** | **V** | **L** | **D** | **K** | **E** | **T** | **K** | **K** | **K** | **D** | **N** | **V** | **I** | **S** | **A** | **N** | **A** | **A** | **N** | **T** | **I** | **A** | **T** | **D** | **L** | **L** | **G** | **S** | **V** | **E** | **D** | **P** | **S** | **G** | **Y** | **V** | **Y** | **N** | **M** | **Y** | **N** | **P** | **N** | **F** | **S** | **L** | **A** | **A** | **K** | **T** | **G** | **T** | **A** | **E** | **I** | **K** | **D** | **K** | **Q** | **D** | **T** | **D** | **G** | **K** | **E** | **N** | **S** | **F** | **L** | **L** | **T** | **L** | **D** | **R** | **S** | **N** | **N** | **K** | **F** | **L** | **T** | **M** | **I** | **M** | **V** | **E** | **N** | **S** | **G** | **E** | **N** | **G** | **S** | **A** | **T** | **D** | **I** | **S** | **K** | **P** | **L** | **I** | **D** | **Y** | **L** | **E** | **A** | **T** | **I** | **K** |
| **N54564** | **-** | **-** | **-** | **-** | **-** | **-** | **-** | **-** | **-** | **-** | **-** | **-** | **-** | **-** | **-** | **-** | **-** | **-** | **-** | **-** | **-** | **-** | **-** | **-** | **-** | **-** | **-** | **-** | **-** | **-** | **-** | **-** | **-** | **-** | **-** | **-** | **Q** | **W** | **Q** | **A** | **K** | **Q** | **E** | **L** | **A** | **E** | **A** | **K** | **K** | **T** | **A** | **T** | **T** | **F** | **L** | **N** | **V** | **L** | **S** | **K** | **Q** | **E** | **F** | **D** | **K** | **L** | **P** | **S** | **V** | **V** | **Q** | **E** | **A** | **S** | **L** | **K** | **K** | **N** | **G** | **Y** | **D** | **T** | **K** | **S** | **V** | **V** | **E** | **K** | **Y** | **Q** | **A** | **I** | **Y** | **S** | **G** | **I** | **Q** | **A** | **E** | **G** | **V** | **K** | **A** | **S** | **D** | **V** | **Q** | **V** | **K** | **K** | **A** | **K** | **D** | **N** | **Q** | **Y** | **T** | **F** | **T** | **Y** | **K** | **L** | **S** | **M** | **S** | **T** | **P** | **L** | **G** | **E** | **M** | **K** | **D** | **L** | **S** | **Y** | **Q** | **S** | **S** | **I** | **A** | **K** | **K** | **G** | **D** | **T** | **Y** | **Q** | **I** | **A** | **W** | **K** | **P** | **S** | **L** | **I** | **F** | **P** | **D** | **M** | **S** | **G** | **N** | **D** | **K** | **I** | **S** | **I** | **Q** | **V** | **D** | **N** | **A** | **K** | **R** | **G** | **E** | **I** | **V** | **D** | **R** | **N** | **G** | **S** | **G** | **L** | **A** | **I** | **N** | **K** | **V** | **F** | **D** | **E** | **V** | **G** | **V** | **V** | **P** | **G** | **K** | **L** | **G** | **S** | **G** | **A** | **E** | **K** | **T** | **A** | **N** | **I** | **K** | **A** | **F** | **S** | **D** | **K** | **F** | **G** | **V** | **S** | **V** | **D** | **E** | **I** | **N** | **Q** | **K** | **L** | **S** | **Q** | **G** | **W** | **V** | **Q** | **A** | **D** | **S** | **F** | **V** | **P** | **I** | **T** | **V** | **A** | **S** | **E** | **P** | **V** | **T** | **E** | **L** | **P** | **T** | **G** | **A** | **A** | **T** | **K** | **G** | **T** | **E** | **S** | **R** | **Y** | **Y** | **P** | **L** | **G** | **E** | **A** | **A** | **A** | **Q** | **L** | **I** | **G** | **Y** | **T** | **G** | **T** | **I** | **T** | **A** | **E** | **D** | **I** | **E** | **K** | **N** | **P** | **E** | **L** | **S** | **S** | **T** | **G** | **V** | **I** | **G** | **K** | **T** | **G** | **L** | **E** | **R** | **A** | **F** | **D** | **K** | **E** | **L** | **R** | **G** | **Q** | **D** | **G** | **G** | **S** | **L** | **V** | **I** | **L** | **D** | **D** | **K** | **E** | **N** | **V** | **K** | **K** | **A** | **L** | **Q** | **T** | **K** | **E** | **K** | **K** | **D** | **G** | **Q** | **T** | **I** | **K** | **L** | **T** | **I** | **D** | **S** | **G** | **V** | **Q** | **Q** | **Q** | **A** | **F** | **A** | **I** | **F** | **D** | **K** | **R** | **P** | **G** | **S** | **A** | **V** | **I** | **T** | **D** | **P** | **Q** | **K** | **G** | **D** | **L** | **L** | **A** | **T** | **V** | **S** | **S** | **P** | **S** | **Y** | **D** | **P** | **N** | **K** | **M** | **A** | **N** | **G** | **I** | **S** | **Q** | **K** | **E** | **Y** | **D** | **A** | **Y** | **N** | **N** | **N** | **K** | **D** | **L** | **P** | **F** | **T** | **A** | **R** | **F** | **A** | **T** | **G** | **Y** | **A** | **P** | **G** | **S** | **T** | **F** | **K** | **T** | **I** | **T** | **G** | **A** | **I** | **G** | **L** | **D** | **A** | **G** | **T** | **L** | **K** | **P** | **D** | **E** | **E** | **L** | **E** | **I** | **N** | **G** | **L** | **K** | **W** | **Q** | **K** | **D** | **K** | **S** | **W** | **G** | **G** | **Y** | **F** | **A** | **T** | **R** | **V** | **K** | **E** | **A** | **S** | **P** | **V** | **N** | **L** | **R** | **T** | **A** | **L** | **V** | **N** | **S** | **D** | **N** | **I** | **Y** | **F** | **A** | **Q** | **Q** | **T** | **L** | **R** | **M** | **G** | **E** | **D** | **K** | **F** | **R** | **A** | **G** | **L** | **N** | **K** | **F** | **I** | **F** | **G** | **E** | **E** | **L** | **D** | **L** | **P** | **I** | **A** | **M** | **T** | **P** | **A** | **Q** | **I** | **S** | **N** | **E** | **D** | **K** | **F** | **N** | **S** | **E** | **I** | **L** | **L** | **A** | **D** | **T** | **G** | **Y** | **G** | **Q** | **G** | **Q** | **L** | **L** | **I** | **S** | **P** | **I** | **Q** | **Q** | **A** | **T** | **M** | **Y** | **S** | **V** | **F** | **Q** | **N** | **N** | **G** | **T** | **L** | **V** | **Y** | **P** | **K** | **L** | **V** | **L** | **D** | **K** | **E** | **T** | **K** | **K** | **K** | **D** | **N** | **V** | **I** | **S** | **A** | **N** | **A** | **A** | **N** | **T** | **I** | **A** | **T** | **D** | **L** | **L** | **G** | **S** | **V** | **E** | **D** | **P** | **S** | **G** | **Y** | **V** | **Y** | **N** | **M** | **Y** | **N** | **P** | **N** | **F** | **S** | **L** | **A** | **A** | **K** | **T** | **G** | **T** | **A** | **E** | **I** | **K** | **D** | **K** | **Q** | **D** | **T** | **D** | **G** | **K** | **E** | **N** | **S** | **F** | **L** | **L** | **T** | **L** | **D** | **R** | **S** | **N** | **N** | **K** | **F** | **L** | **T** | **M** | **I** | **M** | **V** | **E** | **N** | **S** | **G** | **E** | **N** | **G** | **S** | **A** | **T** | **D** | **I** | **S** | **K** | **P** | **L** | **I** | **D** | **Y** | **L** | **E** | **A** | **T** | **I** | **K** | **-** | **-** | **-** | **-** | **-** | **-** | **-** | **-** | **-** | **-** | **-** | **-** | **-** | **-** | **-** | **-** | **-** | **-** | **-** | **-** | **-** | **-** | **-** | **-** | **-** | **-** | **-** | **-** | **-** | **-** | **-** | **-** | **-** | **-** | **-** | **-** | **Q** | **W** | **Q** | **A** | **K** | **Q** | **E** | **L** | **A** | **E** | **A** | **K** | **K** | **T** | **A** | **T** | **T** | **F** | **L** | **N** | **V** | **L** | **S** | **K** | **Q** | **E** | **F** | **D** | **K** | **L** | **P** | **S** | **V** | **V** | **Q** | **E** | **A** | **S** | **L** | **K** | **K** | **N** | **G** | **Y** | **D** | **T** | **K** | **S** | **V** | **V** | **E** | **K** | **Y** | **Q** | **A** | **I** | **Y** | **S** | **G** | **I** | **Q** | **A** | **E** | **G** | **V** | **K** | **A** | **S** | **D** | **V** | **Q** | **V** | **K** | **K** | **A** | **K** | **D** | **N** | **Q** | **Y** | **T** | **F** | **T** | **Y** | **K** | **L** | **S** | **M** | **S** | **T** | **P** | **L** | **G** | **E** | **M** | **K** | **D** | **L** | **S** | **Y** | **Q** | **S** | **S** | **I** | **A** | **K** | **K** | **G** | **D** | **T** | **Y** | **Q** | **I** | **A** | **W** | **K** | **P** | **S** | **L** | **I** | **F** | **P** | **D** | **M** | **S** | **G** | **N** | **D** | **K** | **I** | **S** | **I** | **Q** | **V** | **D** | **N** | **A** | **K** | **R** | **G** | **E** | **I** | **V** | **D** | **R** | **N** | **G** | **S** | **G** | **L** | **A** | **I** | **N** | **K** | **V** | **F** | **D** | **E** | **V** | **G** | **V** | **V** | **P** | **G** | **K** | **L** | **G** | **S** | **G** | **A** | **E** | **K** | **T** | **A** | **N** | **I** | **K** | **A** | **F** | **S** | **D** | **K** | **F** | **G** | **V** | **S** | **V** | **D** | **E** | **I** | **N** | **Q** | **K** | **L** | **S** | **Q** | **G** | **W** | **V** | **Q** | **A** | **D** | **S** | **F** | **V** | **P** | **I** | **T** | **V** | **A** | **S** | **E** | **P** | **V** | **T** | **E** | **L** | **P** | **T** | **G** | **A** | **A** | **T** | **K** | **G** | **T** | **E** | **S** | **R** | **Y** | **Y** | **P** | **L** | **G** | **E** | **A** | **A** | **A** | **Q** | **L** | **I** | **G** | **Y** | **T** | **G** | **T** | **I** | **T** | **A** | **E** | **D** | **I** | **E** | **K** | **N** | **P** | **E** | **L** | **S** | **S** | **T** | **G** | **V** | **I** | **G** | **K** | **T** | **G** | **L** | **E** | **R** | **A** | **F** | **D** | **K** | **E** | **L** | **R** | **G** | **Q** | **D** | **G** | **G** | **S** | **L** | **V** | **I** | **L** | **D** | **D** | **K** | **E** | **N** | **V** | **K** | **K** | **A** | **L** | **Q** | **T** | **K** | **E** | **K** | **K** | **D** | **G** | **Q** | **T** | **I** | **K** | **L** | **T** | **I** | **D** | **S** | **G** | **V** | **Q** | **Q** | **Q** | **A** | **F** | **A** | **I** | **F** | **D** | **K** | **R** | **P** | **G** | **S** | **A** | **V** | **I** | **T** | **D** | **P** | **Q** | **K** | **G** | **D** | **L** | **L** | **A** | **T** | **V** | **S** | **S** | **P** | **S** | **Y** | **D** | **P** | **N** | **K** | **M** | **A** | **N** | **G** | **I** | **S** | **Q** | **K** | **E** | **Y** | **D** | **A** | **Y** | **N** | **N** | **N** | **K** | **D** | **L** | **P** | **F** | **T** | **A** | **R** | **F** | **A** | **T** | **G** | **Y** | **A** | **P** | **G** | **S** | **T** | **F** | **K** | **T** | **I** | **T** | **G** | **A** | **I** | **G** | **L** | **D** | **A** | **G** | **T** | **L** | **K** | **P** | **D** | **E** | **E** | **L** | **E** | **I** | **N** | **G** | **L** | **K** | **W** | **Q** | **K** | **D** | **K** | **S** | **W** | **G** | **G** | **Y** | **F** | **A** | **T** | **R** | **V** | **K** | **E** | **A** | **S** | **P** | **V** | **N** | **L** | **R** | **T** | **A** | **L** | **V** | **N** | **S** | **D** | **N** | **I** | **Y** | **F** | **A** | **Q** | **Q** | **T** | **L** | **R** | **M** | **G** | **E** | **D** | **K** | **F** | **R** | **A** | **G** | **L** | **N** | **K** | **F** | **I** | **F** | **G** | **E** | **E** | **L** | **D** | **L** | **P** | **I** | **A** | **M** | **T** | **P** | **A** | **Q** | **I** | **S** | **N** | **E** | **D** | **K** | **F** | **N** | **S** | **E** | **I** | **L** | **L** | **A** | **D** | **T** | **G** | **Y** | **G** | **Q** | **G** | **Q** | **L** | **L** | **I** | **S** | **P** | **I** | **Q** | **Q** | **A** | **T** | **M** | **Y** | **S** | **V** | **F** | **Q** | **N** | **N** | **G** | **T** | **L** | **V** | **Y** | **P** | **K** | **L** | **V** | **L** | **D** | **K** | **E** | **T** | **K** | **K** | **K** | **D** | **N** | **V** | **I** | **S** | **A** | **N** | **A** | **A** | **N** | **T** | **I** | **A** | **T** | **D** | **L** | **L** | **G** | **S** | **V** | **E** | **D** | **P** | **S** | **G** | **Y** | **V** | **Y** | **N** | **M** | **Y** | **N** | **P** | **N** | **F** | **S** | **L** | **A** | **A** | **K** | **T** | **G** | **T** | **A** | **E** | **I** | **K** | **D** | **K** | **Q** | **D** | **T** | **D** | **G** | **K** | **E** | **N** | **S** | **F** | **L** | **L** | **T** | **L** | **D** | **R** | **S** | **N** | **N** | **K** | **F** | **L** | **T** | **M** | **I** | **M** | **V** | **E** | **N** | **S** | **G** | **E** | **N** | **G** | **S** | **A** | **T** | **D** | **I** | **S** | **K** | **P** | **L** | **I** | **D** | **Y** | **L** | **E** | **A** | **T** | **I** | **K** |
| **N52789** | **-** | **-** | **-** | **-** | **-** | **-** | **-** | **-** | **-** | **-** | **-** | **-** | **-** | **-** | **-** | **-** | **-** | **-** | **-** | **-** | **-** | **-** | **-** | **-** | **-** | **-** | **-** | **-** | **-** | **-** | **-** | **-** | **-** | **-** | **-** | **-** | **Q** | **W** | **Q** | **A** | **K** | **Q** | **E** | **L** | **A** | **E** | **A** | **K** | **K** | **T** | **A** | **N** | **T** | **F** | **L** | **N** | **V** | **L** | **S** | **K** | **Q** | **E** | **F** | **D** | **K** | **L** | **P** | **S** | **V** | **V** | **Q** | **E** | **S** | **S** | **L** | **K** | **K** | **N** | **G** | **Y** | **D** | **T** | **K** | **S** | **V** | **V** | **E** | **K** | **Y** | **Q** | **A** | **I** | **Y** | **S** | **G** | **I** | **Q** | **A** | **E** | **G** | **V** | **K** | **A** | **S** | **D** | **V** | **Q** | **V** | **K** | **K** | **A** | **K** | **D** | **N** | **Q** | **Y** | **T** | **F** | **T** | **Y** | **K** | **L** | **S** | **M** | **S** | **T** | **P** | **L** | **G** | **E** | **M** | **K** | **D** | **L** | **S** | **Y** | **Q** | **S** | **S** | **I** | **A** | **K** | **K** | **G** | **D** | **T** | **Y** | **Q** | **I** | **S** | **W** | **K** | **P** | **S** | **L** | **I** | **F** | **P** | **D** | **M** | **S** | **G** | **N** | **D** | **K** | **I** | **S** | **I** | **Q** | **V** | **D** | **N** | **A** | **K** | **R** | **G** | **E** | **I** | **V** | **D** | **R** | **N** | **G** | **S** | **G** | **L** | **A** | **I** | **N** | **K** | **V** | **F** | **D** | **E** | **V** | **G** | **V** | **V** | **P** | **G** | **K** | **L** | **G** | **S** | **G** | **A** | **E** | **K** | **T** | **A** | **N** | **I** | **K** | **A** | **F** | **S** | **D** | **K** | **F** | **G** | **V** | **S** | **V** | **D** | **E** | **I** | **N** | **Q** | **K** | **L** | **S** | **Q** | **G** | **W** | **V** | **Q** | **A** | **D** | **S** | **F** | **V** | **P** | **I** | **T** | **V** | **A** | **S** | **E** | **P** | **V** | **T** | **E** | **L** | **P** | **T** | **G** | **A** | **A** | **T** | **K** | **D** | **T** | **E** | **S** | **R** | **Y** | **Y** | **P** | **L** | **G** | **E** | **A** | **A** | **A** | **Q** | **L** | **I** | **G** | **Y** | **T** | **G** | **T** | **I** | **T** | **A** | **E** | **D** | **I** | **E** | **K** | **N** | **P** | **E** | **L** | **S** | **S** | **T** | **G** | **V** | **I** | **G** | **K** | **T** | **G** | **L** | **E** | **R** | **A** | **F** | **D** | **K** | **E** | **L** | **R** | **G** | **Q** | **D** | **G** | **G** | **S** | **L** | **V** | **I** | **L** | **D** | **D** | **K** | **E** | **N** | **V** | **K** | **K** | **A** | **L** | **Q** | **T** | **K** | **E** | **K** | **K** | **D** | **G** | **Q** | **T** | **I** | **K** | **L** | **T** | **I** | **D** | **S** | **G** | **V** | **Q** | **Q** | **Q** | **A** | **F** | **A** | **I** | **F** | **D** | **K** | **R** | **P** | **G** | **S** | **A** | **V** | **I** | **T** | **D** | **P** | **Q** | **K** | **G** | **D** | **L** | **L** | **A** | **T** | **V** | **S** | **S** | **P** | **S** | **Y** | **D** | **P** | **N** | **K** | **M** | **A** | **N** | **G** | **I** | **S** | **Q** | **K** | **E** | **Y** | **D** | **A** | **Y** | **N** | **N** | **N** | **K** | **D** | **L** | **P** | **F** | **T** | **A** | **R** | **F** | **A** | **T** | **G** | **Y** | **A** | **P** | **G** | **S** | **T** | **F** | **K** | **T** | **I** | **T** | **G** | **A** | **I** | **G** | **L** | **D** | **A** | **G** | **T** | **L** | **K** | **P** | **D** | **E** | **E** | **L** | **E** | **I** | **N** | **G** | **L** | **K** | **W** | **Q** | **K** | **D** | **K** | **S** | **W** | **G** | **G** | **Y** | **F** | **A** | **T** | **R** | **V** | **K** | **E** | **A** | **S** | **P** | **V** | **N** | **L** | **R** | **T** | **A** | **L** | **V** | **N** | **S** | **D** | **N** | **I** | **Y** | **F** | **A** | **Q** | **Q** | **T** | **L** | **R** | **M** | **G** | **E** | **D** | **K** | **F** | **R** | **A** | **G** | **L** | **N** | **K** | **F** | **I** | **F** | **G** | **E** | **E** | **L** | **D** | **L** | **P** | **I** | **A** | **M** | **T** | **P** | **A** | **Q** | **I** | **S** | **N** | **E** | **D** | **K** | **F** | **N** | **S** | **E** | **I** | **L** | **L** | **A** | **D** | **T** | **G** | **Y** | **G** | **Q** | **G** | **Q** | **L** | **L** | **I** | **S** | **P** | **I** | **Q** | **Q** | **A** | **T** | **M** | **Y** | **S** | **V** | **F** | **Q** | **N** | **N** | **G** | **T** | **L** | **V** | **Y** | **P** | **K** | **L** | **V** | **L** | **D** | **K** | **E** | **T** | **K** | **K** | **K** | **D** | **N** | **V** | **I** | **S** | **A** | **N** | **A** | **A** | **N** | **T** | **I** | **A** | **T** | **D** | **L** | **L** | **G** | **S** | **V** | **E** | **D** | **P** | **S** | **G** | **Y** | **V** | **Y** | **N** | **M** | **Y** | **N** | **P** | **N** | **F** | **S** | **L** | **A** | **A** | **K** | **T** | **G** | **T** | **A** | **E** | **I** | **K** | **D** | **K** | **Q** | **D** | **T** | **D** | **G** | **K** | **E** | **N** | **S** | **F** | **L** | **L** | **T** | **L** | **D** | **R** | **S** | **N** | **N** | **K** | **F** | **L** | **T** | **M** | **I** | **M** | **V** | **E** | **N** | **S** | **G** | **E** | **N** | **G** | **S** | **A** | **T** | **D** | **I** | **S** | **K** | **P** | **L** | **I** | **D** | **Y** | **L** | **E** | **A** | **T** | **I** | **K** | **-** | **-** | **-** | **-** | **-** | **-** | **-** | **-** | **-** | **-** | **-** | **-** | **-** | **-** | **-** | **-** | **-** | **-** | **-** | **-** | **-** | **-** | **-** | **-** | **-** | **-** | **-** | **-** | **-** | **-** | **-** | **-** | **-** | **-** | **-** | **-** | **Q** | **W** | **Q** | **A** | **K** | **Q** | **E** | **L** | **A** | **E** | **A** | **K** | **K** | **T** | **A** | **N** | **T** | **F** | **L** | **N** | **V** | **L** | **S** | **K** | **Q** | **E** | **F** | **D** | **K** | **L** | **P** | **S** | **V** | **V** | **Q** | **E** | **S** | **S** | **L** | **K** | **K** | **N** | **G** | **Y** | **D** | **T** | **K** | **S** | **V** | **V** | **E** | **K** | **Y** | **Q** | **A** | **I** | **Y** | **S** | **G** | **I** | **Q** | **A** | **E** | **G** | **V** | **K** | **A** | **S** | **D** | **V** | **Q** | **V** | **K** | **K** | **A** | **K** | **D** | **N** | **Q** | **Y** | **T** | **F** | **T** | **Y** | **K** | **L** | **S** | **M** | **S** | **T** | **P** | **L** | **G** | **E** | **M** | **K** | **D** | **L** | **S** | **Y** | **Q** | **S** | **S** | **I** | **A** | **K** | **K** | **G** | **D** | **T** | **Y** | **Q** | **I** | **S** | **W** | **K** | **P** | **S** | **L** | **I** | **F** | **P** | **D** | **M** | **S** | **G** | **N** | **D** | **K** | **I** | **S** | **I** | **Q** | **V** | **D** | **N** | **A** | **K** | **R** | **G** | **E** | **I** | **V** | **D** | **R** | **N** | **G** | **S** | **G** | **L** | **A** | **I** | **N** | **K** | **V** | **F** | **D** | **E** | **V** | **G** | **V** | **V** | **P** | **G** | **K** | **L** | **G** | **S** | **G** | **A** | **E** | **K** | **T** | **A** | **N** | **I** | **K** | **A** | **F** | **S** | **D** | **K** | **F** | **G** | **V** | **S** | **V** | **D** | **E** | **I** | **N** | **Q** | **K** | **L** | **S** | **Q** | **G** | **W** | **V** | **Q** | **A** | **D** | **S** | **F** | **V** | **P** | **I** | **T** | **V** | **A** | **S** | **E** | **P** | **V** | **T** | **E** | **L** | **P** | **T** | **G** | **A** | **A** | **T** | **K** | **D** | **T** | **E** | **S** | **R** | **Y** | **Y** | **P** | **L** | **G** | **E** | **A** | **A** | **A** | **Q** | **L** | **I** | **G** | **Y** | **T** | **G** | **T** | **I** | **T** | **A** | **E** | **D** | **I** | **E** | **K** | **N** | **P** | **E** | **L** | **S** | **S** | **T** | **G** | **V** | **I** | **G** | **K** | **T** | **G** | **L** | **E** | **R** | **A** | **F** | **D** | **K** | **E** | **L** | **R** | **G** | **Q** | **D** | **G** | **G** | **S** | **L** | **V** | **I** | **L** | **D** | **D** | **K** | **E** | **N** | **V** | **K** | **K** | **A** | **L** | **Q** | **T** | **K** | **E** | **K** | **K** | **D** | **G** | **Q** | **T** | **I** | **K** | **L** | **T** | **I** | **D** | **S** | **G** | **V** | **Q** | **Q** | **Q** | **A** | **F** | **A** | **I** | **F** | **D** | **K** | **R** | **P** | **G** | **S** | **A** | **V** | **I** | **T** | **D** | **P** | **Q** | **K** | **G** | **D** | **L** | **L** | **A** | **T** | **V** | **S** | **S** | **P** | **S** | **Y** | **D** | **P** | **N** | **K** | **M** | **A** | **N** | **G** | **I** | **S** | **Q** | **K** | **E** | **Y** | **D** | **A** | **Y** | **N** | **N** | **N** | **K** | **D** | **L** | **P** | **F** | **T** | **A** | **R** | **F** | **A** | **T** | **G** | **Y** | **A** | **P** | **G** | **S** | **T** | **F** | **K** | **T** | **I** | **T** | **G** | **A** | **I** | **G** | **L** | **D** | **A** | **G** | **T** | **L** | **K** | **P** | **D** | **E** | **E** | **L** | **E** | **I** | **N** | **G** | **L** | **K** | **W** | **Q** | **K** | **D** | **K** | **S** | **W** | **G** | **G** | **Y** | **F** | **A** | **T** | **R** | **V** | **K** | **E** | **A** | **S** | **P** | **V** | **N** | **L** | **R** | **T** | **A** | **L** | **V** | **N** | **S** | **D** | **N** | **I** | **Y** | **F** | **A** | **Q** | **Q** | **T** | **L** | **R** | **M** | **G** | **E** | **D** | **K** | **F** | **R** | **A** | **G** | **L** | **N** | **K** | **F** | **I** | **F** | **G** | **E** | **E** | **L** | **D** | **L** | **P** | **I** | **A** | **M** | **T** | **P** | **A** | **Q** | **I** | **S** | **N** | **E** | **D** | **K** | **F** | **N** | **S** | **E** | **I** | **L** | **L** | **A** | **D** | **T** | **G** | **Y** | **G** | **Q** | **G** | **Q** | **L** | **L** | **I** | **S** | **P** | **I** | **Q** | **Q** | **A** | **T** | **M** | **Y** | **S** | **V** | **F** | **Q** | **N** | **N** | **G** | **T** | **L** | **V** | **Y** | **P** | **K** | **L** | **V** | **L** | **D** | **K** | **E** | **T** | **K** | **K** | **K** | **D** | **N** | **V** | **I** | **S** | **A** | **N** | **A** | **A** | **N** | **T** | **I** | **A** | **T** | **D** | **L** | **L** | **G** | **S** | **V** | **E** | **D** | **P** | **S** | **G** | **Y** | **V** | **Y** | **N** | **M** | **Y** | **N** | **P** | **N** | **F** | **S** | **L** | **A** | **A** | **K** | **T** | **G** | **T** | **A** | **E** | **I** | **K** | **D** | **K** | **Q** | **D** | **T** | **D** | **G** | **K** | **E** | **N** | **S** | **F** | **L** | **L** | **T** | **L** | **D** | **R** | **S** | **N** | **N** | **K** | **F** | **L** | **T** | **M** | **I** | **M** | **V** | **E** | **N** | **S** | **G** | **E** | **N** | **G** | **S** | **A** | **T** | **D** | **I** | **S** | **K** | **P** | **L** | **I** | **D** | **Y** | **L** | **E** | **A** | **T** | **I** | **K** |
| **N59963F** | **-** | **-** | **-** | **-** | **-** | **-** | **-** | **-** | **-** | **-** | **-** | **-** | **-** | **-** | **-** | **-** | **-** | **-** | **-** | **-** | **-** | **-** | **-** | **-** | **-** | **-** | **-** | **-** | **-** | **-** | **-** | **-** | **-** | **-** | **-** | **-** | **Q** | **W** | **Q** | **A** | **K** | **Q** | **E** | **L** | **A** | **E** | **A** | **K** | **K** | **T** | **A** | **T** | **T** | **F** | **L** | **N** | **V** | **L** | **T** | **K** | **Q** | **K** | **F** | **D** | **K** | **L** | **P** | **S** | **V** | **V** | **Q** | **E** | **A** | **S** | **L** | **K** | **K** | **N** | **G** | **Y** | **D** | **T** | **K** | **S** | **V** | **V** | **E** | **K** | **Y** | **Q** | **A** | **I** | **Y** | **S** | **G** | **I** | **Q** | **A** | **E** | **G** | **V** | **K** | **A** | **S** | **D** | **V** | **Q** | **V** | **K** | **K** | **A** | **K** | **D** | **N** | **Q** | **Y** | **T** | **F** | **T** | **Y** | **K** | **L** | **S** | **M** | **S** | **T** | **P** | **L** | **G** | **E** | **M** | **K** | **D** | **L** | **S** | **Y** | **Q** | **S** | **S** | **I** | **A** | **K** | **K** | **G** | **D** | **T** | **Y** | **Q** | **I** | **A** | **W** | **K** | **P** | **S** | **L** | **I** | **F** | **P** | **D** | **M** | **S** | **G** | **N** | **D** | **K** | **I** | **S** | **I** | **Q** | **V** | **D** | **N** | **A** | **K** | **R** | **G** | **E** | **I** | **V** | **D** | **R** | **N** | **G** | **S** | **G** | **L** | **A** | **I** | **N** | **K** | **V** | **F** | **D** | **E** | **V** | **G** | **V** | **V** | **P** | **G** | **K** | **L** | **G** | **S** | **G** | **A** | **E** | **K** | **T** | **A** | **N** | **I** | **K** | **A** | **F** | **S** | **D** | **K** | **F** | **G** | **V** | **S** | **V** | **D** | **E** | **I** | **N** | **Q** | **K** | **L** | **S** | **Q** | **G** | **W** | **V** | **Q** | **A** | **D** | **S** | **F** | **V** | **P** | **I** | **T** | **V** | **A** | **S** | **E** | **P** | **V** | **T** | **E** | **L** | **P** | **T** | **G** | **A** | **A** | **T** | **K** | **D** | **T** | **E** | **S** | **R** | **Y** | **Y** | **P** | **L** | **G** | **E** | **A** | **A** | **A** | **Q** | **L** | **I** | **G** | **Y** | **T** | **G** | **T** | **I** | **T** | **A** | **E** | **D** | **I** | **K** | **K** | **N** | **P** | **E** | **L** | **S** | **S** | **T** | **G** | **V** | **I** | **G** | **K** | **T** | **G** | **L** | **E** | **R** | **A** | **F** | **D** | **K** | **E** | **L** | **R** | **G** | **Q** | **D** | **G** | **G** | **S** | **L** | **V** | **I** | **L** | **D** | **D** | **K** | **E** | **N** | **V** | **K** | **K** | **A** | **L** | **Q** | **T** | **K** | **E** | **K** | **K** | **D** | **G** | **Q** | **T** | **I** | **K** | **L** | **T** | **I** | **D** | **S** | **G** | **V** | **Q** | **Q** | **Q** | **A** | **F** | **A** | **I** | **F** | **D** | **K** | **R** | **P** | **G** | **S** | **A** | **V** | **I** | **T** | **D** | **P** | **Q** | **K** | **G** | **D** | **L** | **L** | **A** | **T** | **V** | **S** | **S** | **P** | **S** | **Y** | **D** | **P** | **N** | **K** | **M** | **A** | **N** | **G** | **I** | **S** | **Q** | **K** | **E** | **Y** | **D** | **A** | **Y** | **N** | **N** | **N** | **K** | **D** | **L** | **P** | **F** | **T** | **A** | **R** | **F** | **A** | **T** | **G** | **Y** | **A** | **P** | **G** | **S** | **T** | **F** | **K** | **T** | **I** | **T** | **G** | **A** | **I** | **G** | **L** | **D** | **T** | **G** | **T** | **L** | **K** | **P** | **D** | **E** | **E** | **L** | **E** | **I** | **N** | **G** | **L** | **K** | **W** | **Q** | **K** | **D** | **K** | **S** | **W** | **G** | **G** | **Y** | **F** | **A** | **T** | **R** | **V** | **K** | **E** | **A** | **S** | **P** | **V** | **N** | **L** | **R** | **T** | **A** | **L** | **V** | **N** | **S** | **D** | **N** | **I** | **Y** | **F** | **A** | **Q** | **Q** | **T** | **L** | **R** | **M** | **G** | **E** | **D** | **K** | **F** | **R** | **A** | **G** | **L** | **N** | **K** | **F** | **I** | **F** | **G** | **E** | **E** | **L** | **D** | **L** | **P** | **I** | **A** | **M** | **T** | **P** | **A** | **Q** | **I** | **S** | **N** | **E** | **D** | **K** | **F** | **N** | **S** | **E** | **I** | **L** | **L** | **A** | **D** | **T** | **G** | **Y** | **G** | **Q** | **G** | **Q** | **L** | **L** | **I** | **S** | **P** | **I** | **Q** | **Q** | **A** | **T** | **M** | **Y** | **S** | **V** | **F** | **Q** | **N** | **N** | **G** | **T** | **L** | **V** | **Y** | **P** | **K** | **L** | **V** | **L** | **D** | **K** | **E** | **T** | **K** | **K** | **K** | **D** | **N** | **V** | **I** | **S** | **A** | **N** | **A** | **A** | **N** | **T** | **I** | **A** | **T** | **D** | **L** | **L** | **G** | **S** | **V** | **E** | **D** | **P** | **S** | **G** | **Y** | **V** | **Y** | **N** | **M** | **Y** | **N** | **P** | **N** | **F** | **S** | **L** | **A** | **A** | **K** | **T** | **G** | **T** | **A** | **E** | **I** | **K** | **D** | **K** | **Q** | **D** | **T** | **D** | **G** | **K** | **E** | **N** | **S** | **F** | **L** | **L** | **T** | **L** | **D** | **R** | **S** | **N** | **N** | **K** | **F** | **L** | **T** | **M** | **I** | **M** | **V** | **E** | **N** | **S** | **G** | **E** | **N** | **G** | **S** | **A** | **T** | **D** | **I** | **S** | **K** | **P** | **L** | **I** | **D** | **Y** | **L** | **E** | **A** | **T** | **I** | **K** | **-** | **-** | **-** | **-** | **-** | **-** | **-** | **-** | **-** | **-** | **-** | **-** | **-** | **-** | **-** | **-** | **-** | **-** | **-** | **-** | **-** | **-** | **-** | **-** | **-** | **-** | **-** | **-** | **-** | **-** | **-** | **-** | **-** | **-** | **-** | **-** | **Q** | **W** | **Q** | **A** | **K** | **Q** | **E** | **L** | **A** | **E** | **A** | **K** | **K** | **T** | **A** | **T** | **T** | **F** | **L** | **N** | **V** | **L** | **T** | **K** | **Q** | **K** | **F** | **D** | **K** | **L** | **P** | **S** | **V** | **V** | **Q** | **E** | **A** | **S** | **L** | **K** | **K** | **N** | **G** | **Y** | **D** | **T** | **K** | **S** | **V** | **V** | **E** | **K** | **Y** | **Q** | **A** | **I** | **Y** | **S** | **G** | **I** | **Q** | **A** | **E** | **G** | **V** | **K** | **A** | **S** | **D** | **V** | **Q** | **V** | **K** | **K** | **A** | **K** | **D** | **N** | **Q** | **Y** | **T** | **F** | **T** | **Y** | **K** | **L** | **S** | **M** | **S** | **T** | **P** | **L** | **G** | **E** | **M** | **K** | **D** | **L** | **S** | **Y** | **Q** | **S** | **S** | **I** | **A** | **K** | **K** | **G** | **D** | **T** | **Y** | **Q** | **I** | **A** | **W** | **K** | **P** | **S** | **L** | **I** | **F** | **P** | **D** | **M** | **S** | **G** | **N** | **D** | **K** | **I** | **S** | **I** | **Q** | **V** | **D** | **N** | **A** | **K** | **R** | **G** | **E** | **I** | **V** | **D** | **R** | **N** | **G** | **S** | **G** | **L** | **A** | **I** | **N** | **K** | **V** | **F** | **D** | **E** | **V** | **G** | **V** | **V** | **P** | **G** | **K** | **L** | **G** | **S** | **G** | **A** | **E** | **K** | **T** | **A** | **N** | **I** | **K** | **A** | **F** | **S** | **D** | **K** | **F** | **G** | **V** | **S** | **V** | **D** | **E** | **I** | **N** | **Q** | **K** | **L** | **S** | **Q** | **G** | **W** | **V** | **Q** | **A** | **D** | **S** | **F** | **V** | **P** | **I** | **T** | **V** | **A** | **S** | **E** | **P** | **V** | **T** | **E** | **L** | **P** | **T** | **G** | **A** | **A** | **T** | **K** | **D** | **T** | **E** | **S** | **R** | **Y** | **Y** | **P** | **L** | **G** | **E** | **A** | **A** | **A** | **Q** | **L** | **I** | **G** | **Y** | **T** | **G** | **T** | **I** | **T** | **A** | **E** | **D** | **I** | **K** | **K** | **N** | **P** | **E** | **L** | **S** | **S** | **T** | **G** | **V** | **I** | **G** | **K** | **T** | **G** | **L** | **E** | **R** | **A** | **F** | **D** | **K** | **E** | **L** | **R** | **G** | **Q** | **D** | **G** | **G** | **S** | **L** | **V** | **I** | **L** | **D** | **D** | **K** | **E** | **N** | **V** | **K** | **K** | **A** | **L** | **Q** | **T** | **K** | **E** | **K** | **K** | **D** | **G** | **Q** | **T** | **I** | **K** | **L** | **T** | **I** | **D** | **S** | **G** | **V** | **Q** | **Q** | **Q** | **A** | **F** | **A** | **I** | **F** | **D** | **K** | **R** | **P** | **G** | **S** | **A** | **V** | **I** | **T** | **D** | **P** | **Q** | **K** | **G** | **D** | **L** | **L** | **A** | **T** | **V** | **S** | **S** | **P** | **S** | **Y** | **D** | **P** | **N** | **K** | **M** | **A** | **N** | **G** | **I** | **S** | **Q** | **K** | **E** | **Y** | **D** | **A** | **Y** | **N** | **N** | **N** | **K** | **D** | **L** | **P** | **F** | **T** | **A** | **R** | **F** | **A** | **T** | **G** | **Y** | **A** | **P** | **G** | **S** | **T** | **F** | **K** | **T** | **I** | **T** | **G** | **A** | **I** | **G** | **L** | **D** | **T** | **G** | **T** | **L** | **K** | **P** | **D** | **E** | **E** | **L** | **E** | **I** | **N** | **G** | **L** | **K** | **W** | **Q** | **K** | **D** | **K** | **S** | **W** | **G** | **G** | **Y** | **F** | **A** | **T** | **R** | **V** | **K** | **E** | **A** | **S** | **P** | **V** | **N** | **L** | **R** | **T** | **A** | **L** | **V** | **N** | **S** | **D** | **N** | **I** | **Y** | **F** | **A** | **Q** | **Q** | **T** | **L** | **R** | **M** | **G** | **E** | **D** | **K** | **F** | **R** | **A** | **G** | **L** | **N** | **K** | **F** | **I** | **F** | **G** | **E** | **E** | **L** | **D** | **L** | **P** | **I** | **A** | **M** | **T** | **P** | **A** | **Q** | **I** | **S** | **N** | **E** | **D** | **K** | **F** | **N** | **S** | **E** | **I** | **L** | **L** | **A** | **D** | **T** | **G** | **Y** | **G** | **Q** | **G** | **Q** | **L** | **L** | **I** | **S** | **P** | **I** | **Q** | **Q** | **A** | **T** | **M** | **Y** | **S** | **V** | **F** | **Q** | **N** | **N** | **G** | **T** | **L** | **V** | **Y** | **P** | **K** | **L** | **V** | **L** | **D** | **K** | **E** | **T** | **K** | **K** | **K** | **D** | **N** | **V** | **I** | **S** | **A** | **N** | **A** | **A** | **N** | **T** | **I** | **A** | **T** | **D** | **L** | **L** | **G** | **S** | **V** | **E** | **D** | **P** | **S** | **G** | **Y** | **V** | **Y** | **N** | **M** | **Y** | **N** | **P** | **N** | **F** | **S** | **L** | **A** | **A** | **K** | **T** | **G** | **T** | **A** | **E** | **I** | **K** | **D** | **K** | **Q** | **D** | **T** | **D** | **G** | **K** | **E** | **N** | **S** | **F** | **L** | **L** | **T** | **L** | **D** | **R** | **S** | **N** | **N** | **K** | **F** | **L** | **T** | **M** | **I** | **M** | **V** | **E** | **N** | **S** | **G** | **E** | **N** | **G** | **S** | **A** | **T** | **D** | **I** | **S** | **K** | **P** | **L** | **I** | **D** | **Y** | **L** | **E** | **A** | **T** | **I** | **K** |
| **N52564** | **-** | **-** | **-** | **-** | **-** | **-** | **-** | **-** | **-** | **-** | **-** | **-** | **-** | **-** | **-** | **-** | **-** | **-** | **-** | **-** | **-** | **-** | **-** | **-** | **-** | **-** | **-** | **-** | **-** | **-** | **-** | **-** | **-** | **-** | **-** | **-** | **Q** | **W** | **Q** | **A** | **K** | **Q** | **E** | **L** | **A** | **E** | **A** | **K** | **K** | **T** | **A** | **T** | **T** | **F** | **L** | **N** | **V** | **L** | **S** | **K** | **Q** | **E** | **F** | **D** | **K** | **L** | **P** | **S** | **V** | **V** | **Q** | **E** | **A** | **S** | **L** | **K** | **K** | **N** | **G** | **Y** | **D** | **T** | **K** | **S** | **V** | **V** | **E** | **K** | **Y** | **Q** | **A** | **I** | **Y** | **S** | **G** | **I** | **Q** | **A** | **E** | **G** | **V** | **K** | **A** | **S** | **D** | **V** | **Q** | **V** | **K** | **K** | **A** | **K** | **D** | **N** | **Q** | **Y** | **T** | **F** | **T** | **Y** | **K** | **L** | **S** | **M** | **S** | **T** | **P** | **L** | **G** | **E** | **M** | **K** | **D** | **L** | **S** | **Y** | **Q** | **S** | **S** | **I** | **A** | **K** | **K** | **G** | **D** | **T** | **Y** | **Q** | **I** | **A** | **W** | **K** | **P** | **S** | **L** | **I** | **F** | **P** | **D** | **M** | **S** | **G** | **N** | **D** | **K** | **I** | **S** | **I** | **Q** | **V** | **D** | **N** | **A** | **K** | **R** | **G** | **E** | **I** | **V** | **D** | **R** | **N** | **G** | **S** | **G** | **L** | **A** | **I** | **N** | **K** | **V** | **F** | **D** | **E** | **V** | **G** | **V** | **V** | **P** | **G** | **K** | **L** | **G** | **S** | **G** | **A** | **E** | **K** | **T** | **A** | **N** | **I** | **K** | **A** | **F** | **S** | **D** | **K** | **F** | **G** | **V** | **S** | **V** | **D** | **E** | **I** | **N** | **Q** | **K** | **L** | **S** | **Q** | **G** | **W** | **V** | **Q** | **A** | **D** | **S** | **F** | **V** | **P** | **I** | **T** | **V** | **A** | **S** | **E** | **P** | **V** | **T** | **E** | **L** | **P** | **T** | **G** | **A** | **A** | **T** | **K** | **D** | **T** | **E** | **S** | **R** | **Y** | **Y** | **P** | **L** | **G** | **E** | **A** | **A** | **A** | **Q** | **L** | **I** | **G** | **Y** | **T** | **G** | **T** | **I** | **T** | **A** | **E** | **D** | **I** | **E** | **K** | **N** | **P** | **E** | **L** | **S** | **S** | **T** | **G** | **V** | **I** | **G** | **K** | **T** | **G** | **L** | **E** | **R** | **A** | **F** | **D** | **K** | **E** | **L** | **R** | **G** | **Q** | **D** | **G** | **G** | **S** | **L** | **V** | **I** | **L** | **D** | **D** | **K** | **E** | **N** | **V** | **K** | **K** | **A** | **L** | **Q** | **T** | **K** | **E** | **K** | **K** | **D** | **G** | **Q** | **T** | **I** | **K** | **L** | **T** | **I** | **D** | **S** | **G** | **V** | **Q** | **Q** | **Q** | **A** | **F** | **A** | **I** | **F** | **D** | **K** | **R** | **P** | **G** | **S** | **A** | **V** | **I** | **T** | **D** | **P** | **Q** | **K** | **G** | **D** | **L** | **L** | **A** | **T** | **V** | **S** | **S** | **P** | **S** | **Y** | **D** | **P** | **N** | **K** | **M** | **A** | **N** | **G** | **I** | **S** | **Q** | **K** | **E** | **Y** | **D** | **A** | **Y** | **N** | **N** | **N** | **K** | **D** | **L** | **P** | **F** | **T** | **A** | **R** | **F** | **A** | **T** | **G** | **Y** | **A** | **P** | **G** | **S** | **T** | **F** | **K** | **T** | **I** | **T** | **G** | **A** | **I** | **G** | **L** | **D** | **A** | **G** | **T** | **L** | **K** | **P** | **D** | **E** | **E** | **L** | **E** | **I** | **N** | **G** | **L** | **K** | **W** | **Q** | **K** | **D** | **K** | **S** | **W** | **G** | **G** | **Y** | **F** | **A** | **T** | **R** | **V** | **K** | **E** | **A** | **S** | **P** | **V** | **N** | **L** | **R** | **T** | **A** | **L** | **V** | **N** | **S** | **D** | **N** | **I** | **Y** | **F** | **A** | **Q** | **Q** | **T** | **L** | **R** | **M** | **G** | **E** | **D** | **K** | **F** | **R** | **A** | **G** | **L** | **N** | **K** | **F** | **I** | **F** | **G** | **E** | **E** | **L** | **D** | **L** | **P** | **I** | **A** | **M** | **T** | **P** | **A** | **Q** | **I** | **S** | **N** | **E** | **D** | **K** | **F** | **N** | **S** | **E** | **I** | **L** | **L** | **A** | **D** | **T** | **G** | **Y** | **G** | **Q** | **G** | **Q** | **L** | **L** | **I** | **S** | **P** | **I** | **Q** | **Q** | **A** | **T** | **M** | **Y** | **S** | **V** | **F** | **Q** | **N** | **N** | **G** | **T** | **L** | **V** | **Y** | **P** | **K** | **L** | **V** | **L** | **D** | **K** | **E** | **T** | **K** | **K** | **K** | **D** | **N** | **V** | **I** | **S** | **A** | **N** | **A** | **A** | **N** | **T** | **I** | **A** | **T** | **D** | **L** | **L** | **G** | **S** | **V** | **E** | **D** | **P** | **S** | **G** | **Y** | **V** | **Y** | **N** | **M** | **Y** | **N** | **P** | **N** | **F** | **S** | **L** | **A** | **A** | **K** | **T** | **G** | **T** | **A** | **E** | **I** | **K** | **D** | **K** | **Q** | **D** | **T** | **D** | **G** | **K** | **E** | **N** | **S** | **F** | **L** | **L** | **T** | **L** | **D** | **R** | **S** | **N** | **N** | **K** | **F** | **L** | **T** | **M** | **I** | **M** | **V** | **E** | **N** | **S** | **G** | **E** | **N** | **G** | **S** | **A** | **T** | **D** | **I** | **S** | **K** | **P** | **L** | **I** | **D** | **Y** | **L** | **E** | **A** | **T** | **I** | **K** | **-** | **-** | **-** | **-** | **-** | **-** | **-** | **-** | **-** | **-** | **-** | **-** | **-** | **-** | **-** | **-** | **-** | **-** | **-** | **-** | **-** | **-** | **-** | **-** | **-** | **-** | **-** | **-** | **-** | **-** | **-** | **-** | **-** | **-** | **-** | **-** | **Q** | **W** | **Q** | **A** | **K** | **Q** | **E** | **L** | **A** | **E** | **A** | **K** | **K** | **T** | **A** | **T** | **T** | **F** | **L** | **N** | **V** | **L** | **S** | **K** | **Q** | **E** | **F** | **D** | **K** | **L** | **P** | **S** | **V** | **V** | **Q** | **E** | **A** | **S** | **L** | **K** | **K** | **N** | **G** | **Y** | **D** | **T** | **K** | **S** | **V** | **V** | **E** | **K** | **Y** | **Q** | **A** | **I** | **Y** | **S** | **G** | **I** | **Q** | **A** | **E** | **G** | **V** | **K** | **A** | **S** | **D** | **V** | **Q** | **V** | **K** | **K** | **A** | **K** | **D** | **N** | **Q** | **Y** | **T** | **F** | **T** | **Y** | **K** | **L** | **S** | **M** | **S** | **T** | **P** | **L** | **G** | **E** | **M** | **K** | **D** | **L** | **S** | **Y** | **Q** | **S** | **S** | **I** | **A** | **K** | **K** | **G** | **D** | **T** | **Y** | **Q** | **I** | **A** | **W** | **K** | **P** | **S** | **L** | **I** | **F** | **P** | **D** | **M** | **S** | **G** | **N** | **D** | **K** | **I** | **S** | **I** | **Q** | **V** | **D** | **N** | **A** | **K** | **R** | **G** | **E** | **I** | **V** | **D** | **R** | **N** | **G** | **S** | **G** | **L** | **A** | **I** | **N** | **K** | **V** | **F** | **D** | **E** | **V** | **G** | **V** | **V** | **P** | **G** | **K** | **L** | **G** | **S** | **G** | **A** | **E** | **K** | **T** | **A** | **N** | **I** | **K** | **A** | **F** | **S** | **D** | **K** | **F** | **G** | **V** | **S** | **V** | **D** | **E** | **I** | **N** | **Q** | **K** | **L** | **S** | **Q** | **G** | **W** | **V** | **Q** | **A** | **D** | **S** | **F** | **V** | **P** | **I** | **T** | **V** | **A** | **S** | **E** | **P** | **V** | **T** | **E** | **L** | **P** | **T** | **G** | **A** | **A** | **T** | **K** | **D** | **T** | **E** | **S** | **R** | **Y** | **Y** | **P** | **L** | **G** | **E** | **A** | **A** | **A** | **Q** | **L** | **I** | **G** | **Y** | **T** | **G** | **T** | **I** | **T** | **A** | **E** | **D** | **I** | **E** | **K** | **N** | **P** | **E** | **L** | **S** | **S** | **T** | **G** | **V** | **I** | **G** | **K** | **T** | **G** | **L** | **E** | **R** | **A** | **F** | **D** | **K** | **E** | **L** | **R** | **G** | **Q** | **D** | **G** | **G** | **S** | **L** | **V** | **I** | **L** | **D** | **D** | **K** | **E** | **N** | **V** | **K** | **K** | **A** | **L** | **Q** | **T** | **K** | **E** | **K** | **K** | **D** | **G** | **Q** | **T** | **I** | **K** | **L** | **T** | **I** | **D** | **S** | **G** | **V** | **Q** | **Q** | **Q** | **A** | **F** | **A** | **I** | **F** | **D** | **K** | **R** | **P** | **G** | **S** | **A** | **V** | **I** | **T** | **D** | **P** | **Q** | **K** | **G** | **D** | **L** | **L** | **A** | **T** | **V** | **S** | **S** | **P** | **S** | **Y** | **D** | **P** | **N** | **K** | **M** | **A** | **N** | **G** | **I** | **S** | **Q** | **K** | **E** | **Y** | **D** | **A** | **Y** | **N** | **N** | **N** | **K** | **D** | **L** | **P** | **F** | **T** | **A** | **R** | **F** | **A** | **T** | **G** | **Y** | **A** | **P** | **G** | **S** | **T** | **F** | **K** | **T** | **I** | **T** | **G** | **A** | **I** | **G** | **L** | **D** | **A** | **G** | **T** | **L** | **K** | **P** | **D** | **E** | **E** | **L** | **E** | **I** | **N** | **G** | **L** | **K** | **W** | **Q** | **K** | **D** | **K** | **S** | **W** | **G** | **G** | **Y** | **F** | **A** | **T** | **R** | **V** | **K** | **E** | **A** | **S** | **P** | **V** | **N** | **L** | **R** | **T** | **A** | **L** | **V** | **N** | **S** | **D** | **N** | **I** | **Y** | **F** | **A** | **Q** | **Q** | **T** | **L** | **R** | **M** | **G** | **E** | **D** | **K** | **F** | **R** | **A** | **G** | **L** | **N** | **K** | **F** | **I** | **F** | **G** | **E** | **E** | **L** | **D** | **L** | **P** | **I** | **A** | **M** | **T** | **P** | **A** | **Q** | **I** | **S** | **N** | **E** | **D** | **K** | **F** | **N** | **S** | **E** | **I** | **L** | **L** | **A** | **D** | **T** | **G** | **Y** | **G** | **Q** | **G** | **Q** | **L** | **L** | **I** | **S** | **P** | **I** | **Q** | **Q** | **A** | **T** | **M** | **Y** | **S** | **V** | **F** | **Q** | **N** | **N** | **G** | **T** | **L** | **V** | **Y** | **P** | **K** | **L** | **V** | **L** | **D** | **K** | **E** | **T** | **K** | **K** | **K** | **D** | **N** | **V** | **I** | **S** | **A** | **N** | **A** | **A** | **N** | **T** | **I** | **A** | **T** | **D** | **L** | **L** | **G** | **S** | **V** | **E** | **D** | **P** | **S** | **G** | **Y** | **V** | **Y** | **N** | **M** | **Y** | **N** | **P** | **N** | **F** | **S** | **L** | **A** | **A** | **K** | **T** | **G** | **T** | **A** | **E** | **I** | **K** | **D** | **K** | **Q** | **D** | **T** | **D** | **G** | **K** | **E** | **N** | **S** | **F** | **L** | **L** | **T** | **L** | **D** | **R** | **S** | **N** | **N** | **K** | **F** | **L** | **T** | **M** | **I** | **M** | **V** | **E** | **N** | **S** | **G** | **E** | **N** | **G** | **S** | **A** | **T** | **D** | **I** | **S** | **K** | **P** | **L** | **I** | **D** | **Y** | **L** | **E** | **A** | **T** | **I** | **K** |
| **N53418** | **-** | **-** | **-** | **-** | **-** | **-** | **-** | **-** | **-** | **-** | **-** | **-** | **-** | **-** | **-** | **-** | **-** | **-** | **-** | **-** | **-** | **-** | **-** | **-** | **-** | **-** | **-** | **-** | **-** | **-** | **-** | **-** | **-** | **-** | **-** | **-** | **Q** | **W** | **Q** | **A** | **K** | **Q** | **E** | **L** | **A** | **E** | **A** | **K** | **K** | **T** | **A** | **T** | **T** | **F** | **L** | **N** | **V** | **L** | **S** | **K** | **Q** | **E** | **F** | **D** | **K** | **L** | **P** | **S** | **V** | **V** | **Q** | **E** | **A** | **S** | **L** | **K** | **K** | **N** | **G** | **Y** | **D** | **T** | **K** | **S** | **V** | **V** | **E** | **K** | **Y** | **Q** | **A** | **I** | **Y** | **S** | **G** | **I** | **Q** | **A** | **E** | **G** | **V** | **K** | **A** | **S** | **D** | **V** | **Q** | **V** | **K** | **K** | **A** | **K** | **D** | **N** | **Q** | **Y** | **T** | **F** | **T** | **Y** | **K** | **L** | **S** | **M** | **S** | **T** | **P** | **L** | **G** | **E** | **M** | **K** | **D** | **L** | **S** | **Y** | **Q** | **S** | **S** | **I** | **A** | **K** | **K** | **G** | **D** | **T** | **Y** | **Q** | **I** | **A** | **W** | **K** | **P** | **S** | **L** | **I** | **F** | **P** | **D** | **M** | **S** | **G** | **N** | **D** | **K** | **I** | **S** | **I** | **Q** | **V** | **D** | **N** | **A** | **K** | **R** | **G** | **E** | **I** | **V** | **D** | **R** | **N** | **G** | **S** | **G** | **L** | **A** | **I** | **N** | **K** | **V** | **F** | **D** | **E** | **V** | **G** | **V** | **V** | **P** | **G** | **K** | **L** | **G** | **S** | **G** | **A** | **E** | **K** | **T** | **A** | **N** | **I** | **K** | **A** | **F** | **S** | **D** | **K** | **F** | **G** | **V** | **S** | **V** | **D** | **E** | **I** | **N** | **Q** | **K** | **L** | **S** | **Q** | **G** | **W** | **V** | **Q** | **A** | **D** | **S** | **F** | **V** | **P** | **I** | **T** | **V** | **A** | **S** | **E** | **P** | **V** | **T** | **E** | **L** | **P** | **T** | **G** | **A** | **A** | **T** | **K** | **D** | **T** | **E** | **S** | **R** | **Y** | **Y** | **P** | **L** | **G** | **E** | **A** | **A** | **A** | **Q** | **L** | **I** | **G** | **Y** | **T** | **G** | **T** | **I** | **T** | **A** | **E** | **D** | **I** | **E** | **K** | **N** | **P** | **E** | **L** | **S** | **S** | **T** | **G** | **V** | **I** | **G** | **K** | **T** | **G** | **L** | **E** | **R** | **A** | **F** | **D** | **K** | **E** | **L** | **R** | **G** | **Q** | **D** | **G** | **G** | **S** | **L** | **V** | **I** | **L** | **D** | **D** | **K** | **E** | **N** | **V** | **K** | **K** | **A** | **L** | **Q** | **T** | **K** | **E** | **K** | **K** | **D** | **G** | **Q** | **T** | **I** | **K** | **L** | **T** | **I** | **D** | **S** | **G** | **V** | **Q** | **Q** | **Q** | **A** | **F** | **A** | **I** | **F** | **D** | **K** | **R** | **P** | **G** | **S** | **A** | **V** | **I** | **T** | **D** | **P** | **Q** | **K** | **G** | **D** | **L** | **L** | **A** | **T** | **V** | **S** | **S** | **P** | **S** | **Y** | **D** | **P** | **N** | **K** | **M** | **A** | **N** | **G** | **I** | **S** | **Q** | **K** | **E** | **Y** | **D** | **A** | **Y** | **N** | **N** | **N** | **K** | **D** | **L** | **P** | **F** | **T** | **A** | **R** | **F** | **A** | **T** | **G** | **Y** | **A** | **P** | **G** | **S** | **T** | **F** | **K** | **T** | **I** | **T** | **G** | **A** | **I** | **G** | **L** | **D** | **A** | **G** | **T** | **L** | **K** | **P** | **D** | **E** | **E** | **L** | **E** | **I** | **N** | **G** | **L** | **K** | **W** | **Q** | **K** | **D** | **K** | **S** | **W** | **G** | **G** | **Y** | **F** | **A** | **T** | **R** | **V** | **K** | **E** | **A** | **S** | **P** | **V** | **N** | **L** | **R** | **T** | **A** | **L** | **V** | **N** | **S** | **D** | **N** | **I** | **Y** | **F** | **A** | **Q** | **Q** | **T** | **L** | **R** | **M** | **G** | **E** | **D** | **K** | **F** | **R** | **A** | **G** | **L** | **N** | **K** | **F** | **I** | **F** | **G** | **E** | **E** | **L** | **D** | **L** | **P** | **I** | **A** | **M** | **T** | **P** | **A** | **Q** | **I** | **S** | **N** | **E** | **D** | **K** | **F** | **N** | **S** | **E** | **I** | **L** | **L** | **A** | **D** | **T** | **G** | **Y** | **G** | **Q** | **G** | **Q** | **L** | **L** | **I** | **S** | **P** | **I** | **Q** | **Q** | **A** | **T** | **M** | **Y** | **S** | **V** | **F** | **Q** | **N** | **N** | **G** | **T** | **L** | **V** | **Y** | **P** | **K** | **L** | **V** | **L** | **D** | **K** | **E** | **T** | **K** | **K** | **K** | **D** | **N** | **V** | **I** | **S** | **A** | **N** | **A** | **A** | **N** | **T** | **I** | **A** | **T** | **D** | **L** | **L** | **G** | **S** | **V** | **E** | **D** | **P** | **S** | **G** | **Y** | **V** | **Y** | **N** | **M** | **Y** | **N** | **P** | **N** | **F** | **S** | **L** | **A** | **A** | **K** | **T** | **G** | **T** | **A** | **E** | **I** | **K** | **D** | **K** | **Q** | **D** | **T** | **D** | **G** | **K** | **E** | **N** | **S** | **F** | **L** | **L** | **T** | **L** | **D** | **R** | **S** | **N** | **N** | **K** | **F** | **L** | **T** | **M** | **I** | **M** | **V** | **E** | **N** | **S** | **G** | **E** | **N** | **G** | **S** | **A** | **T** | **D** | **I** | **S** | **K** | **P** | **L** | **I** | **D** | **Y** | **L** | **E** | **A** | **T** | **I** | **K** | **-** | **-** | **-** | **-** | **-** | **-** | **-** | **-** | **-** | **-** | **-** | **-** | **-** | **-** | **-** | **-** | **-** | **-** | **-** | **-** | **-** | **-** | **-** | **-** | **-** | **-** | **-** | **-** | **-** | **-** | **-** | **-** | **-** | **-** | **-** | **-** | **Q** | **W** | **Q** | **A** | **K** | **Q** | **E** | **L** | **A** | **E** | **A** | **K** | **K** | **T** | **A** | **T** | **T** | **F** | **L** | **N** | **V** | **L** | **S** | **K** | **Q** | **E** | **F** | **D** | **K** | **L** | **P** | **S** | **V** | **V** | **Q** | **E** | **A** | **S** | **L** | **K** | **K** | **N** | **G** | **Y** | **D** | **T** | **K** | **S** | **V** | **V** | **E** | **K** | **Y** | **Q** | **A** | **I** | **Y** | **S** | **G** | **I** | **Q** | **A** | **E** | **G** | **V** | **K** | **A** | **S** | **D** | **V** | **Q** | **V** | **K** | **K** | **A** | **K** | **D** | **N** | **Q** | **Y** | **T** | **F** | **T** | **Y** | **K** | **L** | **S** | **M** | **S** | **T** | **P** | **L** | **G** | **E** | **M** | **K** | **D** | **L** | **S** | **Y** | **Q** | **S** | **S** | **I** | **A** | **K** | **K** | **G** | **D** | **T** | **Y** | **Q** | **I** | **A** | **W** | **K** | **P** | **S** | **L** | **I** | **F** | **P** | **D** | **M** | **S** | **G** | **N** | **D** | **K** | **I** | **S** | **I** | **Q** | **V** | **D** | **N** | **A** | **K** | **R** | **G** | **E** | **I** | **V** | **D** | **R** | **N** | **G** | **S** | **G** | **L** | **A** | **I** | **N** | **K** | **V** | **F** | **D** | **E** | **V** | **G** | **V** | **V** | **P** | **G** | **K** | **L** | **G** | **S** | **G** | **A** | **E** | **K** | **T** | **A** | **N** | **I** | **K** | **A** | **F** | **S** | **D** | **K** | **F** | **G** | **V** | **S** | **V** | **D** | **E** | **I** | **N** | **Q** | **K** | **L** | **S** | **Q** | **G** | **W** | **V** | **Q** | **A** | **D** | **S** | **F** | **V** | **P** | **I** | **T** | **V** | **A** | **S** | **E** | **P** | **V** | **T** | **E** | **L** | **P** | **T** | **G** | **A** | **A** | **T** | **K** | **D** | **T** | **E** | **S** | **R** | **Y** | **Y** | **P** | **L** | **G** | **E** | **A** | **A** | **A** | **Q** | **L** | **I** | **G** | **Y** | **T** | **G** | **T** | **I** | **T** | **A** | **E** | **D** | **I** | **E** | **K** | **N** | **P** | **E** | **L** | **S** | **S** | **T** | **G** | **V** | **I** | **G** | **K** | **T** | **G** | **L** | **E** | **R** | **A** | **F** | **D** | **K** | **E** | **L** | **R** | **G** | **Q** | **D** | **G** | **G** | **S** | **L** | **V** | **I** | **L** | **D** | **D** | **K** | **E** | **N** | **V** | **K** | **K** | **A** | **L** | **Q** | **T** | **K** | **E** | **K** | **K** | **D** | **G** | **Q** | **T** | **I** | **K** | **L** | **T** | **I** | **D** | **S** | **G** | **V** | **Q** | **Q** | **Q** | **A** | **F** | **A** | **I** | **F** | **D** | **K** | **R** | **P** | **G** | **S** | **A** | **V** | **I** | **T** | **D** | **P** | **Q** | **K** | **G** | **D** | **L** | **L** | **A** | **T** | **V** | **S** | **S** | **P** | **S** | **Y** | **D** | **P** | **N** | **K** | **M** | **A** | **N** | **G** | **I** | **S** | **Q** | **K** | **E** | **Y** | **D** | **A** | **Y** | **N** | **N** | **N** | **K** | **D** | **L** | **P** | **F** | **T** | **A** | **R** | **F** | **A** | **T** | **G** | **Y** | **A** | **P** | **G** | **S** | **T** | **F** | **K** | **T** | **I** | **T** | **G** | **A** | **I** | **G** | **L** | **D** | **A** | **G** | **T** | **L** | **K** | **P** | **D** | **E** | **E** | **L** | **E** | **I** | **N** | **G** | **L** | **K** | **W** | **Q** | **K** | **D** | **K** | **S** | **W** | **G** | **G** | **Y** | **F** | **A** | **T** | **R** | **V** | **K** | **E** | **A** | **S** | **P** | **V** | **N** | **L** | **R** | **T** | **A** | **L** | **V** | **N** | **S** | **D** | **N** | **I** | **Y** | **F** | **A** | **Q** | **Q** | **T** | **L** | **R** | **M** | **G** | **E** | **D** | **K** | **F** | **R** | **A** | **G** | **L** | **N** | **K** | **F** | **I** | **F** | **G** | **E** | **E** | **L** | **D** | **L** | **P** | **I** | **A** | **M** | **T** | **P** | **A** | **Q** | **I** | **S** | **N** | **E** | **D** | **K** | **F** | **N** | **S** | **E** | **I** | **L** | **L** | **A** | **D** | **T** | **G** | **Y** | **G** | **Q** | **G** | **Q** | **L** | **L** | **I** | **S** | **P** | **I** | **Q** | **Q** | **A** | **T** | **M** | **Y** | **S** | **V** | **F** | **Q** | **N** | **N** | **G** | **T** | **L** | **V** | **Y** | **P** | **K** | **L** | **V** | **L** | **D** | **K** | **E** | **T** | **K** | **K** | **K** | **D** | **N** | **V** | **I** | **S** | **A** | **N** | **A** | **A** | **N** | **T** | **I** | **A** | **T** | **D** | **L** | **L** | **G** | **S** | **V** | **E** | **D** | **P** | **S** | **G** | **Y** | **V** | **Y** | **N** | **M** | **Y** | **N** | **P** | **N** | **F** | **S** | **L** | **A** | **A** | **K** | **T** | **G** | **T** | **A** | **E** | **I** | **K** | **D** | **K** | **Q** | **D** | **T** | **D** | **G** | **K** | **E** | **N** | **S** | **F** | **L** | **L** | **T** | **L** | **D** | **R** | **S** | **N** | **N** | **K** | **F** | **L** | **T** | **M** | **I** | **M** | **V** | **E** | **N** | **S** | **G** | **E** | **N** | **G** | **S** | **A** | **T** | **D** | **I** | **S** | **K** | **P** | **L** | **I** | **D** | **Y** | **L** | **E** | **A** | **T** | **I** | **K** |
| **N59689F** | **-** | **-** | **-** | **-** | **-** | **-** | **-** | **-** | **-** | **-** | **-** | **-** | **-** | **-** | **-** | **-** | **-** | **-** | **-** | **-** | **-** | **-** | **-** | **-** | **-** | **-** | **-** | **-** | **-** | **-** | **-** | **-** | **-** | **-** | **-** | **-** | **Q** | **W** | **Q** | **A** | **K** | **Q** | **E** | **L** | **A** | **E** | **A** | **K** | **K** | **T** | **A** | **T** | **T** | **F** | **L** | **N** | **V** | **L** | **S** | **K** | **Q** | **E** | **F** | **D** | **K** | **L** | **P** | **S** | **V** | **V** | **Q** | **E** | **A** | **S** | **L** | **K** | **K** | **N** | **G** | **Y** | **D** | **T** | **K** | **S** | **V** | **V** | **E** | **K** | **Y** | **Q** | **A** | **I** | **Y** | **S** | **G** | **I** | **Q** | **A** | **E** | **G** | **V** | **K** | **A** | **S** | **D** | **V** | **Q** | **V** | **K** | **K** | **A** | **K** | **D** | **N** | **Q** | **Y** | **T** | **F** | **T** | **Y** | **K** | **L** | **S** | **M** | **S** | **T** | **P** | **L** | **G** | **E** | **M** | **K** | **D** | **L** | **S** | **Y** | **Q** | **S** | **S** | **I** | **A** | **K** | **K** | **G** | **D** | **T** | **Y** | **Q** | **I** | **A** | **W** | **K** | **P** | **S** | **L** | **I** | **F** | **P** | **D** | **M** | **S** | **G** | **N** | **D** | **K** | **I** | **S** | **I** | **Q** | **V** | **D** | **N** | **A** | **K** | **R** | **G** | **E** | **I** | **V** | **D** | **R** | **N** | **G** | **S** | **G** | **L** | **A** | **I** | **N** | **K** | **V** | **F** | **D** | **E** | **V** | **G** | **V** | **V** | **P** | **G** | **K** | **L** | **G** | **S** | **G** | **A** | **E** | **K** | **T** | **A** | **N** | **I** | **K** | **A** | **F** | **S** | **D** | **K** | **F** | **G** | **V** | **S** | **V** | **D** | **E** | **I** | **N** | **Q** | **K** | **L** | **S** | **Q** | **G** | **W** | **V** | **Q** | **A** | **D** | **S** | **F** | **V** | **P** | **I** | **T** | **V** | **A** | **S** | **E** | **P** | **V** | **T** | **E** | **L** | **P** | **T** | **G** | **A** | **A** | **T** | **K** | **D** | **T** | **E** | **S** | **R** | **Y** | **Y** | **P** | **L** | **G** | **E** | **A** | **A** | **A** | **Q** | **L** | **I** | **G** | **Y** | **T** | **G** | **T** | **I** | **T** | **A** | **E** | **D** | **I** | **E** | **K** | **N** | **P** | **E** | **L** | **S** | **S** | **T** | **G** | **V** | **I** | **G** | **K** | **T** | **G** | **L** | **E** | **R** | **A** | **F** | **D** | **K** | **E** | **L** | **R** | **G** | **Q** | **D** | **G** | **G** | **S** | **L** | **V** | **I** | **L** | **D** | **D** | **K** | **E** | **N** | **V** | **K** | **K** | **A** | **L** | **Q** | **T** | **K** | **E** | **K** | **K** | **D** | **G** | **Q** | **T** | **I** | **K** | **L** | **T** | **I** | **D** | **S** | **G** | **V** | **Q** | **Q** | **Q** | **A** | **F** | **A** | **I** | **F** | **D** | **K** | **R** | **P** | **G** | **S** | **A** | **V** | **I** | **T** | **D** | **P** | **Q** | **K** | **G** | **D** | **L** | **L** | **A** | **T** | **V** | **S** | **S** | **P** | **S** | **Y** | **D** | **P** | **N** | **K** | **M** | **A** | **N** | **G** | **I** | **S** | **Q** | **K** | **E** | **Y** | **D** | **A** | **Y** | **N** | **N** | **N** | **K** | **D** | **L** | **P** | **F** | **T** | **A** | **R** | **F** | **A** | **T** | **G** | **Y** | **A** | **P** | **G** | **S** | **T** | **F** | **K** | **T** | **I** | **T** | **G** | **A** | **I** | **G** | **L** | **D** | **A** | **G** | **T** | **L** | **K** | **P** | **D** | **E** | **E** | **L** | **E** | **I** | **N** | **G** | **L** | **K** | **W** | **Q** | **K** | **D** | **K** | **S** | **W** | **G** | **G** | **Y** | **F** | **A** | **T** | **R** | **V** | **K** | **E** | **A** | **S** | **P** | **V** | **N** | **L** | **R** | **T** | **A** | **L** | **V** | **N** | **S** | **D** | **N** | **I** | **Y** | **F** | **A** | **Q** | **Q** | **T** | **L** | **R** | **M** | **G** | **E** | **D** | **K** | **F** | **R** | **A** | **G** | **L** | **N** | **K** | **F** | **I** | **F** | **G** | **E** | **E** | **L** | **D** | **L** | **P** | **I** | **A** | **M** | **T** | **P** | **A** | **Q** | **I** | **S** | **N** | **E** | **D** | **K** | **F** | **N** | **S** | **E** | **I** | **L** | **L** | **A** | **D** | **T** | **G** | **Y** | **G** | **Q** | **G** | **Q** | **L** | **L** | **I** | **S** | **P** | **I** | **Q** | **Q** | **A** | **T** | **M** | **Y** | **S** | **V** | **F** | **Q** | **N** | **N** | **G** | **T** | **L** | **V** | **Y** | **P** | **K** | **L** | **V** | **L** | **D** | **K** | **E** | **T** | **K** | **K** | **K** | **D** | **N** | **V** | **I** | **S** | **A** | **N** | **A** | **A** | **N** | **T** | **I** | **A** | **T** | **D** | **L** | **L** | **G** | **S** | **V** | **E** | **D** | **P** | **S** | **G** | **Y** | **V** | **Y** | **N** | **M** | **Y** | **N** | **P** | **N** | **F** | **S** | **L** | **A** | **A** | **K** | **T** | **G** | **T** | **A** | **E** | **I** | **K** | **D** | **K** | **Q** | **D** | **T** | **D** | **G** | **K** | **E** | **N** | **S** | **F** | **L** | **L** | **T** | **L** | **D** | **R** | **S** | **N** | **N** | **K** | **F** | **L** | **T** | **M** | **I** | **M** | **V** | **E** | **N** | **S** | **G** | **E** | **N** | **G** | **S** | **A** | **T** | **D** | **I** | **S** | **K** | **P** | **L** | **I** | **D** | **Y** | **L** | **E** | **A** | **T** | **I** | **K** | **-** | **-** | **-** | **-** | **-** | **-** | **-** | **-** | **-** | **-** | **-** | **-** | **-** | **-** | **-** | **-** | **-** | **-** | **-** | **-** | **-** | **-** | **-** | **-** | **-** | **-** | **-** | **-** | **-** | **-** | **-** | **-** | **-** | **-** | **-** | **-** | **Q** | **W** | **Q** | **A** | **K** | **Q** | **E** | **L** | **A** | **E** | **A** | **K** | **K** | **T** | **A** | **T** | **T** | **F** | **L** | **N** | **V** | **L** | **S** | **K** | **Q** | **E** | **F** | **D** | **K** | **L** | **P** | **S** | **V** | **V** | **Q** | **E** | **A** | **S** | **L** | **K** | **K** | **N** | **G** | **Y** | **D** | **T** | **K** | **S** | **V** | **V** | **E** | **K** | **Y** | **Q** | **A** | **I** | **Y** | **S** | **G** | **I** | **Q** | **A** | **E** | **G** | **V** | **K** | **A** | **S** | **D** | **V** | **Q** | **V** | **K** | **K** | **A** | **K** | **D** | **N** | **Q** | **Y** | **T** | **F** | **T** | **Y** | **K** | **L** | **S** | **M** | **S** | **T** | **P** | **L** | **G** | **E** | **M** | **K** | **D** | **L** | **S** | **Y** | **Q** | **S** | **S** | **I** | **A** | **K** | **K** | **G** | **D** | **T** | **Y** | **Q** | **I** | **A** | **W** | **K** | **P** | **S** | **L** | **I** | **F** | **P** | **D** | **M** | **S** | **G** | **N** | **D** | **K** | **I** | **S** | **I** | **Q** | **V** | **D** | **N** | **A** | **K** | **R** | **G** | **E** | **I** | **V** | **D** | **R** | **N** | **G** | **S** | **G** | **L** | **A** | **I** | **N** | **K** | **V** | **F** | **D** | **E** | **V** | **G** | **V** | **V** | **P** | **G** | **K** | **L** | **G** | **S** | **G** | **A** | **E** | **K** | **T** | **A** | **N** | **I** | **K** | **A** | **F** | **S** | **D** | **K** | **F** | **G** | **V** | **S** | **V** | **D** | **E** | **I** | **N** | **Q** | **K** | **L** | **S** | **Q** | **G** | **W** | **V** | **Q** | **A** | **D** | **S** | **F** | **V** | **P** | **I** | **T** | **V** | **A** | **S** | **E** | **P** | **V** | **T** | **E** | **L** | **P** | **T** | **G** | **A** | **A** | **T** | **K** | **D** | **T** | **E** | **S** | **R** | **Y** | **Y** | **P** | **L** | **G** | **E** | **A** | **A** | **A** | **Q** | **L** | **I** | **G** | **Y** | **T** | **G** | **T** | **I** | **T** | **A** | **E** | **D** | **I** | **E** | **K** | **N** | **P** | **E** | **L** | **S** | **S** | **T** | **G** | **V** | **I** | **G** | **K** | **T** | **G** | **L** | **E** | **R** | **A** | **F** | **D** | **K** | **E** | **L** | **R** | **G** | **Q** | **D** | **G** | **G** | **S** | **L** | **V** | **I** | **L** | **D** | **D** | **K** | **E** | **N** | **V** | **K** | **K** | **A** | **L** | **Q** | **T** | **K** | **E** | **K** | **K** | **D** | **G** | **Q** | **T** | **I** | **K** | **L** | **T** | **I** | **D** | **S** | **G** | **V** | **Q** | **Q** | **Q** | **A** | **F** | **A** | **I** | **F** | **D** | **K** | **R** | **P** | **G** | **S** | **A** | **V** | **I** | **T** | **D** | **P** | **Q** | **K** | **G** | **D** | **L** | **L** | **A** | **T** | **V** | **S** | **S** | **P** | **S** | **Y** | **D** | **P** | **N** | **K** | **M** | **A** | **N** | **G** | **I** | **S** | **Q** | **K** | **E** | **Y** | **D** | **A** | **Y** | **N** | **N** | **N** | **K** | **D** | **L** | **P** | **F** | **T** | **A** | **R** | **F** | **A** | **T** | **G** | **Y** | **A** | **P** | **G** | **S** | **T** | **F** | **K** | **T** | **I** | **T** | **G** | **A** | **I** | **G** | **L** | **D** | **A** | **G** | **T** | **L** | **K** | **P** | **D** | **E** | **E** | **L** | **E** | **I** | **N** | **G** | **L** | **K** | **W** | **Q** | **K** | **D** | **K** | **S** | **W** | **G** | **G** | **Y** | **F** | **A** | **T** | **R** | **V** | **K** | **E** | **A** | **S** | **P** | **V** | **N** | **L** | **R** | **T** | **A** | **L** | **V** | **N** | **S** | **D** | **N** | **I** | **Y** | **F** | **A** | **Q** | **Q** | **T** | **L** | **R** | **M** | **G** | **E** | **D** | **K** | **F** | **R** | **A** | **G** | **L** | **N** | **K** | **F** | **I** | **F** | **G** | **E** | **E** | **L** | **D** | **L** | **P** | **I** | **A** | **M** | **T** | **P** | **A** | **Q** | **I** | **S** | **N** | **E** | **D** | **K** | **F** | **N** | **S** | **E** | **I** | **L** | **L** | **A** | **D** | **T** | **G** | **Y** | **G** | **Q** | **G** | **Q** | **L** | **L** | **I** | **S** | **P** | **I** | **Q** | **Q** | **A** | **T** | **M** | **Y** | **S** | **V** | **F** | **Q** | **N** | **N** | **G** | **T** | **L** | **V** | **Y** | **P** | **K** | **L** | **V** | **L** | **D** | **K** | **E** | **T** | **K** | **K** | **K** | **D** | **N** | **V** | **I** | **S** | **A** | **N** | **A** | **A** | **N** | **T** | **I** | **A** | **T** | **D** | **L** | **L** | **G** | **S** | **V** | **E** | **D** | **P** | **S** | **G** | **Y** | **V** | **Y** | **N** | **M** | **Y** | **N** | **P** | **N** | **F** | **S** | **L** | **A** | **A** | **K** | **T** | **G** | **T** | **A** | **E** | **I** | **K** | **D** | **K** | **Q** | **D** | **T** | **D** | **G** | **K** | **E** | **N** | **S** | **F** | **L** | **L** | **T** | **L** | **D** | **R** | **S** | **N** | **N** | **K** | **F** | **L** | **T** | **M** | **I** | **M** | **V** | **E** | **N** | **S** | **G** | **E** | **N** | **G** | **S** | **A** | **T** | **D** | **I** | **S** | **K** | **P** | **L** | **I** | **D** | **Y** | **L** | **E** | **A** | **T** | **I** | **K** |
| **N54121** | **-** | **-** | **-** | **-** | **-** | **-** | **-** | **-** | **-** | **-** | **-** | **-** | **-** | **-** | **-** | **-** | **-** | **-** | **-** | **-** | **-** | **-** | **-** | **-** | **-** | **-** | **-** | **-** | **-** | **-** | **-** | **-** | **-** | **-** | **-** | **-** | **Q** | **W** | **Q** | **A** | **K** | **Q** | **E** | **L** | **A** | **E** | **A** | **K** | **K** | **T** | **A** | **T** | **T** | **F** | **L** | **N** | **V** | **L** | **T** | **K** | **Q** | **K** | **F** | **D** | **K** | **L** | **P** | **S** | **V** | **V** | **Q** | **E** | **A** | **S** | **L** | **K** | **K** | **N** | **G** | **Y** | **D** | **T** | **K** | **S** | **V** | **V** | **E** | **K** | **Y** | **Q** | **A** | **I** | **Y** | **S** | **G** | **I** | **Q** | **A** | **E** | **G** | **V** | **K** | **A** | **S** | **D** | **V** | **Q** | **V** | **K** | **K** | **A** | **K** | **D** | **N** | **Q** | **Y** | **T** | **F** | **T** | **Y** | **K** | **L** | **S** | **M** | **S** | **T** | **P** | **L** | **G** | **E** | **M** | **K** | **D** | **L** | **S** | **Y** | **Q** | **S** | **S** | **I** | **A** | **K** | **K** | **G** | **D** | **T** | **Y** | **Q** | **I** | **A** | **W** | **K** | **P** | **S** | **L** | **I** | **F** | **P** | **D** | **M** | **S** | **G** | **N** | **D** | **K** | **I** | **S** | **I** | **Q** | **V** | **D** | **N** | **A** | **K** | **R** | **G** | **E** | **I** | **V** | **D** | **R** | **N** | **G** | **S** | **G** | **L** | **A** | **I** | **N** | **K** | **V** | **F** | **D** | **E** | **V** | **G** | **V** | **V** | **P** | **G** | **K** | **L** | **G** | **S** | **G** | **A** | **E** | **K** | **T** | **A** | **N** | **I** | **K** | **A** | **F** | **S** | **D** | **K** | **F** | **G** | **V** | **S** | **V** | **D** | **E** | **I** | **N** | **Q** | **K** | **L** | **S** | **Q** | **G** | **W** | **V** | **Q** | **A** | **D** | **S** | **F** | **V** | **P** | **I** | **T** | **V** | **A** | **S** | **E** | **P** | **V** | **T** | **E** | **L** | **P** | **T** | **G** | **A** | **A** | **T** | **K** | **D** | **T** | **E** | **S** | **R** | **Y** | **Y** | **P** | **L** | **G** | **E** | **A** | **A** | **A** | **Q** | **L** | **I** | **G** | **Y** | **T** | **G** | **T** | **I** | **T** | **A** | **E** | **D** | **I** | **K** | **K** | **N** | **P** | **E** | **L** | **S** | **S** | **T** | **G** | **V** | **I** | **G** | **K** | **T** | **G** | **L** | **E** | **R** | **A** | **F** | **D** | **K** | **E** | **L** | **R** | **G** | **Q** | **D** | **G** | **G** | **S** | **L** | **V** | **I** | **L** | **D** | **D** | **K** | **E** | **N** | **V** | **K** | **K** | **A** | **L** | **Q** | **T** | **K** | **E** | **K** | **K** | **D** | **G** | **Q** | **T** | **I** | **K** | **L** | **T** | **I** | **D** | **S** | **G** | **V** | **Q** | **Q** | **Q** | **A** | **F** | **A** | **I** | **F** | **D** | **K** | **R** | **P** | **G** | **S** | **A** | **V** | **I** | **T** | **D** | **P** | **Q** | **K** | **G** | **D** | **L** | **L** | **A** | **T** | **V** | **S** | **S** | **P** | **S** | **Y** | **D** | **P** | **N** | **K** | **M** | **A** | **N** | **G** | **I** | **S** | **Q** | **K** | **E** | **Y** | **D** | **A** | **Y** | **N** | **N** | **N** | **K** | **D** | **L** | **P** | **F** | **T** | **A** | **R** | **F** | **A** | **T** | **G** | **Y** | **A** | **P** | **G** | **S** | **T** | **F** | **K** | **T** | **I** | **T** | **G** | **A** | **I** | **G** | **L** | **D** | **T** | **G** | **T** | **L** | **K** | **P** | **D** | **E** | **E** | **L** | **E** | **I** | **N** | **G** | **L** | **K** | **W** | **Q** | **K** | **D** | **K** | **S** | **W** | **G** | **G** | **Y** | **F** | **A** | **T** | **R** | **V** | **K** | **E** | **A** | **S** | **P** | **V** | **N** | **L** | **R** | **T** | **A** | **L** | **V** | **N** | **S** | **D** | **N** | **I** | **Y** | **F** | **A** | **Q** | **Q** | **T** | **L** | **R** | **M** | **G** | **E** | **D** | **K** | **F** | **R** | **A** | **G** | **L** | **N** | **K** | **F** | **I** | **F** | **G** | **E** | **E** | **L** | **D** | **L** | **P** | **I** | **A** | **M** | **T** | **P** | **A** | **Q** | **I** | **S** | **N** | **E** | **D** | **K** | **F** | **N** | **S** | **E** | **I** | **L** | **L** | **A** | **D** | **T** | **G** | **Y** | **G** | **Q** | **G** | **Q** | **L** | **L** | **I** | **S** | **P** | **I** | **Q** | **Q** | **A** | **T** | **M** | **Y** | **S** | **V** | **F** | **Q** | **N** | **N** | **G** | **T** | **L** | **V** | **Y** | **P** | **K** | **L** | **V** | **L** | **D** | **K** | **E** | **T** | **K** | **K** | **K** | **D** | **N** | **V** | **I** | **S** | **A** | **N** | **A** | **A** | **N** | **T** | **I** | **A** | **T** | **D** | **L** | **L** | **G** | **S** | **V** | **E** | **D** | **P** | **S** | **G** | **Y** | **V** | **Y** | **N** | **M** | **Y** | **N** | **P** | **N** | **F** | **S** | **L** | **A** | **A** | **K** | **T** | **G** | **T** | **A** | **E** | **I** | **K** | **D** | **K** | **Q** | **D** | **T** | **D** | **G** | **K** | **E** | **N** | **S** | **F** | **L** | **L** | **T** | **L** | **D** | **R** | **S** | **N** | **N** | **K** | **F** | **L** | **T** | **M** | **I** | **M** | **V** | **E** | **N** | **S** | **G** | **E** | **N** | **G** | **S** | **A** | **T** | **D** | **I** | **S** | **K** | **P** | **L** | **I** | **D** | **Y** | **L** | **E** | **A** | **T** | **I** | **K** | **-** | **-** | **-** | **-** | **-** | **-** | **-** | **-** | **-** | **-** | **-** | **-** | **-** | **-** | **-** | **-** | **-** | **-** | **-** | **-** | **-** | **-** | **-** | **-** | **-** | **-** | **-** | **-** | **-** | **-** | **-** | **-** | **-** | **-** | **-** | **-** | **Q** | **W** | **Q** | **A** | **K** | **Q** | **E** | **L** | **A** | **E** | **A** | **K** | **K** | **T** | **A** | **T** | **T** | **F** | **L** | **N** | **V** | **L** | **T** | **K** | **Q** | **K** | **F** | **D** | **K** | **L** | **P** | **S** | **V** | **V** | **Q** | **E** | **A** | **S** | **L** | **K** | **K** | **N** | **G** | **Y** | **D** | **T** | **K** | **S** | **V** | **V** | **E** | **K** | **Y** | **Q** | **A** | **I** | **Y** | **S** | **G** | **I** | **Q** | **A** | **E** | **G** | **V** | **K** | **A** | **S** | **D** | **V** | **Q** | **V** | **K** | **K** | **A** | **K** | **D** | **N** | **Q** | **Y** | **T** | **F** | **T** | **Y** | **K** | **L** | **S** | **M** | **S** | **T** | **P** | **L** | **G** | **E** | **M** | **K** | **D** | **L** | **S** | **Y** | **Q** | **S** | **S** | **I** | **A** | **K** | **K** | **G** | **D** | **T** | **Y** | **Q** | **I** | **A** | **W** | **K** | **P** | **S** | **L** | **I** | **F** | **P** | **D** | **M** | **S** | **G** | **N** | **D** | **K** | **I** | **S** | **I** | **Q** | **V** | **D** | **N** | **A** | **K** | **R** | **G** | **E** | **I** | **V** | **D** | **R** | **N** | **G** | **S** | **G** | **L** | **A** | **I** | **N** | **K** | **V** | **F** | **D** | **E** | **V** | **G** | **V** | **V** | **P** | **G** | **K** | **L** | **G** | **S** | **G** | **A** | **E** | **K** | **T** | **A** | **N** | **I** | **K** | **A** | **F** | **S** | **D** | **K** | **F** | **G** | **V** | **S** | **V** | **D** | **E** | **I** | **N** | **Q** | **K** | **L** | **S** | **Q** | **G** | **W** | **V** | **Q** | **A** | **D** | **S** | **F** | **V** | **P** | **I** | **T** | **V** | **A** | **S** | **E** | **P** | **V** | **T** | **E** | **L** | **P** | **T** | **G** | **A** | **A** | **T** | **K** | **D** | **T** | **E** | **S** | **R** | **Y** | **Y** | **P** | **L** | **G** | **E** | **A** | **A** | **A** | **Q** | **L** | **I** | **G** | **Y** | **T** | **G** | **T** | **I** | **T** | **A** | **E** | **D** | **I** | **K** | **K** | **N** | **P** | **E** | **L** | **S** | **S** | **T** | **G** | **V** | **I** | **G** | **K** | **T** | **G** | **L** | **E** | **R** | **A** | **F** | **D** | **K** | **E** | **L** | **R** | **G** | **Q** | **D** | **G** | **G** | **S** | **L** | **V** | **I** | **L** | **D** | **D** | **K** | **E** | **N** | **V** | **K** | **K** | **A** | **L** | **Q** | **T** | **K** | **E** | **K** | **K** | **D** | **G** | **Q** | **T** | **I** | **K** | **L** | **T** | **I** | **D** | **S** | **G** | **V** | **Q** | **Q** | **Q** | **A** | **F** | **A** | **I** | **F** | **D** | **K** | **R** | **P** | **G** | **S** | **A** | **V** | **I** | **T** | **D** | **P** | **Q** | **K** | **G** | **D** | **L** | **L** | **A** | **T** | **V** | **S** | **S** | **P** | **S** | **Y** | **D** | **P** | **N** | **K** | **M** | **A** | **N** | **G** | **I** | **S** | **Q** | **K** | **E** | **Y** | **D** | **A** | **Y** | **N** | **N** | **N** | **K** | **D** | **L** | **P** | **F** | **T** | **A** | **R** | **F** | **A** | **T** | **G** | **Y** | **A** | **P** | **G** | **S** | **T** | **F** | **K** | **T** | **I** | **T** | **G** | **A** | **I** | **G** | **L** | **D** | **T** | **G** | **T** | **L** | **K** | **P** | **D** | **E** | **E** | **L** | **E** | **I** | **N** | **G** | **L** | **K** | **W** | **Q** | **K** | **D** | **K** | **S** | **W** | **G** | **G** | **Y** | **F** | **A** | **T** | **R** | **V** | **K** | **E** | **A** | **S** | **P** | **V** | **N** | **L** | **R** | **T** | **A** | **L** | **V** | **N** | **S** | **D** | **N** | **I** | **Y** | **F** | **A** | **Q** | **Q** | **T** | **L** | **R** | **M** | **G** | **E** | **D** | **K** | **F** | **R** | **A** | **G** | **L** | **N** | **K** | **F** | **I** | **F** | **G** | **E** | **E** | **L** | **D** | **L** | **P** | **I** | **A** | **M** | **T** | **P** | **A** | **Q** | **I** | **S** | **N** | **E** | **D** | **K** | **F** | **N** | **S** | **E** | **I** | **L** | **L** | **A** | **D** | **T** | **G** | **Y** | **G** | **Q** | **G** | **Q** | **L** | **L** | **I** | **S** | **P** | **I** | **Q** | **Q** | **A** | **T** | **M** | **Y** | **S** | **V** | **F** | **Q** | **N** | **N** | **G** | **T** | **L** | **V** | **Y** | **P** | **K** | **L** | **V** | **L** | **D** | **K** | **E** | **T** | **K** | **K** | **K** | **D** | **N** | **V** | **I** | **S** | **A** | **N** | **A** | **A** | **N** | **T** | **I** | **A** | **T** | **D** | **L** | **L** | **G** | **S** | **V** | **E** | **D** | **P** | **S** | **G** | **Y** | **V** | **Y** | **N** | **M** | **Y** | **N** | **P** | **N** | **F** | **S** | **L** | **A** | **A** | **K** | **T** | **G** | **T** | **A** | **E** | **I** | **K** | **D** | **K** | **Q** | **D** | **T** | **D** | **G** | **K** | **E** | **N** | **S** | **F** | **L** | **L** | **T** | **L** | **D** | **R** | **S** | **N** | **N** | **K** | **F** | **L** | **T** | **M** | **I** | **M** | **V** | **E** | **N** | **S** | **G** | **E** | **N** | **G** | **S** | **A** | **T** | **D** | **I** | **S** | **K** | **P** | **L** | **I** | **D** | **Y** | **L** | **E** | **A** | **T** | **I** | **K** |
| **N59836F** | **-** | **-** | **-** | **-** | **-** | **-** | **-** | **-** | **-** | **-** | **-** | **-** | **-** | **-** | **-** | **-** | **-** | **-** | **-** | **-** | **-** | **-** | **-** | **-** | **-** | **-** | **-** | **-** | **-** | **-** | **-** | **-** | **-** | **-** | **-** | **-** | **Q** | **W** | **Q** | **A** | **K** | **Q** | **E** | **L** | **A** | **E** | **A** | **K** | **K** | **T** | **A** | **T** | **T** | **F** | **L** | **N** | **V** | **L** | **S** | **K** | **Q** | **E** | **F** | **D** | **K** | **L** | **P** | **S** | **V** | **V** | **Q** | **E** | **A** | **S** | **L** | **K** | **K** | **N** | **G** | **Y** | **D** | **T** | **K** | **S** | **V** | **V** | **E** | **K** | **Y** | **Q** | **A** | **I** | **Y** | **S** | **G** | **I** | **Q** | **A** | **E** | **G** | **V** | **K** | **A** | **S** | **D** | **V** | **Q** | **V** | **K** | **K** | **A** | **K** | **D** | **N** | **Q** | **Y** | **T** | **F** | **T** | **Y** | **K** | **L** | **S** | **M** | **S** | **T** | **P** | **L** | **G** | **E** | **M** | **K** | **D** | **L** | **S** | **Y** | **Q** | **S** | **S** | **I** | **A** | **K** | **K** | **G** | **D** | **T** | **Y** | **Q** | **I** | **A** | **W** | **K** | **P** | **S** | **L** | **I** | **F** | **P** | **D** | **M** | **S** | **G** | **N** | **D** | **K** | **I** | **S** | **I** | **Q** | **V** | **D** | **N** | **A** | **K** | **R** | **G** | **E** | **I** | **V** | **D** | **R** | **N** | **G** | **S** | **G** | **L** | **A** | **I** | **N** | **K** | **V** | **F** | **D** | **E** | **V** | **G** | **V** | **V** | **P** | **G** | **K** | **L** | **G** | **S** | **G** | **A** | **E** | **K** | **T** | **A** | **N** | **I** | **K** | **A** | **F** | **S** | **D** | **K** | **F** | **G** | **V** | **S** | **V** | **D** | **E** | **I** | **N** | **Q** | **K** | **L** | **S** | **Q** | **G** | **W** | **V** | **Q** | **A** | **D** | **S** | **F** | **V** | **P** | **I** | **T** | **V** | **A** | **S** | **E** | **P** | **V** | **T** | **E** | **L** | **P** | **T** | **G** | **A** | **A** | **T** | **K** | **D** | **T** | **E** | **S** | **R** | **Y** | **Y** | **P** | **L** | **G** | **E** | **A** | **A** | **A** | **Q** | **L** | **I** | **G** | **Y** | **T** | **G** | **T** | **I** | **T** | **A** | **E** | **D** | **I** | **E** | **K** | **N** | **P** | **E** | **L** | **S** | **S** | **T** | **G** | **V** | **I** | **G** | **K** | **T** | **G** | **L** | **E** | **R** | **A** | **F** | **D** | **K** | **E** | **L** | **R** | **G** | **Q** | **D** | **G** | **G** | **S** | **L** | **V** | **I** | **L** | **D** | **D** | **K** | **E** | **N** | **V** | **K** | **K** | **A** | **L** | **Q** | **T** | **K** | **E** | **K** | **K** | **D** | **G** | **Q** | **T** | **I** | **K** | **L** | **T** | **I** | **D** | **S** | **G** | **V** | **Q** | **Q** | **Q** | **A** | **F** | **A** | **I** | **F** | **D** | **K** | **R** | **P** | **G** | **S** | **A** | **V** | **I** | **T** | **D** | **P** | **Q** | **K** | **G** | **D** | **L** | **L** | **A** | **T** | **V** | **S** | **S** | **P** | **S** | **Y** | **D** | **P** | **N** | **K** | **M** | **A** | **N** | **G** | **I** | **S** | **Q** | **K** | **E** | **Y** | **D** | **A** | **Y** | **N** | **N** | **N** | **K** | **D** | **L** | **P** | **F** | **T** | **A** | **R** | **F** | **A** | **T** | **G** | **Y** | **A** | **P** | **G** | **S** | **T** | **F** | **K** | **T** | **I** | **T** | **G** | **A** | **I** | **G** | **L** | **D** | **A** | **G** | **T** | **L** | **K** | **P** | **D** | **E** | **E** | **L** | **E** | **I** | **N** | **G** | **L** | **K** | **W** | **Q** | **K** | **D** | **K** | **S** | **W** | **G** | **G** | **Y** | **F** | **A** | **T** | **R** | **V** | **K** | **E** | **A** | **S** | **P** | **V** | **N** | **L** | **R** | **T** | **A** | **L** | **V** | **N** | **S** | **D** | **N** | **I** | **Y** | **F** | **A** | **Q** | **Q** | **T** | **L** | **R** | **M** | **G** | **E** | **D** | **K** | **F** | **R** | **A** | **G** | **L** | **N** | **K** | **F** | **I** | **F** | **G** | **E** | **E** | **L** | **D** | **L** | **P** | **I** | **A** | **M** | **T** | **P** | **A** | **Q** | **I** | **S** | **N** | **E** | **D** | **K** | **F** | **N** | **S** | **E** | **I** | **L** | **L** | **A** | **D** | **T** | **G** | **Y** | **G** | **Q** | **G** | **Q** | **L** | **L** | **I** | **S** | **P** | **I** | **Q** | **Q** | **A** | **T** | **M** | **Y** | **S** | **V** | **F** | **Q** | **N** | **N** | **G** | **T** | **L** | **V** | **Y** | **P** | **K** | **L** | **V** | **L** | **D** | **K** | **E** | **T** | **K** | **K** | **K** | **D** | **N** | **V** | **I** | **S** | **A** | **N** | **A** | **A** | **N** | **T** | **I** | **A** | **T** | **D** | **L** | **L** | **G** | **S** | **V** | **E** | **D** | **P** | **S** | **G** | **Y** | **V** | **Y** | **N** | **M** | **Y** | **N** | **P** | **N** | **F** | **S** | **L** | **A** | **A** | **K** | **T** | **G** | **T** | **A** | **E** | **I** | **K** | **D** | **K** | **Q** | **D** | **T** | **D** | **G** | **K** | **E** | **N** | **S** | **F** | **L** | **L** | **T** | **L** | **D** | **R** | **S** | **N** | **N** | **K** | **F** | **L** | **T** | **M** | **I** | **M** | **V** | **E** | **N** | **S** | **G** | **E** | **N** | **G** | **S** | **A** | **T** | **D** | **I** | **S** | **K** | **P** | **L** | **I** | **D** | **Y** | **L** | **E** | **A** | **T** | **I** | **K** | **-** | **-** | **-** | **-** | **-** | **-** | **-** | **-** | **-** | **-** | **-** | **-** | **-** | **-** | **-** | **-** | **-** | **-** | **-** | **-** | **-** | **-** | **-** | **-** | **-** | **-** | **-** | **-** | **-** | **-** | **-** | **-** | **-** | **-** | **-** | **-** | **Q** | **W** | **Q** | **A** | **K** | **Q** | **E** | **L** | **A** | **E** | **A** | **K** | **K** | **T** | **A** | **T** | **T** | **F** | **L** | **N** | **V** | **L** | **S** | **K** | **Q** | **E** | **F** | **D** | **K** | **L** | **P** | **S** | **V** | **V** | **Q** | **E** | **A** | **S** | **L** | **K** | **K** | **N** | **G** | **Y** | **D** | **T** | **K** | **S** | **V** | **V** | **E** | **K** | **Y** | **Q** | **A** | **I** | **Y** | **S** | **G** | **I** | **Q** | **A** | **E** | **G** | **V** | **K** | **A** | **S** | **D** | **V** | **Q** | **V** | **K** | **K** | **A** | **K** | **D** | **N** | **Q** | **Y** | **T** | **F** | **T** | **Y** | **K** | **L** | **S** | **M** | **S** | **T** | **P** | **L** | **G** | **E** | **M** | **K** | **D** | **L** | **S** | **Y** | **Q** | **S** | **S** | **I** | **A** | **K** | **K** | **G** | **D** | **T** | **Y** | **Q** | **I** | **A** | **W** | **K** | **P** | **S** | **L** | **I** | **F** | **P** | **D** | **M** | **S** | **G** | **N** | **D** | **K** | **I** | **S** | **I** | **Q** | **V** | **D** | **N** | **A** | **K** | **R** | **G** | **E** | **I** | **V** | **D** | **R** | **N** | **G** | **S** | **G** | **L** | **A** | **I** | **N** | **K** | **V** | **F** | **D** | **E** | **V** | **G** | **V** | **V** | **P** | **G** | **K** | **L** | **G** | **S** | **G** | **A** | **E** | **K** | **T** | **A** | **N** | **I** | **K** | **A** | **F** | **S** | **D** | **K** | **F** | **G** | **V** | **S** | **V** | **D** | **E** | **I** | **N** | **Q** | **K** | **L** | **S** | **Q** | **G** | **W** | **V** | **Q** | **A** | **D** | **S** | **F** | **V** | **P** | **I** | **T** | **V** | **A** | **S** | **E** | **P** | **V** | **T** | **E** | **L** | **P** | **T** | **G** | **A** | **A** | **T** | **K** | **D** | **T** | **E** | **S** | **R** | **Y** | **Y** | **P** | **L** | **G** | **E** | **A** | **A** | **A** | **Q** | **L** | **I** | **G** | **Y** | **T** | **G** | **T** | **I** | **T** | **A** | **E** | **D** | **I** | **E** | **K** | **N** | **P** | **E** | **L** | **S** | **S** | **T** | **G** | **V** | **I** | **G** | **K** | **T** | **G** | **L** | **E** | **R** | **A** | **F** | **D** | **K** | **E** | **L** | **R** | **G** | **Q** | **D** | **G** | **G** | **S** | **L** | **V** | **I** | **L** | **D** | **D** | **K** | **E** | **N** | **V** | **K** | **K** | **A** | **L** | **Q** | **T** | **K** | **E** | **K** | **K** | **D** | **G** | **Q** | **T** | **I** | **K** | **L** | **T** | **I** | **D** | **S** | **G** | **V** | **Q** | **Q** | **Q** | **A** | **F** | **A** | **I** | **F** | **D** | **K** | **R** | **P** | **G** | **S** | **A** | **V** | **I** | **T** | **D** | **P** | **Q** | **K** | **G** | **D** | **L** | **L** | **A** | **T** | **V** | **S** | **S** | **P** | **S** | **Y** | **D** | **P** | **N** | **K** | **M** | **A** | **N** | **G** | **I** | **S** | **Q** | **K** | **E** | **Y** | **D** | **A** | **Y** | **N** | **N** | **N** | **K** | **D** | **L** | **P** | **F** | **T** | **A** | **R** | **F** | **A** | **T** | **G** | **Y** | **A** | **P** | **G** | **S** | **T** | **F** | **K** | **T** | **I** | **T** | **G** | **A** | **I** | **G** | **L** | **D** | **A** | **G** | **T** | **L** | **K** | **P** | **D** | **E** | **E** | **L** | **E** | **I** | **N** | **G** | **L** | **K** | **W** | **Q** | **K** | **D** | **K** | **S** | **W** | **G** | **G** | **Y** | **F** | **A** | **T** | **R** | **V** | **K** | **E** | **A** | **S** | **P** | **V** | **N** | **L** | **R** | **T** | **A** | **L** | **V** | **N** | **S** | **D** | **N** | **I** | **Y** | **F** | **A** | **Q** | **Q** | **T** | **L** | **R** | **M** | **G** | **E** | **D** | **K** | **F** | **R** | **A** | **G** | **L** | **N** | **K** | **F** | **I** | **F** | **G** | **E** | **E** | **L** | **D** | **L** | **P** | **I** | **A** | **M** | **T** | **P** | **A** | **Q** | **I** | **S** | **N** | **E** | **D** | **K** | **F** | **N** | **S** | **E** | **I** | **L** | **L** | **A** | **D** | **T** | **G** | **Y** | **G** | **Q** | **G** | **Q** | **L** | **L** | **I** | **S** | **P** | **I** | **Q** | **Q** | **A** | **T** | **M** | **Y** | **S** | **V** | **F** | **Q** | **N** | **N** | **G** | **T** | **L** | **V** | **Y** | **P** | **K** | **L** | **V** | **L** | **D** | **K** | **E** | **T** | **K** | **K** | **K** | **D** | **N** | **V** | **I** | **S** | **A** | **N** | **A** | **A** | **N** | **T** | **I** | **A** | **T** | **D** | **L** | **L** | **G** | **S** | **V** | **E** | **D** | **P** | **S** | **G** | **Y** | **V** | **Y** | **N** | **M** | **Y** | **N** | **P** | **N** | **F** | **S** | **L** | **A** | **A** | **K** | **T** | **G** | **T** | **A** | **E** | **I** | **K** | **D** | **K** | **Q** | **D** | **T** | **D** | **G** | **K** | **E** | **N** | **S** | **F** | **L** | **L** | **T** | **L** | **D** | **R** | **S** | **N** | **N** | **K** | **F** | **L** | **T** | **M** | **I** | **M** | **V** | **E** | **N** | **S** | **G** | **E** | **N** | **G** | **S** | **A** | **T** | **D** | **I** | **S** | **K** | **P** | **L** | **I** | **D** | **Y** | **L** | **E** | **A** | **T** | **I** | **K** |
| **N59936F** | **-** | **-** | **-** | **-** | **-** | **-** | **-** | **-** | **-** | **-** | **-** | **-** | **-** | **-** | **-** | **-** | **-** | **-** | **-** | **-** | **-** | **-** | **-** | **-** | **-** | **-** | **-** | **-** | **-** | **-** | **-** | **-** | **-** | **-** | **-** | **-** | **Q** | **W** | **Q** | **A** | **K** | **Q** | **E** | **L** | **A** | **E** | **A** | **K** | **K** | **T** | **A** | **N** | **T** | **F** | **L** | **N** | **V** | **L** | **S** | **K** | **Q** | **E** | **F** | **D** | **K** | **L** | **P** | **S** | **V** | **V** | **Q** | **E** | **S** | **S** | **L** | **K** | **K** | **N** | **G** | **Y** | **D** | **T** | **K** | **S** | **V** | **V** | **E** | **K** | **Y** | **Q** | **A** | **I** | **Y** | **S** | **G** | **I** | **Q** | **A** | **E** | **G** | **V** | **K** | **A** | **S** | **D** | **V** | **Q** | **V** | **K** | **K** | **A** | **K** | **D** | **N** | **Q** | **Y** | **T** | **F** | **T** | **Y** | **K** | **L** | **S** | **M** | **S** | **T** | **P** | **L** | **G** | **E** | **M** | **K** | **D** | **L** | **S** | **Y** | **Q** | **S** | **S** | **I** | **A** | **K** | **K** | **G** | **D** | **T** | **Y** | **Q** | **I** | **S** | **W** | **K** | **P** | **S** | **L** | **I** | **F** | **P** | **D** | **M** | **S** | **G** | **N** | **D** | **K** | **I** | **S** | **I** | **Q** | **V** | **D** | **N** | **A** | **K** | **R** | **G** | **E** | **I** | **V** | **D** | **R** | **N** | **G** | **S** | **G** | **L** | **A** | **I** | **N** | **K** | **V** | **F** | **D** | **E** | **V** | **G** | **V** | **V** | **P** | **G** | **K** | **L** | **G** | **S** | **G** | **A** | **E** | **K** | **T** | **A** | **N** | **I** | **K** | **A** | **F** | **S** | **D** | **K** | **F** | **G** | **V** | **S** | **V** | **D** | **E** | **I** | **N** | **Q** | **K** | **L** | **S** | **Q** | **G** | **W** | **V** | **Q** | **A** | **D** | **S** | **F** | **V** | **P** | **I** | **T** | **V** | **A** | **S** | **E** | **P** | **V** | **T** | **E** | **L** | **P** | **T** | **G** | **A** | **A** | **T** | **K** | **D** | **T** | **E** | **S** | **R** | **Y** | **Y** | **P** | **L** | **G** | **E** | **A** | **A** | **A** | **Q** | **L** | **I** | **G** | **Y** | **T** | **G** | **T** | **I** | **T** | **A** | **E** | **D** | **I** | **E** | **K** | **N** | **P** | **E** | **L** | **S** | **S** | **T** | **G** | **V** | **I** | **G** | **K** | **T** | **G** | **L** | **E** | **R** | **A** | **F** | **D** | **K** | **E** | **L** | **R** | **G** | **Q** | **D** | **G** | **G** | **S** | **L** | **V** | **I** | **L** | **D** | **D** | **K** | **E** | **N** | **V** | **K** | **K** | **A** | **L** | **Q** | **T** | **K** | **E** | **K** | **K** | **D** | **G** | **Q** | **T** | **I** | **K** | **L** | **T** | **I** | **D** | **S** | **G** | **V** | **Q** | **Q** | **Q** | **A** | **F** | **A** | **I** | **F** | **D** | **K** | **R** | **P** | **G** | **S** | **A** | **V** | **I** | **T** | **D** | **P** | **Q** | **K** | **G** | **D** | **L** | **L** | **A** | **T** | **V** | **S** | **S** | **P** | **S** | **Y** | **D** | **P** | **N** | **K** | **M** | **A** | **N** | **G** | **I** | **S** | **Q** | **K** | **E** | **Y** | **D** | **A** | **Y** | **N** | **N** | **N** | **K** | **D** | **L** | **P** | **F** | **T** | **A** | **R** | **F** | **A** | **T** | **G** | **Y** | **A** | **P** | **G** | **S** | **T** | **F** | **K** | **T** | **I** | **T** | **G** | **A** | **I** | **G** | **L** | **D** | **A** | **G** | **T** | **L** | **K** | **P** | **D** | **E** | **E** | **L** | **E** | **I** | **N** | **G** | **L** | **K** | **W** | **Q** | **K** | **D** | **K** | **S** | **W** | **G** | **G** | **Y** | **F** | **A** | **T** | **R** | **V** | **K** | **E** | **A** | **S** | **P** | **V** | **N** | **L** | **R** | **T** | **A** | **L** | **V** | **N** | **S** | **D** | **N** | **I** | **Y** | **F** | **A** | **Q** | **Q** | **T** | **L** | **R** | **M** | **G** | **E** | **D** | **K** | **F** | **R** | **A** | **G** | **L** | **N** | **K** | **F** | **I** | **F** | **G** | **E** | **E** | **L** | **D** | **L** | **P** | **I** | **A** | **M** | **T** | **P** | **A** | **Q** | **I** | **S** | **N** | **E** | **D** | **K** | **F** | **N** | **S** | **E** | **I** | **L** | **L** | **A** | **D** | **T** | **G** | **Y** | **G** | **Q** | **G** | **Q** | **L** | **L** | **I** | **S** | **P** | **I** | **Q** | **Q** | **A** | **T** | **M** | **Y** | **S** | **V** | **F** | **Q** | **N** | **N** | **G** | **T** | **L** | **V** | **Y** | **P** | **K** | **L** | **V** | **L** | **D** | **K** | **E** | **T** | **K** | **K** | **K** | **D** | **N** | **V** | **I** | **S** | **A** | **N** | **A** | **A** | **N** | **T** | **I** | **A** | **T** | **D** | **L** | **L** | **G** | **S** | **V** | **E** | **D** | **P** | **S** | **G** | **Y** | **V** | **Y** | **N** | **M** | **Y** | **N** | **P** | **N** | **F** | **S** | **L** | **A** | **A** | **K** | **T** | **G** | **T** | **A** | **E** | **I** | **K** | **D** | **K** | **Q** | **D** | **T** | **D** | **G** | **K** | **E** | **N** | **S** | **F** | **L** | **L** | **T** | **L** | **D** | **R** | **S** | **N** | **N** | **K** | **F** | **L** | **T** | **M** | **I** | **M** | **V** | **E** | **N** | **S** | **G** | **E** | **N** | **G** | **S** | **A** | **T** | **D** | **I** | **S** | **K** | **P** | **L** | **I** | **D** | **Y** | **L** | **E** | **A** | **T** | **I** | **K** | **-** | **-** | **-** | **-** | **-** | **-** | **-** | **-** | **-** | **-** | **-** | **-** | **-** | **-** | **-** | **-** | **-** | **-** | **-** | **-** | **-** | **-** | **-** | **-** | **-** | **-** | **-** | **-** | **-** | **-** | **-** | **-** | **-** | **-** | **-** | **-** | **Q** | **W** | **Q** | **A** | **K** | **Q** | **E** | **L** | **A** | **E** | **A** | **K** | **K** | **T** | **A** | **N** | **T** | **F** | **L** | **N** | **V** | **L** | **S** | **K** | **Q** | **E** | **F** | **D** | **K** | **L** | **P** | **S** | **V** | **V** | **Q** | **E** | **S** | **S** | **L** | **K** | **K** | **N** | **G** | **Y** | **D** | **T** | **K** | **S** | **V** | **V** | **E** | **K** | **Y** | **Q** | **A** | **I** | **Y** | **S** | **G** | **I** | **Q** | **A** | **E** | **G** | **V** | **K** | **A** | **S** | **D** | **V** | **Q** | **V** | **K** | **K** | **A** | **K** | **D** | **N** | **Q** | **Y** | **T** | **F** | **T** | **Y** | **K** | **L** | **S** | **M** | **S** | **T** | **P** | **L** | **G** | **E** | **M** | **K** | **D** | **L** | **S** | **Y** | **Q** | **S** | **S** | **I** | **A** | **K** | **K** | **G** | **D** | **T** | **Y** | **Q** | **I** | **S** | **W** | **K** | **P** | **S** | **L** | **I** | **F** | **P** | **D** | **M** | **S** | **G** | **N** | **D** | **K** | **I** | **S** | **I** | **Q** | **V** | **D** | **N** | **A** | **K** | **R** | **G** | **E** | **I** | **V** | **D** | **R** | **N** | **G** | **S** | **G** | **L** | **A** | **I** | **N** | **K** | **V** | **F** | **D** | **E** | **V** | **G** | **V** | **V** | **P** | **G** | **K** | **L** | **G** | **S** | **G** | **A** | **E** | **K** | **T** | **A** | **N** | **I** | **K** | **A** | **F** | **S** | **D** | **K** | **F** | **G** | **V** | **S** | **V** | **D** | **E** | **I** | **N** | **Q** | **K** | **L** | **S** | **Q** | **G** | **W** | **V** | **Q** | **A** | **D** | **S** | **F** | **V** | **P** | **I** | **T** | **V** | **A** | **S** | **E** | **P** | **V** | **T** | **E** | **L** | **P** | **T** | **G** | **A** | **A** | **T** | **K** | **D** | **T** | **E** | **S** | **R** | **Y** | **Y** | **P** | **L** | **G** | **E** | **A** | **A** | **A** | **Q** | **L** | **I** | **G** | **Y** | **T** | **G** | **T** | **I** | **T** | **A** | **E** | **D** | **I** | **E** | **K** | **N** | **P** | **E** | **L** | **S** | **S** | **T** | **G** | **V** | **I** | **G** | **K** | **T** | **G** | **L** | **E** | **R** | **A** | **F** | **D** | **K** | **E** | **L** | **R** | **G** | **Q** | **D** | **G** | **G** | **S** | **L** | **V** | **I** | **L** | **D** | **D** | **K** | **E** | **N** | **V** | **K** | **K** | **A** | **L** | **Q** | **T** | **K** | **E** | **K** | **K** | **D** | **G** | **Q** | **T** | **I** | **K** | **L** | **T** | **I** | **D** | **S** | **G** | **V** | **Q** | **Q** | **Q** | **A** | **F** | **A** | **I** | **F** | **D** | **K** | **R** | **P** | **G** | **S** | **A** | **V** | **I** | **T** | **D** | **P** | **Q** | **K** | **G** | **D** | **L** | **L** | **A** | **T** | **V** | **S** | **S** | **P** | **S** | **Y** | **D** | **P** | **N** | **K** | **M** | **A** | **N** | **G** | **I** | **S** | **Q** | **K** | **E** | **Y** | **D** | **A** | **Y** | **N** | **N** | **N** | **K** | **D** | **L** | **P** | **F** | **T** | **A** | **R** | **F** | **A** | **T** | **G** | **Y** | **A** | **P** | **G** | **S** | **T** | **F** | **K** | **T** | **I** | **T** | **G** | **A** | **I** | **G** | **L** | **D** | **A** | **G** | **T** | **L** | **K** | **P** | **D** | **E** | **E** | **L** | **E** | **I** | **N** | **G** | **L** | **K** | **W** | **Q** | **K** | **D** | **K** | **S** | **W** | **G** | **G** | **Y** | **F** | **A** | **T** | **R** | **V** | **K** | **E** | **A** | **S** | **P** | **V** | **N** | **L** | **R** | **T** | **A** | **L** | **V** | **N** | **S** | **D** | **N** | **I** | **Y** | **F** | **A** | **Q** | **Q** | **T** | **L** | **R** | **M** | **G** | **E** | **D** | **K** | **F** | **R** | **A** | **G** | **L** | **N** | **K** | **F** | **I** | **F** | **G** | **E** | **E** | **L** | **D** | **L** | **P** | **I** | **A** | **M** | **T** | **P** | **A** | **Q** | **I** | **S** | **N** | **E** | **D** | **K** | **F** | **N** | **S** | **E** | **I** | **L** | **L** | **A** | **D** | **T** | **G** | **Y** | **G** | **Q** | **G** | **Q** | **L** | **L** | **I** | **S** | **P** | **I** | **Q** | **Q** | **A** | **T** | **M** | **Y** | **S** | **V** | **F** | **Q** | **N** | **N** | **G** | **T** | **L** | **V** | **Y** | **P** | **K** | **L** | **V** | **L** | **D** | **K** | **E** | **T** | **K** | **K** | **K** | **D** | **N** | **V** | **I** | **S** | **A** | **N** | **A** | **A** | **N** | **T** | **I** | **A** | **T** | **D** | **L** | **L** | **G** | **S** | **V** | **E** | **D** | **P** | **S** | **G** | **Y** | **V** | **Y** | **N** | **M** | **Y** | **N** | **P** | **N** | **F** | **S** | **L** | **A** | **A** | **K** | **T** | **G** | **T** | **A** | **E** | **I** | **K** | **D** | **K** | **Q** | **D** | **T** | **D** | **G** | **K** | **E** | **N** | **S** | **F** | **L** | **L** | **T** | **L** | **D** | **R** | **S** | **N** | **N** | **K** | **F** | **L** | **T** | **M** | **I** | **M** | **V** | **E** | **N** | **S** | **G** | **E** | **N** | **G** | **S** | **A** | **T** | **D** | **I** | **S** | **K** | **P** | **L** | **I** | **D** | **Y** | **L** | **E** | **A** | **T** | **I** | **K** |
| **N59454F** | **-** | **-** | **-** | **-** | **-** | **-** | **-** | **-** | **-** | **-** | **-** | **-** | **-** | **-** | **-** | **-** | **-** | **-** | **-** | **-** | **-** | **-** | **-** | **-** | **-** | **-** | **-** | **-** | **-** | **-** | **-** | **-** | **-** | **-** | **-** | **-** | **Q** | **W** | **Q** | **A** | **K** | **Q** | **E** | **L** | **A** | **E** | **A** | **K** | **K** | **T** | **A** | **T** | **E** | **F** | **L** | **N** | **V** | **L** | **S** | **K** | **Q** | **E** | **F** | **D** | **K** | **L** | **P** | **S** | **V** | **V** | **Q** | **E** | **A** | **S** | **L** | **K** | **K** | **N** | **G** | **Y** | **D** | **T** | **K** | **S** | **V** | **V** | **E** | **K** | **Y** | **Q** | **A** | **I** | **Y** | **S** | **G** | **I** | **Q** | **A** | **E** | **G** | **V** | **K** | **A** | **S** | **D** | **V** | **Q** | **V** | **K** | **K** | **A** | **K** | **D** | **N** | **Q** | **Y** | **T** | **F** | **T** | **Y** | **K** | **L** | **S** | **M** | **S** | **T** | **P** | **L** | **G** | **E** | **M** | **K** | **D** | **L** | **S** | **Y** | **Q** | **S** | **S** | **I** | **A** | **K** | **K** | **G** | **D** | **T** | **Y** | **Q** | **I** | **A** | **W** | **K** | **P** | **S** | **L** | **I** | **F** | **P** | **D** | **M** | **S** | **G** | **N** | **D** | **K** | **I** | **S** | **I** | **Q** | **V** | **D** | **N** | **A** | **K** | **R** | **G** | **E** | **I** | **V** | **D** | **R** | **N** | **G** | **S** | **G** | **L** | **A** | **I** | **N** | **K** | **V** | **F** | **D** | **E** | **V** | **G** | **V** | **V** | **P** | **G** | **K** | **L** | **G** | **S** | **G** | **A** | **E** | **K** | **T** | **A** | **N** | **I** | **K** | **A** | **F** | **S** | **D** | **K** | **F** | **G** | **V** | **S** | **V** | **D** | **E** | **I** | **N** | **Q** | **K** | **L** | **S** | **Q** | **G** | **W** | **V** | **Q** | **A** | **D** | **S** | **F** | **V** | **P** | **I** | **T** | **V** | **A** | **S** | **E** | **P** | **V** | **T** | **E** | **L** | **P** | **T** | **G** | **A** | **A** | **T** | **K** | **D** | **T** | **E** | **S** | **R** | **Y** | **Y** | **P** | **L** | **G** | **E** | **A** | **A** | **A** | **Q** | **L** | **I** | **G** | **Y** | **T** | **G** | **T** | **I** | **T** | **A** | **E** | **D** | **I** | **E** | **K** | **N** | **P** | **E** | **L** | **S** | **S** | **T** | **G** | **V** | **I** | **G** | **K** | **T** | **G** | **L** | **E** | **R** | **A** | **F** | **D** | **K** | **E** | **L** | **R** | **G** | **Q** | **D** | **G** | **G** | **S** | **L** | **V** | **I** | **L** | **D** | **D** | **K** | **E** | **N** | **V** | **K** | **K** | **A** | **L** | **Q** | **T** | **K** | **E** | **K** | **K** | **D** | **G** | **Q** | **T** | **I** | **K** | **L** | **T** | **I** | **D** | **S** | **G** | **V** | **Q** | **Q** | **Q** | **A** | **F** | **A** | **I** | **F** | **D** | **K** | **R** | **P** | **G** | **S** | **A** | **V** | **I** | **T** | **D** | **P** | **Q** | **K** | **G** | **D** | **L** | **L** | **A** | **T** | **V** | **S** | **S** | **P** | **S** | **Y** | **D** | **P** | **N** | **K** | **M** | **A** | **N** | **G** | **I** | **S** | **Q** | **K** | **E** | **Y** | **D** | **A** | **Y** | **N** | **N** | **N** | **K** | **D** | **L** | **P** | **F** | **T** | **A** | **R** | **F** | **A** | **T** | **G** | **Y** | **A** | **P** | **G** | **S** | **T** | **F** | **K** | **T** | **I** | **T** | **G** | **A** | **I** | **G** | **L** | **D** | **A** | **G** | **T** | **L** | **K** | **P** | **D** | **E** | **E** | **L** | **E** | **I** | **N** | **G** | **L** | **K** | **W** | **Q** | **K** | **D** | **K** | **S** | **W** | **G** | **G** | **Y** | **F** | **A** | **T** | **R** | **V** | **K** | **E** | **A** | **S** | **P** | **V** | **N** | **L** | **R** | **T** | **A** | **L** | **V** | **N** | **S** | **D** | **N** | **I** | **Y** | **F** | **A** | **Q** | **Q** | **T** | **L** | **R** | **M** | **G** | **E** | **D** | **K** | **F** | **R** | **A** | **G** | **L** | **N** | **K** | **F** | **I** | **F** | **G** | **E** | **E** | **L** | **D** | **L** | **P** | **I** | **A** | **M** | **T** | **P** | **A** | **Q** | **I** | **S** | **N** | **E** | **D** | **K** | **F** | **N** | **S** | **E** | **I** | **L** | **L** | **A** | **D** | **T** | **G** | **Y** | **G** | **Q** | **G** | **Q** | **L** | **L** | **I** | **S** | **P** | **I** | **Q** | **Q** | **A** | **T** | **M** | **Y** | **S** | **V** | **F** | **Q** | **N** | **N** | **G** | **T** | **L** | **V** | **Y** | **P** | **K** | **I** | **V** | **L** | **D** | **K** | **E** | **T** | **K** | **K** | **K** | **D** | **N** | **V** | **I** | **S** | **A** | **N** | **A** | **A** | **N** | **T** | **I** | **A** | **T** | **D** | **L** | **L** | **G** | **S** | **V** | **E** | **D** | **P** | **S** | **G** | **Y** | **V** | **Y** | **N** | **M** | **Y** | **N** | **P** | **N** | **F** | **S** | **L** | **A** | **A** | **K** | **T** | **G** | **T** | **A** | **E** | **I** | **K** | **D** | **K** | **Q** | **D** | **T** | **D** | **G** | **K** | **E** | **N** | **S** | **F** | **L** | **L** | **T** | **L** | **D** | **R** | **S** | **N** | **N** | **K** | **F** | **L** | **T** | **M** | **I** | **M** | **V** | **E** | **N** | **S** | **G** | **E** | **N** | **G** | **S** | **A** | **T** | **D** | **I** | **S** | **K** | **P** | **L** | **I** | **D** | **Y** | **L** | **E** | **A** | **T** | **I** | **K** | **-** | **-** | **-** | **-** | **-** | **-** | **-** | **-** | **-** | **-** | **-** | **-** | **-** | **-** | **-** | **-** | **-** | **-** | **-** | **-** | **-** | **-** | **-** | **-** | **-** | **-** | **-** | **-** | **-** | **-** | **-** | **-** | **-** | **-** | **-** | **-** | **Q** | **W** | **Q** | **A** | **K** | **Q** | **E** | **L** | **A** | **E** | **A** | **K** | **K** | **T** | **A** | **T** | **E** | **F** | **L** | **N** | **V** | **L** | **S** | **K** | **Q** | **E** | **F** | **D** | **K** | **L** | **P** | **S** | **V** | **V** | **Q** | **E** | **A** | **S** | **L** | **K** | **K** | **N** | **G** | **Y** | **D** | **T** | **K** | **S** | **V** | **V** | **E** | **K** | **Y** | **Q** | **A** | **I** | **Y** | **S** | **G** | **I** | **Q** | **A** | **E** | **G** | **V** | **K** | **A** | **S** | **D** | **V** | **Q** | **V** | **K** | **K** | **A** | **K** | **D** | **N** | **Q** | **Y** | **T** | **F** | **T** | **Y** | **K** | **L** | **S** | **M** | **S** | **T** | **P** | **L** | **G** | **E** | **M** | **K** | **D** | **L** | **S** | **Y** | **Q** | **S** | **S** | **I** | **A** | **K** | **K** | **G** | **D** | **T** | **Y** | **Q** | **I** | **A** | **W** | **K** | **P** | **S** | **L** | **I** | **F** | **P** | **D** | **M** | **S** | **G** | **N** | **D** | **K** | **I** | **S** | **I** | **Q** | **V** | **D** | **N** | **A** | **K** | **R** | **G** | **E** | **I** | **V** | **D** | **R** | **N** | **G** | **S** | **G** | **L** | **A** | **I** | **N** | **K** | **V** | **F** | **D** | **E** | **V** | **G** | **V** | **V** | **P** | **G** | **K** | **L** | **G** | **S** | **G** | **A** | **E** | **K** | **T** | **A** | **N** | **I** | **K** | **A** | **F** | **S** | **D** | **K** | **F** | **G** | **V** | **S** | **V** | **D** | **E** | **I** | **N** | **Q** | **K** | **L** | **S** | **Q** | **G** | **W** | **V** | **Q** | **A** | **D** | **S** | **F** | **V** | **P** | **I** | **T** | **V** | **A** | **S** | **E** | **P** | **V** | **T** | **E** | **L** | **P** | **T** | **G** | **A** | **A** | **T** | **K** | **D** | **T** | **E** | **S** | **R** | **Y** | **Y** | **P** | **L** | **G** | **E** | **A** | **A** | **A** | **Q** | **L** | **I** | **G** | **Y** | **T** | **G** | **T** | **I** | **T** | **A** | **E** | **D** | **I** | **E** | **K** | **N** | **P** | **E** | **L** | **S** | **S** | **T** | **G** | **V** | **I** | **G** | **K** | **T** | **G** | **L** | **E** | **R** | **A** | **F** | **D** | **K** | **E** | **L** | **R** | **G** | **Q** | **D** | **G** | **G** | **S** | **L** | **V** | **I** | **L** | **D** | **D** | **K** | **E** | **N** | **V** | **K** | **K** | **A** | **L** | **Q** | **T** | **K** | **E** | **K** | **K** | **D** | **G** | **Q** | **T** | **I** | **K** | **L** | **T** | **I** | **D** | **S** | **G** | **V** | **Q** | **Q** | **Q** | **A** | **F** | **A** | **I** | **F** | **D** | **K** | **R** | **P** | **G** | **S** | **A** | **V** | **I** | **T** | **D** | **P** | **Q** | **K** | **G** | **D** | **L** | **L** | **A** | **T** | **V** | **S** | **S** | **P** | **S** | **Y** | **D** | **P** | **N** | **K** | **M** | **A** | **N** | **G** | **I** | **S** | **Q** | **K** | **E** | **Y** | **D** | **A** | **Y** | **N** | **N** | **N** | **K** | **D** | **L** | **P** | **F** | **T** | **A** | **R** | **F** | **A** | **T** | **G** | **Y** | **A** | **P** | **G** | **S** | **T** | **F** | **K** | **T** | **I** | **T** | **G** | **A** | **I** | **G** | **L** | **D** | **A** | **G** | **T** | **L** | **K** | **P** | **D** | **E** | **E** | **L** | **E** | **I** | **N** | **G** | **L** | **K** | **W** | **Q** | **K** | **D** | **K** | **S** | **W** | **G** | **G** | **Y** | **F** | **A** | **T** | **R** | **V** | **K** | **E** | **A** | **S** | **P** | **V** | **N** | **L** | **R** | **T** | **A** | **L** | **V** | **N** | **S** | **D** | **N** | **I** | **Y** | **F** | **A** | **Q** | **Q** | **T** | **L** | **R** | **M** | **G** | **E** | **D** | **K** | **F** | **R** | **A** | **G** | **L** | **N** | **K** | **F** | **I** | **F** | **G** | **E** | **E** | **L** | **D** | **L** | **P** | **I** | **A** | **M** | **T** | **P** | **A** | **Q** | **I** | **S** | **N** | **E** | **D** | **K** | **F** | **N** | **S** | **E** | **I** | **L** | **L** | **A** | **D** | **T** | **G** | **Y** | **G** | **Q** | **G** | **Q** | **L** | **L** | **I** | **S** | **P** | **I** | **Q** | **Q** | **A** | **T** | **M** | **Y** | **S** | **V** | **F** | **Q** | **N** | **N** | **G** | **T** | **L** | **V** | **Y** | **P** | **K** | **I** | **V** | **L** | **D** | **K** | **E** | **T** | **K** | **K** | **K** | **D** | **N** | **V** | **I** | **S** | **A** | **N** | **A** | **A** | **N** | **T** | **I** | **A** | **T** | **D** | **L** | **L** | **G** | **S** | **V** | **E** | **D** | **P** | **S** | **G** | **Y** | **V** | **Y** | **N** | **M** | **Y** | **N** | **P** | **N** | **F** | **S** | **L** | **A** | **A** | **K** | **T** | **G** | **T** | **A** | **E** | **I** | **K** | **D** | **K** | **Q** | **D** | **T** | **D** | **G** | **K** | **E** | **N** | **S** | **F** | **L** | **L** | **T** | **L** | **D** | **R** | **S** | **N** | **N** | **K** | **F** | **L** | **T** | **M** | **I** | **M** | **V** | **E** | **N** | **S** | **G** | **E** | **N** | **G** | **S** | **A** | **T** | **D** | **I** | **S** | **K** | **P** | **L** | **I** | **D** | **Y** | **L** | **E** | **A** | **T** | **I** | **K** |
| **N55753** | **-** | **-** | **-** | **-** | **-** | **-** | **-** | **-** | **-** | **-** | **-** | **-** | **-** | **-** | **-** | **-** | **-** | **-** | **-** | **-** | **-** | **-** | **-** | **-** | **-** | **-** | **-** | **-** | **-** | **-** | **-** | **-** | **-** | **-** | **-** | **-** | **Q** | **W** | **Q** | **A** | **K** | **Q** | **E** | **L** | **A** | **E** | **A** | **K** | **K** | **T** | **A** | **T** | **T** | **F** | **L** | **N** | **V** | **L** | **S** | **K** | **Q** | **E** | **F** | **D** | **K** | **L** | **P** | **S** | **V** | **V** | **Q** | **E** | **A** | **S** | **L** | **K** | **K** | **N** | **G** | **Y** | **D** | **T** | **K** | **S** | **V** | **V** | **E** | **K** | **Y** | **Q** | **A** | **I** | **Y** | **S** | **G** | **I** | **Q** | **A** | **E** | **G** | **V** | **K** | **A** | **S** | **D** | **V** | **Q** | **V** | **K** | **K** | **A** | **K** | **D** | **N** | **Q** | **Y** | **T** | **F** | **T** | **Y** | **K** | **L** | **S** | **M** | **S** | **T** | **P** | **L** | **G** | **E** | **M** | **K** | **D** | **L** | **S** | **Y** | **Q** | **S** | **S** | **I** | **D** | **K** | **K** | **G** | **D** | **T** | **Y** | **Q** | **I** | **A** | **W** | **K** | **P** | **S** | **L** | **I** | **F** | **P** | **D** | **M** | **S** | **G** | **N** | **D** | **K** | **I** | **S** | **I** | **Q** | **V** | **D** | **N** | **A** | **K** | **R** | **G** | **E** | **I** | **V** | **D** | **R** | **N** | **G** | **S** | **G** | **L** | **A** | **I** | **N** | **K** | **V** | **F** | **D** | **E** | **V** | **G** | **V** | **V** | **P** | **S** | **K** | **L** | **G** | **S** | **G** | **A** | **E** | **K** | **A** | **A** | **N** | **I** | **K** | **A** | **F** | **S** | **D** | **K** | **F** | **G** | **V** | **S** | **V** | **D** | **E** | **I** | **N** | **Q** | **K** | **L** | **S** | **Q** | **G** | **W** | **V** | **Q** | **A** | **D** | **S** | **F** | **V** | **P** | **I** | **T** | **V** | **A** | **S** | **E** | **P** | **V** | **T** | **E** | **L** | **P** | **T** | **G** | **A** | **A** | **T** | **K** | **D** | **T** | **E** | **S** | **R** | **Y** | **Y** | **P** | **L** | **G** | **E** | **A** | **A** | **A** | **Q** | **L** | **I** | **G** | **Y** | **T** | **G** | **T** | **I** | **T** | **A** | **E** | **D** | **I** | **K** | **K** | **N** | **P** | **E** | **L** | **S** | **S** | **T** | **G** | **V** | **I** | **G** | **K** | **T** | **G** | **L** | **E** | **R** | **A** | **F** | **D** | **K** | **E** | **L** | **R** | **G** | **Q** | **D** | **G** | **G** | **S** | **L** | **V** | **I** | **L** | **D** | **D** | **K** | **E** | **N** | **V** | **K** | **K** | **A** | **L** | **Q** | **T** | **K** | **E** | **K** | **K** | **D** | **G** | **Q** | **T** | **I** | **K** | **L** | **T** | **I** | **D** | **S** | **G** | **V** | **Q** | **Q** | **Q** | **A** | **F** | **A** | **I** | **F** | **D** | **K** | **R** | **P** | **G** | **S** | **A** | **V** | **I** | **T** | **D** | **P** | **Q** | **K** | **G** | **D** | **L** | **L** | **A** | **T** | **V** | **S** | **S** | **P** | **S** | **Y** | **D** | **P** | **N** | **K** | **M** | **A** | **N** | **G** | **I** | **S** | **Q** | **K** | **E** | **Y** | **D** | **A** | **Y** | **N** | **N** | **N** | **K** | **D** | **L** | **P** | **F** | **T** | **A** | **R** | **F** | **A** | **T** | **G** | **Y** | **A** | **P** | **G** | **S** | **T** | **F** | **K** | **T** | **I** | **T** | **G** | **A** | **I** | **G** | **L** | **D** | **A** | **G** | **T** | **L** | **K** | **P** | **D** | **E** | **E** | **L** | **E** | **I** | **N** | **G** | **L** | **K** | **W** | **Q** | **K** | **D** | **K** | **S** | **W** | **G** | **G** | **Y** | **F** | **A** | **T** | **R** | **V** | **K** | **E** | **A** | **S** | **P** | **V** | **N** | **L** | **R** | **T** | **A** | **L** | **V** | **N** | **S** | **D** | **N** | **I** | **Y** | **F** | **A** | **Q** | **Q** | **T** | **L** | **R** | **M** | **G** | **E** | **D** | **K** | **F** | **R** | **A** | **G** | **L** | **N** | **K** | **F** | **I** | **F** | **G** | **E** | **E** | **L** | **D** | **L** | **P** | **I** | **A** | **M** | **T** | **P** | **A** | **Q** | **I** | **S** | **N** | **E** | **D** | **K** | **F** | **N** | **S** | **E** | **I** | **L** | **L** | **A** | **D** | **T** | **G** | **Y** | **G** | **Q** | **G** | **Q** | **L** | **L** | **I** | **S** | **P** | **I** | **Q** | **Q** | **A** | **T** | **M** | **Y** | **S** | **V** | **F** | **Q** | **N** | **N** | **G** | **T** | **L** | **V** | **Y** | **P** | **K** | **L** | **V** | **L** | **D** | **K** | **E** | **T** | **K** | **K** | **K** | **D** | **N** | **V** | **I** | **S** | **A** | **N** | **A** | **A** | **N** | **T** | **I** | **A** | **T** | **D** | **L** | **L** | **G** | **S** | **V** | **E** | **D** | **P** | **S** | **G** | **Y** | **V** | **Y** | **N** | **M** | **Y** | **N** | **P** | **N** | **F** | **S** | **L** | **A** | **A** | **K** | **T** | **G** | **T** | **A** | **E** | **I** | **K** | **D** | **K** | **Q** | **D** | **T** | **D** | **G** | **K** | **E** | **N** | **S** | **F** | **L** | **L** | **T** | **L** | **D** | **R** | **S** | **N** | **N** | **K** | **F** | **L** | **T** | **M** | **I** | **M** | **V** | **E** | **N** | **S** | **G** | **E** | **N** | **G** | **S** | **A** | **T** | **D** | **I** | **S** | **K** | **P** | **L** | **I** | **D** | **Y** | **L** | **E** | **A** | **T** | **I** | **K** | **-** | **-** | **-** | **-** | **-** | **-** | **-** | **-** | **-** | **-** | **-** | **-** | **-** | **-** | **-** | **-** | **-** | **-** | **-** | **-** | **-** | **-** | **-** | **-** | **-** | **-** | **-** | **-** | **-** | **-** | **-** | **-** | **-** | **-** | **-** | **-** | **Q** | **W** | **Q** | **A** | **K** | **Q** | **E** | **L** | **A** | **E** | **A** | **K** | **K** | **T** | **A** | **T** | **T** | **F** | **L** | **N** | **V** | **L** | **S** | **K** | **Q** | **E** | **F** | **D** | **K** | **L** | **P** | **S** | **V** | **V** | **Q** | **E** | **A** | **S** | **L** | **K** | **K** | **N** | **G** | **Y** | **D** | **T** | **K** | **S** | **V** | **V** | **E** | **K** | **Y** | **Q** | **A** | **I** | **Y** | **S** | **G** | **I** | **Q** | **A** | **E** | **G** | **V** | **K** | **A** | **S** | **D** | **V** | **Q** | **V** | **K** | **K** | **A** | **K** | **D** | **N** | **Q** | **Y** | **T** | **F** | **T** | **Y** | **K** | **L** | **S** | **M** | **S** | **T** | **P** | **L** | **G** | **E** | **M** | **K** | **D** | **L** | **S** | **Y** | **Q** | **S** | **S** | **I** | **D** | **K** | **K** | **G** | **D** | **T** | **Y** | **Q** | **I** | **A** | **W** | **K** | **P** | **S** | **L** | **I** | **F** | **P** | **D** | **M** | **S** | **G** | **N** | **D** | **K** | **I** | **S** | **I** | **Q** | **V** | **D** | **N** | **A** | **K** | **R** | **G** | **E** | **I** | **V** | **D** | **R** | **N** | **G** | **S** | **G** | **L** | **A** | **I** | **N** | **K** | **V** | **F** | **D** | **E** | **V** | **G** | **V** | **V** | **P** | **S** | **K** | **L** | **G** | **S** | **G** | **A** | **E** | **K** | **A** | **A** | **N** | **I** | **K** | **A** | **F** | **S** | **D** | **K** | **F** | **G** | **V** | **S** | **V** | **D** | **E** | **I** | **N** | **Q** | **K** | **L** | **S** | **Q** | **G** | **W** | **V** | **Q** | **A** | **D** | **S** | **F** | **V** | **P** | **I** | **T** | **V** | **A** | **S** | **E** | **P** | **V** | **T** | **E** | **L** | **P** | **T** | **G** | **A** | **A** | **T** | **K** | **D** | **T** | **E** | **S** | **R** | **Y** | **Y** | **P** | **L** | **G** | **E** | **A** | **A** | **A** | **Q** | **L** | **I** | **G** | **Y** | **T** | **G** | **T** | **I** | **T** | **A** | **E** | **D** | **I** | **K** | **K** | **N** | **P** | **E** | **L** | **S** | **S** | **T** | **G** | **V** | **I** | **G** | **K** | **T** | **G** | **L** | **E** | **R** | **A** | **F** | **D** | **K** | **E** | **L** | **R** | **G** | **Q** | **D** | **G** | **G** | **S** | **L** | **V** | **I** | **L** | **D** | **D** | **K** | **E** | **N** | **V** | **K** | **K** | **A** | **L** | **Q** | **T** | **K** | **E** | **K** | **K** | **D** | **G** | **Q** | **T** | **I** | **K** | **L** | **T** | **I** | **D** | **S** | **G** | **V** | **Q** | **Q** | **Q** | **A** | **F** | **A** | **I** | **F** | **D** | **K** | **R** | **P** | **G** | **S** | **A** | **V** | **I** | **T** | **D** | **P** | **Q** | **K** | **G** | **D** | **L** | **L** | **A** | **T** | **V** | **S** | **S** | **P** | **S** | **Y** | **D** | **P** | **N** | **K** | **M** | **A** | **N** | **G** | **I** | **S** | **Q** | **K** | **E** | **Y** | **D** | **A** | **Y** | **N** | **N** | **N** | **K** | **D** | **L** | **P** | **F** | **T** | **A** | **R** | **F** | **A** | **T** | **G** | **Y** | **A** | **P** | **G** | **S** | **T** | **F** | **K** | **T** | **I** | **T** | **G** | **A** | **I** | **G** | **L** | **D** | **A** | **G** | **T** | **L** | **K** | **P** | **D** | **E** | **E** | **L** | **E** | **I** | **N** | **G** | **L** | **K** | **W** | **Q** | **K** | **D** | **K** | **S** | **W** | **G** | **G** | **Y** | **F** | **A** | **T** | **R** | **V** | **K** | **E** | **A** | **S** | **P** | **V** | **N** | **L** | **R** | **T** | **A** | **L** | **V** | **N** | **S** | **D** | **N** | **I** | **Y** | **F** | **A** | **Q** | **Q** | **T** | **L** | **R** | **M** | **G** | **E** | **D** | **K** | **F** | **R** | **A** | **G** | **L** | **N** | **K** | **F** | **I** | **F** | **G** | **E** | **E** | **L** | **D** | **L** | **P** | **I** | **A** | **M** | **T** | **P** | **A** | **Q** | **I** | **S** | **N** | **E** | **D** | **K** | **F** | **N** | **S** | **E** | **I** | **L** | **L** | **A** | **D** | **T** | **G** | **Y** | **G** | **Q** | **G** | **Q** | **L** | **L** | **I** | **S** | **P** | **I** | **Q** | **Q** | **A** | **T** | **M** | **Y** | **S** | **V** | **F** | **Q** | **N** | **N** | **G** | **T** | **L** | **V** | **Y** | **P** | **K** | **L** | **V** | **L** | **D** | **K** | **E** | **T** | **K** | **K** | **K** | **D** | **N** | **V** | **I** | **S** | **A** | **N** | **A** | **A** | **N** | **T** | **I** | **A** | **T** | **D** | **L** | **L** | **G** | **S** | **V** | **E** | **D** | **P** | **S** | **G** | **Y** | **V** | **Y** | **N** | **M** | **Y** | **N** | **P** | **N** | **F** | **S** | **L** | **A** | **A** | **K** | **T** | **G** | **T** | **A** | **E** | **I** | **K** | **D** | **K** | **Q** | **D** | **T** | **D** | **G** | **K** | **E** | **N** | **S** | **F** | **L** | **L** | **T** | **L** | **D** | **R** | **S** | **N** | **N** | **K** | **F** | **L** | **T** | **M** | **I** | **M** | **V** | **E** | **N** | **S** | **G** | **E** | **N** | **G** | **S** | **A** | **T** | **D** | **I** | **S** | **K** | **P** | **L** | **I** | **D** | **Y** | **L** | **E** | **A** | **T** | **I** | **K** |
| **N60100F** | **-** | **-** | **-** | **-** | **-** | **-** | **-** | **-** | **-** | **-** | **-** | **-** | **-** | **-** | **-** | **-** | **-** | **-** | **-** | **-** | **-** | **-** | **-** | **-** | **-** | **-** | **-** | **-** | **-** | **-** | **-** | **-** | **-** | **-** | **-** | **-** | **Q** | **W** | **Q** | **A** | **K** | **Q** | **E** | **L** | **A** | **E** | **A** | **K** | **K** | **T** | **A** | **N** | **T** | **F** | **L** | **N** | **V** | **L** | **S** | **K** | **Q** | **E** | **F** | **D** | **K** | **L** | **P** | **S** | **V** | **V** | **Q** | **E** | **A** | **S** | **L** | **K** | **K** | **N** | **G** | **Y** | **D** | **T** | **K** | **S** | **V** | **V** | **E** | **K** | **Y** | **Q** | **A** | **I** | **Y** | **S** | **G** | **I** | **Q** | **A** | **E** | **G** | **V** | **K** | **A** | **S** | **D** | **V** | **Q** | **V** | **K** | **K** | **A** | **K** | **D** | **N** | **Q** | **Y** | **T** | **F** | **T** | **Y** | **K** | **L** | **S** | **M** | **S** | **T** | **P** | **L** | **G** | **E** | **M** | **K** | **D** | **L** | **S** | **Y** | **Q** | **S** | **S** | **I** | **A** | **K** | **K** | **G** | **D** | **T** | **Y** | **Q** | **I** | **A** | **W** | **K** | **P** | **S** | **L** | **I** | **F** | **P** | **D** | **M** | **S** | **G** | **N** | **D** | **K** | **I** | **S** | **I** | **Q** | **V** | **D** | **N** | **A** | **K** | **R** | **G** | **E** | **I** | **V** | **D** | **R** | **N** | **G** | **S** | **G** | **L** | **A** | **I** | **N** | **K** | **V** | **F** | **D** | **E** | **V** | **G** | **V** | **V** | **P** | **G** | **K** | **L** | **G** | **S** | **G** | **A** | **E** | **K** | **T** | **A** | **N** | **I** | **K** | **A** | **F** | **S** | **D** | **K** | **F** | **G** | **V** | **S** | **V** | **D** | **E** | **I** | **N** | **Q** | **K** | **L** | **S** | **Q** | **G** | **W** | **V** | **Q** | **A** | **D** | **S** | **F** | **V** | **P** | **I** | **T** | **V** | **A** | **S** | **E** | **P** | **V** | **T** | **E** | **L** | **P** | **T** | **G** | **A** | **A** | **T** | **K** | **D** | **T** | **E** | **S** | **R** | **Y** | **Y** | **P** | **L** | **G** | **E** | **A** | **A** | **A** | **Q** | **L** | **I** | **G** | **Y** | **T** | **G** | **T** | **I** | **T** | **A** | **E** | **D** | **I** | **E** | **K** | **N** | **P** | **E** | **L** | **S** | **S** | **T** | **G** | **V** | **I** | **G** | **K** | **T** | **G** | **L** | **E** | **R** | **A** | **F** | **D** | **K** | **E** | **L** | **R** | **G** | **Q** | **D** | **G** | **G** | **S** | **L** | **V** | **I** | **L** | **D** | **D** | **K** | **E** | **N** | **V** | **K** | **K** | **A** | **L** | **Q** | **T** | **K** | **E** | **K** | **K** | **D** | **G** | **Q** | **T** | **I** | **K** | **L** | **T** | **I** | **D** | **S** | **G** | **V** | **Q** | **Q** | **Q** | **A** | **F** | **A** | **I** | **F** | **D** | **K** | **R** | **P** | **G** | **S** | **A** | **V** | **I** | **T** | **D** | **P** | **Q** | **K** | **G** | **D** | **L** | **L** | **A** | **T** | **V** | **S** | **S** | **P** | **S** | **Y** | **D** | **P** | **N** | **K** | **M** | **A** | **N** | **G** | **I** | **S** | **Q** | **K** | **D** | **Y** | **D** | **A** | **Y** | **N** | **N** | **N** | **K** | **D** | **L** | **P** | **F** | **T** | **A** | **R** | **F** | **A** | **T** | **G** | **Y** | **A** | **P** | **G** | **S** | **T** | **F** | **K** | **T** | **I** | **T** | **G** | **A** | **I** | **G** | **L** | **D** | **A** | **G** | **T** | **L** | **K** | **P** | **D** | **E** | **E** | **L** | **E** | **I** | **N** | **G** | **L** | **K** | **W** | **Q** | **K** | **D** | **K** | **S** | **W** | **G** | **G** | **Y** | **F** | **A** | **T** | **R** | **V** | **K** | **E** | **A** | **S** | **P** | **V** | **N** | **L** | **R** | **T** | **A** | **L** | **V** | **N** | **S** | **D** | **N** | **I** | **Y** | **F** | **A** | **Q** | **Q** | **T** | **L** | **R** | **M** | **G** | **E** | **D** | **K** | **F** | **R** | **A** | **G** | **L** | **N** | **K** | **F** | **I** | **F** | **G** | **E** | **E** | **L** | **D** | **L** | **P** | **I** | **A** | **M** | **T** | **P** | **A** | **Q** | **I** | **S** | **N** | **E** | **D** | **K** | **F** | **N** | **S** | **E** | **I** | **L** | **L** | **A** | **D** | **T** | **G** | **Y** | **G** | **Q** | **G** | **Q** | **L** | **L** | **I** | **S** | **P** | **I** | **Q** | **Q** | **A** | **T** | **M** | **Y** | **S** | **V** | **F** | **Q** | **N** | **N** | **G** | **T** | **L** | **V** | **Y** | **P** | **K** | **L** | **V** | **L** | **D** | **K** | **E** | **T** | **K** | **K** | **K** | **D** | **N** | **V** | **I** | **S** | **A** | **N** | **A** | **A** | **N** | **T** | **I** | **A** | **T** | **D** | **L** | **L** | **G** | **S** | **V** | **E** | **D** | **P** | **S** | **G** | **Y** | **V** | **Y** | **N** | **M** | **Y** | **N** | **P** | **N** | **F** | **S** | **L** | **A** | **A** | **K** | **T** | **G** | **T** | **A** | **E** | **I** | **K** | **D** | **K** | **Q** | **D** | **T** | **D** | **G** | **K** | **E** | **N** | **S** | **F** | **L** | **L** | **T** | **L** | **D** | **R** | **S** | **N** | **N** | **K** | **F** | **L** | **T** | **M** | **I** | **M** | **V** | **E** | **N** | **S** | **G** | **E** | **N** | **G** | **S** | **A** | **T** | **D** | **I** | **S** | **K** | **P** | **L** | **I** | **D** | **Y** | **L** | **E** | **A** | **T** | **I** | **K** | **-** | **-** | **-** | **-** | **-** | **-** | **-** | **-** | **-** | **-** | **-** | **-** | **-** | **-** | **-** | **-** | **-** | **-** | **-** | **-** | **-** | **-** | **-** | **-** | **-** | **-** | **-** | **-** | **-** | **-** | **-** | **-** | **-** | **-** | **-** | **-** | **Q** | **W** | **Q** | **A** | **K** | **Q** | **E** | **L** | **A** | **E** | **A** | **K** | **K** | **T** | **A** | **N** | **T** | **F** | **L** | **N** | **V** | **L** | **S** | **K** | **Q** | **E** | **F** | **D** | **K** | **L** | **P** | **S** | **V** | **V** | **Q** | **E** | **A** | **S** | **L** | **K** | **K** | **N** | **G** | **Y** | **D** | **T** | **K** | **S** | **V** | **V** | **E** | **K** | **Y** | **Q** | **A** | **I** | **Y** | **S** | **G** | **I** | **Q** | **A** | **E** | **G** | **V** | **K** | **A** | **S** | **D** | **V** | **Q** | **V** | **K** | **K** | **A** | **K** | **D** | **N** | **Q** | **Y** | **T** | **F** | **T** | **Y** | **K** | **L** | **S** | **M** | **S** | **T** | **P** | **L** | **G** | **E** | **M** | **K** | **D** | **L** | **S** | **Y** | **Q** | **S** | **S** | **I** | **A** | **K** | **K** | **G** | **D** | **T** | **Y** | **Q** | **I** | **A** | **W** | **K** | **P** | **S** | **L** | **I** | **F** | **P** | **D** | **M** | **S** | **G** | **N** | **D** | **K** | **I** | **S** | **I** | **Q** | **V** | **D** | **N** | **A** | **K** | **R** | **G** | **E** | **I** | **V** | **D** | **R** | **N** | **G** | **S** | **G** | **L** | **A** | **I** | **N** | **K** | **V** | **F** | **D** | **E** | **V** | **G** | **V** | **V** | **P** | **G** | **K** | **L** | **G** | **S** | **G** | **A** | **E** | **K** | **T** | **A** | **N** | **I** | **K** | **A** | **F** | **S** | **D** | **K** | **F** | **G** | **V** | **S** | **V** | **D** | **E** | **I** | **N** | **Q** | **K** | **L** | **S** | **Q** | **G** | **W** | **V** | **Q** | **A** | **D** | **S** | **F** | **V** | **P** | **I** | **T** | **V** | **A** | **S** | **E** | **P** | **V** | **T** | **E** | **L** | **P** | **T** | **G** | **A** | **A** | **T** | **K** | **D** | **T** | **E** | **S** | **R** | **Y** | **Y** | **P** | **L** | **G** | **E** | **A** | **A** | **A** | **Q** | **L** | **I** | **G** | **Y** | **T** | **G** | **T** | **I** | **T** | **A** | **E** | **D** | **I** | **E** | **K** | **N** | **P** | **E** | **L** | **S** | **S** | **T** | **G** | **V** | **I** | **G** | **K** | **T** | **G** | **L** | **E** | **R** | **A** | **F** | **D** | **K** | **E** | **L** | **R** | **G** | **Q** | **D** | **G** | **G** | **S** | **L** | **V** | **I** | **L** | **D** | **D** | **K** | **E** | **N** | **V** | **K** | **K** | **A** | **L** | **Q** | **T** | **K** | **E** | **K** | **K** | **D** | **G** | **Q** | **T** | **I** | **K** | **L** | **T** | **I** | **D** | **S** | **G** | **V** | **Q** | **Q** | **Q** | **A** | **F** | **A** | **I** | **F** | **D** | **K** | **R** | **P** | **G** | **S** | **A** | **V** | **I** | **T** | **D** | **P** | **Q** | **K** | **G** | **D** | **L** | **L** | **A** | **T** | **V** | **S** | **S** | **P** | **S** | **Y** | **D** | **P** | **N** | **K** | **M** | **A** | **N** | **G** | **I** | **S** | **Q** | **K** | **D** | **Y** | **D** | **A** | **Y** | **N** | **N** | **N** | **K** | **D** | **L** | **P** | **F** | **T** | **A** | **R** | **F** | **A** | **T** | **G** | **Y** | **A** | **P** | **G** | **S** | **T** | **F** | **K** | **T** | **I** | **T** | **G** | **A** | **I** | **G** | **L** | **D** | **A** | **G** | **T** | **L** | **K** | **P** | **D** | **E** | **E** | **L** | **E** | **I** | **N** | **G** | **L** | **K** | **W** | **Q** | **K** | **D** | **K** | **S** | **W** | **G** | **G** | **Y** | **F** | **A** | **T** | **R** | **V** | **K** | **E** | **A** | **S** | **P** | **V** | **N** | **L** | **R** | **T** | **A** | **L** | **V** | **N** | **S** | **D** | **N** | **I** | **Y** | **F** | **A** | **Q** | **Q** | **T** | **L** | **R** | **M** | **G** | **E** | **D** | **K** | **F** | **R** | **A** | **G** | **L** | **N** | **K** | **F** | **I** | **F** | **G** | **E** | **E** | **L** | **D** | **L** | **P** | **I** | **A** | **M** | **T** | **P** | **A** | **Q** | **I** | **S** | **N** | **E** | **D** | **K** | **F** | **N** | **S** | **E** | **I** | **L** | **L** | **A** | **D** | **T** | **G** | **Y** | **G** | **Q** | **G** | **Q** | **L** | **L** | **I** | **S** | **P** | **I** | **Q** | **Q** | **A** | **T** | **M** | **Y** | **S** | **V** | **F** | **Q** | **N** | **N** | **G** | **T** | **L** | **V** | **Y** | **P** | **K** | **L** | **V** | **L** | **D** | **K** | **E** | **T** | **K** | **K** | **K** | **D** | **N** | **V** | **I** | **S** | **A** | **N** | **A** | **A** | **N** | **T** | **I** | **A** | **T** | **D** | **L** | **L** | **G** | **S** | **V** | **E** | **D** | **P** | **S** | **G** | **Y** | **V** | **Y** | **N** | **M** | **Y** | **N** | **P** | **N** | **F** | **S** | **L** | **A** | **A** | **K** | **T** | **G** | **T** | **A** | **E** | **I** | **K** | **D** | **K** | **Q** | **D** | **T** | **D** | **G** | **K** | **E** | **N** | **S** | **F** | **L** | **L** | **T** | **L** | **D** | **R** | **S** | **N** | **N** | **K** | **F** | **L** | **T** | **M** | **I** | **M** | **V** | **E** | **N** | **S** | **G** | **E** | **N** | **G** | **S** | **A** | **T** | **D** | **I** | **S** | **K** | **P** | **L** | **I** | **D** | **Y** | **L** | **E** | **A** | **T** | **I** | **K** |
| **N54156** | **-** | **-** | **-** | **-** | **-** | **-** | **-** | **-** | **-** | **-** | **-** | **-** | **-** | **-** | **-** | **-** | **-** | **-** | **-** | **-** | **-** | **-** | **-** | **-** | **-** | **-** | **-** | **-** | **-** | **-** | **-** | **-** | **-** | **-** | **-** | **-** | **Q** | **W** | **Q** | **A** | **K** | **Q** | **E** | **L** | **A** | **E** | **A** | **K** | **K** | **T** | **A** | **T** | **T** | **F** | **L** | **N** | **V** | **L** | **S** | **K** | **Q** | **E** | **F** | **D** | **K** | **L** | **P** | **S** | **V** | **V** | **Q** | **E** | **A** | **S** | **L** | **K** | **K** | **N** | **G** | **Y** | **D** | **T** | **K** | **S** | **V** | **V** | **E** | **K** | **Y** | **Q** | **A** | **I** | **Y** | **S** | **G** | **I** | **Q** | **A** | **E** | **G** | **V** | **K** | **A** | **S** | **D** | **V** | **Q** | **V** | **K** | **K** | **A** | **K** | **D** | **N** | **Q** | **Y** | **T** | **F** | **T** | **Y** | **K** | **L** | **S** | **M** | **S** | **T** | **P** | **L** | **G** | **E** | **M** | **K** | **D** | **L** | **S** | **Y** | **Q** | **S** | **S** | **I** | **A** | **K** | **K** | **G** | **D** | **T** | **Y** | **Q** | **I** | **A** | **W** | **K** | **P** | **S** | **L** | **I** | **F** | **P** | **D** | **M** | **S** | **G** | **N** | **D** | **K** | **I** | **S** | **I** | **Q** | **V** | **D** | **N** | **A** | **K** | **R** | **G** | **E** | **I** | **V** | **D** | **R** | **N** | **G** | **S** | **G** | **L** | **A** | **I** | **N** | **K** | **V** | **F** | **D** | **E** | **V** | **G** | **V** | **V** | **P** | **G** | **K** | **L** | **G** | **S** | **G** | **A** | **E** | **K** | **T** | **A** | **N** | **I** | **K** | **A** | **F** | **S** | **D** | **K** | **F** | **G** | **V** | **S** | **V** | **D** | **E** | **I** | **N** | **Q** | **K** | **L** | **S** | **Q** | **G** | **W** | **V** | **Q** | **A** | **D** | **S** | **F** | **V** | **P** | **I** | **T** | **V** | **A** | **S** | **E** | **P** | **V** | **T** | **E** | **L** | **P** | **T** | **G** | **A** | **A** | **T** | **K** | **D** | **T** | **E** | **S** | **R** | **Y** | **Y** | **P** | **L** | **G** | **E** | **A** | **A** | **A** | **Q** | **L** | **I** | **G** | **Y** | **T** | **G** | **T** | **I** | **T** | **A** | **E** | **D** | **I** | **E** | **K** | **N** | **P** | **E** | **L** | **S** | **S** | **T** | **G** | **V** | **I** | **G** | **K** | **T** | **G** | **L** | **E** | **R** | **A** | **F** | **D** | **K** | **E** | **L** | **R** | **G** | **Q** | **D** | **G** | **G** | **S** | **L** | **V** | **I** | **L** | **D** | **D** | **K** | **E** | **N** | **V** | **K** | **K** | **A** | **L** | **Q** | **T** | **K** | **E** | **K** | **K** | **D** | **G** | **Q** | **T** | **I** | **K** | **L** | **T** | **I** | **D** | **S** | **G** | **V** | **Q** | **Q** | **Q** | **A** | **F** | **A** | **I** | **F** | **D** | **K** | **R** | **P** | **G** | **S** | **A** | **V** | **I** | **T** | **D** | **P** | **Q** | **K** | **G** | **D** | **L** | **L** | **A** | **T** | **V** | **S** | **S** | **P** | **S** | **Y** | **D** | **P** | **N** | **K** | **M** | **A** | **N** | **G** | **I** | **S** | **Q** | **K** | **E** | **Y** | **D** | **A** | **Y** | **N** | **N** | **N** | **K** | **D** | **L** | **P** | **F** | **T** | **A** | **R** | **F** | **A** | **T** | **G** | **Y** | **A** | **P** | **G** | **S** | **T** | **F** | **K** | **T** | **I** | **T** | **G** | **A** | **I** | **G** | **L** | **D** | **A** | **G** | **T** | **L** | **K** | **P** | **D** | **E** | **E** | **L** | **E** | **I** | **N** | **G** | **L** | **K** | **W** | **Q** | **K** | **D** | **K** | **S** | **W** | **G** | **G** | **Y** | **F** | **A** | **T** | **R** | **V** | **K** | **E** | **A** | **S** | **P** | **V** | **N** | **L** | **R** | **T** | **A** | **L** | **V** | **N** | **S** | **D** | **N** | **I** | **Y** | **F** | **A** | **Q** | **Q** | **T** | **L** | **R** | **M** | **G** | **E** | **D** | **K** | **F** | **R** | **A** | **G** | **L** | **N** | **K** | **F** | **I** | **F** | **G** | **E** | **E** | **L** | **D** | **L** | **P** | **I** | **A** | **M** | **T** | **P** | **A** | **Q** | **I** | **S** | **N** | **E** | **D** | **K** | **F** | **N** | **S** | **E** | **I** | **L** | **L** | **A** | **D** | **T** | **G** | **Y** | **G** | **Q** | **G** | **Q** | **L** | **L** | **I** | **S** | **P** | **I** | **Q** | **Q** | **A** | **T** | **M** | **Y** | **S** | **V** | **F** | **Q** | **N** | **N** | **G** | **T** | **L** | **V** | **Y** | **P** | **K** | **L** | **V** | **L** | **D** | **K** | **E** | **T** | **K** | **K** | **K** | **D** | **N** | **V** | **I** | **S** | **A** | **N** | **A** | **A** | **N** | **T** | **I** | **A** | **T** | **D** | **L** | **L** | **G** | **S** | **V** | **E** | **D** | **P** | **S** | **G** | **Y** | **V** | **Y** | **N** | **M** | **Y** | **N** | **P** | **N** | **F** | **S** | **L** | **A** | **A** | **K** | **T** | **G** | **T** | **A** | **E** | **I** | **K** | **D** | **K** | **Q** | **D** | **T** | **D** | **G** | **K** | **E** | **N** | **S** | **F** | **L** | **L** | **T** | **L** | **D** | **R** | **S** | **N** | **N** | **K** | **F** | **L** | **T** | **M** | **I** | **M** | **V** | **E** | **N** | **S** | **G** | **E** | **N** | **G** | **S** | **A** | **T** | **D** | **I** | **S** | **K** | **P** | **L** | **I** | **D** | **Y** | **L** | **E** | **A** | **T** | **I** | **K** | **-** | **-** | **-** | **-** | **-** | **-** | **-** | **-** | **-** | **-** | **-** | **-** | **-** | **-** | **-** | **-** | **-** | **-** | **-** | **-** | **-** | **-** | **-** | **-** | **-** | **-** | **-** | **-** | **-** | **-** | **-** | **-** | **-** | **-** | **-** | **-** | **Q** | **W** | **Q** | **A** | **K** | **Q** | **E** | **L** | **A** | **E** | **A** | **K** | **K** | **T** | **A** | **T** | **T** | **F** | **L** | **N** | **V** | **L** | **S** | **K** | **Q** | **E** | **F** | **D** | **K** | **L** | **P** | **S** | **V** | **V** | **Q** | **E** | **A** | **S** | **L** | **K** | **K** | **N** | **G** | **Y** | **D** | **T** | **K** | **S** | **V** | **V** | **E** | **K** | **Y** | **Q** | **A** | **I** | **Y** | **S** | **G** | **I** | **Q** | **A** | **E** | **G** | **V** | **K** | **A** | **S** | **D** | **V** | **Q** | **V** | **K** | **K** | **A** | **K** | **D** | **N** | **Q** | **Y** | **T** | **F** | **T** | **Y** | **K** | **L** | **S** | **M** | **S** | **T** | **P** | **L** | **G** | **E** | **M** | **K** | **D** | **L** | **S** | **Y** | **Q** | **S** | **S** | **I** | **A** | **K** | **K** | **G** | **D** | **T** | **Y** | **Q** | **I** | **A** | **W** | **K** | **P** | **S** | **L** | **I** | **F** | **P** | **D** | **M** | **S** | **G** | **N** | **D** | **K** | **I** | **S** | **I** | **Q** | **V** | **D** | **N** | **A** | **K** | **R** | **G** | **E** | **I** | **V** | **D** | **R** | **N** | **G** | **S** | **G** | **L** | **A** | **I** | **N** | **K** | **V** | **F** | **D** | **E** | **V** | **G** | **V** | **V** | **P** | **G** | **K** | **L** | **G** | **S** | **G** | **A** | **E** | **K** | **T** | **A** | **N** | **I** | **K** | **A** | **F** | **S** | **D** | **K** | **F** | **G** | **V** | **S** | **V** | **D** | **E** | **I** | **N** | **Q** | **K** | **L** | **S** | **Q** | **G** | **W** | **V** | **Q** | **A** | **D** | **S** | **F** | **V** | **P** | **I** | **T** | **V** | **A** | **S** | **E** | **P** | **V** | **T** | **E** | **L** | **P** | **T** | **G** | **A** | **A** | **T** | **K** | **D** | **T** | **E** | **S** | **R** | **Y** | **Y** | **P** | **L** | **G** | **E** | **A** | **A** | **A** | **Q** | **L** | **I** | **G** | **Y** | **T** | **G** | **T** | **I** | **T** | **A** | **E** | **D** | **I** | **E** | **K** | **N** | **P** | **E** | **L** | **S** | **S** | **T** | **G** | **V** | **I** | **G** | **K** | **T** | **G** | **L** | **E** | **R** | **A** | **F** | **D** | **K** | **E** | **L** | **R** | **G** | **Q** | **D** | **G** | **G** | **S** | **L** | **V** | **I** | **L** | **D** | **D** | **K** | **E** | **N** | **V** | **K** | **K** | **A** | **L** | **Q** | **T** | **K** | **E** | **K** | **K** | **D** | **G** | **Q** | **T** | **I** | **K** | **L** | **T** | **I** | **D** | **S** | **G** | **V** | **Q** | **Q** | **Q** | **A** | **F** | **A** | **I** | **F** | **D** | **K** | **R** | **P** | **G** | **S** | **A** | **V** | **I** | **T** | **D** | **P** | **Q** | **K** | **G** | **D** | **L** | **L** | **A** | **T** | **V** | **S** | **S** | **P** | **S** | **Y** | **D** | **P** | **N** | **K** | **M** | **A** | **N** | **G** | **I** | **S** | **Q** | **K** | **E** | **Y** | **D** | **A** | **Y** | **N** | **N** | **N** | **K** | **D** | **L** | **P** | **F** | **T** | **A** | **R** | **F** | **A** | **T** | **G** | **Y** | **A** | **P** | **G** | **S** | **T** | **F** | **K** | **T** | **I** | **T** | **G** | **A** | **I** | **G** | **L** | **D** | **A** | **G** | **T** | **L** | **K** | **P** | **D** | **E** | **E** | **L** | **E** | **I** | **N** | **G** | **L** | **K** | **W** | **Q** | **K** | **D** | **K** | **S** | **W** | **G** | **G** | **Y** | **F** | **A** | **T** | **R** | **V** | **K** | **E** | **A** | **S** | **P** | **V** | **N** | **L** | **R** | **T** | **A** | **L** | **V** | **N** | **S** | **D** | **N** | **I** | **Y** | **F** | **A** | **Q** | **Q** | **T** | **L** | **R** | **M** | **G** | **E** | **D** | **K** | **F** | **R** | **A** | **G** | **L** | **N** | **K** | **F** | **I** | **F** | **G** | **E** | **E** | **L** | **D** | **L** | **P** | **I** | **A** | **M** | **T** | **P** | **A** | **Q** | **I** | **S** | **N** | **E** | **D** | **K** | **F** | **N** | **S** | **E** | **I** | **L** | **L** | **A** | **D** | **T** | **G** | **Y** | **G** | **Q** | **G** | **Q** | **L** | **L** | **I** | **S** | **P** | **I** | **Q** | **Q** | **A** | **T** | **M** | **Y** | **S** | **V** | **F** | **Q** | **N** | **N** | **G** | **T** | **L** | **V** | **Y** | **P** | **K** | **L** | **V** | **L** | **D** | **K** | **E** | **T** | **K** | **K** | **K** | **D** | **N** | **V** | **I** | **S** | **A** | **N** | **A** | **A** | **N** | **T** | **I** | **A** | **T** | **D** | **L** | **L** | **G** | **S** | **V** | **E** | **D** | **P** | **S** | **G** | **Y** | **V** | **Y** | **N** | **M** | **Y** | **N** | **P** | **N** | **F** | **S** | **L** | **A** | **A** | **K** | **T** | **G** | **T** | **A** | **E** | **I** | **K** | **D** | **K** | **Q** | **D** | **T** | **D** | **G** | **K** | **E** | **N** | **S** | **F** | **L** | **L** | **T** | **L** | **D** | **R** | **S** | **N** | **N** | **K** | **F** | **L** | **T** | **M** | **I** | **M** | **V** | **E** | **N** | **S** | **G** | **E** | **N** | **G** | **S** | **A** | **T** | **D** | **I** | **S** | **K** | **P** | **L** | **I** | **D** | **Y** | **L** | **E** | **A** | **T** | **I** | **K** |
| **N53420** | **M** | **E** | **R** | **S** | **N** | **R** | **N** | **K** | **K** | **S** | **S** | **K** | **K** | **P** | **L** | **I** | **L** | **G** | **V** | **S** | **A** | **L** | **V** | **L** | **I** | **A** | **A** | **V** | **G** | **G** | **G** | **Y** | **Y** | **A** | **Y** | **S** | **Q** | **W** | **Q** | **A** | **K** | **Q** | **E** | **L** | **A** | **E** | **A** | **K** | **K** | **T** | **A** | **N** | **T** | **F** | **L** | **N** | **V** | **L** | **S** | **K** | **Q** | **E** | **F** | **D** | **K** | **L** | **P** | **S** | **V** | **V** | **Q** | **E** | **T** | **S** | **L** | **K** | **K** | **N** | **G** | **Y** | **D** | **T** | **K** | **S** | **V** | **V** | **E** | **K** | **Y** | **Q** | **A** | **I** | **Y** | **S** | **G** | **I** | **Q** | **A** | **E** | **G** | **V** | **K** | **A** | **S** | **D** | **V** | **Q** | **V** | **K** | **K** | **G** | **K** | **D** | **N** | **Q** | **Y** | **T** | **F** | **T** | **Y** | **K** | **L** | **S** | **M** | **S** | **T** | **P** | **L** | **G** | **E** | **M** | **K** | **D** | **L** | **S** | **Y** | **Q** | **S** | **S** | **I** | **A** | **K** | **K** | **G** | **D** | **N** | **Y** | **Q** | **I** | **A** | **W** | **A** | **P** | **S** | **L** | **I** | **F** | **P** | **D** | **M** | **S** | **G** | **N** | **D** | **K** | **I** | **S** | **I** | **Q** | **V** | **D** | **N** | **A** | **K** | **R** | **G** | **E** | **I** | **V** | **D** | **R** | **N** | **G** | **S** | **G** | **L** | **A** | **I** | **N** | **K** | **V** | **F** | **D** | **E** | **V** | **G** | **V** | **V** | **P** | **G** | **K** | **L** | **G** | **S** | **G** | **A** | **E** | **K** | **T** | **A** | **N** | **I** | **K** | **A** | **F** | **S** | **D** | **K** | **F** | **G** | **V** | **S** | **V** | **D** | **E** | **I** | **N** | **Q** | **K** | **L** | **S** | **Q** | **G** | **W** | **V** | **Q** | **A** | **D** | **S** | **F** | **V** | **P** | **I** | **T** | **V** | **A** | **S** | **E** | **P** | **V** | **T** | **E** | **L** | **P** | **T** | **G** | **A** | **A** | **T** | **K** | **D** | **T** | **E** | **S** | **R** | **Y** | **Y** | **P** | **L** | **G** | **E** | **A** | **A** | **A** | **Q** | **L** | **I** | **G** | **Y** | **T** | **G** | **T** | **I** | **T** | **A** | **E** | **D** | **I** | **E** | **K** | **N** | **P** | **E** | **L** | **S** | **S** | **T** | **G** | **V** | **I** | **G** | **K** | **T** | **G** | **L** | **E** | **R** | **A** | **F** | **D** | **K** | **E** | **L** | **R** | **G** | **Q** | **D** | **G** | **G** | **S** | **L** | **V** | **I** | **L** | **D** | **D** | **K** | **E** | **N** | **V** | **K** | **K** | **A** | **L** | **Q** | **T** | **K** | **E** | **K** | **K** | **D** | **G** | **Q** | **T** | **I** | **K** | **L** | **T** | **I** | **D** | **S** | **G** | **V** | **Q** | **Q** | **Q** | **A** | **F** | **A** | **I** | **F** | **D** | **K** | **R** | **P** | **G** | **S** | **A** | **V** | **I** | **T** | **D** | **P** | **Q** | **K** | **G** | **D** | **L** | **L** | **A** | **T** | **V** | **S** | **S** | **P** | **S** | **Y** | **D** | **P** | **N** | **K** | **M** | **A** | **N** | **G** | **I** | **S** | **Q** | **K** | **E** | **Y** | **D** | **A** | **Y** | **N** | **N** | **N** | **K** | **D** | **L** | **P** | **F** | **T** | **A** | **R** | **F** | **A** | **T** | **G** | **Y** | **A** | **P** | **G** | **S** | **T** | **F** | **K** | **T** | **I** | **T** | **G** | **A** | **I** | **G** | **L** | **D** | **A** | **G** | **T** | **L** | **K** | **P** | **D** | **E** | **E** | **L** | **E** | **I** | **N** | **G** | **L** | **K** | **W** | **Q** | **K** | **D** | **K** | **S** | **W** | **G** | **G** | **Y** | **F** | **A** | **T** | **R** | **V** | **K** | **E** | **A** | **S** | **P** | **V** | **N** | **L** | **R** | **T** | **A** | **L** | **V** | **N** | **S** | **D** | **N** | **I** | **Y** | **F** | **A** | **Q** | **Q** | **T** | **L** | **R** | **M** | **G** | **E** | **D** | **K** | **F** | **R** | **A** | **G** | **L** | **N** | **K** | **F** | **I** | **F** | **G** | **E** | **E** | **L** | **D** | **L** | **P** | **I** | **A** | **M** | **T** | **P** | **A** | **Q** | **I** | **S** | **N** | **E** | **D** | **K** | **F** | **N** | **S** | **E** | **I** | **L** | **L** | **A** | **D** | **T** | **G** | **Y** | **G** | **Q** | **G** | **Q** | **L** | **L** | **I** | **S** | **P** | **I** | **Q** | **Q** | **A** | **T** | **M** | **Y** | **S** | **V** | **F** | **Q** | **N** | **N** | **G** | **T** | **L** | **V** | **Y** | **P** | **K** | **L** | **V** | **L** | **E** | **K** | **E** | **T** | **K** | **K** | **K** | **D** | **N** | **V** | **I** | **S** | **A** | **N** | **A** | **A** | **N** | **T** | **I** | **A** | **T** | **D** | **L** | **L** | **G** | **S** | **V** | **E** | **D** | **P** | **S** | **G** | **Y** | **V** | **Y** | **N** | **M** | **Y** | **N** | **P** | **N** | **F** | **S** | **L** | **A** | **A** | **K** | **T** | **G** | **T** | **A** | **E** | **I** | **K** | **D** | **K** | **Q** | **D** | **T** | **D** | **G** | **K** | **E** | **N** | **S** | **F** | **L** | **L** | **T** | **L** | **D** | **R** | **S** | **N** | **N** | **K** | **F** | **L** | **T** | **M** | **I** | **M** | **V** | **E** | **N** | **S** | **G** | **E** | **N** | **G** | **S** | **A** | **T** | **D** | **I** | **S** | **K** | **P** | **L** | **I** | **D** | **Y** | **L** | **E** | **A** | **T** | **I** | **K** | **M** | **E** | **R** | **S** | **N** | **R** | **N** | **K** | **K** | **S** | **S** | **K** | **K** | **P** | **L** | **I** | **L** | **G** | **V** | **S** | **A** | **L** | **V** | **L** | **I** | **A** | **A** | **V** | **G** | **G** | **G** | **Y** | **Y** | **A** | **Y** | **S** | **Q** | **W** | **Q** | **A** | **K** | **Q** | **E** | **L** | **A** | **E** | **A** | **K** | **K** | **T** | **A** | **N** | **T** | **F** | **L** | **N** | **V** | **L** | **S** | **K** | **Q** | **E** | **F** | **D** | **K** | **L** | **P** | **S** | **V** | **V** | **Q** | **E** | **T** | **S** | **L** | **K** | **K** | **N** | **G** | **Y** | **D** | **T** | **K** | **S** | **V** | **V** | **E** | **K** | **Y** | **Q** | **A** | **I** | **Y** | **S** | **G** | **I** | **Q** | **A** | **E** | **G** | **V** | **K** | **A** | **S** | **D** | **V** | **Q** | **V** | **K** | **K** | **G** | **K** | **D** | **N** | **Q** | **Y** | **T** | **F** | **T** | **Y** | **K** | **L** | **S** | **M** | **S** | **T** | **P** | **L** | **G** | **E** | **M** | **K** | **D** | **L** | **S** | **Y** | **Q** | **S** | **S** | **I** | **A** | **K** | **K** | **G** | **D** | **N** | **Y** | **Q** | **I** | **A** | **W** | **A** | **P** | **S** | **L** | **I** | **F** | **P** | **D** | **M** | **S** | **G** | **N** | **D** | **K** | **I** | **S** | **I** | **Q** | **V** | **D** | **N** | **A** | **K** | **R** | **G** | **E** | **I** | **V** | **D** | **R** | **N** | **G** | **S** | **G** | **L** | **A** | **I** | **N** | **K** | **V** | **F** | **D** | **E** | **V** | **G** | **V** | **V** | **P** | **G** | **K** | **L** | **G** | **S** | **G** | **A** | **E** | **K** | **T** | **A** | **N** | **I** | **K** | **A** | **F** | **S** | **D** | **K** | **F** | **G** | **V** | **S** | **V** | **D** | **E** | **I** | **N** | **Q** | **K** | **L** | **S** | **Q** | **G** | **W** | **V** | **Q** | **A** | **D** | **S** | **F** | **V** | **P** | **I** | **T** | **V** | **A** | **S** | **E** | **P** | **V** | **T** | **E** | **L** | **P** | **T** | **G** | **A** | **A** | **T** | **K** | **D** | **T** | **E** | **S** | **R** | **Y** | **Y** | **P** | **L** | **G** | **E** | **A** | **A** | **A** | **Q** | **L** | **I** | **G** | **Y** | **T** | **G** | **T** | **I** | **T** | **A** | **E** | **D** | **I** | **E** | **K** | **N** | **P** | **E** | **L** | **S** | **S** | **T** | **G** | **V** | **I** | **G** | **K** | **T** | **G** | **L** | **E** | **R** | **A** | **F** | **D** | **K** | **E** | **L** | **R** | **G** | **Q** | **D** | **G** | **G** | **S** | **L** | **V** | **I** | **L** | **D** | **D** | **K** | **E** | **N** | **V** | **K** | **K** | **A** | **L** | **Q** | **T** | **K** | **E** | **K** | **K** | **D** | **G** | **Q** | **T** | **I** | **K** | **L** | **T** | **I** | **D** | **S** | **G** | **V** | **Q** | **Q** | **Q** | **A** | **F** | **A** | **I** | **F** | **D** | **K** | **R** | **P** | **G** | **S** | **A** | **V** | **I** | **T** | **D** | **P** | **Q** | **K** | **G** | **D** | **L** | **L** | **A** | **T** | **V** | **S** | **S** | **P** | **S** | **Y** | **D** | **P** | **N** | **K** | **M** | **A** | **N** | **G** | **I** | **S** | **Q** | **K** | **E** | **Y** | **D** | **A** | **Y** | **N** | **N** | **N** | **K** | **D** | **L** | **P** | **F** | **T** | **A** | **R** | **F** | **A** | **T** | **G** | **Y** | **A** | **P** | **G** | **S** | **T** | **F** | **K** | **T** | **I** | **T** | **G** | **A** | **I** | **G** | **L** | **D** | **A** | **G** | **T** | **L** | **K** | **P** | **D** | **E** | **E** | **L** | **E** | **I** | **N** | **G** | **L** | **K** | **W** | **Q** | **K** | **D** | **K** | **S** | **W** | **G** | **G** | **Y** | **F** | **A** | **T** | **R** | **V** | **K** | **E** | **A** | **S** | **P** | **V** | **N** | **L** | **R** | **T** | **A** | **L** | **V** | **N** | **S** | **D** | **N** | **I** | **Y** | **F** | **A** | **Q** | **Q** | **T** | **L** | **R** | **M** | **G** | **E** | **D** | **K** | **F** | **R** | **A** | **G** | **L** | **N** | **K** | **F** | **I** | **F** | **G** | **E** | **E** | **L** | **D** | **L** | **P** | **I** | **A** | **M** | **T** | **P** | **A** | **Q** | **I** | **S** | **N** | **E** | **D** | **K** | **F** | **N** | **S** | **E** | **I** | **L** | **L** | **A** | **D** | **T** | **G** | **Y** | **G** | **Q** | **G** | **Q** | **L** | **L** | **I** | **S** | **P** | **I** | **Q** | **Q** | **A** | **T** | **M** | **Y** | **S** | **V** | **F** | **Q** | **N** | **N** | **G** | **T** | **L** | **V** | **Y** | **P** | **K** | **L** | **V** | **L** | **E** | **K** | **E** | **T** | **K** | **K** | **K** | **D** | **N** | **V** | **I** | **S** | **A** | **N** | **A** | **A** | **N** | **T** | **I** | **A** | **T** | **D** | **L** | **L** | **G** | **S** | **V** | **E** | **D** | **P** | **S** | **G** | **Y** | **V** | **Y** | **N** | **M** | **Y** | **N** | **P** | **N** | **F** | **S** | **L** | **A** | **A** | **K** | **T** | **G** | **T** | **A** | **E** | **I** | **K** | **D** | **K** | **Q** | **D** | **T** | **D** | **G** | **K** | **E** | **N** | **S** | **F** | **L** | **L** | **T** | **L** | **D** | **R** | **S** | **N** | **N** | **K** | **F** | **L** | **T** | **M** | **I** | **M** | **V** | **E** | **N** | **S** | **G** | **E** | **N** | **G** | **S** | **A** | **T** | **D** | **I** | **S** | **K** | **P** | **L** | **I** | **D** | **Y** | **L** | **E** | **A** | **T** | **I** | **K** |
| **N55338** | **-** | **-** | **-** | **-** | **-** | **-** | **-** | **-** | **-** | **-** | **-** | **-** | **-** | **-** | **-** | **-** | **-** | **-** | **-** | **-** | **-** | **-** | **-** | **-** | **-** | **-** | **-** | **-** | **-** | **-** | **-** | **-** | **-** | **-** | **-** | **-** | **Q** | **W** | **Q** | **A** | **K** | **Q** | **E** | **L** | **A** | **E** | **A** | **K** | **K** | **T** | **A** | **T** | **T** | **F** | **L** | **N** | **V** | **L** | **S** | **K** | **Q** | **E** | **F** | **D** | **K** | **L** | **P** | **S** | **V** | **V** | **Q** | **E** | **A** | **S** | **L** | **K** | **K** | **N** | **G** | **Y** | **D** | **T** | **K** | **S** | **V** | **V** | **E** | **K** | **Y** | **Q** | **A** | **I** | **Y** | **S** | **G** | **I** | **Q** | **A** | **E** | **G** | **V** | **K** | **A** | **S** | **D** | **V** | **Q** | **V** | **K** | **K** | **A** | **K** | **D** | **N** | **Q** | **Y** | **T** | **F** | **T** | **Y** | **K** | **L** | **S** | **M** | **S** | **T** | **P** | **L** | **G** | **E** | **M** | **K** | **D** | **L** | **S** | **Y** | **Q** | **S** | **S** | **I** | **A** | **K** | **K** | **G** | **D** | **T** | **Y** | **Q** | **I** | **A** | **W** | **K** | **P** | **S** | **L** | **I** | **F** | **P** | **D** | **M** | **S** | **G** | **N** | **D** | **K** | **I** | **S** | **I** | **Q** | **V** | **D** | **N** | **A** | **K** | **R** | **G** | **E** | **I** | **V** | **D** | **R** | **N** | **G** | **S** | **G** | **L** | **A** | **I** | **N** | **K** | **V** | **F** | **D** | **E** | **V** | **G** | **V** | **V** | **P** | **G** | **K** | **L** | **G** | **S** | **G** | **A** | **E** | **K** | **T** | **A** | **N** | **I** | **K** | **A** | **F** | **S** | **D** | **K** | **F** | **G** | **V** | **S** | **V** | **D** | **E** | **I** | **N** | **Q** | **K** | **L** | **S** | **Q** | **G** | **W** | **V** | **Q** | **A** | **D** | **S** | **F** | **V** | **P** | **I** | **T** | **V** | **A** | **S** | **E** | **P** | **V** | **T** | **E** | **L** | **P** | **T** | **G** | **A** | **A** | **T** | **K** | **D** | **T** | **E** | **S** | **R** | **Y** | **Y** | **P** | **L** | **G** | **E** | **A** | **A** | **A** | **Q** | **L** | **I** | **G** | **Y** | **T** | **G** | **T** | **I** | **T** | **A** | **E** | **D** | **I** | **E** | **K** | **N** | **P** | **E** | **L** | **S** | **S** | **T** | **G** | **V** | **I** | **G** | **K** | **T** | **G** | **L** | **E** | **R** | **A** | **F** | **D** | **K** | **E** | **L** | **R** | **G** | **Q** | **D** | **G** | **G** | **S** | **L** | **V** | **I** | **L** | **D** | **D** | **K** | **E** | **N** | **V** | **K** | **K** | **A** | **L** | **Q** | **T** | **K** | **E** | **K** | **K** | **D** | **G** | **Q** | **T** | **I** | **K** | **L** | **T** | **I** | **D** | **S** | **G** | **V** | **Q** | **Q** | **Q** | **A** | **F** | **A** | **I** | **F** | **D** | **K** | **R** | **P** | **G** | **S** | **A** | **V** | **I** | **T** | **D** | **P** | **Q** | **K** | **G** | **D** | **L** | **L** | **A** | **T** | **V** | **S** | **S** | **P** | **S** | **Y** | **D** | **P** | **N** | **K** | **M** | **A** | **N** | **G** | **I** | **S** | **Q** | **K** | **E** | **Y** | **D** | **A** | **Y** | **N** | **N** | **N** | **K** | **D** | **L** | **P** | **F** | **T** | **A** | **R** | **F** | **A** | **T** | **G** | **Y** | **A** | **P** | **G** | **S** | **T** | **F** | **K** | **T** | **I** | **T** | **G** | **A** | **I** | **G** | **L** | **D** | **A** | **G** | **T** | **L** | **K** | **P** | **D** | **E** | **E** | **L** | **E** | **I** | **N** | **G** | **L** | **K** | **W** | **Q** | **K** | **D** | **K** | **S** | **W** | **G** | **G** | **Y** | **F** | **A** | **T** | **R** | **V** | **K** | **E** | **A** | **S** | **P** | **V** | **N** | **L** | **R** | **T** | **A** | **L** | **V** | **N** | **S** | **D** | **N** | **I** | **Y** | **F** | **A** | **Q** | **Q** | **T** | **L** | **R** | **M** | **G** | **E** | **D** | **K** | **F** | **R** | **A** | **G** | **L** | **N** | **K** | **F** | **I** | **F** | **G** | **E** | **E** | **L** | **D** | **L** | **P** | **I** | **A** | **M** | **T** | **P** | **A** | **Q** | **I** | **S** | **N** | **E** | **D** | **K** | **F** | **N** | **S** | **E** | **I** | **L** | **L** | **A** | **D** | **T** | **G** | **Y** | **G** | **Q** | **G** | **Q** | **L** | **L** | **I** | **S** | **P** | **I** | **Q** | **Q** | **A** | **T** | **M** | **Y** | **S** | **V** | **F** | **Q** | **N** | **N** | **G** | **T** | **L** | **V** | **Y** | **P** | **K** | **L** | **V** | **L** | **D** | **K** | **E** | **T** | **K** | **K** | **K** | **D** | **N** | **V** | **I** | **S** | **A** | **N** | **A** | **A** | **N** | **T** | **I** | **A** | **T** | **D** | **L** | **L** | **G** | **S** | **V** | **E** | **D** | **P** | **S** | **G** | **Y** | **V** | **Y** | **N** | **M** | **Y** | **N** | **P** | **N** | **F** | **S** | **L** | **A** | **A** | **K** | **T** | **G** | **T** | **A** | **E** | **I** | **K** | **D** | **K** | **Q** | **D** | **T** | **D** | **G** | **K** | **E** | **N** | **S** | **F** | **L** | **L** | **T** | **L** | **D** | **R** | **S** | **N** | **N** | **K** | **F** | **L** | **T** | **M** | **I** | **M** | **V** | **E** | **N** | **S** | **G** | **E** | **N** | **G** | **S** | **A** | **T** | **D** | **I** | **S** | **K** | **P** | **L** | **I** | **D** | **Y** | **L** | **E** | **A** | **T** | **I** | **K** | **-** | **-** | **-** | **-** | **-** | **-** | **-** | **-** | **-** | **-** | **-** | **-** | **-** | **-** | **-** | **-** | **-** | **-** | **-** | **-** | **-** | **-** | **-** | **-** | **-** | **-** | **-** | **-** | **-** | **-** | **-** | **-** | **-** | **-** | **-** | **-** | **Q** | **W** | **Q** | **A** | **K** | **Q** | **E** | **L** | **A** | **E** | **A** | **K** | **K** | **T** | **A** | **T** | **T** | **F** | **L** | **N** | **V** | **L** | **S** | **K** | **Q** | **E** | **F** | **D** | **K** | **L** | **P** | **S** | **V** | **V** | **Q** | **E** | **A** | **S** | **L** | **K** | **K** | **N** | **G** | **Y** | **D** | **T** | **K** | **S** | **V** | **V** | **E** | **K** | **Y** | **Q** | **A** | **I** | **Y** | **S** | **G** | **I** | **Q** | **A** | **E** | **G** | **V** | **K** | **A** | **S** | **D** | **V** | **Q** | **V** | **K** | **K** | **A** | **K** | **D** | **N** | **Q** | **Y** | **T** | **F** | **T** | **Y** | **K** | **L** | **S** | **M** | **S** | **T** | **P** | **L** | **G** | **E** | **M** | **K** | **D** | **L** | **S** | **Y** | **Q** | **S** | **S** | **I** | **A** | **K** | **K** | **G** | **D** | **T** | **Y** | **Q** | **I** | **A** | **W** | **K** | **P** | **S** | **L** | **I** | **F** | **P** | **D** | **M** | **S** | **G** | **N** | **D** | **K** | **I** | **S** | **I** | **Q** | **V** | **D** | **N** | **A** | **K** | **R** | **G** | **E** | **I** | **V** | **D** | **R** | **N** | **G** | **S** | **G** | **L** | **A** | **I** | **N** | **K** | **V** | **F** | **D** | **E** | **V** | **G** | **V** | **V** | **P** | **G** | **K** | **L** | **G** | **S** | **G** | **A** | **E** | **K** | **T** | **A** | **N** | **I** | **K** | **A** | **F** | **S** | **D** | **K** | **F** | **G** | **V** | **S** | **V** | **D** | **E** | **I** | **N** | **Q** | **K** | **L** | **S** | **Q** | **G** | **W** | **V** | **Q** | **A** | **D** | **S** | **F** | **V** | **P** | **I** | **T** | **V** | **A** | **S** | **E** | **P** | **V** | **T** | **E** | **L** | **P** | **T** | **G** | **A** | **A** | **T** | **K** | **D** | **T** | **E** | **S** | **R** | **Y** | **Y** | **P** | **L** | **G** | **E** | **A** | **A** | **A** | **Q** | **L** | **I** | **G** | **Y** | **T** | **G** | **T** | **I** | **T** | **A** | **E** | **D** | **I** | **E** | **K** | **N** | **P** | **E** | **L** | **S** | **S** | **T** | **G** | **V** | **I** | **G** | **K** | **T** | **G** | **L** | **E** | **R** | **A** | **F** | **D** | **K** | **E** | **L** | **R** | **G** | **Q** | **D** | **G** | **G** | **S** | **L** | **V** | **I** | **L** | **D** | **D** | **K** | **E** | **N** | **V** | **K** | **K** | **A** | **L** | **Q** | **T** | **K** | **E** | **K** | **K** | **D** | **G** | **Q** | **T** | **I** | **K** | **L** | **T** | **I** | **D** | **S** | **G** | **V** | **Q** | **Q** | **Q** | **A** | **F** | **A** | **I** | **F** | **D** | **K** | **R** | **P** | **G** | **S** | **A** | **V** | **I** | **T** | **D** | **P** | **Q** | **K** | **G** | **D** | **L** | **L** | **A** | **T** | **V** | **S** | **S** | **P** | **S** | **Y** | **D** | **P** | **N** | **K** | **M** | **A** | **N** | **G** | **I** | **S** | **Q** | **K** | **E** | **Y** | **D** | **A** | **Y** | **N** | **N** | **N** | **K** | **D** | **L** | **P** | **F** | **T** | **A** | **R** | **F** | **A** | **T** | **G** | **Y** | **A** | **P** | **G** | **S** | **T** | **F** | **K** | **T** | **I** | **T** | **G** | **A** | **I** | **G** | **L** | **D** | **A** | **G** | **T** | **L** | **K** | **P** | **D** | **E** | **E** | **L** | **E** | **I** | **N** | **G** | **L** | **K** | **W** | **Q** | **K** | **D** | **K** | **S** | **W** | **G** | **G** | **Y** | **F** | **A** | **T** | **R** | **V** | **K** | **E** | **A** | **S** | **P** | **V** | **N** | **L** | **R** | **T** | **A** | **L** | **V** | **N** | **S** | **D** | **N** | **I** | **Y** | **F** | **A** | **Q** | **Q** | **T** | **L** | **R** | **M** | **G** | **E** | **D** | **K** | **F** | **R** | **A** | **G** | **L** | **N** | **K** | **F** | **I** | **F** | **G** | **E** | **E** | **L** | **D** | **L** | **P** | **I** | **A** | **M** | **T** | **P** | **A** | **Q** | **I** | **S** | **N** | **E** | **D** | **K** | **F** | **N** | **S** | **E** | **I** | **L** | **L** | **A** | **D** | **T** | **G** | **Y** | **G** | **Q** | **G** | **Q** | **L** | **L** | **I** | **S** | **P** | **I** | **Q** | **Q** | **A** | **T** | **M** | **Y** | **S** | **V** | **F** | **Q** | **N** | **N** | **G** | **T** | **L** | **V** | **Y** | **P** | **K** | **L** | **V** | **L** | **D** | **K** | **E** | **T** | **K** | **K** | **K** | **D** | **N** | **V** | **I** | **S** | **A** | **N** | **A** | **A** | **N** | **T** | **I** | **A** | **T** | **D** | **L** | **L** | **G** | **S** | **V** | **E** | **D** | **P** | **S** | **G** | **Y** | **V** | **Y** | **N** | **M** | **Y** | **N** | **P** | **N** | **F** | **S** | **L** | **A** | **A** | **K** | **T** | **G** | **T** | **A** | **E** | **I** | **K** | **D** | **K** | **Q** | **D** | **T** | **D** | **G** | **K** | **E** | **N** | **S** | **F** | **L** | **L** | **T** | **L** | **D** | **R** | **S** | **N** | **N** | **K** | **F** | **L** | **T** | **M** | **I** | **M** | **V** | **E** | **N** | **S** | **G** | **E** | **N** | **G** | **S** | **A** | **T** | **D** | **I** | **S** | **K** | **P** | **L** | **I** | **D** | **Y** | **L** | **E** | **A** | **T** | **I** | **K** |
| **N52587** | **M** | **E** | **R** | **S** | **N** | **R** | **N** | **K** | **K** | **S** | **S** | **K** | **K** | **P** | **L** | **I** | **L** | **G** | **V** | **S** | **A** | **L** | **V** | **L** | **I** | **A** | **A** | **V** | **G** | **G** | **G** | **Y** | **Y** | **A** | **Y** | **S** | **Q** | **W** | **Q** | **A** | **K** | **Q** | **E** | **L** | **A** | **E** | **A** | **K** | **K** | **T** | **A** | **N** | **T** | **F** | **L** | **N** | **V** | **L** | **S** | **K** | **Q** | **E** | **F** | **D** | **K** | **L** | **P** | **S** | **V** | **V** | **Q** | **E** | **T** | **S** | **L** | **K** | **K** | **N** | **G** | **Y** | **D** | **T** | **K** | **S** | **V** | **V** | **E** | **K** | **Y** | **Q** | **A** | **I** | **Y** | **S** | **G** | **I** | **Q** | **A** | **E** | **G** | **V** | **K** | **A** | **S** | **D** | **V** | **Q** | **V** | **K** | **K** | **G** | **K** | **D** | **N** | **Q** | **Y** | **T** | **F** | **T** | **Y** | **K** | **L** | **S** | **M** | **S** | **T** | **P** | **L** | **G** | **E** | **M** | **K** | **D** | **L** | **S** | **Y** | **Q** | **S** | **S** | **I** | **A** | **K** | **K** | **G** | **D** | **N** | **Y** | **Q** | **I** | **A** | **W** | **A** | **P** | **S** | **L** | **I** | **F** | **P** | **D** | **M** | **S** | **G** | **N** | **D** | **K** | **I** | **S** | **I** | **Q** | **V** | **D** | **N** | **A** | **K** | **R** | **G** | **E** | **I** | **V** | **D** | **R** | **N** | **G** | **S** | **G** | **L** | **A** | **I** | **N** | **K** | **V** | **F** | **D** | **E** | **V** | **G** | **V** | **V** | **P** | **G** | **K** | **L** | **G** | **S** | **G** | **A** | **E** | **K** | **T** | **A** | **N** | **I** | **K** | **A** | **F** | **S** | **D** | **K** | **F** | **G** | **V** | **S** | **V** | **D** | **E** | **I** | **N** | **Q** | **K** | **L** | **S** | **Q** | **G** | **W** | **V** | **Q** | **A** | **D** | **S** | **F** | **V** | **P** | **I** | **T** | **V** | **A** | **S** | **E** | **P** | **V** | **T** | **E** | **L** | **P** | **T** | **G** | **A** | **A** | **T** | **K** | **D** | **T** | **E** | **S** | **R** | **Y** | **Y** | **P** | **L** | **G** | **E** | **A** | **A** | **A** | **Q** | **L** | **I** | **G** | **Y** | **T** | **G** | **T** | **I** | **T** | **A** | **E** | **D** | **I** | **E** | **K** | **N** | **P** | **E** | **L** | **S** | **S** | **T** | **G** | **V** | **I** | **G** | **K** | **T** | **G** | **L** | **E** | **R** | **A** | **F** | **D** | **K** | **E** | **L** | **R** | **G** | **Q** | **D** | **G** | **G** | **S** | **L** | **V** | **I** | **L** | **D** | **D** | **K** | **E** | **N** | **V** | **K** | **K** | **A** | **L** | **Q** | **T** | **K** | **E** | **K** | **K** | **D** | **G** | **Q** | **T** | **I** | **K** | **L** | **T** | **I** | **D** | **S** | **G** | **V** | **Q** | **Q** | **Q** | **A** | **F** | **A** | **I** | **F** | **D** | **K** | **R** | **P** | **G** | **S** | **A** | **V** | **I** | **T** | **D** | **P** | **Q** | **K** | **G** | **D** | **L** | **L** | **A** | **T** | **V** | **S** | **S** | **P** | **S** | **Y** | **D** | **P** | **N** | **K** | **M** | **A** | **N** | **G** | **I** | **S** | **Q** | **K** | **E** | **Y** | **D** | **A** | **Y** | **N** | **N** | **N** | **K** | **D** | **L** | **P** | **F** | **T** | **A** | **R** | **F** | **A** | **T** | **G** | **Y** | **A** | **P** | **G** | **S** | **T** | **F** | **K** | **T** | **I** | **T** | **G** | **A** | **I** | **G** | **L** | **D** | **A** | **G** | **T** | **L** | **K** | **P** | **D** | **E** | **E** | **L** | **E** | **I** | **N** | **G** | **L** | **K** | **W** | **Q** | **K** | **D** | **K** | **S** | **W** | **G** | **G** | **Y** | **F** | **A** | **T** | **R** | **V** | **K** | **E** | **A** | **S** | **P** | **V** | **N** | **L** | **R** | **T** | **A** | **L** | **V** | **N** | **S** | **D** | **N** | **I** | **Y** | **F** | **A** | **Q** | **Q** | **T** | **L** | **R** | **M** | **G** | **E** | **D** | **K** | **F** | **R** | **A** | **G** | **L** | **N** | **K** | **F** | **I** | **F** | **G** | **E** | **E** | **L** | **D** | **L** | **P** | **I** | **A** | **M** | **T** | **P** | **A** | **Q** | **I** | **S** | **N** | **E** | **D** | **K** | **F** | **N** | **S** | **E** | **I** | **L** | **L** | **A** | **D** | **T** | **G** | **Y** | **G** | **Q** | **G** | **Q** | **L** | **L** | **I** | **S** | **P** | **I** | **Q** | **Q** | **A** | **T** | **M** | **Y** | **S** | **V** | **F** | **Q** | **N** | **N** | **G** | **T** | **L** | **V** | **Y** | **P** | **K** | **L** | **V** | **L** | **E** | **K** | **E** | **T** | **K** | **K** | **K** | **D** | **N** | **V** | **I** | **S** | **A** | **N** | **A** | **A** | **N** | **T** | **I** | **A** | **T** | **D** | **L** | **L** | **G** | **S** | **V** | **E** | **D** | **P** | **S** | **G** | **Y** | **V** | **Y** | **N** | **M** | **Y** | **N** | **P** | **N** | **F** | **S** | **L** | **A** | **A** | **K** | **T** | **G** | **T** | **A** | **E** | **I** | **K** | **D** | **K** | **Q** | **D** | **T** | **D** | **G** | **K** | **E** | **N** | **S** | **F** | **L** | **L** | **T** | **L** | **D** | **R** | **S** | **N** | **N** | **K** | **F** | **L** | **T** | **M** | **I** | **M** | **V** | **E** | **N** | **S** | **G** | **E** | **N** | **G** | **S** | **A** | **T** | **D** | **I** | **S** | **K** | **P** | **L** | **I** | **D** | **Y** | **L** | **E** | **A** | **T** | **I** | **K** | **M** | **E** | **R** | **S** | **N** | **R** | **N** | **K** | **K** | **S** | **S** | **K** | **K** | **P** | **L** | **I** | **L** | **G** | **V** | **S** | **A** | **L** | **V** | **L** | **I** | **A** | **A** | **V** | **G** | **G** | **G** | **Y** | **Y** | **A** | **Y** | **S** | **Q** | **W** | **Q** | **A** | **K** | **Q** | **E** | **L** | **A** | **E** | **A** | **K** | **K** | **T** | **A** | **N** | **T** | **F** | **L** | **N** | **V** | **L** | **S** | **K** | **Q** | **E** | **F** | **D** | **K** | **L** | **P** | **S** | **V** | **V** | **Q** | **E** | **T** | **S** | **L** | **K** | **K** | **N** | **G** | **Y** | **D** | **T** | **K** | **S** | **V** | **V** | **E** | **K** | **Y** | **Q** | **A** | **I** | **Y** | **S** | **G** | **I** | **Q** | **A** | **E** | **G** | **V** | **K** | **A** | **S** | **D** | **V** | **Q** | **V** | **K** | **K** | **G** | **K** | **D** | **N** | **Q** | **Y** | **T** | **F** | **T** | **Y** | **K** | **L** | **S** | **M** | **S** | **T** | **P** | **L** | **G** | **E** | **M** | **K** | **D** | **L** | **S** | **Y** | **Q** | **S** | **S** | **I** | **A** | **K** | **K** | **G** | **D** | **N** | **Y** | **Q** | **I** | **A** | **W** | **A** | **P** | **S** | **L** | **I** | **F** | **P** | **D** | **M** | **S** | **G** | **N** | **D** | **K** | **I** | **S** | **I** | **Q** | **V** | **D** | **N** | **A** | **K** | **R** | **G** | **E** | **I** | **V** | **D** | **R** | **N** | **G** | **S** | **G** | **L** | **A** | **I** | **N** | **K** | **V** | **F** | **D** | **E** | **V** | **G** | **V** | **V** | **P** | **G** | **K** | **L** | **G** | **S** | **G** | **A** | **E** | **K** | **T** | **A** | **N** | **I** | **K** | **A** | **F** | **S** | **D** | **K** | **F** | **G** | **V** | **S** | **V** | **D** | **E** | **I** | **N** | **Q** | **K** | **L** | **S** | **Q** | **G** | **W** | **V** | **Q** | **A** | **D** | **S** | **F** | **V** | **P** | **I** | **T** | **V** | **A** | **S** | **E** | **P** | **V** | **T** | **E** | **L** | **P** | **T** | **G** | **A** | **A** | **T** | **K** | **D** | **T** | **E** | **S** | **R** | **Y** | **Y** | **P** | **L** | **G** | **E** | **A** | **A** | **A** | **Q** | **L** | **I** | **G** | **Y** | **T** | **G** | **T** | **I** | **T** | **A** | **E** | **D** | **I** | **E** | **K** | **N** | **P** | **E** | **L** | **S** | **S** | **T** | **G** | **V** | **I** | **G** | **K** | **T** | **G** | **L** | **E** | **R** | **A** | **F** | **D** | **K** | **E** | **L** | **R** | **G** | **Q** | **D** | **G** | **G** | **S** | **L** | **V** | **I** | **L** | **D** | **D** | **K** | **E** | **N** | **V** | **K** | **K** | **A** | **L** | **Q** | **T** | **K** | **E** | **K** | **K** | **D** | **G** | **Q** | **T** | **I** | **K** | **L** | **T** | **I** | **D** | **S** | **G** | **V** | **Q** | **Q** | **Q** | **A** | **F** | **A** | **I** | **F** | **D** | **K** | **R** | **P** | **G** | **S** | **A** | **V** | **I** | **T** | **D** | **P** | **Q** | **K** | **G** | **D** | **L** | **L** | **A** | **T** | **V** | **S** | **S** | **P** | **S** | **Y** | **D** | **P** | **N** | **K** | **M** | **A** | **N** | **G** | **I** | **S** | **Q** | **K** | **E** | **Y** | **D** | **A** | **Y** | **N** | **N** | **N** | **K** | **D** | **L** | **P** | **F** | **T** | **A** | **R** | **F** | **A** | **T** | **G** | **Y** | **A** | **P** | **G** | **S** | **T** | **F** | **K** | **T** | **I** | **T** | **G** | **A** | **I** | **G** | **L** | **D** | **A** | **G** | **T** | **L** | **K** | **P** | **D** | **E** | **E** | **L** | **E** | **I** | **N** | **G** | **L** | **K** | **W** | **Q** | **K** | **D** | **K** | **S** | **W** | **G** | **G** | **Y** | **F** | **A** | **T** | **R** | **V** | **K** | **E** | **A** | **S** | **P** | **V** | **N** | **L** | **R** | **T** | **A** | **L** | **V** | **N** | **S** | **D** | **N** | **I** | **Y** | **F** | **A** | **Q** | **Q** | **T** | **L** | **R** | **M** | **G** | **E** | **D** | **K** | **F** | **R** | **A** | **G** | **L** | **N** | **K** | **F** | **I** | **F** | **G** | **E** | **E** | **L** | **D** | **L** | **P** | **I** | **A** | **M** | **T** | **P** | **A** | **Q** | **I** | **S** | **N** | **E** | **D** | **K** | **F** | **N** | **S** | **E** | **I** | **L** | **L** | **A** | **D** | **T** | **G** | **Y** | **G** | **Q** | **G** | **Q** | **L** | **L** | **I** | **S** | **P** | **I** | **Q** | **Q** | **A** | **T** | **M** | **Y** | **S** | **V** | **F** | **Q** | **N** | **N** | **G** | **T** | **L** | **V** | **Y** | **P** | **K** | **L** | **V** | **L** | **E** | **K** | **E** | **T** | **K** | **K** | **K** | **D** | **N** | **V** | **I** | **S** | **A** | **N** | **A** | **A** | **N** | **T** | **I** | **A** | **T** | **D** | **L** | **L** | **G** | **S** | **V** | **E** | **D** | **P** | **S** | **G** | **Y** | **V** | **Y** | **N** | **M** | **Y** | **N** | **P** | **N** | **F** | **S** | **L** | **A** | **A** | **K** | **T** | **G** | **T** | **A** | **E** | **I** | **K** | **D** | **K** | **Q** | **D** | **T** | **D** | **G** | **K** | **E** | **N** | **S** | **F** | **L** | **L** | **T** | **L** | **D** | **R** | **S** | **N** | **N** | **K** | **F** | **L** | **T** | **M** | **I** | **M** | **V** | **E** | **N** | **S** | **G** | **E** | **N** | **G** | **S** | **A** | **T** | **D** | **I** | **S** | **K** | **P** | **L** | **I** | **D** | **Y** | **L** | **E** | **A** | **T** | **I** | **K** |
| **N53434** | **-** | **-** | **-** | **-** | **-** | **-** | **-** | **-** | **-** | **-** | **-** | **-** | **-** | **-** | **-** | **-** | **-** | **-** | **-** | **-** | **-** | **-** | **-** | **-** | **-** | **-** | **-** | **-** | **-** | **-** | **-** | **-** | **-** | **-** | **-** | **-** | **Q** | **W** | **Q** | **A** | **K** | **Q** | **E** | **L** | **A** | **E** | **A** | **K** | **K** | **T** | **A** | **T** | **T** | **F** | **L** | **N** | **V** | **L** | **S** | **K** | **Q** | **E** | **F** | **D** | **K** | **L** | **P** | **S** | **V** | **V** | **Q** | **E** | **A** | **S** | **L** | **K** | **K** | **N** | **G** | **Y** | **D** | **T** | **K** | **S** | **V** | **V** | **E** | **K** | **Y** | **Q** | **A** | **I** | **Y** | **S** | **G** | **I** | **Q** | **A** | **E** | **G** | **V** | **K** | **A** | **S** | **D** | **V** | **Q** | **V** | **K** | **K** | **A** | **K** | **D** | **N** | **Q** | **Y** | **T** | **F** | **T** | **Y** | **K** | **L** | **S** | **M** | **S** | **T** | **P** | **L** | **G** | **E** | **M** | **K** | **D** | **L** | **S** | **Y** | **Q** | **S** | **S** | **I** | **A** | **K** | **K** | **G** | **D** | **T** | **Y** | **Q** | **I** | **A** | **W** | **K** | **P** | **S** | **L** | **I** | **F** | **P** | **D** | **M** | **S** | **G** | **N** | **D** | **K** | **I** | **S** | **I** | **Q** | **V** | **D** | **N** | **A** | **K** | **R** | **G** | **E** | **I** | **V** | **D** | **R** | **N** | **G** | **S** | **G** | **L** | **A** | **I** | **N** | **K** | **V** | **F** | **D** | **E** | **V** | **G** | **V** | **V** | **P** | **G** | **K** | **L** | **G** | **S** | **G** | **A** | **E** | **K** | **T** | **A** | **N** | **I** | **K** | **A** | **F** | **S** | **D** | **K** | **F** | **G** | **V** | **S** | **V** | **D** | **E** | **I** | **N** | **Q** | **K** | **L** | **S** | **Q** | **G** | **W** | **V** | **Q** | **A** | **D** | **S** | **F** | **V** | **P** | **I** | **T** | **V** | **A** | **S** | **E** | **P** | **V** | **T** | **E** | **L** | **P** | **T** | **G** | **A** | **A** | **T** | **K** | **D** | **T** | **E** | **S** | **R** | **Y** | **Y** | **P** | **L** | **G** | **E** | **A** | **A** | **A** | **Q** | **L** | **I** | **G** | **Y** | **T** | **G** | **T** | **I** | **T** | **A** | **E** | **D** | **I** | **E** | **K** | **N** | **P** | **E** | **L** | **S** | **S** | **T** | **G** | **V** | **I** | **G** | **K** | **T** | **G** | **L** | **E** | **R** | **A** | **F** | **D** | **K** | **E** | **L** | **R** | **G** | **Q** | **D** | **G** | **G** | **S** | **L** | **V** | **I** | **L** | **D** | **D** | **K** | **E** | **N** | **V** | **K** | **K** | **A** | **L** | **Q** | **T** | **K** | **E** | **K** | **K** | **D** | **G** | **Q** | **T** | **I** | **K** | **L** | **T** | **I** | **D** | **S** | **G** | **V** | **Q** | **Q** | **Q** | **A** | **F** | **A** | **I** | **F** | **D** | **K** | **R** | **P** | **G** | **S** | **A** | **V** | **I** | **T** | **D** | **P** | **Q** | **K** | **G** | **D** | **L** | **L** | **A** | **T** | **V** | **S** | **S** | **P** | **S** | **Y** | **D** | **P** | **N** | **K** | **M** | **A** | **N** | **G** | **I** | **S** | **Q** | **K** | **E** | **Y** | **D** | **A** | **Y** | **N** | **N** | **N** | **K** | **D** | **L** | **P** | **F** | **T** | **A** | **R** | **F** | **A** | **T** | **G** | **Y** | **A** | **P** | **G** | **S** | **T** | **F** | **K** | **T** | **I** | **T** | **G** | **A** | **I** | **G** | **L** | **D** | **A** | **G** | **T** | **L** | **K** | **P** | **D** | **E** | **E** | **L** | **E** | **I** | **N** | **G** | **L** | **K** | **W** | **Q** | **K** | **D** | **K** | **S** | **W** | **G** | **G** | **Y** | **F** | **A** | **T** | **R** | **V** | **K** | **E** | **A** | **S** | **P** | **V** | **N** | **L** | **R** | **T** | **A** | **L** | **V** | **N** | **S** | **D** | **N** | **I** | **Y** | **F** | **A** | **Q** | **Q** | **T** | **L** | **R** | **M** | **G** | **E** | **D** | **K** | **F** | **R** | **A** | **G** | **L** | **N** | **K** | **F** | **I** | **F** | **G** | **E** | **E** | **L** | **D** | **L** | **P** | **I** | **A** | **M** | **T** | **P** | **A** | **Q** | **I** | **S** | **N** | **E** | **D** | **K** | **F** | **N** | **S** | **E** | **I** | **L** | **L** | **A** | **D** | **T** | **G** | **Y** | **G** | **Q** | **G** | **Q** | **L** | **L** | **I** | **S** | **P** | **I** | **Q** | **Q** | **A** | **T** | **M** | **Y** | **S** | **V** | **F** | **Q** | **N** | **N** | **G** | **T** | **L** | **V** | **Y** | **P** | **K** | **L** | **V** | **L** | **D** | **K** | **E** | **T** | **K** | **K** | **K** | **D** | **N** | **V** | **I** | **S** | **A** | **N** | **A** | **A** | **N** | **T** | **I** | **A** | **T** | **D** | **L** | **L** | **G** | **S** | **V** | **E** | **D** | **P** | **S** | **G** | **Y** | **V** | **Y** | **N** | **M** | **Y** | **N** | **P** | **N** | **F** | **S** | **L** | **A** | **A** | **K** | **T** | **G** | **T** | **A** | **E** | **I** | **K** | **D** | **K** | **Q** | **D** | **T** | **D** | **G** | **K** | **E** | **N** | **S** | **F** | **L** | **L** | **T** | **L** | **D** | **R** | **S** | **N** | **N** | **K** | **F** | **L** | **T** | **M** | **I** | **M** | **V** | **E** | **N** | **S** | **G** | **E** | **N** | **G** | **S** | **A** | **T** | **D** | **I** | **S** | **K** | **P** | **L** | **I** | **D** | **Y** | **L** | **E** | **A** | **T** | **I** | **K** | **-** | **-** | **-** | **-** | **-** | **-** | **-** | **-** | **-** | **-** | **-** | **-** | **-** | **-** | **-** | **-** | **-** | **-** | **-** | **-** | **-** | **-** | **-** | **-** | **-** | **-** | **-** | **-** | **-** | **-** | **-** | **-** | **-** | **-** | **-** | **-** | **Q** | **W** | **Q** | **A** | **K** | **Q** | **E** | **L** | **A** | **E** | **A** | **K** | **K** | **T** | **A** | **T** | **T** | **F** | **L** | **N** | **V** | **L** | **S** | **K** | **Q** | **E** | **F** | **D** | **K** | **L** | **P** | **S** | **V** | **V** | **Q** | **E** | **A** | **S** | **L** | **K** | **K** | **N** | **G** | **Y** | **D** | **T** | **K** | **S** | **V** | **V** | **E** | **K** | **Y** | **Q** | **A** | **I** | **Y** | **S** | **G** | **I** | **Q** | **A** | **E** | **G** | **V** | **K** | **A** | **S** | **D** | **V** | **Q** | **V** | **K** | **K** | **A** | **K** | **D** | **N** | **Q** | **Y** | **T** | **F** | **T** | **Y** | **K** | **L** | **S** | **M** | **S** | **T** | **P** | **L** | **G** | **E** | **M** | **K** | **D** | **L** | **S** | **Y** | **Q** | **S** | **S** | **I** | **A** | **K** | **K** | **G** | **D** | **T** | **Y** | **Q** | **I** | **A** | **W** | **K** | **P** | **S** | **L** | **I** | **F** | **P** | **D** | **M** | **S** | **G** | **N** | **D** | **K** | **I** | **S** | **I** | **Q** | **V** | **D** | **N** | **A** | **K** | **R** | **G** | **E** | **I** | **V** | **D** | **R** | **N** | **G** | **S** | **G** | **L** | **A** | **I** | **N** | **K** | **V** | **F** | **D** | **E** | **V** | **G** | **V** | **V** | **P** | **G** | **K** | **L** | **G** | **S** | **G** | **A** | **E** | **K** | **T** | **A** | **N** | **I** | **K** | **A** | **F** | **S** | **D** | **K** | **F** | **G** | **V** | **S** | **V** | **D** | **E** | **I** | **N** | **Q** | **K** | **L** | **S** | **Q** | **G** | **W** | **V** | **Q** | **A** | **D** | **S** | **F** | **V** | **P** | **I** | **T** | **V** | **A** | **S** | **E** | **P** | **V** | **T** | **E** | **L** | **P** | **T** | **G** | **A** | **A** | **T** | **K** | **D** | **T** | **E** | **S** | **R** | **Y** | **Y** | **P** | **L** | **G** | **E** | **A** | **A** | **A** | **Q** | **L** | **I** | **G** | **Y** | **T** | **G** | **T** | **I** | **T** | **A** | **E** | **D** | **I** | **E** | **K** | **N** | **P** | **E** | **L** | **S** | **S** | **T** | **G** | **V** | **I** | **G** | **K** | **T** | **G** | **L** | **E** | **R** | **A** | **F** | **D** | **K** | **E** | **L** | **R** | **G** | **Q** | **D** | **G** | **G** | **S** | **L** | **V** | **I** | **L** | **D** | **D** | **K** | **E** | **N** | **V** | **K** | **K** | **A** | **L** | **Q** | **T** | **K** | **E** | **K** | **K** | **D** | **G** | **Q** | **T** | **I** | **K** | **L** | **T** | **I** | **D** | **S** | **G** | **V** | **Q** | **Q** | **Q** | **A** | **F** | **A** | **I** | **F** | **D** | **K** | **R** | **P** | **G** | **S** | **A** | **V** | **I** | **T** | **D** | **P** | **Q** | **K** | **G** | **D** | **L** | **L** | **A** | **T** | **V** | **S** | **S** | **P** | **S** | **Y** | **D** | **P** | **N** | **K** | **M** | **A** | **N** | **G** | **I** | **S** | **Q** | **K** | **E** | **Y** | **D** | **A** | **Y** | **N** | **N** | **N** | **K** | **D** | **L** | **P** | **F** | **T** | **A** | **R** | **F** | **A** | **T** | **G** | **Y** | **A** | **P** | **G** | **S** | **T** | **F** | **K** | **T** | **I** | **T** | **G** | **A** | **I** | **G** | **L** | **D** | **A** | **G** | **T** | **L** | **K** | **P** | **D** | **E** | **E** | **L** | **E** | **I** | **N** | **G** | **L** | **K** | **W** | **Q** | **K** | **D** | **K** | **S** | **W** | **G** | **G** | **Y** | **F** | **A** | **T** | **R** | **V** | **K** | **E** | **A** | **S** | **P** | **V** | **N** | **L** | **R** | **T** | **A** | **L** | **V** | **N** | **S** | **D** | **N** | **I** | **Y** | **F** | **A** | **Q** | **Q** | **T** | **L** | **R** | **M** | **G** | **E** | **D** | **K** | **F** | **R** | **A** | **G** | **L** | **N** | **K** | **F** | **I** | **F** | **G** | **E** | **E** | **L** | **D** | **L** | **P** | **I** | **A** | **M** | **T** | **P** | **A** | **Q** | **I** | **S** | **N** | **E** | **D** | **K** | **F** | **N** | **S** | **E** | **I** | **L** | **L** | **A** | **D** | **T** | **G** | **Y** | **G** | **Q** | **G** | **Q** | **L** | **L** | **I** | **S** | **P** | **I** | **Q** | **Q** | **A** | **T** | **M** | **Y** | **S** | **V** | **F** | **Q** | **N** | **N** | **G** | **T** | **L** | **V** | **Y** | **P** | **K** | **L** | **V** | **L** | **D** | **K** | **E** | **T** | **K** | **K** | **K** | **D** | **N** | **V** | **I** | **S** | **A** | **N** | **A** | **A** | **N** | **T** | **I** | **A** | **T** | **D** | **L** | **L** | **G** | **S** | **V** | **E** | **D** | **P** | **S** | **G** | **Y** | **V** | **Y** | **N** | **M** | **Y** | **N** | **P** | **N** | **F** | **S** | **L** | **A** | **A** | **K** | **T** | **G** | **T** | **A** | **E** | **I** | **K** | **D** | **K** | **Q** | **D** | **T** | **D** | **G** | **K** | **E** | **N** | **S** | **F** | **L** | **L** | **T** | **L** | **D** | **R** | **S** | **N** | **N** | **K** | **F** | **L** | **T** | **M** | **I** | **M** | **V** | **E** | **N** | **S** | **G** | **E** | **N** | **G** | **S** | **A** | **T** | **D** | **I** | **S** | **K** | **P** | **L** | **I** | **D** | **Y** | **L** | **E** | **A** | **T** | **I** | **K** |
 **SNP position** | . | . | . | . | . | . | . | . | . | . | . | . | . | . | . | . | . | . | . | . | . | . | . | . | . | . | . | . | . | . | . | . | . | . | . | . | . | . | . | . | . | . | . | . | . | . | . | . | . | 50 | . | 52 | 53 | . | . | . | . | . | 59 | . | . | 62 | . | . | . | . | . | . | . | 70 | . | . | 73 | . | . | . | . | . | . | . | . | . | . | . | . | . | . | . | . | . | . | . | . | . | . | . | . | . | . | 100 | . | . | . | . | . | . | . | . | . | . | 111 | . | . | . | . | . | . | . | 119 | . | . | . | . | . | . | . | . | . | . | . | . | . | . | . | . | . | . | . | . | . | 141 | . | . | . | . | 146 | . | . | . | 150 | . | 152 | . | . | . | . | . | . | . | . | . | . | . | . | . | 166 | . | . | . | . | . | . | . | . | . | . | . | . | . | . | . | . | . | . | . | . | 187 | . | . | . | . | . | . | . | . | . | . | . | . | 200 | . | . | . | . | . | . | . | . | 209 | . | . | . | . | . | . | . | . | 218 | . | . | . | . | 223 | . | . | . | . | 228 | . | . | . | . | . | . | . | . | . | . | . | . | . | . | 243 | . | . | . | . | . | . | . | . | . | . | . | . | . | . | . | . | . | 261 | . | . | . | . | . | . | . | . | . | . | . | . | . | . | . | . | . | . | . | . | . | . | . | . | 286 | . | . | 289 | . | . | . | . | . | . | . | . | . | . | . | . | . | . | . | . | . | . | . | . | . | . | . | . | . | . | . | . | . | . | . | . | . | . | . | . | . | . | . | . | . | . | . | . | . | . | . | . | 338 | . | . | . | . | . | . | . | . | . | . | . | . | . | . | . | . | . | . | . | . | . | . | . | . | . | . | 365 | . | . | . | 369 | . | . | . | . | . | . | . | . | . | . | . | . | . | . | . | . | . | . | . | . | . | . | . | . | . | . | . | . | . | . | 400 | . | . | . | . | . | . | . | . | . | . | . | . | . | . | . | . | . | . | . | . | . | . | . | . | . | . | . | . | . | . | . | . | . | . | . | . | 437 | . | . | . | . | . | . | . | . | . | . | . | . | . | . | . | . | . | . | . | . | . | . | . | . | . | . | . | . | . | . | . | . | . | . | . | . | . | 475 | . | . | . | . | . | . | . | . | . | . | . | . | . | . | . | . | . | . | . | . | . | . | . | . | . | . | . | . | . | . | . | . | . | . | . | . | . | . | . | . | . | . | . | . | 520 | . | . | . | . | . | . | . | . | . | . | . | . | . | . | . | . | . | . | . | . | . | . | . | . | . | . | . | . | . | . | . | . | . | . | . | . | . | . | . | . | . | . | . | . | . | . | . | . | . | 570 | . | . | 573 | . | 575 | 576 | 577 | . | . | . | . | 582 | . | . | . | . | . | . | . | . | . | . | . | . | . | . | . | . | . | . | . | . | . | . | 605 | . | . | . | . | . | . | . | . | . | . | . | . | . | . | . | . | . | 623 | . | . | . | . | . | . | . | . | 632 | . | . | . | . | . | . | . | . | . | . | . | . | . | . | . | . | . | . | . | . | . | . | . | . | . | . | . | . | . | . | . | . | . | . | . | . | . | . | . | . | . | . | . | . | . | . | . | . |

|  |  |
| --- | --- |
|  |

|  |  |
| --- | --- |
|  |

|  |  |
| --- | --- |
| **Legend** | |
  | **Reference Sequence** |

  |
|  | **Validated SNPs** |
|  | **Non-validated SNPs** |
|  | **New SNPs** |
|  | **GAPs** |
|  |  |

  
  |
|  |  |
