# Supplementary material for: sraX: A Novel Comprehensive Resistome Analysis Tool
Source: Front Microbiol. 2020 Feb 5;11:52. doi: 10.3389/fmicb.2020.00052 (PMC7025521; doi:10.3389/fmicb.2020.00052)
Supplement: Supplementary file 4 [file Data_Sheet_3.ZIP › parC/parC_dna.html]

 
 
 
 

   parC   

    
    
    Definition:  Not indicated  
    
    
    Reference organism:  Not indicated     
    
     Summary Data     Parameter    Value (n)     Total number of genomes  101    MSA length  2451    Total number of SNPs  152    SNP density (# SNPs/MSA length)  0.062      
    
    
    
 
    Enterococci (sraxID_P5emqHy6qk7D)    
   A   
   T   
   G   
   G   
   A   
   A   
   A   
   A   
   T   
   C   
   G   
   A   
   C   
   A   
   A   
   G   
   A   
   A   
   G   
   T   
   A   
   C   
   A   
   A   
   G   
   A   
   A   
   T   
   T   
   A   
   A   
   C   
   T   
   T   
   T   
   A   
   G   
   A   
   A   
   G   
   A   
   A   
   G   
   T   
   G   
   A   
   T   
   G   
   G   
   G   
   T   
   G   
   A   
   C   
   C   
   G   
   C   
   T   
   T   
   C   
   G   
   G   
   A   
   C   
   G   
   T   
   T   
   A   
   T   
   T   
   C   
   C   
   A   
   A   
   A   
   T   
   A   
   T   
   A   
   T   
   T   
   A   
   T   
    C    
   C   
   A   
    A    
   G   
   A   
   A   
   C   
   G   
   T   
   G   
   C   
   A   
   C   
   T   
   G   
   C   
   C   
   T   
   G   
   A   
   T   
   A   
   T   
    C    
   C   
   G   
   T   
   G   
   A   
   T   
   G   
   G   
   A   
   T   
   T   
   G   
   A   
   A   
    G    
   C   
   C   
   A   
   G   
   T   
    G    
   C   
   A   
   A   
   C   
   G   
   C   
   C   
   G   
   G   
   A   
   T   
   A   
   T   
   T   
   A   
   T   
   T   
    T    
   G   
   C   
    C    
   A   
   T   
   G   
   A   
   A   
   C   
   A   
   A   
   A   
   G   
   A   
   T   
   G   
   G   
   C   
   A   
   A   
   T   
   A   
   C   
   G   
   T   
   A   
   T   
   G   
   A   
   C   
   A   
   A   
   A   
   G   
   G   
   A   
   T   
   T   
    C    
   C   
   G   
   T   
   A   
   A   
   A   
   T   
   C   
   A   
   G   
   C   
   A   
   A   
   A   
   A   
   T   
   C   
   T   
   G   
   T   
   C   
   G   
   G   
   A   
   A   
   A   
   T   
   A   
   T   
   C   
   A   
   T   
   G   
   G   
   G   
    G    
   A   
   A   
   T   
   T   
   A   
   T   
   C   
   A   
   T   
   C   
   C   
   T   
   C   
   A   
   T   
   G   
   G   
   T   
   G   
   A   
   C   
   A   
   G   
   C   
   A   
   G   
   T   
   A   
   T   
   T   
   T   
   A   
   T   
   G   
   A   
   A   
   G   
   C   
   G   
   A   
   T   
   G   
   G   
   T   
   C   
   C   
   G   
   C   
   A   
   T   
   G   
   A   
   G   
   C   
   C   
   A   
   A   
   G   
   A   
    C    
   T   
   G   
   G   
   A   
   A   
   G   
   T   
   T   
   G   
   C   
   G   
   G   
   G   
   A   
   A   
   G   
   T   
   A   
   C   
   T   
   G   
   A   
   T   
   C   
   G   
   A   
    A    
   A   
   T   
   G   
   C   
   A   
   C   
   G   
   G   
    G    
   A   
   A   
   T   
   A   
   A   
    C    
   G   
   G   
   G   
   A   
   G   
   T   
   A   
   T   
   G   
   G   
   A   
   C   
   G   
   G   
   T   
   G   
   A   
   T   
   C   
   C   
   A   
   C   
   C   
   T   
   G   
   C   
   T   
   G   
   C   
   G   
   A   
   T   
   G   
   C   
   G   
   T   
   T   
   A   
   T   
   A   
   C   
   T   
   G   
   A   
   A   
   G   
   C   
   C   
   A   
   G   
    A    
   T   
   T   
   A   
   T   
   C   
   C   
   A   
   A   
   G   
   T   
   T   
   A   
   A   
   G   
   T   
   G   
   G   
   A   
   G   
   A   
   G   
   A   
   T   
   G   
    C    
   T   
   A   
    G    
   C   
   A   
   G   
   A   
   T   
   A   
   T   
   C   
   G   
   A   
    G    
   A   
   A   
   A   
   G   
   A   
   A   
   A   
   C   
   A   
   G   
   T   
    C    
   G   
   A   
   T   
   C   
   T   
   C   
   G   
   T   
   G   
   T   
   G   
   G   
   A   
   A   
   T   
   T   
   T   
   T   
   G   
   A   
   T   
   G   
   A   
   T   
   A   
   C   
   A   
   G   
   A   
   A   
   A   
   A   
   A   
   G   
   A   
   A   
   C   
   C   
   A   
   A   
   C   
   A   
   G   
   T   
   C   
   C   
   T   
   T   
   C   
   C   
   A   
   G   
   C   
   T   
   C   
   G   
   A   
   T   
   A   
   C   
   C   
   C   
   A   
   A   
   A   
   C   
   C   
   T   
   T   
   C   
   T   
    C    
   G   
   T   
   C   
   A   
   A   
   T   
   G   
   G   
   A   
   T   
   C   
   G   
   A   
   C   
   A   
   G   
   G   
   T   
   A   
   T   
   A   
   T   
   C   
   T   
   G   
   C   
   T   
   G   
   G   
   A   
   T   
   A   
   T   
   G   
   C   
    A    
   A   
   C   
   A   
   G   
   A   
   A   
   A   
   T   
   C   
   C   
   C   
   G   
   A   
   C   
    T    
   C   
   A   
   T   
   A   
   A   
   C   
   T   
   T   
   A   
   G   
   C   
   T   
   G   
   A   
   A   
   G   
   T   
   G   
   A   
   T   
    C    
   G   
   A   
    T    
   G   
   G   
   C   
   A   
   C   
   A   
   A   
   T   
   C   
   T   
   A   
   T   
   A   
   T   
   G   
   A   
   T   
   C   
   G   
   A   
   T   
   C   
   A   
   T   
   C   
   C   
   A   
   C   
   A   
   A   
   G   
   C   
   G   
   A   
   G   
   C   
   C   
   T   
   T   
   G   
   A   
   A   
   A   
   A   
   A   
   T   
   T   
   G   
   A   
   T   
   G   
   G   
   A   
   A   
   T   
   A   
   T   
   A   
   T   
   T   
   C   
   C   
   T   
   G   
   G   
   A   
   C   
   C   
   T   
   G   
   A   
   C   
   T   
   T   
    T    
   C   
   C   
   A   
   A   
   C   
   A   
   G   
   G   
   A   
   G   
   G   
    G    
   A   
   T   
   C   
   T   
   T   
   G   
   C   
   A   
   A   
   G   
   G   
    A    
   A   
   A   
   A   
   G   
   A   
   T   
   G   
   A   
   A   
   A   
   T   
   C   
   A   
   A   
   A   
   A   
   A   
   A   
   G   
   C   
   T   
   T   
   A   
   T   
   G   
   A   
   A   
   A   
   C   
   A   
   G   
   G   
    A    
   C   
   G   
   A   
   G   
   G   
   A   
   A   
   A   
   A   
   G   
   T   
   T   
   A   
    T    
   T   
   C   
   T   
   T   
   C   
   G   
   T   
   T   
   C   
   G   
   A   
   A   
   A   
   A   
   C   
   A   
   A   
   A   
   G   
   A   
   T   
   A   
   G   
   A   
    G    
   G   
   C   
   A   
   A   
   T   
   G   
   A   
   A   
   G   
   G   
   G   
    T    
   A   
   A   
    T    
   A   
   A   
   A   
   C   
   A   
   G   
   C   
   A   
   A   
   A   
   T   
   C   
   G   
   T   
   G   
   A   
   T   
   C   
   A   
   C   
    A    
   G   
   A   
   A   
   A   
   T   
   C   
   C   
   C   
   A   
   T   
   A   
   T   
   G   
   A   
   A   
   G   
   T   
   A   
   A   
   A   
   T   
   A   
   A   
   A   
   G   
   C   
   G   
   A   
   C   
    C    
   T   
   T   
   G   
   G   
   T   
   G   
   A   
   A   
   A   
   A   
   A   
   A   
   A   
   T   
   G   
   G   
   A   
   T   
   G   
   A   
   G   
   A   
   T   
   C   
   C   
   G   
   T   
   T   
   T   
   G   
   A   
   A   
   T   
   A   
   A   
   G   
   A   
   A   
   A   
   A   
   T   
   C   
   G   
   A   
    T    
   G   
   G   
   A   
   A   
   T   
   A   
   G   
   C   
   T   
   G   
   A   
   A   
   G   
   T   
    A    
   C   
   G   
   A   
   G   
   A   
   T   
   G   
   A   
   A   
   A   
   G   
   T   
   G   
   A   
   T   
    A    
   G   
    A    
   A   
   C   
    A    
   G   
   G   
   T   
   C   
   T   
   G   
   C   
   A   
   A   
   A   
   T   
   T   
   G   
   T   
   C   
   G   
   T   
    C    
   G   
   A   
   A   
   T   
   T   
   G   
   A   
   A   
   A   
   A   
   A   
   A   
   G   
   A   
    C    
   G   
   C   
   C   
   A   
   A   
    T    
   G   
   C   
   T   
   C   
   A   
   A   
   G   
   G   
    A    
   A   
   T   
   T   
   T   
   T   
   G   
   A   
   A   
   T   
   T   
   A   
    T    
   T   
   T   
   G   
   T   
   T   
   C   
   A   
   A   
   A   
   A   
   A   
   C   
   A   
   C   
   T   
   G   
   A   
   G   
    C    
   T   
   A   
   C   
   A   
   G   
   A   
   T   
   C   
   A   
   A   
   T   
   T   
   A   
   C   
   A   
   A   
   T   
   T   
   T   
    C    
   A   
   A   
   T   
   A   
   T   
   G   
   G   
   T   
   C   
   G   
   C   
   T   
   A   
   T   
   C   
   G   
   A   
    C    
   C   
   A   
   T   
   A   
   T   
   G   
   A   
   C   
   T   
   C   
   C   
   G   
   C   
   A   
    T    
   C   
   A   
   A   
   G   
   T   
   T   
   G   
   G   
   A   
   C   
   T   
   G   
   A   
   A   
   A   
   G   
   A   
   C   
   A   
   T   
   T   
   T   
   T   
   A   
   A   
   G   
   A   
   A   
   G   
   T   
   T   
   A   
   T   
   A   
   T   
   C   
   G   
   A   
   G   
   C   
   A   
   T   
   C   
   G   
   A   
   A   
   A   
   G   
   C   
   A   
   A   
   G   
   T   
   C   
   A   
   T   
   A   
   A   
   C   
   A   
   A   
   A   
   A   
   C   
   G   
    T    
   A   
   G   
    T    
   C   
   A   
   A   
   T   
   T   
   C   
   G   
   A   
   T   
   T   
   T   
   A   
   G   
   C   
   C   
   A   
   A   
   A   
   G   
   C   
   T   
   C   
   A   
   A   
   A   
   A   
   A   
    A    
   G   
   A   
   C   
   A   
   G   
   C   
   A   
   T   
   A   
   T   
   C   
   G   
   T   
    A    
   G   
   A   
   A   
   G   
   G   
    A    
    T    
   T   
   G   
   A   
   T   
   G   
   A   
   A   
   A   
   G   
   C   
   T   
   T   
   T   
   A   
   T   
   C   
   G   
   A   
   T   
   T   
   C   
   T   
   G   
   G   
   A   
   T   
   G   
   A   
   A   
   G   
   T   
   A   
   A   
   T   
   C   
   G   
    C    
   A   
   A   
   C   
   G   
   A   
   T   
   T   
   C   
   G   
   T   
   G   
   A   
   A   
   A   
   G   
   T   
   A   
   A   
   A   
   G   
   A   
   C   
   A   
   A   
   A   
   A   
   A   
   A   
   G   
   A   
    C    
   G   
   C   
    C    
   A   
   A   
   A   
   A   
   A   
   G   
   A   
   A   
   T   
   T   
   T   
   A   
   G   
   T   
   C   
   G   
   A   
    C    
   G   
   T   
    C    
   T   
   T   
    T    
   C   
   A   
   A   
   T   
   T   
   T   
   A   
   C   
   A   
   G   
   A   
   A   
   G   
   A   
   A   
   C   
   A   
   A   
   G   
   C   
   A   
   G   
   A   
   A   
   G   
   C   
   A   
   A   
   T   
    T    
   G   
   T   
   C   
   A   
   C   
   A   
   T   
   T   
   G   
   C   
   A   
   A   
    T    
   T   
   A   
   T   
   A   
    C    
   C   
   G   
   T   
   T   
   T   
   G   
   A   
   C   
   G   
   A   
   A   
    C    
   A   
   C   
    G    
   G   
   A   
   T   
   A   
   T   
   C   
   A   
   C   
   A   
   G   
   A   
   G   
   C   
   T   
   T   
   C   
   A   
   A   
   A   
   A   
   G   
   G   
   A   
   A   
    T    
   C   
    C    
   G   
   A   
   A   
   A   
    G    
   C   
   T   
   T   
   G   
   A   
   T   
   T   
   G   
   C   
    G    
   C   
   A   
   A   
   A   
   T   
   C   
   A   
   C   
   A   
   G   
   A   
   G   
   T   
   T   
    G    
   A   
   A   
   T   
   A   
   A   
   A   
   A   
   T   
   C   
   C   
   T   
   T   
   T   
   C   
   A   
   A   
   A   
    T    
   G   
   A   
   C   
   A   
   A   
   A   
   G   
   A   
    G    
   C   
   T   
   T   
   T   
   T   
    C    
   T   
   C   
   T   
   G   
   T   
   T   
   A   
   T   
   G   
   A   
   A   
   A   
   A   
   A   
   A   
   G   
   A   
   A   
   T   
   T   
   G   
   C   
   G   
   A   
   G   
   A   
   A   
   G   
   T   
   G   
   A   
   A   
   A   
   A   
   A   
   A   
   A   
   A   
   T   
   T   
   A   
    T    
   T   
   C   
   T   
   T   
   C   
   C   
   G   
   C   
   T   
   C   
   G   
   T   
   C   
    T    
    A    
   A   
   C   
   G   
   A   
   C   
   A   
   A   
   T   
   C   
    G    
   A   
   A   
   G   
   A   
   C   
   G   
   A   
   A   
   A   
   T   
   C   
   G   
   A   
   A   
   G   
   A   
   A   
   A   
   T   
   C   
   A   
   A   
   A   
   A   
   T   
    T    
   G   
   A   
   T   
   A   
   C   
   G   
   C   
   A   
   A   
    G    
   T   
    C    
   T   
   T   
   A   
   G   
   T   
   G   
   G   
   C   
    A    
   C   
   A   
   A   
   G   
   A   
   A   
   G   
   A   
   C   
   G   
   T   
   A   
   A   
   T   
    T    
   G   
   T   
   C   
   A   
   G   
   T   
   G   
   T   
    G    
   A   
   C   
    G    
   C   
   G   
   A   
   G   
   A   
   A   
   G   
   G   
   C   
   T   
   A   
   T   
   G   
   T   
   G   
   A   
   A   
   G   
   C   
   G   
   A   
   A   
   C   
   A   
   A   
   G   
   C   
   C   
   T   
   T   
   C   
   G   
   T   
   T   
   C   
    T    
   T   
   A   
   T   
   A   
   G   
   T   
   G   
   C   
   T   
   T   
   C   
    C    
   A   
   A   
   A   
   C   
   C   
   A   
   G   
   A   
   A   
   G   
   A   
   A   
   A   
   T   
   C   
   G   
   G   
   G   
   A   
   T   
   G   
   C   
   G   
   A   
   G   
   A   
   A   
   G   
   G   
   C   
   G   
   A   
    C    
   T   
   A   
   C   
   T   
   T   
   G   
   C   
   T   
    A    
   T   
   A   
   C   
    T    
   C   
   A   
   G   
   G   
   T   
   G   
   A   
   A   
   T   
   T   
    G    
   A   
   G   
    T    
   A   
   C   
   A   
    T    
   T   
   G   
   G   
   A   
   C   
   C   
   A   
   T   
   G   
   T   
   T   
   C   
   T   
   T   
   C   
   T   
   G   
   A   
   T   
    C    
   A   
   C   
    A    
   A   
   A   
   T   
   A   
   A   
   A   
   G   
   C   
   A   
   A   
   A   
   T   
   G   
   T   
   G   
   A   
   T   
   C   
   T   
   A   
   C   
   C   
   G   
    C    
   C   
   C   
   T   
   G   
   T   
    C    
   C   
   A   
   T   
   G   
   A   
   A   
   C   
   T   
   A   
   C   
   C   
    C    
   G   
   A   
   T   
   T   
   T   
   G   
   A   
   A   
   A   
   T   
   G   
   G   
   A   
   A   
   A   
   G   
   A   
   T   
   G   
   C   
   T   
   G   
   G   
    T    
   G   
   A   
    G    
   C   
   A   
    C    
   A   
   T   
   T   
   T   
   C   
   T   
   C   
   A   
   A   
   A   
   C   
   A   
   A   
   T   
   C   
   T   
   C   
   C   
   A   
   A   
   T   
   C   
   T   
   G   
   T   
   C   
   T   
   G   
   T   
   A   
   G   
   A   
    C    
   G   
   A   
   A   
   T   
   C   
   G   
   A   
   T   
    T    
   T   
   T   
   A   
   G   
   C   
   T   
   G   
   T   
   T   
   T   
   T   
    C    
   C   
   C   
   A   
   T   
   A   
   T   
   C   
   A   
   A   
   A   
   A   
   A   
   A   
   T   
    T    
   C   
   A   
   A   
   G   
   C   
   A   
   G   
   A   
    G    
   A   
   A   
   A   
   A   
   C   
   A   
   T   
   T   
   T   
   G   
   T   
    A    
   T   
   T   
   C   
   A   
   T   
   C   
   A   
   G   
   C   
   A   
   A   
   G   
   A   
   A   
   T   
   G   
   G   
   A   
   T   
   T   
    G    
    A    
   T   
   C   
   A   
   A   
   G   
   C   
   A   
   A   
   A   
   C   
   A   
   C   
   G   
   A   
   A   
   T   
   G   
   A   
   C   
   A   
   G   
   A   
   G   
   T   
   T   
   C   
   G   
   A   
   A   
   C   
   C   
   A   
   T   
   G   
   G   
   C   
   G   
   C   
   A   
   C   
   C   
   T   
   A   
    T    
   A   
   A   
   G   
   A   
   G   
   C   
   C   
   G   
    T    
   C   
   C   
   G   
   C   
   T   
   C   
   A   
   G   
   C   
   T   
   G   
   T   
   A   
   T   
   G   
   A   
   A   
   G   
   C   
   T   
   A   
   A   
   A   
   G   
   G   
   C   
   G   
   G   
   A   
   A   
   G   
   A   
   A   
   G   
   A   
   T   
   G   
   A   
   A   
   C   
   T   
    G    
   A   
   T   
   T   
   G   
   C   
   T   
   G   
   T   
    G    
   T   
   A   
   T   
   C   
   T   
   G   
   A   
   A   
   A   
   G   
   A   
   A   
   G   
   A   
   T   
   C   
    A    
   G   
   C   
   C   
   A   
   G   
   G   
   C   
   T   
   T   
   A   
   G   
   A   
   T   
   G   
   T   
    T    
   T   
   T   
   C   
   T   
   T   
   A   
   G   
   T   
   A   
   A   
   C   
   G   
    C    
   A   
   T   
   C   
   A   
   A   
   G   
   G   
   A   
   A   
   T   
   G   
   G   
   G   
    T    
   T   
   T   
   G   
   C   
   G   
   C   
   T   
    A    
   T   
   C   
   C   
   T   
   T   
   T   
   A   
   A   
    C    
   A   
   G   
   A   
   T   
   G   
   T   
   T   
   C   
   C   
   C   
   G   
   T   
   T   
   G   
   T   
   T   
   G   
   G   
   A   
   G   
   C   
   A   
   A   
   A   
   G   
   G   
   C   
   A   
   G   
    C    
   T   
   G   
   G   
   A   
   G   
   T   
   G   
   A   
   A   
   G   
   T   
   C   
   T   
   A   
   T   
   G   
   A   
   A   
   T   
   T   
   T   
   G   
   A   
   A   
   A   
   G   
   A   
   T   
   G   
   G   
   A   
   G   
   A   
   T   
   A   
   G   
   G   
    A    
   T   
   T   
    A    
   T   
   C   
   A   
   A   
    C    
   G   
   G   
    G    
   C   
   T   
    G    
   C   
   T   
   T   
   G   
   T   
   G   
   T   
   T   
   A   
   G   
   C   
   A   
   G   
   A   
    A    
   G   
   G   
    T    
   G   
   A   
   T   
   A   
   C   
   G   
   C   
   C   
    G    
   A   
   T   
   C   
   G   
   T   
   A   
   A   
   T   
   C   
   G   
   T   
   T   
   A   
   C   
   T   
   C   
   A   
   G   
   C   
   G   
   A   
   G   
   G   
   A   
   G   
   C   
    T    
   G   
   T   
    T    
   A   
   A   
   A   
   C   
   G   
   C   
   A   
   T   
   G   
   C   
   T   
   T   
   G   
   C   
   T   
   C   
   A   
   A   
   G   
   A   
   A   
   A   
   T   
   C   
   A   
   G   
   C   
   C   
   A   
   A   
   A   
   C   
    T    
   T   
   C   
   A   
   C   
   G   
   T   
   G   
   C   
   G   
   A   
   A   
   G   
   C   
   G   
   G   
   G   
   G   
   A   
   G   
   T   
   G   
   A   
   C   
   G   
   G   
   T   
   C   
   A   
   T   
   G   
   C   
   G   
   T   
   G   
   A   
   G   
   C   
   T   
   G   
   A   
   A   
   A   
   A   
   A   
   A   
   A   
   A   
   T   
   C   
   C   
    G    
   C   
   A   
    C    
   C   
   G   
    T    
   A   
   T   
   T   
   A   
   T   
   C   
   T   
   A   
   T   
   A   
   T   
   G   
   T   
   C   
   G   
   G   
   A   
   A   
   G   
   G   
   A   
   A   
   G   
   A   
   T   
   C   
   A   
   A   
   A   
   A   
   G   
   A   
   A   
   A   
   T   
   A   
   A   
   C   
   C   
   T   
   T   
   G   
   A   
   T   
   G   
   A   
   A   
   T   
   C   
   A   
   A   
   A   
   A   
   A   
   G   
   G   
    G    
   C   
   A   
    G    
   C   
   A   
   G   
    T    
   T   
   A   
   A   
   C   
   G   
   A   
   T   
   T   
   G   
   A   
    C    
   C   
   C   
   A   
   A   
   C   
    T    
   G   
   A   
   T   
   T   
   T   
    C    
   C   
   C   
   T   
   A   
   T   
   A   
   G   
   G   
   T   
   G   
   A   
   T   
   C   
   G   
   A   
   A   
   C   
   A   
   T   
   C   
   G   
   A   
   A   
   T   
   G   
   G   
    A    
   T   
   C   
    A    
   T   
   T   
   T   
   G   
   C   
    G    
   A   
   T   
   G   
   G   
   A   
    T    
   G   
   A   
   G   
   A   
   A   
   A   
   A   
   A   
   A   
   G   
   G   
   T   
   G   
   G   
   A   
   G   
   A   
   A   
   G   
   T   
   C   
   A   
   T   
   C   
   G   
   A   
   A   
   G   
   T   
    T    
   A   
    T    
   C   
   G   
   A   
   T   
   G   
   C   
   T   
   C   
   C   
   T   
   G   
   A   
   G   
    A    
   T   
   T   
   T   
   T   
    C    
   A   
   T   
    C    
   G   
   A   
    G    
    T    
   C   
    G    
   T   
   A   
   A   
 

 
   N60045F   
   A   
   T   
   G   
   G   
   A   
   A   
   A   
   A   
   T   
   C   
   G   
   A   
   C   
   A   
   A   
   G   
   A   
   A   
   G   
   T   
   A   
   C   
   A   
   A   
   G   
   A   
   A   
   T   
   T   
   A   
   A   
   C   
   T   
   T   
   T   
   A   
   G   
   A   
   A   
   G   
   A   
   A   
   G   
   T   
   G   
   A   
   T   
   G   
   G   
   G   
   T   
   G   
   A   
   C   
   C   
   G   
   C   
   T   
   T   
   C   
   G   
   G   
   A   
   C   
   G   
   T   
   T   
   A   
   T   
   T   
   C   
   C   
   A   
   A   
   A   
   T   
   A   
   T   
   A   
   T   
   T   
   A   
   T   
    C    
   C   
   A   
    A    
   G   
   A   
   A   
   C   
   G   
   T   
   G   
   C   
   A   
   C   
   T   
   G   
   C   
   C   
   T   
   G   
   A   
   T   
   A   
   T   
    C    
   C   
   G   
   T   
   G   
   A   
   T   
   G   
   G   
   A   
   T   
   T   
   G   
   A   
   A   
    G    
   C   
   C   
   A   
   G   
   T   
    G    
   C   
   A   
   A   
   C   
   G   
   C   
   C   
   G   
   G   
   A   
   T   
   A   
   T   
   T   
   A   
   T   
   T   
    C    
   G   
   C   
    T    
   A   
   T   
   G   
   A   
   A   
   C   
   A   
   A   
   A   
   G   
   A   
   T   
   G   
   G   
   C   
   A   
   A   
   T   
   A   
   C   
   G   
   T   
   A   
   T   
   G   
   A   
   C   
   A   
   A   
   A   
   G   
   G   
   A   
   T   
   T   
    C    
   C   
   G   
   T   
   A   
   A   
   A   
   T   
   C   
   A   
   G   
   C   
   A   
   A   
   A   
   A   
   T   
   C   
   T   
   G   
   T   
   C   
   G   
   G   
   A   
   A   
   A   
   T   
   A   
   T   
   C   
   A   
   T   
   G   
   G   
   G   
    G    
   A   
   A   
   T   
   T   
   A   
   T   
   C   
   A   
   T   
   C   
   C   
   T   
   C   
   A   
   T   
   G   
   G   
   T   
   G   
   A   
   C   
   A   
   G   
   C   
   A   
   G   
   T   
   A   
   T   
   T   
   T   
   A   
   T   
   G   
   A   
   A   
   G   
   C   
   G   
   A   
   T   
   G   
   G   
   T   
   C   
   C   
   G   
   C   
   A   
   T   
   G   
   A   
   G   
   C   
   C   
   A   
   A   
   G   
   A   
    C    
   T   
   G   
   G   
   A   
   A   
   G   
   T   
   T   
   G   
   C   
   G   
   G   
   G   
   A   
   A   
   G   
   T   
   A   
   C   
   T   
   G   
   A   
   T   
   C   
   G   
   A   
    A    
   A   
   T   
   G   
   C   
   A   
   C   
   G   
   G   
    G    
   A   
   A   
   T   
   A   
   A   
    C    
   G   
   G   
   G   
   A   
   G   
   T   
   A   
   T   
   G   
   G   
   A   
   C   
   G   
   G   
   T   
   G   
   A   
   T   
   C   
   C   
   A   
   C   
   C   
   T   
   G   
   C   
   T   
   G   
   C   
   G   
   A   
   T   
   G   
   C   
   G   
   T   
   T   
   A   
   T   
   A   
   C   
   T   
   G   
   A   
   A   
   G   
   C   
   C   
   A   
   G   
    A    
   T   
   T   
   A   
   T   
   C   
   C   
   A   
   A   
   G   
   T   
   T   
   A   
   A   
   G   
   T   
   G   
   G   
   A   
   G   
   A   
   G   
   A   
   T   
   G   
    C    
   T   
   A   
    G    
   C   
   A   
   G   
   A   
   T   
   A   
   T   
   C   
   G   
   A   
    A    
   A   
   A   
   A   
   G   
   A   
   A   
   A   
   C   
   A   
   G   
   T   
    C    
   G   
   A   
   T   
   C   
   T   
   C   
   G   
   T   
   G   
   T   
   G   
   G   
   A   
   A   
   T   
   T   
   T   
   T   
   G   
   A   
   T   
   G   
   A   
   T   
   A   
   C   
   A   
   G   
   A   
   A   
   A   
   A   
   A   
   G   
   A   
   A   
   C   
   C   
   A   
   A   
   C   
   A   
   G   
   T   
   C   
   C   
   T   
   T   
   C   
   C   
   A   
   G   
   C   
   T   
   C   
   G   
   A   
   T   
   A   
   C   
   C   
   C   
   A   
   A   
   A   
   C   
   C   
   T   
   T   
   C   
   T   
    C    
   G   
   T   
   C   
   A   
   A   
   T   
   G   
   G   
   A   
   T   
   C   
   G   
   A   
   C   
   A   
   G   
   G   
   T   
   A   
   T   
   A   
   T   
   C   
   T   
   G   
   C   
   T   
   G   
   G   
   A   
   T   
   A   
   T   
   G   
   C   
    A    
   A   
   C   
   A   
   G   
   A   
   A   
   A   
   T   
   C   
   C   
   C   
   G   
   A   
   C   
    T    
   C   
   A   
   T   
   A   
   A   
   C   
   T   
   T   
   A   
   G   
   C   
   T   
   G   
   A   
   A   
   G   
   T   
   G   
   A   
   T   
    C    
   G   
   A   
    T    
   G   
   G   
   C   
   A   
   C   
   A   
   A   
   T   
   C   
   T   
   A   
   T   
   A   
   T   
   G   
   A   
   T   
   C   
   G   
   A   
   T   
   C   
   A   
   T   
   C   
   C   
   A   
   C   
   A   
   A   
   G   
   C   
   G   
   A   
   G   
   C   
   C   
   T   
   T   
   G   
   A   
   A   
   A   
   A   
   A   
   T   
   T   
   G   
   A   
   T   
   G   
   G   
   A   
   A   
   T   
   A   
   T   
   A   
   T   
   T   
   C   
   C   
   T   
   G   
   G   
   A   
   C   
   C   
   T   
   G   
   A   
   C   
   T   
   T   
    T    
   C   
   C   
   A   
   A   
   C   
   A   
   G   
   G   
   A   
   G   
   G   
    G    
   A   
   T   
   C   
   T   
   T   
   G   
   C   
   A   
   A   
   G   
   G   
    A    
   A   
   A   
   A   
   G   
   A   
   T   
   G   
   A   
   A   
   A   
   T   
   C   
   A   
   A   
   A   
   A   
   A   
   A   
   G   
   C   
   T   
   T   
   A   
   T   
   G   
   A   
   A   
   A   
   C   
   A   
   G   
   G   
    G    
   C   
   G   
   A   
   G   
   G   
   A   
   A   
   A   
   A   
   G   
   T   
   T   
   A   
    T    
   T   
   C   
   T   
   T   
   C   
   G   
   T   
   T   
   C   
   G   
   A   
   A   
   A   
   A   
   C   
   A   
   A   
   A   
   G   
   A   
   T   
   A   
   G   
   A   
    A    
   G   
   C   
   A   
   A   
   T   
   G   
   A   
   A   
   G   
   G   
   G   
    T    
   A   
   A   
    T    
   A   
   A   
   A   
   C   
   A   
   G   
   C   
   A   
   A   
   A   
   T   
   C   
   G   
   T   
   G   
   A   
   T   
   C   
   A   
   C   
    A    
   G   
   A   
   A   
   A   
   T   
   C   
   C   
   C   
   A   
   T   
   A   
   T   
   G   
   A   
   A   
   G   
   T   
   A   
   A   
   A   
   T   
   A   
   A   
   A   
   G   
   C   
   G   
   A   
   C   
    C    
   T   
   T   
   G   
   G   
   T   
   G   
   A   
   A   
   A   
   A   
   A   
   A   
   A   
   T   
   G   
   G   
   A   
   T   
   G   
   A   
   G   
   A   
   T   
   C   
   C   
   G   
   T   
   T   
   T   
   G   
   A   
   A   
   T   
   A   
   A   
   G   
   A   
   A   
   A   
   A   
   T   
   C   
   G   
   A   
    T    
   G   
   G   
   A   
   A   
   T   
   A   
   G   
   C   
   T   
   G   
   A   
   A   
   G   
   T   
    A    
   C   
   G   
   A   
   G   
   A   
   T   
   G   
   A   
   A   
   A   
   G   
   T   
   G   
   A   
   T   
    A    
   G   
    A    
   A   
   C   
    A    
   G   
   G   
   T   
   C   
   T   
   G   
   C   
   A   
   A   
   A   
   T   
   T   
   G   
   T   
   C   
   G   
   T   
    C    
   G   
   A   
   A   
   T   
   T   
   G   
   A   
   A   
   A   
   A   
   A   
   A   
   G   
   A   
    C    
   G   
   C   
   C   
   A   
   A   
    T    
   G   
   C   
   T   
   C   
   A   
   A   
   G   
   G   
    A    
   A   
   T   
   T   
   T   
   T   
   G   
   A   
   A   
   T   
   T   
   A   
    T    
   T   
   T   
   G   
   T   
   T   
   C   
   A   
   A   
   A   
   A   
   A   
   C   
   A   
   C   
   T   
   G   
   A   
   G   
    C    
   T   
   A   
   C   
   A   
   G   
   A   
   T   
   C   
   A   
   A   
   T   
   T   
   A   
   C   
   A   
   A   
   T   
   T   
   T   
    C    
   A   
   A   
   T   
   A   
   T   
   G   
   G   
   T   
   C   
   G   
   C   
   T   
   A   
   T   
   C   
   G   
   A   
    C    
   C   
   A   
   T   
   A   
   T   
   G   
   A   
   C   
   T   
   C   
   C   
   G   
   C   
   A   
    T    
   C   
   A   
   A   
   G   
   T   
   T   
   G   
   G   
   A   
   C   
   T   
   G   
   A   
   A   
   A   
   G   
   A   
   C   
   A   
   T   
   T   
   T   
   T   
   A   
   A   
   G   
   A   
   A   
   G   
   T   
   T   
   A   
   T   
   A   
   T   
   C   
   G   
   A   
   G   
   C   
   A   
   T   
   C   
   G   
   A   
   A   
   A   
   G   
   C   
   A   
   A   
   G   
   T   
   C   
   A   
   T   
   A   
   A   
   C   
   A   
   A   
   A   
   A   
   C   
   G   
    T    
   A   
   G   
    T    
   C   
   A   
   A   
   T   
   T   
   C   
   G   
   A   
   T   
   T   
   T   
   A   
   G   
   C   
   C   
   A   
   A   
   A   
   G   
   C   
   T   
   C   
   A   
   A   
   A   
   A   
   A   
    A    
   G   
   A   
   C   
   A   
   G   
   C   
   A   
   T   
   A   
   T   
   C   
   G   
   T   
    A    
   G   
   A   
   A   
   G   
   G   
    A    
    T    
   T   
   G   
   A   
   T   
   G   
   A   
   A   
   A   
   G   
   C   
   T   
   T   
   T   
   A   
   T   
   C   
   G   
   A   
   T   
   T   
   C   
   T   
   G   
   G   
   A   
   T   
   G   
   A   
   A   
   G   
   T   
   A   
   A   
   T   
   C   
   G   
    C    
   A   
   A   
   C   
   G   
   A   
   T   
   T   
   C   
   G   
   T   
   G   
   A   
   A   
   A   
   G   
   T   
   A   
   A   
   A   
   G   
   A   
   C   
   A   
   A   
   A   
   A   
   A   
   A   
   G   
   A   
    C    
   G   
   C   
    C    
   A   
   A   
   A   
   A   
   A   
   G   
   A   
   A   
   T   
   T   
   T   
   A   
   G   
   T   
   C   
   G   
   A   
    C    
   G   
   T   
    C    
   T   
   T   
    T    
   C   
   A   
   A   
   T   
   T   
   T   
   A   
   C   
   A   
   G   
   A   
   A   
   G   
   A   
   A   
   C   
   A   
   A   
   G   
   C   
   A   
   G   
   A   
   A   
   G   
   C   
   A   
   A   
   T   
    T    
   G   
   T   
   C   
   A   
   C   
   A   
   T   
   T   
   G   
   C   
   A   
   A   
    T    
   T   
   A   
   T   
   A   
    C    
   C   
   G   
   T   
   T   
   T   
   G   
   A   
   C   
   G   
   A   
   A   
    C    
   A   
   C   
    G    
   G   
   A   
   T   
   A   
   T   
   C   
   A   
   C   
   A   
   G   
   A   
   G   
   C   
   T   
   T   
   C   
   A   
   A   
   A   
   A   
   G   
   G   
   A   
   A   
    T    
   C   
    C    
   G   
   A   
   A   
   A   
    G    
   C   
   T   
   T   
   G   
   A   
   T   
   T   
   G   
   C   
    G    
   C   
   A   
   A   
   A   
   T   
   C   
   A   
   C   
   A   
   G   
   A   
   G   
   T   
   T   
    G    
   A   
   A   
   T   
   A   
   A   
   A   
   A   
   T   
   C   
   C   
   T   
   T   
   T   
   C   
   A   
   A   
   A   
    T    
   G   
   A   
   C   
   A   
   A   
   A   
   G   
   A   
    G    
   C   
   T   
   T   
   T   
   T   
    C    
   T   
   C   
   T   
   G   
   T   
   T   
   A   
   T   
   G   
   A   
   A   
   A   
   A   
   A   
   A   
   G   
   A   
   A   
   T   
   T   
   G   
   C   
   G   
   A   
   G   
   A   
   A   
   G   
   T   
   G   
   A   
   A   
   A   
   A   
   A   
   A   
   A   
   A   
   T   
   T   
   A   
    T    
   T   
   C   
   T   
   T   
   C   
   C   
   G   
   C   
   T   
   C   
   G   
   T   
   C   
    T    
    A    
   A   
   C   
   G   
   A   
   C   
   A   
   A   
   T   
   C   
    G    
   A   
   A   
   G   
   A   
   C   
   G   
   A   
   A   
   A   
   T   
   C   
   G   
   A   
   A   
   G   
   A   
   A   
   A   
   T   
   C   
   A   
   A   
   A   
   A   
   T   
    C    
   G   
   A   
   T   
   A   
   C   
   G   
   C   
   A   
   A   
    G    
   T   
    T    
   T   
   T   
   A   
   G   
   T   
   G   
   G   
   C   
    G    
   C   
   A   
   A   
   G   
   A   
   A   
   G   
   A   
   C   
   G   
   T   
   A   
   A   
   T   
    C    
   G   
   T   
   C   
   A   
   G   
   T   
   G   
   T   
    G    
   A   
   C   
    G    
   C   
   G   
   A   
   G   
   A   
   A   
   G   
   G   
   C   
   T   
   A   
   T   
   G   
   T   
   G   
   A   
   A   
   G   
   C   
   G   
   A   
   A   
   C   
   A   
   A   
   G   
   C   
   C   
   T   
   T   
   C   
   G   
   T   
   T   
   C   
    T    
   T   
   A   
   T   
   A   
   G   
   T   
   G   
   C   
   T   
   T   
   C   
    G    
   A   
   A   
   A   
   C   
   C   
   A   
   G   
   A   
   A   
   G   
   A   
   A   
   A   
   T   
   C   
   G   
   G   
   G   
   A   
   T   
   G   
   C   
   G   
   A   
   G   
   A   
   A   
   G   
   G   
   C   
   G   
   A   
    C    
   T   
   A   
   C   
   T   
   T   
   G   
   C   
   T   
    G    
   T   
   A   
   C   
    T    
   C   
   A   
   G   
   G   
   T   
   G   
   A   
   A   
   T   
   T   
    G    
   A   
   G   
    C    
   A   
   C   
   A   
    T    
   T   
   G   
   G   
   A   
   C   
   C   
   A   
   T   
   G   
   T   
   T   
   C   
   T   
   T   
   C   
   T   
   G   
   A   
   T   
    T    
   A   
   C   
    G    
   A   
   A   
   T   
   A   
   A   
   A   
   G   
   C   
   A   
   A   
   A   
   T   
   G   
   T   
   G   
   A   
   T   
   C   
   T   
   A   
   C   
   C   
   G   
    T    
   C   
   C   
   T   
   G   
   T   
    T    
   C   
   A   
   T   
   G   
   A   
   A   
   C   
   T   
   A   
   C   
   C   
    C    
   G   
   A   
   T   
   T   
   T   
   G   
   A   
   A   
   A   
   T   
   G   
   G   
   A   
   A   
   A   
   G   
   A   
   T   
   G   
   C   
   T   
   G   
   G   
    C    
   G   
   A   
    A    
   C   
   A   
    T    
   A   
   T   
   T   
   T   
   C   
   T   
   C   
   A   
   A   
   A   
   C   
   A   
   A   
   T   
   C   
   T   
   C   
   C   
   A   
   A   
   T   
   C   
   T   
   G   
   T   
   C   
   T   
   G   
   T   
   A   
   G   
   A   
    T    
   G   
   A   
   A   
   T   
   C   
   G   
   A   
   T   
    C    
   T   
   T   
   A   
   G   
   C   
   T   
   G   
   T   
   T   
   T   
   T   
    T    
   C   
   C   
   A   
   T   
   A   
   T   
   C   
   A   
   A   
   A   
   A   
   A   
   A   
   T   
    T    
   C   
   A   
   A   
   G   
   C   
   A   
   G   
   A   
    G    
   A   
   A   
   A   
   A   
   C   
   A   
   T   
   T   
   T   
   G   
   T   
    A    
   T   
   T   
   C   
   A   
   T   
   C   
   A   
   G   
   C   
   A   
   A   
   G   
   A   
   A   
   T   
   G   
   G   
   A   
   T   
   T   
    G    
    A    
   T   
   C   
   A   
   A   
   G   
   C   
   A   
   A   
   A   
   C   
   A   
   C   
   G   
   A   
   A   
   T   
   G   
   A   
   C   
   A   
   G   
   A   
   G   
   T   
   T   
   C   
   G   
   A   
   A   
   C   
   C   
   A   
   T   
   G   
   G   
   C   
   G   
   C   
   A   
   C   
   C   
   T   
   A   
    T    
   A   
   A   
   G   
   A   
   G   
   C   
   C   
   G   
    T    
   C   
   C   
   G   
   C   
   T   
   C   
   A   
   G   
   C   
   T   
   G   
   T   
   A   
   T   
   G   
   A   
   A   
   G   
   C   
   T   
   A   
   A   
   A   
   G   
   G   
   C   
   G   
   G   
   A   
   A   
   G   
   A   
   A   
   G   
   A   
   T   
   G   
   A   
   A   
   C   
   T   
    G    
   A   
   T   
   T   
   G   
   C   
   T   
   G   
   T   
    G    
   T   
   A   
   T   
   C   
   T   
   G   
   A   
   A   
   A   
   G   
   A   
   A   
   G   
   A   
   T   
   C   
    G    
   G   
   C   
   C   
   A   
   G   
   G   
   C   
   T   
   T   
   A   
   G   
   A   
   T   
   G   
   T   
    T    
   T   
   T   
   C   
   T   
   T   
   A   
   G   
   T   
   A   
   A   
   C   
   G   
    T    
   A   
   T   
   C   
   A   
   A   
   G   
   G   
   A   
   A   
   T   
   G   
   G   
   G   
    T    
   T   
   T   
   G   
   C   
   G   
   C   
   T   
    A    
   T   
   C   
   C   
   T   
   T   
   T   
   A   
   A   
    C    
   A   
   G   
   A   
   T   
   G   
   T   
   T   
   C   
   C   
   C   
   G   
   T   
   T   
   G   
   T   
   T   
   G   
   G   
   A   
   G   
   C   
   A   
   A   
   A   
   G   
   G   
   C   
   A   
   G   
    C    
   T   
   G   
   G   
   A   
   G   
   T   
   G   
   A   
   A   
   G   
   T   
   C   
   T   
   A   
   T   
   G   
   A   
   A   
   T   
   T   
   T   
   G   
   A   
   A   
   A   
   G   
   A   
   T   
   G   
   G   
   A   
   G   
   A   
   T   
   A   
   G   
   G   
    A    
   T   
   T   
    A    
   T   
   C   
   A   
   A   
    C    
   G   
   G   
    G    
   C   
   T   
    G    
   C   
   T   
   T   
   G   
   T   
   G   
   T   
   T   
   A   
   G   
   C   
   A   
   G   
   A   
    A    
   G   
   G   
    T    
   G   
   A   
   T   
   A   
   C   
   G   
   C   
   C   
    G    
   A   
   T   
   C   
   G   
   T   
   A   
   A   
   T   
   C   
   G   
   T   
   T   
   A   
   C   
   T   
   C   
   A   
   G   
   C   
   G   
   A   
   G   
   G   
   A   
   G   
   C   
    T    
   G   
   T   
    T    
   A   
   A   
   A   
   C   
   G   
   C   
   A   
   T   
   G   
   C   
   T   
   T   
   G   
   C   
   T   
   C   
   A   
   A   
   G   
   A   
   A   
   A   
   T   
   C   
   A   
   G   
   C   
   C   
   A   
   A   
   A   
   C   
    T    
   T   
   C   
   A   
   C   
   G   
   T   
   G   
   C   
   G   
   A   
   A   
   G   
   C   
   G   
   G   
   G   
   G   
   A   
   G   
   T   
   G   
   A   
   C   
   G   
   G   
   T   
   C   
   A   
   T   
   G   
   C   
   G   
   T   
   G   
   A   
   G   
   C   
   T   
   G   
   A   
   A   
   A   
   A   
   A   
   A   
   A   
   A   
   T   
   C   
   C   
    T    
   C   
   A   
    T    
   C   
   G   
    C    
   A   
   T   
   T   
   A   
   T   
   C   
   T   
   A   
   T   
   A   
   T   
   G   
   T   
   C   
   G   
   G   
   A   
   A   
   G   
   G   
   A   
   A   
   G   
   A   
   T   
   C   
   A   
   A   
   A   
   A   
   G   
   A   
   A   
   A   
   T   
   A   
   A   
   C   
   C   
   T   
   T   
   G   
   A   
   T   
   G   
   A   
   A   
   T   
   C   
   A   
   A   
   A   
   A   
   A   
   G   
   G   
    G    
   C   
   A   
    G    
   C   
   A   
   G   
    T    
   T   
   A   
   A   
   C   
   G   
   A   
   T   
   T   
   G   
   A   
    C    
   C   
   C   
   A   
   A   
   C   
    T    
   G   
   A   
   T   
   T   
   T   
    C    
   C   
   C   
   T   
   A   
   T   
   A   
   G   
   G   
   T   
   G   
   A   
   T   
   C   
   G   
   A   
   A   
   C   
   A   
   T   
   C   
   G   
   A   
   A   
   T   
   G   
   G   
    A    
   T   
   C   
    A    
   T   
   T   
   T   
   G   
   C   
    G    
   A   
   T   
   G   
   G   
   A   
    T    
   G   
   A   
   G   
   A   
   A   
   A   
   A   
   A   
   A   
   G   
   G   
   T   
   G   
   G   
   A   
   G   
   A   
   A   
   G   
   T   
   C   
   A   
   T   
   C   
   G   
   A   
   A   
   G   
   T   
    T    
   A   
    T    
   C   
   G   
   A   
   T   
   G   
   C   
   T   
   C   
   C   
   T   
   G   
   A   
   G   
    A    
   T   
   T   
   T   
   T   
    C    
   A   
   T   
    C    
   G   
   A   
    G    
    T    
   C   
    G    
   -   
   -   
   -   
 

 
   N60190F   
   A   
   T   
   G   
   G   
   A   
   A   
   A   
   A   
   T   
   C   
   G   
   A   
   C   
   A   
   A   
   G   
   A   
   A   
   G   
   T   
   A   
   C   
   A   
   A   
   G   
   A   
   A   
   T   
   T   
   A   
   A   
   C   
   T   
   T   
   T   
   A   
   G   
   A   
   A   
   G   
   A   
   A   
   G   
   T   
   G   
   A   
   T   
   G   
   G   
   G   
   T   
   G   
   A   
   C   
   C   
   G   
   C   
   T   
   T   
   C   
   G   
   G   
   A   
   C   
   G   
   T   
   T   
   A   
   T   
   T   
   C   
   C   
   A   
   A   
   A   
   T   
   A   
   T   
   A   
   T   
   T   
   A   
   T   
    C    
   C   
   A   
    A    
   G   
   A   
   A   
   C   
   G   
   T   
   G   
   C   
   A   
   C   
   T   
   G   
   C   
   C   
   T   
   G   
   A   
   T   
   A   
   T   
    C    
   C   
   G   
   T   
   G   
   A   
   T   
   G   
   G   
   A   
   T   
   T   
   G   
   A   
   A   
    A    
   C   
   C   
   A   
   G   
   T   
    G    
   C   
   A   
   A   
   C   
   G   
   C   
   C   
   G   
   G   
   A   
   T   
   A   
   T   
   T   
   A   
   T   
   T   
    T    
   G   
   C   
    C    
   A   
   T   
   G   
   A   
   A   
   C   
   A   
   A   
   A   
   G   
   A   
   T   
   G   
   G   
   C   
   A   
   A   
   T   
   A   
   C   
   G   
   T   
   A   
   T   
   G   
   A   
   C   
   A   
   A   
   A   
   G   
   G   
   A   
   T   
   T   
    C    
   C   
   G   
   T   
   A   
   A   
   A   
   T   
   C   
   A   
   G   
   C   
   A   
   A   
   A   
   A   
   T   
   C   
   T   
   G   
   T   
   C   
   G   
   G   
   A   
   A   
   A   
   T   
   A   
   T   
   C   
   A   
   T   
   G   
   G   
   G   
    G    
   A   
   A   
   T   
   T   
   A   
   T   
   C   
   A   
   T   
   C   
   C   
   T   
   C   
   A   
   T   
   G   
   G   
   T   
   G   
   A   
   C   
   A   
   G   
   C   
   A   
   G   
   T   
   A   
   T   
   T   
   T   
   A   
   T   
   G   
   A   
   A   
   G   
   C   
   G   
   A   
   T   
   G   
   G   
   T   
   C   
   C   
   G   
   C   
   A   
   T   
   G   
   A   
   G   
   C   
   C   
   A   
   A   
   G   
   A   
    T    
   T   
   G   
   G   
   A   
   A   
   G   
   T   
   T   
   G   
   C   
   G   
   G   
   G   
   A   
   A   
   G   
   T   
   A   
   C   
   T   
   G   
   A   
   T   
   C   
   G   
   A   
    A    
   A   
   T   
   G   
   C   
   A   
   C   
   G   
   G   
    G    
   A   
   A   
   T   
   A   
   A   
    T    
   G   
   G   
   G   
   A   
   G   
   T   
   A   
   T   
   G   
   G   
   A   
   C   
   G   
   G   
   T   
   G   
   A   
   T   
   C   
   C   
   A   
   C   
   C   
   T   
   G   
   C   
   T   
   G   
   C   
   G   
   A   
   T   
   G   
   C   
   G   
   T   
   T   
   A   
   T   
   A   
   C   
   T   
   G   
   A   
   A   
   G   
   C   
   C   
   A   
   G   
    A    
   T   
   T   
   A   
   T   
   C   
   C   
   A   
   A   
   G   
   T   
   T   
   A   
   A   
   G   
   T   
   G   
   G   
   A   
   G   
   A   
   G   
   A   
   T   
   G   
    C    
   T   
   A   
    G    
   C   
   A   
   G   
   A   
   T   
   A   
   T   
   C   
   G   
   A   
    A    
   A   
   A   
   A   
   G   
   A   
   A   
   A   
   C   
   A   
   G   
   T   
    C    
   G   
   A   
   T   
   C   
   T   
   C   
   G   
   T   
   G   
   T   
   G   
   G   
   A   
   A   
   T   
   T   
   T   
   T   
   G   
   A   
   T   
   G   
   A   
   T   
   A   
   C   
   A   
   G   
   A   
   A   
   A   
   A   
   A   
   G   
   A   
   A   
   C   
   C   
   A   
   A   
   C   
   A   
   G   
   T   
   C   
   C   
   T   
   T   
   C   
   C   
   A   
   G   
   C   
   T   
   C   
   G   
   A   
   T   
   A   
   C   
   C   
   C   
   A   
   A   
   A   
   C   
   C   
   T   
   T   
   C   
   T   
    A    
   G   
   T   
   C   
   A   
   A   
   T   
   G   
   G   
   A   
   T   
   C   
   G   
   A   
   C   
   A   
   G   
   G   
   T   
   A   
   T   
   A   
   T   
   C   
   T   
   G   
   C   
   T   
   G   
   G   
   A   
   T   
   A   
   T   
   G   
   C   
    G    
   A   
   C   
   A   
   G   
   A   
   A   
   A   
   T   
   C   
   C   
   C   
   G   
   A   
   C   
    T    
   C   
   A   
   T   
   A   
   A   
   C   
   T   
   T   
   A   
   G   
   C   
   T   
   G   
   A   
   A   
   G   
   T   
   G   
   A   
   T   
    C    
   G   
   A   
    C    
   G   
   G   
   C   
   A   
   C   
   A   
   A   
   T   
   C   
   T   
   A   
   T   
   A   
   T   
   G   
   A   
   T   
   C   
   G   
   A   
   T   
   C   
   A   
   T   
   C   
   C   
   A   
   C   
   A   
   A   
   G   
   C   
   G   
   A   
   G   
   C   
   C   
   T   
   T   
   G   
   A   
   A   
   A   
   A   
   A   
   T   
   T   
   G   
   A   
   T   
   G   
   G   
   A   
   A   
   T   
   A   
   T   
   A   
   T   
   T   
   C   
   C   
   T   
   G   
   G   
   A   
   C   
   C   
   T   
   G   
   A   
   C   
   T   
   T   
    T    
   C   
   C   
   A   
   A   
   C   
   A   
   G   
   G   
   A   
   G   
   G   
    G    
   A   
   T   
   C   
   T   
   T   
   G   
   C   
   A   
   A   
   G   
   G   
    G    
   A   
   A   
   A   
   G   
   A   
   T   
   G   
   A   
   A   
   A   
   T   
   C   
   A   
   A   
   A   
   A   
   A   
   A   
   G   
   C   
   T   
   T   
   A   
   T   
   G   
   A   
   A   
   A   
   C   
   A   
   G   
   G   
    G    
   C   
   G   
   A   
   G   
   G   
   A   
   A   
   A   
   A   
   G   
   T   
   T   
   A   
    T    
   T   
   C   
   T   
   T   
   C   
   G   
   T   
   T   
   C   
   G   
   A   
   A   
   A   
   A   
   C   
   A   
   A   
   A   
   G   
   A   
   T   
   A   
   G   
   A   
    A    
   G   
   C   
   A   
   A   
   T   
   G   
   A   
   A   
   G   
   G   
   G   
    C    
   A   
   A   
    T    
   A   
   A   
   A   
   C   
   A   
   G   
   C   
   A   
   A   
   A   
   T   
   C   
   G   
   T   
   G   
   A   
   T   
   C   
   A   
   C   
    T    
   G   
   A   
   A   
   A   
   T   
   C   
   C   
   C   
   A   
   T   
   A   
   T   
   G   
   A   
   A   
   G   
   T   
   A   
   A   
   A   
   T   
   A   
   A   
   A   
   G   
   C   
   G   
   A   
   C   
    G    
   T   
   T   
   G   
   G   
   T   
   G   
   A   
   A   
   A   
   A   
   A   
   A   
   A   
   T   
   G   
   G   
   A   
   T   
   G   
   A   
   G   
   A   
   T   
   C   
   C   
   G   
   T   
   T   
   T   
   G   
   A   
   A   
   T   
   A   
   A   
   G   
   A   
   A   
   A   
   A   
   T   
   C   
   G   
   A   
    T    
   G   
   G   
   A   
   A   
   T   
   A   
   G   
   C   
   T   
   G   
   A   
   A   
   G   
   T   
    A    
   C   
   G   
   A   
   G   
   A   
   T   
   G   
   A   
   A   
   A   
   G   
   T   
   G   
   A   
   T   
    C    
   G   
    A    
   A   
   C   
    T    
   G   
   G   
   T   
   C   
   T   
   G   
   C   
   A   
   A   
   A   
   T   
   T   
   G   
   T   
   C   
   G   
   T   
    T    
   G   
   A   
   A   
   T   
   T   
   G   
   A   
   A   
   A   
   A   
   A   
   A   
   G   
   A   
    T    
   G   
   C   
   C   
   A   
   A   
    T    
   G   
   C   
   T   
   C   
   A   
   A   
   G   
   G   
    A    
   A   
   T   
   T   
   T   
   T   
   G   
   A   
   A   
   T   
   T   
   A   
    T    
   T   
   T   
   G   
   T   
   T   
   C   
   A   
   A   
   A   
   A   
   A   
   C   
   A   
   C   
   T   
   G   
   A   
   G   
    C    
   T   
   A   
   C   
   A   
   G   
   A   
   T   
   C   
   A   
   A   
   T   
   T   
   A   
   C   
   A   
   A   
   T   
   T   
   T   
    T    
   A   
   A   
   T   
   A   
   T   
   G   
   G   
   T   
   C   
   G   
   C   
   T   
   A   
   T   
   C   
   G   
   A   
    T    
   C   
   A   
   T   
   A   
   T   
   G   
   A   
   C   
   T   
   C   
   C   
   G   
   C   
   A   
    C    
   C   
   A   
   A   
   G   
   T   
   T   
   G   
   G   
   A   
   C   
   T   
   G   
   A   
   A   
   A   
   G   
   A   
   C   
   A   
   T   
   T   
   T   
   T   
   A   
   A   
   G   
   A   
   A   
   G   
   T   
   T   
   A   
   T   
   A   
   T   
   C   
   G   
   A   
   G   
   C   
   A   
   T   
   C   
   G   
   A   
   A   
   A   
   G   
   C   
   A   
   A   
   G   
   T   
   C   
   A   
   T   
   A   
   A   
   C   
   A   
   A   
   A   
   A   
   C   
   G   
    C    
   A   
   G   
    C    
   C   
   A   
   A   
   T   
   T   
   C   
   G   
   A   
   T   
   T   
   T   
   A   
   G   
   C   
   C   
   A   
   A   
   A   
   G   
   C   
   T   
   C   
   A   
   A   
   A   
   A   
   A   
    A    
   G   
   A   
   C   
   A   
   G   
   C   
   A   
   T   
   A   
   T   
   C   
   G   
   T   
    G    
   G   
   A   
   A   
   G   
   G   
    C    
    C    
   T   
   G   
   A   
   T   
   G   
   A   
   A   
   A   
   G   
   C   
   T   
   T   
   T   
   A   
   T   
   C   
   G   
   A   
   T   
   T   
   C   
   T   
   G   
   G   
   A   
   T   
   G   
   A   
   A   
   G   
   T   
   A   
   A   
   T   
   C   
   G   
    C    
   A   
   A   
   C   
   G   
   A   
   T   
   T   
   C   
   G   
   T   
   G   
   A   
   A   
   A   
   G   
   T   
   A   
   A   
   A   
   G   
   A   
   C   
   A   
   A   
   A   
   A   
   A   
   A   
   G   
   A   
    T    
   G   
   C   
    T    
   A   
   A   
   A   
   A   
   A   
   G   
   A   
   A   
   T   
   T   
   T   
   A   
   G   
   T   
   C   
   G   
   A   
    T    
   G   
   T   
    T    
   T   
   T   
    C    
   C   
   A   
   A   
   T   
   T   
   T   
   A   
   C   
   A   
   G   
   A   
   A   
   G   
   A   
   A   
   C   
   A   
   A   
   G   
   C   
   A   
   G   
   A   
   A   
   G   
   C   
   A   
   A   
   T   
    C    
   G   
   T   
   C   
   A   
   C   
   A   
   T   
   T   
   G   
   C   
   A   
   A   
    C    
   T   
   A   
   T   
   A   
    C    
   C   
   G   
   T   
   T   
   T   
   G   
   A   
   C   
   G   
   A   
   A   
    C    
   A   
   C   
    A    
   G   
   A   
   T   
   A   
   T   
   C   
   A   
   C   
   A   
   G   
   A   
   G   
   C   
   T   
   T   
   C   
   A   
   A   
   A   
   A   
   G   
   G   
   A   
   A   
    G    
   C   
    C    
   G   
   A   
   A   
   A   
    G    
   C   
   T   
   T   
   G   
   A   
   T   
   T   
   G   
   C   
    A    
   C   
   A   
   A   
   A   
   T   
   C   
   A   
   C   
   A   
   G   
   A   
   G   
   T   
   T   
    G    
   A   
   A   
   T   
   A   
   A   
   A   
   A   
   T   
   C   
   C   
   T   
   T   
   T   
   C   
   A   
   A   
   A   
    C    
   G   
   A   
   C   
   A   
   A   
   A   
   G   
   A   
    G    
   C   
   T   
   T   
   T   
   T   
    C    
   T   
   C   
   T   
   G   
   T   
   T   
   A   
   T   
   G   
   A   
   A   
   A   
   A   
   A   
   A   
   G   
   A   
   A   
   T   
   T   
   G   
   C   
   G   
   A   
   G   
   A   
   A   
   G   
   T   
   G   
   A   
   A   
   A   
   A   
   A   
   A   
   A   
   A   
   T   
   T   
   A   
    C    
   T   
   C   
   T   
   T   
   C   
   C   
   G   
   C   
   T   
   C   
   G   
   T   
   C   
    T    
    G    
   A   
   C   
   G   
   A   
   C   
   A   
   A   
   T   
   C   
    C    
   A   
   A   
   G   
   A   
   C   
   G   
   A   
   A   
   A   
   T   
   C   
   G   
   A   
   A   
   G   
   A   
   A   
   A   
   T   
   C   
   A   
   A   
   A   
   A   
   T   
    C    
   G   
   A   
   T   
   A   
   C   
   G   
   C   
   A   
   A   
    G    
   T   
    T    
   T   
   T   
   A   
   G   
   T   
   G   
   G   
   C   
    G    
   C   
   A   
   A   
   G   
   A   
   A   
   G   
   A   
   C   
   G   
   T   
   A   
   A   
   T   
    C    
   G   
   T   
   C   
   A   
   G   
   T   
   G   
   T   
    G    
   A   
   C   
    G    
   C   
   G   
   A   
   G   
   A   
   A   
   G   
   G   
   C   
   T   
   A   
   T   
   G   
   T   
   G   
   A   
   A   
   G   
   C   
   G   
   A   
   A   
   C   
   A   
   A   
   G   
   C   
   C   
   T   
   T   
   C   
   G   
   T   
   T   
   C   
    T    
   T   
   A   
   T   
   A   
   G   
   T   
   G   
   C   
   T   
   T   
   C   
    G    
   A   
   A   
   A   
   C   
   C   
   A   
   G   
   A   
   A   
   G   
   A   
   A   
   A   
   T   
   C   
   G   
   G   
   G   
   A   
   T   
   G   
   C   
   G   
   A   
   G   
   A   
   A   
   G   
   G   
   C   
   G   
   A   
    T    
   T   
   A   
   C   
   T   
   T   
   G   
   C   
   T   
    G    
   T   
   A   
   C   
    T    
   C   
   A   
   G   
   G   
   T   
   G   
   A   
   A   
   T   
   T   
    G    
   A   
   G   
    C    
   A   
   C   
   A   
    T    
   T   
   G   
   G   
   A   
   C   
   C   
   A   
   T   
   G   
   T   
   T   
   C   
   T   
   T   
   C   
   T   
   G   
   A   
   T   
    T    
   A   
   C   
    G    
   A   
   A   
   T   
   A   
   A   
   A   
   G   
   C   
   A   
   A   
   A   
   T   
   G   
   T   
   G   
   A   
   T   
   C   
   T   
   A   
   C   
   C   
   G   
    T    
   C   
   C   
   T   
   G   
   T   
    T    
   C   
   A   
   T   
   G   
   A   
   A   
   C   
   T   
   A   
   C   
   C   
    C    
   G   
   A   
   T   
   T   
   T   
   G   
   A   
   A   
   A   
   T   
   G   
   G   
   A   
   A   
   A   
   G   
   A   
   T   
   G   
   C   
   T   
   G   
   G   
    C    
   G   
   A   
    A    
   C   
   A   
    C    
   A   
   T   
   T   
   T   
   C   
   T   
   C   
   A   
   A   
   A   
   C   
   A   
   A   
   T   
   C   
   T   
   C   
   C   
   A   
   A   
   T   
   C   
   T   
   G   
   T   
   C   
   T   
   G   
   T   
   A   
   G   
   A   
    T    
   G   
   A   
   A   
   T   
   C   
   G   
   A   
   T   
    C    
   T   
   T   
   A   
   G   
   C   
   T   
   G   
   T   
   T   
   T   
   T   
    C    
   C   
   C   
   A   
   T   
   A   
   T   
   C   
   A   
   A   
   A   
   A   
   A   
   A   
   T   
    C    
   C   
   A   
   A   
   G   
   C   
   A   
   G   
   A   
    A    
   A   
   A   
   A   
   A   
   C   
   A   
   T   
   T   
   T   
   G   
   T   
    A    
   T   
   T   
   C   
   A   
   T   
   C   
   A   
   G   
   C   
   A   
   A   
   G   
   A   
   A   
   T   
   G   
   G   
   A   
   T   
   T   
    G    
    A    
   T   
   C   
   A   
   A   
   G   
   C   
   A   
   A   
   A   
   C   
   A   
   C   
   G   
   A   
   A   
   T   
   G   
   A   
   C   
   A   
   G   
   A   
   G   
   T   
   T   
   C   
   G   
   A   
   A   
   C   
   C   
   A   
   T   
   G   
   G   
   C   
   G   
   C   
   A   
   C   
   C   
   T   
   A   
    T    
   A   
   A   
   G   
   A   
   G   
   C   
   C   
   G   
    T    
   C   
   C   
   G   
   C   
   T   
   C   
   A   
   G   
   C   
   T   
   G   
   T   
   A   
   T   
   G   
   A   
   A   
   G   
   C   
   T   
   A   
   A   
   A   
   G   
   G   
   C   
   G   
   G   
   A   
   A   
   G   
   A   
   A   
   G   
   A   
   T   
   G   
   A   
   A   
   C   
   T   
    T    
   A   
   T   
   T   
   G   
   C   
   T   
   G   
   T   
    A    
   T   
   A   
   T   
   C   
   T   
   G   
   A   
   A   
   A   
   G   
   A   
   A   
   G   
   A   
   T   
   C   
    A    
   G   
   C   
   C   
   A   
   G   
   G   
   C   
   T   
   T   
   A   
   G   
   A   
   T   
   G   
   T   
    G    
   T   
   T   
   C   
   T   
   T   
   A   
   G   
   T   
   A   
   A   
   C   
   G   
    C    
   A   
   T   
   C   
   A   
   A   
   G   
   G   
   A   
   A   
   T   
   G   
   G   
   G   
    C    
   T   
   T   
   G   
   C   
   G   
   C   
   T   
    A    
   T   
   C   
   C   
   T   
   T   
   T   
   A   
   A   
    C    
   A   
   G   
   A   
   T   
   G   
   T   
   T   
   C   
   C   
   C   
   G   
   T   
   T   
   G   
   T   
   T   
   G   
   G   
   A   
   G   
   C   
   A   
   A   
   A   
   G   
   G   
   C   
   A   
   G   
    C    
   T   
   G   
   G   
   A   
   G   
   T   
   G   
   A   
   A   
   G   
   T   
   C   
   T   
   A   
   T   
   G   
   A   
   A   
   T   
   T   
   T   
   G   
   A   
   A   
   A   
   G   
   A   
   T   
   G   
   G   
   A   
   G   
   A   
   T   
   A   
   G   
   G   
    A    
   T   
   T   
    G    
   T   
   C   
   A   
   A   
    T    
   G   
   G   
    G    
   C   
   T   
    G    
   C   
   T   
   T   
   G   
   T   
   G   
   T   
   T   
   A   
   G   
   C   
   A   
   G   
   A   
    C    
   G   
   G   
    A    
   G   
   A   
   T   
   A   
   C   
   G   
   C   
   C   
    A    
   A   
   T   
   C   
   G   
   T   
   A   
   A   
   T   
   C   
   G   
   T   
   T   
   A   
   C   
   T   
   C   
   A   
   G   
   C   
   G   
   A   
   G   
   G   
   A   
   G   
   C   
    C    
   G   
   T   
    G    
   A   
   A   
   A   
   C   
   G   
   C   
   A   
   T   
   G   
   C   
   T   
   T   
   G   
   C   
   T   
   C   
   A   
   A   
   G   
   A   
   A   
   A   
   T   
   C   
   A   
   G   
   C   
   C   
   A   
   A   
   A   
   C   
    T    
   T   
   C   
   A   
   C   
   G   
   T   
   G   
   C   
   G   
   A   
   A   
   G   
   C   
   G   
   G   
   G   
   G   
   A   
   G   
   T   
   G   
   A   
   C   
   G   
   G   
   T   
   C   
   A   
   T   
   G   
   C   
   G   
   T   
   G   
   A   
   G   
   C   
   T   
   G   
   A   
   A   
   A   
   A   
   A   
   A   
   A   
   A   
   T   
   C   
   C   
    G    
   C   
   A   
    C    
   C   
   G   
    T    
   A   
   T   
   T   
   A   
   T   
   C   
   T   
   A   
   T   
   A   
   T   
   G   
   T   
   C   
   G   
   G   
   A   
   A   
   G   
   G   
   A   
   A   
   G   
   A   
   T   
   C   
   A   
   A   
   A   
   A   
   G   
   A   
   A   
   A   
   T   
   A   
   A   
   C   
   C   
   T   
   T   
   G   
   A   
   T   
   G   
   A   
   A   
   T   
   C   
   A   
   A   
   A   
   A   
   A   
   G   
   G   
    A    
   C   
   A   
    A    
   C   
   A   
   G   
    A    
   T   
   A   
   A   
   C   
   G   
   A   
   T   
   T   
   G   
   A   
    T    
   C   
   C   
   A   
   A   
   C   
    G    
   G   
   A   
   T   
   T   
   T   
    T    
   C   
   C   
   T   
   A   
   T   
   A   
   G   
   G   
   T   
   G   
   A   
   T   
   C   
   G   
   A   
   A   
   C   
   A   
   T   
   C   
   G   
   A   
   A   
   T   
   G   
   G   
    T    
   T   
   C   
    G    
   T   
   T   
   T   
   G   
   C   
    T    
   A   
   T   
   G   
   G   
   A   
    C    
   G   
   A   
   G   
   A   
   A   
   A   
   A   
   A   
   A   
   G   
   G   
   T   
   G   
   G   
   A   
   G   
   A   
   A   
   G   
   T   
   C   
   A   
   T   
   C   
   G   
   A   
   A   
   G   
   T   
    T    
   A   
    T    
   C   
   G   
   A   
   T   
   G   
   C   
   T   
   C   
   C   
   T   
   G   
   A   
   G   
    A    
   T   
   T   
   T   
   T   
    C    
   A   
   T   
    T    
   G   
   A   
    A    
    T    
   C   
    A    
   -   
   -   
   -   
 

 
   N52751   
   A   
   T   
   G   
   G   
   A   
   A   
   A   
   A   
   T   
   C   
   G   
   A   
   C   
   A   
   A   
   G   
   A   
   A   
   G   
   T   
   A   
   C   
   A   
   A   
   G   
   A   
   A   
   T   
   T   
   A   
   A   
   C   
   T   
   T   
   T   
   A   
   G   
   A   
   A   
   G   
   A   
   A   
   G   
   T   
   G   
   A   
   T   
   G   
   G   
   G   
   T   
   G   
   A   
   C   
   C   
   G   
   C   
   T   
   T   
   C   
   G   
   G   
   A   
   C   
   G   
   T   
   T   
   A   
   T   
   T   
   C   
   C   
   A   
   A   
   A   
   T   
   A   
   T   
   A   
   T   
   T   
   A   
   T   
    C    
   C   
   A   
    A    
   G   
   A   
   A   
   C   
   G   
   T   
   G   
   C   
   A   
   C   
   T   
   G   
   C   
   C   
   T   
   G   
   A   
   T   
   A   
   T   
    C    
   C   
   G   
   T   
   G   
   A   
   T   
   G   
   G   
   A   
   T   
   T   
   G   
   A   
   A   
    G    
   C   
   C   
   A   
   G   
   T   
    G    
   C   
   A   
   A   
   C   
   G   
   C   
   C   
   G   
   G   
   A   
   T   
   A   
   T   
   T   
   A   
   T   
   T   
    T    
   G   
   C   
    C    
   A   
   T   
   G   
   A   
   A   
   C   
   A   
   A   
   A   
   G   
   A   
   T   
   G   
   G   
   C   
   A   
   A   
   T   
   A   
   C   
   G   
   T   
   A   
   T   
   G   
   A   
   C   
   A   
   A   
   A   
   G   
   G   
   A   
   T   
   T   
    C    
   C   
   G   
   T   
   A   
   A   
   A   
   T   
   C   
   A   
   G   
   C   
   A   
   A   
   A   
   A   
   T   
   C   
   T   
   G   
   T   
   C   
   G   
   G   
   A   
   A   
   A   
   T   
   A   
   T   
   C   
   A   
   T   
   G   
   G   
   G   
    G    
   A   
   A   
   T   
   T   
   A   
   T   
   C   
   A   
   T   
   C   
   C   
   T   
   C   
   A   
   T   
   G   
   G   
   T   
   G   
   A   
   C   
   A   
   G   
   C   
   A   
   G   
   T   
   A   
   T   
   T   
   T   
   A   
   T   
   G   
   A   
   A   
   G   
   C   
   G   
   A   
   T   
   G   
   G   
   T   
   C   
   C   
   G   
   C   
   A   
   T   
   G   
   A   
   G   
   C   
   C   
   A   
   A   
   G   
   A   
    C    
   T   
   G   
   G   
   A   
   A   
   G   
   T   
   T   
   G   
   C   
   G   
   G   
   G   
   A   
   A   
   G   
   T   
   A   
   C   
   T   
   G   
   A   
   T   
   C   
   G   
   A   
    A    
   A   
   T   
   G   
   C   
   A   
   C   
   G   
   G   
    G    
   A   
   A   
   T   
   A   
   A   
    C    
   G   
   G   
   G   
   A   
   G   
   T   
   A   
   T   
   G   
   G   
   A   
   C   
   G   
   G   
   T   
   G   
   A   
   T   
   C   
   C   
   A   
   C   
   C   
   T   
   G   
   C   
   T   
   G   
   C   
   G   
   A   
   T   
   G   
   C   
   G   
   T   
   T   
   A   
   T   
   A   
   C   
   T   
   G   
   A   
   A   
   G   
   C   
   C   
   A   
   G   
    A    
   T   
   T   
   A   
   T   
   C   
   C   
   A   
   A   
   G   
   T   
   T   
   A   
   A   
   G   
   T   
   G   
   G   
   A   
   G   
   A   
   G   
   A   
   T   
   G   
    C    
   T   
   A   
    G    
   C   
   A   
   G   
   A   
   T   
   A   
   T   
   C   
   G   
   A   
    A    
   A   
   A   
   A   
   G   
   A   
   A   
   A   
   C   
   A   
   G   
   T   
    C    
   G   
   A   
   T   
   C   
   T   
   C   
   G   
   T   
   G   
   T   
   G   
   G   
   A   
   A   
   T   
   T   
   T   
   T   
   G   
   A   
   T   
   G   
   A   
   T   
   A   
   C   
   A   
   G   
   A   
   A   
   A   
   A   
   A   
   G   
   A   
   A   
   C   
   C   
   A   
   A   
   C   
   A   
   G   
   T   
   C   
   C   
   T   
   T   
   C   
   C   
   A   
   G   
   C   
   T   
   C   
   G   
   A   
   T   
   A   
   C   
   C   
   C   
   A   
   A   
   A   
   C   
   C   
   T   
   T   
   C   
   T   
    C    
   G   
   T   
   C   
   A   
   A   
   T   
   G   
   G   
   A   
   T   
   C   
   G   
   A   
   C   
   A   
   G   
   G   
   T   
   A   
   T   
   A   
   T   
   C   
   T   
   G   
   C   
   T   
   G   
   G   
   A   
   T   
   A   
   T   
   G   
   C   
    A    
   A   
   C   
   A   
   G   
   A   
   A   
   A   
   T   
   C   
   C   
   C   
   G   
   A   
   C   
    T    
   C   
   A   
   T   
   A   
   A   
   C   
   T   
   T   
   A   
   G   
   C   
   T   
   G   
   A   
   A   
   G   
   T   
   G   
   A   
   T   
    T    
   G   
   A   
    T    
   G   
   G   
   C   
   A   
   C   
   A   
   A   
   T   
   C   
   T   
   A   
   T   
   A   
   T   
   G   
   A   
   T   
   C   
   G   
   A   
   T   
   C   
   A   
   T   
   C   
   C   
   A   
   C   
   A   
   A   
   G   
   C   
   G   
   A   
   G   
   C   
   C   
   T   
   T   
   G   
   A   
   A   
   A   
   A   
   A   
   T   
   T   
   G   
   A   
   T   
   G   
   G   
   A   
   A   
   T   
   A   
   T   
   A   
   T   
   T   
   C   
   C   
   T   
   G   
   G   
   A   
   C   
   C   
   T   
   G   
   A   
   C   
   T   
   T   
    T    
   C   
   C   
   A   
   A   
   C   
   A   
   G   
   G   
   A   
   G   
   G   
    G    
   A   
   T   
   C   
   T   
   T   
   G   
   C   
   A   
   A   
   G   
   G   
    A    
   A   
   A   
   A   
   G   
   A   
   T   
   G   
   A   
   A   
   A   
   T   
   C   
   A   
   A   
   A   
   A   
   A   
   A   
   G   
   C   
   T   
   T   
   A   
   T   
   G   
   A   
   A   
   A   
   C   
   A   
   G   
   G   
    G    
   C   
   G   
   A   
   G   
   G   
   A   
   A   
   A   
   A   
   G   
   T   
   T   
   A   
    T    
   T   
   C   
   T   
   T   
   C   
   G   
   T   
   T   
   C   
   G   
   A   
   A   
   A   
   A   
   C   
   A   
   A   
   A   
   G   
   A   
   T   
   A   
   G   
   A   
    G    
   G   
   C   
   A   
   A   
   T   
   G   
   A   
   A   
   G   
   G   
   G   
    T    
   A   
   A   
    T    
   A   
   A   
   A   
   C   
   A   
   G   
   C   
   A   
   A   
   A   
   T   
   C   
   G   
   T   
   G   
   A   
   T   
   C   
   A   
   C   
    A    
   G   
   A   
   A   
   A   
   T   
   C   
   C   
   C   
   A   
   T   
   A   
   T   
   G   
   A   
   A   
   G   
   T   
   A   
   A   
   A   
   T   
   A   
   A   
   A   
   G   
   C   
   G   
   A   
   C   
    C    
   T   
   T   
   G   
   G   
   T   
   G   
   A   
   A   
   A   
   A   
   A   
   A   
   A   
   T   
   G   
   G   
   A   
   T   
   G   
   A   
   G   
   A   
   T   
   C   
   C   
   G   
   T   
   T   
   T   
   G   
   A   
   A   
   T   
   A   
   A   
   G   
   A   
   A   
   A   
   A   
   T   
   C   
   G   
   A   
    T    
   G   
   G   
   A   
   A   
   T   
   A   
   G   
   C   
   T   
   G   
   A   
   A   
   G   
   T   
    A    
   C   
   G   
   A   
   G   
   A   
   T   
   G   
   A   
   A   
   A   
   G   
   T   
   G   
   A   
   T   
    A    
   G   
    A    
   A   
   C   
    A    
   G   
   G   
   T   
   C   
   T   
   G   
   C   
   A   
   A   
   A   
   T   
   T   
   G   
   T   
   C   
   G   
   T   
    C    
   G   
   A   
   A   
   T   
   T   
   G   
   A   
   A   
   A   
   A   
   A   
   A   
   G   
   A   
    C    
   G   
   C   
   C   
   A   
   A   
    T    
   G   
   C   
   T   
   C   
   A   
   A   
   G   
   G   
    A    
   A   
   T   
   T   
   T   
   T   
   G   
   A   
   A   
   T   
   T   
   A   
    T    
   T   
   T   
   G   
   T   
   T   
   C   
   A   
   A   
   A   
   A   
   A   
   C   
   A   
   C   
   T   
   G   
   A   
   G   
    C    
   T   
   A   
   C   
   A   
   G   
   A   
   T   
   C   
   A   
   A   
   T   
   T   
   A   
   C   
   A   
   A   
   T   
   T   
   T   
    C    
   A   
   A   
   T   
   A   
   T   
   G   
   G   
   T   
   C   
   G   
   C   
   T   
   A   
   T   
   C   
   G   
   A   
    C    
   C   
   A   
   T   
   A   
   T   
   G   
   A   
   C   
   T   
   C   
   C   
   G   
   C   
   A   
    T    
   C   
   A   
   A   
   G   
   T   
   T   
   G   
   G   
   A   
   C   
   T   
   G   
   A   
   A   
   A   
   G   
   A   
   C   
   A   
   T   
   T   
   T   
   T   
   A   
   A   
   G   
   A   
   A   
   G   
   T   
   T   
   A   
   T   
   A   
   T   
   C   
   G   
   A   
   G   
   C   
   A   
   T   
   C   
   G   
   A   
   A   
   A   
   G   
   C   
   A   
   A   
   G   
   T   
   C   
   A   
   T   
   A   
   A   
   C   
   A   
   A   
   A   
   A   
   C   
   G   
    T    
   A   
   G   
    T    
   C   
   A   
   A   
   T   
   T   
   C   
   G   
   A   
   T   
   T   
   T   
   A   
   G   
   C   
   C   
   A   
   A   
   A   
   G   
   C   
   T   
   C   
   A   
   A   
   A   
   A   
   A   
    A    
   G   
   A   
   C   
   A   
   G   
   C   
   A   
   T   
   A   
   T   
   C   
   G   
   T   
    A    
   G   
   A   
   A   
   G   
   G   
    A    
    T    
   T   
   G   
   A   
   T   
   G   
   A   
   A   
   A   
   G   
   C   
   T   
   T   
   T   
   A   
   T   
   C   
   G   
   A   
   T   
   T   
   C   
   T   
   G   
   G   
   A   
   T   
   G   
   A   
   A   
   G   
   T   
   A   
   A   
   T   
   C   
   G   
    T    
   A   
   A   
   C   
   G   
   A   
   T   
   T   
   C   
   G   
   T   
   G   
   A   
   A   
   A   
   G   
   T   
   A   
   A   
   A   
   G   
   A   
   C   
   A   
   A   
   A   
   A   
   A   
   A   
   G   
   A   
    C    
   G   
   C   
    C    
   A   
   A   
   A   
   A   
   A   
   G   
   A   
   A   
   T   
   T   
   T   
   A   
   G   
   T   
   C   
   G   
   A   
    C    
   G   
   T   
    C    
   T   
   T   
    T    
   C   
   A   
   A   
   T   
   T   
   T   
   A   
   C   
   A   
   G   
   A   
   A   
   G   
   A   
   A   
   C   
   A   
   A   
   G   
   C   
   A   
   G   
   A   
   A   
   G   
   C   
   A   
   A   
   T   
    T    
   G   
   T   
   C   
   A   
   C   
   A   
   T   
   T   
   G   
   C   
   A   
   A   
    T    
   T   
   A   
   T   
   A   
    C    
   C   
   G   
   T   
   T   
   T   
   G   
   A   
   C   
   G   
   A   
   A   
    C    
   A   
   C   
    G    
   G   
   A   
   T   
   A   
   T   
   C   
   A   
   C   
   A   
   G   
   A   
   G   
   C   
   T   
   T   
   C   
   A   
   A   
   A   
   A   
   G   
   G   
   A   
   A   
    T    
   C   
    C    
   G   
   A   
   A   
   A   
    G    
   C   
   T   
   T   
   G   
   A   
   T   
   T   
   G   
   C   
    G    
   C   
   A   
   A   
   A   
   T   
   C   
   A   
   C   
   A   
   G   
   A   
   G   
   T   
   T   
    G    
   A   
   A   
   T   
   A   
   A   
   A   
   A   
   T   
   C   
   C   
   T   
   T   
   T   
   C   
   A   
   A   
   A   
    T    
   G   
   A   
   C   
   A   
   A   
   A   
   G   
   A   
    G    
   C   
   T   
   T   
   T   
   T   
    C    
   T   
   C   
   T   
   G   
   T   
   T   
   A   
   T   
   G   
   A   
   A   
   A   
   A   
   A   
   A   
   G   
   A   
   A   
   T   
   T   
   G   
   C   
   G   
   A   
   G   
   A   
   A   
   G   
   T   
   G   
   A   
   A   
   A   
   A   
   A   
   A   
   A   
   A   
   T   
   T   
   A   
    T    
   T   
   C   
   T   
   T   
   C   
   C   
   G   
   C   
   T   
   C   
   G   
   T   
   C   
    T    
    A    
   A   
   C   
   G   
   A   
   C   
   A   
   A   
   T   
   C   
    G    
   A   
   A   
   G   
   A   
   C   
   G   
   A   
   A   
   A   
   T   
   C   
   G   
   A   
   A   
   G   
   A   
   A   
   A   
   T   
   C   
   A   
   A   
   A   
   A   
   T   
    T    
   G   
   A   
   T   
   A   
   C   
   G   
   C   
   A   
   A   
    G    
   T   
    C    
   T   
   T   
   A   
   G   
   T   
   G   
   G   
   C   
    A    
   C   
   A   
   A   
   G   
   A   
   A   
   G   
   A   
   C   
   G   
   T   
   A   
   A   
   T   
    T    
   G   
   T   
   C   
   A   
   G   
   T   
   G   
   T   
    G    
   A   
   C   
    G    
   C   
   G   
   A   
   G   
   A   
   A   
   G   
   G   
   C   
   T   
   A   
   T   
   G   
   T   
   G   
   A   
   A   
   G   
   C   
   G   
   A   
   A   
   C   
   A   
   A   
   G   
   C   
   C   
   T   
   T   
   C   
   G   
   T   
   T   
   C   
    T    
   T   
   A   
   T   
   A   
   G   
   T   
   G   
   C   
   T   
   T   
   C   
    C    
   A   
   A   
   A   
   C   
   C   
   A   
   G   
   A   
   A   
   G   
   A   
   A   
   A   
   T   
   C   
   G   
   G   
   G   
   A   
   T   
   G   
   C   
   G   
   A   
   G   
   A   
   A   
   G   
   G   
   C   
   G   
   A   
    C    
   T   
   A   
   C   
   T   
   T   
   G   
   C   
   T   
    A    
   T   
   A   
   C   
    T    
   C   
   A   
   G   
   G   
   T   
   G   
   A   
   A   
   T   
   T   
    G    
   A   
   G   
    T    
   A   
   C   
   A   
    T    
   T   
   G   
   G   
   A   
   C   
   C   
   A   
   T   
   G   
   T   
   T   
   C   
   T   
   T   
   C   
   T   
   G   
   A   
   T   
    C    
   A   
   C   
    A    
   A   
   A   
   T   
   A   
   A   
   A   
   G   
   C   
   A   
   A   
   A   
   T   
   G   
   T   
   G   
   A   
   T   
   C   
   T   
   A   
   C   
   C   
   G   
    C    
   C   
   C   
   T   
   G   
   T   
    C    
   C   
   A   
   T   
   G   
   A   
   A   
   C   
   T   
   A   
   C   
   C   
    C    
   G   
   A   
   T   
   T   
   T   
   G   
   A   
   A   
   A   
   T   
   G   
   G   
   A   
   A   
   A   
   G   
   A   
   T   
   G   
   C   
   T   
   G   
   G   
    T    
   G   
   A   
    G    
   C   
   A   
    C    
   A   
   T   
   T   
   T   
   C   
   T   
   C   
   A   
   A   
   A   
   C   
   A   
   A   
   T   
   C   
   T   
   C   
   C   
   A   
   A   
   T   
   C   
   T   
   G   
   T   
   C   
   T   
   G   
   T   
   A   
   G   
   A   
    C    
   G   
   A   
   A   
   T   
   C   
   G   
   A   
   T   
    T    
   T   
   T   
   A   
   G   
   C   
   T   
   G   
   T   
   T   
   T   
   T   
    C    
   C   
   C   
   A   
   T   
   A   
   T   
   C   
   A   
   A   
   A   
   A   
   A   
   A   
   T   
    T    
   C   
   A   
   A   
   G   
   C   
   A   
   G   
   A   
    G    
   A   
   A   
   A   
   A   
   C   
   A   
   T   
   T   
   T   
   G   
   T   
    A    
   T   
   T   
   C   
   A   
   T   
   C   
   A   
   G   
   C   
   A   
   A   
   G   
   A   
   A   
   T   
   G   
   G   
   A   
   T   
   T   
    G    
    A    
   T   
   C   
   A   
   A   
   G   
   C   
   A   
   A   
   A   
   C   
   A   
   C   
   G   
   A   
   A   
   T   
   G   
   A   
   C   
   A   
   G   
   A   
   G   
   T   
   T   
   C   
   G   
   A   
   A   
   C   
   C   
   A   
   T   
   G   
   G   
   C   
   G   
   C   
   A   
   C   
   C   
   T   
   A   
    T    
   A   
   A   
   G   
   A   
   G   
   C   
   C   
   G   
    T    
   C   
   C   
   G   
   C   
   T   
   C   
   A   
   G   
   C   
   T   
   G   
   T   
   A   
   T   
   G   
   A   
   A   
   G   
   C   
   T   
   A   
   A   
   A   
   G   
   G   
   C   
   G   
   G   
   A   
   A   
   G   
   A   
   A   
   G   
   A   
   T   
   G   
   A   
   A   
   C   
   T   
    G    
   A   
   T   
   T   
   G   
   C   
   T   
   G   
   T   
    G    
   T   
   A   
   T   
   C   
   T   
   G   
   A   
   A   
   A   
   G   
   A   
   A   
   G   
   A   
   T   
   C   
    A    
   G   
   C   
   C   
   A   
   G   
   G   
   C   
   T   
   T   
   A   
   G   
   A   
   T   
   G   
   T   
    T    
   T   
   T   
   C   
   T   
   T   
   A   
   G   
   T   
   A   
   A   
   C   
   G   
    C    
   A   
   T   
   C   
   A   
   A   
   G   
   G   
   A   
   A   
   T   
   G   
   G   
   G   
    T    
   T   
   T   
   G   
   C   
   G   
   C   
   T   
    A    
   T   
   C   
   C   
   T   
   T   
   T   
   A   
   A   
    C    
   A   
   G   
   A   
   T   
   G   
   T   
   T   
   C   
   C   
   C   
   G   
   T   
   T   
   G   
   T   
   T   
   G   
   G   
   A   
   G   
   C   
   A   
   A   
   A   
   G   
   G   
   C   
   A   
   G   
    C    
   T   
   G   
   G   
   A   
   G   
   T   
   G   
   A   
   A   
   G   
   T   
   C   
   T   
   A   
   T   
   G   
   A   
   A   
   T   
   T   
   T   
   G   
   A   
   A   
   A   
   G   
   A   
   T   
   G   
   G   
   A   
   G   
   A   
   T   
   A   
   G   
   G   
    A    
   T   
   T   
    A    
   T   
   C   
   A   
   A   
    C    
   G   
   G   
    G    
   C   
   T   
    G    
   C   
   T   
   T   
   G   
   T   
   G   
   T   
   T   
   A   
   G   
   C   
   A   
   G   
   A   
    A    
   G   
   G   
    T    
   G   
   A   
   T   
   A   
   C   
   G   
   C   
   C   
    G    
   A   
   T   
   C   
   G   
   T   
   A   
   A   
   T   
   C   
   G   
   T   
   T   
   A   
   C   
   T   
   C   
   A   
   G   
   C   
   G   
   A   
   G   
   G   
   A   
   G   
   C   
    T    
   G   
   T   
    T    
   A   
   A   
   A   
   C   
   G   
   C   
   A   
   T   
   G   
   C   
   T   
   T   
   G   
   C   
   T   
   C   
   A   
   A   
   G   
   A   
   A   
   A   
   T   
   C   
   A   
   G   
   C   
   C   
   A   
   A   
   A   
   C   
    T    
   T   
   C   
   A   
   C   
   G   
   T   
   G   
   C   
   G   
   A   
   A   
   G   
   C   
   G   
   G   
   G   
   G   
   A   
   G   
   T   
   G   
   A   
   C   
   G   
   G   
   T   
   C   
   A   
   T   
   G   
   C   
   G   
   T   
   G   
   A   
   G   
   C   
   T   
   G   
   A   
   A   
   A   
   A   
   A   
   A   
   A   
   A   
   T   
   C   
   C   
    G    
   C   
   A   
    C    
   C   
   G   
    T    
   A   
   T   
   T   
   A   
   T   
   C   
   T   
   A   
   T   
   A   
   T   
   G   
   T   
   C   
   G   
   G   
   A   
   A   
   G   
   G   
   A   
   A   
   G   
   A   
   T   
   C   
   A   
   A   
   A   
   A   
   G   
   A   
   A   
   A   
   T   
   A   
   A   
   C   
   C   
   T   
   T   
   G   
   A   
   T   
   G   
   A   
   A   
   T   
   C   
   A   
   A   
   A   
   A   
   A   
   G   
   G   
    G    
   C   
   A   
    G    
   C   
   A   
   G   
    T    
   T   
   A   
   A   
   C   
   G   
   A   
   T   
   T   
   G   
   A   
    C    
   C   
   C   
   A   
   A   
   C   
    T    
   G   
   A   
   T   
   T   
   T   
    C    
   C   
   C   
   T   
   A   
   T   
   A   
   G   
   G   
   T   
   G   
   A   
   T   
   C   
   G   
   A   
   A   
   C   
   A   
   T   
   C   
   G   
   A   
   A   
   T   
   G   
   G   
    A    
   T   
   C   
    A    
   T   
   T   
   T   
   G   
   C   
    G    
   A   
   T   
   G   
   G   
   A   
    T    
   G   
   A   
   G   
   A   
   A   
   A   
   A   
   A   
   A   
   G   
   G   
   T   
   G   
   G   
   A   
   G   
   A   
   A   
   G   
   T   
   C   
   A   
   T   
   C   
   G   
   A   
   A   
   G   
   T   
    T    
   A   
    T    
   C   
   G   
   A   
   T   
   G   
   C   
   T   
   C   
   C   
   T   
   G   
   A   
   G   
    A    
   T   
   T   
   T   
   T   
    C    
   A   
   T   
    C    
   G   
   A   
    G    
    T    
   C   
    G    
   -   
   -   
   -   
 

 
   N54671   
   A   
   T   
   G   
   G   
   A   
   A   
   A   
   A   
   T   
   C   
   G   
   A   
   C   
   A   
   A   
   G   
   A   
   A   
   G   
   T   
   A   
   C   
   A   
   A   
   G   
   A   
   A   
   T   
   T   
   A   
   A   
   C   
   T   
   T   
   T   
   A   
   G   
   A   
   A   
   G   
   A   
   A   
   G   
   T   
   G   
   A   
   T   
   G   
   G   
   G   
   T   
   G   
   A   
   C   
   C   
   G   
   C   
   T   
   T   
   C   
   G   
   G   
   A   
   C   
   G   
   T   
   T   
   A   
   T   
   T   
   C   
   C   
   A   
   A   
   A   
   T   
   A   
   T   
   A   
   T   
   T   
   A   
   T   
    C    
   C   
   A   
    A    
   G   
   A   
   A   
   C   
   G   
   T   
   G   
   C   
   A   
   C   
   T   
   G   
   C   
   C   
   T   
   G   
   A   
   T   
   A   
   T   
    C    
   C   
   G   
   T   
   G   
   A   
   T   
   G   
   G   
   A   
   T   
   T   
   G   
   A   
   A   
    G    
   C   
   C   
   A   
   G   
   T   
    G    
   C   
   A   
   A   
   C   
   G   
   C   
   C   
   G   
   G   
   A   
   T   
   A   
   T   
   T   
   A   
   T   
   T   
    T    
   G   
   C   
    C    
   A   
   T   
   G   
   A   
   A   
   C   
   A   
   A   
   A   
   G   
   A   
   T   
   G   
   G   
   C   
   A   
   A   
   T   
   A   
   C   
   G   
   T   
   A   
   T   
   G   
   A   
   C   
   A   
   A   
   A   
   G   
   G   
   A   
   T   
   T   
    C    
   C   
   G   
   T   
   A   
   A   
   A   
   T   
   C   
   A   
   G   
   C   
   A   
   A   
   A   
   A   
   T   
   C   
   T   
   G   
   T   
   C   
   G   
   G   
   A   
   A   
   A   
   T   
   A   
   T   
   C   
   A   
   T   
   G   
   G   
   G   
    G    
   A   
   A   
   T   
   T   
   A   
   T   
   C   
   A   
   T   
   C   
   C   
   T   
   C   
   A   
   T   
   G   
   G   
   T   
   G   
   A   
   C   
   A   
   G   
   C   
   A   
   G   
   T   
   A   
   T   
   T   
   T   
   A   
   T   
   G   
   A   
   A   
   G   
   C   
   G   
   A   
   T   
   G   
   G   
   T   
   C   
   C   
   G   
   C   
   A   
   T   
   G   
   A   
   G   
   C   
   C   
   A   
   A   
   G   
   A   
    C    
   T   
   G   
   G   
   A   
   A   
   G   
   T   
   T   
   G   
   C   
   G   
   G   
   G   
   A   
   A   
   G   
   T   
   A   
   C   
   T   
   G   
   A   
   T   
   C   
   G   
   A   
    A    
   A   
   T   
   G   
   C   
   A   
   C   
   G   
   G   
    G    
   A   
   A   
   T   
   A   
   A   
    C    
   G   
   G   
   G   
   A   
   G   
   T   
   A   
   T   
   G   
   G   
   A   
   C   
   G   
   G   
   T   
   G   
   A   
   T   
   C   
   C   
   A   
   C   
   C   
   T   
   G   
   C   
   T   
   G   
   C   
   G   
   A   
   T   
   G   
   C   
   G   
   T   
   T   
   A   
   T   
   A   
   C   
   T   
   G   
   A   
   A   
   G   
   C   
   C   
   A   
   G   
    A    
   T   
   T   
   A   
   T   
   C   
   C   
   A   
   A   
   G   
   T   
   T   
   A   
   A   
   G   
   T   
   G   
   G   
   A   
   G   
   A   
   G   
   A   
   T   
   G   
    C    
   T   
   A   
    G    
   C   
   A   
   G   
   A   
   T   
   A   
   T   
   C   
   G   
   A   
    A    
   A   
   A   
   A   
   G   
   A   
   A   
   A   
   C   
   A   
   G   
   T   
    C    
   G   
   A   
   T   
   C   
   T   
   C   
   G   
   T   
   G   
   T   
   G   
   G   
   A   
   A   
   T   
   T   
   T   
   T   
   G   
   A   
   T   
   G   
   A   
   T   
   A   
   C   
   A   
   G   
   A   
   A   
   A   
   A   
   A   
   G   
   A   
   A   
   C   
   C   
   A   
   A   
   C   
   A   
   G   
   T   
   C   
   C   
   T   
   T   
   C   
   C   
   A   
   G   
   C   
   T   
   C   
   G   
   A   
   T   
   A   
   C   
   C   
   C   
   A   
   A   
   A   
   C   
   C   
   T   
   T   
   C   
   T   
    C    
   G   
   T   
   C   
   A   
   A   
   T   
   G   
   G   
   A   
   T   
   C   
   G   
   A   
   C   
   A   
   G   
   G   
   T   
   A   
   T   
   A   
   T   
   C   
   T   
   G   
   C   
   T   
   G   
   G   
   A   
   T   
   A   
   T   
   G   
   C   
    A    
   A   
   C   
   A   
   G   
   A   
   A   
   A   
   T   
   C   
   C   
   C   
   G   
   A   
   C   
    T    
   C   
   A   
   T   
   A   
   A   
   C   
   T   
   T   
   A   
   G   
   C   
   T   
   G   
   A   
   A   
   G   
   T   
   G   
   A   
   T   
    T    
   G   
   A   
    T    
   G   
   G   
   C   
   A   
   C   
   A   
   A   
   T   
   C   
   T   
   A   
   T   
   A   
   T   
   G   
   A   
   T   
   C   
   G   
   A   
   T   
   C   
   A   
   T   
   C   
   C   
   A   
   C   
   A   
   A   
   G   
   C   
   G   
   A   
   G   
   C   
   C   
   T   
   T   
   G   
   A   
   A   
   A   
   A   
   A   
   T   
   T   
   G   
   A   
   T   
   G   
   G   
   A   
   A   
   T   
   A   
   T   
   A   
   T   
   T   
   C   
   C   
   T   
   G   
   G   
   A   
   C   
   C   
   T   
   G   
   A   
   C   
   T   
   T   
    T    
   C   
   C   
   A   
   A   
   C   
   A   
   G   
   G   
   A   
   G   
   G   
    G    
   A   
   T   
   C   
   T   
   T   
   G   
   C   
   A   
   A   
   G   
   G   
    A    
   A   
   A   
   A   
   G   
   A   
   T   
   G   
   A   
   A   
   A   
   T   
   C   
   A   
   A   
   A   
   A   
   A   
   A   
   G   
   C   
   T   
   T   
   A   
   T   
   G   
   A   
   A   
   A   
   C   
   A   
   G   
   G   
    G    
   C   
   G   
   A   
   G   
   G   
   A   
   A   
   A   
   A   
   G   
   T   
   T   
   A   
    T    
   T   
   C   
   T   
   T   
   C   
   G   
   T   
   T   
   C   
   G   
   A   
   A   
   A   
   A   
   C   
   A   
   A   
   A   
   G   
   A   
   T   
   A   
   G   
   A   
    G    
   G   
   C   
   A   
   A   
   T   
   G   
   A   
   A   
   G   
   G   
   G   
    T    
   A   
   A   
    T    
   A   
   A   
   A   
   C   
   A   
   G   
   C   
   A   
   A   
   A   
   T   
   C   
   G   
   T   
   G   
   A   
   T   
   C   
   A   
   C   
    A    
   G   
   A   
   A   
   A   
   T   
   C   
   C   
   C   
   A   
   T   
   A   
   T   
   G   
   A   
   A   
   G   
   T   
   A   
   A   
   A   
   T   
   A   
   A   
   A   
   G   
   C   
   G   
   A   
   C   
    C    
   T   
   T   
   G   
   G   
   T   
   G   
   A   
   A   
   A   
   A   
   A   
   A   
   A   
   T   
   G   
   G   
   A   
   T   
   G   
   A   
   G   
   A   
   T   
   C   
   C   
   G   
   T   
   T   
   T   
   G   
   A   
   A   
   T   
   A   
   A   
   G   
   A   
   A   
   A   
   A   
   T   
   C   
   G   
   A   
    T    
   G   
   G   
   A   
   A   
   T   
   A   
   G   
   C   
   T   
   G   
   A   
   A   
   G   
   T   
    A    
   C   
   G   
   A   
   G   
   A   
   T   
   G   
   A   
   A   
   A   
   G   
   T   
   G   
   A   
   T   
    A    
   G   
    A    
   A   
   C   
    A    
   G   
   G   
   T   
   C   
   T   
   G   
   C   
   A   
   A   
   A   
   T   
   T   
   G   
   T   
   C   
   G   
   T   
    C    
   G   
   A   
   A   
   T   
   T   
   G   
   A   
   A   
   A   
   A   
   A   
   A   
   G   
   A   
    C    
   G   
   C   
   C   
   A   
   A   
    T    
   G   
   C   
   T   
   C   
   A   
   A   
   G   
   G   
    A    
   A   
   T   
   T   
   T   
   T   
   G   
   A   
   A   
   T   
   T   
   A   
    T    
   T   
   T   
   G   
   T   
   T   
   C   
   A   
   A   
   A   
   A   
   A   
   C   
   A   
   C   
   T   
   G   
   A   
   G   
    C    
   T   
   A   
   C   
   A   
   G   
   A   
   T   
   C   
   A   
   A   
   T   
   T   
   A   
   C   
   A   
   A   
   T   
   T   
   T   
    C    
   A   
   A   
   T   
   A   
   T   
   G   
   G   
   T   
   C   
   G   
   C   
   T   
   A   
   T   
   C   
   G   
   A   
    C    
   C   
   A   
   T   
   A   
   T   
   G   
   A   
   C   
   T   
   C   
   C   
   G   
   C   
   A   
    T    
   C   
   A   
   A   
   G   
   T   
   T   
   G   
   G   
   A   
   C   
   T   
   G   
   A   
   A   
   A   
   G   
   A   
   C   
   A   
   T   
   T   
   T   
   T   
   A   
   A   
   G   
   A   
   A   
   G   
   T   
   T   
   A   
   T   
   A   
   T   
   C   
   G   
   A   
   G   
   C   
   A   
   T   
   C   
   G   
   A   
   A   
   A   
   G   
   C   
   A   
   A   
   G   
   T   
   C   
   A   
   T   
   A   
   A   
   C   
   A   
   A   
   A   
   A   
   C   
   G   
    T    
   A   
   G   
    T    
   C   
   A   
   A   
   T   
   T   
   C   
   G   
   A   
   T   
   T   
   T   
   A   
   G   
   C   
   C   
   A   
   A   
   A   
   G   
   C   
   T   
   C   
   A   
   A   
   A   
   A   
   A   
    A    
   G   
   A   
   C   
   A   
   G   
   C   
   A   
   T   
   A   
   T   
   C   
   G   
   T   
    A    
   G   
   A   
   A   
   G   
   G   
    A    
    T    
   T   
   G   
   A   
   T   
   G   
   A   
   A   
   A   
   G   
   C   
   T   
   T   
   T   
   A   
   T   
   C   
   G   
   A   
   T   
   T   
   C   
   T   
   G   
   G   
   A   
   T   
   G   
   A   
   A   
   G   
   T   
   A   
   A   
   T   
   C   
   G   
    T    
   A   
   A   
   C   
   G   
   A   
   T   
   T   
   C   
   G   
   T   
   G   
   A   
   A   
   A   
   G   
   T   
   A   
   A   
   A   
   G   
   A   
   C   
   A   
   A   
   A   
   A   
   A   
   A   
   G   
   A   
    C    
   G   
   C   
    C    
   A   
   A   
   A   
   A   
   A   
   G   
   A   
   A   
   T   
   T   
   T   
   A   
   G   
   T   
   C   
   G   
   A   
    C    
   G   
   T   
    C    
   T   
   T   
    T    
   C   
   A   
   A   
   T   
   T   
   T   
   A   
   C   
   A   
   G   
   A   
   A   
   G   
   A   
   A   
   C   
   A   
   A   
   G   
   C   
   A   
   G   
   A   
   A   
   G   
   C   
   A   
   A   
   T   
    T    
   G   
   T   
   C   
   A   
   C   
   A   
   T   
   T   
   G   
   C   
   A   
   A   
    T    
   T   
   A   
   T   
   A   
    C    
   C   
   G   
   T   
   T   
   T   
   G   
   A   
   C   
   G   
   A   
   A   
    C    
   A   
   C   
    G    
   G   
   A   
   T   
   A   
   T   
   C   
   A   
   C   
   A   
   G   
   A   
   G   
   C   
   T   
   T   
   C   
   A   
   A   
   A   
   A   
   G   
   G   
   A   
   A   
    T    
   C   
    C    
   G   
   A   
   A   
   A   
    G    
   C   
   T   
   T   
   G   
   A   
   T   
   T   
   G   
   C   
    G    
   C   
   A   
   A   
   A   
   T   
   C   
   A   
   C   
   A   
   G   
   A   
   G   
   T   
   T   
    G    
   A   
   A   
   T   
   A   
   A   
   A   
   A   
   T   
   C   
   C   
   T   
   T   
   T   
   C   
   A   
   A   
   A   
    T    
   G   
   A   
   C   
   A   
   A   
   A   
   G   
   A   
    G    
   C   
   T   
   T   
   T   
   T   
    C    
   T   
   C   
   T   
   G   
   T   
   T   
   A   
   T   
   G   
   A   
   A   
   A   
   A   
   A   
   A   
   G   
   A   
   A   
   T   
   T   
   G   
   C   
   G   
   A   
   G   
   A   
   A   
   G   
   T   
   G   
   A   
   A   
   A   
   A   
   A   
   A   
   A   
   A   
   T   
   T   
   A   
    T    
   T   
   C   
   T   
   T   
   C   
   C   
   G   
   C   
   T   
   C   
   G   
   T   
   C   
    T    
    A    
   A   
   C   
   G   
   A   
   C   
   A   
   A   
   T   
   C   
    G    
   A   
   A   
   G   
   A   
   C   
   G   
   A   
   A   
   A   
   T   
   C   
   G   
   A   
   A   
   G   
   A   
   A   
   A   
   T   
   C   
   A   
   A   
   A   
   A   
   T   
    T    
   G   
   A   
   T   
   A   
   C   
   G   
   C   
   A   
   A   
    G    
   T   
    C    
   T   
   T   
   A   
   G   
   T   
   G   
   G   
   C   
    A    
   C   
   A   
   A   
   G   
   A   
   A   
   G   
   A   
   C   
   G   
   T   
   A   
   A   
   T   
    T    
   G   
   T   
   C   
   A   
   G   
   T   
   G   
   T   
    G    
   A   
   C   
    G    
   C   
   G   
   A   
   G   
   A   
   A   
   G   
   G   
   C   
   T   
   A   
   T   
   G   
   T   
   G   
   A   
   A   
   G   
   C   
   G   
   A   
   A   
   C   
   A   
   A   
   G   
   C   
   C   
   T   
   T   
   C   
   G   
   T   
   T   
   C   
    T    
   T   
   A   
   T   
   A   
   G   
   T   
   G   
   C   
   T   
   T   
   C   
    C    
   A   
   A   
   A   
   C   
   C   
   A   
   G   
   A   
   A   
   G   
   A   
   A   
   A   
   T   
   C   
   G   
   G   
   G   
   A   
   T   
   G   
   C   
   G   
   A   
   G   
   A   
   A   
   G   
   G   
   C   
   G   
   A   
    C    
   T   
   A   
   C   
   T   
   T   
   G   
   C   
   T   
    A    
   T   
   A   
   C   
    T    
   C   
   A   
   G   
   G   
   T   
   G   
   A   
   A   
   T   
   T   
    G    
   A   
   G   
    T    
   A   
   C   
   A   
    A    
   T   
   G   
   G   
   A   
   C   
   C   
   A   
   T   
   G   
   T   
   T   
   C   
   T   
   T   
   C   
   T   
   G   
   A   
   T   
    C    
   A   
   C   
    A    
   A   
   A   
   T   
   A   
   A   
   A   
   G   
   C   
   A   
   A   
   A   
   T   
   G   
   T   
   G   
   A   
   T   
   C   
   T   
   A   
   C   
   C   
   G   
    C    
   C   
   C   
   T   
   G   
   T   
    C    
   C   
   A   
   T   
   G   
   A   
   A   
   C   
   T   
   A   
   C   
   C   
    C    
   G   
   A   
   T   
   T   
   T   
   G   
   A   
   A   
   A   
   T   
   G   
   G   
   A   
   A   
   A   
   G   
   A   
   T   
   G   
   C   
   T   
   G   
   G   
    T    
   G   
   A   
    G    
   C   
   A   
    C    
   A   
   T   
   T   
   T   
   C   
   T   
   C   
   A   
   A   
   A   
   C   
   A   
   A   
   T   
   C   
   T   
   C   
   C   
   A   
   A   
   T   
   C   
   T   
   G   
   T   
   C   
   T   
   G   
   T   
   A   
   G   
   A   
    C    
   G   
   A   
   A   
   T   
   C   
   G   
   A   
   T   
    T    
   T   
   T   
   A   
   G   
   C   
   T   
   G   
   T   
   T   
   T   
   T   
    C    
   C   
   C   
   A   
   T   
   A   
   T   
   C   
   A   
   A   
   A   
   A   
   A   
   A   
   T   
    T    
   C   
   A   
   A   
   G   
   C   
   A   
   G   
   A   
    G    
   A   
   A   
   A   
   A   
   C   
   A   
   T   
   T   
   T   
   G   
   T   
    A    
   T   
   T   
   C   
   A   
   T   
   C   
   A   
   G   
   C   
   A   
   A   
   G   
   A   
   A   
   T   
   G   
   G   
   A   
   T   
   T   
    G    
    A    
   T   
   C   
   A   
   A   
   G   
   C   
   A   
   A   
   A   
   C   
   A   
   C   
   G   
   A   
   A   
   T   
   G   
   A   
   C   
   A   
   G   
   A   
   G   
   T   
   T   
   C   
   G   
   A   
   A   
   C   
   C   
   A   
   T   
   G   
   G   
   C   
   G   
   C   
   A   
   C   
   C   
   T   
   A   
    T    
   A   
   A   
   G   
   A   
   G   
   C   
   C   
   G   
    T    
   C   
   C   
   G   
   C   
   T   
   C   
   A   
   G   
   C   
   T   
   G   
   T   
   A   
   T   
   G   
   A   
   A   
   G   
   C   
   T   
   A   
   A   
   A   
   G   
   G   
   C   
   G   
   G   
   A   
   A   
   G   
   A   
   A   
   G   
   A   
   T   
   G   
   A   
   A   
   C   
   T   
    G    
   A   
   T   
   T   
   G   
   C   
   T   
   G   
   T   
    G    
   T   
   A   
   T   
   C   
   T   
   G   
   A   
   A   
   A   
   G   
   A   
   A   
   G   
   A   
   T   
   C   
    A    
   G   
   C   
   C   
   A   
   G   
   G   
   C   
   T   
   T   
   A   
   G   
   A   
   T   
   G   
   T   
    T    
   T   
   T   
   C   
   T   
   T   
   A   
   G   
   T   
   A   
   A   
   C   
   G   
    C    
   A   
   T   
   C   
   A   
   A   
   G   
   G   
   A   
   A   
   T   
   G   
   G   
   G   
    T    
   T   
   T   
   G   
   C   
   G   
   C   
   T   
    A    
   T   
   C   
   C   
   T   
   T   
   T   
   A   
   A   
    C    
   A   
   G   
   A   
   T   
   G   
   T   
   T   
   C   
   C   
   C   
   G   
   T   
   T   
   G   
   T   
   T   
   G   
   G   
   A   
   G   
   C   
   A   
   A   
   A   
   G   
   G   
   C   
   A   
   G   
    C    
   T   
   G   
   G   
   A   
   G   
   T   
   G   
   A   
   A   
   G   
   T   
   C   
   T   
   A   
   T   
   G   
   A   
   A   
   T   
   T   
   T   
   G   
   A   
   A   
   A   
   G   
   A   
   T   
   G   
   G   
   A   
   G   
   A   
   T   
   A   
   G   
   G   
    A    
   T   
   T   
    A    
   T   
   C   
   A   
   A   
    C    
   G   
   G   
    G    
   C   
   T   
    G    
   C   
   T   
   T   
   G   
   T   
   G   
   T   
   T   
   A   
   G   
   C   
   A   
   G   
   A   
    A    
   G   
   G   
    T    
   G   
   A   
   T   
   A   
   C   
   G   
   C   
   C   
    G    
   A   
   T   
   C   
   G   
   T   
   A   
   A   
   T   
   C   
   G   
   T   
   T   
   A   
   C   
   T   
   C   
   A   
   G   
   C   
   G   
   A   
   G   
   G   
   A   
   G   
   C   
    T    
   G   
   T   
    T    
   A   
   A   
   A   
   C   
   G   
   C   
   A   
   T   
   G   
   C   
   T   
   T   
   G   
   C   
   T   
   C   
   A   
   A   
   G   
   A   
   A   
   A   
   T   
   C   
   A   
   G   
   C   
   C   
   A   
   A   
   A   
   C   
    T    
   T   
   C   
   A   
   C   
   G   
   T   
   G   
   C   
   G   
   A   
   A   
   G   
   C   
   G   
   G   
   G   
   G   
   A   
   G   
   T   
   G   
   A   
   C   
   G   
   G   
   T   
   C   
   A   
   T   
   G   
   C   
   G   
   T   
   G   
   A   
   G   
   C   
   T   
   G   
   A   
   A   
   A   
   A   
   A   
   A   
   A   
   A   
   T   
   C   
   C   
    G    
   C   
   A   
    C    
   C   
   G   
    T    
   A   
   T   
   T   
   A   
   T   
   C   
   T   
   A   
   T   
   A   
   T   
   G   
   T   
   C   
   G   
   G   
   A   
   A   
   G   
   G   
   A   
   A   
   G   
   A   
   T   
   C   
   A   
   A   
   A   
   A   
   G   
   A   
   A   
   A   
   T   
   A   
   A   
   C   
   C   
   T   
   T   
   G   
   A   
   T   
   G   
   A   
   A   
   T   
   C   
   A   
   A   
   A   
   A   
   A   
   G   
   G   
    G    
   C   
   A   
    G    
   C   
   A   
   G   
    T    
   T   
   A   
   A   
   C   
   G   
   A   
   T   
   T   
   G   
   A   
    C    
   C   
   C   
   A   
   A   
   C   
    T    
   G   
   A   
   T   
   T   
   T   
    C    
   C   
   C   
   T   
   A   
   T   
   A   
   G   
   G   
   T   
   G   
   A   
   T   
   C   
   G   
   A   
   A   
   C   
   A   
   T   
   C   
   G   
   A   
   A   
   T   
   G   
   G   
    A    
   T   
   C   
    A    
   T   
   T   
   T   
   G   
   C   
    G    
   A   
   T   
   G   
   G   
   A   
    T    
   G   
   A   
   G   
   A   
   A   
   A   
   A   
   A   
   A   
   G   
   G   
   T   
   G   
   G   
   A   
   G   
   A   
   A   
   G   
   T   
   C   
   A   
   T   
   C   
   G   
   A   
   A   
   G   
   T   
    T    
   A   
    T    
   C   
   G   
   A   
   T   
   G   
   C   
   T   
   C   
   C   
   T   
   G   
   A   
   G   
    A    
   T   
   T   
   T   
   T   
    C    
   A   
   T   
    C    
   G   
   A   
    G    
    T    
   C   
    G    
   -   
   -   
   -   
 

 
   N55072   
   A   
   T   
   G   
   G   
   A   
   A   
   A   
   A   
   T   
   C   
   G   
   A   
   C   
   A   
   A   
   G   
   A   
   A   
   G   
   T   
   A   
   C   
   A   
   A   
   G   
   A   
   A   
   T   
   T   
   A   
   A   
   C   
   T   
   T   
   T   
   A   
   G   
   A   
   A   
   G   
   A   
   A   
   G   
   T   
   G   
   A   
   T   
   G   
   G   
   G   
   T   
   G   
   A   
   C   
   C   
   G   
   C   
   T   
   T   
   C   
   G   
   G   
   A   
   C   
   G   
   T   
   T   
   A   
   T   
   T   
   C   
   C   
   A   
   A   
   A   
   T   
   A   
   T   
   A   
   T   
   T   
   A   
   T   
    C    
   C   
   A   
    A    
   G   
   A   
   A   
   C   
   G   
   T   
   G   
   C   
   A   
   C   
   T   
   G   
   C   
   C   
   T   
   G   
   A   
   T   
   A   
   T   
    C    
   C   
   G   
   T   
   G   
   A   
   T   
   G   
   G   
   A   
   T   
   T   
   G   
   A   
   A   
    G    
   C   
   C   
   A   
   G   
   T   
    G    
   C   
   A   
   A   
   C   
   G   
   C   
   C   
   G   
   G   
   A   
   T   
   A   
   T   
   T   
   A   
   T   
   T   
    T    
   G   
   C   
    C    
   A   
   T   
   G   
   A   
   A   
   C   
   A   
   A   
   A   
   G   
   A   
   T   
   G   
   G   
   C   
   A   
   A   
   T   
   A   
   C   
   G   
   T   
   A   
   T   
   G   
   A   
   C   
   A   
   A   
   A   
   G   
   G   
   A   
   T   
   T   
    C    
   C   
   G   
   T   
   A   
   A   
   A   
   T   
   C   
   A   
   G   
   C   
   A   
   A   
   A   
   A   
   T   
   C   
   T   
   G   
   T   
   C   
   G   
   G   
   A   
   A   
   A   
   T   
   A   
   T   
   C   
   A   
   T   
   G   
   G   
   G   
    G    
   A   
   A   
   T   
   T   
   A   
   T   
   C   
   A   
   T   
   C   
   C   
   T   
   C   
   A   
   T   
   G   
   G   
   T   
   G   
   A   
   C   
   A   
   G   
   C   
   A   
   G   
   T   
   A   
   T   
   T   
   T   
   A   
   T   
   G   
   A   
   A   
   G   
   C   
   G   
   A   
   T   
   G   
   G   
   T   
   C   
   C   
   G   
   C   
   A   
   T   
   G   
   A   
   G   
   C   
   C   
   A   
   A   
   G   
   A   
    C    
   T   
   G   
   G   
   A   
   A   
   G   
   T   
   T   
   G   
   C   
   G   
   G   
   G   
   A   
   A   
   G   
   T   
   A   
   C   
   T   
   G   
   A   
   T   
   C   
   G   
   A   
    A    
   A   
   T   
   G   
   C   
   A   
   C   
   G   
   G   
    G    
   A   
   A   
   T   
   A   
   A   
    C    
   G   
   G   
   G   
   A   
   G   
   T   
   A   
   T   
   G   
   G   
   A   
   C   
   G   
   G   
   T   
   G   
   A   
   T   
   C   
   C   
   A   
   C   
   C   
   T   
   G   
   C   
   T   
   G   
   C   
   G   
   A   
   T   
   G   
   C   
   G   
   T   
   T   
   A   
   T   
   A   
   C   
   T   
   G   
   A   
   A   
   G   
   C   
   C   
   A   
   G   
    A    
   T   
   T   
   A   
   T   
   C   
   C   
   A   
   A   
   G   
   T   
   T   
   A   
   A   
   G   
   T   
   G   
   G   
   A   
   G   
   A   
   G   
   A   
   T   
   G   
    C    
   T   
   A   
    G    
   C   
   A   
   G   
   A   
   T   
   A   
   T   
   C   
   G   
   A   
    A    
   A   
   A   
   A   
   G   
   A   
   A   
   A   
   C   
   A   
   G   
   T   
    C    
   G   
   A   
   T   
   C   
   T   
   C   
   G   
   T   
   G   
   T   
   G   
   G   
   A   
   A   
   T   
   T   
   T   
   T   
   G   
   A   
   T   
   G   
   A   
   T   
   A   
   C   
   A   
   G   
   A   
   A   
   A   
   A   
   A   
   G   
   A   
   A   
   C   
   C   
   A   
   A   
   C   
   A   
   G   
   T   
   C   
   C   
   T   
   T   
   C   
   C   
   A   
   G   
   C   
   T   
   C   
   G   
   A   
   T   
   A   
   C   
   C   
   C   
   A   
   A   
   A   
   C   
   C   
   T   
   T   
   C   
   T   
    C    
   G   
   T   
   C   
   A   
   A   
   T   
   G   
   G   
   A   
   T   
   C   
   G   
   A   
   C   
   A   
   G   
   G   
   T   
   A   
   T   
   A   
   T   
   C   
   T   
   G   
   C   
   T   
   G   
   G   
   A   
   T   
   A   
   T   
   G   
   C   
    A    
   A   
   C   
   A   
   G   
   A   
   A   
   A   
   T   
   C   
   C   
   C   
   G   
   A   
   C   
    T    
   C   
   A   
   T   
   A   
   A   
   C   
   T   
   T   
   A   
   G   
   C   
   T   
   G   
   A   
   A   
   G   
   T   
   G   
   A   
   T   
    C    
   G   
   A   
    T    
   G   
   G   
   C   
   A   
   C   
   A   
   A   
   T   
   C   
   T   
   A   
   T   
   A   
   T   
   G   
   A   
   T   
   C   
   G   
   A   
   T   
   C   
   A   
   T   
   C   
   C   
   A   
   C   
   A   
   A   
   G   
   C   
   G   
   A   
   G   
   C   
   C   
   T   
   T   
   G   
   A   
   A   
   A   
   A   
   A   
   T   
   T   
   G   
   A   
   T   
   G   
   G   
   A   
   A   
   T   
   A   
   T   
   A   
   T   
   T   
   C   
   C   
   T   
   G   
   G   
   A   
   C   
   C   
   T   
   G   
   A   
   C   
   T   
   T   
    T    
   C   
   C   
   A   
   A   
   C   
   A   
   G   
   G   
   A   
   G   
   G   
    G    
   A   
   T   
   C   
   T   
   T   
   G   
   C   
   A   
   A   
   G   
   G   
    A    
   A   
   A   
   A   
   G   
   A   
   T   
   G   
   A   
   A   
   A   
   T   
   C   
   A   
   A   
   A   
   A   
   A   
   A   
   G   
   C   
   T   
   T   
   A   
   T   
   G   
   A   
   A   
   A   
   C   
   A   
   G   
   G   
    G    
   C   
   G   
   A   
   G   
   G   
   A   
   A   
   A   
   A   
   G   
   T   
   T   
   A   
    T    
   T   
   C   
   T   
   T   
   C   
   G   
   T   
   T   
   C   
   G   
   A   
   A   
   A   
   A   
   C   
   A   
   A   
   A   
   G   
   A   
   T   
   A   
   G   
   A   
    G    
   G   
   C   
   A   
   A   
   T   
   G   
   A   
   A   
   G   
   G   
   G   
    T    
   A   
   A   
    T    
   A   
   A   
   A   
   C   
   A   
   G   
   C   
   A   
   A   
   A   
   T   
   C   
   G   
   T   
   G   
   A   
   T   
   C   
   A   
   C   
    A    
   G   
   A   
   A   
   A   
   T   
   C   
   C   
   C   
   A   
   T   
   A   
   T   
   G   
   A   
   A   
   G   
   T   
   A   
   A   
   A   
   T   
   A   
   A   
   A   
   G   
   C   
   G   
   A   
   C   
    C    
   T   
   T   
   G   
   G   
   T   
   G   
   A   
   A   
   A   
   A   
   A   
   A   
   A   
   T   
   G   
   G   
   A   
   T   
   G   
   A   
   G   
   A   
   T   
   C   
   C   
   G   
   T   
   T   
   T   
   G   
   A   
   A   
   T   
   A   
   A   
   G   
   A   
   A   
   A   
   A   
   T   
   C   
   G   
   A   
    T    
   G   
   G   
   A   
   A   
   T   
   A   
   G   
   C   
   T   
   G   
   A   
   A   
   G   
   T   
    A    
   C   
   G   
   A   
   G   
   A   
   T   
   G   
   A   
   A   
   A   
   G   
   T   
   G   
   A   
   T   
    A    
   G   
    A    
   A   
   C   
    A    
   G   
   G   
   T   
   C   
   T   
   G   
   C   
   A   
   A   
   A   
   T   
   T   
   G   
   T   
   C   
   G   
   T   
    C    
   G   
   A   
   A   
   T   
   T   
   G   
   A   
   A   
   A   
   A   
   A   
   A   
   G   
   A   
    C    
   G   
   C   
   C   
   A   
   A   
    T    
   G   
   C   
   T   
   C   
   A   
   A   
   G   
   G   
    A    
   A   
   T   
   T   
   T   
   T   
   G   
   A   
   A   
   T   
   T   
   A   
    T    
   T   
   T   
   G   
   T   
   T   
   C   
   A   
   A   
   A   
   A   
   A   
   C   
   A   
   C   
   T   
   G   
   A   
   G   
    C    
   T   
   A   
   C   
   A   
   G   
   A   
   T   
   C   
   A   
   A   
   T   
   T   
   A   
   C   
   A   
   A   
   T   
   T   
   T   
    C    
   A   
   A   
   T   
   A   
   T   
   G   
   G   
   T   
   C   
   G   
   C   
   T   
   A   
   T   
   C   
   G   
   A   
    C    
   C   
   A   
   T   
   A   
   T   
   G   
   A   
   C   
   T   
   C   
   C   
   G   
   C   
   A   
    T    
   C   
   A   
   A   
   G   
   T   
   T   
   G   
   G   
   A   
   C   
   T   
   G   
   A   
   A   
   A   
   G   
   A   
   C   
   A   
   T   
   T   
   T   
   T   
   A   
   A   
   G   
   A   
   A   
   G   
   T   
   T   
   A   
   T   
   A   
   T   
   C   
   G   
   A   
   G   
   C   
   A   
   T   
   C   
   G   
   A   
   A   
   A   
   G   
   C   
   A   
   A   
   G   
   T   
   C   
   A   
   T   
   A   
   A   
   C   
   A   
   A   
   A   
   A   
   C   
   G   
    T    
   A   
   G   
    T    
   C   
   A   
   A   
   T   
   T   
   C   
   G   
   A   
   T   
   T   
   T   
   A   
   G   
   C   
   C   
   A   
   A   
   A   
   G   
   C   
   T   
   C   
   A   
   A   
   A   
   A   
   A   
    A    
   G   
   A   
   C   
   A   
   G   
   C   
   A   
   T   
   A   
   T   
   C   
   G   
   T   
    A    
   G   
   A   
   A   
   G   
   G   
    A    
    T    
   T   
   G   
   A   
   T   
   G   
   A   
   A   
   A   
   G   
   C   
   T   
   T   
   T   
   A   
   T   
   C   
   G   
   A   
   T   
   T   
   C   
   T   
   G   
   G   
   A   
   T   
   G   
   A   
   A   
   G   
   T   
   A   
   A   
   T   
   C   
   G   
    C    
   A   
   A   
   C   
   G   
   A   
   T   
   T   
   C   
   G   
   T   
   G   
   A   
   A   
   A   
   G   
   T   
   A   
   A   
   A   
   G   
   A   
   C   
   A   
   A   
   A   
   A   
   A   
   A   
   G   
   A   
    C    
   G   
   C   
    C    
   A   
   A   
   A   
   A   
   A   
   G   
   A   
   A   
   T   
   T   
   T   
   A   
   G   
   T   
   C   
   G   
   A   
    C    
   G   
   T   
    C    
   T   
   T   
    T    
   C   
   A   
   A   
   T   
   T   
   T   
   A   
   C   
   A   
   G   
   A   
   A   
   G   
   A   
   A   
   C   
   A   
   A   
   G   
   C   
   A   
   G   
   A   
   A   
   G   
   C   
   A   
   A   
   T   
    T    
   G   
   T   
   C   
   A   
   C   
   A   
   T   
   T   
   G   
   C   
   A   
   A   
    T    
   T   
   A   
   T   
   A   
    C    
   C   
   G   
   T   
   T   
   T   
   G   
   A   
   C   
   G   
   A   
   A   
    C    
   A   
   C   
    G    
   G   
   A   
   T   
   A   
   T   
   C   
   A   
   C   
   A   
   G   
   A   
   G   
   C   
   T   
   T   
   C   
   A   
   A   
   A   
   A   
   G   
   G   
   A   
   A   
    T    
   C   
    C    
   G   
   A   
   A   
   A   
    G    
   C   
   T   
   T   
   G   
   A   
   T   
   T   
   G   
   C   
    G    
   C   
   A   
   A   
   A   
   T   
   C   
   A   
   C   
   A   
   G   
   A   
   G   
   T   
   T   
    G    
   A   
   A   
   T   
   A   
   A   
   A   
   A   
   T   
   C   
   C   
   T   
   T   
   T   
   C   
   A   
   A   
   A   
    T    
   G   
   A   
   C   
   A   
   A   
   A   
   G   
   A   
    G    
   C   
   T   
   T   
   T   
   T   
    C    
   T   
   C   
   T   
   G   
   T   
   T   
   A   
   T   
   G   
   A   
   A   
   A   
   A   
   A   
   A   
   G   
   A   
   A   
   T   
   T   
   G   
   C   
   G   
   A   
   G   
   A   
   A   
   G   
   T   
   G   
   A   
   A   
   A   
   A   
   A   
   A   
   A   
   A   
   T   
   T   
   A   
    T    
   T   
   C   
   T   
   T   
   C   
   C   
   G   
   C   
   T   
   C   
   G   
   T   
   C   
    T    
    A    
   A   
   C   
   G   
   A   
   C   
   A   
   A   
   T   
   C   
    G    
   A   
   A   
   G   
   A   
   C   
   G   
   A   
   A   
   A   
   T   
   C   
   G   
   A   
   A   
   G   
   A   
   A   
   A   
   T   
   C   
   A   
   A   
   A   
   A   
   T   
    T    
   G   
   A   
   T   
   A   
   C   
   G   
   C   
   A   
   A   
    G    
   T   
    C    
   T   
   T   
   A   
   G   
   T   
   G   
   G   
   C   
    A    
   C   
   A   
   A   
   G   
   A   
   A   
   G   
   A   
   C   
   G   
   T   
   A   
   A   
   T   
    T    
   G   
   T   
   C   
   A   
   G   
   T   
   G   
   T   
    G    
   A   
   C   
    G    
   C   
   G   
   A   
   G   
   A   
   A   
   G   
   G   
   C   
   T   
   A   
   T   
   G   
   T   
   G   
   A   
   A   
   G   
   C   
   G   
   A   
   A   
   C   
   A   
   A   
   G   
   C   
   C   
   T   
   T   
   C   
   G   
   T   
   T   
   C   
    T    
   T   
   A   
   T   
   A   
   G   
   T   
   G   
   C   
   T   
   T   
   C   
    C    
   A   
   A   
   A   
   C   
   C   
   A   
   G   
   A   
   A   
   G   
   A   
   A   
   A   
   T   
   C   
   G   
   G   
   G   
   A   
   T   
   G   
   C   
   G   
   A   
   G   
   A   
   A   
   G   
   G   
   C   
   G   
   A   
    C    
   T   
   A   
   C   
   T   
   T   
   G   
   C   
   T   
    A    
   T   
   A   
   C   
    T    
   C   
   A   
   G   
   G   
   T   
   G   
   A   
   A   
   T   
   T   
    G    
   A   
   G   
    T    
   A   
   C   
   A   
    T    
   T   
   G   
   G   
   A   
   C   
   C   
   A   
   T   
   G   
   T   
   T   
   C   
   T   
   T   
   C   
   T   
   G   
   A   
   T   
    C    
   A   
   C   
    A    
   A   
   A   
   T   
   A   
   A   
   A   
   G   
   C   
   A   
   A   
   A   
   T   
   G   
   T   
   G   
   A   
   T   
   C   
   T   
   A   
   C   
   C   
   G   
    C    
   C   
   C   
   T   
   G   
   T   
    C    
   C   
   A   
   T   
   G   
   A   
   A   
   C   
   T   
   A   
   C   
   C   
    C    
   G   
   A   
   T   
   T   
   T   
   G   
   A   
   A   
   A   
   T   
   G   
   G   
   A   
   A   
   A   
   G   
   A   
   T   
   G   
   C   
   T   
   G   
   G   
    T    
   G   
   A   
    G    
   C   
   A   
    C    
   A   
   T   
   T   
   T   
   C   
   T   
   C   
   A   
   A   
   A   
   C   
   A   
   A   
   T   
   C   
   T   
   C   
   C   
   A   
   A   
   T   
   C   
   T   
   G   
   T   
   C   
   T   
   G   
   T   
   A   
   G   
   A   
    C    
   G   
   A   
   A   
   T   
   C   
   G   
   A   
   T   
    T    
   T   
   T   
   A   
   G   
   C   
   T   
   G   
   T   
   T   
   T   
   T   
    C    
   C   
   C   
   A   
   T   
   A   
   T   
   C   
   A   
   A   
   A   
   A   
   A   
   A   
   T   
    T    
   C   
   A   
   A   
   G   
   C   
   A   
   G   
   A   
    G    
   A   
   A   
   A   
   A   
   C   
   A   
   T   
   T   
   T   
   G   
   T   
    A    
   T   
   T   
   C   
   A   
   T   
   C   
   A   
   G   
   C   
   A   
   A   
   G   
   A   
   A   
   T   
   G   
   G   
   A   
   T   
   T   
    G    
    A    
   T   
   C   
   A   
   A   
   G   
   C   
   A   
   A   
   A   
   C   
   A   
   C   
   G   
   A   
   A   
   T   
   G   
   A   
   C   
   A   
   G   
   A   
   G   
   T   
   T   
   C   
   G   
   A   
   A   
   C   
   C   
   A   
   T   
   G   
   G   
   C   
   G   
   C   
   A   
   C   
   C   
   T   
   A   
    T    
   A   
   A   
   G   
   A   
   G   
   C   
   C   
   G   
    T    
   C   
   C   
   G   
   C   
   T   
   C   
   A   
   G   
   C   
   T   
   G   
   T   
   A   
   T   
   G   
   A   
   A   
   G   
   C   
   T   
   A   
   A   
   A   
   G   
   G   
   C   
   G   
   G   
   A   
   A   
   G   
   A   
   A   
   G   
   A   
   T   
   G   
   A   
   A   
   C   
   T   
    G    
   A   
   T   
   T   
   G   
   C   
   T   
   G   
   T   
    G    
   T   
   A   
   T   
   C   
   T   
   G   
   A   
   A   
   A   
   G   
   A   
   A   
   G   
   A   
   T   
   C   
    A    
   G   
   C   
   C   
   A   
   G   
   G   
   C   
   T   
   T   
   A   
   G   
   A   
   T   
   G   
   T   
    T    
   T   
   T   
   C   
   T   
   T   
   A   
   G   
   T   
   A   
   A   
   C   
   G   
    C    
   A   
   T   
   C   
   A   
   A   
   G   
   G   
   A   
   A   
   T   
   G   
   G   
   G   
    T    
   T   
   T   
   G   
   C   
   G   
   C   
   T   
    A    
   T   
   C   
   C   
   T   
   T   
   T   
   A   
   A   
    C    
   A   
   G   
   A   
   T   
   G   
   T   
   T   
   C   
   C   
   C   
   G   
   T   
   T   
   G   
   T   
   T   
   G   
   G   
   A   
   G   
   C   
   A   
   A   
   A   
   G   
   G   
   C   
   A   
   G   
    C    
   T   
   G   
   G   
   A   
   G   
   T   
   G   
   A   
   A   
   G   
   T   
   C   
   T   
   A   
   T   
   G   
   A   
   A   
   T   
   T   
   T   
   G   
   A   
   A   
   A   
   G   
   A   
   T   
   G   
   G   
   A   
   G   
   A   
   T   
   A   
   G   
   G   
    A    
   T   
   T   
    A    
   T   
   C   
   A   
   A   
    C    
   G   
   G   
    G    
   C   
   T   
    G    
   C   
   T   
   T   
   G   
   T   
   G   
   T   
   T   
   A   
   G   
   C   
   A   
   G   
   A   
    A    
   G   
   G   
    T    
   G   
   A   
   T   
   A   
   C   
   G   
   C   
   C   
    G    
   A   
   T   
   C   
   G   
   T   
   A   
   A   
   T   
   C   
   G   
   T   
   T   
   A   
   C   
   T   
   C   
   A   
   G   
   C   
   G   
   A   
   G   
   G   
   A   
   G   
   C   
    T    
   G   
   T   
    T    
   A   
   A   
   A   
   C   
   G   
   C   
   A   
   T   
   G   
   C   
   T   
   T   
   G   
   C   
   T   
   C   
   A   
   A   
   G   
   A   
   A   
   A   
   T   
   C   
   A   
   G   
   C   
   C   
   A   
   A   
   A   
   C   
    T    
   T   
   C   
   A   
   C   
   G   
   T   
   G   
   C   
   G   
   A   
   A   
   G   
   C   
   G   
   G   
   G   
   G   
   A   
   G   
   T   
   G   
   A   
   C   
   G   
   G   
   T   
   C   
   A   
   T   
   G   
   C   
   G   
   T   
   G   
   A   
   G   
   C   
   T   
   G   
   A   
   A   
   A   
   A   
   A   
   A   
   A   
   A   
   T   
   C   
   C   
    G    
   C   
   A   
    C    
   C   
   G   
    T    
   A   
   T   
   T   
   A   
   T   
   C   
   T   
   A   
   T   
   A   
   T   
   G   
   T   
   C   
   G   
   G   
   A   
   A   
   G   
   G   
   A   
   A   
   G   
   A   
   T   
   C   
   A   
   A   
   A   
   A   
   G   
   A   
   A   
   A   
   T   
   A   
   A   
   C   
   C   
   T   
   T   
   G   
   A   
   T   
   G   
   A   
   A   
   T   
   C   
   A   
   A   
   A   
   A   
   A   
   G   
   G   
    G    
   C   
   A   
    G    
   C   
   A   
   G   
    T    
   T   
   A   
   A   
   C   
   G   
   A   
   T   
   T   
   G   
   A   
    C    
   C   
   C   
   A   
   A   
   C   
    T    
   G   
   A   
   T   
   T   
   T   
    C    
   C   
   C   
   T   
   A   
   T   
   A   
   G   
   G   
   T   
   G   
   A   
   T   
   C   
   G   
   A   
   A   
   C   
   A   
   T   
   C   
   G   
   A   
   A   
   T   
   G   
   G   
    A    
   T   
   C   
    A    
   T   
   T   
   T   
   G   
   C   
    G    
   A   
   T   
   G   
   G   
   A   
    T    
   G   
   A   
   G   
   A   
   A   
   A   
   A   
   A   
   A   
   G   
   G   
   T   
   G   
   G   
   A   
   G   
   A   
   A   
   G   
   T   
   C   
   A   
   T   
   C   
   G   
   A   
   A   
   G   
   T   
    T    
   A   
    T    
   C   
   G   
   A   
   T   
   G   
   C   
   T   
   C   
   C   
   T   
   G   
   A   
   G   
    A    
   T   
   T   
   T   
   T   
    C    
   A   
   T   
    C    
   G   
   A   
    G    
    T    
   C   
    G    
   -   
   -   
   -   
 

 
   N53702   
   A   
   T   
   G   
   G   
   A   
   A   
   A   
   A   
   T   
   C   
   G   
   A   
   C   
   A   
   A   
   G   
   A   
   A   
   G   
   T   
   A   
   C   
   A   
   A   
   G   
   A   
   A   
   T   
   T   
   A   
   A   
   C   
   T   
   T   
   T   
   A   
   G   
   A   
   A   
   G   
   A   
   A   
   G   
   T   
   G   
   A   
   T   
   G   
   G   
   G   
   T   
   G   
   A   
   C   
   C   
   G   
   C   
   T   
   T   
   C   
   G   
   G   
   A   
   C   
   G   
   T   
   T   
   A   
   T   
   T   
   C   
   C   
   A   
   A   
   A   
   T   
   A   
   T   
   A   
   T   
   T   
   A   
   T   
    C    
   C   
   A   
    A    
   G   
   A   
   A   
   C   
   G   
   T   
   G   
   C   
   A   
   C   
   T   
   G   
   C   
   C   
   T   
   G   
   A   
   T   
   A   
   T   
    T    
   C   
   G   
   T   
   G   
   A   
   T   
   G   
   G   
   A   
   T   
   T   
   G   
   A   
   A   
    G    
   C   
   C   
   A   
   G   
   T   
    G    
   C   
   A   
   A   
   C   
   G   
   C   
   C   
   G   
   G   
   A   
   T   
   A   
   T   
   T   
   A   
   T   
   T   
    T    
   G   
   C   
    C    
   A   
   T   
   G   
   A   
   A   
   C   
   A   
   A   
   A   
   G   
   A   
   T   
   G   
   G   
   C   
   A   
   A   
   T   
   A   
   C   
   G   
   T   
   A   
   T   
   G   
   A   
   C   
   A   
   A   
   A   
   G   
   G   
   A   
   T   
   T   
    C    
   C   
   G   
   T   
   A   
   A   
   A   
   T   
   C   
   A   
   G   
   C   
   A   
   A   
   A   
   A   
   T   
   C   
   T   
   G   
   T   
   C   
   G   
   G   
   A   
   A   
   A   
   T   
   A   
   T   
   C   
   A   
   T   
   G   
   G   
   G   
    G    
   A   
   A   
   T   
   T   
   A   
   T   
   C   
   A   
   T   
   C   
   C   
   T   
   C   
   A   
   T   
   G   
   G   
   T   
   G   
   A   
   C   
   A   
   G   
   C   
   A   
   G   
   T   
   A   
   T   
   T   
   T   
   A   
   T   
   G   
   A   
   A   
   G   
   C   
   G   
   A   
   T   
   G   
   G   
   T   
   C   
   C   
   G   
   C   
   A   
   T   
   G   
   A   
   G   
   C   
   C   
   A   
   A   
   G   
   A   
    C    
   T   
   G   
   G   
   A   
   A   
   G   
   T   
   T   
   G   
   C   
   G   
   G   
   G   
   A   
   A   
   G   
   T   
   A   
   C   
   T   
   G   
   A   
   T   
   C   
   G   
   A   
    A    
   A   
   T   
   G   
   C   
   A   
   C   
   G   
   G   
    G    
   A   
   A   
   T   
   A   
   A   
    C    
   G   
   G   
   G   
   A   
   G   
   T   
   A   
   T   
   G   
   G   
   A   
   C   
   G   
   G   
   T   
   G   
   A   
   T   
   C   
   C   
   A   
   C   
   C   
   T   
   G   
   C   
   T   
   G   
   C   
   G   
   A   
   T   
   G   
   C   
   G   
   T   
   T   
   A   
   T   
   A   
   C   
   T   
   G   
   A   
   A   
   G   
   C   
   C   
   A   
   G   
    A    
   T   
   T   
   A   
   T   
   C   
   C   
   A   
   A   
   G   
   T   
   T   
   A   
   A   
   G   
   T   
   G   
   G   
   A   
   G   
   A   
   G   
   A   
   T   
   G   
    C    
   T   
   A   
    G    
   C   
   A   
   G   
   A   
   T   
   A   
   T   
   C   
   G   
   A   
    A    
   A   
   A   
   A   
   G   
   A   
   A   
   A   
   C   
   A   
   G   
   T   
    C    
   G   
   A   
   T   
   C   
   T   
   C   
   G   
   T   
   G   
   T   
   G   
   G   
   A   
   A   
   T   
   T   
   T   
   T   
   G   
   A   
   T   
   G   
   A   
   T   
   A   
   C   
   A   
   G   
   A   
   A   
   A   
   A   
   A   
   G   
   A   
   A   
   C   
   C   
   A   
   A   
   C   
   A   
   G   
   T   
   C   
   C   
   T   
   T   
   C   
   C   
   A   
   G   
   C   
   T   
   C   
   G   
   A   
   T   
   A   
   C   
   C   
   C   
   A   
   A   
   A   
   C   
   C   
   T   
   T   
   C   
   T   
    C    
   G   
   T   
   C   
   A   
   A   
   T   
   G   
   G   
   A   
   T   
   C   
   G   
   A   
   C   
   A   
   G   
   G   
   T   
   A   
   T   
   A   
   T   
   C   
   T   
   G   
   C   
   T   
   G   
   G   
   A   
   T   
   A   
   T   
   G   
   C   
    A    
   A   
   C   
   A   
   G   
   A   
   A   
   A   
   T   
   C   
   C   
   C   
   G   
   A   
   C   
    T    
   C   
   A   
   T   
   A   
   A   
   C   
   T   
   T   
   A   
   G   
   C   
   T   
   G   
   A   
   A   
   G   
   T   
   G   
   A   
   T   
    C    
   G   
   A   
    T    
   G   
   G   
   C   
   A   
   C   
   A   
   A   
   T   
   C   
   T   
   A   
   T   
   A   
   T   
   G   
   A   
   T   
   C   
   G   
   A   
   T   
   C   
   A   
   T   
   C   
   C   
   A   
   C   
   A   
   A   
   G   
   C   
   G   
   A   
   G   
   C   
   C   
   T   
   T   
   G   
   A   
   A   
   A   
   A   
   A   
   T   
   T   
   G   
   A   
   T   
   G   
   G   
   A   
   A   
   T   
   A   
   T   
   A   
   T   
   T   
   C   
   C   
   T   
   G   
   G   
   A   
   C   
   C   
   T   
   G   
   A   
   C   
   T   
   T   
    T    
   C   
   C   
   A   
   A   
   C   
   A   
   G   
   G   
   A   
   G   
   G   
    G    
   A   
   T   
   C   
   T   
   T   
   G   
   C   
   A   
   A   
   G   
   G   
    A    
   A   
   A   
   A   
   G   
   A   
   T   
   G   
   A   
   A   
   A   
   T   
   C   
   A   
   A   
   A   
   A   
   A   
   A   
   G   
   C   
   T   
   T   
   A   
   T   
   G   
   A   
   A   
   A   
   C   
   A   
   G   
   G   
    G    
   C   
   G   
   A   
   G   
   G   
   A   
   A   
   A   
   A   
   G   
   T   
   T   
   A   
    T    
   T   
   C   
   T   
   T   
   C   
   G   
   T   
   T   
   C   
   G   
   A   
   A   
   A   
   A   
   C   
   A   
   A   
   A   
   G   
   A   
   T   
   A   
   G   
   A   
    A    
   G   
   C   
   A   
   A   
   T   
   G   
   A   
   A   
   G   
   G   
   G   
    T    
   A   
   A   
    T    
   A   
   A   
   A   
   C   
   A   
   G   
   C   
   A   
   A   
   A   
   T   
   C   
   G   
   T   
   G   
   A   
   T   
   C   
   A   
   C   
    A    
   G   
   A   
   A   
   A   
   T   
   C   
   C   
   C   
   A   
   T   
   A   
   T   
   G   
   A   
   A   
   G   
   T   
   A   
   A   
   A   
   T   
   A   
   A   
   A   
   G   
   C   
   G   
   A   
   C   
    C    
   T   
   T   
   G   
   G   
   T   
   G   
   A   
   A   
   A   
   A   
   A   
   A   
   A   
   T   
   G   
   G   
   A   
   T   
   G   
   A   
   G   
   A   
   T   
   C   
   C   
   G   
   T   
   T   
   T   
   G   
   A   
   A   
   T   
   A   
   A   
   G   
   A   
   A   
   A   
   A   
   T   
   C   
   G   
   A   
    T    
   G   
   G   
   A   
   A   
   T   
   A   
   G   
   C   
   T   
   G   
   A   
   A   
   G   
   T   
    A    
   C   
   G   
   A   
   G   
   A   
   T   
   G   
   A   
   A   
   A   
   G   
   T   
   G   
   A   
   T   
    A    
   G   
    A    
   A   
   C   
    A    
   G   
   G   
   T   
   C   
   T   
   G   
   C   
   A   
   A   
   A   
   T   
   T   
   G   
   T   
   C   
   G   
   T   
    C    
   G   
   A   
   A   
   T   
   T   
   G   
   A   
   A   
   A   
   A   
   A   
   A   
   G   
   A   
    C    
   G   
   C   
   C   
   A   
   A   
    T    
   G   
   C   
   T   
   C   
   A   
   A   
   G   
   G   
    A    
   A   
   T   
   T   
   T   
   T   
   G   
   A   
   A   
   T   
   T   
   A   
    T    
   T   
   T   
   G   
   T   
   T   
   C   
   A   
   A   
   A   
   A   
   A   
   C   
   A   
   C   
   T   
   G   
   A   
   G   
    C    
   T   
   A   
   C   
   A   
   G   
   A   
   T   
   C   
   A   
   A   
   T   
   T   
   A   
   C   
   A   
   A   
   T   
   T   
   T   
    C    
   A   
   A   
   T   
   A   
   T   
   G   
   G   
   T   
   C   
   G   
   C   
   T   
   A   
   T   
   C   
   G   
   A   
    C    
   C   
   A   
   T   
   A   
   T   
   G   
   A   
   C   
   T   
   C   
   C   
   G   
   C   
   A   
    T    
   C   
   A   
   A   
   G   
   T   
   T   
   G   
   G   
   A   
   C   
   T   
   G   
   A   
   A   
   A   
   G   
   A   
   C   
   A   
   T   
   T   
   T   
   T   
   A   
   A   
   G   
   A   
   A   
   G   
   T   
   T   
   A   
   T   
   A   
   T   
   C   
   G   
   A   
   G   
   C   
   A   
   T   
   C   
   G   
   A   
   A   
   A   
   G   
   C   
   A   
   A   
   G   
   T   
   C   
   A   
   T   
   A   
   A   
   C   
   A   
   A   
   A   
   A   
   C   
   G   
    T    
   A   
   G   
    T    
   C   
   A   
   A   
   T   
   T   
   C   
   G   
   A   
   T   
   T   
   T   
   A   
   G   
   C   
   C   
   A   
   A   
   A   
   G   
   C   
   T   
   C   
   A   
   A   
   A   
   A   
   A   
    A    
   G   
   A   
   C   
   A   
   G   
   C   
   A   
   T   
   A   
   T   
   C   
   G   
   T   
    A    
   G   
   A   
   A   
   G   
   G   
    A    
    T    
   T   
   G   
   A   
   T   
   G   
   A   
   A   
   A   
   G   
   C   
   T   
   T   
   T   
   A   
   T   
   C   
   G   
   A   
   T   
   T   
   C   
   T   
   G   
   G   
   A   
   T   
   G   
   A   
   A   
   G   
   T   
   A   
   A   
   T   
   C   
   G   
    C    
   A   
   A   
   C   
   G   
   A   
   T   
   T   
   C   
   G   
   T   
   G   
   A   
   A   
   A   
   G   
   T   
   A   
   A   
   A   
   G   
   A   
   C   
   A   
   A   
   A   
   A   
   A   
   A   
   G   
   A   
    C    
   G   
   C   
    C    
   A   
   A   
   A   
   A   
   A   
   G   
   A   
   A   
   T   
   T   
   T   
   A   
   G   
   T   
   C   
   G   
   A   
    C    
   G   
   T   
    C    
   T   
   T   
    T    
   C   
   A   
   A   
   T   
   T   
   T   
   A   
   C   
   A   
   G   
   A   
   A   
   G   
   A   
   A   
   C   
   A   
   A   
   G   
   C   
   A   
   G   
   A   
   A   
   G   
   C   
   A   
   A   
   T   
    T    
   G   
   T   
   C   
   A   
   C   
   A   
   T   
   T   
   G   
   C   
   A   
   A   
    T    
   T   
   A   
   T   
   A   
    C    
   C   
   G   
   T   
   T   
   T   
   G   
   A   
   C   
   G   
   A   
   A   
    C    
   A   
   C   
    G    
   G   
   A   
   T   
   A   
   T   
   C   
   A   
   C   
   A   
   G   
   A   
   G   
   C   
   T   
   T   
   C   
   A   
   A   
   A   
   A   
   G   
   G   
   A   
   A   
    T    
   C   
    C    
   G   
   A   
   A   
   A   
    G    
   C   
   T   
   T   
   G   
   A   
   T   
   T   
   G   
   C   
    G    
   C   
   A   
   A   
   A   
   T   
   C   
   A   
   C   
   A   
   G   
   A   
   G   
   T   
   T   
    G    
   A   
   A   
   T   
   A   
   A   
   A   
   A   
   T   
   C   
   C   
   T   
   T   
   T   
   C   
   A   
   A   
   A   
    T    
   G   
   A   
   C   
   A   
   A   
   A   
   G   
   A   
    G    
   C   
   T   
   T   
   T   
   T   
    C    
   T   
   C   
   T   
   G   
   T   
   T   
   A   
   T   
   G   
   A   
   A   
   A   
   A   
   A   
   A   
   G   
   A   
   A   
   T   
   T   
   G   
   C   
   G   
   A   
   G   
   A   
   A   
   G   
   T   
   G   
   A   
   A   
   A   
   A   
   A   
   A   
   A   
   A   
   T   
   T   
   A   
    T    
   T   
   C   
   T   
   T   
   C   
   C   
   G   
   C   
   T   
   C   
   G   
   T   
   C   
    T    
    A    
   A   
   C   
   G   
   A   
   C   
   A   
   A   
   T   
   C   
    G    
   A   
   A   
   G   
   A   
   C   
   G   
   A   
   A   
   A   
   T   
   C   
   G   
   A   
   A   
   G   
   A   
   A   
   A   
   T   
   C   
   A   
   A   
   A   
   A   
   T   
    T    
   G   
   A   
   T   
   A   
   C   
   G   
   C   
   A   
   A   
    G    
   T   
    C    
   T   
   T   
   A   
   G   
   T   
   G   
   G   
   C   
    A    
   C   
   A   
   A   
   G   
   A   
   A   
   G   
   A   
   C   
   G   
   T   
   A   
   A   
   T   
    T    
   G   
   T   
   C   
   A   
   G   
   T   
   G   
   T   
    G    
   A   
   C   
    G    
   C   
   G   
   A   
   G   
   A   
   A   
   G   
   G   
   C   
   T   
   A   
   T   
   G   
   T   
   G   
   A   
   A   
   G   
   C   
   G   
   A   
   A   
   C   
   A   
   A   
   G   
   C   
   C   
   T   
   T   
   C   
   G   
   T   
   T   
   C   
    T    
   T   
   A   
   T   
   A   
   G   
   T   
   G   
   C   
   T   
   T   
   C   
    C    
   A   
   A   
   A   
   C   
   C   
   A   
   G   
   A   
   A   
   G   
   A   
   A   
   A   
   T   
   C   
   G   
   G   
   G   
   A   
   T   
   G   
   C   
   G   
   A   
   G   
   A   
   A   
   G   
   G   
   C   
   G   
   A   
    C    
   T   
   A   
   C   
   T   
   T   
   G   
   C   
   T   
    A    
   T   
   A   
   C   
    T    
   C   
   A   
   G   
   G   
   T   
   G   
   A   
   A   
   T   
   T   
    G    
   A   
   G   
    T    
   A   
   C   
   A   
    T    
   T   
   G   
   G   
   A   
   C   
   C   
   A   
   T   
   G   
   T   
   T   
   C   
   T   
   T   
   C   
   T   
   G   
   A   
   T   
    C    
   A   
   C   
    A    
   A   
   A   
   T   
   A   
   A   
   A   
   G   
   C   
   A   
   A   
   A   
   T   
   G   
   T   
   G   
   A   
   T   
   C   
   T   
   A   
   C   
   C   
   G   
    C    
   C   
   C   
   T   
   G   
   T   
    C    
   C   
   A   
   T   
   G   
   A   
   A   
   C   
   T   
   A   
   C   
   C   
    C    
   G   
   A   
   T   
   T   
   T   
   G   
   A   
   A   
   A   
   T   
   G   
   G   
   A   
   A   
   A   
   G   
   A   
   T   
   G   
   C   
   T   
   G   
   G   
    T    
   G   
   A   
    G    
   C   
   A   
    C    
   A   
   T   
   T   
   T   
   C   
   T   
   C   
   A   
   A   
   A   
   C   
   A   
   A   
   T   
   C   
   T   
   C   
   C   
   A   
   A   
   T   
   C   
   T   
   G   
   T   
   C   
   T   
   G   
   T   
   A   
   G   
   A   
    C    
   G   
   A   
   A   
   T   
   C   
   G   
   A   
   T   
    T    
   T   
   T   
   A   
   G   
   C   
   T   
   G   
   T   
   T   
   T   
   T   
    C    
   C   
   C   
   A   
   T   
   A   
   T   
   C   
   A   
   A   
   A   
   A   
   A   
   A   
   T   
    T    
   C   
   A   
   A   
   G   
   C   
   A   
   G   
   A   
    G    
   A   
   A   
   A   
   A   
   C   
   A   
   T   
   T   
   T   
   G   
   T   
    A    
   T   
   T   
   C   
   A   
   T   
   C   
   A   
   G   
   C   
   A   
   A   
   G   
   A   
   A   
   T   
   G   
   G   
   A   
   T   
   T   
    G    
    A    
   T   
   C   
   A   
   A   
   G   
   C   
   A   
   A   
   A   
   C   
   A   
   C   
   G   
   A   
   A   
   T   
   G   
   A   
   C   
   A   
   G   
   A   
   G   
   T   
   T   
   C   
   G   
   A   
   A   
   C   
   C   
   A   
   T   
   G   
   G   
   C   
   G   
   C   
   A   
   C   
   C   
   T   
   A   
    T    
   A   
   A   
   G   
   A   
   G   
   C   
   C   
   G   
    T    
   C   
   C   
   G   
   C   
   T   
   C   
   A   
   G   
   C   
   T   
   G   
   T   
   A   
   T   
   G   
   A   
   A   
   G   
   C   
   T   
   A   
   A   
   A   
   G   
   G   
   C   
   G   
   G   
   A   
   A   
   G   
   A   
   A   
   G   
   A   
   T   
   G   
   A   
   A   
   C   
   T   
    G    
   A   
   T   
   T   
   G   
   C   
   T   
   G   
   T   
    G    
   T   
   A   
   T   
   C   
   T   
   G   
   A   
   A   
   A   
   G   
   A   
   A   
   G   
   A   
   T   
   C   
    A    
   G   
   C   
   C   
   A   
   G   
   G   
   C   
   T   
   T   
   A   
   G   
   A   
   T   
   G   
   T   
    T    
   T   
   T   
   C   
   T   
   T   
   A   
   G   
   T   
   A   
   A   
   C   
   G   
    C    
   A   
   T   
   C   
   A   
   A   
   G   
   G   
   A   
   A   
   T   
   G   
   G   
   G   
    T    
   T   
   T   
   G   
   C   
   G   
   C   
   T   
    A    
   T   
   C   
   C   
   T   
   T   
   T   
   A   
   A   
    C    
   A   
   G   
   A   
   T   
   G   
   T   
   T   
   C   
   C   
   C   
   G   
   T   
   T   
   G   
   T   
   T   
   G   
   G   
   A   
   G   
   C   
   A   
   A   
   A   
   G   
   G   
   C   
   A   
   G   
    C    
   T   
   G   
   G   
   A   
   G   
   T   
   G   
   A   
   A   
   G   
   T   
   C   
   T   
   A   
   T   
   G   
   A   
   A   
   T   
   T   
   T   
   G   
   A   
   A   
   A   
   G   
   A   
   T   
   G   
   G   
   A   
   G   
   A   
   T   
   A   
   G   
   G   
    A    
   T   
   T   
    A    
   T   
   C   
   A   
   A   
    C    
   G   
   G   
    G    
   C   
   T   
    A    
   C   
   T   
   T   
   G   
   T   
   G   
   T   
   T   
   A   
   G   
   C   
   A   
   G   
   A   
    A    
   G   
   G   
    T    
   G   
   A   
   T   
   A   
   C   
   G   
   C   
   C   
    G    
   A   
   T   
   C   
   G   
   T   
   A   
   A   
   T   
   C   
   G   
   T   
   T   
   A   
   C   
   T   
   C   
   A   
   G   
   C   
   G   
   A   
   G   
   G   
   A   
   G   
   C   
    T    
   G   
   T   
    T    
   A   
   A   
   A   
   C   
   G   
   C   
   A   
   T   
   G   
   C   
   T   
   T   
   G   
   C   
   T   
   C   
   A   
   A   
   G   
   A   
   A   
   A   
   T   
   C   
   A   
   G   
   C   
   C   
   A   
   A   
   A   
   C   
    T    
   T   
   C   
   A   
   C   
   G   
   T   
   G   
   C   
   G   
   A   
   A   
   G   
   C   
   G   
   G   
   G   
   G   
   A   
   G   
   T   
   G   
   A   
   C   
   G   
   G   
   T   
   C   
   A   
   T   
   G   
   C   
   G   
   T   
   G   
   A   
   G   
   C   
   T   
   G   
   A   
   A   
   A   
   A   
   A   
   A   
   A   
   A   
   T   
   C   
   C   
    G    
   C   
   A   
    C    
   C   
   G   
    T    
   A   
   T   
   T   
   A   
   T   
   C   
   T   
   A   
   T   
   A   
   T   
   G   
   T   
   C   
   G   
   G   
   A   
   A   
   G   
   G   
   A   
   A   
   G   
   A   
   T   
   C   
   A   
   A   
   A   
   A   
   G   
   A   
   A   
   A   
   T   
   A   
   A   
   C   
   C   
   T   
   T   
   G   
   A   
   T   
   G   
   A   
   A   
   T   
   C   
   A   
   A   
   A   
   A   
   A   
   G   
   G   
    G    
   C   
   A   
    G    
   C   
   A   
   G   
    T    
   T   
   A   
   A   
   C   
   G   
   A   
   T   
   T   
   G   
   A   
    C    
   C   
   C   
   A   
   A   
   C   
    T    
   G   
   A   
   T   
   T   
   T   
    C    
   C   
   C   
   T   
   A   
   T   
   A   
   G   
   G   
   T   
   G   
   A   
   T   
   C   
   G   
   A   
   A   
   C   
   A   
   T   
   C   
   G   
   A   
   A   
   T   
   G   
   G   
    A    
   T   
   C   
    A    
   T   
   T   
   T   
   G   
   C   
    G    
   A   
   T   
   G   
   G   
   A   
    T    
   G   
   A   
   G   
   A   
   A   
   A   
   A   
   A   
   A   
   G   
   G   
   T   
   G   
   G   
   A   
   G   
   A   
   A   
   G   
   T   
   C   
   A   
   T   
   C   
   G   
   A   
   A   
   G   
   T   
    T    
   A   
    T    
   C   
   G   
   A   
   T   
   G   
   C   
   T   
   C   
   C   
   T   
   G   
   A   
   G   
    A    
   T   
   T   
   T   
   T   
    C    
   A   
   T   
    C    
   G   
   A   
    G    
    T    
   C   
    G    
   -   
   -   
   -   
 

 
   N52636   
   A   
   T   
   G   
   G   
   A   
   A   
   A   
   A   
   T   
   C   
   G   
   A   
   C   
   A   
   A   
   G   
   A   
   A   
   G   
   T   
   A   
   C   
   A   
   A   
   G   
   A   
   A   
   T   
   T   
   A   
   A   
   C   
   T   
   T   
   T   
   A   
   G   
   A   
   A   
   G   
   A   
   A   
   G   
   T   
   G   
   A   
   T   
   G   
   G   
   G   
   T   
   G   
   A   
   C   
   C   
   G   
   C   
   T   
   T   
   C   
   G   
   G   
   A   
   C   
   G   
   T   
   T   
   A   
   T   
   T   
   C   
   C   
   A   
   A   
   A   
   T   
   A   
   T   
   A   
   T   
   T   
   A   
   T   
    C    
   C   
   A   
    A    
   G   
   A   
   A   
   C   
   G   
   T   
   G   
   C   
   A   
   C   
   T   
   G   
   C   
   C   
   T   
   G   
   A   
   T   
   A   
   T   
    C    
   C   
   G   
   T   
   G   
   A   
   T   
   G   
   G   
   A   
   T   
   T   
   G   
   A   
   A   
    G    
   C   
   C   
   A   
   G   
   T   
    G    
   C   
   A   
   A   
   C   
   G   
   C   
   C   
   G   
   G   
   A   
   T   
   A   
   T   
   T   
   A   
   T   
   T   
    T    
   G   
   C   
    C    
   A   
   T   
   G   
   A   
   A   
   C   
   A   
   A   
   A   
   G   
   A   
   T   
   G   
   G   
   C   
   A   
   A   
   T   
   A   
   C   
   G   
   T   
   A   
   T   
   G   
   A   
   C   
   A   
   A   
   A   
   G   
   G   
   A   
   T   
   T   
    C    
   C   
   G   
   T   
   A   
   A   
   A   
   T   
   C   
   A   
   G   
   C   
   A   
   A   
   A   
   A   
   T   
   C   
   T   
   G   
   T   
   C   
   G   
   G   
   A   
   A   
   A   
   T   
   A   
   T   
   C   
   A   
   T   
   G   
   G   
   G   
    G    
   A   
   A   
   T   
   T   
   A   
   T   
   C   
   A   
   T   
   C   
   C   
   T   
   C   
   A   
   T   
   G   
   G   
   T   
   G   
   A   
   C   
   A   
   G   
   C   
   A   
   G   
   T   
   A   
   T   
   T   
   T   
   A   
   T   
   G   
   A   
   A   
   G   
   C   
   G   
   A   
   T   
   G   
   G   
   T   
   C   
   C   
   G   
   C   
   A   
   T   
   G   
   A   
   G   
   C   
   C   
   A   
   A   
   G   
   A   
    C    
   T   
   G   
   G   
   A   
   A   
   G   
   T   
   T   
   G   
   C   
   G   
   G   
   G   
   A   
   A   
   G   
   T   
   A   
   C   
   T   
   G   
   A   
   T   
   C   
   G   
   A   
    A    
   A   
   T   
   G   
   C   
   A   
   C   
   G   
   G   
    G    
   A   
   A   
   T   
   A   
   A   
    C    
   G   
   G   
   G   
   A   
   G   
   T   
   A   
   T   
   G   
   G   
   A   
   C   
   G   
   G   
   T   
   G   
   A   
   T   
   C   
   C   
   A   
   C   
   C   
   T   
   G   
   C   
   T   
   G   
   C   
   G   
   A   
   T   
   G   
   C   
   G   
   T   
   T   
   A   
   T   
   A   
   C   
   T   
   G   
   A   
   A   
   G   
   C   
   C   
   A   
   G   
    G    
   T   
   T   
   A   
   T   
   C   
   C   
   A   
   A   
   G   
   T   
   T   
   A   
   A   
   G   
   T   
   G   
   G   
   A   
   G   
   A   
   G   
   A   
   T   
   G   
    C    
   T   
   A   
    G    
   C   
   A   
   G   
   A   
   T   
   A   
   T   
   C   
   G   
   A   
    A    
   A   
   A   
   A   
   G   
   A   
   A   
   A   
   C   
   A   
   G   
   T   
    C    
   G   
   A   
   T   
   C   
   T   
   C   
   G   
   T   
   G   
   T   
   G   
   G   
   A   
   A   
   T   
   T   
   T   
   T   
   G   
   A   
   T   
   G   
   A   
   T   
   A   
   C   
   A   
   G   
   A   
   A   
   A   
   A   
   A   
   G   
   A   
   A   
   C   
   C   
   A   
   A   
   C   
   A   
   G   
   T   
   C   
   C   
   T   
   T   
   C   
   C   
   A   
   G   
   C   
   T   
   C   
   G   
   A   
   T   
   A   
   C   
   C   
   C   
   A   
   A   
   A   
   C   
   C   
   T   
   T   
   C   
   T   
    A    
   G   
   T   
   C   
   A   
   A   
   T   
   G   
   G   
   A   
   T   
   C   
   G   
   A   
   C   
   A   
   G   
   G   
   T   
   A   
   T   
   A   
   T   
   C   
   T   
   G   
   C   
   T   
   G   
   G   
   A   
   T   
   A   
   T   
   G   
   C   
    G    
   A   
   C   
   A   
   G   
   A   
   A   
   A   
   T   
   C   
   C   
   C   
   G   
   A   
   C   
    T    
   C   
   A   
   T   
   A   
   A   
   C   
   T   
   T   
   A   
   G   
   C   
   T   
   G   
   A   
   A   
   G   
   T   
   G   
   A   
   T   
    C    
   G   
   A   
    C    
   G   
   G   
   C   
   A   
   C   
   A   
   A   
   T   
   C   
   T   
   A   
   T   
   A   
   T   
   G   
   A   
   T   
   C   
   G   
   A   
   T   
   C   
   A   
   T   
   C   
   C   
   A   
   C   
   A   
   A   
   G   
   C   
   G   
   A   
   G   
   C   
   C   
   T   
   T   
   G   
   A   
   A   
   A   
   A   
   A   
   T   
   T   
   G   
   A   
   T   
   G   
   G   
   A   
   A   
   T   
   A   
   T   
   A   
   T   
   T   
   C   
   C   
   T   
   G   
   G   
   A   
   C   
   C   
   T   
   G   
   A   
   C   
   T   
   T   
    T    
   C   
   C   
   A   
   A   
   C   
   A   
   G   
   G   
   A   
   G   
   G   
    G    
   A   
   T   
   C   
   T   
   T   
   G   
   C   
   A   
   A   
   G   
   G   
    A    
   A   
   A   
   A   
   G   
   A   
   T   
   G   
   A   
   A   
   A   
   T   
   C   
   A   
   A   
   A   
   A   
   A   
   A   
   G   
   C   
   T   
   T   
   A   
   T   
   G   
   A   
   A   
   A   
   C   
   A   
   G   
   G   
    G    
   C   
   G   
   A   
   G   
   G   
   A   
   A   
   A   
   A   
   G   
   T   
   T   
   A   
    T    
   T   
   C   
   T   
   T   
   C   
   G   
   T   
   T   
   C   
   G   
   A   
   A   
   A   
   A   
   C   
   A   
   A   
   A   
   G   
   A   
   T   
   A   
   G   
   A   
    A    
   G   
   C   
   A   
   A   
   T   
   G   
   A   
   A   
   G   
   G   
   G   
    T    
   A   
   A   
    T    
   A   
   A   
   A   
   C   
   A   
   G   
   C   
   A   
   A   
   A   
   T   
   C   
   G   
   T   
   G   
   A   
   T   
   C   
   A   
   C   
    A    
   G   
   A   
   A   
   A   
   T   
   C   
   C   
   C   
   A   
   T   
   A   
   T   
   G   
   A   
   A   
   G   
   T   
   A   
   A   
   A   
   T   
   A   
   A   
   A   
   G   
   C   
   G   
   A   
   C   
    C    
   T   
   T   
   G   
   G   
   T   
   G   
   A   
   A   
   A   
   A   
   A   
   A   
   A   
   T   
   G   
   G   
   A   
   T   
   G   
   A   
   G   
   A   
   T   
   C   
   C   
   G   
   T   
   T   
   T   
   G   
   A   
   A   
   T   
   A   
   A   
   G   
   A   
   A   
   A   
   A   
   T   
   C   
   G   
   A   
    T    
   G   
   G   
   A   
   A   
   T   
   A   
   G   
   C   
   T   
   G   
   A   
   A   
   G   
   T   
    A    
   C   
   G   
   A   
   G   
   A   
   T   
   G   
   A   
   A   
   A   
   G   
   T   
   G   
   A   
   T   
    A    
   G   
    A    
   A   
   C   
    A    
   G   
   G   
   T   
   C   
   T   
   G   
   C   
   A   
   A   
   A   
   T   
   T   
   G   
   T   
   C   
   G   
   T   
    C    
   G   
   A   
   A   
   T   
   T   
   G   
   A   
   A   
   A   
   A   
   A   
   A   
   G   
   A   
    C    
   G   
   C   
   C   
   A   
   A   
    T    
   G   
   C   
   T   
   C   
   A   
   A   
   G   
   G   
    A    
   A   
   T   
   T   
   T   
   T   
   G   
   A   
   A   
   T   
   T   
   A   
    T    
   T   
   T   
   G   
   T   
   T   
   C   
   A   
   A   
   A   
   A   
   A   
   C   
   A   
   C   
   T   
   G   
   A   
   G   
    C    
   T   
   A   
   C   
   A   
   G   
   A   
   T   
   C   
   A   
   A   
   T   
   T   
   A   
   C   
   A   
   A   
   T   
   T   
   T   
    C    
   A   
   A   
   T   
   A   
   T   
   G   
   G   
   T   
   C   
   G   
   C   
   T   
   A   
   T   
   C   
   G   
   A   
    C    
   C   
   A   
   T   
   A   
   T   
   G   
   A   
   C   
   T   
   C   
   C   
   G   
   C   
   A   
    T    
   C   
   A   
   A   
   G   
   T   
   T   
   G   
   G   
   A   
   C   
   T   
   G   
   A   
   A   
   A   
   G   
   A   
   C   
   A   
   T   
   T   
   T   
   T   
   A   
   A   
   G   
   A   
   A   
   G   
   T   
   T   
   A   
   T   
   A   
   T   
   C   
   G   
   A   
   G   
   C   
   A   
   T   
   C   
   G   
   A   
   A   
   A   
   G   
   C   
   A   
   A   
   G   
   T   
   C   
   A   
   T   
   A   
   A   
   C   
   A   
   A   
   A   
   A   
   C   
   G   
    T    
   A   
   G   
    T    
   C   
   A   
   A   
   T   
   T   
   C   
   G   
   A   
   T   
   T   
   T   
   A   
   G   
   C   
   C   
   A   
   A   
   A   
   G   
   C   
   T   
   C   
   A   
   A   
   A   
   A   
   A   
    A    
   G   
   A   
   C   
   A   
   G   
   C   
   A   
   T   
   A   
   T   
   C   
   G   
   T   
    A    
   G   
   A   
   A   
   G   
   G   
    A    
    T    
   T   
   G   
   A   
   T   
   G   
   A   
   A   
   A   
   G   
   C   
   T   
   T   
   T   
   A   
   T   
   C   
   G   
   A   
   T   
   T   
   C   
   T   
   G   
   G   
   A   
   T   
   G   
   A   
   A   
   G   
   T   
   A   
   A   
   T   
   C   
   G   
    C    
   A   
   A   
   C   
   G   
   A   
   T   
   T   
   C   
   G   
   T   
   G   
   A   
   A   
   A   
   G   
   T   
   A   
   A   
   A   
   G   
   A   
   C   
   A   
   A   
   A   
   A   
   A   
   A   
   G   
   A   
    C    
   G   
   C   
    C    
   A   
   A   
   A   
   A   
   A   
   G   
   A   
   A   
   T   
   T   
   T   
   A   
   G   
   T   
   C   
   G   
   A   
    C    
   G   
   T   
    C    
   T   
   T   
    T    
   C   
   A   
   A   
   T   
   T   
   T   
   A   
   C   
   A   
   G   
   A   
   A   
   G   
   A   
   A   
   C   
   A   
   A   
   G   
   C   
   A   
   G   
   A   
   A   
   G   
   C   
   A   
   A   
   T   
    T    
   G   
   T   
   C   
   A   
   C   
   A   
   T   
   T   
   G   
   C   
   A   
   A   
    T    
   T   
   A   
   T   
   A   
    C    
   C   
   G   
   T   
   T   
   T   
   G   
   A   
   C   
   G   
   A   
   A   
    C    
   A   
   C   
    G    
   G   
   A   
   T   
   A   
   T   
   C   
   A   
   C   
   A   
   G   
   A   
   G   
   C   
   T   
   T   
   C   
   A   
   A   
   A   
   A   
   G   
   G   
   A   
   A   
    T    
   C   
    C    
   G   
   A   
   A   
   A   
    G    
   C   
   T   
   T   
   G   
   A   
   T   
   T   
   G   
   C   
    G    
   C   
   A   
   A   
   A   
   T   
   C   
   A   
   C   
   A   
   G   
   A   
   G   
   T   
   T   
    G    
   A   
   A   
   T   
   A   
   A   
   A   
   A   
   T   
   C   
   C   
   T   
   T   
   T   
   C   
   A   
   A   
   A   
    T    
   G   
   A   
   C   
   A   
   A   
   A   
   G   
   A   
    G    
   C   
   T   
   T   
   T   
   T   
    C    
   T   
   C   
   T   
   G   
   T   
   T   
   A   
   T   
   G   
   A   
   A   
   A   
   A   
   A   
   A   
   G   
   A   
   A   
   T   
   T   
   G   
   C   
   G   
   A   
   G   
   A   
   A   
   G   
   T   
   G   
   A   
   A   
   A   
   A   
   A   
   A   
   A   
   A   
   T   
   T   
   A   
    T    
   T   
   C   
   T   
   T   
   C   
   C   
   G   
   C   
   T   
   C   
   G   
   T   
   C   
    T    
    A    
   A   
   C   
   G   
   A   
   C   
   A   
   A   
   T   
   C   
    G    
   A   
   A   
   G   
   A   
   C   
   G   
   A   
   A   
   A   
   T   
   C   
   G   
   A   
   A   
   G   
   A   
   A   
   A   
   T   
   C   
   A   
   A   
   A   
   A   
   T   
    T    
   G   
   A   
   T   
   A   
   C   
   G   
   C   
   A   
   A   
    G    
   T   
    C    
   T   
   T   
   A   
   G   
   T   
   G   
   G   
   C   
    A    
   C   
   A   
   A   
   G   
   A   
   A   
   G   
   A   
   C   
   G   
   T   
   A   
   A   
   T   
    T    
   G   
   T   
   C   
   A   
   G   
   T   
   G   
   T   
    G    
   A   
   C   
    G    
   C   
   G   
   A   
   G   
   A   
   A   
   G   
   G   
   C   
   T   
   A   
   T   
   G   
   T   
   G   
   A   
   A   
   G   
   C   
   G   
   A   
   A   
   C   
   A   
   A   
   G   
   C   
   C   
   T   
   T   
   C   
   G   
   T   
   T   
   C   
    T    
   T   
   A   
   T   
   A   
   G   
   T   
   G   
   C   
   T   
   T   
   C   
    C    
   A   
   A   
   A   
   C   
   C   
   A   
   G   
   A   
   A   
   G   
   A   
   A   
   A   
   T   
   C   
   G   
   G   
   G   
   A   
   T   
   G   
   C   
   G   
   A   
   G   
   A   
   A   
   G   
   G   
   C   
   G   
   A   
    C    
   T   
   A   
   C   
   T   
   T   
   G   
   C   
   T   
    A    
   T   
   A   
   C   
    T    
   C   
   A   
   G   
   G   
   T   
   G   
   A   
   A   
   T   
   T   
    G    
   A   
   G   
    T    
   A   
   C   
   A   
    T    
   T   
   G   
   G   
   A   
   C   
   C   
   A   
   T   
   G   
   T   
   T   
   C   
   T   
   T   
   C   
   T   
   G   
   A   
   T   
    C    
   A   
   C   
    A    
   A   
   A   
   T   
   A   
   A   
   A   
   G   
   C   
   A   
   A   
   A   
   T   
   G   
   T   
   G   
   A   
   T   
   C   
   T   
   A   
   C   
   C   
   G   
    C    
   C   
   C   
   T   
   G   
   T   
    C    
   C   
   A   
   T   
   G   
   A   
   A   
   C   
   T   
   A   
   C   
   C   
    C    
   G   
   A   
   T   
   T   
   T   
   G   
   A   
   A   
   A   
   T   
   G   
   G   
   A   
   A   
   A   
   G   
   A   
   T   
   G   
   C   
   T   
   G   
   G   
    T    
   G   
   A   
    G    
   C   
   A   
    C    
   A   
   T   
   T   
   T   
   C   
   T   
   C   
   A   
   A   
   A   
   C   
   A   
   A   
   T   
   C   
   T   
   C   
   C   
   A   
   A   
   T   
   C   
   T   
   G   
   T   
   C   
   T   
   G   
   T   
   A   
   G   
   A   
    C    
   G   
   A   
   A   
   T   
   C   
   G   
   A   
   T   
    T    
   T   
   T   
   A   
   G   
   C   
   T   
   G   
   T   
   T   
   T   
   T   
    C    
   C   
   C   
   A   
   T   
   A   
   T   
   C   
   A   
   A   
   A   
   A   
   A   
   A   
   T   
    T    
   C   
   A   
   A   
   G   
   C   
   A   
   G   
   A   
    G    
   A   
   A   
   A   
   A   
   C   
   A   
   T   
   T   
   T   
   G   
   T   
    A    
   T   
   T   
   C   
   A   
   T   
   C   
   A   
   G   
   C   
   A   
   A   
   G   
   A   
   A   
   T   
   G   
   G   
   A   
   T   
   T   
    G    
    A    
   T   
   C   
   A   
   A   
   G   
   C   
   A   
   A   
   A   
   C   
   A   
   C   
   G   
   A   
   A   
   T   
   G   
   A   
   C   
   A   
   G   
   A   
   G   
   T   
   T   
   C   
   G   
   A   
   A   
   C   
   C   
   A   
   T   
   G   
   G   
   C   
   G   
   C   
   A   
   C   
   C   
   T   
   A   
    T    
   A   
   A   
   G   
   A   
   G   
   C   
   C   
   G   
    T    
   C   
   C   
   G   
   C   
   T   
   C   
   A   
   G   
   C   
   T   
   G   
   T   
   A   
   T   
   G   
   A   
   A   
   G   
   C   
   T   
   A   
   A   
   A   
   G   
   G   
   C   
   G   
   G   
   A   
   A   
   G   
   A   
   A   
   G   
   A   
   T   
   G   
   A   
   A   
   C   
   T   
    G    
   A   
   T   
   T   
   G   
   C   
   T   
   G   
   T   
    G    
   T   
   A   
   T   
   C   
   T   
   G   
   A   
   A   
   A   
   G   
   A   
   A   
   G   
   A   
   T   
   C   
    A    
   G   
   C   
   C   
   A   
   G   
   G   
   C   
   T   
   T   
   A   
   G   
   A   
   T   
   G   
   T   
    T    
   T   
   T   
   C   
   T   
   T   
   A   
   G   
   T   
   A   
   A   
   C   
   G   
    C    
   A   
   T   
   C   
   A   
   A   
   G   
   G   
   A   
   A   
   T   
   G   
   G   
   G   
    T    
   T   
   T   
   G   
   C   
   G   
   C   
   T   
    A    
   T   
   C   
   C   
   T   
   T   
   T   
   A   
   A   
    C    
   A   
   G   
   A   
   T   
   G   
   T   
   T   
   C   
   C   
   C   
   G   
   T   
   T   
   G   
   T   
   T   
   G   
   G   
   A   
   G   
   C   
   A   
   A   
   A   
   G   
   G   
   C   
   A   
   G   
    C    
   T   
   G   
   G   
   A   
   G   
   T   
   G   
   A   
   A   
   G   
   T   
   C   
   T   
   A   
   T   
   G   
   A   
   A   
   T   
   T   
   T   
   G   
   A   
   A   
   A   
   G   
   A   
   T   
   G   
   G   
   A   
   G   
   A   
   T   
   A   
   G   
   G   
    A    
   T   
   T   
    A    
   T   
   C   
   A   
   A   
    C    
   G   
   G   
    G    
   C   
   T   
    G    
   C   
   T   
   T   
   G   
   T   
   G   
   T   
   T   
   A   
   G   
   C   
   A   
   G   
   A   
    A    
   G   
   G   
    T    
   G   
   A   
   T   
   A   
   C   
   G   
   C   
   C   
    G    
   A   
   T   
   C   
   G   
   T   
   A   
   A   
   T   
   C   
   G   
   T   
   T   
   A   
   C   
   T   
   C   
   A   
   G   
   C   
   G   
   A   
   G   
   G   
   A   
   G   
   C   
    T    
   G   
   T   
    T    
   A   
   A   
   A   
   C   
   G   
   C   
   A   
   T   
   G   
   C   
   T   
   T   
   G   
   C   
   T   
   C   
   A   
   A   
   G   
   A   
   A   
   A   
   T   
   C   
   A   
   G   
   C   
   C   
   A   
   A   
   A   
   C   
    T    
   T   
   C   
   A   
   C   
   G   
   T   
   G   
   C   
   G   
   A   
   A   
   G   
   C   
   G   
   G   
   G   
   G   
   A   
   G   
   T   
   G   
   A   
   C   
   G   
   G   
   T   
   C   
   A   
   T   
   G   
   C   
   G   
   T   
   G   
   A   
   G   
   C   
   T   
   G   
   A   
   A   
   A   
   A   
   A   
   A   
   A   
   A   
   T   
   C   
   C   
    G    
   C   
   A   
    C    
   C   
   G   
    T    
   A   
   T   
   T   
   A   
   T   
   C   
   T   
   A   
   T   
   A   
   T   
   G   
   T   
   C   
   G   
   G   
   A   
   A   
   G   
   G   
   A   
   A   
   G   
   A   
   T   
   C   
   A   
   A   
   A   
   A   
   G   
   A   
   A   
   A   
   T   
   A   
   A   
   C   
   C   
   T   
   T   
   G   
   A   
   T   
   G   
   A   
   A   
   T   
   C   
   A   
   A   
   A   
   A   
   A   
   G   
   G   
    G    
   C   
   A   
    G    
   C   
   A   
   G   
    T    
   T   
   A   
   A   
   C   
   G   
   A   
   T   
   T   
   G   
   A   
    C    
   C   
   C   
   A   
   A   
   C   
    T    
   G   
   A   
   T   
   T   
   T   
    C    
   C   
   C   
   T   
   A   
   T   
   A   
   G   
   G   
   T   
   G   
   A   
   T   
   C   
   G   
   A   
   A   
   C   
   A   
   T   
   C   
   G   
   A   
   A   
   T   
   G   
   G   
    A    
   T   
   C   
    A    
   T   
   T   
   T   
   G   
   C   
    G    
   A   
   T   
   G   
   G   
   A   
    T    
   G   
   A   
   G   
   A   
   A   
   A   
   A   
   A   
   A   
   G   
   G   
   T   
   G   
   G   
   A   
   G   
   A   
   A   
   G   
   T   
   C   
   A   
   T   
   C   
   G   
   A   
   A   
   G   
   T   
    T    
   A   
    T    
   C   
   G   
   A   
   T   
   G   
   C   
   T   
   C   
   C   
   T   
   G   
   A   
   G   
    A    
   T   
   T   
   T   
   T   
    C    
   A   
   T   
    C    
   G   
   A   
    G    
    T    
   C   
    G    
   -   
   -   
   -   
 

 
   N52732   
   A   
   T   
   G   
   G   
   A   
   A   
   A   
   A   
   T   
   C   
   G   
   A   
   C   
   A   
   A   
   G   
   A   
   A   
   G   
   T   
   A   
   C   
   A   
   A   
   G   
   A   
   A   
   T   
   T   
   A   
   A   
   C   
   T   
   T   
   T   
   A   
   G   
   A   
   A   
   G   
   A   
   A   
   G   
   T   
   G   
   A   
   T   
   G   
   G   
   G   
   T   
   G   
   A   
   C   
   C   
   G   
   C   
   T   
   T   
   C   
   G   
   G   
   A   
   C   
   G   
   T   
   T   
   A   
   T   
   T   
   C   
   C   
   A   
   A   
   A   
   T   
   A   
   T   
   A   
   T   
   T   
   A   
   T   
    C    
   C   
   A   
    A    
   G   
   A   
   A   
   C   
   G   
   T   
   G   
   C   
   A   
   C   
   T   
   G   
   C   
   C   
   T   
   G   
   A   
   T   
   A   
   T   
    C    
   C   
   G   
   T   
   G   
   A   
   T   
   G   
   G   
   A   
   T   
   T   
   G   
   A   
   A   
    A    
   C   
   C   
   A   
   G   
   T   
    G    
   C   
   A   
   A   
   C   
   G   
   C   
   C   
   G   
   G   
   A   
   T   
   A   
   T   
   T   
   A   
   T   
   T   
    T    
   G   
   C   
    C    
   A   
   T   
   G   
   A   
   A   
   C   
   A   
   A   
   A   
   G   
   A   
   T   
   G   
   G   
   C   
   A   
   A   
   T   
   A   
   C   
   G   
   T   
   A   
   T   
   G   
   A   
   C   
   A   
   A   
   A   
   G   
   G   
   A   
   T   
   T   
    C    
   C   
   G   
   T   
   A   
   A   
   A   
   T   
   C   
   A   
   G   
   C   
   A   
   A   
   A   
   A   
   T   
   C   
   T   
   G   
   T   
   C   
   G   
   G   
   A   
   A   
   A   
   T   
   A   
   T   
   C   
   A   
   T   
   G   
   G   
   G   
    A    
   A   
   A   
   T   
   T   
   A   
   T   
   C   
   A   
   T   
   C   
   C   
   T   
   C   
   A   
   T   
   G   
   G   
   T   
   G   
   A   
   C   
   A   
   G   
   C   
   A   
   G   
   T   
   A   
   T   
   T   
   T   
   A   
   T   
   G   
   A   
   A   
   G   
   C   
   G   
   A   
   T   
   G   
   G   
   T   
   C   
   C   
   G   
   C   
   A   
   T   
   G   
   A   
   G   
   C   
   C   
   A   
   A   
   G   
   A   
    T    
   T   
   G   
   G   
   A   
   A   
   G   
   T   
   T   
   G   
   C   
   G   
   G   
   G   
   A   
   A   
   G   
   T   
   A   
   C   
   T   
   G   
   A   
   T   
   C   
   G   
   A   
    G    
   A   
   T   
   G   
   C   
   A   
   C   
   G   
   G   
    A    
   A   
   A   
   T   
   A   
   A   
    C    
   G   
   G   
   G   
   A   
   G   
   T   
   A   
   T   
   G   
   G   
   A   
   C   
   G   
   G   
   T   
   G   
   A   
   T   
   C   
   C   
   A   
   C   
   C   
   T   
   G   
   C   
   T   
   G   
   C   
   G   
   A   
   T   
   G   
   C   
   G   
   T   
   T   
   A   
   T   
   A   
   C   
   T   
   G   
   A   
   A   
   G   
   C   
   C   
   A   
   G   
    A    
   T   
   T   
   A   
   T   
   C   
   C   
   A   
   A   
   G   
   T   
   T   
   A   
   A   
   G   
   T   
   G   
   G   
   A   
   G   
   A   
   G   
   A   
   T   
   G   
    C    
   T   
   A   
    G    
   C   
   A   
   G   
   A   
   T   
   A   
   T   
   C   
   G   
   A   
    A    
   A   
   A   
   A   
   G   
   A   
   A   
   A   
   C   
   A   
   G   
   T   
    T    
   G   
   A   
   T   
   C   
   T   
   C   
   G   
   T   
   G   
   T   
   G   
   G   
   A   
   A   
   T   
   T   
   T   
   T   
   G   
   A   
   T   
   G   
   A   
   T   
   A   
   C   
   A   
   G   
   A   
   A   
   A   
   A   
   A   
   G   
   A   
   A   
   C   
   C   
   A   
   A   
   C   
   A   
   G   
   T   
   C   
   C   
   T   
   T   
   C   
   C   
   A   
   G   
   C   
   T   
   C   
   G   
   A   
   T   
   A   
   C   
   C   
   C   
   A   
   A   
   A   
   C   
   C   
   T   
   T   
   C   
   T   
    A    
   G   
   T   
   C   
   A   
   A   
   T   
   G   
   G   
   A   
   T   
   C   
   G   
   A   
   C   
   A   
   G   
   G   
   T   
   A   
   T   
   A   
   T   
   C   
   T   
   G   
   C   
   T   
   G   
   G   
   A   
   T   
   A   
   T   
   G   
   C   
    G    
   A   
   C   
   A   
   G   
   A   
   A   
   A   
   T   
   C   
   C   
   C   
   G   
   A   
   C   
    G    
   C   
   A   
   T   
   A   
   A   
   C   
   T   
   T   
   A   
   G   
   C   
   T   
   G   
   A   
   A   
   G   
   T   
   G   
   A   
   T   
    C    
   G   
   A   
    C    
   G   
   G   
   C   
   A   
   C   
   A   
   A   
   T   
   C   
   T   
   A   
   T   
   A   
   T   
   G   
   A   
   T   
   C   
   G   
   A   
   T   
   C   
   A   
   T   
   C   
   C   
   A   
   C   
   A   
   A   
   G   
   C   
   G   
   A   
   G   
   C   
   C   
   T   
   T   
   G   
   A   
   A   
   A   
   A   
   A   
   T   
   T   
   G   
   A   
   T   
   G   
   G   
   A   
   A   
   T   
   A   
   T   
   A   
   T   
   T   
   C   
   C   
   T   
   G   
   G   
   A   
   C   
   C   
   T   
   G   
   A   
   C   
   T   
   T   
    T    
   C   
   C   
   A   
   A   
   C   
   A   
   G   
   G   
   A   
   G   
   G   
    G    
   A   
   T   
   C   
   T   
   T   
   G   
   C   
   A   
   A   
   G   
   G   
    A    
   A   
   A   
   A   
   G   
   A   
   T   
   G   
   A   
   A   
   A   
   T   
   C   
   A   
   A   
   A   
   A   
   A   
   A   
   G   
   C   
   T   
   T   
   A   
   T   
   G   
   A   
   A   
   A   
   C   
   A   
   G   
   G   
    G    
   C   
   G   
   A   
   G   
   G   
   A   
   A   
   A   
   A   
   G   
   T   
   T   
   A   
    T    
   T   
   C   
   T   
   T   
   C   
   G   
   T   
   T   
   C   
   G   
   A   
   A   
   A   
   A   
   C   
   A   
   A   
   A   
   G   
   A   
   T   
   A   
   G   
   A   
    A    
   G   
   C   
   A   
   A   
   T   
   G   
   A   
   A   
   G   
   G   
   G   
    C    
   A   
   A   
    C    
   A   
   A   
   A   
   C   
   A   
   G   
   C   
   A   
   A   
   A   
   T   
   C   
   G   
   T   
   G   
   A   
   T   
   C   
   A   
   C   
    T    
   G   
   A   
   A   
   A   
   T   
   C   
   C   
   C   
   A   
   T   
   A   
   T   
   G   
   A   
   A   
   G   
   T   
   A   
   A   
   A   
   T   
   A   
   A   
   A   
   G   
   C   
   G   
   A   
   C   
    A    
   T   
   T   
   G   
   G   
   T   
   G   
   A   
   A   
   A   
   A   
   A   
   A   
   A   
   T   
   G   
   G   
   A   
   T   
   G   
   A   
   G   
   A   
   T   
   C   
   C   
   G   
   T   
   T   
   T   
   G   
   A   
   A   
   T   
   A   
   A   
   G   
   A   
   A   
   A   
   A   
   T   
   C   
   G   
   A   
    C    
   G   
   G   
   A   
   A   
   T   
   A   
   G   
   C   
   T   
   G   
   A   
   A   
   G   
   T   
    G    
   C   
   G   
   A   
   G   
   A   
   T   
   G   
   A   
   A   
   A   
   G   
   T   
   G   
   A   
   T   
    C    
   G   
    A    
   A   
   C   
    T    
   G   
   G   
   T   
   C   
   T   
   G   
   C   
   A   
   A   
   A   
   T   
   T   
   G   
   T   
   C   
   G   
   T   
    T    
   G   
   A   
   A   
   T   
   T   
   G   
   A   
   A   
   A   
   A   
   A   
   A   
   G   
   A   
    C    
   G   
   C   
   C   
   A   
   A   
    T    
   G   
   C   
   T   
   C   
   A   
   A   
   G   
   G   
    A    
   A   
   T   
   T   
   T   
   T   
   G   
   A   
   A   
   T   
   T   
   A   
    T    
   T   
   T   
   G   
   T   
   T   
   C   
   A   
   A   
   A   
   A   
   A   
   C   
   A   
   C   
   T   
   G   
   A   
   G   
    C    
   T   
   A   
   C   
   A   
   G   
   A   
   T   
   C   
   A   
   A   
   T   
   T   
   A   
   C   
   A   
   A   
   T   
   T   
   T   
    C    
   A   
   A   
   T   
   A   
   T   
   G   
   G   
   T   
   C   
   G   
   C   
   T   
   A   
   T   
   C   
   G   
   A   
    T    
   C   
   A   
   T   
   A   
   T   
   G   
   A   
   C   
   T   
   C   
   C   
   G   
   C   
   A   
    T    
   C   
   A   
   A   
   G   
   T   
   T   
   G   
   G   
   A   
   C   
   T   
   G   
   A   
   A   
   A   
   G   
   A   
   C   
   A   
   T   
   T   
   T   
   T   
   A   
   A   
   G   
   A   
   A   
   G   
   T   
   T   
   A   
   T   
   A   
   T   
   C   
   G   
   A   
   G   
   C   
   A   
   T   
   C   
   G   
   A   
   A   
   A   
   G   
   C   
   A   
   A   
   G   
   T   
   C   
   A   
   T   
   A   
   A   
   C   
   A   
   A   
   A   
   A   
   C   
   G   
    C    
   A   
   G   
    C    
   C   
   A   
   A   
   T   
   T   
   C   
   G   
   A   
   T   
   T   
   T   
   A   
   G   
   C   
   C   
   A   
   A   
   A   
   G   
   C   
   T   
   C   
   A   
   A   
   A   
   A   
   A   
    A    
   G   
   A   
   C   
   A   
   G   
   C   
   A   
   T   
   A   
   T   
   C   
   G   
   T   
    G    
   G   
   A   
   A   
   G   
   G   
    C    
    C    
   T   
   G   
   A   
   T   
   G   
   A   
   A   
   A   
   G   
   C   
   T   
   T   
   T   
   A   
   T   
   C   
   G   
   A   
   T   
   T   
   C   
   T   
   G   
   G   
   A   
   T   
   G   
   A   
   A   
   G   
   T   
   A   
   A   
   T   
   C   
   G   
    C    
   A   
   A   
   C   
   G   
   A   
   T   
   T   
   C   
   G   
   T   
   G   
   A   
   A   
   A   
   G   
   T   
   A   
   A   
   A   
   G   
   A   
   C   
   A   
   A   
   A   
   A   
   A   
   A   
   G   
   A   
    C    
   G   
   C   
    C    
   A   
   A   
   A   
   A   
   A   
   G   
   A   
   A   
   T   
   T   
   T   
   A   
   G   
   T   
   C   
   G   
   A   
    C    
   G   
   T   
    T    
   T   
   T   
    C    
   C   
   A   
   A   
   T   
   T   
   T   
   A   
   C   
   A   
   G   
   A   
   A   
   G   
   A   
   A   
   C   
   A   
   A   
   G   
   C   
   A   
   G   
   A   
   A   
   G   
   C   
   A   
   A   
   T   
    C    
   G   
   T   
   C   
   A   
   C   
   A   
   T   
   T   
   G   
   C   
   A   
   A   
    C    
   T   
   A   
   T   
   A   
    T    
   C   
   G   
   T   
   T   
   T   
   G   
   A   
   C   
   G   
   A   
   A   
    C    
   A   
   C   
    A    
   G   
   A   
   T   
   A   
   T   
   C   
   A   
   C   
   A   
   G   
   A   
   G   
   C   
   T   
   T   
   C   
   A   
   A   
   A   
   A   
   G   
   G   
   A   
   A   
    T    
   C   
    C    
   G   
   A   
   A   
   A   
    G    
   C   
   T   
   T   
   G   
   A   
   T   
   T   
   G   
   C   
    A    
   C   
   A   
   A   
   A   
   T   
   C   
   A   
   C   
   A   
   G   
   A   
   G   
   T   
   T   
    G    
   A   
   A   
   T   
   A   
   A   
   A   
   A   
   T   
   C   
   C   
   T   
   T   
   T   
   C   
   A   
   A   
   A   
    C    
   G   
   A   
   C   
   A   
   A   
   A   
   G   
   A   
    G    
   C   
   T   
   T   
   T   
   T   
    C    
   T   
   C   
   T   
   G   
   T   
   T   
   A   
   T   
   G   
   A   
   A   
   A   
   A   
   A   
   A   
   G   
   A   
   A   
   T   
   T   
   G   
   C   
   G   
   A   
   G   
   A   
   A   
   G   
   T   
   G   
   A   
   A   
   A   
   A   
   A   
   A   
   A   
   A   
   T   
   T   
   A   
    C    
   T   
   C   
   T   
   T   
   C   
   C   
   G   
   C   
   T   
   C   
   G   
   T   
   C   
    T    
    G    
   A   
   C   
   G   
   A   
   C   
   A   
   A   
   T   
   C   
    G    
   A   
   A   
   G   
   A   
   C   
   G   
   A   
   A   
   A   
   T   
   C   
   G   
   A   
   A   
   G   
   A   
   A   
   A   
   T   
   C   
   A   
   A   
   A   
   A   
   T   
    C    
   G   
   A   
   T   
   A   
   C   
   G   
   C   
   A   
   A   
    G    
   T   
    T    
   T   
   T   
   A   
   G   
   T   
   G   
   G   
   C   
    G    
   C   
   A   
   A   
   G   
   A   
   A   
   G   
   A   
   C   
   G   
   T   
   A   
   A   
   T   
    C    
   G   
   T   
   C   
   A   
   G   
   T   
   G   
   T   
    G    
   A   
   C   
    A    
   C   
   G   
   A   
   G   
   A   
   A   
   G   
   G   
   C   
   T   
   A   
   T   
   G   
   T   
   G   
   A   
   A   
   G   
   C   
   G   
   A   
   A   
   C   
   A   
   A   
   G   
   C   
   C   
   T   
   T   
   C   
   G   
   T   
   T   
   C   
    T    
   T   
   A   
   T   
   A   
   G   
   T   
   G   
   C   
   T   
   T   
   C   
    G    
   A   
   A   
   A   
   C   
   C   
   A   
   G   
   A   
   A   
   G   
   A   
   A   
   A   
   T   
   C   
   G   
   G   
   G   
   A   
   T   
   G   
   C   
   G   
   A   
   G   
   A   
   A   
   G   
   G   
   C   
   G   
   A   
    C    
   T   
   A   
   C   
   T   
   T   
   G   
   C   
   T   
    G    
   T   
   A   
   C   
    T    
   C   
   A   
   G   
   G   
   T   
   G   
   A   
   A   
   T   
   T   
    G    
   A   
   G   
    C    
   A   
   C   
   A   
    T    
   T   
   G   
   G   
   A   
   C   
   C   
   A   
   T   
   G   
   T   
   T   
   C   
   T   
   T   
   C   
   T   
   G   
   A   
   T   
    T    
   A   
   C   
    G    
   A   
   A   
   T   
   A   
   A   
   A   
   G   
   C   
   A   
   A   
   A   
   T   
   G   
   T   
   G   
   A   
   T   
   C   
   T   
   A   
   C   
   C   
   G   
    T    
   C   
   C   
   T   
   G   
   T   
    T    
   C   
   A   
   T   
   G   
   A   
   A   
   C   
   T   
   A   
   C   
   C   
    C    
   G   
   A   
   T   
   T   
   T   
   G   
   A   
   A   
   A   
   T   
   G   
   G   
   A   
   A   
   A   
   G   
   A   
   T   
   G   
   C   
   T   
   G   
   G   
    C    
   G   
   A   
    A    
   C   
   A   
    C    
   A   
   T   
   T   
   T   
   C   
   T   
   C   
   A   
   A   
   A   
   C   
   A   
   A   
   T   
   C   
   T   
   C   
   C   
   A   
   A   
   T   
   C   
   T   
   G   
   T   
   C   
   T   
   G   
   T   
   A   
   G   
   A   
    T    
   G   
   A   
   A   
   T   
   C   
   G   
   A   
   T   
    C    
   T   
   T   
   A   
   G   
   C   
   T   
   G   
   T   
   T   
   T   
   T   
    C    
   C   
   C   
   A   
   T   
   A   
   T   
   C   
   A   
   A   
   A   
   A   
   A   
   A   
   T   
    C    
   C   
   A   
   A   
   G   
   C   
   A   
   G   
   A   
    G    
   A   
   A   
   A   
   A   
   C   
   A   
   T   
   T   
   T   
   G   
   T   
    A    
   T   
   T   
   C   
   A   
   T   
   C   
   A   
   G   
   C   
   A   
   A   
   G   
   A   
   A   
   T   
   G   
   G   
   A   
   T   
   T   
    G    
    A    
   T   
   C   
   A   
   A   
   G   
   C   
   A   
   A   
   A   
   C   
   A   
   C   
   G   
   A   
   A   
   T   
   G   
   A   
   C   
   A   
   G   
   A   
   G   
   T   
   T   
   C   
   G   
   A   
   A   
   C   
   C   
   A   
   T   
   G   
   G   
   C   
   G   
   C   
   A   
   C   
   C   
   T   
   A   
    C    
   A   
   A   
   G   
   A   
   G   
   C   
   C   
   G   
    T    
   C   
   C   
   G   
   C   
   T   
   C   
   A   
   G   
   C   
   T   
   G   
   T   
   A   
   T   
   G   
   A   
   A   
   G   
   C   
   T   
   A   
   A   
   A   
   G   
   G   
   C   
   G   
   G   
   A   
   A   
   G   
   A   
   A   
   G   
   A   
   T   
   G   
   A   
   A   
   C   
   T   
    T    
   A   
   T   
   T   
   G   
   C   
   T   
   G   
   T   
    A    
   T   
   A   
   T   
   C   
   T   
   G   
   A   
   A   
   A   
   G   
   A   
   A   
   G   
   A   
   T   
   C   
    A    
   G   
   C   
   C   
   A   
   G   
   G   
   C   
   T   
   T   
   A   
   G   
   A   
   T   
   G   
   T   
    G    
   T   
   T   
   C   
   T   
   T   
   A   
   G   
   T   
   A   
   A   
   C   
   G   
    C    
   A   
   T   
   C   
   A   
   A   
   G   
   G   
   A   
   A   
   T   
   G   
   G   
   G   
    C    
   T   
   T   
   G   
   C   
   G   
   C   
   T   
    A    
   T   
   C   
   C   
   T   
   T   
   T   
   A   
   A   
    C    
   A   
   G   
   A   
   T   
   G   
   T   
   T   
   C   
   C   
   C   
   G   
   T   
   T   
   G   
   T   
   T   
   G   
   G   
   A   
   G   
   C   
   A   
   A   
   A   
   G   
   G   
   C   
   A   
   G   
    C    
   T   
   G   
   G   
   A   
   G   
   T   
   G   
   A   
   A   
   G   
   T   
   C   
   T   
   A   
   T   
   G   
   A   
   A   
   T   
   T   
   T   
   G   
   A   
   A   
   A   
   G   
   A   
   T   
   G   
   G   
   A   
   G   
   A   
   T   
   A   
   G   
   G   
    A    
   T   
   T   
    G    
   T   
   C   
   A   
   A   
    T    
   G   
   G   
    A    
   C   
   T   
    G    
   C   
   T   
   T   
   G   
   T   
   G   
   T   
   T   
   A   
   G   
   C   
   A   
   G   
   A   
    A    
   G   
   G   
    A    
   G   
   A   
   T   
   A   
   C   
   G   
   C   
   C   
    G    
   A   
   T   
   C   
   G   
   T   
   A   
   A   
   T   
   C   
   G   
   T   
   T   
   A   
   C   
   T   
   C   
   A   
   G   
   C   
   G   
   A   
   G   
   G   
   A   
   G   
   C   
    T    
   G   
   T   
    G    
   A   
   A   
   A   
   C   
   G   
   C   
   A   
   T   
   G   
   C   
   T   
   T   
   G   
   C   
   T   
   C   
   A   
   A   
   G   
   A   
   A   
   A   
   T   
   C   
   A   
   G   
   C   
   C   
   A   
   A   
   A   
   C   
    T    
   T   
   C   
   A   
   C   
   G   
   T   
   G   
   C   
   G   
   A   
   A   
   G   
   C   
   G   
   G   
   G   
   G   
   A   
   G   
   T   
   G   
   A   
   C   
   G   
   G   
   T   
   C   
   A   
   T   
   G   
   C   
   G   
   T   
   G   
   A   
   G   
   C   
   T   
   G   
   A   
   A   
   A   
   A   
   A   
   A   
   A   
   A   
   T   
   C   
   C   
    G    
   C   
   A   
    C    
   C   
   G   
    C    
   A   
   T   
   T   
   A   
   T   
   C   
   T   
   A   
   T   
   A   
   T   
   G   
   T   
   C   
   G   
   G   
   A   
   A   
   G   
   G   
   A   
   A   
   G   
   A   
   T   
   C   
   A   
   A   
   A   
   A   
   G   
   A   
   A   
   A   
   T   
   A   
   A   
   C   
   C   
   T   
   T   
   G   
   A   
   T   
   G   
   A   
   A   
   T   
   C   
   A   
   A   
   A   
   A   
   A   
   G   
   G   
    A    
   C   
   A   
    A    
   C   
   A   
   G   
    A    
   T   
   A   
   A   
   C   
   G   
   A   
   T   
   T   
   G   
   A   
    T    
   C   
   C   
   A   
   A   
   C   
    G    
   G   
   A   
   T   
   T   
   T   
    T    
   C   
   C   
   T   
   A   
   T   
   A   
   G   
   G   
   T   
   G   
   A   
   T   
   C   
   G   
   A   
   A   
   C   
   A   
   T   
   C   
   G   
   A   
   A   
   T   
   G   
   G   
    T    
   T   
   C   
    G    
   T   
   T   
   T   
   G   
   C   
    T    
   A   
   T   
   G   
   G   
   A   
    C    
   G   
   A   
   G   
   A   
   A   
   A   
   A   
   A   
   A   
   G   
   G   
   T   
   G   
   G   
   A   
   G   
   A   
   A   
   G   
   T   
   C   
   A   
   T   
   C   
   G   
   A   
   A   
   G   
   T   
    T    
   A   
    T    
   C   
   G   
   A   
   T   
   G   
   C   
   T   
   C   
   C   
   T   
   G   
   A   
   G   
    A    
   T   
   T   
   T   
   T   
    C    
   A   
   T   
    C    
   G   
   A   
    A    
    T    
   C   
    A    
   -   
   -   
   -   
 

 
   N54150   
   A   
   T   
   G   
   G   
   A   
   A   
   A   
   A   
   T   
   C   
   G   
   A   
   C   
   A   
   A   
   G   
   A   
   A   
   G   
   T   
   A   
   C   
   A   
   A   
   G   
   A   
   A   
   T   
   T   
   A   
   A   
   C   
   T   
   T   
   T   
   A   
   G   
   A   
   A   
   G   
   A   
   A   
   G   
   T   
   G   
   A   
   T   
   G   
   G   
   G   
   T   
   G   
   A   
   C   
   C   
   G   
   C   
   T   
   T   
   C   
   G   
   G   
   A   
   C   
   G   
   T   
   T   
   A   
   T   
   T   
   C   
   C   
   A   
   A   
   A   
   T   
   A   
   T   
   A   
   T   
   T   
   A   
   T   
    C    
   C   
   A   
    A    
   G   
   A   
   A   
   C   
   G   
   T   
   G   
   C   
   A   
   C   
   T   
   G   
   C   
   C   
   T   
   G   
   A   
   T   
   A   
   T   
    C    
   C   
   G   
   T   
   G   
   A   
   T   
   G   
   G   
   A   
   T   
   T   
   G   
   A   
   A   
    G    
   C   
   C   
   A   
   G   
   T   
    G    
   C   
   A   
   A   
   C   
   G   
   C   
   C   
   G   
   G   
   A   
   T   
   A   
   T   
   T   
   A   
   T   
   T   
    T    
   G   
   C   
    C    
   A   
   T   
   G   
   A   
   A   
   C   
   A   
   A   
   A   
   G   
   A   
   T   
   G   
   G   
   C   
   A   
   A   
   T   
   A   
   C   
   G   
   T   
   A   
   T   
   G   
   A   
   C   
   A   
   A   
   A   
   G   
   G   
   A   
   T   
   T   
    C    
   C   
   G   
   T   
   A   
   A   
   A   
   T   
   C   
   A   
   G   
   C   
   A   
   A   
   A   
   A   
   T   
   C   
   T   
   G   
   T   
   C   
   G   
   G   
   A   
   A   
   A   
   T   
   A   
   T   
   C   
   A   
   T   
   G   
   G   
   G   
    G    
   A   
   A   
   T   
   T   
   A   
   T   
   C   
   A   
   T   
   C   
   C   
   T   
   C   
   A   
   T   
   G   
   G   
   T   
   G   
   A   
   C   
   A   
   G   
   C   
   A   
   G   
   T   
   A   
   T   
   T   
   T   
   A   
   T   
   G   
   A   
   A   
   G   
   C   
   G   
   A   
   T   
   G   
   G   
   T   
   C   
   C   
   G   
   C   
   A   
   T   
   G   
   A   
   G   
   C   
   C   
   A   
   A   
   G   
   A   
    C    
   T   
   G   
   G   
   A   
   A   
   G   
   T   
   T   
   G   
   C   
   G   
   G   
   G   
   A   
   A   
   G   
   T   
   A   
   C   
   T   
   G   
   A   
   T   
   C   
   G   
   A   
    A    
   A   
   T   
   G   
   C   
   A   
   C   
   G   
   G   
    G    
   A   
   A   
   T   
   A   
   A   
    C    
   G   
   G   
   G   
   A   
   G   
   T   
   A   
   T   
   G   
   G   
   A   
   C   
   G   
   G   
   T   
   G   
   A   
   T   
   C   
   C   
   A   
   C   
   C   
   T   
   G   
   C   
   T   
   G   
   C   
   G   
   A   
   T   
   G   
   C   
   G   
   T   
   T   
   A   
   T   
   A   
   C   
   T   
   G   
   A   
   A   
   G   
   C   
   C   
   A   
   G   
    G    
   T   
   T   
   A   
   T   
   C   
   C   
   A   
   A   
   G   
   T   
   T   
   A   
   A   
   G   
   T   
   G   
   G   
   A   
   G   
   A   
   G   
   A   
   T   
   G   
    C    
   T   
   A   
    G    
   C   
   A   
   G   
   A   
   T   
   A   
   T   
   C   
   G   
   A   
    A    
   A   
   A   
   A   
   G   
   A   
   A   
   A   
   C   
   A   
   G   
   T   
    C    
   G   
   A   
   T   
   C   
   T   
   C   
   G   
   T   
   G   
   T   
   G   
   G   
   A   
   A   
   T   
   T   
   T   
   T   
   G   
   A   
   T   
   G   
   A   
   T   
   A   
   C   
   A   
   G   
   A   
   A   
   A   
   A   
   A   
   G   
   A   
   A   
   C   
   C   
   A   
   A   
   C   
   A   
   G   
   T   
   C   
   C   
   T   
   T   
   C   
   C   
   A   
   G   
   C   
   T   
   C   
   G   
   A   
   T   
   A   
   C   
   C   
   C   
   A   
   A   
   A   
   C   
   C   
   T   
   T   
   C   
   T   
    A    
   G   
   T   
   C   
   A   
   A   
   T   
   G   
   G   
   A   
   T   
   C   
   G   
   A   
   C   
   A   
   G   
   G   
   T   
   A   
   T   
   A   
   T   
   C   
   T   
   G   
   C   
   T   
   G   
   G   
   A   
   T   
   A   
   T   
   G   
   C   
    G    
   A   
   C   
   A   
   G   
   A   
   A   
   A   
   T   
   C   
   C   
   C   
   G   
   A   
   C   
    T    
   C   
   A   
   T   
   A   
   A   
   C   
   T   
   T   
   A   
   G   
   C   
   T   
   G   
   A   
   A   
   G   
   T   
   G   
   A   
   T   
    C    
   G   
   A   
    C    
   G   
   G   
   C   
   A   
   C   
   A   
   A   
   T   
   C   
   T   
   A   
   T   
   A   
   T   
   G   
   A   
   T   
   C   
   G   
   A   
   T   
   C   
   A   
   T   
   C   
   C   
   A   
   C   
   A   
   A   
   G   
   C   
   G   
   A   
   G   
   C   
   C   
   T   
   T   
   G   
   A   
   A   
   A   
   A   
   A   
   T   
   T   
   G   
   A   
   T   
   G   
   G   
   A   
   A   
   T   
   A   
   T   
   A   
   T   
   T   
   C   
   C   
   T   
   G   
   G   
   A   
   C   
   C   
   T   
   G   
   A   
   C   
   T   
   T   
    T    
   C   
   C   
   A   
   A   
   C   
   A   
   G   
   G   
   A   
   G   
   G   
    G    
   A   
   T   
   C   
   T   
   T   
   G   
   C   
   A   
   A   
   G   
   G   
    A    
   A   
   A   
   A   
   G   
   A   
   T   
   G   
   A   
   A   
   A   
   T   
   C   
   A   
   A   
   A   
   A   
   A   
   A   
   G   
   C   
   T   
   T   
   A   
   T   
   G   
   A   
   A   
   A   
   C   
   A   
   G   
   G   
    G    
   C   
   G   
   A   
   G   
   G   
   A   
   A   
   A   
   A   
   G   
   T   
   T   
   A   
    T    
   T   
   C   
   T   
   T   
   C   
   G   
   T   
   T   
   C   
   G   
   A   
   A   
   A   
   A   
   C   
   A   
   A   
   A   
   G   
   A   
   T   
   A   
   G   
   A   
    A    
   G   
   C   
   A   
   A   
   T   
   G   
   A   
   A   
   G   
   G   
   G   
    T    
   A   
   A   
    T    
   A   
   A   
   A   
   C   
   A   
   G   
   C   
   A   
   A   
   A   
   T   
   C   
   G   
   T   
   G   
   A   
   T   
   C   
   A   
   C   
    A    
   G   
   A   
   A   
   A   
   T   
   C   
   C   
   C   
   A   
   T   
   A   
   T   
   G   
   A   
   A   
   G   
   T   
   A   
   A   
   A   
   T   
   A   
   A   
   A   
   G   
   C   
   G   
   A   
   C   
    C    
   T   
   T   
   G   
   G   
   T   
   G   
   A   
   A   
   A   
   A   
   A   
   A   
   A   
   T   
   G   
   G   
   A   
   T   
   G   
   A   
   G   
   A   
   T   
   C   
   C   
   G   
   T   
   T   
   T   
   G   
   A   
   A   
   T   
   A   
   A   
   G   
   A   
   A   
   A   
   A   
   T   
   C   
   G   
   A   
    T    
   G   
   G   
   A   
   A   
   T   
   A   
   G   
   C   
   T   
   G   
   A   
   A   
   G   
   T   
    A    
   C   
   G   
   A   
   G   
   A   
   T   
   G   
   A   
   A   
   A   
   G   
   T   
   G   
   A   
   T   
    A    
   G   
    A    
   A   
   C   
    A    
   G   
   G   
   T   
   C   
   T   
   G   
   C   
   A   
   A   
   A   
   T   
   T   
   G   
   T   
   C   
   G   
   T   
    C    
   G   
   A   
   A   
   T   
   T   
   G   
   A   
   A   
   A   
   A   
   A   
   A   
   G   
   A   
    C    
   G   
   C   
   C   
   A   
   A   
    T    
   G   
   C   
   T   
   C   
   A   
   A   
   G   
   G   
    A    
   A   
   T   
   T   
   T   
   T   
   G   
   A   
   A   
   T   
   T   
   A   
    T    
   T   
   T   
   G   
   T   
   T   
   C   
   A   
   A   
   A   
   A   
   A   
   C   
   A   
   C   
   T   
   G   
   A   
   G   
    C    
   T   
   A   
   C   
   A   
   G   
   A   
   T   
   C   
   A   
   A   
   T   
   T   
   A   
   C   
   A   
   A   
   T   
   T   
   T   
    C    
   A   
   A   
   T   
   A   
   T   
   G   
   G   
   T   
   C   
   G   
   C   
   T   
   A   
   T   
   C   
   G   
   A   
    C    
   C   
   A   
   T   
   A   
   T   
   G   
   A   
   C   
   T   
   C   
   C   
   G   
   C   
   A   
    T    
   C   
   A   
   A   
   G   
   T   
   T   
   G   
   G   
   A   
   C   
   T   
   G   
   A   
   A   
   A   
   G   
   A   
   C   
   A   
   T   
   T   
   T   
   T   
   A   
   A   
   G   
   A   
   A   
   G   
   T   
   T   
   A   
   T   
   A   
   T   
   C   
   G   
   A   
   G   
   C   
   A   
   T   
   C   
   G   
   A   
   A   
   A   
   G   
   C   
   A   
   A   
   G   
   T   
   C   
   A   
   T   
   A   
   A   
   C   
   A   
   A   
   A   
   A   
   C   
   G   
    T    
   A   
   G   
    T    
   C   
   A   
   A   
   T   
   T   
   C   
   G   
   A   
   T   
   T   
   T   
   A   
   G   
   C   
   C   
   A   
   A   
   A   
   G   
   C   
   T   
   C   
   A   
   A   
   A   
   A   
   A   
    A    
   G   
   A   
   C   
   A   
   G   
   C   
   A   
   T   
   A   
   T   
   C   
   G   
   T   
    A    
   G   
   A   
   A   
   G   
   G   
    A    
    T    
   T   
   G   
   A   
   T   
   G   
   A   
   A   
   A   
   G   
   C   
   T   
   T   
   T   
   A   
   T   
   C   
   G   
   A   
   T   
   T   
   C   
   T   
   G   
   G   
   A   
   T   
   G   
   A   
   A   
   G   
   T   
   A   
   A   
   T   
   C   
   G   
    C    
   A   
   A   
   C   
   G   
   A   
   T   
   T   
   C   
   G   
   T   
   G   
   A   
   A   
   A   
   G   
   T   
   A   
   A   
   A   
   G   
   A   
   C   
   A   
   A   
   A   
   A   
   A   
   A   
   G   
   A   
    C    
   G   
   C   
    C    
   A   
   A   
   A   
   A   
   A   
   G   
   A   
   A   
   T   
   T   
   T   
   A   
   G   
   T   
   C   
   G   
   A   
    C    
   G   
   T   
    C    
   T   
   T   
    T    
   C   
   A   
   A   
   T   
   T   
   T   
   A   
   C   
   A   
   G   
   A   
   A   
   G   
   A   
   A   
   C   
   A   
   A   
   G   
   C   
   A   
   G   
   A   
   A   
   G   
   C   
   A   
   A   
   T   
    T    
   G   
   T   
   C   
   A   
   C   
   A   
   T   
   T   
   G   
   C   
   A   
   A   
    T    
   T   
   A   
   T   
   A   
    C    
   C   
   G   
   T   
   T   
   T   
   G   
   A   
   C   
   G   
   A   
   A   
    C    
   A   
   C   
    G    
   G   
   A   
   T   
   A   
   T   
   C   
   A   
   C   
   A   
   G   
   A   
   G   
   C   
   T   
   T   
   C   
   A   
   A   
   A   
   A   
   G   
   G   
   A   
   A   
    T    
   C   
    C    
   G   
   A   
   A   
   A   
    G    
   C   
   T   
   T   
   G   
   A   
   T   
   T   
   G   
   C   
    G    
   C   
   A   
   A   
   A   
   T   
   C   
   A   
   C   
   A   
   G   
   A   
   G   
   T   
   T   
    G    
   A   
   A   
   T   
   A   
   A   
   A   
   A   
   T   
   C   
   C   
   T   
   T   
   T   
   C   
   A   
   A   
   A   
    T    
   G   
   A   
   C   
   A   
   A   
   A   
   G   
   A   
    G    
   C   
   T   
   T   
   T   
   T   
    C    
   T   
   C   
   T   
   G   
   T   
   T   
   A   
   T   
   G   
   A   
   A   
   A   
   A   
   A   
   A   
   G   
   A   
   A   
   T   
   T   
   G   
   C   
   G   
   A   
   G   
   A   
   A   
   G   
   T   
   G   
   A   
   A   
   A   
   A   
   A   
   A   
   A   
   A   
   T   
   T   
   A   
    T    
   T   
   C   
   T   
   T   
   C   
   C   
   G   
   C   
   T   
   C   
   G   
   T   
   C   
    T    
    A    
   A   
   C   
   G   
   A   
   C   
   A   
   A   
   T   
   C   
    G    
   A   
   A   
   G   
   A   
   C   
   G   
   A   
   A   
   A   
   T   
   C   
   G   
   A   
   A   
   G   
   A   
   A   
   A   
   T   
   C   
   A   
   A   
   A   
   A   
   T   
    T    
   G   
   A   
   T   
   A   
   C   
   G   
   C   
   A   
   A   
    G    
   T   
    C    
   T   
   T   
   A   
   G   
   T   
   G   
   G   
   C   
    A    
   C   
   A   
   A   
   G   
   A   
   A   
   G   
   A   
   C   
   G   
   T   
   A   
   A   
   T   
    T    
   G   
   T   
   C   
   A   
   G   
   T   
   G   
   T   
    G    
   A   
   C   
    G    
   C   
   G   
   A   
   G   
   A   
   A   
   G   
   G   
   C   
   T   
   A   
   T   
   G   
   T   
   G   
   A   
   A   
   G   
   C   
   G   
   A   
   A   
   C   
   A   
   A   
   G   
   C   
   C   
   T   
   T   
   C   
   G   
   T   
   T   
   C   
    T    
   T   
   A   
   T   
   A   
   G   
   T   
   G   
   C   
   T   
   T   
   C   
    C    
   A   
   A   
   A   
   C   
   C   
   A   
   G   
   A   
   A   
   G   
   A   
   A   
   A   
   T   
   C   
   G   
   G   
   G   
   A   
   T   
   G   
   C   
   G   
   A   
   G   
   A   
   A   
   G   
   G   
   C   
   G   
   A   
    C    
   T   
   A   
   C   
   T   
   T   
   G   
   C   
   T   
    A    
   T   
   A   
   C   
    T    
   C   
   A   
   G   
   G   
   T   
   G   
   A   
   A   
   T   
   T   
    G    
   A   
   G   
    T    
   A   
   C   
   A   
    T    
   T   
   G   
   G   
   A   
   C   
   C   
   A   
   T   
   G   
   T   
   T   
   C   
   T   
   T   
   C   
   T   
   G   
   A   
   T   
    C    
   A   
   C   
    A    
   A   
   A   
   T   
   A   
   A   
   A   
   G   
   C   
   A   
   A   
   A   
   T   
   G   
   T   
   G   
   A   
   T   
   C   
   T   
   A   
   C   
   C   
   G   
    C    
   C   
   C   
   T   
   G   
   T   
    C    
   C   
   A   
   T   
   G   
   A   
   A   
   C   
   T   
   A   
   C   
   C   
    C    
   G   
   A   
   T   
   T   
   T   
   G   
   A   
   A   
   A   
   T   
   G   
   G   
   A   
   A   
   A   
   G   
   A   
   T   
   G   
   C   
   T   
   G   
   G   
    T    
   G   
   A   
    G    
   C   
   A   
    C    
   A   
   T   
   T   
   T   
   C   
   T   
   C   
   A   
   A   
   A   
   C   
   A   
   A   
   T   
   C   
   T   
   C   
   C   
   A   
   A   
   T   
   C   
   T   
   G   
   T   
   C   
   T   
   G   
   T   
   A   
   G   
   A   
    C    
   G   
   A   
   A   
   T   
   C   
   G   
   A   
   T   
    T    
   T   
   T   
   A   
   G   
   C   
   T   
   G   
   T   
   T   
   T   
   T   
    C    
   C   
   C   
   A   
   T   
   A   
   T   
   C   
   A   
   A   
   A   
   A   
   A   
   A   
   T   
    T    
   C   
   A   
   A   
   G   
   C   
   A   
   G   
   A   
    G    
   A   
   A   
   A   
   A   
   C   
   A   
   T   
   T   
   T   
   G   
   T   
    A    
   T   
   T   
   C   
   A   
   T   
   C   
   A   
   G   
   C   
   A   
   A   
   G   
   A   
   A   
   T   
   G   
   G   
   A   
   T   
   T   
    G    
    A    
   T   
   C   
   A   
   A   
   G   
   C   
   A   
   A   
   A   
   C   
   A   
   C   
   G   
   A   
   A   
   T   
   G   
   A   
   C   
   A   
   G   
   A   
   G   
   T   
   T   
   C   
   G   
   A   
   A   
   C   
   C   
   A   
   T   
   G   
   G   
   C   
   G   
   C   
   A   
   C   
   C   
   T   
   A   
    T    
   A   
   A   
   G   
   A   
   G   
   C   
   C   
   G   
    T    
   C   
   C   
   G   
   C   
   T   
   C   
   A   
   G   
   C   
   T   
   G   
   T   
   A   
   T   
   G   
   A   
   A   
   G   
   C   
   T   
   A   
   A   
   A   
   G   
   G   
   C   
   G   
   G   
   A   
   A   
   G   
   A   
   A   
   G   
   A   
   T   
   G   
   A   
   A   
   C   
   T   
    G    
   A   
   T   
   T   
   G   
   C   
   T   
   G   
   T   
    G    
   T   
   A   
   T   
   C   
   T   
   G   
   A   
   A   
   A   
   G   
   A   
   A   
   G   
   A   
   T   
   C   
    A    
   G   
   C   
   C   
   A   
   G   
   G   
   C   
   T   
   T   
   A   
   G   
   A   
   T   
   G   
   T   
    T    
   T   
   T   
   C   
   T   
   T   
   A   
   G   
   T   
   A   
   A   
   C   
   G   
    C    
   A   
   T   
   C   
   A   
   A   
   G   
   G   
   A   
   A   
   T   
   G   
   G   
   G   
    T    
   T   
   T   
   G   
   C   
   G   
   C   
   T   
    A    
   T   
   C   
   C   
   T   
   T   
   T   
   A   
   A   
    C    
   A   
   G   
   A   
   T   
   G   
   T   
   T   
   C   
   C   
   C   
   G   
   T   
   T   
   G   
   T   
   T   
   G   
   G   
   A   
   G   
   C   
   A   
   A   
   A   
   G   
   G   
   C   
   A   
   G   
    C    
   T   
   G   
   G   
   A   
   G   
   T   
   G   
   A   
   A   
   G   
   T   
   C   
   T   
   A   
   T   
   G   
   A   
   A   
   T   
   T   
   T   
   G   
   A   
   A   
   A   
   G   
   A   
   T   
   G   
   G   
   A   
   G   
   A   
   T   
   A   
   G   
   G   
    A    
   T   
   T   
    A    
   T   
   C   
   A   
   A   
    C    
   G   
   G   
    G    
   C   
   T   
    G    
   C   
   T   
   T   
   G   
   T   
   G   
   T   
   T   
   A   
   G   
   C   
   A   
   G   
   A   
    A    
   G   
   G   
    T    
   G   
   A   
   T   
   A   
   C   
   G   
   C   
   C   
    G    
   A   
   T   
   C   
   G   
   T   
   A   
   A   
   T   
   C   
   G   
   T   
   T   
   A   
   C   
   T   
   C   
   A   
   G   
   C   
   G   
   A   
   G   
   G   
   A   
   G   
   C   
    T    
   G   
   T   
    T    
   A   
   A   
   A   
   C   
   G   
   C   
   A   
   T   
   G   
   C   
   T   
   T   
   G   
   C   
   T   
   C   
   A   
   A   
   G   
   A   
   A   
   A   
   T   
   C   
   A   
   G   
   C   
   C   
   A   
   A   
   A   
   C   
    T    
   T   
   C   
   A   
   C   
   G   
   T   
   G   
   C   
   G   
   A   
   A   
   G   
   C   
   G   
   G   
   G   
   G   
   A   
   G   
   T   
   G   
   A   
   C   
   G   
   G   
   T   
   C   
   A   
   T   
   G   
   C   
   G   
   T   
   G   
   A   
   G   
   C   
   T   
   G   
   A   
   A   
   A   
   A   
   A   
   A   
   A   
   A   
   T   
   C   
   C   
    G    
   C   
   A   
    C    
   C   
   G   
    T    
   A   
   T   
   T   
   A   
   T   
   C   
   T   
   A   
   T   
   A   
   T   
   G   
   T   
   C   
   G   
   G   
   A   
   A   
   G   
   G   
   A   
   A   
   G   
   A   
   T   
   C   
   A   
   A   
   A   
   A   
   G   
   A   
   A   
   A   
   T   
   A   
   A   
   C   
   C   
   T   
   T   
   G   
   A   
   T   
   G   
   A   
   A   
   T   
   C   
   A   
   A   
   A   
   A   
   A   
   G   
   G   
    G    
   C   
   A   
    G    
   C   
   A   
   G   
    T    
   T   
   A   
   A   
   C   
   G   
   A   
   T   
   T   
   G   
   A   
    C    
   C   
   C   
   A   
   A   
   C   
    T    
   G   
   A   
   T   
   T   
   T   
    C    
   C   
   C   
   T   
   A   
   T   
   A   
   G   
   G   
   T   
   G   
   A   
   T   
   C   
   G   
   A   
   A   
   C   
   A   
   T   
   C   
   G   
   A   
   A   
   T   
   G   
   G   
    A    
   T   
   C   
    A    
   T   
   T   
   T   
   G   
   C   
    G    
   A   
   T   
   G   
   G   
   A   
    T    
   G   
   A   
   G   
   A   
   A   
   A   
   A   
   A   
   A   
   G   
   G   
   T   
   G   
   G   
   A   
   G   
   A   
   A   
   G   
   T   
   C   
   A   
   T   
   C   
   G   
   A   
   A   
   G   
   T   
    T    
   A   
    T    
   C   
   G   
   A   
   T   
   G   
   C   
   T   
   C   
   C   
   T   
   G   
   A   
   G   
    A    
   T   
   T   
   T   
   T   
    C    
   A   
   T   
    C    
   G   
   A   
    G    
    T    
   C   
    G    
   -   
   -   
   -   
 

 
   N60463F   
   A   
   T   
   G   
   G   
   A   
   A   
   A   
   A   
   T   
   C   
   G   
   A   
   C   
   A   
   A   
   G   
   A   
   A   
   G   
   T   
   A   
   C   
   A   
   A   
   G   
   A   
   A   
   T   
   T   
   A   
   A   
   C   
   T   
   T   
   T   
   A   
   G   
   A   
   A   
   G   
   A   
   A   
   G   
   T   
   G   
   A   
   T   
   G   
   G   
   G   
   T   
   G   
   A   
   C   
   C   
   G   
   C   
   T   
   T   
   C   
   G   
   G   
   A   
   C   
   G   
   T   
   T   
   A   
   T   
   T   
   C   
   C   
   A   
   A   
   A   
   T   
   A   
   T   
   A   
   T   
   T   
   A   
   T   
    C    
   C   
   A   
    A    
   G   
   A   
   A   
   C   
   G   
   T   
   G   
   C   
   A   
   C   
   T   
   G   
   C   
   C   
   T   
   G   
   A   
   T   
   A   
   T   
    T    
   C   
   G   
   T   
   G   
   A   
   T   
   G   
   G   
   A   
   T   
   T   
   G   
   A   
   A   
    G    
   C   
   C   
   A   
   G   
   T   
    G    
   C   
   A   
   A   
   C   
   G   
   C   
   C   
   G   
   G   
   A   
   T   
   A   
   T   
   T   
   A   
   T   
   T   
    T    
   G   
   C   
    C    
   A   
   T   
   G   
   A   
   A   
   C   
   A   
   A   
   A   
   G   
   A   
   T   
   G   
   G   
   C   
   A   
   A   
   T   
   A   
   C   
   G   
   T   
   A   
   T   
   G   
   A   
   C   
   A   
   A   
   A   
   G   
   G   
   A   
   T   
   T   
    C    
   C   
   G   
   T   
   A   
   A   
   A   
   T   
   C   
   A   
   G   
   C   
   A   
   A   
   A   
   A   
   T   
   C   
   T   
   G   
   T   
   C   
   G   
   G   
   A   
   A   
   A   
   T   
   A   
   T   
   C   
   A   
   T   
   G   
   G   
   G   
    G    
   A   
   A   
   T   
   T   
   A   
   T   
   C   
   A   
   T   
   C   
   C   
   T   
   C   
   A   
   T   
   G   
   G   
   T   
   G   
   A   
   C   
   A   
   G   
   C   
   A   
   G   
   T   
   A   
   T   
   T   
   T   
   A   
   T   
   G   
   A   
   A   
   G   
   C   
   G   
   A   
   T   
   G   
   G   
   T   
   C   
   C   
   G   
   C   
   A   
   T   
   G   
   A   
   G   
   C   
   C   
   A   
   A   
   G   
   A   
    C    
   T   
   G   
   G   
   A   
   A   
   G   
   T   
   T   
   G   
   C   
   G   
   G   
   G   
   A   
   A   
   G   
   T   
   A   
   C   
   T   
   G   
   A   
   T   
   C   
   G   
   A   
    A    
   A   
   T   
   G   
   C   
   A   
   C   
   G   
   G   
    G    
   A   
   A   
   T   
   A   
   A   
    C    
   G   
   G   
   G   
   A   
   G   
   T   
   A   
   T   
   G   
   G   
   A   
   C   
   G   
   G   
   T   
   G   
   A   
   T   
   C   
   C   
   A   
   C   
   C   
   T   
   G   
   C   
   T   
   G   
   C   
   G   
   A   
   T   
   G   
   C   
   G   
   T   
   T   
   A   
   T   
   A   
   C   
   T   
   G   
   A   
   A   
   G   
   C   
   C   
   A   
   G   
    A    
   T   
   T   
   A   
   T   
   C   
   C   
   A   
   A   
   G   
   T   
   T   
   A   
   A   
   G   
   T   
   G   
   G   
   A   
   G   
   A   
   G   
   A   
   T   
   G   
    C    
   T   
   A   
    G    
   C   
   A   
   G   
   A   
   T   
   A   
   T   
   C   
   G   
   A   
    A    
   A   
   A   
   A   
   G   
   A   
   A   
   A   
   C   
   A   
   G   
   T   
    C    
   G   
   A   
   T   
   C   
   T   
   C   
   G   
   T   
   G   
   T   
   G   
   G   
   A   
   A   
   T   
   T   
   T   
   T   
   G   
   A   
   T   
   G   
   A   
   T   
   A   
   C   
   A   
   G   
   A   
   A   
   A   
   A   
   A   
   G   
   A   
   A   
   C   
   C   
   A   
   A   
   C   
   A   
   G   
   T   
   C   
   C   
   T   
   T   
   C   
   C   
   A   
   G   
   C   
   T   
   C   
   G   
   A   
   T   
   A   
   C   
   C   
   C   
   A   
   A   
   A   
   C   
   C   
   T   
   T   
   C   
   T   
    C    
   G   
   T   
   C   
   A   
   A   
   T   
   G   
   G   
   A   
   T   
   C   
   G   
   A   
   C   
   A   
   G   
   G   
   T   
   A   
   T   
   A   
   T   
   C   
   T   
   G   
   C   
   T   
   G   
   G   
   A   
   T   
   A   
   T   
   G   
   C   
    A    
   A   
   C   
   A   
   G   
   A   
   A   
   A   
   T   
   C   
   C   
   C   
   G   
   A   
   C   
    T    
   C   
   A   
   T   
   A   
   A   
   C   
   T   
   T   
   A   
   G   
   C   
   T   
   G   
   A   
   A   
   G   
   T   
   G   
   A   
   T   
    C    
   G   
   A   
    T    
   G   
   G   
   C   
   A   
   C   
   A   
   A   
   T   
   C   
   T   
   A   
   T   
   A   
   T   
   G   
   A   
   T   
   C   
   G   
   A   
   T   
   C   
   A   
   T   
   C   
   C   
   A   
   C   
   A   
   A   
   G   
   C   
   G   
   A   
   G   
   C   
   C   
   T   
   T   
   G   
   A   
   A   
   A   
   A   
   A   
   T   
   T   
   G   
   A   
   T   
   G   
   G   
   A   
   A   
   T   
   A   
   T   
   A   
   T   
   T   
   C   
   C   
   T   
   G   
   G   
   A   
   C   
   C   
   T   
   G   
   A   
   C   
   T   
   T   
    T    
   C   
   C   
   A   
   A   
   C   
   A   
   G   
   G   
   A   
   G   
   G   
    G    
   A   
   T   
   C   
   T   
   T   
   G   
   C   
   A   
   A   
   G   
   G   
    A    
   A   
   A   
   A   
   G   
   A   
   T   
   G   
   A   
   A   
   A   
   T   
   C   
   A   
   A   
   A   
   A   
   A   
   A   
   G   
   C   
   T   
   T   
   A   
   T   
   G   
   A   
   A   
   A   
   C   
   A   
   G   
   G   
    G    
   C   
   G   
   A   
   G   
   G   
   A   
   A   
   A   
   A   
   G   
   T   
   T   
   A   
    T    
   T   
   C   
   T   
   T   
   C   
   G   
   T   
   T   
   C   
   G   
   A   
   A   
   A   
   A   
   C   
   A   
   A   
   A   
   G   
   A   
   T   
   A   
   G   
   A   
    A    
   G   
   C   
   A   
   A   
   T   
   G   
   A   
   A   
   G   
   G   
   G   
    T    
   A   
   A   
    T    
   A   
   A   
   A   
   C   
   A   
   G   
   C   
   A   
   A   
   A   
   T   
   C   
   G   
   T   
   G   
   A   
   T   
   C   
   A   
   C   
    A    
   G   
   A   
   A   
   A   
   T   
   C   
   C   
   C   
   A   
   T   
   A   
   T   
   G   
   A   
   A   
   G   
   T   
   A   
   A   
   A   
   T   
   A   
   A   
   A   
   G   
   C   
   G   
   A   
   C   
    C    
   T   
   T   
   G   
   G   
   T   
   G   
   A   
   A   
   A   
   A   
   A   
   A   
   A   
   T   
   G   
   G   
   A   
   T   
   G   
   A   
   G   
   A   
   T   
   C   
   C   
   G   
   T   
   T   
   T   
   G   
   A   
   A   
   T   
   A   
   A   
   G   
   A   
   A   
   A   
   A   
   T   
   C   
   G   
   A   
    T    
   G   
   G   
   A   
   A   
   T   
   A   
   G   
   C   
   T   
   G   
   A   
   A   
   G   
   T   
    A    
   C   
   G   
   A   
   G   
   A   
   T   
   G   
   A   
   A   
   A   
   G   
   T   
   G   
   A   
   T   
    A    
   G   
    A    
   A   
   C   
    A    
   G   
   G   
   T   
   C   
   T   
   G   
   C   
   A   
   A   
   A   
   T   
   T   
   G   
   T   
   C   
   G   
   T   
    C    
   G   
   A   
   A   
   T   
   T   
   G   
   A   
   A   
   A   
   A   
   A   
   A   
   G   
   A   
    C    
   G   
   C   
   C   
   A   
   A   
    T    
   G   
   C   
   T   
   C   
   A   
   A   
   G   
   G   
    A    
   A   
   T   
   T   
   T   
   T   
   G   
   A   
   A   
   T   
   T   
   A   
    T    
   T   
   T   
   G   
   T   
   T   
   C   
   A   
   A   
   A   
   A   
   A   
   C   
   A   
   C   
   T   
   G   
   A   
   G   
    C    
   T   
   A   
   C   
   A   
   G   
   A   
   T   
   C   
   A   
   A   
   T   
   T   
   A   
   C   
   A   
   A   
   T   
   T   
   T   
    C    
   A   
   A   
   T   
   A   
   T   
   G   
   G   
   T   
   C   
   G   
   C   
   T   
   A   
   T   
   C   
   G   
   A   
    C    
   C   
   A   
   T   
   A   
   T   
   G   
   A   
   C   
   T   
   C   
   C   
   G   
   C   
   A   
    T    
   C   
   A   
   A   
   G   
   T   
   T   
   G   
   G   
   A   
   C   
   T   
   G   
   A   
   A   
   A   
   G   
   A   
   C   
   A   
   T   
   T   
   T   
   T   
   A   
   A   
   G   
   A   
   A   
   G   
   T   
   T   
   A   
   T   
   A   
   T   
   C   
   G   
   A   
   G   
   C   
   A   
   T   
   C   
   G   
   A   
   A   
   A   
   G   
   C   
   A   
   A   
   G   
   T   
   C   
   A   
   T   
   A   
   A   
   C   
   A   
   A   
   A   
   A   
   C   
   G   
    T    
   A   
   G   
    T    
   C   
   A   
   A   
   T   
   T   
   C   
   G   
   A   
   T   
   T   
   T   
   A   
   G   
   C   
   C   
   A   
   A   
   A   
   G   
   C   
   T   
   C   
   A   
   A   
   A   
   A   
   A   
    A    
   G   
   A   
   C   
   A   
   G   
   C   
   A   
   T   
   A   
   T   
   C   
   G   
   T   
    A    
   G   
   A   
   A   
   G   
   G   
    A    
    T    
   T   
   G   
   A   
   T   
   G   
   A   
   A   
   A   
   G   
   C   
   T   
   T   
   T   
   A   
   T   
   C   
   G   
   A   
   T   
   T   
   C   
   T   
   G   
   G   
   A   
   T   
   G   
   A   
   A   
   G   
   T   
   A   
   A   
   T   
   C   
   G   
    C    
   A   
   A   
   C   
   G   
   A   
   T   
   T   
   C   
   G   
   T   
   G   
   A   
   A   
   A   
   G   
   T   
   A   
   A   
   A   
   G   
   A   
   C   
   A   
   A   
   A   
   A   
   A   
   A   
   G   
   A   
    C    
   G   
   C   
    C    
   A   
   A   
   A   
   A   
   A   
   G   
   A   
   A   
   T   
   T   
   T   
   A   
   G   
   T   
   C   
   G   
   A   
    C    
   G   
   T   
    C    
   T   
   T   
    T    
   C   
   A   
   A   
   T   
   T   
   T   
   A   
   C   
   A   
   G   
   A   
   A   
   G   
   A   
   A   
   C   
   A   
   A   
   G   
   C   
   A   
   G   
   A   
   A   
   G   
   C   
   A   
   A   
   T   
    T    
   G   
   T   
   C   
   A   
   C   
   A   
   T   
   T   
   G   
   C   
   A   
   A   
    T    
   T   
   A   
   T   
   A   
    C    
   C   
   G   
   T   
   T   
   T   
   G   
   A   
   C   
   G   
   A   
   A   
    C    
   A   
   C   
    G    
   G   
   A   
   T   
   A   
   T   
   C   
   A   
   C   
   A   
   G   
   A   
   G   
   C   
   T   
   T   
   C   
   A   
   A   
   A   
   A   
   G   
   G   
   A   
   A   
    T    
   C   
    C    
   G   
   A   
   A   
   A   
    G    
   C   
   T   
   T   
   G   
   A   
   T   
   T   
   G   
   C   
    G    
   C   
   A   
   A   
   A   
   T   
   C   
   A   
   C   
   A   
   G   
   A   
   G   
   T   
   T   
    G    
   A   
   A   
   T   
   A   
   A   
   A   
   A   
   T   
   C   
   C   
   T   
   T   
   T   
   C   
   A   
   A   
   A   
    T    
   G   
   A   
   C   
   A   
   A   
   A   
   G   
   A   
    G    
   C   
   T   
   T   
   T   
   T   
    C    
   T   
   C   
   T   
   G   
   T   
   T   
   A   
   T   
   G   
   A   
   A   
   A   
   A   
   A   
   A   
   G   
   A   
   A   
   T   
   T   
   G   
   C   
   G   
   A   
   G   
   A   
   A   
   G   
   T   
   G   
   A   
   A   
   A   
   A   
   A   
   A   
   A   
   A   
   T   
   T   
   A   
    T    
   T   
   C   
   T   
   T   
   C   
   C   
   G   
   C   
   T   
   C   
   G   
   T   
   C   
    T    
    A    
   A   
   C   
   G   
   A   
   C   
   A   
   A   
   T   
   C   
    G    
   A   
   A   
   G   
   A   
   C   
   G   
   A   
   A   
   A   
   T   
   C   
   G   
   A   
   A   
   G   
   A   
   A   
   A   
   T   
   C   
   A   
   A   
   A   
   A   
   T   
    T    
   G   
   A   
   T   
   A   
   C   
   G   
   C   
   A   
   A   
    G    
   T   
    C    
   T   
   T   
   A   
   G   
   T   
   G   
   G   
   C   
    A    
   C   
   A   
   A   
   G   
   A   
   A   
   G   
   A   
   C   
   G   
   T   
   A   
   A   
   T   
    T    
   G   
   T   
   C   
   A   
   G   
   T   
   G   
   T   
    G    
   A   
   C   
    G    
   C   
   G   
   A   
   G   
   A   
   A   
   G   
   G   
   C   
   T   
   A   
   T   
   G   
   T   
   G   
   A   
   A   
   G   
   C   
   G   
   A   
   A   
   C   
   A   
   A   
   G   
   C   
   C   
   T   
   T   
   C   
   G   
   T   
   T   
   C   
    T    
   T   
   A   
   T   
   A   
   G   
   T   
   G   
   C   
   T   
   T   
   C   
    C    
   A   
   A   
   A   
   C   
   C   
   A   
   G   
   A   
   A   
   G   
   A   
   A   
   A   
   T   
   C   
   G   
   G   
   G   
   A   
   T   
   G   
   C   
   G   
   A   
   G   
   A   
   A   
   G   
   G   
   C   
   G   
   A   
    C    
   T   
   A   
   C   
   T   
   T   
   G   
   C   
   T   
    A    
   T   
   A   
   C   
    T    
   C   
   A   
   G   
   G   
   T   
   G   
   A   
   A   
   T   
   T   
    G    
   A   
   G   
    T    
   A   
   C   
   A   
    T    
   T   
   G   
   G   
   A   
   C   
   C   
   A   
   T   
   G   
   T   
   T   
   C   
   T   
   T   
   C   
   T   
   G   
   A   
   T   
    C    
   A   
   C   
    A    
   A   
   A   
   T   
   A   
   A   
   A   
   G   
   C   
   A   
   A   
   A   
   T   
   G   
   T   
   G   
   A   
   T   
   C   
   T   
   A   
   C   
   C   
   G   
    C    
   C   
   C   
   T   
   G   
   T   
    C    
   C   
   A   
   T   
   G   
   A   
   A   
   C   
   T   
   A   
   C   
   C   
    C    
   G   
   A   
   T   
   T   
   T   
   G   
   A   
   A   
   A   
   T   
   G   
   G   
   A   
   A   
   A   
   G   
   A   
   T   
   G   
   C   
   T   
   G   
   G   
    T    
   G   
   A   
    G    
   C   
   A   
    C    
   A   
   T   
   T   
   T   
   C   
   T   
   C   
   A   
   A   
   A   
   C   
   A   
   A   
   T   
   C   
   T   
   C   
   C   
   A   
   A   
   T   
   C   
   T   
   G   
   T   
   C   
   T   
   G   
   T   
   A   
   G   
   A   
    C    
   G   
   A   
   A   
   T   
   C   
   G   
   A   
   T   
    T    
   T   
   T   
   A   
   G   
   C   
   T   
   G   
   T   
   T   
   T   
   T   
    C    
   C   
   C   
   A   
   T   
   A   
   T   
   C   
   A   
   A   
   A   
   A   
   A   
   A   
   T   
    T    
   C   
   A   
   A   
   G   
   C   
   A   
   G   
   A   
    G    
   A   
   A   
   A   
   A   
   C   
   A   
   T   
   T   
   T   
   G   
   T   
    A    
   T   
   T   
   C   
   A   
   T   
   C   
   A   
   G   
   C   
   A   
   A   
   G   
   A   
   A   
   T   
   G   
   G   
   A   
   T   
   T   
    G    
    A    
   T   
   C   
   A   
   A   
   G   
   C   
   A   
   A   
   A   
   C   
   A   
   C   
   G   
   A   
   A   
   T   
   G   
   A   
   C   
   A   
   G   
   A   
   G   
   T   
   T   
   C   
   G   
   A   
   A   
   C   
   C   
   A   
   T   
   G   
   G   
   C   
   G   
   C   
   A   
   C   
   C   
   T   
   A   
    T    
   A   
   A   
   G   
   A   
   G   
   C   
   C   
   G   
    T    
   C   
   C   
   G   
   C   
   T   
   C   
   A   
   G   
   C   
   T   
   G   
   T   
   A   
   T   
   G   
   A   
   A   
   G   
   C   
   T   
   A   
   A   
   A   
   G   
   G   
   C   
   G   
   G   
   A   
   A   
   G   
   A   
   A   
   G   
   A   
   T   
   G   
   A   
   A   
   C   
   T   
    G    
   A   
   T   
   T   
   G   
   C   
   T   
   G   
   T   
    G    
   T   
   A   
   T   
   C   
   T   
   G   
   A   
   A   
   A   
   G   
   A   
   A   
   G   
   A   
   T   
   C   
    A    
   G   
   C   
   C   
   A   
   G   
   G   
   C   
   T   
   T   
   A   
   G   
   A   
   T   
   G   
   T   
    T    
   T   
   T   
   C   
   T   
   T   
   A   
   G   
   T   
   A   
   A   
   C   
   G   
    C    
   A   
   T   
   C   
   A   
   A   
   G   
   G   
   A   
   A   
   T   
   G   
   G   
   G   
    T    
   T   
   T   
   G   
   C   
   G   
   C   
   T   
    A    
   T   
   C   
   C   
   T   
   T   
   T   
   A   
   A   
    C    
   A   
   G   
   A   
   T   
   G   
   T   
   T   
   C   
   C   
   C   
   G   
   T   
   T   
   G   
   T   
   T   
   G   
   G   
   A   
   G   
   C   
   A   
   A   
   A   
   G   
   G   
   C   
   A   
   G   
    C    
   T   
   G   
   G   
   A   
   G   
   T   
   G   
   A   
   A   
   G   
   T   
   C   
   T   
   A   
   T   
   G   
   A   
   A   
   T   
   T   
   T   
   G   
   A   
   A   
   A   
   G   
   A   
   T   
   G   
   G   
   A   
   G   
   A   
   T   
   A   
   G   
   G   
    A    
   T   
   T   
    A    
   T   
   C   
   A   
   A   
    C    
   G   
   G   
    G    
   C   
   T   
    A    
   C   
   T   
   T   
   G   
   T   
   G   
   T   
   T   
   A   
   G   
   C   
   A   
   G   
   A   
    A    
   G   
   G   
    T    
   G   
   A   
   T   
   A   
   C   
   G   
   C   
   C   
    G    
   A   
   T   
   C   
   G   
   T   
   A   
   A   
   T   
   C   
   G   
   T   
   T   
   A   
   C   
   T   
   C   
   A   
   G   
   C   
   G   
   A   
   G   
   G   
   A   
   G   
   C   
    T    
   G   
   T   
    T    
   A   
   A   
   A   
   C   
   G   
   C   
   A   
   T   
   G   
   C   
   T   
   T   
   G   
   C   
   T   
   C   
   A   
   A   
   G   
   A   
   A   
   A   
   T   
   C   
   A   
   G   
   C   
   C   
   A   
   A   
   A   
   C   
    T    
   T   
   C   
   A   
   C   
   G   
   T   
   G   
   C   
   G   
   A   
   A   
   G   
   C   
   G   
   G   
   G   
   G   
   A   
   G   
   T   
   G   
   A   
   C   
   G   
   G   
   T   
   C   
   A   
   T   
   G   
   C   
   G   
   T   
   G   
   A   
   G   
   C   
   T   
   G   
   A   
   A   
   A   
   A   
   A   
   A   
   A   
   A   
   T   
   C   
   C   
    G    
   C   
   A   
    C    
   C   
   G   
    T    
   A   
   T   
   T   
   A   
   T   
   C   
   T   
   A   
   T   
   A   
   T   
   G   
   T   
   C   
   G   
   G   
   A   
   A   
   G   
   G   
   A   
   A   
   G   
   A   
   T   
   C   
   A   
   A   
   A   
   A   
   G   
   A   
   A   
   A   
   T   
   A   
   A   
   C   
   C   
   T   
   T   
   G   
   A   
   T   
   G   
   A   
   A   
   T   
   C   
   A   
   A   
   A   
   A   
   A   
   G   
   G   
    G    
   C   
   A   
    G    
   C   
   A   
   G   
    T    
   T   
   A   
   A   
   C   
   G   
   A   
   T   
   T   
   G   
   A   
    C    
   C   
   C   
   A   
   A   
   C   
    T    
   G   
   A   
   T   
   T   
   T   
    C    
   C   
   C   
   T   
   A   
   T   
   A   
   G   
   G   
   T   
   G   
   A   
   T   
   C   
   G   
   A   
   A   
   C   
   A   
   T   
   C   
   G   
   A   
   A   
   T   
   G   
   G   
    A    
   T   
   C   
    A    
   T   
   T   
   T   
   G   
   C   
    G    
   A   
   T   
   G   
   G   
   A   
    T    
   G   
   A   
   G   
   A   
   A   
   A   
   A   
   A   
   A   
   G   
   G   
   T   
   G   
   G   
   A   
   G   
   A   
   A   
   G   
   T   
   C   
   A   
   T   
   C   
   G   
   A   
   A   
   G   
   T   
    T    
   A   
    T    
   C   
   G   
   A   
   T   
   G   
   C   
   T   
   C   
   C   
   T   
   G   
   A   
   G   
    A    
   T   
   T   
   T   
   T   
    C    
   A   
   T   
    C    
   G   
   A   
    G    
    T    
   C   
    G    
   -   
   -   
   -   
 

 
   N59947F   
   A   
   T   
   G   
   G   
   A   
   A   
   A   
   A   
   T   
   C   
   G   
   A   
   C   
   A   
   A   
   G   
   A   
   A   
   G   
   T   
   A   
   C   
   A   
   A   
   G   
   A   
   A   
   T   
   T   
   A   
   A   
   C   
   T   
   T   
   T   
   A   
   G   
   A   
   A   
   G   
   A   
   A   
   G   
   T   
   G   
   A   
   T   
   G   
   G   
   G   
   T   
   G   
   A   
   C   
   C   
   G   
   C   
   T   
   T   
   C   
   G   
   G   
   A   
   C   
   G   
   T   
   T   
   A   
   T   
   T   
   C   
   C   
   A   
   A   
   A   
   T   
   A   
   T   
   A   
   T   
   T   
   A   
   T   
    A    
   C   
   A   
    A    
   G   
   A   
   A   
   C   
   G   
   T   
   G   
   C   
   A   
   C   
   T   
   G   
   C   
   C   
   T   
   G   
   A   
   T   
   A   
   T   
    C    
   C   
   G   
   T   
   G   
   A   
   T   
   G   
   G   
   A   
   T   
   T   
   G   
   A   
   A   
    A    
   C   
   C   
   A   
   G   
   T   
    G    
   C   
   A   
   A   
   C   
   G   
   C   
   C   
   G   
   G   
   A   
   T   
   A   
   T   
   T   
   A   
   T   
   T   
    T    
   G   
   C   
    C    
   A   
   T   
   G   
   A   
   A   
   C   
   A   
   A   
   A   
   G   
   A   
   T   
   G   
   G   
   C   
   A   
   A   
   T   
   A   
   C   
   G   
   T   
   A   
   T   
   G   
   A   
   C   
   A   
   A   
   A   
   G   
   G   
   A   
   T   
   T   
    C    
   C   
   G   
   T   
   A   
   A   
   A   
   T   
   C   
   A   
   G   
   C   
   A   
   A   
   A   
   A   
   T   
   C   
   T   
   G   
   T   
   C   
   G   
   G   
   A   
   A   
   A   
   T   
   A   
   T   
   C   
   A   
   T   
   G   
   G   
   G   
    A    
   A   
   A   
   T   
   T   
   A   
   T   
   C   
   A   
   T   
   C   
   C   
   T   
   C   
   A   
   T   
   G   
   G   
   T   
   G   
   A   
   C   
   A   
   G   
   C   
   A   
   G   
   T   
   A   
   T   
   T   
   T   
   A   
   T   
   G   
   A   
   A   
   G   
   C   
   G   
   A   
   T   
   G   
   G   
   T   
   C   
   C   
   G   
   C   
   A   
   T   
   G   
   A   
   G   
   C   
   C   
   A   
   A   
   G   
   A   
    T    
   T   
   G   
   G   
   A   
   A   
   G   
   T   
   T   
   G   
   C   
   G   
   G   
   G   
   A   
   A   
   G   
   T   
   A   
   C   
   T   
   G   
   A   
   T   
   C   
   G   
   A   
    A    
   A   
   T   
   G   
   C   
   A   
   C   
   G   
   G   
    G    
   A   
   A   
   T   
   A   
   A   
    T    
   G   
   G   
   G   
   A   
   G   
   T   
   A   
   T   
   G   
   G   
   A   
   C   
   G   
   G   
   T   
   G   
   A   
   T   
   C   
   C   
   A   
   C   
   C   
   T   
   G   
   C   
   T   
   G   
   C   
   G   
   A   
   T   
   G   
   C   
   G   
   T   
   T   
   A   
   T   
   A   
   C   
   T   
   G   
   A   
   A   
   G   
   C   
   C   
   A   
   G   
    A    
   T   
   T   
   A   
   T   
   C   
   C   
   A   
   A   
   G   
   T   
   T   
   A   
   A   
   G   
   T   
   G   
   G   
   A   
   G   
   A   
   G   
   A   
   T   
   G   
    C    
   T   
   A   
    G    
   C   
   A   
   G   
   A   
   T   
   A   
   T   
   C   
   G   
   A   
    A    
   A   
   A   
   A   
   G   
   A   
   A   
   A   
   C   
   A   
   G   
   T   
    C    
   G   
   A   
   T   
   C   
   T   
   C   
   G   
   T   
   G   
   T   
   G   
   G   
   A   
   A   
   T   
   T   
   T   
   T   
   G   
   A   
   T   
   G   
   A   
   T   
   A   
   C   
   A   
   G   
   A   
   A   
   A   
   A   
   A   
   G   
   A   
   A   
   C   
   C   
   A   
   A   
   C   
   A   
   G   
   T   
   C   
   C   
   T   
   T   
   C   
   C   
   A   
   G   
   C   
   T   
   C   
   G   
   A   
   T   
   A   
   C   
   C   
   C   
   A   
   A   
   A   
   C   
   C   
   T   
   T   
   C   
   T   
    A    
   G   
   T   
   C   
   A   
   A   
   T   
   G   
   G   
   A   
   T   
   C   
   G   
   A   
   C   
   A   
   G   
   G   
   T   
   A   
   T   
   A   
   T   
   C   
   T   
   G   
   C   
   T   
   G   
   G   
   A   
   T   
   A   
   T   
   G   
   C   
    G    
   A   
   C   
   A   
   G   
   A   
   A   
   A   
   T   
   C   
   C   
   C   
   G   
   A   
   C   
    T    
   C   
   A   
   T   
   A   
   A   
   C   
   T   
   T   
   A   
   G   
   C   
   T   
   G   
   A   
   A   
   G   
   T   
   G   
   A   
   T   
    C    
   G   
   A   
    C    
   G   
   G   
   C   
   A   
   C   
   A   
   A   
   T   
   C   
   T   
   A   
   T   
   A   
   T   
   G   
   A   
   T   
   C   
   G   
   A   
   T   
   C   
   A   
   T   
   C   
   C   
   A   
   C   
   A   
   A   
   G   
   C   
   G   
   A   
   G   
   C   
   C   
   T   
   T   
   G   
   A   
   A   
   A   
   A   
   A   
   T   
   T   
   G   
   A   
   T   
   G   
   G   
   A   
   A   
   T   
   A   
   T   
   A   
   T   
   T   
   C   
   C   
   T   
   G   
   G   
   A   
   C   
   C   
   T   
   G   
   A   
   C   
   T   
   T   
    T    
   C   
   C   
   A   
   A   
   C   
   A   
   G   
   G   
   A   
   G   
   G   
    G    
   A   
   T   
   C   
   T   
   T   
   G   
   C   
   A   
   A   
   G   
   G   
    G    
   A   
   A   
   A   
   G   
   A   
   T   
   G   
   A   
   A   
   A   
   T   
   C   
   A   
   A   
   A   
   A   
   A   
   A   
   G   
   C   
   T   
   T   
   A   
   T   
   G   
   A   
   A   
   A   
   C   
   A   
   G   
   G   
    G    
   C   
   G   
   A   
   G   
   G   
   A   
   A   
   A   
   A   
   G   
   T   
   T   
   A   
    T    
   T   
   C   
   T   
   T   
   C   
   G   
   T   
   T   
   C   
   G   
   A   
   A   
   A   
   A   
   C   
   A   
   A   
   A   
   G   
   A   
   T   
   A   
   G   
   A   
    A    
   G   
   C   
   A   
   A   
   T   
   G   
   A   
   A   
   G   
   G   
   G   
    C    
   A   
   A   
    T    
   A   
   A   
   A   
   C   
   A   
   G   
   C   
   A   
   A   
   A   
   T   
   C   
   G   
   T   
   G   
   A   
   T   
   C   
   A   
   C   
    T    
   G   
   A   
   A   
   A   
   T   
   C   
   C   
   C   
   A   
   T   
   A   
   T   
   G   
   A   
   A   
   G   
   T   
   A   
   A   
   A   
   T   
   A   
   A   
   A   
   G   
   C   
   G   
   A   
   C   
    G    
   T   
   T   
   G   
   G   
   T   
   G   
   A   
   A   
   A   
   A   
   A   
   A   
   A   
   T   
   G   
   G   
   A   
   T   
   G   
   A   
   G   
   A   
   T   
   C   
   C   
   G   
   T   
   T   
   T   
   G   
   A   
   A   
   T   
   A   
   A   
   G   
   A   
   A   
   A   
   A   
   T   
   C   
   G   
   A   
    T    
   G   
   G   
   A   
   A   
   T   
   A   
   G   
   C   
   T   
   G   
   A   
   A   
   G   
   T   
    A    
   C   
   G   
   A   
   G   
   A   
   T   
   G   
   A   
   A   
   A   
   G   
   T   
   G   
   A   
   T   
    C    
   G   
    A    
   A   
   C   
    T    
   G   
   G   
   T   
   C   
   T   
   G   
   C   
   A   
   A   
   A   
   T   
   T   
   G   
   T   
   C   
   G   
   T   
    T    
   G   
   A   
   A   
   T   
   T   
   G   
   A   
   A   
   A   
   A   
   A   
   A   
   G   
   A   
    T    
   G   
   C   
   C   
   A   
   A   
    T    
   G   
   C   
   T   
   C   
   A   
   A   
   G   
   G   
    A    
   A   
   T   
   T   
   T   
   T   
   G   
   A   
   A   
   T   
   T   
   A   
    T    
   T   
   T   
   G   
   T   
   T   
   C   
   A   
   A   
   A   
   A   
   A   
   C   
   A   
   C   
   T   
   G   
   A   
   G   
    C    
   T   
   A   
   C   
   A   
   G   
   A   
   T   
   C   
   A   
   A   
   T   
   T   
   A   
   C   
   A   
   A   
   T   
   T   
   T   
    C    
   A   
   A   
   T   
   A   
   T   
   G   
   G   
   T   
   C   
   G   
   C   
   T   
   A   
   T   
   C   
   G   
   A   
    T    
   C   
   A   
   T   
   A   
   T   
   G   
   A   
   C   
   T   
   C   
   C   
   G   
   C   
   A   
    C    
   C   
   A   
   A   
   G   
   T   
   T   
   G   
   G   
   A   
   C   
   T   
   G   
   A   
   A   
   A   
   G   
   A   
   C   
   A   
   T   
   T   
   T   
   T   
   A   
   A   
   G   
   A   
   A   
   G   
   T   
   T   
   A   
   T   
   A   
   T   
   C   
   G   
   A   
   G   
   C   
   A   
   T   
   C   
   G   
   A   
   A   
   A   
   G   
   C   
   A   
   A   
   G   
   T   
   C   
   A   
   T   
   A   
   A   
   C   
   A   
   A   
   A   
   A   
   C   
   G   
    C    
   A   
   G   
    C    
   C   
   A   
   A   
   T   
   T   
   C   
   G   
   A   
   T   
   T   
   T   
   A   
   G   
   C   
   C   
   A   
   A   
   A   
   G   
   C   
   T   
   C   
   A   
   A   
   A   
   A   
   A   
    A    
   G   
   A   
   C   
   A   
   G   
   C   
   A   
   T   
   A   
   T   
   C   
   G   
   T   
    G    
   G   
   A   
   A   
   G   
   G   
    C    
    C    
   T   
   G   
   A   
   T   
   G   
   A   
   A   
   A   
   G   
   C   
   T   
   T   
   T   
   A   
   T   
   C   
   G   
   A   
   T   
   T   
   C   
   T   
   G   
   G   
   A   
   T   
   G   
   A   
   A   
   G   
   T   
   A   
   A   
   T   
   C   
   G   
    C    
   A   
   A   
   C   
   G   
   A   
   T   
   T   
   C   
   G   
   T   
   G   
   A   
   A   
   A   
   G   
   T   
   A   
   A   
   A   
   G   
   A   
   C   
   A   
   A   
   A   
   A   
   A   
   A   
   G   
   A   
    T    
   G   
   C   
    T    
   A   
   A   
   A   
   A   
   A   
   G   
   A   
   A   
   T   
   T   
   T   
   A   
   G   
   T   
   C   
   G   
   A   
    T    
   G   
   T   
    T    
   T   
   T   
    C    
   C   
   A   
   A   
   T   
   T   
   T   
   A   
   C   
   A   
   G   
   A   
   A   
   G   
   A   
   A   
   C   
   A   
   A   
   G   
   C   
   A   
   G   
   A   
   A   
   G   
   C   
   A   
   A   
   T   
    C    
   G   
   T   
   C   
   A   
   C   
   A   
   T   
   T   
   G   
   C   
   A   
   A   
    C    
   T   
   A   
   T   
   A   
    C    
   C   
   G   
   T   
   T   
   T   
   G   
   A   
   C   
   G   
   A   
   A   
    C    
   A   
   C   
    A    
   G   
   A   
   T   
   A   
   T   
   C   
   A   
   C   
   A   
   G   
   A   
   G   
   C   
   T   
   T   
   C   
   A   
   A   
   A   
   A   
   G   
   G   
   A   
   A   
    G    
   C   
    C    
   G   
   A   
   A   
   A   
    G    
   C   
   T   
   T   
   G   
   A   
   T   
   T   
   G   
   C   
    A    
   C   
   A   
   A   
   A   
   T   
   C   
   A   
   C   
   A   
   G   
   A   
   G   
   T   
   T   
    G    
   A   
   A   
   T   
   A   
   A   
   A   
   A   
   T   
   C   
   C   
   T   
   T   
   T   
   C   
   A   
   A   
   A   
    C    
   G   
   A   
   C   
   A   
   A   
   A   
   G   
   A   
    A    
   C   
   T   
   T   
   T   
   T   
    C    
   T   
   C   
   T   
   G   
   T   
   T   
   A   
   T   
   G   
   A   
   A   
   A   
   A   
   A   
   A   
   G   
   A   
   A   
   T   
   T   
   G   
   C   
   G   
   A   
   G   
   A   
   A   
   G   
   T   
   G   
   A   
   A   
   A   
   A   
   A   
   A   
   A   
   A   
   T   
   T   
   A   
    C    
   T   
   C   
   T   
   T   
   C   
   C   
   G   
   C   
   T   
   C   
   G   
   T   
   C   
    T    
    G    
   A   
   C   
   G   
   A   
   C   
   A   
   A   
   T   
   C   
    C    
   A   
   A   
   G   
   A   
   C   
   G   
   A   
   A   
   A   
   T   
   C   
   G   
   A   
   A   
   G   
   A   
   A   
   A   
   T   
   C   
   A   
   A   
   A   
   A   
   T   
    C    
   G   
   A   
   T   
   A   
   C   
   G   
   C   
   A   
   A   
    G    
   T   
    T    
   T   
   T   
   A   
   G   
   T   
   G   
   G   
   C   
    G    
   C   
   A   
   A   
   G   
   A   
   A   
   G   
   A   
   C   
   G   
   T   
   A   
   A   
   T   
    C    
   G   
   T   
   C   
   A   
   G   
   T   
   G   
   T   
    G    
   A   
   C   
    G    
   C   
   G   
   A   
   G   
   A   
   A   
   G   
   G   
   C   
   T   
   A   
   T   
   G   
   T   
   G   
   A   
   A   
   G   
   C   
   G   
   A   
   A   
   C   
   A   
   A   
   G   
   C   
   C   
   T   
   T   
   C   
   G   
   T   
   T   
   C   
    T    
   T   
   A   
   T   
   A   
   G   
   T   
   G   
   C   
   T   
   T   
   C   
    G    
   A   
   A   
   A   
   C   
   C   
   A   
   G   
   A   
   A   
   G   
   A   
   A   
   A   
   T   
   C   
   G   
   G   
   G   
   A   
   T   
   G   
   C   
   G   
   A   
   G   
   A   
   A   
   G   
   G   
   C   
   G   
   A   
    T    
   T   
   A   
   C   
   T   
   T   
   G   
   C   
   T   
    G    
   T   
   A   
   C   
    T    
   C   
   A   
   G   
   G   
   T   
   G   
   A   
   A   
   T   
   T   
    G    
   A   
   G   
    C    
   A   
   C   
   A   
    T    
   T   
   G   
   G   
   A   
   C   
   C   
   A   
   T   
   G   
   T   
   T   
   C   
   T   
   T   
   C   
   T   
   G   
   A   
   T   
    T    
   A   
   C   
    G    
   A   
   A   
   T   
   A   
   A   
   A   
   G   
   C   
   A   
   A   
   A   
   T   
   G   
   T   
   G   
   A   
   T   
   C   
   T   
   A   
   C   
   C   
   G   
    T    
   C   
   C   
   T   
   G   
   T   
    T    
   C   
   A   
   T   
   G   
   A   
   A   
   C   
   T   
   A   
   C   
   C   
    C    
   G   
   A   
   T   
   T   
   T   
   G   
   A   
   A   
   A   
   T   
   G   
   G   
   A   
   A   
   A   
   G   
   A   
   T   
   G   
   C   
   T   
   G   
   G   
    C    
   G   
   A   
    A    
   C   
   A   
    C    
   A   
   T   
   T   
   T   
   C   
   T   
   C   
   A   
   A   
   A   
   C   
   A   
   A   
   T   
   C   
   T   
   C   
   C   
   A   
   A   
   T   
   C   
   T   
   G   
   T   
   C   
   T   
   G   
   T   
   A   
   G   
   A   
    T    
   G   
   A   
   A   
   T   
   C   
   G   
   A   
   T   
    C    
   T   
   T   
   A   
   G   
   C   
   T   
   G   
   T   
   T   
   T   
   T   
    C    
   C   
   C   
   A   
   T   
   A   
   T   
   C   
   A   
   A   
   A   
   A   
   A   
   A   
   T   
    C    
   C   
   A   
   A   
   G   
   C   
   A   
   G   
   A   
    A    
   A   
   A   
   A   
   A   
   C   
   A   
   T   
   T   
   T   
   G   
   T   
    A    
   T   
   T   
   C   
   A   
   T   
   C   
   A   
   G   
   C   
   A   
   A   
   G   
   A   
   A   
   T   
   G   
   G   
   A   
   T   
   T   
    G    
    A    
   T   
   C   
   A   
   A   
   G   
   C   
   A   
   A   
   A   
   C   
   A   
   C   
   G   
   A   
   A   
   T   
   G   
   A   
   C   
   A   
   G   
   A   
   G   
   T   
   T   
   C   
   G   
   A   
   A   
   C   
   C   
   A   
   T   
   G   
   G   
   C   
   G   
   C   
   A   
   C   
   C   
   T   
   A   
    T    
   A   
   A   
   G   
   A   
   G   
   C   
   C   
   G   
    T    
   C   
   C   
   G   
   C   
   T   
   C   
   A   
   G   
   C   
   T   
   G   
   T   
   A   
   T   
   G   
   A   
   A   
   G   
   C   
   T   
   A   
   A   
   A   
   G   
   G   
   C   
   G   
   G   
   A   
   A   
   G   
   A   
   A   
   G   
   A   
   T   
   G   
   A   
   A   
   C   
   T   
    T    
   A   
   T   
   T   
   G   
   C   
   T   
   G   
   T   
    A    
   T   
   A   
   T   
   C   
   T   
   G   
   A   
   A   
   A   
   G   
   A   
   A   
   G   
   A   
   T   
   C   
    A    
   G   
   C   
   C   
   A   
   G   
   G   
   C   
   T   
   T   
   A   
   G   
   A   
   T   
   G   
   T   
    G    
   T   
   T   
   C   
   T   
   T   
   A   
   G   
   T   
   A   
   A   
   C   
   G   
    C    
   A   
   T   
   C   
   A   
   A   
   G   
   G   
   A   
   A   
   T   
   G   
   G   
   G   
    C    
   T   
   T   
   G   
   C   
   G   
   C   
   T   
    A    
   T   
   C   
   C   
   T   
   T   
   T   
   A   
   A   
    C    
   A   
   G   
   A   
   T   
   G   
   T   
   T   
   C   
   C   
   C   
   G   
   T   
   T   
   G   
   T   
   T   
   G   
   G   
   A   
   G   
   C   
   A   
   A   
   A   
   G   
   G   
   C   
   A   
   G   
    C    
   T   
   G   
   G   
   A   
   G   
   T   
   G   
   A   
   A   
   G   
   T   
   C   
   T   
   A   
   T   
   G   
   A   
   A   
   T   
   T   
   T   
   G   
   A   
   A   
   A   
   G   
   A   
   T   
   G   
   G   
   A   
   G   
   A   
   T   
   A   
   G   
   G   
    A    
   T   
   T   
    G    
   T   
   C   
   A   
   A   
    T    
   G   
   G   
    G    
   C   
   T   
    G    
   C   
   T   
   T   
   G   
   T   
   G   
   T   
   T   
   A   
   G   
   C   
   A   
   G   
   A   
    C    
   G   
   G   
    A    
   G   
   A   
   T   
   A   
   C   
   G   
   C   
   C   
    A    
   A   
   T   
   C   
   G   
   T   
   A   
   A   
   T   
   C   
   G   
   T   
   T   
   A   
   C   
   T   
   C   
   A   
   G   
   C   
   G   
   A   
   G   
   G   
   A   
   G   
   C   
    C    
   G   
   T   
    G    
   A   
   A   
   A   
   C   
   G   
   C   
   A   
   T   
   G   
   C   
   T   
   T   
   G   
   C   
   T   
   C   
   A   
   A   
   G   
   A   
   A   
   A   
   T   
   C   
   A   
   G   
   C   
   C   
   A   
   A   
   A   
   C   
    G    
   T   
   C   
   A   
   C   
   G   
   T   
   G   
   C   
   G   
   A   
   A   
   G   
   C   
   G   
   G   
   G   
   G   
   A   
   G   
   T   
   G   
   A   
   C   
   G   
   G   
   T   
   C   
   A   
   T   
   G   
   C   
   G   
   T   
   G   
   A   
   G   
   C   
   T   
   G   
   A   
   A   
   A   
   A   
   A   
   A   
   A   
   A   
   T   
   C   
   C   
    G    
   C   
   A   
    C    
   C   
   G   
    T    
   A   
   T   
   T   
   A   
   T   
   C   
   T   
   A   
   T   
   A   
   T   
   G   
   T   
   C   
   G   
   G   
   A   
   A   
   G   
   G   
   A   
   A   
   G   
   A   
   T   
   C   
   A   
   A   
   A   
   A   
   G   
   A   
   A   
   A   
   T   
   A   
   A   
   C   
   C   
   T   
   T   
   G   
   A   
   T   
   G   
   A   
   A   
   T   
   C   
   A   
   A   
   A   
   A   
   A   
   G   
   G   
    A    
   C   
   A   
    A    
   C   
   A   
   G   
    A    
   T   
   A   
   A   
   C   
   G   
   A   
   T   
   T   
   G   
   A   
    T    
   C   
   C   
   A   
   A   
   C   
    G    
   G   
   A   
   T   
   T   
   T   
    T    
   C   
   C   
   T   
   A   
   T   
   A   
   G   
   G   
   T   
   G   
   A   
   T   
   C   
   G   
   A   
   A   
   C   
   A   
   T   
   C   
   G   
   A   
   A   
   T   
   G   
   G   
    T    
   T   
   C   
    G    
   T   
   T   
   T   
   G   
   C   
    T    
   A   
   T   
   G   
   G   
   A   
    C    
   G   
   A   
   G   
   A   
   A   
   A   
   A   
   A   
   A   
   G   
   G   
   T   
   G   
   G   
   A   
   G   
   A   
   A   
   G   
   T   
   C   
   A   
   T   
   C   
   G   
   A   
   A   
   G   
   T   
    T    
   A   
    T    
   C   
   G   
   A   
   T   
   G   
   C   
   T   
   C   
   C   
   T   
   G   
   A   
   G   
    A    
   T   
   T   
   T   
   T   
    C    
   A   
   T   
    T    
   G   
   A   
    A    
    T    
   C   
    A    
   -   
   -   
   -   
 

 
   N59653F   
   A   
   T   
   G   
   G   
   A   
   A   
   A   
   A   
   T   
   C   
   G   
   A   
   C   
   A   
   A   
   G   
   A   
   A   
   G   
   T   
   A   
   C   
   A   
   A   
   G   
   A   
   A   
   T   
   T   
   A   
   A   
   C   
   T   
   T   
   T   
   A   
   G   
   A   
   A   
   G   
   A   
   A   
   G   
   T   
   G   
   A   
   T   
   G   
   G   
   G   
   T   
   G   
   A   
   C   
   C   
   G   
   C   
   T   
   T   
   C   
   G   
   G   
   A   
   C   
   G   
   T   
   T   
   A   
   T   
   T   
   C   
   C   
   A   
   A   
   A   
   T   
   A   
   T   
   A   
   T   
   T   
   A   
   T   
    C    
   C   
   A   
    A    
   G   
   A   
   A   
   C   
   G   
   T   
   G   
   C   
   A   
   C   
   T   
   G   
   C   
   C   
   T   
   G   
   A   
   T   
   A   
   T   
    C    
   C   
   G   
   T   
   G   
   A   
   T   
   G   
   G   
   A   
   T   
   T   
   G   
   A   
   A   
    A    
   C   
   C   
   A   
   G   
   T   
    G    
   C   
   A   
   A   
   C   
   G   
   C   
   C   
   G   
   G   
   A   
   T   
   A   
   T   
   T   
   A   
   T   
   T   
    T    
   G   
   C   
    C    
   A   
   T   
   G   
   A   
   A   
   C   
   A   
   A   
   A   
   G   
   A   
   T   
   G   
   G   
   C   
   A   
   A   
   T   
   A   
   C   
   G   
   T   
   A   
   T   
   G   
   A   
   C   
   A   
   A   
   A   
   G   
   G   
   A   
   T   
   T   
    C    
   C   
   G   
   T   
   A   
   A   
   A   
   T   
   C   
   A   
   G   
   C   
   A   
   A   
   A   
   A   
   T   
   C   
   T   
   G   
   T   
   C   
   G   
   G   
   A   
   A   
   A   
   T   
   A   
   T   
   C   
   A   
   T   
   G   
   G   
   G   
    G    
   A   
   A   
   T   
   T   
   A   
   T   
   C   
   A   
   T   
   C   
   C   
   T   
   C   
   A   
   T   
   G   
   G   
   T   
   G   
   A   
   C   
   A   
   G   
   C   
   A   
   G   
   T   
   A   
   T   
   T   
   T   
   A   
   T   
   G   
   A   
   A   
   G   
   C   
   G   
   A   
   T   
   G   
   G   
   T   
   C   
   C   
   G   
   C   
   A   
   T   
   G   
   A   
   G   
   C   
   C   
   A   
   A   
   G   
   A   
    T    
   T   
   G   
   G   
   A   
   A   
   G   
   T   
   T   
   G   
   C   
   G   
   G   
   G   
   A   
   A   
   G   
   T   
   A   
   C   
   T   
   G   
   A   
   T   
   C   
   G   
   A   
    A    
   A   
   T   
   G   
   C   
   A   
   C   
   G   
   G   
    G    
   A   
   A   
   T   
   A   
   A   
    T    
   G   
   G   
   G   
   A   
   G   
   T   
   A   
   T   
   G   
   G   
   A   
   C   
   G   
   G   
   T   
   G   
   A   
   T   
   C   
   C   
   A   
   C   
   C   
   T   
   G   
   C   
   T   
   G   
   C   
   G   
   A   
   T   
   G   
   C   
   G   
   T   
   T   
   A   
   T   
   A   
   C   
   T   
   G   
   A   
   A   
   G   
   C   
   C   
   A   
   G   
    A    
   T   
   T   
   A   
   T   
   C   
   C   
   A   
   A   
   G   
   T   
   T   
   A   
   A   
   G   
   T   
   G   
   G   
   A   
   G   
   A   
   G   
   A   
   T   
   G   
    C    
   T   
   A   
    G    
   C   
   A   
   G   
   A   
   T   
   A   
   T   
   C   
   G   
   A   
    A    
   A   
   A   
   A   
   G   
   A   
   A   
   A   
   C   
   A   
   G   
   T   
    C    
   G   
   A   
   T   
   C   
   T   
   C   
   G   
   T   
   G   
   T   
   G   
   G   
   A   
   A   
   T   
   T   
   T   
   T   
   G   
   A   
   T   
   G   
   A   
   T   
   A   
   C   
   A   
   G   
   A   
   A   
   A   
   A   
   A   
   G   
   A   
   A   
   C   
   C   
   A   
   A   
   C   
   A   
   G   
   T   
   C   
   C   
   T   
   T   
   C   
   C   
   A   
   G   
   C   
   T   
   C   
   G   
   A   
   T   
   A   
   C   
   C   
   C   
   A   
   A   
   A   
   C   
   C   
   T   
   T   
   C   
   T   
    A    
   G   
   T   
   C   
   A   
   A   
   T   
   G   
   G   
   A   
   T   
   C   
   G   
   A   
   C   
   A   
   G   
   G   
   T   
   A   
   T   
   A   
   T   
   C   
   T   
   G   
   C   
   T   
   G   
   G   
   A   
   T   
   A   
   T   
   G   
   C   
    G    
   A   
   C   
   A   
   G   
   A   
   A   
   A   
   T   
   C   
   C   
   C   
   G   
   A   
   C   
    T    
   C   
   A   
   T   
   A   
   A   
   C   
   T   
   T   
   A   
   G   
   C   
   T   
   G   
   A   
   A   
   G   
   T   
   G   
   A   
   T   
    C    
   G   
   A   
    C    
   G   
   G   
   C   
   A   
   C   
   A   
   A   
   T   
   C   
   T   
   A   
   T   
   A   
   T   
   G   
   A   
   T   
   C   
   G   
   A   
   T   
   C   
   A   
   T   
   C   
   C   
   A   
   C   
   A   
   A   
   G   
   C   
   G   
   A   
   G   
   C   
   C   
   T   
   T   
   G   
   A   
   A   
   A   
   A   
   A   
   T   
   T   
   G   
   A   
   T   
   G   
   G   
   A   
   A   
   T   
   A   
   T   
   A   
   T   
   T   
   C   
   C   
   T   
   G   
   G   
   A   
   C   
   C   
   T   
   G   
   A   
   C   
   T   
   T   
    T    
   C   
   C   
   A   
   A   
   C   
   A   
   G   
   G   
   A   
   G   
   G   
    G    
   A   
   T   
   C   
   T   
   T   
   G   
   C   
   A   
   A   
   G   
   G   
    G    
   A   
   A   
   A   
   G   
   A   
   T   
   G   
   A   
   A   
   A   
   T   
   C   
   A   
   A   
   A   
   A   
   A   
   A   
   G   
   C   
   T   
   T   
   A   
   T   
   G   
   A   
   A   
   A   
   C   
   A   
   G   
   G   
    G    
   C   
   G   
   A   
   G   
   G   
   A   
   A   
   A   
   A   
   G   
   T   
   T   
   A   
    T    
   T   
   C   
   T   
   T   
   C   
   G   
   T   
   T   
   C   
   G   
   A   
   A   
   A   
   A   
   C   
   A   
   A   
   A   
   G   
   A   
   T   
   A   
   G   
   A   
    A    
   G   
   C   
   A   
   A   
   T   
   G   
   A   
   A   
   G   
   G   
   G   
    C    
   A   
   A   
    T    
   A   
   A   
   A   
   C   
   A   
   G   
   C   
   A   
   A   
   A   
   T   
   C   
   G   
   T   
   G   
   A   
   T   
   C   
   A   
   C   
    T    
   G   
   A   
   A   
   A   
   T   
   C   
   C   
   C   
   A   
   T   
   A   
   T   
   G   
   A   
   A   
   G   
   T   
   A   
   A   
   A   
   T   
   A   
   A   
   A   
   G   
   C   
   G   
   A   
   C   
    G    
   T   
   T   
   G   
   G   
   T   
   G   
   A   
   A   
   A   
   A   
   A   
   A   
   A   
   T   
   G   
   G   
   A   
   T   
   G   
   A   
   G   
   A   
   T   
   C   
   C   
   G   
   T   
   T   
   T   
   G   
   A   
   A   
   T   
   A   
   A   
   G   
   A   
   A   
   A   
   A   
   T   
   C   
   G   
   A   
    T    
   G   
   G   
   A   
   A   
   T   
   A   
   G   
   C   
   T   
   G   
   A   
   A   
   G   
   T   
    A    
   C   
   G   
   A   
   G   
   A   
   T   
   G   
   A   
   A   
   A   
   G   
   T   
   G   
   A   
   T   
    C    
   G   
    A    
   A   
   C   
    T    
   G   
   G   
   T   
   C   
   T   
   G   
   C   
   A   
   A   
   A   
   T   
   T   
   G   
   T   
   C   
   G   
   T   
    T    
   G   
   A   
   A   
   T   
   T   
   G   
   A   
   A   
   A   
   A   
   A   
   A   
   G   
   A   
    T    
   G   
   C   
   C   
   A   
   A   
    T    
   G   
   C   
   T   
   C   
   A   
   A   
   G   
   G   
    A    
   A   
   T   
   T   
   T   
   T   
   G   
   A   
   A   
   T   
   T   
   A   
    T    
   T   
   T   
   G   
   T   
   T   
   C   
   A   
   A   
   A   
   A   
   A   
   C   
   A   
   C   
   T   
   G   
   A   
   G   
    C    
   T   
   A   
   C   
   A   
   G   
   A   
   T   
   C   
   A   
   A   
   T   
   T   
   A   
   C   
   A   
   A   
   T   
   T   
   T   
    T    
   A   
   A   
   T   
   A   
   T   
   G   
   G   
   T   
   C   
   G   
   C   
   T   
   A   
   T   
   C   
   G   
   A   
    T    
   C   
   A   
   T   
   A   
   T   
   G   
   A   
   C   
   T   
   C   
   C   
   G   
   C   
   A   
    C    
   C   
   A   
   A   
   G   
   T   
   T   
   G   
   G   
   A   
   C   
   T   
   G   
   A   
   A   
   A   
   G   
   A   
   C   
   A   
   T   
   T   
   T   
   T   
   A   
   A   
   G   
   A   
   A   
   G   
   T   
   T   
   A   
   T   
   A   
   T   
   C   
   G   
   A   
   G   
   C   
   A   
   T   
   C   
   G   
   A   
   A   
   A   
   G   
   C   
   A   
   A   
   G   
   T   
   C   
   A   
   T   
   A   
   A   
   C   
   A   
   A   
   A   
   A   
   C   
   G   
    C    
   A   
   G   
    C    
   C   
   A   
   A   
   T   
   T   
   C   
   G   
   A   
   T   
   T   
   T   
   A   
   G   
   C   
   C   
   A   
   A   
   A   
   G   
   C   
   T   
   C   
   A   
   A   
   A   
   A   
   A   
    A    
   G   
   A   
   C   
   A   
   G   
   C   
   A   
   T   
   A   
   T   
   C   
   G   
   T   
    G    
   G   
   A   
   A   
   G   
   G   
    C    
    C    
   T   
   G   
   A   
   T   
   G   
   A   
   A   
   A   
   G   
   C   
   T   
   T   
   T   
   A   
   T   
   C   
   G   
   A   
   T   
   T   
   C   
   T   
   G   
   G   
   A   
   T   
   G   
   A   
   A   
   G   
   T   
   A   
   A   
   T   
   C   
   G   
    C    
   A   
   A   
   C   
   G   
   A   
   T   
   T   
   C   
   G   
   T   
   G   
   A   
   A   
   A   
   G   
   T   
   A   
   A   
   A   
   G   
   A   
   C   
   A   
   A   
   A   
   A   
   A   
   A   
   G   
   A   
    T    
   G   
   C   
    T    
   A   
   A   
   A   
   A   
   A   
   G   
   A   
   A   
   T   
   T   
   T   
   A   
   G   
   T   
   C   
   G   
   A   
    T    
   G   
   T   
    T    
   T   
   T   
    C    
   C   
   A   
   A   
   T   
   T   
   T   
   A   
   C   
   A   
   G   
   A   
   A   
   G   
   A   
   A   
   C   
   A   
   A   
   G   
   C   
   A   
   G   
   A   
   A   
   G   
   C   
   A   
   A   
   T   
    C    
   G   
   T   
   C   
   A   
   C   
   A   
   T   
   T   
   G   
   C   
   A   
   A   
    C    
   T   
   A   
   T   
   A   
    C    
   C   
   G   
   T   
   T   
   T   
   G   
   A   
   C   
   G   
   A   
   A   
    C    
   A   
   C   
    A    
   G   
   A   
   T   
   A   
   T   
   C   
   A   
   C   
   A   
   G   
   A   
   G   
   C   
   T   
   T   
   C   
   A   
   A   
   A   
   A   
   G   
   G   
   A   
   A   
    G    
   C   
    C    
   G   
   A   
   A   
   A   
    G    
   C   
   T   
   T   
   G   
   A   
   T   
   T   
   G   
   C   
    A    
   C   
   A   
   A   
   A   
   T   
   C   
   A   
   C   
   A   
   G   
   A   
   G   
   T   
   T   
    G    
   A   
   A   
   T   
   A   
   A   
   A   
   A   
   T   
   C   
   C   
   T   
   T   
   T   
   C   
   A   
   A   
   A   
    C    
   G   
   A   
   C   
   A   
   A   
   A   
   G   
   A   
    G    
   C   
   T   
   T   
   T   
   T   
    C    
   T   
   C   
   T   
   G   
   T   
   T   
   A   
   T   
   G   
   A   
   A   
   A   
   A   
   A   
   A   
   G   
   A   
   A   
   T   
   T   
   G   
   C   
   G   
   A   
   G   
   A   
   A   
   G   
   T   
   G   
   A   
   A   
   A   
   A   
   A   
   A   
   A   
   A   
   T   
   T   
   A   
    C    
   T   
   C   
   T   
   T   
   C   
   C   
   G   
   C   
   T   
   C   
   G   
   T   
   C   
    T    
    G    
   A   
   C   
   G   
   A   
   C   
   A   
   A   
   T   
   C   
    C    
   A   
   A   
   G   
   A   
   C   
   G   
   A   
   A   
   A   
   T   
   C   
   G   
   A   
   A   
   G   
   A   
   A   
   A   
   T   
   C   
   A   
   A   
   A   
   A   
   T   
    C    
   G   
   A   
   T   
   A   
   C   
   G   
   C   
   A   
   A   
    G    
   T   
    T    
   T   
   T   
   A   
   G   
   T   
   G   
   G   
   C   
    G    
   C   
   A   
   A   
   G   
   A   
   A   
   G   
   A   
   C   
   G   
   T   
   A   
   A   
   T   
    C    
   G   
   T   
   C   
   A   
   G   
   T   
   G   
   T   
    G    
   A   
   C   
    G    
   C   
   G   
   A   
   G   
   A   
   A   
   G   
   G   
   C   
   T   
   A   
   T   
   G   
   T   
   G   
   A   
   A   
   G   
   C   
   G   
   A   
   A   
   C   
   A   
   A   
   G   
   C   
   C   
   T   
   T   
   C   
   G   
   T   
   T   
   C   
    T    
   T   
   A   
   T   
   A   
   G   
   T   
   G   
   C   
   T   
   T   
   C   
    G    
   A   
   A   
   A   
   C   
   C   
   A   
   G   
   A   
   A   
   G   
   A   
   A   
   A   
   T   
   C   
   G   
   G   
   G   
   A   
   T   
   G   
   C   
   G   
   A   
   G   
   A   
   A   
   G   
   G   
   C   
   G   
   A   
    T    
   T   
   A   
   C   
   T   
   T   
   G   
   C   
   T   
    G    
   T   
   A   
   C   
    A    
   C   
   A   
   G   
   G   
   T   
   G   
   A   
   A   
   T   
   T   
    G    
   A   
   G   
    C    
   A   
   C   
   A   
    T    
   T   
   G   
   G   
   A   
   C   
   C   
   A   
   T   
   G   
   T   
   T   
   C   
   T   
   T   
   C   
   T   
   G   
   A   
   T   
    T    
   A   
   C   
    G    
   A   
   A   
   T   
   A   
   A   
   A   
   G   
   C   
   A   
   A   
   A   
   T   
   G   
   T   
   G   
   A   
   T   
   C   
   T   
   A   
   C   
   C   
   G   
    T    
   C   
   C   
   T   
   G   
   T   
    T    
   C   
   A   
   T   
   G   
   A   
   A   
   C   
   T   
   A   
   C   
   C   
    C    
   G   
   A   
   T   
   T   
   T   
   G   
   A   
   A   
   A   
   T   
   G   
   G   
   A   
   A   
   A   
   G   
   A   
   T   
   G   
   C   
   T   
   G   
   G   
    C    
   G   
   A   
    A    
   C   
   A   
    C    
   A   
   T   
   T   
   T   
   C   
   T   
   C   
   A   
   A   
   A   
   C   
   A   
   A   
   T   
   C   
   T   
   C   
   C   
   A   
   A   
   T   
   C   
   T   
   G   
   T   
   C   
   T   
   G   
   T   
   A   
   G   
   A   
    T    
   G   
   A   
   A   
   T   
   C   
   G   
   A   
   T   
    C    
   T   
   T   
   A   
   G   
   C   
   T   
   G   
   T   
   T   
   T   
   T   
    C    
   C   
   C   
   A   
   T   
   A   
   T   
   C   
   A   
   A   
   A   
   A   
   A   
   A   
   T   
    C    
   C   
   A   
   A   
   G   
   C   
   A   
   G   
   A   
    A    
   A   
   A   
   A   
   A   
   C   
   A   
   T   
   T   
   T   
   G   
   T   
    A    
   T   
   T   
   C   
   A   
   T   
   C   
   A   
   G   
   C   
   A   
   A   
   G   
   A   
   A   
   T   
   G   
   G   
   A   
   T   
   T   
    G    
    A    
   T   
   C   
   A   
   A   
   G   
   C   
   A   
   A   
   A   
   C   
   A   
   C   
   G   
   A   
   A   
   T   
   G   
   A   
   C   
   A   
   G   
   A   
   G   
   T   
   T   
   C   
   G   
   A   
   A   
   C   
   C   
   A   
   T   
   G   
   G   
   C   
   G   
   C   
   A   
   C   
   C   
   T   
   A   
    T    
   A   
   A   
   G   
   A   
   G   
   C   
   C   
   G   
    T    
   C   
   C   
   G   
   C   
   T   
   C   
   A   
   G   
   C   
   T   
   G   
   T   
   A   
   T   
   G   
   A   
   A   
   G   
   C   
   T   
   A   
   A   
   A   
   G   
   G   
   C   
   G   
   G   
   A   
   A   
   G   
   A   
   A   
   G   
   A   
   T   
   G   
   A   
   A   
   C   
   T   
    T    
   A   
   T   
   T   
   G   
   C   
   T   
   G   
   T   
    A    
   T   
   A   
   T   
   C   
   T   
   G   
   A   
   A   
   A   
   G   
   A   
   A   
   G   
   A   
   T   
   C   
    A    
   G   
   C   
   C   
   A   
   G   
   G   
   C   
   T   
   T   
   A   
   G   
   A   
   T   
   G   
   T   
    G    
   T   
   T   
   C   
   T   
   T   
   A   
   G   
   T   
   A   
   A   
   C   
   G   
    C    
   A   
   T   
   C   
   A   
   A   
   G   
   G   
   A   
   A   
   T   
   G   
   G   
   G   
    C    
   T   
   T   
   G   
   C   
   G   
   C   
   T   
    A    
   T   
   C   
   C   
   T   
   T   
   T   
   A   
   A   
    C    
   A   
   G   
   A   
   T   
   G   
   T   
   T   
   C   
   C   
   C   
   G   
   T   
   T   
   G   
   T   
   T   
   G   
   G   
   A   
   G   
   C   
   A   
   A   
   A   
   G   
   G   
   C   
   A   
   G   
    C    
   T   
   G   
   G   
   A   
   G   
   T   
   G   
   A   
   A   
   G   
   T   
   C   
   T   
   A   
   T   
   G   
   A   
   A   
   T   
   T   
   T   
   G   
   A   
   A   
   A   
   G   
   A   
   T   
   G   
   G   
   A   
   G   
   A   
   T   
   A   
   G   
   G   
    A    
   T   
   T   
    G    
   T   
   C   
   A   
   A   
    T    
   G   
   G   
    G    
   C   
   T   
    G    
   C   
   T   
   T   
   G   
   T   
   G   
   T   
   T   
   A   
   G   
   C   
   A   
   G   
   A   
    C    
   G   
   G   
    A    
   G   
   A   
   T   
   A   
   C   
   G   
   C   
   C   
    A    
   A   
   T   
   C   
   G   
   T   
   A   
   A   
   T   
   C   
   G   
   T   
   T   
   A   
   C   
   T   
   C   
   A   
   G   
   C   
   G   
   A   
   G   
   G   
   A   
   G   
   C   
    C    
   G   
   T   
    G    
   A   
   A   
   A   
   C   
   G   
   C   
   A   
   T   
   G   
   C   
   T   
   T   
   G   
   C   
   T   
   C   
   A   
   A   
   G   
   A   
   A   
   A   
   T   
   C   
   A   
   G   
   C   
   C   
   A   
   A   
   A   
   C   
    T    
   T   
   C   
   A   
   C   
   G   
   T   
   G   
   C   
   G   
   A   
   A   
   G   
   C   
   G   
   G   
   G   
   G   
   A   
   G   
   T   
   G   
   A   
   C   
   G   
   G   
   T   
   C   
   A   
   T   
   G   
   C   
   G   
   T   
   G   
   A   
   G   
   C   
   T   
   G   
   A   
   A   
   A   
   A   
   A   
   A   
   A   
   A   
   T   
   C   
   C   
    G    
   C   
   A   
    C    
   C   
   G   
    T    
   A   
   T   
   T   
   A   
   T   
   C   
   T   
   A   
   T   
   A   
   T   
   G   
   T   
   C   
   G   
   G   
   A   
   A   
   G   
   G   
   A   
   A   
   G   
   A   
   T   
   C   
   A   
   A   
   A   
   A   
   G   
   A   
   A   
   A   
   T   
   A   
   A   
   C   
   C   
   T   
   T   
   G   
   A   
   T   
   G   
   A   
   A   
   T   
   C   
   A   
   A   
   A   
   A   
   A   
   G   
   G   
    A    
   C   
   A   
    A    
   C   
   A   
   G   
    A    
   T   
   A   
   A   
   C   
   G   
   A   
   T   
   T   
   G   
   A   
    T    
   C   
   C   
   A   
   A   
   C   
    G    
   G   
   A   
   T   
   T   
   T   
    T    
   C   
   C   
   T   
   A   
   T   
   A   
   G   
   G   
   T   
   G   
   A   
   T   
   C   
   G   
   A   
   A   
   C   
   A   
   T   
   C   
   G   
   A   
   A   
   T   
   G   
   G   
    T    
   T   
   C   
    G    
   T   
   T   
   T   
   G   
   C   
    T    
   A   
   T   
   G   
   G   
   A   
    C    
   G   
   A   
   G   
   A   
   A   
   A   
   A   
   A   
   A   
   G   
   G   
   T   
   G   
   G   
   A   
   G   
   A   
   A   
   G   
   T   
   C   
   A   
   T   
   C   
   G   
   A   
   A   
   G   
   T   
    T    
   A   
    T    
   C   
   G   
   A   
   T   
   G   
   C   
   T   
   C   
   C   
   T   
   G   
   A   
   G   
    A    
   T   
   T   
   T   
   T   
    C    
   A   
   T   
    T    
   G   
   A   
    A    
    T    
   C   
    A    
   -   
   -   
   -   
 

 
   N55299   
   A   
   T   
   G   
   G   
   A   
   A   
   A   
   A   
   T   
   C   
   G   
   A   
   C   
   A   
   A   
   G   
   A   
   A   
   G   
   T   
   A   
   C   
   A   
   A   
   G   
   A   
   A   
   T   
   T   
   A   
   A   
   C   
   T   
   T   
   T   
   A   
   G   
   A   
   A   
   G   
   A   
   A   
   G   
   T   
   G   
   A   
   T   
   G   
   G   
   G   
   T   
   G   
   A   
   C   
   C   
   G   
   C   
   T   
   T   
   C   
   G   
   G   
   A   
   C   
   G   
   T   
   T   
   A   
   T   
   T   
   C   
   C   
   A   
   A   
   A   
   T   
   A   
   T   
   A   
   T   
   T   
   A   
   T   
    C    
   C   
   A   
    A    
   G   
   A   
   A   
   C   
   G   
   T   
   G   
   C   
   A   
   C   
   T   
   G   
   C   
   C   
   T   
   G   
   A   
   T   
   A   
   T   
    C    
   C   
   G   
   T   
   G   
   A   
   T   
   G   
   G   
   A   
   T   
   T   
   G   
   A   
   A   
    G    
   C   
   C   
   A   
   G   
   T   
    G    
   C   
   A   
   A   
   C   
   G   
   C   
   C   
   G   
   G   
   A   
   T   
   A   
   T   
   T   
   A   
   T   
   T   
    T    
   G   
   C   
    C    
   A   
   T   
   G   
   A   
   A   
   C   
   A   
   A   
   A   
   G   
   A   
   T   
   G   
   G   
   C   
   A   
   A   
   T   
   A   
   C   
   G   
   T   
   A   
   T   
   G   
   A   
   C   
   A   
   A   
   A   
   G   
   G   
   A   
   T   
   T   
    C    
   C   
   G   
   T   
   A   
   A   
   A   
   T   
   C   
   A   
   G   
   C   
   A   
   A   
   A   
   A   
   T   
   C   
   T   
   G   
   T   
   C   
   G   
   G   
   A   
   A   
   A   
   T   
   A   
   T   
   C   
   A   
   T   
   G   
   G   
   G   
    G    
   A   
   A   
   T   
   T   
   A   
   T   
   C   
   A   
   T   
   C   
   C   
   T   
   C   
   A   
   T   
   G   
   G   
   T   
   G   
   A   
   C   
   A   
   G   
   C   
   A   
   G   
   T   
   A   
   T   
   T   
   T   
   A   
   T   
   G   
   A   
   A   
   G   
   C   
   G   
   A   
   T   
   G   
   G   
   T   
   C   
   C   
   G   
   C   
   A   
   T   
   G   
   A   
   G   
   C   
   C   
   A   
   A   
   G   
   A   
    C    
   T   
   G   
   G   
   A   
   A   
   G   
   T   
   T   
   G   
   C   
   G   
   G   
   G   
   A   
   A   
   G   
   T   
   A   
   C   
   T   
   G   
   A   
   T   
   C   
   G   
   A   
    A    
   A   
   T   
   G   
   C   
   A   
   C   
   G   
   G   
    G    
   A   
   A   
   T   
   A   
   A   
    C    
   G   
   G   
   G   
   A   
   G   
   T   
   A   
   T   
   G   
   G   
   A   
   C   
   G   
   G   
   T   
   G   
   A   
   T   
   C   
   C   
   A   
   C   
   C   
   T   
   G   
   C   
   T   
   G   
   C   
   G   
   A   
   T   
   G   
   C   
   G   
   T   
   T   
   A   
   T   
   A   
   C   
   T   
   G   
   A   
   A   
   G   
   C   
   C   
   A   
   G   
    A    
   T   
   T   
   A   
   T   
   C   
   C   
   A   
   A   
   G   
   T   
   T   
   A   
   A   
   G   
   T   
   G   
   G   
   A   
   G   
   A   
   G   
   A   
   T   
   G   
    C    
   T   
   A   
    G    
   C   
   A   
   G   
   A   
   T   
   A   
   T   
   C   
   G   
   A   
    A    
   A   
   A   
   A   
   G   
   A   
   A   
   A   
   C   
   A   
   G   
   T   
    C    
   G   
   A   
   T   
   C   
   T   
   C   
   G   
   T   
   G   
   T   
   G   
   G   
   A   
   A   
   T   
   T   
   T   
   T   
   G   
   A   
   T   
   G   
   A   
   T   
   A   
   C   
   A   
   G   
   A   
   A   
   A   
   A   
   A   
   G   
   A   
   A   
   C   
   C   
   A   
   A   
   C   
   A   
   G   
   T   
   C   
   C   
   T   
   T   
   C   
   C   
   A   
   G   
   C   
   T   
   C   
   G   
   A   
   T   
   A   
   C   
   C   
   C   
   A   
   A   
   A   
   C   
   C   
   T   
   T   
   C   
   T   
    C    
   G   
   T   
   C   
   A   
   A   
   T   
   G   
   G   
   A   
   T   
   C   
   G   
   A   
   C   
   A   
   G   
   G   
   T   
   A   
   T   
   A   
   T   
   C   
   T   
   G   
   C   
   T   
   G   
   G   
   A   
   T   
   A   
   T   
   G   
   C   
    A    
   A   
   C   
   A   
   G   
   A   
   A   
   A   
   T   
   C   
   C   
   C   
   G   
   A   
   C   
    T    
   C   
   A   
   T   
   A   
   A   
   C   
   T   
   T   
   A   
   G   
   C   
   T   
   G   
   A   
   A   
   G   
   T   
   G   
   A   
   T   
    C    
   G   
   A   
    T    
   G   
   G   
   C   
   A   
   C   
   A   
   A   
   T   
   C   
   T   
   A   
   T   
   A   
   T   
   G   
   A   
   T   
   C   
   G   
   A   
   T   
   C   
   A   
   T   
   C   
   C   
   A   
   C   
   A   
   A   
   G   
   C   
   G   
   A   
   G   
   C   
   C   
   T   
   T   
   G   
   A   
   A   
   A   
   A   
   A   
   T   
   T   
   G   
   A   
   T   
   G   
   G   
   A   
   A   
   T   
   A   
   T   
   A   
   T   
   T   
   C   
   C   
   T   
   G   
   G   
   A   
   C   
   C   
   T   
   G   
   A   
   C   
   T   
   T   
    T    
   C   
   C   
   A   
   A   
   C   
   A   
   G   
   G   
   A   
   G   
   G   
    G    
   A   
   T   
   C   
   T   
   T   
   G   
   C   
   A   
   A   
   G   
   G   
    A    
   A   
   A   
   A   
   G   
   A   
   T   
   G   
   A   
   A   
   A   
   T   
   C   
   A   
   A   
   A   
   A   
   A   
   A   
   G   
   C   
   T   
   T   
   A   
   T   
   G   
   A   
   A   
   A   
   C   
   A   
   G   
   G   
    G    
   C   
   G   
   A   
   G   
   G   
   A   
   A   
   A   
   A   
   G   
   T   
   T   
   A   
    T    
   T   
   C   
   T   
   T   
   C   
   G   
   T   
   T   
   C   
   G   
   A   
   A   
   A   
   A   
   C   
   A   
   A   
   A   
   G   
   A   
   T   
   A   
   G   
   A   
    G    
   G   
   C   
   A   
   A   
   T   
   G   
   A   
   A   
   G   
   G   
   G   
    T    
   A   
   A   
    T    
   A   
   A   
   A   
   C   
   A   
   G   
   C   
   A   
   A   
   A   
   T   
   C   
   G   
   T   
   G   
   A   
   T   
   C   
   A   
   C   
    A    
   G   
   A   
   A   
   A   
   T   
   C   
   C   
   C   
   A   
   T   
   A   
   T   
   G   
   A   
   A   
   G   
   T   
   A   
   A   
   A   
   T   
   A   
   A   
   A   
   G   
   C   
   G   
   A   
   C   
    C    
   T   
   T   
   G   
   G   
   T   
   G   
   A   
   A   
   A   
   A   
   A   
   A   
   A   
   T   
   G   
   G   
   A   
   T   
   G   
   A   
   G   
   A   
   T   
   C   
   C   
   G   
   T   
   T   
   T   
   G   
   A   
   A   
   T   
   A   
   A   
   G   
   A   
   A   
   A   
   A   
   T   
   C   
   G   
   A   
    T    
   G   
   G   
   A   
   A   
   T   
   A   
   G   
   C   
   T   
   G   
   A   
   A   
   G   
   T   
    A    
   C   
   G   
   A   
   G   
   A   
   T   
   G   
   A   
   A   
   A   
   G   
   T   
   G   
   A   
   T   
    A    
   G   
    A    
   A   
   C   
    A    
   G   
   G   
   T   
   C   
   T   
   G   
   C   
   A   
   A   
   A   
   T   
   T   
   G   
   T   
   C   
   G   
   T   
    C    
   G   
   A   
   A   
   T   
   T   
   G   
   A   
   A   
   A   
   A   
   A   
   A   
   G   
   A   
    C    
   G   
   C   
   C   
   A   
   A   
    T    
   G   
   C   
   T   
   C   
   A   
   A   
   G   
   G   
    A    
   A   
   T   
   T   
   T   
   T   
   G   
   A   
   A   
   T   
   T   
   A   
    T    
   T   
   T   
   G   
   T   
   T   
   C   
   A   
   A   
   A   
   A   
   A   
   C   
   A   
   C   
   T   
   G   
   A   
   G   
    C    
   T   
   A   
   C   
   A   
   G   
   A   
   T   
   C   
   A   
   A   
   T   
   T   
   A   
   C   
   A   
   A   
   T   
   T   
   T   
    C    
   A   
   A   
   T   
   A   
   T   
   G   
   G   
   T   
   C   
   G   
   C   
   T   
   A   
   T   
   C   
   G   
   A   
    C    
   C   
   A   
   T   
   A   
   T   
   G   
   A   
   C   
   T   
   C   
   C   
   G   
   C   
   A   
    T    
   C   
   A   
   A   
   G   
   T   
   T   
   G   
   G   
   A   
   C   
   T   
   G   
   A   
   A   
   A   
   G   
   A   
   C   
   A   
   T   
   T   
   T   
   T   
   A   
   A   
   G   
   A   
   A   
   G   
   T   
   T   
   A   
   T   
   A   
   T   
   C   
   G   
   A   
   G   
   C   
   A   
   T   
   C   
   G   
   A   
   A   
   A   
   G   
   C   
   A   
   A   
   G   
   T   
   C   
   A   
   T   
   A   
   A   
   C   
   A   
   A   
   A   
   A   
   C   
   G   
    T    
   A   
   G   
    T    
   C   
   A   
   A   
   T   
   T   
   C   
   G   
   A   
   T   
   T   
   T   
   A   
   G   
   C   
   C   
   A   
   A   
   A   
   G   
   C   
   T   
   C   
   A   
   A   
   A   
   A   
   A   
    A    
   G   
   A   
   C   
   A   
   G   
   C   
   A   
   T   
   A   
   T   
   C   
   G   
   T   
    A    
   G   
   A   
   A   
   G   
   G   
    A    
    T    
   T   
   G   
   A   
   T   
   G   
   A   
   A   
   A   
   G   
   C   
   T   
   T   
   T   
   A   
   T   
   C   
   G   
   A   
   T   
   T   
   C   
   T   
   G   
   G   
   A   
   T   
   G   
   A   
   A   
   G   
   T   
   A   
   A   
   T   
   C   
   G   
    C    
   A   
   A   
   C   
   G   
   A   
   T   
   T   
   C   
   G   
   T   
   G   
   A   
   A   
   A   
   G   
   T   
   A   
   A   
   A   
   G   
   A   
   C   
   A   
   A   
   A   
   A   
   A   
   A   
   G   
   A   
    C    
   G   
   C   
    C    
   A   
   A   
   A   
   A   
   A   
   G   
   A   
   A   
   T   
   T   
   T   
   A   
   G   
   T   
   C   
   G   
   A   
    C    
   G   
   T   
    C    
   T   
   T   
    T    
   C   
   A   
   A   
   T   
   T   
   T   
   A   
   C   
   A   
   G   
   A   
   A   
   G   
   A   
   A   
   C   
   A   
   A   
   G   
   C   
   A   
   G   
   A   
   A   
   G   
   C   
   A   
   A   
   T   
    T    
   G   
   T   
   C   
   A   
   C   
   A   
   T   
   T   
   G   
   C   
   A   
   A   
    T    
   T   
   A   
   T   
   A   
    C    
   C   
   G   
   T   
   T   
   T   
   G   
   A   
   C   
   G   
   A   
   A   
    C    
   A   
   C   
    G    
   G   
   A   
   T   
   A   
   T   
   C   
   A   
   C   
   A   
   G   
   A   
   G   
   C   
   T   
   T   
   C   
   A   
   A   
   A   
   A   
   G   
   G   
   A   
   A   
    T    
   C   
    C    
   G   
   A   
   A   
   A   
    G    
   C   
   T   
   T   
   G   
   A   
   T   
   T   
   G   
   C   
    G    
   C   
   A   
   A   
   A   
   T   
   C   
   A   
   C   
   A   
   G   
   A   
   G   
   T   
   T   
    G    
   A   
   A   
   T   
   A   
   A   
   A   
   A   
   T   
   C   
   C   
   T   
   T   
   T   
   C   
   A   
   A   
   A   
    T    
   G   
   A   
   C   
   A   
   A   
   A   
   G   
   A   
    G    
   C   
   T   
   T   
   T   
   T   
    C    
   T   
   C   
   T   
   G   
   T   
   T   
   A   
   T   
   G   
   A   
   A   
   A   
   A   
   A   
   A   
   G   
   A   
   A   
   T   
   T   
   G   
   C   
   G   
   A   
   G   
   A   
   A   
   G   
   T   
   G   
   A   
   A   
   A   
   A   
   A   
   A   
   A   
   A   
   T   
   T   
   A   
    T    
   T   
   C   
   T   
   T   
   C   
   C   
   G   
   C   
   T   
   C   
   G   
   T   
   C   
    A    
    A    
   A   
   C   
   G   
   A   
   C   
   A   
   A   
   T   
   C   
    G    
   A   
   A   
   G   
   A   
   C   
   G   
   A   
   A   
   A   
   T   
   C   
   G   
   A   
   A   
   G   
   A   
   A   
   A   
   T   
   C   
   A   
   A   
   A   
   A   
   T   
    T    
   G   
   A   
   T   
   A   
   C   
   G   
   C   
   A   
   A   
    G    
   T   
    C    
   T   
   T   
   A   
   G   
   T   
   G   
   G   
   C   
    A    
   C   
   A   
   A   
   G   
   A   
   A   
   G   
   A   
   C   
   G   
   T   
   A   
   A   
   T   
    T    
   G   
   T   
   C   
   A   
   G   
   T   
   G   
   T   
    G    
   A   
   C   
    G    
   C   
   G   
   A   
   G   
   A   
   A   
   G   
   G   
   C   
   T   
   A   
   T   
   G   
   T   
   G   
   A   
   A   
   G   
   C   
   G   
   A   
   A   
   C   
   A   
   A   
   G   
   C   
   C   
   T   
   T   
   C   
   G   
   T   
   T   
   C   
    T    
   T   
   A   
   T   
   A   
   G   
   T   
   G   
   C   
   T   
   T   
   C   
    C    
   A   
   A   
   A   
   C   
   C   
   A   
   G   
   A   
   A   
   G   
   A   
   A   
   A   
   T   
   C   
   G   
   G   
   G   
   A   
   T   
   G   
   C   
   G   
   A   
   G   
   A   
   A   
   G   
   G   
   C   
   G   
   A   
    C    
   T   
   A   
   C   
   T   
   T   
   G   
   C   
   T   
    A    
   T   
   A   
   C   
    T    
   C   
   A   
   G   
   G   
   T   
   G   
   A   
   A   
   T   
   T   
    G    
   A   
   G   
    T    
   A   
   C   
   A   
    T    
   T   
   G   
   G   
   A   
   C   
   C   
   A   
   T   
   G   
   T   
   T   
   C   
   T   
   T   
   C   
   T   
   G   
   A   
   T   
    C    
   A   
   C   
    A    
   A   
   A   
   T   
   A   
   A   
   A   
   G   
   C   
   A   
   A   
   A   
   T   
   G   
   T   
   G   
   A   
   T   
   C   
   T   
   A   
   C   
   C   
   G   
    C    
   C   
   C   
   T   
   G   
   T   
    C    
   C   
   A   
   T   
   G   
   A   
   A   
   C   
   T   
   A   
   C   
   C   
    C    
   G   
   A   
   T   
   T   
   T   
   G   
   A   
   A   
   A   
   T   
   G   
   G   
   A   
   A   
   A   
   G   
   A   
   T   
   G   
   C   
   T   
   G   
   G   
    T    
   G   
   A   
    G    
   C   
   A   
    C    
   A   
   T   
   T   
   T   
   C   
   T   
   C   
   A   
   A   
   A   
   C   
   A   
   A   
   T   
   C   
   T   
   C   
   C   
   A   
   A   
   T   
   C   
   T   
   G   
   T   
   C   
   T   
   G   
   T   
   A   
   G   
   A   
    C    
   G   
   A   
   A   
   T   
   C   
   G   
   A   
   T   
    T    
   T   
   T   
   A   
   G   
   C   
   T   
   G   
   T   
   T   
   T   
   T   
    C    
   C   
   C   
   A   
   T   
   A   
   T   
   C   
   A   
   A   
   A   
   A   
   A   
   A   
   T   
    T    
   C   
   A   
   A   
   G   
   C   
   A   
   G   
   A   
    G    
   A   
   A   
   A   
   A   
   C   
   A   
   T   
   T   
   T   
   G   
   T   
    A    
   T   
   T   
   C   
   A   
   T   
   C   
   A   
   G   
   C   
   A   
   A   
   G   
   A   
   A   
   T   
   G   
   G   
   A   
   T   
   T   
    G    
    A    
   T   
   C   
   A   
   A   
   G   
   C   
   A   
   A   
   A   
   C   
   A   
   C   
   G   
   A   
   A   
   T   
   G   
   A   
   C   
   A   
   G   
   A   
   G   
   T   
   T   
   C   
   G   
   A   
   A   
   C   
   C   
   A   
   T   
   G   
   G   
   C   
   G   
   C   
   A   
   C   
   C   
   T   
   A   
    T    
   A   
   A   
   G   
   A   
   G   
   C   
   C   
   G   
    T    
   C   
   C   
   G   
   C   
   T   
   C   
   A   
   G   
   C   
   T   
   G   
   T   
   A   
   T   
   G   
   A   
   A   
   G   
   C   
   T   
   A   
   A   
   A   
   G   
   G   
   C   
   G   
   G   
   A   
   A   
   G   
   A   
   A   
   G   
   A   
   T   
   G   
   A   
   A   
   C   
   T   
    G    
   A   
   T   
   T   
   G   
   C   
   T   
   G   
   T   
    G    
   T   
   A   
   T   
   C   
   T   
   G   
   A   
   A   
   A   
   G   
   A   
   A   
   G   
   A   
   T   
   C   
    A    
   G   
   C   
   C   
   A   
   G   
   G   
   C   
   T   
   T   
   A   
   G   
   A   
   T   
   G   
   T   
    T    
   T   
   T   
   C   
   T   
   T   
   A   
   G   
   T   
   A   
   A   
   C   
   G   
    C    
   A   
   T   
   C   
   A   
   A   
   G   
   G   
   A   
   A   
   T   
   G   
   G   
   G   
    T    
   T   
   T   
   G   
   C   
   G   
   C   
   T   
    A    
   T   
   C   
   C   
   T   
   T   
   T   
   A   
   A   
    C    
   A   
   G   
   A   
   T   
   G   
   T   
   T   
   C   
   C   
   C   
   G   
   T   
   T   
   G   
   T   
   T   
   G   
   G   
   A   
   G   
   C   
   A   
   A   
   A   
   G   
   G   
   C   
   A   
   G   
    C    
   T   
   G   
   G   
   A   
   G   
   T   
   G   
   A   
   A   
   G   
   T   
   C   
   T   
   A   
   T   
   G   
   A   
   A   
   T   
   T   
   T   
   G   
   A   
   A   
   A   
   G   
   A   
   T   
   G   
   G   
   A   
   G   
   A   
   T   
   A   
   G   
   G   
    A    
   T   
   T   
    A    
   T   
   C   
   A   
   A   
    C    
   G   
   G   
    G    
   C   
   T   
    G    
   C   
   T   
   T   
   G   
   T   
   G   
   T   
   T   
   A   
   G   
   C   
   A   
   G   
   A   
    A    
   G   
   G   
    T    
   G   
   A   
   T   
   A   
   C   
   G   
   C   
   C   
    G    
   A   
   T   
   C   
   G   
   T   
   A   
   A   
   T   
   C   
   G   
   T   
   T   
   A   
   C   
   T   
   C   
   A   
   G   
   C   
   G   
   A   
   G   
   G   
   A   
   G   
   C   
    T    
   G   
   T   
    T    
   A   
   A   
   A   
   C   
   G   
   C   
   A   
   T   
   G   
   C   
   T   
   T   
   G   
   C   
   T   
   C   
   A   
   A   
   G   
   A   
   A   
   A   
   T   
   C   
   A   
   G   
   C   
   C   
   A   
   A   
   A   
   C   
    T    
   T   
   C   
   A   
   C   
   G   
   T   
   G   
   C   
   G   
   A   
   A   
   G   
   C   
   G   
   G   
   G   
   G   
   A   
   G   
   T   
   G   
   A   
   C   
   G   
   G   
   T   
   C   
   A   
   T   
   G   
   C   
   G   
   T   
   G   
   A   
   G   
   C   
   T   
   G   
   A   
   A   
   A   
   A   
   A   
   A   
   A   
   A   
   T   
   C   
   C   
    G    
   C   
   A   
    C    
   C   
   G   
    T    
   A   
   T   
   T   
   A   
   T   
   C   
   T   
   A   
   T   
   A   
   T   
   G   
   T   
   C   
   G   
   G   
   A   
   A   
   G   
   G   
   A   
   A   
   G   
   A   
   T   
   C   
   A   
   A   
   A   
   A   
   G   
   A   
   A   
   A   
   T   
   A   
   A   
   C   
   C   
   T   
   T   
   G   
   A   
   T   
   G   
   A   
   A   
   T   
   C   
   A   
   A   
   A   
   A   
   A   
   G   
   G   
    G    
   C   
   A   
    G    
   C   
   A   
   G   
    T    
   T   
   A   
   A   
   C   
   G   
   A   
   T   
   T   
   G   
   A   
    C    
   C   
   C   
   A   
   A   
   C   
    T    
   G   
   A   
   T   
   T   
   T   
    C    
   C   
   C   
   T   
   A   
   T   
   A   
   G   
   G   
   T   
   G   
   A   
   T   
   C   
   G   
   A   
   A   
   C   
   A   
   T   
   C   
   G   
   A   
   A   
   T   
   G   
   G   
    A    
   T   
   C   
    A    
   T   
   T   
   T   
   G   
   C   
    G    
   A   
   T   
   G   
   G   
   A   
    T    
   G   
   A   
   G   
   A   
   A   
   A   
   A   
   A   
   A   
   G   
   G   
   T   
   G   
   G   
   A   
   G   
   A   
   A   
   G   
   T   
   C   
   A   
   T   
   C   
   G   
   A   
   A   
   G   
   T   
    T    
   A   
    T    
   C   
   G   
   A   
   T   
   G   
   C   
   T   
   C   
   C   
   T   
   G   
   A   
   G   
    A    
   T   
   T   
   T   
   T   
    C    
   A   
   T   
    C    
   G   
   A   
    G    
    T    
   C   
    G    
   -   
   -   
   -   
 

 
   N60417F   
   A   
   T   
   G   
   G   
   A   
   A   
   A   
   A   
   T   
   C   
   G   
   A   
   C   
   A   
   A   
   G   
   A   
   A   
   G   
   T   
   A   
   C   
   A   
   A   
   G   
   A   
   A   
   T   
   T   
   A   
   A   
   C   
   T   
   T   
   T   
   A   
   G   
   A   
   A   
   G   
   A   
   A   
   G   
   T   
   G   
   A   
   T   
   G   
   G   
   G   
   T   
   G   
   A   
   C   
   C   
   G   
   C   
   T   
   T   
   C   
   G   
   G   
   A   
   C   
   G   
   T   
   T   
   A   
   T   
   T   
   C   
   C   
   A   
   A   
   A   
   T   
   A   
   T   
   A   
   T   
   T   
   A   
   T   
    C    
   C   
   A   
    A    
   G   
   A   
   A   
   C   
   G   
   T   
   G   
   C   
   A   
   C   
   T   
   G   
   C   
   C   
   T   
   G   
   A   
   T   
   A   
   T   
    C    
   C   
   G   
   T   
   G   
   A   
   T   
   G   
   G   
   A   
   T   
   T   
   G   
   A   
   A   
    A    
   C   
   C   
   A   
   G   
   T   
    A    
   C   
   A   
   A   
   C   
   G   
   C   
   C   
   G   
   G   
   A   
   T   
   A   
   T   
   T   
   A   
   T   
   T   
    T    
   G   
   C   
    C    
   A   
   T   
   G   
   A   
   A   
   C   
   A   
   A   
   A   
   G   
   A   
   T   
   G   
   G   
   C   
   A   
   A   
   T   
   A   
   C   
   G   
   T   
   A   
   T   
   G   
   A   
   C   
   A   
   A   
   A   
   G   
   G   
   A   
   T   
   T   
    C    
   C   
   G   
   T   
   A   
   A   
   A   
   T   
   C   
   A   
   G   
   C   
   A   
   A   
   A   
   A   
   T   
   C   
   T   
   G   
   T   
   C   
   G   
   G   
   A   
   A   
   A   
   T   
   A   
   T   
   C   
   A   
   T   
   G   
   G   
   G   
    G    
   A   
   A   
   T   
   T   
   A   
   T   
   C   
   A   
   T   
   C   
   C   
   T   
   C   
   A   
   T   
   G   
   G   
   T   
   G   
   A   
   C   
   A   
   G   
   C   
   A   
   G   
   T   
   A   
   T   
   T   
   T   
   A   
   T   
   G   
   A   
   A   
   G   
   C   
   G   
   A   
   T   
   G   
   G   
   T   
   C   
   C   
   G   
   C   
   A   
   T   
   G   
   A   
   G   
   C   
   C   
   A   
   A   
   G   
   A   
    T    
   T   
   G   
   G   
   A   
   A   
   G   
   T   
   T   
   G   
   C   
   G   
   G   
   G   
   A   
   A   
   G   
   T   
   A   
   C   
   T   
   G   
   A   
   T   
   C   
   G   
   A   
    G    
   A   
   T   
   G   
   C   
   A   
   C   
   G   
   G   
    A    
   A   
   A   
   T   
   A   
   A   
    C    
   G   
   G   
   G   
   A   
   G   
   T   
   A   
   T   
   G   
   G   
   A   
   C   
   G   
   G   
   T   
   G   
   A   
   T   
   C   
   C   
   A   
   C   
   C   
   T   
   G   
   C   
   T   
   G   
   C   
   G   
   A   
   T   
   G   
   C   
   G   
   T   
   T   
   A   
   T   
   A   
   C   
   T   
   G   
   A   
   A   
   G   
   C   
   C   
   A   
   G   
    A    
   T   
   T   
   A   
   T   
   C   
   C   
   A   
   A   
   G   
   T   
   T   
   A   
   A   
   G   
   T   
   G   
   G   
   A   
   G   
   A   
   G   
   A   
   T   
   G   
    C    
   T   
   A   
    G    
   C   
   A   
   G   
   A   
   T   
   A   
   T   
   C   
   G   
   A   
    A    
   A   
   A   
   A   
   G   
   A   
   A   
   A   
   C   
   A   
   G   
   T   
    C    
   G   
   A   
   T   
   C   
   T   
   C   
   G   
   T   
   G   
   T   
   G   
   G   
   A   
   A   
   T   
   T   
   T   
   T   
   G   
   A   
   T   
   G   
   A   
   T   
   A   
   C   
   A   
   G   
   A   
   A   
   A   
   A   
   A   
   G   
   A   
   A   
   C   
   C   
   A   
   A   
   C   
   A   
   G   
   T   
   C   
   C   
   T   
   T   
   C   
   C   
   A   
   G   
   C   
   T   
   C   
   G   
   A   
   T   
   A   
   C   
   C   
   C   
   A   
   A   
   A   
   C   
   C   
   T   
   T   
   C   
   T   
    A    
   G   
   T   
   C   
   A   
   A   
   T   
   G   
   G   
   A   
   T   
   C   
   G   
   A   
   C   
   A   
   G   
   G   
   T   
   A   
   T   
   A   
   T   
   C   
   T   
   G   
   C   
   T   
   G   
   G   
   A   
   T   
   A   
   T   
   G   
   C   
    G    
   A   
   C   
   A   
   G   
   A   
   A   
   A   
   T   
   C   
   C   
   C   
   G   
   A   
   C   
    T    
   C   
   A   
   T   
   A   
   A   
   C   
   T   
   T   
   A   
   G   
   C   
   T   
   G   
   A   
   A   
   G   
   T   
   G   
   A   
   T   
    C    
   G   
   A   
    C    
   G   
   G   
   C   
   A   
   C   
   A   
   A   
   T   
   C   
   T   
   A   
   T   
   A   
   T   
   G   
   A   
   T   
   C   
   G   
   A   
   T   
   C   
   A   
   T   
   C   
   C   
   A   
   C   
   A   
   A   
   G   
   C   
   G   
   A   
   G   
   C   
   C   
   T   
   T   
   G   
   A   
   A   
   A   
   A   
   A   
   T   
   T   
   G   
   A   
   T   
   G   
   G   
   A   
   A   
   T   
   A   
   T   
   A   
   T   
   T   
   C   
   C   
   T   
   G   
   G   
   A   
   C   
   C   
   T   
   G   
   A   
   C   
   T   
   T   
    C    
   C   
   C   
   A   
   A   
   C   
   A   
   G   
   G   
   A   
   G   
   G   
    G    
   A   
   T   
   C   
   T   
   T   
   G   
   C   
   A   
   A   
   G   
   G   
    A    
   A   
   A   
   A   
   G   
   A   
   T   
   G   
   A   
   A   
   A   
   T   
   C   
   A   
   A   
   A   
   A   
   A   
   A   
   G   
   C   
   T   
   T   
   A   
   T   
   G   
   A   
   A   
   A   
   C   
   A   
   G   
   G   
    G    
   C   
   G   
   A   
   G   
   G   
   A   
   A   
   A   
   A   
   G   
   T   
   T   
   A   
    T    
   T   
   C   
   T   
   T   
   C   
   G   
   T   
   T   
   C   
   G   
   A   
   A   
   A   
   A   
   C   
   A   
   A   
   A   
   G   
   A   
   T   
   A   
   G   
   A   
    A    
   G   
   C   
   A   
   A   
   T   
   G   
   A   
   A   
   G   
   G   
   G   
    C    
   A   
   A   
    T    
   A   
   A   
   A   
   C   
   A   
   G   
   C   
   A   
   A   
   A   
   T   
   C   
   G   
   T   
   G   
   A   
   T   
   C   
   A   
   C   
    T    
   G   
   A   
   A   
   A   
   T   
   C   
   C   
   C   
   A   
   T   
   A   
   T   
   G   
   A   
   A   
   G   
   T   
   A   
   A   
   A   
   T   
   A   
   A   
   A   
   G   
   C   
   G   
   A   
   C   
    A    
   T   
   T   
   G   
   G   
   T   
   G   
   A   
   A   
   A   
   A   
   A   
   A   
   A   
   T   
   G   
   G   
   A   
   T   
   G   
   A   
   G   
   A   
   T   
   C   
   C   
   G   
   T   
   T   
   T   
   G   
   A   
   A   
   T   
   A   
   A   
   G   
   A   
   A   
   A   
   A   
   T   
   C   
   G   
   A   
    C    
   G   
   G   
   A   
   A   
   T   
   A   
   G   
   C   
   T   
   G   
   A   
   A   
   G   
   T   
    G    
   C   
   G   
   A   
   G   
   A   
   T   
   G   
   A   
   A   
   A   
   G   
   T   
   G   
   A   
   T   
    C    
   G   
    A    
   A   
   C   
    T    
   G   
   G   
   T   
   C   
   T   
   G   
   C   
   A   
   A   
   A   
   T   
   T   
   G   
   T   
   C   
   G   
   T   
    T    
   G   
   A   
   A   
   T   
   T   
   G   
   A   
   A   
   A   
   A   
   A   
   A   
   G   
   A   
    C    
   G   
   C   
   C   
   A   
   A   
    T    
   G   
   C   
   T   
   C   
   A   
   A   
   G   
   G   
    A    
   A   
   T   
   T   
   T   
   T   
   G   
   A   
   A   
   T   
   T   
   A   
    T    
   T   
   T   
   G   
   T   
   T   
   C   
   A   
   A   
   A   
   A   
   A   
   C   
   A   
   C   
   T   
   G   
   A   
   G   
    C    
   T   
   A   
   C   
   A   
   G   
   A   
   T   
   C   
   A   
   A   
   T   
   T   
   A   
   C   
   A   
   A   
   T   
   T   
   T   
    C    
   A   
   A   
   T   
   A   
   T   
   G   
   G   
   T   
   C   
   G   
   C   
   T   
   A   
   T   
   C   
   G   
   A   
    T    
   C   
   A   
   T   
   A   
   T   
   G   
   A   
   C   
   T   
   C   
   C   
   G   
   C   
   A   
    T    
   C   
   A   
   A   
   G   
   T   
   T   
   G   
   G   
   A   
   C   
   T   
   G   
   A   
   A   
   A   
   G   
   A   
   C   
   A   
   T   
   T   
   T   
   T   
   A   
   A   
   G   
   A   
   A   
   G   
   T   
   T   
   A   
   T   
   A   
   T   
   C   
   G   
   A   
   G   
   C   
   A   
   T   
   C   
   G   
   A   
   A   
   A   
   G   
   C   
   A   
   A   
   G   
   T   
   C   
   A   
   T   
   A   
   A   
   C   
   A   
   A   
   A   
   A   
   C   
   G   
    C    
   A   
   G   
    C    
   C   
   A   
   A   
   T   
   T   
   C   
   G   
   A   
   T   
   T   
   T   
   A   
   G   
   C   
   C   
   A   
   A   
   A   
   G   
   C   
   T   
   C   
   A   
   A   
   A   
   A   
   A   
    A    
   G   
   A   
   C   
   A   
   G   
   C   
   A   
   T   
   A   
   T   
   C   
   G   
   T   
    G    
   G   
   A   
   A   
   G   
   G   
    C    
    C    
   T   
   G   
   A   
   T   
   G   
   A   
   A   
   A   
   G   
   C   
   T   
   T   
   T   
   A   
   T   
   C   
   G   
   A   
   T   
   T   
   C   
   T   
   G   
   G   
   A   
   T   
   G   
   A   
   A   
   G   
   T   
   A   
   A   
   T   
   C   
   G   
    C    
   A   
   A   
   C   
   G   
   A   
   T   
   T   
   C   
   G   
   T   
   G   
   A   
   A   
   A   
   G   
   T   
   A   
   A   
   A   
   G   
   A   
   C   
   A   
   A   
   A   
   A   
   A   
   A   
   G   
   A   
    C    
   G   
   C   
    C    
   A   
   A   
   A   
   A   
   A   
   G   
   A   
   A   
   T   
   T   
   T   
   A   
   G   
   T   
   C   
   G   
   A   
    C    
   G   
   T   
    T    
   T   
   T   
    C    
   C   
   A   
   A   
   T   
   T   
   T   
   A   
   C   
   A   
   G   
   A   
   A   
   G   
   A   
   A   
   C   
   A   
   A   
   G   
   C   
   A   
   G   
   A   
   A   
   G   
   C   
   A   
   A   
   T   
    C    
   G   
   T   
   C   
   A   
   C   
   A   
   T   
   T   
   G   
   C   
   A   
   A   
    C    
   T   
   A   
   T   
   A   
    T    
   C   
   G   
   T   
   T   
   T   
   G   
   A   
   C   
   G   
   A   
   A   
    C    
   A   
   C   
    A    
   G   
   A   
   T   
   A   
   T   
   C   
   A   
   C   
   A   
   G   
   A   
   G   
   C   
   T   
   T   
   C   
   A   
   A   
   A   
   A   
   G   
   G   
   A   
   A   
    T    
   C   
    C    
   G   
   A   
   A   
   A   
    G    
   C   
   T   
   T   
   G   
   A   
   T   
   T   
   G   
   C   
    A    
   C   
   A   
   A   
   A   
   T   
   C   
   A   
   C   
   A   
   G   
   A   
   G   
   T   
   T   
    A    
   A   
   A   
   T   
   A   
   A   
   A   
   A   
   T   
   C   
   C   
   T   
   T   
   T   
   C   
   A   
   A   
   A   
    C    
   G   
   A   
   C   
   A   
   A   
   A   
   G   
   A   
    G    
   C   
   T   
   T   
   T   
   T   
    C    
   T   
   C   
   T   
   G   
   T   
   T   
   A   
   T   
   G   
   A   
   A   
   A   
   A   
   A   
   A   
   G   
   A   
   A   
   T   
   T   
   G   
   C   
   G   
   A   
   G   
   A   
   A   
   G   
   T   
   G   
   A   
   A   
   A   
   A   
   A   
   A   
   A   
   A   
   T   
   T   
   A   
    C    
   T   
   C   
   T   
   T   
   C   
   C   
   G   
   C   
   T   
   C   
   G   
   T   
   C   
    T    
    G    
   A   
   C   
   G   
   A   
   C   
   A   
   A   
   T   
   C   
    G    
   A   
   A   
   G   
   A   
   C   
   G   
   A   
   A   
   A   
   T   
   C   
   G   
   A   
   A   
   G   
   A   
   A   
   A   
   T   
   C   
   A   
   A   
   A   
   A   
   T   
    C    
   G   
   A   
   T   
   A   
   C   
   G   
   C   
   A   
   A   
    G    
   T   
    T    
   T   
   T   
   A   
   G   
   T   
   G   
   G   
   C   
    G    
   C   
   A   
   A   
   G   
   A   
   A   
   G   
   A   
   C   
   G   
   T   
   A   
   A   
   T   
    C    
   G   
   T   
   C   
   A   
   G   
   T   
   G   
   T   
    G    
   A   
   C   
    A    
   C   
   G   
   A   
   G   
   A   
   A   
   G   
   G   
   C   
   T   
   A   
   T   
   G   
   T   
   G   
   A   
   A   
   G   
   C   
   G   
   A   
   A   
   C   
   A   
   A   
   G   
   C   
   C   
   T   
   T   
   C   
   G   
   T   
   T   
   C   
    T    
   T   
   A   
   T   
   A   
   G   
   T   
   G   
   C   
   T   
   T   
   C   
    G    
   A   
   A   
   A   
   C   
   C   
   A   
   G   
   A   
   A   
   G   
   A   
   A   
   A   
   T   
   C   
   G   
   G   
   G   
   A   
   T   
   G   
   C   
   G   
   A   
   G   
   A   
   A   
   G   
   G   
   C   
   G   
   A   
    C    
   T   
   A   
   C   
   T   
   T   
   G   
   C   
   T   
    G    
   T   
   A   
   C   
    T    
   C   
   A   
   G   
   G   
   T   
   G   
   A   
   A   
   T   
   T   
    G    
   A   
   G   
    C    
   A   
   C   
   A   
    T    
   T   
   G   
   G   
   A   
   C   
   C   
   A   
   T   
   G   
   T   
   T   
   C   
   T   
   T   
   C   
   T   
   G   
   A   
   T   
    T    
   A   
   C   
    G    
   A   
   A   
   T   
   A   
   A   
   A   
   G   
   C   
   A   
   A   
   A   
   T   
   G   
   T   
   G   
   A   
   T   
   C   
   T   
   A   
   C   
   C   
   G   
    T    
   C   
   C   
   T   
   G   
   T   
    T    
   C   
   A   
   T   
   G   
   A   
   A   
   C   
   T   
   A   
   C   
   C   
    C    
   G   
   A   
   T   
   T   
   T   
   G   
   A   
   A   
   A   
   T   
   G   
   G   
   A   
   A   
   A   
   G   
   A   
   T   
   G   
   C   
   T   
   G   
   G   
    C    
   G   
   A   
    A    
   C   
   A   
    C    
   A   
   T   
   T   
   T   
   C   
   T   
   C   
   A   
   A   
   A   
   C   
   A   
   A   
   T   
   C   
   T   
   C   
   C   
   A   
   A   
   T   
   C   
   T   
   G   
   T   
   C   
   T   
   G   
   T   
   A   
   G   
   A   
    T    
   G   
   A   
   A   
   T   
   C   
   G   
   A   
   T   
    C    
   T   
   T   
   A   
   G   
   C   
   T   
   G   
   T   
   T   
   T   
   T   
    C    
   C   
   C   
   A   
   T   
   A   
   T   
   C   
   A   
   A   
   A   
   A   
   A   
   A   
   T   
    C    
   C   
   A   
   A   
   G   
   C   
   A   
   G   
   A   
    G    
   A   
   A   
   A   
   A   
   C   
   A   
   T   
   T   
   T   
   G   
   T   
    A    
   T   
   T   
   C   
   A   
   T   
   C   
   A   
   G   
   C   
   A   
   A   
   G   
   A   
   A   
   T   
   G   
   G   
   A   
   T   
   T   
    G    
    A    
   T   
   C   
   A   
   A   
   G   
   C   
   A   
   A   
   A   
   C   
   A   
   C   
   G   
   A   
   A   
   T   
   G   
   A   
   C   
   A   
   G   
   A   
   G   
   T   
   T   
   C   
   G   
   A   
   A   
   C   
   C   
   A   
   T   
   G   
   G   
   C   
   G   
   C   
   A   
   C   
   C   
   T   
   A   
    C    
   A   
   A   
   G   
   A   
   G   
   C   
   C   
   G   
    T    
   C   
   C   
   G   
   C   
   T   
   C   
   A   
   G   
   C   
   T   
   G   
   T   
   A   
   T   
   G   
   A   
   A   
   G   
   C   
   T   
   A   
   A   
   A   
   G   
   G   
   C   
   G   
   G   
   A   
   A   
   G   
   A   
   A   
   G   
   A   
   T   
   G   
   A   
   A   
   C   
   T   
    T    
   A   
   T   
   T   
   G   
   C   
   T   
   G   
   T   
    A    
   T   
   A   
   T   
   C   
   T   
   G   
   A   
   A   
   A   
   G   
   A   
   A   
   G   
   A   
   T   
   C   
    A    
   G   
   C   
   C   
   A   
   G   
   G   
   C   
   T   
   T   
   A   
   G   
   A   
   T   
   G   
   T   
    G    
   T   
   T   
   C   
   T   
   T   
   A   
   G   
   T   
   A   
   A   
   C   
   G   
    C    
   A   
   T   
   C   
   A   
   A   
   G   
   G   
   A   
   A   
   T   
   G   
   G   
   G   
    C    
   T   
   T   
   G   
   C   
   G   
   C   
   T   
    A    
   T   
   C   
   C   
   T   
   T   
   T   
   A   
   A   
    C    
   A   
   G   
   A   
   T   
   G   
   T   
   T   
   C   
   C   
   C   
   G   
   T   
   T   
   G   
   T   
   T   
   G   
   G   
   A   
   G   
   C   
   A   
   A   
   A   
   G   
   G   
   C   
   A   
   G   
    C    
   T   
   G   
   G   
   A   
   G   
   T   
   G   
   A   
   A   
   G   
   T   
   C   
   T   
   A   
   T   
   G   
   A   
   A   
   T   
   T   
   T   
   G   
   A   
   A   
   A   
   G   
   A   
   T   
   G   
   G   
   A   
   G   
   A   
   T   
   A   
   G   
   G   
    A    
   T   
   T   
    G    
   T   
   C   
   A   
   A   
    T    
   G   
   G   
    A    
   C   
   T   
    G    
   C   
   T   
   T   
   G   
   T   
   G   
   T   
   T   
   A   
   G   
   C   
   A   
   G   
   A   
    C    
   G   
   G   
    A    
   G   
   A   
   T   
   A   
   C   
   G   
   C   
   C   
    G    
   A   
   T   
   C   
   G   
   T   
   A   
   A   
   T   
   C   
   G   
   T   
   T   
   A   
   C   
   T   
   C   
   A   
   G   
   C   
   G   
   A   
   G   
   G   
   A   
   G   
   C   
    T    
   G   
   T   
    G    
   A   
   A   
   A   
   C   
   G   
   C   
   A   
   T   
   G   
   C   
   T   
   T   
   G   
   C   
   T   
   C   
   A   
   A   
   G   
   A   
   A   
   A   
   T   
   C   
   A   
   G   
   C   
   C   
   A   
   A   
   A   
   C   
    T    
   T   
   C   
   A   
   C   
   G   
   T   
   G   
   C   
   G   
   A   
   A   
   G   
   C   
   G   
   G   
   G   
   G   
   A   
   G   
   T   
   G   
   A   
   C   
   G   
   G   
   T   
   C   
   A   
   T   
   G   
   C   
   G   
   T   
   G   
   A   
   G   
   C   
   T   
   G   
   A   
   A   
   A   
   A   
   A   
   A   
   A   
   A   
   T   
   C   
   C   
    G    
   C   
   A   
    C    
   C   
   G   
    C    
   A   
   T   
   T   
   A   
   T   
   C   
   T   
   A   
   T   
   A   
   T   
   G   
   T   
   C   
   G   
   G   
   A   
   A   
   G   
   G   
   A   
   A   
   G   
   A   
   T   
   C   
   A   
   A   
   A   
   A   
   G   
   A   
   A   
   A   
   T   
   A   
   A   
   C   
   C   
   T   
   T   
   G   
   A   
   T   
   G   
   A   
   A   
   T   
   C   
   A   
   A   
   A   
   A   
   A   
   G   
   G   
    A    
   C   
   A   
    A    
   C   
   A   
   G   
    A    
   T   
   A   
   A   
   C   
   G   
   A   
   T   
   T   
   G   
   A   
    T    
   C   
   C   
   A   
   A   
   C   
    G    
   G   
   A   
   T   
   T   
   T   
    T    
   C   
   C   
   T   
   A   
   T   
   A   
   G   
   G   
   T   
   G   
   A   
   T   
   C   
   G   
   A   
   A   
   C   
   A   
   T   
   C   
   G   
   A   
   A   
   T   
   G   
   G   
    T    
   T   
   C   
    G    
   T   
   T   
   T   
   G   
   C   
    T    
   A   
   T   
   G   
   G   
   A   
    C    
   G   
   A   
   G   
   A   
   A   
   A   
   A   
   A   
   A   
   G   
   G   
   T   
   G   
   G   
   A   
   G   
   A   
   A   
   G   
   T   
   C   
   A   
   T   
   C   
   G   
   A   
   A   
   G   
   T   
    T    
   A   
    T    
   C   
   G   
   A   
   T   
   G   
   C   
   T   
   C   
   C   
   T   
   G   
   A   
   G   
    A    
   T   
   T   
   T   
   T   
    C    
   A   
   T   
    C    
   G   
   A   
    A    
    T    
   C   
    A    
   -   
   -   
   -   
 

 
   N53479   
   A   
   T   
   G   
   G   
   A   
   A   
   A   
   A   
   T   
   C   
   G   
   A   
   C   
   A   
   A   
   G   
   A   
   A   
   G   
   T   
   A   
   C   
   A   
   A   
   G   
   A   
   A   
   T   
   T   
   A   
   A   
   C   
   T   
   T   
   T   
   A   
   G   
   A   
   A   
   G   
   A   
   A   
   G   
   T   
   G   
   A   
   T   
   G   
   G   
   G   
   T   
   G   
   A   
   C   
   C   
   G   
   C   
   T   
   T   
   C   
   G   
   G   
   A   
   C   
   G   
   T   
   T   
   A   
   T   
   T   
   C   
   C   
   A   
   A   
   A   
   T   
   A   
   T   
   A   
   T   
   T   
   A   
   T   
    C    
   C   
   A   
    A    
   G   
   A   
   A   
   C   
   G   
   T   
   G   
   C   
   A   
   C   
   T   
   G   
   C   
   C   
   T   
   G   
   A   
   T   
   A   
   T   
    C    
   C   
   G   
   T   
   G   
   A   
   T   
   G   
   G   
   A   
   T   
   T   
   G   
   A   
   A   
    G    
   C   
   C   
   A   
   G   
   T   
    G    
   C   
   A   
   A   
   C   
   G   
   C   
   C   
   G   
   G   
   A   
   T   
   A   
   T   
   T   
   A   
   T   
   T   
    T    
   G   
   C   
    C    
   A   
   T   
   G   
   A   
   A   
   C   
   A   
   A   
   A   
   G   
   A   
   T   
   G   
   G   
   C   
   A   
   A   
   T   
   A   
   C   
   G   
   T   
   A   
   T   
   G   
   A   
   C   
   A   
   A   
   A   
   G   
   G   
   A   
   T   
   T   
    T    
   C   
   G   
   T   
   A   
   A   
   A   
   T   
   C   
   A   
   G   
   C   
   A   
   A   
   A   
   A   
   T   
   C   
   T   
   G   
   T   
   C   
   G   
   G   
   A   
   A   
   A   
   T   
   A   
   T   
   C   
   A   
   T   
   G   
   G   
   G   
    G    
   A   
   A   
   T   
   T   
   A   
   T   
   C   
   A   
   T   
   C   
   C   
   T   
   C   
   A   
   T   
   G   
   G   
   T   
   G   
   A   
   C   
   A   
   G   
   C   
   A   
   G   
   T   
   A   
   T   
   T   
   T   
   A   
   T   
   G   
   A   
   A   
   G   
   C   
   G   
   A   
   T   
   G   
   G   
   T   
   C   
   C   
   G   
   C   
   A   
   T   
   G   
   A   
   G   
   C   
   C   
   A   
   A   
   G   
   A   
    C    
   T   
   G   
   G   
   A   
   A   
   G   
   T   
   T   
   G   
   C   
   G   
   G   
   G   
   A   
   A   
   G   
   T   
   A   
   C   
   T   
   G   
   A   
   T   
   C   
   G   
   A   
    A    
   A   
   T   
   G   
   C   
   A   
   C   
   G   
   G   
    G    
   A   
   A   
   T   
   A   
   A   
    C    
   G   
   G   
   G   
   A   
   G   
   T   
   A   
   T   
   G   
   G   
   A   
   C   
   G   
   G   
   T   
   G   
   A   
   T   
   C   
   C   
   A   
   C   
   C   
   T   
   G   
   C   
   T   
   G   
   C   
   G   
   A   
   T   
   G   
   C   
   G   
   T   
   T   
   A   
   T   
   A   
   C   
   T   
   G   
   A   
   A   
   G   
   C   
   C   
   A   
   G   
    A    
   T   
   T   
   A   
   T   
   C   
   C   
   A   
   A   
   G   
   T   
   T   
   A   
   A   
   G   
   T   
   G   
   G   
   A   
   G   
   A   
   G   
   A   
   T   
   G   
    C    
   T   
   A   
    G    
   C   
   A   
   G   
   A   
   T   
   A   
   T   
   C   
   G   
   A   
    G    
   A   
   A   
   A   
   G   
   A   
   A   
   A   
   C   
   A   
   G   
   T   
    C    
   G   
   A   
   T   
   C   
   T   
   C   
   G   
   T   
   G   
   T   
   G   
   G   
   A   
   A   
   T   
   T   
   T   
   T   
   G   
   A   
   T   
   G   
   A   
   T   
   A   
   C   
   A   
   G   
   A   
   A   
   A   
   A   
   A   
   G   
   A   
   A   
   C   
   C   
   A   
   A   
   C   
   A   
   G   
   T   
   C   
   C   
   T   
   T   
   C   
   C   
   A   
   G   
   C   
   T   
   C   
   G   
   A   
   T   
   A   
   C   
   C   
   C   
   A   
   A   
   A   
   C   
   C   
   T   
   T   
   C   
   T   
    C    
   G   
   T   
   C   
   A   
   A   
   T   
   G   
   G   
   A   
   T   
   C   
   G   
   A   
   C   
   A   
   G   
   G   
   T   
   A   
   T   
   A   
   T   
   C   
   T   
   G   
   C   
   T   
   G   
   G   
   A   
   T   
   A   
   T   
   G   
   C   
    A    
   A   
   C   
   A   
   G   
   A   
   A   
   A   
   T   
   C   
   C   
   C   
   G   
   A   
   C   
    T    
   C   
   A   
   T   
   A   
   A   
   C   
   T   
   T   
   A   
   G   
   C   
   T   
   G   
   A   
   A   
   G   
   T   
   G   
   A   
   T   
    C    
   G   
   A   
    T    
   G   
   G   
   C   
   A   
   C   
   A   
   A   
   T   
   C   
   T   
   A   
   T   
   A   
   T   
   G   
   A   
   T   
   C   
   G   
   A   
   T   
   C   
   A   
   T   
   C   
   C   
   A   
   C   
   A   
   A   
   G   
   C   
   G   
   A   
   G   
   C   
   C   
   T   
   T   
   G   
   A   
   A   
   A   
   A   
   A   
   T   
   T   
   G   
   A   
   T   
   G   
   G   
   A   
   A   
   T   
   A   
   T   
   A   
   T   
   T   
   C   
   C   
   T   
   G   
   G   
   A   
   C   
   C   
   T   
   G   
   A   
   C   
   T   
   T   
    T    
   C   
   C   
   A   
   A   
   C   
   A   
   G   
   G   
   A   
   G   
   G   
    G    
   A   
   T   
   C   
   T   
   T   
   G   
   C   
   A   
   A   
   G   
   G   
    A    
   A   
   A   
   A   
   G   
   A   
   T   
   G   
   A   
   A   
   A   
   T   
   C   
   A   
   A   
   A   
   A   
   A   
   A   
   G   
   C   
   T   
   T   
   A   
   T   
   G   
   A   
   A   
   A   
   C   
   A   
   G   
   G   
    G    
   C   
   G   
   A   
   G   
   G   
   A   
   A   
   A   
   A   
   G   
   T   
   T   
   A   
    T    
   T   
   C   
   T   
   T   
   C   
   G   
   T   
   T   
   C   
   G   
   A   
   A   
   A   
   A   
   C   
   A   
   A   
   A   
   G   
   A   
   T   
   A   
   G   
   A   
    G    
   G   
   C   
   A   
   A   
   T   
   G   
   A   
   A   
   G   
   G   
   G   
    T    
   A   
   A   
    T    
   A   
   A   
   A   
   C   
   A   
   G   
   C   
   A   
   A   
   A   
   T   
   C   
   G   
   T   
   G   
   A   
   T   
   C   
   A   
   C   
    A    
   G   
   A   
   A   
   A   
   T   
   C   
   C   
   C   
   A   
   T   
   A   
   T   
   G   
   A   
   A   
   G   
   T   
   A   
   A   
   A   
   T   
   A   
   A   
   A   
   G   
   C   
   G   
   A   
   C   
    C    
   T   
   T   
   G   
   G   
   T   
   G   
   A   
   A   
   A   
   A   
   A   
   A   
   A   
   T   
   G   
   G   
   A   
   T   
   G   
   A   
   G   
   A   
   T   
   C   
   C   
   G   
   T   
   T   
   T   
   G   
   A   
   A   
   T   
   A   
   A   
   G   
   A   
   A   
   A   
   A   
   T   
   C   
   G   
   A   
    T    
   G   
   G   
   A   
   A   
   T   
   A   
   G   
   C   
   T   
   G   
   A   
   A   
   G   
   T   
    A    
   C   
   G   
   A   
   G   
   A   
   T   
   G   
   A   
   A   
   A   
   G   
   T   
   G   
   A   
   T   
    A    
   G   
    A    
   A   
   C   
    A    
   G   
   G   
   T   
   C   
   T   
   G   
   C   
   A   
   A   
   A   
   T   
   T   
   G   
   T   
   C   
   G   
   T   
    C    
   G   
   A   
   A   
   T   
   T   
   G   
   A   
   A   
   A   
   A   
   A   
   A   
   G   
   A   
    C    
   G   
   C   
   C   
   A   
   A   
    T    
   G   
   C   
   T   
   C   
   A   
   A   
   G   
   G   
    A    
   A   
   T   
   T   
   T   
   T   
   G   
   A   
   A   
   T   
   T   
   A   
    T    
   T   
   T   
   G   
   T   
   T   
   C   
   A   
   A   
   A   
   A   
   A   
   C   
   A   
   C   
   T   
   G   
   A   
   G   
    C    
   T   
   A   
   C   
   A   
   G   
   A   
   T   
   C   
   A   
   A   
   T   
   T   
   A   
   C   
   A   
   A   
   T   
   T   
   T   
    C    
   A   
   A   
   T   
   A   
   T   
   G   
   G   
   T   
   C   
   G   
   C   
   T   
   A   
   T   
   C   
   G   
   A   
    C    
   C   
   A   
   T   
   A   
   T   
   G   
   A   
   C   
   T   
   C   
   C   
   G   
   C   
   A   
    T    
   C   
   A   
   A   
   G   
   T   
   T   
   G   
   G   
   A   
   C   
   T   
   G   
   A   
   A   
   A   
   G   
   A   
   C   
   A   
   T   
   T   
   T   
   T   
   A   
   A   
   G   
   A   
   A   
   G   
   T   
   T   
   A   
   T   
   A   
   T   
   C   
   G   
   A   
   G   
   C   
   A   
   T   
   C   
   G   
   A   
   A   
   A   
   G   
   C   
   A   
   A   
   G   
   T   
   C   
   A   
   T   
   A   
   A   
   C   
   A   
   A   
   A   
   A   
   C   
   G   
    T    
   A   
   G   
    T    
   C   
   A   
   A   
   T   
   T   
   C   
   G   
   A   
   T   
   T   
   T   
   A   
   G   
   C   
   C   
   A   
   A   
   A   
   G   
   C   
   T   
   C   
   A   
   A   
   A   
   A   
   A   
    A    
   G   
   A   
   C   
   A   
   G   
   C   
   A   
   T   
   A   
   T   
   C   
   G   
   T   
    A    
   G   
   A   
   A   
   G   
   G   
    A    
    T    
   T   
   G   
   A   
   T   
   G   
   A   
   A   
   A   
   G   
   C   
   T   
   T   
   T   
   A   
   T   
   C   
   G   
   A   
   T   
   T   
   C   
   T   
   G   
   G   
   A   
   T   
   G   
   A   
   A   
   G   
   T   
   A   
   A   
   T   
   C   
   G   
    C    
   A   
   A   
   C   
   G   
   A   
   T   
   T   
   C   
   G   
   T   
   G   
   A   
   A   
   A   
   G   
   T   
   A   
   A   
   A   
   G   
   A   
   C   
   A   
   A   
   A   
   A   
   A   
   A   
   G   
   A   
    C    
   G   
   C   
    C    
   A   
   A   
   A   
   A   
   A   
   G   
   A   
   A   
   T   
   T   
   T   
   A   
   G   
   T   
   C   
   G   
   A   
    C    
   G   
   T   
    C    
   T   
   T   
    T    
   C   
   A   
   A   
   T   
   T   
   T   
   A   
   C   
   A   
   G   
   A   
   A   
   G   
   A   
   A   
   C   
   A   
   A   
   G   
   C   
   A   
   G   
   A   
   A   
   G   
   C   
   A   
   A   
   T   
    T    
   G   
   T   
   C   
   A   
   C   
   A   
   T   
   T   
   G   
   C   
   A   
   A   
    T    
   T   
   A   
   T   
   A   
    C    
   C   
   G   
   T   
   T   
   T   
   G   
   A   
   C   
   G   
   A   
   A   
    C    
   A   
   C   
    G    
   G   
   A   
   T   
   A   
   T   
   C   
   A   
   C   
   A   
   G   
   A   
   G   
   C   
   T   
   T   
   C   
   A   
   A   
   A   
   A   
   G   
   G   
   A   
   A   
    T    
   C   
    C    
   G   
   A   
   A   
   A   
    G    
   C   
   T   
   T   
   G   
   A   
   T   
   T   
   G   
   C   
    G    
   C   
   A   
   A   
   A   
   T   
   C   
   A   
   C   
   A   
   G   
   A   
   G   
   T   
   T   
    G    
   A   
   A   
   T   
   A   
   A   
   A   
   A   
   T   
   C   
   C   
   T   
   T   
   T   
   C   
   A   
   A   
   A   
    T    
   G   
   A   
   C   
   A   
   A   
   A   
   G   
   A   
    G    
   C   
   T   
   T   
   T   
   T   
    C    
   T   
   C   
   T   
   G   
   T   
   T   
   A   
   T   
   G   
   A   
   A   
   A   
   A   
   A   
   A   
   G   
   A   
   A   
   T   
   T   
   G   
   C   
   G   
   A   
   G   
   A   
   A   
   G   
   T   
   G   
   A   
   A   
   A   
   A   
   A   
   A   
   A   
   A   
   T   
   T   
   A   
    T    
   T   
   C   
   T   
   T   
   C   
   C   
   G   
   C   
   T   
   C   
   G   
   T   
   C   
    T    
    A    
   A   
   C   
   G   
   A   
   C   
   A   
   A   
   T   
   C   
    G    
   A   
   A   
   G   
   A   
   C   
   G   
   A   
   A   
   A   
   T   
   C   
   G   
   A   
   A   
   G   
   A   
   A   
   A   
   T   
   C   
   A   
   A   
   A   
   A   
   T   
    T    
   G   
   A   
   T   
   A   
   C   
   G   
   C   
   A   
   A   
    G    
   T   
    C    
   T   
   T   
   A   
   G   
   T   
   G   
   G   
   C   
    A    
   C   
   A   
   A   
   G   
   A   
   A   
   G   
   A   
   C   
   G   
   T   
   A   
   A   
   T   
    T    
   G   
   T   
   C   
   A   
   G   
   T   
   G   
   T   
    G    
   A   
   C   
    G    
   C   
   G   
   A   
   G   
   A   
   A   
   G   
   G   
   C   
   T   
   A   
   T   
   G   
   T   
   G   
   A   
   A   
   G   
   C   
   G   
   A   
   A   
   C   
   A   
   A   
   G   
   C   
   C   
   T   
   T   
   C   
   G   
   T   
   T   
   C   
    T    
   T   
   A   
   T   
   A   
   G   
   T   
   G   
   C   
   T   
   T   
   C   
    C    
   A   
   A   
   A   
   C   
   C   
   A   
   G   
   A   
   A   
   G   
   A   
   A   
   A   
   T   
   C   
   G   
   G   
   G   
   A   
   T   
   G   
   C   
   G   
   A   
   G   
   A   
   A   
   G   
   G   
   C   
   G   
   A   
    C    
   T   
   A   
   C   
   T   
   T   
   G   
   C   
   T   
    A    
   T   
   A   
   C   
    T    
   C   
   A   
   G   
   G   
   T   
   G   
   A   
   A   
   T   
   T   
    G    
   A   
   G   
    T    
   A   
   C   
   A   
    T    
   T   
   G   
   G   
   A   
   C   
   C   
   A   
   T   
   G   
   T   
   T   
   C   
   T   
   T   
   C   
   T   
   G   
   A   
   T   
    C    
   A   
   C   
    A    
   A   
   A   
   T   
   A   
   A   
   A   
   G   
   C   
   A   
   A   
   A   
   T   
   G   
   T   
   G   
   A   
   T   
   C   
   T   
   A   
   C   
   C   
   G   
    C    
   C   
   C   
   T   
   G   
   T   
    C    
   C   
   A   
   T   
   G   
   A   
   A   
   C   
   T   
   A   
   C   
   C   
    C    
   G   
   A   
   T   
   T   
   T   
   G   
   A   
   A   
   A   
   T   
   G   
   G   
   A   
   A   
   A   
   G   
   A   
   T   
   G   
   C   
   T   
   G   
   G   
    T    
   G   
   A   
    G    
   C   
   A   
    C    
   A   
   T   
   T   
   T   
   C   
   T   
   C   
   A   
   A   
   A   
   C   
   A   
   A   
   T   
   C   
   T   
   C   
   C   
   A   
   A   
   T   
   C   
   T   
   G   
   T   
   C   
   T   
   G   
   T   
   A   
   G   
   A   
    C    
   G   
   A   
   A   
   T   
   C   
   G   
   A   
   T   
    T    
   T   
   T   
   A   
   G   
   C   
   T   
   G   
   T   
   T   
   T   
   T   
    C    
   C   
   C   
   A   
   T   
   A   
   T   
   C   
   A   
   A   
   A   
   A   
   A   
   A   
   T   
    T    
   C   
   A   
   A   
   G   
   C   
   A   
   G   
   A   
    G    
   A   
   A   
   A   
   A   
   C   
   A   
   T   
   T   
   T   
   G   
   T   
    A    
   T   
   T   
   C   
   A   
   T   
   C   
   A   
   G   
   C   
   A   
   A   
   G   
   A   
   A   
   T   
   G   
   G   
   A   
   T   
   T   
    G    
    A    
   T   
   C   
   A   
   A   
   G   
   C   
   A   
   A   
   A   
   C   
   A   
   C   
   G   
   A   
   A   
   T   
   G   
   A   
   C   
   A   
   G   
   A   
   G   
   T   
   T   
   C   
   G   
   A   
   A   
   C   
   C   
   A   
   T   
   G   
   G   
   C   
   G   
   C   
   A   
   C   
   C   
   T   
   A   
    T    
   A   
   A   
   G   
   A   
   G   
   C   
   C   
   G   
    T    
   C   
   C   
   G   
   C   
   T   
   C   
   A   
   G   
   C   
   T   
   G   
   T   
   A   
   T   
   G   
   A   
   A   
   G   
   C   
   T   
   A   
   A   
   A   
   G   
   G   
   C   
   G   
   G   
   A   
   A   
   G   
   A   
   A   
   G   
   A   
   T   
   G   
   A   
   A   
   C   
   T   
    G    
   A   
   T   
   T   
   G   
   C   
   T   
   G   
   T   
    G    
   T   
   A   
   T   
   C   
   T   
   G   
   A   
   A   
   A   
   G   
   A   
   A   
   G   
   A   
   T   
   C   
    A    
   G   
   C   
   C   
   A   
   G   
   G   
   C   
   T   
   T   
   A   
   G   
   A   
   T   
   G   
   T   
    T    
   T   
   T   
   C   
   T   
   T   
   A   
   G   
   T   
   A   
   A   
   C   
   G   
    C    
   A   
   T   
   C   
   A   
   A   
   G   
   G   
   A   
   A   
   T   
   G   
   G   
   G   
    T    
   T   
   T   
   G   
   C   
   G   
   C   
   T   
    A    
   T   
   C   
   C   
   T   
   T   
   T   
   A   
   A   
    C    
   A   
   G   
   A   
   T   
   G   
   T   
   T   
   C   
   C   
   C   
   G   
   T   
   T   
   G   
   T   
   T   
   G   
   G   
   A   
   G   
   C   
   A   
   A   
   A   
   G   
   G   
   C   
   A   
   G   
    C    
   T   
   G   
   G   
   A   
   G   
   T   
   G   
   A   
   A   
   G   
   T   
   C   
   T   
   A   
   T   
   G   
   A   
   A   
   T   
   T   
   T   
   G   
   A   
   A   
   A   
   G   
   A   
   T   
   G   
   G   
   A   
   G   
   A   
   T   
   A   
   G   
   G   
    A    
   T   
   T   
    A    
   T   
   C   
   A   
   A   
    C    
   G   
   G   
    G    
   C   
   T   
    G    
   C   
   T   
   T   
   G   
   T   
   G   
   T   
   T   
   A   
   G   
   C   
   A   
   G   
   A   
    A    
   G   
   G   
    T    
   G   
   A   
   T   
   A   
   C   
   G   
   C   
   C   
    G    
   A   
   T   
   C   
   G   
   T   
   A   
   A   
   T   
   C   
   G   
   T   
   T   
   A   
   C   
   T   
   C   
   A   
   G   
   C   
   G   
   A   
   G   
   G   
   A   
   G   
   C   
    T    
   G   
   T   
    T    
   A   
   A   
   A   
   C   
   G   
   C   
   A   
   T   
   G   
   C   
   T   
   T   
   G   
   C   
   T   
   C   
   A   
   A   
   G   
   A   
   A   
   A   
   T   
   C   
   A   
   G   
   C   
   C   
   A   
   A   
   A   
   C   
    T    
   T   
   C   
   A   
   C   
   G   
   T   
   G   
   C   
   G   
   A   
   A   
   G   
   C   
   G   
   G   
   G   
   G   
   A   
   G   
   T   
   G   
   A   
   C   
   G   
   G   
   T   
   C   
   A   
   T   
   G   
   C   
   G   
   T   
   G   
   A   
   G   
   C   
   T   
   G   
   A   
   A   
   A   
   A   
   A   
   A   
   A   
   A   
   T   
   C   
   C   
    G    
   C   
   A   
    C    
   C   
   G   
    T    
   A   
   T   
   T   
   A   
   T   
   C   
   T   
   A   
   T   
   A   
   T   
   G   
   T   
   C   
   G   
   G   
   A   
   A   
   G   
   G   
   A   
   A   
   G   
   A   
   T   
   C   
   A   
   A   
   A   
   A   
   G   
   A   
   A   
   A   
   T   
   A   
   A   
   C   
   C   
   T   
   T   
   G   
   A   
   T   
   G   
   A   
   A   
   T   
   C   
   A   
   A   
   A   
   A   
   A   
   G   
   G   
    G    
   C   
   A   
    G    
   C   
   A   
   G   
    T    
   T   
   A   
   A   
   C   
   G   
   A   
   T   
   T   
   G   
   A   
    C    
   C   
   C   
   A   
   A   
   C   
    T    
   G   
   A   
   T   
   T   
   T   
    C    
   C   
   C   
   T   
   A   
   T   
   A   
   G   
   G   
   T   
   G   
   A   
   T   
   C   
   G   
   A   
   A   
   C   
   A   
   T   
   C   
   G   
   A   
   A   
   T   
   G   
   G   
    A    
   T   
   C   
    A    
   T   
   T   
   T   
   G   
   C   
    G    
   A   
   T   
   G   
   G   
   A   
    T    
   G   
   A   
   G   
   A   
   A   
   A   
   A   
   A   
   A   
   G   
   G   
   T   
   G   
   G   
   A   
   G   
   A   
   A   
   G   
   T   
   C   
   A   
   T   
   C   
   G   
   A   
   A   
   G   
   T   
    T    
   A   
    T    
   C   
   G   
   A   
   T   
   G   
   C   
   T   
   C   
   C   
   T   
   G   
   A   
   G   
    A    
   T   
   T   
   T   
   T   
    C    
   A   
   T   
    C    
   G   
   A   
    G    
    T    
   C   
    G    
   -   
   -   
   -   
 

 
   N60336F   
   A   
   T   
   G   
   G   
   A   
   A   
   A   
   A   
   T   
   C   
   G   
   A   
   C   
   A   
   A   
   G   
   A   
   A   
   G   
   T   
   A   
   C   
   A   
   A   
   G   
   A   
   A   
   T   
   T   
   A   
   A   
   C   
   T   
   T   
   T   
   A   
   G   
   A   
   A   
   G   
   A   
   A   
   G   
   T   
   G   
   A   
   T   
   G   
   G   
   G   
   T   
   G   
   A   
   C   
   C   
   G   
   C   
   T   
   T   
   C   
   G   
   G   
   A   
   C   
   G   
   T   
   T   
   A   
   T   
   T   
   C   
   C   
   A   
   A   
   A   
   T   
   A   
   T   
   A   
   T   
   T   
   A   
   T   
    C    
   C   
   A   
    A    
   G   
   A   
   A   
   C   
   G   
   T   
   G   
   C   
   A   
   C   
   T   
   G   
   C   
   C   
   T   
   G   
   A   
   T   
   A   
   T   
    C    
   C   
   G   
   T   
   G   
   A   
   T   
   G   
   G   
   A   
   T   
   T   
   G   
   A   
   A   
    G    
   C   
   C   
   A   
   G   
   T   
    G    
   C   
   A   
   A   
   C   
   G   
   C   
   C   
   G   
   G   
   A   
   T   
   A   
   T   
   T   
   A   
   T   
   T   
    T    
   G   
   C   
    C    
   A   
   T   
   G   
   A   
   A   
   C   
   A   
   A   
   A   
   G   
   A   
   T   
   G   
   G   
   C   
   A   
   A   
   T   
   A   
   C   
   G   
   T   
   A   
   T   
   G   
   A   
   C   
   A   
   A   
   A   
   G   
   G   
   A   
   T   
   T   
    C    
   C   
   G   
   T   
   A   
   A   
   A   
   T   
   C   
   A   
   G   
   C   
   A   
   A   
   A   
   A   
   T   
   C   
   T   
   G   
   T   
   C   
   G   
   G   
   A   
   A   
   A   
   T   
   A   
   T   
   C   
   A   
   T   
   G   
   G   
   G   
    G    
   A   
   A   
   T   
   T   
   A   
   T   
   C   
   A   
   T   
   C   
   C   
   T   
   C   
   A   
   T   
   G   
   G   
   T   
   G   
   A   
   C   
   A   
   G   
   C   
   A   
   G   
   T   
   A   
   T   
   T   
   T   
   A   
   T   
   G   
   A   
   A   
   G   
   C   
   G   
   A   
   T   
   G   
   G   
   T   
   C   
   C   
   G   
   C   
   A   
   T   
   G   
   A   
   G   
   C   
   C   
   A   
   A   
   G   
   A   
    C    
   T   
   G   
   G   
   A   
   A   
   G   
   T   
   T   
   G   
   C   
   G   
   G   
   G   
   A   
   A   
   G   
   T   
   A   
   C   
   T   
   G   
   A   
   T   
   C   
   G   
   A   
    A    
   A   
   T   
   G   
   C   
   A   
   C   
   G   
   G   
    G    
   A   
   A   
   T   
   A   
   A   
    C    
   G   
   G   
   G   
   A   
   G   
   T   
   A   
   T   
   G   
   G   
   A   
   C   
   G   
   G   
   T   
   G   
   A   
   T   
   C   
   C   
   A   
   C   
   C   
   T   
   G   
   C   
   T   
   G   
   C   
   G   
   A   
   T   
   G   
   C   
   G   
   T   
   T   
   A   
   T   
   A   
   C   
   T   
   G   
   A   
   A   
   G   
   C   
   C   
   A   
   G   
    A    
   T   
   T   
   A   
   T   
   C   
   C   
   A   
   A   
   G   
   T   
   T   
   A   
   A   
   G   
   T   
   G   
   G   
   A   
   G   
   A   
   G   
   A   
   T   
   G   
    C    
   T   
   A   
    G    
   C   
   A   
   G   
   A   
   T   
   A   
   T   
   C   
   G   
   A   
    A    
   A   
   A   
   A   
   G   
   A   
   A   
   A   
   C   
   A   
   G   
   T   
    C    
   G   
   A   
   T   
   C   
   T   
   C   
   G   
   T   
   G   
   T   
   G   
   G   
   A   
   A   
   T   
   T   
   T   
   T   
   G   
   A   
   T   
   G   
   A   
   T   
   A   
   C   
   A   
   G   
   A   
   A   
   A   
   A   
   A   
   G   
   A   
   A   
   C   
   C   
   A   
   A   
   C   
   A   
   G   
   T   
   C   
   C   
   T   
   T   
   C   
   C   
   A   
   G   
   C   
   T   
   C   
   G   
   A   
   T   
   A   
   C   
   C   
   C   
   A   
   A   
   A   
   C   
   C   
   T   
   T   
   C   
   T   
    C    
   G   
   T   
   C   
   A   
   A   
   T   
   G   
   G   
   A   
   T   
   C   
   G   
   A   
   C   
   A   
   G   
   G   
   T   
   A   
   T   
   A   
   T   
   C   
   T   
   G   
   C   
   T   
   G   
   G   
   A   
   T   
   A   
   T   
   G   
   C   
    A    
   A   
   C   
   A   
   G   
   A   
   A   
   A   
   T   
   C   
   C   
   C   
   G   
   A   
   C   
    T    
   C   
   A   
   T   
   A   
   A   
   C   
   T   
   T   
   A   
   G   
   C   
   T   
   G   
   A   
   A   
   G   
   T   
   G   
   A   
   T   
    C    
   G   
   A   
    T    
   G   
   G   
   C   
   A   
   C   
   A   
   A   
   T   
   C   
   T   
   A   
   T   
   A   
   T   
   G   
   A   
   T   
   C   
   G   
   A   
   T   
   C   
   A   
   T   
   C   
   C   
   A   
   C   
   A   
   A   
   G   
   C   
   G   
   A   
   G   
   C   
   C   
   T   
   T   
   G   
   A   
   A   
   A   
   A   
   A   
   T   
   T   
   G   
   A   
   T   
   G   
   G   
   A   
   A   
   T   
   A   
   T   
   A   
   T   
   T   
   C   
   C   
   T   
   G   
   G   
   A   
   C   
   C   
   T   
   G   
   A   
   C   
   T   
   T   
    T    
   C   
   C   
   A   
   A   
   C   
   A   
   G   
   G   
   A   
   G   
   G   
    G    
   A   
   T   
   C   
   T   
   T   
   G   
   C   
   A   
   A   
   G   
   G   
    A    
   A   
   A   
   A   
   G   
   A   
   T   
   G   
   A   
   A   
   A   
   T   
   C   
   A   
   A   
   A   
   A   
   A   
   A   
   G   
   C   
   T   
   T   
   A   
   T   
   G   
   A   
   A   
   A   
   C   
   A   
   G   
   G   
    G    
   C   
   G   
   A   
   G   
   G   
   A   
   A   
   A   
   A   
   G   
   T   
   T   
   A   
    T    
   T   
   C   
   T   
   T   
   C   
   G   
   T   
   T   
   C   
   G   
   A   
   A   
   A   
   A   
   C   
   A   
   A   
   A   
   G   
   A   
   T   
   A   
   G   
   A   
    G    
   G   
   C   
   A   
   A   
   T   
   G   
   A   
   A   
   G   
   G   
   G   
    T    
   A   
   A   
    T    
   A   
   A   
   A   
   C   
   A   
   G   
   C   
   A   
   A   
   A   
   T   
   C   
   G   
   T   
   G   
   A   
   T   
   C   
   A   
   C   
    A    
   G   
   A   
   A   
   A   
   T   
   C   
   C   
   C   
   A   
   T   
   A   
   T   
   G   
   A   
   A   
   G   
   T   
   A   
   A   
   A   
   T   
   A   
   A   
   A   
   G   
   C   
   G   
   A   
   C   
    C    
   T   
   T   
   G   
   G   
   T   
   G   
   A   
   A   
   A   
   A   
   A   
   A   
   A   
   T   
   G   
   G   
   A   
   T   
   G   
   A   
   G   
   A   
   T   
   C   
   C   
   G   
   T   
   T   
   T   
   G   
   A   
   A   
   T   
   A   
   A   
   G   
   A   
   A   
   A   
   A   
   T   
   C   
   G   
   A   
    T    
   G   
   G   
   A   
   A   
   T   
   A   
   G   
   C   
   T   
   G   
   A   
   A   
   G   
   T   
    A    
   C   
   G   
   A   
   G   
   A   
   T   
   G   
   A   
   A   
   A   
   G   
   T   
   G   
   A   
   T   
    A    
   G   
    A    
   A   
   C   
    A    
   G   
   G   
   T   
   C   
   T   
   G   
   C   
   A   
   A   
   A   
   T   
   T   
   G   
   T   
   C   
   G   
   T   
    C    
   G   
   A   
   A   
   T   
   T   
   G   
   A   
   A   
   A   
   A   
   A   
   A   
   G   
   A   
    C    
   G   
   C   
   C   
   A   
   A   
    T    
   G   
   C   
   T   
   C   
   A   
   A   
   G   
   G   
    A    
   A   
   T   
   T   
   T   
   T   
   G   
   A   
   A   
   T   
   T   
   A   
    T    
   T   
   T   
   G   
   T   
   T   
   C   
   A   
   A   
   A   
   A   
   A   
   C   
   A   
   C   
   T   
   G   
   A   
   G   
    C    
   T   
   A   
   C   
   A   
   G   
   A   
   T   
   C   
   A   
   A   
   T   
   T   
   A   
   C   
   A   
   A   
   T   
   T   
   T   
    C    
   A   
   A   
   T   
   A   
   T   
   G   
   G   
   T   
   C   
   G   
   C   
   T   
   A   
   T   
   C   
   G   
   A   
    C    
   C   
   A   
   T   
   A   
   T   
   G   
   A   
   C   
   T   
   C   
   C   
   G   
   C   
   A   
    T    
   C   
   A   
   A   
   G   
   T   
   T   
   G   
   G   
   A   
   C   
   T   
   G   
   A   
   A   
   A   
   G   
   A   
   C   
   A   
   T   
   T   
   T   
   T   
   A   
   A   
   G   
   A   
   A   
   G   
   T   
   T   
   A   
   T   
   A   
   T   
   C   
   G   
   A   
   G   
   C   
   A   
   T   
   C   
   G   
   A   
   A   
   A   
   G   
   C   
   A   
   A   
   G   
   T   
   C   
   A   
   T   
   A   
   A   
   C   
   A   
   A   
   A   
   A   
   C   
   G   
    T    
   A   
   G   
    T    
   C   
   A   
   A   
   T   
   T   
   C   
   G   
   A   
   T   
   T   
   T   
   A   
   G   
   C   
   C   
   A   
   A   
   A   
   G   
   C   
   T   
   C   
   A   
   A   
   A   
   A   
   A   
    A    
   G   
   A   
   C   
   A   
   G   
   C   
   A   
   T   
   A   
   T   
   C   
   G   
   T   
    A    
   G   
   A   
   A   
   G   
   G   
    A    
    T    
   T   
   G   
   A   
   T   
   G   
   A   
   A   
   A   
   G   
   C   
   T   
   T   
   T   
   A   
   T   
   C   
   G   
   A   
   T   
   T   
   C   
   T   
   G   
   G   
   A   
   T   
   G   
   A   
   A   
   G   
   T   
   A   
   A   
   T   
   C   
   G   
    C    
   A   
   A   
   C   
   G   
   A   
   T   
   T   
   C   
   G   
   T   
   G   
   A   
   A   
   A   
   G   
   T   
   A   
   A   
   A   
   G   
   A   
   C   
   A   
   A   
   A   
   A   
   A   
   A   
   G   
   A   
    C    
   G   
   C   
    C    
   A   
   A   
   A   
   A   
   A   
   G   
   A   
   A   
   T   
   T   
   T   
   A   
   G   
   T   
   C   
   G   
   A   
    C    
   G   
   T   
    C    
   T   
   T   
    T    
   C   
   A   
   A   
   T   
   T   
   T   
   A   
   C   
   A   
   G   
   A   
   A   
   G   
   A   
   A   
   C   
   A   
   A   
   G   
   C   
   A   
   G   
   A   
   A   
   G   
   C   
   A   
   A   
   T   
    T    
   G   
   T   
   C   
   A   
   C   
   A   
   T   
   T   
   G   
   C   
   A   
   A   
    T    
   T   
   A   
   T   
   A   
    C    
   C   
   G   
   T   
   T   
   T   
   G   
   A   
   C   
   G   
   A   
   A   
    C    
   A   
   C   
    G    
   G   
   A   
   T   
   A   
   T   
   C   
   A   
   C   
   A   
   G   
   A   
   G   
   C   
   T   
   T   
   C   
   A   
   A   
   A   
   A   
   G   
   G   
   A   
   A   
    T    
   C   
    C    
   G   
   A   
   A   
   A   
    G    
   C   
   T   
   T   
   G   
   A   
   T   
   T   
   G   
   C   
    G    
   C   
   A   
   A   
   A   
   T   
   C   
   A   
   C   
   A   
   G   
   A   
   G   
   T   
   T   
    G    
   A   
   A   
   T   
   A   
   A   
   A   
   A   
   T   
   C   
   C   
   T   
   T   
   T   
   C   
   A   
   A   
   A   
    T    
   G   
   A   
   C   
   A   
   A   
   A   
   G   
   A   
    G    
   C   
   T   
   T   
   T   
   T   
    C    
   T   
   C   
   T   
   G   
   T   
   T   
   A   
   T   
   G   
   A   
   A   
   A   
   A   
   A   
   A   
   G   
   A   
   A   
   T   
   T   
   G   
   C   
   G   
   A   
   G   
   A   
   A   
   G   
   T   
   G   
   A   
   A   
   A   
   A   
   A   
   A   
   A   
   A   
   T   
   T   
   A   
    T    
   T   
   C   
   T   
   T   
   C   
   C   
   G   
   C   
   T   
   C   
   G   
   T   
   C   
    T    
    A    
   A   
   C   
   G   
   A   
   C   
   A   
   A   
   T   
   C   
    G    
   A   
   A   
   G   
   A   
   C   
   G   
   A   
   A   
   A   
   T   
   C   
   G   
   A   
   A   
   G   
   A   
   A   
   A   
   T   
   C   
   A   
   A   
   A   
   A   
   T   
    T    
   G   
   A   
   T   
   A   
   C   
   G   
   C   
   A   
   A   
    G    
   T   
    C    
   T   
   T   
   A   
   G   
   T   
   G   
   G   
   C   
    A    
   C   
   A   
   A   
   G   
   A   
   A   
   G   
   A   
   C   
   G   
   T   
   A   
   A   
   T   
    T    
   G   
   T   
   C   
   A   
   G   
   T   
   G   
   T   
    G    
   A   
   C   
    G    
   C   
   G   
   A   
   G   
   A   
   A   
   G   
   G   
   C   
   T   
   A   
   T   
   G   
   T   
   G   
   A   
   A   
   G   
   C   
   G   
   A   
   A   
   C   
   A   
   A   
   G   
   C   
   C   
   T   
   T   
   C   
   G   
   T   
   T   
   C   
    T    
   T   
   A   
   T   
   A   
   G   
   T   
   G   
   C   
   T   
   T   
   C   
    C    
   A   
   A   
   A   
   C   
   C   
   A   
   G   
   A   
   A   
   G   
   A   
   A   
   A   
   T   
   C   
   G   
   G   
   G   
   A   
   T   
   G   
   C   
   G   
   A   
   G   
   A   
   A   
   G   
   G   
   C   
   G   
   A   
    C    
   T   
   A   
   C   
   T   
   T   
   G   
   C   
   T   
    A    
   T   
   A   
   C   
    T    
   C   
   A   
   G   
   G   
   T   
   G   
   A   
   A   
   T   
   T   
    G    
   A   
   G   
    T    
   A   
   C   
   A   
    T    
   T   
   G   
   G   
   A   
   C   
   C   
   A   
   T   
   G   
   T   
   T   
   C   
   T   
   T   
   C   
   T   
   G   
   A   
   T   
    C    
   A   
   C   
    A    
   A   
   A   
   T   
   A   
   A   
   A   
   G   
   C   
   A   
   A   
   A   
   T   
   G   
   T   
   G   
   A   
   T   
   C   
   T   
   A   
   C   
   C   
   G   
    C    
   C   
   C   
   T   
   G   
   T   
    C    
   C   
   A   
   T   
   G   
   A   
   A   
   C   
   T   
   A   
   C   
   C   
    C    
   G   
   A   
   T   
   T   
   T   
   G   
   A   
   A   
   A   
   T   
   G   
   G   
   A   
   A   
   A   
   G   
   A   
   T   
   G   
   C   
   T   
   G   
   G   
    T    
   G   
   A   
    G    
   C   
   A   
    C    
   A   
   T   
   T   
   T   
   C   
   T   
   C   
   A   
   A   
   A   
   C   
   A   
   A   
   T   
   C   
   T   
   C   
   C   
   A   
   A   
   T   
   C   
   T   
   G   
   T   
   C   
   T   
   G   
   T   
   A   
   G   
   A   
    C    
   G   
   A   
   A   
   T   
   C   
   G   
   A   
   T   
    T    
   T   
   T   
   A   
   G   
   C   
   T   
   G   
   T   
   T   
   T   
   T   
    C    
   C   
   C   
   A   
   T   
   A   
   T   
   C   
   A   
   A   
   A   
   A   
   A   
   A   
   T   
    T    
   C   
   A   
   A   
   G   
   C   
   A   
   G   
   A   
    G    
   A   
   A   
   A   
   A   
   C   
   A   
   T   
   T   
   T   
   G   
   T   
    A    
   T   
   T   
   C   
   A   
   T   
   C   
   A   
   G   
   C   
   A   
   A   
   G   
   A   
   A   
   T   
   G   
   G   
   A   
   T   
   T   
    G    
    A    
   T   
   C   
   A   
   A   
   G   
   C   
   A   
   A   
   A   
   C   
   A   
   C   
   G   
   A   
   A   
   T   
   G   
   A   
   C   
   A   
   G   
   A   
   G   
   T   
   T   
   C   
   G   
   A   
   A   
   C   
   C   
   A   
   T   
   G   
   G   
   C   
   G   
   C   
   A   
   C   
   C   
   T   
   A   
    T    
   A   
   A   
   G   
   A   
   G   
   C   
   C   
   G   
    T    
   C   
   C   
   G   
   C   
   T   
   C   
   A   
   G   
   C   
   T   
   G   
   T   
   A   
   T   
   G   
   A   
   A   
   G   
   C   
   T   
   A   
   A   
   A   
   G   
   G   
   C   
   G   
   G   
   A   
   A   
   G   
   A   
   A   
   G   
   A   
   T   
   G   
   A   
   A   
   C   
   T   
    G    
   A   
   T   
   T   
   G   
   C   
   T   
   G   
   T   
    G    
   T   
   A   
   T   
   C   
   T   
   G   
   A   
   A   
   A   
   G   
   A   
   A   
   G   
   A   
   T   
   C   
    A    
   G   
   C   
   C   
   A   
   G   
   G   
   C   
   T   
   T   
   A   
   G   
   A   
   T   
   G   
   T   
    T    
   T   
   T   
   C   
   T   
   T   
   A   
   G   
   T   
   A   
   A   
   C   
   G   
    C    
   A   
   T   
   C   
   A   
   A   
   G   
   G   
   A   
   A   
   T   
   G   
   G   
   G   
    T    
   T   
   T   
   G   
   C   
   G   
   C   
   T   
    A    
   T   
   C   
   C   
   T   
   T   
   T   
   A   
   A   
    C    
   A   
   G   
   A   
   T   
   G   
   T   
   T   
   C   
   C   
   C   
   G   
   T   
   T   
   G   
   T   
   T   
   G   
   G   
   A   
   G   
   C   
   A   
   A   
   A   
   G   
   G   
   C   
   A   
   G   
    C    
   T   
   G   
   G   
   A   
   G   
   T   
   G   
   A   
   A   
   G   
   T   
   C   
   T   
   A   
   T   
   G   
   A   
   A   
   T   
   T   
   T   
   G   
   A   
   A   
   A   
   G   
   A   
   T   
   G   
   G   
   A   
   G   
   A   
   T   
   A   
   G   
   G   
    A    
   T   
   T   
    A    
   T   
   C   
   A   
   A   
    C    
   G   
   G   
    G    
   C   
   T   
    G    
   C   
   T   
   T   
   G   
   T   
   G   
   T   
   T   
   A   
   G   
   C   
   A   
   G   
   A   
    A    
   G   
   G   
    T    
   G   
   A   
   T   
   A   
   C   
   G   
   C   
   C   
    G    
   A   
   T   
   C   
   G   
   T   
   A   
   A   
   T   
   C   
   G   
   T   
   T   
   A   
   C   
   T   
   C   
   A   
   G   
   C   
   G   
   A   
   G   
   G   
   A   
   G   
   C   
    T    
   G   
   T   
    T    
   A   
   A   
   A   
   C   
   G   
   C   
   A   
   T   
   G   
   C   
   T   
   T   
   G   
   C   
   T   
   C   
   A   
   A   
   G   
   A   
   A   
   A   
   T   
   C   
   A   
   G   
   C   
   C   
   A   
   A   
   A   
   C   
    T    
   T   
   C   
   A   
   C   
   G   
   T   
   G   
   C   
   G   
   A   
   A   
   G   
   C   
   G   
   G   
   G   
   G   
   A   
   G   
   T   
   G   
   A   
   C   
   G   
   G   
   T   
   C   
   A   
   T   
   G   
   C   
   G   
   T   
   G   
   A   
   G   
   C   
   T   
   G   
   A   
   A   
   A   
   A   
   A   
   A   
   A   
   A   
   T   
   C   
   C   
    G    
   C   
   A   
    C    
   C   
   G   
    T    
   A   
   T   
   T   
   A   
   T   
   C   
   T   
   A   
   T   
   A   
   T   
   G   
   T   
   C   
   G   
   G   
   A   
   A   
   G   
   G   
   A   
   A   
   G   
   A   
   T   
   C   
   A   
   A   
   A   
   A   
   G   
   A   
   A   
   A   
   T   
   A   
   A   
   C   
   C   
   T   
   T   
   G   
   A   
   T   
   G   
   A   
   A   
   T   
   C   
   A   
   A   
   A   
   A   
   A   
   G   
   G   
    G    
   C   
   A   
    G    
   C   
   A   
   G   
    T    
   T   
   A   
   A   
   C   
   G   
   A   
   T   
   T   
   G   
   A   
    C    
   C   
   C   
   A   
   A   
   C   
    T    
   G   
   A   
   T   
   T   
   T   
    C    
   C   
   C   
   T   
   A   
   T   
   A   
   G   
   G   
   T   
   G   
   A   
   T   
   C   
   G   
   A   
   A   
   C   
   A   
   T   
   C   
   G   
   A   
   A   
   T   
   G   
   G   
    A    
   T   
   C   
    A    
   T   
   T   
   T   
   G   
   C   
    G    
   A   
   T   
   G   
   G   
   A   
    T    
   G   
   A   
   G   
   A   
   A   
   A   
   A   
   A   
   A   
   G   
   G   
   T   
   G   
   G   
   A   
   G   
   A   
   A   
   G   
   T   
   C   
   A   
   T   
   C   
   G   
   A   
   A   
   G   
   T   
    T    
   A   
    T    
   C   
   G   
   A   
   T   
   G   
   C   
   T   
   C   
   C   
   T   
   G   
   A   
   G   
    A    
   T   
   T   
   T   
   T   
    C    
   A   
   T   
    C    
   G   
   A   
    G    
    T    
   C   
    G    
   -   
   -   
   -   
 

 
   N52629   
   A   
   T   
   G   
   G   
   A   
   A   
   A   
   A   
   T   
   C   
   G   
   A   
   C   
   A   
   A   
   G   
   A   
   A   
   G   
   T   
   A   
   C   
   A   
   A   
   G   
   A   
   A   
   T   
   T   
   A   
   A   
   C   
   T   
   T   
   T   
   A   
   G   
   A   
   A   
   G   
   A   
   A   
   G   
   T   
   G   
   A   
   T   
   G   
   G   
   G   
   T   
   G   
   A   
   C   
   C   
   G   
   C   
   T   
   T   
   C   
   G   
   G   
   A   
   C   
   G   
   T   
   T   
   A   
   T   
   T   
   C   
   C   
   A   
   A   
   A   
   T   
   A   
   T   
   A   
   T   
   T   
   A   
   T   
    C    
   C   
   A   
    G    
   G   
   A   
   A   
   C   
   G   
   T   
   G   
   C   
   A   
   C   
   T   
   G   
   C   
   C   
   T   
   G   
   A   
   T   
   A   
   T   
    C    
   C   
   G   
   T   
   G   
   A   
   T   
   G   
   G   
   A   
   T   
   T   
   G   
   A   
   A   
    G    
   C   
   C   
   A   
   G   
   T   
    G    
   C   
   A   
   A   
   C   
   G   
   C   
   C   
   G   
   G   
   A   
   T   
   A   
   T   
   T   
   A   
   T   
   T   
    T    
   G   
   C   
    C    
   A   
   T   
   G   
   A   
   A   
   C   
   A   
   A   
   A   
   G   
   A   
   T   
   G   
   G   
   C   
   A   
   A   
   T   
   A   
   C   
   G   
   T   
   A   
   T   
   G   
   A   
   C   
   A   
   A   
   A   
   G   
   G   
   A   
   T   
   T   
    C    
   C   
   G   
   T   
   A   
   A   
   A   
   T   
   C   
   A   
   G   
   C   
   A   
   A   
   A   
   A   
   T   
   C   
   T   
   G   
   T   
   C   
   G   
   G   
   A   
   A   
   A   
   T   
   A   
   T   
   C   
   A   
   T   
   G   
   G   
   G   
    G    
   A   
   A   
   T   
   T   
   A   
   T   
   C   
   A   
   T   
   C   
   C   
   T   
   C   
   A   
   T   
   G   
   G   
   T   
   G   
   A   
   C   
   A   
   G   
   C   
   A   
   G   
   T   
   A   
   T   
   T   
   T   
   A   
   T   
   G   
   A   
   A   
   G   
   C   
   G   
   A   
   T   
   G   
   G   
   T   
   C   
   C   
   G   
   C   
   A   
   T   
   G   
   A   
   G   
   C   
   C   
   A   
   A   
   G   
   A   
    C    
   T   
   G   
   G   
   A   
   A   
   G   
   T   
   T   
   G   
   C   
   G   
   G   
   G   
   A   
   A   
   G   
   T   
   A   
   C   
   T   
   G   
   A   
   T   
   C   
   G   
   A   
    A    
   A   
   T   
   G   
   C   
   A   
   C   
   G   
   G   
    G    
   A   
   A   
   T   
   A   
   A   
    C    
   G   
   G   
   G   
   A   
   G   
   T   
   A   
   T   
   G   
   G   
   A   
   C   
   G   
   G   
   T   
   G   
   A   
   T   
   C   
   C   
   A   
   C   
   C   
   T   
   G   
   C   
   T   
   G   
   C   
   G   
   A   
   T   
   G   
   C   
   G   
   T   
   T   
   A   
   T   
   A   
   C   
   T   
   G   
   A   
   A   
   G   
   C   
   C   
   A   
   G   
    A    
   T   
   T   
   A   
   T   
   C   
   C   
   A   
   A   
   G   
   T   
   T   
   A   
   A   
   G   
   T   
   G   
   G   
   A   
   G   
   A   
   G   
   A   
   T   
   G   
    C    
   T   
   A   
    G    
   C   
   A   
   G   
   A   
   T   
   A   
   T   
   C   
   G   
   A   
    A    
   A   
   A   
   A   
   G   
   A   
   A   
   A   
   C   
   A   
   G   
   T   
    C    
   G   
   A   
   T   
   C   
   T   
   C   
   G   
   T   
   G   
   T   
   G   
   G   
   A   
   A   
   T   
   T   
   T   
   T   
   G   
   A   
   T   
   G   
   A   
   T   
   A   
   C   
   A   
   G   
   A   
   A   
   A   
   A   
   A   
   G   
   A   
   A   
   C   
   C   
   A   
   A   
   C   
   A   
   G   
   T   
   C   
   C   
   T   
   T   
   C   
   C   
   A   
   G   
   C   
   T   
   C   
   G   
   A   
   T   
   A   
   C   
   C   
   C   
   A   
   A   
   A   
   C   
   C   
   T   
   T   
   C   
   T   
    C    
   G   
   T   
   C   
   A   
   A   
   T   
   G   
   G   
   A   
   T   
   C   
   G   
   A   
   C   
   A   
   G   
   G   
   T   
   A   
   T   
   A   
   T   
   C   
   T   
   G   
   C   
   T   
   G   
   G   
   A   
   T   
   A   
   T   
   G   
   C   
    A    
   A   
   C   
   A   
   G   
   A   
   A   
   A   
   T   
   C   
   C   
   C   
   G   
   A   
   C   
    T    
   C   
   A   
   T   
   A   
   A   
   C   
   T   
   T   
   A   
   G   
   C   
   T   
   G   
   A   
   A   
   G   
   T   
   G   
   A   
   T   
    C    
   G   
   A   
    T    
   G   
   G   
   C   
   A   
   C   
   A   
   A   
   T   
   C   
   T   
   A   
   T   
   A   
   T   
   G   
   A   
   T   
   C   
   G   
   A   
   T   
   C   
   A   
   T   
   C   
   C   
   A   
   C   
   A   
   A   
   G   
   C   
   G   
   A   
   G   
   C   
   C   
   T   
   T   
   G   
   A   
   A   
   A   
   A   
   A   
   T   
   T   
   G   
   A   
   T   
   G   
   G   
   A   
   A   
   T   
   A   
   T   
   A   
   T   
   T   
   C   
   C   
   T   
   G   
   G   
   A   
   C   
   C   
   T   
   G   
   A   
   C   
   T   
   T   
    T    
   C   
   C   
   A   
   A   
   C   
   A   
   G   
   G   
   A   
   G   
   G   
    G    
   A   
   T   
   C   
   T   
   T   
   G   
   C   
   A   
   A   
   G   
   G   
    A    
   A   
   A   
   A   
   G   
   A   
   T   
   G   
   A   
   A   
   A   
   T   
   C   
   A   
   A   
   A   
   A   
   A   
   A   
   G   
   C   
   T   
   T   
   A   
   T   
   G   
   A   
   A   
   A   
   C   
   A   
   G   
   G   
    G    
   C   
   G   
   A   
   G   
   G   
   A   
   A   
   A   
   A   
   G   
   T   
   T   
   A   
    T    
   T   
   C   
   T   
   T   
   C   
   G   
   T   
   T   
   C   
   G   
   A   
   A   
   A   
   A   
   C   
   A   
   A   
   A   
   G   
   A   
   T   
   A   
   G   
   A   
    A    
   G   
   C   
   A   
   A   
   T   
   G   
   A   
   A   
   G   
   G   
   G   
    T    
   A   
   A   
    T    
   A   
   A   
   A   
   C   
   A   
   G   
   C   
   A   
   A   
   A   
   T   
   C   
   G   
   T   
   G   
   A   
   T   
   C   
   A   
   C   
    A    
   G   
   A   
   A   
   A   
   T   
   C   
   C   
   C   
   A   
   T   
   A   
   T   
   G   
   A   
   A   
   G   
   T   
   A   
   A   
   A   
   T   
   A   
   A   
   A   
   G   
   C   
   G   
   A   
   C   
    C    
   T   
   T   
   G   
   G   
   T   
   G   
   A   
   A   
   A   
   A   
   A   
   A   
   A   
   T   
   G   
   G   
   A   
   T   
   G   
   A   
   G   
   A   
   T   
   C   
   C   
   G   
   T   
   T   
   T   
   G   
   A   
   A   
   T   
   A   
   A   
   G   
   A   
   A   
   A   
   A   
   T   
   C   
   G   
   A   
    T    
   G   
   G   
   A   
   A   
   T   
   A   
   G   
   C   
   T   
   G   
   A   
   A   
   G   
   T   
    A    
   C   
   G   
   A   
   G   
   A   
   T   
   G   
   A   
   A   
   A   
   G   
   T   
   G   
   A   
   T   
    A    
   G   
    A    
   A   
   C   
    A    
   G   
   G   
   T   
   C   
   T   
   G   
   C   
   A   
   A   
   A   
   T   
   T   
   G   
   T   
   C   
   G   
   T   
    C    
   G   
   A   
   A   
   T   
   T   
   G   
   A   
   A   
   A   
   A   
   A   
   A   
   G   
   A   
    C    
   G   
   C   
   C   
   A   
   A   
    T    
   G   
   C   
   T   
   C   
   A   
   A   
   G   
   G   
    A    
   A   
   T   
   T   
   T   
   T   
   G   
   A   
   A   
   T   
   T   
   A   
    T    
   T   
   T   
   G   
   T   
   T   
   C   
   A   
   A   
   A   
   A   
   A   
   C   
   A   
   C   
   T   
   G   
   A   
   G   
    C    
   T   
   A   
   C   
   A   
   G   
   A   
   T   
   C   
   A   
   A   
   T   
   T   
   A   
   C   
   A   
   A   
   T   
   T   
   T   
    C    
   A   
   A   
   T   
   A   
   T   
   G   
   G   
   T   
   C   
   G   
   C   
   T   
   A   
   T   
   C   
   G   
   A   
    C    
   C   
   A   
   T   
   A   
   T   
   G   
   A   
   C   
   T   
   C   
   C   
   G   
   C   
   A   
    T    
   C   
   A   
   A   
   G   
   T   
   T   
   G   
   G   
   A   
   C   
   T   
   G   
   A   
   A   
   A   
   G   
   A   
   C   
   A   
   T   
   T   
   T   
   T   
   A   
   A   
   G   
   A   
   A   
   G   
   T   
   T   
   A   
   T   
   A   
   T   
   C   
   G   
   A   
   G   
   C   
   A   
   T   
   C   
   G   
   A   
   A   
   A   
   G   
   C   
   A   
   A   
   G   
   T   
   C   
   A   
   T   
   A   
   A   
   C   
   A   
   A   
   A   
   A   
   C   
   G   
    T    
   A   
   G   
    T    
   C   
   A   
   A   
   T   
   T   
   C   
   G   
   A   
   T   
   T   
   T   
   A   
   G   
   C   
   C   
   A   
   A   
   A   
   G   
   C   
   T   
   C   
   A   
   A   
   A   
   A   
   A   
    A    
   G   
   A   
   C   
   A   
   G   
   C   
   A   
   T   
   A   
   T   
   C   
   G   
   T   
    A    
   G   
   A   
   A   
   G   
   G   
    A    
    T    
   T   
   G   
   A   
   T   
   G   
   A   
   A   
   A   
   G   
   C   
   T   
   T   
   T   
   A   
   T   
   C   
   G   
   A   
   T   
   T   
   C   
   T   
   G   
   G   
   A   
   T   
   G   
   A   
   A   
   G   
   T   
   A   
   A   
   T   
   C   
   G   
    C    
   A   
   A   
   C   
   G   
   A   
   T   
   T   
   C   
   G   
   T   
   G   
   A   
   A   
   A   
   G   
   T   
   A   
   A   
   A   
   G   
   A   
   C   
   A   
   A   
   A   
   A   
   A   
   A   
   G   
   A   
    C    
   G   
   C   
    C    
   A   
   A   
   A   
   A   
   A   
   G   
   A   
   A   
   T   
   T   
   T   
   A   
   G   
   T   
   C   
   G   
   A   
    C    
   G   
   T   
    C    
   T   
   T   
    T    
   C   
   A   
   A   
   T   
   T   
   T   
   A   
   C   
   A   
   G   
   A   
   A   
   G   
   A   
   A   
   C   
   A   
   A   
   G   
   C   
   A   
   G   
   A   
   A   
   G   
   C   
   A   
   A   
   T   
    T    
   G   
   T   
   C   
   A   
   C   
   A   
   T   
   T   
   G   
   C   
   A   
   A   
    T    
   T   
   A   
   T   
   A   
    C    
   C   
   G   
   T   
   T   
   T   
   G   
   A   
   C   
   G   
   A   
   A   
    C    
   A   
   C   
    G    
   G   
   A   
   T   
   A   
   T   
   C   
   A   
   C   
   A   
   G   
   A   
   G   
   C   
   T   
   T   
   C   
   A   
   A   
   A   
   A   
   G   
   G   
   A   
   A   
    T    
   C   
    C    
   G   
   A   
   A   
   A   
    G    
   C   
   T   
   T   
   G   
   A   
   T   
   T   
   G   
   C   
    G    
   C   
   A   
   A   
   A   
   T   
   C   
   A   
   C   
   A   
   G   
   A   
   G   
   T   
   T   
    G    
   A   
   A   
   T   
   A   
   A   
   A   
   A   
   T   
   C   
   C   
   T   
   T   
   T   
   C   
   A   
   A   
   A   
    T    
   G   
   A   
   C   
   A   
   A   
   A   
   G   
   A   
    G    
   C   
   T   
   T   
   T   
   T   
    C    
   T   
   C   
   T   
   G   
   T   
   T   
   A   
   T   
   G   
   A   
   A   
   A   
   A   
   A   
   A   
   G   
   A   
   A   
   T   
   T   
   G   
   C   
   G   
   A   
   G   
   A   
   A   
   G   
   T   
   G   
   A   
   A   
   A   
   A   
   A   
   A   
   A   
   A   
   T   
   T   
   A   
    T    
   T   
   C   
   T   
   T   
   C   
   C   
   G   
   C   
   T   
   C   
   G   
   T   
   C   
    T    
    A    
   A   
   C   
   G   
   A   
   C   
   A   
   A   
   T   
   C   
    G    
   A   
   A   
   G   
   A   
   C   
   G   
   A   
   A   
   A   
   T   
   C   
   G   
   A   
   A   
   G   
   A   
   A   
   A   
   T   
   C   
   A   
   A   
   A   
   A   
   T   
    T    
   G   
   A   
   T   
   A   
   C   
   G   
   C   
   A   
   A   
    G    
   T   
    C    
   T   
   T   
   A   
   G   
   T   
   G   
   G   
   C   
    A    
   C   
   A   
   A   
   G   
   A   
   A   
   G   
   A   
   C   
   G   
   T   
   A   
   A   
   T   
    T    
   G   
   T   
   C   
   A   
   G   
   T   
   G   
   T   
    A    
   A   
   C   
    G    
   C   
   G   
   A   
   G   
   A   
   A   
   G   
   G   
   C   
   T   
   A   
   T   
   G   
   T   
   G   
   A   
   A   
   G   
   C   
   G   
   A   
   A   
   C   
   A   
   A   
   G   
   C   
   C   
   T   
   T   
   C   
   G   
   T   
   T   
   C   
    T    
   T   
   A   
   T   
   A   
   G   
   T   
   G   
   C   
   T   
   T   
   C   
    C    
   A   
   A   
   A   
   C   
   C   
   A   
   G   
   A   
   A   
   G   
   A   
   A   
   A   
   T   
   C   
   G   
   G   
   G   
   A   
   T   
   G   
   C   
   G   
   A   
   G   
   A   
   A   
   G   
   G   
   C   
   G   
   A   
    C    
   T   
   A   
   C   
   T   
   T   
   G   
   C   
   T   
    A    
   T   
   A   
   C   
    T    
   C   
   A   
   G   
   G   
   T   
   G   
   A   
   A   
   T   
   T   
    G    
   A   
   G   
    T    
   A   
   C   
   A   
    T    
   T   
   G   
   G   
   A   
   C   
   C   
   A   
   T   
   G   
   T   
   T   
   C   
   T   
   T   
   C   
   T   
   G   
   A   
   T   
    C    
   A   
   C   
    A    
   A   
   A   
   T   
   A   
   A   
   A   
   G   
   C   
   A   
   A   
   A   
   T   
   G   
   T   
   G   
   A   
   T   
   C   
   T   
   A   
   C   
   C   
   G   
    C    
   C   
   C   
   T   
   G   
   T   
    C    
   C   
   A   
   T   
   G   
   A   
   A   
   C   
   T   
   A   
   C   
   C   
    C    
   G   
   A   
   T   
   T   
   T   
   G   
   A   
   A   
   A   
   T   
   G   
   G   
   A   
   A   
   A   
   G   
   A   
   T   
   G   
   C   
   T   
   G   
   G   
    T    
   G   
   A   
    G    
   C   
   A   
    C    
   A   
   T   
   T   
   T   
   C   
   T   
   C   
   A   
   A   
   A   
   C   
   A   
   A   
   T   
   C   
   T   
   C   
   C   
   A   
   A   
   T   
   C   
   T   
   G   
   T   
   C   
   T   
   G   
   T   
   A   
   G   
   A   
    C    
   G   
   A   
   A   
   T   
   C   
   G   
   A   
   T   
    T    
   T   
   T   
   A   
   G   
   C   
   T   
   G   
   T   
   T   
   T   
   T   
    C    
   C   
   C   
   A   
   T   
   A   
   T   
   C   
   A   
   A   
   A   
   A   
   A   
   A   
   T   
    T    
   C   
   A   
   A   
   G   
   C   
   A   
   G   
   A   
    G    
   A   
   A   
   A   
   A   
   C   
   A   
   T   
   T   
   T   
   G   
   T   
    A    
   T   
   T   
   C   
   A   
   T   
   C   
   A   
   G   
   C   
   A   
   A   
   G   
   A   
   A   
   T   
   G   
   G   
   A   
   T   
   T   
    G    
    A    
   T   
   C   
   A   
   A   
   G   
   C   
   A   
   A   
   A   
   C   
   A   
   C   
   G   
   A   
   A   
   T   
   G   
   A   
   C   
   A   
   G   
   A   
   G   
   T   
   T   
   C   
   G   
   A   
   A   
   C   
   C   
   A   
   T   
   G   
   G   
   C   
   G   
   C   
   A   
   C   
   C   
   T   
   A   
    T    
   A   
   A   
   G   
   A   
   G   
   C   
   C   
   G   
    T    
   C   
   C   
   G   
   C   
   T   
   C   
   A   
   G   
   C   
   T   
   G   
   T   
   A   
   T   
   G   
   A   
   A   
   G   
   C   
   T   
   A   
   A   
   A   
   G   
   G   
   C   
   G   
   G   
   A   
   A   
   G   
   A   
   A   
   G   
   A   
   T   
   G   
   A   
   A   
   C   
   T   
    G    
   A   
   T   
   T   
   G   
   C   
   T   
   G   
   T   
    G    
   T   
   A   
   T   
   C   
   T   
   G   
   A   
   A   
   A   
   G   
   A   
   A   
   G   
   A   
   T   
   C   
    A    
   G   
   C   
   C   
   A   
   G   
   G   
   C   
   T   
   T   
   A   
   G   
   A   
   T   
   G   
   T   
    T    
   T   
   T   
   C   
   T   
   T   
   A   
   G   
   T   
   A   
   A   
   C   
   G   
    C    
   A   
   T   
   C   
   A   
   A   
   G   
   G   
   A   
   A   
   T   
   G   
   G   
   G   
    T    
   T   
   T   
   G   
   C   
   G   
   C   
   T   
    A    
   T   
   C   
   C   
   T   
   T   
   T   
   A   
   A   
    C    
   A   
   G   
   A   
   T   
   G   
   T   
   T   
   C   
   C   
   C   
   G   
   T   
   T   
   G   
   T   
   T   
   G   
   G   
   A   
   G   
   C   
   A   
   A   
   A   
   G   
   G   
   C   
   A   
   G   
    C    
   T   
   G   
   G   
   A   
   G   
   T   
   G   
   A   
   A   
   G   
   T   
   C   
   T   
   A   
   T   
   G   
   A   
   A   
   T   
   T   
   T   
   G   
   A   
   A   
   A   
   G   
   A   
   T   
   G   
   G   
   A   
   G   
   A   
   T   
   A   
   G   
   G   
    A    
   T   
   T   
    A    
   T   
   C   
   A   
   A   
    C    
   G   
   G   
    G    
   C   
   T   
    G    
   C   
   T   
   T   
   G   
   T   
   G   
   T   
   T   
   A   
   G   
   C   
   A   
   G   
   A   
    A    
   G   
   G   
    T    
   G   
   A   
   T   
   A   
   C   
   G   
   C   
   C   
    G    
   A   
   T   
   C   
   G   
   T   
   A   
   A   
   T   
   C   
   G   
   T   
   T   
   A   
   C   
   T   
   C   
   A   
   G   
   C   
   G   
   A   
   G   
   G   
   A   
   G   
   C   
    T    
   G   
   T   
    T    
   A   
   A   
   A   
   C   
   G   
   C   
   A   
   T   
   G   
   C   
   T   
   T   
   G   
   C   
   T   
   C   
   A   
   A   
   G   
   A   
   A   
   A   
   T   
   C   
   A   
   G   
   C   
   C   
   A   
   A   
   A   
   C   
    T    
   T   
   C   
   A   
   C   
   G   
   T   
   G   
   C   
   G   
   A   
   A   
   G   
   C   
   G   
   G   
   G   
   G   
   A   
   G   
   T   
   G   
   A   
   C   
   G   
   G   
   T   
   C   
   A   
   T   
   G   
   C   
   G   
   T   
   G   
   A   
   G   
   C   
   T   
   G   
   A   
   A   
   A   
   A   
   A   
   A   
   A   
   A   
   T   
   C   
   C   
    G    
   C   
   A   
    C    
   C   
   G   
    T    
   A   
   T   
   T   
   A   
   T   
   C   
   T   
   A   
   T   
   A   
   T   
   G   
   T   
   C   
   G   
   G   
   A   
   A   
   G   
   G   
   A   
   A   
   G   
   A   
   T   
   C   
   A   
   A   
   A   
   A   
   G   
   A   
   A   
   A   
   T   
   A   
   A   
   C   
   C   
   T   
   T   
   G   
   A   
   T   
   G   
   A   
   A   
   T   
   C   
   A   
   A   
   A   
   A   
   A   
   G   
   G   
    G    
   C   
   A   
    G    
   C   
   A   
   G   
    T    
   T   
   A   
   A   
   C   
   G   
   A   
   T   
   T   
   G   
   A   
    C    
   C   
   C   
   A   
   A   
   C   
    T    
   G   
   A   
   T   
   T   
   T   
    C    
   C   
   C   
   T   
   A   
   T   
   A   
   G   
   G   
   T   
   G   
   A   
   T   
   C   
   G   
   A   
   A   
   C   
   A   
   T   
   C   
   G   
   A   
   A   
   T   
   G   
   G   
    A    
   T   
   C   
    A    
   T   
   T   
   T   
   G   
   C   
    G    
   A   
   T   
   G   
   G   
   A   
    T    
   G   
   A   
   G   
   A   
   A   
   A   
   A   
   A   
   A   
   G   
   G   
   T   
   G   
   G   
   A   
   G   
   A   
   A   
   G   
   T   
   C   
   A   
   T   
   C   
   G   
   A   
   A   
   G   
   T   
    T    
   A   
    T    
   C   
   G   
   A   
   T   
   G   
   C   
   T   
   C   
   C   
   T   
   G   
   A   
   G   
    A    
   T   
   T   
   T   
   T   
    C    
   A   
   T   
    C    
   G   
   A   
    G    
    T    
   C   
    G    
   -   
   -   
   -   
 

 
   N60519F   
   A   
   T   
   G   
   G   
   A   
   A   
   A   
   A   
   T   
   C   
   G   
   A   
   C   
   A   
   A   
   G   
   A   
   A   
   G   
   T   
   A   
   C   
   A   
   A   
   G   
   A   
   A   
   T   
   T   
   A   
   A   
   C   
   T   
   T   
   T   
   A   
   G   
   A   
   A   
   G   
   A   
   A   
   G   
   T   
   G   
   A   
   T   
   G   
   G   
   G   
   T   
   G   
   A   
   C   
   C   
   G   
   C   
   T   
   T   
   C   
   G   
   G   
   A   
   C   
   G   
   T   
   T   
   A   
   T   
   T   
   C   
   C   
   A   
   A   
   A   
   T   
   A   
   T   
   A   
   T   
   T   
   A   
   T   
    C    
   C   
   A   
    A    
   G   
   A   
   A   
   C   
   G   
   T   
   G   
   C   
   A   
   C   
   T   
   G   
   C   
   C   
   T   
   G   
   A   
   T   
   A   
   T   
    C    
   C   
   G   
   T   
   G   
   A   
   T   
   G   
   G   
   A   
   T   
   T   
   G   
   A   
   A   
    G    
   C   
   C   
   A   
   G   
   T   
    G    
   C   
   A   
   A   
   C   
   G   
   C   
   C   
   G   
   G   
   A   
   T   
   A   
   T   
   T   
   A   
   T   
   T   
    T    
   G   
   C   
    C    
   A   
   T   
   G   
   A   
   A   
   C   
   A   
   A   
   A   
   G   
   A   
   T   
   G   
   G   
   C   
   A   
   A   
   T   
   A   
   C   
   G   
   T   
   A   
   T   
   G   
   A   
   C   
   A   
   A   
   A   
   G   
   G   
   A   
   T   
   T   
    C    
   C   
   G   
   T   
   A   
   A   
   A   
   T   
   C   
   A   
   G   
   C   
   A   
   A   
   A   
   A   
   T   
   C   
   T   
   G   
   T   
   C   
   G   
   G   
   A   
   A   
   A   
   T   
   A   
   T   
   C   
   A   
   T   
   G   
   G   
   G   
    G    
   A   
   A   
   T   
   T   
   A   
   T   
   C   
   A   
   T   
   C   
   C   
   T   
   C   
   A   
   T   
   G   
   G   
   T   
   G   
   A   
   C   
   A   
   G   
   C   
   A   
   G   
   T   
   A   
   T   
   T   
   T   
   A   
   T   
   G   
   A   
   A   
   G   
   C   
   G   
   A   
   T   
   G   
   G   
   T   
   C   
   C   
   G   
   C   
   A   
   T   
   G   
   A   
   G   
   C   
   C   
   A   
   A   
   G   
   A   
    C    
   T   
   G   
   G   
   A   
   A   
   G   
   T   
   T   
   G   
   C   
   G   
   G   
   G   
   A   
   A   
   G   
   T   
   A   
   C   
   T   
   G   
   A   
   T   
   C   
   G   
   A   
    A    
   A   
   T   
   G   
   C   
   A   
   C   
   G   
   G   
    G    
   A   
   A   
   T   
   A   
   A   
    C    
   G   
   G   
   G   
   A   
   G   
   T   
   A   
   T   
   G   
   G   
   A   
   C   
   G   
   G   
   T   
   G   
   A   
   T   
   C   
   C   
   A   
   C   
   C   
   T   
   G   
   C   
   T   
   G   
   C   
   G   
   A   
   T   
   G   
   C   
   G   
   T   
   T   
   A   
   T   
   A   
   C   
   T   
   G   
   A   
   A   
   G   
   C   
   C   
   A   
   G   
    A    
   T   
   T   
   A   
   T   
   C   
   C   
   A   
   A   
   G   
   T   
   T   
   A   
   A   
   G   
   T   
   G   
   G   
   A   
   G   
   A   
   G   
   A   
   T   
   G   
    C    
   T   
   A   
    G    
   C   
   A   
   G   
   A   
   T   
   A   
   T   
   C   
   G   
   A   
    A    
   A   
   A   
   A   
   G   
   A   
   A   
   A   
   C   
   A   
   G   
   T   
    C    
   G   
   A   
   T   
   C   
   T   
   C   
   G   
   T   
   G   
   T   
   G   
   G   
   A   
   A   
   T   
   T   
   T   
   T   
   G   
   A   
   T   
   G   
   A   
   T   
   A   
   C   
   A   
   G   
   A   
   A   
   A   
   A   
   A   
   G   
   A   
   A   
   C   
   C   
   A   
   A   
   C   
   A   
   G   
   T   
   C   
   C   
   T   
   T   
   C   
   C   
   A   
   G   
   C   
   T   
   C   
   G   
   A   
   T   
   A   
   C   
   C   
   C   
   A   
   A   
   A   
   C   
   C   
   T   
   T   
   C   
   T   
    C    
   G   
   T   
   C   
   A   
   A   
   T   
   G   
   G   
   A   
   T   
   C   
   G   
   A   
   C   
   A   
   G   
   G   
   T   
   A   
   T   
   A   
   T   
   C   
   T   
   G   
   C   
   T   
   G   
   G   
   A   
   T   
   A   
   T   
   G   
   C   
    A    
   A   
   C   
   A   
   G   
   A   
   A   
   A   
   T   
   C   
   C   
   C   
   G   
   A   
   C   
    T    
   C   
   A   
   T   
   A   
   A   
   C   
   T   
   T   
   A   
   G   
   C   
   T   
   G   
   A   
   A   
   G   
   T   
   G   
   A   
   T   
    C    
   G   
   A   
    T    
   G   
   G   
   C   
   A   
   C   
   A   
   A   
   T   
   C   
   T   
   A   
   T   
   A   
   T   
   G   
   A   
   T   
   C   
   G   
   A   
   T   
   C   
   A   
   T   
   C   
   C   
   A   
   C   
   A   
   A   
   G   
   C   
   G   
   A   
   G   
   C   
   C   
   T   
   T   
   G   
   A   
   A   
   A   
   A   
   A   
   T   
   T   
   G   
   A   
   T   
   G   
   G   
   A   
   A   
   T   
   A   
   T   
   A   
   T   
   T   
   C   
   C   
   T   
   G   
   G   
   A   
   C   
   C   
   T   
   G   
   A   
   C   
   T   
   T   
    T    
   C   
   C   
   A   
   A   
   C   
   A   
   G   
   G   
   A   
   G   
   G   
    G    
   A   
   T   
   C   
   T   
   T   
   G   
   C   
   A   
   A   
   G   
   G   
    A    
   A   
   A   
   A   
   G   
   A   
   T   
   G   
   A   
   A   
   A   
   T   
   C   
   A   
   A   
   A   
   A   
   A   
   A   
   G   
   C   
   T   
   T   
   A   
   T   
   G   
   A   
   A   
   A   
   C   
   A   
   G   
   G   
    G    
   C   
   G   
   A   
   G   
   G   
   A   
   A   
   A   
   A   
   G   
   T   
   T   
   A   
    T    
   T   
   C   
   T   
   T   
   C   
   G   
   T   
   T   
   C   
   G   
   A   
   A   
   A   
   A   
   C   
   A   
   A   
   A   
   G   
   A   
   T   
   A   
   G   
   A   
    A    
   G   
   C   
   A   
   A   
   T   
   G   
   A   
   A   
   G   
   G   
   G   
    T    
   A   
   A   
    T    
   A   
   A   
   A   
   C   
   A   
   G   
   C   
   A   
   A   
   A   
   T   
   C   
   G   
   T   
   G   
   A   
   T   
   C   
   A   
   C   
    A    
   G   
   A   
   A   
   A   
   T   
   C   
   C   
   C   
   A   
   T   
   A   
   T   
   G   
   A   
   A   
   G   
   T   
   A   
   A   
   A   
   T   
   A   
   A   
   A   
   G   
   C   
   G   
   A   
   C   
    C    
   T   
   T   
   G   
   G   
   T   
   G   
   A   
   A   
   A   
   A   
   A   
   A   
   A   
   T   
   G   
   G   
   A   
   T   
   G   
   A   
   G   
   A   
   T   
   C   
   C   
   G   
   T   
   T   
   T   
   G   
   A   
   A   
   T   
   A   
   A   
   G   
   A   
   A   
   A   
   A   
   T   
   C   
   G   
   A   
    T    
   G   
   G   
   A   
   A   
   T   
   A   
   G   
   C   
   T   
   G   
   A   
   A   
   G   
   T   
    A    
   C   
   G   
   A   
   G   
   A   
   T   
   G   
   A   
   A   
   A   
   G   
   T   
   G   
   A   
   T   
    A    
   G   
    A    
   A   
   C   
    A    
   G   
   G   
   T   
   C   
   T   
   G   
   C   
   A   
   A   
   A   
   T   
   T   
   G   
   T   
   C   
   G   
   T   
    C    
   G   
   A   
   A   
   T   
   T   
   G   
   A   
   A   
   A   
   A   
   A   
   A   
   G   
   A   
    C    
   G   
   C   
   C   
   A   
   A   
    T    
   G   
   C   
   T   
   C   
   A   
   A   
   G   
   G   
    A    
   A   
   T   
   T   
   T   
   T   
   G   
   A   
   A   
   T   
   T   
   A   
    T    
   T   
   T   
   G   
   T   
   T   
   C   
   A   
   A   
   A   
   A   
   A   
   C   
   A   
   C   
   T   
   G   
   A   
   G   
    C    
   T   
   A   
   C   
   A   
   G   
   A   
   T   
   C   
   A   
   A   
   T   
   T   
   A   
   C   
   A   
   A   
   T   
   T   
   T   
    C    
   A   
   A   
   T   
   A   
   T   
   G   
   G   
   T   
   C   
   G   
   C   
   T   
   A   
   T   
   C   
   G   
   A   
    C    
   C   
   A   
   T   
   A   
   T   
   G   
   A   
   C   
   T   
   C   
   C   
   G   
   C   
   A   
    T    
   C   
   A   
   A   
   G   
   T   
   T   
   G   
   G   
   A   
   C   
   T   
   G   
   A   
   A   
   A   
   G   
   A   
   C   
   A   
   T   
   T   
   T   
   T   
   A   
   A   
   G   
   A   
   A   
   G   
   T   
   T   
   A   
   T   
   A   
   T   
   C   
   G   
   A   
   G   
   C   
   A   
   T   
   C   
   G   
   A   
   A   
   A   
   G   
   C   
   A   
   A   
   G   
   T   
   C   
   A   
   T   
   A   
   A   
   C   
   A   
   A   
   A   
   A   
   C   
   G   
    T    
   A   
   G   
    T    
   C   
   A   
   A   
   T   
   T   
   C   
   G   
   A   
   T   
   T   
   T   
   A   
   G   
   C   
   C   
   A   
   A   
   A   
   G   
   C   
   T   
   C   
   A   
   A   
   A   
   A   
   A   
    A    
   G   
   A   
   C   
   A   
   G   
   C   
   A   
   T   
   A   
   T   
   C   
   G   
   T   
    A    
   G   
   A   
   A   
   G   
   G   
    A    
    T    
   T   
   G   
   A   
   T   
   G   
   A   
   A   
   A   
   G   
   C   
   T   
   T   
   T   
   A   
   T   
   C   
   G   
   A   
   T   
   T   
   C   
   T   
   G   
   G   
   A   
   T   
   G   
   A   
   A   
   G   
   T   
   A   
   A   
   T   
   C   
   G   
    C    
   A   
   A   
   C   
   G   
   A   
   T   
   T   
   C   
   G   
   T   
   G   
   A   
   A   
   A   
   G   
   T   
   A   
   A   
   A   
   G   
   A   
   C   
   A   
   A   
   A   
   A   
   A   
   A   
   G   
   A   
    C    
   G   
   C   
    C    
   A   
   A   
   A   
   A   
   A   
   G   
   A   
   A   
   T   
   T   
   T   
   A   
   G   
   T   
   C   
   G   
   A   
    C    
   G   
   T   
    C    
   T   
   T   
    T    
   C   
   A   
   A   
   T   
   T   
   T   
   A   
   C   
   A   
   G   
   A   
   A   
   G   
   A   
   A   
   C   
   A   
   A   
   G   
   C   
   A   
   G   
   A   
   A   
   G   
   C   
   A   
   A   
   T   
    T    
   G   
   T   
   C   
   A   
   C   
   A   
   T   
   T   
   G   
   C   
   A   
   A   
    T    
   T   
   A   
   T   
   A   
    C    
   C   
   G   
   T   
   T   
   T   
   G   
   A   
   C   
   G   
   A   
   A   
    C    
   A   
   C   
    G    
   G   
   A   
   T   
   A   
   T   
   C   
   A   
   C   
   A   
   G   
   A   
   G   
   C   
   T   
   T   
   C   
   A   
   A   
   A   
   A   
   G   
   G   
   A   
   A   
    T    
   C   
    C    
   G   
   A   
   A   
   A   
    G    
   C   
   T   
   T   
   G   
   A   
   T   
   T   
   G   
   C   
    G    
   C   
   A   
   A   
   A   
   T   
   C   
   A   
   C   
   A   
   G   
   A   
   G   
   T   
   T   
    G    
   A   
   A   
   T   
   A   
   A   
   A   
   A   
   T   
   C   
   C   
   T   
   T   
   T   
   C   
   A   
   A   
   A   
    T    
   G   
   A   
   C   
   A   
   A   
   A   
   G   
   A   
    G    
   C   
   T   
   T   
   T   
   T   
    C    
   T   
   C   
   T   
   G   
   T   
   T   
   A   
   T   
   G   
   A   
   A   
   A   
   A   
   A   
   A   
   G   
   A   
   A   
   T   
   T   
   G   
   C   
   G   
   A   
   G   
   A   
   A   
   G   
   T   
   G   
   A   
   A   
   A   
   A   
   A   
   A   
   A   
   A   
   T   
   T   
   A   
    T    
   T   
   C   
   T   
   T   
   C   
   C   
   G   
   C   
   T   
   C   
   G   
   T   
   C   
    T    
    A    
   A   
   C   
   G   
   A   
   C   
   A   
   A   
   T   
   C   
    G    
   A   
   A   
   G   
   A   
   C   
   G   
   A   
   A   
   A   
   T   
   C   
   G   
   A   
   A   
   G   
   A   
   A   
   A   
   T   
   C   
   A   
   A   
   A   
   A   
   T   
    T    
   G   
   A   
   T   
   A   
   C   
   G   
   C   
   A   
   A   
    A    
   T   
    C    
   T   
   T   
   A   
   G   
   T   
   G   
   G   
   C   
    A    
   C   
   A   
   A   
   G   
   A   
   A   
   G   
   A   
   C   
   G   
   T   
   A   
   A   
   T   
    T    
   G   
   T   
   C   
   A   
   G   
   T   
   G   
   T   
    G    
   A   
   C   
    G    
   C   
   G   
   A   
   G   
   A   
   A   
   G   
   G   
   C   
   T   
   A   
   T   
   G   
   T   
   G   
   A   
   A   
   G   
   C   
   G   
   A   
   A   
   C   
   A   
   A   
   G   
   C   
   C   
   T   
   T   
   C   
   G   
   T   
   T   
   C   
    T    
   T   
   A   
   T   
   A   
   G   
   T   
   G   
   C   
   T   
   T   
   C   
    C    
   A   
   A   
   A   
   C   
   C   
   A   
   G   
   A   
   A   
   G   
   A   
   A   
   A   
   T   
   C   
   G   
   G   
   G   
   A   
   T   
   G   
   C   
   G   
   A   
   G   
   A   
   A   
   G   
   G   
   C   
   G   
   A   
    C    
   T   
   A   
   C   
   T   
   T   
   G   
   C   
   T   
    A    
   T   
   A   
   C   
    T    
   C   
   A   
   G   
   G   
   T   
   G   
   A   
   A   
   T   
   T   
    G    
   A   
   G   
    T    
   A   
   C   
   A   
    T    
   T   
   G   
   G   
   A   
   C   
   C   
   A   
   T   
   G   
   T   
   T   
   C   
   T   
   T   
   C   
   T   
   G   
   A   
   T   
    C    
   A   
   C   
    A    
   A   
   A   
   T   
   A   
   A   
   A   
   G   
   C   
   A   
   A   
   A   
   T   
   G   
   T   
   G   
   A   
   T   
   C   
   T   
   A   
   C   
   C   
   G   
    C    
   C   
   C   
   T   
   G   
   T   
    C    
   C   
   A   
   T   
   G   
   A   
   A   
   C   
   T   
   A   
   C   
   C   
    T    
   G   
   A   
   T   
   T   
   T   
   G   
   A   
   A   
   A   
   T   
   G   
   G   
   A   
   A   
   A   
   G   
   A   
   T   
   G   
   C   
   T   
   G   
   G   
    T    
   G   
   A   
    G    
   C   
   A   
    C    
   A   
   T   
   T   
   T   
   C   
   T   
   C   
   A   
   A   
   A   
   C   
   A   
   A   
   T   
   C   
   T   
   C   
   C   
   A   
   A   
   T   
   C   
   T   
   G   
   T   
   C   
   T   
   G   
   T   
   A   
   G   
   A   
    C    
   G   
   A   
   A   
   T   
   C   
   G   
   A   
   T   
    T    
   T   
   T   
   A   
   G   
   C   
   T   
   G   
   T   
   T   
   T   
   T   
    C    
   C   
   C   
   A   
   T   
   A   
   T   
   C   
   A   
   A   
   A   
   A   
   A   
   A   
   T   
    C    
   C   
   A   
   A   
   G   
   C   
   A   
   G   
   A   
    G    
   A   
   A   
   A   
   A   
   C   
   A   
   T   
   T   
   T   
   G   
   T   
    A    
   T   
   T   
   C   
   A   
   T   
   C   
   A   
   G   
   C   
   A   
   A   
   G   
   A   
   A   
   T   
   G   
   G   
   A   
   T   
   T   
    G    
    A    
   T   
   C   
   A   
   A   
   G   
   C   
   A   
   A   
   A   
   C   
   A   
   C   
   G   
   A   
   A   
   T   
   G   
   A   
   C   
   A   
   G   
   A   
   G   
   T   
   T   
   C   
   G   
   A   
   A   
   C   
   C   
   A   
   T   
   G   
   G   
   C   
   G   
   C   
   A   
   C   
   C   
   T   
   A   
    T    
   A   
   A   
   G   
   A   
   G   
   C   
   C   
   G   
    T    
   C   
   C   
   G   
   C   
   T   
   C   
   A   
   G   
   C   
   T   
   G   
   T   
   A   
   T   
   G   
   A   
   A   
   G   
   C   
   T   
   A   
   A   
   A   
   G   
   G   
   C   
   G   
   G   
   A   
   A   
   G   
   A   
   A   
   G   
   A   
   T   
   G   
   A   
   A   
   C   
   T   
    G    
   A   
   T   
   T   
   G   
   C   
   T   
   G   
   T   
    G    
   T   
   A   
   T   
   C   
   T   
   G   
   A   
   A   
   A   
   G   
   A   
   A   
   G   
   A   
   T   
   C   
    A    
   G   
   C   
   C   
   A   
   G   
   G   
   C   
   T   
   T   
   A   
   G   
   A   
   T   
   G   
   T   
    T    
   T   
   T   
   C   
   T   
   T   
   A   
   G   
   T   
   A   
   A   
   C   
   G   
    C    
   A   
   T   
   C   
   A   
   A   
   G   
   G   
   A   
   A   
   T   
   G   
   G   
   G   
    T    
   T   
   T   
   G   
   C   
   G   
   C   
   T   
    A    
   T   
   C   
   C   
   T   
   T   
   T   
   A   
   A   
    C    
   A   
   G   
   A   
   T   
   G   
   T   
   T   
   C   
   C   
   C   
   G   
   T   
   T   
   G   
   T   
   T   
   G   
   G   
   A   
   G   
   C   
   A   
   A   
   A   
   G   
   G   
   C   
   A   
   G   
    C    
   T   
   G   
   G   
   A   
   G   
   T   
   G   
   A   
   A   
   G   
   T   
   C   
   T   
   A   
   T   
   G   
   A   
   A   
   T   
   T   
   T   
   G   
   A   
   A   
   A   
   G   
   A   
   T   
   G   
   G   
   A   
   G   
   A   
   T   
   A   
   G   
   G   
    A    
   T   
   T   
    A    
   T   
   C   
   A   
   A   
    C    
   G   
   G   
    G    
   C   
   T   
    G    
   C   
   T   
   T   
   G   
   T   
   G   
   T   
   T   
   A   
   G   
   C   
   A   
   G   
   A   
    A    
   G   
   G   
    T    
   G   
   A   
   T   
   A   
   C   
   G   
   C   
   C   
    G    
   A   
   T   
   C   
   G   
   T   
   A   
   A   
   T   
   C   
   G   
   T   
   T   
   A   
   C   
   T   
   C   
   A   
   G   
   C   
   G   
   A   
   G   
   G   
   A   
   G   
   C   
    T    
   G   
   T   
    T    
   A   
   A   
   A   
   C   
   G   
   C   
   A   
   T   
   G   
   C   
   T   
   T   
   G   
   C   
   T   
   C   
   A   
   A   
   G   
   A   
   A   
   A   
   T   
   C   
   A   
   G   
   C   
   C   
   A   
   A   
   A   
   C   
    T    
   T   
   C   
   A   
   C   
   G   
   T   
   G   
   C   
   G   
   A   
   A   
   G   
   C   
   G   
   G   
   G   
   G   
   A   
   G   
   T   
   G   
   A   
   C   
   G   
   G   
   T   
   C   
   A   
   T   
   G   
   C   
   G   
   T   
   G   
   A   
   G   
   C   
   T   
   G   
   A   
   A   
   A   
   A   
   A   
   A   
   A   
   A   
   T   
   C   
   C   
    G    
   C   
   A   
    C    
   C   
   G   
    T    
   A   
   T   
   T   
   A   
   T   
   C   
   T   
   A   
   T   
   A   
   T   
   G   
   T   
   C   
   G   
   G   
   A   
   A   
   G   
   G   
   A   
   A   
   G   
   A   
   T   
   C   
   A   
   A   
   A   
   A   
   G   
   A   
   A   
   A   
   T   
   A   
   A   
   C   
   C   
   T   
   T   
   G   
   A   
   T   
   G   
   A   
   A   
   T   
   C   
   A   
   A   
   A   
   A   
   A   
   G   
   G   
    G    
   C   
   A   
    G    
   C   
   A   
   G   
    T    
   T   
   A   
   A   
   C   
   G   
   A   
   T   
   T   
   G   
   A   
    C    
   C   
   C   
   A   
   A   
   C   
    T    
   G   
   A   
   T   
   T   
   T   
    C    
   C   
   C   
   T   
   A   
   T   
   A   
   G   
   G   
   T   
   G   
   A   
   T   
   C   
   G   
   A   
   A   
   C   
   A   
   T   
   C   
   G   
   A   
   A   
   T   
   G   
   G   
    A    
   T   
   C   
    A    
   T   
   T   
   T   
   G   
   C   
    G    
   A   
   T   
   G   
   G   
   A   
    T    
   G   
   A   
   G   
   A   
   A   
   A   
   A   
   A   
   A   
   G   
   G   
   T   
   G   
   G   
   A   
   G   
   A   
   A   
   G   
   T   
   C   
   A   
   T   
   C   
   G   
   A   
   A   
   G   
   T   
    C    
   A   
    A    
   C   
   G   
   A   
   T   
   G   
   C   
   T   
   C   
   C   
   T   
   G   
   A   
   G   
    A    
   T   
   T   
   T   
   T   
    C    
   A   
   T   
    C    
   G   
   A   
    G    
    T    
   C   
    G    
   -   
   -   
   -   
 

 
   N54152   
   A   
   T   
   G   
   G   
   A   
   A   
   A   
   A   
   T   
   C   
   G   
   A   
   C   
   A   
   A   
   G   
   A   
   A   
   G   
   T   
   A   
   C   
   A   
   A   
   G   
   A   
   A   
   T   
   T   
   A   
   A   
   C   
   T   
   T   
   T   
   A   
   G   
   A   
   A   
   G   
   A   
   A   
   G   
   T   
   G   
   A   
   T   
   G   
   G   
   G   
   T   
   G   
   A   
   C   
   C   
   G   
   C   
   T   
   T   
   C   
   G   
   G   
   A   
   C   
   G   
   T   
   T   
   A   
   T   
   T   
   C   
   C   
   A   
   A   
   A   
   T   
   A   
   T   
   A   
   T   
   T   
   A   
   T   
    C    
   C   
   A   
    A    
   G   
   A   
   A   
   C   
   G   
   T   
   G   
   C   
   A   
   C   
   T   
   G   
   C   
   C   
   T   
   G   
   A   
   T   
   A   
   T   
    C    
   C   
   G   
   T   
   G   
   A   
   T   
   G   
   G   
   A   
   T   
   T   
   G   
   A   
   A   
    G    
   C   
   C   
   A   
   G   
   T   
    G    
   C   
   A   
   A   
   C   
   G   
   C   
   C   
   G   
   G   
   A   
   T   
   A   
   T   
   T   
   A   
   T   
   T   
    T    
   G   
   C   
    C    
   A   
   T   
   G   
   A   
   A   
   C   
   A   
   A   
   A   
   G   
   A   
   T   
   G   
   G   
   C   
   A   
   A   
   T   
   A   
   C   
   G   
   T   
   A   
   T   
   G   
   A   
   C   
   A   
   A   
   A   
   G   
   G   
   A   
   T   
   T   
    C    
   C   
   G   
   T   
   A   
   A   
   A   
   T   
   C   
   A   
   G   
   C   
   A   
   A   
   A   
   A   
   T   
   C   
   T   
   G   
   T   
   C   
   G   
   G   
   A   
   A   
   A   
   T   
   A   
   T   
   C   
   A   
   T   
   G   
   G   
   G   
    G    
   A   
   A   
   T   
   T   
   A   
   T   
   C   
   A   
   T   
   C   
   C   
   T   
   C   
   A   
   T   
   G   
   G   
   T   
   G   
   A   
   C   
   A   
   G   
   C   
   A   
   G   
   T   
   A   
   T   
   T   
   T   
   A   
   T   
   G   
   A   
   A   
   G   
   C   
   G   
   A   
   T   
   G   
   G   
   T   
   C   
   C   
   G   
   C   
   A   
   T   
   G   
   A   
   G   
   C   
   C   
   A   
   A   
   G   
   A   
    C    
   T   
   G   
   G   
   A   
   A   
   G   
   T   
   T   
   G   
   C   
   G   
   G   
   G   
   A   
   A   
   G   
   T   
   A   
   C   
   T   
   G   
   A   
   T   
   C   
   G   
   A   
    A    
   A   
   T   
   G   
   C   
   A   
   C   
   G   
   G   
    G    
   A   
   A   
   T   
   A   
   A   
    C    
   G   
   G   
   G   
   A   
   G   
   T   
   A   
   T   
   G   
   G   
   A   
   C   
   G   
   G   
   T   
   G   
   A   
   T   
   C   
   C   
   A   
   C   
   C   
   T   
   G   
   C   
   T   
   G   
   C   
   G   
   A   
   T   
   G   
   C   
   G   
   T   
   T   
   A   
   T   
   A   
   C   
   T   
   G   
   A   
   A   
   G   
   C   
   C   
   A   
   G   
    A    
   T   
   T   
   A   
   T   
   C   
   C   
   A   
   A   
   G   
   T   
   T   
   A   
   A   
   G   
   T   
   G   
   G   
   A   
   G   
   A   
   G   
   A   
   T   
   G   
    C    
   T   
   A   
    G    
   C   
   A   
   G   
   A   
   T   
   A   
   T   
   C   
   G   
   A   
    A    
   A   
   A   
   A   
   G   
   A   
   A   
   A   
   C   
   A   
   G   
   T   
    C    
   G   
   A   
   T   
   C   
   T   
   C   
   G   
   T   
   G   
   T   
   G   
   G   
   A   
   A   
   T   
   T   
   T   
   T   
   G   
   A   
   T   
   G   
   A   
   T   
   A   
   C   
   A   
   G   
   A   
   A   
   A   
   A   
   A   
   G   
   A   
   A   
   C   
   C   
   A   
   A   
   C   
   A   
   G   
   T   
   C   
   C   
   T   
   T   
   C   
   C   
   A   
   G   
   C   
   T   
   C   
   G   
   A   
   T   
   A   
   C   
   C   
   C   
   A   
   A   
   A   
   C   
   C   
   T   
   T   
   C   
   T   
    C    
   G   
   T   
   C   
   A   
   A   
   T   
   G   
   G   
   A   
   T   
   C   
   G   
   A   
   C   
   A   
   G   
   G   
   T   
   A   
   T   
   A   
   T   
   C   
   T   
   G   
   C   
   T   
   G   
   G   
   A   
   T   
   A   
   T   
   G   
   C   
    A    
   A   
   C   
   A   
   G   
   A   
   A   
   A   
   T   
   C   
   C   
   C   
   G   
   A   
   C   
    T    
   C   
   A   
   T   
   A   
   A   
   C   
   T   
   T   
   A   
   G   
   C   
   T   
   G   
   A   
   A   
   G   
   T   
   G   
   A   
   T   
    C    
   G   
   A   
    T    
   G   
   G   
   C   
   A   
   C   
   A   
   A   
   T   
   C   
   T   
   A   
   T   
   A   
   T   
   G   
   A   
   T   
   C   
   G   
   A   
   T   
   C   
   A   
   T   
   C   
   C   
   A   
   C   
   A   
   A   
   G   
   C   
   G   
   A   
   G   
   C   
   C   
   T   
   T   
   G   
   A   
   A   
   A   
   A   
   A   
   T   
   T   
   G   
   A   
   T   
   G   
   G   
   A   
   A   
   T   
   A   
   T   
   A   
   T   
   T   
   C   
   C   
   T   
   G   
   G   
   A   
   C   
   C   
   T   
   G   
   A   
   C   
   T   
   T   
    T    
   C   
   C   
   A   
   A   
   C   
   A   
   G   
   G   
   A   
   G   
   G   
    G    
   A   
   T   
   C   
   T   
   T   
   G   
   C   
   A   
   A   
   G   
   G   
    A    
   A   
   A   
   A   
   G   
   A   
   T   
   G   
   A   
   A   
   A   
   T   
   C   
   A   
   A   
   A   
   A   
   A   
   A   
   G   
   C   
   T   
   T   
   A   
   T   
   G   
   A   
   A   
   A   
   C   
   A   
   G   
   G   
    G    
   C   
   G   
   A   
   G   
   G   
   A   
   A   
   A   
   A   
   G   
   T   
   T   
   A   
    T    
   T   
   C   
   T   
   T   
   C   
   G   
   T   
   T   
   C   
   G   
   A   
   A   
   A   
   A   
   C   
   A   
   A   
   A   
   G   
   A   
   T   
   A   
   G   
   A   
    A    
   G   
   C   
   A   
   A   
   T   
   G   
   A   
   A   
   G   
   G   
   G   
    T    
   A   
   A   
    T    
   A   
   A   
   A   
   C   
   A   
   G   
   C   
   A   
   A   
   A   
   T   
   C   
   G   
   T   
   G   
   A   
   T   
   C   
   A   
   C   
    A    
   G   
   A   
   A   
   A   
   T   
   C   
   C   
   C   
   A   
   T   
   A   
   T   
   G   
   A   
   A   
   G   
   T   
   A   
   A   
   A   
   T   
   A   
   A   
   A   
   G   
   C   
   G   
   A   
   C   
    C    
   T   
   T   
   G   
   G   
   T   
   G   
   A   
   A   
   A   
   A   
   A   
   A   
   A   
   T   
   G   
   G   
   A   
   T   
   G   
   A   
   G   
   A   
   T   
   C   
   C   
   G   
   T   
   T   
   T   
   G   
   A   
   A   
   T   
   A   
   A   
   G   
   A   
   A   
   A   
   A   
   T   
   C   
   G   
   A   
    T    
   G   
   G   
   A   
   A   
   T   
   A   
   G   
   C   
   T   
   G   
   A   
   A   
   G   
   T   
    A    
   C   
   G   
   A   
   G   
   A   
   T   
   G   
   A   
   A   
   A   
   G   
   T   
   G   
   A   
   T   
    A    
   G   
    A    
   A   
   C   
    A    
   G   
   G   
   T   
   C   
   T   
   G   
   C   
   A   
   A   
   A   
   T   
   T   
   G   
   T   
   C   
   G   
   T   
    C    
   G   
   A   
   A   
   T   
   T   
   G   
   A   
   A   
   A   
   A   
   A   
   A   
   G   
   A   
    C    
   G   
   C   
   C   
   A   
   A   
    T    
   G   
   C   
   T   
   C   
   A   
   A   
   G   
   G   
    A    
   A   
   T   
   T   
   T   
   T   
   G   
   A   
   A   
   T   
   T   
   A   
    T    
   T   
   T   
   G   
   T   
   T   
   C   
   A   
   A   
   A   
   A   
   A   
   C   
   A   
   C   
   T   
   G   
   A   
   G   
    C    
   T   
   A   
   C   
   A   
   G   
   A   
   T   
   C   
   A   
   A   
   T   
   T   
   A   
   C   
   A   
   A   
   T   
   T   
   T   
    C    
   A   
   A   
   T   
   A   
   T   
   G   
   G   
   T   
   C   
   G   
   C   
   T   
   A   
   T   
   C   
   G   
   A   
    C    
   C   
   A   
   T   
   A   
   T   
   G   
   A   
   C   
   T   
   C   
   C   
   G   
   C   
   A   
    T    
   C   
   A   
   A   
   G   
   T   
   T   
   G   
   G   
   A   
   C   
   T   
   G   
   A   
   A   
   A   
   G   
   A   
   C   
   A   
   T   
   T   
   T   
   T   
   A   
   A   
   G   
   A   
   A   
   G   
   T   
   T   
   A   
   T   
   A   
   T   
   C   
   G   
   A   
   G   
   C   
   A   
   T   
   C   
   G   
   A   
   A   
   A   
   G   
   C   
   A   
   A   
   G   
   T   
   C   
   A   
   T   
   A   
   A   
   C   
   A   
   A   
   A   
   A   
   C   
   G   
    T    
   A   
   G   
    T    
   C   
   A   
   A   
   T   
   T   
   C   
   G   
   A   
   T   
   T   
   T   
   A   
   G   
   C   
   C   
   A   
   A   
   A   
   G   
   C   
   T   
   C   
   A   
   A   
   A   
   A   
   A   
    A    
   G   
   A   
   C   
   A   
   G   
   C   
   A   
   T   
   A   
   T   
   C   
   G   
   T   
    A    
   G   
   A   
   A   
   G   
   G   
    A    
    T    
   T   
   G   
   A   
   T   
   G   
   A   
   A   
   A   
   G   
   C   
   T   
   T   
   T   
   A   
   T   
   C   
   G   
   A   
   T   
   T   
   C   
   T   
   G   
   G   
   A   
   T   
   G   
   A   
   A   
   G   
   T   
   A   
   A   
   T   
   C   
   G   
    C    
   A   
   A   
   C   
   G   
   A   
   T   
   T   
   C   
   G   
   T   
   G   
   A   
   A   
   A   
   G   
   T   
   A   
   A   
   A   
   G   
   A   
   C   
   A   
   A   
   A   
   A   
   A   
   A   
   G   
   A   
    C    
   G   
   C   
    C    
   A   
   A   
   A   
   A   
   A   
   G   
   A   
   A   
   T   
   T   
   T   
   A   
   G   
   T   
   C   
   G   
   A   
    C    
   G   
   T   
    C    
   T   
   T   
    T    
   C   
   A   
   A   
   T   
   T   
   T   
   A   
   C   
   A   
   G   
   A   
   A   
   G   
   A   
   A   
   C   
   A   
   A   
   G   
   C   
   A   
   G   
   A   
   A   
   G   
   C   
   A   
   A   
   T   
    T    
   G   
   T   
   C   
   A   
   C   
   A   
   T   
   T   
   G   
   C   
   A   
   A   
    T    
   T   
   A   
   T   
   A   
    C    
   C   
   G   
   T   
   T   
   T   
   G   
   A   
   C   
   G   
   A   
   A   
    C    
   A   
   C   
    G    
   G   
   A   
   T   
   A   
   T   
   C   
   A   
   C   
   A   
   G   
   A   
   G   
   C   
   T   
   T   
   C   
   A   
   A   
   A   
   A   
   G   
   G   
   A   
   A   
    T    
   C   
    C    
   G   
   A   
   A   
   A   
    G    
   C   
   T   
   T   
   G   
   A   
   T   
   T   
   G   
   C   
    G    
   C   
   A   
   A   
   A   
   T   
   C   
   A   
   C   
   A   
   G   
   A   
   G   
   T   
   T   
    G    
   A   
   A   
   T   
   A   
   A   
   A   
   A   
   T   
   C   
   C   
   T   
   T   
   T   
   C   
   A   
   A   
   A   
    T    
   G   
   A   
   C   
   A   
   A   
   A   
   G   
   A   
    G    
   C   
   T   
   T   
   T   
   T   
    C    
   T   
   C   
   T   
   G   
   T   
   T   
   A   
   T   
   G   
   A   
   A   
   A   
   A   
   A   
   A   
   G   
   A   
   A   
   T   
   T   
   G   
   C   
   G   
   A   
   G   
   A   
   A   
   G   
   T   
   G   
   A   
   A   
   A   
   A   
   A   
   A   
   A   
   A   
   T   
   T   
   A   
    T    
   T   
   C   
   T   
   T   
   C   
   C   
   G   
   C   
   T   
   C   
   G   
   T   
   C   
    T    
    A    
   A   
   C   
   G   
   A   
   C   
   A   
   A   
   T   
   C   
    G    
   A   
   A   
   G   
   A   
   C   
   G   
   A   
   A   
   A   
   T   
   C   
   G   
   A   
   A   
   G   
   A   
   A   
   A   
   T   
   C   
   A   
   A   
   A   
   A   
   T   
    T    
   G   
   A   
   T   
   A   
   C   
   G   
   C   
   A   
   A   
    G    
   T   
    C    
   T   
   T   
   A   
   G   
   T   
   G   
   G   
   C   
    A    
   C   
   A   
   A   
   G   
   A   
   A   
   G   
   A   
   C   
   G   
   T   
   A   
   A   
   T   
    T    
   G   
   T   
   C   
   A   
   G   
   T   
   G   
   T   
    G    
   A   
   C   
    G    
   C   
   G   
   A   
   G   
   A   
   A   
   G   
   G   
   C   
   T   
   A   
   T   
   G   
   T   
   G   
   A   
   A   
   G   
   C   
   G   
   A   
   A   
   C   
   A   
   A   
   G   
   C   
   C   
   T   
   T   
   C   
   G   
   T   
   T   
   C   
    T    
   T   
   A   
   T   
   A   
   G   
   T   
   G   
   C   
   T   
   T   
   C   
    C    
   A   
   A   
   A   
   C   
   C   
   A   
   G   
   A   
   A   
   G   
   A   
   A   
   A   
   T   
   C   
   G   
   G   
   G   
   A   
   T   
   G   
   C   
   G   
   A   
   G   
   A   
   A   
   G   
   G   
   C   
   G   
   A   
    C    
   T   
   A   
   C   
   T   
   T   
   G   
   C   
   T   
    A    
   T   
   A   
   C   
    T    
   C   
   A   
   G   
   G   
   T   
   G   
   A   
   A   
   T   
   T   
    G    
   A   
   G   
    T    
   A   
   C   
   A   
    T    
   T   
   G   
   G   
   A   
   C   
   C   
   A   
   T   
   G   
   T   
   T   
   C   
   T   
   T   
   C   
   T   
   G   
   A   
   T   
    C    
   A   
   C   
    A    
   A   
   A   
   T   
   A   
   A   
   A   
   G   
   C   
   A   
   A   
   A   
   T   
   G   
   T   
   G   
   A   
   T   
   C   
   T   
   A   
   C   
   C   
   G   
    C    
   C   
   C   
   T   
   G   
   T   
    C    
   C   
   A   
   T   
   G   
   A   
   A   
   C   
   T   
   A   
   C   
   C   
    C    
   G   
   A   
   T   
   T   
   T   
   G   
   A   
   A   
   A   
   T   
   G   
   G   
   A   
   A   
   A   
   G   
   A   
   T   
   G   
   C   
   T   
   G   
   G   
    T    
   G   
   A   
    G    
   C   
   A   
    C    
   A   
   T   
   T   
   T   
   C   
   T   
   C   
   A   
   A   
   A   
   C   
   A   
   A   
   T   
   C   
   T   
   C   
   C   
   A   
   A   
   T   
   C   
   T   
   G   
   T   
   C   
   T   
   G   
   T   
   A   
   G   
   A   
    C    
   G   
   A   
   A   
   T   
   C   
   G   
   A   
   T   
    T    
   T   
   T   
   A   
   G   
   C   
   T   
   G   
   T   
   T   
   T   
   T   
    C    
   C   
   C   
   A   
   T   
   A   
   T   
   C   
   A   
   A   
   A   
   A   
   A   
   A   
   T   
    T    
   C   
   A   
   A   
   G   
   C   
   A   
   G   
   A   
    G    
   A   
   A   
   A   
   A   
   C   
   A   
   T   
   T   
   T   
   G   
   T   
    A    
   T   
   T   
   C   
   A   
   T   
   C   
   A   
   G   
   C   
   A   
   A   
   G   
   A   
   A   
   T   
   G   
   G   
   A   
   T   
   T   
    G    
    A    
   T   
   C   
   A   
   A   
   G   
   C   
   A   
   A   
   A   
   C   
   A   
   C   
   G   
   A   
   A   
   T   
   G   
   A   
   C   
   A   
   G   
   A   
   G   
   T   
   T   
   C   
   G   
   A   
   A   
   C   
   C   
   A   
   T   
   G   
   G   
   C   
   G   
   C   
   A   
   C   
   C   
   T   
   A   
    T    
   A   
   A   
   G   
   A   
   G   
   C   
   C   
   G   
    T    
   C   
   C   
   G   
   C   
   T   
   C   
   A   
   G   
   C   
   T   
   G   
   T   
   A   
   T   
   G   
   A   
   A   
   G   
   C   
   T   
   A   
   A   
   A   
   G   
   G   
   C   
   G   
   G   
   A   
   A   
   G   
   A   
   A   
   G   
   A   
   T   
   G   
   A   
   A   
   C   
   T   
    G    
   A   
   T   
   T   
   G   
   C   
   T   
   G   
   T   
    G    
   T   
   A   
   T   
   C   
   T   
   G   
   A   
   A   
   A   
   G   
   A   
   A   
   G   
   A   
   T   
   C   
    A    
   G   
   C   
   C   
   A   
   G   
   G   
   C   
   T   
   T   
   A   
   G   
   A   
   T   
   G   
   T   
    T    
   T   
   T   
   C   
   T   
   T   
   A   
   G   
   T   
   A   
   A   
   C   
   G   
    C    
   A   
   T   
   C   
   A   
   A   
   G   
   G   
   A   
   A   
   T   
   G   
   G   
   G   
    T    
   T   
   T   
   G   
   C   
   G   
   C   
   T   
    A    
   T   
   C   
   C   
   T   
   T   
   T   
   A   
   A   
    C    
   A   
   G   
   A   
   T   
   G   
   T   
   T   
   C   
   C   
   C   
   G   
   T   
   T   
   G   
   T   
   T   
   G   
   G   
   A   
   G   
   C   
   A   
   A   
   A   
   G   
   G   
   C   
   A   
   G   
    C    
   T   
   G   
   G   
   A   
   G   
   T   
   G   
   A   
   A   
   G   
   T   
   C   
   T   
   A   
   T   
   G   
   A   
   A   
   T   
   T   
   T   
   G   
   A   
   A   
   A   
   G   
   A   
   T   
   G   
   G   
   A   
   G   
   A   
   T   
   A   
   G   
   G   
    A    
   T   
   T   
    A    
   T   
   C   
   A   
   A   
    C    
   G   
   G   
    G    
   C   
   T   
    A    
   C   
   T   
   T   
   G   
   T   
   G   
   T   
   T   
   A   
   G   
   C   
   A   
   G   
   A   
    A    
   G   
   G   
    T    
   G   
   A   
   T   
   A   
   C   
   G   
   C   
   C   
    G    
   A   
   T   
   C   
   G   
   T   
   A   
   A   
   T   
   C   
   G   
   T   
   T   
   A   
   C   
   T   
   C   
   A   
   G   
   C   
   G   
   A   
   G   
   G   
   A   
   G   
   C   
    T    
   G   
   T   
    T    
   A   
   A   
   A   
   C   
   G   
   C   
   A   
   T   
   G   
   C   
   T   
   T   
   G   
   C   
   T   
   C   
   A   
   A   
   G   
   A   
   A   
   A   
   T   
   C   
   A   
   G   
   C   
   C   
   A   
   A   
   A   
   C   
    T    
   T   
   C   
   A   
   C   
   G   
   T   
   G   
   C   
   G   
   A   
   A   
   G   
   C   
   G   
   G   
   G   
   G   
   A   
   G   
   T   
   G   
   A   
   C   
   G   
   G   
   T   
   C   
   A   
   T   
   G   
   C   
   G   
   T   
   G   
   A   
   G   
   C   
   T   
   G   
   A   
   A   
   A   
   A   
   A   
   A   
   A   
   A   
   T   
   C   
   C   
    G    
   C   
   A   
    C    
   C   
   G   
    T    
   A   
   T   
   T   
   A   
   T   
   C   
   T   
   A   
   T   
   A   
   T   
   G   
   T   
   C   
   G   
   G   
   A   
   A   
   G   
   G   
   A   
   A   
   G   
   A   
   T   
   C   
   A   
   A   
   A   
   A   
   G   
   A   
   A   
   A   
   T   
   A   
   A   
   C   
   C   
   T   
   T   
   G   
   A   
   T   
   G   
   A   
   A   
   T   
   C   
   A   
   A   
   A   
   A   
   A   
   G   
   G   
    G    
   C   
   A   
    G    
   C   
   A   
   G   
    T    
   T   
   A   
   A   
   C   
   G   
   A   
   T   
   T   
   G   
   A   
    C    
   C   
   C   
   A   
   A   
   C   
    T    
   G   
   A   
   T   
   T   
   T   
    C    
   C   
   C   
   T   
   A   
   T   
   A   
   G   
   G   
   T   
   G   
   A   
   T   
   C   
   G   
   A   
   A   
   C   
   A   
   T   
   C   
   G   
   A   
   A   
   T   
   G   
   G   
    A    
   T   
   C   
    A    
   T   
   T   
   T   
   G   
   C   
    G    
   A   
   T   
   G   
   G   
   A   
    T    
   G   
   A   
   G   
   A   
   A   
   A   
   A   
   A   
   A   
   G   
   G   
   T   
   G   
   G   
   A   
   G   
   A   
   A   
   G   
   T   
   C   
   A   
   T   
   C   
   G   
   A   
   A   
   G   
   T   
    T    
   A   
    T    
   C   
   G   
   A   
   T   
   G   
   C   
   T   
   C   
   C   
   T   
   G   
   A   
   G   
    A    
   T   
   T   
   T   
   T   
    C    
   A   
   T   
    C    
   G   
   A   
    G    
    T    
   C   
    G    
   -   
   -   
   -   
 

 
   N60324F   
   A   
   T   
   G   
   G   
   A   
   A   
   A   
   A   
   T   
   C   
   G   
   A   
   C   
   A   
   A   
   G   
   A   
   A   
   G   
   T   
   A   
   C   
   A   
   A   
   G   
   A   
   A   
   T   
   T   
   A   
   A   
   C   
   T   
   T   
   T   
   A   
   G   
   A   
   A   
   G   
   A   
   A   
   G   
   T   
   G   
   A   
   T   
   G   
   G   
   G   
   T   
   G   
   A   
   C   
   C   
   G   
   C   
   T   
   T   
   C   
   G   
   G   
   A   
   C   
   G   
   T   
   T   
   A   
   T   
   T   
   C   
   C   
   A   
   A   
   A   
   T   
   A   
   T   
   A   
   T   
   T   
   A   
   T   
    C    
   C   
   A   
    A    
   G   
   A   
   A   
   C   
   G   
   T   
   G   
   C   
   A   
   C   
   T   
   G   
   C   
   C   
   T   
   G   
   A   
   T   
   A   
   T   
    C    
   C   
   G   
   T   
   G   
   A   
   T   
   G   
   G   
   A   
   T   
   T   
   G   
   A   
   A   
    G    
   C   
   C   
   A   
   G   
   T   
    G    
   C   
   A   
   A   
   C   
   G   
   C   
   C   
   G   
   G   
   A   
   T   
   A   
   T   
   T   
   A   
   T   
   T   
    T    
   G   
   C   
    C    
   A   
   T   
   G   
   A   
   A   
   C   
   A   
   A   
   A   
   G   
   A   
   T   
   G   
   G   
   C   
   A   
   A   
   T   
   A   
   C   
   G   
   T   
   A   
   T   
   G   
   A   
   C   
   A   
   A   
   A   
   G   
   G   
   A   
   T   
   T   
    C    
   C   
   G   
   T   
   A   
   A   
   A   
   T   
   C   
   A   
   G   
   C   
   A   
   A   
   A   
   A   
   T   
   C   
   T   
   G   
   T   
   C   
   G   
   G   
   A   
   A   
   A   
   T   
   A   
   T   
   C   
   A   
   T   
   G   
   G   
   G   
    G    
   A   
   A   
   T   
   T   
   A   
   T   
   C   
   A   
   T   
   C   
   C   
   T   
   C   
   A   
   T   
   G   
   G   
   T   
   G   
   A   
   C   
   A   
   G   
   C   
   A   
   G   
   T   
   A   
   T   
   T   
   T   
   A   
   T   
   G   
   A   
   A   
   G   
   C   
   G   
   A   
   T   
   G   
   G   
   T   
   C   
   C   
   G   
   C   
   A   
   T   
   G   
   A   
   G   
   C   
   C   
   A   
   A   
   G   
   A   
    C    
   T   
   G   
   G   
   A   
   A   
   G   
   T   
   T   
   G   
   C   
   G   
   G   
   G   
   A   
   A   
   G   
   T   
   A   
   C   
   T   
   G   
   A   
   T   
   C   
   G   
   A   
    A    
   A   
   T   
   G   
   C   
   A   
   C   
   G   
   G   
    G    
   A   
   A   
   T   
   A   
   A   
    C    
   G   
   G   
   G   
   A   
   G   
   T   
   A   
   T   
   G   
   G   
   A   
   C   
   G   
   G   
   T   
   G   
   A   
   T   
   C   
   C   
   A   
   C   
   C   
   T   
   G   
   C   
   T   
   G   
   C   
   G   
   A   
   T   
   G   
   C   
   G   
   T   
   T   
   A   
   T   
   A   
   C   
   T   
   G   
   A   
   A   
   G   
   C   
   C   
   A   
   G   
    A    
   T   
   T   
   A   
   T   
   C   
   C   
   A   
   A   
   G   
   T   
   T   
   A   
   A   
   G   
   T   
   G   
   G   
   A   
   G   
   A   
   G   
   A   
   T   
   G   
    C    
   T   
   A   
    G    
   C   
   A   
   G   
   A   
   T   
   A   
   T   
   C   
   G   
   A   
    A    
   A   
   A   
   A   
   G   
   A   
   A   
   A   
   C   
   A   
   G   
   T   
    C    
   G   
   A   
   T   
   C   
   T   
   C   
   G   
   T   
   G   
   T   
   G   
   G   
   A   
   A   
   T   
   T   
   T   
   T   
   G   
   A   
   T   
   G   
   A   
   T   
   A   
   C   
   A   
   G   
   A   
   A   
   A   
   A   
   A   
   G   
   A   
   A   
   C   
   C   
   A   
   A   
   C   
   A   
   G   
   T   
   C   
   C   
   T   
   T   
   C   
   C   
   A   
   G   
   C   
   T   
   C   
   G   
   A   
   T   
   A   
   C   
   C   
   C   
   A   
   A   
   A   
   C   
   C   
   T   
   T   
   C   
   T   
    C    
   G   
   T   
   C   
   A   
   A   
   T   
   G   
   G   
   A   
   T   
   C   
   G   
   A   
   C   
   A   
   G   
   G   
   T   
   A   
   T   
   A   
   T   
   C   
   T   
   G   
   C   
   T   
   G   
   G   
   A   
   T   
   A   
   T   
   G   
   C   
    A    
   A   
   C   
   A   
   G   
   A   
   A   
   A   
   T   
   C   
   C   
   C   
   G   
   A   
   C   
    T    
   C   
   A   
   T   
   A   
   A   
   C   
   T   
   T   
   A   
   G   
   C   
   T   
   G   
   A   
   A   
   G   
   T   
   G   
   A   
   T   
    T    
   G   
   A   
    T    
   G   
   G   
   C   
   A   
   C   
   A   
   A   
   T   
   C   
   T   
   A   
   T   
   A   
   T   
   G   
   A   
   T   
   C   
   G   
   A   
   T   
   C   
   A   
   T   
   C   
   C   
   A   
   C   
   A   
   A   
   G   
   C   
   G   
   A   
   G   
   C   
   C   
   T   
   T   
   G   
   A   
   A   
   A   
   A   
   A   
   T   
   T   
   G   
   A   
   T   
   G   
   G   
   A   
   A   
   T   
   A   
   T   
   A   
   T   
   T   
   C   
   C   
   T   
   G   
   G   
   A   
   C   
   C   
   T   
   G   
   A   
   C   
   T   
   T   
    T    
   C   
   C   
   A   
   A   
   C   
   A   
   G   
   G   
   A   
   G   
   G   
    G    
   A   
   T   
   C   
   T   
   T   
   G   
   C   
   A   
   A   
   G   
   G   
    A    
   A   
   A   
   A   
   G   
   A   
   T   
   G   
   A   
   A   
   A   
   T   
   C   
   A   
   A   
   A   
   A   
   A   
   A   
   G   
   C   
   T   
   T   
   A   
   T   
   G   
   A   
   A   
   A   
   C   
   A   
   G   
   G   
    G    
   C   
   G   
   A   
   G   
   G   
   A   
   A   
   A   
   A   
   G   
   T   
   T   
   A   
    T    
   T   
   C   
   T   
   T   
   C   
   G   
   T   
   T   
   C   
   G   
   A   
   A   
   A   
   A   
   C   
   A   
   A   
   A   
   G   
   A   
   T   
   A   
   G   
   A   
    G    
   G   
   C   
   A   
   A   
   T   
   G   
   A   
   A   
   G   
   G   
   G   
    T    
   A   
   A   
    T    
   A   
   A   
   A   
   C   
   A   
   G   
   C   
   A   
   A   
   A   
   T   
   C   
   G   
   T   
   G   
   A   
   T   
   C   
   A   
   C   
    A    
   G   
   A   
   A   
   A   
   T   
   C   
   C   
   C   
   A   
   T   
   A   
   T   
   G   
   A   
   A   
   G   
   T   
   A   
   A   
   A   
   T   
   A   
   A   
   A   
   G   
   C   
   G   
   A   
   C   
    C    
   T   
   T   
   G   
   G   
   T   
   G   
   A   
   A   
   A   
   A   
   A   
   A   
   A   
   T   
   G   
   G   
   A   
   T   
   G   
   A   
   G   
   A   
   T   
   C   
   C   
   G   
   T   
   T   
   T   
   G   
   A   
   A   
   T   
   A   
   A   
   G   
   A   
   A   
   A   
   A   
   T   
   C   
   G   
   A   
    T    
   G   
   G   
   A   
   A   
   T   
   A   
   G   
   C   
   T   
   G   
   A   
   A   
   G   
   T   
    A    
   C   
   G   
   A   
   G   
   A   
   T   
   G   
   A   
   A   
   A   
   G   
   T   
   G   
   A   
   T   
    A    
   G   
    A    
   A   
   C   
    A    
   G   
   G   
   T   
   C   
   T   
   G   
   C   
   A   
   A   
   A   
   T   
   T   
   G   
   T   
   C   
   G   
   T   
    C    
   G   
   A   
   A   
   T   
   T   
   G   
   A   
   A   
   A   
   A   
   A   
   A   
   G   
   A   
    C    
   G   
   C   
   C   
   A   
   A   
    T    
   G   
   C   
   T   
   C   
   A   
   A   
   G   
   G   
    A    
   A   
   T   
   T   
   T   
   T   
   G   
   A   
   A   
   T   
   T   
   A   
    T    
   T   
   T   
   G   
   T   
   T   
   C   
   A   
   A   
   A   
   A   
   A   
   C   
   A   
   C   
   T   
   G   
   A   
   G   
    C    
   T   
   A   
   C   
   A   
   G   
   A   
   T   
   C   
   A   
   A   
   T   
   T   
   A   
   C   
   A   
   A   
   T   
   T   
   T   
    C    
   A   
   A   
   T   
   A   
   T   
   G   
   G   
   T   
   C   
   G   
   C   
   T   
   A   
   T   
   C   
   G   
   A   
    C    
   C   
   A   
   T   
   A   
   T   
   G   
   A   
   C   
   T   
   C   
   C   
   G   
   C   
   A   
    T    
   C   
   A   
   A   
   G   
   T   
   T   
   G   
   G   
   A   
   C   
   T   
   G   
   A   
   A   
   A   
   G   
   A   
   C   
   A   
   T   
   T   
   T   
   T   
   A   
   A   
   G   
   A   
   A   
   G   
   T   
   T   
   A   
   T   
   A   
   T   
   C   
   G   
   A   
   G   
   C   
   A   
   T   
   C   
   G   
   A   
   A   
   A   
   G   
   C   
   A   
   A   
   G   
   T   
   C   
   A   
   T   
   A   
   A   
   C   
   A   
   A   
   A   
   A   
   C   
   G   
    T    
   A   
   G   
    T    
   C   
   A   
   A   
   T   
   T   
   C   
   G   
   A   
   T   
   T   
   T   
   A   
   G   
   C   
   C   
   A   
   A   
   A   
   G   
   C   
   T   
   C   
   A   
   A   
   A   
   A   
   A   
    A    
   G   
   A   
   C   
   A   
   G   
   C   
   A   
   T   
   A   
   T   
   C   
   G   
   T   
    A    
   G   
   A   
   A   
   G   
   G   
    A    
    T    
   T   
   G   
   A   
   T   
   G   
   A   
   A   
   A   
   G   
   C   
   T   
   T   
   T   
   A   
   T   
   C   
   G   
   A   
   T   
   T   
   C   
   T   
   G   
   G   
   A   
   T   
   G   
   A   
   A   
   G   
   T   
   A   
   A   
   T   
   C   
   G   
    T    
   A   
   A   
   C   
   G   
   A   
   T   
   T   
   C   
   G   
   T   
   G   
   A   
   A   
   A   
   G   
   T   
   A   
   A   
   A   
   G   
   A   
   C   
   A   
   A   
   A   
   A   
   A   
   A   
   G   
   A   
    C    
   G   
   C   
    C    
   A   
   A   
   A   
   A   
   A   
   G   
   A   
   A   
   T   
   T   
   T   
   A   
   G   
   T   
   C   
   G   
   A   
    C    
   G   
   T   
    C    
   T   
   T   
    T    
   C   
   A   
   A   
   T   
   T   
   T   
   A   
   C   
   A   
   G   
   A   
   A   
   G   
   A   
   A   
   C   
   A   
   A   
   G   
   C   
   A   
   G   
   A   
   A   
   G   
   C   
   A   
   A   
   T   
    T    
   G   
   T   
   C   
   A   
   C   
   A   
   T   
   T   
   G   
   C   
   A   
   A   
    T    
   T   
   A   
   T   
   A   
    C    
   C   
   G   
   T   
   T   
   T   
   G   
   A   
   C   
   G   
   A   
   A   
    C    
   A   
   C   
    G    
   G   
   A   
   T   
   A   
   T   
   C   
   A   
   C   
   A   
   G   
   A   
   G   
   C   
   T   
   T   
   C   
   A   
   A   
   A   
   A   
   G   
   G   
   A   
   A   
    T    
   C   
    C    
   G   
   A   
   A   
   A   
    G    
   C   
   T   
   T   
   G   
   A   
   T   
   T   
   G   
   C   
    G    
   C   
   A   
   A   
   A   
   T   
   C   
   A   
   C   
   A   
   G   
   A   
   G   
   T   
   T   
    G    
   A   
   A   
   T   
   A   
   A   
   A   
   A   
   T   
   C   
   C   
   T   
   T   
   T   
   C   
   A   
   A   
   A   
    T    
   G   
   A   
   C   
   A   
   A   
   A   
   G   
   A   
    G    
   C   
   T   
   T   
   T   
   T   
    C    
   T   
   C   
   T   
   G   
   T   
   T   
   A   
   T   
   G   
   A   
   A   
   A   
   A   
   A   
   A   
   G   
   A   
   A   
   T   
   T   
   G   
   C   
   G   
   A   
   G   
   A   
   A   
   G   
   T   
   G   
   A   
   A   
   A   
   A   
   A   
   A   
   A   
   A   
   T   
   T   
   A   
    T    
   T   
   C   
   T   
   T   
   C   
   C   
   G   
   C   
   T   
   C   
   G   
   T   
   C   
    T    
    A    
   A   
   C   
   G   
   A   
   C   
   A   
   A   
   T   
   C   
    G    
   A   
   A   
   G   
   A   
   C   
   G   
   A   
   A   
   A   
   T   
   C   
   G   
   A   
   A   
   G   
   A   
   A   
   A   
   T   
   C   
   A   
   A   
   A   
   A   
   T   
    T    
   G   
   A   
   T   
   A   
   C   
   G   
   C   
   A   
   A   
    G    
   T   
    C    
   T   
   T   
   A   
   G   
   T   
   G   
   G   
   C   
    A    
   C   
   A   
   A   
   G   
   A   
   A   
   G   
   A   
   C   
   G   
   T   
   A   
   A   
   T   
    T    
   G   
   T   
   C   
   A   
   G   
   T   
   G   
   T   
    G    
   A   
   C   
    G    
   C   
   G   
   A   
   G   
   A   
   A   
   G   
   G   
   C   
   T   
   A   
   T   
   G   
   T   
   G   
   A   
   A   
   G   
   C   
   G   
   A   
   A   
   C   
   A   
   A   
   G   
   C   
   C   
   T   
   T   
   C   
   G   
   T   
   T   
   C   
    T    
   T   
   A   
   T   
   A   
   G   
   T   
   G   
   C   
   T   
   T   
   C   
    C    
   A   
   A   
   A   
   C   
   C   
   A   
   G   
   A   
   A   
   G   
   A   
   A   
   A   
   T   
   C   
   G   
   G   
   G   
   A   
   T   
   G   
   C   
   G   
   A   
   G   
   A   
   A   
   G   
   G   
   C   
   G   
   A   
    C    
   T   
   A   
   C   
   T   
   T   
   G   
   C   
   T   
    A    
   T   
   A   
   C   
    T    
   C   
   A   
   G   
   G   
   T   
   G   
   A   
   A   
   T   
   T   
    G    
   A   
   G   
    T    
   A   
   C   
   A   
    T    
   T   
   G   
   G   
   A   
   C   
   C   
   A   
   T   
   G   
   T   
   T   
   C   
   T   
   T   
   C   
   T   
   G   
   A   
   T   
    C    
   A   
   C   
    A    
   A   
   A   
   T   
   A   
   A   
   A   
   G   
   C   
   A   
   A   
   A   
   T   
   G   
   T   
   G   
   A   
   T   
   C   
   T   
   A   
   C   
   C   
   G   
    C    
   C   
   C   
   T   
   G   
   T   
    C    
   C   
   A   
   T   
   G   
   A   
   A   
   C   
   T   
   A   
   C   
   C   
    C    
   G   
   A   
   T   
   T   
   T   
   G   
   A   
   A   
   A   
   T   
   G   
   G   
   A   
   A   
   A   
   G   
   A   
   T   
   G   
   C   
   T   
   G   
   G   
    T    
   G   
   A   
    G    
   C   
   A   
    C    
   A   
   T   
   T   
   T   
   C   
   T   
   C   
   A   
   A   
   A   
   C   
   A   
   A   
   T   
   C   
   T   
   C   
   C   
   A   
   A   
   T   
   C   
   T   
   G   
   T   
   C   
   T   
   G   
   T   
   A   
   G   
   A   
    C    
   G   
   A   
   A   
   T   
   C   
   G   
   A   
   T   
    T    
   T   
   T   
   A   
   G   
   C   
   T   
   G   
   T   
   T   
   T   
   T   
    C    
   C   
   C   
   A   
   T   
   A   
   T   
   C   
   A   
   A   
   A   
   A   
   A   
   A   
   T   
    T    
   C   
   A   
   A   
   G   
   C   
   A   
   G   
   A   
    G    
   A   
   A   
   A   
   A   
   C   
   A   
   T   
   T   
   T   
   G   
   T   
    A    
   T   
   T   
   C   
   A   
   T   
   C   
   A   
   G   
   C   
   A   
   A   
   G   
   A   
   A   
   T   
   G   
   G   
   A   
   T   
   T   
    G    
    A    
   T   
   C   
   A   
   A   
   G   
   C   
   A   
   A   
   A   
   C   
   A   
   C   
   G   
   A   
   A   
   T   
   G   
   A   
   C   
   A   
   G   
   A   
   G   
   T   
   T   
   C   
   G   
   A   
   A   
   C   
   C   
   A   
   T   
   G   
   G   
   C   
   G   
   C   
   A   
   C   
   C   
   T   
   A   
    T    
   A   
   A   
   G   
   A   
   G   
   C   
   C   
   G   
    T    
   C   
   C   
   G   
   C   
   T   
   C   
   A   
   G   
   C   
   T   
   G   
   T   
   A   
   T   
   G   
   A   
   A   
   G   
   C   
   T   
   A   
   A   
   A   
   G   
   G   
   C   
   G   
   G   
   A   
   A   
   G   
   A   
   A   
   G   
   A   
   T   
   G   
   A   
   A   
   C   
   T   
    G    
   A   
   T   
   T   
   G   
   C   
   T   
   G   
   T   
    G    
   T   
   A   
   T   
   C   
   T   
   G   
   A   
   A   
   A   
   G   
   A   
   A   
   G   
   A   
   T   
   C   
    A    
   G   
   C   
   C   
   A   
   G   
   G   
   C   
   T   
   T   
   A   
   G   
   A   
   T   
   G   
   T   
    T    
   T   
   T   
   C   
   T   
   T   
   A   
   G   
   T   
   A   
   A   
   C   
   G   
    C    
   A   
   T   
   C   
   A   
   A   
   G   
   G   
   A   
   A   
   T   
   G   
   G   
   G   
    T    
   T   
   T   
   G   
   C   
   G   
   C   
   T   
    A    
   T   
   C   
   C   
   T   
   T   
   T   
   A   
   A   
    C    
   A   
   G   
   A   
   T   
   G   
   T   
   T   
   C   
   C   
   C   
   G   
   T   
   T   
   G   
   T   
   T   
   G   
   G   
   A   
   G   
   C   
   A   
   A   
   A   
   G   
   G   
   C   
   A   
   G   
    C    
   T   
   G   
   G   
   A   
   G   
   T   
   G   
   A   
   A   
   G   
   T   
   C   
   T   
   A   
   T   
   G   
   A   
   A   
   T   
   T   
   T   
   G   
   A   
   A   
   A   
   G   
   A   
   T   
   G   
   G   
   A   
   G   
   A   
   T   
   A   
   G   
   G   
    A    
   T   
   T   
    A    
   T   
   C   
   A   
   A   
    C    
   G   
   G   
    G    
   C   
   T   
    G    
   C   
   T   
   T   
   G   
   T   
   G   
   T   
   T   
   A   
   G   
   C   
   A   
   G   
   A   
    A    
   G   
   G   
    T    
   G   
   A   
   T   
   A   
   C   
   G   
   C   
   C   
    G    
   A   
   T   
   C   
   G   
   T   
   A   
   A   
   T   
   C   
   G   
   T   
   T   
   A   
   C   
   T   
   C   
   A   
   G   
   C   
   G   
   A   
   G   
   G   
   A   
   G   
   C   
    T    
   G   
   T   
    T    
   A   
   A   
   A   
   C   
   G   
   C   
   A   
   T   
   G   
   C   
   T   
   T   
   G   
   C   
   T   
   C   
   A   
   A   
   G   
   A   
   A   
   A   
   T   
   C   
   A   
   G   
   C   
   C   
   A   
   A   
   A   
   C   
    T    
   T   
   C   
   A   
   C   
   G   
   T   
   G   
   C   
   G   
   A   
   A   
   G   
   C   
   G   
   G   
   G   
   G   
   A   
   G   
   T   
   G   
   A   
   C   
   G   
   G   
   T   
   C   
   A   
   T   
   G   
   C   
   G   
   T   
   G   
   A   
   G   
   C   
   T   
   G   
   A   
   A   
   A   
   A   
   A   
   A   
   A   
   A   
   T   
   C   
   C   
    G    
   C   
   A   
    C    
   C   
   G   
    T    
   A   
   T   
   T   
   A   
   T   
   C   
   T   
   A   
   T   
   A   
   T   
   G   
   T   
   C   
   G   
   G   
   A   
   A   
   G   
   G   
   A   
   A   
   G   
   A   
   T   
   C   
   A   
   A   
   A   
   A   
   G   
   A   
   A   
   A   
   T   
   A   
   A   
   C   
   C   
   T   
   T   
   G   
   A   
   T   
   G   
   A   
   A   
   T   
   C   
   A   
   A   
   A   
   A   
   A   
   G   
   G   
    G    
   C   
   A   
    G    
   C   
   A   
   G   
    T    
   T   
   A   
   A   
   C   
   G   
   A   
   T   
   T   
   G   
   A   
    C    
   C   
   C   
   A   
   A   
   C   
    T    
   G   
   A   
   T   
   T   
   T   
    C    
   C   
   C   
   T   
   A   
   T   
   A   
   G   
   G   
   T   
   G   
   A   
   T   
   C   
   G   
   A   
   A   
   C   
   A   
   T   
   C   
   G   
   A   
   A   
   T   
   G   
   G   
    A    
   T   
   C   
    A    
   T   
   T   
   T   
   G   
   C   
    G    
   A   
   T   
   G   
   G   
   A   
    T    
   G   
   A   
   G   
   A   
   A   
   A   
   A   
   A   
   A   
   G   
   G   
   T   
   G   
   G   
   A   
   G   
   A   
   A   
   G   
   T   
   C   
   A   
   T   
   C   
   G   
   A   
   A   
   G   
   T   
    T    
   A   
    T    
   C   
   G   
   A   
   T   
   G   
   C   
   T   
   C   
   C   
   T   
   G   
   A   
   G   
    A    
   T   
   T   
   T   
   T   
    C    
   A   
   T   
    C    
   G   
   A   
    G    
    T    
   C   
    G    
   -   
   -   
   -   
 

 
   N52702   
   A   
   T   
   G   
   G   
   A   
   A   
   A   
   A   
   T   
   C   
   G   
   A   
   C   
   A   
   A   
   G   
   A   
   A   
   G   
   T   
   A   
   C   
   A   
   A   
   G   
   A   
   A   
   T   
   T   
   A   
   A   
   C   
   T   
   T   
   T   
   A   
   G   
   A   
   A   
   G   
   A   
   A   
   G   
   T   
   G   
   A   
   T   
   G   
   G   
   G   
   T   
   G   
   A   
   C   
   C   
   G   
   C   
   T   
   T   
   C   
   G   
   G   
   A   
   C   
   G   
   T   
   T   
   A   
   T   
   T   
   C   
   C   
   A   
   A   
   A   
   T   
   A   
   T   
   A   
   T   
   T   
   A   
   T   
    C    
   C   
   A   
    A    
   G   
   A   
   A   
   C   
   G   
   T   
   G   
   C   
   A   
   C   
   T   
   G   
   C   
   C   
   T   
   G   
   A   
   T   
   A   
   T   
    C    
   C   
   G   
   T   
   G   
   A   
   T   
   G   
   G   
   A   
   T   
   T   
   G   
   A   
   A   
    G    
   C   
   C   
   A   
   G   
   T   
    G    
   C   
   A   
   A   
   C   
   G   
   C   
   C   
   G   
   G   
   A   
   T   
   A   
   T   
   T   
   A   
   T   
   T   
    T    
   G   
   C   
    C    
   A   
   T   
   G   
   A   
   A   
   C   
   A   
   A   
   A   
   G   
   A   
   T   
   G   
   G   
   C   
   A   
   A   
   T   
   A   
   C   
   G   
   T   
   A   
   T   
   G   
   A   
   C   
   A   
   A   
   A   
   G   
   G   
   A   
   T   
   T   
    C    
   C   
   G   
   T   
   A   
   A   
   A   
   T   
   C   
   A   
   G   
   C   
   A   
   A   
   A   
   A   
   T   
   C   
   T   
   G   
   T   
   C   
   G   
   G   
   A   
   A   
   A   
   T   
   A   
   T   
   C   
   A   
   T   
   G   
   G   
   G   
    G    
   A   
   A   
   T   
   T   
   A   
   T   
   C   
   A   
   T   
   C   
   C   
   T   
   C   
   A   
   T   
   G   
   G   
   T   
   G   
   A   
   C   
   A   
   G   
   C   
   A   
   G   
   T   
   A   
   T   
   T   
   T   
   A   
   T   
   G   
   A   
   A   
   G   
   C   
   G   
   A   
   T   
   G   
   G   
   T   
   C   
   C   
   G   
   C   
   A   
   T   
   G   
   A   
   G   
   C   
   C   
   A   
   A   
   G   
   A   
    C    
   T   
   G   
   G   
   A   
   A   
   G   
   T   
   T   
   G   
   C   
   G   
   G   
   G   
   A   
   A   
   G   
   T   
   A   
   C   
   T   
   G   
   A   
   T   
   C   
   G   
   A   
    A    
   A   
   T   
   G   
   C   
   A   
   C   
   G   
   G   
    G    
   A   
   A   
   T   
   A   
   A   
    C    
   G   
   G   
   G   
   A   
   G   
   T   
   A   
   T   
   G   
   G   
   A   
   C   
   G   
   G   
   T   
   G   
   A   
   T   
   C   
   C   
   A   
   C   
   C   
   T   
   G   
   C   
   T   
   G   
   C   
   G   
   A   
   T   
   G   
   C   
   G   
   T   
   T   
   A   
   T   
   A   
   C   
   T   
   G   
   A   
   A   
   G   
   C   
   C   
   A   
   G   
    A    
   T   
   T   
   A   
   T   
   C   
   C   
   A   
   A   
   G   
   T   
   T   
   A   
   A   
   G   
   T   
   G   
   G   
   A   
   G   
   A   
   G   
   A   
   T   
   G   
    C    
   T   
   A   
    G    
   C   
   A   
   G   
   A   
   T   
   A   
   T   
   C   
   G   
   A   
    A    
   A   
   A   
   A   
   G   
   A   
   A   
   A   
   C   
   A   
   G   
   T   
    C    
   G   
   A   
   T   
   C   
   T   
   C   
   G   
   T   
   G   
   T   
   G   
   G   
   A   
   A   
   T   
   T   
   T   
   T   
   G   
   A   
   T   
   G   
   A   
   T   
   A   
   C   
   A   
   G   
   A   
   A   
   A   
   A   
   A   
   G   
   A   
   A   
   C   
   C   
   A   
   A   
   C   
   A   
   G   
   T   
   C   
   C   
   T   
   T   
   C   
   C   
   A   
   G   
   C   
   T   
   C   
   G   
   A   
   T   
   A   
   C   
   C   
   C   
   A   
   A   
   A   
   C   
   C   
   T   
   T   
   C   
   T   
    C    
   G   
   T   
   C   
   A   
   A   
   T   
   G   
   G   
   A   
   T   
   C   
   G   
   A   
   C   
   A   
   G   
   G   
   T   
   A   
   T   
   A   
   T   
   C   
   T   
   G   
   C   
   T   
   G   
   G   
   A   
   T   
   A   
   T   
   G   
   C   
    A    
   A   
   C   
   A   
   G   
   A   
   A   
   A   
   T   
   C   
   C   
   C   
   G   
   A   
   C   
    T    
   C   
   A   
   T   
   A   
   A   
   C   
   T   
   T   
   A   
   G   
   C   
   T   
   G   
   A   
   A   
   G   
   T   
   G   
   A   
   T   
    T    
   G   
   A   
    T    
   G   
   G   
   C   
   A   
   C   
   A   
   A   
   T   
   C   
   T   
   A   
   T   
   A   
   T   
   G   
   A   
   T   
   C   
   G   
   A   
   T   
   C   
   A   
   T   
   C   
   C   
   A   
   C   
   A   
   A   
   G   
   C   
   G   
   A   
   G   
   C   
   C   
   T   
   T   
   G   
   A   
   A   
   A   
   A   
   A   
   T   
   T   
   G   
   A   
   T   
   G   
   G   
   A   
   A   
   T   
   A   
   T   
   A   
   T   
   T   
   C   
   C   
   T   
   G   
   G   
   A   
   C   
   C   
   T   
   G   
   A   
   C   
   T   
   T   
    T    
   C   
   C   
   A   
   A   
   C   
   A   
   G   
   G   
   A   
   G   
   G   
    G    
   A   
   T   
   C   
   T   
   T   
   G   
   C   
   A   
   A   
   G   
   G   
    A    
   A   
   A   
   A   
   G   
   A   
   T   
   G   
   A   
   A   
   A   
   T   
   C   
   A   
   A   
   A   
   A   
   A   
   A   
   G   
   C   
   T   
   T   
   A   
   T   
   G   
   A   
   A   
   A   
   C   
   A   
   G   
   G   
    G    
   C   
   G   
   A   
   G   
   G   
   A   
   A   
   A   
   A   
   G   
   T   
   T   
   A   
    T    
   T   
   C   
   T   
   T   
   C   
   G   
   T   
   T   
   C   
   G   
   A   
   A   
   A   
   A   
   C   
   A   
   A   
   A   
   G   
   A   
   T   
   A   
   G   
   A   
    G    
   G   
   C   
   A   
   A   
   T   
   G   
   A   
   A   
   G   
   G   
   G   
    T    
   A   
   A   
    T    
   A   
   A   
   A   
   C   
   A   
   G   
   C   
   A   
   A   
   A   
   T   
   C   
   G   
   T   
   G   
   A   
   T   
   C   
   A   
   C   
    A    
   G   
   A   
   A   
   A   
   T   
   C   
   C   
   C   
   A   
   T   
   A   
   T   
   G   
   A   
   A   
   G   
   T   
   A   
   A   
   A   
   T   
   A   
   A   
   A   
   G   
   C   
   G   
   A   
   C   
    C    
   T   
   T   
   G   
   G   
   T   
   G   
   A   
   A   
   A   
   A   
   A   
   A   
   A   
   T   
   G   
   G   
   A   
   T   
   G   
   A   
   G   
   A   
   T   
   C   
   C   
   G   
   T   
   T   
   T   
   G   
   A   
   A   
   T   
   A   
   A   
   G   
   A   
   A   
   A   
   A   
   T   
   C   
   G   
   A   
    T    
   G   
   G   
   A   
   A   
   T   
   A   
   G   
   C   
   T   
   G   
   A   
   A   
   G   
   T   
    A    
   C   
   G   
   A   
   G   
   A   
   T   
   G   
   A   
   A   
   A   
   G   
   T   
   G   
   A   
   T   
    A    
   G   
    A    
   A   
   C   
    A    
   G   
   G   
   T   
   C   
   T   
   G   
   C   
   A   
   A   
   A   
   T   
   T   
   G   
   T   
   C   
   G   
   T   
    C    
   G   
   A   
   A   
   T   
   T   
   G   
   A   
   A   
   A   
   A   
   A   
   A   
   G   
   A   
    C    
   G   
   C   
   C   
   A   
   A   
    T    
   G   
   C   
   T   
   C   
   A   
   A   
   G   
   G   
    A    
   A   
   T   
   T   
   T   
   T   
   G   
   A   
   A   
   T   
   T   
   A   
    T    
   T   
   T   
   G   
   T   
   T   
   C   
   A   
   A   
   A   
   A   
   A   
   C   
   A   
   C   
   T   
   G   
   A   
   G   
    C    
   T   
   A   
   C   
   A   
   G   
   A   
   T   
   C   
   A   
   A   
   T   
   T   
   A   
   C   
   A   
   A   
   T   
   T   
   T   
    C    
   A   
   A   
   T   
   A   
   T   
   G   
   G   
   T   
   C   
   G   
   C   
   T   
   A   
   T   
   C   
   G   
   A   
    C    
   C   
   A   
   T   
   A   
   T   
   G   
   A   
   C   
   T   
   C   
   C   
   G   
   C   
   A   
    T    
   C   
   A   
   A   
   G   
   T   
   T   
   G   
   G   
   A   
   C   
   T   
   G   
   A   
   A   
   A   
   G   
   A   
   C   
   A   
   T   
   T   
   T   
   T   
   A   
   A   
   G   
   A   
   A   
   G   
   T   
   T   
   A   
   T   
   A   
   T   
   C   
   G   
   A   
   G   
   C   
   A   
   T   
   C   
   G   
   A   
   A   
   A   
   G   
   C   
   A   
   A   
   G   
   T   
   C   
   A   
   T   
   A   
   A   
   C   
   A   
   A   
   A   
   A   
   C   
   G   
    T    
   A   
   G   
    T    
   C   
   A   
   A   
   T   
   T   
   C   
   G   
   A   
   T   
   T   
   T   
   A   
   G   
   C   
   C   
   A   
   A   
   A   
   G   
   C   
   T   
   C   
   A   
   A   
   A   
   A   
   A   
    A    
   G   
   A   
   C   
   A   
   G   
   C   
   A   
   T   
   A   
   T   
   C   
   G   
   T   
    A    
   G   
   A   
   A   
   G   
   G   
    A    
    T    
   T   
   G   
   A   
   T   
   G   
   A   
   A   
   A   
   G   
   C   
   T   
   T   
   T   
   A   
   T   
   C   
   G   
   A   
   T   
   T   
   C   
   T   
   G   
   G   
   A   
   T   
   G   
   A   
   A   
   G   
   T   
   A   
   A   
   T   
   C   
   G   
    T    
   A   
   A   
   C   
   G   
   A   
   T   
   T   
   C   
   G   
   T   
   G   
   A   
   A   
   A   
   G   
   T   
   A   
   A   
   A   
   G   
   A   
   C   
   A   
   A   
   A   
   A   
   A   
   A   
   G   
   A   
    C    
   G   
   C   
    C    
   A   
   A   
   A   
   A   
   A   
   G   
   A   
   A   
   T   
   T   
   T   
   A   
   G   
   T   
   C   
   G   
   A   
    C    
   G   
   T   
    C    
   T   
   T   
    T    
   C   
   A   
   A   
   T   
   T   
   T   
   A   
   C   
   A   
   G   
   A   
   A   
   G   
   A   
   A   
   C   
   A   
   A   
   G   
   C   
   A   
   G   
   A   
   A   
   G   
   C   
   A   
   A   
   T   
    T    
   G   
   T   
   C   
   A   
   C   
   A   
   T   
   T   
   G   
   C   
   A   
   A   
    T    
   T   
   A   
   T   
   A   
    C    
   C   
   G   
   T   
   T   
   T   
   G   
   A   
   C   
   G   
   A   
   A   
    C    
   A   
   C   
    G    
   G   
   A   
   T   
   A   
   T   
   C   
   A   
   C   
   A   
   G   
   A   
   G   
   C   
   T   
   T   
   C   
   A   
   A   
   A   
   A   
   G   
   G   
   A   
   A   
    T    
   C   
    C    
   G   
   A   
   A   
   A   
    G    
   C   
   T   
   T   
   G   
   A   
   T   
   T   
   G   
   C   
    G    
   C   
   A   
   A   
   A   
   T   
   C   
   A   
   C   
   A   
   G   
   A   
   G   
   T   
   T   
    G    
   A   
   A   
   T   
   A   
   A   
   A   
   A   
   T   
   C   
   C   
   T   
   T   
   T   
   C   
   A   
   A   
   A   
    T    
   G   
   A   
   C   
   A   
   A   
   A   
   G   
   A   
    G    
   C   
   T   
   T   
   T   
   T   
    C    
   T   
   C   
   T   
   G   
   T   
   T   
   A   
   T   
   G   
   A   
   A   
   A   
   A   
   A   
   A   
   G   
   A   
   A   
   T   
   T   
   G   
   C   
   G   
   A   
   G   
   A   
   A   
   G   
   T   
   G   
   A   
   A   
   A   
   A   
   A   
   A   
   A   
   A   
   T   
   T   
   A   
    T    
   T   
   C   
   T   
   T   
   C   
   C   
   G   
   C   
   T   
   C   
   G   
   T   
   C   
    T    
    A    
   A   
   C   
   G   
   A   
   C   
   A   
   A   
   T   
   C   
    G    
   A   
   A   
   G   
   A   
   C   
   G   
   A   
   A   
   A   
   T   
   C   
   G   
   A   
   A   
   G   
   A   
   A   
   A   
   T   
   C   
   A   
   A   
   A   
   A   
   T   
    T    
   G   
   A   
   T   
   A   
   C   
   G   
   C   
   A   
   A   
    G    
   T   
    C    
   T   
   T   
   A   
   G   
   T   
   G   
   G   
   C   
    A    
   C   
   A   
   A   
   G   
   A   
   A   
   G   
   A   
   C   
   G   
   T   
   A   
   A   
   T   
    T    
   G   
   T   
   C   
   A   
   G   
   T   
   G   
   T   
    G    
   A   
   C   
    G    
   C   
   G   
   A   
   G   
   A   
   A   
   G   
   G   
   C   
   T   
   A   
   T   
   G   
   T   
   G   
   A   
   A   
   G   
   C   
   G   
   A   
   A   
   C   
   A   
   A   
   G   
   C   
   C   
   T   
   T   
   C   
   G   
   T   
   T   
   C   
    T    
   T   
   A   
   T   
   A   
   G   
   T   
   G   
   C   
   T   
   T   
   C   
    C    
   A   
   A   
   A   
   C   
   C   
   A   
   G   
   A   
   A   
   G   
   A   
   A   
   A   
   T   
   C   
   G   
   G   
   G   
   A   
   T   
   G   
   C   
   G   
   A   
   G   
   A   
   A   
   G   
   G   
   C   
   G   
   A   
    C    
   T   
   A   
   C   
   T   
   T   
   G   
   C   
   T   
    A    
   T   
   A   
   C   
    T    
   C   
   A   
   G   
   G   
   T   
   G   
   A   
   A   
   T   
   T   
    G    
   A   
   G   
    T    
   A   
   C   
   A   
    T    
   T   
   G   
   G   
   A   
   C   
   C   
   A   
   T   
   G   
   T   
   T   
   C   
   T   
   T   
   C   
   T   
   G   
   A   
   T   
    C    
   A   
   C   
    A    
   A   
   A   
   T   
   A   
   A   
   A   
   G   
   C   
   A   
   A   
   A   
   T   
   G   
   T   
   G   
   A   
   T   
   C   
   T   
   A   
   C   
   C   
   G   
    C    
   C   
   C   
   T   
   G   
   T   
    C    
   C   
   A   
   T   
   G   
   A   
   A   
   C   
   T   
   A   
   C   
   C   
    C    
   G   
   A   
   T   
   T   
   T   
   G   
   A   
   A   
   A   
   T   
   G   
   G   
   A   
   A   
   A   
   G   
   A   
   T   
   G   
   C   
   T   
   G   
   G   
    T    
   G   
   A   
    G    
   C   
   A   
    C    
   A   
   T   
   T   
   T   
   C   
   T   
   C   
   A   
   A   
   A   
   C   
   A   
   A   
   T   
   C   
   T   
   C   
   C   
   A   
   A   
   T   
   C   
   T   
   G   
   T   
   C   
   T   
   G   
   T   
   A   
   G   
   A   
    C    
   G   
   A   
   A   
   T   
   C   
   G   
   A   
   T   
    T    
   T   
   T   
   A   
   G   
   C   
   T   
   G   
   T   
   T   
   T   
   T   
    C    
   C   
   C   
   A   
   T   
   A   
   T   
   C   
   A   
   A   
   A   
   A   
   A   
   A   
   T   
    T    
   C   
   A   
   A   
   G   
   C   
   A   
   G   
   A   
    G    
   A   
   A   
   A   
   A   
   C   
   A   
   T   
   T   
   T   
   G   
   T   
    A    
   T   
   T   
   C   
   A   
   T   
   C   
   A   
   G   
   C   
   A   
   A   
   G   
   A   
   A   
   T   
   G   
   G   
   A   
   T   
   T   
    G    
    A    
   T   
   C   
   A   
   A   
   G   
   C   
   A   
   A   
   A   
   C   
   A   
   C   
   G   
   A   
   A   
   T   
   G   
   A   
   C   
   A   
   G   
   A   
   G   
   T   
   T   
   C   
   G   
   A   
   A   
   C   
   C   
   A   
   T   
   G   
   G   
   C   
   G   
   C   
   A   
   C   
   C   
   T   
   A   
    T    
   A   
   A   
   G   
   A   
   G   
   C   
   C   
   G   
    T    
   C   
   C   
   G   
   C   
   T   
   C   
   A   
   G   
   C   
   T   
   G   
   T   
   A   
   T   
   G   
   A   
   A   
   G   
   C   
   T   
   A   
   A   
   A   
   G   
   G   
   C   
   G   
   G   
   A   
   A   
   G   
   A   
   A   
   G   
   A   
   T   
   G   
   A   
   A   
   C   
   T   
    G    
   A   
   T   
   T   
   G   
   C   
   T   
   G   
   T   
    G    
   T   
   A   
   T   
   C   
   T   
   G   
   A   
   A   
   A   
   G   
   A   
   A   
   G   
   A   
   T   
   C   
    A    
   G   
   C   
   C   
   A   
   G   
   G   
   C   
   T   
   T   
   A   
   G   
   A   
   T   
   G   
   T   
    T    
   T   
   T   
   C   
   T   
   T   
   A   
   G   
   T   
   A   
   A   
   C   
   G   
    C    
   A   
   T   
   C   
   A   
   A   
   G   
   G   
   A   
   A   
   T   
   G   
   G   
   G   
    T    
   T   
   T   
   G   
   C   
   G   
   C   
   T   
    A    
   T   
   C   
   C   
   T   
   T   
   T   
   A   
   A   
    C    
   A   
   G   
   A   
   T   
   G   
   T   
   T   
   C   
   C   
   C   
   G   
   T   
   T   
   G   
   T   
   T   
   G   
   G   
   A   
   G   
   C   
   A   
   A   
   A   
   G   
   G   
   C   
   A   
   G   
    C    
   T   
   G   
   G   
   A   
   G   
   T   
   G   
   A   
   A   
   G   
   T   
   C   
   T   
   A   
   T   
   G   
   A   
   A   
   T   
   T   
   T   
   G   
   A   
   A   
   A   
   G   
   A   
   T   
   G   
   G   
   A   
   G   
   A   
   T   
   A   
   G   
   G   
    A    
   T   
   T   
    A    
   T   
   C   
   A   
   A   
    C    
   G   
   G   
    G    
   C   
   T   
    G    
   C   
   T   
   T   
   G   
   T   
   G   
   T   
   T   
   A   
   G   
   C   
   A   
   G   
   A   
    A    
   G   
   G   
    T    
   G   
   A   
   T   
   A   
   C   
   G   
   C   
   C   
    G    
   A   
   T   
   C   
   G   
   T   
   A   
   A   
   T   
   C   
   G   
   T   
   T   
   A   
   C   
   T   
   C   
   A   
   G   
   C   
   G   
   A   
   G   
   G   
   A   
   G   
   C   
    T    
   G   
   T   
    T    
   A   
   A   
   A   
   C   
   G   
   C   
   A   
   T   
   G   
   C   
   T   
   T   
   G   
   C   
   T   
   C   
   A   
   A   
   G   
   A   
   A   
   A   
   T   
   C   
   A   
   G   
   C   
   C   
   A   
   A   
   A   
   C   
    T    
   T   
   C   
   A   
   C   
   G   
   T   
   G   
   C   
   G   
   A   
   A   
   G   
   C   
   G   
   G   
   G   
   G   
   A   
   G   
   T   
   G   
   A   
   C   
   G   
   G   
   T   
   C   
   A   
   T   
   G   
   C   
   G   
   T   
   G   
   A   
   G   
   C   
   T   
   G   
   A   
   A   
   A   
   A   
   A   
   A   
   A   
   A   
   T   
   C   
   C   
    G    
   C   
   A   
    C    
   C   
   G   
    T    
   A   
   T   
   T   
   A   
   T   
   C   
   T   
   A   
   T   
   A   
   T   
   G   
   T   
   C   
   G   
   G   
   A   
   A   
   G   
   G   
   A   
   A   
   G   
   A   
   T   
   C   
   A   
   A   
   A   
   A   
   G   
   A   
   A   
   A   
   T   
   A   
   A   
   C   
   C   
   T   
   T   
   G   
   A   
   T   
   G   
   A   
   A   
   T   
   C   
   A   
   A   
   A   
   A   
   A   
   G   
   G   
    G    
   C   
   A   
    G    
   C   
   A   
   G   
    T    
   T   
   A   
   A   
   C   
   G   
   A   
   T   
   T   
   G   
   A   
    C    
   C   
   C   
   A   
   A   
   C   
    T    
   G   
   A   
   T   
   T   
   T   
    C    
   C   
   C   
   T   
   A   
   T   
   A   
   G   
   G   
   T   
   G   
   A   
   T   
   C   
   G   
   A   
   A   
   C   
   A   
   T   
   C   
   G   
   A   
   A   
   T   
   G   
   G   
    A    
   T   
   C   
    A    
   T   
   T   
   T   
   G   
   C   
    G    
   A   
   T   
   G   
   G   
   A   
    T    
   G   
   A   
   G   
   A   
   A   
   A   
   A   
   A   
   A   
   G   
   G   
   T   
   G   
   G   
   A   
   G   
   A   
   A   
   G   
   T   
   C   
   A   
   T   
   C   
   G   
   A   
   A   
   G   
   T   
    T    
   A   
    T    
   C   
   G   
   A   
   T   
   G   
   C   
   T   
   C   
   C   
   T   
   G   
   A   
   G   
    A    
   T   
   T   
   T   
   T   
    C    
   A   
   T   
    C    
   G   
   A   
    G    
    T    
   C   
    G    
   -   
   -   
   -   
 

 
   N60252F   
   A   
   T   
   G   
   G   
   A   
   A   
   A   
   A   
   T   
   C   
   G   
   A   
   C   
   A   
   A   
   G   
   A   
   A   
   G   
   T   
   A   
   C   
   A   
   A   
   G   
   A   
   A   
   T   
   T   
   A   
   A   
   C   
   T   
   T   
   T   
   A   
   G   
   A   
   A   
   G   
   A   
   A   
   G   
   T   
   G   
   A   
   T   
   G   
   G   
   G   
   T   
   G   
   A   
   C   
   C   
   G   
   C   
   T   
   T   
   C   
   G   
   G   
   A   
   C   
   G   
   T   
   T   
   A   
   T   
   T   
   C   
   C   
   A   
   A   
   A   
   T   
   A   
   T   
   A   
   T   
   T   
   A   
   T   
    C    
   C   
   A   
    A    
   G   
   A   
   A   
   C   
   G   
   T   
   G   
   C   
   A   
   C   
   T   
   G   
   C   
   C   
   T   
   G   
   A   
   T   
   A   
   T   
    C    
   C   
   G   
   T   
   G   
   A   
   T   
   G   
   G   
   A   
   T   
   T   
   G   
   A   
   A   
    G    
   C   
   C   
   A   
   G   
   T   
    G    
   C   
   A   
   A   
   C   
   G   
   C   
   C   
   G   
   G   
   A   
   T   
   A   
   T   
   T   
   A   
   T   
   T   
    T    
   G   
   C   
    C    
   A   
   T   
   G   
   A   
   A   
   C   
   A   
   A   
   A   
   G   
   A   
   T   
   G   
   G   
   C   
   A   
   A   
   T   
   A   
   C   
   G   
   T   
   A   
   T   
   G   
   A   
   C   
   A   
   A   
   A   
   G   
   G   
   A   
   T   
   T   
    C    
   C   
   G   
   T   
   A   
   A   
   A   
   T   
   C   
   A   
   G   
   C   
   A   
   A   
   A   
   A   
   T   
   C   
   T   
   G   
   T   
   C   
   G   
   G   
   A   
   A   
   A   
   T   
   A   
   T   
   C   
   A   
   T   
   G   
   G   
   G   
    G    
   A   
   A   
   T   
   T   
   A   
   T   
   C   
   A   
   T   
   C   
   C   
   T   
   C   
   A   
   T   
   G   
   G   
   T   
   G   
   A   
   C   
   A   
   G   
   C   
   A   
   G   
   T   
   A   
   T   
   T   
   T   
   A   
   T   
   G   
   A   
   A   
   G   
   C   
   G   
   A   
   T   
   G   
   G   
   T   
   C   
   C   
   G   
   C   
   A   
   T   
   G   
   A   
   G   
   C   
   C   
   A   
   A   
   G   
   A   
    C    
   T   
   G   
   G   
   A   
   A   
   G   
   T   
   T   
   G   
   C   
   G   
   G   
   G   
   A   
   A   
   G   
   T   
   A   
   C   
   T   
   G   
   A   
   T   
   C   
   G   
   A   
    A    
   A   
   T   
   G   
   C   
   A   
   C   
   G   
   G   
    G    
   A   
   A   
   T   
   A   
   A   
    C    
   G   
   G   
   G   
   A   
   G   
   T   
   A   
   T   
   G   
   G   
   A   
   C   
   G   
   G   
   T   
   G   
   A   
   T   
   C   
   C   
   A   
   C   
   C   
   T   
   G   
   C   
   T   
   G   
   C   
   G   
   A   
   T   
   G   
   C   
   G   
   T   
   T   
   A   
   T   
   A   
   C   
   T   
   G   
   A   
   A   
   G   
   C   
   C   
   A   
   G   
    A    
   T   
   T   
   A   
   T   
   C   
   C   
   A   
   A   
   G   
   T   
   T   
   A   
   A   
   G   
   T   
   G   
   G   
   A   
   G   
   A   
   G   
   A   
   T   
   G   
    C    
   T   
   A   
    G    
   C   
   A   
   G   
   A   
   T   
   A   
   T   
   C   
   G   
   A   
    A    
   A   
   A   
   A   
   G   
   A   
   A   
   A   
   C   
   A   
   G   
   T   
    C    
   G   
   A   
   T   
   C   
   T   
   C   
   G   
   T   
   G   
   T   
   G   
   G   
   A   
   A   
   T   
   T   
   T   
   T   
   G   
   A   
   T   
   G   
   A   
   T   
   A   
   C   
   A   
   G   
   A   
   A   
   A   
   A   
   A   
   G   
   A   
   A   
   C   
   C   
   A   
   A   
   C   
   A   
   G   
   T   
   C   
   C   
   T   
   T   
   C   
   C   
   A   
   G   
   C   
   T   
   C   
   G   
   A   
   T   
   A   
   C   
   C   
   C   
   A   
   A   
   A   
   C   
   C   
   T   
   T   
   C   
   T   
    C    
   G   
   T   
   C   
   A   
   A   
   T   
   G   
   G   
   A   
   T   
   C   
   G   
   A   
   C   
   A   
   G   
   G   
   T   
   A   
   T   
   A   
   T   
   C   
   T   
   G   
   C   
   T   
   G   
   G   
   A   
   T   
   A   
   T   
   G   
   C   
    A    
   A   
   C   
   A   
   G   
   A   
   A   
   A   
   T   
   C   
   C   
   C   
   G   
   A   
   C   
    T    
   C   
   A   
   T   
   A   
   A   
   C   
   T   
   T   
   A   
   G   
   C   
   T   
   G   
   A   
   A   
   G   
   T   
   G   
   A   
   T   
    C    
   G   
   A   
    T    
   G   
   G   
   C   
   A   
   C   
   A   
   A   
   T   
   C   
   T   
   A   
   T   
   A   
   T   
   G   
   A   
   T   
   C   
   G   
   A   
   T   
   C   
   A   
   T   
   C   
   C   
   A   
   C   
   A   
   A   
   G   
   C   
   G   
   A   
   G   
   C   
   C   
   T   
   T   
   G   
   A   
   A   
   A   
   A   
   A   
   T   
   T   
   G   
   A   
   T   
   G   
   G   
   A   
   A   
   T   
   A   
   T   
   A   
   T   
   T   
   C   
   C   
   T   
   G   
   G   
   A   
   C   
   C   
   T   
   G   
   A   
   C   
   T   
   T   
    T    
   C   
   C   
   A   
   A   
   C   
   A   
   G   
   G   
   A   
   G   
   G   
    G    
   A   
   T   
   C   
   T   
   T   
   G   
   C   
   A   
   A   
   G   
   G   
    A    
   A   
   A   
   A   
   G   
   A   
   T   
   G   
   A   
   A   
   A   
   T   
   C   
   A   
   A   
   A   
   A   
   A   
   A   
   G   
   C   
   T   
   T   
   A   
   T   
   G   
   A   
   A   
   A   
   C   
   A   
   G   
   G   
    G    
   C   
   G   
   A   
   G   
   G   
   A   
   A   
   A   
   A   
   G   
   T   
   T   
   A   
    T    
   T   
   C   
   T   
   T   
   C   
   G   
   T   
   T   
   C   
   G   
   A   
   A   
   A   
   A   
   C   
   A   
   A   
   A   
   G   
   A   
   T   
   A   
   G   
   A   
    G    
   G   
   C   
   A   
   A   
   T   
   G   
   A   
   A   
   G   
   G   
   G   
    T    
   A   
   A   
    T    
   A   
   A   
   A   
   C   
   A   
   G   
   C   
   A   
   A   
   A   
   T   
   C   
   G   
   T   
   G   
   A   
   T   
   C   
   A   
   C   
    A    
   G   
   A   
   A   
   A   
   T   
   C   
   C   
   C   
   A   
   T   
   A   
   T   
   G   
   A   
   A   
   G   
   T   
   A   
   A   
   A   
   T   
   A   
   A   
   A   
   G   
   C   
   G   
   A   
   C   
    C    
   T   
   T   
   G   
   G   
   T   
   G   
   A   
   A   
   A   
   A   
   A   
   A   
   A   
   T   
   G   
   G   
   A   
   T   
   G   
   A   
   G   
   A   
   T   
   C   
   C   
   G   
   T   
   T   
   T   
   G   
   A   
   A   
   T   
   A   
   A   
   G   
   A   
   A   
   A   
   A   
   T   
   C   
   G   
   A   
    T    
   G   
   G   
   A   
   A   
   T   
   A   
   G   
   C   
   T   
   G   
   A   
   A   
   G   
   T   
    A    
   C   
   G   
   A   
   G   
   A   
   T   
   G   
   A   
   A   
   A   
   G   
   T   
   G   
   A   
   T   
    A    
   G   
    A    
   A   
   C   
    A    
   G   
   G   
   T   
   C   
   T   
   G   
   C   
   A   
   A   
   A   
   T   
   T   
   G   
   T   
   C   
   G   
   T   
    C    
   G   
   A   
   A   
   T   
   T   
   G   
   A   
   A   
   A   
   A   
   A   
   A   
   G   
   A   
    C    
   G   
   C   
   C   
   A   
   A   
    T    
   G   
   C   
   T   
   C   
   A   
   A   
   G   
   G   
    A    
   A   
   T   
   T   
   T   
   T   
   G   
   A   
   A   
   T   
   T   
   A   
    T    
   T   
   T   
   G   
   T   
   T   
   C   
   A   
   A   
   A   
   A   
   A   
   C   
   A   
   C   
   T   
   G   
   A   
   G   
    C    
   T   
   A   
   C   
   A   
   G   
   A   
   T   
   C   
   A   
   A   
   T   
   T   
   A   
   C   
   A   
   A   
   T   
   T   
   T   
    C    
   A   
   A   
   T   
   A   
   T   
   G   
   G   
   T   
   C   
   G   
   C   
   T   
   A   
   T   
   C   
   G   
   A   
    C    
   C   
   A   
   T   
   A   
   T   
   G   
   A   
   C   
   T   
   C   
   C   
   G   
   C   
   A   
    T    
   C   
   A   
   A   
   G   
   T   
   T   
   G   
   G   
   A   
   C   
   T   
   G   
   A   
   A   
   A   
   G   
   A   
   C   
   A   
   T   
   T   
   T   
   T   
   A   
   A   
   G   
   A   
   A   
   G   
   T   
   T   
   A   
   T   
   A   
   T   
   C   
   G   
   A   
   G   
   C   
   A   
   T   
   C   
   G   
   A   
   A   
   A   
   G   
   C   
   A   
   A   
   G   
   T   
   C   
   A   
   T   
   A   
   A   
   C   
   A   
   A   
   A   
   A   
   C   
   G   
    T    
   A   
   G   
    T    
   C   
   A   
   A   
   T   
   T   
   C   
   G   
   A   
   T   
   T   
   T   
   A   
   G   
   C   
   C   
   A   
   A   
   A   
   G   
   C   
   T   
   C   
   A   
   A   
   A   
   A   
   A   
    A    
   G   
   A   
   C   
   A   
   G   
   C   
   A   
   T   
   A   
   T   
   C   
   G   
   T   
    A    
   G   
   A   
   A   
   G   
   G   
    A    
    T    
   T   
   G   
   A   
   T   
   G   
   A   
   A   
   A   
   G   
   C   
   T   
   T   
   T   
   A   
   T   
   C   
   G   
   A   
   T   
   T   
   C   
   T   
   G   
   G   
   A   
   T   
   G   
   A   
   A   
   G   
   T   
   A   
   A   
   T   
   C   
   G   
    C    
   A   
   A   
   C   
   G   
   A   
   T   
   T   
   C   
   G   
   T   
   G   
   A   
   A   
   A   
   G   
   T   
   A   
   A   
   A   
   G   
   A   
   C   
   A   
   A   
   A   
   A   
   A   
   A   
   G   
   A   
    C    
   G   
   C   
    C    
   A   
   A   
   A   
   A   
   A   
   G   
   A   
   A   
   T   
   T   
   T   
   A   
   G   
   T   
   C   
   G   
   A   
    C    
   G   
   T   
    C    
   T   
   T   
    T    
   C   
   A   
   A   
   T   
   T   
   T   
   A   
   C   
   A   
   G   
   A   
   A   
   G   
   A   
   A   
   C   
   A   
   A   
   G   
   C   
   A   
   G   
   A   
   A   
   G   
   C   
   A   
   A   
   T   
    T    
   G   
   T   
   C   
   A   
   C   
   A   
   T   
   T   
   G   
   C   
   A   
   A   
    T    
   T   
   A   
   T   
   A   
    C    
   C   
   G   
   T   
   T   
   T   
   G   
   A   
   C   
   G   
   A   
   A   
    C    
   A   
   C   
    G    
   G   
   A   
   T   
   A   
   T   
   C   
   A   
   C   
   A   
   G   
   A   
   G   
   C   
   T   
   T   
   C   
   A   
   A   
   A   
   A   
   G   
   G   
   A   
   A   
    T    
   C   
    C    
   G   
   A   
   A   
   A   
    G    
   C   
   T   
   T   
   G   
   A   
   T   
   T   
   G   
   C   
    G    
   C   
   A   
   A   
   A   
   T   
   C   
   A   
   C   
   A   
   G   
   A   
   G   
   T   
   T   
    G    
   A   
   A   
   T   
   A   
   A   
   A   
   A   
   T   
   C   
   C   
   T   
   T   
   T   
   C   
   A   
   A   
   A   
    T    
   G   
   A   
   C   
   A   
   A   
   A   
   G   
   A   
    G    
   C   
   T   
   T   
   T   
   T   
    C    
   T   
   C   
   T   
   G   
   T   
   T   
   A   
   T   
   G   
   A   
   A   
   A   
   A   
   A   
   A   
   G   
   A   
   A   
   T   
   T   
   G   
   C   
   G   
   A   
   G   
   A   
   A   
   G   
   T   
   G   
   A   
   A   
   A   
   A   
   A   
   A   
   A   
   A   
   T   
   T   
   A   
    T    
   T   
   C   
   T   
   T   
   C   
   C   
   G   
   C   
   T   
   C   
   G   
   T   
   C   
    T    
    A    
   A   
   C   
   G   
   A   
   C   
   A   
   A   
   T   
   C   
    G    
   A   
   A   
   G   
   A   
   C   
   G   
   A   
   A   
   A   
   T   
   C   
   G   
   A   
   A   
   G   
   A   
   A   
   A   
   T   
   C   
   A   
   A   
   A   
   A   
   T   
    T    
   G   
   A   
   T   
   A   
   C   
   G   
   C   
   A   
   A   
    G    
   T   
    C    
   T   
   T   
   A   
   G   
   T   
   G   
   G   
   C   
    A    
   C   
   A   
   A   
   G   
   A   
   A   
   G   
   A   
   C   
   G   
   T   
   A   
   A   
   T   
    T    
   G   
   T   
   C   
   A   
   G   
   T   
   G   
   T   
    G    
   A   
   C   
    G    
   C   
   G   
   A   
   G   
   A   
   A   
   G   
   G   
   C   
   T   
   A   
   T   
   G   
   T   
   G   
   A   
   A   
   G   
   C   
   G   
   A   
   A   
   C   
   A   
   A   
   G   
   C   
   C   
   T   
   T   
   C   
   G   
   T   
   T   
   C   
    T    
   T   
   A   
   T   
   A   
   G   
   T   
   G   
   C   
   T   
   T   
   C   
    C    
   A   
   A   
   A   
   C   
   C   
   A   
   G   
   A   
   A   
   G   
   A   
   A   
   A   
   T   
   C   
   G   
   G   
   G   
   A   
   T   
   G   
   C   
   G   
   A   
   G   
   A   
   A   
   G   
   G   
   C   
   G   
   A   
    C    
   T   
   A   
   C   
   T   
   T   
   G   
   C   
   T   
    A    
   T   
   A   
   C   
    T    
   C   
   A   
   G   
   G   
   T   
   G   
   A   
   A   
   T   
   T   
    G    
   A   
   G   
    T    
   A   
   C   
   A   
    T    
   T   
   G   
   G   
   A   
   C   
   C   
   A   
   T   
   G   
   T   
   T   
   C   
   T   
   T   
   C   
   T   
   G   
   A   
   T   
    C    
   A   
   C   
    A    
   A   
   A   
   T   
   A   
   A   
   A   
   G   
   C   
   A   
   A   
   A   
   T   
   G   
   T   
   G   
   A   
   T   
   C   
   T   
   A   
   C   
   C   
   G   
    C    
   C   
   C   
   T   
   G   
   T   
    C    
   C   
   A   
   T   
   G   
   A   
   A   
   C   
   T   
   A   
   C   
   C   
    C    
   G   
   A   
   T   
   T   
   T   
   G   
   A   
   A   
   A   
   T   
   G   
   G   
   A   
   A   
   A   
   G   
   A   
   T   
   G   
   C   
   T   
   G   
   G   
    T    
   G   
   A   
    G    
   C   
   A   
    C    
   A   
   T   
   T   
   T   
   C   
   T   
   C   
   A   
   A   
   A   
   C   
   A   
   A   
   T   
   C   
   T   
   C   
   C   
   A   
   A   
   T   
   C   
   T   
   G   
   T   
   C   
   T   
   G   
   T   
   A   
   G   
   A   
    C    
   G   
   A   
   A   
   T   
   C   
   G   
   A   
   T   
    T    
   T   
   T   
   A   
   G   
   C   
   T   
   G   
   T   
   T   
   T   
   T   
    C    
   C   
   C   
   A   
   T   
   A   
   T   
   C   
   A   
   A   
   A   
   A   
   A   
   A   
   T   
    T    
   C   
   A   
   A   
   G   
   C   
   A   
   G   
   A   
    G    
   A   
   A   
   A   
   A   
   C   
   A   
   T   
   T   
   T   
   G   
   T   
    A    
   T   
   T   
   C   
   A   
   T   
   C   
   A   
   G   
   C   
   A   
   A   
   G   
   A   
   A   
   T   
   G   
   G   
   A   
   T   
   T   
    G    
    A    
   T   
   C   
   A   
   A   
   G   
   C   
   A   
   A   
   A   
   C   
   A   
   C   
   G   
   A   
   A   
   T   
   G   
   A   
   C   
   A   
   G   
   A   
   G   
   T   
   T   
   C   
   G   
   A   
   A   
   C   
   C   
   A   
   T   
   G   
   G   
   C   
   G   
   C   
   A   
   C   
   C   
   T   
   A   
    T    
   A   
   A   
   G   
   A   
   G   
   C   
   C   
   G   
    T    
   C   
   C   
   G   
   C   
   T   
   C   
   A   
   G   
   C   
   T   
   G   
   T   
   A   
   T   
   G   
   A   
   A   
   G   
   C   
   T   
   A   
   A   
   A   
   G   
   G   
   C   
   G   
   G   
   A   
   A   
   G   
   A   
   A   
   G   
   A   
   T   
   G   
   A   
   A   
   C   
   T   
    G    
   A   
   T   
   T   
   G   
   C   
   T   
   G   
   T   
    G    
   T   
   A   
   T   
   C   
   T   
   G   
   A   
   A   
   A   
   G   
   A   
   A   
   G   
   A   
   T   
   C   
    A    
   G   
   C   
   C   
   A   
   G   
   G   
   C   
   T   
   T   
   A   
   G   
   A   
   T   
   G   
   T   
    T    
   T   
   T   
   C   
   T   
   T   
   A   
   G   
   T   
   A   
   A   
   C   
   G   
    C    
   A   
   T   
   C   
   A   
   A   
   G   
   G   
   A   
   A   
   T   
   G   
   G   
   G   
    T    
   T   
   T   
   G   
   C   
   G   
   C   
   T   
    G    
   T   
   C   
   C   
   T   
   T   
   T   
   A   
   A   
    C    
   A   
   G   
   A   
   T   
   G   
   T   
   T   
   C   
   C   
   C   
   G   
   T   
   T   
   G   
   T   
   T   
   G   
   G   
   A   
   G   
   C   
   A   
   A   
   A   
   G   
   G   
   C   
   A   
   G   
    C    
   T   
   G   
   G   
   A   
   G   
   T   
   G   
   A   
   A   
   G   
   T   
   C   
   T   
   A   
   T   
   G   
   A   
   A   
   T   
   T   
   T   
   G   
   A   
   A   
   A   
   G   
   A   
   T   
   G   
   G   
   A   
   G   
   A   
   T   
   A   
   G   
   G   
    A    
   T   
   T   
    A    
   T   
   C   
   A   
   A   
    C    
   G   
   G   
    G    
   C   
   T   
    G    
   C   
   T   
   T   
   G   
   T   
   G   
   T   
   T   
   A   
   G   
   C   
   A   
   G   
   A   
    A    
   G   
   G   
    T    
   G   
   A   
   T   
   A   
   C   
   G   
   C   
   C   
    G    
   A   
   T   
   C   
   G   
   T   
   A   
   A   
   T   
   C   
   G   
   T   
   T   
   A   
   C   
   T   
   C   
   A   
   G   
   C   
   G   
   A   
   G   
   G   
   A   
   G   
   C   
    T    
   G   
   T   
    T    
   A   
   A   
   A   
   C   
   G   
   C   
   A   
   T   
   G   
   C   
   T   
   T   
   G   
   C   
   T   
   C   
   A   
   A   
   G   
   A   
   A   
   A   
   T   
   C   
   A   
   G   
   C   
   C   
   A   
   A   
   A   
   C   
    T    
   T   
   C   
   A   
   C   
   G   
   T   
   G   
   C   
   G   
   A   
   A   
   G   
   C   
   G   
   G   
   G   
   G   
   A   
   G   
   T   
   G   
   A   
   C   
   G   
   G   
   T   
   C   
   A   
   T   
   G   
   C   
   G   
   T   
   G   
   A   
   G   
   C   
   T   
   G   
   A   
   A   
   A   
   A   
   A   
   A   
   A   
   A   
   T   
   C   
   C   
    G    
   C   
   A   
    C    
   C   
   G   
    T    
   A   
   T   
   T   
   A   
   T   
   C   
   T   
   A   
   T   
   A   
   T   
   G   
   T   
   C   
   G   
   G   
   A   
   A   
   G   
   G   
   A   
   A   
   G   
   A   
   T   
   C   
   A   
   A   
   A   
   A   
   G   
   A   
   A   
   A   
   T   
   A   
   A   
   C   
   C   
   T   
   T   
   G   
   A   
   T   
   G   
   A   
   A   
   T   
   C   
   A   
   A   
   A   
   A   
   A   
   G   
   G   
    G    
   C   
   A   
    G    
   C   
   A   
   G   
    T    
   T   
   A   
   A   
   C   
   G   
   A   
   T   
   T   
   G   
   A   
    C    
   C   
   C   
   A   
   A   
   C   
    T    
   G   
   A   
   T   
   T   
   T   
    C    
   C   
   C   
   T   
   A   
   T   
   A   
   G   
   G   
   T   
   G   
   A   
   T   
   C   
   G   
   A   
   A   
   C   
   A   
   T   
   C   
   G   
   A   
   A   
   T   
   G   
   G   
    A    
   T   
   C   
    A    
   T   
   T   
   T   
   G   
   C   
    G    
   A   
   T   
   G   
   G   
   A   
    T    
   G   
   A   
   G   
   A   
   A   
   A   
   A   
   A   
   A   
   G   
   G   
   T   
   G   
   G   
   A   
   G   
   A   
   A   
   G   
   T   
   C   
   A   
   T   
   C   
   G   
   A   
   A   
   G   
   T   
    T    
   A   
    T    
   C   
   G   
   A   
   T   
   G   
   C   
   T   
   C   
   C   
   T   
   G   
   A   
   G   
    A    
   T   
   T   
   T   
   T   
    C    
   A   
   T   
    C    
   G   
   A   
    G    
    T    
   C   
    G    
   -   
   -   
   -   
 

 
   N59531F   
   A   
   T   
   G   
   G   
   A   
   A   
   A   
   A   
   T   
   C   
   G   
   A   
   C   
   A   
   A   
   G   
   A   
   A   
   G   
   T   
   A   
   C   
   A   
   A   
   G   
   A   
   A   
   T   
   T   
   A   
   A   
   C   
   T   
   T   
   T   
   A   
   G   
   A   
   A   
   G   
   A   
   A   
   G   
   T   
   G   
   A   
   T   
   G   
   G   
   G   
   T   
   G   
   A   
   C   
   C   
   G   
   C   
   T   
   T   
   C   
   G   
   G   
   A   
   C   
   G   
   T   
   T   
   A   
   T   
   T   
   C   
   C   
   A   
   A   
   A   
   T   
   A   
   T   
   A   
   T   
   T   
   A   
   T   
    C    
   C   
   A   
    A    
   G   
   A   
   A   
   C   
   G   
   T   
   G   
   C   
   A   
   C   
   T   
   G   
   C   
   C   
   T   
   G   
   A   
   T   
   A   
   T   
    C    
   C   
   G   
   T   
   G   
   A   
   T   
   G   
   G   
   A   
   T   
   T   
   G   
   A   
   A   
    A    
   C   
   C   
   A   
   G   
   T   
    A    
   C   
   A   
   A   
   C   
   G   
   C   
   C   
   G   
   G   
   A   
   T   
   A   
   T   
   T   
   A   
   T   
   T   
    T    
   G   
   C   
    C    
   A   
   T   
   G   
   A   
   A   
   C   
   A   
   A   
   A   
   G   
   A   
   T   
   G   
   G   
   C   
   A   
   A   
   T   
   A   
   C   
   G   
   T   
   A   
   T   
   G   
   A   
   C   
   A   
   A   
   A   
   G   
   G   
   A   
   T   
   T   
    C    
   C   
   G   
   T   
   A   
   A   
   A   
   T   
   C   
   A   
   G   
   C   
   A   
   A   
   A   
   A   
   T   
   C   
   T   
   G   
   T   
   C   
   G   
   G   
   A   
   A   
   A   
   T   
   A   
   T   
   C   
   A   
   T   
   G   
   G   
   G   
    G    
   A   
   A   
   T   
   T   
   A   
   T   
   C   
   A   
   T   
   C   
   C   
   T   
   C   
   A   
   T   
   G   
   G   
   T   
   G   
   A   
   C   
   A   
   G   
   C   
   A   
   G   
   T   
   A   
   T   
   T   
   T   
   A   
   T   
   G   
   A   
   A   
   G   
   C   
   G   
   A   
   T   
   G   
   G   
   T   
   C   
   C   
   G   
   C   
   A   
   T   
   G   
   A   
   G   
   C   
   C   
   A   
   A   
   G   
   A   
    T    
   T   
   G   
   G   
   A   
   A   
   G   
   T   
   T   
   G   
   C   
   G   
   G   
   G   
   A   
   A   
   G   
   T   
   A   
   C   
   T   
   G   
   A   
   T   
   C   
   G   
   A   
    G    
   A   
   T   
   G   
   C   
   A   
   C   
   G   
   G   
    A    
   A   
   A   
   T   
   A   
   A   
    C    
   G   
   G   
   G   
   A   
   G   
   T   
   A   
   T   
   G   
   G   
   A   
   C   
   G   
   G   
   T   
   G   
   A   
   T   
   C   
   C   
   A   
   C   
   C   
   T   
   G   
   C   
   T   
   G   
   C   
   G   
   A   
   T   
   G   
   C   
   G   
   T   
   T   
   A   
   T   
   A   
   C   
   T   
   G   
   A   
   A   
   G   
   C   
   C   
   A   
   G   
    A    
   T   
   T   
   A   
   T   
   C   
   C   
   A   
   A   
   G   
   T   
   T   
   A   
   A   
   G   
   T   
   G   
   G   
   A   
   G   
   A   
   G   
   A   
   T   
   G   
    C    
   T   
   A   
    G    
   C   
   A   
   G   
   A   
   T   
   A   
   T   
   C   
   G   
   A   
    A    
   A   
   A   
   A   
   G   
   A   
   A   
   A   
   C   
   A   
   G   
   T   
    T    
   G   
   A   
   T   
   C   
   T   
   C   
   G   
   T   
   G   
   T   
   G   
   G   
   A   
   A   
   T   
   T   
   T   
   T   
   G   
   A   
   T   
   G   
   A   
   T   
   A   
   C   
   A   
   G   
   A   
   A   
   A   
   A   
   A   
   G   
   A   
   A   
   C   
   C   
   A   
   A   
   C   
   A   
   G   
   T   
   C   
   C   
   T   
   T   
   C   
   C   
   A   
   G   
   C   
   T   
   C   
   G   
   A   
   T   
   A   
   C   
   C   
   C   
   A   
   A   
   A   
   C   
   C   
   T   
   T   
   C   
   T   
    A    
   G   
   T   
   C   
   A   
   A   
   T   
   G   
   G   
   A   
   T   
   C   
   G   
   A   
   C   
   A   
   G   
   G   
   T   
   A   
   T   
   A   
   T   
   C   
   T   
   G   
   C   
   T   
   G   
   G   
   A   
   T   
   A   
   T   
   G   
   C   
    G    
   A   
   C   
   A   
   G   
   A   
   A   
   A   
   T   
   C   
   C   
   C   
   G   
   A   
   C   
    G    
   C   
   A   
   T   
   A   
   A   
   C   
   T   
   T   
   A   
   G   
   C   
   T   
   G   
   A   
   A   
   G   
   T   
   G   
   A   
   T   
    C    
   G   
   A   
    C    
   G   
   G   
   C   
   A   
   C   
   A   
   A   
   T   
   C   
   T   
   A   
   T   
   A   
   T   
   G   
   A   
   T   
   C   
   G   
   A   
   T   
   C   
   A   
   T   
   C   
   C   
   A   
   C   
   A   
   A   
   G   
   C   
   G   
   A   
   G   
   C   
   C   
   T   
   T   
   G   
   A   
   A   
   A   
   A   
   A   
   T   
   T   
   G   
   A   
   T   
   G   
   G   
   A   
   A   
   T   
   A   
   T   
   A   
   T   
   T   
   C   
   C   
   T   
   G   
   G   
   A   
   C   
   C   
   T   
   G   
   A   
   C   
   T   
   T   
    T    
   C   
   C   
   A   
   A   
   C   
   A   
   G   
   G   
   A   
   G   
   G   
    G    
   A   
   T   
   C   
   T   
   T   
   G   
   C   
   A   
   A   
   G   
   G   
    A    
   A   
   A   
   A   
   G   
   A   
   T   
   G   
   A   
   A   
   A   
   T   
   C   
   A   
   A   
   A   
   A   
   A   
   A   
   G   
   C   
   T   
   T   
   A   
   T   
   G   
   A   
   A   
   A   
   C   
   A   
   G   
   G   
    G    
   C   
   G   
   A   
   G   
   G   
   A   
   A   
   A   
   A   
   G   
   T   
   T   
   A   
    A    
   T   
   C   
   T   
   T   
   C   
   G   
   T   
   T   
   C   
   G   
   A   
   A   
   A   
   A   
   C   
   A   
   A   
   A   
   G   
   A   
   T   
   A   
   G   
   A   
    A    
   G   
   C   
   A   
   A   
   T   
   G   
   A   
   A   
   G   
   G   
   G   
    C    
   A   
   A   
    T    
   A   
   A   
   A   
   C   
   A   
   G   
   C   
   A   
   A   
   A   
   T   
   C   
   G   
   T   
   G   
   A   
   T   
   C   
   A   
   C   
    T    
   G   
   A   
   A   
   A   
   T   
   C   
   C   
   C   
   A   
   T   
   A   
   T   
   G   
   A   
   A   
   G   
   T   
   A   
   A   
   A   
   T   
   A   
   A   
   A   
   G   
   C   
   G   
   A   
   C   
    A    
   T   
   T   
   G   
   G   
   T   
   G   
   A   
   A   
   A   
   A   
   A   
   A   
   A   
   T   
   G   
   G   
   A   
   T   
   G   
   A   
   G   
   A   
   T   
   C   
   C   
   G   
   T   
   T   
   T   
   G   
   A   
   A   
   T   
   A   
   A   
   G   
   A   
   A   
   A   
   A   
   T   
   C   
   G   
   A   
    T    
   G   
   G   
   A   
   A   
   T   
   A   
   G   
   C   
   T   
   G   
   A   
   A   
   G   
   T   
    A    
   C   
   G   
   A   
   G   
   A   
   T   
   G   
   A   
   A   
   A   
   G   
   T   
   G   
   A   
   T   
    C    
   G   
    A    
   A   
   C   
    T    
   G   
   G   
   T   
   C   
   T   
   G   
   C   
   A   
   A   
   A   
   T   
   T   
   G   
   T   
   C   
   G   
   T   
    T    
   G   
   A   
   A   
   T   
   T   
   G   
   A   
   A   
   A   
   A   
   A   
   A   
   G   
   A   
    C    
   G   
   C   
   C   
   A   
   A   
    T    
   G   
   C   
   T   
   C   
   A   
   A   
   G   
   G   
    A    
   A   
   T   
   T   
   T   
   T   
   G   
   A   
   A   
   T   
   T   
   A   
    C    
   T   
   T   
   G   
   T   
   T   
   C   
   A   
   A   
   A   
   A   
   A   
   C   
   A   
   C   
   T   
   G   
   A   
   G   
    C    
   T   
   A   
   C   
   A   
   G   
   A   
   T   
   C   
   A   
   A   
   T   
   T   
   A   
   C   
   A   
   A   
   T   
   T   
   T   
    C    
   A   
   A   
   T   
   A   
   T   
   G   
   G   
   T   
   C   
   G   
   C   
   T   
   A   
   T   
   C   
   G   
   A   
    T    
   C   
   A   
   T   
   A   
   T   
   G   
   A   
   C   
   T   
   C   
   C   
   G   
   C   
   A   
    T    
   C   
   A   
   A   
   G   
   T   
   T   
   G   
   G   
   A   
   C   
   T   
   G   
   A   
   A   
   A   
   G   
   A   
   C   
   A   
   T   
   T   
   T   
   T   
   A   
   A   
   G   
   A   
   A   
   G   
   T   
   T   
   A   
   T   
   A   
   T   
   C   
   G   
   A   
   G   
   C   
   A   
   T   
   C   
   G   
   A   
   A   
   A   
   G   
   C   
   A   
   A   
   G   
   T   
   C   
   A   
   T   
   A   
   A   
   C   
   A   
   A   
   A   
   A   
   C   
   G   
    C    
   A   
   G   
    C    
   C   
   A   
   A   
   T   
   T   
   C   
   G   
   A   
   T   
   T   
   T   
   A   
   G   
   C   
   C   
   A   
   A   
   A   
   G   
   C   
   T   
   C   
   A   
   A   
   A   
   A   
   A   
    A    
   G   
   A   
   C   
   A   
   G   
   C   
   A   
   T   
   A   
   T   
   C   
   G   
   T   
    G    
   G   
   A   
   A   
   G   
   G   
    C    
    C    
   T   
   G   
   A   
   T   
   G   
   A   
   A   
   A   
   G   
   C   
   T   
   T   
   T   
   A   
   T   
   C   
   G   
   A   
   T   
   T   
   C   
   T   
   G   
   G   
   A   
   T   
   G   
   A   
   A   
   G   
   T   
   A   
   A   
   T   
   C   
   G   
    C    
   A   
   A   
   C   
   G   
   A   
   T   
   T   
   C   
   G   
   T   
   G   
   A   
   A   
   A   
   G   
   T   
   A   
   A   
   A   
   G   
   A   
   C   
   A   
   A   
   A   
   A   
   A   
   A   
   G   
   A   
    C    
   G   
   C   
    C    
   A   
   A   
   A   
   A   
   A   
   G   
   A   
   A   
   T   
   T   
   T   
   A   
   G   
   T   
   C   
   G   
   A   
    C    
   G   
   T   
    T    
   T   
   T   
    C    
   C   
   A   
   A   
   T   
   T   
   T   
   A   
   C   
   A   
   G   
   A   
   A   
   G   
   A   
   A   
   C   
   A   
   A   
   G   
   C   
   A   
   G   
   A   
   A   
   G   
   C   
   A   
   A   
   T   
    C    
   G   
   T   
   C   
   A   
   C   
   A   
   T   
   T   
   G   
   C   
   A   
   A   
    C    
   T   
   A   
   T   
   A   
    T    
   C   
   G   
   T   
   T   
   T   
   G   
   A   
   C   
   G   
   A   
   A   
    T    
   A   
   C   
    A    
   G   
   A   
   T   
   A   
   T   
   C   
   A   
   C   
   A   
   G   
   A   
   G   
   C   
   T   
   T   
   C   
   A   
   A   
   A   
   A   
   G   
   G   
   A   
   A   
    T    
   C   
    T    
   G   
   A   
   A   
   A   
    G    
   C   
   T   
   T   
   G   
   A   
   T   
   T   
   G   
   C   
    A    
   C   
   A   
   A   
   A   
   T   
   C   
   A   
   C   
   A   
   G   
   A   
   G   
   T   
   T   
    G    
   A   
   A   
   T   
   A   
   A   
   A   
   A   
   T   
   C   
   C   
   T   
   T   
   T   
   C   
   A   
   A   
   A   
    C    
   G   
   A   
   C   
   A   
   A   
   A   
   G   
   A   
    G    
   C   
   T   
   T   
   T   
   T   
    C    
   T   
   C   
   T   
   G   
   T   
   T   
   A   
   T   
   G   
   A   
   A   
   A   
   A   
   A   
   A   
   G   
   A   
   A   
   T   
   T   
   G   
   C   
   G   
   A   
   G   
   A   
   A   
   G   
   T   
   G   
   A   
   A   
   A   
   A   
   A   
   A   
   A   
   A   
   T   
   T   
   A   
    C    
   T   
   C   
   T   
   T   
   C   
   C   
   G   
   C   
   T   
   C   
   G   
   T   
   C   
    T    
    G    
   A   
   C   
   G   
   A   
   C   
   A   
   A   
   T   
   C   
    G    
   A   
   A   
   G   
   A   
   C   
   G   
   A   
   A   
   A   
   T   
   C   
   G   
   A   
   A   
   G   
   A   
   A   
   A   
   T   
   C   
   A   
   A   
   A   
   A   
   T   
    C    
   G   
   A   
   T   
   A   
   C   
   G   
   C   
   A   
   A   
    G    
   T   
    T    
   T   
   T   
   A   
   G   
   T   
   G   
   G   
   C   
    G    
   C   
   A   
   A   
   G   
   A   
   A   
   G   
   A   
   C   
   G   
   T   
   A   
   A   
   T   
    C    
   G   
   T   
   C   
   A   
   G   
   T   
   G   
   T   
    G    
   A   
   C   
    A    
   C   
   G   
   A   
   G   
   A   
   A   
   G   
   G   
   C   
   T   
   A   
   T   
   G   
   T   
   G   
   A   
   A   
   G   
   C   
   G   
   A   
   A   
   C   
   A   
   A   
   G   
   C   
   C   
   T   
   T   
   C   
   G   
   T   
   T   
   C   
    T    
   T   
   A   
   T   
   A   
   G   
   T   
   G   
   C   
   T   
   T   
   C   
    G    
   A   
   A   
   A   
   C   
   C   
   A   
   G   
   A   
   A   
   G   
   A   
   A   
   A   
   T   
   C   
   G   
   G   
   G   
   A   
   T   
   G   
   C   
   G   
   A   
   G   
   A   
   A   
   G   
   G   
   C   
   G   
   A   
    C    
   T   
   A   
   C   
   T   
   T   
   G   
   C   
   T   
    G    
   T   
   A   
   C   
    T    
   C   
   A   
   G   
   G   
   T   
   G   
   A   
   A   
   T   
   T   
    G    
   A   
   G   
    C    
   A   
   C   
   A   
    T    
   T   
   G   
   G   
   A   
   C   
   C   
   A   
   T   
   G   
   T   
   T   
   C   
   T   
   T   
   C   
   T   
   G   
   A   
   T   
    T    
   A   
   C   
    G    
   A   
   A   
   T   
   A   
   A   
   A   
   G   
   C   
   A   
   A   
   A   
   T   
   G   
   T   
   G   
   A   
   T   
   C   
   T   
   A   
   C   
   C   
   G   
    T    
   C   
   C   
   T   
   G   
   T   
    T    
   C   
   A   
   T   
   G   
   A   
   A   
   C   
   T   
   A   
   C   
   C   
    C    
   G   
   A   
   T   
   T   
   T   
   G   
   A   
   A   
   A   
   T   
   G   
   G   
   A   
   A   
   A   
   G   
   A   
   T   
   G   
   C   
   T   
   G   
   G   
    C    
   G   
   A   
    A    
   C   
   A   
    C    
   A   
   T   
   T   
   T   
   C   
   T   
   C   
   A   
   A   
   A   
   C   
   A   
   A   
   T   
   C   
   T   
   C   
   C   
   A   
   A   
   T   
   C   
   T   
   G   
   T   
   C   
   T   
   G   
   T   
   A   
   G   
   A   
    T    
   G   
   A   
   A   
   T   
   C   
   G   
   A   
   T   
    C    
   T   
   T   
   A   
   G   
   C   
   T   
   G   
   T   
   T   
   T   
   T   
    C    
   C   
   C   
   A   
   T   
   A   
   T   
   C   
   A   
   A   
   A   
   A   
   A   
   A   
   T   
    C    
   C   
   A   
   A   
   G   
   C   
   A   
   G   
   A   
    G    
   A   
   A   
   A   
   A   
   C   
   A   
   T   
   T   
   T   
   G   
   T   
    A    
   T   
   T   
   C   
   A   
   T   
   C   
   A   
   G   
   C   
   A   
   A   
   G   
   A   
   A   
   T   
   G   
   G   
   A   
   T   
   T   
    G    
    A    
   T   
   C   
   A   
   A   
   G   
   C   
   A   
   A   
   A   
   C   
   A   
   C   
   G   
   A   
   A   
   T   
   G   
   A   
   C   
   A   
   G   
   A   
   G   
   T   
   T   
   C   
   G   
   A   
   A   
   C   
   C   
   A   
   T   
   G   
   G   
   C   
   G   
   C   
   A   
   C   
   C   
   T   
   A   
    C    
   A   
   A   
   G   
   A   
   G   
   C   
   C   
   G   
    T    
   C   
   C   
   G   
   C   
   T   
   C   
   A   
   G   
   C   
   T   
   G   
   T   
   A   
   T   
   G   
   A   
   A   
   G   
   C   
   T   
   A   
   A   
   A   
   G   
   G   
   C   
   G   
   G   
   A   
   A   
   G   
   A   
   A   
   G   
   A   
   T   
   G   
   A   
   A   
   C   
   T   
    T    
   A   
   T   
   T   
   G   
   C   
   T   
   G   
   T   
    A    
   T   
   A   
   T   
   C   
   T   
   G   
   A   
   A   
   A   
   G   
   A   
   A   
   G   
   A   
   T   
   C   
    A    
   G   
   C   
   C   
   A   
   G   
   G   
   C   
   T   
   T   
   A   
   G   
   A   
   T   
   G   
   T   
    G    
   T   
   T   
   C   
   T   
   T   
   A   
   G   
   T   
   A   
   A   
   C   
   G   
    C    
   A   
   T   
   C   
   A   
   A   
   G   
   G   
   A   
   A   
   T   
   G   
   G   
   G   
    C    
   T   
   T   
   G   
   C   
   G   
   C   
   T   
    A    
   T   
   C   
   C   
   T   
   T   
   T   
   A   
   A   
    C    
   A   
   G   
   A   
   T   
   G   
   T   
   T   
   C   
   C   
   C   
   G   
   T   
   T   
   G   
   T   
   T   
   G   
   G   
   A   
   G   
   C   
   A   
   A   
   A   
   G   
   G   
   C   
   A   
   G   
    C    
   T   
   G   
   G   
   A   
   G   
   T   
   G   
   A   
   A   
   G   
   T   
   C   
   T   
   A   
   T   
   G   
   A   
   A   
   T   
   T   
   T   
   G   
   A   
   A   
   A   
   G   
   A   
   T   
   G   
   G   
   A   
   G   
   A   
   T   
   A   
   G   
   G   
    A    
   T   
   T   
    G    
   T   
   C   
   A   
   A   
    T    
   G   
   G   
    G    
   C   
   T   
    G    
   C   
   T   
   T   
   G   
   T   
   G   
   T   
   T   
   A   
   G   
   C   
   A   
   G   
   A   
    C    
   G   
   G   
    A    
   G   
   A   
   T   
   A   
   C   
   G   
   C   
   C   
    G    
   A   
   T   
   C   
   G   
   T   
   A   
   A   
   T   
   C   
   G   
   T   
   T   
   A   
   C   
   T   
   C   
   A   
   G   
   C   
   G   
   A   
   G   
   G   
   A   
   G   
   C   
    T    
   G   
   T   
    G    
   A   
   A   
   A   
   C   
   G   
   C   
   A   
   T   
   G   
   C   
   T   
   T   
   G   
   C   
   T   
   C   
   A   
   A   
   G   
   A   
   A   
   A   
   T   
   C   
   A   
   G   
   C   
   C   
   A   
   A   
   A   
   C   
    T    
   T   
   C   
   A   
   C   
   G   
   T   
   G   
   C   
   G   
   A   
   A   
   G   
   C   
   G   
   G   
   G   
   G   
   A   
   G   
   T   
   G   
   A   
   C   
   G   
   G   
   T   
   C   
   A   
   T   
   G   
   C   
   G   
   T   
   G   
   A   
   G   
   C   
   T   
   G   
   A   
   A   
   A   
   A   
   A   
   A   
   A   
   A   
   T   
   C   
   C   
    G    
   C   
   A   
    C    
   C   
   G   
    C    
   A   
   T   
   T   
   A   
   T   
   C   
   T   
   A   
   T   
   A   
   T   
   G   
   T   
   C   
   G   
   G   
   A   
   A   
   G   
   G   
   A   
   A   
   G   
   A   
   T   
   C   
   A   
   A   
   A   
   A   
   G   
   A   
   A   
   A   
   T   
   A   
   A   
   C   
   C   
   T   
   T   
   G   
   A   
   T   
   G   
   A   
   A   
   T   
   C   
   A   
   A   
   A   
   A   
   A   
   G   
   G   
    A    
   C   
   A   
    A    
   C   
   A   
   G   
    A    
   T   
   A   
   A   
   C   
   G   
   A   
   T   
   T   
   G   
   A   
    T    
   C   
   C   
   A   
   A   
   C   
    G    
   G   
   A   
   T   
   T   
   T   
    T    
   C   
   C   
   T   
   A   
   T   
   A   
   G   
   G   
   T   
   G   
   A   
   T   
   C   
   G   
   A   
   A   
   C   
   A   
   T   
   C   
   G   
   A   
   A   
   T   
   G   
   G   
    T    
   T   
   C   
    G    
   T   
   T   
   T   
   G   
   C   
    T    
   A   
   T   
   G   
   G   
   A   
    C    
   G   
   A   
   G   
   A   
   A   
   A   
   A   
   A   
   A   
   G   
   G   
   T   
   G   
   G   
   A   
   G   
   A   
   A   
   G   
   T   
   C   
   A   
   T   
   C   
   G   
   A   
   A   
   G   
   T   
    T    
   A   
    T    
   C   
   G   
   A   
   T   
   G   
   C   
   T   
   C   
   C   
   T   
   G   
   A   
   G   
    A    
   T   
   T   
   T   
   T   
    C    
   A   
   T   
    C    
   G   
   A   
    A    
    T    
   C   
    A    
   -   
   -   
   -   
 

 
   N52769   
   A   
   T   
   G   
   G   
   A   
   A   
   A   
   A   
   T   
   C   
   G   
   A   
   C   
   A   
   A   
   G   
   A   
   A   
   G   
   T   
   A   
   C   
   A   
   A   
   G   
   A   
   A   
   T   
   T   
   A   
   A   
   C   
   T   
   T   
   T   
   A   
   G   
   A   
   A   
   G   
   A   
   A   
   G   
   T   
   G   
   A   
   T   
   G   
   G   
   G   
   T   
   G   
   A   
   C   
   C   
   G   
   C   
   T   
   T   
   C   
   G   
   G   
   A   
   C   
   G   
   T   
   T   
   A   
   T   
   T   
   C   
   C   
   A   
   A   
   A   
   T   
   A   
   T   
   A   
   T   
   T   
   A   
   T   
    C    
   C   
   A   
    A    
   G   
   A   
   A   
   C   
   G   
   T   
   G   
   C   
   A   
   C   
   T   
   G   
   C   
   C   
   T   
   G   
   A   
   T   
   A   
   T   
    C    
   C   
   G   
   T   
   G   
   A   
   T   
   G   
   G   
   A   
   T   
   T   
   G   
   A   
   A   
    A    
   C   
   C   
   A   
   G   
   T   
    G    
   C   
   A   
   A   
   C   
   G   
   C   
   C   
   G   
   G   
   A   
   T   
   A   
   T   
   T   
   A   
   T   
   T   
    T    
   G   
   C   
    C    
   A   
   T   
   G   
   A   
   A   
   C   
   A   
   A   
   A   
   G   
   A   
   T   
   G   
   G   
   C   
   A   
   A   
   T   
   A   
   C   
   G   
   T   
   A   
   T   
   G   
   A   
   C   
   A   
   A   
   A   
   G   
   G   
   A   
   T   
   T   
    C    
   C   
   G   
   T   
   A   
   A   
   A   
   T   
   C   
   A   
   G   
   C   
   A   
   A   
   A   
   A   
   T   
   C   
   T   
   G   
   T   
   C   
   G   
   G   
   A   
   A   
   A   
   T   
   A   
   T   
   C   
   A   
   T   
   G   
   G   
   G   
    A    
   A   
   A   
   T   
   T   
   A   
   T   
   C   
   A   
   T   
   C   
   C   
   T   
   C   
   A   
   T   
   G   
   G   
   T   
   G   
   A   
   C   
   A   
   G   
   C   
   A   
   G   
   T   
   A   
   T   
   T   
   T   
   A   
   T   
   G   
   A   
   A   
   G   
   C   
   G   
   A   
   T   
   G   
   G   
   T   
   C   
   C   
   G   
   C   
   A   
   T   
   G   
   A   
   G   
   C   
   C   
   A   
   A   
   G   
   A   
    T    
   T   
   G   
   G   
   A   
   A   
   G   
   T   
   T   
   G   
   C   
   G   
   G   
   G   
   A   
   A   
   G   
   T   
   A   
   C   
   T   
   G   
   A   
   T   
   C   
   G   
   A   
    G    
   A   
   T   
   G   
   C   
   A   
   C   
   G   
   G   
    A    
   A   
   A   
   T   
   A   
   A   
    C    
   G   
   G   
   G   
   A   
   G   
   T   
   A   
   T   
   G   
   G   
   A   
   C   
   G   
   G   
   T   
   G   
   A   
   T   
   C   
   C   
   A   
   C   
   C   
   T   
   G   
   C   
   T   
   G   
   C   
   G   
   A   
   T   
   G   
   C   
   G   
   T   
   T   
   A   
   T   
   A   
   C   
   T   
   G   
   A   
   A   
   G   
   C   
   C   
   A   
   G   
    A    
   T   
   T   
   A   
   T   
   C   
   C   
   A   
   A   
   G   
   T   
   T   
   A   
   A   
   G   
   T   
   G   
   G   
   A   
   G   
   A   
   G   
   A   
   T   
   G   
    C    
   T   
   A   
    G    
   C   
   A   
   G   
   A   
   T   
   A   
   T   
   C   
   G   
   A   
    A    
   A   
   A   
   A   
   G   
   A   
   A   
   A   
   C   
   A   
   G   
   T   
    T    
   G   
   A   
   T   
   C   
   T   
   C   
   G   
   T   
   G   
   T   
   G   
   G   
   A   
   A   
   T   
   T   
   T   
   T   
   G   
   A   
   T   
   G   
   A   
   T   
   A   
   C   
   A   
   G   
   A   
   A   
   A   
   A   
   A   
   G   
   A   
   A   
   C   
   C   
   A   
   A   
   C   
   A   
   G   
   T   
   C   
   C   
   T   
   T   
   C   
   C   
   A   
   G   
   C   
   T   
   C   
   G   
   A   
   T   
   A   
   C   
   C   
   C   
   A   
   A   
   A   
   C   
   C   
   T   
   T   
   C   
   T   
    A    
   G   
   T   
   C   
   A   
   A   
   T   
   G   
   G   
   A   
   T   
   C   
   G   
   A   
   C   
   A   
   G   
   G   
   T   
   A   
   T   
   A   
   T   
   C   
   T   
   G   
   C   
   T   
   G   
   G   
   A   
   T   
   A   
   T   
   G   
   C   
    G    
   A   
   C   
   A   
   G   
   A   
   A   
   A   
   T   
   C   
   C   
   C   
   G   
   A   
   C   
    G    
   C   
   A   
   T   
   A   
   A   
   C   
   T   
   T   
   A   
   G   
   C   
   T   
   G   
   A   
   A   
   G   
   T   
   G   
   A   
   T   
    C    
   G   
   A   
    C    
   G   
   G   
   C   
   A   
   C   
   A   
   A   
   T   
   C   
   T   
   A   
   T   
   A   
   T   
   G   
   A   
   T   
   C   
   G   
   A   
   T   
   C   
   A   
   T   
   C   
   C   
   A   
   C   
   A   
   A   
   G   
   C   
   G   
   A   
   G   
   C   
   C   
   T   
   T   
   G   
   A   
   A   
   A   
   A   
   A   
   T   
   T   
   G   
   A   
   T   
   G   
   G   
   A   
   A   
   T   
   A   
   T   
   A   
   T   
   T   
   C   
   C   
   T   
   G   
   G   
   A   
   C   
   C   
   T   
   G   
   A   
   C   
   T   
   T   
    T    
   C   
   C   
   A   
   A   
   C   
   A   
   G   
   G   
   A   
   G   
   G   
    G    
   A   
   T   
   C   
   T   
   T   
   G   
   C   
   A   
   A   
   G   
   G   
    A    
   A   
   A   
   A   
   G   
   A   
   T   
   G   
   A   
   A   
   A   
   T   
   C   
   A   
   A   
   A   
   A   
   A   
   A   
   G   
   C   
   T   
   T   
   A   
   T   
   G   
   A   
   A   
   A   
   C   
   A   
   G   
   G   
    G    
   C   
   G   
   A   
   G   
   G   
   A   
   A   
   A   
   A   
   G   
   T   
   T   
   A   
    T    
   T   
   C   
   T   
   T   
   C   
   G   
   T   
   T   
   C   
   G   
   A   
   A   
   A   
   A   
   C   
   A   
   A   
   A   
   G   
   A   
   T   
   A   
   G   
   A   
    A    
   G   
   C   
   A   
   A   
   T   
   G   
   A   
   A   
   G   
   G   
   G   
    C    
   A   
   A   
    C    
   A   
   A   
   A   
   C   
   A   
   G   
   C   
   A   
   A   
   A   
   T   
   C   
   G   
   T   
   G   
   A   
   T   
   C   
   A   
   C   
    T    
   G   
   A   
   A   
   A   
   T   
   C   
   C   
   C   
   A   
   T   
   A   
   T   
   G   
   A   
   A   
   G   
   T   
   A   
   A   
   A   
   T   
   A   
   A   
   A   
   G   
   C   
   G   
   A   
   C   
    A    
   T   
   T   
   G   
   G   
   T   
   G   
   A   
   A   
   A   
   A   
   A   
   A   
   A   
   T   
   G   
   G   
   A   
   T   
   G   
   A   
   G   
   A   
   T   
   C   
   C   
   G   
   T   
   T   
   T   
   G   
   A   
   A   
   T   
   A   
   A   
   G   
   A   
   A   
   A   
   A   
   T   
   C   
   G   
   A   
    T    
   G   
   G   
   A   
   A   
   T   
   A   
   G   
   C   
   T   
   G   
   A   
   A   
   G   
   T   
    A    
   C   
   G   
   A   
   G   
   A   
   T   
   G   
   A   
   A   
   A   
   G   
   T   
   G   
   A   
   T   
    C    
   G   
    G    
   A   
   C   
    T    
   G   
   G   
   T   
   C   
   T   
   G   
   C   
   A   
   A   
   A   
   T   
   T   
   G   
   T   
   C   
   G   
   T   
    T    
   G   
   A   
   A   
   T   
   T   
   G   
   A   
   A   
   A   
   A   
   A   
   A   
   G   
   A   
    C    
   G   
   C   
   C   
   A   
   A   
    C    
   G   
   C   
   T   
   C   
   A   
   A   
   G   
   G   
    T    
   A   
   T   
   T   
   T   
   T   
   G   
   A   
   A   
   T   
   T   
   A   
    T    
   T   
   T   
   G   
   T   
   T   
   C   
   A   
   A   
   A   
   A   
   A   
   C   
   A   
   C   
   T   
   G   
   A   
   G   
    C    
   T   
   A   
   C   
   A   
   G   
   A   
   T   
   C   
   A   
   A   
   T   
   T   
   A   
   C   
   A   
   A   
   T   
   T   
   T   
    C    
   A   
   A   
   T   
   A   
   T   
   G   
   G   
   T   
   C   
   G   
   C   
   T   
   A   
   T   
   C   
   G   
   A   
    T    
   C   
   A   
   T   
   A   
   T   
   G   
   A   
   C   
   T   
   C   
   C   
   G   
   C   
   A   
    T    
   C   
   A   
   A   
   G   
   T   
   T   
   G   
   G   
   A   
   C   
   T   
   G   
   A   
   A   
   A   
   G   
   A   
   C   
   A   
   T   
   T   
   T   
   T   
   A   
   A   
   G   
   A   
   A   
   G   
   T   
   T   
   A   
   T   
   A   
   T   
   C   
   G   
   A   
   G   
   C   
   A   
   T   
   C   
   G   
   A   
   A   
   A   
   G   
   C   
   A   
   A   
   G   
   T   
   C   
   A   
   T   
   A   
   A   
   C   
   A   
   A   
   A   
   A   
   C   
   G   
    C    
   A   
   G   
    C    
   C   
   A   
   A   
   T   
   T   
   C   
   G   
   A   
   T   
   T   
   T   
   A   
   G   
   C   
   C   
   A   
   A   
   A   
   G   
   C   
   T   
   C   
   A   
   A   
   A   
   A   
   A   
    A    
   G   
   A   
   C   
   A   
   G   
   C   
   A   
   T   
   A   
   T   
   C   
   G   
   T   
    G    
   G   
   A   
   A   
   G   
   G   
    C    
    C    
   T   
   G   
   A   
   T   
   G   
   A   
   A   
   A   
   G   
   C   
   T   
   T   
   T   
   A   
   T   
   C   
   G   
   A   
   T   
   T   
   C   
   T   
   G   
   G   
   A   
   T   
   G   
   A   
   A   
   G   
   T   
   A   
   A   
   T   
   C   
   G   
    C    
   A   
   A   
   C   
   G   
   A   
   T   
   T   
   C   
   G   
   T   
   G   
   A   
   A   
   A   
   G   
   T   
   A   
   A   
   A   
   G   
   A   
   C   
   A   
   A   
   A   
   A   
   A   
   A   
   G   
   A   
    C    
   G   
   C   
    C    
   A   
   A   
   A   
   A   
   A   
   G   
   A   
   A   
   T   
   T   
   T   
   A   
   G   
   T   
   C   
   G   
   A   
    C    
   G   
   T   
    T    
   T   
   T   
    C    
   C   
   A   
   A   
   T   
   T   
   T   
   A   
   C   
   A   
   G   
   A   
   A   
   G   
   A   
   A   
   C   
   A   
   A   
   G   
   C   
   A   
   G   
   A   
   A   
   G   
   C   
   A   
   A   
   T   
    C    
   G   
   T   
   C   
   A   
   C   
   A   
   T   
   T   
   G   
   C   
   A   
   A   
    C    
   T   
   A   
   T   
   A   
    T    
   C   
   G   
   T   
   T   
   T   
   G   
   A   
   C   
   G   
   A   
   A   
    C    
   A   
   C   
    A    
   G   
   A   
   T   
   A   
   T   
   C   
   A   
   C   
   A   
   G   
   A   
   G   
   C   
   T   
   T   
   C   
   A   
   A   
   A   
   A   
   G   
   G   
   A   
   A   
    T    
   C   
    T    
   G   
   A   
   A   
   A   
    G    
   C   
   T   
   T   
   G   
   A   
   T   
   T   
   G   
   C   
    A    
   C   
   A   
   A   
   A   
   T   
   C   
   A   
   C   
   A   
   G   
   A   
   G   
   T   
   T   
    G    
   A   
   A   
   T   
   A   
   A   
   A   
   A   
   T   
   C   
   C   
   T   
   T   
   T   
   C   
   A   
   A   
   A   
    C    
   G   
   A   
   C   
   A   
   A   
   A   
   G   
   A   
    G    
   C   
   T   
   T   
   T   
   T   
    C    
   T   
   C   
   T   
   G   
   T   
   T   
   A   
   T   
   G   
   A   
   A   
   A   
   A   
   A   
   A   
   G   
   A   
   A   
   T   
   T   
   G   
   C   
   G   
   A   
   G   
   A   
   A   
   G   
   T   
   G   
   A   
   A   
   A   
   A   
   A   
   A   
   A   
   A   
   T   
   T   
   A   
    C    
   T   
   C   
   T   
   T   
   C   
   C   
   G   
   C   
   T   
   C   
   G   
   T   
   C   
    T    
    G    
   A   
   C   
   G   
   A   
   C   
   A   
   A   
   T   
   C   
    G    
   A   
   A   
   G   
   A   
   C   
   G   
   A   
   A   
   A   
   T   
   C   
   G   
   A   
   A   
   G   
   A   
   A   
   A   
   T   
   C   
   A   
   A   
   A   
   A   
   T   
    C    
   G   
   A   
   T   
   A   
   C   
   G   
   C   
   A   
   A   
    G    
   T   
    T    
   T   
   T   
   A   
   G   
   T   
   G   
   G   
   C   
    G    
   C   
   A   
   A   
   G   
   A   
   A   
   G   
   A   
   C   
   G   
   T   
   A   
   A   
   T   
    C    
   G   
   T   
   C   
   A   
   G   
   T   
   G   
   T   
    G    
   A   
   C   
    A    
   C   
   G   
   A   
   G   
   A   
   A   
   G   
   G   
   C   
   T   
   A   
   T   
   G   
   T   
   G   
   A   
   A   
   G   
   C   
   G   
   A   
   A   
   C   
   A   
   A   
   G   
   C   
   C   
   T   
   T   
   C   
   G   
   T   
   T   
   C   
    T    
   T   
   A   
   T   
   A   
   G   
   T   
   G   
   C   
   T   
   T   
   C   
    C    
   A   
   A   
   A   
   C   
   C   
   A   
   G   
   A   
   A   
   G   
   A   
   A   
   A   
   T   
   C   
   G   
   G   
   G   
   A   
   T   
   G   
   C   
   G   
   A   
   G   
   A   
   A   
   G   
   G   
   C   
   G   
   A   
    C    
   T   
   A   
   C   
   T   
   T   
   G   
   C   
   T   
    G    
   T   
   A   
   C   
    T    
   C   
   A   
   G   
   G   
   T   
   G   
   A   
   A   
   T   
   T   
    G    
   A   
   G   
    C    
   A   
   C   
   A   
    T    
   T   
   G   
   G   
   A   
   C   
   C   
   A   
   T   
   G   
   T   
   T   
   C   
   T   
   T   
   C   
   T   
   G   
   A   
   T   
    T    
   A   
   C   
    G    
   A   
   A   
   T   
   A   
   A   
   A   
   G   
   C   
   A   
   A   
   A   
   T   
   G   
   T   
   G   
   A   
   T   
   C   
   T   
   A   
   C   
   C   
   G   
    T    
   C   
   C   
   T   
   G   
   T   
    T    
   C   
   A   
   T   
   G   
   A   
   A   
   C   
   T   
   A   
   C   
   C   
    C    
   G   
   A   
   T   
   T   
   T   
   G   
   A   
   A   
   A   
   T   
   G   
   G   
   A   
   A   
   A   
   G   
   A   
   T   
   G   
   C   
   T   
   G   
   G   
    C    
   G   
   A   
    A    
   C   
   A   
    C    
   A   
   T   
   T   
   T   
   C   
   T   
   C   
   A   
   A   
   A   
   C   
   A   
   A   
   T   
   C   
   T   
   C   
   C   
   A   
   A   
   T   
   C   
   T   
   G   
   T   
   C   
   T   
   G   
   T   
   A   
   G   
   A   
    T    
   G   
   A   
   A   
   T   
   C   
   G   
   A   
   T   
    C    
   T   
   T   
   A   
   G   
   C   
   T   
   G   
   T   
   T   
   T   
   T   
    C    
   C   
   C   
   A   
   T   
   A   
   T   
   C   
   A   
   A   
   A   
   A   
   A   
   A   
   T   
    C    
   C   
   A   
   A   
   G   
   C   
   A   
   G   
   A   
    G    
   A   
   A   
   A   
   A   
   C   
   A   
   T   
   T   
   T   
   G   
   T   
    T    
   T   
   T   
   C   
   A   
   T   
   C   
   A   
   G   
   C   
   A   
   A   
   G   
   A   
   A   
   T   
   G   
   G   
   A   
   T   
   T   
    G    
    A    
   T   
   C   
   A   
   A   
   G   
   C   
   A   
   A   
   A   
   C   
   A   
   C   
   G   
   A   
   A   
   T   
   G   
   A   
   C   
   A   
   G   
   A   
   G   
   T   
   T   
   C   
   G   
   A   
   A   
   C   
   C   
   A   
   T   
   G   
   G   
   C   
   G   
   C   
   A   
   C   
   C   
   T   
   A   
    C    
   A   
   A   
   G   
   A   
   G   
   C   
   C   
   G   
    T    
   C   
   C   
   G   
   C   
   T   
   C   
   A   
   G   
   C   
   T   
   G   
   T   
   A   
   T   
   G   
   A   
   A   
   G   
   C   
   T   
   A   
   A   
   A   
   G   
   G   
   C   
   G   
   G   
   A   
   A   
   G   
   A   
   A   
   G   
   A   
   T   
   G   
   A   
   A   
   C   
   T   
    T    
   A   
   T   
   T   
   G   
   C   
   T   
   G   
   T   
    A    
   T   
   A   
   T   
   C   
   T   
   G   
   A   
   A   
   A   
   G   
   A   
   A   
   G   
   A   
   T   
   C   
    A    
   G   
   C   
   C   
   A   
   G   
   G   
   C   
   T   
   T   
   A   
   G   
   A   
   T   
   G   
   T   
    G    
   T   
   T   
   C   
   T   
   T   
   A   
   G   
   T   
   A   
   A   
   C   
   G   
    C    
   A   
   T   
   C   
   A   
   A   
   G   
   G   
   A   
   A   
   T   
   G   
   G   
   G   
    C    
   T   
   T   
   G   
   C   
   G   
   C   
   T   
    A    
   T   
   C   
   C   
   T   
   T   
   T   
   A   
   A   
    C    
   A   
   G   
   A   
   T   
   G   
   T   
   T   
   C   
   C   
   C   
   G   
   T   
   T   
   G   
   T   
   T   
   G   
   G   
   A   
   G   
   C   
   A   
   A   
   A   
   G   
   G   
   C   
   A   
   G   
    C    
   T   
   G   
   G   
   A   
   G   
   T   
   G   
   A   
   A   
   G   
   T   
   C   
   T   
   A   
   T   
   G   
   A   
   A   
   T   
   T   
   T   
   G   
   A   
   A   
   A   
   G   
   A   
   T   
   G   
   G   
   A   
   G   
   A   
   T   
   A   
   G   
   G   
    A    
   T   
   T   
    G    
   T   
   C   
   A   
   A   
    T    
   G   
   G   
    G    
   C   
   T   
    G    
   C   
   T   
   T   
   G   
   T   
   G   
   T   
   T   
   A   
   G   
   C   
   A   
   G   
   A   
    C    
   G   
   G   
    A    
   G   
   A   
   T   
   A   
   C   
   G   
   C   
   C   
    G    
   A   
   T   
   C   
   G   
   T   
   A   
   A   
   T   
   C   
   G   
   T   
   T   
   A   
   C   
   T   
   C   
   A   
   G   
   C   
   G   
   A   
   G   
   G   
   A   
   G   
   C   
    T    
   G   
   T   
    G    
   A   
   A   
   A   
   C   
   G   
   C   
   A   
   T   
   G   
   C   
   T   
   T   
   G   
   C   
   T   
   C   
   A   
   A   
   G   
   A   
   A   
   A   
   T   
   C   
   A   
   G   
   C   
   C   
   A   
   A   
   A   
   C   
    T    
   T   
   C   
   A   
   C   
   G   
   T   
   G   
   C   
   G   
   A   
   A   
   G   
   C   
   G   
   G   
   G   
   G   
   A   
   G   
   T   
   G   
   A   
   C   
   G   
   G   
   T   
   C   
   A   
   T   
   G   
   C   
   G   
   T   
   G   
   A   
   G   
   C   
   T   
   G   
   A   
   A   
   A   
   A   
   A   
   A   
   A   
   A   
   T   
   C   
   C   
    G    
   C   
   A   
    C    
   C   
   G   
    C    
   A   
   T   
   T   
   A   
   T   
   C   
   T   
   A   
   T   
   A   
   T   
   G   
   T   
   C   
   G   
   G   
   A   
   A   
   G   
   G   
   A   
   A   
   G   
   A   
   T   
   C   
   A   
   A   
   A   
   A   
   G   
   A   
   A   
   A   
   T   
   A   
   A   
   C   
   C   
   T   
   T   
   G   
   A   
   T   
   G   
   A   
   A   
   T   
   C   
   A   
   A   
   A   
   A   
   A   
   G   
   G   
    A    
   C   
   A   
    A    
   C   
   A   
   G   
    A    
   T   
   A   
   A   
   C   
   G   
   A   
   T   
   T   
   G   
   A   
    T    
   C   
   C   
   A   
   A   
   C   
    G    
   G   
   A   
   T   
   T   
   T   
    T    
   C   
   C   
   T   
   A   
   T   
   A   
   G   
   G   
   T   
   G   
   A   
   T   
   C   
   G   
   A   
   A   
   C   
   A   
   T   
   C   
   G   
   A   
   A   
   T   
   G   
   G   
    T    
   T   
   C   
    G    
   T   
   T   
   T   
   G   
   C   
    T    
   A   
   T   
   G   
   G   
   A   
    C    
   G   
   A   
   G   
   A   
   A   
   A   
   A   
   A   
   A   
   G   
   G   
   T   
   G   
   G   
   A   
   G   
   A   
   A   
   G   
   T   
   C   
   A   
   T   
   C   
   G   
   A   
   A   
   G   
   T   
    T    
   A   
    T    
   C   
   G   
   A   
   T   
   G   
   C   
   T   
   C   
   C   
   T   
   G   
   A   
   G   
    A    
   T   
   T   
   T   
   T   
    C    
   A   
   T   
    C    
   G   
   A   
    A    
    T    
   C   
    A    
   -   
   -   
   -   
 

 
   N54546   
   A   
   T   
   G   
   G   
   A   
   A   
   A   
   A   
   T   
   C   
   G   
   A   
   C   
   A   
   A   
   G   
   A   
   A   
   G   
   T   
   A   
   C   
   A   
   A   
   G   
   A   
   A   
   T   
   T   
   A   
   A   
   C   
   T   
   T   
   T   
   A   
   G   
   A   
   A   
   G   
   A   
   A   
   G   
   T   
   G   
   A   
   T   
   G   
   G   
   G   
   T   
   G   
   A   
   C   
   C   
   G   
   C   
   T   
   T   
   C   
   G   
   G   
   A   
   C   
   G   
   T   
   T   
   A   
   T   
   T   
   C   
   C   
   A   
   A   
   A   
   T   
   A   
   T   
   A   
   T   
   T   
   A   
   T   
    C    
   C   
   A   
    A    
   G   
   A   
   A   
   C   
   G   
   T   
   G   
   C   
   A   
   C   
   T   
   G   
   C   
   C   
   T   
   G   
   A   
   T   
   A   
   T   
    C    
   C   
   G   
   T   
   G   
   A   
   T   
   G   
   G   
   A   
   T   
   T   
   G   
   A   
   A   
    G    
   C   
   C   
   A   
   G   
   T   
    G    
   C   
   A   
   A   
   C   
   G   
   C   
   C   
   G   
   G   
   A   
   T   
   A   
   T   
   T   
   A   
   T   
   T   
    T    
   G   
   C   
    C    
   A   
   T   
   G   
   A   
   A   
   C   
   A   
   A   
   A   
   G   
   A   
   T   
   G   
   G   
   C   
   A   
   A   
   T   
   A   
   C   
   G   
   T   
   A   
   T   
   G   
   A   
   C   
   A   
   A   
   A   
   G   
   G   
   A   
   T   
   T   
    C    
   C   
   G   
   T   
   A   
   A   
   A   
   T   
   C   
   A   
   G   
   C   
   A   
   A   
   A   
   A   
   T   
   C   
   T   
   G   
   T   
   C   
   G   
   G   
   A   
   A   
   A   
   T   
   A   
   T   
   C   
   A   
   T   
   G   
   G   
   G   
    G    
   A   
   A   
   T   
   T   
   A   
   T   
   C   
   A   
   T   
   C   
   C   
   T   
   C   
   A   
   T   
   G   
   G   
   T   
   G   
   A   
   C   
   A   
   G   
   C   
   A   
   G   
   T   
   A   
   T   
   T   
   T   
   A   
   T   
   G   
   A   
   A   
   G   
   C   
   G   
   A   
   T   
   G   
   G   
   T   
   C   
   C   
   G   
   C   
   A   
   T   
   G   
   A   
   G   
   C   
   C   
   A   
   A   
   G   
   A   
    C    
   T   
   G   
   G   
   A   
   A   
   G   
   T   
   T   
   G   
   C   
   G   
   G   
   G   
   A   
   A   
   G   
   T   
   A   
   C   
   T   
   G   
   A   
   T   
   C   
   G   
   A   
    A    
   A   
   T   
   G   
   C   
   A   
   C   
   G   
   G   
    G    
   A   
   A   
   T   
   A   
   A   
    C    
   G   
   G   
   G   
   A   
   G   
   T   
   A   
   T   
   G   
   G   
   A   
   C   
   G   
   G   
   T   
   G   
   A   
   T   
   C   
   C   
   A   
   C   
   C   
   T   
   G   
   C   
   T   
   G   
   C   
   G   
   A   
   T   
   G   
   C   
   G   
   T   
   T   
   A   
   T   
   A   
   C   
   T   
   G   
   A   
   A   
   G   
   C   
   C   
   A   
   G   
    A    
   T   
   T   
   A   
   T   
   C   
   C   
   A   
   A   
   G   
   T   
   T   
   A   
   A   
   G   
   T   
   G   
   G   
   A   
   G   
   A   
   G   
   A   
   T   
   G   
    C    
   T   
   A   
    G    
   C   
   A   
   G   
   A   
   T   
   A   
   T   
   C   
   G   
   A   
    A    
   A   
   A   
   A   
   G   
   A   
   A   
   A   
   C   
   A   
   G   
   T   
    C    
   G   
   A   
   T   
   C   
   T   
   C   
   G   
   T   
   G   
   T   
   G   
   G   
   A   
   A   
   T   
   T   
   T   
   T   
   G   
   A   
   T   
   G   
   A   
   T   
   A   
   C   
   A   
   G   
   A   
   A   
   A   
   A   
   A   
   G   
   A   
   A   
   C   
   C   
   A   
   A   
   C   
   A   
   G   
   T   
   C   
   C   
   T   
   T   
   C   
   C   
   A   
   G   
   C   
   T   
   C   
   G   
   A   
   T   
   A   
   C   
   C   
   C   
   A   
   A   
   A   
   C   
   C   
   T   
   T   
   C   
   T   
    C    
   G   
   T   
   C   
   A   
   A   
   T   
   G   
   G   
   A   
   T   
   C   
   G   
   A   
   C   
   A   
   G   
   G   
   T   
   A   
   T   
   A   
   T   
   C   
   T   
   G   
   C   
   T   
   G   
   G   
   A   
   T   
   A   
   T   
   G   
   C   
    A    
   A   
   C   
   A   
   G   
   A   
   A   
   A   
   T   
   C   
   C   
   C   
   G   
   A   
   C   
    T    
   C   
   A   
   T   
   A   
   A   
   C   
   T   
   T   
   A   
   G   
   C   
   T   
   G   
   A   
   A   
   G   
   T   
   G   
   A   
   T   
    T    
   G   
   A   
    T    
   G   
   G   
   C   
   A   
   C   
   A   
   A   
   T   
   C   
   T   
   A   
   T   
   A   
   T   
   G   
   A   
   T   
   C   
   G   
   A   
   T   
   C   
   A   
   T   
   C   
   C   
   A   
   C   
   A   
   A   
   G   
   C   
   G   
   A   
   G   
   C   
   C   
   T   
   T   
   G   
   A   
   A   
   A   
   A   
   A   
   T   
   T   
   G   
   A   
   T   
   G   
   G   
   A   
   A   
   T   
   A   
   T   
   A   
   T   
   T   
   C   
   C   
   T   
   G   
   G   
   A   
   C   
   C   
   T   
   G   
   A   
   C   
   T   
   T   
    T    
   C   
   C   
   A   
   A   
   C   
   A   
   G   
   G   
   A   
   G   
   G   
    G    
   A   
   T   
   C   
   T   
   T   
   G   
   C   
   A   
   A   
   G   
   G   
    A    
   A   
   A   
   A   
   G   
   A   
   T   
   G   
   A   
   A   
   A   
   T   
   C   
   A   
   A   
   A   
   A   
   A   
   A   
   G   
   C   
   T   
   T   
   A   
   T   
   G   
   A   
   A   
   A   
   C   
   A   
   G   
   G   
    G    
   C   
   G   
   A   
   G   
   G   
   A   
   A   
   A   
   A   
   G   
   T   
   T   
   A   
    T    
   T   
   C   
   T   
   T   
   C   
   G   
   T   
   T   
   C   
   G   
   A   
   A   
   A   
   A   
   C   
   A   
   A   
   A   
   G   
   A   
   T   
   A   
   G   
   A   
    G    
   G   
   C   
   A   
   A   
   T   
   G   
   A   
   A   
   G   
   G   
   G   
    T    
   A   
   A   
    T    
   A   
   A   
   A   
   C   
   A   
   G   
   C   
   A   
   A   
   A   
   T   
   C   
   G   
   T   
   G   
   A   
   T   
   C   
   A   
   C   
    A    
   G   
   A   
   A   
   A   
   T   
   C   
   C   
   C   
   A   
   T   
   A   
   T   
   G   
   A   
   A   
   G   
   T   
   A   
   A   
   A   
   T   
   A   
   A   
   A   
   G   
   C   
   G   
   A   
   C   
    C    
   T   
   T   
   G   
   G   
   T   
   G   
   A   
   A   
   A   
   A   
   A   
   A   
   A   
   T   
   G   
   G   
   A   
   T   
   G   
   A   
   G   
   A   
   T   
   C   
   C   
   G   
   T   
   T   
   T   
   G   
   A   
   A   
   T   
   A   
   A   
   G   
   A   
   A   
   A   
   A   
   T   
   C   
   G   
   A   
    T    
   G   
   G   
   A   
   A   
   T   
   A   
   G   
   C   
   T   
   G   
   A   
   A   
   G   
   T   
    A    
   C   
   G   
   A   
   G   
   A   
   T   
   G   
   A   
   A   
   A   
   G   
   T   
   G   
   A   
   T   
    A    
   G   
    A    
   A   
   C   
    A    
   G   
   G   
   T   
   C   
   T   
   G   
   C   
   A   
   A   
   A   
   T   
   T   
   G   
   T   
   C   
   G   
   T   
    C    
   G   
   A   
   A   
   T   
   T   
   G   
   A   
   A   
   A   
   A   
   A   
   A   
   G   
   A   
    C    
   G   
   C   
   C   
   A   
   A   
    T    
   G   
   C   
   T   
   C   
   A   
   A   
   G   
   G   
    A    
   A   
   T   
   T   
   T   
   T   
   G   
   A   
   A   
   T   
   T   
   A   
    T    
   T   
   T   
   G   
   T   
   T   
   C   
   A   
   A   
   A   
   A   
   A   
   C   
   A   
   C   
   T   
   G   
   A   
   G   
    C    
   T   
   A   
   C   
   A   
   G   
   A   
   T   
   C   
   A   
   A   
   T   
   T   
   A   
   C   
   A   
   A   
   T   
   T   
   T   
    C    
   A   
   A   
   T   
   A   
   T   
   G   
   G   
   T   
   C   
   G   
   C   
   T   
   A   
   T   
   C   
   G   
   A   
    C    
   C   
   A   
   T   
   A   
   T   
   G   
   A   
   C   
   T   
   C   
   C   
   G   
   C   
   A   
    T    
   C   
   A   
   A   
   G   
   T   
   T   
   G   
   G   
   A   
   C   
   T   
   G   
   A   
   A   
   A   
   G   
   A   
   C   
   A   
   T   
   T   
   T   
   T   
   A   
   A   
   G   
   A   
   A   
   G   
   T   
   T   
   A   
   T   
   A   
   T   
   C   
   G   
   A   
   G   
   C   
   A   
   T   
   C   
   G   
   A   
   A   
   A   
   G   
   C   
   A   
   A   
   G   
   T   
   C   
   A   
   T   
   A   
   A   
   C   
   A   
   A   
   A   
   A   
   C   
   G   
    T    
   A   
   G   
    T    
   C   
   A   
   A   
   T   
   T   
   C   
   G   
   A   
   T   
   T   
   T   
   A   
   G   
   C   
   C   
   A   
   A   
   A   
   G   
   C   
   T   
   C   
   A   
   A   
   A   
   A   
   A   
    A    
   G   
   A   
   C   
   A   
   G   
   C   
   A   
   T   
   A   
   T   
   C   
   G   
   T   
    A    
   G   
   A   
   A   
   G   
   G   
    A    
    T    
   T   
   G   
   A   
   T   
   G   
   A   
   A   
   A   
   G   
   C   
   T   
   T   
   T   
   A   
   T   
   C   
   G   
   A   
   T   
   T   
   C   
   T   
   G   
   G   
   A   
   T   
   G   
   A   
   A   
   G   
   T   
   A   
   A   
   T   
   C   
   G   
    T    
   A   
   A   
   C   
   G   
   A   
   T   
   T   
   C   
   G   
   T   
   G   
   A   
   A   
   A   
   G   
   T   
   A   
   A   
   A   
   G   
   A   
   C   
   A   
   A   
   A   
   A   
   A   
   A   
   G   
   A   
    C    
   G   
   C   
    C    
   A   
   A   
   A   
   A   
   A   
   G   
   A   
   A   
   T   
   T   
   T   
   A   
   G   
   T   
   C   
   G   
   A   
    C    
   G   
   T   
    C    
   T   
   T   
    T    
   C   
   A   
   A   
   T   
   T   
   T   
   A   
   C   
   A   
   G   
   A   
   A   
   G   
   A   
   A   
   C   
   A   
   A   
   G   
   C   
   A   
   G   
   A   
   A   
   G   
   C   
   A   
   A   
   T   
    T    
   G   
   T   
   C   
   A   
   C   
   A   
   T   
   T   
   G   
   C   
   A   
   A   
    T    
   T   
   A   
   T   
   A   
    C    
   C   
   G   
   T   
   T   
   T   
   G   
   A   
   C   
   G   
   A   
   A   
    C    
   A   
   C   
    G    
   G   
   A   
   T   
   A   
   T   
   C   
   A   
   C   
   A   
   G   
   A   
   G   
   C   
   T   
   T   
   C   
   A   
   A   
   A   
   A   
   G   
   G   
   A   
   A   
    T    
   C   
    C    
   G   
   A   
   A   
   A   
    G    
   C   
   T   
   T   
   G   
   A   
   T   
   T   
   G   
   C   
    G    
   C   
   A   
   A   
   A   
   T   
   C   
   A   
   C   
   A   
   G   
   A   
   G   
   T   
   T   
    G    
   A   
   A   
   T   
   A   
   A   
   A   
   A   
   T   
   C   
   C   
   T   
   T   
   T   
   C   
   A   
   A   
   A   
    T    
   G   
   A   
   C   
   A   
   A   
   A   
   G   
   A   
    G    
   C   
   T   
   T   
   T   
   T   
    C    
   T   
   C   
   T   
   G   
   T   
   T   
   A   
   T   
   G   
   A   
   A   
   A   
   A   
   A   
   A   
   G   
   A   
   A   
   T   
   T   
   G   
   C   
   G   
   A   
   G   
   A   
   A   
   G   
   T   
   G   
   A   
   A   
   A   
   A   
   A   
   A   
   A   
   A   
   T   
   T   
   A   
    T    
   T   
   C   
   T   
   T   
   C   
   C   
   G   
   C   
   T   
   C   
   G   
   T   
   C   
    T    
    A    
   A   
   C   
   G   
   A   
   C   
   A   
   A   
   T   
   C   
    G    
   A   
   A   
   G   
   A   
   C   
   G   
   A   
   A   
   A   
   T   
   C   
   G   
   A   
   A   
   G   
   A   
   A   
   A   
   T   
   C   
   A   
   A   
   A   
   A   
   T   
    T    
   G   
   A   
   T   
   A   
   C   
   G   
   C   
   A   
   A   
    G    
   T   
    C    
   T   
   T   
   A   
   G   
   T   
   G   
   G   
   C   
    A    
   C   
   A   
   A   
   G   
   A   
   A   
   G   
   A   
   C   
   G   
   T   
   A   
   A   
   T   
    T    
   G   
   T   
   C   
   A   
   G   
   T   
   G   
   T   
    G    
   A   
   C   
    G    
   C   
   G   
   A   
   G   
   A   
   A   
   G   
   G   
   C   
   T   
   A   
   T   
   G   
   T   
   G   
   A   
   A   
   G   
   C   
   G   
   A   
   A   
   C   
   A   
   A   
   G   
   C   
   C   
   T   
   T   
   C   
   G   
   T   
   T   
   C   
    T    
   T   
   A   
   T   
   A   
   G   
   T   
   G   
   C   
   T   
   T   
   C   
    C    
   A   
   A   
   A   
   C   
   C   
   A   
   G   
   A   
   A   
   G   
   A   
   A   
   A   
   T   
   C   
   G   
   G   
   G   
   A   
   T   
   G   
   C   
   G   
   A   
   G   
   A   
   A   
   G   
   G   
   C   
   G   
   A   
    C    
   T   
   A   
   C   
   T   
   T   
   G   
   C   
   T   
    A    
   T   
   A   
   C   
    T    
   C   
   A   
   G   
   G   
   T   
   G   
   A   
   A   
   T   
   T   
    G    
   A   
   G   
    T    
   A   
   C   
   A   
    T    
   T   
   G   
   G   
   A   
   C   
   C   
   A   
   T   
   G   
   T   
   T   
   C   
   T   
   T   
   C   
   T   
   G   
   A   
   T   
    C    
   A   
   C   
    A    
   A   
   A   
   T   
   A   
   A   
   A   
   G   
   C   
   A   
   A   
   A   
   T   
   G   
   T   
   G   
   A   
   T   
   C   
   T   
   A   
   C   
   C   
   G   
    C    
   C   
   C   
   T   
   G   
   T   
    C    
   C   
   A   
   T   
   G   
   A   
   A   
   C   
   T   
   A   
   C   
   C   
    C    
   G   
   A   
   T   
   T   
   T   
   G   
   A   
   A   
   A   
   T   
   G   
   G   
   A   
   A   
   A   
   G   
   A   
   T   
   G   
   C   
   T   
   G   
   G   
    T    
   G   
   A   
    G    
   C   
   A   
    C    
   A   
   T   
   T   
   T   
   C   
   T   
   C   
   A   
   A   
   A   
   C   
   A   
   A   
   T   
   C   
   T   
   C   
   C   
   A   
   A   
   T   
   C   
   T   
   G   
   T   
   C   
   T   
   G   
   T   
   A   
   G   
   A   
    C    
   G   
   A   
   A   
   T   
   C   
   G   
   A   
   T   
    T    
   T   
   T   
   A   
   G   
   C   
   T   
   G   
   T   
   T   
   T   
   T   
    C    
   C   
   C   
   A   
   T   
   A   
   T   
   C   
   A   
   A   
   A   
   A   
   A   
   A   
   T   
    T    
   C   
   A   
   A   
   G   
   C   
   A   
   G   
   A   
    G    
   A   
   A   
   A   
   A   
   C   
   A   
   T   
   T   
   T   
   G   
   T   
    A    
   T   
   T   
   C   
   A   
   T   
   C   
   A   
   G   
   C   
   A   
   A   
   G   
   A   
   A   
   T   
   G   
   G   
   A   
   T   
   T   
    G    
    A    
   T   
   C   
   A   
   A   
   G   
   C   
   A   
   A   
   A   
   C   
   A   
   C   
   G   
   A   
   A   
   T   
   G   
   A   
   C   
   A   
   G   
   A   
   G   
   T   
   T   
   C   
   G   
   A   
   A   
   C   
   C   
   A   
   T   
   G   
   G   
   C   
   G   
   C   
   A   
   C   
   C   
   T   
   A   
    T    
   A   
   A   
   G   
   A   
   G   
   C   
   C   
   G   
    T    
   C   
   C   
   G   
   C   
   T   
   C   
   A   
   G   
   C   
   T   
   G   
   T   
   A   
   T   
   G   
   A   
   A   
   G   
   C   
   T   
   A   
   A   
   A   
   G   
   G   
   C   
   G   
   G   
   A   
   A   
   G   
   A   
   A   
   G   
   A   
   T   
   G   
   A   
   A   
   C   
   T   
    G    
   A   
   T   
   T   
   G   
   C   
   T   
   G   
   T   
    G    
   T   
   A   
   T   
   C   
   T   
   G   
   A   
   A   
   A   
   G   
   A   
   A   
   G   
   A   
   T   
   C   
    A    
   G   
   C   
   C   
   A   
   G   
   G   
   C   
   T   
   T   
   A   
   G   
   A   
   T   
   G   
   T   
    T    
   T   
   T   
   C   
   T   
   T   
   A   
   G   
   T   
   A   
   A   
   C   
   G   
    C    
   A   
   T   
   C   
   A   
   A   
   G   
   G   
   A   
   A   
   T   
   G   
   G   
   G   
    T    
   T   
   T   
   G   
   C   
   G   
   C   
   T   
    A    
   T   
   C   
   C   
   T   
   T   
   T   
   A   
   A   
    C    
   A   
   G   
   A   
   T   
   G   
   T   
   T   
   C   
   C   
   C   
   G   
   T   
   T   
   G   
   T   
   T   
   G   
   G   
   A   
   G   
   C   
   A   
   A   
   A   
   G   
   G   
   C   
   A   
   G   
    C    
   T   
   G   
   G   
   A   
   G   
   T   
   G   
   A   
   A   
   G   
   T   
   C   
   T   
   A   
   T   
   G   
   A   
   A   
   T   
   T   
   T   
   G   
   A   
   A   
   A   
   G   
   A   
   T   
   G   
   G   
   A   
   G   
   A   
   T   
   A   
   G   
   G   
    A    
   T   
   T   
    A    
   T   
   C   
   A   
   A   
    C    
   G   
   G   
    G    
   C   
   T   
    G    
   C   
   T   
   T   
   G   
   T   
   G   
   T   
   T   
   A   
   G   
   C   
   A   
   G   
   A   
    A    
   G   
   G   
    T    
   G   
   A   
   T   
   A   
   C   
   G   
   C   
   C   
    G    
   A   
   T   
   C   
   G   
   T   
   A   
   A   
   T   
   C   
   G   
   T   
   T   
   A   
   C   
   T   
   C   
   A   
   G   
   C   
   G   
   A   
   G   
   G   
   A   
   G   
   C   
    T    
   G   
   T   
    T    
   A   
   A   
   A   
   C   
   G   
   C   
   A   
   T   
   G   
   C   
   T   
   T   
   G   
   C   
   T   
   C   
   A   
   A   
   G   
   A   
   A   
   A   
   T   
   C   
   A   
   G   
   C   
   C   
   A   
   A   
   A   
   C   
    T    
   T   
   C   
   A   
   C   
   G   
   T   
   G   
   C   
   G   
   A   
   A   
   G   
   C   
   G   
   G   
   G   
   G   
   A   
   G   
   T   
   G   
   A   
   C   
   G   
   G   
   T   
   C   
   A   
   T   
   G   
   C   
   G   
   T   
   G   
   A   
   G   
   C   
   T   
   G   
   A   
   A   
   A   
   A   
   A   
   A   
   A   
   A   
   T   
   C   
   C   
    G    
   C   
   A   
    C    
   C   
   G   
    T    
   A   
   T   
   T   
   A   
   T   
   C   
   T   
   A   
   T   
   A   
   T   
   G   
   T   
   C   
   G   
   G   
   A   
   A   
   G   
   G   
   A   
   A   
   G   
   A   
   T   
   C   
   A   
   A   
   A   
   A   
   G   
   A   
   A   
   A   
   T   
   A   
   A   
   C   
   C   
   T   
   T   
   G   
   A   
   T   
   G   
   A   
   A   
   T   
   C   
   A   
   A   
   A   
   A   
   A   
   G   
   G   
    G    
   C   
   A   
    G    
   C   
   A   
   G   
    T    
   T   
   A   
   A   
   C   
   G   
   A   
   T   
   T   
   G   
   A   
    C    
   C   
   C   
   A   
   A   
   C   
    T    
   G   
   A   
   T   
   T   
   T   
    C    
   C   
   C   
   T   
   A   
   T   
   A   
   G   
   G   
   T   
   G   
   A   
   T   
   C   
   G   
   A   
   A   
   C   
   A   
   T   
   C   
   G   
   A   
   A   
   T   
   G   
   G   
    A    
   T   
   C   
    A    
   T   
   T   
   T   
   G   
   C   
    G    
   A   
   T   
   G   
   G   
   A   
    T    
   G   
   A   
   G   
   A   
   A   
   A   
   A   
   A   
   A   
   G   
   G   
   T   
   G   
   G   
   A   
   G   
   A   
   A   
   G   
   T   
   C   
   A   
   T   
   C   
   G   
   A   
   A   
   G   
   T   
    T    
   A   
    T    
   C   
   G   
   A   
   T   
   G   
   C   
   T   
   C   
   C   
   T   
   G   
   A   
   G   
    A    
   T   
   T   
   T   
   T   
    C    
   A   
   T   
    C    
   G   
   A   
    G    
    T    
   C   
    G    
   -   
   -   
   -   
 

 
   N52829   
   A   
   T   
   G   
   G   
   A   
   A   
   A   
   A   
   T   
   C   
   G   
   A   
   C   
   A   
   A   
   G   
   A   
   A   
   G   
   T   
   A   
   C   
   A   
   A   
   G   
   A   
   A   
   T   
   T   
   A   
   A   
   C   
   T   
   T   
   T   
   A   
   G   
   A   
   A   
   G   
   A   
   A   
   G   
   T   
   G   
   A   
   T   
   G   
   G   
   G   
   T   
   G   
   A   
   C   
   C   
   G   
   C   
   T   
   T   
   C   
   G   
   G   
   A   
   C   
   G   
   T   
   T   
   A   
   T   
   T   
   C   
   C   
   A   
   A   
   A   
   T   
   A   
   T   
   A   
   T   
   T   
   A   
   T   
    C    
   C   
   A   
    A    
   G   
   A   
   A   
   C   
   G   
   T   
   G   
   C   
   A   
   C   
   T   
   G   
   C   
   C   
   T   
   G   
   A   
   T   
   A   
   T   
    C    
   C   
   G   
   T   
   G   
   A   
   T   
   G   
   G   
   A   
   T   
   T   
   G   
   A   
   A   
    G    
   C   
   C   
   A   
   G   
   T   
    G    
   C   
   A   
   A   
   C   
   G   
   C   
   C   
   G   
   G   
   A   
   T   
   A   
   T   
   T   
   A   
   T   
   T   
    T    
   G   
   C   
    C    
   A   
   T   
   G   
   A   
   A   
   C   
   A   
   A   
   A   
   G   
   A   
   T   
   G   
   G   
   C   
   A   
   A   
   T   
   A   
   C   
   G   
   T   
   A   
   T   
   G   
   A   
   C   
   A   
   A   
   A   
   G   
   G   
   A   
   T   
   T   
    C    
   C   
   G   
   T   
   A   
   A   
   A   
   T   
   C   
   A   
   G   
   C   
   A   
   A   
   A   
   A   
   T   
   C   
   T   
   G   
   T   
   C   
   G   
   G   
   A   
   A   
   A   
   T   
   A   
   T   
   C   
   A   
   T   
   G   
   G   
   G   
    G    
   A   
   A   
   T   
   T   
   A   
   T   
   C   
   A   
   T   
   C   
   C   
   T   
   C   
   A   
   T   
   G   
   G   
   T   
   G   
   A   
   C   
   A   
   G   
   C   
   A   
   G   
   T   
   A   
   T   
   T   
   T   
   A   
   T   
   G   
   A   
   A   
   G   
   C   
   G   
   A   
   T   
   G   
   G   
   T   
   C   
   C   
   G   
   C   
   A   
   T   
   G   
   A   
   G   
   C   
   C   
   A   
   A   
   G   
   A   
    C    
   T   
   G   
   G   
   A   
   A   
   G   
   T   
   T   
   G   
   C   
   G   
   G   
   G   
   A   
   A   
   G   
   T   
   A   
   C   
   T   
   G   
   A   
   T   
   C   
   G   
   A   
    A    
   A   
   T   
   G   
   C   
   A   
   C   
   G   
   G   
    G    
   A   
   A   
   T   
   A   
   A   
    C    
   G   
   G   
   G   
   A   
   G   
   T   
   A   
   T   
   G   
   G   
   A   
   C   
   G   
   G   
   T   
   G   
   A   
   T   
   C   
   C   
   A   
   C   
   C   
   T   
   G   
   C   
   T   
   G   
   C   
   G   
   A   
   T   
   G   
   C   
   G   
   T   
   T   
   A   
   T   
   A   
   C   
   T   
   G   
   A   
   A   
   G   
   C   
   C   
   A   
   G   
    A    
   T   
   T   
   A   
   T   
   C   
   C   
   A   
   A   
   G   
   T   
   T   
   A   
   A   
   G   
   T   
   G   
   G   
   A   
   G   
   A   
   G   
   A   
   T   
   G   
    C    
   T   
   A   
    G    
   C   
   A   
   G   
   A   
   T   
   A   
   T   
   C   
   G   
   A   
    A    
   A   
   A   
   A   
   G   
   A   
   A   
   A   
   C   
   A   
   G   
   T   
    C    
   G   
   A   
   T   
   C   
   T   
   C   
   G   
   T   
   G   
   T   
   G   
   G   
   A   
   A   
   T   
   T   
   T   
   T   
   G   
   A   
   T   
   G   
   A   
   T   
   A   
   C   
   A   
   G   
   A   
   A   
   A   
   A   
   A   
   G   
   A   
   A   
   C   
   C   
   A   
   A   
   C   
   A   
   G   
   T   
   C   
   C   
   T   
   T   
   C   
   C   
   A   
   G   
   C   
   T   
   C   
   G   
   A   
   T   
   A   
   C   
   C   
   C   
   A   
   A   
   A   
   C   
   C   
   T   
   T   
   C   
   T   
    C    
   G   
   T   
   C   
   A   
   A   
   T   
   G   
   G   
   A   
   T   
   C   
   G   
   A   
   C   
   A   
   G   
   G   
   T   
   A   
   T   
   A   
   T   
   C   
   T   
   G   
   C   
   T   
   G   
   G   
   A   
   T   
   A   
   T   
   G   
   C   
    A    
   A   
   C   
   A   
   G   
   A   
   A   
   A   
   T   
   C   
   C   
   C   
   G   
   A   
   C   
    T    
   C   
   A   
   T   
   A   
   A   
   C   
   T   
   T   
   A   
   G   
   C   
   T   
   G   
   A   
   A   
   G   
   T   
   G   
   A   
   T   
    T    
   G   
   A   
    T    
   G   
   G   
   C   
   A   
   C   
   A   
   A   
   T   
   C   
   T   
   A   
   T   
   A   
   T   
   G   
   A   
   T   
   C   
   G   
   A   
   T   
   C   
   A   
   T   
   C   
   C   
   A   
   C   
   A   
   A   
   G   
   C   
   G   
   A   
   G   
   C   
   C   
   T   
   T   
   G   
   A   
   A   
   A   
   A   
   A   
   T   
   T   
   G   
   A   
   T   
   G   
   G   
   A   
   A   
   T   
   A   
   T   
   A   
   T   
   T   
   C   
   C   
   T   
   G   
   G   
   A   
   C   
   C   
   T   
   G   
   A   
   C   
   T   
   T   
    T    
   C   
   C   
   A   
   A   
   C   
   A   
   G   
   G   
   A   
   G   
   G   
    G    
   A   
   T   
   C   
   T   
   T   
   G   
   C   
   A   
   A   
   G   
   G   
    A    
   A   
   A   
   A   
   G   
   A   
   T   
   G   
   A   
   A   
   A   
   T   
   C   
   A   
   A   
   A   
   A   
   A   
   A   
   G   
   C   
   T   
   T   
   A   
   T   
   G   
   A   
   A   
   A   
   C   
   A   
   G   
   G   
    G    
   C   
   G   
   A   
   G   
   G   
   A   
   A   
   A   
   A   
   G   
   T   
   T   
   A   
    T    
   T   
   C   
   T   
   T   
   C   
   G   
   T   
   T   
   C   
   G   
   A   
   A   
   A   
   A   
   C   
   A   
   A   
   A   
   G   
   A   
   T   
   A   
   G   
   A   
    G    
   G   
   C   
   A   
   A   
   T   
   G   
   A   
   A   
   G   
   G   
   G   
    T    
   A   
   A   
    T    
   A   
   A   
   A   
   C   
   A   
   G   
   C   
   A   
   A   
   A   
   T   
   C   
   G   
   T   
   G   
   A   
   T   
   C   
   A   
   C   
    A    
   G   
   A   
   A   
   A   
   T   
   C   
   C   
   C   
   A   
   T   
   A   
   T   
   G   
   A   
   A   
   G   
   T   
   A   
   A   
   A   
   T   
   A   
   A   
   A   
   G   
   C   
   G   
   A   
   C   
    C    
   T   
   T   
   G   
   G   
   T   
   G   
   A   
   A   
   A   
   A   
   A   
   A   
   A   
   T   
   G   
   G   
   A   
   T   
   G   
   A   
   G   
   A   
   T   
   C   
   C   
   G   
   T   
   T   
   T   
   G   
   A   
   A   
   T   
   A   
   A   
   G   
   A   
   A   
   A   
   A   
   T   
   C   
   G   
   A   
    T    
   G   
   G   
   A   
   A   
   T   
   A   
   G   
   C   
   T   
   G   
   A   
   A   
   G   
   T   
    A    
   C   
   G   
   A   
   G   
   A   
   T   
   G   
   A   
   A   
   A   
   G   
   T   
   G   
   A   
   T   
    A    
   G   
    A    
   A   
   C   
    A    
   G   
   G   
   T   
   C   
   T   
   G   
   C   
   A   
   A   
   A   
   T   
   T   
   G   
   T   
   C   
   G   
   T   
    C    
   G   
   A   
   A   
   T   
   T   
   G   
   A   
   A   
   A   
   A   
   A   
   A   
   G   
   A   
    C    
   G   
   C   
   C   
   A   
   A   
    T    
   G   
   C   
   T   
   C   
   A   
   A   
   G   
   G   
    A    
   A   
   T   
   T   
   T   
   T   
   G   
   A   
   A   
   T   
   T   
   A   
    T    
   T   
   T   
   G   
   T   
   T   
   C   
   A   
   A   
   A   
   A   
   A   
   C   
   A   
   C   
   T   
   G   
   A   
   G   
    C    
   T   
   A   
   C   
   A   
   G   
   A   
   T   
   C   
   A   
   A   
   T   
   T   
   A   
   C   
   A   
   A   
   T   
   T   
   T   
    C    
   A   
   A   
   T   
   A   
   T   
   G   
   G   
   T   
   C   
   G   
   C   
   T   
   A   
   T   
   C   
   G   
   A   
    C    
   C   
   A   
   T   
   A   
   T   
   G   
   A   
   C   
   T   
   C   
   C   
   G   
   C   
   A   
    T    
   C   
   A   
   A   
   G   
   T   
   T   
   G   
   G   
   A   
   C   
   T   
   G   
   A   
   A   
   A   
   G   
   A   
   C   
   A   
   T   
   T   
   T   
   T   
   A   
   A   
   G   
   A   
   A   
   G   
   T   
   T   
   A   
   T   
   A   
   T   
   C   
   G   
   A   
   G   
   C   
   A   
   T   
   C   
   G   
   A   
   A   
   A   
   G   
   C   
   A   
   A   
   G   
   T   
   C   
   A   
   T   
   A   
   A   
   C   
   A   
   A   
   A   
   A   
   C   
   G   
    T    
   A   
   G   
    T    
   C   
   A   
   A   
   T   
   T   
   C   
   G   
   A   
   T   
   T   
   T   
   A   
   G   
   C   
   C   
   A   
   A   
   A   
   G   
   C   
   T   
   C   
   A   
   A   
   A   
   A   
   A   
    A    
   G   
   A   
   C   
   A   
   G   
   C   
   A   
   T   
   A   
   T   
   C   
   G   
   T   
    A    
   G   
   A   
   A   
   G   
   G   
    A    
    T    
   T   
   G   
   A   
   T   
   G   
   A   
   A   
   A   
   G   
   C   
   T   
   T   
   T   
   A   
   T   
   C   
   G   
   A   
   T   
   T   
   C   
   T   
   G   
   G   
   A   
   T   
   G   
   A   
   A   
   G   
   T   
   A   
   A   
   T   
   C   
   G   
    T    
   A   
   A   
   C   
   G   
   A   
   T   
   T   
   C   
   G   
   T   
   G   
   A   
   A   
   A   
   G   
   T   
   A   
   A   
   A   
   G   
   A   
   C   
   A   
   A   
   A   
   A   
   A   
   A   
   G   
   A   
    C    
   G   
   C   
    C    
   A   
   A   
   A   
   A   
   A   
   G   
   A   
   A   
   T   
   T   
   T   
   A   
   G   
   T   
   C   
   G   
   A   
    C    
   G   
   T   
    C    
   T   
   T   
    T    
   C   
   A   
   A   
   T   
   T   
   T   
   A   
   C   
   A   
   G   
   A   
   A   
   G   
   A   
   A   
   C   
   A   
   A   
   G   
   C   
   A   
   G   
   A   
   A   
   G   
   C   
   A   
   A   
   T   
    T    
   G   
   T   
   C   
   A   
   C   
   A   
   T   
   T   
   G   
   C   
   A   
   A   
    T    
   T   
   A   
   T   
   A   
    C    
   C   
   G   
   T   
   T   
   T   
   G   
   A   
   C   
   G   
   A   
   A   
    C    
   A   
   C   
    G    
   G   
   A   
   T   
   A   
   T   
   C   
   A   
   C   
   A   
   G   
   A   
   G   
   C   
   T   
   T   
   C   
   A   
   A   
   A   
   A   
   G   
   G   
   A   
   A   
    T    
   C   
    C    
   G   
   A   
   A   
   A   
    G    
   C   
   T   
   T   
   G   
   A   
   T   
   T   
   G   
   C   
    G    
   C   
   A   
   A   
   A   
   T   
   C   
   A   
   C   
   A   
   G   
   A   
   G   
   T   
   T   
    G    
   A   
   A   
   T   
   A   
   A   
   A   
   A   
   T   
   C   
   C   
   T   
   T   
   T   
   C   
   A   
   A   
   A   
    T    
   G   
   A   
   C   
   A   
   A   
   A   
   G   
   A   
    G    
   C   
   T   
   T   
   T   
   T   
    C    
   T   
   C   
   T   
   G   
   T   
   T   
   A   
   T   
   G   
   A   
   A   
   A   
   A   
   A   
   A   
   G   
   A   
   A   
   T   
   T   
   G   
   C   
   G   
   A   
   G   
   A   
   A   
   G   
   T   
   G   
   A   
   A   
   A   
   A   
   A   
   A   
   A   
   A   
   T   
   T   
   A   
    T    
   T   
   C   
   T   
   T   
   C   
   C   
   G   
   C   
   T   
   C   
   G   
   T   
   C   
    T    
    A    
   A   
   C   
   G   
   A   
   C   
   A   
   A   
   T   
   C   
    G    
   A   
   A   
   G   
   A   
   C   
   G   
   A   
   A   
   A   
   T   
   C   
   G   
   A   
   A   
   G   
   A   
   A   
   A   
   T   
   C   
   A   
   A   
   A   
   A   
   T   
    T    
   G   
   A   
   T   
   A   
   C   
   G   
   C   
   A   
   A   
    G    
   T   
    C    
   T   
   T   
   A   
   G   
   T   
   G   
   G   
   C   
    A    
   C   
   A   
   A   
   G   
   A   
   A   
   G   
   A   
   C   
   G   
   T   
   A   
   A   
   T   
    T    
   G   
   T   
   C   
   A   
   G   
   T   
   G   
   T   
    G    
   A   
   C   
    G    
   C   
   G   
   A   
   G   
   A   
   A   
   G   
   G   
   C   
   T   
   A   
   T   
   G   
   T   
   G   
   A   
   A   
   G   
   C   
   G   
   A   
   A   
   C   
   A   
   A   
   G   
   C   
   C   
   T   
   T   
   C   
   G   
   T   
   T   
   C   
    T    
   T   
   A   
   T   
   A   
   G   
   T   
   G   
   C   
   T   
   T   
   C   
    C    
   A   
   A   
   A   
   C   
   C   
   A   
   G   
   A   
   A   
   G   
   A   
   A   
   A   
   T   
   C   
   G   
   G   
   G   
   A   
   T   
   G   
   C   
   G   
   A   
   G   
   A   
   A   
   G   
   G   
   C   
   G   
   A   
    C    
   T   
   A   
   C   
   T   
   T   
   G   
   C   
   T   
    A    
   T   
   A   
   C   
    T    
   C   
   A   
   G   
   G   
   T   
   G   
   A   
   A   
   T   
   T   
    G    
   A   
   G   
    T    
   A   
   C   
   A   
    T    
   T   
   G   
   G   
   A   
   C   
   C   
   A   
   T   
   G   
   T   
   T   
   C   
   T   
   T   
   C   
   T   
   G   
   A   
   T   
    C    
   A   
   C   
    A    
   A   
   A   
   T   
   A   
   A   
   A   
   G   
   C   
   A   
   A   
   A   
   T   
   G   
   T   
   G   
   A   
   T   
   C   
   T   
   A   
   C   
   C   
   G   
    C    
   C   
   C   
   T   
   G   
   T   
    C    
   C   
   A   
   T   
   G   
   A   
   A   
   C   
   T   
   A   
   C   
   C   
    C    
   G   
   A   
   T   
   T   
   T   
   G   
   A   
   A   
   A   
   T   
   G   
   G   
   A   
   A   
   A   
   G   
   A   
   T   
   G   
   C   
   T   
   G   
   G   
    T    
   G   
   A   
    G    
   C   
   A   
    C    
   A   
   T   
   T   
   T   
   C   
   T   
   C   
   A   
   A   
   A   
   C   
   A   
   A   
   T   
   C   
   T   
   C   
   C   
   A   
   A   
   T   
   C   
   T   
   G   
   T   
   C   
   T   
   G   
   T   
   A   
   G   
   A   
    C    
   G   
   A   
   A   
   T   
   C   
   G   
   A   
   T   
    T    
   T   
   T   
   A   
   G   
   C   
   T   
   G   
   T   
   T   
   T   
   T   
    C    
   C   
   C   
   A   
   T   
   A   
   T   
   C   
   A   
   A   
   A   
   A   
   A   
   A   
   T   
    T    
   C   
   A   
   A   
   G   
   C   
   A   
   G   
   A   
    G    
   A   
   A   
   A   
   A   
   C   
   A   
   T   
   T   
   T   
   G   
   T   
    A    
   T   
   T   
   C   
   A   
   T   
   C   
   A   
   G   
   C   
   A   
   A   
   G   
   A   
   A   
   T   
   G   
   G   
   A   
   T   
   T   
    G    
    A    
   T   
   C   
   A   
   A   
   G   
   C   
   A   
   A   
   A   
   C   
   A   
   C   
   G   
   A   
   A   
   T   
   G   
   A   
   C   
   A   
   G   
   A   
   G   
   T   
   T   
   C   
   G   
   A   
   A   
   C   
   C   
   A   
   T   
   G   
   G   
   C   
   G   
   C   
   A   
   C   
   C   
   T   
   A   
    T    
   A   
   A   
   G   
   A   
   G   
   C   
   C   
   G   
    T    
   C   
   C   
   G   
   C   
   T   
   C   
   A   
   G   
   C   
   T   
   G   
   T   
   A   
   T   
   G   
   A   
   A   
   G   
   C   
   T   
   A   
   A   
   A   
   G   
   G   
   C   
   G   
   G   
   A   
   A   
   G   
   A   
   A   
   G   
   A   
   T   
   G   
   A   
   A   
   C   
   T   
    G    
   A   
   T   
   T   
   G   
   C   
   T   
   G   
   T   
    G    
   T   
   A   
   T   
   C   
   T   
   G   
   A   
   A   
   A   
   G   
   A   
   A   
   G   
   A   
   T   
   C   
    A    
   G   
   C   
   C   
   A   
   G   
   G   
   C   
   T   
   T   
   A   
   G   
   A   
   T   
   G   
   T   
    T    
   T   
   T   
   C   
   T   
   T   
   A   
   G   
   T   
   A   
   A   
   C   
   G   
    C    
   A   
   T   
   C   
   A   
   A   
   G   
   G   
   A   
   A   
   T   
   G   
   G   
   G   
    T    
   T   
   T   
   G   
   C   
   G   
   C   
   T   
    A    
   T   
   C   
   C   
   T   
   T   
   T   
   A   
   A   
    C    
   A   
   G   
   A   
   T   
   G   
   T   
   T   
   C   
   C   
   C   
   G   
   T   
   T   
   G   
   T   
   T   
   G   
   G   
   A   
   G   
   C   
   A   
   A   
   A   
   G   
   G   
   C   
   A   
   G   
    T    
   T   
   G   
   G   
   A   
   G   
   T   
   G   
   A   
   A   
   G   
   T   
   C   
   T   
   A   
   T   
   G   
   A   
   A   
   T   
   T   
   T   
   G   
   A   
   A   
   A   
   G   
   A   
   T   
   G   
   G   
   A   
   G   
   A   
   T   
   A   
   G   
   G   
    A    
   T   
   T   
    A    
   T   
   C   
   A   
   A   
    C    
   G   
   G   
    G    
   C   
   T   
    G    
   C   
   T   
   T   
   G   
   T   
   G   
   T   
   T   
   A   
   G   
   C   
   A   
   G   
   A   
    A    
   G   
   G   
    T    
   G   
   A   
   T   
   A   
   C   
   G   
   C   
   C   
    G    
   A   
   T   
   C   
   G   
   T   
   A   
   A   
   T   
   C   
   G   
   T   
   T   
   A   
   C   
   T   
   C   
   A   
   G   
   C   
   G   
   A   
   G   
   G   
   A   
   G   
   C   
    T    
   G   
   T   
    T    
   A   
   A   
   A   
   C   
   G   
   C   
   A   
   T   
   G   
   C   
   T   
   T   
   G   
   C   
   T   
   C   
   A   
   A   
   G   
   A   
   A   
   A   
   T   
   C   
   A   
   G   
   C   
   C   
   A   
   A   
   A   
   C   
    T    
   T   
   C   
   A   
   C   
   G   
   T   
   G   
   C   
   G   
   A   
   A   
   G   
   C   
   G   
   G   
   G   
   G   
   A   
   G   
   T   
   G   
   A   
   C   
   G   
   G   
   T   
   C   
   A   
   T   
   G   
   C   
   G   
   T   
   G   
   A   
   G   
   C   
   T   
   G   
   A   
   A   
   A   
   A   
   A   
   A   
   A   
   A   
   T   
   C   
   C   
    G    
   C   
   A   
    C    
   C   
   G   
    T    
   A   
   T   
   T   
   A   
   T   
   C   
   T   
   A   
   T   
   A   
   T   
   G   
   T   
   C   
   G   
   G   
   A   
   A   
   G   
   G   
   A   
   A   
   G   
   A   
   T   
   C   
   A   
   A   
   A   
   A   
   G   
   A   
   A   
   A   
   T   
   A   
   A   
   C   
   C   
   T   
   T   
   G   
   A   
   T   
   G   
   A   
   A   
   T   
   C   
   A   
   A   
   A   
   A   
   A   
   G   
   G   
    G    
   C   
   A   
    G    
   C   
   A   
   G   
    T    
   T   
   A   
   A   
   C   
   G   
   A   
   T   
   T   
   G   
   A   
    C    
   C   
   C   
   A   
   A   
   C   
    T    
   G   
   A   
   T   
   T   
   T   
    C    
   C   
   C   
   T   
   A   
   T   
   A   
   G   
   G   
   T   
   G   
   A   
   T   
   C   
   G   
   A   
   A   
   C   
   A   
   T   
   C   
   G   
   A   
   A   
   T   
   G   
   G   
    A    
   T   
   C   
    A    
   T   
   T   
   T   
   G   
   C   
    G    
   A   
   T   
   G   
   G   
   A   
    T    
   G   
   A   
   G   
   A   
   A   
   A   
   A   
   A   
   A   
   G   
   G   
   T   
   G   
   G   
   A   
   G   
   A   
   A   
   G   
   T   
   C   
   A   
   T   
   C   
   G   
   A   
   A   
   G   
   T   
    T    
   A   
    T    
   C   
   G   
   A   
   T   
   G   
   C   
   T   
   C   
   C   
   T   
   G   
   A   
   G   
    A    
   T   
   T   
   T   
   T   
    C    
   A   
   T   
    C    
   G   
   A   
    G    
    T    
   C   
    G    
   -   
   -   
   -   
 

 
   N60048F   
   A   
   T   
   G   
   G   
   A   
   A   
   A   
   A   
   T   
   C   
   G   
   A   
   C   
   A   
   A   
   G   
   A   
   A   
   G   
   T   
   A   
   C   
   A   
   A   
   G   
   A   
   A   
   T   
   T   
   A   
   A   
   C   
   T   
   T   
   T   
   A   
   G   
   A   
   A   
   G   
   A   
   A   
   G   
   T   
   G   
   A   
   T   
   G   
   G   
   G   
   T   
   G   
   A   
   C   
   C   
   G   
   C   
   T   
   T   
   C   
   G   
   G   
   A   
   C   
   G   
   T   
   T   
   A   
   T   
   T   
   C   
   C   
   A   
   A   
   A   
   T   
   A   
   T   
   A   
   T   
   T   
   A   
   T   
    C    
   C   
   A   
    A    
   G   
   A   
   A   
   C   
   G   
   T   
   G   
   C   
   A   
   C   
   T   
   G   
   C   
   C   
   T   
   G   
   A   
   T   
   A   
   T   
    C    
   C   
   G   
   T   
   G   
   A   
   T   
   G   
   G   
   A   
   T   
   T   
   G   
   A   
   A   
    G    
   C   
   C   
   A   
   G   
   T   
    G    
   C   
   A   
   A   
   C   
   G   
   C   
   C   
   G   
   G   
   A   
   T   
   A   
   T   
   T   
   A   
   T   
   T   
    C    
   G   
   C   
    T    
   A   
   T   
   G   
   A   
   A   
   C   
   A   
   A   
   A   
   G   
   A   
   T   
   G   
   G   
   C   
   A   
   A   
   T   
   A   
   C   
   G   
   T   
   A   
   T   
   G   
   A   
   C   
   A   
   A   
   A   
   G   
   G   
   A   
   T   
   T   
    C    
   C   
   G   
   T   
   A   
   A   
   A   
   T   
   C   
   A   
   G   
   C   
   A   
   A   
   A   
   A   
   T   
   C   
   T   
   G   
   T   
   C   
   G   
   G   
   A   
   A   
   A   
   T   
   A   
   T   
   C   
   A   
   T   
   G   
   G   
   G   
    G    
   A   
   A   
   T   
   T   
   A   
   T   
   C   
   A   
   T   
   C   
   C   
   T   
   C   
   A   
   T   
   G   
   G   
   T   
   G   
   A   
   C   
   A   
   G   
   C   
   A   
   G   
   T   
   A   
   T   
   T   
   T   
   A   
   T   
   G   
   A   
   A   
   G   
   C   
   G   
   A   
   T   
   G   
   G   
   T   
   C   
   C   
   G   
   C   
   A   
   T   
   G   
   A   
   G   
   C   
   C   
   A   
   A   
   G   
   A   
    C    
   T   
   G   
   G   
   A   
   A   
   G   
   T   
   T   
   G   
   C   
   G   
   G   
   G   
   A   
   A   
   G   
   T   
   A   
   C   
   T   
   G   
   A   
   T   
   C   
   G   
   A   
    A    
   A   
   T   
   G   
   C   
   A   
   C   
   G   
   G   
    G    
   A   
   A   
   T   
   A   
   A   
    C    
   G   
   G   
   G   
   A   
   G   
   T   
   A   
   T   
   G   
   G   
   A   
   C   
   G   
   G   
   T   
   G   
   A   
   T   
   C   
   C   
   A   
   C   
   C   
   T   
   G   
   C   
   T   
   G   
   C   
   G   
   A   
   T   
   G   
   C   
   G   
   T   
   T   
   A   
   T   
   A   
   C   
   T   
   G   
   A   
   A   
   G   
   C   
   C   
   A   
   G   
    A    
   T   
   T   
   A   
   T   
   C   
   C   
   A   
   A   
   G   
   T   
   T   
   A   
   A   
   G   
   T   
   G   
   G   
   A   
   G   
   A   
   G   
   A   
   T   
   G   
    C    
   T   
   A   
    G    
   C   
   A   
   G   
   A   
   T   
   A   
   T   
   C   
   G   
   A   
    A    
   A   
   A   
   A   
   G   
   A   
   A   
   A   
   C   
   A   
   G   
   T   
    C    
   G   
   A   
   T   
   C   
   T   
   C   
   G   
   T   
   G   
   T   
   G   
   G   
   A   
   A   
   T   
   T   
   T   
   T   
   G   
   A   
   T   
   G   
   A   
   T   
   A   
   C   
   A   
   G   
   A   
   A   
   A   
   A   
   A   
   G   
   A   
   A   
   C   
   C   
   A   
   A   
   C   
   A   
   G   
   T   
   C   
   C   
   T   
   T   
   C   
   C   
   A   
   G   
   C   
   T   
   C   
   G   
   A   
   T   
   A   
   C   
   C   
   C   
   A   
   A   
   A   
   C   
   C   
   T   
   T   
   C   
   T   
    C    
   G   
   T   
   C   
   A   
   A   
   T   
   G   
   G   
   A   
   T   
   C   
   G   
   A   
   C   
   A   
   G   
   G   
   T   
   A   
   T   
   A   
   T   
   C   
   T   
   G   
   C   
   T   
   G   
   G   
   A   
   T   
   A   
   T   
   G   
   C   
    A    
   A   
   C   
   A   
   G   
   A   
   A   
   A   
   T   
   C   
   C   
   C   
   G   
   A   
   C   
    T    
   C   
   A   
   T   
   A   
   A   
   C   
   T   
   T   
   A   
   G   
   C   
   T   
   G   
   A   
   A   
   G   
   T   
   G   
   A   
   T   
    C    
   G   
   A   
    T    
   G   
   G   
   C   
   A   
   C   
   A   
   A   
   T   
   C   
   T   
   A   
   T   
   A   
   T   
   G   
   A   
   T   
   C   
   G   
   A   
   T   
   C   
   A   
   T   
   C   
   C   
   A   
   C   
   A   
   A   
   G   
   C   
   G   
   A   
   G   
   C   
   C   
   T   
   T   
   G   
   A   
   A   
   A   
   A   
   A   
   T   
   T   
   G   
   A   
   T   
   G   
   G   
   A   
   A   
   T   
   A   
   T   
   A   
   T   
   T   
   C   
   C   
   T   
   G   
   G   
   A   
   C   
   C   
   T   
   G   
   A   
   C   
   T   
   T   
    T    
   C   
   C   
   A   
   A   
   C   
   A   
   G   
   G   
   A   
   G   
   G   
    G    
   A   
   T   
   C   
   T   
   T   
   G   
   C   
   A   
   A   
   G   
   G   
    A    
   A   
   A   
   A   
   G   
   A   
   T   
   G   
   A   
   A   
   A   
   T   
   C   
   A   
   A   
   A   
   A   
   A   
   A   
   G   
   C   
   T   
   T   
   A   
   T   
   G   
   A   
   A   
   A   
   C   
   A   
   G   
   G   
    G    
   C   
   G   
   A   
   G   
   G   
   A   
   A   
   A   
   A   
   G   
   T   
   T   
   A   
    T    
   T   
   C   
   T   
   T   
   C   
   G   
   T   
   T   
   C   
   G   
   A   
   A   
   A   
   A   
   C   
   A   
   A   
   A   
   G   
   A   
   T   
   A   
   G   
   A   
    A    
   G   
   C   
   A   
   A   
   T   
   G   
   A   
   A   
   G   
   G   
   G   
    T    
   A   
   A   
    T    
   A   
   A   
   A   
   C   
   A   
   G   
   C   
   A   
   A   
   A   
   T   
   C   
   G   
   T   
   G   
   A   
   T   
   C   
   A   
   C   
    A    
   G   
   A   
   A   
   A   
   T   
   C   
   C   
   C   
   A   
   T   
   A   
   T   
   G   
   A   
   A   
   G   
   T   
   A   
   A   
   A   
   T   
   A   
   A   
   A   
   G   
   C   
   G   
   A   
   C   
    C    
   T   
   T   
   G   
   G   
   T   
   G   
   A   
   A   
   A   
   A   
   A   
   A   
   A   
   T   
   G   
   G   
   A   
   T   
   G   
   A   
   G   
   A   
   T   
   C   
   C   
   G   
   T   
   T   
   T   
   G   
   A   
   A   
   T   
   A   
   A   
   G   
   A   
   A   
   A   
   A   
   T   
   C   
   G   
   A   
    T    
   G   
   G   
   A   
   A   
   T   
   A   
   G   
   C   
   T   
   G   
   A   
   A   
   G   
   T   
    A    
   C   
   G   
   A   
   G   
   A   
   T   
   G   
   A   
   A   
   A   
   G   
   T   
   G   
   A   
   T   
    A    
   G   
    A    
   A   
   C   
    A    
   G   
   G   
   T   
   C   
   T   
   G   
   C   
   A   
   A   
   A   
   T   
   T   
   G   
   T   
   C   
   G   
   T   
    C    
   G   
   A   
   A   
   T   
   T   
   G   
   A   
   A   
   A   
   A   
   A   
   A   
   G   
   A   
    C    
   G   
   C   
   C   
   A   
   A   
    T    
   G   
   C   
   T   
   C   
   A   
   A   
   G   
   G   
    A    
   A   
   T   
   T   
   T   
   T   
   G   
   A   
   A   
   T   
   T   
   A   
    T    
   T   
   T   
   G   
   T   
   T   
   C   
   A   
   A   
   A   
   A   
   A   
   C   
   A   
   C   
   T   
   G   
   A   
   G   
    C    
   T   
   A   
   C   
   A   
   G   
   A   
   T   
   C   
   A   
   A   
   T   
   T   
   A   
   C   
   A   
   A   
   T   
   T   
   T   
    C    
   A   
   A   
   T   
   A   
   T   
   G   
   G   
   T   
   C   
   G   
   C   
   T   
   A   
   T   
   C   
   G   
   A   
    C    
   C   
   A   
   T   
   A   
   T   
   G   
   A   
   C   
   T   
   C   
   C   
   G   
   C   
   A   
    T    
   C   
   A   
   A   
   G   
   T   
   T   
   G   
   G   
   A   
   C   
   T   
   G   
   A   
   A   
   A   
   G   
   A   
   C   
   A   
   T   
   T   
   T   
   T   
   A   
   A   
   G   
   A   
   A   
   G   
   T   
   T   
   A   
   T   
   A   
   T   
   C   
   G   
   A   
   G   
   C   
   A   
   T   
   C   
   G   
   A   
   A   
   A   
   G   
   C   
   A   
   A   
   G   
   T   
   C   
   A   
   T   
   A   
   A   
   C   
   A   
   A   
   A   
   A   
   C   
   G   
    T    
   A   
   G   
    T    
   C   
   A   
   A   
   T   
   T   
   C   
   G   
   A   
   T   
   T   
   T   
   A   
   G   
   C   
   C   
   A   
   A   
   A   
   G   
   C   
   T   
   C   
   A   
   A   
   A   
   A   
   A   
    A    
   G   
   A   
   C   
   A   
   G   
   C   
   A   
   T   
   A   
   T   
   C   
   G   
   T   
    A    
   G   
   A   
   A   
   G   
   G   
    A    
    T    
   T   
   G   
   A   
   T   
   G   
   A   
   A   
   A   
   G   
   C   
   T   
   T   
   T   
   A   
   T   
   C   
   G   
   A   
   T   
   T   
   C   
   T   
   G   
   G   
   A   
   T   
   G   
   A   
   A   
   G   
   T   
   A   
   A   
   T   
   C   
   G   
    C    
   A   
   A   
   C   
   G   
   A   
   T   
   T   
   C   
   G   
   T   
   G   
   A   
   A   
   A   
   G   
   T   
   A   
   A   
   A   
   G   
   A   
   C   
   A   
   A   
   A   
   A   
   A   
   A   
   G   
   A   
    C    
   G   
   C   
    C    
   A   
   A   
   A   
   A   
   A   
   G   
   A   
   A   
   T   
   T   
   T   
   A   
   G   
   T   
   C   
   G   
   A   
    C    
   G   
   T   
    C    
   T   
   T   
    T    
   C   
   A   
   A   
   T   
   T   
   T   
   A   
   C   
   A   
   G   
   A   
   A   
   G   
   A   
   A   
   C   
   A   
   A   
   G   
   C   
   A   
   G   
   A   
   A   
   G   
   C   
   A   
   A   
   T   
    T    
   G   
   T   
   C   
   A   
   C   
   A   
   T   
   T   
   G   
   C   
   A   
   A   
    T    
   T   
   A   
   T   
   A   
    C    
   C   
   G   
   T   
   T   
   T   
   G   
   A   
   C   
   G   
   A   
   A   
    C    
   A   
   C   
    G    
   G   
   A   
   T   
   A   
   T   
   C   
   A   
   C   
   A   
   G   
   A   
   G   
   C   
   T   
   T   
   C   
   A   
   A   
   A   
   A   
   G   
   G   
   A   
   A   
    T    
   C   
    C    
   G   
   A   
   A   
   A   
    G    
   C   
   T   
   T   
   G   
   A   
   T   
   T   
   G   
   C   
    G    
   C   
   A   
   A   
   A   
   T   
   C   
   A   
   C   
   A   
   G   
   A   
   G   
   T   
   T   
    G    
   A   
   A   
   T   
   A   
   A   
   A   
   A   
   T   
   C   
   C   
   T   
   T   
   T   
   C   
   A   
   A   
   A   
    T    
   G   
   A   
   C   
   A   
   A   
   A   
   G   
   A   
    G    
   C   
   T   
   T   
   T   
   T   
    C    
   T   
   C   
   T   
   G   
   T   
   T   
   A   
   T   
   G   
   A   
   A   
   A   
   A   
   A   
   A   
   G   
   A   
   A   
   T   
   T   
   G   
   C   
   G   
   A   
   G   
   A   
   A   
   G   
   T   
   G   
   A   
   A   
   A   
   A   
   A   
   A   
   A   
   A   
   T   
   T   
   A   
    T    
   T   
   C   
   T   
   T   
   C   
   C   
   G   
   C   
   T   
   C   
   G   
   T   
   C   
    T    
    A    
   A   
   C   
   G   
   A   
   C   
   A   
   A   
   T   
   C   
    G    
   A   
   A   
   G   
   A   
   C   
   G   
   A   
   A   
   A   
   T   
   C   
   G   
   A   
   A   
   G   
   A   
   A   
   A   
   T   
   C   
   A   
   A   
   A   
   A   
   T   
    C    
   G   
   A   
   T   
   A   
   C   
   G   
   C   
   A   
   A   
    G    
   T   
    T    
   T   
   T   
   A   
   G   
   T   
   G   
   G   
   C   
    G    
   C   
   A   
   A   
   G   
   A   
   A   
   G   
   A   
   C   
   G   
   T   
   A   
   A   
   T   
    C    
   G   
   T   
   C   
   A   
   G   
   T   
   G   
   T   
    G    
   A   
   C   
    G    
   C   
   G   
   A   
   G   
   A   
   A   
   G   
   G   
   C   
   T   
   A   
   T   
   G   
   T   
   G   
   A   
   A   
   G   
   C   
   G   
   A   
   A   
   C   
   A   
   A   
   G   
   C   
   C   
   T   
   T   
   C   
   G   
   T   
   T   
   C   
    T    
   T   
   A   
   T   
   A   
   G   
   T   
   G   
   C   
   T   
   T   
   C   
    G    
   A   
   A   
   A   
   C   
   C   
   A   
   G   
   A   
   A   
   G   
   A   
   A   
   A   
   T   
   C   
   G   
   G   
   G   
   A   
   T   
   G   
   C   
   G   
   A   
   G   
   A   
   A   
   G   
   G   
   C   
   G   
   A   
    C    
   T   
   A   
   C   
   T   
   T   
   G   
   C   
   T   
    G    
   T   
   A   
   C   
    T    
   C   
   A   
   G   
   G   
   T   
   G   
   A   
   A   
   T   
   T   
    G    
   A   
   G   
    C    
   A   
   C   
   A   
    T    
   T   
   G   
   G   
   A   
   C   
   C   
   A   
   T   
   G   
   T   
   T   
   C   
   T   
   T   
   C   
   T   
   G   
   A   
   T   
    T    
   A   
   C   
    G    
   A   
   A   
   T   
   A   
   A   
   A   
   G   
   C   
   A   
   A   
   A   
   T   
   G   
   T   
   G   
   A   
   T   
   C   
   T   
   A   
   C   
   C   
   G   
    T    
   C   
   C   
   T   
   G   
   T   
    T    
   C   
   A   
   T   
   G   
   A   
   A   
   C   
   T   
   A   
   C   
   C   
    C    
   G   
   A   
   T   
   T   
   T   
   G   
   A   
   A   
   A   
   T   
   G   
   G   
   A   
   A   
   A   
   G   
   A   
   T   
   G   
   C   
   T   
   G   
   G   
    C    
   G   
   A   
    A    
   C   
   A   
    T    
   A   
   T   
   T   
   T   
   C   
   T   
   C   
   A   
   A   
   A   
   C   
   A   
   A   
   T   
   C   
   T   
   C   
   C   
   A   
   A   
   T   
   C   
   T   
   G   
   T   
   C   
   T   
   G   
   T   
   A   
   G   
   A   
    T    
   G   
   A   
   A   
   T   
   C   
   G   
   A   
   T   
    C    
   T   
   T   
   A   
   G   
   C   
   T   
   G   
   T   
   T   
   T   
   T   
    T    
   C   
   C   
   A   
   T   
   A   
   T   
   C   
   A   
   A   
   A   
   A   
   A   
   A   
   T   
    T    
   C   
   A   
   A   
   G   
   C   
   A   
   G   
   A   
    G    
   A   
   A   
   A   
   A   
   C   
   A   
   T   
   T   
   T   
   G   
   T   
    A    
   T   
   T   
   C   
   A   
   T   
   C   
   A   
   G   
   C   
   A   
   A   
   G   
   A   
   A   
   T   
   G   
   G   
   A   
   T   
   T   
    G    
    A    
   T   
   C   
   A   
   A   
   G   
   C   
   A   
   A   
   A   
   C   
   A   
   C   
   G   
   A   
   A   
   T   
   G   
   A   
   C   
   A   
   G   
   A   
   G   
   T   
   T   
   C   
   G   
   A   
   A   
   C   
   C   
   A   
   T   
   G   
   G   
   C   
   G   
   C   
   A   
   C   
   C   
   T   
   A   
    T    
   A   
   A   
   G   
   A   
   G   
   C   
   C   
   G   
    T    
   C   
   C   
   G   
   C   
   T   
   C   
   A   
   G   
   C   
   T   
   G   
   T   
   A   
   T   
   G   
   A   
   A   
   G   
   C   
   T   
   A   
   A   
   A   
   G   
   G   
   C   
   G   
   G   
   A   
   A   
   G   
   A   
   A   
   G   
   A   
   T   
   G   
   A   
   A   
   C   
   T   
    G    
   A   
   T   
   T   
   G   
   C   
   T   
   G   
   T   
    G    
   T   
   A   
   T   
   C   
   T   
   G   
   A   
   A   
   A   
   G   
   A   
   A   
   G   
   A   
   T   
   C   
    G    
   G   
   C   
   C   
   A   
   G   
   G   
   C   
   T   
   T   
   A   
   G   
   A   
   T   
   G   
   T   
    T    
   T   
   T   
   C   
   T   
   T   
   A   
   G   
   T   
   A   
   A   
   C   
   G   
    T    
   A   
   T   
   C   
   A   
   A   
   G   
   G   
   A   
   A   
   T   
   G   
   G   
   G   
    T    
   T   
   T   
   G   
   C   
   G   
   C   
   T   
    A    
   T   
   C   
   C   
   T   
   T   
   T   
   A   
   A   
    C    
   A   
   G   
   A   
   T   
   G   
   T   
   T   
   C   
   C   
   C   
   G   
   T   
   T   
   G   
   T   
   T   
   G   
   G   
   A   
   G   
   C   
   A   
   A   
   A   
   G   
   G   
   C   
   A   
   G   
    C    
   T   
   G   
   G   
   A   
   G   
   T   
   G   
   A   
   A   
   G   
   T   
   C   
   T   
   A   
   T   
   G   
   A   
   A   
   T   
   T   
   T   
   G   
   A   
   A   
   A   
   G   
   A   
   T   
   G   
   G   
   A   
   G   
   A   
   T   
   A   
   G   
   G   
    A    
   T   
   T   
    A    
   T   
   C   
   A   
   A   
    C    
   G   
   G   
    G    
   C   
   T   
    G    
   C   
   T   
   T   
   G   
   T   
   G   
   T   
   T   
   A   
   G   
   C   
   A   
   G   
   A   
    A    
   G   
   G   
    T    
   G   
   A   
   T   
   A   
   C   
   G   
   C   
   C   
    G    
   A   
   T   
   C   
   G   
   T   
   A   
   A   
   T   
   C   
   G   
   T   
   T   
   A   
   C   
   T   
   C   
   A   
   G   
   C   
   G   
   A   
   G   
   G   
   A   
   G   
   C   
    T    
   G   
   T   
    T    
   A   
   A   
   A   
   C   
   G   
   C   
   A   
   T   
   G   
   C   
   T   
   T   
   G   
   C   
   T   
   C   
   A   
   A   
   G   
   A   
   A   
   A   
   T   
   C   
   A   
   G   
   C   
   C   
   A   
   A   
   A   
   C   
    T    
   T   
   C   
   A   
   C   
   G   
   T   
   G   
   C   
   G   
   A   
   A   
   G   
   C   
   G   
   G   
   G   
   G   
   A   
   G   
   T   
   G   
   A   
   C   
   G   
   G   
   T   
   C   
   A   
   T   
   G   
   C   
   G   
   T   
   G   
   A   
   G   
   C   
   T   
   G   
   A   
   A   
   A   
   A   
   A   
   A   
   A   
   A   
   T   
   C   
   C   
    T    
   C   
   A   
    T    
   C   
   G   
    C    
   A   
   T   
   T   
   A   
   T   
   C   
   T   
   A   
   T   
   A   
   T   
   G   
   T   
   C   
   G   
   G   
   A   
   A   
   G   
   G   
   A   
   A   
   G   
   A   
   T   
   C   
   A   
   A   
   A   
   A   
   G   
   A   
   A   
   A   
   T   
   A   
   A   
   C   
   C   
   T   
   T   
   G   
   A   
   T   
   G   
   A   
   A   
   T   
   C   
   A   
   A   
   A   
   A   
   A   
   G   
   G   
    G    
   C   
   A   
    G    
   C   
   A   
   G   
    T    
   T   
   A   
   A   
   C   
   G   
   A   
   T   
   T   
   G   
   A   
    C    
   C   
   C   
   A   
   A   
   C   
    T    
   G   
   A   
   T   
   T   
   T   
    C    
   C   
   C   
   T   
   A   
   T   
   A   
   G   
   G   
   T   
   G   
   A   
   T   
   C   
   G   
   A   
   A   
   C   
   A   
   T   
   C   
   G   
   A   
   A   
   T   
   G   
   G   
    A    
   T   
   C   
    A    
   T   
   T   
   T   
   G   
   C   
    G    
   A   
   T   
   G   
   G   
   A   
    T    
   G   
   A   
   G   
   A   
   A   
   A   
   A   
   A   
   A   
   G   
   G   
   T   
   G   
   G   
   A   
   G   
   A   
   A   
   G   
   T   
   C   
   A   
   T   
   C   
   G   
   A   
   A   
   G   
   T   
    T    
   A   
    T    
   C   
   G   
   A   
   T   
   G   
   C   
   T   
   C   
   C   
   T   
   G   
   A   
   G   
    A    
   T   
   T   
   T   
   T   
    C    
   A   
   T   
    C    
   G   
   A   
    G    
    T    
   C   
    G    
   -   
   -   
   -   
 

 
   N59589F   
   A   
   T   
   G   
   G   
   A   
   A   
   A   
   A   
   T   
   C   
   G   
   A   
   C   
   A   
   A   
   G   
   A   
   A   
   G   
   T   
   A   
   C   
   A   
   A   
   G   
   A   
   A   
   T   
   T   
   A   
   A   
   C   
   T   
   T   
   T   
   A   
   G   
   A   
   A   
   G   
   A   
   A   
   G   
   T   
   G   
   A   
   T   
   G   
   G   
   G   
   T   
   G   
   A   
   C   
   C   
   G   
   C   
   T   
   T   
   C   
   G   
   G   
   A   
   C   
   G   
   T   
   T   
   A   
   T   
   T   
   C   
   C   
   A   
   A   
   A   
   T   
   A   
   T   
   A   
   T   
   T   
   A   
   T   
    C    
   C   
   A   
    A    
   G   
   A   
   A   
   C   
   G   
   T   
   G   
   C   
   A   
   C   
   T   
   G   
   C   
   C   
   T   
   G   
   A   
   T   
   A   
   T   
    C    
   C   
   G   
   T   
   G   
   A   
   T   
   G   
   G   
   A   
   T   
   T   
   G   
   A   
   A   
    A    
   C   
   C   
   A   
   G   
   T   
    G    
   C   
   A   
   A   
   C   
   G   
   C   
   C   
   G   
   G   
   A   
   T   
   A   
   T   
   T   
   A   
   T   
   T   
    T    
   G   
   C   
    C    
   A   
   T   
   G   
   A   
   A   
   C   
   A   
   A   
   A   
   G   
   A   
   T   
   G   
   G   
   C   
   A   
   A   
   T   
   A   
   C   
   G   
   T   
   A   
   T   
   G   
   A   
   C   
   A   
   A   
   A   
   G   
   G   
   A   
   T   
   T   
    C    
   C   
   G   
   T   
   A   
   A   
   A   
   T   
   C   
   A   
   G   
   C   
   A   
   A   
   A   
   A   
   T   
   C   
   T   
   G   
   T   
   C   
   G   
   G   
   A   
   A   
   A   
   T   
   A   
   T   
   C   
   A   
   T   
   G   
   G   
   G   
    G    
   A   
   A   
   T   
   T   
   A   
   T   
   C   
   A   
   T   
   C   
   C   
   T   
   C   
   A   
   T   
   G   
   G   
   T   
   G   
   A   
   C   
   A   
   G   
   C   
   A   
   G   
   T   
   A   
   T   
   T   
   T   
   A   
   T   
   G   
   A   
   A   
   G   
   C   
   G   
   A   
   T   
   G   
   G   
   T   
   C   
   C   
   G   
   C   
   A   
   T   
   G   
   A   
   G   
   C   
   C   
   A   
   A   
   G   
   A   
    T    
   T   
   G   
   G   
   A   
   A   
   G   
   T   
   T   
   G   
   C   
   G   
   G   
   G   
   A   
   A   
   G   
   T   
   A   
   C   
   T   
   G   
   A   
   T   
   C   
   G   
   A   
    A    
   A   
   T   
   G   
   C   
   A   
   C   
   G   
   G   
    G    
   A   
   A   
   T   
   A   
   A   
    T    
   G   
   G   
   G   
   A   
   G   
   T   
   A   
   T   
   G   
   G   
   A   
   C   
   G   
   G   
   T   
   G   
   A   
   T   
   C   
   C   
   A   
   C   
   C   
   T   
   G   
   C   
   T   
   G   
   C   
   G   
   A   
   T   
   G   
   C   
   G   
   T   
   T   
   A   
   T   
   A   
   C   
   T   
   G   
   A   
   A   
   G   
   C   
   C   
   A   
   G   
    A    
   T   
   T   
   A   
   T   
   C   
   C   
   A   
   A   
   G   
   T   
   T   
   A   
   A   
   G   
   T   
   G   
   G   
   A   
   G   
   A   
   G   
   A   
   T   
   G   
    C    
   T   
   A   
    G    
   C   
   A   
   G   
   A   
   T   
   A   
   T   
   C   
   G   
   A   
    A    
   A   
   A   
   A   
   G   
   A   
   A   
   A   
   C   
   A   
   G   
   T   
    C    
   G   
   A   
   T   
   C   
   T   
   C   
   G   
   T   
   G   
   T   
   G   
   G   
   A   
   A   
   T   
   T   
   T   
   T   
   G   
   A   
   T   
   G   
   A   
   T   
   A   
   C   
   A   
   G   
   A   
   A   
   A   
   A   
   A   
   G   
   A   
   A   
   C   
   C   
   A   
   A   
   C   
   A   
   G   
   T   
   C   
   C   
   T   
   T   
   C   
   C   
   A   
   G   
   C   
   T   
   C   
   G   
   A   
   T   
   A   
   C   
   C   
   C   
   A   
   A   
   A   
   C   
   C   
   T   
   T   
   C   
   T   
    A    
   G   
   T   
   C   
   A   
   A   
   T   
   G   
   G   
   A   
   T   
   C   
   G   
   A   
   C   
   A   
   G   
   G   
   T   
   A   
   T   
   A   
   T   
   C   
   T   
   G   
   C   
   T   
   G   
   G   
   A   
   T   
   A   
   T   
   G   
   C   
    G    
   A   
   C   
   A   
   G   
   A   
   A   
   A   
   T   
   C   
   C   
   C   
   G   
   A   
   C   
    T    
   C   
   A   
   T   
   A   
   A   
   C   
   T   
   T   
   A   
   G   
   C   
   T   
   G   
   A   
   A   
   G   
   T   
   G   
   A   
   T   
    C    
   G   
   A   
    C    
   G   
   G   
   C   
   A   
   C   
   A   
   A   
   T   
   C   
   T   
   A   
   T   
   A   
   T   
   G   
   A   
   T   
   C   
   G   
   A   
   T   
   C   
   A   
   T   
   C   
   C   
   A   
   C   
   A   
   A   
   G   
   C   
   G   
   A   
   G   
   C   
   C   
   T   
   T   
   G   
   A   
   A   
   A   
   A   
   A   
   T   
   T   
   G   
   A   
   T   
   G   
   G   
   A   
   A   
   T   
   A   
   T   
   A   
   T   
   T   
   C   
   C   
   T   
   G   
   G   
   A   
   C   
   C   
   T   
   G   
   A   
   C   
   T   
   T   
    T    
   C   
   C   
   A   
   A   
   C   
   A   
   G   
   G   
   A   
   G   
   G   
    G    
   A   
   T   
   C   
   T   
   T   
   G   
   C   
   A   
   A   
   G   
   G   
    G    
   A   
   A   
   A   
   G   
   A   
   T   
   G   
   A   
   A   
   A   
   T   
   C   
   A   
   A   
   A   
   A   
   A   
   A   
   G   
   C   
   T   
   T   
   A   
   T   
   G   
   A   
   A   
   A   
   C   
   A   
   G   
   G   
    G    
   C   
   G   
   A   
   G   
   G   
   A   
   A   
   A   
   A   
   G   
   T   
   T   
   A   
    T    
   T   
   C   
   T   
   T   
   C   
   G   
   T   
   T   
   C   
   G   
   A   
   A   
   A   
   A   
   C   
   A   
   A   
   A   
   G   
   A   
   T   
   A   
   G   
   A   
    A    
   G   
   C   
   A   
   A   
   T   
   G   
   A   
   A   
   G   
   G   
   G   
    C    
   A   
   A   
    T    
   A   
   A   
   A   
   C   
   A   
   G   
   C   
   A   
   A   
   A   
   T   
   C   
   G   
   T   
   G   
   A   
   T   
   C   
   A   
   C   
    T    
   G   
   A   
   A   
   A   
   T   
   C   
   C   
   C   
   A   
   T   
   A   
   T   
   G   
   A   
   A   
   G   
   T   
   A   
   A   
   A   
   T   
   A   
   A   
   A   
   G   
   C   
   G   
   A   
   C   
    G    
   T   
   T   
   G   
   G   
   T   
   G   
   A   
   A   
   A   
   A   
   A   
   A   
   A   
   T   
   G   
   G   
   A   
   T   
   G   
   A   
   G   
   A   
   T   
   C   
   C   
   G   
   T   
   T   
   T   
   G   
   A   
   A   
   T   
   A   
   A   
   G   
   A   
   A   
   A   
   A   
   T   
   C   
   G   
   A   
    T    
   G   
   G   
   A   
   A   
   T   
   A   
   G   
   C   
   T   
   G   
   A   
   A   
   G   
   T   
    A    
   C   
   G   
   A   
   G   
   A   
   T   
   G   
   A   
   A   
   A   
   G   
   T   
   G   
   A   
   T   
    C    
   G   
    A    
   A   
   C   
    T    
   G   
   G   
   T   
   C   
   T   
   G   
   C   
   A   
   A   
   A   
   T   
   T   
   G   
   T   
   C   
   G   
   T   
    T    
   G   
   A   
   A   
   T   
   T   
   G   
   A   
   A   
   A   
   A   
   A   
   A   
   G   
   A   
    T    
   G   
   C   
   C   
   A   
   A   
    T    
   G   
   C   
   T   
   C   
   A   
   A   
   G   
   G   
    A    
   A   
   T   
   T   
   T   
   T   
   G   
   A   
   A   
   T   
   T   
   A   
    T    
   T   
   T   
   G   
   T   
   T   
   C   
   A   
   A   
   A   
   A   
   A   
   C   
   A   
   C   
   T   
   G   
   A   
   G   
    C    
   T   
   A   
   C   
   A   
   G   
   A   
   T   
   C   
   A   
   A   
   T   
   T   
   A   
   C   
   A   
   A   
   T   
   T   
   T   
    C    
   A   
   A   
   T   
   A   
   T   
   G   
   G   
   T   
   C   
   G   
   C   
   T   
   A   
   T   
   C   
   G   
   A   
    T    
   C   
   A   
   T   
   A   
   T   
   G   
   A   
   C   
   T   
   C   
   C   
   G   
   C   
   A   
    C    
   C   
   A   
   A   
   G   
   T   
   T   
   G   
   G   
   A   
   C   
   T   
   G   
   A   
   A   
   A   
   G   
   A   
   C   
   A   
   T   
   T   
   T   
   T   
   A   
   A   
   G   
   A   
   A   
   G   
   T   
   T   
   A   
   T   
   A   
   T   
   C   
   G   
   A   
   G   
   C   
   A   
   T   
   C   
   G   
   A   
   A   
   A   
   G   
   C   
   A   
   A   
   G   
   T   
   C   
   A   
   T   
   A   
   A   
   C   
   A   
   A   
   A   
   A   
   C   
   G   
    C    
   A   
   G   
    C    
   C   
   A   
   A   
   T   
   T   
   C   
   G   
   A   
   T   
   T   
   T   
   A   
   G   
   C   
   C   
   A   
   A   
   A   
   G   
   C   
   T   
   C   
   A   
   A   
   A   
   A   
   A   
    A    
   G   
   A   
   C   
   A   
   G   
   C   
   A   
   T   
   A   
   T   
   C   
   G   
   T   
    G    
   G   
   A   
   A   
   G   
   G   
    C    
    C    
   T   
   G   
   A   
   T   
   G   
   A   
   A   
   A   
   G   
   C   
   T   
   T   
   T   
   A   
   T   
   C   
   G   
   A   
   T   
   T   
   C   
   T   
   G   
   G   
   A   
   T   
   G   
   A   
   A   
   G   
   T   
   A   
   A   
   T   
   C   
   G   
    C    
   A   
   A   
   C   
   G   
   A   
   T   
   T   
   C   
   G   
   T   
   G   
   A   
   A   
   A   
   G   
   T   
   A   
   A   
   A   
   G   
   A   
   C   
   A   
   A   
   A   
   A   
   A   
   A   
   G   
   A   
    T    
   G   
   C   
    T    
   A   
   A   
   A   
   A   
   A   
   G   
   A   
   A   
   T   
   T   
   T   
   A   
   G   
   T   
   C   
   G   
   A   
    T    
   G   
   T   
    T    
   T   
   T   
    C    
   C   
   A   
   A   
   T   
   T   
   T   
   A   
   C   
   A   
   G   
   A   
   A   
   G   
   A   
   A   
   C   
   A   
   A   
   G   
   C   
   A   
   G   
   A   
   A   
   G   
   C   
   A   
   A   
   T   
    C    
   G   
   T   
   C   
   A   
   C   
   A   
   T   
   T   
   G   
   C   
   A   
   A   
    C    
   T   
   A   
   T   
   A   
    C    
   C   
   G   
   T   
   T   
   T   
   G   
   A   
   C   
   G   
   A   
   A   
    C    
   A   
   C   
    A    
   G   
   A   
   T   
   A   
   T   
   C   
   A   
   C   
   A   
   G   
   A   
   G   
   C   
   T   
   T   
   C   
   A   
   A   
   A   
   A   
   G   
   G   
   A   
   A   
    G    
   C   
    C    
   G   
   A   
   A   
   A   
    G    
   C   
   T   
   T   
   G   
   A   
   T   
   T   
   G   
   C   
    A    
   C   
   A   
   A   
   A   
   T   
   C   
   A   
   C   
   A   
   G   
   A   
   G   
   T   
   T   
    G    
   A   
   A   
   T   
   A   
   A   
   A   
   A   
   T   
   C   
   C   
   T   
   T   
   T   
   C   
   A   
   A   
   A   
    C    
   G   
   A   
   C   
   A   
   A   
   A   
   G   
   A   
    G    
   C   
   T   
   T   
   T   
   T   
    C    
   T   
   C   
   T   
   G   
   T   
   T   
   A   
   T   
   G   
   A   
   A   
   A   
   A   
   A   
   A   
   G   
   A   
   A   
   T   
   T   
   G   
   C   
   G   
   A   
   G   
   A   
   A   
   G   
   T   
   G   
   A   
   A   
   A   
   A   
   A   
   A   
   A   
   A   
   T   
   T   
   A   
    C    
   T   
   C   
   T   
   T   
   C   
   C   
   G   
   C   
   T   
   C   
   G   
   T   
   C   
    T    
    G    
   A   
   C   
   G   
   A   
   C   
   A   
   A   
   T   
   C   
    C    
   A   
   A   
   G   
   A   
   C   
   G   
   A   
   A   
   A   
   T   
   C   
   G   
   A   
   A   
   G   
   A   
   A   
   A   
   T   
   C   
   A   
   A   
   A   
   A   
   T   
    C    
   G   
   A   
   T   
   A   
   C   
   G   
   C   
   A   
   A   
    G    
   T   
    T    
   T   
   T   
   A   
   G   
   T   
   G   
   G   
   C   
    G    
   C   
   A   
   A   
   G   
   A   
   A   
   G   
   A   
   C   
   G   
   T   
   A   
   A   
   T   
    C    
   G   
   T   
   C   
   A   
   G   
   T   
   G   
   T   
    G    
   A   
   C   
    G    
   C   
   G   
   A   
   G   
   A   
   A   
   G   
   G   
   C   
   T   
   A   
   T   
   G   
   T   
   G   
   A   
   A   
   G   
   C   
   G   
   A   
   A   
   C   
   A   
   A   
   G   
   C   
   C   
   T   
   T   
   C   
   G   
   T   
   T   
   C   
    T    
   T   
   A   
   T   
   A   
   G   
   T   
   G   
   C   
   T   
   T   
   C   
    G    
   A   
   A   
   A   
   C   
   C   
   A   
   G   
   A   
   A   
   G   
   A   
   A   
   A   
   T   
   C   
   G   
   G   
   G   
   A   
   T   
   G   
   C   
   G   
   A   
   G   
   A   
   A   
   G   
   G   
   C   
   G   
   A   
    T    
   T   
   A   
   C   
   T   
   T   
   G   
   C   
   T   
    G    
   T   
   A   
   C   
    T    
   C   
   A   
   G   
   G   
   T   
   G   
   A   
   A   
   T   
   T   
    G    
   A   
   G   
    C    
   A   
   C   
   A   
    T    
   T   
   G   
   G   
   A   
   C   
   C   
   A   
   T   
   G   
   T   
   T   
   C   
   T   
   T   
   C   
   T   
   G   
   A   
   T   
    T    
   A   
   C   
    G    
   A   
   A   
   T   
   A   
   A   
   A   
   G   
   C   
   A   
   A   
   A   
   T   
   G   
   T   
   G   
   A   
   T   
   C   
   T   
   A   
   C   
   C   
   G   
    T    
   C   
   C   
   T   
   G   
   T   
    T    
   C   
   A   
   T   
   G   
   A   
   A   
   C   
   T   
   A   
   C   
   C   
    C    
   G   
   A   
   T   
   T   
   T   
   G   
   A   
   A   
   A   
   T   
   G   
   G   
   A   
   A   
   A   
   G   
   A   
   T   
   G   
   C   
   T   
   G   
   G   
    C    
   G   
   A   
    A    
   C   
   A   
    C    
   A   
   T   
   T   
   T   
   C   
   T   
   C   
   A   
   A   
   A   
   C   
   A   
   A   
   T   
   C   
   T   
   C   
   C   
   A   
   A   
   T   
   C   
   T   
   G   
   T   
   C   
   T   
   G   
   T   
   A   
   G   
   A   
    T    
   G   
   A   
   A   
   T   
   C   
   G   
   A   
   T   
    C    
   T   
   T   
   A   
   G   
   C   
   T   
   G   
   T   
   T   
   T   
   T   
    C    
   C   
   C   
   A   
   T   
   A   
   T   
   C   
   A   
   A   
   A   
   A   
   A   
   A   
   T   
    C    
   C   
   A   
   A   
   G   
   C   
   A   
   G   
   A   
    A    
   A   
   A   
   A   
   A   
   C   
   A   
   T   
   T   
   T   
   G   
   T   
    A    
   T   
   T   
   C   
   A   
   T   
   C   
   A   
   G   
   C   
   A   
   A   
   G   
   A   
   A   
   T   
   G   
   G   
   A   
   T   
   T   
    G    
    A    
   T   
   C   
   A   
   A   
   G   
   C   
   A   
   A   
   A   
   C   
   A   
   C   
   G   
   A   
   A   
   T   
   G   
   A   
   C   
   A   
   G   
   A   
   G   
   T   
   T   
   C   
   G   
   A   
   A   
   C   
   C   
   A   
   T   
   G   
   G   
   C   
   G   
   C   
   A   
   C   
   C   
   T   
   A   
    T    
   A   
   A   
   G   
   A   
   G   
   C   
   C   
   G   
    T    
   C   
   C   
   G   
   C   
   T   
   C   
   A   
   G   
   C   
   T   
   G   
   T   
   A   
   T   
   G   
   A   
   A   
   G   
   C   
   T   
   A   
   A   
   A   
   G   
   G   
   C   
   G   
   G   
   A   
   A   
   G   
   A   
   A   
   G   
   A   
   T   
   G   
   A   
   A   
   C   
   T   
    T    
   A   
   T   
   T   
   G   
   C   
   T   
   G   
   T   
    A    
   T   
   A   
   T   
   C   
   T   
   G   
   A   
   A   
   A   
   G   
   A   
   A   
   G   
   A   
   T   
   C   
    A    
   G   
   C   
   C   
   A   
   G   
   G   
   C   
   T   
   T   
   A   
   G   
   A   
   T   
   G   
   T   
    G    
   T   
   T   
   C   
   T   
   T   
   A   
   G   
   T   
   A   
   A   
   C   
   G   
    C    
   A   
   T   
   C   
   A   
   A   
   G   
   G   
   A   
   A   
   T   
   G   
   G   
   G   
    C    
   T   
   T   
   G   
   C   
   G   
   C   
   T   
    A    
   T   
   C   
   C   
   T   
   T   
   T   
   A   
   A   
    C    
   A   
   G   
   A   
   T   
   G   
   T   
   T   
   C   
   C   
   C   
   G   
   T   
   T   
   G   
   T   
   T   
   G   
   G   
   A   
   G   
   C   
   A   
   A   
   A   
   G   
   G   
   C   
   A   
   G   
    C    
   T   
   G   
   G   
   A   
   G   
   T   
   G   
   A   
   A   
   G   
   T   
   C   
   T   
   A   
   T   
   G   
   A   
   A   
   T   
   T   
   T   
   G   
   A   
   A   
   A   
   G   
   A   
   T   
   G   
   G   
   A   
   G   
   A   
   T   
   A   
   G   
   G   
    A    
   T   
   T   
    G    
   T   
   C   
   A   
   A   
    T    
   G   
   G   
    G    
   C   
   T   
    G    
   C   
   T   
   T   
   G   
   T   
   G   
   T   
   T   
   A   
   G   
   C   
   A   
   G   
   A   
    C    
   G   
   G   
    A    
   G   
   A   
   T   
   A   
   C   
   G   
   C   
   C   
    A    
   A   
   T   
   C   
   G   
   T   
   A   
   A   
   T   
   C   
   G   
   T   
   T   
   A   
   C   
   T   
   C   
   A   
   G   
   C   
   G   
   A   
   G   
   G   
   A   
   G   
   C   
    C    
   G   
   T   
    G    
   A   
   A   
   A   
   C   
   G   
   C   
   A   
   T   
   G   
   C   
   T   
   T   
   G   
   C   
   T   
   C   
   A   
   A   
   G   
   A   
   A   
   A   
   T   
   C   
   A   
   G   
   C   
   C   
   A   
   A   
   A   
   C   
    T    
   T   
   C   
   A   
   C   
   G   
   T   
   G   
   C   
   G   
   A   
   A   
   G   
   C   
   G   
   G   
   G   
   G   
   A   
   G   
   T   
   G   
   A   
   C   
   G   
   G   
   T   
   C   
   A   
   T   
   G   
   C   
   G   
   T   
   G   
   A   
   G   
   C   
   T   
   G   
   A   
   A   
   A   
   A   
   A   
   A   
   A   
   A   
   T   
   C   
   C   
    G    
   C   
   A   
    C    
   C   
   G   
    T    
   A   
   T   
   T   
   A   
   T   
   C   
   T   
   A   
   T   
   A   
   T   
   G   
   T   
   C   
   G   
   G   
   A   
   A   
   G   
   G   
   A   
   A   
   G   
   A   
   T   
   C   
   A   
   A   
   A   
   A   
   G   
   A   
   A   
   A   
   T   
   A   
   A   
   C   
   C   
   T   
   T   
   G   
   A   
   T   
   G   
   A   
   A   
   T   
   C   
   A   
   A   
   A   
   A   
   A   
   G   
   G   
    A    
   C   
   A   
    A    
   C   
   A   
   G   
    A    
   T   
   A   
   A   
   C   
   G   
   A   
   T   
   T   
   G   
   A   
    T    
   C   
   C   
   A   
   A   
   C   
    G    
   G   
   A   
   T   
   T   
   T   
    T    
   C   
   C   
   T   
   A   
   T   
   A   
   G   
   G   
   T   
   G   
   A   
   T   
   C   
   G   
   A   
   A   
   C   
   A   
   T   
   C   
   G   
   A   
   A   
   T   
   G   
   G   
    T    
   T   
   C   
    G    
   T   
   T   
   T   
   G   
   C   
    T    
   A   
   T   
   G   
   G   
   A   
    C    
   G   
   A   
   G   
   A   
   A   
   A   
   A   
   A   
   A   
   G   
   G   
   T   
   G   
   G   
   A   
   G   
   A   
   A   
   G   
   T   
   C   
   A   
   T   
   C   
   G   
   A   
   A   
   G   
   T   
    T    
   A   
    T    
   C   
   G   
   A   
   T   
   G   
   C   
   T   
   C   
   C   
   T   
   G   
   A   
   G   
    G    
   T   
   T   
   T   
   T   
    C    
   A   
   T   
    T    
   G   
   A   
    A    
    T    
   C   
    A    
   -   
   -   
   -   
 

 
   N54107   
   A   
   T   
   G   
   G   
   A   
   A   
   A   
   A   
   T   
   C   
   G   
   A   
   C   
   A   
   A   
   G   
   A   
   A   
   G   
   T   
   A   
   C   
   A   
   A   
   G   
   A   
   A   
   T   
   T   
   A   
   A   
   C   
   T   
   T   
   T   
   A   
   G   
   A   
   A   
   G   
   A   
   A   
   G   
   T   
   G   
   A   
   T   
   G   
   G   
   G   
   T   
   G   
   A   
   C   
   C   
   G   
   C   
   T   
   T   
   C   
   G   
   G   
   A   
   C   
   G   
   T   
   T   
   A   
   T   
   T   
   C   
   C   
   A   
   A   
   A   
   T   
   A   
   T   
   A   
   T   
   T   
   A   
   T   
    C    
   C   
   A   
    A    
   G   
   A   
   A   
   C   
   G   
   T   
   G   
   C   
   A   
   C   
   T   
   G   
   C   
   C   
   T   
   G   
   A   
   T   
   A   
   T   
    C    
   C   
   G   
   T   
   G   
   A   
   T   
   G   
   G   
   A   
   T   
   T   
   G   
   A   
   A   
    G    
   C   
   C   
   A   
   G   
   T   
    G    
   C   
   A   
   A   
   C   
   G   
   C   
   C   
   G   
   G   
   A   
   T   
   A   
   T   
   T   
   A   
   T   
   T   
    T    
   G   
   C   
    C    
   A   
   T   
   G   
   A   
   A   
   C   
   A   
   A   
   A   
   G   
   A   
   T   
   G   
   G   
   C   
   A   
   A   
   T   
   A   
   C   
   G   
   T   
   A   
   T   
   G   
   A   
   C   
   A   
   A   
   A   
   G   
   G   
   A   
   T   
   T   
    C    
   C   
   G   
   T   
   A   
   A   
   A   
   T   
   C   
   A   
   G   
   C   
   A   
   A   
   A   
   A   
   T   
   C   
   T   
   G   
   T   
   C   
   G   
   G   
   A   
   A   
   A   
   T   
   A   
   T   
   C   
   A   
   T   
   G   
   G   
   G   
    G    
   A   
   A   
   T   
   T   
   A   
   T   
   C   
   A   
   T   
   C   
   C   
   T   
   C   
   A   
   T   
   G   
   G   
   T   
   G   
   A   
   C   
   A   
   G   
   C   
   A   
   G   
   T   
   A   
   T   
   T   
   T   
   A   
   T   
   G   
   A   
   A   
   G   
   C   
   G   
   A   
   T   
   G   
   G   
   T   
   C   
   C   
   G   
   C   
   A   
   T   
   G   
   A   
   G   
   C   
   C   
   A   
   A   
   G   
   A   
    C    
   T   
   G   
   G   
   A   
   A   
   G   
   T   
   T   
   G   
   C   
   G   
   G   
   G   
   A   
   A   
   G   
   T   
   A   
   C   
   T   
   G   
   A   
   T   
   C   
   G   
   A   
    A    
   A   
   T   
   G   
   C   
   A   
   C   
   G   
   G   
    G    
   A   
   A   
   T   
   A   
   A   
    C    
   G   
   G   
   G   
   A   
   G   
   T   
   A   
   T   
   G   
   G   
   A   
   C   
   G   
   G   
   T   
   G   
   A   
   T   
   C   
   C   
   A   
   C   
   C   
   T   
   G   
   C   
   T   
   G   
   C   
   G   
   A   
   T   
   G   
   C   
   G   
   T   
   T   
   A   
   T   
   A   
   C   
   T   
   G   
   A   
   A   
   G   
   C   
   C   
   A   
   G   
    A    
   T   
   T   
   A   
   T   
   C   
   C   
   A   
   A   
   G   
   T   
   T   
   A   
   A   
   G   
   T   
   G   
   G   
   A   
   G   
   A   
   G   
   A   
   T   
   G   
    C    
   T   
   A   
    G    
   C   
   A   
   G   
   A   
   T   
   A   
   T   
   C   
   G   
   A   
    A    
   A   
   A   
   A   
   G   
   A   
   A   
   A   
   C   
   A   
   G   
   T   
    C    
   G   
   A   
   T   
   C   
   T   
   C   
   G   
   T   
   G   
   T   
   G   
   G   
   A   
   A   
   T   
   T   
   T   
   T   
   G   
   A   
   T   
   G   
   A   
   T   
   A   
   C   
   A   
   G   
   A   
   A   
   A   
   A   
   A   
   G   
   A   
   A   
   C   
   C   
   A   
   A   
   C   
   A   
   G   
   T   
   C   
   C   
   T   
   T   
   C   
   C   
   A   
   G   
   C   
   T   
   C   
   G   
   A   
   T   
   A   
   C   
   C   
   C   
   A   
   A   
   A   
   C   
   C   
   T   
   T   
   C   
   T   
    C    
   G   
   T   
   C   
   A   
   A   
   T   
   G   
   G   
   A   
   T   
   C   
   G   
   A   
   C   
   A   
   G   
   G   
   T   
   A   
   T   
   A   
   T   
   C   
   T   
   G   
   C   
   T   
   G   
   G   
   A   
   T   
   A   
   T   
   G   
   C   
    A    
   A   
   C   
   A   
   G   
   A   
   A   
   A   
   T   
   C   
   C   
   C   
   G   
   A   
   C   
    T    
   C   
   A   
   T   
   A   
   A   
   C   
   T   
   T   
   A   
   G   
   C   
   T   
   G   
   A   
   A   
   G   
   T   
   G   
   A   
   T   
    T    
   G   
   A   
    T    
   G   
   G   
   C   
   A   
   C   
   A   
   A   
   T   
   C   
   T   
   A   
   T   
   A   
   T   
   G   
   A   
   T   
   C   
   G   
   A   
   T   
   C   
   A   
   T   
   C   
   C   
   A   
   C   
   A   
   A   
   G   
   C   
   G   
   A   
   G   
   C   
   C   
   T   
   T   
   G   
   A   
   A   
   A   
   A   
   A   
   T   
   T   
   G   
   A   
   T   
   G   
   G   
   A   
   A   
   T   
   A   
   T   
   A   
   T   
   T   
   C   
   C   
   T   
   G   
   G   
   A   
   C   
   C   
   T   
   G   
   A   
   C   
   T   
   T   
    T    
   C   
   C   
   A   
   A   
   C   
   A   
   G   
   G   
   A   
   G   
   G   
    G    
   A   
   T   
   C   
   T   
   T   
   G   
   C   
   A   
   A   
   G   
   G   
    A    
   A   
   A   
   A   
   G   
   A   
   T   
   G   
   A   
   A   
   A   
   T   
   C   
   A   
   A   
   A   
   A   
   A   
   A   
   G   
   C   
   T   
   T   
   A   
   T   
   G   
   A   
   A   
   A   
   C   
   A   
   G   
   G   
    G    
   C   
   G   
   A   
   G   
   G   
   A   
   A   
   A   
   A   
   G   
   T   
   T   
   A   
    T    
   T   
   C   
   T   
   T   
   C   
   G   
   T   
   T   
   C   
   G   
   A   
   A   
   A   
   A   
   C   
   A   
   A   
   A   
   G   
   A   
   T   
   A   
   G   
   A   
    G    
   G   
   C   
   A   
   A   
   T   
   G   
   A   
   A   
   G   
   G   
   G   
    T    
   A   
   A   
    T    
   A   
   A   
   A   
   C   
   A   
   G   
   C   
   A   
   A   
   A   
   T   
   C   
   G   
   T   
   G   
   A   
   T   
   C   
   A   
   C   
    A    
   G   
   A   
   A   
   A   
   T   
   C   
   C   
   C   
   A   
   T   
   A   
   T   
   G   
   A   
   A   
   G   
   T   
   A   
   A   
   A   
   T   
   A   
   A   
   A   
   G   
   C   
   G   
   A   
   C   
    C    
   T   
   T   
   G   
   G   
   T   
   G   
   A   
   A   
   A   
   A   
   A   
   A   
   A   
   T   
   G   
   G   
   A   
   T   
   G   
   A   
   G   
   A   
   T   
   C   
   C   
   G   
   T   
   T   
   T   
   G   
   A   
   A   
   T   
   A   
   A   
   G   
   A   
   A   
   A   
   A   
   T   
   C   
   G   
   A   
    T    
   G   
   G   
   A   
   A   
   T   
   A   
   G   
   C   
   T   
   G   
   A   
   A   
   G   
   T   
    A    
   C   
   G   
   A   
   G   
   A   
   T   
   G   
   A   
   A   
   A   
   G   
   T   
   G   
   A   
   T   
    A    
   G   
    A    
   A   
   C   
    A    
   G   
   G   
   T   
   C   
   T   
   G   
   C   
   A   
   A   
   A   
   T   
   T   
   G   
   T   
   C   
   G   
   T   
    C    
   G   
   A   
   A   
   T   
   T   
   G   
   A   
   A   
   A   
   A   
   A   
   A   
   G   
   A   
    C    
   G   
   C   
   C   
   A   
   A   
    T    
   G   
   C   
   T   
   C   
   A   
   A   
   G   
   G   
    A    
   A   
   T   
   T   
   T   
   T   
   G   
   A   
   A   
   T   
   T   
   A   
    T    
   T   
   T   
   G   
   T   
   T   
   C   
   A   
   A   
   A   
   A   
   A   
   C   
   A   
   C   
   T   
   G   
   A   
   G   
    C    
   T   
   A   
   C   
   A   
   G   
   A   
   T   
   C   
   A   
   A   
   T   
   T   
   A   
   C   
   A   
   A   
   T   
   T   
   T   
    C    
   A   
   A   
   T   
   A   
   T   
   G   
   G   
   T   
   C   
   G   
   C   
   T   
   A   
   T   
   C   
   G   
   A   
    C    
   C   
   A   
   T   
   A   
   T   
   G   
   A   
   C   
   T   
   C   
   C   
   G   
   C   
   A   
    T    
   C   
   A   
   A   
   G   
   T   
   T   
   G   
   G   
   A   
   C   
   T   
   G   
   A   
   A   
   A   
   G   
   A   
   C   
   A   
   T   
   T   
   T   
   T   
   A   
   A   
   G   
   A   
   A   
   G   
   T   
   T   
   A   
   T   
   A   
   T   
   C   
   G   
   A   
   G   
   C   
   A   
   T   
   C   
   G   
   A   
   A   
   A   
   G   
   C   
   A   
   A   
   G   
   T   
   C   
   A   
   T   
   A   
   A   
   C   
   A   
   A   
   A   
   A   
   C   
   G   
    T    
   A   
   G   
    T    
   C   
   A   
   A   
   T   
   T   
   C   
   G   
   A   
   T   
   T   
   T   
   A   
   G   
   C   
   C   
   A   
   A   
   A   
   G   
   C   
   T   
   C   
   A   
   A   
   A   
   A   
   A   
    A    
   G   
   A   
   C   
   A   
   G   
   C   
   A   
   T   
   A   
   T   
   C   
   G   
   T   
    A    
   G   
   A   
   A   
   G   
   G   
    A    
    T    
   T   
   G   
   A   
   T   
   G   
   A   
   A   
   A   
   G   
   C   
   T   
   T   
   T   
   A   
   T   
   C   
   G   
   A   
   T   
   T   
   C   
   T   
   G   
   G   
   A   
   T   
   G   
   A   
   A   
   G   
   T   
   A   
   A   
   T   
   C   
   G   
    T    
   A   
   A   
   C   
   G   
   A   
   T   
   T   
   C   
   G   
   T   
   G   
   A   
   A   
   A   
   G   
   T   
   A   
   A   
   A   
   G   
   A   
   C   
   A   
   A   
   A   
   A   
   A   
   A   
   G   
   A   
    C    
   G   
   C   
    C    
   A   
   A   
   A   
   A   
   A   
   G   
   A   
   A   
   T   
   T   
   T   
   A   
   G   
   T   
   C   
   G   
   A   
    C    
   G   
   T   
    C    
   T   
   T   
    T    
   C   
   A   
   A   
   T   
   T   
   T   
   A   
   C   
   A   
   G   
   A   
   A   
   G   
   A   
   A   
   C   
   A   
   A   
   G   
   C   
   A   
   G   
   A   
   A   
   G   
   C   
   A   
   A   
   T   
    T    
   G   
   T   
   C   
   A   
   C   
   A   
   T   
   T   
   G   
   C   
   A   
   A   
    T    
   T   
   A   
   T   
   A   
    C    
   C   
   G   
   T   
   T   
   T   
   G   
   A   
   C   
   G   
   A   
   A   
    C    
   A   
   C   
    G    
   G   
   A   
   T   
   A   
   T   
   C   
   A   
   C   
   A   
   G   
   A   
   G   
   C   
   T   
   T   
   C   
   A   
   A   
   A   
   A   
   G   
   G   
   A   
   A   
    T    
   C   
    C    
   G   
   A   
   A   
   A   
    G    
   C   
   T   
   T   
   G   
   A   
   T   
   T   
   G   
   C   
    G    
   C   
   A   
   A   
   A   
   T   
   C   
   A   
   C   
   A   
   G   
   A   
   G   
   T   
   T   
    G    
   A   
   A   
   T   
   A   
   A   
   A   
   A   
   T   
   C   
   C   
   T   
   T   
   T   
   C   
   A   
   A   
   A   
    T    
   G   
   A   
   C   
   A   
   A   
   A   
   G   
   A   
    G    
   C   
   T   
   T   
   T   
   T   
    C    
   T   
   C   
   T   
   G   
   T   
   T   
   A   
   T   
   G   
   A   
   A   
   A   
   A   
   A   
   A   
   G   
   A   
   A   
   T   
   T   
   G   
   C   
   G   
   A   
   G   
   A   
   A   
   G   
   T   
   G   
   A   
   A   
   A   
   A   
   A   
   A   
   A   
   A   
   T   
   T   
   A   
    T    
   T   
   C   
   T   
   T   
   C   
   C   
   G   
   C   
   T   
   C   
   G   
   T   
   C   
    T    
    A    
   A   
   C   
   G   
   A   
   C   
   A   
   A   
   T   
   C   
    G    
   A   
   A   
   G   
   A   
   C   
   G   
   A   
   A   
   A   
   T   
   C   
   G   
   A   
   A   
   G   
   A   
   A   
   A   
   T   
   C   
   A   
   A   
   A   
   A   
   T   
    T    
   G   
   A   
   T   
   A   
   C   
   G   
   C   
   A   
   A   
    G    
   T   
    C    
   T   
   T   
   A   
   G   
   T   
   G   
   G   
   C   
    A    
   C   
   A   
   A   
   G   
   A   
   A   
   G   
   A   
   C   
   G   
   T   
   A   
   A   
   T   
    T    
   G   
   T   
   C   
   A   
   G   
   T   
   G   
   T   
    G    
   A   
   C   
    G    
   C   
   G   
   A   
   G   
   A   
   A   
   G   
   G   
   C   
   T   
   A   
   T   
   G   
   T   
   G   
   A   
   A   
   G   
   C   
   G   
   A   
   A   
   C   
   A   
   A   
   G   
   C   
   C   
   T   
   T   
   C   
   G   
   T   
   T   
   C   
    T    
   T   
   A   
   T   
   A   
   G   
   T   
   G   
   C   
   T   
   T   
   C   
    C    
   A   
   A   
   A   
   C   
   C   
   A   
   G   
   A   
   A   
   G   
   A   
   A   
   A   
   T   
   C   
   G   
   G   
   G   
   A   
   T   
   G   
   C   
   G   
   A   
   G   
   A   
   A   
   G   
   G   
   C   
   G   
   A   
    C    
   T   
   A   
   C   
   T   
   T   
   G   
   C   
   T   
    A    
   T   
   A   
   C   
    T    
   C   
   A   
   G   
   G   
   T   
   G   
   A   
   A   
   T   
   T   
    G    
   A   
   G   
    T    
   A   
   C   
   A   
    T    
   T   
   G   
   G   
   A   
   C   
   C   
   A   
   T   
   G   
   T   
   T   
   C   
   T   
   T   
   C   
   T   
   G   
   A   
   T   
    C    
   A   
   C   
    A    
   A   
   A   
   T   
   A   
   A   
   A   
   G   
   C   
   A   
   A   
   A   
   T   
   G   
   T   
   G   
   A   
   T   
   C   
   T   
   A   
   C   
   C   
   G   
    C    
   C   
   C   
   T   
   G   
   T   
    C    
   C   
   A   
   T   
   G   
   A   
   A   
   C   
   T   
   A   
   C   
   C   
    C    
   G   
   A   
   T   
   T   
   T   
   G   
   A   
   A   
   A   
   T   
   G   
   G   
   A   
   A   
   A   
   G   
   A   
   T   
   G   
   C   
   T   
   G   
   G   
    T    
   G   
   A   
    G    
   C   
   A   
    C    
   A   
   T   
   T   
   T   
   C   
   T   
   C   
   A   
   A   
   A   
   C   
   A   
   A   
   T   
   C   
   T   
   C   
   C   
   A   
   A   
   T   
   C   
   T   
   G   
   T   
   C   
   T   
   G   
   T   
   A   
   G   
   A   
    C    
   G   
   A   
   A   
   T   
   C   
   G   
   A   
   T   
    T    
   T   
   T   
   A   
   G   
   C   
   T   
   G   
   T   
   T   
   T   
   T   
    C    
   C   
   C   
   A   
   T   
   A   
   T   
   C   
   A   
   A   
   A   
   A   
   A   
   A   
   T   
    T    
   C   
   A   
   A   
   G   
   C   
   A   
   G   
   A   
    G    
   A   
   A   
   A   
   A   
   C   
   A   
   T   
   T   
   T   
   G   
   T   
    A    
   T   
   T   
   C   
   A   
   T   
   C   
   A   
   G   
   C   
   A   
   A   
   G   
   A   
   A   
   T   
   G   
   G   
   A   
   T   
   T   
    G    
    A    
   T   
   C   
   A   
   A   
   G   
   C   
   A   
   A   
   A   
   C   
   A   
   C   
   G   
   A   
   A   
   T   
   G   
   A   
   C   
   A   
   G   
   A   
   G   
   T   
   T   
   C   
   G   
   A   
   A   
   C   
   C   
   A   
   T   
   G   
   G   
   C   
   G   
   C   
   A   
   C   
   C   
   T   
   A   
    T    
   A   
   A   
   G   
   A   
   G   
   C   
   C   
   G   
    T    
   C   
   C   
   G   
   C   
   T   
   C   
   A   
   G   
   C   
   T   
   G   
   T   
   A   
   T   
   G   
   A   
   A   
   G   
   C   
   T   
   A   
   A   
   A   
   G   
   G   
   C   
   G   
   G   
   A   
   A   
   G   
   A   
   A   
   G   
   A   
   T   
   G   
   A   
   A   
   C   
   T   
    G    
   A   
   T   
   T   
   G   
   C   
   T   
   G   
   T   
    G    
   T   
   A   
   T   
   C   
   T   
   G   
   A   
   A   
   A   
   G   
   A   
   A   
   G   
   A   
   T   
   C   
    A    
   G   
   C   
   C   
   A   
   G   
   G   
   C   
   T   
   T   
   A   
   G   
   A   
   T   
   G   
   T   
    T    
   T   
   T   
   C   
   T   
   T   
   A   
   G   
   T   
   A   
   A   
   C   
   G   
    C    
   A   
   T   
   C   
   A   
   A   
   G   
   G   
   A   
   A   
   T   
   G   
   G   
   G   
    T    
   T   
   T   
   G   
   C   
   G   
   C   
   T   
    A    
   T   
   C   
   C   
   T   
   T   
   T   
   A   
   A   
    C    
   A   
   G   
   A   
   T   
   G   
   T   
   T   
   C   
   C   
   C   
   G   
   T   
   T   
   G   
   T   
   T   
   G   
   G   
   A   
   G   
   C   
   A   
   A   
   A   
   G   
   G   
   C   
   A   
   G   
    C    
   T   
   G   
   G   
   A   
   G   
   T   
   G   
   A   
   A   
   G   
   T   
   C   
   T   
   A   
   T   
   G   
   A   
   A   
   T   
   T   
   T   
   G   
   A   
   A   
   A   
   G   
   A   
   T   
   G   
   G   
   A   
   G   
   A   
   T   
   A   
   G   
   G   
    A    
   T   
   T   
    A    
   T   
   C   
   A   
   A   
    C    
   G   
   G   
    G    
   C   
   T   
    G    
   C   
   T   
   T   
   G   
   T   
   G   
   T   
   T   
   A   
   G   
   C   
   A   
   G   
   A   
    A    
   G   
   G   
    T    
   G   
   A   
   T   
   A   
   C   
   G   
   C   
   C   
    G    
   A   
   T   
   C   
   G   
   T   
   A   
   A   
   T   
   C   
   G   
   T   
   T   
   A   
   C   
   T   
   C   
   A   
   G   
   C   
   G   
   A   
   G   
   G   
   A   
   G   
   C   
    T    
   G   
   T   
    T    
   A   
   A   
   A   
   C   
   G   
   C   
   A   
   T   
   G   
   C   
   T   
   T   
   G   
   C   
   T   
   C   
   A   
   A   
   G   
   A   
   A   
   A   
   T   
   C   
   A   
   G   
   C   
   C   
   A   
   A   
   A   
   C   
    T    
   T   
   C   
   A   
   C   
   G   
   T   
   G   
   C   
   G   
   A   
   A   
   G   
   C   
   G   
   G   
   G   
   G   
   A   
   G   
   T   
   G   
   A   
   C   
   G   
   G   
   T   
   C   
   A   
   T   
   G   
   C   
   G   
   T   
   G   
   A   
   G   
   C   
   T   
   G   
   A   
   A   
   A   
   A   
   A   
   A   
   A   
   A   
   T   
   C   
   C   
    G    
   C   
   A   
    C    
   C   
   G   
    T    
   A   
   T   
   T   
   A   
   T   
   C   
   T   
   A   
   T   
   A   
   T   
   G   
   T   
   C   
   G   
   G   
   A   
   A   
   G   
   G   
   A   
   A   
   G   
   A   
   T   
   C   
   A   
   A   
   A   
   A   
   G   
   A   
   A   
   A   
   T   
   A   
   A   
   C   
   C   
   T   
   T   
   G   
   A   
   T   
   G   
   A   
   A   
   T   
   C   
   A   
   A   
   A   
   A   
   A   
   G   
   G   
    G    
   C   
   A   
    G    
   C   
   A   
   G   
    T    
   T   
   A   
   A   
   C   
   G   
   A   
   T   
   T   
   G   
   A   
    C    
   C   
   C   
   A   
   A   
   C   
    T    
   G   
   A   
   T   
   T   
   T   
    C    
   C   
   C   
   T   
   A   
   T   
   A   
   G   
   G   
   T   
   G   
   A   
   T   
   C   
   G   
   A   
   A   
   C   
   A   
   T   
   C   
   G   
   A   
   A   
   T   
   G   
   G   
    A    
   T   
   C   
    A    
   T   
   T   
   T   
   G   
   C   
    G    
   A   
   T   
   G   
   G   
   A   
    T    
   G   
   A   
   G   
   A   
   A   
   A   
   A   
   A   
   A   
   G   
   G   
   T   
   G   
   G   
   A   
   G   
   A   
   A   
   G   
   T   
   C   
   A   
   T   
   C   
   G   
   A   
   A   
   G   
   T   
    T    
   A   
    T    
   C   
   G   
   A   
   T   
   G   
   C   
   T   
   C   
   C   
   T   
   G   
   A   
   G   
    A    
   T   
   T   
   T   
   T   
    C    
   A   
   T   
    C    
   G   
   A   
    G    
    T    
   C   
    G    
   -   
   -   
   -   
 

 
   N54035   
   A   
   T   
   G   
   G   
   A   
   A   
   A   
   A   
   T   
   C   
   G   
   A   
   C   
   A   
   A   
   G   
   A   
   A   
   G   
   T   
   A   
   C   
   A   
   A   
   G   
   A   
   A   
   T   
   T   
   A   
   A   
   C   
   T   
   T   
   T   
   A   
   G   
   A   
   A   
   G   
   A   
   A   
   G   
   T   
   G   
   A   
   T   
   G   
   G   
   G   
   T   
   G   
   A   
   C   
   C   
   G   
   C   
   T   
   T   
   C   
   G   
   G   
   A   
   C   
   G   
   T   
   T   
   A   
   T   
   T   
   C   
   C   
   A   
   A   
   A   
   T   
   A   
   T   
   A   
   T   
   T   
   A   
   T   
    C    
   C   
   A   
    A    
   G   
   A   
   A   
   C   
   G   
   T   
   G   
   C   
   A   
   C   
   T   
   G   
   C   
   C   
   T   
   G   
   A   
   T   
   A   
   T   
    C    
   C   
   G   
   T   
   G   
   A   
   T   
   G   
   G   
   A   
   T   
   T   
   G   
   A   
   A   
    G    
   C   
   C   
   A   
   G   
   T   
    G    
   C   
   A   
   A   
   C   
   G   
   C   
   C   
   G   
   G   
   A   
   T   
   A   
   T   
   T   
   A   
   T   
   T   
    C    
   G   
   C   
    T    
   A   
   T   
   G   
   A   
   A   
   C   
   A   
   A   
   A   
   G   
   A   
   T   
   G   
   G   
   C   
   A   
   A   
   T   
   A   
   C   
   G   
   T   
   A   
   T   
   G   
   A   
   C   
   A   
   A   
   A   
   G   
   G   
   A   
   T   
   T   
    C    
   C   
   G   
   T   
   A   
   A   
   A   
   T   
   C   
   A   
   G   
   C   
   A   
   A   
   A   
   A   
   T   
   C   
   T   
   G   
   T   
   C   
   G   
   G   
   A   
   A   
   A   
   T   
   A   
   T   
   C   
   A   
   T   
   G   
   G   
   G   
    G    
   A   
   A   
   T   
   T   
   A   
   T   
   C   
   A   
   T   
   C   
   C   
   T   
   C   
   A   
   T   
   G   
   G   
   T   
   G   
   A   
   C   
   A   
   G   
   C   
   A   
   G   
   T   
   A   
   T   
   T   
   T   
   A   
   T   
   G   
   A   
   A   
   G   
   C   
   G   
   A   
   T   
   G   
   G   
   T   
   C   
   C   
   G   
   C   
   A   
   T   
   G   
   A   
   G   
   C   
   C   
   A   
   A   
   G   
   A   
    C    
   T   
   G   
   G   
   A   
   A   
   G   
   T   
   T   
   G   
   C   
   G   
   G   
   G   
   A   
   A   
   G   
   T   
   A   
   C   
   T   
   G   
   A   
   T   
   C   
   G   
   A   
    A    
   A   
   T   
   G   
   C   
   A   
   C   
   G   
   G   
    G    
   A   
   A   
   T   
   A   
   A   
    C    
   G   
   G   
   G   
   A   
   G   
   T   
   A   
   T   
   G   
   G   
   A   
   C   
   G   
   G   
   T   
   G   
   A   
   T   
   C   
   C   
   A   
   C   
   C   
   T   
   G   
   C   
   T   
   G   
   C   
   G   
   A   
   T   
   G   
   C   
   G   
   T   
   T   
   A   
   T   
   A   
   C   
   T   
   G   
   A   
   A   
   G   
   C   
   C   
   A   
   G   
    A    
   T   
   T   
   A   
   T   
   C   
   C   
   A   
   A   
   G   
   T   
   T   
   A   
   A   
   G   
   T   
   G   
   G   
   A   
   G   
   A   
   G   
   A   
   T   
   G   
    C    
   T   
   A   
    G    
   C   
   A   
   G   
   A   
   T   
   A   
   T   
   C   
   G   
   A   
    A    
   A   
   A   
   A   
   G   
   A   
   A   
   A   
   C   
   A   
   G   
   T   
    C    
   G   
   A   
   T   
   C   
   T   
   C   
   G   
   T   
   G   
   T   
   G   
   G   
   A   
   A   
   T   
   T   
   T   
   T   
   G   
   A   
   T   
   G   
   A   
   T   
   A   
   C   
   A   
   G   
   A   
   A   
   A   
   A   
   A   
   G   
   A   
   A   
   C   
   C   
   A   
   A   
   C   
   A   
   G   
   T   
   C   
   C   
   T   
   T   
   C   
   C   
   A   
   G   
   C   
   T   
   C   
   G   
   A   
   T   
   A   
   C   
   C   
   C   
   A   
   A   
   A   
   C   
   C   
   T   
   T   
   C   
   T   
    C    
   G   
   T   
   C   
   A   
   A   
   T   
   G   
   G   
   A   
   T   
   C   
   G   
   A   
   C   
   A   
   G   
   G   
   T   
   A   
   T   
   A   
   T   
   C   
   T   
   G   
   C   
   T   
   G   
   G   
   A   
   T   
   A   
   T   
   G   
   C   
    A    
   A   
   C   
   A   
   G   
   A   
   A   
   A   
   T   
   C   
   C   
   C   
   G   
   A   
   C   
    T    
   C   
   A   
   T   
   A   
   A   
   C   
   T   
   T   
   A   
   G   
   C   
   T   
   G   
   A   
   A   
   G   
   T   
   G   
   A   
   T   
    C    
   G   
   A   
    T    
   G   
   G   
   C   
   A   
   C   
   A   
   A   
   T   
   C   
   T   
   A   
   T   
   A   
   T   
   G   
   A   
   T   
   C   
   G   
   A   
   T   
   C   
   A   
   T   
   C   
   C   
   A   
   C   
   A   
   A   
   G   
   C   
   G   
   A   
   G   
   C   
   C   
   T   
   T   
   G   
   A   
   A   
   A   
   A   
   A   
   T   
   T   
   G   
   A   
   T   
   G   
   G   
   A   
   A   
   T   
   A   
   T   
   A   
   T   
   T   
   C   
   C   
   T   
   G   
   G   
   A   
   C   
   C   
   T   
   G   
   A   
   C   
   T   
   T   
    T    
   C   
   C   
   A   
   A   
   C   
   A   
   G   
   G   
   A   
   G   
   G   
    G    
   A   
   T   
   C   
   T   
   T   
   G   
   C   
   A   
   A   
   G   
   G   
    A    
   A   
   A   
   A   
   G   
   A   
   T   
   G   
   A   
   A   
   A   
   T   
   C   
   A   
   A   
   A   
   A   
   A   
   A   
   G   
   C   
   T   
   T   
   A   
   T   
   G   
   A   
   A   
   A   
   C   
   A   
   G   
   G   
    G    
   C   
   G   
   A   
   G   
   G   
   A   
   A   
   A   
   A   
   G   
   T   
   T   
   A   
    T    
   T   
   C   
   T   
   T   
   C   
   G   
   T   
   T   
   C   
   G   
   A   
   A   
   A   
   A   
   C   
   A   
   A   
   A   
   G   
   A   
   T   
   A   
   G   
   A   
    A    
   G   
   C   
   A   
   A   
   T   
   G   
   A   
   A   
   G   
   G   
   G   
    T    
   A   
   A   
    T    
   A   
   A   
   A   
   C   
   A   
   G   
   C   
   A   
   A   
   A   
   T   
   C   
   G   
   T   
   G   
   A   
   T   
   C   
   A   
   C   
    A    
   G   
   A   
   A   
   A   
   T   
   C   
   C   
   C   
   A   
   T   
   A   
   T   
   G   
   A   
   A   
   G   
   T   
   A   
   A   
   A   
   T   
   A   
   A   
   A   
   G   
   C   
   G   
   A   
   C   
    C    
   T   
   T   
   G   
   G   
   T   
   G   
   A   
   A   
   A   
   A   
   A   
   A   
   A   
   T   
   G   
   G   
   A   
   T   
   G   
   A   
   G   
   A   
   T   
   C   
   C   
   G   
   T   
   T   
   T   
   G   
   A   
   A   
   T   
   A   
   A   
   G   
   A   
   A   
   A   
   A   
   T   
   C   
   G   
   A   
    T    
   G   
   G   
   A   
   A   
   T   
   A   
   G   
   C   
   T   
   G   
   A   
   A   
   G   
   T   
    A    
   C   
   G   
   A   
   G   
   A   
   T   
   G   
   A   
   A   
   A   
   G   
   T   
   G   
   A   
   T   
    A    
   G   
    A    
   A   
   C   
    A    
   G   
   G   
   T   
   C   
   T   
   G   
   C   
   A   
   A   
   A   
   T   
   T   
   G   
   T   
   C   
   G   
   T   
    C    
   G   
   A   
   A   
   T   
   T   
   G   
   A   
   A   
   A   
   A   
   A   
   A   
   G   
   A   
    C    
   G   
   C   
   C   
   A   
   A   
    T    
   G   
   C   
   T   
   C   
   A   
   A   
   G   
   G   
    A    
   A   
   T   
   T   
   T   
   T   
   G   
   A   
   A   
   T   
   T   
   A   
    T    
   T   
   T   
   G   
   T   
   T   
   C   
   A   
   A   
   A   
   A   
   A   
   C   
   A   
   C   
   T   
   G   
   A   
   G   
    C    
   T   
   A   
   C   
   A   
   G   
   A   
   T   
   C   
   A   
   A   
   T   
   T   
   A   
   C   
   A   
   A   
   T   
   T   
   T   
    C    
   A   
   A   
   T   
   A   
   T   
   G   
   G   
   T   
   C   
   G   
   C   
   T   
   A   
   T   
   C   
   G   
   A   
    C    
   C   
   A   
   T   
   A   
   T   
   G   
   A   
   C   
   T   
   C   
   C   
   G   
   C   
   A   
    T    
   C   
   A   
   A   
   G   
   T   
   T   
   G   
   G   
   A   
   C   
   T   
   G   
   A   
   A   
   A   
   G   
   A   
   C   
   A   
   T   
   T   
   T   
   T   
   A   
   A   
   G   
   A   
   A   
   G   
   T   
   T   
   A   
   T   
   A   
   T   
   C   
   G   
   A   
   G   
   C   
   A   
   T   
   C   
   G   
   A   
   A   
   A   
   G   
   C   
   A   
   A   
   G   
   T   
   C   
   A   
   T   
   A   
   A   
   C   
   A   
   A   
   A   
   A   
   C   
   G   
    T    
   A   
   G   
    T    
   C   
   A   
   A   
   T   
   T   
   C   
   G   
   A   
   T   
   T   
   T   
   A   
   G   
   C   
   C   
   A   
   A   
   A   
   G   
   C   
   T   
   C   
   A   
   A   
   A   
   A   
   A   
    A    
   G   
   A   
   C   
   A   
   G   
   C   
   A   
   T   
   A   
   T   
   C   
   G   
   T   
    A    
   G   
   A   
   A   
   G   
   G   
    A    
    T    
   T   
   G   
   A   
   T   
   G   
   A   
   A   
   A   
   G   
   C   
   T   
   T   
   T   
   A   
   T   
   C   
   G   
   A   
   T   
   T   
   C   
   T   
   G   
   G   
   A   
   T   
   G   
   A   
   A   
   G   
   T   
   A   
   A   
   T   
   C   
   G   
    C    
   A   
   A   
   C   
   G   
   A   
   T   
   T   
   C   
   G   
   T   
   G   
   A   
   A   
   A   
   G   
   T   
   A   
   A   
   A   
   G   
   A   
   C   
   A   
   A   
   A   
   A   
   A   
   A   
   G   
   A   
    C    
   G   
   C   
    C    
   A   
   A   
   A   
   A   
   A   
   G   
   A   
   A   
   T   
   T   
   T   
   A   
   G   
   T   
   C   
   G   
   A   
    C    
   G   
   T   
    C    
   T   
   T   
    T    
   C   
   A   
   A   
   T   
   T   
   T   
   A   
   C   
   A   
   G   
   A   
   A   
   G   
   A   
   A   
   C   
   A   
   A   
   G   
   C   
   A   
   G   
   A   
   A   
   G   
   C   
   A   
   A   
   T   
    T    
   G   
   T   
   C   
   A   
   C   
   A   
   T   
   T   
   G   
   C   
   A   
   A   
    T    
   T   
   A   
   T   
   A   
    C    
   C   
   G   
   T   
   T   
   T   
   G   
   A   
   C   
   G   
   A   
   A   
    C    
   A   
   C   
    G    
   G   
   A   
   T   
   A   
   T   
   C   
   A   
   C   
   A   
   G   
   A   
   G   
   C   
   T   
   T   
   C   
   A   
   A   
   A   
   A   
   G   
   G   
   A   
   A   
    T    
   C   
    C    
   G   
   A   
   A   
   A   
    G    
   C   
   T   
   T   
   G   
   A   
   T   
   T   
   G   
   C   
    G    
   C   
   A   
   A   
   A   
   T   
   C   
   A   
   C   
   A   
   G   
   A   
   G   
   T   
   T   
    G    
   A   
   A   
   T   
   A   
   A   
   A   
   A   
   T   
   C   
   C   
   T   
   T   
   T   
   C   
   A   
   A   
   A   
    T    
   G   
   A   
   C   
   A   
   A   
   A   
   G   
   A   
    G    
   C   
   T   
   T   
   T   
   T   
    C    
   T   
   C   
   T   
   G   
   T   
   T   
   A   
   T   
   G   
   A   
   A   
   A   
   A   
   A   
   A   
   G   
   A   
   A   
   T   
   T   
   G   
   C   
   G   
   A   
   G   
   A   
   A   
   G   
   T   
   G   
   A   
   A   
   A   
   A   
   A   
   A   
   A   
   A   
   T   
   T   
   A   
    T    
   T   
   C   
   T   
   T   
   C   
   C   
   G   
   C   
   T   
   C   
   G   
   T   
   C   
    T    
    A    
   A   
   C   
   G   
   A   
   C   
   A   
   A   
   T   
   C   
    G    
   A   
   A   
   G   
   A   
   C   
   G   
   A   
   A   
   A   
   T   
   C   
   G   
   A   
   A   
   G   
   A   
   A   
   A   
   T   
   C   
   A   
   A   
   A   
   A   
   T   
    C    
   G   
   A   
   T   
   A   
   C   
   G   
   C   
   A   
   A   
    G    
   T   
    T    
   T   
   T   
   A   
   G   
   T   
   G   
   G   
   C   
    G    
   C   
   A   
   A   
   G   
   A   
   A   
   G   
   A   
   C   
   G   
   T   
   A   
   A   
   T   
    C    
   G   
   T   
   C   
   A   
   G   
   T   
   G   
   T   
    G    
   A   
   C   
    G    
   C   
   G   
   A   
   G   
   A   
   A   
   G   
   G   
   C   
   T   
   A   
   T   
   G   
   T   
   G   
   A   
   A   
   G   
   C   
   G   
   A   
   A   
   C   
   A   
   A   
   G   
   C   
   C   
   T   
   T   
   C   
   G   
   T   
   T   
   C   
    T    
   T   
   A   
   T   
   A   
   G   
   T   
   G   
   C   
   T   
   T   
   C   
    G    
   A   
   A   
   A   
   C   
   C   
   A   
   G   
   A   
   A   
   G   
   A   
   A   
   A   
   T   
   C   
   G   
   G   
   G   
   A   
   T   
   G   
   C   
   G   
   A   
   G   
   A   
   A   
   G   
   G   
   C   
   G   
   A   
    C    
   T   
   A   
   C   
   T   
   T   
   G   
   C   
   T   
    G    
   T   
   A   
   C   
    T    
   C   
   A   
   G   
   G   
   T   
   G   
   A   
   A   
   T   
   T   
    G    
   A   
   G   
    C    
   A   
   C   
   A   
    T    
   T   
   G   
   G   
   A   
   C   
   C   
   A   
   T   
   G   
   T   
   T   
   C   
   T   
   T   
   C   
   T   
   G   
   A   
   T   
    T    
   A   
   C   
    G    
   A   
   A   
   T   
   A   
   A   
   A   
   G   
   C   
   A   
   A   
   A   
   T   
   G   
   T   
   G   
   A   
   T   
   C   
   T   
   A   
   C   
   C   
   G   
    T    
   C   
   C   
   T   
   G   
   T   
    T    
   C   
   A   
   T   
   G   
   A   
   A   
   C   
   T   
   A   
   C   
   C   
    C    
   G   
   A   
   T   
   T   
   T   
   G   
   A   
   A   
   A   
   T   
   G   
   G   
   A   
   A   
   A   
   G   
   A   
   T   
   G   
   C   
   T   
   G   
   G   
    C    
   G   
   A   
    A    
   C   
   A   
    T    
   A   
   T   
   T   
   T   
   C   
   T   
   C   
   A   
   A   
   A   
   C   
   A   
   A   
   T   
   C   
   T   
   C   
   C   
   A   
   A   
   T   
   C   
   T   
   G   
   T   
   C   
   T   
   G   
   T   
   A   
   G   
   A   
    T    
   G   
   A   
   A   
   T   
   C   
   G   
   A   
   T   
    C    
   T   
   T   
   A   
   G   
   C   
   T   
   G   
   T   
   T   
   T   
   T   
    T    
   C   
   C   
   A   
   T   
   A   
   T   
   C   
   A   
   A   
   A   
   A   
   A   
   A   
   T   
    T    
   C   
   A   
   A   
   G   
   C   
   A   
   G   
   A   
    G    
   A   
   A   
   A   
   A   
   C   
   A   
   T   
   T   
   T   
   G   
   T   
    A    
   T   
   T   
   C   
   A   
   T   
   C   
   A   
   G   
   C   
   A   
   A   
   G   
   A   
   A   
   T   
   G   
   G   
   A   
   T   
   T   
    G    
    A    
   T   
   C   
   A   
   A   
   G   
   C   
   A   
   A   
   A   
   C   
   A   
   C   
   G   
   A   
   A   
   T   
   G   
   A   
   C   
   A   
   G   
   A   
   G   
   T   
   T   
   C   
   G   
   A   
   A   
   C   
   C   
   A   
   T   
   G   
   G   
   C   
   G   
   C   
   A   
   C   
   C   
   T   
   A   
    T    
   A   
   A   
   G   
   A   
   G   
   C   
   C   
   G   
    T    
   C   
   C   
   G   
   C   
   T   
   C   
   A   
   G   
   C   
   T   
   G   
   T   
   A   
   T   
   G   
   A   
   A   
   G   
   C   
   T   
   A   
   A   
   A   
   G   
   G   
   C   
   G   
   G   
   A   
   A   
   G   
   A   
   A   
   G   
   A   
   T   
   G   
   A   
   A   
   C   
   T   
    G    
   A   
   T   
   T   
   G   
   C   
   T   
   G   
   T   
    G    
   T   
   A   
   T   
   C   
   T   
   G   
   A   
   A   
   A   
   G   
   A   
   A   
   G   
   A   
   T   
   C   
    G    
   G   
   C   
   C   
   A   
   G   
   G   
   C   
   T   
   T   
   A   
   G   
   A   
   T   
   G   
   T   
    T    
   T   
   T   
   C   
   T   
   T   
   A   
   G   
   T   
   A   
   A   
   C   
   G   
    T    
   A   
   T   
   C   
   A   
   A   
   G   
   G   
   A   
   A   
   T   
   G   
   G   
   G   
    T    
   T   
   T   
   G   
   C   
   G   
   C   
   T   
    A    
   T   
   C   
   C   
   T   
   T   
   T   
   A   
   A   
    C    
   A   
   G   
   A   
   T   
   G   
   T   
   T   
   C   
   C   
   C   
   G   
   T   
   T   
   G   
   T   
   T   
   G   
   G   
   A   
   G   
   C   
   A   
   A   
   A   
   G   
   G   
   C   
   A   
   G   
    C    
   T   
   G   
   G   
   A   
   G   
   T   
   G   
   A   
   A   
   G   
   T   
   C   
   T   
   A   
   T   
   G   
   A   
   A   
   T   
   T   
   T   
   G   
   A   
   A   
   A   
   G   
   A   
   T   
   G   
   G   
   A   
   G   
   A   
   T   
   A   
   G   
   G   
    A    
   T   
   T   
    A    
   T   
   C   
   A   
   A   
    C    
   G   
   G   
    G    
   C   
   T   
    G    
   C   
   T   
   T   
   G   
   T   
   G   
   T   
   T   
   A   
   G   
   C   
   A   
   G   
   A   
    A    
   G   
   G   
    T    
   G   
   A   
   T   
   A   
   C   
   G   
   C   
   C   
    G    
   A   
   T   
   C   
   G   
   T   
   A   
   A   
   T   
   C   
   G   
   T   
   T   
   A   
   C   
   T   
   C   
   A   
   G   
   C   
   G   
   A   
   G   
   G   
   A   
   G   
   C   
    T    
   G   
   T   
    T    
   A   
   A   
   A   
   C   
   G   
   C   
   A   
   T   
   G   
   C   
   T   
   T   
   G   
   C   
   T   
   C   
   A   
   A   
   G   
   A   
   A   
   A   
   T   
   C   
   A   
   G   
   C   
   C   
   A   
   A   
   A   
   C   
    T    
   T   
   C   
   A   
   C   
   G   
   T   
   G   
   C   
   G   
   A   
   A   
   G   
   C   
   G   
   G   
   G   
   G   
   A   
   G   
   T   
   G   
   A   
   C   
   G   
   G   
   T   
   C   
   A   
   T   
   G   
   C   
   G   
   T   
   G   
   A   
   G   
   C   
   T   
   G   
   A   
   A   
   A   
   A   
   A   
   A   
   A   
   A   
   T   
   C   
   C   
    T    
   C   
   A   
    T    
   C   
   G   
    C    
   A   
   T   
   T   
   A   
   T   
   C   
   T   
   A   
   T   
   A   
   T   
   G   
   T   
   C   
   G   
   G   
   A   
   A   
   G   
   G   
   A   
   A   
   G   
   A   
   T   
   C   
   A   
   A   
   A   
   A   
   G   
   A   
   A   
   A   
   T   
   A   
   A   
   C   
   C   
   T   
   T   
   G   
   A   
   T   
   G   
   A   
   A   
   T   
   C   
   A   
   A   
   A   
   A   
   A   
   G   
   G   
    G    
   C   
   A   
    G    
   C   
   A   
   G   
    T    
   T   
   A   
   A   
   C   
   G   
   A   
   T   
   T   
   G   
   A   
    C    
   C   
   C   
   A   
   A   
   C   
    T    
   G   
   A   
   T   
   T   
   T   
    C    
   C   
   C   
   T   
   A   
   T   
   A   
   G   
   G   
   T   
   G   
   A   
   T   
   C   
   G   
   A   
   A   
   C   
   A   
   T   
   C   
   G   
   A   
   A   
   T   
   G   
   G   
    A    
   T   
   C   
    A    
   T   
   T   
   T   
   G   
   C   
    G    
   A   
   T   
   G   
   G   
   A   
    T    
   G   
   A   
   G   
   A   
   A   
   A   
   A   
   A   
   A   
   G   
   G   
   T   
   G   
   G   
   A   
   G   
   A   
   A   
   G   
   T   
   C   
   A   
   T   
   C   
   G   
   A   
   A   
   G   
   T   
    T    
   A   
    T    
   C   
   G   
   A   
   T   
   G   
   C   
   T   
   C   
   C   
   T   
   G   
   A   
   G   
    A    
   T   
   T   
   T   
   T   
    C    
   A   
   T   
    C    
   G   
   A   
    G    
    T    
   C   
    G    
   -   
   -   
   -   
 

 
   N60357F   
   A   
   T   
   G   
   G   
   A   
   A   
   A   
   A   
   T   
   C   
   G   
   A   
   C   
   A   
   A   
   G   
   A   
   A   
   G   
   T   
   A   
   C   
   A   
   A   
   G   
   A   
   A   
   T   
   T   
   A   
   A   
   C   
   T   
   T   
   T   
   A   
   G   
   A   
   A   
   G   
   A   
   A   
   G   
   T   
   G   
   A   
   T   
   G   
   G   
   G   
   T   
   G   
   A   
   C   
   C   
   G   
   C   
   T   
   T   
   C   
   G   
   G   
   A   
   C   
   G   
   T   
   T   
   A   
   T   
   T   
   C   
   C   
   A   
   A   
   A   
   T   
   A   
   T   
   A   
   T   
   T   
   A   
   T   
    C    
   C   
   A   
    A    
   G   
   A   
   A   
   C   
   G   
   T   
   G   
   C   
   A   
   C   
   T   
   G   
   C   
   C   
   T   
   G   
   A   
   T   
   A   
   T   
    C    
   C   
   G   
   T   
   G   
   A   
   T   
   G   
   G   
   A   
   T   
   T   
   G   
   A   
   A   
    G    
   C   
   C   
   A   
   G   
   T   
    G    
   C   
   A   
   A   
   C   
   G   
   C   
   C   
   G   
   G   
   A   
   T   
   A   
   T   
   T   
   A   
   T   
   T   
    T    
   G   
   C   
    C    
   A   
   T   
   G   
   A   
   A   
   C   
   A   
   A   
   A   
   G   
   A   
   T   
   G   
   G   
   C   
   A   
   A   
   T   
   A   
   C   
   G   
   T   
   A   
   T   
   G   
   A   
   C   
   A   
   A   
   A   
   G   
   G   
   A   
   T   
   T   
    C    
   C   
   G   
   T   
   A   
   A   
   A   
   T   
   C   
   A   
   G   
   C   
   A   
   A   
   A   
   A   
   T   
   C   
   T   
   G   
   T   
   C   
   G   
   G   
   A   
   A   
   A   
   T   
   A   
   T   
   C   
   A   
   T   
   G   
   G   
   G   
    G    
   A   
   A   
   T   
   T   
   A   
   T   
   C   
   A   
   T   
   C   
   C   
   T   
   C   
   A   
   T   
   G   
   G   
   T   
   G   
   A   
   C   
   A   
   G   
   C   
   A   
   G   
   T   
   A   
   T   
   T   
   T   
   A   
   T   
   G   
   A   
   A   
   G   
   C   
   G   
   A   
   T   
   G   
   G   
   T   
   C   
   C   
   G   
   C   
   A   
   T   
   G   
   A   
   G   
   C   
   C   
   A   
   A   
   G   
   A   
    C    
   T   
   G   
   G   
   A   
   A   
   G   
   T   
   T   
   G   
   C   
   G   
   G   
   G   
   A   
   A   
   G   
   T   
   A   
   C   
   T   
   G   
   A   
   T   
   C   
   G   
   A   
    A    
   A   
   T   
   G   
   C   
   A   
   C   
   G   
   G   
    G    
   A   
   A   
   T   
   A   
   A   
    C    
   G   
   G   
   G   
   A   
   G   
   T   
   A   
   T   
   G   
   G   
   A   
   C   
   G   
   G   
   T   
   G   
   A   
   T   
   C   
   C   
   A   
   C   
   C   
   T   
   G   
   C   
   T   
   G   
   C   
   G   
   A   
   T   
   G   
   C   
   G   
   T   
   T   
   A   
   T   
   A   
   C   
   T   
   G   
   A   
   A   
   G   
   C   
   C   
   A   
   G   
    A    
   T   
   T   
   A   
   T   
   C   
   C   
   A   
   A   
   G   
   T   
   T   
   A   
   A   
   G   
   T   
   G   
   G   
   A   
   G   
   A   
   G   
   A   
   T   
   G   
    C    
   T   
   A   
    G    
   C   
   A   
   G   
   A   
   T   
   A   
   T   
   C   
   G   
   A   
    A    
   A   
   A   
   A   
   G   
   A   
   A   
   A   
   C   
   A   
   G   
   T   
    C    
   G   
   A   
   T   
   C   
   T   
   C   
   G   
   T   
   G   
   T   
   G   
   G   
   A   
   A   
   T   
   T   
   T   
   T   
   G   
   A   
   T   
   G   
   A   
   T   
   A   
   C   
   A   
   G   
   A   
   A   
   A   
   A   
   A   
   G   
   A   
   A   
   C   
   C   
   A   
   A   
   C   
   A   
   G   
   T   
   C   
   C   
   T   
   T   
   C   
   C   
   A   
   G   
   C   
   T   
   C   
   G   
   A   
   T   
   A   
   C   
   C   
   C   
   A   
   A   
   A   
   C   
   C   
   T   
   T   
   C   
   T   
    C    
   G   
   T   
   C   
   A   
   A   
   T   
   G   
   G   
   A   
   T   
   C   
   G   
   A   
   C   
   A   
   G   
   G   
   T   
   A   
   T   
   A   
   T   
   C   
   T   
   G   
   C   
   T   
   G   
   G   
   A   
   T   
   A   
   T   
   G   
   C   
    A    
   A   
   C   
   A   
   G   
   A   
   A   
   A   
   T   
   C   
   C   
   C   
   G   
   A   
   C   
    T    
   C   
   A   
   T   
   A   
   A   
   C   
   T   
   T   
   A   
   G   
   C   
   T   
   G   
   A   
   A   
   G   
   T   
   G   
   A   
   T   
    T    
   G   
   A   
    T    
   G   
   G   
   C   
   A   
   C   
   A   
   A   
   T   
   C   
   T   
   A   
   T   
   A   
   T   
   G   
   A   
   T   
   C   
   G   
   A   
   T   
   C   
   A   
   T   
   C   
   C   
   A   
   C   
   A   
   A   
   G   
   C   
   G   
   A   
   G   
   C   
   C   
   T   
   T   
   G   
   A   
   A   
   A   
   A   
   A   
   T   
   T   
   G   
   A   
   T   
   G   
   G   
   A   
   A   
   T   
   A   
   T   
   A   
   T   
   T   
   C   
   C   
   T   
   G   
   G   
   A   
   C   
   C   
   T   
   G   
   A   
   C   
   T   
   T   
    T    
   C   
   C   
   A   
   A   
   C   
   A   
   G   
   G   
   A   
   G   
   G   
    G    
   A   
   T   
   C   
   T   
   T   
   G   
   C   
   A   
   A   
   G   
   G   
    A    
   A   
   A   
   A   
   G   
   A   
   T   
   G   
   A   
   A   
   A   
   T   
   C   
   A   
   A   
   A   
   A   
   A   
   A   
   G   
   C   
   T   
   T   
   A   
   T   
   G   
   A   
   A   
   A   
   C   
   A   
   G   
   G   
    G    
   C   
   G   
   A   
   G   
   G   
   A   
   A   
   A   
   A   
   G   
   T   
   T   
   A   
    T    
   T   
   C   
   T   
   T   
   C   
   G   
   T   
   T   
   C   
   G   
   A   
   A   
   A   
   A   
   C   
   A   
   A   
   A   
   G   
   A   
   T   
   A   
   G   
   A   
    G    
   G   
   C   
   A   
   A   
   T   
   G   
   A   
   A   
   G   
   G   
   G   
    T    
   A   
   A   
    T    
   A   
   A   
   A   
   C   
   A   
   G   
   C   
   A   
   A   
   A   
   T   
   C   
   G   
   T   
   G   
   A   
   T   
   C   
   A   
   C   
    A    
   G   
   A   
   A   
   A   
   T   
   C   
   C   
   C   
   A   
   T   
   A   
   T   
   G   
   A   
   A   
   G   
   T   
   A   
   A   
   A   
   T   
   A   
   A   
   A   
   G   
   C   
   G   
   A   
   C   
    C    
   T   
   T   
   G   
   G   
   T   
   G   
   A   
   A   
   A   
   A   
   A   
   A   
   A   
   T   
   G   
   G   
   A   
   T   
   G   
   A   
   G   
   A   
   T   
   C   
   C   
   G   
   T   
   T   
   T   
   G   
   A   
   A   
   T   
   A   
   A   
   G   
   A   
   A   
   A   
   A   
   T   
   C   
   G   
   A   
    T    
   G   
   G   
   A   
   A   
   T   
   A   
   G   
   C   
   T   
   G   
   A   
   A   
   G   
   T   
    A    
   C   
   G   
   A   
   G   
   A   
   T   
   G   
   A   
   A   
   A   
   G   
   T   
   G   
   A   
   T   
    A    
   G   
    A    
   A   
   C   
    A    
   G   
   G   
   T   
   C   
   T   
   G   
   C   
   A   
   A   
   A   
   T   
   T   
   G   
   T   
   C   
   G   
   T   
    C    
   G   
   A   
   A   
   T   
   T   
   G   
   A   
   A   
   A   
   A   
   A   
   A   
   G   
   A   
    C    
   G   
   C   
   C   
   A   
   A   
    T    
   G   
   C   
   T   
   C   
   A   
   A   
   G   
   G   
    A    
   A   
   T   
   T   
   T   
   T   
   G   
   A   
   A   
   T   
   T   
   A   
    T    
   T   
   T   
   G   
   T   
   T   
   C   
   A   
   A   
   A   
   A   
   A   
   C   
   A   
   C   
   T   
   G   
   A   
   G   
    C    
   T   
   A   
   C   
   A   
   G   
   A   
   T   
   C   
   A   
   A   
   T   
   T   
   A   
   C   
   A   
   A   
   T   
   T   
   T   
    C    
   A   
   A   
   T   
   A   
   T   
   G   
   G   
   T   
   C   
   G   
   C   
   T   
   A   
   T   
   C   
   G   
   A   
    C    
   C   
   A   
   T   
   A   
   T   
   G   
   A   
   C   
   T   
   C   
   C   
   G   
   C   
   A   
    T    
   C   
   A   
   A   
   G   
   T   
   T   
   G   
   G   
   A   
   C   
   T   
   G   
   A   
   A   
   A   
   G   
   A   
   C   
   A   
   T   
   T   
   T   
   T   
   A   
   A   
   G   
   A   
   A   
   G   
   T   
   T   
   A   
   T   
   A   
   T   
   C   
   G   
   A   
   G   
   C   
   A   
   T   
   C   
   G   
   A   
   A   
   A   
   G   
   C   
   A   
   A   
   G   
   T   
   C   
   A   
   T   
   A   
   A   
   C   
   A   
   A   
   A   
   A   
   C   
   G   
    T    
   A   
   G   
    T    
   C   
   A   
   A   
   T   
   T   
   C   
   G   
   A   
   T   
   T   
   T   
   A   
   G   
   C   
   C   
   A   
   A   
   A   
   G   
   C   
   T   
   C   
   A   
   A   
   A   
   A   
   A   
    A    
   G   
   A   
   C   
   A   
   G   
   C   
   A   
   T   
   A   
   T   
   C   
   G   
   T   
    A    
   G   
   A   
   A   
   G   
   G   
    A    
    T    
   T   
   G   
   A   
   T   
   G   
   A   
   A   
   A   
   G   
   C   
   T   
   T   
   T   
   A   
   T   
   C   
   G   
   A   
   T   
   T   
   C   
   T   
   G   
   G   
   A   
   T   
   G   
   A   
   A   
   G   
   T   
   A   
   A   
   T   
   C   
   G   
    T    
   A   
   A   
   C   
   G   
   A   
   T   
   T   
   C   
   G   
   T   
   G   
   A   
   A   
   A   
   G   
   T   
   A   
   A   
   A   
   G   
   A   
   C   
   A   
   A   
   A   
   A   
   A   
   A   
   G   
   A   
    C    
   G   
   C   
    C    
   A   
   A   
   A   
   A   
   A   
   G   
   A   
   A   
   T   
   T   
   T   
   A   
   G   
   T   
   C   
   G   
   A   
    C    
   G   
   T   
    C    
   T   
   T   
    T    
   C   
   A   
   A   
   T   
   T   
   T   
   A   
   C   
   A   
   G   
   A   
   A   
   G   
   A   
   A   
   C   
   A   
   A   
   G   
   C   
   A   
   G   
   A   
   A   
   G   
   C   
   A   
   A   
   T   
    T    
   G   
   T   
   C   
   A   
   C   
   A   
   T   
   T   
   G   
   C   
   A   
   A   
    T    
   T   
   A   
   T   
   A   
    C    
   C   
   G   
   T   
   T   
   T   
   G   
   A   
   C   
   G   
   A   
   A   
    C    
   A   
   C   
    G    
   G   
   A   
   T   
   A   
   T   
   C   
   A   
   C   
   A   
   G   
   A   
   G   
   C   
   T   
   T   
   C   
   A   
   A   
   A   
   A   
   G   
   G   
   A   
   A   
    T    
   C   
    C    
   G   
   A   
   A   
   A   
    G    
   C   
   T   
   T   
   G   
   A   
   T   
   T   
   G   
   C   
    G    
   C   
   A   
   A   
   A   
   T   
   C   
   A   
   C   
   A   
   G   
   A   
   G   
   T   
   T   
    G    
   A   
   A   
   T   
   A   
   A   
   A   
   A   
   T   
   C   
   C   
   T   
   T   
   T   
   C   
   A   
   A   
   A   
    T    
   G   
   A   
   C   
   A   
   A   
   A   
   G   
   A   
    G    
   C   
   T   
   T   
   T   
   T   
    C    
   T   
   C   
   T   
   G   
   T   
   T   
   A   
   T   
   G   
   A   
   A   
   A   
   A   
   A   
   A   
   G   
   A   
   A   
   T   
   T   
   G   
   C   
   G   
   A   
   G   
   A   
   A   
   G   
   T   
   G   
   A   
   A   
   A   
   A   
   A   
   A   
   A   
   A   
   T   
   T   
   A   
    T    
   T   
   C   
   T   
   T   
   C   
   C   
   G   
   C   
   T   
   C   
   G   
   T   
   C   
    T    
    A    
   A   
   C   
   G   
   A   
   C   
   A   
   A   
   T   
   C   
    G    
   A   
   A   
   G   
   A   
   C   
   G   
   A   
   A   
   A   
   T   
   C   
   G   
   A   
   A   
   G   
   A   
   A   
   A   
   T   
   C   
   A   
   A   
   A   
   A   
   T   
    T    
   G   
   A   
   T   
   A   
   C   
   G   
   C   
   A   
   A   
    G    
   T   
    C    
   T   
   T   
   A   
   G   
   T   
   G   
   G   
   C   
    A    
   C   
   A   
   A   
   G   
   A   
   A   
   G   
   A   
   C   
   G   
   T   
   A   
   A   
   T   
    T    
   G   
   T   
   C   
   A   
   G   
   T   
   G   
   T   
    G    
   A   
   C   
    G    
   C   
   G   
   A   
   G   
   A   
   A   
   G   
   G   
   C   
   T   
   A   
   T   
   G   
   T   
   G   
   A   
   A   
   G   
   C   
   G   
   A   
   A   
   C   
   A   
   A   
   G   
   C   
   C   
   T   
   T   
   C   
   G   
   T   
   T   
   C   
    T    
   T   
   A   
   T   
   A   
   G   
   T   
   G   
   C   
   T   
   T   
   C   
    C    
   A   
   A   
   A   
   C   
   C   
   A   
   G   
   A   
   A   
   G   
   A   
   A   
   A   
   T   
   C   
   G   
   G   
   G   
   A   
   T   
   G   
   C   
   G   
   A   
   G   
   A   
   A   
   G   
   G   
   C   
   G   
   A   
    C    
   T   
   A   
   C   
   T   
   T   
   G   
   C   
   T   
    A    
   T   
   A   
   C   
    T    
   C   
   A   
   G   
   G   
   T   
   G   
   A   
   A   
   T   
   T   
    G    
   A   
   G   
    T    
   A   
   C   
   A   
    T    
   T   
   G   
   G   
   A   
   C   
   C   
   A   
   T   
   G   
   T   
   T   
   C   
   T   
   T   
   C   
   T   
   G   
   A   
   T   
    C    
   A   
   C   
    A    
   A   
   A   
   T   
   A   
   A   
   A   
   G   
   C   
   A   
   A   
   A   
   T   
   G   
   T   
   G   
   A   
   T   
   C   
   T   
   A   
   C   
   C   
   G   
    C    
   C   
   C   
   T   
   G   
   T   
    C    
   C   
   A   
   T   
   G   
   A   
   A   
   C   
   T   
   A   
   C   
   C   
    C    
   G   
   A   
   T   
   T   
   T   
   G   
   A   
   A   
   A   
   T   
   G   
   G   
   A   
   A   
   A   
   G   
   A   
   T   
   G   
   C   
   T   
   G   
   G   
    T    
   G   
   A   
    G    
   C   
   A   
    C    
   A   
   T   
   T   
   T   
   C   
   T   
   C   
   A   
   A   
   A   
   C   
   A   
   A   
   T   
   C   
   T   
   C   
   C   
   A   
   A   
   T   
   C   
   T   
   G   
   T   
   C   
   T   
   G   
   T   
   A   
   G   
   A   
    C    
   G   
   A   
   A   
   T   
   C   
   G   
   A   
   T   
    T    
   T   
   T   
   A   
   G   
   C   
   T   
   G   
   T   
   T   
   T   
   T   
    C    
   C   
   C   
   A   
   T   
   A   
   T   
   C   
   A   
   A   
   A   
   A   
   A   
   A   
   T   
    T    
   C   
   A   
   A   
   G   
   C   
   A   
   G   
   A   
    G    
   A   
   A   
   A   
   A   
   C   
   A   
   T   
   T   
   T   
   G   
   T   
    A    
   T   
   T   
   C   
   A   
   T   
   C   
   A   
   G   
   C   
   A   
   A   
   G   
   A   
   A   
   T   
   G   
   G   
   A   
   T   
   T   
    G    
    A    
   T   
   C   
   A   
   A   
   G   
   C   
   A   
   A   
   A   
   C   
   A   
   C   
   G   
   A   
   A   
   T   
   G   
   A   
   C   
   A   
   G   
   A   
   G   
   T   
   T   
   C   
   G   
   A   
   A   
   C   
   C   
   A   
   T   
   G   
   G   
   C   
   G   
   C   
   A   
   C   
   C   
   T   
   A   
    T    
   A   
   A   
   G   
   A   
   G   
   C   
   C   
   G   
    T    
   C   
   C   
   G   
   C   
   T   
   C   
   A   
   G   
   C   
   T   
   G   
   T   
   A   
   T   
   G   
   A   
   A   
   G   
   C   
   T   
   A   
   A   
   A   
   G   
   G   
   C   
   G   
   G   
   A   
   A   
   G   
   A   
   A   
   G   
   A   
   T   
   G   
   A   
   A   
   C   
   T   
    G    
   A   
   T   
   T   
   G   
   C   
   T   
   G   
   T   
    G    
   T   
   A   
   T   
   C   
   T   
   G   
   A   
   A   
   A   
   G   
   A   
   A   
   G   
   A   
   T   
   C   
    A    
   G   
   C   
   C   
   A   
   G   
   G   
   C   
   T   
   T   
   A   
   G   
   A   
   T   
   G   
   T   
    T    
   T   
   T   
   C   
   T   
   T   
   A   
   G   
   T   
   A   
   A   
   C   
   G   
    C    
   A   
   T   
   C   
   A   
   A   
   G   
   G   
   A   
   A   
   T   
   G   
   G   
   G   
    T    
   T   
   T   
   G   
   C   
   G   
   C   
   T   
    A    
   T   
   C   
   C   
   T   
   T   
   T   
   A   
   A   
    C    
   A   
   G   
   A   
   T   
   G   
   T   
   T   
   C   
   C   
   C   
   G   
   T   
   T   
   G   
   T   
   T   
   G   
   G   
   A   
   G   
   C   
   A   
   A   
   A   
   G   
   G   
   C   
   A   
   G   
    C    
   T   
   G   
   G   
   A   
   G   
   T   
   G   
   A   
   A   
   G   
   T   
   C   
   T   
   A   
   T   
   G   
   A   
   A   
   T   
   T   
   T   
   G   
   A   
   A   
   A   
   G   
   A   
   T   
   G   
   G   
   A   
   G   
   A   
   T   
   A   
   G   
   G   
    A    
   T   
   T   
    A    
   T   
   C   
   A   
   A   
    C    
   G   
   G   
    G    
   C   
   T   
    G    
   C   
   T   
   T   
   G   
   T   
   G   
   T   
   T   
   A   
   G   
   C   
   A   
   G   
   A   
    A    
   G   
   G   
    T    
   G   
   A   
   T   
   A   
   C   
   G   
   C   
   C   
    G    
   A   
   T   
   C   
   G   
   T   
   A   
   A   
   T   
   C   
   G   
   T   
   T   
   A   
   C   
   T   
   C   
   A   
   G   
   C   
   G   
   A   
   G   
   G   
   A   
   G   
   C   
    T    
   G   
   T   
    T    
   A   
   A   
   A   
   C   
   G   
   C   
   A   
   T   
   G   
   C   
   T   
   T   
   G   
   C   
   T   
   C   
   A   
   A   
   G   
   A   
   A   
   A   
   T   
   C   
   A   
   G   
   C   
   C   
   A   
   A   
   A   
   C   
    T    
   T   
   C   
   A   
   C   
   G   
   T   
   G   
   C   
   G   
   A   
   A   
   G   
   C   
   G   
   G   
   G   
   G   
   A   
   G   
   T   
   G   
   A   
   C   
   G   
   G   
   T   
   C   
   A   
   T   
   G   
   C   
   G   
   T   
   G   
   A   
   G   
   C   
   T   
   G   
   A   
   A   
   A   
   A   
   A   
   A   
   A   
   A   
   T   
   C   
   C   
    G    
   C   
   A   
    C    
   C   
   G   
    T    
   A   
   T   
   T   
   A   
   T   
   C   
   T   
   A   
   T   
   A   
   T   
   G   
   T   
   C   
   G   
   G   
   A   
   A   
   G   
   G   
   A   
   A   
   G   
   A   
   T   
   C   
   A   
   A   
   A   
   A   
   G   
   A   
   A   
   A   
   T   
   A   
   A   
   C   
   C   
   T   
   T   
   G   
   A   
   T   
   G   
   A   
   A   
   T   
   C   
   A   
   A   
   A   
   A   
   A   
   G   
   G   
    G    
   C   
   A   
    G    
   C   
   A   
   G   
    T    
   T   
   A   
   A   
   C   
   G   
   A   
   T   
   T   
   G   
   A   
    C    
   C   
   C   
   A   
   A   
   C   
    T    
   G   
   A   
   T   
   T   
   T   
    C    
   C   
   C   
   T   
   A   
   T   
   A   
   G   
   G   
   T   
   G   
   A   
   T   
   C   
   G   
   A   
   A   
   C   
   A   
   T   
   C   
   G   
   A   
   A   
   T   
   G   
   G   
    A    
   T   
   C   
    A    
   T   
   T   
   T   
   G   
   C   
    G    
   A   
   T   
   G   
   G   
   A   
    T    
   G   
   A   
   G   
   A   
   A   
   A   
   A   
   A   
   A   
   G   
   G   
   T   
   G   
   G   
   A   
   G   
   A   
   A   
   G   
   T   
   C   
   A   
   T   
   C   
   G   
   A   
   A   
   G   
   T   
    T    
   A   
    T    
   C   
   G   
   A   
   T   
   G   
   C   
   T   
   C   
   C   
   T   
   G   
   A   
   G   
    A    
   T   
   T   
   T   
   T   
    C    
   A   
   T   
    C    
   G   
   A   
    G    
    T    
   C   
    G    
   -   
   -   
   -   
 

 
   N53548   
   A   
   T   
   G   
   G   
   A   
   A   
   A   
   A   
   T   
   C   
   G   
   A   
   C   
   A   
   A   
   G   
   A   
   A   
   G   
   T   
   A   
   C   
   A   
   A   
   G   
   A   
   A   
   T   
   T   
   A   
   A   
   C   
   T   
   T   
   T   
   A   
   G   
   A   
   A   
   G   
   A   
   A   
   G   
   T   
   G   
   A   
   T   
   G   
   G   
   G   
   T   
   G   
   A   
   C   
   C   
   G   
   C   
   T   
   T   
   C   
   G   
   G   
   A   
   C   
   G   
   T   
   T   
   A   
   T   
   T   
   C   
   C   
   A   
   A   
   A   
   T   
   A   
   T   
   A   
   T   
   T   
   A   
   T   
    C    
   C   
   A   
    A    
   G   
   A   
   A   
   C   
   G   
   T   
   G   
   C   
   A   
   C   
   T   
   G   
   C   
   C   
   T   
   G   
   A   
   T   
   A   
   T   
    C    
   C   
   G   
   T   
   G   
   A   
   T   
   G   
   G   
   A   
   T   
   T   
   G   
   A   
   A   
    G    
   C   
   C   
   A   
   G   
   T   
    G    
   C   
   A   
   A   
   C   
   G   
   C   
   C   
   G   
   G   
   A   
   T   
   A   
   T   
   T   
   A   
   T   
   T   
    T    
   G   
   C   
    C    
   A   
   T   
   G   
   A   
   A   
   C   
   A   
   A   
   A   
   G   
   A   
   T   
   G   
   G   
   C   
   A   
   A   
   T   
   A   
   C   
   G   
   T   
   A   
   T   
   G   
   A   
   C   
   A   
   A   
   A   
   G   
   G   
   A   
   T   
   T   
    C    
   C   
   G   
   T   
   A   
   A   
   A   
   T   
   C   
   A   
   G   
   C   
   A   
   A   
   A   
   A   
   T   
   C   
   T   
   G   
   T   
   C   
   G   
   G   
   A   
   A   
   A   
   T   
   A   
   T   
   C   
   A   
   T   
   G   
   G   
   G   
    G    
   A   
   A   
   T   
   T   
   A   
   T   
   C   
   A   
   T   
   C   
   C   
   T   
   C   
   A   
   T   
   G   
   G   
   T   
   G   
   A   
   C   
   A   
   G   
   C   
   A   
   G   
   T   
   A   
   T   
   T   
   T   
   A   
   T   
   G   
   A   
   A   
   G   
   C   
   G   
   A   
   T   
   G   
   G   
   T   
   C   
   C   
   G   
   C   
   A   
   T   
   G   
   A   
   G   
   C   
   C   
   A   
   A   
   G   
   A   
    C    
   T   
   G   
   G   
   A   
   A   
   G   
   T   
   T   
   G   
   C   
   G   
   G   
   G   
   A   
   A   
   G   
   T   
   A   
   C   
   T   
   G   
   A   
   T   
   C   
   G   
   A   
    A    
   A   
   T   
   G   
   C   
   A   
   C   
   G   
   G   
    G    
   A   
   A   
   T   
   A   
   A   
    C    
   G   
   G   
   G   
   A   
   G   
   T   
   A   
   T   
   G   
   G   
   A   
   C   
   G   
   G   
   T   
   G   
   A   
   T   
   C   
   C   
   A   
   C   
   C   
   T   
   G   
   C   
   T   
   G   
   C   
   G   
   A   
   T   
   G   
   C   
   G   
   T   
   T   
   A   
   T   
   A   
   C   
   T   
   G   
   A   
   A   
   G   
   C   
   C   
   A   
   G   
    A    
   T   
   T   
   A   
   T   
   C   
   C   
   A   
   A   
   G   
   T   
   T   
   A   
   A   
   G   
   T   
   G   
   G   
   A   
   G   
   A   
   G   
   A   
   T   
   G   
    C    
   T   
   A   
    G    
   C   
   A   
   G   
   A   
   T   
   A   
   T   
   C   
   G   
   A   
    A    
   A   
   A   
   A   
   G   
   A   
   A   
   A   
   C   
   A   
   G   
   T   
    C    
   G   
   A   
   T   
   C   
   T   
   C   
   G   
   T   
   G   
   T   
   G   
   G   
   A   
   A   
   T   
   T   
   T   
   T   
   G   
   A   
   T   
   G   
   A   
   T   
   A   
   C   
   A   
   G   
   A   
   A   
   A   
   A   
   A   
   G   
   A   
   A   
   C   
   C   
   A   
   A   
   C   
   A   
   G   
   T   
   C   
   C   
   T   
   T   
   C   
   C   
   A   
   G   
   C   
   T   
   C   
   G   
   A   
   T   
   A   
   C   
   C   
   C   
   A   
   A   
   A   
   C   
   C   
   T   
   T   
   C   
   T   
    C    
   G   
   T   
   C   
   A   
   A   
   T   
   G   
   G   
   A   
   T   
   C   
   G   
   A   
   C   
   A   
   G   
   G   
   T   
   A   
   T   
   A   
   T   
   C   
   T   
   G   
   C   
   T   
   G   
   G   
   A   
   T   
   A   
   T   
   G   
   C   
    A    
   A   
   C   
   A   
   G   
   A   
   A   
   A   
   T   
   C   
   C   
   C   
   G   
   A   
   C   
    T    
   C   
   A   
   T   
   A   
   A   
   C   
   T   
   T   
   A   
   G   
   C   
   T   
   G   
   A   
   A   
   G   
   T   
   G   
   A   
   T   
    C    
   G   
   A   
    T    
   G   
   G   
   C   
   A   
   C   
   A   
   A   
   T   
   C   
   T   
   A   
   T   
   A   
   T   
   G   
   A   
   T   
   C   
   G   
   A   
   T   
   C   
   A   
   T   
   C   
   C   
   A   
   C   
   A   
   A   
   G   
   C   
   G   
   A   
   G   
   C   
   C   
   T   
   T   
   G   
   A   
   A   
   A   
   A   
   A   
   T   
   T   
   G   
   A   
   T   
   G   
   G   
   A   
   A   
   T   
   A   
   T   
   A   
   T   
   T   
   C   
   C   
   T   
   G   
   G   
   A   
   C   
   C   
   T   
   G   
   A   
   C   
   T   
   T   
    T    
   C   
   C   
   A   
   A   
   C   
   A   
   G   
   G   
   A   
   G   
   G   
    G    
   A   
   T   
   C   
   T   
   T   
   G   
   C   
   A   
   A   
   G   
   G   
    A    
   A   
   A   
   A   
   G   
   A   
   T   
   G   
   A   
   A   
   A   
   T   
   C   
   A   
   A   
   A   
   A   
   A   
   A   
   G   
   C   
   T   
   T   
   A   
   T   
   G   
   A   
   A   
   A   
   C   
   A   
   G   
   G   
    G    
   C   
   G   
   A   
   G   
   G   
   A   
   A   
   A   
   A   
   G   
   T   
   T   
   A   
    T    
   T   
   C   
   T   
   T   
   C   
   G   
   T   
   T   
   C   
   G   
   A   
   A   
   A   
   A   
   C   
   A   
   A   
   A   
   G   
   A   
   T   
   A   
   G   
   A   
    G    
   G   
   C   
   A   
   A   
   T   
   G   
   A   
   A   
   G   
   G   
   G   
    T    
   A   
   A   
    T    
   A   
   A   
   A   
   C   
   A   
   G   
   C   
   A   
   A   
   A   
   T   
   C   
   G   
   T   
   G   
   A   
   T   
   C   
   A   
   C   
    A    
   G   
   A   
   A   
   A   
   T   
   C   
   C   
   C   
   A   
   T   
   A   
   T   
   G   
   A   
   A   
   G   
   T   
   A   
   A   
   A   
   T   
   A   
   A   
   A   
   G   
   C   
   G   
   A   
   C   
    C    
   T   
   T   
   G   
   G   
   T   
   G   
   A   
   A   
   A   
   A   
   A   
   A   
   A   
   T   
   G   
   G   
   A   
   T   
   G   
   A   
   G   
   A   
   T   
   C   
   C   
   G   
   T   
   T   
   T   
   G   
   A   
   A   
   T   
   A   
   A   
   G   
   A   
   A   
   A   
   A   
   T   
   C   
   G   
   A   
    T    
   G   
   G   
   A   
   A   
   T   
   A   
   G   
   C   
   T   
   G   
   A   
   A   
   G   
   T   
    A    
   C   
   G   
   A   
   G   
   A   
   T   
   G   
   A   
   A   
   A   
   G   
   T   
   G   
   A   
   T   
    A    
   G   
    A    
   A   
   C   
    A    
   G   
   G   
   T   
   C   
   T   
   G   
   C   
   A   
   A   
   A   
   T   
   T   
   G   
   T   
   C   
   G   
   T   
    C    
   G   
   A   
   A   
   T   
   T   
   G   
   A   
   A   
   A   
   A   
   A   
   A   
   G   
   A   
    C    
   G   
   C   
   C   
   A   
   A   
    T    
   G   
   C   
   T   
   C   
   A   
   A   
   G   
   G   
    A    
   A   
   T   
   T   
   T   
   T   
   G   
   A   
   A   
   T   
   T   
   A   
    T    
   T   
   T   
   G   
   T   
   T   
   C   
   A   
   A   
   A   
   A   
   A   
   C   
   A   
   C   
   T   
   G   
   A   
   G   
    C    
   T   
   A   
   C   
   A   
   G   
   A   
   T   
   C   
   A   
   A   
   T   
   T   
   A   
   C   
   A   
   A   
   T   
   T   
   T   
    C    
   A   
   A   
   T   
   A   
   T   
   G   
   G   
   T   
   C   
   G   
   C   
   T   
   A   
   T   
   C   
   G   
   A   
    C    
   C   
   A   
   T   
   A   
   T   
   G   
   A   
   C   
   T   
   C   
   C   
   G   
   C   
   A   
    T    
   C   
   A   
   A   
   G   
   T   
   T   
   G   
   G   
   A   
   C   
   T   
   G   
   A   
   A   
   A   
   G   
   A   
   C   
   A   
   T   
   T   
   T   
   T   
   A   
   A   
   G   
   A   
   A   
   G   
   T   
   T   
   A   
   T   
   A   
   T   
   C   
   G   
   A   
   G   
   C   
   A   
   T   
   C   
   G   
   A   
   A   
   A   
   G   
   C   
   A   
   A   
   G   
   T   
   C   
   A   
   T   
   A   
   A   
   C   
   A   
   A   
   A   
   A   
   C   
   G   
    T    
   A   
   G   
    T    
   C   
   A   
   A   
   T   
   T   
   C   
   G   
   A   
   T   
   T   
   T   
   A   
   G   
   C   
   C   
   A   
   A   
   A   
   G   
   C   
   T   
   C   
   A   
   A   
   A   
   A   
   A   
    A    
   G   
   A   
   C   
   A   
   G   
   C   
   A   
   T   
   A   
   T   
   C   
   G   
   T   
    A    
   G   
   A   
   A   
   G   
   G   
    A    
    T    
   T   
   G   
   A   
   T   
   G   
   A   
   A   
   A   
   G   
   C   
   T   
   T   
   T   
   A   
   T   
   C   
   G   
   A   
   T   
   T   
   C   
   T   
   G   
   G   
   A   
   T   
   G   
   A   
   A   
   G   
   T   
   A   
   A   
   T   
   C   
   G   
    C    
   A   
   A   
   C   
   G   
   A   
   T   
   T   
   C   
   G   
   T   
   G   
   A   
   A   
   A   
   G   
   T   
   A   
   A   
   A   
   G   
   A   
   C   
   A   
   A   
   A   
   A   
   A   
   A   
   G   
   A   
    C    
   G   
   C   
    C    
   A   
   A   
   A   
   A   
   A   
   G   
   A   
   A   
   T   
   T   
   T   
   A   
   G   
   T   
   C   
   G   
   A   
    C    
   G   
   T   
    C    
   T   
   T   
    T    
   C   
   A   
   A   
   T   
   T   
   T   
   A   
   C   
   A   
   G   
   A   
   A   
   G   
   A   
   A   
   C   
   A   
   A   
   G   
   C   
   A   
   G   
   A   
   A   
   G   
   C   
   A   
   A   
   T   
    T    
   G   
   T   
   C   
   A   
   C   
   A   
   T   
   T   
   G   
   C   
   A   
   A   
    T    
   T   
   A   
   T   
   A   
    C    
   C   
   G   
   T   
   T   
   T   
   G   
   A   
   C   
   G   
   A   
   A   
    C    
   A   
   C   
    G    
   G   
   A   
   T   
   A   
   T   
   C   
   A   
   C   
   A   
   G   
   A   
   G   
   C   
   T   
   T   
   C   
   A   
   A   
   A   
   A   
   G   
   G   
   A   
   A   
    T    
   C   
    C    
   G   
   A   
   A   
   A   
    G    
   C   
   T   
   T   
   G   
   A   
   T   
   T   
   G   
   C   
    G    
   C   
   A   
   A   
   A   
   T   
   C   
   A   
   C   
   A   
   G   
   A   
   G   
   T   
   T   
    G    
   A   
   A   
   T   
   A   
   A   
   A   
   A   
   T   
   C   
   C   
   T   
   T   
   T   
   C   
   A   
   A   
   A   
    T    
   G   
   A   
   C   
   A   
   A   
   A   
   G   
   A   
    G    
   C   
   T   
   T   
   T   
   T   
    C    
   T   
   C   
   T   
   G   
   T   
   T   
   A   
   T   
   G   
   A   
   A   
   A   
   A   
   A   
   A   
   G   
   A   
   A   
   T   
   T   
   G   
   C   
   G   
   A   
   G   
   A   
   A   
   G   
   T   
   G   
   A   
   A   
   A   
   A   
   A   
   A   
   A   
   A   
   T   
   T   
   A   
    T    
   T   
   C   
   T   
   T   
   C   
   C   
   G   
   C   
   T   
   C   
   G   
   T   
   C   
    T    
    A    
   A   
   C   
   G   
   A   
   C   
   A   
   A   
   T   
   C   
    G    
   A   
   A   
   G   
   A   
   C   
   G   
   A   
   A   
   A   
   T   
   C   
   G   
   A   
   A   
   G   
   A   
   A   
   A   
   T   
   C   
   A   
   A   
   A   
   A   
   T   
    T    
   G   
   A   
   T   
   A   
   C   
   G   
   C   
   A   
   A   
    G    
   T   
    C    
   T   
   T   
   A   
   G   
   T   
   G   
   G   
   C   
    A    
   C   
   A   
   A   
   G   
   A   
   A   
   G   
   A   
   C   
   G   
   T   
   A   
   A   
   T   
    T    
   G   
   T   
   C   
   A   
   G   
   T   
   G   
   T   
    G    
   A   
   C   
    G    
   C   
   G   
   A   
   G   
   A   
   A   
   G   
   G   
   C   
   T   
   A   
   T   
   G   
   T   
   G   
   A   
   A   
   G   
   C   
   G   
   A   
   A   
   C   
   A   
   A   
   G   
   C   
   C   
   T   
   T   
   C   
   G   
   T   
   T   
   C   
    T    
   T   
   A   
   T   
   A   
   G   
   T   
   G   
   C   
   T   
   T   
   C   
    C    
   A   
   A   
   A   
   C   
   C   
   A   
   G   
   A   
   A   
   G   
   A   
   A   
   A   
   T   
   C   
   G   
   G   
   G   
   A   
   T   
   G   
   C   
   G   
   A   
   G   
   A   
   A   
   G   
   G   
   C   
   G   
   A   
    C    
   T   
   A   
   C   
   T   
   T   
   G   
   C   
   T   
    A    
   T   
   A   
   C   
    T    
   C   
   A   
   G   
   G   
   T   
   G   
   A   
   A   
   T   
   T   
    G    
   A   
   G   
    T    
   A   
   C   
   A   
    T    
   T   
   G   
   G   
   A   
   C   
   C   
   A   
   T   
   G   
   T   
   T   
   C   
   T   
   T   
   C   
   T   
   G   
   A   
   T   
    C    
   A   
   C   
    A    
   A   
   A   
   T   
   A   
   A   
   A   
   G   
   C   
   A   
   A   
   A   
   T   
   G   
   T   
   G   
   A   
   T   
   C   
   T   
   A   
   C   
   C   
   G   
    C    
   C   
   C   
   T   
   G   
   T   
    C    
   C   
   A   
   T   
   G   
   A   
   A   
   C   
   T   
   A   
   C   
   C   
    C    
   G   
   A   
   T   
   T   
   T   
   G   
   A   
   A   
   A   
   T   
   G   
   G   
   A   
   A   
   A   
   G   
   A   
   T   
   G   
   C   
   T   
   G   
   G   
    T    
   G   
   A   
    G    
   C   
   A   
    C    
   A   
   T   
   T   
   T   
   C   
   T   
   C   
   A   
   A   
   A   
   C   
   A   
   A   
   T   
   C   
   T   
   C   
   C   
   A   
   A   
   T   
   C   
   T   
   G   
   T   
   C   
   T   
   G   
   T   
   A   
   G   
   A   
    C    
   G   
   A   
   A   
   T   
   C   
   G   
   A   
   T   
    T    
   T   
   T   
   A   
   G   
   C   
   T   
   G   
   T   
   T   
   T   
   T   
    C    
   C   
   C   
   A   
   T   
   A   
   T   
   C   
   A   
   A   
   A   
   A   
   A   
   A   
   T   
    T    
   C   
   A   
   A   
   G   
   C   
   A   
   G   
   A   
    G    
   A   
   A   
   A   
   A   
   C   
   A   
   T   
   T   
   T   
   G   
   T   
    A    
   T   
   T   
   C   
   A   
   T   
   C   
   A   
   G   
   C   
   A   
   A   
   G   
   A   
   A   
   T   
   G   
   G   
   A   
   T   
   T   
    G    
    A    
   T   
   C   
   A   
   A   
   G   
   C   
   A   
   A   
   A   
   C   
   A   
   C   
   G   
   A   
   A   
   T   
   G   
   A   
   C   
   A   
   G   
   A   
   G   
   T   
   T   
   C   
   G   
   A   
   A   
   C   
   C   
   A   
   T   
   G   
   G   
   C   
   G   
   C   
   A   
   C   
   C   
   T   
   A   
    T    
   A   
   A   
   G   
   A   
   G   
   C   
   C   
   G   
    T    
   C   
   C   
   G   
   C   
   T   
   C   
   A   
   G   
   C   
   T   
   G   
   T   
   A   
   T   
   G   
   A   
   A   
   G   
   C   
   T   
   A   
   A   
   A   
   G   
   G   
   C   
   G   
   G   
   A   
   A   
   G   
   A   
   A   
   G   
   A   
   T   
   G   
   A   
   A   
   C   
   T   
    G    
   A   
   T   
   T   
   G   
   C   
   T   
   G   
   T   
    G    
   T   
   A   
   T   
   C   
   T   
   G   
   A   
   A   
   A   
   G   
   A   
   A   
   G   
   A   
   T   
   C   
    A    
   G   
   C   
   C   
   A   
   G   
   G   
   C   
   T   
   T   
   A   
   G   
   A   
   T   
   G   
   T   
    T    
   T   
   T   
   C   
   T   
   T   
   A   
   G   
   T   
   A   
   A   
   C   
   G   
    C    
   A   
   T   
   C   
   A   
   A   
   G   
   G   
   A   
   A   
   T   
   G   
   G   
   G   
    T    
   T   
   T   
   G   
   C   
   G   
   C   
   T   
    A    
   T   
   C   
   C   
   T   
   T   
   T   
   A   
   A   
    C    
   A   
   G   
   A   
   T   
   G   
   T   
   T   
   C   
   C   
   C   
   G   
   T   
   T   
   G   
   T   
   T   
   G   
   G   
   A   
   G   
   C   
   A   
   A   
   A   
   G   
   G   
   C   
   A   
   G   
    C    
   T   
   G   
   G   
   A   
   G   
   T   
   G   
   A   
   A   
   G   
   T   
   C   
   T   
   A   
   T   
   G   
   A   
   A   
   T   
   T   
   T   
   G   
   A   
   A   
   A   
   G   
   A   
   T   
   G   
   G   
   A   
   G   
   A   
   T   
   A   
   G   
   G   
    A    
   T   
   T   
    A    
   T   
   C   
   A   
   A   
    C    
   G   
   G   
    G    
   C   
   T   
    G    
   C   
   T   
   T   
   G   
   T   
   G   
   T   
   T   
   A   
   G   
   C   
   A   
   G   
   A   
    A    
   G   
   G   
    T    
   G   
   A   
   T   
   A   
   C   
   G   
   C   
   C   
    G    
   A   
   T   
   C   
   G   
   T   
   A   
   A   
   T   
   C   
   G   
   T   
   T   
   A   
   C   
   T   
   C   
   A   
   G   
   C   
   G   
   A   
   G   
   G   
   A   
   G   
   C   
    T    
   G   
   T   
    T    
   A   
   A   
   A   
   C   
   G   
   C   
   A   
   T   
   G   
   C   
   T   
   T   
   G   
   C   
   T   
   C   
   A   
   A   
   G   
   A   
   A   
   A   
   T   
   C   
   A   
   G   
   C   
   C   
   A   
   A   
   A   
   C   
    T    
   T   
   C   
   A   
   C   
   G   
   T   
   G   
   C   
   G   
   A   
   A   
   G   
   C   
   G   
   G   
   G   
   G   
   A   
   G   
   T   
   G   
   A   
   C   
   G   
   G   
   T   
   C   
   A   
   T   
   G   
   C   
   G   
   T   
   G   
   A   
   G   
   C   
   T   
   G   
   A   
   A   
   A   
   A   
   A   
   A   
   A   
   A   
   T   
   C   
   C   
    G    
   C   
   A   
    C    
   C   
   G   
    T    
   A   
   T   
   T   
   A   
   T   
   C   
   T   
   A   
   T   
   A   
   T   
   G   
   T   
   C   
   G   
   G   
   A   
   A   
   G   
   G   
   A   
   A   
   G   
   A   
   T   
   C   
   A   
   A   
   A   
   A   
   G   
   A   
   A   
   A   
   T   
   A   
   A   
   C   
   C   
   T   
   T   
   G   
   A   
   T   
   G   
   A   
   A   
   T   
   C   
   A   
   A   
   A   
   A   
   A   
   G   
   G   
    G    
   C   
   A   
    G    
   C   
   A   
   G   
    T    
   T   
   A   
   A   
   C   
   G   
   A   
   T   
   T   
   G   
   A   
    C    
   C   
   C   
   A   
   A   
   C   
    T    
   G   
   A   
   T   
   T   
   T   
    C    
   C   
   C   
   T   
   A   
   T   
   A   
   G   
   G   
   T   
   G   
   A   
   T   
   C   
   G   
   A   
   A   
   C   
   A   
   T   
   C   
   G   
   A   
   A   
   T   
   G   
   G   
    A    
   T   
   C   
    A    
   T   
   T   
   T   
   G   
   C   
    G    
   A   
   T   
   G   
   G   
   A   
    T    
   G   
   A   
   G   
   A   
   A   
   A   
   A   
   A   
   A   
   G   
   G   
   T   
   G   
   G   
   A   
   G   
   A   
   A   
   G   
   T   
   C   
   A   
   T   
   C   
   G   
   A   
   A   
   G   
   T   
    T    
   A   
    T    
   C   
   G   
   A   
   T   
   G   
   C   
   T   
   C   
   C   
   T   
   G   
   A   
   G   
    A    
   T   
   T   
   T   
   T   
    C    
   A   
   T   
    C    
   G   
   A   
    G    
    T    
   C   
    G    
   -   
   -   
   -   
 

 
   N55279   
   A   
   T   
   G   
   G   
   A   
   A   
   A   
   A   
   T   
   C   
   G   
   A   
   C   
   A   
   A   
   G   
   A   
   A   
   G   
   T   
   A   
   C   
   A   
   A   
   G   
   A   
   A   
   T   
   T   
   A   
   A   
   C   
   T   
   T   
   T   
   A   
   G   
   A   
   A   
   G   
   A   
   A   
   G   
   T   
   G   
   A   
   T   
   G   
   G   
   G   
   T   
   G   
   A   
   C   
   C   
   G   
   C   
   T   
   T   
   C   
   G   
   G   
   A   
   C   
   G   
   T   
   T   
   A   
   T   
   T   
   C   
   C   
   A   
   A   
   A   
   T   
   A   
   T   
   A   
   T   
   T   
   A   
   T   
    C    
   C   
   A   
    A    
   G   
   A   
   A   
   C   
   G   
   T   
   G   
   C   
   A   
   C   
   T   
   G   
   C   
   C   
   T   
   G   
   A   
   T   
   A   
   T   
    C    
   C   
   G   
   T   
   G   
   A   
   T   
   G   
   G   
   A   
   T   
   T   
   G   
   A   
   A   
    A    
   C   
   C   
   A   
   G   
   T   
    G    
   C   
   A   
   A   
   C   
   G   
   C   
   C   
   G   
   G   
   A   
   T   
   A   
   T   
   T   
   A   
   T   
   T   
    T    
   G   
   C   
    C    
   A   
   T   
   G   
   A   
   A   
   C   
   A   
   A   
   A   
   G   
   A   
   T   
   G   
   G   
   C   
   A   
   A   
   T   
   A   
   C   
   G   
   T   
   A   
   T   
   G   
   A   
   C   
   A   
   A   
   A   
   G   
   G   
   A   
   T   
   T   
    C    
   C   
   G   
   T   
   A   
   A   
   A   
   T   
   C   
   A   
   G   
   C   
   A   
   A   
   A   
   A   
   T   
   C   
   T   
   G   
   T   
   C   
   G   
   G   
   A   
   A   
   A   
   T   
   A   
   T   
   C   
   A   
   T   
   G   
   G   
   G   
    G    
   A   
   A   
   T   
   T   
   A   
   T   
   C   
   A   
   T   
   C   
   C   
   T   
   C   
   A   
   T   
   G   
   G   
   T   
   G   
   A   
   C   
   A   
   G   
   C   
   A   
   G   
   T   
   A   
   T   
   T   
   T   
   A   
   T   
   G   
   A   
   A   
   G   
   C   
   G   
   A   
   T   
   G   
   G   
   T   
   C   
   C   
   G   
   C   
   A   
   T   
   G   
   A   
   G   
   C   
   C   
   A   
   A   
   G   
   A   
    T    
   T   
   G   
   G   
   A   
   A   
   G   
   T   
   T   
   G   
   C   
   G   
   G   
   G   
   A   
   A   
   G   
   T   
   A   
   C   
   T   
   G   
   A   
   T   
   C   
   G   
   A   
    A    
   A   
   T   
   G   
   C   
   A   
   C   
   G   
   G   
    G    
   A   
   A   
   T   
   A   
   A   
    T    
   G   
   G   
   G   
   A   
   G   
   T   
   A   
   T   
   G   
   G   
   A   
   C   
   G   
   G   
   T   
   G   
   A   
   T   
   C   
   C   
   A   
   C   
   C   
   T   
   G   
   C   
   T   
   G   
   C   
   G   
   A   
   T   
   G   
   C   
   G   
   T   
   T   
   A   
   T   
   A   
   C   
   T   
   G   
   A   
   A   
   G   
   C   
   C   
   A   
   G   
    A    
   T   
   T   
   A   
   T   
   C   
   C   
   A   
   A   
   G   
   T   
   T   
   A   
   A   
   G   
   T   
   G   
   G   
   A   
   G   
   A   
   G   
   A   
   T   
   G   
    C    
   T   
   A   
    G    
   C   
   A   
   G   
   A   
   T   
   A   
   T   
   C   
   G   
   A   
    A    
   A   
   A   
   A   
   G   
   A   
   A   
   A   
   C   
   A   
   G   
   T   
    C    
   G   
   A   
   T   
   C   
   T   
   C   
   G   
   T   
   G   
   T   
   G   
   G   
   A   
   A   
   T   
   T   
   T   
   T   
   G   
   A   
   T   
   G   
   A   
   T   
   A   
   C   
   A   
   G   
   A   
   A   
   A   
   A   
   A   
   G   
   A   
   A   
   C   
   C   
   A   
   A   
   C   
   A   
   G   
   T   
   C   
   C   
   T   
   T   
   C   
   C   
   A   
   G   
   C   
   T   
   C   
   G   
   A   
   T   
   A   
   C   
   C   
   C   
   A   
   A   
   A   
   C   
   C   
   T   
   T   
   C   
   T   
    A    
   G   
   T   
   C   
   A   
   A   
   T   
   G   
   G   
   A   
   T   
   C   
   G   
   A   
   C   
   A   
   G   
   G   
   T   
   A   
   T   
   A   
   T   
   C   
   T   
   G   
   C   
   T   
   G   
   G   
   A   
   T   
   A   
   T   
   G   
   C   
    G    
   A   
   C   
   A   
   G   
   A   
   A   
   A   
   T   
   C   
   C   
   C   
   G   
   A   
   C   
    T    
   C   
   A   
   T   
   A   
   A   
   C   
   T   
   T   
   A   
   G   
   C   
   T   
   G   
   A   
   A   
   G   
   T   
   G   
   A   
   T   
    C    
   G   
   A   
    C    
   G   
   G   
   C   
   A   
   C   
   A   
   A   
   T   
   C   
   T   
   A   
   T   
   A   
   T   
   G   
   A   
   T   
   C   
   G   
   A   
   T   
   C   
   A   
   T   
   C   
   C   
   A   
   C   
   A   
   A   
   G   
   C   
   G   
   A   
   G   
   C   
   C   
   T   
   T   
   G   
   A   
   A   
   A   
   A   
   A   
   T   
   T   
   G   
   A   
   T   
   G   
   G   
   A   
   A   
   T   
   A   
   T   
   A   
   T   
   T   
   C   
   C   
   T   
   G   
   G   
   A   
   C   
   C   
   T   
   G   
   A   
   C   
   T   
   T   
    T    
   C   
   C   
   A   
   A   
   C   
   A   
   G   
   G   
   A   
   G   
   G   
    A    
   A   
   T   
   C   
   T   
   T   
   G   
   C   
   A   
   A   
   G   
   G   
    G    
   A   
   A   
   A   
   G   
   A   
   T   
   G   
   A   
   A   
   A   
   T   
   C   
   A   
   A   
   A   
   A   
   A   
   A   
   G   
   C   
   T   
   T   
   A   
   T   
   G   
   A   
   A   
   A   
   C   
   A   
   G   
   G   
    G    
   C   
   G   
   A   
   G   
   G   
   A   
   A   
   A   
   A   
   G   
   T   
   T   
   A   
    T    
   T   
   C   
   T   
   T   
   C   
   G   
   T   
   T   
   C   
   G   
   A   
   A   
   A   
   A   
   C   
   A   
   A   
   A   
   G   
   A   
   T   
   A   
   G   
   A   
    A    
   G   
   C   
   A   
   A   
   T   
   G   
   A   
   A   
   G   
   G   
   G   
    C    
   A   
   A   
    T    
   A   
   A   
   A   
   C   
   A   
   G   
   C   
   A   
   A   
   A   
   T   
   C   
   G   
   T   
   G   
   A   
   T   
   C   
   A   
   C   
    T    
   G   
   A   
   A   
   A   
   T   
   C   
   C   
   C   
   A   
   T   
   A   
   T   
   G   
   A   
   A   
   G   
   T   
   A   
   A   
   A   
   T   
   A   
   A   
   A   
   G   
   C   
   G   
   A   
   C   
    G    
   T   
   T   
   G   
   G   
   T   
   G   
   A   
   A   
   A   
   A   
   A   
   A   
   A   
   T   
   G   
   G   
   A   
   T   
   G   
   A   
   G   
   A   
   T   
   C   
   C   
   G   
   T   
   T   
   T   
   G   
   A   
   A   
   T   
   A   
   A   
   G   
   A   
   A   
   A   
   A   
   T   
   C   
   G   
   A   
    T    
   G   
   G   
   A   
   A   
   T   
   A   
   G   
   C   
   T   
   G   
   A   
   A   
   G   
   T   
    A    
   C   
   G   
   A   
   G   
   A   
   T   
   G   
   A   
   A   
   A   
   G   
   T   
   G   
   A   
   T   
    C    
   G   
    A    
   A   
   C   
    T    
   G   
   G   
   T   
   C   
   T   
   G   
   C   
   A   
   A   
   A   
   T   
   T   
   G   
   T   
   C   
   G   
   T   
    T    
   G   
   A   
   A   
   T   
   T   
   G   
   A   
   A   
   A   
   A   
   A   
   A   
   G   
   A   
    T    
   G   
   C   
   C   
   A   
   A   
    T    
   G   
   C   
   T   
   C   
   A   
   A   
   G   
   G   
    A    
   A   
   T   
   T   
   T   
   T   
   G   
   A   
   A   
   T   
   T   
   A   
    T    
   T   
   T   
   G   
   T   
   T   
   C   
   A   
   A   
   A   
   A   
   A   
   C   
   A   
   C   
   T   
   G   
   A   
   G   
    C    
   T   
   A   
   C   
   A   
   G   
   A   
   T   
   C   
   A   
   A   
   T   
   T   
   A   
   C   
   A   
   A   
   T   
   T   
   T   
    C    
   A   
   A   
   T   
   A   
   T   
   G   
   G   
   T   
   C   
   G   
   C   
   T   
   A   
   T   
   C   
   G   
   A   
    T    
   C   
   A   
   T   
   A   
   T   
   G   
   A   
   C   
   T   
   C   
   C   
   G   
   C   
   A   
    C    
   C   
   A   
   A   
   G   
   T   
   T   
   G   
   G   
   A   
   C   
   T   
   G   
   A   
   A   
   A   
   G   
   A   
   C   
   A   
   T   
   T   
   T   
   T   
   A   
   A   
   G   
   A   
   A   
   G   
   T   
   T   
   A   
   T   
   A   
   T   
   C   
   G   
   A   
   G   
   C   
   A   
   T   
   C   
   G   
   A   
   A   
   A   
   G   
   C   
   A   
   A   
   G   
   T   
   C   
   A   
   T   
   A   
   A   
   C   
   A   
   A   
   A   
   A   
   C   
   G   
    C    
   A   
   G   
    C    
   C   
   A   
   A   
   T   
   T   
   C   
   G   
   A   
   T   
   T   
   T   
   A   
   G   
   C   
   C   
   A   
   A   
   A   
   G   
   C   
   T   
   C   
   A   
   A   
   A   
   A   
   A   
    C    
   G   
   A   
   C   
   A   
   G   
   C   
   A   
   T   
   A   
   T   
   C   
   G   
   T   
    G    
   G   
   A   
   A   
   G   
   G   
    C    
    C    
   T   
   G   
   A   
   T   
   G   
   A   
   A   
   A   
   G   
   C   
   T   
   T   
   T   
   A   
   T   
   C   
   G   
   A   
   T   
   T   
   C   
   T   
   G   
   G   
   A   
   T   
   G   
   A   
   A   
   G   
   T   
   A   
   A   
   T   
   C   
   G   
    C    
   A   
   A   
   C   
   G   
   A   
   T   
   T   
   C   
   G   
   T   
   G   
   A   
   A   
   A   
   G   
   T   
   A   
   A   
   A   
   G   
   A   
   C   
   A   
   A   
   A   
   A   
   A   
   A   
   G   
   A   
    C    
   G   
   C   
    T    
   A   
   A   
   A   
   A   
   A   
   G   
   A   
   A   
   T   
   T   
   T   
   A   
   G   
   T   
   C   
   G   
   A   
    T    
   G   
   T   
    C    
   T   
   T   
    C    
   C   
   A   
   A   
   T   
   T   
   T   
   A   
   C   
   A   
   G   
   A   
   A   
   G   
   A   
   A   
   C   
   A   
   A   
   G   
   C   
   A   
   G   
   A   
   A   
   G   
   C   
   A   
   A   
   T   
    C    
   G   
   T   
   C   
   A   
   C   
   A   
   T   
   T   
   G   
   C   
   A   
   A   
    C    
   T   
   A   
   T   
   A   
    C    
   C   
   G   
   T   
   T   
   T   
   G   
   A   
   C   
   G   
   A   
   A   
    C    
   A   
   C   
    A    
   G   
   A   
   T   
   A   
   T   
   C   
   A   
   C   
   A   
   G   
   A   
   G   
   C   
   T   
   T   
   C   
   A   
   A   
   A   
   A   
   G   
   G   
   A   
   A   
    G    
   C   
    C    
   G   
   A   
   A   
   A   
    G    
   C   
   T   
   T   
   G   
   A   
   T   
   T   
   G   
   C   
    A    
   C   
   A   
   A   
   A   
   T   
   C   
   A   
   C   
   A   
   G   
   A   
   G   
   T   
   T   
    G    
   A   
   A   
   T   
   A   
   A   
   A   
   A   
   T   
   C   
   C   
   T   
   T   
   T   
   C   
   A   
   A   
   A   
    C    
   G   
   A   
   C   
   A   
   A   
   A   
   G   
   A   
    G    
   C   
   T   
   T   
   T   
   T   
    C    
   T   
   C   
   T   
   G   
   T   
   T   
   A   
   T   
   G   
   A   
   A   
   A   
   A   
   A   
   A   
   G   
   A   
   A   
   T   
   T   
   G   
   C   
   G   
   A   
   G   
   A   
   A   
   G   
   T   
   G   
   A   
   A   
   A   
   A   
   A   
   A   
   A   
   A   
   T   
   T   
   A   
    C    
   T   
   C   
   T   
   T   
   C   
   C   
   G   
   C   
   T   
   C   
   G   
   T   
   C   
    T    
    G    
   A   
   C   
   G   
   A   
   C   
   A   
   A   
   T   
   C   
    C    
   A   
   A   
   G   
   A   
   C   
   G   
   A   
   A   
   A   
   T   
   C   
   G   
   A   
   A   
   G   
   A   
   A   
   A   
   T   
   C   
   A   
   A   
   A   
   A   
   T   
    C    
   G   
   A   
   T   
   A   
   C   
   G   
   C   
   A   
   A   
    G    
   T   
    T    
   T   
   T   
   A   
   G   
   T   
   G   
   G   
   C   
    G    
   C   
   A   
   A   
   G   
   A   
   A   
   G   
   A   
   C   
   G   
   T   
   A   
   A   
   T   
    C    
   G   
   T   
   C   
   A   
   G   
   T   
   G   
   T   
    G    
   A   
   C   
    G    
   C   
   G   
   A   
   G   
   A   
   A   
   G   
   G   
   C   
   T   
   A   
   T   
   G   
   T   
   G   
   A   
   A   
   G   
   C   
   G   
   A   
   A   
   C   
   A   
   A   
   G   
   C   
   C   
   T   
   T   
   C   
   G   
   T   
   T   
   C   
    C    
   T   
   A   
   T   
   A   
   G   
   T   
   G   
   C   
   T   
   T   
   C   
    G    
   A   
   A   
   A   
   C   
   C   
   A   
   G   
   A   
   A   
   G   
   A   
   A   
   A   
   T   
   C   
   G   
   G   
   G   
   A   
   T   
   G   
   C   
   G   
   A   
   G   
   A   
   A   
   G   
   G   
   C   
   G   
   A   
    T    
   T   
   A   
   C   
   T   
   T   
   G   
   C   
   T   
    G    
   T   
   A   
   C   
    T    
   C   
   A   
   G   
   G   
   T   
   G   
   A   
   A   
   T   
   T   
    G    
   A   
   G   
    C    
   A   
   C   
   A   
    T    
   T   
   G   
   G   
   A   
   C   
   C   
   A   
   T   
   G   
   T   
   T   
   C   
   T   
   T   
   C   
   T   
   G   
   A   
   T   
    T    
   A   
   C   
    G    
   A   
   A   
   T   
   A   
   A   
   A   
   G   
   C   
   A   
   A   
   A   
   T   
   G   
   T   
   G   
   A   
   T   
   C   
   T   
   A   
   C   
   C   
   G   
    T    
   C   
   C   
   T   
   G   
   T   
    T    
   C   
   A   
   T   
   G   
   A   
   A   
   C   
   T   
   A   
   C   
   C   
    C    
   G   
   A   
   T   
   T   
   T   
   G   
   A   
   A   
   A   
   T   
   G   
   G   
   A   
   A   
   A   
   G   
   A   
   T   
   G   
   C   
   T   
   G   
   G   
    C    
   G   
   A   
    A    
   C   
   A   
    C    
   A   
   T   
   T   
   T   
   C   
   T   
   C   
   A   
   A   
   A   
   C   
   A   
   A   
   T   
   C   
   T   
   C   
   C   
   A   
   A   
   T   
   C   
   T   
   G   
   T   
   C   
   T   
   G   
   T   
   A   
   G   
   A   
    T    
   G   
   A   
   A   
   T   
   C   
   G   
   A   
   T   
    C    
   T   
   T   
   A   
   G   
   C   
   T   
   G   
   T   
   T   
   T   
   T   
    C    
   C   
   C   
   A   
   T   
   A   
   T   
   C   
   A   
   A   
   A   
   A   
   A   
   A   
   T   
    C    
   C   
   A   
   A   
   G   
   C   
   A   
   G   
   A   
    A    
   A   
   A   
   A   
   A   
   C   
   A   
   T   
   T   
   T   
   G   
   T   
    A    
   T   
   T   
   C   
   A   
   T   
   C   
   A   
   G   
   C   
   A   
   A   
   G   
   A   
   A   
   T   
   G   
   G   
   A   
   T   
   T   
    A    
    A    
   T   
   C   
   A   
   A   
   G   
   C   
   A   
   A   
   A   
   C   
   A   
   C   
   G   
   A   
   A   
   T   
   G   
   A   
   C   
   A   
   G   
   A   
   G   
   T   
   T   
   C   
   G   
   A   
   A   
   C   
   C   
   A   
   T   
   G   
   G   
   C   
   G   
   C   
   A   
   C   
   C   
   T   
   A   
    T    
   A   
   A   
   G   
   A   
   G   
   C   
   C   
   G   
    T    
   C   
   C   
   G   
   C   
   T   
   C   
   A   
   G   
   C   
   T   
   G   
   T   
   A   
   T   
   G   
   A   
   A   
   G   
   C   
   T   
   A   
   A   
   A   
   G   
   G   
   C   
   G   
   G   
   A   
   A   
   G   
   A   
   A   
   G   
   A   
   T   
   G   
   A   
   A   
   C   
   T   
    T    
   A   
   T   
   T   
   G   
   C   
   T   
   G   
   T   
    A    
   T   
   A   
   T   
   C   
   T   
   G   
   A   
   A   
   A   
   G   
   A   
   A   
   G   
   A   
   T   
   C   
    A    
   G   
   C   
   C   
   A   
   G   
   G   
   C   
   T   
   T   
   A   
   G   
   A   
   T   
   G   
   T   
    G    
   T   
   T   
   C   
   T   
   T   
   A   
   G   
   T   
   A   
   A   
   C   
   G   
    T    
   A   
   T   
   C   
   A   
   A   
   G   
   G   
   A   
   A   
   T   
   G   
   G   
   G   
    C    
   T   
   T   
   G   
   C   
   G   
   C   
   T   
    A    
   T   
   C   
   C   
   T   
   T   
   T   
   A   
   A   
    C    
   A   
   G   
   A   
   T   
   G   
   T   
   T   
   C   
   C   
   C   
   G   
   T   
   T   
   G   
   T   
   T   
   G   
   G   
   A   
   G   
   C   
   A   
   A   
   A   
   G   
   G   
   C   
   A   
   G   
    C    
   T   
   G   
   G   
   A   
   G   
   T   
   G   
   A   
   A   
   G   
   T   
   C   
   T   
   A   
   T   
   G   
   A   
   A   
   T   
   T   
   T   
   G   
   A   
   A   
   A   
   G   
   A   
   T   
   G   
   G   
   A   
   G   
   A   
   T   
   A   
   G   
   G   
    A    
   T   
   T   
    G    
   T   
   C   
   A   
   A   
    T    
   G   
   G   
    G    
   C   
   T   
    G    
   C   
   T   
   T   
   G   
   T   
   G   
   T   
   T   
   A   
   G   
   C   
   A   
   G   
   A   
    C    
   G   
   G   
    A    
   G   
   A   
   T   
   A   
   C   
   G   
   C   
   C   
    A    
   A   
   T   
   C   
   G   
   T   
   A   
   A   
   T   
   C   
   G   
   T   
   T   
   A   
   C   
   T   
   C   
   A   
   G   
   C   
   G   
   A   
   G   
   G   
   A   
   G   
   C   
    C    
   G   
   T   
    G    
   A   
   A   
   A   
   C   
   G   
   C   
   A   
   T   
   G   
   C   
   T   
   T   
   G   
   C   
   T   
   C   
   A   
   A   
   G   
   A   
   A   
   A   
   T   
   C   
   A   
   G   
   C   
   C   
   A   
   A   
   A   
   C   
    T    
   T   
   C   
   A   
   C   
   G   
   T   
   G   
   C   
   G   
   A   
   A   
   G   
   C   
   G   
   G   
   G   
   G   
   A   
   G   
   T   
   G   
   A   
   C   
   G   
   G   
   T   
   C   
   A   
   T   
   G   
   C   
   G   
   T   
   G   
   A   
   G   
   C   
   T   
   G   
   A   
   A   
   A   
   A   
   A   
   A   
   A   
   A   
   T   
   C   
   C   
    G    
   C   
   A   
    C    
   C   
   G   
    T    
   A   
   T   
   T   
   A   
   T   
   C   
   T   
   A   
   T   
   A   
   T   
   G   
   T   
   C   
   G   
   G   
   A   
   A   
   G   
   G   
   A   
   A   
   G   
   A   
   T   
   C   
   A   
   A   
   A   
   A   
   G   
   A   
   A   
   A   
   T   
   A   
   A   
   C   
   C   
   T   
   T   
   G   
   A   
   T   
   G   
   A   
   A   
   T   
   C   
   A   
   A   
   A   
   A   
   A   
   G   
   G   
    A    
   C   
   A   
    A    
   C   
   A   
   G   
    A    
   T   
   A   
   A   
   C   
   G   
   A   
   T   
   T   
   G   
   A   
    T    
   C   
   C   
   A   
   A   
   C   
    G    
   G   
   A   
   T   
   T   
   T   
    T    
   C   
   C   
   T   
   A   
   T   
   A   
   G   
   G   
   T   
   G   
   A   
   T   
   C   
   G   
   A   
   A   
   C   
   A   
   T   
   C   
   G   
   A   
   A   
   T   
   G   
   G   
    T    
   T   
   C   
    G    
   T   
   T   
   T   
   G   
   C   
    T    
   A   
   T   
   G   
   G   
   A   
    C    
   G   
   A   
   G   
   A   
   A   
   A   
   A   
   A   
   A   
   G   
   G   
   T   
   G   
   G   
   A   
   G   
   A   
   A   
   G   
   T   
   C   
   A   
   T   
   C   
   G   
   A   
   A   
   G   
   T   
    T    
   A   
    T    
   C   
   G   
   A   
   T   
   G   
   C   
   T   
   C   
   C   
   T   
   G   
   A   
   G   
    A    
   T   
   T   
   T   
   T   
    C    
   A   
   T   
    T    
   G   
   A   
    A    
    T    
   C   
    A    
   -   
   -   
   -   
 

 
   N59503F   
   A   
   T   
   G   
   G   
   A   
   A   
   A   
   A   
   T   
   C   
   G   
   A   
   C   
   A   
   A   
   G   
   A   
   A   
   G   
   T   
   A   
   C   
   A   
   A   
   G   
   A   
   A   
   T   
   T   
   A   
   A   
   C   
   T   
   T   
   T   
   A   
   G   
   A   
   A   
   G   
   A   
   A   
   G   
   T   
   G   
   A   
   T   
   G   
   G   
   G   
   T   
   G   
   A   
   C   
   C   
   G   
   C   
   T   
   T   
   C   
   G   
   G   
   A   
   C   
   G   
   T   
   T   
   A   
   T   
   T   
   C   
   C   
   A   
   A   
   A   
   T   
   A   
   T   
   A   
   T   
   T   
   A   
   T   
    C    
   C   
   A   
    A    
   G   
   A   
   A   
   C   
   G   
   T   
   G   
   C   
   A   
   C   
   T   
   G   
   C   
   C   
   T   
   G   
   A   
   T   
   A   
   T   
    T    
   C   
   G   
   T   
   G   
   A   
   T   
   G   
   G   
   A   
   T   
   T   
   G   
   A   
   A   
    G    
   C   
   C   
   A   
   G   
   T   
    G    
   C   
   A   
   A   
   C   
   G   
   C   
   C   
   G   
   G   
   A   
   T   
   A   
   T   
   T   
   A   
   T   
   T   
    T    
   G   
   C   
    C    
   A   
   T   
   G   
   A   
   A   
   C   
   A   
   A   
   A   
   G   
   A   
   T   
   G   
   G   
   C   
   A   
   A   
   T   
   A   
   C   
   G   
   T   
   A   
   T   
   G   
   A   
   C   
   A   
   A   
   A   
   G   
   G   
   A   
   T   
   T   
    C    
   C   
   G   
   T   
   A   
   A   
   A   
   T   
   C   
   A   
   G   
   C   
   A   
   A   
   A   
   A   
   T   
   C   
   T   
   G   
   T   
   C   
   G   
   G   
   A   
   A   
   A   
   T   
   A   
   T   
   C   
   A   
   T   
   G   
   G   
   G   
    G    
   A   
   A   
   T   
   T   
   A   
   T   
   C   
   A   
   T   
   C   
   C   
   T   
   C   
   A   
   T   
   G   
   G   
   T   
   G   
   A   
   C   
   A   
   G   
   C   
   A   
   G   
   T   
   A   
   T   
   T   
   T   
   A   
   T   
   G   
   A   
   A   
   G   
   C   
   G   
   A   
   T   
   G   
   G   
   T   
   C   
   C   
   G   
   C   
   A   
   T   
   G   
   A   
   G   
   C   
   C   
   A   
   A   
   G   
   A   
    C    
   T   
   G   
   G   
   A   
   A   
   G   
   T   
   T   
   G   
   C   
   G   
   G   
   G   
   A   
   A   
   G   
   T   
   A   
   C   
   T   
   G   
   A   
   T   
   C   
   G   
   A   
    A    
   A   
   T   
   G   
   C   
   A   
   C   
   G   
   G   
    G    
   A   
   A   
   T   
   A   
   A   
    C    
   G   
   G   
   G   
   A   
   G   
   T   
   A   
   T   
   G   
   G   
   A   
   C   
   G   
   G   
   T   
   G   
   A   
   T   
   C   
   C   
   A   
   C   
   C   
   T   
   G   
   C   
   T   
   G   
   C   
   G   
   A   
   T   
   G   
   C   
   G   
   T   
   T   
   A   
   T   
   A   
   C   
   T   
   G   
   A   
   A   
   G   
   C   
   C   
   A   
   G   
    A    
   T   
   T   
   A   
   T   
   C   
   C   
   A   
   A   
   G   
   T   
   T   
   A   
   A   
   G   
   T   
   G   
   G   
   A   
   G   
   A   
   G   
   A   
   T   
   G   
    C    
   T   
   A   
    G    
   C   
   A   
   G   
   A   
   T   
   A   
   T   
   C   
   G   
   A   
    A    
   A   
   A   
   A   
   G   
   A   
   A   
   A   
   C   
   A   
   G   
   T   
    C    
   G   
   A   
   T   
   C   
   T   
   C   
   G   
   T   
   G   
   T   
   G   
   G   
   A   
   A   
   T   
   T   
   T   
   T   
   G   
   A   
   T   
   G   
   A   
   T   
   A   
   C   
   A   
   G   
   A   
   A   
   A   
   A   
   A   
   G   
   A   
   A   
   C   
   C   
   A   
   A   
   C   
   A   
   G   
   T   
   C   
   C   
   T   
   T   
   C   
   C   
   A   
   G   
   C   
   T   
   C   
   G   
   A   
   T   
   A   
   C   
   C   
   C   
   A   
   A   
   A   
   C   
   C   
   T   
   T   
   C   
   T   
    C    
   G   
   T   
   C   
   A   
   A   
   T   
   G   
   G   
   A   
   T   
   C   
   G   
   A   
   C   
   A   
   G   
   G   
   T   
   A   
   T   
   A   
   T   
   C   
   T   
   G   
   C   
   T   
   G   
   G   
   A   
   T   
   A   
   T   
   G   
   C   
    A    
   A   
   C   
   A   
   G   
   A   
   A   
   A   
   T   
   C   
   C   
   C   
   G   
   A   
   C   
    T    
   C   
   A   
   T   
   A   
   A   
   C   
   T   
   T   
   A   
   G   
   C   
   T   
   G   
   A   
   A   
   G   
   T   
   G   
   A   
   T   
    C    
   G   
   A   
    T    
   G   
   G   
   C   
   A   
   C   
   A   
   A   
   T   
   C   
   T   
   A   
   T   
   A   
   T   
   G   
   A   
   T   
   C   
   G   
   A   
   T   
   C   
   A   
   T   
   C   
   C   
   A   
   C   
   A   
   A   
   G   
   C   
   G   
   A   
   G   
   C   
   C   
   T   
   T   
   G   
   A   
   A   
   A   
   A   
   A   
   T   
   T   
   G   
   A   
   T   
   G   
   G   
   A   
   A   
   T   
   A   
   T   
   A   
   T   
   T   
   C   
   C   
   T   
   G   
   G   
   A   
   C   
   C   
   T   
   G   
   A   
   C   
   T   
   T   
    T    
   C   
   C   
   A   
   A   
   C   
   A   
   G   
   G   
   A   
   G   
   G   
    G    
   A   
   T   
   C   
   T   
   T   
   G   
   C   
   A   
   A   
   G   
   G   
    A    
   A   
   A   
   A   
   G   
   A   
   T   
   G   
   A   
   A   
   A   
   T   
   C   
   A   
   A   
   A   
   A   
   A   
   A   
   G   
   C   
   T   
   T   
   A   
   T   
   G   
   A   
   A   
   A   
   C   
   A   
   G   
   G   
    G    
   C   
   G   
   A   
   G   
   G   
   A   
   A   
   A   
   A   
   G   
   T   
   T   
   A   
    T    
   T   
   C   
   T   
   T   
   C   
   G   
   T   
   T   
   C   
   G   
   A   
   A   
   A   
   A   
   C   
   A   
   A   
   A   
   G   
   A   
   T   
   A   
   G   
   A   
    A    
   G   
   C   
   A   
   A   
   T   
   G   
   A   
   A   
   G   
   G   
   G   
    T    
   A   
   A   
    T    
   A   
   A   
   A   
   C   
   A   
   G   
   C   
   A   
   A   
   A   
   T   
   C   
   G   
   T   
   G   
   A   
   T   
   C   
   A   
   C   
    A    
   G   
   A   
   A   
   A   
   T   
   C   
   C   
   C   
   A   
   T   
   A   
   T   
   G   
   A   
   A   
   G   
   T   
   A   
   A   
   A   
   T   
   A   
   A   
   A   
   G   
   C   
   G   
   A   
   C   
    C    
   T   
   T   
   G   
   G   
   T   
   G   
   A   
   A   
   A   
   A   
   A   
   A   
   A   
   T   
   G   
   G   
   A   
   T   
   G   
   A   
   G   
   A   
   T   
   C   
   C   
   G   
   T   
   T   
   T   
   G   
   A   
   A   
   T   
   A   
   A   
   G   
   A   
   A   
   A   
   A   
   T   
   C   
   G   
   A   
    T    
   G   
   G   
   A   
   A   
   T   
   A   
   G   
   C   
   T   
   G   
   A   
   A   
   G   
   T   
    A    
   C   
   G   
   A   
   G   
   A   
   T   
   G   
   A   
   A   
   A   
   G   
   T   
   G   
   A   
   T   
    A    
   G   
    A    
   A   
   C   
    A    
   G   
   G   
   T   
   C   
   T   
   G   
   C   
   A   
   A   
   A   
   T   
   T   
   G   
   T   
   C   
   G   
   T   
    C    
   G   
   A   
   A   
   T   
   T   
   G   
   A   
   A   
   A   
   A   
   A   
   A   
   G   
   A   
    C    
   G   
   C   
   C   
   A   
   A   
    T    
   G   
   C   
   T   
   C   
   A   
   A   
   G   
   G   
    A    
   A   
   T   
   T   
   T   
   T   
   G   
   A   
   A   
   T   
   T   
   A   
    T    
   T   
   T   
   G   
   T   
   T   
   C   
   A   
   A   
   A   
   A   
   A   
   C   
   A   
   C   
   T   
   G   
   A   
   G   
    C    
   T   
   A   
   C   
   A   
   G   
   A   
   T   
   C   
   A   
   A   
   T   
   T   
   A   
   C   
   A   
   A   
   T   
   T   
   T   
    C    
   A   
   A   
   T   
   A   
   T   
   G   
   G   
   T   
   C   
   G   
   C   
   T   
   A   
   T   
   C   
   G   
   A   
    C    
   C   
   A   
   T   
   A   
   T   
   G   
   A   
   C   
   T   
   C   
   C   
   G   
   C   
   A   
    T    
   C   
   A   
   A   
   G   
   T   
   T   
   G   
   G   
   A   
   C   
   T   
   G   
   A   
   A   
   A   
   G   
   A   
   C   
   A   
   T   
   T   
   T   
   T   
   A   
   A   
   G   
   A   
   A   
   G   
   T   
   T   
   A   
   T   
   A   
   T   
   C   
   G   
   A   
   G   
   C   
   A   
   T   
   C   
   G   
   A   
   A   
   A   
   G   
   C   
   A   
   A   
   G   
   T   
   C   
   A   
   T   
   A   
   A   
   C   
   A   
   A   
   A   
   A   
   C   
   G   
    T    
   A   
   G   
    T    
   C   
   A   
   A   
   T   
   T   
   C   
   G   
   A   
   T   
   T   
   T   
   A   
   G   
   C   
   C   
   A   
   A   
   A   
   G   
   C   
   T   
   C   
   A   
   A   
   A   
   A   
   A   
    A    
   G   
   A   
   C   
   A   
   G   
   C   
   A   
   T   
   A   
   T   
   C   
   G   
   T   
    A    
   G   
   A   
   A   
   G   
   G   
    A    
    T    
   T   
   G   
   A   
   T   
   G   
   A   
   A   
   A   
   G   
   C   
   T   
   T   
   T   
   A   
   T   
   C   
   G   
   A   
   T   
   T   
   C   
   T   
   G   
   G   
   A   
   T   
   G   
   A   
   A   
   G   
   T   
   A   
   A   
   T   
   C   
   G   
    C    
   A   
   A   
   C   
   G   
   A   
   T   
   T   
   C   
   G   
   T   
   G   
   A   
   A   
   A   
   G   
   T   
   A   
   A   
   A   
   G   
   A   
   C   
   A   
   A   
   A   
   A   
   A   
   A   
   G   
   A   
    C    
   G   
   C   
    C    
   A   
   A   
   A   
   A   
   A   
   G   
   A   
   A   
   T   
   T   
   T   
   A   
   G   
   T   
   C   
   G   
   A   
    C    
   G   
   T   
    C    
   T   
   T   
    T    
   C   
   A   
   A   
   T   
   T   
   T   
   A   
   C   
   A   
   G   
   A   
   A   
   G   
   A   
   A   
   C   
   A   
   A   
   G   
   C   
   A   
   G   
   A   
   A   
   G   
   C   
   A   
   A   
   T   
    T    
   G   
   T   
   C   
   A   
   C   
   A   
   T   
   T   
   G   
   C   
   A   
   A   
    T    
   T   
   A   
   T   
   A   
    C    
   C   
   G   
   T   
   T   
   T   
   G   
   A   
   C   
   G   
   A   
   A   
    C    
   A   
   C   
    G    
   G   
   A   
   T   
   A   
   T   
   C   
   A   
   C   
   A   
   G   
   A   
   G   
   C   
   T   
   T   
   C   
   A   
   A   
   A   
   A   
   G   
   G   
   A   
   A   
    T    
   C   
    C    
   G   
   A   
   A   
   A   
    G    
   C   
   T   
   T   
   G   
   A   
   T   
   T   
   G   
   C   
    G    
   C   
   A   
   A   
   A   
   T   
   C   
   A   
   C   
   A   
   G   
   A   
   G   
   T   
   T   
    G    
   A   
   A   
   T   
   A   
   A   
   A   
   A   
   T   
   C   
   C   
   T   
   T   
   T   
   C   
   A   
   A   
   A   
    T    
   G   
   A   
   C   
   A   
   A   
   A   
   G   
   A   
    G    
   C   
   T   
   T   
   T   
   T   
    C    
   T   
   C   
   T   
   G   
   T   
   T   
   A   
   T   
   G   
   A   
   A   
   A   
   A   
   A   
   A   
   G   
   A   
   A   
   T   
   T   
   G   
   C   
   G   
   A   
   G   
   A   
   A   
   G   
   T   
   G   
   A   
   A   
   A   
   A   
   A   
   A   
   A   
   A   
   T   
   T   
   A   
    T    
   T   
   C   
   T   
   T   
   C   
   C   
   G   
   C   
   T   
   C   
   G   
   T   
   C   
    T    
    A    
   A   
   C   
   G   
   A   
   C   
   A   
   A   
   T   
   C   
    G    
   A   
   A   
   G   
   A   
   C   
   G   
   A   
   A   
   A   
   T   
   C   
   G   
   A   
   A   
   G   
   A   
   A   
   A   
   T   
   C   
   A   
   A   
   A   
   A   
   T   
    T    
   G   
   A   
   T   
   A   
   C   
   G   
   C   
   A   
   A   
    G    
   T   
    C    
   T   
   T   
   A   
   G   
   T   
   G   
   G   
   C   
    A    
   C   
   A   
   A   
   G   
   A   
   A   
   G   
   A   
   C   
   G   
   T   
   A   
   A   
   T   
    T    
   G   
   T   
   C   
   A   
   G   
   T   
   G   
   T   
    G    
   A   
   C   
    G    
   C   
   G   
   A   
   G   
   A   
   A   
   G   
   G   
   C   
   T   
   A   
   T   
   G   
   T   
   G   
   A   
   A   
   G   
   C   
   G   
   A   
   A   
   C   
   A   
   A   
   G   
   C   
   C   
   T   
   T   
   C   
   G   
   T   
   T   
   C   
    T    
   T   
   A   
   T   
   A   
   G   
   T   
   G   
   C   
   T   
   T   
   C   
    C    
   A   
   A   
   A   
   C   
   C   
   A   
   G   
   A   
   A   
   G   
   A   
   A   
   A   
   T   
   C   
   G   
   G   
   G   
   A   
   T   
   G   
   C   
   G   
   A   
   G   
   A   
   A   
   G   
   G   
   C   
   G   
   A   
    C    
   T   
   A   
   C   
   T   
   T   
   G   
   C   
   T   
    A    
   T   
   A   
   C   
    T    
   C   
   A   
   G   
   G   
   T   
   G   
   A   
   A   
   T   
   T   
    G    
   A   
   G   
    T    
   A   
   C   
   A   
    T    
   T   
   G   
   G   
   A   
   C   
   C   
   A   
   T   
   G   
   T   
   T   
   C   
   T   
   T   
   C   
   T   
   G   
   A   
   T   
    C    
   A   
   C   
    A    
   A   
   A   
   T   
   A   
   A   
   A   
   G   
   C   
   A   
   A   
   A   
   T   
   G   
   T   
   G   
   A   
   T   
   C   
   T   
   A   
   C   
   C   
   G   
    C    
   C   
   C   
   T   
   G   
   T   
    C    
   C   
   A   
   T   
   G   
   A   
   A   
   C   
   T   
   A   
   C   
   C   
    C    
   G   
   A   
   T   
   T   
   T   
   G   
   A   
   A   
   A   
   T   
   G   
   G   
   A   
   A   
   A   
   G   
   A   
   T   
   G   
   C   
   T   
   G   
   G   
    T    
   G   
   A   
    G    
   C   
   A   
    C    
   A   
   T   
   T   
   T   
   C   
   T   
   C   
   A   
   A   
   A   
   C   
   A   
   A   
   T   
   C   
   T   
   C   
   C   
   A   
   A   
   T   
   C   
   T   
   G   
   T   
   C   
   T   
   G   
   T   
   A   
   G   
   A   
    C    
   G   
   A   
   A   
   T   
   C   
   G   
   A   
   T   
    T    
   T   
   T   
   A   
   G   
   C   
   T   
   G   
   T   
   T   
   T   
   T   
    C    
   C   
   C   
   A   
   T   
   A   
   T   
   C   
   A   
   A   
   A   
   A   
   A   
   A   
   T   
    T    
   C   
   A   
   A   
   G   
   C   
   A   
   G   
   A   
    G    
   A   
   A   
   A   
   A   
   C   
   A   
   T   
   T   
   T   
   G   
   T   
    A    
   T   
   T   
   C   
   A   
   T   
   C   
   A   
   G   
   C   
   A   
   A   
   G   
   A   
   A   
   T   
   G   
   G   
   A   
   T   
   T   
    G    
    A    
   T   
   C   
   A   
   A   
   G   
   C   
   A   
   A   
   A   
   C   
   A   
   C   
   G   
   A   
   A   
   T   
   G   
   A   
   C   
   A   
   G   
   A   
   G   
   T   
   T   
   C   
   G   
   A   
   A   
   C   
   C   
   A   
   T   
   G   
   G   
   C   
   G   
   C   
   A   
   C   
   C   
   T   
   A   
    T    
   A   
   A   
   G   
   A   
   G   
   C   
   C   
   G   
    T    
   C   
   C   
   G   
   C   
   T   
   C   
   A   
   G   
   C   
   T   
   G   
   T   
   A   
   T   
   G   
   A   
   A   
   G   
   C   
   T   
   A   
   A   
   A   
   G   
   G   
   C   
   G   
   G   
   A   
   A   
   G   
   A   
   A   
   G   
   A   
   T   
   G   
   A   
   A   
   C   
   T   
    G    
   A   
   T   
   T   
   G   
   C   
   T   
   G   
   T   
    G    
   T   
   A   
   T   
   C   
   T   
   G   
   A   
   A   
   A   
   G   
   A   
   A   
   G   
   A   
   T   
   C   
    A    
   G   
   C   
   C   
   A   
   G   
   G   
   C   
   T   
   T   
   A   
   G   
   A   
   T   
   G   
   T   
    T    
   T   
   T   
   C   
   T   
   T   
   A   
   G   
   T   
   A   
   A   
   C   
   G   
    C    
   A   
   T   
   C   
   A   
   A   
   G   
   G   
   A   
   A   
   T   
   G   
   G   
   G   
    T    
   T   
   T   
   G   
   C   
   G   
   C   
   T   
    A    
   T   
   C   
   C   
   T   
   T   
   T   
   A   
   A   
    C    
   A   
   G   
   A   
   T   
   G   
   T   
   T   
   C   
   C   
   C   
   G   
   T   
   T   
   G   
   T   
   T   
   G   
   G   
   A   
   G   
   C   
   A   
   A   
   A   
   G   
   G   
   C   
   A   
   G   
    C    
   T   
   G   
   G   
   A   
   G   
   T   
   G   
   A   
   A   
   G   
   T   
   C   
   T   
   A   
   T   
   G   
   A   
   A   
   T   
   T   
   T   
   G   
   A   
   A   
   A   
   G   
   A   
   T   
   G   
   G   
   A   
   G   
   A   
   T   
   A   
   G   
   G   
    A    
   T   
   T   
    A    
   T   
   C   
   A   
   A   
    C    
   G   
   G   
    G    
   C   
   T   
    A    
   C   
   T   
   T   
   G   
   T   
   G   
   T   
   T   
   A   
   G   
   C   
   A   
   G   
   A   
    A    
   G   
   G   
    T    
   G   
   A   
   T   
   A   
   C   
   G   
   C   
   C   
    G    
   A   
   T   
   C   
   G   
   T   
   A   
   A   
   T   
   C   
   G   
   T   
   T   
   A   
   C   
   T   
   C   
   A   
   G   
   C   
   G   
   A   
   G   
   G   
   A   
   G   
   C   
    T    
   G   
   T   
    T    
   A   
   A   
   A   
   C   
   G   
   C   
   A   
   T   
   G   
   C   
   T   
   T   
   G   
   C   
   T   
   C   
   A   
   A   
   G   
   A   
   A   
   A   
   T   
   C   
   A   
   G   
   C   
   C   
   A   
   A   
   A   
   C   
    T    
   T   
   C   
   A   
   C   
   G   
   T   
   G   
   C   
   G   
   A   
   A   
   G   
   C   
   G   
   G   
   G   
   G   
   A   
   G   
   T   
   G   
   A   
   C   
   G   
   G   
   T   
   C   
   A   
   T   
   G   
   C   
   G   
   T   
   G   
   A   
   G   
   C   
   T   
   G   
   A   
   A   
   A   
   A   
   A   
   A   
   A   
   A   
   T   
   C   
   C   
    G    
   C   
   A   
    C    
   C   
   G   
    T    
   A   
   T   
   T   
   A   
   T   
   C   
   T   
   A   
   T   
   A   
   T   
   G   
   T   
   C   
   G   
   G   
   A   
   A   
   G   
   G   
   A   
   A   
   G   
   A   
   T   
   C   
   A   
   A   
   A   
   A   
   G   
   A   
   A   
   A   
   T   
   A   
   A   
   C   
   C   
   T   
   T   
   G   
   A   
   T   
   G   
   A   
   A   
   T   
   C   
   A   
   A   
   A   
   A   
   A   
   G   
   G   
    G    
   C   
   A   
    G    
   C   
   A   
   G   
    T    
   T   
   A   
   A   
   C   
   G   
   A   
   T   
   T   
   G   
   A   
    C    
   C   
   C   
   A   
   A   
   C   
    T    
   G   
   A   
   T   
   T   
   T   
    C    
   C   
   C   
   T   
   A   
   T   
   A   
   G   
   G   
   T   
   G   
   A   
   T   
   C   
   G   
   A   
   A   
   C   
   A   
   T   
   C   
   G   
   A   
   A   
   T   
   G   
   G   
    A    
   T   
   C   
    A    
   T   
   T   
   T   
   G   
   C   
    G    
   A   
   T   
   G   
   G   
   A   
    T    
   G   
   A   
   G   
   A   
   A   
   A   
   A   
   A   
   A   
   G   
   G   
   T   
   G   
   G   
   A   
   G   
   A   
   A   
   G   
   T   
   C   
   A   
   T   
   C   
   G   
   A   
   A   
   G   
   T   
    T    
   A   
    T    
   C   
   G   
   A   
   T   
   G   
   C   
   T   
   C   
   C   
   T   
   G   
   A   
   G   
    A    
   T   
   T   
   T   
   T   
    C    
   A   
   T   
    C    
   G   
   A   
    G    
    T    
   C   
    G    
   -   
   -   
   -   
 

 
   N59624F   
   A   
   T   
   G   
   G   
   A   
   A   
   A   
   A   
   T   
   C   
   G   
   A   
   C   
   A   
   A   
   G   
   A   
   A   
   G   
   T   
   A   
   C   
   A   
   A   
   G   
   A   
   A   
   T   
   T   
   A   
   A   
   C   
   T   
   T   
   T   
   A   
   G   
   A   
   A   
   G   
   A   
   A   
   G   
   T   
   G   
   A   
   T   
   G   
   G   
   G   
   T   
   G   
   A   
   C   
   C   
   G   
   C   
   T   
   T   
   C   
   G   
   G   
   A   
   C   
   G   
   T   
   T   
   A   
   T   
   T   
   C   
   C   
   A   
   A   
   A   
   T   
   A   
   T   
   A   
   T   
   T   
   A   
   T   
    C    
   C   
   A   
    A    
   G   
   A   
   A   
   C   
   G   
   T   
   G   
   C   
   A   
   C   
   T   
   G   
   C   
   C   
   T   
   G   
   A   
   T   
   A   
   T   
    C    
   C   
   G   
   T   
   G   
   A   
   T   
   G   
   G   
   A   
   T   
   T   
   G   
   A   
   A   
    A    
   C   
   C   
   A   
   G   
   T   
    G    
   C   
   A   
   A   
   C   
   G   
   C   
   C   
   G   
   G   
   A   
   T   
   A   
   T   
   T   
   A   
   T   
   T   
    T    
   G   
   C   
    C    
   A   
   T   
   G   
   A   
   A   
   C   
   A   
   A   
   A   
   G   
   A   
   T   
   G   
   G   
   C   
   A   
   A   
   T   
   A   
   C   
   G   
   T   
   A   
   T   
   G   
   A   
   C   
   A   
   A   
   A   
   G   
   G   
   A   
   T   
   T   
    C    
   C   
   G   
   T   
   A   
   A   
   A   
   T   
   C   
   A   
   G   
   C   
   A   
   A   
   A   
   A   
   T   
   C   
   T   
   G   
   T   
   C   
   G   
   G   
   A   
   A   
   A   
   T   
   A   
   T   
   C   
   A   
   T   
   G   
   G   
   G   
    G    
   A   
   A   
   T   
   T   
   A   
   T   
   C   
   A   
   T   
   C   
   C   
   T   
   C   
   A   
   T   
   G   
   G   
   T   
   G   
   A   
   C   
   A   
   G   
   C   
   A   
   G   
   T   
   A   
   T   
   T   
   T   
   A   
   T   
   G   
   A   
   A   
   G   
   C   
   G   
   A   
   T   
   G   
   G   
   T   
   C   
   C   
   G   
   C   
   A   
   T   
   G   
   A   
   G   
   C   
   C   
   A   
   A   
   G   
   A   
    T    
   T   
   G   
   G   
   A   
   A   
   G   
   T   
   T   
   G   
   C   
   G   
   G   
   G   
   A   
   A   
   G   
   T   
   A   
   C   
   T   
   G   
   A   
   T   
   C   
   G   
   A   
    A    
   A   
   T   
   G   
   C   
   A   
   C   
   G   
   G   
    G    
   A   
   A   
   T   
   A   
   A   
    T    
   G   
   G   
   G   
   A   
   G   
   T   
   A   
   T   
   G   
   G   
   A   
   C   
   G   
   G   
   T   
   G   
   A   
   T   
   C   
   C   
   A   
   C   
   C   
   T   
   G   
   C   
   T   
   G   
   C   
   G   
   A   
   T   
   G   
   C   
   G   
   T   
   T   
   A   
   T   
   A   
   C   
   T   
   G   
   A   
   A   
   G   
   C   
   C   
   A   
   G   
    A    
   T   
   T   
   A   
   T   
   C   
   C   
   A   
   A   
   G   
   T   
   T   
   A   
   A   
   G   
   T   
   G   
   G   
   A   
   G   
   A   
   G   
   A   
   T   
   G   
    C    
   T   
   A   
    G    
   C   
   A   
   G   
   A   
   T   
   A   
   T   
   C   
   G   
   A   
    A    
   A   
   A   
   A   
   G   
   A   
   A   
   A   
   C   
   A   
   G   
   T   
    C    
   G   
   A   
   T   
   C   
   T   
   C   
   G   
   T   
   G   
   T   
   G   
   G   
   A   
   A   
   T   
   T   
   T   
   T   
   G   
   A   
   T   
   G   
   A   
   T   
   A   
   C   
   A   
   G   
   A   
   A   
   A   
   A   
   A   
   G   
   A   
   A   
   C   
   C   
   A   
   A   
   C   
   A   
   G   
   T   
   C   
   C   
   T   
   T   
   C   
   C   
   A   
   G   
   C   
   T   
   C   
   G   
   A   
   T   
   A   
   C   
   C   
   C   
   A   
   A   
   A   
   C   
   C   
   T   
   T   
   C   
   T   
    A    
   G   
   T   
   C   
   A   
   A   
   T   
   G   
   G   
   A   
   T   
   C   
   G   
   A   
   C   
   A   
   G   
   G   
   T   
   A   
   T   
   A   
   T   
   C   
   T   
   G   
   C   
   T   
   G   
   G   
   A   
   T   
   A   
   T   
   G   
   C   
    G    
   A   
   C   
   A   
   G   
   A   
   A   
   A   
   T   
   C   
   C   
   C   
   G   
   A   
   C   
    T    
   C   
   A   
   T   
   A   
   A   
   C   
   T   
   T   
   A   
   G   
   C   
   T   
   G   
   A   
   A   
   G   
   T   
   G   
   A   
   T   
    C    
   G   
   A   
    C    
   G   
   G   
   C   
   A   
   C   
   A   
   A   
   T   
   C   
   T   
   A   
   T   
   A   
   T   
   G   
   A   
   T   
   C   
   G   
   A   
   T   
   C   
   A   
   T   
   C   
   C   
   A   
   C   
   A   
   A   
   G   
   C   
   G   
   A   
   G   
   C   
   C   
   T   
   T   
   G   
   A   
   A   
   A   
   A   
   A   
   T   
   T   
   G   
   A   
   T   
   G   
   G   
   A   
   A   
   T   
   A   
   T   
   A   
   T   
   T   
   C   
   C   
   T   
   G   
   G   
   A   
   C   
   C   
   T   
   G   
   A   
   C   
   T   
   T   
    T    
   C   
   C   
   A   
   A   
   C   
   A   
   G   
   G   
   A   
   G   
   G   
    G    
   A   
   T   
   C   
   T   
   T   
   G   
   C   
   A   
   A   
   G   
   G   
    G    
   A   
   A   
   A   
   G   
   A   
   T   
   G   
   A   
   A   
   A   
   T   
   C   
   A   
   A   
   A   
   A   
   A   
   A   
   G   
   C   
   T   
   T   
   A   
   T   
   G   
   A   
   A   
   A   
   C   
   A   
   G   
   G   
    G    
   C   
   G   
   A   
   G   
   G   
   A   
   A   
   A   
   A   
   G   
   T   
   T   
   A   
    T    
   T   
   C   
   T   
   T   
   C   
   G   
   T   
   T   
   C   
   G   
   A   
   A   
   A   
   A   
   C   
   A   
   A   
   A   
   G   
   A   
   T   
   A   
   G   
   A   
    A    
   G   
   C   
   A   
   A   
   T   
   G   
   A   
   A   
   G   
   G   
   G   
    C    
   A   
   A   
    T    
   A   
   A   
   A   
   C   
   A   
   G   
   C   
   A   
   A   
   A   
   T   
   C   
   G   
   T   
   G   
   A   
   T   
   C   
   A   
   C   
    T    
   G   
   A   
   A   
   A   
   T   
   C   
   C   
   C   
   A   
   T   
   A   
   T   
   G   
   A   
   A   
   G   
   T   
   A   
   A   
   A   
   T   
   A   
   A   
   A   
   G   
   C   
   G   
   A   
   C   
    G    
   T   
   T   
   G   
   G   
   T   
   G   
   A   
   A   
   A   
   A   
   A   
   A   
   A   
   T   
   G   
   G   
   A   
   T   
   G   
   A   
   G   
   A   
   T   
   C   
   C   
   G   
   T   
   T   
   T   
   G   
   A   
   A   
   T   
   A   
   A   
   G   
   A   
   A   
   A   
   A   
   T   
   C   
   G   
   A   
    T    
   G   
   G   
   A   
   A   
   T   
   A   
   G   
   C   
   T   
   G   
   A   
   A   
   G   
   T   
    A    
   C   
   G   
   A   
   G   
   A   
   T   
   G   
   A   
   A   
   A   
   G   
   T   
   G   
   A   
   T   
    C    
   G   
    A    
   A   
   C   
    T    
   G   
   G   
   T   
   C   
   T   
   G   
   C   
   A   
   A   
   A   
   T   
   T   
   G   
   T   
   C   
   G   
   T   
    T    
   G   
   A   
   A   
   T   
   T   
   G   
   A   
   A   
   A   
   A   
   A   
   A   
   G   
   A   
    T    
   G   
   C   
   C   
   A   
   A   
    T    
   G   
   C   
   T   
   C   
   A   
   A   
   G   
   G   
    A    
   A   
   T   
   T   
   T   
   T   
   G   
   A   
   A   
   T   
   T   
   A   
    T    
   T   
   T   
   G   
   T   
   T   
   C   
   A   
   A   
   A   
   A   
   A   
   C   
   A   
   C   
   T   
   G   
   A   
   G   
    C    
   T   
   A   
   C   
   A   
   G   
   A   
   T   
   C   
   A   
   A   
   T   
   T   
   A   
   C   
   A   
   A   
   T   
   T   
   T   
    C    
   A   
   A   
   T   
   A   
   T   
   G   
   G   
   T   
   C   
   G   
   C   
   T   
   A   
   T   
   C   
   G   
   A   
    T    
   C   
   A   
   T   
   A   
   T   
   G   
   A   
   C   
   T   
   C   
   C   
   G   
   C   
   A   
    C    
   C   
   A   
   A   
   G   
   T   
   T   
   G   
   G   
   A   
   C   
   T   
   G   
   A   
   A   
   A   
   G   
   A   
   C   
   A   
   T   
   T   
   T   
   T   
   A   
   A   
   G   
   A   
   A   
   G   
   T   
   T   
   A   
   T   
   A   
   T   
   C   
   G   
   A   
   G   
   C   
   A   
   T   
   C   
   G   
   A   
   A   
   A   
   G   
   C   
   A   
   A   
   G   
   T   
   C   
   A   
   T   
   A   
   A   
   C   
   A   
   A   
   A   
   A   
   C   
   G   
    C    
   A   
   G   
    C    
   C   
   A   
   A   
   T   
   T   
   C   
   G   
   A   
   T   
   T   
   T   
   A   
   G   
   C   
   C   
   A   
   A   
   A   
   G   
   C   
   T   
   C   
   A   
   A   
   A   
   A   
   A   
    A    
   G   
   A   
   C   
   A   
   G   
   C   
   A   
   T   
   A   
   T   
   C   
   G   
   T   
    G    
   G   
   A   
   A   
   G   
   G   
    C    
    C    
   T   
   G   
   A   
   T   
   G   
   A   
   A   
   A   
   G   
   C   
   T   
   T   
   T   
   A   
   T   
   C   
   G   
   A   
   T   
   T   
   C   
   T   
   G   
   G   
   A   
   T   
   G   
   A   
   A   
   G   
   T   
   A   
   A   
   T   
   C   
   G   
    C    
   A   
   A   
   C   
   G   
   A   
   T   
   T   
   C   
   G   
   T   
   G   
   A   
   A   
   A   
   G   
   T   
   A   
   A   
   A   
   G   
   A   
   C   
   A   
   A   
   A   
   A   
   A   
   A   
   G   
   A   
    T    
   G   
   C   
    T    
   A   
   A   
   A   
   A   
   A   
   G   
   A   
   A   
   T   
   T   
   T   
   A   
   G   
   T   
   C   
   G   
   A   
    T    
   G   
   T   
    T    
   T   
   T   
    C    
   C   
   A   
   A   
   T   
   T   
   T   
   A   
   C   
   A   
   G   
   A   
   A   
   G   
   A   
   A   
   C   
   A   
   A   
   G   
   C   
   A   
   G   
   A   
   A   
   G   
   C   
   A   
   A   
   T   
    C    
   G   
   T   
   C   
   A   
   C   
   A   
   T   
   T   
   G   
   C   
   A   
   A   
    C    
   T   
   A   
   T   
   A   
    C    
   C   
   G   
   T   
   T   
   T   
   G   
   A   
   C   
   G   
   A   
   A   
    C    
   A   
   C   
    A    
   G   
   A   
   T   
   A   
   T   
   C   
   A   
   C   
   A   
   G   
   A   
   G   
   C   
   T   
   T   
   C   
   A   
   A   
   A   
   A   
   G   
   G   
   A   
   A   
    G    
   C   
    C    
   G   
   A   
   A   
   A   
    G    
   C   
   T   
   T   
   G   
   A   
   T   
   T   
   G   
   C   
    A    
   C   
   A   
   A   
   A   
   T   
   C   
   A   
   C   
   A   
   G   
   A   
   G   
   T   
   T   
    G    
   A   
   A   
   T   
   A   
   A   
   A   
   A   
   T   
   C   
   C   
   T   
   T   
   T   
   C   
   A   
   A   
   A   
    C    
   G   
   A   
   C   
   A   
   A   
   A   
   G   
   A   
    G    
   C   
   T   
   T   
   T   
   T   
    C    
   T   
   C   
   T   
   G   
   T   
   T   
   A   
   T   
   G   
   A   
   A   
   A   
   A   
   A   
   A   
   G   
   A   
   A   
   T   
   T   
   G   
   C   
   G   
   A   
   G   
   A   
   A   
   G   
   T   
   G   
   A   
   A   
   A   
   A   
   A   
   A   
   A   
   A   
   T   
   T   
   A   
    C    
   T   
   C   
   T   
   T   
   C   
   C   
   G   
   C   
   T   
   C   
   G   
   T   
   C   
    T    
    G    
   A   
   C   
   G   
   A   
   C   
   A   
   A   
   T   
   C   
    C    
   A   
   A   
   G   
   A   
   C   
   G   
   A   
   A   
   A   
   T   
   C   
   G   
   A   
   A   
   G   
   A   
   A   
   A   
   T   
   C   
   A   
   A   
   A   
   A   
   T   
    C    
   G   
   A   
   T   
   A   
   C   
   G   
   C   
   A   
   A   
    G    
   T   
    T    
   T   
   T   
   A   
   G   
   T   
   G   
   G   
   C   
    G    
   C   
   A   
   A   
   G   
   A   
   A   
   G   
   A   
   C   
   G   
   T   
   A   
   A   
   T   
    C    
   G   
   T   
   C   
   A   
   G   
   T   
   G   
   T   
    G    
   A   
   C   
    G    
   C   
   G   
   A   
   G   
   A   
   A   
   G   
   G   
   C   
   T   
   A   
   T   
   G   
   T   
   G   
   A   
   A   
   G   
   C   
   G   
   A   
   A   
   C   
   A   
   A   
   G   
   C   
   C   
   T   
   T   
   C   
   G   
   T   
   T   
   C   
    T    
   T   
   A   
   T   
   A   
   G   
   T   
   G   
   C   
   T   
   T   
   C   
    G    
   A   
   A   
   A   
   C   
   C   
   A   
   G   
   A   
   A   
   G   
   A   
   A   
   A   
   T   
   C   
   G   
   G   
   G   
   A   
   T   
   G   
   C   
   G   
   A   
   G   
   A   
   A   
   G   
   G   
   C   
   G   
   A   
    T    
   T   
   A   
   C   
   T   
   T   
   G   
   C   
   T   
    G    
   T   
   A   
   C   
    T    
   C   
   A   
   G   
   G   
   T   
   G   
   A   
   A   
   T   
   T   
    G    
   A   
   G   
    C    
   A   
   C   
   A   
    T    
   T   
   G   
   G   
   A   
   C   
   C   
   A   
   T   
   G   
   T   
   T   
   C   
   T   
   T   
   C   
   T   
   G   
   A   
   T   
    T    
   A   
   C   
    G    
   A   
   A   
   T   
   A   
   A   
   A   
   G   
   C   
   A   
   A   
   A   
   T   
   G   
   T   
   G   
   A   
   T   
   C   
   T   
   A   
   C   
   C   
   G   
    T    
   C   
   C   
   T   
   G   
   T   
    T    
   C   
   A   
   T   
   G   
   A   
   A   
   C   
   T   
   A   
   C   
   C   
    C    
   G   
   A   
   T   
   T   
   T   
   G   
   A   
   A   
   A   
   T   
   G   
   G   
   A   
   A   
   A   
   G   
   A   
   T   
   G   
   C   
   T   
   G   
   G   
    C    
   G   
   A   
    A    
   C   
   A   
    C    
   A   
   T   
   T   
   T   
   C   
   T   
   C   
   A   
   A   
   A   
   C   
   A   
   A   
   T   
   C   
   T   
   C   
   C   
   A   
   A   
   T   
   C   
   T   
   G   
   T   
   C   
   T   
   G   
   T   
   A   
   G   
   A   
    T    
   G   
   A   
   A   
   T   
   C   
   G   
   A   
   T   
    C    
   T   
   T   
   A   
   G   
   C   
   T   
   G   
   T   
   T   
   T   
   T   
    C    
   C   
   C   
   A   
   T   
   A   
   T   
   C   
   A   
   A   
   A   
   A   
   A   
   A   
   T   
    C    
   C   
   A   
   A   
   G   
   C   
   A   
   G   
   A   
    A    
   A   
   A   
   A   
   A   
   C   
   A   
   T   
   T   
   T   
   G   
   T   
    A    
   T   
   T   
   C   
   A   
   T   
   C   
   A   
   G   
   C   
   A   
   A   
   G   
   A   
   A   
   T   
   G   
   G   
   A   
   T   
   T   
    G    
    A    
   T   
   C   
   A   
   A   
   G   
   C   
   A   
   A   
   A   
   C   
   A   
   C   
   G   
   A   
   A   
   T   
   G   
   A   
   C   
   A   
   G   
   A   
   G   
   T   
   T   
   C   
   G   
   A   
   A   
   C   
   C   
   A   
   T   
   G   
   G   
   C   
   G   
   C   
   A   
   C   
   C   
   T   
   A   
    T    
   A   
   A   
   G   
   A   
   G   
   C   
   C   
   G   
    T    
   C   
   C   
   G   
   C   
   T   
   C   
   A   
   G   
   C   
   T   
   G   
   T   
   A   
   T   
   G   
   A   
   A   
   G   
   C   
   T   
   A   
   A   
   A   
   G   
   G   
   C   
   G   
   G   
   A   
   A   
   G   
   A   
   A   
   G   
   A   
   T   
   G   
   A   
   A   
   C   
   T   
    T    
   A   
   T   
   T   
   G   
   C   
   T   
   G   
   T   
    A    
   T   
   A   
   T   
   C   
   T   
   G   
   A   
   A   
   A   
   G   
   A   
   A   
   G   
   A   
   T   
   C   
    A    
   G   
   C   
   C   
   A   
   G   
   G   
   C   
   T   
   T   
   A   
   G   
   A   
   T   
   G   
   T   
    G    
   T   
   T   
   C   
   T   
   T   
   A   
   G   
   T   
   A   
   A   
   C   
   G   
    C    
   A   
   T   
   C   
   A   
   A   
   G   
   G   
   A   
   A   
   T   
   G   
   G   
   G   
    C    
   T   
   T   
   G   
   C   
   G   
   C   
   T   
    A    
   T   
   C   
   C   
   T   
   T   
   T   
   A   
   A   
    C    
   A   
   G   
   A   
   T   
   G   
   T   
   T   
   C   
   C   
   C   
   G   
   T   
   T   
   G   
   T   
   T   
   G   
   G   
   A   
   G   
   C   
   A   
   A   
   A   
   G   
   G   
   C   
   A   
   G   
    C    
   T   
   G   
   G   
   A   
   G   
   T   
   G   
   A   
   A   
   G   
   T   
   C   
   T   
   A   
   T   
   G   
   A   
   A   
   T   
   T   
   T   
   G   
   A   
   A   
   A   
   G   
   A   
   T   
   G   
   G   
   A   
   G   
   A   
   T   
   A   
   G   
   G   
    A    
   T   
   T   
    G    
   T   
   C   
   A   
   A   
    T    
   G   
   G   
    G    
   C   
   T   
    G    
   C   
   T   
   T   
   G   
   T   
   G   
   T   
   T   
   A   
   G   
   C   
   A   
   G   
   A   
    C    
   G   
   G   
    A    
   G   
   A   
   T   
   A   
   C   
   G   
   C   
   C   
    A    
   A   
   T   
   C   
   G   
   T   
   A   
   A   
   T   
   C   
   G   
   T   
   T   
   A   
   C   
   T   
   C   
   A   
   G   
   C   
   G   
   A   
   G   
   G   
   A   
   G   
   C   
    C    
   G   
   T   
    G    
   A   
   A   
   A   
   C   
   G   
   C   
   A   
   T   
   G   
   C   
   T   
   T   
   G   
   C   
   T   
   C   
   A   
   A   
   G   
   A   
   A   
   A   
   T   
   C   
   A   
   G   
   C   
   C   
   A   
   A   
   A   
   C   
    T    
   T   
   C   
   A   
   C   
   G   
   T   
   G   
   C   
   G   
   A   
   A   
   G   
   C   
   G   
   G   
   G   
   G   
   A   
   G   
   T   
   G   
   A   
   C   
   G   
   G   
   T   
   C   
   A   
   T   
   G   
   C   
   G   
   T   
   G   
   A   
   G   
   C   
   T   
   G   
   A   
   A   
   A   
   A   
   A   
   A   
   A   
   A   
   T   
   C   
   C   
    G    
   C   
   A   
    C    
   C   
   G   
    T    
   A   
   T   
   T   
   A   
   T   
   C   
   T   
   A   
   T   
   A   
   T   
   G   
   T   
   C   
   G   
   G   
   A   
   A   
   G   
   G   
   A   
   A   
   G   
   A   
   T   
   C   
   A   
   A   
   A   
   A   
   G   
   A   
   A   
   A   
   T   
   A   
   A   
   C   
   C   
   T   
   T   
   G   
   A   
   T   
   G   
   A   
   A   
   T   
   C   
   A   
   A   
   A   
   A   
   A   
   G   
   G   
    A    
   C   
   A   
    A    
   C   
   A   
   G   
    A    
   T   
   A   
   A   
   C   
   G   
   A   
   T   
   T   
   G   
   A   
    T    
   C   
   C   
   A   
   A   
   C   
    G    
   G   
   A   
   T   
   T   
   T   
    T    
   C   
   C   
   T   
   A   
   T   
   A   
   G   
   G   
   T   
   G   
   A   
   T   
   C   
   G   
   A   
   A   
   C   
   A   
   T   
   C   
   G   
   A   
   A   
   T   
   G   
   G   
    T    
   T   
   C   
    G    
   T   
   T   
   T   
   G   
   C   
    T    
   A   
   T   
   G   
   G   
   A   
    C    
   G   
   A   
   G   
   A   
   A   
   A   
   A   
   A   
   A   
   G   
   G   
   T   
   G   
   G   
   A   
   G   
   A   
   A   
   G   
   T   
   C   
   A   
   T   
   C   
   G   
   A   
   A   
   G   
   T   
    T    
   A   
    T    
   C   
   G   
   A   
   T   
   G   
   C   
   T   
   C   
   C   
   T   
   G   
   A   
   G   
    G    
   T   
   T   
   T   
   T   
    C    
   A   
   T   
    T    
   G   
   A   
    A    
    T    
   C   
    A    
   -   
   -   
   -   
 

 
   N60260F   
   A   
   T   
   G   
   G   
   A   
   A   
   A   
   A   
   T   
   C   
   G   
   A   
   C   
   A   
   A   
   G   
   A   
   A   
   G   
   T   
   A   
   C   
   A   
   A   
   G   
   A   
   A   
   T   
   T   
   A   
   A   
   C   
   T   
   T   
   T   
   A   
   G   
   A   
   A   
   G   
   A   
   A   
   G   
   T   
   G   
   A   
   T   
   G   
   G   
   G   
   T   
   G   
   A   
   C   
   C   
   G   
   C   
   T   
   T   
   C   
   G   
   G   
   A   
   C   
   G   
   T   
   T   
   A   
   T   
   T   
   C   
   C   
   A   
   A   
   A   
   T   
   A   
   T   
   A   
   T   
   T   
   A   
   T   
    C    
   C   
   A   
    A    
   G   
   A   
   A   
   C   
   G   
   T   
   G   
   C   
   A   
   C   
   T   
   G   
   C   
   C   
   T   
   G   
   A   
   T   
   A   
   T   
    C    
   C   
   G   
   T   
   G   
   A   
   T   
   G   
   G   
   A   
   T   
   T   
   G   
   A   
   A   
    G    
   C   
   C   
   A   
   G   
   T   
    G    
   C   
   A   
   A   
   C   
   G   
   C   
   C   
   G   
   G   
   A   
   T   
   A   
   T   
   T   
   A   
   T   
   T   
    T    
   G   
   C   
    C    
   A   
   T   
   G   
   A   
   A   
   C   
   A   
   A   
   A   
   G   
   A   
   T   
   G   
   G   
   C   
   A   
   A   
   T   
   A   
   C   
   G   
   T   
   A   
   T   
   G   
   A   
   C   
   A   
   A   
   A   
   G   
   G   
   A   
   T   
   T   
    C    
   C   
   G   
   T   
   A   
   A   
   A   
   T   
   C   
   A   
   G   
   C   
   A   
   A   
   A   
   A   
   T   
   C   
   T   
   G   
   T   
   C   
   G   
   G   
   A   
   A   
   A   
   T   
   A   
   T   
   C   
   A   
   T   
   G   
   G   
   G   
    G    
   A   
   A   
   T   
   T   
   A   
   T   
   C   
   A   
   T   
   C   
   C   
   T   
   C   
   A   
   T   
   G   
   G   
   T   
   G   
   A   
   C   
   A   
   G   
   C   
   A   
   G   
   T   
   A   
   T   
   T   
   T   
   A   
   T   
   G   
   A   
   A   
   G   
   C   
   G   
   A   
   T   
   G   
   G   
   T   
   C   
   C   
   G   
   C   
   A   
   T   
   G   
   A   
   G   
   C   
   C   
   A   
   A   
   G   
   A   
    C    
   T   
   G   
   G   
   A   
   A   
   G   
   T   
   T   
   G   
   C   
   G   
   G   
   G   
   A   
   A   
   G   
   T   
   A   
   C   
   T   
   G   
   A   
   T   
   C   
   G   
   A   
    A    
   A   
   T   
   G   
   C   
   A   
   C   
   G   
   G   
    G    
   A   
   A   
   T   
   A   
   A   
    C    
   G   
   G   
   G   
   A   
   G   
   T   
   A   
   T   
   G   
   G   
   A   
   C   
   G   
   G   
   T   
   G   
   A   
   T   
   C   
   C   
   A   
   C   
   C   
   T   
   G   
   C   
   T   
   G   
   C   
   G   
   A   
   T   
   G   
   C   
   G   
   T   
   T   
   A   
   T   
   A   
   C   
   T   
   G   
   A   
   A   
   G   
   C   
   C   
   A   
   G   
    A    
   T   
   T   
   A   
   T   
   C   
   C   
   A   
   A   
   G   
   T   
   T   
   A   
   A   
   G   
   T   
   G   
   G   
   A   
   G   
   A   
   G   
   A   
   T   
   G   
    C    
   T   
   A   
    G    
   C   
   A   
   G   
   A   
   T   
   A   
   T   
   C   
   G   
   A   
    A    
   A   
   A   
   A   
   G   
   A   
   A   
   A   
   C   
   A   
   G   
   T   
    C    
   G   
   A   
   T   
   C   
   T   
   C   
   G   
   T   
   G   
   T   
   G   
   G   
   A   
   A   
   T   
   T   
   T   
   T   
   G   
   A   
   T   
   G   
   A   
   T   
   A   
   C   
   A   
   G   
   A   
   A   
   A   
   A   
   A   
   G   
   A   
   A   
   C   
   C   
   A   
   A   
   C   
   A   
   G   
   T   
   C   
   C   
   T   
   T   
   C   
   C   
   A   
   G   
   C   
   T   
   C   
   G   
   A   
   T   
   A   
   C   
   C   
   C   
   A   
   A   
   A   
   C   
   C   
   T   
   T   
   C   
   T   
    C    
   G   
   T   
   C   
   A   
   A   
   T   
   G   
   G   
   A   
   T   
   C   
   G   
   A   
   C   
   A   
   G   
   G   
   T   
   A   
   T   
   A   
   T   
   C   
   T   
   G   
   C   
   T   
   G   
   G   
   A   
   T   
   A   
   T   
   G   
   C   
    A    
   A   
   C   
   A   
   G   
   A   
   A   
   A   
   T   
   C   
   C   
   C   
   G   
   A   
   C   
    T    
   C   
   A   
   T   
   A   
   A   
   C   
   T   
   T   
   A   
   G   
   C   
   T   
   G   
   A   
   A   
   G   
   T   
   G   
   A   
   T   
    C    
   G   
   A   
    T    
   G   
   G   
   C   
   A   
   C   
   A   
   A   
   T   
   C   
   T   
   A   
   T   
   A   
   T   
   G   
   A   
   T   
   C   
   G   
   A   
   T   
   C   
   A   
   T   
   C   
   C   
   A   
   C   
   A   
   A   
   G   
   C   
   G   
   A   
   G   
   C   
   C   
   T   
   T   
   G   
   A   
   A   
   A   
   A   
   A   
   T   
   T   
   G   
   A   
   T   
   G   
   G   
   A   
   A   
   T   
   A   
   T   
   A   
   T   
   T   
   C   
   C   
   T   
   G   
   G   
   A   
   C   
   C   
   T   
   G   
   A   
   C   
   T   
   T   
    T    
   C   
   C   
   A   
   A   
   C   
   A   
   G   
   G   
   A   
   G   
   G   
    G    
   A   
   T   
   C   
   T   
   T   
   G   
   C   
   A   
   A   
   G   
   G   
    A    
   A   
   A   
   A   
   G   
   A   
   T   
   G   
   A   
   A   
   A   
   T   
   C   
   A   
   A   
   A   
   A   
   A   
   A   
   G   
   C   
   T   
   T   
   A   
   T   
   G   
   A   
   A   
   A   
   C   
   A   
   G   
   G   
    G    
   C   
   G   
   A   
   G   
   G   
   A   
   A   
   A   
   A   
   G   
   T   
   T   
   A   
    T    
   T   
   C   
   T   
   T   
   C   
   G   
   T   
   T   
   C   
   G   
   A   
   A   
   A   
   A   
   C   
   A   
   A   
   A   
   G   
   A   
   T   
   A   
   G   
   A   
    G    
   G   
   C   
   A   
   A   
   T   
   G   
   A   
   A   
   G   
   G   
   G   
    T    
   A   
   A   
    T    
   A   
   A   
   A   
   C   
   A   
   G   
   C   
   A   
   A   
   A   
   T   
   C   
   G   
   T   
   G   
   A   
   T   
   C   
   A   
   C   
    A    
   G   
   A   
   A   
   A   
   T   
   C   
   C   
   C   
   A   
   T   
   A   
   T   
   G   
   A   
   A   
   G   
   T   
   A   
   A   
   A   
   T   
   A   
   A   
   A   
   G   
   C   
   G   
   A   
   C   
    C    
   T   
   T   
   G   
   G   
   T   
   G   
   A   
   A   
   A   
   A   
   A   
   A   
   A   
   T   
   G   
   G   
   A   
   T   
   G   
   A   
   G   
   A   
   T   
   C   
   C   
   G   
   T   
   T   
   T   
   G   
   A   
   A   
   T   
   A   
   A   
   G   
   A   
   A   
   A   
   A   
   T   
   C   
   G   
   A   
    T    
   G   
   G   
   A   
   A   
   T   
   A   
   G   
   C   
   T   
   G   
   A   
   A   
   G   
   T   
    A    
   C   
   G   
   A   
   G   
   A   
   T   
   G   
   A   
   A   
   A   
   G   
   T   
   G   
   A   
   T   
    A    
   G   
    A    
   A   
   C   
    A    
   G   
   G   
   T   
   C   
   T   
   G   
   C   
   A   
   A   
   A   
   T   
   T   
   G   
   T   
   C   
   G   
   T   
    C    
   G   
   A   
   A   
   T   
   T   
   G   
   A   
   A   
   A   
   A   
   A   
   A   
   G   
   A   
    C    
   G   
   C   
   C   
   A   
   A   
    T    
   G   
   C   
   T   
   C   
   A   
   A   
   G   
   G   
    A    
   A   
   T   
   T   
   T   
   T   
   G   
   A   
   A   
   T   
   T   
   A   
    T    
   T   
   T   
   G   
   T   
   T   
   C   
   A   
   A   
   A   
   A   
   A   
   C   
   A   
   C   
   T   
   G   
   A   
   G   
    C    
   T   
   A   
   C   
   A   
   G   
   A   
   T   
   C   
   A   
   A   
   T   
   T   
   A   
   C   
   A   
   A   
   T   
   T   
   T   
    C    
   A   
   A   
   T   
   A   
   T   
   G   
   G   
   T   
   C   
   G   
   C   
   T   
   A   
   T   
   C   
   G   
   A   
    C    
   C   
   A   
   T   
   A   
   T   
   G   
   A   
   C   
   T   
   C   
   C   
   G   
   C   
   A   
    T    
   C   
   A   
   A   
   G   
   T   
   T   
   G   
   G   
   A   
   C   
   T   
   G   
   A   
   A   
   A   
   G   
   A   
   C   
   A   
   T   
   T   
   T   
   T   
   A   
   A   
   G   
   A   
   A   
   G   
   T   
   T   
   A   
   T   
   A   
   T   
   C   
   G   
   A   
   G   
   C   
   A   
   T   
   C   
   G   
   A   
   A   
   A   
   G   
   C   
   A   
   A   
   G   
   T   
   C   
   A   
   T   
   A   
   A   
   C   
   A   
   A   
   A   
   A   
   C   
   G   
    T    
   A   
   G   
    T    
   C   
   A   
   A   
   T   
   T   
   C   
   G   
   A   
   T   
   T   
   T   
   A   
   G   
   C   
   C   
   A   
   A   
   A   
   G   
   C   
   T   
   C   
   A   
   A   
   A   
   A   
   A   
    A    
   G   
   A   
   C   
   A   
   G   
   C   
   A   
   T   
   A   
   T   
   C   
   G   
   T   
    A    
   G   
   A   
   A   
   G   
   G   
    A    
    T    
   T   
   G   
   A   
   T   
   G   
   A   
   A   
   A   
   G   
   C   
   T   
   T   
   T   
   A   
   T   
   C   
   G   
   A   
   T   
   T   
   C   
   T   
   G   
   G   
   A   
   T   
   G   
   A   
   A   
   G   
   T   
   A   
   A   
   T   
   C   
   G   
    C    
   A   
   A   
   C   
   G   
   A   
   T   
   T   
   C   
   G   
   T   
   G   
   A   
   A   
   A   
   G   
   T   
   A   
   A   
   A   
   G   
   A   
   C   
   A   
   A   
   A   
   A   
   A   
   A   
   G   
   A   
    C    
   G   
   C   
    C    
   A   
   A   
   A   
   A   
   A   
   G   
   A   
   A   
   T   
   T   
   T   
   A   
   G   
   T   
   C   
   G   
   A   
    C    
   G   
   T   
    C    
   T   
   T   
    T    
   C   
   A   
   A   
   T   
   T   
   T   
   A   
   C   
   A   
   G   
   A   
   A   
   G   
   A   
   A   
   C   
   A   
   A   
   G   
   C   
   A   
   G   
   A   
   A   
   G   
   C   
   A   
   A   
   T   
    T    
   G   
   T   
   C   
   A   
   C   
   A   
   T   
   T   
   G   
   C   
   A   
   A   
    T    
   T   
   A   
   T   
   A   
    C    
   C   
   G   
   T   
   T   
   T   
   G   
   A   
   C   
   G   
   A   
   A   
    C    
   A   
   C   
    G    
   G   
   A   
   T   
   A   
   T   
   C   
   A   
   C   
   A   
   G   
   A   
   G   
   C   
   T   
   T   
   C   
   A   
   A   
   A   
   A   
   G   
   G   
   A   
   A   
    T    
   C   
    C    
   G   
   A   
   A   
   A   
    G    
   C   
   T   
   T   
   G   
   A   
   T   
   T   
   G   
   C   
    G    
   C   
   A   
   A   
   A   
   T   
   C   
   A   
   C   
   A   
   G   
   A   
   G   
   T   
   T   
    G    
   A   
   A   
   T   
   A   
   A   
   A   
   A   
   T   
   C   
   C   
   T   
   T   
   T   
   C   
   A   
   A   
   A   
    T    
   G   
   A   
   C   
   A   
   A   
   A   
   G   
   A   
    G    
   C   
   T   
   T   
   T   
   T   
    C    
   T   
   C   
   T   
   G   
   T   
   T   
   A   
   T   
   G   
   A   
   A   
   A   
   A   
   A   
   A   
   G   
   A   
   A   
   T   
   T   
   G   
   C   
   G   
   A   
   G   
   A   
   A   
   G   
   T   
   G   
   A   
   A   
   A   
   A   
   A   
   A   
   A   
   A   
   T   
   T   
   A   
    T    
   T   
   C   
   T   
   T   
   C   
   C   
   G   
   C   
   T   
   C   
   G   
   T   
   C   
    T    
    A    
   A   
   C   
   G   
   A   
   C   
   A   
   A   
   T   
   C   
    G    
   A   
   A   
   G   
   A   
   C   
   G   
   A   
   A   
   A   
   T   
   C   
   G   
   A   
   A   
   G   
   A   
   A   
   A   
   T   
   C   
   A   
   A   
   A   
   A   
   T   
    T    
   G   
   A   
   T   
   A   
   C   
   G   
   C   
   A   
   A   
    G    
   T   
    C    
   T   
   T   
   A   
   G   
   T   
   G   
   G   
   C   
    A    
   C   
   A   
   A   
   G   
   A   
   A   
   G   
   A   
   C   
   G   
   T   
   A   
   A   
   T   
    T    
   G   
   T   
   C   
   A   
   G   
   T   
   G   
   T   
    G    
   A   
   C   
    G    
   C   
   G   
   A   
   G   
   A   
   A   
   G   
   G   
   C   
   T   
   A   
   T   
   G   
   T   
   G   
   A   
   A   
   G   
   C   
   G   
   A   
   A   
   C   
   A   
   A   
   G   
   C   
   C   
   T   
   T   
   C   
   G   
   T   
   T   
   C   
    T    
   T   
   A   
   T   
   A   
   G   
   T   
   G   
   C   
   T   
   T   
   C   
    C    
   A   
   A   
   A   
   C   
   C   
   A   
   G   
   A   
   A   
   G   
   A   
   A   
   A   
   T   
   C   
   G   
   G   
   G   
   A   
   T   
   G   
   C   
   G   
   A   
   G   
   A   
   A   
   G   
   G   
   C   
   G   
   A   
    C    
   T   
   A   
   C   
   T   
   T   
   G   
   C   
   T   
    A    
   T   
   A   
   C   
    T    
   C   
   A   
   G   
   G   
   T   
   G   
   A   
   A   
   T   
   T   
    G    
   A   
   G   
    T    
   A   
   C   
   A   
    T    
   T   
   G   
   G   
   A   
   C   
   C   
   A   
   T   
   G   
   T   
   T   
   C   
   T   
   T   
   C   
   T   
   G   
   A   
   T   
    C    
   A   
   C   
    A    
   A   
   A   
   T   
   A   
   A   
   A   
   G   
   C   
   A   
   A   
   A   
   T   
   G   
   T   
   G   
   A   
   T   
   C   
   T   
   A   
   C   
   C   
   G   
    C    
   C   
   C   
   T   
   G   
   T   
    C    
   C   
   A   
   T   
   G   
   A   
   A   
   C   
   T   
   A   
   C   
   C   
    C    
   G   
   A   
   T   
   T   
   T   
   G   
   A   
   A   
   A   
   T   
   G   
   G   
   A   
   A   
   A   
   G   
   A   
   T   
   G   
   C   
   T   
   G   
   G   
    T    
   G   
   A   
    G    
   C   
   A   
    C    
   A   
   T   
   T   
   T   
   C   
   T   
   C   
   A   
   A   
   A   
   C   
   A   
   A   
   T   
   C   
   T   
   C   
   C   
   A   
   A   
   T   
   C   
   T   
   G   
   T   
   C   
   T   
   G   
   T   
   A   
   G   
   A   
    C    
   G   
   A   
   A   
   T   
   C   
   G   
   A   
   T   
    T    
   T   
   T   
   A   
   G   
   C   
   T   
   G   
   T   
   T   
   T   
   T   
    C    
   C   
   C   
   A   
   T   
   A   
   T   
   C   
   A   
   A   
   A   
   A   
   A   
   A   
   T   
    T    
   C   
   A   
   A   
   G   
   C   
   A   
   G   
   A   
    G    
   A   
   A   
   A   
   A   
   C   
   A   
   T   
   T   
   T   
   G   
   T   
    A    
   T   
   T   
   C   
   A   
   T   
   C   
   A   
   G   
   C   
   A   
   A   
   G   
   A   
   A   
   T   
   G   
   G   
   A   
   T   
   T   
    G    
    A    
   T   
   C   
   A   
   A   
   G   
   C   
   A   
   A   
   A   
   C   
   A   
   C   
   G   
   A   
   A   
   T   
   G   
   A   
   C   
   A   
   G   
   A   
   G   
   T   
   T   
   C   
   G   
   A   
   A   
   C   
   C   
   A   
   T   
   G   
   G   
   C   
   G   
   C   
   A   
   C   
   C   
   T   
   A   
    T    
   A   
   A   
   G   
   A   
   G   
   C   
   C   
   G   
    T    
   C   
   C   
   G   
   C   
   T   
   C   
   A   
   G   
   C   
   T   
   G   
   T   
   A   
   T   
   G   
   A   
   A   
   G   
   C   
   T   
   A   
   A   
   A   
   G   
   G   
   C   
   G   
   G   
   A   
   A   
   G   
   A   
   A   
   G   
   A   
   T   
   G   
   A   
   A   
   C   
   T   
    G    
   A   
   T   
   T   
   G   
   C   
   T   
   G   
   T   
    G    
   T   
   A   
   T   
   C   
   T   
   G   
   A   
   A   
   A   
   G   
   A   
   A   
   G   
   A   
   T   
   C   
    A    
   G   
   C   
   C   
   A   
   G   
   G   
   C   
   T   
   T   
   A   
   G   
   A   
   T   
   G   
   T   
    T    
   T   
   T   
   C   
   T   
   T   
   A   
   G   
   T   
   A   
   A   
   C   
   G   
    C    
   A   
   T   
   C   
   A   
   A   
   G   
   G   
   A   
   A   
   T   
   G   
   G   
   G   
    T    
   T   
   T   
   G   
   C   
   G   
   C   
   T   
    A    
   T   
   C   
   C   
   T   
   T   
   T   
   A   
   A   
    C    
   A   
   G   
   A   
   T   
   G   
   T   
   T   
   C   
   C   
   C   
   G   
   T   
   T   
   G   
   T   
   T   
   G   
   G   
   A   
   G   
   C   
   A   
   A   
   A   
   G   
   G   
   C   
   A   
   G   
    C    
   T   
   G   
   G   
   A   
   G   
   T   
   G   
   A   
   A   
   G   
   T   
   C   
   T   
   A   
   T   
   G   
   A   
   A   
   T   
   T   
   T   
   G   
   A   
   A   
   A   
   G   
   A   
   T   
   G   
   G   
   A   
   G   
   A   
   T   
   A   
   G   
   G   
    A    
   T   
   T   
    A    
   T   
   C   
   A   
   A   
    C    
   G   
   G   
    G    
   C   
   T   
    G    
   C   
   T   
   T   
   G   
   T   
   G   
   T   
   T   
   A   
   G   
   C   
   A   
   G   
   A   
    A    
   G   
   G   
    T    
   G   
   A   
   T   
   A   
   C   
   G   
   C   
   C   
    G    
   A   
   T   
   C   
   G   
   T   
   A   
   A   
   T   
   C   
   G   
   T   
   T   
   A   
   C   
   T   
   C   
   A   
   G   
   C   
   G   
   A   
   G   
   G   
   A   
   G   
   C   
    T    
   G   
   T   
    T    
   A   
   A   
   A   
   C   
   G   
   C   
   A   
   T   
   G   
   C   
   T   
   T   
   G   
   C   
   T   
   C   
   A   
   A   
   G   
   A   
   A   
   A   
   T   
   C   
   A   
   G   
   C   
   C   
   A   
   A   
   A   
   C   
    T    
   T   
   C   
   A   
   C   
   G   
   T   
   G   
   C   
   G   
   A   
   A   
   G   
   C   
   G   
   G   
   G   
   G   
   A   
   G   
   T   
   G   
   A   
   C   
   G   
   G   
   T   
   C   
   A   
   T   
   G   
   C   
   G   
   T   
   G   
   A   
   G   
   C   
   T   
   G   
   A   
   A   
   A   
   A   
   A   
   A   
   A   
   A   
   T   
   C   
   C   
    G    
   C   
   A   
    C    
   C   
   G   
    T    
   A   
   T   
   T   
   A   
   T   
   C   
   T   
   A   
   T   
   A   
   T   
   G   
   T   
   C   
   G   
   G   
   A   
   A   
   G   
   G   
   A   
   A   
   G   
   A   
   T   
   C   
   A   
   A   
   A   
   A   
   G   
   A   
   A   
   A   
   T   
   A   
   A   
   C   
   C   
   T   
   T   
   G   
   A   
   T   
   G   
   A   
   A   
   T   
   C   
   A   
   A   
   A   
   A   
   A   
   G   
   G   
    G    
   C   
   A   
    G    
   C   
   A   
   G   
    T    
   T   
   A   
   A   
   C   
   G   
   A   
   T   
   T   
   G   
   A   
    C    
   C   
   C   
   A   
   A   
   C   
    T    
   G   
   A   
   T   
   T   
   T   
    C    
   C   
   C   
   T   
   A   
   T   
   A   
   G   
   G   
   T   
   G   
   A   
   T   
   C   
   G   
   A   
   A   
   C   
   A   
   T   
   C   
   G   
   A   
   A   
   T   
   G   
   G   
    A    
   T   
   C   
    A    
   T   
   T   
   T   
   G   
   C   
    G    
   A   
   T   
   G   
   G   
   A   
    T    
   G   
   A   
   G   
   A   
   A   
   A   
   A   
   A   
   A   
   G   
   G   
   T   
   G   
   G   
   A   
   G   
   A   
   A   
   G   
   T   
   C   
   A   
   T   
   C   
   G   
   A   
   A   
   G   
   T   
    T    
   A   
    T    
   C   
   G   
   A   
   T   
   G   
   C   
   T   
   C   
   C   
   T   
   G   
   A   
   G   
    A    
   T   
   T   
   T   
   T   
    C    
   A   
   T   
    C    
   G   
   A   
    G    
    T    
   C   
    G    
   -   
   -   
   -   
 

 
   N54551   
   A   
   T   
   G   
   G   
   A   
   A   
   A   
   A   
   T   
   C   
   G   
   A   
   C   
   A   
   A   
   G   
   A   
   A   
   G   
   T   
   A   
   C   
   A   
   A   
   G   
   A   
   A   
   T   
   T   
   A   
   A   
   C   
   T   
   T   
   T   
   A   
   G   
   A   
   A   
   G   
   A   
   A   
   G   
   T   
   G   
   A   
   T   
   G   
   G   
   G   
   T   
   G   
   A   
   C   
   C   
   G   
   C   
   T   
   T   
   C   
   G   
   G   
   A   
   C   
   G   
   T   
   T   
   A   
   T   
   T   
   C   
   C   
   A   
   A   
   A   
   T   
   A   
   T   
   A   
   T   
   T   
   A   
   T   
    C    
   C   
   A   
    A    
   G   
   A   
   A   
   C   
   G   
   T   
   G   
   C   
   A   
   C   
   T   
   G   
   C   
   C   
   T   
   G   
   A   
   T   
   A   
   T   
    T    
   C   
   G   
   T   
   G   
   A   
   T   
   G   
   G   
   A   
   T   
   T   
   G   
   A   
   A   
    G    
   C   
   C   
   A   
   G   
   T   
    G    
   C   
   A   
   A   
   C   
   G   
   C   
   C   
   G   
   G   
   A   
   T   
   A   
   T   
   T   
   A   
   T   
   T   
    T    
   G   
   C   
    C    
   A   
   T   
   G   
   A   
   A   
   C   
   A   
   A   
   A   
   G   
   A   
   T   
   G   
   G   
   C   
   A   
   A   
   T   
   A   
   C   
   G   
   T   
   A   
   T   
   G   
   A   
   C   
   A   
   A   
   A   
   G   
   G   
   A   
   T   
   T   
    C    
   C   
   G   
   T   
   A   
   A   
   A   
   T   
   C   
   A   
   G   
   C   
   A   
   A   
   A   
   A   
   T   
   C   
   T   
   G   
   T   
   C   
   G   
   G   
   A   
   A   
   A   
   T   
   A   
   T   
   C   
   A   
   T   
   G   
   G   
   G   
    G    
   A   
   A   
   T   
   T   
   A   
   T   
   C   
   A   
   T   
   C   
   C   
   T   
   C   
   A   
   T   
   G   
   G   
   T   
   G   
   A   
   C   
   A   
   G   
   C   
   A   
   G   
   T   
   A   
   T   
   T   
   T   
   A   
   T   
   G   
   A   
   A   
   G   
   C   
   G   
   A   
   T   
   G   
   G   
   T   
   C   
   C   
   G   
   C   
   A   
   T   
   G   
   A   
   G   
   C   
   C   
   A   
   A   
   G   
   A   
    C    
   T   
   G   
   G   
   A   
   A   
   G   
   T   
   T   
   G   
   C   
   G   
   G   
   G   
   A   
   A   
   G   
   T   
   A   
   C   
   T   
   G   
   A   
   T   
   C   
   G   
   A   
    A    
   A   
   T   
   G   
   C   
   A   
   C   
   G   
   G   
    G    
   A   
   A   
   T   
   A   
   A   
    C    
   G   
   G   
   G   
   A   
   G   
   T   
   A   
   T   
   G   
   G   
   A   
   C   
   G   
   G   
   T   
   G   
   A   
   T   
   C   
   C   
   A   
   C   
   C   
   T   
   G   
   C   
   T   
   G   
   C   
   G   
   A   
   T   
   G   
   C   
   G   
   T   
   T   
   A   
   T   
   A   
   C   
   T   
   G   
   A   
   A   
   G   
   C   
   C   
   A   
   G   
    A    
   T   
   T   
   A   
   T   
   C   
   C   
   A   
   A   
   G   
   T   
   T   
   A   
   A   
   G   
   T   
   G   
   G   
   A   
   G   
   A   
   G   
   A   
   T   
   G   
    C    
   T   
   A   
    G    
   C   
   A   
   G   
   A   
   T   
   A   
   T   
   C   
   G   
   A   
    A    
   A   
   A   
   A   
   G   
   A   
   A   
   A   
   C   
   A   
   G   
   T   
    C    
   G   
   A   
   T   
   C   
   T   
   C   
   G   
   T   
   G   
   T   
   G   
   G   
   A   
   A   
   T   
   T   
   T   
   T   
   G   
   A   
   T   
   G   
   A   
   T   
   A   
   C   
   A   
   G   
   A   
   A   
   A   
   A   
   A   
   G   
   A   
   A   
   C   
   C   
   A   
   A   
   C   
   A   
   G   
   T   
   C   
   C   
   T   
   T   
   C   
   C   
   A   
   G   
   C   
   T   
   C   
   G   
   A   
   T   
   A   
   C   
   C   
   C   
   A   
   A   
   A   
   C   
   C   
   T   
   T   
   C   
   T   
    C    
   G   
   T   
   C   
   A   
   A   
   T   
   G   
   G   
   A   
   T   
   C   
   G   
   A   
   C   
   A   
   G   
   G   
   T   
   A   
   T   
   A   
   T   
   C   
   T   
   G   
   C   
   T   
   G   
   G   
   A   
   T   
   A   
   T   
   G   
   C   
    A    
   A   
   C   
   A   
   G   
   A   
   A   
   A   
   T   
   C   
   C   
   C   
   G   
   A   
   C   
    T    
   C   
   A   
   T   
   A   
   A   
   C   
   T   
   T   
   A   
   G   
   C   
   T   
   G   
   A   
   A   
   G   
   T   
   G   
   A   
   T   
    C    
   G   
   A   
    T    
   G   
   G   
   C   
   A   
   C   
   A   
   A   
   T   
   C   
   T   
   A   
   T   
   A   
   T   
   G   
   A   
   T   
   C   
   G   
   A   
   T   
   C   
   A   
   T   
   C   
   C   
   A   
   C   
   A   
   A   
   G   
   C   
   G   
   A   
   G   
   C   
   C   
   T   
   T   
   G   
   A   
   A   
   A   
   A   
   A   
   T   
   T   
   G   
   A   
   T   
   G   
   G   
   A   
   A   
   T   
   A   
   T   
   A   
   T   
   T   
   C   
   C   
   T   
   G   
   G   
   A   
   C   
   C   
   T   
   G   
   A   
   C   
   T   
   T   
    T    
   C   
   C   
   A   
   A   
   C   
   A   
   G   
   G   
   A   
   G   
   G   
    G    
   A   
   T   
   C   
   T   
   T   
   G   
   C   
   A   
   A   
   G   
   G   
    A    
   A   
   A   
   A   
   G   
   A   
   T   
   G   
   A   
   A   
   A   
   T   
   C   
   A   
   A   
   A   
   A   
   A   
   A   
   G   
   C   
   T   
   T   
   A   
   T   
   G   
   A   
   A   
   A   
   C   
   A   
   G   
   G   
    G    
   C   
   G   
   A   
   G   
   G   
   A   
   A   
   A   
   A   
   G   
   T   
   T   
   A   
    T    
   T   
   C   
   T   
   T   
   C   
   G   
   T   
   T   
   C   
   G   
   A   
   A   
   A   
   A   
   C   
   A   
   A   
   A   
   G   
   A   
   T   
   A   
   G   
   A   
    A    
   G   
   C   
   A   
   A   
   T   
   G   
   A   
   A   
   G   
   G   
   G   
    T    
   A   
   A   
    T    
   A   
   A   
   A   
   C   
   A   
   G   
   C   
   A   
   A   
   A   
   T   
   C   
   G   
   T   
   G   
   A   
   T   
   C   
   A   
   C   
    A    
   G   
   A   
   A   
   A   
   T   
   C   
   C   
   C   
   A   
   T   
   A   
   T   
   G   
   A   
   A   
   G   
   T   
   A   
   A   
   A   
   T   
   A   
   A   
   A   
   G   
   C   
   G   
   A   
   C   
    C    
   T   
   T   
   G   
   G   
   T   
   G   
   A   
   A   
   A   
   A   
   A   
   A   
   A   
   T   
   G   
   G   
   A   
   T   
   G   
   A   
   G   
   A   
   T   
   C   
   C   
   G   
   T   
   T   
   T   
   G   
   A   
   A   
   T   
   A   
   A   
   G   
   A   
   A   
   A   
   A   
   T   
   C   
   G   
   A   
    T    
   G   
   G   
   A   
   A   
   T   
   A   
   G   
   C   
   T   
   G   
   A   
   A   
   G   
   T   
    A    
   C   
   G   
   A   
   G   
   A   
   T   
   G   
   A   
   A   
   A   
   G   
   T   
   G   
   A   
   T   
    A    
   G   
    A    
   A   
   C   
    A    
   G   
   G   
   T   
   C   
   T   
   G   
   C   
   A   
   A   
   A   
   T   
   T   
   G   
   T   
   C   
   G   
   T   
    C    
   G   
   A   
   A   
   T   
   T   
   G   
   A   
   A   
   A   
   A   
   A   
   A   
   G   
   A   
    C    
   G   
   C   
   C   
   A   
   A   
    T    
   G   
   C   
   T   
   C   
   A   
   A   
   G   
   G   
    A    
   A   
   T   
   T   
   T   
   T   
   G   
   A   
   A   
   T   
   T   
   A   
    T    
   T   
   T   
   G   
   T   
   T   
   C   
   A   
   A   
   A   
   A   
   A   
   C   
   A   
   C   
   T   
   G   
   A   
   G   
    C    
   T   
   A   
   C   
   A   
   G   
   A   
   T   
   C   
   A   
   A   
   T   
   T   
   A   
   C   
   A   
   A   
   T   
   T   
   T   
    C    
   A   
   A   
   T   
   A   
   T   
   G   
   G   
   T   
   C   
   G   
   C   
   T   
   A   
   T   
   C   
   G   
   A   
    C    
   C   
   A   
   T   
   A   
   T   
   G   
   A   
   C   
   T   
   C   
   C   
   G   
   C   
   A   
    T    
   C   
   A   
   A   
   G   
   T   
   T   
   G   
   G   
   A   
   C   
   T   
   G   
   A   
   A   
   A   
   G   
   A   
   C   
   A   
   T   
   T   
   T   
   T   
   A   
   A   
   G   
   A   
   A   
   G   
   T   
   T   
   A   
   T   
   A   
   T   
   C   
   G   
   A   
   G   
   C   
   A   
   T   
   C   
   G   
   A   
   A   
   A   
   G   
   C   
   A   
   A   
   G   
   T   
   C   
   A   
   T   
   A   
   A   
   C   
   A   
   A   
   A   
   A   
   C   
   G   
    T    
   A   
   G   
    T    
   C   
   A   
   A   
   T   
   T   
   C   
   G   
   A   
   T   
   T   
   T   
   A   
   G   
   C   
   C   
   A   
   A   
   A   
   G   
   C   
   T   
   C   
   A   
   A   
   A   
   A   
   A   
    A    
   G   
   A   
   C   
   A   
   G   
   C   
   A   
   T   
   A   
   T   
   C   
   G   
   T   
    A    
   G   
   A   
   A   
   G   
   G   
    A    
    T    
   T   
   G   
   A   
   T   
   G   
   A   
   A   
   A   
   G   
   C   
   T   
   T   
   T   
   A   
   T   
   C   
   G   
   A   
   T   
   T   
   C   
   T   
   G   
   G   
   A   
   T   
   G   
   A   
   A   
   G   
   T   
   A   
   A   
   T   
   C   
   G   
    C    
   A   
   A   
   C   
   G   
   A   
   T   
   T   
   C   
   G   
   T   
   G   
   A   
   A   
   A   
   G   
   T   
   A   
   A   
   A   
   G   
   A   
   C   
   A   
   A   
   A   
   A   
   A   
   A   
   G   
   A   
    C    
   G   
   C   
    C    
   A   
   A   
   A   
   A   
   A   
   G   
   A   
   A   
   T   
   T   
   T   
   A   
   G   
   T   
   C   
   G   
   A   
    C    
   G   
   T   
    C    
   T   
   T   
    T    
   C   
   A   
   A   
   T   
   T   
   T   
   A   
   C   
   A   
   G   
   A   
   A   
   G   
   A   
   A   
   C   
   A   
   A   
   G   
   C   
   A   
   G   
   A   
   A   
   G   
   C   
   A   
   A   
   T   
    T    
   G   
   T   
   C   
   A   
   C   
   A   
   T   
   T   
   G   
   C   
   A   
   A   
    T    
   T   
   A   
   T   
   A   
    C    
   C   
   G   
   T   
   T   
   T   
   G   
   A   
   C   
   G   
   A   
   A   
    C    
   A   
   C   
    G    
   G   
   A   
   T   
   A   
   T   
   C   
   A   
   C   
   A   
   G   
   A   
   G   
   C   
   T   
   T   
   C   
   A   
   A   
   A   
   A   
   G   
   G   
   A   
   A   
    T    
   C   
    C    
   G   
   A   
   A   
   A   
    G    
   C   
   T   
   T   
   G   
   A   
   T   
   T   
   G   
   C   
    G    
   C   
   A   
   A   
   A   
   T   
   C   
   A   
   C   
   A   
   G   
   A   
   G   
   T   
   T   
    G    
   A   
   A   
   T   
   A   
   A   
   A   
   A   
   T   
   C   
   C   
   T   
   T   
   T   
   C   
   A   
   A   
   A   
    T    
   G   
   A   
   C   
   A   
   A   
   A   
   G   
   A   
    G    
   C   
   T   
   T   
   T   
   T   
    C    
   T   
   C   
   T   
   G   
   T   
   T   
   A   
   T   
   G   
   A   
   A   
   A   
   A   
   A   
   A   
   G   
   A   
   A   
   T   
   T   
   G   
   C   
   G   
   A   
   G   
   A   
   A   
   G   
   T   
   G   
   A   
   A   
   A   
   A   
   A   
   A   
   A   
   A   
   T   
   T   
   A   
    T    
   T   
   C   
   T   
   T   
   C   
   C   
   G   
   C   
   T   
   C   
   G   
   T   
   C   
    T    
    A    
   A   
   C   
   G   
   A   
   C   
   A   
   A   
   T   
   C   
    G    
   A   
   A   
   G   
   A   
   C   
   G   
   A   
   A   
   A   
   T   
   C   
   G   
   A   
   A   
   G   
   A   
   A   
   A   
   T   
   C   
   A   
   A   
   A   
   A   
   T   
    T    
   G   
   A   
   T   
   A   
   C   
   G   
   C   
   A   
   A   
    G    
   T   
    C    
   T   
   T   
   A   
   G   
   T   
   G   
   G   
   C   
    A    
   C   
   A   
   A   
   G   
   A   
   A   
   G   
   A   
   C   
   G   
   T   
   A   
   A   
   T   
    T    
   G   
   T   
   C   
   A   
   G   
   T   
   G   
   T   
    G    
   A   
   C   
    G    
   C   
   G   
   A   
   G   
   A   
   A   
   G   
   G   
   C   
   T   
   A   
   T   
   G   
   T   
   G   
   A   
   A   
   G   
   C   
   G   
   A   
   A   
   C   
   A   
   A   
   G   
   C   
   C   
   T   
   T   
   C   
   G   
   T   
   T   
   C   
    T    
   T   
   A   
   T   
   A   
   G   
   T   
   G   
   C   
   T   
   T   
   C   
    C    
   A   
   A   
   A   
   C   
   C   
   A   
   G   
   A   
   A   
   G   
   A   
   A   
   A   
   T   
   C   
   G   
   G   
   G   
   A   
   T   
   G   
   C   
   G   
   A   
   G   
   A   
   A   
   G   
   G   
   C   
   G   
   A   
    C    
   T   
   A   
   C   
   T   
   T   
   G   
   C   
   T   
    A    
   T   
   A   
   C   
    T    
   C   
   A   
   G   
   G   
   T   
   G   
   A   
   A   
   T   
   T   
    G    
   A   
   G   
    T    
   A   
   C   
   A   
    T    
   T   
   G   
   G   
   A   
   C   
   C   
   A   
   T   
   G   
   T   
   T   
   C   
   T   
   T   
   C   
   T   
   G   
   A   
   T   
    C    
   A   
   C   
    A    
   A   
   A   
   T   
   A   
   A   
   A   
   G   
   C   
   A   
   A   
   A   
   T   
   G   
   T   
   G   
   A   
   T   
   C   
   T   
   A   
   C   
   C   
   G   
    C    
   C   
   C   
   T   
   G   
   T   
    C    
   C   
   A   
   T   
   G   
   A   
   A   
   C   
   T   
   A   
   C   
   C   
    C    
   G   
   A   
   T   
   T   
   T   
   G   
   A   
   A   
   A   
   T   
   G   
   G   
   A   
   A   
   A   
   G   
   A   
   T   
   G   
   C   
   T   
   G   
   G   
    T    
   G   
   A   
    G    
   C   
   A   
    C    
   A   
   T   
   T   
   T   
   C   
   T   
   C   
   A   
   A   
   A   
   C   
   A   
   A   
   T   
   C   
   T   
   C   
   C   
   A   
   A   
   T   
   C   
   T   
   G   
   T   
   C   
   T   
   G   
   T   
   A   
   G   
   A   
    C    
   G   
   A   
   A   
   T   
   C   
   G   
   A   
   T   
    T    
   T   
   T   
   A   
   G   
   C   
   T   
   G   
   T   
   T   
   T   
   T   
    C    
   C   
   C   
   A   
   T   
   A   
   T   
   C   
   A   
   A   
   A   
   A   
   A   
   A   
   T   
    T    
   C   
   A   
   A   
   G   
   C   
   A   
   G   
   A   
    G    
   A   
   A   
   A   
   A   
   C   
   A   
   T   
   T   
   T   
   G   
   T   
    A    
   T   
   T   
   C   
   A   
   T   
   C   
   A   
   G   
   C   
   A   
   A   
   G   
   A   
   A   
   T   
   G   
   G   
   A   
   T   
   T   
    G    
    A    
   T   
   C   
   A   
   A   
   G   
   C   
   A   
   A   
   A   
   C   
   A   
   C   
   G   
   A   
   A   
   T   
   G   
   A   
   C   
   A   
   G   
   A   
   G   
   T   
   T   
   C   
   G   
   A   
   A   
   C   
   C   
   A   
   T   
   G   
   G   
   C   
   G   
   C   
   A   
   C   
   C   
   T   
   A   
    T    
   A   
   A   
   G   
   A   
   G   
   C   
   C   
   G   
    T    
   C   
   C   
   G   
   C   
   T   
   C   
   A   
   G   
   C   
   T   
   G   
   T   
   A   
   T   
   G   
   A   
   A   
   G   
   C   
   T   
   A   
   A   
   A   
   G   
   G   
   C   
   G   
   G   
   A   
   A   
   G   
   A   
   A   
   G   
   A   
   T   
   G   
   A   
   A   
   C   
   T   
    G    
   A   
   T   
   T   
   G   
   C   
   T   
   G   
   T   
    G    
   T   
   A   
   T   
   C   
   T   
   G   
   A   
   A   
   A   
   G   
   A   
   A   
   G   
   A   
   T   
   C   
    A    
   G   
   C   
   C   
   A   
   G   
   G   
   C   
   T   
   T   
   A   
   G   
   A   
   T   
   G   
   T   
    T    
   T   
   T   
   C   
   T   
   T   
   A   
   G   
   T   
   A   
   A   
   C   
   G   
    C    
   A   
   T   
   C   
   A   
   A   
   G   
   G   
   A   
   A   
   T   
   G   
   G   
   G   
    T    
   T   
   T   
   G   
   C   
   G   
   C   
   T   
    A    
   T   
   C   
   C   
   T   
   T   
   T   
   A   
   A   
    C    
   A   
   G   
   A   
   T   
   G   
   T   
   T   
   C   
   C   
   C   
   G   
   T   
   T   
   G   
   T   
   T   
   G   
   G   
   A   
   G   
   C   
   A   
   A   
   A   
   G   
   G   
   C   
   A   
   G   
    C    
   T   
   G   
   G   
   A   
   G   
   T   
   G   
   A   
   A   
   G   
   T   
   C   
   T   
   A   
   T   
   G   
   A   
   A   
   T   
   T   
   T   
   G   
   A   
   A   
   A   
   G   
   A   
   T   
   G   
   G   
   A   
   G   
   A   
   T   
   A   
   G   
   G   
    A    
   T   
   T   
    A    
   T   
   C   
   A   
   A   
    C    
   G   
   G   
    G    
   C   
   T   
    A    
   C   
   T   
   T   
   G   
   T   
   G   
   T   
   T   
   A   
   G   
   C   
   A   
   G   
   A   
    A    
   G   
   G   
    T    
   G   
   A   
   T   
   A   
   C   
   G   
   C   
   C   
    G    
   A   
   T   
   C   
   G   
   T   
   A   
   A   
   T   
   C   
   G   
   T   
   T   
   A   
   C   
   T   
   C   
   A   
   G   
   C   
   G   
   A   
   G   
   G   
   A   
   G   
   C   
    T    
   G   
   T   
    T    
   A   
   A   
   A   
   C   
   G   
   C   
   A   
   T   
   G   
   C   
   T   
   T   
   G   
   C   
   T   
   C   
   A   
   A   
   G   
   A   
   A   
   A   
   T   
   C   
   A   
   G   
   C   
   C   
   A   
   A   
   A   
   C   
    T    
   T   
   C   
   A   
   C   
   G   
   T   
   G   
   C   
   G   
   A   
   A   
   G   
   C   
   G   
   G   
   G   
   G   
   A   
   G   
   T   
   G   
   A   
   C   
   G   
   G   
   T   
   C   
   A   
   T   
   G   
   C   
   G   
   T   
   G   
   A   
   G   
   C   
   T   
   G   
   A   
   A   
   A   
   A   
   A   
   A   
   A   
   A   
   T   
   C   
   C   
    G    
   C   
   A   
    C    
   C   
   G   
    T    
   A   
   T   
   T   
   A   
   T   
   C   
   T   
   A   
   T   
   A   
   T   
   G   
   T   
   C   
   G   
   G   
   A   
   A   
   G   
   G   
   A   
   A   
   G   
   A   
   T   
   C   
   A   
   A   
   A   
   A   
   G   
   A   
   A   
   A   
   T   
   A   
   A   
   C   
   C   
   T   
   T   
   G   
   A   
   T   
   G   
   A   
   A   
   T   
   C   
   A   
   A   
   A   
   A   
   A   
   G   
   G   
    G    
   C   
   A   
    G    
   C   
   A   
   G   
    T    
   T   
   A   
   A   
   C   
   G   
   A   
   T   
   T   
   G   
   A   
    C    
   C   
   C   
   A   
   A   
   C   
    T    
   G   
   A   
   T   
   T   
   T   
    C    
   C   
   C   
   T   
   A   
   T   
   A   
   G   
   G   
   T   
   G   
   A   
   T   
   C   
   G   
   A   
   A   
   C   
   A   
   T   
   C   
   G   
   A   
   A   
   T   
   G   
   G   
    A    
   T   
   C   
    A    
   T   
   T   
   T   
   G   
   C   
    G    
   A   
   T   
   G   
   G   
   A   
    T    
   G   
   A   
   G   
   A   
   A   
   A   
   A   
   A   
   A   
   G   
   G   
   T   
   G   
   G   
   A   
   G   
   A   
   A   
   G   
   T   
   C   
   A   
   T   
   C   
   G   
   A   
   A   
   G   
   T   
    T    
   A   
    T    
   C   
   G   
   A   
   T   
   G   
   C   
   T   
   C   
   C   
   T   
   G   
   A   
   G   
    A    
   T   
   T   
   T   
   T   
    C    
   A   
   T   
    C    
   G   
   A   
    G    
    T    
   C   
    G    
   -   
   -   
   -   
 

 
   N60299F   
   A   
   T   
   G   
   G   
   A   
   A   
   A   
   A   
   T   
   C   
   G   
   A   
   C   
   A   
   A   
   G   
   A   
   A   
   G   
   T   
   A   
   C   
   A   
   A   
   G   
   A   
   A   
   T   
   T   
   A   
   A   
   C   
   T   
   T   
   T   
   A   
   G   
   A   
   A   
   G   
   A   
   A   
   G   
   T   
   G   
   A   
   T   
   G   
   G   
   G   
   T   
   G   
   A   
   C   
   C   
   G   
   C   
   T   
   T   
   C   
   G   
   G   
   A   
   C   
   G   
   T   
   T   
   A   
   T   
   T   
   C   
   C   
   A   
   A   
   A   
   T   
   A   
   T   
   A   
   T   
   T   
   A   
   T   
    C    
   C   
   A   
    A    
   G   
   A   
   A   
   C   
   G   
   T   
   G   
   C   
   A   
   C   
   T   
   G   
   C   
   C   
   T   
   G   
   A   
   T   
   A   
   T   
    C    
   C   
   G   
   T   
   G   
   A   
   T   
   G   
   G   
   A   
   T   
   T   
   G   
   A   
   A   
    G    
   C   
   C   
   A   
   G   
   T   
    G    
   C   
   A   
   A   
   C   
   G   
   C   
   C   
   G   
   G   
   A   
   T   
   A   
   T   
   T   
   A   
   T   
   T   
    T    
   G   
   C   
    C    
   A   
   T   
   G   
   A   
   A   
   C   
   A   
   A   
   A   
   G   
   A   
   T   
   G   
   G   
   C   
   A   
   A   
   T   
   A   
   C   
   G   
   T   
   A   
   T   
   G   
   A   
   C   
   A   
   A   
   A   
   G   
   G   
   A   
   T   
   T   
    C    
   C   
   G   
   T   
   A   
   A   
   A   
   T   
   C   
   A   
   G   
   C   
   A   
   A   
   A   
   A   
   T   
   C   
   T   
   G   
   T   
   C   
   G   
   G   
   A   
   A   
   A   
   T   
   A   
   T   
   C   
   A   
   T   
   G   
   G   
   G   
    G    
   A   
   A   
   T   
   T   
   A   
   T   
   C   
   A   
   T   
   C   
   C   
   T   
   C   
   A   
   T   
   G   
   G   
   T   
   G   
   A   
   C   
   A   
   G   
   C   
   A   
   G   
   T   
   A   
   T   
   T   
   T   
   A   
   T   
   G   
   A   
   A   
   G   
   C   
   G   
   A   
   T   
   G   
   G   
   T   
   C   
   C   
   G   
   C   
   A   
   T   
   G   
   A   
   G   
   C   
   C   
   A   
   A   
   G   
   A   
    C    
   T   
   G   
   G   
   A   
   A   
   G   
   T   
   T   
   G   
   C   
   G   
   G   
   G   
   A   
   A   
   G   
   T   
   A   
   C   
   T   
   G   
   A   
   T   
   C   
   G   
   A   
    A    
   A   
   T   
   G   
   C   
   A   
   C   
   G   
   G   
    G    
   A   
   A   
   T   
   A   
   A   
    C    
   G   
   G   
   G   
   A   
   G   
   T   
   A   
   T   
   G   
   G   
   A   
   C   
   G   
   G   
   T   
   G   
   A   
   T   
   C   
   C   
   A   
   C   
   C   
   T   
   G   
   C   
   T   
   G   
   C   
   G   
   A   
   T   
   G   
   C   
   G   
   T   
   T   
   A   
   T   
   A   
   C   
   T   
   G   
   A   
   A   
   G   
   C   
   C   
   A   
   G   
    A    
   T   
   T   
   A   
   T   
   C   
   C   
   A   
   A   
   G   
   T   
   T   
   A   
   A   
   G   
   T   
   G   
   G   
   A   
   G   
   A   
   G   
   A   
   T   
   G   
    C    
   T   
   A   
    G    
   C   
   A   
   G   
   A   
   T   
   A   
   T   
   C   
   G   
   A   
    A    
   A   
   A   
   A   
   G   
   A   
   A   
   A   
   C   
   A   
   G   
   T   
    C    
   G   
   A   
   T   
   C   
   T   
   C   
   G   
   T   
   G   
   T   
   G   
   G   
   A   
   A   
   T   
   T   
   T   
   T   
   G   
   A   
   T   
   G   
   A   
   T   
   A   
   C   
   A   
   G   
   A   
   A   
   A   
   A   
   A   
   G   
   A   
   A   
   C   
   C   
   A   
   A   
   C   
   A   
   G   
   T   
   C   
   C   
   T   
   T   
   C   
   C   
   A   
   G   
   C   
   T   
   C   
   G   
   A   
   T   
   A   
   C   
   C   
   C   
   A   
   A   
   A   
   C   
   C   
   T   
   T   
   C   
   T   
    C    
   G   
   T   
   C   
   A   
   A   
   T   
   G   
   G   
   A   
   T   
   C   
   G   
   A   
   C   
   A   
   G   
   G   
   T   
   A   
   T   
   A   
   T   
   C   
   T   
   G   
   C   
   T   
   G   
   G   
   A   
   T   
   A   
   T   
   G   
   C   
    A    
   A   
   C   
   A   
   G   
   A   
   A   
   A   
   T   
   C   
   C   
   C   
   G   
   A   
   C   
    T    
   C   
   A   
   T   
   A   
   A   
   C   
   T   
   T   
   A   
   G   
   C   
   T   
   G   
   A   
   A   
   G   
   T   
   G   
   A   
   T   
    T    
   G   
   A   
    T    
   G   
   G   
   C   
   A   
   C   
   A   
   A   
   T   
   C   
   T   
   A   
   T   
   A   
   T   
   G   
   A   
   T   
   C   
   G   
   A   
   T   
   C   
   A   
   T   
   C   
   C   
   A   
   C   
   A   
   A   
   G   
   C   
   G   
   A   
   G   
   C   
   C   
   T   
   T   
   G   
   A   
   A   
   A   
   A   
   A   
   T   
   T   
   G   
   A   
   T   
   G   
   G   
   A   
   A   
   T   
   A   
   T   
   A   
   T   
   T   
   C   
   C   
   T   
   G   
   G   
   A   
   C   
   C   
   T   
   G   
   A   
   C   
   T   
   T   
    T    
   C   
   C   
   A   
   A   
   C   
   A   
   G   
   G   
   A   
   G   
   G   
    G    
   A   
   T   
   C   
   T   
   T   
   G   
   C   
   A   
   A   
   G   
   G   
    A    
   A   
   A   
   A   
   G   
   A   
   T   
   G   
   A   
   A   
   A   
   T   
   C   
   A   
   A   
   A   
   A   
   A   
   A   
   G   
   C   
   T   
   T   
   A   
   T   
   G   
   A   
   A   
   A   
   C   
   A   
   G   
   G   
    G    
   C   
   G   
   A   
   G   
   G   
   A   
   A   
   A   
   A   
   G   
   T   
   T   
   A   
    T    
   T   
   C   
   T   
   T   
   C   
   G   
   T   
   T   
   C   
   G   
   A   
   A   
   A   
   A   
   C   
   A   
   A   
   A   
   G   
   A   
   T   
   A   
   G   
   A   
    G    
   G   
   C   
   A   
   A   
   T   
   G   
   A   
   A   
   G   
   G   
   G   
    T    
   A   
   A   
    T    
   A   
   A   
   A   
   C   
   A   
   G   
   C   
   A   
   A   
   A   
   T   
   C   
   G   
   T   
   G   
   A   
   T   
   C   
   A   
   C   
    A    
   G   
   A   
   A   
   A   
   T   
   C   
   C   
   C   
   A   
   T   
   A   
   T   
   G   
   A   
   A   
   G   
   T   
   A   
   A   
   A   
   T   
   A   
   A   
   A   
   G   
   C   
   G   
   A   
   C   
    C    
   T   
   T   
   G   
   G   
   T   
   G   
   A   
   A   
   A   
   A   
   A   
   A   
   A   
   T   
   G   
   G   
   A   
   T   
   G   
   A   
   G   
   A   
   T   
   C   
   C   
   G   
   T   
   T   
   T   
   G   
   A   
   A   
   T   
   A   
   A   
   G   
   A   
   A   
   A   
   A   
   T   
   C   
   G   
   A   
    T    
   G   
   G   
   A   
   A   
   T   
   A   
   G   
   C   
   T   
   G   
   A   
   A   
   G   
   T   
    A    
   C   
   G   
   A   
   G   
   A   
   T   
   G   
   A   
   A   
   A   
   G   
   T   
   G   
   A   
   T   
    A    
   G   
    A    
   A   
   C   
    A    
   G   
   G   
   T   
   C   
   T   
   G   
   C   
   A   
   A   
   A   
   T   
   T   
   G   
   T   
   C   
   G   
   T   
    C    
   G   
   A   
   A   
   T   
   T   
   G   
   A   
   A   
   A   
   A   
   A   
   A   
   G   
   A   
    C    
   G   
   C   
   C   
   A   
   A   
    T    
   G   
   C   
   T   
   C   
   A   
   A   
   G   
   G   
    A    
   A   
   T   
   T   
   T   
   T   
   G   
   A   
   A   
   T   
   T   
   A   
    T    
   T   
   T   
   G   
   T   
   T   
   C   
   A   
   A   
   A   
   A   
   A   
   C   
   A   
   C   
   T   
   G   
   A   
   G   
    C    
   T   
   A   
   C   
   A   
   G   
   A   
   T   
   C   
   A   
   A   
   T   
   T   
   A   
   C   
   A   
   A   
   T   
   T   
   T   
    C    
   A   
   A   
   T   
   A   
   T   
   G   
   G   
   T   
   C   
   G   
   C   
   T   
   A   
   T   
   C   
   G   
   A   
    C    
   C   
   A   
   T   
   A   
   T   
   G   
   A   
   C   
   T   
   C   
   C   
   G   
   C   
   A   
    T    
   C   
   A   
   A   
   G   
   T   
   T   
   G   
   G   
   A   
   C   
   T   
   G   
   A   
   A   
   A   
   G   
   A   
   C   
   A   
   T   
   T   
   T   
   T   
   A   
   A   
   G   
   A   
   A   
   G   
   T   
   T   
   A   
   T   
   A   
   T   
   C   
   G   
   A   
   G   
   C   
   A   
   T   
   C   
   G   
   A   
   A   
   A   
   G   
   C   
   A   
   A   
   G   
   T   
   C   
   A   
   T   
   A   
   A   
   C   
   A   
   A   
   A   
   A   
   C   
   G   
    T    
   A   
   G   
    T    
   C   
   A   
   A   
   T   
   T   
   C   
   G   
   A   
   T   
   T   
   T   
   A   
   G   
   C   
   C   
   A   
   A   
   A   
   G   
   C   
   T   
   C   
   A   
   A   
   A   
   A   
   A   
    A    
   G   
   A   
   C   
   A   
   G   
   C   
   A   
   T   
   A   
   T   
   C   
   G   
   T   
    A    
   G   
   A   
   A   
   G   
   G   
    A    
    T    
   T   
   G   
   A   
   T   
   G   
   A   
   A   
   A   
   G   
   C   
   T   
   T   
   T   
   A   
   T   
   C   
   G   
   A   
   T   
   T   
   C   
   T   
   G   
   G   
   A   
   T   
   G   
   A   
   A   
   G   
   T   
   A   
   A   
   T   
   C   
   G   
    T    
   A   
   A   
   C   
   G   
   A   
   T   
   T   
   C   
   G   
   T   
   G   
   A   
   A   
   A   
   G   
   T   
   A   
   A   
   A   
   G   
   A   
   C   
   A   
   A   
   A   
   A   
   A   
   A   
   G   
   A   
    C    
   G   
   C   
    C    
   A   
   A   
   A   
   A   
   A   
   G   
   A   
   A   
   T   
   T   
   T   
   A   
   G   
   T   
   C   
   G   
   A   
    C    
   G   
   T   
    C    
   T   
   T   
    T    
   C   
   A   
   A   
   T   
   T   
   T   
   A   
   C   
   A   
   G   
   A   
   A   
   G   
   A   
   A   
   C   
   A   
   A   
   G   
   C   
   A   
   G   
   A   
   A   
   G   
   C   
   A   
   A   
   T   
    T    
   G   
   T   
   C   
   A   
   C   
   A   
   T   
   T   
   G   
   C   
   A   
   A   
    T    
   T   
   A   
   T   
   A   
    C    
   C   
   G   
   T   
   T   
   T   
   G   
   A   
   C   
   G   
   A   
   A   
    C    
   A   
   C   
    G    
   G   
   A   
   T   
   A   
   T   
   C   
   A   
   C   
   A   
   G   
   A   
   G   
   C   
   T   
   T   
   C   
   A   
   A   
   A   
   A   
   G   
   G   
   A   
   A   
    T    
   C   
    C    
   G   
   A   
   A   
   A   
    G    
   C   
   T   
   T   
   G   
   A   
   T   
   T   
   G   
   C   
    G    
   C   
   A   
   A   
   A   
   T   
   C   
   A   
   C   
   A   
   G   
   A   
   G   
   T   
   T   
    G    
   A   
   A   
   T   
   A   
   A   
   A   
   A   
   T   
   C   
   C   
   T   
   T   
   T   
   C   
   A   
   A   
   A   
    T    
   G   
   A   
   C   
   A   
   A   
   A   
   G   
   A   
    G    
   C   
   T   
   T   
   T   
   T   
    C    
   T   
   C   
   T   
   G   
   T   
   T   
   A   
   T   
   G   
   A   
   A   
   A   
   A   
   A   
   A   
   G   
   A   
   A   
   T   
   T   
   G   
   C   
   G   
   A   
   G   
   A   
   A   
   G   
   T   
   G   
   A   
   A   
   A   
   A   
   A   
   A   
   A   
   A   
   T   
   T   
   A   
    T    
   T   
   C   
   T   
   T   
   C   
   C   
   G   
   C   
   T   
   C   
   G   
   T   
   C   
    T    
    A    
   A   
   C   
   G   
   A   
   C   
   A   
   A   
   T   
   C   
    G    
   A   
   A   
   G   
   A   
   C   
   G   
   A   
   A   
   A   
   T   
   C   
   G   
   A   
   A   
   G   
   A   
   A   
   A   
   T   
   C   
   A   
   A   
   A   
   A   
   T   
    T    
   G   
   A   
   T   
   A   
   C   
   G   
   C   
   A   
   A   
    G    
   T   
    C    
   T   
   T   
   A   
   G   
   T   
   G   
   G   
   C   
    A    
   C   
   A   
   A   
   G   
   A   
   A   
   G   
   A   
   C   
   G   
   T   
   A   
   A   
   T   
    T    
   G   
   T   
   C   
   A   
   G   
   T   
   G   
   T   
    G    
   A   
   C   
    G    
   C   
   G   
   A   
   G   
   A   
   A   
   G   
   G   
   C   
   T   
   A   
   T   
   G   
   T   
   G   
   A   
   A   
   G   
   C   
   G   
   A   
   A   
   C   
   A   
   A   
   G   
   C   
   C   
   T   
   T   
   C   
   G   
   T   
   T   
   C   
    T    
   T   
   A   
   T   
   A   
   G   
   T   
   G   
   C   
   T   
   T   
   C   
    C    
   A   
   A   
   A   
   C   
   C   
   A   
   G   
   A   
   A   
   G   
   A   
   A   
   A   
   T   
   C   
   G   
   G   
   G   
   A   
   T   
   G   
   C   
   G   
   A   
   G   
   A   
   A   
   G   
   G   
   C   
   G   
   A   
    C    
   T   
   A   
   C   
   T   
   T   
   G   
   C   
   T   
    A    
   T   
   A   
   C   
    T    
   C   
   A   
   G   
   G   
   T   
   G   
   A   
   A   
   T   
   T   
    G    
   A   
   G   
    T    
   A   
   C   
   A   
    T    
   T   
   G   
   G   
   A   
   C   
   C   
   A   
   T   
   G   
   T   
   T   
   C   
   T   
   T   
   C   
   T   
   G   
   A   
   T   
    C    
   A   
   C   
    A    
   A   
   A   
   T   
   A   
   A   
   A   
   G   
   C   
   A   
   A   
   A   
   T   
   G   
   T   
   G   
   A   
   T   
   C   
   T   
   A   
   C   
   C   
   G   
    C    
   C   
   C   
   T   
   G   
   T   
    C    
   C   
   A   
   T   
   G   
   A   
   A   
   C   
   T   
   A   
   C   
   C   
    C    
   G   
   A   
   T   
   T   
   T   
   G   
   A   
   A   
   A   
   T   
   G   
   G   
   A   
   A   
   A   
   G   
   A   
   T   
   G   
   C   
   T   
   G   
   G   
    T    
   G   
   A   
    G    
   C   
   A   
    C    
   A   
   T   
   T   
   T   
   C   
   T   
   C   
   A   
   A   
   A   
   C   
   A   
   A   
   T   
   C   
   T   
   C   
   C   
   A   
   A   
   T   
   C   
   T   
   G   
   T   
   C   
   T   
   G   
   T   
   A   
   G   
   A   
    C    
   G   
   A   
   A   
   T   
   C   
   G   
   A   
   T   
    T    
   T   
   T   
   A   
   G   
   C   
   T   
   G   
   T   
   T   
   T   
   T   
    C    
   C   
   C   
   A   
   T   
   A   
   T   
   C   
   A   
   A   
   A   
   A   
   A   
   A   
   T   
    T    
   C   
   A   
   A   
   G   
   C   
   A   
   G   
   A   
    G    
   A   
   A   
   A   
   A   
   C   
   A   
   T   
   T   
   T   
   G   
   T   
    A    
   T   
   T   
   C   
   A   
   T   
   C   
   A   
   G   
   C   
   A   
   A   
   G   
   A   
   A   
   T   
   G   
   G   
   A   
   T   
   T   
    G    
    A    
   T   
   C   
   A   
   A   
   G   
   C   
   A   
   A   
   A   
   C   
   A   
   C   
   G   
   A   
   A   
   T   
   G   
   A   
   C   
   A   
   G   
   A   
   G   
   T   
   T   
   C   
   G   
   A   
   A   
   C   
   C   
   A   
   T   
   G   
   G   
   C   
   G   
   C   
   A   
   C   
   C   
   T   
   A   
    T    
   A   
   A   
   G   
   A   
   G   
   C   
   C   
   G   
    T    
   C   
   C   
   G   
   C   
   T   
   C   
   A   
   G   
   C   
   T   
   G   
   T   
   A   
   T   
   G   
   A   
   A   
   G   
   C   
   T   
   A   
   A   
   A   
   G   
   G   
   C   
   G   
   G   
   A   
   A   
   G   
   A   
   A   
   G   
   A   
   T   
   G   
   A   
   A   
   C   
   T   
    G    
   A   
   T   
   T   
   G   
   C   
   T   
   G   
   T   
    G    
   T   
   A   
   T   
   C   
   T   
   G   
   A   
   A   
   A   
   G   
   A   
   A   
   G   
   A   
   T   
   C   
    A    
   G   
   C   
   C   
   A   
   G   
   G   
   C   
   T   
   T   
   A   
   G   
   A   
   T   
   G   
   T   
    T    
   T   
   T   
   C   
   T   
   T   
   A   
   G   
   T   
   A   
   A   
   C   
   G   
    C    
   A   
   T   
   C   
   A   
   A   
   G   
   G   
   A   
   A   
   T   
   G   
   G   
   G   
    T    
   T   
   T   
   G   
   C   
   G   
   C   
   T   
    A    
   T   
   C   
   C   
   T   
   T   
   T   
   A   
   A   
    C    
   A   
   G   
   A   
   T   
   G   
   T   
   T   
   C   
   C   
   C   
   G   
   T   
   T   
   G   
   T   
   T   
   G   
   G   
   A   
   G   
   C   
   A   
   A   
   A   
   G   
   G   
   C   
   A   
   G   
    C    
   T   
   G   
   G   
   A   
   G   
   T   
   G   
   A   
   A   
   G   
   T   
   C   
   T   
   A   
   T   
   G   
   A   
   A   
   T   
   T   
   T   
   G   
   A   
   A   
   A   
   G   
   A   
   T   
   G   
   G   
   A   
   G   
   A   
   T   
   A   
   G   
   G   
    A    
   T   
   T   
    A    
   T   
   C   
   A   
   A   
    C    
   G   
   G   
    G    
   C   
   T   
    G    
   C   
   T   
   T   
   G   
   T   
   G   
   T   
   T   
   A   
   G   
   C   
   A   
   G   
   A   
    A    
   G   
   G   
    T    
   G   
   A   
   T   
   A   
   C   
   G   
   C   
   C   
    G    
   A   
   T   
   C   
   G   
   T   
   A   
   A   
   T   
   C   
   G   
   T   
   T   
   A   
   C   
   T   
   C   
   A   
   G   
   C   
   G   
   A   
   G   
   G   
   A   
   G   
   C   
    T    
   G   
   T   
    T    
   A   
   A   
   A   
   C   
   G   
   C   
   A   
   T   
   G   
   C   
   T   
   T   
   G   
   C   
   T   
   C   
   A   
   A   
   G   
   A   
   A   
   A   
   T   
   C   
   A   
   G   
   C   
   C   
   A   
   A   
   A   
   C   
    T    
   T   
   C   
   A   
   C   
   G   
   T   
   G   
   C   
   G   
   A   
   A   
   G   
   C   
   G   
   G   
   G   
   G   
   A   
   G   
   T   
   G   
   A   
   C   
   G   
   G   
   T   
   C   
   A   
   T   
   G   
   C   
   G   
   T   
   G   
   A   
   G   
   C   
   T   
   G   
   A   
   A   
   A   
   A   
   A   
   A   
   A   
   A   
   T   
   C   
   C   
    G    
   C   
   A   
    C    
   C   
   G   
    T    
   A   
   T   
   T   
   A   
   T   
   C   
   T   
   A   
   T   
   A   
   T   
   G   
   T   
   C   
   G   
   G   
   A   
   A   
   G   
   G   
   A   
   A   
   G   
   A   
   T   
   C   
   A   
   A   
   A   
   A   
   G   
   A   
   A   
   A   
   T   
   A   
   A   
   C   
   C   
   T   
   T   
   G   
   A   
   T   
   G   
   A   
   A   
   T   
   C   
   A   
   A   
   A   
   A   
   A   
   G   
   G   
    G    
   C   
   A   
    G    
   C   
   A   
   G   
    T    
   T   
   A   
   A   
   C   
   G   
   A   
   T   
   T   
   G   
   A   
    C    
   C   
   C   
   A   
   A   
   C   
    T    
   G   
   A   
   T   
   T   
   T   
    C    
   C   
   C   
   T   
   A   
   T   
   A   
   G   
   G   
   T   
   G   
   A   
   T   
   C   
   G   
   A   
   A   
   C   
   A   
   T   
   C   
   G   
   A   
   A   
   T   
   G   
   G   
    A    
   T   
   C   
    A    
   T   
   T   
   T   
   G   
   C   
    G    
   A   
   T   
   G   
   G   
   A   
    T    
   G   
   A   
   G   
   A   
   A   
   A   
   A   
   A   
   A   
   G   
   G   
   T   
   G   
   G   
   A   
   G   
   A   
   A   
   G   
   T   
   C   
   A   
   T   
   C   
   G   
   A   
   A   
   G   
   T   
    T    
   A   
    T    
   C   
   G   
   A   
   T   
   G   
   C   
   T   
   C   
   C   
   T   
   G   
   A   
   G   
    A    
   T   
   T   
   T   
   T   
    C    
   A   
   T   
    C    
   G   
   A   
    G    
    T    
   C   
    G    
   -   
   -   
   -   
 

 
   N59943F   
   A   
   T   
   G   
   G   
   A   
   A   
   A   
   A   
   T   
   C   
   G   
   A   
   C   
   A   
   A   
   G   
   A   
   A   
   G   
   T   
   A   
   C   
   A   
   A   
   G   
   A   
   A   
   T   
   T   
   A   
   A   
   C   
   T   
   T   
   T   
   A   
   G   
   A   
   A   
   G   
   A   
   A   
   G   
   T   
   G   
   A   
   T   
   G   
   G   
   G   
   T   
   G   
   A   
   C   
   C   
   G   
   C   
   T   
   T   
   C   
   G   
   G   
   A   
   C   
   G   
   T   
   T   
   A   
   T   
   T   
   C   
   C   
   A   
   A   
   A   
   T   
   A   
   T   
   A   
   T   
   T   
   A   
   T   
    C    
   C   
   A   
    A    
   G   
   A   
   A   
   C   
   G   
   T   
   G   
   C   
   A   
   C   
   T   
   G   
   C   
   C   
   T   
   G   
   A   
   T   
   A   
   T   
    C    
   C   
   G   
   T   
   G   
   A   
   T   
   G   
   G   
   A   
   T   
   T   
   G   
   A   
   A   
    A    
   C   
   C   
   A   
   G   
   T   
    A    
   C   
   A   
   A   
   C   
   G   
   C   
   C   
   G   
   G   
   A   
   T   
   A   
   T   
   T   
   A   
   T   
   T   
    T    
   G   
   C   
    C    
   A   
   T   
   G   
   A   
   A   
   C   
   A   
   A   
   A   
   G   
   A   
   T   
   G   
   G   
   C   
   A   
   A   
   T   
   A   
   C   
   G   
   T   
   A   
   T   
   G   
   A   
   C   
   A   
   A   
   A   
   G   
   G   
   A   
   T   
   T   
    C    
   C   
   G   
   T   
   A   
   A   
   A   
   T   
   C   
   A   
   G   
   C   
   A   
   A   
   A   
   A   
   T   
   C   
   T   
   G   
   T   
   C   
   G   
   G   
   A   
   A   
   A   
   T   
   A   
   T   
   C   
   A   
   T   
   G   
   G   
   G   
    G    
   A   
   A   
   T   
   T   
   A   
   T   
   C   
   A   
   T   
   C   
   C   
   T   
   C   
   A   
   T   
   G   
   G   
   T   
   G   
   A   
   C   
   A   
   G   
   C   
   A   
   G   
   T   
   A   
   T   
   T   
   T   
   A   
   T   
   G   
   A   
   A   
   G   
   C   
   G   
   A   
   T   
   G   
   G   
   T   
   C   
   C   
   G   
   C   
   A   
   T   
   G   
   A   
   G   
   C   
   C   
   A   
   A   
   G   
   A   
    T    
   T   
   G   
   G   
   A   
   A   
   G   
   T   
   T   
   G   
   C   
   G   
   G   
   G   
   A   
   A   
   G   
   T   
   A   
   C   
   T   
   G   
   A   
   T   
   C   
   G   
   A   
    G    
   A   
   T   
   G   
   C   
   A   
   C   
   G   
   G   
    A    
   A   
   A   
   T   
   A   
   A   
    C    
   G   
   G   
   G   
   A   
   G   
   T   
   A   
   T   
   G   
   G   
   A   
   C   
   G   
   G   
   T   
   G   
   A   
   T   
   C   
   C   
   A   
   C   
   C   
   T   
   G   
   C   
   T   
   G   
   C   
   G   
   A   
   T   
   G   
   C   
   G   
   T   
   T   
   A   
   T   
   A   
   C   
   T   
   G   
   A   
   A   
   G   
   C   
   C   
   A   
   G   
    A    
   T   
   T   
   A   
   T   
   C   
   C   
   A   
   A   
   G   
   T   
   T   
   A   
   A   
   G   
   T   
   G   
   G   
   A   
   G   
   A   
   G   
   A   
   T   
   G   
    C    
   T   
   A   
    G    
   C   
   A   
   G   
   A   
   T   
   A   
   T   
   C   
   G   
   A   
    A    
   A   
   A   
   A   
   G   
   A   
   A   
   A   
   C   
   A   
   G   
   T   
    C    
   G   
   A   
   T   
   C   
   T   
   C   
   G   
   T   
   G   
   T   
   G   
   G   
   A   
   A   
   T   
   T   
   T   
   T   
   G   
   A   
   T   
   G   
   A   
   T   
   A   
   C   
   A   
   G   
   A   
   A   
   A   
   A   
   A   
   G   
   A   
   A   
   C   
   C   
   A   
   A   
   C   
   A   
   G   
   T   
   C   
   C   
   T   
   T   
   C   
   C   
   A   
   G   
   C   
   T   
   C   
   G   
   A   
   T   
   A   
   C   
   C   
   C   
   A   
   A   
   A   
   C   
   C   
   T   
   T   
   C   
   T   
    A    
   G   
   T   
   C   
   A   
   A   
   T   
   G   
   G   
   A   
   T   
   C   
   G   
   A   
   C   
   A   
   G   
   G   
   T   
   A   
   T   
   A   
   T   
   C   
   T   
   G   
   C   
   T   
   G   
   G   
   A   
   T   
   A   
   T   
   G   
   C   
    G    
   A   
   C   
   A   
   G   
   A   
   A   
   A   
   T   
   C   
   C   
   C   
   G   
   A   
   C   
    T    
   C   
   A   
   T   
   A   
   A   
   C   
   T   
   T   
   A   
   G   
   C   
   T   
   G   
   A   
   A   
   G   
   T   
   G   
   A   
   T   
    C    
   G   
   A   
    C    
   G   
   G   
   C   
   A   
   C   
   A   
   A   
   T   
   C   
   T   
   A   
   T   
   A   
   T   
   G   
   A   
   T   
   C   
   G   
   A   
   T   
   C   
   A   
   T   
   C   
   C   
   A   
   C   
   A   
   A   
   G   
   C   
   G   
   A   
   G   
   C   
   C   
   T   
   T   
   G   
   A   
   A   
   A   
   A   
   A   
   T   
   T   
   G   
   A   
   T   
   G   
   G   
   A   
   A   
   T   
   A   
   T   
   A   
   T   
   T   
   C   
   C   
   T   
   G   
   G   
   A   
   C   
   C   
   T   
   G   
   A   
   C   
   T   
   T   
    C    
   C   
   C   
   A   
   A   
   C   
   A   
   G   
   G   
   A   
   G   
   G   
    G    
   A   
   T   
   C   
   T   
   T   
   G   
   C   
   A   
   A   
   G   
   G   
    A    
   A   
   A   
   A   
   G   
   A   
   T   
   G   
   A   
   A   
   A   
   T   
   C   
   A   
   A   
   A   
   A   
   A   
   A   
   G   
   C   
   T   
   T   
   A   
   T   
   G   
   A   
   A   
   A   
   C   
   A   
   G   
   G   
    G    
   C   
   G   
   A   
   G   
   G   
   A   
   A   
   A   
   A   
   G   
   T   
   T   
   A   
    T    
   T   
   C   
   T   
   T   
   C   
   G   
   T   
   T   
   C   
   G   
   A   
   A   
   A   
   A   
   C   
   A   
   A   
   A   
   G   
   A   
   T   
   A   
   G   
   A   
    A    
   G   
   C   
   A   
   A   
   T   
   G   
   A   
   A   
   G   
   G   
   G   
    C    
   A   
   A   
    T    
   A   
   A   
   A   
   C   
   A   
   G   
   C   
   A   
   A   
   A   
   T   
   C   
   G   
   T   
   G   
   A   
   T   
   C   
   A   
   C   
    T    
   G   
   A   
   A   
   A   
   T   
   C   
   C   
   C   
   A   
   T   
   A   
   T   
   G   
   A   
   A   
   G   
   T   
   A   
   A   
   A   
   T   
   A   
   A   
   A   
   G   
   C   
   G   
   A   
   C   
    A    
   T   
   T   
   G   
   G   
   T   
   G   
   A   
   A   
   A   
   A   
   A   
   A   
   A   
   T   
   G   
   G   
   A   
   T   
   G   
   A   
   G   
   A   
   T   
   C   
   C   
   G   
   T   
   T   
   T   
   G   
   A   
   A   
   T   
   A   
   A   
   G   
   A   
   A   
   A   
   A   
   T   
   C   
   G   
   A   
    C    
   G   
   G   
   A   
   A   
   T   
   A   
   G   
   C   
   T   
   G   
   A   
   A   
   G   
   T   
    G    
   C   
   G   
   A   
   G   
   A   
   T   
   G   
   A   
   A   
   A   
   G   
   T   
   G   
   A   
   T   
    C    
   G   
    A    
   A   
   C   
    T    
   G   
   G   
   T   
   C   
   T   
   G   
   C   
   A   
   A   
   A   
   T   
   T   
   G   
   T   
   C   
   G   
   T   
    T    
   G   
   A   
   A   
   T   
   T   
   G   
   A   
   A   
   A   
   A   
   A   
   A   
   G   
   A   
    C    
   G   
   C   
   C   
   A   
   A   
    T    
   G   
   C   
   T   
   C   
   A   
   A   
   G   
   G   
    A    
   A   
   T   
   T   
   T   
   T   
   G   
   A   
   A   
   T   
   T   
   A   
    T    
   T   
   T   
   G   
   T   
   T   
   C   
   A   
   A   
   A   
   A   
   A   
   C   
   A   
   C   
   T   
   G   
   A   
   G   
    C    
   T   
   A   
   C   
   A   
   G   
   A   
   T   
   C   
   A   
   A   
   T   
   T   
   A   
   C   
   A   
   A   
   T   
   T   
   T   
    C    
   A   
   A   
   T   
   A   
   T   
   G   
   G   
   T   
   C   
   G   
   C   
   T   
   A   
   T   
   C   
   G   
   A   
    T    
   C   
   A   
   T   
   A   
   T   
   G   
   A   
   C   
   T   
   C   
   C   
   G   
   C   
   A   
    T    
   C   
   A   
   A   
   G   
   T   
   T   
   G   
   G   
   A   
   C   
   T   
   G   
   A   
   A   
   A   
   G   
   A   
   C   
   A   
   T   
   T   
   T   
   T   
   A   
   A   
   G   
   A   
   A   
   G   
   T   
   T   
   A   
   T   
   A   
   T   
   C   
   G   
   A   
   G   
   C   
   A   
   T   
   C   
   G   
   A   
   A   
   A   
   G   
   C   
   A   
   A   
   G   
   T   
   C   
   A   
   T   
   A   
   A   
   C   
   A   
   A   
   A   
   A   
   C   
   G   
    C    
   A   
   G   
    C    
   C   
   A   
   A   
   T   
   T   
   C   
   G   
   A   
   T   
   T   
   T   
   A   
   G   
   C   
   C   
   A   
   A   
   A   
   G   
   C   
   T   
   C   
   A   
   A   
   A   
   A   
   A   
    A    
   G   
   A   
   C   
   A   
   G   
   C   
   A   
   T   
   A   
   T   
   C   
   G   
   T   
    G    
   G   
   A   
   A   
   G   
   G   
    C    
    C    
   T   
   G   
   A   
   T   
   G   
   A   
   A   
   A   
   G   
   C   
   T   
   T   
   T   
   A   
   T   
   C   
   G   
   A   
   T   
   T   
   C   
   T   
   G   
   G   
   A   
   T   
   G   
   A   
   A   
   G   
   T   
   A   
   A   
   T   
   C   
   G   
    C    
   A   
   A   
   C   
   G   
   A   
   T   
   T   
   C   
   G   
   T   
   G   
   A   
   A   
   A   
   G   
   T   
   A   
   A   
   A   
   G   
   A   
   C   
   A   
   A   
   A   
   A   
   A   
   A   
   G   
   A   
    C    
   G   
   C   
    C    
   A   
   A   
   A   
   A   
   A   
   G   
   A   
   A   
   T   
   T   
   T   
   A   
   G   
   T   
   C   
   G   
   A   
    C    
   G   
   T   
    T    
   T   
   T   
    C    
   C   
   A   
   A   
   T   
   T   
   T   
   A   
   C   
   A   
   G   
   A   
   A   
   G   
   A   
   A   
   C   
   A   
   A   
   G   
   C   
   A   
   G   
   A   
   A   
   G   
   C   
   A   
   A   
   T   
    C    
   G   
   T   
   C   
   A   
   C   
   A   
   T   
   T   
   G   
   C   
   A   
   A   
    C    
   T   
   A   
   T   
   A   
    T    
   C   
   G   
   T   
   T   
   T   
   G   
   A   
   C   
   G   
   A   
   A   
    C    
   A   
   C   
    A    
   G   
   A   
   T   
   A   
   T   
   C   
   A   
   C   
   A   
   G   
   A   
   G   
   C   
   T   
   T   
   C   
   A   
   A   
   A   
   A   
   G   
   G   
   A   
   A   
    T    
   C   
    C    
   G   
   A   
   A   
   A   
    G    
   C   
   T   
   T   
   G   
   A   
   T   
   T   
   G   
   C   
    A    
   C   
   A   
   A   
   A   
   T   
   C   
   A   
   C   
   A   
   G   
   A   
   G   
   T   
   T   
    A    
   A   
   A   
   T   
   A   
   A   
   A   
   A   
   T   
   C   
   C   
   T   
   T   
   T   
   C   
   A   
   A   
   A   
    C    
   G   
   A   
   C   
   A   
   A   
   A   
   G   
   A   
    G    
   C   
   T   
   T   
   T   
   T   
    C    
   T   
   C   
   T   
   G   
   T   
   T   
   A   
   T   
   G   
   A   
   A   
   A   
   A   
   A   
   A   
   G   
   A   
   A   
   T   
   T   
   G   
   C   
   G   
   A   
   G   
   A   
   A   
   G   
   T   
   G   
   A   
   A   
   A   
   A   
   A   
   A   
   A   
   A   
   T   
   T   
   A   
    C    
   T   
   C   
   T   
   T   
   C   
   C   
   G   
   C   
   T   
   C   
   G   
   T   
   C   
    T    
    G    
   A   
   C   
   G   
   A   
   C   
   A   
   A   
   T   
   C   
    G    
   A   
   A   
   G   
   A   
   C   
   G   
   A   
   A   
   A   
   T   
   C   
   G   
   A   
   A   
   G   
   A   
   A   
   A   
   T   
   C   
   A   
   A   
   A   
   A   
   T   
    C    
   G   
   A   
   T   
   A   
   C   
   G   
   C   
   A   
   A   
    G    
   T   
    T    
   T   
   T   
   A   
   G   
   T   
   G   
   G   
   C   
    G    
   C   
   A   
   A   
   G   
   A   
   A   
   G   
   A   
   C   
   G   
   T   
   A   
   A   
   T   
    C    
   G   
   T   
   C   
   A   
   G   
   T   
   G   
   T   
    G    
   A   
   C   
    A    
   C   
   G   
   A   
   G   
   A   
   A   
   G   
   G   
   C   
   T   
   A   
   T   
   G   
   T   
   G   
   A   
   A   
   G   
   C   
   G   
   A   
   A   
   C   
   A   
   A   
   G   
   C   
   C   
   T   
   T   
   C   
   G   
   T   
   T   
   C   
    T    
   T   
   A   
   T   
   A   
   G   
   T   
   G   
   C   
   T   
   T   
   C   
    G    
   A   
   A   
   A   
   C   
   C   
   A   
   G   
   A   
   A   
   G   
   A   
   A   
   A   
   T   
   C   
   G   
   G   
   G   
   A   
   T   
   G   
   C   
   G   
   A   
   G   
   A   
   A   
   G   
   G   
   C   
   G   
   A   
    C    
   T   
   A   
   C   
   T   
   T   
   G   
   C   
   T   
    G    
   T   
   A   
   C   
    T    
   C   
   A   
   G   
   G   
   T   
   G   
   A   
   A   
   T   
   T   
    G    
   A   
   G   
    C    
   A   
   C   
   A   
    T    
   T   
   G   
   G   
   A   
   C   
   C   
   A   
   T   
   G   
   T   
   T   
   C   
   T   
   T   
   C   
   T   
   G   
   A   
   T   
    T    
   A   
   C   
    G    
   A   
   A   
   T   
   A   
   A   
   A   
   G   
   C   
   A   
   A   
   A   
   T   
   G   
   T   
   G   
   A   
   T   
   C   
   T   
   A   
   C   
   C   
   G   
    T    
   C   
   C   
   T   
   G   
   T   
    T    
   C   
   A   
   T   
   G   
   A   
   A   
   C   
   T   
   A   
   C   
   C   
    C    
   G   
   A   
   T   
   T   
   T   
   G   
   A   
   A   
   A   
   T   
   G   
   G   
   A   
   A   
   A   
   G   
   A   
   T   
   G   
   C   
   T   
   G   
   G   
    C    
   G   
   A   
    A    
   C   
   A   
    C    
   A   
   T   
   T   
   T   
   C   
   T   
   C   
   A   
   A   
   A   
   C   
   A   
   A   
   T   
   C   
   T   
   C   
   C   
   A   
   A   
   T   
   C   
   T   
   G   
   T   
   C   
   T   
   G   
   T   
   A   
   G   
   A   
    T    
   G   
   A   
   A   
   T   
   C   
   G   
   A   
   T   
    C    
   T   
   T   
   A   
   G   
   C   
   T   
   G   
   T   
   T   
   T   
   T   
    C    
   C   
   C   
   A   
   T   
   A   
   T   
   C   
   A   
   A   
   A   
   A   
   A   
   A   
   T   
    C    
   C   
   A   
   A   
   G   
   C   
   A   
   G   
   A   
    G    
   A   
   A   
   A   
   A   
   C   
   A   
   T   
   T   
   T   
   G   
   T   
    A    
   T   
   T   
   C   
   A   
   T   
   C   
   A   
   G   
   C   
   A   
   A   
   G   
   A   
   A   
   T   
   G   
   G   
   A   
   T   
   T   
    G    
    A    
   T   
   C   
   A   
   A   
   G   
   C   
   A   
   A   
   A   
   C   
   A   
   C   
   G   
   A   
   A   
   T   
   G   
   A   
   C   
   A   
   G   
   A   
   G   
   T   
   T   
   C   
   G   
   A   
   A   
   C   
   C   
   A   
   T   
   G   
   G   
   C   
   G   
   C   
   A   
   C   
   C   
   T   
   A   
    C    
   A   
   A   
   G   
   A   
   G   
   C   
   C   
   G   
    T    
   C   
   C   
   G   
   C   
   T   
   C   
   A   
   G   
   C   
   T   
   G   
   T   
   A   
   T   
   G   
   A   
   A   
   G   
   C   
   T   
   A   
   A   
   A   
   G   
   G   
   C   
   G   
   G   
   A   
   A   
   G   
   A   
   A   
   G   
   A   
   T   
   G   
   A   
   A   
   C   
   T   
    T    
   A   
   T   
   T   
   G   
   C   
   T   
   G   
   T   
    A    
   T   
   A   
   T   
   C   
   T   
   G   
   A   
   A   
   A   
   G   
   A   
   A   
   G   
   A   
   T   
   C   
    A    
   G   
   C   
   C   
   A   
   G   
   G   
   C   
   T   
   T   
   A   
   G   
   A   
   T   
   G   
   T   
    G    
   T   
   T   
   C   
   T   
   T   
   A   
   G   
   T   
   A   
   A   
   C   
   G   
    C    
   A   
   T   
   C   
   A   
   A   
   G   
   G   
   A   
   A   
   T   
   G   
   G   
   G   
    C    
   T   
   T   
   G   
   C   
   G   
   C   
   T   
    A    
   T   
   C   
   C   
   T   
   T   
   T   
   A   
   A   
    C    
   A   
   G   
   A   
   T   
   G   
   T   
   T   
   C   
   C   
   C   
   G   
   T   
   T   
   G   
   T   
   T   
   G   
   G   
   A   
   G   
   C   
   A   
   A   
   A   
   G   
   G   
   C   
   A   
   G   
    C    
   T   
   G   
   G   
   A   
   G   
   T   
   G   
   A   
   A   
   G   
   T   
   C   
   T   
   A   
   T   
   G   
   A   
   A   
   T   
   T   
   T   
   G   
   A   
   A   
   A   
   G   
   A   
   T   
   G   
   G   
   A   
   G   
   A   
   T   
   A   
   G   
   G   
    A    
   T   
   T   
    G    
   T   
   C   
   A   
   A   
    T    
   G   
   G   
    A    
   C   
   T   
    G    
   C   
   T   
   T   
   G   
   T   
   G   
   T   
   T   
   A   
   G   
   C   
   A   
   G   
   A   
    C    
   G   
   G   
    A    
   G   
   A   
   T   
   A   
   C   
   G   
   C   
   C   
    G    
   A   
   T   
   C   
   G   
   T   
   A   
   A   
   T   
   C   
   G   
   T   
   T   
   A   
   C   
   T   
   C   
   A   
   G   
   C   
   G   
   A   
   G   
   G   
   A   
   G   
   C   
    T    
   G   
   T   
    G    
   A   
   A   
   A   
   C   
   G   
   C   
   A   
   T   
   G   
   C   
   T   
   T   
   G   
   C   
   T   
   C   
   A   
   A   
   G   
   A   
   A   
   A   
   T   
   C   
   A   
   G   
   C   
   C   
   A   
   A   
   A   
   C   
    T    
   T   
   C   
   A   
   C   
   G   
   T   
   G   
   C   
   G   
   A   
   A   
   G   
   C   
   G   
   G   
   G   
   G   
   A   
   G   
   T   
   G   
   A   
   C   
   G   
   G   
   T   
   C   
   A   
   T   
   G   
   C   
   G   
   T   
   G   
   A   
   G   
   C   
   T   
   G   
   A   
   A   
   A   
   A   
   A   
   A   
   A   
   A   
   T   
   C   
   C   
    G    
   C   
   A   
    C    
   C   
   G   
    C    
   A   
   T   
   T   
   A   
   T   
   C   
   T   
   A   
   T   
   A   
   T   
   G   
   T   
   C   
   G   
   G   
   A   
   A   
   G   
   G   
   A   
   A   
   G   
   A   
   T   
   C   
   A   
   A   
   A   
   A   
   G   
   A   
   A   
   A   
   T   
   A   
   A   
   C   
   C   
   T   
   T   
   G   
   A   
   T   
   G   
   A   
   A   
   T   
   C   
   A   
   A   
   A   
   A   
   A   
   G   
   G   
    A    
   C   
   A   
    A    
   C   
   A   
   G   
    A    
   T   
   A   
   A   
   C   
   G   
   A   
   T   
   T   
   G   
   A   
    T    
   C   
   C   
   A   
   A   
   C   
    G    
   G   
   A   
   T   
   T   
   T   
    T    
   C   
   C   
   T   
   A   
   T   
   A   
   G   
   G   
   T   
   G   
   A   
   T   
   C   
   G   
   A   
   A   
   C   
   A   
   T   
   C   
   G   
   A   
   A   
   T   
   G   
   G   
    T    
   T   
   C   
    G    
   T   
   T   
   T   
   G   
   C   
    T    
   A   
   T   
   G   
   G   
   A   
    C    
   G   
   A   
   G   
   A   
   A   
   A   
   A   
   A   
   A   
   G   
   G   
   T   
   G   
   G   
   A   
   G   
   A   
   A   
   G   
   T   
   C   
   A   
   T   
   C   
   G   
   A   
   A   
   G   
   T   
    T    
   A   
    T    
   C   
   G   
   A   
   T   
   G   
   C   
   T   
   C   
   C   
   T   
   G   
   A   
   G   
    A    
   T   
   T   
   T   
   T   
    C    
   A   
   T   
    C    
   G   
   A   
    A    
    T    
   C   
    A    
   -   
   -   
   -   
 

 
   N55229   
   A   
   T   
   G   
   G   
   A   
   A   
   A   
   A   
   T   
   C   
   G   
   A   
   C   
   A   
   A   
   G   
   A   
   A   
   G   
   T   
   A   
   C   
   A   
   A   
   G   
   A   
   A   
   T   
   T   
   A   
   A   
   C   
   T   
   T   
   T   
   A   
   G   
   A   
   A   
   G   
   A   
   A   
   G   
   T   
   G   
   A   
   T   
   G   
   G   
   G   
   T   
   G   
   A   
   C   
   C   
   G   
   C   
   T   
   T   
   C   
   G   
   G   
   A   
   C   
   G   
   T   
   T   
   A   
   T   
   T   
   C   
   C   
   A   
   A   
   A   
   T   
   A   
   T   
   A   
   T   
   T   
   A   
   T   
    C    
   C   
   A   
    A    
   G   
   A   
   A   
   C   
   G   
   T   
   G   
   C   
   A   
   C   
   T   
   G   
   C   
   C   
   T   
   G   
   A   
   T   
   A   
   T   
    C    
   C   
   G   
   T   
   G   
   A   
   T   
   G   
   G   
   A   
   T   
   T   
   G   
   A   
   A   
    G    
   C   
   C   
   A   
   G   
   T   
    G    
   C   
   A   
   A   
   C   
   G   
   C   
   C   
   G   
   G   
   A   
   T   
   A   
   T   
   T   
   A   
   T   
   T   
    T    
   G   
   C   
    C    
   A   
   T   
   G   
   A   
   A   
   C   
   A   
   A   
   A   
   G   
   A   
   T   
   G   
   G   
   C   
   A   
   A   
   T   
   A   
   C   
   G   
   T   
   A   
   T   
   G   
   A   
   C   
   A   
   A   
   A   
   G   
   G   
   A   
   T   
   T   
    C    
   C   
   G   
   T   
   A   
   A   
   A   
   T   
   C   
   A   
   G   
   C   
   A   
   A   
   A   
   A   
   T   
   C   
   T   
   G   
   T   
   C   
   G   
   G   
   A   
   A   
   A   
   T   
   A   
   T   
   C   
   A   
   T   
   G   
   G   
   G   
    G    
   A   
   A   
   T   
   T   
   A   
   T   
   C   
   A   
   T   
   C   
   C   
   T   
   C   
   A   
   T   
   G   
   G   
   T   
   G   
   A   
   C   
   A   
   G   
   C   
   A   
   G   
   T   
   A   
   T   
   T   
   T   
   A   
   T   
   G   
   A   
   A   
   G   
   C   
   G   
   A   
   T   
   G   
   G   
   T   
   C   
   C   
   G   
   C   
   A   
   T   
   G   
   A   
   G   
   C   
   C   
   A   
   A   
   G   
   A   
    C    
   T   
   G   
   G   
   A   
   A   
   G   
   T   
   T   
   G   
   C   
   G   
   G   
   G   
   A   
   A   
   G   
   T   
   A   
   C   
   T   
   G   
   A   
   T   
   C   
   G   
   A   
    A    
   A   
   T   
   G   
   C   
   A   
   C   
   G   
   G   
    G    
   A   
   A   
   T   
   A   
   A   
    C    
   G   
   G   
   G   
   A   
   G   
   T   
   A   
   T   
   G   
   G   
   A   
   C   
   G   
   G   
   T   
   G   
   A   
   T   
   C   
   C   
   A   
   C   
   C   
   T   
   G   
   C   
   T   
   G   
   C   
   G   
   A   
   T   
   G   
   C   
   G   
   T   
   T   
   A   
   T   
   A   
   C   
   T   
   G   
   A   
   A   
   G   
   C   
   C   
   A   
   G   
    A    
   T   
   T   
   A   
   T   
   C   
   C   
   A   
   A   
   G   
   T   
   T   
   A   
   A   
   G   
   T   
   G   
   G   
   A   
   G   
   A   
   G   
   A   
   T   
   G   
    C    
   T   
   A   
    T    
   C   
   A   
   G   
   A   
   T   
   A   
   T   
   C   
   G   
   A   
    A    
   A   
   A   
   A   
   G   
   A   
   A   
   A   
   C   
   A   
   G   
   T   
    C    
   G   
   A   
   T   
   C   
   T   
   C   
   G   
   T   
   G   
   T   
   G   
   G   
   A   
   A   
   T   
   T   
   T   
   T   
   G   
   A   
   T   
   G   
   A   
   T   
   A   
   C   
   A   
   G   
   A   
   A   
   A   
   A   
   A   
   G   
   A   
   A   
   C   
   C   
   A   
   A   
   C   
   A   
   G   
   T   
   C   
   C   
   T   
   T   
   C   
   C   
   A   
   G   
   C   
   T   
   C   
   G   
   A   
   T   
   A   
   C   
   C   
   C   
   A   
   A   
   A   
   C   
   C   
   T   
   T   
   C   
   T   
    C    
   G   
   T   
   C   
   A   
   A   
   T   
   G   
   G   
   A   
   T   
   C   
   G   
   A   
   C   
   A   
   G   
   G   
   T   
   A   
   T   
   A   
   T   
   C   
   T   
   G   
   C   
   T   
   G   
   G   
   A   
   T   
   A   
   T   
   G   
   C   
    A    
   A   
   C   
   A   
   G   
   A   
   A   
   A   
   T   
   C   
   C   
   C   
   G   
   A   
   C   
    T    
   C   
   A   
   T   
   A   
   A   
   C   
   T   
   T   
   A   
   G   
   C   
   T   
   G   
   A   
   A   
   G   
   T   
   G   
   A   
   T   
    C    
   G   
   A   
    T    
   G   
   G   
   C   
   A   
   C   
   A   
   A   
   T   
   C   
   T   
   A   
   T   
   A   
   T   
   G   
   A   
   T   
   C   
   G   
   A   
   T   
   C   
   A   
   T   
   C   
   C   
   A   
   C   
   A   
   A   
   G   
   C   
   G   
   A   
   G   
   C   
   C   
   T   
   T   
   G   
   A   
   A   
   A   
   A   
   A   
   T   
   T   
   G   
   A   
   T   
   G   
   G   
   A   
   A   
   T   
   A   
   T   
   A   
   T   
   T   
   C   
   C   
   T   
   G   
   G   
   A   
   C   
   C   
   T   
   G   
   A   
   C   
   T   
   T   
    T    
   C   
   C   
   A   
   A   
   C   
   A   
   G   
   G   
   A   
   G   
   G   
    G    
   A   
   T   
   C   
   T   
   T   
   G   
   C   
   A   
   A   
   G   
   G   
    A    
   A   
   A   
   A   
   G   
   A   
   T   
   G   
   A   
   A   
   A   
   T   
   C   
   A   
   A   
   A   
   A   
   A   
   A   
   G   
   C   
   T   
   T   
   A   
   T   
   G   
   A   
   A   
   A   
   C   
   A   
   G   
   G   
    G    
   C   
   G   
   A   
   G   
   G   
   A   
   A   
   A   
   A   
   G   
   T   
   T   
   A   
    T    
   T   
   C   
   T   
   T   
   C   
   G   
   T   
   T   
   C   
   G   
   A   
   A   
   A   
   A   
   C   
   A   
   A   
   A   
   G   
   A   
   T   
   A   
   G   
   A   
    G    
   G   
   C   
   A   
   A   
   T   
   G   
   A   
   A   
   G   
   G   
   G   
    T    
   A   
   A   
    T    
   A   
   A   
   A   
   C   
   A   
   G   
   C   
   A   
   A   
   A   
   T   
   C   
   G   
   T   
   G   
   A   
   T   
   C   
   A   
   C   
    A    
   G   
   A   
   A   
   A   
   T   
   C   
   C   
   C   
   A   
   T   
   A   
   T   
   G   
   A   
   A   
   G   
   T   
   A   
   A   
   A   
   T   
   A   
   A   
   A   
   G   
   C   
   G   
   A   
   C   
    C    
   T   
   T   
   G   
   G   
   T   
   G   
   A   
   A   
   A   
   A   
   A   
   A   
   A   
   T   
   G   
   G   
   A   
   T   
   G   
   A   
   G   
   A   
   T   
   C   
   C   
   G   
   T   
   T   
   T   
   G   
   A   
   A   
   T   
   A   
   A   
   G   
   A   
   A   
   A   
   A   
   T   
   C   
   G   
   A   
    T    
   G   
   G   
   A   
   A   
   T   
   A   
   G   
   C   
   T   
   G   
   A   
   A   
   G   
   T   
    A    
   C   
   G   
   A   
   G   
   A   
   T   
   G   
   A   
   A   
   A   
   G   
   T   
   G   
   A   
   T   
    A    
   G   
    A    
   A   
   C   
    A    
   G   
   G   
   T   
   C   
   T   
   G   
   C   
   A   
   A   
   A   
   T   
   T   
   G   
   T   
   C   
   G   
   T   
    C    
   G   
   A   
   A   
   T   
   T   
   G   
   A   
   A   
   A   
   A   
   A   
   A   
   G   
   A   
    C    
   G   
   C   
   C   
   A   
   A   
    T    
   G   
   C   
   T   
   C   
   A   
   A   
   G   
   G   
    A    
   A   
   T   
   T   
   T   
   T   
   G   
   A   
   A   
   T   
   T   
   A   
    T    
   T   
   T   
   G   
   T   
   T   
   C   
   A   
   A   
   A   
   A   
   A   
   C   
   A   
   C   
   T   
   G   
   A   
   G   
    C    
   T   
   A   
   C   
   A   
   G   
   A   
   T   
   C   
   A   
   A   
   T   
   T   
   A   
   C   
   A   
   A   
   T   
   T   
   T   
    C    
   A   
   A   
   T   
   A   
   T   
   G   
   G   
   T   
   C   
   G   
   C   
   T   
   A   
   T   
   C   
   G   
   A   
    C    
   C   
   A   
   T   
   A   
   T   
   G   
   A   
   C   
   T   
   C   
   C   
   G   
   C   
   A   
    T    
   C   
   A   
   A   
   G   
   T   
   T   
   G   
   G   
   A   
   C   
   T   
   G   
   A   
   A   
   A   
   G   
   A   
   C   
   A   
   T   
   T   
   T   
   T   
   A   
   A   
   G   
   A   
   A   
   G   
   T   
   T   
   A   
   T   
   A   
   T   
   C   
   G   
   A   
   G   
   C   
   A   
   T   
   C   
   G   
   A   
   A   
   A   
   G   
   C   
   A   
   A   
   G   
   T   
   C   
   A   
   T   
   A   
   A   
   C   
   A   
   A   
   A   
   A   
   C   
   G   
    T    
   A   
   G   
    T    
   C   
   A   
   A   
   T   
   T   
   C   
   G   
   A   
   T   
   T   
   T   
   A   
   G   
   C   
   C   
   A   
   A   
   A   
   G   
   C   
   T   
   C   
   A   
   A   
   A   
   A   
   A   
    A    
   G   
   A   
   C   
   A   
   G   
   C   
   A   
   T   
   A   
   T   
   C   
   G   
   T   
    A    
   G   
   A   
   A   
   G   
   G   
    A    
    T    
   T   
   G   
   A   
   T   
   G   
   A   
   A   
   A   
   G   
   C   
   T   
   T   
   T   
   A   
   T   
   C   
   G   
   A   
   T   
   T   
   C   
   T   
   G   
   G   
   A   
   T   
   G   
   A   
   A   
   G   
   T   
   A   
   A   
   T   
   C   
   G   
    C    
   A   
   A   
   C   
   G   
   A   
   T   
   T   
   C   
   G   
   T   
   G   
   A   
   A   
   A   
   G   
   T   
   A   
   A   
   A   
   G   
   A   
   C   
   A   
   A   
   A   
   A   
   A   
   A   
   G   
   A   
    C    
   G   
   C   
    C    
   A   
   A   
   A   
   A   
   A   
   G   
   A   
   A   
   T   
   T   
   T   
   A   
   G   
   T   
   C   
   G   
   A   
    C    
   G   
   T   
    C    
   T   
   T   
    T    
   C   
   A   
   A   
   T   
   T   
   T   
   A   
   C   
   A   
   G   
   A   
   A   
   G   
   A   
   A   
   C   
   A   
   A   
   G   
   C   
   A   
   G   
   A   
   A   
   G   
   C   
   A   
   A   
   T   
    T    
   G   
   T   
   C   
   A   
   C   
   A   
   T   
   T   
   G   
   C   
   A   
   A   
    T    
   T   
   A   
   T   
   A   
    C    
   C   
   G   
   T   
   T   
   T   
   G   
   A   
   C   
   G   
   A   
   A   
    C    
   A   
   C   
    G    
   G   
   A   
   T   
   A   
   T   
   C   
   A   
   C   
   A   
   G   
   A   
   G   
   C   
   T   
   T   
   C   
   A   
   A   
   A   
   A   
   G   
   G   
   A   
   A   
    T    
   C   
    C    
   G   
   A   
   A   
   A   
    G    
   C   
   T   
   T   
   G   
   A   
   T   
   T   
   G   
   C   
    G    
   C   
   A   
   A   
   A   
   T   
   C   
   A   
   C   
   A   
   G   
   A   
   G   
   T   
   T   
    G    
   A   
   A   
   T   
   A   
   A   
   A   
   A   
   T   
   C   
   C   
   T   
   T   
   T   
   C   
   A   
   A   
   A   
    T    
   G   
   A   
   C   
   A   
   A   
   A   
   G   
   A   
    G    
   C   
   T   
   T   
   T   
   T   
    C    
   T   
   C   
   T   
   G   
   T   
   T   
   A   
   T   
   G   
   A   
   A   
   A   
   A   
   A   
   A   
   G   
   A   
   A   
   T   
   T   
   G   
   C   
   G   
   A   
   G   
   A   
   A   
   G   
   T   
   G   
   A   
   A   
   A   
   A   
   A   
   A   
   A   
   A   
   T   
   T   
   A   
    T    
   T   
   C   
   T   
   T   
   C   
   C   
   G   
   C   
   T   
   C   
   G   
   T   
   C   
    T    
    A    
   A   
   C   
   G   
   A   
   C   
   A   
   A   
   T   
   C   
    G    
   A   
   A   
   G   
   A   
   C   
   G   
   A   
   A   
   A   
   T   
   C   
   G   
   A   
   A   
   G   
   A   
   A   
   A   
   T   
   C   
   A   
   A   
   A   
   A   
   T   
    T    
   G   
   A   
   T   
   A   
   C   
   G   
   C   
   A   
   A   
    G    
   T   
    C    
   T   
   T   
   A   
   G   
   T   
   G   
   G   
   C   
    A    
   C   
   A   
   A   
   G   
   A   
   A   
   G   
   A   
   C   
   G   
   T   
   A   
   A   
   T   
    T    
   G   
   T   
   C   
   A   
   G   
   T   
   G   
   T   
    G    
   A   
   C   
    G    
   C   
   G   
   A   
   G   
   A   
   A   
   G   
   G   
   C   
   T   
   A   
   T   
   G   
   T   
   G   
   A   
   A   
   G   
   C   
   G   
   A   
   A   
   C   
   A   
   A   
   G   
   C   
   C   
   T   
   T   
   C   
   G   
   T   
   T   
   C   
    T    
   T   
   A   
   T   
   A   
   G   
   T   
   G   
   C   
   T   
   T   
   C   
    C    
   A   
   A   
   A   
   C   
   C   
   A   
   G   
   A   
   A   
   G   
   A   
   A   
   A   
   T   
   C   
   G   
   G   
   G   
   A   
   T   
   G   
   C   
   G   
   A   
   G   
   A   
   A   
   G   
   G   
   C   
   G   
   A   
    C    
   T   
   A   
   C   
   T   
   T   
   G   
   C   
   T   
    A    
   T   
   A   
   C   
    T    
   C   
   A   
   G   
   G   
   T   
   G   
   A   
   A   
   T   
   T   
    G    
   A   
   G   
    T    
   A   
   C   
   A   
    T    
   T   
   G   
   G   
   A   
   C   
   C   
   A   
   T   
   G   
   T   
   T   
   C   
   T   
   T   
   C   
   T   
   G   
   A   
   T   
    C    
   A   
   C   
    A    
   A   
   A   
   T   
   A   
   A   
   A   
   G   
   C   
   A   
   A   
   A   
   T   
   G   
   T   
   G   
   A   
   T   
   C   
   T   
   A   
   C   
   C   
   G   
    C    
   C   
   C   
   T   
   G   
   T   
    C    
   C   
   A   
   T   
   G   
   A   
   A   
   C   
   T   
   A   
   C   
   C   
    C    
   G   
   A   
   T   
   T   
   T   
   G   
   A   
   A   
   A   
   T   
   G   
   G   
   A   
   A   
   A   
   G   
   A   
   T   
   G   
   C   
   T   
   G   
   G   
    T    
   G   
   A   
    G    
   C   
   A   
    C    
   A   
   T   
   T   
   T   
   C   
   T   
   C   
   A   
   A   
   A   
   C   
   A   
   A   
   T   
   C   
   T   
   C   
   C   
   A   
   A   
   T   
   C   
   T   
   G   
   T   
   C   
   T   
   G   
   T   
   A   
   G   
   A   
    C    
   G   
   A   
   A   
   T   
   C   
   G   
   A   
   T   
    T    
   T   
   T   
   A   
   G   
   C   
   T   
   G   
   T   
   T   
   T   
   T   
    C    
   C   
   C   
   A   
   T   
   A   
   T   
   C   
   A   
   A   
   A   
   A   
   A   
   A   
   T   
    T    
   C   
   A   
   A   
   G   
   C   
   A   
   G   
   A   
    G    
   A   
   A   
   A   
   A   
   C   
   A   
   T   
   T   
   T   
   G   
   T   
    A    
   T   
   T   
   C   
   A   
   T   
   C   
   A   
   G   
   C   
   A   
   A   
   G   
   A   
   A   
   T   
   G   
   G   
   A   
   T   
   T   
    G    
    A    
   T   
   C   
   A   
   A   
   G   
   C   
   A   
   A   
   A   
   C   
   A   
   C   
   G   
   A   
   A   
   T   
   G   
   A   
   C   
   A   
   G   
   A   
   G   
   T   
   T   
   C   
   G   
   A   
   A   
   C   
   C   
   A   
   T   
   G   
   G   
   C   
   G   
   C   
   A   
   C   
   C   
   T   
   A   
    T    
   A   
   A   
   G   
   A   
   G   
   C   
   C   
   G   
    T    
   C   
   C   
   G   
   C   
   T   
   C   
   A   
   G   
   C   
   T   
   G   
   T   
   A   
   T   
   G   
   A   
   A   
   G   
   C   
   T   
   A   
   A   
   A   
   G   
   G   
   C   
   G   
   G   
   A   
   A   
   G   
   A   
   A   
   G   
   A   
   T   
   G   
   A   
   A   
   C   
   T   
    G    
   A   
   T   
   T   
   G   
   C   
   T   
   G   
   T   
    G    
   T   
   A   
   T   
   C   
   T   
   G   
   A   
   A   
   A   
   G   
   A   
   A   
   G   
   A   
   T   
   C   
    A    
   G   
   C   
   C   
   A   
   G   
   G   
   C   
   T   
   T   
   A   
   G   
   A   
   T   
   G   
   T   
    T    
   T   
   T   
   C   
   T   
   T   
   A   
   G   
   T   
   A   
   A   
   C   
   G   
    C    
   A   
   T   
   C   
   A   
   A   
   G   
   G   
   A   
   A   
   T   
   G   
   G   
   G   
    T    
   T   
   T   
   G   
   C   
   G   
   C   
   T   
    A    
   T   
   C   
   C   
   T   
   T   
   T   
   A   
   A   
    C    
   A   
   G   
   A   
   T   
   G   
   T   
   T   
   C   
   C   
   C   
   G   
   T   
   T   
   G   
   T   
   T   
   G   
   G   
   A   
   G   
   C   
   A   
   A   
   A   
   G   
   G   
   C   
   A   
   G   
    C    
   T   
   G   
   G   
   A   
   G   
   T   
   G   
   A   
   A   
   G   
   T   
   C   
   T   
   A   
   T   
   G   
   A   
   A   
   T   
   T   
   T   
   G   
   A   
   A   
   A   
   G   
   A   
   T   
   G   
   G   
   A   
   G   
   A   
   T   
   A   
   G   
   G   
    A    
   T   
   T   
    A    
   T   
   C   
   A   
   A   
    C    
   G   
   G   
    G    
   C   
   T   
    G    
   C   
   T   
   T   
   G   
   T   
   G   
   T   
   T   
   A   
   G   
   C   
   A   
   G   
   A   
    A    
   G   
   G   
    T    
   G   
   A   
   T   
   A   
   C   
   G   
   C   
   C   
    G    
   A   
   T   
   C   
   G   
   T   
   A   
   A   
   T   
   C   
   G   
   T   
   T   
   A   
   C   
   T   
   C   
   A   
   G   
   C   
   G   
   A   
   G   
   G   
   A   
   G   
   C   
    T    
   G   
   T   
    T    
   A   
   A   
   A   
   C   
   G   
   C   
   A   
   T   
   G   
   C   
   T   
   T   
   G   
   C   
   T   
   C   
   A   
   A   
   G   
   A   
   A   
   A   
   T   
   C   
   A   
   G   
   C   
   C   
   A   
   A   
   A   
   C   
    T    
   T   
   C   
   A   
   C   
   G   
   T   
   G   
   C   
   G   
   A   
   A   
   G   
   C   
   G   
   G   
   G   
   G   
   A   
   G   
   T   
   G   
   A   
   C   
   G   
   G   
   T   
   C   
   A   
   T   
   G   
   C   
   G   
   T   
   G   
   A   
   G   
   C   
   T   
   G   
   A   
   A   
   A   
   A   
   A   
   A   
   A   
   A   
   T   
   C   
   C   
    G    
   C   
   A   
    C    
   C   
   G   
    T    
   A   
   T   
   T   
   A   
   T   
   C   
   T   
   A   
   T   
   A   
   T   
   G   
   T   
   C   
   G   
   G   
   A   
   A   
   G   
   G   
   A   
   A   
   G   
   A   
   T   
   C   
   A   
   A   
   A   
   A   
   G   
   A   
   A   
   A   
   T   
   A   
   A   
   C   
   C   
   T   
   T   
   G   
   A   
   T   
   G   
   A   
   A   
   T   
   C   
   A   
   A   
   A   
   A   
   A   
   G   
   G   
    G    
   C   
   A   
    G    
   C   
   A   
   G   
    T    
   T   
   A   
   A   
   C   
   G   
   A   
   T   
   T   
   G   
   A   
    C    
   C   
   C   
   A   
   A   
   C   
    T    
   G   
   A   
   T   
   T   
   T   
    C    
   C   
   C   
   T   
   A   
   T   
   A   
   G   
   G   
   T   
   G   
   A   
   T   
   C   
   G   
   A   
   A   
   C   
   A   
   T   
   C   
   G   
   A   
   A   
   T   
   G   
   G   
    A    
   T   
   C   
    A    
   T   
   T   
   T   
   G   
   C   
    G    
   A   
   T   
   G   
   G   
   A   
    T    
   G   
   A   
   G   
   A   
   A   
   A   
   A   
   A   
   A   
   G   
   G   
   T   
   G   
   G   
   A   
   G   
   A   
   A   
   G   
   T   
   C   
   A   
   T   
   C   
   G   
   A   
   A   
   G   
   T   
    T    
   A   
    T    
   C   
   G   
   A   
   T   
   G   
   C   
   T   
   C   
   C   
   T   
   G   
   A   
   G   
    A    
   T   
   T   
   T   
   T   
    C    
   A   
   T   
    C    
   G   
   A   
    G    
    T    
   C   
    G    
   -   
   -   
   -   
 

 
   N59891F   
   A   
   T   
   G   
   G   
   A   
   A   
   A   
   A   
   T   
   C   
   G   
   A   
   C   
   A   
   A   
   G   
   A   
   A   
   G   
   T   
   A   
   C   
   A   
   A   
   G   
   A   
   A   
   T   
   T   
   A   
   A   
   C   
   T   
   T   
   T   
   A   
   G   
   A   
   A   
   G   
   A   
   A   
   G   
   T   
   G   
   A   
   T   
   G   
   G   
   G   
   T   
   G   
   A   
   C   
   C   
   G   
   C   
   T   
   T   
   C   
   G   
   G   
   A   
   C   
   G   
   T   
   T   
   A   
   T   
   T   
   C   
   C   
   A   
   A   
   A   
   T   
   A   
   T   
   A   
   T   
   T   
   A   
   T   
    C    
   C   
   A   
    A    
   G   
   A   
   A   
   C   
   G   
   T   
   G   
   C   
   A   
   C   
   T   
   G   
   C   
   C   
   T   
   G   
   A   
   T   
   A   
   T   
    C    
   C   
   G   
   T   
   G   
   A   
   T   
   G   
   G   
   A   
   T   
   T   
   G   
   A   
   A   
    G    
   C   
   C   
   A   
   G   
   T   
    G    
   C   
   A   
   A   
   C   
   G   
   C   
   C   
   G   
   G   
   A   
   T   
   A   
   T   
   T   
   A   
   T   
   T   
    T    
   G   
   C   
    C    
   A   
   T   
   G   
   A   
   A   
   C   
   A   
   A   
   A   
   G   
   A   
   T   
   G   
   G   
   C   
   A   
   A   
   T   
   A   
   C   
   G   
   T   
   A   
   T   
   G   
   A   
   C   
   A   
   A   
   A   
   G   
   G   
   A   
   T   
   T   
    C    
   C   
   G   
   T   
   A   
   A   
   A   
   T   
   C   
   A   
   G   
   C   
   A   
   A   
   A   
   A   
   T   
   C   
   T   
   G   
   T   
   C   
   G   
   G   
   A   
   A   
   A   
   T   
   A   
   T   
   C   
   A   
   T   
   G   
   G   
   G   
    G    
   A   
   A   
   T   
   T   
   A   
   T   
   C   
   A   
   T   
   C   
   C   
   T   
   C   
   A   
   T   
   G   
   G   
   T   
   G   
   A   
   C   
   A   
   G   
   C   
   A   
   G   
   T   
   A   
   T   
   T   
   T   
   A   
   T   
   G   
   A   
   A   
   G   
   C   
   G   
   A   
   T   
   G   
   G   
   T   
   C   
   C   
   G   
   C   
   A   
   T   
   G   
   A   
   G   
   C   
   C   
   A   
   A   
   G   
   A   
    C    
   T   
   G   
   G   
   A   
   A   
   G   
   T   
   T   
   G   
   C   
   G   
   G   
   G   
   A   
   A   
   G   
   T   
   A   
   C   
   T   
   G   
   A   
   T   
   C   
   G   
   A   
    A    
   A   
   T   
   G   
   C   
   A   
   C   
   G   
   G   
    G    
   A   
   A   
   T   
   A   
   A   
    C    
   G   
   G   
   G   
   A   
   G   
   T   
   A   
   T   
   G   
   G   
   A   
   C   
   G   
   G   
   T   
   G   
   A   
   T   
   C   
   C   
   A   
   C   
   C   
   T   
   G   
   C   
   T   
   G   
   C   
   G   
   A   
   T   
   G   
   C   
   G   
   T   
   T   
   A   
   T   
   A   
   C   
   T   
   G   
   A   
   A   
   G   
   C   
   C   
   A   
   G   
    A    
   T   
   T   
   A   
   T   
   C   
   C   
   A   
   A   
   G   
   T   
   T   
   A   
   A   
   G   
   T   
   G   
   G   
   A   
   G   
   A   
   G   
   A   
   T   
   G   
    C    
   T   
   A   
    G    
   C   
   A   
   G   
   A   
   T   
   A   
   T   
   C   
   G   
   A   
    A    
   A   
   A   
   A   
   G   
   A   
   A   
   A   
   C   
   A   
   G   
   T   
    C    
   G   
   A   
   T   
   C   
   T   
   C   
   G   
   T   
   G   
   T   
   G   
   G   
   A   
   A   
   T   
   T   
   T   
   T   
   G   
   A   
   T   
   G   
   A   
   T   
   A   
   C   
   A   
   G   
   A   
   A   
   A   
   A   
   A   
   G   
   A   
   A   
   C   
   C   
   A   
   A   
   C   
   A   
   G   
   T   
   C   
   C   
   T   
   T   
   C   
   C   
   A   
   G   
   C   
   T   
   C   
   G   
   A   
   T   
   A   
   C   
   C   
   C   
   A   
   A   
   A   
   C   
   C   
   T   
   T   
   C   
   T   
    C    
   G   
   T   
   C   
   A   
   A   
   T   
   G   
   G   
   A   
   T   
   C   
   G   
   A   
   C   
   A   
   G   
   G   
   T   
   A   
   T   
   A   
   T   
   C   
   T   
   G   
   C   
   T   
   G   
   G   
   A   
   T   
   A   
   T   
   G   
   C   
    A    
   A   
   C   
   A   
   G   
   A   
   A   
   A   
   T   
   C   
   C   
   C   
   G   
   A   
   C   
    T    
   C   
   A   
   T   
   A   
   A   
   C   
   T   
   T   
   A   
   G   
   C   
   T   
   G   
   A   
   A   
   G   
   T   
   G   
   A   
   T   
    C    
   G   
   A   
    T    
   G   
   G   
   C   
   A   
   C   
   A   
   A   
   T   
   C   
   T   
   A   
   T   
   A   
   T   
   G   
   A   
   T   
   C   
   G   
   A   
   T   
   C   
   A   
   T   
   C   
   C   
   A   
   C   
   A   
   A   
   G   
   C   
   G   
   A   
   G   
   C   
   C   
   T   
   T   
   G   
   A   
   A   
   A   
   A   
   A   
   T   
   T   
   G   
   A   
   T   
   G   
   G   
   A   
   A   
   T   
   A   
   T   
   A   
   T   
   T   
   C   
   C   
   T   
   G   
   G   
   A   
   C   
   C   
   T   
   G   
   A   
   C   
   T   
   T   
    T    
   C   
   C   
   A   
   A   
   C   
   A   
   G   
   G   
   A   
   G   
   G   
    G    
   A   
   T   
   C   
   T   
   T   
   G   
   C   
   A   
   A   
   G   
   G   
    A    
   A   
   A   
   A   
   G   
   A   
   T   
   G   
   A   
   A   
   A   
   T   
   C   
   A   
   A   
   A   
   A   
   A   
   A   
   G   
   C   
   T   
   T   
   A   
   T   
   G   
   A   
   A   
   A   
   C   
   A   
   G   
   G   
    G    
   C   
   G   
   A   
   G   
   G   
   A   
   A   
   A   
   A   
   G   
   T   
   T   
   A   
    T    
   T   
   C   
   T   
   T   
   C   
   G   
   T   
   T   
   C   
   G   
   A   
   A   
   A   
   A   
   C   
   A   
   A   
   A   
   G   
   A   
   T   
   A   
   G   
   A   
    G    
   G   
   C   
   A   
   A   
   T   
   G   
   A   
   A   
   G   
   G   
   G   
    T    
   A   
   A   
    T    
   A   
   A   
   A   
   C   
   A   
   G   
   C   
   A   
   A   
   A   
   T   
   C   
   G   
   T   
   G   
   A   
   T   
   C   
   A   
   C   
    A    
   G   
   A   
   A   
   A   
   T   
   C   
   C   
   C   
   A   
   T   
   A   
   T   
   G   
   A   
   A   
   G   
   T   
   A   
   A   
   A   
   T   
   A   
   A   
   A   
   G   
   C   
   G   
   A   
   C   
    C    
   T   
   T   
   G   
   G   
   T   
   G   
   A   
   A   
   A   
   A   
   A   
   A   
   A   
   T   
   G   
   G   
   A   
   T   
   G   
   A   
   G   
   A   
   T   
   C   
   C   
   G   
   T   
   T   
   T   
   G   
   A   
   A   
   T   
   A   
   A   
   G   
   A   
   A   
   A   
   A   
   T   
   C   
   G   
   A   
    T    
   G   
   G   
   A   
   A   
   T   
   A   
   G   
   C   
   T   
   G   
   A   
   A   
   G   
   T   
    A    
   C   
   G   
   A   
   G   
   A   
   T   
   G   
   A   
   A   
   A   
   G   
   T   
   G   
   A   
   T   
    A    
   G   
    A    
   A   
   C   
    A    
   G   
   G   
   T   
   C   
   T   
   G   
   C   
   A   
   A   
   A   
   T   
   T   
   G   
   T   
   C   
   G   
   T   
    C    
   G   
   A   
   A   
   T   
   T   
   G   
   A   
   A   
   A   
   A   
   A   
   A   
   G   
   A   
    C    
   G   
   C   
   C   
   A   
   A   
    T    
   G   
   C   
   T   
   C   
   A   
   A   
   G   
   G   
    A    
   A   
   T   
   T   
   T   
   T   
   G   
   A   
   A   
   T   
   T   
   A   
    T    
   T   
   T   
   G   
   T   
   T   
   C   
   A   
   A   
   A   
   A   
   A   
   C   
   A   
   C   
   T   
   G   
   A   
   G   
    C    
   T   
   A   
   C   
   A   
   G   
   A   
   T   
   C   
   A   
   A   
   T   
   T   
   A   
   C   
   A   
   A   
   T   
   T   
   T   
    C    
   A   
   A   
   T   
   A   
   T   
   G   
   G   
   T   
   C   
   G   
   C   
   T   
   A   
   T   
   C   
   G   
   A   
    C    
   C   
   A   
   T   
   A   
   T   
   G   
   A   
   C   
   T   
   C   
   C   
   G   
   C   
   A   
    T    
   C   
   A   
   A   
   G   
   T   
   T   
   G   
   G   
   A   
   C   
   T   
   G   
   A   
   A   
   A   
   G   
   A   
   C   
   A   
   T   
   T   
   T   
   T   
   A   
   A   
   G   
   A   
   A   
   G   
   T   
   T   
   A   
   T   
   A   
   T   
   C   
   G   
   A   
   G   
   C   
   A   
   T   
   C   
   G   
   A   
   A   
   A   
   G   
   C   
   A   
   A   
   G   
   T   
   C   
   A   
   T   
   A   
   A   
   C   
   A   
   A   
   A   
   A   
   C   
   G   
    T    
   A   
   G   
    T    
   C   
   A   
   A   
   T   
   T   
   C   
   G   
   A   
   T   
   T   
   T   
   A   
   G   
   C   
   C   
   A   
   A   
   A   
   G   
   C   
   T   
   C   
   A   
   A   
   A   
   A   
   A   
    A    
   G   
   A   
   C   
   A   
   G   
   C   
   A   
   T   
   A   
   T   
   C   
   G   
   T   
    A    
   G   
   A   
   A   
   G   
   G   
    A    
    T    
   T   
   G   
   A   
   T   
   G   
   A   
   A   
   A   
   G   
   C   
   T   
   T   
   T   
   A   
   T   
   C   
   G   
   A   
   T   
   T   
   C   
   T   
   G   
   G   
   A   
   T   
   G   
   A   
   A   
   G   
   T   
   A   
   A   
   T   
   C   
   G   
    C    
   A   
   A   
   C   
   G   
   A   
   T   
   T   
   C   
   G   
   T   
   G   
   A   
   A   
   A   
   G   
   T   
   A   
   A   
   A   
   G   
   A   
   C   
   A   
   A   
   A   
   A   
   A   
   A   
   G   
   A   
    C    
   G   
   C   
    C    
   A   
   A   
   A   
   A   
   A   
   G   
   A   
   A   
   T   
   T   
   T   
   A   
   G   
   T   
   C   
   G   
   A   
    C    
   G   
   T   
    C    
   T   
   T   
    T    
   C   
   A   
   A   
   T   
   T   
   T   
   A   
   C   
   A   
   G   
   A   
   A   
   G   
   A   
   A   
   C   
   A   
   A   
   G   
   C   
   A   
   G   
   A   
   A   
   G   
   C   
   A   
   A   
   T   
    T    
   G   
   T   
   C   
   A   
   C   
   A   
   T   
   T   
   G   
   C   
   A   
   A   
    T    
   T   
   A   
   T   
   A   
    C    
   C   
   G   
   T   
   T   
   T   
   G   
   A   
   C   
   G   
   A   
   A   
    C    
   A   
   C   
    G    
   G   
   A   
   T   
   A   
   T   
   C   
   A   
   C   
   A   
   G   
   A   
   G   
   C   
   T   
   T   
   C   
   A   
   A   
   A   
   A   
   G   
   G   
   A   
   A   
    T    
   C   
    C    
   G   
   A   
   A   
   A   
    G    
   C   
   T   
   T   
   G   
   A   
   T   
   T   
   G   
   C   
    G    
   C   
   A   
   A   
   A   
   T   
   C   
   A   
   C   
   A   
   G   
   A   
   G   
   T   
   T   
    G    
   A   
   A   
   T   
   A   
   A   
   A   
   A   
   T   
   C   
   C   
   T   
   T   
   T   
   C   
   A   
   A   
   A   
    T    
   G   
   A   
   C   
   A   
   A   
   A   
   G   
   A   
    G    
   C   
   T   
   T   
   T   
   T   
    C    
   T   
   C   
   T   
   G   
   T   
   T   
   A   
   T   
   G   
   A   
   A   
   A   
   A   
   A   
   A   
   G   
   A   
   A   
   T   
   T   
   G   
   C   
   G   
   A   
   G   
   A   
   A   
   G   
   T   
   G   
   A   
   A   
   A   
   A   
   A   
   A   
   A   
   A   
   T   
   T   
   A   
    T    
   T   
   C   
   T   
   T   
   C   
   C   
   G   
   C   
   T   
   C   
   G   
   T   
   C   
    T    
    A    
   A   
   C   
   G   
   A   
   C   
   A   
   A   
   T   
   C   
    G    
   A   
   A   
   G   
   A   
   C   
   G   
   A   
   A   
   A   
   T   
   C   
   G   
   A   
   A   
   G   
   A   
   A   
   A   
   T   
   C   
   A   
   A   
   A   
   A   
   T   
    T    
   G   
   A   
   T   
   A   
   C   
   G   
   C   
   A   
   A   
    G    
   T   
    C    
   T   
   T   
   A   
   G   
   T   
   G   
   G   
   C   
    A    
   C   
   A   
   A   
   G   
   A   
   A   
   G   
   A   
   C   
   G   
   T   
   A   
   A   
   T   
    T    
   G   
   T   
   C   
   A   
   G   
   T   
   G   
   T   
    G    
   A   
   C   
    G    
   C   
   G   
   A   
   G   
   A   
   A   
   G   
   G   
   C   
   T   
   A   
   T   
   G   
   T   
   G   
   A   
   A   
   G   
   C   
   G   
   A   
   A   
   C   
   A   
   A   
   G   
   C   
   C   
   T   
   T   
   C   
   G   
   T   
   T   
   C   
    T    
   T   
   A   
   T   
   A   
   G   
   T   
   G   
   C   
   T   
   T   
   C   
    C    
   A   
   A   
   A   
   C   
   C   
   A   
   G   
   A   
   A   
   G   
   A   
   A   
   A   
   T   
   C   
   G   
   G   
   G   
   A   
   T   
   G   
   C   
   G   
   A   
   G   
   A   
   A   
   G   
   G   
   C   
   G   
   A   
    C    
   T   
   A   
   C   
   T   
   T   
   G   
   C   
   T   
    A    
   T   
   A   
   C   
    T    
   C   
   A   
   G   
   G   
   T   
   G   
   A   
   A   
   T   
   T   
    G    
   A   
   G   
    T    
   A   
   C   
   A   
    T    
   T   
   G   
   G   
   A   
   C   
   C   
   A   
   T   
   G   
   T   
   T   
   C   
   T   
   T   
   C   
   T   
   G   
   A   
   T   
    C    
   A   
   C   
    A    
   A   
   A   
   T   
   A   
   A   
   A   
   G   
   C   
   A   
   A   
   A   
   T   
   G   
   T   
   G   
   A   
   T   
   C   
   T   
   A   
   C   
   C   
   G   
    C    
   C   
   C   
   T   
   G   
   T   
    C    
   C   
   A   
   T   
   G   
   A   
   A   
   C   
   T   
   A   
   C   
   C   
    C    
   G   
   A   
   T   
   T   
   T   
   G   
   A   
   A   
   A   
   T   
   G   
   G   
   A   
   A   
   A   
   G   
   A   
   T   
   G   
   C   
   T   
   G   
   G   
    T    
   G   
   A   
    G    
   C   
   A   
    C    
   A   
   T   
   T   
   T   
   C   
   T   
   C   
   A   
   A   
   A   
   C   
   A   
   A   
   T   
   C   
   T   
   C   
   C   
   A   
   A   
   T   
   C   
   T   
   G   
   T   
   C   
   T   
   G   
   T   
   A   
   G   
   A   
    C    
   G   
   A   
   A   
   T   
   C   
   G   
   A   
   T   
    T    
   T   
   T   
   A   
   G   
   C   
   T   
   G   
   T   
   T   
   T   
   T   
    C    
   C   
   C   
   A   
   T   
   A   
   T   
   C   
   A   
   A   
   A   
   A   
   A   
   A   
   T   
    T    
   C   
   A   
   A   
   G   
   C   
   A   
   G   
   A   
    G    
   A   
   A   
   A   
   A   
   C   
   A   
   T   
   T   
   T   
   G   
   T   
    A    
   T   
   T   
   C   
   A   
   T   
   C   
   A   
   G   
   C   
   A   
   A   
   G   
   A   
   A   
   T   
   G   
   G   
   A   
   T   
   T   
    G    
    A    
   T   
   C   
   A   
   A   
   G   
   C   
   A   
   A   
   A   
   C   
   A   
   C   
   G   
   A   
   A   
   T   
   G   
   A   
   C   
   A   
   G   
   A   
   G   
   T   
   T   
   C   
   G   
   A   
   A   
   C   
   C   
   A   
   T   
   G   
   G   
   C   
   G   
   C   
   A   
   C   
   C   
   T   
   A   
    T    
   A   
   A   
   G   
   A   
   G   
   C   
   C   
   G   
    T    
   C   
   C   
   G   
   C   
   T   
   C   
   A   
   G   
   C   
   T   
   G   
   T   
   A   
   T   
   G   
   A   
   A   
   G   
   C   
   T   
   A   
   A   
   A   
   G   
   G   
   C   
   G   
   G   
   A   
   A   
   G   
   A   
   A   
   G   
   A   
   T   
   G   
   A   
   A   
   C   
   T   
    G    
   A   
   T   
   T   
   G   
   C   
   T   
   G   
   T   
    G    
   T   
   A   
   T   
   C   
   T   
   G   
   A   
   A   
   A   
   G   
   A   
   A   
   G   
   A   
   T   
   C   
    A    
   G   
   C   
   C   
   A   
   G   
   G   
   C   
   T   
   T   
   A   
   G   
   A   
   T   
   G   
   T   
    T    
   T   
   T   
   C   
   T   
   T   
   A   
   G   
   T   
   A   
   A   
   C   
   G   
    C    
   A   
   T   
   C   
   A   
   A   
   G   
   G   
   A   
   A   
   T   
   G   
   G   
   G   
    T    
   T   
   T   
   G   
   C   
   G   
   C   
   T   
    G    
   T   
   C   
   C   
   T   
   T   
   T   
   A   
   A   
    C    
   A   
   G   
   A   
   T   
   G   
   T   
   T   
   C   
   C   
   C   
   G   
   T   
   T   
   G   
   T   
   T   
   G   
   G   
   A   
   G   
   C   
   A   
   A   
   A   
   G   
   G   
   C   
   A   
   G   
    C    
   T   
   G   
   G   
   A   
   G   
   T   
   G   
   A   
   A   
   G   
   T   
   C   
   T   
   A   
   T   
   G   
   A   
   A   
   T   
   T   
   T   
   G   
   A   
   A   
   A   
   G   
   A   
   T   
   G   
   G   
   A   
   G   
   A   
   T   
   A   
   G   
   G   
    A    
   T   
   T   
    A    
   T   
   C   
   A   
   A   
    C    
   G   
   G   
    G    
   C   
   T   
    G    
   C   
   T   
   T   
   G   
   T   
   G   
   T   
   T   
   A   
   G   
   C   
   A   
   G   
   A   
    A    
   G   
   G   
    T    
   G   
   A   
   T   
   A   
   C   
   G   
   C   
   C   
    G    
   A   
   T   
   C   
   G   
   T   
   A   
   A   
   T   
   C   
   G   
   T   
   T   
   A   
   C   
   T   
   C   
   A   
   G   
   C   
   G   
   A   
   G   
   G   
   A   
   G   
   C   
    T    
   G   
   T   
    T    
   A   
   A   
   A   
   C   
   G   
   C   
   A   
   T   
   G   
   C   
   T   
   T   
   G   
   C   
   T   
   C   
   A   
   A   
   G   
   A   
   A   
   A   
   T   
   C   
   A   
   G   
   C   
   C   
   A   
   A   
   A   
   C   
    T    
   T   
   C   
   A   
   C   
   G   
   T   
   G   
   C   
   G   
   A   
   A   
   G   
   C   
   G   
   G   
   G   
   G   
   A   
   G   
   T   
   G   
   A   
   C   
   G   
   G   
   T   
   C   
   A   
   T   
   G   
   C   
   G   
   T   
   G   
   A   
   G   
   C   
   T   
   G   
   A   
   A   
   A   
   A   
   A   
   A   
   A   
   A   
   T   
   C   
   C   
    G    
   C   
   A   
    C    
   C   
   G   
    T    
   A   
   T   
   T   
   A   
   T   
   C   
   T   
   A   
   T   
   A   
   T   
   G   
   T   
   C   
   G   
   G   
   A   
   A   
   G   
   G   
   A   
   A   
   G   
   A   
   T   
   C   
   A   
   A   
   A   
   A   
   G   
   A   
   A   
   A   
   T   
   A   
   A   
   C   
   C   
   T   
   T   
   G   
   A   
   T   
   G   
   A   
   A   
   T   
   C   
   A   
   A   
   A   
   A   
   A   
   G   
   G   
    G    
   C   
   A   
    G    
   C   
   A   
   G   
    T    
   T   
   A   
   A   
   C   
   G   
   A   
   T   
   T   
   G   
   A   
    C    
   C   
   C   
   A   
   A   
   C   
    T    
   G   
   A   
   T   
   T   
   T   
    C    
   C   
   C   
   T   
   A   
   T   
   A   
   G   
   G   
   T   
   G   
   A   
   T   
   C   
   G   
   A   
   A   
   C   
   A   
   T   
   C   
   G   
   A   
   A   
   T   
   G   
   G   
    A    
   T   
   C   
    A    
   T   
   T   
   T   
   G   
   C   
    G    
   A   
   T   
   G   
   G   
   A   
    T    
   G   
   A   
   G   
   A   
   A   
   A   
   A   
   A   
   A   
   G   
   G   
   T   
   G   
   G   
   A   
   G   
   A   
   A   
   G   
   T   
   C   
   A   
   T   
   C   
   G   
   A   
   A   
   G   
   T   
    T    
   A   
    T    
   C   
   G   
   A   
   T   
   G   
   C   
   T   
   C   
   C   
   T   
   G   
   A   
   G   
    A    
   T   
   T   
   T   
   T   
    C    
   A   
   T   
    C    
   G   
   A   
    G    
    T    
   C   
    G    
   -   
   -   
   -   
 

 
   N55655   
   A   
   T   
   G   
   G   
   A   
   A   
   A   
   A   
   T   
   C   
   G   
   A   
   C   
   A   
   A   
   G   
   A   
   A   
   G   
   T   
   A   
   C   
   A   
   A   
   G   
   A   
   A   
   T   
   T   
   A   
   A   
   C   
   T   
   T   
   T   
   A   
   G   
   A   
   A   
   G   
   A   
   A   
   G   
   T   
   G   
   A   
   T   
   G   
   G   
   G   
   T   
   G   
   A   
   C   
   C   
   G   
   C   
   T   
   T   
   C   
   G   
   G   
   A   
   C   
   G   
   T   
   T   
   A   
   T   
   T   
   C   
   C   
   A   
   A   
   A   
   T   
   A   
   T   
   A   
   T   
   T   
   A   
   T   
    C    
   C   
   A   
    A    
   G   
   A   
   A   
   C   
   G   
   T   
   G   
   C   
   A   
   C   
   T   
   G   
   C   
   C   
   T   
   G   
   A   
   T   
   A   
   T   
    C    
   C   
   G   
   T   
   G   
   A   
   T   
   G   
   G   
   A   
   T   
   T   
   G   
   A   
   A   
    G    
   C   
   C   
   A   
   G   
   T   
    G    
   C   
   A   
   A   
   C   
   G   
   C   
   C   
   G   
   G   
   A   
   T   
   A   
   T   
   T   
   A   
   T   
   T   
    T    
   G   
   C   
    C    
   A   
   T   
   G   
   A   
   A   
   C   
   A   
   A   
   A   
   G   
   A   
   T   
   G   
   G   
   C   
   A   
   A   
   T   
   A   
   C   
   G   
   T   
   A   
   T   
   G   
   A   
   C   
   A   
   A   
   A   
   G   
   G   
   A   
   T   
   T   
    C    
   C   
   G   
   T   
   A   
   A   
   A   
   T   
   C   
   A   
   G   
   C   
   A   
   A   
   A   
   A   
   T   
   C   
   T   
   G   
   T   
   C   
   G   
   G   
   A   
   A   
   A   
   T   
   A   
   T   
   C   
   A   
   T   
   G   
   G   
   G   
    G    
   A   
   A   
   T   
   T   
   A   
   T   
   C   
   A   
   T   
   C   
   C   
   T   
   C   
   A   
   T   
   G   
   G   
   T   
   G   
   A   
   C   
   A   
   G   
   C   
   A   
   G   
   T   
   A   
   T   
   T   
   T   
   A   
   T   
   G   
   A   
   A   
   G   
   C   
   G   
   A   
   T   
   G   
   G   
   T   
   C   
   C   
   G   
   C   
   A   
   T   
   G   
   A   
   G   
   C   
   C   
   A   
   A   
   G   
   A   
    C    
   T   
   G   
   G   
   A   
   A   
   G   
   T   
   T   
   G   
   C   
   G   
   G   
   G   
   A   
   A   
   G   
   T   
   A   
   C   
   T   
   G   
   A   
   T   
   C   
   G   
   A   
    A    
   A   
   T   
   G   
   C   
   A   
   C   
   G   
   G   
    G    
   A   
   A   
   T   
   A   
   A   
    C    
   G   
   G   
   G   
   A   
   G   
   T   
   A   
   T   
   G   
   G   
   A   
   C   
   G   
   G   
   T   
   G   
   A   
   T   
   C   
   C   
   A   
   C   
   C   
   T   
   G   
   C   
   T   
   G   
   C   
   G   
   A   
   T   
   G   
   C   
   G   
   T   
   T   
   A   
   T   
   A   
   C   
   T   
   G   
   A   
   A   
   G   
   C   
   C   
   A   
   G   
    A    
   T   
   T   
   A   
   T   
   C   
   C   
   A   
   A   
   G   
   T   
   T   
   A   
   A   
   G   
   T   
   G   
   G   
   A   
   G   
   A   
   G   
   A   
   T   
   G   
    C    
   T   
   A   
    G    
   C   
   A   
   G   
   A   
   T   
   A   
   T   
   C   
   G   
   A   
    A    
   A   
   A   
   A   
   G   
   A   
   A   
   A   
   C   
   A   
   G   
   T   
    C    
   G   
   A   
   T   
   C   
   T   
   C   
   G   
   T   
   G   
   T   
   G   
   G   
   A   
   A   
   T   
   T   
   T   
   T   
   G   
   A   
   T   
   G   
   A   
   T   
   A   
   C   
   A   
   G   
   A   
   A   
   A   
   A   
   A   
   G   
   A   
   A   
   C   
   C   
   A   
   A   
   C   
   A   
   G   
   T   
   C   
   C   
   T   
   T   
   C   
   C   
   A   
   G   
   C   
   T   
   C   
   G   
   A   
   T   
   A   
   C   
   C   
   C   
   A   
   A   
   A   
   C   
   C   
   T   
   T   
   C   
   T   
    C    
   G   
   T   
   C   
   A   
   A   
   T   
   G   
   G   
   A   
   T   
   C   
   G   
   A   
   C   
   A   
   G   
   G   
   T   
   A   
   T   
   A   
   T   
   C   
   T   
   G   
   C   
   T   
   G   
   G   
   A   
   T   
   A   
   T   
   G   
   C   
    A    
   A   
   C   
   A   
   G   
   A   
   A   
   A   
   T   
   C   
   C   
   C   
   G   
   A   
   C   
    T    
   C   
   A   
   T   
   A   
   A   
   C   
   T   
   T   
   A   
   G   
   C   
   T   
   G   
   A   
   A   
   G   
   T   
   G   
   A   
   T   
    T    
   G   
   A   
    T    
   G   
   G   
   C   
   A   
   C   
   A   
   A   
   T   
   C   
   T   
   A   
   T   
   A   
   T   
   G   
   A   
   T   
   C   
   G   
   A   
   T   
   C   
   A   
   T   
   C   
   C   
   A   
   C   
   A   
   A   
   G   
   C   
   G   
   A   
   G   
   C   
   C   
   T   
   T   
   G   
   A   
   A   
   A   
   A   
   A   
   T   
   T   
   G   
   A   
   T   
   G   
   G   
   A   
   A   
   T   
   A   
   T   
   A   
   T   
   T   
   C   
   C   
   T   
   G   
   G   
   A   
   C   
   C   
   T   
   G   
   A   
   C   
   T   
   T   
    T    
   C   
   C   
   A   
   A   
   C   
   A   
   G   
   G   
   A   
   G   
   G   
    G    
   A   
   T   
   C   
   T   
   T   
   G   
   C   
   A   
   A   
   G   
   G   
    A    
   A   
   A   
   A   
   G   
   A   
   T   
   G   
   A   
   A   
   A   
   T   
   C   
   A   
   A   
   A   
   A   
   A   
   A   
   G   
   C   
   T   
   T   
   A   
   T   
   G   
   A   
   A   
   A   
   C   
   A   
   G   
   G   
    G    
   C   
   G   
   A   
   G   
   G   
   A   
   A   
   A   
   A   
   G   
   T   
   T   
   A   
    T    
   T   
   C   
   T   
   T   
   C   
   G   
   T   
   T   
   C   
   G   
   A   
   A   
   A   
   A   
   C   
   A   
   A   
   A   
   G   
   A   
   T   
   A   
   G   
   A   
    G    
   G   
   C   
   A   
   A   
   T   
   G   
   A   
   A   
   G   
   G   
   G   
    T    
   A   
   A   
    T    
   A   
   A   
   A   
   C   
   A   
   G   
   C   
   A   
   A   
   A   
   T   
   C   
   G   
   T   
   G   
   A   
   T   
   C   
   A   
   C   
    A    
   G   
   A   
   A   
   A   
   T   
   C   
   C   
   C   
   A   
   T   
   A   
   T   
   G   
   A   
   A   
   G   
   T   
   A   
   A   
   A   
   T   
   A   
   A   
   A   
   G   
   C   
   G   
   A   
   C   
    C    
   T   
   T   
   G   
   G   
   T   
   G   
   A   
   A   
   A   
   A   
   A   
   A   
   A   
   T   
   G   
   G   
   A   
   T   
   G   
   A   
   G   
   A   
   T   
   C   
   C   
   G   
   T   
   T   
   T   
   G   
   A   
   A   
   T   
   A   
   A   
   G   
   A   
   A   
   A   
   A   
   T   
   C   
   G   
   A   
    T    
   G   
   G   
   A   
   A   
   T   
   A   
   G   
   C   
   T   
   G   
   A   
   A   
   G   
   T   
    A    
   C   
   G   
   A   
   G   
   A   
   T   
   G   
   A   
   A   
   A   
   G   
   T   
   G   
   A   
   T   
    A    
   G   
    A    
   A   
   C   
    A    
   G   
   G   
   T   
   C   
   T   
   G   
   C   
   A   
   A   
   A   
   T   
   T   
   G   
   T   
   C   
   G   
   T   
    C    
   G   
   A   
   A   
   T   
   T   
   G   
   A   
   A   
   A   
   A   
   A   
   A   
   G   
   A   
    C    
   G   
   C   
   C   
   A   
   A   
    T    
   G   
   C   
   T   
   C   
   A   
   A   
   G   
   G   
    A    
   A   
   T   
   T   
   T   
   T   
   G   
   A   
   A   
   T   
   T   
   A   
    T    
   T   
   T   
   G   
   T   
   T   
   C   
   A   
   A   
   A   
   A   
   A   
   C   
   A   
   C   
   T   
   G   
   A   
   G   
    C    
   T   
   A   
   C   
   A   
   G   
   A   
   T   
   C   
   A   
   A   
   T   
   T   
   A   
   C   
   A   
   A   
   T   
   T   
   T   
    C    
   A   
   A   
   T   
   A   
   T   
   G   
   G   
   T   
   C   
   G   
   C   
   T   
   A   
   T   
   C   
   G   
   A   
    C    
   C   
   A   
   T   
   A   
   T   
   G   
   A   
   C   
   T   
   C   
   C   
   G   
   C   
   A   
    T    
   C   
   A   
   A   
   G   
   T   
   T   
   G   
   G   
   A   
   C   
   T   
   G   
   A   
   A   
   A   
   G   
   A   
   C   
   A   
   T   
   T   
   T   
   T   
   A   
   A   
   G   
   A   
   A   
   G   
   T   
   T   
   A   
   T   
   A   
   T   
   C   
   G   
   A   
   G   
   C   
   A   
   T   
   C   
   G   
   A   
   A   
   A   
   G   
   C   
   A   
   A   
   G   
   T   
   C   
   A   
   T   
   A   
   A   
   C   
   A   
   A   
   A   
   A   
   C   
   G   
    T    
   A   
   G   
    T    
   C   
   A   
   A   
   T   
   T   
   C   
   G   
   A   
   T   
   T   
   T   
   A   
   G   
   C   
   C   
   A   
   A   
   A   
   G   
   C   
   T   
   C   
   A   
   A   
   A   
   A   
   A   
    A    
   G   
   A   
   C   
   A   
   G   
   C   
   A   
   T   
   A   
   T   
   C   
   G   
   T   
    A    
   G   
   A   
   A   
   G   
   G   
    A    
    T    
   T   
   G   
   A   
   T   
   G   
   A   
   A   
   A   
   G   
   C   
   T   
   T   
   T   
   A   
   T   
   C   
   G   
   A   
   T   
   T   
   C   
   T   
   G   
   G   
   A   
   T   
   G   
   A   
   A   
   G   
   T   
   A   
   A   
   T   
   C   
   G   
    T    
   A   
   A   
   C   
   G   
   A   
   T   
   T   
   C   
   G   
   T   
   G   
   A   
   A   
   A   
   G   
   T   
   A   
   A   
   A   
   G   
   A   
   C   
   A   
   A   
   A   
   A   
   A   
   A   
   G   
   A   
    C    
   G   
   C   
    C    
   A   
   A   
   A   
   A   
   A   
   G   
   A   
   A   
   T   
   T   
   T   
   A   
   G   
   T   
   C   
   G   
   A   
    C    
   G   
   T   
    C    
   T   
   T   
    T    
   C   
   A   
   A   
   T   
   T   
   T   
   A   
   C   
   A   
   G   
   A   
   A   
   G   
   A   
   A   
   C   
   A   
   A   
   G   
   C   
   A   
   G   
   A   
   A   
   G   
   C   
   A   
   A   
   T   
    T    
   G   
   T   
   C   
   A   
   C   
   A   
   T   
   T   
   G   
   C   
   A   
   A   
    T    
   T   
   A   
   T   
   A   
    C    
   C   
   G   
   T   
   T   
   T   
   G   
   A   
   C   
   G   
   A   
   A   
    C    
   A   
   C   
    G    
   G   
   A   
   T   
   A   
   T   
   C   
   A   
   C   
   A   
   G   
   A   
   G   
   C   
   T   
   T   
   C   
   A   
   A   
   A   
   A   
   G   
   G   
   A   
   A   
    T    
   C   
    C    
   G   
   A   
   A   
   A   
    G    
   C   
   T   
   T   
   G   
   A   
   T   
   T   
   G   
   C   
    G    
   C   
   A   
   A   
   A   
   T   
   C   
   A   
   C   
   A   
   G   
   A   
   G   
   T   
   T   
    G    
   A   
   A   
   T   
   A   
   A   
   A   
   A   
   T   
   C   
   C   
   T   
   T   
   T   
   C   
   A   
   A   
   A   
    T    
   G   
   A   
   C   
   A   
   A   
   A   
   G   
   A   
    G    
   C   
   T   
   T   
   T   
   T   
    C    
   T   
   C   
   T   
   G   
   T   
   T   
   A   
   T   
   G   
   A   
   A   
   A   
   A   
   A   
   A   
   G   
   A   
   A   
   T   
   T   
   G   
   C   
   G   
   A   
   G   
   A   
   A   
   G   
   T   
   G   
   A   
   A   
   A   
   A   
   A   
   A   
   A   
   A   
   T   
   T   
   A   
    T    
   T   
   C   
   T   
   T   
   C   
   C   
   G   
   C   
   T   
   C   
   G   
   T   
   C   
    T    
    A    
   A   
   C   
   G   
   A   
   C   
   A   
   A   
   T   
   C   
    G    
   A   
   A   
   G   
   A   
   C   
   G   
   A   
   A   
   A   
   T   
   C   
   G   
   A   
   A   
   G   
   A   
   A   
   A   
   T   
   C   
   A   
   A   
   A   
   A   
   T   
    T    
   G   
   A   
   T   
   A   
   C   
   G   
   C   
   A   
   A   
    G    
   T   
    C    
   T   
   T   
   A   
   G   
   T   
   G   
   G   
   C   
    A    
   C   
   A   
   A   
   G   
   A   
   A   
   G   
   A   
   C   
   G   
   T   
   A   
   A   
   T   
    T    
   G   
   T   
   C   
   A   
   G   
   T   
   G   
   T   
    G    
   A   
   C   
    G    
   C   
   G   
   A   
   G   
   A   
   A   
   G   
   G   
   C   
   T   
   A   
   T   
   G   
   T   
   G   
   A   
   A   
   G   
   C   
   G   
   A   
   A   
   C   
   A   
   A   
   G   
   C   
   C   
   T   
   T   
   C   
   G   
   T   
   T   
   C   
    T    
   T   
   A   
   T   
   A   
   G   
   T   
   G   
   C   
   T   
   T   
   C   
    C    
   A   
   A   
   A   
   C   
   C   
   A   
   G   
   A   
   A   
   G   
   A   
   A   
   A   
   T   
   C   
   G   
   G   
   G   
   A   
   T   
   G   
   C   
   G   
   A   
   G   
   A   
   A   
   G   
   G   
   C   
   G   
   A   
    C    
   T   
   A   
   C   
   T   
   T   
   G   
   C   
   T   
    A    
   T   
   A   
   C   
    T    
   C   
   A   
   G   
   G   
   T   
   G   
   A   
   A   
   T   
   T   
    G    
   A   
   G   
    T    
   A   
   C   
   A   
    T    
   T   
   G   
   G   
   A   
   C   
   C   
   A   
   T   
   G   
   T   
   T   
   C   
   T   
   T   
   C   
   T   
   G   
   A   
   T   
    C    
   A   
   C   
    A    
   A   
   A   
   T   
   A   
   A   
   A   
   G   
   C   
   A   
   A   
   A   
   T   
   G   
   T   
   G   
   A   
   T   
   C   
   T   
   A   
   C   
   C   
   G   
    C    
   C   
   C   
   T   
   G   
   T   
    C    
   C   
   A   
   T   
   G   
   A   
   A   
   C   
   T   
   A   
   C   
   C   
    C    
   G   
   A   
   T   
   T   
   T   
   G   
   A   
   A   
   A   
   T   
   G   
   G   
   A   
   A   
   A   
   G   
   A   
   T   
   G   
   C   
   T   
   G   
   G   
    T    
   G   
   A   
    G    
   C   
   A   
    C    
   A   
   T   
   T   
   T   
   C   
   T   
   C   
   A   
   A   
   A   
   C   
   A   
   A   
   T   
   C   
   T   
   C   
   C   
   A   
   A   
   T   
   C   
   T   
   G   
   T   
   C   
   T   
   G   
   T   
   A   
   G   
   A   
    C    
   G   
   A   
   A   
   T   
   C   
   G   
   A   
   T   
    T    
   T   
   T   
   A   
   G   
   C   
   T   
   G   
   T   
   T   
   T   
   T   
    C    
   C   
   C   
   A   
   T   
   A   
   T   
   C   
   A   
   A   
   A   
   A   
   A   
   A   
   T   
    T    
   C   
   A   
   A   
   G   
   C   
   A   
   G   
   A   
    G    
   A   
   A   
   A   
   A   
   C   
   A   
   T   
   T   
   T   
   G   
   T   
    A    
   T   
   T   
   C   
   A   
   T   
   C   
   A   
   G   
   C   
   A   
   A   
   G   
   A   
   A   
   T   
   G   
   G   
   A   
   T   
   T   
    G    
    A    
   T   
   C   
   A   
   A   
   G   
   C   
   A   
   A   
   A   
   C   
   A   
   C   
   G   
   A   
   A   
   T   
   G   
   A   
   C   
   A   
   G   
   A   
   G   
   T   
   T   
   C   
   G   
   A   
   A   
   C   
   C   
   A   
   T   
   G   
   G   
   C   
   G   
   C   
   A   
   C   
   C   
   T   
   A   
    T    
   A   
   A   
   G   
   A   
   G   
   C   
   C   
   G   
    T    
   C   
   C   
   G   
   C   
   T   
   C   
   A   
   G   
   C   
   T   
   G   
   T   
   A   
   T   
   G   
   A   
   A   
   G   
   C   
   T   
   A   
   A   
   A   
   G   
   G   
   C   
   G   
   G   
   A   
   A   
   G   
   A   
   A   
   G   
   A   
   T   
   G   
   A   
   A   
   C   
   T   
    G    
   A   
   T   
   T   
   G   
   C   
   T   
   G   
   T   
    G    
   T   
   A   
   T   
   C   
   T   
   G   
   A   
   A   
   A   
   G   
   A   
   A   
   G   
   A   
   T   
   C   
    A    
   G   
   C   
   C   
   A   
   G   
   G   
   C   
   T   
   T   
   A   
   G   
   A   
   T   
   G   
   T   
    T    
   T   
   T   
   C   
   T   
   T   
   A   
   G   
   T   
   A   
   A   
   C   
   G   
    C    
   A   
   T   
   C   
   A   
   A   
   G   
   G   
   A   
   A   
   T   
   G   
   G   
   G   
    T    
   T   
   T   
   G   
   C   
   G   
   C   
   T   
    A    
   T   
   C   
   C   
   T   
   T   
   T   
   A   
   A   
    C    
   A   
   G   
   A   
   T   
   G   
   T   
   T   
   C   
   C   
   C   
   G   
   T   
   T   
   G   
   T   
   T   
   G   
   G   
   A   
   G   
   C   
   A   
   A   
   A   
   G   
   G   
   C   
   A   
   G   
    C    
   T   
   G   
   G   
   A   
   G   
   T   
   G   
   A   
   A   
   G   
   T   
   C   
   T   
   A   
   T   
   G   
   A   
   A   
   T   
   T   
   T   
   G   
   A   
   A   
   A   
   G   
   A   
   T   
   G   
   G   
   A   
   G   
   A   
   T   
   A   
   G   
   G   
    A    
   T   
   T   
    A    
   T   
   C   
   A   
   A   
    C    
   G   
   G   
    G    
   C   
   T   
    G    
   C   
   T   
   T   
   G   
   T   
   G   
   T   
   T   
   A   
   G   
   C   
   A   
   G   
   A   
    A    
   G   
   G   
    T    
   G   
   A   
   T   
   A   
   C   
   G   
   C   
   C   
    G    
   A   
   T   
   C   
   G   
   T   
   A   
   A   
   T   
   C   
   G   
   T   
   T   
   A   
   C   
   T   
   C   
   A   
   G   
   C   
   G   
   A   
   G   
   G   
   A   
   G   
   C   
    T    
   G   
   T   
    T    
   A   
   A   
   A   
   C   
   G   
   C   
   A   
   T   
   G   
   C   
   T   
   T   
   G   
   C   
   T   
   C   
   A   
   A   
   G   
   A   
   A   
   A   
   T   
   C   
   A   
   G   
   C   
   C   
   A   
   A   
   A   
   C   
    T    
   T   
   C   
   A   
   C   
   G   
   T   
   G   
   C   
   G   
   A   
   A   
   G   
   C   
   G   
   G   
   G   
   G   
   A   
   G   
   T   
   G   
   A   
   C   
   G   
   G   
   T   
   C   
   A   
   T   
   G   
   C   
   G   
   T   
   G   
   A   
   G   
   C   
   T   
   G   
   A   
   A   
   A   
   A   
   A   
   A   
   A   
   A   
   T   
   C   
   C   
    G    
   C   
   A   
    C    
   C   
   G   
    T    
   A   
   T   
   T   
   A   
   T   
   C   
   T   
   A   
   T   
   A   
   T   
   G   
   T   
   C   
   G   
   G   
   A   
   A   
   G   
   G   
   A   
   A   
   G   
   A   
   T   
   C   
   A   
   A   
   A   
   A   
   G   
   A   
   A   
   A   
   T   
   A   
   A   
   C   
   C   
   T   
   T   
   G   
   A   
   T   
   G   
   A   
   A   
   T   
   C   
   A   
   A   
   A   
   A   
   A   
   G   
   G   
    G    
   C   
   A   
    G    
   C   
   A   
   G   
    T    
   T   
   A   
   A   
   C   
   G   
   A   
   T   
   T   
   G   
   A   
    C    
   C   
   C   
   A   
   A   
   C   
    T    
   G   
   A   
   T   
   T   
   T   
    C    
   C   
   C   
   T   
   A   
   T   
   A   
   G   
   G   
   T   
   G   
   A   
   T   
   C   
   G   
   A   
   A   
   C   
   A   
   T   
   C   
   G   
   A   
   A   
   T   
   G   
   G   
    A    
   T   
   C   
    A    
   T   
   T   
   T   
   G   
   C   
    G    
   A   
   T   
   G   
   G   
   A   
    T    
   G   
   A   
   G   
   A   
   A   
   A   
   A   
   A   
   A   
   G   
   G   
   T   
   G   
   G   
   A   
   G   
   A   
   A   
   G   
   T   
   C   
   A   
   T   
   C   
   G   
   A   
   A   
   G   
   T   
    T    
   A   
    T    
   C   
   G   
   A   
   T   
   G   
   C   
   T   
   C   
   C   
   T   
   G   
   A   
   G   
    A    
   T   
   T   
   T   
   T   
    C    
   A   
   T   
    C    
   G   
   A   
    G    
    T    
   C   
    G    
   -   
   -   
   -   
 

 
   N52656   
   A   
   T   
   G   
   G   
   A   
   A   
   A   
   A   
   T   
   C   
   G   
   A   
   C   
   A   
   A   
   G   
   A   
   A   
   G   
   T   
   A   
   C   
   A   
   A   
   G   
   A   
   A   
   T   
   T   
   A   
   A   
   C   
   T   
   T   
   T   
   A   
   G   
   A   
   A   
   G   
   A   
   A   
   G   
   T   
   G   
   A   
   T   
   G   
   G   
   G   
   T   
   G   
   A   
   C   
   C   
   G   
   C   
   T   
   T   
   C   
   G   
   G   
   A   
   C   
   G   
   T   
   T   
   A   
   T   
   T   
   C   
   C   
   A   
   A   
   A   
   T   
   A   
   T   
   A   
   T   
   T   
   A   
   T   
    C    
   C   
   A   
    A    
   G   
   A   
   A   
   C   
   G   
   T   
   G   
   C   
   A   
   C   
   T   
   G   
   C   
   C   
   T   
   G   
   A   
   T   
   A   
   T   
    C    
   C   
   G   
   T   
   G   
   A   
   T   
   G   
   G   
   A   
   T   
   T   
   G   
   A   
   A   
    A    
   C   
   C   
   A   
   G   
   T   
    A    
   C   
   A   
   A   
   C   
   G   
   C   
   C   
   G   
   G   
   A   
   T   
   A   
   T   
   T   
   A   
   T   
   T   
    T    
   G   
   C   
    C    
   A   
   T   
   G   
   A   
   A   
   C   
   A   
   A   
   A   
   G   
   A   
   T   
   G   
   G   
   C   
   A   
   A   
   T   
   A   
   C   
   G   
   T   
   A   
   T   
   G   
   A   
   C   
   A   
   A   
   A   
   G   
   G   
   A   
   T   
   T   
    C    
   C   
   G   
   T   
   A   
   A   
   A   
   T   
   C   
   A   
   G   
   C   
   A   
   A   
   A   
   A   
   T   
   C   
   T   
   G   
   T   
   C   
   G   
   G   
   A   
   A   
   A   
   T   
   A   
   T   
   C   
   A   
   T   
   G   
   G   
   G   
    G    
   A   
   A   
   T   
   T   
   A   
   T   
   C   
   A   
   T   
   C   
   C   
   T   
   C   
   A   
   T   
   G   
   G   
   T   
   G   
   A   
   C   
   A   
   G   
   C   
   A   
   G   
   T   
   A   
   T   
   T   
   T   
   A   
   T   
   G   
   A   
   A   
   G   
   C   
   G   
   A   
   T   
   G   
   G   
   T   
   C   
   C   
   G   
   C   
   A   
   T   
   G   
   A   
   G   
   C   
   C   
   A   
   A   
   G   
   A   
    T    
   T   
   G   
   G   
   A   
   A   
   G   
   T   
   T   
   G   
   C   
   G   
   G   
   G   
   A   
   A   
   G   
   T   
   A   
   C   
   T   
   G   
   A   
   T   
   C   
   G   
   A   
    G    
   A   
   T   
   G   
   C   
   A   
   C   
   G   
   G   
    A    
   A   
   A   
   T   
   A   
   A   
    C    
   G   
   G   
   G   
   A   
   G   
   T   
   A   
   T   
   G   
   G   
   A   
   C   
   G   
   G   
   T   
   G   
   A   
   T   
   C   
   C   
   A   
   C   
   C   
   T   
   G   
   C   
   T   
   G   
   C   
   G   
   A   
   T   
   G   
   C   
   G   
   T   
   T   
   A   
   T   
   A   
   C   
   T   
   G   
   A   
   A   
   G   
   C   
   C   
   A   
   G   
    A    
   T   
   T   
   A   
   T   
   C   
   C   
   A   
   A   
   G   
   T   
   T   
   A   
   A   
   G   
   T   
   G   
   G   
   A   
   G   
   A   
   G   
   A   
   T   
   G   
    C    
   T   
   A   
    G    
   C   
   A   
   G   
   A   
   T   
   A   
   T   
   C   
   G   
   A   
    A    
   A   
   A   
   A   
   G   
   A   
   A   
   A   
   C   
   A   
   G   
   T   
    C    
   G   
   A   
   T   
   C   
   T   
   C   
   G   
   T   
   G   
   T   
   G   
   G   
   A   
   A   
   T   
   T   
   T   
   T   
   G   
   A   
   T   
   G   
   A   
   T   
   A   
   C   
   A   
   G   
   A   
   A   
   A   
   A   
   A   
   G   
   A   
   A   
   C   
   C   
   A   
   A   
   C   
   A   
   G   
   T   
   C   
   C   
   T   
   T   
   C   
   C   
   A   
   G   
   C   
   T   
   C   
   G   
   A   
   T   
   A   
   C   
   C   
   C   
   A   
   A   
   A   
   C   
   C   
   T   
   T   
   C   
   T   
    A    
   G   
   T   
   C   
   A   
   A   
   T   
   G   
   G   
   A   
   T   
   C   
   G   
   A   
   C   
   A   
   G   
   G   
   T   
   A   
   T   
   A   
   T   
   C   
   T   
   G   
   C   
   T   
   G   
   G   
   A   
   T   
   A   
   T   
   G   
   C   
    G    
   A   
   C   
   A   
   G   
   A   
   A   
   A   
   T   
   C   
   C   
   C   
   G   
   A   
   C   
    T    
   C   
   A   
   T   
   A   
   A   
   C   
   T   
   T   
   A   
   G   
   C   
   T   
   G   
   A   
   A   
   G   
   T   
   G   
   A   
   T   
    C    
   G   
   A   
    C    
   G   
   G   
   C   
   A   
   C   
   A   
   A   
   T   
   C   
   T   
   A   
   T   
   A   
   T   
   G   
   A   
   T   
   C   
   G   
   A   
   T   
   C   
   A   
   T   
   C   
   C   
   A   
   C   
   A   
   A   
   G   
   C   
   G   
   A   
   G   
   C   
   C   
   T   
   T   
   G   
   A   
   A   
   A   
   A   
   A   
   T   
   T   
   G   
   A   
   T   
   G   
   G   
   A   
   A   
   T   
   A   
   T   
   A   
   T   
   T   
   C   
   C   
   T   
   G   
   G   
   A   
   C   
   C   
   T   
   G   
   A   
   C   
   T   
   T   
    C    
   C   
   C   
   A   
   A   
   C   
   A   
   G   
   G   
   A   
   G   
   G   
    G    
   A   
   T   
   C   
   T   
   T   
   G   
   C   
   A   
   A   
   G   
   G   
    A    
   A   
   A   
   A   
   G   
   A   
   T   
   G   
   A   
   A   
   A   
   T   
   C   
   A   
   A   
   A   
   A   
   A   
   A   
   G   
   C   
   T   
   T   
   A   
   T   
   G   
   A   
   A   
   A   
   C   
   A   
   G   
   G   
    G    
   C   
   G   
   A   
   G   
   G   
   A   
   A   
   A   
   A   
   G   
   T   
   T   
   A   
    T    
   T   
   C   
   T   
   T   
   C   
   G   
   T   
   T   
   C   
   G   
   A   
   A   
   A   
   A   
   C   
   A   
   A   
   A   
   G   
   A   
   T   
   A   
   G   
   A   
    A    
   G   
   C   
   A   
   A   
   T   
   G   
   A   
   A   
   G   
   G   
   G   
    C    
   A   
   A   
    T    
   A   
   A   
   A   
   C   
   A   
   G   
   C   
   A   
   A   
   A   
   T   
   C   
   G   
   T   
   G   
   A   
   T   
   C   
   A   
   C   
    T    
   G   
   A   
   A   
   A   
   T   
   C   
   C   
   C   
   A   
   T   
   A   
   T   
   G   
   A   
   A   
   G   
   T   
   A   
   A   
   A   
   T   
   A   
   A   
   A   
   G   
   C   
   G   
   A   
   C   
    A    
   T   
   T   
   G   
   G   
   T   
   G   
   A   
   A   
   A   
   A   
   A   
   A   
   A   
   T   
   G   
   G   
   A   
   T   
   G   
   A   
   G   
   A   
   T   
   C   
   C   
   G   
   T   
   T   
   T   
   G   
   A   
   A   
   T   
   A   
   A   
   G   
   A   
   A   
   A   
   A   
   T   
   C   
   G   
   A   
    C    
   G   
   G   
   A   
   A   
   T   
   A   
   G   
   C   
   T   
   G   
   A   
   A   
   G   
   T   
    G    
   C   
   G   
   A   
   G   
   A   
   T   
   G   
   A   
   A   
   A   
   G   
   T   
   G   
   A   
   T   
    C    
   G   
    A    
   A   
   C   
    T    
   G   
   G   
   T   
   C   
   T   
   G   
   C   
   A   
   A   
   A   
   T   
   T   
   G   
   T   
   C   
   G   
   T   
    T    
   G   
   A   
   A   
   T   
   T   
   G   
   A   
   A   
   A   
   A   
   A   
   A   
   G   
   A   
    C    
   G   
   C   
   C   
   A   
   A   
    T    
   G   
   C   
   T   
   C   
   A   
   A   
   G   
   G   
    A    
   A   
   T   
   T   
   T   
   T   
   G   
   A   
   A   
   T   
   T   
   A   
    T    
   T   
   T   
   G   
   T   
   T   
   C   
   A   
   A   
   A   
   A   
   A   
   C   
   A   
   C   
   T   
   G   
   A   
   G   
    C    
   T   
   A   
   C   
   A   
   G   
   A   
   T   
   C   
   A   
   A   
   T   
   T   
   A   
   C   
   A   
   A   
   T   
   T   
   T   
    C    
   A   
   A   
   T   
   A   
   T   
   G   
   G   
   T   
   C   
   G   
   C   
   T   
   A   
   T   
   C   
   G   
   A   
    T    
   C   
   A   
   T   
   A   
   T   
   G   
   A   
   C   
   T   
   C   
   C   
   G   
   C   
   A   
    T    
   C   
   A   
   A   
   G   
   T   
   T   
   G   
   G   
   A   
   C   
   T   
   G   
   A   
   A   
   A   
   G   
   A   
   C   
   A   
   T   
   T   
   T   
   T   
   A   
   A   
   G   
   A   
   A   
   G   
   T   
   T   
   A   
   T   
   A   
   T   
   C   
   G   
   A   
   G   
   C   
   A   
   T   
   C   
   G   
   A   
   A   
   A   
   G   
   C   
   A   
   A   
   G   
   T   
   C   
   A   
   T   
   A   
   A   
   C   
   A   
   A   
   A   
   A   
   C   
   G   
    C    
   A   
   G   
    C    
   C   
   A   
   A   
   T   
   T   
   C   
   G   
   A   
   T   
   T   
   T   
   A   
   G   
   C   
   C   
   A   
   A   
   A   
   G   
   C   
   T   
   C   
   A   
   A   
   A   
   A   
   A   
    A    
   G   
   A   
   C   
   A   
   G   
   C   
   A   
   T   
   A   
   T   
   C   
   G   
   T   
    G    
   G   
   A   
   A   
   G   
   G   
    C    
    C    
   T   
   G   
   A   
   T   
   G   
   A   
   A   
   A   
   G   
   C   
   T   
   T   
   T   
   A   
   T   
   C   
   G   
   A   
   T   
   T   
   C   
   T   
   G   
   G   
   A   
   T   
   G   
   A   
   A   
   G   
   T   
   A   
   A   
   T   
   C   
   G   
    C    
   A   
   A   
   C   
   G   
   A   
   T   
   T   
   C   
   G   
   T   
   G   
   A   
   A   
   A   
   G   
   T   
   A   
   A   
   A   
   G   
   A   
   C   
   A   
   A   
   A   
   A   
   A   
   A   
   G   
   A   
    C    
   G   
   C   
    C    
   A   
   A   
   A   
   A   
   A   
   G   
   A   
   A   
   T   
   T   
   T   
   A   
   G   
   T   
   C   
   G   
   A   
    C    
   G   
   T   
    T    
   T   
   T   
    C    
   C   
   A   
   A   
   T   
   T   
   T   
   A   
   C   
   A   
   G   
   A   
   A   
   G   
   A   
   A   
   C   
   A   
   A   
   G   
   C   
   A   
   G   
   A   
   A   
   G   
   C   
   A   
   A   
   T   
    C    
   G   
   T   
   C   
   A   
   C   
   A   
   T   
   T   
   G   
   C   
   A   
   A   
    C    
   T   
   A   
   T   
   A   
    T    
   C   
   G   
   T   
   T   
   T   
   G   
   A   
   C   
   G   
   A   
   A   
    C    
   A   
   C   
    A    
   G   
   A   
   T   
   A   
   T   
   C   
   A   
   C   
   A   
   G   
   A   
   G   
   C   
   T   
   T   
   C   
   A   
   A   
   A   
   A   
   G   
   G   
   A   
   A   
    T    
   C   
    C    
   G   
   A   
   A   
   A   
    G    
   C   
   T   
   T   
   G   
   A   
   T   
   T   
   G   
   C   
    A    
   C   
   A   
   A   
   A   
   T   
   C   
   A   
   C   
   A   
   G   
   A   
   G   
   T   
   T   
    G    
   A   
   A   
   T   
   A   
   A   
   A   
   A   
   T   
   C   
   C   
   T   
   T   
   T   
   C   
   A   
   A   
   A   
    C    
   G   
   A   
   C   
   A   
   A   
   A   
   G   
   A   
    G    
   C   
   T   
   T   
   T   
   T   
    C    
   T   
   C   
   T   
   G   
   T   
   T   
   A   
   T   
   G   
   A   
   A   
   A   
   A   
   A   
   A   
   G   
   A   
   A   
   T   
   T   
   G   
   C   
   G   
   A   
   G   
   A   
   A   
   G   
   T   
   G   
   A   
   A   
   A   
   A   
   A   
   A   
   A   
   A   
   T   
   T   
   A   
    C    
   T   
   C   
   T   
   T   
   C   
   C   
   G   
   C   
   T   
   C   
   G   
   T   
   C   
    T    
    G    
   A   
   C   
   G   
   A   
   C   
   A   
   A   
   T   
   C   
    G    
   A   
   A   
   G   
   A   
   C   
   G   
   A   
   A   
   A   
   T   
   C   
   G   
   A   
   A   
   G   
   A   
   A   
   A   
   T   
   C   
   A   
   A   
   A   
   A   
   T   
    C    
   G   
   A   
   T   
   A   
   C   
   G   
   C   
   A   
   A   
    G    
   T   
    T    
   T   
   T   
   A   
   G   
   T   
   G   
   G   
   C   
    G    
   C   
   A   
   A   
   G   
   A   
   A   
   G   
   A   
   C   
   G   
   T   
   A   
   A   
   T   
    C    
   G   
   T   
   C   
   A   
   G   
   T   
   G   
   T   
    G    
   A   
   C   
    A    
   C   
   G   
   A   
   G   
   A   
   A   
   G   
   G   
   C   
   T   
   A   
   T   
   G   
   T   
   G   
   A   
   A   
   G   
   C   
   G   
   A   
   A   
   C   
   A   
   A   
   G   
   C   
   C   
   T   
   T   
   C   
   G   
   T   
   T   
   C   
    T    
   T   
   A   
   T   
   A   
   G   
   T   
   G   
   C   
   T   
   T   
   C   
    G    
   A   
   A   
   A   
   C   
   C   
   A   
   G   
   A   
   A   
   G   
   A   
   A   
   A   
   T   
   C   
   G   
   G   
   G   
   A   
   T   
   G   
   C   
   G   
   A   
   G   
   A   
   A   
   G   
   G   
   C   
   G   
   A   
    C    
   T   
   A   
   C   
   T   
   T   
   G   
   C   
   T   
    G    
   T   
   A   
   C   
    T    
   C   
   A   
   G   
   G   
   T   
   G   
   A   
   A   
   T   
   T   
    G    
   A   
   G   
    C    
   A   
   C   
   A   
    T    
   T   
   G   
   G   
   A   
   C   
   C   
   A   
   T   
   G   
   T   
   T   
   C   
   T   
   T   
   C   
   T   
   G   
   A   
   T   
    T    
   A   
   C   
    G    
   A   
   A   
   T   
   A   
   A   
   A   
   G   
   C   
   A   
   A   
   A   
   T   
   G   
   T   
   G   
   A   
   T   
   C   
   T   
   A   
   C   
   C   
   G   
    T    
   C   
   C   
   T   
   G   
   T   
    T    
   C   
   A   
   T   
   G   
   A   
   A   
   C   
   T   
   A   
   C   
   C   
    C    
   G   
   A   
   T   
   T   
   T   
   G   
   A   
   A   
   A   
   T   
   G   
   G   
   A   
   A   
   A   
   G   
   A   
   T   
   G   
   C   
   T   
   G   
   G   
    C    
   G   
   A   
    A    
   C   
   A   
    C    
   A   
   T   
   T   
   T   
   C   
   T   
   C   
   A   
   A   
   A   
   C   
   A   
   A   
   T   
   C   
   T   
   C   
   C   
   A   
   A   
   T   
   C   
   T   
   G   
   T   
   C   
   T   
   G   
   T   
   A   
   G   
   A   
    T    
   G   
   A   
   A   
   T   
   C   
   G   
   A   
   T   
    C    
   T   
   T   
   A   
   G   
   C   
   T   
   G   
   T   
   T   
   T   
   T   
    C    
   C   
   C   
   A   
   T   
   A   
   T   
   C   
   A   
   A   
   A   
   A   
   A   
   A   
   T   
    C    
   C   
   A   
   A   
   G   
   C   
   A   
   G   
   A   
    G    
   A   
   A   
   A   
   A   
   C   
   A   
   T   
   T   
   T   
   G   
   T   
    A    
   T   
   T   
   C   
   A   
   T   
   C   
   A   
   G   
   C   
   A   
   A   
   G   
   A   
   A   
   T   
   G   
   G   
   A   
   T   
   T   
    G    
    A    
   T   
   C   
   A   
   A   
   G   
   C   
   A   
   A   
   A   
   C   
   A   
   C   
   G   
   A   
   A   
   T   
   G   
   A   
   C   
   A   
   G   
   A   
   G   
   T   
   T   
   C   
   G   
   A   
   A   
   C   
   C   
   A   
   T   
   G   
   G   
   C   
   G   
   C   
   A   
   C   
   C   
   T   
   A   
    C    
   A   
   A   
   G   
   A   
   G   
   C   
   C   
   G   
    T    
   C   
   C   
   G   
   C   
   T   
   C   
   A   
   G   
   C   
   T   
   G   
   T   
   A   
   T   
   G   
   A   
   A   
   G   
   C   
   T   
   A   
   A   
   A   
   G   
   G   
   C   
   G   
   G   
   A   
   A   
   G   
   A   
   A   
   G   
   A   
   T   
   G   
   A   
   A   
   C   
   T   
    T    
   A   
   T   
   T   
   G   
   C   
   T   
   G   
   T   
    A    
   T   
   A   
   T   
   C   
   T   
   G   
   A   
   A   
   A   
   G   
   A   
   A   
   G   
   A   
   T   
   C   
    A    
   G   
   C   
   C   
   A   
   G   
   G   
   C   
   T   
   T   
   A   
   G   
   A   
   T   
   G   
   T   
    G    
   T   
   T   
   C   
   T   
   T   
   A   
   G   
   T   
   A   
   A   
   C   
   G   
    C    
   A   
   T   
   C   
   A   
   A   
   G   
   G   
   A   
   A   
   T   
   G   
   G   
   G   
    C    
   T   
   T   
   G   
   C   
   G   
   C   
   T   
    A    
   T   
   C   
   C   
   T   
   T   
   T   
   A   
   A   
    C    
   A   
   G   
   A   
   T   
   G   
   T   
   T   
   C   
   C   
   C   
   G   
   T   
   T   
   G   
   T   
   T   
   G   
   G   
   A   
   G   
   C   
   A   
   A   
   A   
   G   
   G   
   C   
   A   
   G   
    C    
   T   
   G   
   G   
   A   
   G   
   T   
   G   
   A   
   A   
   G   
   T   
   C   
   T   
   A   
   T   
   G   
   A   
   A   
   T   
   T   
   T   
   G   
   A   
   A   
   A   
   G   
   A   
   T   
   G   
   G   
   A   
   G   
   A   
   T   
   A   
   G   
   G   
    G    
   T   
   T   
    G    
   T   
   C   
   A   
   A   
    T    
   G   
   G   
    A    
   C   
   T   
    G    
   C   
   T   
   T   
   G   
   T   
   G   
   T   
   T   
   A   
   G   
   C   
   A   
   G   
   A   
    C    
   G   
   G   
    A    
   G   
   A   
   T   
   A   
   C   
   G   
   C   
   C   
    G    
   A   
   T   
   C   
   G   
   T   
   A   
   A   
   T   
   C   
   G   
   T   
   T   
   A   
   C   
   T   
   C   
   A   
   G   
   C   
   G   
   A   
   G   
   G   
   A   
   G   
   C   
    T    
   G   
   T   
    G    
   A   
   A   
   A   
   C   
   G   
   C   
   A   
   T   
   G   
   C   
   T   
   T   
   G   
   C   
   T   
   C   
   A   
   A   
   G   
   A   
   A   
   A   
   T   
   C   
   A   
   G   
   C   
   C   
   A   
   A   
   A   
   C   
    T    
   T   
   C   
   A   
   C   
   G   
   T   
   G   
   C   
   G   
   A   
   A   
   G   
   C   
   G   
   G   
   G   
   G   
   A   
   G   
   T   
   G   
   A   
   C   
   G   
   G   
   T   
   C   
   A   
   T   
   G   
   C   
   G   
   T   
   G   
   A   
   G   
   C   
   T   
   G   
   A   
   A   
   A   
   A   
   A   
   A   
   A   
   A   
   T   
   C   
   C   
    G    
   C   
   A   
    C    
   C   
   G   
    C    
   A   
   T   
   T   
   A   
   T   
   C   
   T   
   A   
   T   
   A   
   T   
   G   
   T   
   C   
   G   
   G   
   A   
   A   
   G   
   G   
   A   
   A   
   G   
   A   
   T   
   C   
   A   
   A   
   A   
   A   
   G   
   A   
   A   
   A   
   T   
   A   
   A   
   C   
   C   
   T   
   T   
   G   
   A   
   T   
   G   
   A   
   A   
   T   
   C   
   A   
   A   
   A   
   A   
   A   
   G   
   G   
    A    
   C   
   A   
    A    
   C   
   A   
   G   
    A    
   T   
   A   
   A   
   C   
   G   
   A   
   T   
   T   
   G   
   A   
    T    
   C   
   C   
   A   
   A   
   C   
    G    
   G   
   A   
   T   
   T   
   T   
    T    
   C   
   C   
   T   
   A   
   T   
   A   
   G   
   G   
   T   
   G   
   A   
   T   
   C   
   G   
   A   
   A   
   C   
   A   
   T   
   C   
   G   
   A   
   A   
   T   
   G   
   G   
    T    
   T   
   C   
    G    
   T   
   T   
   T   
   G   
   C   
    T    
   A   
   T   
   G   
   G   
   A   
    C    
   G   
   A   
   G   
   A   
   A   
   A   
   A   
   A   
   A   
   G   
   G   
   T   
   G   
   G   
   A   
   G   
   A   
   A   
   G   
   T   
   C   
   A   
   T   
   C   
   G   
   A   
   A   
   G   
   T   
    T    
   A   
    T    
   C   
   G   
   A   
   T   
   G   
   C   
   T   
   C   
   C   
   T   
   G   
   A   
   G   
    A    
   T   
   T   
   T   
   T   
    C    
   A   
   T   
    C    
   G   
   A   
    A    
    T    
   C   
    A    
   -   
   -   
   -   
 

 
   N54155   
   A   
   T   
   G   
   G   
   A   
   A   
   A   
   A   
   T   
   C   
   G   
   A   
   C   
   A   
   A   
   G   
   A   
   A   
   G   
   T   
   A   
   C   
   A   
   A   
   G   
   A   
   A   
   T   
   T   
   A   
   A   
   C   
   T   
   T   
   T   
   A   
   G   
   A   
   A   
   G   
   A   
   A   
   G   
   T   
   G   
   A   
   T   
   G   
   G   
   G   
   T   
   G   
   A   
   C   
   C   
   G   
   C   
   T   
   T   
   C   
   G   
   G   
   A   
   C   
   G   
   T   
   T   
   A   
   T   
   T   
   C   
   C   
   A   
   A   
   A   
   T   
   A   
   T   
   A   
   T   
   T   
   A   
   T   
    C    
   C   
   A   
    A    
   G   
   A   
   A   
   C   
   G   
   T   
   G   
   C   
   A   
   C   
   T   
   G   
   C   
   C   
   T   
   G   
   A   
   T   
   A   
   T   
    C    
   C   
   G   
   T   
   G   
   A   
   T   
   G   
   G   
   A   
   T   
   T   
   G   
   A   
   A   
    G    
   C   
   C   
   A   
   G   
   T   
    G    
   C   
   A   
   A   
   C   
   G   
   C   
   C   
   G   
   G   
   A   
   T   
   A   
   T   
   T   
   A   
   T   
   T   
    T    
   G   
   C   
    C    
   A   
   T   
   G   
   A   
   A   
   C   
   A   
   A   
   A   
   G   
   A   
   T   
   G   
   G   
   C   
   A   
   A   
   T   
   A   
   C   
   G   
   T   
   A   
   T   
   G   
   A   
   C   
   A   
   A   
   A   
   G   
   G   
   A   
   T   
   T   
    C    
   C   
   G   
   T   
   A   
   A   
   A   
   T   
   C   
   A   
   G   
   C   
   A   
   A   
   A   
   A   
   T   
   C   
   T   
   G   
   T   
   C   
   G   
   G   
   A   
   A   
   A   
   T   
   A   
   T   
   C   
   A   
   T   
   G   
   G   
   G   
    G    
   A   
   A   
   T   
   T   
   A   
   T   
   C   
   A   
   T   
   C   
   C   
   T   
   C   
   A   
   T   
   G   
   G   
   T   
   G   
   A   
   C   
   A   
   G   
   C   
   A   
   G   
   T   
   A   
   T   
   T   
   T   
   A   
   T   
   G   
   A   
   A   
   G   
   C   
   G   
   A   
   T   
   G   
   G   
   T   
   C   
   C   
   G   
   C   
   A   
   T   
   G   
   A   
   G   
   C   
   C   
   A   
   A   
   G   
   A   
    C    
   T   
   G   
   G   
   A   
   A   
   G   
   T   
   T   
   G   
   C   
   G   
   G   
   G   
   A   
   A   
   G   
   T   
   A   
   C   
   T   
   G   
   A   
   T   
   C   
   G   
   A   
    A    
   A   
   T   
   G   
   C   
   A   
   C   
   G   
   G   
    G    
   A   
   A   
   T   
   A   
   A   
    C    
   G   
   G   
   G   
   A   
   G   
   T   
   A   
   T   
   G   
   G   
   A   
   C   
   G   
   G   
   T   
   G   
   A   
   T   
   C   
   C   
   A   
   C   
   C   
   T   
   G   
   C   
   T   
   G   
   C   
   G   
   A   
   T   
   G   
   C   
   G   
   T   
   T   
   A   
   T   
   A   
   C   
   T   
   G   
   A   
   A   
   G   
   C   
   C   
   A   
   G   
    A    
   T   
   T   
   A   
   T   
   C   
   C   
   A   
   A   
   G   
   T   
   T   
   A   
   A   
   G   
   T   
   G   
   G   
   A   
   G   
   A   
   G   
   A   
   T   
   G   
    C    
   T   
   A   
    G    
   C   
   A   
   G   
   A   
   T   
   A   
   T   
   C   
   G   
   A   
    A    
   A   
   A   
   A   
   G   
   A   
   A   
   A   
   C   
   A   
   G   
   T   
    C    
   G   
   A   
   T   
   C   
   T   
   C   
   G   
   T   
   G   
   T   
   G   
   G   
   A   
   A   
   T   
   T   
   T   
   T   
   G   
   A   
   T   
   G   
   A   
   T   
   A   
   C   
   A   
   G   
   A   
   A   
   A   
   A   
   A   
   G   
   A   
   A   
   C   
   C   
   A   
   A   
   C   
   A   
   G   
   T   
   C   
   C   
   T   
   T   
   C   
   C   
   A   
   G   
   C   
   T   
   C   
   G   
   A   
   T   
   A   
   C   
   C   
   C   
   A   
   A   
   A   
   C   
   C   
   T   
   T   
   C   
   T   
    C    
   G   
   T   
   C   
   A   
   A   
   T   
   G   
   G   
   A   
   T   
   C   
   G   
   A   
   C   
   A   
   G   
   G   
   T   
   A   
   T   
   A   
   T   
   C   
   T   
   G   
   C   
   T   
   G   
   G   
   A   
   T   
   A   
   T   
   G   
   C   
    A    
   A   
   C   
   A   
   G   
   A   
   A   
   A   
   T   
   C   
   C   
   C   
   G   
   A   
   C   
    T    
   C   
   A   
   T   
   A   
   A   
   C   
   T   
   T   
   A   
   G   
   C   
   T   
   G   
   A   
   A   
   G   
   T   
   G   
   A   
   T   
    T    
   G   
   A   
    T    
   G   
   G   
   C   
   A   
   C   
   A   
   A   
   T   
   C   
   T   
   A   
   T   
   A   
   T   
   G   
   A   
   T   
   C   
   G   
   A   
   T   
   C   
   A   
   T   
   C   
   C   
   A   
   C   
   A   
   A   
   G   
   C   
   G   
   A   
   G   
   C   
   C   
   T   
   T   
   G   
   A   
   A   
   A   
   A   
   A   
   T   
   T   
   G   
   A   
   T   
   G   
   G   
   A   
   A   
   T   
   A   
   T   
   A   
   T   
   T   
   C   
   C   
   T   
   G   
   G   
   A   
   C   
   C   
   T   
   G   
   A   
   C   
   T   
   T   
    T    
   C   
   C   
   A   
   A   
   C   
   A   
   G   
   G   
   A   
   G   
   G   
    G    
   A   
   T   
   C   
   T   
   T   
   G   
   C   
   A   
   A   
   G   
   G   
    A    
   A   
   A   
   A   
   G   
   A   
   T   
   G   
   A   
   A   
   A   
   T   
   C   
   A   
   A   
   A   
   A   
   A   
   A   
   G   
   C   
   T   
   T   
   A   
   T   
   G   
   A   
   A   
   A   
   C   
   A   
   G   
   G   
    G    
   C   
   G   
   A   
   G   
   G   
   A   
   A   
   A   
   A   
   G   
   T   
   T   
   A   
    T    
   T   
   C   
   T   
   T   
   C   
   G   
   T   
   T   
   C   
   G   
   A   
   A   
   A   
   A   
   C   
   A   
   A   
   A   
   G   
   A   
   T   
   A   
   G   
   A   
    G    
   G   
   C   
   A   
   A   
   T   
   G   
   A   
   A   
   G   
   G   
   G   
    T    
   A   
   A   
    T    
   A   
   A   
   A   
   C   
   A   
   G   
   C   
   A   
   A   
   A   
   T   
   C   
   G   
   T   
   G   
   A   
   T   
   C   
   A   
   C   
    A    
   G   
   A   
   A   
   A   
   T   
   C   
   C   
   C   
   A   
   T   
   A   
   T   
   G   
   A   
   A   
   G   
   T   
   A   
   A   
   A   
   T   
   A   
   A   
   A   
   G   
   C   
   G   
   A   
   C   
    C    
   T   
   T   
   G   
   G   
   T   
   G   
   A   
   A   
   A   
   A   
   A   
   A   
   A   
   T   
   G   
   G   
   A   
   T   
   G   
   A   
   G   
   A   
   T   
   C   
   C   
   G   
   T   
   T   
   T   
   G   
   A   
   A   
   T   
   A   
   A   
   G   
   A   
   A   
   A   
   A   
   T   
   C   
   G   
   A   
    T    
   G   
   G   
   A   
   A   
   T   
   A   
   G   
   C   
   T   
   G   
   A   
   A   
   G   
   T   
    A    
   C   
   G   
   A   
   G   
   A   
   T   
   G   
   A   
   A   
   A   
   G   
   T   
   G   
   A   
   T   
    A    
   G   
    A    
   A   
   C   
    A    
   G   
   G   
   T   
   C   
   T   
   G   
   C   
   A   
   A   
   A   
   T   
   T   
   G   
   T   
   C   
   G   
   T   
    C    
   G   
   A   
   A   
   T   
   T   
   G   
   A   
   A   
   A   
   A   
   A   
   A   
   G   
   A   
    C    
   G   
   C   
   C   
   A   
   A   
    T    
   G   
   C   
   T   
   C   
   A   
   A   
   G   
   G   
    A    
   A   
   T   
   T   
   T   
   T   
   G   
   A   
   A   
   T   
   T   
   A   
    T    
   T   
   T   
   G   
   T   
   T   
   C   
   A   
   A   
   A   
   A   
   A   
   C   
   A   
   C   
   T   
   G   
   A   
   G   
    C    
   T   
   A   
   C   
   A   
   G   
   A   
   T   
   C   
   A   
   A   
   T   
   T   
   A   
   C   
   A   
   A   
   T   
   T   
   T   
    C    
   A   
   A   
   T   
   A   
   T   
   G   
   G   
   T   
   C   
   G   
   C   
   T   
   A   
   T   
   C   
   G   
   A   
    C    
   C   
   A   
   T   
   A   
   T   
   G   
   A   
   C   
   T   
   C   
   C   
   G   
   C   
   A   
    T    
   C   
   A   
   A   
   G   
   T   
   T   
   G   
   G   
   A   
   C   
   T   
   G   
   A   
   A   
   A   
   G   
   A   
   C   
   A   
   T   
   T   
   T   
   T   
   A   
   A   
   G   
   A   
   A   
   G   
   T   
   T   
   A   
   T   
   A   
   T   
   C   
   G   
   A   
   G   
   C   
   A   
   T   
   C   
   G   
   A   
   A   
   A   
   G   
   C   
   A   
   A   
   G   
   T   
   C   
   A   
   T   
   A   
   A   
   C   
   A   
   A   
   A   
   A   
   C   
   G   
    T    
   A   
   G   
    T    
   C   
   A   
   A   
   T   
   T   
   C   
   G   
   A   
   T   
   T   
   T   
   A   
   G   
   C   
   C   
   A   
   A   
   A   
   G   
   C   
   T   
   C   
   A   
   A   
   A   
   A   
   A   
    A    
   G   
   A   
   C   
   A   
   G   
   C   
   A   
   T   
   A   
   T   
   C   
   G   
   T   
    A    
   G   
   A   
   A   
   G   
   G   
    A    
    T    
   T   
   G   
   A   
   T   
   G   
   A   
   A   
   A   
   G   
   C   
   T   
   T   
   T   
   A   
   T   
   C   
   G   
   A   
   T   
   T   
   C   
   T   
   G   
   G   
   A   
   T   
   G   
   A   
   A   
   G   
   T   
   A   
   A   
   T   
   C   
   G   
    T    
   A   
   A   
   C   
   G   
   A   
   T   
   T   
   C   
   G   
   T   
   G   
   A   
   A   
   A   
   G   
   T   
   A   
   A   
   A   
   G   
   A   
   C   
   A   
   A   
   A   
   A   
   A   
   A   
   G   
   A   
    C    
   G   
   C   
    C    
   A   
   A   
   A   
   A   
   A   
   G   
   A   
   A   
   T   
   T   
   T   
   A   
   G   
   T   
   C   
   G   
   A   
    C    
   G   
   T   
    C    
   T   
   T   
    T    
   C   
   A   
   A   
   T   
   T   
   T   
   A   
   C   
   A   
   G   
   A   
   A   
   G   
   A   
   A   
   C   
   A   
   A   
   G   
   C   
   A   
   G   
   A   
   A   
   G   
   C   
   A   
   A   
   T   
    T    
   G   
   T   
   C   
   A   
   C   
   A   
   T   
   T   
   G   
   C   
   A   
   A   
    T    
   T   
   A   
   T   
   A   
    C    
   C   
   G   
   T   
   T   
   T   
   G   
   A   
   C   
   G   
   A   
   A   
    C    
   A   
   C   
    G    
   G   
   A   
   T   
   A   
   T   
   C   
   A   
   C   
   A   
   G   
   A   
   G   
   C   
   T   
   T   
   C   
   A   
   A   
   A   
   A   
   G   
   G   
   A   
   A   
    T    
   C   
    C    
   G   
   A   
   A   
   A   
    G    
   C   
   T   
   T   
   G   
   A   
   T   
   T   
   G   
   C   
    G    
   C   
   A   
   A   
   A   
   T   
   C   
   A   
   C   
   A   
   G   
   A   
   G   
   T   
   T   
    G    
   A   
   A   
   T   
   A   
   A   
   A   
   A   
   T   
   C   
   C   
   T   
   T   
   T   
   C   
   A   
   A   
   A   
    T    
   G   
   A   
   C   
   A   
   A   
   A   
   G   
   A   
    G    
   C   
   T   
   T   
   T   
   T   
    C    
   T   
   C   
   T   
   G   
   T   
   T   
   A   
   T   
   G   
   A   
   A   
   A   
   A   
   A   
   A   
   G   
   A   
   A   
   T   
   T   
   G   
   C   
   G   
   A   
   G   
   A   
   A   
   G   
   T   
   G   
   A   
   A   
   A   
   A   
   A   
   A   
   A   
   A   
   T   
   T   
   A   
    T    
   T   
   C   
   T   
   T   
   C   
   C   
   G   
   C   
   T   
   C   
   G   
   T   
   C   
    T    
    A    
   A   
   C   
   G   
   A   
   C   
   A   
   A   
   T   
   C   
    G    
   A   
   A   
   G   
   A   
   C   
   G   
   A   
   A   
   A   
   T   
   C   
   G   
   A   
   A   
   G   
   A   
   A   
   A   
   T   
   C   
   A   
   A   
   A   
   A   
   T   
    T    
   G   
   A   
   T   
   A   
   C   
   G   
   C   
   A   
   A   
    G    
   T   
    C    
   T   
   T   
   A   
   G   
   T   
   G   
   G   
   C   
    A    
   C   
   A   
   A   
   G   
   A   
   A   
   G   
   A   
   C   
   G   
   T   
   A   
   A   
   T   
    T    
   G   
   T   
   C   
   A   
   G   
   T   
   G   
   T   
    G    
   A   
   C   
    G    
   C   
   G   
   A   
   G   
   A   
   A   
   G   
   G   
   C   
   T   
   A   
   T   
   G   
   T   
   G   
   A   
   A   
   G   
   C   
   G   
   A   
   A   
   C   
   A   
   A   
   G   
   C   
   C   
   T   
   T   
   C   
   G   
   T   
   T   
   C   
    T    
   T   
   A   
   T   
   A   
   G   
   T   
   G   
   C   
   T   
   T   
   C   
    C    
   A   
   A   
   A   
   C   
   C   
   A   
   G   
   A   
   A   
   G   
   A   
   A   
   A   
   T   
   C   
   G   
   G   
   G   
   A   
   T   
   G   
   C   
   G   
   A   
   G   
   A   
   A   
   G   
   G   
   C   
   G   
   A   
    C    
   T   
   A   
   C   
   T   
   T   
   G   
   C   
   T   
    A    
   T   
   A   
   C   
    T    
   C   
   A   
   G   
   G   
   T   
   G   
   A   
   A   
   T   
   T   
    G    
   A   
   G   
    T    
   A   
   C   
   A   
    T    
   T   
   G   
   G   
   A   
   C   
   C   
   A   
   T   
   G   
   T   
   T   
   C   
   T   
   T   
   C   
   T   
   G   
   A   
   T   
    C    
   A   
   C   
    A    
   A   
   A   
   T   
   A   
   A   
   A   
   G   
   C   
   A   
   A   
   A   
   T   
   G   
   T   
   G   
   A   
   T   
   C   
   T   
   A   
   C   
   C   
   G   
    C    
   C   
   C   
   T   
   G   
   T   
    C    
   C   
   A   
   T   
   G   
   A   
   A   
   C   
   T   
   A   
   C   
   C   
    C    
   G   
   A   
   T   
   T   
   T   
   G   
   A   
   A   
   A   
   T   
   G   
   G   
   A   
   A   
   A   
   G   
   A   
   T   
   G   
   C   
   T   
   G   
   G   
    T    
   G   
   A   
    G    
   C   
   A   
    C    
   A   
   T   
   T   
   T   
   C   
   T   
   C   
   A   
   A   
   A   
   C   
   A   
   A   
   T   
   C   
   T   
   C   
   C   
   A   
   A   
   T   
   C   
   T   
   G   
   T   
   C   
   T   
   G   
   T   
   A   
   G   
   A   
    C    
   G   
   A   
   A   
   T   
   C   
   G   
   A   
   T   
    T    
   T   
   T   
   A   
   G   
   C   
   T   
   G   
   T   
   T   
   T   
   T   
    C    
   C   
   C   
   A   
   T   
   A   
   T   
   C   
   A   
   A   
   A   
   A   
   A   
   A   
   T   
    T    
   C   
   A   
   A   
   G   
   C   
   A   
   G   
   A   
    G    
   A   
   A   
   A   
   A   
   C   
   A   
   T   
   T   
   T   
   G   
   T   
    A    
   T   
   T   
   C   
   A   
   T   
   C   
   A   
   G   
   C   
   A   
   A   
   G   
   A   
   A   
   T   
   G   
   G   
   A   
   T   
   T   
    G    
    A    
   T   
   C   
   A   
   A   
   G   
   C   
   A   
   A   
   A   
   C   
   A   
   C   
   G   
   A   
   A   
   T   
   G   
   A   
   C   
   A   
   G   
   A   
   G   
   T   
   T   
   C   
   G   
   A   
   A   
   C   
   C   
   A   
   T   
   G   
   G   
   C   
   G   
   C   
   A   
   C   
   C   
   T   
   A   
    T    
   A   
   A   
   G   
   A   
   G   
   C   
   C   
   G   
    T    
   C   
   C   
   G   
   C   
   T   
   C   
   A   
   G   
   C   
   T   
   G   
   T   
   A   
   T   
   G   
   A   
   A   
   G   
   C   
   T   
   A   
   A   
   A   
   G   
   G   
   C   
   G   
   G   
   A   
   A   
   G   
   A   
   A   
   G   
   A   
   T   
   G   
   A   
   A   
   C   
   T   
    G    
   A   
   T   
   T   
   G   
   C   
   T   
   G   
   T   
    G    
   T   
   A   
   T   
   C   
   T   
   G   
   A   
   A   
   A   
   G   
   A   
   A   
   G   
   A   
   T   
   C   
    A    
   G   
   C   
   C   
   A   
   G   
   G   
   C   
   T   
   T   
   A   
   G   
   A   
   T   
   G   
   T   
    T    
   T   
   T   
   C   
   T   
   T   
   A   
   G   
   T   
   A   
   A   
   C   
   G   
    C    
   A   
   T   
   C   
   A   
   A   
   G   
   G   
   A   
   A   
   T   
   G   
   G   
   G   
    T    
   T   
   T   
   G   
   C   
   G   
   C   
   T   
    A    
   T   
   C   
   C   
   T   
   T   
   T   
   A   
   A   
    C    
   A   
   G   
   A   
   T   
   G   
   T   
   T   
   C   
   C   
   C   
   G   
   T   
   T   
   G   
   T   
   T   
   G   
   G   
   A   
   G   
   C   
   A   
   A   
   A   
   G   
   G   
   C   
   A   
   G   
    C    
   T   
   G   
   G   
   A   
   G   
   T   
   G   
   A   
   A   
   G   
   T   
   C   
   T   
   A   
   T   
   G   
   A   
   A   
   T   
   T   
   T   
   G   
   A   
   A   
   A   
   G   
   A   
   T   
   G   
   G   
   A   
   G   
   A   
   T   
   A   
   G   
   G   
    A    
   T   
   T   
    A    
   T   
   C   
   A   
   A   
    C    
   G   
   G   
    G    
   C   
   T   
    G    
   C   
   T   
   T   
   G   
   T   
   G   
   T   
   T   
   A   
   G   
   C   
   A   
   G   
   A   
    A    
   G   
   G   
    T    
   G   
   A   
   T   
   A   
   C   
   G   
   C   
   C   
    G    
   A   
   T   
   C   
   G   
   T   
   A   
   A   
   T   
   C   
   G   
   T   
   T   
   A   
   C   
   T   
   C   
   A   
   G   
   C   
   G   
   A   
   G   
   G   
   A   
   G   
   C   
    T    
   G   
   T   
    T    
   A   
   A   
   A   
   C   
   G   
   C   
   A   
   T   
   G   
   C   
   T   
   T   
   G   
   C   
   T   
   C   
   A   
   A   
   G   
   A   
   A   
   A   
   T   
   C   
   A   
   G   
   C   
   C   
   A   
   A   
   A   
   C   
    T    
   T   
   C   
   A   
   C   
   G   
   T   
   G   
   C   
   G   
   A   
   A   
   G   
   C   
   G   
   G   
   G   
   G   
   A   
   G   
   T   
   G   
   A   
   C   
   G   
   G   
   T   
   C   
   A   
   T   
   G   
   C   
   G   
   T   
   G   
   A   
   G   
   C   
   T   
   G   
   A   
   A   
   A   
   A   
   A   
   A   
   A   
   A   
   T   
   C   
   C   
    G    
   C   
   A   
    C    
   C   
   G   
    T    
   A   
   T   
   T   
   A   
   T   
   C   
   T   
   A   
   T   
   A   
   T   
   G   
   T   
   C   
   G   
   G   
   A   
   A   
   G   
   G   
   A   
   A   
   G   
   A   
   T   
   C   
   A   
   A   
   A   
   A   
   G   
   A   
   A   
   A   
   T   
   A   
   A   
   C   
   C   
   T   
   T   
   G   
   A   
   T   
   G   
   A   
   A   
   T   
   C   
   A   
   A   
   A   
   A   
   A   
   G   
   G   
    G    
   C   
   A   
    G    
   C   
   A   
   G   
    T    
   T   
   A   
   A   
   C   
   G   
   A   
   T   
   T   
   G   
   A   
    C    
   C   
   C   
   A   
   A   
   C   
    T    
   G   
   A   
   T   
   T   
   T   
    C    
   C   
   C   
   T   
   A   
   T   
   A   
   G   
   G   
   T   
   G   
   A   
   T   
   C   
   G   
   A   
   A   
   C   
   A   
   T   
   C   
   G   
   A   
   A   
   T   
   G   
   G   
    A    
   T   
   C   
    A    
   T   
   T   
   T   
   G   
   C   
    G    
   A   
   T   
   G   
   G   
   A   
    T    
   G   
   A   
   G   
   A   
   A   
   A   
   A   
   A   
   A   
   G   
   G   
   T   
   G   
   G   
   A   
   G   
   A   
   A   
   G   
   T   
   C   
   A   
   T   
   C   
   G   
   A   
   A   
   G   
   T   
    T    
   A   
    T    
   C   
   G   
   A   
   T   
   G   
   C   
   T   
   C   
   C   
   T   
   G   
   A   
   G   
    A    
   T   
   T   
   T   
   T   
    C    
   A   
   T   
    C    
   G   
   A   
    G    
    T    
   C   
    G    
   -   
   -   
   -   
 

 
   N55337   
   A   
   T   
   G   
   G   
   A   
   A   
   A   
   A   
   T   
   C   
   G   
   A   
   C   
   A   
   A   
   G   
   A   
   A   
   G   
   T   
   A   
   C   
   A   
   A   
   G   
   A   
   A   
   T   
   T   
   A   
   A   
   C   
   T   
   T   
   T   
   A   
   G   
   A   
   A   
   G   
   A   
   A   
   G   
   T   
   G   
   A   
   T   
   G   
   G   
   G   
   T   
   G   
   A   
   C   
   C   
   G   
   C   
   T   
   T   
   C   
   G   
   G   
   A   
   C   
   G   
   T   
   T   
   A   
   T   
   T   
   C   
   C   
   A   
   A   
   A   
   T   
   A   
   T   
   A   
   T   
   T   
   A   
   T   
    C    
   C   
   A   
    A    
   G   
   A   
   A   
   C   
   G   
   T   
   G   
   C   
   A   
   C   
   T   
   G   
   C   
   C   
   T   
   G   
   A   
   T   
   A   
   T   
    C    
   C   
   G   
   T   
   G   
   A   
   T   
   G   
   G   
   A   
   T   
   T   
   G   
   A   
   A   
    G    
   C   
   C   
   A   
   G   
   T   
    G    
   C   
   A   
   A   
   C   
   G   
   C   
   C   
   G   
   G   
   A   
   T   
   A   
   T   
   T   
   A   
   T   
   T   
    T    
   G   
   C   
    C    
   A   
   T   
   G   
   A   
   A   
   C   
   A   
   A   
   A   
   G   
   A   
   T   
   G   
   G   
   C   
   A   
   A   
   T   
   A   
   C   
   G   
   T   
   A   
   T   
   G   
   A   
   C   
   A   
   A   
   A   
   G   
   G   
   A   
   T   
   T   
    C    
   C   
   G   
   T   
   A   
   A   
   A   
   T   
   C   
   A   
   G   
   C   
   A   
   A   
   A   
   A   
   T   
   C   
   T   
   G   
   T   
   C   
   G   
   G   
   A   
   A   
   A   
   T   
   A   
   T   
   C   
   A   
   T   
   G   
   G   
   G   
    G    
   A   
   A   
   T   
   T   
   A   
   T   
   C   
   A   
   T   
   C   
   C   
   T   
   C   
   A   
   T   
   G   
   G   
   T   
   G   
   A   
   C   
   A   
   G   
   C   
   A   
   G   
   T   
   A   
   T   
   T   
   T   
   A   
   T   
   G   
   A   
   A   
   G   
   C   
   G   
   A   
   T   
   G   
   G   
   T   
   C   
   C   
   G   
   C   
   A   
   T   
   G   
   A   
   G   
   C   
   C   
   A   
   A   
   G   
   A   
    C    
   T   
   G   
   G   
   A   
   A   
   G   
   T   
   T   
   G   
   C   
   G   
   G   
   G   
   A   
   A   
   G   
   T   
   A   
   C   
   T   
   G   
   A   
   T   
   C   
   G   
   A   
    A    
   A   
   T   
   G   
   C   
   A   
   C   
   G   
   G   
    G    
   A   
   A   
   T   
   A   
   A   
    C    
   G   
   G   
   G   
   A   
   G   
   T   
   A   
   T   
   G   
   G   
   A   
   C   
   G   
   G   
   T   
   G   
   A   
   T   
   C   
   C   
   A   
   C   
   C   
   T   
   G   
   C   
   T   
   G   
   C   
   G   
   A   
   T   
   G   
   C   
   G   
   T   
   T   
   A   
   T   
   A   
   C   
   T   
   G   
   A   
   A   
   G   
   C   
   C   
   A   
   G   
    A    
   T   
   T   
   A   
   T   
   C   
   C   
   A   
   A   
   G   
   T   
   T   
   A   
   A   
   G   
   T   
   G   
   G   
   A   
   G   
   A   
   G   
   A   
   T   
   G   
    C    
   T   
   A   
    G    
   C   
   A   
   G   
   A   
   T   
   A   
   T   
   C   
   G   
   A   
    A    
   A   
   A   
   A   
   G   
   A   
   A   
   A   
   C   
   A   
   G   
   T   
    C    
   G   
   A   
   T   
   C   
   T   
   C   
   G   
   T   
   G   
   T   
   G   
   G   
   A   
   A   
   T   
   T   
   T   
   T   
   G   
   A   
   T   
   G   
   A   
   T   
   A   
   C   
   A   
   G   
   A   
   A   
   A   
   A   
   A   
   G   
   A   
   A   
   C   
   C   
   A   
   A   
   C   
   A   
   G   
   T   
   C   
   C   
   T   
   T   
   C   
   C   
   A   
   G   
   C   
   T   
   C   
   G   
   A   
   T   
   A   
   C   
   C   
   C   
   A   
   A   
   A   
   C   
   C   
   T   
   T   
   C   
   T   
    C    
   G   
   T   
   C   
   A   
   A   
   T   
   G   
   G   
   A   
   T   
   C   
   G   
   A   
   C   
   A   
   G   
   G   
   T   
   A   
   T   
   A   
   T   
   C   
   T   
   G   
   C   
   T   
   G   
   G   
   A   
   T   
   A   
   T   
   G   
   C   
    A    
   A   
   C   
   A   
   G   
   A   
   A   
   A   
   T   
   C   
   C   
   C   
   G   
   A   
   C   
    T    
   C   
   A   
   T   
   A   
   A   
   C   
   T   
   T   
   A   
   G   
   C   
   T   
   G   
   A   
   A   
   G   
   T   
   G   
   A   
   T   
    T    
   G   
   A   
    T    
   G   
   G   
   C   
   A   
   C   
   A   
   A   
   T   
   C   
   T   
   A   
   T   
   A   
   T   
   G   
   A   
   T   
   C   
   G   
   A   
   T   
   C   
   A   
   T   
   C   
   C   
   A   
   C   
   A   
   A   
   G   
   C   
   G   
   A   
   G   
   C   
   C   
   T   
   T   
   G   
   A   
   A   
   A   
   A   
   A   
   T   
   T   
   G   
   A   
   T   
   G   
   G   
   A   
   A   
   T   
   A   
   T   
   A   
   T   
   T   
   C   
   C   
   T   
   G   
   G   
   A   
   C   
   C   
   T   
   G   
   A   
   C   
   T   
   T   
    T    
   C   
   C   
   A   
   A   
   C   
   A   
   G   
   G   
   A   
   G   
   G   
    G    
   A   
   T   
   C   
   T   
   T   
   G   
   C   
   A   
   A   
   G   
   G   
    A    
   A   
   A   
   A   
   G   
   A   
   T   
   G   
   A   
   A   
   A   
   T   
   C   
   A   
   A   
   A   
   A   
   A   
   A   
   G   
   C   
   T   
   T   
   A   
   T   
   G   
   A   
   A   
   A   
   C   
   A   
   G   
   G   
    G    
   C   
   G   
   A   
   G   
   G   
   A   
   A   
   A   
   A   
   G   
   T   
   T   
   A   
    T    
   T   
   C   
   T   
   T   
   C   
   G   
   T   
   T   
   C   
   G   
   A   
   A   
   A   
   A   
   C   
   A   
   A   
   A   
   G   
   A   
   T   
   A   
   G   
   A   
    G    
   G   
   C   
   A   
   A   
   T   
   G   
   A   
   A   
   G   
   G   
   G   
    T    
   A   
   A   
    T    
   A   
   A   
   A   
   C   
   A   
   G   
   C   
   A   
   A   
   A   
   T   
   C   
   G   
   T   
   G   
   A   
   T   
   C   
   A   
   C   
    A    
   G   
   A   
   A   
   A   
   T   
   C   
   C   
   C   
   A   
   T   
   A   
   T   
   G   
   A   
   A   
   G   
   T   
   A   
   A   
   A   
   T   
   A   
   A   
   A   
   G   
   C   
   G   
   A   
   C   
    C    
   T   
   T   
   G   
   G   
   T   
   G   
   A   
   A   
   A   
   A   
   A   
   A   
   A   
   T   
   G   
   G   
   A   
   T   
   G   
   A   
   G   
   A   
   T   
   C   
   C   
   G   
   T   
   T   
   T   
   G   
   A   
   A   
   T   
   A   
   A   
   G   
   A   
   A   
   A   
   A   
   T   
   C   
   G   
   A   
    T    
   G   
   G   
   A   
   A   
   T   
   A   
   G   
   C   
   T   
   G   
   A   
   A   
   G   
   T   
    A    
   C   
   G   
   A   
   G   
   A   
   T   
   G   
   A   
   A   
   A   
   G   
   T   
   G   
   A   
   T   
    A    
   G   
    A    
   A   
   C   
    A    
   G   
   G   
   T   
   C   
   T   
   G   
   C   
   A   
   A   
   A   
   T   
   T   
   G   
   T   
   C   
   G   
   T   
    C    
   G   
   A   
   A   
   T   
   T   
   G   
   A   
   A   
   A   
   A   
   A   
   A   
   G   
   A   
    C    
   G   
   C   
   C   
   A   
   A   
    T    
   G   
   C   
   T   
   C   
   A   
   A   
   G   
   G   
    A    
   A   
   T   
   T   
   T   
   T   
   G   
   A   
   A   
   T   
   T   
   A   
    T    
   T   
   T   
   G   
   T   
   T   
   C   
   A   
   A   
   A   
   A   
   A   
   C   
   A   
   C   
   T   
   G   
   A   
   G   
    C    
   T   
   A   
   C   
   A   
   G   
   A   
   T   
   C   
   A   
   A   
   T   
   T   
   A   
   C   
   A   
   A   
   T   
   T   
   T   
    C    
   A   
   A   
   T   
   A   
   T   
   G   
   G   
   T   
   C   
   G   
   C   
   T   
   A   
   T   
   C   
   G   
   A   
    C    
   C   
   A   
   T   
   A   
   T   
   G   
   A   
   C   
   T   
   C   
   C   
   G   
   C   
   A   
    T    
   C   
   A   
   A   
   G   
   T   
   T   
   G   
   G   
   A   
   C   
   T   
   G   
   A   
   A   
   A   
   G   
   A   
   C   
   A   
   T   
   T   
   T   
   T   
   A   
   A   
   G   
   A   
   A   
   G   
   T   
   T   
   A   
   T   
   A   
   T   
   C   
   G   
   A   
   G   
   C   
   A   
   T   
   C   
   G   
   A   
   A   
   A   
   G   
   C   
   A   
   A   
   G   
   T   
   C   
   A   
   T   
   A   
   A   
   C   
   A   
   A   
   A   
   A   
   C   
   G   
    T    
   A   
   G   
    T    
   C   
   A   
   A   
   T   
   T   
   C   
   G   
   A   
   T   
   T   
   T   
   A   
   G   
   C   
   C   
   A   
   A   
   A   
   G   
   C   
   T   
   C   
   A   
   A   
   A   
   A   
   A   
    A    
   G   
   A   
   C   
   A   
   G   
   C   
   A   
   T   
   A   
   T   
   C   
   G   
   T   
    A    
   G   
   A   
   A   
   G   
   G   
    A    
    T    
   T   
   G   
   A   
   T   
   G   
   A   
   A   
   A   
   G   
   C   
   T   
   T   
   T   
   A   
   T   
   C   
   G   
   A   
   T   
   T   
   C   
   T   
   G   
   G   
   A   
   T   
   G   
   A   
   A   
   G   
   T   
   A   
   A   
   T   
   C   
   G   
    T    
   A   
   A   
   C   
   G   
   A   
   T   
   T   
   C   
   G   
   T   
   G   
   A   
   A   
   A   
   G   
   T   
   A   
   A   
   A   
   G   
   A   
   C   
   A   
   A   
   A   
   A   
   A   
   A   
   G   
   A   
    C    
   G   
   C   
    C    
   A   
   A   
   A   
   A   
   A   
   G   
   A   
   A   
   T   
   T   
   T   
   A   
   G   
   T   
   C   
   G   
   A   
    C    
   G   
   T   
    C    
   T   
   T   
    T    
   C   
   A   
   A   
   T   
   T   
   T   
   A   
   C   
   A   
   G   
   A   
   A   
   G   
   A   
   A   
   C   
   A   
   A   
   G   
   C   
   A   
   G   
   A   
   A   
   G   
   C   
   A   
   A   
   T   
    T    
   G   
   T   
   C   
   A   
   C   
   A   
   T   
   T   
   G   
   C   
   A   
   A   
    T    
   T   
   A   
   T   
   A   
    C    
   C   
   G   
   T   
   T   
   T   
   G   
   A   
   C   
   G   
   A   
   A   
    C    
   A   
   C   
    G    
   G   
   A   
   T   
   A   
   T   
   C   
   A   
   C   
   A   
   G   
   A   
   G   
   C   
   T   
   T   
   C   
   A   
   A   
   A   
   A   
   G   
   G   
   A   
   A   
    T    
   C   
    C    
   G   
   A   
   A   
   A   
    G    
   C   
   T   
   T   
   G   
   A   
   T   
   T   
   G   
   C   
    G    
   C   
   A   
   A   
   A   
   T   
   C   
   A   
   C   
   A   
   G   
   A   
   G   
   T   
   T   
    G    
   A   
   A   
   T   
   A   
   A   
   A   
   A   
   T   
   C   
   C   
   T   
   T   
   T   
   C   
   A   
   A   
   A   
    T    
   G   
   A   
   C   
   A   
   A   
   A   
   G   
   A   
    G    
   C   
   T   
   T   
   T   
   T   
    C    
   T   
   C   
   T   
   G   
   T   
   T   
   A   
   T   
   G   
   A   
   A   
   A   
   A   
   A   
   A   
   G   
   A   
   A   
   T   
   T   
   G   
   C   
   G   
   A   
   G   
   A   
   A   
   G   
   T   
   G   
   A   
   A   
   A   
   A   
   A   
   A   
   A   
   A   
   T   
   T   
   A   
    T    
   T   
   C   
   T   
   T   
   C   
   C   
   G   
   C   
   T   
   C   
   G   
   T   
   C   
    T    
    A    
   A   
   C   
   G   
   A   
   C   
   A   
   A   
   T   
   C   
    G    
   A   
   A   
   G   
   A   
   C   
   G   
   A   
   A   
   A   
   T   
   C   
   G   
   A   
   A   
   G   
   A   
   A   
   A   
   T   
   C   
   A   
   A   
   A   
   A   
   T   
    T    
   G   
   A   
   T   
   A   
   C   
   G   
   C   
   A   
   A   
    G    
   T   
    C    
   T   
   T   
   A   
   G   
   T   
   G   
   G   
   C   
    A    
   C   
   A   
   A   
   G   
   A   
   A   
   G   
   A   
   C   
   G   
   T   
   A   
   A   
   T   
    T    
   G   
   T   
   C   
   A   
   G   
   T   
   G   
   T   
    G    
   A   
   C   
    G    
   C   
   G   
   A   
   G   
   A   
   A   
   G   
   G   
   C   
   T   
   A   
   T   
   G   
   T   
   G   
   A   
   A   
   G   
   C   
   G   
   A   
   A   
   C   
   A   
   A   
   G   
   C   
   C   
   T   
   T   
   C   
   G   
   T   
   T   
   C   
    T    
   T   
   A   
   T   
   A   
   G   
   T   
   G   
   C   
   T   
   T   
   C   
    C    
   A   
   A   
   A   
   C   
   C   
   A   
   G   
   A   
   A   
   G   
   A   
   A   
   A   
   T   
   C   
   G   
   G   
   G   
   A   
   T   
   G   
   C   
   G   
   A   
   G   
   A   
   A   
   G   
   G   
   C   
   G   
   A   
    C    
   T   
   A   
   C   
   T   
   T   
   G   
   C   
   T   
    A    
   T   
   A   
   C   
    T    
   C   
   A   
   G   
   G   
   T   
   G   
   A   
   A   
   T   
   T   
    G    
   A   
   G   
    T    
   A   
   C   
   A   
    T    
   T   
   G   
   G   
   A   
   C   
   C   
   A   
   T   
   G   
   T   
   T   
   C   
   T   
   T   
   C   
   T   
   G   
   A   
   T   
    C    
   A   
   C   
    A    
   A   
   A   
   T   
   A   
   A   
   A   
   G   
   C   
   A   
   A   
   A   
   T   
   G   
   T   
   G   
   A   
   T   
   C   
   T   
   A   
   C   
   C   
   G   
    C    
   C   
   C   
   T   
   G   
   T   
    C    
   C   
   A   
   T   
   G   
   A   
   A   
   C   
   T   
   A   
   C   
   C   
    C    
   G   
   A   
   T   
   T   
   T   
   G   
   A   
   A   
   A   
   T   
   G   
   G   
   A   
   A   
   A   
   G   
   A   
   T   
   G   
   C   
   T   
   G   
   G   
    T    
   G   
   A   
    G    
   C   
   A   
    C    
   A   
   T   
   T   
   T   
   C   
   T   
   C   
   A   
   A   
   A   
   C   
   A   
   A   
   T   
   C   
   T   
   C   
   C   
   A   
   A   
   T   
   C   
   T   
   G   
   T   
   C   
   T   
   G   
   T   
   A   
   G   
   A   
    C    
   G   
   A   
   A   
   T   
   C   
   G   
   A   
   T   
    T    
   T   
   T   
   A   
   G   
   C   
   T   
   G   
   T   
   T   
   T   
   T   
    C    
   C   
   C   
   A   
   T   
   A   
   T   
   C   
   A   
   A   
   A   
   A   
   A   
   A   
   T   
    T    
   C   
   A   
   A   
   G   
   C   
   A   
   G   
   A   
    G    
   A   
   A   
   A   
   A   
   C   
   A   
   T   
   T   
   T   
   G   
   T   
    A    
   T   
   T   
   C   
   A   
   T   
   C   
   A   
   G   
   C   
   A   
   A   
   G   
   A   
   A   
   T   
   G   
   G   
   A   
   T   
   T   
    G    
    A    
   T   
   C   
   A   
   A   
   G   
   C   
   A   
   A   
   A   
   C   
   A   
   C   
   G   
   A   
   A   
   T   
   G   
   A   
   C   
   A   
   G   
   A   
   G   
   T   
   T   
   C   
   G   
   A   
   A   
   C   
   C   
   A   
   T   
   G   
   G   
   C   
   G   
   C   
   A   
   C   
   C   
   T   
   A   
    T    
   A   
   A   
   G   
   A   
   G   
   C   
   C   
   G   
    T    
   C   
   C   
   G   
   C   
   T   
   C   
   A   
   G   
   C   
   T   
   G   
   T   
   A   
   T   
   G   
   A   
   A   
   G   
   C   
   T   
   A   
   A   
   A   
   G   
   G   
   C   
   G   
   G   
   A   
   A   
   G   
   A   
   A   
   G   
   A   
   T   
   G   
   A   
   A   
   C   
   T   
    G    
   A   
   T   
   T   
   G   
   C   
   T   
   G   
   T   
    G    
   T   
   A   
   T   
   C   
   T   
   G   
   A   
   A   
   A   
   G   
   A   
   A   
   G   
   A   
   T   
   C   
    A    
   G   
   C   
   C   
   A   
   G   
   G   
   C   
   T   
   T   
   A   
   G   
   A   
   T   
   G   
   T   
    T    
   T   
   T   
   C   
   T   
   T   
   A   
   G   
   T   
   A   
   A   
   C   
   G   
    C    
   A   
   T   
   C   
   A   
   A   
   G   
   G   
   A   
   A   
   T   
   G   
   G   
   G   
    T    
   T   
   T   
   G   
   C   
   G   
   C   
   T   
    A    
   T   
   C   
   C   
   T   
   T   
   T   
   A   
   A   
    C    
   A   
   G   
   A   
   T   
   G   
   T   
   T   
   C   
   C   
   C   
   G   
   T   
   T   
   G   
   T   
   T   
   G   
   G   
   A   
   G   
   C   
   A   
   A   
   A   
   G   
   G   
   C   
   A   
   G   
    C    
   T   
   G   
   G   
   A   
   G   
   T   
   G   
   A   
   A   
   G   
   T   
   C   
   T   
   A   
   T   
   G   
   A   
   A   
   T   
   T   
   T   
   G   
   A   
   A   
   A   
   G   
   A   
   T   
   G   
   G   
   A   
   G   
   A   
   T   
   A   
   G   
   G   
    A    
   T   
   T   
    A    
   T   
   C   
   A   
   A   
    C    
   G   
   G   
    G    
   C   
   T   
    G    
   C   
   T   
   T   
   G   
   T   
   G   
   T   
   T   
   A   
   G   
   C   
   A   
   G   
   A   
    A    
   G   
   G   
    T    
   G   
   A   
   T   
   A   
   C   
   G   
   C   
   C   
    G    
   A   
   T   
   C   
   G   
   T   
   A   
   A   
   T   
   C   
   G   
   T   
   T   
   A   
   C   
   T   
   C   
   A   
   G   
   C   
   G   
   A   
   G   
   G   
   A   
   G   
   C   
    T    
   G   
   T   
    T    
   A   
   A   
   A   
   C   
   G   
   C   
   A   
   T   
   G   
   C   
   T   
   T   
   G   
   C   
   T   
   C   
   A   
   A   
   G   
   A   
   A   
   A   
   T   
   C   
   A   
   G   
   C   
   C   
   A   
   A   
   A   
   C   
    T    
   T   
   C   
   A   
   C   
   G   
   T   
   G   
   C   
   G   
   A   
   A   
   G   
   C   
   G   
   G   
   G   
   G   
   A   
   G   
   T   
   G   
   A   
   C   
   G   
   G   
   T   
   C   
   A   
   T   
   G   
   C   
   G   
   T   
   G   
   A   
   G   
   C   
   T   
   G   
   A   
   A   
   A   
   A   
   A   
   A   
   A   
   A   
   T   
   C   
   C   
    G    
   C   
   A   
    C    
   C   
   G   
    T    
   A   
   T   
   T   
   A   
   T   
   C   
   T   
   A   
   T   
   A   
   T   
   G   
   T   
   C   
   G   
   G   
   A   
   A   
   G   
   G   
   A   
   A   
   G   
   A   
   T   
   C   
   A   
   A   
   A   
   A   
   G   
   A   
   A   
   A   
   T   
   A   
   A   
   C   
   C   
   T   
   T   
   G   
   A   
   T   
   G   
   A   
   A   
   T   
   C   
   A   
   A   
   A   
   A   
   A   
   G   
   G   
    G    
   C   
   A   
    G    
   C   
   A   
   G   
    T    
   T   
   A   
   A   
   C   
   G   
   A   
   T   
   T   
   G   
   A   
    C    
   C   
   C   
   A   
   A   
   C   
    T    
   G   
   A   
   T   
   T   
   T   
    C    
   C   
   C   
   T   
   A   
   T   
   A   
   G   
   G   
   T   
   G   
   A   
   T   
   C   
   G   
   A   
   A   
   C   
   A   
   T   
   C   
   G   
   A   
   A   
   T   
   G   
   G   
    A    
   T   
   C   
    A    
   T   
   T   
   T   
   G   
   C   
    G    
   A   
   T   
   G   
   G   
   A   
    T    
   G   
   A   
   G   
   A   
   A   
   A   
   A   
   A   
   A   
   G   
   G   
   T   
   G   
   G   
   A   
   G   
   A   
   A   
   G   
   T   
   C   
   A   
   T   
   C   
   G   
   A   
   A   
   G   
   T   
    T    
   A   
    T    
   C   
   G   
   A   
   T   
   G   
   C   
   T   
   C   
   C   
   T   
   G   
   A   
   G   
    A    
   T   
   T   
   T   
   T   
    C    
   A   
   T   
    C    
   G   
   A   
    G    
    T    
   C   
    G    
   -   
   -   
   -   
 

 
   N54567   
   A   
   T   
   G   
   G   
   A   
   A   
   A   
   A   
   T   
   C   
   G   
   A   
   C   
   A   
   A   
   G   
   A   
   A   
   G   
   T   
   A   
   C   
   A   
   A   
   G   
   A   
   A   
   T   
   T   
   A   
   A   
   C   
   T   
   T   
   T   
   A   
   G   
   A   
   A   
   G   
   A   
   A   
   G   
   T   
   G   
   A   
   T   
   G   
   G   
   G   
   T   
   G   
   A   
   C   
   C   
   G   
   C   
   T   
   T   
   C   
   G   
   G   
   A   
   C   
   G   
   T   
   T   
   A   
   T   
   T   
   C   
   C   
   A   
   A   
   A   
   T   
   A   
   T   
   A   
   T   
   T   
   A   
   T   
    C    
   C   
   A   
    A    
   G   
   A   
   A   
   C   
   G   
   T   
   G   
   C   
   A   
   C   
   T   
   G   
   C   
   C   
   T   
   G   
   A   
   T   
   A   
   T   
    C    
   C   
   G   
   T   
   G   
   A   
   T   
   G   
   G   
   A   
   T   
   T   
   G   
   A   
   A   
    G    
   C   
   C   
   A   
   G   
   T   
    G    
   C   
   A   
   A   
   C   
   G   
   C   
   C   
   G   
   G   
   A   
   T   
   A   
   T   
   T   
   A   
   T   
   T   
    T    
   G   
   C   
    C    
   A   
   T   
   G   
   A   
   A   
   C   
   A   
   A   
   A   
   G   
   A   
   T   
   G   
   G   
   C   
   A   
   A   
   T   
   A   
   C   
   G   
   T   
   A   
   T   
   G   
   A   
   C   
   A   
   A   
   A   
   G   
   G   
   A   
   T   
   T   
    C    
   C   
   G   
   T   
   A   
   A   
   A   
   T   
   C   
   A   
   G   
   C   
   A   
   A   
   A   
   A   
   T   
   C   
   T   
   G   
   T   
   C   
   G   
   G   
   A   
   A   
   A   
   T   
   A   
   T   
   C   
   A   
   T   
   G   
   G   
   G   
    G    
   A   
   A   
   T   
   T   
   A   
   T   
   C   
   A   
   T   
   C   
   C   
   T   
   C   
   A   
   T   
   G   
   G   
   T   
   G   
   A   
   C   
   A   
   G   
   C   
   A   
   G   
   T   
   A   
   T   
   T   
   T   
   A   
   T   
   G   
   A   
   A   
   G   
   C   
   G   
   A   
   T   
   G   
   G   
   T   
   C   
   C   
   G   
   C   
   A   
   T   
   G   
   A   
   G   
   C   
   C   
   A   
   A   
   G   
   A   
    C    
   T   
   G   
   G   
   A   
   A   
   G   
   T   
   T   
   G   
   C   
   G   
   G   
   G   
   A   
   A   
   G   
   T   
   A   
   C   
   T   
   G   
   A   
   T   
   C   
   G   
   A   
    A    
   A   
   T   
   G   
   C   
   A   
   C   
   G   
   G   
    G    
   A   
   A   
   T   
   A   
   A   
    C    
   G   
   G   
   G   
   A   
   G   
   T   
   A   
   T   
   G   
   G   
   A   
   C   
   G   
   G   
   T   
   G   
   A   
   T   
   C   
   C   
   A   
   C   
   C   
   T   
   G   
   C   
   T   
   G   
   C   
   G   
   A   
   T   
   G   
   C   
   G   
   T   
   T   
   A   
   T   
   A   
   C   
   T   
   G   
   A   
   A   
   G   
   C   
   C   
   A   
   G   
    A    
   T   
   T   
   A   
   T   
   C   
   C   
   A   
   A   
   G   
   T   
   T   
   A   
   A   
   G   
   T   
   G   
   G   
   A   
   G   
   A   
   G   
   A   
   T   
   G   
    C    
   T   
   A   
    G    
   C   
   A   
   G   
   A   
   T   
   A   
   T   
   C   
   G   
   A   
    A    
   A   
   A   
   A   
   G   
   A   
   A   
   A   
   C   
   A   
   G   
   T   
    C    
   G   
   A   
   T   
   C   
   T   
   C   
   G   
   T   
   G   
   T   
   G   
   G   
   A   
   A   
   T   
   T   
   T   
   T   
   G   
   A   
   T   
   G   
   A   
   T   
   A   
   C   
   A   
   G   
   A   
   A   
   A   
   A   
   A   
   G   
   A   
   A   
   C   
   C   
   A   
   A   
   C   
   A   
   G   
   T   
   C   
   C   
   T   
   T   
   C   
   C   
   A   
   G   
   C   
   T   
   C   
   G   
   A   
   T   
   A   
   C   
   C   
   C   
   A   
   A   
   A   
   C   
   C   
   T   
   T   
   C   
   T   
    C    
   G   
   T   
   C   
   A   
   A   
   T   
   G   
   G   
   A   
   T   
   C   
   G   
   A   
   C   
   A   
   G   
   G   
   T   
   A   
   T   
   A   
   T   
   C   
   T   
   G   
   C   
   T   
   G   
   G   
   A   
   T   
   A   
   T   
   G   
   C   
    A    
   A   
   C   
   A   
   G   
   A   
   A   
   A   
   T   
   C   
   C   
   C   
   G   
   A   
   C   
    T    
   C   
   A   
   T   
   A   
   A   
   C   
   T   
   T   
   A   
   G   
   C   
   T   
   G   
   A   
   A   
   G   
   T   
   G   
   A   
   T   
    T    
   G   
   A   
    T    
   G   
   G   
   C   
   A   
   C   
   A   
   A   
   T   
   C   
   T   
   A   
   T   
   A   
   T   
   G   
   A   
   T   
   C   
   G   
   A   
   T   
   C   
   A   
   T   
   C   
   C   
   A   
   C   
   A   
   A   
   G   
   C   
   G   
   A   
   G   
   C   
   C   
   T   
   T   
   G   
   A   
   A   
   A   
   A   
   A   
   T   
   T   
   G   
   A   
   T   
   G   
   G   
   A   
   A   
   T   
   A   
   T   
   A   
   T   
   T   
   C   
   C   
   T   
   G   
   G   
   A   
   C   
   C   
   T   
   G   
   A   
   C   
   T   
   T   
    T    
   C   
   C   
   A   
   A   
   C   
   A   
   G   
   G   
   A   
   G   
   G   
    G    
   A   
   T   
   C   
   T   
   T   
   G   
   C   
   A   
   A   
   G   
   G   
    A    
   A   
   A   
   A   
   G   
   A   
   T   
   G   
   A   
   A   
   A   
   T   
   C   
   A   
   A   
   A   
   A   
   A   
   A   
   G   
   C   
   T   
   T   
   A   
   T   
   G   
   A   
   A   
   A   
   C   
   A   
   G   
   G   
    G    
   C   
   G   
   A   
   G   
   G   
   A   
   A   
   A   
   A   
   G   
   T   
   T   
   A   
    T    
   T   
   C   
   T   
   T   
   C   
   G   
   T   
   T   
   C   
   G   
   A   
   A   
   A   
   A   
   C   
   A   
   A   
   A   
   G   
   A   
   T   
   A   
   G   
   A   
    G    
   G   
   C   
   A   
   A   
   T   
   G   
   A   
   A   
   G   
   G   
   G   
    T    
   A   
   A   
    T    
   A   
   A   
   A   
   C   
   A   
   G   
   C   
   A   
   A   
   A   
   T   
   C   
   G   
   T   
   G   
   A   
   T   
   C   
   A   
   C   
    A    
   G   
   A   
   A   
   A   
   T   
   C   
   C   
   C   
   A   
   T   
   A   
   T   
   G   
   A   
   A   
   G   
   T   
   A   
   A   
   A   
   T   
   A   
   A   
   A   
   G   
   C   
   G   
   A   
   C   
    C    
   T   
   T   
   G   
   G   
   T   
   G   
   A   
   A   
   A   
   A   
   A   
   A   
   A   
   T   
   G   
   G   
   A   
   T   
   G   
   A   
   G   
   A   
   T   
   C   
   C   
   G   
   T   
   T   
   T   
   G   
   A   
   A   
   T   
   A   
   A   
   G   
   A   
   A   
   A   
   A   
   T   
   C   
   G   
   A   
    T    
   G   
   G   
   A   
   A   
   T   
   A   
   G   
   C   
   T   
   G   
   A   
   A   
   G   
   T   
    A    
   C   
   G   
   A   
   G   
   A   
   T   
   G   
   A   
   A   
   A   
   G   
   T   
   G   
   A   
   T   
    A    
   G   
    A    
   A   
   C   
    A    
   G   
   G   
   T   
   C   
   T   
   G   
   C   
   A   
   A   
   A   
   T   
   T   
   G   
   T   
   C   
   G   
   T   
    C    
   G   
   A   
   A   
   T   
   T   
   G   
   A   
   A   
   A   
   A   
   A   
   A   
   G   
   A   
    C    
   G   
   C   
   C   
   A   
   A   
    T    
   G   
   C   
   T   
   C   
   A   
   A   
   G   
   G   
    A    
   A   
   T   
   T   
   T   
   T   
   G   
   A   
   A   
   T   
   T   
   A   
    T    
   T   
   T   
   G   
   T   
   T   
   C   
   A   
   A   
   A   
   A   
   A   
   C   
   A   
   C   
   T   
   G   
   A   
   G   
    C    
   T   
   A   
   C   
   A   
   G   
   A   
   T   
   C   
   A   
   A   
   T   
   T   
   A   
   C   
   A   
   A   
   T   
   T   
   T   
    C    
   A   
   A   
   T   
   A   
   T   
   G   
   G   
   T   
   C   
   G   
   C   
   T   
   A   
   T   
   C   
   G   
   A   
    C    
   C   
   A   
   T   
   A   
   T   
   G   
   A   
   C   
   T   
   C   
   C   
   G   
   C   
   A   
    T    
   C   
   A   
   A   
   G   
   T   
   T   
   G   
   G   
   A   
   C   
   T   
   G   
   A   
   A   
   A   
   G   
   A   
   C   
   A   
   T   
   T   
   T   
   T   
   A   
   A   
   G   
   A   
   A   
   G   
   T   
   T   
   A   
   T   
   A   
   T   
   C   
   G   
   A   
   G   
   C   
   A   
   T   
   C   
   G   
   A   
   A   
   A   
   G   
   C   
   A   
   A   
   G   
   T   
   C   
   A   
   T   
   A   
   A   
   C   
   A   
   A   
   A   
   A   
   C   
   G   
    T    
   A   
   G   
    T    
   C   
   A   
   A   
   T   
   T   
   C   
   G   
   A   
   T   
   T   
   T   
   A   
   G   
   C   
   C   
   A   
   A   
   A   
   G   
   C   
   T   
   C   
   A   
   A   
   A   
   A   
   A   
    A    
   G   
   A   
   C   
   A   
   G   
   C   
   A   
   T   
   A   
   T   
   C   
   G   
   T   
    A    
   G   
   A   
   A   
   G   
   G   
    A    
    T    
   T   
   G   
   A   
   T   
   G   
   A   
   A   
   A   
   G   
   C   
   T   
   T   
   T   
   A   
   T   
   C   
   G   
   A   
   T   
   T   
   C   
   T   
   G   
   G   
   A   
   T   
   G   
   A   
   A   
   G   
   T   
   A   
   A   
   T   
   C   
   G   
    T    
   A   
   A   
   C   
   G   
   A   
   T   
   T   
   C   
   G   
   T   
   G   
   A   
   A   
   A   
   G   
   T   
   A   
   A   
   A   
   G   
   A   
   C   
   A   
   A   
   A   
   A   
   A   
   A   
   G   
   A   
    C    
   G   
   C   
    C    
   A   
   A   
   A   
   A   
   A   
   G   
   A   
   A   
   T   
   T   
   T   
   A   
   G   
   T   
   C   
   G   
   A   
    C    
   G   
   T   
    C    
   T   
   T   
    T    
   C   
   A   
   A   
   T   
   T   
   T   
   A   
   C   
   A   
   G   
   A   
   A   
   G   
   A   
   A   
   C   
   A   
   A   
   G   
   C   
   A   
   G   
   A   
   A   
   G   
   C   
   A   
   A   
   T   
    T    
   G   
   T   
   C   
   A   
   C   
   A   
   T   
   T   
   G   
   C   
   A   
   A   
    T    
   T   
   A   
   T   
   A   
    C    
   C   
   G   
   T   
   T   
   T   
   G   
   A   
   C   
   G   
   A   
   A   
    C    
   A   
   C   
    G    
   G   
   A   
   T   
   A   
   T   
   C   
   A   
   C   
   A   
   G   
   A   
   G   
   C   
   T   
   T   
   C   
   A   
   A   
   A   
   A   
   G   
   G   
   A   
   A   
    T    
   C   
    C    
   G   
   A   
   A   
   A   
    G    
   C   
   T   
   T   
   G   
   A   
   T   
   T   
   G   
   C   
    G    
   C   
   A   
   A   
   A   
   T   
   C   
   A   
   C   
   A   
   G   
   A   
   G   
   T   
   T   
    G    
   A   
   A   
   T   
   A   
   A   
   A   
   A   
   T   
   C   
   C   
   T   
   T   
   T   
   C   
   A   
   A   
   A   
    T    
   G   
   A   
   C   
   A   
   A   
   A   
   G   
   A   
    G    
   C   
   T   
   T   
   T   
   T   
    C    
   T   
   C   
   T   
   G   
   T   
   T   
   A   
   T   
   G   
   A   
   A   
   A   
   A   
   A   
   A   
   G   
   A   
   A   
   T   
   T   
   G   
   C   
   G   
   A   
   G   
   A   
   A   
   G   
   T   
   G   
   A   
   A   
   A   
   A   
   A   
   A   
   A   
   A   
   T   
   T   
   A   
    T    
   T   
   C   
   T   
   T   
   C   
   C   
   G   
   C   
   T   
   C   
   G   
   T   
   C   
    T    
    A    
   A   
   C   
   G   
   A   
   C   
   A   
   A   
   T   
   C   
    G    
   A   
   A   
   G   
   A   
   C   
   G   
   A   
   A   
   A   
   T   
   C   
   G   
   A   
   A   
   G   
   A   
   A   
   A   
   T   
   C   
   A   
   A   
   A   
   A   
   T   
    T    
   G   
   A   
   T   
   A   
   C   
   G   
   C   
   A   
   A   
    G    
   T   
    C    
   T   
   T   
   A   
   G   
   T   
   G   
   G   
   C   
    A    
   C   
   A   
   A   
   G   
   A   
   A   
   G   
   A   
   C   
   G   
   T   
   A   
   A   
   T   
    T    
   G   
   T   
   C   
   A   
   G   
   T   
   G   
   T   
    G    
   A   
   C   
    G    
   C   
   G   
   A   
   G   
   A   
   A   
   G   
   G   
   C   
   T   
   A   
   T   
   G   
   T   
   G   
   A   
   A   
   G   
   C   
   G   
   A   
   A   
   C   
   A   
   A   
   G   
   C   
   C   
   T   
   T   
   C   
   G   
   T   
   T   
   C   
    T    
   T   
   A   
   T   
   A   
   G   
   T   
   G   
   C   
   T   
   T   
   C   
    C    
   A   
   A   
   A   
   C   
   C   
   A   
   G   
   A   
   A   
   G   
   A   
   A   
   A   
   T   
   C   
   G   
   G   
   G   
   A   
   T   
   G   
   C   
   G   
   A   
   G   
   A   
   A   
   G   
   G   
   C   
   G   
   A   
    C    
   T   
   A   
   C   
   T   
   T   
   G   
   C   
   T   
    A    
   T   
   A   
   C   
    T    
   C   
   A   
   G   
   G   
   T   
   G   
   A   
   A   
   T   
   T   
    G    
   A   
   G   
    T    
   A   
   C   
   A   
    T    
   T   
   G   
   G   
   A   
   C   
   C   
   A   
   T   
   G   
   T   
   T   
   C   
   T   
   T   
   C   
   T   
   G   
   A   
   T   
    C    
   A   
   C   
    A    
   A   
   A   
   T   
   A   
   A   
   A   
   G   
   C   
   A   
   A   
   A   
   T   
   G   
   T   
   G   
   A   
   T   
   C   
   T   
   A   
   C   
   C   
   G   
    C    
   C   
   C   
   T   
   G   
   T   
    C    
   C   
   A   
   T   
   G   
   A   
   A   
   C   
   T   
   A   
   C   
   C   
    C    
   G   
   A   
   T   
   T   
   T   
   G   
   A   
   A   
   A   
   T   
   G   
   G   
   A   
   A   
   A   
   G   
   A   
   T   
   G   
   C   
   T   
   G   
   G   
    T    
   G   
   A   
    G    
   C   
   A   
    C    
   A   
   T   
   T   
   T   
   C   
   T   
   C   
   A   
   A   
   A   
   C   
   A   
   A   
   T   
   C   
   T   
   C   
   C   
   A   
   A   
   T   
   C   
   T   
   G   
   T   
   C   
   T   
   G   
   T   
   A   
   G   
   A   
    C    
   G   
   A   
   A   
   T   
   C   
   G   
   A   
   T   
    T    
   T   
   T   
   A   
   G   
   C   
   T   
   G   
   T   
   T   
   T   
   T   
    C    
   C   
   C   
   A   
   T   
   A   
   T   
   C   
   A   
   A   
   A   
   A   
   A   
   A   
   T   
    T    
   C   
   A   
   A   
   G   
   C   
   A   
   G   
   A   
    G    
   A   
   A   
   A   
   A   
   C   
   A   
   T   
   T   
   T   
   G   
   T   
    A    
   T   
   T   
   C   
   A   
   T   
   C   
   A   
   G   
   C   
   A   
   A   
   G   
   A   
   A   
   T   
   G   
   G   
   A   
   T   
   T   
    G    
    A    
   T   
   C   
   A   
   A   
   G   
   C   
   A   
   A   
   A   
   C   
   A   
   C   
   G   
   A   
   A   
   T   
   G   
   A   
   C   
   A   
   G   
   A   
   G   
   T   
   T   
   C   
   G   
   A   
   A   
   C   
   C   
   A   
   T   
   G   
   G   
   C   
   G   
   C   
   A   
   C   
   C   
   T   
   A   
    T    
   A   
   A   
   G   
   A   
   G   
   C   
   C   
   G   
    T    
   C   
   C   
   G   
   C   
   T   
   C   
   A   
   G   
   C   
   T   
   G   
   T   
   A   
   T   
   G   
   A   
   A   
   G   
   C   
   T   
   A   
   A   
   A   
   G   
   G   
   C   
   G   
   G   
   A   
   A   
   G   
   A   
   A   
   G   
   A   
   T   
   G   
   A   
   A   
   C   
   T   
    G    
   A   
   T   
   T   
   G   
   C   
   T   
   G   
   T   
    G    
   T   
   A   
   T   
   C   
   T   
   G   
   A   
   A   
   A   
   G   
   A   
   A   
   G   
   A   
   T   
   C   
    A    
   G   
   C   
   C   
   A   
   G   
   G   
   C   
   T   
   T   
   A   
   G   
   A   
   T   
   G   
   T   
    T    
   T   
   T   
   C   
   T   
   T   
   A   
   G   
   T   
   A   
   A   
   C   
   G   
    C    
   A   
   T   
   C   
   A   
   A   
   G   
   G   
   A   
   A   
   T   
   G   
   G   
   G   
    T    
   T   
   T   
   G   
   C   
   G   
   C   
   T   
    A    
   T   
   C   
   C   
   T   
   T   
   T   
   A   
   A   
    C    
   A   
   G   
   A   
   T   
   G   
   T   
   T   
   C   
   C   
   C   
   G   
   T   
   T   
   G   
   T   
   T   
   G   
   G   
   A   
   G   
   C   
   A   
   A   
   A   
   G   
   G   
   C   
   A   
   G   
    C    
   T   
   G   
   G   
   A   
   G   
   T   
   G   
   A   
   A   
   G   
   T   
   C   
   T   
   A   
   T   
   G   
   A   
   A   
   T   
   T   
   T   
   G   
   A   
   A   
   A   
   G   
   A   
   T   
   G   
   G   
   A   
   G   
   A   
   T   
   A   
   G   
   G   
    A    
   T   
   T   
    A    
   T   
   C   
   A   
   A   
    C    
   G   
   G   
    G    
   C   
   T   
    G    
   C   
   T   
   T   
   G   
   T   
   G   
   T   
   T   
   A   
   G   
   C   
   A   
   G   
   A   
    A    
   G   
   G   
    T    
   G   
   A   
   T   
   A   
   C   
   G   
   C   
   C   
    G    
   A   
   T   
   C   
   G   
   T   
   A   
   A   
   T   
   C   
   G   
   T   
   T   
   A   
   C   
   T   
   C   
   A   
   G   
   C   
   G   
   A   
   G   
   G   
   A   
   G   
   C   
    T    
   G   
   T   
    T    
   A   
   A   
   A   
   C   
   G   
   C   
   A   
   T   
   G   
   C   
   T   
   T   
   G   
   C   
   T   
   C   
   A   
   A   
   G   
   A   
   A   
   A   
   T   
   C   
   A   
   G   
   C   
   C   
   A   
   A   
   A   
   C   
    T    
   T   
   C   
   A   
   C   
   G   
   T   
   G   
   C   
   G   
   A   
   A   
   G   
   C   
   G   
   G   
   G   
   G   
   A   
   G   
   T   
   G   
   A   
   C   
   G   
   G   
   T   
   C   
   A   
   T   
   G   
   C   
   G   
   T   
   G   
   A   
   G   
   C   
   T   
   G   
   A   
   A   
   A   
   A   
   A   
   A   
   A   
   A   
   T   
   C   
   C   
    G    
   C   
   A   
    C    
   C   
   G   
    T    
   A   
   T   
   T   
   A   
   T   
   C   
   T   
   A   
   T   
   A   
   T   
   G   
   T   
   C   
   G   
   G   
   A   
   A   
   G   
   G   
   A   
   A   
   G   
   A   
   T   
   C   
   A   
   A   
   A   
   A   
   G   
   A   
   A   
   A   
   T   
   A   
   A   
   C   
   C   
   T   
   T   
   G   
   A   
   T   
   G   
   A   
   A   
   T   
   C   
   A   
   A   
   A   
   A   
   A   
   G   
   G   
    G    
   C   
   A   
    G    
   C   
   A   
   G   
    T    
   T   
   A   
   A   
   C   
   G   
   A   
   T   
   T   
   G   
   A   
    C    
   C   
   C   
   A   
   A   
   C   
    T    
   G   
   A   
   T   
   T   
   T   
    C    
   C   
   C   
   T   
   A   
   T   
   A   
   G   
   G   
   T   
   G   
   A   
   T   
   C   
   G   
   A   
   A   
   C   
   A   
   T   
   C   
   G   
   A   
   A   
   T   
   G   
   G   
    A    
   T   
   C   
    A    
   T   
   T   
   T   
   G   
   C   
    G    
   A   
   T   
   G   
   G   
   A   
    T    
   G   
   A   
   G   
   A   
   A   
   A   
   A   
   A   
   A   
   G   
   G   
   T   
   G   
   G   
   A   
   G   
   A   
   A   
   G   
   T   
   C   
   A   
   T   
   C   
   G   
   A   
   A   
   G   
   T   
    T    
   A   
    T    
   C   
   G   
   A   
   T   
   G   
   C   
   T   
   C   
   C   
   T   
   G   
   A   
   G   
    A    
   T   
   T   
   T   
   T   
    C    
   A   
   T   
    C    
   G   
   A   
    G    
    T    
   C   
    G    
   -   
   -   
   -   
 

 
   N52459   
   A   
   T   
   G   
   G   
   A   
   A   
   A   
   A   
   T   
   C   
   G   
   A   
   C   
   A   
   A   
   G   
   A   
   A   
   G   
   T   
   A   
   C   
   A   
   A   
   G   
   A   
   A   
   T   
   T   
   A   
   A   
   C   
   T   
   T   
   T   
   A   
   G   
   A   
   A   
   G   
   A   
   A   
   G   
   T   
   G   
   A   
   T   
   G   
   G   
   G   
   T   
   G   
   A   
   C   
   C   
   G   
   C   
   T   
   T   
   C   
   G   
   G   
   A   
   C   
   G   
   T   
   T   
   A   
   T   
   T   
   C   
   C   
   A   
   A   
   A   
   T   
   A   
   T   
   A   
   T   
   T   
   A   
   T   
    C    
   C   
   A   
    A    
   G   
   A   
   A   
   C   
   G   
   T   
   G   
   C   
   A   
   C   
   T   
   G   
   C   
   C   
   T   
   G   
   A   
   T   
   A   
   T   
    T    
   C   
   G   
   T   
   G   
   A   
   T   
   G   
   G   
   A   
   T   
   T   
   G   
   A   
   A   
    G    
   C   
   C   
   A   
   G   
   T   
    G    
   C   
   A   
   A   
   C   
   G   
   C   
   C   
   G   
   G   
   A   
   T   
   A   
   T   
   T   
   A   
   T   
   T   
    T    
   G   
   C   
    C    
   A   
   T   
   G   
   A   
   A   
   C   
   A   
   A   
   A   
   G   
   A   
   T   
   G   
   G   
   C   
   A   
   A   
   T   
   A   
   C   
   G   
   T   
   A   
   T   
   G   
   A   
   C   
   A   
   A   
   A   
   G   
   G   
   A   
   T   
   T   
    C    
   C   
   G   
   T   
   A   
   A   
   A   
   T   
   C   
   A   
   G   
   C   
   A   
   A   
   A   
   A   
   T   
   C   
   T   
   G   
   T   
   C   
   G   
   G   
   A   
   A   
   A   
   T   
   A   
   T   
   C   
   A   
   T   
   G   
   G   
   G   
    G    
   A   
   A   
   T   
   T   
   A   
   T   
   C   
   A   
   T   
   C   
   C   
   T   
   C   
   A   
   T   
   G   
   G   
   T   
   G   
   A   
   C   
   A   
   G   
   C   
   A   
   G   
   T   
   A   
   T   
   T   
   T   
   A   
   T   
   G   
   A   
   A   
   G   
   C   
   G   
   A   
   T   
   G   
   G   
   T   
   C   
   C   
   G   
   C   
   A   
   T   
   G   
   A   
   G   
   C   
   C   
   A   
   A   
   G   
   A   
    C    
   T   
   G   
   G   
   A   
   A   
   G   
   T   
   T   
   G   
   C   
   G   
   G   
   G   
   A   
   A   
   G   
   T   
   A   
   C   
   T   
   G   
   A   
   T   
   C   
   G   
   A   
    A    
   A   
   T   
   G   
   C   
   A   
   C   
   G   
   G   
    G    
   A   
   A   
   T   
   A   
   A   
    C    
   G   
   G   
   G   
   A   
   G   
   T   
   A   
   T   
   G   
   G   
   A   
   C   
   G   
   G   
   T   
   G   
   A   
   T   
   C   
   C   
   A   
   C   
   C   
   T   
   G   
   C   
   T   
   G   
   C   
   G   
   A   
   T   
   G   
   C   
   G   
   T   
   T   
   A   
   T   
   A   
   C   
   T   
   G   
   A   
   A   
   G   
   C   
   C   
   A   
   G   
    A    
   T   
   T   
   A   
   T   
   C   
   C   
   A   
   A   
   G   
   T   
   T   
   A   
   A   
   G   
   T   
   G   
   G   
   A   
   G   
   A   
   G   
   A   
   T   
   G   
    C    
   T   
   A   
    G    
   C   
   A   
   G   
   A   
   T   
   A   
   T   
   C   
   G   
   A   
    A    
   A   
   A   
   A   
   G   
   A   
   A   
   A   
   C   
   A   
   G   
   T   
    C    
   G   
   A   
   T   
   C   
   T   
   C   
   G   
   T   
   G   
   T   
   G   
   G   
   A   
   A   
   T   
   T   
   T   
   T   
   G   
   A   
   T   
   G   
   A   
   T   
   A   
   C   
   A   
   G   
   A   
   A   
   A   
   A   
   A   
   G   
   A   
   A   
   C   
   C   
   A   
   A   
   C   
   A   
   G   
   T   
   C   
   C   
   T   
   T   
   C   
   C   
   A   
   G   
   C   
   T   
   C   
   G   
   A   
   T   
   A   
   C   
   C   
   C   
   A   
   A   
   A   
   C   
   C   
   T   
   T   
   C   
   T   
    C    
   G   
   T   
   C   
   A   
   A   
   T   
   G   
   G   
   A   
   T   
   C   
   G   
   A   
   C   
   A   
   G   
   G   
   T   
   A   
   T   
   A   
   T   
   C   
   T   
   G   
   C   
   T   
   G   
   G   
   A   
   T   
   A   
   T   
   G   
   C   
    A    
   A   
   C   
   A   
   G   
   A   
   A   
   A   
   T   
   C   
   C   
   C   
   G   
   A   
   C   
    T    
   C   
   A   
   T   
   A   
   A   
   C   
   T   
   T   
   A   
   G   
   C   
   T   
   G   
   A   
   A   
   G   
   T   
   G   
   A   
   T   
    C    
   G   
   A   
    T    
   G   
   G   
   C   
   A   
   C   
   A   
   A   
   T   
   C   
   T   
   A   
   T   
   A   
   T   
   G   
   A   
   T   
   C   
   G   
   A   
   T   
   C   
   A   
   T   
   C   
   C   
   A   
   C   
   A   
   A   
   G   
   C   
   G   
   A   
   G   
   C   
   C   
   T   
   T   
   G   
   A   
   A   
   A   
   A   
   A   
   T   
   T   
   G   
   A   
   T   
   G   
   G   
   A   
   A   
   T   
   A   
   T   
   A   
   T   
   T   
   C   
   C   
   T   
   G   
   G   
   A   
   C   
   C   
   T   
   G   
   A   
   C   
   T   
   T   
    T    
   C   
   C   
   A   
   A   
   C   
   A   
   G   
   G   
   A   
   G   
   G   
    G    
   A   
   T   
   C   
   T   
   T   
   G   
   C   
   A   
   A   
   G   
   G   
    A    
   A   
   A   
   A   
   G   
   A   
   T   
   G   
   A   
   A   
   A   
   T   
   C   
   A   
   A   
   A   
   A   
   A   
   A   
   G   
   C   
   T   
   T   
   A   
   T   
   G   
   A   
   A   
   A   
   C   
   A   
   G   
   G   
    G    
   C   
   G   
   A   
   G   
   G   
   A   
   A   
   A   
   A   
   G   
   T   
   T   
   A   
    T    
   T   
   C   
   T   
   T   
   C   
   G   
   T   
   T   
   C   
   G   
   A   
   A   
   A   
   A   
   C   
   A   
   A   
   A   
   G   
   A   
   T   
   A   
   G   
   A   
    A    
   G   
   C   
   A   
   A   
   T   
   G   
   A   
   A   
   G   
   G   
   G   
    T    
   A   
   A   
    T    
   A   
   A   
   A   
   C   
   A   
   G   
   C   
   A   
   A   
   A   
   T   
   C   
   G   
   T   
   G   
   A   
   T   
   C   
   A   
   C   
    A    
   G   
   A   
   A   
   A   
   T   
   C   
   C   
   C   
   A   
   T   
   A   
   T   
   G   
   A   
   A   
   G   
   T   
   A   
   A   
   A   
   T   
   A   
   A   
   A   
   G   
   C   
   G   
   A   
   C   
    C    
   T   
   T   
   G   
   G   
   T   
   G   
   A   
   A   
   A   
   A   
   A   
   A   
   A   
   T   
   G   
   G   
   A   
   T   
   G   
   A   
   G   
   A   
   T   
   C   
   C   
   G   
   T   
   T   
   T   
   G   
   A   
   A   
   T   
   A   
   A   
   G   
   A   
   A   
   A   
   A   
   T   
   C   
   G   
   A   
    T    
   G   
   G   
   A   
   A   
   T   
   A   
   G   
   C   
   T   
   G   
   A   
   A   
   G   
   T   
    A    
   C   
   G   
   A   
   G   
   A   
   T   
   G   
   A   
   A   
   A   
   G   
   T   
   G   
   A   
   T   
    A    
   G   
    A    
   A   
   C   
    A    
   G   
   G   
   T   
   C   
   T   
   G   
   C   
   A   
   A   
   A   
   T   
   T   
   G   
   T   
   C   
   G   
   T   
    C    
   G   
   A   
   A   
   T   
   T   
   G   
   A   
   A   
   A   
   A   
   A   
   A   
   G   
   A   
    C    
   G   
   C   
   C   
   A   
   A   
    T    
   G   
   C   
   T   
   C   
   A   
   A   
   G   
   G   
    A    
   A   
   T   
   T   
   T   
   T   
   G   
   A   
   A   
   T   
   T   
   A   
    T    
   T   
   T   
   G   
   T   
   T   
   C   
   A   
   A   
   A   
   A   
   A   
   C   
   A   
   C   
   T   
   G   
   A   
   G   
    C    
   T   
   A   
   C   
   A   
   G   
   A   
   T   
   C   
   A   
   A   
   T   
   T   
   A   
   C   
   A   
   A   
   T   
   T   
   T   
    C    
   A   
   A   
   T   
   A   
   T   
   G   
   G   
   T   
   C   
   G   
   C   
   T   
   A   
   T   
   C   
   G   
   A   
    C    
   C   
   A   
   T   
   A   
   T   
   G   
   A   
   C   
   T   
   C   
   C   
   G   
   C   
   A   
    T    
   C   
   A   
   A   
   G   
   T   
   T   
   G   
   G   
   A   
   C   
   T   
   G   
   A   
   A   
   A   
   G   
   A   
   C   
   A   
   T   
   T   
   T   
   T   
   A   
   A   
   G   
   A   
   A   
   G   
   T   
   T   
   A   
   T   
   A   
   T   
   C   
   G   
   A   
   G   
   C   
   A   
   T   
   C   
   G   
   A   
   A   
   A   
   G   
   C   
   A   
   A   
   G   
   T   
   C   
   A   
   T   
   A   
   A   
   C   
   A   
   A   
   A   
   A   
   C   
   G   
    T    
   A   
   G   
    T    
   C   
   A   
   A   
   T   
   T   
   C   
   G   
   A   
   T   
   T   
   T   
   A   
   G   
   C   
   C   
   A   
   A   
   A   
   G   
   C   
   T   
   C   
   A   
   A   
   A   
   A   
   A   
    A    
   G   
   A   
   C   
   A   
   G   
   C   
   A   
   T   
   A   
   T   
   C   
   G   
   T   
    A    
   G   
   A   
   A   
   G   
   G   
    A    
    T    
   T   
   G   
   A   
   T   
   G   
   A   
   A   
   A   
   G   
   C   
   T   
   T   
   T   
   A   
   T   
   C   
   G   
   A   
   T   
   T   
   C   
   T   
   G   
   G   
   A   
   T   
   G   
   A   
   A   
   G   
   T   
   A   
   A   
   T   
   C   
   G   
    C    
   A   
   A   
   C   
   G   
   A   
   T   
   T   
   C   
   G   
   T   
   G   
   A   
   A   
   A   
   G   
   T   
   A   
   A   
   A   
   G   
   A   
   C   
   A   
   A   
   A   
   A   
   A   
   A   
   G   
   A   
    C    
   G   
   C   
    C    
   A   
   A   
   A   
   A   
   A   
   G   
   A   
   A   
   T   
   T   
   T   
   A   
   G   
   T   
   C   
   G   
   A   
    C    
   G   
   T   
    C    
   T   
   T   
    T    
   C   
   A   
   A   
   T   
   T   
   T   
   A   
   C   
   A   
   G   
   A   
   A   
   G   
   A   
   A   
   C   
   A   
   A   
   G   
   C   
   A   
   G   
   A   
   A   
   G   
   C   
   A   
   A   
   T   
    T    
   G   
   T   
   C   
   A   
   C   
   A   
   T   
   T   
   G   
   C   
   A   
   A   
    T    
   T   
   A   
   T   
   A   
    C    
   C   
   G   
   T   
   T   
   T   
   G   
   A   
   C   
   G   
   A   
   A   
    C    
   A   
   C   
    G    
   G   
   A   
   T   
   A   
   T   
   C   
   A   
   C   
   A   
   G   
   A   
   G   
   C   
   T   
   T   
   C   
   A   
   A   
   A   
   A   
   G   
   G   
   A   
   A   
    T    
   C   
    C    
   G   
   A   
   A   
   A   
    G    
   C   
   T   
   T   
   G   
   A   
   T   
   T   
   G   
   C   
    G    
   C   
   A   
   A   
   A   
   T   
   C   
   A   
   C   
   A   
   G   
   A   
   G   
   T   
   T   
    G    
   A   
   A   
   T   
   A   
   A   
   A   
   A   
   T   
   C   
   C   
   T   
   T   
   T   
   C   
   A   
   A   
   A   
    T    
   G   
   A   
   C   
   A   
   A   
   A   
   G   
   A   
    G    
   C   
   T   
   T   
   T   
   T   
    C    
   T   
   C   
   T   
   G   
   T   
   T   
   A   
   T   
   G   
   A   
   A   
   A   
   A   
   A   
   A   
   G   
   A   
   A   
   T   
   T   
   G   
   C   
   G   
   A   
   G   
   A   
   A   
   G   
   T   
   G   
   A   
   A   
   A   
   A   
   A   
   A   
   A   
   A   
   T   
   T   
   A   
    T    
   T   
   C   
   T   
   T   
   C   
   C   
   G   
   C   
   T   
   C   
   G   
   T   
   C   
    T    
    A    
   A   
   C   
   G   
   A   
   C   
   A   
   A   
   T   
   C   
    G    
   A   
   A   
   G   
   A   
   C   
   G   
   A   
   A   
   A   
   T   
   C   
   G   
   A   
   A   
   G   
   A   
   A   
   A   
   T   
   C   
   A   
   A   
   A   
   A   
   T   
    T    
   G   
   A   
   T   
   A   
   C   
   G   
   C   
   A   
   A   
    G    
   T   
    C    
   T   
   T   
   A   
   G   
   T   
   G   
   G   
   C   
    A    
   C   
   A   
   A   
   G   
   A   
   A   
   G   
   A   
   C   
   G   
   T   
   A   
   A   
   T   
    T    
   G   
   T   
   C   
   A   
   G   
   T   
   G   
   T   
    G    
   A   
   C   
    G    
   C   
   G   
   A   
   G   
   A   
   A   
   G   
   G   
   C   
   T   
   A   
   T   
   G   
   T   
   G   
   A   
   A   
   G   
   C   
   G   
   A   
   A   
   C   
   A   
   A   
   G   
   C   
   C   
   T   
   T   
   C   
   G   
   T   
   T   
   C   
    T    
   T   
   A   
   T   
   A   
   G   
   T   
   G   
   C   
   T   
   T   
   C   
    C    
   A   
   A   
   A   
   C   
   C   
   A   
   G   
   A   
   A   
   G   
   A   
   A   
   A   
   T   
   C   
   G   
   G   
   G   
   A   
   T   
   G   
   C   
   G   
   A   
   G   
   A   
   A   
   G   
   G   
   C   
   G   
   A   
    C    
   T   
   A   
   C   
   T   
   T   
   G   
   C   
   T   
    A    
   T   
   A   
   C   
    T    
   C   
   A   
   G   
   G   
   T   
   G   
   A   
   A   
   T   
   T   
    G    
   A   
   G   
    T    
   A   
   C   
   A   
    T    
   T   
   G   
   G   
   A   
   C   
   C   
   A   
   T   
   G   
   T   
   T   
   C   
   T   
   T   
   C   
   T   
   G   
   A   
   T   
    C    
   A   
   C   
    A    
   A   
   A   
   T   
   A   
   A   
   A   
   G   
   C   
   A   
   A   
   A   
   T   
   G   
   T   
   G   
   A   
   T   
   C   
   T   
   A   
   C   
   C   
   G   
    C    
   C   
   C   
   T   
   G   
   T   
    C    
   C   
   A   
   T   
   G   
   A   
   A   
   C   
   T   
   A   
   C   
   C   
    C    
   G   
   A   
   T   
   T   
   T   
   G   
   A   
   A   
   A   
   T   
   G   
   G   
   A   
   A   
   A   
   G   
   A   
   T   
   G   
   C   
   T   
   G   
   G   
    T    
   G   
   A   
    G    
   C   
   A   
    C    
   A   
   T   
   T   
   T   
   C   
   T   
   C   
   A   
   A   
   A   
   C   
   A   
   A   
   T   
   C   
   T   
   C   
   C   
   A   
   A   
   T   
   C   
   T   
   G   
   T   
   C   
   T   
   G   
   T   
   A   
   G   
   A   
    C    
   G   
   A   
   A   
   T   
   C   
   G   
   A   
   T   
    T    
   T   
   T   
   A   
   G   
   C   
   T   
   G   
   T   
   T   
   T   
   T   
    C    
   C   
   C   
   A   
   T   
   A   
   T   
   C   
   A   
   A   
   A   
   A   
   A   
   A   
   T   
    T    
   C   
   A   
   A   
   G   
   C   
   A   
   G   
   A   
    G    
   A   
   A   
   A   
   A   
   C   
   A   
   T   
   T   
   T   
   G   
   T   
    A    
   T   
   T   
   C   
   A   
   T   
   C   
   A   
   G   
   C   
   A   
   A   
   G   
   A   
   A   
   T   
   G   
   G   
   A   
   T   
   T   
    G    
    A    
   T   
   C   
   A   
   A   
   G   
   C   
   A   
   A   
   A   
   C   
   A   
   C   
   G   
   A   
   A   
   T   
   G   
   A   
   C   
   A   
   G   
   A   
   G   
   T   
   T   
   C   
   G   
   A   
   A   
   C   
   C   
   A   
   T   
   G   
   G   
   C   
   G   
   C   
   A   
   C   
   C   
   T   
   A   
    T    
   A   
   A   
   G   
   A   
   G   
   C   
   C   
   G   
    T    
   C   
   C   
   G   
   C   
   T   
   C   
   A   
   G   
   C   
   T   
   G   
   T   
   A   
   T   
   G   
   A   
   A   
   G   
   C   
   T   
   A   
   A   
   A   
   G   
   G   
   C   
   G   
   G   
   A   
   A   
   G   
   A   
   A   
   G   
   A   
   T   
   G   
   A   
   A   
   C   
   T   
    G    
   A   
   T   
   T   
   G   
   C   
   T   
   G   
   T   
    G    
   T   
   A   
   T   
   C   
   T   
   G   
   A   
   A   
   A   
   G   
   A   
   A   
   G   
   A   
   T   
   C   
    A    
   G   
   C   
   C   
   A   
   G   
   G   
   C   
   T   
   T   
   A   
   G   
   A   
   T   
   G   
   T   
    T    
   T   
   T   
   C   
   T   
   T   
   A   
   G   
   T   
   A   
   A   
   C   
   G   
    C    
   A   
   T   
   C   
   A   
   A   
   G   
   G   
   A   
   A   
   T   
   G   
   G   
   G   
    T    
   T   
   T   
   G   
   C   
   G   
   C   
   T   
    A    
   T   
   C   
   C   
   T   
   T   
   T   
   A   
   A   
    C    
   A   
   G   
   A   
   T   
   G   
   T   
   T   
   C   
   C   
   C   
   G   
   T   
   T   
   G   
   T   
   T   
   G   
   G   
   A   
   G   
   C   
   A   
   A   
   A   
   G   
   G   
   C   
   A   
   G   
    C    
   T   
   G   
   G   
   A   
   G   
   T   
   G   
   A   
   A   
   G   
   T   
   C   
   T   
   A   
   T   
   G   
   A   
   A   
   T   
   T   
   T   
   G   
   A   
   A   
   A   
   G   
   A   
   T   
   G   
   G   
   A   
   G   
   A   
   T   
   A   
   G   
   G   
    A    
   T   
   T   
    A    
   T   
   C   
   A   
   A   
    C    
   G   
   G   
    G    
   C   
   T   
    A    
   C   
   T   
   T   
   G   
   T   
   G   
   T   
   T   
   A   
   G   
   C   
   A   
   G   
   A   
    A    
   G   
   G   
    T    
   G   
   A   
   T   
   A   
   C   
   G   
   C   
   C   
    G    
   A   
   T   
   C   
   G   
   T   
   A   
   A   
   T   
   C   
   G   
   T   
   T   
   A   
   C   
   T   
   C   
   A   
   G   
   C   
   G   
   A   
   G   
   G   
   A   
   G   
   C   
    T    
   G   
   T   
    T    
   A   
   A   
   A   
   C   
   G   
   C   
   A   
   T   
   G   
   C   
   T   
   T   
   G   
   C   
   T   
   C   
   A   
   A   
   G   
   A   
   A   
   A   
   T   
   C   
   A   
   G   
   C   
   C   
   A   
   A   
   A   
   C   
    T    
   T   
   C   
   A   
   C   
   G   
   T   
   G   
   C   
   G   
   A   
   A   
   G   
   C   
   G   
   G   
   G   
   G   
   A   
   G   
   T   
   G   
   A   
   C   
   G   
   G   
   T   
   C   
   A   
   T   
   G   
   C   
   G   
   T   
   G   
   A   
   G   
   C   
   T   
   G   
   A   
   A   
   A   
   A   
   A   
   A   
   A   
   A   
   T   
   C   
   C   
    G    
   C   
   A   
    C    
   C   
   G   
    T    
   A   
   T   
   T   
   A   
   T   
   C   
   T   
   A   
   T   
   A   
   T   
   G   
   T   
   C   
   G   
   G   
   A   
   A   
   G   
   G   
   A   
   A   
   G   
   A   
   T   
   C   
   A   
   A   
   A   
   A   
   G   
   A   
   A   
   A   
   T   
   A   
   A   
   C   
   C   
   T   
   T   
   G   
   A   
   T   
   G   
   A   
   A   
   T   
   C   
   A   
   A   
   A   
   A   
   A   
   G   
   G   
    G    
   C   
   A   
    G    
   C   
   A   
   G   
    T    
   T   
   A   
   A   
   C   
   G   
   A   
   T   
   T   
   G   
   A   
    C    
   C   
   C   
   A   
   A   
   C   
    T    
   G   
   A   
   T   
   T   
   T   
    C    
   C   
   C   
   T   
   A   
   T   
   A   
   G   
   G   
   T   
   G   
   A   
   T   
   C   
   G   
   A   
   A   
   C   
   A   
   T   
   C   
   G   
   A   
   A   
   T   
   G   
   G   
    A    
   T   
   C   
    A    
   T   
   T   
   T   
   G   
   C   
    G    
   A   
   T   
   G   
   G   
   A   
    T    
   G   
   A   
   G   
   A   
   A   
   A   
   A   
   A   
   A   
   G   
   G   
   T   
   G   
   G   
   A   
   G   
   A   
   A   
   G   
   T   
   C   
   A   
   T   
   C   
   G   
   A   
   A   
   G   
   T   
    T    
   A   
    T    
   C   
   G   
   A   
   T   
   G   
   C   
   T   
   C   
   C   
   T   
   G   
   A   
   G   
    A    
   T   
   T   
   T   
   T   
    C    
   A   
   T   
    C    
   G   
   A   
    G    
    T    
   C   
    G    
   -   
   -   
   -   
 

 
   N53804   
   A   
   T   
   G   
   G   
   A   
   A   
   A   
   A   
   T   
   C   
   G   
   A   
   C   
   A   
   A   
   G   
   A   
   A   
   G   
   T   
   A   
   C   
   A   
   A   
   G   
   A   
   A   
   T   
   T   
   A   
   A   
   C   
   T   
   T   
   T   
   A   
   G   
   A   
   A   
   G   
   A   
   A   
   G   
   T   
   G   
   A   
   T   
   G   
   G   
   G   
   T   
   G   
   A   
   C   
   C   
   G   
   C   
   T   
   T   
   C   
   G   
   G   
   A   
   C   
   G   
   T   
   T   
   A   
   T   
   T   
   C   
   C   
   A   
   A   
   A   
   T   
   A   
   T   
   A   
   T   
   T   
   A   
   T   
    C    
   C   
   A   
    A    
   G   
   A   
   A   
   C   
   G   
   T   
   G   
   C   
   A   
   C   
   T   
   G   
   C   
   C   
   T   
   G   
   A   
   T   
   A   
   T   
    C    
   C   
   G   
   T   
   G   
   A   
   T   
   G   
   G   
   A   
   T   
   T   
   G   
   A   
   A   
    G    
   C   
   C   
   A   
   G   
   T   
    G    
   C   
   A   
   A   
   C   
   G   
   C   
   C   
   G   
   G   
   A   
   T   
   A   
   T   
   T   
   A   
   T   
   T   
    T    
   G   
   C   
    C    
   A   
   T   
   G   
   A   
   A   
   C   
   A   
   A   
   A   
   G   
   A   
   T   
   G   
   G   
   C   
   A   
   A   
   T   
   A   
   C   
   G   
   T   
   A   
   T   
   G   
   A   
   C   
   A   
   A   
   A   
   G   
   G   
   A   
   T   
   T   
    C    
   C   
   G   
   T   
   A   
   A   
   A   
   T   
   C   
   A   
   G   
   C   
   A   
   A   
   A   
   A   
   T   
   C   
   T   
   G   
   T   
   C   
   G   
   G   
   A   
   A   
   A   
   T   
   A   
   T   
   C   
   A   
   T   
   G   
   G   
   G   
    G    
   A   
   A   
   T   
   T   
   A   
   T   
   C   
   A   
   T   
   C   
   C   
   T   
   C   
   A   
   T   
   G   
   G   
   T   
   G   
   A   
   C   
   A   
   G   
   C   
   A   
   G   
   T   
   A   
   T   
   T   
   T   
   A   
   T   
   G   
   A   
   A   
   G   
   C   
   G   
   A   
   T   
   G   
   G   
   T   
   C   
   C   
   G   
   C   
   A   
   T   
   G   
   A   
   G   
   C   
   C   
   A   
   A   
   G   
   A   
    C    
   T   
   G   
   G   
   A   
   A   
   G   
   T   
   T   
   G   
   C   
   G   
   G   
   G   
   A   
   A   
   G   
   T   
   A   
   C   
   T   
   G   
   A   
   T   
   C   
   G   
   A   
    A    
   A   
   T   
   G   
   C   
   A   
   C   
   G   
   G   
    G    
   A   
   A   
   T   
   A   
   A   
    C    
   G   
   G   
   G   
   A   
   G   
   T   
   A   
   T   
   G   
   G   
   A   
   C   
   G   
   G   
   T   
   G   
   A   
   T   
   C   
   C   
   A   
   C   
   C   
   T   
   G   
   C   
   T   
   G   
   C   
   G   
   A   
   T   
   G   
   C   
   G   
   T   
   T   
   A   
   T   
   A   
   C   
   T   
   G   
   A   
   A   
   G   
   C   
   C   
   A   
   G   
    A    
   T   
   T   
   A   
   T   
   C   
   C   
   A   
   A   
   G   
   T   
   T   
   A   
   A   
   G   
   T   
   G   
   G   
   A   
   G   
   A   
   G   
   A   
   T   
   G   
    C    
   T   
   A   
    G    
   C   
   A   
   G   
   A   
   T   
   A   
   T   
   C   
   G   
   A   
    A    
   A   
   A   
   A   
   G   
   A   
   A   
   A   
   C   
   A   
   G   
   T   
    C    
   G   
   A   
   T   
   C   
   T   
   C   
   G   
   T   
   G   
   T   
   G   
   G   
   A   
   A   
   T   
   T   
   T   
   T   
   G   
   A   
   T   
   G   
   A   
   T   
   A   
   C   
   A   
   G   
   A   
   A   
   A   
   A   
   A   
   G   
   A   
   A   
   C   
   C   
   A   
   A   
   C   
   A   
   G   
   T   
   C   
   C   
   T   
   T   
   C   
   C   
   A   
   G   
   C   
   T   
   C   
   G   
   A   
   T   
   A   
   C   
   C   
   C   
   A   
   A   
   A   
   C   
   C   
   T   
   T   
   C   
   T   
    C    
   G   
   T   
   C   
   A   
   A   
   T   
   G   
   G   
   A   
   T   
   C   
   G   
   A   
   C   
   A   
   G   
   G   
   T   
   A   
   T   
   A   
   T   
   C   
   T   
   G   
   C   
   T   
   G   
   G   
   A   
   T   
   A   
   T   
   G   
   C   
    A    
   A   
   C   
   A   
   G   
   A   
   A   
   A   
   T   
   C   
   C   
   C   
   G   
   A   
   C   
    T    
   C   
   A   
   T   
   A   
   A   
   C   
   T   
   T   
   A   
   G   
   C   
   T   
   G   
   A   
   A   
   G   
   T   
   G   
   A   
   T   
    C    
   G   
   A   
    T    
   G   
   G   
   C   
   A   
   C   
   A   
   A   
   T   
   C   
   T   
   A   
   T   
   A   
   T   
   G   
   A   
   T   
   C   
   G   
   A   
   T   
   C   
   A   
   T   
   C   
   C   
   A   
   C   
   A   
   A   
   G   
   C   
   G   
   A   
   G   
   C   
   C   
   T   
   T   
   G   
   A   
   A   
   A   
   A   
   A   
   T   
   T   
   G   
   A   
   T   
   G   
   G   
   A   
   A   
   T   
   A   
   T   
   A   
   T   
   T   
   C   
   C   
   T   
   G   
   G   
   A   
   C   
   C   
   T   
   G   
   A   
   C   
   T   
   T   
    T    
   C   
   C   
   A   
   A   
   C   
   A   
   G   
   G   
   A   
   G   
   G   
    G    
   A   
   T   
   C   
   T   
   T   
   G   
   C   
   A   
   A   
   G   
   G   
    A    
   A   
   A   
   A   
   G   
   A   
   T   
   G   
   A   
   A   
   A   
   T   
   C   
   A   
   A   
   A   
   A   
   A   
   A   
   G   
   C   
   T   
   T   
   A   
   T   
   G   
   A   
   A   
   A   
   C   
   A   
   G   
   G   
    G    
   C   
   G   
   A   
   G   
   G   
   A   
   A   
   A   
   A   
   G   
   T   
   T   
   A   
    T    
   T   
   C   
   T   
   T   
   C   
   G   
   T   
   T   
   C   
   G   
   A   
   A   
   A   
   A   
   C   
   A   
   A   
   A   
   G   
   A   
   T   
   A   
   G   
   A   
    G    
   G   
   C   
   A   
   A   
   T   
   G   
   A   
   A   
   G   
   G   
   G   
    T    
   A   
   A   
    T    
   A   
   A   
   A   
   C   
   A   
   G   
   C   
   A   
   A   
   A   
   T   
   C   
   G   
   T   
   G   
   A   
   T   
   C   
   A   
   C   
    A    
   G   
   A   
   A   
   A   
   T   
   C   
   C   
   C   
   A   
   T   
   A   
   T   
   G   
   A   
   A   
   G   
   T   
   A   
   A   
   A   
   T   
   A   
   A   
   A   
   G   
   C   
   G   
   A   
   C   
    C    
   T   
   T   
   G   
   G   
   T   
   G   
   A   
   A   
   A   
   A   
   A   
   A   
   A   
   T   
   G   
   G   
   A   
   T   
   G   
   A   
   G   
   A   
   T   
   C   
   C   
   G   
   T   
   T   
   T   
   G   
   A   
   A   
   T   
   A   
   A   
   G   
   A   
   A   
   A   
   A   
   T   
   C   
   G   
   A   
    T    
   G   
   G   
   A   
   A   
   T   
   A   
   G   
   C   
   T   
   G   
   A   
   A   
   G   
   T   
    A    
   C   
   G   
   A   
   G   
   A   
   T   
   G   
   A   
   A   
   A   
   G   
   T   
   G   
   A   
   T   
    A    
   G   
    A    
   A   
   C   
    A    
   G   
   G   
   T   
   C   
   T   
   G   
   C   
   A   
   A   
   A   
   T   
   T   
   G   
   T   
   C   
   G   
   T   
    C    
   G   
   A   
   A   
   T   
   T   
   G   
   A   
   A   
   A   
   A   
   A   
   A   
   G   
   A   
    C    
   G   
   C   
   C   
   A   
   A   
    T    
   G   
   C   
   T   
   C   
   A   
   A   
   G   
   G   
    A    
   A   
   T   
   T   
   T   
   T   
   G   
   A   
   A   
   T   
   T   
   A   
    T    
   T   
   T   
   G   
   T   
   T   
   C   
   A   
   A   
   A   
   A   
   A   
   C   
   A   
   C   
   T   
   G   
   A   
   G   
    C    
   T   
   A   
   C   
   A   
   G   
   A   
   T   
   C   
   A   
   A   
   T   
   T   
   A   
   C   
   A   
   A   
   T   
   T   
   T   
    C    
   A   
   A   
   T   
   A   
   T   
   G   
   G   
   T   
   C   
   G   
   C   
   T   
   A   
   T   
   C   
   G   
   A   
    C    
   C   
   A   
   T   
   A   
   T   
   G   
   A   
   C   
   T   
   C   
   C   
   G   
   C   
   A   
    T    
   C   
   A   
   A   
   G   
   T   
   T   
   G   
   G   
   A   
   C   
   T   
   G   
   A   
   A   
   A   
   G   
   A   
   C   
   A   
   T   
   T   
   T   
   T   
   A   
   A   
   G   
   A   
   A   
   G   
   T   
   T   
   A   
   T   
   A   
   T   
   C   
   G   
   A   
   G   
   C   
   A   
   T   
   C   
   G   
   A   
   A   
   A   
   G   
   C   
   A   
   A   
   G   
   T   
   C   
   A   
   T   
   A   
   A   
   C   
   A   
   A   
   A   
   A   
   C   
   G   
    T    
   A   
   G   
    T    
   C   
   A   
   A   
   T   
   T   
   C   
   G   
   A   
   T   
   T   
   T   
   A   
   G   
   C   
   C   
   A   
   A   
   A   
   G   
   C   
   T   
   C   
   A   
   A   
   A   
   A   
   A   
    A    
   G   
   A   
   C   
   A   
   G   
   C   
   A   
   T   
   A   
   T   
   C   
   G   
   T   
    A    
   G   
   A   
   A   
   G   
   G   
    A    
    T    
   T   
   G   
   A   
   T   
   G   
   A   
   A   
   A   
   G   
   C   
   T   
   T   
   T   
   A   
   T   
   C   
   G   
   A   
   T   
   T   
   C   
   T   
   G   
   G   
   A   
   T   
   G   
   A   
   A   
   G   
   T   
   A   
   A   
   T   
   C   
   G   
    C    
   A   
   A   
   C   
   G   
   A   
   T   
   T   
   C   
   G   
   T   
   G   
   A   
   A   
   A   
   G   
   T   
   A   
   A   
   A   
   G   
   A   
   C   
   A   
   A   
   A   
   A   
   A   
   A   
   G   
   A   
    C    
   G   
   C   
    C    
   A   
   A   
   A   
   A   
   A   
   G   
   A   
   A   
   T   
   T   
   T   
   A   
   G   
   T   
   C   
   G   
   A   
    C    
   G   
   T   
    C    
   T   
   T   
    T    
   C   
   A   
   A   
   T   
   T   
   T   
   A   
   C   
   A   
   G   
   A   
   A   
   G   
   A   
   A   
   C   
   A   
   A   
   G   
   C   
   A   
   G   
   A   
   A   
   G   
   C   
   A   
   A   
   T   
    T    
   G   
   T   
   C   
   A   
   C   
   A   
   T   
   T   
   G   
   C   
   A   
   A   
    T    
   T   
   A   
   T   
   A   
    C    
   C   
   G   
   T   
   T   
   T   
   G   
   A   
   C   
   G   
   A   
   A   
    C    
   A   
   C   
    G    
   G   
   A   
   T   
   A   
   T   
   C   
   A   
   C   
   A   
   G   
   A   
   G   
   C   
   T   
   T   
   C   
   A   
   A   
   A   
   A   
   G   
   G   
   A   
   A   
    T    
   C   
    C    
   G   
   A   
   A   
   A   
    G    
   C   
   T   
   T   
   G   
   A   
   T   
   T   
   G   
   C   
    G    
   C   
   A   
   A   
   A   
   T   
   C   
   A   
   C   
   A   
   G   
   A   
   G   
   T   
   T   
    G    
   A   
   A   
   T   
   A   
   A   
   A   
   A   
   T   
   C   
   C   
   T   
   T   
   T   
   C   
   A   
   A   
   A   
    T    
   G   
   A   
   C   
   A   
   A   
   A   
   G   
   A   
    G    
   C   
   T   
   T   
   T   
   T   
    C    
   T   
   C   
   T   
   G   
   T   
   T   
   A   
   T   
   G   
   A   
   A   
   A   
   A   
   A   
   A   
   G   
   A   
   A   
   T   
   T   
   G   
   C   
   G   
   A   
   G   
   A   
   A   
   G   
   T   
   G   
   A   
   A   
   A   
   A   
   A   
   A   
   A   
   A   
   T   
   T   
   A   
    T    
   T   
   C   
   T   
   T   
   C   
   C   
   G   
   C   
   T   
   C   
   G   
   T   
   C   
    T    
    A    
   A   
   C   
   G   
   A   
   C   
   A   
   A   
   T   
   C   
    G    
   A   
   A   
   G   
   A   
   C   
   G   
   A   
   A   
   A   
   T   
   C   
   G   
   A   
   A   
   G   
   A   
   A   
   A   
   T   
   C   
   A   
   A   
   A   
   A   
   T   
    T    
   G   
   A   
   T   
   A   
   C   
   G   
   C   
   A   
   A   
    G    
   T   
    C    
   T   
   T   
   A   
   G   
   T   
   G   
   G   
   C   
    A    
   C   
   A   
   A   
   G   
   A   
   A   
   G   
   A   
   C   
   G   
   T   
   A   
   A   
   T   
    T    
   G   
   T   
   C   
   A   
   G   
   T   
   G   
   T   
    G    
   A   
   C   
    G    
   C   
   G   
   A   
   G   
   A   
   A   
   G   
   G   
   C   
   T   
   A   
   T   
   G   
   T   
   G   
   A   
   A   
   G   
   C   
   G   
   A   
   A   
   C   
   A   
   A   
   G   
   C   
   C   
   T   
   T   
   C   
   G   
   T   
   T   
   C   
    T    
   T   
   A   
   T   
   A   
   G   
   T   
   G   
   C   
   T   
   T   
   C   
    C    
   A   
   A   
   A   
   C   
   C   
   A   
   G   
   A   
   A   
   G   
   A   
   A   
   A   
   T   
   C   
   G   
   G   
   G   
   A   
   T   
   G   
   C   
   G   
   A   
   G   
   A   
   A   
   G   
   G   
   C   
   G   
   A   
    C    
   T   
   A   
   C   
   T   
   T   
   G   
   C   
   T   
    A    
   T   
   A   
   C   
    T    
   C   
   A   
   G   
   G   
   T   
   G   
   A   
   A   
   T   
   T   
    G    
   A   
   G   
    T    
   A   
   C   
   A   
    T    
   T   
   G   
   G   
   A   
   C   
   C   
   A   
   T   
   G   
   T   
   T   
   C   
   T   
   T   
   C   
   T   
   G   
   A   
   T   
    C    
   A   
   C   
    A    
   A   
   A   
   T   
   A   
   A   
   A   
   G   
   C   
   A   
   A   
   A   
   T   
   G   
   T   
   G   
   A   
   T   
   C   
   T   
   A   
   C   
   C   
   G   
    C    
   C   
   C   
   T   
   G   
   T   
    C    
   C   
   A   
   T   
   G   
   A   
   A   
   C   
   T   
   A   
   C   
   C   
    C    
   G   
   A   
   T   
   T   
   T   
   G   
   A   
   A   
   A   
   T   
   G   
   G   
   A   
   A   
   A   
   G   
   A   
   T   
   G   
   C   
   T   
   G   
   G   
    T    
   G   
   A   
    G    
   C   
   A   
    C    
   A   
   T   
   T   
   T   
   C   
   T   
   C   
   A   
   A   
   A   
   C   
   A   
   A   
   T   
   C   
   T   
   C   
   C   
   A   
   A   
   T   
   C   
   T   
   G   
   T   
   C   
   T   
   G   
   T   
   A   
   G   
   A   
    C    
   G   
   A   
   A   
   T   
   C   
   G   
   A   
   T   
    T    
   T   
   T   
   A   
   G   
   C   
   T   
   G   
   T   
   T   
   T   
   T   
    C    
   C   
   C   
   A   
   T   
   A   
   T   
   C   
   A   
   A   
   A   
   A   
   A   
   A   
   T   
    T    
   C   
   A   
   A   
   G   
   C   
   A   
   G   
   A   
    G    
   A   
   A   
   A   
   A   
   C   
   A   
   T   
   T   
   T   
   G   
   T   
    A    
   T   
   T   
   C   
   A   
   T   
   C   
   A   
   G   
   C   
   A   
   A   
   G   
   A   
   A   
   T   
   G   
   G   
   A   
   T   
   T   
    G    
    A    
   T   
   C   
   A   
   A   
   G   
   C   
   A   
   A   
   A   
   C   
   A   
   C   
   G   
   A   
   A   
   T   
   G   
   A   
   C   
   A   
   G   
   A   
   G   
   T   
   T   
   C   
   G   
   A   
   A   
   C   
   C   
   A   
   T   
   G   
   G   
   C   
   G   
   C   
   A   
   C   
   C   
   T   
   A   
    T    
   A   
   A   
   G   
   A   
   G   
   C   
   C   
   G   
    T    
   C   
   C   
   G   
   C   
   T   
   C   
   A   
   G   
   C   
   T   
   G   
   T   
   A   
   T   
   G   
   A   
   A   
   G   
   C   
   T   
   A   
   A   
   A   
   G   
   G   
   C   
   G   
   G   
   A   
   A   
   G   
   A   
   A   
   G   
   A   
   T   
   G   
   A   
   A   
   C   
   T   
    G    
   A   
   T   
   T   
   G   
   C   
   T   
   G   
   T   
    G    
   T   
   A   
   T   
   C   
   T   
   G   
   A   
   A   
   A   
   G   
   A   
   A   
   G   
   A   
   T   
   C   
    A    
   G   
   C   
   C   
   A   
   G   
   G   
   C   
   T   
   T   
   A   
   G   
   A   
   T   
   G   
   T   
    T    
   T   
   T   
   C   
   T   
   T   
   A   
   G   
   T   
   A   
   A   
   C   
   G   
    C    
   A   
   T   
   C   
   A   
   A   
   G   
   G   
   A   
   A   
   T   
   G   
   G   
   G   
    T    
   T   
   T   
   G   
   C   
   G   
   C   
   T   
    G    
   T   
   C   
   C   
   T   
   T   
   T   
   A   
   A   
    C    
   A   
   G   
   A   
   T   
   G   
   T   
   T   
   C   
   C   
   C   
   G   
   T   
   T   
   G   
   T   
   T   
   G   
   G   
   A   
   G   
   C   
   A   
   A   
   A   
   G   
   G   
   C   
   A   
   G   
    C    
   T   
   G   
   G   
   A   
   G   
   T   
   G   
   A   
   A   
   G   
   T   
   C   
   T   
   A   
   T   
   G   
   A   
   A   
   T   
   T   
   T   
   G   
   A   
   A   
   A   
   G   
   A   
   T   
   G   
   G   
   A   
   G   
   A   
   T   
   A   
   G   
   G   
    A    
   T   
   T   
    A    
   T   
   C   
   A   
   A   
    C    
   G   
   G   
    G    
   C   
   T   
    G    
   C   
   T   
   T   
   G   
   T   
   G   
   T   
   T   
   A   
   G   
   C   
   A   
   G   
   A   
    A    
   G   
   G   
    T    
   G   
   A   
   T   
   A   
   C   
   G   
   C   
   C   
    G    
   A   
   T   
   C   
   G   
   T   
   A   
   A   
   T   
   C   
   G   
   T   
   T   
   A   
   C   
   T   
   C   
   A   
   G   
   C   
   G   
   A   
   G   
   G   
   A   
   G   
   C   
    T    
   G   
   T   
    T    
   A   
   A   
   A   
   C   
   G   
   C   
   A   
   T   
   G   
   C   
   T   
   T   
   G   
   C   
   T   
   C   
   A   
   A   
   G   
   A   
   A   
   A   
   T   
   C   
   A   
   G   
   C   
   C   
   A   
   A   
   A   
   C   
    T    
   T   
   C   
   A   
   C   
   G   
   T   
   G   
   C   
   G   
   A   
   A   
   G   
   C   
   G   
   G   
   G   
   G   
   A   
   G   
   T   
   G   
   A   
   C   
   G   
   G   
   T   
   C   
   A   
   T   
   G   
   C   
   G   
   T   
   G   
   A   
   G   
   C   
   T   
   G   
   A   
   A   
   A   
   A   
   A   
   A   
   A   
   A   
   T   
   C   
   C   
    G    
   C   
   A   
    C    
   C   
   G   
    T    
   A   
   T   
   T   
   A   
   T   
   C   
   T   
   A   
   T   
   A   
   T   
   G   
   T   
   C   
   G   
   G   
   A   
   A   
   G   
   G   
   A   
   A   
   G   
   A   
   T   
   C   
   A   
   A   
   A   
   A   
   G   
   A   
   A   
   A   
   T   
   A   
   A   
   C   
   C   
   T   
   T   
   G   
   A   
   T   
   G   
   A   
   A   
   T   
   C   
   A   
   A   
   A   
   A   
   A   
   G   
   G   
    G    
   C   
   A   
    G    
   C   
   A   
   G   
    T    
   T   
   A   
   A   
   C   
   G   
   A   
   T   
   T   
   G   
   A   
    C    
   C   
   C   
   A   
   A   
   C   
    T    
   G   
   A   
   T   
   T   
   T   
    C    
   C   
   C   
   T   
   A   
   T   
   A   
   G   
   G   
   T   
   G   
   A   
   T   
   C   
   G   
   A   
   A   
   C   
   A   
   T   
   C   
   G   
   A   
   A   
   T   
   G   
   G   
    A    
   T   
   C   
    A    
   T   
   T   
   T   
   G   
   C   
    G    
   A   
   T   
   G   
   G   
   A   
    T    
   G   
   A   
   G   
   A   
   A   
   A   
   A   
   A   
   A   
   G   
   G   
   T   
   G   
   G   
   A   
   G   
   A   
   A   
   G   
   T   
   C   
   A   
   T   
   C   
   G   
   A   
   A   
   G   
   T   
    T    
   A   
    T    
   C   
   G   
   A   
   T   
   G   
   C   
   T   
   C   
   C   
   T   
   G   
   A   
   G   
    A    
   T   
   T   
   T   
   T   
    C    
   A   
   T   
    C    
   G   
   A   
    G    
    T    
   C   
    G    
   -   
   -   
   -   
 

 
   N60200F   
   A   
   T   
   G   
   G   
   A   
   A   
   A   
   A   
   T   
   C   
   G   
   A   
   C   
   A   
   A   
   G   
   A   
   A   
   G   
   T   
   A   
   C   
   A   
   A   
   G   
   A   
   A   
   T   
   T   
   A   
   A   
   C   
   T   
   T   
   T   
   A   
   G   
   A   
   A   
   G   
   A   
   A   
   G   
   T   
   G   
   A   
   T   
   G   
   G   
   G   
   T   
   G   
   A   
   C   
   C   
   G   
   C   
   T   
   T   
   C   
   G   
   G   
   A   
   C   
   G   
   T   
   T   
   A   
   T   
   T   
   C   
   C   
   A   
   A   
   A   
   T   
   A   
   T   
   A   
   T   
   T   
   A   
   T   
    C    
   C   
   A   
    A    
   G   
   A   
   A   
   C   
   G   
   T   
   G   
   C   
   A   
   C   
   T   
   G   
   C   
   C   
   T   
   G   
   A   
   T   
   A   
   T   
    C    
   C   
   G   
   T   
   G   
   A   
   T   
   G   
   G   
   A   
   T   
   T   
   G   
   A   
   A   
    G    
   C   
   C   
   A   
   G   
   T   
    G    
   C   
   A   
   A   
   C   
   G   
   C   
   C   
   G   
   G   
   A   
   T   
   A   
   T   
   T   
   A   
   T   
   T   
    T    
   G   
   C   
    C    
   A   
   T   
   G   
   A   
   A   
   C   
   A   
   A   
   A   
   G   
   A   
   T   
   G   
   G   
   C   
   A   
   A   
   T   
   A   
   C   
   G   
   T   
   A   
   T   
   G   
   A   
   C   
   A   
   A   
   A   
   G   
   G   
   A   
   T   
   T   
    C    
   C   
   G   
   T   
   A   
   A   
   A   
   T   
   C   
   A   
   G   
   C   
   A   
   A   
   A   
   A   
   T   
   C   
   T   
   G   
   T   
   C   
   G   
   G   
   A   
   A   
   A   
   T   
   A   
   T   
   C   
   A   
   T   
   G   
   G   
   G   
    G    
   A   
   A   
   T   
   T   
   A   
   T   
   C   
   A   
   T   
   C   
   C   
   T   
   C   
   A   
   T   
   G   
   G   
   T   
   G   
   A   
   C   
   A   
   G   
   C   
   A   
   G   
   T   
   A   
   T   
   T   
   T   
   A   
   T   
   G   
   A   
   A   
   G   
   C   
   G   
   A   
   T   
   G   
   G   
   T   
   C   
   C   
   G   
   C   
   A   
   T   
   G   
   A   
   G   
   C   
   C   
   A   
   A   
   G   
   A   
    C    
   T   
   G   
   G   
   A   
   A   
   G   
   T   
   T   
   G   
   C   
   G   
   G   
   G   
   A   
   A   
   G   
   T   
   A   
   C   
   T   
   G   
   A   
   T   
   C   
   G   
   A   
    A    
   A   
   T   
   G   
   C   
   A   
   C   
   G   
   G   
    G    
   A   
   A   
   T   
   A   
   A   
    C    
   G   
   G   
   G   
   A   
   G   
   T   
   A   
   T   
   G   
   G   
   A   
   C   
   G   
   G   
   T   
   G   
   A   
   T   
   C   
   C   
   A   
   C   
   C   
   T   
   G   
   C   
   T   
   G   
   C   
   G   
   A   
   T   
   G   
   C   
   G   
   T   
   T   
   A   
   T   
   A   
   C   
   T   
   G   
   A   
   A   
   G   
   C   
   C   
   A   
   G   
    A    
   T   
   T   
   A   
   T   
   C   
   C   
   A   
   A   
   G   
   T   
   T   
   A   
   A   
   G   
   T   
   G   
   G   
   A   
   G   
   A   
   G   
   A   
   T   
   G   
    C    
   T   
   A   
    G    
   C   
   A   
   G   
   A   
   T   
   A   
   T   
   C   
   G   
   A   
    A    
   A   
   A   
   A   
   G   
   A   
   A   
   A   
   C   
   A   
   G   
   T   
    C    
   G   
   A   
   T   
   C   
   T   
   C   
   G   
   T   
   G   
   T   
   G   
   G   
   A   
   A   
   T   
   T   
   T   
   T   
   G   
   A   
   T   
   G   
   A   
   T   
   A   
   C   
   A   
   G   
   A   
   A   
   A   
   A   
   A   
   G   
   A   
   A   
   C   
   C   
   A   
   A   
   C   
   A   
   G   
   T   
   C   
   C   
   T   
   T   
   C   
   C   
   A   
   G   
   C   
   T   
   C   
   G   
   A   
   T   
   A   
   C   
   C   
   C   
   A   
   A   
   A   
   C   
   C   
   T   
   T   
   C   
   T   
    C    
   G   
   T   
   C   
   A   
   A   
   T   
   G   
   G   
   A   
   T   
   C   
   G   
   A   
   C   
   A   
   G   
   G   
   T   
   A   
   T   
   A   
   T   
   C   
   T   
   G   
   C   
   T   
   G   
   G   
   A   
   T   
   A   
   T   
   G   
   C   
    A    
   A   
   C   
   A   
   G   
   A   
   A   
   A   
   T   
   C   
   C   
   C   
   G   
   A   
   C   
    T    
   C   
   A   
   T   
   A   
   A   
   C   
   T   
   T   
   A   
   G   
   C   
   T   
   G   
   A   
   A   
   G   
   T   
   G   
   A   
   T   
    T    
   G   
   A   
    T    
   G   
   G   
   C   
   A   
   C   
   A   
   A   
   T   
   C   
   T   
   A   
   T   
   A   
   T   
   G   
   A   
   T   
   C   
   G   
   A   
   T   
   C   
   A   
   T   
   C   
   C   
   A   
   C   
   A   
   A   
   G   
   C   
   G   
   A   
   G   
   C   
   C   
   T   
   T   
   G   
   A   
   A   
   A   
   A   
   A   
   T   
   T   
   G   
   A   
   T   
   G   
   G   
   A   
   A   
   T   
   A   
   T   
   A   
   T   
   T   
   C   
   C   
   T   
   G   
   G   
   A   
   C   
   C   
   T   
   G   
   A   
   C   
   T   
   T   
    T    
   C   
   C   
   A   
   A   
   C   
   A   
   G   
   G   
   A   
   G   
   G   
    G    
   A   
   T   
   C   
   T   
   T   
   G   
   C   
   A   
   A   
   G   
   G   
    A    
   A   
   A   
   A   
   G   
   A   
   T   
   G   
   A   
   A   
   A   
   T   
   C   
   A   
   A   
   A   
   A   
   A   
   A   
   G   
   C   
   T   
   T   
   A   
   T   
   G   
   A   
   A   
   A   
   C   
   A   
   G   
   G   
    G    
   C   
   G   
   A   
   G   
   G   
   A   
   A   
   A   
   A   
   G   
   T   
   T   
   A   
    T    
   T   
   C   
   T   
   T   
   C   
   G   
   T   
   T   
   C   
   G   
   A   
   A   
   A   
   A   
   C   
   A   
   A   
   A   
   G   
   A   
   T   
   A   
   G   
   A   
    G    
   G   
   C   
   A   
   A   
   T   
   G   
   A   
   A   
   G   
   G   
   G   
    T    
   A   
   A   
    T    
   A   
   A   
   A   
   C   
   A   
   G   
   C   
   A   
   A   
   A   
   T   
   C   
   G   
   T   
   G   
   A   
   T   
   C   
   A   
   C   
    A    
   G   
   A   
   A   
   A   
   T   
   C   
   C   
   C   
   A   
   T   
   A   
   T   
   G   
   A   
   A   
   G   
   T   
   A   
   A   
   A   
   T   
   A   
   A   
   A   
   G   
   C   
   G   
   A   
   C   
    C    
   T   
   T   
   G   
   G   
   T   
   G   
   A   
   A   
   A   
   A   
   A   
   A   
   A   
   T   
   G   
   G   
   A   
   T   
   G   
   A   
   G   
   A   
   T   
   C   
   C   
   G   
   T   
   T   
   T   
   G   
   A   
   A   
   T   
   A   
   A   
   G   
   A   
   A   
   A   
   A   
   T   
   C   
   G   
   A   
    T    
   G   
   G   
   A   
   A   
   T   
   A   
   G   
   C   
   T   
   G   
   A   
   A   
   G   
   T   
    A    
   C   
   G   
   A   
   G   
   A   
   T   
   G   
   A   
   A   
   A   
   G   
   T   
   G   
   A   
   T   
    A    
   G   
    A    
   A   
   C   
    A    
   G   
   G   
   T   
   C   
   T   
   G   
   C   
   A   
   A   
   A   
   T   
   T   
   G   
   T   
   C   
   G   
   T   
    C    
   G   
   A   
   A   
   T   
   T   
   G   
   A   
   A   
   A   
   A   
   A   
   A   
   G   
   A   
    C    
   G   
   C   
   C   
   A   
   A   
    T    
   G   
   C   
   T   
   C   
   A   
   A   
   G   
   G   
    A    
   A   
   T   
   T   
   T   
   T   
   G   
   A   
   A   
   T   
   T   
   A   
    T    
   T   
   T   
   G   
   T   
   T   
   C   
   A   
   A   
   A   
   A   
   A   
   C   
   A   
   C   
   T   
   G   
   A   
   G   
    C    
   T   
   A   
   C   
   A   
   G   
   A   
   T   
   C   
   A   
   A   
   T   
   T   
   A   
   C   
   A   
   A   
   T   
   T   
   T   
    C    
   A   
   A   
   T   
   A   
   T   
   G   
   G   
   T   
   C   
   G   
   C   
   T   
   A   
   T   
   C   
   G   
   A   
    C    
   C   
   A   
   T   
   A   
   T   
   G   
   A   
   C   
   T   
   C   
   C   
   G   
   C   
   A   
    T    
   C   
   A   
   A   
   G   
   T   
   T   
   G   
   G   
   A   
   C   
   T   
   G   
   A   
   A   
   A   
   G   
   A   
   C   
   A   
   T   
   T   
   T   
   T   
   A   
   A   
   G   
   A   
   A   
   G   
   T   
   T   
   A   
   T   
   A   
   T   
   C   
   G   
   A   
   G   
   C   
   A   
   T   
   C   
   G   
   A   
   A   
   A   
   G   
   C   
   A   
   A   
   G   
   T   
   C   
   A   
   T   
   A   
   A   
   C   
   A   
   A   
   A   
   A   
   C   
   G   
    T    
   A   
   G   
    T    
   C   
   A   
   A   
   T   
   T   
   C   
   G   
   A   
   T   
   T   
   T   
   A   
   G   
   C   
   C   
   A   
   A   
   A   
   G   
   C   
   T   
   C   
   A   
   A   
   A   
   A   
   A   
    A    
   G   
   A   
   C   
   A   
   G   
   C   
   A   
   T   
   A   
   T   
   C   
   G   
   T   
    A    
   G   
   A   
   A   
   G   
   G   
    A    
    T    
   T   
   G   
   A   
   T   
   G   
   A   
   A   
   A   
   G   
   C   
   T   
   T   
   T   
   A   
   T   
   C   
   G   
   A   
   T   
   T   
   C   
   T   
   G   
   G   
   A   
   T   
   G   
   A   
   A   
   G   
   T   
   A   
   A   
   T   
   C   
   G   
    T    
   A   
   A   
   C   
   G   
   A   
   T   
   T   
   C   
   G   
   T   
   G   
   A   
   A   
   A   
   G   
   T   
   A   
   A   
   A   
   G   
   A   
   C   
   A   
   A   
   A   
   A   
   A   
   A   
   G   
   A   
    C    
   G   
   C   
    C    
   A   
   A   
   A   
   A   
   A   
   G   
   A   
   A   
   T   
   T   
   T   
   A   
   G   
   T   
   C   
   G   
   A   
    C    
   G   
   T   
    C    
   T   
   T   
    T    
   C   
   A   
   A   
   T   
   T   
   T   
   A   
   C   
   A   
   G   
   A   
   A   
   G   
   A   
   A   
   C   
   A   
   A   
   G   
   C   
   A   
   G   
   A   
   A   
   G   
   C   
   A   
   A   
   T   
    T    
   G   
   T   
   C   
   A   
   C   
   A   
   T   
   T   
   G   
   C   
   A   
   A   
    T    
   T   
   A   
   T   
   A   
    C    
   C   
   G   
   T   
   T   
   T   
   G   
   A   
   C   
   G   
   A   
   A   
    C    
   A   
   C   
    G    
   G   
   A   
   T   
   A   
   T   
   C   
   A   
   C   
   A   
   G   
   A   
   G   
   C   
   T   
   T   
   C   
   A   
   A   
   A   
   A   
   G   
   G   
   A   
   A   
    T    
   C   
    C    
   G   
   A   
   A   
   A   
    G    
   C   
   T   
   T   
   G   
   A   
   T   
   T   
   G   
   C   
    G    
   C   
   A   
   A   
   A   
   T   
   C   
   A   
   C   
   A   
   G   
   A   
   G   
   T   
   T   
    G    
   A   
   A   
   T   
   A   
   A   
   A   
   A   
   T   
   C   
   C   
   T   
   T   
   T   
   C   
   A   
   A   
   A   
    T    
   G   
   A   
   C   
   A   
   A   
   A   
   G   
   A   
    G    
   C   
   T   
   T   
   T   
   T   
    C    
   T   
   C   
   T   
   G   
   T   
   T   
   A   
   T   
   G   
   A   
   A   
   A   
   A   
   A   
   A   
   G   
   A   
   A   
   T   
   T   
   G   
   C   
   G   
   A   
   G   
   A   
   A   
   G   
   T   
   G   
   A   
   A   
   A   
   A   
   A   
   A   
   A   
   A   
   T   
   T   
   A   
    T    
   T   
   C   
   T   
   T   
   C   
   C   
   G   
   C   
   T   
   C   
   G   
   T   
   C   
    T    
    A    
   A   
   C   
   G   
   A   
   C   
   A   
   A   
   T   
   C   
    G    
   A   
   A   
   G   
   A   
   C   
   G   
   A   
   A   
   A   
   T   
   C   
   G   
   A   
   A   
   G   
   A   
   A   
   A   
   T   
   C   
   A   
   A   
   A   
   A   
   T   
    T    
   G   
   A   
   T   
   A   
   C   
   G   
   C   
   A   
   A   
    G    
   T   
    C    
   T   
   T   
   A   
   G   
   T   
   G   
   G   
   C   
    A    
   C   
   A   
   A   
   G   
   A   
   A   
   G   
   A   
   C   
   G   
   T   
   A   
   A   
   T   
    T    
   G   
   T   
   C   
   A   
   G   
   T   
   G   
   T   
    G    
   A   
   C   
    G    
   C   
   G   
   A   
   G   
   A   
   A   
   G   
   G   
   C   
   T   
   A   
   T   
   G   
   T   
   G   
   A   
   A   
   G   
   C   
   G   
   A   
   A   
   C   
   A   
   A   
   G   
   C   
   C   
   T   
   T   
   C   
   G   
   T   
   T   
   C   
    T    
   T   
   A   
   T   
   A   
   G   
   T   
   G   
   C   
   T   
   T   
   C   
    C    
   A   
   A   
   A   
   C   
   C   
   A   
   G   
   A   
   A   
   G   
   A   
   A   
   A   
   T   
   C   
   G   
   G   
   G   
   A   
   T   
   G   
   C   
   G   
   A   
   G   
   A   
   A   
   G   
   G   
   C   
   G   
   A   
    C    
   T   
   A   
   C   
   T   
   T   
   G   
   C   
   T   
    A    
   T   
   A   
   C   
    T    
   C   
   A   
   G   
   G   
   T   
   G   
   A   
   A   
   T   
   T   
    G    
   A   
   G   
    T    
   A   
   C   
   A   
    T    
   T   
   G   
   G   
   A   
   C   
   C   
   A   
   T   
   G   
   T   
   T   
   C   
   T   
   T   
   C   
   T   
   G   
   A   
   T   
    C    
   A   
   C   
    A    
   A   
   A   
   T   
   A   
   A   
   A   
   G   
   C   
   A   
   A   
   A   
   T   
   G   
   T   
   G   
   A   
   T   
   C   
   T   
   A   
   C   
   C   
   G   
    C    
   C   
   C   
   T   
   G   
   T   
    C    
   C   
   A   
   T   
   G   
   A   
   A   
   C   
   T   
   A   
   C   
   C   
    C    
   G   
   A   
   T   
   T   
   T   
   G   
   A   
   A   
   A   
   T   
   G   
   G   
   A   
   A   
   A   
   G   
   A   
   T   
   G   
   C   
   T   
   G   
   G   
    T    
   G   
   A   
    G    
   C   
   A   
    C    
   A   
   T   
   T   
   T   
   C   
   T   
   C   
   A   
   A   
   A   
   C   
   A   
   A   
   T   
   C   
   T   
   C   
   C   
   A   
   A   
   T   
   C   
   T   
   G   
   T   
   C   
   T   
   G   
   T   
   A   
   G   
   A   
    C    
   G   
   A   
   A   
   T   
   C   
   G   
   A   
   T   
    T    
   T   
   T   
   A   
   G   
   C   
   T   
   G   
   T   
   T   
   T   
   T   
    C    
   C   
   C   
   A   
   T   
   A   
   T   
   C   
   A   
   A   
   A   
   A   
   A   
   A   
   T   
    T    
   C   
   A   
   A   
   G   
   C   
   A   
   G   
   A   
    G    
   A   
   A   
   A   
   A   
   C   
   A   
   T   
   T   
   T   
   G   
   T   
    A    
   T   
   T   
   C   
   A   
   T   
   C   
   A   
   G   
   C   
   A   
   A   
   G   
   A   
   A   
   T   
   G   
   G   
   A   
   T   
   T   
    G    
    A    
   T   
   C   
   A   
   A   
   G   
   C   
   A   
   A   
   A   
   C   
   A   
   C   
   G   
   A   
   A   
   T   
   G   
   A   
   C   
   A   
   G   
   A   
   G   
   T   
   T   
   C   
   G   
   A   
   A   
   C   
   C   
   A   
   T   
   G   
   G   
   C   
   G   
   C   
   A   
   C   
   C   
   T   
   A   
    T    
   A   
   A   
   G   
   A   
   G   
   C   
   C   
   G   
    T    
   C   
   C   
   G   
   C   
   T   
   C   
   A   
   G   
   C   
   T   
   G   
   T   
   A   
   T   
   G   
   A   
   A   
   G   
   C   
   T   
   A   
   A   
   A   
   G   
   G   
   C   
   G   
   G   
   A   
   A   
   G   
   A   
   A   
   G   
   A   
   T   
   G   
   A   
   A   
   C   
   T   
    G    
   A   
   T   
   T   
   G   
   C   
   T   
   G   
   T   
    G    
   T   
   A   
   T   
   C   
   T   
   G   
   A   
   A   
   A   
   G   
   A   
   A   
   G   
   A   
   T   
   C   
    A    
   G   
   C   
   C   
   A   
   G   
   G   
   C   
   T   
   T   
   A   
   G   
   A   
   T   
   G   
   T   
    T    
   T   
   T   
   C   
   T   
   T   
   A   
   G   
   T   
   A   
   A   
   C   
   G   
    C    
   A   
   T   
   C   
   A   
   A   
   G   
   G   
   A   
   A   
   T   
   G   
   G   
   G   
    T    
   T   
   T   
   G   
   C   
   G   
   C   
   T   
    A    
   T   
   C   
   C   
   T   
   T   
   T   
   A   
   A   
    C    
   A   
   G   
   A   
   T   
   G   
   T   
   T   
   C   
   C   
   C   
   G   
   T   
   T   
   G   
   T   
   T   
   G   
   G   
   A   
   G   
   C   
   A   
   A   
   A   
   G   
   G   
   C   
   A   
   G   
    C    
   T   
   G   
   G   
   A   
   G   
   T   
   G   
   A   
   A   
   G   
   T   
   C   
   T   
   A   
   T   
   G   
   A   
   A   
   T   
   T   
   T   
   G   
   A   
   A   
   A   
   G   
   A   
   T   
   G   
   G   
   A   
   G   
   A   
   T   
   A   
   G   
   G   
    A    
   T   
   T   
    A    
   T   
   C   
   A   
   A   
    C    
   G   
   G   
    G    
   C   
   T   
    G    
   C   
   T   
   T   
   G   
   T   
   G   
   T   
   T   
   A   
   G   
   C   
   A   
   G   
   A   
    A    
   G   
   G   
    T    
   G   
   A   
   T   
   A   
   C   
   G   
   C   
   C   
    G    
   A   
   T   
   C   
   G   
   T   
   A   
   A   
   T   
   C   
   G   
   T   
   T   
   A   
   C   
   T   
   C   
   A   
   G   
   C   
   G   
   A   
   G   
   G   
   A   
   G   
   C   
    T    
   G   
   T   
    T    
   A   
   A   
   A   
   C   
   G   
   C   
   A   
   T   
   G   
   C   
   T   
   T   
   G   
   C   
   T   
   C   
   A   
   A   
   G   
   A   
   A   
   A   
   T   
   C   
   A   
   G   
   C   
   C   
   A   
   A   
   A   
   C   
    T    
   T   
   C   
   A   
   C   
   G   
   T   
   G   
   C   
   G   
   A   
   A   
   G   
   C   
   G   
   G   
   G   
   G   
   A   
   G   
   T   
   G   
   A   
   C   
   G   
   G   
   T   
   C   
   A   
   T   
   G   
   C   
   G   
   T   
   G   
   A   
   G   
   C   
   T   
   G   
   A   
   A   
   A   
   A   
   A   
   A   
   A   
   A   
   T   
   C   
   C   
    G    
   C   
   A   
    C    
   C   
   G   
    T    
   A   
   T   
   T   
   A   
   T   
   C   
   T   
   A   
   T   
   A   
   T   
   G   
   T   
   C   
   G   
   G   
   A   
   A   
   G   
   G   
   A   
   A   
   G   
   A   
   T   
   C   
   A   
   A   
   A   
   A   
   G   
   A   
   A   
   A   
   T   
   A   
   A   
   C   
   C   
   T   
   T   
   G   
   A   
   T   
   G   
   A   
   A   
   T   
   C   
   A   
   A   
   A   
   A   
   A   
   G   
   G   
    G    
   C   
   A   
    G    
   C   
   A   
   G   
    T    
   T   
   A   
   A   
   C   
   G   
   A   
   T   
   T   
   G   
   A   
    C    
   C   
   C   
   A   
   A   
   C   
    T    
   G   
   A   
   T   
   T   
   T   
    C    
   C   
   C   
   T   
   A   
   T   
   A   
   G   
   G   
   T   
   G   
   A   
   T   
   C   
   G   
   A   
   A   
   C   
   A   
   T   
   C   
   G   
   A   
   A   
   T   
   G   
   G   
    A    
   T   
   C   
    A    
   T   
   T   
   T   
   G   
   C   
    G    
   A   
   T   
   G   
   G   
   A   
    T    
   G   
   A   
   G   
   A   
   A   
   A   
   A   
   A   
   A   
   G   
   G   
   T   
   G   
   G   
   A   
   G   
   A   
   A   
   G   
   T   
   C   
   A   
   T   
   C   
   G   
   A   
   A   
   G   
   T   
    T    
   A   
    T    
   C   
   G   
   A   
   T   
   G   
   C   
   T   
   C   
   C   
   T   
   G   
   A   
   G   
    A    
   T   
   T   
   T   
   T   
    C    
   A   
   T   
    C    
   G   
   A   
    G    
    T    
   C   
    G    
   -   
   -   
   -   
 

 
   N53655   
   A   
   T   
   G   
   G   
   A   
   A   
   A   
   A   
   T   
   C   
   G   
   A   
   C   
   A   
   A   
   G   
   A   
   A   
   G   
   T   
   A   
   C   
   A   
   A   
   G   
   A   
   A   
   T   
   T   
   A   
   A   
   C   
   T   
   T   
   T   
   A   
   G   
   A   
   A   
   G   
   A   
   A   
   G   
   T   
   G   
   A   
   T   
   G   
   G   
   G   
   T   
   G   
   A   
   C   
   C   
   G   
   C   
   T   
   T   
   C   
   G   
   G   
   A   
   C   
   G   
   T   
   T   
   A   
   T   
   T   
   C   
   C   
   A   
   A   
   A   
   T   
   A   
   T   
   A   
   T   
   T   
   A   
   T   
    C    
   C   
   A   
    A    
   G   
   A   
   A   
   C   
   G   
   T   
   G   
   C   
   A   
   C   
   T   
   G   
   C   
   C   
   T   
   G   
   A   
   T   
   A   
   T   
    C    
   C   
   G   
   T   
   G   
   A   
   T   
   G   
   G   
   A   
   T   
   T   
   G   
   A   
   A   
    G    
   C   
   C   
   A   
   G   
   T   
    G    
   C   
   A   
   A   
   C   
   G   
   C   
   C   
   G   
   G   
   A   
   T   
   A   
   T   
   T   
   A   
   T   
   T   
    T    
   G   
   C   
    C    
   A   
   T   
   G   
   A   
   A   
   C   
   A   
   A   
   A   
   G   
   A   
   T   
   G   
   G   
   C   
   A   
   A   
   T   
   A   
   C   
   G   
   T   
   A   
   T   
   G   
   A   
   C   
   A   
   A   
   A   
   G   
   G   
   A   
   T   
   T   
    C    
   C   
   G   
   T   
   A   
   A   
   A   
   T   
   C   
   A   
   G   
   C   
   A   
   A   
   A   
   A   
   T   
   C   
   T   
   G   
   T   
   C   
   G   
   G   
   A   
   A   
   A   
   T   
   A   
   T   
   C   
   A   
   T   
   G   
   G   
   G   
    G    
   A   
   A   
   T   
   T   
   A   
   T   
   C   
   A   
   T   
   C   
   C   
   T   
   C   
   A   
   T   
   G   
   G   
   T   
   G   
   A   
   C   
   A   
   G   
   C   
   A   
   G   
   T   
   A   
   T   
   T   
   T   
   A   
   T   
   G   
   A   
   A   
   G   
   C   
   G   
   A   
   T   
   G   
   G   
   T   
   C   
   C   
   G   
   C   
   A   
   T   
   G   
   A   
   G   
   C   
   C   
   A   
   A   
   G   
   A   
    C    
   T   
   G   
   G   
   A   
   A   
   G   
   T   
   T   
   G   
   C   
   G   
   G   
   G   
   A   
   A   
   G   
   T   
   A   
   C   
   T   
   G   
   A   
   T   
   C   
   G   
   A   
    A    
   A   
   T   
   G   
   C   
   A   
   C   
   G   
   G   
    G    
   A   
   A   
   T   
   A   
   A   
    C    
   G   
   G   
   G   
   A   
   G   
   T   
   A   
   T   
   G   
   G   
   A   
   C   
   G   
   G   
   T   
   G   
   A   
   T   
   C   
   C   
   A   
   C   
   C   
   T   
   G   
   C   
   T   
   G   
   C   
   G   
   A   
   T   
   G   
   C   
   G   
   T   
   T   
   A   
   T   
   A   
   C   
   T   
   G   
   A   
   A   
   G   
   C   
   C   
   A   
   G   
    A    
   T   
   T   
   A   
   T   
   C   
   C   
   A   
   A   
   G   
   T   
   T   
   A   
   A   
   G   
   T   
   G   
   G   
   A   
   G   
   A   
   G   
   A   
   T   
   G   
    C    
   T   
   A   
    G    
   C   
   A   
   G   
   A   
   T   
   A   
   T   
   C   
   G   
   A   
    A    
   A   
   A   
   A   
   G   
   A   
   A   
   A   
   C   
   A   
   G   
   T   
    C    
   G   
   A   
   T   
   C   
   T   
   C   
   G   
   T   
   G   
   T   
   G   
   G   
   A   
   A   
   T   
   T   
   T   
   T   
   G   
   A   
   T   
   G   
   A   
   T   
   A   
   C   
   A   
   G   
   A   
   A   
   A   
   A   
   A   
   G   
   A   
   A   
   C   
   C   
   A   
   A   
   C   
   A   
   G   
   T   
   C   
   C   
   T   
   T   
   C   
   C   
   A   
   G   
   C   
   T   
   C   
   G   
   A   
   T   
   A   
   C   
   C   
   C   
   A   
   A   
   A   
   C   
   C   
   T   
   T   
   C   
   T   
    C    
   G   
   T   
   C   
   A   
   A   
   T   
   G   
   G   
   A   
   T   
   C   
   G   
   A   
   C   
   A   
   G   
   G   
   T   
   A   
   T   
   A   
   T   
   C   
   T   
   G   
   C   
   T   
   G   
   G   
   A   
   T   
   A   
   T   
   G   
   C   
    A    
   A   
   C   
   A   
   G   
   A   
   A   
   A   
   T   
   C   
   C   
   C   
   G   
   A   
   C   
    T    
   C   
   A   
   T   
   A   
   A   
   C   
   T   
   T   
   A   
   G   
   C   
   T   
   G   
   A   
   A   
   G   
   T   
   G   
   A   
   T   
    T    
   G   
   A   
    T    
   G   
   G   
   C   
   A   
   C   
   A   
   A   
   T   
   C   
   T   
   A   
   T   
   A   
   T   
   G   
   A   
   T   
   C   
   G   
   A   
   T   
   C   
   A   
   T   
   C   
   C   
   A   
   C   
   A   
   A   
   G   
   C   
   G   
   A   
   G   
   C   
   C   
   T   
   T   
   G   
   A   
   A   
   A   
   A   
   A   
   T   
   T   
   G   
   A   
   T   
   G   
   G   
   A   
   A   
   T   
   A   
   T   
   A   
   T   
   T   
   C   
   C   
   T   
   G   
   G   
   A   
   C   
   C   
   T   
   G   
   A   
   C   
   T   
   T   
    T    
   C   
   C   
   A   
   A   
   C   
   A   
   G   
   G   
   A   
   G   
   G   
    G    
   A   
   T   
   C   
   T   
   T   
   G   
   C   
   A   
   A   
   G   
   G   
    A    
   A   
   A   
   A   
   G   
   A   
   T   
   G   
   A   
   A   
   A   
   T   
   C   
   A   
   A   
   A   
   A   
   A   
   A   
   G   
   C   
   T   
   T   
   A   
   T   
   G   
   A   
   A   
   A   
   C   
   A   
   G   
   G   
    G    
   C   
   G   
   A   
   G   
   G   
   A   
   A   
   A   
   A   
   G   
   T   
   T   
   A   
    T    
   T   
   C   
   T   
   T   
   C   
   G   
   T   
   T   
   C   
   G   
   A   
   A   
   A   
   A   
   C   
   A   
   A   
   A   
   G   
   A   
   T   
   A   
   G   
   A   
    G    
   G   
   C   
   A   
   A   
   T   
   G   
   A   
   A   
   G   
   G   
   G   
    T    
   A   
   A   
    T    
   A   
   A   
   A   
   C   
   A   
   G   
   C   
   A   
   A   
   A   
   T   
   C   
   G   
   T   
   G   
   A   
   T   
   C   
   A   
   C   
    A    
   G   
   A   
   A   
   A   
   T   
   C   
   C   
   C   
   A   
   T   
   A   
   T   
   G   
   A   
   A   
   G   
   T   
   A   
   A   
   A   
   T   
   A   
   A   
   A   
   G   
   C   
   G   
   A   
   C   
    C    
   T   
   T   
   G   
   G   
   T   
   G   
   A   
   A   
   A   
   A   
   A   
   A   
   A   
   T   
   G   
   G   
   A   
   T   
   G   
   A   
   G   
   A   
   T   
   C   
   C   
   G   
   T   
   T   
   T   
   G   
   A   
   A   
   T   
   A   
   A   
   G   
   A   
   A   
   A   
   A   
   T   
   C   
   G   
   A   
    T    
   G   
   G   
   A   
   A   
   T   
   A   
   G   
   C   
   T   
   G   
   A   
   A   
   G   
   T   
    A    
   C   
   G   
   A   
   G   
   A   
   T   
   G   
   A   
   A   
   A   
   G   
   T   
   G   
   A   
   T   
    A    
   G   
    A    
   A   
   C   
    A    
   G   
   G   
   T   
   C   
   T   
   G   
   C   
   A   
   A   
   A   
   T   
   T   
   G   
   T   
   C   
   G   
   T   
    C    
   G   
   A   
   A   
   T   
   T   
   G   
   A   
   A   
   A   
   A   
   A   
   A   
   G   
   A   
    C    
   G   
   C   
   C   
   A   
   A   
    T    
   G   
   C   
   T   
   C   
   A   
   A   
   G   
   G   
    A    
   A   
   T   
   T   
   T   
   T   
   G   
   A   
   A   
   T   
   T   
   A   
    T    
   T   
   T   
   G   
   T   
   T   
   C   
   A   
   A   
   A   
   A   
   A   
   C   
   A   
   C   
   T   
   G   
   A   
   G   
    C    
   T   
   A   
   C   
   A   
   G   
   A   
   T   
   C   
   A   
   A   
   T   
   T   
   A   
   C   
   A   
   A   
   T   
   T   
   T   
    C    
   A   
   A   
   T   
   A   
   T   
   G   
   G   
   T   
   C   
   G   
   C   
   T   
   A   
   T   
   C   
   G   
   A   
    C    
   C   
   A   
   T   
   A   
   T   
   G   
   A   
   C   
   T   
   C   
   C   
   G   
   C   
   A   
    T    
   C   
   A   
   A   
   G   
   T   
   T   
   G   
   G   
   A   
   C   
   T   
   G   
   A   
   A   
   A   
   G   
   A   
   C   
   A   
   T   
   T   
   T   
   T   
   A   
   A   
   G   
   A   
   A   
   G   
   T   
   T   
   A   
   T   
   A   
   T   
   C   
   G   
   A   
   G   
   C   
   A   
   T   
   C   
   G   
   A   
   A   
   A   
   G   
   C   
   A   
   A   
   G   
   T   
   C   
   A   
   T   
   A   
   A   
   C   
   A   
   A   
   A   
   A   
   C   
   G   
    T    
   A   
   G   
    T    
   C   
   A   
   A   
   T   
   T   
   C   
   G   
   A   
   T   
   T   
   T   
   A   
   G   
   C   
   C   
   A   
   A   
   A   
   G   
   C   
   T   
   C   
   A   
   A   
   A   
   A   
   A   
    A    
   G   
   A   
   C   
   A   
   G   
   C   
   A   
   T   
   A   
   T   
   C   
   G   
   T   
    A    
   G   
   A   
   A   
   G   
   G   
    A    
    T    
   T   
   G   
   A   
   T   
   G   
   A   
   A   
   A   
   G   
   C   
   T   
   T   
   T   
   A   
   T   
   C   
   G   
   A   
   T   
   T   
   C   
   T   
   G   
   G   
   A   
   T   
   G   
   A   
   A   
   G   
   T   
   A   
   A   
   T   
   C   
   G   
    T    
   A   
   A   
   C   
   G   
   A   
   T   
   T   
   C   
   G   
   T   
   G   
   A   
   A   
   A   
   G   
   T   
   A   
   A   
   A   
   G   
   A   
   C   
   A   
   A   
   A   
   A   
   A   
   A   
   G   
   A   
    C    
   G   
   C   
    C    
   A   
   A   
   A   
   A   
   A   
   G   
   A   
   A   
   T   
   T   
   T   
   A   
   G   
   T   
   C   
   G   
   A   
    C    
   G   
   T   
    C    
   T   
   T   
    T    
   C   
   A   
   A   
   T   
   T   
   T   
   A   
   C   
   A   
   G   
   A   
   A   
   G   
   A   
   A   
   C   
   A   
   A   
   G   
   C   
   A   
   G   
   A   
   A   
   G   
   C   
   A   
   A   
   T   
    T    
   G   
   T   
   C   
   A   
   C   
   A   
   T   
   T   
   G   
   C   
   A   
   A   
    T    
   T   
   A   
   T   
   A   
    C    
   C   
   G   
   T   
   T   
   T   
   G   
   A   
   C   
   G   
   A   
   A   
    C    
   A   
   C   
    G    
   G   
   A   
   T   
   A   
   T   
   C   
   A   
   C   
   A   
   G   
   A   
   G   
   C   
   T   
   T   
   C   
   A   
   A   
   A   
   A   
   G   
   G   
   A   
   A   
    T    
   C   
    C    
   G   
   A   
   A   
   A   
    G    
   C   
   T   
   T   
   G   
   A   
   T   
   T   
   G   
   C   
    G    
   C   
   A   
   A   
   A   
   T   
   C   
   A   
   C   
   A   
   G   
   A   
   G   
   T   
   T   
    G    
   A   
   A   
   T   
   A   
   A   
   A   
   A   
   T   
   C   
   C   
   T   
   T   
   T   
   C   
   A   
   A   
   A   
    T    
   G   
   A   
   C   
   A   
   A   
   A   
   G   
   A   
    G    
   C   
   T   
   T   
   T   
   T   
    C    
   T   
   C   
   T   
   G   
   T   
   T   
   A   
   T   
   G   
   A   
   A   
   A   
   A   
   A   
   A   
   G   
   A   
   A   
   T   
   T   
   G   
   C   
   G   
   A   
   G   
   A   
   A   
   G   
   T   
   G   
   A   
   A   
   A   
   A   
   A   
   A   
   A   
   A   
   T   
   T   
   A   
    T    
   T   
   C   
   T   
   T   
   C   
   C   
   G   
   C   
   T   
   C   
   G   
   T   
   C   
    T    
    A    
   A   
   C   
   G   
   A   
   C   
   A   
   A   
   T   
   C   
    G    
   A   
   A   
   G   
   A   
   C   
   G   
   A   
   A   
   A   
   T   
   C   
   G   
   A   
   A   
   G   
   A   
   A   
   A   
   T   
   C   
   A   
   A   
   A   
   A   
   T   
    T    
   G   
   A   
   T   
   A   
   C   
   G   
   C   
   A   
   A   
    G    
   T   
    C    
   T   
   T   
   A   
   G   
   T   
   G   
   G   
   C   
    A    
   C   
   A   
   A   
   G   
   A   
   A   
   G   
   A   
   C   
   G   
   T   
   A   
   A   
   T   
    T    
   G   
   T   
   C   
   A   
   G   
   T   
   G   
   T   
    G    
   A   
   C   
    G    
   C   
   G   
   A   
   G   
   A   
   A   
   G   
   G   
   C   
   T   
   A   
   T   
   G   
   T   
   G   
   A   
   A   
   G   
   C   
   G   
   A   
   A   
   C   
   A   
   A   
   G   
   C   
   C   
   T   
   T   
   C   
   G   
   T   
   T   
   C   
    T    
   T   
   A   
   T   
   A   
   G   
   T   
   G   
   C   
   T   
   T   
   C   
    C    
   A   
   A   
   A   
   C   
   C   
   A   
   G   
   A   
   A   
   G   
   A   
   A   
   A   
   T   
   C   
   G   
   G   
   G   
   A   
   T   
   G   
   C   
   G   
   A   
   G   
   A   
   A   
   G   
   G   
   C   
   G   
   A   
    C    
   T   
   A   
   C   
   T   
   T   
   G   
   C   
   T   
    A    
   T   
   A   
   C   
    T    
   C   
   A   
   G   
   G   
   T   
   G   
   A   
   A   
   T   
   T   
    G    
   A   
   G   
    T    
   A   
   C   
   A   
    T    
   T   
   G   
   G   
   A   
   C   
   C   
   A   
   T   
   G   
   T   
   T   
   C   
   T   
   T   
   C   
   T   
   G   
   A   
   T   
    C    
   A   
   C   
    A    
   A   
   A   
   T   
   A   
   A   
   A   
   G   
   C   
   A   
   A   
   A   
   T   
   G   
   T   
   G   
   A   
   T   
   C   
   T   
   A   
   C   
   C   
   G   
    C    
   C   
   C   
   T   
   G   
   T   
    C    
   C   
   A   
   T   
   G   
   A   
   A   
   C   
   T   
   A   
   C   
   C   
    C    
   G   
   A   
   T   
   T   
   T   
   G   
   A   
   A   
   A   
   T   
   G   
   G   
   A   
   A   
   A   
   G   
   A   
   T   
   G   
   C   
   T   
   G   
   G   
    T    
   G   
   A   
    G    
   C   
   A   
    C    
   A   
   T   
   T   
   T   
   C   
   T   
   C   
   A   
   A   
   A   
   C   
   A   
   A   
   T   
   C   
   T   
   C   
   C   
   A   
   A   
   T   
   C   
   T   
   G   
   T   
   C   
   T   
   G   
   T   
   A   
   G   
   A   
    C    
   G   
   A   
   A   
   T   
   C   
   G   
   A   
   T   
    T    
   T   
   T   
   A   
   G   
   C   
   T   
   G   
   T   
   T   
   T   
   T   
    C    
   C   
   C   
   A   
   T   
   A   
   T   
   C   
   A   
   A   
   A   
   A   
   A   
   A   
   T   
    T    
   C   
   A   
   A   
   G   
   C   
   A   
   G   
   A   
    G    
   A   
   A   
   A   
   A   
   C   
   A   
   T   
   T   
   T   
   G   
   T   
    A    
   T   
   T   
   C   
   A   
   T   
   C   
   A   
   G   
   C   
   A   
   A   
   G   
   A   
   A   
   T   
   G   
   G   
   A   
   T   
   T   
    G    
    A    
   T   
   C   
   A   
   A   
   G   
   C   
   A   
   A   
   A   
   C   
   A   
   C   
   G   
   A   
   A   
   T   
   G   
   A   
   C   
   A   
   G   
   A   
   G   
   T   
   T   
   C   
   G   
   A   
   A   
   C   
   C   
   A   
   T   
   G   
   G   
   C   
   G   
   C   
   A   
   C   
   C   
   T   
   A   
    T    
   A   
   A   
   G   
   A   
   G   
   C   
   C   
   G   
    T    
   C   
   C   
   G   
   C   
   T   
   C   
   A   
   G   
   C   
   T   
   G   
   T   
   A   
   T   
   G   
   A   
   A   
   G   
   C   
   T   
   A   
   A   
   A   
   G   
   G   
   C   
   G   
   G   
   A   
   A   
   G   
   A   
   A   
   G   
   A   
   T   
   G   
   A   
   A   
   C   
   T   
    G    
   A   
   T   
   T   
   G   
   C   
   T   
   G   
   T   
    G    
   T   
   A   
   T   
   C   
   T   
   G   
   A   
   A   
   A   
   G   
   A   
   A   
   G   
   A   
   T   
   C   
    A    
   G   
   C   
   C   
   A   
   G   
   G   
   C   
   T   
   T   
   A   
   G   
   A   
   T   
   G   
   T   
    T    
   T   
   T   
   C   
   T   
   T   
   A   
   G   
   T   
   A   
   A   
   C   
   G   
    C    
   A   
   T   
   C   
   A   
   A   
   G   
   G   
   A   
   A   
   T   
   G   
   G   
   G   
    T    
   T   
   T   
   G   
   C   
   G   
   C   
   T   
    A    
   T   
   C   
   C   
   T   
   T   
   T   
   A   
   A   
    C    
   A   
   G   
   A   
   T   
   G   
   T   
   T   
   C   
   C   
   C   
   G   
   T   
   T   
   G   
   T   
   T   
   G   
   G   
   A   
   G   
   C   
   A   
   A   
   A   
   G   
   G   
   C   
   A   
   G   
    C    
   T   
   G   
   G   
   A   
   G   
   T   
   G   
   A   
   A   
   G   
   T   
   C   
   T   
   A   
   T   
   G   
   A   
   A   
   T   
   T   
   T   
   G   
   A   
   A   
   A   
   G   
   A   
   T   
   G   
   G   
   A   
   G   
   A   
   T   
   A   
   G   
   G   
    A    
   T   
   T   
    A    
   T   
   C   
   A   
   A   
    C    
   G   
   G   
    G    
   C   
   T   
    G    
   C   
   T   
   T   
   G   
   T   
   G   
   T   
   T   
   A   
   G   
   C   
   A   
   G   
   A   
    A    
   G   
   G   
    T    
   G   
   A   
   T   
   A   
   C   
   G   
   C   
   C   
    G    
   A   
   T   
   C   
   G   
   T   
   A   
   A   
   T   
   C   
   G   
   T   
   T   
   A   
   C   
   T   
   C   
   A   
   G   
   C   
   G   
   A   
   G   
   G   
   A   
   G   
   C   
    T    
   G   
   T   
    T    
   A   
   A   
   A   
   C   
   G   
   C   
   A   
   T   
   G   
   C   
   T   
   T   
   G   
   C   
   T   
   C   
   A   
   A   
   G   
   A   
   A   
   A   
   T   
   C   
   A   
   G   
   C   
   C   
   A   
   A   
   A   
   C   
    T    
   T   
   C   
   A   
   C   
   G   
   T   
   G   
   C   
   G   
   A   
   A   
   G   
   C   
   G   
   G   
   G   
   G   
   A   
   G   
   T   
   G   
   A   
   C   
   G   
   G   
   T   
   C   
   A   
   T   
   G   
   C   
   G   
   T   
   G   
   A   
   G   
   C   
   T   
   G   
   A   
   A   
   A   
   A   
   A   
   A   
   A   
   A   
   T   
   C   
   C   
    G    
   C   
   A   
    C    
   C   
   G   
    T    
   A   
   T   
   T   
   A   
   T   
   C   
   T   
   A   
   T   
   A   
   T   
   G   
   T   
   C   
   G   
   G   
   A   
   A   
   G   
   G   
   A   
   A   
   G   
   A   
   T   
   C   
   A   
   A   
   A   
   A   
   G   
   A   
   A   
   A   
   T   
   A   
   A   
   C   
   C   
   T   
   T   
   G   
   A   
   T   
   G   
   A   
   A   
   T   
   C   
   A   
   A   
   A   
   A   
   A   
   G   
   G   
    G    
   C   
   A   
    G    
   C   
   A   
   G   
    T    
   T   
   A   
   A   
   C   
   G   
   A   
   T   
   T   
   G   
   A   
    C    
   C   
   C   
   A   
   A   
   C   
    T    
   G   
   A   
   T   
   T   
   T   
    C    
   C   
   C   
   T   
   A   
   T   
   A   
   G   
   G   
   T   
   G   
   A   
   T   
   C   
   G   
   A   
   A   
   C   
   A   
   T   
   C   
   G   
   A   
   A   
   T   
   G   
   G   
    A    
   T   
   C   
    A    
   T   
   T   
   T   
   G   
   C   
    G    
   A   
   T   
   G   
   G   
   A   
    T    
   G   
   A   
   G   
   A   
   A   
   A   
   A   
   A   
   A   
   G   
   G   
   T   
   G   
   G   
   A   
   G   
   A   
   A   
   G   
   T   
   C   
   A   
   T   
   C   
   G   
   A   
   A   
   G   
   T   
    T    
   A   
    T    
   C   
   G   
   A   
   T   
   G   
   C   
   T   
   C   
   C   
   T   
   G   
   A   
   G   
    A    
   T   
   T   
   T   
   T   
    C    
   A   
   T   
    C    
   G   
   A   
    G    
    T    
   C   
    G    
   -   
   -   
   -   
 

 
   N54550   
   A   
   T   
   G   
   G   
   A   
   A   
   A   
   A   
   T   
   C   
   G   
   A   
   C   
   A   
   A   
   G   
   A   
   A   
   G   
   T   
   A   
   C   
   A   
   A   
   G   
   A   
   A   
   T   
   T   
   A   
   A   
   C   
   T   
   T   
   T   
   A   
   G   
   A   
   A   
   G   
   A   
   A   
   G   
   T   
   G   
   A   
   T   
   G   
   G   
   G   
   T   
   G   
   A   
   C   
   C   
   G   
   C   
   T   
   T   
   C   
   G   
   G   
   A   
   C   
   G   
   T   
   T   
   A   
   T   
   T   
   C   
   C   
   A   
   A   
   A   
   T   
   A   
   T   
   A   
   T   
   T   
   A   
   T   
    C    
   C   
   A   
    A    
   G   
   A   
   A   
   C   
   G   
   T   
   G   
   C   
   A   
   C   
   T   
   G   
   C   
   C   
   T   
   G   
   A   
   T   
   A   
   T   
    T    
   C   
   G   
   T   
   G   
   A   
   T   
   G   
   G   
   A   
   T   
   T   
   G   
   A   
   A   
    G    
   C   
   C   
   A   
   G   
   T   
    G    
   C   
   A   
   A   
   C   
   G   
   C   
   C   
   G   
   G   
   A   
   T   
   A   
   T   
   T   
   A   
   T   
   T   
    T    
   G   
   C   
    C    
   A   
   T   
   G   
   A   
   A   
   C   
   A   
   A   
   A   
   G   
   A   
   T   
   G   
   G   
   C   
   A   
   A   
   T   
   A   
   C   
   G   
   T   
   A   
   T   
   G   
   A   
   C   
   A   
   A   
   A   
   G   
   G   
   A   
   T   
   T   
    C    
   C   
   G   
   T   
   A   
   A   
   A   
   T   
   C   
   A   
   G   
   C   
   A   
   A   
   A   
   A   
   T   
   C   
   T   
   G   
   T   
   C   
   G   
   G   
   A   
   A   
   A   
   T   
   A   
   T   
   C   
   A   
   T   
   G   
   G   
   G   
    G    
   A   
   A   
   T   
   T   
   A   
   T   
   C   
   A   
   T   
   C   
   C   
   T   
   C   
   A   
   T   
   G   
   G   
   T   
   G   
   A   
   C   
   A   
   G   
   C   
   A   
   G   
   T   
   A   
   T   
   T   
   T   
   A   
   T   
   G   
   A   
   A   
   G   
   C   
   G   
   A   
   T   
   G   
   G   
   T   
   C   
   C   
   G   
   C   
   A   
   T   
   G   
   A   
   G   
   C   
   C   
   A   
   A   
   G   
   A   
    C    
   T   
   G   
   G   
   A   
   A   
   G   
   T   
   T   
   G   
   C   
   G   
   G   
   G   
   A   
   A   
   G   
   T   
   A   
   C   
   T   
   G   
   A   
   T   
   C   
   G   
   A   
    A    
   A   
   T   
   G   
   C   
   A   
   C   
   G   
   G   
    G    
   A   
   A   
   T   
   A   
   A   
    C    
   G   
   G   
   G   
   A   
   G   
   T   
   A   
   T   
   G   
   G   
   A   
   C   
   G   
   G   
   T   
   G   
   A   
   T   
   C   
   C   
   A   
   C   
   C   
   T   
   G   
   C   
   T   
   G   
   C   
   G   
   A   
   T   
   G   
   C   
   G   
   T   
   T   
   A   
   T   
   A   
   C   
   T   
   G   
   A   
   A   
   G   
   C   
   C   
   A   
   G   
    A    
   T   
   T   
   A   
   T   
   C   
   C   
   A   
   A   
   G   
   T   
   T   
   A   
   A   
   G   
   T   
   G   
   G   
   A   
   G   
   A   
   G   
   A   
   T   
   G   
    C    
   T   
   A   
    G    
   C   
   A   
   G   
   A   
   T   
   A   
   T   
   C   
   G   
   A   
    A    
   A   
   A   
   A   
   G   
   A   
   A   
   A   
   C   
   A   
   G   
   T   
    C    
   G   
   A   
   T   
   C   
   T   
   C   
   G   
   T   
   G   
   T   
   G   
   G   
   A   
   A   
   T   
   T   
   T   
   T   
   G   
   A   
   T   
   G   
   A   
   T   
   A   
   C   
   A   
   G   
   A   
   A   
   A   
   A   
   A   
   G   
   A   
   A   
   C   
   C   
   A   
   A   
   C   
   A   
   G   
   T   
   C   
   C   
   T   
   T   
   C   
   C   
   A   
   G   
   C   
   T   
   C   
   G   
   A   
   T   
   A   
   C   
   C   
   C   
   A   
   A   
   A   
   C   
   C   
   T   
   T   
   C   
   T   
    C    
   G   
   T   
   C   
   A   
   A   
   T   
   G   
   G   
   A   
   T   
   C   
   G   
   A   
   C   
   A   
   G   
   G   
   T   
   A   
   T   
   A   
   T   
   C   
   T   
   G   
   C   
   T   
   G   
   G   
   A   
   T   
   A   
   T   
   G   
   C   
    A    
   A   
   C   
   A   
   G   
   A   
   A   
   A   
   T   
   C   
   C   
   C   
   G   
   A   
   C   
    T    
   C   
   A   
   T   
   A   
   A   
   C   
   T   
   T   
   A   
   G   
   C   
   T   
   G   
   A   
   A   
   G   
   T   
   G   
   A   
   T   
    C    
   G   
   A   
    T    
   G   
   G   
   C   
   A   
   C   
   A   
   A   
   T   
   C   
   T   
   A   
   T   
   A   
   T   
   G   
   A   
   T   
   C   
   G   
   A   
   T   
   C   
   A   
   T   
   C   
   C   
   A   
   C   
   A   
   A   
   G   
   C   
   G   
   A   
   G   
   C   
   C   
   T   
   T   
   G   
   A   
   A   
   A   
   A   
   A   
   T   
   T   
   G   
   A   
   T   
   G   
   G   
   A   
   A   
   T   
   A   
   T   
   A   
   T   
   T   
   C   
   C   
   T   
   G   
   G   
   A   
   C   
   C   
   T   
   G   
   A   
   C   
   T   
   T   
    T    
   C   
   C   
   A   
   A   
   C   
   A   
   G   
   G   
   A   
   G   
   G   
    G    
   A   
   T   
   C   
   T   
   T   
   G   
   C   
   A   
   A   
   G   
   G   
    A    
   A   
   A   
   A   
   G   
   A   
   T   
   G   
   A   
   A   
   A   
   T   
   C   
   A   
   A   
   A   
   A   
   A   
   A   
   G   
   C   
   T   
   T   
   A   
   T   
   G   
   A   
   A   
   A   
   C   
   A   
   G   
   G   
    G    
   C   
   G   
   A   
   G   
   G   
   A   
   A   
   A   
   A   
   G   
   T   
   T   
   A   
    T    
   T   
   C   
   T   
   T   
   C   
   G   
   T   
   T   
   C   
   G   
   A   
   A   
   A   
   A   
   C   
   A   
   A   
   A   
   G   
   A   
   T   
   A   
   G   
   A   
    A    
   G   
   C   
   A   
   A   
   T   
   G   
   A   
   A   
   G   
   G   
   G   
    T    
   A   
   A   
    T    
   A   
   A   
   A   
   C   
   A   
   G   
   C   
   A   
   A   
   A   
   T   
   C   
   G   
   T   
   G   
   A   
   T   
   C   
   A   
   C   
    A    
   G   
   A   
   A   
   A   
   T   
   C   
   C   
   C   
   A   
   T   
   A   
   T   
   G   
   A   
   A   
   G   
   T   
   A   
   A   
   A   
   T   
   A   
   A   
   A   
   G   
   C   
   G   
   A   
   C   
    C    
   T   
   T   
   G   
   G   
   T   
   G   
   A   
   A   
   A   
   A   
   A   
   A   
   A   
   T   
   G   
   G   
   A   
   T   
   G   
   A   
   G   
   A   
   T   
   C   
   C   
   G   
   T   
   T   
   T   
   G   
   A   
   A   
   T   
   A   
   A   
   G   
   A   
   A   
   A   
   A   
   T   
   C   
   G   
   A   
    T    
   G   
   G   
   A   
   A   
   T   
   A   
   G   
   C   
   T   
   G   
   A   
   A   
   G   
   T   
    A    
   C   
   G   
   A   
   G   
   A   
   T   
   G   
   A   
   A   
   A   
   G   
   T   
   G   
   A   
   T   
    A    
   G   
    A    
   A   
   C   
    A    
   G   
   G   
   T   
   C   
   T   
   G   
   C   
   A   
   A   
   A   
   T   
   T   
   G   
   T   
   C   
   G   
   T   
    C    
   G   
   A   
   A   
   T   
   T   
   G   
   A   
   A   
   A   
   A   
   A   
   A   
   G   
   A   
    C    
   G   
   C   
   C   
   A   
   A   
    T    
   G   
   C   
   T   
   C   
   A   
   A   
   G   
   G   
    A    
   A   
   T   
   T   
   T   
   T   
   G   
   A   
   A   
   T   
   T   
   A   
    T    
   T   
   T   
   G   
   T   
   T   
   C   
   A   
   A   
   A   
   A   
   A   
   C   
   A   
   C   
   T   
   G   
   A   
   G   
    C    
   T   
   A   
   C   
   A   
   G   
   A   
   T   
   C   
   A   
   A   
   T   
   T   
   A   
   C   
   A   
   A   
   T   
   T   
   T   
    C    
   A   
   A   
   T   
   A   
   T   
   G   
   G   
   T   
   C   
   G   
   C   
   T   
   A   
   T   
   C   
   G   
   A   
    C    
   C   
   A   
   T   
   A   
   T   
   G   
   A   
   C   
   T   
   C   
   C   
   G   
   C   
   A   
    T    
   C   
   A   
   A   
   G   
   T   
   T   
   G   
   G   
   A   
   C   
   T   
   G   
   A   
   A   
   A   
   G   
   A   
   C   
   A   
   T   
   T   
   T   
   T   
   A   
   A   
   G   
   A   
   A   
   G   
   T   
   T   
   A   
   T   
   A   
   T   
   C   
   G   
   A   
   G   
   C   
   A   
   T   
   C   
   G   
   A   
   A   
   A   
   G   
   C   
   A   
   A   
   G   
   T   
   C   
   A   
   T   
   A   
   A   
   C   
   A   
   A   
   A   
   A   
   C   
   G   
    T    
   A   
   G   
    T    
   C   
   A   
   A   
   T   
   T   
   C   
   G   
   A   
   T   
   T   
   T   
   A   
   G   
   C   
   C   
   A   
   A   
   A   
   G   
   C   
   T   
   C   
   A   
   A   
   A   
   A   
   A   
    A    
   G   
   A   
   C   
   A   
   G   
   C   
   A   
   T   
   A   
   T   
   C   
   G   
   T   
    A    
   G   
   A   
   A   
   G   
   G   
    A    
    T    
   T   
   G   
   A   
   T   
   G   
   A   
   A   
   A   
   G   
   C   
   T   
   T   
   T   
   A   
   T   
   C   
   G   
   A   
   T   
   T   
   C   
   T   
   G   
   G   
   A   
   T   
   G   
   A   
   A   
   G   
   T   
   A   
   A   
   T   
   C   
   G   
    C    
   A   
   A   
   C   
   G   
   A   
   T   
   T   
   C   
   G   
   T   
   G   
   A   
   A   
   A   
   G   
   T   
   A   
   A   
   A   
   G   
   A   
   C   
   A   
   A   
   A   
   A   
   A   
   A   
   G   
   A   
    C    
   G   
   C   
    C    
   A   
   A   
   A   
   A   
   A   
   G   
   A   
   A   
   T   
   T   
   T   
   A   
   G   
   T   
   C   
   G   
   A   
    C    
   G   
   T   
    C    
   T   
   T   
    T    
   C   
   A   
   A   
   T   
   T   
   T   
   A   
   C   
   A   
   G   
   A   
   A   
   G   
   A   
   A   
   C   
   A   
   A   
   G   
   C   
   A   
   G   
   A   
   A   
   G   
   C   
   A   
   A   
   T   
    T    
   G   
   T   
   C   
   A   
   C   
   A   
   T   
   T   
   G   
   C   
   A   
   A   
    T    
   T   
   A   
   T   
   A   
    C    
   C   
   G   
   T   
   T   
   T   
   G   
   A   
   C   
   G   
   A   
   A   
    C    
   A   
   C   
    G    
   G   
   A   
   T   
   A   
   T   
   C   
   A   
   C   
   A   
   G   
   A   
   G   
   C   
   T   
   T   
   C   
   A   
   A   
   A   
   A   
   G   
   G   
   A   
   A   
    T    
   C   
    C    
   G   
   A   
   A   
   A   
    G    
   C   
   T   
   T   
   G   
   A   
   T   
   T   
   G   
   C   
    G    
   C   
   A   
   A   
   A   
   T   
   C   
   A   
   C   
   A   
   G   
   A   
   G   
   T   
   T   
    G    
   A   
   A   
   T   
   A   
   A   
   A   
   A   
   T   
   C   
   C   
   T   
   T   
   T   
   C   
   A   
   A   
   A   
    T    
   G   
   A   
   C   
   A   
   A   
   A   
   G   
   A   
    G    
   C   
   T   
   T   
   T   
   T   
    C    
   T   
   C   
   T   
   G   
   T   
   T   
   A   
   T   
   G   
   A   
   A   
   A   
   A   
   A   
   A   
   G   
   A   
   A   
   T   
   T   
   G   
   C   
   G   
   A   
   G   
   A   
   A   
   G   
   T   
   G   
   A   
   A   
   A   
   A   
   A   
   A   
   A   
   A   
   T   
   T   
   A   
    T    
   T   
   C   
   T   
   T   
   C   
   C   
   G   
   C   
   T   
   C   
   G   
   T   
   C   
    T    
    A    
   A   
   C   
   G   
   A   
   C   
   A   
   A   
   T   
   C   
    G    
   A   
   A   
   G   
   A   
   C   
   G   
   A   
   A   
   A   
   T   
   C   
   G   
   A   
   A   
   G   
   A   
   A   
   A   
   T   
   C   
   A   
   A   
   A   
   A   
   T   
    T    
   G   
   A   
   T   
   A   
   C   
   G   
   C   
   A   
   A   
    G    
   T   
    C    
   T   
   T   
   A   
   G   
   T   
   G   
   G   
   C   
    A    
   C   
   A   
   A   
   G   
   A   
   A   
   G   
   A   
   C   
   G   
   T   
   A   
   A   
   T   
    T    
   G   
   T   
   C   
   A   
   G   
   T   
   G   
   T   
    G    
   A   
   C   
    G    
   C   
   G   
   A   
   G   
   A   
   A   
   G   
   G   
   C   
   T   
   A   
   T   
   G   
   T   
   G   
   A   
   A   
   G   
   C   
   G   
   A   
   A   
   C   
   A   
   A   
   G   
   C   
   C   
   T   
   T   
   C   
   G   
   T   
   T   
   C   
    T    
   T   
   A   
   T   
   A   
   G   
   T   
   G   
   C   
   T   
   T   
   C   
    C    
   A   
   A   
   A   
   C   
   C   
   A   
   G   
   A   
   A   
   G   
   A   
   A   
   A   
   T   
   C   
   G   
   G   
   G   
   A   
   T   
   G   
   C   
   G   
   A   
   G   
   A   
   A   
   G   
   G   
   C   
   G   
   A   
    C    
   T   
   A   
   C   
   T   
   T   
   G   
   C   
   T   
    A    
   T   
   A   
   C   
    T    
   C   
   A   
   G   
   G   
   T   
   G   
   A   
   A   
   T   
   T   
    G    
   A   
   G   
    T    
   A   
   C   
   A   
    T    
   T   
   G   
   G   
   A   
   C   
   C   
   A   
   T   
   G   
   T   
   T   
   C   
   T   
   T   
   C   
   T   
   G   
   A   
   T   
    C    
   A   
   C   
    A    
   A   
   A   
   T   
   A   
   A   
   A   
   G   
   C   
   A   
   A   
   A   
   T   
   G   
   T   
   G   
   A   
   T   
   C   
   T   
   A   
   C   
   C   
   G   
    C    
   C   
   C   
   T   
   G   
   T   
    C    
   C   
   A   
   T   
   G   
   A   
   A   
   C   
   T   
   A   
   C   
   C   
    C    
   G   
   A   
   T   
   T   
   T   
   G   
   A   
   A   
   A   
   T   
   G   
   G   
   A   
   A   
   A   
   G   
   A   
   T   
   G   
   C   
   T   
   G   
   G   
    T    
   G   
   A   
    G    
   C   
   A   
    C    
   A   
   T   
   T   
   T   
   C   
   T   
   C   
   A   
   A   
   A   
   C   
   A   
   A   
   T   
   C   
   T   
   C   
   C   
   A   
   A   
   T   
   C   
   T   
   G   
   T   
   C   
   T   
   G   
   T   
   A   
   G   
   A   
    C    
   G   
   A   
   A   
   T   
   C   
   G   
   A   
   T   
    T    
   T   
   T   
   A   
   G   
   C   
   T   
   G   
   T   
   T   
   T   
   T   
    C    
   C   
   C   
   A   
   T   
   A   
   T   
   C   
   A   
   A   
   A   
   A   
   A   
   A   
   T   
    T    
   C   
   A   
   A   
   G   
   C   
   A   
   G   
   A   
    G    
   A   
   A   
   A   
   A   
   C   
   A   
   T   
   T   
   T   
   G   
   T   
    A    
   T   
   T   
   C   
   A   
   T   
   C   
   A   
   G   
   C   
   A   
   A   
   G   
   A   
   A   
   T   
   G   
   G   
   A   
   T   
   T   
    G    
    A    
   T   
   C   
   A   
   A   
   G   
   C   
   A   
   A   
   A   
   C   
   A   
   C   
   G   
   A   
   A   
   T   
   G   
   A   
   C   
   A   
   G   
   A   
   G   
   T   
   T   
   C   
   G   
   A   
   A   
   C   
   C   
   A   
   T   
   G   
   G   
   C   
   G   
   C   
   A   
   C   
   C   
   T   
   A   
    T    
   A   
   A   
   G   
   A   
   G   
   C   
   C   
   G   
    T    
   C   
   C   
   G   
   C   
   T   
   C   
   A   
   G   
   C   
   T   
   G   
   T   
   A   
   T   
   G   
   A   
   A   
   G   
   C   
   T   
   A   
   A   
   A   
   G   
   G   
   C   
   G   
   G   
   A   
   A   
   G   
   A   
   A   
   G   
   A   
   T   
   G   
   A   
   A   
   C   
   T   
    G    
   A   
   T   
   T   
   G   
   C   
   T   
   G   
   T   
    G    
   T   
   A   
   T   
   C   
   T   
   G   
   A   
   A   
   A   
   G   
   A   
   A   
   G   
   A   
   T   
   C   
    A    
   G   
   C   
   C   
   A   
   G   
   G   
   C   
   T   
   T   
   A   
   G   
   A   
   T   
   G   
   T   
    T    
   T   
   T   
   C   
   T   
   T   
   A   
   G   
   T   
   A   
   A   
   C   
   G   
    C    
   A   
   T   
   C   
   A   
   A   
   G   
   G   
   A   
   A   
   T   
   G   
   G   
   G   
    T    
   T   
   T   
   G   
   C   
   G   
   C   
   T   
    A    
   T   
   C   
   C   
   T   
   T   
   T   
   A   
   A   
    C    
   A   
   G   
   A   
   T   
   G   
   T   
   T   
   C   
   C   
   C   
   G   
   T   
   T   
   G   
   T   
   T   
   G   
   G   
   A   
   G   
   C   
   A   
   A   
   A   
   G   
   G   
   C   
   A   
   G   
    C    
   T   
   G   
   G   
   A   
   G   
   T   
   G   
   A   
   A   
   G   
   T   
   C   
   T   
   A   
   T   
   G   
   A   
   A   
   T   
   T   
   T   
   G   
   A   
   A   
   A   
   G   
   A   
   T   
   G   
   G   
   A   
   G   
   A   
   T   
   A   
   G   
   G   
    A    
   T   
   T   
    A    
   T   
   C   
   A   
   A   
    C    
   G   
   G   
    G    
   C   
   T   
    A    
   C   
   T   
   T   
   G   
   T   
   G   
   T   
   T   
   A   
   G   
   C   
   A   
   G   
   A   
    A    
   G   
   G   
    T    
   G   
   A   
   T   
   A   
   C   
   G   
   C   
   C   
    G    
   A   
   T   
   C   
   G   
   T   
   A   
   A   
   T   
   C   
   G   
   T   
   T   
   A   
   C   
   T   
   C   
   A   
   G   
   C   
   G   
   A   
   G   
   G   
   A   
   G   
   C   
    T    
   G   
   T   
    T    
   A   
   A   
   A   
   C   
   G   
   C   
   A   
   T   
   G   
   C   
   T   
   T   
   G   
   C   
   T   
   C   
   A   
   A   
   G   
   A   
   A   
   A   
   T   
   C   
   A   
   G   
   C   
   C   
   A   
   A   
   A   
   C   
    T    
   T   
   C   
   A   
   C   
   G   
   T   
   G   
   C   
   G   
   A   
   A   
   G   
   C   
   G   
   G   
   G   
   G   
   A   
   G   
   T   
   G   
   A   
   C   
   G   
   G   
   T   
   C   
   A   
   T   
   G   
   C   
   G   
   T   
   G   
   A   
   G   
   C   
   T   
   G   
   A   
   A   
   A   
   A   
   A   
   A   
   A   
   A   
   T   
   C   
   C   
    G    
   C   
   A   
    C    
   C   
   G   
    T    
   A   
   T   
   T   
   A   
   T   
   C   
   T   
   A   
   T   
   A   
   T   
   G   
   T   
   C   
   G   
   G   
   A   
   A   
   G   
   G   
   A   
   A   
   G   
   A   
   T   
   C   
   A   
   A   
   A   
   A   
   G   
   A   
   A   
   A   
   T   
   A   
   A   
   C   
   C   
   T   
   T   
   G   
   A   
   T   
   G   
   A   
   A   
   T   
   C   
   A   
   A   
   A   
   A   
   A   
   G   
   G   
    G    
   C   
   A   
    G    
   C   
   A   
   G   
    T    
   T   
   A   
   A   
   C   
   G   
   A   
   T   
   T   
   G   
   A   
    C    
   C   
   C   
   A   
   A   
   C   
    T    
   G   
   A   
   T   
   T   
   T   
    C    
   C   
   C   
   T   
   A   
   T   
   A   
   G   
   G   
   T   
   G   
   A   
   T   
   C   
   G   
   A   
   A   
   C   
   A   
   T   
   C   
   G   
   A   
   A   
   T   
   G   
   G   
    A    
   T   
   C   
    A    
   T   
   T   
   T   
   G   
   C   
    G    
   A   
   T   
   G   
   G   
   A   
    T    
   G   
   A   
   G   
   A   
   A   
   A   
   A   
   A   
   A   
   G   
   G   
   T   
   G   
   G   
   A   
   G   
   A   
   A   
   G   
   T   
   C   
   A   
   T   
   C   
   G   
   A   
   A   
   G   
   T   
    T    
   A   
    T    
   C   
   G   
   A   
   T   
   G   
   C   
   T   
   C   
   C   
   T   
   G   
   A   
   G   
    A    
   T   
   T   
   T   
   T   
    C    
   A   
   T   
    C    
   G   
   A   
    G    
    T    
   C   
    G    
   -   
   -   
   -   
 

 
   N60363F   
   A   
   T   
   G   
   G   
   A   
   A   
   A   
   A   
   T   
   C   
   G   
   A   
   C   
   A   
   A   
   G   
   A   
   A   
   G   
   T   
   A   
   C   
   A   
   A   
   G   
   A   
   A   
   T   
   T   
   A   
   A   
   C   
   T   
   T   
   T   
   A   
   G   
   A   
   A   
   G   
   A   
   A   
   G   
   T   
   G   
   A   
   T   
   G   
   G   
   G   
   T   
   G   
   A   
   C   
   C   
   G   
   C   
   T   
   T   
   C   
   G   
   G   
   A   
   C   
   G   
   T   
   T   
   A   
   T   
   T   
   C   
   C   
   A   
   A   
   A   
   T   
   A   
   T   
   A   
   T   
   T   
   A   
   T   
    C    
   C   
   A   
    A    
   G   
   A   
   A   
   C   
   G   
   T   
   G   
   C   
   A   
   C   
   T   
   G   
   C   
   C   
   T   
   G   
   A   
   T   
   A   
   T   
    T    
   C   
   G   
   T   
   G   
   A   
   T   
   G   
   G   
   A   
   T   
   T   
   G   
   A   
   A   
    G    
   C   
   C   
   A   
   G   
   T   
    G    
   C   
   A   
   A   
   C   
   G   
   C   
   C   
   G   
   G   
   A   
   T   
   A   
   T   
   T   
   A   
   T   
   T   
    T    
   G   
   C   
    C    
   A   
   T   
   G   
   A   
   A   
   C   
   A   
   A   
   A   
   G   
   A   
   T   
   G   
   G   
   C   
   A   
   A   
   T   
   A   
   C   
   G   
   T   
   A   
   T   
   G   
   A   
   C   
   A   
   A   
   A   
   G   
   G   
   A   
   T   
   T   
    C    
   C   
   G   
   T   
   A   
   A   
   A   
   T   
   C   
   A   
   G   
   C   
   A   
   A   
   A   
   A   
   T   
   C   
   T   
   G   
   T   
   C   
   G   
   G   
   A   
   A   
   A   
   T   
   A   
   T   
   C   
   A   
   T   
   G   
   G   
   G   
    G    
   A   
   A   
   T   
   T   
   A   
   T   
   C   
   A   
   T   
   C   
   C   
   T   
   C   
   A   
   T   
   G   
   G   
   T   
   G   
   A   
   C   
   A   
   G   
   C   
   A   
   G   
   T   
   A   
   T   
   T   
   T   
   A   
   T   
   G   
   A   
   A   
   G   
   C   
   G   
   A   
   T   
   G   
   G   
   T   
   C   
   C   
   G   
   C   
   A   
   T   
   G   
   A   
   G   
   C   
   C   
   A   
   A   
   G   
   A   
    C    
   T   
   G   
   G   
   A   
   A   
   G   
   T   
   T   
   G   
   C   
   G   
   G   
   G   
   A   
   A   
   G   
   T   
   A   
   C   
   T   
   G   
   A   
   T   
   C   
   G   
   A   
    A    
   A   
   T   
   G   
   C   
   A   
   C   
   G   
   G   
    G    
   A   
   A   
   T   
   A   
   A   
    C    
   G   
   G   
   G   
   A   
   G   
   T   
   A   
   T   
   G   
   G   
   A   
   C   
   G   
   G   
   T   
   G   
   A   
   T   
   C   
   C   
   A   
   C   
   C   
   T   
   G   
   C   
   T   
   G   
   C   
   G   
   A   
   T   
   G   
   C   
   G   
   T   
   T   
   A   
   T   
   A   
   C   
   T   
   G   
   A   
   A   
   G   
   C   
   C   
   A   
   G   
    A    
   T   
   T   
   A   
   T   
   C   
   C   
   A   
   A   
   G   
   T   
   T   
   A   
   A   
   G   
   T   
   G   
   G   
   A   
   G   
   A   
   G   
   A   
   T   
   G   
    C    
   T   
   A   
    G    
   C   
   A   
   G   
   A   
   T   
   A   
   T   
   C   
   G   
   A   
    A    
   A   
   A   
   A   
   G   
   A   
   A   
   A   
   C   
   A   
   G   
   T   
    C    
   G   
   A   
   T   
   C   
   T   
   C   
   G   
   T   
   G   
   T   
   G   
   G   
   A   
   A   
   T   
   T   
   T   
   T   
   G   
   A   
   T   
   G   
   A   
   T   
   A   
   C   
   A   
   G   
   A   
   A   
   A   
   A   
   A   
   G   
   A   
   A   
   C   
   C   
   A   
   A   
   C   
   A   
   G   
   T   
   C   
   C   
   T   
   T   
   C   
   C   
   A   
   G   
   C   
   T   
   C   
   G   
   A   
   T   
   A   
   C   
   C   
   C   
   A   
   A   
   A   
   C   
   C   
   T   
   T   
   C   
   T   
    C    
   G   
   T   
   C   
   A   
   A   
   T   
   G   
   G   
   A   
   T   
   C   
   G   
   A   
   C   
   A   
   G   
   G   
   T   
   A   
   T   
   A   
   T   
   C   
   T   
   G   
   C   
   T   
   G   
   G   
   A   
   T   
   A   
   T   
   G   
   C   
    A    
   A   
   C   
   A   
   G   
   A   
   A   
   A   
   T   
   C   
   C   
   C   
   G   
   A   
   C   
    T    
   C   
   A   
   T   
   A   
   A   
   C   
   T   
   T   
   A   
   G   
   C   
   T   
   G   
   A   
   A   
   G   
   T   
   G   
   A   
   T   
    C    
   G   
   A   
    T    
   G   
   G   
   C   
   A   
   C   
   A   
   A   
   T   
   C   
   T   
   A   
   T   
   A   
   T   
   G   
   A   
   T   
   C   
   G   
   A   
   T   
   C   
   A   
   T   
   C   
   C   
   A   
   C   
   A   
   A   
   G   
   C   
   G   
   A   
   G   
   C   
   C   
   T   
   T   
   G   
   A   
   A   
   A   
   A   
   A   
   T   
   T   
   G   
   A   
   T   
   G   
   G   
   A   
   A   
   T   
   A   
   T   
   A   
   T   
   T   
   C   
   C   
   T   
   G   
   G   
   A   
   C   
   C   
   T   
   G   
   A   
   C   
   T   
   T   
    T    
   C   
   C   
   A   
   A   
   C   
   A   
   G   
   G   
   A   
   G   
   G   
    G    
   A   
   T   
   C   
   T   
   T   
   G   
   C   
   A   
   A   
   G   
   G   
    A    
   A   
   A   
   A   
   G   
   A   
   T   
   G   
   A   
   A   
   A   
   T   
   C   
   A   
   A   
   A   
   A   
   A   
   A   
   G   
   C   
   T   
   T   
   A   
   T   
   G   
   A   
   A   
   A   
   C   
   A   
   G   
   G   
    G    
   C   
   G   
   A   
   G   
   G   
   A   
   A   
   A   
   A   
   G   
   T   
   T   
   A   
    T    
   T   
   C   
   T   
   T   
   C   
   G   
   T   
   T   
   C   
   G   
   A   
   A   
   A   
   A   
   C   
   A   
   A   
   A   
   G   
   A   
   T   
   A   
   G   
   A   
    A    
   G   
   C   
   A   
   A   
   T   
   G   
   A   
   A   
   G   
   G   
   G   
    T    
   A   
   A   
    T    
   A   
   A   
   A   
   C   
   A   
   G   
   C   
   A   
   A   
   A   
   T   
   C   
   G   
   T   
   G   
   A   
   T   
   C   
   A   
   C   
    A    
   G   
   A   
   A   
   A   
   T   
   C   
   C   
   C   
   A   
   T   
   A   
   T   
   G   
   A   
   A   
   G   
   T   
   A   
   A   
   A   
   T   
   A   
   A   
   A   
   G   
   C   
   G   
   A   
   C   
    C    
   T   
   T   
   G   
   G   
   T   
   G   
   A   
   A   
   A   
   A   
   A   
   A   
   A   
   T   
   G   
   G   
   A   
   T   
   G   
   A   
   G   
   A   
   T   
   C   
   C   
   G   
   T   
   T   
   T   
   G   
   A   
   A   
   T   
   A   
   A   
   G   
   A   
   A   
   A   
   A   
   T   
   C   
   G   
   A   
    T    
   G   
   G   
   A   
   A   
   T   
   A   
   G   
   C   
   T   
   G   
   A   
   A   
   G   
   T   
    A    
   C   
   G   
   A   
   G   
   A   
   T   
   G   
   A   
   A   
   A   
   G   
   T   
   G   
   A   
   T   
    A    
   G   
    A    
   A   
   C   
    A    
   G   
   G   
   T   
   C   
   T   
   G   
   C   
   A   
   A   
   A   
   T   
   T   
   G   
   T   
   C   
   G   
   T   
    C    
   G   
   A   
   A   
   T   
   T   
   G   
   A   
   A   
   A   
   A   
   A   
   A   
   G   
   A   
    C    
   G   
   C   
   C   
   A   
   A   
    T    
   G   
   C   
   T   
   C   
   A   
   A   
   G   
   G   
    A    
   A   
   T   
   T   
   T   
   T   
   G   
   A   
   A   
   T   
   T   
   A   
    T    
   T   
   T   
   G   
   T   
   T   
   C   
   A   
   A   
   A   
   A   
   A   
   C   
   A   
   C   
   T   
   G   
   A   
   G   
    C    
   T   
   A   
   C   
   A   
   G   
   A   
   T   
   C   
   A   
   A   
   T   
   T   
   A   
   C   
   A   
   A   
   T   
   T   
   T   
    C    
   A   
   A   
   T   
   A   
   T   
   G   
   G   
   T   
   C   
   G   
   C   
   T   
   A   
   T   
   C   
   G   
   A   
    C    
   C   
   A   
   T   
   A   
   T   
   G   
   A   
   C   
   T   
   C   
   C   
   G   
   C   
   A   
    T    
   C   
   A   
   A   
   G   
   T   
   T   
   G   
   G   
   A   
   C   
   T   
   G   
   A   
   A   
   A   
   G   
   A   
   C   
   A   
   T   
   T   
   T   
   T   
   A   
   A   
   G   
   A   
   A   
   G   
   T   
   T   
   A   
   T   
   A   
   T   
   C   
   G   
   A   
   G   
   C   
   A   
   T   
   C   
   G   
   A   
   A   
   A   
   G   
   C   
   A   
   A   
   G   
   T   
   C   
   A   
   T   
   A   
   A   
   C   
   A   
   A   
   A   
   A   
   C   
   G   
    T    
   A   
   G   
    T    
   C   
   A   
   A   
   T   
   T   
   C   
   G   
   A   
   T   
   T   
   T   
   A   
   G   
   C   
   C   
   A   
   A   
   A   
   G   
   C   
   T   
   C   
   A   
   A   
   A   
   A   
   A   
    A    
   G   
   A   
   C   
   A   
   G   
   C   
   A   
   T   
   A   
   T   
   C   
   G   
   T   
    A    
   G   
   A   
   A   
   G   
   G   
    A    
    T    
   T   
   G   
   A   
   T   
   G   
   A   
   A   
   A   
   G   
   C   
   T   
   T   
   T   
   A   
   T   
   C   
   G   
   A   
   T   
   T   
   C   
   T   
   G   
   G   
   A   
   T   
   G   
   A   
   A   
   G   
   T   
   A   
   A   
   T   
   C   
   G   
    C    
   A   
   A   
   C   
   G   
   A   
   T   
   T   
   C   
   G   
   T   
   G   
   A   
   A   
   A   
   G   
   T   
   A   
   A   
   A   
   G   
   A   
   C   
   A   
   A   
   A   
   A   
   A   
   A   
   G   
   A   
    C    
   G   
   C   
    C    
   A   
   A   
   A   
   A   
   A   
   G   
   A   
   A   
   T   
   T   
   T   
   A   
   G   
   T   
   C   
   G   
   A   
    C    
   G   
   T   
    C    
   T   
   T   
    T    
   C   
   A   
   A   
   T   
   T   
   T   
   A   
   C   
   A   
   G   
   A   
   A   
   G   
   A   
   A   
   C   
   A   
   A   
   G   
   C   
   A   
   G   
   A   
   A   
   G   
   C   
   A   
   A   
   T   
    T    
   G   
   T   
   C   
   A   
   C   
   A   
   T   
   T   
   G   
   C   
   A   
   A   
    T    
   T   
   A   
   T   
   A   
    C    
   C   
   G   
   T   
   T   
   T   
   G   
   A   
   C   
   G   
   A   
   A   
    C    
   A   
   C   
    G    
   G   
   A   
   T   
   A   
   T   
   C   
   A   
   C   
   A   
   G   
   A   
   G   
   C   
   T   
   T   
   C   
   A   
   A   
   A   
   A   
   G   
   G   
   A   
   A   
    T    
   C   
    C    
   G   
   A   
   A   
   A   
    G    
   C   
   T   
   T   
   G   
   A   
   T   
   T   
   G   
   C   
    G    
   C   
   A   
   A   
   A   
   T   
   C   
   A   
   C   
   A   
   G   
   A   
   G   
   T   
   T   
    G    
   A   
   A   
   T   
   A   
   A   
   A   
   A   
   T   
   C   
   C   
   T   
   T   
   T   
   C   
   A   
   A   
   A   
    T    
   G   
   A   
   C   
   A   
   A   
   A   
   G   
   A   
    G    
   C   
   T   
   T   
   T   
   T   
    C    
   T   
   C   
   T   
   G   
   T   
   T   
   A   
   T   
   G   
   A   
   A   
   A   
   A   
   A   
   A   
   G   
   A   
   A   
   T   
   T   
   G   
   C   
   G   
   A   
   G   
   A   
   A   
   G   
   T   
   G   
   A   
   A   
   A   
   A   
   A   
   A   
   A   
   A   
   T   
   T   
   A   
    T    
   T   
   C   
   T   
   T   
   C   
   C   
   G   
   C   
   T   
   C   
   G   
   T   
   C   
    T    
    A    
   A   
   C   
   G   
   A   
   C   
   A   
   A   
   T   
   C   
    G    
   A   
   A   
   G   
   A   
   C   
   G   
   A   
   A   
   A   
   T   
   C   
   G   
   A   
   A   
   G   
   A   
   A   
   A   
   T   
   C   
   A   
   A   
   A   
   A   
   T   
    T    
   G   
   A   
   T   
   A   
   C   
   G   
   C   
   A   
   A   
    G    
   T   
    C    
   T   
   T   
   A   
   G   
   T   
   G   
   G   
   C   
    A    
   C   
   A   
   A   
   G   
   A   
   A   
   G   
   A   
   C   
   G   
   T   
   A   
   A   
   T   
    T    
   G   
   T   
   C   
   A   
   G   
   T   
   G   
   T   
    G    
   A   
   C   
    G    
   C   
   G   
   A   
   G   
   A   
   A   
   G   
   G   
   C   
   T   
   A   
   T   
   G   
   T   
   G   
   A   
   A   
   G   
   C   
   G   
   A   
   A   
   C   
   A   
   A   
   G   
   C   
   C   
   T   
   T   
   C   
   G   
   T   
   T   
   C   
    T    
   T   
   A   
   T   
   A   
   G   
   T   
   G   
   C   
   T   
   T   
   C   
    C    
   A   
   A   
   A   
   C   
   C   
   A   
   G   
   A   
   A   
   G   
   A   
   A   
   A   
   T   
   C   
   G   
   G   
   G   
   A   
   T   
   G   
   C   
   G   
   A   
   G   
   A   
   A   
   G   
   G   
   C   
   G   
   A   
    C    
   T   
   A   
   C   
   T   
   T   
   G   
   C   
   T   
    A    
   T   
   A   
   C   
    T    
   C   
   A   
   G   
   G   
   T   
   G   
   A   
   A   
   T   
   T   
    G    
   A   
   G   
    T    
   A   
   C   
   A   
    T    
   T   
   G   
   G   
   A   
   C   
   C   
   A   
   T   
   G   
   T   
   T   
   C   
   T   
   T   
   C   
   T   
   G   
   A   
   T   
    C    
   A   
   C   
    A    
   A   
   A   
   T   
   A   
   A   
   A   
   G   
   C   
   A   
   A   
   A   
   T   
   G   
   T   
   G   
   A   
   T   
   C   
   T   
   A   
   C   
   C   
   G   
    C    
   C   
   C   
   T   
   G   
   T   
    C    
   C   
   A   
   T   
   G   
   A   
   A   
   C   
   T   
   A   
   C   
   C   
    C    
   G   
   A   
   T   
   T   
   T   
   G   
   A   
   A   
   A   
   T   
   G   
   G   
   A   
   A   
   A   
   G   
   A   
   T   
   G   
   C   
   T   
   G   
   G   
    T    
   G   
   A   
    G    
   C   
   A   
    C    
   A   
   T   
   T   
   T   
   C   
   T   
   C   
   A   
   A   
   A   
   C   
   A   
   A   
   T   
   C   
   T   
   C   
   C   
   A   
   A   
   T   
   C   
   T   
   G   
   T   
   C   
   T   
   G   
   T   
   A   
   G   
   A   
    C    
   G   
   A   
   A   
   T   
   C   
   G   
   A   
   T   
    T    
   T   
   T   
   A   
   G   
   C   
   T   
   G   
   T   
   T   
   T   
   T   
    C    
   C   
   C   
   A   
   T   
   A   
   T   
   C   
   A   
   A   
   A   
   A   
   A   
   A   
   T   
    T    
   C   
   A   
   A   
   G   
   C   
   A   
   G   
   A   
    G    
   A   
   A   
   A   
   A   
   C   
   A   
   T   
   T   
   T   
   G   
   T   
    A    
   T   
   T   
   C   
   A   
   T   
   C   
   A   
   G   
   C   
   A   
   A   
   G   
   A   
   A   
   T   
   G   
   G   
   A   
   T   
   T   
    G    
    A    
   T   
   C   
   A   
   A   
   G   
   C   
   A   
   A   
   A   
   C   
   A   
   C   
   G   
   A   
   A   
   T   
   G   
   A   
   C   
   A   
   G   
   A   
   G   
   T   
   T   
   C   
   G   
   A   
   A   
   C   
   C   
   A   
   T   
   G   
   G   
   C   
   G   
   C   
   A   
   C   
   C   
   T   
   A   
    T    
   A   
   A   
   G   
   A   
   G   
   C   
   C   
   G   
    T    
   C   
   C   
   G   
   C   
   T   
   C   
   A   
   G   
   C   
   T   
   G   
   T   
   A   
   T   
   G   
   A   
   A   
   G   
   C   
   T   
   A   
   A   
   A   
   G   
   G   
   C   
   G   
   G   
   A   
   A   
   G   
   A   
   A   
   G   
   A   
   T   
   G   
   A   
   A   
   C   
   T   
    G    
   A   
   T   
   T   
   G   
   C   
   T   
   G   
   T   
    G    
   T   
   A   
   T   
   C   
   T   
   G   
   A   
   A   
   A   
   G   
   A   
   A   
   G   
   A   
   T   
   C   
    A    
   G   
   C   
   C   
   A   
   G   
   G   
   C   
   T   
   T   
   A   
   G   
   A   
   T   
   G   
   T   
    T    
   T   
   T   
   C   
   T   
   T   
   A   
   G   
   T   
   A   
   A   
   C   
   G   
    C    
   A   
   T   
   C   
   A   
   A   
   G   
   G   
   A   
   A   
   T   
   G   
   G   
   G   
    T    
   T   
   T   
   G   
   C   
   G   
   C   
   T   
    A    
   T   
   C   
   C   
   T   
   T   
   T   
   A   
   A   
    A    
   A   
   G   
   A   
   T   
   G   
   T   
   T   
   C   
   C   
   C   
   G   
   T   
   T   
   G   
   T   
   T   
   G   
   G   
   A   
   G   
   C   
   A   
   A   
   A   
   G   
   G   
   C   
   A   
   G   
    C    
   T   
   G   
   G   
   A   
   G   
   T   
   G   
   A   
   A   
   G   
   T   
   C   
   T   
   A   
   T   
   G   
   A   
   A   
   T   
   T   
   T   
   G   
   A   
   A   
   A   
   G   
   A   
   T   
   G   
   G   
   A   
   G   
   A   
   T   
   A   
   G   
   G   
    A    
   T   
   T   
    A    
   T   
   C   
   A   
   A   
    C    
   G   
   G   
    G    
   C   
   T   
    A    
   C   
   T   
   T   
   G   
   T   
   G   
   T   
   T   
   A   
   G   
   C   
   A   
   G   
   A   
    A    
   G   
   G   
    T    
   G   
   A   
   T   
   A   
   C   
   G   
   C   
   C   
    G    
   A   
   T   
   C   
   G   
   T   
   A   
   A   
   T   
   C   
   G   
   T   
   T   
   A   
   C   
   T   
   C   
   A   
   G   
   C   
   G   
   A   
   G   
   G   
   A   
   G   
   C   
    T    
   G   
   T   
    T    
   A   
   A   
   A   
   C   
   G   
   C   
   A   
   T   
   G   
   C   
   T   
   T   
   G   
   C   
   T   
   C   
   A   
   A   
   G   
   A   
   A   
   A   
   T   
   C   
   A   
   G   
   C   
   C   
   A   
   A   
   A   
   C   
    T    
   T   
   C   
   A   
   C   
   G   
   T   
   G   
   C   
   G   
   A   
   A   
   G   
   C   
   G   
   G   
   G   
   G   
   A   
   G   
   T   
   G   
   A   
   C   
   G   
   G   
   T   
   C   
   A   
   T   
   G   
   C   
   G   
   T   
   G   
   A   
   G   
   C   
   T   
   G   
   A   
   A   
   A   
   A   
   A   
   A   
   A   
   A   
   T   
   C   
   C   
    G    
   C   
   A   
    C    
   C   
   G   
    T    
   A   
   T   
   T   
   A   
   T   
   C   
   T   
   A   
   T   
   A   
   T   
   G   
   T   
   C   
   G   
   G   
   A   
   A   
   G   
   G   
   A   
   A   
   G   
   A   
   T   
   C   
   A   
   A   
   A   
   A   
   G   
   A   
   A   
   A   
   T   
   A   
   A   
   C   
   C   
   T   
   T   
   G   
   A   
   T   
   G   
   A   
   A   
   T   
   C   
   A   
   A   
   A   
   A   
   A   
   G   
   G   
    G    
   C   
   A   
    G    
   C   
   A   
   G   
    T    
   T   
   A   
   A   
   C   
   G   
   A   
   T   
   T   
   G   
   A   
    C    
   C   
   C   
   A   
   A   
   C   
    T    
   G   
   A   
   T   
   T   
   T   
    C    
   C   
   C   
   T   
   A   
   T   
   A   
   G   
   G   
   T   
   G   
   A   
   T   
   C   
   G   
   A   
   A   
   C   
   A   
   T   
   C   
   G   
   A   
   A   
   T   
   G   
   G   
    A    
   T   
   C   
    A    
   T   
   T   
   T   
   G   
   C   
    G    
   A   
   T   
   G   
   G   
   A   
    T    
   G   
   A   
   G   
   A   
   A   
   A   
   A   
   A   
   A   
   G   
   G   
   T   
   G   
   G   
   A   
   G   
   A   
   A   
   G   
   T   
   C   
   A   
   T   
   C   
   G   
   A   
   A   
   G   
   T   
    T    
   A   
    T    
   C   
   G   
   A   
   T   
   G   
   C   
   T   
   C   
   C   
   T   
   G   
   A   
   G   
    A    
   T   
   T   
   T   
   T   
    C    
   A   
   T   
    C    
   G   
   A   
    G    
    T    
   C   
    G    
   -   
   -   
   -   
 

 
   N59504F   
   A   
   T   
   G   
   G   
   A   
   A   
   A   
   A   
   T   
   C   
   G   
   A   
   C   
   A   
   A   
   G   
   A   
   A   
   G   
   T   
   A   
   C   
   A   
   A   
   G   
   A   
   A   
   T   
   T   
   A   
   A   
   C   
   T   
   T   
   T   
   A   
   G   
   A   
   A   
   G   
   A   
   A   
   G   
   T   
   G   
   A   
   T   
   G   
   G   
   G   
   T   
   G   
   A   
   C   
   C   
   G   
   C   
   T   
   T   
   C   
   G   
   G   
   A   
   C   
   G   
   T   
   T   
   A   
   T   
   T   
   C   
   C   
   A   
   A   
   A   
   T   
   A   
   T   
   A   
   T   
   T   
   A   
   T   
    C    
   C   
   A   
    A    
   G   
   A   
   A   
   C   
   G   
   T   
   G   
   C   
   A   
   C   
   T   
   G   
   C   
   C   
   T   
   G   
   A   
   T   
   A   
   T   
    C    
   C   
   G   
   T   
   G   
   A   
   T   
   G   
   G   
   A   
   T   
   T   
   G   
   A   
   A   
    G    
   C   
   C   
   A   
   G   
   T   
    G    
   C   
   A   
   A   
   C   
   G   
   C   
   C   
   G   
   G   
   A   
   T   
   A   
   T   
   T   
   A   
   T   
   T   
    T    
   G   
   C   
    C    
   A   
   T   
   G   
   A   
   A   
   C   
   A   
   A   
   A   
   G   
   A   
   T   
   G   
   G   
   C   
   A   
   A   
   T   
   A   
   C   
   G   
   T   
   A   
   T   
   G   
   A   
   C   
   A   
   A   
   A   
   G   
   G   
   A   
   T   
   T   
    C    
   C   
   G   
   T   
   A   
   A   
   A   
   T   
   C   
   A   
   G   
   C   
   A   
   A   
   A   
   A   
   T   
   C   
   T   
   G   
   T   
   C   
   G   
   G   
   A   
   A   
   A   
   T   
   A   
   T   
   C   
   A   
   T   
   G   
   G   
   G   
    G    
   A   
   A   
   T   
   T   
   A   
   T   
   C   
   A   
   T   
   C   
   C   
   T   
   C   
   A   
   T   
   G   
   G   
   T   
   G   
   A   
   C   
   A   
   G   
   C   
   A   
   G   
   T   
   A   
   T   
   T   
   T   
   A   
   T   
   G   
   A   
   A   
   G   
   C   
   G   
   A   
   T   
   G   
   G   
   T   
   C   
   C   
   G   
   C   
   A   
   T   
   G   
   A   
   G   
   C   
   C   
   A   
   A   
   G   
   A   
    C    
   T   
   G   
   G   
   A   
   A   
   G   
   T   
   T   
   G   
   C   
   G   
   G   
   G   
   A   
   A   
   G   
   T   
   A   
   C   
   T   
   G   
   A   
   T   
   C   
   G   
   A   
    A    
   A   
   T   
   G   
   C   
   A   
   C   
   G   
   G   
    G    
   A   
   A   
   T   
   A   
   A   
    C    
   G   
   G   
   G   
   A   
   G   
   T   
   A   
   T   
   G   
   G   
   A   
   C   
   G   
   G   
   T   
   G   
   A   
   T   
   C   
   C   
   A   
   C   
   C   
   T   
   G   
   C   
   T   
   G   
   C   
   G   
   A   
   T   
   G   
   C   
   G   
   T   
   T   
   A   
   T   
   A   
   C   
   T   
   G   
   A   
   A   
   G   
   C   
   C   
   A   
   G   
    A    
   T   
   T   
   A   
   T   
   C   
   C   
   A   
   A   
   G   
   T   
   T   
   A   
   A   
   G   
   T   
   G   
   G   
   A   
   G   
   A   
   G   
   A   
   T   
   G   
    T    
   T   
   A   
    G    
   C   
   A   
   G   
   A   
   T   
   A   
   T   
   C   
   G   
   A   
    A    
   A   
   A   
   A   
   G   
   A   
   A   
   A   
   C   
   A   
   G   
   T   
    C    
   G   
   A   
   T   
   C   
   T   
   C   
   G   
   T   
   G   
   T   
   G   
   G   
   A   
   A   
   T   
   T   
   T   
   T   
   G   
   A   
   T   
   G   
   A   
   T   
   A   
   C   
   A   
   G   
   A   
   A   
   A   
   A   
   A   
   G   
   A   
   A   
   C   
   C   
   A   
   A   
   C   
   A   
   G   
   T   
   C   
   C   
   T   
   T   
   C   
   C   
   A   
   G   
   C   
   T   
   C   
   G   
   A   
   T   
   A   
   C   
   C   
   C   
   A   
   A   
   A   
   C   
   C   
   T   
   T   
   C   
   T   
    C    
   G   
   T   
   C   
   A   
   A   
   T   
   G   
   G   
   A   
   T   
   C   
   G   
   A   
   C   
   A   
   G   
   G   
   T   
   A   
   T   
   A   
   T   
   C   
   T   
   G   
   C   
   T   
   G   
   G   
   A   
   T   
   A   
   T   
   G   
   C   
    A    
   A   
   C   
   A   
   G   
   A   
   A   
   A   
   T   
   C   
   C   
   C   
   G   
   A   
   C   
    T    
   C   
   A   
   T   
   A   
   A   
   C   
   T   
   T   
   A   
   G   
   C   
   T   
   G   
   A   
   A   
   G   
   T   
   G   
   A   
   T   
    C    
   G   
   A   
    T    
   G   
   G   
   C   
   A   
   C   
   A   
   A   
   T   
   C   
   T   
   A   
   T   
   A   
   T   
   G   
   A   
   T   
   C   
   G   
   A   
   T   
   C   
   A   
   T   
   C   
   C   
   A   
   C   
   A   
   A   
   G   
   C   
   G   
   A   
   G   
   C   
   C   
   T   
   T   
   G   
   A   
   A   
   A   
   A   
   A   
   T   
   T   
   G   
   A   
   T   
   G   
   G   
   A   
   A   
   T   
   A   
   T   
   A   
   T   
   T   
   C   
   C   
   T   
   G   
   G   
   A   
   C   
   C   
   T   
   G   
   A   
   C   
   T   
   T   
    T    
   C   
   C   
   A   
   A   
   C   
   A   
   G   
   G   
   A   
   G   
   G   
    G    
   A   
   T   
   C   
   T   
   T   
   G   
   C   
   A   
   A   
   G   
   G   
    A    
   A   
   A   
   A   
   G   
   A   
   T   
   G   
   A   
   A   
   A   
   T   
   C   
   A   
   A   
   A   
   A   
   A   
   A   
   G   
   C   
   T   
   T   
   A   
   T   
   G   
   A   
   A   
   A   
   C   
   A   
   G   
   G   
    G    
   C   
   G   
   A   
   G   
   G   
   A   
   A   
   A   
   A   
   G   
   T   
   T   
   A   
    T    
   T   
   C   
   T   
   T   
   C   
   G   
   T   
   T   
   C   
   G   
   A   
   A   
   A   
   A   
   C   
   A   
   A   
   A   
   G   
   A   
   T   
   A   
   G   
   A   
    G    
   G   
   C   
   A   
   A   
   T   
   G   
   A   
   A   
   G   
   G   
   G   
    T    
   A   
   A   
    T    
   A   
   A   
   A   
   C   
   A   
   G   
   C   
   A   
   A   
   A   
   T   
   C   
   G   
   T   
   G   
   A   
   T   
   C   
   A   
   C   
    A    
   G   
   A   
   A   
   A   
   T   
   C   
   C   
   C   
   A   
   T   
   A   
   T   
   G   
   A   
   A   
   G   
   T   
   A   
   A   
   A   
   T   
   A   
   A   
   A   
   G   
   C   
   G   
   A   
   C   
    C    
   T   
   T   
   G   
   G   
   T   
   G   
   A   
   A   
   A   
   A   
   A   
   A   
   A   
   T   
   G   
   G   
   A   
   T   
   G   
   A   
   G   
   A   
   T   
   C   
   C   
   G   
   T   
   T   
   T   
   G   
   A   
   A   
   T   
   A   
   A   
   G   
   A   
   A   
   A   
   A   
   T   
   C   
   G   
   A   
    T    
   G   
   G   
   A   
   A   
   T   
   A   
   G   
   C   
   T   
   G   
   A   
   A   
   G   
   T   
    A    
   C   
   G   
   A   
   G   
   A   
   T   
   G   
   A   
   A   
   A   
   G   
   T   
   G   
   A   
   T   
    A    
   G   
    A    
   A   
   C   
    A    
   G   
   G   
   T   
   C   
   T   
   G   
   C   
   A   
   A   
   A   
   T   
   T   
   G   
   T   
   C   
   G   
   T   
    C    
   G   
   A   
   A   
   T   
   T   
   G   
   A   
   A   
   A   
   A   
   A   
   A   
   G   
   A   
    C    
   G   
   C   
   C   
   A   
   A   
    T    
   G   
   C   
   T   
   C   
   A   
   A   
   G   
   G   
    A    
   A   
   T   
   T   
   T   
   T   
   G   
   A   
   A   
   T   
   T   
   A   
    T    
   T   
   T   
   G   
   T   
   T   
   C   
   A   
   A   
   A   
   A   
   A   
   C   
   A   
   C   
   T   
   G   
   A   
   G   
    C    
   T   
   A   
   C   
   A   
   G   
   A   
   T   
   C   
   A   
   A   
   T   
   T   
   A   
   C   
   A   
   A   
   T   
   T   
   T   
    C    
   A   
   A   
   T   
   A   
   T   
   G   
   G   
   T   
   C   
   G   
   C   
   T   
   A   
   T   
   C   
   G   
   A   
    C    
   C   
   A   
   T   
   A   
   T   
   G   
   A   
   C   
   T   
   C   
   C   
   G   
   C   
   A   
    T    
   C   
   A   
   A   
   G   
   T   
   T   
   G   
   G   
   A   
   C   
   T   
   G   
   A   
   A   
   A   
   G   
   A   
   C   
   A   
   T   
   T   
   T   
   T   
   A   
   A   
   G   
   A   
   A   
   G   
   T   
   T   
   A   
   T   
   A   
   T   
   C   
   G   
   A   
   G   
   C   
   A   
   T   
   C   
   G   
   A   
   A   
   A   
   G   
   C   
   A   
   A   
   G   
   T   
   C   
   A   
   T   
   A   
   A   
   C   
   A   
   A   
   A   
   A   
   C   
   G   
    T    
   A   
   G   
    T    
   C   
   A   
   A   
   T   
   T   
   C   
   G   
   A   
   T   
   T   
   T   
   A   
   G   
   C   
   C   
   A   
   A   
   A   
   G   
   C   
   T   
   C   
   A   
   A   
   A   
   A   
   A   
    A    
   G   
   A   
   C   
   A   
   G   
   C   
   A   
   T   
   A   
   T   
   C   
   G   
   T   
    A    
   G   
   A   
   A   
   G   
   G   
    A    
    T    
   T   
   G   
   A   
   T   
   G   
   A   
   A   
   A   
   G   
   C   
   T   
   T   
   T   
   A   
   T   
   C   
   G   
   A   
   T   
   T   
   C   
   T   
   G   
   G   
   A   
   T   
   G   
   A   
   A   
   G   
   T   
   A   
   A   
   T   
   C   
   G   
    C    
   A   
   A   
   C   
   G   
   A   
   T   
   T   
   C   
   G   
   T   
   G   
   A   
   A   
   A   
   G   
   T   
   A   
   A   
   A   
   G   
   A   
   C   
   A   
   A   
   A   
   A   
   A   
   A   
   G   
   A   
    C    
   G   
   C   
    C    
   A   
   A   
   A   
   A   
   A   
   G   
   A   
   A   
   T   
   T   
   T   
   A   
   G   
   T   
   C   
   G   
   A   
    C    
   G   
   T   
    C    
   T   
   T   
    T    
   C   
   A   
   A   
   T   
   T   
   T   
   A   
   C   
   A   
   G   
   A   
   A   
   G   
   A   
   A   
   C   
   A   
   A   
   G   
   C   
   A   
   G   
   A   
   A   
   G   
   C   
   A   
   A   
   T   
    T    
   G   
   T   
   C   
   A   
   C   
   A   
   T   
   T   
   G   
   C   
   A   
   A   
    T    
   T   
   A   
   T   
   A   
    C    
   C   
   G   
   T   
   T   
   T   
   G   
   A   
   C   
   G   
   A   
   A   
    C    
   A   
   C   
    G    
   G   
   A   
   T   
   A   
   T   
   C   
   A   
   C   
   A   
   G   
   A   
   G   
   C   
   T   
   T   
   C   
   A   
   A   
   A   
   A   
   G   
   G   
   A   
   A   
    T    
   C   
    C    
   G   
   A   
   A   
   A   
    G    
   C   
   T   
   T   
   G   
   A   
   T   
   T   
   G   
   C   
    G    
   C   
   A   
   A   
   A   
   T   
   C   
   A   
   C   
   A   
   G   
   A   
   G   
   T   
   T   
    G    
   A   
   A   
   T   
   A   
   A   
   A   
   A   
   T   
   C   
   C   
   T   
   T   
   T   
   C   
   A   
   A   
   A   
    T    
   G   
   A   
   C   
   A   
   A   
   A   
   G   
   A   
    G    
   C   
   T   
   T   
   T   
   T   
    C    
   T   
   C   
   T   
   G   
   T   
   T   
   A   
   T   
   G   
   A   
   A   
   A   
   A   
   A   
   A   
   G   
   A   
   A   
   T   
   T   
   G   
   C   
   G   
   A   
   G   
   A   
   A   
   G   
   T   
   G   
   A   
   A   
   A   
   A   
   A   
   A   
   A   
   A   
   T   
   T   
   A   
    T    
   T   
   C   
   T   
   T   
   C   
   C   
   G   
   C   
   T   
   C   
   G   
   T   
   C   
    T    
    A    
   A   
   C   
   G   
   A   
   C   
   A   
   A   
   T   
   C   
    G    
   A   
   A   
   G   
   A   
   C   
   G   
   A   
   A   
   A   
   T   
   C   
   G   
   A   
   A   
   G   
   A   
   A   
   A   
   T   
   C   
   A   
   A   
   A   
   A   
   T   
    T    
   G   
   A   
   T   
   A   
   C   
   G   
   C   
   A   
   A   
    G    
   T   
    C    
   T   
   T   
   A   
   G   
   T   
   G   
   G   
   C   
    A    
   C   
   A   
   A   
   G   
   A   
   A   
   G   
   A   
   C   
   G   
   T   
   A   
   A   
   T   
    T    
   G   
   T   
   C   
   A   
   G   
   T   
   G   
   T   
    G    
   A   
   C   
    G    
   C   
   G   
   A   
   G   
   A   
   A   
   G   
   G   
   C   
   T   
   A   
   T   
   G   
   T   
   G   
   A   
   A   
   G   
   C   
   G   
   A   
   A   
   C   
   A   
   A   
   G   
   C   
   C   
   T   
   T   
   C   
   G   
   T   
   T   
   C   
    T    
   T   
   A   
   T   
   A   
   G   
   T   
   G   
   C   
   T   
   T   
   C   
    C    
   A   
   A   
   A   
   C   
   C   
   A   
   G   
   A   
   A   
   G   
   A   
   A   
   A   
   T   
   C   
   G   
   G   
   G   
   A   
   T   
   G   
   C   
   G   
   A   
   G   
   A   
   A   
   G   
   G   
   C   
   G   
   A   
    C    
   T   
   A   
   C   
   T   
   T   
   G   
   C   
   T   
    A    
   T   
   A   
   C   
    T    
   C   
   A   
   G   
   G   
   T   
   G   
   A   
   A   
   T   
   T   
    G    
   A   
   G   
    T    
   A   
   C   
   A   
    T    
   T   
   G   
   G   
   A   
   C   
   C   
   A   
   T   
   G   
   T   
   T   
   C   
   T   
   T   
   C   
   T   
   G   
   A   
   T   
    C    
   A   
   C   
    A    
   A   
   A   
   T   
   A   
   A   
   A   
   G   
   C   
   A   
   A   
   A   
   T   
   G   
   T   
   G   
   A   
   T   
   C   
   T   
   A   
   C   
   C   
   G   
    C    
   C   
   C   
   T   
   G   
   T   
    C    
   C   
   A   
   T   
   G   
   A   
   A   
   C   
   T   
   A   
   C   
   C   
    C    
   G   
   A   
   T   
   T   
   T   
   G   
   A   
   A   
   A   
   T   
   G   
   G   
   A   
   A   
   A   
   G   
   A   
   T   
   G   
   C   
   T   
   G   
   G   
    T    
   G   
   A   
    G    
   C   
   A   
    C    
   A   
   T   
   T   
   T   
   C   
   T   
   C   
   A   
   A   
   A   
   C   
   A   
   A   
   T   
   C   
   T   
   C   
   C   
   A   
   A   
   T   
   C   
   T   
   G   
   T   
   C   
   T   
   G   
   T   
   A   
   G   
   A   
    C    
   G   
   A   
   A   
   T   
   C   
   G   
   A   
   T   
    T    
   T   
   T   
   A   
   G   
   C   
   T   
   G   
   T   
   T   
   T   
   T   
    C    
   C   
   C   
   A   
   T   
   A   
   T   
   C   
   A   
   A   
   A   
   A   
   A   
   A   
   T   
    T    
   C   
   A   
   A   
   G   
   C   
   A   
   G   
   A   
    G    
   A   
   A   
   A   
   A   
   C   
   A   
   T   
   T   
   T   
   G   
   T   
    A    
   T   
   T   
   C   
   A   
   T   
   C   
   A   
   G   
   C   
   A   
   A   
   G   
   A   
   A   
   T   
   G   
   G   
   A   
   T   
   T   
    G    
    A    
   T   
   C   
   A   
   A   
   G   
   C   
   A   
   A   
   A   
   C   
   A   
   C   
   G   
   A   
   A   
   T   
   G   
   A   
   C   
   A   
   G   
   A   
   G   
   T   
   T   
   C   
   G   
   A   
   A   
   C   
   C   
   A   
   T   
   G   
   G   
   C   
   G   
   C   
   A   
   C   
   C   
   T   
   A   
    T    
   A   
   A   
   G   
   A   
   G   
   C   
   C   
   G   
    T    
   C   
   C   
   G   
   C   
   T   
   C   
   A   
   G   
   C   
   T   
   G   
   T   
   A   
   T   
   G   
   A   
   A   
   G   
   C   
   T   
   A   
   A   
   A   
   G   
   G   
   C   
   G   
   G   
   A   
   A   
   G   
   A   
   A   
   G   
   A   
   T   
   G   
   A   
   A   
   C   
   T   
    G    
   A   
   T   
   T   
   G   
   C   
   T   
   G   
   T   
    G    
   T   
   A   
   T   
   C   
   T   
   G   
   A   
   A   
   A   
   G   
   A   
   A   
   G   
   A   
   T   
   C   
    A    
   G   
   C   
   C   
   A   
   G   
   G   
   C   
   T   
   T   
   A   
   G   
   A   
   T   
   G   
   T   
    T    
   T   
   T   
   C   
   T   
   T   
   A   
   G   
   T   
   A   
   A   
   C   
   G   
    C    
   A   
   T   
   C   
   A   
   A   
   G   
   G   
   A   
   A   
   T   
   G   
   G   
   G   
    T    
   T   
   T   
   G   
   C   
   G   
   C   
   T   
    A    
   T   
   C   
   C   
   T   
   T   
   T   
   A   
   A   
    C    
   A   
   G   
   A   
   T   
   G   
   T   
   T   
   C   
   C   
   C   
   G   
   T   
   T   
   G   
   T   
   T   
   G   
   G   
   A   
   G   
   C   
   A   
   A   
   A   
   G   
   G   
   C   
   A   
   G   
    C    
   T   
   G   
   G   
   A   
   G   
   T   
   G   
   A   
   A   
   G   
   T   
   C   
   T   
   A   
   T   
   G   
   A   
   A   
   T   
   T   
   T   
   G   
   A   
   A   
   A   
   G   
   A   
   T   
   G   
   G   
   A   
   G   
   A   
   T   
   A   
   G   
   G   
    A    
   T   
   T   
    A    
   T   
   C   
   A   
   A   
    C    
   G   
   G   
    G    
   C   
   T   
    G    
   C   
   T   
   T   
   G   
   T   
   G   
   T   
   T   
   A   
   G   
   C   
   A   
   G   
   A   
    A    
   G   
   G   
    T    
   G   
   A   
   T   
   A   
   C   
   G   
   C   
   C   
    G    
   A   
   T   
   C   
   G   
   T   
   A   
   A   
   T   
   C   
   G   
   T   
   T   
   A   
   C   
   T   
   C   
   A   
   G   
   C   
   G   
   A   
   G   
   G   
   A   
   G   
   C   
    T    
   G   
   T   
    T    
   A   
   A   
   A   
   C   
   G   
   C   
   A   
   T   
   G   
   C   
   T   
   T   
   G   
   C   
   T   
   C   
   A   
   A   
   G   
   A   
   A   
   A   
   T   
   C   
   A   
   G   
   C   
   C   
   A   
   A   
   A   
   C   
    T    
   T   
   C   
   A   
   C   
   G   
   T   
   G   
   C   
   G   
   A   
   A   
   G   
   C   
   G   
   G   
   G   
   G   
   A   
   G   
   T   
   G   
   A   
   C   
   G   
   G   
   T   
   C   
   A   
   T   
   G   
   C   
   G   
   T   
   G   
   A   
   G   
   C   
   T   
   G   
   A   
   A   
   A   
   A   
   A   
   A   
   A   
   A   
   T   
   C   
   C   
    G    
   C   
   A   
    C    
   C   
   G   
    T    
   A   
   T   
   T   
   A   
   T   
   C   
   T   
   A   
   T   
   A   
   T   
   G   
   T   
   C   
   G   
   G   
   A   
   A   
   G   
   G   
   A   
   A   
   G   
   A   
   T   
   C   
   A   
   A   
   A   
   A   
   G   
   A   
   A   
   A   
   T   
   A   
   A   
   C   
   C   
   T   
   T   
   G   
   A   
   T   
   G   
   A   
   A   
   T   
   C   
   A   
   A   
   A   
   A   
   A   
   G   
   G   
    G    
   C   
   A   
    G    
   C   
   A   
   G   
    T    
   T   
   A   
   A   
   C   
   G   
   A   
   T   
   T   
   G   
   A   
    C    
   C   
   C   
   A   
   A   
   C   
    T    
   G   
   A   
   T   
   T   
   T   
    C    
   C   
   C   
   T   
   A   
   T   
   A   
   G   
   G   
   T   
   G   
   A   
   T   
   C   
   G   
   A   
   A   
   C   
   A   
   T   
   C   
   G   
   A   
   A   
   T   
   G   
   G   
    A    
   T   
   C   
    A    
   T   
   T   
   T   
   G   
   C   
    G    
   A   
   T   
   G   
   G   
   A   
    T    
   G   
   A   
   G   
   A   
   A   
   A   
   A   
   A   
   A   
   G   
   G   
   T   
   G   
   G   
   A   
   G   
   A   
   A   
   G   
   T   
   C   
   A   
   T   
   C   
   G   
   A   
   A   
   G   
   T   
    T    
   A   
    T    
   C   
   G   
   A   
   T   
   G   
   C   
   T   
   C   
   C   
   T   
   G   
   A   
   G   
    A    
   T   
   T   
   T   
   T   
    C    
   A   
   T   
    C    
   G   
   A   
    G    
    T    
   C   
    G    
   -   
   -   
   -   
 

 
   N60001F   
   A   
   T   
   G   
   G   
   A   
   A   
   A   
   A   
   T   
   C   
   G   
   A   
   C   
   A   
   A   
   G   
   A   
   A   
   G   
   T   
   A   
   C   
   A   
   A   
   G   
   A   
   A   
   T   
   T   
   A   
   A   
   C   
   T   
   T   
   T   
   A   
   G   
   A   
   A   
   G   
   A   
   A   
   G   
   T   
   G   
   A   
   T   
   G   
   G   
   G   
   T   
   G   
   A   
   C   
   C   
   G   
   C   
   T   
   T   
   C   
   G   
   G   
   A   
   C   
   G   
   T   
   T   
   A   
   T   
   T   
   C   
   C   
   A   
   A   
   A   
   T   
   A   
   T   
   A   
   T   
   T   
   A   
   T   
    C    
   C   
   A   
    A    
   G   
   A   
   A   
   C   
   G   
   T   
   G   
   C   
   A   
   C   
   T   
   G   
   C   
   C   
   T   
   G   
   A   
   T   
   A   
   T   
    C    
   C   
   G   
   T   
   G   
   A   
   T   
   G   
   G   
   A   
   T   
   T   
   G   
   A   
   A   
    A    
   C   
   C   
   A   
   G   
   T   
    G    
   C   
   A   
   A   
   C   
   G   
   C   
   C   
   G   
   G   
   A   
   T   
   A   
   T   
   T   
   A   
   T   
   T   
    T    
   G   
   C   
    C    
   A   
   T   
   G   
   A   
   A   
   C   
   A   
   A   
   A   
   G   
   A   
   T   
   G   
   G   
   C   
   A   
   A   
   T   
   A   
   C   
   G   
   T   
   A   
   T   
   G   
   A   
   C   
   A   
   A   
   A   
   G   
   G   
   A   
   T   
   T   
    C    
   C   
   G   
   T   
   A   
   A   
   A   
   T   
   C   
   A   
   G   
   C   
   A   
   A   
   A   
   A   
   T   
   C   
   T   
   G   
   T   
   C   
   G   
   G   
   A   
   A   
   A   
   T   
   A   
   T   
   C   
   A   
   T   
   G   
   G   
   G   
    A    
   A   
   A   
   T   
   T   
   A   
   T   
   C   
   A   
   T   
   C   
   C   
   T   
   C   
   A   
   T   
   G   
   G   
   T   
   G   
   A   
   C   
   A   
   G   
   C   
   A   
   G   
   T   
   A   
   T   
   T   
   T   
   A   
   T   
   G   
   A   
   A   
   G   
   C   
   G   
   A   
   T   
   G   
   G   
   T   
   C   
   C   
   G   
   C   
   A   
   T   
   G   
   A   
   G   
   C   
   C   
   A   
   A   
   G   
   A   
    T    
   T   
   G   
   G   
   A   
   A   
   G   
   T   
   T   
   G   
   C   
   G   
   G   
   G   
   A   
   A   
   G   
   T   
   A   
   C   
   T   
   G   
   A   
   T   
   C   
   G   
   A   
    A    
   A   
   T   
   G   
   C   
   A   
   C   
   G   
   G   
    G    
   A   
   A   
   T   
   A   
   A   
    T    
   G   
   G   
   G   
   A   
   G   
   T   
   A   
   T   
   G   
   G   
   A   
   C   
   G   
   G   
   T   
   G   
   A   
   T   
   C   
   C   
   A   
   C   
   C   
   T   
   G   
   C   
   T   
   G   
   C   
   G   
   A   
   T   
   G   
   C   
   G   
   T   
   T   
   A   
   T   
   A   
   C   
   T   
   G   
   A   
   A   
   G   
   C   
   C   
   A   
   G   
    A    
   T   
   T   
   A   
   T   
   C   
   C   
   A   
   A   
   G   
   T   
   T   
   A   
   A   
   G   
   T   
   G   
   G   
   A   
   G   
   A   
   G   
   A   
   T   
   G   
    C    
   T   
   A   
    G    
   C   
   A   
   G   
   A   
   T   
   A   
   T   
   C   
   G   
   A   
    A    
   A   
   A   
   A   
   G   
   A   
   A   
   A   
   C   
   A   
   G   
   T   
    C    
   G   
   A   
   T   
   C   
   T   
   C   
   G   
   T   
   G   
   T   
   G   
   G   
   A   
   A   
   T   
   T   
   T   
   T   
   G   
   A   
   T   
   G   
   A   
   T   
   A   
   C   
   A   
   G   
   A   
   A   
   A   
   A   
   A   
   G   
   A   
   A   
   C   
   C   
   A   
   A   
   C   
   A   
   G   
   T   
   C   
   C   
   T   
   T   
   C   
   C   
   A   
   G   
   C   
   T   
   C   
   G   
   A   
   T   
   A   
   C   
   C   
   C   
   A   
   A   
   A   
   C   
   C   
   T   
   T   
   C   
   T   
    A    
   G   
   T   
   C   
   A   
   A   
   T   
   G   
   G   
   A   
   T   
   C   
   G   
   A   
   C   
   A   
   G   
   G   
   T   
   A   
   T   
   A   
   T   
   C   
   T   
   G   
   C   
   T   
   G   
   G   
   A   
   T   
   A   
   T   
   G   
   C   
    G    
   A   
   C   
   A   
   G   
   A   
   A   
   A   
   T   
   C   
   C   
   C   
   G   
   A   
   C   
    T    
   C   
   A   
   T   
   A   
   A   
   C   
   T   
   T   
   A   
   G   
   C   
   T   
   G   
   A   
   A   
   G   
   T   
   G   
   A   
   T   
    C    
   G   
   A   
    C    
   G   
   G   
   C   
   A   
   C   
   A   
   A   
   T   
   C   
   T   
   A   
   T   
   A   
   T   
   G   
   A   
   T   
   C   
   G   
   A   
   T   
   C   
   A   
   T   
   C   
   C   
   A   
   C   
   A   
   A   
   G   
   C   
   G   
   A   
   G   
   C   
   C   
   T   
   T   
   G   
   A   
   A   
   A   
   A   
   A   
   T   
   T   
   G   
   A   
   T   
   G   
   G   
   A   
   A   
   T   
   A   
   T   
   A   
   T   
   T   
   C   
   C   
   T   
   G   
   G   
   A   
   C   
   C   
   T   
   G   
   A   
   C   
   T   
   T   
    T    
   C   
   C   
   A   
   A   
   C   
   A   
   G   
   G   
   A   
   G   
   G   
    G    
   A   
   T   
   C   
   T   
   T   
   G   
   C   
   A   
   A   
   G   
   G   
    G    
   A   
   A   
   A   
   G   
   A   
   T   
   G   
   A   
   A   
   A   
   T   
   C   
   A   
   A   
   A   
   A   
   A   
   A   
   G   
   C   
   T   
   T   
   A   
   T   
   G   
   A   
   A   
   A   
   C   
   A   
   G   
   G   
    G    
   C   
   G   
   A   
   G   
   G   
   A   
   A   
   A   
   A   
   G   
   T   
   T   
   A   
    T    
   T   
   C   
   T   
   T   
   C   
   G   
   T   
   T   
   C   
   G   
   A   
   A   
   A   
   A   
   C   
   A   
   A   
   A   
   G   
   A   
   T   
   A   
   G   
   A   
    A    
   G   
   C   
   A   
   A   
   T   
   G   
   A   
   A   
   G   
   G   
   G   
    C    
   A   
   A   
    T    
   A   
   A   
   A   
   C   
   A   
   G   
   C   
   A   
   A   
   A   
   T   
   C   
   G   
   T   
   G   
   A   
   T   
   C   
   A   
   C   
    T    
   G   
   A   
   A   
   A   
   T   
   C   
   C   
   C   
   A   
   T   
   A   
   T   
   G   
   A   
   A   
   G   
   T   
   A   
   A   
   A   
   T   
   A   
   A   
   A   
   G   
   C   
   G   
   A   
   C   
    G    
   T   
   T   
   G   
   G   
   T   
   G   
   A   
   A   
   A   
   A   
   A   
   A   
   A   
   T   
   G   
   G   
   A   
   T   
   G   
   A   
   G   
   A   
   T   
   C   
   C   
   G   
   T   
   T   
   T   
   G   
   A   
   A   
   T   
   A   
   A   
   G   
   A   
   A   
   A   
   A   
   T   
   C   
   G   
   A   
    T    
   G   
   G   
   A   
   A   
   T   
   A   
   G   
   C   
   T   
   G   
   A   
   A   
   G   
   T   
    A    
   C   
   G   
   A   
   G   
   A   
   T   
   G   
   A   
   A   
   A   
   G   
   T   
   G   
   A   
   T   
    C    
   G   
    A    
   A   
   C   
    T    
   G   
   G   
   T   
   C   
   T   
   G   
   C   
   A   
   A   
   A   
   T   
   T   
   G   
   T   
   C   
   G   
   T   
    T    
   G   
   A   
   A   
   T   
   T   
   G   
   A   
   A   
   A   
   A   
   A   
   A   
   G   
   A   
    T    
   G   
   C   
   C   
   A   
   A   
    T    
   G   
   C   
   T   
   C   
   A   
   A   
   G   
   G   
    A    
   A   
   T   
   T   
   T   
   T   
   G   
   A   
   A   
   T   
   T   
   A   
    T    
   T   
   T   
   G   
   T   
   T   
   C   
   A   
   A   
   A   
   A   
   A   
   C   
   A   
   C   
   T   
   G   
   A   
   G   
    C    
   T   
   A   
   C   
   A   
   G   
   A   
   T   
   C   
   A   
   A   
   T   
   T   
   A   
   C   
   A   
   A   
   T   
   T   
   T   
    C    
   A   
   A   
   T   
   A   
   T   
   G   
   G   
   T   
   C   
   G   
   C   
   T   
   A   
   T   
   C   
   G   
   A   
    T    
   C   
   A   
   T   
   A   
   T   
   G   
   A   
   C   
   T   
   C   
   C   
   G   
   C   
   A   
    C    
   C   
   A   
   A   
   G   
   T   
   T   
   G   
   G   
   A   
   C   
   T   
   G   
   A   
   A   
   A   
   G   
   A   
   C   
   A   
   T   
   T   
   T   
   T   
   A   
   A   
   G   
   A   
   A   
   G   
   T   
   T   
   A   
   T   
   A   
   T   
   C   
   G   
   A   
   G   
   C   
   A   
   T   
   C   
   G   
   A   
   A   
   A   
   G   
   C   
   A   
   A   
   G   
   T   
   C   
   A   
   T   
   A   
   A   
   C   
   A   
   A   
   A   
   A   
   C   
   G   
    C    
   A   
   G   
    C    
   C   
   A   
   A   
   T   
   T   
   C   
   G   
   A   
   T   
   T   
   T   
   A   
   G   
   C   
   C   
   A   
   A   
   A   
   G   
   C   
   T   
   C   
   A   
   A   
   A   
   A   
   A   
    A    
   G   
   A   
   C   
   A   
   G   
   C   
   A   
   T   
   A   
   T   
   C   
   G   
   T   
    G    
   G   
   A   
   A   
   G   
   G   
    C    
    C    
   T   
   G   
   A   
   T   
   G   
   A   
   A   
   A   
   G   
   C   
   T   
   T   
   T   
   A   
   T   
   C   
   G   
   A   
   T   
   T   
   C   
   T   
   G   
   G   
   A   
   T   
   G   
   A   
   A   
   G   
   T   
   A   
   A   
   T   
   C   
   G   
    C    
   A   
   A   
   C   
   G   
   A   
   T   
   T   
   C   
   G   
   T   
   G   
   A   
   A   
   A   
   G   
   T   
   A   
   A   
   A   
   G   
   A   
   C   
   A   
   A   
   A   
   A   
   A   
   A   
   G   
   A   
    T    
   G   
   C   
    T    
   A   
   A   
   A   
   A   
   A   
   G   
   A   
   A   
   T   
   T   
   T   
   A   
   G   
   T   
   C   
   G   
   A   
    T    
   G   
   T   
    T    
   T   
   T   
    C    
   C   
   A   
   A   
   T   
   T   
   T   
   A   
   C   
   A   
   G   
   A   
   A   
   G   
   A   
   A   
   C   
   A   
   A   
   G   
   C   
   A   
   G   
   A   
   A   
   G   
   C   
   A   
   A   
   T   
    C    
   G   
   T   
   C   
   A   
   C   
   A   
   T   
   T   
   G   
   C   
   A   
   A   
    C    
   T   
   A   
   T   
   A   
    C    
   C   
   G   
   T   
   T   
   T   
   G   
   A   
   C   
   G   
   A   
   A   
    C    
   A   
   C   
    A    
   G   
   A   
   T   
   A   
   T   
   C   
   A   
   C   
   A   
   G   
   A   
   G   
   C   
   T   
   T   
   C   
   A   
   A   
   A   
   A   
   G   
   G   
   A   
   A   
    G    
   C   
    C    
   G   
   A   
   A   
   A   
    G    
   C   
   T   
   T   
   G   
   A   
   T   
   T   
   G   
   C   
    A    
   C   
   A   
   A   
   A   
   T   
   C   
   A   
   C   
   A   
   G   
   A   
   G   
   T   
   T   
    G    
   A   
   A   
   T   
   A   
   A   
   A   
   A   
   T   
   C   
   C   
   T   
   T   
   T   
   C   
   A   
   A   
   A   
    C    
   G   
   A   
   C   
   A   
   A   
   A   
   G   
   A   
    G    
   C   
   T   
   T   
   T   
   T   
    C    
   T   
   C   
   T   
   G   
   T   
   T   
   A   
   T   
   G   
   A   
   A   
   A   
   A   
   A   
   A   
   G   
   A   
   A   
   T   
   T   
   G   
   C   
   G   
   A   
   G   
   A   
   A   
   G   
   T   
   G   
   A   
   A   
   A   
   A   
   A   
   A   
   A   
   A   
   T   
   T   
   A   
    C    
   T   
   C   
   T   
   T   
   C   
   C   
   G   
   C   
   T   
   C   
   G   
   T   
   C   
    T    
    G    
   A   
   C   
   G   
   A   
   C   
   A   
   A   
   T   
   C   
    C    
   A   
   A   
   G   
   A   
   C   
   G   
   A   
   A   
   A   
   T   
   C   
   G   
   A   
   A   
   G   
   A   
   A   
   A   
   T   
   C   
   A   
   A   
   A   
   A   
   T   
    C    
   G   
   A   
   T   
   A   
   C   
   G   
   C   
   A   
   A   
    G    
   T   
    T    
   T   
   T   
   A   
   G   
   T   
   G   
   G   
   C   
    G    
   C   
   A   
   A   
   G   
   A   
   A   
   G   
   A   
   C   
   G   
   T   
   A   
   A   
   T   
    C    
   G   
   T   
   C   
   A   
   G   
   T   
   G   
   T   
    G    
   A   
   C   
    G    
   C   
   G   
   A   
   G   
   A   
   A   
   G   
   G   
   C   
   T   
   A   
   T   
   G   
   T   
   G   
   A   
   A   
   G   
   C   
   G   
   A   
   A   
   C   
   A   
   A   
   G   
   C   
   C   
   T   
   T   
   C   
   G   
   T   
   T   
   C   
    T    
   T   
   A   
   T   
   A   
   G   
   T   
   G   
   C   
   T   
   T   
   C   
    G    
   A   
   A   
   A   
   C   
   C   
   A   
   G   
   A   
   A   
   G   
   A   
   A   
   A   
   T   
   C   
   G   
   G   
   G   
   A   
   T   
   G   
   C   
   G   
   A   
   G   
   A   
   A   
   G   
   G   
   C   
   G   
   A   
    T    
   T   
   A   
   C   
   T   
   T   
   G   
   C   
   T   
    G    
   T   
   A   
   C   
    T    
   C   
   A   
   G   
   G   
   T   
   G   
   A   
   A   
   T   
   T   
    G    
   A   
   G   
    C    
   A   
   C   
   A   
    T    
   T   
   G   
   G   
   A   
   C   
   C   
   A   
   T   
   G   
   T   
   T   
   C   
   T   
   T   
   C   
   T   
   G   
   A   
   T   
    T    
   A   
   C   
    G    
   A   
   A   
   T   
   A   
   A   
   A   
   G   
   C   
   A   
   A   
   A   
   T   
   G   
   T   
   G   
   A   
   T   
   C   
   T   
   A   
   C   
   C   
   G   
    T    
   C   
   C   
   T   
   G   
   T   
    T    
   C   
   A   
   T   
   G   
   A   
   A   
   C   
   T   
   A   
   C   
   C   
    C    
   G   
   A   
   T   
   T   
   T   
   G   
   A   
   A   
   A   
   T   
   G   
   G   
   A   
   A   
   A   
   G   
   A   
   T   
   G   
   C   
   T   
   G   
   G   
    C    
   G   
   A   
    A    
   C   
   A   
    C    
   A   
   T   
   T   
   T   
   C   
   T   
   C   
   A   
   A   
   A   
   C   
   A   
   A   
   T   
   C   
   T   
   C   
   C   
   A   
   A   
   T   
   C   
   T   
   G   
   T   
   C   
   T   
   G   
   T   
   A   
   G   
   A   
    T    
   G   
   A   
   A   
   T   
   C   
   G   
   A   
   T   
    C    
   T   
   T   
   A   
   G   
   C   
   T   
   G   
   T   
   T   
   T   
   T   
    C    
   C   
   C   
   A   
   T   
   A   
   T   
   C   
   A   
   A   
   A   
   A   
   A   
   A   
   T   
    C    
   C   
   A   
   A   
   G   
   C   
   A   
   G   
   A   
    A    
   A   
   A   
   A   
   A   
   C   
   A   
   T   
   T   
   T   
   G   
   T   
    A    
   T   
   T   
   C   
   A   
   T   
   C   
   A   
   G   
   C   
   A   
   A   
   G   
   A   
   A   
   T   
   G   
   G   
   A   
   T   
   T   
    G    
    A    
   T   
   C   
   A   
   A   
   G   
   C   
   A   
   A   
   A   
   C   
   A   
   C   
   G   
   A   
   A   
   T   
   G   
   A   
   C   
   A   
   G   
   A   
   G   
   T   
   T   
   C   
   G   
   A   
   A   
   C   
   C   
   A   
   T   
   G   
   G   
   C   
   G   
   C   
   A   
   C   
   C   
   T   
   A   
    T    
   A   
   A   
   G   
   A   
   G   
   C   
   C   
   G   
    T    
   C   
   C   
   G   
   C   
   T   
   C   
   A   
   G   
   C   
   T   
   G   
   T   
   A   
   T   
   G   
   A   
   A   
   G   
   C   
   T   
   A   
   A   
   A   
   G   
   G   
   C   
   G   
   G   
   A   
   A   
   G   
   A   
   A   
   G   
   A   
   T   
   G   
   A   
   A   
   C   
   T   
    T    
   A   
   T   
   T   
   G   
   C   
   T   
   G   
   T   
    A    
   T   
   A   
   T   
   C   
   T   
   G   
   A   
   A   
   A   
   G   
   A   
   A   
   G   
   A   
   T   
   C   
    A    
   G   
   C   
   C   
   A   
   G   
   G   
   C   
   T   
   T   
   A   
   G   
   A   
   T   
   G   
   T   
    G    
   T   
   T   
   C   
   T   
   T   
   A   
   G   
   T   
   A   
   A   
   C   
   G   
    C    
   A   
   T   
   C   
   A   
   A   
   G   
   G   
   A   
   A   
   T   
   G   
   G   
   G   
    C    
   T   
   T   
   G   
   C   
   G   
   C   
   T   
    A    
   T   
   C   
   C   
   T   
   T   
   T   
   A   
   A   
    C    
   A   
   G   
   A   
   T   
   G   
   T   
   T   
   C   
   C   
   C   
   G   
   T   
   T   
   G   
   T   
   T   
   G   
   G   
   A   
   G   
   C   
   A   
   A   
   A   
   G   
   G   
   C   
   A   
   G   
    C    
   T   
   G   
   G   
   A   
   G   
   T   
   G   
   A   
   A   
   G   
   T   
   C   
   T   
   A   
   T   
   G   
   A   
   A   
   T   
   T   
   T   
   G   
   A   
   A   
   A   
   G   
   A   
   T   
   G   
   G   
   A   
   G   
   A   
   T   
   A   
   G   
   G   
    A    
   T   
   T   
    G    
   T   
   C   
   A   
   A   
    T    
   G   
   G   
    G    
   C   
   T   
    G    
   C   
   T   
   T   
   G   
   T   
   G   
   T   
   T   
   A   
   G   
   C   
   A   
   G   
   A   
    C    
   G   
   G   
    A    
   G   
   A   
   T   
   A   
   C   
   G   
   C   
   C   
    A    
   A   
   T   
   C   
   G   
   T   
   A   
   A   
   T   
   C   
   G   
   T   
   T   
   A   
   C   
   T   
   C   
   A   
   G   
   C   
   G   
   A   
   G   
   G   
   A   
   G   
   C   
    C    
   G   
   T   
    G    
   A   
   A   
   A   
   C   
   G   
   C   
   A   
   T   
   G   
   C   
   T   
   T   
   G   
   C   
   T   
   C   
   A   
   A   
   G   
   A   
   A   
   A   
   T   
   C   
   A   
   G   
   C   
   C   
   A   
   A   
   A   
   C   
    T    
   T   
   C   
   A   
   C   
   G   
   T   
   G   
   C   
   G   
   A   
   A   
   G   
   C   
   G   
   G   
   G   
   G   
   A   
   G   
   T   
   G   
   A   
   C   
   G   
   G   
   T   
   C   
   A   
   T   
   G   
   C   
   G   
   T   
   G   
   A   
   G   
   C   
   T   
   G   
   A   
   A   
   A   
   A   
   A   
   A   
   A   
   A   
   T   
   C   
   C   
    G    
   C   
   A   
    C    
   C   
   G   
    T    
   A   
   T   
   T   
   A   
   T   
   C   
   T   
   A   
   T   
   A   
   T   
   G   
   T   
   C   
   G   
   G   
   A   
   A   
   G   
   G   
   A   
   A   
   G   
   A   
   T   
   C   
   A   
   A   
   A   
   A   
   G   
   A   
   A   
   A   
   T   
   A   
   A   
   C   
   C   
   T   
   T   
   G   
   A   
   T   
   G   
   A   
   A   
   T   
   C   
   A   
   A   
   A   
   A   
   A   
   G   
   G   
    A    
   C   
   A   
    A    
   C   
   A   
   G   
    A    
   T   
   A   
   A   
   C   
   G   
   A   
   T   
   T   
   G   
   A   
    T    
   C   
   C   
   A   
   A   
   C   
    G    
   G   
   A   
   T   
   T   
   T   
    T    
   C   
   C   
   T   
   A   
   T   
   A   
   G   
   G   
   T   
   G   
   A   
   T   
   C   
   G   
   A   
   A   
   C   
   A   
   T   
   C   
   G   
   A   
   A   
   T   
   G   
   G   
    T    
   T   
   C   
    G    
   T   
   T   
   T   
   G   
   C   
    T    
   A   
   T   
   G   
   G   
   A   
    C    
   G   
   A   
   G   
   A   
   A   
   A   
   A   
   A   
   A   
   G   
   G   
   T   
   G   
   G   
   A   
   G   
   A   
   A   
   G   
   T   
   C   
   A   
   T   
   C   
   G   
   A   
   A   
   G   
   T   
    T    
   A   
    T    
   C   
   G   
   A   
   T   
   G   
   C   
   T   
   C   
   C   
   T   
   G   
   A   
   G   
    A    
   T   
   T   
   T   
   T   
    C    
   A   
   T   
    T    
   G   
   A   
    A    
    T    
   C   
    A    
   -   
   -   
   -   
 

 
   N59542F   
   A   
   T   
   G   
   G   
   A   
   A   
   A   
   A   
   T   
   C   
   G   
   A   
   C   
   A   
   A   
   G   
   A   
   A   
   G   
   T   
   A   
   C   
   A   
   A   
   G   
   A   
   A   
   T   
   T   
   A   
   A   
   C   
   T   
   T   
   T   
   A   
   G   
   A   
   A   
   G   
   A   
   A   
   G   
   T   
   G   
   A   
   T   
   G   
   G   
   G   
   T   
   G   
   A   
   C   
   C   
   G   
   C   
   T   
   T   
   C   
   G   
   G   
   A   
   C   
   G   
   T   
   T   
   A   
   T   
   T   
   C   
   C   
   A   
   A   
   A   
   T   
   A   
   T   
   A   
   T   
   T   
   A   
   T   
    C    
   C   
   A   
    A    
   G   
   A   
   A   
   C   
   G   
   T   
   G   
   C   
   A   
   C   
   T   
   G   
   C   
   C   
   T   
   G   
   A   
   T   
   A   
   T   
    C    
   C   
   G   
   T   
   G   
   A   
   T   
   G   
   G   
   A   
   T   
   T   
   G   
   A   
   A   
    G    
   C   
   C   
   A   
   G   
   T   
    G    
   C   
   A   
   A   
   C   
   G   
   C   
   C   
   G   
   G   
   A   
   T   
   A   
   T   
   T   
   A   
   T   
   T   
    T    
   G   
   C   
    C    
   A   
   T   
   G   
   A   
   A   
   C   
   A   
   A   
   A   
   G   
   A   
   T   
   G   
   G   
   C   
   A   
   A   
   T   
   A   
   C   
   G   
   T   
   A   
   T   
   G   
   A   
   C   
   A   
   A   
   A   
   G   
   G   
   A   
   T   
   T   
    C    
   C   
   G   
   T   
   A   
   A   
   A   
   T   
   C   
   A   
   G   
   C   
   A   
   A   
   A   
   A   
   T   
   C   
   T   
   G   
   T   
   C   
   G   
   G   
   A   
   A   
   A   
   T   
   A   
   T   
   C   
   A   
   T   
   G   
   G   
   G   
    G    
   A   
   A   
   T   
   T   
   A   
   T   
   C   
   A   
   T   
   C   
   C   
   T   
   C   
   A   
   T   
   G   
   G   
   T   
   G   
   A   
   C   
   A   
   G   
   C   
   A   
   G   
   T   
   A   
   T   
   T   
   T   
   A   
   T   
   G   
   A   
   A   
   G   
   C   
   G   
   A   
   T   
   G   
   G   
   T   
   C   
   C   
   G   
   C   
   A   
   T   
   G   
   A   
   G   
   C   
   C   
   A   
   A   
   G   
   A   
    C    
   T   
   G   
   G   
   A   
   A   
   G   
   T   
   T   
   G   
   C   
   G   
   G   
   G   
   A   
   A   
   G   
   T   
   A   
   C   
   T   
   G   
   A   
   T   
   C   
   G   
   A   
    A    
   A   
   T   
   G   
   C   
   A   
   C   
   G   
   G   
    G    
   A   
   A   
   T   
   A   
   A   
    C    
   G   
   G   
   G   
   A   
   G   
   T   
   A   
   T   
   G   
   G   
   A   
   C   
   G   
   G   
   T   
   G   
   A   
   T   
   C   
   C   
   A   
   C   
   C   
   T   
   G   
   C   
   T   
   G   
   C   
   G   
   A   
   T   
   G   
   C   
   G   
   T   
   T   
   A   
   T   
   A   
   C   
   T   
   G   
   A   
   A   
   G   
   C   
   C   
   A   
   G   
    A    
   T   
   T   
   A   
   T   
   C   
   C   
   A   
   A   
   G   
   T   
   T   
   A   
   A   
   G   
   T   
   G   
   G   
   A   
   G   
   A   
   G   
   A   
   T   
   G   
    C    
   T   
   A   
    G    
   C   
   A   
   G   
   A   
   T   
   A   
   T   
   C   
   G   
   A   
    A    
   A   
   A   
   A   
   G   
   A   
   A   
   A   
   C   
   A   
   G   
   T   
    C    
   G   
   A   
   T   
   C   
   T   
   C   
   G   
   T   
   G   
   T   
   G   
   G   
   A   
   A   
   T   
   T   
   T   
   T   
   G   
   A   
   T   
   G   
   A   
   T   
   A   
   C   
   A   
   G   
   A   
   A   
   A   
   A   
   A   
   G   
   A   
   A   
   C   
   C   
   A   
   A   
   C   
   A   
   G   
   T   
   C   
   C   
   T   
   T   
   C   
   C   
   A   
   G   
   C   
   T   
   C   
   G   
   A   
   T   
   A   
   C   
   C   
   C   
   A   
   A   
   A   
   C   
   C   
   T   
   T   
   C   
   T   
    C    
   G   
   T   
   C   
   A   
   A   
   T   
   G   
   G   
   A   
   T   
   C   
   G   
   A   
   C   
   A   
   G   
   G   
   T   
   A   
   T   
   A   
   T   
   C   
   T   
   G   
   C   
   T   
   G   
   G   
   A   
   T   
   A   
   T   
   G   
   C   
    A    
   A   
   C   
   A   
   G   
   A   
   A   
   A   
   T   
   C   
   C   
   C   
   G   
   A   
   C   
    T    
   C   
   A   
   T   
   A   
   A   
   C   
   T   
   T   
   A   
   G   
   C   
   T   
   G   
   A   
   A   
   G   
   T   
   G   
   A   
   T   
    C    
   G   
   A   
    T    
   G   
   G   
   C   
   A   
   C   
   A   
   A   
   T   
   C   
   T   
   A   
   T   
   A   
   T   
   G   
   A   
   T   
   C   
   G   
   A   
   T   
   C   
   A   
   T   
   C   
   C   
   A   
   C   
   A   
   A   
   G   
   C   
   G   
   A   
   G   
   C   
   C   
   T   
   T   
   G   
   A   
   A   
   A   
   A   
   A   
   T   
   T   
   G   
   A   
   T   
   G   
   G   
   A   
   A   
   T   
   A   
   T   
   A   
   T   
   T   
   C   
   C   
   T   
   G   
   G   
   A   
   C   
   C   
   T   
   G   
   A   
   C   
   T   
   T   
    T    
   C   
   C   
   A   
   A   
   C   
   A   
   G   
   G   
   A   
   G   
   G   
    G    
   A   
   T   
   C   
   T   
   T   
   G   
   C   
   A   
   A   
   G   
   G   
    A    
   A   
   A   
   A   
   G   
   A   
   T   
   G   
   A   
   A   
   A   
   T   
   C   
   A   
   A   
   A   
   A   
   A   
   A   
   G   
   C   
   T   
   T   
   A   
   T   
   G   
   A   
   A   
   A   
   C   
   A   
   G   
   G   
    G    
   C   
   G   
   A   
   G   
   G   
   A   
   A   
   A   
   A   
   G   
   T   
   T   
   A   
    T    
   T   
   C   
   T   
   T   
   C   
   G   
   T   
   T   
   C   
   G   
   A   
   A   
   A   
   A   
   C   
   A   
   A   
   A   
   G   
   A   
   T   
   A   
   G   
   A   
    G    
   G   
   C   
   A   
   A   
   T   
   G   
   A   
   A   
   G   
   G   
   G   
    T    
   A   
   A   
    T    
   A   
   A   
   A   
   C   
   A   
   G   
   C   
   A   
   A   
   A   
   T   
   C   
   G   
   T   
   G   
   A   
   T   
   C   
   A   
   C   
    A    
   G   
   A   
   A   
   A   
   T   
   C   
   C   
   C   
   A   
   T   
   A   
   T   
   G   
   A   
   A   
   G   
   T   
   A   
   A   
   A   
   T   
   A   
   A   
   A   
   G   
   C   
   G   
   A   
   C   
    C    
   T   
   T   
   G   
   G   
   T   
   G   
   A   
   A   
   A   
   A   
   A   
   A   
   A   
   T   
   G   
   G   
   A   
   T   
   G   
   A   
   G   
   A   
   T   
   C   
   C   
   G   
   T   
   T   
   T   
   G   
   A   
   A   
   T   
   A   
   A   
   G   
   A   
   A   
   A   
   A   
   T   
   C   
   G   
   A   
    T    
   G   
   G   
   A   
   A   
   T   
   A   
   G   
   C   
   T   
   G   
   A   
   A   
   G   
   T   
    A    
   C   
   G   
   A   
   G   
   A   
   T   
   G   
   A   
   A   
   A   
   G   
   T   
   G   
   A   
   T   
    A    
   G   
    A    
   A   
   C   
    A    
   G   
   G   
   T   
   C   
   T   
   G   
   C   
   A   
   A   
   A   
   T   
   T   
   G   
   T   
   C   
   G   
   T   
    C    
   G   
   A   
   A   
   T   
   T   
   G   
   A   
   A   
   A   
   A   
   A   
   A   
   G   
   A   
    C    
   G   
   C   
   C   
   A   
   A   
    T    
   G   
   C   
   T   
   C   
   A   
   A   
   G   
   G   
    A    
   A   
   T   
   T   
   T   
   T   
   G   
   A   
   A   
   T   
   T   
   A   
    T    
   T   
   T   
   G   
   T   
   T   
   C   
   A   
   A   
   A   
   A   
   A   
   C   
   A   
   C   
   T   
   G   
   A   
   G   
    C    
   T   
   A   
   C   
   A   
   G   
   A   
   T   
   C   
   A   
   A   
   T   
   T   
   A   
   C   
   A   
   A   
   T   
   T   
   T   
    C    
   A   
   A   
   T   
   A   
   T   
   G   
   G   
   T   
   C   
   G   
   C   
   T   
   A   
   T   
   C   
   G   
   A   
    C    
   C   
   A   
   T   
   A   
   T   
   G   
   A   
   C   
   T   
   C   
   C   
   G   
   C   
   A   
    T    
   C   
   A   
   A   
   G   
   T   
   T   
   G   
   G   
   A   
   C   
   T   
   G   
   A   
   A   
   A   
   G   
   A   
   C   
   A   
   T   
   T   
   T   
   T   
   A   
   A   
   G   
   A   
   A   
   G   
   T   
   T   
   A   
   T   
   A   
   T   
   C   
   G   
   A   
   G   
   C   
   A   
   T   
   C   
   G   
   A   
   A   
   A   
   G   
   C   
   A   
   A   
   G   
   T   
   C   
   A   
   T   
   A   
   A   
   C   
   A   
   A   
   A   
   A   
   C   
   G   
    T    
   A   
   G   
    T    
   C   
   A   
   A   
   T   
   T   
   C   
   G   
   A   
   T   
   T   
   T   
   A   
   G   
   C   
   C   
   A   
   A   
   A   
   G   
   C   
   T   
   C   
   A   
   A   
   A   
   A   
   A   
    A    
   G   
   A   
   C   
   A   
   G   
   C   
   A   
   T   
   A   
   T   
   C   
   G   
   T   
    A    
   G   
   A   
   A   
   G   
   G   
    A    
    T    
   T   
   G   
   A   
   T   
   G   
   A   
   A   
   A   
   G   
   C   
   T   
   T   
   T   
   A   
   T   
   C   
   G   
   A   
   T   
   T   
   C   
   T   
   G   
   G   
   A   
   T   
   G   
   A   
   A   
   G   
   T   
   A   
   A   
   T   
   C   
   G   
    C    
   A   
   A   
   C   
   G   
   A   
   T   
   T   
   C   
   G   
   T   
   G   
   A   
   A   
   A   
   G   
   T   
   A   
   A   
   A   
   G   
   A   
   C   
   A   
   A   
   A   
   A   
   A   
   A   
   G   
   A   
    C    
   G   
   C   
    C    
   A   
   A   
   A   
   A   
   A   
   G   
   A   
   A   
   T   
   T   
   T   
   A   
   G   
   T   
   C   
   G   
   A   
    C    
   G   
   T   
    C    
   T   
   T   
    T    
   C   
   A   
   A   
   T   
   T   
   T   
   A   
   C   
   A   
   G   
   A   
   A   
   G   
   A   
   A   
   C   
   A   
   A   
   G   
   C   
   A   
   G   
   A   
   A   
   G   
   C   
   A   
   A   
   T   
    T    
   G   
   T   
   C   
   A   
   C   
   A   
   T   
   T   
   G   
   C   
   A   
   A   
    T    
   T   
   A   
   T   
   A   
    C    
   C   
   G   
   T   
   T   
   T   
   G   
   A   
   C   
   G   
   A   
   A   
    C    
   A   
   C   
    G    
   G   
   A   
   T   
   A   
   T   
   C   
   A   
   C   
   A   
   G   
   A   
   G   
   C   
   T   
   T   
   C   
   A   
   A   
   A   
   A   
   G   
   G   
   A   
   A   
    T    
   C   
    C    
   G   
   A   
   A   
   A   
    G    
   C   
   T   
   T   
   G   
   A   
   T   
   T   
   G   
   C   
    G    
   C   
   A   
   A   
   A   
   T   
   C   
   A   
   C   
   A   
   G   
   A   
   G   
   T   
   T   
    G    
   A   
   A   
   T   
   A   
   A   
   A   
   A   
   T   
   C   
   C   
   T   
   T   
   T   
   C   
   A   
   A   
   A   
    T    
   G   
   A   
   C   
   A   
   A   
   A   
   G   
   A   
    G    
   C   
   T   
   T   
   T   
   T   
    C    
   T   
   C   
   T   
   G   
   T   
   T   
   A   
   T   
   G   
   A   
   A   
   A   
   A   
   A   
   A   
   G   
   A   
   A   
   T   
   T   
   G   
   C   
   G   
   A   
   G   
   A   
   A   
   G   
   T   
   G   
   A   
   A   
   A   
   A   
   A   
   A   
   A   
   A   
   T   
   T   
   A   
    T    
   T   
   C   
   T   
   T   
   C   
   C   
   G   
   C   
   T   
   C   
   G   
   T   
   C   
    T    
    A    
   A   
   C   
   G   
   A   
   C   
   A   
   A   
   T   
   C   
    G    
   A   
   A   
   G   
   A   
   C   
   G   
   A   
   A   
   A   
   T   
   C   
   G   
   A   
   A   
   G   
   A   
   A   
   A   
   T   
   C   
   A   
   A   
   A   
   A   
   T   
    T    
   G   
   A   
   T   
   A   
   C   
   G   
   C   
   A   
   A   
    G    
   T   
    C    
   T   
   T   
   A   
   G   
   T   
   G   
   G   
   C   
    A    
   C   
   A   
   A   
   G   
   A   
   A   
   G   
   A   
   C   
   G   
   T   
   A   
   A   
   T   
    T    
   G   
   T   
   C   
   A   
   G   
   T   
   G   
   T   
    G    
   A   
   C   
    G    
   C   
   G   
   A   
   G   
   A   
   A   
   G   
   G   
   C   
   T   
   A   
   T   
   G   
   T   
   G   
   A   
   A   
   G   
   C   
   G   
   A   
   A   
   C   
   A   
   A   
   G   
   C   
   C   
   T   
   T   
   C   
   G   
   T   
   T   
   C   
    T    
   T   
   A   
   T   
   A   
   G   
   T   
   G   
   C   
   T   
   T   
   C   
    C    
   A   
   A   
   A   
   C   
   C   
   A   
   G   
   A   
   A   
   G   
   A   
   A   
   A   
   T   
   C   
   G   
   G   
   G   
   A   
   T   
   G   
   C   
   G   
   A   
   G   
   A   
   A   
   G   
   G   
   C   
   G   
   A   
    C    
   T   
   A   
   C   
   T   
   T   
   G   
   C   
   T   
    A    
   T   
   A   
   C   
    T    
   C   
   A   
   G   
   G   
   T   
   G   
   A   
   A   
   T   
   T   
    G    
   A   
   G   
    T    
   A   
   C   
   A   
    T    
   T   
   G   
   G   
   A   
   C   
   C   
   A   
   T   
   G   
   T   
   T   
   C   
   T   
   T   
   C   
   T   
   G   
   A   
   T   
    C    
   A   
   C   
    A    
   A   
   A   
   T   
   A   
   A   
   A   
   G   
   C   
   A   
   A   
   A   
   T   
   G   
   T   
   G   
   A   
   T   
   C   
   T   
   A   
   C   
   C   
   G   
    C    
   C   
   C   
   T   
   G   
   T   
    C    
   C   
   A   
   T   
   G   
   A   
   A   
   C   
   T   
   A   
   C   
   C   
    C    
   G   
   A   
   T   
   T   
   T   
   G   
   A   
   A   
   A   
   T   
   G   
   G   
   A   
   A   
   A   
   G   
   A   
   T   
   G   
   C   
   T   
   G   
   G   
    T    
   G   
   A   
    G    
   C   
   A   
    C    
   A   
   T   
   T   
   T   
   C   
   T   
   C   
   A   
   A   
   A   
   C   
   A   
   A   
   T   
   C   
   T   
   C   
   C   
   A   
   A   
   T   
   C   
   T   
   G   
   T   
   C   
   T   
   G   
   T   
   A   
   G   
   A   
    C    
   G   
   A   
   A   
   T   
   C   
   G   
   A   
   T   
    T    
   T   
   T   
   A   
   G   
   C   
   T   
   G   
   T   
   T   
   T   
   T   
    C    
   C   
   C   
   A   
   T   
   A   
   T   
   C   
   A   
   A   
   A   
   A   
   A   
   A   
   T   
    T    
   C   
   A   
   A   
   G   
   C   
   A   
   G   
   A   
    G    
   A   
   A   
   A   
   A   
   C   
   A   
   T   
   T   
   T   
   G   
   T   
    A    
   T   
   T   
   C   
   A   
   T   
   C   
   A   
   G   
   C   
   A   
   A   
   G   
   A   
   A   
   T   
   G   
   G   
   A   
   T   
   T   
    G    
    A    
   T   
   C   
   A   
   A   
   G   
   C   
   A   
   A   
   A   
   C   
   A   
   C   
   G   
   A   
   A   
   T   
   G   
   A   
   C   
   A   
   G   
   A   
   G   
   T   
   T   
   C   
   G   
   A   
   A   
   C   
   C   
   A   
   T   
   G   
   G   
   C   
   G   
   C   
   A   
   C   
   C   
   T   
   A   
    T    
   A   
   A   
   G   
   A   
   G   
   C   
   C   
   G   
    T    
   C   
   C   
   G   
   C   
   T   
   C   
   A   
   G   
   C   
   T   
   G   
   T   
   A   
   T   
   G   
   A   
   A   
   G   
   C   
   T   
   A   
   A   
   A   
   G   
   G   
   C   
   G   
   G   
   A   
   A   
   G   
   A   
   A   
   G   
   A   
   T   
   G   
   A   
   A   
   C   
   T   
    G    
   A   
   T   
   T   
   G   
   C   
   T   
   G   
   T   
    G    
   T   
   A   
   T   
   C   
   T   
   G   
   A   
   A   
   A   
   G   
   A   
   A   
   G   
   A   
   T   
   C   
    A    
   G   
   C   
   C   
   A   
   G   
   G   
   C   
   T   
   T   
   A   
   G   
   A   
   T   
   G   
   T   
    T    
   T   
   T   
   C   
   T   
   T   
   A   
   G   
   T   
   A   
   A   
   C   
   G   
    C    
   A   
   T   
   C   
   A   
   A   
   G   
   G   
   A   
   A   
   T   
   G   
   G   
   G   
    T    
   T   
   T   
   G   
   C   
   G   
   C   
   T   
    A    
   T   
   C   
   C   
   T   
   T   
   T   
   A   
   A   
    C    
   A   
   G   
   A   
   T   
   G   
   T   
   T   
   C   
   C   
   C   
   G   
   T   
   T   
   G   
   T   
   T   
   G   
   G   
   A   
   G   
   C   
   A   
   A   
   A   
   G   
   G   
   C   
   A   
   G   
    C    
   T   
   G   
   G   
   A   
   G   
   T   
   G   
   A   
   A   
   G   
   T   
   C   
   T   
   A   
   T   
   G   
   A   
   A   
   T   
   T   
   T   
   G   
   A   
   A   
   A   
   G   
   A   
   T   
   G   
   G   
   A   
   G   
   A   
   T   
   A   
   G   
   G   
    A    
   T   
   T   
    A    
   T   
   C   
   A   
   A   
    C    
   G   
   G   
    G    
   C   
   T   
    G    
   C   
   T   
   T   
   G   
   T   
   G   
   T   
   T   
   A   
   G   
   C   
   A   
   G   
   A   
    A    
   G   
   G   
    T    
   G   
   A   
   T   
   A   
   C   
   G   
   C   
   C   
    G    
   A   
   T   
   C   
   G   
   T   
   A   
   A   
   T   
   C   
   G   
   T   
   T   
   A   
   C   
   T   
   C   
   A   
   G   
   C   
   G   
   A   
   G   
   G   
   A   
   G   
   C   
    T    
   G   
   T   
    T    
   A   
   A   
   A   
   C   
   G   
   C   
   A   
   T   
   G   
   C   
   T   
   T   
   G   
   C   
   T   
   C   
   A   
   A   
   G   
   A   
   A   
   A   
   T   
   C   
   A   
   G   
   C   
   C   
   A   
   A   
   A   
   C   
    T    
   T   
   C   
   A   
   C   
   G   
   T   
   G   
   C   
   G   
   A   
   A   
   G   
   C   
   G   
   G   
   G   
   G   
   A   
   G   
   T   
   G   
   A   
   C   
   G   
   G   
   T   
   C   
   A   
   T   
   G   
   C   
   G   
   T   
   G   
   A   
   G   
   C   
   T   
   G   
   A   
   A   
   A   
   A   
   A   
   A   
   A   
   A   
   T   
   C   
   C   
    G    
   C   
   A   
    C    
   C   
   G   
    T    
   A   
   T   
   T   
   A   
   T   
   C   
   T   
   A   
   T   
   A   
   T   
   G   
   T   
   C   
   G   
   G   
   A   
   A   
   G   
   G   
   A   
   A   
   G   
   A   
   T   
   C   
   A   
   A   
   A   
   A   
   G   
   A   
   A   
   A   
   T   
   A   
   A   
   C   
   C   
   T   
   T   
   G   
   A   
   T   
   G   
   A   
   A   
   T   
   C   
   A   
   A   
   A   
   A   
   A   
   G   
   G   
    G    
   C   
   A   
    G    
   C   
   A   
   G   
    T    
   T   
   A   
   A   
   C   
   G   
   A   
   T   
   T   
   G   
   A   
    C    
   C   
   C   
   A   
   A   
   C   
    T    
   G   
   A   
   T   
   T   
   T   
    C    
   C   
   C   
   T   
   A   
   T   
   A   
   G   
   G   
   T   
   G   
   A   
   T   
   C   
   G   
   A   
   A   
   C   
   A   
   T   
   C   
   G   
   A   
   A   
   T   
   G   
   G   
    A    
   T   
   C   
    A    
   T   
   T   
   T   
   G   
   C   
    G    
   A   
   T   
   G   
   G   
   A   
    T    
   G   
   A   
   G   
   A   
   A   
   A   
   A   
   A   
   A   
   G   
   G   
   T   
   G   
   G   
   A   
   G   
   A   
   A   
   G   
   T   
   C   
   A   
   T   
   C   
   G   
   A   
   A   
   G   
   T   
    T    
   A   
    T    
   C   
   G   
   A   
   T   
   G   
   C   
   T   
   C   
   C   
   T   
   G   
   A   
   G   
    A    
   T   
   T   
   T   
   T   
    C    
   A   
   T   
    C    
   G   
   A   
    G    
    T    
   C   
    G    
   -   
   -   
   -   
 

 
   N59745F   
   A   
   T   
   G   
   G   
   A   
   A   
   A   
   A   
   T   
   C   
   G   
   A   
   C   
   A   
   A   
   G   
   A   
   A   
   G   
   T   
   A   
   C   
   A   
   A   
   G   
   A   
   A   
   T   
   T   
   A   
   A   
   C   
   T   
   T   
   T   
   A   
   G   
   A   
   A   
   G   
   A   
   A   
   G   
   T   
   G   
   A   
   T   
   G   
   G   
   G   
   T   
   G   
   A   
   C   
   C   
   G   
   C   
   T   
   T   
   C   
   G   
   G   
   A   
   C   
   G   
   T   
   T   
   A   
   T   
   T   
   C   
   C   
   A   
   A   
   A   
   T   
   A   
   T   
   A   
   T   
   T   
   A   
   T   
    C    
   C   
   A   
    A    
   G   
   A   
   A   
   C   
   G   
   T   
   G   
   C   
   A   
   C   
   T   
   G   
   C   
   C   
   T   
   G   
   A   
   T   
   A   
   T   
    C    
   C   
   G   
   T   
   G   
   A   
   T   
   G   
   G   
   A   
   T   
   T   
   G   
   A   
   A   
    A    
   C   
   C   
   A   
   G   
   T   
    G    
   C   
   A   
   A   
   C   
   G   
   C   
   C   
   G   
   G   
   A   
   T   
   A   
   T   
   T   
   A   
   T   
   T   
    T    
   G   
   C   
    C    
   A   
   T   
   G   
   A   
   A   
   C   
   A   
   A   
   A   
   G   
   A   
   T   
   G   
   G   
   C   
   A   
   A   
   T   
   A   
   C   
   G   
   T   
   A   
   T   
   G   
   A   
   C   
   A   
   A   
   A   
   G   
   G   
   A   
   T   
   T   
    C    
   C   
   G   
   T   
   A   
   A   
   A   
   T   
   C   
   A   
   G   
   C   
   A   
   A   
   A   
   A   
   T   
   C   
   T   
   G   
   T   
   C   
   G   
   G   
   A   
   A   
   A   
   T   
   A   
   T   
   C   
   A   
   T   
   G   
   G   
   G   
    G    
   A   
   A   
   T   
   T   
   A   
   T   
   C   
   A   
   T   
   C   
   C   
   T   
   C   
   A   
   T   
   G   
   G   
   T   
   G   
   A   
   C   
   A   
   G   
   C   
   A   
   G   
   T   
   A   
   T   
   T   
   T   
   A   
   T   
   G   
   A   
   A   
   G   
   C   
   G   
   A   
   T   
   G   
   G   
   T   
   C   
   C   
   G   
   C   
   A   
   T   
   G   
   A   
   G   
   C   
   C   
   A   
   A   
   G   
   A   
    T    
   T   
   G   
   G   
   A   
   A   
   G   
   T   
   T   
   G   
   C   
   G   
   G   
   G   
   A   
   A   
   G   
   T   
   A   
   C   
   T   
   G   
   A   
   T   
   C   
   G   
   A   
    A    
   A   
   T   
   G   
   C   
   A   
   C   
   G   
   G   
    G    
   A   
   A   
   T   
   A   
   A   
    T    
   G   
   G   
   G   
   A   
   G   
   T   
   A   
   T   
   G   
   G   
   A   
   C   
   G   
   G   
   T   
   G   
   A   
   T   
   C   
   C   
   A   
   C   
   C   
   T   
   G   
   C   
   T   
   G   
   C   
   G   
   A   
   T   
   G   
   C   
   G   
   T   
   T   
   A   
   T   
   A   
   C   
   T   
   G   
   A   
   A   
   G   
   C   
   C   
   A   
   G   
    A    
   T   
   T   
   A   
   T   
   C   
   C   
   A   
   A   
   G   
   T   
   T   
   A   
   A   
   G   
   T   
   G   
   G   
   A   
   G   
   A   
   G   
   A   
   T   
   G   
    C    
   T   
   A   
    G    
   C   
   A   
   G   
   A   
   T   
   A   
   T   
   C   
   G   
   A   
    A    
   A   
   A   
   A   
   G   
   A   
   A   
   A   
   C   
   A   
   G   
   T   
    C    
   G   
   A   
   T   
   C   
   T   
   C   
   G   
   T   
   G   
   T   
   G   
   G   
   A   
   A   
   T   
   T   
   T   
   T   
   G   
   A   
   T   
   G   
   A   
   T   
   A   
   C   
   A   
   G   
   A   
   A   
   A   
   A   
   A   
   G   
   A   
   A   
   C   
   C   
   A   
   A   
   C   
   A   
   G   
   T   
   C   
   C   
   T   
   T   
   C   
   C   
   A   
   G   
   C   
   T   
   C   
   G   
   A   
   T   
   A   
   C   
   C   
   C   
   A   
   A   
   A   
   C   
   C   
   T   
   T   
   C   
   T   
    A    
   G   
   T   
   C   
   A   
   A   
   T   
   G   
   G   
   A   
   T   
   C   
   G   
   A   
   C   
   A   
   G   
   G   
   T   
   A   
   T   
   A   
   T   
   C   
   T   
   G   
   C   
   T   
   G   
   G   
   A   
   T   
   A   
   T   
   G   
   C   
    G    
   A   
   C   
   A   
   G   
   A   
   A   
   A   
   T   
   C   
   C   
   C   
   G   
   A   
   C   
    T    
   C   
   A   
   T   
   A   
   A   
   C   
   T   
   T   
   A   
   G   
   C   
   T   
   G   
   A   
   A   
   G   
   T   
   G   
   A   
   T   
    C    
   G   
   A   
    C    
   G   
   G   
   C   
   A   
   C   
   A   
   A   
   T   
   C   
   T   
   A   
   T   
   A   
   T   
   G   
   A   
   T   
   C   
   G   
   A   
   T   
   C   
   A   
   T   
   C   
   C   
   A   
   C   
   A   
   A   
   G   
   C   
   G   
   A   
   G   
   C   
   C   
   T   
   T   
   G   
   A   
   A   
   A   
   A   
   A   
   T   
   T   
   G   
   A   
   T   
   G   
   G   
   A   
   A   
   T   
   A   
   T   
   A   
   T   
   T   
   C   
   C   
   T   
   G   
   G   
   A   
   C   
   C   
   T   
   G   
   A   
   C   
   T   
   T   
    T    
   C   
   C   
   A   
   A   
   C   
   A   
   G   
   G   
   A   
   G   
   G   
    G    
   A   
   T   
   C   
   T   
   T   
   G   
   C   
   A   
   A   
   G   
   G   
    G    
   A   
   A   
   A   
   G   
   A   
   T   
   G   
   A   
   A   
   A   
   T   
   C   
   A   
   A   
   A   
   A   
   A   
   A   
   G   
   C   
   T   
   T   
   A   
   T   
   G   
   A   
   A   
   A   
   C   
   A   
   G   
   G   
    G    
   C   
   G   
   A   
   G   
   G   
   A   
   A   
   A   
   A   
   G   
   T   
   T   
   A   
    T    
   T   
   C   
   T   
   T   
   C   
   G   
   T   
   T   
   C   
   G   
   A   
   A   
   A   
   A   
   C   
   A   
   A   
   A   
   G   
   A   
   T   
   A   
   G   
   A   
    A    
   G   
   C   
   A   
   A   
   T   
   G   
   A   
   A   
   G   
   G   
   G   
    C    
   A   
   A   
    T    
   A   
   A   
   A   
   C   
   A   
   G   
   C   
   A   
   A   
   A   
   T   
   C   
   G   
   T   
   G   
   A   
   T   
   C   
   A   
   C   
    T    
   G   
   A   
   A   
   A   
   T   
   C   
   C   
   C   
   A   
   T   
   A   
   T   
   G   
   A   
   A   
   G   
   T   
   A   
   A   
   A   
   T   
   A   
   A   
   A   
   G   
   C   
   G   
   A   
   C   
    G    
   T   
   T   
   G   
   G   
   T   
   G   
   A   
   A   
   A   
   A   
   A   
   A   
   A   
   T   
   G   
   G   
   A   
   T   
   G   
   A   
   G   
   A   
   T   
   C   
   C   
   G   
   T   
   T   
   T   
   G   
   A   
   A   
   T   
   A   
   A   
   G   
   A   
   A   
   A   
   A   
   T   
   C   
   G   
   A   
    T    
   G   
   G   
   A   
   A   
   T   
   A   
   G   
   C   
   T   
   G   
   A   
   A   
   G   
   T   
    A    
   C   
   G   
   A   
   G   
   A   
   T   
   G   
   A   
   A   
   A   
   G   
   T   
   G   
   A   
   T   
    C    
   G   
    A    
   A   
   C   
    T    
   G   
   G   
   T   
   C   
   T   
   G   
   C   
   A   
   A   
   A   
   T   
   T   
   G   
   T   
   C   
   G   
   T   
    T    
   G   
   A   
   A   
   T   
   T   
   G   
   A   
   A   
   A   
   A   
   A   
   A   
   G   
   A   
    T    
   G   
   C   
   C   
   A   
   A   
    T    
   G   
   C   
   T   
   C   
   A   
   A   
   G   
   G   
    A    
   A   
   T   
   T   
   T   
   T   
   G   
   A   
   A   
   T   
   T   
   A   
    T    
   T   
   T   
   G   
   T   
   T   
   C   
   A   
   A   
   A   
   A   
   A   
   C   
   A   
   C   
   T   
   G   
   A   
   G   
    C    
   T   
   A   
   C   
   A   
   G   
   A   
   T   
   C   
   A   
   A   
   T   
   T   
   A   
   C   
   A   
   A   
   T   
   T   
   T   
    T    
   A   
   A   
   T   
   A   
   T   
   G   
   G   
   T   
   C   
   G   
   C   
   T   
   A   
   T   
   C   
   G   
   A   
    T    
   C   
   A   
   T   
   A   
   T   
   G   
   A   
   C   
   T   
   C   
   C   
   G   
   C   
   A   
    C    
   C   
   A   
   A   
   G   
   T   
   T   
   G   
   G   
   A   
   C   
   T   
   G   
   A   
   A   
   A   
   G   
   A   
   C   
   A   
   T   
   T   
   T   
   T   
   A   
   A   
   G   
   A   
   A   
   G   
   T   
   T   
   A   
   T   
   A   
   T   
   C   
   G   
   A   
   G   
   C   
   A   
   T   
   C   
   G   
   A   
   A   
   A   
   G   
   C   
   A   
   A   
   G   
   T   
   C   
   A   
   T   
   A   
   A   
   C   
   A   
   A   
   A   
   A   
   C   
   G   
    C    
   A   
   G   
    C    
   C   
   A   
   A   
   T   
   T   
   C   
   G   
   A   
   T   
   T   
   T   
   A   
   G   
   C   
   C   
   A   
   A   
   A   
   G   
   C   
   T   
   C   
   A   
   A   
   A   
   A   
   A   
    A    
   G   
   A   
   C   
   A   
   G   
   C   
   A   
   T   
   A   
   T   
   C   
   G   
   T   
    G    
   G   
   A   
   A   
   G   
   G   
    C    
    C    
   T   
   G   
   A   
   T   
   G   
   A   
   A   
   A   
   G   
   C   
   T   
   T   
   T   
   A   
   T   
   C   
   G   
   A   
   T   
   T   
   C   
   T   
   G   
   G   
   A   
   T   
   G   
   A   
   A   
   G   
   T   
   A   
   A   
   T   
   C   
   G   
    C    
   A   
   A   
   C   
   G   
   A   
   T   
   T   
   C   
   G   
   T   
   G   
   A   
   A   
   A   
   G   
   T   
   A   
   A   
   A   
   G   
   A   
   C   
   A   
   A   
   A   
   A   
   A   
   A   
   G   
   A   
    T    
   G   
   C   
    T    
   A   
   A   
   A   
   A   
   A   
   G   
   A   
   A   
   T   
   T   
   T   
   A   
   G   
   T   
   C   
   G   
   A   
    T    
   G   
   T   
    T    
   T   
   T   
    C    
   C   
   A   
   A   
   T   
   T   
   T   
   A   
   C   
   A   
   G   
   A   
   A   
   G   
   A   
   A   
   C   
   A   
   A   
   G   
   C   
   A   
   G   
   A   
   A   
   G   
   C   
   A   
   A   
   T   
    C    
   G   
   T   
   C   
   A   
   C   
   A   
   T   
   T   
   G   
   C   
   A   
   A   
    C    
   T   
   A   
   T   
   A   
    C    
   C   
   G   
   T   
   T   
   T   
   G   
   A   
   C   
   G   
   A   
   A   
    C    
   A   
   C   
    A    
   G   
   A   
   T   
   A   
   T   
   C   
   A   
   C   
   A   
   G   
   A   
   G   
   C   
   T   
   T   
   C   
   A   
   A   
   A   
   A   
   G   
   G   
   A   
   A   
    G    
   C   
    C    
   G   
   A   
   A   
   A   
    G    
   C   
   T   
   T   
   G   
   A   
   T   
   T   
   G   
   C   
    A    
   C   
   A   
   A   
   A   
   T   
   C   
   A   
   C   
   A   
   G   
   A   
   G   
   T   
   T   
    G    
   A   
   A   
   T   
   A   
   A   
   A   
   A   
   T   
   C   
   C   
   T   
   T   
   T   
   C   
   A   
   A   
   A   
    C    
   G   
   A   
   C   
   A   
   A   
   A   
   G   
   A   
    G    
   C   
   T   
   T   
   T   
   T   
    C    
   T   
   C   
   T   
   G   
   T   
   T   
   A   
   T   
   G   
   A   
   A   
   A   
   A   
   A   
   A   
   G   
   A   
   A   
   T   
   T   
   G   
   C   
   G   
   A   
   G   
   A   
   A   
   G   
   T   
   G   
   A   
   A   
   A   
   A   
   A   
   A   
   A   
   A   
   T   
   T   
   A   
    C    
   T   
   C   
   T   
   T   
   C   
   C   
   G   
   C   
   T   
   C   
   G   
   T   
   C   
    T    
    G    
   A   
   C   
   G   
   A   
   C   
   A   
   A   
   T   
   C   
    C    
   A   
   A   
   G   
   A   
   C   
   G   
   A   
   A   
   A   
   T   
   C   
   G   
   A   
   A   
   G   
   A   
   A   
   A   
   T   
   C   
   A   
   A   
   A   
   A   
   T   
    C    
   G   
   A   
   T   
   A   
   C   
   G   
   C   
   A   
   A   
    G    
   T   
    T    
   T   
   T   
   A   
   G   
   T   
   G   
   G   
   C   
    G    
   C   
   A   
   A   
   G   
   A   
   A   
   G   
   A   
   C   
   G   
   T   
   A   
   A   
   T   
    C    
   G   
   T   
   C   
   A   
   G   
   T   
   G   
   T   
    G    
   A   
   C   
    G    
   C   
   G   
   A   
   G   
   A   
   A   
   G   
   G   
   C   
   T   
   A   
   T   
   G   
   T   
   G   
   A   
   A   
   G   
   C   
   G   
   A   
   A   
   C   
   A   
   A   
   G   
   C   
   C   
   T   
   T   
   C   
   G   
   T   
   T   
   C   
    T    
   T   
   A   
   T   
   A   
   G   
   T   
   G   
   C   
   T   
   T   
   C   
    G    
   A   
   A   
   A   
   C   
   C   
   A   
   G   
   A   
   A   
   G   
   A   
   A   
   A   
   T   
   C   
   G   
   G   
   G   
   A   
   T   
   G   
   C   
   G   
   A   
   G   
   A   
   A   
   G   
   G   
   C   
   G   
   A   
    T    
   T   
   A   
   C   
   T   
   T   
   G   
   C   
   T   
    G    
   T   
   A   
   C   
    T    
   C   
   A   
   G   
   G   
   T   
   G   
   A   
   A   
   T   
   T   
    G    
   A   
   G   
    C    
   A   
   C   
   A   
    T    
   T   
   G   
   G   
   A   
   C   
   C   
   A   
   T   
   G   
   T   
   T   
   C   
   T   
   T   
   C   
   T   
   G   
   A   
   T   
    T    
   A   
   C   
    G    
   A   
   A   
   T   
   A   
   A   
   A   
   G   
   C   
   A   
   A   
   A   
   T   
   G   
   T   
   G   
   A   
   T   
   C   
   T   
   A   
   C   
   C   
   G   
    T    
   C   
   C   
   T   
   G   
   T   
    T    
   C   
   A   
   T   
   G   
   A   
   A   
   C   
   T   
   A   
   C   
   C   
    C    
   G   
   A   
   T   
   T   
   T   
   G   
   A   
   A   
   A   
   T   
   G   
   G   
   A   
   A   
   A   
   G   
   A   
   T   
   G   
   C   
   T   
   G   
   G   
    C    
   G   
   A   
    A    
   C   
   A   
    C    
   A   
   T   
   T   
   T   
   C   
   T   
   C   
   A   
   A   
   A   
   C   
   A   
   A   
   T   
   C   
   T   
   C   
   C   
   A   
   A   
   T   
   C   
   T   
   G   
   T   
   C   
   T   
   G   
   T   
   A   
   G   
   A   
    T    
   G   
   A   
   A   
   T   
   C   
   G   
   A   
   T   
    C    
   T   
   T   
   A   
   G   
   C   
   T   
   G   
   T   
   T   
   T   
   T   
    C    
   C   
   C   
   A   
   T   
   A   
   T   
   C   
   A   
   A   
   A   
   A   
   A   
   A   
   T   
    C    
   C   
   A   
   A   
   G   
   C   
   A   
   G   
   A   
    A    
   A   
   A   
   A   
   A   
   C   
   A   
   T   
   T   
   T   
   G   
   T   
    A    
   T   
   T   
   C   
   A   
   T   
   C   
   A   
   G   
   C   
   A   
   A   
   G   
   A   
   A   
   T   
   G   
   G   
   A   
   T   
   T   
    G    
    A    
   T   
   C   
   A   
   A   
   G   
   C   
   A   
   A   
   A   
   C   
   A   
   C   
   G   
   A   
   A   
   T   
   G   
   A   
   C   
   A   
   G   
   A   
   G   
   T   
   T   
   C   
   G   
   A   
   A   
   C   
   C   
   A   
   T   
   G   
   G   
   C   
   G   
   C   
   A   
   C   
   C   
   T   
   A   
    T    
   A   
   A   
   G   
   A   
   G   
   C   
   C   
   G   
    T    
   C   
   C   
   G   
   C   
   T   
   C   
   A   
   G   
   C   
   T   
   G   
   T   
   A   
   T   
   G   
   A   
   A   
   G   
   C   
   T   
   A   
   A   
   A   
   G   
   G   
   C   
   G   
   G   
   A   
   A   
   G   
   A   
   A   
   G   
   A   
   T   
   G   
   A   
   A   
   C   
   T   
    T    
   A   
   T   
   T   
   G   
   C   
   T   
   G   
   T   
    A    
   T   
   A   
   T   
   C   
   T   
   G   
   A   
   A   
   A   
   G   
   A   
   A   
   G   
   A   
   T   
   C   
    A    
   G   
   C   
   C   
   A   
   G   
   G   
   C   
   T   
   T   
   A   
   G   
   A   
   T   
   G   
   T   
    G    
   T   
   T   
   C   
   T   
   T   
   A   
   G   
   T   
   A   
   A   
   C   
   G   
    C    
   A   
   T   
   C   
   A   
   A   
   G   
   G   
   A   
   A   
   T   
   G   
   G   
   G   
    C    
   T   
   T   
   G   
   C   
   G   
   C   
   T   
    A    
   T   
   C   
   C   
   T   
   T   
   T   
   A   
   A   
    C    
   A   
   G   
   A   
   T   
   G   
   T   
   T   
   C   
   C   
   C   
   G   
   T   
   T   
   G   
   T   
   T   
   G   
   G   
   A   
   G   
   C   
   A   
   A   
   A   
   G   
   G   
   C   
   A   
   G   
    C    
   T   
   G   
   G   
   A   
   G   
   T   
   G   
   A   
   A   
   G   
   T   
   C   
   T   
   A   
   T   
   G   
   A   
   A   
   T   
   T   
   T   
   G   
   A   
   A   
   A   
   G   
   A   
   T   
   G   
   G   
   A   
   G   
   A   
   T   
   A   
   G   
   G   
    A    
   T   
   T   
    G    
   T   
   C   
   A   
   A   
    T    
   G   
   G   
    G    
   C   
   T   
    G    
   C   
   T   
   T   
   G   
   T   
   G   
   T   
   T   
   A   
   G   
   C   
   A   
   G   
   A   
    C    
   G   
   G   
    A    
   G   
   A   
   T   
   A   
   C   
   G   
   C   
   C   
    A    
   A   
   T   
   C   
   G   
   T   
   A   
   A   
   T   
   C   
   G   
   T   
   T   
   A   
   C   
   T   
   C   
   A   
   G   
   C   
   G   
   A   
   G   
   G   
   A   
   G   
   C   
    C    
   G   
   T   
    G    
   A   
   A   
   A   
   C   
   G   
   C   
   A   
   T   
   G   
   C   
   T   
   T   
   G   
   C   
   T   
   C   
   A   
   A   
   G   
   A   
   A   
   A   
   T   
   C   
   A   
   G   
   C   
   C   
   A   
   A   
   A   
   C   
    T    
   T   
   C   
   A   
   C   
   G   
   T   
   G   
   C   
   G   
   A   
   A   
   G   
   C   
   G   
   G   
   G   
   G   
   A   
   G   
   T   
   G   
   A   
   C   
   G   
   G   
   T   
   C   
   A   
   T   
   G   
   C   
   G   
   T   
   G   
   A   
   G   
   C   
   T   
   G   
   A   
   A   
   A   
   A   
   A   
   A   
   A   
   A   
   T   
   C   
   C   
    G    
   C   
   A   
    C    
   C   
   G   
    T    
   A   
   T   
   T   
   A   
   T   
   C   
   T   
   A   
   T   
   A   
   T   
   G   
   T   
   C   
   G   
   G   
   A   
   A   
   G   
   G   
   A   
   A   
   G   
   A   
   T   
   C   
   A   
   A   
   A   
   A   
   G   
   A   
   A   
   A   
   T   
   A   
   A   
   C   
   C   
   T   
   T   
   G   
   A   
   T   
   G   
   A   
   A   
   T   
   C   
   A   
   A   
   A   
   A   
   A   
   G   
   G   
    A    
   C   
   A   
    A    
   C   
   A   
   G   
    A    
   T   
   A   
   A   
   C   
   G   
   A   
   T   
   T   
   G   
   A   
    T    
   C   
   C   
   A   
   A   
   C   
    G    
   G   
   A   
   T   
   T   
   T   
    T    
   C   
   C   
   T   
   A   
   T   
   A   
   G   
   G   
   T   
   G   
   A   
   T   
   C   
   G   
   A   
   A   
   C   
   A   
   T   
   C   
   G   
   A   
   A   
   T   
   G   
   G   
    T    
   T   
   C   
    G    
   T   
   T   
   T   
   G   
   C   
    T    
   A   
   T   
   G   
   G   
   A   
    C    
   G   
   A   
   G   
   A   
   A   
   A   
   A   
   A   
   A   
   G   
   G   
   T   
   G   
   G   
   A   
   G   
   A   
   A   
   G   
   T   
   C   
   A   
   T   
   C   
   G   
   A   
   A   
   G   
   T   
    T    
   A   
    T    
   C   
   G   
   A   
   T   
   G   
   C   
   T   
   C   
   C   
   T   
   G   
   A   
   G   
    A    
   T   
   T   
   T   
   T   
    C    
   A   
   T   
    T    
   G   
   A   
    A    
    T    
   C   
    A    
   -   
   -   
   -   
 

 
   N60365F   
   A   
   T   
   G   
   G   
   A   
   A   
   A   
   A   
   T   
   C   
   G   
   A   
   C   
   A   
   A   
   G   
   A   
   A   
   G   
   T   
   A   
   C   
   A   
   A   
   G   
   A   
   A   
   T   
   T   
   A   
   A   
   C   
   T   
   T   
   T   
   A   
   G   
   A   
   A   
   G   
   A   
   A   
   G   
   T   
   G   
   A   
   T   
   G   
   G   
   G   
   T   
   G   
   A   
   C   
   C   
   G   
   C   
   T   
   T   
   C   
   G   
   G   
   A   
   C   
   G   
   T   
   T   
   A   
   T   
   T   
   C   
   C   
   A   
   A   
   A   
   T   
   A   
   T   
   A   
   T   
   T   
   A   
   T   
    C    
   C   
   A   
    A    
   G   
   A   
   A   
   C   
   G   
   T   
   G   
   C   
   A   
   C   
   T   
   G   
   C   
   C   
   T   
   G   
   A   
   T   
   A   
   T   
    C    
   C   
   G   
   T   
   G   
   A   
   T   
   G   
   G   
   A   
   T   
   T   
   G   
   A   
   A   
    G    
   C   
   C   
   A   
   G   
   T   
    G    
   C   
   A   
   A   
   C   
   G   
   C   
   C   
   G   
   G   
   A   
   T   
   A   
   T   
   T   
   A   
   T   
   T   
    T    
   G   
   C   
    C    
   A   
   T   
   G   
   A   
   A   
   C   
   A   
   A   
   A   
   G   
   A   
   T   
   G   
   G   
   C   
   A   
   A   
   T   
   A   
   C   
   G   
   T   
   A   
   T   
   G   
   A   
   C   
   A   
   A   
   A   
   G   
   G   
   A   
   T   
   T   
    C    
   C   
   G   
   T   
   A   
   A   
   A   
   T   
   C   
   A   
   G   
   C   
   A   
   A   
   A   
   A   
   T   
   C   
   T   
   G   
   T   
   C   
   G   
   G   
   A   
   A   
   A   
   T   
   A   
   T   
   C   
   A   
   T   
   G   
   G   
   G   
    G    
   A   
   A   
   T   
   T   
   A   
   T   
   C   
   A   
   T   
   C   
   C   
   T   
   C   
   A   
   T   
   G   
   G   
   T   
   G   
   A   
   C   
   A   
   G   
   C   
   A   
   G   
   T   
   A   
   T   
   T   
   T   
   A   
   T   
   G   
   A   
   A   
   G   
   C   
   G   
   A   
   T   
   G   
   G   
   T   
   C   
   C   
   G   
   C   
   A   
   T   
   G   
   A   
   G   
   C   
   C   
   A   
   A   
   G   
   A   
    C    
   T   
   G   
   G   
   A   
   A   
   G   
   T   
   T   
   G   
   C   
   G   
   G   
   G   
   A   
   A   
   G   
   T   
   A   
   C   
   T   
   G   
   A   
   T   
   C   
   G   
   A   
    A    
   A   
   T   
   G   
   C   
   A   
   C   
   G   
   G   
    G    
   A   
   A   
   T   
   A   
   A   
    C    
   G   
   G   
   G   
   A   
   G   
   T   
   A   
   T   
   G   
   G   
   A   
   C   
   G   
   G   
   T   
   G   
   A   
   T   
   C   
   C   
   A   
   C   
   C   
   T   
   G   
   C   
   T   
   G   
   C   
   G   
   A   
   T   
   G   
   C   
   G   
   T   
   T   
   A   
   T   
   A   
   C   
   T   
   G   
   A   
   A   
   G   
   C   
   C   
   A   
   G   
    A    
   T   
   T   
   A   
   T   
   C   
   C   
   A   
   A   
   G   
   T   
   T   
   A   
   A   
   G   
   T   
   G   
   G   
   A   
   G   
   A   
   G   
   A   
   T   
   G   
    C    
   T   
   A   
    G    
   C   
   A   
   G   
   A   
   T   
   A   
   T   
   C   
   G   
   A   
    A    
   A   
   A   
   A   
   G   
   A   
   A   
   A   
   C   
   A   
   G   
   T   
    C    
   G   
   A   
   T   
   C   
   T   
   C   
   G   
   T   
   G   
   T   
   G   
   G   
   A   
   A   
   T   
   T   
   T   
   T   
   G   
   A   
   T   
   G   
   A   
   T   
   A   
   C   
   A   
   G   
   A   
   A   
   A   
   A   
   A   
   G   
   A   
   A   
   C   
   C   
   A   
   A   
   C   
   A   
   G   
   T   
   C   
   C   
   T   
   T   
   C   
   C   
   A   
   G   
   C   
   T   
   C   
   G   
   A   
   T   
   A   
   C   
   C   
   C   
   A   
   A   
   A   
   C   
   C   
   T   
   T   
   C   
   T   
    C    
   G   
   T   
   C   
   A   
   A   
   T   
   G   
   G   
   A   
   T   
   C   
   G   
   A   
   C   
   A   
   G   
   G   
   T   
   A   
   T   
   A   
   T   
   C   
   T   
   G   
   C   
   T   
   G   
   G   
   A   
   T   
   A   
   T   
   G   
   C   
    A    
   A   
   C   
   A   
   G   
   A   
   A   
   A   
   T   
   C   
   C   
   C   
   G   
   A   
   C   
    T    
   C   
   A   
   T   
   A   
   A   
   C   
   T   
   T   
   A   
   G   
   C   
   T   
   G   
   A   
   A   
   G   
   T   
   G   
   A   
   T   
    C    
   G   
   A   
    T    
   G   
   G   
   C   
   A   
   C   
   A   
   A   
   T   
   C   
   T   
   A   
   T   
   A   
   T   
   G   
   A   
   T   
   C   
   G   
   A   
   T   
   C   
   A   
   T   
   C   
   C   
   A   
   C   
   A   
   A   
   G   
   C   
   G   
   A   
   G   
   C   
   C   
   T   
   T   
   G   
   A   
   A   
   A   
   A   
   A   
   T   
   T   
   G   
   A   
   T   
   G   
   G   
   A   
   A   
   T   
   A   
   T   
   A   
   T   
   T   
   C   
   C   
   T   
   G   
   G   
   A   
   C   
   C   
   T   
   G   
   A   
   C   
   T   
   T   
    T    
   C   
   C   
   A   
   A   
   C   
   A   
   G   
   G   
   A   
   G   
   G   
    G    
   A   
   T   
   C   
   T   
   T   
   G   
   C   
   A   
   A   
   G   
   G   
    A    
   A   
   A   
   A   
   G   
   A   
   T   
   G   
   A   
   A   
   A   
   T   
   C   
   A   
   A   
   A   
   A   
   A   
   A   
   G   
   C   
   T   
   T   
   A   
   T   
   G   
   A   
   A   
   A   
   C   
   A   
   G   
   G   
    G    
   C   
   G   
   A   
   G   
   G   
   A   
   A   
   A   
   A   
   G   
   T   
   T   
   A   
    T    
   T   
   C   
   T   
   T   
   C   
   G   
   T   
   T   
   C   
   G   
   A   
   A   
   A   
   A   
   C   
   A   
   A   
   A   
   G   
   A   
   T   
   A   
   G   
   A   
    G    
   G   
   C   
   A   
   A   
   T   
   G   
   A   
   A   
   G   
   G   
   G   
    T    
   A   
   A   
    T    
   A   
   A   
   A   
   C   
   A   
   G   
   C   
   A   
   A   
   A   
   T   
   C   
   G   
   T   
   G   
   A   
   T   
   C   
   A   
   C   
    A    
   G   
   A   
   A   
   A   
   T   
   C   
   C   
   C   
   A   
   T   
   A   
   T   
   G   
   A   
   A   
   G   
   T   
   A   
   A   
   A   
   T   
   A   
   A   
   A   
   G   
   C   
   G   
   A   
   C   
    C    
   T   
   T   
   G   
   G   
   T   
   G   
   A   
   A   
   A   
   A   
   A   
   A   
   A   
   T   
   G   
   G   
   A   
   T   
   G   
   A   
   G   
   A   
   T   
   C   
   C   
   G   
   T   
   T   
   T   
   G   
   A   
   A   
   T   
   A   
   A   
   G   
   A   
   A   
   A   
   A   
   T   
   C   
   G   
   A   
    T    
   G   
   G   
   A   
   A   
   T   
   A   
   G   
   C   
   T   
   G   
   A   
   A   
   G   
   T   
    A    
   C   
   G   
   A   
   G   
   A   
   T   
   G   
   A   
   A   
   A   
   G   
   T   
   G   
   A   
   T   
    A    
   G   
    A    
   A   
   C   
    A    
   G   
   G   
   T   
   C   
   T   
   G   
   C   
   A   
   A   
   A   
   T   
   T   
   G   
   T   
   C   
   G   
   T   
    C    
   G   
   A   
   A   
   T   
   T   
   G   
   A   
   A   
   A   
   A   
   A   
   A   
   G   
   A   
    C    
   G   
   C   
   C   
   A   
   A   
    T    
   G   
   C   
   T   
   C   
   A   
   A   
   G   
   G   
    A    
   A   
   T   
   T   
   T   
   T   
   G   
   A   
   A   
   T   
   T   
   A   
    T    
   T   
   T   
   G   
   T   
   T   
   C   
   A   
   A   
   A   
   A   
   A   
   C   
   A   
   C   
   T   
   G   
   A   
   G   
    C    
   T   
   A   
   C   
   A   
   G   
   A   
   T   
   C   
   A   
   A   
   T   
   T   
   A   
   C   
   A   
   A   
   T   
   T   
   T   
    C    
   A   
   A   
   T   
   A   
   T   
   G   
   G   
   T   
   C   
   G   
   C   
   T   
   A   
   T   
   C   
   G   
   A   
    C    
   C   
   A   
   T   
   A   
   T   
   G   
   A   
   C   
   T   
   C   
   C   
   G   
   C   
   A   
    T    
   C   
   A   
   A   
   G   
   T   
   T   
   G   
   G   
   A   
   C   
   T   
   G   
   A   
   A   
   A   
   G   
   A   
   C   
   A   
   T   
   T   
   T   
   T   
   A   
   A   
   G   
   A   
   A   
   G   
   T   
   T   
   A   
   T   
   A   
   T   
   C   
   G   
   A   
   G   
   C   
   A   
   T   
   C   
   G   
   A   
   A   
   A   
   G   
   C   
   A   
   A   
   G   
   T   
   C   
   A   
   T   
   A   
   A   
   C   
   A   
   A   
   A   
   A   
   C   
   G   
    T    
   A   
   G   
    T    
   C   
   A   
   A   
   T   
   T   
   C   
   G   
   A   
   T   
   T   
   T   
   A   
   G   
   C   
   C   
   A   
   A   
   A   
   G   
   C   
   T   
   C   
   A   
   A   
   A   
   A   
   A   
    A    
   G   
   A   
   C   
   A   
   G   
   C   
   A   
   T   
   A   
   T   
   C   
   G   
   T   
    A    
   G   
   A   
   A   
   G   
   G   
    A    
    T    
   T   
   G   
   A   
   T   
   G   
   A   
   A   
   A   
   G   
   C   
   T   
   T   
   T   
   A   
   T   
   C   
   G   
   A   
   T   
   T   
   C   
   T   
   G   
   G   
   A   
   T   
   G   
   A   
   A   
   G   
   T   
   A   
   A   
   T   
   C   
   G   
    C    
   A   
   A   
   C   
   G   
   A   
   T   
   T   
   C   
   G   
   T   
   G   
   A   
   A   
   A   
   G   
   T   
   A   
   A   
   A   
   G   
   A   
   C   
   A   
   A   
   A   
   A   
   A   
   A   
   G   
   A   
    C    
   G   
   C   
    C    
   A   
   A   
   A   
   A   
   A   
   G   
   A   
   A   
   T   
   T   
   T   
   A   
   G   
   T   
   C   
   G   
   A   
    C    
   G   
   T   
    C    
   T   
   T   
    T    
   C   
   A   
   A   
   T   
   T   
   T   
   A   
   C   
   A   
   G   
   A   
   A   
   G   
   A   
   A   
   C   
   A   
   A   
   G   
   C   
   A   
   G   
   A   
   A   
   G   
   C   
   A   
   A   
   T   
    T    
   G   
   T   
   C   
   A   
   C   
   A   
   T   
   T   
   G   
   C   
   A   
   A   
    T    
   T   
   A   
   T   
   A   
    C    
   C   
   G   
   T   
   T   
   T   
   G   
   A   
   C   
   G   
   A   
   A   
    C    
   A   
   C   
    G    
   G   
   A   
   T   
   A   
   T   
   C   
   A   
   C   
   A   
   G   
   A   
   G   
   C   
   T   
   T   
   C   
   A   
   A   
   A   
   A   
   G   
   G   
   A   
   A   
    T    
   C   
    C    
   G   
   A   
   A   
   A   
    G    
   C   
   T   
   T   
   G   
   A   
   T   
   T   
   G   
   C   
    G    
   C   
   A   
   A   
   A   
   T   
   C   
   A   
   C   
   A   
   G   
   A   
   G   
   T   
   T   
    G    
   A   
   A   
   T   
   A   
   A   
   A   
   A   
   T   
   C   
   C   
   T   
   T   
   T   
   C   
   A   
   A   
   A   
    T    
   G   
   A   
   C   
   A   
   A   
   A   
   G   
   A   
    G    
   C   
   T   
   T   
   T   
   T   
    C    
   T   
   C   
   T   
   G   
   T   
   T   
   A   
   T   
   G   
   A   
   A   
   A   
   A   
   A   
   A   
   G   
   A   
   A   
   T   
   T   
   G   
   C   
   G   
   A   
   G   
   A   
   A   
   G   
   T   
   G   
   A   
   A   
   A   
   A   
   A   
   A   
   A   
   A   
   T   
   T   
   A   
    T    
   T   
   C   
   T   
   T   
   C   
   C   
   G   
   C   
   T   
   C   
   G   
   T   
   C   
    T    
    A    
   A   
   C   
   G   
   A   
   C   
   A   
   A   
   T   
   C   
    G    
   A   
   A   
   G   
   A   
   C   
   G   
   A   
   A   
   A   
   T   
   C   
   G   
   A   
   A   
   G   
   A   
   A   
   A   
   T   
   C   
   A   
   A   
   A   
   A   
   T   
    T    
   G   
   A   
   T   
   A   
   C   
   G   
   C   
   A   
   A   
    G    
   T   
    C    
   T   
   T   
   A   
   G   
   T   
   G   
   G   
   C   
    A    
   C   
   A   
   A   
   G   
   A   
   A   
   G   
   A   
   C   
   G   
   T   
   A   
   A   
   T   
    T    
   G   
   T   
   C   
   A   
   G   
   T   
   G   
   T   
    G    
   A   
   C   
    G    
   C   
   G   
   A   
   G   
   A   
   A   
   G   
   G   
   C   
   T   
   A   
   T   
   G   
   T   
   G   
   A   
   A   
   G   
   C   
   G   
   A   
   A   
   C   
   A   
   A   
   G   
   C   
   C   
   T   
   T   
   C   
   G   
   T   
   T   
   C   
    T    
   T   
   A   
   T   
   A   
   G   
   T   
   G   
   C   
   T   
   T   
   C   
    C    
   A   
   A   
   A   
   C   
   C   
   A   
   G   
   A   
   A   
   G   
   A   
   A   
   A   
   T   
   C   
   G   
   G   
   G   
   A   
   T   
   G   
   C   
   G   
   A   
   G   
   A   
   A   
   G   
   G   
   C   
   G   
   A   
    C    
   T   
   A   
   C   
   T   
   T   
   G   
   C   
   T   
    A    
   T   
   A   
   C   
    T    
   C   
   A   
   G   
   G   
   T   
   G   
   A   
   A   
   T   
   T   
    G    
   A   
   G   
    T    
   A   
   C   
   A   
    T    
   T   
   G   
   G   
   A   
   C   
   C   
   A   
   T   
   G   
   T   
   T   
   C   
   T   
   T   
   C   
   T   
   G   
   A   
   T   
    C    
   A   
   C   
    A    
   A   
   A   
   T   
   A   
   A   
   A   
   G   
   C   
   A   
   A   
   A   
   T   
   G   
   T   
   G   
   A   
   T   
   C   
   T   
   A   
   C   
   C   
   G   
    C    
   C   
   C   
   T   
   G   
   T   
    C    
   C   
   A   
   T   
   G   
   A   
   A   
   C   
   T   
   A   
   C   
   C   
    C    
   G   
   A   
   T   
   T   
   T   
   G   
   A   
   A   
   A   
   T   
   G   
   G   
   A   
   A   
   A   
   G   
   A   
   T   
   G   
   C   
   T   
   G   
   G   
    T    
   G   
   A   
    G    
   C   
   A   
    C    
   A   
   T   
   T   
   T   
   C   
   T   
   C   
   A   
   A   
   A   
   C   
   A   
   A   
   T   
   C   
   T   
   C   
   C   
   A   
   A   
   T   
   C   
   T   
   G   
   T   
   C   
   T   
   G   
   T   
   A   
   G   
   A   
    C    
   G   
   A   
   A   
   T   
   C   
   G   
   A   
   T   
    T    
   T   
   T   
   A   
   G   
   C   
   T   
   G   
   T   
   T   
   T   
   T   
    C    
   C   
   C   
   A   
   T   
   A   
   T   
   C   
   A   
   A   
   A   
   A   
   A   
   A   
   T   
    T    
   C   
   A   
   A   
   G   
   C   
   A   
   G   
   A   
    G    
   A   
   A   
   A   
   A   
   C   
   A   
   T   
   T   
   T   
   G   
   T   
    A    
   T   
   T   
   C   
   A   
   T   
   C   
   A   
   G   
   C   
   A   
   A   
   G   
   A   
   A   
   T   
   G   
   G   
   A   
   T   
   T   
    G    
    A    
   T   
   C   
   A   
   A   
   G   
   C   
   A   
   A   
   A   
   C   
   A   
   C   
   G   
   A   
   A   
   T   
   G   
   A   
   C   
   A   
   G   
   A   
   G   
   T   
   T   
   C   
   G   
   A   
   A   
   C   
   C   
   A   
   T   
   G   
   G   
   C   
   G   
   C   
   A   
   C   
   C   
   T   
   A   
    T    
   A   
   A   
   G   
   A   
   G   
   C   
   C   
   G   
    T    
   C   
   C   
   G   
   C   
   T   
   C   
   A   
   G   
   C   
   T   
   G   
   T   
   A   
   T   
   G   
   A   
   A   
   G   
   C   
   T   
   A   
   A   
   A   
   G   
   G   
   C   
   G   
   G   
   A   
   A   
   G   
   A   
   A   
   G   
   A   
   T   
   G   
   A   
   A   
   C   
   T   
    G    
   A   
   T   
   T   
   G   
   C   
   T   
   G   
   T   
    G    
   T   
   A   
   T   
   C   
   T   
   G   
   A   
   A   
   A   
   G   
   A   
   A   
   G   
   A   
   T   
   C   
    A    
   G   
   C   
   C   
   A   
   G   
   G   
   C   
   T   
   T   
   A   
   G   
   A   
   T   
   G   
   T   
    T    
   T   
   T   
   C   
   T   
   T   
   A   
   G   
   T   
   A   
   A   
   C   
   G   
    C    
   A   
   T   
   C   
   A   
   A   
   G   
   G   
   A   
   A   
   T   
   G   
   G   
   G   
    T    
   T   
   T   
   G   
   C   
   G   
   C   
   T   
    A    
   T   
   C   
   C   
   T   
   T   
   T   
   A   
   A   
    C    
   A   
   G   
   A   
   T   
   G   
   T   
   T   
   C   
   C   
   C   
   G   
   T   
   T   
   G   
   T   
   T   
   G   
   G   
   A   
   G   
   C   
   A   
   A   
   A   
   G   
   G   
   C   
   A   
   G   
    C    
   T   
   G   
   G   
   A   
   G   
   T   
   G   
   A   
   A   
   G   
   T   
   C   
   T   
   A   
   T   
   G   
   A   
   A   
   T   
   T   
   T   
   G   
   A   
   A   
   A   
   G   
   A   
   T   
   G   
   G   
   A   
   G   
   A   
   T   
   A   
   G   
   G   
    A    
   T   
   T   
    A    
   T   
   C   
   A   
   A   
    C    
   G   
   G   
    G    
   C   
   T   
    G    
   C   
   T   
   T   
   G   
   T   
   G   
   T   
   T   
   A   
   G   
   C   
   A   
   G   
   A   
    A    
   G   
   G   
    T    
   G   
   A   
   T   
   A   
   C   
   G   
   C   
   C   
    G    
   A   
   T   
   C   
   G   
   T   
   A   
   A   
   T   
   C   
   G   
   T   
   T   
   A   
   C   
   T   
   C   
   A   
   G   
   C   
   G   
   A   
   G   
   G   
   A   
   G   
   C   
    T    
   G   
   T   
    T    
   A   
   A   
   A   
   C   
   G   
   C   
   A   
   T   
   G   
   C   
   T   
   T   
   G   
   C   
   T   
   C   
   A   
   A   
   G   
   A   
   A   
   A   
   T   
   C   
   A   
   G   
   C   
   C   
   A   
   A   
   A   
   C   
    T    
   T   
   C   
   A   
   C   
   G   
   T   
   G   
   C   
   G   
   A   
   A   
   G   
   C   
   G   
   G   
   G   
   G   
   A   
   G   
   T   
   G   
   A   
   C   
   G   
   G   
   T   
   C   
   A   
   T   
   G   
   C   
   G   
   T   
   G   
   A   
   G   
   C   
   T   
   G   
   A   
   A   
   A   
   A   
   A   
   A   
   A   
   A   
   T   
   C   
   C   
    G    
   C   
   A   
    C    
   C   
   G   
    T    
   A   
   T   
   T   
   A   
   T   
   C   
   T   
   A   
   T   
   A   
   T   
   G   
   T   
   C   
   G   
   G   
   A   
   A   
   G   
   G   
   A   
   A   
   G   
   A   
   T   
   C   
   A   
   A   
   A   
   A   
   G   
   A   
   A   
   A   
   T   
   A   
   A   
   C   
   C   
   T   
   T   
   G   
   A   
   T   
   G   
   A   
   A   
   T   
   C   
   A   
   A   
   A   
   A   
   A   
   G   
   G   
    G    
   C   
   A   
    G    
   C   
   A   
   G   
    T    
   T   
   A   
   A   
   C   
   G   
   A   
   T   
   T   
   G   
   A   
    C    
   C   
   C   
   A   
   A   
   C   
    T    
   G   
   A   
   T   
   T   
   T   
    C    
   C   
   C   
   T   
   A   
   T   
   A   
   G   
   G   
   T   
   G   
   A   
   T   
   C   
   G   
   A   
   A   
   C   
   A   
   T   
   C   
   G   
   A   
   A   
   T   
   G   
   G   
    A    
   T   
   C   
    A    
   T   
   T   
   T   
   G   
   C   
    G    
   A   
   T   
   G   
   G   
   A   
    T    
   G   
   A   
   G   
   A   
   A   
   A   
   A   
   A   
   A   
   G   
   G   
   T   
   G   
   G   
   A   
   G   
   A   
   A   
   G   
   T   
   C   
   A   
   T   
   C   
   G   
   A   
   A   
   G   
   T   
    T    
   A   
    T    
   C   
   G   
   A   
   T   
   G   
   C   
   T   
   C   
   C   
   T   
   G   
   A   
   G   
    A    
   T   
   T   
   T   
   T   
    C    
   A   
   T   
    C    
   G   
   A   
    G    
    T    
   C   
    G    
   -   
   -   
   -   
 

 
   N54538   
   A   
   T   
   G   
   G   
   A   
   A   
   A   
   A   
   T   
   C   
   G   
   A   
   C   
   A   
   A   
   G   
   A   
   A   
   G   
   T   
   A   
   C   
   A   
   A   
   G   
   A   
   A   
   T   
   T   
   A   
   A   
   C   
   T   
   T   
   T   
   A   
   G   
   A   
   A   
   G   
   A   
   A   
   G   
   T   
   G   
   A   
   T   
   G   
   G   
   G   
   T   
   G   
   A   
   C   
   C   
   G   
   C   
   T   
   T   
   C   
   G   
   G   
   A   
   C   
   G   
   T   
   T   
   A   
   T   
   T   
   C   
   C   
   A   
   A   
   A   
   T   
   A   
   T   
   A   
   T   
   T   
   A   
   T   
    C    
   C   
   A   
    A    
   G   
   A   
   A   
   C   
   G   
   T   
   G   
   C   
   A   
   C   
   T   
   G   
   C   
   C   
   T   
   G   
   A   
   T   
   A   
   T   
    C    
   C   
   G   
   T   
   G   
   A   
   T   
   G   
   G   
   A   
   T   
   T   
   G   
   A   
   A   
    G    
   C   
   C   
   A   
   G   
   T   
    G    
   C   
   A   
   A   
   C   
   G   
   C   
   C   
   G   
   G   
   A   
   T   
   A   
   T   
   T   
   A   
   T   
   T   
    T    
   G   
   C   
    C    
   A   
   T   
   G   
   A   
   A   
   C   
   A   
   A   
   A   
   G   
   A   
   T   
   G   
   G   
   C   
   A   
   A   
   T   
   A   
   C   
   G   
   T   
   A   
   T   
   G   
   A   
   C   
   A   
   A   
   A   
   G   
   G   
   A   
   T   
   T   
    C    
   C   
   G   
   T   
   A   
   A   
   A   
   T   
   C   
   A   
   G   
   C   
   A   
   A   
   A   
   A   
   T   
   C   
   T   
   G   
   T   
   C   
   G   
   G   
   A   
   A   
   A   
   T   
   A   
   T   
   C   
   A   
   T   
   G   
   G   
   G   
    G    
   A   
   A   
   T   
   T   
   A   
   T   
   C   
   A   
   T   
   C   
   C   
   T   
   C   
   A   
   T   
   G   
   G   
   T   
   G   
   A   
   C   
   A   
   G   
   C   
   A   
   G   
   T   
   A   
   T   
   T   
   T   
   A   
   T   
   G   
   A   
   A   
   G   
   C   
   G   
   A   
   T   
   G   
   G   
   T   
   C   
   C   
   G   
   C   
   A   
   T   
   G   
   A   
   G   
   C   
   C   
   A   
   A   
   G   
   A   
    C    
   T   
   G   
   G   
   A   
   A   
   G   
   T   
   T   
   G   
   C   
   G   
   G   
   G   
   A   
   A   
   G   
   T   
   A   
   C   
   T   
   G   
   A   
   T   
   C   
   G   
   A   
    A    
   A   
   T   
   G   
   C   
   A   
   C   
   G   
   G   
    G    
   A   
   A   
   T   
   A   
   A   
    C    
   G   
   G   
   G   
   A   
   G   
   T   
   A   
   T   
   G   
   G   
   A   
   C   
   G   
   G   
   T   
   G   
   A   
   T   
   C   
   C   
   A   
   C   
   C   
   T   
   G   
   C   
   T   
   G   
   C   
   G   
   A   
   T   
   G   
   C   
   G   
   T   
   T   
   A   
   T   
   A   
   C   
   T   
   G   
   A   
   A   
   G   
   C   
   C   
   A   
   G   
    A    
   T   
   T   
   A   
   T   
   C   
   C   
   A   
   A   
   G   
   T   
   T   
   A   
   A   
   G   
   T   
   G   
   G   
   A   
   G   
   A   
   G   
   A   
   T   
   G   
    C    
   T   
   A   
    G    
   C   
   A   
   G   
   A   
   T   
   A   
   T   
   C   
   G   
   A   
    A    
   A   
   A   
   A   
   G   
   A   
   A   
   A   
   C   
   A   
   G   
   T   
    C    
   G   
   A   
   T   
   C   
   T   
   C   
   G   
   T   
   G   
   T   
   G   
   G   
   A   
   A   
   T   
   T   
   T   
   T   
   G   
   A   
   T   
   G   
   A   
   T   
   A   
   C   
   A   
   G   
   A   
   A   
   A   
   A   
   A   
   G   
   A   
   A   
   C   
   C   
   A   
   A   
   C   
   A   
   G   
   T   
   C   
   C   
   T   
   T   
   C   
   C   
   A   
   G   
   C   
   T   
   C   
   G   
   A   
   T   
   A   
   C   
   C   
   C   
   A   
   A   
   A   
   C   
   C   
   T   
   T   
   C   
   T   
    C    
   G   
   T   
   C   
   A   
   A   
   T   
   G   
   G   
   A   
   T   
   C   
   G   
   A   
   C   
   A   
   G   
   G   
   T   
   A   
   T   
   A   
   T   
   C   
   T   
   G   
   C   
   T   
   G   
   G   
   A   
   T   
   A   
   T   
   G   
   C   
    A    
   A   
   C   
   A   
   G   
   A   
   A   
   A   
   T   
   C   
   C   
   C   
   G   
   A   
   C   
    T    
   C   
   A   
   T   
   A   
   A   
   C   
   T   
   T   
   A   
   G   
   C   
   T   
   G   
   A   
   A   
   G   
   T   
   G   
   A   
   T   
    T    
   G   
   A   
    T    
   G   
   G   
   C   
   A   
   C   
   A   
   A   
   T   
   C   
   T   
   A   
   T   
   A   
   T   
   G   
   A   
   T   
   C   
   G   
   A   
   T   
   C   
   A   
   T   
   C   
   C   
   A   
   C   
   A   
   A   
   G   
   C   
   G   
   A   
   G   
   C   
   C   
   T   
   T   
   G   
   A   
   A   
   A   
   A   
   A   
   T   
   T   
   G   
   A   
   T   
   G   
   G   
   A   
   A   
   T   
   A   
   T   
   A   
   T   
   T   
   C   
   C   
   T   
   G   
   G   
   A   
   C   
   C   
   T   
   G   
   A   
   C   
   T   
   T   
    T    
   C   
   C   
   A   
   A   
   C   
   A   
   G   
   G   
   A   
   G   
   G   
    G    
   A   
   T   
   C   
   T   
   T   
   G   
   C   
   A   
   A   
   G   
   G   
    A    
   A   
   A   
   A   
   G   
   A   
   T   
   G   
   A   
   A   
   A   
   T   
   C   
   A   
   A   
   A   
   A   
   A   
   A   
   G   
   C   
   T   
   T   
   A   
   T   
   G   
   A   
   A   
   A   
   C   
   A   
   G   
   G   
    G    
   C   
   G   
   A   
   G   
   G   
   A   
   A   
   A   
   A   
   G   
   T   
   T   
   A   
    T    
   T   
   C   
   T   
   T   
   C   
   G   
   T   
   T   
   C   
   G   
   A   
   A   
   A   
   A   
   C   
   A   
   A   
   A   
   G   
   A   
   T   
   A   
   G   
   A   
    G    
   G   
   C   
   A   
   A   
   T   
   G   
   A   
   A   
   G   
   G   
   G   
    T    
   A   
   A   
    T    
   A   
   A   
   A   
   C   
   A   
   G   
   C   
   A   
   A   
   A   
   T   
   C   
   G   
   T   
   G   
   A   
   T   
   C   
   A   
   C   
    A    
   G   
   A   
   A   
   A   
   T   
   C   
   C   
   C   
   A   
   T   
   A   
   T   
   G   
   A   
   A   
   G   
   T   
   A   
   A   
   A   
   T   
   A   
   A   
   A   
   G   
   C   
   G   
   A   
   C   
    C    
   T   
   T   
   G   
   G   
   T   
   G   
   A   
   A   
   A   
   A   
   A   
   A   
   A   
   T   
   G   
   G   
   A   
   T   
   G   
   A   
   G   
   A   
   T   
   C   
   C   
   G   
   T   
   T   
   T   
   G   
   A   
   A   
   T   
   A   
   A   
   G   
   A   
   A   
   A   
   A   
   T   
   C   
   G   
   A   
    T    
   G   
   G   
   A   
   A   
   T   
   A   
   G   
   C   
   T   
   G   
   A   
   A   
   G   
   T   
    A    
   C   
   G   
   A   
   G   
   A   
   T   
   G   
   A   
   A   
   A   
   G   
   T   
   G   
   A   
   T   
    A    
   G   
    A    
   A   
   C   
    A    
   G   
   G   
   T   
   C   
   T   
   G   
   C   
   A   
   A   
   A   
   T   
   T   
   G   
   T   
   C   
   G   
   T   
    C    
   G   
   A   
   A   
   T   
   T   
   G   
   A   
   A   
   A   
   A   
   A   
   A   
   G   
   A   
    C    
   G   
   C   
   C   
   A   
   A   
    T    
   G   
   C   
   T   
   C   
   A   
   A   
   G   
   G   
    A    
   A   
   T   
   T   
   T   
   T   
   G   
   A   
   A   
   T   
   T   
   A   
    T    
   T   
   T   
   G   
   T   
   T   
   C   
   A   
   A   
   A   
   A   
   A   
   C   
   A   
   C   
   T   
   G   
   A   
   G   
    C    
   T   
   A   
   C   
   A   
   G   
   A   
   T   
   C   
   A   
   A   
   T   
   T   
   A   
   C   
   A   
   A   
   T   
   T   
   T   
    C    
   A   
   A   
   T   
   A   
   T   
   G   
   G   
   T   
   C   
   G   
   C   
   T   
   A   
   T   
   C   
   G   
   A   
    C    
   C   
   A   
   T   
   A   
   T   
   G   
   A   
   C   
   T   
   C   
   C   
   G   
   C   
   A   
    T    
   C   
   A   
   A   
   G   
   T   
   T   
   G   
   G   
   A   
   C   
   T   
   G   
   A   
   A   
   A   
   G   
   A   
   C   
   A   
   T   
   T   
   T   
   T   
   A   
   A   
   G   
   A   
   A   
   G   
   T   
   T   
   A   
   T   
   A   
   T   
   C   
   G   
   A   
   G   
   C   
   A   
   T   
   C   
   G   
   A   
   A   
   A   
   G   
   C   
   A   
   A   
   G   
   T   
   C   
   A   
   T   
   A   
   A   
   C   
   A   
   A   
   A   
   A   
   C   
   G   
    T    
   A   
   G   
    T    
   C   
   A   
   A   
   T   
   T   
   C   
   G   
   A   
   T   
   T   
   T   
   A   
   G   
   C   
   C   
   A   
   A   
   A   
   G   
   C   
   T   
   C   
   A   
   A   
   A   
   A   
   A   
    A    
   G   
   A   
   C   
   A   
   G   
   C   
   A   
   T   
   A   
   T   
   C   
   G   
   T   
    A    
   G   
   A   
   A   
   G   
   G   
    A    
    T    
   T   
   G   
   A   
   T   
   G   
   A   
   A   
   A   
   G   
   C   
   T   
   T   
   T   
   A   
   T   
   C   
   G   
   A   
   T   
   T   
   C   
   T   
   G   
   G   
   A   
   T   
   G   
   A   
   A   
   G   
   T   
   A   
   A   
   T   
   C   
   G   
    T    
   A   
   A   
   C   
   G   
   A   
   T   
   T   
   C   
   G   
   T   
   G   
   A   
   A   
   A   
   G   
   T   
   A   
   A   
   A   
   G   
   A   
   C   
   A   
   A   
   A   
   A   
   A   
   A   
   G   
   A   
    C    
   G   
   C   
    C    
   A   
   A   
   A   
   A   
   A   
   G   
   A   
   A   
   T   
   T   
   T   
   A   
   G   
   T   
   C   
   G   
   A   
    C    
   G   
   T   
    C    
   T   
   T   
    T    
   C   
   A   
   A   
   T   
   T   
   T   
   A   
   C   
   A   
   G   
   A   
   A   
   G   
   A   
   A   
   C   
   A   
   A   
   G   
   C   
   A   
   G   
   A   
   A   
   G   
   C   
   A   
   A   
   T   
    T    
   G   
   T   
   C   
   A   
   C   
   A   
   T   
   T   
   G   
   C   
   A   
   A   
    T    
   T   
   A   
   T   
   A   
    C    
   C   
   G   
   T   
   T   
   T   
   G   
   A   
   C   
   G   
   A   
   A   
    C    
   A   
   C   
    G    
   G   
   A   
   T   
   A   
   T   
   C   
   A   
   C   
   A   
   G   
   A   
   G   
   C   
   T   
   T   
   C   
   A   
   A   
   A   
   A   
   G   
   G   
   A   
   A   
    T    
   C   
    C    
   G   
   A   
   A   
   A   
    G    
   C   
   T   
   T   
   G   
   A   
   T   
   T   
   G   
   C   
    G    
   C   
   A   
   A   
   A   
   T   
   C   
   A   
   C   
   A   
   G   
   A   
   G   
   T   
   T   
    G    
   A   
   A   
   T   
   A   
   A   
   A   
   A   
   T   
   C   
   C   
   T   
   T   
   T   
   C   
   A   
   A   
   A   
    T    
   G   
   A   
   C   
   A   
   A   
   A   
   G   
   A   
    G    
   C   
   T   
   T   
   T   
   T   
    C    
   T   
   C   
   T   
   G   
   T   
   T   
   A   
   T   
   G   
   A   
   A   
   A   
   A   
   A   
   A   
   G   
   A   
   A   
   T   
   T   
   G   
   C   
   G   
   A   
   G   
   A   
   A   
   G   
   T   
   G   
   A   
   A   
   A   
   A   
   A   
   A   
   A   
   A   
   T   
   T   
   A   
    T    
   T   
   C   
   T   
   T   
   C   
   C   
   G   
   C   
   T   
   C   
   G   
   T   
   C   
    T    
    A    
   A   
   C   
   G   
   A   
   C   
   A   
   A   
   T   
   C   
    G    
   A   
   A   
   G   
   A   
   C   
   G   
   A   
   A   
   A   
   T   
   C   
   G   
   A   
   A   
   G   
   A   
   A   
   A   
   T   
   C   
   A   
   A   
   A   
   A   
   T   
    T    
   G   
   A   
   T   
   A   
   C   
   G   
   C   
   A   
   A   
    G    
   T   
    C    
   T   
   T   
   A   
   G   
   T   
   G   
   G   
   C   
    A    
   C   
   A   
   A   
   G   
   A   
   A   
   G   
   A   
   C   
   G   
   T   
   A   
   A   
   T   
    T    
   G   
   T   
   C   
   A   
   G   
   T   
   G   
   T   
    G    
   A   
   C   
    G    
   C   
   G   
   A   
   G   
   A   
   A   
   G   
   G   
   C   
   T   
   A   
   T   
   G   
   T   
   G   
   A   
   A   
   G   
   C   
   G   
   A   
   A   
   C   
   A   
   A   
   G   
   C   
   C   
   T   
   T   
   C   
   G   
   T   
   T   
   C   
    T    
   T   
   A   
   T   
   A   
   G   
   T   
   G   
   C   
   T   
   T   
   C   
    C    
   A   
   A   
   A   
   C   
   C   
   A   
   G   
   A   
   A   
   G   
   A   
   A   
   A   
   T   
   C   
   G   
   G   
   G   
   A   
   T   
   G   
   C   
   G   
   A   
   G   
   A   
   A   
   G   
   G   
   C   
   G   
   A   
    C    
   T   
   A   
   C   
   T   
   T   
   G   
   C   
   T   
    A    
   T   
   A   
   C   
    T    
   C   
   A   
   G   
   G   
   T   
   G   
   A   
   A   
   T   
   T   
    G    
   A   
   G   
    T    
   A   
   C   
   A   
    T    
   T   
   G   
   G   
   A   
   C   
   C   
   A   
   T   
   G   
   T   
   T   
   C   
   T   
   T   
   C   
   T   
   G   
   A   
   T   
    C    
   A   
   C   
    A    
   A   
   A   
   T   
   A   
   A   
   A   
   G   
   C   
   A   
   A   
   A   
   T   
   G   
   T   
   G   
   A   
   T   
   C   
   T   
   A   
   C   
   C   
   G   
    C    
   C   
   C   
   T   
   G   
   T   
    C    
   C   
   A   
   T   
   G   
   A   
   A   
   C   
   T   
   A   
   C   
   C   
    C    
   G   
   A   
   T   
   T   
   T   
   G   
   A   
   A   
   A   
   T   
   G   
   G   
   A   
   A   
   A   
   G   
   A   
   T   
   G   
   C   
   T   
   G   
   G   
    T    
   G   
   A   
    G    
   C   
   A   
    C    
   A   
   T   
   T   
   T   
   C   
   T   
   C   
   A   
   A   
   A   
   C   
   A   
   A   
   T   
   C   
   T   
   C   
   C   
   A   
   A   
   T   
   C   
   T   
   G   
   T   
   C   
   T   
   G   
   T   
   A   
   G   
   A   
    C    
   G   
   A   
   A   
   T   
   C   
   G   
   A   
   T   
    T    
   T   
   T   
   A   
   G   
   C   
   T   
   G   
   T   
   T   
   T   
   T   
    C    
   C   
   C   
   A   
   T   
   A   
   T   
   C   
   A   
   A   
   A   
   A   
   A   
   A   
   T   
    T    
   C   
   A   
   A   
   G   
   C   
   A   
   G   
   A   
    G    
   A   
   A   
   A   
   A   
   C   
   A   
   T   
   T   
   T   
   G   
   T   
    A    
   T   
   T   
   C   
   A   
   T   
   C   
   A   
   G   
   C   
   A   
   A   
   G   
   A   
   A   
   T   
   G   
   G   
   A   
   T   
   T   
    G    
    A    
   T   
   C   
   A   
   A   
   G   
   C   
   A   
   A   
   A   
   C   
   A   
   C   
   G   
   A   
   A   
   T   
   G   
   A   
   C   
   A   
   G   
   A   
   G   
   T   
   T   
   C   
   G   
   A   
   A   
   C   
   C   
   A   
   T   
   G   
   G   
   C   
   G   
   C   
   A   
   C   
   C   
   T   
   A   
    T    
   A   
   A   
   G   
   A   
   G   
   C   
   C   
   G   
    T    
   C   
   C   
   G   
   C   
   T   
   C   
   A   
   G   
   C   
   T   
   G   
   T   
   A   
   T   
   G   
   A   
   A   
   G   
   C   
   T   
   A   
   A   
   A   
   G   
   G   
   C   
   G   
   G   
   A   
   A   
   G   
   A   
   A   
   G   
   A   
   T   
   G   
   A   
   A   
   C   
   T   
    G    
   A   
   T   
   T   
   G   
   C   
   T   
   G   
   T   
    G    
   T   
   A   
   T   
   C   
   T   
   G   
   A   
   A   
   A   
   G   
   A   
   A   
   G   
   A   
   T   
   C   
    A    
   G   
   C   
   C   
   A   
   G   
   G   
   C   
   T   
   T   
   A   
   G   
   A   
   T   
   G   
   T   
    T    
   T   
   T   
   C   
   T   
   T   
   A   
   G   
   T   
   A   
   A   
   C   
   G   
    C    
   A   
   T   
   C   
   A   
   A   
   G   
   G   
   A   
   A   
   T   
   G   
   G   
   G   
    T    
   T   
   T   
   G   
   C   
   G   
   C   
   T   
    A    
   T   
   C   
   C   
   T   
   T   
   T   
   A   
   A   
    C    
   A   
   G   
   A   
   T   
   G   
   T   
   T   
   C   
   C   
   C   
   G   
   T   
   T   
   G   
   T   
   T   
   G   
   G   
   A   
   G   
   C   
   A   
   A   
   A   
   G   
   G   
   C   
   A   
   G   
    C    
   T   
   G   
   G   
   A   
   G   
   T   
   G   
   A   
   A   
   G   
   T   
   C   
   T   
   A   
   T   
   G   
   A   
   A   
   T   
   T   
   T   
   G   
   A   
   A   
   A   
   G   
   A   
   T   
   G   
   G   
   A   
   G   
   A   
   T   
   A   
   G   
   G   
    A    
   T   
   T   
    A    
   T   
   C   
   A   
   A   
    C    
   G   
   G   
    G    
   C   
   T   
    G    
   C   
   T   
   T   
   G   
   T   
   G   
   T   
   T   
   A   
   G   
   C   
   A   
   G   
   A   
    A    
   G   
   G   
    T    
   G   
   A   
   T   
   A   
   C   
   G   
   C   
   C   
    G    
   A   
   T   
   C   
   G   
   T   
   A   
   A   
   T   
   C   
   G   
   T   
   T   
   A   
   C   
   T   
   C   
   A   
   G   
   C   
   G   
   A   
   G   
   G   
   A   
   G   
   C   
    T    
   G   
   T   
    T    
   A   
   A   
   A   
   C   
   G   
   C   
   A   
   T   
   G   
   C   
   T   
   T   
   G   
   C   
   T   
   C   
   A   
   A   
   G   
   A   
   A   
   A   
   T   
   C   
   A   
   G   
   C   
   C   
   A   
   A   
   A   
   C   
    T    
   T   
   C   
   A   
   C   
   G   
   T   
   G   
   C   
   G   
   A   
   A   
   G   
   C   
   G   
   G   
   G   
   G   
   A   
   G   
   T   
   G   
   A   
   C   
   G   
   G   
   T   
   C   
   A   
   T   
   G   
   C   
   G   
   T   
   G   
   A   
   G   
   C   
   T   
   G   
   A   
   A   
   A   
   A   
   A   
   A   
   A   
   A   
   T   
   C   
   C   
    G    
   C   
   A   
    C    
   C   
   G   
    T    
   A   
   T   
   T   
   A   
   T   
   C   
   T   
   A   
   T   
   A   
   T   
   G   
   T   
   C   
   G   
   G   
   A   
   A   
   G   
   G   
   A   
   A   
   G   
   A   
   T   
   C   
   A   
   A   
   A   
   A   
   G   
   A   
   A   
   A   
   T   
   A   
   A   
   C   
   C   
   T   
   T   
   G   
   A   
   T   
   G   
   A   
   A   
   T   
   C   
   A   
   A   
   A   
   A   
   A   
   G   
   G   
    G    
   C   
   A   
    G    
   C   
   A   
   G   
    T    
   T   
   A   
   A   
   C   
   G   
   A   
   T   
   T   
   G   
   A   
    C    
   C   
   C   
   A   
   A   
   C   
    T    
   G   
   A   
   T   
   T   
   T   
    C    
   C   
   C   
   T   
   A   
   T   
   A   
   G   
   G   
   T   
   G   
   A   
   T   
   C   
   G   
   A   
   A   
   C   
   A   
   T   
   C   
   G   
   A   
   A   
   T   
   G   
   G   
    A    
   T   
   C   
    A    
   T   
   T   
   T   
   G   
   C   
    G    
   A   
   T   
   G   
   G   
   A   
    T    
   G   
   A   
   G   
   A   
   A   
   A   
   A   
   A   
   A   
   G   
   G   
   T   
   G   
   G   
   A   
   G   
   A   
   A   
   G   
   T   
   C   
   A   
   T   
   C   
   G   
   A   
   A   
   G   
   T   
    T    
   A   
    T    
   C   
   G   
   A   
   T   
   G   
   C   
   T   
   C   
   C   
   T   
   G   
   A   
   G   
    A    
   T   
   T   
   T   
   T   
    C    
   A   
   T   
    C    
   G   
   A   
    G    
    T    
   C   
    G    
   -   
   -   
   -   
 

 
   N60425F   
   A   
   T   
   G   
   G   
   A   
   A   
   A   
   A   
   T   
   C   
   G   
   A   
   C   
   A   
   A   
   G   
   A   
   A   
   G   
   T   
   A   
   C   
   A   
   A   
   G   
   A   
   A   
   T   
   T   
   A   
   A   
   C   
   T   
   T   
   T   
   A   
   G   
   A   
   A   
   G   
   A   
   A   
   G   
   T   
   G   
   A   
   T   
   G   
   G   
   G   
   T   
   G   
   A   
   C   
   C   
   G   
   C   
   T   
   T   
   C   
   G   
   G   
   A   
   C   
   G   
   T   
   T   
   A   
   T   
   T   
   C   
   C   
   A   
   A   
   A   
   T   
   A   
   T   
   A   
   T   
   T   
   A   
   T   
    C    
   C   
   A   
    A    
   G   
   A   
   A   
   C   
   G   
   T   
   G   
   C   
   A   
   C   
   T   
   G   
   C   
   C   
   T   
   G   
   A   
   T   
   A   
   T   
    C    
   C   
   G   
   T   
   G   
   A   
   T   
   G   
   G   
   A   
   T   
   T   
   G   
   A   
   A   
    G    
   C   
   C   
   A   
   G   
   T   
    G    
   C   
   A   
   A   
   C   
   G   
   C   
   C   
   G   
   G   
   A   
   T   
   A   
   T   
   T   
   A   
   T   
   T   
    C    
   G   
   C   
    T    
   A   
   T   
   G   
   A   
   A   
   C   
   A   
   A   
   A   
   G   
   A   
   T   
   G   
   G   
   C   
   A   
   A   
   T   
   A   
   C   
   G   
   T   
   A   
   T   
   G   
   A   
   C   
   A   
   A   
   A   
   G   
   G   
   A   
   T   
   T   
    C    
   C   
   G   
   T   
   A   
   A   
   A   
   T   
   C   
   A   
   G   
   C   
   A   
   A   
   A   
   A   
   T   
   C   
   T   
   G   
   T   
   C   
   G   
   G   
   A   
   A   
   A   
   T   
   A   
   T   
   C   
   A   
   T   
   G   
   G   
   G   
    G    
   A   
   A   
   T   
   T   
   A   
   T   
   C   
   A   
   T   
   C   
   C   
   T   
   C   
   A   
   T   
   G   
   G   
   T   
   G   
   A   
   C   
   A   
   G   
   C   
   A   
   G   
   T   
   A   
   T   
   T   
   T   
   A   
   T   
   G   
   A   
   A   
   G   
   C   
   G   
   A   
   T   
   G   
   G   
   T   
   C   
   C   
   G   
   C   
   A   
   T   
   G   
   A   
   G   
   C   
   C   
   A   
   A   
   G   
   A   
    C    
   T   
   G   
   G   
   A   
   A   
   G   
   T   
   T   
   G   
   C   
   G   
   G   
   G   
   A   
   A   
   G   
   T   
   A   
   C   
   T   
   G   
   A   
   T   
   C   
   G   
   A   
    A    
   A   
   T   
   G   
   C   
   A   
   C   
   G   
   G   
    G    
   A   
   A   
   T   
   A   
   A   
    C    
   G   
   G   
   G   
   A   
   G   
   T   
   A   
   T   
   G   
   G   
   A   
   C   
   G   
   G   
   T   
   G   
   A   
   T   
   C   
   C   
   A   
   C   
   C   
   T   
   G   
   C   
   T   
   G   
   C   
   G   
   A   
   T   
   G   
   C   
   G   
   T   
   T   
   A   
   T   
   A   
   C   
   T   
   G   
   A   
   A   
   G   
   C   
   C   
   A   
   G   
    A    
   T   
   T   
   A   
   T   
   C   
   C   
   A   
   A   
   G   
   T   
   T   
   A   
   A   
   G   
   T   
   G   
   G   
   A   
   G   
   A   
   G   
   A   
   T   
   G   
    C    
   T   
   A   
    G    
   C   
   A   
   G   
   A   
   T   
   A   
   T   
   C   
   G   
   A   
    A    
   A   
   A   
   A   
   G   
   A   
   A   
   A   
   C   
   A   
   G   
   T   
    C    
   G   
   A   
   T   
   C   
   T   
   C   
   G   
   T   
   G   
   T   
   G   
   G   
   A   
   A   
   T   
   T   
   T   
   T   
   G   
   A   
   T   
   G   
   A   
   T   
   A   
   C   
   A   
   G   
   A   
   A   
   A   
   A   
   A   
   G   
   A   
   A   
   C   
   C   
   A   
   A   
   C   
   A   
   G   
   T   
   C   
   C   
   T   
   T   
   C   
   C   
   A   
   G   
   C   
   T   
   C   
   G   
   A   
   T   
   A   
   C   
   C   
   C   
   A   
   A   
   A   
   C   
   C   
   T   
   T   
   C   
   T   
    C    
   G   
   T   
   C   
   A   
   A   
   T   
   G   
   G   
   A   
   T   
   C   
   G   
   A   
   C   
   A   
   G   
   G   
   T   
   A   
   T   
   A   
   T   
   C   
   T   
   G   
   C   
   T   
   G   
   G   
   A   
   T   
   A   
   T   
   G   
   C   
    A    
   A   
   C   
   A   
   G   
   A   
   A   
   A   
   T   
   C   
   C   
   C   
   G   
   A   
   C   
    T    
   C   
   A   
   T   
   A   
   A   
   C   
   T   
   T   
   A   
   G   
   C   
   T   
   G   
   A   
   A   
   G   
   T   
   G   
   A   
   T   
    C    
   G   
   A   
    T    
   G   
   G   
   C   
   A   
   C   
   A   
   A   
   T   
   C   
   T   
   A   
   T   
   A   
   T   
   G   
   A   
   T   
   C   
   G   
   A   
   T   
   C   
   A   
   T   
   C   
   C   
   A   
   C   
   A   
   A   
   G   
   C   
   G   
   A   
   G   
   C   
   C   
   T   
   T   
   G   
   A   
   A   
   A   
   A   
   A   
   T   
   T   
   G   
   A   
   T   
   G   
   G   
   A   
   A   
   T   
   A   
   T   
   A   
   T   
   T   
   C   
   C   
   T   
   G   
   G   
   A   
   C   
   C   
   T   
   G   
   A   
   C   
   T   
   T   
    T    
   C   
   C   
   A   
   A   
   C   
   A   
   G   
   G   
   A   
   G   
   G   
    G    
   A   
   T   
   C   
   T   
   T   
   G   
   C   
   A   
   A   
   G   
   G   
    A    
   A   
   A   
   A   
   G   
   A   
   T   
   G   
   A   
   A   
   A   
   T   
   C   
   A   
   A   
   A   
   A   
   A   
   A   
   G   
   C   
   T   
   T   
   A   
   T   
   G   
   A   
   A   
   A   
   C   
   A   
   G   
   G   
    G    
   C   
   G   
   A   
   G   
   G   
   A   
   A   
   A   
   A   
   G   
   T   
   T   
   A   
    T    
   T   
   C   
   T   
   T   
   C   
   G   
   T   
   T   
   C   
   G   
   A   
   A   
   A   
   A   
   C   
   A   
   A   
   A   
   G   
   A   
   T   
   A   
   G   
   A   
    A    
   G   
   C   
   A   
   A   
   T   
   G   
   A   
   A   
   G   
   G   
   G   
    T    
   A   
   A   
    T    
   A   
   A   
   A   
   C   
   A   
   G   
   C   
   A   
   A   
   A   
   T   
   C   
   G   
   T   
   G   
   A   
   T   
   C   
   A   
   C   
    A    
   G   
   A   
   A   
   A   
   T   
   C   
   C   
   C   
   A   
   T   
   A   
   T   
   G   
   A   
   A   
   G   
   T   
   A   
   A   
   A   
   T   
   A   
   A   
   A   
   G   
   C   
   G   
   A   
   C   
    C    
   T   
   T   
   G   
   G   
   T   
   G   
   A   
   A   
   A   
   A   
   A   
   A   
   A   
   T   
   G   
   G   
   A   
   T   
   G   
   A   
   G   
   A   
   T   
   C   
   C   
   G   
   T   
   T   
   T   
   G   
   A   
   A   
   T   
   A   
   A   
   G   
   A   
   A   
   A   
   A   
   T   
   C   
   G   
   A   
    T    
   G   
   G   
   A   
   A   
   T   
   A   
   G   
   C   
   T   
   G   
   A   
   A   
   G   
   T   
    A    
   C   
   G   
   A   
   G   
   A   
   T   
   G   
   A   
   A   
   A   
   G   
   T   
   G   
   A   
   T   
    A    
   G   
    A    
   A   
   C   
    A    
   G   
   G   
   T   
   C   
   T   
   G   
   C   
   A   
   A   
   A   
   T   
   T   
   G   
   T   
   C   
   G   
   T   
    C    
   G   
   A   
   A   
   T   
   T   
   G   
   A   
   A   
   A   
   A   
   A   
   A   
   G   
   A   
    C    
   G   
   C   
   C   
   A   
   A   
    T    
   G   
   C   
   T   
   C   
   A   
   A   
   G   
   G   
    A    
   A   
   T   
   T   
   T   
   T   
   G   
   A   
   A   
   T   
   T   
   A   
    T    
   T   
   T   
   G   
   T   
   T   
   C   
   A   
   A   
   A   
   A   
   A   
   C   
   A   
   C   
   T   
   G   
   A   
   G   
    C    
   T   
   A   
   C   
   A   
   G   
   A   
   T   
   C   
   A   
   A   
   T   
   T   
   A   
   C   
   A   
   A   
   T   
   T   
   T   
    C    
   A   
   A   
   T   
   A   
   T   
   G   
   G   
   T   
   C   
   G   
   C   
   T   
   A   
   T   
   C   
   G   
   A   
    C    
   C   
   A   
   T   
   A   
   T   
   G   
   A   
   C   
   T   
   C   
   C   
   G   
   C   
   A   
    T    
   C   
   A   
   A   
   G   
   T   
   T   
   G   
   G   
   A   
   C   
   T   
   G   
   A   
   A   
   A   
   G   
   A   
   C   
   A   
   T   
   T   
   T   
   T   
   A   
   A   
   G   
   A   
   A   
   G   
   T   
   T   
   A   
   T   
   A   
   T   
   C   
   G   
   A   
   G   
   C   
   A   
   T   
   C   
   G   
   A   
   A   
   A   
   G   
   C   
   A   
   A   
   G   
   T   
   C   
   A   
   T   
   A   
   A   
   C   
   A   
   A   
   A   
   A   
   C   
   G   
    T    
   A   
   G   
    T    
   C   
   A   
   A   
   T   
   T   
   C   
   G   
   A   
   T   
   T   
   T   
   A   
   G   
   C   
   C   
   A   
   A   
   A   
   G   
   C   
   T   
   C   
   A   
   A   
   A   
   A   
   A   
    A    
   G   
   A   
   C   
   A   
   G   
   C   
   A   
   T   
   A   
   T   
   C   
   G   
   T   
    A    
   G   
   A   
   A   
   G   
   G   
    A    
    T    
   T   
   G   
   A   
   T   
   G   
   A   
   A   
   A   
   G   
   C   
   T   
   T   
   T   
   A   
   T   
   C   
   G   
   A   
   T   
   T   
   C   
   T   
   G   
   G   
   A   
   T   
   G   
   A   
   A   
   G   
   T   
   A   
   A   
   T   
   C   
   G   
    C    
   A   
   A   
   C   
   G   
   A   
   T   
   T   
   C   
   G   
   T   
   G   
   A   
   A   
   A   
   G   
   T   
   A   
   A   
   A   
   G   
   A   
   C   
   A   
   A   
   A   
   A   
   A   
   A   
   G   
   A   
    C    
   G   
   C   
    C    
   A   
   A   
   A   
   A   
   A   
   G   
   A   
   A   
   T   
   T   
   T   
   A   
   G   
   T   
   C   
   G   
   A   
    C    
   G   
   T   
    C    
   T   
   T   
    T    
   C   
   A   
   A   
   T   
   T   
   T   
   A   
   C   
   A   
   G   
   A   
   A   
   G   
   A   
   A   
   C   
   A   
   A   
   G   
   C   
   A   
   G   
   A   
   A   
   G   
   C   
   A   
   A   
   T   
    T    
   G   
   T   
   C   
   A   
   C   
   A   
   T   
   T   
   G   
   C   
   A   
   A   
    T    
   T   
   A   
   T   
   A   
    C    
   C   
   G   
   T   
   T   
   T   
   G   
   A   
   C   
   G   
   A   
   A   
    C    
   A   
   C   
    G    
   G   
   A   
   T   
   A   
   T   
   C   
   A   
   C   
   A   
   G   
   A   
   G   
   C   
   T   
   T   
   C   
   A   
   A   
   A   
   A   
   G   
   G   
   A   
   A   
    T    
   C   
    C    
   G   
   A   
   A   
   A   
    G    
   C   
   T   
   T   
   G   
   A   
   T   
   T   
   G   
   C   
    G    
   C   
   A   
   A   
   A   
   T   
   C   
   A   
   C   
   A   
   G   
   A   
   G   
   T   
   T   
    G    
   A   
   A   
   T   
   A   
   A   
   A   
   A   
   T   
   C   
   C   
   T   
   T   
   T   
   C   
   A   
   A   
   A   
    T    
   G   
   A   
   C   
   A   
   A   
   A   
   G   
   A   
    G    
   C   
   T   
   T   
   T   
   T   
    C    
   T   
   C   
   T   
   G   
   T   
   T   
   A   
   T   
   G   
   A   
   A   
   A   
   A   
   A   
   A   
   G   
   A   
   A   
   T   
   T   
   G   
   C   
   G   
   A   
   G   
   A   
   A   
   G   
   T   
   G   
   A   
   A   
   A   
   A   
   A   
   A   
   A   
   A   
   T   
   T   
   A   
    T    
   T   
   C   
   T   
   T   
   C   
   C   
   G   
   C   
   T   
   C   
   G   
   T   
   C   
    T    
    A    
   A   
   C   
   G   
   A   
   C   
   A   
   A   
   T   
   C   
    G    
   A   
   A   
   G   
   A   
   C   
   G   
   A   
   A   
   A   
   T   
   C   
   G   
   A   
   A   
   G   
   A   
   A   
   A   
   T   
   C   
   A   
   A   
   A   
   A   
   T   
    C    
   G   
   A   
   T   
   A   
   C   
   G   
   C   
   A   
   A   
    G    
   T   
    T    
   T   
   T   
   A   
   G   
   T   
   G   
   G   
   C   
    G    
   C   
   A   
   A   
   G   
   A   
   A   
   G   
   A   
   C   
   G   
   T   
   A   
   A   
   T   
    C    
   G   
   T   
   C   
   A   
   G   
   T   
   G   
   T   
    G    
   A   
   C   
    G    
   C   
   G   
   A   
   G   
   A   
   A   
   G   
   G   
   C   
   T   
   A   
   T   
   G   
   T   
   G   
   A   
   A   
   G   
   C   
   G   
   A   
   A   
   C   
   A   
   A   
   G   
   C   
   C   
   T   
   T   
   C   
   G   
   T   
   T   
   C   
    T    
   T   
   A   
   T   
   A   
   G   
   T   
   G   
   C   
   T   
   T   
   C   
    G    
   A   
   A   
   A   
   C   
   C   
   A   
   G   
   A   
   A   
   G   
   A   
   A   
   A   
   T   
   C   
   G   
   G   
   G   
   A   
   T   
   G   
   C   
   G   
   A   
   G   
   A   
   A   
   G   
   G   
   C   
   G   
   A   
    C    
   T   
   A   
   C   
   T   
   T   
   G   
   C   
   T   
    G    
   T   
   A   
   C   
    T    
   C   
   A   
   G   
   G   
   T   
   G   
   A   
   A   
   T   
   T   
    G    
   A   
   G   
    C    
   A   
   C   
   A   
    T    
   T   
   G   
   G   
   A   
   C   
   C   
   A   
   T   
   G   
   T   
   T   
   C   
   T   
   T   
   C   
   T   
   G   
   A   
   T   
    T    
   A   
   C   
    G    
   A   
   A   
   T   
   A   
   A   
   A   
   G   
   C   
   A   
   A   
   A   
   T   
   G   
   T   
   G   
   A   
   T   
   C   
   T   
   A   
   C   
   C   
   G   
    T    
   C   
   C   
   T   
   G   
   T   
    T    
   C   
   A   
   T   
   G   
   A   
   A   
   C   
   T   
   A   
   C   
   C   
    C    
   G   
   A   
   T   
   T   
   T   
   G   
   A   
   A   
   A   
   T   
   G   
   G   
   A   
   A   
   A   
   G   
   A   
   T   
   G   
   C   
   T   
   G   
   G   
    C    
   G   
   A   
    A    
   C   
   A   
    T    
   A   
   T   
   T   
   T   
   C   
   T   
   C   
   A   
   A   
   A   
   C   
   A   
   A   
   T   
   C   
   T   
   C   
   C   
   A   
   A   
   T   
   C   
   T   
   G   
   T   
   C   
   T   
   G   
   T   
   A   
   G   
   A   
    T    
   G   
   A   
   A   
   T   
   C   
   G   
   A   
   T   
    C    
   T   
   T   
   A   
   G   
   C   
   T   
   G   
   T   
   T   
   T   
   T   
    T    
   C   
   C   
   A   
   T   
   A   
   T   
   C   
   A   
   A   
   A   
   A   
   A   
   A   
   T   
    T    
   C   
   A   
   A   
   G   
   C   
   A   
   G   
   A   
    G    
   A   
   A   
   A   
   A   
   C   
   A   
   T   
   T   
   T   
   G   
   T   
    A    
   T   
   T   
   C   
   A   
   T   
   C   
   A   
   G   
   C   
   A   
   A   
   G   
   A   
   A   
   T   
   G   
   G   
   A   
   T   
   T   
    G    
    A    
   T   
   C   
   A   
   A   
   G   
   C   
   A   
   A   
   A   
   C   
   A   
   C   
   G   
   A   
   A   
   T   
   G   
   A   
   C   
   A   
   G   
   A   
   G   
   T   
   T   
   C   
   G   
   A   
   A   
   C   
   C   
   A   
   T   
   G   
   G   
   C   
   G   
   C   
   A   
   C   
   C   
   T   
   A   
    T    
   A   
   A   
   G   
   A   
   G   
   C   
   C   
   G   
    T    
   C   
   C   
   G   
   C   
   T   
   C   
   A   
   G   
   C   
   T   
   G   
   T   
   A   
   T   
   G   
   A   
   A   
   G   
   C   
   T   
   A   
   A   
   A   
   G   
   G   
   C   
   G   
   G   
   A   
   A   
   G   
   A   
   A   
   G   
   A   
   T   
   G   
   A   
   A   
   C   
   T   
    G    
   A   
   T   
   T   
   G   
   C   
   T   
   G   
   T   
    G    
   T   
   A   
   T   
   C   
   T   
   G   
   A   
   A   
   A   
   G   
   A   
   A   
   G   
   A   
   T   
   C   
    G    
   G   
   C   
   C   
   A   
   G   
   G   
   C   
   T   
   T   
   A   
   G   
   A   
   T   
   G   
   T   
    T    
   T   
   T   
   C   
   T   
   T   
   A   
   G   
   T   
   A   
   A   
   C   
   G   
    T    
   A   
   T   
   C   
   A   
   A   
   G   
   G   
   A   
   A   
   T   
   G   
   G   
   G   
    T    
   T   
   T   
   G   
   C   
   G   
   C   
   T   
    A    
   T   
   C   
   C   
   T   
   T   
   T   
   A   
   A   
    C    
   A   
   G   
   A   
   T   
   G   
   T   
   T   
   C   
   C   
   C   
   G   
   T   
   T   
   G   
   T   
   T   
   G   
   G   
   A   
   G   
   C   
   A   
   A   
   A   
   G   
   G   
   C   
   A   
   G   
    C    
   T   
   G   
   G   
   A   
   G   
   T   
   G   
   A   
   A   
   G   
   T   
   C   
   T   
   A   
   T   
   G   
   A   
   A   
   T   
   T   
   T   
   G   
   A   
   A   
   A   
   G   
   A   
   T   
   G   
   G   
   A   
   G   
   A   
   T   
   A   
   G   
   G   
    A    
   T   
   T   
    A    
   T   
   C   
   A   
   A   
    C    
   G   
   G   
    G    
   C   
   T   
    G    
   C   
   T   
   T   
   G   
   T   
   G   
   T   
   T   
   A   
   G   
   C   
   A   
   G   
   A   
    A    
   G   
   G   
    T    
   G   
   A   
   T   
   A   
   C   
   G   
   C   
   C   
    G    
   A   
   T   
   C   
   G   
   T   
   A   
   A   
   T   
   C   
   G   
   T   
   T   
   A   
   C   
   T   
   C   
   A   
   G   
   C   
   G   
   A   
   G   
   G   
   A   
   G   
   C   
    T    
   G   
   T   
    T    
   A   
   A   
   A   
   C   
   G   
   C   
   A   
   T   
   G   
   C   
   T   
   T   
   G   
   C   
   T   
   C   
   A   
   A   
   G   
   A   
   A   
   A   
   T   
   C   
   A   
   G   
   C   
   C   
   A   
   A   
   A   
   C   
    T    
   T   
   C   
   A   
   C   
   G   
   T   
   G   
   C   
   G   
   A   
   A   
   G   
   C   
   G   
   G   
   G   
   G   
   A   
   G   
   T   
   G   
   A   
   C   
   G   
   G   
   T   
   C   
   A   
   T   
   G   
   C   
   G   
   T   
   G   
   A   
   G   
   C   
   T   
   G   
   A   
   A   
   A   
   A   
   A   
   A   
   A   
   A   
   T   
   C   
   C   
    T    
   C   
   A   
    T    
   C   
   G   
    C    
   A   
   T   
   T   
   A   
   T   
   C   
   T   
   A   
   T   
   A   
   T   
   G   
   T   
   C   
   G   
   G   
   A   
   A   
   G   
   G   
   A   
   A   
   G   
   A   
   T   
   C   
   A   
   A   
   A   
   A   
   G   
   A   
   A   
   A   
   T   
   A   
   A   
   C   
   C   
   T   
   T   
   G   
   A   
   T   
   G   
   A   
   A   
   T   
   C   
   A   
   A   
   A   
   A   
   A   
   G   
   G   
    G    
   C   
   A   
    G    
   C   
   A   
   G   
    T    
   T   
   A   
   A   
   C   
   G   
   A   
   T   
   T   
   G   
   A   
    C    
   C   
   C   
   A   
   A   
   C   
    T    
   G   
   A   
   T   
   T   
   T   
    C    
   C   
   C   
   T   
   A   
   T   
   A   
   G   
   G   
   T   
   G   
   A   
   T   
   C   
   G   
   A   
   A   
   C   
   A   
   T   
   C   
   G   
   A   
   A   
   T   
   G   
   G   
    A    
   T   
   C   
    A    
   T   
   T   
   T   
   G   
   C   
    G    
   A   
   T   
   G   
   G   
   A   
    T    
   G   
   A   
   G   
   A   
   A   
   A   
   A   
   A   
   A   
   G   
   G   
   T   
   G   
   G   
   A   
   G   
   A   
   A   
   G   
   T   
   C   
   A   
   T   
   C   
   G   
   A   
   A   
   G   
   T   
    T    
   A   
    T    
   C   
   G   
   A   
   T   
   G   
   C   
   T   
   C   
   C   
   T   
   G   
   A   
   G   
    A    
   T   
   T   
   T   
   T   
    C    
   A   
   T   
    C    
   G   
   A   
    G    
    T    
   C   
    G    
   -   
   -   
   -   
 

 
   N53805   
   A   
   T   
   G   
   G   
   A   
   A   
   A   
   A   
   T   
   C   
   G   
   A   
   C   
   A   
   A   
   G   
   A   
   A   
   G   
   T   
   A   
   C   
   A   
   A   
   G   
   A   
   A   
   T   
   T   
   A   
   A   
   C   
   T   
   T   
   T   
   A   
   G   
   A   
   A   
   G   
   A   
   A   
   G   
   T   
   G   
   A   
   T   
   G   
   G   
   G   
   T   
   G   
   A   
   C   
   C   
   G   
   C   
   T   
   T   
   C   
   G   
   G   
   A   
   C   
   G   
   T   
   T   
   A   
   T   
   T   
   C   
   C   
   A   
   A   
   A   
   T   
   A   
   T   
   A   
   T   
   T   
   A   
   T   
    C    
   C   
   A   
    A    
   G   
   A   
   A   
   C   
   G   
   T   
   G   
   C   
   A   
   C   
   T   
   G   
   C   
   C   
   T   
   G   
   A   
   T   
   A   
   T   
    C    
   C   
   G   
   T   
   G   
   A   
   T   
   G   
   G   
   A   
   T   
   T   
   G   
   A   
   A   
    G    
   C   
   C   
   A   
   G   
   T   
    G    
   C   
   A   
   A   
   C   
   G   
   C   
   C   
   G   
   G   
   A   
   T   
   A   
   T   
   T   
   A   
   T   
   T   
    T    
   G   
   C   
    C    
   A   
   T   
   G   
   A   
   A   
   C   
   A   
   A   
   A   
   G   
   A   
   T   
   G   
   G   
   C   
   A   
   A   
   T   
   A   
   C   
   G   
   T   
   A   
   T   
   G   
   A   
   C   
   A   
   A   
   A   
   G   
   G   
   A   
   T   
   T   
    C    
   C   
   G   
   T   
   A   
   A   
   A   
   T   
   C   
   A   
   G   
   C   
   A   
   A   
   A   
   A   
   T   
   C   
   T   
   G   
   T   
   C   
   G   
   G   
   A   
   A   
   A   
   T   
   A   
   T   
   C   
   A   
   T   
   G   
   G   
   G   
    G    
   A   
   A   
   T   
   T   
   A   
   T   
   C   
   A   
   T   
   C   
   C   
   T   
   C   
   A   
   T   
   G   
   G   
   T   
   G   
   A   
   C   
   A   
   G   
   C   
   A   
   G   
   T   
   A   
   T   
   T   
   T   
   A   
   T   
   G   
   A   
   A   
   G   
   C   
   G   
   A   
   T   
   G   
   G   
   T   
   C   
   C   
   G   
   C   
   A   
   T   
   G   
   A   
   G   
   C   
   C   
   A   
   A   
   G   
   A   
    C    
   T   
   G   
   G   
   A   
   A   
   G   
   T   
   T   
   G   
   C   
   G   
   G   
   G   
   A   
   A   
   G   
   T   
   A   
   C   
   T   
   G   
   A   
   T   
   C   
   G   
   A   
    A    
   A   
   T   
   G   
   C   
   A   
   C   
   G   
   G   
    G    
   A   
   A   
   T   
   A   
   A   
    C    
   G   
   G   
   G   
   A   
   G   
   T   
   A   
   T   
   G   
   G   
   A   
   C   
   G   
   G   
   T   
   G   
   A   
   T   
   C   
   C   
   A   
   C   
   C   
   T   
   G   
   C   
   T   
   G   
   C   
   G   
   A   
   T   
   G   
   C   
   G   
   T   
   T   
   A   
   T   
   A   
   C   
   T   
   G   
   A   
   A   
   G   
   C   
   C   
   A   
   G   
    A    
   T   
   T   
   A   
   T   
   C   
   C   
   A   
   A   
   G   
   T   
   T   
   A   
   A   
   G   
   T   
   G   
   G   
   A   
   G   
   A   
   G   
   A   
   T   
   G   
    C    
   T   
   A   
    G    
   C   
   A   
   G   
   A   
   T   
   A   
   T   
   C   
   G   
   A   
    A    
   A   
   A   
   A   
   G   
   A   
   A   
   A   
   C   
   A   
   G   
   T   
    C    
   G   
   A   
   T   
   C   
   T   
   C   
   G   
   T   
   G   
   T   
   G   
   G   
   A   
   A   
   T   
   T   
   T   
   T   
   G   
   A   
   T   
   G   
   A   
   T   
   A   
   C   
   A   
   G   
   A   
   A   
   A   
   A   
   A   
   G   
   A   
   A   
   C   
   C   
   A   
   A   
   C   
   A   
   G   
   T   
   C   
   C   
   T   
   T   
   C   
   C   
   A   
   G   
   C   
   T   
   C   
   G   
   A   
   T   
   A   
   C   
   C   
   C   
   A   
   A   
   A   
   C   
   C   
   T   
   T   
   C   
   T   
    C    
   G   
   T   
   C   
   A   
   A   
   T   
   G   
   G   
   A   
   T   
   C   
   G   
   A   
   C   
   A   
   G   
   G   
   T   
   A   
   T   
   A   
   T   
   C   
   T   
   G   
   C   
   T   
   G   
   G   
   A   
   T   
   A   
   T   
   G   
   C   
    A    
   A   
   C   
   A   
   G   
   A   
   A   
   A   
   T   
   C   
   C   
   C   
   G   
   A   
   C   
    T    
   C   
   A   
   T   
   A   
   A   
   C   
   T   
   T   
   A   
   G   
   C   
   T   
   G   
   A   
   A   
   G   
   T   
   G   
   A   
   T   
    T    
   G   
   A   
    T    
   G   
   G   
   C   
   A   
   C   
   A   
   A   
   T   
   C   
   T   
   A   
   T   
   A   
   T   
   G   
   A   
   T   
   C   
   G   
   A   
   T   
   C   
   A   
   T   
   C   
   C   
   A   
   C   
   A   
   A   
   G   
   C   
   G   
   A   
   G   
   C   
   C   
   T   
   T   
   G   
   A   
   A   
   A   
   A   
   A   
   T   
   T   
   G   
   A   
   T   
   G   
   G   
   A   
   A   
   T   
   A   
   T   
   A   
   T   
   T   
   C   
   C   
   T   
   G   
   G   
   A   
   C   
   C   
   T   
   G   
   A   
   C   
   T   
   T   
    T    
   C   
   C   
   A   
   A   
   C   
   A   
   G   
   G   
   A   
   G   
   G   
    G    
   A   
   T   
   C   
   T   
   T   
   G   
   C   
   A   
   A   
   G   
   G   
    A    
   A   
   A   
   A   
   G   
   A   
   T   
   G   
   A   
   A   
   A   
   T   
   C   
   A   
   A   
   A   
   A   
   A   
   A   
   G   
   C   
   T   
   T   
   A   
   T   
   G   
   A   
   A   
   A   
   C   
   A   
   G   
   G   
    G    
   C   
   G   
   A   
   G   
   G   
   A   
   A   
   A   
   A   
   G   
   T   
   T   
   A   
    T    
   T   
   C   
   T   
   T   
   C   
   G   
   T   
   T   
   C   
   G   
   A   
   A   
   A   
   A   
   C   
   A   
   A   
   A   
   G   
   A   
   T   
   A   
   G   
   A   
    G    
   G   
   C   
   A   
   A   
   T   
   G   
   A   
   A   
   G   
   G   
   G   
    T    
   A   
   A   
    T    
   A   
   A   
   A   
   C   
   A   
   G   
   C   
   A   
   A   
   A   
   T   
   C   
   G   
   T   
   G   
   A   
   T   
   C   
   A   
   C   
    A    
   G   
   A   
   A   
   A   
   T   
   C   
   C   
   C   
   A   
   T   
   A   
   T   
   G   
   A   
   A   
   G   
   T   
   A   
   A   
   A   
   T   
   A   
   A   
   A   
   G   
   C   
   G   
   A   
   C   
    C    
   T   
   T   
   G   
   G   
   T   
   G   
   A   
   A   
   A   
   A   
   A   
   A   
   A   
   T   
   G   
   G   
   A   
   T   
   G   
   A   
   G   
   A   
   T   
   C   
   C   
   G   
   T   
   T   
   T   
   G   
   A   
   A   
   T   
   A   
   A   
   G   
   A   
   A   
   A   
   A   
   T   
   C   
   G   
   A   
    T    
   G   
   G   
   A   
   A   
   T   
   A   
   G   
   C   
   T   
   G   
   A   
   A   
   G   
   T   
    A    
   C   
   G   
   A   
   G   
   A   
   T   
   G   
   A   
   A   
   A   
   G   
   T   
   G   
   A   
   T   
    A    
   G   
    A    
   A   
   C   
    A    
   G   
   G   
   T   
   C   
   T   
   G   
   C   
   A   
   A   
   A   
   T   
   T   
   G   
   T   
   C   
   G   
   T   
    C    
   G   
   A   
   A   
   T   
   T   
   G   
   A   
   A   
   A   
   A   
   A   
   A   
   G   
   A   
    C    
   G   
   C   
   C   
   A   
   A   
    T    
   G   
   C   
   T   
   C   
   A   
   A   
   G   
   G   
    A    
   A   
   T   
   T   
   T   
   T   
   G   
   A   
   A   
   T   
   T   
   A   
    T    
   T   
   T   
   G   
   T   
   T   
   C   
   A   
   A   
   A   
   A   
   A   
   C   
   A   
   C   
   T   
   G   
   A   
   G   
    C    
   T   
   A   
   C   
   A   
   G   
   A   
   T   
   C   
   A   
   A   
   T   
   T   
   A   
   C   
   A   
   A   
   T   
   T   
   T   
    C    
   A   
   A   
   T   
   A   
   T   
   G   
   G   
   T   
   C   
   G   
   C   
   T   
   A   
   T   
   C   
   G   
   A   
    C    
   C   
   A   
   T   
   A   
   T   
   G   
   A   
   C   
   T   
   C   
   C   
   G   
   C   
   A   
    T    
   C   
   A   
   A   
   G   
   T   
   T   
   G   
   G   
   A   
   C   
   T   
   G   
   A   
   A   
   A   
   G   
   A   
   C   
   A   
   T   
   T   
   T   
   T   
   A   
   A   
   G   
   A   
   A   
   G   
   T   
   T   
   A   
   T   
   A   
   T   
   C   
   G   
   A   
   G   
   C   
   A   
   T   
   C   
   G   
   A   
   A   
   A   
   G   
   C   
   A   
   A   
   G   
   T   
   C   
   A   
   T   
   A   
   A   
   C   
   A   
   A   
   A   
   A   
   C   
   G   
    T    
   A   
   G   
    T    
   C   
   A   
   A   
   T   
   T   
   C   
   G   
   A   
   T   
   T   
   T   
   A   
   G   
   C   
   C   
   A   
   A   
   A   
   G   
   C   
   T   
   C   
   A   
   A   
   A   
   A   
   A   
    A    
   G   
   A   
   C   
   A   
   G   
   C   
   A   
   T   
   A   
   T   
   C   
   G   
   T   
    A    
   G   
   A   
   A   
   G   
   G   
    A    
    T    
   T   
   G   
   A   
   T   
   G   
   A   
   A   
   A   
   G   
   C   
   T   
   T   
   T   
   A   
   T   
   C   
   G   
   A   
   T   
   T   
   C   
   T   
   G   
   G   
   A   
   T   
   G   
   A   
   A   
   G   
   T   
   A   
   A   
   T   
   C   
   G   
    T    
   A   
   A   
   C   
   G   
   A   
   T   
   T   
   C   
   G   
   T   
   G   
   A   
   A   
   A   
   G   
   T   
   A   
   A   
   A   
   G   
   A   
   C   
   A   
   A   
   A   
   A   
   A   
   A   
   G   
   A   
    C    
   G   
   C   
    C    
   A   
   A   
   A   
   A   
   A   
   G   
   A   
   A   
   T   
   T   
   T   
   A   
   G   
   T   
   C   
   G   
   A   
    C    
   G   
   T   
    C    
   T   
   T   
    T    
   C   
   A   
   A   
   T   
   T   
   T   
   A   
   C   
   A   
   G   
   A   
   A   
   G   
   A   
   A   
   C   
   A   
   A   
   G   
   C   
   A   
   G   
   A   
   A   
   G   
   C   
   A   
   A   
   T   
    T    
   G   
   T   
   C   
   A   
   C   
   A   
   T   
   T   
   G   
   C   
   A   
   A   
    T    
   T   
   A   
   T   
   A   
    C    
   C   
   G   
   T   
   T   
   T   
   G   
   A   
   C   
   G   
   A   
   A   
    C    
   A   
   C   
    G    
   G   
   A   
   T   
   A   
   T   
   C   
   A   
   C   
   A   
   G   
   A   
   G   
   C   
   T   
   T   
   C   
   A   
   A   
   A   
   A   
   G   
   G   
   A   
   A   
    T    
   C   
    C    
   G   
   A   
   A   
   A   
    G    
   C   
   T   
   T   
   G   
   A   
   T   
   T   
   G   
   C   
    G    
   C   
   A   
   A   
   A   
   T   
   C   
   A   
   C   
   A   
   G   
   A   
   G   
   T   
   T   
    G    
   A   
   A   
   T   
   A   
   A   
   A   
   A   
   T   
   C   
   C   
   T   
   T   
   T   
   C   
   A   
   A   
   A   
    T    
   G   
   A   
   C   
   A   
   A   
   A   
   G   
   A   
    G    
   C   
   T   
   T   
   T   
   T   
    C    
   T   
   C   
   T   
   G   
   T   
   T   
   A   
   T   
   G   
   A   
   A   
   A   
   A   
   A   
   A   
   G   
   A   
   A   
   T   
   T   
   G   
   C   
   G   
   A   
   G   
   A   
   A   
   G   
   T   
   G   
   A   
   A   
   A   
   A   
   A   
   A   
   A   
   A   
   T   
   T   
   A   
    T    
   T   
   C   
   T   
   T   
   C   
   C   
   G   
   C   
   T   
   C   
   G   
   T   
   C   
    T    
    A    
   A   
   C   
   G   
   A   
   C   
   A   
   A   
   T   
   C   
    G    
   A   
   A   
   G   
   A   
   C   
   G   
   A   
   A   
   A   
   T   
   C   
   G   
   A   
   A   
   G   
   A   
   A   
   A   
   T   
   C   
   A   
   A   
   A   
   A   
   T   
    T    
   G   
   A   
   T   
   A   
   C   
   G   
   C   
   A   
   A   
    G    
   T   
    C    
   T   
   T   
   A   
   G   
   T   
   G   
   G   
   C   
    A    
   C   
   A   
   A   
   G   
   A   
   A   
   G   
   A   
   C   
   G   
   T   
   A   
   A   
   T   
    T    
   G   
   T   
   C   
   A   
   G   
   T   
   G   
   T   
    G    
   A   
   C   
    G    
   C   
   G   
   A   
   G   
   A   
   A   
   G   
   G   
   C   
   T   
   A   
   T   
   G   
   T   
   G   
   A   
   A   
   G   
   C   
   G   
   A   
   A   
   C   
   A   
   A   
   G   
   C   
   C   
   T   
   T   
   C   
   G   
   T   
   T   
   C   
    T    
   T   
   A   
   T   
   A   
   G   
   T   
   G   
   C   
   T   
   T   
   C   
    C    
   A   
   A   
   A   
   C   
   C   
   A   
   G   
   A   
   A   
   G   
   A   
   A   
   A   
   T   
   C   
   G   
   G   
   G   
   A   
   T   
   G   
   C   
   G   
   A   
   G   
   A   
   A   
   G   
   G   
   C   
   G   
   A   
    C    
   T   
   A   
   C   
   T   
   T   
   G   
   C   
   T   
    A    
   T   
   A   
   C   
    T    
   C   
   A   
   G   
   G   
   T   
   G   
   A   
   A   
   T   
   T   
    G    
   A   
   G   
    T    
   A   
   C   
   A   
    T    
   T   
   G   
   G   
   A   
   C   
   C   
   A   
   T   
   G   
   T   
   T   
   C   
   T   
   T   
   C   
   T   
   G   
   A   
   T   
    C    
   A   
   C   
    A    
   A   
   A   
   T   
   A   
   A   
   A   
   G   
   C   
   A   
   A   
   A   
   T   
   G   
   T   
   G   
   A   
   T   
   C   
   T   
   A   
   C   
   C   
   G   
    C    
   C   
   C   
   T   
   G   
   T   
    C    
   C   
   A   
   T   
   G   
   A   
   A   
   C   
   T   
   A   
   C   
   C   
    C    
   G   
   A   
   T   
   T   
   T   
   G   
   A   
   A   
   A   
   T   
   G   
   G   
   A   
   A   
   A   
   G   
   A   
   T   
   G   
   C   
   T   
   G   
   G   
    T    
   G   
   A   
    G    
   C   
   A   
    C    
   A   
   T   
   T   
   T   
   C   
   T   
   C   
   A   
   A   
   A   
   C   
   A   
   A   
   T   
   C   
   T   
   C   
   C   
   A   
   A   
   T   
   C   
   T   
   G   
   T   
   C   
   T   
   G   
   T   
   A   
   G   
   A   
    C    
   G   
   A   
   A   
   T   
   C   
   G   
   A   
   T   
    T    
   T   
   T   
   A   
   G   
   C   
   T   
   G   
   T   
   T   
   T   
   T   
    C    
   C   
   C   
   A   
   T   
   A   
   T   
   C   
   A   
   A   
   A   
   A   
   A   
   A   
   T   
    T    
   C   
   A   
   A   
   G   
   C   
   A   
   G   
   A   
    G    
   A   
   A   
   A   
   A   
   C   
   A   
   T   
   T   
   T   
   G   
   T   
    A    
   T   
   T   
   C   
   A   
   T   
   C   
   A   
   G   
   C   
   A   
   A   
   G   
   A   
   A   
   T   
   G   
   G   
   A   
   T   
   T   
    G    
    A    
   T   
   C   
   A   
   A   
   G   
   C   
   A   
   A   
   A   
   C   
   A   
   C   
   G   
   A   
   A   
   T   
   G   
   A   
   C   
   A   
   G   
   A   
   G   
   T   
   T   
   C   
   G   
   A   
   A   
   C   
   C   
   A   
   T   
   G   
   G   
   C   
   G   
   C   
   A   
   C   
   C   
   T   
   A   
    T    
   A   
   A   
   G   
   A   
   G   
   C   
   C   
   G   
    T    
   C   
   C   
   G   
   C   
   T   
   C   
   A   
   G   
   C   
   T   
   G   
   T   
   A   
   T   
   G   
   A   
   A   
   G   
   C   
   T   
   A   
   A   
   A   
   G   
   G   
   C   
   G   
   G   
   A   
   A   
   G   
   A   
   A   
   G   
   A   
   T   
   G   
   A   
   A   
   C   
   T   
    G    
   A   
   T   
   T   
   G   
   C   
   T   
   G   
   T   
    G    
   T   
   A   
   T   
   C   
   T   
   G   
   A   
   A   
   A   
   G   
   A   
   A   
   G   
   A   
   T   
   C   
    A    
   G   
   C   
   C   
   A   
   G   
   G   
   C   
   T   
   T   
   A   
   G   
   A   
   T   
   G   
   T   
    T    
   T   
   T   
   C   
   T   
   T   
   A   
   G   
   T   
   A   
   A   
   C   
   G   
    C    
   A   
   T   
   C   
   A   
   A   
   G   
   G   
   A   
   A   
   T   
   G   
   G   
   G   
    T    
   T   
   T   
   G   
   C   
   G   
   C   
   T   
    A    
   T   
   C   
   C   
   T   
   T   
   T   
   A   
   A   
    C    
   A   
   G   
   A   
   T   
   G   
   T   
   T   
   C   
   C   
   C   
   G   
   T   
   T   
   G   
   T   
   T   
   G   
   G   
   A   
   G   
   C   
   A   
   A   
   A   
   G   
   G   
   C   
   A   
   G   
    C    
   T   
   G   
   G   
   A   
   G   
   T   
   G   
   A   
   A   
   G   
   T   
   C   
   T   
   A   
   T   
   G   
   A   
   A   
   T   
   T   
   T   
   G   
   A   
   A   
   A   
   G   
   A   
   T   
   G   
   G   
   A   
   G   
   A   
   T   
   A   
   G   
   G   
    A    
   T   
   T   
    A    
   T   
   C   
   A   
   A   
    C    
   G   
   G   
    G    
   C   
   T   
    G    
   C   
   T   
   T   
   G   
   T   
   G   
   T   
   T   
   A   
   G   
   C   
   A   
   G   
   A   
    A    
   G   
   G   
    T    
   G   
   A   
   T   
   A   
   C   
   G   
   C   
   C   
    G    
   A   
   T   
   C   
   G   
   T   
   A   
   A   
   T   
   C   
   G   
   T   
   T   
   A   
   C   
   T   
   C   
   A   
   G   
   C   
   G   
   A   
   G   
   G   
   A   
   G   
   C   
    T    
   G   
   T   
    T    
   A   
   A   
   A   
   C   
   G   
   C   
   A   
   T   
   G   
   C   
   T   
   T   
   G   
   C   
   T   
   C   
   A   
   A   
   G   
   A   
   A   
   A   
   T   
   C   
   A   
   G   
   C   
   C   
   A   
   A   
   A   
   C   
    T    
   T   
   C   
   A   
   C   
   G   
   T   
   G   
   C   
   G   
   A   
   A   
   G   
   C   
   G   
   G   
   G   
   G   
   A   
   G   
   T   
   G   
   A   
   C   
   G   
   G   
   T   
   C   
   A   
   T   
   G   
   C   
   G   
   T   
   G   
   A   
   G   
   C   
   T   
   G   
   A   
   A   
   A   
   A   
   A   
   A   
   A   
   A   
   T   
   C   
   C   
    G    
   C   
   A   
    C    
   C   
   G   
    T    
   A   
   T   
   T   
   A   
   T   
   C   
   T   
   A   
   T   
   A   
   T   
   G   
   T   
   C   
   G   
   G   
   A   
   A   
   G   
   G   
   A   
   A   
   G   
   A   
   T   
   C   
   A   
   A   
   A   
   A   
   G   
   A   
   A   
   A   
   T   
   A   
   A   
   C   
   C   
   T   
   T   
   G   
   A   
   T   
   G   
   A   
   A   
   T   
   C   
   A   
   A   
   A   
   A   
   A   
   G   
   G   
    G    
   C   
   A   
    G    
   C   
   A   
   G   
    T    
   T   
   A   
   A   
   C   
   G   
   A   
   T   
   T   
   G   
   A   
    C    
   C   
   C   
   A   
   A   
   C   
    T    
   G   
   A   
   T   
   T   
   T   
    C    
   C   
   C   
   T   
   A   
   T   
   A   
   G   
   G   
   T   
   G   
   A   
   T   
   C   
   G   
   A   
   A   
   C   
   A   
   T   
   C   
   G   
   A   
   A   
   T   
   G   
   G   
    A    
   T   
   C   
    A    
   T   
   T   
   T   
   G   
   C   
    G    
   A   
   T   
   G   
   G   
   A   
    T    
   G   
   A   
   G   
   A   
   A   
   A   
   A   
   A   
   A   
   G   
   G   
   T   
   G   
   G   
   A   
   G   
   A   
   A   
   G   
   T   
   C   
   A   
   T   
   C   
   G   
   A   
   A   
   G   
   T   
    T    
   A   
    T    
   C   
   G   
   A   
   T   
   G   
   C   
   T   
   C   
   C   
   T   
   G   
   A   
   G   
    A    
   T   
   T   
   T   
   T   
    C    
   A   
   T   
    C    
   G   
   A   
    G    
    T    
   C   
    G    
   -   
   -   
   -   
 

 
   N53595   
   A   
   T   
   G   
   G   
   A   
   A   
   A   
   A   
   T   
   C   
   G   
   A   
   C   
   A   
   A   
   G   
   A   
   A   
   G   
   T   
   A   
   C   
   A   
   A   
   G   
   A   
   A   
   T   
   T   
   A   
   A   
   C   
   T   
   T   
   T   
   A   
   G   
   A   
   A   
   G   
   A   
   A   
   G   
   T   
   G   
   A   
   T   
   G   
   G   
   G   
   T   
   G   
   A   
   C   
   C   
   G   
   C   
   T   
   T   
   C   
   G   
   G   
   A   
   C   
   G   
   T   
   T   
   A   
   T   
   T   
   C   
   C   
   A   
   A   
   A   
   T   
   A   
   T   
   A   
   T   
   T   
   A   
   T   
    C    
   C   
   A   
    A    
   G   
   A   
   A   
   C   
   G   
   T   
   G   
   C   
   A   
   C   
   T   
   G   
   C   
   C   
   T   
   G   
   A   
   T   
   A   
   T   
    C    
   C   
   G   
   T   
   G   
   A   
   T   
   G   
   G   
   A   
   T   
   T   
   G   
   A   
   A   
    G    
   C   
   C   
   A   
   G   
   T   
    G    
   C   
   A   
   A   
   C   
   G   
   C   
   C   
   G   
   G   
   A   
   T   
   A   
   T   
   T   
   A   
   T   
   T   
    T    
   G   
   C   
    C    
   A   
   T   
   G   
   A   
   A   
   C   
   A   
   A   
   A   
   G   
   A   
   T   
   G   
   G   
   C   
   A   
   A   
   T   
   A   
   C   
   G   
   T   
   A   
   T   
   G   
   A   
   C   
   A   
   A   
   A   
   G   
   G   
   A   
   T   
   T   
    C    
   C   
   G   
   T   
   A   
   A   
   A   
   T   
   C   
   A   
   G   
   C   
   A   
   A   
   A   
   A   
   T   
   C   
   T   
   G   
   T   
   C   
   G   
   G   
   A   
   A   
   A   
   T   
   A   
   T   
   C   
   A   
   T   
   G   
   G   
   G   
    G    
   A   
   A   
   T   
   T   
   A   
   T   
   C   
   A   
   T   
   C   
   C   
   T   
   C   
   A   
   T   
   G   
   G   
   T   
   G   
   A   
   C   
   A   
   G   
   C   
   A   
   G   
   T   
   A   
   T   
   T   
   T   
   A   
   T   
   G   
   A   
   A   
   G   
   C   
   G   
   A   
   T   
   G   
   G   
   T   
   C   
   C   
   G   
   C   
   A   
   T   
   G   
   A   
   G   
   C   
   C   
   A   
   A   
   G   
   A   
    C    
   T   
   G   
   G   
   A   
   A   
   G   
   T   
   T   
   G   
   C   
   G   
   G   
   G   
   A   
   A   
   G   
   T   
   A   
   C   
   T   
   G   
   A   
   T   
   C   
   G   
   A   
    A    
   A   
   T   
   G   
   C   
   A   
   C   
   G   
   G   
    G    
   A   
   A   
   T   
   A   
   A   
    C    
   G   
   G   
   G   
   A   
   G   
   T   
   A   
   T   
   G   
   G   
   A   
   C   
   G   
   G   
   T   
   G   
   A   
   T   
   C   
   C   
   A   
   C   
   C   
   T   
   G   
   C   
   T   
   G   
   C   
   G   
   A   
   T   
   G   
   C   
   G   
   T   
   T   
   A   
   T   
   A   
   C   
   T   
   G   
   A   
   A   
   G   
   C   
   C   
   A   
   G   
    A    
   T   
   T   
   A   
   T   
   C   
   C   
   A   
   A   
   G   
   T   
   T   
   A   
   A   
   G   
   T   
   G   
   G   
   A   
   G   
   A   
   G   
   A   
   T   
   G   
    C    
   T   
   A   
    G    
   C   
   A   
   G   
   A   
   T   
   A   
   T   
   C   
   G   
   A   
    A    
   A   
   A   
   A   
   G   
   A   
   A   
   A   
   C   
   A   
   G   
   T   
    C    
   G   
   A   
   T   
   C   
   T   
   C   
   G   
   T   
   G   
   T   
   G   
   G   
   A   
   A   
   T   
   T   
   T   
   T   
   G   
   A   
   T   
   G   
   A   
   T   
   A   
   C   
   A   
   G   
   A   
   A   
   A   
   A   
   A   
   G   
   A   
   A   
   C   
   C   
   A   
   A   
   C   
   A   
   G   
   T   
   C   
   C   
   T   
   T   
   C   
   C   
   A   
   G   
   C   
   T   
   C   
   G   
   A   
   T   
   A   
   C   
   C   
   C   
   A   
   A   
   A   
   C   
   C   
   T   
   T   
   C   
   T   
    C    
   G   
   T   
   C   
   A   
   A   
   T   
   G   
   G   
   A   
   T   
   C   
   G   
   A   
   C   
   A   
   G   
   G   
   T   
   A   
   T   
   A   
   T   
   C   
   T   
   G   
   C   
   T   
   G   
   G   
   A   
   T   
   A   
   T   
   G   
   C   
    A    
   A   
   C   
   A   
   G   
   A   
   A   
   A   
   T   
   C   
   C   
   C   
   G   
   A   
   C   
    T    
   C   
   A   
   T   
   A   
   A   
   C   
   T   
   T   
   A   
   G   
   C   
   T   
   G   
   A   
   A   
   G   
   T   
   G   
   A   
   T   
    T    
   G   
   A   
    T    
   G   
   G   
   C   
   A   
   C   
   A   
   A   
   T   
   C   
   T   
   A   
   T   
   A   
   T   
   G   
   A   
   T   
   C   
   G   
   A   
   T   
   C   
   A   
   T   
   C   
   C   
   A   
   C   
   A   
   A   
   G   
   C   
   G   
   A   
   G   
   C   
   C   
   T   
   T   
   G   
   A   
   A   
   A   
   A   
   A   
   T   
   T   
   G   
   A   
   T   
   G   
   G   
   A   
   A   
   T   
   A   
   T   
   A   
   T   
   T   
   C   
   C   
   T   
   G   
   G   
   A   
   C   
   C   
   T   
   G   
   A   
   C   
   T   
   T   
    T    
   C   
   C   
   A   
   A   
   C   
   A   
   G   
   G   
   A   
   G   
   G   
    G    
   A   
   T   
   C   
   T   
   T   
   G   
   C   
   A   
   A   
   G   
   G   
    A    
   A   
   A   
   A   
   G   
   A   
   T   
   G   
   A   
   A   
   A   
   T   
   C   
   A   
   A   
   A   
   A   
   A   
   A   
   G   
   C   
   T   
   T   
   A   
   T   
   G   
   A   
   A   
   A   
   C   
   A   
   G   
   G   
    G    
   C   
   G   
   A   
   G   
   G   
   A   
   A   
   A   
   A   
   G   
   T   
   T   
   A   
    T    
   T   
   C   
   T   
   T   
   C   
   G   
   T   
   T   
   C   
   G   
   A   
   A   
   A   
   A   
   C   
   A   
   A   
   A   
   G   
   A   
   T   
   A   
   G   
   A   
    G    
   G   
   C   
   A   
   A   
   T   
   G   
   A   
   A   
   G   
   G   
   G   
    T    
   A   
   A   
    T    
   A   
   A   
   A   
   C   
   A   
   G   
   C   
   A   
   A   
   A   
   T   
   C   
   G   
   T   
   G   
   A   
   T   
   C   
   A   
   C   
    A    
   G   
   A   
   A   
   A   
   T   
   C   
   C   
   C   
   A   
   T   
   A   
   T   
   G   
   A   
   A   
   G   
   T   
   A   
   A   
   A   
   T   
   A   
   A   
   A   
   G   
   C   
   G   
   A   
   C   
    C    
   T   
   T   
   G   
   G   
   T   
   G   
   A   
   A   
   A   
   A   
   A   
   A   
   A   
   T   
   G   
   G   
   A   
   T   
   G   
   A   
   G   
   A   
   T   
   C   
   C   
   G   
   T   
   T   
   T   
   G   
   A   
   A   
   T   
   A   
   A   
   G   
   A   
   A   
   A   
   A   
   T   
   C   
   G   
   A   
    T    
   G   
   G   
   A   
   A   
   T   
   A   
   G   
   C   
   T   
   G   
   A   
   A   
   G   
   T   
    A    
   C   
   G   
   A   
   G   
   A   
   T   
   G   
   A   
   A   
   A   
   G   
   T   
   G   
   A   
   T   
    A    
   G   
    A    
   A   
   C   
    A    
   G   
   G   
   T   
   C   
   T   
   G   
   C   
   A   
   A   
   A   
   T   
   T   
   G   
   T   
   C   
   G   
   T   
    C    
   G   
   A   
   A   
   T   
   T   
   G   
   A   
   A   
   A   
   A   
   A   
   A   
   G   
   A   
    C    
   G   
   C   
   C   
   A   
   A   
    T    
   G   
   C   
   T   
   C   
   A   
   A   
   G   
   G   
    A    
   A   
   T   
   T   
   T   
   T   
   G   
   A   
   A   
   T   
   T   
   A   
    T    
   T   
   T   
   G   
   T   
   T   
   C   
   A   
   A   
   A   
   A   
   A   
   C   
   A   
   C   
   T   
   G   
   A   
   G   
    C    
   T   
   A   
   C   
   A   
   G   
   A   
   T   
   C   
   A   
   A   
   T   
   T   
   A   
   C   
   A   
   A   
   T   
   T   
   T   
    C    
   A   
   A   
   T   
   A   
   T   
   G   
   G   
   T   
   C   
   G   
   C   
   T   
   A   
   T   
   C   
   G   
   A   
    C    
   C   
   A   
   T   
   A   
   T   
   G   
   A   
   C   
   T   
   C   
   C   
   G   
   C   
   A   
    T    
   C   
   A   
   A   
   G   
   T   
   T   
   G   
   G   
   A   
   C   
   T   
   G   
   A   
   A   
   A   
   G   
   A   
   C   
   A   
   T   
   T   
   T   
   T   
   A   
   A   
   G   
   A   
   A   
   G   
   T   
   T   
   A   
   T   
   A   
   T   
   C   
   G   
   A   
   G   
   C   
   A   
   T   
   C   
   G   
   A   
   A   
   A   
   G   
   C   
   A   
   A   
   G   
   T   
   C   
   A   
   T   
   A   
   A   
   C   
   A   
   A   
   A   
   A   
   C   
   G   
    T    
   A   
   G   
    T    
   C   
   A   
   A   
   T   
   T   
   C   
   G   
   A   
   T   
   T   
   T   
   A   
   G   
   C   
   C   
   A   
   A   
   A   
   G   
   C   
   T   
   C   
   A   
   A   
   A   
   A   
   A   
    A    
   G   
   A   
   C   
   A   
   G   
   C   
   A   
   T   
   A   
   T   
   C   
   G   
   T   
    A    
   G   
   A   
   A   
   G   
   G   
    A    
    T    
   T   
   G   
   A   
   T   
   G   
   A   
   A   
   A   
   G   
   C   
   T   
   T   
   T   
   A   
   T   
   C   
   G   
   A   
   T   
   T   
   C   
   T   
   G   
   G   
   A   
   T   
   G   
   A   
   A   
   G   
   T   
   A   
   A   
   T   
   C   
   G   
    T    
   A   
   A   
   C   
   G   
   A   
   T   
   T   
   C   
   G   
   T   
   G   
   A   
   A   
   A   
   G   
   T   
   A   
   A   
   A   
   G   
   A   
   C   
   A   
   A   
   A   
   A   
   A   
   A   
   G   
   A   
    C    
   G   
   C   
    C    
   A   
   A   
   A   
   A   
   A   
   G   
   A   
   A   
   T   
   T   
   T   
   A   
   G   
   T   
   C   
   G   
   A   
    C    
   G   
   T   
    C    
   T   
   T   
    T    
   C   
   A   
   A   
   T   
   T   
   T   
   A   
   C   
   A   
   G   
   A   
   A   
   G   
   A   
   A   
   C   
   A   
   A   
   G   
   C   
   A   
   G   
   A   
   A   
   G   
   C   
   A   
   A   
   T   
    T    
   G   
   T   
   C   
   A   
   C   
   A   
   T   
   T   
   G   
   C   
   A   
   A   
    T    
   T   
   A   
   T   
   A   
    C    
   C   
   G   
   T   
   T   
   T   
   G   
   A   
   C   
   G   
   A   
   A   
    C    
   A   
   C   
    G    
   G   
   A   
   T   
   A   
   T   
   C   
   A   
   C   
   A   
   G   
   A   
   G   
   C   
   T   
   T   
   C   
   A   
   A   
   A   
   A   
   G   
   G   
   A   
   A   
    T    
   C   
    C    
   G   
   A   
   A   
   A   
    G    
   C   
   T   
   T   
   G   
   A   
   T   
   T   
   G   
   C   
    G    
   C   
   A   
   A   
   A   
   T   
   C   
   A   
   C   
   A   
   G   
   A   
   G   
   T   
   T   
    G    
   A   
   A   
   T   
   A   
   A   
   A   
   A   
   T   
   C   
   C   
   T   
   T   
   T   
   C   
   A   
   A   
   A   
    T    
   G   
   A   
   C   
   A   
   A   
   A   
   G   
   A   
    G    
   C   
   T   
   T   
   T   
   T   
    C    
   T   
   C   
   T   
   G   
   T   
   T   
   A   
   T   
   G   
   A   
   A   
   A   
   A   
   A   
   A   
   G   
   A   
   A   
   T   
   T   
   G   
   C   
   G   
   A   
   G   
   A   
   A   
   G   
   T   
   G   
   A   
   A   
   A   
   A   
   A   
   A   
   A   
   A   
   T   
   T   
   A   
    T    
   T   
   C   
   T   
   T   
   C   
   C   
   G   
   C   
   T   
   C   
   G   
   T   
   C   
    T    
    A    
   A   
   C   
   G   
   A   
   C   
   A   
   A   
   T   
   C   
    G    
   A   
   A   
   G   
   A   
   C   
   G   
   A   
   A   
   A   
   T   
   C   
   G   
   A   
   A   
   G   
   A   
   A   
   A   
   T   
   C   
   A   
   A   
   A   
   A   
   T   
    T    
   G   
   A   
   T   
   A   
   C   
   G   
   C   
   A   
   A   
    G    
   T   
    C    
   T   
   T   
   A   
   G   
   T   
   G   
   G   
   C   
    A    
   C   
   A   
   A   
   G   
   A   
   A   
   G   
   A   
   C   
   G   
   T   
   A   
   A   
   T   
    T    
   G   
   T   
   C   
   A   
   G   
   T   
   G   
   T   
    G    
   A   
   C   
    G    
   C   
   G   
   A   
   G   
   A   
   A   
   G   
   G   
   C   
   T   
   A   
   T   
   G   
   T   
   G   
   A   
   A   
   G   
   C   
   G   
   A   
   A   
   C   
   A   
   A   
   G   
   C   
   C   
   T   
   T   
   C   
   G   
   T   
   T   
   C   
    T    
   T   
   A   
   T   
   A   
   G   
   T   
   G   
   C   
   T   
   T   
   C   
    C    
   A   
   A   
   A   
   C   
   C   
   A   
   G   
   A   
   A   
   G   
   A   
   A   
   A   
   T   
   C   
   G   
   G   
   G   
   A   
   T   
   G   
   C   
   G   
   A   
   G   
   A   
   A   
   G   
   G   
   C   
   G   
   A   
    C    
   T   
   A   
   C   
   T   
   T   
   G   
   C   
   T   
    A    
   T   
   A   
   C   
    T    
   C   
   A   
   G   
   G   
   T   
   G   
   A   
   A   
   T   
   T   
    G    
   A   
   G   
    T    
   A   
   C   
   A   
    T    
   T   
   G   
   G   
   A   
   C   
   C   
   A   
   T   
   G   
   T   
   T   
   C   
   T   
   T   
   C   
   T   
   G   
   A   
   T   
    C    
   A   
   C   
    A    
   A   
   A   
   T   
   A   
   A   
   A   
   G   
   C   
   A   
   A   
   A   
   T   
   G   
   T   
   G   
   A   
   T   
   C   
   T   
   A   
   C   
   C   
   G   
    C    
   C   
   C   
   T   
   G   
   T   
    C    
   C   
   A   
   T   
   G   
   A   
   A   
   C   
   T   
   A   
   C   
   C   
    C    
   G   
   A   
   T   
   T   
   T   
   G   
   A   
   A   
   A   
   T   
   G   
   G   
   A   
   A   
   A   
   G   
   A   
   T   
   G   
   C   
   T   
   G   
   G   
    T    
   G   
   A   
    G    
   C   
   A   
    C    
   A   
   T   
   T   
   T   
   C   
   T   
   C   
   A   
   A   
   A   
   C   
   A   
   A   
   T   
   C   
   T   
   C   
   C   
   A   
   A   
   T   
   C   
   T   
   G   
   T   
   C   
   T   
   G   
   T   
   A   
   G   
   A   
    C    
   G   
   A   
   A   
   T   
   C   
   G   
   A   
   T   
    T    
   T   
   T   
   A   
   G   
   C   
   T   
   G   
   T   
   T   
   T   
   T   
    C    
   C   
   C   
   A   
   T   
   A   
   T   
   C   
   A   
   A   
   A   
   A   
   A   
   A   
   T   
    T    
   C   
   A   
   A   
   G   
   C   
   A   
   G   
   A   
    G    
   A   
   A   
   A   
   A   
   C   
   A   
   T   
   T   
   T   
   G   
   T   
    A    
   T   
   T   
   C   
   A   
   T   
   C   
   A   
   G   
   C   
   A   
   A   
   G   
   A   
   A   
   T   
   G   
   G   
   A   
   T   
   T   
    G    
    A    
   T   
   C   
   A   
   A   
   G   
   C   
   A   
   A   
   A   
   C   
   A   
   C   
   G   
   A   
   A   
   T   
   G   
   A   
   C   
   A   
   G   
   A   
   G   
   T   
   T   
   C   
   G   
   A   
   A   
   C   
   C   
   A   
   T   
   G   
   G   
   C   
   G   
   C   
   A   
   C   
   C   
   T   
   A   
    T    
   A   
   A   
   G   
   A   
   G   
   C   
   C   
   G   
    T    
   C   
   C   
   G   
   C   
   T   
   C   
   A   
   G   
   C   
   T   
   G   
   T   
   A   
   T   
   G   
   A   
   A   
   G   
   C   
   T   
   A   
   A   
   A   
   G   
   G   
   C   
   G   
   G   
   A   
   A   
   G   
   A   
   A   
   G   
   A   
   T   
   G   
   A   
   A   
   C   
   T   
    G    
   A   
   T   
   T   
   G   
   C   
   T   
   G   
   T   
    G    
   T   
   A   
   T   
   C   
   T   
   G   
   A   
   A   
   A   
   G   
   A   
   A   
   G   
   A   
   T   
   C   
    A    
   G   
   C   
   C   
   A   
   G   
   G   
   C   
   T   
   T   
   A   
   G   
   A   
   T   
   G   
   T   
    T    
   T   
   T   
   C   
   T   
   T   
   A   
   G   
   T   
   A   
   A   
   C   
   G   
    C    
   A   
   T   
   C   
   A   
   A   
   G   
   G   
   A   
   A   
   T   
   G   
   G   
   G   
    T    
   T   
   T   
   G   
   C   
   G   
   C   
   T   
    A    
   T   
   C   
   C   
   T   
   T   
   T   
   A   
   A   
    C    
   A   
   G   
   A   
   T   
   G   
   T   
   T   
   C   
   C   
   C   
   G   
   T   
   T   
   G   
   T   
   T   
   G   
   G   
   A   
   G   
   C   
   A   
   A   
   A   
   G   
   G   
   C   
   A   
   G   
    C    
   T   
   G   
   G   
   A   
   G   
   T   
   G   
   A   
   A   
   G   
   T   
   C   
   T   
   A   
   T   
   G   
   A   
   A   
   T   
   T   
   T   
   G   
   A   
   A   
   A   
   G   
   A   
   T   
   G   
   G   
   A   
   G   
   A   
   T   
   A   
   G   
   G   
    A    
   T   
   T   
    A    
   T   
   C   
   A   
   A   
    C    
   G   
   G   
    G    
   C   
   T   
    G    
   C   
   T   
   T   
   G   
   T   
   G   
   T   
   T   
   A   
   G   
   C   
   A   
   G   
   A   
    A    
   G   
   G   
    T    
   G   
   A   
   T   
   A   
   C   
   G   
   C   
   C   
    G    
   A   
   T   
   C   
   G   
   T   
   A   
   A   
   T   
   C   
   G   
   T   
   T   
   A   
   C   
   T   
   C   
   A   
   G   
   C   
   G   
   A   
   G   
   G   
   A   
   G   
   C   
    T    
   G   
   T   
    T    
   A   
   A   
   A   
   C   
   G   
   C   
   A   
   T   
   G   
   C   
   T   
   T   
   G   
   C   
   T   
   C   
   A   
   A   
   G   
   A   
   A   
   A   
   T   
   C   
   A   
   G   
   C   
   C   
   A   
   A   
   A   
   C   
    T    
   T   
   C   
   A   
   C   
   G   
   T   
   G   
   C   
   G   
   A   
   A   
   G   
   C   
   G   
   G   
   G   
   G   
   A   
   G   
   T   
   G   
   A   
   C   
   G   
   G   
   T   
   C   
   A   
   T   
   G   
   C   
   G   
   T   
   G   
   A   
   G   
   C   
   T   
   G   
   A   
   A   
   A   
   A   
   A   
   A   
   A   
   A   
   T   
   C   
   C   
    G    
   C   
   A   
    C    
   C   
   G   
    T    
   A   
   T   
   T   
   A   
   T   
   C   
   T   
   A   
   T   
   A   
   T   
   G   
   T   
   C   
   G   
   G   
   A   
   A   
   G   
   G   
   A   
   A   
   G   
   A   
   T   
   C   
   A   
   A   
   A   
   A   
   G   
   A   
   A   
   A   
   T   
   A   
   A   
   C   
   C   
   T   
   T   
   G   
   A   
   T   
   G   
   A   
   A   
   T   
   C   
   A   
   A   
   A   
   A   
   A   
   G   
   G   
    G    
   C   
   A   
    G    
   C   
   A   
   G   
    T    
   T   
   A   
   A   
   C   
   G   
   A   
   T   
   T   
   G   
   A   
    C    
   C   
   C   
   A   
   A   
   C   
    T    
   G   
   A   
   T   
   T   
   T   
    C    
   C   
   C   
   T   
   A   
   T   
   A   
   G   
   G   
   T   
   G   
   A   
   T   
   C   
   G   
   A   
   A   
   C   
   A   
   T   
   C   
   G   
   A   
   A   
   T   
   G   
   G   
    A    
   T   
   C   
    A    
   T   
   T   
   T   
   G   
   C   
    G    
   A   
   T   
   G   
   G   
   A   
    T    
   G   
   A   
   G   
   A   
   A   
   A   
   A   
   A   
   A   
   G   
   G   
   T   
   G   
   G   
   A   
   G   
   A   
   A   
   G   
   T   
   C   
   A   
   T   
   C   
   G   
   A   
   A   
   G   
   T   
    T    
   A   
    T    
   C   
   G   
   A   
   T   
   G   
   C   
   T   
   C   
   C   
   T   
   G   
   A   
   G   
    A    
   T   
   T   
   T   
   T   
    C    
   A   
   T   
    C    
   G   
   A   
    G    
    T    
   C   
    G    
   -   
   -   
   -   
 

 
   N59567F   
   A   
   T   
   G   
   G   
   A   
   A   
   A   
   A   
   T   
   C   
   G   
   A   
   C   
   A   
   A   
   G   
   A   
   A   
   G   
   T   
   A   
   C   
   A   
   A   
   G   
   A   
   A   
   T   
   T   
   A   
   A   
   C   
   T   
   T   
   T   
   A   
   G   
   A   
   A   
   G   
   A   
   A   
   G   
   T   
   G   
   A   
   T   
   G   
   G   
   G   
   T   
   G   
   A   
   C   
   C   
   G   
   C   
   T   
   T   
   C   
   G   
   G   
   A   
   C   
   G   
   T   
   T   
   A   
   T   
   T   
   C   
   C   
   A   
   A   
   A   
   T   
   A   
   T   
   A   
   T   
   T   
   A   
   T   
    C    
   C   
   A   
    A    
   G   
   A   
   A   
   C   
   G   
   T   
   G   
   C   
   A   
   C   
   T   
   G   
   C   
   C   
   T   
   G   
   A   
   T   
   A   
   T   
    T    
   C   
   G   
   T   
   G   
   A   
   T   
   G   
   G   
   A   
   T   
   T   
   G   
   A   
   A   
    G    
   C   
   C   
   A   
   G   
   T   
    G    
   C   
   A   
   A   
   C   
   G   
   C   
   C   
   G   
   G   
   A   
   T   
   A   
   T   
   T   
   A   
   T   
   T   
    T    
   G   
   C   
    C    
   A   
   T   
   G   
   A   
   A   
   C   
   A   
   A   
   A   
   G   
   A   
   T   
   G   
   G   
   C   
   A   
   A   
   T   
   A   
   C   
   G   
   T   
   A   
   T   
   G   
   A   
   C   
   A   
   A   
   A   
   G   
   G   
   A   
   T   
   T   
    C    
   C   
   G   
   T   
   A   
   A   
   A   
   T   
   C   
   A   
   G   
   C   
   A   
   A   
   A   
   A   
   T   
   C   
   T   
   G   
   T   
   C   
   G   
   G   
   A   
   A   
   A   
   T   
   A   
   T   
   C   
   A   
   T   
   G   
   G   
   G   
    G    
   A   
   A   
   T   
   T   
   A   
   T   
   C   
   A   
   T   
   C   
   C   
   T   
   C   
   A   
   T   
   G   
   G   
   T   
   G   
   A   
   C   
   A   
   G   
   C   
   A   
   G   
   T   
   A   
   T   
   T   
   T   
   A   
   T   
   G   
   A   
   A   
   G   
   C   
   G   
   A   
   T   
   G   
   G   
   T   
   C   
   C   
   G   
   C   
   A   
   T   
   G   
   A   
   G   
   C   
   C   
   A   
   A   
   G   
   A   
    C    
   T   
   G   
   G   
   A   
   A   
   G   
   T   
   T   
   G   
   C   
   G   
   G   
   G   
   A   
   A   
   G   
   T   
   A   
   C   
   T   
   G   
   A   
   T   
   C   
   G   
   A   
    A    
   A   
   T   
   G   
   C   
   A   
   C   
   G   
   G   
    G    
   A   
   A   
   T   
   A   
   A   
    C    
   G   
   G   
   G   
   A   
   G   
   T   
   A   
   T   
   G   
   G   
   A   
   C   
   G   
   G   
   T   
   G   
   A   
   T   
   C   
   C   
   A   
   C   
   C   
   T   
   G   
   C   
   T   
   G   
   C   
   G   
   A   
   T   
   G   
   C   
   G   
   T   
   T   
   A   
   T   
   A   
   C   
   T   
   G   
   A   
   A   
   G   
   C   
   C   
   A   
   G   
    A    
   T   
   T   
   A   
   T   
   C   
   C   
   A   
   A   
   G   
   T   
   T   
   A   
   A   
   G   
   T   
   G   
   G   
   A   
   G   
   A   
   G   
   A   
   T   
   G   
    C    
   T   
   A   
    G    
   C   
   A   
   G   
   A   
   T   
   A   
   T   
   C   
   G   
   A   
    A    
   A   
   A   
   A   
   G   
   A   
   A   
   A   
   C   
   A   
   G   
   T   
    C    
   G   
   A   
   T   
   C   
   T   
   C   
   G   
   T   
   G   
   T   
   G   
   G   
   A   
   A   
   T   
   T   
   T   
   T   
   G   
   A   
   T   
   G   
   A   
   T   
   A   
   C   
   A   
   G   
   A   
   A   
   A   
   A   
   A   
   G   
   A   
   A   
   C   
   C   
   A   
   A   
   C   
   A   
   G   
   T   
   C   
   C   
   T   
   T   
   C   
   C   
   A   
   G   
   C   
   T   
   C   
   G   
   A   
   T   
   A   
   C   
   C   
   C   
   A   
   A   
   A   
   C   
   C   
   T   
   T   
   C   
   T   
    C    
   G   
   T   
   C   
   A   
   A   
   T   
   G   
   G   
   A   
   T   
   C   
   G   
   A   
   C   
   A   
   G   
   G   
   T   
   A   
   T   
   A   
   T   
   C   
   T   
   G   
   C   
   T   
   G   
   G   
   A   
   T   
   A   
   T   
   G   
   C   
    A    
   A   
   C   
   A   
   G   
   A   
   A   
   A   
   T   
   C   
   C   
   C   
   G   
   A   
   C   
    T    
   C   
   A   
   T   
   A   
   A   
   C   
   T   
   T   
   A   
   G   
   C   
   T   
   G   
   A   
   A   
   G   
   T   
   G   
   A   
   T   
    C    
   G   
   A   
    T    
   G   
   G   
   C   
   A   
   C   
   A   
   A   
   T   
   C   
   T   
   A   
   T   
   A   
   T   
   G   
   A   
   T   
   C   
   G   
   A   
   T   
   C   
   A   
   T   
   C   
   C   
   A   
   C   
   A   
   A   
   G   
   C   
   G   
   A   
   G   
   C   
   C   
   T   
   T   
   G   
   A   
   A   
   A   
   A   
   A   
   T   
   T   
   G   
   A   
   T   
   G   
   G   
   A   
   A   
   T   
   A   
   T   
   A   
   T   
   T   
   C   
   C   
   T   
   G   
   G   
   A   
   C   
   C   
   T   
   G   
   A   
   C   
   T   
   T   
    T    
   C   
   C   
   A   
   A   
   C   
   A   
   G   
   G   
   A   
   G   
   G   
    G    
   A   
   T   
   C   
   T   
   T   
   G   
   C   
   A   
   A   
   G   
   G   
    A    
   A   
   A   
   A   
   G   
   A   
   T   
   G   
   A   
   A   
   A   
   T   
   C   
   A   
   A   
   A   
   A   
   A   
   A   
   G   
   C   
   T   
   T   
   A   
   T   
   G   
   A   
   A   
   A   
   C   
   A   
   G   
   G   
    G    
   C   
   G   
   A   
   G   
   G   
   A   
   A   
   A   
   A   
   G   
   T   
   T   
   A   
    T    
   T   
   C   
   T   
   T   
   C   
   G   
   T   
   T   
   C   
   G   
   A   
   A   
   A   
   A   
   C   
   A   
   A   
   A   
   G   
   A   
   T   
   A   
   G   
   A   
    A    
   G   
   C   
   A   
   A   
   T   
   G   
   A   
   A   
   G   
   G   
   G   
    T    
   A   
   A   
    T    
   A   
   A   
   A   
   C   
   A   
   G   
   C   
   A   
   A   
   A   
   T   
   C   
   G   
   T   
   G   
   A   
   T   
   C   
   A   
   C   
    A    
   G   
   A   
   A   
   A   
   T   
   C   
   C   
   C   
   A   
   T   
   A   
   T   
   G   
   A   
   A   
   G   
   T   
   A   
   A   
   A   
   T   
   A   
   A   
   A   
   G   
   C   
   G   
   A   
   C   
    C    
   T   
   T   
   G   
   G   
   T   
   G   
   A   
   A   
   A   
   A   
   A   
   A   
   A   
   T   
   G   
   G   
   A   
   T   
   G   
   A   
   G   
   A   
   T   
   C   
   C   
   G   
   T   
   T   
   T   
   G   
   A   
   A   
   T   
   A   
   A   
   G   
   A   
   A   
   A   
   A   
   T   
   C   
   G   
   A   
    T    
   G   
   G   
   A   
   A   
   T   
   A   
   G   
   C   
   T   
   G   
   A   
   A   
   G   
   T   
    A    
   C   
   G   
   A   
   G   
   A   
   T   
   G   
   A   
   A   
   A   
   G   
   T   
   G   
   A   
   T   
    A    
   G   
    A    
   A   
   C   
    A    
   G   
   G   
   T   
   C   
   T   
   G   
   C   
   A   
   A   
   A   
   T   
   T   
   G   
   T   
   C   
   G   
   T   
    C    
   G   
   A   
   A   
   T   
   T   
   G   
   A   
   A   
   A   
   A   
   A   
   A   
   G   
   A   
    C    
   G   
   C   
   C   
   A   
   A   
    T    
   G   
   C   
   T   
   C   
   A   
   A   
   G   
   G   
    A    
   A   
   T   
   T   
   T   
   T   
   G   
   A   
   A   
   T   
   T   
   A   
    T    
   T   
   T   
   G   
   T   
   T   
   C   
   A   
   A   
   A   
   A   
   A   
   C   
   A   
   C   
   T   
   G   
   A   
   G   
    C    
   T   
   A   
   C   
   A   
   G   
   A   
   T   
   C   
   A   
   A   
   T   
   T   
   A   
   C   
   A   
   A   
   T   
   T   
   T   
    C    
   A   
   A   
   T   
   A   
   T   
   G   
   G   
   T   
   C   
   G   
   C   
   T   
   A   
   T   
   C   
   G   
   A   
    C    
   C   
   A   
   T   
   A   
   T   
   G   
   A   
   C   
   T   
   C   
   C   
   G   
   C   
   A   
    T    
   C   
   A   
   A   
   G   
   T   
   T   
   G   
   G   
   A   
   C   
   T   
   G   
   A   
   A   
   A   
   G   
   A   
   C   
   A   
   T   
   T   
   T   
   T   
   A   
   A   
   G   
   A   
   A   
   G   
   T   
   T   
   A   
   T   
   A   
   T   
   C   
   G   
   A   
   G   
   C   
   A   
   T   
   C   
   G   
   A   
   A   
   A   
   G   
   C   
   A   
   A   
   G   
   T   
   C   
   A   
   T   
   A   
   A   
   C   
   A   
   A   
   A   
   A   
   C   
   G   
    T    
   A   
   G   
    T    
   C   
   A   
   A   
   T   
   T   
   C   
   G   
   A   
   T   
   T   
   T   
   A   
   G   
   C   
   C   
   A   
   A   
   A   
   G   
   C   
   T   
   C   
   A   
   A   
   A   
   A   
   A   
    A    
   G   
   A   
   C   
   A   
   G   
   C   
   A   
   T   
   A   
   T   
   C   
   G   
   T   
    A    
   G   
   A   
   A   
   G   
   G   
    A    
    T    
   T   
   G   
   A   
   T   
   G   
   A   
   A   
   A   
   G   
   C   
   T   
   T   
   T   
   A   
   T   
   C   
   G   
   A   
   T   
   T   
   C   
   T   
   G   
   G   
   A   
   T   
   G   
   A   
   A   
   G   
   T   
   A   
   A   
   T   
   C   
   G   
    C    
   A   
   A   
   C   
   G   
   A   
   T   
   T   
   C   
   G   
   T   
   G   
   A   
   A   
   A   
   G   
   T   
   A   
   A   
   A   
   G   
   A   
   C   
   A   
   A   
   A   
   A   
   A   
   A   
   G   
   A   
    C    
   G   
   C   
    C    
   A   
   A   
   A   
   A   
   A   
   G   
   A   
   A   
   T   
   T   
   T   
   A   
   G   
   T   
   C   
   G   
   A   
    C    
   G   
   T   
    C    
   T   
   T   
    T    
   C   
   A   
   A   
   T   
   T   
   T   
   A   
   C   
   A   
   G   
   A   
   A   
   G   
   A   
   A   
   C   
   A   
   A   
   G   
   C   
   A   
   G   
   A   
   A   
   G   
   C   
   A   
   A   
   T   
    T    
   G   
   T   
   C   
   A   
   C   
   A   
   T   
   T   
   G   
   C   
   A   
   A   
    T    
   T   
   A   
   T   
   A   
    C    
   C   
   G   
   T   
   T   
   T   
   G   
   A   
   C   
   G   
   A   
   A   
    C    
   A   
   C   
    G    
   G   
   A   
   T   
   A   
   T   
   C   
   A   
   C   
   A   
   G   
   A   
   G   
   C   
   T   
   T   
   C   
   A   
   A   
   A   
   A   
   G   
   G   
   A   
   A   
    T    
   C   
    C    
   G   
   A   
   A   
   A   
    G    
   C   
   T   
   T   
   G   
   A   
   T   
   T   
   G   
   C   
    G    
   C   
   A   
   A   
   A   
   T   
   C   
   A   
   C   
   A   
   G   
   A   
   G   
   T   
   T   
    G    
   A   
   A   
   T   
   A   
   A   
   A   
   A   
   T   
   C   
   C   
   T   
   T   
   T   
   C   
   A   
   A   
   A   
    T    
   G   
   A   
   C   
   A   
   A   
   A   
   G   
   A   
    G    
   C   
   T   
   T   
   T   
   T   
    C    
   T   
   C   
   T   
   G   
   T   
   T   
   A   
   T   
   G   
   A   
   A   
   A   
   A   
   A   
   A   
   G   
   A   
   A   
   T   
   T   
   G   
   C   
   G   
   A   
   G   
   A   
   A   
   G   
   T   
   G   
   A   
   A   
   A   
   A   
   A   
   A   
   A   
   A   
   T   
   T   
   A   
    T    
   T   
   C   
   T   
   T   
   C   
   C   
   G   
   C   
   T   
   C   
   G   
   T   
   C   
    T    
    A    
   A   
   C   
   G   
   A   
   C   
   A   
   A   
   T   
   C   
    G    
   A   
   A   
   G   
   A   
   C   
   G   
   A   
   A   
   A   
   T   
   C   
   G   
   A   
   A   
   G   
   A   
   A   
   A   
   T   
   C   
   A   
   A   
   A   
   A   
   T   
    T    
   G   
   A   
   T   
   A   
   C   
   G   
   C   
   A   
   A   
    G    
   T   
    C    
   T   
   T   
   A   
   G   
   T   
   G   
   G   
   C   
    A    
   C   
   A   
   A   
   G   
   A   
   A   
   G   
   A   
   C   
   G   
   T   
   A   
   A   
   T   
    T    
   G   
   T   
   C   
   A   
   G   
   T   
   G   
   T   
    G    
   A   
   C   
    G    
   C   
   G   
   A   
   G   
   A   
   A   
   G   
   G   
   C   
   T   
   A   
   T   
   G   
   T   
   G   
   A   
   A   
   G   
   C   
   G   
   A   
   A   
   C   
   A   
   A   
   G   
   C   
   C   
   T   
   T   
   C   
   G   
   T   
   T   
   C   
    T    
   T   
   A   
   T   
   A   
   G   
   T   
   G   
   C   
   T   
   T   
   C   
    C    
   A   
   A   
   A   
   C   
   C   
   A   
   G   
   A   
   A   
   G   
   A   
   A   
   A   
   T   
   C   
   G   
   G   
   G   
   A   
   T   
   G   
   C   
   G   
   A   
   G   
   A   
   A   
   G   
   G   
   C   
   G   
   A   
    C    
   T   
   A   
   C   
   T   
   T   
   G   
   C   
   T   
    A    
   T   
   A   
   C   
    T    
   C   
   A   
   G   
   G   
   T   
   G   
   A   
   A   
   T   
   T   
    G    
   A   
   G   
    T    
   A   
   C   
   A   
    T    
   T   
   G   
   G   
   A   
   C   
   C   
   A   
   T   
   G   
   T   
   T   
   C   
   T   
   T   
   C   
   T   
   G   
   A   
   T   
    C    
   A   
   C   
    A    
   A   
   A   
   T   
   A   
   A   
   A   
   G   
   C   
   A   
   A   
   A   
   T   
   G   
   T   
   G   
   A   
   T   
   C   
   T   
   A   
   C   
   C   
   G   
    C    
   C   
   C   
   T   
   G   
   T   
    C    
   C   
   A   
   T   
   G   
   A   
   A   
   C   
   T   
   A   
   C   
   C   
    C    
   G   
   A   
   T   
   T   
   T   
   G   
   A   
   A   
   A   
   T   
   G   
   G   
   A   
   A   
   A   
   G   
   A   
   T   
   G   
   C   
   T   
   G   
   G   
    T    
   G   
   A   
    G    
   C   
   A   
    C    
   A   
   T   
   T   
   T   
   C   
   T   
   C   
   A   
   A   
   A   
   C   
   A   
   A   
   T   
   C   
   T   
   C   
   C   
   A   
   A   
   T   
   C   
   T   
   G   
   T   
   C   
   T   
   G   
   T   
   A   
   G   
   A   
    C    
   G   
   A   
   A   
   T   
   C   
   G   
   A   
   T   
    T    
   T   
   T   
   A   
   G   
   C   
   T   
   G   
   T   
   T   
   T   
   T   
    C    
   C   
   C   
   A   
   T   
   A   
   T   
   C   
   A   
   A   
   A   
   A   
   A   
   A   
   T   
    T    
   C   
   A   
   A   
   G   
   C   
   A   
   G   
   A   
    G    
   A   
   A   
   A   
   A   
   C   
   A   
   T   
   T   
   T   
   G   
   T   
    A    
   T   
   T   
   C   
   A   
   T   
   C   
   A   
   G   
   C   
   A   
   A   
   G   
   A   
   A   
   T   
   G   
   G   
   A   
   T   
   T   
    G    
    A    
   T   
   C   
   A   
   A   
   G   
   C   
   A   
   A   
   A   
   C   
   A   
   C   
   G   
   A   
   A   
   T   
   G   
   A   
   C   
   A   
   G   
   A   
   G   
   T   
   T   
   C   
   G   
   A   
   A   
   C   
   C   
   A   
   T   
   G   
   G   
   C   
   G   
   C   
   A   
   C   
   C   
   T   
   A   
    T    
   A   
   A   
   G   
   A   
   G   
   C   
   C   
   G   
    T    
   C   
   C   
   G   
   C   
   T   
   C   
   A   
   G   
   C   
   T   
   G   
   T   
   A   
   T   
   G   
   A   
   A   
   G   
   C   
   T   
   A   
   A   
   A   
   G   
   G   
   C   
   G   
   G   
   A   
   A   
   G   
   A   
   A   
   G   
   A   
   T   
   G   
   A   
   A   
   C   
   T   
    G    
   A   
   T   
   T   
   G   
   C   
   T   
   G   
   T   
    G    
   T   
   A   
   T   
   C   
   T   
   G   
   A   
   A   
   A   
   G   
   A   
   A   
   G   
   A   
   T   
   C   
    A    
   G   
   C   
   C   
   A   
   G   
   G   
   C   
   T   
   T   
   A   
   G   
   A   
   T   
   G   
   T   
    T    
   T   
   T   
   C   
   T   
   T   
   A   
   G   
   T   
   A   
   A   
   C   
   G   
    C    
   A   
   T   
   C   
   A   
   A   
   G   
   G   
   A   
   A   
   T   
   G   
   G   
   G   
    T    
   T   
   T   
   G   
   C   
   G   
   C   
   T   
    A    
   T   
   C   
   C   
   T   
   T   
   T   
   A   
   A   
    C    
   A   
   G   
   A   
   T   
   G   
   T   
   T   
   C   
   C   
   C   
   G   
   T   
   T   
   G   
   T   
   T   
   G   
   G   
   A   
   G   
   C   
   A   
   A   
   A   
   G   
   G   
   C   
   A   
   G   
    C    
   T   
   G   
   G   
   A   
   G   
   T   
   G   
   A   
   A   
   G   
   T   
   C   
   T   
   A   
   T   
   G   
   A   
   A   
   T   
   T   
   T   
   G   
   A   
   A   
   A   
   G   
   A   
   T   
   G   
   G   
   A   
   G   
   A   
   T   
   A   
   G   
   G   
    A    
   T   
   T   
    A    
   T   
   C   
   A   
   A   
    C    
   G   
   G   
    G    
   C   
   T   
    A    
   C   
   T   
   T   
   G   
   T   
   G   
   T   
   T   
   A   
   G   
   C   
   A   
   G   
   A   
    A    
   G   
   G   
    T    
   G   
   A   
   T   
   A   
   C   
   G   
   C   
   C   
    G    
   A   
   T   
   C   
   G   
   T   
   A   
   A   
   T   
   C   
   G   
   T   
   T   
   A   
   C   
   T   
   C   
   A   
   G   
   C   
   G   
   A   
   G   
   G   
   A   
   G   
   C   
    T    
   G   
   T   
    T    
   A   
   A   
   A   
   C   
   G   
   C   
   A   
   T   
   G   
   C   
   T   
   T   
   G   
   C   
   T   
   C   
   A   
   A   
   G   
   A   
   A   
   A   
   T   
   C   
   A   
   G   
   C   
   C   
   A   
   A   
   A   
   C   
    T    
   T   
   C   
   A   
   C   
   G   
   T   
   G   
   C   
   G   
   A   
   A   
   G   
   C   
   G   
   G   
   G   
   G   
   A   
   G   
   T   
   G   
   A   
   C   
   G   
   G   
   T   
   C   
   A   
   T   
   G   
   C   
   G   
   T   
   G   
   A   
   G   
   C   
   T   
   G   
   A   
   A   
   A   
   A   
   A   
   A   
   A   
   A   
   T   
   C   
   C   
    G    
   C   
   A   
    C    
   C   
   G   
    T    
   A   
   T   
   T   
   A   
   T   
   C   
   T   
   A   
   T   
   A   
   T   
   G   
   T   
   C   
   G   
   G   
   A   
   A   
   G   
   G   
   A   
   A   
   G   
   A   
   T   
   C   
   A   
   A   
   A   
   A   
   G   
   A   
   A   
   A   
   T   
   A   
   A   
   C   
   C   
   T   
   T   
   G   
   A   
   T   
   G   
   A   
   A   
   T   
   C   
   A   
   A   
   A   
   A   
   A   
   G   
   G   
    G    
   C   
   A   
    G    
   C   
   A   
   G   
    T    
   T   
   A   
   A   
   C   
   G   
   A   
   T   
   T   
   G   
   A   
    C    
   C   
   C   
   A   
   A   
   C   
    T    
   G   
   A   
   T   
   T   
   T   
    C    
   C   
   C   
   T   
   A   
   T   
   A   
   G   
   G   
   T   
   G   
   A   
   T   
   C   
   G   
   A   
   A   
   C   
   A   
   T   
   C   
   G   
   A   
   A   
   T   
   G   
   G   
    A    
   T   
   C   
    A    
   T   
   T   
   T   
   G   
   C   
    G    
   A   
   T   
   G   
   G   
   A   
    T    
   G   
   A   
   G   
   A   
   A   
   A   
   A   
   A   
   A   
   G   
   G   
   T   
   G   
   G   
   A   
   G   
   A   
   A   
   G   
   T   
   C   
   A   
   T   
   C   
   G   
   A   
   A   
   G   
   T   
    T    
   A   
    T    
   C   
   G   
   A   
   T   
   G   
   C   
   T   
   C   
   C   
   T   
   G   
   A   
   G   
    A    
   T   
   T   
   T   
   T   
    C    
   A   
   T   
    C    
   G   
   A   
    G    
    T    
   C   
    G    
   -   
   -   
   -   
 

 
   N54519   
   A   
   T   
   G   
   G   
   A   
   A   
   A   
   A   
   T   
   C   
   G   
   A   
   C   
   A   
   A   
   G   
   A   
   A   
   G   
   T   
   A   
   C   
   A   
   A   
   G   
   A   
   A   
   T   
   T   
   A   
   A   
   C   
   T   
   T   
   T   
   A   
   G   
   A   
   A   
   G   
   A   
   A   
   G   
   T   
   G   
   A   
   T   
   G   
   G   
   G   
   T   
   G   
   A   
   C   
   C   
   G   
   C   
   T   
   T   
   C   
   G   
   G   
   A   
   C   
   G   
   T   
   T   
   A   
   T   
   T   
   C   
   C   
   A   
   A   
   A   
   T   
   A   
   T   
   A   
   T   
   T   
   A   
   T   
    C    
   C   
   A   
    A    
   G   
   A   
   A   
   C   
   G   
   T   
   G   
   C   
   A   
   C   
   T   
   G   
   C   
   C   
   T   
   G   
   A   
   T   
   A   
   T   
    C    
   C   
   G   
   T   
   G   
   A   
   T   
   G   
   G   
   A   
   T   
   T   
   G   
   A   
   A   
    A    
   C   
   C   
   A   
   G   
   T   
    G    
   C   
   A   
   A   
   C   
   G   
   C   
   C   
   G   
   G   
   A   
   T   
   A   
   T   
   T   
   A   
   T   
   T   
    T    
   G   
   C   
    C    
   A   
   T   
   G   
   A   
   A   
   C   
   A   
   A   
   A   
   G   
   A   
   T   
   G   
   G   
   C   
   A   
   A   
   T   
   A   
   C   
   G   
   T   
   A   
   T   
   G   
   A   
   C   
   A   
   A   
   A   
   G   
   G   
   A   
   T   
   T   
    C    
   C   
   G   
   T   
   A   
   A   
   A   
   T   
   C   
   A   
   G   
   C   
   A   
   A   
   A   
   A   
   T   
   C   
   T   
   G   
   T   
   C   
   G   
   G   
   A   
   A   
   A   
   T   
   A   
   T   
   C   
   A   
   T   
   G   
   G   
   G   
    A    
   A   
   A   
   T   
   T   
   A   
   T   
   C   
   A   
   T   
   C   
   C   
   T   
   C   
   A   
   T   
   G   
   G   
   T   
   G   
   A   
   C   
   A   
   G   
   C   
   A   
   G   
   T   
   A   
   T   
   T   
   T   
   A   
   T   
   G   
   A   
   A   
   G   
   C   
   G   
   A   
   T   
   G   
   G   
   T   
   C   
   C   
   G   
   C   
   A   
   T   
   G   
   A   
   G   
   C   
   C   
   A   
   A   
   G   
   A   
    T    
   T   
   G   
   G   
   A   
   A   
   G   
   T   
   T   
   G   
   C   
   G   
   G   
   G   
   A   
   A   
   G   
   T   
   A   
   C   
   T   
   G   
   A   
   T   
   C   
   G   
   A   
    G    
   A   
   T   
   G   
   C   
   A   
   C   
   G   
   G   
    A    
   A   
   A   
   T   
   A   
   A   
    C    
   G   
   G   
   G   
   A   
   G   
   T   
   A   
   T   
   G   
   G   
   A   
   C   
   G   
   G   
   T   
   G   
   A   
   T   
   C   
   C   
   A   
   C   
   C   
   T   
   G   
   C   
   T   
   G   
   C   
   G   
   A   
   T   
   G   
   C   
   G   
   T   
   T   
   A   
   T   
   A   
   C   
   T   
   G   
   A   
   A   
   G   
   C   
   C   
   A   
   G   
    A    
   T   
   T   
   A   
   T   
   C   
   C   
   A   
   A   
   G   
   T   
   T   
   A   
   A   
   G   
   T   
   G   
   G   
   A   
   G   
   A   
   G   
   A   
   T   
   G   
    C    
   T   
   A   
    G    
   C   
   A   
   G   
   A   
   T   
   A   
   T   
   C   
   G   
   A   
    A    
   A   
   A   
   A   
   G   
   A   
   A   
   A   
   C   
   A   
   G   
   T   
    T    
   G   
   A   
   T   
   C   
   T   
   C   
   G   
   T   
   G   
   T   
   G   
   G   
   A   
   A   
   T   
   T   
   T   
   T   
   G   
   A   
   T   
   G   
   A   
   T   
   A   
   C   
   A   
   G   
   A   
   A   
   A   
   A   
   A   
   G   
   A   
   A   
   C   
   C   
   A   
   A   
   C   
   A   
   G   
   T   
   C   
   C   
   T   
   T   
   C   
   C   
   A   
   G   
   C   
   T   
   C   
   G   
   A   
   T   
   A   
   C   
   C   
   C   
   A   
   A   
   A   
   C   
   C   
   T   
   T   
   C   
   T   
    A    
   G   
   T   
   C   
   A   
   A   
   T   
   G   
   G   
   A   
   T   
   C   
   G   
   A   
   C   
   A   
   G   
   G   
   T   
   A   
   T   
   A   
   T   
   C   
   T   
   G   
   C   
   T   
   G   
   G   
   A   
   T   
   A   
   T   
   G   
   C   
    G    
   A   
   C   
   A   
   G   
   A   
   A   
   A   
   T   
   C   
   C   
   C   
   G   
   A   
   C   
    G    
   C   
   A   
   T   
   A   
   A   
   C   
   T   
   T   
   A   
   G   
   C   
   T   
   G   
   A   
   A   
   G   
   T   
   G   
   A   
   T   
    C    
   G   
   A   
    C    
   G   
   G   
   C   
   A   
   C   
   A   
   A   
   T   
   C   
   T   
   A   
   T   
   A   
   T   
   G   
   A   
   T   
   C   
   G   
   A   
   T   
   C   
   A   
   T   
   C   
   C   
   A   
   C   
   A   
   A   
   G   
   C   
   G   
   A   
   G   
   C   
   C   
   T   
   T   
   G   
   A   
   A   
   A   
   A   
   A   
   T   
   T   
   G   
   A   
   T   
   G   
   G   
   A   
   A   
   T   
   A   
   T   
   A   
   T   
   T   
   C   
   C   
   T   
   G   
   G   
   A   
   C   
   C   
   T   
   G   
   A   
   C   
   T   
   T   
    T    
   C   
   C   
   A   
   A   
   C   
   A   
   G   
   G   
   A   
   G   
   G   
    G    
   A   
   T   
   C   
   T   
   T   
   G   
   C   
   A   
   A   
   G   
   G   
    A    
   A   
   A   
   A   
   G   
   A   
   T   
   G   
   A   
   A   
   A   
   T   
   C   
   A   
   A   
   A   
   A   
   A   
   A   
   G   
   C   
   T   
   T   
   A   
   T   
   G   
   A   
   A   
   A   
   C   
   A   
   G   
   G   
    G    
   C   
   G   
   A   
   G   
   G   
   A   
   A   
   A   
   A   
   G   
   T   
   T   
   A   
    T    
   T   
   C   
   T   
   T   
   C   
   G   
   T   
   T   
   C   
   G   
   A   
   A   
   A   
   A   
   C   
   A   
   A   
   A   
   G   
   A   
   T   
   A   
   G   
   A   
    A    
   G   
   C   
   A   
   A   
   T   
   G   
   A   
   A   
   G   
   G   
   G   
    C    
   A   
   A   
    C    
   A   
   A   
   A   
   C   
   A   
   G   
   C   
   A   
   A   
   A   
   T   
   C   
   G   
   T   
   G   
   A   
   T   
   C   
   A   
   C   
    T    
   G   
   A   
   A   
   A   
   T   
   C   
   C   
   C   
   A   
   T   
   A   
   T   
   G   
   A   
   A   
   G   
   T   
   A   
   A   
   A   
   T   
   A   
   A   
   A   
   G   
   C   
   G   
   A   
   C   
    A    
   T   
   T   
   G   
   G   
   T   
   G   
   A   
   A   
   A   
   A   
   A   
   A   
   A   
   T   
   G   
   G   
   A   
   T   
   G   
   A   
   G   
   A   
   T   
   C   
   C   
   G   
   T   
   T   
   T   
   G   
   A   
   A   
   T   
   A   
   A   
   G   
   A   
   A   
   A   
   A   
   T   
   C   
   G   
   A   
    C    
   G   
   G   
   A   
   A   
   T   
   A   
   G   
   C   
   T   
   G   
   A   
   A   
   G   
   T   
    G    
   C   
   G   
   A   
   G   
   A   
   T   
   G   
   A   
   A   
   A   
   G   
   T   
   G   
   A   
   T   
    C    
   G   
    A    
   A   
   C   
    T    
   G   
   G   
   T   
   C   
   T   
   G   
   C   
   A   
   A   
   A   
   T   
   T   
   G   
   T   
   C   
   G   
   T   
    T    
   G   
   A   
   A   
   T   
   T   
   G   
   A   
   A   
   A   
   A   
   A   
   A   
   G   
   A   
    C    
   G   
   C   
   C   
   A   
   A   
    T    
   G   
   C   
   T   
   C   
   A   
   A   
   G   
   G   
    A    
   A   
   T   
   T   
   T   
   T   
   G   
   A   
   A   
   T   
   T   
   A   
    T    
   T   
   T   
   G   
   T   
   T   
   C   
   A   
   A   
   A   
   A   
   A   
   C   
   A   
   C   
   T   
   G   
   A   
   G   
    C    
   T   
   A   
   C   
   A   
   G   
   A   
   T   
   C   
   A   
   A   
   T   
   T   
   A   
   C   
   A   
   A   
   T   
   T   
   T   
    C    
   A   
   A   
   T   
   A   
   T   
   G   
   G   
   T   
   C   
   G   
   C   
   T   
   A   
   T   
   C   
   G   
   A   
    T    
   C   
   A   
   T   
   A   
   T   
   G   
   A   
   C   
   T   
   C   
   C   
   G   
   C   
   A   
    T    
   C   
   A   
   A   
   G   
   T   
   T   
   G   
   G   
   A   
   C   
   T   
   G   
   A   
   A   
   A   
   G   
   A   
   C   
   A   
   T   
   T   
   T   
   T   
   A   
   A   
   G   
   A   
   A   
   G   
   T   
   T   
   A   
   T   
   A   
   T   
   C   
   G   
   A   
   G   
   C   
   A   
   T   
   C   
   G   
   A   
   A   
   A   
   G   
   C   
   A   
   A   
   G   
   T   
   C   
   A   
   T   
   A   
   A   
   C   
   A   
   A   
   A   
   A   
   C   
   G   
    C    
   A   
   G   
    C    
   C   
   A   
   A   
   T   
   T   
   C   
   G   
   A   
   T   
   T   
   T   
   A   
   G   
   C   
   C   
   A   
   A   
   A   
   G   
   C   
   T   
   C   
   A   
   A   
   A   
   A   
   A   
    A    
   G   
   A   
   C   
   A   
   G   
   C   
   A   
   T   
   A   
   T   
   C   
   G   
   T   
    G    
   G   
   A   
   A   
   G   
   G   
    C    
    C    
   T   
   G   
   A   
   T   
   G   
   A   
   A   
   A   
   G   
   C   
   T   
   T   
   T   
   A   
   T   
   C   
   G   
   A   
   T   
   T   
   C   
   T   
   G   
   G   
   A   
   T   
   G   
   A   
   A   
   G   
   T   
   A   
   A   
   T   
   C   
   G   
    C    
   A   
   A   
   C   
   G   
   A   
   T   
   T   
   C   
   G   
   T   
   G   
   A   
   A   
   A   
   G   
   T   
   A   
   A   
   A   
   G   
   A   
   C   
   A   
   A   
   A   
   A   
   A   
   A   
   G   
   A   
    C    
   G   
   C   
    C    
   A   
   A   
   A   
   A   
   A   
   G   
   A   
   A   
   T   
   T   
   T   
   A   
   G   
   T   
   C   
   G   
   A   
    C    
   G   
   T   
    T    
   T   
   T   
    C    
   C   
   A   
   A   
   T   
   T   
   T   
   A   
   C   
   A   
   G   
   A   
   A   
   G   
   A   
   A   
   C   
   A   
   A   
   G   
   C   
   A   
   G   
   A   
   A   
   G   
   C   
   A   
   A   
   T   
    C    
   G   
   T   
   C   
   A   
   C   
   A   
   T   
   T   
   G   
   C   
   A   
   A   
    C    
   T   
   A   
   T   
   A   
    T    
   C   
   G   
   T   
   T   
   T   
   G   
   A   
   C   
   G   
   A   
   A   
    C    
   A   
   C   
    A    
   G   
   A   
   T   
   A   
   T   
   C   
   A   
   C   
   A   
   G   
   A   
   G   
   C   
   T   
   T   
   C   
   A   
   A   
   A   
   A   
   G   
   G   
   A   
   A   
    T    
   C   
    C    
   G   
   A   
   A   
   A   
    G    
   C   
   T   
   T   
   G   
   A   
   T   
   T   
   G   
   C   
    A    
   C   
   A   
   A   
   A   
   T   
   C   
   A   
   C   
   A   
   G   
   A   
   G   
   T   
   T   
    G    
   A   
   A   
   T   
   A   
   A   
   A   
   A   
   T   
   C   
   C   
   T   
   T   
   T   
   C   
   A   
   A   
   A   
    C    
   G   
   A   
   C   
   A   
   A   
   A   
   G   
   A   
    G    
   C   
   T   
   T   
   T   
   T   
    C    
   T   
   C   
   T   
   G   
   T   
   T   
   A   
   T   
   G   
   A   
   A   
   A   
   A   
   A   
   A   
   G   
   A   
   A   
   T   
   T   
   G   
   C   
   G   
   A   
   G   
   A   
   A   
   G   
   T   
   G   
   A   
   A   
   A   
   A   
   A   
   A   
   A   
   A   
   T   
   T   
   A   
    C    
   T   
   C   
   T   
   T   
   C   
   C   
   G   
   C   
   T   
   C   
   G   
   T   
   C   
    T    
    G    
   A   
   C   
   G   
   A   
   C   
   A   
   A   
   T   
   C   
    G    
   A   
   A   
   G   
   A   
   C   
   G   
   A   
   A   
   A   
   T   
   C   
   G   
   A   
   A   
   G   
   A   
   A   
   A   
   T   
   C   
   A   
   A   
   A   
   A   
   T   
    C    
   G   
   A   
   T   
   A   
   C   
   G   
   C   
   A   
   A   
    G    
   T   
    T    
   T   
   T   
   A   
   G   
   T   
   G   
   G   
   C   
    G    
   C   
   A   
   A   
   G   
   A   
   A   
   G   
   A   
   C   
   G   
   T   
   A   
   A   
   T   
    C    
   G   
   T   
   C   
   A   
   G   
   T   
   G   
   T   
    G    
   A   
   C   
    A    
   C   
   G   
   A   
   G   
   A   
   A   
   G   
   G   
   C   
   T   
   A   
   T   
   G   
   T   
   G   
   A   
   A   
   G   
   C   
   G   
   A   
   A   
   C   
   A   
   A   
   G   
   C   
   C   
   T   
   T   
   C   
   G   
   T   
   T   
   C   
    T    
   T   
   A   
   T   
   A   
   G   
   T   
   G   
   C   
   T   
   T   
   C   
    G    
   A   
   A   
   A   
   C   
   C   
   A   
   G   
   A   
   A   
   G   
   A   
   A   
   A   
   T   
   C   
   G   
   G   
   G   
   A   
   T   
   G   
   C   
   G   
   A   
   G   
   A   
   A   
   G   
   G   
   C   
   G   
   A   
    C    
   T   
   A   
   C   
   T   
   T   
   G   
   C   
   T   
    G    
   T   
   A   
   C   
    T    
   C   
   A   
   G   
   G   
   T   
   G   
   A   
   A   
   T   
   T   
    G    
   A   
   G   
    C    
   A   
   C   
   A   
    T    
   T   
   G   
   G   
   A   
   C   
   C   
   A   
   T   
   G   
   T   
   T   
   C   
   T   
   T   
   C   
   T   
   G   
   A   
   T   
    T    
   A   
   C   
    G    
   A   
   A   
   T   
   A   
   A   
   A   
   G   
   C   
   A   
   A   
   A   
   T   
   G   
   T   
   G   
   A   
   T   
   C   
   T   
   A   
   C   
   C   
   G   
    T    
   C   
   C   
   T   
   G   
   T   
    T    
   C   
   A   
   T   
   G   
   A   
   A   
   C   
   T   
   A   
   C   
   C   
    C    
   G   
   A   
   T   
   T   
   T   
   G   
   A   
   A   
   A   
   T   
   G   
   G   
   A   
   A   
   A   
   G   
   A   
   T   
   G   
   C   
   T   
   G   
   G   
    C    
   G   
   A   
    A    
   C   
   A   
    C    
   A   
   T   
   T   
   T   
   C   
   T   
   C   
   A   
   A   
   A   
   C   
   A   
   A   
   T   
   C   
   T   
   C   
   C   
   A   
   A   
   T   
   C   
   T   
   G   
   T   
   C   
   T   
   G   
   T   
   A   
   G   
   A   
    T    
   G   
   A   
   A   
   T   
   C   
   G   
   A   
   T   
    C    
   T   
   T   
   A   
   G   
   C   
   T   
   G   
   T   
   T   
   T   
   T   
    C    
   C   
   C   
   A   
   T   
   A   
   T   
   C   
   A   
   A   
   A   
   A   
   A   
   A   
   T   
    C    
   C   
   A   
   A   
   G   
   C   
   A   
   G   
   A   
    G    
   A   
   A   
   A   
   A   
   C   
   A   
   T   
   T   
   T   
   G   
   T   
    A    
   T   
   T   
   C   
   A   
   T   
   C   
   A   
   G   
   C   
   A   
   A   
   G   
   A   
   A   
   T   
   G   
   G   
   A   
   T   
   T   
    G    
    A    
   T   
   C   
   A   
   A   
   G   
   C   
   A   
   A   
   A   
   C   
   A   
   C   
   G   
   A   
   A   
   T   
   G   
   A   
   C   
   A   
   G   
   A   
   G   
   T   
   T   
   C   
   G   
   A   
   A   
   C   
   C   
   A   
   T   
   G   
   G   
   C   
   G   
   C   
   A   
   C   
   C   
   T   
   A   
    C    
   A   
   A   
   G   
   A   
   G   
   C   
   C   
   G   
    T    
   C   
   C   
   G   
   C   
   T   
   C   
   A   
   G   
   C   
   T   
   G   
   T   
   A   
   T   
   G   
   A   
   A   
   G   
   C   
   T   
   A   
   A   
   A   
   G   
   G   
   C   
   G   
   G   
   A   
   A   
   G   
   A   
   A   
   G   
   A   
   T   
   G   
   A   
   A   
   C   
   T   
    T    
   A   
   T   
   T   
   G   
   C   
   T   
   G   
   T   
    A    
   T   
   A   
   T   
   C   
   T   
   G   
   A   
   A   
   A   
   G   
   A   
   A   
   G   
   A   
   T   
   C   
    A    
   G   
   C   
   C   
   A   
   G   
   G   
   C   
   T   
   T   
   A   
   G   
   A   
   T   
   G   
   T   
    G    
   T   
   T   
   C   
   T   
   T   
   A   
   G   
   T   
   A   
   A   
   C   
   G   
    C    
   A   
   T   
   C   
   A   
   A   
   G   
   G   
   A   
   A   
   T   
   G   
   G   
   G   
    C    
   T   
   T   
   G   
   C   
   G   
   C   
   T   
    A    
   T   
   C   
   C   
   T   
   T   
   T   
   A   
   A   
    C    
   A   
   G   
   A   
   T   
   G   
   T   
   T   
   C   
   C   
   C   
   G   
   T   
   T   
   G   
   T   
   T   
   G   
   G   
   A   
   G   
   C   
   A   
   A   
   A   
   G   
   G   
   C   
   A   
   G   
    C    
   T   
   G   
   G   
   A   
   G   
   T   
   G   
   A   
   A   
   G   
   T   
   C   
   T   
   A   
   T   
   G   
   A   
   A   
   T   
   T   
   T   
   G   
   A   
   A   
   A   
   G   
   A   
   T   
   G   
   G   
   A   
   G   
   A   
   T   
   A   
   G   
   G   
    A    
   T   
   T   
    G    
   T   
   C   
   A   
   A   
    T    
   G   
   G   
    A    
   C   
   T   
    G    
   C   
   T   
   T   
   G   
   T   
   G   
   T   
   T   
   A   
   G   
   C   
   A   
   G   
   A   
    C    
   G   
   G   
    A    
   G   
   A   
   T   
   A   
   C   
   G   
   C   
   C   
    G    
   A   
   T   
   C   
   G   
   T   
   A   
   A   
   T   
   C   
   G   
   T   
   T   
   A   
   C   
   T   
   C   
   A   
   G   
   C   
   G   
   A   
   G   
   G   
   A   
   G   
   C   
    T    
   G   
   T   
    G    
   A   
   A   
   A   
   C   
   G   
   C   
   A   
   T   
   G   
   C   
   T   
   T   
   G   
   C   
   T   
   C   
   A   
   A   
   G   
   A   
   A   
   A   
   T   
   C   
   A   
   G   
   C   
   C   
   A   
   A   
   A   
   C   
    T    
   T   
   C   
   A   
   C   
   G   
   T   
   G   
   C   
   G   
   A   
   A   
   G   
   C   
   G   
   G   
   G   
   G   
   A   
   G   
   T   
   G   
   A   
   C   
   G   
   G   
   T   
   C   
   A   
   T   
   G   
   C   
   G   
   T   
   G   
   A   
   G   
   C   
   T   
   G   
   A   
   A   
   A   
   A   
   A   
   A   
   A   
   A   
   T   
   C   
   C   
    G    
   C   
   A   
    C    
   C   
   G   
    C    
   A   
   T   
   T   
   A   
   T   
   C   
   T   
   A   
   T   
   A   
   T   
   G   
   T   
   C   
   G   
   G   
   A   
   A   
   G   
   G   
   A   
   A   
   G   
   A   
   T   
   C   
   A   
   A   
   A   
   A   
   G   
   A   
   A   
   A   
   T   
   A   
   A   
   C   
   C   
   T   
   T   
   G   
   A   
   T   
   G   
   A   
   A   
   T   
   C   
   A   
   A   
   A   
   A   
   A   
   G   
   G   
    A    
   C   
   A   
    A    
   C   
   A   
   G   
    A    
   T   
   A   
   A   
   C   
   G   
   A   
   T   
   T   
   G   
   A   
    T    
   C   
   C   
   A   
   A   
   C   
    G    
   G   
   A   
   T   
   T   
   T   
    T    
   C   
   C   
   T   
   A   
   T   
   A   
   G   
   G   
   T   
   G   
   A   
   T   
   C   
   G   
   A   
   A   
   C   
   A   
   T   
   C   
   G   
   A   
   A   
   T   
   G   
   G   
    T    
   T   
   C   
    G    
   T   
   T   
   T   
   G   
   C   
    T    
   A   
   T   
   G   
   G   
   A   
    C    
   G   
   A   
   G   
   A   
   A   
   A   
   A   
   A   
   A   
   G   
   G   
   T   
   G   
   G   
   A   
   G   
   A   
   A   
   G   
   T   
   C   
   A   
   T   
   C   
   G   
   A   
   A   
   G   
   T   
    T    
   A   
    T    
   C   
   G   
   A   
   T   
   G   
   C   
   T   
   C   
   C   
   T   
   G   
   A   
   G   
    A    
   T   
   T   
   T   
   T   
    C    
   A   
   T   
    C    
   G   
   A   
    A    
    T    
   C   
    A    
   -   
   -   
   -   
 

 
   N52643   
   A   
   T   
   G   
   G   
   A   
   A   
   A   
   A   
   T   
   C   
   G   
   A   
   C   
   A   
   A   
   G   
   A   
   A   
   G   
   T   
   A   
   C   
   A   
   A   
   G   
   A   
   A   
   T   
   T   
   A   
   A   
   C   
   T   
   T   
   T   
   A   
   G   
   A   
   A   
   G   
   A   
   A   
   G   
   T   
   G   
   A   
   T   
   G   
   G   
   G   
   T   
   G   
   A   
   C   
   C   
   G   
   C   
   T   
   T   
   C   
   G   
   G   
   A   
   C   
   G   
   T   
   T   
   A   
   T   
   T   
   C   
   C   
   A   
   A   
   A   
   T   
   A   
   T   
   A   
   T   
   T   
   A   
   T   
    C    
   C   
   A   
    A    
   G   
   A   
   A   
   C   
   G   
   T   
   G   
   C   
   A   
   C   
   T   
   G   
   C   
   C   
   T   
   G   
   A   
   T   
   A   
   T   
    T    
   C   
   G   
   T   
   G   
   A   
   T   
   G   
   G   
   A   
   T   
   T   
   G   
   A   
   A   
    G    
   C   
   C   
   A   
   G   
   T   
    G    
   C   
   A   
   A   
   C   
   G   
   C   
   C   
   G   
   G   
   A   
   T   
   A   
   T   
   T   
   A   
   T   
   T   
    T    
   G   
   C   
    C    
   A   
   T   
   G   
   A   
   A   
   C   
   A   
   A   
   A   
   G   
   A   
   T   
   G   
   G   
   C   
   A   
   A   
   T   
   A   
   C   
   G   
   T   
   A   
   T   
   G   
   A   
   C   
   A   
   A   
   A   
   G   
   G   
   A   
   T   
   T   
    C    
   C   
   G   
   T   
   A   
   A   
   A   
   T   
   C   
   A   
   G   
   C   
   A   
   A   
   A   
   A   
   T   
   C   
   T   
   G   
   T   
   C   
   G   
   G   
   A   
   A   
   A   
   T   
   A   
   T   
   C   
   A   
   T   
   G   
   G   
   G   
    G    
   A   
   A   
   T   
   T   
   A   
   T   
   C   
   A   
   T   
   C   
   C   
   T   
   C   
   A   
   T   
   G   
   G   
   T   
   G   
   A   
   C   
   A   
   G   
   C   
   A   
   G   
   T   
   A   
   T   
   T   
   T   
   A   
   T   
   G   
   A   
   A   
   G   
   C   
   G   
   A   
   T   
   G   
   G   
   T   
   C   
   C   
   G   
   C   
   A   
   T   
   G   
   A   
   G   
   C   
   C   
   A   
   A   
   G   
   A   
    C    
   T   
   G   
   G   
   A   
   A   
   G   
   T   
   T   
   G   
   C   
   G   
   G   
   G   
   A   
   A   
   G   
   T   
   A   
   C   
   T   
   G   
   A   
   T   
   C   
   G   
   A   
    A    
   A   
   T   
   G   
   C   
   A   
   C   
   G   
   G   
    G    
   A   
   A   
   T   
   A   
   A   
    C    
   G   
   G   
   G   
   A   
   G   
   T   
   A   
   T   
   G   
   G   
   A   
   C   
   G   
   G   
   T   
   G   
   A   
   T   
   C   
   C   
   A   
   C   
   C   
   T   
   G   
   C   
   T   
   G   
   C   
   G   
   A   
   T   
   G   
   C   
   G   
   T   
   T   
   A   
   T   
   A   
   C   
   T   
   G   
   A   
   A   
   G   
   C   
   C   
   A   
   G   
    A    
   T   
   T   
   A   
   T   
   C   
   C   
   A   
   A   
   G   
   T   
   T   
   A   
   A   
   G   
   T   
   G   
   G   
   A   
   G   
   A   
   G   
   A   
   T   
   G   
    C    
   T   
   A   
    G    
   C   
   A   
   G   
   A   
   T   
   A   
   T   
   C   
   G   
   A   
    A    
   A   
   A   
   A   
   G   
   A   
   A   
   A   
   C   
   A   
   G   
   T   
    C    
   G   
   A   
   T   
   C   
   T   
   C   
   G   
   T   
   G   
   T   
   G   
   G   
   A   
   A   
   T   
   T   
   T   
   T   
   G   
   A   
   T   
   G   
   A   
   T   
   A   
   C   
   A   
   G   
   A   
   A   
   A   
   A   
   A   
   G   
   A   
   A   
   C   
   C   
   A   
   A   
   C   
   A   
   G   
   T   
   C   
   C   
   T   
   T   
   C   
   C   
   A   
   G   
   C   
   T   
   C   
   G   
   A   
   T   
   A   
   C   
   C   
   C   
   A   
   A   
   A   
   C   
   C   
   T   
   T   
   C   
   T   
    C    
   G   
   T   
   C   
   A   
   A   
   T   
   G   
   G   
   A   
   T   
   C   
   G   
   A   
   C   
   A   
   G   
   G   
   T   
   A   
   T   
   A   
   T   
   C   
   T   
   G   
   C   
   T   
   G   
   G   
   A   
   T   
   A   
   T   
   G   
   C   
    A    
   A   
   C   
   A   
   G   
   A   
   A   
   A   
   T   
   C   
   C   
   C   
   G   
   A   
   C   
    T    
   C   
   A   
   T   
   A   
   A   
   C   
   T   
   T   
   A   
   G   
   C   
   T   
   G   
   A   
   A   
   G   
   T   
   G   
   A   
   T   
    C    
   G   
   A   
    T    
   G   
   G   
   C   
   A   
   C   
   A   
   A   
   T   
   C   
   T   
   A   
   T   
   A   
   T   
   G   
   A   
   T   
   C   
   G   
   A   
   T   
   C   
   A   
   T   
   C   
   C   
   A   
   C   
   A   
   A   
   G   
   C   
   G   
   A   
   G   
   C   
   C   
   T   
   T   
   G   
   A   
   A   
   A   
   A   
   A   
   T   
   T   
   G   
   A   
   T   
   G   
   G   
   A   
   A   
   T   
   A   
   T   
   A   
   T   
   T   
   C   
   C   
   T   
   G   
   G   
   A   
   C   
   C   
   T   
   G   
   A   
   C   
   T   
   T   
    T    
   C   
   C   
   A   
   A   
   C   
   A   
   G   
   G   
   A   
   G   
   G   
    G    
   A   
   T   
   C   
   T   
   T   
   G   
   C   
   A   
   A   
   G   
   G   
    A    
   A   
   A   
   A   
   G   
   A   
   T   
   G   
   A   
   A   
   A   
   T   
   C   
   A   
   A   
   A   
   A   
   A   
   A   
   G   
   C   
   T   
   T   
   A   
   T   
   G   
   A   
   A   
   A   
   C   
   A   
   G   
   G   
    G    
   C   
   G   
   A   
   G   
   G   
   A   
   A   
   A   
   A   
   G   
   T   
   T   
   A   
    T    
   T   
   C   
   T   
   T   
   C   
   G   
   T   
   T   
   C   
   G   
   A   
   A   
   A   
   A   
   C   
   A   
   A   
   A   
   G   
   A   
   T   
   A   
   G   
   A   
    A    
   G   
   C   
   A   
   A   
   T   
   G   
   A   
   A   
   G   
   G   
   G   
    T    
   A   
   A   
    T    
   A   
   A   
   A   
   C   
   A   
   G   
   C   
   A   
   A   
   A   
   T   
   C   
   G   
   T   
   G   
   A   
   T   
   C   
   A   
   C   
    A    
   G   
   A   
   A   
   A   
   T   
   C   
   C   
   C   
   A   
   T   
   A   
   T   
   G   
   A   
   A   
   G   
   T   
   A   
   A   
   A   
   T   
   A   
   A   
   A   
   G   
   C   
   G   
   A   
   C   
    C    
   T   
   T   
   G   
   G   
   T   
   G   
   A   
   A   
   A   
   A   
   A   
   A   
   A   
   T   
   G   
   G   
   A   
   T   
   G   
   A   
   G   
   A   
   T   
   C   
   C   
   G   
   T   
   T   
   T   
   G   
   A   
   A   
   T   
   A   
   A   
   G   
   A   
   A   
   A   
   A   
   T   
   C   
   G   
   A   
    T    
   G   
   G   
   A   
   A   
   T   
   A   
   G   
   C   
   T   
   G   
   A   
   A   
   G   
   T   
    A    
   C   
   G   
   A   
   G   
   A   
   T   
   G   
   A   
   A   
   A   
   G   
   T   
   G   
   A   
   T   
    A    
   G   
    A    
   A   
   C   
    A    
   G   
   G   
   T   
   C   
   T   
   G   
   C   
   A   
   A   
   A   
   T   
   T   
   G   
   T   
   C   
   G   
   T   
    C    
   G   
   A   
   A   
   T   
   T   
   G   
   A   
   A   
   A   
   A   
   A   
   A   
   G   
   A   
    C    
   G   
   C   
   C   
   A   
   A   
    T    
   G   
   C   
   T   
   C   
   A   
   A   
   G   
   G   
    A    
   A   
   T   
   T   
   T   
   T   
   G   
   A   
   A   
   T   
   T   
   A   
    T    
   T   
   T   
   G   
   T   
   T   
   C   
   A   
   A   
   A   
   A   
   A   
   C   
   A   
   C   
   T   
   G   
   A   
   G   
    C    
   T   
   A   
   C   
   A   
   G   
   A   
   T   
   C   
   A   
   A   
   T   
   T   
   A   
   C   
   A   
   A   
   T   
   T   
   T   
    C    
   A   
   A   
   T   
   A   
   T   
   G   
   G   
   T   
   C   
   G   
   C   
   T   
   A   
   T   
   C   
   G   
   A   
    C    
   C   
   A   
   T   
   A   
   T   
   G   
   A   
   C   
   T   
   C   
   C   
   G   
   C   
   A   
    T    
   C   
   A   
   A   
   G   
   T   
   T   
   G   
   G   
   A   
   C   
   T   
   G   
   A   
   A   
   A   
   G   
   A   
   C   
   A   
   T   
   T   
   T   
   T   
   A   
   A   
   G   
   A   
   A   
   G   
   T   
   T   
   A   
   T   
   A   
   T   
   C   
   G   
   A   
   G   
   C   
   A   
   T   
   C   
   G   
   A   
   A   
   A   
   G   
   C   
   A   
   A   
   G   
   T   
   C   
   A   
   T   
   A   
   A   
   C   
   A   
   A   
   A   
   A   
   C   
   G   
    T    
   A   
   G   
    T    
   C   
   A   
   A   
   T   
   T   
   C   
   G   
   A   
   T   
   T   
   T   
   A   
   G   
   C   
   C   
   A   
   A   
   A   
   G   
   C   
   T   
   C   
   A   
   A   
   A   
   A   
   A   
    A    
   G   
   A   
   C   
   A   
   G   
   C   
   A   
   T   
   A   
   T   
   C   
   G   
   T   
    A    
   G   
   A   
   A   
   G   
   G   
    A    
    T    
   T   
   G   
   A   
   T   
   G   
   A   
   A   
   A   
   G   
   C   
   T   
   T   
   T   
   A   
   T   
   C   
   G   
   A   
   T   
   T   
   C   
   T   
   G   
   G   
   A   
   T   
   G   
   A   
   A   
   G   
   T   
   A   
   A   
   T   
   C   
   G   
    C    
   A   
   A   
   C   
   G   
   A   
   T   
   T   
   C   
   G   
   T   
   G   
   A   
   A   
   A   
   G   
   T   
   A   
   A   
   A   
   G   
   A   
   C   
   A   
   A   
   A   
   A   
   A   
   A   
   G   
   A   
    C    
   G   
   C   
    C    
   A   
   A   
   A   
   A   
   A   
   G   
   A   
   A   
   T   
   T   
   T   
   A   
   G   
   T   
   C   
   G   
   A   
    C    
   G   
   T   
    C    
   T   
   T   
    T    
   C   
   A   
   A   
   T   
   T   
   T   
   A   
   C   
   A   
   G   
   A   
   A   
   G   
   A   
   A   
   C   
   A   
   A   
   G   
   C   
   A   
   G   
   A   
   A   
   G   
   C   
   A   
   A   
   T   
    T    
   G   
   T   
   C   
   A   
   C   
   A   
   T   
   T   
   G   
   C   
   A   
   A   
    T    
   T   
   A   
   T   
   A   
    C    
   C   
   G   
   T   
   T   
   T   
   G   
   A   
   C   
   G   
   A   
   A   
    C    
   A   
   C   
    G    
   G   
   A   
   T   
   A   
   T   
   C   
   A   
   C   
   A   
   G   
   A   
   G   
   C   
   T   
   T   
   C   
   A   
   A   
   A   
   A   
   G   
   G   
   A   
   A   
    T    
   C   
    C    
   G   
   A   
   A   
   A   
    G    
   C   
   T   
   T   
   G   
   A   
   T   
   T   
   G   
   C   
    G    
   C   
   A   
   A   
   A   
   T   
   C   
   A   
   C   
   A   
   G   
   A   
   G   
   T   
   T   
    G    
   A   
   A   
   T   
   A   
   A   
   A   
   A   
   T   
   C   
   C   
   T   
   T   
   T   
   C   
   A   
   A   
   A   
    T    
   G   
   A   
   C   
   A   
   A   
   A   
   G   
   A   
    G    
   C   
   T   
   T   
   T   
   T   
    C    
   T   
   C   
   T   
   G   
   T   
   T   
   A   
   T   
   G   
   A   
   A   
   A   
   A   
   A   
   A   
   G   
   A   
   A   
   T   
   T   
   G   
   C   
   G   
   A   
   G   
   A   
   A   
   G   
   T   
   G   
   A   
   A   
   A   
   A   
   A   
   A   
   A   
   A   
   T   
   T   
   A   
    T    
   T   
   C   
   T   
   T   
   C   
   C   
   G   
   C   
   T   
   C   
   G   
   T   
   C   
    T    
    A    
   A   
   C   
   G   
   A   
   C   
   A   
   A   
   T   
   C   
    G    
   A   
   A   
   G   
   A   
   C   
   G   
   A   
   A   
   A   
   T   
   C   
   G   
   A   
   A   
   G   
   A   
   A   
   A   
   T   
   C   
   A   
   A   
   A   
   A   
   T   
    T    
   G   
   A   
   T   
   A   
   C   
   G   
   C   
   A   
   A   
    G    
   T   
    C    
   T   
   T   
   A   
   G   
   T   
   G   
   G   
   C   
    A    
   C   
   A   
   A   
   G   
   A   
   A   
   G   
   A   
   C   
   G   
   T   
   A   
   A   
   T   
    T    
   G   
   T   
   C   
   A   
   G   
   T   
   G   
   T   
    G    
   A   
   C   
    G    
   C   
   G   
   A   
   G   
   A   
   A   
   G   
   G   
   C   
   T   
   A   
   T   
   G   
   T   
   G   
   A   
   A   
   G   
   C   
   G   
   A   
   A   
   C   
   A   
   A   
   G   
   C   
   C   
   T   
   T   
   C   
   G   
   T   
   T   
   C   
    T    
   T   
   A   
   T   
   A   
   G   
   T   
   G   
   C   
   T   
   T   
   C   
    C    
   A   
   A   
   A   
   C   
   C   
   A   
   G   
   A   
   A   
   G   
   A   
   A   
   A   
   T   
   C   
   G   
   G   
   G   
   A   
   T   
   G   
   C   
   G   
   A   
   G   
   A   
   A   
   G   
   G   
   C   
   G   
   A   
    C    
   T   
   A   
   C   
   T   
   T   
   G   
   C   
   T   
    A    
   T   
   A   
   C   
    T    
   C   
   A   
   G   
   G   
   T   
   G   
   A   
   A   
   T   
   T   
    G    
   A   
   G   
    T    
   A   
   C   
   A   
    T    
   T   
   G   
   G   
   A   
   C   
   C   
   A   
   T   
   G   
   T   
   T   
   C   
   T   
   T   
   C   
   T   
   G   
   A   
   T   
    C    
   A   
   C   
    A    
   A   
   A   
   T   
   A   
   A   
   A   
   G   
   C   
   A   
   A   
   A   
   T   
   G   
   T   
   G   
   A   
   T   
   C   
   T   
   A   
   C   
   C   
   G   
    C    
   C   
   C   
   T   
   G   
   T   
    C    
   C   
   A   
   T   
   G   
   A   
   A   
   C   
   T   
   A   
   C   
   C   
    C    
   G   
   A   
   T   
   T   
   T   
   G   
   A   
   A   
   A   
   T   
   G   
   G   
   A   
   A   
   A   
   G   
   A   
   T   
   G   
   C   
   T   
   G   
   G   
    T    
   G   
   A   
    G    
   C   
   A   
    C    
   A   
   T   
   T   
   T   
   C   
   T   
   C   
   A   
   A   
   A   
   C   
   A   
   A   
   T   
   C   
   T   
   C   
   C   
   A   
   A   
   T   
   C   
   T   
   G   
   T   
   C   
   T   
   G   
   T   
   A   
   G   
   A   
    C    
   G   
   A   
   A   
   T   
   C   
   G   
   A   
   T   
    T    
   T   
   T   
   A   
   G   
   C   
   T   
   G   
   T   
   T   
   T   
   T   
    C    
   C   
   C   
   A   
   T   
   A   
   T   
   C   
   A   
   A   
   A   
   A   
   A   
   A   
   T   
    T    
   C   
   A   
   A   
   G   
   C   
   A   
   G   
   A   
    G    
   A   
   A   
   A   
   A   
   C   
   A   
   T   
   T   
   T   
   G   
   T   
    A    
   T   
   T   
   C   
   A   
   T   
   C   
   A   
   G   
   C   
   A   
   A   
   G   
   A   
   A   
   T   
   G   
   G   
   A   
   T   
   T   
    G    
    A    
   T   
   C   
   A   
   A   
   G   
   C   
   A   
   A   
   A   
   C   
   A   
   C   
   G   
   A   
   A   
   T   
   G   
   A   
   C   
   A   
   G   
   A   
   G   
   T   
   T   
   C   
   G   
   A   
   A   
   C   
   C   
   A   
   T   
   G   
   G   
   C   
   G   
   C   
   A   
   C   
   C   
   T   
   A   
    T    
   A   
   A   
   G   
   A   
   G   
   C   
   C   
   G   
    T    
   C   
   C   
   G   
   C   
   T   
   C   
   A   
   G   
   C   
   T   
   G   
   T   
   A   
   T   
   G   
   A   
   A   
   G   
   C   
   T   
   A   
   A   
   A   
   G   
   G   
   C   
   G   
   G   
   A   
   A   
   G   
   A   
   A   
   G   
   A   
   T   
   G   
   A   
   A   
   C   
   T   
    G    
   A   
   T   
   T   
   G   
   C   
   T   
   G   
   T   
    G    
   T   
   A   
   T   
   C   
   T   
   G   
   A   
   A   
   A   
   G   
   A   
   A   
   G   
   A   
   T   
   C   
    A    
   G   
   C   
   C   
   A   
   G   
   G   
   C   
   T   
   T   
   A   
   G   
   A   
   T   
   G   
   T   
    T    
   T   
   T   
   C   
   T   
   T   
   A   
   G   
   T   
   A   
   A   
   C   
   G   
    C    
   A   
   T   
   C   
   A   
   A   
   G   
   G   
   A   
   A   
   T   
   G   
   G   
   G   
    T    
   T   
   T   
   G   
   C   
   G   
   C   
   T   
    A    
   T   
   C   
   C   
   T   
   T   
   T   
   A   
   A   
    C    
   A   
   G   
   A   
   T   
   G   
   T   
   T   
   C   
   C   
   C   
   G   
   T   
   T   
   G   
   T   
   T   
   G   
   G   
   A   
   G   
   C   
   A   
   A   
   A   
   G   
   G   
   C   
   A   
   G   
    C    
   T   
   G   
   G   
   A   
   G   
   T   
   G   
   A   
   A   
   G   
   T   
   C   
   T   
   A   
   T   
   G   
   A   
   A   
   T   
   T   
   T   
   G   
   A   
   A   
   A   
   G   
   A   
   T   
   G   
   G   
   A   
   G   
   A   
   T   
   A   
   G   
   G   
    A    
   T   
   T   
    A    
   T   
   C   
   A   
   A   
    C    
   G   
   G   
    G    
   C   
   T   
    A    
   C   
   T   
   T   
   G   
   T   
   G   
   T   
   T   
   A   
   G   
   C   
   A   
   G   
   A   
    A    
   G   
   G   
    T    
   G   
   A   
   T   
   A   
   C   
   G   
   C   
   C   
    G    
   A   
   T   
   C   
   G   
   T   
   A   
   A   
   T   
   C   
   G   
   T   
   T   
   A   
   C   
   T   
   C   
   A   
   G   
   C   
   G   
   A   
   G   
   G   
   A   
   G   
   C   
    T    
   G   
   T   
    T    
   A   
   A   
   A   
   C   
   G   
   C   
   A   
   T   
   G   
   C   
   T   
   T   
   G   
   C   
   T   
   C   
   A   
   A   
   G   
   A   
   A   
   A   
   T   
   C   
   A   
   G   
   C   
   C   
   A   
   A   
   A   
   C   
    T    
   T   
   C   
   A   
   C   
   G   
   T   
   G   
   C   
   G   
   A   
   A   
   G   
   C   
   G   
   G   
   G   
   G   
   A   
   G   
   T   
   G   
   A   
   C   
   G   
   G   
   T   
   C   
   A   
   T   
   G   
   C   
   G   
   T   
   G   
   A   
   G   
   C   
   T   
   G   
   A   
   A   
   A   
   A   
   A   
   A   
   A   
   A   
   T   
   C   
   C   
    G    
   C   
   A   
    C    
   C   
   G   
    T    
   A   
   T   
   T   
   A   
   T   
   C   
   T   
   A   
   T   
   A   
   T   
   G   
   T   
   C   
   G   
   G   
   A   
   A   
   G   
   G   
   A   
   A   
   G   
   A   
   T   
   C   
   A   
   A   
   A   
   A   
   G   
   A   
   A   
   A   
   T   
   A   
   A   
   C   
   C   
   T   
   T   
   G   
   A   
   T   
   G   
   A   
   A   
   T   
   C   
   A   
   A   
   A   
   A   
   A   
   G   
   G   
    G    
   C   
   A   
    G    
   C   
   A   
   G   
    T    
   T   
   A   
   A   
   C   
   G   
   A   
   T   
   T   
   G   
   A   
    C    
   C   
   C   
   A   
   A   
   C   
    T    
   G   
   A   
   T   
   T   
   T   
    C    
   C   
   C   
   T   
   A   
   T   
   A   
   G   
   G   
   T   
   G   
   A   
   T   
   C   
   G   
   A   
   A   
   C   
   A   
   T   
   C   
   G   
   A   
   A   
   T   
   G   
   G   
    A    
   T   
   C   
    A    
   T   
   T   
   T   
   G   
   C   
    G    
   A   
   T   
   G   
   G   
   A   
    T    
   G   
   A   
   G   
   A   
   A   
   A   
   A   
   A   
   A   
   G   
   G   
   T   
   G   
   G   
   A   
   G   
   A   
   A   
   G   
   T   
   C   
   A   
   T   
   C   
   G   
   A   
   A   
   G   
   T   
    T    
   A   
    T    
   C   
   G   
   A   
   T   
   G   
   C   
   T   
   C   
   C   
   T   
   G   
   A   
   G   
    A    
   T   
   T   
   T   
   T   
    C    
   A   
   T   
    C    
   G   
   A   
    G    
    T    
   C   
    G    
   -   
   -   
   -   
 

 
   N59754F   
   A   
   T   
   G   
   G   
   A   
   A   
   A   
   A   
   T   
   C   
   G   
   A   
   C   
   A   
   A   
   G   
   A   
   A   
   G   
   T   
   A   
   C   
   A   
   A   
   G   
   A   
   A   
   T   
   T   
   A   
   A   
   C   
   T   
   T   
   T   
   A   
   G   
   A   
   A   
   G   
   A   
   A   
   G   
   T   
   G   
   A   
   T   
   G   
   G   
   G   
   T   
   G   
   A   
   C   
   C   
   G   
   C   
   T   
   T   
   C   
   G   
   G   
   A   
   C   
   G   
   T   
   T   
   A   
   T   
   T   
   C   
   C   
   A   
   A   
   A   
   T   
   A   
   T   
   A   
   T   
   T   
   A   
   T   
    C    
   C   
   A   
    A    
   G   
   A   
   A   
   C   
   G   
   T   
   G   
   C   
   A   
   C   
   T   
   G   
   C   
   C   
   T   
   G   
   A   
   T   
   A   
   T   
    C    
   C   
   G   
   T   
   G   
   A   
   T   
   G   
   G   
   A   
   T   
   T   
   G   
   A   
   A   
    G    
   C   
   C   
   A   
   G   
   T   
    G    
   C   
   A   
   A   
   C   
   G   
   C   
   C   
   G   
   G   
   A   
   T   
   A   
   T   
   T   
   A   
   T   
   T   
    T    
   G   
   C   
    C    
   A   
   T   
   G   
   A   
   A   
   C   
   A   
   A   
   A   
   G   
   A   
   T   
   G   
   G   
   C   
   A   
   A   
   T   
   A   
   C   
   G   
   T   
   A   
   T   
   G   
   A   
   C   
   A   
   A   
   A   
   G   
   G   
   A   
   T   
   T   
    C    
   C   
   G   
   T   
   A   
   A   
   A   
   T   
   C   
   A   
   G   
   C   
   A   
   A   
   A   
   A   
   T   
   C   
   T   
   G   
   T   
   C   
   G   
   G   
   A   
   A   
   A   
   T   
   A   
   T   
   C   
   A   
   T   
   G   
   G   
   G   
    G    
   A   
   A   
   T   
   T   
   A   
   T   
   C   
   A   
   T   
   C   
   C   
   T   
   C   
   A   
   T   
   G   
   G   
   T   
   G   
   A   
   C   
   A   
   G   
   C   
   A   
   G   
   T   
   A   
   T   
   T   
   T   
   A   
   T   
   G   
   A   
   A   
   G   
   C   
   G   
   A   
   T   
   G   
   G   
   T   
   C   
   C   
   G   
   C   
   A   
   T   
   G   
   A   
   G   
   C   
   C   
   A   
   A   
   G   
   A   
    C    
   T   
   G   
   G   
   A   
   A   
   G   
   T   
   T   
   G   
   C   
   G   
   G   
   G   
   A   
   A   
   G   
   T   
   A   
   C   
   T   
   G   
   A   
   T   
   C   
   G   
   A   
    A    
   A   
   T   
   G   
   C   
   A   
   C   
   G   
   G   
    G    
   A   
   A   
   T   
   A   
   A   
    C    
   G   
   G   
   G   
   A   
   G   
   T   
   A   
   T   
   G   
   G   
   A   
   C   
   G   
   G   
   T   
   G   
   A   
   T   
   C   
   C   
   A   
   C   
   C   
   T   
   G   
   C   
   T   
   G   
   C   
   G   
   A   
   T   
   G   
   C   
   G   
   T   
   T   
   A   
   T   
   A   
   C   
   T   
   G   
   A   
   A   
   G   
   C   
   C   
   A   
   G   
    A    
   T   
   T   
   A   
   T   
   C   
   C   
   A   
   A   
   G   
   T   
   T   
   A   
   A   
   G   
   T   
   G   
   G   
   A   
   G   
   A   
   G   
   A   
   T   
   G   
    C    
   T   
   A   
    G    
   C   
   A   
   G   
   A   
   T   
   A   
   T   
   C   
   G   
   A   
    A    
   A   
   A   
   A   
   G   
   A   
   A   
   A   
   C   
   A   
   G   
   T   
    C    
   G   
   A   
   T   
   C   
   T   
   C   
   G   
   T   
   G   
   T   
   G   
   G   
   A   
   A   
   T   
   T   
   T   
   T   
   G   
   A   
   T   
   G   
   A   
   T   
   A   
   C   
   A   
   G   
   A   
   A   
   A   
   A   
   A   
   G   
   A   
   A   
   C   
   C   
   A   
   A   
   C   
   A   
   G   
   T   
   C   
   C   
   T   
   T   
   C   
   C   
   A   
   G   
   C   
   T   
   C   
   G   
   A   
   T   
   A   
   C   
   C   
   C   
   A   
   A   
   A   
   C   
   C   
   T   
   T   
   C   
   T   
    C    
   G   
   T   
   C   
   A   
   A   
   T   
   G   
   G   
   A   
   T   
   C   
   G   
   A   
   C   
   A   
   G   
   G   
   T   
   A   
   T   
   A   
   T   
   C   
   T   
   G   
   C   
   T   
   G   
   G   
   A   
   T   
   A   
   T   
   G   
   C   
    A    
   A   
   C   
   A   
   G   
   A   
   A   
   A   
   T   
   C   
   C   
   C   
   G   
   A   
   C   
    T    
   C   
   A   
   T   
   A   
   A   
   C   
   T   
   T   
   A   
   G   
   C   
   T   
   G   
   A   
   A   
   G   
   T   
   G   
   A   
   T   
    T    
   G   
   A   
    T    
   G   
   G   
   C   
   A   
   C   
   A   
   A   
   T   
   C   
   T   
   A   
   T   
   A   
   T   
   G   
   A   
   T   
   C   
   G   
   A   
   T   
   C   
   A   
   T   
   C   
   C   
   A   
   C   
   A   
   A   
   G   
   C   
   G   
   A   
   G   
   C   
   C   
   T   
   T   
   G   
   A   
   A   
   A   
   A   
   A   
   T   
   T   
   G   
   A   
   T   
   G   
   G   
   A   
   A   
   T   
   A   
   T   
   A   
   T   
   T   
   C   
   C   
   T   
   G   
   G   
   A   
   C   
   C   
   T   
   G   
   A   
   C   
   T   
   T   
    T    
   C   
   C   
   A   
   A   
   C   
   A   
   G   
   G   
   A   
   G   
   G   
    G    
   A   
   T   
   C   
   T   
   T   
   G   
   C   
   A   
   A   
   G   
   G   
    A    
   A   
   A   
   A   
   G   
   A   
   T   
   G   
   A   
   A   
   A   
   T   
   C   
   A   
   A   
   A   
   A   
   A   
   A   
   G   
   C   
   T   
   T   
   A   
   T   
   G   
   A   
   A   
   A   
   C   
   A   
   G   
   G   
    G    
   C   
   G   
   A   
   G   
   G   
   A   
   A   
   A   
   A   
   G   
   T   
   T   
   A   
    T    
   T   
   C   
   T   
   T   
   C   
   G   
   T   
   T   
   C   
   G   
   A   
   A   
   A   
   A   
   C   
   A   
   A   
   A   
   G   
   A   
   T   
   A   
   G   
   A   
    G    
   G   
   C   
   A   
   A   
   T   
   G   
   A   
   A   
   G   
   G   
   G   
    T    
   A   
   A   
    T    
   A   
   A   
   A   
   C   
   A   
   G   
   C   
   A   
   A   
   A   
   T   
   C   
   G   
   T   
   G   
   A   
   T   
   C   
   A   
   C   
    A    
   G   
   A   
   A   
   A   
   T   
   C   
   C   
   C   
   A   
   T   
   A   
   T   
   G   
   A   
   A   
   G   
   T   
   A   
   A   
   A   
   T   
   A   
   A   
   A   
   G   
   C   
   G   
   A   
   C   
    C    
   T   
   T   
   G   
   G   
   T   
   G   
   A   
   A   
   A   
   A   
   A   
   A   
   A   
   T   
   G   
   G   
   A   
   T   
   G   
   A   
   G   
   A   
   T   
   C   
   C   
   G   
   T   
   T   
   T   
   G   
   A   
   A   
   T   
   A   
   A   
   G   
   A   
   A   
   A   
   A   
   T   
   C   
   G   
   A   
    T    
   G   
   G   
   A   
   A   
   T   
   A   
   G   
   C   
   T   
   G   
   A   
   A   
   G   
   T   
    A    
   C   
   G   
   A   
   G   
   A   
   T   
   G   
   A   
   A   
   A   
   G   
   T   
   G   
   A   
   T   
    A    
   G   
    A    
   A   
   C   
    A    
   G   
   G   
   T   
   C   
   T   
   G   
   C   
   A   
   A   
   A   
   T   
   T   
   G   
   T   
   C   
   G   
   T   
    C    
   G   
   A   
   A   
   T   
   T   
   G   
   A   
   A   
   A   
   A   
   A   
   A   
   G   
   A   
    C    
   G   
   C   
   C   
   A   
   A   
    T    
   G   
   C   
   T   
   C   
   A   
   A   
   G   
   G   
    A    
   A   
   T   
   T   
   T   
   T   
   G   
   A   
   A   
   T   
   T   
   A   
    T    
   T   
   T   
   G   
   T   
   T   
   C   
   A   
   A   
   A   
   A   
   A   
   C   
   A   
   C   
   T   
   G   
   A   
   G   
    C    
   T   
   A   
   C   
   A   
   G   
   A   
   T   
   C   
   A   
   A   
   T   
   T   
   A   
   C   
   A   
   A   
   T   
   T   
   T   
    C    
   A   
   A   
   T   
   A   
   T   
   G   
   G   
   T   
   C   
   G   
   C   
   T   
   A   
   T   
   C   
   G   
   A   
    C    
   C   
   A   
   T   
   A   
   T   
   G   
   A   
   C   
   T   
   C   
   C   
   G   
   C   
   A   
    T    
   C   
   A   
   A   
   G   
   T   
   T   
   G   
   G   
   A   
   C   
   T   
   G   
   A   
   A   
   A   
   G   
   A   
   C   
   A   
   T   
   T   
   T   
   T   
   A   
   A   
   G   
   A   
   A   
   G   
   T   
   T   
   A   
   T   
   A   
   T   
   C   
   G   
   A   
   G   
   C   
   A   
   T   
   C   
   G   
   A   
   A   
   A   
   G   
   C   
   A   
   A   
   G   
   T   
   C   
   A   
   T   
   A   
   A   
   C   
   A   
   A   
   A   
   A   
   C   
   G   
    T    
   A   
   G   
    T    
   C   
   A   
   A   
   T   
   T   
   C   
   G   
   A   
   T   
   T   
   T   
   A   
   G   
   C   
   C   
   A   
   A   
   A   
   G   
   C   
   T   
   C   
   A   
   A   
   A   
   A   
   A   
    A    
   G   
   A   
   C   
   A   
   G   
   C   
   A   
   T   
   A   
   T   
   C   
   G   
   T   
    A    
   G   
   A   
   A   
   G   
   G   
    A    
    T    
   T   
   G   
   A   
   T   
   G   
   A   
   A   
   A   
   G   
   C   
   T   
   T   
   T   
   A   
   T   
   C   
   G   
   A   
   T   
   T   
   C   
   T   
   G   
   G   
   A   
   T   
   G   
   A   
   A   
   G   
   T   
   A   
   A   
   T   
   C   
   G   
    T    
   A   
   A   
   C   
   G   
   A   
   T   
   T   
   C   
   G   
   T   
   G   
   A   
   A   
   A   
   G   
   T   
   A   
   A   
   A   
   G   
   A   
   C   
   A   
   A   
   A   
   A   
   A   
   A   
   G   
   A   
    C    
   G   
   C   
    C    
   A   
   A   
   A   
   A   
   A   
   G   
   A   
   A   
   T   
   T   
   T   
   A   
   G   
   T   
   C   
   G   
   A   
    C    
   G   
   T   
    C    
   T   
   T   
    T    
   C   
   A   
   A   
   T   
   T   
   T   
   A   
   C   
   A   
   G   
   A   
   A   
   G   
   A   
   A   
   C   
   A   
   A   
   G   
   C   
   A   
   G   
   A   
   A   
   G   
   C   
   A   
   A   
   T   
    T    
   G   
   T   
   C   
   A   
   C   
   A   
   T   
   T   
   G   
   C   
   A   
   A   
    T    
   T   
   A   
   T   
   A   
    C    
   C   
   G   
   T   
   T   
   T   
   G   
   A   
   C   
   G   
   A   
   A   
    C    
   A   
   C   
    G    
   G   
   A   
   T   
   A   
   T   
   C   
   A   
   C   
   A   
   G   
   A   
   G   
   C   
   T   
   T   
   C   
   A   
   A   
   A   
   A   
   G   
   G   
   A   
   A   
    T    
   C   
    C    
   G   
   A   
   A   
   A   
    G    
   C   
   T   
   T   
   G   
   A   
   T   
   T   
   G   
   C   
    G    
   C   
   A   
   A   
   A   
   T   
   C   
   A   
   C   
   A   
   G   
   A   
   G   
   T   
   T   
    G    
   A   
   A   
   T   
   A   
   A   
   A   
   A   
   T   
   C   
   C   
   T   
   T   
   T   
   C   
   A   
   A   
   A   
    T    
   G   
   A   
   C   
   A   
   A   
   A   
   G   
   A   
    G    
   C   
   T   
   T   
   T   
   T   
    C    
   T   
   C   
   T   
   G   
   T   
   T   
   A   
   T   
   G   
   A   
   A   
   A   
   A   
   A   
   A   
   G   
   A   
   A   
   T   
   T   
   G   
   C   
   G   
   A   
   G   
   A   
   A   
   G   
   T   
   G   
   A   
   A   
   A   
   A   
   A   
   A   
   A   
   A   
   T   
   T   
   A   
    T    
   T   
   C   
   T   
   T   
   C   
   C   
   G   
   C   
   T   
   C   
   G   
   T   
   C   
    T    
    A    
   A   
   C   
   G   
   A   
   C   
   A   
   A   
   T   
   C   
    G    
   A   
   A   
   G   
   A   
   C   
   G   
   A   
   A   
   A   
   T   
   C   
   G   
   A   
   A   
   G   
   A   
   A   
   A   
   T   
   C   
   A   
   A   
   A   
   A   
   T   
    T    
   G   
   A   
   T   
   A   
   C   
   G   
   C   
   A   
   A   
    G    
   T   
    C    
   T   
   T   
   A   
   G   
   T   
   G   
   G   
   C   
    A    
   C   
   A   
   A   
   G   
   A   
   A   
   G   
   A   
   C   
   G   
   T   
   A   
   A   
   T   
    T    
   G   
   T   
   C   
   A   
   G   
   T   
   G   
   T   
    G    
   A   
   C   
    G    
   C   
   G   
   A   
   G   
   A   
   A   
   G   
   G   
   C   
   T   
   A   
   T   
   G   
   T   
   G   
   A   
   A   
   G   
   C   
   G   
   A   
   A   
   C   
   A   
   A   
   G   
   C   
   C   
   T   
   T   
   C   
   G   
   T   
   T   
   C   
    T    
   T   
   A   
   T   
   A   
   G   
   T   
   G   
   C   
   T   
   T   
   C   
    C    
   A   
   A   
   A   
   C   
   C   
   A   
   G   
   A   
   A   
   G   
   A   
   A   
   A   
   T   
   C   
   G   
   G   
   G   
   A   
   T   
   G   
   C   
   G   
   A   
   G   
   A   
   A   
   G   
   G   
   C   
   G   
   A   
    C    
   T   
   A   
   C   
   T   
   T   
   G   
   C   
   T   
    A    
   T   
   A   
   C   
    T    
   C   
   A   
   G   
   G   
   T   
   G   
   A   
   A   
   T   
   T   
    G    
   A   
   G   
    T    
   A   
   C   
   A   
    T    
   T   
   G   
   G   
   A   
   C   
   C   
   A   
   T   
   G   
   T   
   T   
   C   
   T   
   T   
   C   
   T   
   G   
   A   
   T   
    C    
   A   
   C   
    A    
   A   
   A   
   T   
   A   
   A   
   A   
   G   
   C   
   A   
   A   
   A   
   T   
   G   
   T   
   G   
   A   
   T   
   C   
   T   
   A   
   C   
   C   
   G   
    C    
   C   
   C   
   T   
   G   
   T   
    C    
   C   
   A   
   T   
   G   
   A   
   A   
   C   
   T   
   A   
   C   
   C   
    C    
   G   
   A   
   T   
   T   
   T   
   G   
   A   
   A   
   A   
   T   
   G   
   G   
   A   
   A   
   A   
   G   
   A   
   T   
   G   
   C   
   T   
   G   
   G   
    T    
   G   
   A   
    G    
   C   
   A   
    C    
   A   
   T   
   T   
   T   
   C   
   T   
   C   
   A   
   A   
   A   
   C   
   A   
   A   
   T   
   C   
   T   
   C   
   C   
   A   
   A   
   T   
   C   
   T   
   G   
   T   
   C   
   T   
   G   
   T   
   A   
   G   
   A   
    C    
   G   
   A   
   A   
   T   
   C   
   G   
   A   
   T   
    T    
   T   
   T   
   A   
   G   
   C   
   T   
   G   
   T   
   T   
   T   
   T   
    C    
   C   
   C   
   A   
   T   
   A   
   T   
   C   
   A   
   A   
   A   
   A   
   A   
   A   
   T   
    T    
   C   
   A   
   A   
   G   
   C   
   A   
   G   
   A   
    G    
   A   
   A   
   A   
   A   
   C   
   A   
   T   
   T   
   T   
   G   
   T   
    A    
   T   
   T   
   C   
   A   
   T   
   C   
   A   
   G   
   C   
   A   
   A   
   G   
   A   
   A   
   T   
   G   
   G   
   A   
   T   
   T   
    G    
    A    
   T   
   C   
   A   
   A   
   G   
   C   
   A   
   A   
   A   
   C   
   A   
   C   
   G   
   A   
   A   
   T   
   G   
   A   
   C   
   A   
   G   
   A   
   G   
   T   
   T   
   C   
   G   
   A   
   A   
   C   
   C   
   A   
   T   
   G   
   G   
   C   
   G   
   C   
   A   
   C   
   C   
   T   
   A   
    T    
   A   
   A   
   G   
   A   
   G   
   C   
   C   
   G   
    T    
   C   
   C   
   G   
   C   
   T   
   C   
   A   
   G   
   C   
   T   
   G   
   T   
   A   
   T   
   G   
   A   
   A   
   G   
   C   
   T   
   A   
   A   
   A   
   G   
   G   
   C   
   G   
   G   
   A   
   A   
   G   
   A   
   A   
   G   
   A   
   T   
   G   
   A   
   A   
   C   
   T   
    G    
   A   
   T   
   T   
   G   
   C   
   T   
   G   
   T   
    G    
   T   
   A   
   T   
   C   
   T   
   G   
   A   
   A   
   A   
   G   
   A   
   A   
   G   
   A   
   T   
   C   
    A    
   G   
   C   
   C   
   A   
   G   
   G   
   C   
   T   
   T   
   A   
   G   
   A   
   T   
   G   
   T   
    T    
   T   
   T   
   C   
   T   
   T   
   A   
   G   
   T   
   A   
   A   
   C   
   G   
    C    
   A   
   T   
   C   
   A   
   A   
   G   
   G   
   A   
   A   
   T   
   G   
   G   
   G   
    T    
   T   
   T   
   G   
   C   
   G   
   C   
   T   
    A    
   T   
   C   
   C   
   T   
   T   
   T   
   A   
   A   
    C    
   A   
   G   
   A   
   T   
   G   
   T   
   T   
   C   
   C   
   C   
   G   
   T   
   T   
   G   
   T   
   T   
   G   
   G   
   A   
   G   
   C   
   A   
   A   
   A   
   G   
   G   
   C   
   A   
   G   
    C    
   T   
   G   
   G   
   A   
   G   
   T   
   G   
   A   
   A   
   G   
   T   
   C   
   T   
   A   
   T   
   G   
   A   
   A   
   T   
   T   
   T   
   G   
   A   
   A   
   A   
   G   
   A   
   T   
   G   
   G   
   A   
   G   
   A   
   T   
   A   
   G   
   G   
    A    
   T   
   T   
    A    
   T   
   C   
   A   
   A   
    C    
   G   
   G   
    G    
   C   
   T   
    G    
   C   
   T   
   T   
   G   
   T   
   G   
   T   
   T   
   A   
   G   
   C   
   A   
   G   
   A   
    A    
   G   
   G   
    T    
   G   
   A   
   T   
   A   
   C   
   G   
   C   
   C   
    G    
   A   
   T   
   C   
   G   
   T   
   A   
   A   
   T   
   C   
   G   
   T   
   T   
   A   
   C   
   T   
   C   
   A   
   G   
   C   
   G   
   A   
   G   
   G   
   A   
   G   
   C   
    T    
   G   
   T   
    T    
   A   
   A   
   A   
   C   
   G   
   C   
   A   
   T   
   G   
   C   
   T   
   T   
   G   
   C   
   T   
   C   
   A   
   A   
   G   
   A   
   A   
   A   
   T   
   C   
   A   
   G   
   C   
   C   
   A   
   A   
   A   
   C   
    T    
   T   
   C   
   A   
   C   
   G   
   T   
   G   
   C   
   G   
   A   
   A   
   G   
   C   
   G   
   G   
   G   
   G   
   A   
   G   
   T   
   G   
   A   
   C   
   G   
   G   
   T   
   C   
   A   
   T   
   G   
   C   
   G   
   T   
   G   
   A   
   G   
   C   
   T   
   G   
   A   
   A   
   A   
   A   
   A   
   A   
   A   
   A   
   T   
   C   
   C   
    G    
   C   
   A   
    C    
   C   
   G   
    T    
   A   
   T   
   T   
   A   
   T   
   C   
   T   
   A   
   T   
   A   
   T   
   G   
   T   
   C   
   G   
   G   
   A   
   A   
   G   
   G   
   A   
   A   
   G   
   A   
   T   
   C   
   A   
   A   
   A   
   A   
   G   
   A   
   A   
   A   
   T   
   A   
   A   
   C   
   C   
   T   
   T   
   G   
   A   
   T   
   G   
   A   
   A   
   T   
   C   
   A   
   A   
   A   
   A   
   A   
   G   
   G   
    G    
   C   
   A   
    G    
   C   
   A   
   G   
    T    
   T   
   A   
   A   
   C   
   G   
   A   
   T   
   T   
   G   
   A   
    C    
   C   
   C   
   A   
   A   
   C   
    T    
   G   
   A   
   T   
   T   
   T   
    C    
   C   
   C   
   T   
   A   
   T   
   A   
   G   
   G   
   T   
   G   
   A   
   T   
   C   
   G   
   A   
   A   
   C   
   A   
   T   
   C   
   G   
   A   
   A   
   T   
   G   
   G   
    A    
   T   
   C   
    A    
   T   
   T   
   T   
   G   
   C   
    G    
   A   
   T   
   G   
   G   
   A   
    T    
   G   
   A   
   G   
   A   
   A   
   A   
   A   
   A   
   A   
   G   
   G   
   T   
   G   
   G   
   A   
   G   
   A   
   A   
   G   
   T   
   C   
   A   
   T   
   C   
   G   
   A   
   A   
   G   
   T   
    T    
   A   
    T    
   C   
   G   
   A   
   T   
   G   
   C   
   T   
   C   
   C   
   T   
   G   
   A   
   G   
    A    
   T   
   T   
   T   
   T   
    C    
   A   
   T   
    C    
   G   
   A   
    G    
    T    
   C   
    G    
   -   
   -   
   -   
 

 
   N52824   
   A   
   T   
   G   
   G   
   A   
   A   
   A   
   A   
   T   
   C   
   G   
   A   
   C   
   A   
   A   
   G   
   A   
   A   
   G   
   T   
   A   
   C   
   A   
   A   
   G   
   A   
   A   
   T   
   T   
   A   
   A   
   C   
   T   
   T   
   T   
   A   
   G   
   A   
   A   
   G   
   A   
   A   
   G   
   T   
   G   
   A   
   T   
   G   
   G   
   G   
   T   
   G   
   A   
   C   
   C   
   G   
   C   
   T   
   T   
   C   
   G   
   G   
   A   
   C   
   G   
   T   
   T   
   A   
   T   
   T   
   C   
   C   
   A   
   A   
   A   
   T   
   A   
   T   
   A   
   T   
   T   
   A   
   T   
    C    
   C   
   A   
    A    
   G   
   A   
   A   
   C   
   G   
   T   
   G   
   C   
   A   
   C   
   T   
   G   
   C   
   C   
   T   
   G   
   A   
   T   
   A   
   T   
    C    
   C   
   G   
   T   
   G   
   A   
   T   
   G   
   G   
   A   
   T   
   T   
   G   
   A   
   A   
    G    
   C   
   C   
   A   
   G   
   T   
    G    
   C   
   A   
   A   
   C   
   G   
   C   
   C   
   G   
   G   
   A   
   T   
   A   
   T   
   T   
   A   
   T   
   T   
    T    
   G   
   C   
    C    
   A   
   T   
   G   
   A   
   A   
   C   
   A   
   A   
   A   
   G   
   A   
   T   
   G   
   G   
   C   
   A   
   A   
   T   
   A   
   C   
   G   
   T   
   A   
   T   
   G   
   A   
   C   
   A   
   A   
   A   
   G   
   G   
   A   
   T   
   T   
    C    
   C   
   G   
   T   
   A   
   A   
   A   
   T   
   C   
   A   
   G   
   C   
   A   
   A   
   A   
   A   
   T   
   C   
   T   
   G   
   T   
   C   
   G   
   G   
   A   
   A   
   A   
   T   
   A   
   T   
   C   
   A   
   T   
   G   
   G   
   G   
    G    
   A   
   A   
   T   
   T   
   A   
   T   
   C   
   A   
   T   
   C   
   C   
   T   
   C   
   A   
   T   
   G   
   G   
   T   
   G   
   A   
   C   
   A   
   G   
   C   
   A   
   G   
   T   
   A   
   T   
   T   
   T   
   A   
   T   
   G   
   A   
   A   
   G   
   C   
   G   
   A   
   T   
   G   
   G   
   T   
   C   
   C   
   G   
   C   
   A   
   T   
   G   
   A   
   G   
   C   
   C   
   A   
   A   
   G   
   A   
    C    
   T   
   G   
   G   
   A   
   A   
   G   
   T   
   T   
   G   
   C   
   G   
   G   
   G   
   A   
   A   
   G   
   T   
   A   
   C   
   T   
   G   
   A   
   T   
   C   
   G   
   A   
    A    
   A   
   T   
   G   
   C   
   A   
   C   
   G   
   G   
    G    
   A   
   A   
   T   
   A   
   A   
    C    
   G   
   G   
   G   
   A   
   G   
   T   
   A   
   T   
   G   
   G   
   A   
   C   
   G   
   G   
   T   
   G   
   A   
   T   
   C   
   C   
   A   
   C   
   C   
   T   
   G   
   C   
   T   
   G   
   C   
   G   
   A   
   T   
   G   
   C   
   G   
   T   
   T   
   A   
   T   
   A   
   C   
   T   
   G   
   A   
   A   
   G   
   C   
   C   
   A   
   G   
    A    
   T   
   T   
   A   
   T   
   C   
   C   
   A   
   A   
   G   
   T   
   T   
   A   
   A   
   G   
   T   
   G   
   G   
   A   
   G   
   A   
   G   
   A   
   T   
   G   
    C    
   T   
   A   
    G    
   C   
   A   
   G   
   A   
   T   
   A   
   T   
   C   
   G   
   A   
    A    
   A   
   A   
   A   
   G   
   A   
   A   
   A   
   C   
   A   
   G   
   T   
    C    
   G   
   A   
   T   
   C   
   T   
   C   
   G   
   T   
   G   
   T   
   G   
   G   
   A   
   A   
   T   
   T   
   T   
   T   
   G   
   A   
   T   
   G   
   A   
   T   
   A   
   C   
   A   
   G   
   A   
   A   
   A   
   A   
   A   
   G   
   A   
   A   
   C   
   C   
   A   
   A   
   C   
   A   
   G   
   T   
   C   
   C   
   T   
   T   
   C   
   C   
   A   
   G   
   C   
   T   
   C   
   G   
   A   
   T   
   A   
   C   
   C   
   C   
   A   
   A   
   A   
   C   
   C   
   T   
   T   
   C   
   T   
    C    
   G   
   T   
   C   
   A   
   A   
   T   
   G   
   G   
   A   
   T   
   C   
   G   
   A   
   C   
   A   
   G   
   G   
   T   
   A   
   T   
   A   
   T   
   C   
   T   
   G   
   C   
   T   
   G   
   G   
   A   
   T   
   A   
   T   
   G   
   C   
    A    
   A   
   C   
   A   
   G   
   A   
   A   
   A   
   T   
   C   
   C   
   C   
   G   
   A   
   C   
    T    
   C   
   A   
   T   
   A   
   A   
   C   
   T   
   T   
   A   
   G   
   C   
   T   
   G   
   A   
   A   
   G   
   T   
   G   
   A   
   T   
    T    
   G   
   A   
    T    
   G   
   G   
   C   
   A   
   C   
   A   
   A   
   T   
   C   
   T   
   A   
   T   
   A   
   T   
   G   
   A   
   T   
   C   
   G   
   A   
   T   
   C   
   A   
   T   
   C   
   C   
   A   
   C   
   A   
   A   
   G   
   C   
   G   
   A   
   G   
   C   
   C   
   T   
   T   
   G   
   A   
   A   
   A   
   A   
   A   
   T   
   T   
   G   
   A   
   T   
   G   
   G   
   A   
   A   
   T   
   A   
   T   
   A   
   T   
   T   
   C   
   C   
   T   
   G   
   G   
   A   
   C   
   C   
   T   
   G   
   A   
   C   
   T   
   T   
    T    
   C   
   C   
   A   
   A   
   C   
   A   
   G   
   G   
   A   
   G   
   G   
    G    
   A   
   T   
   C   
   T   
   T   
   G   
   C   
   A   
   A   
   G   
   G   
    A    
   A   
   A   
   A   
   G   
   A   
   T   
   G   
   A   
   A   
   A   
   T   
   C   
   A   
   A   
   A   
   A   
   A   
   A   
   G   
   C   
   T   
   T   
   A   
   T   
   G   
   A   
   A   
   A   
   C   
   A   
   G   
   G   
    G    
   C   
   G   
   A   
   G   
   G   
   A   
   A   
   A   
   A   
   G   
   T   
   T   
   A   
    T    
   T   
   C   
   T   
   T   
   C   
   G   
   T   
   T   
   C   
   G   
   A   
   A   
   A   
   A   
   C   
   A   
   A   
   A   
   G   
   A   
   T   
   A   
   G   
   A   
    G    
   G   
   C   
   A   
   A   
   T   
   G   
   A   
   A   
   G   
   G   
   G   
    T    
   A   
   A   
    T    
   A   
   A   
   A   
   C   
   A   
   G   
   C   
   A   
   A   
   A   
   T   
   C   
   G   
   T   
   G   
   A   
   T   
   C   
   A   
   C   
    A    
   G   
   A   
   A   
   A   
   T   
   C   
   C   
   C   
   A   
   T   
   A   
   T   
   G   
   A   
   A   
   G   
   T   
   A   
   A   
   A   
   T   
   A   
   A   
   A   
   G   
   C   
   G   
   A   
   C   
    C    
   T   
   T   
   G   
   G   
   T   
   G   
   A   
   A   
   A   
   A   
   A   
   A   
   A   
   T   
   G   
   G   
   A   
   T   
   G   
   A   
   G   
   A   
   T   
   C   
   C   
   G   
   T   
   T   
   T   
   G   
   A   
   A   
   T   
   A   
   A   
   G   
   A   
   A   
   A   
   A   
   T   
   C   
   G   
   A   
    T    
   G   
   G   
   A   
   A   
   T   
   A   
   G   
   C   
   T   
   G   
   A   
   A   
   G   
   T   
    A    
   C   
   G   
   A   
   G   
   A   
   T   
   G   
   A   
   A   
   A   
   G   
   T   
   G   
   A   
   T   
    A    
   G   
    A    
   A   
   C   
    A    
   G   
   G   
   T   
   C   
   T   
   G   
   C   
   A   
   A   
   A   
   T   
   T   
   G   
   T   
   C   
   G   
   T   
    C    
   G   
   A   
   A   
   T   
   T   
   G   
   A   
   A   
   A   
   A   
   A   
   A   
   G   
   A   
    C    
   G   
   C   
   C   
   A   
   A   
    T    
   G   
   C   
   T   
   C   
   A   
   A   
   G   
   G   
    A    
   A   
   T   
   T   
   T   
   T   
   G   
   A   
   A   
   T   
   T   
   A   
    T    
   T   
   T   
   G   
   T   
   T   
   C   
   A   
   A   
   A   
   A   
   A   
   C   
   A   
   C   
   T   
   G   
   A   
   G   
    C    
   T   
   A   
   C   
   A   
   G   
   A   
   T   
   C   
   A   
   A   
   T   
   T   
   A   
   C   
   A   
   A   
   T   
   T   
   T   
    C    
   A   
   A   
   T   
   A   
   T   
   G   
   G   
   T   
   C   
   G   
   C   
   T   
   A   
   T   
   C   
   G   
   A   
    C    
   C   
   A   
   T   
   A   
   T   
   G   
   A   
   C   
   T   
   C   
   C   
   G   
   C   
   A   
    T    
   C   
   A   
   A   
   G   
   T   
   T   
   G   
   G   
   A   
   C   
   T   
   G   
   A   
   A   
   A   
   G   
   A   
   C   
   A   
   T   
   T   
   T   
   T   
   A   
   A   
   G   
   A   
   A   
   G   
   T   
   T   
   A   
   T   
   A   
   T   
   C   
   G   
   A   
   G   
   C   
   A   
   T   
   C   
   G   
   A   
   A   
   A   
   G   
   C   
   A   
   A   
   G   
   T   
   C   
   A   
   T   
   A   
   A   
   C   
   A   
   A   
   A   
   A   
   C   
   G   
    T    
   A   
   G   
    T    
   C   
   A   
   A   
   T   
   T   
   C   
   G   
   A   
   T   
   T   
   T   
   A   
   G   
   C   
   C   
   A   
   A   
   A   
   G   
   C   
   T   
   C   
   A   
   A   
   A   
   A   
   A   
    A    
   G   
   A   
   C   
   A   
   G   
   C   
   A   
   T   
   A   
   T   
   C   
   G   
   T   
    A    
   G   
   A   
   A   
   G   
   G   
    A    
    T    
   T   
   G   
   A   
   T   
   G   
   A   
   A   
   A   
   G   
   C   
   T   
   T   
   T   
   A   
   T   
   C   
   G   
   A   
   T   
   T   
   C   
   T   
   G   
   G   
   A   
   T   
   G   
   A   
   A   
   G   
   T   
   A   
   A   
   T   
   C   
   G   
    T    
   A   
   A   
   C   
   G   
   A   
   T   
   T   
   C   
   G   
   T   
   G   
   A   
   A   
   A   
   G   
   T   
   A   
   A   
   A   
   G   
   A   
   C   
   A   
   A   
   A   
   A   
   A   
   A   
   G   
   A   
    C    
   G   
   C   
    C    
   A   
   A   
   A   
   A   
   A   
   G   
   A   
   A   
   T   
   T   
   T   
   A   
   G   
   T   
   C   
   G   
   A   
    C    
   G   
   T   
    C    
   T   
   T   
    T    
   C   
   A   
   A   
   T   
   T   
   T   
   A   
   C   
   A   
   G   
   A   
   A   
   G   
   A   
   A   
   C   
   A   
   A   
   G   
   C   
   A   
   G   
   A   
   A   
   G   
   C   
   A   
   A   
   T   
    T    
   G   
   T   
   C   
   A   
   C   
   A   
   T   
   T   
   G   
   C   
   A   
   A   
    T    
   T   
   A   
   T   
   A   
    C    
   C   
   G   
   T   
   T   
   T   
   G   
   A   
   C   
   G   
   A   
   A   
    C    
   A   
   C   
    G    
   G   
   A   
   T   
   A   
   T   
   C   
   A   
   C   
   A   
   G   
   A   
   G   
   C   
   T   
   T   
   C   
   A   
   A   
   A   
   A   
   G   
   G   
   A   
   A   
    T    
   C   
    C    
   G   
   A   
   A   
   A   
    G    
   C   
   T   
   T   
   G   
   A   
   T   
   T   
   G   
   C   
    G    
   C   
   A   
   A   
   A   
   T   
   C   
   A   
   C   
   A   
   G   
   A   
   G   
   T   
   T   
    G    
   A   
   A   
   T   
   A   
   A   
   A   
   A   
   T   
   C   
   C   
   T   
   T   
   T   
   C   
   A   
   A   
   A   
    T    
   G   
   A   
   C   
   A   
   A   
   A   
   G   
   A   
    G    
   C   
   T   
   T   
   T   
   T   
    C    
   T   
   C   
   T   
   G   
   T   
   T   
   A   
   T   
   G   
   A   
   A   
   A   
   A   
   A   
   A   
   G   
   A   
   A   
   T   
   T   
   G   
   C   
   G   
   A   
   G   
   A   
   A   
   G   
   T   
   G   
   A   
   A   
   A   
   A   
   A   
   A   
   A   
   A   
   T   
   T   
   A   
    T    
   T   
   C   
   T   
   T   
   C   
   C   
   G   
   C   
   T   
   C   
   G   
   T   
   C   
    T    
    A    
   A   
   C   
   G   
   A   
   C   
   A   
   A   
   T   
   C   
    G    
   A   
   A   
   G   
   A   
   C   
   G   
   A   
   A   
   A   
   T   
   C   
   G   
   A   
   A   
   G   
   A   
   A   
   A   
   T   
   C   
   A   
   A   
   A   
   A   
   T   
    T    
   G   
   A   
   T   
   A   
   C   
   G   
   C   
   A   
   A   
    G    
   T   
    C    
   T   
   T   
   A   
   G   
   T   
   G   
   G   
   C   
    A    
   C   
   A   
   A   
   G   
   A   
   A   
   G   
   A   
   C   
   G   
   T   
   A   
   A   
   T   
    T    
   G   
   T   
   C   
   A   
   G   
   T   
   G   
   T   
    G    
   A   
   C   
    G    
   C   
   G   
   A   
   G   
   A   
   A   
   G   
   G   
   C   
   T   
   A   
   T   
   G   
   T   
   G   
   A   
   A   
   G   
   C   
   G   
   A   
   A   
   C   
   A   
   A   
   G   
   C   
   C   
   T   
   T   
   C   
   G   
   T   
   T   
   C   
    T    
   T   
   A   
   T   
   A   
   G   
   T   
   G   
   C   
   T   
   T   
   C   
    C    
   A   
   A   
   A   
   C   
   C   
   A   
   G   
   A   
   A   
   G   
   A   
   A   
   A   
   T   
   C   
   G   
   G   
   G   
   A   
   T   
   G   
   C   
   G   
   A   
   G   
   A   
   A   
   G   
   G   
   C   
   G   
   A   
    C    
   T   
   A   
   C   
   T   
   T   
   G   
   C   
   T   
    A    
   T   
   A   
   C   
    T    
   C   
   A   
   G   
   G   
   T   
   G   
   A   
   A   
   T   
   T   
    G    
   A   
   G   
    T    
   A   
   C   
   A   
    T    
   T   
   G   
   G   
   A   
   C   
   C   
   A   
   T   
   G   
   T   
   T   
   C   
   T   
   T   
   C   
   T   
   G   
   A   
   T   
    C    
   A   
   C   
    A    
   A   
   A   
   T   
   A   
   A   
   A   
   G   
   C   
   A   
   A   
   A   
   T   
   G   
   T   
   G   
   A   
   T   
   C   
   T   
   A   
   C   
   C   
   G   
    C    
   C   
   C   
   T   
   G   
   T   
    C    
   C   
   A   
   T   
   G   
   A   
   A   
   C   
   T   
   A   
   C   
   C   
    C    
   G   
   A   
   T   
   T   
   T   
   G   
   A   
   A   
   A   
   T   
   G   
   G   
   A   
   A   
   A   
   G   
   A   
   T   
   G   
   C   
   T   
   G   
   G   
    T    
   G   
   A   
    G    
   C   
   A   
    C    
   A   
   T   
   T   
   T   
   C   
   T   
   C   
   A   
   A   
   A   
   C   
   A   
   A   
   T   
   C   
   T   
   C   
   C   
   A   
   A   
   T   
   C   
   T   
   G   
   T   
   C   
   T   
   G   
   T   
   A   
   G   
   A   
    C    
   G   
   A   
   A   
   T   
   C   
   G   
   A   
   T   
    T    
   T   
   T   
   A   
   G   
   C   
   T   
   G   
   T   
   T   
   T   
   T   
    C    
   C   
   C   
   A   
   T   
   A   
   T   
   C   
   A   
   A   
   A   
   A   
   A   
   A   
   T   
    T    
   C   
   A   
   A   
   G   
   C   
   A   
   G   
   A   
    G    
   A   
   A   
   A   
   A   
   C   
   A   
   T   
   T   
   T   
   G   
   T   
    A    
   T   
   T   
   C   
   A   
   T   
   C   
   A   
   G   
   C   
   A   
   A   
   G   
   A   
   A   
   T   
   G   
   G   
   A   
   T   
   T   
    G    
    A    
   T   
   C   
   A   
   A   
   G   
   C   
   A   
   A   
   A   
   C   
   A   
   C   
   G   
   A   
   A   
   T   
   G   
   A   
   C   
   A   
   G   
   A   
   G   
   T   
   T   
   C   
   G   
   A   
   A   
   C   
   C   
   A   
   T   
   G   
   G   
   C   
   G   
   C   
   A   
   C   
   C   
   T   
   A   
    T    
   A   
   A   
   G   
   A   
   G   
   C   
   C   
   G   
    T    
   C   
   C   
   G   
   C   
   T   
   C   
   A   
   G   
   C   
   T   
   G   
   T   
   A   
   T   
   G   
   A   
   A   
   G   
   C   
   T   
   A   
   A   
   A   
   G   
   G   
   C   
   G   
   G   
   A   
   A   
   G   
   A   
   A   
   G   
   A   
   T   
   G   
   A   
   A   
   C   
   T   
    G    
   A   
   T   
   T   
   G   
   C   
   T   
   G   
   T   
    G    
   T   
   A   
   T   
   C   
   T   
   G   
   A   
   A   
   A   
   G   
   A   
   A   
   G   
   A   
   T   
   C   
    A    
   G   
   C   
   C   
   A   
   G   
   G   
   C   
   T   
   T   
   A   
   G   
   A   
   T   
   G   
   T   
    T    
   T   
   T   
   C   
   T   
   T   
   A   
   G   
   T   
   A   
   A   
   C   
   G   
    C    
   A   
   T   
   C   
   A   
   A   
   G   
   G   
   A   
   A   
   T   
   G   
   G   
   G   
    T    
   T   
   T   
   G   
   C   
   G   
   C   
   T   
    A    
   T   
   C   
   C   
   T   
   T   
   T   
   A   
   A   
    C    
   A   
   G   
   A   
   T   
   G   
   T   
   T   
   C   
   C   
   C   
   G   
   T   
   T   
   G   
   T   
   T   
   G   
   G   
   A   
   G   
   C   
   A   
   A   
   A   
   G   
   G   
   C   
   A   
   G   
    C    
   T   
   G   
   G   
   A   
   G   
   T   
   G   
   A   
   A   
   G   
   T   
   C   
   T   
   A   
   T   
   G   
   A   
   A   
   T   
   T   
   T   
   G   
   A   
   A   
   A   
   G   
   A   
   T   
   G   
   G   
   A   
   G   
   A   
   T   
   A   
   G   
   G   
    A    
   T   
   T   
    A    
   T   
   C   
   A   
   A   
    C    
   G   
   G   
    G    
   C   
   T   
    G    
   C   
   T   
   T   
   G   
   T   
   G   
   T   
   T   
   A   
   G   
   C   
   A   
   G   
   A   
    A    
   G   
   G   
    T    
   G   
   A   
   T   
   A   
   C   
   G   
   C   
   C   
    G    
   A   
   T   
   C   
   G   
   T   
   A   
   A   
   T   
   C   
   G   
   T   
   T   
   A   
   C   
   T   
   C   
   A   
   G   
   C   
   G   
   A   
   G   
   G   
   A   
   G   
   C   
    T    
   G   
   T   
    T    
   A   
   A   
   A   
   C   
   G   
   C   
   A   
   T   
   G   
   C   
   T   
   T   
   G   
   C   
   T   
   C   
   A   
   A   
   G   
   A   
   A   
   A   
   T   
   C   
   A   
   G   
   C   
   C   
   A   
   A   
   A   
   C   
    T    
   T   
   C   
   A   
   C   
   G   
   T   
   G   
   C   
   G   
   A   
   A   
   G   
   C   
   G   
   G   
   G   
   G   
   A   
   G   
   T   
   G   
   A   
   C   
   G   
   G   
   T   
   C   
   A   
   T   
   G   
   C   
   G   
   T   
   G   
   A   
   G   
   C   
   T   
   G   
   A   
   A   
   A   
   A   
   A   
   A   
   A   
   A   
   T   
   C   
   C   
    G    
   C   
   A   
    C    
   C   
   G   
    T    
   A   
   T   
   T   
   A   
   T   
   C   
   T   
   A   
   T   
   A   
   T   
   G   
   T   
   C   
   G   
   G   
   A   
   A   
   G   
   G   
   A   
   A   
   G   
   A   
   T   
   C   
   A   
   A   
   A   
   A   
   G   
   A   
   A   
   A   
   T   
   A   
   A   
   C   
   C   
   T   
   T   
   G   
   A   
   T   
   G   
   A   
   A   
   T   
   C   
   A   
   A   
   A   
   A   
   A   
   G   
   G   
    G    
   C   
   A   
    G    
   C   
   A   
   G   
    T    
   T   
   A   
   A   
   C   
   G   
   A   
   T   
   T   
   G   
   A   
    C    
   C   
   C   
   A   
   A   
   C   
    T    
   G   
   A   
   T   
   T   
   T   
    C    
   C   
   C   
   T   
   A   
   T   
   A   
   G   
   G   
   T   
   G   
   A   
   T   
   C   
   G   
   A   
   A   
   C   
   A   
   T   
   C   
   G   
   A   
   A   
   T   
   G   
   G   
    A    
   T   
   C   
    A    
   T   
   T   
   T   
   G   
   C   
    G    
   A   
   T   
   G   
   G   
   A   
    T    
   G   
   A   
   G   
   A   
   A   
   A   
   A   
   A   
   A   
   G   
   G   
   T   
   G   
   G   
   A   
   G   
   A   
   A   
   G   
   T   
   C   
   A   
   T   
   C   
   G   
   A   
   A   
   G   
   T   
    T    
   A   
    T    
   C   
   G   
   A   
   T   
   G   
   C   
   T   
   C   
   C   
   T   
   G   
   A   
   G   
    A    
   T   
   T   
   T   
   T   
    C    
   A   
   T   
    C    
   G   
   A   
    G    
    T    
   C   
    G    
   -   
   -   
   -   
 

 
   N54560   
   A   
   T   
   G   
   G   
   A   
   A   
   A   
   A   
   T   
   C   
   G   
   A   
   C   
   A   
   A   
   G   
   A   
   A   
   G   
   T   
   A   
   C   
   A   
   A   
   G   
   A   
   A   
   T   
   T   
   A   
   A   
   C   
   T   
   T   
   T   
   A   
   G   
   A   
   A   
   G   
   A   
   A   
   G   
   T   
   G   
   A   
   T   
   G   
   G   
   G   
   T   
   G   
   A   
   C   
   C   
   G   
   C   
   T   
   T   
   C   
   G   
   G   
   A   
   C   
   G   
   T   
   T   
   A   
   T   
   T   
   C   
   C   
   A   
   A   
   A   
   T   
   A   
   T   
   A   
   T   
   T   
   A   
   T   
    C    
   C   
   A   
    A    
   G   
   A   
   A   
   C   
   G   
   T   
   G   
   C   
   A   
   C   
   T   
   G   
   C   
   C   
   T   
   G   
   A   
   T   
   A   
   T   
    C    
   C   
   G   
   T   
   G   
   A   
   T   
   G   
   G   
   A   
   T   
   T   
   G   
   A   
   A   
    G    
   C   
   C   
   A   
   G   
   T   
    G    
   C   
   A   
   A   
   C   
   G   
   C   
   C   
   G   
   G   
   A   
   T   
   A   
   T   
   T   
   A   
   T   
   T   
    C    
   G   
   C   
    T    
   A   
   T   
   G   
   A   
   A   
   C   
   A   
   A   
   A   
   G   
   A   
   T   
   G   
   G   
   C   
   A   
   A   
   T   
   A   
   C   
   G   
   T   
   A   
   T   
   G   
   A   
   C   
   A   
   A   
   A   
   G   
   G   
   A   
   T   
   T   
    C    
   C   
   G   
   T   
   A   
   A   
   A   
   T   
   C   
   A   
   G   
   C   
   A   
   A   
   A   
   A   
   T   
   C   
   T   
   G   
   T   
   C   
   G   
   G   
   A   
   A   
   A   
   T   
   A   
   T   
   C   
   A   
   T   
   G   
   G   
   G   
    G    
   A   
   A   
   T   
   T   
   A   
   T   
   C   
   A   
   T   
   C   
   C   
   T   
   C   
   A   
   T   
   G   
   G   
   T   
   G   
   A   
   C   
   A   
   G   
   C   
   A   
   G   
   T   
   A   
   T   
   T   
   T   
   A   
   T   
   G   
   A   
   A   
   G   
   C   
   G   
   A   
   T   
   G   
   G   
   T   
   C   
   C   
   G   
   C   
   A   
   T   
   G   
   A   
   G   
   C   
   C   
   A   
   A   
   G   
   A   
    C    
   T   
   G   
   G   
   A   
   A   
   G   
   T   
   T   
   G   
   C   
   G   
   G   
   G   
   A   
   A   
   G   
   T   
   A   
   C   
   T   
   G   
   A   
   T   
   C   
   G   
   A   
    A    
   A   
   T   
   G   
   C   
   A   
   C   
   G   
   G   
    G    
   A   
   A   
   T   
   A   
   A   
    C    
   G   
   G   
   G   
   A   
   G   
   T   
   A   
   T   
   G   
   G   
   A   
   C   
   G   
   G   
   T   
   G   
   A   
   T   
   C   
   C   
   A   
   C   
   C   
   T   
   G   
   C   
   T   
   G   
   C   
   G   
   A   
   T   
   G   
   C   
   G   
   T   
   T   
   A   
   T   
   A   
   C   
   T   
   G   
   A   
   A   
   G   
   C   
   C   
   A   
   G   
    A    
   T   
   T   
   A   
   T   
   C   
   C   
   A   
   A   
   G   
   T   
   T   
   A   
   A   
   G   
   T   
   G   
   G   
   A   
   G   
   A   
   G   
   A   
   T   
   G   
    C    
   T   
   A   
    G    
   C   
   A   
   G   
   A   
   T   
   A   
   T   
   C   
   G   
   A   
    A    
   A   
   A   
   A   
   G   
   A   
   A   
   A   
   C   
   A   
   G   
   T   
    C    
   G   
   A   
   T   
   C   
   T   
   C   
   G   
   T   
   G   
   T   
   G   
   G   
   A   
   A   
   T   
   T   
   T   
   T   
   G   
   A   
   T   
   G   
   A   
   T   
   A   
   C   
   A   
   G   
   A   
   A   
   A   
   A   
   A   
   G   
   A   
   A   
   C   
   C   
   A   
   A   
   C   
   A   
   G   
   T   
   C   
   C   
   T   
   T   
   C   
   C   
   A   
   G   
   C   
   T   
   C   
   G   
   A   
   T   
   A   
   C   
   C   
   C   
   A   
   A   
   A   
   C   
   C   
   T   
   T   
   C   
   T   
    C    
   G   
   T   
   C   
   A   
   A   
   T   
   G   
   G   
   A   
   T   
   C   
   G   
   A   
   C   
   A   
   G   
   G   
   T   
   A   
   T   
   A   
   T   
   C   
   T   
   G   
   C   
   T   
   G   
   G   
   A   
   T   
   A   
   T   
   G   
   C   
    A    
   A   
   C   
   A   
   G   
   A   
   A   
   A   
   T   
   C   
   C   
   C   
   G   
   A   
   C   
    T    
   C   
   A   
   T   
   A   
   A   
   C   
   T   
   T   
   A   
   G   
   C   
   T   
   G   
   A   
   A   
   G   
   T   
   G   
   A   
   T   
    C    
   G   
   A   
    T    
   G   
   G   
   C   
   A   
   C   
   A   
   A   
   T   
   C   
   T   
   A   
   T   
   A   
   T   
   G   
   A   
   T   
   C   
   G   
   A   
   T   
   C   
   A   
   T   
   C   
   C   
   A   
   C   
   A   
   A   
   G   
   C   
   G   
   A   
   G   
   C   
   C   
   T   
   T   
   G   
   A   
   A   
   A   
   A   
   A   
   T   
   T   
   G   
   A   
   T   
   G   
   G   
   A   
   A   
   T   
   A   
   T   
   A   
   T   
   T   
   C   
   C   
   T   
   G   
   G   
   A   
   C   
   C   
   T   
   G   
   A   
   C   
   T   
   T   
    T    
   C   
   C   
   A   
   A   
   C   
   A   
   G   
   G   
   A   
   G   
   G   
    G    
   A   
   T   
   C   
   T   
   T   
   G   
   C   
   A   
   A   
   G   
   G   
    A    
   A   
   A   
   A   
   G   
   A   
   T   
   G   
   A   
   A   
   A   
   T   
   C   
   A   
   A   
   A   
   A   
   A   
   A   
   G   
   C   
   T   
   T   
   A   
   T   
   G   
   A   
   A   
   A   
   C   
   A   
   G   
   G   
    G    
   C   
   G   
   A   
   G   
   G   
   A   
   A   
   A   
   A   
   G   
   T   
   T   
   A   
    T    
   T   
   C   
   T   
   T   
   C   
   G   
   T   
   T   
   C   
   G   
   A   
   A   
   A   
   A   
   C   
   A   
   A   
   A   
   G   
   A   
   T   
   A   
   G   
   A   
    A    
   G   
   C   
   A   
   A   
   T   
   G   
   A   
   A   
   G   
   G   
   G   
    T    
   A   
   A   
    T    
   A   
   A   
   A   
   C   
   A   
   G   
   C   
   A   
   A   
   A   
   T   
   C   
   G   
   T   
   G   
   A   
   T   
   C   
   A   
   C   
    A    
   G   
   A   
   A   
   A   
   T   
   C   
   C   
   C   
   A   
   T   
   A   
   T   
   G   
   A   
   A   
   G   
   T   
   A   
   A   
   A   
   T   
   A   
   A   
   A   
   G   
   C   
   G   
   A   
   C   
    C    
   T   
   T   
   G   
   G   
   T   
   G   
   A   
   A   
   A   
   A   
   A   
   A   
   A   
   T   
   G   
   G   
   A   
   T   
   G   
   A   
   G   
   A   
   T   
   C   
   C   
   G   
   T   
   T   
   T   
   G   
   A   
   A   
   T   
   A   
   A   
   G   
   A   
   A   
   A   
   A   
   T   
   C   
   G   
   A   
    T    
   G   
   G   
   A   
   A   
   T   
   A   
   G   
   C   
   T   
   G   
   A   
   A   
   G   
   T   
    A    
   C   
   G   
   A   
   G   
   A   
   T   
   G   
   A   
   A   
   A   
   G   
   T   
   G   
   A   
   T   
    A    
   G   
    A    
   A   
   C   
    A    
   G   
   G   
   T   
   C   
   T   
   G   
   C   
   A   
   A   
   A   
   T   
   T   
   G   
   T   
   C   
   G   
   T   
    C    
   G   
   A   
   A   
   T   
   T   
   G   
   A   
   A   
   A   
   A   
   A   
   A   
   G   
   A   
    C    
   G   
   C   
   C   
   A   
   A   
    T    
   G   
   C   
   T   
   C   
   A   
   A   
   G   
   G   
    A    
   A   
   T   
   T   
   T   
   T   
   G   
   A   
   A   
   T   
   T   
   A   
    T    
   T   
   T   
   G   
   T   
   T   
   C   
   A   
   A   
   A   
   A   
   A   
   C   
   A   
   C   
   T   
   G   
   A   
   G   
    C    
   T   
   A   
   C   
   A   
   G   
   A   
   T   
   C   
   A   
   A   
   T   
   T   
   A   
   C   
   A   
   A   
   T   
   T   
   T   
    C    
   A   
   A   
   T   
   A   
   T   
   G   
   G   
   T   
   C   
   G   
   C   
   T   
   A   
   T   
   C   
   G   
   A   
    C    
   C   
   A   
   T   
   A   
   T   
   G   
   A   
   C   
   T   
   C   
   C   
   G   
   C   
   A   
    T    
   C   
   A   
   A   
   G   
   T   
   T   
   G   
   G   
   A   
   C   
   T   
   G   
   A   
   A   
   A   
   G   
   A   
   C   
   A   
   T   
   T   
   T   
   T   
   A   
   A   
   G   
   A   
   A   
   G   
   T   
   T   
   A   
   T   
   A   
   T   
   C   
   G   
   A   
   G   
   C   
   A   
   T   
   C   
   G   
   A   
   A   
   A   
   G   
   C   
   A   
   A   
   G   
   T   
   C   
   A   
   T   
   A   
   A   
   C   
   A   
   A   
   A   
   A   
   C   
   G   
    T    
   A   
   G   
    T    
   C   
   A   
   A   
   T   
   T   
   C   
   G   
   A   
   T   
   T   
   T   
   A   
   G   
   C   
   C   
   A   
   A   
   A   
   G   
   C   
   T   
   C   
   A   
   A   
   A   
   A   
   A   
    A    
   G   
   A   
   C   
   A   
   G   
   C   
   A   
   T   
   A   
   T   
   C   
   G   
   T   
    A    
   G   
   A   
   A   
   G   
   G   
    A    
    T    
   T   
   G   
   A   
   T   
   G   
   A   
   A   
   A   
   G   
   C   
   T   
   T   
   T   
   A   
   T   
   C   
   G   
   A   
   T   
   T   
   C   
   T   
   G   
   G   
   A   
   T   
   G   
   A   
   A   
   G   
   T   
   A   
   A   
   T   
   C   
   G   
    C    
   A   
   A   
   C   
   G   
   A   
   T   
   T   
   C   
   G   
   T   
   G   
   A   
   A   
   A   
   G   
   T   
   A   
   A   
   A   
   G   
   A   
   C   
   A   
   A   
   A   
   A   
   A   
   A   
   G   
   A   
    C    
   G   
   C   
    C    
   A   
   A   
   A   
   A   
   A   
   G   
   A   
   A   
   T   
   T   
   T   
   A   
   G   
   T   
   C   
   G   
   A   
    C    
   G   
   T   
    C    
   T   
   T   
    T    
   C   
   A   
   A   
   T   
   T   
   T   
   A   
   C   
   A   
   G   
   A   
   A   
   G   
   A   
   A   
   C   
   A   
   A   
   G   
   C   
   A   
   G   
   A   
   A   
   G   
   C   
   A   
   A   
   T   
    T    
   G   
   T   
   C   
   A   
   C   
   A   
   T   
   T   
   G   
   C   
   A   
   A   
    T    
   T   
   A   
   T   
   A   
    C    
   C   
   G   
   T   
   T   
   T   
   G   
   A   
   C   
   G   
   A   
   A   
    C    
   A   
   C   
    G    
   G   
   A   
   T   
   A   
   T   
   C   
   A   
   C   
   A   
   G   
   A   
   G   
   C   
   T   
   T   
   C   
   A   
   A   
   A   
   A   
   G   
   G   
   A   
   A   
    T    
   C   
    C    
   G   
   A   
   A   
   A   
    G    
   C   
   T   
   T   
   G   
   A   
   T   
   T   
   G   
   C   
    G    
   C   
   A   
   A   
   A   
   T   
   C   
   A   
   C   
   A   
   G   
   A   
   G   
   T   
   T   
    G    
   A   
   A   
   T   
   A   
   A   
   A   
   A   
   T   
   C   
   C   
   T   
   T   
   T   
   C   
   A   
   A   
   A   
    T    
   G   
   A   
   C   
   A   
   A   
   A   
   G   
   A   
    G    
   C   
   T   
   T   
   T   
   T   
    C    
   T   
   C   
   T   
   G   
   T   
   T   
   A   
   T   
   G   
   A   
   A   
   A   
   A   
   A   
   A   
   G   
   A   
   A   
   T   
   T   
   G   
   C   
   G   
   A   
   G   
   A   
   A   
   G   
   T   
   G   
   A   
   A   
   A   
   A   
   A   
   A   
   A   
   A   
   T   
   T   
   A   
    T    
   T   
   C   
   T   
   T   
   C   
   C   
   G   
   C   
   T   
   C   
   G   
   T   
   C   
    T    
    A    
   A   
   C   
   G   
   A   
   C   
   A   
   A   
   T   
   C   
    G    
   A   
   A   
   G   
   A   
   C   
   G   
   A   
   A   
   A   
   T   
   C   
   G   
   A   
   A   
   G   
   A   
   A   
   A   
   T   
   C   
   A   
   A   
   A   
   A   
   T   
    C    
   G   
   A   
   T   
   A   
   C   
   G   
   C   
   A   
   A   
    G    
   T   
    T    
   T   
   T   
   A   
   G   
   T   
   G   
   G   
   C   
    G    
   C   
   A   
   A   
   G   
   A   
   A   
   G   
   A   
   C   
   G   
   T   
   A   
   A   
   T   
    C    
   G   
   T   
   C   
   A   
   G   
   T   
   G   
   T   
    G    
   A   
   C   
    G    
   C   
   G   
   A   
   G   
   A   
   A   
   G   
   G   
   C   
   T   
   A   
   T   
   G   
   T   
   G   
   A   
   A   
   G   
   C   
   G   
   A   
   A   
   C   
   A   
   A   
   G   
   C   
   C   
   T   
   T   
   C   
   G   
   T   
   T   
   C   
    T    
   T   
   A   
   T   
   A   
   G   
   T   
   G   
   C   
   T   
   T   
   C   
    G    
   A   
   A   
   A   
   C   
   C   
   A   
   G   
   A   
   A   
   G   
   A   
   A   
   A   
   T   
   C   
   G   
   G   
   G   
   A   
   T   
   G   
   C   
   G   
   A   
   G   
   A   
   A   
   G   
   G   
   C   
   G   
   A   
    C    
   T   
   A   
   C   
   T   
   T   
   G   
   C   
   T   
    G    
   T   
   A   
   C   
    T    
   C   
   A   
   G   
   G   
   T   
   G   
   A   
   A   
   T   
   T   
    G    
   A   
   G   
    C    
   A   
   C   
   A   
    T    
   T   
   G   
   G   
   A   
   C   
   C   
   A   
   T   
   G   
   T   
   T   
   C   
   T   
   T   
   C   
   T   
   G   
   A   
   T   
    T    
   A   
   C   
    G    
   A   
   A   
   T   
   A   
   A   
   A   
   G   
   C   
   A   
   A   
   A   
   T   
   G   
   T   
   G   
   A   
   T   
   C   
   T   
   A   
   C   
   C   
   G   
    T    
   C   
   C   
   T   
   G   
   T   
    T    
   C   
   A   
   T   
   G   
   A   
   A   
   C   
   T   
   A   
   C   
   C   
    C    
   G   
   A   
   T   
   T   
   T   
   G   
   A   
   A   
   A   
   T   
   G   
   G   
   A   
   A   
   A   
   G   
   A   
   T   
   G   
   C   
   T   
   G   
   G   
    C    
   G   
   A   
    A    
   C   
   A   
    C    
   A   
   T   
   T   
   T   
   C   
   T   
   C   
   A   
   A   
   A   
   C   
   A   
   A   
   T   
   C   
   T   
   C   
   C   
   A   
   A   
   T   
   C   
   T   
   G   
   T   
   C   
   T   
   G   
   T   
   A   
   G   
   A   
    T    
   G   
   A   
   A   
   T   
   C   
   G   
   A   
   T   
    C    
   T   
   T   
   A   
   G   
   C   
   T   
   G   
   T   
   T   
   T   
   T   
    T    
   C   
   C   
   A   
   T   
   A   
   T   
   C   
   A   
   A   
   A   
   A   
   A   
   A   
   T   
    T    
   C   
   A   
   A   
   G   
   C   
   A   
   G   
   A   
    G    
   A   
   A   
   A   
   A   
   C   
   A   
   T   
   T   
   T   
   G   
   T   
    A    
   T   
   T   
   C   
   A   
   T   
   C   
   A   
   G   
   C   
   A   
   A   
   G   
   A   
   A   
   T   
   G   
   G   
   A   
   T   
   T   
    G    
    A    
   T   
   C   
   A   
   A   
   G   
   C   
   A   
   A   
   A   
   C   
   A   
   C   
   G   
   A   
   A   
   T   
   G   
   A   
   C   
   A   
   G   
   A   
   G   
   T   
   T   
   C   
   G   
   A   
   A   
   C   
   C   
   A   
   T   
   G   
   G   
   C   
   G   
   C   
   A   
   C   
   C   
   T   
   A   
    T    
   A   
   A   
   G   
   A   
   G   
   C   
   C   
   G   
    T    
   C   
   C   
   G   
   C   
   T   
   C   
   A   
   G   
   C   
   T   
   G   
   T   
   A   
   T   
   G   
   A   
   A   
   G   
   C   
   T   
   A   
   A   
   A   
   G   
   G   
   C   
   G   
   G   
   A   
   A   
   G   
   A   
   A   
   G   
   A   
   T   
   G   
   A   
   A   
   C   
   T   
    G    
   A   
   T   
   T   
   G   
   C   
   T   
   G   
   T   
    G    
   T   
   A   
   T   
   C   
   T   
   G   
   A   
   A   
   A   
   G   
   A   
   A   
   G   
   A   
   T   
   C   
    G    
   G   
   C   
   C   
   A   
   G   
   G   
   C   
   T   
   T   
   A   
   G   
   A   
   T   
   G   
   T   
    T    
   T   
   T   
   C   
   T   
   T   
   A   
   G   
   T   
   A   
   A   
   C   
   G   
    T    
   A   
   T   
   C   
   A   
   A   
   G   
   G   
   A   
   A   
   T   
   G   
   G   
   G   
    T    
   T   
   T   
   G   
   C   
   G   
   C   
   T   
    A    
   T   
   C   
   C   
   T   
   T   
   T   
   A   
   A   
    C    
   A   
   G   
   A   
   T   
   G   
   T   
   T   
   C   
   C   
   C   
   G   
   T   
   T   
   G   
   T   
   T   
   G   
   G   
   A   
   G   
   C   
   A   
   A   
   A   
   G   
   G   
   C   
   A   
   G   
    C    
   T   
   G   
   G   
   A   
   G   
   T   
   G   
   A   
   A   
   G   
   T   
   C   
   T   
   A   
   T   
   G   
   A   
   A   
   T   
   T   
   T   
   G   
   A   
   A   
   A   
   G   
   A   
   T   
   G   
   G   
   A   
   G   
   A   
   T   
   A   
   G   
   G   
    A    
   T   
   T   
    A    
   T   
   C   
   A   
   A   
    C    
   G   
   G   
    G    
   C   
   T   
    G    
   C   
   T   
   T   
   G   
   T   
   G   
   T   
   T   
   A   
   G   
   C   
   A   
   G   
   A   
    A    
   G   
   G   
    T    
   G   
   A   
   T   
   A   
   C   
   G   
   C   
   C   
    G    
   A   
   T   
   C   
   G   
   T   
   A   
   A   
   T   
   C   
   G   
   T   
   T   
   A   
   C   
   T   
   C   
   A   
   G   
   C   
   G   
   A   
   G   
   G   
   A   
   G   
   C   
    T    
   G   
   T   
    T    
   A   
   A   
   A   
   C   
   G   
   C   
   A   
   T   
   G   
   C   
   T   
   T   
   G   
   C   
   T   
   C   
   A   
   A   
   G   
   A   
   A   
   A   
   T   
   C   
   A   
   G   
   C   
   C   
   A   
   A   
   A   
   C   
    T    
   T   
   C   
   A   
   C   
   G   
   T   
   G   
   C   
   G   
   A   
   A   
   G   
   C   
   G   
   G   
   G   
   G   
   A   
   G   
   T   
   G   
   A   
   C   
   G   
   G   
   T   
   C   
   A   
   T   
   G   
   C   
   G   
   T   
   G   
   A   
   G   
   C   
   T   
   G   
   A   
   A   
   A   
   A   
   A   
   A   
   A   
   A   
   T   
   C   
   C   
    T    
   C   
   A   
    T    
   C   
   G   
    C    
   A   
   T   
   T   
   A   
   T   
   C   
   T   
   A   
   T   
   A   
   T   
   G   
   T   
   C   
   G   
   G   
   A   
   A   
   G   
   G   
   A   
   A   
   G   
   A   
   T   
   C   
   A   
   A   
   A   
   A   
   G   
   A   
   A   
   A   
   T   
   A   
   A   
   C   
   C   
   T   
   T   
   G   
   A   
   T   
   G   
   A   
   A   
   T   
   C   
   A   
   A   
   A   
   A   
   A   
   G   
   G   
    G    
   C   
   A   
    G    
   C   
   A   
   G   
    T    
   T   
   A   
   A   
   C   
   G   
   A   
   T   
   T   
   G   
   A   
    C    
   C   
   C   
   A   
   A   
   C   
    T    
   G   
   A   
   T   
   T   
   T   
    C    
   C   
   C   
   T   
   A   
   T   
   A   
   G   
   G   
   T   
   G   
   A   
   T   
   C   
   G   
   A   
   A   
   C   
   A   
   T   
   C   
   G   
   A   
   A   
   T   
   G   
   G   
    A    
   T   
   C   
    A    
   T   
   T   
   T   
   G   
   C   
    G    
   A   
   T   
   G   
   G   
   A   
    T    
   G   
   A   
   G   
   A   
   A   
   A   
   A   
   A   
   A   
   G   
   G   
   T   
   G   
   G   
   A   
   G   
   A   
   A   
   G   
   T   
   C   
   A   
   T   
   C   
   G   
   A   
   A   
   G   
   T   
    T    
   A   
    T    
   C   
   G   
   A   
   T   
   G   
   C   
   T   
   C   
   C   
   T   
   G   
   A   
   G   
    A    
   T   
   T   
   T   
   T   
    C    
   A   
   T   
    C    
   G   
   A   
    G    
    T    
   C   
    G    
   -   
   -   
   -   
 

 
   N60449F   
   A   
   T   
   G   
   G   
   A   
   A   
   A   
   A   
   T   
   C   
   G   
   A   
   C   
   A   
   A   
   G   
   A   
   A   
   G   
   T   
   A   
   C   
   A   
   A   
   G   
   A   
   A   
   T   
   T   
   A   
   A   
   C   
   T   
   T   
   T   
   A   
   G   
   A   
   A   
   G   
   A   
   A   
   G   
   T   
   G   
   A   
   T   
   G   
   G   
   G   
   T   
   G   
   A   
   C   
   C   
   G   
   C   
   T   
   T   
   C   
   G   
   G   
   A   
   C   
   G   
   T   
   T   
   A   
   T   
   T   
   C   
   C   
   A   
   A   
   A   
   T   
   A   
   T   
   A   
   T   
   T   
   A   
   T   
    C    
   C   
   A   
    A    
   G   
   A   
   A   
   C   
   G   
   T   
   G   
   C   
   A   
   C   
   T   
   G   
   C   
   C   
   T   
   G   
   A   
   T   
   A   
   T   
    C    
   C   
   G   
   T   
   G   
   A   
   T   
   G   
   G   
   A   
   T   
   T   
   G   
   A   
   A   
    G    
   C   
   C   
   A   
   G   
   T   
    G    
   C   
   A   
   A   
   C   
   G   
   C   
   C   
   G   
   G   
   A   
   T   
   A   
   T   
   T   
   A   
   T   
   T   
    T    
   G   
   C   
    C    
   A   
   T   
   G   
   A   
   A   
   C   
   A   
   A   
   A   
   G   
   A   
   T   
   G   
   G   
   C   
   A   
   A   
   T   
   A   
   C   
   G   
   T   
   A   
   T   
   G   
   A   
   C   
   A   
   A   
   A   
   G   
   G   
   A   
   T   
   T   
    C    
   C   
   G   
   T   
   A   
   A   
   A   
   T   
   C   
   A   
   G   
   C   
   A   
   A   
   A   
   A   
   T   
   C   
   T   
   G   
   T   
   C   
   G   
   G   
   A   
   A   
   A   
   T   
   A   
   T   
   C   
   A   
   T   
   G   
   G   
   G   
    G    
   A   
   A   
   T   
   T   
   A   
   T   
   C   
   A   
   T   
   C   
   C   
   T   
   C   
   A   
   T   
   G   
   G   
   T   
   G   
   A   
   C   
   A   
   G   
   C   
   A   
   G   
   T   
   A   
   T   
   T   
   T   
   A   
   T   
   G   
   A   
   A   
   G   
   C   
   G   
   A   
   T   
   G   
   G   
   T   
   C   
   C   
   G   
   C   
   A   
   T   
   G   
   A   
   G   
   C   
   C   
   A   
   A   
   G   
   A   
    C    
   T   
   G   
   G   
   A   
   A   
   G   
   T   
   T   
   G   
   C   
   G   
   G   
   G   
   A   
   A   
   G   
   T   
   A   
   C   
   T   
   G   
   A   
   T   
   C   
   G   
   A   
    A    
   A   
   T   
   G   
   C   
   A   
   C   
   G   
   G   
    G    
   A   
   A   
   T   
   A   
   A   
    C    
   G   
   G   
   G   
   A   
   G   
   T   
   A   
   T   
   G   
   G   
   A   
   C   
   G   
   G   
   T   
   G   
   A   
   T   
   C   
   C   
   A   
   C   
   C   
   T   
   G   
   C   
   T   
   G   
   C   
   G   
   A   
   T   
   G   
   C   
   G   
   T   
   T   
   A   
   T   
   A   
   C   
   T   
   G   
   A   
   A   
   G   
   C   
   C   
   A   
   G   
    A    
   T   
   T   
   A   
   T   
   C   
   C   
   A   
   A   
   G   
   T   
   T   
   A   
   A   
   G   
   T   
   G   
   G   
   A   
   G   
   A   
   G   
   A   
   T   
   G   
    C    
   T   
   A   
    G    
   C   
   A   
   G   
   A   
   T   
   A   
   T   
   C   
   G   
   A   
    A    
   A   
   A   
   A   
   G   
   A   
   A   
   A   
   C   
   A   
   G   
   T   
    C    
   G   
   A   
   T   
   C   
   T   
   C   
   G   
   T   
   G   
   T   
   G   
   G   
   A   
   A   
   T   
   T   
   T   
   T   
   G   
   A   
   T   
   G   
   A   
   T   
   A   
   C   
   A   
   G   
   A   
   A   
   A   
   A   
   A   
   G   
   A   
   A   
   C   
   C   
   A   
   A   
   C   
   A   
   G   
   T   
   C   
   C   
   T   
   T   
   C   
   C   
   A   
   G   
   C   
   T   
   C   
   G   
   A   
   T   
   A   
   C   
   C   
   C   
   A   
   A   
   A   
   C   
   C   
   T   
   T   
   C   
   T   
    C    
   G   
   T   
   C   
   A   
   A   
   T   
   G   
   G   
   A   
   T   
   C   
   G   
   A   
   C   
   A   
   G   
   G   
   T   
   A   
   T   
   A   
   T   
   C   
   T   
   G   
   C   
   T   
   G   
   G   
   A   
   T   
   A   
   T   
   G   
   C   
    A    
   A   
   C   
   A   
   G   
   A   
   A   
   A   
   T   
   C   
   C   
   C   
   G   
   A   
   C   
    T    
   C   
   A   
   T   
   A   
   A   
   C   
   T   
   T   
   A   
   G   
   C   
   T   
   G   
   A   
   A   
   G   
   T   
   G   
   A   
   T   
    C    
   G   
   A   
    T    
   G   
   G   
   C   
   A   
   C   
   A   
   A   
   T   
   C   
   T   
   A   
   T   
   A   
   T   
   G   
   A   
   T   
   C   
   G   
   A   
   T   
   C   
   A   
   T   
   C   
   C   
   A   
   C   
   A   
   A   
   G   
   C   
   G   
   A   
   G   
   C   
   C   
   T   
   T   
   G   
   A   
   A   
   A   
   A   
   A   
   T   
   T   
   G   
   A   
   T   
   G   
   G   
   A   
   A   
   T   
   A   
   T   
   A   
   T   
   T   
   C   
   C   
   T   
   G   
   G   
   A   
   C   
   C   
   T   
   G   
   A   
   C   
   T   
   T   
    T    
   C   
   C   
   A   
   A   
   C   
   A   
   G   
   G   
   A   
   G   
   G   
    G    
   A   
   T   
   C   
   T   
   T   
   G   
   C   
   A   
   A   
   G   
   G   
    A    
   A   
   A   
   A   
   G   
   A   
   T   
   G   
   A   
   A   
   A   
   T   
   C   
   A   
   A   
   A   
   A   
   A   
   A   
   G   
   C   
   T   
   T   
   A   
   T   
   G   
   A   
   A   
   A   
   C   
   A   
   G   
   G   
    G    
   C   
   G   
   A   
   G   
   G   
   A   
   A   
   A   
   A   
   G   
   T   
   T   
   A   
    T    
   T   
   C   
   T   
   T   
   C   
   G   
   T   
   T   
   C   
   G   
   A   
   A   
   A   
   A   
   C   
   A   
   A   
   A   
   G   
   A   
   T   
   A   
   G   
   A   
    G    
   G   
   C   
   A   
   A   
   T   
   G   
   A   
   A   
   G   
   G   
   G   
    T    
   A   
   A   
    T    
   A   
   A   
   A   
   C   
   A   
   G   
   C   
   A   
   A   
   A   
   T   
   C   
   G   
   T   
   G   
   A   
   T   
   C   
   A   
   C   
    A    
   G   
   A   
   A   
   A   
   T   
   C   
   C   
   C   
   A   
   T   
   A   
   T   
   G   
   A   
   A   
   G   
   T   
   A   
   A   
   A   
   T   
   A   
   A   
   A   
   G   
   C   
   G   
   A   
   C   
    C    
   T   
   T   
   G   
   G   
   T   
   G   
   A   
   A   
   A   
   A   
   A   
   A   
   A   
   T   
   G   
   G   
   A   
   T   
   G   
   A   
   G   
   A   
   T   
   C   
   C   
   G   
   T   
   T   
   T   
   G   
   A   
   A   
   T   
   A   
   A   
   G   
   A   
   A   
   A   
   A   
   T   
   C   
   G   
   A   
    T    
   G   
   G   
   A   
   A   
   T   
   A   
   G   
   C   
   T   
   G   
   A   
   A   
   G   
   T   
    A    
   C   
   G   
   A   
   G   
   A   
   T   
   G   
   A   
   A   
   A   
   G   
   T   
   G   
   A   
   T   
    A    
   G   
    A    
   A   
   C   
    A    
   G   
   G   
   T   
   C   
   T   
   G   
   C   
   A   
   A   
   A   
   T   
   T   
   G   
   T   
   C   
   G   
   T   
    C    
   G   
   A   
   A   
   T   
   T   
   G   
   A   
   A   
   A   
   A   
   A   
   A   
   G   
   A   
    C    
   G   
   C   
   C   
   A   
   A   
    T    
   G   
   C   
   T   
   C   
   A   
   A   
   G   
   G   
    A    
   A   
   T   
   T   
   T   
   T   
   G   
   A   
   A   
   T   
   T   
   A   
    T    
   T   
   T   
   G   
   T   
   T   
   C   
   A   
   A   
   A   
   A   
   A   
   C   
   A   
   C   
   T   
   G   
   A   
   G   
    C    
   T   
   A   
   C   
   A   
   G   
   A   
   T   
   C   
   A   
   A   
   T   
   T   
   A   
   C   
   A   
   A   
   T   
   T   
   T   
    C    
   A   
   A   
   T   
   A   
   T   
   G   
   G   
   T   
   C   
   G   
   C   
   T   
   A   
   T   
   C   
   G   
   A   
    C    
   C   
   A   
   T   
   A   
   T   
   G   
   A   
   C   
   T   
   C   
   C   
   G   
   C   
   A   
    T    
   C   
   A   
   A   
   G   
   T   
   T   
   G   
   G   
   A   
   C   
   T   
   G   
   A   
   A   
   A   
   G   
   A   
   C   
   A   
   T   
   T   
   T   
   T   
   A   
   A   
   G   
   A   
   A   
   G   
   T   
   T   
   A   
   T   
   A   
   T   
   C   
   G   
   A   
   G   
   C   
   A   
   T   
   C   
   G   
   A   
   A   
   A   
   G   
   C   
   A   
   A   
   G   
   T   
   C   
   A   
   T   
   A   
   A   
   C   
   A   
   A   
   A   
   A   
   C   
   G   
    T    
   A   
   G   
    T    
   C   
   A   
   A   
   T   
   T   
   C   
   G   
   A   
   T   
   T   
   T   
   A   
   G   
   C   
   C   
   A   
   A   
   A   
   G   
   C   
   T   
   C   
   A   
   A   
   A   
   A   
   A   
    A    
   G   
   A   
   C   
   A   
   G   
   C   
   A   
   T   
   A   
   T   
   C   
   G   
   T   
    A    
   G   
   A   
   A   
   G   
   G   
    A    
    T    
   T   
   G   
   A   
   T   
   G   
   A   
   A   
   A   
   G   
   C   
   T   
   T   
   T   
   A   
   T   
   C   
   G   
   A   
   T   
   T   
   C   
   T   
   G   
   G   
   A   
   T   
   G   
   A   
   A   
   G   
   T   
   A   
   A   
   T   
   C   
   G   
    C    
   A   
   A   
   C   
   G   
   A   
   T   
   T   
   C   
   G   
   T   
   G   
   A   
   A   
   A   
   G   
   T   
   A   
   A   
   A   
   G   
   A   
   C   
   A   
   A   
   A   
   A   
   A   
   A   
   G   
   A   
    C    
   G   
   C   
    C    
   A   
   A   
   A   
   A   
   A   
   G   
   A   
   A   
   T   
   T   
   T   
   A   
   G   
   T   
   C   
   G   
   A   
    C    
   G   
   T   
    C    
   T   
   T   
    T    
   C   
   A   
   A   
   T   
   T   
   T   
   A   
   C   
   A   
   G   
   A   
   A   
   G   
   A   
   A   
   C   
   A   
   A   
   G   
   C   
   A   
   G   
   A   
   A   
   G   
   C   
   A   
   A   
   T   
    T    
   G   
   T   
   C   
   A   
   C   
   A   
   T   
   T   
   G   
   C   
   A   
   A   
    T    
   T   
   A   
   T   
   A   
    C    
   C   
   G   
   T   
   T   
   T   
   G   
   A   
   C   
   G   
   A   
   A   
    C    
   A   
   C   
    G    
   G   
   A   
   T   
   A   
   T   
   C   
   A   
   C   
   A   
   G   
   A   
   G   
   C   
   T   
   T   
   C   
   A   
   A   
   A   
   A   
   G   
   G   
   A   
   A   
    T    
   C   
    C    
   G   
   A   
   A   
   A   
    G    
   C   
   T   
   T   
   G   
   A   
   T   
   T   
   G   
   C   
    G    
   C   
   A   
   A   
   A   
   T   
   C   
   A   
   C   
   A   
   G   
   A   
   G   
   T   
   T   
    G    
   A   
   A   
   T   
   A   
   A   
   A   
   A   
   T   
   C   
   C   
   T   
   T   
   T   
   C   
   A   
   A   
   A   
    T    
   G   
   A   
   C   
   A   
   A   
   A   
   G   
   A   
    G    
   C   
   T   
   T   
   T   
   T   
    C    
   T   
   C   
   T   
   G   
   T   
   T   
   A   
   T   
   G   
   A   
   A   
   A   
   A   
   A   
   A   
   G   
   A   
   A   
   T   
   T   
   G   
   C   
   G   
   A   
   G   
   A   
   A   
   G   
   T   
   G   
   A   
   A   
   A   
   A   
   A   
   A   
   A   
   A   
   T   
   T   
   A   
    T    
   T   
   C   
   T   
   T   
   C   
   C   
   G   
   C   
   T   
   C   
   G   
   T   
   C   
    T    
    A    
   A   
   C   
   G   
   A   
   C   
   A   
   A   
   T   
   C   
    G    
   A   
   A   
   G   
   A   
   C   
   G   
   A   
   A   
   A   
   T   
   C   
   G   
   A   
   A   
   G   
   A   
   A   
   A   
   T   
   C   
   A   
   A   
   A   
   A   
   T   
    T    
   G   
   A   
   T   
   A   
   C   
   G   
   C   
   A   
   A   
    G    
   T   
    C    
   T   
   T   
   A   
   G   
   T   
   G   
   G   
   C   
    A    
   C   
   A   
   A   
   G   
   A   
   A   
   G   
   A   
   C   
   G   
   T   
   A   
   A   
   T   
    T    
   G   
   T   
   C   
   A   
   G   
   T   
   G   
   T   
    G    
   A   
   C   
    G    
   C   
   G   
   A   
   G   
   A   
   A   
   G   
   G   
   C   
   T   
   A   
   T   
   G   
   T   
   G   
   A   
   A   
   G   
   C   
   G   
   A   
   A   
   C   
   A   
   A   
   G   
   C   
   C   
   T   
   T   
   C   
   G   
   T   
   T   
   C   
    T    
   T   
   A   
   T   
   A   
   G   
   T   
   G   
   C   
   T   
   T   
   C   
    C    
   A   
   A   
   A   
   C   
   C   
   A   
   G   
   A   
   A   
   G   
   A   
   A   
   A   
   T   
   C   
   G   
   G   
   G   
   A   
   T   
   G   
   C   
   G   
   A   
   G   
   A   
   A   
   G   
   G   
   C   
   G   
   A   
    C    
   T   
   A   
   C   
   T   
   T   
   G   
   C   
   T   
    A    
   T   
   A   
   C   
    T    
   C   
   A   
   G   
   G   
   T   
   G   
   A   
   A   
   T   
   T   
    G    
   A   
   G   
    T    
   A   
   C   
   A   
    T    
   T   
   G   
   G   
   A   
   C   
   C   
   A   
   T   
   G   
   T   
   T   
   C   
   T   
   T   
   C   
   T   
   G   
   A   
   T   
    C    
   A   
   C   
    A    
   A   
   A   
   T   
   A   
   A   
   A   
   G   
   C   
   A   
   A   
   A   
   T   
   G   
   T   
   G   
   A   
   T   
   C   
   T   
   A   
   C   
   C   
   G   
    C    
   C   
   C   
   T   
   G   
   T   
    C    
   C   
   A   
   T   
   G   
   A   
   A   
   C   
   T   
   A   
   C   
   C   
    C    
   G   
   A   
   T   
   T   
   T   
   G   
   A   
   A   
   A   
   T   
   G   
   G   
   A   
   A   
   A   
   G   
   A   
   T   
   G   
   C   
   T   
   G   
   G   
    T    
   G   
   A   
    G    
   C   
   A   
    C    
   A   
   T   
   T   
   T   
   C   
   T   
   C   
   A   
   A   
   A   
   C   
   A   
   A   
   T   
   C   
   T   
   C   
   C   
   A   
   A   
   T   
   C   
   T   
   G   
   T   
   C   
   T   
   G   
   T   
   A   
   G   
   A   
    C    
   G   
   A   
   A   
   T   
   C   
   G   
   A   
   T   
    T    
   T   
   T   
   A   
   G   
   C   
   T   
   G   
   T   
   T   
   T   
   T   
    C    
   C   
   C   
   A   
   T   
   A   
   T   
   C   
   A   
   A   
   A   
   A   
   A   
   A   
   T   
    T    
   C   
   A   
   A   
   G   
   C   
   A   
   G   
   A   
    G    
   A   
   A   
   A   
   A   
   C   
   A   
   T   
   T   
   T   
   G   
   T   
    A    
   T   
   T   
   C   
   A   
   T   
   C   
   A   
   G   
   C   
   A   
   A   
   G   
   A   
   A   
   T   
   G   
   G   
   A   
   T   
   T   
    G    
    A    
   T   
   C   
   A   
   A   
   G   
   C   
   A   
   A   
   A   
   C   
   A   
   C   
   G   
   A   
   A   
   T   
   G   
   A   
   C   
   A   
   G   
   A   
   G   
   T   
   T   
   C   
   G   
   A   
   A   
   C   
   C   
   A   
   T   
   G   
   G   
   C   
   G   
   C   
   A   
   C   
   C   
   T   
   A   
    T    
   A   
   A   
   G   
   A   
   G   
   C   
   C   
   G   
    T    
   C   
   C   
   G   
   C   
   T   
   C   
   A   
   G   
   C   
   T   
   G   
   T   
   A   
   T   
   G   
   A   
   A   
   G   
   C   
   T   
   A   
   A   
   A   
   G   
   G   
   C   
   G   
   G   
   A   
   A   
   G   
   A   
   A   
   G   
   A   
   T   
   G   
   A   
   A   
   C   
   T   
    G    
   A   
   T   
   T   
   G   
   C   
   T   
   G   
   T   
    G    
   T   
   A   
   T   
   C   
   T   
   G   
   A   
   A   
   A   
   G   
   A   
   A   
   G   
   A   
   T   
   C   
    A    
   G   
   C   
   C   
   A   
   G   
   G   
   C   
   T   
   T   
   A   
   G   
   A   
   T   
   G   
   T   
    T    
   T   
   T   
   C   
   T   
   T   
   A   
   G   
   T   
   A   
   A   
   C   
   G   
    C    
   A   
   T   
   C   
   A   
   A   
   G   
   G   
   A   
   A   
   T   
   G   
   G   
   G   
    T    
   T   
   T   
   G   
   C   
   G   
   C   
   T   
    G    
   T   
   C   
   C   
   T   
   T   
   T   
   A   
   A   
    C    
   A   
   G   
   A   
   T   
   G   
   T   
   T   
   C   
   C   
   C   
   G   
   T   
   T   
   G   
   T   
   T   
   G   
   G   
   A   
   G   
   C   
   A   
   A   
   A   
   G   
   G   
   C   
   A   
   G   
    C    
   T   
   G   
   G   
   A   
   G   
   T   
   G   
   A   
   A   
   G   
   T   
   C   
   T   
   A   
   T   
   G   
   A   
   A   
   T   
   T   
   T   
   G   
   A   
   A   
   A   
   G   
   A   
   T   
   G   
   G   
   A   
   G   
   A   
   T   
   A   
   G   
   G   
    A    
   T   
   T   
    A    
   T   
   C   
   A   
   A   
    C    
   G   
   G   
    G    
   C   
   T   
    G    
   C   
   T   
   T   
   G   
   T   
   G   
   T   
   T   
   A   
   G   
   C   
   A   
   G   
   A   
    A    
   G   
   G   
    T    
   G   
   A   
   T   
   A   
   C   
   G   
   C   
   C   
    G    
   A   
   T   
   C   
   G   
   T   
   A   
   A   
   T   
   C   
   G   
   T   
   T   
   A   
   C   
   T   
   C   
   A   
   G   
   C   
   G   
   A   
   G   
   G   
   A   
   G   
   C   
    T    
   G   
   T   
    T    
   A   
   A   
   A   
   C   
   G   
   C   
   A   
   T   
   G   
   C   
   T   
   T   
   G   
   C   
   T   
   C   
   A   
   A   
   G   
   A   
   A   
   A   
   T   
   C   
   A   
   G   
   C   
   C   
   A   
   A   
   A   
   C   
    T    
   T   
   C   
   A   
   C   
   G   
   T   
   G   
   C   
   G   
   A   
   A   
   G   
   C   
   G   
   G   
   G   
   G   
   A   
   G   
   T   
   G   
   A   
   C   
   G   
   G   
   T   
   C   
   A   
   T   
   G   
   C   
   G   
   T   
   G   
   A   
   G   
   C   
   T   
   G   
   A   
   A   
   A   
   A   
   A   
   A   
   A   
   A   
   T   
   C   
   C   
    G    
   C   
   A   
    C    
   C   
   G   
    T    
   A   
   T   
   T   
   A   
   T   
   C   
   T   
   A   
   T   
   A   
   T   
   G   
   T   
   C   
   G   
   G   
   A   
   A   
   G   
   G   
   A   
   A   
   G   
   A   
   T   
   C   
   A   
   A   
   A   
   A   
   G   
   A   
   A   
   A   
   T   
   A   
   A   
   C   
   C   
   T   
   T   
   G   
   A   
   T   
   G   
   A   
   A   
   T   
   C   
   A   
   A   
   A   
   A   
   A   
   G   
   G   
    G    
   C   
   A   
    G    
   C   
   A   
   G   
    T    
   T   
   A   
   A   
   C   
   G   
   A   
   T   
   T   
   G   
   A   
    C    
   C   
   C   
   A   
   A   
   C   
    T    
   G   
   A   
   T   
   T   
   T   
    C    
   C   
   C   
   T   
   A   
   T   
   A   
   G   
   G   
   T   
   G   
   A   
   T   
   C   
   G   
   A   
   A   
   C   
   A   
   T   
   C   
   G   
   A   
   A   
   T   
   G   
   G   
    A    
   T   
   C   
    A    
   T   
   T   
   T   
   G   
   C   
    G    
   A   
   T   
   G   
   G   
   A   
    T    
   G   
   A   
   G   
   A   
   A   
   A   
   A   
   A   
   A   
   G   
   G   
   T   
   G   
   G   
   A   
   G   
   A   
   A   
   G   
   T   
   C   
   A   
   T   
   C   
   G   
   A   
   A   
   G   
   T   
    T    
   A   
    T    
   C   
   G   
   A   
   T   
   G   
   C   
   T   
   C   
   C   
   T   
   G   
   A   
   G   
    A    
   T   
   T   
   T   
   T   
    C    
   A   
   T   
    C    
   G   
   A   
    G    
    T    
   C   
    G    
   -   
   -   
   -   
 

 
   N55317   
   A   
   T   
   G   
   G   
   A   
   A   
   A   
   A   
   T   
   C   
   G   
   A   
   C   
   A   
   A   
   G   
   A   
   A   
   G   
   T   
   A   
   C   
   A   
   A   
   G   
   A   
   A   
   T   
   T   
   A   
   A   
   C   
   T   
   T   
   T   
   A   
   G   
   A   
   A   
   G   
   A   
   A   
   G   
   T   
   G   
   A   
   T   
   G   
   G   
   G   
   T   
   G   
   A   
   C   
   C   
   G   
   C   
   T   
   T   
   C   
   G   
   G   
   A   
   C   
   G   
   T   
   T   
   A   
   T   
   T   
   C   
   C   
   A   
   A   
   A   
   T   
   A   
   T   
   A   
   T   
   T   
   A   
   T   
    C    
   C   
   A   
    A    
   G   
   A   
   A   
   C   
   G   
   T   
   G   
   C   
   A   
   C   
   T   
   G   
   C   
   C   
   T   
   G   
   A   
   T   
   A   
   T   
    C    
   C   
   G   
   T   
   G   
   A   
   T   
   G   
   G   
   A   
   T   
   T   
   G   
   A   
   A   
    A    
   C   
   C   
   A   
   G   
   T   
    G    
   C   
   A   
   A   
   C   
   G   
   C   
   C   
   G   
   G   
   A   
   T   
   A   
   T   
   T   
   A   
   T   
   T   
    T    
   G   
   C   
    C    
   A   
   T   
   G   
   A   
   A   
   C   
   A   
   A   
   A   
   G   
   A   
   T   
   G   
   G   
   C   
   A   
   A   
   T   
   A   
   C   
   G   
   T   
   A   
   T   
   G   
   A   
   C   
   A   
   A   
   A   
   G   
   G   
   A   
   T   
   T   
    C    
   C   
   G   
   T   
   A   
   A   
   A   
   T   
   C   
   A   
   G   
   C   
   A   
   A   
   A   
   A   
   T   
   C   
   T   
   G   
   T   
   C   
   G   
   G   
   A   
   A   
   A   
   T   
   A   
   T   
   C   
   A   
   T   
   G   
   G   
   G   
    A    
   A   
   A   
   T   
   T   
   A   
   T   
   C   
   A   
   T   
   C   
   C   
   T   
   C   
   A   
   T   
   G   
   G   
   T   
   G   
   A   
   C   
   A   
   G   
   C   
   A   
   G   
   T   
   A   
   T   
   T   
   T   
   A   
   T   
   G   
   A   
   A   
   G   
   C   
   G   
   A   
   T   
   G   
   G   
   T   
   C   
   C   
   G   
   C   
   A   
   T   
   G   
   A   
   G   
   C   
   C   
   A   
   A   
   G   
   A   
    T    
   T   
   G   
   G   
   A   
   A   
   G   
   T   
   T   
   G   
   C   
   G   
   G   
   G   
   A   
   A   
   G   
   T   
   A   
   C   
   T   
   G   
   A   
   T   
   C   
   G   
   A   
    G    
   A   
   T   
   G   
   C   
   A   
   C   
   G   
   G   
    A    
   A   
   A   
   T   
   A   
   A   
    C    
   G   
   G   
   G   
   A   
   G   
   T   
   A   
   T   
   G   
   G   
   A   
   C   
   G   
   G   
   T   
   G   
   A   
   T   
   C   
   C   
   A   
   C   
   C   
   T   
   G   
   C   
   T   
   G   
   C   
   G   
   A   
   T   
   G   
   C   
   G   
   T   
   T   
   A   
   T   
   A   
   C   
   T   
   G   
   A   
   A   
   G   
   C   
   C   
   A   
   G   
    A    
   T   
   T   
   A   
   T   
   C   
   C   
   A   
   A   
   G   
   T   
   T   
   A   
   A   
   G   
   T   
   G   
   G   
   A   
   G   
   A   
   G   
   A   
   T   
   G   
    C    
   T   
   A   
    G    
   C   
   A   
   G   
   A   
   T   
   A   
   T   
   C   
   G   
   A   
    A    
   A   
   A   
   A   
   G   
   A   
   A   
   A   
   C   
   A   
   G   
   T   
    T    
   G   
   A   
   T   
   C   
   T   
   C   
   G   
   T   
   G   
   T   
   G   
   G   
   A   
   A   
   T   
   T   
   T   
   T   
   G   
   A   
   T   
   G   
   A   
   T   
   A   
   C   
   A   
   G   
   A   
   A   
   A   
   A   
   A   
   G   
   A   
   A   
   C   
   C   
   A   
   A   
   C   
   A   
   G   
   T   
   C   
   C   
   T   
   T   
   C   
   C   
   A   
   G   
   C   
   T   
   C   
   G   
   A   
   T   
   A   
   C   
   C   
   C   
   A   
   A   
   A   
   C   
   C   
   T   
   T   
   C   
   T   
    A    
   G   
   T   
   C   
   A   
   A   
   T   
   G   
   G   
   A   
   T   
   C   
   G   
   A   
   C   
   A   
   G   
   G   
   T   
   A   
   T   
   A   
   T   
   C   
   T   
   G   
   C   
   T   
   G   
   G   
   A   
   T   
   A   
   T   
   G   
   C   
    G    
   A   
   C   
   A   
   G   
   A   
   A   
   A   
   T   
   C   
   C   
   C   
   G   
   A   
   C   
    G    
   C   
   A   
   T   
   A   
   A   
   C   
   T   
   T   
   A   
   G   
   C   
   T   
   G   
   A   
   A   
   G   
   T   
   G   
   A   
   T   
    C    
   G   
   A   
    C    
   G   
   G   
   C   
   A   
   C   
   A   
   A   
   T   
   C   
   T   
   A   
   T   
   A   
   T   
   G   
   A   
   T   
   C   
   G   
   A   
   T   
   C   
   A   
   T   
   C   
   C   
   A   
   C   
   A   
   A   
   G   
   C   
   G   
   A   
   G   
   C   
   C   
   T   
   T   
   G   
   A   
   A   
   A   
   A   
   A   
   T   
   T   
   G   
   A   
   T   
   G   
   G   
   A   
   A   
   T   
   A   
   T   
   A   
   T   
   T   
   C   
   C   
   T   
   G   
   G   
   A   
   C   
   C   
   T   
   G   
   A   
   C   
   T   
   T   
    T    
   C   
   C   
   A   
   A   
   C   
   A   
   G   
   G   
   A   
   G   
   G   
    G    
   A   
   T   
   C   
   T   
   T   
   G   
   C   
   A   
   A   
   G   
   G   
    A    
   A   
   A   
   A   
   G   
   A   
   T   
   G   
   A   
   A   
   A   
   T   
   C   
   A   
   A   
   A   
   A   
   A   
   A   
   G   
   C   
   T   
   T   
   A   
   T   
   G   
   A   
   A   
   A   
   C   
   A   
   G   
   G   
    G    
   C   
   G   
   A   
   G   
   G   
   A   
   A   
   A   
   A   
   G   
   T   
   T   
   A   
    T    
   T   
   C   
   T   
   T   
   C   
   G   
   T   
   T   
   C   
   G   
   A   
   A   
   A   
   A   
   C   
   A   
   A   
   A   
   G   
   A   
   T   
   A   
   G   
   A   
    A    
   G   
   C   
   A   
   A   
   T   
   G   
   A   
   A   
   G   
   G   
   G   
    C    
   A   
   A   
    C    
   A   
   A   
   A   
   C   
   A   
   G   
   C   
   A   
   A   
   A   
   T   
   C   
   G   
   T   
   G   
   A   
   T   
   C   
   A   
   C   
    T    
   G   
   A   
   A   
   A   
   T   
   C   
   C   
   C   
   A   
   T   
   A   
   T   
   G   
   A   
   A   
   G   
   T   
   A   
   A   
   A   
   T   
   A   
   A   
   A   
   G   
   C   
   G   
   A   
   C   
    A    
   T   
   T   
   G   
   G   
   T   
   G   
   A   
   A   
   A   
   A   
   A   
   A   
   A   
   T   
   G   
   G   
   A   
   T   
   G   
   A   
   G   
   A   
   T   
   C   
   C   
   G   
   T   
   T   
   T   
   G   
   A   
   A   
   T   
   A   
   A   
   G   
   A   
   A   
   A   
   A   
   T   
   C   
   G   
   A   
    T    
   G   
   G   
   A   
   A   
   T   
   A   
   G   
   C   
   T   
   G   
   A   
   A   
   G   
   T   
    A    
   C   
   G   
   A   
   G   
   A   
   T   
   G   
   A   
   A   
   A   
   G   
   T   
   G   
   A   
   T   
    C    
   G   
    G    
   A   
   C   
    T    
   G   
   G   
   T   
   C   
   T   
   G   
   C   
   A   
   A   
   A   
   T   
   T   
   G   
   T   
   C   
   G   
   T   
    T    
   G   
   A   
   A   
   T   
   T   
   G   
   A   
   A   
   A   
   A   
   A   
   A   
   G   
   A   
    C    
   G   
   C   
   C   
   A   
   A   
    C    
   G   
   C   
   T   
   C   
   A   
   A   
   G   
   G   
    T    
   A   
   T   
   T   
   T   
   T   
   G   
   A   
   A   
   T   
   T   
   A   
    T    
   T   
   T   
   G   
   T   
   T   
   C   
   A   
   A   
   A   
   A   
   A   
   C   
   A   
   C   
   T   
   G   
   A   
   G   
    C    
   T   
   A   
   C   
   A   
   G   
   A   
   T   
   C   
   A   
   A   
   T   
   T   
   A   
   C   
   A   
   A   
   T   
   T   
   T   
    C    
   A   
   A   
   T   
   A   
   T   
   G   
   G   
   T   
   C   
   G   
   C   
   T   
   A   
   T   
   C   
   G   
   A   
    T    
   C   
   A   
   T   
   A   
   T   
   G   
   A   
   C   
   T   
   C   
   C   
   G   
   C   
   A   
    T    
   C   
   A   
   A   
   G   
   T   
   T   
   G   
   G   
   A   
   C   
   T   
   G   
   A   
   A   
   A   
   G   
   A   
   C   
   A   
   T   
   T   
   T   
   T   
   A   
   A   
   G   
   A   
   A   
   G   
   T   
   T   
   A   
   T   
   A   
   T   
   C   
   G   
   A   
   G   
   C   
   A   
   T   
   C   
   G   
   A   
   A   
   A   
   G   
   C   
   A   
   A   
   G   
   T   
   C   
   A   
   T   
   A   
   A   
   C   
   A   
   A   
   A   
   A   
   C   
   G   
    C    
   A   
   G   
    C    
   C   
   A   
   A   
   T   
   T   
   C   
   G   
   A   
   T   
   T   
   T   
   A   
   G   
   C   
   C   
   A   
   A   
   A   
   G   
   C   
   T   
   C   
   A   
   A   
   A   
   A   
   A   
    A    
   G   
   A   
   C   
   A   
   G   
   C   
   A   
   T   
   A   
   T   
   C   
   G   
   T   
    G    
   G   
   A   
   A   
   G   
   G   
    C    
    C    
   T   
   G   
   A   
   T   
   G   
   A   
   A   
   A   
   G   
   C   
   T   
   T   
   T   
   A   
   T   
   C   
   G   
   A   
   T   
   T   
   C   
   T   
   G   
   G   
   A   
   T   
   G   
   A   
   A   
   G   
   T   
   A   
   A   
   T   
   C   
   G   
    C    
   A   
   A   
   C   
   G   
   A   
   T   
   T   
   C   
   G   
   T   
   G   
   A   
   A   
   A   
   G   
   T   
   A   
   A   
   A   
   G   
   A   
   C   
   A   
   A   
   A   
   A   
   A   
   A   
   G   
   A   
    C    
   G   
   C   
    C    
   A   
   A   
   A   
   A   
   A   
   G   
   A   
   A   
   T   
   T   
   T   
   A   
   G   
   T   
   C   
   G   
   A   
    C    
   G   
   T   
    T    
   T   
   T   
    C    
   C   
   A   
   A   
   T   
   T   
   T   
   A   
   C   
   A   
   G   
   A   
   A   
   G   
   A   
   A   
   C   
   A   
   A   
   G   
   C   
   A   
   G   
   A   
   A   
   G   
   C   
   A   
   A   
   T   
    C    
   G   
   T   
   C   
   A   
   C   
   A   
   T   
   T   
   G   
   C   
   A   
   A   
    C    
   T   
   A   
   T   
   A   
    T    
   C   
   G   
   T   
   T   
   T   
   G   
   A   
   C   
   G   
   A   
   A   
    C    
   A   
   C   
    A    
   G   
   A   
   T   
   A   
   T   
   C   
   A   
   C   
   A   
   G   
   A   
   G   
   C   
   T   
   T   
   C   
   A   
   A   
   A   
   A   
   G   
   G   
   A   
   A   
    T    
   C   
    T    
   G   
   A   
   A   
   A   
    G    
   C   
   T   
   T   
   G   
   A   
   T   
   T   
   G   
   C   
    A    
   C   
   A   
   A   
   A   
   T   
   C   
   A   
   C   
   A   
   G   
   A   
   G   
   T   
   T   
    G    
   A   
   A   
   T   
   A   
   A   
   A   
   A   
   T   
   C   
   C   
   T   
   T   
   T   
   C   
   A   
   A   
   A   
    C    
   G   
   A   
   C   
   A   
   A   
   A   
   G   
   A   
    G    
   C   
   T   
   T   
   T   
   T   
    C    
   T   
   C   
   T   
   G   
   T   
   T   
   A   
   T   
   G   
   A   
   A   
   A   
   A   
   A   
   A   
   G   
   A   
   A   
   T   
   T   
   G   
   C   
   G   
   A   
   G   
   A   
   A   
   G   
   T   
   G   
   A   
   A   
   A   
   A   
   A   
   A   
   A   
   A   
   T   
   T   
   A   
    C    
   T   
   C   
   T   
   T   
   C   
   C   
   G   
   C   
   T   
   C   
   G   
   T   
   C   
    T    
    G    
   A   
   C   
   G   
   A   
   C   
   A   
   A   
   T   
   C   
    G    
   A   
   A   
   G   
   A   
   C   
   G   
   A   
   A   
   A   
   T   
   C   
   G   
   A   
   A   
   G   
   A   
   A   
   A   
   T   
   C   
   A   
   A   
   A   
   A   
   T   
    C    
   G   
   A   
   T   
   A   
   C   
   G   
   C   
   A   
   A   
    G    
   T   
    T    
   T   
   T   
   A   
   G   
   T   
   G   
   G   
   C   
    G    
   C   
   A   
   A   
   G   
   A   
   A   
   G   
   A   
   C   
   G   
   T   
   A   
   A   
   T   
    C    
   G   
   T   
   C   
   A   
   G   
   T   
   G   
   T   
    G    
   A   
   C   
    A    
   C   
   G   
   A   
   G   
   A   
   A   
   G   
   G   
   C   
   T   
   A   
   T   
   G   
   T   
   G   
   A   
   A   
   G   
   C   
   G   
   A   
   A   
   C   
   A   
   A   
   G   
   C   
   C   
   T   
   T   
   C   
   G   
   T   
   T   
   C   
    T    
   T   
   A   
   T   
   A   
   G   
   T   
   G   
   C   
   T   
   T   
   C   
    C    
   A   
   A   
   A   
   C   
   C   
   A   
   G   
   A   
   A   
   G   
   A   
   A   
   A   
   T   
   C   
   G   
   G   
   G   
   A   
   T   
   G   
   C   
   G   
   A   
   G   
   A   
   A   
   G   
   G   
   C   
   G   
   A   
    C    
   T   
   A   
   C   
   T   
   T   
   G   
   C   
   T   
    G    
   T   
   A   
   C   
    T    
   C   
   A   
   G   
   G   
   T   
   G   
   A   
   A   
   T   
   T   
    G    
   A   
   G   
    C    
   A   
   C   
   A   
    T    
   T   
   G   
   G   
   A   
   C   
   C   
   A   
   T   
   G   
   T   
   T   
   C   
   T   
   T   
   C   
   T   
   G   
   A   
   T   
    T    
   A   
   C   
    G    
   A   
   A   
   T   
   A   
   A   
   A   
   G   
   C   
   A   
   A   
   A   
   T   
   G   
   T   
   G   
   A   
   T   
   C   
   T   
   A   
   C   
   C   
   G   
    T    
   C   
   C   
   T   
   G   
   T   
    T    
   C   
   A   
   T   
   G   
   A   
   A   
   C   
   T   
   A   
   C   
   C   
    C    
   G   
   A   
   T   
   T   
   T   
   G   
   A   
   A   
   A   
   T   
   G   
   G   
   A   
   A   
   A   
   G   
   A   
   T   
   G   
   C   
   T   
   G   
   G   
    C    
   G   
   A   
    A    
   C   
   A   
    C    
   A   
   T   
   T   
   T   
   C   
   T   
   C   
   A   
   A   
   A   
   C   
   A   
   A   
   T   
   C   
   T   
   C   
   C   
   A   
   A   
   T   
   C   
   T   
   G   
   T   
   C   
   T   
   G   
   T   
   A   
   G   
   A   
    T    
   G   
   A   
   A   
   T   
   C   
   G   
   A   
   T   
    C    
   T   
   T   
   A   
   G   
   C   
   T   
   G   
   T   
   T   
   T   
   T   
    C    
   C   
   C   
   A   
   T   
   A   
   T   
   C   
   A   
   A   
   A   
   A   
   A   
   A   
   T   
    C    
   C   
   A   
   A   
   G   
   C   
   A   
   G   
   A   
    G    
   A   
   A   
   A   
   A   
   C   
   A   
   T   
   T   
   T   
   G   
   T   
    T    
   T   
   T   
   C   
   A   
   T   
   C   
   A   
   G   
   C   
   A   
   A   
   G   
   A   
   A   
   T   
   G   
   G   
   A   
   T   
   T   
    G    
    A    
   T   
   C   
   A   
   A   
   G   
   C   
   A   
   A   
   A   
   C   
   A   
   C   
   G   
   A   
   A   
   T   
   G   
   A   
   C   
   A   
   G   
   A   
   G   
   T   
   T   
   C   
   G   
   A   
   A   
   C   
   C   
   A   
   T   
   G   
   G   
   C   
   G   
   C   
   A   
   C   
   C   
   T   
   A   
    C    
   A   
   A   
   G   
   A   
   G   
   C   
   C   
   G   
    T    
   C   
   C   
   G   
   C   
   T   
   C   
   A   
   G   
   C   
   T   
   G   
   T   
   A   
   T   
   G   
   A   
   A   
   G   
   C   
   T   
   A   
   A   
   A   
   G   
   G   
   C   
   G   
   G   
   A   
   A   
   G   
   A   
   A   
   G   
   A   
   T   
   G   
   A   
   A   
   C   
   T   
    T    
   A   
   T   
   T   
   G   
   C   
   T   
   G   
   T   
    A    
   T   
   A   
   T   
   C   
   T   
   G   
   A   
   A   
   A   
   G   
   A   
   A   
   G   
   A   
   T   
   C   
    A    
   G   
   C   
   C   
   A   
   G   
   G   
   C   
   T   
   T   
   A   
   G   
   A   
   T   
   G   
   T   
    G    
   T   
   T   
   C   
   T   
   T   
   A   
   G   
   T   
   A   
   A   
   C   
   G   
    C    
   A   
   T   
   C   
   A   
   A   
   G   
   G   
   A   
   A   
   T   
   G   
   G   
   G   
    C    
   T   
   T   
   G   
   C   
   G   
   C   
   T   
    A    
   T   
   C   
   C   
   T   
   T   
   T   
   A   
   A   
    C    
   A   
   G   
   A   
   T   
   G   
   T   
   T   
   C   
   C   
   C   
   G   
   T   
   T   
   G   
   T   
   T   
   G   
   G   
   A   
   G   
   C   
   A   
   A   
   A   
   G   
   G   
   C   
   A   
   G   
    C    
   T   
   G   
   G   
   A   
   G   
   T   
   G   
   A   
   A   
   G   
   T   
   C   
   T   
   A   
   T   
   G   
   A   
   A   
   T   
   T   
   T   
   G   
   A   
   A   
   A   
   G   
   A   
   T   
   G   
   G   
   A   
   G   
   A   
   T   
   A   
   G   
   G   
    A    
   T   
   T   
    G    
   T   
   C   
   A   
   A   
    T    
   G   
   G   
    G    
   C   
   T   
    G    
   C   
   T   
   T   
   G   
   T   
   G   
   T   
   T   
   A   
   G   
   C   
   A   
   G   
   A   
    C    
   G   
   G   
    A    
   G   
   A   
   T   
   A   
   C   
   G   
   C   
   C   
    G    
   A   
   T   
   C   
   G   
   T   
   A   
   A   
   T   
   C   
   G   
   T   
   T   
   A   
   C   
   T   
   C   
   A   
   G   
   C   
   G   
   A   
   G   
   G   
   A   
   G   
   C   
    T    
   G   
   T   
    G    
   A   
   A   
   A   
   C   
   G   
   C   
   A   
   T   
   G   
   C   
   T   
   T   
   G   
   C   
   T   
   C   
   A   
   A   
   G   
   A   
   A   
   A   
   T   
   C   
   A   
   G   
   C   
   C   
   A   
   A   
   A   
   C   
    T    
   T   
   C   
   A   
   C   
   G   
   T   
   G   
   C   
   G   
   A   
   A   
   G   
   C   
   G   
   G   
   G   
   G   
   A   
   G   
   T   
   G   
   A   
   C   
   G   
   G   
   T   
   C   
   A   
   T   
   G   
   C   
   G   
   T   
   G   
   A   
   G   
   C   
   T   
   G   
   A   
   A   
   A   
   A   
   A   
   A   
   A   
   A   
   T   
   C   
   C   
    G    
   C   
   A   
    C    
   C   
   G   
    C    
   A   
   T   
   T   
   A   
   T   
   C   
   T   
   A   
   T   
   A   
   T   
   G   
   T   
   C   
   G   
   G   
   A   
   A   
   G   
   G   
   A   
   A   
   G   
   A   
   T   
   C   
   A   
   A   
   A   
   A   
   G   
   A   
   A   
   A   
   T   
   A   
   A   
   C   
   C   
   T   
   T   
   G   
   A   
   T   
   G   
   A   
   A   
   T   
   C   
   A   
   A   
   A   
   A   
   A   
   G   
   G   
    A    
   C   
   A   
    A    
   C   
   A   
   G   
    A    
   T   
   A   
   A   
   C   
   G   
   A   
   T   
   T   
   G   
   A   
    T    
   C   
   C   
   A   
   A   
   C   
    G    
   G   
   A   
   T   
   T   
   T   
    T    
   C   
   C   
   T   
   A   
   T   
   A   
   G   
   G   
   T   
   G   
   A   
   T   
   C   
   G   
   A   
   A   
   C   
   A   
   T   
   C   
   G   
   A   
   A   
   T   
   G   
   G   
    T    
   T   
   C   
    G    
   T   
   T   
   T   
   G   
   C   
    T    
   A   
   T   
   G   
   G   
   A   
    C    
   G   
   A   
   G   
   A   
   A   
   A   
   A   
   A   
   A   
   G   
   G   
   T   
   G   
   G   
   A   
   G   
   A   
   A   
   G   
   T   
   C   
   A   
   T   
   C   
   G   
   A   
   A   
   G   
   T   
    T    
   A   
    T    
   C   
   G   
   A   
   T   
   G   
   C   
   T   
   C   
   C   
   T   
   G   
   A   
   G   
    A    
   T   
   T   
   T   
   T   
    C    
   A   
   T   
    C    
   G   
   A   
    A    
    T    
   C   
    A    
   -   
   -   
   -   
 

 
   N59469F   
   A   
   T   
   G   
   G   
   A   
   A   
   A   
   A   
   T   
   C   
   G   
   A   
   C   
   A   
   A   
   G   
   A   
   A   
   G   
   T   
   A   
   C   
   A   
   A   
   G   
   A   
   A   
   T   
   T   
   A   
   A   
   C   
   T   
   T   
   T   
   A   
   G   
   A   
   A   
   G   
   A   
   A   
   G   
   T   
   G   
   A   
   T   
   G   
   G   
   G   
   T   
   G   
   A   
   C   
   C   
   G   
   C   
   T   
   T   
   C   
   G   
   G   
   A   
   C   
   G   
   T   
   T   
   A   
   T   
   T   
   C   
   C   
   A   
   A   
   A   
   T   
   A   
   T   
   A   
   T   
   T   
   A   
   T   
    C    
   C   
   A   
    A    
   G   
   A   
   A   
   C   
   G   
   T   
   G   
   C   
   A   
   C   
   T   
   G   
   C   
   C   
   T   
   G   
   A   
   T   
   A   
   T   
    C    
   C   
   G   
   T   
   G   
   A   
   T   
   G   
   G   
   A   
   T   
   T   
   G   
   A   
   A   
    G    
   C   
   C   
   A   
   G   
   T   
    G    
   C   
   A   
   A   
   C   
   G   
   C   
   C   
   G   
   G   
   A   
   T   
   A   
   T   
   T   
   A   
   T   
   T   
    C    
   G   
   C   
    T    
   A   
   T   
   G   
   A   
   A   
   C   
   A   
   A   
   A   
   G   
   A   
   T   
   G   
   G   
   C   
   A   
   A   
   T   
   A   
   C   
   G   
   T   
   A   
   T   
   G   
   A   
   C   
   A   
   A   
   A   
   G   
   G   
   A   
   T   
   T   
    C    
   C   
   G   
   T   
   A   
   A   
   A   
   T   
   C   
   A   
   G   
   C   
   A   
   A   
   A   
   A   
   T   
   C   
   T   
   G   
   T   
   C   
   G   
   G   
   A   
   A   
   A   
   T   
   A   
   T   
   C   
   A   
   T   
   G   
   G   
   G   
    G    
   A   
   A   
   T   
   T   
   A   
   T   
   C   
   A   
   T   
   C   
   C   
   T   
   C   
   A   
   T   
   G   
   G   
   T   
   G   
   A   
   C   
   A   
   G   
   C   
   A   
   G   
   T   
   A   
   T   
   T   
   T   
   A   
   T   
   G   
   A   
   A   
   G   
   C   
   G   
   A   
   T   
   G   
   G   
   T   
   C   
   C   
   G   
   C   
   A   
   T   
   G   
   A   
   G   
   C   
   C   
   A   
   A   
   G   
   A   
    C    
   T   
   G   
   G   
   A   
   A   
   G   
   T   
   T   
   G   
   C   
   G   
   G   
   G   
   A   
   A   
   G   
   T   
   A   
   C   
   T   
   G   
   A   
   T   
   C   
   G   
   A   
    A    
   A   
   T   
   G   
   C   
   A   
   C   
   G   
   G   
    G    
   A   
   A   
   T   
   A   
   A   
    C    
   G   
   G   
   G   
   A   
   G   
   T   
   A   
   T   
   G   
   G   
   A   
   C   
   G   
   G   
   T   
   G   
   A   
   T   
   C   
   C   
   A   
   C   
   C   
   T   
   G   
   C   
   T   
   G   
   C   
   G   
   A   
   T   
   G   
   C   
   G   
   T   
   T   
   A   
   T   
   A   
   C   
   T   
   G   
   A   
   A   
   G   
   C   
   C   
   A   
   G   
    A    
   T   
   T   
   A   
   T   
   C   
   C   
   A   
   A   
   G   
   T   
   T   
   A   
   A   
   G   
   T   
   G   
   G   
   A   
   G   
   A   
   G   
   A   
   T   
   G   
    C    
   T   
   A   
    G    
   C   
   A   
   G   
   A   
   T   
   A   
   T   
   C   
   G   
   A   
    A    
   A   
   A   
   A   
   G   
   A   
   A   
   A   
   C   
   A   
   G   
   T   
    C    
   G   
   A   
   T   
   C   
   T   
   C   
   G   
   T   
   G   
   T   
   G   
   G   
   A   
   A   
   T   
   T   
   T   
   T   
   G   
   A   
   T   
   G   
   A   
   T   
   A   
   C   
   A   
   G   
   A   
   A   
   A   
   A   
   A   
   G   
   A   
   A   
   C   
   C   
   A   
   A   
   C   
   A   
   G   
   T   
   C   
   C   
   T   
   T   
   C   
   C   
   A   
   G   
   C   
   T   
   C   
   G   
   A   
   T   
   A   
   C   
   C   
   C   
   A   
   A   
   A   
   C   
   C   
   T   
   T   
   C   
   T   
    C    
   G   
   T   
   C   
   A   
   A   
   T   
   G   
   G   
   A   
   T   
   C   
   G   
   A   
   C   
   A   
   G   
   G   
   T   
   A   
   T   
   A   
   T   
   C   
   T   
   G   
   C   
   T   
   G   
   G   
   A   
   T   
   A   
   T   
   G   
   C   
    A    
   A   
   C   
   A   
   G   
   A   
   A   
   A   
   T   
   C   
   C   
   C   
   G   
   A   
   C   
    T    
   C   
   A   
   T   
   A   
   A   
   C   
   T   
   T   
   A   
   G   
   C   
   T   
   G   
   A   
   A   
   G   
   T   
   G   
   A   
   T   
    C    
   G   
   A   
    T    
   G   
   G   
   C   
   A   
   C   
   A   
   A   
   T   
   C   
   T   
   A   
   T   
   A   
   T   
   G   
   A   
   T   
   C   
   G   
   A   
   T   
   C   
   A   
   T   
   C   
   C   
   A   
   C   
   A   
   A   
   G   
   C   
   G   
   A   
   G   
   C   
   C   
   T   
   T   
   G   
   A   
   A   
   A   
   A   
   A   
   T   
   T   
   G   
   A   
   T   
   G   
   G   
   A   
   A   
   T   
   A   
   T   
   A   
   T   
   T   
   C   
   C   
   T   
   G   
   G   
   A   
   C   
   C   
   T   
   G   
   A   
   C   
   T   
   T   
    T    
   C   
   C   
   A   
   A   
   C   
   A   
   G   
   G   
   A   
   G   
   G   
    G    
   A   
   T   
   C   
   T   
   T   
   G   
   C   
   A   
   A   
   G   
   G   
    A    
   A   
   A   
   A   
   G   
   A   
   T   
   G   
   A   
   A   
   A   
   T   
   C   
   A   
   A   
   A   
   A   
   A   
   A   
   G   
   C   
   T   
   T   
   A   
   T   
   G   
   A   
   A   
   A   
   C   
   A   
   G   
   G   
    G    
   C   
   G   
   A   
   G   
   G   
   A   
   A   
   A   
   A   
   G   
   T   
   T   
   A   
    T    
   T   
   C   
   T   
   T   
   C   
   G   
   T   
   T   
   C   
   G   
   A   
   A   
   A   
   A   
   C   
   A   
   A   
   A   
   G   
   A   
   T   
   A   
   G   
   A   
    A    
   G   
   C   
   A   
   A   
   T   
   G   
   A   
   A   
   G   
   G   
   G   
    T    
   A   
   A   
    T    
   A   
   A   
   A   
   C   
   A   
   G   
   C   
   A   
   A   
   A   
   T   
   C   
   G   
   T   
   G   
   A   
   T   
   C   
   A   
   C   
    A    
   G   
   A   
   A   
   A   
   T   
   C   
   C   
   C   
   A   
   T   
   A   
   T   
   G   
   A   
   A   
   G   
   T   
   A   
   A   
   A   
   T   
   A   
   A   
   A   
   G   
   C   
   G   
   A   
   C   
    C    
   T   
   T   
   G   
   G   
   T   
   G   
   A   
   A   
   A   
   A   
   A   
   A   
   A   
   T   
   G   
   G   
   A   
   T   
   G   
   A   
   G   
   A   
   T   
   C   
   C   
   G   
   T   
   T   
   T   
   G   
   A   
   A   
   T   
   A   
   A   
   G   
   A   
   A   
   A   
   A   
   T   
   C   
   G   
   A   
    T    
   G   
   G   
   A   
   A   
   T   
   A   
   G   
   C   
   T   
   G   
   A   
   A   
   G   
   T   
    A    
   C   
   G   
   A   
   G   
   A   
   T   
   G   
   A   
   A   
   A   
   G   
   T   
   G   
   A   
   T   
    A    
   G   
    A    
   A   
   C   
    A    
   G   
   G   
   T   
   C   
   T   
   G   
   C   
   A   
   A   
   A   
   T   
   T   
   G   
   T   
   C   
   G   
   T   
    C    
   G   
   A   
   A   
   T   
   T   
   G   
   A   
   A   
   A   
   A   
   A   
   A   
   G   
   A   
    C    
   G   
   C   
   C   
   A   
   A   
    T    
   G   
   C   
   T   
   C   
   A   
   A   
   G   
   G   
    A    
   A   
   T   
   T   
   T   
   T   
   G   
   A   
   A   
   T   
   T   
   A   
    T    
   T   
   T   
   G   
   T   
   T   
   C   
   A   
   A   
   A   
   A   
   A   
   C   
   A   
   C   
   T   
   G   
   A   
   G   
    C    
   T   
   A   
   C   
   A   
   G   
   A   
   T   
   C   
   A   
   A   
   T   
   T   
   A   
   C   
   A   
   A   
   T   
   T   
   T   
    C    
   A   
   A   
   T   
   A   
   T   
   G   
   G   
   T   
   C   
   G   
   C   
   T   
   A   
   T   
   C   
   G   
   A   
    C    
   C   
   A   
   T   
   A   
   T   
   G   
   A   
   C   
   T   
   C   
   C   
   G   
   C   
   A   
    T    
   C   
   A   
   A   
   G   
   T   
   T   
   G   
   G   
   A   
   C   
   T   
   G   
   A   
   A   
   A   
   G   
   A   
   C   
   A   
   T   
   T   
   T   
   T   
   A   
   A   
   G   
   A   
   A   
   G   
   T   
   T   
   A   
   T   
   A   
   T   
   C   
   G   
   A   
   G   
   C   
   A   
   T   
   C   
   G   
   A   
   A   
   A   
   G   
   C   
   A   
   A   
   G   
   T   
   C   
   A   
   T   
   A   
   A   
   C   
   A   
   A   
   A   
   A   
   C   
   G   
    T    
   A   
   G   
    T    
   C   
   A   
   A   
   T   
   T   
   C   
   G   
   A   
   T   
   T   
   T   
   A   
   G   
   C   
   C   
   A   
   A   
   A   
   G   
   C   
   T   
   C   
   A   
   A   
   A   
   A   
   A   
    A    
   G   
   A   
   C   
   A   
   G   
   C   
   A   
   T   
   A   
   T   
   C   
   G   
   T   
    A    
   G   
   A   
   A   
   G   
   G   
    A    
    T    
   T   
   G   
   A   
   T   
   G   
   A   
   A   
   A   
   G   
   C   
   T   
   T   
   T   
   A   
   T   
   C   
   G   
   A   
   T   
   T   
   C   
   T   
   G   
   G   
   A   
   T   
   G   
   A   
   A   
   G   
   T   
   A   
   A   
   T   
   C   
   G   
    C    
   A   
   A   
   C   
   G   
   A   
   T   
   T   
   C   
   G   
   T   
   G   
   A   
   A   
   A   
   G   
   T   
   A   
   A   
   A   
   G   
   A   
   C   
   A   
   A   
   A   
   A   
   A   
   A   
   G   
   A   
    C    
   G   
   C   
    C    
   A   
   A   
   A   
   A   
   A   
   G   
   A   
   A   
   T   
   T   
   T   
   A   
   G   
   T   
   C   
   G   
   A   
    C    
   G   
   T   
    C    
   T   
   T   
    T    
   C   
   A   
   A   
   T   
   T   
   T   
   A   
   C   
   A   
   G   
   A   
   A   
   G   
   A   
   A   
   C   
   A   
   A   
   G   
   C   
   A   
   G   
   A   
   A   
   G   
   C   
   A   
   A   
   T   
    T    
   G   
   T   
   C   
   A   
   C   
   A   
   T   
   T   
   G   
   C   
   A   
   A   
    T    
   T   
   A   
   T   
   A   
    C    
   C   
   G   
   T   
   T   
   T   
   G   
   A   
   C   
   G   
   A   
   A   
    C    
   A   
   C   
    G    
   G   
   A   
   T   
   A   
   T   
   C   
   A   
   C   
   A   
   G   
   A   
   G   
   C   
   T   
   T   
   C   
   A   
   A   
   A   
   A   
   G   
   G   
   A   
   A   
    T    
   C   
    C    
   G   
   A   
   A   
   A   
    G    
   C   
   T   
   T   
   G   
   A   
   T   
   T   
   G   
   C   
    G    
   C   
   A   
   A   
   A   
   T   
   C   
   A   
   C   
   A   
   G   
   A   
   G   
   T   
   T   
    G    
   A   
   A   
   T   
   A   
   A   
   A   
   A   
   T   
   C   
   C   
   T   
   T   
   T   
   C   
   A   
   A   
   A   
    T    
   G   
   A   
   C   
   A   
   A   
   A   
   G   
   A   
    G    
   C   
   T   
   T   
   T   
   T   
    C    
   T   
   C   
   T   
   G   
   T   
   T   
   A   
   T   
   G   
   A   
   A   
   A   
   A   
   A   
   A   
   G   
   A   
   A   
   T   
   T   
   G   
   C   
   G   
   A   
   G   
   A   
   A   
   G   
   T   
   G   
   A   
   A   
   A   
   A   
   A   
   A   
   A   
   A   
   T   
   T   
   A   
    T    
   T   
   C   
   T   
   T   
   C   
   C   
   G   
   C   
   T   
   C   
   G   
   T   
   C   
    T    
    A    
   A   
   C   
   G   
   A   
   C   
   A   
   A   
   T   
   C   
    G    
   A   
   A   
   G   
   A   
   C   
   G   
   A   
   A   
   A   
   T   
   C   
   G   
   A   
   A   
   G   
   A   
   A   
   A   
   T   
   C   
   A   
   A   
   A   
   A   
   T   
    C    
   G   
   A   
   T   
   A   
   C   
   G   
   C   
   A   
   A   
    G    
   T   
    T    
   T   
   T   
   A   
   G   
   T   
   G   
   G   
   C   
    G    
   C   
   A   
   A   
   G   
   A   
   A   
   G   
   A   
   C   
   G   
   T   
   A   
   A   
   T   
    C    
   G   
   T   
   C   
   A   
   G   
   T   
   G   
   T   
    G    
   A   
   C   
    G    
   C   
   G   
   A   
   G   
   A   
   A   
   G   
   G   
   C   
   T   
   A   
   T   
   G   
   T   
   G   
   A   
   A   
   G   
   C   
   G   
   A   
   A   
   C   
   A   
   A   
   G   
   C   
   C   
   T   
   T   
   C   
   G   
   T   
   T   
   C   
    T    
   T   
   A   
   T   
   A   
   G   
   T   
   G   
   C   
   T   
   T   
   C   
    G    
   A   
   A   
   A   
   C   
   C   
   A   
   G   
   A   
   A   
   G   
   A   
   A   
   A   
   T   
   C   
   G   
   G   
   G   
   A   
   T   
   G   
   C   
   G   
   A   
   G   
   A   
   A   
   G   
   G   
   C   
   G   
   A   
    C    
   T   
   A   
   C   
   T   
   T   
   G   
   C   
   T   
    G    
   T   
   A   
   C   
    T    
   C   
   A   
   G   
   G   
   T   
   G   
   A   
   A   
   T   
   T   
    G    
   A   
   G   
    C    
   A   
   C   
   A   
    T    
   T   
   G   
   G   
   A   
   C   
   C   
   A   
   T   
   G   
   T   
   T   
   C   
   T   
   T   
   C   
   T   
   G   
   A   
   T   
    T    
   A   
   C   
    G    
   A   
   A   
   T   
   A   
   A   
   A   
   G   
   C   
   A   
   A   
   A   
   T   
   G   
   T   
   G   
   A   
   T   
   C   
   T   
   A   
   C   
   C   
   G   
    T    
   C   
   C   
   T   
   G   
   T   
    T    
   C   
   A   
   T   
   G   
   A   
   A   
   C   
   T   
   A   
   C   
   C   
    C    
   G   
   A   
   T   
   T   
   T   
   G   
   A   
   A   
   A   
   T   
   G   
   G   
   A   
   A   
   A   
   G   
   A   
   T   
   G   
   C   
   T   
   G   
   G   
    C    
   G   
   A   
    A    
   C   
   A   
    T    
   A   
   T   
   T   
   T   
   C   
   T   
   C   
   A   
   A   
   A   
   C   
   A   
   A   
   T   
   C   
   T   
   C   
   C   
   A   
   A   
   T   
   C   
   T   
   G   
   T   
   C   
   T   
   G   
   T   
   A   
   G   
   A   
    T    
   G   
   A   
   A   
   T   
   C   
   G   
   A   
   T   
    C    
   T   
   T   
   A   
   G   
   C   
   T   
   G   
   T   
   T   
   T   
   T   
    T    
   C   
   C   
   A   
   T   
   A   
   T   
   C   
   A   
   A   
   A   
   A   
   A   
   A   
   T   
    T    
   C   
   A   
   A   
   G   
   C   
   A   
   G   
   A   
    G    
   A   
   A   
   A   
   A   
   C   
   A   
   T   
   T   
   T   
   G   
   T   
    A    
   T   
   T   
   C   
   A   
   T   
   C   
   A   
   G   
   C   
   A   
   A   
   G   
   A   
   A   
   T   
   G   
   G   
   A   
   T   
   T   
    G    
    A    
   T   
   C   
   A   
   A   
   G   
   C   
   A   
   A   
   A   
   C   
   A   
   C   
   G   
   A   
   A   
   T   
   G   
   A   
   C   
   A   
   G   
   A   
   G   
   T   
   T   
   C   
   G   
   A   
   A   
   C   
   C   
   A   
   T   
   G   
   G   
   C   
   G   
   C   
   A   
   C   
   C   
   T   
   A   
    T    
   A   
   A   
   G   
   A   
   G   
   C   
   C   
   G   
    T    
   C   
   C   
   G   
   C   
   T   
   C   
   A   
   G   
   C   
   T   
   G   
   T   
   A   
   T   
   G   
   A   
   A   
   G   
   C   
   T   
   A   
   A   
   A   
   G   
   G   
   C   
   G   
   G   
   A   
   A   
   G   
   A   
   A   
   G   
   A   
   T   
   G   
   A   
   A   
   C   
   T   
    G    
   A   
   T   
   T   
   G   
   C   
   T   
   G   
   T   
    G    
   T   
   A   
   T   
   C   
   T   
   G   
   A   
   A   
   A   
   G   
   A   
   A   
   G   
   A   
   T   
   C   
    G    
   G   
   C   
   C   
   A   
   G   
   G   
   C   
   T   
   T   
   A   
   G   
   A   
   T   
   G   
   T   
    T    
   T   
   T   
   C   
   T   
   T   
   A   
   G   
   T   
   A   
   A   
   C   
   G   
    T    
   A   
   T   
   C   
   A   
   A   
   G   
   G   
   A   
   A   
   T   
   G   
   G   
   G   
    T    
   T   
   T   
   G   
   C   
   G   
   C   
   T   
    A    
   T   
   C   
   C   
   T   
   T   
   T   
   A   
   A   
    C    
   A   
   G   
   A   
   T   
   G   
   T   
   T   
   C   
   C   
   C   
   G   
   T   
   T   
   G   
   T   
   T   
   G   
   G   
   A   
   G   
   C   
   A   
   A   
   A   
   G   
   G   
   C   
   A   
   G   
    C    
   T   
   G   
   G   
   A   
   G   
   T   
   G   
   A   
   A   
   G   
   T   
   C   
   T   
   A   
   T   
   G   
   A   
   A   
   T   
   T   
   T   
   G   
   A   
   A   
   A   
   G   
   A   
   T   
   G   
   G   
   A   
   G   
   A   
   T   
   A   
   G   
   G   
    A    
   T   
   T   
    A    
   T   
   C   
   A   
   A   
    C    
   G   
   G   
    G    
   C   
   T   
    G    
   C   
   T   
   T   
   G   
   T   
   G   
   T   
   T   
   A   
   G   
   C   
   A   
   G   
   A   
    A    
   G   
   G   
    T    
   G   
   A   
   T   
   A   
   C   
   G   
   C   
   C   
    G    
   A   
   T   
   C   
   G   
   T   
   A   
   A   
   T   
   C   
   G   
   T   
   T   
   A   
   C   
   T   
   C   
   A   
   G   
   C   
   G   
   A   
   G   
   G   
   A   
   G   
   C   
    T    
   G   
   T   
    T    
   A   
   A   
   A   
   C   
   G   
   C   
   A   
   T   
   G   
   C   
   T   
   T   
   G   
   C   
   T   
   C   
   A   
   A   
   G   
   A   
   A   
   A   
   T   
   C   
   A   
   G   
   C   
   C   
   A   
   A   
   A   
   C   
    T    
   T   
   C   
   A   
   C   
   G   
   T   
   G   
   C   
   G   
   A   
   A   
   G   
   C   
   G   
   G   
   G   
   G   
   A   
   G   
   T   
   G   
   A   
   C   
   G   
   G   
   T   
   C   
   A   
   T   
   G   
   C   
   G   
   T   
   G   
   A   
   G   
   C   
   T   
   G   
   A   
   A   
   A   
   A   
   A   
   A   
   A   
   A   
   T   
   C   
   C   
    T    
   C   
   A   
    T    
   C   
   G   
    C    
   A   
   T   
   T   
   A   
   T   
   C   
   T   
   A   
   T   
   A   
   T   
   G   
   T   
   C   
   G   
   G   
   A   
   A   
   G   
   G   
   A   
   A   
   G   
   A   
   T   
   C   
   A   
   A   
   A   
   A   
   G   
   A   
   A   
   A   
   T   
   A   
   A   
   C   
   C   
   T   
   T   
   G   
   A   
   T   
   G   
   A   
   A   
   T   
   C   
   A   
   A   
   A   
   A   
   A   
   G   
   G   
    G    
   C   
   A   
    G    
   C   
   A   
   G   
    T    
   T   
   A   
   A   
   C   
   G   
   A   
   T   
   T   
   G   
   A   
    C    
   C   
   C   
   A   
   A   
   C   
    T    
   G   
   A   
   T   
   T   
   T   
    C    
   C   
   C   
   T   
   A   
   T   
   A   
   G   
   G   
   T   
   G   
   A   
   T   
   C   
   G   
   A   
   A   
   C   
   A   
   T   
   C   
   G   
   A   
   A   
   T   
   G   
   G   
    A    
   T   
   C   
    A    
   T   
   T   
   T   
   G   
   C   
    G    
   A   
   T   
   G   
   G   
   A   
    T    
   G   
   A   
   G   
   A   
   A   
   A   
   A   
   A   
   A   
   G   
   G   
   T   
   G   
   G   
   A   
   G   
   A   
   A   
   G   
   T   
   C   
   A   
   T   
   C   
   G   
   A   
   A   
   G   
   T   
    T    
   A   
    T    
   C   
   G   
   A   
   T   
   G   
   C   
   T   
   C   
   C   
   T   
   G   
   A   
   G   
    A    
   T   
   T   
   T   
   T   
    C    
   A   
   T   
    C    
   G   
   A   
    G    
    T    
   C   
    G    
   -   
   -   
   -   
 

 
   N59951F   
   A   
   T   
   G   
   G   
   A   
   A   
   A   
   A   
   T   
   C   
   G   
   A   
   C   
   A   
   A   
   G   
   A   
   A   
   G   
   T   
   A   
   C   
   A   
   A   
   G   
   A   
   A   
   T   
   T   
   A   
   A   
   C   
   T   
   T   
   T   
   A   
   G   
   A   
   A   
   G   
   A   
   A   
   G   
   T   
   G   
   A   
   T   
   G   
   G   
   G   
   T   
   G   
   A   
   C   
   C   
   G   
   C   
   T   
   T   
   C   
   G   
   G   
   A   
   C   
   G   
   T   
   T   
   A   
   T   
   T   
   C   
   C   
   A   
   A   
   A   
   T   
   A   
   T   
   A   
   T   
   T   
   A   
   T   
    C    
   C   
   A   
    A    
   G   
   A   
   A   
   C   
   G   
   T   
   G   
   C   
   A   
   C   
   T   
   G   
   C   
   C   
   T   
   G   
   A   
   T   
   A   
   T   
    C    
   C   
   G   
   T   
   G   
   A   
   T   
   G   
   G   
   A   
   T   
   T   
   G   
   A   
   A   
    G    
   C   
   C   
   A   
   G   
   T   
    G    
   C   
   A   
   A   
   C   
   G   
   C   
   C   
   G   
   G   
   A   
   T   
   A   
   T   
   T   
   A   
   T   
   T   
    C    
   G   
   C   
    T    
   A   
   T   
   G   
   A   
   A   
   C   
   A   
   A   
   A   
   G   
   A   
   T   
   G   
   G   
   C   
   A   
   A   
   T   
   A   
   C   
   G   
   T   
   A   
   T   
   G   
   A   
   C   
   A   
   A   
   A   
   G   
   G   
   A   
   T   
   T   
    C    
   C   
   G   
   T   
   A   
   A   
   A   
   T   
   C   
   A   
   G   
   C   
   A   
   A   
   A   
   A   
   T   
   C   
   T   
   G   
   T   
   C   
   G   
   G   
   A   
   A   
   A   
   T   
   A   
   T   
   C   
   A   
   T   
   G   
   G   
   G   
    G    
   A   
   A   
   T   
   T   
   A   
   T   
   C   
   A   
   T   
   C   
   C   
   T   
   C   
   A   
   T   
   G   
   G   
   T   
   G   
   A   
   C   
   A   
   G   
   C   
   A   
   G   
   T   
   A   
   T   
   T   
   T   
   A   
   T   
   G   
   A   
   A   
   G   
   C   
   G   
   A   
   T   
   G   
   G   
   T   
   C   
   C   
   G   
   C   
   A   
   T   
   G   
   A   
   G   
   C   
   C   
   A   
   A   
   G   
   A   
    C    
   T   
   G   
   G   
   A   
   A   
   G   
   T   
   T   
   G   
   C   
   G   
   G   
   G   
   A   
   A   
   G   
   T   
   A   
   C   
   T   
   G   
   A   
   T   
   C   
   G   
   A   
    A    
   A   
   T   
   G   
   C   
   A   
   C   
   G   
   G   
    G    
   A   
   A   
   T   
   A   
   A   
    C    
   G   
   G   
   G   
   A   
   G   
   T   
   A   
   T   
   G   
   G   
   A   
   C   
   G   
   G   
   T   
   G   
   A   
   T   
   C   
   C   
   A   
   C   
   C   
   T   
   G   
   C   
   T   
   G   
   C   
   G   
   A   
   T   
   G   
   C   
   G   
   T   
   T   
   A   
   T   
   A   
   C   
   T   
   G   
   A   
   A   
   G   
   C   
   C   
   A   
   G   
    A    
   T   
   T   
   A   
   T   
   C   
   C   
   A   
   A   
   G   
   T   
   T   
   A   
   A   
   G   
   T   
   G   
   G   
   A   
   G   
   A   
   G   
   A   
   T   
   G   
    C    
   T   
   A   
    G    
   C   
   A   
   G   
   A   
   T   
   A   
   T   
   C   
   G   
   A   
    A    
   A   
   A   
   A   
   G   
   A   
   A   
   A   
   C   
   A   
   G   
   T   
    C    
   G   
   A   
   T   
   C   
   T   
   C   
   G   
   T   
   G   
   T   
   G   
   G   
   A   
   A   
   T   
   T   
   T   
   T   
   G   
   A   
   T   
   G   
   A   
   T   
   A   
   C   
   A   
   G   
   A   
   A   
   A   
   A   
   A   
   G   
   A   
   A   
   C   
   C   
   A   
   A   
   C   
   A   
   G   
   T   
   C   
   C   
   T   
   T   
   C   
   C   
   A   
   G   
   C   
   T   
   C   
   G   
   A   
   T   
   A   
   C   
   C   
   C   
   A   
   A   
   A   
   C   
   C   
   T   
   T   
   C   
   T   
    C    
   G   
   T   
   C   
   A   
   A   
   T   
   G   
   G   
   A   
   T   
   C   
   G   
   A   
   C   
   A   
   G   
   G   
   T   
   A   
   T   
   A   
   T   
   C   
   T   
   G   
   C   
   T   
   G   
   G   
   A   
   T   
   A   
   T   
   G   
   C   
    A    
   A   
   C   
   A   
   G   
   A   
   A   
   A   
   T   
   C   
   C   
   C   
   G   
   A   
   C   
    T    
   C   
   A   
   T   
   A   
   A   
   C   
   T   
   T   
   A   
   G   
   C   
   T   
   G   
   A   
   A   
   G   
   T   
   G   
   A   
   T   
    C    
   G   
   A   
    T    
   G   
   G   
   C   
   A   
   C   
   A   
   A   
   T   
   C   
   T   
   A   
   T   
   A   
   T   
   G   
   A   
   T   
   C   
   G   
   A   
   T   
   C   
   A   
   T   
   C   
   C   
   A   
   C   
   A   
   A   
   G   
   C   
   G   
   A   
   G   
   C   
   C   
   T   
   T   
   G   
   A   
   A   
   A   
   A   
   A   
   T   
   T   
   G   
   A   
   T   
   G   
   G   
   A   
   A   
   T   
   A   
   T   
   A   
   T   
   T   
   C   
   C   
   T   
   G   
   G   
   A   
   C   
   C   
   T   
   G   
   A   
   C   
   T   
   T   
    T    
   C   
   C   
   A   
   A   
   C   
   A   
   G   
   G   
   A   
   G   
   G   
    G    
   A   
   T   
   C   
   T   
   T   
   G   
   C   
   A   
   A   
   G   
   G   
    A    
   A   
   A   
   A   
   G   
   A   
   T   
   G   
   A   
   A   
   A   
   T   
   C   
   A   
   A   
   A   
   A   
   A   
   A   
   G   
   C   
   T   
   T   
   A   
   T   
   G   
   A   
   A   
   A   
   C   
   A   
   G   
   G   
    G    
   C   
   G   
   A   
   G   
   G   
   A   
   A   
   A   
   A   
   G   
   T   
   T   
   A   
    T    
   T   
   C   
   T   
   T   
   C   
   G   
   T   
   T   
   C   
   G   
   A   
   A   
   A   
   A   
   C   
   A   
   A   
   A   
   G   
   A   
   T   
   A   
   G   
   A   
    A    
   G   
   C   
   A   
   A   
   T   
   G   
   A   
   A   
   G   
   G   
   G   
    T    
   A   
   A   
    T    
   A   
   A   
   A   
   C   
   A   
   G   
   C   
   A   
   A   
   A   
   T   
   C   
   G   
   T   
   G   
   A   
   T   
   C   
   A   
   C   
    A    
   G   
   A   
   A   
   A   
   T   
   C   
   C   
   C   
   A   
   T   
   A   
   T   
   G   
   A   
   A   
   G   
   T   
   A   
   A   
   A   
   T   
   A   
   A   
   A   
   G   
   C   
   G   
   A   
   C   
    C    
   T   
   T   
   G   
   G   
   T   
   G   
   A   
   A   
   A   
   A   
   A   
   A   
   A   
   T   
   G   
   G   
   A   
   T   
   G   
   A   
   G   
   A   
   T   
   C   
   C   
   G   
   T   
   T   
   T   
   G   
   A   
   A   
   T   
   A   
   A   
   G   
   A   
   A   
   A   
   A   
   T   
   C   
   G   
   A   
    T    
   G   
   G   
   A   
   A   
   T   
   A   
   G   
   C   
   T   
   G   
   A   
   A   
   G   
   T   
    A    
   C   
   G   
   A   
   G   
   A   
   T   
   G   
   A   
   A   
   A   
   G   
   T   
   G   
   A   
   T   
    A    
   G   
    A    
   A   
   C   
    A    
   G   
   G   
   T   
   C   
   T   
   G   
   C   
   A   
   A   
   A   
   T   
   T   
   G   
   T   
   C   
   G   
   T   
    C    
   G   
   A   
   A   
   T   
   T   
   G   
   A   
   A   
   A   
   A   
   A   
   A   
   G   
   A   
    C    
   G   
   C   
   C   
   A   
   A   
    T    
   G   
   C   
   T   
   C   
   A   
   A   
   G   
   G   
    A    
   A   
   T   
   T   
   T   
   T   
   G   
   A   
   A   
   T   
   T   
   A   
    T    
   T   
   T   
   G   
   T   
   T   
   C   
   A   
   A   
   A   
   A   
   A   
   C   
   A   
   C   
   T   
   G   
   A   
   G   
    C    
   T   
   A   
   C   
   A   
   G   
   A   
   T   
   C   
   A   
   A   
   T   
   T   
   A   
   C   
   A   
   A   
   T   
   T   
   T   
    C    
   A   
   A   
   T   
   A   
   T   
   G   
   G   
   T   
   C   
   G   
   C   
   T   
   A   
   T   
   C   
   G   
   A   
    C    
   C   
   A   
   T   
   A   
   T   
   G   
   A   
   C   
   T   
   C   
   C   
   G   
   C   
   A   
    T    
   C   
   A   
   A   
   G   
   T   
   T   
   G   
   G   
   A   
   C   
   T   
   G   
   A   
   A   
   A   
   G   
   A   
   C   
   A   
   T   
   T   
   T   
   T   
   A   
   A   
   G   
   A   
   A   
   G   
   T   
   T   
   A   
   T   
   A   
   T   
   C   
   G   
   A   
   G   
   C   
   A   
   T   
   C   
   G   
   A   
   A   
   A   
   G   
   C   
   A   
   A   
   G   
   T   
   C   
   A   
   T   
   A   
   A   
   C   
   A   
   A   
   A   
   A   
   C   
   G   
    T    
   A   
   G   
    T    
   C   
   A   
   A   
   T   
   T   
   C   
   G   
   A   
   T   
   T   
   T   
   A   
   G   
   C   
   C   
   A   
   A   
   A   
   G   
   C   
   T   
   C   
   A   
   A   
   A   
   A   
   A   
    A    
   G   
   A   
   C   
   A   
   G   
   C   
   A   
   T   
   A   
   T   
   C   
   G   
   T   
    A    
   G   
   A   
   A   
   G   
   G   
    A    
    T    
   T   
   G   
   A   
   T   
   G   
   A   
   A   
   A   
   G   
   C   
   T   
   T   
   T   
   A   
   T   
   C   
   G   
   A   
   T   
   T   
   C   
   T   
   G   
   G   
   A   
   T   
   G   
   A   
   A   
   G   
   T   
   A   
   A   
   T   
   C   
   G   
    C    
   A   
   A   
   C   
   G   
   A   
   T   
   T   
   C   
   G   
   T   
   G   
   A   
   A   
   A   
   G   
   T   
   A   
   A   
   A   
   G   
   A   
   C   
   A   
   A   
   A   
   A   
   A   
   A   
   G   
   A   
    C    
   G   
   C   
    C    
   A   
   A   
   A   
   A   
   A   
   G   
   A   
   A   
   T   
   T   
   T   
   A   
   G   
   T   
   C   
   G   
   A   
    C    
   G   
   T   
    C    
   T   
   T   
    T    
   C   
   A   
   A   
   T   
   T   
   T   
   A   
   C   
   A   
   G   
   A   
   A   
   G   
   A   
   A   
   C   
   A   
   A   
   G   
   C   
   A   
   G   
   A   
   A   
   G   
   C   
   A   
   A   
   T   
    T    
   G   
   T   
   C   
   A   
   C   
   A   
   T   
   T   
   G   
   C   
   A   
   A   
    T    
   T   
   A   
   T   
   A   
    C    
   C   
   G   
   T   
   T   
   T   
   G   
   A   
   C   
   G   
   A   
   A   
    C    
   A   
   C   
    G    
   G   
   A   
   T   
   A   
   T   
   C   
   A   
   C   
   A   
   G   
   A   
   G   
   C   
   T   
   T   
   C   
   A   
   A   
   A   
   A   
   G   
   G   
   A   
   A   
    T    
   C   
    C    
   G   
   A   
   A   
   A   
    G    
   C   
   T   
   T   
   G   
   A   
   T   
   T   
   G   
   C   
    G    
   C   
   A   
   A   
   A   
   T   
   C   
   A   
   C   
   A   
   G   
   A   
   G   
   T   
   T   
    G    
   A   
   A   
   T   
   A   
   A   
   A   
   A   
   T   
   C   
   C   
   T   
   T   
   T   
   C   
   A   
   A   
   A   
    T    
   G   
   A   
   C   
   A   
   A   
   A   
   G   
   A   
    G    
   C   
   T   
   T   
   T   
   T   
    C    
   T   
   C   
   T   
   G   
   T   
   T   
   A   
   T   
   G   
   A   
   A   
   A   
   A   
   A   
   A   
   G   
   A   
   A   
   T   
   T   
   G   
   C   
   G   
   A   
   G   
   A   
   A   
   G   
   T   
   G   
   A   
   A   
   A   
   A   
   A   
   A   
   A   
   A   
   T   
   T   
   A   
    T    
   T   
   C   
   T   
   T   
   C   
   C   
   G   
   C   
   T   
   C   
   G   
   T   
   C   
    T    
    A    
   A   
   C   
   G   
   A   
   C   
   A   
   A   
   T   
   C   
    G    
   A   
   A   
   G   
   A   
   C   
   G   
   A   
   A   
   A   
   T   
   C   
   G   
   A   
   A   
   G   
   A   
   A   
   A   
   T   
   C   
   A   
   A   
   A   
   A   
   T   
    C    
   G   
   A   
   T   
   A   
   C   
   G   
   C   
   A   
   A   
    G    
   T   
    T    
   T   
   T   
   A   
   G   
   T   
   G   
   G   
   C   
    G    
   C   
   A   
   A   
   G   
   A   
   A   
   G   
   A   
   C   
   G   
   T   
   A   
   A   
   T   
    C    
   G   
   T   
   C   
   A   
   G   
   T   
   G   
   T   
    G    
   A   
   C   
    G    
   C   
   G   
   A   
   G   
   A   
   A   
   G   
   G   
   C   
   T   
   A   
   T   
   G   
   T   
   G   
   A   
   A   
   G   
   C   
   G   
   A   
   A   
   C   
   A   
   A   
   G   
   C   
   C   
   T   
   T   
   C   
   G   
   T   
   T   
   C   
    T    
   T   
   A   
   T   
   A   
   G   
   T   
   G   
   C   
   T   
   T   
   C   
    G    
   A   
   A   
   A   
   C   
   C   
   A   
   G   
   A   
   A   
   G   
   A   
   A   
   A   
   T   
   C   
   G   
   G   
   G   
   A   
   T   
   G   
   C   
   G   
   A   
   G   
   A   
   A   
   G   
   G   
   C   
   G   
   A   
    C    
   T   
   A   
   C   
   T   
   T   
   G   
   C   
   T   
    G    
   T   
   A   
   C   
    T    
   C   
   A   
   G   
   G   
   T   
   G   
   A   
   A   
   T   
   T   
    G    
   A   
   G   
    C    
   A   
   C   
   A   
    T    
   T   
   G   
   G   
   A   
   C   
   C   
   A   
   T   
   G   
   T   
   T   
   C   
   T   
   T   
   C   
   T   
   G   
   A   
   T   
    T    
   A   
   C   
    G    
   A   
   A   
   T   
   A   
   A   
   A   
   G   
   C   
   A   
   A   
   A   
   T   
   G   
   T   
   G   
   A   
   T   
   C   
   T   
   A   
   C   
   C   
   G   
    T    
   C   
   C   
   T   
   G   
   T   
    T    
   C   
   A   
   T   
   G   
   A   
   A   
   C   
   T   
   A   
   C   
   C   
    C    
   G   
   A   
   T   
   T   
   T   
   G   
   A   
   A   
   A   
   T   
   G   
   G   
   A   
   A   
   A   
   G   
   A   
   T   
   G   
   C   
   T   
   G   
   G   
    C    
   G   
   A   
    A    
   C   
   A   
    T    
   A   
   T   
   T   
   T   
   C   
   T   
   C   
   A   
   A   
   A   
   C   
   A   
   A   
   T   
   C   
   T   
   C   
   C   
   A   
   A   
   T   
   C   
   T   
   G   
   T   
   C   
   T   
   G   
   T   
   A   
   G   
   A   
    T    
   G   
   A   
   A   
   T   
   C   
   G   
   A   
   T   
    C    
   T   
   T   
   A   
   G   
   C   
   T   
   G   
   T   
   T   
   T   
   T   
    T    
   C   
   C   
   A   
   T   
   A   
   T   
   C   
   A   
   A   
   A   
   A   
   A   
   A   
   T   
    T    
   C   
   A   
   A   
   G   
   C   
   A   
   G   
   A   
    G    
   A   
   A   
   A   
   A   
   C   
   A   
   T   
   T   
   T   
   G   
   T   
    A    
   T   
   T   
   C   
   A   
   T   
   C   
   A   
   G   
   C   
   A   
   A   
   G   
   A   
   A   
   T   
   G   
   G   
   A   
   T   
   T   
    G    
    A    
   T   
   C   
   A   
   A   
   G   
   C   
   A   
   A   
   A   
   C   
   A   
   C   
   G   
   A   
   A   
   T   
   G   
   A   
   C   
   A   
   G   
   A   
   G   
   T   
   T   
   C   
   G   
   A   
   A   
   C   
   C   
   A   
   T   
   G   
   G   
   C   
   G   
   C   
   A   
   C   
   C   
   T   
   A   
    T    
   A   
   A   
   G   
   A   
   G   
   C   
   C   
   G   
    T    
   C   
   C   
   G   
   C   
   T   
   C   
   A   
   G   
   C   
   T   
   G   
   T   
   A   
   T   
   G   
   A   
   A   
   G   
   C   
   T   
   A   
   A   
   A   
   G   
   G   
   C   
   G   
   G   
   A   
   A   
   G   
   A   
   A   
   G   
   A   
   T   
   G   
   A   
   A   
   C   
   T   
    G    
   A   
   T   
   T   
   G   
   C   
   T   
   G   
   T   
    G    
   T   
   A   
   T   
   C   
   T   
   G   
   A   
   A   
   A   
   G   
   A   
   A   
   G   
   A   
   T   
   C   
    G    
   G   
   C   
   C   
   A   
   G   
   G   
   C   
   T   
   T   
   A   
   G   
   A   
   T   
   G   
   T   
    T    
   T   
   T   
   C   
   T   
   T   
   A   
   G   
   T   
   A   
   A   
   C   
   G   
    T    
   A   
   T   
   C   
   A   
   A   
   G   
   G   
   A   
   A   
   T   
   G   
   G   
   G   
    T    
   T   
   T   
   G   
   C   
   G   
   C   
   T   
    A    
   T   
   C   
   C   
   T   
   T   
   T   
   A   
   A   
    C    
   A   
   G   
   A   
   T   
   G   
   T   
   T   
   C   
   C   
   C   
   G   
   T   
   T   
   G   
   T   
   T   
   G   
   G   
   A   
   G   
   C   
   A   
   A   
   A   
   G   
   G   
   C   
   A   
   G   
    C    
   T   
   G   
   G   
   A   
   G   
   T   
   G   
   A   
   A   
   G   
   T   
   C   
   T   
   A   
   T   
   G   
   A   
   A   
   T   
   T   
   T   
   G   
   A   
   A   
   A   
   G   
   A   
   T   
   G   
   G   
   A   
   G   
   A   
   T   
   A   
   G   
   G   
    A    
   T   
   T   
    A    
   T   
   C   
   A   
   A   
    C    
   G   
   G   
    G    
   C   
   T   
    G    
   C   
   T   
   T   
   G   
   T   
   G   
   T   
   T   
   A   
   G   
   C   
   A   
   G   
   A   
    A    
   G   
   G   
    T    
   G   
   A   
   T   
   A   
   C   
   G   
   C   
   C   
    G    
   A   
   T   
   C   
   G   
   T   
   A   
   A   
   T   
   C   
   G   
   T   
   T   
   A   
   C   
   T   
   C   
   A   
   G   
   C   
   G   
   A   
   G   
   G   
   A   
   G   
   C   
    T    
   G   
   T   
    T    
   A   
   A   
   A   
   C   
   G   
   C   
   A   
   T   
   G   
   C   
   T   
   T   
   G   
   C   
   T   
   C   
   A   
   A   
   G   
   A   
   A   
   A   
   T   
   C   
   A   
   G   
   C   
   C   
   A   
   A   
   A   
   C   
    T    
   T   
   C   
   A   
   C   
   G   
   T   
   G   
   C   
   G   
   A   
   A   
   G   
   C   
   G   
   G   
   G   
   G   
   A   
   G   
   T   
   G   
   A   
   C   
   G   
   G   
   T   
   C   
   A   
   T   
   G   
   C   
   G   
   T   
   G   
   A   
   G   
   C   
   T   
   G   
   A   
   A   
   A   
   A   
   A   
   A   
   A   
   A   
   T   
   C   
   C   
    T    
   C   
   A   
    T    
   C   
   G   
    C    
   A   
   T   
   T   
   A   
   T   
   C   
   T   
   A   
   T   
   A   
   T   
   G   
   T   
   C   
   G   
   G   
   A   
   A   
   G   
   G   
   A   
   A   
   G   
   A   
   T   
   C   
   A   
   A   
   A   
   A   
   G   
   A   
   A   
   A   
   T   
   A   
   A   
   C   
   C   
   T   
   T   
   G   
   A   
   T   
   G   
   A   
   A   
   T   
   C   
   A   
   A   
   A   
   A   
   A   
   G   
   G   
    G    
   C   
   A   
    G    
   C   
   A   
   G   
    T    
   T   
   A   
   A   
   C   
   G   
   A   
   T   
   T   
   G   
   A   
    C    
   C   
   C   
   A   
   A   
   C   
    T    
   G   
   A   
   T   
   T   
   T   
    C    
   C   
   C   
   T   
   A   
   T   
   A   
   G   
   G   
   T   
   G   
   A   
   T   
   C   
   G   
   A   
   A   
   C   
   A   
   T   
   C   
   G   
   A   
   A   
   T   
   G   
   G   
    A    
   T   
   C   
    A    
   T   
   T   
   T   
   G   
   C   
    G    
   A   
   T   
   G   
   G   
   A   
    T    
   G   
   A   
   G   
   A   
   A   
   A   
   A   
   A   
   A   
   G   
   G   
   T   
   G   
   G   
   A   
   G   
   A   
   A   
   G   
   T   
   C   
   A   
   T   
   C   
   G   
   A   
   A   
   G   
   T   
    T    
   A   
    T    
   C   
   G   
   A   
   T   
   G   
   C   
   T   
   C   
   C   
   T   
   G   
   A   
   G   
    A    
   T   
   T   
   T   
   T   
    C    
   A   
   T   
    C    
   G   
   A   
    G    
    T    
   C   
    G    
   -   
   -   
   -   
 

 
   N55077   
   A   
   T   
   G   
   G   
   A   
   A   
   A   
   A   
   T   
   C   
   G   
   A   
   C   
   A   
   A   
   G   
   A   
   A   
   G   
   T   
   A   
   C   
   A   
   A   
   G   
   A   
   A   
   T   
   T   
   A   
   A   
   C   
   T   
   T   
   T   
   A   
   G   
   A   
   A   
   G   
   A   
   A   
   G   
   T   
   G   
   A   
   T   
   G   
   G   
   G   
   T   
   G   
   A   
   C   
   C   
   G   
   C   
   T   
   T   
   C   
   G   
   G   
   A   
   C   
   G   
   T   
   T   
   A   
   T   
   T   
   C   
   C   
   A   
   A   
   A   
   T   
   A   
   T   
   A   
   T   
   T   
   A   
   T   
    C    
   C   
   A   
    A    
   G   
   A   
   A   
   C   
   G   
   T   
   G   
   C   
   A   
   C   
   T   
   G   
   C   
   C   
   T   
   G   
   A   
   T   
   A   
   T   
    C    
   C   
   G   
   T   
   G   
   A   
   T   
   G   
   G   
   A   
   T   
   T   
   G   
   A   
   A   
    G    
   C   
   C   
   A   
   G   
   T   
    G    
   C   
   A   
   A   
   C   
   G   
   C   
   C   
   G   
   G   
   A   
   T   
   A   
   T   
   T   
   A   
   T   
   T   
    T    
   G   
   C   
    C    
   A   
   T   
   G   
   A   
   A   
   C   
   A   
   A   
   A   
   G   
   A   
   T   
   G   
   G   
   C   
   A   
   A   
   T   
   A   
   C   
   G   
   T   
   A   
   T   
   G   
   A   
   C   
   A   
   A   
   A   
   G   
   G   
   A   
   T   
   T   
    C    
   C   
   G   
   T   
   A   
   A   
   A   
   T   
   C   
   A   
   G   
   C   
   A   
   A   
   A   
   A   
   T   
   C   
   T   
   G   
   T   
   C   
   G   
   G   
   A   
   A   
   A   
   T   
   A   
   T   
   C   
   A   
   T   
   G   
   G   
   G   
    G    
   A   
   A   
   T   
   T   
   A   
   T   
   C   
   A   
   T   
   C   
   C   
   T   
   C   
   A   
   T   
   G   
   G   
   T   
   G   
   A   
   C   
   A   
   G   
   C   
   A   
   G   
   T   
   A   
   T   
   T   
   T   
   A   
   T   
   G   
   A   
   A   
   G   
   C   
   G   
   A   
   T   
   G   
   G   
   T   
   C   
   C   
   G   
   C   
   A   
   T   
   G   
   A   
   G   
   C   
   C   
   A   
   A   
   G   
   A   
    C    
   T   
   G   
   G   
   A   
   A   
   G   
   T   
   T   
   G   
   C   
   G   
   G   
   G   
   A   
   A   
   G   
   T   
   A   
   C   
   T   
   G   
   A   
   T   
   C   
   G   
   A   
    A    
   A   
   T   
   G   
   C   
   A   
   C   
   G   
   G   
    G    
   A   
   A   
   T   
   A   
   A   
    C    
   G   
   G   
   G   
   A   
   G   
   T   
   A   
   T   
   G   
   G   
   A   
   C   
   G   
   G   
   T   
   G   
   A   
   T   
   C   
   C   
   A   
   C   
   C   
   T   
   G   
   C   
   T   
   G   
   C   
   G   
   A   
   T   
   G   
   C   
   G   
   T   
   T   
   A   
   T   
   A   
   C   
   T   
   G   
   A   
   A   
   G   
   C   
   C   
   A   
   G   
    A    
   T   
   T   
   A   
   T   
   C   
   C   
   A   
   A   
   G   
   T   
   T   
   A   
   A   
   G   
   T   
   G   
   G   
   A   
   G   
   A   
   G   
   A   
   T   
   G   
    C    
   T   
   A   
    G    
   C   
   A   
   G   
   A   
   T   
   A   
   T   
   C   
   G   
   A   
    A    
   A   
   A   
   A   
   G   
   A   
   A   
   A   
   C   
   A   
   G   
   T   
    C    
   G   
   A   
   T   
   C   
   T   
   C   
   G   
   T   
   G   
   T   
   G   
   G   
   A   
   A   
   T   
   T   
   T   
   T   
   G   
   A   
   T   
   G   
   A   
   T   
   A   
   C   
   A   
   G   
   A   
   A   
   A   
   A   
   A   
   G   
   A   
   A   
   C   
   C   
   A   
   A   
   C   
   A   
   G   
   T   
   C   
   C   
   T   
   T   
   C   
   C   
   A   
   G   
   C   
   T   
   C   
   G   
   A   
   T   
   A   
   C   
   C   
   C   
   A   
   A   
   A   
   C   
   C   
   T   
   T   
   C   
   T   
    C    
   G   
   T   
   C   
   A   
   A   
   T   
   G   
   G   
   A   
   T   
   C   
   G   
   A   
   C   
   A   
   G   
   G   
   T   
   A   
   T   
   A   
   T   
   C   
   T   
   G   
   C   
   T   
   G   
   G   
   A   
   T   
   A   
   T   
   G   
   C   
    A    
   A   
   C   
   A   
   G   
   A   
   A   
   A   
   T   
   C   
   C   
   C   
   G   
   A   
   C   
    T    
   C   
   A   
   T   
   A   
   A   
   C   
   T   
   T   
   A   
   G   
   C   
   T   
   G   
   A   
   A   
   G   
   T   
   G   
   A   
   T   
    C    
   G   
   A   
    T    
   G   
   G   
   C   
   A   
   C   
   A   
   A   
   T   
   C   
   T   
   A   
   T   
   A   
   T   
   G   
   A   
   T   
   C   
   G   
   A   
   T   
   C   
   A   
   T   
   C   
   C   
   A   
   C   
   A   
   A   
   G   
   C   
   G   
   A   
   G   
   C   
   C   
   T   
   T   
   G   
   A   
   A   
   A   
   A   
   A   
   T   
   T   
   G   
   A   
   T   
   G   
   G   
   A   
   A   
   T   
   A   
   T   
   A   
   T   
   T   
   C   
   C   
   T   
   G   
   G   
   A   
   C   
   C   
   T   
   G   
   A   
   C   
   T   
   T   
    T    
   C   
   C   
   A   
   A   
   C   
   A   
   G   
   G   
   A   
   G   
   G   
    G    
   A   
   T   
   C   
   T   
   T   
   G   
   C   
   A   
   A   
   G   
   G   
    A    
   A   
   A   
   A   
   G   
   A   
   T   
   G   
   A   
   A   
   A   
   T   
   C   
   A   
   A   
   A   
   A   
   A   
   A   
   G   
   C   
   T   
   T   
   A   
   T   
   G   
   A   
   A   
   A   
   C   
   A   
   G   
   G   
    G    
   C   
   G   
   A   
   G   
   G   
   A   
   A   
   A   
   A   
   G   
   T   
   T   
   A   
    T    
   T   
   C   
   T   
   T   
   C   
   G   
   T   
   T   
   C   
   G   
   A   
   A   
   A   
   A   
   C   
   A   
   A   
   A   
   G   
   A   
   T   
   A   
   G   
   A   
    G    
   G   
   C   
   A   
   A   
   T   
   G   
   A   
   A   
   G   
   G   
   G   
    T    
   A   
   A   
    T    
   A   
   A   
   A   
   C   
   A   
   G   
   C   
   A   
   A   
   A   
   T   
   C   
   G   
   T   
   G   
   A   
   T   
   C   
   A   
   C   
    A    
   G   
   A   
   A   
   A   
   T   
   C   
   C   
   C   
   A   
   T   
   A   
   T   
   G   
   A   
   A   
   G   
   T   
   A   
   A   
   A   
   T   
   A   
   A   
   A   
   G   
   C   
   G   
   A   
   C   
    C    
   T   
   T   
   G   
   G   
   T   
   G   
   A   
   A   
   A   
   A   
   A   
   A   
   A   
   T   
   G   
   G   
   A   
   T   
   G   
   A   
   G   
   A   
   T   
   C   
   C   
   G   
   T   
   T   
   T   
   G   
   A   
   A   
   T   
   A   
   A   
   G   
   A   
   A   
   A   
   A   
   T   
   C   
   G   
   A   
    T    
   G   
   G   
   A   
   A   
   T   
   A   
   G   
   C   
   T   
   G   
   A   
   A   
   G   
   T   
    A    
   C   
   G   
   A   
   G   
   A   
   T   
   G   
   A   
   A   
   A   
   G   
   T   
   G   
   A   
   T   
    A    
   G   
    A    
   A   
   C   
    A    
   G   
   G   
   T   
   C   
   T   
   G   
   C   
   A   
   A   
   A   
   T   
   T   
   G   
   T   
   C   
   G   
   T   
    C    
   G   
   A   
   A   
   T   
   T   
   G   
   A   
   A   
   A   
   A   
   A   
   A   
   G   
   A   
    C    
   G   
   C   
   C   
   A   
   A   
    T    
   G   
   C   
   T   
   C   
   A   
   A   
   G   
   G   
    A    
   A   
   T   
   T   
   T   
   T   
   G   
   A   
   A   
   T   
   T   
   A   
    T    
   T   
   T   
   G   
   T   
   T   
   C   
   A   
   A   
   A   
   A   
   A   
   C   
   A   
   C   
   T   
   G   
   A   
   G   
    C    
   T   
   A   
   C   
   A   
   G   
   A   
   T   
   C   
   A   
   A   
   T   
   T   
   A   
   C   
   A   
   A   
   T   
   T   
   T   
    C    
   A   
   A   
   T   
   A   
   T   
   G   
   G   
   T   
   C   
   G   
   C   
   T   
   A   
   T   
   C   
   G   
   A   
    C    
   C   
   A   
   T   
   A   
   T   
   G   
   A   
   C   
   T   
   C   
   C   
   G   
   C   
   A   
    T    
   C   
   A   
   A   
   G   
   T   
   T   
   G   
   G   
   A   
   C   
   T   
   G   
   A   
   A   
   A   
   G   
   A   
   C   
   A   
   T   
   T   
   T   
   T   
   A   
   A   
   G   
   A   
   A   
   G   
   T   
   T   
   A   
   T   
   A   
   T   
   C   
   G   
   A   
   G   
   C   
   A   
   T   
   C   
   G   
   A   
   A   
   A   
   G   
   C   
   A   
   A   
   G   
   T   
   C   
   A   
   T   
   A   
   A   
   C   
   A   
   A   
   A   
   A   
   C   
   G   
    T    
   A   
   G   
    T    
   C   
   A   
   A   
   T   
   T   
   C   
   G   
   A   
   T   
   T   
   T   
   A   
   G   
   C   
   C   
   A   
   A   
   A   
   G   
   C   
   T   
   C   
   A   
   A   
   A   
   A   
   A   
    A    
   G   
   A   
   C   
   A   
   G   
   C   
   A   
   T   
   A   
   T   
   C   
   G   
   T   
    A    
   G   
   A   
   A   
   G   
   G   
    A    
    T    
   T   
   G   
   A   
   T   
   G   
   A   
   A   
   A   
   G   
   C   
   T   
   T   
   T   
   A   
   T   
   C   
   G   
   A   
   T   
   T   
   C   
   T   
   G   
   G   
   A   
   T   
   G   
   A   
   A   
   G   
   T   
   A   
   A   
   T   
   C   
   G   
    C    
   A   
   A   
   C   
   G   
   A   
   T   
   T   
   C   
   G   
   T   
   G   
   A   
   A   
   A   
   G   
   T   
   A   
   A   
   A   
   G   
   A   
   C   
   A   
   A   
   A   
   A   
   A   
   A   
   G   
   A   
    C    
   G   
   C   
    C    
   A   
   A   
   A   
   A   
   A   
   G   
   A   
   A   
   T   
   T   
   T   
   A   
   G   
   T   
   C   
   G   
   A   
    C    
   G   
   T   
    C    
   T   
   T   
    T    
   C   
   A   
   A   
   T   
   T   
   T   
   A   
   C   
   A   
   G   
   A   
   A   
   G   
   A   
   A   
   C   
   A   
   A   
   G   
   C   
   A   
   G   
   A   
   A   
   G   
   C   
   A   
   A   
   T   
    T    
   G   
   T   
   C   
   A   
   C   
   A   
   T   
   T   
   G   
   C   
   A   
   A   
    T    
   T   
   A   
   T   
   A   
    C    
   C   
   G   
   T   
   T   
   T   
   G   
   A   
   C   
   G   
   A   
   A   
    C    
   A   
   C   
    G    
   G   
   A   
   T   
   A   
   T   
   C   
   A   
   C   
   A   
   G   
   A   
   G   
   C   
   T   
   T   
   C   
   A   
   A   
   A   
   A   
   G   
   G   
   A   
   A   
    T    
   C   
    C    
   G   
   A   
   A   
   A   
    G    
   C   
   T   
   T   
   G   
   A   
   T   
   T   
   G   
   C   
    G    
   C   
   A   
   A   
   A   
   T   
   C   
   A   
   C   
   A   
   G   
   A   
   G   
   T   
   T   
    G    
   A   
   A   
   T   
   A   
   A   
   A   
   A   
   T   
   C   
   C   
   T   
   T   
   T   
   C   
   A   
   A   
   A   
    T    
   G   
   A   
   C   
   A   
   A   
   A   
   G   
   A   
    G    
   C   
   T   
   T   
   T   
   T   
    C    
   T   
   C   
   T   
   G   
   T   
   T   
   A   
   T   
   G   
   A   
   A   
   A   
   A   
   A   
   A   
   G   
   A   
   A   
   T   
   T   
   G   
   C   
   G   
   A   
   G   
   A   
   A   
   G   
   T   
   G   
   A   
   A   
   A   
   A   
   A   
   A   
   A   
   A   
   T   
   T   
   A   
    T    
   T   
   C   
   T   
   T   
   C   
   C   
   G   
   C   
   T   
   C   
   G   
   T   
   C   
    T    
    A    
   A   
   C   
   G   
   A   
   C   
   A   
   A   
   T   
   C   
    G    
   A   
   A   
   G   
   A   
   C   
   G   
   A   
   A   
   A   
   T   
   C   
   G   
   A   
   A   
   G   
   A   
   A   
   A   
   T   
   C   
   A   
   A   
   A   
   A   
   T   
    T    
   G   
   A   
   T   
   A   
   C   
   G   
   C   
   A   
   A   
    G    
   T   
    C    
   T   
   T   
   A   
   G   
   T   
   G   
   G   
   C   
    A    
   C   
   A   
   A   
   G   
   A   
   A   
   G   
   A   
   C   
   G   
   T   
   A   
   A   
   T   
    T    
   G   
   T   
   C   
   A   
   G   
   T   
   G   
   T   
    G    
   A   
   C   
    G    
   C   
   G   
   A   
   G   
   A   
   A   
   G   
   G   
   C   
   T   
   A   
   T   
   G   
   T   
   G   
   A   
   A   
   G   
   C   
   G   
   A   
   A   
   C   
   A   
   A   
   G   
   C   
   C   
   T   
   T   
   C   
   G   
   T   
   T   
   C   
    T    
   T   
   A   
   T   
   A   
   G   
   T   
   G   
   C   
   T   
   T   
   C   
    C    
   A   
   A   
   A   
   C   
   C   
   A   
   G   
   A   
   A   
   G   
   A   
   A   
   A   
   T   
   C   
   G   
   G   
   G   
   A   
   T   
   G   
   C   
   G   
   A   
   G   
   A   
   A   
   G   
   G   
   C   
   G   
   A   
    C    
   T   
   A   
   C   
   T   
   T   
   G   
   C   
   T   
    A    
   T   
   A   
   C   
    T    
   C   
   A   
   G   
   G   
   T   
   G   
   A   
   A   
   T   
   T   
    G    
   A   
   G   
    T    
   A   
   C   
   A   
    T    
   T   
   G   
   G   
   A   
   C   
   C   
   A   
   T   
   G   
   T   
   T   
   C   
   T   
   T   
   C   
   T   
   G   
   A   
   T   
    C    
   A   
   C   
    A    
   A   
   A   
   T   
   A   
   A   
   A   
   G   
   C   
   A   
   A   
   A   
   T   
   G   
   T   
   G   
   A   
   T   
   C   
   T   
   A   
   C   
   C   
   G   
    C    
   C   
   C   
   T   
   G   
   T   
    C    
   C   
   A   
   T   
   G   
   A   
   A   
   C   
   T   
   A   
   C   
   C   
    C    
   G   
   A   
   T   
   T   
   T   
   G   
   A   
   A   
   A   
   T   
   G   
   G   
   A   
   A   
   A   
   G   
   A   
   T   
   G   
   C   
   T   
   G   
   G   
    T    
   G   
   A   
    G    
   C   
   A   
    C    
   A   
   T   
   T   
   T   
   C   
   T   
   C   
   A   
   A   
   A   
   C   
   A   
   A   
   T   
   C   
   T   
   C   
   C   
   A   
   A   
   T   
   C   
   T   
   G   
   T   
   C   
   T   
   G   
   T   
   A   
   G   
   A   
    C    
   G   
   A   
   A   
   T   
   C   
   G   
   A   
   T   
    T    
   T   
   T   
   A   
   G   
   C   
   T   
   G   
   T   
   T   
   T   
   T   
    C    
   C   
   C   
   A   
   T   
   A   
   T   
   C   
   A   
   A   
   A   
   A   
   A   
   A   
   T   
    T    
   C   
   A   
   A   
   G   
   C   
   A   
   G   
   A   
    G    
   A   
   A   
   A   
   A   
   C   
   A   
   T   
   T   
   T   
   G   
   T   
    A    
   T   
   T   
   C   
   A   
   T   
   C   
   A   
   G   
   C   
   A   
   A   
   G   
   A   
   A   
   T   
   G   
   G   
   A   
   T   
   T   
    G    
    A    
   T   
   C   
   A   
   A   
   G   
   C   
   A   
   A   
   A   
   C   
   A   
   C   
   G   
   A   
   A   
   T   
   G   
   A   
   C   
   A   
   G   
   A   
   G   
   T   
   T   
   C   
   G   
   A   
   A   
   C   
   C   
   A   
   T   
   G   
   G   
   C   
   G   
   C   
   A   
   C   
   C   
   T   
   A   
    T    
   A   
   A   
   G   
   A   
   G   
   C   
   C   
   G   
    T    
   C   
   C   
   G   
   C   
   T   
   C   
   A   
   G   
   C   
   T   
   G   
   T   
   A   
   T   
   G   
   A   
   A   
   G   
   C   
   T   
   A   
   A   
   A   
   G   
   G   
   C   
   G   
   G   
   A   
   A   
   G   
   A   
   A   
   G   
   A   
   T   
   G   
   A   
   A   
   C   
   T   
    G    
   A   
   T   
   T   
   G   
   C   
   T   
   G   
   T   
    G    
   T   
   A   
   T   
   C   
   T   
   G   
   A   
   A   
   A   
   G   
   A   
   A   
   G   
   A   
   T   
   C   
    A    
   G   
   C   
   C   
   A   
   G   
   G   
   C   
   T   
   T   
   A   
   G   
   A   
   T   
   G   
   T   
    T    
   T   
   T   
   C   
   T   
   T   
   A   
   G   
   T   
   A   
   A   
   C   
   G   
    C    
   A   
   T   
   C   
   A   
   A   
   G   
   G   
   A   
   A   
   T   
   G   
   G   
   G   
    T    
   T   
   T   
   G   
   C   
   G   
   C   
   T   
    A    
   T   
   C   
   C   
   T   
   T   
   T   
   A   
   A   
    C    
   A   
   G   
   A   
   T   
   G   
   T   
   T   
   C   
   C   
   C   
   G   
   T   
   T   
   G   
   T   
   T   
   G   
   G   
   A   
   G   
   C   
   A   
   A   
   A   
   G   
   G   
   C   
   A   
   G   
    C    
   T   
   G   
   G   
   A   
   G   
   T   
   G   
   A   
   A   
   G   
   T   
   C   
   T   
   A   
   T   
   G   
   A   
   A   
   T   
   T   
   T   
   G   
   A   
   A   
   A   
   G   
   A   
   T   
   G   
   G   
   A   
   G   
   A   
   T   
   A   
   G   
   G   
    A    
   T   
   T   
    A    
   T   
   C   
   A   
   A   
    C    
   G   
   G   
    G    
   C   
   T   
    G    
   C   
   T   
   T   
   G   
   T   
   G   
   T   
   T   
   A   
   G   
   C   
   A   
   G   
   A   
    A    
   G   
   G   
    T    
   G   
   A   
   T   
   A   
   C   
   G   
   C   
   C   
    G    
   A   
   T   
   C   
   G   
   T   
   A   
   A   
   T   
   C   
   G   
   T   
   T   
   A   
   C   
   T   
   C   
   A   
   G   
   C   
   G   
   A   
   G   
   G   
   A   
   G   
   C   
    T    
   G   
   T   
    T    
   A   
   A   
   A   
   C   
   G   
   C   
   A   
   T   
   G   
   C   
   T   
   T   
   G   
   C   
   T   
   C   
   A   
   A   
   G   
   A   
   A   
   A   
   T   
   C   
   A   
   G   
   C   
   C   
   A   
   A   
   A   
   C   
    T    
   T   
   C   
   A   
   C   
   G   
   T   
   G   
   C   
   G   
   A   
   A   
   G   
   C   
   G   
   G   
   G   
   G   
   A   
   G   
   T   
   G   
   A   
   C   
   G   
   G   
   T   
   C   
   A   
   T   
   G   
   C   
   G   
   T   
   G   
   A   
   G   
   C   
   T   
   G   
   A   
   A   
   A   
   A   
   A   
   A   
   A   
   A   
   T   
   C   
   C   
    G    
   C   
   A   
    C    
   C   
   G   
    T    
   A   
   T   
   T   
   A   
   T   
   C   
   T   
   A   
   T   
   A   
   T   
   G   
   T   
   C   
   G   
   G   
   A   
   A   
   G   
   G   
   A   
   A   
   G   
   A   
   T   
   C   
   A   
   A   
   A   
   A   
   G   
   A   
   A   
   A   
   T   
   A   
   A   
   C   
   C   
   T   
   T   
   G   
   A   
   T   
   G   
   A   
   A   
   T   
   C   
   A   
   A   
   A   
   A   
   A   
   G   
   G   
    G    
   C   
   A   
    G    
   C   
   A   
   G   
    T    
   T   
   A   
   A   
   C   
   G   
   A   
   T   
   T   
   G   
   A   
    C    
   C   
   C   
   A   
   A   
   C   
    T    
   G   
   A   
   T   
   T   
   T   
    C    
   C   
   C   
   T   
   A   
   T   
   A   
   G   
   G   
   T   
   G   
   A   
   T   
   C   
   G   
   A   
   A   
   C   
   A   
   T   
   C   
   G   
   A   
   A   
   T   
   G   
   G   
    A    
   T   
   C   
    A    
   T   
   T   
   T   
   G   
   C   
    G    
   A   
   T   
   G   
   G   
   A   
    T    
   G   
   A   
   G   
   A   
   A   
   A   
   A   
   A   
   A   
   G   
   G   
   T   
   G   
   G   
   A   
   G   
   A   
   A   
   G   
   T   
   C   
   A   
   T   
   C   
   G   
   A   
   A   
   G   
   T   
    T    
   A   
    T    
   C   
   G   
   A   
   T   
   G   
   C   
   T   
   C   
   C   
   T   
   G   
   A   
   G   
    A    
   T   
   T   
   T   
   T   
    C    
   A   
   T   
    C    
   G   
   A   
    G    
    T    
   C   
    G    
   -   
   -   
   -   
 

 
   N52690   
   A   
   T   
   G   
   G   
   A   
   A   
   A   
   A   
   T   
   C   
   G   
   A   
   C   
   A   
   A   
   G   
   A   
   A   
   G   
   T   
   A   
   C   
   A   
   A   
   G   
   A   
   A   
   T   
   T   
   A   
   A   
   C   
   T   
   T   
   T   
   A   
   G   
   A   
   A   
   G   
   A   
   A   
   G   
   T   
   G   
   A   
   T   
   G   
   G   
   G   
   T   
   G   
   A   
   C   
   C   
   G   
   C   
   T   
   T   
   C   
   G   
   G   
   A   
   C   
   G   
   T   
   T   
   A   
   T   
   T   
   C   
   C   
   A   
   A   
   A   
   T   
   A   
   T   
   A   
   T   
   T   
   A   
   T   
    C    
   C   
   A   
    A    
   G   
   A   
   A   
   C   
   G   
   T   
   G   
   C   
   A   
   C   
   T   
   G   
   C   
   C   
   T   
   G   
   A   
   T   
   A   
   T   
    C    
   C   
   G   
   T   
   G   
   A   
   T   
   G   
   G   
   A   
   T   
   T   
   G   
   A   
   A   
    G    
   C   
   C   
   A   
   G   
   T   
    G    
   C   
   A   
   A   
   C   
   G   
   C   
   C   
   G   
   G   
   A   
   T   
   A   
   T   
   T   
   A   
   T   
   T   
    T    
   G   
   C   
    A    
   A   
   T   
   G   
   A   
   A   
   C   
   A   
   A   
   A   
   G   
   A   
   T   
   G   
   G   
   C   
   A   
   A   
   T   
   A   
   C   
   G   
   T   
   A   
   T   
   G   
   A   
   C   
   A   
   A   
   A   
   G   
   G   
   A   
   T   
   T   
    C    
   C   
   G   
   T   
   A   
   A   
   A   
   T   
   C   
   A   
   G   
   C   
   A   
   A   
   A   
   A   
   T   
   C   
   T   
   G   
   T   
   C   
   G   
   G   
   A   
   A   
   A   
   T   
   A   
   T   
   C   
   A   
   T   
   G   
   G   
   G   
    G    
   A   
   A   
   T   
   T   
   A   
   T   
   C   
   A   
   T   
   C   
   C   
   T   
   C   
   A   
   T   
   G   
   G   
   T   
   G   
   A   
   C   
   A   
   G   
   C   
   A   
   G   
   T   
   A   
   T   
   T   
   T   
   A   
   T   
   G   
   A   
   A   
   G   
   C   
   G   
   A   
   T   
   G   
   G   
   T   
   C   
   C   
   G   
   C   
   A   
   T   
   G   
   A   
   G   
   C   
   C   
   A   
   A   
   G   
   A   
    C    
   T   
   G   
   G   
   A   
   A   
   G   
   T   
   T   
   G   
   C   
   G   
   G   
   G   
   A   
   A   
   G   
   T   
   A   
   C   
   T   
   G   
   A   
   T   
   C   
   G   
   A   
    A    
   A   
   T   
   G   
   C   
   A   
   C   
   G   
   G   
    G    
   A   
   A   
   T   
   A   
   A   
    C    
   G   
   G   
   G   
   A   
   G   
   T   
   A   
   T   
   G   
   G   
   A   
   C   
   G   
   G   
   T   
   G   
   A   
   T   
   C   
   C   
   A   
   C   
   C   
   T   
   G   
   C   
   T   
   G   
   C   
   G   
   A   
   T   
   G   
   C   
   G   
   T   
   T   
   A   
   T   
   A   
   C   
   T   
   G   
   A   
   A   
   G   
   C   
   C   
   A   
   G   
    A    
   T   
   T   
   A   
   T   
   C   
   C   
   A   
   A   
   G   
   T   
   T   
   A   
   A   
   G   
   T   
   G   
   G   
   A   
   G   
   A   
   G   
   A   
   T   
   G   
    C    
   T   
   A   
    G    
   C   
   A   
   G   
   A   
   T   
   A   
   T   
   C   
   G   
   A   
    A    
   A   
   A   
   A   
   G   
   A   
   A   
   A   
   C   
   A   
   G   
   T   
    C    
   G   
   A   
   T   
   C   
   T   
   C   
   G   
   T   
   G   
   T   
   G   
   G   
   A   
   A   
   T   
   T   
   T   
   T   
   G   
   A   
   T   
   G   
   A   
   T   
   A   
   C   
   A   
   G   
   A   
   A   
   A   
   A   
   A   
   G   
   A   
   A   
   C   
   C   
   A   
   A   
   C   
   A   
   G   
   T   
   C   
   C   
   T   
   T   
   C   
   C   
   A   
   G   
   C   
   T   
   C   
   G   
   A   
   T   
   A   
   C   
   C   
   C   
   A   
   A   
   A   
   C   
   C   
   T   
   T   
   C   
   T   
    C    
   G   
   T   
   C   
   A   
   A   
   T   
   G   
   G   
   A   
   T   
   C   
   G   
   A   
   C   
   A   
   G   
   G   
   T   
   A   
   T   
   A   
   T   
   C   
   T   
   G   
   C   
   T   
   G   
   G   
   A   
   T   
   A   
   T   
   G   
   C   
    A    
   A   
   C   
   A   
   G   
   A   
   A   
   A   
   T   
   C   
   C   
   C   
   G   
   A   
   C   
    T    
   C   
   A   
   T   
   A   
   A   
   C   
   T   
   T   
   A   
   G   
   C   
   T   
   G   
   A   
   A   
   G   
   T   
   G   
   A   
   T   
    C    
   G   
   A   
    T    
   G   
   G   
   C   
   A   
   C   
   A   
   A   
   T   
   C   
   T   
   A   
   T   
   A   
   T   
   G   
   A   
   T   
   C   
   G   
   A   
   T   
   C   
   A   
   T   
   C   
   C   
   A   
   C   
   A   
   A   
   G   
   C   
   G   
   A   
   G   
   C   
   C   
   T   
   T   
   G   
   A   
   A   
   A   
   A   
   A   
   T   
   T   
   G   
   A   
   T   
   G   
   G   
   A   
   A   
   T   
   A   
   T   
   A   
   T   
   T   
   C   
   C   
   T   
   G   
   G   
   A   
   C   
   C   
   T   
   G   
   A   
   C   
   T   
   T   
    T    
   C   
   C   
   A   
   A   
   C   
   A   
   G   
   G   
   A   
   G   
   G   
    G    
   A   
   T   
   C   
   T   
   T   
   G   
   C   
   A   
   A   
   G   
   G   
    A    
   A   
   A   
   A   
   G   
   A   
   T   
   G   
   A   
   A   
   A   
   T   
   C   
   A   
   A   
   A   
   A   
   A   
   A   
   G   
   C   
   T   
   T   
   A   
   T   
   G   
   A   
   A   
   A   
   C   
   A   
   G   
   G   
    G    
   C   
   G   
   A   
   G   
   G   
   A   
   A   
   A   
   A   
   G   
   T   
   T   
   A   
    T    
   T   
   C   
   T   
   T   
   C   
   G   
   T   
   T   
   C   
   G   
   A   
   A   
   A   
   A   
   C   
   A   
   A   
   A   
   G   
   A   
   T   
   A   
   G   
   A   
    A    
   G   
   C   
   A   
   A   
   T   
   G   
   A   
   A   
   G   
   G   
   G   
    T    
   A   
   A   
    T    
   A   
   A   
   A   
   C   
   A   
   G   
   C   
   A   
   A   
   A   
   T   
   C   
   G   
   T   
   G   
   A   
   T   
   C   
   A   
   C   
    A    
   G   
   A   
   A   
   A   
   T   
   C   
   C   
   C   
   A   
   T   
   A   
   T   
   G   
   A   
   A   
   G   
   T   
   A   
   A   
   A   
   T   
   A   
   A   
   A   
   G   
   C   
   G   
   A   
   C   
    C    
   T   
   T   
   G   
   G   
   T   
   G   
   A   
   A   
   A   
   A   
   A   
   A   
   A   
   T   
   G   
   G   
   A   
   T   
   G   
   A   
   G   
   A   
   T   
   C   
   C   
   G   
   T   
   T   
   T   
   G   
   A   
   A   
   T   
   A   
   A   
   G   
   A   
   A   
   A   
   A   
   T   
   C   
   G   
   A   
    T    
   G   
   G   
   A   
   A   
   T   
   A   
   G   
   C   
   T   
   G   
   A   
   A   
   G   
   T   
    A    
   C   
   G   
   A   
   G   
   A   
   T   
   G   
   A   
   A   
   A   
   G   
   T   
   G   
   A   
   T   
    A    
   G   
    A    
   A   
   C   
    A    
   G   
   G   
   T   
   C   
   T   
   G   
   C   
   A   
   A   
   A   
   T   
   T   
   G   
   T   
   C   
   G   
   T   
    C    
   G   
   A   
   A   
   T   
   T   
   G   
   A   
   A   
   A   
   A   
   A   
   A   
   G   
   A   
    C    
   G   
   C   
   C   
   A   
   A   
    T    
   G   
   C   
   T   
   C   
   A   
   A   
   G   
   G   
    A    
   A   
   T   
   T   
   T   
   T   
   G   
   A   
   A   
   T   
   T   
   A   
    T    
   T   
   T   
   G   
   T   
   T   
   C   
   A   
   A   
   A   
   A   
   A   
   C   
   A   
   C   
   T   
   G   
   A   
   G   
    C    
   T   
   A   
   C   
   A   
   G   
   A   
   T   
   C   
   A   
   A   
   T   
   T   
   A   
   C   
   A   
   A   
   T   
   T   
   T   
    C    
   A   
   A   
   T   
   A   
   T   
   G   
   G   
   T   
   C   
   G   
   C   
   T   
   A   
   T   
   C   
   G   
   A   
    C    
   C   
   A   
   T   
   A   
   T   
   G   
   A   
   C   
   T   
   C   
   C   
   G   
   C   
   A   
    T    
   C   
   A   
   A   
   G   
   T   
   T   
   G   
   G   
   A   
   C   
   T   
   G   
   A   
   A   
   A   
   G   
   A   
   C   
   A   
   T   
   T   
   T   
   T   
   A   
   A   
   G   
   A   
   A   
   G   
   T   
   T   
   A   
   T   
   A   
   T   
   C   
   G   
   A   
   G   
   C   
   A   
   T   
   C   
   G   
   A   
   A   
   A   
   G   
   C   
   A   
   A   
   G   
   T   
   C   
   A   
   T   
   A   
   A   
   C   
   A   
   A   
   A   
   A   
   C   
   G   
    T    
   A   
   G   
    T    
   C   
   A   
   A   
   T   
   T   
   C   
   G   
   A   
   T   
   T   
   T   
   A   
   G   
   C   
   C   
   A   
   A   
   A   
   G   
   C   
   T   
   C   
   A   
   A   
   A   
   A   
   A   
    A    
   G   
   A   
   C   
   A   
   G   
   C   
   A   
   T   
   A   
   T   
   C   
   G   
   T   
    A    
   G   
   A   
   A   
   G   
   G   
    A    
    T    
   T   
   G   
   A   
   T   
   G   
   A   
   A   
   A   
   G   
   C   
   T   
   T   
   T   
   A   
   T   
   C   
   G   
   A   
   T   
   T   
   C   
   T   
   G   
   G   
   A   
   T   
   G   
   A   
   A   
   G   
   T   
   A   
   A   
   T   
   C   
   G   
    C    
   A   
   A   
   C   
   G   
   A   
   T   
   T   
   C   
   G   
   T   
   G   
   A   
   A   
   A   
   G   
   T   
   A   
   A   
   A   
   G   
   A   
   C   
   A   
   A   
   A   
   A   
   A   
   A   
   G   
   A   
    C    
   G   
   C   
    C    
   A   
   A   
   A   
   A   
   A   
   G   
   A   
   A   
   T   
   T   
   T   
   A   
   G   
   T   
   C   
   G   
   A   
    C    
   G   
   T   
    C    
   T   
   T   
    T    
   C   
   A   
   A   
   T   
   T   
   T   
   A   
   C   
   A   
   G   
   A   
   A   
   G   
   A   
   A   
   C   
   A   
   A   
   G   
   C   
   A   
   G   
   A   
   A   
   G   
   C   
   A   
   A   
   T   
    T    
   G   
   T   
   C   
   A   
   C   
   A   
   T   
   T   
   G   
   C   
   A   
   A   
    T    
   T   
   A   
   T   
   A   
    C    
   C   
   G   
   T   
   T   
   T   
   G   
   A   
   C   
   G   
   A   
   A   
    C    
   A   
   C   
    G    
   G   
   A   
   T   
   A   
   T   
   C   
   A   
   C   
   A   
   G   
   A   
   G   
   C   
   T   
   T   
   C   
   A   
   A   
   A   
   A   
   G   
   G   
   A   
   A   
    T    
   C   
    C    
   G   
   A   
   A   
   A   
    G    
   C   
   T   
   T   
   G   
   A   
   T   
   T   
   G   
   C   
    G    
   C   
   A   
   A   
   A   
   T   
   C   
   A   
   C   
   A   
   G   
   A   
   G   
   T   
   T   
    G    
   A   
   A   
   T   
   A   
   A   
   A   
   A   
   T   
   C   
   C   
   T   
   T   
   T   
   C   
   A   
   A   
   A   
    T    
   G   
   A   
   C   
   A   
   A   
   A   
   G   
   A   
    G    
   C   
   T   
   T   
   T   
   T   
    C    
   T   
   C   
   T   
   G   
   T   
   T   
   A   
   T   
   G   
   A   
   A   
   A   
   A   
   A   
   A   
   G   
   A   
   A   
   T   
   T   
   G   
   C   
   G   
   A   
   G   
   A   
   A   
   G   
   T   
   G   
   A   
   A   
   A   
   A   
   A   
   A   
   A   
   A   
   T   
   T   
   A   
    T    
   T   
   C   
   T   
   T   
   C   
   C   
   G   
   C   
   T   
   C   
   G   
   T   
   C   
    T    
    A    
   A   
   C   
   G   
   A   
   C   
   A   
   A   
   T   
   C   
    G    
   A   
   A   
   G   
   A   
   C   
   G   
   A   
   A   
   A   
   T   
   C   
   G   
   A   
   A   
   G   
   A   
   A   
   A   
   T   
   C   
   A   
   A   
   A   
   A   
   T   
    T    
   G   
   A   
   T   
   A   
   C   
   G   
   C   
   A   
   A   
    G    
   T   
    C    
   T   
   T   
   A   
   G   
   T   
   G   
   G   
   C   
    A    
   C   
   A   
   A   
   G   
   A   
   A   
   G   
   A   
   C   
   G   
   T   
   A   
   A   
   T   
    T    
   G   
   T   
   C   
   A   
   G   
   T   
   G   
   T   
    G    
   A   
   C   
    G    
   C   
   G   
   A   
   G   
   A   
   A   
   G   
   G   
   C   
   T   
   A   
   T   
   G   
   T   
   G   
   A   
   A   
   G   
   C   
   G   
   A   
   A   
   C   
   A   
   A   
   G   
   C   
   C   
   T   
   T   
   C   
   G   
   T   
   T   
   C   
    T    
   T   
   A   
   T   
   A   
   G   
   T   
   G   
   C   
   T   
   T   
   C   
    C    
   A   
   A   
   A   
   C   
   C   
   A   
   G   
   A   
   A   
   G   
   A   
   A   
   A   
   T   
   C   
   G   
   G   
   G   
   A   
   T   
   G   
   C   
   G   
   A   
   G   
   A   
   A   
   G   
   G   
   C   
   G   
   A   
    C    
   T   
   A   
   C   
   T   
   T   
   G   
   C   
   T   
    A    
   T   
   A   
   C   
    T    
   C   
   A   
   G   
   G   
   T   
   G   
   A   
   A   
   T   
   T   
    G    
   A   
   G   
    T    
   A   
   C   
   A   
    T    
   T   
   G   
   G   
   A   
   C   
   C   
   A   
   T   
   G   
   T   
   T   
   C   
   T   
   T   
   C   
   T   
   G   
   A   
   T   
    C    
   A   
   C   
    A    
   A   
   A   
   T   
   A   
   A   
   A   
   G   
   C   
   A   
   A   
   A   
   T   
   G   
   T   
   G   
   A   
   T   
   C   
   T   
   A   
   C   
   C   
   G   
    C    
   C   
   C   
   T   
   G   
   T   
    C    
   C   
   A   
   T   
   G   
   A   
   A   
   C   
   T   
   A   
   C   
   C   
    C    
   G   
   A   
   T   
   T   
   T   
   G   
   A   
   A   
   A   
   T   
   G   
   G   
   A   
   A   
   A   
   G   
   A   
   T   
   G   
   C   
   T   
   G   
   G   
    T    
   G   
   A   
    G    
   C   
   A   
    C    
   A   
   T   
   T   
   T   
   C   
   T   
   C   
   A   
   A   
   A   
   C   
   A   
   A   
   T   
   C   
   T   
   C   
   C   
   A   
   A   
   T   
   C   
   T   
   G   
   T   
   C   
   T   
   G   
   T   
   A   
   G   
   A   
    C    
   G   
   A   
   A   
   T   
   C   
   G   
   A   
   T   
    T    
   T   
   T   
   A   
   G   
   C   
   T   
   G   
   T   
   T   
   T   
   T   
    C    
   C   
   C   
   A   
   T   
   A   
   T   
   C   
   A   
   A   
   A   
   A   
   A   
   A   
   T   
    T    
   C   
   A   
   A   
   G   
   C   
   A   
   G   
   A   
    G    
   A   
   A   
   A   
   A   
   C   
   A   
   T   
   T   
   T   
   G   
   T   
    A    
   T   
   T   
   C   
   A   
   T   
   C   
   A   
   G   
   C   
   A   
   A   
   G   
   A   
   A   
   T   
   G   
   G   
   A   
   T   
   T   
    G    
    A    
   T   
   C   
   A   
   A   
   G   
   C   
   A   
   A   
   A   
   C   
   A   
   C   
   G   
   A   
   A   
   T   
   G   
   A   
   C   
   A   
   G   
   A   
   G   
   T   
   T   
   C   
   G   
   A   
   A   
   C   
   C   
   A   
   T   
   G   
   G   
   C   
   G   
   C   
   A   
   C   
   C   
   T   
   A   
    T    
   A   
   A   
   G   
   A   
   G   
   C   
   C   
   G   
    T    
   C   
   C   
   G   
   C   
   T   
   C   
   A   
   G   
   C   
   T   
   G   
   T   
   A   
   T   
   G   
   A   
   A   
   G   
   C   
   T   
   A   
   A   
   A   
   G   
   G   
   C   
   G   
   G   
   A   
   A   
   G   
   A   
   A   
   G   
   A   
   T   
   G   
   A   
   A   
   C   
   T   
    G    
   A   
   T   
   T   
   G   
   C   
   T   
   G   
   T   
    G    
   T   
   A   
   T   
   C   
   T   
   G   
   A   
   A   
   A   
   G   
   A   
   A   
   G   
   A   
   T   
   C   
    A    
   G   
   C   
   C   
   A   
   G   
   G   
   C   
   T   
   T   
   A   
   G   
   A   
   T   
   G   
   T   
    T    
   T   
   T   
   C   
   T   
   T   
   A   
   G   
   T   
   A   
   A   
   C   
   G   
    C    
   A   
   T   
   C   
   A   
   A   
   G   
   G   
   A   
   A   
   T   
   G   
   G   
   G   
    T    
   T   
   T   
   G   
   C   
   G   
   C   
   T   
    A    
   T   
   C   
   C   
   T   
   T   
   T   
   A   
   A   
    C    
   A   
   G   
   A   
   T   
   G   
   T   
   T   
   C   
   C   
   C   
   G   
   T   
   T   
   G   
   T   
   T   
   G   
   G   
   A   
   G   
   C   
   A   
   A   
   A   
   G   
   G   
   C   
   A   
   G   
    C    
   T   
   G   
   G   
   A   
   G   
   T   
   G   
   A   
   A   
   G   
   T   
   C   
   T   
   A   
   T   
   G   
   A   
   A   
   T   
   T   
   T   
   G   
   A   
   A   
   A   
   G   
   A   
   T   
   G   
   G   
   A   
   G   
   A   
   T   
   A   
   G   
   G   
    A    
   T   
   T   
    A    
   T   
   C   
   A   
   A   
    C    
   G   
   G   
    G    
   C   
   T   
    G    
   C   
   T   
   T   
   G   
   T   
   G   
   T   
   T   
   A   
   G   
   C   
   A   
   G   
   A   
    A    
   G   
   G   
    T    
   G   
   A   
   T   
   A   
   C   
   G   
   C   
   C   
    G    
   A   
   T   
   C   
   G   
   T   
   A   
   A   
   T   
   C   
   G   
   T   
   T   
   A   
   C   
   T   
   C   
   A   
   G   
   C   
   G   
   A   
   G   
   G   
   A   
   G   
   C   
    T    
   G   
   T   
    T    
   A   
   A   
   A   
   C   
   G   
   C   
   A   
   T   
   G   
   C   
   T   
   T   
   G   
   C   
   T   
   C   
   A   
   A   
   G   
   A   
   A   
   A   
   T   
   C   
   A   
   G   
   C   
   C   
   A   
   A   
   A   
   C   
    T    
   T   
   C   
   A   
   C   
   G   
   T   
   G   
   C   
   G   
   A   
   A   
   G   
   C   
   G   
   G   
   G   
   G   
   A   
   G   
   T   
   G   
   A   
   C   
   G   
   G   
   T   
   C   
   A   
   T   
   G   
   C   
   G   
   T   
   G   
   A   
   G   
   C   
   T   
   G   
   A   
   A   
   A   
   A   
   A   
   A   
   A   
   A   
   T   
   C   
   C   
    G    
   C   
   A   
    C    
   C   
   G   
    T    
   A   
   T   
   T   
   A   
   T   
   C   
   T   
   A   
   T   
   A   
   T   
   G   
   T   
   C   
   G   
   G   
   A   
   A   
   G   
   G   
   A   
   A   
   G   
   A   
   T   
   C   
   A   
   A   
   A   
   A   
   G   
   A   
   A   
   A   
   T   
   A   
   A   
   C   
   C   
   T   
   T   
   G   
   A   
   T   
   G   
   A   
   A   
   T   
   C   
   A   
   A   
   A   
   A   
   A   
   G   
   G   
    G    
   C   
   A   
    G    
   C   
   A   
   G   
    T    
   T   
   A   
   A   
   C   
   G   
   A   
   T   
   T   
   G   
   A   
    C    
   C   
   C   
   A   
   A   
   C   
    T    
   G   
   A   
   T   
   T   
   T   
    C    
   C   
   C   
   T   
   A   
   T   
   A   
   G   
   G   
   T   
   G   
   A   
   T   
   C   
   G   
   A   
   A   
   C   
   A   
   T   
   C   
   G   
   A   
   A   
   T   
   G   
   G   
    A    
   T   
   C   
    A    
   T   
   T   
   T   
   G   
   C   
    G    
   A   
   T   
   G   
   G   
   A   
    T    
   G   
   A   
   G   
   A   
   A   
   A   
   A   
   A   
   A   
   G   
   G   
   T   
   G   
   G   
   A   
   G   
   A   
   A   
   G   
   T   
   C   
   A   
   T   
   C   
   G   
   A   
   A   
   G   
   T   
    T    
   A   
    T    
   C   
   G   
   A   
   T   
   G   
   C   
   T   
   C   
   C   
   T   
   G   
   A   
   G   
    A    
   T   
   T   
   T   
   T   
    T    
   A   
   T   
    C    
   G   
   A   
    G    
    T    
   C   
    G    
   -   
   -   
   -   
 

 
   N59907F   
   A   
   T   
   G   
   G   
   A   
   A   
   A   
   A   
   T   
   C   
   G   
   A   
   C   
   A   
   A   
   G   
   A   
   A   
   G   
   T   
   A   
   C   
   A   
   A   
   G   
   A   
   A   
   T   
   T   
   A   
   A   
   C   
   T   
   T   
   T   
   A   
   G   
   A   
   A   
   G   
   A   
   A   
   G   
   T   
   G   
   A   
   T   
   G   
   G   
   G   
   T   
   G   
   A   
   C   
   C   
   G   
   C   
   T   
   T   
   C   
   G   
   G   
   A   
   C   
   G   
   T   
   T   
   A   
   T   
   T   
   C   
   C   
   A   
   A   
   A   
   T   
   A   
   T   
   A   
   T   
   T   
   A   
   T   
    C    
   C   
   A   
    A    
   G   
   A   
   A   
   C   
   G   
   T   
   G   
   C   
   A   
   C   
   T   
   G   
   C   
   C   
   T   
   G   
   A   
   T   
   A   
   T   
    C    
   C   
   G   
   T   
   G   
   A   
   T   
   G   
   G   
   A   
   T   
   T   
   G   
   A   
   A   
    G    
   C   
   C   
   A   
   G   
   T   
    G    
   C   
   A   
   A   
   C   
   G   
   C   
   C   
   G   
   G   
   A   
   T   
   A   
   T   
   T   
   A   
   T   
   T   
    C    
   G   
   C   
    T    
   A   
   T   
   G   
   A   
   A   
   C   
   A   
   A   
   A   
   G   
   A   
   T   
   G   
   G   
   C   
   A   
   A   
   T   
   A   
   C   
   G   
   T   
   A   
   T   
   G   
   A   
   C   
   A   
   A   
   A   
   G   
   G   
   A   
   T   
   T   
    C    
   C   
   G   
   T   
   A   
   A   
   A   
   T   
   C   
   A   
   G   
   C   
   A   
   A   
   A   
   A   
   T   
   C   
   T   
   G   
   T   
   C   
   G   
   G   
   A   
   A   
   A   
   T   
   A   
   T   
   C   
   A   
   T   
   G   
   G   
   G   
    G    
   A   
   A   
   T   
   T   
   A   
   T   
   C   
   A   
   T   
   C   
   C   
   T   
   C   
   A   
   T   
   G   
   G   
   T   
   G   
   A   
   C   
   A   
   G   
   C   
   A   
   G   
   T   
   A   
   T   
   T   
   T   
   A   
   T   
   G   
   A   
   A   
   G   
   C   
   G   
   A   
   T   
   G   
   G   
   T   
   C   
   C   
   G   
   C   
   A   
   T   
   G   
   A   
   G   
   C   
   C   
   A   
   A   
   G   
   A   
    C    
   T   
   G   
   G   
   A   
   A   
   G   
   T   
   T   
   G   
   C   
   G   
   G   
   G   
   A   
   A   
   G   
   T   
   A   
   C   
   T   
   G   
   A   
   T   
   C   
   G   
   A   
    A    
   A   
   T   
   G   
   C   
   A   
   C   
   G   
   G   
    G    
   A   
   A   
   T   
   A   
   A   
    C    
   G   
   G   
   G   
   A   
   G   
   T   
   A   
   T   
   G   
   G   
   A   
   C   
   G   
   G   
   T   
   G   
   A   
   T   
   C   
   C   
   A   
   C   
   C   
   T   
   G   
   C   
   T   
   G   
   C   
   G   
   A   
   T   
   G   
   C   
   G   
   T   
   T   
   A   
   T   
   A   
   C   
   T   
   G   
   A   
   A   
   G   
   C   
   C   
   A   
   G   
    A    
   T   
   T   
   A   
   T   
   C   
   C   
   A   
   A   
   G   
   T   
   T   
   A   
   A   
   G   
   T   
   G   
   G   
   A   
   G   
   A   
   G   
   A   
   T   
   G   
    C    
   T   
   A   
    G    
   C   
   A   
   G   
   A   
   T   
   A   
   T   
   C   
   G   
   A   
    A    
   A   
   A   
   A   
   G   
   A   
   A   
   A   
   C   
   A   
   G   
   T   
    C    
   G   
   A   
   T   
   C   
   T   
   C   
   G   
   T   
   G   
   T   
   G   
   G   
   A   
   A   
   T   
   T   
   T   
   T   
   G   
   A   
   T   
   G   
   A   
   T   
   A   
   C   
   A   
   G   
   A   
   A   
   A   
   A   
   A   
   G   
   A   
   A   
   C   
   C   
   A   
   A   
   C   
   A   
   G   
   T   
   C   
   C   
   T   
   T   
   C   
   C   
   A   
   G   
   C   
   T   
   C   
   G   
   A   
   T   
   A   
   C   
   C   
   C   
   A   
   A   
   A   
   C   
   C   
   T   
   T   
   C   
   T   
    C    
   G   
   T   
   C   
   A   
   A   
   T   
   G   
   G   
   A   
   T   
   C   
   G   
   A   
   C   
   A   
   G   
   G   
   T   
   A   
   T   
   A   
   T   
   C   
   T   
   G   
   C   
   T   
   G   
   G   
   A   
   T   
   A   
   T   
   G   
   C   
    A    
   A   
   C   
   A   
   G   
   A   
   A   
   A   
   T   
   C   
   C   
   C   
   G   
   A   
   C   
    T    
   C   
   A   
   T   
   A   
   A   
   C   
   T   
   T   
   A   
   G   
   C   
   T   
   G   
   A   
   A   
   G   
   T   
   G   
   A   
   T   
    C    
   G   
   A   
    T    
   G   
   G   
   C   
   A   
   C   
   A   
   A   
   T   
   C   
   T   
   A   
   T   
   A   
   T   
   G   
   A   
   T   
   C   
   G   
   A   
   T   
   C   
   A   
   T   
   C   
   C   
   A   
   C   
   A   
   A   
   G   
   C   
   G   
   A   
   G   
   C   
   C   
   T   
   T   
   G   
   A   
   A   
   A   
   A   
   A   
   T   
   T   
   G   
   A   
   T   
   G   
   G   
   A   
   A   
   T   
   A   
   T   
   A   
   T   
   T   
   C   
   C   
   T   
   G   
   G   
   A   
   C   
   C   
   T   
   G   
   A   
   C   
   T   
   T   
    T    
   C   
   C   
   A   
   A   
   C   
   A   
   G   
   G   
   A   
   G   
   G   
    G    
   A   
   T   
   C   
   T   
   T   
   G   
   C   
   A   
   A   
   G   
   G   
    A    
   A   
   A   
   A   
   G   
   A   
   T   
   G   
   A   
   A   
   A   
   T   
   C   
   A   
   A   
   A   
   A   
   A   
   A   
   G   
   C   
   T   
   T   
   A   
   T   
   G   
   A   
   A   
   A   
   C   
   A   
   G   
   G   
    G    
   C   
   G   
   A   
   G   
   G   
   A   
   A   
   A   
   A   
   G   
   T   
   T   
   A   
    T    
   T   
   C   
   T   
   T   
   C   
   G   
   T   
   T   
   C   
   G   
   A   
   A   
   A   
   A   
   C   
   A   
   A   
   A   
   G   
   A   
   T   
   A   
   G   
   A   
    A    
   G   
   C   
   A   
   A   
   T   
   G   
   A   
   A   
   G   
   G   
   G   
    T    
   A   
   A   
    T    
   A   
   A   
   A   
   C   
   A   
   G   
   C   
   A   
   A   
   A   
   T   
   C   
   G   
   T   
   G   
   A   
   T   
   C   
   A   
   C   
    A    
   G   
   A   
   A   
   A   
   T   
   C   
   C   
   C   
   A   
   T   
   A   
   T   
   G   
   A   
   A   
   G   
   T   
   A   
   A   
   A   
   T   
   A   
   A   
   A   
   G   
   C   
   G   
   A   
   C   
    C    
   T   
   T   
   G   
   G   
   T   
   G   
   A   
   A   
   A   
   A   
   A   
   A   
   A   
   T   
   G   
   G   
   A   
   T   
   G   
   A   
   G   
   A   
   T   
   C   
   C   
   G   
   T   
   T   
   T   
   G   
   A   
   A   
   T   
   A   
   A   
   G   
   A   
   A   
   A   
   A   
   T   
   C   
   G   
   A   
    T    
   G   
   G   
   A   
   A   
   T   
   A   
   G   
   C   
   T   
   G   
   A   
   A   
   G   
   T   
    A    
   C   
   G   
   A   
   G   
   A   
   T   
   G   
   A   
   A   
   A   
   G   
   T   
   G   
   A   
   T   
    A    
   G   
    A    
   A   
   C   
    A    
   G   
   G   
   T   
   C   
   T   
   G   
   C   
   A   
   A   
   A   
   T   
   T   
   G   
   T   
   C   
   G   
   T   
    C    
   G   
   A   
   A   
   T   
   T   
   G   
   A   
   A   
   A   
   A   
   A   
   A   
   G   
   A   
    C    
   G   
   C   
   C   
   A   
   A   
    T    
   G   
   C   
   T   
   C   
   A   
   A   
   G   
   G   
    A    
   A   
   T   
   T   
   T   
   T   
   G   
   A   
   A   
   T   
   T   
   A   
    T    
   T   
   T   
   G   
   T   
   T   
   C   
   A   
   A   
   A   
   A   
   A   
   C   
   A   
   C   
   T   
   G   
   A   
   G   
    C    
   T   
   A   
   C   
   A   
   G   
   A   
   T   
   C   
   A   
   A   
   T   
   T   
   A   
   C   
   A   
   A   
   T   
   T   
   T   
    C    
   A   
   A   
   T   
   A   
   T   
   G   
   G   
   T   
   C   
   G   
   C   
   T   
   A   
   T   
   C   
   G   
   A   
    C    
   C   
   A   
   T   
   A   
   T   
   G   
   A   
   C   
   T   
   C   
   C   
   G   
   C   
   A   
    T    
   C   
   A   
   A   
   G   
   T   
   T   
   G   
   G   
   A   
   C   
   T   
   G   
   A   
   A   
   A   
   G   
   A   
   C   
   A   
   T   
   T   
   T   
   T   
   A   
   A   
   G   
   A   
   A   
   G   
   T   
   T   
   A   
   T   
   A   
   T   
   C   
   G   
   A   
   G   
   C   
   A   
   T   
   C   
   G   
   A   
   A   
   A   
   G   
   C   
   A   
   A   
   G   
   T   
   C   
   A   
   T   
   A   
   A   
   C   
   A   
   A   
   A   
   A   
   C   
   G   
    T    
   A   
   G   
    T    
   C   
   A   
   A   
   T   
   T   
   C   
   G   
   A   
   T   
   T   
   T   
   A   
   G   
   C   
   C   
   A   
   A   
   A   
   G   
   C   
   T   
   C   
   A   
   A   
   A   
   A   
   A   
    A    
   G   
   A   
   C   
   A   
   G   
   C   
   A   
   T   
   A   
   T   
   C   
   G   
   T   
    A    
   G   
   A   
   A   
   G   
   G   
    A    
    T    
   T   
   G   
   A   
   T   
   G   
   A   
   A   
   A   
   G   
   C   
   T   
   T   
   T   
   A   
   T   
   C   
   G   
   A   
   T   
   T   
   C   
   T   
   G   
   G   
   A   
   T   
   G   
   A   
   A   
   G   
   T   
   A   
   A   
   T   
   C   
   G   
    C    
   A   
   A   
   C   
   G   
   A   
   T   
   T   
   C   
   G   
   T   
   G   
   A   
   A   
   A   
   G   
   T   
   A   
   A   
   A   
   G   
   A   
   C   
   A   
   A   
   A   
   A   
   A   
   A   
   G   
   A   
    C    
   G   
   C   
    C    
   A   
   A   
   A   
   A   
   A   
   G   
   A   
   A   
   T   
   T   
   T   
   A   
   G   
   T   
   C   
   G   
   A   
    C    
   G   
   T   
    C    
   T   
   T   
    T    
   C   
   A   
   A   
   T   
   T   
   T   
   A   
   C   
   A   
   G   
   A   
   A   
   G   
   A   
   A   
   C   
   A   
   A   
   G   
   C   
   A   
   G   
   A   
   A   
   G   
   C   
   A   
   A   
   T   
    T    
   G   
   T   
   C   
   A   
   C   
   A   
   T   
   T   
   G   
   C   
   A   
   A   
    T    
   T   
   A   
   T   
   A   
    C    
   C   
   G   
   T   
   T   
   T   
   G   
   A   
   C   
   G   
   A   
   A   
    C    
   A   
   C   
    G    
   G   
   A   
   T   
   A   
   T   
   C   
   A   
   C   
   A   
   G   
   A   
   G   
   C   
   T   
   T   
   C   
   A   
   A   
   A   
   A   
   G   
   G   
   A   
   A   
    T    
   C   
    C    
   G   
   A   
   A   
   A   
    G    
   C   
   T   
   T   
   G   
   A   
   T   
   T   
   G   
   C   
    G    
   C   
   A   
   A   
   A   
   T   
   C   
   A   
   C   
   A   
   G   
   A   
   G   
   T   
   T   
    G    
   A   
   A   
   T   
   A   
   A   
   A   
   A   
   T   
   C   
   C   
   T   
   T   
   T   
   C   
   A   
   A   
   A   
    T    
   G   
   A   
   C   
   A   
   A   
   A   
   G   
   A   
    G    
   C   
   T   
   T   
   T   
   T   
    C    
   T   
   C   
   T   
   G   
   T   
   T   
   A   
   T   
   G   
   A   
   A   
   A   
   A   
   A   
   A   
   G   
   A   
   A   
   T   
   T   
   G   
   C   
   G   
   A   
   G   
   A   
   A   
   G   
   T   
   G   
   A   
   A   
   A   
   A   
   A   
   A   
   A   
   A   
   T   
   T   
   A   
    T    
   T   
   C   
   T   
   T   
   C   
   C   
   G   
   C   
   T   
   C   
   G   
   T   
   C   
    T    
    A    
   A   
   C   
   G   
   A   
   C   
   A   
   A   
   T   
   C   
    G    
   A   
   A   
   G   
   A   
   C   
   G   
   A   
   A   
   A   
   T   
   C   
   G   
   A   
   A   
   G   
   A   
   A   
   A   
   T   
   C   
   A   
   A   
   A   
   A   
   T   
    C    
   G   
   A   
   T   
   A   
   C   
   G   
   C   
   A   
   A   
    G    
   T   
    T    
   T   
   T   
   A   
   G   
   T   
   G   
   G   
   C   
    G    
   C   
   A   
   A   
   G   
   A   
   A   
   G   
   A   
   C   
   G   
   T   
   A   
   A   
   T   
    C    
   G   
   T   
   C   
   A   
   G   
   T   
   G   
   T   
    G    
   A   
   C   
    G    
   C   
   G   
   A   
   G   
   A   
   A   
   G   
   G   
   C   
   T   
   A   
   T   
   G   
   T   
   G   
   A   
   A   
   G   
   C   
   G   
   A   
   A   
   C   
   A   
   A   
   G   
   C   
   C   
   T   
   T   
   C   
   G   
   T   
   T   
   C   
    T    
   T   
   A   
   T   
   A   
   G   
   T   
   G   
   C   
   T   
   T   
   C   
    G    
   A   
   A   
   A   
   C   
   C   
   A   
   G   
   A   
   A   
   G   
   A   
   A   
   A   
   T   
   C   
   G   
   G   
   G   
   A   
   T   
   G   
   C   
   G   
   A   
   G   
   A   
   A   
   G   
   G   
   C   
   G   
   A   
    C    
   T   
   A   
   C   
   T   
   T   
   G   
   C   
   T   
    G    
   T   
   A   
   C   
    T    
   C   
   A   
   G   
   G   
   T   
   G   
   A   
   A   
   T   
   T   
    G    
   A   
   G   
    C    
   A   
   C   
   A   
    T    
   T   
   G   
   G   
   A   
   C   
   C   
   A   
   T   
   G   
   T   
   T   
   C   
   T   
   T   
   C   
   T   
   G   
   A   
   T   
    T    
   A   
   C   
    G    
   A   
   A   
   T   
   A   
   A   
   A   
   G   
   C   
   A   
   A   
   A   
   T   
   G   
   T   
   G   
   A   
   T   
   C   
   T   
   A   
   C   
   C   
   G   
    T    
   C   
   C   
   T   
   G   
   T   
    T    
   C   
   A   
   T   
   G   
   A   
   A   
   C   
   T   
   A   
   C   
   C   
    C    
   G   
   A   
   T   
   T   
   T   
   G   
   A   
   A   
   A   
   T   
   G   
   G   
   A   
   A   
   A   
   G   
   A   
   T   
   G   
   C   
   T   
   G   
   G   
    C    
   G   
   A   
    A    
   C   
   A   
    T    
   A   
   T   
   T   
   T   
   C   
   T   
   C   
   A   
   A   
   A   
   C   
   A   
   A   
   T   
   C   
   T   
   C   
   C   
   A   
   A   
   T   
   C   
   T   
   G   
   T   
   C   
   T   
   G   
   T   
   A   
   G   
   A   
    T    
   G   
   A   
   A   
   T   
   C   
   G   
   A   
   T   
    C    
   T   
   T   
   A   
   G   
   C   
   T   
   G   
   T   
   T   
   T   
   T   
    T    
   C   
   C   
   A   
   T   
   A   
   T   
   C   
   A   
   A   
   A   
   A   
   A   
   A   
   T   
    T    
   C   
   A   
   A   
   G   
   C   
   A   
   G   
   A   
    G    
   A   
   A   
   A   
   A   
   C   
   A   
   T   
   T   
   T   
   G   
   T   
    A    
   T   
   T   
   C   
   A   
   T   
   C   
   A   
   G   
   C   
   A   
   A   
   G   
   A   
   A   
   T   
   G   
   G   
   A   
   T   
   T   
    G    
    A    
   T   
   C   
   A   
   A   
   G   
   C   
   A   
   A   
   A   
   C   
   A   
   C   
   G   
   A   
   A   
   T   
   G   
   A   
   C   
   A   
   G   
   A   
   G   
   T   
   T   
   C   
   G   
   A   
   A   
   C   
   C   
   A   
   T   
   G   
   G   
   C   
   G   
   C   
   A   
   C   
   C   
   T   
   A   
    T    
   A   
   A   
   G   
   A   
   G   
   C   
   C   
   G   
    T    
   C   
   C   
   G   
   C   
   T   
   C   
   A   
   G   
   C   
   T   
   G   
   T   
   A   
   T   
   G   
   A   
   A   
   G   
   C   
   T   
   A   
   A   
   A   
   G   
   G   
   C   
   G   
   G   
   A   
   A   
   G   
   A   
   A   
   G   
   A   
   T   
   G   
   A   
   A   
   C   
   T   
    G    
   A   
   T   
   T   
   G   
   C   
   T   
   G   
   T   
    G    
   T   
   A   
   T   
   C   
   T   
   G   
   A   
   A   
   A   
   G   
   A   
   A   
   G   
   A   
   T   
   C   
    G    
   G   
   C   
   C   
   A   
   G   
   G   
   C   
   T   
   T   
   A   
   G   
   A   
   T   
   G   
   T   
    T    
   T   
   T   
   C   
   T   
   T   
   A   
   G   
   T   
   A   
   A   
   C   
   G   
    T    
   A   
   T   
   C   
   A   
   A   
   G   
   G   
   A   
   A   
   T   
   G   
   G   
   G   
    T    
   T   
   T   
   G   
   C   
   G   
   C   
   T   
    A    
   T   
   C   
   C   
   T   
   T   
   T   
   A   
   A   
    C    
   A   
   G   
   A   
   T   
   G   
   T   
   T   
   C   
   C   
   C   
   G   
   T   
   T   
   G   
   T   
   T   
   G   
   G   
   A   
   G   
   C   
   A   
   A   
   A   
   G   
   G   
   C   
   A   
   G   
    C    
   T   
   G   
   G   
   A   
   G   
   T   
   G   
   A   
   A   
   G   
   T   
   C   
   T   
   A   
   T   
   G   
   A   
   A   
   T   
   T   
   T   
   G   
   A   
   A   
   A   
   G   
   A   
   T   
   G   
   G   
   A   
   G   
   A   
   T   
   A   
   G   
   G   
    A    
   T   
   T   
    A    
   T   
   C   
   A   
   A   
    C    
   G   
   G   
    G    
   C   
   T   
    G    
   C   
   T   
   T   
   G   
   T   
   G   
   T   
   T   
   A   
   G   
   C   
   A   
   G   
   A   
    A    
   G   
   G   
    T    
   G   
   A   
   T   
   A   
   C   
   G   
   C   
   C   
    G    
   A   
   T   
   C   
   G   
   T   
   A   
   A   
   T   
   C   
   G   
   T   
   T   
   A   
   C   
   T   
   C   
   A   
   G   
   C   
   G   
   A   
   G   
   G   
   A   
   G   
   C   
    T    
   G   
   T   
    T    
   A   
   A   
   A   
   C   
   G   
   C   
   A   
   T   
   G   
   C   
   T   
   T   
   G   
   C   
   T   
   C   
   A   
   A   
   G   
   A   
   A   
   A   
   T   
   C   
   A   
   G   
   C   
   C   
   A   
   A   
   A   
   C   
    T    
   T   
   C   
   A   
   C   
   G   
   T   
   G   
   C   
   G   
   A   
   A   
   G   
   C   
   G   
   G   
   G   
   G   
   A   
   G   
   T   
   G   
   A   
   C   
   G   
   G   
   T   
   C   
   A   
   T   
   G   
   C   
   G   
   T   
   G   
   A   
   G   
   C   
   T   
   G   
   A   
   A   
   A   
   A   
   A   
   A   
   A   
   A   
   T   
   C   
   C   
    T    
   C   
   A   
    T    
   C   
   G   
    C    
   A   
   T   
   T   
   A   
   T   
   C   
   T   
   A   
   T   
   A   
   T   
   G   
   T   
   C   
   G   
   G   
   A   
   A   
   G   
   G   
   A   
   A   
   G   
   A   
   T   
   C   
   A   
   A   
   A   
   A   
   G   
   A   
   A   
   A   
   T   
   A   
   A   
   C   
   C   
   T   
   T   
   G   
   A   
   T   
   G   
   A   
   A   
   T   
   C   
   A   
   A   
   A   
   A   
   A   
   G   
   G   
    G    
   C   
   A   
    G    
   C   
   A   
   G   
    T    
   T   
   A   
   A   
   C   
   G   
   A   
   T   
   T   
   G   
   A   
    C    
   C   
   C   
   A   
   A   
   C   
    T    
   G   
   A   
   T   
   T   
   T   
    C    
   C   
   C   
   T   
   A   
   T   
   A   
   G   
   G   
   T   
   G   
   A   
   T   
   C   
   G   
   A   
   A   
   C   
   A   
   T   
   C   
   G   
   A   
   A   
   T   
   G   
   G   
    A    
   T   
   C   
    A    
   T   
   T   
   T   
   G   
   C   
    G    
   A   
   T   
   G   
   G   
   A   
    T    
   G   
   A   
   G   
   A   
   A   
   A   
   A   
   A   
   A   
   G   
   G   
   T   
   G   
   G   
   A   
   G   
   A   
   A   
   G   
   T   
   C   
   A   
   T   
   C   
   G   
   A   
   A   
   G   
   T   
    T    
   A   
    T    
   C   
   G   
   A   
   T   
   G   
   C   
   T   
   C   
   C   
   T   
   G   
   A   
   G   
    A    
   T   
   T   
   T   
   T   
    C    
   A   
   T   
    C    
   G   
   A   
    G    
    T    
   C   
    G    
   -   
   -   
   -   
 

 
   N60061F   
   A   
   T   
   G   
   G   
   A   
   A   
   A   
   A   
   T   
   C   
   G   
   A   
   C   
   A   
   A   
   G   
   A   
   A   
   G   
   T   
   A   
   C   
   A   
   A   
   G   
   A   
   A   
   T   
   T   
   A   
   A   
   C   
   T   
   T   
   T   
   A   
   G   
   A   
   A   
   G   
   A   
   A   
   G   
   T   
   G   
   A   
   T   
   G   
   G   
   G   
   T   
   G   
   A   
   C   
   C   
   G   
   C   
   T   
   T   
   C   
   G   
   G   
   A   
   C   
   G   
   T   
   T   
   A   
   T   
   T   
   C   
   C   
   A   
   A   
   A   
   T   
   A   
   T   
   A   
   T   
   T   
   A   
   T   
    C    
   C   
   A   
    A    
   G   
   A   
   A   
   C   
   G   
   T   
   G   
   C   
   A   
   C   
   T   
   G   
   C   
   C   
   T   
   G   
   A   
   T   
   A   
   T   
    C    
   C   
   G   
   T   
   G   
   A   
   T   
   G   
   G   
   A   
   T   
   T   
   G   
   A   
   A   
    G    
   C   
   C   
   A   
   G   
   T   
    G    
   C   
   A   
   A   
   C   
   G   
   C   
   C   
   G   
   G   
   A   
   T   
   A   
   T   
   T   
   A   
   T   
   T   
    C    
   G   
   C   
    T    
   A   
   T   
   G   
   A   
   A   
   C   
   A   
   A   
   A   
   G   
   A   
   T   
   G   
   G   
   C   
   A   
   A   
   T   
   A   
   C   
   G   
   T   
   A   
   T   
   G   
   A   
   C   
   A   
   A   
   A   
   G   
   G   
   A   
   T   
   T   
    C    
   C   
   G   
   T   
   A   
   A   
   A   
   T   
   C   
   A   
   G   
   C   
   A   
   A   
   A   
   A   
   T   
   C   
   T   
   G   
   T   
   C   
   G   
   G   
   A   
   A   
   A   
   T   
   A   
   T   
   C   
   A   
   T   
   G   
   G   
   G   
    G    
   A   
   A   
   T   
   T   
   A   
   T   
   C   
   A   
   T   
   C   
   C   
   T   
   C   
   A   
   T   
   G   
   G   
   T   
   G   
   A   
   C   
   A   
   G   
   C   
   A   
   G   
   T   
   A   
   T   
   T   
   T   
   A   
   T   
   G   
   A   
   A   
   G   
   C   
   G   
   A   
   T   
   G   
   G   
   T   
   C   
   C   
   G   
   C   
   A   
   T   
   G   
   A   
   G   
   C   
   C   
   A   
   A   
   G   
   A   
    C    
   T   
   G   
   G   
   A   
   A   
   G   
   T   
   T   
   G   
   C   
   G   
   G   
   G   
   A   
   A   
   G   
   T   
   A   
   C   
   T   
   G   
   A   
   T   
   C   
   G   
   A   
    A    
   A   
   T   
   G   
   C   
   A   
   C   
   G   
   G   
    G    
   A   
   A   
   T   
   A   
   A   
    C    
   G   
   G   
   G   
   A   
   G   
   T   
   A   
   T   
   G   
   G   
   A   
   C   
   G   
   G   
   T   
   G   
   A   
   T   
   C   
   C   
   A   
   C   
   C   
   T   
   G   
   C   
   T   
   G   
   C   
   G   
   A   
   T   
   G   
   C   
   G   
   T   
   T   
   A   
   T   
   A   
   C   
   T   
   G   
   A   
   A   
   G   
   C   
   C   
   A   
   G   
    A    
   T   
   T   
   A   
   T   
   C   
   C   
   A   
   A   
   G   
   T   
   T   
   A   
   A   
   G   
   T   
   G   
   G   
   A   
   G   
   A   
   G   
   A   
   T   
   G   
    C    
   T   
   A   
    G    
   C   
   A   
   G   
   A   
   T   
   A   
   T   
   C   
   G   
   A   
    A    
   A   
   A   
   A   
   G   
   A   
   A   
   A   
   C   
   A   
   G   
   T   
    C    
   G   
   A   
   T   
   C   
   T   
   C   
   G   
   T   
   G   
   T   
   G   
   G   
   A   
   A   
   T   
   T   
   T   
   T   
   G   
   A   
   T   
   G   
   A   
   T   
   A   
   C   
   A   
   G   
   A   
   A   
   A   
   A   
   A   
   G   
   A   
   A   
   C   
   C   
   A   
   A   
   C   
   A   
   G   
   T   
   C   
   C   
   T   
   T   
   C   
   C   
   A   
   G   
   C   
   T   
   C   
   G   
   A   
   T   
   A   
   C   
   C   
   C   
   A   
   A   
   A   
   C   
   C   
   T   
   T   
   C   
   T   
    C    
   G   
   T   
   C   
   A   
   A   
   T   
   G   
   G   
   A   
   T   
   C   
   G   
   A   
   C   
   A   
   G   
   G   
   T   
   A   
   T   
   A   
   T   
   C   
   T   
   G   
   C   
   T   
   G   
   G   
   A   
   T   
   A   
   T   
   G   
   C   
    A    
   A   
   C   
   A   
   G   
   A   
   A   
   A   
   T   
   C   
   C   
   C   
   G   
   A   
   C   
    T    
   C   
   A   
   T   
   A   
   A   
   C   
   T   
   T   
   A   
   G   
   C   
   T   
   G   
   A   
   A   
   G   
   T   
   G   
   A   
   T   
    C    
   G   
   A   
    T    
   G   
   G   
   C   
   A   
   C   
   A   
   A   
   T   
   C   
   T   
   A   
   T   
   A   
   T   
   G   
   A   
   T   
   C   
   G   
   A   
   T   
   C   
   A   
   T   
   C   
   C   
   A   
   C   
   A   
   A   
   G   
   C   
   G   
   A   
   G   
   C   
   C   
   T   
   T   
   G   
   A   
   A   
   A   
   A   
   A   
   T   
   T   
   G   
   A   
   T   
   G   
   G   
   A   
   A   
   T   
   A   
   T   
   A   
   T   
   T   
   C   
   C   
   T   
   G   
   G   
   A   
   C   
   C   
   T   
   G   
   A   
   C   
   T   
   T   
    T    
   C   
   C   
   A   
   A   
   C   
   A   
   G   
   G   
   A   
   G   
   G   
    G    
   A   
   T   
   C   
   T   
   T   
   G   
   C   
   A   
   A   
   G   
   G   
    A    
   A   
   A   
   A   
   G   
   A   
   T   
   G   
   A   
   A   
   A   
   T   
   C   
   A   
   A   
   A   
   A   
   A   
   A   
   G   
   C   
   T   
   T   
   A   
   T   
   G   
   A   
   A   
   A   
   C   
   A   
   G   
   G   
    G    
   C   
   G   
   A   
   G   
   G   
   A   
   A   
   A   
   A   
   G   
   T   
   T   
   A   
    T    
   T   
   C   
   T   
   T   
   C   
   G   
   T   
   T   
   C   
   G   
   A   
   A   
   A   
   A   
   C   
   A   
   A   
   A   
   G   
   A   
   T   
   A   
   G   
   A   
    A    
   G   
   C   
   A   
   A   
   T   
   G   
   A   
   A   
   G   
   G   
   G   
    T    
   A   
   A   
    T    
   A   
   A   
   A   
   C   
   A   
   G   
   C   
   A   
   A   
   A   
   T   
   C   
   G   
   T   
   G   
   A   
   T   
   C   
   A   
   C   
    A    
   G   
   A   
   A   
   A   
   T   
   C   
   C   
   C   
   A   
   T   
   A   
   T   
   G   
   A   
   A   
   G   
   T   
   A   
   A   
   A   
   T   
   A   
   A   
   A   
   G   
   C   
   G   
   A   
   C   
    C    
   T   
   T   
   G   
   G   
   T   
   G   
   A   
   A   
   A   
   A   
   A   
   A   
   A   
   T   
   G   
   G   
   A   
   T   
   G   
   A   
   G   
   A   
   T   
   C   
   C   
   G   
   T   
   T   
   T   
   G   
   A   
   A   
   T   
   A   
   A   
   G   
   A   
   A   
   A   
   A   
   T   
   C   
   G   
   A   
    T    
   G   
   G   
   A   
   A   
   T   
   A   
   G   
   C   
   T   
   G   
   A   
   A   
   G   
   T   
    A    
   C   
   G   
   A   
   G   
   A   
   T   
   G   
   A   
   A   
   A   
   G   
   T   
   G   
   A   
   T   
    A    
   G   
    A    
   A   
   C   
    A    
   G   
   G   
   T   
   C   
   T   
   G   
   C   
   A   
   A   
   A   
   T   
   T   
   G   
   T   
   C   
   G   
   T   
    C    
   G   
   A   
   A   
   T   
   T   
   G   
   A   
   A   
   A   
   A   
   A   
   A   
   G   
   A   
    C    
   G   
   C   
   C   
   A   
   A   
    T    
   G   
   C   
   T   
   C   
   A   
   A   
   G   
   G   
    A    
   A   
   T   
   T   
   T   
   T   
   G   
   A   
   A   
   T   
   T   
   A   
    T    
   T   
   T   
   G   
   T   
   T   
   C   
   A   
   A   
   A   
   A   
   A   
   C   
   A   
   C   
   T   
   G   
   A   
   G   
    C    
   T   
   A   
   C   
   A   
   G   
   A   
   T   
   C   
   A   
   A   
   T   
   T   
   A   
   C   
   A   
   A   
   T   
   T   
   T   
    C    
   A   
   A   
   T   
   A   
   T   
   G   
   G   
   T   
   C   
   G   
   C   
   T   
   A   
   T   
   C   
   G   
   A   
    C    
   C   
   A   
   T   
   A   
   T   
   G   
   A   
   C   
   T   
   C   
   C   
   G   
   C   
   A   
    T    
   C   
   A   
   A   
   G   
   T   
   T   
   G   
   G   
   A   
   C   
   T   
   G   
   A   
   A   
   A   
   G   
   A   
   C   
   A   
   T   
   T   
   T   
   T   
   A   
   A   
   G   
   A   
   A   
   G   
   T   
   T   
   A   
   T   
   A   
   T   
   C   
   G   
   A   
   G   
   C   
   A   
   T   
   C   
   G   
   A   
   A   
   A   
   G   
   C   
   A   
   A   
   G   
   T   
   C   
   A   
   T   
   A   
   A   
   C   
   A   
   A   
   A   
   A   
   C   
   G   
    T    
   A   
   G   
    T    
   C   
   A   
   A   
   T   
   T   
   C   
   G   
   A   
   T   
   T   
   T   
   A   
   G   
   C   
   C   
   A   
   A   
   A   
   G   
   C   
   T   
   C   
   A   
   A   
   A   
   A   
   A   
    A    
   G   
   A   
   C   
   A   
   G   
   C   
   A   
   T   
   A   
   T   
   C   
   G   
   T   
    A    
   G   
   A   
   A   
   G   
   G   
    A    
    T    
   T   
   G   
   A   
   T   
   G   
   A   
   A   
   A   
   G   
   C   
   T   
   T   
   T   
   A   
   T   
   C   
   G   
   A   
   T   
   T   
   C   
   T   
   G   
   G   
   A   
   T   
   G   
   A   
   A   
   G   
   T   
   A   
   A   
   T   
   C   
   G   
    C    
   A   
   A   
   C   
   G   
   A   
   T   
   T   
   C   
   G   
   T   
   G   
   A   
   A   
   A   
   G   
   T   
   A   
   A   
   A   
   G   
   A   
   C   
   A   
   A   
   A   
   A   
   A   
   A   
   G   
   A   
    C    
   G   
   C   
    C    
   A   
   A   
   A   
   A   
   A   
   G   
   A   
   A   
   T   
   T   
   T   
   A   
   G   
   T   
   C   
   G   
   A   
    C    
   G   
   T   
    C    
   T   
   T   
    T    
   C   
   A   
   A   
   T   
   T   
   T   
   A   
   C   
   A   
   G   
   A   
   A   
   G   
   A   
   A   
   C   
   A   
   A   
   G   
   C   
   A   
   G   
   A   
   A   
   G   
   C   
   A   
   A   
   T   
    T    
   G   
   T   
   C   
   A   
   C   
   A   
   T   
   T   
   G   
   C   
   A   
   A   
    T    
   T   
   A   
   T   
   A   
    C    
   C   
   G   
   T   
   T   
   T   
   G   
   A   
   C   
   G   
   A   
   A   
    C    
   A   
   C   
    G    
   G   
   A   
   T   
   A   
   T   
   C   
   A   
   C   
   A   
   G   
   A   
   G   
   C   
   T   
   T   
   C   
   A   
   A   
   A   
   A   
   G   
   G   
   A   
   A   
    T    
   C   
    C    
   G   
   A   
   A   
   A   
    G    
   C   
   T   
   T   
   G   
   A   
   T   
   T   
   G   
   C   
    G    
   C   
   A   
   A   
   A   
   T   
   C   
   A   
   C   
   A   
   G   
   A   
   G   
   T   
   T   
    G    
   A   
   A   
   T   
   A   
   A   
   A   
   A   
   T   
   C   
   C   
   T   
   T   
   T   
   C   
   A   
   A   
   A   
    T    
   G   
   A   
   C   
   A   
   A   
   A   
   G   
   A   
    G    
   C   
   T   
   T   
   T   
   T   
    C    
   T   
   C   
   T   
   G   
   T   
   T   
   A   
   T   
   G   
   A   
   A   
   A   
   A   
   A   
   A   
   G   
   A   
   A   
   T   
   T   
   G   
   C   
   G   
   A   
   G   
   A   
   A   
   G   
   T   
   G   
   A   
   A   
   A   
   A   
   A   
   A   
   A   
   A   
   T   
   T   
   A   
    T    
   T   
   C   
   T   
   T   
   C   
   C   
   G   
   C   
   T   
   C   
   G   
   T   
   C   
    T    
    A    
   A   
   C   
   G   
   A   
   C   
   A   
   A   
   T   
   C   
    G    
   A   
   A   
   G   
   A   
   C   
   G   
   A   
   A   
   A   
   T   
   C   
   G   
   A   
   A   
   G   
   A   
   A   
   A   
   T   
   C   
   A   
   A   
   A   
   A   
   T   
    C    
   G   
   A   
   T   
   A   
   C   
   G   
   C   
   A   
   A   
    G    
   T   
    T    
   T   
   T   
   A   
   G   
   T   
   G   
   G   
   C   
    G    
   C   
   A   
   A   
   G   
   A   
   A   
   G   
   A   
   C   
   G   
   T   
   A   
   A   
   T   
    C    
   G   
   T   
   C   
   A   
   G   
   T   
   G   
   T   
    G    
   A   
   C   
    G    
   C   
   G   
   A   
   G   
   A   
   A   
   G   
   G   
   C   
   T   
   A   
   T   
   G   
   T   
   G   
   A   
   A   
   G   
   C   
   G   
   A   
   A   
   C   
   A   
   A   
   G   
   C   
   C   
   T   
   T   
   C   
   G   
   T   
   T   
   C   
    T    
   T   
   A   
   T   
   A   
   G   
   T   
   G   
   C   
   T   
   T   
   C   
    G    
   A   
   A   
   A   
   C   
   C   
   A   
   G   
   A   
   A   
   G   
   A   
   A   
   A   
   T   
   C   
   G   
   G   
   G   
   A   
   T   
   G   
   C   
   G   
   A   
   G   
   A   
   A   
   G   
   G   
   C   
   G   
   A   
    C    
   T   
   A   
   C   
   T   
   T   
   G   
   C   
   T   
    G    
   T   
   A   
   C   
    T    
   C   
   A   
   G   
   G   
   T   
   G   
   A   
   A   
   T   
   T   
    G    
   A   
   G   
    C    
   A   
   C   
   A   
    T    
   T   
   G   
   G   
   A   
   C   
   C   
   A   
   T   
   G   
   T   
   T   
   C   
   T   
   T   
   C   
   T   
   G   
   A   
   T   
    T    
   A   
   C   
    G    
   A   
   A   
   T   
   A   
   A   
   A   
   G   
   C   
   A   
   A   
   A   
   T   
   G   
   T   
   G   
   A   
   T   
   C   
   T   
   A   
   C   
   C   
   G   
    T    
   C   
   C   
   T   
   G   
   T   
    T    
   C   
   A   
   T   
   G   
   A   
   A   
   C   
   T   
   A   
   C   
   C   
    C    
   G   
   A   
   T   
   T   
   T   
   G   
   A   
   A   
   A   
   T   
   G   
   G   
   A   
   A   
   A   
   G   
   A   
   T   
   G   
   C   
   T   
   G   
   G   
    C    
   G   
   A   
    A    
   C   
   A   
    T    
   A   
   T   
   T   
   T   
   C   
   T   
   C   
   A   
   A   
   A   
   C   
   A   
   A   
   T   
   C   
   T   
   C   
   C   
   A   
   A   
   T   
   C   
   T   
   G   
   T   
   C   
   T   
   G   
   T   
   A   
   G   
   A   
    T    
   G   
   A   
   A   
   T   
   C   
   G   
   A   
   T   
    C    
   T   
   T   
   A   
   G   
   C   
   T   
   G   
   T   
   T   
   T   
   T   
    T    
   C   
   C   
   A   
   T   
   A   
   T   
   C   
   A   
   A   
   A   
   A   
   A   
   A   
   T   
    T    
   C   
   A   
   A   
   G   
   C   
   A   
   G   
   A   
    G    
   A   
   A   
   A   
   A   
   C   
   A   
   T   
   T   
   T   
   G   
   T   
    A    
   T   
   T   
   C   
   A   
   T   
   C   
   A   
   G   
   C   
   A   
   A   
   G   
   A   
   A   
   T   
   G   
   G   
   A   
   T   
   T   
    G    
    A    
   T   
   C   
   A   
   A   
   G   
   C   
   A   
   A   
   A   
   C   
   A   
   C   
   G   
   A   
   A   
   T   
   G   
   A   
   C   
   A   
   G   
   A   
   G   
   T   
   T   
   C   
   G   
   A   
   A   
   C   
   C   
   A   
   T   
   G   
   G   
   C   
   G   
   C   
   A   
   C   
   C   
   T   
   A   
    T    
   A   
   A   
   G   
   A   
   G   
   C   
   C   
   G   
    T    
   C   
   C   
   G   
   C   
   T   
   C   
   A   
   G   
   C   
   T   
   G   
   T   
   A   
   T   
   G   
   A   
   A   
   G   
   C   
   T   
   A   
   A   
   A   
   G   
   G   
   C   
   G   
   G   
   A   
   A   
   G   
   A   
   A   
   G   
   A   
   T   
   G   
   A   
   A   
   C   
   T   
    G    
   A   
   T   
   T   
   G   
   C   
   T   
   G   
   T   
    G    
   T   
   A   
   T   
   C   
   T   
   G   
   A   
   A   
   A   
   G   
   A   
   A   
   G   
   A   
   T   
   C   
    G    
   G   
   C   
   C   
   A   
   G   
   G   
   C   
   T   
   T   
   A   
   G   
   A   
   T   
   G   
   T   
    T    
   T   
   T   
   C   
   T   
   T   
   A   
   G   
   T   
   A   
   A   
   C   
   G   
    T    
   A   
   T   
   C   
   A   
   A   
   G   
   G   
   A   
   A   
   T   
   G   
   G   
   G   
    T    
   T   
   T   
   G   
   C   
   G   
   C   
   T   
    A    
   T   
   C   
   C   
   T   
   T   
   T   
   A   
   A   
    C    
   A   
   G   
   A   
   T   
   G   
   T   
   T   
   C   
   C   
   C   
   G   
   T   
   T   
   G   
   T   
   T   
   G   
   G   
   A   
   G   
   C   
   A   
   A   
   A   
   G   
   G   
   C   
   A   
   G   
    C    
   T   
   G   
   G   
   A   
   G   
   T   
   G   
   A   
   A   
   G   
   T   
   C   
   T   
   A   
   T   
   G   
   A   
   A   
   T   
   T   
   T   
   G   
   A   
   A   
   A   
   G   
   A   
   T   
   G   
   G   
   A   
   G   
   A   
   T   
   A   
   G   
   G   
    A    
   T   
   T   
    A    
   T   
   C   
   A   
   A   
    C    
   G   
   G   
    G    
   C   
   T   
    G    
   C   
   T   
   T   
   G   
   T   
   G   
   T   
   T   
   A   
   G   
   C   
   A   
   G   
   A   
    A    
   G   
   G   
    T    
   G   
   A   
   T   
   A   
   C   
   G   
   C   
   C   
    G    
   A   
   T   
   C   
   G   
   T   
   A   
   A   
   T   
   C   
   G   
   T   
   T   
   A   
   C   
   T   
   C   
   A   
   G   
   C   
   G   
   A   
   G   
   G   
   A   
   G   
   C   
    T    
   G   
   T   
    T    
   A   
   A   
   A   
   C   
   G   
   C   
   A   
   T   
   G   
   C   
   T   
   T   
   G   
   C   
   T   
   C   
   A   
   A   
   G   
   A   
   A   
   A   
   T   
   C   
   A   
   G   
   C   
   C   
   A   
   A   
   A   
   C   
    T    
   T   
   C   
   A   
   C   
   G   
   T   
   G   
   C   
   G   
   A   
   A   
   G   
   C   
   G   
   G   
   G   
   G   
   A   
   G   
   T   
   G   
   A   
   C   
   G   
   G   
   T   
   C   
   A   
   T   
   G   
   C   
   G   
   T   
   G   
   A   
   G   
   C   
   T   
   G   
   A   
   A   
   A   
   A   
   A   
   A   
   A   
   A   
   T   
   C   
   C   
    T    
   C   
   A   
    T    
   C   
   G   
    C    
   A   
   T   
   T   
   A   
   T   
   C   
   T   
   A   
   T   
   A   
   T   
   G   
   T   
   C   
   G   
   G   
   A   
   A   
   G   
   G   
   A   
   A   
   G   
   A   
   T   
   C   
   A   
   A   
   A   
   A   
   G   
   A   
   A   
   A   
   T   
   A   
   A   
   C   
   C   
   T   
   T   
   G   
   A   
   T   
   G   
   A   
   A   
   T   
   C   
   A   
   A   
   A   
   A   
   A   
   G   
   G   
    G    
   C   
   A   
    G    
   C   
   A   
   G   
    T    
   T   
   A   
   A   
   C   
   G   
   A   
   T   
   T   
   G   
   A   
    C    
   C   
   C   
   A   
   A   
   C   
    T    
   G   
   A   
   T   
   T   
   T   
    C    
   C   
   C   
   T   
   A   
   T   
   A   
   G   
   G   
   T   
   G   
   A   
   T   
   C   
   G   
   A   
   A   
   C   
   A   
   T   
   C   
   G   
   A   
   A   
   T   
   G   
   G   
    A    
   T   
   C   
    A    
   T   
   T   
   T   
   G   
   C   
    G    
   A   
   T   
   G   
   G   
   A   
    T    
   G   
   A   
   G   
   A   
   A   
   A   
   A   
   A   
   A   
   G   
   G   
   T   
   G   
   G   
   A   
   G   
   A   
   A   
   G   
   T   
   C   
   A   
   T   
   C   
   G   
   A   
   A   
   G   
   T   
    T    
   A   
    T    
   C   
   G   
   A   
   T   
   G   
   C   
   T   
   C   
   C   
   T   
   G   
   A   
   G   
    A    
   T   
   T   
   T   
   T   
    C    
   A   
   T   
    C    
   G   
   A   
    G    
    T    
   C   
    G    
   -   
   -   
   -   
 

 
   N59779F   
   A   
   T   
   G   
   G   
   A   
   A   
   A   
   A   
   T   
   C   
   G   
   A   
   C   
   A   
   A   
   G   
   A   
   A   
   G   
   T   
   A   
   C   
   A   
   A   
   G   
   A   
   A   
   T   
   T   
   A   
   A   
   C   
   T   
   T   
   T   
   A   
   G   
   A   
   A   
   G   
   A   
   A   
   G   
   T   
   G   
   A   
   T   
   G   
   G   
   G   
   T   
   G   
   A   
   C   
   C   
   G   
   C   
   T   
   T   
   C   
   G   
   G   
   A   
   C   
   G   
   T   
   T   
   A   
   T   
   T   
   C   
   C   
   A   
   A   
   A   
   T   
   A   
   T   
   A   
   T   
   T   
   A   
   T   
    C    
   C   
   A   
    A    
   G   
   A   
   A   
   C   
   G   
   T   
   G   
   C   
   A   
   C   
   T   
   G   
   C   
   C   
   T   
   G   
   A   
   T   
   A   
   T   
    C    
   C   
   G   
   T   
   G   
   A   
   T   
   G   
   G   
   A   
   T   
   T   
   G   
   A   
   A   
    G    
   C   
   C   
   A   
   G   
   T   
    G    
   C   
   A   
   A   
   C   
   G   
   C   
   C   
   G   
   G   
   A   
   T   
   A   
   T   
   T   
   A   
   T   
   T   
    T    
   G   
   C   
    C    
   A   
   T   
   G   
   A   
   A   
   C   
   A   
   A   
   A   
   G   
   A   
   T   
   G   
   G   
   C   
   A   
   A   
   T   
   A   
   C   
   G   
   T   
   A   
   T   
   G   
   A   
   C   
   A   
   A   
   A   
   G   
   G   
   A   
   T   
   T   
    C    
   C   
   G   
   T   
   A   
   A   
   A   
   T   
   C   
   A   
   G   
   C   
   A   
   A   
   A   
   A   
   T   
   C   
   T   
   G   
   T   
   C   
   G   
   G   
   A   
   A   
   A   
   T   
   A   
   T   
   C   
   A   
   T   
   G   
   G   
   G   
    G    
   A   
   A   
   T   
   T   
   A   
   T   
   C   
   A   
   T   
   C   
   C   
   T   
   C   
   A   
   T   
   G   
   G   
   T   
   G   
   A   
   C   
   A   
   G   
   C   
   A   
   G   
   T   
   A   
   T   
   T   
   T   
   A   
   T   
   G   
   A   
   A   
   G   
   C   
   G   
   A   
   T   
   G   
   G   
   T   
   C   
   C   
   G   
   C   
   A   
   T   
   G   
   A   
   G   
   C   
   C   
   A   
   A   
   G   
   A   
    C    
   T   
   G   
   G   
   A   
   A   
   G   
   T   
   T   
   G   
   C   
   G   
   G   
   G   
   A   
   A   
   G   
   T   
   A   
   C   
   T   
   G   
   A   
   T   
   C   
   G   
   A   
    A    
   A   
   T   
   G   
   C   
   A   
   C   
   G   
   G   
    G    
   A   
   A   
   T   
   A   
   A   
    C    
   G   
   G   
   G   
   A   
   G   
   T   
   A   
   T   
   G   
   G   
   A   
   C   
   G   
   G   
   T   
   G   
   A   
   T   
   C   
   C   
   A   
   C   
   C   
   T   
   G   
   C   
   T   
   G   
   C   
   G   
   A   
   T   
   G   
   C   
   G   
   T   
   T   
   A   
   T   
   A   
   C   
   T   
   G   
   A   
   A   
   G   
   C   
   C   
   A   
   G   
    A    
   T   
   T   
   A   
   T   
   C   
   C   
   A   
   A   
   G   
   T   
   T   
   A   
   A   
   G   
   T   
   G   
   G   
   A   
   G   
   A   
   G   
   A   
   T   
   G   
    C    
   T   
   A   
    G    
   C   
   A   
   G   
   A   
   T   
   A   
   T   
   C   
   G   
   A   
    A    
   A   
   A   
   A   
   G   
   A   
   A   
   A   
   C   
   A   
   G   
   T   
    C    
   G   
   A   
   T   
   C   
   T   
   C   
   G   
   T   
   G   
   T   
   G   
   G   
   A   
   A   
   T   
   T   
   T   
   T   
   G   
   A   
   T   
   G   
   A   
   T   
   A   
   C   
   A   
   G   
   A   
   A   
   A   
   A   
   A   
   G   
   A   
   A   
   C   
   C   
   A   
   A   
   C   
   A   
   G   
   T   
   C   
   C   
   T   
   T   
   C   
   C   
   A   
   G   
   C   
   T   
   C   
   G   
   A   
   T   
   A   
   C   
   C   
   C   
   A   
   A   
   A   
   C   
   C   
   T   
   T   
   C   
   T   
    C    
   G   
   T   
   C   
   A   
   A   
   T   
   G   
   G   
   A   
   T   
   C   
   G   
   A   
   C   
   A   
   G   
   G   
   T   
   A   
   T   
   A   
   T   
   C   
   T   
   G   
   C   
   T   
   G   
   G   
   A   
   T   
   A   
   T   
   G   
   C   
    A    
   A   
   C   
   A   
   G   
   A   
   A   
   A   
   T   
   C   
   C   
   C   
   G   
   A   
   C   
    T    
   C   
   A   
   T   
   A   
   A   
   C   
   T   
   T   
   A   
   G   
   C   
   T   
   G   
   A   
   A   
   G   
   T   
   G   
   A   
   T   
    C    
   G   
   A   
    T    
   G   
   G   
   C   
   A   
   C   
   A   
   A   
   T   
   C   
   T   
   A   
   T   
   A   
   T   
   G   
   A   
   T   
   C   
   G   
   A   
   T   
   C   
   A   
   T   
   C   
   C   
   A   
   C   
   A   
   A   
   G   
   C   
   G   
   A   
   G   
   C   
   C   
   T   
   T   
   G   
   A   
   A   
   A   
   A   
   A   
   T   
   T   
   G   
   A   
   T   
   G   
   G   
   A   
   A   
   T   
   A   
   T   
   A   
   T   
   T   
   C   
   C   
   T   
   G   
   G   
   A   
   C   
   C   
   T   
   G   
   A   
   C   
   T   
   T   
    T    
   C   
   C   
   A   
   A   
   C   
   A   
   G   
   G   
   A   
   G   
   G   
    G    
   A   
   T   
   C   
   T   
   T   
   G   
   C   
   A   
   A   
   G   
   G   
    A    
   A   
   A   
   A   
   G   
   A   
   T   
   G   
   A   
   A   
   A   
   T   
   C   
   A   
   A   
   A   
   A   
   A   
   A   
   G   
   C   
   T   
   T   
   A   
   T   
   G   
   A   
   A   
   A   
   C   
   A   
   G   
   G   
    G    
   C   
   G   
   A   
   G   
   G   
   A   
   A   
   A   
   A   
   G   
   T   
   T   
   A   
    T    
   T   
   C   
   T   
   T   
   C   
   G   
   T   
   T   
   C   
   G   
   A   
   A   
   A   
   A   
   C   
   A   
   A   
   A   
   G   
   A   
   T   
   A   
   G   
   A   
    G    
   G   
   C   
   A   
   A   
   T   
   G   
   A   
   A   
   G   
   G   
   G   
    T    
   A   
   A   
    T    
   A   
   A   
   A   
   C   
   A   
   G   
   C   
   A   
   A   
   A   
   T   
   C   
   G   
   T   
   G   
   A   
   T   
   C   
   A   
   C   
    A    
   G   
   A   
   A   
   A   
   T   
   C   
   C   
   C   
   A   
   T   
   A   
   T   
   G   
   A   
   A   
   G   
   T   
   A   
   A   
   A   
   T   
   A   
   A   
   A   
   G   
   C   
   G   
   A   
   C   
    C    
   T   
   T   
   G   
   G   
   T   
   G   
   A   
   A   
   A   
   A   
   A   
   A   
   A   
   T   
   G   
   G   
   A   
   T   
   G   
   A   
   G   
   A   
   T   
   C   
   C   
   G   
   T   
   T   
   T   
   G   
   A   
   A   
   T   
   A   
   A   
   G   
   A   
   A   
   A   
   A   
   T   
   C   
   G   
   A   
    T    
   G   
   G   
   A   
   A   
   T   
   A   
   G   
   C   
   T   
   G   
   A   
   A   
   G   
   T   
    A    
   C   
   G   
   A   
   G   
   A   
   T   
   G   
   A   
   A   
   A   
   G   
   T   
   G   
   A   
   T   
    A    
   G   
    A    
   A   
   C   
    A    
   G   
   G   
   T   
   C   
   T   
   G   
   C   
   A   
   A   
   A   
   T   
   T   
   G   
   T   
   C   
   G   
   T   
    C    
   G   
   A   
   A   
   T   
   T   
   G   
   A   
   A   
   A   
   A   
   A   
   A   
   G   
   A   
    C    
   G   
   C   
   C   
   A   
   A   
    T    
   G   
   C   
   T   
   C   
   A   
   A   
   G   
   G   
    A    
   A   
   T   
   T   
   T   
   T   
   G   
   A   
   A   
   T   
   T   
   A   
    T    
   T   
   T   
   G   
   T   
   T   
   C   
   A   
   A   
   A   
   A   
   A   
   C   
   A   
   C   
   T   
   G   
   A   
   G   
    C    
   T   
   A   
   C   
   A   
   G   
   A   
   T   
   C   
   A   
   A   
   T   
   T   
   A   
   C   
   A   
   A   
   T   
   T   
   T   
    C    
   A   
   A   
   T   
   A   
   T   
   G   
   G   
   T   
   C   
   G   
   C   
   T   
   A   
   T   
   C   
   G   
   A   
    C    
   C   
   A   
   T   
   A   
   T   
   G   
   A   
   C   
   T   
   C   
   C   
   G   
   C   
   A   
    T    
   C   
   A   
   A   
   G   
   T   
   T   
   G   
   G   
   A   
   C   
   T   
   G   
   A   
   A   
   A   
   G   
   A   
   C   
   A   
   T   
   T   
   T   
   T   
   A   
   A   
   G   
   A   
   A   
   G   
   T   
   T   
   A   
   T   
   A   
   T   
   C   
   G   
   A   
   G   
   C   
   A   
   T   
   C   
   G   
   A   
   A   
   A   
   G   
   C   
   A   
   A   
   G   
   T   
   C   
   A   
   T   
   A   
   A   
   C   
   A   
   A   
   A   
   A   
   C   
   G   
    T    
   A   
   G   
    T    
   C   
   A   
   A   
   T   
   T   
   C   
   G   
   A   
   T   
   T   
   T   
   A   
   G   
   C   
   C   
   A   
   A   
   A   
   G   
   C   
   T   
   C   
   A   
   A   
   A   
   A   
   A   
    A    
   G   
   A   
   C   
   A   
   G   
   C   
   A   
   T   
   A   
   T   
   C   
   G   
   T   
    A    
   G   
   A   
   A   
   G   
   G   
    A    
    T    
   T   
   G   
   A   
   T   
   G   
   A   
   A   
   A   
   G   
   C   
   T   
   T   
   T   
   A   
   T   
   C   
   G   
   A   
   T   
   T   
   C   
   T   
   G   
   G   
   A   
   T   
   G   
   A   
   A   
   G   
   T   
   A   
   A   
   T   
   C   
   G   
    C    
   A   
   A   
   C   
   G   
   A   
   T   
   T   
   C   
   G   
   T   
   G   
   A   
   A   
   A   
   G   
   T   
   A   
   A   
   A   
   G   
   A   
   C   
   A   
   A   
   A   
   A   
   A   
   A   
   G   
   A   
    C    
   G   
   C   
    C    
   A   
   A   
   A   
   A   
   A   
   G   
   A   
   A   
   T   
   T   
   T   
   A   
   G   
   T   
   C   
   G   
   A   
    C    
   G   
   T   
    C    
   T   
   T   
    T    
   C   
   A   
   A   
   T   
   T   
   T   
   A   
   C   
   A   
   G   
   A   
   A   
   G   
   A   
   A   
   C   
   A   
   A   
   G   
   C   
   A   
   G   
   A   
   A   
   G   
   C   
   A   
   A   
   T   
    T    
   G   
   T   
   C   
   A   
   C   
   A   
   T   
   T   
   G   
   C   
   A   
   A   
    T    
   T   
   A   
   T   
   A   
    C    
   C   
   G   
   T   
   T   
   T   
   G   
   A   
   C   
   G   
   A   
   A   
    C    
   A   
   C   
    G    
   G   
   A   
   T   
   A   
   T   
   C   
   A   
   C   
   A   
   G   
   A   
   G   
   C   
   T   
   T   
   C   
   A   
   A   
   A   
   A   
   G   
   G   
   A   
   A   
    T    
   C   
    C    
   G   
   A   
   A   
   A   
    G    
   C   
   T   
   T   
   G   
   A   
   T   
   T   
   G   
   C   
    G    
   C   
   A   
   A   
   A   
   T   
   C   
   A   
   C   
   A   
   G   
   A   
   G   
   T   
   T   
    G    
   A   
   A   
   T   
   A   
   A   
   A   
   A   
   T   
   C   
   C   
   T   
   T   
   T   
   C   
   A   
   A   
   A   
    T    
   G   
   A   
   C   
   A   
   A   
   A   
   G   
   A   
    G    
   C   
   T   
   T   
   T   
   T   
    C    
   T   
   C   
   T   
   G   
   T   
   T   
   A   
   T   
   G   
   A   
   A   
   A   
   A   
   A   
   A   
   G   
   A   
   A   
   T   
   T   
   G   
   C   
   G   
   A   
   G   
   A   
   A   
   G   
   T   
   G   
   A   
   A   
   A   
   A   
   A   
   A   
   A   
   A   
   T   
   T   
   A   
    T    
   T   
   C   
   T   
   T   
   C   
   C   
   G   
   C   
   T   
   C   
   G   
   T   
   C   
    T    
    A    
   A   
   C   
   G   
   A   
   C   
   A   
   A   
   T   
   C   
    G    
   A   
   A   
   G   
   A   
   C   
   G   
   A   
   A   
   A   
   T   
   C   
   G   
   A   
   A   
   G   
   A   
   A   
   A   
   T   
   C   
   A   
   A   
   A   
   A   
   T   
    T    
   G   
   A   
   T   
   A   
   C   
   G   
   C   
   A   
   A   
    G    
   T   
    C    
   T   
   T   
   A   
   G   
   T   
   G   
   G   
   C   
    A    
   C   
   A   
   A   
   G   
   A   
   A   
   G   
   A   
   C   
   G   
   T   
   A   
   A   
   T   
    T    
   G   
   T   
   C   
   A   
   G   
   T   
   G   
   T   
    G    
   A   
   C   
    G    
   C   
   G   
   A   
   G   
   A   
   A   
   G   
   G   
   C   
   T   
   A   
   T   
   G   
   T   
   G   
   A   
   A   
   G   
   C   
   G   
   A   
   A   
   C   
   A   
   A   
   G   
   C   
   C   
   T   
   T   
   C   
   G   
   T   
   T   
   C   
    T    
   T   
   A   
   T   
   A   
   G   
   T   
   G   
   C   
   T   
   T   
   C   
    C    
   A   
   A   
   A   
   C   
   C   
   A   
   G   
   A   
   A   
   G   
   A   
   A   
   A   
   T   
   C   
   G   
   G   
   G   
   A   
   T   
   G   
   C   
   G   
   A   
   G   
   A   
   A   
   G   
   G   
   C   
   G   
   A   
    C    
   T   
   A   
   C   
   T   
   T   
   G   
   C   
   T   
    A    
   T   
   A   
   C   
    T    
   C   
   A   
   G   
   G   
   T   
   G   
   A   
   A   
   T   
   T   
    T    
   A   
   G   
    T    
   A   
   C   
   A   
    T    
   T   
   G   
   G   
   A   
   C   
   C   
   A   
   T   
   G   
   T   
   T   
   C   
   T   
   T   
   C   
   T   
   G   
   A   
   T   
    C    
   A   
   C   
    A    
   A   
   A   
   T   
   A   
   A   
   A   
   G   
   C   
   A   
   A   
   A   
   T   
   G   
   T   
   G   
   A   
   T   
   C   
   T   
   A   
   C   
   C   
   G   
    C    
   C   
   C   
   T   
   G   
   T   
    C    
   C   
   A   
   T   
   G   
   A   
   A   
   C   
   T   
   A   
   C   
   C   
    C    
   G   
   A   
   T   
   T   
   T   
   G   
   A   
   A   
   A   
   T   
   G   
   G   
   A   
   A   
   A   
   G   
   A   
   T   
   G   
   C   
   T   
   G   
   G   
    T    
   G   
   A   
    G    
   C   
   A   
    C    
   A   
   T   
   T   
   T   
   C   
   T   
   C   
   A   
   A   
   A   
   C   
   A   
   A   
   T   
   C   
   T   
   C   
   C   
   A   
   A   
   T   
   C   
   T   
   G   
   T   
   C   
   T   
   G   
   T   
   A   
   G   
   A   
    C    
   G   
   A   
   A   
   T   
   C   
   G   
   A   
   T   
    T    
   T   
   T   
   A   
   G   
   C   
   T   
   G   
   T   
   T   
   T   
   T   
    C    
   C   
   C   
   A   
   T   
   A   
   T   
   C   
   A   
   A   
   A   
   A   
   A   
   A   
   T   
    T    
   C   
   A   
   A   
   G   
   C   
   A   
   G   
   A   
    G    
   A   
   A   
   A   
   A   
   C   
   A   
   T   
   T   
   T   
   G   
   T   
    A    
   T   
   T   
   C   
   A   
   T   
   C   
   A   
   G   
   C   
   A   
   A   
   G   
   A   
   A   
   T   
   G   
   G   
   A   
   T   
   T   
    G    
    A    
   T   
   C   
   A   
   A   
   G   
   C   
   A   
   A   
   A   
   C   
   A   
   C   
   G   
   A   
   A   
   T   
   G   
   A   
   C   
   A   
   G   
   A   
   G   
   T   
   T   
   C   
   G   
   A   
   A   
   C   
   C   
   A   
   T   
   G   
   G   
   C   
   G   
   C   
   A   
   C   
   C   
   T   
   A   
    T    
   A   
   A   
   G   
   A   
   G   
   C   
   C   
   G   
    T    
   C   
   C   
   G   
   C   
   T   
   C   
   A   
   G   
   C   
   T   
   G   
   T   
   A   
   T   
   G   
   A   
   A   
   G   
   C   
   T   
   A   
   A   
   A   
   G   
   G   
   C   
   G   
   G   
   A   
   A   
   G   
   A   
   A   
   G   
   A   
   T   
   G   
   A   
   A   
   C   
   T   
    G    
   A   
   T   
   T   
   G   
   C   
   T   
   G   
   T   
    G    
   T   
   A   
   T   
   C   
   T   
   G   
   A   
   A   
   A   
   G   
   A   
   A   
   G   
   A   
   T   
   C   
    A    
   G   
   C   
   C   
   A   
   G   
   G   
   C   
   T   
   T   
   A   
   G   
   A   
   T   
   G   
   T   
    T    
   T   
   T   
   C   
   T   
   T   
   A   
   G   
   T   
   A   
   A   
   C   
   G   
    C    
   A   
   T   
   C   
   A   
   A   
   G   
   G   
   A   
   A   
   T   
   G   
   G   
   G   
    T    
   T   
   T   
   G   
   C   
   G   
   C   
   T   
    A    
   T   
   C   
   C   
   T   
   T   
   T   
   A   
   A   
    C    
   A   
   G   
   A   
   T   
   G   
   T   
   T   
   C   
   C   
   C   
   G   
   T   
   T   
   G   
   T   
   T   
   G   
   G   
   A   
   G   
   C   
   A   
   A   
   A   
   G   
   G   
   C   
   A   
   G   
    C    
   T   
   G   
   G   
   A   
   G   
   T   
   G   
   A   
   A   
   G   
   T   
   C   
   T   
   A   
   T   
   G   
   A   
   A   
   T   
   T   
   T   
   G   
   A   
   A   
   A   
   G   
   A   
   T   
   G   
   G   
   A   
   G   
   A   
   T   
   A   
   G   
   G   
    A    
   T   
   T   
    A    
   T   
   C   
   A   
   A   
    C    
   G   
   G   
    G    
   C   
   T   
    G    
   C   
   T   
   T   
   G   
   T   
   G   
   T   
   T   
   A   
   G   
   C   
   A   
   G   
   A   
    A    
   G   
   G   
    T    
   G   
   A   
   T   
   A   
   C   
   G   
   C   
   C   
    G    
   A   
   T   
   C   
   G   
   T   
   A   
   A   
   T   
   C   
   G   
   T   
   T   
   A   
   C   
   T   
   C   
   A   
   G   
   C   
   G   
   A   
   G   
   G   
   A   
   G   
   C   
    T    
   G   
   T   
    T    
   A   
   A   
   A   
   C   
   G   
   C   
   A   
   T   
   G   
   C   
   T   
   T   
   G   
   C   
   T   
   C   
   A   
   A   
   G   
   A   
   A   
   A   
   T   
   C   
   A   
   G   
   C   
   C   
   A   
   A   
   A   
   C   
    T    
   T   
   C   
   A   
   C   
   G   
   T   
   G   
   C   
   G   
   A   
   A   
   G   
   C   
   G   
   G   
   G   
   G   
   A   
   G   
   T   
   G   
   A   
   C   
   G   
   G   
   T   
   C   
   A   
   T   
   G   
   C   
   G   
   T   
   G   
   A   
   G   
   C   
   T   
   G   
   A   
   A   
   A   
   A   
   A   
   A   
   A   
   A   
   T   
   C   
   C   
    G    
   C   
   A   
    C    
   C   
   G   
    T    
   A   
   T   
   T   
   A   
   T   
   C   
   T   
   A   
   T   
   A   
   T   
   G   
   T   
   C   
   G   
   G   
   A   
   A   
   G   
   G   
   A   
   A   
   G   
   A   
   T   
   C   
   A   
   A   
   A   
   A   
   G   
   A   
   A   
   A   
   T   
   A   
   A   
   C   
   C   
   T   
   T   
   G   
   A   
   T   
   G   
   A   
   A   
   T   
   C   
   A   
   A   
   A   
   A   
   A   
   G   
   G   
    G    
   C   
   A   
    G    
   C   
   A   
   G   
    T    
   T   
   A   
   A   
   C   
   G   
   A   
   T   
   T   
   G   
   A   
    C    
   C   
   C   
   A   
   A   
   C   
    T    
   G   
   A   
   T   
   T   
   T   
    C    
   C   
   C   
   T   
   A   
   T   
   A   
   G   
   G   
   T   
   G   
   A   
   T   
   C   
   G   
   A   
   A   
   C   
   A   
   T   
   C   
   G   
   A   
   A   
   T   
   G   
   G   
    A    
   T   
   C   
    A    
   T   
   T   
   T   
   G   
   C   
    G    
   A   
   T   
   G   
   G   
   A   
    T    
   G   
   A   
   G   
   A   
   A   
   A   
   A   
   A   
   A   
   G   
   G   
   T   
   G   
   G   
   A   
   G   
   A   
   A   
   G   
   T   
   C   
   A   
   T   
   C   
   G   
   A   
   A   
   G   
   T   
    T    
   A   
    T    
   C   
   G   
   A   
   T   
   G   
   C   
   T   
   C   
   C   
   T   
   G   
   A   
   G   
    A    
   T   
   T   
   T   
   T   
    C    
   A   
   T   
    C    
   G   
   A   
    G    
    T    
   C   
    G    
   -   
   -   
   -   
 

 
   N59439F   
   A   
   T   
   G   
   G   
   A   
   A   
   A   
   A   
   T   
   C   
   G   
   A   
   C   
   A   
   A   
   G   
   A   
   A   
   G   
   T   
   A   
   C   
   A   
   A   
   G   
   A   
   A   
   T   
   T   
   A   
   A   
   C   
   T   
   T   
   T   
   A   
   G   
   A   
   A   
   G   
   A   
   A   
   G   
   T   
   G   
   A   
   T   
   G   
   G   
   G   
   T   
   G   
   A   
   C   
   C   
   G   
   C   
   T   
   T   
   C   
   G   
   G   
   A   
   C   
   G   
   T   
   T   
   A   
   T   
   T   
   C   
   C   
   A   
   A   
   A   
   T   
   A   
   T   
   A   
   T   
   T   
   A   
   T   
    C    
   C   
   A   
    A    
   G   
   A   
   A   
   C   
   G   
   T   
   G   
   C   
   A   
   C   
   T   
   G   
   C   
   C   
   T   
   G   
   A   
   T   
   A   
   T   
    C    
   C   
   G   
   T   
   G   
   A   
   T   
   G   
   G   
   A   
   T   
   T   
   G   
   A   
   A   
    G    
   C   
   C   
   A   
   G   
   T   
    G    
   C   
   A   
   A   
   C   
   G   
   C   
   C   
   G   
   G   
   A   
   T   
   A   
   T   
   T   
   A   
   T   
   T   
    T    
   G   
   C   
    C    
   A   
   T   
   G   
   A   
   A   
   C   
   A   
   A   
   A   
   G   
   A   
   T   
   G   
   G   
   C   
   A   
   A   
   T   
   A   
   C   
   G   
   T   
   A   
   T   
   G   
   A   
   C   
   A   
   A   
   A   
   G   
   G   
   A   
   T   
   T   
    C    
   C   
   G   
   T   
   A   
   A   
   A   
   T   
   C   
   A   
   G   
   C   
   A   
   A   
   A   
   A   
   T   
   C   
   T   
   G   
   T   
   C   
   G   
   G   
   A   
   A   
   A   
   T   
   A   
   T   
   C   
   A   
   T   
   G   
   G   
   G   
    G    
   A   
   A   
   T   
   T   
   A   
   T   
   C   
   A   
   T   
   C   
   C   
   T   
   C   
   A   
   T   
   G   
   G   
   T   
   G   
   A   
   C   
   A   
   G   
   C   
   A   
   G   
   T   
   A   
   T   
   T   
   T   
   A   
   T   
   G   
   A   
   A   
   G   
   C   
   G   
   A   
   T   
   G   
   G   
   T   
   C   
   C   
   G   
   C   
   A   
   T   
   G   
   A   
   G   
   C   
   C   
   A   
   A   
   G   
   A   
    C    
   T   
   G   
   G   
   A   
   A   
   G   
   T   
   T   
   G   
   C   
   G   
   G   
   G   
   A   
   A   
   G   
   T   
   A   
   C   
   T   
   G   
   A   
   T   
   C   
   G   
   A   
    A    
   A   
   T   
   G   
   C   
   A   
   C   
   G   
   G   
    G    
   A   
   A   
   T   
   A   
   A   
    C    
   G   
   G   
   G   
   A   
   G   
   T   
   A   
   T   
   G   
   G   
   A   
   C   
   G   
   G   
   T   
   G   
   A   
   T   
   C   
   C   
   A   
   C   
   C   
   T   
   G   
   C   
   T   
   G   
   C   
   G   
   A   
   T   
   G   
   C   
   G   
   T   
   T   
   A   
   T   
   A   
   C   
   T   
   G   
   A   
   A   
   G   
   C   
   C   
   A   
   G   
    A    
   T   
   T   
   A   
   T   
   C   
   C   
   A   
   A   
   G   
   T   
   T   
   A   
   A   
   G   
   T   
   G   
   G   
   A   
   G   
   A   
   G   
   A   
   T   
   G   
    C    
   T   
   A   
    G    
   C   
   A   
   G   
   A   
   T   
   A   
   T   
   C   
   G   
   A   
    A    
   A   
   A   
   A   
   G   
   A   
   A   
   A   
   C   
   A   
   G   
   T   
    C    
   G   
   A   
   T   
   C   
   T   
   C   
   G   
   T   
   G   
   T   
   G   
   G   
   A   
   A   
   T   
   T   
   T   
   T   
   G   
   A   
   T   
   G   
   A   
   T   
   A   
   C   
   A   
   G   
   A   
   A   
   A   
   A   
   A   
   G   
   A   
   A   
   C   
   C   
   A   
   A   
   C   
   A   
   G   
   T   
   C   
   C   
   T   
   T   
   C   
   C   
   A   
   G   
   C   
   T   
   C   
   G   
   A   
   T   
   A   
   C   
   C   
   C   
   A   
   A   
   A   
   C   
   C   
   T   
   T   
   C   
   T   
    C    
   G   
   T   
   C   
   A   
   A   
   T   
   G   
   G   
   A   
   T   
   C   
   G   
   A   
   C   
   A   
   G   
   G   
   T   
   A   
   T   
   A   
   T   
   C   
   T   
   G   
   C   
   T   
   G   
   G   
   A   
   T   
   A   
   T   
   G   
   C   
    A    
   A   
   C   
   A   
   G   
   A   
   A   
   A   
   T   
   C   
   C   
   C   
   G   
   A   
   C   
    T    
   C   
   A   
   T   
   A   
   A   
   C   
   T   
   T   
   A   
   G   
   C   
   T   
   G   
   A   
   A   
   G   
   T   
   G   
   A   
   T   
    C    
   G   
   A   
    T    
   G   
   G   
   C   
   A   
   C   
   A   
   A   
   T   
   C   
   T   
   A   
   T   
   A   
   T   
   G   
   A   
   T   
   C   
   G   
   A   
   T   
   C   
   A   
   T   
   C   
   C   
   A   
   C   
   A   
   A   
   G   
   C   
   G   
   A   
   G   
   C   
   C   
   T   
   T   
   G   
   A   
   A   
   A   
   A   
   A   
   T   
   T   
   G   
   A   
   T   
   G   
   G   
   A   
   A   
   T   
   A   
   T   
   A   
   T   
   T   
   C   
   C   
   T   
   G   
   G   
   A   
   C   
   C   
   T   
   G   
   A   
   C   
   T   
   T   
    T    
   C   
   C   
   A   
   A   
   C   
   A   
   G   
   G   
   A   
   G   
   G   
    G    
   A   
   T   
   C   
   T   
   T   
   G   
   C   
   A   
   A   
   G   
   G   
    A    
   A   
   A   
   A   
   G   
   A   
   T   
   G   
   A   
   A   
   A   
   T   
   C   
   A   
   A   
   A   
   A   
   A   
   A   
   G   
   C   
   T   
   T   
   A   
   T   
   G   
   A   
   A   
   A   
   C   
   A   
   G   
   G   
    G    
   C   
   G   
   A   
   G   
   G   
   A   
   A   
   A   
   A   
   G   
   T   
   T   
   A   
    T    
   T   
   C   
   T   
   T   
   C   
   G   
   T   
   T   
   C   
   G   
   A   
   A   
   A   
   A   
   C   
   A   
   A   
   A   
   G   
   A   
   T   
   A   
   G   
   A   
    G    
   G   
   C   
   A   
   A   
   T   
   G   
   A   
   A   
   G   
   G   
   G   
    T    
   A   
   A   
    T    
   A   
   A   
   A   
   C   
   A   
   G   
   C   
   A   
   A   
   A   
   T   
   C   
   G   
   T   
   G   
   A   
   T   
   C   
   A   
   C   
    A    
   G   
   A   
   A   
   A   
   T   
   C   
   C   
   C   
   A   
   T   
   A   
   T   
   G   
   A   
   A   
   G   
   T   
   A   
   A   
   A   
   T   
   A   
   A   
   A   
   G   
   C   
   G   
   A   
   C   
    C    
   T   
   T   
   G   
   G   
   T   
   G   
   A   
   A   
   A   
   A   
   A   
   A   
   A   
   T   
   G   
   G   
   A   
   T   
   G   
   A   
   G   
   A   
   T   
   C   
   C   
   G   
   T   
   T   
   T   
   G   
   A   
   A   
   T   
   A   
   A   
   G   
   A   
   A   
   A   
   A   
   T   
   C   
   G   
   A   
    T    
   G   
   G   
   A   
   A   
   T   
   A   
   G   
   C   
   T   
   G   
   A   
   A   
   G   
   T   
    A    
   C   
   G   
   A   
   G   
   A   
   T   
   G   
   A   
   A   
   A   
   G   
   T   
   G   
   A   
   T   
    A    
   G   
    A    
   A   
   C   
    A    
   G   
   G   
   T   
   C   
   T   
   G   
   C   
   A   
   A   
   A   
   T   
   T   
   G   
   T   
   C   
   G   
   T   
    C    
   G   
   A   
   A   
   T   
   T   
   G   
   A   
   A   
   A   
   A   
   A   
   A   
   G   
   A   
    C    
   G   
   C   
   C   
   A   
   A   
    T    
   G   
   C   
   T   
   C   
   A   
   A   
   G   
   G   
    A    
   A   
   T   
   T   
   T   
   T   
   G   
   A   
   A   
   T   
   T   
   A   
    T    
   T   
   T   
   G   
   T   
   T   
   C   
   A   
   A   
   A   
   A   
   A   
   C   
   A   
   C   
   T   
   G   
   A   
   G   
    C    
   T   
   A   
   C   
   A   
   G   
   A   
   T   
   C   
   A   
   A   
   T   
   T   
   A   
   C   
   A   
   A   
   T   
   T   
   T   
    C    
   A   
   A   
   T   
   A   
   T   
   G   
   G   
   T   
   C   
   G   
   C   
   T   
   A   
   T   
   C   
   G   
   A   
    C    
   C   
   A   
   T   
   A   
   T   
   G   
   A   
   C   
   T   
   C   
   C   
   G   
   C   
   A   
    T    
   C   
   A   
   A   
   G   
   T   
   T   
   G   
   G   
   A   
   C   
   T   
   G   
   A   
   A   
   A   
   G   
   A   
   C   
   A   
   T   
   T   
   T   
   T   
   A   
   A   
   G   
   A   
   A   
   G   
   T   
   T   
   A   
   T   
   A   
   T   
   C   
   G   
   A   
   G   
   C   
   A   
   T   
   C   
   G   
   A   
   A   
   A   
   G   
   C   
   A   
   A   
   G   
   T   
   C   
   A   
   T   
   A   
   A   
   C   
   A   
   A   
   A   
   A   
   C   
   G   
    T    
   A   
   G   
    T    
   C   
   A   
   A   
   T   
   T   
   C   
   G   
   A   
   T   
   T   
   T   
   A   
   G   
   C   
   C   
   A   
   A   
   A   
   G   
   C   
   T   
   C   
   A   
   A   
   A   
   A   
   A   
    A    
   G   
   A   
   C   
   A   
   G   
   C   
   A   
   T   
   A   
   T   
   C   
   G   
   T   
    A    
   G   
   A   
   A   
   G   
   G   
    A    
    T    
   T   
   G   
   A   
   T   
   G   
   A   
   A   
   A   
   G   
   C   
   T   
   T   
   T   
   A   
   T   
   C   
   G   
   A   
   T   
   T   
   C   
   T   
   G   
   G   
   A   
   T   
   G   
   A   
   A   
   G   
   T   
   A   
   A   
   T   
   C   
   G   
    C    
   A   
   A   
   C   
   G   
   A   
   T   
   T   
   C   
   G   
   T   
   G   
   A   
   A   
   A   
   G   
   T   
   A   
   A   
   A   
   G   
   A   
   C   
   A   
   A   
   A   
   A   
   A   
   A   
   G   
   A   
    C    
   G   
   C   
    C    
   A   
   A   
   A   
   A   
   A   
   G   
   A   
   A   
   T   
   T   
   T   
   A   
   G   
   T   
   C   
   G   
   A   
    C    
   G   
   T   
    C    
   T   
   T   
    T    
   C   
   A   
   A   
   T   
   T   
   T   
   A   
   C   
   A   
   G   
   A   
   A   
   G   
   A   
   A   
   C   
   A   
   A   
   G   
   C   
   A   
   G   
   A   
   A   
   G   
   C   
   A   
   A   
   T   
    T    
   G   
   T   
   C   
   A   
   C   
   A   
   T   
   T   
   G   
   C   
   A   
   A   
    T    
   T   
   A   
   T   
   A   
    C    
   C   
   G   
   T   
   T   
   T   
   G   
   A   
   C   
   G   
   A   
   A   
    C    
   A   
   C   
    G    
   G   
   A   
   T   
   A   
   T   
   C   
   A   
   C   
   A   
   G   
   A   
   G   
   C   
   T   
   T   
   C   
   A   
   A   
   A   
   A   
   G   
   G   
   A   
   A   
    T    
   C   
    C    
   G   
   A   
   A   
   A   
    G    
   C   
   T   
   T   
   G   
   A   
   T   
   T   
   G   
   C   
    G    
   C   
   A   
   A   
   A   
   T   
   C   
   A   
   C   
   A   
   G   
   A   
   G   
   T   
   T   
    G    
   A   
   A   
   T   
   A   
   A   
   A   
   A   
   T   
   C   
   C   
   T   
   T   
   T   
   C   
   A   
   A   
   A   
    T    
   G   
   A   
   C   
   A   
   A   
   A   
   G   
   A   
    G    
   C   
   T   
   T   
   T   
   T   
    C    
   T   
   C   
   T   
   G   
   T   
   T   
   A   
   T   
   G   
   A   
   A   
   A   
   A   
   A   
   A   
   G   
   A   
   A   
   T   
   T   
   G   
   C   
   G   
   A   
   G   
   A   
   A   
   G   
   T   
   G   
   A   
   A   
   A   
   A   
   A   
   A   
   A   
   A   
   T   
   T   
   A   
    T    
   T   
   C   
   T   
   T   
   C   
   C   
   G   
   C   
   T   
   C   
   G   
   T   
   C   
    T    
    A    
   A   
   C   
   G   
   A   
   C   
   A   
   A   
   T   
   C   
    G    
   A   
   A   
   G   
   A   
   C   
   G   
   A   
   A   
   A   
   T   
   C   
   G   
   A   
   A   
   G   
   A   
   A   
   A   
   T   
   C   
   A   
   A   
   A   
   A   
   T   
    T    
   G   
   A   
   T   
   A   
   C   
   G   
   C   
   A   
   A   
    G    
   T   
    C    
   T   
   T   
   A   
   G   
   T   
   G   
   G   
   C   
    A    
   C   
   A   
   A   
   G   
   A   
   A   
   G   
   A   
   C   
   G   
   T   
   A   
   A   
   T   
    T    
   G   
   T   
   C   
   A   
   G   
   T   
   G   
   T   
    G    
   A   
   C   
    G    
   C   
   G   
   A   
   G   
   A   
   A   
   G   
   G   
   C   
   T   
   A   
   T   
   G   
   T   
   G   
   A   
   A   
   G   
   C   
   G   
   A   
   A   
   C   
   A   
   A   
   G   
   C   
   C   
   T   
   T   
   C   
   G   
   T   
   T   
   C   
    T    
   T   
   A   
   T   
   A   
   G   
   T   
   G   
   C   
   T   
   T   
   C   
    C    
   A   
   A   
   A   
   C   
   C   
   A   
   G   
   A   
   A   
   G   
   A   
   A   
   A   
   T   
   C   
   G   
   G   
   G   
   A   
   T   
   G   
   C   
   G   
   A   
   G   
   A   
   A   
   G   
   G   
   C   
   G   
   A   
    C    
   T   
   A   
   C   
   T   
   T   
   G   
   C   
   T   
    A    
   T   
   A   
   C   
    T    
   C   
   A   
   G   
   G   
   T   
   G   
   A   
   A   
   T   
   T   
    G    
   A   
   G   
    T    
   A   
   C   
   A   
    T    
   T   
   G   
   G   
   A   
   C   
   C   
   A   
   T   
   G   
   T   
   T   
   C   
   T   
   T   
   C   
   T   
   G   
   A   
   T   
    C    
   A   
   C   
    A    
   A   
   A   
   T   
   A   
   A   
   A   
   G   
   C   
   A   
   A   
   A   
   T   
   G   
   T   
   G   
   A   
   T   
   C   
   T   
   A   
   C   
   C   
   G   
    C    
   C   
   C   
   T   
   G   
   T   
    C    
   C   
   A   
   T   
   G   
   A   
   A   
   C   
   T   
   A   
   C   
   C   
    C    
   G   
   A   
   T   
   T   
   T   
   G   
   A   
   A   
   A   
   T   
   G   
   G   
   A   
   A   
   A   
   G   
   A   
   T   
   G   
   C   
   T   
   G   
   G   
    T    
   G   
   A   
    G    
   C   
   A   
    C    
   A   
   T   
   T   
   T   
   C   
   T   
   C   
   A   
   A   
   A   
   C   
   A   
   A   
   T   
   C   
   T   
   C   
   C   
   A   
   A   
   T   
   C   
   T   
   G   
   T   
   C   
   T   
   G   
   T   
   A   
   G   
   A   
    C    
   G   
   A   
   A   
   T   
   C   
   G   
   A   
   T   
    T    
   T   
   T   
   A   
   G   
   C   
   T   
   G   
   T   
   T   
   T   
   T   
    C    
   C   
   C   
   A   
   T   
   A   
   T   
   C   
   A   
   A   
   A   
   A   
   A   
   A   
   T   
    T    
   C   
   A   
   A   
   G   
   C   
   A   
   G   
   A   
    G    
   A   
   A   
   A   
   A   
   C   
   A   
   T   
   T   
   T   
   G   
   T   
    A    
   T   
   T   
   C   
   A   
   T   
   C   
   A   
   G   
   C   
   A   
   A   
   G   
   A   
   A   
   T   
   G   
   G   
   A   
   T   
   T   
    G    
    A    
   T   
   C   
   A   
   A   
   G   
   C   
   A   
   A   
   A   
   C   
   A   
   C   
   G   
   A   
   A   
   T   
   G   
   A   
   C   
   A   
   G   
   A   
   G   
   T   
   T   
   C   
   G   
   A   
   A   
   C   
   C   
   A   
   T   
   G   
   G   
   C   
   G   
   C   
   A   
   C   
   C   
   T   
   A   
    T    
   A   
   A   
   G   
   A   
   G   
   C   
   C   
   G   
    T    
   C   
   C   
   G   
   C   
   T   
   C   
   A   
   G   
   C   
   T   
   G   
   T   
   A   
   T   
   G   
   A   
   A   
   G   
   C   
   T   
   A   
   A   
   A   
   G   
   G   
   C   
   G   
   G   
   A   
   A   
   G   
   A   
   A   
   G   
   A   
   T   
   G   
   A   
   A   
   C   
   T   
    G    
   A   
   T   
   T   
   G   
   C   
   T   
   G   
   T   
    G    
   T   
   A   
   T   
   C   
   T   
   G   
   A   
   A   
   A   
   G   
   A   
   A   
   G   
   A   
   T   
   C   
    A    
   G   
   C   
   C   
   A   
   G   
   G   
   C   
   T   
   T   
   A   
   G   
   A   
   T   
   G   
   T   
    T    
   T   
   T   
   C   
   T   
   T   
   A   
   G   
   T   
   A   
   A   
   C   
   G   
    C    
   A   
   T   
   C   
   A   
   A   
   G   
   G   
   A   
   A   
   T   
   G   
   G   
   G   
    T    
   T   
   T   
   G   
   C   
   G   
   C   
   T   
    A    
   T   
   C   
   C   
   T   
   T   
   T   
   A   
   A   
    C    
   A   
   G   
   A   
   T   
   G   
   T   
   T   
   C   
   C   
   C   
   G   
   T   
   T   
   G   
   T   
   T   
   G   
   G   
   A   
   G   
   C   
   A   
   A   
   A   
   G   
   G   
   C   
   A   
   G   
    C    
   T   
   G   
   G   
   A   
   G   
   T   
   G   
   A   
   A   
   G   
   T   
   C   
   T   
   A   
   T   
   G   
   A   
   A   
   T   
   T   
   T   
   G   
   A   
   A   
   A   
   G   
   A   
   T   
   G   
   G   
   A   
   G   
   A   
   T   
   A   
   G   
   G   
    A    
   T   
   T   
    A    
   T   
   C   
   A   
   A   
    C    
   G   
   G   
    G    
   C   
   T   
    G    
   C   
   T   
   T   
   G   
   T   
   G   
   T   
   T   
   A   
   G   
   C   
   A   
   G   
   A   
    A    
   G   
   G   
    T    
   G   
   A   
   T   
   A   
   C   
   G   
   C   
   C   
    G    
   A   
   T   
   C   
   G   
   T   
   A   
   A   
   T   
   C   
   G   
   T   
   T   
   A   
   C   
   T   
   C   
   A   
   G   
   C   
   G   
   A   
   G   
   G   
   A   
   G   
   C   
    T    
   G   
   T   
    T    
   A   
   A   
   A   
   C   
   G   
   C   
   A   
   T   
   G   
   C   
   T   
   T   
   G   
   C   
   T   
   C   
   A   
   A   
   G   
   A   
   A   
   A   
   T   
   C   
   A   
   G   
   C   
   C   
   A   
   A   
   A   
   C   
    T    
   T   
   C   
   A   
   C   
   G   
   T   
   G   
   C   
   G   
   A   
   A   
   G   
   C   
   G   
   G   
   G   
   G   
   A   
   G   
   T   
   G   
   A   
   C   
   G   
   G   
   T   
   C   
   A   
   T   
   G   
   C   
   G   
   T   
   G   
   A   
   G   
   C   
   T   
   G   
   A   
   A   
   A   
   A   
   A   
   A   
   A   
   A   
   T   
   C   
   C   
    G    
   C   
   A   
    C    
   C   
   G   
    T    
   A   
   T   
   T   
   A   
   T   
   C   
   T   
   A   
   T   
   A   
   T   
   G   
   T   
   C   
   G   
   G   
   A   
   A   
   G   
   G   
   A   
   A   
   G   
   A   
   T   
   C   
   A   
   A   
   A   
   A   
   G   
   A   
   A   
   A   
   T   
   A   
   A   
   C   
   C   
   T   
   T   
   G   
   A   
   T   
   G   
   A   
   A   
   T   
   C   
   A   
   A   
   A   
   A   
   A   
   G   
   G   
    G    
   C   
   A   
    G    
   C   
   A   
   G   
    T    
   T   
   A   
   A   
   C   
   G   
   A   
   T   
   T   
   G   
   A   
    C    
   C   
   C   
   A   
   A   
   C   
    T    
   G   
   A   
   T   
   T   
   T   
    C    
   C   
   C   
   T   
   A   
   T   
   A   
   G   
   G   
   T   
   G   
   A   
   T   
   C   
   G   
   A   
   A   
   C   
   A   
   T   
   C   
   G   
   A   
   A   
   T   
   G   
   G   
    A    
   T   
   C   
    A    
   T   
   T   
   T   
   G   
   C   
    G    
   A   
   T   
   G   
   G   
   A   
    T    
   G   
   A   
   G   
   A   
   A   
   A   
   A   
   A   
   A   
   G   
   G   
   T   
   G   
   G   
   A   
   G   
   A   
   A   
   G   
   T   
   C   
   A   
   T   
   C   
   G   
   A   
   A   
   G   
   T   
    T    
   A   
    T    
   C   
   G   
   A   
   T   
   G   
   C   
   T   
   C   
   C   
   T   
   G   
   A   
   G   
    A    
   T   
   T   
   T   
   T   
    C    
   A   
   T   
    C    
   G   
   A   
    G    
    T    
   C   
    G    
   -   
   -   
   -   
 

 
   N59711F   
   A   
   T   
   G   
   G   
   A   
   A   
   A   
   A   
   T   
   C   
   G   
   A   
   C   
   A   
   A   
   G   
   A   
   A   
   G   
   T   
   A   
   C   
   A   
   A   
   G   
   A   
   A   
   T   
   T   
   A   
   A   
   C   
   T   
   T   
   T   
   A   
   G   
   A   
   A   
   G   
   A   
   A   
   G   
   T   
   G   
   A   
   T   
   G   
   G   
   G   
   T   
   G   
   A   
   C   
   C   
   G   
   C   
   T   
   T   
   C   
   G   
   G   
   A   
   C   
   G   
   T   
   T   
   A   
   T   
   T   
   C   
   C   
   A   
   A   
   A   
   T   
   A   
   T   
   A   
   T   
   T   
   A   
   T   
    C    
   C   
   A   
    A    
   G   
   A   
   A   
   C   
   G   
   T   
   G   
   C   
   A   
   C   
   T   
   G   
   C   
   C   
   T   
   G   
   A   
   T   
   A   
   T   
    C    
   C   
   G   
   T   
   G   
   A   
   T   
   G   
   G   
   A   
   T   
   T   
   G   
   A   
   A   
    A    
   C   
   C   
   A   
   G   
   T   
    G    
   C   
   A   
   A   
   C   
   G   
   C   
   C   
   G   
   G   
   A   
   T   
   A   
   T   
   T   
   A   
   T   
   T   
    T    
   G   
   C   
    C    
   A   
   T   
   G   
   A   
   A   
   C   
   A   
   A   
   A   
   G   
   A   
   T   
   G   
   G   
   C   
   A   
   A   
   T   
   A   
   C   
   G   
   T   
   A   
   T   
   G   
   A   
   C   
   A   
   A   
   A   
   G   
   G   
   A   
   T   
   T   
    C    
   C   
   G   
   T   
   A   
   A   
   A   
   T   
   C   
   A   
   G   
   C   
   A   
   A   
   A   
   A   
   T   
   C   
   T   
   G   
   T   
   C   
   G   
   G   
   A   
   A   
   A   
   T   
   A   
   T   
   C   
   A   
   T   
   G   
   G   
   G   
    G    
   A   
   A   
   T   
   T   
   A   
   T   
   C   
   A   
   T   
   C   
   C   
   T   
   C   
   A   
   T   
   G   
   G   
   T   
   G   
   A   
   C   
   A   
   G   
   C   
   A   
   G   
   T   
   A   
   T   
   T   
   T   
   A   
   T   
   G   
   A   
   A   
   G   
   C   
   G   
   A   
   T   
   G   
   G   
   T   
   C   
   C   
   G   
   C   
   A   
   T   
   G   
   A   
   G   
   C   
   C   
   A   
   A   
   G   
   A   
    T    
   T   
   G   
   G   
   A   
   A   
   G   
   T   
   T   
   G   
   C   
   G   
   G   
   G   
   A   
   A   
   G   
   T   
   A   
   C   
   T   
   G   
   A   
   T   
   C   
   G   
   A   
    A    
   A   
   T   
   G   
   C   
   A   
   C   
   G   
   G   
    G    
   A   
   A   
   T   
   A   
   A   
    T    
   G   
   G   
   G   
   A   
   G   
   T   
   A   
   T   
   G   
   G   
   A   
   C   
   G   
   G   
   T   
   G   
   A   
   T   
   C   
   C   
   A   
   C   
   C   
   T   
   G   
   C   
   T   
   G   
   C   
   G   
   A   
   T   
   G   
   C   
   G   
   T   
   T   
   A   
   T   
   A   
   C   
   T   
   G   
   A   
   A   
   G   
   C   
   C   
   A   
   G   
    A    
   T   
   T   
   A   
   T   
   C   
   C   
   A   
   A   
   G   
   T   
   T   
   A   
   A   
   G   
   T   
   G   
   G   
   A   
   G   
   A   
   G   
   A   
   T   
   G   
    C    
   T   
   A   
    G    
   C   
   A   
   G   
   A   
   T   
   A   
   T   
   C   
   G   
   A   
    A    
   A   
   A   
   A   
   G   
   A   
   A   
   A   
   C   
   A   
   G   
   T   
    C    
   G   
   A   
   T   
   C   
   T   
   C   
   G   
   T   
   G   
   T   
   G   
   G   
   A   
   A   
   T   
   T   
   T   
   T   
   G   
   A   
   T   
   G   
   A   
   T   
   A   
   C   
   A   
   G   
   A   
   A   
   A   
   A   
   A   
   G   
   A   
   A   
   C   
   C   
   A   
   A   
   C   
   A   
   G   
   T   
   C   
   C   
   T   
   T   
   C   
   C   
   A   
   G   
   C   
   T   
   C   
   G   
   A   
   T   
   A   
   C   
   C   
   C   
   A   
   A   
   A   
   C   
   C   
   T   
   T   
   C   
   T   
    A    
   G   
   T   
   C   
   A   
   A   
   T   
   G   
   G   
   A   
   T   
   C   
   G   
   A   
   C   
   A   
   G   
   G   
   T   
   A   
   T   
   A   
   T   
   C   
   T   
   G   
   C   
   T   
   G   
   G   
   A   
   T   
   A   
   T   
   G   
   C   
    G    
   A   
   C   
   A   
   G   
   A   
   A   
   A   
   T   
   C   
   C   
   C   
   G   
   A   
   C   
    T    
   C   
   A   
   T   
   A   
   A   
   C   
   T   
   T   
   A   
   G   
   C   
   T   
   G   
   A   
   A   
   G   
   T   
   G   
   A   
   T   
    C    
   G   
   A   
    C    
   G   
   G   
   C   
   A   
   C   
   A   
   A   
   T   
   C   
   T   
   A   
   T   
   A   
   T   
   G   
   A   
   T   
   C   
   G   
   A   
   T   
   C   
   A   
   T   
   C   
   C   
   A   
   C   
   A   
   A   
   G   
   C   
   G   
   A   
   G   
   C   
   C   
   T   
   T   
   G   
   A   
   A   
   A   
   A   
   A   
   T   
   T   
   G   
   A   
   T   
   G   
   G   
   A   
   A   
   T   
   A   
   T   
   A   
   T   
   T   
   C   
   C   
   T   
   G   
   G   
   A   
   C   
   C   
   T   
   G   
   A   
   C   
   T   
   T   
    T    
   C   
   C   
   A   
   A   
   C   
   A   
   G   
   G   
   A   
   G   
   G   
    G    
   A   
   T   
   C   
   T   
   T   
   G   
   C   
   A   
   A   
   G   
   G   
    G    
   A   
   A   
   A   
   G   
   A   
   T   
   G   
   A   
   A   
   A   
   T   
   C   
   A   
   A   
   A   
   A   
   A   
   A   
   G   
   C   
   T   
   T   
   A   
   T   
   G   
   A   
   A   
   A   
   C   
   A   
   G   
   G   
    G    
   C   
   G   
   A   
   G   
   G   
   A   
   A   
   A   
   A   
   G   
   T   
   T   
   A   
    T    
   T   
   C   
   T   
   T   
   C   
   G   
   T   
   T   
   C   
   G   
   A   
   A   
   A   
   A   
   C   
   A   
   A   
   A   
   G   
   A   
   T   
   A   
   G   
   A   
    A    
   G   
   C   
   A   
   A   
   T   
   G   
   A   
   A   
   G   
   G   
   G   
    C    
   A   
   A   
    T    
   A   
   A   
   A   
   C   
   A   
   G   
   C   
   A   
   A   
   A   
   T   
   C   
   G   
   T   
   G   
   A   
   T   
   C   
   A   
   C   
    T    
   G   
   A   
   A   
   A   
   T   
   C   
   C   
   C   
   A   
   T   
   A   
   T   
   G   
   A   
   A   
   G   
   T   
   A   
   A   
   A   
   T   
   A   
   A   
   A   
   G   
   C   
   G   
   A   
   C   
    G    
   T   
   T   
   G   
   G   
   T   
   G   
   A   
   A   
   A   
   A   
   A   
   A   
   A   
   T   
   G   
   G   
   A   
   T   
   G   
   A   
   G   
   A   
   T   
   C   
   C   
   G   
   T   
   T   
   T   
   G   
   A   
   A   
   T   
   A   
   A   
   G   
   A   
   A   
   A   
   A   
   T   
   C   
   G   
   A   
    T    
   G   
   G   
   A   
   A   
   T   
   A   
   G   
   C   
   T   
   G   
   A   
   A   
   G   
   T   
    A    
   C   
   G   
   A   
   G   
   A   
   T   
   G   
   A   
   A   
   A   
   G   
   T   
   G   
   A   
   T   
    C    
   G   
    A    
   A   
   C   
    T    
   G   
   G   
   T   
   C   
   T   
   G   
   C   
   A   
   A   
   A   
   T   
   T   
   G   
   T   
   C   
   G   
   T   
    T    
   G   
   A   
   A   
   T   
   T   
   G   
   A   
   A   
   A   
   A   
   A   
   A   
   G   
   A   
    T    
   G   
   C   
   C   
   A   
   A   
    T    
   G   
   C   
   T   
   C   
   A   
   A   
   G   
   G   
    A    
   A   
   T   
   T   
   T   
   T   
   G   
   A   
   A   
   T   
   T   
   A   
    T    
   T   
   T   
   G   
   T   
   T   
   C   
   A   
   A   
   A   
   A   
   A   
   C   
   A   
   C   
   T   
   G   
   A   
   G   
    C    
   T   
   A   
   C   
   A   
   G   
   A   
   T   
   C   
   A   
   A   
   T   
   T   
   A   
   C   
   A   
   A   
   T   
   T   
   T   
    C    
   A   
   A   
   T   
   A   
   T   
   G   
   G   
   T   
   C   
   G   
   C   
   T   
   A   
   T   
   C   
   G   
   A   
    T    
   C   
   A   
   T   
   A   
   T   
   G   
   A   
   C   
   T   
   C   
   C   
   G   
   C   
   A   
    C    
   C   
   A   
   A   
   G   
   T   
   T   
   G   
   G   
   A   
   C   
   T   
   G   
   A   
   A   
   A   
   G   
   A   
   C   
   A   
   T   
   T   
   T   
   T   
   A   
   A   
   G   
   A   
   A   
   G   
   T   
   T   
   A   
   T   
   A   
   T   
   C   
   G   
   A   
   G   
   C   
   A   
   T   
   C   
   G   
   A   
   A   
   A   
   G   
   C   
   A   
   A   
   G   
   T   
   C   
   A   
   T   
   A   
   A   
   C   
   A   
   A   
   A   
   A   
   C   
   G   
    C    
   A   
   G   
    C    
   C   
   A   
   A   
   T   
   T   
   C   
   G   
   A   
   T   
   T   
   T   
   A   
   G   
   C   
   C   
   A   
   A   
   A   
   G   
   C   
   T   
   C   
   A   
   A   
   A   
   A   
   A   
    A    
   G   
   A   
   C   
   A   
   G   
   C   
   A   
   T   
   A   
   T   
   C   
   G   
   T   
    G    
   G   
   A   
   A   
   G   
   G   
    C    
    C    
   T   
   G   
   A   
   T   
   G   
   A   
   A   
   A   
   G   
   C   
   T   
   T   
   T   
   A   
   T   
   C   
   G   
   A   
   T   
   T   
   C   
   T   
   G   
   G   
   A   
   T   
   G   
   A   
   A   
   G   
   T   
   A   
   A   
   T   
   C   
   G   
    C    
   A   
   A   
   C   
   G   
   A   
   T   
   T   
   C   
   G   
   T   
   G   
   A   
   A   
   A   
   G   
   T   
   A   
   A   
   A   
   G   
   A   
   C   
   A   
   A   
   A   
   A   
   A   
   A   
   G   
   A   
    C    
   G   
   C   
    C    
   A   
   A   
   A   
   A   
   A   
   G   
   A   
   A   
   T   
   T   
   T   
   A   
   G   
   T   
   C   
   G   
   A   
    T    
   G   
   T   
    C    
   T   
   T   
    C    
   C   
   A   
   A   
   T   
   T   
   T   
   A   
   C   
   A   
   G   
   A   
   A   
   G   
   A   
   A   
   C   
   A   
   A   
   G   
   C   
   A   
   G   
   A   
   A   
   G   
   C   
   A   
   A   
   T   
    C    
   G   
   T   
   C   
   A   
   C   
   A   
   T   
   T   
   G   
   C   
   A   
   A   
    C    
   T   
   A   
   T   
   A   
    C    
   C   
   G   
   T   
   T   
   T   
   G   
   A   
   C   
   G   
   A   
   A   
    C    
   A   
   C   
    G    
   G   
   A   
   T   
   A   
   T   
   C   
   A   
   C   
   A   
   G   
   A   
   G   
   C   
   T   
   T   
   C   
   A   
   A   
   A   
   A   
   G   
   G   
   A   
   A   
    T    
   C   
    C    
   G   
   A   
   A   
   A   
    A    
   C   
   T   
   T   
   G   
   A   
   T   
   T   
   G   
   C   
    A    
   C   
   A   
   A   
   A   
   T   
   C   
   A   
   C   
   A   
   G   
   A   
   G   
   T   
   T   
    G    
   A   
   A   
   T   
   A   
   A   
   A   
   A   
   T   
   C   
   C   
   T   
   T   
   T   
   C   
   A   
   A   
   A   
    C    
   G   
   A   
   C   
   A   
   A   
   A   
   G   
   A   
    G    
   C   
   T   
   T   
   T   
   T   
    T    
   T   
   C   
   T   
   G   
   T   
   T   
   A   
   T   
   G   
   A   
   A   
   A   
   A   
   A   
   A   
   G   
   A   
   A   
   T   
   T   
   G   
   C   
   G   
   A   
   G   
   A   
   A   
   G   
   T   
   G   
   A   
   A   
   A   
   A   
   A   
   A   
   A   
   A   
   T   
   T   
   A   
    C    
   T   
   C   
   T   
   T   
   C   
   C   
   G   
   C   
   T   
   C   
   G   
   T   
   C   
    T    
    G    
   A   
   C   
   G   
   A   
   C   
   A   
   A   
   T   
   C   
    C    
   A   
   A   
   G   
   A   
   C   
   G   
   A   
   A   
   A   
   T   
   C   
   G   
   A   
   A   
   G   
   A   
   A   
   A   
   T   
   C   
   A   
   A   
   A   
   A   
   T   
    C    
   G   
   A   
   T   
   A   
   C   
   G   
   C   
   A   
   A   
    G    
   T   
    T    
   T   
   T   
   A   
   G   
   T   
   G   
   G   
   C   
    G    
   C   
   A   
   A   
   G   
   A   
   A   
   G   
   A   
   C   
   G   
   T   
   A   
   A   
   T   
    C    
   G   
   T   
   C   
   A   
   G   
   T   
   G   
   T   
    G    
   A   
   C   
    G    
   C   
   G   
   A   
   G   
   A   
   A   
   G   
   G   
   C   
   T   
   A   
   T   
   G   
   T   
   G   
   A   
   A   
   G   
   C   
   G   
   A   
   A   
   C   
   A   
   A   
   G   
   C   
   C   
   T   
   T   
   C   
   G   
   T   
   T   
   C   
    C    
   T   
   A   
   T   
   A   
   G   
   T   
   G   
   C   
   T   
   T   
   C   
    G    
   A   
   A   
   A   
   C   
   C   
   A   
   G   
   A   
   A   
   G   
   A   
   A   
   A   
   T   
   C   
   G   
   G   
   G   
   A   
   T   
   G   
   C   
   G   
   A   
   G   
   A   
   A   
   G   
   G   
   C   
   G   
   A   
    T    
   T   
   A   
   C   
   T   
   T   
   G   
   C   
   T   
    G    
   T   
   A   
   C   
    T    
   C   
   A   
   G   
   G   
   T   
   G   
   A   
   A   
   T   
   T   
    G    
   A   
   G   
    C    
   A   
   C   
   A   
    T    
   T   
   G   
   G   
   A   
   C   
   C   
   A   
   T   
   G   
   T   
   T   
   C   
   T   
   T   
   C   
   T   
   G   
   A   
   T   
    T    
   A   
   C   
    G    
   A   
   A   
   T   
   A   
   A   
   A   
   G   
   C   
   A   
   A   
   A   
   T   
   G   
   T   
   G   
   A   
   T   
   C   
   T   
   A   
   C   
   C   
   G   
    T    
   C   
   C   
   T   
   G   
   T   
    T    
   C   
   A   
   T   
   G   
   A   
   A   
   C   
   T   
   A   
   C   
   C   
    C    
   G   
   A   
   T   
   T   
   T   
   G   
   A   
   A   
   A   
   T   
   G   
   G   
   A   
   A   
   A   
   G   
   A   
   T   
   G   
   C   
   T   
   G   
   G   
    C    
   G   
   A   
    A    
   C   
   A   
    C    
   A   
   T   
   T   
   T   
   C   
   T   
   C   
   A   
   A   
   A   
   C   
   A   
   A   
   T   
   C   
   T   
   C   
   C   
   A   
   A   
   T   
   C   
   T   
   G   
   T   
   C   
   T   
   G   
   T   
   A   
   G   
   A   
    T    
   G   
   A   
   A   
   T   
   C   
   G   
   A   
   T   
    C    
   T   
   T   
   A   
   G   
   C   
   T   
   G   
   T   
   T   
   T   
   T   
    C    
   C   
   C   
   A   
   T   
   A   
   T   
   C   
   A   
   A   
   A   
   A   
   A   
   A   
   T   
    C    
   C   
   A   
   A   
   G   
   C   
   A   
   G   
   A   
    A    
   A   
   A   
   A   
   A   
   C   
   A   
   T   
   T   
   T   
   G   
   T   
    A    
   T   
   T   
   C   
   A   
   T   
   C   
   A   
   G   
   C   
   A   
   A   
   G   
   A   
   A   
   T   
   G   
   G   
   A   
   T   
   T   
    G    
    A    
   T   
   C   
   A   
   A   
   G   
   C   
   A   
   A   
   A   
   C   
   A   
   C   
   G   
   A   
   A   
   T   
   G   
   A   
   C   
   A   
   G   
   A   
   G   
   T   
   T   
   C   
   G   
   A   
   A   
   C   
   C   
   A   
   T   
   G   
   G   
   C   
   G   
   C   
   A   
   C   
   C   
   T   
   A   
    T    
   A   
   A   
   G   
   A   
   G   
   C   
   C   
   G   
    T    
   C   
   C   
   G   
   C   
   T   
   C   
   A   
   G   
   C   
   T   
   G   
   T   
   A   
   T   
   G   
   A   
   A   
   G   
   C   
   T   
   A   
   A   
   A   
   G   
   G   
   C   
   G   
   G   
   A   
   A   
   G   
   A   
   A   
   G   
   A   
   T   
   G   
   A   
   A   
   C   
   T   
    T    
   A   
   T   
   T   
   G   
   C   
   T   
   G   
   T   
    A    
   T   
   A   
   T   
   C   
   T   
   G   
   A   
   A   
   A   
   G   
   A   
   A   
   G   
   A   
   T   
   C   
    A    
   G   
   C   
   C   
   A   
   G   
   G   
   C   
   T   
   T   
   A   
   G   
   A   
   T   
   G   
   T   
    G    
   T   
   T   
   C   
   T   
   T   
   A   
   G   
   T   
   A   
   A   
   C   
   G   
    C    
   A   
   T   
   C   
   A   
   A   
   G   
   G   
   A   
   A   
   T   
   G   
   G   
   G   
    C    
   T   
   T   
   G   
   C   
   G   
   C   
   T   
    A    
   T   
   C   
   C   
   T   
   T   
   T   
   A   
   A   
    C    
   A   
   G   
   A   
   T   
   G   
   T   
   T   
   C   
   C   
   C   
   G   
   T   
   T   
   G   
   T   
   T   
   G   
   G   
   A   
   G   
   C   
   A   
   A   
   A   
   G   
   G   
   C   
   A   
   G   
    C    
   T   
   G   
   G   
   A   
   G   
   T   
   G   
   A   
   A   
   G   
   T   
   C   
   T   
   A   
   T   
   G   
   A   
   A   
   T   
   T   
   T   
   G   
   A   
   A   
   A   
   G   
   A   
   T   
   G   
   G   
   A   
   G   
   A   
   T   
   A   
   G   
   G   
    A    
   T   
   T   
    G    
   T   
   C   
   A   
   A   
    T    
   G   
   G   
    G    
   C   
   T   
    G    
   C   
   T   
   T   
   G   
   T   
   G   
   T   
   T   
   A   
   G   
   C   
   A   
   G   
   A   
    C    
   G   
   G   
    A    
   G   
   A   
   T   
   A   
   C   
   G   
   C   
   C   
    A    
   A   
   T   
   C   
   G   
   T   
   A   
   A   
   T   
   C   
   G   
   T   
   T   
   A   
   C   
   T   
   C   
   A   
   G   
   C   
   G   
   A   
   G   
   G   
   A   
   G   
   C   
    C    
   G   
   T   
    G    
   A   
   A   
   A   
   C   
   G   
   C   
   A   
   T   
   G   
   C   
   T   
   T   
   G   
   C   
   T   
   C   
   A   
   A   
   G   
   A   
   A   
   A   
   T   
   C   
   A   
   G   
   C   
   C   
   A   
   A   
   A   
   C   
    T    
   T   
   C   
   A   
   C   
   G   
   T   
   G   
   C   
   G   
   A   
   A   
   G   
   C   
   G   
   G   
   G   
   G   
   A   
   G   
   T   
   G   
   A   
   C   
   G   
   G   
   T   
   C   
   A   
   T   
   G   
   C   
   G   
   T   
   G   
   A   
   G   
   C   
   T   
   G   
   A   
   A   
   A   
   A   
   A   
   A   
   A   
   A   
   T   
   C   
   C   
    G    
   C   
   A   
    C    
   C   
   G   
    T    
   A   
   T   
   T   
   A   
   T   
   C   
   T   
   A   
   T   
   A   
   T   
   G   
   T   
   C   
   G   
   G   
   A   
   A   
   G   
   G   
   A   
   A   
   G   
   A   
   T   
   C   
   A   
   A   
   A   
   A   
   G   
   A   
   A   
   A   
   T   
   A   
   A   
   C   
   C   
   T   
   T   
   G   
   A   
   T   
   G   
   A   
   A   
   T   
   C   
   A   
   A   
   A   
   A   
   A   
   G   
   G   
    A    
   C   
   A   
    A    
   C   
   A   
   G   
    A    
   T   
   A   
   A   
   C   
   G   
   A   
   T   
   T   
   G   
   A   
    T    
   C   
   C   
   A   
   A   
   C   
    G    
   G   
   A   
   T   
   T   
   T   
    T    
   C   
   C   
   T   
   A   
   T   
   A   
   G   
   G   
   T   
   G   
   A   
   T   
   C   
   G   
   A   
   A   
   C   
   A   
   T   
   C   
   G   
   A   
   A   
   T   
   G   
   G   
    T    
   T   
   C   
    G    
   T   
   T   
   T   
   G   
   C   
    T    
   A   
   T   
   G   
   G   
   A   
    C    
   G   
   A   
   G   
   A   
   A   
   A   
   A   
   A   
   A   
   G   
   G   
   T   
   G   
   G   
   A   
   G   
   A   
   A   
   G   
   T   
   C   
   A   
   T   
   C   
   G   
   A   
   A   
   G   
   T   
    T    
   A   
    T    
   C   
   G   
   A   
   T   
   G   
   C   
   T   
   C   
   C   
   T   
   G   
   A   
   G   
    A    
   T   
   T   
   T   
   T   
    C    
   A   
   T   
    T    
   G   
   A   
    A    
    A    
   C   
    A    
   -   
   -   
   -   
 

 
   N54190   
   A   
   T   
   G   
   G   
   A   
   A   
   A   
   A   
   T   
   C   
   G   
   A   
   C   
   A   
   A   
   G   
   A   
   A   
   G   
   T   
   A   
   C   
   A   
   A   
   G   
   A   
   A   
   T   
   T   
   A   
   A   
   C   
   T   
   T   
   T   
   A   
   G   
   A   
   A   
   G   
   A   
   A   
   G   
   T   
   G   
   A   
   T   
   G   
   G   
   G   
   T   
   G   
   A   
   C   
   C   
   G   
   C   
   T   
   T   
   C   
   G   
   G   
   A   
   C   
   G   
   T   
   T   
   A   
   T   
   T   
   C   
   C   
   A   
   A   
   A   
   T   
   A   
   T   
   A   
   T   
   T   
   A   
   T   
    C    
   C   
   A   
    A    
   G   
   A   
   A   
   C   
   G   
   T   
   G   
   C   
   A   
   C   
   T   
   G   
   C   
   C   
   T   
   G   
   A   
   T   
   A   
   T   
    C    
   C   
   G   
   T   
   G   
   A   
   T   
   G   
   G   
   A   
   T   
   T   
   G   
   A   
   A   
    G    
   C   
   C   
   A   
   G   
   T   
    G    
   C   
   A   
   A   
   C   
   G   
   C   
   C   
   G   
   G   
   A   
   T   
   A   
   T   
   T   
   A   
   T   
   T   
    T    
   G   
   C   
    C    
   A   
   T   
   G   
   A   
   A   
   C   
   A   
   A   
   A   
   G   
   A   
   T   
   G   
   G   
   C   
   A   
   A   
   T   
   A   
   C   
   G   
   T   
   A   
   T   
   G   
   A   
   C   
   A   
   A   
   A   
   G   
   G   
   A   
   T   
   T   
    C    
   C   
   G   
   T   
   A   
   A   
   A   
   T   
   C   
   A   
   G   
   C   
   A   
   A   
   A   
   A   
   T   
   C   
   T   
   G   
   T   
   C   
   G   
   G   
   A   
   A   
   A   
   T   
   A   
   T   
   C   
   A   
   T   
   G   
   G   
   G   
    G    
   A   
   A   
   T   
   T   
   A   
   T   
   C   
   A   
   T   
   C   
   C   
   T   
   C   
   A   
   T   
   G   
   G   
   T   
   G   
   A   
   C   
   A   
   G   
   C   
   A   
   G   
   T   
   A   
   T   
   T   
   T   
   A   
   T   
   G   
   A   
   A   
   G   
   C   
   G   
   A   
   T   
   G   
   G   
   T   
   C   
   C   
   G   
   C   
   A   
   T   
   G   
   A   
   G   
   C   
   C   
   A   
   A   
   G   
   A   
    C    
   T   
   G   
   G   
   A   
   A   
   G   
   T   
   T   
   G   
   C   
   G   
   G   
   G   
   A   
   A   
   G   
   T   
   A   
   C   
   T   
   G   
   A   
   T   
   C   
   G   
   A   
    A    
   A   
   T   
   G   
   C   
   A   
   C   
   G   
   G   
    G    
   A   
   A   
   T   
   A   
   A   
    C    
   G   
   G   
   G   
   A   
   G   
   T   
   A   
   T   
   G   
   G   
   A   
   C   
   G   
   G   
   T   
   G   
   A   
   T   
   C   
   C   
   A   
   C   
   C   
   T   
   G   
   C   
   T   
   G   
   C   
   G   
   A   
   T   
   G   
   C   
   G   
   T   
   T   
   A   
   T   
   A   
   C   
   T   
   G   
   A   
   A   
   G   
   C   
   C   
   A   
   G   
    A    
   T   
   T   
   A   
   T   
   C   
   C   
   A   
   A   
   G   
   T   
   T   
   A   
   A   
   G   
   T   
   G   
   G   
   A   
   G   
   A   
   G   
   A   
   T   
   G   
    C    
   T   
   A   
    G    
   C   
   A   
   G   
   A   
   T   
   A   
   T   
   C   
   G   
   A   
    A    
   A   
   A   
   A   
   G   
   A   
   A   
   A   
   C   
   A   
   G   
   T   
    C    
   G   
   A   
   T   
   C   
   T   
   C   
   G   
   T   
   G   
   T   
   G   
   G   
   A   
   A   
   T   
   T   
   T   
   T   
   G   
   A   
   T   
   G   
   A   
   T   
   A   
   C   
   A   
   G   
   A   
   A   
   A   
   A   
   A   
   G   
   A   
   A   
   C   
   C   
   A   
   A   
   C   
   A   
   G   
   T   
   C   
   C   
   T   
   T   
   C   
   C   
   A   
   G   
   C   
   T   
   C   
   G   
   A   
   T   
   A   
   C   
   C   
   C   
   A   
   A   
   A   
   C   
   C   
   T   
   T   
   C   
   T   
    C    
   G   
   T   
   C   
   A   
   A   
   T   
   G   
   G   
   A   
   T   
   C   
   G   
   A   
   C   
   A   
   G   
   G   
   T   
   A   
   T   
   A   
   T   
   C   
   T   
   G   
   C   
   T   
   G   
   G   
   A   
   T   
   A   
   T   
   G   
   C   
    A    
   A   
   C   
   A   
   G   
   A   
   A   
   A   
   T   
   C   
   C   
   C   
   G   
   A   
   C   
    T    
   C   
   A   
   T   
   A   
   A   
   C   
   T   
   T   
   A   
   G   
   C   
   T   
   G   
   A   
   A   
   G   
   T   
   G   
   A   
   T   
    T    
   G   
   A   
    T    
   G   
   G   
   C   
   A   
   C   
   A   
   A   
   T   
   C   
   T   
   A   
   T   
   A   
   T   
   G   
   A   
   T   
   C   
   G   
   A   
   T   
   C   
   A   
   T   
   C   
   C   
   A   
   C   
   A   
   A   
   G   
   C   
   G   
   A   
   G   
   C   
   C   
   T   
   T   
   G   
   A   
   A   
   A   
   A   
   A   
   T   
   T   
   G   
   A   
   T   
   G   
   G   
   A   
   A   
   T   
   A   
   T   
   A   
   T   
   T   
   C   
   C   
   T   
   G   
   G   
   A   
   C   
   C   
   T   
   G   
   A   
   C   
   T   
   T   
    T    
   C   
   C   
   A   
   A   
   C   
   A   
   G   
   G   
   A   
   G   
   G   
    G    
   A   
   T   
   C   
   T   
   T   
   G   
   C   
   A   
   A   
   G   
   G   
    A    
   A   
   A   
   A   
   G   
   A   
   T   
   G   
   A   
   A   
   A   
   T   
   C   
   A   
   A   
   A   
   A   
   A   
   A   
   G   
   C   
   T   
   T   
   A   
   T   
   G   
   A   
   A   
   A   
   C   
   A   
   G   
   G   
    G    
   C   
   G   
   A   
   G   
   G   
   A   
   A   
   A   
   A   
   G   
   T   
   T   
   A   
    T    
   T   
   C   
   T   
   T   
   C   
   G   
   T   
   T   
   C   
   G   
   A   
   A   
   A   
   A   
   C   
   A   
   A   
   A   
   G   
   A   
   T   
   A   
   G   
   A   
    G    
   G   
   C   
   A   
   A   
   T   
   G   
   A   
   A   
   G   
   G   
   G   
    T    
   A   
   A   
    T    
   A   
   A   
   A   
   C   
   A   
   G   
   C   
   A   
   A   
   A   
   T   
   C   
   G   
   T   
   G   
   A   
   T   
   C   
   A   
   C   
    A    
   G   
   A   
   A   
   A   
   T   
   C   
   C   
   C   
   A   
   T   
   A   
   T   
   G   
   A   
   A   
   G   
   T   
   A   
   A   
   A   
   T   
   A   
   A   
   A   
   G   
   C   
   G   
   A   
   C   
    C    
   T   
   T   
   G   
   G   
   T   
   G   
   A   
   A   
   A   
   A   
   A   
   A   
   A   
   T   
   G   
   G   
   A   
   T   
   G   
   A   
   G   
   A   
   T   
   C   
   C   
   G   
   T   
   T   
   T   
   G   
   A   
   A   
   T   
   A   
   A   
   G   
   A   
   A   
   A   
   A   
   T   
   C   
   G   
   A   
    T    
   G   
   G   
   A   
   A   
   T   
   A   
   G   
   C   
   T   
   G   
   A   
   A   
   G   
   T   
    A    
   C   
   G   
   A   
   G   
   A   
   T   
   G   
   A   
   A   
   A   
   G   
   T   
   G   
   A   
   T   
    A    
   G   
    A    
   A   
   C   
    A    
   G   
   G   
   T   
   C   
   T   
   G   
   C   
   A   
   A   
   A   
   T   
   T   
   G   
   T   
   C   
   G   
   T   
    C    
   G   
   A   
   A   
   T   
   T   
   G   
   A   
   A   
   A   
   A   
   A   
   A   
   G   
   A   
    C    
   G   
   C   
   C   
   A   
   A   
    T    
   G   
   C   
   T   
   C   
   A   
   A   
   G   
   G   
    A    
   A   
   T   
   T   
   T   
   T   
   G   
   A   
   A   
   T   
   T   
   A   
    T    
   T   
   T   
   G   
   T   
   T   
   C   
   A   
   A   
   A   
   A   
   A   
   C   
   A   
   C   
   T   
   G   
   A   
   G   
    C    
   T   
   A   
   C   
   A   
   G   
   A   
   T   
   C   
   A   
   A   
   T   
   T   
   A   
   C   
   A   
   A   
   T   
   T   
   T   
    C    
   A   
   A   
   T   
   A   
   T   
   G   
   G   
   T   
   C   
   G   
   C   
   T   
   A   
   T   
   C   
   G   
   A   
    C    
   C   
   A   
   T   
   A   
   T   
   G   
   A   
   C   
   T   
   C   
   C   
   G   
   C   
   A   
    T    
   C   
   A   
   A   
   G   
   T   
   T   
   G   
   G   
   A   
   C   
   T   
   G   
   A   
   A   
   A   
   G   
   A   
   C   
   A   
   T   
   T   
   T   
   T   
   A   
   A   
   G   
   A   
   A   
   G   
   T   
   T   
   A   
   T   
   A   
   T   
   C   
   G   
   A   
   G   
   C   
   A   
   T   
   C   
   G   
   A   
   A   
   A   
   G   
   C   
   A   
   A   
   G   
   T   
   C   
   A   
   T   
   A   
   A   
   C   
   A   
   A   
   A   
   A   
   C   
   G   
    T    
   A   
   G   
    T    
   C   
   A   
   A   
   T   
   T   
   C   
   G   
   A   
   T   
   T   
   T   
   A   
   G   
   C   
   C   
   A   
   A   
   A   
   G   
   C   
   T   
   C   
   A   
   A   
   A   
   A   
   A   
    A    
   G   
   A   
   C   
   A   
   G   
   C   
   A   
   T   
   A   
   T   
   C   
   G   
   T   
    A    
   G   
   A   
   A   
   G   
   G   
    A    
    T    
   T   
   G   
   A   
   T   
   G   
   A   
   A   
   A   
   G   
   C   
   T   
   T   
   T   
   A   
   T   
   C   
   G   
   A   
   T   
   T   
   C   
   T   
   G   
   G   
   A   
   T   
   G   
   A   
   A   
   G   
   T   
   A   
   A   
   T   
   C   
   G   
    T    
   A   
   A   
   C   
   G   
   A   
   T   
   T   
   C   
   G   
   T   
   G   
   A   
   A   
   A   
   G   
   T   
   A   
   A   
   A   
   G   
   A   
   C   
   A   
   A   
   A   
   A   
   A   
   A   
   G   
   A   
    C    
   G   
   C   
    C    
   A   
   A   
   A   
   A   
   A   
   G   
   A   
   A   
   T   
   T   
   T   
   A   
   G   
   T   
   C   
   G   
   A   
    C    
   G   
   T   
    C    
   T   
   T   
    T    
   C   
   A   
   A   
   T   
   T   
   T   
   A   
   C   
   A   
   G   
   A   
   A   
   G   
   A   
   A   
   C   
   A   
   A   
   G   
   C   
   A   
   G   
   A   
   A   
   G   
   C   
   A   
   A   
   T   
    T    
   G   
   T   
   C   
   A   
   C   
   A   
   T   
   T   
   G   
   C   
   A   
   A   
    T    
   T   
   A   
   T   
   A   
    C    
   C   
   G   
   T   
   T   
   T   
   G   
   A   
   C   
   G   
   A   
   A   
    C    
   A   
   C   
    G    
   G   
   A   
   T   
   A   
   T   
   C   
   A   
   C   
   A   
   G   
   A   
   G   
   C   
   T   
   T   
   C   
   A   
   A   
   A   
   A   
   G   
   G   
   A   
   A   
    T    
   C   
    C    
   G   
   A   
   A   
   A   
    G    
   C   
   T   
   T   
   G   
   A   
   T   
   T   
   G   
   C   
    G    
   C   
   A   
   A   
   A   
   T   
   C   
   A   
   C   
   A   
   G   
   A   
   G   
   T   
   T   
    G    
   A   
   A   
   T   
   A   
   A   
   A   
   A   
   T   
   C   
   C   
   T   
   T   
   T   
   C   
   A   
   A   
   A   
    T    
   G   
   A   
   C   
   A   
   A   
   A   
   G   
   A   
    G    
   C   
   T   
   T   
   T   
   T   
    C    
   T   
   C   
   T   
   G   
   T   
   T   
   A   
   T   
   G   
   A   
   A   
   A   
   A   
   A   
   A   
   G   
   A   
   A   
   T   
   T   
   G   
   C   
   G   
   A   
   G   
   A   
   A   
   G   
   T   
   G   
   A   
   A   
   A   
   A   
   A   
   A   
   A   
   A   
   T   
   T   
   A   
    T    
   T   
   C   
   T   
   T   
   C   
   C   
   G   
   C   
   T   
   C   
   G   
   T   
   C   
    T    
    A    
   A   
   C   
   G   
   A   
   C   
   A   
   A   
   T   
   C   
    G    
   A   
   A   
   G   
   A   
   C   
   G   
   A   
   A   
   A   
   T   
   C   
   G   
   A   
   A   
   G   
   A   
   A   
   A   
   T   
   C   
   A   
   A   
   A   
   A   
   T   
    T    
   G   
   A   
   T   
   A   
   C   
   G   
   C   
   A   
   A   
    G    
   T   
    C    
   T   
   T   
   A   
   G   
   T   
   G   
   G   
   C   
    A    
   C   
   A   
   A   
   G   
   A   
   A   
   G   
   A   
   C   
   G   
   T   
   A   
   A   
   T   
    T    
   G   
   T   
   C   
   A   
   G   
   T   
   G   
   T   
    G    
   A   
   C   
    G    
   C   
   G   
   A   
   G   
   A   
   A   
   G   
   G   
   C   
   T   
   A   
   T   
   G   
   T   
   G   
   A   
   A   
   G   
   C   
   G   
   A   
   A   
   C   
   A   
   A   
   G   
   C   
   C   
   T   
   T   
   C   
   G   
   T   
   T   
   C   
    T    
   T   
   A   
   T   
   A   
   G   
   T   
   G   
   C   
   T   
   T   
   C   
    C    
   A   
   A   
   A   
   C   
   C   
   A   
   G   
   A   
   A   
   G   
   A   
   A   
   A   
   T   
   C   
   G   
   G   
   G   
   A   
   T   
   G   
   C   
   G   
   A   
   G   
   A   
   A   
   G   
   G   
   C   
   G   
   A   
    C    
   T   
   A   
   C   
   T   
   T   
   G   
   C   
   T   
    A    
   T   
   A   
   C   
    T    
   C   
   A   
   G   
   G   
   T   
   G   
   A   
   A   
   T   
   T   
    G    
   A   
   G   
    T    
   A   
   C   
   A   
    T    
   T   
   G   
   G   
   A   
   C   
   C   
   A   
   T   
   G   
   T   
   T   
   C   
   T   
   T   
   C   
   T   
   G   
   A   
   T   
    C    
   A   
   C   
    A    
   A   
   A   
   T   
   A   
   A   
   A   
   G   
   C   
   A   
   A   
   A   
   T   
   G   
   T   
   G   
   A   
   T   
   C   
   T   
   A   
   C   
   C   
   G   
    C    
   C   
   C   
   T   
   G   
   T   
    C    
   C   
   A   
   T   
   G   
   A   
   A   
   C   
   T   
   A   
   C   
   C   
    C    
   G   
   A   
   T   
   T   
   T   
   G   
   A   
   A   
   A   
   T   
   G   
   G   
   A   
   A   
   A   
   G   
   A   
   T   
   G   
   C   
   T   
   G   
   G   
    T    
   G   
   A   
    G    
   C   
   A   
    C    
   A   
   T   
   T   
   T   
   C   
   T   
   C   
   A   
   A   
   A   
   C   
   A   
   A   
   T   
   C   
   T   
   C   
   C   
   A   
   A   
   T   
   C   
   T   
   G   
   T   
   C   
   T   
   G   
   T   
   A   
   G   
   A   
    C    
   G   
   A   
   A   
   T   
   C   
   G   
   A   
   T   
    T    
   T   
   T   
   A   
   G   
   C   
   T   
   G   
   T   
   T   
   T   
   T   
    C    
   C   
   C   
   A   
   T   
   A   
   T   
   C   
   A   
   A   
   A   
   A   
   A   
   A   
   T   
    T    
   C   
   A   
   A   
   G   
   C   
   A   
   G   
   A   
    G    
   A   
   A   
   A   
   A   
   C   
   A   
   T   
   T   
   T   
   G   
   T   
    A    
   T   
   T   
   C   
   A   
   T   
   C   
   A   
   G   
   C   
   A   
   A   
   G   
   A   
   A   
   T   
   G   
   G   
   A   
   T   
   T   
    G    
    A    
   T   
   C   
   A   
   A   
   G   
   C   
   A   
   A   
   A   
   C   
   A   
   C   
   G   
   A   
   A   
   T   
   G   
   A   
   C   
   A   
   G   
   A   
   G   
   T   
   T   
   C   
   G   
   A   
   A   
   C   
   C   
   A   
   T   
   G   
   G   
   C   
   G   
   C   
   A   
   C   
   C   
   T   
   A   
    T    
   A   
   A   
   G   
   A   
   G   
   C   
   C   
   G   
    T    
   C   
   C   
   G   
   C   
   T   
   C   
   A   
   G   
   C   
   T   
   G   
   T   
   A   
   T   
   G   
   A   
   A   
   G   
   C   
   T   
   A   
   A   
   A   
   G   
   G   
   C   
   G   
   G   
   A   
   A   
   G   
   A   
   A   
   G   
   A   
   T   
   G   
   A   
   A   
   C   
   T   
    G    
   A   
   T   
   T   
   G   
   C   
   T   
   G   
   T   
    G    
   T   
   A   
   T   
   C   
   T   
   G   
   A   
   A   
   A   
   G   
   A   
   A   
   G   
   A   
   T   
   C   
    A    
   G   
   C   
   C   
   A   
   G   
   G   
   C   
   T   
   T   
   A   
   G   
   A   
   T   
   G   
   T   
    T    
   T   
   T   
   C   
   T   
   T   
   A   
   G   
   T   
   A   
   A   
   C   
   G   
    C    
   A   
   T   
   C   
   A   
   A   
   G   
   G   
   A   
   A   
   T   
   G   
   G   
   G   
    T    
   T   
   T   
   G   
   C   
   G   
   C   
   T   
    A    
   T   
   C   
   C   
   T   
   T   
   T   
   A   
   A   
    C    
   A   
   G   
   A   
   T   
   G   
   T   
   T   
   C   
   C   
   C   
   G   
   T   
   T   
   G   
   T   
   T   
   G   
   G   
   A   
   G   
   C   
   A   
   A   
   A   
   G   
   G   
   C   
   A   
   G   
    C    
   T   
   G   
   G   
   A   
   G   
   T   
   G   
   A   
   A   
   G   
   T   
   C   
   T   
   A   
   T   
   G   
   A   
   A   
   T   
   T   
   T   
   G   
   A   
   A   
   A   
   G   
   A   
   T   
   G   
   G   
   A   
   G   
   A   
   T   
   A   
   G   
   G   
    A    
   T   
   T   
    A    
   T   
   C   
   A   
   A   
    C    
   G   
   G   
    G    
   C   
   T   
    G    
   C   
   T   
   T   
   G   
   T   
   G   
   T   
   T   
   A   
   G   
   C   
   A   
   G   
   A   
    A    
   G   
   G   
    T    
   G   
   A   
   T   
   A   
   C   
   G   
   C   
   C   
    G    
   A   
   T   
   C   
   G   
   T   
   A   
   A   
   T   
   C   
   G   
   T   
   T   
   A   
   C   
   T   
   C   
   A   
   G   
   C   
   G   
   A   
   G   
   G   
   A   
   G   
   C   
    T    
   G   
   T   
    T    
   A   
   A   
   A   
   C   
   G   
   C   
   A   
   T   
   G   
   C   
   T   
   T   
   G   
   C   
   T   
   C   
   A   
   A   
   G   
   A   
   A   
   A   
   T   
   C   
   A   
   G   
   C   
   C   
   A   
   A   
   A   
   C   
    T    
   T   
   C   
   A   
   C   
   G   
   T   
   G   
   C   
   G   
   A   
   A   
   G   
   C   
   G   
   G   
   G   
   G   
   A   
   G   
   T   
   G   
   A   
   C   
   G   
   G   
   T   
   C   
   A   
   T   
   G   
   C   
   G   
   T   
   G   
   A   
   G   
   C   
   T   
   G   
   A   
   A   
   A   
   A   
   A   
   A   
   A   
   A   
   T   
   C   
   C   
    G    
   C   
   A   
    C    
   C   
   G   
    T    
   A   
   T   
   T   
   A   
   T   
   C   
   T   
   A   
   T   
   A   
   T   
   G   
   T   
   C   
   G   
   G   
   A   
   A   
   G   
   G   
   A   
   A   
   G   
   A   
   T   
   C   
   A   
   A   
   A   
   A   
   G   
   A   
   A   
   A   
   T   
   A   
   A   
   C   
   C   
   T   
   T   
   G   
   A   
   T   
   G   
   A   
   A   
   T   
   C   
   A   
   A   
   A   
   A   
   A   
   G   
   G   
    G    
   C   
   A   
    G    
   C   
   A   
   G   
    T    
   T   
   A   
   A   
   C   
   G   
   A   
   T   
   T   
   G   
   A   
    C    
   C   
   C   
   A   
   A   
   C   
    T    
   G   
   A   
   T   
   T   
   T   
    C    
   C   
   C   
   T   
   A   
   T   
   A   
   G   
   G   
   T   
   G   
   A   
   T   
   C   
   G   
   A   
   A   
   C   
   A   
   T   
   C   
   G   
   A   
   A   
   T   
   G   
   G   
    A    
   T   
   C   
    A    
   T   
   T   
   T   
   G   
   C   
    G    
   A   
   T   
   G   
   G   
   A   
    T    
   G   
   A   
   G   
   A   
   A   
   A   
   A   
   A   
   A   
   G   
   G   
   T   
   G   
   G   
   A   
   G   
   A   
   A   
   G   
   T   
   C   
   A   
   T   
   C   
   G   
   A   
   A   
   G   
   T   
    T    
   A   
    T    
   C   
   G   
   A   
   T   
   G   
   C   
   T   
   C   
   C   
   T   
   G   
   A   
   G   
    A    
   T   
   T   
   T   
   T   
    C    
   A   
   T   
    C    
   G   
   A   
    G    
    T    
   C   
    G    
   -   
   -   
   -   
 

 
   N59492F   
   A   
   T   
   G   
   G   
   A   
   A   
   A   
   A   
   T   
   C   
   G   
   A   
   C   
   A   
   A   
   G   
   A   
   A   
   G   
   T   
   A   
   C   
   A   
   A   
   G   
   A   
   A   
   T   
   T   
   A   
   A   
   C   
   T   
   T   
   T   
   A   
   G   
   A   
   A   
   G   
   A   
   A   
   G   
   T   
   G   
   A   
   T   
   G   
   G   
   G   
   T   
   G   
   A   
   C   
   C   
   G   
   C   
   T   
   T   
   C   
   G   
   G   
   A   
   C   
   G   
   T   
   T   
   A   
   T   
   T   
   C   
   C   
   A   
   A   
   A   
   T   
   A   
   T   
   A   
   T   
   T   
   A   
   T   
    C    
   C   
   A   
    A    
   G   
   A   
   A   
   C   
   G   
   T   
   G   
   C   
   A   
   C   
   T   
   G   
   C   
   C   
   T   
   G   
   A   
   T   
   A   
   T   
    T    
   C   
   G   
   T   
   G   
   A   
   T   
   G   
   G   
   A   
   T   
   T   
   G   
   A   
   A   
    G    
   C   
   C   
   A   
   G   
   T   
    G    
   C   
   A   
   A   
   C   
   G   
   C   
   C   
   G   
   G   
   A   
   T   
   A   
   T   
   T   
   A   
   T   
   T   
    T    
   G   
   C   
    C    
   A   
   T   
   G   
   A   
   A   
   C   
   A   
   A   
   A   
   G   
   A   
   T   
   G   
   G   
   C   
   A   
   A   
   T   
   A   
   C   
   G   
   T   
   A   
   T   
   G   
   A   
   C   
   A   
   A   
   A   
   G   
   G   
   A   
   T   
   T   
    C    
   C   
   G   
   T   
   A   
   A   
   A   
   T   
   C   
   A   
   G   
   C   
   A   
   A   
   A   
   A   
   T   
   C   
   T   
   G   
   T   
   C   
   G   
   G   
   A   
   A   
   A   
   T   
   A   
   T   
   C   
   A   
   T   
   G   
   G   
   G   
    G    
   A   
   A   
   T   
   T   
   A   
   T   
   C   
   A   
   T   
   C   
   C   
   T   
   C   
   A   
   T   
   G   
   G   
   T   
   G   
   A   
   C   
   A   
   G   
   C   
   A   
   G   
   T   
   A   
   T   
   T   
   T   
   A   
   T   
   G   
   A   
   A   
   G   
   C   
   G   
   A   
   T   
   G   
   G   
   T   
   C   
   C   
   G   
   C   
   A   
   T   
   G   
   A   
   G   
   C   
   C   
   A   
   A   
   G   
   A   
    C    
   T   
   G   
   G   
   A   
   A   
   G   
   T   
   T   
   G   
   C   
   G   
   G   
   G   
   A   
   A   
   G   
   T   
   A   
   C   
   T   
   G   
   A   
   T   
   C   
   G   
   A   
    A    
   A   
   T   
   G   
   C   
   A   
   C   
   G   
   G   
    G    
   A   
   A   
   T   
   A   
   A   
    C    
   G   
   G   
   G   
   A   
   G   
   T   
   A   
   T   
   G   
   G   
   A   
   C   
   G   
   G   
   T   
   G   
   A   
   T   
   C   
   C   
   A   
   C   
   C   
   T   
   G   
   C   
   T   
   G   
   C   
   G   
   A   
   T   
   G   
   C   
   G   
   T   
   T   
   A   
   T   
   A   
   C   
   T   
   G   
   A   
   A   
   G   
   C   
   C   
   A   
   G   
    A    
   T   
   T   
   A   
   T   
   C   
   C   
   A   
   A   
   G   
   T   
   T   
   A   
   A   
   G   
   T   
   G   
   G   
   A   
   G   
   A   
   G   
   A   
   T   
   G   
    C    
   T   
   A   
    G    
   C   
   A   
   G   
   A   
   T   
   A   
   T   
   C   
   G   
   A   
    A    
   A   
   A   
   A   
   G   
   A   
   A   
   A   
   C   
   A   
   G   
   T   
    C    
   G   
   A   
   T   
   C   
   T   
   C   
   G   
   T   
   G   
   T   
   G   
   G   
   A   
   A   
   T   
   T   
   T   
   T   
   G   
   A   
   T   
   G   
   A   
   T   
   A   
   C   
   A   
   G   
   A   
   A   
   A   
   A   
   A   
   G   
   A   
   A   
   C   
   C   
   A   
   A   
   C   
   A   
   G   
   T   
   C   
   C   
   T   
   T   
   C   
   C   
   A   
   G   
   C   
   T   
   C   
   G   
   A   
   T   
   A   
   C   
   C   
   C   
   A   
   A   
   A   
   C   
   C   
   T   
   T   
   C   
   T   
    C    
   G   
   T   
   C   
   A   
   A   
   T   
   G   
   G   
   A   
   T   
   C   
   G   
   A   
   C   
   A   
   G   
   G   
   T   
   A   
   T   
   A   
   T   
   C   
   T   
   G   
   C   
   T   
   G   
   G   
   A   
   T   
   A   
   T   
   G   
   C   
    A    
   A   
   C   
   A   
   G   
   A   
   A   
   A   
   T   
   C   
   C   
   C   
   G   
   A   
   C   
    T    
   C   
   A   
   T   
   A   
   A   
   C   
   T   
   T   
   A   
   G   
   C   
   T   
   G   
   A   
   A   
   G   
   T   
   G   
   A   
   T   
    C    
   G   
   A   
    T    
   G   
   G   
   C   
   A   
   C   
   A   
   A   
   T   
   C   
   T   
   A   
   T   
   A   
   T   
   G   
   A   
   T   
   C   
   G   
   A   
   T   
   C   
   A   
   T   
   C   
   C   
   A   
   C   
   A   
   A   
   G   
   C   
   G   
   A   
   G   
   C   
   C   
   T   
   T   
   G   
   A   
   A   
   A   
   A   
   A   
   T   
   T   
   G   
   A   
   T   
   G   
   G   
   A   
   A   
   T   
   A   
   T   
   A   
   T   
   T   
   C   
   C   
   T   
   G   
   G   
   A   
   C   
   C   
   T   
   G   
   A   
   C   
   T   
   T   
    T    
   C   
   C   
   A   
   A   
   C   
   A   
   G   
   G   
   A   
   G   
   G   
    G    
   A   
   T   
   C   
   T   
   T   
   G   
   C   
   A   
   A   
   G   
   G   
    A    
   A   
   A   
   A   
   G   
   A   
   T   
   G   
   A   
   A   
   A   
   T   
   C   
   A   
   A   
   A   
   A   
   A   
   A   
   G   
   C   
   T   
   T   
   A   
   T   
   G   
   A   
   A   
   A   
   C   
   A   
   G   
   G   
    G    
   C   
   G   
   A   
   G   
   G   
   A   
   A   
   A   
   A   
   G   
   T   
   T   
   A   
    T    
   T   
   C   
   T   
   T   
   C   
   G   
   T   
   T   
   C   
   G   
   A   
   A   
   A   
   A   
   C   
   A   
   A   
   A   
   G   
   A   
   T   
   A   
   G   
   A   
    A    
   G   
   C   
   A   
   A   
   T   
   G   
   A   
   A   
   G   
   G   
   G   
    T    
   A   
   A   
    T    
   A   
   A   
   A   
   C   
   A   
   G   
   C   
   A   
   A   
   A   
   T   
   C   
   G   
   T   
   G   
   A   
   T   
   C   
   A   
   C   
    A    
   G   
   A   
   A   
   A   
   T   
   C   
   C   
   C   
   A   
   T   
   A   
   T   
   G   
   A   
   A   
   G   
   T   
   A   
   A   
   A   
   T   
   A   
   A   
   A   
   G   
   C   
   G   
   A   
   C   
    C    
   T   
   T   
   G   
   G   
   T   
   G   
   A   
   A   
   A   
   A   
   A   
   A   
   A   
   T   
   G   
   G   
   A   
   T   
   G   
   A   
   G   
   A   
   T   
   C   
   C   
   G   
   T   
   T   
   T   
   G   
   A   
   A   
   T   
   A   
   A   
   G   
   A   
   A   
   A   
   A   
   T   
   C   
   G   
   A   
    T    
   G   
   G   
   A   
   A   
   T   
   A   
   G   
   C   
   T   
   G   
   A   
   A   
   G   
   T   
    A    
   C   
   G   
   A   
   G   
   A   
   T   
   G   
   A   
   A   
   A   
   G   
   T   
   G   
   A   
   T   
    A    
   G   
    A    
   A   
   C   
    A    
   G   
   G   
   T   
   C   
   T   
   G   
   C   
   A   
   A   
   A   
   T   
   T   
   G   
   T   
   C   
   G   
   T   
    C    
   G   
   A   
   A   
   T   
   T   
   G   
   A   
   A   
   A   
   A   
   A   
   A   
   G   
   A   
    C    
   G   
   C   
   C   
   A   
   A   
    T    
   G   
   C   
   T   
   C   
   A   
   A   
   G   
   G   
    A    
   A   
   T   
   T   
   T   
   T   
   G   
   A   
   A   
   T   
   T   
   A   
    T    
   T   
   T   
   G   
   T   
   T   
   C   
   A   
   A   
   A   
   A   
   A   
   C   
   A   
   C   
   T   
   G   
   A   
   G   
    C    
   T   
   A   
   C   
   A   
   G   
   A   
   T   
   C   
   A   
   A   
   T   
   T   
   A   
   C   
   A   
   A   
   T   
   T   
   T   
    C    
   A   
   A   
   T   
   A   
   T   
   G   
   G   
   T   
   C   
   G   
   C   
   T   
   A   
   T   
   C   
   G   
   A   
    C    
   C   
   A   
   T   
   A   
   T   
   G   
   A   
   C   
   T   
   C   
   C   
   G   
   C   
   A   
    T    
   C   
   A   
   A   
   G   
   T   
   T   
   G   
   G   
   A   
   C   
   T   
   G   
   A   
   A   
   A   
   G   
   A   
   C   
   A   
   T   
   T   
   T   
   T   
   A   
   A   
   G   
   A   
   A   
   G   
   T   
   T   
   A   
   T   
   A   
   T   
   C   
   G   
   A   
   G   
   C   
   A   
   T   
   C   
   G   
   A   
   A   
   A   
   G   
   C   
   A   
   A   
   G   
   T   
   C   
   A   
   T   
   A   
   A   
   C   
   A   
   A   
   A   
   A   
   C   
   G   
    T    
   A   
   G   
    T    
   C   
   A   
   A   
   T   
   T   
   C   
   G   
   A   
   T   
   T   
   T   
   A   
   G   
   C   
   C   
   A   
   A   
   A   
   G   
   C   
   T   
   C   
   A   
   A   
   A   
   A   
   A   
    A    
   G   
   A   
   C   
   A   
   G   
   C   
   A   
   T   
   A   
   T   
   C   
   G   
   T   
    A    
   G   
   A   
   A   
   G   
   G   
    A    
    T    
   T   
   G   
   A   
   T   
   G   
   A   
   A   
   A   
   G   
   C   
   T   
   T   
   T   
   A   
   T   
   C   
   G   
   A   
   T   
   T   
   C   
   T   
   G   
   G   
   A   
   T   
   G   
   A   
   A   
   G   
   T   
   A   
   A   
   T   
   C   
   G   
    C    
   A   
   A   
   C   
   G   
   A   
   T   
   T   
   C   
   G   
   T   
   G   
   A   
   A   
   A   
   G   
   T   
   A   
   A   
   A   
   G   
   A   
   C   
   A   
   A   
   A   
   A   
   A   
   A   
   G   
   A   
    C    
   G   
   C   
    C    
   A   
   A   
   A   
   A   
   A   
   G   
   A   
   A   
   T   
   T   
   T   
   A   
   G   
   T   
   C   
   G   
   A   
    C    
   G   
   T   
    C    
   T   
   T   
    T    
   C   
   A   
   A   
   T   
   T   
   T   
   A   
   C   
   A   
   G   
   A   
   A   
   G   
   A   
   A   
   C   
   A   
   A   
   G   
   C   
   A   
   G   
   A   
   A   
   G   
   C   
   A   
   A   
   T   
    T    
   G   
   T   
   C   
   A   
   C   
   A   
   T   
   T   
   G   
   C   
   A   
   A   
    T    
   T   
   A   
   T   
   A   
    C    
   C   
   G   
   T   
   T   
   T   
   G   
   A   
   C   
   G   
   A   
   A   
    C    
   A   
   C   
    G    
   G   
   A   
   T   
   A   
   T   
   C   
   A   
   C   
   A   
   G   
   A   
   G   
   C   
   T   
   T   
   C   
   A   
   A   
   A   
   A   
   G   
   G   
   A   
   A   
    T    
   C   
    C    
   G   
   A   
   A   
   A   
    G    
   C   
   T   
   T   
   G   
   A   
   T   
   T   
   G   
   C   
    G    
   C   
   A   
   A   
   A   
   T   
   C   
   A   
   C   
   A   
   G   
   A   
   G   
   T   
   T   
    G    
   A   
   A   
   T   
   A   
   A   
   A   
   A   
   T   
   C   
   C   
   T   
   T   
   T   
   C   
   A   
   A   
   A   
    T    
   G   
   A   
   C   
   A   
   A   
   A   
   G   
   A   
    G    
   C   
   T   
   T   
   T   
   T   
    C    
   T   
   C   
   T   
   G   
   T   
   T   
   A   
   T   
   G   
   A   
   A   
   A   
   A   
   A   
   A   
   G   
   A   
   A   
   T   
   T   
   G   
   C   
   G   
   A   
   G   
   A   
   A   
   G   
   T   
   G   
   A   
   A   
   A   
   A   
   A   
   A   
   A   
   A   
   T   
   T   
   A   
    T    
   T   
   C   
   T   
   T   
   C   
   C   
   G   
   C   
   T   
   C   
   G   
   T   
   C   
    T    
    A    
   A   
   C   
   G   
   A   
   C   
   A   
   A   
   T   
   C   
    G    
   A   
   A   
   G   
   A   
   C   
   G   
   A   
   A   
   A   
   T   
   C   
   G   
   A   
   A   
   G   
   A   
   A   
   A   
   T   
   C   
   A   
   A   
   A   
   A   
   T   
    T    
   G   
   A   
   T   
   A   
   C   
   G   
   C   
   A   
   A   
    G    
   T   
    C    
   T   
   T   
   A   
   G   
   T   
   G   
   G   
   C   
    A    
   C   
   A   
   A   
   G   
   A   
   A   
   G   
   A   
   C   
   G   
   T   
   A   
   A   
   T   
    T    
   G   
   T   
   C   
   A   
   G   
   T   
   G   
   T   
    G    
   A   
   C   
    G    
   C   
   G   
   A   
   G   
   A   
   A   
   G   
   G   
   C   
   T   
   A   
   T   
   G   
   T   
   G   
   A   
   A   
   G   
   C   
   G   
   A   
   A   
   C   
   A   
   A   
   G   
   C   
   C   
   T   
   T   
   C   
   G   
   T   
   T   
   C   
    T    
   T   
   A   
   T   
   A   
   G   
   T   
   G   
   C   
   T   
   T   
   C   
    C    
   A   
   A   
   A   
   C   
   C   
   A   
   G   
   A   
   A   
   G   
   A   
   A   
   A   
   T   
   C   
   G   
   G   
   G   
   A   
   T   
   G   
   C   
   G   
   A   
   G   
   A   
   A   
   G   
   G   
   C   
   G   
   A   
    C    
   T   
   A   
   C   
   T   
   T   
   G   
   C   
   T   
    A    
   T   
   A   
   C   
    T    
   C   
   A   
   G   
   G   
   T   
   G   
   A   
   A   
   T   
   T   
    G    
   A   
   G   
    T    
   A   
   C   
   A   
    T    
   T   
   G   
   G   
   A   
   C   
   C   
   A   
   T   
   G   
   T   
   T   
   C   
   T   
   T   
   C   
   T   
   G   
   A   
   T   
    C    
   A   
   C   
    A    
   A   
   A   
   T   
   A   
   A   
   A   
   G   
   C   
   A   
   A   
   A   
   T   
   G   
   T   
   G   
   A   
   T   
   C   
   T   
   A   
   C   
   C   
   G   
    C    
   C   
   C   
   T   
   G   
   T   
    C    
   C   
   A   
   T   
   G   
   A   
   A   
   C   
   T   
   A   
   C   
   C   
    C    
   G   
   A   
   T   
   T   
   T   
   G   
   A   
   A   
   A   
   T   
   G   
   G   
   A   
   A   
   A   
   G   
   A   
   T   
   G   
   C   
   T   
   G   
   G   
    T    
   G   
   A   
    G    
   C   
   A   
    C    
   A   
   T   
   T   
   T   
   C   
   T   
   C   
   A   
   A   
   A   
   C   
   A   
   A   
   T   
   C   
   T   
   C   
   C   
   A   
   A   
   T   
   C   
   T   
   G   
   T   
   C   
   T   
   G   
   T   
   A   
   G   
   A   
    C    
   G   
   A   
   A   
   T   
   C   
   G   
   A   
   T   
    T    
   T   
   T   
   A   
   G   
   C   
   T   
   G   
   T   
   T   
   T   
   T   
    C    
   C   
   C   
   A   
   T   
   A   
   T   
   C   
   A   
   A   
   A   
   A   
   A   
   A   
   T   
    T    
   C   
   A   
   A   
   G   
   C   
   A   
   G   
   A   
    G    
   A   
   A   
   A   
   A   
   C   
   A   
   T   
   T   
   T   
   G   
   T   
    A    
   T   
   T   
   C   
   A   
   T   
   C   
   A   
   G   
   C   
   A   
   A   
   G   
   A   
   A   
   T   
   G   
   G   
   A   
   T   
   T   
    G    
    A    
   T   
   C   
   A   
   A   
   G   
   C   
   A   
   A   
   A   
   C   
   A   
   C   
   G   
   A   
   A   
   T   
   G   
   A   
   C   
   A   
   G   
   A   
   G   
   T   
   T   
   C   
   G   
   A   
   A   
   C   
   C   
   A   
   T   
   G   
   G   
   C   
   G   
   C   
   A   
   C   
   C   
   T   
   A   
    T    
   A   
   A   
   G   
   A   
   G   
   C   
   C   
   G   
    T    
   C   
   C   
   G   
   C   
   T   
   C   
   A   
   G   
   C   
   T   
   G   
   T   
   A   
   T   
   G   
   A   
   A   
   G   
   C   
   T   
   A   
   A   
   A   
   G   
   G   
   C   
   G   
   G   
   A   
   A   
   G   
   A   
   A   
   G   
   A   
   T   
   G   
   A   
   A   
   C   
   T   
    G    
   A   
   T   
   T   
   G   
   C   
   T   
   G   
   T   
    G    
   T   
   A   
   T   
   C   
   T   
   G   
   A   
   A   
   A   
   G   
   A   
   A   
   G   
   A   
   T   
   C   
    A    
   G   
   C   
   C   
   A   
   G   
   G   
   C   
   T   
   T   
   A   
   G   
   A   
   T   
   G   
   T   
    T    
   T   
   T   
   C   
   T   
   T   
   A   
   G   
   T   
   A   
   A   
   C   
   G   
    C    
   A   
   T   
   C   
   A   
   A   
   G   
   G   
   A   
   A   
   T   
   G   
   G   
   G   
    T    
   T   
   T   
   G   
   C   
   G   
   C   
   T   
    A    
   T   
   C   
   C   
   T   
   T   
   T   
   A   
   A   
    C    
   A   
   G   
   A   
   T   
   G   
   T   
   T   
   C   
   C   
   C   
   G   
   T   
   T   
   G   
   T   
   T   
   G   
   G   
   A   
   G   
   C   
   A   
   A   
   A   
   G   
   G   
   C   
   A   
   G   
    C    
   T   
   G   
   G   
   A   
   G   
   T   
   G   
   A   
   A   
   G   
   T   
   C   
   T   
   A   
   T   
   G   
   A   
   A   
   T   
   T   
   T   
   G   
   A   
   A   
   A   
   G   
   A   
   T   
   G   
   G   
   A   
   G   
   A   
   T   
   A   
   G   
   G   
    A    
   T   
   T   
    A    
   T   
   C   
   A   
   A   
    C    
   G   
   G   
    G    
   C   
   T   
    A    
   C   
   T   
   T   
   G   
   T   
   G   
   T   
   T   
   A   
   G   
   C   
   A   
   G   
   A   
    A    
   G   
   G   
    T    
   G   
   A   
   T   
   A   
   C   
   G   
   C   
   C   
    G    
   A   
   T   
   C   
   G   
   T   
   A   
   A   
   T   
   C   
   G   
   T   
   T   
   A   
   C   
   T   
   C   
   A   
   G   
   C   
   G   
   A   
   G   
   G   
   A   
   G   
   C   
    T    
   G   
   T   
    T    
   A   
   A   
   A   
   C   
   G   
   C   
   A   
   T   
   G   
   C   
   T   
   T   
   G   
   C   
   T   
   C   
   A   
   A   
   G   
   A   
   A   
   A   
   T   
   C   
   A   
   G   
   C   
   C   
   A   
   A   
   A   
   C   
    T    
   T   
   C   
   A   
   C   
   G   
   T   
   G   
   C   
   G   
   A   
   A   
   G   
   C   
   G   
   G   
   G   
   G   
   A   
   G   
   T   
   G   
   A   
   C   
   G   
   G   
   T   
   C   
   A   
   T   
   G   
   C   
   G   
   T   
   G   
   A   
   G   
   C   
   T   
   G   
   A   
   A   
   A   
   A   
   A   
   A   
   A   
   A   
   T   
   C   
   C   
    G    
   C   
   A   
    C    
   C   
   G   
    T    
   A   
   T   
   T   
   A   
   T   
   C   
   T   
   A   
   T   
   A   
   T   
   G   
   T   
   C   
   G   
   G   
   A   
   A   
   G   
   G   
   A   
   A   
   G   
   A   
   T   
   C   
   A   
   A   
   A   
   A   
   G   
   A   
   A   
   A   
   T   
   A   
   A   
   C   
   C   
   T   
   T   
   G   
   A   
   T   
   G   
   A   
   A   
   T   
   C   
   A   
   A   
   A   
   A   
   A   
   G   
   G   
    G    
   C   
   A   
    G    
   C   
   A   
   G   
    T    
   T   
   A   
   A   
   C   
   G   
   A   
   T   
   T   
   G   
   A   
    C    
   C   
   C   
   A   
   A   
   C   
    T    
   G   
   A   
   T   
   T   
   T   
    C    
   C   
   C   
   T   
   A   
   T   
   A   
   G   
   G   
   T   
   G   
   A   
   T   
   C   
   G   
   A   
   A   
   C   
   A   
   T   
   C   
   G   
   A   
   A   
   T   
   G   
   G   
    A    
   T   
   C   
    A    
   T   
   T   
   T   
   G   
   C   
    G    
   A   
   T   
   G   
   G   
   A   
    T    
   G   
   A   
   G   
   A   
   A   
   A   
   A   
   A   
   A   
   G   
   G   
   T   
   G   
   G   
   A   
   G   
   A   
   A   
   G   
   T   
   C   
   A   
   T   
   C   
   G   
   A   
   A   
   G   
   T   
    T    
   A   
    T    
   C   
   G   
   A   
   T   
   G   
   C   
   T   
   C   
   C   
   T   
   G   
   A   
   G   
    A    
   T   
   T   
   T   
   T   
    C    
   A   
   T   
    C    
   G   
   A   
    G    
    T    
   C   
    G    
   -   
   -   
   -   
 

 
   N55728   
   A   
   T   
   G   
   G   
   A   
   A   
   A   
   A   
   T   
   C   
   G   
   A   
   C   
   A   
   A   
   G   
   A   
   A   
   G   
   T   
   A   
   C   
   A   
   A   
   G   
   A   
   A   
   T   
   T   
   A   
   A   
   C   
   T   
   T   
   T   
   A   
   G   
   A   
   A   
   G   
   A   
   A   
   G   
   T   
   G   
   A   
   T   
   G   
   G   
   G   
   T   
   G   
   A   
   C   
   C   
   G   
   C   
   T   
   T   
   C   
   G   
   G   
   A   
   C   
   G   
   T   
   T   
   A   
   T   
   T   
   C   
   C   
   A   
   A   
   A   
   T   
   A   
   T   
   A   
   T   
   T   
   A   
   T   
    C    
   C   
   A   
    A    
   G   
   A   
   A   
   C   
   G   
   T   
   G   
   C   
   A   
   C   
   T   
   G   
   C   
   C   
   T   
   G   
   A   
   T   
   A   
   T   
    C    
   C   
   G   
   T   
   G   
   A   
   T   
   G   
   G   
   A   
   T   
   T   
   G   
   A   
   A   
    G    
   C   
   C   
   A   
   G   
   T   
    G    
   C   
   A   
   A   
   C   
   G   
   C   
   C   
   G   
   G   
   A   
   T   
   A   
   T   
   T   
   A   
   T   
   T   
    T    
   G   
   C   
    C    
   A   
   T   
   G   
   A   
   A   
   C   
   A   
   A   
   A   
   G   
   A   
   T   
   G   
   G   
   C   
   A   
   A   
   T   
   A   
   C   
   G   
   T   
   A   
   T   
   G   
   A   
   C   
   A   
   A   
   A   
   G   
   G   
   A   
   T   
   T   
    C    
   C   
   G   
   T   
   A   
   A   
   A   
   T   
   C   
   A   
   G   
   C   
   A   
   A   
   A   
   A   
   T   
   C   
   T   
   G   
   T   
   C   
   G   
   G   
   A   
   A   
   A   
   T   
   A   
   T   
   C   
   A   
   T   
   G   
   G   
   G   
    G    
   A   
   A   
   T   
   T   
   A   
   T   
   C   
   A   
   T   
   C   
   C   
   T   
   C   
   A   
   T   
   G   
   G   
   T   
   G   
   A   
   C   
   A   
   G   
   C   
   A   
   G   
   T   
   A   
   T   
   T   
   T   
   A   
   T   
   G   
   A   
   A   
   G   
   C   
   G   
   A   
   T   
   G   
   G   
   T   
   C   
   C   
   G   
   C   
   A   
   T   
   G   
   A   
   G   
   C   
   C   
   A   
   A   
   G   
   A   
    C    
   T   
   G   
   G   
   A   
   A   
   G   
   T   
   T   
   G   
   C   
   G   
   G   
   G   
   A   
   A   
   G   
   T   
   A   
   C   
   T   
   G   
   A   
   T   
   C   
   G   
   A   
    A    
   A   
   T   
   G   
   C   
   A   
   C   
   G   
   G   
    G    
   A   
   A   
   T   
   A   
   A   
    C    
   G   
   G   
   G   
   A   
   G   
   T   
   A   
   T   
   G   
   G   
   A   
   C   
   G   
   G   
   T   
   G   
   A   
   T   
   C   
   C   
   A   
   C   
   C   
   T   
   G   
   C   
   T   
   G   
   C   
   G   
   A   
   T   
   G   
   C   
   G   
   T   
   T   
   A   
   T   
   A   
   C   
   T   
   G   
   A   
   A   
   G   
   C   
   C   
   A   
   G   
    A    
   T   
   T   
   A   
   T   
   C   
   C   
   A   
   A   
   G   
   T   
   T   
   A   
   A   
   G   
   T   
   G   
   G   
   A   
   G   
   A   
   G   
   A   
   T   
   G   
    C    
   T   
   A   
    G    
   C   
   A   
   G   
   A   
   T   
   A   
   T   
   C   
   G   
   A   
    A    
   A   
   A   
   A   
   G   
   A   
   A   
   A   
   C   
   A   
   G   
   T   
    C    
   G   
   A   
   T   
   C   
   T   
   C   
   G   
   T   
   G   
   T   
   G   
   G   
   A   
   A   
   T   
   T   
   T   
   T   
   G   
   A   
   T   
   G   
   A   
   T   
   A   
   C   
   A   
   G   
   A   
   A   
   A   
   A   
   A   
   G   
   A   
   A   
   C   
   C   
   A   
   A   
   C   
   A   
   G   
   T   
   C   
   C   
   T   
   T   
   C   
   C   
   A   
   G   
   C   
   T   
   C   
   G   
   A   
   T   
   A   
   C   
   C   
   C   
   A   
   A   
   A   
   C   
   C   
   T   
   T   
   C   
   T   
    C    
   G   
   T   
   C   
   A   
   A   
   T   
   G   
   G   
   A   
   T   
   C   
   G   
   A   
   C   
   A   
   G   
   G   
   T   
   A   
   T   
   A   
   T   
   C   
   T   
   G   
   C   
   T   
   G   
   G   
   A   
   T   
   A   
   T   
   G   
   C   
    A    
   A   
   C   
   A   
   G   
   A   
   A   
   A   
   T   
   C   
   C   
   C   
   G   
   A   
   C   
    T    
   C   
   A   
   T   
   A   
   A   
   C   
   T   
   T   
   A   
   G   
   C   
   T   
   G   
   A   
   A   
   G   
   T   
   G   
   A   
   T   
    T    
   G   
   A   
    T    
   G   
   G   
   C   
   A   
   C   
   A   
   A   
   T   
   C   
   T   
   A   
   T   
   A   
   T   
   G   
   A   
   T   
   C   
   G   
   A   
   T   
   C   
   A   
   T   
   C   
   C   
   A   
   C   
   A   
   A   
   G   
   C   
   G   
   A   
   G   
   C   
   C   
   T   
   T   
   G   
   A   
   A   
   A   
   A   
   A   
   T   
   T   
   G   
   A   
   T   
   G   
   G   
   A   
   A   
   T   
   A   
   T   
   A   
   T   
   T   
   C   
   C   
   T   
   G   
   G   
   A   
   C   
   C   
   T   
   G   
   A   
   C   
   T   
   T   
    T    
   C   
   C   
   A   
   A   
   C   
   A   
   G   
   G   
   A   
   G   
   G   
    G    
   A   
   T   
   C   
   T   
   T   
   G   
   C   
   A   
   A   
   G   
   G   
    A    
   A   
   A   
   A   
   G   
   A   
   T   
   G   
   A   
   A   
   A   
   T   
   C   
   A   
   A   
   A   
   A   
   A   
   A   
   G   
   C   
   T   
   T   
   A   
   T   
   G   
   A   
   A   
   A   
   C   
   A   
   G   
   G   
    G    
   C   
   G   
   A   
   G   
   G   
   A   
   A   
   A   
   A   
   G   
   T   
   T   
   A   
    T    
   T   
   C   
   T   
   T   
   C   
   G   
   T   
   T   
   C   
   G   
   A   
   A   
   A   
   A   
   C   
   A   
   A   
   A   
   G   
   A   
   T   
   A   
   G   
   A   
    G    
   G   
   C   
   A   
   A   
   T   
   G   
   A   
   A   
   G   
   G   
   G   
    T    
   A   
   A   
    T    
   A   
   A   
   A   
   C   
   A   
   G   
   C   
   A   
   A   
   A   
   T   
   C   
   G   
   T   
   G   
   A   
   T   
   C   
   A   
   C   
    A    
   G   
   A   
   A   
   A   
   T   
   C   
   C   
   C   
   A   
   T   
   A   
   T   
   G   
   A   
   A   
   G   
   T   
   A   
   A   
   A   
   T   
   A   
   A   
   A   
   G   
   C   
   G   
   A   
   C   
    C    
   T   
   T   
   G   
   G   
   T   
   G   
   A   
   A   
   A   
   A   
   A   
   A   
   A   
   T   
   G   
   G   
   A   
   T   
   G   
   A   
   G   
   A   
   T   
   C   
   C   
   G   
   T   
   T   
   T   
   G   
   A   
   A   
   T   
   A   
   A   
   G   
   A   
   A   
   A   
   A   
   T   
   C   
   G   
   A   
    T    
   G   
   G   
   A   
   A   
   T   
   A   
   G   
   C   
   T   
   G   
   A   
   A   
   G   
   T   
    A    
   C   
   G   
   A   
   G   
   A   
   T   
   G   
   A   
   A   
   A   
   G   
   T   
   G   
   A   
   T   
    A    
   G   
    A    
   A   
   C   
    A    
   G   
   G   
   T   
   C   
   T   
   G   
   C   
   A   
   A   
   A   
   T   
   T   
   G   
   T   
   C   
   G   
   T   
    C    
   G   
   A   
   A   
   T   
   T   
   G   
   A   
   A   
   A   
   A   
   A   
   A   
   G   
   A   
    C    
   G   
   C   
   C   
   A   
   A   
    T    
   G   
   C   
   T   
   C   
   A   
   A   
   G   
   G   
    A    
   A   
   T   
   T   
   T   
   T   
   G   
   A   
   A   
   T   
   T   
   A   
    T    
   T   
   T   
   G   
   T   
   T   
   C   
   A   
   A   
   A   
   A   
   A   
   C   
   A   
   C   
   T   
   G   
   A   
   G   
    C    
   T   
   A   
   C   
   A   
   G   
   A   
   T   
   C   
   A   
   A   
   T   
   T   
   A   
   C   
   A   
   A   
   T   
   T   
   T   
    C    
   A   
   A   
   T   
   A   
   T   
   G   
   G   
   T   
   C   
   G   
   C   
   T   
   A   
   T   
   C   
   G   
   A   
    C    
   C   
   A   
   T   
   A   
   T   
   G   
   A   
   C   
   T   
   C   
   C   
   G   
   C   
   A   
    T    
   C   
   A   
   A   
   G   
   T   
   T   
   G   
   G   
   A   
   C   
   T   
   G   
   A   
   A   
   A   
   G   
   A   
   C   
   A   
   T   
   T   
   T   
   T   
   A   
   A   
   G   
   A   
   A   
   G   
   T   
   T   
   A   
   T   
   A   
   T   
   C   
   G   
   A   
   G   
   C   
   A   
   T   
   C   
   G   
   A   
   A   
   A   
   G   
   C   
   A   
   A   
   G   
   T   
   C   
   A   
   T   
   A   
   A   
   C   
   A   
   A   
   A   
   A   
   C   
   G   
    T    
   A   
   G   
    T    
   C   
   A   
   A   
   T   
   T   
   C   
   G   
   A   
   T   
   T   
   T   
   A   
   G   
   C   
   C   
   A   
   A   
   A   
   G   
   C   
   T   
   C   
   A   
   A   
   A   
   A   
   A   
    A    
   G   
   A   
   C   
   A   
   G   
   C   
   A   
   T   
   A   
   T   
   C   
   G   
   T   
    A    
   G   
   A   
   A   
   G   
   G   
    A    
    T    
   T   
   G   
   A   
   T   
   G   
   A   
   A   
   A   
   G   
   C   
   T   
   T   
   T   
   A   
   T   
   C   
   G   
   A   
   T   
   T   
   C   
   T   
   G   
   G   
   A   
   T   
   G   
   A   
   A   
   G   
   T   
   A   
   A   
   T   
   C   
   G   
    T    
   A   
   A   
   C   
   G   
   A   
   T   
   T   
   C   
   G   
   T   
   G   
   A   
   A   
   A   
   G   
   T   
   A   
   A   
   A   
   G   
   A   
   C   
   A   
   A   
   A   
   A   
   A   
   A   
   G   
   A   
    C    
   G   
   C   
    C    
   A   
   A   
   A   
   A   
   A   
   G   
   A   
   A   
   T   
   T   
   T   
   A   
   G   
   T   
   C   
   G   
   A   
    C    
   G   
   T   
    C    
   T   
   T   
    T    
   C   
   A   
   A   
   T   
   T   
   T   
   A   
   C   
   A   
   G   
   A   
   A   
   G   
   A   
   A   
   C   
   A   
   A   
   G   
   C   
   A   
   G   
   A   
   A   
   G   
   C   
   A   
   A   
   T   
    T    
   G   
   T   
   C   
   A   
   C   
   A   
   T   
   T   
   G   
   C   
   A   
   A   
    T    
   T   
   A   
   T   
   A   
    C    
   C   
   G   
   T   
   T   
   T   
   G   
   A   
   C   
   G   
   A   
   A   
    C    
   A   
   C   
    G    
   G   
   A   
   T   
   A   
   T   
   C   
   A   
   C   
   A   
   G   
   A   
   G   
   C   
   T   
   T   
   C   
   A   
   A   
   A   
   A   
   G   
   G   
   A   
   A   
    T    
   C   
    C    
   G   
   A   
   A   
   A   
    G    
   C   
   T   
   T   
   G   
   A   
   T   
   T   
   G   
   C   
    G    
   C   
   A   
   A   
   A   
   T   
   C   
   A   
   C   
   A   
   G   
   A   
   G   
   T   
   T   
    G    
   A   
   A   
   T   
   A   
   A   
   A   
   A   
   T   
   C   
   C   
   T   
   T   
   T   
   C   
   A   
   A   
   A   
    T    
   G   
   A   
   C   
   A   
   A   
   A   
   G   
   A   
    G    
   C   
   T   
   T   
   T   
   T   
    C    
   T   
   C   
   T   
   G   
   T   
   T   
   A   
   T   
   G   
   A   
   A   
   A   
   A   
   A   
   A   
   G   
   A   
   A   
   T   
   T   
   G   
   C   
   G   
   A   
   G   
   A   
   A   
   G   
   T   
   G   
   A   
   A   
   A   
   A   
   A   
   A   
   A   
   A   
   T   
   T   
   A   
    T    
   T   
   C   
   T   
   T   
   C   
   C   
   G   
   C   
   T   
   C   
   G   
   T   
   C   
    T    
    A    
   A   
   C   
   G   
   A   
   C   
   A   
   A   
   T   
   C   
    G    
   A   
   A   
   G   
   A   
   C   
   G   
   A   
   A   
   A   
   T   
   C   
   G   
   A   
   A   
   G   
   A   
   A   
   A   
   T   
   C   
   A   
   A   
   A   
   A   
   T   
    T    
   G   
   A   
   T   
   A   
   C   
   G   
   C   
   A   
   A   
    G    
   T   
    C    
   T   
   T   
   A   
   G   
   T   
   G   
   G   
   C   
    A    
   C   
   A   
   A   
   G   
   A   
   A   
   G   
   A   
   C   
   G   
   T   
   A   
   A   
   T   
    T    
   G   
   T   
   C   
   A   
   G   
   T   
   G   
   T   
    G    
   A   
   C   
    G    
   C   
   G   
   A   
   G   
   A   
   A   
   G   
   G   
   C   
   T   
   A   
   T   
   G   
   T   
   G   
   A   
   A   
   G   
   C   
   G   
   A   
   A   
   C   
   A   
   A   
   G   
   C   
   C   
   T   
   T   
   C   
   G   
   T   
   T   
   C   
    T    
   T   
   A   
   T   
   A   
   G   
   T   
   G   
   C   
   T   
   T   
   C   
    C    
   A   
   A   
   A   
   C   
   C   
   A   
   G   
   A   
   A   
   G   
   A   
   A   
   A   
   T   
   C   
   G   
   G   
   G   
   A   
   T   
   G   
   C   
   G   
   A   
   G   
   A   
   A   
   G   
   G   
   C   
   G   
   A   
    C    
   T   
   A   
   C   
   T   
   T   
   G   
   C   
   T   
    A    
   T   
   A   
   C   
    T    
   C   
   A   
   G   
   G   
   T   
   G   
   A   
   A   
   T   
   T   
    G    
   A   
   G   
    T    
   A   
   C   
   A   
    A    
   T   
   G   
   G   
   A   
   C   
   C   
   A   
   T   
   G   
   T   
   T   
   C   
   T   
   T   
   C   
   T   
   G   
   A   
   T   
    C    
   A   
   C   
    A    
   A   
   A   
   T   
   A   
   A   
   A   
   G   
   C   
   A   
   A   
   A   
   T   
   G   
   T   
   G   
   A   
   T   
   C   
   T   
   A   
   C   
   C   
   G   
    C    
   C   
   C   
   T   
   G   
   T   
    C    
   C   
   A   
   T   
   G   
   A   
   A   
   C   
   T   
   A   
   C   
   C   
    C    
   G   
   A   
   T   
   T   
   T   
   G   
   A   
   A   
   A   
   T   
   G   
   G   
   A   
   A   
   A   
   G   
   A   
   T   
   G   
   C   
   T   
   G   
   G   
    T    
   G   
   A   
    G    
   C   
   A   
    C    
   A   
   T   
   T   
   T   
   C   
   T   
   C   
   A   
   A   
   A   
   C   
   A   
   A   
   T   
   C   
   T   
   C   
   C   
   A   
   A   
   T   
   C   
   T   
   G   
   T   
   C   
   T   
   G   
   T   
   A   
   G   
   A   
    C    
   G   
   A   
   A   
   T   
   C   
   G   
   A   
   T   
    T    
   T   
   T   
   A   
   G   
   C   
   T   
   G   
   T   
   T   
   T   
   T   
    C    
   C   
   C   
   A   
   T   
   A   
   T   
   C   
   A   
   A   
   A   
   A   
   A   
   A   
   T   
    T    
   C   
   A   
   A   
   G   
   C   
   A   
   G   
   A   
    G    
   A   
   A   
   A   
   A   
   C   
   A   
   T   
   T   
   T   
   G   
   T   
    A    
   T   
   T   
   C   
   A   
   T   
   C   
   A   
   G   
   C   
   A   
   A   
   G   
   A   
   A   
   T   
   G   
   G   
   A   
   T   
   T   
    G    
    A    
   T   
   C   
   A   
   A   
   G   
   C   
   A   
   A   
   A   
   C   
   A   
   C   
   G   
   A   
   A   
   T   
   G   
   A   
   C   
   A   
   G   
   A   
   G   
   T   
   T   
   C   
   G   
   A   
   A   
   C   
   C   
   A   
   T   
   G   
   G   
   C   
   G   
   C   
   A   
   C   
   C   
   T   
   A   
    T    
   A   
   A   
   G   
   A   
   G   
   C   
   C   
   G   
    T    
   C   
   C   
   G   
   C   
   T   
   C   
   A   
   G   
   C   
   T   
   G   
   T   
   A   
   T   
   G   
   A   
   A   
   G   
   C   
   T   
   A   
   A   
   A   
   G   
   G   
   C   
   G   
   G   
   A   
   A   
   G   
   A   
   A   
   G   
   A   
   T   
   G   
   A   
   A   
   C   
   T   
    G    
   A   
   T   
   T   
   G   
   C   
   T   
   G   
   T   
    G    
   T   
   A   
   T   
   C   
   T   
   G   
   A   
   A   
   A   
   G   
   A   
   A   
   G   
   A   
   T   
   C   
    A    
   G   
   C   
   C   
   A   
   G   
   G   
   C   
   T   
   T   
   A   
   G   
   A   
   T   
   G   
   T   
    T    
   T   
   T   
   C   
   T   
   T   
   A   
   G   
   T   
   A   
   A   
   C   
   G   
    C    
   A   
   T   
   C   
   A   
   A   
   G   
   G   
   A   
   A   
   T   
   G   
   G   
   G   
    T    
   T   
   T   
   G   
   C   
   G   
   C   
   T   
    A    
   T   
   C   
   C   
   T   
   T   
   T   
   A   
   A   
    C    
   A   
   G   
   A   
   T   
   G   
   T   
   T   
   C   
   C   
   C   
   G   
   T   
   T   
   G   
   T   
   T   
   G   
   G   
   A   
   G   
   C   
   A   
   A   
   A   
   G   
   G   
   C   
   A   
   G   
    C    
   T   
   G   
   G   
   A   
   G   
   T   
   G   
   A   
   A   
   G   
   T   
   C   
   T   
   A   
   T   
   G   
   A   
   A   
   T   
   T   
   T   
   G   
   A   
   A   
   A   
   G   
   A   
   T   
   G   
   G   
   A   
   G   
   A   
   T   
   A   
   G   
   G   
    A    
   T   
   T   
    A    
   T   
   C   
   A   
   A   
    C    
   G   
   G   
    G    
   C   
   T   
    G    
   C   
   T   
   T   
   G   
   T   
   G   
   T   
   T   
   A   
   G   
   C   
   A   
   G   
   A   
    A    
   G   
   G   
    T    
   G   
   A   
   T   
   A   
   C   
   G   
   C   
   C   
    G    
   A   
   T   
   C   
   G   
   T   
   A   
   A   
   T   
   C   
   G   
   T   
   T   
   A   
   C   
   T   
   C   
   A   
   G   
   C   
   G   
   A   
   G   
   G   
   A   
   G   
   C   
    T    
   G   
   T   
    T    
   A   
   A   
   A   
   C   
   G   
   C   
   A   
   T   
   G   
   C   
   T   
   T   
   G   
   C   
   T   
   C   
   A   
   A   
   G   
   A   
   A   
   A   
   T   
   C   
   A   
   G   
   C   
   C   
   A   
   A   
   A   
   C   
    T    
   T   
   C   
   A   
   C   
   G   
   T   
   G   
   C   
   G   
   A   
   A   
   G   
   C   
   G   
   G   
   G   
   G   
   A   
   G   
   T   
   G   
   A   
   C   
   G   
   G   
   T   
   C   
   A   
   T   
   G   
   C   
   G   
   T   
   G   
   A   
   G   
   C   
   T   
   G   
   A   
   A   
   A   
   A   
   A   
   A   
   A   
   A   
   T   
   C   
   C   
    G    
   C   
   A   
    C    
   C   
   G   
    T    
   A   
   T   
   T   
   A   
   T   
   C   
   T   
   A   
   T   
   A   
   T   
   G   
   T   
   C   
   G   
   G   
   A   
   A   
   G   
   G   
   A   
   A   
   G   
   A   
   T   
   C   
   A   
   A   
   A   
   A   
   G   
   A   
   A   
   A   
   T   
   A   
   A   
   C   
   C   
   T   
   T   
   G   
   A   
   T   
   G   
   A   
   A   
   T   
   C   
   A   
   A   
   A   
   A   
   A   
   G   
   G   
    G    
   C   
   A   
    G    
   C   
   A   
   G   
    T    
   T   
   A   
   A   
   C   
   G   
   A   
   T   
   T   
   G   
   A   
    C    
   C   
   C   
   A   
   A   
   C   
    T    
   G   
   A   
   T   
   T   
   T   
    C    
   C   
   C   
   T   
   A   
   T   
   A   
   G   
   G   
   T   
   G   
   A   
   T   
   C   
   G   
   A   
   A   
   C   
   A   
   T   
   C   
   G   
   A   
   A   
   T   
   G   
   G   
    A    
   T   
   C   
    A    
   T   
   T   
   T   
   G   
   C   
    G    
   A   
   T   
   G   
   G   
   A   
    T    
   G   
   A   
   G   
   A   
   A   
   A   
   A   
   A   
   A   
   G   
   G   
   T   
   G   
   G   
   A   
   G   
   A   
   A   
   G   
   T   
   C   
   A   
   T   
   C   
   G   
   A   
   A   
   G   
   T   
    T    
   A   
    T    
   C   
   G   
   A   
   T   
   G   
   C   
   T   
   C   
   C   
   T   
   G   
   A   
   G   
    A    
   T   
   T   
   T   
   T   
    C    
   A   
   T   
    C    
   G   
   A   
    G    
    T    
   C   
    G    
   -   
   -   
   -   
 

 
   N52649   
   A   
   T   
   G   
   G   
   A   
   A   
   A   
   A   
   T   
   C   
   G   
   A   
   C   
   A   
   A   
   G   
   A   
   A   
   G   
   T   
   A   
   C   
   A   
   A   
   G   
   A   
   A   
   T   
   T   
   A   
   A   
   C   
   T   
   T   
   T   
   A   
   G   
   A   
   A   
   G   
   A   
   A   
   G   
   T   
   G   
   A   
   T   
   G   
   G   
   G   
   T   
   G   
   A   
   C   
   C   
   G   
   C   
   T   
   T   
   C   
   G   
   G   
   A   
   C   
   G   
   T   
   T   
   A   
   T   
   T   
   C   
   C   
   A   
   A   
   A   
   T   
   A   
   T   
   A   
   T   
   T   
   A   
   T   
    C    
   C   
   A   
    A    
   G   
   A   
   A   
   C   
   G   
   T   
   G   
   C   
   A   
   C   
   T   
   G   
   C   
   C   
   T   
   G   
   A   
   T   
   A   
   T   
    C    
   C   
   G   
   T   
   G   
   A   
   T   
   G   
   G   
   A   
   T   
   T   
   G   
   A   
   A   
    G    
   C   
   C   
   A   
   G   
   T   
    G    
   C   
   A   
   A   
   C   
   G   
   C   
   C   
   G   
   G   
   A   
   T   
   A   
   T   
   T   
   A   
   T   
   T   
    T    
   G   
   C   
    C    
   A   
   T   
   G   
   A   
   A   
   C   
   A   
   A   
   A   
   G   
   A   
   T   
   G   
   G   
   C   
   A   
   A   
   T   
   A   
   C   
   G   
   T   
   A   
   T   
   G   
   A   
   C   
   A   
   A   
   A   
   G   
   G   
   A   
   T   
   T   
    C    
   C   
   G   
   T   
   A   
   A   
   A   
   T   
   C   
   A   
   G   
   C   
   A   
   A   
   A   
   A   
   T   
   C   
   T   
   G   
   T   
   C   
   G   
   G   
   A   
   A   
   A   
   T   
   A   
   T   
   C   
   A   
   T   
   G   
   G   
   G   
    G    
   A   
   A   
   T   
   T   
   A   
   T   
   C   
   A   
   T   
   C   
   C   
   T   
   C   
   A   
   T   
   G   
   G   
   T   
   G   
   A   
   C   
   A   
   G   
   C   
   A   
   G   
   T   
   A   
   T   
   T   
   T   
   A   
   T   
   G   
   A   
   A   
   G   
   C   
   G   
   A   
   T   
   G   
   G   
   T   
   C   
   C   
   G   
   C   
   A   
   T   
   G   
   A   
   G   
   C   
   C   
   A   
   A   
   G   
   A   
    C    
   T   
   G   
   G   
   A   
   A   
   G   
   T   
   T   
   G   
   C   
   G   
   G   
   G   
   A   
   A   
   G   
   T   
   A   
   C   
   T   
   G   
   A   
   T   
   C   
   G   
   A   
    A    
   A   
   T   
   G   
   C   
   A   
   C   
   G   
   G   
    G    
   A   
   A   
   T   
   A   
   A   
    C    
   G   
   G   
   G   
   A   
   G   
   T   
   A   
   T   
   G   
   G   
   A   
   C   
   G   
   G   
   T   
   G   
   A   
   T   
   C   
   C   
   A   
   C   
   C   
   T   
   G   
   C   
   T   
   G   
   C   
   G   
   A   
   T   
   G   
   C   
   G   
   T   
   T   
   A   
   T   
   A   
   C   
   T   
   G   
   A   
   A   
   G   
   C   
   C   
   A   
   G   
    A    
   T   
   T   
   A   
   T   
   C   
   C   
   A   
   A   
   G   
   T   
   T   
   A   
   A   
   G   
   T   
   G   
   G   
   A   
   G   
   A   
   G   
   A   
   T   
   G   
    C    
   T   
   A   
    G    
   C   
   A   
   G   
   A   
   T   
   A   
   T   
   C   
   G   
   A   
    A    
   A   
   A   
   A   
   G   
   A   
   A   
   A   
   C   
   A   
   G   
   T   
    C    
   G   
   A   
   T   
   C   
   T   
   C   
   G   
   T   
   G   
   T   
   G   
   G   
   A   
   A   
   T   
   T   
   T   
   T   
   G   
   A   
   T   
   G   
   A   
   T   
   A   
   C   
   A   
   G   
   A   
   A   
   A   
   A   
   A   
   G   
   A   
   A   
   C   
   C   
   A   
   A   
   C   
   A   
   G   
   T   
   C   
   C   
   T   
   T   
   C   
   C   
   A   
   G   
   C   
   T   
   C   
   G   
   A   
   T   
   A   
   C   
   C   
   C   
   A   
   A   
   A   
   C   
   C   
   T   
   T   
   C   
   T   
    C    
   G   
   T   
   C   
   A   
   A   
   T   
   G   
   G   
   A   
   T   
   C   
   G   
   A   
   C   
   A   
   G   
   G   
   T   
   A   
   T   
   A   
   T   
   C   
   T   
   G   
   C   
   T   
   G   
   G   
   A   
   T   
   A   
   T   
   G   
   C   
    A    
   A   
   C   
   A   
   G   
   A   
   A   
   A   
   T   
   C   
   C   
   C   
   G   
   A   
   C   
    T    
   C   
   A   
   T   
   A   
   A   
   C   
   T   
   T   
   A   
   G   
   C   
   T   
   G   
   A   
   A   
   G   
   T   
   G   
   A   
   T   
    C    
   G   
   A   
    T    
   G   
   G   
   C   
   A   
   C   
   A   
   A   
   T   
   C   
   T   
   A   
   T   
   A   
   T   
   G   
   A   
   T   
   C   
   G   
   A   
   T   
   C   
   A   
   T   
   C   
   C   
   A   
   C   
   A   
   A   
   G   
   C   
   G   
   A   
   G   
   C   
   C   
   T   
   T   
   G   
   A   
   A   
   A   
   A   
   A   
   T   
   T   
   G   
   A   
   T   
   G   
   G   
   A   
   A   
   T   
   A   
   T   
   A   
   T   
   T   
   C   
   C   
   T   
   G   
   G   
   A   
   C   
   C   
   T   
   G   
   A   
   C   
   T   
   T   
    T    
   C   
   C   
   A   
   A   
   C   
   A   
   G   
   G   
   A   
   G   
   G   
    G    
   A   
   T   
   C   
   T   
   T   
   G   
   C   
   A   
   A   
   G   
   G   
    A    
   A   
   A   
   A   
   G   
   A   
   T   
   G   
   A   
   A   
   A   
   T   
   C   
   A   
   A   
   A   
   A   
   A   
   A   
   G   
   C   
   T   
   T   
   A   
   T   
   G   
   A   
   A   
   A   
   C   
   A   
   G   
   G   
    G    
   C   
   G   
   A   
   G   
   G   
   A   
   A   
   A   
   A   
   G   
   T   
   T   
   A   
    T    
   T   
   C   
   T   
   T   
   C   
   G   
   T   
   T   
   C   
   G   
   A   
   A   
   A   
   A   
   C   
   A   
   A   
   A   
   G   
   A   
   T   
   A   
   G   
   A   
    G    
   G   
   C   
   A   
   A   
   T   
   G   
   A   
   A   
   G   
   G   
   G   
    T    
   A   
   A   
    T    
   A   
   A   
   A   
   C   
   A   
   G   
   C   
   A   
   A   
   A   
   T   
   C   
   G   
   T   
   G   
   A   
   T   
   C   
   A   
   C   
    A    
   G   
   A   
   A   
   A   
   T   
   C   
   C   
   C   
   A   
   T   
   A   
   T   
   G   
   A   
   A   
   G   
   T   
   A   
   A   
   A   
   T   
   A   
   A   
   A   
   G   
   C   
   G   
   A   
   C   
    C    
   T   
   T   
   G   
   G   
   T   
   G   
   A   
   A   
   A   
   A   
   A   
   A   
   A   
   T   
   G   
   G   
   A   
   T   
   G   
   A   
   G   
   A   
   T   
   C   
   C   
   G   
   T   
   T   
   T   
   G   
   A   
   A   
   T   
   A   
   A   
   G   
   A   
   A   
   A   
   A   
   T   
   C   
   G   
   A   
    T    
   G   
   G   
   A   
   A   
   T   
   A   
   G   
   C   
   T   
   G   
   A   
   A   
   G   
   T   
    A    
   C   
   G   
   A   
   G   
   A   
   T   
   G   
   A   
   A   
   A   
   G   
   T   
   G   
   A   
   T   
    A    
   G   
    A    
   A   
   C   
    A    
   G   
   G   
   T   
   C   
   T   
   G   
   C   
   A   
   A   
   A   
   T   
   T   
   G   
   T   
   C   
   G   
   T   
    C    
   G   
   A   
   A   
   T   
   T   
   G   
   A   
   A   
   A   
   A   
   A   
   A   
   G   
   A   
    C    
   G   
   C   
   C   
   A   
   A   
    T    
   G   
   C   
   T   
   C   
   A   
   A   
   G   
   G   
    A    
   A   
   T   
   T   
   T   
   T   
   G   
   A   
   A   
   T   
   T   
   A   
    T    
   T   
   T   
   G   
   T   
   T   
   C   
   A   
   A   
   A   
   A   
   A   
   C   
   A   
   C   
   T   
   G   
   A   
   G   
    T    
   T   
   A   
   C   
   A   
   G   
   A   
   T   
   C   
   A   
   A   
   T   
   T   
   A   
   C   
   A   
   A   
   T   
   T   
   T   
    C    
   A   
   A   
   T   
   A   
   T   
   G   
   G   
   T   
   C   
   G   
   C   
   T   
   A   
   T   
   C   
   G   
   A   
    C    
   C   
   A   
   T   
   A   
   T   
   G   
   A   
   C   
   T   
   C   
   C   
   G   
   C   
   A   
    T    
   C   
   A   
   A   
   G   
   T   
   T   
   G   
   G   
   A   
   C   
   T   
   G   
   A   
   A   
   A   
   G   
   A   
   C   
   A   
   T   
   T   
   T   
   T   
   A   
   A   
   G   
   A   
   A   
   G   
   T   
   T   
   A   
   T   
   A   
   T   
   C   
   G   
   A   
   G   
   C   
   A   
   T   
   C   
   G   
   A   
   A   
   A   
   G   
   C   
   A   
   A   
   G   
   T   
   C   
   A   
   T   
   A   
   A   
   C   
   A   
   A   
   A   
   A   
   C   
   G   
    T    
   A   
   G   
    T    
   C   
   A   
   A   
   T   
   T   
   C   
   G   
   A   
   T   
   T   
   T   
   A   
   G   
   C   
   C   
   A   
   A   
   A   
   G   
   C   
   T   
   C   
   A   
   A   
   A   
   A   
   A   
    A    
   G   
   A   
   C   
   A   
   G   
   C   
   A   
   T   
   A   
   T   
   C   
   G   
   T   
    A    
   G   
   A   
   A   
   G   
   G   
    A    
    T    
   T   
   G   
   A   
   T   
   G   
   A   
   A   
   A   
   G   
   C   
   T   
   T   
   T   
   A   
   T   
   C   
   G   
   A   
   T   
   T   
   C   
   T   
   G   
   G   
   A   
   T   
   G   
   A   
   A   
   G   
   T   
   A   
   A   
   T   
   C   
   G   
    C    
   A   
   A   
   C   
   G   
   A   
   T   
   T   
   C   
   G   
   T   
   G   
   A   
   A   
   A   
   G   
   T   
   A   
   A   
   A   
   G   
   A   
   C   
   A   
   A   
   A   
   A   
   A   
   A   
   G   
   A   
    C    
   G   
   C   
    C    
   A   
   A   
   A   
   A   
   A   
   G   
   A   
   A   
   T   
   T   
   T   
   A   
   G   
   T   
   C   
   G   
   A   
    C    
   G   
   T   
    C    
   T   
   T   
    T    
   C   
   A   
   A   
   T   
   T   
   T   
   A   
   C   
   A   
   G   
   A   
   A   
   G   
   A   
   A   
   C   
   A   
   A   
   G   
   C   
   A   
   G   
   A   
   A   
   G   
   C   
   A   
   A   
   T   
    T    
   G   
   T   
   C   
   A   
   C   
   A   
   T   
   T   
   G   
   C   
   A   
   A   
    T    
   T   
   A   
   T   
   A   
    C    
   C   
   G   
   T   
   T   
   T   
   G   
   A   
   C   
   G   
   A   
   A   
    C    
   A   
   C   
    G    
   G   
   A   
   T   
   A   
   T   
   C   
   A   
   C   
   A   
   G   
   A   
   G   
   C   
   T   
   T   
   C   
   A   
   A   
   A   
   A   
   G   
   G   
   A   
   A   
    T    
   C   
    C    
   G   
   A   
   A   
   A   
    G    
   C   
   T   
   T   
   G   
   A   
   T   
   T   
   G   
   C   
    G    
   C   
   A   
   A   
   A   
   T   
   C   
   A   
   C   
   A   
   G   
   A   
   G   
   T   
   T   
    G    
   A   
   A   
   T   
   A   
   A   
   A   
   A   
   T   
   C   
   C   
   T   
   T   
   T   
   C   
   A   
   A   
   A   
    T    
   G   
   A   
   C   
   A   
   A   
   A   
   G   
   A   
    G    
   C   
   T   
   T   
   T   
   T   
    C    
   T   
   C   
   T   
   G   
   T   
   T   
   A   
   T   
   G   
   A   
   A   
   A   
   A   
   A   
   A   
   G   
   A   
   A   
   T   
   T   
   G   
   C   
   G   
   A   
   G   
   A   
   A   
   G   
   T   
   G   
   A   
   A   
   A   
   A   
   A   
   A   
   A   
   A   
   T   
   T   
   A   
    T    
   T   
   C   
   T   
   T   
   C   
   C   
   G   
   C   
   T   
   C   
   G   
   T   
   C   
    T    
    A    
   A   
   C   
   G   
   A   
   C   
   A   
   A   
   T   
   C   
    G    
   A   
   A   
   G   
   A   
   C   
   G   
   A   
   A   
   A   
   T   
   C   
   G   
   A   
   A   
   G   
   A   
   A   
   A   
   T   
   C   
   A   
   A   
   A   
   A   
   T   
    T    
   G   
   A   
   T   
   A   
   C   
   G   
   C   
   A   
   A   
    G    
   T   
    C    
   T   
   T   
   A   
   G   
   T   
   G   
   G   
   C   
    A    
   C   
   A   
   A   
   G   
   A   
   A   
   G   
   A   
   C   
   G   
   T   
   A   
   A   
   T   
    T    
   G   
   T   
   C   
   A   
   G   
   T   
   G   
   T   
    G    
   A   
   C   
    G    
   C   
   G   
   A   
   G   
   A   
   A   
   G   
   G   
   C   
   T   
   A   
   T   
   G   
   T   
   G   
   A   
   A   
   G   
   C   
   G   
   A   
   A   
   C   
   A   
   A   
   G   
   C   
   C   
   T   
   T   
   C   
   G   
   T   
   T   
   C   
    T    
   T   
   A   
   T   
   A   
   G   
   T   
   G   
   C   
   T   
   T   
   C   
    C    
   A   
   A   
   A   
   C   
   C   
   A   
   G   
   A   
   A   
   G   
   A   
   A   
   A   
   T   
   C   
   G   
   G   
   G   
   A   
   T   
   G   
   C   
   G   
   A   
   G   
   A   
   A   
   G   
   G   
   C   
   G   
   A   
    C    
   T   
   A   
   C   
   T   
   T   
   G   
   C   
   T   
    A    
   T   
   A   
   C   
    T    
   C   
   A   
   G   
   G   
   T   
   G   
   A   
   A   
   T   
   T   
    G    
   A   
   G   
    T    
   A   
   C   
   A   
    T    
   T   
   G   
   G   
   A   
   C   
   C   
   A   
   T   
   G   
   T   
   T   
   C   
   T   
   T   
   C   
   T   
   G   
   A   
   T   
    C    
   A   
   C   
    A    
   A   
   A   
   T   
   A   
   A   
   A   
   G   
   C   
   A   
   A   
   A   
   T   
   G   
   T   
   G   
   A   
   T   
   C   
   T   
   A   
   C   
   C   
   G   
    C    
   C   
   C   
   T   
   G   
   T   
    C    
   C   
   A   
   T   
   G   
   A   
   A   
   C   
   T   
   A   
   C   
   C   
    C    
   G   
   A   
   T   
   T   
   T   
   G   
   A   
   A   
   A   
   T   
   G   
   G   
   A   
   A   
   A   
   G   
   A   
   T   
   G   
   C   
   T   
   G   
   G   
    T    
   G   
   A   
    G    
   C   
   A   
    C    
   A   
   T   
   T   
   T   
   C   
   T   
   C   
   A   
   A   
   A   
   C   
   A   
   A   
   T   
   C   
   T   
   C   
   C   
   A   
   A   
   T   
   C   
   T   
   G   
   T   
   C   
   T   
   G   
   T   
   A   
   G   
   A   
    C    
   G   
   A   
   A   
   T   
   C   
   G   
   A   
   T   
    T    
   T   
   T   
   A   
   G   
   C   
   T   
   G   
   T   
   T   
   T   
   T   
    C    
   C   
   C   
   A   
   T   
   A   
   T   
   C   
   A   
   A   
   A   
   A   
   A   
   A   
   T   
    T    
   C   
   A   
   A   
   G   
   C   
   A   
   G   
   A   
    G    
   A   
   A   
   A   
   A   
   C   
   A   
   T   
   T   
   T   
   G   
   T   
    A    
   T   
   T   
   C   
   A   
   T   
   C   
   A   
   G   
   C   
   A   
   A   
   G   
   A   
   A   
   T   
   G   
   G   
   A   
   T   
   T   
    G    
    A    
   T   
   C   
   A   
   A   
   G   
   C   
   A   
   A   
   A   
   C   
   A   
   C   
   G   
   A   
   A   
   T   
   G   
   A   
   C   
   A   
   G   
   A   
   G   
   T   
   T   
   C   
   G   
   A   
   A   
   C   
   C   
   A   
   T   
   G   
   G   
   C   
   G   
   C   
   A   
   C   
   C   
   T   
   A   
    T    
   A   
   A   
   G   
   A   
   G   
   C   
   C   
   G   
    T    
   C   
   C   
   G   
   C   
   T   
   C   
   A   
   G   
   C   
   T   
   G   
   T   
   A   
   T   
   G   
   A   
   A   
   G   
   C   
   T   
   A   
   A   
   A   
   G   
   G   
   C   
   G   
   G   
   A   
   A   
   G   
   A   
   A   
   G   
   A   
   T   
   G   
   A   
   A   
   C   
   T   
    G    
   A   
   T   
   T   
   G   
   C   
   T   
   G   
   T   
    G    
   T   
   A   
   T   
   C   
   T   
   G   
   A   
   A   
   A   
   G   
   A   
   A   
   G   
   A   
   T   
   C   
    A    
   G   
   C   
   C   
   A   
   G   
   G   
   C   
   T   
   T   
   A   
   G   
   A   
   T   
   G   
   T   
    T    
   T   
   T   
   C   
   T   
   T   
   A   
   G   
   T   
   A   
   A   
   C   
   G   
    C    
   A   
   T   
   C   
   A   
   A   
   G   
   G   
   A   
   A   
   T   
   G   
   G   
   G   
    T    
   T   
   T   
   G   
   C   
   G   
   C   
   T   
    A    
   T   
   C   
   C   
   T   
   T   
   T   
   A   
   A   
    C    
   A   
   G   
   A   
   T   
   G   
   T   
   T   
   C   
   C   
   C   
   G   
   T   
   T   
   G   
   T   
   T   
   G   
   G   
   A   
   G   
   C   
   A   
   A   
   A   
   G   
   G   
   C   
   A   
   G   
    C    
   T   
   G   
   G   
   A   
   G   
   T   
   G   
   A   
   A   
   G   
   T   
   C   
   T   
   A   
   T   
   G   
   A   
   A   
   T   
   T   
   T   
   G   
   A   
   A   
   A   
   G   
   A   
   T   
   G   
   G   
   A   
   G   
   A   
   T   
   A   
   G   
   G   
    A    
   T   
   T   
    A    
   T   
   C   
   A   
   A   
    C    
   G   
   G   
    G    
   C   
   T   
    G    
   C   
   T   
   T   
   G   
   T   
   G   
   T   
   T   
   A   
   G   
   C   
   A   
   G   
   A   
    A    
   G   
   G   
    T    
   G   
   A   
   T   
   A   
   C   
   G   
   C   
   C   
    G    
   A   
   T   
   C   
   G   
   T   
   A   
   A   
   T   
   C   
   G   
   T   
   T   
   A   
   C   
   T   
   C   
   A   
   G   
   C   
   G   
   A   
   G   
   G   
   A   
   G   
   C   
    T    
   G   
   T   
    T    
   A   
   A   
   A   
   C   
   G   
   C   
   A   
   T   
   G   
   C   
   T   
   T   
   G   
   C   
   T   
   C   
   A   
   A   
   G   
   A   
   A   
   A   
   T   
   C   
   A   
   G   
   C   
   C   
   A   
   A   
   A   
   C   
    T    
   T   
   C   
   A   
   C   
   G   
   T   
   G   
   C   
   G   
   A   
   A   
   G   
   C   
   G   
   G   
   G   
   G   
   A   
   G   
   T   
   G   
   A   
   C   
   G   
   G   
   T   
   C   
   A   
   T   
   G   
   C   
   G   
   T   
   G   
   A   
   G   
   C   
   T   
   G   
   A   
   A   
   A   
   A   
   A   
   A   
   A   
   A   
   T   
   C   
   C   
    G    
   C   
   A   
    C    
   C   
   G   
    T    
   A   
   T   
   T   
   A   
   T   
   C   
   T   
   A   
   T   
   A   
   T   
   G   
   T   
   C   
   G   
   G   
   A   
   A   
   G   
   G   
   A   
   A   
   G   
   A   
   T   
   C   
   A   
   A   
   A   
   A   
   G   
   A   
   A   
   A   
   T   
   A   
   A   
   C   
   C   
   T   
   T   
   G   
   A   
   T   
   G   
   A   
   A   
   T   
   C   
   A   
   A   
   A   
   A   
   A   
   G   
   G   
    G    
   C   
   A   
    G    
   C   
   A   
   G   
    T    
   T   
   A   
   A   
   C   
   G   
   A   
   T   
   T   
   G   
   A   
    C    
   C   
   C   
   A   
   A   
   C   
    T    
   G   
   A   
   T   
   T   
   T   
    C    
   C   
   C   
   T   
   A   
   T   
   A   
   G   
   G   
   T   
   G   
   A   
   T   
   C   
   G   
   A   
   A   
   C   
   A   
   T   
   C   
   G   
   A   
   A   
   T   
   G   
   G   
    A    
   T   
   C   
    A    
   T   
   T   
   T   
   G   
   C   
    G    
   A   
   T   
   G   
   G   
   A   
    T    
   G   
   A   
   G   
   A   
   A   
   A   
   A   
   A   
   A   
   G   
   G   
   T   
   G   
   G   
   A   
   G   
   A   
   A   
   G   
   T   
   C   
   A   
   T   
   C   
   G   
   A   
   A   
   G   
   T   
    T    
   A   
    T    
   C   
   G   
   A   
   T   
   G   
   C   
   T   
   C   
   C   
   T   
   G   
   A   
   G   
    A    
   T   
   T   
   T   
   T   
    C    
   A   
   T   
    C    
   G   
   A   
    G    
    T    
   C   
    G    
   -   
   -   
   -   
 

 
   N53559   
   A   
   T   
   G   
   G   
   A   
   A   
   A   
   A   
   T   
   C   
   G   
   A   
   C   
   A   
   A   
   G   
   A   
   A   
   G   
   T   
   A   
   C   
   A   
   A   
   G   
   A   
   A   
   T   
   T   
   A   
   A   
   C   
   T   
   T   
   T   
   A   
   G   
   A   
   A   
   G   
   A   
   A   
   G   
   T   
   G   
   A   
   T   
   G   
   G   
   G   
   T   
   G   
   A   
   C   
   C   
   G   
   C   
   T   
   T   
   C   
   G   
   G   
   A   
   C   
   G   
   T   
   T   
   A   
   T   
   T   
   C   
   C   
   A   
   A   
   A   
   T   
   A   
   T   
   A   
   T   
   T   
   A   
   T   
    C    
   C   
   A   
    A    
   G   
   A   
   A   
   C   
   G   
   T   
   G   
   C   
   A   
   C   
   T   
   G   
   C   
   C   
   T   
   G   
   A   
   T   
   A   
   T   
    C    
   C   
   G   
   T   
   G   
   A   
   T   
   G   
   G   
   A   
   T   
   T   
   G   
   A   
   A   
    G    
   C   
   C   
   A   
   G   
   T   
    G    
   C   
   A   
   A   
   C   
   G   
   C   
   C   
   G   
   G   
   A   
   T   
   A   
   T   
   T   
   A   
   T   
   T   
    T    
   G   
   C   
    C    
   A   
   T   
   G   
   A   
   A   
   C   
   A   
   A   
   A   
   G   
   A   
   T   
   G   
   G   
   C   
   A   
   A   
   T   
   A   
   C   
   G   
   T   
   A   
   T   
   G   
   A   
   C   
   A   
   A   
   A   
   G   
   G   
   A   
   T   
   T   
    C    
   C   
   G   
   T   
   A   
   A   
   A   
   T   
   C   
   A   
   G   
   C   
   A   
   A   
   A   
   A   
   T   
   C   
   T   
   G   
   T   
   C   
   G   
   G   
   A   
   A   
   A   
   T   
   A   
   T   
   C   
   A   
   T   
   G   
   G   
   G   
    G    
   A   
   A   
   T   
   T   
   A   
   T   
   C   
   A   
   T   
   C   
   C   
   T   
   C   
   A   
   T   
   G   
   G   
   T   
   G   
   A   
   C   
   A   
   G   
   C   
   A   
   G   
   T   
   A   
   T   
   T   
   T   
   A   
   T   
   G   
   A   
   A   
   G   
   C   
   G   
   A   
   T   
   G   
   G   
   T   
   C   
   C   
   G   
   C   
   A   
   T   
   G   
   A   
   G   
   C   
   C   
   A   
   A   
   G   
   A   
    C    
   T   
   G   
   G   
   A   
   A   
   G   
   T   
   T   
   G   
   C   
   G   
   G   
   G   
   A   
   A   
   G   
   T   
   A   
   C   
   T   
   G   
   A   
   T   
   C   
   G   
   A   
    A    
   A   
   T   
   G   
   C   
   A   
   C   
   G   
   G   
    G    
   A   
   A   
   T   
   A   
   A   
    C    
   G   
   G   
   G   
   A   
   G   
   T   
   A   
   T   
   G   
   G   
   A   
   C   
   G   
   G   
   T   
   G   
   A   
   T   
   C   
   C   
   A   
   C   
   C   
   T   
   G   
   C   
   T   
   G   
   C   
   G   
   A   
   T   
   G   
   C   
   G   
   T   
   T   
   A   
   T   
   A   
   C   
   T   
   G   
   A   
   A   
   G   
   C   
   C   
   A   
   G   
    A    
   T   
   T   
   A   
   T   
   C   
   C   
   A   
   A   
   G   
   T   
   T   
   A   
   A   
   G   
   T   
   G   
   G   
   A   
   G   
   A   
   G   
   A   
   T   
   G   
    C    
   T   
   A   
    G    
   C   
   A   
   G   
   A   
   T   
   A   
   T   
   C   
   G   
   A   
    A    
   A   
   A   
   A   
   G   
   A   
   A   
   A   
   C   
   A   
   G   
   T   
    C    
   G   
   A   
   T   
   C   
   T   
   C   
   G   
   T   
   G   
   T   
   G   
   G   
   A   
   A   
   T   
   T   
   T   
   T   
   G   
   A   
   T   
   G   
   A   
   T   
   A   
   C   
   A   
   G   
   A   
   A   
   A   
   A   
   A   
   G   
   A   
   A   
   C   
   C   
   A   
   A   
   C   
   A   
   G   
   T   
   C   
   C   
   T   
   T   
   C   
   C   
   A   
   G   
   C   
   T   
   C   
   G   
   A   
   T   
   A   
   C   
   C   
   C   
   A   
   A   
   A   
   C   
   C   
   T   
   T   
   C   
   T   
    C    
   G   
   T   
   C   
   A   
   A   
   T   
   G   
   G   
   A   
   T   
   C   
   G   
   A   
   C   
   A   
   G   
   G   
   T   
   A   
   T   
   A   
   T   
   C   
   T   
   G   
   C   
   T   
   G   
   G   
   A   
   T   
   A   
   T   
   G   
   C   
    A    
   A   
   C   
   A   
   G   
   A   
   A   
   A   
   T   
   C   
   C   
   C   
   G   
   A   
   C   
    T    
   C   
   A   
   T   
   A   
   A   
   C   
   T   
   T   
   A   
   G   
   C   
   T   
   G   
   A   
   A   
   G   
   T   
   G   
   A   
   T   
    T    
   G   
   A   
    T    
   G   
   G   
   C   
   A   
   C   
   A   
   A   
   T   
   C   
   T   
   A   
   T   
   A   
   T   
   G   
   A   
   T   
   C   
   G   
   A   
   T   
   C   
   A   
   T   
   C   
   C   
   A   
   C   
   A   
   A   
   G   
   C   
   G   
   A   
   G   
   C   
   C   
   T   
   T   
   G   
   A   
   A   
   A   
   A   
   A   
   T   
   T   
   G   
   A   
   T   
   G   
   G   
   A   
   A   
   T   
   A   
   T   
   A   
   T   
   T   
   C   
   C   
   T   
   G   
   G   
   A   
   C   
   C   
   T   
   G   
   A   
   C   
   T   
   T   
    T    
   C   
   C   
   A   
   A   
   C   
   A   
   G   
   G   
   A   
   G   
   G   
    G    
   A   
   T   
   C   
   T   
   T   
   G   
   C   
   A   
   A   
   G   
   G   
    A    
   A   
   A   
   A   
   G   
   A   
   T   
   G   
   A   
   A   
   A   
   T   
   C   
   A   
   A   
   A   
   A   
   A   
   A   
   G   
   C   
   T   
   T   
   A   
   T   
   G   
   A   
   A   
   A   
   C   
   A   
   G   
   G   
    G    
   C   
   G   
   A   
   G   
   G   
   A   
   A   
   A   
   A   
   G   
   T   
   T   
   A   
    T    
   T   
   C   
   T   
   T   
   C   
   G   
   T   
   T   
   C   
   G   
   A   
   A   
   A   
   A   
   C   
   A   
   A   
   A   
   G   
   A   
   T   
   A   
   G   
   A   
    G    
   G   
   C   
   A   
   A   
   T   
   G   
   A   
   A   
   G   
   G   
   G   
    T    
   A   
   A   
    T    
   A   
   A   
   A   
   C   
   A   
   G   
   C   
   A   
   A   
   A   
   T   
   C   
   G   
   T   
   G   
   A   
   T   
   C   
   A   
   C   
    A    
   G   
   A   
   A   
   A   
   T   
   C   
   C   
   C   
   A   
   T   
   A   
   T   
   G   
   A   
   A   
   G   
   T   
   A   
   A   
   A   
   T   
   A   
   A   
   A   
   G   
   C   
   G   
   A   
   C   
    C    
   T   
   T   
   G   
   G   
   T   
   G   
   A   
   A   
   A   
   A   
   A   
   A   
   A   
   T   
   G   
   G   
   A   
   T   
   G   
   A   
   G   
   A   
   T   
   C   
   C   
   G   
   T   
   T   
   T   
   G   
   A   
   A   
   T   
   A   
   A   
   G   
   A   
   A   
   A   
   A   
   T   
   C   
   G   
   A   
    T    
   G   
   G   
   A   
   A   
   T   
   A   
   G   
   C   
   T   
   G   
   A   
   A   
   G   
   T   
    A    
   C   
   G   
   A   
   G   
   A   
   T   
   G   
   A   
   A   
   A   
   G   
   T   
   G   
   A   
   T   
    A    
   G   
    A    
   A   
   C   
    A    
   G   
   G   
   T   
   C   
   T   
   G   
   C   
   A   
   A   
   A   
   T   
   T   
   G   
   T   
   C   
   G   
   T   
    C    
   G   
   A   
   A   
   T   
   T   
   G   
   A   
   A   
   A   
   A   
   A   
   A   
   G   
   A   
    C    
   G   
   C   
   C   
   A   
   A   
    T    
   G   
   C   
   T   
   C   
   A   
   A   
   G   
   G   
    A    
   A   
   T   
   T   
   T   
   T   
   G   
   A   
   A   
   T   
   T   
   A   
    T    
   T   
   T   
   G   
   T   
   T   
   C   
   A   
   A   
   A   
   A   
   A   
   C   
   A   
   C   
   T   
   G   
   A   
   G   
    C    
   T   
   A   
   C   
   A   
   G   
   A   
   T   
   C   
   A   
   A   
   T   
   T   
   A   
   C   
   A   
   A   
   T   
   T   
   T   
    C    
   A   
   A   
   T   
   A   
   T   
   G   
   G   
   T   
   C   
   G   
   C   
   T   
   A   
   T   
   C   
   G   
   A   
    C    
   C   
   A   
   T   
   A   
   T   
   G   
   A   
   C   
   T   
   C   
   C   
   G   
   C   
   A   
    T    
   C   
   A   
   A   
   G   
   T   
   T   
   G   
   G   
   A   
   C   
   T   
   G   
   A   
   A   
   A   
   G   
   A   
   C   
   A   
   T   
   T   
   T   
   T   
   A   
   A   
   G   
   A   
   A   
   G   
   T   
   T   
   A   
   T   
   A   
   T   
   C   
   G   
   A   
   G   
   C   
   A   
   T   
   C   
   G   
   A   
   A   
   A   
   G   
   C   
   A   
   A   
   G   
   T   
   C   
   A   
   T   
   A   
   A   
   C   
   A   
   A   
   A   
   A   
   C   
   G   
    T    
   A   
   G   
    T    
   C   
   A   
   A   
   T   
   T   
   C   
   G   
   A   
   T   
   T   
   T   
   A   
   G   
   C   
   C   
   A   
   A   
   A   
   G   
   C   
   T   
   C   
   A   
   A   
   A   
   A   
   A   
    A    
   G   
   A   
   C   
   A   
   G   
   C   
   A   
   T   
   A   
   T   
   C   
   G   
   T   
    A    
   G   
   A   
   A   
   G   
   G   
    A    
    T    
   T   
   G   
   A   
   T   
   G   
   A   
   A   
   A   
   G   
   C   
   T   
   T   
   T   
   A   
   T   
   C   
   G   
   A   
   T   
   T   
   C   
   T   
   G   
   G   
   A   
   T   
   G   
   A   
   A   
   G   
   T   
   A   
   A   
   T   
   C   
   G   
    T    
   A   
   A   
   C   
   G   
   A   
   T   
   T   
   C   
   G   
   T   
   G   
   A   
   A   
   A   
   G   
   T   
   A   
   A   
   A   
   G   
   A   
   C   
   A   
   A   
   A   
   A   
   A   
   A   
   G   
   A   
    C    
   G   
   C   
    C    
   A   
   A   
   A   
   A   
   A   
   G   
   A   
   A   
   T   
   T   
   T   
   A   
   G   
   T   
   C   
   G   
   A   
    C    
   G   
   T   
    C    
   T   
   T   
    T    
   C   
   A   
   A   
   T   
   T   
   T   
   A   
   C   
   A   
   G   
   A   
   A   
   G   
   A   
   A   
   C   
   A   
   A   
   G   
   C   
   A   
   G   
   A   
   A   
   G   
   C   
   A   
   A   
   T   
    T    
   G   
   T   
   C   
   A   
   C   
   A   
   T   
   T   
   G   
   C   
   A   
   A   
    T    
   T   
   A   
   T   
   A   
    C    
   C   
   G   
   T   
   T   
   T   
   G   
   A   
   C   
   G   
   A   
   A   
    C    
   A   
   C   
    G    
   G   
   A   
   T   
   A   
   T   
   C   
   A   
   C   
   A   
   G   
   A   
   G   
   C   
   T   
   T   
   C   
   A   
   A   
   A   
   A   
   G   
   G   
   A   
   A   
    T    
   C   
    C    
   G   
   A   
   A   
   A   
    G    
   C   
   T   
   T   
   G   
   A   
   T   
   T   
   G   
   C   
    G    
   C   
   A   
   A   
   A   
   T   
   C   
   A   
   C   
   A   
   G   
   A   
   G   
   T   
   T   
    G    
   A   
   A   
   T   
   A   
   A   
   A   
   A   
   T   
   C   
   C   
   T   
   T   
   T   
   C   
   A   
   A   
   A   
    T    
   G   
   A   
   C   
   A   
   A   
   A   
   G   
   A   
    G    
   C   
   T   
   T   
   T   
   T   
    C    
   T   
   C   
   T   
   G   
   T   
   T   
   A   
   T   
   G   
   A   
   A   
   A   
   A   
   A   
   A   
   G   
   A   
   A   
   T   
   T   
   G   
   C   
   G   
   A   
   G   
   A   
   A   
   G   
   T   
   G   
   A   
   A   
   A   
   A   
   A   
   A   
   A   
   A   
   T   
   T   
   A   
    T    
   T   
   C   
   T   
   T   
   C   
   C   
   G   
   C   
   T   
   C   
   G   
   T   
   C   
    T    
    A    
   A   
   C   
   G   
   A   
   C   
   A   
   A   
   T   
   C   
    G    
   A   
   A   
   G   
   A   
   C   
   G   
   A   
   A   
   A   
   T   
   C   
   G   
   A   
   A   
   G   
   A   
   A   
   A   
   T   
   C   
   A   
   A   
   A   
   A   
   T   
    T    
   G   
   A   
   T   
   A   
   C   
   G   
   C   
   A   
   A   
    G    
   T   
    C    
   T   
   T   
   A   
   G   
   T   
   G   
   G   
   C   
    A    
   C   
   A   
   A   
   G   
   A   
   A   
   G   
   A   
   C   
   G   
   T   
   A   
   A   
   T   
    T    
   G   
   T   
   C   
   A   
   G   
   T   
   G   
   T   
    G    
   A   
   C   
    G    
   C   
   G   
   A   
   G   
   A   
   A   
   G   
   G   
   C   
   T   
   A   
   T   
   G   
   T   
   G   
   A   
   A   
   G   
   C   
   G   
   A   
   A   
   C   
   A   
   A   
   G   
   C   
   C   
   T   
   T   
   C   
   G   
   T   
   T   
   C   
    T    
   T   
   A   
   T   
   A   
   G   
   T   
   G   
   C   
   T   
   T   
   C   
    C    
   A   
   A   
   A   
   C   
   C   
   A   
   G   
   A   
   A   
   G   
   A   
   A   
   A   
   T   
   C   
   G   
   G   
   G   
   A   
   T   
   G   
   C   
   G   
   A   
   G   
   A   
   A   
   G   
   G   
   C   
   G   
   A   
    C    
   T   
   A   
   C   
   T   
   T   
   G   
   C   
   T   
    A    
   T   
   A   
   C   
    T    
   C   
   A   
   G   
   G   
   T   
   G   
   A   
   A   
   T   
   T   
    G    
   A   
   G   
    T    
   A   
   C   
   A   
    T    
   T   
   G   
   G   
   A   
   C   
   C   
   A   
   T   
   G   
   T   
   T   
   C   
   T   
   T   
   C   
   T   
   G   
   A   
   T   
    C    
   A   
   C   
    A    
   A   
   A   
   T   
   A   
   A   
   A   
   G   
   C   
   A   
   A   
   A   
   T   
   G   
   T   
   G   
   A   
   T   
   C   
   T   
   A   
   C   
   C   
   G   
    C    
   C   
   C   
   T   
   G   
   T   
    C    
   C   
   A   
   T   
   G   
   A   
   A   
   C   
   T   
   A   
   C   
   C   
    C    
   G   
   A   
   T   
   T   
   T   
   G   
   A   
   A   
   A   
   T   
   G   
   G   
   A   
   A   
   A   
   G   
   A   
   T   
   G   
   C   
   T   
   G   
   G   
    T    
   G   
   A   
    G    
   C   
   A   
    C    
   A   
   T   
   T   
   T   
   C   
   T   
   C   
   A   
   A   
   A   
   C   
   A   
   A   
   T   
   C   
   T   
   C   
   C   
   A   
   A   
   T   
   C   
   T   
   G   
   T   
   C   
   T   
   G   
   T   
   A   
   G   
   A   
    C    
   G   
   A   
   A   
   T   
   C   
   G   
   A   
   T   
    T    
   T   
   T   
   A   
   G   
   C   
   T   
   G   
   T   
   T   
   T   
   T   
    C    
   C   
   C   
   A   
   T   
   A   
   T   
   C   
   A   
   A   
   A   
   A   
   A   
   A   
   T   
    T    
   C   
   A   
   A   
   G   
   C   
   A   
   G   
   A   
    G    
   A   
   A   
   A   
   A   
   C   
   A   
   T   
   T   
   T   
   G   
   T   
    A    
   T   
   T   
   C   
   A   
   T   
   C   
   A   
   G   
   C   
   A   
   A   
   G   
   A   
   A   
   T   
   G   
   G   
   A   
   T   
   T   
    G    
    A    
   T   
   C   
   A   
   A   
   G   
   C   
   A   
   A   
   A   
   C   
   A   
   C   
   G   
   A   
   A   
   T   
   G   
   A   
   C   
   A   
   G   
   A   
   G   
   T   
   T   
   C   
   G   
   A   
   A   
   C   
   C   
   A   
   T   
   G   
   G   
   C   
   G   
   C   
   A   
   C   
   C   
   T   
   A   
    T    
   A   
   A   
   G   
   A   
   G   
   C   
   C   
   G   
    T    
   C   
   C   
   G   
   C   
   T   
   C   
   A   
   G   
   C   
   T   
   G   
   T   
   A   
   T   
   G   
   A   
   A   
   G   
   C   
   T   
   A   
   A   
   A   
   G   
   G   
   C   
   G   
   G   
   A   
   A   
   G   
   A   
   A   
   G   
   A   
   T   
   G   
   A   
   A   
   C   
   T   
    G    
   A   
   T   
   T   
   G   
   C   
   T   
   G   
   T   
    G    
   T   
   A   
   T   
   C   
   T   
   G   
   A   
   A   
   A   
   G   
   A   
   A   
   G   
   A   
   T   
   C   
    A    
   G   
   C   
   C   
   A   
   G   
   G   
   C   
   T   
   T   
   A   
   G   
   A   
   T   
   G   
   T   
    T    
   T   
   T   
   C   
   T   
   T   
   A   
   G   
   T   
   A   
   A   
   C   
   G   
    C    
   A   
   T   
   C   
   A   
   A   
   G   
   G   
   A   
   A   
   T   
   G   
   G   
   G   
    T    
   T   
   T   
   G   
   C   
   G   
   C   
   T   
    A    
   T   
   C   
   C   
   T   
   T   
   T   
   A   
   A   
    C    
   A   
   G   
   A   
   T   
   G   
   T   
   T   
   C   
   C   
   C   
   G   
   T   
   T   
   G   
   T   
   T   
   G   
   G   
   A   
   G   
   C   
   A   
   A   
   A   
   G   
   G   
   C   
   A   
   G   
    C    
   T   
   G   
   G   
   A   
   G   
   T   
   G   
   A   
   A   
   G   
   T   
   C   
   T   
   A   
   T   
   G   
   A   
   A   
   T   
   T   
   T   
   G   
   A   
   A   
   A   
   G   
   A   
   T   
   G   
   G   
   A   
   G   
   A   
   T   
   A   
   G   
   G   
    A    
   T   
   T   
    A    
   T   
   C   
   A   
   A   
    C    
   G   
   G   
    G    
   C   
   T   
    G    
   C   
   T   
   T   
   G   
   T   
   G   
   T   
   T   
   A   
   G   
   C   
   A   
   G   
   A   
    A    
   G   
   G   
    T    
   G   
   A   
   T   
   A   
   C   
   G   
   C   
   C   
    G    
   A   
   T   
   C   
   G   
   T   
   A   
   A   
   T   
   C   
   G   
   T   
   T   
   A   
   C   
   T   
   C   
   A   
   G   
   C   
   G   
   A   
   G   
   G   
   A   
   G   
   C   
    T    
   G   
   T   
    T    
   A   
   A   
   A   
   C   
   G   
   C   
   A   
   T   
   G   
   C   
   T   
   T   
   G   
   C   
   T   
   C   
   A   
   A   
   G   
   A   
   A   
   A   
   T   
   C   
   A   
   G   
   C   
   C   
   A   
   A   
   A   
   C   
    T    
   T   
   C   
   A   
   C   
   G   
   T   
   G   
   C   
   G   
   A   
   A   
   G   
   C   
   G   
   G   
   G   
   G   
   A   
   G   
   T   
   G   
   A   
   C   
   G   
   G   
   T   
   C   
   A   
   T   
   G   
   C   
   G   
   T   
   G   
   A   
   G   
   C   
   T   
   G   
   A   
   A   
   A   
   A   
   A   
   A   
   A   
   A   
   T   
   C   
   C   
    G    
   C   
   A   
    C    
   C   
   G   
    T    
   A   
   T   
   T   
   A   
   T   
   C   
   T   
   A   
   T   
   A   
   T   
   G   
   T   
   C   
   G   
   G   
   A   
   A   
   G   
   G   
   A   
   A   
   G   
   A   
   T   
   C   
   A   
   A   
   A   
   A   
   G   
   A   
   A   
   A   
   T   
   A   
   A   
   C   
   C   
   T   
   T   
   G   
   A   
   T   
   G   
   A   
   A   
   T   
   C   
   A   
   A   
   A   
   A   
   A   
   G   
   G   
    G    
   C   
   A   
    G    
   C   
   A   
   G   
    T    
   T   
   A   
   A   
   C   
   G   
   A   
   T   
   T   
   G   
   A   
    C    
   C   
   C   
   A   
   A   
   C   
    T    
   G   
   A   
   T   
   T   
   T   
    C    
   C   
   C   
   T   
   A   
   T   
   A   
   G   
   G   
   T   
   G   
   A   
   T   
   C   
   G   
   A   
   A   
   C   
   A   
   T   
   C   
   G   
   A   
   A   
   T   
   G   
   G   
    A    
   T   
   C   
    A    
   T   
   T   
   T   
   G   
   C   
    G    
   A   
   T   
   G   
   G   
   A   
    T    
   G   
   A   
   G   
   A   
   A   
   A   
   A   
   A   
   A   
   G   
   G   
   T   
   G   
   G   
   A   
   G   
   A   
   A   
   G   
   T   
   C   
   A   
   T   
   C   
   G   
   A   
   A   
   G   
   T   
    T    
   A   
    T    
   C   
   G   
   A   
   T   
   G   
   C   
   T   
   C   
   C   
   T   
   G   
   A   
   G   
    A    
   T   
   T   
   T   
   T   
    C    
   A   
   T   
    C    
   G   
   A   
    G    
    T    
   C   
    G    
   -   
   -   
   -   
 

 
   N59435F   
   A   
   T   
   G   
   G   
   A   
   A   
   A   
   A   
   T   
   C   
   G   
   A   
   C   
   A   
   A   
   G   
   A   
   A   
   G   
   T   
   A   
   C   
   A   
   A   
   G   
   A   
   A   
   T   
   T   
   A   
   A   
   C   
   T   
   T   
   T   
   A   
   G   
   A   
   A   
   G   
   A   
   A   
   G   
   T   
   G   
   A   
   T   
   G   
   G   
   G   
   T   
   G   
   A   
   C   
   C   
   G   
   C   
   T   
   T   
   C   
   G   
   G   
   A   
   C   
   G   
   T   
   T   
   A   
   T   
   T   
   C   
   C   
   A   
   A   
   A   
   T   
   A   
   T   
   A   
   T   
   T   
   A   
   T   
    C    
   C   
   A   
    A    
   G   
   A   
   A   
   C   
   G   
   T   
   G   
   C   
   A   
   C   
   T   
   G   
   C   
   C   
   T   
   G   
   A   
   T   
   A   
   T   
    T    
   C   
   G   
   T   
   G   
   A   
   T   
   G   
   G   
   A   
   T   
   T   
   G   
   A   
   A   
    G    
   C   
   C   
   A   
   G   
   T   
    G    
   C   
   A   
   A   
   C   
   G   
   C   
   C   
   G   
   G   
   A   
   T   
   A   
   T   
   T   
   A   
   T   
   T   
    T    
   G   
   C   
    C    
   A   
   T   
   G   
   A   
   A   
   C   
   A   
   A   
   A   
   G   
   A   
   T   
   G   
   G   
   C   
   A   
   A   
   T   
   A   
   C   
   G   
   T   
   A   
   T   
   G   
   A   
   C   
   A   
   A   
   A   
   G   
   G   
   A   
   T   
   T   
    C    
   C   
   G   
   T   
   A   
   A   
   A   
   T   
   C   
   A   
   G   
   C   
   A   
   A   
   A   
   A   
   T   
   C   
   T   
   G   
   T   
   C   
   G   
   G   
   A   
   A   
   A   
   T   
   A   
   T   
   C   
   A   
   T   
   G   
   G   
   G   
    G    
   A   
   A   
   T   
   T   
   A   
   T   
   C   
   A   
   T   
   C   
   C   
   T   
   C   
   A   
   T   
   G   
   G   
   T   
   G   
   A   
   C   
   A   
   G   
   C   
   A   
   G   
   T   
   A   
   T   
   T   
   T   
   A   
   T   
   G   
   A   
   A   
   G   
   C   
   G   
   A   
   T   
   G   
   G   
   T   
   C   
   C   
   G   
   C   
   A   
   T   
   G   
   A   
   G   
   C   
   C   
   A   
   A   
   G   
   A   
    C    
   T   
   G   
   G   
   A   
   A   
   G   
   T   
   T   
   G   
   C   
   G   
   G   
   G   
   A   
   A   
   G   
   T   
   A   
   C   
   T   
   G   
   A   
   T   
   C   
   G   
   A   
    A    
   A   
   T   
   G   
   C   
   A   
   C   
   G   
   G   
    G    
   A   
   A   
   T   
   A   
   A   
    C    
   G   
   G   
   G   
   A   
   G   
   T   
   A   
   T   
   G   
   G   
   A   
   C   
   G   
   G   
   T   
   G   
   A   
   T   
   C   
   C   
   A   
   C   
   C   
   T   
   G   
   C   
   T   
   G   
   C   
   G   
   A   
   T   
   G   
   C   
   G   
   T   
   T   
   A   
   T   
   A   
   C   
   T   
   G   
   A   
   A   
   G   
   C   
   C   
   A   
   G   
    A    
   T   
   T   
   A   
   T   
   C   
   C   
   A   
   A   
   G   
   T   
   T   
   A   
   A   
   G   
   T   
   G   
   G   
   A   
   G   
   A   
   G   
   A   
   T   
   G   
    C    
   T   
   A   
    G    
   C   
   A   
   G   
   A   
   T   
   A   
   T   
   C   
   G   
   A   
    A    
   A   
   A   
   A   
   G   
   A   
   A   
   A   
   C   
   A   
   G   
   T   
    C    
   G   
   A   
   T   
   C   
   T   
   C   
   G   
   T   
   G   
   T   
   G   
   G   
   A   
   A   
   T   
   T   
   T   
   T   
   G   
   A   
   T   
   G   
   A   
   T   
   A   
   C   
   A   
   G   
   A   
   A   
   A   
   A   
   A   
   G   
   A   
   A   
   C   
   C   
   A   
   A   
   C   
   A   
   G   
   T   
   C   
   C   
   T   
   T   
   C   
   C   
   A   
   G   
   C   
   T   
   C   
   G   
   A   
   T   
   A   
   C   
   C   
   C   
   A   
   A   
   A   
   C   
   C   
   T   
   T   
   C   
   T   
    C    
   G   
   T   
   C   
   A   
   A   
   T   
   G   
   G   
   A   
   T   
   C   
   G   
   A   
   C   
   A   
   G   
   G   
   T   
   A   
   T   
   A   
   T   
   C   
   T   
   G   
   C   
   T   
   G   
   G   
   A   
   T   
   A   
   T   
   G   
   C   
    A    
   A   
   C   
   A   
   G   
   A   
   A   
   A   
   T   
   C   
   C   
   C   
   G   
   A   
   C   
    T    
   C   
   A   
   T   
   A   
   A   
   C   
   T   
   T   
   A   
   G   
   C   
   T   
   G   
   A   
   A   
   G   
   T   
   G   
   A   
   T   
    C    
   G   
   A   
    T    
   G   
   G   
   C   
   A   
   C   
   A   
   A   
   T   
   C   
   T   
   A   
   T   
   A   
   T   
   G   
   A   
   T   
   C   
   G   
   A   
   T   
   C   
   A   
   T   
   C   
   C   
   A   
   C   
   A   
   A   
   G   
   C   
   G   
   A   
   G   
   C   
   C   
   T   
   T   
   G   
   A   
   A   
   A   
   A   
   A   
   T   
   T   
   G   
   A   
   T   
   G   
   G   
   A   
   A   
   T   
   A   
   T   
   A   
   T   
   T   
   C   
   C   
   T   
   G   
   G   
   A   
   C   
   C   
   T   
   G   
   A   
   C   
   T   
   T   
    T    
   C   
   C   
   A   
   A   
   C   
   A   
   G   
   G   
   A   
   G   
   G   
    G    
   A   
   T   
   C   
   T   
   T   
   G   
   C   
   A   
   A   
   G   
   G   
    A    
   A   
   A   
   A   
   G   
   A   
   T   
   G   
   A   
   A   
   A   
   T   
   C   
   A   
   A   
   A   
   A   
   A   
   A   
   G   
   C   
   T   
   T   
   A   
   T   
   G   
   A   
   A   
   A   
   C   
   A   
   G   
   G   
    G    
   C   
   G   
   A   
   G   
   G   
   A   
   A   
   A   
   A   
   G   
   T   
   T   
   A   
    T    
   T   
   C   
   T   
   T   
   C   
   G   
   T   
   T   
   C   
   G   
   A   
   A   
   A   
   A   
   C   
   A   
   A   
   A   
   G   
   A   
   T   
   A   
   G   
   A   
    A    
   G   
   C   
   A   
   A   
   T   
   G   
   A   
   A   
   G   
   G   
   G   
    T    
   A   
   A   
    T    
   A   
   A   
   A   
   C   
   A   
   G   
   C   
   A   
   A   
   A   
   T   
   C   
   G   
   T   
   G   
   A   
   T   
   C   
   A   
   C   
    A    
   G   
   A   
   A   
   A   
   T   
   C   
   C   
   C   
   A   
   T   
   A   
   T   
   G   
   A   
   A   
   G   
   T   
   A   
   A   
   A   
   T   
   A   
   A   
   A   
   G   
   C   
   G   
   A   
   C   
    C    
   T   
   T   
   G   
   G   
   T   
   G   
   A   
   A   
   A   
   A   
   A   
   A   
   A   
   T   
   G   
   G   
   A   
   T   
   G   
   A   
   G   
   A   
   T   
   C   
   C   
   G   
   T   
   T   
   T   
   G   
   A   
   A   
   T   
   A   
   A   
   G   
   A   
   A   
   A   
   A   
   T   
   C   
   G   
   A   
    T    
   G   
   G   
   A   
   A   
   T   
   A   
   G   
   C   
   T   
   G   
   A   
   A   
   G   
   T   
    A    
   C   
   G   
   A   
   G   
   A   
   T   
   G   
   A   
   A   
   A   
   G   
   T   
   G   
   A   
   T   
    A    
   G   
    A    
   A   
   C   
    A    
   G   
   G   
   T   
   C   
   T   
   G   
   C   
   A   
   A   
   A   
   T   
   T   
   G   
   T   
   C   
   G   
   T   
    C    
   G   
   A   
   A   
   T   
   T   
   G   
   A   
   A   
   A   
   A   
   A   
   A   
   G   
   A   
    C    
   G   
   C   
   C   
   A   
   A   
    T    
   G   
   C   
   T   
   C   
   A   
   A   
   G   
   G   
    A    
   A   
   T   
   T   
   T   
   T   
   G   
   A   
   A   
   T   
   T   
   A   
    T    
   T   
   T   
   G   
   T   
   T   
   C   
   A   
   A   
   A   
   A   
   A   
   C   
   A   
   C   
   T   
   G   
   A   
   G   
    C    
   T   
   A   
   C   
   A   
   G   
   A   
   T   
   C   
   A   
   A   
   T   
   T   
   A   
   C   
   A   
   A   
   T   
   T   
   T   
    C    
   A   
   A   
   T   
   A   
   T   
   G   
   G   
   T   
   C   
   G   
   C   
   T   
   A   
   T   
   C   
   G   
   A   
    C    
   C   
   A   
   T   
   A   
   T   
   G   
   A   
   C   
   T   
   C   
   C   
   G   
   C   
   A   
    T    
   C   
   A   
   A   
   G   
   T   
   T   
   G   
   G   
   A   
   C   
   T   
   G   
   A   
   A   
   A   
   G   
   A   
   C   
   A   
   T   
   T   
   T   
   T   
   A   
   A   
   G   
   A   
   A   
   G   
   T   
   T   
   A   
   T   
   A   
   T   
   C   
   G   
   A   
   G   
   C   
   A   
   T   
   C   
   G   
   A   
   A   
   A   
   G   
   C   
   A   
   A   
   G   
   T   
   C   
   A   
   T   
   A   
   A   
   C   
   A   
   A   
   A   
   A   
   C   
   G   
    T    
   A   
   G   
    T    
   C   
   A   
   A   
   T   
   T   
   C   
   G   
   A   
   T   
   T   
   T   
   A   
   G   
   C   
   C   
   A   
   A   
   A   
   G   
   C   
   T   
   C   
   A   
   A   
   A   
   A   
   A   
    A    
   G   
   A   
   C   
   A   
   G   
   C   
   A   
   T   
   A   
   T   
   C   
   G   
   T   
    A    
   G   
   A   
   A   
   G   
   G   
    A    
    T    
   T   
   G   
   A   
   T   
   G   
   A   
   A   
   A   
   G   
   C   
   T   
   T   
   T   
   A   
   T   
   C   
   G   
   A   
   T   
   T   
   C   
   T   
   G   
   G   
   A   
   T   
   G   
   A   
   A   
   G   
   T   
   A   
   A   
   T   
   C   
   G   
    C    
   A   
   A   
   C   
   G   
   A   
   T   
   T   
   C   
   G   
   T   
   G   
   A   
   A   
   A   
   G   
   T   
   A   
   A   
   A   
   G   
   A   
   C   
   A   
   A   
   A   
   A   
   A   
   A   
   G   
   A   
    C    
   G   
   C   
    C    
   A   
   A   
   A   
   A   
   A   
   G   
   A   
   A   
   T   
   T   
   T   
   A   
   G   
   T   
   C   
   G   
   A   
    C    
   G   
   T   
    C    
   T   
   T   
    T    
   C   
   A   
   A   
   T   
   T   
   T   
   A   
   C   
   A   
   G   
   A   
   A   
   G   
   A   
   A   
   C   
   A   
   A   
   G   
   C   
   A   
   G   
   A   
   A   
   G   
   C   
   A   
   A   
   T   
    T    
   G   
   T   
   C   
   A   
   C   
   A   
   T   
   T   
   G   
   C   
   A   
   A   
    T    
   T   
   A   
   T   
   A   
    C    
   C   
   G   
   T   
   T   
   T   
   G   
   A   
   C   
   G   
   A   
   A   
    C    
   A   
   C   
    G    
   G   
   A   
   T   
   A   
   T   
   C   
   A   
   C   
   A   
   G   
   A   
   G   
   C   
   T   
   T   
   C   
   A   
   A   
   A   
   A   
   G   
   G   
   A   
   A   
    T    
   C   
    C    
   G   
   A   
   A   
   A   
    G    
   C   
   T   
   T   
   G   
   A   
   T   
   T   
   G   
   C   
    G    
   C   
   A   
   A   
   A   
   T   
   C   
   A   
   C   
   A   
   G   
   A   
   G   
   T   
   T   
    G    
   A   
   A   
   T   
   A   
   A   
   A   
   A   
   T   
   C   
   C   
   T   
   T   
   T   
   C   
   A   
   A   
   A   
    T    
   G   
   A   
   C   
   A   
   A   
   A   
   G   
   A   
    G    
   C   
   T   
   T   
   T   
   T   
    C    
   T   
   C   
   T   
   G   
   T   
   T   
   A   
   T   
   G   
   A   
   A   
   A   
   A   
   A   
   A   
   G   
   A   
   A   
   T   
   T   
   G   
   C   
   G   
   A   
   G   
   A   
   A   
   G   
   T   
   G   
   A   
   A   
   A   
   A   
   A   
   A   
   A   
   A   
   T   
   T   
   A   
    T    
   T   
   C   
   T   
   T   
   C   
   C   
   G   
   C   
   T   
   C   
   G   
   T   
   C   
    T    
    A    
   A   
   C   
   G   
   A   
   C   
   A   
   A   
   T   
   C   
    G    
   A   
   A   
   G   
   A   
   C   
   G   
   A   
   A   
   A   
   T   
   C   
   G   
   A   
   A   
   G   
   A   
   A   
   A   
   T   
   C   
   A   
   A   
   A   
   A   
   T   
    T    
   G   
   A   
   T   
   A   
   C   
   G   
   C   
   A   
   A   
    G    
   T   
    C    
   T   
   T   
   A   
   G   
   T   
   G   
   G   
   C   
    A    
   C   
   A   
   A   
   G   
   A   
   A   
   G   
   A   
   C   
   G   
   T   
   A   
   A   
   T   
    T    
   G   
   T   
   C   
   A   
   G   
   T   
   G   
   T   
    G    
   A   
   C   
    G    
   C   
   G   
   A   
   G   
   A   
   A   
   G   
   G   
   C   
   T   
   A   
   T   
   G   
   T   
   G   
   A   
   A   
   G   
   C   
   G   
   A   
   A   
   C   
   A   
   A   
   G   
   C   
   C   
   T   
   T   
   C   
   G   
   T   
   T   
   C   
    T    
   T   
   A   
   T   
   A   
   G   
   T   
   G   
   C   
   T   
   T   
   C   
    C    
   A   
   A   
   A   
   C   
   C   
   A   
   G   
   A   
   A   
   G   
   A   
   A   
   A   
   T   
   C   
   G   
   G   
   G   
   A   
   T   
   G   
   C   
   G   
   A   
   G   
   A   
   A   
   G   
   G   
   C   
   G   
   A   
    C    
   T   
   A   
   C   
   T   
   T   
   G   
   C   
   T   
    A    
   T   
   A   
   C   
    T    
   C   
   A   
   G   
   G   
   T   
   G   
   A   
   A   
   T   
   T   
    G    
   A   
   G   
    T    
   A   
   C   
   A   
    T    
   T   
   G   
   G   
   A   
   C   
   C   
   A   
   T   
   G   
   T   
   T   
   C   
   T   
   T   
   C   
   T   
   G   
   A   
   T   
    C    
   A   
   C   
    A    
   A   
   A   
   T   
   A   
   A   
   A   
   G   
   C   
   A   
   A   
   A   
   T   
   G   
   T   
   G   
   A   
   T   
   C   
   T   
   A   
   C   
   C   
   G   
    C    
   C   
   C   
   T   
   G   
   T   
    C    
   C   
   A   
   T   
   G   
   A   
   A   
   C   
   T   
   A   
   C   
   C   
    C    
   G   
   A   
   T   
   T   
   T   
   G   
   A   
   A   
   A   
   T   
   G   
   G   
   A   
   A   
   A   
   G   
   A   
   T   
   G   
   C   
   T   
   G   
   G   
    T    
   G   
   A   
    G    
   C   
   A   
    C    
   A   
   T   
   T   
   T   
   C   
   T   
   C   
   A   
   A   
   A   
   C   
   A   
   A   
   T   
   C   
   T   
   C   
   C   
   A   
   A   
   T   
   C   
   T   
   G   
   T   
   C   
   T   
   G   
   T   
   A   
   G   
   A   
    C    
   G   
   A   
   A   
   T   
   C   
   G   
   A   
   T   
    T    
   T   
   T   
   A   
   G   
   C   
   T   
   G   
   T   
   T   
   T   
   T   
    C    
   C   
   C   
   A   
   T   
   A   
   T   
   C   
   A   
   A   
   A   
   A   
   A   
   A   
   T   
    T    
   C   
   A   
   A   
   G   
   C   
   A   
   G   
   A   
    G    
   A   
   A   
   A   
   A   
   C   
   A   
   T   
   T   
   T   
   G   
   T   
    A    
   T   
   T   
   C   
   A   
   T   
   C   
   A   
   G   
   C   
   A   
   A   
   G   
   A   
   A   
   T   
   G   
   G   
   A   
   T   
   T   
    G    
    A    
   T   
   C   
   A   
   A   
   G   
   C   
   A   
   A   
   A   
   C   
   A   
   C   
   G   
   A   
   A   
   T   
   G   
   A   
   C   
   A   
   G   
   A   
   G   
   T   
   T   
   C   
   G   
   A   
   A   
   C   
   C   
   A   
   T   
   G   
   G   
   C   
   G   
   C   
   A   
   C   
   C   
   T   
   A   
    T    
   A   
   A   
   G   
   A   
   G   
   C   
   C   
   G   
    T    
   C   
   C   
   G   
   C   
   T   
   C   
   A   
   G   
   C   
   T   
   G   
   T   
   A   
   T   
   G   
   A   
   A   
   G   
   C   
   T   
   A   
   A   
   A   
   G   
   G   
   C   
   G   
   G   
   A   
   A   
   G   
   A   
   A   
   G   
   A   
   T   
   G   
   A   
   A   
   C   
   T   
    G    
   A   
   T   
   T   
   G   
   C   
   T   
   G   
   T   
    G    
   T   
   A   
   T   
   C   
   T   
   G   
   A   
   A   
   A   
   G   
   A   
   A   
   G   
   A   
   T   
   C   
    A    
   G   
   C   
   C   
   A   
   G   
   G   
   C   
   T   
   T   
   A   
   G   
   A   
   T   
   G   
   T   
    T    
   T   
   T   
   C   
   T   
   T   
   A   
   G   
   T   
   A   
   A   
   C   
   G   
    C    
   A   
   T   
   C   
   A   
   A   
   G   
   G   
   A   
   A   
   T   
   G   
   G   
   G   
    T    
   T   
   T   
   G   
   C   
   G   
   C   
   T   
    A    
   T   
   C   
   C   
   T   
   T   
   T   
   A   
   A   
    C    
   A   
   G   
   A   
   T   
   G   
   T   
   T   
   C   
   C   
   C   
   G   
   T   
   T   
   G   
   T   
   T   
   G   
   G   
   A   
   G   
   C   
   A   
   A   
   A   
   G   
   G   
   C   
   A   
   G   
    C    
   T   
   G   
   G   
   A   
   G   
   T   
   G   
   A   
   A   
   G   
   T   
   C   
   T   
   A   
   T   
   G   
   A   
   A   
   T   
   T   
   T   
   G   
   A   
   A   
   A   
   G   
   A   
   T   
   G   
   G   
   A   
   G   
   A   
   T   
   A   
   G   
   G   
    A    
   T   
   T   
    A    
   T   
   C   
   A   
   A   
    C    
   G   
   G   
    G    
   C   
   T   
    A    
   C   
   T   
   T   
   G   
   T   
   G   
   T   
   T   
   A   
   G   
   C   
   A   
   G   
   A   
    A    
   G   
   G   
    T    
   G   
   A   
   T   
   A   
   C   
   G   
   C   
   C   
    G    
   A   
   T   
   C   
   G   
   T   
   A   
   A   
   T   
   C   
   G   
   T   
   T   
   A   
   C   
   T   
   C   
   A   
   G   
   C   
   G   
   A   
   G   
   G   
   A   
   G   
   C   
    T    
   G   
   T   
    T    
   A   
   A   
   A   
   C   
   G   
   C   
   A   
   T   
   G   
   C   
   T   
   T   
   G   
   C   
   T   
   C   
   A   
   A   
   G   
   A   
   A   
   A   
   T   
   C   
   A   
   G   
   C   
   C   
   A   
   A   
   A   
   C   
    T    
   T   
   C   
   A   
   C   
   G   
   T   
   G   
   C   
   G   
   A   
   A   
   G   
   C   
   G   
   G   
   G   
   G   
   A   
   G   
   T   
   G   
   A   
   C   
   G   
   G   
   T   
   C   
   A   
   T   
   G   
   C   
   G   
   T   
   G   
   A   
   G   
   C   
   T   
   G   
   A   
   A   
   A   
   A   
   A   
   A   
   A   
   A   
   T   
   C   
   C   
    G    
   C   
   A   
    C    
   C   
   G   
    T    
   A   
   T   
   T   
   A   
   T   
   C   
   T   
   A   
   T   
   A   
   T   
   G   
   T   
   C   
   G   
   G   
   A   
   A   
   G   
   G   
   A   
   A   
   G   
   A   
   T   
   C   
   A   
   A   
   A   
   A   
   G   
   A   
   A   
   A   
   T   
   A   
   A   
   C   
   C   
   T   
   T   
   G   
   A   
   T   
   G   
   A   
   A   
   T   
   C   
   A   
   A   
   A   
   A   
   A   
   G   
   G   
    G    
   C   
   A   
    G    
   C   
   A   
   G   
    T    
   T   
   A   
   A   
   C   
   G   
   A   
   T   
   T   
   G   
   A   
    C    
   C   
   C   
   A   
   A   
   C   
    T    
   G   
   A   
   T   
   T   
   T   
    C    
   C   
   C   
   T   
   A   
   T   
   A   
   G   
   G   
   T   
   G   
   A   
   T   
   C   
   G   
   A   
   A   
   C   
   A   
   T   
   C   
   G   
   A   
   A   
   T   
   G   
   G   
    A    
   T   
   C   
    A    
   T   
   T   
   T   
   G   
   C   
    G    
   A   
   T   
   G   
   G   
   A   
    T    
   G   
   A   
   G   
   A   
   A   
   A   
   A   
   A   
   A   
   G   
   G   
   T   
   G   
   G   
   A   
   G   
   A   
   A   
   G   
   T   
   C   
   A   
   T   
   C   
   G   
   A   
   A   
   G   
   T   
    T    
   A   
    T    
   C   
   G   
   A   
   T   
   G   
   C   
   T   
   C   
   C   
   T   
   G   
   A   
   G   
    A    
   T   
   T   
   T   
   T   
    C    
   A   
   T   
    C    
   G   
   A   
    G    
    T    
   C   
    G    
   -   
   -   
   -   
 

 
   N59484F   
   A   
   T   
   G   
   G   
   A   
   A   
   A   
   A   
   T   
   C   
   G   
   A   
   C   
   A   
   A   
   G   
   A   
   A   
   G   
   T   
   A   
   C   
   A   
   A   
   G   
   A   
   A   
   T   
   T   
   A   
   A   
   C   
   T   
   T   
   T   
   A   
   G   
   A   
   A   
   G   
   A   
   A   
   G   
   T   
   G   
   A   
   T   
   G   
   G   
   G   
   T   
   G   
   A   
   C   
   C   
   G   
   C   
   T   
   T   
   C   
   G   
   G   
   A   
   C   
   G   
   T   
   T   
   A   
   T   
   T   
   C   
   C   
   A   
   A   
   A   
   T   
   A   
   T   
   A   
   T   
   T   
   A   
   T   
    C    
   C   
   A   
    A    
   G   
   A   
   A   
   C   
   G   
   T   
   G   
   C   
   A   
   C   
   T   
   G   
   C   
   C   
   T   
   G   
   A   
   T   
   A   
   T   
    T    
   C   
   G   
   T   
   G   
   A   
   T   
   G   
   G   
   A   
   T   
   T   
   G   
   A   
   A   
    G    
   C   
   C   
   A   
   G   
   T   
    G    
   C   
   A   
   A   
   C   
   G   
   C   
   C   
   G   
   G   
   A   
   T   
   A   
   T   
   T   
   A   
   T   
   T   
    T    
   G   
   C   
    C    
   A   
   T   
   G   
   A   
   A   
   C   
   A   
   A   
   A   
   G   
   A   
   T   
   G   
   G   
   C   
   A   
   A   
   T   
   A   
   C   
   G   
   T   
   A   
   T   
   G   
   A   
   C   
   A   
   A   
   A   
   G   
   G   
   A   
   T   
   T   
    C    
   C   
   G   
   T   
   A   
   A   
   A   
   T   
   C   
   A   
   G   
   C   
   A   
   A   
   A   
   A   
   T   
   C   
   T   
   G   
   T   
   C   
   G   
   G   
   A   
   A   
   A   
   T   
   A   
   T   
   C   
   A   
   T   
   G   
   G   
   G   
    G    
   A   
   A   
   T   
   T   
   A   
   T   
   C   
   A   
   T   
   C   
   C   
   T   
   C   
   A   
   T   
   G   
   G   
   T   
   G   
   A   
   C   
   A   
   G   
   C   
   A   
   G   
   T   
   A   
   T   
   T   
   T   
   A   
   T   
   G   
   A   
   A   
   G   
   C   
   G   
   A   
   T   
   G   
   G   
   T   
   C   
   C   
   G   
   C   
   A   
   T   
   G   
   A   
   G   
   C   
   C   
   A   
   A   
   G   
   A   
    C    
   T   
   G   
   G   
   A   
   A   
   G   
   T   
   T   
   G   
   C   
   G   
   G   
   G   
   A   
   A   
   G   
   T   
   A   
   C   
   T   
   G   
   A   
   T   
   C   
   G   
   A   
    A    
   A   
   T   
   G   
   C   
   A   
   C   
   G   
   G   
    G    
   A   
   A   
   T   
   A   
   A   
    C    
   G   
   G   
   G   
   A   
   G   
   T   
   A   
   T   
   G   
   G   
   A   
   C   
   G   
   G   
   T   
   G   
   A   
   T   
   C   
   C   
   A   
   C   
   C   
   T   
   G   
   C   
   T   
   G   
   C   
   G   
   A   
   T   
   G   
   C   
   G   
   T   
   T   
   A   
   T   
   A   
   C   
   T   
   G   
   A   
   A   
   G   
   C   
   C   
   A   
   G   
    A    
   T   
   T   
   A   
   T   
   C   
   C   
   A   
   A   
   G   
   T   
   T   
   A   
   A   
   G   
   T   
   G   
   G   
   A   
   G   
   A   
   G   
   A   
   T   
   G   
    C    
   T   
   A   
    G    
   C   
   A   
   G   
   A   
   T   
   A   
   T   
   C   
   G   
   A   
    A    
   A   
   A   
   A   
   G   
   A   
   A   
   A   
   C   
   A   
   G   
   T   
    C    
   G   
   A   
   T   
   C   
   T   
   C   
   G   
   T   
   G   
   T   
   G   
   G   
   A   
   A   
   T   
   T   
   T   
   T   
   G   
   A   
   T   
   G   
   A   
   T   
   A   
   C   
   A   
   G   
   A   
   A   
   A   
   A   
   A   
   G   
   A   
   A   
   C   
   C   
   A   
   A   
   C   
   A   
   G   
   T   
   C   
   C   
   T   
   T   
   C   
   C   
   A   
   G   
   C   
   T   
   C   
   G   
   A   
   T   
   A   
   C   
   C   
   C   
   A   
   A   
   A   
   C   
   C   
   T   
   T   
   C   
   T   
    C    
   G   
   T   
   C   
   A   
   A   
   T   
   G   
   G   
   A   
   T   
   C   
   G   
   A   
   C   
   A   
   G   
   G   
   T   
   A   
   T   
   A   
   T   
   C   
   T   
   G   
   C   
   T   
   G   
   G   
   A   
   T   
   A   
   T   
   G   
   C   
    A    
   A   
   C   
   A   
   G   
   A   
   A   
   A   
   T   
   C   
   C   
   C   
   G   
   A   
   C   
    T    
   C   
   A   
   T   
   A   
   A   
   C   
   T   
   T   
   A   
   G   
   C   
   T   
   G   
   A   
   A   
   G   
   T   
   G   
   A   
   T   
    C    
   G   
   A   
    T    
   G   
   G   
   C   
   A   
   C   
   A   
   A   
   T   
   C   
   T   
   A   
   T   
   A   
   T   
   G   
   A   
   T   
   C   
   G   
   A   
   T   
   C   
   A   
   T   
   C   
   C   
   A   
   C   
   A   
   A   
   G   
   C   
   G   
   A   
   G   
   C   
   C   
   T   
   T   
   G   
   A   
   A   
   A   
   A   
   A   
   T   
   T   
   G   
   A   
   T   
   G   
   G   
   A   
   A   
   T   
   A   
   T   
   A   
   T   
   T   
   C   
   C   
   T   
   G   
   G   
   A   
   C   
   C   
   T   
   G   
   A   
   C   
   T   
   T   
    T    
   C   
   C   
   A   
   A   
   C   
   A   
   G   
   G   
   A   
   G   
   G   
    G    
   A   
   T   
   C   
   T   
   T   
   G   
   C   
   A   
   A   
   G   
   G   
    A    
   A   
   A   
   A   
   G   
   A   
   T   
   G   
   A   
   A   
   A   
   T   
   C   
   A   
   A   
   A   
   A   
   A   
   A   
   G   
   C   
   T   
   T   
   A   
   T   
   G   
   A   
   A   
   A   
   C   
   A   
   G   
   G   
    G    
   C   
   G   
   A   
   G   
   G   
   A   
   A   
   A   
   A   
   G   
   T   
   T   
   A   
    T    
   T   
   C   
   T   
   T   
   C   
   G   
   T   
   T   
   C   
   G   
   A   
   A   
   A   
   A   
   C   
   A   
   A   
   A   
   G   
   A   
   T   
   A   
   G   
   A   
    A    
   G   
   C   
   A   
   A   
   T   
   G   
   A   
   A   
   G   
   G   
   G   
    T    
   A   
   A   
    T    
   A   
   A   
   A   
   C   
   A   
   G   
   C   
   A   
   A   
   A   
   T   
   C   
   G   
   T   
   G   
   A   
   T   
   C   
   A   
   C   
    A    
   G   
   A   
   A   
   A   
   T   
   C   
   C   
   C   
   A   
   T   
   A   
   T   
   G   
   A   
   A   
   G   
   T   
   A   
   A   
   A   
   T   
   A   
   A   
   A   
   G   
   C   
   G   
   A   
   C   
    C    
   T   
   T   
   G   
   G   
   T   
   G   
   A   
   A   
   A   
   A   
   A   
   A   
   A   
   T   
   G   
   G   
   A   
   T   
   G   
   A   
   G   
   A   
   T   
   C   
   C   
   G   
   T   
   T   
   T   
   G   
   A   
   A   
   T   
   A   
   A   
   G   
   A   
   A   
   A   
   A   
   T   
   C   
   G   
   A   
    T    
   G   
   G   
   A   
   A   
   T   
   A   
   G   
   C   
   T   
   G   
   A   
   A   
   G   
   T   
    A    
   C   
   G   
   A   
   G   
   A   
   T   
   G   
   A   
   A   
   A   
   G   
   T   
   G   
   A   
   T   
    A    
   G   
    A    
   A   
   C   
    A    
   G   
   G   
   T   
   C   
   T   
   G   
   C   
   A   
   A   
   A   
   T   
   T   
   G   
   T   
   C   
   G   
   T   
    C    
   G   
   A   
   A   
   T   
   T   
   G   
   A   
   A   
   A   
   A   
   A   
   A   
   G   
   A   
    C    
   G   
   C   
   C   
   A   
   A   
    T    
   G   
   C   
   T   
   C   
   A   
   A   
   G   
   G   
    A    
   A   
   T   
   T   
   T   
   T   
   G   
   A   
   A   
   T   
   T   
   A   
    T    
   T   
   T   
   G   
   T   
   T   
   C   
   A   
   A   
   A   
   A   
   A   
   C   
   A   
   C   
   T   
   G   
   A   
   G   
    C    
   T   
   A   
   C   
   A   
   G   
   A   
   T   
   C   
   A   
   A   
   T   
   T   
   A   
   C   
   A   
   A   
   T   
   T   
   T   
    C    
   A   
   A   
   T   
   A   
   T   
   G   
   G   
   T   
   C   
   G   
   C   
   T   
   A   
   T   
   C   
   G   
   A   
    C    
   C   
   A   
   T   
   A   
   T   
   G   
   A   
   C   
   T   
   C   
   C   
   G   
   C   
   A   
    T    
   C   
   A   
   A   
   G   
   T   
   T   
   G   
   G   
   A   
   C   
   T   
   G   
   A   
   A   
   A   
   G   
   A   
   C   
   A   
   T   
   T   
   T   
   T   
   A   
   A   
   G   
   A   
   A   
   G   
   T   
   T   
   A   
   T   
   A   
   T   
   C   
   G   
   A   
   G   
   C   
   A   
   T   
   C   
   G   
   A   
   A   
   A   
   G   
   C   
   A   
   A   
   G   
   T   
   C   
   A   
   T   
   A   
   A   
   C   
   A   
   A   
   A   
   A   
   C   
   G   
    T    
   A   
   G   
    T    
   C   
   A   
   A   
   T   
   T   
   C   
   G   
   A   
   T   
   T   
   T   
   A   
   G   
   C   
   C   
   A   
   A   
   A   
   G   
   C   
   T   
   C   
   A   
   A   
   A   
   A   
   A   
    A    
   G   
   A   
   C   
   A   
   G   
   C   
   A   
   T   
   A   
   T   
   C   
   G   
   T   
    A    
   G   
   A   
   A   
   G   
   G   
    A    
    T    
   T   
   G   
   A   
   T   
   G   
   A   
   A   
   A   
   G   
   C   
   T   
   T   
   T   
   A   
   T   
   C   
   G   
   A   
   T   
   T   
   C   
   T   
   G   
   G   
   A   
   T   
   G   
   A   
   A   
   G   
   T   
   A   
   A   
   T   
   C   
   G   
    C    
   A   
   A   
   C   
   G   
   A   
   T   
   T   
   C   
   G   
   T   
   G   
   A   
   A   
   A   
   G   
   T   
   A   
   A   
   A   
   G   
   A   
   C   
   A   
   A   
   A   
   A   
   A   
   A   
   G   
   A   
    C    
   G   
   C   
    C    
   A   
   A   
   A   
   A   
   A   
   G   
   A   
   A   
   T   
   T   
   T   
   A   
   G   
   T   
   C   
   G   
   A   
    C    
   G   
   T   
    C    
   T   
   T   
    T    
   C   
   A   
   A   
   T   
   T   
   T   
   A   
   C   
   A   
   G   
   A   
   A   
   G   
   A   
   A   
   C   
   A   
   A   
   G   
   C   
   A   
   G   
   A   
   A   
   G   
   C   
   A   
   A   
   T   
    T    
   G   
   T   
   C   
   A   
   C   
   A   
   T   
   T   
   G   
   C   
   A   
   A   
    T    
   T   
   A   
   T   
   A   
    C    
   C   
   G   
   T   
   T   
   T   
   G   
   A   
   C   
   G   
   A   
   A   
    C    
   A   
   C   
    G    
   G   
   A   
   T   
   A   
   T   
   C   
   A   
   C   
   A   
   G   
   A   
   G   
   C   
   T   
   T   
   C   
   A   
   A   
   A   
   A   
   G   
   G   
   A   
   A   
    T    
   C   
    C    
   G   
   A   
   A   
   A   
    G    
   C   
   T   
   T   
   G   
   A   
   T   
   T   
   G   
   C   
    G    
   C   
   A   
   A   
   A   
   T   
   C   
   A   
   C   
   A   
   G   
   A   
   G   
   T   
   T   
    G    
   A   
   A   
   T   
   A   
   A   
   A   
   A   
   T   
   C   
   C   
   T   
   T   
   T   
   C   
   A   
   A   
   A   
    T    
   G   
   A   
   C   
   A   
   A   
   A   
   G   
   A   
    G    
   C   
   T   
   T   
   T   
   T   
    C    
   T   
   C   
   T   
   G   
   T   
   T   
   A   
   T   
   G   
   A   
   A   
   A   
   A   
   A   
   A   
   G   
   A   
   A   
   T   
   T   
   G   
   C   
   G   
   A   
   G   
   A   
   A   
   G   
   T   
   G   
   A   
   A   
   A   
   A   
   A   
   A   
   A   
   A   
   T   
   T   
   A   
    T    
   T   
   C   
   T   
   T   
   C   
   C   
   G   
   C   
   T   
   C   
   G   
   T   
   C   
    T    
    A    
   A   
   C   
   G   
   A   
   C   
   A   
   A   
   T   
   C   
    G    
   A   
   A   
   G   
   A   
   C   
   G   
   A   
   A   
   A   
   T   
   C   
   G   
   A   
   A   
   G   
   A   
   A   
   A   
   T   
   C   
   A   
   A   
   A   
   A   
   T   
    T    
   G   
   A   
   T   
   A   
   C   
   G   
   C   
   A   
   A   
    G    
   T   
    C    
   T   
   T   
   A   
   G   
   T   
   G   
   G   
   C   
    A    
   C   
   A   
   A   
   G   
   A   
   A   
   G   
   A   
   C   
   G   
   T   
   A   
   A   
   T   
    T    
   G   
   T   
   C   
   A   
   G   
   T   
   G   
   T   
    G    
   A   
   C   
    G    
   C   
   G   
   A   
   G   
   A   
   A   
   G   
   G   
   C   
   T   
   A   
   T   
   G   
   T   
   G   
   A   
   A   
   G   
   C   
   G   
   A   
   A   
   C   
   A   
   A   
   G   
   C   
   C   
   T   
   T   
   C   
   G   
   T   
   T   
   C   
    T    
   T   
   A   
   T   
   A   
   G   
   T   
   G   
   C   
   T   
   T   
   C   
    C    
   A   
   A   
   A   
   C   
   C   
   A   
   G   
   A   
   A   
   G   
   A   
   A   
   A   
   T   
   C   
   G   
   G   
   G   
   A   
   T   
   G   
   C   
   G   
   A   
   G   
   A   
   A   
   G   
   G   
   C   
   G   
   A   
    C    
   T   
   A   
   C   
   T   
   T   
   G   
   C   
   T   
    A    
   T   
   A   
   C   
    T    
   C   
   A   
   G   
   G   
   T   
   G   
   A   
   A   
   T   
   T   
    G    
   A   
   G   
    T    
   A   
   C   
   A   
    T    
   T   
   G   
   G   
   A   
   C   
   C   
   A   
   T   
   G   
   T   
   T   
   C   
   T   
   T   
   C   
   T   
   G   
   A   
   T   
    C    
   A   
   C   
    A    
   A   
   A   
   T   
   A   
   A   
   A   
   G   
   C   
   A   
   A   
   A   
   T   
   G   
   T   
   G   
   A   
   T   
   C   
   T   
   A   
   C   
   C   
   G   
    C    
   C   
   C   
   T   
   G   
   T   
    C    
   C   
   A   
   T   
   G   
   A   
   A   
   C   
   T   
   A   
   C   
   C   
    C    
   G   
   A   
   T   
   T   
   T   
   G   
   A   
   A   
   A   
   T   
   G   
   G   
   A   
   A   
   A   
   G   
   A   
   T   
   G   
   C   
   T   
   G   
   G   
    T    
   G   
   A   
    G    
   C   
   A   
    C    
   A   
   T   
   T   
   T   
   C   
   T   
   C   
   A   
   A   
   A   
   C   
   A   
   A   
   T   
   C   
   T   
   C   
   C   
   A   
   A   
   T   
   C   
   T   
   G   
   T   
   C   
   T   
   G   
   T   
   A   
   G   
   A   
    C    
   G   
   A   
   A   
   T   
   C   
   G   
   A   
   T   
    T    
   T   
   T   
   A   
   G   
   C   
   T   
   G   
   T   
   T   
   T   
   T   
    C    
   C   
   C   
   A   
   T   
   A   
   T   
   C   
   A   
   A   
   A   
   A   
   A   
   A   
   T   
    T    
   C   
   A   
   A   
   G   
   C   
   A   
   G   
   A   
    G    
   A   
   A   
   A   
   A   
   C   
   A   
   T   
   T   
   T   
   G   
   T   
    A    
   T   
   T   
   C   
   A   
   T   
   C   
   A   
   G   
   C   
   A   
   A   
   G   
   A   
   A   
   T   
   G   
   G   
   A   
   T   
   T   
    G    
    A    
   T   
   C   
   A   
   A   
   G   
   C   
   A   
   A   
   A   
   C   
   A   
   C   
   G   
   A   
   A   
   T   
   G   
   A   
   C   
   A   
   G   
   A   
   G   
   T   
   T   
   C   
   G   
   A   
   A   
   C   
   C   
   A   
   T   
   G   
   G   
   C   
   G   
   C   
   A   
   C   
   C   
   T   
   A   
    T    
   A   
   A   
   G   
   A   
   G   
   C   
   C   
   G   
    T    
   C   
   C   
   G   
   C   
   T   
   C   
   A   
   G   
   C   
   T   
   G   
   T   
   A   
   T   
   G   
   A   
   A   
   G   
   C   
   T   
   A   
   A   
   A   
   G   
   G   
   C   
   G   
   G   
   A   
   A   
   G   
   A   
   A   
   G   
   A   
   T   
   G   
   A   
   A   
   C   
   T   
    G    
   A   
   T   
   T   
   G   
   C   
   T   
   G   
   T   
    G    
   T   
   A   
   T   
   C   
   T   
   G   
   A   
   A   
   A   
   G   
   A   
   A   
   G   
   A   
   T   
   C   
    A    
   G   
   C   
   C   
   A   
   G   
   G   
   C   
   T   
   T   
   A   
   G   
   A   
   T   
   G   
   T   
    T    
   T   
   T   
   C   
   T   
   T   
   A   
   G   
   T   
   A   
   A   
   C   
   G   
    C    
   A   
   T   
   C   
   A   
   A   
   G   
   G   
   A   
   A   
   T   
   G   
   G   
   G   
    T    
   T   
   T   
   G   
   C   
   G   
   C   
   T   
    A    
   T   
   C   
   C   
   T   
   T   
   T   
   A   
   A   
    C    
   A   
   G   
   A   
   T   
   G   
   T   
   T   
   C   
   C   
   C   
   G   
   T   
   T   
   G   
   T   
   T   
   G   
   G   
   A   
   G   
   C   
   A   
   A   
   A   
   G   
   G   
   C   
   A   
   G   
    C    
   T   
   G   
   G   
   A   
   G   
   T   
   G   
   A   
   A   
   G   
   T   
   C   
   T   
   A   
   T   
   G   
   A   
   A   
   T   
   T   
   T   
   G   
   A   
   A   
   A   
   G   
   A   
   T   
   G   
   G   
   A   
   G   
   A   
   T   
   A   
   G   
   G   
    A    
   T   
   T   
    A    
   T   
   C   
   A   
   A   
    C    
   G   
   G   
    G    
   C   
   T   
    A    
   C   
   T   
   T   
   G   
   T   
   G   
   T   
   T   
   A   
   G   
   C   
   A   
   G   
   A   
    A    
   G   
   G   
    T    
   G   
   A   
   T   
   A   
   C   
   G   
   C   
   C   
    G    
   A   
   T   
   C   
   G   
   T   
   A   
   A   
   T   
   C   
   G   
   T   
   T   
   A   
   C   
   T   
   C   
   A   
   G   
   C   
   G   
   A   
   G   
   G   
   A   
   G   
   C   
    T    
   G   
   T   
    T    
   A   
   A   
   A   
   C   
   G   
   C   
   A   
   T   
   G   
   C   
   T   
   T   
   G   
   C   
   T   
   C   
   A   
   A   
   G   
   A   
   A   
   A   
   T   
   C   
   A   
   G   
   C   
   C   
   A   
   A   
   A   
   C   
    T    
   T   
   C   
   A   
   C   
   G   
   T   
   G   
   C   
   G   
   A   
   A   
   G   
   C   
   G   
   G   
   G   
   G   
   A   
   G   
   T   
   G   
   A   
   C   
   G   
   G   
   T   
   C   
   A   
   T   
   G   
   C   
   G   
   T   
   G   
   A   
   G   
   C   
   T   
   G   
   A   
   A   
   A   
   A   
   A   
   A   
   A   
   A   
   T   
   C   
   C   
    G    
   C   
   A   
    C    
   C   
   G   
    T    
   A   
   T   
   T   
   A   
   T   
   C   
   T   
   A   
   T   
   A   
   T   
   G   
   T   
   C   
   G   
   G   
   A   
   A   
   G   
   G   
   A   
   A   
   G   
   A   
   T   
   C   
   A   
   A   
   A   
   A   
   G   
   A   
   A   
   A   
   T   
   A   
   A   
   C   
   C   
   T   
   T   
   G   
   A   
   T   
   G   
   A   
   A   
   T   
   C   
   A   
   A   
   A   
   A   
   A   
   G   
   G   
    G    
   C   
   A   
    G    
   C   
   A   
   G   
    T    
   T   
   A   
   A   
   C   
   G   
   A   
   T   
   T   
   G   
   A   
    C    
   C   
   C   
   A   
   A   
   C   
    T    
   G   
   A   
   T   
   T   
   T   
    C    
   C   
   C   
   T   
   A   
   T   
   A   
   G   
   G   
   T   
   G   
   A   
   T   
   C   
   G   
   A   
   A   
   C   
   A   
   T   
   C   
   G   
   A   
   A   
   T   
   G   
   G   
    A    
   T   
   C   
    A    
   T   
   T   
   T   
   G   
   C   
    G    
   A   
   T   
   G   
   G   
   A   
    T    
   G   
   A   
   G   
   A   
   A   
   A   
   A   
   A   
   A   
   G   
   G   
   T   
   G   
   G   
   A   
   G   
   A   
   A   
   G   
   T   
   C   
   A   
   T   
   C   
   G   
   A   
   A   
   G   
   T   
    T    
   A   
    T    
   C   
   G   
   A   
   T   
   G   
   C   
   T   
   C   
   C   
   T   
   G   
   A   
   G   
    A    
   T   
   T   
   T   
   T   
    C    
   A   
   T   
    C    
   G   
   A   
    G    
    T    
   C   
    G    
   -   
   -   
   -   
 

 
   N55290   
   A   
   T   
   G   
   G   
   A   
   A   
   A   
   A   
   T   
   C   
   G   
   A   
   C   
   A   
   A   
   G   
   A   
   A   
   G   
   T   
   A   
   C   
   A   
   A   
   G   
   A   
   A   
   T   
   T   
   A   
   A   
   C   
   T   
   T   
   T   
   A   
   G   
   A   
   A   
   G   
   A   
   A   
   G   
   T   
   G   
   A   
   T   
   G   
   G   
   G   
   T   
   G   
   A   
   C   
   C   
   G   
   C   
   T   
   T   
   C   
   G   
   G   
   A   
   C   
   G   
   T   
   T   
   A   
   T   
   T   
   C   
   C   
   A   
   A   
   A   
   T   
   A   
   T   
   A   
   T   
   T   
   A   
   T   
    C    
   C   
   A   
    A    
   G   
   A   
   A   
   C   
   G   
   T   
   G   
   C   
   A   
   C   
   T   
   G   
   C   
   C   
   T   
   G   
   A   
   T   
   A   
   T   
    C    
   C   
   G   
   T   
   G   
   A   
   T   
   G   
   G   
   A   
   T   
   T   
   G   
   A   
   A   
    A    
   C   
   C   
   A   
   G   
   T   
    G    
   C   
   A   
   A   
   C   
   G   
   C   
   C   
   G   
   G   
   A   
   T   
   A   
   T   
   T   
   A   
   T   
   T   
    T    
   G   
   C   
    C    
   A   
   T   
   G   
   A   
   A   
   C   
   A   
   A   
   A   
   G   
   A   
   T   
   G   
   G   
   C   
   A   
   A   
   T   
   A   
   C   
   G   
   T   
   A   
   T   
   G   
   A   
   C   
   A   
   A   
   A   
   G   
   G   
   A   
   T   
   T   
    C    
   C   
   G   
   T   
   A   
   A   
   A   
   T   
   C   
   A   
   G   
   C   
   A   
   A   
   A   
   A   
   T   
   C   
   T   
   G   
   T   
   C   
   G   
   G   
   A   
   A   
   A   
   T   
   A   
   T   
   C   
   A   
   T   
   G   
   G   
   G   
    A    
   A   
   A   
   T   
   T   
   A   
   T   
   C   
   A   
   T   
   C   
   C   
   T   
   C   
   A   
   T   
   G   
   G   
   T   
   G   
   A   
   C   
   A   
   G   
   C   
   A   
   G   
   T   
   A   
   T   
   T   
   T   
   A   
   T   
   G   
   A   
   A   
   G   
   C   
   G   
   A   
   T   
   G   
   G   
   T   
   C   
   C   
   G   
   C   
   A   
   T   
   G   
   A   
   G   
   C   
   C   
   A   
   A   
   G   
   A   
    T    
   T   
   G   
   G   
   A   
   A   
   G   
   T   
   T   
   G   
   C   
   G   
   G   
   G   
   A   
   A   
   G   
   T   
   A   
   C   
   T   
   G   
   A   
   T   
   C   
   G   
   A   
    G    
   A   
   T   
   G   
   C   
   A   
   C   
   G   
   G   
    A    
   A   
   A   
   T   
   A   
   A   
    C    
   G   
   G   
   G   
   A   
   G   
   T   
   A   
   T   
   G   
   G   
   A   
   C   
   G   
   G   
   T   
   G   
   A   
   T   
   C   
   C   
   A   
   C   
   C   
   T   
   G   
   C   
   T   
   G   
   C   
   G   
   A   
   T   
   G   
   C   
   G   
   T   
   T   
   A   
   T   
   A   
   C   
   T   
   G   
   A   
   A   
   G   
   C   
   C   
   A   
   G   
    A    
   T   
   T   
   A   
   T   
   C   
   C   
   A   
   A   
   G   
   T   
   T   
   A   
   A   
   G   
   T   
   G   
   G   
   A   
   G   
   A   
   G   
   A   
   T   
   G   
    C    
   T   
   A   
    G    
   C   
   A   
   G   
   A   
   T   
   A   
   T   
   C   
   G   
   A   
    A    
   A   
   A   
   A   
   G   
   A   
   A   
   A   
   C   
   A   
   G   
   T   
    T    
   G   
   A   
   T   
   C   
   T   
   C   
   G   
   T   
   G   
   T   
   G   
   G   
   A   
   A   
   T   
   T   
   T   
   T   
   G   
   A   
   T   
   G   
   A   
   T   
   A   
   C   
   A   
   G   
   A   
   A   
   A   
   A   
   A   
   G   
   A   
   A   
   C   
   C   
   A   
   A   
   C   
   A   
   G   
   T   
   C   
   C   
   T   
   T   
   C   
   C   
   A   
   G   
   C   
   T   
   C   
   G   
   A   
   T   
   A   
   C   
   C   
   C   
   A   
   A   
   A   
   C   
   C   
   T   
   T   
   C   
   T   
    A    
   G   
   T   
   C   
   A   
   A   
   T   
   G   
   G   
   A   
   T   
   C   
   G   
   A   
   C   
   A   
   G   
   G   
   T   
   A   
   T   
   A   
   T   
   C   
   T   
   G   
   C   
   T   
   G   
   G   
   A   
   T   
   A   
   T   
   G   
   C   
    G    
   A   
   C   
   A   
   G   
   A   
   A   
   A   
   T   
   C   
   C   
   C   
   G   
   A   
   C   
    G    
   C   
   A   
   T   
   A   
   A   
   C   
   T   
   T   
   A   
   G   
   C   
   T   
   G   
   A   
   A   
   G   
   T   
   G   
   A   
   T   
    C    
   G   
   A   
    C    
   G   
   G   
   C   
   A   
   C   
   A   
   A   
   T   
   C   
   T   
   A   
   T   
   A   
   T   
   G   
   A   
   T   
   C   
   G   
   A   
   T   
   C   
   A   
   T   
   C   
   C   
   A   
   C   
   A   
   A   
   G   
   C   
   G   
   A   
   G   
   C   
   C   
   T   
   T   
   G   
   A   
   A   
   A   
   A   
   A   
   T   
   T   
   G   
   A   
   T   
   G   
   G   
   A   
   A   
   T   
   A   
   T   
   A   
   T   
   T   
   C   
   C   
   T   
   G   
   G   
   A   
   C   
   C   
   T   
   G   
   A   
   C   
   T   
   T   
    T    
   C   
   C   
   A   
   A   
   C   
   A   
   G   
   G   
   A   
   G   
   G   
    G    
   A   
   T   
   C   
   T   
   T   
   G   
   C   
   A   
   A   
   G   
   G   
    A    
   A   
   A   
   A   
   G   
   A   
   T   
   G   
   A   
   A   
   A   
   T   
   C   
   A   
   A   
   A   
   A   
   A   
   A   
   G   
   C   
   T   
   T   
   A   
   T   
   G   
   A   
   A   
   A   
   C   
   A   
   G   
   G   
    G    
   C   
   G   
   A   
   G   
   G   
   A   
   A   
   A   
   A   
   G   
   T   
   T   
   A   
    T    
   T   
   C   
   T   
   T   
   C   
   G   
   T   
   T   
   C   
   G   
   A   
   A   
   A   
   A   
   C   
   A   
   A   
   A   
   G   
   A   
   T   
   A   
   G   
   A   
    A    
   G   
   C   
   A   
   A   
   T   
   G   
   A   
   A   
   G   
   G   
   G   
    C    
   A   
   A   
    C    
   A   
   A   
   A   
   C   
   A   
   G   
   C   
   A   
   A   
   A   
   T   
   C   
   G   
   T   
   G   
   A   
   T   
   C   
   A   
   C   
    T    
   G   
   A   
   A   
   A   
   T   
   C   
   C   
   C   
   A   
   T   
   A   
   T   
   G   
   A   
   A   
   G   
   T   
   A   
   A   
   A   
   T   
   A   
   A   
   A   
   G   
   C   
   G   
   A   
   C   
    A    
   T   
   T   
   G   
   G   
   T   
   G   
   A   
   A   
   A   
   A   
   A   
   A   
   A   
   T   
   G   
   G   
   A   
   T   
   G   
   A   
   G   
   A   
   T   
   C   
   C   
   G   
   T   
   T   
   T   
   G   
   A   
   A   
   T   
   A   
   A   
   G   
   A   
   A   
   A   
   A   
   T   
   C   
   G   
   A   
    T    
   G   
   G   
   A   
   A   
   T   
   A   
   G   
   C   
   T   
   G   
   A   
   A   
   G   
   T   
    A    
   C   
   G   
   A   
   G   
   A   
   T   
   G   
   A   
   A   
   A   
   G   
   T   
   G   
   A   
   T   
    C    
   G   
    G    
   A   
   C   
    T    
   G   
   G   
   T   
   C   
   T   
   G   
   C   
   A   
   A   
   A   
   T   
   T   
   G   
   T   
   C   
   G   
   T   
    T    
   G   
   A   
   A   
   T   
   T   
   G   
   A   
   A   
   A   
   A   
   A   
   A   
   G   
   A   
    C    
   G   
   C   
   C   
   A   
   A   
    C    
   G   
   C   
   T   
   C   
   A   
   A   
   G   
   G   
    T    
   A   
   T   
   T   
   T   
   T   
   G   
   A   
   A   
   T   
   T   
   A   
    T    
   T   
   T   
   G   
   T   
   T   
   C   
   A   
   A   
   A   
   A   
   A   
   C   
   A   
   C   
   T   
   G   
   A   
   G   
    C    
   T   
   A   
   C   
   A   
   G   
   A   
   T   
   C   
   A   
   A   
   T   
   T   
   A   
   C   
   A   
   A   
   T   
   T   
   T   
    C    
   A   
   A   
   T   
   A   
   T   
   G   
   G   
   T   
   C   
   G   
   C   
   T   
   A   
   T   
   C   
   G   
   A   
    T    
   C   
   A   
   T   
   A   
   T   
   G   
   A   
   C   
   T   
   C   
   C   
   G   
   C   
   A   
    T    
   C   
   A   
   A   
   G   
   T   
   T   
   G   
   G   
   A   
   C   
   T   
   G   
   A   
   A   
   A   
   G   
   A   
   C   
   A   
   T   
   T   
   T   
   T   
   A   
   A   
   G   
   A   
   A   
   G   
   T   
   T   
   A   
   T   
   A   
   T   
   C   
   G   
   A   
   G   
   C   
   A   
   T   
   C   
   G   
   A   
   A   
   A   
   G   
   C   
   A   
   A   
   G   
   T   
   C   
   A   
   T   
   A   
   A   
   C   
   A   
   A   
   A   
   A   
   C   
   G   
    C    
   A   
   G   
    C    
   C   
   A   
   A   
   T   
   T   
   C   
   G   
   A   
   T   
   T   
   T   
   A   
   G   
   C   
   C   
   A   
   A   
   A   
   G   
   C   
   T   
   C   
   A   
   A   
   A   
   A   
   A   
    A    
   G   
   A   
   C   
   A   
   G   
   C   
   A   
   T   
   A   
   T   
   C   
   G   
   T   
    G    
   G   
   A   
   A   
   G   
   G   
    C    
    C    
   T   
   G   
   A   
   T   
   G   
   A   
   A   
   A   
   G   
   C   
   T   
   T   
   T   
   A   
   T   
   C   
   G   
   A   
   T   
   T   
   C   
   T   
   G   
   G   
   A   
   T   
   G   
   A   
   A   
   G   
   T   
   A   
   A   
   T   
   C   
   G   
    C    
   A   
   A   
   C   
   G   
   A   
   T   
   T   
   C   
   G   
   T   
   G   
   A   
   A   
   A   
   G   
   T   
   A   
   A   
   A   
   G   
   A   
   C   
   A   
   A   
   A   
   A   
   A   
   A   
   G   
   A   
    C    
   G   
   C   
    C    
   A   
   A   
   A   
   A   
   A   
   G   
   A   
   A   
   T   
   T   
   T   
   A   
   G   
   T   
   C   
   G   
   A   
    C    
   G   
   T   
    T    
   T   
   T   
    C    
   C   
   A   
   A   
   T   
   T   
   T   
   A   
   C   
   A   
   G   
   A   
   A   
   G   
   A   
   A   
   C   
   A   
   A   
   G   
   C   
   A   
   G   
   A   
   A   
   G   
   C   
   A   
   A   
   T   
    C    
   G   
   T   
   C   
   A   
   C   
   A   
   T   
   T   
   G   
   C   
   A   
   A   
    C    
   T   
   A   
   T   
   A   
    T    
   C   
   G   
   T   
   T   
   T   
   G   
   A   
   C   
   G   
   A   
   A   
    C    
   A   
   C   
    A    
   G   
   A   
   T   
   A   
   T   
   C   
   A   
   C   
   A   
   G   
   A   
   G   
   C   
   T   
   T   
   C   
   A   
   A   
   A   
   A   
   G   
   G   
   A   
   A   
    T    
   C   
    T    
   G   
   A   
   A   
   A   
    G    
   C   
   T   
   T   
   G   
   A   
   T   
   T   
   G   
   C   
    A    
   C   
   A   
   A   
   A   
   T   
   C   
   A   
   C   
   A   
   G   
   A   
   G   
   T   
   T   
    G    
   A   
   A   
   T   
   A   
   A   
   A   
   A   
   T   
   C   
   C   
   T   
   T   
   T   
   C   
   A   
   A   
   A   
    C    
   G   
   A   
   C   
   A   
   A   
   A   
   G   
   A   
    G    
   C   
   T   
   T   
   T   
   T   
    C    
   T   
   C   
   T   
   G   
   T   
   T   
   A   
   T   
   G   
   A   
   A   
   A   
   A   
   A   
   A   
   G   
   A   
   A   
   T   
   T   
   G   
   C   
   G   
   A   
   G   
   A   
   A   
   G   
   T   
   G   
   A   
   A   
   A   
   A   
   A   
   A   
   A   
   A   
   T   
   T   
   A   
    C    
   T   
   C   
   T   
   T   
   C   
   C   
   G   
   C   
   T   
   C   
   G   
   T   
   C   
    T    
    G    
   A   
   C   
   G   
   A   
   C   
   A   
   A   
   T   
   C   
    G    
   A   
   A   
   G   
   A   
   C   
   G   
   A   
   A   
   A   
   T   
   C   
   G   
   A   
   A   
   G   
   A   
   A   
   A   
   T   
   C   
   A   
   A   
   A   
   A   
   T   
    C    
   G   
   A   
   T   
   A   
   C   
   G   
   C   
   A   
   A   
    G    
   T   
    T    
   T   
   T   
   A   
   G   
   T   
   G   
   G   
   C   
    G    
   C   
   A   
   A   
   G   
   A   
   A   
   G   
   A   
   C   
   G   
   T   
   A   
   A   
   T   
    C    
   G   
   T   
   C   
   A   
   G   
   T   
   G   
   T   
    G    
   A   
   C   
    A    
   C   
   G   
   A   
   G   
   A   
   A   
   G   
   G   
   C   
   T   
   A   
   T   
   G   
   T   
   G   
   A   
   A   
   G   
   C   
   G   
   A   
   A   
   C   
   A   
   A   
   G   
   C   
   C   
   T   
   T   
   C   
   G   
   T   
   T   
   C   
    T    
   T   
   A   
   T   
   A   
   G   
   T   
   G   
   C   
   T   
   T   
   C   
    C    
   A   
   A   
   A   
   C   
   C   
   A   
   G   
   A   
   A   
   G   
   A   
   A   
   A   
   T   
   C   
   G   
   G   
   G   
   A   
   T   
   G   
   C   
   G   
   A   
   G   
   A   
   A   
   G   
   G   
   C   
   G   
   A   
    C    
   T   
   A   
   C   
   T   
   T   
   G   
   C   
   T   
    G    
   T   
   A   
   C   
    T    
   C   
   A   
   G   
   G   
   T   
   G   
   A   
   A   
   T   
   T   
    G    
   A   
   G   
    C    
   A   
   C   
   A   
    T    
   T   
   G   
   G   
   A   
   C   
   C   
   A   
   T   
   G   
   T   
   T   
   C   
   T   
   T   
   C   
   T   
   G   
   A   
   T   
    T    
   A   
   C   
    G    
   A   
   A   
   T   
   A   
   A   
   A   
   G   
   C   
   A   
   A   
   A   
   T   
   G   
   T   
   G   
   A   
   T   
   C   
   T   
   A   
   C   
   C   
   G   
    T    
   C   
   C   
   T   
   G   
   T   
    T    
   C   
   A   
   T   
   G   
   A   
   A   
   C   
   T   
   A   
   C   
   C   
    C    
   G   
   A   
   T   
   T   
   T   
   G   
   A   
   A   
   A   
   T   
   G   
   G   
   A   
   A   
   A   
   G   
   A   
   T   
   G   
   C   
   T   
   G   
   G   
    C    
   G   
   A   
    A    
   C   
   A   
    C    
   A   
   T   
   T   
   T   
   C   
   T   
   C   
   A   
   A   
   A   
   C   
   A   
   A   
   T   
   C   
   T   
   C   
   C   
   A   
   A   
   T   
   C   
   T   
   G   
   T   
   C   
   T   
   G   
   T   
   A   
   G   
   A   
    T    
   G   
   A   
   A   
   T   
   C   
   G   
   A   
   T   
    C    
   T   
   T   
   A   
   G   
   C   
   T   
   G   
   T   
   T   
   T   
   T   
    C    
   C   
   C   
   A   
   T   
   A   
   T   
   C   
   A   
   A   
   A   
   A   
   A   
   A   
   T   
    C    
   C   
   A   
   A   
   G   
   C   
   A   
   G   
   A   
    G    
   A   
   A   
   A   
   A   
   C   
   A   
   T   
   T   
   T   
   G   
   T   
    T    
   T   
   T   
   C   
   A   
   T   
   C   
   A   
   G   
   C   
   A   
   A   
   G   
   A   
   A   
   T   
   G   
   G   
   A   
   T   
   T   
    G    
    A    
   T   
   C   
   A   
   A   
   G   
   C   
   A   
   A   
   A   
   C   
   A   
   C   
   G   
   A   
   A   
   T   
   G   
   A   
   C   
   A   
   G   
   A   
   G   
   T   
   T   
   C   
   G   
   A   
   A   
   C   
   C   
   A   
   T   
   G   
   G   
   C   
   G   
   C   
   A   
   C   
   C   
   T   
   A   
    C    
   A   
   A   
   G   
   A   
   G   
   C   
   C   
   G   
    T    
   C   
   C   
   G   
   C   
   T   
   C   
   A   
   G   
   C   
   T   
   G   
   T   
   A   
   T   
   G   
   A   
   A   
   G   
   C   
   T   
   A   
   A   
   A   
   G   
   G   
   C   
   G   
   G   
   A   
   A   
   G   
   A   
   A   
   G   
   A   
   T   
   G   
   A   
   A   
   C   
   T   
    T    
   A   
   T   
   T   
   G   
   C   
   T   
   G   
   T   
    A    
   T   
   A   
   T   
   C   
   T   
   G   
   A   
   A   
   A   
   G   
   A   
   A   
   G   
   A   
   T   
   C   
    A    
   G   
   C   
   C   
   A   
   G   
   G   
   C   
   T   
   T   
   A   
   G   
   A   
   T   
   G   
   T   
    G    
   T   
   T   
   C   
   T   
   T   
   A   
   G   
   T   
   A   
   A   
   C   
   G   
    C    
   A   
   T   
   C   
   A   
   A   
   G   
   G   
   A   
   A   
   T   
   G   
   G   
   G   
    C    
   T   
   T   
   G   
   C   
   G   
   C   
   T   
    A    
   T   
   C   
   C   
   T   
   T   
   T   
   A   
   A   
    C    
   A   
   G   
   A   
   T   
   G   
   T   
   T   
   C   
   C   
   C   
   G   
   T   
   T   
   G   
   T   
   T   
   G   
   G   
   A   
   G   
   C   
   A   
   A   
   A   
   G   
   G   
   C   
   A   
   G   
    C    
   T   
   G   
   G   
   A   
   G   
   T   
   G   
   A   
   A   
   G   
   T   
   C   
   T   
   A   
   T   
   G   
   A   
   A   
   T   
   T   
   T   
   G   
   A   
   A   
   A   
   G   
   A   
   T   
   G   
   G   
   A   
   G   
   A   
   T   
   A   
   G   
   G   
    A    
   T   
   T   
    G    
   T   
   C   
   A   
   A   
    T    
   G   
   G   
    G    
   C   
   T   
    G    
   C   
   T   
   T   
   G   
   T   
   G   
   T   
   T   
   A   
   G   
   C   
   A   
   G   
   A   
    C    
   G   
   G   
    A    
   G   
   A   
   T   
   A   
   C   
   G   
   C   
   C   
    G    
   A   
   T   
   C   
   G   
   T   
   A   
   A   
   T   
   C   
   G   
   T   
   T   
   A   
   C   
   T   
   C   
   A   
   G   
   C   
   G   
   A   
   G   
   G   
   A   
   G   
   C   
    T    
   G   
   T   
    G    
   A   
   A   
   A   
   C   
   G   
   C   
   A   
   T   
   G   
   C   
   T   
   T   
   G   
   C   
   T   
   C   
   A   
   A   
   G   
   A   
   A   
   A   
   T   
   C   
   A   
   G   
   C   
   C   
   A   
   A   
   A   
   C   
    T    
   T   
   C   
   A   
   C   
   G   
   T   
   G   
   C   
   G   
   A   
   A   
   G   
   C   
   G   
   G   
   G   
   G   
   A   
   G   
   T   
   G   
   A   
   C   
   G   
   G   
   T   
   C   
   A   
   T   
   G   
   C   
   G   
   T   
   G   
   A   
   G   
   C   
   T   
   G   
   A   
   A   
   A   
   A   
   A   
   A   
   A   
   A   
   T   
   C   
   C   
    G    
   C   
   A   
    C    
   C   
   G   
    C    
   A   
   T   
   T   
   A   
   T   
   C   
   T   
   A   
   T   
   A   
   T   
   G   
   T   
   C   
   G   
   G   
   A   
   A   
   G   
   G   
   A   
   A   
   G   
   A   
   T   
   C   
   A   
   A   
   A   
   A   
   G   
   A   
   A   
   A   
   T   
   A   
   A   
   C   
   C   
   T   
   T   
   G   
   A   
   T   
   G   
   A   
   A   
   T   
   C   
   A   
   A   
   A   
   A   
   A   
   G   
   G   
    A    
   C   
   A   
    A    
   C   
   A   
   G   
    A    
   T   
   A   
   A   
   C   
   G   
   A   
   T   
   T   
   G   
   A   
    T    
   C   
   C   
   A   
   A   
   C   
    G    
   G   
   A   
   T   
   T   
   T   
    T    
   C   
   C   
   T   
   A   
   T   
   A   
   G   
   G   
   T   
   G   
   A   
   T   
   C   
   G   
   A   
   A   
   C   
   A   
   T   
   C   
   G   
   A   
   A   
   T   
   G   
   G   
    T    
   T   
   C   
    G    
   T   
   T   
   T   
   G   
   C   
    T    
   A   
   T   
   G   
   G   
   A   
    C    
   G   
   A   
   G   
   A   
   A   
   A   
   A   
   A   
   A   
   G   
   G   
   T   
   G   
   G   
   A   
   G   
   A   
   A   
   G   
   T   
   C   
   A   
   T   
   C   
   G   
   A   
   A   
   G   
   T   
    T    
   A   
    T    
   C   
   G   
   A   
   T   
   G   
   C   
   T   
   C   
   C   
   T   
   G   
   A   
   G   
    A    
   T   
   T   
   T   
   T   
    C    
   A   
   T   
    C    
   G   
   A   
    A    
    T    
   C   
    A    
   -   
   -   
   -   
 

 
   N52777   
   A   
   T   
   G   
   G   
   A   
   A   
   A   
   A   
   T   
   C   
   G   
   A   
   C   
   A   
   A   
   G   
   A   
   A   
   G   
   T   
   A   
   C   
   A   
   A   
   G   
   A   
   A   
   T   
   T   
   A   
   A   
   C   
   T   
   T   
   T   
   A   
   G   
   A   
   A   
   G   
   A   
   A   
   G   
   T   
   G   
   A   
   T   
   G   
   G   
   G   
   T   
   G   
   A   
   C   
   C   
   G   
   C   
   T   
   T   
   C   
   G   
   G   
   A   
   C   
   G   
   T   
   T   
   A   
   T   
   T   
   C   
   C   
   A   
   A   
   A   
   T   
   A   
   T   
   A   
   T   
   T   
   A   
   T   
    C    
   C   
   A   
    A    
   G   
   A   
   A   
   C   
   G   
   T   
   G   
   C   
   A   
   C   
   T   
   G   
   C   
   C   
   T   
   G   
   A   
   T   
   A   
   T   
    C    
   C   
   G   
   T   
   G   
   A   
   T   
   G   
   G   
   A   
   T   
   T   
   G   
   A   
   A   
    G    
   C   
   C   
   A   
   G   
   T   
    G    
   C   
   A   
   A   
   C   
   G   
   C   
   C   
   G   
   G   
   A   
   T   
   A   
   T   
   T   
   A   
   T   
   T   
    T    
   G   
   C   
    C    
   A   
   T   
   G   
   A   
   A   
   C   
   A   
   A   
   A   
   G   
   A   
   T   
   G   
   G   
   C   
   A   
   A   
   T   
   A   
   C   
   G   
   T   
   A   
   T   
   G   
   A   
   C   
   A   
   A   
   A   
   G   
   G   
   A   
   T   
   T   
    C    
   C   
   G   
   T   
   A   
   A   
   A   
   T   
   C   
   A   
   G   
   C   
   A   
   A   
   A   
   A   
   T   
   C   
   T   
   G   
   T   
   C   
   G   
   G   
   A   
   A   
   A   
   T   
   A   
   T   
   C   
   A   
   T   
   G   
   G   
   G   
    G    
   A   
   A   
   T   
   T   
   A   
   T   
   C   
   A   
   T   
   C   
   C   
   T   
   C   
   A   
   T   
   G   
   G   
   T   
   G   
   A   
   C   
   A   
   G   
   C   
   A   
   G   
   T   
   A   
   T   
   T   
   T   
   A   
   T   
   G   
   A   
   A   
   G   
   C   
   G   
   A   
   T   
   G   
   G   
   T   
   C   
   C   
   G   
   C   
   A   
   T   
   G   
   A   
   G   
   C   
   C   
   A   
   A   
   G   
   A   
    C    
   T   
   G   
   G   
   A   
   A   
   G   
   T   
   T   
   G   
   C   
   G   
   G   
   G   
   A   
   A   
   G   
   T   
   A   
   C   
   T   
   G   
   A   
   T   
   C   
   G   
   A   
    A    
   A   
   T   
   G   
   C   
   A   
   C   
   G   
   G   
    G    
   A   
   A   
   T   
   A   
   A   
    C    
   G   
   G   
   G   
   A   
   G   
   T   
   A   
   T   
   G   
   G   
   A   
   C   
   G   
   G   
   T   
   G   
   A   
   T   
   C   
   C   
   A   
   C   
   C   
   T   
   G   
   C   
   T   
   G   
   C   
   G   
   A   
   T   
   G   
   C   
   G   
   T   
   T   
   A   
   T   
   A   
   C   
   T   
   G   
   A   
   A   
   G   
   C   
   C   
   A   
   G   
    A    
   T   
   T   
   A   
   T   
   C   
   C   
   A   
   A   
   G   
   T   
   T   
   A   
   A   
   G   
   T   
   G   
   G   
   A   
   G   
   A   
   G   
   A   
   T   
   G   
    C    
   T   
   A   
    G    
   C   
   A   
   G   
   A   
   T   
   A   
   T   
   C   
   G   
   A   
    A    
   A   
   A   
   A   
   G   
   A   
   A   
   A   
   C   
   A   
   G   
   T   
    C    
   G   
   A   
   T   
   C   
   T   
   C   
   G   
   T   
   G   
   T   
   G   
   G   
   A   
   A   
   T   
   T   
   T   
   T   
   G   
   A   
   T   
   G   
   A   
   T   
   A   
   C   
   A   
   G   
   A   
   A   
   A   
   A   
   A   
   G   
   A   
   A   
   C   
   C   
   A   
   A   
   C   
   A   
   G   
   T   
   C   
   C   
   T   
   T   
   C   
   C   
   A   
   G   
   C   
   T   
   C   
   G   
   A   
   T   
   A   
   C   
   C   
   C   
   A   
   A   
   A   
   C   
   C   
   T   
   T   
   C   
   T   
    C    
   G   
   T   
   C   
   A   
   A   
   T   
   G   
   G   
   A   
   T   
   C   
   G   
   A   
   C   
   A   
   G   
   G   
   T   
   A   
   T   
   A   
   T   
   C   
   T   
   G   
   C   
   T   
   G   
   G   
   A   
   T   
   A   
   T   
   G   
   C   
    A    
   A   
   C   
   A   
   G   
   A   
   A   
   A   
   T   
   C   
   C   
   C   
   G   
   A   
   C   
    T    
   C   
   A   
   T   
   A   
   A   
   C   
   T   
   T   
   A   
   G   
   C   
   T   
   G   
   A   
   A   
   G   
   T   
   G   
   A   
   T   
    C    
   G   
   A   
    T    
   G   
   G   
   C   
   A   
   C   
   A   
   A   
   T   
   C   
   T   
   A   
   T   
   A   
   T   
   G   
   A   
   T   
   C   
   G   
   A   
   T   
   C   
   A   
   T   
   C   
   C   
   A   
   C   
   A   
   A   
   G   
   C   
   G   
   A   
   G   
   C   
   C   
   T   
   T   
   G   
   A   
   A   
   A   
   A   
   A   
   T   
   T   
   G   
   A   
   T   
   G   
   G   
   A   
   A   
   T   
   A   
   T   
   A   
   T   
   T   
   C   
   C   
   T   
   G   
   G   
   A   
   C   
   C   
   T   
   G   
   A   
   C   
   T   
   T   
    T    
   C   
   C   
   A   
   A   
   C   
   A   
   G   
   G   
   A   
   G   
   G   
    G    
   A   
   T   
   C   
   T   
   T   
   G   
   C   
   A   
   A   
   G   
   G   
    A    
   A   
   A   
   A   
   G   
   A   
   T   
   G   
   A   
   A   
   A   
   T   
   C   
   A   
   A   
   A   
   A   
   A   
   A   
   G   
   C   
   T   
   T   
   A   
   T   
   G   
   A   
   A   
   A   
   C   
   A   
   G   
   G   
    G    
   C   
   G   
   A   
   G   
   G   
   A   
   A   
   A   
   A   
   G   
   T   
   T   
   A   
    T    
   T   
   C   
   T   
   T   
   C   
   G   
   T   
   T   
   C   
   G   
   A   
   A   
   A   
   A   
   C   
   A   
   A   
   A   
   G   
   A   
   T   
   A   
   G   
   A   
    A    
   G   
   C   
   A   
   A   
   T   
   G   
   A   
   A   
   G   
   G   
   G   
    T    
   A   
   A   
    T    
   A   
   A   
   A   
   C   
   A   
   G   
   C   
   A   
   A   
   A   
   T   
   C   
   G   
   T   
   G   
   A   
   T   
   C   
   A   
   C   
    A    
   G   
   A   
   A   
   A   
   T   
   C   
   C   
   C   
   A   
   T   
   A   
   T   
   G   
   A   
   A   
   G   
   T   
   A   
   A   
   A   
   T   
   A   
   A   
   A   
   G   
   C   
   G   
   A   
   C   
    C    
   T   
   T   
   G   
   G   
   T   
   G   
   A   
   A   
   A   
   A   
   A   
   A   
   A   
   T   
   G   
   G   
   A   
   T   
   G   
   A   
   G   
   A   
   T   
   C   
   C   
   G   
   T   
   T   
   T   
   G   
   A   
   A   
   T   
   A   
   A   
   G   
   A   
   A   
   A   
   A   
   T   
   C   
   G   
   A   
    T    
   G   
   G   
   A   
   A   
   T   
   A   
   G   
   C   
   T   
   G   
   A   
   A   
   G   
   T   
    A    
   C   
   G   
   A   
   G   
   A   
   T   
   G   
   A   
   A   
   A   
   G   
   T   
   G   
   A   
   T   
    A    
   G   
    A    
   A   
   C   
    A    
   G   
   G   
   T   
   C   
   T   
   G   
   C   
   A   
   A   
   A   
   T   
   T   
   G   
   T   
   C   
   G   
   T   
    C    
   G   
   A   
   A   
   T   
   T   
   G   
   A   
   A   
   A   
   A   
   A   
   A   
   G   
   A   
    C    
   G   
   C   
   C   
   A   
   A   
    T    
   G   
   C   
   T   
   C   
   A   
   A   
   G   
   G   
    A    
   A   
   T   
   T   
   T   
   T   
   G   
   A   
   A   
   T   
   T   
   A   
    T    
   T   
   T   
   G   
   T   
   T   
   C   
   A   
   A   
   A   
   A   
   A   
   C   
   A   
   C   
   T   
   G   
   A   
   G   
    C    
   T   
   A   
   C   
   A   
   G   
   A   
   T   
   C   
   A   
   A   
   T   
   T   
   A   
   C   
   A   
   A   
   T   
   T   
   T   
    C    
   A   
   A   
   T   
   A   
   T   
   G   
   G   
   T   
   C   
   G   
   C   
   T   
   A   
   T   
   C   
   G   
   A   
    C    
   C   
   A   
   T   
   A   
   T   
   G   
   A   
   C   
   T   
   C   
   C   
   G   
   C   
   A   
    T    
   C   
   A   
   A   
   G   
   T   
   T   
   G   
   G   
   A   
   C   
   T   
   G   
   A   
   A   
   A   
   G   
   A   
   C   
   A   
   T   
   T   
   T   
   T   
   A   
   A   
   G   
   A   
   A   
   G   
   T   
   T   
   A   
   T   
   A   
   T   
   C   
   G   
   A   
   G   
   C   
   A   
   T   
   C   
   G   
   A   
   A   
   A   
   G   
   C   
   A   
   A   
   G   
   T   
   C   
   A   
   T   
   A   
   A   
   C   
   A   
   A   
   A   
   A   
   C   
   G   
    T    
   A   
   G   
    T    
   C   
   A   
   A   
   T   
   T   
   C   
   G   
   A   
   T   
   T   
   T   
   A   
   G   
   C   
   C   
   A   
   A   
   A   
   G   
   C   
   T   
   C   
   A   
   A   
   A   
   A   
   A   
    A    
   G   
   A   
   C   
   A   
   G   
   C   
   A   
   T   
   A   
   T   
   C   
   G   
   T   
    A    
   G   
   A   
   A   
   G   
   G   
    A    
    T    
   T   
   G   
   A   
   T   
   G   
   A   
   A   
   A   
   G   
   C   
   T   
   T   
   T   
   A   
   T   
   C   
   G   
   A   
   T   
   T   
   C   
   T   
   G   
   G   
   A   
   T   
   G   
   A   
   A   
   G   
   T   
   A   
   A   
   T   
   C   
   G   
    C    
   A   
   A   
   C   
   G   
   A   
   T   
   T   
   C   
   G   
   T   
   G   
   A   
   A   
   A   
   G   
   T   
   A   
   A   
   A   
   G   
   A   
   C   
   A   
   A   
   A   
   A   
   A   
   A   
   G   
   A   
    C    
   G   
   C   
    C    
   A   
   A   
   A   
   A   
   A   
   G   
   A   
   A   
   T   
   T   
   T   
   A   
   G   
   T   
   C   
   G   
   A   
    C    
   G   
   T   
    C    
   T   
   T   
    T    
   C   
   A   
   A   
   T   
   T   
   T   
   A   
   C   
   A   
   G   
   A   
   A   
   G   
   A   
   A   
   C   
   A   
   A   
   G   
   C   
   A   
   G   
   A   
   A   
   G   
   C   
   A   
   A   
   T   
    T    
   G   
   T   
   C   
   A   
   C   
   A   
   T   
   T   
   G   
   C   
   A   
   A   
    T    
   T   
   A   
   T   
   A   
    C    
   C   
   G   
   T   
   T   
   T   
   G   
   A   
   C   
   G   
   A   
   A   
    C    
   A   
   C   
    G    
   G   
   A   
   T   
   A   
   T   
   C   
   A   
   C   
   A   
   G   
   A   
   G   
   C   
   T   
   T   
   C   
   A   
   A   
   A   
   A   
   G   
   G   
   A   
   A   
    T    
   C   
    C    
   G   
   A   
   A   
   A   
    G    
   C   
   T   
   T   
   G   
   A   
   T   
   T   
   G   
   C   
    G    
   C   
   A   
   A   
   A   
   T   
   C   
   A   
   C   
   A   
   G   
   A   
   G   
   T   
   T   
    G    
   A   
   A   
   T   
   A   
   A   
   A   
   A   
   T   
   C   
   C   
   T   
   T   
   T   
   C   
   A   
   A   
   A   
    T    
   G   
   A   
   C   
   A   
   A   
   A   
   G   
   A   
    G    
   C   
   T   
   T   
   T   
   T   
    C    
   T   
   C   
   T   
   G   
   T   
   T   
   A   
   T   
   G   
   A   
   A   
   A   
   A   
   A   
   A   
   G   
   A   
   A   
   T   
   T   
   G   
   C   
   G   
   A   
   G   
   A   
   A   
   G   
   T   
   G   
   A   
   A   
   A   
   A   
   A   
   A   
   A   
   A   
   T   
   T   
   A   
    T    
   T   
   C   
   T   
   T   
   C   
   C   
   G   
   C   
   T   
   C   
   G   
   T   
   C   
    T    
    A    
   A   
   C   
   G   
   A   
   C   
   A   
   A   
   T   
   C   
    G    
   A   
   A   
   G   
   A   
   C   
   G   
   A   
   A   
   A   
   T   
   C   
   G   
   A   
   A   
   G   
   A   
   A   
   A   
   T   
   C   
   A   
   A   
   A   
   A   
   T   
    T    
   G   
   A   
   T   
   A   
   C   
   G   
   C   
   A   
   A   
    G    
   T   
    C    
   T   
   T   
   A   
   G   
   T   
   G   
   G   
   C   
    A    
   C   
   A   
   A   
   G   
   A   
   A   
   G   
   A   
   C   
   G   
   T   
   A   
   A   
   T   
    T    
   G   
   T   
   C   
   A   
   G   
   T   
   G   
   T   
    G    
   A   
   C   
    G    
   C   
   G   
   A   
   G   
   A   
   A   
   G   
   G   
   C   
   T   
   A   
   T   
   G   
   T   
   G   
   A   
   A   
   G   
   C   
   G   
   A   
   A   
   C   
   A   
   A   
   G   
   C   
   C   
   T   
   T   
   C   
   G   
   T   
   T   
   C   
    T    
   T   
   A   
   T   
   A   
   G   
   T   
   G   
   C   
   T   
   T   
   C   
    C    
   A   
   A   
   A   
   C   
   C   
   A   
   G   
   A   
   A   
   G   
   A   
   A   
   A   
   T   
   C   
   G   
   G   
   G   
   A   
   T   
   G   
   C   
   G   
   A   
   G   
   A   
   A   
   G   
   G   
   C   
   G   
   A   
    C    
   T   
   A   
   C   
   T   
   T   
   G   
   C   
   T   
    A    
   T   
   A   
   C   
    T    
   C   
   A   
   G   
   G   
   T   
   G   
   A   
   A   
   T   
   T   
    G    
   A   
   G   
    T    
   A   
   C   
   A   
    T    
   T   
   G   
   G   
   A   
   C   
   C   
   A   
   T   
   G   
   T   
   T   
   C   
   T   
   T   
   C   
   T   
   G   
   A   
   T   
    C    
   A   
   C   
    A    
   A   
   A   
   T   
   A   
   A   
   A   
   G   
   C   
   A   
   A   
   A   
   T   
   G   
   T   
   G   
   A   
   T   
   C   
   T   
   A   
   C   
   C   
   G   
    C    
   C   
   C   
   T   
   G   
   T   
    C    
   C   
   A   
   T   
   G   
   A   
   A   
   C   
   T   
   A   
   C   
   C   
    C    
   G   
   A   
   T   
   T   
   T   
   G   
   A   
   A   
   A   
   T   
   G   
   G   
   A   
   A   
   A   
   G   
   A   
   T   
   G   
   C   
   T   
   G   
   G   
    T    
   G   
   A   
    G    
   C   
   A   
    C    
   A   
   T   
   T   
   T   
   C   
   T   
   C   
   A   
   A   
   A   
   C   
   A   
   A   
   T   
   C   
   T   
   C   
   C   
   A   
   A   
   T   
   C   
   T   
   G   
   T   
   C   
   T   
   G   
   T   
   A   
   G   
   A   
    C    
   G   
   A   
   A   
   T   
   C   
   G   
   A   
   T   
    T    
   T   
   T   
   A   
   G   
   C   
   T   
   G   
   T   
   T   
   T   
   T   
    C    
   C   
   C   
   A   
   T   
   A   
   T   
   C   
   A   
   A   
   A   
   A   
   A   
   A   
   T   
    T    
   C   
   A   
   A   
   G   
   C   
   A   
   G   
   A   
    G    
   A   
   A   
   A   
   A   
   C   
   A   
   T   
   T   
   T   
   G   
   T   
    A    
   T   
   T   
   C   
   A   
   T   
   C   
   A   
   G   
   C   
   A   
   A   
   G   
   A   
   A   
   T   
   G   
   G   
   A   
   T   
   T   
    G    
    A    
   T   
   C   
   A   
   A   
   G   
   C   
   A   
   A   
   A   
   C   
   A   
   C   
   G   
   A   
   A   
   T   
   G   
   A   
   C   
   A   
   G   
   A   
   G   
   T   
   T   
   C   
   G   
   A   
   A   
   C   
   C   
   A   
   T   
   G   
   G   
   C   
   G   
   C   
   A   
   C   
   C   
   T   
   A   
    T    
   A   
   A   
   G   
   A   
   G   
   C   
   C   
   G   
    T    
   C   
   C   
   G   
   C   
   T   
   C   
   A   
   G   
   C   
   T   
   G   
   T   
   A   
   T   
   G   
   A   
   A   
   G   
   C   
   T   
   A   
   A   
   A   
   G   
   G   
   C   
   G   
   G   
   A   
   A   
   G   
   A   
   A   
   G   
   A   
   T   
   G   
   A   
   A   
   C   
   T   
    G    
   A   
   T   
   T   
   G   
   C   
   T   
   G   
   T   
    G    
   T   
   A   
   T   
   C   
   T   
   G   
   A   
   A   
   A   
   G   
   A   
   A   
   G   
   A   
   T   
   C   
    A    
   G   
   C   
   C   
   A   
   G   
   G   
   C   
   T   
   T   
   A   
   G   
   A   
   T   
   G   
   T   
    T    
   T   
   T   
   C   
   T   
   T   
   A   
   G   
   T   
   A   
   A   
   C   
   G   
    C    
   A   
   T   
   C   
   A   
   A   
   G   
   G   
   A   
   A   
   T   
   G   
   G   
   G   
    T    
   T   
   T   
   G   
   C   
   G   
   C   
   T   
    A    
   T   
   C   
   C   
   T   
   T   
   T   
   A   
   A   
    C    
   A   
   G   
   A   
   T   
   G   
   T   
   T   
   C   
   C   
   C   
   G   
   T   
   T   
   G   
   T   
   T   
   G   
   G   
   A   
   G   
   C   
   A   
   A   
   A   
   G   
   G   
   C   
   A   
   G   
    C    
   T   
   G   
   G   
   A   
   G   
   T   
   G   
   A   
   A   
   G   
   T   
   C   
   T   
   A   
   T   
   G   
   A   
   A   
   T   
   T   
   T   
   G   
   A   
   A   
   A   
   G   
   A   
   T   
   G   
   G   
   A   
   G   
   A   
   T   
   A   
   G   
   G   
    A    
   T   
   T   
    A    
   T   
   C   
   A   
   A   
    C    
   G   
   G   
    G    
   C   
   T   
    A    
   C   
   T   
   T   
   G   
   T   
   G   
   T   
   T   
   A   
   G   
   C   
   A   
   G   
   A   
    A    
   G   
   G   
    T    
   G   
   A   
   T   
   A   
   C   
   G   
   C   
   C   
    G    
   A   
   T   
   C   
   G   
   T   
   A   
   A   
   T   
   C   
   G   
   T   
   T   
   A   
   C   
   T   
   C   
   A   
   G   
   C   
   G   
   A   
   G   
   G   
   A   
   G   
   C   
    T    
   G   
   T   
    T    
   A   
   A   
   A   
   C   
   G   
   C   
   A   
   T   
   G   
   C   
   T   
   T   
   G   
   C   
   T   
   C   
   A   
   A   
   G   
   A   
   A   
   A   
   T   
   C   
   A   
   G   
   C   
   C   
   A   
   A   
   A   
   C   
    T    
   T   
   C   
   A   
   C   
   G   
   T   
   G   
   C   
   G   
   A   
   A   
   G   
   C   
   G   
   G   
   G   
   G   
   A   
   G   
   T   
   G   
   A   
   C   
   G   
   G   
   T   
   C   
   A   
   T   
   G   
   C   
   G   
   T   
   G   
   A   
   G   
   C   
   T   
   G   
   A   
   A   
   A   
   A   
   A   
   A   
   A   
   A   
   T   
   C   
   C   
    G    
   C   
   A   
    C    
   C   
   G   
    T    
   A   
   T   
   T   
   A   
   T   
   C   
   T   
   A   
   T   
   A   
   T   
   G   
   T   
   C   
   G   
   G   
   A   
   A   
   G   
   G   
   A   
   A   
   G   
   A   
   T   
   C   
   A   
   A   
   A   
   A   
   G   
   A   
   A   
   A   
   T   
   A   
   A   
   C   
   C   
   T   
   T   
   G   
   A   
   T   
   G   
   A   
   A   
   T   
   C   
   A   
   A   
   A   
   A   
   A   
   G   
   G   
    G    
   C   
   A   
    G    
   C   
   A   
   G   
    T    
   T   
   A   
   A   
   C   
   G   
   A   
   T   
   T   
   G   
   A   
    C    
   C   
   C   
   A   
   A   
   C   
    T    
   G   
   A   
   T   
   T   
   T   
    C    
   C   
   C   
   T   
   A   
   T   
   A   
   G   
   G   
   T   
   G   
   A   
   T   
   C   
   G   
   A   
   A   
   C   
   A   
   T   
   C   
   G   
   A   
   A   
   T   
   G   
   G   
    A    
   T   
   C   
    A    
   T   
   T   
   T   
   G   
   C   
    G    
   A   
   T   
   G   
   G   
   A   
    T    
   G   
   A   
   G   
   A   
   A   
   A   
   A   
   A   
   A   
   G   
   G   
   T   
   G   
   G   
   A   
   G   
   A   
   A   
   G   
   T   
   C   
   A   
   T   
   C   
   G   
   A   
   A   
   G   
   T   
    T    
   A   
    T    
   C   
   G   
   A   
   T   
   G   
   C   
   T   
   C   
   C   
   T   
   G   
   A   
   G   
    A    
   T   
   T   
   T   
   T   
    C    
   A   
   T   
    C    
   G   
   A   
    G    
    T    
   C   
    G    
   -   
   -   
   -   
 

 
   N54599   
   A   
   T   
   G   
   G   
   A   
   A   
   A   
   A   
   T   
   C   
   G   
   A   
   C   
   A   
   A   
   G   
   A   
   A   
   G   
   T   
   A   
   C   
   A   
   A   
   G   
   A   
   A   
   T   
   T   
   A   
   A   
   C   
   T   
   T   
   T   
   A   
   G   
   A   
   A   
   G   
   A   
   A   
   G   
   T   
   G   
   A   
   T   
   G   
   G   
   G   
   T   
   G   
   A   
   C   
   C   
   G   
   C   
   T   
   T   
   C   
   G   
   G   
   A   
   C   
   G   
   T   
   T   
   A   
   T   
   T   
   C   
   C   
   A   
   A   
   A   
   T   
   A   
   T   
   A   
   T   
   T   
   A   
   T   
    C    
   C   
   A   
    A    
   G   
   A   
   A   
   C   
   G   
   T   
   G   
   C   
   A   
   C   
   T   
   G   
   C   
   C   
   T   
   G   
   A   
   T   
   A   
   T   
    C    
   C   
   G   
   T   
   G   
   A   
   T   
   G   
   G   
   A   
   T   
   T   
   G   
   A   
   A   
    A    
   C   
   C   
   A   
   G   
   T   
    G    
   C   
   A   
   A   
   C   
   G   
   C   
   C   
   G   
   G   
   A   
   T   
   A   
   T   
   T   
   A   
   T   
   T   
    T    
   G   
   C   
    C    
   A   
   T   
   G   
   A   
   A   
   C   
   A   
   A   
   A   
   G   
   A   
   T   
   G   
   G   
   C   
   A   
   A   
   T   
   A   
   C   
   G   
   T   
   A   
   T   
   G   
   A   
   C   
   A   
   A   
   A   
   G   
   G   
   A   
   T   
   T   
    C    
   C   
   G   
   T   
   A   
   A   
   A   
   T   
   C   
   A   
   G   
   C   
   A   
   A   
   A   
   A   
   T   
   C   
   T   
   G   
   T   
   C   
   G   
   G   
   A   
   A   
   A   
   T   
   A   
   T   
   C   
   A   
   T   
   G   
   G   
   G   
    A    
   A   
   A   
   T   
   T   
   A   
   T   
   C   
   A   
   T   
   C   
   C   
   T   
   C   
   A   
   T   
   G   
   G   
   T   
   G   
   A   
   C   
   A   
   G   
   C   
   A   
   G   
   T   
   A   
   T   
   T   
   T   
   A   
   T   
   G   
   A   
   A   
   G   
   C   
   G   
   A   
   T   
   G   
   G   
   T   
   C   
   C   
   G   
   C   
   A   
   T   
   G   
   A   
   G   
   C   
   C   
   A   
   A   
   G   
   A   
    T    
   T   
   G   
   G   
   A   
   A   
   G   
   T   
   T   
   G   
   C   
   G   
   G   
   G   
   A   
   A   
   G   
   T   
   A   
   C   
   T   
   G   
   A   
   T   
   C   
   G   
   A   
    G    
   A   
   T   
   G   
   C   
   A   
   C   
   G   
   G   
    A    
   A   
   A   
   T   
   A   
   A   
    C    
   G   
   G   
   G   
   A   
   G   
   T   
   A   
   T   
   G   
   G   
   A   
   C   
   G   
   G   
   T   
   G   
   A   
   T   
   C   
   C   
   A   
   C   
   C   
   T   
   G   
   C   
   T   
   G   
   C   
   G   
   A   
   T   
   G   
   C   
   G   
   T   
   T   
   A   
   T   
   A   
   C   
   T   
   G   
   A   
   A   
   G   
   C   
   C   
   A   
   G   
    A    
   T   
   T   
   A   
   T   
   C   
   C   
   A   
   A   
   G   
   T   
   T   
   A   
   A   
   G   
   T   
   G   
   G   
   A   
   G   
   A   
   G   
   A   
   T   
   G   
    C    
   T   
   A   
    G    
   C   
   A   
   G   
   A   
   T   
   A   
   T   
   C   
   G   
   A   
    A    
   A   
   A   
   A   
   G   
   A   
   A   
   A   
   C   
   A   
   G   
   T   
    T    
   G   
   A   
   T   
   C   
   T   
   C   
   G   
   T   
   G   
   T   
   G   
   G   
   A   
   A   
   T   
   T   
   T   
   T   
   G   
   A   
   T   
   G   
   A   
   T   
   A   
   C   
   A   
   G   
   A   
   A   
   A   
   A   
   A   
   G   
   A   
   A   
   C   
   C   
   A   
   A   
   C   
   A   
   G   
   T   
   C   
   C   
   T   
   T   
   C   
   C   
   A   
   G   
   C   
   T   
   C   
   G   
   A   
   T   
   A   
   C   
   C   
   C   
   A   
   A   
   A   
   C   
   C   
   T   
   T   
   C   
   T   
    A    
   G   
   T   
   C   
   A   
   A   
   T   
   G   
   G   
   A   
   T   
   C   
   G   
   A   
   C   
   A   
   G   
   G   
   T   
   A   
   T   
   A   
   T   
   C   
   T   
   G   
   C   
   T   
   G   
   G   
   A   
   T   
   A   
   T   
   G   
   C   
    G    
   A   
   C   
   A   
   G   
   A   
   A   
   A   
   T   
   C   
   C   
   C   
   G   
   A   
   C   
    G    
   C   
   A   
   T   
   A   
   A   
   C   
   T   
   T   
   A   
   G   
   C   
   T   
   G   
   A   
   A   
   G   
   T   
   G   
   A   
   T   
    C    
   G   
   A   
    C    
   G   
   G   
   C   
   A   
   C   
   A   
   A   
   T   
   C   
   T   
   A   
   T   
   A   
   T   
   G   
   A   
   T   
   C   
   G   
   A   
   T   
   C   
   A   
   T   
   C   
   C   
   A   
   C   
   A   
   A   
   G   
   C   
   G   
   A   
   G   
   C   
   C   
   T   
   T   
   G   
   A   
   A   
   A   
   A   
   A   
   T   
   T   
   G   
   A   
   T   
   G   
   G   
   A   
   A   
   T   
   A   
   T   
   A   
   T   
   T   
   C   
   C   
   T   
   G   
   G   
   A   
   C   
   C   
   T   
   G   
   A   
   C   
   T   
   T   
    T    
   C   
   C   
   A   
   A   
   C   
   A   
   G   
   G   
   A   
   G   
   G   
    G    
   A   
   T   
   C   
   T   
   T   
   G   
   C   
   A   
   A   
   G   
   G   
    A    
   A   
   A   
   A   
   G   
   A   
   T   
   G   
   A   
   A   
   A   
   T   
   C   
   A   
   A   
   A   
   A   
   A   
   A   
   G   
   C   
   T   
   T   
   A   
   T   
   G   
   A   
   A   
   A   
   C   
   A   
   G   
   G   
    G    
   C   
   G   
   A   
   G   
   G   
   A   
   A   
   A   
   A   
   G   
   T   
   T   
   A   
    T    
   T   
   C   
   T   
   T   
   C   
   G   
   T   
   T   
   C   
   G   
   A   
   A   
   A   
   A   
   C   
   A   
   A   
   A   
   G   
   A   
   T   
   A   
   G   
   A   
    A    
   G   
   C   
   A   
   A   
   T   
   G   
   A   
   A   
   G   
   G   
   G   
    C    
   A   
   A   
    C    
   A   
   A   
   A   
   C   
   A   
   G   
   C   
   A   
   A   
   A   
   T   
   C   
   G   
   T   
   G   
   A   
   T   
   C   
   A   
   C   
    T    
   G   
   A   
   A   
   A   
   T   
   C   
   C   
   C   
   A   
   T   
   A   
   T   
   G   
   A   
   A   
   G   
   T   
   A   
   A   
   A   
   T   
   A   
   A   
   A   
   G   
   C   
   G   
   A   
   C   
    A    
   T   
   T   
   G   
   G   
   T   
   G   
   A   
   A   
   A   
   A   
   A   
   A   
   A   
   T   
   G   
   G   
   A   
   T   
   G   
   A   
   G   
   A   
   T   
   C   
   C   
   G   
   T   
   T   
   T   
   G   
   A   
   A   
   T   
   A   
   A   
   G   
   A   
   A   
   A   
   A   
   T   
   C   
   G   
   A   
    T    
   G   
   G   
   A   
   A   
   T   
   A   
   G   
   C   
   T   
   G   
   A   
   A   
   G   
   T   
    A    
   C   
   G   
   A   
   G   
   A   
   T   
   G   
   A   
   A   
   A   
   G   
   T   
   G   
   A   
   T   
    C    
   G   
    G    
   A   
   C   
    T    
   G   
   G   
   T   
   C   
   T   
   G   
   C   
   A   
   A   
   A   
   T   
   T   
   G   
   T   
   C   
   G   
   T   
    T    
   G   
   A   
   A   
   T   
   T   
   G   
   A   
   A   
   A   
   A   
   A   
   A   
   G   
   A   
    C    
   G   
   C   
   C   
   A   
   A   
    C    
   G   
   C   
   T   
   C   
   A   
   A   
   G   
   G   
    T    
   A   
   T   
   T   
   T   
   T   
   G   
   A   
   A   
   T   
   T   
   A   
    T    
   T   
   T   
   G   
   T   
   T   
   C   
   A   
   A   
   A   
   A   
   A   
   C   
   A   
   C   
   T   
   G   
   A   
   G   
    C    
   T   
   A   
   C   
   A   
   G   
   A   
   T   
   C   
   A   
   A   
   T   
   T   
   A   
   C   
   A   
   A   
   T   
   T   
   T   
    C    
   A   
   A   
   T   
   A   
   T   
   G   
   G   
   T   
   C   
   G   
   C   
   T   
   A   
   T   
   C   
   G   
   A   
    T    
   C   
   A   
   T   
   A   
   T   
   G   
   A   
   C   
   T   
   C   
   C   
   G   
   C   
   A   
    T    
   C   
   A   
   A   
   G   
   T   
   T   
   G   
   G   
   A   
   C   
   T   
   G   
   A   
   A   
   A   
   G   
   A   
   C   
   A   
   T   
   T   
   T   
   T   
   A   
   A   
   G   
   A   
   A   
   G   
   T   
   T   
   A   
   T   
   A   
   T   
   C   
   G   
   A   
   G   
   C   
   A   
   T   
   C   
   G   
   A   
   A   
   A   
   G   
   C   
   A   
   A   
   G   
   T   
   C   
   A   
   T   
   A   
   A   
   C   
   A   
   A   
   A   
   A   
   C   
   G   
    C    
   A   
   G   
    C    
   C   
   A   
   A   
   T   
   T   
   C   
   G   
   A   
   T   
   T   
   T   
   A   
   G   
   C   
   C   
   A   
   A   
   A   
   G   
   C   
   T   
   C   
   A   
   A   
   A   
   A   
   A   
    A    
   G   
   A   
   C   
   A   
   G   
   C   
   A   
   T   
   A   
   T   
   C   
   G   
   T   
    G    
   G   
   A   
   A   
   G   
   G   
    C    
    C    
   T   
   G   
   A   
   T   
   G   
   A   
   A   
   A   
   G   
   C   
   T   
   T   
   T   
   A   
   T   
   C   
   G   
   A   
   T   
   T   
   C   
   T   
   G   
   G   
   A   
   T   
   G   
   A   
   A   
   G   
   T   
   A   
   A   
   T   
   C   
   G   
    C    
   A   
   A   
   C   
   G   
   A   
   T   
   T   
   C   
   G   
   T   
   G   
   A   
   A   
   A   
   G   
   T   
   A   
   A   
   A   
   G   
   A   
   C   
   A   
   A   
   A   
   A   
   A   
   A   
   G   
   A   
    C    
   G   
   C   
    C    
   A   
   A   
   A   
   A   
   A   
   G   
   A   
   A   
   T   
   T   
   T   
   A   
   G   
   T   
   C   
   G   
   A   
    C    
   G   
   T   
    T    
   T   
   T   
    C    
   C   
   A   
   A   
   T   
   T   
   T   
   A   
   C   
   A   
   G   
   A   
   A   
   G   
   A   
   A   
   C   
   A   
   A   
   G   
   C   
   A   
   G   
   A   
   A   
   G   
   C   
   A   
   A   
   T   
    C    
   G   
   T   
   C   
   A   
   C   
   A   
   T   
   T   
   G   
   C   
   A   
   A   
    C    
   T   
   A   
   T   
   A   
    T    
   C   
   G   
   T   
   T   
   T   
   G   
   A   
   C   
   G   
   A   
   A   
    C    
   A   
   C   
    A    
   G   
   A   
   T   
   A   
   T   
   C   
   A   
   C   
   A   
   G   
   A   
   G   
   C   
   T   
   T   
   C   
   A   
   A   
   A   
   A   
   G   
   G   
   A   
   A   
    T    
   C   
    T    
   G   
   A   
   A   
   A   
    G    
   C   
   T   
   T   
   G   
   A   
   T   
   T   
   G   
   C   
    A    
   C   
   A   
   A   
   A   
   T   
   C   
   A   
   C   
   A   
   G   
   A   
   G   
   T   
   T   
    G    
   A   
   A   
   T   
   A   
   A   
   A   
   A   
   T   
   C   
   C   
   T   
   T   
   T   
   C   
   A   
   A   
   A   
    C    
   G   
   A   
   C   
   A   
   A   
   A   
   G   
   A   
    G    
   C   
   T   
   T   
   T   
   T   
    C    
   T   
   C   
   T   
   G   
   T   
   T   
   A   
   T   
   G   
   A   
   A   
   A   
   A   
   A   
   A   
   G   
   A   
   A   
   T   
   T   
   G   
   C   
   G   
   A   
   G   
   A   
   A   
   G   
   T   
   G   
   A   
   A   
   A   
   A   
   A   
   A   
   A   
   A   
   T   
   T   
   A   
    C    
   T   
   C   
   T   
   T   
   C   
   C   
   G   
   C   
   T   
   C   
   G   
   T   
   C   
    T    
    G    
   A   
   C   
   G   
   A   
   C   
   A   
   A   
   T   
   C   
    G    
   A   
   A   
   G   
   A   
   C   
   G   
   A   
   A   
   A   
   T   
   C   
   G   
   A   
   A   
   G   
   A   
   A   
   A   
   T   
   C   
   A   
   A   
   A   
   A   
   T   
    C    
   G   
   A   
   T   
   A   
   C   
   G   
   C   
   A   
   A   
    G    
   T   
    T    
   T   
   T   
   A   
   G   
   T   
   G   
   G   
   C   
    G    
   C   
   A   
   A   
   G   
   A   
   A   
   G   
   A   
   C   
   G   
   T   
   A   
   A   
   T   
    C    
   G   
   T   
   C   
   A   
   G   
   T   
   G   
   T   
    G    
   A   
   C   
    A    
   C   
   G   
   A   
   G   
   A   
   A   
   G   
   G   
   C   
   T   
   A   
   T   
   G   
   T   
   G   
   A   
   A   
   G   
   C   
   G   
   A   
   A   
   C   
   A   
   A   
   G   
   C   
   C   
   T   
   T   
   C   
   G   
   T   
   T   
   C   
    T    
   T   
   A   
   T   
   A   
   G   
   T   
   G   
   C   
   T   
   T   
   C   
    C    
   A   
   A   
   A   
   C   
   C   
   A   
   G   
   A   
   A   
   G   
   A   
   A   
   A   
   T   
   C   
   G   
   G   
   G   
   A   
   T   
   G   
   C   
   G   
   A   
   G   
   A   
   A   
   G   
   G   
   C   
   G   
   A   
    C    
   T   
   A   
   C   
   T   
   T   
   G   
   C   
   T   
    G    
   T   
   A   
   C   
    T    
   C   
   A   
   G   
   G   
   T   
   G   
   A   
   A   
   T   
   T   
    G    
   A   
   G   
    C    
   A   
   C   
   A   
    T    
   T   
   G   
   G   
   A   
   C   
   C   
   A   
   T   
   G   
   T   
   T   
   C   
   T   
   T   
   C   
   T   
   G   
   A   
   T   
    T    
   A   
   C   
    G    
   A   
   A   
   T   
   A   
   A   
   A   
   G   
   C   
   A   
   A   
   A   
   T   
   G   
   T   
   G   
   A   
   T   
   C   
   T   
   A   
   C   
   C   
   G   
    T    
   C   
   C   
   T   
   G   
   T   
    T    
   C   
   A   
   T   
   G   
   A   
   A   
   C   
   T   
   A   
   C   
   C   
    C    
   G   
   A   
   T   
   T   
   T   
   G   
   A   
   A   
   A   
   T   
   G   
   G   
   A   
   A   
   A   
   G   
   A   
   T   
   G   
   C   
   T   
   G   
   G   
    C    
   G   
   A   
    A    
   C   
   A   
    C    
   A   
   T   
   T   
   T   
   C   
   T   
   C   
   A   
   A   
   A   
   C   
   A   
   A   
   T   
   C   
   T   
   C   
   C   
   A   
   A   
   T   
   C   
   T   
   G   
   T   
   C   
   T   
   G   
   T   
   A   
   G   
   A   
    T    
   G   
   A   
   A   
   T   
   C   
   G   
   A   
   T   
    C    
   T   
   T   
   A   
   G   
   C   
   T   
   G   
   T   
   T   
   T   
   T   
    C    
   C   
   C   
   A   
   T   
   A   
   T   
   C   
   A   
   A   
   A   
   A   
   A   
   A   
   T   
    C    
   C   
   A   
   A   
   G   
   C   
   A   
   G   
   A   
    G    
   A   
   A   
   A   
   A   
   C   
   A   
   T   
   T   
   T   
   G   
   T   
    T    
   T   
   T   
   C   
   A   
   T   
   C   
   A   
   G   
   C   
   A   
   A   
   G   
   A   
   A   
   T   
   G   
   G   
   A   
   T   
   T   
    G    
    G    
   T   
   C   
   A   
   A   
   G   
   C   
   A   
   A   
   A   
   C   
   A   
   C   
   G   
   A   
   A   
   T   
   G   
   A   
   C   
   A   
   G   
   A   
   G   
   T   
   T   
   C   
   G   
   A   
   A   
   C   
   C   
   A   
   T   
   G   
   G   
   C   
   G   
   C   
   A   
   C   
   C   
   T   
   A   
    C    
   A   
   A   
   G   
   A   
   G   
   C   
   C   
   G   
    T    
   C   
   C   
   G   
   C   
   T   
   C   
   A   
   G   
   C   
   T   
   G   
   T   
   A   
   T   
   G   
   A   
   A   
   G   
   C   
   T   
   A   
   A   
   A   
   G   
   G   
   C   
   G   
   G   
   A   
   A   
   G   
   A   
   A   
   G   
   A   
   T   
   G   
   A   
   A   
   C   
   T   
    T    
   A   
   T   
   T   
   G   
   C   
   T   
   G   
   T   
    A    
   T   
   A   
   T   
   C   
   T   
   G   
   A   
   A   
   A   
   G   
   A   
   A   
   G   
   A   
   T   
   C   
    A    
   G   
   C   
   C   
   A   
   G   
   G   
   C   
   T   
   T   
   A   
   G   
   A   
   T   
   G   
   T   
    G    
   T   
   T   
   C   
   T   
   T   
   A   
   G   
   T   
   A   
   A   
   C   
   G   
    C    
   A   
   T   
   C   
   A   
   A   
   G   
   G   
   A   
   A   
   T   
   G   
   G   
   G   
    C    
   T   
   T   
   G   
   C   
   G   
   C   
   T   
    A    
   T   
   C   
   C   
   T   
   T   
   T   
   A   
   A   
    C    
   A   
   G   
   A   
   T   
   G   
   T   
   T   
   C   
   C   
   C   
   G   
   T   
   T   
   G   
   T   
   T   
   G   
   G   
   A   
   G   
   C   
   A   
   A   
   A   
   G   
   G   
   C   
   A   
   G   
    C    
   T   
   G   
   G   
   A   
   G   
   T   
   G   
   A   
   A   
   G   
   T   
   C   
   T   
   A   
   T   
   G   
   A   
   A   
   T   
   T   
   T   
   G   
   A   
   A   
   A   
   G   
   A   
   T   
   G   
   G   
   A   
   G   
   A   
   T   
   A   
   G   
   G   
    A    
   T   
   T   
    G    
   T   
   C   
   A   
   A   
    T    
   G   
   G   
    G    
   C   
   T   
    G    
   C   
   T   
   T   
   G   
   T   
   G   
   T   
   T   
   A   
   G   
   C   
   A   
   G   
   A   
    C    
   G   
   G   
    A    
   G   
   A   
   T   
   A   
   C   
   G   
   C   
   C   
    G    
   A   
   T   
   C   
   G   
   T   
   A   
   A   
   T   
   C   
   G   
   T   
   T   
   A   
   C   
   T   
   C   
   A   
   G   
   C   
   G   
   A   
   G   
   G   
   A   
   G   
   C   
    T    
   G   
   T   
    G    
   A   
   A   
   A   
   C   
   G   
   C   
   A   
   T   
   G   
   C   
   T   
   T   
   G   
   C   
   T   
   C   
   A   
   A   
   G   
   A   
   A   
   A   
   T   
   C   
   A   
   G   
   C   
   C   
   A   
   A   
   A   
   C   
    T    
   T   
   C   
   A   
   C   
   G   
   T   
   G   
   C   
   G   
   A   
   A   
   G   
   C   
   G   
   G   
   G   
   G   
   A   
   G   
   T   
   G   
   A   
   C   
   G   
   G   
   T   
   C   
   A   
   T   
   G   
   C   
   G   
   T   
   G   
   A   
   G   
   C   
   T   
   G   
   A   
   A   
   A   
   A   
   A   
   A   
   A   
   A   
   T   
   C   
   C   
    G    
   C   
   A   
    C    
   C   
   G   
    C    
   A   
   T   
   T   
   A   
   T   
   C   
   T   
   A   
   T   
   A   
   T   
   G   
   T   
   C   
   G   
   G   
   A   
   A   
   G   
   G   
   A   
   A   
   G   
   A   
   T   
   C   
   A   
   A   
   A   
   A   
   G   
   A   
   A   
   A   
   T   
   A   
   A   
   C   
   C   
   T   
   T   
   G   
   A   
   T   
   G   
   A   
   A   
   T   
   C   
   A   
   A   
   A   
   A   
   A   
   G   
   G   
    A    
   C   
   A   
    A    
   C   
   A   
   G   
    A    
   T   
   A   
   A   
   C   
   G   
   A   
   T   
   T   
   G   
   A   
    T    
   C   
   C   
   A   
   A   
   C   
    G    
   G   
   A   
   T   
   T   
   T   
    T    
   C   
   C   
   T   
   A   
   T   
   A   
   G   
   G   
   T   
   G   
   A   
   T   
   C   
   G   
   A   
   A   
   C   
   A   
   T   
   C   
   G   
   A   
   A   
   T   
   G   
   G   
    T    
   T   
   C   
    G    
   T   
   T   
   T   
   G   
   C   
    T    
   A   
   T   
   G   
   G   
   A   
    C    
   G   
   A   
   G   
   A   
   A   
   A   
   A   
   A   
   A   
   G   
   G   
   T   
   G   
   G   
   A   
   G   
   A   
   A   
   G   
   T   
   C   
   A   
   T   
   C   
   G   
   A   
   A   
   G   
   T   
    T    
   A   
    T    
   C   
   G   
   A   
   T   
   G   
   C   
   T   
   C   
   C   
   T   
   G   
   A   
   G   
    A    
   T   
   T   
   T   
   T   
    C    
   A   
   T   
    C    
   G   
   A   
    A    
    T    
   C   
    A    
   -   
   -   
   -   
 

 
   N54070   
   A   
   T   
   G   
   G   
   A   
   A   
   A   
   A   
   T   
   C   
   G   
   A   
   C   
   A   
   A   
   G   
   A   
   A   
   G   
   T   
   A   
   C   
   A   
   A   
   G   
   A   
   A   
   T   
   T   
   A   
   A   
   C   
   T   
   T   
   T   
   A   
   G   
   A   
   A   
   G   
   A   
   A   
   G   
   T   
   G   
   A   
   T   
   G   
   G   
   G   
   T   
   G   
   A   
   C   
   C   
   G   
   C   
   T   
   T   
   C   
   G   
   G   
   A   
   C   
   G   
   T   
   T   
   A   
   T   
   T   
   C   
   C   
   A   
   A   
   A   
   T   
   A   
   T   
   A   
   T   
   T   
   A   
   T   
    C    
   C   
   A   
    A    
   G   
   A   
   A   
   C   
   G   
   T   
   G   
   C   
   A   
   C   
   T   
   G   
   C   
   C   
   T   
   G   
   A   
   T   
   A   
   T   
    T    
   C   
   G   
   T   
   G   
   A   
   T   
   G   
   G   
   A   
   T   
   T   
   G   
   A   
   A   
    G    
   C   
   C   
   A   
   G   
   T   
    G    
   C   
   A   
   A   
   C   
   G   
   C   
   C   
   G   
   G   
   A   
   T   
   A   
   T   
   T   
   A   
   T   
   T   
    T    
   G   
   C   
    C    
   A   
   T   
   G   
   A   
   A   
   C   
   A   
   A   
   A   
   G   
   A   
   T   
   G   
   G   
   C   
   A   
   A   
   T   
   A   
   C   
   G   
   T   
   A   
   T   
   G   
   A   
   C   
   A   
   A   
   A   
   G   
   G   
   A   
   T   
   T   
    C    
   C   
   G   
   T   
   A   
   A   
   A   
   T   
   C   
   A   
   G   
   C   
   A   
   A   
   A   
   A   
   T   
   C   
   T   
   G   
   T   
   C   
   G   
   G   
   A   
   A   
   A   
   T   
   A   
   T   
   C   
   A   
   T   
   G   
   G   
   G   
    G    
   A   
   A   
   T   
   T   
   A   
   T   
   C   
   A   
   T   
   C   
   C   
   T   
   C   
   A   
   T   
   G   
   G   
   T   
   G   
   A   
   C   
   A   
   G   
   C   
   A   
   G   
   T   
   A   
   T   
   T   
   T   
   A   
   T   
   G   
   A   
   A   
   G   
   C   
   G   
   A   
   T   
   G   
   G   
   T   
   C   
   C   
   G   
   C   
   A   
   T   
   G   
   A   
   G   
   C   
   C   
   A   
   A   
   G   
   A   
    C    
   T   
   G   
   G   
   A   
   A   
   G   
   T   
   T   
   G   
   C   
   G   
   G   
   G   
   A   
   A   
   G   
   T   
   A   
   C   
   T   
   G   
   A   
   T   
   C   
   G   
   A   
    A    
   A   
   T   
   G   
   C   
   A   
   C   
   G   
   G   
    G    
   A   
   A   
   T   
   A   
   A   
    C    
   G   
   G   
   G   
   A   
   G   
   T   
   A   
   T   
   G   
   G   
   A   
   C   
   G   
   G   
   T   
   G   
   A   
   T   
   C   
   C   
   A   
   C   
   C   
   T   
   G   
   C   
   T   
   G   
   C   
   G   
   A   
   T   
   G   
   C   
   G   
   T   
   T   
   A   
   T   
   A   
   C   
   T   
   G   
   A   
   A   
   G   
   C   
   C   
   A   
   G   
    A    
   T   
   T   
   A   
   T   
   C   
   C   
   A   
   A   
   G   
   T   
   T   
   A   
   A   
   G   
   T   
   G   
   G   
   A   
   G   
   A   
   G   
   A   
   T   
   G   
    C    
   T   
   A   
    G    
   C   
   A   
   G   
   A   
   T   
   A   
   T   
   C   
   G   
   A   
    A    
   A   
   A   
   A   
   G   
   A   
   A   
   A   
   C   
   A   
   G   
   T   
    C    
   G   
   A   
   T   
   C   
   T   
   C   
   G   
   T   
   G   
   T   
   G   
   G   
   A   
   A   
   T   
   T   
   T   
   T   
   G   
   A   
   T   
   G   
   A   
   T   
   A   
   C   
   A   
   G   
   A   
   A   
   A   
   A   
   A   
   G   
   A   
   A   
   C   
   C   
   A   
   A   
   C   
   A   
   G   
   T   
   C   
   C   
   T   
   T   
   C   
   C   
   A   
   G   
   C   
   T   
   C   
   G   
   A   
   T   
   A   
   C   
   C   
   C   
   A   
   A   
   A   
   C   
   C   
   T   
   T   
   C   
   T   
    C    
   G   
   T   
   C   
   A   
   A   
   T   
   G   
   G   
   A   
   T   
   C   
   G   
   A   
   C   
   A   
   G   
   G   
   T   
   A   
   T   
   A   
   T   
   C   
   T   
   G   
   C   
   T   
   G   
   G   
   A   
   T   
   A   
   T   
   G   
   C   
    A    
   A   
   C   
   A   
   G   
   A   
   A   
   A   
   T   
   C   
   C   
   C   
   G   
   A   
   C   
    T    
   C   
   A   
   T   
   A   
   A   
   C   
   T   
   T   
   A   
   G   
   C   
   T   
   G   
   A   
   A   
   G   
   T   
   G   
   A   
   T   
    C    
   G   
   A   
    T    
   G   
   G   
   C   
   A   
   C   
   A   
   A   
   T   
   C   
   T   
   A   
   T   
   A   
   T   
   G   
   A   
   T   
   C   
   G   
   A   
   T   
   C   
   A   
   T   
   C   
   C   
   A   
   C   
   A   
   A   
   G   
   C   
   G   
   A   
   G   
   C   
   C   
   T   
   T   
   G   
   A   
   A   
   A   
   A   
   A   
   T   
   T   
   G   
   A   
   T   
   G   
   G   
   A   
   A   
   T   
   A   
   T   
   A   
   T   
   T   
   C   
   C   
   T   
   G   
   G   
   A   
   C   
   C   
   T   
   G   
   A   
   C   
   T   
   T   
    T    
   C   
   C   
   A   
   A   
   C   
   A   
   G   
   G   
   A   
   G   
   G   
    G    
   A   
   T   
   C   
   T   
   T   
   G   
   C   
   A   
   A   
   G   
   G   
    A    
   A   
   A   
   A   
   G   
   A   
   T   
   G   
   A   
   A   
   A   
   T   
   C   
   A   
   A   
   A   
   A   
   A   
   A   
   G   
   C   
   T   
   T   
   A   
   T   
   G   
   A   
   A   
   A   
   C   
   A   
   G   
   G   
    G    
   C   
   G   
   A   
   G   
   G   
   A   
   A   
   A   
   A   
   G   
   T   
   T   
   A   
    T    
   T   
   C   
   T   
   T   
   C   
   G   
   T   
   T   
   C   
   G   
   A   
   A   
   A   
   A   
   C   
   A   
   A   
   A   
   G   
   A   
   T   
   A   
   G   
   A   
    A    
   G   
   C   
   A   
   A   
   T   
   G   
   A   
   A   
   G   
   G   
   G   
    T    
   A   
   A   
    T    
   A   
   A   
   A   
   C   
   A   
   G   
   C   
   A   
   A   
   A   
   T   
   C   
   G   
   T   
   G   
   A   
   T   
   C   
   A   
   C   
    A    
   G   
   A   
   A   
   A   
   T   
   C   
   C   
   C   
   A   
   T   
   A   
   T   
   G   
   A   
   A   
   G   
   T   
   A   
   A   
   A   
   T   
   A   
   A   
   A   
   G   
   C   
   G   
   A   
   C   
    C    
   T   
   T   
   G   
   G   
   T   
   G   
   A   
   A   
   A   
   A   
   A   
   A   
   A   
   T   
   G   
   G   
   A   
   T   
   G   
   A   
   G   
   A   
   T   
   C   
   C   
   G   
   T   
   T   
   T   
   G   
   A   
   A   
   T   
   A   
   A   
   G   
   A   
   A   
   A   
   A   
   T   
   C   
   G   
   A   
    T    
   G   
   G   
   A   
   A   
   T   
   A   
   G   
   C   
   T   
   G   
   A   
   A   
   G   
   T   
    A    
   C   
   G   
   A   
   G   
   A   
   T   
   G   
   A   
   A   
   A   
   G   
   T   
   G   
   A   
   T   
    A    
   G   
    A    
   A   
   C   
    A    
   G   
   G   
   T   
   C   
   T   
   G   
   C   
   A   
   A   
   A   
   T   
   T   
   G   
   T   
   C   
   G   
   T   
    C    
   G   
   A   
   A   
   T   
   T   
   G   
   A   
   A   
   A   
   A   
   A   
   A   
   G   
   A   
    C    
   G   
   C   
   C   
   A   
   A   
    T    
   G   
   C   
   T   
   C   
   A   
   A   
   G   
   G   
    A    
   A   
   T   
   T   
   T   
   T   
   G   
   A   
   A   
   T   
   T   
   A   
    T    
   T   
   T   
   G   
   T   
   T   
   C   
   A   
   A   
   A   
   A   
   A   
   C   
   A   
   C   
   T   
   G   
   A   
   G   
    C    
   T   
   A   
   C   
   A   
   G   
   A   
   T   
   C   
   A   
   A   
   T   
   T   
   A   
   C   
   A   
   A   
   T   
   T   
   T   
    C    
   A   
   A   
   T   
   A   
   T   
   G   
   G   
   T   
   C   
   G   
   C   
   T   
   A   
   T   
   C   
   G   
   A   
    C    
   C   
   A   
   T   
   A   
   T   
   G   
   A   
   C   
   T   
   C   
   C   
   G   
   C   
   A   
    T    
   C   
   A   
   A   
   G   
   T   
   T   
   G   
   G   
   A   
   C   
   T   
   G   
   A   
   A   
   A   
   G   
   A   
   C   
   A   
   T   
   T   
   T   
   T   
   A   
   A   
   G   
   A   
   A   
   G   
   T   
   T   
   A   
   T   
   A   
   T   
   C   
   G   
   A   
   G   
   C   
   A   
   T   
   C   
   G   
   A   
   A   
   A   
   G   
   C   
   A   
   A   
   G   
   T   
   C   
   A   
   T   
   A   
   A   
   C   
   A   
   A   
   A   
   A   
   C   
   G   
    T    
   A   
   G   
    T    
   C   
   A   
   A   
   T   
   T   
   C   
   G   
   A   
   T   
   T   
   T   
   A   
   G   
   C   
   C   
   A   
   A   
   A   
   G   
   C   
   T   
   C   
   A   
   A   
   A   
   A   
   A   
    A    
   G   
   A   
   C   
   A   
   G   
   C   
   A   
   T   
   A   
   T   
   C   
   G   
   T   
    A    
   G   
   A   
   A   
   G   
   G   
    A    
    T    
   T   
   G   
   A   
   T   
   G   
   A   
   A   
   A   
   G   
   C   
   T   
   T   
   T   
   A   
   T   
   C   
   G   
   A   
   T   
   T   
   C   
   T   
   G   
   G   
   A   
   T   
   G   
   A   
   A   
   G   
   T   
   A   
   A   
   T   
   C   
   G   
    C    
   A   
   A   
   C   
   G   
   A   
   T   
   T   
   C   
   G   
   T   
   G   
   A   
   A   
   A   
   G   
   T   
   A   
   A   
   A   
   G   
   A   
   C   
   A   
   A   
   A   
   A   
   A   
   A   
   G   
   A   
    C    
   G   
   C   
    C    
   A   
   A   
   A   
   A   
   A   
   G   
   A   
   A   
   T   
   T   
   T   
   A   
   G   
   T   
   C   
   G   
   A   
    C    
   G   
   T   
    C    
   T   
   T   
    T    
   C   
   A   
   A   
   T   
   T   
   T   
   A   
   C   
   A   
   G   
   A   
   A   
   G   
   A   
   A   
   C   
   A   
   A   
   G   
   C   
   A   
   G   
   A   
   A   
   G   
   C   
   A   
   A   
   T   
    T    
   G   
   T   
   C   
   A   
   C   
   A   
   T   
   T   
   G   
   C   
   A   
   A   
    T    
   T   
   A   
   T   
   A   
    C    
   C   
   G   
   T   
   T   
   T   
   G   
   A   
   C   
   G   
   A   
   A   
    C    
   A   
   C   
    G    
   G   
   A   
   T   
   A   
   T   
   C   
   A   
   C   
   A   
   G   
   A   
   G   
   C   
   T   
   T   
   C   
   A   
   A   
   A   
   A   
   G   
   G   
   A   
   A   
    T    
   C   
    C    
   G   
   A   
   A   
   A   
    G    
   C   
   T   
   T   
   G   
   A   
   T   
   T   
   G   
   C   
    G    
   C   
   A   
   A   
   A   
   T   
   C   
   A   
   C   
   A   
   G   
   A   
   G   
   T   
   T   
    G    
   A   
   A   
   T   
   A   
   A   
   A   
   A   
   T   
   C   
   C   
   T   
   T   
   T   
   C   
   A   
   A   
   A   
    T    
   G   
   A   
   C   
   A   
   A   
   A   
   G   
   A   
    G    
   C   
   T   
   T   
   T   
   T   
    C    
   T   
   C   
   T   
   G   
   T   
   T   
   A   
   T   
   G   
   A   
   A   
   A   
   A   
   A   
   A   
   G   
   A   
   A   
   T   
   T   
   G   
   C   
   G   
   A   
   G   
   A   
   A   
   G   
   T   
   G   
   A   
   A   
   A   
   A   
   A   
   A   
   A   
   A   
   T   
   T   
   A   
    T    
   T   
   C   
   T   
   T   
   C   
   C   
   G   
   C   
   T   
   C   
   G   
   T   
   C   
    T    
    A    
   A   
   C   
   G   
   A   
   C   
   A   
   A   
   T   
   C   
    G    
   A   
   A   
   G   
   A   
   C   
   G   
   A   
   A   
   A   
   T   
   C   
   G   
   A   
   A   
   G   
   A   
   A   
   A   
   T   
   C   
   A   
   A   
   A   
   A   
   T   
    T    
   G   
   A   
   T   
   A   
   C   
   G   
   C   
   A   
   A   
    G    
   T   
    C    
   T   
   T   
   A   
   G   
   T   
   G   
   G   
   C   
    A    
   C   
   A   
   A   
   G   
   A   
   A   
   G   
   A   
   C   
   G   
   T   
   A   
   A   
   T   
    T    
   G   
   T   
   C   
   A   
   G   
   T   
   G   
   T   
    G    
   A   
   C   
    G    
   C   
   G   
   A   
   G   
   A   
   A   
   G   
   G   
   C   
   T   
   A   
   T   
   G   
   T   
   G   
   A   
   A   
   G   
   C   
   G   
   A   
   A   
   C   
   A   
   A   
   G   
   C   
   C   
   T   
   T   
   C   
   G   
   T   
   T   
   C   
    T    
   T   
   A   
   T   
   A   
   G   
   T   
   G   
   C   
   T   
   T   
   C   
    C    
   A   
   A   
   A   
   C   
   C   
   A   
   G   
   A   
   A   
   G   
   A   
   A   
   A   
   T   
   C   
   G   
   G   
   G   
   A   
   T   
   G   
   C   
   G   
   A   
   G   
   A   
   A   
   G   
   G   
   C   
   G   
   A   
    C    
   T   
   A   
   C   
   T   
   T   
   G   
   C   
   T   
    A    
   T   
   A   
   C   
    T    
   C   
   A   
   G   
   G   
   T   
   G   
   A   
   A   
   T   
   T   
    G    
   A   
   G   
    T    
   A   
   C   
   A   
    T    
   T   
   G   
   G   
   A   
   C   
   C   
   A   
   T   
   G   
   T   
   T   
   C   
   T   
   T   
   C   
   T   
   G   
   A   
   T   
    C    
   A   
   C   
    A    
   A   
   A   
   T   
   A   
   A   
   A   
   G   
   C   
   A   
   A   
   A   
   T   
   G   
   T   
   G   
   A   
   T   
   C   
   T   
   A   
   C   
   C   
   G   
    C    
   C   
   C   
   T   
   G   
   T   
    C    
   C   
   A   
   T   
   G   
   A   
   A   
   C   
   T   
   A   
   C   
   C   
    C    
   G   
   A   
   T   
   T   
   T   
   G   
   A   
   A   
   A   
   T   
   G   
   G   
   A   
   A   
   A   
   G   
   A   
   T   
   G   
   C   
   T   
   G   
   G   
    T    
   G   
   A   
    G    
   C   
   A   
    C    
   A   
   T   
   T   
   T   
   C   
   T   
   C   
   A   
   A   
   A   
   C   
   A   
   A   
   T   
   C   
   T   
   C   
   C   
   A   
   A   
   T   
   C   
   T   
   G   
   T   
   C   
   T   
   G   
   T   
   A   
   G   
   A   
    C    
   G   
   A   
   A   
   T   
   C   
   G   
   A   
   T   
    T    
   T   
   T   
   A   
   G   
   C   
   T   
   G   
   T   
   T   
   T   
   T   
    C    
   C   
   C   
   A   
   T   
   A   
   T   
   C   
   A   
   A   
   A   
   A   
   A   
   A   
   T   
    T    
   C   
   A   
   A   
   G   
   C   
   A   
   G   
   A   
    G    
   A   
   A   
   A   
   A   
   C   
   A   
   T   
   T   
   T   
   G   
   T   
    A    
   T   
   T   
   C   
   A   
   T   
   C   
   A   
   G   
   C   
   A   
   A   
   G   
   A   
   A   
   T   
   G   
   G   
   A   
   T   
   T   
    G    
    A    
   T   
   C   
   A   
   A   
   G   
   C   
   A   
   A   
   A   
   C   
   A   
   C   
   G   
   A   
   A   
   T   
   G   
   A   
   C   
   A   
   G   
   A   
   G   
   T   
   T   
   C   
   G   
   A   
   A   
   C   
   C   
   A   
   T   
   G   
   G   
   C   
   G   
   C   
   A   
   C   
   C   
   T   
   A   
    T    
   A   
   A   
   G   
   A   
   G   
   C   
   C   
   G   
    T    
   C   
   C   
   G   
   C   
   T   
   C   
   A   
   G   
   C   
   T   
   G   
   T   
   A   
   T   
   G   
   A   
   A   
   G   
   C   
   T   
   A   
   A   
   A   
   G   
   G   
   C   
   G   
   G   
   A   
   A   
   G   
   A   
   A   
   G   
   A   
   T   
   G   
   A   
   A   
   C   
   T   
    G    
   A   
   T   
   T   
   G   
   C   
   T   
   G   
   T   
    G    
   T   
   A   
   T   
   C   
   T   
   G   
   A   
   A   
   A   
   G   
   A   
   A   
   G   
   A   
   T   
   C   
    A    
   G   
   C   
   C   
   A   
   G   
   G   
   C   
   T   
   T   
   A   
   G   
   A   
   T   
   G   
   T   
    T    
   T   
   T   
   C   
   T   
   T   
   A   
   G   
   T   
   A   
   A   
   C   
   G   
    C    
   A   
   T   
   C   
   A   
   A   
   G   
   G   
   A   
   A   
   T   
   G   
   G   
   G   
    T    
   T   
   T   
   G   
   C   
   G   
   C   
   T   
    A    
   T   
   C   
   C   
   T   
   T   
   T   
   A   
   A   
    C    
   A   
   G   
   A   
   T   
   G   
   T   
   T   
   C   
   C   
   C   
   G   
   T   
   T   
   G   
   T   
   T   
   G   
   G   
   A   
   G   
   C   
   A   
   A   
   A   
   G   
   G   
   C   
   A   
   G   
    C    
   T   
   G   
   G   
   A   
   G   
   T   
   G   
   A   
   A   
   G   
   T   
   C   
   T   
   A   
   T   
   G   
   A   
   A   
   T   
   T   
   T   
   G   
   A   
   A   
   A   
   G   
   A   
   T   
   G   
   G   
   A   
   G   
   A   
   T   
   A   
   G   
   G   
    A    
   T   
   T   
    A    
   T   
   C   
   A   
   A   
    C    
   G   
   G   
    G    
   C   
   T   
    A    
   C   
   T   
   T   
   G   
   T   
   G   
   T   
   T   
   A   
   G   
   C   
   A   
   G   
   A   
    A    
   G   
   G   
    T    
   G   
   A   
   T   
   A   
   C   
   G   
   C   
   C   
    G    
   A   
   T   
   C   
   G   
   T   
   A   
   A   
   T   
   C   
   G   
   T   
   T   
   A   
   C   
   T   
   C   
   A   
   G   
   C   
   G   
   A   
   G   
   G   
   A   
   G   
   C   
    T    
   G   
   T   
    T    
   A   
   A   
   A   
   C   
   G   
   C   
   A   
   T   
   G   
   C   
   T   
   T   
   G   
   C   
   T   
   C   
   A   
   A   
   G   
   A   
   A   
   A   
   T   
   C   
   A   
   G   
   C   
   C   
   A   
   A   
   A   
   C   
    T    
   T   
   C   
   A   
   C   
   G   
   T   
   G   
   C   
   G   
   A   
   A   
   G   
   C   
   G   
   G   
   G   
   G   
   A   
   G   
   T   
   G   
   A   
   C   
   G   
   G   
   T   
   C   
   A   
   T   
   G   
   C   
   G   
   T   
   G   
   A   
   G   
   C   
   T   
   G   
   A   
   A   
   A   
   A   
   A   
   A   
   A   
   A   
   T   
   C   
   C   
    G    
   C   
   A   
    C    
   C   
   G   
    T    
   A   
   T   
   T   
   A   
   T   
   C   
   T   
   A   
   T   
   A   
   T   
   G   
   T   
   C   
   G   
   G   
   A   
   A   
   G   
   G   
   A   
   A   
   G   
   A   
   T   
   C   
   A   
   A   
   A   
   A   
   G   
   A   
   A   
   A   
   T   
   A   
   A   
   C   
   C   
   T   
   T   
   G   
   A   
   T   
   G   
   A   
   A   
   T   
   C   
   A   
   A   
   A   
   A   
   A   
   G   
   G   
    G    
   C   
   A   
    G    
   C   
   A   
   G   
    T    
   T   
   A   
   A   
   C   
   G   
   A   
   T   
   T   
   G   
   A   
    C    
   C   
   C   
   A   
   A   
   C   
    T    
   G   
   A   
   T   
   T   
   T   
    C    
   C   
   C   
   T   
   A   
   T   
   A   
   G   
   G   
   T   
   G   
   A   
   T   
   C   
   G   
   A   
   A   
   C   
   A   
   T   
   C   
   G   
   A   
   A   
   T   
   G   
   G   
    A    
   T   
   C   
    A    
   T   
   T   
   T   
   G   
   C   
    G    
   A   
   T   
   G   
   G   
   A   
    T    
   G   
   A   
   G   
   A   
   A   
   A   
   A   
   A   
   A   
   G   
   G   
   T   
   G   
   G   
   A   
   G   
   A   
   A   
   G   
   T   
   C   
   A   
   T   
   C   
   G   
   A   
   A   
   G   
   T   
    T    
   A   
    T    
   C   
   G   
   A   
   T   
   G   
   C   
   T   
   C   
   C   
   T   
   G   
   A   
   G   
    A    
   T   
   T   
   T   
   T   
    C    
   A   
   T   
    C    
   G   
   A   
    G    
    T    
   C   
    G    
   -   
   -   
   -   
 

 
   N60490F   
   A   
   T   
   G   
   G   
   A   
   A   
   A   
   A   
   T   
   C   
   G   
   A   
   C   
   A   
   A   
   G   
   A   
   A   
   G   
   T   
   A   
   C   
   A   
   A   
   G   
   A   
   A   
   T   
   T   
   A   
   A   
   C   
   T   
   T   
   T   
   A   
   G   
   A   
   A   
   G   
   A   
   A   
   G   
   T   
   G   
   A   
   T   
   G   
   G   
   G   
   T   
   G   
   A   
   C   
   C   
   G   
   C   
   T   
   T   
   C   
   G   
   G   
   A   
   C   
   G   
   T   
   T   
   A   
   T   
   T   
   C   
   C   
   A   
   A   
   A   
   T   
   A   
   T   
   A   
   T   
   T   
   A   
   T   
    C    
   C   
   A   
    A    
   G   
   A   
   A   
   C   
   G   
   T   
   G   
   C   
   A   
   C   
   T   
   G   
   C   
   C   
   T   
   G   
   A   
   T   
   A   
   T   
    T    
   C   
   G   
   T   
   G   
   A   
   T   
   G   
   G   
   A   
   T   
   T   
   G   
   A   
   A   
    G    
   C   
   C   
   A   
   G   
   T   
    G    
   C   
   A   
   A   
   C   
   G   
   C   
   C   
   G   
   G   
   A   
   T   
   A   
   T   
   T   
   A   
   T   
   T   
    T    
   G   
   C   
    C    
   A   
   T   
   G   
   A   
   A   
   C   
   A   
   A   
   A   
   G   
   A   
   T   
   G   
   G   
   C   
   A   
   A   
   T   
   A   
   C   
   G   
   T   
   A   
   T   
   G   
   A   
   C   
   A   
   A   
   A   
   G   
   G   
   A   
   T   
   T   
    C    
   C   
   G   
   T   
   A   
   A   
   A   
   T   
   C   
   A   
   G   
   C   
   A   
   A   
   A   
   A   
   T   
   C   
   T   
   G   
   T   
   C   
   G   
   G   
   A   
   A   
   A   
   T   
   A   
   T   
   C   
   A   
   T   
   G   
   G   
   G   
    G    
   A   
   A   
   T   
   T   
   A   
   T   
   C   
   A   
   T   
   C   
   C   
   T   
   C   
   A   
   T   
   G   
   G   
   T   
   G   
   A   
   C   
   A   
   G   
   C   
   A   
   G   
   T   
   A   
   T   
   T   
   T   
   A   
   T   
   G   
   A   
   A   
   G   
   C   
   G   
   A   
   T   
   G   
   G   
   T   
   C   
   C   
   G   
   C   
   A   
   T   
   G   
   A   
   G   
   C   
   C   
   A   
   A   
   G   
   A   
    C    
   T   
   G   
   G   
   A   
   A   
   G   
   T   
   T   
   G   
   C   
   G   
   G   
   G   
   A   
   A   
   G   
   T   
   A   
   C   
   T   
   G   
   A   
   T   
   C   
   G   
   A   
    A    
   A   
   T   
   G   
   C   
   A   
   C   
   G   
   G   
    G    
   A   
   A   
   T   
   A   
   A   
    C    
   G   
   G   
   G   
   A   
   G   
   T   
   A   
   T   
   G   
   G   
   A   
   C   
   G   
   G   
   T   
   G   
   A   
   T   
   C   
   C   
   A   
   C   
   C   
   T   
   G   
   C   
   T   
   G   
   C   
   G   
   A   
   T   
   G   
   C   
   G   
   T   
   T   
   A   
   T   
   A   
   C   
   T   
   G   
   A   
   A   
   G   
   C   
   C   
   A   
   G   
    A    
   T   
   T   
   A   
   T   
   C   
   C   
   A   
   A   
   G   
   T   
   T   
   A   
   A   
   G   
   T   
   G   
   G   
   A   
   G   
   A   
   G   
   A   
   T   
   G   
    C    
   T   
   A   
    G    
   C   
   A   
   G   
   A   
   T   
   A   
   T   
   C   
   G   
   A   
    A    
   A   
   A   
   A   
   G   
   A   
   A   
   A   
   C   
   A   
   G   
   T   
    C    
   G   
   A   
   T   
   C   
   T   
   C   
   G   
   T   
   G   
   T   
   G   
   G   
   A   
   A   
   T   
   T   
   T   
   T   
   G   
   A   
   T   
   G   
   A   
   T   
   A   
   C   
   A   
   G   
   A   
   A   
   A   
   A   
   A   
   G   
   A   
   A   
   C   
   C   
   A   
   A   
   C   
   A   
   G   
   T   
   C   
   C   
   T   
   T   
   C   
   C   
   A   
   G   
   C   
   T   
   C   
   G   
   A   
   T   
   A   
   C   
   C   
   C   
   A   
   A   
   A   
   C   
   C   
   T   
   T   
   C   
   T   
    C    
   G   
   T   
   C   
   A   
   A   
   T   
   G   
   G   
   A   
   T   
   C   
   G   
   A   
   C   
   A   
   G   
   G   
   T   
   A   
   T   
   A   
   T   
   C   
   T   
   G   
   C   
   T   
   G   
   G   
   A   
   T   
   A   
   T   
   G   
   C   
    A    
   A   
   C   
   A   
   G   
   A   
   A   
   A   
   T   
   C   
   C   
   C   
   G   
   A   
   C   
    T    
   C   
   A   
   T   
   A   
   A   
   C   
   T   
   T   
   A   
   G   
   C   
   T   
   G   
   A   
   A   
   G   
   T   
   G   
   A   
   T   
    C    
   G   
   A   
    T    
   G   
   G   
   C   
   A   
   C   
   A   
   A   
   T   
   C   
   T   
   A   
   T   
   A   
   T   
   G   
   A   
   T   
   C   
   G   
   A   
   T   
   C   
   A   
   T   
   C   
   C   
   A   
   C   
   A   
   A   
   G   
   C   
   G   
   A   
   G   
   C   
   C   
   T   
   T   
   G   
   A   
   A   
   A   
   A   
   A   
   T   
   T   
   G   
   A   
   T   
   G   
   G   
   A   
   A   
   T   
   A   
   T   
   A   
   T   
   T   
   C   
   C   
   T   
   G   
   G   
   A   
   C   
   C   
   T   
   G   
   A   
   C   
   T   
   T   
    T    
   C   
   C   
   A   
   A   
   C   
   A   
   G   
   G   
   A   
   G   
   G   
    G    
   A   
   T   
   C   
   T   
   T   
   G   
   C   
   A   
   A   
   G   
   G   
    A    
   A   
   A   
   A   
   G   
   A   
   T   
   G   
   A   
   A   
   A   
   T   
   C   
   A   
   A   
   A   
   A   
   A   
   A   
   G   
   C   
   T   
   T   
   A   
   T   
   G   
   A   
   A   
   A   
   C   
   A   
   G   
   G   
    G    
   C   
   G   
   A   
   G   
   G   
   A   
   A   
   A   
   A   
   G   
   T   
   T   
   A   
    T    
   T   
   C   
   T   
   T   
   C   
   G   
   T   
   T   
   C   
   G   
   A   
   A   
   A   
   A   
   C   
   A   
   A   
   A   
   G   
   A   
   T   
   A   
   G   
   A   
    A    
   G   
   C   
   A   
   A   
   T   
   G   
   A   
   A   
   G   
   G   
   G   
    T    
   A   
   A   
    T    
   A   
   A   
   A   
   C   
   A   
   G   
   C   
   A   
   A   
   A   
   T   
   C   
   G   
   T   
   G   
   A   
   T   
   C   
   A   
   C   
    A    
   G   
   A   
   A   
   A   
   T   
   C   
   C   
   C   
   A   
   T   
   A   
   T   
   G   
   A   
   A   
   G   
   T   
   A   
   A   
   A   
   T   
   A   
   A   
   A   
   G   
   C   
   G   
   A   
   C   
    C    
   T   
   T   
   G   
   G   
   T   
   G   
   A   
   A   
   A   
   A   
   A   
   A   
   A   
   T   
   G   
   G   
   A   
   T   
   G   
   A   
   G   
   A   
   T   
   C   
   C   
   G   
   T   
   T   
   T   
   G   
   A   
   A   
   T   
   A   
   A   
   G   
   A   
   A   
   A   
   A   
   T   
   C   
   G   
   A   
    T    
   G   
   G   
   A   
   A   
   T   
   A   
   G   
   C   
   T   
   G   
   A   
   A   
   G   
   T   
    A    
   C   
   G   
   A   
   G   
   A   
   T   
   G   
   A   
   A   
   A   
   G   
   T   
   G   
   A   
   T   
    A    
   G   
    A    
   A   
   C   
    A    
   G   
   G   
   T   
   C   
   T   
   G   
   C   
   A   
   A   
   A   
   T   
   T   
   G   
   T   
   C   
   G   
   T   
    C    
   G   
   A   
   A   
   T   
   T   
   G   
   A   
   A   
   A   
   A   
   A   
   A   
   G   
   A   
    C    
   G   
   C   
   C   
   A   
   A   
    T    
   G   
   C   
   T   
   C   
   A   
   A   
   G   
   G   
    A    
   A   
   T   
   T   
   T   
   T   
   G   
   A   
   A   
   T   
   T   
   A   
    T    
   T   
   T   
   G   
   T   
   T   
   C   
   A   
   A   
   A   
   A   
   A   
   C   
   A   
   C   
   T   
   G   
   A   
   G   
    C    
   T   
   A   
   C   
   A   
   G   
   A   
   T   
   C   
   A   
   A   
   T   
   T   
   A   
   C   
   A   
   A   
   T   
   T   
   T   
    C    
   A   
   A   
   T   
   A   
   T   
   G   
   G   
   T   
   C   
   G   
   C   
   T   
   A   
   T   
   C   
   G   
   A   
    C    
   C   
   A   
   T   
   A   
   T   
   G   
   A   
   C   
   T   
   C   
   C   
   G   
   C   
   A   
    T    
   C   
   A   
   A   
   G   
   T   
   T   
   G   
   G   
   A   
   C   
   T   
   G   
   A   
   A   
   A   
   G   
   A   
   C   
   A   
   T   
   T   
   T   
   T   
   A   
   A   
   G   
   A   
   A   
   G   
   T   
   T   
   A   
   T   
   A   
   T   
   C   
   G   
   A   
   G   
   C   
   A   
   T   
   C   
   G   
   A   
   A   
   A   
   G   
   C   
   A   
   A   
   G   
   T   
   C   
   A   
   T   
   A   
   A   
   C   
   A   
   A   
   A   
   A   
   C   
   G   
    T    
   A   
   G   
    T    
   C   
   A   
   A   
   T   
   T   
   C   
   G   
   A   
   T   
   T   
   T   
   A   
   G   
   C   
   C   
   A   
   A   
   A   
   G   
   C   
   T   
   C   
   A   
   A   
   A   
   A   
   A   
    A    
   G   
   A   
   C   
   A   
   G   
   C   
   A   
   T   
   A   
   T   
   C   
   G   
   T   
    A    
   G   
   A   
   A   
   G   
   G   
    A    
    T    
   T   
   G   
   A   
   T   
   G   
   A   
   A   
   A   
   G   
   C   
   T   
   T   
   T   
   A   
   T   
   C   
   G   
   A   
   T   
   T   
   C   
   T   
   G   
   G   
   A   
   T   
   G   
   A   
   A   
   G   
   T   
   A   
   A   
   T   
   C   
   G   
    C    
   A   
   A   
   C   
   G   
   A   
   T   
   T   
   C   
   G   
   T   
   G   
   A   
   A   
   A   
   G   
   T   
   A   
   A   
   A   
   G   
   A   
   C   
   A   
   A   
   A   
   A   
   A   
   A   
   G   
   A   
    C    
   G   
   C   
    C    
   A   
   A   
   A   
   A   
   A   
   G   
   A   
   A   
   T   
   T   
   T   
   A   
   G   
   T   
   C   
   G   
   A   
    C    
   G   
   T   
    C    
   T   
   T   
    T    
   C   
   A   
   A   
   T   
   T   
   T   
   A   
   C   
   A   
   G   
   A   
   A   
   G   
   A   
   A   
   C   
   A   
   A   
   G   
   C   
   A   
   G   
   A   
   A   
   G   
   C   
   A   
   A   
   T   
    T    
   G   
   T   
   C   
   A   
   C   
   A   
   T   
   T   
   G   
   C   
   A   
   A   
    T    
   T   
   A   
   T   
   A   
    C    
   C   
   G   
   T   
   T   
   T   
   G   
   A   
   C   
   G   
   A   
   A   
    C    
   A   
   C   
    G    
   G   
   A   
   T   
   A   
   T   
   C   
   A   
   C   
   A   
   G   
   A   
   G   
   C   
   T   
   T   
   C   
   A   
   A   
   A   
   A   
   G   
   G   
   A   
   A   
    T    
   C   
    C    
   G   
   A   
   A   
   A   
    G    
   C   
   T   
   T   
   G   
   A   
   T   
   T   
   G   
   C   
    G    
   C   
   A   
   A   
   A   
   T   
   C   
   A   
   C   
   A   
   G   
   A   
   G   
   T   
   T   
    G    
   A   
   A   
   T   
   A   
   A   
   A   
   A   
   T   
   C   
   C   
   T   
   T   
   T   
   C   
   A   
   A   
   A   
    T    
   G   
   A   
   C   
   A   
   A   
   A   
   G   
   A   
    G    
   C   
   T   
   T   
   T   
   T   
    C    
   T   
   C   
   T   
   G   
   T   
   T   
   A   
   T   
   G   
   A   
   A   
   A   
   A   
   A   
   A   
   G   
   A   
   A   
   T   
   T   
   G   
   C   
   G   
   A   
   G   
   A   
   A   
   G   
   T   
   G   
   A   
   A   
   A   
   A   
   A   
   A   
   A   
   A   
   T   
   T   
   A   
    T    
   T   
   C   
   T   
   T   
   C   
   C   
   G   
   C   
   T   
   C   
   G   
   T   
   C   
    T    
    A    
   A   
   C   
   G   
   A   
   C   
   A   
   A   
   T   
   C   
    G    
   A   
   A   
   G   
   A   
   C   
   G   
   A   
   A   
   A   
   T   
   C   
   G   
   A   
   A   
   G   
   A   
   A   
   A   
   T   
   C   
   A   
   A   
   A   
   A   
   T   
    T    
   G   
   A   
   T   
   A   
   C   
   G   
   C   
   A   
   A   
    G    
   T   
    C    
   T   
   T   
   A   
   G   
   T   
   G   
   G   
   C   
    A    
   C   
   A   
   A   
   G   
   A   
   A   
   G   
   A   
   C   
   G   
   T   
   A   
   A   
   T   
    T    
   G   
   T   
   C   
   A   
   G   
   T   
   G   
   T   
    G    
   A   
   C   
    G    
   C   
   G   
   A   
   G   
   A   
   A   
   G   
   G   
   C   
   T   
   A   
   T   
   G   
   T   
   G   
   A   
   A   
   G   
   C   
   G   
   A   
   A   
   C   
   A   
   A   
   G   
   C   
   C   
   T   
   T   
   C   
   G   
   T   
   T   
   C   
    T    
   T   
   A   
   T   
   A   
   G   
   T   
   G   
   C   
   T   
   T   
   C   
    C    
   A   
   A   
   A   
   C   
   C   
   A   
   G   
   A   
   A   
   G   
   A   
   A   
   A   
   T   
   C   
   G   
   G   
   G   
   A   
   T   
   G   
   C   
   G   
   A   
   G   
   A   
   A   
   G   
   G   
   C   
   G   
   A   
    C    
   T   
   A   
   C   
   T   
   T   
   G   
   C   
   T   
    A    
   T   
   A   
   C   
    T    
   C   
   A   
   G   
   G   
   T   
   G   
   A   
   A   
   T   
   T   
    G    
   A   
   G   
    T    
   A   
   C   
   A   
    T    
   T   
   G   
   G   
   A   
   C   
   C   
   A   
   T   
   G   
   T   
   T   
   C   
   T   
   T   
   C   
   T   
   G   
   A   
   T   
    C    
   A   
   C   
    A    
   A   
   A   
   T   
   A   
   A   
   A   
   G   
   C   
   A   
   A   
   A   
   T   
   G   
   T   
   G   
   A   
   T   
   C   
   T   
   A   
   C   
   C   
   G   
    C    
   C   
   C   
   T   
   G   
   T   
    C    
   C   
   A   
   T   
   G   
   A   
   A   
   C   
   T   
   A   
   C   
   C   
    C    
   G   
   A   
   T   
   T   
   T   
   G   
   A   
   A   
   A   
   T   
   G   
   G   
   A   
   A   
   A   
   G   
   A   
   T   
   G   
   C   
   T   
   G   
   G   
    T    
   G   
   A   
    G    
   C   
   A   
    C    
   A   
   T   
   T   
   T   
   C   
   T   
   C   
   A   
   A   
   A   
   C   
   A   
   A   
   T   
   C   
   T   
   C   
   C   
   A   
   A   
   T   
   C   
   T   
   G   
   T   
   C   
   T   
   G   
   T   
   A   
   G   
   A   
    C    
   G   
   A   
   A   
   T   
   C   
   G   
   A   
   T   
    T    
   T   
   T   
   A   
   G   
   C   
   T   
   G   
   T   
   T   
   T   
   T   
    C    
   C   
   C   
   A   
   T   
   A   
   T   
   C   
   A   
   A   
   A   
   A   
   A   
   A   
   T   
    T    
   C   
   A   
   A   
   G   
   C   
   A   
   G   
   A   
    G    
   A   
   A   
   A   
   A   
   C   
   A   
   T   
   T   
   T   
   G   
   T   
    A    
   T   
   T   
   C   
   A   
   T   
   C   
   A   
   G   
   C   
   A   
   A   
   G   
   A   
   A   
   T   
   G   
   G   
   A   
   T   
   T   
    G    
    A    
   T   
   C   
   A   
   A   
   G   
   C   
   A   
   A   
   A   
   C   
   A   
   C   
   G   
   A   
   A   
   T   
   G   
   A   
   C   
   A   
   G   
   A   
   G   
   T   
   T   
   C   
   G   
   A   
   A   
   C   
   C   
   A   
   T   
   G   
   G   
   C   
   G   
   C   
   A   
   C   
   C   
   T   
   A   
    T    
   A   
   A   
   G   
   A   
   G   
   C   
   C   
   G   
    T    
   C   
   C   
   G   
   C   
   T   
   C   
   A   
   G   
   C   
   T   
   G   
   T   
   A   
   T   
   G   
   A   
   A   
   G   
   C   
   T   
   A   
   A   
   A   
   G   
   G   
   C   
   G   
   G   
   A   
   A   
   G   
   A   
   A   
   G   
   A   
   T   
   G   
   A   
   A   
   C   
   T   
    G    
   A   
   T   
   T   
   G   
   C   
   T   
   G   
   T   
    G    
   T   
   A   
   T   
   C   
   T   
   G   
   A   
   A   
   A   
   G   
   A   
   A   
   G   
   A   
   T   
   C   
    A    
   G   
   C   
   C   
   A   
   G   
   G   
   C   
   T   
   T   
   A   
   G   
   A   
   T   
   G   
   T   
    T    
   T   
   T   
   C   
   T   
   T   
   A   
   G   
   T   
   A   
   A   
   C   
   G   
    C    
   A   
   T   
   C   
   A   
   A   
   G   
   G   
   A   
   A   
   T   
   G   
   G   
   G   
    T    
   T   
   T   
   G   
   C   
   G   
   C   
   T   
    A    
   T   
   C   
   C   
   T   
   T   
   T   
   A   
   A   
    C    
   A   
   G   
   A   
   T   
   G   
   T   
   T   
   C   
   C   
   C   
   G   
   T   
   T   
   G   
   T   
   T   
   G   
   G   
   A   
   G   
   C   
   A   
   A   
   A   
   G   
   G   
   C   
   A   
   G   
    C    
   T   
   G   
   G   
   A   
   G   
   T   
   G   
   A   
   A   
   G   
   T   
   C   
   T   
   A   
   T   
   G   
   A   
   A   
   T   
   T   
   T   
   G   
   A   
   A   
   A   
   G   
   A   
   T   
   G   
   G   
   A   
   G   
   A   
   T   
   A   
   G   
   G   
    A    
   T   
   T   
    A    
   T   
   C   
   A   
   A   
    C    
   G   
   G   
    G    
   C   
   T   
    A    
   C   
   T   
   T   
   G   
   T   
   G   
   T   
   T   
   A   
   G   
   C   
   A   
   G   
   A   
    A    
   G   
   G   
    T    
   G   
   A   
   T   
   A   
   C   
   G   
   C   
   C   
    G    
   A   
   T   
   C   
   G   
   T   
   A   
   A   
   T   
   C   
   G   
   T   
   T   
   A   
   C   
   T   
   C   
   A   
   G   
   C   
   G   
   A   
   G   
   G   
   A   
   G   
   C   
    T    
   G   
   T   
    T    
   A   
   A   
   A   
   C   
   G   
   C   
   A   
   T   
   G   
   C   
   T   
   T   
   G   
   C   
   T   
   C   
   A   
   A   
   G   
   A   
   A   
   A   
   T   
   C   
   A   
   G   
   C   
   C   
   A   
   A   
   A   
   C   
    T    
   T   
   C   
   A   
   C   
   G   
   T   
   G   
   C   
   G   
   A   
   A   
   G   
   C   
   G   
   G   
   G   
   G   
   A   
   G   
   T   
   G   
   A   
   C   
   G   
   G   
   T   
   C   
   A   
   T   
   G   
   C   
   G   
   T   
   G   
   A   
   G   
   C   
   T   
   G   
   A   
   A   
   A   
   A   
   A   
   A   
   A   
   A   
   T   
   C   
   C   
    G    
   C   
   A   
    C    
   C   
   G   
    T    
   A   
   T   
   T   
   A   
   T   
   C   
   T   
   A   
   T   
   A   
   T   
   G   
   T   
   C   
   G   
   G   
   A   
   A   
   G   
   G   
   A   
   A   
   G   
   A   
   T   
   C   
   A   
   A   
   A   
   A   
   G   
   A   
   A   
   A   
   T   
   A   
   A   
   C   
   C   
   T   
   T   
   G   
   A   
   T   
   G   
   A   
   A   
   T   
   C   
   A   
   A   
   A   
   A   
   A   
   G   
   G   
    G    
   C   
   A   
    G    
   C   
   A   
   G   
    T    
   T   
   A   
   A   
   C   
   G   
   A   
   T   
   T   
   G   
   A   
    C    
   C   
   C   
   A   
   A   
   C   
    T    
   G   
   A   
   T   
   T   
   T   
    C    
   C   
   C   
   T   
   A   
   T   
   A   
   G   
   G   
   T   
   G   
   A   
   T   
   C   
   G   
   A   
   A   
   C   
   A   
   T   
   C   
   G   
   A   
   A   
   T   
   G   
   G   
    A    
   T   
   C   
    A    
   T   
   T   
   T   
   G   
   C   
    G    
   A   
   T   
   G   
   G   
   A   
    T    
   G   
   A   
   G   
   A   
   A   
   A   
   A   
   A   
   A   
   G   
   G   
   T   
   G   
   G   
   A   
   G   
   A   
   A   
   G   
   T   
   C   
   A   
   T   
   C   
   G   
   A   
   A   
   G   
   T   
    T    
   A   
    T    
   C   
   G   
   A   
   T   
   G   
   C   
   T   
   C   
   C   
   T   
   G   
   A   
   G   
    A    
   T   
   T   
   T   
   T   
    C    
   A   
   T   
    C    
   G   
   A   
    G    
    T    
   C   
    G    
   -   
   -   
   -   
 

 
   N53439   
   A   
   T   
   G   
   G   
   A   
   A   
   A   
   A   
   T   
   C   
   G   
   A   
   C   
   A   
   A   
   G   
   A   
   A   
   G   
   T   
   A   
   C   
   A   
   A   
   G   
   A   
   A   
   T   
   T   
   A   
   A   
   C   
   T   
   T   
   T   
   A   
   G   
   A   
   A   
   G   
   A   
   A   
   G   
   T   
   G   
   A   
   T   
   G   
   G   
   G   
   T   
   G   
   A   
   C   
   C   
   G   
   C   
   T   
   T   
   C   
   G   
   G   
   A   
   C   
   G   
   T   
   T   
   A   
   T   
   T   
   C   
   C   
   A   
   A   
   A   
   T   
   A   
   T   
   A   
   T   
   T   
   A   
   T   
    C    
   C   
   A   
    A    
   G   
   A   
   A   
   C   
   G   
   T   
   G   
   C   
   A   
   C   
   T   
   G   
   C   
   C   
   T   
   G   
   A   
   T   
   A   
   T   
    C    
   C   
   G   
   T   
   G   
   A   
   T   
   G   
   G   
   A   
   T   
   T   
   G   
   A   
   A   
    G    
   C   
   C   
   A   
   G   
   T   
    G    
   C   
   A   
   A   
   C   
   G   
   C   
   C   
   G   
   G   
   A   
   T   
   A   
   T   
   T   
   A   
   T   
   T   
    T    
   G   
   C   
    C    
   A   
   T   
   G   
   A   
   A   
   C   
   A   
   A   
   A   
   G   
   A   
   T   
   G   
   G   
   C   
   A   
   A   
   T   
   A   
   C   
   G   
   T   
   A   
   T   
   G   
   A   
   C   
   A   
   A   
   A   
   G   
   G   
   A   
   T   
   T   
    C    
   C   
   G   
   T   
   A   
   A   
   A   
   T   
   C   
   A   
   G   
   C   
   A   
   A   
   A   
   A   
   T   
   C   
   T   
   G   
   T   
   C   
   G   
   G   
   A   
   A   
   A   
   T   
   A   
   T   
   C   
   A   
   T   
   G   
   G   
   G   
    G    
   A   
   A   
   T   
   T   
   A   
   T   
   C   
   A   
   T   
   C   
   C   
   T   
   C   
   A   
   T   
   G   
   G   
   T   
   G   
   A   
   C   
   A   
   G   
   C   
   A   
   G   
   T   
   A   
   T   
   T   
   T   
   A   
   T   
   G   
   A   
   A   
   G   
   C   
   G   
   A   
   T   
   G   
   G   
   T   
   C   
   C   
   G   
   C   
   A   
   T   
   G   
   A   
   G   
   C   
   C   
   A   
   A   
   G   
   A   
    C    
   T   
   G   
   G   
   A   
   A   
   G   
   T   
   T   
   G   
   C   
   G   
   G   
   G   
   A   
   A   
   G   
   T   
   A   
   C   
   T   
   G   
   A   
   T   
   C   
   G   
   A   
    A    
   A   
   T   
   G   
   C   
   A   
   C   
   G   
   G   
    G    
   A   
   A   
   T   
   A   
   A   
    C    
   G   
   G   
   G   
   A   
   G   
   T   
   A   
   T   
   G   
   G   
   A   
   C   
   G   
   G   
   T   
   G   
   A   
   T   
   C   
   C   
   A   
   C   
   C   
   T   
   G   
   C   
   T   
   G   
   C   
   G   
   A   
   T   
   G   
   C   
   G   
   T   
   T   
   A   
   T   
   A   
   C   
   T   
   G   
   A   
   A   
   G   
   C   
   C   
   A   
   G   
    A    
   T   
   T   
   A   
   T   
   C   
   C   
   A   
   A   
   G   
   T   
   T   
   A   
   A   
   G   
   T   
   G   
   G   
   A   
   G   
   A   
   G   
   A   
   T   
   G   
    C    
   T   
   A   
    G    
   C   
   A   
   G   
   A   
   T   
   A   
   T   
   C   
   G   
   A   
    A    
   A   
   A   
   A   
   G   
   A   
   A   
   A   
   C   
   A   
   G   
   T   
    C    
   G   
   A   
   T   
   C   
   T   
   C   
   G   
   T   
   G   
   T   
   G   
   G   
   A   
   A   
   T   
   T   
   T   
   T   
   G   
   A   
   T   
   G   
   A   
   T   
   A   
   C   
   A   
   G   
   A   
   A   
   A   
   A   
   A   
   G   
   A   
   A   
   C   
   C   
   A   
   A   
   C   
   A   
   G   
   T   
   C   
   C   
   T   
   T   
   C   
   C   
   A   
   G   
   C   
   T   
   C   
   G   
   A   
   T   
   A   
   C   
   C   
   C   
   A   
   A   
   A   
   C   
   C   
   T   
   T   
   C   
   T   
    C    
   G   
   T   
   C   
   A   
   A   
   T   
   G   
   G   
   A   
   T   
   C   
   G   
   A   
   C   
   A   
   G   
   G   
   T   
   A   
   T   
   A   
   T   
   C   
   T   
   G   
   C   
   T   
   G   
   G   
   A   
   T   
   A   
   T   
   G   
   C   
    A    
   A   
   C   
   A   
   G   
   A   
   A   
   A   
   T   
   C   
   C   
   C   
   G   
   A   
   C   
    T    
   C   
   A   
   T   
   A   
   A   
   C   
   T   
   T   
   A   
   G   
   C   
   T   
   G   
   A   
   A   
   G   
   T   
   G   
   A   
   T   
    T    
   G   
   A   
    T    
   G   
   G   
   C   
   A   
   C   
   A   
   A   
   T   
   C   
   T   
   A   
   T   
   A   
   T   
   G   
   A   
   T   
   C   
   G   
   A   
   T   
   C   
   A   
   T   
   C   
   C   
   A   
   C   
   A   
   A   
   G   
   C   
   G   
   A   
   G   
   C   
   C   
   T   
   T   
   G   
   A   
   A   
   A   
   A   
   A   
   T   
   T   
   G   
   A   
   T   
   G   
   G   
   A   
   A   
   T   
   A   
   T   
   A   
   T   
   T   
   C   
   C   
   T   
   G   
   G   
   A   
   C   
   C   
   T   
   G   
   A   
   C   
   T   
   T   
    T    
   C   
   C   
   A   
   A   
   C   
   A   
   G   
   G   
   A   
   G   
   G   
    G    
   A   
   T   
   C   
   T   
   T   
   G   
   C   
   A   
   A   
   G   
   G   
    A    
   A   
   A   
   A   
   G   
   A   
   T   
   G   
   A   
   A   
   A   
   T   
   C   
   A   
   A   
   A   
   A   
   A   
   A   
   G   
   C   
   T   
   T   
   A   
   T   
   G   
   A   
   A   
   A   
   C   
   A   
   G   
   G   
    G    
   C   
   G   
   A   
   G   
   G   
   A   
   A   
   A   
   A   
   G   
   T   
   T   
   A   
    T    
   T   
   C   
   T   
   T   
   C   
   G   
   T   
   T   
   C   
   G   
   A   
   A   
   A   
   A   
   C   
   A   
   A   
   A   
   G   
   A   
   T   
   A   
   G   
   A   
    G    
   G   
   C   
   A   
   A   
   T   
   G   
   A   
   A   
   G   
   G   
   G   
    T    
   A   
   A   
    T    
   A   
   A   
   A   
   C   
   A   
   G   
   C   
   A   
   A   
   A   
   T   
   C   
   G   
   T   
   G   
   A   
   T   
   C   
   A   
   C   
    A    
   G   
   A   
   A   
   A   
   T   
   C   
   C   
   C   
   A   
   T   
   A   
   T   
   G   
   A   
   A   
   G   
   T   
   A   
   A   
   A   
   T   
   A   
   A   
   A   
   G   
   C   
   G   
   A   
   C   
    C    
   T   
   T   
   G   
   G   
   T   
   G   
   A   
   A   
   A   
   A   
   A   
   A   
   A   
   T   
   G   
   G   
   A   
   T   
   G   
   A   
   G   
   A   
   T   
   C   
   C   
   G   
   T   
   T   
   T   
   G   
   A   
   A   
   T   
   A   
   A   
   G   
   A   
   A   
   A   
   A   
   T   
   C   
   G   
   A   
    T    
   G   
   G   
   A   
   A   
   T   
   A   
   G   
   C   
   T   
   G   
   A   
   A   
   G   
   T   
    A    
   C   
   G   
   A   
   G   
   A   
   T   
   G   
   A   
   A   
   A   
   G   
   T   
   G   
   A   
   T   
    A    
   G   
    A    
   A   
   C   
    A    
   G   
   G   
   T   
   C   
   T   
   G   
   C   
   A   
   A   
   A   
   T   
   T   
   G   
   T   
   C   
   G   
   T   
    C    
   G   
   A   
   A   
   T   
   T   
   G   
   A   
   A   
   A   
   A   
   A   
   A   
   G   
   A   
    C    
   G   
   C   
   C   
   A   
   A   
    T    
   G   
   C   
   T   
   C   
   A   
   A   
   G   
   G   
    A    
   A   
   T   
   T   
   T   
   T   
   G   
   A   
   A   
   T   
   T   
   A   
    T    
   T   
   T   
   G   
   T   
   T   
   C   
   A   
   A   
   A   
   A   
   A   
   C   
   A   
   C   
   T   
   G   
   A   
   G   
    C    
   T   
   A   
   C   
   A   
   G   
   A   
   T   
   C   
   A   
   A   
   T   
   T   
   A   
   C   
   A   
   A   
   T   
   T   
   T   
    C    
   A   
   A   
   T   
   A   
   T   
   G   
   G   
   T   
   C   
   G   
   C   
   T   
   A   
   T   
   C   
   G   
   A   
    C    
   C   
   A   
   T   
   A   
   T   
   G   
   A   
   C   
   T   
   C   
   C   
   G   
   C   
   A   
    T    
   C   
   A   
   A   
   G   
   T   
   T   
   G   
   G   
   A   
   C   
   T   
   G   
   A   
   A   
   A   
   G   
   A   
   C   
   A   
   T   
   T   
   T   
   T   
   A   
   A   
   G   
   A   
   A   
   G   
   T   
   T   
   A   
   T   
   A   
   T   
   C   
   G   
   A   
   G   
   C   
   A   
   T   
   C   
   G   
   A   
   A   
   A   
   G   
   C   
   A   
   A   
   G   
   T   
   C   
   A   
   T   
   A   
   A   
   C   
   A   
   A   
   A   
   A   
   C   
   G   
    T    
   A   
   G   
    T    
   C   
   A   
   A   
   T   
   T   
   C   
   G   
   A   
   T   
   T   
   T   
   A   
   G   
   C   
   C   
   A   
   A   
   A   
   G   
   C   
   T   
   C   
   A   
   A   
   A   
   A   
   A   
    A    
   G   
   A   
   C   
   A   
   G   
   C   
   A   
   T   
   A   
   T   
   C   
   G   
   T   
    A    
   G   
   A   
   A   
   G   
   G   
    A    
    T    
   T   
   G   
   A   
   T   
   G   
   A   
   A   
   A   
   G   
   C   
   T   
   T   
   T   
   A   
   T   
   C   
   G   
   A   
   T   
   T   
   C   
   T   
   G   
   G   
   A   
   T   
   G   
   A   
   A   
   G   
   T   
   A   
   A   
   T   
   C   
   G   
    T    
   A   
   A   
   C   
   G   
   A   
   T   
   T   
   C   
   G   
   T   
   G   
   A   
   A   
   A   
   G   
   T   
   A   
   A   
   A   
   G   
   A   
   C   
   A   
   A   
   A   
   A   
   A   
   A   
   G   
   A   
    C    
   G   
   C   
    C    
   A   
   A   
   A   
   A   
   A   
   G   
   A   
   A   
   T   
   T   
   T   
   A   
   G   
   T   
   C   
   G   
   A   
    C    
   G   
   T   
    C    
   T   
   T   
    T    
   C   
   A   
   A   
   T   
   T   
   T   
   A   
   C   
   A   
   G   
   A   
   A   
   G   
   A   
   A   
   C   
   A   
   A   
   G   
   C   
   A   
   G   
   A   
   A   
   G   
   C   
   A   
   A   
   T   
    T    
   G   
   T   
   C   
   A   
   C   
   A   
   T   
   T   
   G   
   C   
   A   
   A   
    T    
   T   
   A   
   T   
   A   
    C    
   C   
   G   
   T   
   T   
   T   
   G   
   A   
   C   
   G   
   A   
   A   
    C    
   A   
   C   
    G    
   G   
   A   
   T   
   A   
   T   
   C   
   A   
   C   
   A   
   G   
   A   
   G   
   C   
   T   
   T   
   C   
   A   
   A   
   A   
   A   
   G   
   G   
   A   
   A   
    T    
   C   
    C    
   G   
   A   
   A   
   A   
    G    
   C   
   T   
   T   
   G   
   A   
   T   
   T   
   G   
   C   
    G    
   C   
   A   
   A   
   A   
   T   
   C   
   A   
   C   
   A   
   G   
   A   
   G   
   T   
   T   
    G    
   A   
   A   
   T   
   A   
   A   
   A   
   A   
   T   
   C   
   C   
   T   
   T   
   T   
   C   
   A   
   A   
   A   
    T    
   G   
   A   
   C   
   A   
   A   
   A   
   G   
   A   
    G    
   C   
   T   
   T   
   T   
   T   
    C    
   T   
   C   
   T   
   G   
   T   
   T   
   A   
   T   
   G   
   A   
   A   
   A   
   A   
   A   
   A   
   G   
   A   
   A   
   T   
   T   
   G   
   C   
   G   
   A   
   G   
   A   
   A   
   G   
   T   
   G   
   A   
   A   
   A   
   A   
   A   
   A   
   A   
   A   
   T   
   T   
   A   
    T    
   T   
   C   
   T   
   T   
   C   
   C   
   G   
   C   
   T   
   C   
   G   
   T   
   C   
    T    
    A    
   A   
   C   
   G   
   A   
   C   
   A   
   A   
   T   
   C   
    G    
   A   
   A   
   G   
   A   
   C   
   G   
   A   
   A   
   A   
   T   
   C   
   G   
   A   
   A   
   G   
   A   
   A   
   A   
   T   
   C   
   A   
   A   
   A   
   A   
   T   
    T    
   G   
   A   
   T   
   A   
   C   
   G   
   C   
   A   
   A   
    G    
   T   
    C    
   T   
   T   
   A   
   G   
   T   
   G   
   G   
   C   
    A    
   C   
   A   
   A   
   G   
   A   
   A   
   G   
   A   
   C   
   G   
   T   
   A   
   A   
   T   
    T    
   G   
   T   
   C   
   A   
   G   
   T   
   G   
   T   
    G    
   A   
   C   
    G    
   C   
   G   
   A   
   G   
   A   
   A   
   G   
   G   
   C   
   T   
   A   
   T   
   G   
   T   
   G   
   A   
   A   
   G   
   C   
   G   
   A   
   A   
   C   
   A   
   A   
   G   
   C   
   C   
   T   
   T   
   C   
   G   
   T   
   T   
   C   
    T    
   T   
   A   
   T   
   A   
   G   
   T   
   G   
   C   
   T   
   T   
   C   
    C    
   A   
   A   
   A   
   C   
   C   
   A   
   G   
   A   
   A   
   G   
   A   
   A   
   A   
   T   
   C   
   G   
   G   
   G   
   A   
   T   
   G   
   C   
   G   
   A   
   G   
   A   
   A   
   G   
   G   
   C   
   G   
   A   
    C    
   T   
   A   
   C   
   T   
   T   
   G   
   C   
   T   
    A    
   T   
   A   
   C   
    T    
   C   
   A   
   G   
   G   
   T   
   G   
   A   
   A   
   T   
   T   
    G    
   A   
   G   
    T    
   A   
   C   
   A   
    T    
   T   
   G   
   G   
   A   
   C   
   C   
   A   
   T   
   G   
   T   
   T   
   C   
   T   
   T   
   C   
   T   
   G   
   A   
   T   
    C    
   A   
   C   
    A    
   A   
   A   
   T   
   A   
   A   
   A   
   G   
   C   
   A   
   A   
   A   
   T   
   G   
   T   
   G   
   A   
   T   
   C   
   T   
   A   
   C   
   C   
   G   
    C    
   C   
   C   
   T   
   G   
   T   
    C    
   C   
   A   
   T   
   G   
   A   
   A   
   C   
   T   
   A   
   C   
   C   
    C    
   G   
   A   
   T   
   T   
   T   
   G   
   A   
   A   
   A   
   T   
   G   
   G   
   A   
   A   
   A   
   G   
   A   
   T   
   G   
   C   
   T   
   G   
   G   
    T    
   G   
   A   
    G    
   C   
   A   
    C    
   A   
   T   
   T   
   T   
   C   
   T   
   C   
   A   
   A   
   A   
   C   
   A   
   A   
   T   
   C   
   T   
   C   
   C   
   A   
   A   
   T   
   C   
   T   
   G   
   T   
   C   
   T   
   G   
   T   
   A   
   G   
   A   
    C    
   G   
   A   
   A   
   T   
   C   
   G   
   A   
   T   
    T    
   T   
   T   
   A   
   G   
   C   
   T   
   G   
   T   
   T   
   T   
   T   
    C    
   C   
   C   
   A   
   T   
   A   
   T   
   C   
   A   
   A   
   A   
   A   
   A   
   A   
   T   
    T    
   C   
   A   
   A   
   G   
   C   
   A   
   G   
   A   
    G    
   A   
   A   
   A   
   A   
   C   
   A   
   T   
   T   
   T   
   G   
   T   
    A    
   T   
   T   
   C   
   A   
   T   
   C   
   A   
   G   
   C   
   A   
   A   
   G   
   A   
   A   
   T   
   G   
   G   
   A   
   T   
   T   
    G    
    A    
   T   
   C   
   A   
   A   
   G   
   C   
   A   
   A   
   A   
   C   
   A   
   C   
   G   
   A   
   A   
   T   
   G   
   A   
   C   
   A   
   G   
   A   
   G   
   T   
   T   
   C   
   G   
   A   
   A   
   C   
   C   
   A   
   T   
   G   
   G   
   C   
   G   
   C   
   A   
   C   
   C   
   T   
   A   
    T    
   A   
   A   
   G   
   A   
   G   
   C   
   C   
   G   
    T    
   C   
   C   
   G   
   C   
   T   
   C   
   A   
   G   
   C   
   T   
   G   
   T   
   A   
   T   
   G   
   A   
   A   
   G   
   C   
   T   
   A   
   A   
   A   
   G   
   G   
   C   
   G   
   G   
   A   
   A   
   G   
   A   
   A   
   G   
   A   
   T   
   G   
   A   
   A   
   C   
   T   
    G    
   A   
   T   
   T   
   G   
   C   
   T   
   G   
   T   
    G    
   T   
   A   
   T   
   C   
   T   
   G   
   A   
   A   
   A   
   G   
   A   
   A   
   G   
   A   
   T   
   C   
    A    
   G   
   C   
   C   
   A   
   G   
   G   
   C   
   T   
   T   
   A   
   G   
   A   
   T   
   G   
   T   
    T    
   T   
   T   
   C   
   T   
   T   
   A   
   G   
   T   
   A   
   A   
   C   
   G   
    C    
   A   
   T   
   C   
   A   
   A   
   G   
   G   
   A   
   A   
   T   
   G   
   G   
   G   
    T    
   T   
   T   
   G   
   C   
   G   
   C   
   T   
    A    
   T   
   C   
   C   
   T   
   T   
   T   
   A   
   A   
    C    
   A   
   G   
   A   
   T   
   G   
   T   
   T   
   C   
   C   
   C   
   G   
   T   
   T   
   G   
   T   
   T   
   G   
   G   
   A   
   G   
   C   
   A   
   A   
   A   
   G   
   G   
   C   
   A   
   G   
    C    
   T   
   G   
   G   
   A   
   G   
   T   
   G   
   A   
   A   
   G   
   T   
   C   
   T   
   A   
   T   
   G   
   A   
   A   
   T   
   T   
   T   
   G   
   A   
   A   
   A   
   G   
   A   
   T   
   G   
   G   
   A   
   G   
   A   
   T   
   A   
   G   
   G   
    A    
   T   
   T   
    A    
   T   
   C   
   A   
   A   
    C    
   G   
   G   
    G    
   C   
   T   
    G    
   C   
   T   
   T   
   G   
   T   
   G   
   T   
   T   
   A   
   G   
   C   
   A   
   G   
   A   
    A    
   G   
   G   
    T    
   G   
   A   
   T   
   A   
   C   
   G   
   C   
   C   
    G    
   A   
   T   
   C   
   G   
   T   
   A   
   A   
   T   
   C   
   G   
   T   
   T   
   A   
   C   
   T   
   C   
   A   
   G   
   C   
   G   
   A   
   G   
   G   
   A   
   G   
   C   
    T    
   G   
   T   
    T    
   A   
   A   
   A   
   C   
   G   
   C   
   A   
   T   
   G   
   C   
   T   
   T   
   G   
   C   
   T   
   C   
   A   
   A   
   G   
   A   
   A   
   A   
   T   
   C   
   A   
   G   
   C   
   C   
   A   
   A   
   A   
   C   
    T    
   T   
   C   
   A   
   C   
   G   
   T   
   G   
   C   
   G   
   A   
   A   
   G   
   C   
   G   
   G   
   G   
   G   
   A   
   G   
   T   
   G   
   A   
   C   
   G   
   G   
   T   
   C   
   A   
   T   
   G   
   C   
   G   
   T   
   G   
   A   
   G   
   C   
   T   
   G   
   A   
   A   
   A   
   A   
   A   
   A   
   A   
   A   
   T   
   C   
   C   
    G    
   C   
   A   
    C    
   C   
   G   
    T    
   A   
   T   
   T   
   A   
   T   
   C   
   T   
   A   
   T   
   A   
   T   
   G   
   T   
   C   
   G   
   G   
   A   
   A   
   G   
   G   
   A   
   A   
   G   
   A   
   T   
   C   
   A   
   A   
   A   
   A   
   G   
   A   
   A   
   A   
   T   
   A   
   A   
   C   
   C   
   T   
   T   
   G   
   A   
   T   
   G   
   A   
   A   
   T   
   C   
   A   
   A   
   A   
   A   
   A   
   G   
   G   
    G    
   C   
   A   
    G    
   C   
   A   
   G   
    T    
   T   
   A   
   A   
   C   
   G   
   A   
   T   
   T   
   G   
   A   
    C    
   C   
   C   
   A   
   A   
   C   
    T    
   G   
   A   
   T   
   T   
   T   
    C    
   C   
   C   
   T   
   A   
   T   
   A   
   G   
   G   
   T   
   G   
   A   
   T   
   C   
   G   
   A   
   A   
   C   
   A   
   T   
   C   
   G   
   A   
   A   
   T   
   G   
   G   
    A    
   T   
   C   
    A    
   T   
   T   
   T   
   G   
   C   
    G    
   A   
   T   
   G   
   G   
   A   
    T    
   G   
   A   
   G   
   A   
   A   
   A   
   A   
   A   
   A   
   G   
   G   
   T   
   G   
   G   
   A   
   G   
   A   
   A   
   G   
   T   
   C   
   A   
   T   
   C   
   G   
   A   
   A   
   G   
   T   
    T    
   A   
    T    
   C   
   G   
   A   
   T   
   G   
   C   
   T   
   C   
   C   
   T   
   G   
   A   
   G   
    A    
   T   
   T   
   T   
   T   
    C    
   A   
   T   
    C    
   G   
   A   
    G    
    T    
   C   
    G    
   -   
   -   
   -   
 

 
   N59998F   
   A   
   T   
   G   
   G   
   A   
   A   
   A   
   A   
   T   
   C   
   G   
   A   
   C   
   A   
   A   
   G   
   A   
   A   
   G   
   T   
   A   
   C   
   A   
   A   
   G   
   A   
   A   
   T   
   T   
   A   
   A   
   C   
   T   
   T   
   T   
   A   
   G   
   A   
   A   
   G   
   A   
   A   
   G   
   T   
   G   
   A   
   T   
   G   
   G   
   G   
   T   
   G   
   A   
   C   
   C   
   G   
   C   
   T   
   T   
   C   
   G   
   G   
   A   
   C   
   G   
   T   
   T   
   A   
   T   
   T   
   C   
   C   
   A   
   A   
   A   
   T   
   A   
   T   
   A   
   T   
   T   
   A   
   T   
    C    
   C   
   A   
    A    
   G   
   A   
   A   
   C   
   G   
   T   
   G   
   C   
   A   
   C   
   T   
   G   
   C   
   C   
   T   
   G   
   A   
   T   
   A   
   T   
    T    
   C   
   G   
   T   
   G   
   A   
   T   
   G   
   G   
   A   
   T   
   T   
   G   
   A   
   A   
    G    
   C   
   C   
   A   
   G   
   T   
    G    
   C   
   A   
   A   
   C   
   G   
   C   
   C   
   G   
   G   
   A   
   T   
   A   
   T   
   T   
   A   
   T   
   T   
    T    
   G   
   C   
    C    
   A   
   T   
   G   
   A   
   A   
   C   
   A   
   A   
   A   
   G   
   A   
   T   
   G   
   G   
   C   
   A   
   A   
   T   
   A   
   C   
   G   
   T   
   A   
   T   
   G   
   A   
   C   
   A   
   A   
   A   
   G   
   G   
   A   
   T   
   T   
    C    
   C   
   G   
   T   
   A   
   A   
   A   
   T   
   C   
   A   
   G   
   C   
   A   
   A   
   A   
   A   
   T   
   C   
   T   
   G   
   T   
   C   
   G   
   G   
   A   
   A   
   A   
   T   
   A   
   T   
   C   
   A   
   T   
   G   
   G   
   G   
    G    
   A   
   A   
   T   
   T   
   A   
   T   
   C   
   A   
   T   
   C   
   C   
   T   
   C   
   A   
   T   
   G   
   G   
   T   
   G   
   A   
   C   
   A   
   G   
   C   
   A   
   G   
   T   
   A   
   T   
   T   
   T   
   A   
   T   
   G   
   A   
   A   
   G   
   C   
   G   
   A   
   T   
   G   
   G   
   T   
   C   
   C   
   G   
   C   
   A   
   T   
   G   
   A   
   G   
   C   
   C   
   A   
   A   
   G   
   A   
    C    
   T   
   G   
   G   
   A   
   A   
   G   
   T   
   T   
   G   
   C   
   G   
   G   
   G   
   A   
   A   
   G   
   T   
   A   
   C   
   T   
   G   
   A   
   T   
   C   
   G   
   A   
    A    
   A   
   T   
   G   
   C   
   A   
   C   
   G   
   G   
    G    
   A   
   A   
   T   
   A   
   A   
    C    
   G   
   G   
   G   
   A   
   G   
   T   
   A   
   T   
   G   
   G   
   A   
   C   
   G   
   G   
   T   
   G   
   A   
   T   
   C   
   C   
   A   
   C   
   C   
   T   
   G   
   C   
   T   
   G   
   C   
   G   
   A   
   T   
   G   
   C   
   G   
   T   
   T   
   A   
   T   
   A   
   C   
   T   
   G   
   A   
   A   
   G   
   C   
   C   
   A   
   G   
    A    
   T   
   T   
   A   
   T   
   C   
   C   
   A   
   A   
   G   
   T   
   T   
   A   
   A   
   G   
   T   
   G   
   G   
   A   
   G   
   A   
   G   
   A   
   T   
   G   
    C    
   T   
   A   
    G    
   C   
   A   
   G   
   A   
   T   
   A   
   T   
   C   
   G   
   A   
    A    
   A   
   A   
   A   
   G   
   A   
   A   
   A   
   C   
   A   
   G   
   T   
    C    
   G   
   A   
   T   
   C   
   T   
   C   
   G   
   T   
   G   
   T   
   G   
   G   
   A   
   A   
   T   
   T   
   T   
   T   
   G   
   A   
   T   
   G   
   A   
   T   
   A   
   C   
   A   
   G   
   A   
   A   
   A   
   A   
   A   
   G   
   A   
   A   
   C   
   C   
   A   
   A   
   C   
   A   
   G   
   T   
   C   
   C   
   T   
   T   
   C   
   C   
   A   
   G   
   C   
   T   
   C   
   G   
   A   
   T   
   A   
   C   
   C   
   C   
   A   
   A   
   A   
   C   
   C   
   T   
   T   
   C   
   T   
    C    
   G   
   T   
   C   
   A   
   A   
   T   
   G   
   G   
   A   
   T   
   C   
   G   
   A   
   C   
   A   
   G   
   G   
   T   
   A   
   T   
   A   
   T   
   C   
   T   
   G   
   C   
   T   
   G   
   G   
   A   
   T   
   A   
   T   
   G   
   C   
    A    
   A   
   C   
   A   
   G   
   A   
   A   
   A   
   T   
   C   
   C   
   C   
   G   
   A   
   C   
    T    
   C   
   A   
   T   
   A   
   A   
   C   
   T   
   T   
   A   
   G   
   C   
   T   
   G   
   A   
   A   
   G   
   T   
   G   
   A   
   T   
    C    
   G   
   A   
    T    
   G   
   G   
   C   
   A   
   C   
   A   
   A   
   T   
   C   
   T   
   A   
   T   
   A   
   T   
   G   
   A   
   T   
   C   
   G   
   A   
   T   
   C   
   A   
   T   
   C   
   C   
   A   
   C   
   A   
   A   
   G   
   C   
   G   
   A   
   G   
   C   
   C   
   T   
   T   
   G   
   A   
   A   
   A   
   A   
   A   
   T   
   T   
   G   
   A   
   T   
   G   
   G   
   A   
   A   
   T   
   A   
   T   
   A   
   T   
   T   
   C   
   C   
   T   
   G   
   G   
   A   
   C   
   C   
   T   
   G   
   A   
   C   
   T   
   T   
    T    
   C   
   C   
   A   
   A   
   C   
   A   
   G   
   G   
   A   
   G   
   G   
    G    
   A   
   T   
   C   
   T   
   T   
   G   
   C   
   A   
   A   
   G   
   G   
    A    
   A   
   A   
   A   
   G   
   A   
   T   
   G   
   A   
   A   
   A   
   T   
   C   
   A   
   A   
   A   
   A   
   A   
   A   
   G   
   C   
   T   
   T   
   A   
   T   
   G   
   A   
   A   
   A   
   C   
   A   
   G   
   G   
    G    
   C   
   G   
   A   
   G   
   G   
   A   
   A   
   A   
   A   
   G   
   T   
   T   
   A   
    T    
   T   
   C   
   T   
   T   
   C   
   G   
   T   
   T   
   C   
   G   
   A   
   A   
   A   
   A   
   C   
   A   
   A   
   A   
   G   
   A   
   T   
   A   
   G   
   A   
    A    
   G   
   C   
   A   
   A   
   T   
   G   
   A   
   A   
   G   
   G   
   G   
    T    
   A   
   A   
    T    
   A   
   A   
   A   
   C   
   A   
   G   
   C   
   A   
   A   
   A   
   T   
   C   
   G   
   T   
   G   
   A   
   T   
   C   
   A   
   C   
    A    
   G   
   A   
   A   
   A   
   T   
   C   
   C   
   C   
   A   
   T   
   A   
   T   
   G   
   A   
   A   
   G   
   T   
   A   
   A   
   A   
   T   
   A   
   A   
   A   
   G   
   C   
   G   
   A   
   C   
    C    
   T   
   T   
   G   
   G   
   T   
   G   
   A   
   A   
   A   
   A   
   A   
   A   
   A   
   T   
   G   
   G   
   A   
   T   
   G   
   A   
   G   
   A   
   T   
   C   
   C   
   G   
   T   
   T   
   T   
   G   
   A   
   A   
   T   
   A   
   A   
   G   
   A   
   A   
   A   
   A   
   T   
   C   
   G   
   A   
    T    
   G   
   G   
   A   
   A   
   T   
   A   
   G   
   C   
   T   
   G   
   A   
   A   
   G   
   T   
    A    
   C   
   G   
   A   
   G   
   A   
   T   
   G   
   A   
   A   
   A   
   G   
   T   
   G   
   A   
   T   
    A    
   G   
    A    
   A   
   C   
    A    
   G   
   G   
   T   
   C   
   T   
   G   
   C   
   A   
   A   
   A   
   T   
   T   
   G   
   T   
   C   
   G   
   T   
    C    
   G   
   A   
   A   
   T   
   T   
   G   
   A   
   A   
   A   
   A   
   A   
   A   
   G   
   A   
    C    
   G   
   C   
   C   
   A   
   A   
    T    
   G   
   C   
   T   
   C   
   A   
   A   
   G   
   G   
    A    
   A   
   T   
   T   
   T   
   T   
   G   
   A   
   A   
   T   
   T   
   A   
    T    
   T   
   T   
   G   
   T   
   T   
   C   
   A   
   A   
   A   
   A   
   A   
   C   
   A   
   C   
   T   
   G   
   A   
   G   
    C    
   T   
   A   
   C   
   A   
   G   
   A   
   T   
   C   
   A   
   A   
   T   
   T   
   A   
   C   
   A   
   A   
   T   
   T   
   T   
    C    
   A   
   A   
   T   
   A   
   T   
   G   
   G   
   T   
   C   
   G   
   C   
   T   
   A   
   T   
   C   
   G   
   A   
    C    
   C   
   A   
   T   
   A   
   T   
   G   
   A   
   C   
   T   
   C   
   C   
   G   
   C   
   A   
    T    
   C   
   A   
   A   
   G   
   T   
   T   
   G   
   G   
   A   
   C   
   T   
   G   
   A   
   A   
   A   
   G   
   A   
   C   
   A   
   T   
   T   
   T   
   T   
   A   
   A   
   G   
   A   
   A   
   G   
   T   
   T   
   A   
   T   
   A   
   T   
   C   
   G   
   A   
   G   
   C   
   A   
   T   
   C   
   G   
   A   
   A   
   A   
   G   
   C   
   A   
   A   
   G   
   T   
   C   
   A   
   T   
   A   
   A   
   C   
   A   
   A   
   A   
   A   
   C   
   G   
    T    
   A   
   G   
    T    
   C   
   A   
   A   
   T   
   T   
   C   
   G   
   A   
   T   
   T   
   T   
   A   
   G   
   C   
   C   
   A   
   A   
   A   
   G   
   C   
   T   
   C   
   A   
   A   
   A   
   A   
   A   
    A    
   G   
   A   
   C   
   A   
   G   
   C   
   A   
   T   
   A   
   T   
   C   
   G   
   T   
    A    
   G   
   A   
   A   
   G   
   G   
    A    
    T    
   T   
   G   
   A   
   T   
   G   
   A   
   A   
   A   
   G   
   C   
   T   
   T   
   T   
   A   
   T   
   C   
   G   
   A   
   T   
   T   
   C   
   T   
   G   
   G   
   A   
   T   
   G   
   A   
   A   
   G   
   T   
   A   
   A   
   T   
   C   
   G   
    C    
   A   
   A   
   C   
   G   
   A   
   T   
   T   
   C   
   G   
   T   
   G   
   A   
   A   
   A   
   G   
   T   
   A   
   A   
   A   
   G   
   A   
   C   
   A   
   A   
   A   
   A   
   A   
   A   
   G   
   A   
    C    
   G   
   C   
    C    
   A   
   A   
   A   
   A   
   A   
   G   
   A   
   A   
   T   
   T   
   T   
   A   
   G   
   T   
   C   
   G   
   A   
    C    
   G   
   T   
    C    
   T   
   T   
    T    
   C   
   A   
   A   
   T   
   T   
   T   
   A   
   C   
   A   
   G   
   A   
   A   
   G   
   A   
   A   
   C   
   A   
   A   
   G   
   C   
   A   
   G   
   A   
   A   
   G   
   C   
   A   
   A   
   T   
    T    
   G   
   T   
   C   
   A   
   C   
   A   
   T   
   T   
   G   
   C   
   A   
   A   
    T    
   T   
   A   
   T   
   A   
    C    
   C   
   G   
   T   
   T   
   T   
   G   
   A   
   C   
   G   
   A   
   A   
    C    
   A   
   C   
    G    
   G   
   A   
   T   
   A   
   T   
   C   
   A   
   C   
   A   
   G   
   A   
   G   
   C   
   T   
   T   
   C   
   A   
   A   
   A   
   A   
   G   
   G   
   A   
   A   
    T    
   C   
    C    
   G   
   A   
   A   
   A   
    G    
   C   
   T   
   T   
   G   
   A   
   T   
   T   
   G   
   C   
    G    
   C   
   A   
   A   
   A   
   T   
   C   
   A   
   C   
   A   
   G   
   A   
   G   
   T   
   T   
    G    
   A   
   A   
   T   
   A   
   A   
   A   
   A   
   T   
   C   
   C   
   T   
   T   
   T   
   C   
   A   
   A   
   A   
    T    
   G   
   A   
   C   
   A   
   A   
   A   
   G   
   A   
    G    
   C   
   T   
   T   
   T   
   T   
    C    
   T   
   C   
   T   
   G   
   T   
   T   
   A   
   T   
   G   
   A   
   A   
   A   
   A   
   A   
   A   
   G   
   A   
   A   
   T   
   T   
   G   
   C   
   G   
   A   
   G   
   A   
   A   
   G   
   T   
   G   
   A   
   A   
   A   
   A   
   A   
   A   
   A   
   A   
   T   
   T   
   A   
    T    
   T   
   C   
   T   
   T   
   C   
   C   
   G   
   C   
   T   
   C   
   G   
   T   
   C   
    T    
    A    
   A   
   C   
   G   
   A   
   C   
   A   
   A   
   T   
   C   
    G    
   A   
   A   
   G   
   A   
   C   
   G   
   A   
   A   
   A   
   T   
   C   
   G   
   A   
   A   
   G   
   A   
   A   
   A   
   T   
   C   
   A   
   A   
   A   
   A   
   T   
    T    
   G   
   A   
   T   
   A   
   C   
   G   
   C   
   A   
   A   
    G    
   T   
    C    
   T   
   T   
   A   
   G   
   T   
   G   
   G   
   C   
    A    
   C   
   A   
   A   
   G   
   A   
   A   
   G   
   A   
   C   
   G   
   T   
   A   
   A   
   T   
    T    
   G   
   T   
   C   
   A   
   G   
   T   
   G   
   T   
    G    
   A   
   C   
    G    
   C   
   G   
   A   
   G   
   A   
   A   
   G   
   G   
   C   
   T   
   A   
   T   
   G   
   T   
   G   
   A   
   A   
   G   
   C   
   G   
   A   
   A   
   C   
   A   
   A   
   G   
   C   
   C   
   T   
   T   
   C   
   G   
   T   
   T   
   C   
    T    
   T   
   A   
   T   
   A   
   G   
   T   
   G   
   C   
   T   
   T   
   C   
    C    
   A   
   A   
   A   
   C   
   C   
   A   
   G   
   A   
   A   
   G   
   A   
   A   
   A   
   T   
   C   
   G   
   G   
   G   
   A   
   T   
   G   
   C   
   G   
   A   
   G   
   A   
   A   
   G   
   G   
   C   
   G   
   A   
    C    
   T   
   A   
   C   
   T   
   T   
   G   
   C   
   T   
    A    
   T   
   A   
   C   
    T    
   C   
   A   
   G   
   G   
   T   
   G   
   A   
   A   
   T   
   T   
    G    
   A   
   G   
    T    
   A   
   C   
   A   
    T    
   T   
   G   
   G   
   A   
   C   
   C   
   A   
   T   
   G   
   T   
   T   
   C   
   T   
   T   
   C   
   T   
   G   
   A   
   T   
    C    
   A   
   C   
    A    
   A   
   A   
   T   
   A   
   A   
   A   
   G   
   C   
   A   
   A   
   A   
   T   
   G   
   T   
   G   
   A   
   T   
   C   
   T   
   A   
   C   
   C   
   G   
    C    
   C   
   C   
   T   
   G   
   T   
    C    
   C   
   A   
   T   
   G   
   A   
   A   
   C   
   T   
   A   
   C   
   C   
    C    
   G   
   A   
   T   
   T   
   T   
   G   
   A   
   A   
   A   
   T   
   G   
   G   
   A   
   A   
   A   
   G   
   A   
   T   
   G   
   C   
   T   
   G   
   G   
    T    
   G   
   A   
    G    
   C   
   A   
    C    
   A   
   T   
   T   
   T   
   C   
   T   
   C   
   A   
   A   
   A   
   C   
   A   
   A   
   T   
   C   
   T   
   C   
   C   
   A   
   A   
   T   
   C   
   T   
   G   
   T   
   C   
   T   
   G   
   T   
   A   
   G   
   A   
    C    
   G   
   A   
   A   
   T   
   C   
   G   
   A   
   T   
    T    
   T   
   T   
   A   
   G   
   C   
   T   
   G   
   T   
   T   
   T   
   T   
    C    
   C   
   C   
   A   
   T   
   A   
   T   
   C   
   A   
   A   
   A   
   A   
   A   
   A   
   T   
    T    
   C   
   A   
   A   
   G   
   C   
   A   
   G   
   A   
    G    
   A   
   A   
   A   
   A   
   C   
   A   
   T   
   T   
   T   
   G   
   T   
    A    
   T   
   T   
   C   
   A   
   T   
   C   
   A   
   G   
   C   
   A   
   A   
   G   
   A   
   A   
   T   
   G   
   G   
   A   
   T   
   T   
    G    
    A    
   T   
   C   
   A   
   A   
   G   
   C   
   A   
   A   
   A   
   C   
   A   
   C   
   G   
   A   
   A   
   T   
   G   
   A   
   C   
   A   
   G   
   A   
   G   
   T   
   T   
   C   
   G   
   A   
   A   
   C   
   C   
   A   
   T   
   G   
   G   
   C   
   G   
   C   
   A   
   C   
   C   
   T   
   A   
    T    
   A   
   A   
   G   
   A   
   G   
   C   
   C   
   G   
    C    
   C   
   C   
   G   
   C   
   T   
   C   
   A   
   G   
   C   
   T   
   G   
   T   
   A   
   T   
   G   
   A   
   A   
   G   
   C   
   T   
   A   
   A   
   A   
   G   
   G   
   C   
   G   
   G   
   A   
   A   
   G   
   A   
   A   
   G   
   A   
   T   
   G   
   A   
   A   
   C   
   T   
    G    
   A   
   T   
   T   
   G   
   C   
   T   
   G   
   T   
    G    
   T   
   A   
   T   
   C   
   T   
   G   
   A   
   A   
   A   
   G   
   A   
   A   
   G   
   A   
   T   
   C   
    A    
   G   
   C   
   C   
   A   
   G   
   G   
   C   
   T   
   T   
   A   
   G   
   A   
   T   
   G   
   T   
    T    
   T   
   T   
   C   
   T   
   T   
   A   
   G   
   T   
   A   
   A   
   C   
   G   
    C    
   A   
   T   
   C   
   A   
   A   
   G   
   G   
   A   
   A   
   T   
   G   
   G   
   G   
    T    
   T   
   T   
   G   
   C   
   G   
   C   
   T   
    A    
   T   
   C   
   C   
   T   
   T   
   T   
   A   
   A   
    C    
   A   
   G   
   A   
   T   
   G   
   T   
   T   
   C   
   C   
   C   
   G   
   T   
   T   
   G   
   T   
   T   
   G   
   G   
   A   
   G   
   C   
   A   
   A   
   A   
   G   
   G   
   C   
   A   
   G   
    C    
   T   
   G   
   G   
   A   
   G   
   T   
   G   
   A   
   A   
   G   
   T   
   C   
   T   
   A   
   T   
   G   
   A   
   A   
   T   
   T   
   T   
   G   
   A   
   A   
   A   
   G   
   A   
   T   
   G   
   G   
   A   
   G   
   A   
   T   
   A   
   G   
   G   
    A    
   T   
   T   
    A    
   T   
   C   
   A   
   A   
    C    
   G   
   G   
    G    
   C   
   T   
    A    
   C   
   T   
   T   
   G   
   T   
   G   
   T   
   T   
   A   
   G   
   C   
   A   
   G   
   A   
    A    
   G   
   G   
    T    
   G   
   A   
   T   
   A   
   C   
   G   
   C   
   C   
    G    
   A   
   T   
   C   
   G   
   T   
   A   
   A   
   T   
   C   
   G   
   T   
   T   
   A   
   C   
   T   
   C   
   A   
   G   
   C   
   G   
   A   
   G   
   G   
   A   
   G   
   C   
    T    
   G   
   T   
    T    
   A   
   A   
   A   
   C   
   G   
   C   
   A   
   T   
   G   
   C   
   T   
   T   
   G   
   C   
   T   
   C   
   A   
   A   
   G   
   A   
   A   
   A   
   T   
   C   
   A   
   G   
   C   
   C   
   A   
   A   
   A   
   C   
    T    
   T   
   C   
   A   
   C   
   G   
   T   
   G   
   C   
   G   
   A   
   A   
   G   
   C   
   G   
   G   
   G   
   G   
   A   
   G   
   T   
   G   
   A   
   C   
   G   
   G   
   T   
   C   
   A   
   T   
   G   
   C   
   G   
   T   
   G   
   A   
   G   
   C   
   T   
   G   
   A   
   A   
   A   
   A   
   A   
   A   
   A   
   A   
   T   
   C   
   C   
    G    
   C   
   A   
    C    
   C   
   G   
    T    
   A   
   T   
   T   
   A   
   T   
   C   
   T   
   A   
   T   
   A   
   T   
   G   
   T   
   C   
   G   
   G   
   A   
   A   
   G   
   G   
   A   
   A   
   G   
   A   
   T   
   C   
   A   
   A   
   A   
   A   
   G   
   A   
   A   
   A   
   T   
   A   
   A   
   C   
   C   
   T   
   T   
   G   
   A   
   T   
   G   
   A   
   A   
   T   
   C   
   A   
   A   
   A   
   A   
   A   
   G   
   G   
    G    
   C   
   A   
    G    
   C   
   A   
   G   
    T    
   T   
   A   
   A   
   C   
   G   
   A   
   T   
   T   
   G   
   A   
    C    
   C   
   C   
   A   
   A   
   C   
    T    
   G   
   A   
   T   
   T   
   T   
    C    
   C   
   C   
   T   
   A   
   T   
   A   
   G   
   G   
   T   
   G   
   A   
   T   
   C   
   G   
   A   
   A   
   C   
   A   
   T   
   C   
   G   
   A   
   A   
   T   
   G   
   G   
    A    
   T   
   C   
    A    
   T   
   T   
   T   
   G   
   C   
    G    
   A   
   T   
   G   
   G   
   A   
    T    
   G   
   A   
   G   
   A   
   A   
   A   
   A   
   A   
   A   
   G   
   G   
   T   
   G   
   G   
   A   
   G   
   A   
   A   
   G   
   T   
   C   
   A   
   T   
   C   
   G   
   A   
   A   
   G   
   T   
    T    
   A   
    T    
   C   
   G   
   A   
   T   
   G   
   C   
   T   
   C   
   C   
   T   
   G   
   A   
   G   
    A    
   T   
   T   
   T   
   T   
    C    
   A   
   T   
    C    
   G   
   A   
    G    
    T    
   C   
    G    
   -   
   -   
   -   
 

 
   N60123F   
   A   
   T   
   G   
   G   
   A   
   A   
   A   
   A   
   T   
   C   
   G   
   A   
   C   
   A   
   A   
   G   
   A   
   A   
   G   
   T   
   A   
   C   
   A   
   A   
   G   
   A   
   A   
   T   
   T   
   A   
   A   
   C   
   T   
   T   
   T   
   A   
   G   
   A   
   A   
   G   
   A   
   A   
   G   
   T   
   G   
   A   
   T   
   G   
   G   
   G   
   T   
   G   
   A   
   C   
   C   
   G   
   C   
   T   
   T   
   C   
   G   
   G   
   A   
   C   
   G   
   T   
   T   
   A   
   T   
   T   
   C   
   C   
   A   
   A   
   A   
   T   
   A   
   T   
   A   
   T   
   T   
   A   
   T   
    C    
   C   
   A   
    A    
   G   
   A   
   A   
   C   
   G   
   T   
   G   
   C   
   A   
   C   
   T   
   G   
   C   
   C   
   T   
   G   
   A   
   T   
   A   
   T   
    T    
   C   
   G   
   T   
   G   
   A   
   T   
   G   
   G   
   A   
   T   
   T   
   G   
   A   
   A   
    G    
   C   
   C   
   A   
   G   
   T   
    G    
   C   
   A   
   A   
   C   
   G   
   C   
   C   
   G   
   G   
   A   
   T   
   A   
   T   
   T   
   A   
   T   
   T   
    T    
   G   
   C   
    C    
   A   
   T   
   G   
   A   
   A   
   C   
   A   
   A   
   A   
   G   
   A   
   T   
   G   
   G   
   C   
   A   
   A   
   T   
   A   
   C   
   G   
   T   
   A   
   T   
   G   
   A   
   C   
   A   
   A   
   A   
   G   
   G   
   A   
   T   
   T   
    C    
   C   
   G   
   T   
   A   
   A   
   A   
   T   
   C   
   A   
   G   
   C   
   A   
   A   
   A   
   A   
   T   
   C   
   T   
   G   
   T   
   C   
   G   
   G   
   A   
   A   
   A   
   T   
   A   
   T   
   C   
   A   
   T   
   G   
   G   
   G   
    G    
   A   
   A   
   T   
   T   
   A   
   T   
   C   
   A   
   T   
   C   
   C   
   T   
   C   
   A   
   T   
   G   
   G   
   T   
   G   
   A   
   C   
   A   
   G   
   C   
   A   
   G   
   T   
   A   
   T   
   T   
   T   
   A   
   T   
   G   
   A   
   A   
   G   
   C   
   G   
   A   
   T   
   G   
   G   
   T   
   C   
   C   
   G   
   C   
   A   
   T   
   G   
   A   
   G   
   C   
   C   
   A   
   A   
   G   
   A   
    C    
   T   
   G   
   G   
   A   
   A   
   G   
   T   
   T   
   G   
   C   
   G   
   G   
   G   
   A   
   A   
   G   
   T   
   A   
   C   
   T   
   G   
   A   
   T   
   C   
   G   
   A   
    A    
   A   
   T   
   G   
   C   
   A   
   C   
   G   
   G   
    G    
   A   
   A   
   T   
   A   
   A   
    C    
   G   
   G   
   G   
   A   
   G   
   T   
   A   
   T   
   G   
   G   
   A   
   C   
   G   
   G   
   T   
   G   
   A   
   T   
   C   
   C   
   A   
   C   
   C   
   T   
   G   
   C   
   T   
   G   
   C   
   G   
   A   
   T   
   G   
   C   
   G   
   T   
   T   
   A   
   T   
   A   
   C   
   T   
   G   
   A   
   A   
   G   
   C   
   C   
   A   
   G   
    A    
   T   
   T   
   A   
   T   
   C   
   C   
   A   
   A   
   G   
   T   
   T   
   A   
   A   
   G   
   T   
   G   
   G   
   A   
   G   
   A   
   G   
   A   
   T   
   G   
    C    
   T   
   A   
    G    
   C   
   A   
   G   
   A   
   T   
   A   
   T   
   C   
   G   
   A   
    A    
   A   
   A   
   A   
   G   
   A   
   A   
   A   
   C   
   A   
   G   
   T   
    C    
   G   
   A   
   T   
   C   
   T   
   C   
   G   
   T   
   G   
   T   
   G   
   G   
   A   
   A   
   T   
   T   
   T   
   T   
   G   
   A   
   T   
   G   
   A   
   T   
   A   
   C   
   A   
   G   
   A   
   A   
   A   
   A   
   A   
   G   
   A   
   A   
   C   
   C   
   A   
   A   
   C   
   A   
   G   
   T   
   C   
   C   
   T   
   T   
   C   
   C   
   A   
   G   
   C   
   T   
   C   
   G   
   A   
   T   
   A   
   C   
   C   
   C   
   A   
   A   
   A   
   C   
   C   
   T   
   T   
   C   
   T   
    C    
   G   
   T   
   C   
   A   
   A   
   T   
   G   
   G   
   A   
   T   
   C   
   G   
   A   
   C   
   A   
   G   
   G   
   T   
   A   
   T   
   A   
   T   
   C   
   T   
   G   
   C   
   T   
   G   
   G   
   A   
   T   
   A   
   T   
   G   
   C   
    A    
   A   
   C   
   A   
   G   
   A   
   A   
   A   
   T   
   C   
   C   
   C   
   G   
   A   
   C   
    T    
   C   
   A   
   T   
   A   
   A   
   C   
   T   
   T   
   A   
   G   
   C   
   T   
   G   
   A   
   A   
   G   
   T   
   G   
   A   
   T   
    C    
   G   
   A   
    T    
   G   
   G   
   C   
   A   
   C   
   A   
   A   
   T   
   C   
   T   
   A   
   T   
   A   
   T   
   G   
   A   
   T   
   C   
   G   
   A   
   T   
   C   
   A   
   T   
   C   
   C   
   A   
   C   
   A   
   A   
   G   
   C   
   G   
   A   
   G   
   C   
   C   
   T   
   T   
   G   
   A   
   A   
   A   
   A   
   A   
   T   
   T   
   G   
   A   
   T   
   G   
   G   
   A   
   A   
   T   
   A   
   T   
   A   
   T   
   T   
   C   
   C   
   T   
   G   
   G   
   A   
   C   
   C   
   T   
   G   
   A   
   C   
   T   
   T   
    T    
   C   
   C   
   A   
   A   
   C   
   A   
   G   
   G   
   A   
   G   
   G   
    G    
   A   
   T   
   C   
   T   
   T   
   G   
   C   
   A   
   A   
   G   
   G   
    A    
   A   
   A   
   A   
   G   
   A   
   T   
   G   
   A   
   A   
   A   
   T   
   C   
   A   
   A   
   A   
   A   
   A   
   A   
   G   
   C   
   T   
   T   
   A   
   T   
   G   
   A   
   A   
   A   
   C   
   A   
   G   
   G   
    G    
   C   
   G   
   A   
   G   
   G   
   A   
   A   
   A   
   A   
   G   
   T   
   T   
   A   
    T    
   T   
   C   
   T   
   T   
   C   
   G   
   T   
   T   
   C   
   G   
   A   
   A   
   A   
   A   
   C   
   A   
   A   
   A   
   G   
   A   
   T   
   A   
   G   
   A   
    A    
   G   
   C   
   A   
   A   
   T   
   G   
   A   
   A   
   G   
   G   
   G   
    T    
   A   
   A   
    T    
   A   
   A   
   A   
   C   
   A   
   G   
   C   
   A   
   A   
   A   
   T   
   C   
   G   
   T   
   G   
   A   
   T   
   C   
   A   
   C   
    A    
   G   
   A   
   A   
   A   
   T   
   C   
   C   
   C   
   A   
   T   
   A   
   T   
   G   
   A   
   A   
   G   
   T   
   A   
   A   
   A   
   T   
   A   
   A   
   A   
   G   
   C   
   G   
   A   
   C   
    C    
   T   
   T   
   G   
   G   
   T   
   G   
   A   
   A   
   A   
   A   
   A   
   A   
   A   
   T   
   G   
   G   
   A   
   T   
   G   
   A   
   G   
   A   
   T   
   C   
   C   
   G   
   T   
   T   
   T   
   G   
   A   
   A   
   T   
   A   
   A   
   G   
   A   
   A   
   A   
   A   
   T   
   C   
   G   
   A   
    T    
   G   
   G   
   A   
   A   
   T   
   A   
   G   
   C   
   T   
   G   
   A   
   A   
   G   
   T   
    A    
   C   
   G   
   A   
   G   
   A   
   T   
   G   
   A   
   A   
   A   
   G   
   T   
   G   
   A   
   T   
    A    
   G   
    A    
   A   
   C   
    A    
   G   
   G   
   T   
   C   
   T   
   G   
   C   
   A   
   A   
   A   
   T   
   T   
   G   
   T   
   C   
   G   
   T   
    C    
   G   
   A   
   A   
   T   
   T   
   G   
   A   
   A   
   A   
   A   
   A   
   A   
   G   
   A   
    C    
   G   
   C   
   C   
   A   
   A   
    T    
   G   
   C   
   T   
   C   
   A   
   A   
   G   
   G   
    A    
   A   
   T   
   T   
   T   
   T   
   G   
   A   
   A   
   T   
   T   
   A   
    T    
   T   
   T   
   G   
   T   
   T   
   C   
   A   
   A   
   A   
   A   
   A   
   C   
   A   
   C   
   T   
   G   
   A   
   G   
    C    
   T   
   A   
   C   
   A   
   G   
   A   
   T   
   C   
   A   
   A   
   T   
   T   
   A   
   C   
   A   
   A   
   T   
   T   
   T   
    C    
   A   
   A   
   T   
   A   
   T   
   G   
   G   
   T   
   C   
   G   
   C   
   T   
   A   
   T   
   C   
   G   
   A   
    C    
   C   
   A   
   T   
   A   
   T   
   G   
   A   
   C   
   T   
   C   
   C   
   G   
   C   
   A   
    T    
   C   
   A   
   A   
   G   
   T   
   T   
   G   
   G   
   A   
   C   
   T   
   G   
   A   
   A   
   A   
   G   
   A   
   C   
   A   
   T   
   T   
   T   
   T   
   A   
   A   
   G   
   A   
   A   
   G   
   T   
   T   
   A   
   T   
   A   
   T   
   C   
   G   
   A   
   G   
   C   
   A   
   T   
   C   
   G   
   A   
   A   
   A   
   G   
   C   
   A   
   A   
   G   
   T   
   C   
   A   
   T   
   A   
   A   
   C   
   A   
   A   
   A   
   A   
   C   
   G   
    T    
   A   
   G   
    T    
   C   
   A   
   A   
   T   
   T   
   C   
   G   
   A   
   T   
   T   
   T   
   A   
   G   
   C   
   C   
   A   
   A   
   A   
   G   
   C   
   T   
   C   
   A   
   A   
   A   
   A   
   A   
    A    
   G   
   A   
   C   
   A   
   G   
   C   
   A   
   T   
   A   
   T   
   C   
   G   
   T   
    A    
   G   
   A   
   A   
   G   
   G   
    A    
    T    
   T   
   G   
   A   
   T   
   G   
   A   
   A   
   A   
   G   
   C   
   T   
   T   
   T   
   A   
   T   
   C   
   G   
   A   
   T   
   T   
   C   
   T   
   G   
   G   
   A   
   T   
   G   
   A   
   A   
   G   
   T   
   A   
   A   
   T   
   C   
   G   
    C    
   A   
   A   
   C   
   G   
   A   
   T   
   T   
   C   
   G   
   T   
   G   
   A   
   A   
   A   
   G   
   T   
   A   
   A   
   A   
   G   
   A   
   C   
   A   
   A   
   A   
   A   
   A   
   A   
   G   
   A   
    C    
   G   
   C   
    C    
   A   
   A   
   A   
   A   
   A   
   G   
   A   
   A   
   T   
   T   
   T   
   A   
   G   
   T   
   C   
   G   
   A   
    C    
   G   
   T   
    C    
   T   
   T   
    T    
   C   
   A   
   A   
   T   
   T   
   T   
   A   
   C   
   A   
   G   
   A   
   A   
   G   
   A   
   A   
   C   
   A   
   A   
   G   
   C   
   A   
   G   
   A   
   A   
   G   
   C   
   A   
   A   
   T   
    T    
   G   
   T   
   C   
   A   
   C   
   A   
   T   
   T   
   G   
   C   
   A   
   A   
    T    
   T   
   A   
   T   
   A   
    C    
   C   
   G   
   T   
   T   
   T   
   G   
   A   
   C   
   G   
   A   
   A   
    C    
   A   
   C   
    G    
   G   
   A   
   T   
   A   
   T   
   C   
   A   
   C   
   A   
   G   
   A   
   G   
   C   
   T   
   T   
   C   
   A   
   A   
   A   
   A   
   G   
   G   
   A   
   A   
    T    
   C   
    C    
   G   
   A   
   A   
   A   
    G    
   C   
   T   
   T   
   G   
   A   
   T   
   T   
   G   
   C   
    G    
   C   
   A   
   A   
   A   
   T   
   C   
   A   
   C   
   A   
   G   
   A   
   G   
   T   
   T   
    G    
   A   
   A   
   T   
   A   
   A   
   A   
   A   
   T   
   C   
   C   
   T   
   T   
   T   
   C   
   A   
   A   
   A   
    T    
   G   
   A   
   C   
   A   
   A   
   A   
   G   
   A   
    G    
   C   
   T   
   T   
   T   
   T   
    C    
   T   
   C   
   T   
   G   
   T   
   T   
   A   
   T   
   G   
   A   
   A   
   A   
   A   
   A   
   A   
   G   
   A   
   A   
   T   
   T   
   G   
   C   
   G   
   A   
   G   
   A   
   A   
   G   
   T   
   G   
   A   
   A   
   A   
   A   
   A   
   A   
   A   
   A   
   T   
   T   
   A   
    T    
   T   
   C   
   T   
   T   
   C   
   C   
   G   
   C   
   T   
   C   
   G   
   T   
   C   
    T    
    A    
   A   
   C   
   G   
   A   
   C   
   A   
   A   
   T   
   C   
    G    
   A   
   A   
   G   
   A   
   C   
   G   
   A   
   A   
   A   
   T   
   C   
   G   
   A   
   A   
   G   
   A   
   A   
   A   
   T   
   C   
   A   
   A   
   A   
   A   
   T   
    T    
   G   
   A   
   T   
   A   
   C   
   G   
   C   
   A   
   A   
    G    
   T   
    C    
   T   
   T   
   A   
   G   
   T   
   G   
   G   
   C   
    A    
   C   
   A   
   A   
   G   
   A   
   A   
   G   
   A   
   C   
   G   
   T   
   A   
   A   
   T   
    T    
   G   
   T   
   C   
   A   
   G   
   T   
   G   
   T   
    G    
   A   
   C   
    G    
   C   
   G   
   A   
   G   
   A   
   A   
   G   
   G   
   C   
   T   
   A   
   T   
   G   
   T   
   G   
   A   
   A   
   G   
   C   
   G   
   A   
   A   
   C   
   A   
   A   
   G   
   C   
   C   
   T   
   T   
   C   
   G   
   T   
   T   
   C   
    T    
   T   
   A   
   T   
   A   
   G   
   T   
   G   
   C   
   T   
   T   
   C   
    C    
   A   
   A   
   A   
   C   
   C   
   A   
   G   
   A   
   A   
   G   
   A   
   A   
   A   
   T   
   C   
   G   
   G   
   G   
   A   
   T   
   G   
   C   
   G   
   A   
   G   
   A   
   A   
   G   
   G   
   C   
   G   
   A   
    C    
   T   
   A   
   C   
   T   
   T   
   G   
   C   
   T   
    A    
   T   
   A   
   C   
    T    
   C   
   A   
   G   
   G   
   T   
   G   
   A   
   A   
   T   
   T   
    G    
   A   
   G   
    T    
   A   
   C   
   A   
    T    
   T   
   G   
   G   
   A   
   C   
   C   
   A   
   T   
   G   
   T   
   T   
   C   
   T   
   T   
   C   
   T   
   G   
   A   
   T   
    C    
   A   
   C   
    A    
   A   
   A   
   T   
   A   
   A   
   A   
   G   
   C   
   A   
   A   
   A   
   T   
   G   
   T   
   G   
   A   
   T   
   C   
   T   
   A   
   C   
   C   
   G   
    C    
   C   
   C   
   T   
   G   
   T   
    C    
   C   
   A   
   T   
   G   
   A   
   A   
   C   
   T   
   A   
   C   
   C   
    C    
   G   
   A   
   T   
   T   
   T   
   G   
   A   
   A   
   A   
   T   
   G   
   G   
   A   
   A   
   A   
   G   
   A   
   T   
   G   
   C   
   T   
   G   
   G   
    T    
   G   
   A   
    G    
   C   
   A   
    C    
   A   
   T   
   T   
   T   
   C   
   T   
   C   
   A   
   A   
   A   
   C   
   A   
   A   
   T   
   C   
   T   
   C   
   C   
   A   
   A   
   T   
   C   
   T   
   G   
   T   
   C   
   T   
   G   
   T   
   A   
   G   
   A   
    C    
   G   
   A   
   A   
   T   
   C   
   G   
   A   
   T   
    T    
   T   
   T   
   A   
   G   
   C   
   T   
   G   
   T   
   T   
   T   
   T   
    C    
   C   
   C   
   A   
   T   
   A   
   T   
   C   
   A   
   A   
   A   
   A   
   A   
   A   
   T   
    T    
   C   
   A   
   A   
   G   
   C   
   A   
   G   
   A   
    G    
   A   
   A   
   A   
   A   
   C   
   A   
   T   
   T   
   T   
   G   
   T   
    A    
   T   
   T   
   C   
   A   
   T   
   C   
   A   
   G   
   C   
   A   
   A   
   G   
   A   
   A   
   T   
   G   
   G   
   A   
   T   
   T   
    G    
    A    
   T   
   C   
   A   
   A   
   G   
   C   
   A   
   A   
   A   
   C   
   A   
   C   
   G   
   A   
   A   
   T   
   G   
   A   
   C   
   A   
   G   
   A   
   G   
   T   
   T   
   C   
   G   
   A   
   A   
   C   
   C   
   A   
   T   
   G   
   G   
   C   
   G   
   C   
   A   
   C   
   C   
   T   
   A   
    T    
   A   
   A   
   G   
   A   
   G   
   C   
   C   
   G   
    T    
   C   
   C   
   G   
   C   
   T   
   C   
   A   
   G   
   C   
   T   
   G   
   T   
   A   
   T   
   G   
   A   
   A   
   G   
   C   
   T   
   A   
   A   
   A   
   G   
   G   
   C   
   G   
   G   
   A   
   A   
   G   
   A   
   A   
   G   
   A   
   T   
   G   
   A   
   A   
   C   
   T   
    G    
   A   
   T   
   T   
   G   
   C   
   T   
   G   
   T   
    G    
   T   
   A   
   T   
   C   
   T   
   G   
   A   
   A   
   A   
   G   
   A   
   A   
   G   
   A   
   T   
   C   
    A    
   G   
   C   
   C   
   A   
   G   
   G   
   C   
   T   
   T   
   A   
   G   
   A   
   T   
   G   
   T   
    T    
   T   
   T   
   C   
   T   
   T   
   A   
   G   
   T   
   A   
   A   
   C   
   G   
    C    
   A   
   T   
   C   
   A   
   A   
   G   
   G   
   A   
   A   
   T   
   G   
   G   
   G   
    T    
   T   
   T   
   G   
   C   
   G   
   C   
   T   
    A    
   T   
   C   
   C   
   T   
   T   
   T   
   A   
   A   
    C    
   A   
   G   
   A   
   T   
   G   
   T   
   T   
   C   
   C   
   C   
   G   
   T   
   T   
   G   
   T   
   T   
   G   
   G   
   A   
   G   
   C   
   A   
   A   
   A   
   G   
   G   
   C   
   A   
   G   
    C    
   T   
   G   
   G   
   A   
   G   
   T   
   G   
   A   
   A   
   G   
   T   
   C   
   T   
   A   
   T   
   G   
   A   
   A   
   T   
   T   
   T   
   G   
   A   
   A   
   A   
   G   
   A   
   T   
   G   
   G   
   A   
   G   
   A   
   T   
   A   
   G   
   G   
    A    
   T   
   T   
    A    
   T   
   C   
   A   
   A   
    C    
   G   
   G   
    G    
   C   
   T   
    A    
   C   
   T   
   T   
   G   
   T   
   G   
   T   
   T   
   A   
   G   
   C   
   A   
   G   
   A   
    A    
   G   
   G   
    T    
   G   
   A   
   T   
   A   
   C   
   G   
   C   
   C   
    G    
   A   
   T   
   C   
   G   
   T   
   A   
   A   
   T   
   C   
   G   
   T   
   T   
   A   
   C   
   T   
   C   
   A   
   G   
   C   
   G   
   A   
   G   
   G   
   A   
   G   
   C   
    T    
   G   
   T   
    T    
   A   
   A   
   A   
   C   
   G   
   C   
   A   
   T   
   G   
   C   
   T   
   T   
   G   
   C   
   T   
   C   
   A   
   A   
   G   
   A   
   A   
   A   
   T   
   C   
   A   
   G   
   C   
   C   
   A   
   A   
   A   
   C   
    T    
   T   
   C   
   A   
   C   
   G   
   T   
   G   
   C   
   G   
   A   
   A   
   G   
   C   
   G   
   G   
   G   
   G   
   A   
   G   
   T   
   G   
   A   
   C   
   G   
   G   
   T   
   C   
   A   
   T   
   G   
   C   
   G   
   T   
   G   
   A   
   G   
   C   
   T   
   G   
   A   
   A   
   A   
   A   
   A   
   A   
   A   
   A   
   T   
   C   
   C   
    G    
   C   
   A   
    C    
   C   
   G   
    T    
   A   
   T   
   T   
   A   
   T   
   C   
   T   
   A   
   T   
   A   
   T   
   G   
   T   
   C   
   G   
   G   
   A   
   A   
   G   
   G   
   A   
   A   
   G   
   A   
   T   
   C   
   A   
   A   
   A   
   A   
   G   
   A   
   A   
   A   
   T   
   A   
   A   
   C   
   C   
   T   
   T   
   G   
   A   
   T   
   G   
   A   
   A   
   T   
   C   
   A   
   A   
   A   
   A   
   A   
   G   
   G   
    G    
   C   
   A   
    G    
   C   
   A   
   G   
    T    
   T   
   A   
   A   
   C   
   G   
   A   
   T   
   T   
   G   
   A   
    C    
   C   
   C   
   A   
   A   
   C   
    T    
   G   
   A   
   T   
   T   
   T   
    C    
   C   
   C   
   T   
   A   
   T   
   A   
   G   
   G   
   T   
   G   
   A   
   T   
   C   
   G   
   A   
   A   
   C   
   A   
   T   
   C   
   G   
   A   
   A   
   T   
   G   
   G   
    A    
   T   
   C   
    A    
   T   
   T   
   T   
   G   
   C   
    G    
   A   
   T   
   G   
   G   
   A   
    T    
   G   
   A   
   G   
   A   
   A   
   A   
   A   
   A   
   A   
   G   
   G   
   T   
   G   
   G   
   A   
   G   
   A   
   A   
   G   
   T   
   C   
   A   
   T   
   C   
   G   
   A   
   A   
   G   
   T   
    T    
   A   
    T    
   C   
   G   
   A   
   T   
   G   
   C   
   T   
   C   
   C   
   T   
   G   
   A   
   G   
    A    
   T   
   T   
   T   
   T   
    C    
   A   
   T   
    C    
   G   
   A   
    G    
    T    
   C   
    G    
   -   
   -   
   -   
 

 
   N53533   
   A   
   T   
   G   
   G   
   A   
   A   
   A   
   A   
   T   
   C   
   G   
   A   
   C   
   A   
   A   
   G   
   A   
   A   
   G   
   T   
   A   
   C   
   A   
   A   
   G   
   A   
   A   
   T   
   T   
   A   
   A   
   C   
   T   
   T   
   T   
   A   
   G   
   A   
   A   
   G   
   A   
   A   
   G   
   T   
   G   
   A   
   T   
   G   
   G   
   G   
   T   
   G   
   A   
   C   
   C   
   G   
   C   
   T   
   T   
   C   
   G   
   G   
   A   
   C   
   G   
   T   
   T   
   A   
   T   
   T   
   C   
   C   
   A   
   A   
   A   
   T   
   A   
   T   
   A   
   T   
   T   
   A   
   T   
    C    
   C   
   A   
    A    
   G   
   A   
   A   
   C   
   G   
   T   
   G   
   C   
   A   
   C   
   T   
   G   
   C   
   C   
   T   
   G   
   A   
   T   
   A   
   T   
    C    
   C   
   G   
   T   
   G   
   A   
   T   
   G   
   G   
   A   
   T   
   T   
   G   
   A   
   A   
    G    
   C   
   C   
   A   
   G   
   T   
    G    
   C   
   A   
   A   
   C   
   G   
   C   
   C   
   G   
   G   
   A   
   T   
   A   
   T   
   T   
   A   
   T   
   T   
    T    
   G   
   C   
    C    
   A   
   T   
   G   
   A   
   A   
   C   
   A   
   A   
   A   
   G   
   A   
   T   
   G   
   G   
   C   
   A   
   A   
   T   
   A   
   C   
   G   
   T   
   A   
   T   
   G   
   A   
   C   
   A   
   A   
   A   
   G   
   G   
   A   
   T   
   T   
    C    
   C   
   G   
   T   
   A   
   A   
   A   
   T   
   C   
   A   
   G   
   C   
   A   
   A   
   A   
   A   
   T   
   C   
   T   
   G   
   T   
   C   
   G   
   G   
   A   
   A   
   A   
   T   
   A   
   T   
   C   
   A   
   T   
   G   
   G   
   G   
    G    
   A   
   A   
   T   
   T   
   A   
   T   
   C   
   A   
   T   
   C   
   C   
   T   
   C   
   A   
   T   
   G   
   G   
   T   
   G   
   A   
   C   
   A   
   G   
   C   
   A   
   G   
   T   
   A   
   T   
   T   
   T   
   A   
   T   
   G   
   A   
   A   
   G   
   C   
   G   
   A   
   T   
   G   
   G   
   T   
   C   
   C   
   G   
   C   
   A   
   T   
   G   
   A   
   G   
   C   
   C   
   A   
   A   
   G   
   A   
    C    
   T   
   G   
   G   
   A   
   A   
   G   
   T   
   T   
   G   
   C   
   G   
   G   
   G   
   A   
   A   
   G   
   T   
   A   
   C   
   T   
   G   
   A   
   T   
   C   
   G   
   A   
    A    
   A   
   T   
   G   
   C   
   A   
   C   
   G   
   G   
    G    
   A   
   A   
   T   
   A   
   A   
    C    
   G   
   G   
   G   
   A   
   G   
   T   
   A   
   T   
   G   
   G   
   A   
   C   
   G   
   G   
   T   
   G   
   A   
   T   
   C   
   C   
   A   
   C   
   C   
   T   
   G   
   C   
   T   
   G   
   C   
   G   
   A   
   T   
   G   
   C   
   G   
   T   
   T   
   A   
   T   
   A   
   C   
   T   
   G   
   A   
   A   
   G   
   C   
   C   
   A   
   G   
    A    
   T   
   T   
   A   
   T   
   C   
   C   
   A   
   A   
   G   
   T   
   T   
   A   
   A   
   G   
   T   
   G   
   G   
   A   
   G   
   A   
   G   
   A   
   T   
   G   
    C    
   T   
   A   
    G    
   C   
   A   
   G   
   A   
   T   
   A   
   T   
   C   
   G   
   A   
    A    
   A   
   A   
   A   
   G   
   A   
   A   
   A   
   C   
   A   
   G   
   T   
    C    
   G   
   A   
   T   
   C   
   T   
   C   
   G   
   T   
   G   
   T   
   G   
   G   
   A   
   A   
   T   
   T   
   T   
   T   
   G   
   A   
   T   
   G   
   A   
   T   
   A   
   C   
   A   
   G   
   A   
   A   
   A   
   A   
   A   
   G   
   A   
   A   
   C   
   C   
   A   
   A   
   C   
   A   
   G   
   T   
   C   
   C   
   T   
   T   
   C   
   C   
   A   
   G   
   C   
   T   
   C   
   G   
   A   
   T   
   A   
   C   
   C   
   C   
   A   
   A   
   A   
   C   
   C   
   T   
   T   
   C   
   T   
    C    
   G   
   T   
   C   
   A   
   A   
   T   
   G   
   G   
   A   
   T   
   C   
   G   
   A   
   C   
   A   
   G   
   G   
   T   
   A   
   T   
   A   
   T   
   C   
   T   
   G   
   C   
   T   
   G   
   G   
   A   
   T   
   A   
   T   
   G   
   C   
    A    
   A   
   C   
   A   
   G   
   A   
   A   
   A   
   T   
   C   
   C   
   C   
   G   
   A   
   C   
    T    
   C   
   A   
   T   
   A   
   A   
   C   
   T   
   T   
   A   
   G   
   C   
   T   
   G   
   A   
   A   
   G   
   T   
   G   
   A   
   T   
    C    
   G   
   A   
    T    
   G   
   G   
   C   
   A   
   C   
   A   
   A   
   T   
   C   
   T   
   A   
   T   
   A   
   T   
   G   
   A   
   T   
   C   
   G   
   A   
   T   
   C   
   A   
   T   
   C   
   C   
   A   
   C   
   A   
   A   
   G   
   C   
   G   
   A   
   G   
   C   
   C   
   T   
   T   
   G   
   A   
   A   
   A   
   A   
   A   
   T   
   T   
   G   
   A   
   T   
   G   
   G   
   A   
   A   
   T   
   A   
   T   
   A   
   T   
   T   
   C   
   C   
   T   
   G   
   G   
   A   
   C   
   C   
   T   
   G   
   A   
   C   
   T   
   T   
    T    
   C   
   C   
   A   
   A   
   C   
   A   
   G   
   G   
   A   
   G   
   G   
    G    
   A   
   T   
   C   
   T   
   T   
   G   
   C   
   A   
   A   
   G   
   G   
    A    
   A   
   A   
   A   
   G   
   A   
   T   
   G   
   A   
   A   
   A   
   T   
   C   
   A   
   A   
   A   
   A   
   A   
   A   
   G   
   C   
   T   
   T   
   A   
   T   
   G   
   A   
   A   
   A   
   C   
   A   
   G   
   G   
    G    
   C   
   G   
   A   
   G   
   G   
   A   
   A   
   A   
   A   
   G   
   T   
   T   
   A   
    T    
   T   
   C   
   T   
   T   
   C   
   G   
   T   
   T   
   C   
   G   
   A   
   A   
   A   
   A   
   C   
   A   
   A   
   A   
   G   
   A   
   T   
   A   
   G   
   A   
    G    
   G   
   C   
   A   
   A   
   T   
   G   
   A   
   A   
   G   
   G   
   G   
    T    
   A   
   A   
    T    
   A   
   A   
   A   
   C   
   A   
   G   
   C   
   A   
   A   
   A   
   T   
   C   
   G   
   T   
   G   
   A   
   T   
   C   
   A   
   C   
    A    
   G   
   A   
   A   
   A   
   T   
   C   
   C   
   C   
   A   
   T   
   A   
   T   
   G   
   A   
   A   
   G   
   T   
   A   
   A   
   A   
   T   
   A   
   A   
   A   
   G   
   C   
   G   
   A   
   C   
    C    
   T   
   T   
   G   
   G   
   T   
   G   
   A   
   A   
   A   
   A   
   A   
   A   
   A   
   T   
   G   
   G   
   A   
   T   
   G   
   A   
   G   
   A   
   T   
   C   
   C   
   G   
   T   
   T   
   T   
   G   
   A   
   A   
   T   
   A   
   A   
   G   
   A   
   A   
   A   
   A   
   T   
   C   
   G   
   A   
    T    
   G   
   G   
   A   
   A   
   T   
   A   
   G   
   C   
   T   
   G   
   A   
   A   
   G   
   T   
    A    
   C   
   G   
   A   
   G   
   A   
   T   
   G   
   A   
   A   
   A   
   G   
   T   
   G   
   A   
   T   
    A    
   G   
    A    
   A   
   C   
    A    
   G   
   G   
   T   
   C   
   T   
   G   
   C   
   A   
   A   
   A   
   T   
   T   
   G   
   T   
   C   
   G   
   T   
    C    
   G   
   A   
   A   
   T   
   T   
   G   
   A   
   A   
   A   
   A   
   A   
   A   
   G   
   A   
    C    
   G   
   C   
   C   
   A   
   A   
    T    
   G   
   C   
   T   
   C   
   A   
   A   
   G   
   G   
    A    
   A   
   T   
   T   
   T   
   T   
   G   
   A   
   A   
   T   
   T   
   A   
    T    
   T   
   T   
   G   
   T   
   T   
   C   
   A   
   A   
   A   
   A   
   A   
   C   
   A   
   C   
   T   
   G   
   A   
   G   
    C    
   T   
   A   
   C   
   A   
   G   
   A   
   T   
   C   
   A   
   A   
   T   
   T   
   A   
   C   
   A   
   A   
   T   
   T   
   T   
    C    
   A   
   A   
   T   
   A   
   T   
   G   
   G   
   T   
   C   
   G   
   C   
   T   
   A   
   T   
   C   
   G   
   A   
    C    
   C   
   A   
   T   
   A   
   T   
   G   
   A   
   C   
   T   
   C   
   C   
   G   
   C   
   A   
    T    
   C   
   A   
   A   
   G   
   T   
   T   
   G   
   G   
   A   
   C   
   T   
   G   
   A   
   A   
   A   
   G   
   A   
   C   
   A   
   T   
   T   
   T   
   T   
   A   
   A   
   G   
   A   
   A   
   G   
   T   
   T   
   A   
   T   
   A   
   T   
   C   
   G   
   A   
   G   
   C   
   A   
   T   
   C   
   G   
   A   
   A   
   A   
   G   
   C   
   A   
   A   
   G   
   T   
   C   
   A   
   T   
   A   
   A   
   C   
   A   
   A   
   A   
   A   
   C   
   G   
    T    
   A   
   G   
    T    
   C   
   A   
   A   
   T   
   T   
   C   
   G   
   A   
   T   
   T   
   T   
   A   
   G   
   C   
   C   
   A   
   A   
   A   
   G   
   C   
   T   
   C   
   A   
   A   
   A   
   A   
   A   
    A    
   G   
   A   
   C   
   A   
   G   
   C   
   A   
   T   
   A   
   T   
   C   
   G   
   T   
    A    
   G   
   A   
   A   
   G   
   G   
    A    
    T    
   T   
   G   
   A   
   T   
   G   
   A   
   A   
   A   
   G   
   C   
   T   
   T   
   T   
   A   
   T   
   C   
   G   
   A   
   T   
   T   
   C   
   T   
   G   
   G   
   A   
   T   
   G   
   A   
   A   
   G   
   T   
   A   
   A   
   T   
   C   
   G   
    C    
   A   
   A   
   C   
   G   
   A   
   T   
   T   
   C   
   G   
   T   
   G   
   A   
   A   
   A   
   G   
   T   
   A   
   A   
   A   
   G   
   A   
   C   
   A   
   A   
   A   
   A   
   A   
   A   
   G   
   A   
    C    
   G   
   C   
    C    
   A   
   A   
   A   
   A   
   A   
   G   
   A   
   A   
   T   
   T   
   T   
   A   
   G   
   T   
   C   
   G   
   A   
    C    
   G   
   T   
    C    
   T   
   T   
    T    
   C   
   A   
   A   
   T   
   T   
   T   
   A   
   C   
   A   
   G   
   A   
   A   
   G   
   A   
   A   
   C   
   A   
   A   
   G   
   C   
   A   
   G   
   A   
   A   
   G   
   C   
   A   
   A   
   T   
    T    
   G   
   T   
   C   
   A   
   C   
   A   
   T   
   T   
   G   
   C   
   A   
   A   
    T    
   T   
   A   
   T   
   A   
    C    
   C   
   G   
   T   
   T   
   T   
   G   
   A   
   C   
   G   
   A   
   A   
    C    
   A   
   C   
    G    
   G   
   A   
   T   
   A   
   T   
   C   
   A   
   C   
   A   
   G   
   A   
   G   
   C   
   T   
   T   
   C   
   A   
   A   
   A   
   A   
   G   
   G   
   A   
   A   
    T    
   C   
    C    
   G   
   A   
   A   
   A   
    G    
   C   
   T   
   T   
   G   
   A   
   T   
   T   
   G   
   C   
    G    
   C   
   A   
   A   
   A   
   T   
   C   
   A   
   C   
   A   
   G   
   A   
   G   
   T   
   T   
    G    
   A   
   A   
   T   
   A   
   A   
   A   
   A   
   T   
   C   
   C   
   T   
   T   
   T   
   C   
   A   
   A   
   A   
    T    
   G   
   A   
   C   
   A   
   A   
   A   
   G   
   A   
    G    
   C   
   T   
   T   
   T   
   T   
    C    
   T   
   C   
   T   
   G   
   T   
   T   
   A   
   T   
   G   
   A   
   A   
   A   
   A   
   A   
   A   
   G   
   A   
   A   
   T   
   T   
   G   
   C   
   G   
   A   
   G   
   A   
   A   
   G   
   T   
   G   
   A   
   A   
   A   
   A   
   A   
   A   
   A   
   A   
   T   
   T   
   A   
    T    
   T   
   C   
   T   
   T   
   C   
   C   
   G   
   C   
   T   
   C   
   G   
   T   
   C   
    T    
    A    
   A   
   C   
   G   
   A   
   C   
   A   
   A   
   T   
   C   
    G    
   A   
   A   
   G   
   A   
   C   
   G   
   A   
   A   
   A   
   T   
   C   
   G   
   A   
   A   
   G   
   A   
   A   
   A   
   T   
   C   
   A   
   A   
   A   
   A   
   T   
    T    
   G   
   A   
   T   
   A   
   C   
   G   
   C   
   A   
   A   
    G    
   T   
    C    
   T   
   T   
   A   
   G   
   T   
   G   
   G   
   C   
    A    
   C   
   A   
   A   
   G   
   A   
   A   
   G   
   A   
   C   
   G   
   T   
   A   
   A   
   T   
    T    
   G   
   T   
   C   
   A   
   G   
   T   
   G   
   T   
    G    
   A   
   C   
    G    
   C   
   G   
   A   
   G   
   A   
   A   
   G   
   G   
   C   
   T   
   A   
   T   
   G   
   T   
   G   
   A   
   A   
   G   
   C   
   G   
   A   
   A   
   C   
   A   
   A   
   G   
   C   
   C   
   T   
   T   
   C   
   G   
   T   
   T   
   C   
    T    
   T   
   A   
   T   
   A   
   G   
   T   
   G   
   C   
   T   
   T   
   C   
    C    
   A   
   A   
   A   
   C   
   C   
   A   
   G   
   A   
   A   
   G   
   A   
   A   
   A   
   T   
   C   
   G   
   G   
   G   
   A   
   T   
   G   
   C   
   G   
   A   
   G   
   A   
   A   
   G   
   G   
   C   
   G   
   A   
    C    
   T   
   A   
   C   
   T   
   T   
   G   
   C   
   T   
    A    
   T   
   A   
   C   
    T    
   C   
   A   
   G   
   G   
   T   
   G   
   A   
   A   
   T   
   T   
    G    
   A   
   G   
    T    
   A   
   C   
   A   
    T    
   T   
   G   
   G   
   A   
   C   
   C   
   A   
   T   
   G   
   T   
   T   
   C   
   T   
   T   
   C   
   T   
   G   
   A   
   T   
    C    
   A   
   C   
    A    
   A   
   A   
   T   
   A   
   A   
   A   
   G   
   C   
   A   
   A   
   A   
   T   
   G   
   T   
   G   
   A   
   T   
   C   
   T   
   A   
   C   
   C   
   G   
    C    
   C   
   C   
   T   
   G   
   T   
    C    
   C   
   A   
   T   
   G   
   A   
   A   
   C   
   T   
   A   
   C   
   C   
    C    
   G   
   A   
   T   
   T   
   T   
   G   
   A   
   A   
   A   
   T   
   G   
   G   
   A   
   A   
   A   
   G   
   A   
   T   
   G   
   C   
   T   
   G   
   G   
    T    
   G   
   A   
    G    
   C   
   A   
    C    
   A   
   T   
   T   
   T   
   C   
   T   
   C   
   A   
   A   
   A   
   C   
   A   
   A   
   T   
   C   
   T   
   C   
   C   
   A   
   A   
   T   
   C   
   T   
   G   
   T   
   C   
   T   
   G   
   T   
   A   
   G   
   A   
    C    
   G   
   A   
   A   
   T   
   C   
   G   
   A   
   T   
    T    
   T   
   T   
   A   
   G   
   C   
   T   
   G   
   T   
   T   
   T   
   T   
    C    
   C   
   C   
   A   
   T   
   A   
   T   
   C   
   A   
   A   
   A   
   A   
   A   
   A   
   T   
    T    
   C   
   A   
   A   
   G   
   C   
   A   
   G   
   A   
    G    
   A   
   A   
   A   
   A   
   C   
   A   
   T   
   T   
   T   
   G   
   T   
    A    
   T   
   T   
   C   
   A   
   T   
   C   
   A   
   G   
   C   
   A   
   A   
   G   
   A   
   A   
   T   
   G   
   G   
   A   
   T   
   T   
    G    
    A    
   T   
   C   
   A   
   A   
   G   
   C   
   A   
   A   
   A   
   C   
   A   
   C   
   G   
   A   
   A   
   T   
   G   
   A   
   C   
   A   
   G   
   A   
   G   
   T   
   T   
   C   
   G   
   A   
   A   
   C   
   C   
   A   
   T   
   G   
   G   
   C   
   G   
   C   
   A   
   C   
   C   
   T   
   A   
    T    
   A   
   A   
   G   
   A   
   G   
   C   
   C   
   G   
    T    
   C   
   C   
   G   
   C   
   T   
   C   
   A   
   G   
   C   
   T   
   G   
   T   
   A   
   T   
   G   
   A   
   A   
   G   
   C   
   T   
   A   
   A   
   A   
   G   
   G   
   C   
   G   
   G   
   A   
   A   
   G   
   A   
   A   
   G   
   A   
   T   
   G   
   A   
   A   
   C   
   T   
    G    
   A   
   T   
   T   
   G   
   C   
   T   
   G   
   T   
    G    
   T   
   A   
   T   
   C   
   T   
   G   
   A   
   A   
   A   
   G   
   A   
   A   
   G   
   A   
   T   
   C   
    A    
   G   
   C   
   C   
   A   
   G   
   G   
   C   
   T   
   T   
   A   
   G   
   A   
   T   
   G   
   T   
    T    
   T   
   T   
   C   
   T   
   T   
   A   
   G   
   T   
   A   
   A   
   C   
   G   
    C    
   A   
   T   
   C   
   A   
   A   
   G   
   G   
   A   
   A   
   T   
   G   
   G   
   G   
    T    
   T   
   T   
   G   
   C   
   G   
   C   
   T   
    A    
   T   
   C   
   C   
   T   
   T   
   T   
   A   
   A   
    C    
   A   
   G   
   A   
   T   
   G   
   T   
   T   
   C   
   C   
   C   
   G   
   T   
   T   
   G   
   T   
   T   
   G   
   G   
   A   
   G   
   C   
   A   
   A   
   A   
   G   
   G   
   C   
   A   
   G   
    C    
   T   
   G   
   G   
   A   
   G   
   T   
   G   
   A   
   A   
   G   
   T   
   C   
   T   
   A   
   T   
   G   
   A   
   A   
   T   
   T   
   T   
   G   
   A   
   A   
   A   
   G   
   A   
   T   
   G   
   G   
   A   
   G   
   A   
   T   
   A   
   G   
   G   
    A    
   T   
   T   
    A    
   T   
   C   
   A   
   A   
    C    
   G   
   G   
    G    
   C   
   T   
    G    
   C   
   T   
   T   
   G   
   T   
   G   
   T   
   T   
   A   
   G   
   C   
   A   
   G   
   A   
    A    
   G   
   G   
    T    
   G   
   A   
   T   
   A   
   C   
   G   
   C   
   C   
    G    
   A   
   T   
   C   
   G   
   T   
   A   
   A   
   T   
   C   
   G   
   T   
   T   
   A   
   C   
   T   
   C   
   A   
   G   
   C   
   G   
   A   
   G   
   G   
   A   
   G   
   C   
    T    
   G   
   T   
    T    
   A   
   A   
   A   
   C   
   G   
   C   
   A   
   T   
   G   
   C   
   T   
   T   
   G   
   C   
   T   
   C   
   A   
   A   
   G   
   A   
   A   
   A   
   T   
   C   
   A   
   G   
   C   
   C   
   A   
   A   
   A   
   C   
    T    
   T   
   C   
   A   
   C   
   G   
   T   
   G   
   C   
   G   
   A   
   A   
   G   
   C   
   G   
   G   
   G   
   G   
   A   
   G   
   T   
   G   
   A   
   C   
   G   
   G   
   T   
   C   
   A   
   T   
   G   
   C   
   G   
   T   
   G   
   A   
   G   
   C   
   T   
   G   
   A   
   A   
   A   
   A   
   A   
   A   
   A   
   A   
   T   
   C   
   C   
    G    
   C   
   A   
    C    
   C   
   G   
    T    
   A   
   T   
   T   
   A   
   T   
   C   
   T   
   A   
   T   
   A   
   T   
   G   
   T   
   C   
   G   
   G   
   A   
   A   
   G   
   G   
   A   
   A   
   G   
   A   
   T   
   C   
   A   
   A   
   A   
   A   
   G   
   A   
   A   
   A   
   T   
   A   
   A   
   C   
   C   
   T   
   T   
   G   
   A   
   T   
   G   
   A   
   A   
   T   
   C   
   A   
   A   
   A   
   A   
   A   
   G   
   G   
    G    
   C   
   A   
    G    
   C   
   A   
   G   
    T    
   T   
   A   
   A   
   C   
   G   
   A   
   T   
   T   
   G   
   A   
    C    
   C   
   C   
   A   
   A   
   C   
    T    
   G   
   A   
   T   
   T   
   T   
    C    
   C   
   C   
   T   
   A   
   T   
   A   
   G   
   G   
   T   
   G   
   A   
   T   
   C   
   G   
   A   
   A   
   C   
   A   
   T   
   C   
   G   
   A   
   A   
   T   
   G   
   G   
    A    
   T   
   C   
    A    
   T   
   T   
   T   
   G   
   C   
    G    
   A   
   T   
   G   
   G   
   A   
    T    
   G   
   A   
   G   
   A   
   A   
   A   
   A   
   A   
   A   
   G   
   G   
   T   
   G   
   G   
   A   
   G   
   A   
   A   
   G   
   T   
   C   
   A   
   T   
   C   
   G   
   A   
   A   
   G   
   T   
    T    
   A   
    T    
   C   
   G   
   A   
   T   
   G   
   C   
   T   
   C   
   C   
   T   
   G   
   A   
   G   
    A    
   T   
   T   
   T   
   T   
    C    
   A   
   T   
    C    
   G   
   A   
    G    
    T    
   C   
    G    
   -   
   -   
   -   
 

 
   N59513F   
   A   
   T   
   G   
   G   
   A   
   A   
   A   
   A   
   T   
   C   
   G   
   A   
   C   
   A   
   A   
   G   
   A   
   A   
   G   
   T   
   A   
   C   
   A   
   A   
   G   
   A   
   A   
   T   
   T   
   A   
   A   
   C   
   T   
   T   
   T   
   A   
   G   
   A   
   A   
   G   
   A   
   A   
   G   
   T   
   G   
   A   
   T   
   G   
   G   
   G   
   T   
   G   
   A   
   C   
   C   
   G   
   C   
   T   
   T   
   C   
   G   
   G   
   A   
   C   
   G   
   T   
   T   
   A   
   T   
   T   
   C   
   C   
   A   
   A   
   A   
   T   
   A   
   T   
   A   
   T   
   T   
   A   
   T   
    C    
   C   
   A   
    A    
   G   
   A   
   A   
   C   
   G   
   T   
   G   
   C   
   A   
   C   
   T   
   G   
   C   
   C   
   T   
   G   
   A   
   T   
   A   
   T   
    C    
   C   
   G   
   T   
   G   
   A   
   T   
   G   
   G   
   A   
   T   
   T   
   G   
   A   
   A   
    A    
   C   
   C   
   A   
   G   
   T   
    G    
   C   
   A   
   A   
   C   
   G   
   C   
   C   
   G   
   G   
   A   
   T   
   A   
   T   
   T   
   A   
   T   
   T   
    T    
   G   
   C   
    C    
   A   
   T   
   G   
   A   
   A   
   C   
   A   
   A   
   A   
   G   
   A   
   T   
   G   
   G   
   C   
   A   
   A   
   T   
   A   
   C   
   G   
   T   
   A   
   T   
   G   
   A   
   C   
   A   
   A   
   A   
   G   
   G   
   A   
   T   
   T   
    C    
   C   
   G   
   T   
   A   
   A   
   A   
   T   
   C   
   A   
   G   
   C   
   A   
   A   
   A   
   A   
   T   
   C   
   T   
   G   
   T   
   C   
   G   
   G   
   A   
   A   
   A   
   T   
   A   
   T   
   C   
   A   
   T   
   G   
   G   
   G   
    G    
   A   
   A   
   T   
   T   
   A   
   T   
   C   
   A   
   T   
   C   
   C   
   T   
   C   
   A   
   T   
   G   
   G   
   T   
   G   
   A   
   C   
   A   
   G   
   C   
   A   
   G   
   T   
   A   
   T   
   T   
   T   
   A   
   T   
   G   
   A   
   A   
   G   
   C   
   G   
   A   
   T   
   G   
   G   
   T   
   C   
   C   
   G   
   C   
   A   
   T   
   G   
   A   
   G   
   C   
   C   
   A   
   A   
   G   
   A   
    T    
   T   
   G   
   G   
   A   
   A   
   G   
   T   
   T   
   G   
   C   
   G   
   G   
   G   
   A   
   A   
   G   
   T   
   A   
   C   
   T   
   G   
   A   
   T   
   C   
   G   
   A   
    A    
   A   
   T   
   G   
   C   
   A   
   C   
   G   
   G   
    G    
   A   
   A   
   T   
   A   
   A   
    T    
   G   
   G   
   G   
   A   
   G   
   T   
   A   
   T   
   G   
   G   
   A   
   C   
   G   
   G   
   T   
   G   
   A   
   T   
   C   
   C   
   A   
   C   
   C   
   T   
   G   
   C   
   T   
   G   
   C   
   G   
   A   
   T   
   G   
   C   
   G   
   T   
   T   
   A   
   T   
   A   
   C   
   T   
   G   
   A   
   A   
   G   
   C   
   C   
   A   
   G   
    A    
   T   
   T   
   A   
   T   
   C   
   C   
   A   
   A   
   G   
   T   
   T   
   A   
   A   
   G   
   T   
   G   
   G   
   A   
   G   
   A   
   G   
   A   
   T   
   G   
    C    
   T   
   A   
    G    
   C   
   A   
   G   
   A   
   T   
   A   
   T   
   C   
   G   
   A   
    A    
   A   
   A   
   A   
   G   
   A   
   A   
   A   
   C   
   A   
   G   
   T   
    C    
   G   
   A   
   T   
   C   
   T   
   C   
   G   
   T   
   G   
   T   
   G   
   G   
   A   
   A   
   T   
   T   
   T   
   T   
   G   
   A   
   T   
   G   
   A   
   T   
   A   
   C   
   A   
   G   
   A   
   A   
   A   
   A   
   A   
   G   
   A   
   A   
   C   
   C   
   A   
   A   
   C   
   A   
   G   
   T   
   C   
   C   
   T   
   T   
   C   
   C   
   A   
   G   
   C   
   T   
   C   
   G   
   A   
   T   
   A   
   C   
   C   
   C   
   A   
   A   
   A   
   C   
   C   
   T   
   T   
   C   
   T   
    A    
   G   
   T   
   C   
   A   
   A   
   T   
   G   
   G   
   A   
   T   
   C   
   G   
   A   
   C   
   A   
   G   
   G   
   T   
   A   
   T   
   A   
   T   
   C   
   T   
   G   
   C   
   T   
   G   
   G   
   A   
   T   
   A   
   T   
   G   
   C   
    G    
   A   
   C   
   A   
   G   
   A   
   A   
   A   
   T   
   C   
   C   
   C   
   G   
   A   
   C   
    T    
   C   
   A   
   T   
   A   
   A   
   C   
   T   
   T   
   A   
   G   
   C   
   T   
   G   
   A   
   A   
   G   
   T   
   G   
   A   
   T   
    C    
   G   
   A   
    C    
   G   
   G   
   C   
   A   
   C   
   A   
   A   
   T   
   C   
   T   
   A   
   T   
   A   
   T   
   G   
   A   
   T   
   C   
   G   
   A   
   T   
   C   
   A   
   T   
   C   
   C   
   A   
   C   
   A   
   A   
   G   
   C   
   G   
   A   
   G   
   C   
   C   
   T   
   T   
   G   
   A   
   A   
   A   
   A   
   A   
   T   
   T   
   G   
   A   
   T   
   G   
   G   
   A   
   A   
   T   
   A   
   T   
   A   
   T   
   T   
   C   
   C   
   T   
   G   
   G   
   A   
   C   
   C   
   T   
   G   
   A   
   C   
   T   
   T   
    T    
   C   
   C   
   A   
   A   
   C   
   A   
   G   
   G   
   A   
   G   
   G   
    G    
   A   
   T   
   C   
   T   
   T   
   G   
   C   
   A   
   A   
   G   
   G   
    G    
   A   
   A   
   A   
   G   
   A   
   T   
   G   
   A   
   A   
   A   
   T   
   C   
   A   
   A   
   A   
   A   
   A   
   A   
   G   
   C   
   T   
   T   
   A   
   T   
   G   
   A   
   A   
   A   
   C   
   A   
   G   
   G   
    G    
   C   
   G   
   A   
   G   
   G   
   A   
   A   
   A   
   A   
   G   
   T   
   T   
   A   
    T    
   T   
   C   
   T   
   T   
   C   
   G   
   T   
   T   
   C   
   G   
   A   
   A   
   A   
   A   
   C   
   A   
   A   
   A   
   G   
   A   
   T   
   A   
   G   
   A   
    A    
   G   
   C   
   A   
   A   
   T   
   G   
   A   
   A   
   G   
   G   
   G   
    C    
   A   
   A   
    T    
   A   
   A   
   A   
   C   
   A   
   G   
   C   
   A   
   A   
   A   
   T   
   C   
   G   
   T   
   G   
   A   
   T   
   C   
   A   
   C   
    T    
   G   
   A   
   A   
   A   
   T   
   C   
   C   
   C   
   A   
   T   
   A   
   T   
   G   
   A   
   A   
   G   
   T   
   A   
   A   
   A   
   T   
   A   
   A   
   A   
   G   
   C   
   G   
   A   
   C   
    G    
   T   
   T   
   G   
   G   
   T   
   G   
   A   
   A   
   A   
   A   
   A   
   A   
   A   
   T   
   G   
   G   
   A   
   T   
   G   
   A   
   G   
   A   
   T   
   C   
   C   
   G   
   T   
   T   
   T   
   G   
   A   
   A   
   T   
   A   
   A   
   G   
   A   
   A   
   A   
   A   
   T   
   C   
   G   
   A   
    T    
   G   
   G   
   A   
   A   
   T   
   A   
   G   
   C   
   T   
   G   
   A   
   A   
   G   
   T   
    A    
   C   
   G   
   A   
   G   
   A   
   T   
   G   
   A   
   A   
   A   
   G   
   T   
   G   
   A   
   T   
    C    
   G   
    A    
   A   
   C   
    T    
   G   
   G   
   T   
   C   
   T   
   G   
   C   
   A   
   A   
   A   
   T   
   T   
   G   
   T   
   C   
   G   
   T   
    T    
   G   
   A   
   A   
   T   
   T   
   G   
   A   
   A   
   A   
   A   
   A   
   A   
   G   
   A   
    T    
   G   
   C   
   C   
   A   
   A   
    T    
   G   
   C   
   T   
   C   
   A   
   A   
   G   
   G   
    A    
   A   
   T   
   T   
   T   
   T   
   G   
   A   
   A   
   T   
   T   
   A   
    T    
   T   
   T   
   G   
   T   
   T   
   C   
   A   
   A   
   A   
   A   
   A   
   C   
   A   
   C   
   T   
   G   
   A   
   G   
    C    
   T   
   A   
   C   
   A   
   G   
   A   
   T   
   C   
   A   
   A   
   T   
   T   
   A   
   C   
   A   
   A   
   T   
   T   
   T   
    T    
   A   
   A   
   T   
   A   
   T   
   G   
   G   
   T   
   C   
   G   
   C   
   T   
   A   
   T   
   C   
   G   
   A   
    T    
   C   
   A   
   T   
   A   
   T   
   G   
   A   
   C   
   T   
   C   
   C   
   G   
   C   
   A   
    C    
   C   
   A   
   A   
   G   
   T   
   T   
   G   
   G   
   A   
   C   
   T   
   G   
   A   
   A   
   A   
   G   
   A   
   C   
   A   
   T   
   T   
   T   
   T   
   A   
   A   
   G   
   A   
   A   
   G   
   T   
   T   
   A   
   T   
   A   
   T   
   C   
   G   
   A   
   G   
   C   
   A   
   T   
   C   
   G   
   A   
   A   
   A   
   G   
   C   
   A   
   A   
   G   
   T   
   C   
   A   
   T   
   A   
   A   
   C   
   A   
   A   
   A   
   A   
   C   
   G   
    C    
   A   
   G   
    C    
   C   
   A   
   A   
   T   
   T   
   C   
   G   
   A   
   T   
   T   
   T   
   A   
   G   
   C   
   C   
   A   
   A   
   A   
   G   
   C   
   T   
   C   
   A   
   A   
   A   
   A   
   A   
    A    
   G   
   A   
   C   
   A   
   G   
   C   
   A   
   T   
   A   
   T   
   C   
   G   
   T   
    G    
   G   
   A   
   A   
   G   
   G   
    C    
    C    
   T   
   G   
   A   
   T   
   G   
   A   
   A   
   A   
   G   
   C   
   T   
   T   
   T   
   A   
   T   
   C   
   G   
   A   
   T   
   T   
   C   
   T   
   G   
   G   
   A   
   T   
   G   
   A   
   A   
   G   
   T   
   A   
   A   
   T   
   C   
   G   
    C    
   A   
   A   
   C   
   G   
   A   
   T   
   T   
   C   
   G   
   T   
   G   
   A   
   A   
   A   
   G   
   T   
   A   
   A   
   A   
   G   
   A   
   C   
   A   
   A   
   A   
   A   
   A   
   A   
   G   
   A   
    T    
   G   
   C   
    T    
   A   
   A   
   A   
   A   
   A   
   G   
   A   
   A   
   T   
   T   
   T   
   A   
   G   
   T   
   C   
   G   
   A   
    T    
   G   
   T   
    T    
   T   
   T   
    C    
   C   
   A   
   A   
   T   
   T   
   T   
   A   
   C   
   A   
   G   
   A   
   A   
   G   
   A   
   A   
   C   
   A   
   A   
   G   
   C   
   A   
   G   
   A   
   A   
   G   
   C   
   A   
   A   
   T   
    C    
   G   
   T   
   C   
   A   
   C   
   A   
   T   
   T   
   G   
   C   
   A   
   A   
    C    
   T   
   A   
   T   
   A   
    C    
   C   
   G   
   T   
   T   
   T   
   G   
   A   
   C   
   G   
   A   
   A   
    C    
   A   
   C   
    A    
   G   
   A   
   T   
   A   
   T   
   C   
   A   
   C   
   A   
   G   
   A   
   G   
   C   
   T   
   T   
   C   
   A   
   A   
   A   
   A   
   G   
   G   
   A   
   A   
    G    
   C   
    C    
   G   
   A   
   A   
   A   
    G    
   C   
   T   
   T   
   G   
   A   
   T   
   T   
   G   
   C   
    A    
   C   
   A   
   A   
   A   
   T   
   C   
   A   
   C   
   A   
   G   
   A   
   G   
   T   
   T   
    G    
   A   
   A   
   T   
   A   
   A   
   A   
   A   
   T   
   C   
   C   
   T   
   T   
   T   
   C   
   A   
   A   
   A   
    C    
   G   
   A   
   C   
   A   
   A   
   A   
   G   
   A   
    G    
   C   
   T   
   T   
   T   
   T   
    C    
   T   
   C   
   T   
   G   
   T   
   T   
   A   
   T   
   G   
   A   
   A   
   A   
   A   
   A   
   A   
   G   
   A   
   A   
   T   
   T   
   G   
   C   
   G   
   A   
   G   
   A   
   A   
   G   
   T   
   G   
   A   
   A   
   A   
   A   
   A   
   A   
   A   
   A   
   T   
   T   
   A   
    C    
   T   
   C   
   T   
   T   
   C   
   C   
   G   
   C   
   T   
   C   
   G   
   T   
   C   
    T    
    G    
   A   
   C   
   G   
   A   
   C   
   A   
   A   
   T   
   C   
    C    
   A   
   A   
   G   
   A   
   C   
   G   
   A   
   A   
   A   
   T   
   C   
   G   
   A   
   A   
   G   
   A   
   A   
   A   
   T   
   C   
   A   
   A   
   A   
   A   
   T   
    C    
   G   
   A   
   T   
   A   
   C   
   G   
   C   
   A   
   A   
    G    
   T   
    T    
   T   
   T   
   A   
   G   
   T   
   G   
   G   
   C   
    G    
   C   
   A   
   A   
   G   
   A   
   A   
   G   
   A   
   C   
   G   
   T   
   A   
   A   
   T   
    C    
   G   
   T   
   C   
   A   
   G   
   T   
   G   
   T   
    G    
   A   
   C   
    G    
   C   
   G   
   A   
   G   
   A   
   A   
   G   
   G   
   C   
   T   
   A   
   T   
   G   
   T   
   G   
   A   
   A   
   G   
   C   
   G   
   A   
   A   
   C   
   A   
   A   
   G   
   C   
   C   
   T   
   T   
   C   
   G   
   T   
   T   
   C   
    T    
   T   
   A   
   T   
   A   
   G   
   T   
   G   
   C   
   T   
   T   
   C   
    G    
   A   
   A   
   A   
   C   
   C   
   A   
   G   
   A   
   A   
   G   
   A   
   A   
   A   
   T   
   C   
   G   
   G   
   G   
   A   
   T   
   G   
   C   
   G   
   A   
   G   
   A   
   A   
   G   
   G   
   C   
   G   
   A   
    T    
   T   
   A   
   C   
   T   
   T   
   G   
   C   
   T   
    G    
   T   
   A   
   C   
    T    
   C   
   A   
   G   
   G   
   T   
   G   
   A   
   A   
   T   
   T   
    G    
   A   
   G   
    C    
   A   
   C   
   A   
    T    
   T   
   G   
   G   
   A   
   C   
   C   
   A   
   T   
   G   
   T   
   T   
   C   
   T   
   T   
   C   
   T   
   G   
   A   
   T   
    T    
   A   
   C   
    G    
   A   
   A   
   T   
   A   
   A   
   A   
   G   
   C   
   A   
   A   
   A   
   T   
   G   
   T   
   G   
   A   
   T   
   C   
   T   
   A   
   C   
   C   
   G   
    T    
   C   
   C   
   T   
   G   
   T   
    T    
   C   
   A   
   T   
   G   
   A   
   A   
   C   
   T   
   A   
   C   
   C   
    C    
   G   
   A   
   T   
   T   
   T   
   G   
   A   
   A   
   A   
   T   
   G   
   G   
   A   
   A   
   A   
   G   
   A   
   T   
   G   
   C   
   T   
   G   
   G   
    C    
   G   
   A   
    A    
   C   
   A   
    C    
   A   
   T   
   T   
   T   
   C   
   T   
   C   
   A   
   A   
   A   
   C   
   A   
   A   
   T   
   C   
   T   
   C   
   C   
   A   
   A   
   T   
   C   
   T   
   G   
   T   
   C   
   T   
   G   
   T   
   A   
   G   
   A   
    T    
   G   
   A   
   A   
   T   
   C   
   G   
   A   
   T   
    C    
   T   
   T   
   A   
   G   
   C   
   T   
   G   
   T   
   T   
   T   
   T   
    C    
   C   
   C   
   A   
   T   
   A   
   T   
   C   
   A   
   A   
   A   
   A   
   A   
   A   
   T   
    C    
   C   
   A   
   A   
   G   
   C   
   A   
   G   
   A   
    A    
   A   
   A   
   A   
   A   
   C   
   A   
   T   
   T   
   T   
   G   
   T   
    A    
   T   
   T   
   C   
   A   
   T   
   C   
   A   
   G   
   C   
   A   
   A   
   G   
   A   
   A   
   T   
   G   
   G   
   A   
   T   
   T   
    G    
    A    
   T   
   C   
   A   
   A   
   G   
   C   
   A   
   A   
   A   
   C   
   A   
   C   
   G   
   A   
   A   
   T   
   G   
   A   
   C   
   A   
   G   
   A   
   G   
   T   
   T   
   C   
   G   
   A   
   A   
   C   
   C   
   A   
   T   
   G   
   G   
   C   
   G   
   C   
   A   
   C   
   C   
   T   
   A   
    T    
   A   
   A   
   G   
   A   
   G   
   C   
   C   
   G   
    T    
   C   
   C   
   G   
   C   
   T   
   C   
   A   
   G   
   C   
   T   
   G   
   T   
   A   
   T   
   G   
   A   
   A   
   G   
   C   
   T   
   A   
   A   
   A   
   G   
   G   
   C   
   G   
   G   
   A   
   A   
   G   
   A   
   A   
   G   
   A   
   T   
   G   
   A   
   A   
   C   
   T   
    T    
   A   
   T   
   T   
   G   
   C   
   T   
   G   
   T   
    A    
   T   
   A   
   T   
   C   
   T   
   G   
   A   
   A   
   A   
   G   
   A   
   A   
   G   
   A   
   T   
   C   
    A    
   G   
   C   
   C   
   A   
   G   
   G   
   C   
   T   
   T   
   A   
   G   
   A   
   T   
   G   
   T   
    G    
   T   
   T   
   C   
   T   
   T   
   A   
   G   
   T   
   A   
   A   
   C   
   G   
    C    
   A   
   T   
   C   
   A   
   A   
   G   
   G   
   A   
   A   
   T   
   G   
   G   
   G   
    C    
   T   
   T   
   G   
   C   
   G   
   C   
   T   
    A    
   T   
   C   
   C   
   T   
   T   
   T   
   A   
   A   
    C    
   A   
   G   
   A   
   T   
   G   
   T   
   T   
   C   
   C   
   C   
   G   
   T   
   T   
   G   
   T   
   T   
   G   
   G   
   A   
   G   
   C   
   A   
   A   
   A   
   G   
   G   
   C   
   A   
   G   
    C    
   T   
   G   
   G   
   A   
   G   
   T   
   G   
   A   
   A   
   G   
   T   
   C   
   T   
   A   
   T   
   G   
   A   
   A   
   T   
   T   
   T   
   G   
   A   
   A   
   A   
   G   
   A   
   T   
   G   
   G   
   A   
   G   
   A   
   T   
   A   
   G   
   G   
    A    
   T   
   T   
    G    
   T   
   C   
   A   
   A   
    T    
   G   
   G   
    G    
   C   
   T   
    G    
   C   
   T   
   T   
   G   
   T   
   G   
   T   
   T   
   A   
   G   
   C   
   A   
   G   
   A   
    C    
   G   
   G   
    A    
   G   
   A   
   T   
   A   
   C   
   G   
   C   
   C   
    A    
   A   
   T   
   C   
   G   
   T   
   A   
   A   
   T   
   C   
   G   
   T   
   T   
   A   
   C   
   T   
   C   
   A   
   G   
   C   
   G   
   A   
   G   
   G   
   A   
   G   
   C   
    C    
   G   
   T   
    G    
   A   
   A   
   A   
   C   
   G   
   C   
   A   
   T   
   G   
   C   
   T   
   T   
   G   
   C   
   T   
   C   
   A   
   A   
   G   
   A   
   A   
   A   
   T   
   C   
   A   
   G   
   C   
   C   
   A   
   A   
   A   
   C   
    T    
   T   
   C   
   A   
   C   
   G   
   T   
   G   
   C   
   G   
   A   
   A   
   G   
   C   
   G   
   G   
   G   
   G   
   A   
   G   
   T   
   G   
   A   
   C   
   G   
   G   
   T   
   C   
   A   
   T   
   G   
   C   
   G   
   T   
   G   
   A   
   G   
   C   
   T   
   G   
   A   
   A   
   A   
   A   
   A   
   A   
   A   
   A   
   T   
   C   
   C   
    G    
   C   
   A   
    C    
   C   
   G   
    T    
   A   
   T   
   T   
   A   
   T   
   C   
   T   
   A   
   T   
   A   
   T   
   G   
   T   
   C   
   G   
   G   
   A   
   A   
   G   
   G   
   A   
   A   
   G   
   A   
   T   
   C   
   A   
   A   
   A   
   A   
   G   
   A   
   A   
   A   
   T   
   A   
   A   
   C   
   C   
   T   
   T   
   G   
   A   
   T   
   G   
   A   
   A   
   T   
   C   
   A   
   A   
   A   
   A   
   A   
   G   
   G   
    A    
   C   
   A   
    A    
   C   
   A   
   G   
    A    
   T   
   A   
   A   
   C   
   G   
   A   
   T   
   T   
   G   
   A   
    T    
   C   
   C   
   A   
   A   
   C   
    G    
   G   
   A   
   T   
   T   
   T   
    T    
   C   
   C   
   T   
   A   
   T   
   A   
   G   
   G   
   T   
   G   
   A   
   T   
   C   
   G   
   A   
   A   
   C   
   A   
   T   
   C   
   G   
   A   
   A   
   T   
   G   
   G   
    T    
   T   
   C   
    G    
   T   
   T   
   T   
   G   
   C   
    T    
   A   
   T   
   G   
   G   
   A   
    C    
   G   
   A   
   G   
   A   
   A   
   A   
   A   
   A   
   A   
   G   
   G   
   T   
   G   
   G   
   A   
   G   
   A   
   A   
   G   
   T   
   C   
   A   
   T   
   C   
   G   
   A   
   A   
   G   
   T   
    T    
   A   
    T    
   C   
   G   
   A   
   T   
   G   
   C   
   T   
   C   
   C   
   T   
   G   
   A   
   G   
    A    
   T   
   T   
   T   
   T   
    C    
   A   
   T   
    T    
   G   
   A   
    A    
    T    
   C   
    A    
   -   
   -   
   -   
 

 
   N59559F   
   A   
   T   
   G   
   G   
   A   
   A   
   A   
   A   
   T   
   C   
   G   
   A   
   C   
   A   
   A   
   G   
   A   
   A   
   G   
   T   
   A   
   C   
   A   
   A   
   G   
   A   
   A   
   T   
   T   
   A   
   A   
   C   
   T   
   T   
   T   
   A   
   G   
   A   
   A   
   G   
   A   
   A   
   G   
   T   
   G   
   A   
   T   
   G   
   G   
   G   
   T   
   G   
   A   
   C   
   C   
   G   
   C   
   T   
   T   
   C   
   G   
   G   
   A   
   C   
   G   
   T   
   T   
   A   
   T   
   T   
   C   
   C   
   A   
   A   
   A   
   T   
   A   
   T   
   A   
   T   
   T   
   A   
   T   
    C    
   C   
   A   
    A    
   G   
   A   
   A   
   C   
   G   
   T   
   G   
   C   
   A   
   C   
   T   
   G   
   C   
   C   
   T   
   G   
   A   
   T   
   A   
   T   
    C    
   C   
   G   
   T   
   G   
   A   
   T   
   G   
   G   
   A   
   T   
   T   
   G   
   A   
   A   
    A    
   C   
   C   
   A   
   G   
   T   
    A    
   C   
   A   
   A   
   C   
   G   
   C   
   C   
   G   
   G   
   A   
   T   
   A   
   T   
   T   
   A   
   T   
   T   
    T    
   G   
   C   
    C    
   A   
   T   
   G   
   A   
   A   
   C   
   A   
   A   
   A   
   G   
   A   
   T   
   G   
   G   
   C   
   A   
   A   
   T   
   A   
   C   
   G   
   T   
   A   
   T   
   G   
   A   
   C   
   A   
   A   
   A   
   G   
   G   
   A   
   T   
   T   
    C    
   C   
   G   
   T   
   A   
   A   
   A   
   T   
   C   
   A   
   G   
   C   
   A   
   A   
   A   
   A   
   T   
   C   
   T   
   G   
   T   
   C   
   G   
   G   
   A   
   A   
   A   
   T   
   A   
   T   
   C   
   A   
   T   
   G   
   G   
   G   
    G    
   A   
   A   
   T   
   T   
   A   
   T   
   C   
   A   
   T   
   C   
   C   
   T   
   C   
   A   
   T   
   G   
   G   
   T   
   G   
   A   
   C   
   A   
   G   
   C   
   A   
   G   
   T   
   A   
   T   
   T   
   T   
   A   
   T   
   G   
   A   
   A   
   G   
   C   
   G   
   A   
   T   
   G   
   G   
   T   
   C   
   C   
   G   
   C   
   A   
   T   
   G   
   A   
   G   
   C   
   C   
   A   
   A   
   G   
   A   
    T    
   T   
   G   
   G   
   A   
   A   
   G   
   T   
   T   
   G   
   C   
   G   
   G   
   G   
   A   
   A   
   G   
   T   
   A   
   C   
   T   
   G   
   A   
   T   
   C   
   G   
   A   
    G    
   A   
   T   
   G   
   C   
   A   
   C   
   G   
   G   
    A    
   A   
   A   
   T   
   A   
   A   
    C    
   G   
   G   
   G   
   A   
   G   
   T   
   A   
   T   
   G   
   G   
   A   
   C   
   G   
   G   
   T   
   G   
   A   
   T   
   C   
   C   
   A   
   C   
   C   
   T   
   G   
   C   
   T   
   G   
   C   
   G   
   A   
   T   
   G   
   C   
   G   
   T   
   T   
   A   
   T   
   A   
   C   
   T   
   G   
   A   
   A   
   G   
   C   
   C   
   A   
   G   
    A    
   T   
   T   
   A   
   T   
   C   
   C   
   A   
   A   
   G   
   T   
   T   
   A   
   A   
   G   
   T   
   G   
   G   
   A   
   G   
   A   
   G   
   A   
   T   
   G   
    C    
   T   
   A   
    G    
   C   
   A   
   G   
   A   
   T   
   A   
   T   
   C   
   G   
   A   
    A    
   A   
   A   
   A   
   G   
   A   
   A   
   A   
   C   
   A   
   G   
   T   
    C    
   G   
   A   
   T   
   C   
   T   
   C   
   G   
   T   
   G   
   T   
   G   
   G   
   A   
   A   
   T   
   T   
   T   
   T   
   G   
   A   
   T   
   G   
   A   
   T   
   A   
   C   
   A   
   G   
   A   
   A   
   A   
   A   
   A   
   G   
   A   
   A   
   C   
   C   
   A   
   A   
   C   
   A   
   G   
   T   
   C   
   C   
   T   
   T   
   C   
   C   
   A   
   G   
   C   
   T   
   C   
   G   
   A   
   T   
   A   
   C   
   C   
   C   
   A   
   A   
   A   
   C   
   C   
   T   
   T   
   C   
   T   
    A    
   G   
   T   
   C   
   A   
   A   
   T   
   G   
   G   
   A   
   T   
   C   
   G   
   A   
   C   
   A   
   G   
   G   
   T   
   A   
   T   
   A   
   T   
   C   
   T   
   G   
   C   
   T   
   G   
   G   
   A   
   T   
   A   
   T   
   G   
   C   
    G    
   A   
   C   
   A   
   G   
   A   
   A   
   A   
   T   
   C   
   C   
   C   
   G   
   A   
   C   
    T    
   C   
   A   
   T   
   A   
   A   
   C   
   T   
   T   
   A   
   G   
   C   
   T   
   G   
   A   
   A   
   G   
   T   
   G   
   A   
   T   
    C    
   G   
   A   
    C    
   G   
   G   
   C   
   A   
   C   
   A   
   A   
   T   
   C   
   T   
   A   
   T   
   A   
   T   
   G   
   A   
   T   
   C   
   G   
   A   
   T   
   C   
   A   
   T   
   C   
   C   
   A   
   C   
   A   
   A   
   G   
   C   
   G   
   A   
   G   
   C   
   C   
   T   
   T   
   G   
   A   
   A   
   A   
   A   
   A   
   T   
   T   
   G   
   A   
   T   
   G   
   G   
   A   
   A   
   T   
   A   
   T   
   A   
   T   
   T   
   C   
   C   
   T   
   G   
   G   
   A   
   C   
   C   
   T   
   G   
   A   
   C   
   T   
   T   
    C    
   C   
   C   
   A   
   A   
   C   
   A   
   G   
   G   
   A   
   G   
   G   
    G    
   A   
   T   
   C   
   T   
   T   
   G   
   C   
   A   
   A   
   G   
   G   
    A    
   A   
   A   
   A   
   G   
   A   
   T   
   G   
   A   
   A   
   A   
   T   
   C   
   A   
   A   
   A   
   A   
   A   
   A   
   G   
   C   
   T   
   T   
   A   
   T   
   G   
   A   
   A   
   A   
   C   
   A   
   G   
   G   
    G    
   C   
   G   
   A   
   G   
   G   
   A   
   A   
   A   
   A   
   G   
   T   
   T   
   A   
    T    
   T   
   C   
   T   
   T   
   C   
   G   
   T   
   T   
   C   
   G   
   A   
   A   
   A   
   A   
   C   
   A   
   A   
   A   
   G   
   A   
   T   
   A   
   G   
   A   
    A    
   G   
   C   
   A   
   A   
   T   
   G   
   A   
   A   
   G   
   G   
   G   
    C    
   A   
   A   
    T    
   A   
   A   
   A   
   C   
   A   
   G   
   C   
   A   
   A   
   A   
   T   
   C   
   G   
   T   
   G   
   A   
   T   
   C   
   A   
   C   
    T    
   G   
   A   
   A   
   A   
   T   
   C   
   C   
   C   
   A   
   T   
   A   
   T   
   G   
   A   
   A   
   G   
   T   
   A   
   A   
   A   
   T   
   A   
   A   
   A   
   G   
   C   
   G   
   A   
   C   
    A    
   T   
   T   
   G   
   G   
   T   
   G   
   A   
   A   
   A   
   A   
   A   
   A   
   A   
   T   
   G   
   G   
   A   
   T   
   G   
   A   
   G   
   A   
   T   
   C   
   C   
   G   
   T   
   T   
   T   
   G   
   A   
   A   
   T   
   A   
   A   
   G   
   A   
   A   
   A   
   A   
   T   
   C   
   G   
   A   
    C    
   G   
   G   
   A   
   A   
   T   
   A   
   G   
   C   
   T   
   G   
   A   
   A   
   G   
   T   
    G    
   C   
   G   
   A   
   G   
   A   
   T   
   G   
   A   
   A   
   A   
   G   
   T   
   G   
   A   
   T   
    C    
   G   
    A    
   A   
   C   
    T    
   G   
   G   
   T   
   C   
   T   
   G   
   C   
   A   
   A   
   A   
   T   
   T   
   G   
   T   
   C   
   G   
   T   
    T    
   G   
   A   
   A   
   T   
   T   
   G   
   A   
   A   
   A   
   A   
   A   
   A   
   G   
   A   
    C    
   G   
   C   
   C   
   A   
   A   
    T    
   G   
   C   
   T   
   C   
   A   
   A   
   G   
   G   
    A    
   A   
   T   
   T   
   T   
   T   
   G   
   A   
   A   
   T   
   T   
   A   
    T    
   T   
   T   
   G   
   T   
   T   
   C   
   A   
   A   
   A   
   A   
   A   
   C   
   A   
   C   
   T   
   G   
   A   
   G   
    C    
   T   
   A   
   C   
   A   
   G   
   A   
   T   
   C   
   A   
   A   
   T   
   T   
   A   
   C   
   A   
   A   
   T   
   T   
   T   
    C    
   A   
   A   
   T   
   A   
   T   
   G   
   G   
   T   
   C   
   G   
   C   
   T   
   A   
   T   
   C   
   G   
   A   
    T    
   C   
   A   
   T   
   A   
   T   
   G   
   A   
   C   
   T   
   C   
   C   
   G   
   C   
   A   
    T    
   C   
   A   
   A   
   G   
   T   
   T   
   G   
   G   
   A   
   C   
   T   
   G   
   A   
   A   
   A   
   G   
   A   
   C   
   A   
   T   
   T   
   T   
   T   
   A   
   A   
   G   
   A   
   A   
   G   
   T   
   T   
   A   
   T   
   A   
   T   
   C   
   G   
   A   
   G   
   C   
   A   
   T   
   C   
   G   
   A   
   A   
   A   
   G   
   C   
   A   
   A   
   G   
   T   
   C   
   A   
   T   
   A   
   A   
   C   
   A   
   A   
   A   
   A   
   C   
   G   
    C    
   A   
   G   
    C    
   C   
   A   
   A   
   T   
   T   
   C   
   G   
   A   
   T   
   T   
   T   
   A   
   G   
   C   
   C   
   A   
   A   
   A   
   G   
   C   
   T   
   C   
   A   
   A   
   A   
   A   
   A   
    A    
   G   
   A   
   C   
   A   
   G   
   C   
   A   
   T   
   A   
   T   
   C   
   G   
   T   
    G    
   G   
   A   
   A   
   G   
   G   
    C    
    C    
   T   
   G   
   A   
   T   
   G   
   A   
   A   
   A   
   G   
   C   
   T   
   T   
   T   
   A   
   T   
   C   
   G   
   A   
   T   
   T   
   C   
   T   
   G   
   G   
   A   
   T   
   G   
   A   
   A   
   G   
   T   
   A   
   A   
   T   
   C   
   G   
    C    
   A   
   A   
   C   
   G   
   A   
   T   
   T   
   C   
   G   
   T   
   G   
   A   
   A   
   A   
   G   
   T   
   A   
   A   
   A   
   G   
   A   
   C   
   A   
   A   
   A   
   A   
   A   
   A   
   G   
   A   
    C    
   G   
   C   
    C    
   A   
   A   
   A   
   A   
   A   
   G   
   A   
   A   
   T   
   T   
   T   
   A   
   G   
   T   
   C   
   G   
   A   
    C    
   G   
   T   
    T    
   T   
   T   
    C    
   C   
   A   
   A   
   T   
   T   
   T   
   A   
   C   
   A   
   G   
   A   
   A   
   G   
   A   
   A   
   C   
   A   
   A   
   G   
   C   
   A   
   G   
   A   
   A   
   G   
   C   
   A   
   A   
   T   
    C    
   G   
   T   
   C   
   A   
   C   
   A   
   T   
   T   
   G   
   C   
   A   
   A   
    C    
   T   
   A   
   T   
   A   
    T    
   C   
   G   
   T   
   T   
   T   
   G   
   A   
   C   
   G   
   A   
   A   
    C    
   A   
   C   
    A    
   G   
   A   
   T   
   A   
   T   
   C   
   A   
   C   
   A   
   G   
   A   
   G   
   C   
   T   
   T   
   C   
   A   
   A   
   A   
   A   
   G   
   G   
   A   
   A   
    T    
   C   
    C    
   G   
   A   
   A   
   A   
    G    
   C   
   T   
   T   
   G   
   A   
   T   
   T   
   G   
   C   
    A    
   C   
   A   
   A   
   A   
   T   
   C   
   A   
   C   
   A   
   G   
   A   
   G   
   T   
   T   
    G    
   A   
   A   
   T   
   A   
   A   
   A   
   A   
   T   
   C   
   C   
   T   
   T   
   T   
   C   
   A   
   A   
   A   
    C    
   G   
   A   
   C   
   A   
   A   
   A   
   G   
   A   
    G    
   C   
   T   
   T   
   T   
   T   
    C    
   T   
   C   
   T   
   G   
   T   
   T   
   A   
   T   
   G   
   A   
   A   
   A   
   A   
   A   
   A   
   G   
   A   
   A   
   T   
   T   
   G   
   C   
   G   
   A   
   G   
   A   
   A   
   G   
   T   
   G   
   A   
   A   
   A   
   A   
   A   
   A   
   A   
   A   
   T   
   T   
   A   
    C    
   T   
   C   
   T   
   T   
   C   
   C   
   G   
   C   
   T   
   C   
   G   
   T   
   C   
    T    
    G    
   A   
   C   
   G   
   A   
   C   
   A   
   A   
   T   
   C   
    G    
   A   
   A   
   G   
   A   
   C   
   G   
   A   
   A   
   A   
   T   
   C   
   G   
   A   
   A   
   G   
   A   
   A   
   A   
   T   
   C   
   A   
   A   
   A   
   A   
   T   
    C    
   G   
   A   
   T   
   A   
   C   
   G   
   C   
   A   
   A   
    G    
   T   
    T    
   T   
   T   
   A   
   G   
   T   
   G   
   G   
   C   
    G    
   C   
   A   
   A   
   G   
   A   
   A   
   G   
   A   
   C   
   G   
   T   
   A   
   A   
   T   
    C    
   G   
   T   
   C   
   A   
   G   
   T   
   G   
   T   
    G    
   A   
   C   
    A    
   C   
   G   
   A   
   G   
   A   
   A   
   G   
   G   
   C   
   T   
   A   
   T   
   G   
   T   
   G   
   A   
   A   
   G   
   C   
   G   
   A   
   A   
   C   
   A   
   A   
   G   
   C   
   C   
   T   
   T   
   C   
   G   
   T   
   T   
   C   
    T    
   T   
   A   
   T   
   A   
   G   
   T   
   G   
   C   
   T   
   T   
   C   
    G    
   A   
   A   
   A   
   C   
   C   
   A   
   G   
   A   
   A   
   G   
   A   
   A   
   A   
   T   
   C   
   G   
   G   
   G   
   A   
   T   
   G   
   C   
   G   
   A   
   G   
   A   
   A   
   G   
   G   
   C   
   G   
   A   
    C    
   T   
   A   
   C   
   T   
   T   
   G   
   C   
   T   
    G    
   T   
   A   
   C   
    T    
   C   
   A   
   G   
   G   
   T   
   G   
   A   
   A   
   T   
   T   
    G    
   A   
   G   
    C    
   A   
   C   
   A   
    T    
   T   
   G   
   G   
   A   
   C   
   C   
   A   
   T   
   G   
   T   
   T   
   C   
   T   
   T   
   C   
   T   
   G   
   A   
   T   
    T    
   A   
   C   
    G    
   A   
   A   
   T   
   A   
   A   
   A   
   G   
   C   
   A   
   A   
   A   
   T   
   G   
   T   
   G   
   A   
   T   
   C   
   T   
   A   
   C   
   C   
   G   
    T    
   C   
   C   
   T   
   G   
   T   
    T    
   C   
   A   
   T   
   G   
   A   
   A   
   C   
   T   
   A   
   C   
   C   
    C    
   G   
   A   
   T   
   T   
   T   
   G   
   A   
   A   
   A   
   T   
   G   
   G   
   A   
   A   
   A   
   G   
   A   
   T   
   G   
   C   
   T   
   G   
   G   
    C    
   G   
   A   
    A    
   C   
   A   
    C    
   A   
   T   
   T   
   T   
   C   
   T   
   C   
   A   
   A   
   A   
   C   
   A   
   A   
   T   
   C   
   T   
   C   
   C   
   A   
   A   
   T   
   C   
   T   
   G   
   T   
   C   
   T   
   G   
   T   
   A   
   G   
   A   
    T    
   G   
   A   
   A   
   T   
   C   
   G   
   A   
   T   
    C    
   T   
   T   
   A   
   G   
   C   
   T   
   G   
   T   
   T   
   T   
   T   
    C    
   C   
   C   
   A   
   T   
   A   
   T   
   C   
   A   
   A   
   A   
   A   
   A   
   A   
   T   
    C    
   C   
   A   
   A   
   G   
   C   
   A   
   G   
   A   
    G    
   A   
   A   
   A   
   A   
   C   
   A   
   T   
   T   
   T   
   G   
   T   
    A    
   T   
   T   
   C   
   A   
   T   
   C   
   A   
   G   
   C   
   A   
   A   
   G   
   A   
   A   
   T   
   G   
   G   
   A   
   T   
   T   
    G    
    A    
   T   
   C   
   A   
   A   
   G   
   C   
   A   
   A   
   A   
   C   
   A   
   C   
   G   
   A   
   A   
   T   
   G   
   A   
   C   
   A   
   G   
   A   
   G   
   T   
   T   
   C   
   G   
   A   
   A   
   C   
   C   
   A   
   T   
   G   
   G   
   C   
   G   
   C   
   A   
   C   
   C   
   T   
   A   
    C    
   A   
   A   
   G   
   A   
   G   
   C   
   C   
   G   
    T    
   C   
   C   
   G   
   C   
   T   
   C   
   A   
   G   
   C   
   T   
   G   
   T   
   A   
   T   
   G   
   A   
   A   
   G   
   C   
   T   
   A   
   A   
   A   
   G   
   G   
   C   
   G   
   G   
   A   
   A   
   G   
   A   
   A   
   G   
   A   
   T   
   G   
   A   
   A   
   C   
   T   
    T    
   A   
   T   
   T   
   G   
   C   
   T   
   G   
   T   
    A    
   T   
   A   
   T   
   C   
   T   
   G   
   A   
   A   
   A   
   G   
   A   
   A   
   G   
   A   
   T   
   C   
    A    
   G   
   C   
   C   
   A   
   G   
   G   
   C   
   T   
   T   
   A   
   G   
   A   
   T   
   G   
   T   
    G    
   T   
   T   
   C   
   T   
   T   
   A   
   G   
   T   
   A   
   A   
   C   
   G   
    C    
   A   
   T   
   C   
   A   
   A   
   G   
   G   
   A   
   A   
   T   
   G   
   G   
   G   
    C    
   T   
   T   
   G   
   C   
   G   
   C   
   T   
    A    
   T   
   C   
   C   
   T   
   T   
   T   
   A   
   A   
    C    
   A   
   G   
   A   
   T   
   G   
   T   
   T   
   C   
   C   
   C   
   G   
   T   
   T   
   G   
   T   
   T   
   G   
   G   
   A   
   G   
   C   
   A   
   A   
   A   
   G   
   G   
   C   
   A   
   G   
    C    
   T   
   G   
   G   
   A   
   G   
   T   
   G   
   A   
   A   
   G   
   T   
   C   
   T   
   A   
   T   
   G   
   A   
   A   
   T   
   T   
   T   
   G   
   A   
   A   
   A   
   G   
   A   
   T   
   G   
   G   
   A   
   G   
   A   
   T   
   A   
   G   
   G   
    A    
   T   
   T   
    G    
   T   
   C   
   A   
   A   
    T    
   G   
   G   
    A    
   C   
   T   
    G    
   C   
   T   
   T   
   G   
   T   
   G   
   T   
   T   
   A   
   G   
   C   
   A   
   G   
   A   
    C    
   G   
   G   
    A    
   G   
   A   
   T   
   A   
   C   
   G   
   C   
   C   
    G    
   A   
   T   
   C   
   G   
   T   
   A   
   A   
   T   
   C   
   G   
   T   
   T   
   A   
   C   
   T   
   C   
   A   
   G   
   C   
   G   
   A   
   G   
   G   
   A   
   G   
   C   
    T    
   G   
   T   
    G    
   A   
   A   
   A   
   C   
   G   
   C   
   A   
   T   
   G   
   C   
   T   
   T   
   G   
   C   
   T   
   C   
   A   
   A   
   G   
   A   
   A   
   A   
   T   
   C   
   A   
   G   
   C   
   C   
   A   
   A   
   A   
   C   
    T    
   T   
   C   
   A   
   C   
   G   
   T   
   G   
   C   
   G   
   A   
   A   
   G   
   C   
   G   
   G   
   G   
   G   
   A   
   G   
   T   
   G   
   A   
   C   
   G   
   G   
   T   
   C   
   A   
   T   
   G   
   C   
   G   
   T   
   G   
   A   
   G   
   C   
   T   
   G   
   A   
   A   
   A   
   A   
   A   
   A   
   A   
   A   
   T   
   C   
   C   
    G    
   C   
   A   
    C    
   C   
   G   
    C    
   A   
   T   
   T   
   A   
   T   
   C   
   T   
   A   
   T   
   A   
   T   
   G   
   T   
   C   
   G   
   G   
   A   
   A   
   G   
   G   
   A   
   A   
   G   
   A   
   T   
   C   
   A   
   A   
   A   
   A   
   G   
   A   
   A   
   A   
   T   
   A   
   A   
   C   
   C   
   T   
   T   
   G   
   A   
   T   
   G   
   A   
   A   
   T   
   C   
   A   
   A   
   A   
   A   
   A   
   G   
   G   
    A    
   C   
   A   
    A    
   C   
   A   
   G   
    A    
   T   
   A   
   A   
   C   
   G   
   A   
   T   
   T   
   G   
   A   
    T    
   C   
   C   
   A   
   A   
   C   
    G    
   G   
   A   
   T   
   T   
   T   
    T    
   C   
   C   
   T   
   A   
   T   
   A   
   G   
   G   
   T   
   G   
   A   
   T   
   C   
   G   
   A   
   A   
   C   
   A   
   T   
   C   
   G   
   A   
   A   
   T   
   G   
   G   
    T    
   T   
   C   
    G    
   T   
   T   
   T   
   G   
   C   
    T    
   A   
   T   
   G   
   G   
   A   
    C    
   G   
   A   
   G   
   A   
   A   
   A   
   A   
   A   
   A   
   G   
   G   
   T   
   G   
   G   
   A   
   G   
   A   
   A   
   G   
   T   
   C   
   A   
   T   
   C   
   G   
   A   
   A   
   G   
   T   
    T    
   A   
    T    
   C   
   G   
   A   
   T   
   G   
   C   
   T   
   C   
   C   
   T   
   G   
   A   
   G   
    A    
   T   
   T   
   T   
   T   
    C    
   A   
   T   
    C    
   G   
   A   
    A    
    T    
   C   
    A    
   -   
   -   
   -   
 

 
   N59788F   
   A   
   T   
   G   
   G   
   A   
   A   
   A   
   A   
   T   
   C   
   G   
   A   
   C   
   A   
   A   
   G   
   A   
   A   
   G   
   T   
   A   
   C   
   A   
   A   
   G   
   A   
   A   
   T   
   T   
   A   
   A   
   C   
   T   
   T   
   T   
   A   
   G   
   A   
   A   
   G   
   A   
   A   
   G   
   T   
   G   
   A   
   T   
   G   
   G   
   G   
   T   
   G   
   A   
   C   
   C   
   G   
   C   
   T   
   T   
   C   
   G   
   G   
   A   
   C   
   G   
   T   
   T   
   A   
   T   
   T   
   C   
   C   
   A   
   A   
   A   
   T   
   A   
   T   
   A   
   T   
   T   
   A   
   T   
    C    
   C   
   A   
    A    
   G   
   A   
   A   
   C   
   G   
   T   
   G   
   C   
   A   
   C   
   T   
   G   
   C   
   C   
   T   
   G   
   A   
   T   
   A   
   T   
    C    
   C   
   G   
   T   
   G   
   A   
   T   
   G   
   G   
   A   
   T   
   T   
   G   
   A   
   A   
    G    
   C   
   C   
   A   
   G   
   T   
    G    
   C   
   A   
   A   
   C   
   G   
   C   
   C   
   G   
   G   
   A   
   T   
   A   
   T   
   T   
   A   
   T   
   T   
    T    
   G   
   C   
    C    
   A   
   T   
   G   
   A   
   A   
   C   
   A   
   A   
   A   
   G   
   A   
   T   
   G   
   G   
   C   
   A   
   A   
   T   
   A   
   C   
   G   
   T   
   A   
   T   
   G   
   A   
   C   
   A   
   A   
   A   
   G   
   G   
   A   
   T   
   T   
    C    
   C   
   G   
   T   
   A   
   A   
   A   
   T   
   C   
   A   
   G   
   C   
   A   
   A   
   A   
   A   
   T   
   C   
   T   
   G   
   T   
   C   
   G   
   G   
   A   
   A   
   A   
   T   
   A   
   T   
   C   
   A   
   T   
   G   
   G   
   G   
    G    
   A   
   A   
   T   
   T   
   A   
   T   
   C   
   A   
   T   
   C   
   C   
   T   
   C   
   A   
   T   
   G   
   G   
   T   
   G   
   A   
   C   
   A   
   G   
   C   
   A   
   G   
   T   
   A   
   T   
   T   
   T   
   A   
   T   
   G   
   A   
   A   
   G   
   C   
   G   
   A   
   T   
   G   
   G   
   T   
   C   
   C   
   G   
   C   
   A   
   T   
   G   
   A   
   G   
   C   
   C   
   A   
   A   
   G   
   A   
    C    
   T   
   G   
   G   
   A   
   A   
   G   
   T   
   T   
   G   
   C   
   G   
   G   
   G   
   A   
   A   
   G   
   T   
   A   
   C   
   T   
   G   
   A   
   T   
   C   
   G   
   A   
    A    
   A   
   T   
   G   
   C   
   A   
   C   
   G   
   G   
    G    
   A   
   A   
   T   
   A   
   A   
    C    
   G   
   G   
   G   
   A   
   G   
   T   
   A   
   T   
   G   
   G   
   A   
   C   
   G   
   G   
   T   
   G   
   A   
   T   
   C   
   C   
   A   
   C   
   C   
   T   
   G   
   C   
   T   
   G   
   C   
   G   
   A   
   T   
   G   
   C   
   G   
   T   
   T   
   A   
   T   
   A   
   C   
   T   
   G   
   A   
   A   
   G   
   C   
   C   
   A   
   G   
    A    
   T   
   T   
   A   
   T   
   C   
   C   
   A   
   A   
   G   
   T   
   T   
   A   
   A   
   G   
   T   
   G   
   G   
   A   
   G   
   A   
   G   
   A   
   T   
   G   
    C    
   T   
   A   
    G    
   C   
   A   
   G   
   A   
   T   
   A   
   T   
   C   
   G   
   A   
    A    
   A   
   A   
   A   
   G   
   A   
   A   
   A   
   C   
   A   
   G   
   T   
    C    
   G   
   A   
   T   
   C   
   T   
   C   
   G   
   T   
   G   
   T   
   G   
   G   
   A   
   A   
   T   
   T   
   T   
   T   
   G   
   A   
   T   
   G   
   A   
   T   
   A   
   C   
   A   
   G   
   A   
   A   
   A   
   A   
   A   
   G   
   A   
   A   
   C   
   C   
   A   
   A   
   C   
   A   
   G   
   T   
   C   
   C   
   T   
   T   
   C   
   C   
   A   
   G   
   C   
   T   
   C   
   G   
   A   
   T   
   A   
   C   
   C   
   C   
   A   
   A   
   A   
   C   
   C   
   T   
   T   
   C   
   T   
    C    
   G   
   T   
   C   
   A   
   A   
   T   
   G   
   G   
   A   
   T   
   C   
   G   
   A   
   C   
   A   
   G   
   G   
   T   
   A   
   T   
   A   
   T   
   C   
   T   
   G   
   C   
   T   
   G   
   G   
   A   
   T   
   A   
   T   
   G   
   C   
    A    
   A   
   C   
   A   
   G   
   A   
   A   
   A   
   T   
   C   
   C   
   C   
   G   
   A   
   C   
    T    
   C   
   A   
   T   
   A   
   A   
   C   
   T   
   T   
   A   
   G   
   C   
   T   
   G   
   A   
   A   
   G   
   T   
   G   
   A   
   T   
    C    
   G   
   A   
    T    
   G   
   G   
   C   
   A   
   C   
   A   
   A   
   T   
   C   
   T   
   A   
   T   
   A   
   T   
   G   
   A   
   T   
   C   
   G   
   A   
   T   
   C   
   A   
   T   
   C   
   C   
   A   
   C   
   A   
   A   
   G   
   C   
   G   
   A   
   G   
   C   
   C   
   T   
   T   
   G   
   A   
   A   
   A   
   A   
   A   
   T   
   T   
   G   
   A   
   T   
   G   
   G   
   A   
   A   
   T   
   A   
   T   
   A   
   T   
   T   
   C   
   C   
   T   
   G   
   G   
   A   
   C   
   C   
   T   
   G   
   A   
   C   
   T   
   T   
    T    
   C   
   C   
   A   
   A   
   C   
   A   
   G   
   G   
   A   
   G   
   G   
    G    
   A   
   T   
   C   
   T   
   T   
   G   
   C   
   A   
   A   
   G   
   G   
    A    
   A   
   A   
   A   
   G   
   A   
   T   
   G   
   A   
   A   
   A   
   T   
   C   
   A   
   A   
   A   
   A   
   A   
   A   
   G   
   C   
   T   
   T   
   A   
   T   
   G   
   A   
   A   
   A   
   C   
   A   
   G   
   G   
    G    
   C   
   G   
   A   
   G   
   G   
   A   
   A   
   A   
   A   
   G   
   T   
   T   
   A   
    T    
   T   
   C   
   T   
   T   
   C   
   G   
   T   
   T   
   C   
   G   
   A   
   A   
   A   
   A   
   C   
   A   
   A   
   A   
   G   
   A   
   T   
   A   
   G   
   A   
    A    
   G   
   C   
   A   
   A   
   T   
   G   
   A   
   A   
   G   
   G   
   G   
    T    
   A   
   A   
    T    
   A   
   A   
   A   
   C   
   A   
   G   
   C   
   A   
   A   
   A   
   T   
   C   
   G   
   T   
   G   
   A   
   T   
   C   
   A   
   C   
    A    
   G   
   A   
   A   
   A   
   T   
   C   
   C   
   C   
   A   
   T   
   A   
   T   
   G   
   A   
   A   
   G   
   T   
   A   
   A   
   A   
   T   
   A   
   A   
   A   
   G   
   C   
   G   
   A   
   C   
    C    
   T   
   T   
   G   
   G   
   T   
   G   
   A   
   A   
   A   
   A   
   A   
   A   
   A   
   T   
   G   
   G   
   A   
   T   
   G   
   A   
   G   
   A   
   T   
   C   
   C   
   G   
   T   
   T   
   T   
   G   
   A   
   A   
   T   
   A   
   A   
   G   
   A   
   A   
   A   
   A   
   T   
   C   
   G   
   A   
    T    
   G   
   G   
   A   
   A   
   T   
   A   
   G   
   C   
   T   
   G   
   A   
   A   
   G   
   T   
    A    
   C   
   G   
   A   
   G   
   A   
   T   
   G   
   A   
   A   
   A   
   G   
   T   
   G   
   A   
   T   
    A    
   G   
    A    
   A   
   C   
    A    
   G   
   G   
   T   
   C   
   T   
   G   
   C   
   A   
   A   
   A   
   T   
   T   
   G   
   T   
   C   
   G   
   T   
    C    
   G   
   A   
   A   
   T   
   T   
   G   
   A   
   A   
   A   
   A   
   A   
   A   
   G   
   A   
    C    
   G   
   C   
   C   
   A   
   A   
    T    
   G   
   C   
   T   
   C   
   A   
   A   
   G   
   G   
    A    
   A   
   T   
   T   
   T   
   T   
   G   
   A   
   A   
   T   
   T   
   A   
    T    
   T   
   T   
   G   
   T   
   T   
   C   
   A   
   A   
   A   
   A   
   A   
   C   
   A   
   C   
   T   
   G   
   A   
   G   
    C    
   T   
   A   
   C   
   A   
   G   
   A   
   T   
   C   
   A   
   A   
   T   
   T   
   A   
   C   
   A   
   A   
   T   
   T   
   T   
    C    
   A   
   A   
   T   
   A   
   T   
   G   
   G   
   T   
   C   
   G   
   C   
   T   
   A   
   T   
   C   
   G   
   A   
    C    
   C   
   A   
   T   
   A   
   T   
   G   
   A   
   C   
   T   
   C   
   C   
   G   
   C   
   A   
    T    
   C   
   A   
   A   
   G   
   T   
   T   
   G   
   G   
   A   
   C   
   T   
   G   
   A   
   A   
   A   
   G   
   A   
   C   
   A   
   T   
   T   
   T   
   T   
   A   
   A   
   G   
   A   
   A   
   G   
   T   
   T   
   A   
   T   
   A   
   T   
   C   
   G   
   A   
   G   
   C   
   A   
   T   
   C   
   G   
   A   
   A   
   A   
   G   
   C   
   A   
   A   
   G   
   T   
   C   
   A   
   T   
   A   
   A   
   C   
   A   
   A   
   A   
   A   
   C   
   G   
    T    
   A   
   G   
    T    
   C   
   A   
   A   
   T   
   T   
   C   
   G   
   A   
   T   
   T   
   T   
   A   
   G   
   C   
   C   
   A   
   A   
   A   
   G   
   C   
   T   
   C   
   A   
   A   
   A   
   A   
   A   
    A    
   G   
   A   
   C   
   A   
   G   
   C   
   A   
   T   
   A   
   T   
   C   
   G   
   T   
    A    
   G   
   A   
   A   
   G   
   G   
    A    
    T    
   T   
   G   
   A   
   T   
   G   
   A   
   A   
   A   
   G   
   C   
   T   
   T   
   T   
   A   
   T   
   C   
   G   
   A   
   T   
   T   
   C   
   T   
   G   
   G   
   A   
   T   
   G   
   A   
   A   
   G   
   T   
   A   
   A   
   T   
   C   
   G   
    C    
   A   
   A   
   C   
   G   
   A   
   T   
   T   
   C   
   G   
   T   
   G   
   A   
   A   
   A   
   G   
   T   
   A   
   A   
   A   
   G   
   A   
   C   
   A   
   A   
   A   
   A   
   A   
   A   
   G   
   A   
    C    
   G   
   C   
    C    
   A   
   A   
   A   
   A   
   A   
   G   
   A   
   A   
   T   
   T   
   T   
   A   
   G   
   T   
   C   
   G   
   A   
    C    
   G   
   T   
    C    
   T   
   T   
    T    
   C   
   A   
   A   
   T   
   T   
   T   
   A   
   C   
   A   
   G   
   A   
   A   
   G   
   A   
   A   
   C   
   A   
   A   
   G   
   C   
   A   
   G   
   A   
   A   
   G   
   C   
   A   
   A   
   T   
    T    
   G   
   T   
   C   
   A   
   C   
   A   
   T   
   T   
   G   
   C   
   A   
   A   
    T    
   T   
   A   
   T   
   A   
    C    
   C   
   G   
   T   
   T   
   T   
   G   
   A   
   C   
   G   
   A   
   A   
    C    
   A   
   C   
    G    
   G   
   A   
   T   
   A   
   T   
   C   
   A   
   C   
   A   
   G   
   A   
   G   
   C   
   T   
   T   
   C   
   A   
   A   
   A   
   A   
   G   
   G   
   A   
   A   
    T    
   C   
    C    
   G   
   A   
   A   
   A   
    G    
   C   
   T   
   T   
   G   
   A   
   T   
   T   
   G   
   C   
    G    
   C   
   A   
   A   
   A   
   T   
   C   
   A   
   C   
   A   
   G   
   A   
   G   
   T   
   T   
    G    
   A   
   A   
   T   
   A   
   A   
   A   
   A   
   T   
   C   
   C   
   T   
   T   
   T   
   C   
   A   
   A   
   A   
    T    
   G   
   A   
   C   
   A   
   A   
   A   
   G   
   A   
    G    
   C   
   T   
   T   
   T   
   T   
    C    
   T   
   C   
   T   
   G   
   T   
   T   
   A   
   T   
   G   
   A   
   A   
   A   
   A   
   A   
   A   
   G   
   A   
   A   
   T   
   T   
   G   
   C   
   G   
   A   
   G   
   A   
   A   
   G   
   T   
   G   
   A   
   A   
   A   
   A   
   A   
   A   
   A   
   A   
   T   
   T   
   A   
    T    
   T   
   C   
   T   
   T   
   C   
   C   
   G   
   C   
   T   
   C   
   G   
   T   
   C   
    T    
    A    
   A   
   C   
   G   
   A   
   C   
   A   
   A   
   T   
   C   
    G    
   A   
   A   
   G   
   A   
   C   
   G   
   A   
   A   
   A   
   T   
   C   
   G   
   A   
   A   
   G   
   A   
   A   
   A   
   T   
   C   
   A   
   A   
   A   
   A   
   T   
    T    
   G   
   A   
   T   
   A   
   C   
   G   
   C   
   A   
   A   
    A    
   T   
    C    
   T   
   T   
   A   
   G   
   T   
   G   
   G   
   C   
    A    
   C   
   A   
   A   
   G   
   A   
   A   
   G   
   A   
   C   
   G   
   T   
   A   
   A   
   T   
    T    
   G   
   T   
   C   
   A   
   G   
   T   
   G   
   T   
    G    
   A   
   C   
    G    
   C   
   G   
   A   
   G   
   A   
   A   
   G   
   G   
   C   
   T   
   A   
   T   
   G   
   T   
   G   
   A   
   A   
   G   
   C   
   G   
   A   
   A   
   C   
   A   
   A   
   G   
   C   
   C   
   T   
   T   
   C   
   G   
   T   
   T   
   C   
    T    
   T   
   A   
   T   
   A   
   G   
   T   
   G   
   C   
   T   
   T   
   C   
    C    
   A   
   A   
   A   
   C   
   C   
   A   
   G   
   A   
   A   
   G   
   A   
   A   
   A   
   T   
   C   
   G   
   G   
   G   
   A   
   T   
   G   
   C   
   G   
   A   
   G   
   A   
   A   
   G   
   G   
   C   
   G   
   A   
    C    
   T   
   A   
   C   
   T   
   T   
   G   
   C   
   T   
    A    
   T   
   A   
   C   
    T    
   C   
   A   
   G   
   G   
   T   
   G   
   A   
   A   
   T   
   T   
    G    
   A   
   G   
    T    
   A   
   C   
   A   
    T    
   T   
   G   
   G   
   A   
   C   
   C   
   A   
   T   
   G   
   T   
   T   
   C   
   T   
   T   
   C   
   T   
   G   
   A   
   T   
    C    
   A   
   C   
    A    
   A   
   A   
   T   
   A   
   A   
   A   
   G   
   C   
   A   
   A   
   A   
   T   
   G   
   T   
   G   
   A   
   T   
   C   
   T   
   A   
   C   
   C   
   G   
    C    
   C   
   C   
   T   
   G   
   T   
    C    
   C   
   A   
   T   
   G   
   A   
   A   
   C   
   T   
   A   
   C   
   C   
    T    
   G   
   A   
   T   
   T   
   T   
   G   
   A   
   A   
   A   
   T   
   G   
   G   
   A   
   A   
   A   
   G   
   A   
   T   
   G   
   C   
   T   
   G   
   G   
    T    
   G   
   A   
    G    
   C   
   A   
    C    
   A   
   T   
   T   
   T   
   C   
   T   
   C   
   A   
   A   
   A   
   C   
   A   
   A   
   T   
   C   
   T   
   C   
   C   
   A   
   A   
   T   
   C   
   T   
   G   
   T   
   C   
   T   
   G   
   T   
   A   
   G   
   A   
    C    
   G   
   A   
   A   
   T   
   C   
   G   
   A   
   T   
    T    
   T   
   T   
   A   
   G   
   C   
   T   
   G   
   T   
   T   
   T   
   T   
    C    
   C   
   C   
   A   
   T   
   A   
   T   
   C   
   A   
   A   
   A   
   A   
   A   
   A   
   T   
    C    
   C   
   A   
   A   
   G   
   C   
   A   
   G   
   A   
    G    
   A   
   A   
   A   
   A   
   C   
   A   
   T   
   T   
   T   
   G   
   T   
    A    
   T   
   T   
   C   
   A   
   T   
   C   
   A   
   G   
   C   
   A   
   A   
   G   
   A   
   A   
   T   
   G   
   G   
   A   
   T   
   T   
    G    
    A    
   T   
   C   
   A   
   A   
   G   
   C   
   A   
   A   
   A   
   C   
   A   
   C   
   G   
   A   
   A   
   T   
   G   
   A   
   C   
   A   
   G   
   A   
   G   
   T   
   T   
   C   
   G   
   A   
   A   
   C   
   C   
   A   
   T   
   G   
   G   
   C   
   G   
   C   
   A   
   C   
   C   
   T   
   A   
    T    
   A   
   A   
   G   
   A   
   G   
   C   
   C   
   G   
    T    
   C   
   C   
   G   
   C   
   T   
   C   
   A   
   G   
   C   
   T   
   G   
   T   
   A   
   T   
   G   
   A   
   A   
   G   
   C   
   T   
   A   
   A   
   A   
   G   
   G   
   C   
   G   
   G   
   A   
   A   
   G   
   A   
   A   
   G   
   A   
   T   
   G   
   A   
   A   
   C   
   T   
    G    
   A   
   T   
   T   
   G   
   C   
   T   
   G   
   T   
    G    
   T   
   A   
   T   
   C   
   T   
   G   
   A   
   A   
   A   
   G   
   A   
   A   
   G   
   A   
   T   
   C   
    A    
   G   
   C   
   C   
   A   
   G   
   G   
   C   
   T   
   T   
   A   
   G   
   A   
   T   
   G   
   T   
    T    
   T   
   T   
   C   
   T   
   T   
   A   
   G   
   T   
   A   
   A   
   C   
   G   
    C    
   A   
   T   
   C   
   A   
   A   
   G   
   G   
   A   
   A   
   T   
   G   
   G   
   G   
    T    
   T   
   T   
   G   
   C   
   G   
   C   
   T   
    A    
   T   
   C   
   C   
   T   
   T   
   T   
   A   
   A   
    C    
   A   
   G   
   A   
   T   
   G   
   T   
   T   
   C   
   C   
   C   
   G   
   T   
   T   
   G   
   T   
   T   
   G   
   G   
   A   
   G   
   C   
   A   
   A   
   A   
   G   
   G   
   C   
   A   
   G   
    C    
   T   
   G   
   G   
   A   
   G   
   T   
   G   
   A   
   A   
   G   
   T   
   C   
   T   
   A   
   T   
   G   
   A   
   A   
   T   
   T   
   T   
   G   
   A   
   A   
   A   
   G   
   A   
   T   
   G   
   G   
   A   
   G   
   A   
   T   
   A   
   G   
   G   
    A    
   T   
   T   
    A    
   T   
   C   
   A   
   A   
    C    
   G   
   G   
    G    
   C   
   T   
    G    
   C   
   T   
   T   
   G   
   T   
   G   
   T   
   T   
   A   
   G   
   C   
   A   
   G   
   A   
    A    
   G   
   G   
    T    
   G   
   A   
   T   
   A   
   C   
   G   
   C   
   C   
    G    
   A   
   T   
   C   
   G   
   T   
   A   
   A   
   T   
   C   
   G   
   T   
   T   
   A   
   C   
   T   
   C   
   A   
   G   
   C   
   G   
   A   
   G   
   G   
   A   
   G   
   C   
    T    
   G   
   T   
    T    
   A   
   A   
   A   
   C   
   G   
   C   
   A   
   T   
   G   
   C   
   T   
   T   
   G   
   C   
   T   
   C   
   A   
   A   
   G   
   A   
   A   
   A   
   T   
   C   
   A   
   G   
   C   
   C   
   A   
   A   
   A   
   C   
    T    
   T   
   C   
   A   
   C   
   G   
   T   
   G   
   C   
   G   
   A   
   A   
   G   
   C   
   G   
   G   
   G   
   G   
   A   
   G   
   T   
   G   
   A   
   C   
   G   
   G   
   T   
   C   
   A   
   T   
   G   
   C   
   G   
   T   
   G   
   A   
   G   
   C   
   T   
   G   
   A   
   A   
   A   
   A   
   A   
   A   
   A   
   A   
   T   
   C   
   C   
    G    
   C   
   A   
    C    
   C   
   G   
    T    
   A   
   T   
   T   
   A   
   T   
   C   
   T   
   A   
   T   
   A   
   T   
   G   
   T   
   C   
   G   
   G   
   A   
   A   
   G   
   G   
   A   
   A   
   G   
   A   
   T   
   C   
   A   
   A   
   A   
   A   
   G   
   A   
   A   
   A   
   T   
   A   
   A   
   C   
   C   
   T   
   T   
   G   
   A   
   T   
   G   
   A   
   A   
   T   
   C   
   A   
   A   
   A   
   A   
   A   
   G   
   G   
    G    
   C   
   A   
    G    
   C   
   A   
   G   
    T    
   T   
   A   
   A   
   C   
   G   
   A   
   T   
   T   
   G   
   A   
    C    
   C   
   C   
   A   
   A   
   C   
    T    
   G   
   A   
   T   
   T   
   T   
    C    
   C   
   C   
   T   
   A   
   T   
   A   
   G   
   G   
   T   
   G   
   A   
   T   
   C   
   G   
   A   
   A   
   C   
   A   
   T   
   C   
   G   
   A   
   A   
   T   
   G   
   G   
    A    
   T   
   C   
    A    
   T   
   T   
   T   
   G   
   C   
    G    
   A   
   T   
   G   
   G   
   A   
    T    
   G   
   A   
   G   
   A   
   A   
   A   
   A   
   A   
   A   
   G   
   G   
   T   
   G   
   G   
   A   
   G   
   A   
   A   
   G   
   T   
   C   
   A   
   T   
   C   
   G   
   A   
   A   
   G   
   T   
    C    
   A   
    A    
   C   
   G   
   A   
   T   
   G   
   C   
   T   
   C   
   C   
   T   
   G   
   A   
   G   
    A    
   T   
   T   
   T   
   T   
    C    
   A   
   T   
    C    
   G   
   A   
    G    
    T    
   C   
    G    
   -   
   -   
   -   
 

 
   N59517F   
   A   
   T   
   G   
   G   
   A   
   A   
   A   
   A   
   T   
   C   
   G   
   A   
   C   
   A   
   A   
   G   
   A   
   A   
   G   
   T   
   A   
   C   
   A   
   A   
   G   
   A   
   A   
   T   
   T   
   A   
   A   
   C   
   T   
   T   
   T   
   A   
   G   
   A   
   A   
   G   
   A   
   A   
   G   
   T   
   G   
   A   
   T   
   G   
   G   
   G   
   T   
   G   
   A   
   C   
   C   
   G   
   C   
   T   
   T   
   C   
   G   
   G   
   A   
   C   
   G   
   T   
   T   
   A   
   T   
   T   
   C   
   C   
   A   
   A   
   A   
   T   
   A   
   T   
   A   
   T   
   T   
   A   
   T   
    C    
   C   
   A   
    A    
   G   
   A   
   A   
   C   
   G   
   T   
   G   
   C   
   A   
   C   
   T   
   G   
   C   
   C   
   T   
   G   
   A   
   T   
   A   
   T   
    C    
   C   
   G   
   T   
   G   
   A   
   T   
   G   
   G   
   A   
   T   
   T   
   G   
   A   
   A   
    G    
   C   
   C   
   A   
   G   
   T   
    G    
   C   
   A   
   A   
   C   
   G   
   C   
   C   
   G   
   G   
   A   
   T   
   A   
   T   
   T   
   A   
   T   
   T   
    T    
   G   
   C   
    C    
   A   
   T   
   G   
   A   
   A   
   C   
   A   
   A   
   A   
   G   
   A   
   T   
   G   
   G   
   C   
   A   
   A   
   T   
   A   
   C   
   G   
   T   
   A   
   T   
   G   
   A   
   C   
   A   
   A   
   A   
   G   
   G   
   A   
   T   
   T   
    C    
   C   
   G   
   T   
   A   
   A   
   A   
   T   
   C   
   A   
   G   
   C   
   A   
   A   
   A   
   A   
   T   
   C   
   T   
   G   
   T   
   C   
   G   
   G   
   A   
   A   
   A   
   T   
   A   
   T   
   C   
   A   
   T   
   G   
   G   
   G   
    G    
   A   
   A   
   T   
   T   
   A   
   T   
   C   
   A   
   T   
   C   
   C   
   T   
   C   
   A   
   T   
   G   
   G   
   T   
   G   
   A   
   C   
   A   
   G   
   C   
   A   
   G   
   T   
   A   
   T   
   T   
   T   
   A   
   T   
   G   
   A   
   A   
   G   
   C   
   G   
   A   
   T   
   G   
   G   
   T   
   C   
   C   
   G   
   C   
   A   
   T   
   G   
   A   
   G   
   C   
   C   
   A   
   A   
   G   
   A   
    C    
   T   
   G   
   G   
   A   
   A   
   G   
   T   
   T   
   G   
   C   
   G   
   G   
   G   
   A   
   A   
   G   
   T   
   A   
   C   
   T   
   G   
   A   
   T   
   C   
   G   
   A   
    A    
   A   
   T   
   G   
   C   
   A   
   C   
   G   
   G   
    G    
   A   
   A   
   T   
   A   
   A   
    C    
   G   
   G   
   G   
   A   
   G   
   T   
   A   
   T   
   G   
   G   
   A   
   C   
   G   
   G   
   T   
   G   
   A   
   T   
   C   
   C   
   A   
   C   
   C   
   T   
   G   
   C   
   T   
   G   
   C   
   G   
   A   
   T   
   G   
   C   
   G   
   T   
   T   
   A   
   T   
   A   
   C   
   T   
   G   
   A   
   A   
   G   
   C   
   C   
   A   
   G   
    A    
   T   
   T   
   A   
   T   
   C   
   C   
   A   
   A   
   G   
   T   
   T   
   A   
   A   
   G   
   T   
   G   
   G   
   A   
   G   
   A   
   G   
   A   
   T   
   G   
    C    
   T   
   A   
    G    
   C   
   A   
   G   
   A   
   T   
   A   
   T   
   C   
   G   
   A   
    A    
   A   
   A   
   A   
   G   
   A   
   A   
   A   
   C   
   A   
   G   
   T   
    C    
   G   
   A   
   T   
   C   
   T   
   C   
   G   
   T   
   G   
   T   
   G   
   G   
   A   
   A   
   T   
   T   
   T   
   T   
   G   
   A   
   T   
   G   
   A   
   T   
   A   
   C   
   A   
   G   
   A   
   A   
   A   
   A   
   A   
   G   
   A   
   A   
   C   
   C   
   A   
   A   
   C   
   A   
   G   
   T   
   C   
   C   
   T   
   T   
   C   
   C   
   A   
   G   
   C   
   T   
   C   
   G   
   A   
   T   
   A   
   C   
   C   
   C   
   A   
   A   
   A   
   C   
   C   
   T   
   T   
   C   
   T   
    C    
   G   
   T   
   C   
   A   
   A   
   T   
   G   
   G   
   A   
   T   
   C   
   G   
   A   
   C   
   A   
   G   
   G   
   T   
   A   
   T   
   A   
   T   
   C   
   T   
   G   
   C   
   T   
   G   
   G   
   A   
   T   
   A   
   T   
   G   
   C   
    A    
   A   
   C   
   A   
   G   
   A   
   A   
   A   
   T   
   C   
   C   
   C   
   G   
   A   
   C   
    T    
   C   
   A   
   T   
   A   
   A   
   C   
   T   
   T   
   A   
   G   
   C   
   T   
   G   
   A   
   A   
   G   
   T   
   G   
   A   
   T   
    C    
   G   
   A   
    T    
   G   
   G   
   C   
   A   
   C   
   A   
   A   
   T   
   C   
   T   
   A   
   T   
   A   
   T   
   G   
   A   
   T   
   C   
   G   
   A   
   T   
   C   
   A   
   T   
   C   
   C   
   A   
   C   
   A   
   A   
   G   
   C   
   G   
   A   
   G   
   C   
   C   
   T   
   T   
   G   
   A   
   A   
   A   
   A   
   A   
   T   
   T   
   G   
   A   
   T   
   G   
   G   
   A   
   A   
   T   
   A   
   T   
   A   
   T   
   T   
   C   
   C   
   T   
   G   
   G   
   A   
   C   
   C   
   T   
   G   
   A   
   C   
   T   
   T   
    T    
   C   
   C   
   A   
   A   
   C   
   A   
   G   
   G   
   A   
   G   
   G   
    G    
   A   
   T   
   C   
   T   
   T   
   G   
   C   
   A   
   A   
   G   
   G   
    A    
   A   
   A   
   A   
   G   
   A   
   T   
   G   
   A   
   A   
   A   
   T   
   C   
   A   
   A   
   A   
   A   
   A   
   A   
   G   
   C   
   T   
   T   
   A   
   T   
   G   
   A   
   A   
   A   
   C   
   A   
   G   
   G   
    G    
   C   
   G   
   A   
   G   
   G   
   A   
   A   
   A   
   A   
   G   
   T   
   T   
   A   
    T    
   T   
   C   
   T   
   T   
   C   
   G   
   T   
   T   
   C   
   G   
   A   
   A   
   A   
   A   
   C   
   A   
   A   
   A   
   G   
   A   
   T   
   A   
   G   
   A   
    G    
   G   
   C   
   A   
   A   
   T   
   G   
   A   
   A   
   G   
   G   
   G   
    T    
   A   
   A   
    T    
   A   
   A   
   A   
   C   
   A   
   G   
   C   
   A   
   A   
   A   
   T   
   C   
   G   
   T   
   G   
   A   
   T   
   C   
   A   
   C   
    A    
   G   
   A   
   A   
   A   
   T   
   C   
   C   
   C   
   A   
   T   
   A   
   T   
   G   
   A   
   A   
   G   
   T   
   A   
   A   
   A   
   T   
   A   
   A   
   A   
   G   
   C   
   G   
   A   
   C   
    C    
   T   
   T   
   G   
   G   
   T   
   G   
   A   
   A   
   A   
   A   
   A   
   A   
   A   
   T   
   G   
   G   
   A   
   T   
   G   
   A   
   G   
   A   
   T   
   C   
   C   
   G   
   T   
   T   
   T   
   G   
   A   
   A   
   T   
   A   
   A   
   G   
   A   
   A   
   A   
   A   
   T   
   C   
   G   
   A   
    T    
   G   
   G   
   A   
   A   
   T   
   A   
   G   
   C   
   T   
   G   
   A   
   A   
   G   
   T   
    A    
   C   
   G   
   A   
   G   
   A   
   T   
   G   
   A   
   A   
   A   
   G   
   T   
   G   
   A   
   T   
    A    
   G   
    A    
   A   
   C   
    A    
   G   
   G   
   T   
   C   
   T   
   G   
   C   
   A   
   A   
   A   
   T   
   T   
   G   
   T   
   C   
   G   
   T   
    C    
   G   
   A   
   A   
   T   
   T   
   G   
   A   
   A   
   A   
   A   
   A   
   A   
   G   
   A   
    C    
   G   
   C   
   C   
   A   
   A   
    T    
   G   
   C   
   T   
   C   
   A   
   A   
   G   
   G   
    A    
   A   
   T   
   T   
   T   
   T   
   G   
   A   
   A   
   T   
   T   
   A   
    T    
   T   
   T   
   G   
   T   
   T   
   C   
   A   
   A   
   A   
   A   
   A   
   C   
   A   
   C   
   T   
   G   
   A   
   G   
    C    
   T   
   A   
   C   
   A   
   G   
   A   
   T   
   C   
   A   
   A   
   T   
   T   
   A   
   C   
   A   
   A   
   T   
   T   
   T   
    C    
   A   
   A   
   T   
   A   
   T   
   G   
   G   
   T   
   C   
   G   
   C   
   T   
   A   
   T   
   C   
   G   
   A   
    C    
   C   
   A   
   T   
   A   
   T   
   G   
   A   
   C   
   T   
   C   
   C   
   G   
   C   
   A   
    T    
   C   
   A   
   A   
   G   
   T   
   T   
   G   
   G   
   A   
   C   
   T   
   G   
   A   
   A   
   A   
   G   
   A   
   C   
   A   
   T   
   T   
   T   
   T   
   A   
   A   
   G   
   A   
   A   
   G   
   T   
   T   
   A   
   T   
   A   
   T   
   C   
   G   
   A   
   G   
   C   
   A   
   T   
   C   
   G   
   A   
   A   
   A   
   G   
   C   
   A   
   A   
   G   
   T   
   C   
   A   
   T   
   A   
   A   
   C   
   A   
   A   
   A   
   A   
   C   
   G   
    T    
   A   
   G   
    T    
   C   
   A   
   A   
   T   
   T   
   C   
   G   
   A   
   T   
   T   
   T   
   A   
   G   
   C   
   C   
   A   
   A   
   A   
   G   
   C   
   T   
   C   
   A   
   A   
   A   
   A   
   A   
    A    
   G   
   A   
   C   
   A   
   G   
   C   
   A   
   T   
   A   
   T   
   C   
   G   
   T   
    A    
   G   
   A   
   A   
   G   
   G   
    A    
    T    
   T   
   G   
   A   
   T   
   G   
   A   
   A   
   A   
   G   
   C   
   T   
   T   
   T   
   A   
   T   
   C   
   G   
   A   
   T   
   T   
   C   
   T   
   G   
   G   
   A   
   T   
   G   
   A   
   A   
   G   
   T   
   A   
   A   
   T   
   C   
   G   
    C    
   A   
   A   
   C   
   G   
   A   
   T   
   T   
   C   
   G   
   T   
   G   
   A   
   A   
   A   
   G   
   T   
   A   
   A   
   A   
   G   
   A   
   C   
   A   
   A   
   A   
   A   
   A   
   A   
   G   
   A   
    C    
   G   
   C   
    C    
   A   
   A   
   A   
   A   
   A   
   G   
   A   
   A   
   T   
   T   
   T   
   A   
   G   
   T   
   C   
   G   
   A   
    C    
   G   
   T   
    C    
   T   
   T   
    T    
   C   
   A   
   A   
   T   
   T   
   T   
   A   
   C   
   A   
   G   
   A   
   A   
   G   
   A   
   A   
   C   
   A   
   A   
   G   
   C   
   A   
   G   
   A   
   A   
   G   
   C   
   A   
   A   
   T   
    T    
   G   
   T   
   C   
   A   
   C   
   A   
   T   
   T   
   G   
   C   
   A   
   A   
    T    
   T   
   A   
   T   
   A   
    C    
   C   
   G   
   T   
   T   
   T   
   G   
   A   
   C   
   G   
   A   
   A   
    C    
   A   
   C   
    G    
   G   
   A   
   T   
   A   
   T   
   C   
   A   
   C   
   A   
   G   
   A   
   G   
   C   
   T   
   T   
   C   
   A   
   A   
   A   
   A   
   G   
   G   
   A   
   A   
    T    
   C   
    C    
   G   
   A   
   A   
   A   
    G    
   C   
   T   
   T   
   G   
   A   
   T   
   T   
   G   
   C   
    G    
   C   
   A   
   A   
   A   
   T   
   C   
   A   
   C   
   A   
   G   
   A   
   G   
   T   
   T   
    G    
   A   
   A   
   T   
   A   
   A   
   A   
   A   
   T   
   C   
   C   
   T   
   T   
   T   
   C   
   A   
   A   
   A   
    T    
   G   
   A   
   C   
   A   
   A   
   A   
   G   
   A   
    G    
   C   
   T   
   T   
   T   
   T   
    C    
   T   
   C   
   T   
   G   
   T   
   T   
   A   
   T   
   G   
   A   
   A   
   A   
   A   
   A   
   A   
   G   
   A   
   A   
   T   
   T   
   G   
   C   
   G   
   A   
   G   
   A   
   A   
   G   
   T   
   G   
   A   
   A   
   A   
   A   
   A   
   A   
   A   
   A   
   T   
   T   
   A   
    T    
   T   
   C   
   T   
   T   
   C   
   C   
   G   
   C   
   T   
   C   
   G   
   T   
   C   
    T    
    A    
   A   
   C   
   G   
   A   
   C   
   A   
   A   
   T   
   C   
    G    
   A   
   A   
   G   
   A   
   C   
   G   
   A   
   A   
   A   
   T   
   C   
   G   
   A   
   A   
   G   
   A   
   A   
   A   
   T   
   C   
   A   
   A   
   A   
   A   
   T   
    T    
   G   
   A   
   T   
   A   
   C   
   G   
   C   
   A   
   A   
    G    
   T   
    C    
   T   
   T   
   A   
   G   
   T   
   G   
   G   
   C   
    A    
   C   
   A   
   A   
   G   
   A   
   A   
   G   
   A   
   C   
   G   
   T   
   A   
   A   
   T   
    T    
   G   
   T   
   C   
   A   
   G   
   T   
   G   
   T   
    G    
   A   
   C   
    G    
   C   
   G   
   A   
   G   
   A   
   A   
   G   
   G   
   C   
   T   
   A   
   T   
   G   
   T   
   G   
   A   
   A   
   G   
   C   
   G   
   A   
   A   
   C   
   A   
   A   
   G   
   C   
   C   
   T   
   T   
   C   
   G   
   T   
   T   
   C   
    T    
   T   
   A   
   T   
   A   
   G   
   T   
   G   
   C   
   T   
   T   
   C   
    C    
   A   
   A   
   A   
   C   
   C   
   A   
   G   
   A   
   A   
   G   
   A   
   A   
   A   
   T   
   C   
   G   
   G   
   G   
   A   
   T   
   G   
   C   
   G   
   A   
   G   
   A   
   A   
   G   
   G   
   C   
   G   
   A   
    C    
   T   
   A   
   C   
   T   
   T   
   G   
   C   
   T   
    A    
   T   
   A   
   C   
    T    
   C   
   A   
   G   
   G   
   T   
   G   
   A   
   A   
   T   
   T   
    G    
   A   
   G   
    T    
   A   
   C   
   A   
    T    
   T   
   G   
   G   
   A   
   C   
   C   
   A   
   T   
   G   
   T   
   T   
   C   
   T   
   T   
   C   
   T   
   G   
   A   
   T   
    C    
   A   
   C   
    A    
   A   
   A   
   T   
   A   
   A   
   A   
   G   
   C   
   A   
   A   
   A   
   T   
   G   
   T   
   G   
   A   
   T   
   C   
   T   
   A   
   C   
   C   
   G   
    C    
   C   
   C   
   T   
   G   
   T   
    C    
   C   
   A   
   T   
   G   
   A   
   A   
   C   
   T   
   A   
   C   
   C   
    C    
   G   
   A   
   T   
   T   
   T   
   G   
   A   
   A   
   A   
   T   
   G   
   G   
   A   
   A   
   A   
   G   
   A   
   T   
   G   
   C   
   T   
   G   
   G   
    T    
   G   
   A   
    G    
   C   
   A   
    C    
   A   
   T   
   T   
   T   
   C   
   T   
   C   
   A   
   A   
   A   
   C   
   A   
   A   
   T   
   C   
   T   
   C   
   C   
   A   
   A   
   T   
   C   
   T   
   G   
   T   
   C   
   T   
   G   
   T   
   A   
   G   
   A   
    C    
   G   
   A   
   A   
   T   
   C   
   G   
   A   
   T   
    T    
   T   
   T   
   A   
   G   
   C   
   T   
   G   
   T   
   T   
   T   
   T   
    C    
   C   
   C   
   A   
   T   
   A   
   T   
   C   
   A   
   A   
   A   
   A   
   A   
   A   
   T   
    T    
   C   
   A   
   A   
   G   
   C   
   A   
   G   
   A   
    G    
   A   
   A   
   A   
   A   
   C   
   A   
   T   
   T   
   T   
   G   
   T   
    A    
   T   
   T   
   C   
   A   
   T   
   C   
   A   
   G   
   C   
   A   
   A   
   G   
   A   
   A   
   T   
   G   
   G   
   A   
   T   
   T   
    G    
    A    
   T   
   C   
   A   
   A   
   G   
   C   
   A   
   A   
   A   
   C   
   A   
   C   
   G   
   A   
   A   
   T   
   G   
   A   
   C   
   A   
   G   
   A   
   G   
   T   
   T   
   C   
   G   
   A   
   A   
   C   
   C   
   A   
   T   
   G   
   G   
   C   
   G   
   C   
   A   
   C   
   C   
   T   
   A   
    T    
   A   
   A   
   G   
   A   
   G   
   C   
   C   
   G   
    T    
   C   
   C   
   G   
   C   
   T   
   C   
   A   
   G   
   C   
   T   
   G   
   T   
   A   
   T   
   G   
   A   
   A   
   G   
   C   
   T   
   A   
   A   
   A   
   G   
   G   
   C   
   G   
   G   
   A   
   A   
   G   
   A   
   A   
   G   
   A   
   T   
   G   
   A   
   A   
   C   
   T   
    G    
   A   
   T   
   T   
   G   
   C   
   T   
   G   
   T   
    G    
   T   
   A   
   T   
   C   
   T   
   G   
   A   
   A   
   A   
   G   
   A   
   A   
   G   
   A   
   T   
   C   
    A    
   G   
   C   
   C   
   A   
   G   
   G   
   C   
   T   
   T   
   A   
   G   
   A   
   T   
   G   
   T   
    T    
   T   
   T   
   C   
   T   
   T   
   A   
   G   
   T   
   A   
   A   
   C   
   G   
    C    
   A   
   T   
   C   
   A   
   A   
   G   
   G   
   A   
   A   
   T   
   G   
   G   
   G   
    T    
   T   
   T   
   G   
   C   
   G   
   C   
   T   
    A    
   T   
   C   
   C   
   T   
   T   
   T   
   A   
   A   
    C    
   A   
   G   
   A   
   T   
   G   
   T   
   T   
   C   
   C   
   C   
   G   
   T   
   T   
   G   
   T   
   T   
   G   
   G   
   A   
   G   
   C   
   A   
   A   
   A   
   G   
   G   
   C   
   A   
   G   
    C    
   T   
   G   
   G   
   A   
   G   
   T   
   G   
   A   
   A   
   G   
   T   
   C   
   T   
   A   
   T   
   G   
   A   
   A   
   T   
   T   
   T   
   G   
   A   
   A   
   A   
   G   
   A   
   T   
   G   
   G   
   A   
   G   
   A   
   T   
   A   
   G   
   G   
    A    
   T   
   T   
    A    
   T   
   C   
   A   
   A   
    C    
   G   
   G   
    G    
   C   
   T   
    G    
   C   
   T   
   T   
   G   
   T   
   G   
   T   
   T   
   A   
   G   
   C   
   A   
   G   
   A   
    A    
   G   
   G   
    T    
   G   
   A   
   T   
   A   
   C   
   G   
   C   
   C   
    G    
   A   
   T   
   C   
   G   
   T   
   A   
   A   
   T   
   C   
   G   
   T   
   T   
   A   
   C   
   T   
   C   
   A   
   G   
   C   
   G   
   A   
   G   
   G   
   A   
   G   
   C   
    T    
   G   
   T   
    T    
   A   
   A   
   A   
   C   
   G   
   C   
   A   
   T   
   G   
   C   
   T   
   T   
   G   
   C   
   T   
   C   
   A   
   A   
   G   
   A   
   A   
   A   
   T   
   C   
   A   
   G   
   C   
   C   
   A   
   A   
   A   
   C   
    T    
   T   
   C   
   A   
   C   
   G   
   T   
   G   
   C   
   G   
   A   
   A   
   G   
   C   
   G   
   G   
   G   
   G   
   A   
   G   
   T   
   G   
   A   
   C   
   G   
   G   
   T   
   C   
   A   
   T   
   G   
   C   
   G   
   T   
   G   
   A   
   G   
   C   
   T   
   G   
   A   
   A   
   A   
   A   
   A   
   A   
   A   
   A   
   T   
   C   
   C   
    G    
   C   
   A   
    C    
   C   
   G   
    T    
   A   
   T   
   T   
   A   
   T   
   C   
   T   
   A   
   T   
   A   
   T   
   G   
   T   
   C   
   G   
   G   
   A   
   A   
   G   
   G   
   A   
   A   
   G   
   A   
   T   
   C   
   A   
   A   
   A   
   A   
   G   
   A   
   A   
   A   
   T   
   A   
   A   
   C   
   C   
   T   
   T   
   G   
   A   
   T   
   G   
   A   
   A   
   T   
   C   
   A   
   A   
   A   
   A   
   A   
   G   
   G   
    G    
   C   
   A   
    G    
   C   
   A   
   G   
    T    
   T   
   A   
   A   
   C   
   G   
   A   
   T   
   T   
   G   
   A   
    C    
   C   
   C   
   A   
   A   
   C   
    T    
   G   
   A   
   T   
   T   
   T   
    C    
   C   
   C   
   T   
   A   
   T   
   A   
   G   
   G   
   T   
   G   
   A   
   T   
   C   
   G   
   A   
   A   
   C   
   A   
   T   
   C   
   G   
   A   
   A   
   T   
   G   
   G   
    A    
   T   
   C   
    A    
   T   
   T   
   T   
   G   
   C   
    G    
   A   
   T   
   G   
   G   
   A   
    T    
   G   
   A   
   G   
   A   
   A   
   A   
   A   
   A   
   A   
   G   
   G   
   T   
   G   
   G   
   A   
   G   
   A   
   A   
   G   
   T   
   C   
   A   
   T   
   C   
   G   
   A   
   A   
   G   
   T   
    T    
   A   
    T    
   C   
   G   
   A   
   T   
   G   
   C   
   T   
   C   
   C   
   T   
   G   
   A   
   G   
    A    
   T   
   T   
   T   
   T   
    C    
   A   
   T   
    C    
   G   
   A   
    G    
    T    
   C   
    G    
   -   
   -   
   -   
 

 
   N59613F   
   A   
   T   
   G   
   G   
   A   
   A   
   A   
   A   
   T   
   C   
   G   
   A   
   C   
   A   
   A   
   G   
   A   
   A   
   G   
   T   
   A   
   C   
   A   
   A   
   G   
   A   
   A   
   T   
   T   
   A   
   A   
   C   
   T   
   T   
   T   
   A   
   G   
   A   
   A   
   G   
   A   
   A   
   G   
   T   
   G   
   A   
   T   
   G   
   G   
   G   
   T   
   G   
   A   
   C   
   C   
   G   
   C   
   T   
   T   
   C   
   G   
   G   
   A   
   C   
   G   
   T   
   T   
   A   
   T   
   T   
   C   
   C   
   A   
   A   
   A   
   T   
   A   
   T   
   A   
   T   
   T   
   A   
   T   
    A    
   C   
   A   
    A    
   G   
   A   
   A   
   C   
   G   
   T   
   G   
   C   
   A   
   C   
   T   
   G   
   C   
   C   
   T   
   G   
   A   
   T   
   A   
   T   
    C    
   C   
   G   
   T   
   G   
   A   
   T   
   G   
   G   
   A   
   T   
   T   
   G   
   A   
   A   
    A    
   C   
   C   
   A   
   G   
   T   
    G    
   C   
   A   
   A   
   C   
   G   
   C   
   C   
   G   
   G   
   A   
   T   
   A   
   T   
   T   
   A   
   T   
   T   
    T    
   G   
   C   
    C    
   A   
   T   
   G   
   A   
   A   
   C   
   A   
   A   
   A   
   G   
   A   
   T   
   G   
   G   
   C   
   A   
   A   
   T   
   A   
   C   
   G   
   T   
   A   
   T   
   G   
   A   
   C   
   A   
   A   
   A   
   G   
   G   
   A   
   T   
   T   
    C    
   C   
   G   
   T   
   A   
   A   
   A   
   T   
   C   
   A   
   G   
   C   
   A   
   A   
   A   
   A   
   T   
   C   
   T   
   G   
   T   
   C   
   G   
   G   
   A   
   A   
   A   
   T   
   A   
   T   
   C   
   A   
   T   
   G   
   G   
   G   
    A    
   A   
   A   
   T   
   T   
   A   
   T   
   C   
   A   
   T   
   C   
   C   
   T   
   C   
   A   
   T   
   G   
   G   
   T   
   G   
   A   
   C   
   A   
   G   
   C   
   A   
   G   
   T   
   A   
   T   
   T   
   T   
   A   
   T   
   G   
   A   
   A   
   G   
   C   
   G   
   A   
   T   
   G   
   G   
   T   
   C   
   C   
   G   
   C   
   A   
   T   
   G   
   A   
   G   
   C   
   C   
   A   
   A   
   G   
   A   
    T    
   T   
   G   
   G   
   A   
   A   
   G   
   T   
   T   
   G   
   C   
   G   
   G   
   G   
   A   
   A   
   G   
   T   
   A   
   C   
   T   
   G   
   A   
   T   
   C   
   G   
   A   
    A    
   A   
   T   
   G   
   C   
   A   
   C   
   G   
   G   
    G    
   A   
   A   
   T   
   A   
   A   
    T    
   G   
   G   
   G   
   A   
   G   
   T   
   A   
   T   
   G   
   G   
   A   
   C   
   G   
   G   
   T   
   G   
   A   
   T   
   C   
   C   
   A   
   C   
   C   
   T   
   G   
   C   
   T   
   G   
   C   
   G   
   A   
   T   
   G   
   C   
   G   
   T   
   T   
   A   
   T   
   A   
   C   
   T   
   G   
   A   
   A   
   G   
   C   
   C   
   A   
   G   
    A    
   T   
   T   
   A   
   T   
   C   
   C   
   A   
   A   
   G   
   T   
   T   
   A   
   A   
   G   
   T   
   G   
   G   
   A   
   G   
   A   
   G   
   A   
   T   
   G   
    C    
   T   
   A   
    G    
   C   
   A   
   G   
   A   
   T   
   A   
   T   
   C   
   G   
   A   
    A    
   A   
   A   
   A   
   G   
   A   
   A   
   A   
   C   
   A   
   G   
   T   
    C    
   G   
   A   
   T   
   C   
   T   
   C   
   G   
   T   
   G   
   T   
   G   
   G   
   A   
   A   
   T   
   T   
   T   
   T   
   G   
   A   
   T   
   G   
   A   
   T   
   A   
   C   
   A   
   G   
   A   
   A   
   A   
   A   
   A   
   G   
   A   
   A   
   C   
   C   
   A   
   A   
   C   
   A   
   G   
   T   
   C   
   C   
   T   
   T   
   C   
   C   
   A   
   G   
   C   
   T   
   C   
   G   
   A   
   T   
   A   
   C   
   C   
   C   
   A   
   A   
   A   
   C   
   C   
   T   
   T   
   C   
   T   
    A    
   G   
   T   
   C   
   A   
   A   
   T   
   G   
   G   
   A   
   T   
   C   
   G   
   A   
   C   
   A   
   G   
   G   
   T   
   A   
   T   
   A   
   T   
   C   
   T   
   G   
   C   
   T   
   G   
   G   
   A   
   T   
   A   
   T   
   G   
   C   
    G    
   A   
   C   
   A   
   G   
   A   
   A   
   A   
   T   
   C   
   C   
   C   
   G   
   A   
   C   
    T    
   C   
   A   
   T   
   A   
   A   
   C   
   T   
   T   
   A   
   G   
   C   
   T   
   G   
   A   
   A   
   G   
   T   
   G   
   A   
   T   
    C    
   G   
   A   
    C    
   G   
   G   
   C   
   A   
   C   
   A   
   A   
   T   
   C   
   T   
   A   
   T   
   A   
   T   
   G   
   A   
   T   
   C   
   G   
   A   
   T   
   C   
   A   
   T   
   C   
   C   
   A   
   C   
   A   
   A   
   G   
   C   
   G   
   A   
   G   
   C   
   C   
   T   
   T   
   G   
   A   
   A   
   A   
   A   
   A   
   T   
   T   
   G   
   A   
   T   
   G   
   G   
   A   
   A   
   T   
   A   
   T   
   A   
   T   
   T   
   C   
   C   
   T   
   G   
   G   
   A   
   C   
   C   
   T   
   G   
   A   
   C   
   T   
   T   
    T    
   C   
   C   
   A   
   A   
   C   
   A   
   G   
   G   
   A   
   G   
   G   
    G    
   A   
   T   
   C   
   T   
   T   
   G   
   C   
   A   
   A   
   G   
   G   
    G    
   A   
   A   
   A   
   G   
   A   
   T   
   G   
   A   
   A   
   A   
   T   
   C   
   A   
   A   
   A   
   A   
   A   
   A   
   G   
   C   
   T   
   T   
   A   
   T   
   G   
   A   
   A   
   A   
   C   
   A   
   G   
   G   
    G    
   C   
   G   
   A   
   G   
   G   
   A   
   A   
   A   
   A   
   G   
   T   
   T   
   A   
    T    
   T   
   C   
   T   
   T   
   C   
   G   
   T   
   T   
   C   
   G   
   A   
   A   
   A   
   A   
   C   
   A   
   A   
   A   
   G   
   A   
   T   
   A   
   G   
   A   
    A    
   G   
   C   
   A   
   A   
   T   
   G   
   A   
   A   
   G   
   G   
   G   
    C    
   A   
   A   
    T    
   A   
   A   
   A   
   C   
   A   
   G   
   C   
   A   
   A   
   A   
   T   
   C   
   G   
   T   
   G   
   A   
   T   
   C   
   A   
   C   
    T    
   G   
   A   
   A   
   A   
   T   
   C   
   C   
   C   
   A   
   T   
   A   
   T   
   G   
   A   
   A   
   G   
   T   
   A   
   A   
   A   
   T   
   A   
   A   
   A   
   G   
   C   
   G   
   A   
   C   
    G    
   T   
   T   
   G   
   G   
   T   
   G   
   A   
   A   
   A   
   A   
   A   
   A   
   A   
   T   
   G   
   G   
   A   
   T   
   G   
   A   
   G   
   A   
   T   
   C   
   C   
   G   
   T   
   T   
   T   
   G   
   A   
   A   
   T   
   A   
   A   
   G   
   A   
   A   
   A   
   A   
   T   
   C   
   G   
   A   
    T    
   G   
   G   
   A   
   A   
   T   
   A   
   G   
   C   
   T   
   G   
   A   
   A   
   G   
   T   
    A    
   C   
   G   
   A   
   G   
   A   
   T   
   G   
   A   
   A   
   A   
   G   
   T   
   G   
   A   
   T   
    C    
   G   
    A    
   A   
   C   
    T    
   G   
   G   
   T   
   C   
   T   
   G   
   C   
   A   
   A   
   A   
   T   
   T   
   G   
   T   
   C   
   G   
   T   
    T    
   G   
   A   
   A   
   T   
   T   
   G   
   A   
   A   
   A   
   A   
   A   
   A   
   G   
   A   
    T    
   G   
   C   
   C   
   A   
   A   
    T    
   G   
   C   
   T   
   C   
   A   
   A   
   G   
   G   
    A    
   A   
   T   
   T   
   T   
   T   
   G   
   A   
   A   
   T   
   T   
   A   
    T    
   T   
   T   
   G   
   T   
   T   
   C   
   A   
   A   
   A   
   A   
   A   
   C   
   A   
   C   
   T   
   G   
   A   
   G   
    C    
   T   
   A   
   C   
   A   
   G   
   A   
   T   
   C   
   A   
   A   
   T   
   T   
   A   
   C   
   A   
   A   
   T   
   T   
   T   
    C    
   A   
   A   
   T   
   A   
   T   
   G   
   G   
   T   
   C   
   G   
   C   
   T   
   A   
   T   
   C   
   G   
   A   
    T    
   C   
   A   
   T   
   A   
   T   
   G   
   A   
   C   
   T   
   C   
   C   
   G   
   C   
   A   
    C    
   C   
   A   
   A   
   G   
   T   
   T   
   G   
   G   
   A   
   C   
   T   
   G   
   A   
   A   
   A   
   G   
   A   
   C   
   A   
   T   
   T   
   T   
   T   
   A   
   A   
   G   
   A   
   A   
   G   
   T   
   T   
   A   
   T   
   A   
   T   
   C   
   G   
   A   
   G   
   C   
   A   
   T   
   C   
   G   
   A   
   A   
   A   
   G   
   C   
   A   
   A   
   G   
   T   
   C   
   A   
   T   
   A   
   A   
   C   
   A   
   A   
   A   
   A   
   C   
   G   
    C    
   A   
   G   
    C    
   C   
   A   
   A   
   T   
   T   
   C   
   G   
   A   
   T   
   T   
   T   
   A   
   G   
   C   
   C   
   A   
   A   
   A   
   G   
   C   
   T   
   C   
   A   
   A   
   A   
   A   
   A   
    A    
   G   
   A   
   C   
   A   
   G   
   C   
   A   
   T   
   A   
   T   
   C   
   G   
   T   
    G    
   G   
   A   
   A   
   G   
   G   
    C    
    C    
   T   
   G   
   A   
   T   
   G   
   A   
   A   
   A   
   G   
   C   
   T   
   T   
   T   
   A   
   T   
   C   
   G   
   A   
   T   
   T   
   C   
   T   
   G   
   G   
   A   
   T   
   G   
   A   
   A   
   G   
   T   
   A   
   A   
   T   
   C   
   G   
    C    
   A   
   A   
   C   
   G   
   A   
   T   
   T   
   C   
   G   
   T   
   G   
   A   
   A   
   A   
   G   
   T   
   A   
   A   
   A   
   G   
   A   
   C   
   A   
   A   
   A   
   A   
   A   
   A   
   G   
   A   
    T    
   G   
   C   
    T    
   A   
   A   
   A   
   A   
   A   
   G   
   A   
   A   
   T   
   T   
   T   
   A   
   G   
   T   
   C   
   G   
   A   
    T    
   G   
   T   
    T    
   T   
   T   
    C    
   C   
   A   
   A   
   T   
   T   
   T   
   A   
   C   
   A   
   G   
   A   
   A   
   G   
   A   
   A   
   C   
   A   
   A   
   G   
   C   
   A   
   G   
   A   
   A   
   G   
   C   
   A   
   A   
   T   
    C    
   G   
   T   
   C   
   A   
   C   
   A   
   T   
   T   
   G   
   C   
   A   
   A   
    C    
   T   
   A   
   T   
   A   
    C    
   C   
   G   
   T   
   T   
   T   
   G   
   A   
   C   
   G   
   A   
   A   
    C    
   A   
   C   
    A    
   G   
   A   
   T   
   A   
   T   
   C   
   A   
   C   
   A   
   G   
   A   
   G   
   C   
   T   
   T   
   C   
   A   
   A   
   A   
   A   
   G   
   G   
   A   
   A   
    G    
   C   
    C    
   G   
   A   
   A   
   A   
    G    
   C   
   T   
   T   
   G   
   A   
   T   
   T   
   G   
   C   
    A    
   C   
   A   
   A   
   A   
   T   
   C   
   A   
   C   
   A   
   G   
   A   
   G   
   T   
   T   
    G    
   A   
   A   
   T   
   A   
   A   
   A   
   A   
   T   
   C   
   C   
   T   
   T   
   T   
   C   
   A   
   A   
   A   
    C    
   G   
   A   
   C   
   A   
   A   
   A   
   G   
   A   
    A    
   C   
   T   
   T   
   T   
   T   
    C    
   T   
   C   
   T   
   G   
   T   
   T   
   A   
   T   
   G   
   A   
   A   
   A   
   A   
   A   
   A   
   G   
   A   
   A   
   T   
   T   
   G   
   C   
   G   
   A   
   G   
   A   
   A   
   G   
   T   
   G   
   A   
   A   
   A   
   A   
   A   
   A   
   A   
   A   
   T   
   T   
   A   
    C    
   T   
   C   
   T   
   T   
   C   
   C   
   G   
   C   
   T   
   C   
   G   
   T   
   C   
    T    
    G    
   A   
   C   
   G   
   A   
   C   
   A   
   A   
   T   
   C   
    C    
   A   
   A   
   G   
   A   
   C   
   G   
   A   
   A   
   A   
   T   
   C   
   G   
   A   
   A   
   G   
   A   
   A   
   A   
   T   
   C   
   A   
   A   
   A   
   A   
   T   
    C    
   G   
   A   
   T   
   A   
   C   
   G   
   C   
   A   
   A   
    G    
   T   
    T    
   T   
   T   
   A   
   G   
   T   
   G   
   G   
   C   
    G    
   C   
   A   
   A   
   G   
   A   
   A   
   G   
   A   
   C   
   G   
   T   
   A   
   A   
   T   
    C    
   G   
   T   
   C   
   A   
   G   
   T   
   G   
   T   
    G    
   A   
   C   
    G    
   C   
   G   
   A   
   G   
   A   
   A   
   G   
   G   
   C   
   T   
   A   
   T   
   G   
   T   
   G   
   A   
   A   
   G   
   C   
   G   
   A   
   A   
   C   
   A   
   A   
   G   
   C   
   C   
   T   
   T   
   C   
   G   
   T   
   T   
   C   
    T    
   T   
   A   
   T   
   A   
   G   
   T   
   G   
   C   
   T   
   T   
   C   
    G    
   A   
   A   
   A   
   C   
   C   
   A   
   G   
   A   
   A   
   G   
   A   
   A   
   A   
   T   
   C   
   G   
   G   
   G   
   A   
   T   
   G   
   C   
   G   
   A   
   G   
   A   
   A   
   G   
   G   
   C   
   G   
   A   
    T    
   T   
   A   
   C   
   T   
   T   
   G   
   C   
   T   
    G    
   T   
   A   
   C   
    T    
   C   
   A   
   G   
   G   
   T   
   G   
   A   
   A   
   T   
   T   
    G    
   A   
   G   
    C    
   A   
   C   
   A   
    T    
   T   
   G   
   G   
   A   
   C   
   C   
   A   
   T   
   G   
   T   
   T   
   C   
   T   
   T   
   C   
   T   
   G   
   A   
   T   
    T    
   A   
   C   
    G    
   A   
   A   
   T   
   A   
   A   
   A   
   G   
   C   
   A   
   A   
   A   
   T   
   G   
   T   
   G   
   A   
   T   
   C   
   T   
   A   
   C   
   C   
   G   
    T    
   C   
   C   
   T   
   G   
   T   
    T    
   C   
   A   
   T   
   G   
   A   
   A   
   C   
   T   
   A   
   C   
   C   
    C    
   G   
   A   
   T   
   T   
   T   
   G   
   A   
   A   
   A   
   T   
   G   
   G   
   A   
   A   
   A   
   G   
   A   
   T   
   G   
   C   
   T   
   G   
   G   
    C    
   G   
   A   
    A    
   C   
   A   
    C    
   A   
   T   
   T   
   T   
   C   
   T   
   C   
   A   
   A   
   A   
   C   
   A   
   A   
   T   
   C   
   T   
   C   
   C   
   A   
   A   
   T   
   C   
   T   
   G   
   T   
   C   
   T   
   G   
   T   
   A   
   G   
   A   
    T    
   G   
   A   
   A   
   T   
   C   
   G   
   A   
   T   
    C    
   T   
   T   
   A   
   G   
   C   
   T   
   G   
   T   
   T   
   T   
   T   
    C    
   C   
   C   
   A   
   T   
   A   
   T   
   C   
   A   
   A   
   A   
   A   
   A   
   A   
   T   
    C    
   C   
   A   
   A   
   G   
   C   
   A   
   G   
   A   
    A    
   A   
   A   
   A   
   A   
   C   
   A   
   T   
   T   
   T   
   G   
   T   
    A    
   T   
   T   
   C   
   A   
   T   
   C   
   A   
   G   
   C   
   A   
   A   
   G   
   A   
   A   
   T   
   G   
   G   
   A   
   T   
   T   
    G    
    A    
   T   
   C   
   A   
   A   
   G   
   C   
   A   
   A   
   A   
   C   
   A   
   C   
   G   
   A   
   A   
   T   
   G   
   A   
   C   
   A   
   G   
   A   
   G   
   T   
   T   
   C   
   G   
   A   
   A   
   C   
   C   
   A   
   T   
   G   
   G   
   C   
   G   
   C   
   A   
   C   
   C   
   T   
   A   
    T    
   A   
   A   
   G   
   A   
   G   
   C   
   C   
   G   
    T    
   C   
   C   
   G   
   C   
   T   
   C   
   A   
   G   
   C   
   T   
   G   
   T   
   A   
   T   
   G   
   A   
   A   
   G   
   C   
   T   
   A   
   A   
   A   
   G   
   G   
   C   
   G   
   G   
   A   
   A   
   G   
   A   
   A   
   G   
   A   
   T   
   G   
   A   
   A   
   C   
   T   
    T    
   A   
   T   
   T   
   G   
   C   
   T   
   G   
   T   
    A    
   T   
   A   
   T   
   C   
   T   
   G   
   A   
   A   
   A   
   G   
   A   
   A   
   G   
   A   
   T   
   C   
    A    
   G   
   C   
   C   
   A   
   G   
   G   
   C   
   T   
   T   
   A   
   G   
   A   
   T   
   G   
   T   
    G    
   T   
   T   
   C   
   T   
   T   
   A   
   G   
   T   
   A   
   A   
   C   
   G   
    C    
   A   
   T   
   C   
   A   
   A   
   G   
   G   
   A   
   A   
   T   
   G   
   G   
   G   
    C    
   T   
   T   
   G   
   C   
   G   
   C   
   T   
    A    
   T   
   C   
   C   
   T   
   T   
   T   
   A   
   A   
    C    
   A   
   G   
   A   
   T   
   G   
   T   
   T   
   C   
   C   
   C   
   G   
   T   
   T   
   G   
   T   
   T   
   G   
   G   
   A   
   G   
   C   
   A   
   A   
   A   
   G   
   G   
   C   
   A   
   G   
    C    
   T   
   G   
   G   
   A   
   G   
   T   
   G   
   A   
   A   
   G   
   T   
   C   
   T   
   A   
   T   
   G   
   A   
   A   
   T   
   T   
   T   
   G   
   A   
   A   
   A   
   G   
   A   
   T   
   G   
   G   
   A   
   G   
   A   
   T   
   A   
   G   
   G   
    A    
   T   
   T   
    G    
   T   
   C   
   A   
   A   
    T    
   G   
   G   
    G    
   C   
   T   
    G    
   C   
   T   
   T   
   G   
   T   
   G   
   T   
   T   
   A   
   G   
   C   
   A   
   G   
   A   
    C    
   G   
   G   
    A    
   G   
   A   
   T   
   A   
   C   
   G   
   C   
   C   
    A    
   A   
   T   
   C   
   G   
   T   
   A   
   A   
   T   
   C   
   G   
   T   
   T   
   A   
   C   
   T   
   C   
   A   
   G   
   C   
   G   
   A   
   G   
   G   
   A   
   G   
   C   
    C    
   G   
   T   
    G    
   A   
   A   
   A   
   C   
   G   
   C   
   A   
   T   
   G   
   C   
   T   
   T   
   G   
   C   
   T   
   C   
   A   
   A   
   G   
   A   
   A   
   A   
   T   
   C   
   A   
   G   
   C   
   C   
   A   
   A   
   A   
   C   
    G    
   T   
   C   
   A   
   C   
   G   
   T   
   G   
   C   
   G   
   A   
   A   
   G   
   C   
   G   
   G   
   G   
   G   
   A   
   G   
   T   
   G   
   A   
   C   
   G   
   G   
   T   
   C   
   A   
   T   
   G   
   C   
   G   
   T   
   G   
   A   
   G   
   C   
   T   
   G   
   A   
   A   
   A   
   A   
   A   
   A   
   A   
   A   
   T   
   C   
   C   
    G    
   C   
   A   
    C    
   C   
   G   
    T    
   A   
   T   
   T   
   A   
   T   
   C   
   T   
   A   
   T   
   A   
   T   
   G   
   T   
   C   
   G   
   G   
   A   
   A   
   G   
   G   
   A   
   A   
   G   
   A   
   T   
   C   
   A   
   A   
   A   
   A   
   G   
   A   
   A   
   A   
   T   
   A   
   A   
   C   
   C   
   T   
   T   
   G   
   A   
   T   
   G   
   A   
   A   
   T   
   C   
   A   
   A   
   A   
   A   
   A   
   G   
   G   
    A    
   C   
   A   
    A    
   C   
   A   
   G   
    A    
   T   
   A   
   A   
   C   
   G   
   A   
   T   
   T   
   G   
   A   
    T    
   C   
   C   
   A   
   A   
   C   
    G    
   G   
   A   
   T   
   T   
   T   
    T    
   C   
   C   
   T   
   A   
   T   
   A   
   G   
   G   
   T   
   G   
   A   
   T   
   C   
   G   
   A   
   A   
   C   
   A   
   T   
   C   
   G   
   A   
   A   
   T   
   G   
   G   
    T    
   T   
   C   
    G    
   T   
   T   
   T   
   G   
   C   
    T    
   A   
   T   
   G   
   G   
   A   
    C    
   G   
   A   
   G   
   A   
   A   
   A   
   A   
   A   
   A   
   G   
   G   
   T   
   G   
   G   
   A   
   G   
   A   
   A   
   G   
   T   
   C   
   A   
   T   
   C   
   G   
   A   
   A   
   G   
   T   
    T    
   A   
    T    
   C   
   G   
   A   
   T   
   G   
   C   
   T   
   C   
   C   
   T   
   G   
   A   
   G   
    A    
   T   
   T   
   T   
   T   
    C    
   A   
   T   
    T    
   G   
   A   
    A    
    T    
   C   
    A    
   -   
   -   
   -   
 

 
   N60284F   
   A   
   T   
   G   
   G   
   A   
   A   
   A   
   A   
   T   
   C   
   G   
   A   
   C   
   A   
   A   
   G   
   A   
   A   
   G   
   T   
   A   
   C   
   A   
   A   
   G   
   A   
   A   
   T   
   T   
   A   
   A   
   C   
   T   
   T   
   T   
   A   
   G   
   A   
   A   
   G   
   A   
   A   
   G   
   T   
   G   
   A   
   T   
   G   
   G   
   G   
   T   
   G   
   A   
   C   
   C   
   G   
   C   
   T   
   T   
   C   
   G   
   G   
   A   
   C   
   G   
   T   
   T   
   A   
   T   
   T   
   C   
   C   
   A   
   A   
   A   
   T   
   A   
   T   
   A   
   T   
   T   
   A   
   T   
    C    
   C   
   A   
    A    
   G   
   A   
   A   
   C   
   G   
   T   
   G   
   C   
   A   
   C   
   T   
   G   
   C   
   C   
   T   
   G   
   A   
   T   
   A   
   T   
    C    
   C   
   G   
   T   
   G   
   A   
   T   
   G   
   G   
   A   
   T   
   T   
   G   
   A   
   A   
    G    
   C   
   C   
   A   
   G   
   T   
    G    
   C   
   A   
   A   
   C   
   G   
   C   
   C   
   G   
   G   
   A   
   T   
   A   
   T   
   T   
   A   
   T   
   T   
    T    
   G   
   C   
    C    
   A   
   T   
   G   
   A   
   A   
   C   
   A   
   A   
   A   
   G   
   A   
   T   
   G   
   G   
   C   
   A   
   A   
   T   
   A   
   C   
   G   
   T   
   A   
   T   
   G   
   A   
   C   
   A   
   A   
   A   
   G   
   G   
   A   
   T   
   T   
    C    
   C   
   G   
   T   
   A   
   A   
   A   
   T   
   C   
   A   
   G   
   C   
   A   
   A   
   A   
   A   
   T   
   C   
   T   
   G   
   T   
   C   
   G   
   G   
   A   
   A   
   A   
   T   
   A   
   T   
   C   
   A   
   T   
   G   
   G   
   G   
    G    
   A   
   A   
   T   
   T   
   A   
   T   
   C   
   A   
   T   
   C   
   C   
   T   
   C   
   A   
   T   
   G   
   G   
   T   
   G   
   A   
   C   
   A   
   G   
   C   
   A   
   G   
   T   
   A   
   T   
   T   
   T   
   A   
   T   
   G   
   A   
   A   
   G   
   C   
   G   
   A   
   T   
   G   
   G   
   T   
   C   
   C   
   G   
   C   
   A   
   T   
   G   
   A   
   G   
   C   
   C   
   A   
   A   
   G   
   A   
    C    
   T   
   G   
   G   
   A   
   A   
   G   
   T   
   T   
   G   
   C   
   G   
   G   
   G   
   A   
   A   
   G   
   T   
   A   
   C   
   T   
   G   
   A   
   T   
   C   
   G   
   A   
    A    
   A   
   T   
   G   
   C   
   A   
   C   
   G   
   G   
    G    
   A   
   A   
   T   
   A   
   A   
    C    
   G   
   G   
   G   
   A   
   G   
   T   
   A   
   T   
   G   
   G   
   A   
   C   
   G   
   G   
   T   
   G   
   A   
   T   
   C   
   C   
   A   
   C   
   C   
   T   
   G   
   C   
   T   
   G   
   C   
   G   
   A   
   T   
   G   
   C   
   G   
   T   
   T   
   A   
   T   
   A   
   C   
   T   
   G   
   A   
   A   
   G   
   C   
   C   
   A   
   G   
    A    
   T   
   T   
   A   
   T   
   C   
   C   
   A   
   A   
   G   
   T   
   T   
   A   
   A   
   G   
   T   
   G   
   G   
   A   
   G   
   A   
   G   
   A   
   T   
   G   
    C    
   T   
   A   
    G    
   C   
   A   
   G   
   A   
   T   
   A   
   T   
   C   
   G   
   A   
    A    
   A   
   A   
   A   
   G   
   A   
   A   
   A   
   C   
   A   
   G   
   T   
    C    
   G   
   A   
   T   
   C   
   T   
   C   
   G   
   T   
   G   
   T   
   G   
   G   
   A   
   A   
   T   
   T   
   T   
   T   
   G   
   A   
   T   
   G   
   A   
   T   
   A   
   C   
   A   
   G   
   A   
   A   
   A   
   A   
   A   
   G   
   A   
   A   
   C   
   C   
   A   
   A   
   C   
   A   
   G   
   T   
   C   
   C   
   T   
   T   
   C   
   C   
   A   
   G   
   C   
   T   
   C   
   G   
   A   
   T   
   A   
   C   
   C   
   C   
   A   
   A   
   A   
   C   
   C   
   T   
   T   
   C   
   T   
    C    
   G   
   T   
   C   
   A   
   A   
   T   
   G   
   G   
   A   
   T   
   C   
   G   
   A   
   C   
   A   
   G   
   G   
   T   
   A   
   T   
   A   
   T   
   C   
   T   
   G   
   C   
   T   
   G   
   G   
   A   
   T   
   A   
   T   
   G   
   C   
    A    
   A   
   C   
   A   
   G   
   A   
   A   
   A   
   T   
   C   
   C   
   C   
   G   
   A   
   C   
    T    
   C   
   A   
   T   
   A   
   A   
   C   
   T   
   T   
   A   
   G   
   C   
   T   
   G   
   A   
   A   
   G   
   T   
   G   
   A   
   T   
    C    
   G   
   A   
    T    
   G   
   G   
   C   
   A   
   C   
   A   
   A   
   T   
   C   
   T   
   A   
   T   
   A   
   T   
   G   
   A   
   T   
   C   
   G   
   A   
   T   
   C   
   A   
   T   
   C   
   C   
   A   
   C   
   A   
   A   
   G   
   C   
   G   
   A   
   G   
   C   
   C   
   T   
   T   
   G   
   A   
   A   
   A   
   A   
   A   
   T   
   T   
   G   
   A   
   T   
   G   
   G   
   A   
   A   
   T   
   A   
   T   
   A   
   T   
   T   
   C   
   C   
   T   
   G   
   G   
   A   
   C   
   C   
   T   
   G   
   A   
   C   
   T   
   T   
    T    
   C   
   C   
   A   
   A   
   C   
   A   
   G   
   G   
   A   
   G   
   G   
    G    
   A   
   T   
   C   
   T   
   T   
   G   
   C   
   A   
   A   
   G   
   G   
    A    
   A   
   A   
   A   
   G   
   A   
   T   
   G   
   A   
   A   
   A   
   T   
   C   
   A   
   A   
   A   
   A   
   A   
   A   
   G   
   C   
   T   
   T   
   A   
   T   
   G   
   A   
   A   
   A   
   C   
   A   
   G   
   G   
    G    
   C   
   G   
   A   
   G   
   G   
   A   
   A   
   A   
   A   
   G   
   T   
   T   
   A   
    T    
   T   
   C   
   T   
   T   
   C   
   G   
   T   
   T   
   C   
   G   
   A   
   A   
   A   
   A   
   C   
   A   
   A   
   A   
   G   
   A   
   T   
   A   
   G   
   A   
    G    
   G   
   C   
   A   
   A   
   T   
   G   
   A   
   A   
   G   
   G   
   G   
    T    
   A   
   A   
    T    
   A   
   A   
   A   
   C   
   A   
   G   
   C   
   A   
   A   
   A   
   T   
   C   
   G   
   T   
   G   
   A   
   T   
   C   
   A   
   C   
    A    
   G   
   A   
   A   
   A   
   T   
   C   
   C   
   C   
   A   
   T   
   A   
   T   
   G   
   A   
   A   
   G   
   T   
   A   
   A   
   A   
   T   
   A   
   A   
   A   
   G   
   C   
   G   
   A   
   C   
    C    
   T   
   T   
   G   
   G   
   T   
   G   
   A   
   A   
   A   
   A   
   A   
   A   
   A   
   T   
   G   
   G   
   A   
   T   
   G   
   A   
   G   
   A   
   T   
   C   
   C   
   G   
   T   
   T   
   T   
   G   
   A   
   A   
   T   
   A   
   A   
   G   
   A   
   A   
   A   
   A   
   T   
   C   
   G   
   A   
    T    
   G   
   G   
   A   
   A   
   T   
   A   
   G   
   C   
   T   
   G
[truncated: 28,136 more chars]
