# Supplementary material for: sraX: A Novel Comprehensive Resistome Analysis Tool
Source: Front Microbiol. 2020 Feb 5;11:52. doi: 10.3389/fmicb.2020.00052 (PMC7025521; doi:10.3389/fmicb.2020.00052)
Supplement: Supplementary file 4 [file Data_Sheet_3.ZIP › liaS/liaS_Efaecium_aa.html]

# liaS

|  |
| --- |
|  |

|  |
| --- |
|  |

|  |  |
| --- | --- |
| **Definition:** | LiaS is a histidine kinase found in the liaFSR signal transduction pathway. Mutations confer daptomycin resistance. |

|  |
| --- |
|  |

|  |
| --- |
|  |

|  |  |  |
| --- | --- | --- |
| **Reference organism:** | *Enterococcus faecium DO* | [NCBI, CARD] |

|  |
| --- |
|  |

|  |
| --- |
|  |

|  |  |
| --- | --- |
| **Summary Data** | || **Parameter** | **Value_(n)_** || Total number of genomes | 101 |
| MSA length | 355 |
| Total number of SNPs | 20 |
| SNP density (# SNPs/MSA length) | 0.056 |

|  |
| --- |
|  |

|  |
| --- |
|  |

|  |
| --- |
|  |

|  |
| --- |
|  |

|  |  |  |  |  |
| --- | --- | --- | --- | --- |
| **SNPs conferring antibiotic resistance** | | | | || **Reference gene** | **Seq. pos.** | **Ref\_base** | **Alt\_base** | **Confirmation SNP?** |||  |  |  |  |  |
| --- | --- | --- | --- | --- |
| liaS 120 T A **No** | | | | |
||  |  |  |  |  |
| --- | --- | --- | --- | --- |
| liaS 180 A T **No** | | | | |
||  |  |  |  |  |
| --- | --- | --- | --- | --- |
| liaS 192 E G **Yes** | | | | |
||  |  |  |  |  |
| --- | --- | --- | --- | --- |
| liaS 264 H Q **No** | | | | |

|  |
| --- |
|  |

|  |
| --- |
|  |

|  |
| --- |
|  |

|  |  |  |  |  |  |  |  |  |  |  |  |  |  |  |  |  |  |  |  |  |  |  |  |  |  |  |  |  |  |  |  |  |  |  |  |  |  |  |  |  |  |  |  |  |  |  |  |  |  |  |  |  |  |  |  |  |  |  |  |  |  |  |  |  |  |  |  |  |  |  |  |  |  |  |  |  |  |  |  |  |  |  |  |  |  |  |  |  |  |  |  |  |  |  |  |  |  |  |  |  |  |  |  |  |  |  |  |  |  |  |  |  |  |  |  |  |  |  |  |  |  |  |  |  |  |  |  |  |  |  |  |  |  |  |  |  |  |  |  |  |  |  |  |  |  |  |  |  |  |  |  |  |  |  |  |  |  |  |  |  |  |  |  |  |  |  |  |  |  |  |  |  |  |  |  |  |  |  |  |  |  |  |  |  |  |  |  |  |  |  |  |  |  |  |  |  |  |  |  |  |  |  |  |  |  |  |  |  |  |  |  |  |  |  |  |  |  |  |  |  |  |  |  |  |  |  |  |  |  |  |  |  |  |  |  |  |  |  |  |  |  |  |  |  |  |  |  |  |  |  |  |  |  |  |  |  |  |  |  |  |  |  |  |  |  |  |  |  |  |  |  |  |  |  |  |  |  |  |  |  |  |  |  |  |  |  |  |  |  |  |  |  |  |  |  |  |  |  |  |  |  |  |  |  |  |  |  |  |  |  |  |  |  |  |  |  |  |  |  |  |  |  |  |  |  |  |  |  |  |  |  |  |  |  |  |  |  |  |  |  |  |  |  |  |  |  |  |  |  |  |  |  |  |  |  |  |
| --- | --- | --- | --- | --- | --- | --- | --- | --- | --- | --- | --- | --- | --- | --- | --- | --- | --- | --- | --- | --- | --- | --- | --- | --- | --- | --- | --- | --- | --- | --- | --- | --- | --- | --- | --- | --- | --- | --- | --- | --- | --- | --- | --- | --- | --- | --- | --- | --- | --- | --- | --- | --- | --- | --- | --- | --- | --- | --- | --- | --- | --- | --- | --- | --- | --- | --- | --- | --- | --- | --- | --- | --- | --- | --- | --- | --- | --- | --- | --- | --- | --- | --- | --- | --- | --- | --- | --- | --- | --- | --- | --- | --- | --- | --- | --- | --- | --- | --- | --- | --- | --- | --- | --- | --- | --- | --- | --- | --- | --- | --- | --- | --- | --- | --- | --- | --- | --- | --- | --- | --- | --- | --- | --- | --- | --- | --- | --- | --- | --- | --- | --- | --- | --- | --- | --- | --- | --- | --- | --- | --- | --- | --- | --- | --- | --- | --- | --- | --- | --- | --- | --- | --- | --- | --- | --- | --- | --- | --- | --- | --- | --- | --- | --- | --- | --- | --- | --- | --- | --- | --- | --- | --- | --- | --- | --- | --- | --- | --- | --- | --- | --- | --- | --- | --- | --- | --- | --- | --- | --- | --- | --- | --- | --- | --- | --- | --- | --- | --- | --- | --- | --- | --- | --- | --- | --- | --- | --- | --- | --- | --- | --- | --- | --- | --- | --- | --- | --- | --- | --- | --- | --- | --- | --- | --- | --- | --- | --- | --- | --- | --- | --- | --- | --- | --- | --- | --- | --- | --- | --- | --- | --- | --- | --- | --- | --- | --- | --- | --- | --- | --- | --- | --- | --- | --- | --- | --- | --- | --- | --- | --- | --- | --- | --- | --- | --- | --- | --- | --- | --- | --- | --- | --- | --- | --- | --- | --- | --- | --- | --- | --- | --- | --- | --- | --- | --- | --- | --- | --- | --- | --- | --- | --- | --- | --- | --- | --- | --- | --- | --- | --- | --- | --- | --- | --- | --- | --- | --- | --- | --- | --- | --- | --- | --- | --- | --- | --- | --- | --- | --- | --- | --- | --- | --- | --- | --- | --- | --- | --- | --- | --- | --- | --- | --- | --- | --- | --- | --- | --- | --- | --- | --- | --- | --- | --- | --- | --- | --- | --- | --- | --- | --- | --- | --- | --- | --- | --- |
|  || **Enterococcus faecium DO (AFK58561.1)** | **M** | **M** | **G** | **K** | **I** | **S** | **R** | **A** | **M** | **L** | **A** | **V** | **Y** | **S** | **G** | **I** | **A** | **A** | **F** | **L** | **I** | **I** | **L** | **F** | **S** | **L** | **F** | **T** | **Y** | **F** | **Y** | **A** | **S** | **N** | **Q** | **S** | **H** | **W** | **W** | **G** | **E** | **L** | **L** | **R** | **A** | **R** | **L** | **L** | **Y** | **V** | **P** | **L** | **I** | **F** | **H** | **L** | **L** | **A** | **I** | **S** | **L** | **G** | **V** | **G** | **L** | **I** | **V** | **F** | **L** | **L** | **L** | **S** | **L** | **I** | **Q** | **K** | **T** | **K** | **Y** | **G** | **K** | **I** | **E** | **E** | **K** | **L** | **R** | **A** | **L** | **S** | **S** | **G** | **N** | **Y** | **E** | **S** | **K** | **L** | **L** | **L** | **L** | **P** | **I** | **P** | **S** | **A** | **S** | **D** | **D** | **L** | **Y** | **I** | **K** | **D** | **I** | **D** | **K** | **E** | **I** | **T** | **K** | **I** | **K** | **E** | **K** | **M** | **I** | **E** | **V** | **S** | **S** | **E** | **L** | **Q** | **I** | **V** | **T** | **S** | **R** | **P** | **Q** | **Y** | **V** | **D** | **G** | **Q** | **T** | **K** | **E** | **E** | **I** | **L** | **E** | **L** | **E** | **R** | **H** | **R** | **L** | **A** | **R** | **E** | **L** | **H** | **D** | **S** | **V** | **S** | **Q** | **Q** | **L** | **F** | **A** | **A** | **M** | **M** | **M** | **M** | **S** | **A** | **L** | **T** | **E** | **Q** | **A** | **E** | **K** | **S** | **E** | **T** | **P** | **E** | **M** | **F** | **R** | **K** | **Q** | **L** | **K** | **M** | **V** | **A** | **E** | **I** | **I** | **N** | **A** | **S** | **Q** | **S** | **E** | **M** | **R** | **A** | **L** | **L** | **L** | **H** | **L** | **R** | **P** | **V** | **N** | **L** | **E** | **E** | **K** | **S** | **L** | **K** | **Q** | **G** | **I** | **E** | **Q** | **L** | **L** | **K** | **E** | **L** | **Q** | **N** | **K** | **I** | **Q** | **I** | **S** | **L** | **K** | **W** | **D** | **V** | **E** | **D** | **V** | **K** | **L** | **S** | **S** | **S** | **I** | **E** | **D** | **H** | **L** | **F** | **R** | **I** | **V** | **Q** | **E** | **L** | **L** | **S** | **N** | **T** | **L** | **R** | **H** | **A** | **K** | **A** | **N** | **E** | **L** | **E** | **V** | **Y** | **L** | **H** | **K** | **I** | **D** | **N** | **N** | **L** | **L** | **L** | **R** | **I** | **I** | **D** | **D** | **G** | **T** | **G** | **F** | **N** | **M** | **N** | **E** | **T** | **K** | **T** | **G** | **S** | **Y** | **G** | **L** | **N** | **N** | **I** | **K** | **E** | **R** | **V** | **A** | **G** | **I** | **G** | **G** | **T** | **V** | **K** | **I** | **I** | **S** | **F** | **K** | **G** | **Q** | **G** | **T** | **S** | **V** | **E** | **I** | **K** | **V** | **P** | **L** | **M** | **K** | **E** | **A** |
| **N54671** | **M** | **M** | **G** | **K** | **I** | **S** | **R** | **A** | **M** | **L** | **A** | **V** | **Y** | **S** | **G** | **I** | **A** | **A** | **F** | **L** | **I** | **I** | **L** | **F** | **S** | **L** | **F** | **T** | **Y** | **F** | **Y** | **A** | **S** | **N** | **Q** | **S** | **H** | **W** | **W** | **G** | **E** | **L** | **L** | **R** | **A** | **R** | **L** | **L** | **Y** | **V** | **P** | **L** | **I** | **F** | **H** | **L** | **L** | **A** | **I** | **S** | **L** | **G** | **V** | **G** | **L** | **I** | **V** | **F** | **L** | **L** | **L** | **S** | **L** | **I** | **Q** | **K** | **T** | **K** | **Y** | **G** | **K** | **I** | **E** | **E** | **K** | **L** | **R** | **A** | **L** | **S** | **S** | **G** | **N** | **Y** | **E** | **S** | **K** | **L** | **L** | **L** | **L** | **P** | **I** | **P** | **S** | **A** | **S** | **D** | **D** | **L** | **Y** | **I** | **K** | **D** | **I** | **D** | **K** | **E** | **I** | **T** | **K** | **I** | **K** | **E** | **K** | **M** | **I** | **E** | **V** | **S** | **S** | **E** | **L** | **Q** | **I** | **V** | **T** | **S** | **R** | **P** | **Q** | **Y** | **V** | **D** | **G** | **Q** | **T** | **K** | **E** | **E** | **I** | **L** | **E** | **L** | **E** | **R** | **H** | **R** | **L** | **A** | **R** | **E** | **L** | **H** | **D** | **S** | **V** | **S** | **Q** | **Q** | **L** | **F** | **A** | **A** | **M** | **M** | **M** | **M** | **S** | **A** | **L** | **T** | **E** | **Q** | **A** | **E** | **K** | **S** | **E** | **T** | **P** | **E** | **M** | **F** | **R** | **K** | **Q** | **L** | **K** | **M** | **V** | **A** | **E** | **I** | **I** | **N** | **A** | **S** | **Q** | **S** | **E** | **M** | **R** | **A** | **L** | **L** | **L** | **H** | **L** | **R** | **P** | **V** | **N** | **L** | **E** | **E** | **K** | **S** | **L** | **K** | **Q** | **G** | **I** | **E** | **Q** | **L** | **L** | **K** | **E** | **L** | **Q** | **N** | **K** | **I** | **Q** | **I** | **S** | **L** | **K** | **W** | **D** | **V** | **E** | **D** | **V** | **K** | **L** | **S** | **S** | **S** | **I** | **E** | **D** | **H** | **L** | **F** | **R** | **I** | **V** | **Q** | **E** | **L** | **L** | **S** | **N** | **T** | **L** | **R** | **H** | **A** | **K** | **A** | **N** | **E** | **L** | **E** | **V** | **Y** | **L** | **H** | **K** | **I** | **D** | **N** | **N** | **L** | **L** | **L** | **R** | **I** | **I** | **D** | **D** | **G** | **T** | **G** | **F** | **N** | **M** | **N** | **E** | **T** | **K** | **T** | **G** | **S** | **Y** | **G** | **L** | **N** | **N** | **I** | **K** | **E** | **R** | **V** | **A** | **G** | **I** | **G** | **G** | **T** | **V** | **K** | **I** | **I** | **S** | **F** | **K** | **G** | **Q** | **G** | **T** | **S** | **V** | **E** | **I** | **K** | **V** | **P** | **L** | **M** | **K** | **E** | **A** |
| **N60299F** | **M** | **M** | **G** | **R** | **I** | **S** | **K** | **A** | **M** | **L** | **A** | **V** | **Y** | **S** | **G** | **I** | **A** | **A** | **F** | **L** | **I** | **I** | **L** | **F** | **S** | **L** | **F** | **T** | **Y** | **F** | **Y** | **A** | **S** | **N** | **Q** | **S** | **H** | **W** | **W** | **G** | **E** | **L** | **L** | **R** | **A** | **R** | **L** | **L** | **Y** | **V** | **P** | **L** | **I** | **F** | **H** | **L** | **M** | **A** | **I** | **S** | **L** | **G** | **V** | **G** | **L** | **I** | **V** | **F** | **L** | **L** | **L** | **S** | **L** | **I** | **Q** | **K** | **A** | **K** | **Y** | **G** | **K** | **I** | **E** | **E** | **K** | **L** | **R** | **A** | **L** | **S** | **S** | **G** | **N** | **Y** | **E** | **S** | **K** | **L** | **L** | **H** | **Q** | **P** | **I** | **P** | **S** | **A** | **S** | **D** | **D** | **L** | **Y** | **I** | **K** | **D** | **I** | **D** | **M** | **E** | **I** | **T** | **K** | **I** | **K** | **E** | **K** | **M** | **I** | **E** | **V** | **S** | **S** | **E** | **L** | **Q** | **I** | **M** | **T** | **S** | **R** | **P** | **Q** | **Y** | **V** | **D** | **G** | **Q** | **T** | **K** | **E** | **E** | **I** | **L** | **E** | **L** | **E** | **R** | **H** | **R** | **L** | **A** | **R** | **E** | **L** | **H** | **D** | **S** | **V** | **S** | **Q** | **Q** | **L** | **F** | **A** | **A** | **M** | **M** | **M** | **M** | **S** | **A** | **L** | **T** | **E** | **Q** | **A** | **E** | **K** | **S** | **E** | **T** | **P** | **G** | **M** | **F** | **R** | **K** | **Q** | **L** | **K** | **M** | **V** | **A** | **E** | **I** | **I** | **N** | **A** | **S** | **Q** | **S** | **E** | **M** | **R** | **A** | **L** | **L** | **L** | **H** | **L** | **R** | **P** | **V** | **N** | **L** | **E** | **E** | **K** | **S** | **L** | **K** | **Q** | **G** | **I** | **E** | **Q** | **L** | **L** | **K** | **E** | **L** | **Q** | **N** | **K** | **I** | **Q** | **I** | **S** | **L** | **K** | **W** | **D** | **V** | **E** | **D** | **V** | **K** | **L** | **T** | **S** | **S** | **I** | **E** | **D** | **H** | **L** | **F** | **R** | **I** | **V** | **Q** | **E** | **L** | **L** | **S** | **N** | **T** | **L** | **R** | **H** | **A** | **K** | **A** | **N** | **E** | **L** | **E** | **V** | **Y** | **L** | **H** | **K** | **I** | **D** | **N** | **N** | **L** | **L** | **L** | **R** | **I** | **I** | **D** | **D** | **G** | **T** | **G** | **F** | **N** | **M** | **N** | **E** | **T** | **K** | **T** | **G** | **S** | **Y** | **G** | **L** | **N** | **N** | **I** | **K** | **E** | **R** | **V** | **A** | **G** | **I** | **G** | **G** | **T** | **V** | **K** | **I** | **I** | **S** | **F** | **K** | **G** | **Q** | **G** | **T** | **S** | **V** | **E** | **I** | **K** | **V** | **P** | **L** | **M** | **K** | **E** | **A** |
| **N60363F** | **M** | **M** | **G** | **R** | **I** | **S** | **K** | **A** | **M** | **L** | **A** | **V** | **Y** | **S** | **G** | **I** | **A** | **A** | **F** | **L** | **I** | **I** | **L** | **F** | **S** | **L** | **F** | **T** | **Y** | **F** | **Y** | **A** | **S** | **N** | **Q** | **S** | **H** | **W** | **W** | **G** | **E** | **L** | **L** | **R** | **A** | **R** | **L** | **L** | **Y** | **V** | **P** | **L** | **I** | **F** | **H** | **L** | **M** | **A** | **I** | **S** | **L** | **G** | **V** | **G** | **L** | **I** | **V** | **F** | **L** | **L** | **L** | **S** | **L** | **I** | **Q** | **K** | **T** | **K** | **Y** | **G** | **K** | **I** | **E** | **E** | **K** | **L** | **R** | **A** | **L** | **S** | **S** | **G** | **N** | **Y** | **E** | **S** | **K** | **L** | **L** | **H** | **Q** | **P** | **I** | **P** | **S** | **A** | **S** | **D** | **D** | **L** | **Y** | **I** | **K** | **D** | **I** | **D** | **M** | **E** | **I** | **T** | **K** | **I** | **K** | **E** | **K** | **M** | **I** | **E** | **V** | **S** | **S** | **E** | **L** | **Q** | **I** | **M** | **T** | **S** | **R** | **P** | **Q** | **Y** | **V** | **D** | **G** | **Q** | **T** | **K** | **E** | **E** | **I** | **L** | **E** | **L** | **E** | **R** | **H** | **R** | **L** | **A** | **R** | **E** | **L** | **H** | **D** | **S** | **V** | **S** | **Q** | **Q** | **L** | **F** | **A** | **A** | **M** | **M** | **M** | **M** | **S** | **A** | **L** | **T** | **E** | **Q** | **A** | **E** | **K** | **S** | **E** | **T** | **P** | **E** | **M** | **F** | **R** | **K** | **Q** | **L** | **K** | **M** | **V** | **A** | **E** | **I** | **I** | **N** | **A** | **S** | **Q** | **S** | **E** | **M** | **R** | **A** | **L** | **L** | **L** | **H** | **L** | **R** | **P** | **V** | **N** | **L** | **E** | **E** | **K** | **S** | **L** | **K** | **Q** | **G** | **I** | **E** | **Q** | **L** | **L** | **K** | **E** | **L** | **Q** | **N** | **K** | **I** | **Q** | **I** | **S** | **L** | **K** | **W** | **D** | **V** | **E** | **D** | **V** | **K** | **L** | **T** | **S** | **S** | **I** | **E** | **D** | **H** | **L** | **F** | **R** | **I** | **V** | **Q** | **E** | **L** | **L** | **S** | **N** | **T** | **L** | **R** | **H** | **A** | **K** | **A** | **N** | **E** | **L** | **E** | **V** | **Y** | **L** | **H** | **K** | **I** | **D** | **N** | **N** | **L** | **L** | **L** | **R** | **I** | **I** | **D** | **D** | **G** | **T** | **G** | **F** | **N** | **M** | **N** | **E** | **T** | **K** | **T** | **G** | **S** | **Y** | **G** | **L** | **N** | **N** | **I** | **K** | **E** | **R** | **V** | **A** | **G** | **I** | **G** | **G** | **T** | **V** | **K** | **I** | **I** | **S** | **F** | **K** | **G** | **Q** | **G** | **T** | **S** | **V** | **E** | **I** | **K** | **V** | **P** | **L** | **M** | **K** | **E** | **A** |
| **N53548** | **M** | **M** | **G** | **R** | **I** | **S** | **K** | **A** | **M** | **L** | **A** | **V** | **Y** | **S** | **G** | **I** | **A** | **A** | **F** | **L** | **I** | **I** | **L** | **F** | **S** | **L** | **F** | **T** | **Y** | **F** | **Y** | **A** | **S** | **N** | **Q** | **S** | **H** | **W** | **W** | **G** | **E** | **L** | **L** | **R** | **A** | **R** | **L** | **L** | **Y** | **V** | **P** | **L** | **I** | **F** | **H** | **L** | **M** | **A** | **I** | **S** | **L** | **G** | **V** | **G** | **L** | **I** | **V** | **F** | **L** | **L** | **L** | **S** | **L** | **I** | **Q** | **K** | **A** | **K** | **Y** | **G** | **K** | **I** | **E** | **E** | **K** | **L** | **R** | **A** | **L** | **S** | **S** | **G** | **N** | **Y** | **E** | **S** | **K** | **L** | **L** | **H** | **Q** | **P** | **I** | **P** | **S** | **A** | **S** | **D** | **D** | **L** | **Y** | **I** | **K** | **D** | **I** | **D** | **M** | **E** | **I** | **T** | **K** | **I** | **K** | **E** | **K** | **M** | **I** | **E** | **V** | **S** | **S** | **E** | **L** | **Q** | **I** | **M** | **T** | **S** | **R** | **P** | **Q** | **Y** | **V** | **D** | **G** | **Q** | **T** | **K** | **E** | **E** | **I** | **L** | **E** | **L** | **E** | **R** | **H** | **R** | **L** | **A** | **R** | **E** | **L** | **H** | **D** | **S** | **V** | **S** | **Q** | **Q** | **L** | **F** | **A** | **A** | **M** | **M** | **M** | **M** | **S** | **A** | **L** | **T** | **E** | **Q** | **A** | **E** | **K** | **S** | **E** | **T** | **P** | **E** | **M** | **F** | **R** | **K** | **Q** | **L** | **K** | **M** | **V** | **A** | **E** | **I** | **I** | **N** | **A** | **S** | **Q** | **S** | **E** | **M** | **R** | **A** | **L** | **L** | **L** | **H** | **L** | **R** | **P** | **V** | **N** | **L** | **E** | **E** | **K** | **S** | **L** | **K** | **Q** | **G** | **I** | **E** | **Q** | **L** | **L** | **K** | **E** | **L** | **Q** | **N** | **K** | **I** | **Q** | **I** | **S** | **L** | **K** | **W** | **D** | **V** | **E** | **D** | **V** | **K** | **L** | **T** | **S** | **S** | **I** | **E** | **D** | **H** | **L** | **F** | **R** | **I** | **V** | **Q** | **E** | **L** | **L** | **S** | **N** | **T** | **L** | **R** | **H** | **A** | **K** | **A** | **N** | **E** | **L** | **E** | **V** | **Y** | **L** | **H** | **K** | **I** | **D** | **N** | **N** | **L** | **L** | **L** | **R** | **I** | **I** | **D** | **D** | **G** | **T** | **G** | **F** | **N** | **M** | **N** | **E** | **T** | **K** | **T** | **G** | **S** | **Y** | **G** | **L** | **N** | **N** | **I** | **K** | **E** | **R** | **V** | **A** | **G** | **I** | **G** | **G** | **T** | **V** | **K** | **I** | **I** | **S** | **F** | **K** | **G** | **Q** | **G** | **T** | **S** | **V** | **E** | **I** | **K** | **V** | **P** | **L** | **M** | **K** | **E** | **A** |
| **N54551** | **M** | **M** | **G** | **R** | **I** | **S** | **K** | **A** | **M** | **L** | **A** | **V** | **Y** | **S** | **G** | **I** | **A** | **A** | **F** | **L** | **I** | **I** | **L** | **F** | **S** | **L** | **F** | **T** | **Y** | **F** | **Y** | **A** | **S** | **N** | **Q** | **S** | **H** | **W** | **W** | **G** | **E** | **L** | **L** | **R** | **A** | **R** | **L** | **L** | **Y** | **V** | **P** | **L** | **I** | **F** | **H** | **L** | **M** | **A** | **I** | **S** | **L** | **G** | **V** | **G** | **L** | **I** | **V** | **F** | **L** | **L** | **L** | **S** | **L** | **I** | **Q** | **K** | **A** | **K** | **Y** | **G** | **K** | **I** | **E** | **E** | **K** | **L** | **R** | **A** | **L** | **S** | **S** | **G** | **N** | **Y** | **E** | **S** | **K** | **L** | **L** | **H** | **Q** | **P** | **I** | **P** | **S** | **A** | **S** | **D** | **D** | **L** | **Y** | **I** | **K** | **D** | **I** | **D** | **I** | **E** | **I** | **T** | **K** | **I** | **K** | **E** | **K** | **M** | **I** | **E** | **V** | **S** | **S** | **E** | **L** | **Q** | **I** | **M** | **T** | **S** | **R** | **P** | **Q** | **Y** | **V** | **D** | **G** | **Q** | **T** | **K** | **E** | **E** | **I** | **L** | **E** | **L** | **E** | **R** | **H** | **R** | **L** | **A** | **R** | **E** | **L** | **H** | **D** | **S** | **V** | **S** | **Q** | **Q** | **L** | **F** | **A** | **A** | **M** | **M** | **M** | **M** | **S** | **A** | **L** | **T** | **E** | **Q** | **A** | **E** | **K** | **S** | **E** | **T** | **P** | **E** | **M** | **F** | **R** | **K** | **Q** | **L** | **K** | **M** | **V** | **A** | **E** | **I** | **I** | **N** | **A** | **S** | **Q** | **S** | **E** | **M** | **R** | **A** | **L** | **L** | **L** | **H** | **L** | **R** | **P** | **V** | **N** | **L** | **E** | **E** | **K** | **S** | **L** | **K** | **Q** | **G** | **I** | **E** | **Q** | **L** | **L** | **K** | **E** | **L** | **Q** | **N** | **K** | **I** | **Q** | **I** | **S** | **L** | **K** | **W** | **D** | **V** | **E** | **D** | **V** | **K** | **L** | **T** | **S** | **S** | **I** | **E** | **D** | **H** | **L** | **F** | **R** | **I** | **V** | **Q** | **E** | **L** | **L** | **S** | **N** | **T** | **L** | **R** | **H** | **A** | **K** | **A** | **N** | **E** | **L** | **E** | **V** | **Y** | **L** | **H** | **K** | **I** | **D** | **N** | **N** | **L** | **L** | **L** | **R** | **I** | **I** | **D** | **D** | **G** | **T** | **G** | **F** | **N** | **M** | **N** | **E** | **T** | **K** | **T** | **G** | **S** | **Y** | **G** | **L** | **N** | **N** | **I** | **K** | **E** | **R** | **V** | **A** | **G** | **I** | **G** | **G** | **T** | **V** | **K** | **I** | **I** | **S** | **F** | **K** | **G** | **Q** | **G** | **T** | **S** | **V** | **E** | **I** | **K** | **V** | **P** | **L** | **M** | **K** | **E** | **A** |
| **N54567** | **M** | **M** | **G** | **K** | **I** | **S** | **R** | **A** | **M** | **L** | **A** | **V** | **Y** | **S** | **G** | **I** | **A** | **A** | **F** | **L** | **I** | **I** | **L** | **F** | **S** | **L** | **F** | **T** | **Y** | **F** | **Y** | **A** | **S** | **N** | **Q** | **S** | **H** | **W** | **W** | **G** | **E** | **L** | **L** | **R** | **A** | **R** | **L** | **L** | **Y** | **V** | **P** | **L** | **I** | **F** | **H** | **L** | **L** | **A** | **I** | **S** | **L** | **G** | **V** | **G** | **L** | **I** | **V** | **F** | **L** | **L** | **L** | **S** | **L** | **I** | **Q** | **K** | **T** | **K** | **Y** | **G** | **K** | **I** | **E** | **E** | **K** | **L** | **R** | **A** | **L** | **S** | **S** | **G** | **N** | **Y** | **E** | **S** | **K** | **L** | **L** | **L** | **L** | **P** | **I** | **P** | **S** | **A** | **S** | **D** | **D** | **L** | **Y** | **I** | **K** | **D** | **I** | **D** | **K** | **E** | **I** | **T** | **K** | **I** | **K** | **E** | **K** | **M** | **I** | **E** | **V** | **S** | **S** | **E** | **L** | **Q** | **I** | **V** | **T** | **S** | **R** | **P** | **Q** | **Y** | **V** | **D** | **G** | **Q** | **T** | **K** | **E** | **E** | **I** | **L** | **E** | **L** | **E** | **R** | **H** | **R** | **L** | **A** | **R** | **E** | **L** | **H** | **D** | **S** | **V** | **S** | **Q** | **Q** | **L** | **F** | **A** | **A** | **M** | **M** | **M** | **M** | **S** | **A** | **L** | **T** | **E** | **Q** | **A** | **E** | **K** | **S** | **E** | **T** | **P** | **E** | **M** | **F** | **R** | **K** | **Q** | **L** | **K** | **M** | **V** | **A** | **E** | **I** | **I** | **N** | **A** | **S** | **Q** | **S** | **E** | **M** | **R** | **A** | **L** | **L** | **L** | **H** | **L** | **R** | **P** | **V** | **N** | **L** | **E** | **E** | **K** | **S** | **L** | **K** | **Q** | **G** | **I** | **E** | **Q** | **L** | **L** | **K** | **E** | **L** | **Q** | **N** | **K** | **I** | **Q** | **I** | **S** | **L** | **K** | **W** | **D** | **V** | **E** | **D** | **V** | **K** | **L** | **S** | **S** | **S** | **I** | **E** | **D** | **H** | **L** | **F** | **R** | **I** | **V** | **Q** | **E** | **L** | **L** | **S** | **N** | **T** | **L** | **R** | **H** | **A** | **K** | **A** | **N** | **E** | **L** | **E** | **V** | **Y** | **L** | **H** | **K** | **I** | **D** | **N** | **N** | **L** | **L** | **L** | **R** | **I** | **I** | **D** | **D** | **G** | **T** | **G** | **F** | **N** | **M** | **N** | **E** | **T** | **K** | **T** | **G** | **S** | **Y** | **G** | **L** | **N** | **N** | **I** | **K** | **E** | **R** | **V** | **A** | **G** | **I** | **G** | **G** | **T** | **V** | **K** | **I** | **I** | **S** | **F** | **K** | **G** | **Q** | **G** | **T** | **S** | **V** | **E** | **I** | **K** | **V** | **P** | **L** | **M** | **K** | **E** | **A** |
| **N54035** | **M** | **M** | **G** | **R** | **I** | **S** | **K** | **A** | **M** | **L** | **A** | **V** | **Y** | **S** | **G** | **I** | **A** | **A** | **F** | **L** | **I** | **I** | **L** | **F** | **S** | **L** | **F** | **T** | **Y** | **F** | **Y** | **A** | **S** | **N** | **Q** | **S** | **H** | **W** | **W** | **G** | **E** | **L** | **L** | **R** | **A** | **R** | **L** | **L** | **Y** | **V** | **P** | **L** | **I** | **F** | **H** | **L** | **M** | **A** | **I** | **S** | **L** | **G** | **V** | **G** | **L** | **I** | **V** | **F** | **L** | **L** | **L** | **S** | **L** | **I** | **Q** | **K** | **A** | **K** | **Y** | **G** | **K** | **I** | **E** | **E** | **K** | **L** | **R** | **A** | **L** | **S** | **S** | **G** | **N** | **Y** | **E** | **S** | **K** | **L** | **L** | **H** | **Q** | **P** | **I** | **P** | **S** | **T** | **S** | **D** | **D** | **L** | **Y** | **I** | **K** | **D** | **I** | **D** | **M** | **E** | **I** | **T** | **K** | **I** | **K** | **E** | **K** | **M** | **I** | **E** | **V** | **S** | **S** | **E** | **L** | **Q** | **I** | **M** | **T** | **S** | **R** | **P** | **Q** | **Y** | **V** | **D** | **G** | **Q** | **T** | **K** | **E** | **E** | **I** | **L** | **E** | **L** | **E** | **R** | **H** | **R** | **L** | **A** | **R** | **E** | **L** | **H** | **D** | **S** | **V** | **S** | **Q** | **Q** | **L** | **F** | **A** | **A** | **M** | **M** | **M** | **M** | **S** | **A** | **L** | **T** | **E** | **Q** | **A** | **E** | **K** | **S** | **E** | **T** | **P** | **E** | **M** | **F** | **R** | **K** | **Q** | **L** | **K** | **M** | **V** | **A** | **E** | **I** | **I** | **N** | **A** | **S** | **Q** | **S** | **E** | **M** | **R** | **A** | **L** | **L** | **L** | **H** | **L** | **R** | **P** | **V** | **N** | **L** | **E** | **E** | **K** | **S** | **L** | **K** | **Q** | **G** | **I** | **E** | **Q** | **L** | **L** | **K** | **E** | **L** | **Q** | **N** | **K** | **I** | **Q** | **I** | **S** | **L** | **K** | **W** | **D** | **V** | **E** | **D** | **V** | **K** | **L** | **T** | **S** | **S** | **I** | **E** | **D** | **H** | **L** | **F** | **R** | **I** | **V** | **Q** | **E** | **L** | **L** | **S** | **N** | **T** | **L** | **R** | **H** | **A** | **K** | **A** | **N** | **E** | **L** | **E** | **V** | **Y** | **L** | **H** | **K** | **I** | **D** | **N** | **N** | **L** | **L** | **L** | **R** | **I** | **I** | **D** | **D** | **G** | **T** | **G** | **F** | **N** | **M** | **N** | **E** | **T** | **K** | **T** | **G** | **S** | **Y** | **G** | **L** | **N** | **N** | **I** | **K** | **E** | **R** | **V** | **A** | **G** | **I** | **G** | **G** | **T** | **V** | **K** | **I** | **I** | **S** | **F** | **K** | **G** | **Q** | **G** | **T** | **S** | **V** | **E** | **I** | **K** | **V** | **P** | **L** | **M** | **K** | **E** | **A** |
| **N54107** | **M** | **M** | **G** | **R** | **I** | **S** | **K** | **A** | **M** | **L** | **A** | **V** | **Y** | **S** | **G** | **I** | **A** | **A** | **F** | **L** | **I** | **I** | **L** | **F** | **S** | **L** | **F** | **T** | **Y** | **F** | **Y** | **A** | **S** | **N** | **Q** | **S** | **H** | **W** | **W** | **G** | **E** | **L** | **L** | **R** | **A** | **R** | **L** | **L** | **Y** | **V** | **P** | **L** | **I** | **F** | **H** | **L** | **M** | **A** | **I** | **S** | **L** | **G** | **V** | **G** | **L** | **I** | **V** | **F** | **L** | **L** | **L** | **S** | **L** | **I** | **Q** | **K** | **A** | **K** | **Y** | **G** | **K** | **I** | **E** | **E** | **K** | **L** | **R** | **A** | **L** | **S** | **S** | **G** | **N** | **Y** | **E** | **S** | **K** | **L** | **L** | **H** | **Q** | **P** | **I** | **P** | **S** | **A** | **S** | **D** | **D** | **L** | **Y** | **I** | **K** | **D** | **I** | **D** | **M** | **E** | **I** | **T** | **K** | **I** | **K** | **E** | **K** | **M** | **I** | **E** | **V** | **S** | **S** | **E** | **L** | **Q** | **I** | **M** | **T** | **S** | **R** | **P** | **Q** | **Y** | **V** | **D** | **G** | **Q** | **T** | **K** | **E** | **E** | **I** | **L** | **E** | **L** | **E** | **R** | **H** | **R** | **L** | **A** | **R** | **E** | **L** | **H** | **D** | **S** | **V** | **S** | **Q** | **Q** | **L** | **F** | **A** | **A** | **M** | **M** | **M** | **M** | **S** | **A** | **L** | **T** | **E** | **Q** | **A** | **E** | **K** | **S** | **E** | **T** | **P** | **E** | **M** | **F** | **R** | **K** | **Q** | **L** | **K** | **M** | **V** | **A** | **E** | **I** | **I** | **N** | **A** | **S** | **Q** | **S** | **E** | **M** | **R** | **A** | **L** | **L** | **L** | **H** | **L** | **R** | **P** | **V** | **N** | **L** | **E** | **E** | **K** | **S** | **L** | **K** | **Q** | **G** | **I** | **E** | **Q** | **L** | **L** | **K** | **E** | **L** | **Q** | **N** | **K** | **I** | **Q** | **I** | **S** | **L** | **K** | **W** | **D** | **V** | **E** | **D** | **V** | **K** | **L** | **T** | **S** | **S** | **I** | **E** | **D** | **H** | **L** | **F** | **R** | **I** | **V** | **Q** | **E** | **L** | **L** | **S** | **N** | **T** | **L** | **R** | **H** | **A** | **K** | **A** | **N** | **E** | **L** | **E** | **V** | **Y** | **L** | **H** | **K** | **I** | **D** | **N** | **N** | **L** | **L** | **L** | **R** | **I** | **I** | **D** | **D** | **G** | **T** | **G** | **F** | **N** | **M** | **N** | **E** | **T** | **K** | **T** | **G** | **S** | **Y** | **G** | **L** | **N** | **N** | **I** | **K** | **E** | **R** | **V** | **A** | **G** | **I** | **G** | **G** | **T** | **V** | **K** | **I** | **I** | **S** | **F** | **K** | **G** | **Q** | **G** | **T** | **S** | **V** | **E** | **I** | **K** | **V** | **P** | **L** | **M** | **K** | **E** | **A** |
| **N59559F** | **M** | **M** | **G** | **K** | **I** | **S** | **R** | **A** | **M** | **L** | **A** | **V** | **Y** | **S** | **G** | **I** | **A** | **A** | **F** | **L** | **I** | **I** | **L** | **F** | **S** | **L** | **F** | **T** | **Y** | **F** | **Y** | **A** | **S** | **N** | **Q** | **S** | **H** | **W** | **W** | **G** | **E** | **L** | **L** | **R** | **A** | **R** | **L** | **L** | **Y** | **V** | **P** | **L** | **I** | **F** | **H** | **L** | **L** | **A** | **I** | **S** | **L** | **G** | **V** | **G** | **L** | **I** | **V** | **F** | **L** | **L** | **L** | **S** | **L** | **I** | **Q** | **K** | **T** | **K** | **Y** | **G** | **K** | **I** | **E** | **E** | **K** | **L** | **R** | **A** | **L** | **S** | **S** | **G** | **N** | **Y** | **E** | **S** | **K** | **L** | **L** | **L** | **L** | **P** | **I** | **P** | **S** | **A** | **S** | **D** | **D** | **L** | **Y** | **I** | **K** | **D** | **I** | **D** | **K** | **E** | **I** | **T** | **K** | **I** | **K** | **E** | **K** | **M** | **I** | **E** | **V** | **S** | **S** | **E** | **L** | **Q** | **I** | **V** | **T** | **S** | **R** | **P** | **Q** | **Y** | **V** | **D** | **G** | **Q** | **T** | **K** | **E** | **E** | **I** | **L** | **E** | **L** | **E** | **R** | **H** | **R** | **L** | **A** | **R** | **E** | **L** | **H** | **D** | **S** | **V** | **S** | **Q** | **Q** | **L** | **F** | **A** | **A** | **M** | **M** | **M** | **M** | **S** | **A** | **L** | **T** | **E** | **Q** | **A** | **E** | **K** | **S** | **E** | **T** | **P** | **E** | **M** | **F** | **R** | **K** | **Q** | **L** | **K** | **M** | **V** | **A** | **E** | **I** | **I** | **N** | **A** | **S** | **Q** | **S** | **E** | **M** | **R** | **A** | **L** | **L** | **L** | **H** | **L** | **R** | **P** | **V** | **N** | **L** | **E** | **E** | **K** | **S** | **L** | **K** | **Q** | **G** | **I** | **E** | **Q** | **L** | **L** | **K** | **E** | **L** | **Q** | **N** | **K** | **I** | **Q** | **I** | **S** | **L** | **K** | **W** | **D** | **V** | **E** | **D** | **V** | **K** | **L** | **S** | **S** | **S** | **I** | **E** | **D** | **H** | **L** | **F** | **R** | **I** | **V** | **Q** | **E** | **L** | **L** | **S** | **N** | **T** | **L** | **R** | **H** | **A** | **K** | **A** | **N** | **E** | **L** | **E** | **V** | **Y** | **L** | **H** | **K** | **I** | **D** | **N** | **N** | **L** | **L** | **L** | **R** | **I** | **I** | **D** | **D** | **G** | **T** | **G** | **F** | **N** | **M** | **N** | **E** | **T** | **K** | **T** | **G** | **S** | **Y** | **G** | **L** | **N** | **N** | **I** | **K** | **E** | **R** | **V** | **A** | **G** | **I** | **G** | **G** | **T** | **V** | **K** | **I** | **I** | **S** | **F** | **K** | **G** | **Q** | **G** | **T** | **S** | **V** | **E** | **I** | **K** | **V** | **P** | **L** | **M** | **K** | **E** | **A** |
| **N59567F** | **M** | **M** | **G** | **R** | **I** | **S** | **K** | **A** | **M** | **L** | **A** | **V** | **Y** | **S** | **G** | **I** | **A** | **A** | **F** | **L** | **I** | **I** | **L** | **F** | **S** | **L** | **F** | **T** | **Y** | **F** | **Y** | **A** | **S** | **N** | **Q** | **S** | **H** | **W** | **W** | **G** | **E** | **L** | **L** | **R** | **A** | **R** | **L** | **L** | **Y** | **V** | **P** | **L** | **I** | **F** | **H** | **L** | **M** | **A** | **I** | **S** | **L** | **G** | **V** | **G** | **L** | **I** | **V** | **F** | **L** | **L** | **L** | **S** | **L** | **I** | **Q** | **K** | **T** | **K** | **Y** | **G** | **K** | **I** | **E** | **E** | **K** | **L** | **R** | **A** | **L** | **S** | **S** | **G** | **N** | **Y** | **E** | **S** | **K** | **L** | **L** | **H** | **Q** | **P** | **I** | **P** | **S** | **A** | **S** | **D** | **D** | **L** | **Y** | **I** | **K** | **D** | **I** | **D** | **M** | **E** | **I** | **T** | **K** | **I** | **K** | **E** | **K** | **M** | **I** | **E** | **V** | **S** | **S** | **E** | **L** | **Q** | **I** | **M** | **T** | **S** | **R** | **P** | **Q** | **Y** | **V** | **D** | **G** | **Q** | **T** | **K** | **E** | **E** | **I** | **L** | **E** | **L** | **E** | **R** | **H** | **R** | **L** | **A** | **R** | **E** | **L** | **H** | **D** | **S** | **V** | **S** | **Q** | **Q** | **L** | **F** | **A** | **A** | **M** | **M** | **M** | **M** | **S** | **A** | **L** | **T** | **E** | **Q** | **A** | **E** | **K** | **S** | **E** | **T** | **P** | **E** | **M** | **F** | **R** | **K** | **Q** | **L** | **K** | **M** | **V** | **A** | **E** | **I** | **I** | **N** | **A** | **S** | **Q** | **S** | **E** | **M** | **R** | **A** | **L** | **L** | **L** | **H** | **L** | **R** | **P** | **V** | **N** | **L** | **E** | **E** | **K** | **S** | **L** | **K** | **Q** | **G** | **I** | **E** | **Q** | **L** | **L** | **K** | **E** | **L** | **Q** | **N** | **K** | **I** | **Q** | **I** | **S** | **L** | **K** | **W** | **D** | **V** | **E** | **D** | **V** | **K** | **L** | **T** | **S** | **S** | **I** | **E** | **D** | **H** | **L** | **F** | **R** | **I** | **V** | **Q** | **E** | **L** | **L** | **S** | **N** | **T** | **L** | **R** | **H** | **A** | **K** | **A** | **N** | **E** | **L** | **E** | **V** | **Y** | **L** | **H** | **K** | **I** | **D** | **N** | **N** | **L** | **L** | **L** | **R** | **I** | **I** | **D** | **D** | **G** | **T** | **G** | **F** | **N** | **M** | **N** | **E** | **T** | **K** | **T** | **G** | **S** | **Y** | **G** | **L** | **N** | **N** | **I** | **K** | **E** | **R** | **V** | **A** | **G** | **I** | **G** | **G** | **T** | **V** | **K** | **I** | **I** | **S** | **F** | **K** | **G** | **Q** | **G** | **T** | **S** | **V** | **E** | **I** | **K** | **V** | **P** | **L** | **M** | **K** | **E** | **A** |
| **N52629** | **M** | **M** | **G** | **R** | **I** | **S** | **K** | **A** | **M** | **L** | **A** | **V** | **Y** | **S** | **G** | **I** | **A** | **A** | **F** | **L** | **I** | **I** | **L** | **F** | **S** | **L** | **F** | **T** | **Y** | **F** | **Y** | **A** | **S** | **N** | **Q** | **S** | **H** | **W** | **W** | **G** | **E** | **L** | **L** | **R** | **A** | **R** | **L** | **L** | **Y** | **V** | **P** | **L** | **I** | **F** | **H** | **L** | **M** | **A** | **I** | **S** | **L** | **G** | **V** | **G** | **L** | **I** | **V** | **F** | **L** | **L** | **L** | **S** | **L** | **I** | **Q** | **K** | **A** | **K** | **Y** | **G** | **K** | **I** | **E** | **E** | **K** | **L** | **R** | **A** | **L** | **S** | **S** | **G** | **N** | **Y** | **E** | **S** | **K** | **L** | **L** | **H** | **Q** | **P** | **I** | **P** | **S** | **A** | **S** | **D** | **D** | **L** | **Y** | **I** | **K** | **D** | **I** | **D** | **M** | **E** | **I** | **T** | **K** | **I** | **K** | **E** | **K** | **M** | **I** | **E** | **V** | **S** | **S** | **E** | **L** | **Q** | **I** | **M** | **T** | **S** | **R** | **P** | **Q** | **Y** | **V** | **D** | **G** | **Q** | **T** | **K** | **E** | **E** | **I** | **L** | **E** | **L** | **E** | **R** | **H** | **R** | **L** | **A** | **R** | **E** | **L** | **H** | **D** | **S** | **V** | **S** | **Q** | **Q** | **L** | **F** | **A** | **A** | **M** | **M** | **M** | **M** | **S** | **A** | **L** | **T** | **E** | **Q** | **A** | **E** | **K** | **S** | **E** | **T** | **P** | **E** | **M** | **F** | **R** | **K** | **Q** | **L** | **K** | **M** | **V** | **A** | **E** | **I** | **I** | **N** | **A** | **S** | **Q** | **S** | **E** | **M** | **R** | **A** | **L** | **L** | **L** | **H** | **L** | **R** | **P** | **V** | **N** | **L** | **E** | **E** | **K** | **S** | **L** | **K** | **Q** | **G** | **I** | **E** | **Q** | **L** | **L** | **K** | **E** | **L** | **Q** | **N** | **K** | **I** | **Q** | **I** | **S** | **L** | **K** | **W** | **D** | **V** | **E** | **D** | **V** | **K** | **L** | **T** | **S** | **S** | **I** | **E** | **D** | **H** | **L** | **F** | **R** | **I** | **V** | **Q** | **E** | **L** | **L** | **S** | **N** | **T** | **L** | **R** | **H** | **A** | **K** | **A** | **N** | **E** | **L** | **E** | **V** | **Y** | **L** | **H** | **K** | **I** | **D** | **N** | **N** | **L** | **L** | **L** | **R** | **I** | **I** | **D** | **D** | **G** | **T** | **G** | **F** | **N** | **M** | **N** | **E** | **T** | **K** | **T** | **G** | **S** | **Y** | **G** | **L** | **N** | **N** | **I** | **K** | **E** | **R** | **V** | **A** | **G** | **I** | **G** | **G** | **T** | **V** | **K** | **I** | **I** | **S** | **F** | **K** | **G** | **Q** | **G** | **T** | **S** | **V** | **E** | **I** | **K** | **V** | **P** | **L** | **M** | **K** | **E** | **A** |
| **N59907F** | **M** | **M** | **G** | **R** | **I** | **S** | **K** | **A** | **M** | **L** | **A** | **V** | **Y** | **S** | **G** | **I** | **A** | **A** | **F** | **L** | **I** | **I** | **L** | **F** | **S** | **L** | **F** | **T** | **Y** | **F** | **Y** | **A** | **S** | **N** | **Q** | **S** | **H** | **W** | **W** | **G** | **E** | **L** | **L** | **R** | **A** | **R** | **L** | **L** | **Y** | **V** | **P** | **L** | **I** | **F** | **H** | **L** | **M** | **A** | **I** | **S** | **L** | **G** | **V** | **G** | **L** | **I** | **V** | **F** | **L** | **L** | **L** | **S** | **L** | **I** | **Q** | **K** | **A** | **K** | **Y** | **G** | **K** | **I** | **E** | **E** | **K** | **L** | **R** | **A** | **L** | **S** | **S** | **G** | **N** | **Y** | **E** | **S** | **K** | **L** | **L** | **H** | **Q** | **P** | **I** | **P** | **S** | **T** | **S** | **D** | **D** | **L** | **Y** | **I** | **K** | **D** | **I** | **D** | **M** | **E** | **I** | **T** | **K** | **I** | **K** | **E** | **K** | **M** | **I** | **E** | **V** | **S** | **S** | **E** | **L** | **Q** | **I** | **M** | **T** | **S** | **R** | **P** | **Q** | **Y** | **V** | **D** | **G** | **Q** | **T** | **K** | **E** | **E** | **I** | **L** | **E** | **L** | **E** | **R** | **H** | **R** | **L** | **A** | **R** | **E** | **L** | **H** | **D** | **S** | **V** | **S** | **Q** | **Q** | **L** | **F** | **A** | **A** | **M** | **M** | **M** | **M** | **S** | **A** | **L** | **T** | **E** | **Q** | **A** | **E** | **K** | **S** | **E** | **T** | **P** | **E** | **M** | **F** | **R** | **K** | **Q** | **L** | **K** | **M** | **V** | **A** | **E** | **I** | **I** | **N** | **A** | **S** | **Q** | **S** | **E** | **M** | **R** | **A** | **L** | **L** | **L** | **H** | **L** | **R** | **P** | **V** | **N** | **L** | **E** | **E** | **K** | **S** | **L** | **K** | **Q** | **G** | **I** | **E** | **Q** | **L** | **L** | **K** | **E** | **L** | **Q** | **N** | **K** | **I** | **Q** | **I** | **S** | **L** | **K** | **W** | **N** | **V** | **E** | **D** | **V** | **K** | **L** | **T** | **S** | **S** | **I** | **E** | **D** | **H** | **L** | **F** | **R** | **I** | **V** | **Q** | **E** | **L** | **L** | **S** | **N** | **T** | **L** | **R** | **H** | **A** | **K** | **A** | **N** | **E** | **L** | **E** | **V** | **Y** | **L** | **H** | **K** | **I** | **D** | **N** | **N** | **L** | **L** | **L** | **R** | **I** | **I** | **D** | **D** | **G** | **T** | **G** | **F** | **N** | **M** | **N** | **E** | **T** | **K** | **T** | **G** | **S** | **Y** | **G** | **L** | **N** | **N** | **I** | **K** | **E** | **R** | **V** | **A** | **G** | **I** | **G** | **G** | **T** | **V** | **K** | **I** | **I** | **S** | **F** | **K** | **G** | **Q** | **G** | **T** | **S** | **V** | **E** | **I** | **K** | **V** | **P** | **L** | **M** | **K** | **E** | **A** |
| **N60048F** | **M** | **M** | **G** | **R** | **I** | **S** | **K** | **A** | **M** | **L** | **A** | **V** | **Y** | **S** | **G** | **I** | **A** | **A** | **F** | **L** | **I** | **I** | **L** | **F** | **S** | **L** | **F** | **T** | **Y** | **F** | **Y** | **A** | **S** | **N** | **Q** | **S** | **H** | **W** | **W** | **G** | **E** | **L** | **L** | **R** | **A** | **R** | **L** | **L** | **Y** | **V** | **P** | **L** | **I** | **F** | **H** | **L** | **M** | **A** | **I** | **S** | **L** | **G** | **V** | **G** | **L** | **I** | **V** | **F** | **L** | **L** | **L** | **S** | **L** | **I** | **Q** | **K** | **A** | **K** | **Y** | **G** | **K** | **I** | **E** | **E** | **K** | **L** | **R** | **A** | **L** | **S** | **S** | **G** | **N** | **Y** | **E** | **S** | **K** | **L** | **L** | **H** | **Q** | **P** | **I** | **P** | **S** | **T** | **S** | **D** | **D** | **L** | **Y** | **I** | **K** | **D** | **I** | **D** | **M** | **E** | **I** | **T** | **K** | **I** | **K** | **E** | **K** | **M** | **I** | **E** | **V** | **S** | **S** | **E** | **L** | **Q** | **I** | **M** | **T** | **S** | **R** | **P** | **Q** | **Y** | **V** | **D** | **G** | **Q** | **T** | **K** | **E** | **E** | **I** | **L** | **E** | **L** | **E** | **R** | **H** | **R** | **L** | **A** | **R** | **E** | **L** | **H** | **D** | **S** | **V** | **S** | **Q** | **Q** | **L** | **F** | **A** | **A** | **M** | **M** | **M** | **M** | **S** | **A** | **L** | **T** | **E** | **Q** | **A** | **E** | **K** | **S** | **E** | **T** | **P** | **E** | **M** | **F** | **R** | **K** | **Q** | **L** | **K** | **M** | **V** | **A** | **E** | **I** | **I** | **N** | **A** | **S** | **Q** | **S** | **E** | **M** | **R** | **A** | **L** | **L** | **L** | **H** | **L** | **R** | **P** | **V** | **N** | **L** | **E** | **E** | **K** | **S** | **L** | **K** | **Q** | **G** | **I** | **E** | **Q** | **L** | **L** | **K** | **E** | **L** | **Q** | **N** | **K** | **I** | **Q** | **I** | **S** | **L** | **K** | **W** | **D** | **V** | **E** | **D** | **V** | **K** | **L** | **T** | **S** | **S** | **I** | **E** | **D** | **H** | **L** | **F** | **R** | **I** | **V** | **Q** | **E** | **L** | **L** | **S** | **N** | **T** | **L** | **R** | **H** | **A** | **K** | **A** | **N** | **E** | **L** | **E** | **V** | **Y** | **L** | **H** | **K** | **I** | **D** | **N** | **N** | **L** | **L** | **L** | **R** | **I** | **I** | **D** | **D** | **G** | **T** | **G** | **F** | **N** | **M** | **N** | **E** | **T** | **K** | **T** | **G** | **S** | **Y** | **G** | **L** | **N** | **N** | **I** | **K** | **E** | **R** | **V** | **A** | **G** | **I** | **G** | **G** | **T** | **V** | **K** | **I** | **I** | **S** | **F** | **K** | **G** | **Q** | **G** | **T** | **S** | **V** | **E** | **I** | **K** | **V** | **P** | **L** | **M** | **K** | **E** | **A** |
| **N60417F** | **M** | **M** | **G** | **K** | **I** | **S** | **R** | **A** | **M** | **L** | **A** | **V** | **Y** | **S** | **G** | **I** | **A** | **A** | **F** | **L** | **I** | **I** | **L** | **F** | **S** | **L** | **F** | **T** | **Y** | **F** | **Y** | **A** | **S** | **N** | **Q** | **S** | **H** | **W** | **W** | **G** | **E** | **L** | **L** | **R** | **A** | **R** | **L** | **L** | **Y** | **V** | **P** | **L** | **I** | **F** | **H** | **L** | **L** | **A** | **I** | **S** | **L** | **G** | **V** | **G** | **L** | **I** | **V** | **F** | **L** | **L** | **L** | **S** | **L** | **I** | **Q** | **K** | **T** | **K** | **Y** | **G** | **K** | **I** | **E** | **E** | **K** | **L** | **R** | **A** | **L** | **S** | **S** | **G** | **N** | **Y** | **E** | **S** | **K** | **L** | **L** | **L** | **L** | **P** | **I** | **P** | **S** | **A** | **S** | **D** | **D** | **L** | **Y** | **I** | **K** | **D** | **I** | **D** | **K** | **E** | **I** | **T** | **K** | **I** | **K** | **E** | **K** | **M** | **I** | **E** | **V** | **S** | **S** | **E** | **L** | **Q** | **I** | **V** | **T** | **S** | **R** | **P** | **Q** | **Y** | **V** | **D** | **G** | **Q** | **T** | **K** | **E** | **E** | **I** | **L** | **E** | **L** | **E** | **R** | **H** | **R** | **L** | **A** | **R** | **E** | **L** | **H** | **D** | **S** | **V** | **S** | **Q** | **Q** | **L** | **F** | **A** | **A** | **M** | **M** | **M** | **M** | **S** | **A** | **L** | **T** | **E** | **Q** | **A** | **E** | **K** | **S** | **E** | **T** | **P** | **E** | **M** | **F** | **R** | **K** | **Q** | **L** | **K** | **M** | **V** | **A** | **E** | **I** | **I** | **N** | **A** | **S** | **Q** | **S** | **E** | **M** | **R** | **A** | **L** | **L** | **L** | **H** | **L** | **R** | **P** | **V** | **N** | **L** | **E** | **E** | **K** | **S** | **L** | **K** | **Q** | **G** | **I** | **E** | **Q** | **L** | **L** | **K** | **E** | **L** | **Q** | **N** | **K** | **I** | **Q** | **I** | **S** | **L** | **K** | **W** | **D** | **V** | **E** | **D** | **V** | **K** | **L** | **S** | **S** | **S** | **I** | **E** | **D** | **H** | **L** | **F** | **R** | **I** | **V** | **Q** | **E** | **L** | **L** | **S** | **N** | **T** | **L** | **R** | **H** | **A** | **K** | **A** | **N** | **E** | **L** | **E** | **V** | **Y** | **L** | **H** | **K** | **I** | **D** | **N** | **N** | **L** | **L** | **L** | **R** | **I** | **I** | **D** | **D** | **G** | **T** | **G** | **F** | **N** | **M** | **N** | **E** | **T** | **K** | **T** | **G** | **S** | **Y** | **G** | **L** | **N** | **N** | **I** | **K** | **E** | **R** | **V** | **A** | **G** | **I** | **G** | **G** | **T** | **V** | **K** | **I** | **I** | **S** | **F** | **K** | **G** | **Q** | **G** | **T** | **S** | **V** | **E** | **I** | **K** | **V** | **P** | **L** | **M** | **K** | **E** | **A** |
| **N55337** | **M** | **M** | **G** | **R** | **I** | **S** | **K** | **A** | **M** | **L** | **A** | **V** | **Y** | **S** | **G** | **I** | **A** | **A** | **F** | **L** | **I** | **I** | **L** | **F** | **S** | **L** | **F** | **T** | **Y** | **F** | **Y** | **A** | **S** | **N** | **Q** | **R** | **H** | **W** | **W** | **G** | **E** | **L** | **L** | **R** | **A** | **R** | **L** | **L** | **Y** | **V** | **P** | **L** | **I** | **F** | **H** | **L** | **M** | **A** | **I** | **S** | **L** | **G** | **V** | **G** | **L** | **I** | **V** | **F** | **L** | **L** | **L** | **S** | **L** | **I** | **Q** | **K** | **A** | **K** | **Y** | **G** | **K** | **I** | **E** | **E** | **K** | **L** | **R** | **A** | **L** | **S** | **S** | **G** | **N** | **Y** | **E** | **S** | **K** | **L** | **L** | **H** | **Q** | **P** | **I** | **P** | **S** | **A** | **S** | **D** | **D** | **L** | **Y** | **I** | **K** | **D** | **I** | **D** | **M** | **E** | **I** | **T** | **K** | **I** | **K** | **E** | **K** | **M** | **I** | **E** | **V** | **S** | **S** | **E** | **L** | **Q** | **I** | **M** | **T** | **S** | **R** | **P** | **Q** | **Y** | **V** | **D** | **G** | **Q** | **T** | **K** | **E** | **E** | **I** | **L** | **E** | **L** | **E** | **R** | **H** | **R** | **L** | **A** | **R** | **E** | **L** | **H** | **D** | **S** | **V** | **S** | **Q** | **Q** | **L** | **F** | **A** | **A** | **M** | **M** | **M** | **M** | **S** | **A** | **L** | **T** | **E** | **Q** | **A** | **E** | **K** | **S** | **E** | **T** | **P** | **E** | **M** | **F** | **R** | **K** | **Q** | **L** | **K** | **M** | **V** | **A** | **E** | **I** | **I** | **N** | **A** | **S** | **Q** | **S** | **E** | **M** | **R** | **A** | **L** | **L** | **L** | **H** | **L** | **R** | **P** | **V** | **N** | **L** | **E** | **E** | **K** | **S** | **L** | **K** | **Q** | **G** | **I** | **E** | **Q** | **L** | **L** | **K** | **E** | **L** | **Q** | **N** | **K** | **I** | **Q** | **I** | **S** | **L** | **K** | **W** | **D** | **V** | **E** | **D** | **V** | **K** | **L** | **T** | **S** | **S** | **I** | **E** | **D** | **H** | **L** | **F** | **R** | **I** | **V** | **Q** | **E** | **L** | **L** | **S** | **N** | **T** | **L** | **R** | **H** | **A** | **K** | **A** | **N** | **E** | **L** | **E** | **V** | **Y** | **L** | **H** | **K** | **I** | **D** | **N** | **N** | **L** | **L** | **L** | **R** | **I** | **I** | **D** | **D** | **G** | **T** | **G** | **F** | **N** | **M** | **N** | **E** | **T** | **K** | **T** | **G** | **S** | **Y** | **G** | **L** | **N** | **N** | **I** | **K** | **E** | **R** | **V** | **A** | **G** | **I** | **G** | **G** | **T** | **V** | **K** | **I** | **I** | **S** | **F** | **K** | **G** | **Q** | **G** | **T** | **S** | **V** | **E** | **I** | **K** | **V** | **P** | **L** | **M** | **K** | **E** | **A** |
| **N59435F** | **M** | **M** | **G** | **R** | **I** | **S** | **K** | **A** | **M** | **L** | **A** | **V** | **Y** | **S** | **G** | **I** | **A** | **A** | **F** | **L** | **I** | **I** | **L** | **F** | **S** | **L** | **F** | **T** | **Y** | **F** | **Y** | **A** | **S** | **N** | **Q** | **S** | **H** | **W** | **W** | **G** | **E** | **L** | **L** | **R** | **A** | **R** | **L** | **L** | **Y** | **V** | **P** | **L** | **I** | **F** | **H** | **L** | **M** | **A** | **I** | **S** | **L** | **G** | **V** | **G** | **L** | **I** | **V** | **F** | **L** | **L** | **L** | **S** | **L** | **I** | **Q** | **K** | **T** | **K** | **Y** | **G** | **K** | **I** | **E** | **E** | **K** | **L** | **R** | **A** | **L** | **S** | **S** | **G** | **N** | **Y** | **E** | **S** | **K** | **L** | **L** | **H** | **Q** | **P** | **I** | **P** | **S** | **A** | **S** | **D** | **D** | **L** | **Y** | **I** | **K** | **D** | **I** | **D** | **M** | **E** | **I** | **T** | **K** | **I** | **K** | **E** | **K** | **M** | **I** | **E** | **V** | **S** | **S** | **E** | **L** | **Q** | **I** | **M** | **T** | **S** | **R** | **P** | **Q** | **Y** | **V** | **D** | **G** | **Q** | **T** | **K** | **E** | **E** | **I** | **L** | **E** | **L** | **E** | **R** | **H** | **R** | **L** | **A** | **R** | **E** | **L** | **H** | **D** | **S** | **V** | **S** | **Q** | **Q** | **L** | **F** | **A** | **A** | **M** | **M** | **M** | **M** | **S** | **A** | **L** | **T** | **E** | **Q** | **A** | **E** | **K** | **S** | **E** | **T** | **P** | **E** | **M** | **F** | **R** | **K** | **Q** | **L** | **K** | **M** | **V** | **A** | **E** | **I** | **I** | **N** | **A** | **S** | **Q** | **S** | **E** | **M** | **R** | **A** | **L** | **L** | **L** | **H** | **L** | **R** | **P** | **V** | **N** | **L** | **E** | **E** | **K** | **S** | **L** | **K** | **Q** | **G** | **I** | **E** | **Q** | **L** | **L** | **K** | **E** | **L** | **Q** | **N** | **K** | **I** | **Q** | **I** | **S** | **L** | **K** | **W** | **D** | **V** | **E** | **D** | **V** | **K** | **L** | **T** | **S** | **S** | **I** | **E** | **D** | **H** | **L** | **F** | **R** | **I** | **V** | **Q** | **E** | **L** | **L** | **S** | **N** | **T** | **L** | **R** | **H** | **A** | **K** | **A** | **N** | **E** | **L** | **E** | **V** | **Y** | **L** | **H** | **K** | **I** | **D** | **N** | **N** | **L** | **L** | **L** | **R** | **I** | **I** | **D** | **D** | **G** | **T** | **G** | **F** | **N** | **M** | **N** | **E** | **T** | **K** | **T** | **G** | **S** | **Y** | **G** | **L** | **N** | **N** | **I** | **K** | **E** | **R** | **V** | **A** | **G** | **I** | **G** | **G** | **T** | **V** | **K** | **I** | **I** | **S** | **F** | **K** | **G** | **Q** | **G** | **T** | **S** | **V** | **E** | **I** | **K** | **V** | **P** | **L** | **M** | **K** | **E** | **A** |
| **N59943F** | **M** | **M** | **G** | **K** | **I** | **S** | **R** | **A** | **M** | **L** | **A** | **V** | **Y** | **S** | **G** | **I** | **A** | **A** | **F** | **L** | **I** | **I** | **L** | **F** | **S** | **L** | **F** | **T** | **Y** | **F** | **Y** | **A** | **S** | **N** | **Q** | **S** | **H** | **W** | **W** | **G** | **E** | **L** | **L** | **R** | **A** | **R** | **L** | **L** | **Y** | **V** | **P** | **L** | **I** | **F** | **H** | **L** | **L** | **A** | **I** | **S** | **L** | **G** | **V** | **G** | **L** | **I** | **V** | **F** | **L** | **L** | **L** | **S** | **L** | **I** | **Q** | **K** | **T** | **K** | **Y** | **G** | **K** | **I** | **E** | **E** | **K** | **L** | **R** | **A** | **L** | **S** | **S** | **G** | **N** | **Y** | **E** | **S** | **K** | **L** | **L** | **L** | **L** | **P** | **I** | **P** | **S** | **A** | **S** | **D** | **D** | **L** | **Y** | **I** | **K** | **D** | **I** | **D** | **K** | **E** | **I** | **T** | **K** | **I** | **K** | **E** | **K** | **M** | **I** | **E** | **V** | **S** | **S** | **E** | **L** | **Q** | **I** | **V** | **T** | **S** | **R** | **P** | **Q** | **Y** | **V** | **D** | **G** | **Q** | **T** | **K** | **E** | **E** | **I** | **L** | **E** | **L** | **E** | **R** | **H** | **R** | **L** | **A** | **R** | **E** | **L** | **H** | **D** | **S** | **V** | **S** | **Q** | **Q** | **L** | **F** | **A** | **A** | **M** | **M** | **M** | **M** | **S** | **A** | **L** | **T** | **E** | **Q** | **A** | **E** | **K** | **S** | **E** | **T** | **P** | **E** | **M** | **F** | **R** | **K** | **Q** | **L** | **K** | **M** | **V** | **A** | **E** | **I** | **I** | **N** | **A** | **S** | **Q** | **S** | **E** | **M** | **R** | **A** | **L** | **L** | **L** | **H** | **L** | **R** | **P** | **V** | **N** | **L** | **E** | **E** | **K** | **S** | **L** | **K** | **Q** | **G** | **I** | **E** | **Q** | **L** | **L** | **K** | **E** | **L** | **Q** | **N** | **K** | **I** | **Q** | **I** | **S** | **L** | **K** | **W** | **D** | **V** | **E** | **D** | **V** | **K** | **L** | **S** | **S** | **S** | **I** | **E** | **D** | **H** | **L** | **F** | **R** | **I** | **V** | **Q** | **E** | **L** | **L** | **S** | **N** | **T** | **L** | **R** | **H** | **A** | **K** | **A** | **N** | **E** | **L** | **E** | **V** | **Y** | **L** | **H** | **K** | **I** | **D** | **N** | **N** | **L** | **L** | **L** | **R** | **I** | **I** | **D** | **D** | **G** | **T** | **G** | **F** | **N** | **M** | **N** | **E** | **T** | **K** | **T** | **G** | **S** | **Y** | **G** | **L** | **N** | **N** | **I** | **K** | **E** | **R** | **V** | **A** | **G** | **I** | **G** | **G** | **T** | **V** | **K** | **I** | **I** | **S** | **F** | **K** | **G** | **Q** | **G** | **T** | **S** | **V** | **E** | **I** | **K** | **V** | **P** | **L** | **M** | **K** | **E** | **A** |
| **N60365F** | **M** | **M** | **G** | **R** | **I** | **S** | **K** | **A** | **M** | **L** | **A** | **V** | **Y** | **S** | **G** | **I** | **A** | **A** | **F** | **L** | **I** | **I** | **L** | **F** | **S** | **L** | **F** | **T** | **Y** | **F** | **Y** | **A** | **S** | **N** | **Q** | **S** | **H** | **W** | **W** | **G** | **E** | **L** | **L** | **R** | **A** | **R** | **L** | **L** | **Y** | **V** | **P** | **L** | **I** | **F** | **H** | **L** | **M** | **A** | **I** | **S** | **L** | **G** | **V** | **G** | **L** | **I** | **V** | **F** | **L** | **L** | **L** | **S** | **L** | **I** | **Q** | **K** | **A** | **K** | **Y** | **G** | **K** | **I** | **E** | **E** | **K** | **L** | **R** | **A** | **L** | **S** | **S** | **G** | **N** | **Y** | **E** | **S** | **K** | **L** | **L** | **H** | **Q** | **P** | **I** | **P** | **S** | **A** | **S** | **D** | **D** | **L** | **Y** | **I** | **K** | **D** | **I** | **D** | **M** | **E** | **I** | **T** | **K** | **I** | **K** | **E** | **K** | **M** | **I** | **E** | **V** | **S** | **S** | **E** | **L** | **Q** | **I** | **M** | **T** | **S** | **R** | **P** | **Q** | **Y** | **V** | **D** | **G** | **Q** | **T** | **K** | **E** | **E** | **I** | **L** | **E** | **L** | **E** | **R** | **H** | **R** | **L** | **A** | **R** | **E** | **L** | **H** | **D** | **S** | **V** | **S** | **Q** | **Q** | **L** | **F** | **A** | **A** | **M** | **M** | **M** | **M** | **S** | **A** | **L** | **T** | **E** | **Q** | **A** | **E** | **K** | **S** | **E** | **T** | **P** | **E** | **M** | **F** | **R** | **K** | **Q** | **L** | **K** | **M** | **V** | **A** | **E** | **I** | **I** | **N** | **A** | **S** | **Q** | **S** | **E** | **M** | **R** | **A** | **L** | **L** | **L** | **H** | **L** | **R** | **P** | **V** | **N** | **L** | **E** | **E** | **K** | **S** | **L** | **K** | **Q** | **G** | **I** | **E** | **Q** | **L** | **L** | **K** | **E** | **L** | **Q** | **N** | **K** | **I** | **Q** | **I** | **S** | **L** | **K** | **W** | **D** | **V** | **E** | **D** | **V** | **K** | **L** | **T** | **S** | **S** | **I** | **E** | **D** | **H** | **L** | **F** | **R** | **I** | **V** | **Q** | **E** | **L** | **L** | **S** | **N** | **T** | **L** | **R** | **H** | **A** | **K** | **A** | **N** | **E** | **L** | **E** | **V** | **Y** | **L** | **H** | **K** | **I** | **D** | **N** | **N** | **L** | **L** | **L** | **R** | **I** | **I** | **D** | **D** | **G** | **T** | **G** | **F** | **N** | **M** | **N** | **E** | **T** | **K** | **T** | **G** | **S** | **Y** | **G** | **L** | **N** | **N** | **I** | **K** | **E** | **R** | **V** | **A** | **G** | **I** | **G** | **G** | **T** | **V** | **K** | **I** | **I** | **S** | **F** | **K** | **G** | **Q** | **G** | **T** | **S** | **V** | **E** | **I** | **K** | **V** | **P** | **L** | **M** | **K** | **E** | **A** |
| **N60425F** | **M** | **M** | **G** | **R** | **I** | **S** | **K** | **A** | **M** | **L** | **A** | **V** | **Y** | **S** | **G** | **I** | **A** | **A** | **F** | **L** | **I** | **I** | **L** | **F** | **S** | **L** | **F** | **T** | **Y** | **F** | **Y** | **A** | **S** | **N** | **Q** | **S** | **H** | **W** | **W** | **G** | **E** | **L** | **L** | **R** | **A** | **R** | **L** | **L** | **Y** | **V** | **P** | **L** | **I** | **F** | **H** | **L** | **M** | **A** | **I** | **S** | **L** | **G** | **V** | **G** | **L** | **I** | **V** | **F** | **L** | **L** | **L** | **S** | **L** | **I** | **Q** | **K** | **A** | **K** | **Y** | **G** | **K** | **I** | **E** | **E** | **K** | **L** | **R** | **A** | **L** | **S** | **S** | **G** | **N** | **Y** | **E** | **S** | **K** | **L** | **L** | **H** | **Q** | **P** | **I** | **P** | **S** | **T** | **S** | **D** | **D** | **L** | **Y** | **I** | **K** | **D** | **I** | **D** | **M** | **E** | **I** | **T** | **K** | **I** | **K** | **E** | **K** | **M** | **I** | **E** | **V** | **S** | **S** | **E** | **L** | **Q** | **I** | **M** | **T** | **S** | **R** | **P** | **Q** | **Y** | **V** | **D** | **G** | **Q** | **T** | **K** | **E** | **E** | **I** | **L** | **E** | **L** | **E** | **R** | **H** | **R** | **L** | **A** | **R** | **E** | **L** | **H** | **D** | **S** | **V** | **S** | **Q** | **Q** | **L** | **F** | **A** | **A** | **M** | **M** | **M** | **M** | **S** | **A** | **L** | **T** | **E** | **Q** | **A** | **E** | **K** | **S** | **E** | **T** | **P** | **E** | **M** | **F** | **R** | **K** | **Q** | **L** | **K** | **M** | **V** | **A** | **E** | **I** | **I** | **N** | **A** | **S** | **Q** | **S** | **E** | **M** | **R** | **A** | **L** | **L** | **L** | **H** | **L** | **R** | **P** | **V** | **N** | **L** | **E** | **E** | **K** | **S** | **L** | **K** | **Q** | **G** | **I** | **E** | **Q** | **L** | **L** | **K** | **E** | **L** | **Q** | **N** | **K** | **I** | **Q** | **I** | **S** | **L** | **K** | **W** | **D** | **V** | **E** | **D** | **V** | **K** | **L** | **T** | **S** | **S** | **I** | **E** | **D** | **H** | **L** | **F** | **R** | **I** | **V** | **Q** | **E** | **L** | **L** | **S** | **N** | **T** | **L** | **R** | **H** | **A** | **K** | **A** | **N** | **E** | **L** | **E** | **V** | **Y** | **L** | **H** | **K** | **I** | **D** | **N** | **N** | **L** | **L** | **L** | **R** | **I** | **I** | **D** | **D** | **G** | **T** | **G** | **F** | **N** | **M** | **N** | **E** | **T** | **K** | **T** | **G** | **S** | **Y** | **G** | **L** | **N** | **N** | **I** | **K** | **E** | **R** | **V** | **A** | **G** | **I** | **G** | **G** | **T** | **V** | **K** | **I** | **I** | **S** | **F** | **K** | **G** | **Q** | **G** | **T** | **S** | **V** | **E** | **I** | **K** | **V** | **P** | **L** | **M** | **K** | **E** | **A** |
| **N60463F** | **M** | **M** | **G** | **R** | **I** | **S** | **K** | **A** | **M** | **L** | **A** | **V** | **Y** | **S** | **G** | **I** | **A** | **A** | **F** | **L** | **I** | **I** | **L** | **F** | **S** | **L** | **F** | **T** | **Y** | **F** | **Y** | **A** | **S** | **N** | **Q** | **S** | **H** | **W** | **W** | **G** | **E** | **L** | **L** | **R** | **A** | **R** | **L** | **L** | **Y** | **V** | **P** | **L** | **I** | **F** | **H** | **L** | **M** | **A** | **I** | **S** | **L** | **G** | **V** | **G** | **L** | **I** | **V** | **F** | **L** | **L** | **L** | **S** | **L** | **I** | **Q** | **K** | **T** | **K** | **Y** | **G** | **K** | **I** | **E** | **E** | **K** | **L** | **R** | **A** | **L** | **S** | **S** | **G** | **N** | **Y** | **E** | **S** | **K** | **L** | **L** | **H** | **Q** | **P** | **I** | **P** | **S** | **A** | **S** | **D** | **D** | **L** | **Y** | **I** | **K** | **D** | **I** | **D** | **M** | **E** | **I** | **T** | **K** | **I** | **K** | **E** | **K** | **M** | **I** | **E** | **V** | **S** | **S** | **E** | **L** | **Q** | **I** | **M** | **T** | **S** | **R** | **P** | **Q** | **Y** | **V** | **D** | **G** | **Q** | **T** | **K** | **E** | **E** | **I** | **L** | **E** | **L** | **E** | **R** | **H** | **R** | **L** | **A** | **R** | **E** | **L** | **H** | **D** | **S** | **V** | **S** | **Q** | **Q** | **L** | **F** | **A** | **A** | **M** | **M** | **M** | **M** | **S** | **A** | **L** | **T** | **E** | **Q** | **A** | **E** | **K** | **S** | **E** | **T** | **P** | **E** | **M** | **F** | **R** | **K** | **Q** | **L** | **K** | **M** | **V** | **A** | **E** | **I** | **I** | **N** | **A** | **S** | **Q** | **S** | **E** | **M** | **R** | **A** | **L** | **L** | **L** | **H** | **L** | **R** | **P** | **V** | **N** | **L** | **E** | **E** | **K** | **S** | **L** | **K** | **Q** | **G** | **I** | **E** | **Q** | **L** | **L** | **K** | **E** | **L** | **Q** | **N** | **K** | **I** | **Q** | **I** | **S** | **L** | **K** | **W** | **D** | **V** | **E** | **D** | **V** | **K** | **L** | **T** | **S** | **S** | **I** | **E** | **D** | **H** | **L** | **F** | **R** | **I** | **V** | **Q** | **E** | **L** | **L** | **S** | **N** | **T** | **L** | **R** | **H** | **A** | **K** | **A** | **N** | **E** | **L** | **E** | **V** | **Y** | **L** | **H** | **K** | **I** | **D** | **N** | **N** | **L** | **L** | **L** | **R** | **I** | **I** | **D** | **D** | **G** | **T** | **G** | **F** | **N** | **M** | **N** | **E** | **T** | **K** | **T** | **G** | **S** | **Y** | **G** | **L** | **N** | **N** | **I** | **K** | **E** | **R** | **V** | **A** | **G** | **I** | **G** | **G** | **T** | **V** | **K** | **I** | **I** | **S** | **F** | **K** | **G** | **Q** | **G** | **T** | **S** | **V** | **E** | **I** | **K** | **V** | **P** | **L** | **M** | **K** | **E** | **A** |
| **N52649** | **M** | **M** | **G** | **R** | **I** | **S** | **K** | **A** | **M** | **L** | **A** | **V** | **Y** | **S** | **G** | **I** | **A** | **A** | **F** | **L** | **I** | **I** | **L** | **F** | **S** | **L** | **F** | **T** | **Y** | **F** | **Y** | **A** | **S** | **N** | **Q** | **S** | **H** | **W** | **W** | **G** | **E** | **L** | **L** | **R** | **A** | **R** | **L** | **L** | **Y** | **V** | **P** | **L** | **I** | **F** | **H** | **L** | **M** | **A** | **I** | **S** | **L** | **G** | **V** | **G** | **L** | **I** | **V** | **F** | **L** | **L** | **L** | **S** | **L** | **I** | **Q** | **K** | **A** | **K** | **Y** | **G** | **K** | **I** | **E** | **E** | **K** | **L** | **R** | **A** | **L** | **S** | **S** | **G** | **N** | **Y** | **E** | **S** | **K** | **L** | **L** | **H** | **Q** | **P** | **I** | **P** | **S** | **A** | **S** | **D** | **D** | **L** | **Y** | **I** | **K** | **D** | **I** | **D** | **M** | **E** | **I** | **T** | **K** | **I** | **K** | **E** | **K** | **M** | **I** | **E** | **V** | **S** | **S** | **E** | **L** | **Q** | **I** | **M** | **T** | **S** | **R** | **P** | **Q** | **Y** | **V** | **D** | **G** | **Q** | **T** | **K** | **E** | **E** | **I** | **L** | **E** | **L** | **E** | **R** | **H** | **R** | **L** | **A** | **R** | **E** | **L** | **H** | **D** | **S** | **V** | **S** | **Q** | **Q** | **L** | **F** | **A** | **A** | **M** | **M** | **M** | **M** | **S** | **A** | **L** | **T** | **E** | **Q** | **A** | **E** | **K** | **S** | **E** | **T** | **P** | **E** | **M** | **F** | **R** | **K** | **Q** | **L** | **K** | **M** | **V** | **A** | **E** | **I** | **I** | **N** | **A** | **S** | **Q** | **S** | **E** | **M** | **R** | **A** | **L** | **L** | **L** | **H** | **L** | **R** | **P** | **V** | **N** | **L** | **E** | **E** | **K** | **S** | **L** | **K** | **Q** | **G** | **I** | **E** | **Q** | **L** | **L** | **K** | **E** | **L** | **Q** | **N** | **K** | **I** | **Q** | **I** | **S** | **L** | **K** | **W** | **D** | **V** | **E** | **D** | **V** | **K** | **L** | **T** | **S** | **S** | **I** | **E** | **D** | **H** | **L** | **F** | **R** | **I** | **V** | **Q** | **E** | **L** | **L** | **S** | **N** | **T** | **L** | **R** | **H** | **A** | **K** | **A** | **N** | **E** | **L** | **E** | **V** | **Y** | **L** | **H** | **K** | **I** | **D** | **N** | **N** | **L** | **L** | **L** | **R** | **I** | **I** | **D** | **D** | **G** | **T** | **G** | **F** | **N** | **M** | **N** | **E** | **T** | **K** | **T** | **G** | **S** | **Y** | **G** | **L** | **N** | **N** | **I** | **K** | **E** | **R** | **V** | **A** | **G** | **I** | **G** | **G** | **T** | **V** | **K** | **I** | **I** | **S** | **F** | **K** | **G** | **Q** | **G** | **T** | **S** | **V** | **E** | **I** | **K** | **V** | **P** | **L** | **M** | **K** | **E** | **A** |
| **N53533** | **M** | **M** | **G** | **R** | **I** | **S** | **K** | **A** | **M** | **L** | **A** | **V** | **Y** | **S** | **G** | **I** | **A** | **A** | **F** | **L** | **I** | **I** | **L** | **F** | **S** | **L** | **F** | **T** | **Y** | **F** | **Y** | **A** | **S** | **N** | **Q** | **S** | **H** | **W** | **W** | **G** | **E** | **L** | **L** | **R** | **A** | **R** | **L** | **L** | **Y** | **V** | **P** | **L** | **I** | **F** | **H** | **L** | **M** | **A** | **I** | **S** | **L** | **G** | **V** | **G** | **L** | **I** | **V** | **F** | **L** | **L** | **L** | **S** | **L** | **I** | **Q** | **K** | **A** | **K** | **Y** | **G** | **K** | **I** | **E** | **E** | **K** | **L** | **R** | **A** | **L** | **S** | **S** | **G** | **N** | **Y** | **E** | **S** | **K** | **L** | **L** | **H** | **Q** | **P** | **I** | **P** | **S** | **A** | **S** | **D** | **D** | **L** | **Y** | **I** | **K** | **D** | **I** | **D** | **M** | **E** | **I** | **T** | **K** | **I** | **K** | **E** | **K** | **M** | **I** | **E** | **V** | **S** | **S** | **E** | **L** | **Q** | **I** | **M** | **T** | **S** | **R** | **P** | **Q** | **Y** | **V** | **D** | **G** | **Q** | **T** | **K** | **E** | **E** | **I** | **L** | **E** | **L** | **E** | **R** | **H** | **R** | **L** | **A** | **R** | **E** | **L** | **H** | **D** | **S** | **V** | **S** | **Q** | **Q** | **L** | **F** | **A** | **A** | **M** | **M** | **M** | **M** | **S** | **A** | **L** | **T** | **E** | **Q** | **A** | **E** | **K** | **S** | **E** | **T** | **P** | **E** | **M** | **F** | **R** | **K** | **Q** | **L** | **K** | **M** | **V** | **A** | **E** | **I** | **I** | **N** | **A** | **S** | **Q** | **S** | **E** | **M** | **R** | **A** | **L** | **L** | **L** | **H** | **L** | **R** | **P** | **V** | **N** | **L** | **E** | **E** | **K** | **S** | **L** | **K** | **Q** | **G** | **I** | **E** | **Q** | **L** | **L** | **K** | **E** | **L** | **Q** | **N** | **K** | **I** | **Q** | **I** | **S** | **L** | **K** | **W** | **D** | **V** | **E** | **D** | **V** | **K** | **L** | **T** | **S** | **S** | **I** | **E** | **D** | **H** | **L** | **F** | **R** | **I** | **V** | **Q** | **E** | **L** | **L** | **S** | **N** | **T** | **L** | **R** | **H** | **A** | **K** | **A** | **N** | **E** | **L** | **E** | **V** | **Y** | **L** | **H** | **K** | **I** | **D** | **N** | **N** | **L** | **L** | **L** | **R** | **I** | **I** | **D** | **D** | **G** | **T** | **G** | **F** | **N** | **M** | **N** | **E** | **T** | **K** | **T** | **G** | **S** | **Y** | **G** | **L** | **N** | **N** | **I** | **K** | **E** | **R** | **V** | **A** | **G** | **I** | **G** | **G** | **T** | **V** | **K** | **I** | **I** | **S** | **F** | **K** | **G** | **Q** | **G** | **T** | **S** | **V** | **E** | **I** | **K** | **V** | **P** | **L** | **M** | **K** | **E** | **A** |
| **N60284F** | **M** | **M** | **G** | **R** | **I** | **S** | **K** | **A** | **M** | **L** | **A** | **V** | **Y** | **S** | **G** | **I** | **A** | **A** | **F** | **L** | **I** | **I** | **L** | **F** | **S** | **L** | **F** | **T** | **Y** | **F** | **Y** | **A** | **S** | **N** | **Q** | **S** | **H** | **W** | **W** | **G** | **E** | **L** | **L** | **R** | **A** | **R** | **L** | **L** | **Y** | **V** | **P** | **L** | **I** | **F** | **H** | **L** | **M** | **A** | **I** | **S** | **L** | **G** | **V** | **G** | **L** | **I** | **V** | **F** | **L** | **L** | **L** | **S** | **L** | **I** | **Q** | **K** | **A** | **K** | **Y** | **G** | **K** | **I** | **E** | **E** | **K** | **L** | **R** | **A** | **L** | **S** | **S** | **G** | **N** | **Y** | **E** | **S** | **K** | **L** | **L** | **H** | **Q** | **P** | **I** | **P** | **S** | **A** | **S** | **D** | **D** | **L** | **Y** | **I** | **K** | **D** | **I** | **D** | **M** | **E** | **I** | **T** | **K** | **I** | **K** | **E** | **K** | **M** | **I** | **E** | **V** | **S** | **S** | **E** | **L** | **Q** | **I** | **M** | **T** | **S** | **R** | **P** | **Q** | **Y** | **V** | **D** | **G** | **Q** | **T** | **K** | **E** | **E** | **I** | **L** | **E** | **L** | **E** | **R** | **H** | **R** | **L** | **A** | **R** | **E** | **L** | **H** | **D** | **S** | **V** | **S** | **Q** | **Q** | **L** | **F** | **A** | **A** | **M** | **M** | **M** | **M** | **S** | **A** | **L** | **T** | **E** | **Q** | **A** | **E** | **K** | **S** | **E** | **T** | **P** | **E** | **M** | **F** | **R** | **K** | **Q** | **L** | **K** | **M** | **V** | **A** | **E** | **I** | **I** | **N** | **A** | **S** | **Q** | **S** | **E** | **M** | **R** | **A** | **L** | **L** | **L** | **H** | **L** | **R** | **P** | **V** | **N** | **L** | **E** | **E** | **K** | **S** | **L** | **K** | **Q** | **G** | **I** | **E** | **Q** | **L** | **L** | **K** | **E** | **L** | **Q** | **N** | **K** | **I** | **Q** | **I** | **S** | **L** | **K** | **W** | **D** | **V** | **E** | **D** | **V** | **K** | **L** | **T** | **S** | **S** | **I** | **E** | **D** | **H** | **L** | **F** | **R** | **I** | **V** | **Q** | **E** | **L** | **L** | **S** | **N** | **T** | **L** | **R** | **H** | **A** | **K** | **A** | **N** | **E** | **L** | **E** | **V** | **Y** | **L** | **H** | **K** | **I** | **D** | **N** | **N** | **L** | **L** | **L** | **R** | **I** | **I** | **D** | **D** | **G** | **T** | **G** | **F** | **N** | **M** | **N** | **E** | **T** | **K** | **T** | **G** | **S** | **Y** | **G** | **L** | **N** | **N** | **I** | **K** | **E** | **R** | **V** | **A** | **G** | **I** | **G** | **G** | **T** | **V** | **K** | **I** | **I** | **S** | **F** | **K** | **G** | **Q** | **G** | **T** | **S** | **V** | **E** | **I** | **K** | **V** | **P** | **L** | **M** | **K** | **E** | **A** |
| **N52702** | **M** | **M** | **G** | **R** | **I** | **S** | **K** | **A** | **M** | **L** | **A** | **V** | **Y** | **S** | **G** | **I** | **A** | **A** | **F** | **L** | **I** | **I** | **L** | **F** | **S** | **L** | **F** | **T** | **Y** | **F** | **Y** | **A** | **S** | **N** | **Q** | **S** | **H** | **W** | **W** | **G** | **E** | **L** | **L** | **R** | **A** | **R** | **L** | **L** | **Y** | **V** | **P** | **L** | **I** | **F** | **H** | **L** | **M** | **A** | **I** | **S** | **L** | **G** | **V** | **G** | **L** | **I** | **V** | **F** | **L** | **L** | **L** | **S** | **L** | **I** | **Q** | **K** | **A** | **K** | **Y** | **G** | **K** | **I** | **E** | **E** | **K** | **L** | **R** | **A** | **L** | **S** | **S** | **G** | **N** | **Y** | **E** | **S** | **K** | **L** | **L** | **H** | **Q** | **P** | **I** | **P** | **S** | **A** | **S** | **D** | **D** | **L** | **Y** | **I** | **K** | **D** | **I** | **D** | **M** | **E** | **I** | **T** | **K** | **I** | **K** | **E** | **K** | **M** | **I** | **E** | **V** | **S** | **S** | **E** | **L** | **Q** | **I** | **M** | **T** | **S** | **R** | **P** | **Q** | **Y** | **V** | **D** | **G** | **Q** | **T** | **K** | **E** | **E** | **I** | **L** | **E** | **L** | **E** | **R** | **H** | **R** | **L** | **A** | **R** | **E** | **L** | **H** | **D** | **S** | **V** | **S** | **Q** | **Q** | **L** | **F** | **A** | **A** | **M** | **M** | **M** | **M** | **S** | **A** | **L** | **T** | **E** | **Q** | **A** | **E** | **K** | **S** | **E** | **T** | **P** | **E** | **M** | **F** | **R** | **K** | **Q** | **L** | **K** | **M** | **V** | **A** | **E** | **I** | **I** | **N** | **A** | **S** | **Q** | **S** | **E** | **M** | **R** | **A** | **L** | **L** | **L** | **H** | **L** | **R** | **P** | **V** | **N** | **L** | **E** | **E** | **K** | **S** | **L** | **K** | **Q** | **G** | **I** | **E** | **Q** | **L** | **L** | **K** | **E** | **L** | **Q** | **N** | **K** | **I** | **Q** | **I** | **S** | **L** | **K** | **W** | **D** | **V** | **E** | **D** | **V** | **K** | **L** | **T** | **S** | **S** | **I** | **E** | **D** | **H** | **L** | **F** | **R** | **I** | **V** | **Q** | **E** | **L** | **L** | **S** | **N** | **T** | **L** | **R** | **H** | **A** | **K** | **A** | **N** | **E** | **L** | **E** | **V** | **Y** | **L** | **H** | **K** | **I** | **D** | **N** | **N** | **L** | **L** | **L** | **R** | **I** | **I** | **D** | **D** | **G** | **T** | **G** | **F** | **N** | **M** | **N** | **E** | **T** | **K** | **T** | **G** | **S** | **Y** | **G** | **L** | **N** | **N** | **I** | **K** | **E** | **R** | **V** | **A** | **G** | **I** | **G** | **G** | **T** | **V** | **K** | **I** | **I** | **S** | **F** | **K** | **G** | **Q** | **G** | **T** | **S** | **V** | **E** | **I** | **K** | **V** | **P** | **L** | **M** | **K** | **E** | **A** |
| **N54550** | **M** | **M** | **G** | **R** | **I** | **S** | **K** | **A** | **M** | **L** | **A** | **V** | **Y** | **S** | **G** | **I** | **A** | **A** | **F** | **L** | **I** | **I** | **L** | **F** | **S** | **L** | **F** | **T** | **Y** | **F** | **Y** | **A** | **S** | **N** | **Q** | **S** | **H** | **W** | **W** | **G** | **E** | **L** | **L** | **R** | **A** | **R** | **L** | **L** | **Y** | **V** | **P** | **L** | **I** | **F** | **H** | **L** | **M** | **A** | **I** | **S** | **L** | **G** | **V** | **G** | **L** | **I** | **V** | **F** | **L** | **L** | **L** | **S** | **L** | **I** | **Q** | **K** | **T** | **K** | **Y** | **G** | **K** | **I** | **E** | **E** | **K** | **L** | **R** | **A** | **L** | **S** | **S** | **G** | **N** | **Y** | **E** | **S** | **K** | **L** | **L** | **H** | **Q** | **P** | **I** | **P** | **S** | **A** | **S** | **D** | **D** | **L** | **Y** | **I** | **K** | **D** | **I** | **D** | **M** | **E** | **I** | **T** | **K** | **I** | **K** | **E** | **K** | **M** | **I** | **E** | **V** | **S** | **S** | **E** | **L** | **Q** | **I** | **M** | **T** | **S** | **R** | **P** | **Q** | **Y** | **V** | **D** | **G** | **Q** | **T** | **K** | **E** | **E** | **I** | **L** | **E** | **L** | **E** | **R** | **H** | **R** | **L** | **A** | **R** | **E** | **L** | **H** | **D** | **S** | **V** | **S** | **Q** | **Q** | **L** | **F** | **A** | **A** | **M** | **M** | **M** | **M** | **S** | **A** | **L** | **T** | **E** | **Q** | **A** | **E** | **K** | **S** | **E** | **T** | **P** | **E** | **M** | **F** | **R** | **K** | **Q** | **L** | **K** | **M** | **V** | **A** | **E** | **I** | **I** | **N** | **A** | **S** | **Q** | **S** | **E** | **M** | **R** | **A** | **L** | **L** | **L** | **H** | **L** | **R** | **P** | **V** | **N** | **L** | **E** | **E** | **K** | **S** | **L** | **K** | **Q** | **G** | **I** | **E** | **Q** | **L** | **L** | **K** | **E** | **L** | **Q** | **N** | **K** | **I** | **Q** | **I** | **S** | **L** | **K** | **W** | **D** | **V** | **E** | **D** | **V** | **K** | **L** | **T** | **S** | **S** | **I** | **E** | **D** | **H** | **L** | **F** | **R** | **I** | **V** | **Q** | **E** | **L** | **L** | **S** | **N** | **T** | **L** | **R** | **H** | **A** | **K** | **A** | **N** | **E** | **L** | **E** | **V** | **Y** | **L** | **H** | **K** | **I** | **D** | **N** | **N** | **L** | **L** | **L** | **R** | **I** | **I** | **D** | **D** | **G** | **T** | **G** | **F** | **N** | **M** | **N** | **E** | **T** | **K** | **T** | **G** | **S** | **Y** | **G** | **L** | **N** | **N** | **I** | **K** | **E** | **R** | **V** | **A** | **G** | **I** | **G** | **G** | **T** | **V** | **K** | **I** | **I** | **S** | **F** | **K** | **G** | **Q** | **G** | **T** | **S** | **V** | **E** | **I** | **K** | **V** | **P** | **L** | **M** | **K** | **E** | **A** |
| **N55229** | **M** | **M** | **G** | **R** | **I** | **S** | **K** | **A** | **M** | **L** | **A** | **V** | **Y** | **S** | **G** | **I** | **A** | **A** | **F** | **L** | **I** | **I** | **L** | **F** | **S** | **L** | **F** | **T** | **Y** | **F** | **Y** | **A** | **S** | **N** | **Q** | **S** | **H** | **W** | **W** | **G** | **E** | **L** | **L** | **R** | **A** | **R** | **L** | **L** | **Y** | **V** | **P** | **L** | **I** | **F** | **H** | **L** | **M** | **A** | **I** | **S** | **L** | **G** | **V** | **G** | **L** | **I** | **V** | **F** | **L** | **L** | **L** | **S** | **L** | **I** | **Q** | **K** | **A** | **K** | **Y** | **E** | **K** | **I** | **E** | **E** | **K** | **L** | **R** | **A** | **L** | **S** | **S** | **G** | **N** | **Y** | **E** | **S** | **K** | **L** | **L** | **H** | **Q** | **P** | **I** | **P** | **S** | **A** | **S** | **D** | **D** | **L** | **Y** | **I** | **K** | **D** | **I** | **D** | **M** | **E** | **I** | **T** | **K** | **I** | **K** | **E** | **K** | **M** | **I** | **E** | **V** | **S** | **S** | **E** | **L** | **Q** | **I** | **M** | **T** | **S** | **R** | **P** | **Q** | **Y** | **V** | **D** | **G** | **Q** | **T** | **K** | **E** | **E** | **I** | **L** | **E** | **L** | **E** | **R** | **H** | **R** | **L** | **A** | **R** | **E** | **L** | **H** | **D** | **S** | **V** | **S** | **Q** | **Q** | **L** | **F** | **A** | **A** | **M** | **M** | **M** | **M** | **S** | **A** | **L** | **T** | **E** | **Q** | **A** | **E** | **K** | **S** | **E** | **T** | **T** | **E** | **M** | **F** | **R** | **K** | **Q** | **L** | **K** | **M** | **V** | **A** | **E** | **I** | **I** | **N** | **A** | **S** | **Q** | **S** | **E** | **M** | **R** | **A** | **L** | **L** | **L** | **H** | **L** | **R** | **P** | **V** | **N** | **L** | **E** | **E** | **K** | **S** | **L** | **K** | **Q** | **G** | **I** | **E** | **Q** | **L** | **L** | **K** | **E** | **L** | **Q** | **N** | **K** | **I** | **Q** | **I** | **S** | **L** | **K** | **W** | **D** | **V** | **E** | **D** | **V** | **K** | **L** | **T** | **S** | **S** | **I** | **E** | **D** | **H** | **L** | **F** | **R** | **I** | **V** | **Q** | **E** | **L** | **L** | **S** | **N** | **T** | **L** | **R** | **H** | **A** | **K** | **A** | **N** | **E** | **L** | **E** | **V** | **Y** | **L** | **H** | **K** | **I** | **D** | **N** | **N** | **L** | **L** | **L** | **R** | **I** | **I** | **D** | **D** | **G** | **T** | **G** | **F** | **N** | **M** | **N** | **E** | **T** | **K** | **T** | **G** | **S** | **Y** | **G** | **L** | **N** | **N** | **I** | **K** | **E** | **R** | **V** | **A** | **G** | **I** | **G** | **G** | **T** | **V** | **K** | **I** | **I** | **S** | **F** | **K** | **G** | **Q** | **G** | **T** | **S** | **V** | **E** | **I** | **K** | **V** | **P** | **L** | **M** | **K** | **E** | **A** |
| **N59484F** | **M** | **M** | **G** | **R** | **I** | **S** | **K** | **A** | **M** | **L** | **A** | **V** | **Y** | **S** | **G** | **I** | **A** | **A** | **F** | **L** | **I** | **I** | **L** | **F** | **S** | **L** | **F** | **T** | **Y** | **F** | **Y** | **A** | **S** | **N** | **Q** | **S** | **H** | **W** | **W** | **G** | **E** | **L** | **L** | **R** | **A** | **R** | **L** | **L** | **Y** | **V** | **P** | **L** | **I** | **F** | **H** | **L** | **M** | **A** | **I** | **S** | **L** | **G** | **V** | **G** | **L** | **I** | **V** | **F** | **L** | **L** | **L** | **S** | **L** | **I** | **Q** | **K** | **T** | **K** | **Y** | **G** | **K** | **I** | **E** | **E** | **K** | **L** | **R** | **A** | **L** | **S** | **S** | **G** | **N** | **Y** | **E** | **S** | **K** | **L** | **L** | **H** | **Q** | **P** | **I** | **P** | **S** | **A** | **S** | **D** | **D** | **L** | **Y** | **I** | **K** | **D** | **I** | **D** | **M** | **E** | **I** | **T** | **K** | **I** | **K** | **E** | **K** | **M** | **I** | **E** | **V** | **S** | **S** | **E** | **L** | **Q** | **I** | **M** | **T** | **S** | **R** | **P** | **Q** | **Y** | **V** | **D** | **G** | **Q** | **T** | **K** | **E** | **E** | **I** | **L** | **E** | **L** | **E** | **R** | **H** | **R** | **L** | **A** | **R** | **E** | **L** | **H** | **D** | **S** | **V** | **S** | **Q** | **Q** | **L** | **F** | **A** | **A** | **M** | **M** | **M** | **M** | **S** | **A** | **L** | **T** | **E** | **Q** | **A** | **E** | **K** | **S** | **E** | **T** | **P** | **E** | **M** | **F** | **R** | **K** | **Q** | **L** | **K** | **M** | **V** | **A** | **E** | **I** | **I** | **N** | **A** | **S** | **Q** | **S** | **E** | **M** | **R** | **A** | **L** | **L** | **L** | **H** | **L** | **R** | **P** | **V** | **N** | **L** | **E** | **E** | **K** | **S** | **L** | **K** | **Q** | **G** | **I** | **E** | **Q** | **L** | **L** | **K** | **E** | **L** | **Q** | **N** | **K** | **I** | **Q** | **I** | **S** | **L** | **K** | **W** | **D** | **V** | **E** | **D** | **V** | **K** | **L** | **T** | **S** | **S** | **I** | **E** | **D** | **H** | **L** | **F** | **R** | **I** | **V** | **Q** | **E** | **L** | **L** | **S** | **N** | **T** | **L** | **R** | **H** | **A** | **K** | **A** | **N** | **E** | **L** | **E** | **V** | **Y** | **L** | **H** | **K** | **I** | **D** | **N** | **N** | **L** | **L** | **L** | **R** | **I** | **I** | **D** | **D** | **G** | **T** | **G** | **F** | **N** | **M** | **N** | **E** | **T** | **K** | **T** | **G** | **S** | **Y** | **G** | **L** | **N** | **N** | **I** | **K** | **E** | **R** | **V** | **A** | **G** | **I** | **G** | **G** | **T** | **V** | **K** | **I** | **I** | **S** | **F** | **K** | **G** | **Q** | **G** | **T** | **S** | **V** | **E** | **I** | **K** | **V** | **P** | **L** | **M** | **K** | **E** | **A** |
| **N59492F** | **M** | **M** | **G** | **R** | **I** | **S** | **K** | **A** | **M** | **L** | **A** | **V** | **Y** | **S** | **G** | **I** | **A** | **A** | **F** | **L** | **I** | **I** | **L** | **F** | **S** | **L** | **F** | **T** | **Y** | **F** | **Y** | **A** | **S** | **N** | **Q** | **S** | **H** | **W** | **W** | **G** | **E** | **L** | **L** | **R** | **A** | **R** | **L** | **L** | **Y** | **V** | **P** | **L** | **I** | **F** | **H** | **L** | **M** | **A** | **I** | **S** | **L** | **G** | **V** | **G** | **L** | **I** | **V** | **F** | **L** | **L** | **L** | **S** | **L** | **I** | **Q** | **K** | **T** | **K** | **Y** | **G** | **K** | **I** | **E** | **E** | **K** | **L** | **R** | **A** | **L** | **S** | **S** | **G** | **N** | **Y** | **E** | **S** | **K** | **L** | **L** | **H** | **Q** | **P** | **I** | **P** | **S** | **A** | **S** | **D** | **D** | **L** | **Y** | **I** | **K** | **D** | **I** | **D** | **M** | **E** | **I** | **T** | **K** | **I** | **K** | **E** | **K** | **M** | **I** | **E** | **V** | **S** | **S** | **E** | **L** | **Q** | **I** | **M** | **T** | **S** | **R** | **P** | **Q** | **Y** | **V** | **D** | **G** | **Q** | **T** | **K** | **E** | **E** | **I** | **L** | **E** | **L** | **E** | **R** | **H** | **R** | **L** | **A** | **R** | **E** | **L** | **H** | **D** | **S** | **V** | **S** | **Q** | **Q** | **L** | **F** | **A** | **A** | **M** | **M** | **M** | **M** | **S** | **A** | **L** | **T** | **E** | **Q** | **A** | **E** | **K** | **S** | **E** | **T** | **P** | **E** | **M** | **F** | **R** | **K** | **Q** | **L** | **K** | **M** | **V** | **A** | **E** | **I** | **I** | **N** | **A** | **S** | **Q** | **S** | **E** | **M** | **R** | **A** | **L** | **L** | **L** | **H** | **L** | **R** | **P** | **V** | **N** | **L** | **E** | **E** | **K** | **S** | **L** | **K** | **Q** | **G** | **I** | **E** | **Q** | **L** | **L** | **K** | **E** | **L** | **Q** | **N** | **K** | **I** | **Q** | **I** | **S** | **L** | **K** | **W** | **D** | **V** | **E** | **D** | **V** | **K** | **L** | **T** | **S** | **S** | **I** | **E** | **D** | **H** | **L** | **F** | **R** | **I** | **V** | **Q** | **E** | **L** | **L** | **S** | **N** | **T** | **L** | **R** | **H** | **A** | **K** | **A** | **N** | **E** | **L** | **E** | **V** | **Y** | **L** | **H** | **K** | **I** | **D** | **N** | **N** | **L** | **L** | **L** | **R** | **I** | **I** | **D** | **D** | **G** | **T** | **G** | **F** | **N** | **M** | **N** | **E** | **T** | **K** | **T** | **G** | **S** | **Y** | **G** | **L** | **N** | **N** | **I** | **K** | **E** | **R** | **V** | **A** | **G** | **I** | **G** | **G** | **T** | **V** | **K** | **I** | **I** | **S** | **F** | **K** | **G** | **Q** | **G** | **T** | **S** | **V** | **E** | **I** | **K** | **V** | **P** | **L** | **M** | **K** | **E** | **A** |
| **N60200F** | **M** | **M** | **G** | **R** | **I** | **S** | **K** | **A** | **M** | **L** | **A** | **V** | **Y** | **S** | **G** | **I** | **A** | **A** | **F** | **L** | **I** | **I** | **L** | **F** | **S** | **L** | **F** | **T** | **Y** | **F** | **Y** | **A** | **S** | **N** | **Q** | **S** | **H** | **W** | **W** | **G** | **E** | **L** | **L** | **R** | **A** | **R** | **L** | **L** | **Y** | **V** | **P** | **L** | **I** | **F** | **H** | **L** | **M** | **A** | **I** | **S** | **L** | **G** | **V** | **G** | **L** | **I** | **V** | **F** | **L** | **L** | **L** | **S** | **L** | **I** | **Q** | **K** | **A** | **K** | **Y** | **G** | **K** | **I** | **E** | **E** | **K** | **L** | **R** | **A** | **L** | **S** | **S** | **G** | **N** | **Y** | **E** | **S** | **K** | **L** | **L** | **H** | **Q** | **P** | **I** | **P** | **S** | **A** | **S** | **D** | **D** | **L** | **Y** | **I** | **K** | **D** | **I** | **D** | **M** | **E** | **I** | **T** | **K** | **I** | **K** | **E** | **K** | **M** | **I** | **E** | **V** | **S** | **S** | **E** | **L** | **Q** | **I** | **M** | **T** | **S** | **R** | **P** | **Q** | **Y** | **V** | **D** | **G** | **Q** | **T** | **K** | **E** | **E** | **I** | **L** | **E** | **L** | **E** | **R** | **H** | **R** | **L** | **A** | **R** | **E** | **L** | **H** | **D** | **S** | **V** | **S** | **Q** | **Q** | **L** | **F** | **A** | **A** | **M** | **M** | **M** | **M** | **S** | **A** | **L** | **T** | **E** | **Q** | **A** | **E** | **K** | **S** | **E** | **T** | **P** | **E** | **M** | **F** | **R** | **K** | **Q** | **L** | **K** | **M** | **V** | **A** | **E** | **I** | **I** | **N** | **A** | **S** | **Q** | **S** | **E** | **M** | **R** | **A** | **L** | **L** | **L** | **H** | **L** | **R** | **P** | **V** | **N** | **L** | **E** | **E** | **K** | **S** | **L** | **K** | **Q** | **G** | **I** | **E** | **Q** | **L** | **L** | **K** | **E** | **L** | **Q** | **N** | **K** | **I** | **Q** | **I** | **S** | **L** | **K** | **W** | **D** | **V** | **E** | **D** | **V** | **K** | **L** | **T** | **S** | **S** | **I** | **E** | **D** | **H** | **L** | **F** | **R** | **I** | **V** | **Q** | **E** | **L** | **L** | **S** | **N** | **T** | **L** | **R** | **H** | **A** | **K** | **A** | **N** | **E** | **L** | **E** | **V** | **Y** | **L** | **H** | **K** | **I** | **D** | **N** | **N** | **L** | **L** | **L** | **R** | **I** | **I** | **D** | **D** | **G** | **T** | **G** | **F** | **N** | **M** | **N** | **E** | **T** | **K** | **T** | **G** | **S** | **Y** | **G** | **L** | **N** | **N** | **I** | **K** | **E** | **R** | **V** | **A** | **G** | **I** | **G** | **G** | **T** | **V** | **K** | **I** | **I** | **S** | **F** | **K** | **G** | **Q** | **G** | **T** | **S** | **V** | **E** | **I** | **K** | **V** | **P** | **L** | **M** | **K** | **E** | **A** |
| **N55655** | **M** | **M** | **G** | **R** | **I** | **S** | **K** | **A** | **M** | **L** | **A** | **V** | **Y** | **S** | **G** | **I** | **A** | **A** | **F** | **L** | **I** | **I** | **L** | **F** | **S** | **L** | **F** | **T** | **Y** | **F** | **Y** | **A** | **S** | **N** | **Q** | **R** | **H** | **W** | **W** | **G** | **E** | **L** | **L** | **R** | **A** | **R** | **L** | **L** | **Y** | **V** | **P** | **L** | **I** | **F** | **H** | **L** | **M** | **A** | **I** | **S** | **L** | **G** | **V** | **G** | **L** | **I** | **V** | **F** | **L** | **L** | **L** | **S** | **L** | **I** | **Q** | **K** | **A** | **K** | **Y** | **G** | **K** | **I** | **E** | **E** | **K** | **L** | **R** | **A** | **L** | **S** | **S** | **G** | **N** | **Y** | **E** | **S** | **K** | **L** | **L** | **H** | **Q** | **P** | **I** | **P** | **S** | **A** | **S** | **D** | **D** | **L** | **Y** | **I** | **K** | **D** | **I** | **D** | **M** | **E** | **I** | **T** | **K** | **I** | **K** | **E** | **K** | **M** | **I** | **E** | **V** | **S** | **S** | **E** | **L** | **Q** | **I** | **M** | **T** | **S** | **R** | **P** | **Q** | **Y** | **V** | **D** | **G** | **Q** | **T** | **K** | **E** | **E** | **I** | **L** | **E** | **L** | **E** | **R** | **H** | **R** | **L** | **A** | **R** | **E** | **L** | **H** | **D** | **S** | **V** | **S** | **Q** | **Q** | **L** | **F** | **A** | **A** | **M** | **M** | **M** | **M** | **S** | **A** | **L** | **T** | **E** | **Q** | **A** | **E** | **K** | **S** | **E** | **T** | **P** | **E** | **M** | **F** | **R** | **K** | **Q** | **L** | **K** | **M** | **V** | **A** | **E** | **I** | **I** | **N** | **A** | **S** | **Q** | **S** | **E** | **M** | **R** | **A** | **L** | **L** | **L** | **H** | **L** | **R** | **P** | **V** | **N** | **L** | **E** | **E** | **K** | **S** | **L** | **K** | **Q** | **G** | **I** | **E** | **Q** | **L** | **L** | **K** | **E** | **L** | **Q** | **N** | **K** | **I** | **Q** | **I** | **S** | **L** | **K** | **W** | **D** | **V** | **E** | **D** | **V** | **K** | **L** | **T** | **S** | **S** | **I** | **E** | **D** | **H** | **L** | **F** | **R** | **I** | **V** | **Q** | **E** | **L** | **L** | **S** | **N** | **T** | **L** | **R** | **H** | **A** | **K** | **A** | **N** | **E** | **L** | **E** | **V** | **Y** | **L** | **H** | **K** | **I** | **D** | **N** | **N** | **L** | **L** | **L** | **R** | **I** | **I** | **D** | **D** | **G** | **T** | **G** | **F** | **N** | **M** | **N** | **E** | **T** | **K** | **T** | **G** | **S** | **Y** | **G** | **L** | **N** | **N** | **I** | **K** | **E** | **R** | **V** | **A** | **G** | **I** | **G** | **G** | **T** | **V** | **K** | **I** | **I** | **S** | **F** | **K** | **G** | **Q** | **G** | **T** | **S** | **V** | **E** | **I** | **K** | **V** | **P** | **L** | **M** | **K** | **E** | **A** |
| **N52824** | **M** | **M** | **G** | **K** | **I** | **S** | **R** | **A** | **M** | **L** | **A** | **V** | **Y** | **S** | **G** | **I** | **A** | **A** | **F** | **L** | **I** | **I** | **L** | **F** | **S** | **L** | **F** | **T** | **Y** | **F** | **Y** | **A** | **S** | **N** | **Q** | **S** | **H** | **W** | **W** | **G** | **E** | **L** | **L** | **R** | **A** | **R** | **L** | **L** | **Y** | **V** | **P** | **L** | **I** | **F** | **H** | **L** | **L** | **A** | **I** | **S** | **L** | **G** | **V** | **G** | **L** | **I** | **V** | **F** | **L** | **L** | **L** | **S** | **L** | **I** | **Q** | **K** | **T** | **K** | **Y** | **G** | **K** | **I** | **E** | **E** | **K** | **L** | **R** | **A** | **L** | **S** | **S** | **G** | **N** | **Y** | **E** | **S** | **K** | **L** | **L** | **L** | **L** | **P** | **I** | **P** | **S** | **A** | **S** | **D** | **D** | **L** | **Y** | **I** | **K** | **D** | **I** | **D** | **K** | **E** | **I** | **T** | **K** | **I** | **K** | **E** | **K** | **M** | **I** | **E** | **V** | **S** | **S** | **E** | **L** | **Q** | **I** | **V** | **T** | **S** | **R** | **P** | **Q** | **Y** | **V** | **D** | **G** | **Q** | **T** | **K** | **E** | **E** | **I** | **L** | **E** | **L** | **E** | **R** | **H** | **R** | **L** | **A** | **R** | **E** | **L** | **H** | **D** | **S** | **V** | **S** | **Q** | **Q** | **L** | **F** | **A** | **A** | **M** | **M** | **M** | **M** | **S** | **A** | **L** | **T** | **E** | **Q** | **A** | **E** | **K** | **S** | **E** | **T** | **P** | **E** | **M** | **F** | **R** | **K** | **Q** | **L** | **K** | **M** | **V** | **A** | **E** | **I** | **I** | **N** | **A** | **S** | **Q** | **S** | **E** | **M** | **R** | **A** | **L** | **L** | **L** | **H** | **L** | **R** | **P** | **V** | **N** | **L** | **E** | **E** | **K** | **S** | **L** | **K** | **Q** | **G** | **I** | **E** | **Q** | **L** | **L** | **K** | **E** | **L** | **Q** | **N** | **K** | **I** | **Q** | **I** | **S** | **L** | **K** | **W** | **D** | **V** | **E** | **D** | **V** | **K** | **L** | **S** | **S** | **S** | **I** | **E** | **D** | **H** | **L** | **F** | **R** | **I** | **V** | **Q** | **E** | **L** | **L** | **S** | **N** | **T** | **L** | **R** | **H** | **A** | **K** | **A** | **N** | **E** | **L** | **E** | **V** | **Y** | **L** | **H** | **K** | **I** | **D** | **N** | **N** | **L** | **L** | **L** | **R** | **I** | **I** | **D** | **D** | **G** | **T** | **G** | **F** | **N** | **M** | **N** | **E** | **T** | **K** | **T** | **G** | **S** | **Y** | **G** | **L** | **N** | **N** | **I** | **K** | **E** | **R** | **V** | **A** | **G** | **I** | **G** | **G** | **T** | **V** | **K** | **I** | **I** | **S** | **F** | **K** | **G** | **Q** | **G** | **T** | **S** | **V** | **E** | **I** | **K** | **V** | **P** | **L** | **M** | **K** | **E** | **A** |
| **N54599** | **M** | **M** | **G** | **K** | **I** | **S** | **R** | **A** | **M** | **L** | **A** | **V** | **Y** | **S** | **G** | **I** | **A** | **A** | **F** | **L** | **I** | **I** | **L** | **F** | **S** | **L** | **F** | **T** | **Y** | **F** | **Y** | **A** | **S** | **N** | **Q** | **S** | **H** | **W** | **W** | **G** | **E** | **L** | **L** | **R** | **A** | **R** | **L** | **L** | **Y** | **V** | **P** | **L** | **I** | **F** | **H** | **L** | **L** | **A** | **I** | **S** | **L** | **G** | **V** | **G** | **L** | **I** | **V** | **F** | **L** | **L** | **L** | **S** | **L** | **I** | **Q** | **K** | **T** | **K** | **Y** | **G** | **K** | **I** | **E** | **E** | **K** | **L** | **R** | **A** | **L** | **S** | **S** | **G** | **N** | **Y** | **E** | **S** | **K** | **L** | **L** | **L** | **L** | **P** | **I** | **P** | **S** | **A** | **S** | **D** | **D** | **L** | **Y** | **I** | **K** | **D** | **I** | **D** | **K** | **E** | **I** | **T** | **K** | **I** | **K** | **E** | **K** | **M** | **I** | **E** | **V** | **S** | **S** | **E** | **L** | **Q** | **I** | **V** | **T** | **S** | **R** | **P** | **Q** | **Y** | **V** | **D** | **G** | **Q** | **T** | **K** | **E** | **E** | **I** | **L** | **E** | **L** | **E** | **R** | **H** | **R** | **L** | **A** | **R** | **E** | **L** | **H** | **D** | **S** | **V** | **S** | **Q** | **Q** | **L** | **F** | **A** | **A** | **M** | **M** | **M** | **M** | **S** | **A** | **L** | **T** | **E** | **Q** | **A** | **E** | **K** | **S** | **E** | **T** | **P** | **E** | **M** | **F** | **R** | **K** | **Q** | **L** | **K** | **M** | **V** | **A** | **E** | **I** | **I** | **N** | **A** | **S** | **Q** | **S** | **E** | **M** | **R** | **A** | **L** | **L** | **L** | **H** | **L** | **R** | **P** | **V** | **N** | **L** | **E** | **E** | **K** | **S** | **L** | **K** | **Q** | **G** | **I** | **E** | **Q** | **L** | **L** | **K** | **E** | **L** | **Q** | **N** | **K** | **I** | **Q** | **I** | **S** | **L** | **K** | **W** | **D** | **V** | **E** | **D** | **V** | **K** | **L** | **S** | **S** | **S** | **I** | **E** | **D** | **H** | **L** | **F** | **R** | **I** | **V** | **Q** | **E** | **L** | **L** | **S** | **N** | **T** | **L** | **R** | **H** | **A** | **K** | **A** | **N** | **E** | **L** | **E** | **V** | **Y** | **L** | **H** | **K** | **I** | **D** | **N** | **N** | **L** | **L** | **L** | **R** | **I** | **I** | **D** | **D** | **G** | **T** | **G** | **F** | **N** | **M** | **N** | **E** | **T** | **K** | **T** | **G** | **S** | **Y** | **G** | **L** | **N** | **N** | **I** | **K** | **E** | **R** | **V** | **A** | **G** | **I** | **G** | **G** | **T** | **V** | **K** | **I** | **I** | **S** | **F** | **K** | **G** | **Q** | **G** | **T** | **S** | **V** | **E** | **I** | **K** | **V** | **P** | **L** | **M** | **K** | **E** | **A** |
| **N52732** | **M** | **M** | **G** | **K** | **I** | **S** | **R** | **A** | **M** | **L** | **A** | **V** | **Y** | **S** | **G** | **I** | **A** | **A** | **F** | **L** | **I** | **I** | **L** | **F** | **S** | **L** | **F** | **T** | **Y** | **F** | **Y** | **A** | **S** | **N** | **Q** | **S** | **H** | **W** | **W** | **G** | **E** | **L** | **L** | **R** | **A** | **R** | **L** | **L** | **Y** | **V** | **P** | **L** | **I** | **F** | **H** | **L** | **L** | **A** | **I** | **S** | **L** | **G** | **V** | **G** | **L** | **I** | **V** | **F** | **L** | **L** | **L** | **S** | **L** | **I** | **Q** | **K** | **T** | **K** | **Y** | **G** | **K** | **I** | **E** | **E** | **K** | **L** | **R** | **A** | **L** | **S** | **S** | **G** | **N** | **Y** | **E** | **S** | **K** | **L** | **L** | **L** | **L** | **P** | **I** | **P** | **S** | **A** | **S** | **D** | **D** | **L** | **Y** | **I** | **K** | **D** | **I** | **D** | **K** | **E** | **I** | **T** | **K** | **I** | **K** | **E** | **K** | **M** | **I** | **E** | **V** | **S** | **S** | **E** | **L** | **Q** | **I** | **V** | **T** | **S** | **R** | **P** | **Q** | **Y** | **V** | **D** | **G** | **Q** | **T** | **K** | **E** | **E** | **I** | **L** | **E** | **L** | **E** | **R** | **H** | **R** | **L** | **A** | **R** | **E** | **L** | **H** | **D** | **S** | **V** | **S** | **Q** | **Q** | **L** | **F** | **A** | **A** | **M** | **M** | **M** | **M** | **S** | **A** | **L** | **T** | **E** | **Q** | **A** | **E** | **K** | **S** | **E** | **T** | **P** | **E** | **M** | **F** | **R** | **K** | **Q** | **L** | **K** | **M** | **V** | **A** | **E** | **I** | **I** | **N** | **A** | **S** | **Q** | **S** | **E** | **M** | **R** | **A** | **L** | **L** | **L** | **H** | **L** | **R** | **P** | **V** | **N** | **L** | **E** | **E** | **K** | **S** | **L** | **K** | **Q** | **G** | **I** | **E** | **Q** | **L** | **L** | **K** | **E** | **L** | **Q** | **N** | **K** | **I** | **Q** | **I** | **S** | **L** | **K** | **W** | **D** | **V** | **E** | **D** | **V** | **K** | **L** | **S** | **S** | **S** | **I** | **E** | **D** | **H** | **L** | **F** | **R** | **I** | **V** | **Q** | **E** | **L** | **L** | **S** | **N** | **T** | **L** | **R** | **H** | **A** | **K** | **A** | **N** | **E** | **L** | **E** | **V** | **Y** | **L** | **H** | **K** | **I** | **D** | **N** | **N** | **L** | **L** | **L** | **R** | **I** | **I** | **D** | **D** | **G** | **T** | **G** | **F** | **N** | **M** | **N** | **E** | **T** | **K** | **T** | **G** | **S** | **Y** | **G** | **L** | **N** | **N** | **I** | **K** | **E** | **R** | **V** | **A** | **G** | **I** | **G** | **G** | **T** | **V** | **K** | **I** | **I** | **S** | **F** | **K** | **G** | **Q** | **G** | **T** | **S** | **V** | **E** | **I** | **K** | **V** | **P** | **L** | **M** | **K** | **E** | **A** |
| **N60357F** | **M** | **M** | **G** | **R** | **I** | **S** | **K** | **A** | **M** | **L** | **A** | **V** | **Y** | **S** | **G** | **I** | **A** | **A** | **F** | **L** | **I** | **I** | **L** | **F** | **S** | **L** | **F** | **T** | **Y** | **F** | **Y** | **A** | **S** | **N** | **Q** | **S** | **H** | **W** | **W** | **G** | **E** | **L** | **L** | **R** | **A** | **R** | **L** | **L** | **Y** | **V** | **P** | **L** | **I** | **F** | **H** | **L** | **M** | **A** | **I** | **S** | **L** | **G** | **V** | **G** | **L** | **I** | **V** | **F** | **L** | **L** | **L** | **S** | **L** | **I** | **Q** | **K** | **A** | **K** | **Y** | **G** | **K** | **I** | **E** | **E** | **K** | **L** | **R** | **A** | **L** | **S** | **S** | **G** | **N** | **Y** | **E** | **S** | **K** | **L** | **L** | **H** | **Q** | **P** | **I** | **P** | **S** | **A** | **S** | **D** | **D** | **L** | **Y** | **I** | **K** | **D** | **I** | **D** | **M** | **E** | **I** | **T** | **K** | **I** | **K** | **E** | **K** | **M** | **I** | **E** | **V** | **S** | **S** | **E** | **L** | **Q** | **I** | **M** | **T** | **S** | **R** | **P** | **Q** | **Y** | **V** | **D** | **G** | **Q** | **T** | **K** | **E** | **E** | **I** | **L** | **E** | **L** | **E** | **R** | **H** | **R** | **L** | **A** | **R** | **E** | **L** | **H** | **D** | **S** | **V** | **S** | **Q** | **Q** | **L** | **F** | **A** | **A** | **M** | **M** | **M** | **M** | **S** | **A** | **L** | **T** | **E** | **Q** | **A** | **E** | **K** | **S** | **E** | **T** | **P** | **G** | **M** | **F** | **R** | **K** | **Q** | **L** | **K** | **M** | **V** | **A** | **E** | **I** | **I** | **N** | **A** | **S** | **Q** | **S** | **E** | **M** | **R** | **A** | **L** | **L** | **L** | **H** | **L** | **R** | **P** | **V** | **N** | **L** | **E** | **E** | **K** | **S** | **L** | **K** | **Q** | **G** | **I** | **E** | **Q** | **L** | **L** | **K** | **E** | **L** | **Q** | **N** | **K** | **I** | **Q** | **I** | **S** | **L** | **K** | **W** | **D** | **V** | **E** | **D** | **V** | **K** | **L** | **T** | **S** | **S** | **I** | **E** | **D** | **H** | **L** | **F** | **R** | **I** | **V** | **Q** | **E** | **L** | **L** | **S** | **N** | **T** | **L** | **R** | **H** | **A** | **K** | **A** | **N** | **E** | **L** | **E** | **V** | **Y** | **L** | **H** | **K** | **I** | **D** | **N** | **N** | **L** | **L** | **L** | **R** | **I** | **I** | **D** | **D** | **G** | **T** | **G** | **F** | **N** | **M** | **N** | **E** | **T** | **K** | **T** | **G** | **S** | **Y** | **G** | **L** | **N** | **N** | **I** | **K** | **E** | **R** | **V** | **A** | **G** | **I** | **G** | **G** | **T** | **V** | **K** | **I** | **I** | **S** | **F** | **K** | **G** | **Q** | **G** | **T** | **S** | **V** | **E** | **I** | **K** | **V** | **P** | **L** | **M** | **K** | **E** | **A** |
| **N60190F** | **M** | **M** | **G** | **K** | **I** | **S** | **R** | **A** | **M** | **L** | **A** | **V** | **Y** | **S** | **G** | **I** | **A** | **A** | **F** | **L** | **I** | **I** | **L** | **F** | **S** | **L** | **F** | **T** | **Y** | **F** | **Y** | **A** | **S** | **N** | **Q** | **S** | **H** | **W** | **W** | **G** | **E** | **L** | **L** | **R** | **A** | **R** | **L** | **L** | **Y** | **V** | **P** | **L** | **I** | **F** | **H** | **L** | **L** | **A** | **I** | **S** | **L** | **G** | **V** | **G** | **L** | **I** | **V** | **F** | **L** | **L** | **L** | **S** | **L** | **I** | **Q** | **K** | **T** | **K** | **Y** | **G** | **K** | **I** | **E** | **E** | **K** | **L** | **R** | **A** | **L** | **S** | **S** | **G** | **N** | **Y** | **E** | **S** | **K** | **L** | **L** | **L** | **L** | **P** | **I** | **P** | **S** | **A** | **S** | **D** | **D** | **L** | **Y** | **I** | **K** | **D** | **I** | **D** | **K** | **E** | **I** | **T** | **K** | **I** | **K** | **E** | **K** | **M** | **I** | **E** | **V** | **S** | **S** | **E** | **L** | **Q** | **I** | **V** | **T** | **S** | **R** | **P** | **Q** | **Y** | **V** | **D** | **G** | **Q** | **T** | **K** | **E** | **E** | **I** | **L** | **E** | **L** | **E** | **R** | **H** | **R** | **L** | **A** | **R** | **E** | **L** | **H** | **D** | **S** | **V** | **S** | **Q** | **Q** | **L** | **F** | **A** | **A** | **M** | **M** | **M** | **M** | **S** | **A** | **L** | **T** | **E** | **Q** | **A** | **E** | **K** | **S** | **E** | **T** | **P** | **E** | **M** | **F** | **R** | **K** | **Q** | **L** | **K** | **M** | **V** | **A** | **E** | **I** | **I** | **N** | **A** | **S** | **Q** | **S** | **E** | **M** | **R** | **A** | **L** | **L** | **L** | **H** | **L** | **R** | **P** | **V** | **N** | **L** | **E** | **E** | **K** | **S** | **L** | **K** | **Q** | **G** | **I** | **E** | **Q** | **L** | **L** | **K** | **E** | **L** | **Q** | **N** | **K** | **I** | **Q** | **I** | **S** | **L** | **K** | **W** | **D** | **V** | **E** | **D** | **V** | **K** | **L** | **S** | **S** | **S** | **I** | **E** | **D** | **H** | **L** | **F** | **R** | **I** | **V** | **Q** | **E** | **L** | **L** | **S** | **N** | **T** | **L** | **R** | **H** | **A** | **K** | **A** | **N** | **E** | **L** | **E** | **V** | **Y** | **L** | **H** | **K** | **I** | **D** | **N** | **N** | **L** | **L** | **L** | **R** | **I** | **I** | **D** | **D** | **G** | **T** | **G** | **F** | **N** | **M** | **N** | **E** | **T** | **K** | **T** | **G** | **S** | **Y** | **G** | **L** | **N** | **N** | **I** | **K** | **E** | **R** | **V** | **V** | **G** | **I** | **G** | **G** | **T** | **V** | **K** | **I** | **I** | **S** | **F** | **K** | **G** | **Q** | **G** | **T** | **S** | **V** | **E** | **I** | **K** | **V** | **P** | **L** | **M** | **K** | **E** | **A** |
| **N59711F** | **M** | **M** | **G** | **K** | **I** | **S** | **R** | **A** | **M** | **L** | **A** | **V** | **Y** | **S** | **G** | **I** | **A** | **A** | **F** | **L** | **I** | **I** | **L** | **F** | **S** | **L** | **F** | **T** | **Y** | **F** | **Y** | **A** | **S** | **N** | **Q** | **S** | **H** | **W** | **W** | **G** | **E** | **L** | **L** | **R** | **A** | **R** | **L** | **L** | **Y** | **V** | **P** | **L** | **I** | **F** | **H** | **L** | **L** | **A** | **I** | **S** | **L** | **G** | **V** | **G** | **L** | **I** | **V** | **F** | **L** | **L** | **L** | **S** | **L** | **I** | **Q** | **K** | **T** | **K** | **Y** | **G** | **K** | **I** | **E** | **E** | **K** | **L** | **R** | **A** | **L** | **S** | **S** | **G** | **N** | **Y** | **E** | **S** | **K** | **L** | **L** | **L** | **L** | **P** | **I** | **P** | **S** | **A** | **S** | **D** | **D** | **L** | **Y** | **I** | **K** | **D** | **I** | **D** | **K** | **E** | **I** | **T** | **K** | **I** | **K** | **E** | **K** | **M** | **I** | **E** | **V** | **S** | **S** | **E** | **L** | **Q** | **I** | **V** | **T** | **S** | **R** | **P** | **Q** | **Y** | **V** | **D** | **G** | **Q** | **T** | **K** | **E** | **E** | **I** | **L** | **E** | **L** | **E** | **R** | **H** | **R** | **L** | **A** | **R** | **E** | **L** | **H** | **D** | **S** | **V** | **S** | **Q** | **Q** | **L** | **F** | **A** | **A** | **M** | **M** | **M** | **M** | **S** | **A** | **L** | **T** | **E** | **Q** | **A** | **E** | **K** | **S** | **E** | **T** | **P** | **E** | **M** | **F** | **R** | **K** | **Q** | **L** | **K** | **M** | **V** | **A** | **E** | **I** | **I** | **N** | **A** | **S** | **Q** | **S** | **E** | **M** | **R** | **A** | **L** | **L** | **L** | **H** | **L** | **R** | **P** | **V** | **N** | **L** | **E** | **E** | **K** | **S** | **L** | **K** | **Q** | **G** | **I** | **E** | **Q** | **L** | **L** | **K** | **E** | **L** | **Q** | **N** | **K** | **I** | **Q** | **I** | **S** | **L** | **K** | **W** | **D** | **V** | **E** | **D** | **V** | **K** | **L** | **S** | **S** | **S** | **I** | **E** | **D** | **H** | **L** | **F** | **R** | **I** | **V** | **Q** | **E** | **L** | **L** | **S** | **N** | **T** | **L** | **R** | **H** | **A** | **K** | **A** | **N** | **E** | **L** | **E** | **V** | **Y** | **L** | **H** | **K** | **I** | **D** | **N** | **N** | **L** | **L** | **L** | **R** | **I** | **I** | **D** | **D** | **G** | **T** | **G** | **F** | **N** | **M** | **N** | **E** | **T** | **K** | **T** | **G** | **S** | **Y** | **G** | **L** | **N** | **N** | **I** | **K** | **E** | **R** | **V** | **A** | **G** | **I** | **G** | **G** | **T** | **V** | **K** | **I** | **I** | **S** | **F** | **K** | **G** | **Q** | **G** | **T** | **S** | **V** | **E** | **I** | **K** | **V** | **P** | **L** | **M** | **K** | **E** | **A** |
| **N53479** | **M** | **M** | **G** | **R** | **I** | **S** | **K** | **A** | **M** | **L** | **A** | **V** | **Y** | **S** | **G** | **I** | **A** | **A** | **F** | **L** | **I** | **I** | **L** | **F** | **S** | **L** | **F** | **T** | **Y** | **F** | **Y** | **A** | **S** | **N** | **Q** | **S** | **H** | **W** | **W** | **G** | **E** | **L** | **L** | **R** | **A** | **R** | **L** | **L** | **Y** | **V** | **P** | **L** | **I** | **F** | **H** | **L** | **M** | **A** | **I** | **S** | **L** | **G** | **V** | **G** | **L** | **I** | **V** | **F** | **L** | **L** | **L** | **S** | **L** | **I** | **Q** | **K** | **A** | **K** | **Y** | **G** | **K** | **I** | **E** | **E** | **K** | **L** | **R** | **A** | **L** | **S** | **S** | **G** | **N** | **Y** | **E** | **S** | **K** | **L** | **L** | **H** | **Q** | **P** | **I** | **P** | **S** | **A** | **S** | **D** | **D** | **L** | **Y** | **I** | **K** | **D** | **I** | **D** | **M** | **E** | **I** | **T** | **K** | **I** | **K** | **E** | **K** | **M** | **I** | **E** | **V** | **S** | **S** | **E** | **L** | **Q** | **I** | **M** | **T** | **S** | **R** | **P** | **Q** | **Y** | **V** | **D** | **G** | **Q** | **T** | **K** | **E** | **E** | **I** | **L** | **E** | **L** | **E** | **R** | **H** | **R** | **L** | **A** | **R** | **E** | **L** | **H** | **D** | **S** | **V** | **S** | **Q** | **Q** | **L** | **F** | **A** | **A** | **M** | **M** | **M** | **M** | **S** | **A** | **L** | **T** | **E** | **Q** | **A** | **E** | **K** | **S** | **E** | **T** | **P** | **E** | **M** | **F** | **R** | **K** | **Q** | **L** | **K** | **M** | **V** | **A** | **E** | **I** | **I** | **N** | **A** | **S** | **Q** | **S** | **E** | **M** | **R** | **A** | **L** | **L** | **L** | **H** | **L** | **R** | **P** | **V** | **N** | **L** | **E** | **E** | **K** | **S** | **L** | **K** | **Q** | **G** | **I** | **E** | **Q** | **L** | **L** | **K** | **E** | **L** | **Q** | **N** | **K** | **I** | **Q** | **I** | **S** | **L** | **K** | **W** | **D** | **V** | **E** | **D** | **V** | **K** | **L** | **T** | **S** | **S** | **I** | **E** | **D** | **H** | **L** | **F** | **R** | **I** | **V** | **Q** | **E** | **L** | **L** | **S** | **N** | **T** | **L** | **R** | **H** | **A** | **K** | **A** | **N** | **E** | **L** | **E** | **V** | **Y** | **L** | **H** | **K** | **I** | **D** | **N** | **N** | **L** | **L** | **L** | **R** | **I** | **I** | **D** | **D** | **G** | **T** | **G** | **F** | **N** | **M** | **N** | **E** | **T** | **K** | **T** | **G** | **S** | **Y** | **G** | **L** | **N** | **N** | **I** | **K** | **E** | **R** | **V** | **A** | **G** | **I** | **G** | **G** | **T** | **V** | **K** | **I** | **I** | **S** | **F** | **K** | **G** | **Q** | **G** | **T** | **S** | **V** | **E** | **I** | **K** | **V** | **P** | **L** | **M** | **K** | **E** | **A** |
| **N54519** | **M** | **M** | **G** | **K** | **I** | **S** | **R** | **A** | **M** | **L** | **A** | **V** | **Y** | **S** | **G** | **I** | **A** | **A** | **F** | **L** | **I** | **I** | **L** | **F** | **S** | **L** | **F** | **T** | **Y** | **F** | **Y** | **A** | **S** | **N** | **Q** | **S** | **H** | **W** | **W** | **G** | **E** | **L** | **L** | **R** | **A** | **R** | **L** | **L** | **Y** | **V** | **P** | **L** | **I** | **F** | **H** | **L** | **L** | **A** | **I** | **S** | **L** | **G** | **V** | **G** | **L** | **I** | **V** | **F** | **L** | **L** | **L** | **S** | **L** | **I** | **Q** | **K** | **T** | **K** | **Y** | **G** | **K** | **I** | **E** | **E** | **K** | **L** | **R** | **A** | **L** | **S** | **S** | **G** | **N** | **Y** | **E** | **S** | **K** | **L** | **L** | **L** | **L** | **P** | **I** | **P** | **S** | **A** | **S** | **D** | **D** | **L** | **Y** | **I** | **K** | **D** | **I** | **D** | **K** | **E** | **I** | **T** | **K** | **I** | **K** | **E** | **K** | **M** | **I** | **E** | **V** | **S** | **S** | **E** | **L** | **Q** | **I** | **V** | **T** | **S** | **R** | **P** | **Q** | **Y** | **V** | **D** | **G** | **Q** | **T** | **K** | **E** | **E** | **I** | **L** | **E** | **L** | **E** | **R** | **H** | **R** | **L** | **A** | **R** | **E** | **L** | **H** | **D** | **S** | **V** | **S** | **Q** | **Q** | **L** | **F** | **A** | **A** | **M** | **M** | **M** | **M** | **S** | **A** | **L** | **T** | **E** | **Q** | **A** | **E** | **K** | **S** | **E** | **T** | **P** | **E** | **M** | **F** | **R** | **K** | **Q** | **L** | **K** | **M** | **V** | **A** | **E** | **I** | **I** | **N** | **A** | **S** | **Q** | **S** | **E** | **M** | **R** | **A** | **L** | **L** | **L** | **H** | **L** | **R** | **P** | **V** | **N** | **L** | **E** | **E** | **K** | **S** | **L** | **K** | **Q** | **G** | **I** | **E** | **Q** | **L** | **L** | **K** | **E** | **L** | **Q** | **N** | **K** | **I** | **Q** | **I** | **S** | **L** | **K** | **W** | **D** | **V** | **E** | **D** | **V** | **K** | **L** | **S** | **S** | **S** | **I** | **E** | **D** | **H** | **L** | **F** | **R** | **I** | **V** | **Q** | **E** | **L** | **L** | **S** | **N** | **T** | **L** | **R** | **H** | **A** | **K** | **A** | **N** | **E** | **L** | **E** | **V** | **Y** | **L** | **H** | **K** | **I** | **D** | **N** | **N** | **L** | **L** | **L** | **R** | **I** | **I** | **D** | **D** | **G** | **T** | **G** | **F** | **N** | **M** | **N** | **E** | **T** | **K** | **T** | **G** | **S** | **Y** | **G** | **L** | **N** | **N** | **I** | **K** | **E** | **R** | **V** | **A** | **G** | **I** | **G** | **G** | **T** | **V** | **K** | **I** | **I** | **S** | **F** | **K** | **G** | **Q** | **G** | **T** | **S** | **V** | **E** | **I** | **K** | **V** | **P** | **L** | **M** | **K** | **E** | **A** |
| **N52769** | **M** | **M** | **G** | **K** | **I** | **S** | **R** | **A** | **M** | **L** | **A** | **V** | **Y** | **S** | **G** | **I** | **A** | **A** | **F** | **L** | **I** | **I** | **L** | **F** | **S** | **L** | **F** | **T** | **Y** | **F** | **Y** | **A** | **S** | **N** | **Q** | **S** | **H** | **W** | **W** | **G** | **E** | **L** | **L** | **R** | **A** | **R** | **L** | **L** | **Y** | **V** | **P** | **L** | **I** | **F** | **H** | **L** | **L** | **A** | **I** | **S** | **L** | **G** | **V** | **G** | **L** | **I** | **V** | **F** | **L** | **L** | **L** | **S** | **L** | **I** | **Q** | **K** | **T** | **K** | **Y** | **G** | **K** | **I** | **E** | **E** | **K** | **L** | **R** | **A** | **L** | **S** | **S** | **G** | **N** | **Y** | **E** | **S** | **K** | **L** | **L** | **L** | **L** | **P** | **I** | **P** | **S** | **A** | **S** | **D** | **D** | **L** | **Y** | **I** | **K** | **D** | **I** | **D** | **K** | **E** | **I** | **T** | **K** | **I** | **K** | **E** | **K** | **M** | **I** | **E** | **V** | **S** | **S** | **E** | **L** | **Q** | **I** | **V** | **T** | **S** | **R** | **P** | **Q** | **Y** | **V** | **D** | **G** | **Q** | **T** | **K** | **E** | **E** | **I** | **L** | **E** | **L** | **E** | **R** | **H** | **R** | **L** | **A** | **R** | **E** | **L** | **H** | **D** | **S** | **V** | **S** | **Q** | **Q** | **L** | **F** | **A** | **A** | **M** | **M** | **M** | **M** | **S** | **A** | **L** | **T** | **E** | **Q** | **A** | **E** | **K** | **S** | **E** | **T** | **P** | **E** | **M** | **F** | **R** | **K** | **Q** | **L** | **K** | **M** | **V** | **A** | **E** | **I** | **I** | **N** | **A** | **S** | **Q** | **S** | **E** | **M** | **R** | **A** | **L** | **L** | **L** | **H** | **L** | **R** | **P** | **V** | **N** | **L** | **E** | **E** | **K** | **S** | **L** | **K** | **Q** | **G** | **I** | **E** | **Q** | **L** | **L** | **K** | **E** | **L** | **Q** | **N** | **K** | **I** | **Q** | **I** | **S** | **L** | **K** | **W** | **D** | **V** | **E** | **D** | **V** | **K** | **L** | **S** | **S** | **S** | **I** | **E** | **D** | **H** | **L** | **F** | **R** | **I** | **V** | **Q** | **E** | **L** | **L** | **S** | **N** | **T** | **L** | **R** | **H** | **A** | **K** | **A** | **N** | **E** | **L** | **E** | **V** | **Y** | **L** | **H** | **K** | **I** | **D** | **N** | **N** | **L** | **L** | **L** | **R** | **I** | **I** | **D** | **D** | **G** | **T** | **G** | **F** | **N** | **M** | **N** | **E** | **T** | **K** | **T** | **G** | **S** | **Y** | **G** | **L** | **N** | **N** | **I** | **K** | **E** | **R** | **V** | **A** | **G** | **I** | **G** | **G** | **T** | **V** | **K** | **I** | **I** | **S** | **F** | **K** | **G** | **Q** | **G** | **T** | **S** | **V** | **E** | **I** | **K** | **V** | **P** | **L** | **M** | **K** | **E** | **A** |
| **N59624F** | **M** | **M** | **G** | **K** | **I** | **S** | **R** | **A** | **M** | **L** | **A** | **V** | **Y** | **S** | **G** | **I** | **A** | **A** | **F** | **L** | **I** | **I** | **L** | **F** | **S** | **L** | **F** | **T** | **Y** | **F** | **Y** | **A** | **S** | **N** | **Q** | **S** | **H** | **W** | **W** | **G** | **E** | **L** | **L** | **R** | **A** | **R** | **L** | **L** | **Y** | **V** | **P** | **L** | **I** | **F** | **H** | **L** | **L** | **A** | **I** | **S** | **L** | **G** | **V** | **G** | **L** | **I** | **V** | **F** | **L** | **L** | **L** | **S** | **L** | **I** | **Q** | **K** | **T** | **K** | **Y** | **G** | **K** | **I** | **E** | **E** | **K** | **L** | **R** | **A** | **L** | **S** | **S** | **G** | **N** | **Y** | **E** | **S** | **K** | **L** | **L** | **L** | **L** | **P** | **I** | **P** | **S** | **A** | **S** | **D** | **D** | **L** | **Y** | **I** | **K** | **D** | **I** | **D** | **K** | **E** | **I** | **T** | **K** | **I** | **K** | **E** | **K** | **M** | **I** | **E** | **V** | **S** | **S** | **E** | **L** | **Q** | **I** | **V** | **T** | **S** | **R** | **P** | **Q** | **Y** | **V** | **D** | **G** | **Q** | **T** | **K** | **E** | **E** | **I** | **L** | **E** | **L** | **E** | **R** | **H** | **R** | **L** | **A** | **R** | **E** | **L** | **H** | **D** | **S** | **V** | **S** | **Q** | **Q** | **L** | **F** | **A** | **A** | **M** | **M** | **M** | **M** | **S** | **A** | **L** | **T** | **E** | **Q** | **A** | **E** | **K** | **S** | **E** | **T** | **P** | **E** | **M** | **F** | **R** | **K** | **Q** | **L** | **K** | **M** | **V** | **A** | **E** | **I** | **I** | **N** | **A** | **S** | **Q** | **S** | **E** | **M** | **R** | **A** | **L** | **L** | **L** | **H** | **L** | **R** | **P** | **V** | **N** | **L** | **E** | **E** | **K** | **S** | **L** | **K** | **Q** | **G** | **I** | **E** | **Q** | **L** | **L** | **K** | **E** | **L** | **Q** | **N** | **K** | **I** | **Q** | **I** | **S** | **L** | **K** | **W** | **D** | **V** | **E** | **D** | **V** | **K** | **L** | **S** | **S** | **S** | **I** | **E** | **D** | **H** | **L** | **F** | **R** | **I** | **V** | **Q** | **E** | **L** | **L** | **S** | **N** | **T** | **L** | **R** | **H** | **A** | **K** | **A** | **N** | **E** | **L** | **E** | **V** | **Y** | **L** | **H** | **K** | **I** | **D** | **N** | **N** | **L** | **L** | **L** | **R** | **I** | **I** | **D** | **D** | **G** | **T** | **G** | **F** | **N** | **M** | **N** | **E** | **T** | **K** | **T** | **G** | **S** | **Y** | **G** | **L** | **N** | **N** | **I** | **K** | **E** | **R** | **V** | **A** | **G** | **I** | **G** | **G** | **T** | **V** | **K** | **I** | **I** | **S** | **F** | **K** | **G** | **Q** | **G** | **T** | **S** | **V** | **E** | **I** | **K** | **V** | **P** | **L** | **M** | **K** | **E** | **A** |
| **N59542F** | **M** | **M** | **G** | **R** | **I** | **S** | **K** | **A** | **M** | **L** | **A** | **V** | **Y** | **S** | **G** | **I** | **A** | **A** | **F** | **L** | **I** | **I** | **L** | **F** | **S** | **L** | **F** | **T** | **Y** | **F** | **Y** | **A** | **S** | **N** | **Q** | **S** | **H** | **W** | **W** | **G** | **E** | **L** | **L** | **R** | **A** | **R** | **L** | **L** | **Y** | **V** | **P** | **L** | **I** | **F** | **H** | **L** | **M** | **A** | **I** | **S** | **L** | **G** | **V** | **G** | **L** | **I** | **V** | **F** | **L** | **L** | **L** | **S** | **L** | **I** | **Q** | **K** | **A** | **K** | **Y** | **G** | **K** | **I** | **E** | **E** | **K** | **L** | **R** | **A** | **L** | **S** | **S** | **G** | **N** | **Y** | **E** | **S** | **K** | **L** | **L** | **H** | **Q** | **P** | **I** | **P** | **S** | **A** | **S** | **D** | **D** | **L** | **Y** | **I** | **K** | **D** | **I** | **D** | **M** | **E** | **I** | **T** | **K** | **I** | **K** | **E** | **K** | **M** | **I** | **E** | **V** | **S** | **S** | **E** | **L** | **Q** | **I** | **M** | **T** | **S** | **R** | **P** | **Q** | **Y** | **V** | **D** | **G** | **Q** | **T** | **K** | **E** | **E** | **I** | **L** | **E** | **L** | **E** | **R** | **H** | **R** | **L** | **A** | **R** | **E** | **L** | **H** | **D** | **S** | **V** | **S** | **Q** | **Q** | **L** | **F** | **A** | **A** | **M** | **M** | **M** | **M** | **S** | **A** | **L** | **T** | **E** | **Q** | **A** | **E** | **K** | **S** | **E** | **T** | **P** | **E** | **M** | **F** | **R** | **K** | **Q** | **L** | **K** | **M** | **V** | **A** | **E** | **I** | **I** | **N** | **A** | **S** | **Q** | **S** | **E** | **M** | **R** | **A** | **L** | **L** | **L** | **H** | **L** | **R** | **P** | **V** | **N** | **L** | **E** | **E** | **K** | **S** | **L** | **K** | **Q** | **G** | **I** | **E** | **Q** | **L** | **L** | **K** | **E** | **L** | **Q** | **N** | **K** | **I** | **Q** | **I** | **S** | **L** | **K** | **W** | **D** | **V** | **E** | **D** | **V** | **K** | **L** | **T** | **S** | **S** | **I** | **E** | **D** | **H** | **L** | **F** | **R** | **I** | **V** | **Q** | **E** | **L** | **L** | **S** | **N** | **T** | **L** | **R** | **H** | **A** | **K** | **A** | **N** | **E** | **L** | **E** | **V** | **Y** | **L** | **H** | **K** | **I** | **D** | **N** | **N** | **L** | **L** | **L** | **R** | **I** | **I** | **D** | **D** | **G** | **T** | **G** | **F** | **N** | **M** | **N** | **E** | **T** | **K** | **T** | **G** | **S** | **Y** | **G** | **L** | **N** | **N** | **I** | **K** | **E** | **R** | **V** | **A** | **G** | **I** | **G** | **G** | **T** | **V** | **K** | **I** | **I** | **S** | **F** | **K** | **G** | **Q** | **G** | **T** | **S** | **V** | **E** | **I** | **K** | **V** | **P** | **L** | **M** | **K** | **E** | **A** |
| **N60260F** | **M** | **M** | **G** | **R** | **I** | **S** | **K** | **A** | **M** | **L** | **A** | **V** | **Y** | **S** | **G** | **I** | **A** | **A** | **F** | **L** | **I** | **I** | **L** | **F** | **S** | **L** | **F** | **T** | **Y** | **F** | **Y** | **A** | **S** | **N** | **Q** | **S** | **H** | **W** | **W** | **G** | **E** | **L** | **L** | **R** | **A** | **R** | **L** | **L** | **Y** | **V** | **P** | **L** | **I** | **F** | **H** | **L** | **M** | **A** | **I** | **S** | **L** | **G** | **V** | **G** | **L** | **I** | **V** | **F** | **L** | **L** | **L** | **S** | **L** | **I** | **Q** | **K** | **A** | **K** | **Y** | **G** | **K** | **I** | **E** | **E** | **K** | **L** | **R** | **A** | **L** | **S** | **S** | **G** | **N** | **Y** | **E** | **S** | **K** | **L** | **L** | **H** | **Q** | **P** | **I** | **P** | **S** | **A** | **S** | **D** | **D** | **L** | **Y** | **I** | **K** | **D** | **I** | **D** | **M** | **E** | **I** | **T** | **K** | **I** | **K** | **E** | **K** | **M** | **I** | **E** | **V** | **S** | **S** | **E** | **L** | **Q** | **I** | **M** | **T** | **S** | **R** | **P** | **Q** | **Y** | **V** | **D** | **G** | **Q** | **T** | **K** | **E** | **E** | **I** | **L** | **E** | **L** | **E** | **R** | **H** | **R** | **L** | **A** | **R** | **E** | **L** | **H** | **D** | **S** | **V** | **S** | **Q** | **Q** | **L** | **F** | **A** | **A** | **M** | **M** | **M** | **M** | **S** | **A** | **L** | **T** | **E** | **Q** | **A** | **E** | **K** | **S** | **E** | **T** | **P** | **E** | **M** | **F** | **R** | **K** | **Q** | **L** | **K** | **M** | **V** | **A** | **E** | **I** | **I** | **N** | **A** | **S** | **Q** | **S** | **E** | **M** | **R** | **A** | **L** | **L** | **L** | **H** | **L** | **R** | **P** | **V** | **N** | **L** | **E** | **E** | **K** | **S** | **L** | **K** | **Q** | **G** | **I** | **E** | **Q** | **L** | **L** | **K** | **E** | **L** | **Q** | **N** | **K** | **I** | **Q** | **I** | **S** | **L** | **K** | **W** | **D** | **V** | **E** | **D** | **V** | **K** | **L** | **T** | **S** | **S** | **I** | **E** | **D** | **H** | **L** | **F** | **R** | **I** | **V** | **Q** | **E** | **L** | **L** | **S** | **N** | **T** | **L** | **R** | **H** | **A** | **K** | **A** | **N** | **E** | **L** | **E** | **V** | **Y** | **L** | **H** | **K** | **I** | **D** | **N** | **N** | **L** | **L** | **L** | **R** | **I** | **I** | **D** | **D** | **G** | **T** | **G** | **F** | **N** | **M** | **N** | **E** | **T** | **K** | **T** | **G** | **S** | **Y** | **G** | **L** | **N** | **N** | **I** | **K** | **E** | **R** | **V** | **A** | **G** | **I** | **G** | **G** | **T** | **V** | **K** | **I** | **I** | **S** | **F** | **K** | **G** | **Q** | **G** | **T** | **S** | **V** | **E** | **I** | **K** | **V** | **P** | **L** | **M** | **K** | **E** | **A** |
| **N55077** | **M** | **M** | **G** | **R** | **I** | **S** | **K** | **A** | **M** | **L** | **A** | **V** | **Y** | **S** | **G** | **I** | **A** | **A** | **F** | **L** | **I** | **I** | **L** | **F** | **S** | **L** | **F** | **T** | **Y** | **F** | **Y** | **A** | **S** | **N** | **Q** | **S** | **H** | **W** | **W** | **G** | **E** | **L** | **L** | **R** | **A** | **R** | **L** | **L** | **Y** | **V** | **P** | **L** | **I** | **F** | **H** | **L** | **M** | **A** | **I** | **S** | **L** | **G** | **V** | **G** | **L** | **I** | **V** | **F** | **L** | **L** | **L** | **S** | **L** | **I** | **Q** | **K** | **A** | **K** | **Y** | **G** | **K** | **I** | **E** | **E** | **K** | **L** | **R** | **A** | **L** | **S** | **S** | **G** | **N** | **Y** | **E** | **S** | **K** | **L** | **L** | **H** | **Q** | **P** | **I** | **P** | **S** | **A** | **S** | **D** | **D** | **L** | **Y** | **I** | **K** | **D** | **I** | **D** | **M** | **E** | **I** | **T** | **K** | **I** | **K** | **E** | **K** | **M** | **I** | **E** | **V** | **S** | **S** | **E** | **L** | **Q** | **I** | **M** | **T** | **S** | **R** | **P** | **Q** | **Y** | **V** | **D** | **G** | **Q** | **T** | **K** | **E** | **E** | **I** | **L** | **E** | **L** | **E** | **R** | **H** | **R** | **L** | **A** | **R** | **E** | **L** | **H** | **D** | **S** | **V** | **S** | **Q** | **Q** | **L** | **F** | **A** | **A** | **M** | **M** | **M** | **M** | **S** | **A** | **L** | **T** | **E** | **Q** | **A** | **E** | **K** | **S** | **E** | **T** | **P** | **E** | **M** | **F** | **R** | **K** | **Q** | **L** | **K** | **M** | **V** | **A** | **E** | **I** | **I** | **N** | **A** | **S** | **Q** | **S** | **E** | **M** | **R** | **A** | **L** | **L** | **L** | **H** | **L** | **R** | **P** | **V** | **N** | **L** | **E** | **E** | **K** | **S** | **L** | **K** | **Q** | **G** | **I** | **E** | **Q** | **L** | **L** | **K** | **E** | **L** | **Q** | **N** | **K** | **I** | **Q** | **I** | **S** | **L** | **K** | **W** | **D** | **V** | **E** | **D** | **V** | **K** | **L** | **T** | **S** | **S** | **I** | **E** | **D** | **H** | **L** | **F** | **R** | **I** | **V** | **Q** | **E** | **L** | **L** | **S** | **N** | **T** | **L** | **R** | **H** | **A** | **K** | **A** | **N** | **E** | **L** | **E** | **V** | **Y** | **L** | **H** | **K** | **I** | **D** | **N** | **N** | **L** | **L** | **L** | **R** | **I** | **I** | **D** | **D** | **G** | **T** | **G** | **F** | **N** | **M** | **N** | **E** | **T** | **K** | **T** | **G** | **S** | **Y** | **G** | **L** | **N** | **N** | **I** | **K** | **E** | **R** | **V** | **A** | **G** | **I** | **G** | **G** | **T** | **V** | **K** | **I** | **I** | **S** | **F** | **K** | **G** | **Q** | **G** | **T** | **S** | **V** | **E** | **I** | **K** | **V** | **P** | **L** | **M** | **K** | **E** | **A** |
| **N60336F** | **M** | **M** | **G** | **R** | **I** | **S** | **K** | **A** | **M** | **L** | **A** | **V** | **Y** | **S** | **G** | **I** | **A** | **A** | **F** | **L** | **I** | **I** | **L** | **F** | **S** | **L** | **F** | **T** | **Y** | **F** | **Y** | **A** | **S** | **N** | **Q** | **S** | **H** | **W** | **W** | **G** | **E** | **L** | **L** | **R** | **A** | **R** | **L** | **L** | **Y** | **V** | **P** | **L** | **I** | **F** | **H** | **L** | **M** | **A** | **I** | **S** | **L** | **G** | **V** | **G** | **L** | **I** | **V** | **F** | **L** | **L** | **L** | **S** | **L** | **I** | **Q** | **K** | **A** | **K** | **Y** | **G** | **K** | **I** | **E** | **E** | **K** | **L** | **R** | **A** | **L** | **S** | **S** | **G** | **N** | **Y** | **E** | **S** | **K** | **L** | **L** | **H** | **Q** | **P** | **I** | **P** | **S** | **A** | **S** | **D** | **D** | **L** | **Y** | **I** | **K** | **D** | **I** | **D** | **M** | **E** | **I** | **T** | **K** | **I** | **K** | **E** | **K** | **M** | **I** | **E** | **V** | **S** | **S** | **E** | **L** | **Q** | **I** | **M** | **T** | **S** | **R** | **P** | **Q** | **Y** | **V** | **D** | **G** | **Q** | **T** | **K** | **E** | **E** | **I** | **L** | **E** | **L** | **E** | **R** | **H** | **R** | **L** | **A** | **R** | **E** | **L** | **H** | **D** | **S** | **V** | **S** | **Q** | **Q** | **L** | **F** | **A** | **A** | **M** | **M** | **M** | **M** | **S** | **A** | **L** | **T** | **E** | **Q** | **A** | **E** | **K** | **S** | **E** | **T** | **P** | **E** | **M** | **F** | **R** | **K** | **Q** | **L** | **K** | **M** | **V** | **A** | **E** | **I** | **I** | **N** | **A** | **S** | **Q** | **S** | **E** | **M** | **R** | **A** | **L** | **L** | **L** | **H** | **L** | **R** | **P** | **V** | **N** | **L** | **E** | **E** | **K** | **S** | **L** | **K** | **Q** | **G** | **I** | **E** | **Q** | **L** | **L** | **K** | **E** | **L** | **Q** | **N** | **K** | **I** | **Q** | **I** | **S** | **L** | **K** | **W** | **D** | **V** | **E** | **D** | **V** | **K** | **L** | **T** | **S** | **S** | **I** | **E** | **D** | **H** | **L** | **F** | **R** | **I** | **V** | **Q** | **E** | **L** | **L** | **S** | **N** | **T** | **L** | **R** | **H** | **A** | **K** | **A** | **N** | **E** | **L** | **E** | **V** | **Y** | **L** | **H** | **K** | **I** | **D** | **N** | **N** | **L** | **L** | **L** | **R** | **I** | **I** | **D** | **D** | **G** | **T** | **G** | **F** | **N** | **M** | **N** | **E** | **T** | **K** | **T** | **G** | **S** | **Y** | **G** | **L** | **N** | **N** | **I** | **K** | **E** | **R** | **V** | **A** | **G** | **I** | **G** | **G** | **T** | **V** | **K** | **I** | **I** | **S** | **F** | **K** | **G** | **Q** | **G** | **T** | **S** | **V** | **E** | **I** | **K** | **V** | **P** | **L** | **M** | **K** | **E** | **A** |
| **N60045F** | **M** | **M** | **G** | **R** | **I** | **S** | **K** | **A** | **M** | **L** | **A** | **V** | **Y** | **S** | **G** | **I** | **A** | **A** | **F** | **L** | **I** | **I** | **L** | **F** | **S** | **L** | **F** | **T** | **Y** | **F** | **Y** | **A** | **S** | **N** | **Q** | **S** | **H** | **W** | **W** | **G** | **E** | **L** | **L** | **R** | **A** | **R** | **L** | **L** | **Y** | **V** | **P** | **L** | **I** | **F** | **H** | **L** | **M** | **A** | **I** | **S** | **L** | **G** | **V** | **G** | **L** | **I** | **V** | **F** | **L** | **L** | **L** | **S** | **L** | **I** | **Q** | **K** | **A** | **K** | **Y** | **G** | **K** | **I** | **E** | **E** | **K** | **L** | **R** | **A** | **L** | **S** | **S** | **G** | **N** | **Y** | **E** | **S** | **K** | **L** | **L** | **H** | **Q** | **P** | **I** | **P** | **S** | **T** | **S** | **D** | **D** | **L** | **Y** | **I** | **K** | **D** | **I** | **D** | **M** | **E** | **I** | **T** | **K** | **I** | **K** | **E** | **K** | **M** | **I** | **E** | **V** | **S** | **S** | **E** | **L** | **Q** | **I** | **M** | **T** | **S** | **R** | **P** | **Q** | **Y** | **V** | **D** | **G** | **Q** | **T** | **K** | **E** | **E** | **I** | **L** | **E** | **L** | **E** | **R** | **H** | **R** | **L** | **A** | **R** | **E** | **L** | **H** | **D** | **S** | **V** | **S** | **Q** | **Q** | **L** | **F** | **A** | **A** | **M** | **M** | **M** | **M** | **S** | **A** | **L** | **T** | **E** | **Q** | **A** | **E** | **K** | **S** | **E** | **T** | **P** | **E** | **M** | **F** | **R** | **K** | **Q** | **L** | **K** | **M** | **V** | **A** | **E** | **I** | **I** | **N** | **A** | **S** | **Q** | **S** | **E** | **M** | **R** | **A** | **L** | **L** | **L** | **H** | **L** | **R** | **P** | **V** | **N** | **L** | **E** | **E** | **K** | **S** | **L** | **K** | **Q** | **G** | **I** | **E** | **Q** | **L** | **L** | **K** | **E** | **L** | **Q** | **N** | **K** | **I** | **Q** | **I** | **S** | **L** | **K** | **W** | **N** | **V** | **E** | **D** | **V** | **K** | **L** | **T** | **S** | **S** | **I** | **E** | **D** | **H** | **L** | **F** | **R** | **I** | **V** | **Q** | **E** | **L** | **L** | **S** | **N** | **T** | **L** | **R** | **H** | **A** | **K** | **A** | **N** | **E** | **L** | **E** | **V** | **Y** | **L** | **H** | **K** | **I** | **D** | **N** | **N** | **L** | **L** | **L** | **R** | **I** | **I** | **D** | **D** | **G** | **T** | **G** | **F** | **N** | **M** | **N** | **E** | **T** | **K** | **T** | **G** | **S** | **Y** | **G** | **L** | **N** | **N** | **I** | **K** | **E** | **R** | **V** | **A** | **G** | **I** | **G** | **G** | **T** | **V** | **K** | **I** | **I** | **S** | **F** | **K** | **G** | **Q** | **G** | **T** | **S** | **V** | **E** | **I** | **K** | **V** | **P** | **L** | **M** | **K** | **E** | **A** |
| **N53439** | **M** | **M** | **G** | **R** | **I** | **S** | **K** | **A** | **M** | **L** | **A** | **V** | **Y** | **S** | **G** | **I** | **A** | **A** | **F** | **L** | **I** | **I** | **L** | **F** | **S** | **L** | **F** | **T** | **Y** | **F** | **Y** | **A** | **S** | **N** | **Q** | **S** | **H** | **W** | **W** | **G** | **E** | **L** | **L** | **R** | **A** | **R** | **L** | **L** | **Y** | **V** | **P** | **L** | **I** | **F** | **H** | **L** | **M** | **A** | **I** | **S** | **L** | **G** | **V** | **G** | **L** | **I** | **V** | **F** | **L** | **L** | **L** | **S** | **L** | **I** | **Q** | **K** | **A** | **K** | **Y** | **G** | **K** | **I** | **E** | **E** | **K** | **L** | **R** | **A** | **L** | **S** | **S** | **G** | **N** | **Y** | **E** | **S** | **K** | **L** | **L** | **H** | **Q** | **P** | **I** | **P** | **S** | **A** | **S** | **D** | **D** | **L** | **Y** | **I** | **K** | **D** | **I** | **D** | **M** | **E** | **I** | **T** | **K** | **I** | **K** | **E** | **K** | **M** | **I** | **E** | **V** | **S** | **S** | **E** | **L** | **Q** | **I** | **M** | **T** | **S** | **R** | **P** | **Q** | **Y** | **V** | **D** | **G** | **Q** | **T** | **K** | **E** | **E** | **I** | **L** | **E** | **L** | **E** | **R** | **H** | **R** | **L** | **A** | **R** | **E** | **L** | **H** | **D** | **S** | **V** | **S** | **Q** | **Q** | **L** | **F** | **A** | **A** | **M** | **M** | **M** | **M** | **S** | **A** | **L** | **T** | **E** | **Q** | **A** | **E** | **K** | **S** | **E** | **T** | **P** | **G** | **M** | **F** | **R** | **K** | **Q** | **L** | **K** | **M** | **V** | **A** | **E** | **I** | **I** | **N** | **A** | **S** | **Q** | **S** | **E** | **M** | **R** | **A** | **L** | **L** | **L** | **H** | **L** | **R** | **P** | **V** | **N** | **L** | **E** | **E** | **K** | **S** | **L** | **K** | **Q** | **G** | **I** | **E** | **Q** | **L** | **L** | **K** | **E** | **L** | **Q** | **N** | **K** | **I** | **Q** | **I** | **S** | **L** | **K** | **W** | **D** | **V** | **E** | **D** | **V** | **K** | **L** | **T** | **S** | **S** | **I** | **E** | **D** | **H** | **L** | **F** | **R** | **I** | **V** | **Q** | **E** | **L** | **L** | **S** | **N** | **T** | **L** | **R** | **H** | **A** | **K** | **A** | **N** | **E** | **L** | **E** | **V** | **Y** | **L** | **H** | **K** | **I** | **D** | **N** | **N** | **L** | **L** | **L** | **R** | **I** | **I** | **D** | **D** | **G** | **T** | **G** | **F** | **N** | **M** | **N** | **E** | **T** | **K** | **T** | **G** | **S** | **Y** | **G** | **L** | **N** | **N** | **I** | **K** | **E** | **R** | **V** | **A** | **G** | **I** | **G** | **G** | **T** | **V** | **K** | **I** | **I** | **S** | **F** | **K** | **G** | **Q** | **G** | **T** | **S** | **V** | **E** | **I** | **K** | **V** | **P** | **L** | **M** | **K** | **E** | **A** |
| **N60123F** | **M** | **M** | **G** | **R** | **I** | **S** | **K** | **A** | **M** | **L** | **A** | **V** | **Y** | **S** | **G** | **I** | **A** | **A** | **F** | **L** | **I** | **I** | **L** | **F** | **S** | **L** | **F** | **T** | **Y** | **F** | **Y** | **A** | **S** | **N** | **Q** | **S** | **H** | **W** | **W** | **G** | **E** | **L** | **L** | **R** | **A** | **R** | **L** | **L** | **Y** | **V** | **P** | **L** | **I** | **F** | **H** | **L** | **M** | **A** | **I** | **S** | **L** | **G** | **V** | **G** | **L** | **I** | **V** | **F** | **L** | **L** | **L** | **S** | **L** | **I** | **Q** | **K** | **T** | **K** | **Y** | **G** | **K** | **I** | **E** | **E** | **K** | **L** | **R** | **A** | **L** | **S** | **S** | **G** | **N** | **Y** | **E** | **S** | **K** | **L** | **L** | **H** | **Q** | **P** | **I** | **P** | **S** | **A** | **S** | **D** | **D** | **L** | **Y** | **I** | **K** | **D** | **I** | **D** | **M** | **E** | **I** | **T** | **K** | **I** | **K** | **E** | **K** | **M** | **I** | **E** | **V** | **S** | **S** | **E** | **L** | **Q** | **I** | **M** | **T** | **S** | **R** | **P** | **Q** | **Y** | **V** | **D** | **G** | **Q** | **T** | **K** | **E** | **E** | **I** | **L** | **E** | **L** | **E** | **R** | **H** | **R** | **L** | **A** | **R** | **E** | **L** | **H** | **D** | **S** | **V** | **S** | **Q** | **Q** | **L** | **F** | **A** | **A** | **M** | **M** | **M** | **M** | **S** | **A** | **L** | **T** | **E** | **Q** | **A** | **E** | **K** | **S** | **E** | **T** | **P** | **E** | **M** | **F** | **R** | **K** | **Q** | **L** | **K** | **M** | **V** | **A** | **E** | **I** | **I** | **N** | **A** | **S** | **Q** | **S** | **E** | **M** | **R** | **A** | **L** | **L** | **L** | **H** | **L** | **R** | **P** | **V** | **N** | **L** | **E** | **E** | **K** | **S** | **L** | **K** | **Q** | **G** | **I** | **E** | **Q** | **L** | **L** | **K** | **E** | **L** | **Q** | **N** | **K** | **I** | **Q** | **I** | **S** | **L** | **K** | **W** | **D** | **V** | **E** | **D** | **V** | **K** | **L** | **T** | **S** | **S** | **I** | **E** | **D** | **H** | **L** | **F** | **R** | **I** | **V** | **Q** | **E** | **L** | **L** | **S** | **N** | **T** | **L** | **R** | **H** | **A** | **K** | **A** | **N** | **E** | **L** | **E** | **V** | **Y** | **L** | **H** | **K** | **I** | **D** | **N** | **N** | **L** | **L** | **L** | **R** | **I** | **I** | **D** | **D** | **G** | **T** | **G** | **F** | **N** | **M** | **N** | **E** | **T** | **K** | **T** | **G** | **S** | **Y** | **G** | **L** | **N** | **N** | **I** | **K** | **E** | **R** | **V** | **A** | **G** | **I** | **G** | **G** | **T** | **V** | **K** | **I** | **I** | **S** | **F** | **K** | **G** | **Q** | **G** | **T** | **S** | **V** | **E** | **I** | **K** | **V** | **P** | **L** | **M** | **K** | **E** | **A** |
| **N59788F** | **M** | **M** | **G** | **R** | **I** | **S** | **K** | **A** | **M** | **L** | **A** | **V** | **Y** | **S** | **G** | **I** | **A** | **A** | **F** | **L** | **I** | **I** | **L** | **F** | **S** | **L** | **F** | **T** | **Y** | **F** | **Y** | **A** | **S** | **N** | **Q** | **S** | **H** | **W** | **W** | **G** | **E** | **L** | **L** | **R** | **A** | **R** | **L** | **L** | **Y** | **V** | **P** | **L** | **I** | **F** | **H** | **L** | **M** | **A** | **I** | **S** | **L** | **G** | **V** | **G** | **L** | **I** | **V** | **F** | **L** | **L** | **L** | **S** | **L** | **I** | **Q** | **K** | **A** | **K** | **Y** | **G** | **K** | **I** | **E** | **E** | **K** | **L** | **R** | **A** | **L** | **S** | **S** | **G** | **N** | **Y** | **E** | **S** | **K** | **L** | **L** | **H** | **Q** | **P** | **I** | **P** | **S** | **A** | **S** | **D** | **D** | **L** | **Y** | **I** | **K** | **D** | **I** | **D** | **M** | **E** | **I** | **T** | **K** | **I** | **K** | **E** | **K** | **M** | **I** | **E** | **V** | **S** | **S** | **E** | **L** | **Q** | **I** | **M** | **T** | **S** | **R** | **P** | **Q** | **Y** | **V** | **D** | **G** | **Q** | **T** | **K** | **E** | **E** | **I** | **L** | **E** | **L** | **E** | **R** | **H** | **R** | **L** | **A** | **R** | **E** | **L** | **H** | **D** | **S** | **V** | **S** | **Q** | **Q** | **L** | **F** | **A** | **A** | **M** | **M** | **M** | **M** | **S** | **A** | **L** | **T** | **E** | **Q** | **A** | **E** | **K** | **S** | **E** | **T** | **P** | **E** | **M** | **F** | **R** | **K** | **Q** | **L** | **K** | **M** | **V** | **A** | **E** | **I** | **I** | **N** | **A** | **S** | **Q** | **S** | **E** | **M** | **R** | **A** | **L** | **L** | **L** | **H** | **L** | **R** | **P** | **V** | **N** | **L** | **E** | **E** | **K** | **S** | **L** | **K** | **Q** | **G** | **I** | **E** | **Q** | **L** | **L** | **K** | **E** | **L** | **Q** | **N** | **K** | **I** | **Q** | **I** | **S** | **L** | **K** | **W** | **D** | **V** | **E** | **D** | **V** | **K** | **L** | **T** | **S** | **S** | **I** | **E** | **D** | **H** | **L** | **F** | **R** | **I** | **V** | **Q** | **E** | **L** | **L** | **S** | **N** | **T** | **L** | **R** | **H** | **A** | **K** | **A** | **N** | **E** | **L** | **E** | **V** | **Y** | **L** | **H** | **K** | **I** | **D** | **N** | **N** | **L** | **L** | **L** | **R** | **I** | **I** | **D** | **D** | **G** | **T** | **G** | **F** | **N** | **M** | **N** | **E** | **T** | **K** | **T** | **G** | **S** | **Y** | **G** | **L** | **N** | **N** | **I** | **K** | **E** | **R** | **V** | **A** | **G** | **I** | **G** | **G** | **T** | **V** | **K** | **I** | **I** | **S** | **F** | **K** | **G** | **Q** | **G** | **T** | **S** | **V** | **E** | **I** | **K** | **V** | **P** | **L** | **M** | **K** | **E** | **A** |
| **N59891F** | **M** | **M** | **G** | **R** | **I** | **S** | **K** | **A** | **M** | **L** | **A** | **V** | **Y** | **S** | **G** | **I** | **A** | **A** | **F** | **L** | **I** | **I** | **L** | **F** | **S** | **L** | **F** | **T** | **Y** | **F** | **Y** | **A** | **S** | **N** | **Q** | **S** | **H** | **W** | **W** | **G** | **E** | **L** | **L** | **R** | **A** | **R** | **L** | **L** | **Y** | **V** | **P** | **L** | **I** | **F** | **H** | **L** | **M** | **A** | **I** | **S** | **L** | **G** | **V** | **G** | **L** | **I** | **V** | **F** | **L** | **L** | **L** | **S** | **L** | **I** | **Q** | **K** | **A** | **K** | **Y** | **G** | **K** | **I** | **E** | **E** | **K** | **L** | **R** | **A** | **L** | **S** | **S** | **G** | **N** | **Y** | **E** | **S** | **K** | **L** | **L** | **H** | **Q** | **P** | **I** | **P** | **S** | **A** | **S** | **D** | **D** | **L** | **Y** | **I** | **K** | **D** | **I** | **D** | **M** | **E** | **I** | **T** | **K** | **I** | **K** | **E** | **K** | **M** | **I** | **E** | **V** | **S** | **S** | **E** | **L** | **Q** | **I** | **M** | **T** | **S** | **R** | **P** | **Q** | **Y** | **V** | **D** | **G** | **Q** | **T** | **K** | **E** | **E** | **I** | **L** | **E** | **L** | **E** | **R** | **H** | **R** | **L** | **A** | **R** | **E** | **L** | **H** | **D** | **S** | **V** | **S** | **Q** | **Q** | **L** | **F** | **A** | **A** | **M** | **M** | **M** | **M** | **S** | **A** | **L** | **T** | **E** | **Q** | **A** | **E** | **K** | **S** | **E** | **T** | **P** | **E** | **M** | **F** | **R** | **K** | **Q** | **L** | **K** | **M** | **V** | **A** | **E** | **I** | **I** | **N** | **A** | **S** | **Q** | **S** | **E** | **M** | **R** | **A** | **L** | **L** | **L** | **H** | **L** | **R** | **P** | **V** | **N** | **L** | **E** | **E** | **K** | **S** | **L** | **K** | **Q** | **G** | **I** | **E** | **Q** | **L** | **L** | **K** | **E** | **L** | **Q** | **N** | **K** | **I** | **Q** | **I** | **S** | **L** | **K** | **W** | **D** | **V** | **E** | **D** | **V** | **K** | **L** | **T** | **S** | **S** | **I** | **E** | **D** | **H** | **L** | **F** | **R** | **I** | **V** | **Q** | **E** | **L** | **L** | **S** | **N** | **T** | **L** | **R** | **H** | **A** | **K** | **A** | **N** | **E** | **L** | **E** | **V** | **Y** | **L** | **H** | **K** | **I** | **D** | **N** | **N** | **L** | **L** | **L** | **R** | **I** | **I** | **D** | **D** | **G** | **T** | **G** | **F** | **N** | **M** | **N** | **E** | **T** | **K** | **T** | **G** | **S** | **Y** | **G** | **L** | **N** | **N** | **I** | **K** | **E** | **R** | **V** | **A** | **G** | **I** | **G** | **G** | **T** | **V** | **K** | **I** | **I** | **S** | **F** | **K** | **G** | **Q** | **G** | **T** | **S** | **V** | **E** | **I** | **K** | **V** | **P** | **L** | **M** | **K** | **E** | **A** |
| **N60449F** | **M** | **M** | **G** | **R** | **I** | **S** | **K** | **A** | **M** | **L** | **A** | **V** | **Y** | **S** | **G** | **I** | **A** | **A** | **F** | **L** | **I** | **I** | **L** | **F** | **S** | **L** | **F** | **T** | **Y** | **F** | **Y** | **A** | **S** | **N** | **Q** | **S** | **H** | **W** | **W** | **G** | **E** | **L** | **L** | **R** | **A** | **R** | **L** | **L** | **Y** | **V** | **P** | **L** | **I** | **F** | **H** | **L** | **M** | **A** | **I** | **S** | **L** | **G** | **V** | **G** | **L** | **I** | **V** | **F** | **L** | **L** | **L** | **S** | **L** | **I** | **Q** | **K** | **A** | **K** | **Y** | **G** | **K** | **I** | **E** | **E** | **K** | **L** | **R** | **A** | **L** | **S** | **S** | **G** | **N** | **Y** | **E** | **S** | **K** | **L** | **L** | **H** | **Q** | **P** | **I** | **P** | **S** | **A** | **S** | **D** | **D** | **L** | **Y** | **I** | **K** | **D** | **I** | **D** | **M** | **E** | **I** | **T** | **K** | **I** | **K** | **E** | **K** | **M** | **I** | **E** | **V** | **S** | **S** | **E** | **L** | **Q** | **I** | **M** | **T** | **S** | **R** | **P** | **Q** | **Y** | **V** | **D** | **G** | **Q** | **T** | **K** | **E** | **E** | **I** | **L** | **E** | **L** | **E** | **R** | **H** | **R** | **L** | **A** | **R** | **E** | **L** | **H** | **D** | **S** | **V** | **S** | **Q** | **Q** | **L** | **F** | **A** | **A** | **M** | **M** | **M** | **M** | **S** | **A** | **L** | **T** | **E** | **Q** | **A** | **E** | **K** | **S** | **E** | **T** | **P** | **E** | **M** | **F** | **R** | **K** | **Q** | **L** | **K** | **M** | **V** | **A** | **E** | **I** | **I** | **N** | **A** | **S** | **Q** | **S** | **E** | **M** | **R** | **A** | **L** | **L** | **L** | **H** | **L** | **R** | **P** | **V** | **N** | **L** | **E** | **E** | **K** | **S** | **L** | **K** | **Q** | **G** | **I** | **E** | **Q** | **L** | **L** | **K** | **E** | **L** | **Q** | **N** | **K** | **I** | **Q** | **I** | **S** | **L** | **K** | **W** | **D** | **V** | **E** | **D** | **V** | **K** | **L** | **T** | **S** | **S** | **I** | **E** | **D** | **H** | **L** | **F** | **R** | **I** | **V** | **Q** | **E** | **L** | **L** | **S** | **N** | **T** | **L** | **R** | **H** | **A** | **K** | **A** | **N** | **E** | **L** | **E** | **V** | **Y** | **L** | **H** | **K** | **I** | **D** | **N** | **N** | **L** | **L** | **L** | **R** | **I** | **I** | **D** | **D** | **G** | **T** | **G** | **F** | **N** | **M** | **N** | **E** | **T** | **K** | **T** | **G** | **S** | **Y** | **G** | **L** | **N** | **N** | **I** | **K** | **E** | **R** | **V** | **A** | **G** | **I** | **G** | **G** | **T** | **V** | **K** | **I** | **I** | **S** | **F** | **K** | **G** | **Q** | **G** | **T** | **S** | **V** | **E** | **I** | **K** | **V** | **P** | **L** | **M** | **K** | **E** | **A** |
| **N55728** | **M** | **M** | **G** | **K** | **I** | **S** | **R** | **A** | **M** | **L** | **A** | **V** | **Y** | **S** | **G** | **I** | **A** | **A** | **F** | **L** | **I** | **I** | **L** | **F** | **S** | **L** | **F** | **T** | **Y** | **F** | **Y** | **A** | **S** | **N** | **Q** | **S** | **H** | **W** | **W** | **G** | **E** | **L** | **L** | **R** | **A** | **R** | **L** | **L** | **Y** | **V** | **P** | **L** | **I** | **F** | **H** | **L** | **L** | **A** | **I** | **S** | **L** | **G** | **V** | **G** | **L** | **I** | **V** | **F** | **L** | **L** | **L** | **S** | **L** | **I** | **Q** | **K** | **T** | **K** | **Y** | **G** | **K** | **I** | **E** | **E** | **K** | **L** | **R** | **A** | **L** | **S** | **S** | **G** | **N** | **Y** | **E** | **S** | **K** | **L** | **L** | **L** | **L** | **P** | **I** | **P** | **S** | **A** | **S** | **D** | **D** | **L** | **Y** | **I** | **K** | **D** | **I** | **D** | **K** | **E** | **I** | **T** | **K** | **I** | **K** | **E** | **K** | **M** | **I** | **E** | **V** | **S** | **S** | **E** | **L** | **Q** | **I** | **V** | **T** | **S** | **R** | **P** | **Q** | **Y** | **V** | **D** | **G** | **Q** | **T** | **K** | **E** | **E** | **I** | **L** | **E** | **L** | **E** | **R** | **H** | **R** | **L** | **A** | **R** | **E** | **L** | **H** | **D** | **S** | **V** | **S** | **Q** | **Q** | **L** | **F** | **A** | **A** | **M** | **M** | **M** | **M** | **S** | **A** | **L** | **T** | **E** | **Q** | **A** | **E** | **K** | **S** | **E** | **T** | **P** | **E** | **M** | **F** | **R** | **K** | **Q** | **L** | **K** | **M** | **V** | **A** | **E** | **I** | **I** | **N** | **A** | **S** | **Q** | **S** | **E** | **M** | **R** | **A** | **L** | **L** | **L** | **H** | **L** | **R** | **P** | **V** | **N** | **L** | **E** | **E** | **K** | **S** | **L** | **K** | **Q** | **G** | **I** | **E** | **Q** | **L** | **L** | **K** | **E** | **L** | **Q** | **N** | **K** | **I** | **Q** | **I** | **S** | **L** | **K** | **W** | **D** | **V** | **E** | **D** | **V** | **K** | **L** | **S** | **S** | **S** | **I** | **E** | **D** | **H** | **L** | **F** | **R** | **I** | **V** | **Q** | **E** | **L** | **L** | **S** | **N** | **T** | **L** | **R** | **H** | **A** | **K** | **A** | **N** | **E** | **L** | **E** | **V** | **Y** | **L** | **H** | **K** | **I** | **D** | **N** | **N** | **L** | **L** | **L** | **R** | **I** | **I** | **D** | **D** | **G** | **T** | **G** | **F** | **N** | **M** | **N** | **E** | **T** | **K** | **T** | **G** | **S** | **Y** | **G** | **L** | **N** | **N** | **I** | **K** | **E** | **R** | **V** | **A** | **G** | **I** | **G** | **G** | **T** | **V** | **K** | **I** | **I** | **S** | **F** | **K** | **G** | **Q** | **G** | **T** | **S** | **V** | **E** | **I** | **K** | **V** | **P** | **L** | **M** | **K** | **E** | **A** |
| **N55317** | **M** | **M** | **G** | **K** | **I** | **S** | **R** | **A** | **M** | **L** | **A** | **V** | **Y** | **S** | **G** | **I** | **A** | **A** | **F** | **L** | **I** | **I** | **L** | **F** | **S** | **L** | **F** | **T** | **Y** | **F** | **Y** | **A** | **S** | **N** | **Q** | **S** | **H** | **W** | **W** | **G** | **E** | **L** | **L** | **R** | **A** | **R** | **L** | **L** | **Y** | **V** | **P** | **L** | **I** | **F** | **H** | **L** | **L** | **A** | **I** | **S** | **L** | **G** | **V** | **G** | **L** | **I** | **V** | **F** | **L** | **L** | **L** | **S** | **L** | **I** | **Q** | **K** | **T** | **K** | **Y** | **G** | **K** | **I** | **E** | **E** | **K** | **L** | **R** | **A** | **L** | **S** | **S** | **G** | **N** | **Y** | **E** | **S** | **K** | **L** | **L** | **L** | **L** | **P** | **I** | **P** | **S** | **A** | **S** | **D** | **D** | **L** | **Y** | **I** | **K** | **D** | **I** | **D** | **K** | **E** | **I** | **T** | **K** | **I** | **K** | **E** | **K** | **M** | **I** | **E** | **V** | **S** | **S** | **E** | **L** | **Q** | **I** | **V** | **T** | **S** | **R** | **P** | **Q** | **Y** | **V** | **D** | **G** | **Q** | **T** | **K** | **E** | **E** | **I** | **L** | **E** | **L** | **E** | **R** | **H** | **R** | **L** | **A** | **R** | **E** | **L** | **H** | **D** | **S** | **V** | **S** | **Q** | **Q** | **L** | **F** | **A** | **A** | **M** | **M** | **M** | **M** | **S** | **A** | **L** | **T** | **E** | **Q** | **A** | **E** | **K** | **S** | **E** | **T** | **P** | **E** | **M** | **F** | **R** | **K** | **Q** | **L** | **K** | **M** | **V** | **A** | **E** | **I** | **I** | **N** | **A** | **S** | **Q** | **S** | **E** | **M** | **R** | **A** | **L** | **L** | **L** | **H** | **L** | **R** | **P** | **V** | **N** | **L** | **E** | **E** | **K** | **S** | **L** | **K** | **Q** | **G** | **I** | **E** | **Q** | **L** | **L** | **K** | **E** | **L** | **Q** | **N** | **K** | **I** | **Q** | **I** | **S** | **L** | **K** | **W** | **D** | **V** | **E** | **D** | **V** | **K** | **L** | **S** | **S** | **S** | **I** | **E** | **D** | **H** | **L** | **F** | **R** | **I** | **V** | **Q** | **E** | **L** | **L** | **S** | **N** | **T** | **L** | **R** | **H** | **A** | **K** | **A** | **N** | **E** | **L** | **E** | **V** | **Y** | **L** | **H** | **K** | **I** | **D** | **N** | **N** | **L** | **L** | **L** | **R** | **I** | **I** | **D** | **D** | **G** | **T** | **G** | **F** | **N** | **M** | **N** | **E** | **T** | **K** | **T** | **G** | **S** | **Y** | **G** | **L** | **N** | **N** | **I** | **K** | **E** | **R** | **V** | **A** | **G** | **I** | **G** | **G** | **T** | **V** | **K** | **I** | **I** | **S** | **F** | **K** | **G** | **Q** | **G** | **T** | **S** | **V** | **E** | **I** | **K** | **V** | **P** | **L** | **M** | **K** | **E** | **A** |
| **N55072** | **M** | **M** | **G** | **R** | **I** | **S** | **K** | **A** | **M** | **L** | **A** | **V** | **Y** | **S** | **G** | **I** | **A** | **A** | **F** | **L** | **I** | **I** | **L** | **F** | **S** | **L** | **F** | **T** | **Y** | **F** | **Y** | **A** | **S** | **N** | **Q** | **S** | **H** | **W** | **W** | **G** | **E** | **L** | **L** | **R** | **A** | **R** | **L** | **L** | **Y** | **V** | **P** | **L** | **I** | **F** | **H** | **L** | **M** | **A** | **I** | **S** | **L** | **G** | **V** | **G** | **L** | **I** | **V** | **F** | **L** | **L** | **L** | **S** | **L** | **I** | **Q** | **K** | **A** | **K** | **Y** | **G** | **K** | **I** | **E** | **E** | **K** | **L** | **R** | **A** | **L** | **S** | **S** | **G** | **N** | **Y** | **E** | **S** | **K** | **L** | **L** | **H** | **Q** | **P** | **I** | **P** | **S** | **A** | **S** | **D** | **D** | **L** | **Y** | **I** | **K** | **D** | **I** | **D** | **M** | **E** | **I** | **T** | **K** | **I** | **K** | **E** | **K** | **M** | **I** | **E** | **V** | **S** | **S** | **E** | **L** | **Q** | **I** | **M** | **T** | **S** | **R** | **P** | **Q** | **Y** | **V** | **D** | **G** | **Q** | **T** | **K** | **E** | **E** | **I** | **L** | **E** | **L** | **E** | **R** | **H** | **R** | **L** | **A** | **R** | **E** | **L** | **H** | **D** | **S** | **V** | **S** | **Q** | **Q** | **L** | **F** | **A** | **A** | **M** | **M** | **M** | **M** | **S** | **A** | **L** | **T** | **E** | **Q** | **A** | **E** | **K** | **S** | **E** | **T** | **P** | **E** | **M** | **F** | **R** | **K** | **Q** | **L** | **K** | **M** | **V** | **A** | **E** | **I** | **I** | **N** | **A** | **S** | **Q** | **S** | **E** | **M** | **R** | **A** | **L** | **L** | **L** | **H** | **L** | **R** | **P** | **V** | **N** | **L** | **E** | **E** | **K** | **S** | **L** | **K** | **Q** | **G** | **I** | **E** | **Q** | **L** | **L** | **K** | **E** | **L** | **Q** | **N** | **K** | **I** | **Q** | **I** | **S** | **L** | **K** | **W** | **D** | **V** | **E** | **D** | **V** | **K** | **L** | **T** | **S** | **S** | **I** | **E** | **D** | **H** | **L** | **F** | **R** | **I** | **V** | **Q** | **E** | **L** | **L** | **S** | **N** | **T** | **L** | **R** | **H** | **A** | **K** | **A** | **N** | **E** | **L** | **E** | **V** | **Y** | **L** | **H** | **K** | **I** | **D** | **N** | **N** | **L** | **L** | **L** | **R** | **I** | **I** | **D** | **D** | **G** | **T** | **G** | **F** | **N** | **M** | **N** | **E** | **T** | **K** | **T** | **G** | **S** | **Y** | **G** | **L** | **N** | **N** | **I** | **K** | **E** | **R** | **V** | **A** | **G** | **I** | **G** | **G** | **T** | **V** | **K** | **I** | **I** | **S** | **F** | **K** | **G** | **Q** | **G** | **T** | **S** | **V** | **E** | **I** | **K** | **V** | **P** | **L** | **M** | **K** | **E** | **A** |
| **N54152** | **M** | **M** | **G** | **R** | **I** | **S** | **K** | **A** | **M** | **L** | **A** | **V** | **Y** | **S** | **G** | **I** | **A** | **A** | **F** | **L** | **I** | **I** | **L** | **F** | **S** | **L** | **F** | **T** | **Y** | **F** | **Y** | **A** | **S** | **N** | **Q** | **S** | **H** | **W** | **W** | **G** | **E** | **L** | **L** | **R** | **A** | **R** | **L** | **L** | **Y** | **V** | **P** | **L** | **I** | **F** | **H** | **L** | **M** | **A** | **I** | **S** | **L** | **G** | **V** | **G** | **L** | **I** | **V** | **F** | **L** | **L** | **L** | **S** | **L** | **I** | **Q** | **K** | **A** | **K** | **Y** | **G** | **K** | **I** | **E** | **E** | **K** | **L** | **R** | **A** | **L** | **S** | **S** | **G** | **N** | **Y** | **E** | **S** | **K** | **L** | **L** | **H** | **Q** | **P** | **I** | **P** | **S** | **A** | **S** | **D** | **D** | **L** | **Y** | **I** | **K** | **D** | **I** | **D** | **M** | **E** | **I** | **T** | **K** | **I** | **K** | **E** | **K** | **M** | **I** | **E** | **V** | **S** | **S** | **E** | **L** | **Q** | **I** | **M** | **T** | **S** | **R** | **P** | **Q** | **Y** | **V** | **D** | **G** | **Q** | **T** | **K** | **E** | **E** | **I** | **L** | **E** | **L** | **E** | **R** | **H** | **R** | **L** | **A** | **R** | **E** | **L** | **H** | **D** | **S** | **V** | **S** | **Q** | **Q** | **L** | **F** | **A** | **A** | **M** | **M** | **M** | **M** | **S** | **A** | **L** | **T** | **E** | **Q** | **A** | **E** | **K** | **S** | **E** | **T** | **P** | **E** | **M** | **F** | **R** | **K** | **Q** | **L** | **K** | **M** | **V** | **A** | **E** | **I** | **I** | **N** | **A** | **S** | **Q** | **S** | **E** | **M** | **R** | **A** | **L** | **L** | **L** | **H** | **L** | **R** | **P** | **V** | **N** | **L** | **E** | **E** | **K** | **S** | **L** | **K** | **Q** | **G** | **I** | **E** | **Q** | **L** | **L** | **K** | **E** | **L** | **Q** | **N** | **K** | **I** | **Q** | **I** | **S** | **L** | **K** | **W** | **D** | **V** | **E** | **D** | **V** | **K** | **L** | **T** | **S** | **S** | **I** | **E** | **D** | **H** | **L** | **F** | **R** | **I** | **V** | **Q** | **E** | **L** | **L** | **S** | **N** | **T** | **L** | **R** | **H** | **A** | **K** | **A** | **N** | **E** | **L** | **E** | **V** | **Y** | **L** | **H** | **K** | **I** | **D** | **N** | **N** | **L** | **L** | **L** | **R** | **I** | **I** | **D** | **D** | **G** | **T** | **G** | **F** | **N** | **M** | **N** | **E** | **T** | **K** | **T** | **G** | **S** | **Y** | **G** | **L** | **N** | **N** | **I** | **K** | **E** | **R** | **V** | **A** | **G** | **I** | **G** | **G** | **T** | **V** | **K** | **I** | **I** | **S** | **F** | **K** | **G** | **Q** | **G** | **T** | **S** | **V** | **E** | **I** | **K** | **V** | **P** | **L** | **M** | **K** | **E** | **A** |
| **N59517F** | **M** | **M** | **G** | **R** | **I** | **S** | **K** | **A** | **M** | **L** | **A** | **V** | **Y** | **S** | **G** | **I** | **A** | **A** | **F** | **L** | **I** | **I** | **L** | **F** | **S** | **L** | **F** | **T** | **Y** | **F** | **Y** | **A** | **S** | **N** | **Q** | **S** | **H** | **W** | **W** | **G** | **E** | **L** | **L** | **R** | **A** | **R** | **L** | **L** | **Y** | **V** | **P** | **L** | **I** | **F** | **H** | **L** | **M** | **A** | **I** | **S** | **L** | **G** | **V** | **G** | **L** | **I** | **V** | **F** | **L** | **L** | **L** | **S** | **L** | **I** | **Q** | **K** | **A** | **K** | **Y** | **G** | **K** | **I** | **E** | **E** | **K** | **L** | **R** | **A** | **L** | **S** | **S** | **G** | **N** | **Y** | **E** | **S** | **K** | **L** | **L** | **H** | **Q** | **P** | **I** | **P** | **S** | **A** | **S** | **D** | **D** | **L** | **Y** | **I** | **K** | **D** | **I** | **D** | **M** | **E** | **I** | **T** | **K** | **I** | **K** | **E** | **K** | **M** | **I** | **E** | **V** | **S** | **S** | **E** | **L** | **Q** | **I** | **M** | **T** | **S** | **R** | **P** | **Q** | **Y** | **V** | **D** | **G** | **Q** | **T** | **K** | **E** | **E** | **I** | **L** | **E** | **L** | **E** | **R** | **H** | **R** | **L** | **A** | **R** | **E** | **L** | **H** | **D** | **S** | **V** | **S** | **Q** | **Q** | **L** | **F** | **A** | **A** | **M** | **M** | **M** | **M** | **S** | **A** | **L** | **T** | **E** | **Q** | **A** | **E** | **K** | **S** | **E** | **T** | **P** | **E** | **M** | **F** | **R** | **K** | **Q** | **L** | **K** | **M** | **V** | **A** | **E** | **I** | **I** | **N** | **A** | **S** | **Q** | **S** | **E** | **M** | **R** | **A** | **L** | **L** | **L** | **H** | **L** | **R** | **P** | **V** | **N** | **L** | **E** | **E** | **K** | **S** | **L** | **K** | **Q** | **G** | **I** | **E** | **Q** | **L** | **L** | **K** | **E** | **L** | **Q** | **N** | **K** | **I** | **Q** | **I** | **S** | **L** | **K** | **W** | **D** | **V** | **E** | **D** | **V** | **K** | **L** | **T** | **S** | **S** | **I** | **E** | **D** | **H** | **L** | **F** | **R** | **I** | **V** | **Q** | **E** | **L** | **L** | **S** | **N** | **T** | **L** | **R** | **H** | **A** | **K** | **A** | **N** | **E** | **L** | **E** | **V** | **Y** | **L** | **H** | **K** | **I** | **D** | **N** | **N** | **L** | **L** | **L** | **R** | **I** | **I** | **D** | **D** | **G** | **T** | **G** | **F** | **N** | **M** | **N** | **E** | **T** | **K** | **T** | **G** | **S** | **Y** | **G** | **L** | **N** | **N** | **I** | **K** | **E** | **R** | **V** | **A** | **G** | **I** | **G** | **G** | **T** | **V** | **K** | **I** | **I** | **S** | **F** | **K** | **G** | **Q** | **G** | **T** | **S** | **V** | **E** | **I** | **K** | **V** | **P** | **L** | **M** | **K** | **E** | **A** |
| **N52690** | **M** | **M** | **G** | **R** | **I** | **S** | **K** | **A** | **M** | **L** | **A** | **V** | **Y** | **S** | **G** | **I** | **A** | **A** | **F** | **L** | **I** | **I** | **L** | **F** | **S** | **L** | **F** | **T** | **Y** | **F** | **Y** | **A** | **S** | **N** | **Q** | **S** | **H** | **W** | **W** | **G** | **E** | **L** | **L** | **R** | **A** | **R** | **L** | **L** | **Y** | **V** | **P** | **L** | **I** | **F** | **H** | **L** | **M** | **A** | **I** | **S** | **L** | **G** | **V** | **G** | **L** | **I** | **V** | **F** | **L** | **L** | **L** | **S** | **L** | **I** | **Q** | **K** | **A** | **K** | **Y** | **G** | **K** | **I** | **E** | **E** | **K** | **L** | **R** | **A** | **L** | **S** | **S** | **G** | **N** | **Y** | **E** | **S** | **K** | **L** | **L** | **H** | **Q** | **P** | **I** | **P** | **S** | **A** | **S** | **D** | **D** | **L** | **Y** | **I** | **K** | **D** | **I** | **D** | **M** | **E** | **I** | **T** | **K** | **I** | **K** | **E** | **K** | **M** | **I** | **E** | **V** | **S** | **S** | **E** | **L** | **Q** | **I** | **M** | **T** | **S** | **R** | **P** | **Q** | **Y** | **V** | **D** | **G** | **Q** | **T** | **K** | **E** | **E** | **I** | **L** | **E** | **L** | **E** | **R** | **H** | **R** | **L** | **A** | **R** | **E** | **L** | **H** | **D** | **S** | **V** | **S** | **Q** | **Q** | **L** | **F** | **A** | **A** | **M** | **M** | **M** | **M** | **S** | **A** | **L** | **T** | **E** | **Q** | **A** | **E** | **K** | **S** | **E** | **T** | **P** | **E** | **M** | **F** | **R** | **K** | **Q** | **L** | **K** | **M** | **V** | **A** | **E** | **I** | **I** | **N** | **A** | **S** | **Q** | **S** | **E** | **M** | **R** | **A** | **L** | **L** | **L** | **H** | **L** | **R** | **P** | **V** | **N** | **L** | **E** | **E** | **K** | **S** | **L** | **K** | **Q** | **G** | **I** | **E** | **Q** | **L** | **L** | **K** | **E** | **L** | **Q** | **N** | **K** | **I** | **Q** | **I** | **S** | **L** | **K** | **W** | **D** | **V** | **E** | **D** | **V** | **K** | **L** | **T** | **S** | **S** | **I** | **E** | **D** | **H** | **L** | **F** | **R** | **I** | **V** | **Q** | **E** | **L** | **L** | **S** | **N** | **T** | **L** | **R** | **H** | **A** | **K** | **A** | **N** | **E** | **L** | **E** | **V** | **Y** | **L** | **H** | **K** | **I** | **D** | **N** | **N** | **L** | **L** | **L** | **R** | **I** | **I** | **D** | **D** | **G** | **T** | **G** | **F** | **N** | **M** | **N** | **E** | **T** | **K** | **T** | **G** | **S** | **Y** | **G** | **L** | **N** | **N** | **I** | **K** | **E** | **R** | **V** | **A** | **G** | **I** | **G** | **G** | **T** | **V** | **K** | **I** | **I** | **S** | **F** | **K** | **G** | **Q** | **G** | **T** | **S** | **V** | **E** | **I** | **K** | **V** | **P** | **L** | **M** | **K** | **E** | **A** |
| **N52656** | **M** | **M** | **G** | **K** | **I** | **S** | **R** | **A** | **M** | **L** | **A** | **V** | **Y** | **S** | **G** | **I** | **A** | **A** | **F** | **L** | **I** | **I** | **L** | **F** | **S** | **L** | **F** | **T** | **Y** | **F** | **Y** | **A** | **S** | **N** | **Q** | **S** | **H** | **W** | **W** | **G** | **E** | **L** | **L** | **R** | **A** | **R** | **L** | **L** | **Y** | **V** | **P** | **L** | **I** | **F** | **H** | **L** | **L** | **A** | **I** | **S** | **L** | **G** | **V** | **G** | **L** | **I** | **V** | **F** | **L** | **L** | **L** | **S** | **L** | **I** | **Q** | **K** | **T** | **K** | **Y** | **G** | **K** | **I** | **E** | **E** | **K** | **L** | **R** | **A** | **L** | **S** | **S** | **G** | **N** | **Y** | **E** | **S** | **K** | **L** | **L** | **L** | **L** | **P** | **I** | **P** | **S** | **A** | **S** | **D** | **D** | **L** | **Y** | **I** | **K** | **D** | **I** | **D** | **K** | **E** | **I** | **T** | **K** | **I** | **K** | **E** | **K** | **M** | **I** | **E** | **V** | **S** | **S** | **E** | **L** | **Q** | **I** | **V** | **T** | **S** | **R** | **P** | **Q** | **Y** | **V** | **D** | **G** | **Q** | **T** | **K** | **E** | **E** | **I** | **L** | **E** | **L** | **E** | **R** | **H** | **R** | **L** | **A** | **R** | **E** | **L** | **H** | **D** | **S** | **V** | **S** | **Q** | **Q** | **L** | **F** | **A** | **A** | **M** | **M** | **M** | **M** | **S** | **A** | **L** | **T** | **E** | **Q** | **A** | **E** | **K** | **S** | **E** | **T** | **P** | **E** | **M** | **F** | **R** | **K** | **Q** | **L** | **K** | **M** | **V** | **A** | **E** | **I** | **I** | **N** | **A** | **S** | **Q** | **S** | **E** | **M** | **R** | **A** | **L** | **L** | **L** | **H** | **L** | **R** | **P** | **V** | **N** | **L** | **E** | **E** | **K** | **S** | **L** | **K** | **Q** | **G** | **I** | **E** | **Q** | **L** | **L** | **K** | **E** | **L** | **Q** | **N** | **K** | **I** | **Q** | **I** | **S** | **L** | **K** | **W** | **D** | **V** | **E** | **D** | **V** | **K** | **L** | **S** | **S** | **S** | **I** | **E** | **D** | **H** | **L** | **F** | **R** | **I** | **V** | **Q** | **E** | **L** | **L** | **S** | **N** | **T** | **L** | **R** | **H** | **A** | **K** | **A** | **N** | **E** | **L** | **E** | **V** | **Y** | **L** | **H** | **K** | **I** | **D** | **N** | **N** | **L** | **L** | **L** | **R** | **I** | **I** | **D** | **D** | **G** | **T** | **G** | **F** | **N** | **M** | **N** | **E** | **T** | **K** | **T** | **G** | **S** | **Y** | **G** | **L** | **N** | **N** | **I** | **K** | **E** | **R** | **V** | **A** | **G** | **I** | **G** | **G** | **T** | **V** | **K** | **I** | **I** | **S** | **F** | **K** | **G** | **Q** | **G** | **T** | **S** | **V** | **E** | **I** | **K** | **V** | **P** | **L** | **M** | **K** | **E** | **A** |
| **N59653F** | **M** | **M** | **G** | **K** | **I** | **S** | **R** | **A** | **M** | **L** | **A** | **V** | **Y** | **S** | **G** | **I** | **A** | **A** | **F** | **L** | **I** | **I** | **L** | **F** | **S** | **L** | **F** | **T** | **Y** | **F** | **Y** | **A** | **S** | **N** | **Q** | **S** | **H** | **W** | **W** | **G** | **E** | **L** | **L** | **R** | **A** | **R** | **L** | **L** | **Y** | **V** | **P** | **L** | **I** | **F** | **H** | **L** | **L** | **A** | **I** | **S** | **L** | **G** | **V** | **G** | **L** | **I** | **V** | **F** | **L** | **L** | **L** | **S** | **L** | **I** | **Q** | **K** | **T** | **K** | **Y** | **G** | **K** | **I** | **E** | **E** | **K** | **L** | **R** | **A** | **L** | **S** | **S** | **G** | **N** | **Y** | **E** | **S** | **K** | **L** | **L** | **L** | **L** | **P** | **I** | **P** | **S** | **A** | **S** | **D** | **D** | **L** | **Y** | **I** | **K** | **D** | **I** | **D** | **K** | **E** | **I** | **T** | **K** | **I** | **K** | **E** | **K** | **M** | **I** | **E** | **V** | **S** | **S** | **E** | **L** | **Q** | **I** | **V** | **T** | **S** | **R** | **P** | **Q** | **Y** | **V** | **D** | **G** | **Q** | **T** | **K** | **E** | **E** | **I** | **L** | **E** | **L** | **E** | **R** | **H** | **R** | **L** | **A** | **R** | **E** | **L** | **H** | **D** | **S** | **V** | **S** | **Q** | **Q** | **L** | **F** | **A** | **A** | **M** | **M** | **M** | **M** | **S** | **A** | **L** | **T** | **E** | **Q** | **A** | **E** | **K** | **S** | **E** | **T** | **P** | **E** | **M** | **F** | **R** | **K** | **Q** | **L** | **K** | **M** | **V** | **A** | **E** | **I** | **I** | **N** | **A** | **S** | **Q** | **S** | **E** | **M** | **R** | **A** | **L** | **L** | **L** | **H** | **L** | **R** | **P** | **V** | **N** | **L** | **E** | **E** | **K** | **S** | **L** | **K** | **Q** | **G** | **I** | **E** | **Q** | **L** | **L** | **K** | **E** | **L** | **Q** | **N** | **K** | **I** | **Q** | **I** | **S** | **L** | **K** | **W** | **D** | **V** | **E** | **D** | **V** | **K** | **L** | **S** | **S** | **S** | **I** | **E** | **D** | **H** | **L** | **F** | **R** | **I** | **V** | **Q** | **E** | **L** | **L** | **S** | **N** | **T** | **L** | **R** | **H** | **A** | **K** | **A** | **N** | **E** | **L** | **E** | **V** | **Y** | **L** | **H** | **K** | **I** | **D** | **N** | **N** | **L** | **L** | **L** | **R** | **I** | **I** | **D** | **D** | **G** | **T** | **G** | **F** | **N** | **M** | **N** | **E** | **T** | **K** | **T** | **G** | **S** | **Y** | **G** | **L** | **N** | **N** | **I** | **K** | **E** | **R** | **V** | **A** | **G** | **I** | **G** | **G** | **T** | **V** | **K** | **I** | **I** | **S** | **F** | **K** | **G** | **Q** | **G** | **T** | **S** | **V** | **E** | **I** | **K** | **V** | **P** | **L** | **M** | **K** | **E** | **A** |
| **N54150** | **M** | **M** | **G** | **R** | **I** | **S** | **K** | **A** | **M** | **L** | **A** | **V** | **Y** | **S** | **G** | **I** | **A** | **A** | **F** | **L** | **I** | **I** | **L** | **F** | **S** | **L** | **F** | **T** | **Y** | **F** | **Y** | **A** | **S** | **N** | **Q** | **S** | **H** | **W** | **W** | **G** | **E** | **L** | **L** | **R** | **A** | **R** | **L** | **L** | **Y** | **V** | **P** | **L** | **I** | **F** | **H** | **L** | **M** | **A** | **I** | **S** | **L** | **G** | **V** | **G** | **L** | **I** | **V** | **F** | **L** | **L** | **L** | **S** | **L** | **I** | **Q** | **K** | **A** | **K** | **Y** | **G** | **K** | **I** | **E** | **E** | **K** | **L** | **R** | **A** | **L** | **S** | **S** | **G** | **N** | **Y** | **E** | **S** | **K** | **L** | **L** | **H** | **Q** | **P** | **I** | **P** | **S** | **A** | **S** | **D** | **D** | **L** | **Y** | **I** | **K** | **D** | **I** | **D** | **M** | **E** | **I** | **T** | **K** | **I** | **K** | **E** | **K** | **M** | **I** | **E** | **V** | **S** | **S** | **E** | **L** | **Q** | **I** | **M** | **T** | **S** | **R** | **P** | **Q** | **Y** | **V** | **D** | **G** | **Q** | **T** | **K** | **E** | **E** | **I** | **L** | **E** | **L** | **E** | **R** | **H** | **R** | **L** | **A** | **R** | **E** | **L** | **H** | **D** | **S** | **V** | **S** | **Q** | **Q** | **L** | **F** | **A** | **A** | **M** | **M** | **M** | **M** | **S** | **A** | **L** | **T** | **E** | **Q** | **A** | **E** | **K** | **S** | **E** | **T** | **P** | **E** | **M** | **F** | **R** | **K** | **Q** | **L** | **K** | **M** | **V** | **A** | **E** | **I** | **I** | **N** | **A** | **S** | **Q** | **S** | **E** | **M** | **R** | **A** | **L** | **L** | **L** | **H** | **L** | **R** | **P** | **V** | **N** | **L** | **E** | **E** | **K** | **S** | **L** | **K** | **Q** | **G** | **I** | **E** | **Q** | **L** | **L** | **K** | **E** | **L** | **Q** | **N** | **K** | **I** | **Q** | **I** | **S** | **L** | **K** | **W** | **D** | **V** | **E** | **D** | **V** | **K** | **L** | **T** | **S** | **S** | **I** | **E** | **D** | **H** | **L** | **F** | **R** | **I** | **V** | **Q** | **E** | **L** | **L** | **S** | **N** | **T** | **L** | **R** | **H** | **A** | **K** | **A** | **N** | **E** | **L** | **E** | **V** | **Y** | **L** | **H** | **K** | **I** | **D** | **N** | **N** | **L** | **L** | **L** | **R** | **I** | **I** | **D** | **D** | **G** | **T** | **G** | **F** | **N** | **M** | **N** | **E** | **T** | **K** | **T** | **G** | **S** | **Y** | **G** | **L** | **N** | **N** | **I** | **K** | **E** | **R** | **V** | **A** | **G** | **I** | **G** | **G** | **T** | **V** | **K** | **I** | **I** | **S** | **F** | **K** | **G** | **Q** | **G** | **T** | **S** | **V** | **E** | **I** | **K** | **V** | **P** | **L** | **M** | **K** | **E** | **A** |
| **N53805** | **M** | **M** | **G** | **R** | **I** | **S** | **K** | **A** | **M** | **L** | **A** | **V** | **Y** | **S** | **G** | **I** | **A** | **A** | **F** | **L** | **I** | **I** | **L** | **F** | **S** | **L** | **F** | **T** | **Y** | **F** | **Y** | **A** | **S** | **N** | **Q** | **R** | **H** | **W** | **W** | **G** | **E** | **L** | **L** | **R** | **A** | **R** | **L** | **L** | **Y** | **V** | **P** | **L** | **I** | **F** | **H** | **L** | **M** | **A** | **I** | **S** | **L** | **G** | **V** | **G** | **L** | **I** | **V** | **F** | **L** | **L** | **L** | **S** | **L** | **I** | **Q** | **K** | **A** | **K** | **Y** | **G** | **K** | **I** | **E** | **E** | **K** | **L** | **R** | **A** | **L** | **S** | **S** | **G** | **N** | **Y** | **E** | **S** | **K** | **L** | **L** | **H** | **Q** | **P** | **I** | **P** | **S** | **A** | **S** | **D** | **D** | **L** | **Y** | **I** | **K** | **D** | **I** | **D** | **M** | **E** | **I** | **T** | **K** | **I** | **K** | **E** | **K** | **M** | **I** | **E** | **V** | **S** | **S** | **E** | **L** | **Q** | **I** | **M** | **T** | **S** | **R** | **P** | **Q** | **Y** | **V** | **D** | **G** | **Q** | **T** | **K** | **E** | **E** | **I** | **L** | **E** | **L** | **E** | **R** | **H** | **R** | **L** | **A** | **R** | **E** | **L** | **H** | **D** | **S** | **V** | **S** | **Q** | **Q** | **L** | **F** | **A** | **A** | **M** | **M** | **M** | **M** | **S** | **A** | **L** | **T** | **E** | **Q** | **A** | **E** | **K** | **S** | **E** | **T** | **P** | **E** | **M** | **F** | **R** | **K** | **Q** | **L** | **K** | **M** | **V** | **A** | **E** | **I** | **I** | **N** | **A** | **S** | **Q** | **S** | **E** | **M** | **R** | **A** | **L** | **L** | **L** | **H** | **L** | **R** | **P** | **V** | **N** | **L** | **E** | **E** | **K** | **S** | **L** | **K** | **Q** | **G** | **I** | **E** | **Q** | **L** | **L** | **K** | **E** | **L** | **Q** | **N** | **K** | **I** | **Q** | **I** | **S** | **L** | **K** | **W** | **D** | **V** | **E** | **D** | **V** | **K** | **L** | **T** | **S** | **S** | **I** | **E** | **D** | **H** | **L** | **F** | **R** | **I** | **V** | **Q** | **E** | **L** | **L** | **S** | **N** | **T** | **L** | **R** | **H** | **A** | **K** | **A** | **N** | **E** | **L** | **E** | **V** | **Y** | **L** | **H** | **K** | **I** | **D** | **N** | **N** | **L** | **L** | **L** | **R** | **I** | **I** | **D** | **D** | **G** | **T** | **G** | **F** | **N** | **M** | **N** | **E** | **T** | **K** | **T** | **G** | **S** | **Y** | **G** | **L** | **N** | **N** | **I** | **K** | **E** | **R** | **V** | **A** | **G** | **I** | **G** | **G** | **T** | **V** | **K** | **I** | **I** | **S** | **F** | **K** | **G** | **Q** | **G** | **T** | **S** | **V** | **E** | **I** | **K** | **V** | **P** | **L** | **M** | **K** | **E** | **A** |
| **N55279** | **M** | **M** | **G** | **K** | **I** | **S** | **R** | **A** | **M** | **L** | **A** | **V** | **Y** | **S** | **G** | **I** | **A** | **A** | **F** | **L** | **I** | **I** | **L** | **F** | **S** | **L** | **F** | **T** | **Y** | **F** | **Y** | **A** | **S** | **N** | **Q** | **S** | **H** | **W** | **W** | **G** | **E** | **L** | **L** | **R** | **A** | **R** | **L** | **L** | **Y** | **V** | **P** | **L** | **I** | **F** | **H** | **L** | **L** | **A** | **I** | **S** | **L** | **G** | **V** | **G** | **L** | **I** | **V** | **F** | **L** | **L** | **L** | **S** | **L** | **I** | **Q** | **K** | **T** | **K** | **Y** | **G** | **K** | **I** | **E** | **E** | **K** | **L** | **R** | **A** | **L** | **S** | **S** | **G** | **N** | **Y** | **E** | **S** | **K** | **L** | **L** | **L** | **L** | **P** | **I** | **P** | **S** | **A** | **S** | **D** | **D** | **L** | **Y** | **I** | **K** | **D** | **I** | **D** | **K** | **E** | **I** | **T** | **K** | **I** | **K** | **E** | **K** | **M** | **I** | **E** | **V** | **S** | **S** | **E** | **L** | **Q** | **I** | **V** | **T** | **S** | **R** | **P** | **Q** | **Y** | **V** | **D** | **G** | **Q** | **T** | **K** | **E** | **E** | **I** | **L** | **E** | **L** | **E** | **R** | **H** | **R** | **L** | **A** | **R** | **E** | **L** | **H** | **D** | **S** | **V** | **S** | **Q** | **Q** | **L** | **F** | **A** | **A** | **M** | **M** | **M** | **M** | **S** | **A** | **L** | **T** | **E** | **Q** | **A** | **E** | **K** | **S** | **E** | **T** | **P** | **E** | **M** | **F** | **R** | **K** | **Q** | **L** | **K** | **M** | **V** | **A** | **E** | **I** | **I** | **N** | **A** | **S** | **Q** | **S** | **E** | **M** | **R** | **A** | **L** | **L** | **L** | **H** | **L** | **R** | **P** | **V** | **N** | **L** | **E** | **E** | **K** | **S** | **L** | **K** | **Q** | **G** | **I** | **E** | **Q** | **L** | **L** | **K** | **E** | **L** | **Q** | **N** | **K** | **I** | **Q** | **I** | **S** | **L** | **K** | **W** | **D** | **V** | **E** | **D** | **V** | **K** | **L** | **S** | **S** | **S** | **I** | **E** | **D** | **H** | **L** | **F** | **R** | **I** | **V** | **Q** | **E** | **L** | **L** | **S** | **N** | **T** | **L** | **R** | **H** | **A** | **K** | **A** | **N** | **E** | **L** | **E** | **V** | **Y** | **L** | **H** | **K** | **I** | **D** | **N** | **N** | **L** | **L** | **L** | **R** | **I** | **I** | **D** | **D** | **G** | **T** | **G** | **F** | **N** | **M** | **N** | **E** | **T** | **K** | **T** | **G** | **S** | **Y** | **G** | **L** | **N** | **N** | **I** | **K** | **E** | **R** | **V** | **A** | **G** | **I** | **G** | **G** | **T** | **V** | **K** | **I** | **I** | **S** | **F** | **K** | **G** | **Q** | **G** | **T** | **S** | **V** | **E** | **I** | **K** | **V** | **P** | **L** | **M** | **K** | **E** | **A** |
| **N54070** | **M** | **M** | **G** | **R** | **I** | **S** | **K** | **A** | **M** | **L** | **A** | **V** | **Y** | **S** | **G** | **I** | **A** | **A** | **F** | **L** | **I** | **I** | **L** | **F** | **S** | **L** | **F** | **T** | **Y** | **F** | **Y** | **A** | **S** | **N** | **Q** | **S** | **H** | **W** | **W** | **G** | **E** | **L** | **L** | **R** | **A** | **R** | **L** | **L** | **Y** | **V** | **P** | **L** | **I** | **F** | **H** | **L** | **M** | **A** | **I** | **S** | **L** | **G** | **V** | **G** | **L** | **I** | **V** | **F** | **L** | **L** | **L** | **S** | **L** | **I** | **Q** | **K** | **A** | **K** | **Y** | **G** | **K** | **I** | **E** | **E** | **K** | **L** | **R** | **A** | **L** | **S** | **S** | **G** | **N** | **Y** | **E** | **S** | **K** | **L** | **L** | **H** | **Q** | **P** | **I** | **P** | **S** | **A** | **S** | **D** | **D** | **L** | **Y** | **I** | **K** | **D** | **I** | **D** | **I** | **E** | **I** | **T** | **K** | **I** | **K** | **E** | **K** | **M** | **I** | **E** | **V** | **S** | **S** | **E** | **L** | **Q** | **I** | **M** | **T** | **S** | **R** | **P** | **Q** | **Y** | **V** | **D** | **G** | **Q** | **T** | **K** | **E** | **E** | **I** | **L** | **E** | **L** | **E** | **R** | **H** | **R** | **L** | **A** | **R** | **E** | **L** | **H** | **D** | **S** | **V** | **S** | **Q** | **Q** | **L** | **F** | **A** | **A** | **M** | **M** | **M** | **M** | **S** | **A** | **L** | **T** | **E** | **Q** | **A** | **E** | **K** | **S** | **E** | **T** | **P** | **E** | **M** | **F** | **R** | **K** | **Q** | **L** | **K** | **M** | **V** | **A** | **E** | **I** | **I** | **N** | **A** | **S** | **Q** | **S** | **E** | **M** | **R** | **A** | **L** | **L** | **L** | **H** | **L** | **R** | **P** | **V** | **N** | **L** | **E** | **E** | **K** | **S** | **L** | **K** | **Q** | **G** | **I** | **E** | **Q** | **L** | **L** | **K** | **E** | **L** | **Q** | **N** | **K** | **I** | **Q** | **I** | **S** | **L** | **K** | **W** | **D** | **V** | **E** | **D** | **V** | **K** | **L** | **T** | **S** | **S** | **I** | **E** | **D** | **H** | **L** | **F** | **R** | **I** | **V** | **Q** | **E** | **L** | **L** | **S** | **N** | **T** | **L** | **R** | **H** | **A** | **K** | **A** | **N** | **E** | **L** | **E** | **V** | **Y** | **L** | **H** | **K** | **I** | **D** | **N** | **N** | **L** | **L** | **L** | **R** | **I** | **I** | **D** | **D** | **G** | **T** | **G** | **F** | **N** | **M** | **N** | **E** | **T** | **K** | **T** | **G** | **S** | **Y** | **G** | **L** | **N** | **N** | **I** | **K** | **E** | **R** | **V** | **A** | **G** | **I** | **G** | **G** | **T** | **V** | **K** | **I** | **I** | **S** | **F** | **K** | **G** | **Q** | **G** | **T** | **S** | **V** | **E** | **I** | **K** | **V** | **P** | **L** | **M** | **K** | **E** | **A** |
| **N59469F** | **M** | **M** | **G** | **R** | **I** | **S** | **K** | **A** | **M** | **L** | **A** | **V** | **Y** | **S** | **G** | **I** | **A** | **A** | **F** | **L** | **I** | **I** | **L** | **F** | **S** | **L** | **F** | **T** | **Y** | **F** | **Y** | **A** | **S** | **N** | **Q** | **S** | **H** | **W** | **W** | **G** | **E** | **L** | **L** | **R** | **A** | **R** | **L** | **L** | **Y** | **V** | **P** | **L** | **I** | **F** | **H** | **L** | **M** | **A** | **I** | **S** | **L** | **G** | **V** | **G** | **L** | **I** | **V** | **F** | **L** | **L** | **L** | **S** | **L** | **I** | **Q** | **K** | **A** | **K** | **Y** | **G** | **K** | **I** | **E** | **E** | **K** | **L** | **R** | **A** | **L** | **S** | **S** | **G** | **N** | **Y** | **E** | **S** | **K** | **L** | **L** | **H** | **Q** | **P** | **I** | **P** | **S** | **T** | **S** | **D** | **D** | **L** | **Y** | **I** | **K** | **D** | **I** | **D** | **M** | **E** | **I** | **T** | **K** | **I** | **K** | **E** | **K** | **M** | **I** | **E** | **V** | **S** | **S** | **E** | **L** | **Q** | **I** | **M** | **T** | **S** | **R** | **P** | **Q** | **Y** | **V** | **D** | **G** | **Q** | **T** | **K** | **E** | **E** | **I** | **L** | **E** | **L** | **E** | **R** | **H** | **R** | **L** | **A** | **R** | **E** | **L** | **H** | **D** | **S** | **V** | **S** | **Q** | **Q** | **L** | **F** | **A** | **A** | **M** | **M** | **M** | **M** | **S** | **A** | **L** | **T** | **E** | **Q** | **A** | **E** | **K** | **S** | **E** | **T** | **P** | **E** | **M** | **F** | **R** | **K** | **Q** | **L** | **K** | **M** | **V** | **A** | **E** | **I** | **I** | **N** | **A** | **S** | **Q** | **S** | **E** | **M** | **R** | **A** | **L** | **L** | **L** | **H** | **L** | **R** | **P** | **V** | **N** | **L** | **E** | **E** | **K** | **S** | **L** | **K** | **Q** | **G** | **I** | **E** | **Q** | **L** | **L** | **K** | **E** | **L** | **Q** | **N** | **K** | **I** | **Q** | **I** | **S** | **L** | **K** | **W** | **D** | **V** | **E** | **D** | **V** | **K** | **L** | **T** | **S** | **S** | **I** | **E** | **D** | **H** | **L** | **F** | **R** | **I** | **V** | **Q** | **E** | **L** | **L** | **S** | **N** | **T** | **L** | **R** | **H** | **A** | **K** | **A** | **N** | **E** | **L** | **E** | **V** | **Y** | **L** | **H** | **K** | **I** | **D** | **N** | **N** | **L** | **L** | **L** | **R** | **I** | **I** | **D** | **D** | **G** | **T** | **G** | **F** | **N** | **M** | **N** | **E** | **T** | **K** | **T** | **G** | **S** | **Y** | **G** | **L** | **N** | **N** | **I** | **K** | **E** | **R** | **V** | **A** | **G** | **I** | **G** | **G** | **T** | **V** | **K** | **I** | **I** | **S** | **F** | **K** | **G** | **Q** | **G** | **T** | **S** | **V** | **E** | **I** | **K** | **V** | **P** | **L** | **M** | **K** | **E** | **A** |
| **N52751** | **M** | **M** | **G** | **R** | **I** | **S** | **K** | **A** | **M** | **L** | **A** | **V** | **Y** | **S** | **G** | **I** | **A** | **A** | **F** | **L** | **I** | **I** | **L** | **F** | **S** | **L** | **F** | **T** | **Y** | **F** | **Y** | **A** | **S** | **N** | **Q** | **S** | **H** | **W** | **W** | **G** | **E** | **L** | **L** | **R** | **A** | **R** | **L** | **L** | **Y** | **V** | **P** | **L** | **I** | **F** | **H** | **L** | **M** | **A** | **I** | **S** | **L** | **G** | **V** | **G** | **L** | **I** | **V** | **F** | **L** | **L** | **L** | **S** | **L** | **I** | **Q** | **K** | **A** | **K** | **Y** | **G** | **K** | **I** | **E** | **E** | **K** | **L** | **R** | **A** | **L** | **S** | **S** | **G** | **N** | **Y** | **E** | **S** | **K** | **L** | **L** | **H** | **Q** | **P** | **I** | **P** | **S** | **A** | **S** | **D** | **D** | **L** | **Y** | **I** | **K** | **D** | **I** | **D** | **M** | **E** | **I** | **T** | **K** | **I** | **K** | **E** | **K** | **M** | **I** | **E** | **V** | **S** | **S** | **E** | **L** | **Q** | **I** | **M** | **T** | **S** | **R** | **P** | **Q** | **Y** | **V** | **D** | **G** | **Q** | **T** | **K** | **E** | **E** | **I** | **L** | **E** | **L** | **E** | **R** | **H** | **R** | **L** | **A** | **R** | **E** | **L** | **H** | **D** | **S** | **V** | **S** | **Q** | **Q** | **L** | **F** | **A** | **A** | **M** | **M** | **M** | **M** | **S** | **A** | **L** | **T** | **E** | **Q** | **A** | **E** | **K** | **S** | **E** | **T** | **P** | **E** | **M** | **F** | **R** | **K** | **Q** | **L** | **K** | **M** | **V** | **A** | **E** | **I** | **I** | **N** | **A** | **S** | **Q** | **S** | **E** | **M** | **R** | **A** | **L** | **L** | **L** | **H** | **L** | **R** | **P** | **V** | **N** | **L** | **E** | **E** | **K** | **S** | **L** | **K** | **Q** | **G** | **I** | **E** | **Q** | **L** | **L** | **K** | **E** | **L** | **Q** | **N** | **K** | **I** | **Q** | **I** | **S** | **L** | **K** | **W** | **D** | **V** | **E** | **D** | **V** | **K** | **L** | **T** | **S** | **S** | **I** | **E** | **D** | **H** | **L** | **F** | **R** | **I** | **V** | **Q** | **E** | **L** | **L** | **S** | **N** | **T** | **L** | **R** | **H** | **A** | **K** | **A** | **N** | **E** | **L** | **E** | **V** | **Y** | **L** | **H** | **K** | **I** | **D** | **N** | **N** | **L** | **L** | **L** | **R** | **I** | **I** | **D** | **D** | **G** | **T** | **G** | **F** | **N** | **M** | **N** | **E** | **T** | **K** | **T** | **G** | **S** | **Y** | **G** | **L** | **N** | **N** | **I** | **K** | **E** | **R** | **V** | **A** | **G** | **I** | **G** | **G** | **T** | **V** | **K** | **I** | **I** | **S** | **F** | **K** | **G** | **Q** | **G** | **T** | **S** | **V** | **E** | **I** | **K** | **V** | **P** | **L** | **M** | **K** | **E** | **A** |
| **N52829** | **M** | **M** | **G** | **R** | **I** | **S** | **K** | **A** | **M** | **L** | **A** | **V** | **Y** | **S** | **G** | **I** | **A** | **A** | **F** | **L** | **I** | **I** | **L** | **F** | **S** | **L** | **F** | **T** | **Y** | **F** | **Y** | **A** | **S** | **N** | **Q** | **S** | **H** | **W** | **W** | **G** | **E** | **L** | **L** | **R** | **A** | **R** | **L** | **L** | **Y** | **V** | **P** | **L** | **I** | **F** | **H** | **L** | **M** | **A** | **I** | **S** | **L** | **G** | **V** | **G** | **L** | **I** | **V** | **F** | **L** | **L** | **L** | **S** | **L** | **I** | **Q** | **K** | **A** | **K** | **Y** | **G** | **K** | **I** | **E** | **E** | **K** | **L** | **R** | **A** | **L** | **S** | **S** | **G** | **N** | **Y** | **E** | **S** | **K** | **L** | **L** | **H** | **Q** | **P** | **I** | **P** | **S** | **A** | **S** | **D** | **D** | **L** | **Y** | **I** | **K** | **D** | **I** | **D** | **I** | **E** | **I** | **T** | **K** | **I** | **K** | **E** | **K** | **M** | **I** | **E** | **V** | **S** | **S** | **E** | **L** | **Q** | **I** | **M** | **T** | **S** | **R** | **P** | **Q** | **Y** | **V** | **D** | **G** | **Q** | **T** | **K** | **E** | **E** | **I** | **L** | **E** | **L** | **E** | **R** | **H** | **R** | **L** | **A** | **R** | **E** | **L** | **H** | **D** | **S** | **V** | **S** | **Q** | **Q** | **L** | **F** | **A** | **A** | **M** | **M** | **M** | **M** | **S** | **A** | **L** | **T** | **E** | **Q** | **A** | **E** | **K** | **S** | **E** | **T** | **P** | **E** | **M** | **F** | **R** | **K** | **Q** | **L** | **K** | **M** | **V** | **A** | **E** | **I** | **I** | **N** | **A** | **S** | **Q** | **S** | **E** | **M** | **R** | **A** | **L** | **L** | **L** | **H** | **L** | **R** | **P** | **V** | **N** | **L** | **E** | **E** | **K** | **S** | **L** | **K** | **Q** | **G** | **I** | **E** | **Q** | **L** | **L** | **K** | **E** | **L** | **Q** | **N** | **K** | **I** | **Q** | **I** | **S** | **L** | **K** | **W** | **D** | **V** | **E** | **D** | **V** | **K** | **L** | **T** | **S** | **S** | **I** | **E** | **D** | **H** | **L** | **F** | **R** | **I** | **V** | **Q** | **E** | **L** | **L** | **S** | **N** | **T** | **L** | **R** | **H** | **A** | **K** | **A** | **N** | **E** | **L** | **E** | **V** | **Y** | **L** | **H** | **K** | **I** | **D** | **N** | **N** | **L** | **L** | **L** | **R** | **I** | **I** | **D** | **D** | **G** | **T** | **G** | **F** | **N** | **M** | **N** | **E** | **T** | **K** | **T** | **G** | **S** | **Y** | **G** | **L** | **N** | **N** | **I** | **K** | **E** | **R** | **V** | **A** | **G** | **I** | **G** | **G** | **T** | **V** | **K** | **I** | **I** | **S** | **F** | **K** | **G** | **Q** | **G** | **T** | **S** | **V** | **E** | **I** | **K** | **V** | **P** | **L** | **M** | **K** | **E** | **A** |
| **N54560** | **M** | **M** | **G** | **R** | **I** | **S** | **K** | **A** | **M** | **L** | **A** | **V** | **Y** | **S** | **G** | **I** | **A** | **A** | **F** | **L** | **I** | **I** | **L** | **F** | **S** | **L** | **F** | **T** | **Y** | **F** | **Y** | **A** | **S** | **N** | **Q** | **S** | **H** | **W** | **W** | **G** | **E** | **L** | **L** | **R** | **A** | **R** | **L** | **L** | **Y** | **V** | **P** | **L** | **I** | **F** | **H** | **L** | **M** | **A** | **I** | **S** | **L** | **G** | **V** | **G** | **L** | **I** | **V** | **F** | **L** | **L** | **L** | **S** | **L** | **I** | **Q** | **K** | **A** | **K** | **Y** | **G** | **K** | **I** | **E** | **E** | **K** | **L** | **R** | **A** | **L** | **S** | **S** | **G** | **N** | **Y** | **E** | **S** | **K** | **L** | **L** | **H** | **Q** | **P** | **I** | **P** | **S** | **T** | **S** | **D** | **D** | **L** | **Y** | **I** | **K** | **D** | **I** | **D** | **M** | **E** | **I** | **T** | **K** | **I** | **K** | **E** | **K** | **M** | **I** | **E** | **V** | **S** | **S** | **E** | **L** | **Q** | **I** | **M** | **T** | **S** | **R** | **P** | **Q** | **Y** | **V** | **D** | **G** | **Q** | **T** | **K** | **E** | **E** | **I** | **L** | **E** | **L** | **E** | **R** | **H** | **R** | **L** | **A** | **R** | **E** | **L** | **H** | **D** | **S** | **V** | **S** | **Q** | **Q** | **L** | **F** | **A** | **A** | **M** | **M** | **M** | **M** | **S** | **A** | **L** | **T** | **E** | **Q** | **A** | **E** | **K** | **S** | **E** | **T** | **P** | **E** | **M** | **F** | **R** | **K** | **Q** | **L** | **K** | **M** | **V** | **A** | **E** | **I** | **I** | **N** | **A** | **S** | **Q** | **S** | **E** | **M** | **R** | **A** | **L** | **L** | **L** | **H** | **L** | **R** | **P** | **V** | **N** | **L** | **E** | **E** | **K** | **S** | **L** | **K** | **Q** | **G** | **I** | **E** | **Q** | **L** | **L** | **K** | **E** | **L** | **Q** | **N** | **K** | **I** | **Q** | **I** | **S** | **L** | **K** | **W** | **D** | **V** | **E** | **D** | **V** | **K** | **L** | **T** | **S** | **S** | **I** | **E** | **D** | **H** | **L** | **F** | **R** | **I** | **V** | **Q** | **E** | **L** | **L** | **S** | **N** | **T** | **L** | **R** | **H** | **A** | **K** | **A** | **N** | **E** | **L** | **E** | **V** | **Y** | **L** | **H** | **K** | **I** | **D** | **N** | **N** | **L** | **L** | **L** | **R** | **I** | **I** | **D** | **D** | **G** | **T** | **G** | **F** | **N** | **M** | **N** | **E** | **T** | **K** | **T** | **G** | **S** | **Y** | **G** | **L** | **N** | **N** | **I** | **K** | **E** | **R** | **V** | **A** | **G** | **I** | **G** | **G** | **T** | **V** | **K** | **I** | **I** | **S** | **F** | **K** | **G** | **Q** | **G** | **T** | **S** | **V** | **E** | **I** | **K** | **V** | **P** | **L** | **M** | **K** | **E** | **A** |
| **N60490F** | **M** | **M** | **G** | **R** | **I** | **S** | **K** | **A** | **M** | **L** | **A** | **V** | **Y** | **S** | **G** | **I** | **A** | **A** | **F** | **L** | **I** | **I** | **L** | **F** | **S** | **L** | **F** | **T** | **Y** | **F** | **Y** | **A** | **S** | **N** | **Q** | **S** | **H** | **W** | **W** | **G** | **E** | **L** | **L** | **R** | **A** | **R** | **L** | **L** | **Y** | **V** | **P** | **L** | **I** | **F** | **H** | **L** | **M** | **A** | **I** | **S** | **L** | **G** | **V** | **G** | **L** | **I** | **V** | **F** | **L** | **L** | **L** | **S** | **L** | **I** | **Q** | **K** | **T** | **K** | **Y** | **G** | **K** | **I** | **E** | **E** | **K** | **L** | **R** | **A** | **L** | **S** | **S** | **G** | **N** | **Y** | **E** | **S** | **K** | **L** | **L** | **H** | **Q** | **P** | **I** | **P** | **S** | **A** | **S** | **D** | **D** | **L** | **Y** | **I** | **K** | **D** | **I** | **D** | **M** | **E** | **I** | **T** | **K** | **I** | **K** | **E** | **K** | **M** | **I** | **E** | **V** | **S** | **S** | **E** | **L** | **Q** | **I** | **M** | **T** | **S** | **R** | **P** | **Q** | **Y** | **V** | **D** | **G** | **Q** | **T** | **K** | **E** | **E** | **I** | **L** | **E** | **L** | **E** | **R** | **H** | **R** | **L** | **A** | **R** | **E** | **L** | **H** | **D** | **S** | **V** | **S** | **Q** | **Q** | **L** | **F** | **A** | **A** | **M** | **M** | **M** | **M** | **S** | **A** | **L** | **T** | **E** | **Q** | **A** | **E** | **K** | **S** | **E** | **T** | **P** | **E** | **M** | **F** | **R** | **K** | **Q** | **L** | **K** | **M** | **V** | **A** | **E** | **I** | **I** | **N** | **A** | **S** | **Q** | **S** | **E** | **M** | **R** | **A** | **L** | **L** | **L** | **H** | **L** | **R** | **P** | **V** | **N** | **L** | **E** | **E** | **K** | **S** | **L** | **K** | **Q** | **G** | **I** | **E** | **Q** | **L** | **L** | **K** | **E** | **L** | **Q** | **N** | **K** | **I** | **Q** | **I** | **S** | **L** | **K** | **W** | **D** | **V** | **E** | **D** | **V** | **K** | **L** | **T** | **S** | **S** | **I** | **E** | **D** | **H** | **L** | **F** | **R** | **I** | **V** | **Q** | **E** | **L** | **L** | **S** | **N** | **T** | **L** | **R** | **H** | **A** | **K** | **A** | **N** | **E** | **L** | **E** | **V** | **Y** | **L** | **H** | **K** | **I** | **D** | **N** | **N** | **L** | **L** | **L** | **R** | **I** | **I** | **D** | **D** | **G** | **T** | **G** | **F** | **N** | **M** | **N** | **E** | **T** | **K** | **T** | **G** | **S** | **Y** | **G** | **L** | **N** | **N** | **I** | **K** | **E** | **R** | **V** | **A** | **G** | **I** | **G** | **G** | **T** | **V** | **K** | **I** | **I** | **S** | **F** | **K** | **G** | **Q** | **G** | **T** | **S** | **V** | **E** | **I** | **K** | **V** | **P** | **L** | **M** | **K** | **E** | **A** |
| **N59779F** | **M** | **M** | **G** | **R** | **I** | **S** | **K** | **A** | **M** | **L** | **A** | **V** | **Y** | **S** | **G** | **I** | **A** | **A** | **F** | **L** | **I** | **I** | **L** | **F** | **S** | **L** | **F** | **T** | **Y** | **F** | **Y** | **A** | **S** | **N** | **Q** | **S** | **H** | **W** | **W** | **G** | **E** | **L** | **L** | **R** | **A** | **R** | **L** | **L** | **Y** | **V** | **P** | **L** | **I** | **F** | **H** | **L** | **M** | **A** | **I** | **S** | **L** | **G** | **V** | **G** | **L** | **I** | **V** | **F** | **L** | **L** | **L** | **S** | **L** | **I** | **Q** | **K** | **A** | **K** | **Y** | **G** | **K** | **I** | **E** | **E** | **K** | **L** | **R** | **A** | **L** | **S** | **S** | **G** | **N** | **Y** | **E** | **S** | **K** | **L** | **L** | **H** | **Q** | **P** | **I** | **P** | **S** | **A** | **S** | **D** | **D** | **L** | **Y** | **I** | **K** | **D** | **I** | **D** | **M** | **E** | **I** | **T** | **K** | **I** | **K** | **E** | **K** | **M** | **I** | **E** | **V** | **S** | **S** | **E** | **L** | **Q** | **I** | **M** | **T** | **S** | **R** | **P** | **Q** | **Y** | **V** | **D** | **G** | **Q** | **T** | **K** | **E** | **E** | **I** | **L** | **E** | **L** | **E** | **R** | **H** | **R** | **L** | **A** | **R** | **E** | **L** | **H** | **D** | **S** | **V** | **S** | **Q** | **Q** | **L** | **F** | **A** | **A** | **M** | **M** | **M** | **M** | **S** | **A** | **L** | **T** | **E** | **Q** | **A** | **E** | **K** | **S** | **E** | **T** | **P** | **E** | **M** | **F** | **R** | **K** | **Q** | **L** | **K** | **M** | **V** | **A** | **E** | **I** | **I** | **N** | **A** | **S** | **Q** | **S** | **E** | **M** | **R** | **A** | **L** | **L** | **L** | **H** | **L** | **R** | **P** | **V** | **N** | **L** | **E** | **E** | **K** | **S** | **L** | **K** | **Q** | **G** | **I** | **E** | **Q** | **L** | **L** | **K** | **E** | **L** | **Q** | **N** | **K** | **I** | **Q** | **I** | **S** | **L** | **K** | **W** | **D** | **V** | **E** | **D** | **V** | **K** | **L** | **T** | **S** | **S** | **I** | **E** | **D** | **H** | **L** | **F** | **R** | **I** | **V** | **Q** | **E** | **L** | **L** | **S** | **N** | **T** | **L** | **R** | **H** | **A** | **K** | **A** | **N** | **E** | **L** | **E** | **V** | **Y** | **L** | **H** | **K** | **I** | **D** | **N** | **N** | **L** | **L** | **L** | **R** | **I** | **I** | **D** | **D** | **G** | **T** | **G** | **F** | **N** | **M** | **N** | **E** | **T** | **K** | **T** | **G** | **S** | **Y** | **G** | **L** | **N** | **N** | **I** | **K** | **E** | **R** | **V** | **A** | **G** | **I** | **G** | **G** | **T** | **V** | **K** | **I** | **I** | **S** | **F** | **K** | **G** | **Q** | **G** | **T** | **S** | **V** | **E** | **I** | **K** | **V** | **P** | **L** | **M** | **K** | **E** | **A** |
| **N60252F** | **M** | **M** | **G** | **R** | **I** | **S** | **K** | **A** | **M** | **L** | **A** | **V** | **Y** | **S** | **G** | **I** | **A** | **A** | **F** | **L** | **I** | **I** | **L** | **F** | **S** | **L** | **F** | **T** | **Y** | **F** | **Y** | **A** | **S** | **N** | **Q** | **S** | **H** | **W** | **W** | **G** | **E** | **L** | **L** | **R** | **A** | **R** | **L** | **L** | **Y** | **V** | **P** | **L** | **I** | **F** | **H** | **L** | **M** | **A** | **I** | **S** | **L** | **G** | **V** | **G** | **L** | **I** | **V** | **F** | **L** | **L** | **L** | **S** | **L** | **I** | **Q** | **K** | **A** | **K** | **Y** | **G** | **K** | **I** | **E** | **E** | **K** | **L** | **R** | **A** | **L** | **S** | **S** | **G** | **N** | **Y** | **E** | **S** | **K** | **L** | **L** | **H** | **Q** | **P** | **I** | **P** | **S** | **A** | **S** | **D** | **D** | **L** | **Y** | **I** | **K** | **D** | **I** | **D** | **M** | **E** | **I** | **T** | **K** | **I** | **K** | **E** | **K** | **M** | **I** | **E** | **V** | **S** | **S** | **E** | **L** | **Q** | **I** | **M** | **T** | **S** | **R** | **P** | **Q** | **Y** | **V** | **D** | **G** | **Q** | **T** | **K** | **E** | **E** | **I** | **L** | **E** | **L** | **E** | **R** | **H** | **R** | **L** | **A** | **R** | **E** | **L** | **H** | **D** | **S** | **V** | **S** | **Q** | **Q** | **L** | **F** | **A** | **A** | **M** | **M** | **M** | **M** | **S** | **A** | **L** | **T** | **E** | **Q** | **A** | **E** | **K** | **S** | **E** | **T** | **P** | **E** | **M** | **F** | **R** | **K** | **Q** | **L** | **K** | **M** | **V** | **A** | **E** | **I** | **I** | **N** | **A** | **S** | **Q** | **S** | **E** | **M** | **R** | **A** | **L** | **L** | **L** | **H** | **L** | **R** | **P** | **V** | **N** | **L** | **E** | **E** | **K** | **S** | **L** | **K** | **Q** | **G** | **I** | **E** | **Q** | **L** | **L** | **K** | **E** | **L** | **Q** | **N** | **K** | **I** | **Q** | **I** | **S** | **L** | **K** | **W** | **D** | **V** | **E** | **D** | **V** | **K** | **L** | **T** | **S** | **S** | **I** | **E** | **D** | **H** | **L** | **F** | **R** | **I** | **V** | **Q** | **E** | **L** | **L** | **S** | **N** | **T** | **L** | **R** | **H** | **A** | **K** | **A** | **N** | **E** | **L** | **E** | **V** | **Y** | **L** | **H** | **K** | **I** | **D** | **N** | **N** | **L** | **L** | **L** | **R** | **I** | **I** | **D** | **D** | **G** | **T** | **G** | **F** | **N** | **M** | **N** | **E** | **T** | **K** | **T** | **G** | **S** | **Y** | **G** | **L** | **N** | **N** | **I** | **K** | **E** | **R** | **V** | **A** | **G** | **I** | **G** | **G** | **T** | **V** | **K** | **I** | **I** | **S** | **F** | **K** | **G** | **Q** | **G** | **T** | **S** | **V** | **E** | **I** | **K** | **V** | **P** | **L** | **M** | **K** | **E** | **A** |
| **N54155** | **M** | **M** | **G** | **K** | **I** | **S** | **R** | **A** | **M** | **L** | **A** | **V** | **Y** | **S** | **G** | **I** | **A** | **A** | **F** | **L** | **I** | **I** | **L** | **F** | **S** | **L** | **F** | **T** | **Y** | **F** | **Y** | **A** | **S** | **N** | **Q** | **S** | **H** | **W** | **W** | **G** | **E** | **L** | **L** | **R** | **A** | **R** | **L** | **L** | **Y** | **V** | **P** | **L** | **I** | **F** | **H** | **L** | **L** | **A** | **I** | **S** | **L** | **G** | **V** | **G** | **L** | **I** | **V** | **F** | **L** | **L** | **L** | **S** | **L** | **I** | **Q** | **K** | **T** | **K** | **Y** | **G** | **K** | **I** | **E** | **E** | **K** | **L** | **R** | **A** | **L** | **S** | **S** | **G** | **N** | **Y** | **E** | **S** | **K** | **L** | **L** | **L** | **L** | **P** | **I** | **P** | **S** | **A** | **S** | **D** | **D** | **L** | **Y** | **I** | **K** | **D** | **I** | **D** | **K** | **E** | **I** | **T** | **K** | **I** | **K** | **E** | **K** | **M** | **I** | **E** | **V** | **S** | **S** | **E** | **L** | **Q** | **I** | **V** | **T** | **S** | **R** | **P** | **Q** | **Y** | **V** | **D** | **G** | **Q** | **T** | **K** | **E** | **E** | **I** | **L** | **E** | **L** | **E** | **R** | **H** | **R** | **L** | **A** | **R** | **E** | **L** | **H** | **D** | **S** | **V** | **S** | **Q** | **Q** | **L** | **F** | **A** | **A** | **M** | **M** | **M** | **M** | **S** | **A** | **L** | **T** | **E** | **Q** | **A** | **E** | **K** | **S** | **E** | **T** | **P** | **E** | **M** | **F** | **R** | **K** | **Q** | **L** | **K** | **M** | **V** | **A** | **E** | **I** | **I** | **N** | **A** | **S** | **Q** | **S** | **E** | **M** | **R** | **A** | **L** | **L** | **L** | **H** | **L** | **R** | **P** | **V** | **N** | **L** | **E** | **E** | **K** | **S** | **L** | **K** | **Q** | **G** | **I** | **E** | **Q** | **L** | **L** | **K** | **E** | **L** | **Q** | **N** | **K** | **I** | **Q** | **I** | **S** | **L** | **K** | **W** | **D** | **V** | **E** | **D** | **V** | **K** | **L** | **S** | **S** | **S** | **I** | **E** | **D** | **H** | **L** | **F** | **R** | **I** | **V** | **Q** | **E** | **L** | **L** | **S** | **N** | **T** | **L** | **R** | **H** | **A** | **K** | **A** | **N** | **E** | **L** | **E** | **V** | **Y** | **L** | **H** | **K** | **I** | **D** | **N** | **N** | **L** | **L** | **L** | **R** | **I** | **I** | **D** | **D** | **G** | **T** | **G** | **F** | **N** | **M** | **N** | **E** | **T** | **K** | **T** | **G** | **S** | **Y** | **G** | **L** | **N** | **N** | **I** | **K** | **E** | **R** | **V** | **A** | **G** | **I** | **G** | **G** | **T** | **V** | **K** | **I** | **I** | **S** | **F** | **K** | **G** | **Q** | **G** | **T** | **S** | **V** | **E** | **I** | **K** | **V** | **P** | **L** | **M** | **K** | **E** | **A** |
| **N53559** | **M** | **M** | **G** | **R** | **I** | **S** | **K** | **A** | **M** | **L** | **A** | **V** | **Y** | **S** | **G** | **I** | **A** | **A** | **F** | **L** | **I** | **I** | **L** | **F** | **S** | **L** | **F** | **T** | **Y** | **F** | **Y** | **A** | **S** | **N** | **Q** | **S** | **H** | **W** | **W** | **G** | **E** | **L** | **L** | **R** | **A** | **R** | **L** | **L** | **Y** | **V** | **P** | **L** | **I** | **F** | **H** | **L** | **M** | **A** | **I** | **S** | **L** | **G** | **V** | **G** | **L** | **I** | **V** | **F** | **L** | **L** | **L** | **S** | **L** | **I** | **Q** | **K** | **A** | **K** | **Y** | **G** | **K** | **I** | **E** | **E** | **K** | **L** | **R** | **A** | **L** | **S** | **S** | **G** | **N** | **Y** | **E** | **S** | **K** | **L** | **L** | **H** | **Q** | **P** | **I** | **P** | **S** | **A** | **S** | **D** | **D** | **L** | **Y** | **I** | **K** | **D** | **I** | **D** | **I** | **E** | **I** | **T** | **K** | **I** | **K** | **E** | **K** | **M** | **I** | **E** | **V** | **S** | **S** | **E** | **L** | **Q** | **I** | **M** | **T** | **S** | **R** | **P** | **Q** | **Y** | **V** | **D** | **G** | **Q** | **T** | **K** | **E** | **E** | **I** | **L** | **E** | **L** | **E** | **R** | **H** | **R** | **L** | **A** | **R** | **E** | **L** | **H** | **D** | **S** | **V** | **S** | **Q** | **Q** | **L** | **F** | **A** | **A** | **M** | **M** | **M** | **M** | **S** | **A** | **L** | **T** | **E** | **Q** | **A** | **E** | **K** | **S** | **E** | **T** | **P** | **E** | **M** | **F** | **R** | **K** | **Q** | **L** | **K** | **M** | **V** | **A** | **E** | **I** | **I** | **N** | **A** | **S** | **Q** | **S** | **E** | **M** | **R** | **A** | **L** | **L** | **L** | **H** | **L** | **R** | **P** | **V** | **N** | **L** | **E** | **E** | **K** | **S** | **L** | **K** | **Q** | **G** | **I** | **E** | **Q** | **L** | **L** | **K** | **E** | **L** | **Q** | **N** | **K** | **I** | **Q** | **I** | **S** | **L** | **K** | **W** | **D** | **V** | **E** | **D** | **V** | **K** | **L** | **T** | **S** | **S** | **I** | **E** | **D** | **H** | **L** | **F** | **R** | **I** | **V** | **Q** | **E** | **L** | **L** | **S** | **N** | **T** | **L** | **R** | **H** | **A** | **K** | **A** | **N** | **E** | **L** | **E** | **V** | **Y** | **L** | **H** | **K** | **I** | **D** | **N** | **N** | **L** | **L** | **L** | **R** | **I** | **I** | **D** | **D** | **G** | **T** | **G** | **F** | **N** | **M** | **N** | **E** | **T** | **K** | **T** | **G** | **S** | **Y** | **G** | **L** | **N** | **N** | **I** | **K** | **E** | **R** | **V** | **A** | **G** | **I** | **G** | **G** | **T** | **V** | **K** | **I** | **I** | **S** | **F** | **K** | **G** | **Q** | **G** | **T** | **S** | **V** | **E** | **I** | **K** | **V** | **P** | **L** | **M** | **K** | **E** | **A** |
| **N59754F** | **M** | **M** | **G** | **R** | **I** | **S** | **K** | **A** | **M** | **L** | **A** | **V** | **Y** | **S** | **G** | **I** | **A** | **A** | **F** | **L** | **I** | **I** | **L** | **F** | **S** | **L** | **F** | **T** | **Y** | **F** | **Y** | **A** | **S** | **N** | **Q** | **S** | **H** | **W** | **W** | **G** | **E** | **L** | **L** | **R** | **A** | **R** | **L** | **L** | **Y** | **V** | **P** | **L** | **I** | **F** | **H** | **L** | **M** | **A** | **I** | **S** | **L** | **G** | **V** | **G** | **L** | **I** | **V** | **F** | **L** | **L** | **L** | **S** | **L** | **I** | **Q** | **K** | **A** | **K** | **Y** | **G** | **K** | **I** | **E** | **E** | **K** | **L** | **R** | **A** | **L** | **S** | **S** | **G** | **N** | **Y** | **E** | **S** | **K** | **L** | **L** | **H** | **Q** | **P** | **I** | **P** | **S** | **A** | **S** | **D** | **D** | **L** | **Y** | **I** | **K** | **D** | **I** | **D** | **M** | **E** | **I** | **T** | **K** | **I** | **K** | **E** | **K** | **M** | **I** | **E** | **V** | **S** | **S** | **E** | **L** | **Q** | **I** | **M** | **T** | **S** | **R** | **P** | **Q** | **Y** | **V** | **D** | **G** | **Q** | **T** | **K** | **E** | **E** | **I** | **L** | **E** | **L** | **E** | **R** | **H** | **R** | **L** | **A** | **R** | **E** | **L** | **H** | **D** | **S** | **V** | **S** | **Q** | **Q** | **L** | **F** | **A** | **A** | **M** | **M** | **M** | **M** | **S** | **A** | **L** | **T** | **E** | **Q** | **A** | **E** | **K** | **S** | **E** | **T** | **P** | **G** | **M** | **F** | **R** | **K** | **Q** | **L** | **K** | **M** | **V** | **A** | **E** | **I** | **I** | **N** | **A** | **S** | **Q** | **S** | **E** | **M** | **R** | **A** | **L** | **L** | **L** | **H** | **L** | **R** | **P** | **V** | **N** | **L** | **E** | **E** | **K** | **S** | **L** | **K** | **Q** | **G** | **I** | **E** | **Q** | **L** | **L** | **K** | **E** | **L** | **Q** | **N** | **K** | **I** | **Q** | **I** | **S** | **L** | **K** | **W** | **D** | **V** | **E** | **D** | **V** | **K** | **L** | **T** | **S** | **S** | **I** | **E** | **D** | **H** | **L** | **F** | **R** | **I** | **V** | **Q** | **E** | **L** | **L** | **S** | **N** | **T** | **L** | **R** | **H** | **A** | **K** | **A** | **N** | **E** | **L** | **E** | **V** | **Y** | **L** | **H** | **K** | **I** | **D** | **N** | **N** | **L** | **L** | **L** | **R** | **I** | **I** | **D** | **D** | **G** | **T** | **G** | **F** | **N** | **M** | **N** | **E** | **T** | **K** | **T** | **G** | **S** | **Y** | **G** | **L** | **N** | **N** | **I** | **K** | **E** | **R** | **V** | **A** | **G** | **I** | **G** | **G** | **T** | **V** | **K** | **I** | **I** | **S** | **F** | **K** | **G** | **Q** | **G** | **T** | **S** | **V** | **E** | **I** | **K** | **V** | **P** | **L** | **M** | **K** | **E** | **A** |
| **N59951F** | **M** | **M** | **G** | **R** | **I** | **S** | **K** | **A** | **M** | **L** | **A** | **V** | **Y** | **S** | **G** | **I** | **A** | **A** | **F** | **L** | **I** | **I** | **L** | **F** | **S** | **L** | **F** | **T** | **Y** | **F** | **Y** | **A** | **S** | **N** | **Q** | **S** | **H** | **W** | **W** | **G** | **E** | **L** | **L** | **R** | **A** | **R** | **L** | **L** | **Y** | **V** | **P** | **L** | **I** | **F** | **H** | **L** | **M** | **A** | **I** | **S** | **L** | **G** | **V** | **G** | **L** | **I** | **V** | **F** | **L** | **L** | **L** | **S** | **L** | **I** | **Q** | **K** | **A** | **K** | **Y** | **G** | **K** | **I** | **E** | **E** | **K** | **L** | **R** | **A** | **L** | **S** | **S** | **G** | **N** | **Y** | **E** | **S** | **K** | **L** | **L** | **H** | **Q** | **P** | **I** | **P** | **S** | **T** | **S** | **D** | **D** | **L** | **Y** | **I** | **K** | **D** | **I** | **D** | **M** | **E** | **I** | **T** | **K** | **I** | **K** | **E** | **K** | **M** | **I** | **E** | **V** | **S** | **S** | **E** | **L** | **Q** | **I** | **M** | **T** | **S** | **R** | **P** | **Q** | **Y** | **V** | **D** | **G** | **Q** | **T** | **K** | **E** | **E** | **I** | **L** | **E** | **L** | **E** | **R** | **H** | **R** | **L** | **A** | **R** | **E** | **L** | **H** | **D** | **S** | **V** | **S** | **Q** | **Q** | **L** | **F** | **A** | **A** | **M** | **M** | **M** | **M** | **S** | **A** | **L** | **T** | **E** | **Q** | **A** | **E** | **K** | **S** | **E** | **T** | **P** | **E** | **M** | **F** | **R** | **K** | **Q** | **L** | **K** | **M** | **V** | **A** | **E** | **I** | **I** | **N** | **A** | **S** | **Q** | **S** | **E** | **M** | **R** | **A** | **L** | **L** | **L** | **H** | **L** | **R** | **P** | **V** | **N** | **L** | **E** | **E** | **K** | **S** | **L** | **K** | **Q** | **G** | **I** | **E** | **Q** | **L** | **L** | **K** | **E** | **L** | **Q** | **N** | **K** | **I** | **Q** | **I** | **S** | **L** | **K** | **W** | **D** | **V** | **E** | **D** | **V** | **K** | **L** | **T** | **S** | **S** | **I** | **E** | **D** | **H** | **L** | **F** | **R** | **I** | **V** | **Q** | **E** | **L** | **L** | **S** | **N** | **T** | **L** | **R** | **H** | **A** | **K** | **A** | **N** | **E** | **L** | **E** | **V** | **Y** | **L** | **H** | **K** | **I** | **D** | **N** | **N** | **L** | **L** | **L** | **R** | **I** | **I** | **D** | **D** | **G** | **T** | **G** | **F** | **N** | **M** | **N** | **E** | **T** | **K** | **T** | **G** | **S** | **Y** | **G** | **L** | **N** | **N** | **I** | **K** | **E** | **R** | **V** | **A** | **G** | **I** | **G** | **G** | **T** | **V** | **K** | **I** | **I** | **S** | **F** | **K** | **G** | **Q** | **G** | **T** | **S** | **V** | **E** | **I** | **K** | **V** | **P** | **L** | **M** | **K** | **E** | **A** |
| **N55299** | **M** | **M** | **G** | **R** | **I** | **S** | **K** | **A** | **M** | **L** | **A** | **V** | **Y** | **S** | **G** | **I** | **A** | **A** | **F** | **L** | **I** | **I** | **L** | **F** | **S** | **L** | **F** | **T** | **Y** | **F** | **Y** | **A** | **S** | **N** | **Q** | **S** | **H** | **W** | **W** | **G** | **E** | **L** | **L** | **R** | **A** | **R** | **L** | **L** | **Y** | **V** | **P** | **L** | **I** | **F** | **H** | **L** | **M** | **A** | **I** | **S** | **L** | **G** | **V** | **G** | **L** | **I** | **V** | **F** | **L** | **L** | **L** | **S** | **L** | **I** | **Q** | **K** | **A** | **K** | **Y** | **G** | **K** | **I** | **E** | **E** | **K** | **L** | **R** | **A** | **L** | **S** | **S** | **G** | **N** | **Y** | **E** | **S** | **K** | **L** | **L** | **H** | **Q** | **P** | **I** | **P** | **S** | **A** | **S** | **D** | **D** | **L** | **Y** | **I** | **K** | **D** | **I** | **D** | **M** | **E** | **I** | **T** | **K** | **I** | **K** | **E** | **K** | **M** | **I** | **E** | **V** | **S** | **S** | **E** | **L** | **Q** | **I** | **M** | **T** | **S** | **R** | **P** | **Q** | **Y** | **V** | **D** | **G** | **Q** | **T** | **K** | **E** | **E** | **I** | **L** | **E** | **L** | **E** | **R** | **H** | **R** | **L** | **A** | **R** | **E** | **L** | **H** | **D** | **S** | **V** | **S** | **Q** | **Q** | **L** | **F** | **A** | **A** | **M** | **M** | **M** | **M** | **S** | **A** | **L** | **T** | **E** | **Q** | **A** | **E** | **K** | **S** | **E** | **T** | **P** | **E** | **M** | **F** | **R** | **K** | **Q** | **L** | **K** | **M** | **V** | **A** | **E** | **I** | **I** | **N** | **A** | **S** | **Q** | **S** | **E** | **M** | **R** | **A** | **L** | **L** | **L** | **H** | **L** | **R** | **P** | **V** | **N** | **L** | **E** | **E** | **K** | **S** | **L** | **K** | **Q** | **G** | **I** | **E** | **Q** | **L** | **L** | **K** | **E** | **L** | **Q** | **N** | **K** | **I** | **Q** | **I** | **S** | **L** | **K** | **W** | **D** | **V** | **E** | **D** | **V** | **K** | **L** | **T** | **S** | **S** | **I** | **E** | **D** | **H** | **L** | **F** | **R** | **I** | **V** | **Q** | **E** | **L** | **L** | **S** | **N** | **T** | **L** | **R** | **H** | **A** | **K** | **A** | **N** | **E** | **L** | **E** | **V** | **Y** | **L** | **H** | **K** | **I** | **D** | **N** | **N** | **L** | **L** | **L** | **R** | **I** | **I** | **D** | **D** | **G** | **T** | **G** | **F** | **N** | **M** | **N** | **E** | **T** | **K** | **T** | **G** | **S** | **Y** | **G** | **L** | **N** | **N** | **I** | **K** | **E** | **R** | **V** | **A** | **G** | **I** | **G** | **G** | **T** | **V** | **K** | **I** | **I** | **S** | **F** | **K** | **G** | **Q** | **G** | **T** | **S** | **V** | **E** | **I** | **K** | **V** | **P** | **L** | **M** | **K** | **E** | **A** |
| **N59531F** | **M** | **M** | **G** | **K** | **I** | **S** | **R** | **A** | **M** | **L** | **A** | **V** | **Y** | **S** | **G** | **I** | **A** | **A** | **F** | **L** | **I** | **I** | **L** | **F** | **S** | **L** | **F** | **T** | **Y** | **F** | **Y** | **A** | **S** | **N** | **Q** | **S** | **H** | **W** | **W** | **G** | **E** | **L** | **L** | **R** | **A** | **R** | **L** | **L** | **Y** | **V** | **P** | **L** | **I** | **F** | **H** | **L** | **L** | **A** | **I** | **S** | **L** | **G** | **V** | **G** | **L** | **I** | **V** | **F** | **L** | **L** | **L** | **S** | **L** | **I** | **Q** | **K** | **T** | **K** | **Y** | **G** | **K** | **I** | **E** | **E** | **K** | **L** | **R** | **A** | **L** | **S** | **S** | **G** | **N** | **Y** | **E** | **S** | **K** | **L** | **L** | **L** | **L** | **P** | **I** | **P** | **S** | **A** | **S** | **D** | **D** | **L** | **Y** | **I** | **K** | **D** | **I** | **D** | **K** | **E** | **I** | **T** | **R** | **I** | **K** | **E** | **K** | **M** | **I** | **E** | **V** | **S** | **S** | **E** | **L** | **Q** | **I** | **V** | **T** | **S** | **R** | **P** | **Q** | **Y** | **V** | **D** | **G** | **Q** | **T** | **K** | **E** | **E** | **I** | **L** | **E** | **L** | **E** | **R** | **H** | **R** | **L** | **A** | **R** | **E** | **L** | **H** | **D** | **S** | **V** | **S** | **Q** | **Q** | **L** | **F** | **A** | **A** | **M** | **M** | **M** | **M** | **S** | **A** | **L** | **T** | **E** | **Q** | **A** | **E** | **K** | **S** | **E** | **T** | **P** | **E** | **M** | **F** | **R** | **K** | **Q** | **L** | **K** | **M** | **V** | **A** | **E** | **I** | **I** | **N** | **A** | **S** | **Q** | **S** | **E** | **M** | **R** | **A** | **L** | **L** | **L** | **H** | **L** | **R** | **P** | **V** | **N** | **L** | **E** | **E** | **K** | **S** | **L** | **K** | **Q** | **G** | **I** | **E** | **Q** | **L** | **L** | **K** | **E** | **L** | **Q** | **N** | **K** | **I** | **Q** | **I** | **S** | **L** | **K** | **W** | **D** | **V** | **E** | **D** | **V** | **K** | **L** | **S** | **S** | **S** | **I** | **E** | **D** | **H** | **L** | **F** | **R** | **I** | **V** | **Q** | **E** | **L** | **L** | **S** | **N** | **T** | **L** | **R** | **H** | **A** | **K** | **A** | **N** | **E** | **L** | **E** | **V** | **Y** | **L** | **H** | **K** | **I** | **D** | **N** | **N** | **L** | **L** | **L** | **R** | **I** | **I** | **D** | **D** | **G** | **T** | **G** | **F** | **N** | **M** | **N** | **E** | **T** | **K** | **T** | **G** | **S** | **Y** | **G** | **L** | **N** | **N** | **I** | **K** | **E** | **R** | **V** | **A** | **G** | **I** | **G** | **G** | **T** | **V** | **K** | **I** | **I** | **S** | **F** | **K** | **G** | **Q** | **G** | **T** | **S** | **V** | **E** | **I** | **K** | **V** | **P** | **L** | **M** | **K** | **E** | **A** |
| **N59503F** | **M** | **M** | **G** | **R** | **I** | **S** | **K** | **A** | **M** | **L** | **A** | **V** | **Y** | **S** | **G** | **I** | **A** | **A** | **F** | **L** | **I** | **I** | **L** | **F** | **S** | **L** | **F** | **T** | **Y** | **F** | **Y** | **A** | **S** | **N** | **Q** | **S** | **H** | **W** | **W** | **G** | **E** | **L** | **L** | **R** | **A** | **R** | **L** | **L** | **Y** | **V** | **P** | **L** | **I** | **F** | **H** | **L** | **M** | **A** | **I** | **S** | **L** | **G** | **V** | **G** | **L** | **I** | **V** | **F** | **L** | **L** | **L** | **S** | **L** | **I** | **Q** | **K** | **T** | **K** | **Y** | **G** | **K** | **I** | **E** | **E** | **K** | **L** | **R** | **A** | **L** | **S** | **S** | **G** | **N** | **Y** | **E** | **S** | **K** | **L** | **L** | **H** | **Q** | **P** | **I** | **P** | **S** | **A** | **S** | **D** | **D** | **L** | **Y** | **I** | **K** | **D** | **I** | **D** | **M** | **E** | **I** | **T** | **K** | **I** | **K** | **E** | **K** | **M** | **I** | **E** | **V** | **S** | **S** | **E** | **L** | **Q** | **I** | **M** | **T** | **S** | **R** | **P** | **Q** | **Y** | **V** | **D** | **G** | **Q** | **T** | **K** | **E** | **E** | **I** | **L** | **E** | **L** | **E** | **R** | **H** | **R** | **L** | **A** | **R** | **E** | **L** | **H** | **D** | **S** | **V** | **S** | **Q** | **Q** | **L** | **F** | **A** | **A** | **M** | **M** | **M** | **M** | **S** | **A** | **L** | **T** | **E** | **Q** | **A** | **E** | **K** | **S** | **E** | **T** | **P** | **E** | **M** | **F** | **R** | **K** | **Q** | **L** | **K** | **M** | **V** | **A** | **E** | **I** | **I** | **N** | **A** | **S** | **Q** | **S** | **E** | **M** | **R** | **A** | **L** | **L** | **L** | **H** | **L** | **R** | **P** | **V** | **N** | **L** | **E** | **E** | **K** | **S** | **L** | **K** | **Q** | **G** | **I** | **E** | **Q** | **L** | **L** | **K** | **E** | **L** | **Q** | **N** | **K** | **I** | **Q** | **I** | **S** | **L** | **K** | **W** | **D** | **V** | **E** | **D** | **V** | **K** | **L** | **T** | **S** | **S** | **I** | **E** | **D** | **H** | **L** | **F** | **R** | **I** | **V** | **Q** | **E** | **L** | **L** | **S** | **N** | **T** | **L** | **R** | **H** | **A** | **K** | **A** | **N** | **E** | **L** | **E** | **V** | **Y** | **L** | **H** | **K** | **I** | **D** | **N** | **N** | **L** | **L** | **L** | **R** | **I** | **I** | **D** | **D** | **G** | **T** | **G** | **F** | **N** | **M** | **N** | **E** | **T** | **K** | **T** | **G** | **S** | **Y** | **G** | **L** | **N** | **N** | **I** | **K** | **E** | **R** | **V** | **A** | **G** | **I** | **G** | **G** | **T** | **V** | **K** | **I** | **I** | **S** | **F** | **K** | **G** | **Q** | **G** | **T** | **S** | **V** | **E** | **I** | **K** | **V** | **P** | **L** | **M** | **K** | **E** | **A** |
| **N52643** | **M** | **M** | **G** | **R** | **I** | **S** | **K** | **A** | **M** | **L** | **A** | **V** | **Y** | **S** | **G** | **I** | **A** | **A** | **F** | **L** | **I** | **I** | **L** | **F** | **S** | **L** | **F** | **T** | **Y** | **F** | **Y** | **A** | **S** | **N** | **Q** | **S** | **H** | **W** | **W** | **G** | **E** | **L** | **L** | **R** | **A** | **R** | **L** | **L** | **Y** | **V** | **P** | **L** | **I** | **F** | **H** | **L** | **M** | **A** | **I** | **S** | **L** | **G** | **V** | **G** | **L** | **I** | **V** | **F** | **L** | **L** | **L** | **S** | **L** | **I** | **Q** | **K** | **A** | **K** | **Y** | **G** | **K** | **I** | **E** | **E** | **K** | **L** | **R** | **A** | **L** | **S** | **S** | **G** | **N** | **Y** | **E** | **S** | **K** | **L** | **L** | **H** | **Q** | **P** | **I** | **P** | **S** | **A** | **S** | **D** | **D** | **L** | **Y** | **I** | **K** | **D** | **I** | **D** | **I** | **E** | **I** | **T** | **K** | **I** | **K** | **E** | **K** | **M** | **I** | **E** | **V** | **S** | **S** | **E** | **L** | **Q** | **I** | **M** | **T** | **S** | **R** | **P** | **Q** | **Y** | **V** | **D** | **G** | **Q** | **T** | **K** | **E** | **E** | **I** | **L** | **E** | **L** | **E** | **R** | **H** | **R** | **L** | **A** | **R** | **E** | **L** | **H** | **D** | **S** | **V** | **S** | **Q** | **Q** | **L** | **F** | **A** | **A** | **M** | **M** | **M** | **M** | **S** | **A** | **L** | **T** | **E** | **Q** | **A** | **E** | **K** | **S** | **E** | **T** | **P** | **E** | **M** | **F** | **R** | **K** | **Q** | **L** | **K** | **M** | **V** | **A** | **E** | **I** | **I** | **N** | **A** | **S** | **Q** | **S** | **E** | **M** | **R** | **A** | **L** | **L** | **L** | **H** | **L** | **R** | **P** | **V** | **N** | **L** | **E** | **E** | **K** | **S** | **L** | **K** | **Q** | **G** | **I** | **E** | **Q** | **L** | **L** | **K** | **E** | **L** | **Q** | **N** | **K** | **I** | **Q** | **I** | **S** | **L** | **K** | **W** | **D** | **V** | **E** | **D** | **V** | **K** | **L** | **T** | **S** | **S** | **I** | **E** | **D** | **H** | **L** | **F** | **R** | **I** | **V** | **Q** | **E** | **L** | **L** | **S** | **N** | **T** | **L** | **R** | **H** | **A** | **K** | **A** | **N** | **E** | **L** | **E** | **V** | **Y** | **L** | **H** | **K** | **I** | **D** | **N** | **N** | **L** | **L** | **L** | **R** | **I** | **I** | **D** | **D** | **G** | **T** | **G** | **F** | **N** | **M** | **N** | **E** | **T** | **K** | **T** | **G** | **S** | **Y** | **G** | **L** | **N** | **N** | **I** | **K** | **E** | **R** | **V** | **A** | **G** | **I** | **G** | **G** | **T** | **V** | **K** | **I** | **I** | **S** | **F** | **K** | **G** | **Q** | **G** | **T** | **S** | **V** | **E** | **I** | **K** | **V** | **P** | **L** | **M** | **K** | **E** | **A** |
| **N53702** | **M** | **M** | **G** | **K** | **I** | **S** | **R** | **A** | **M** | **L** | **A** | **V** | **Y** | **S** | **G** | **I** | **A** | **A** | **F** | **L** | **I** | **I** | **L** | **F** | **S** | **L** | **F** | **T** | **Y** | **F** | **Y** | **A** | **S** | **N** | **Q** | **S** | **H** | **W** | **W** | **G** | **E** | **L** | **L** | **R** | **A** | **R** | **L** | **L** | **Y** | **V** | **P** | **L** | **I** | **F** | **H** | **L** | **L** | **A** | **I** | **S** | **L** | **G** | **V** | **G** | **L** | **I** | **V** | **F** | **L** | **L** | **L** | **S** | **L** | **I** | **Q** | **K** | **T** | **K** | **Y** | **G** | **K** | **I** | **E** | **E** | **K** | **L** | **R** | **A** | **L** | **S** | **S** | **G** | **N** | **Y** | **E** | **S** | **K** | **L** | **L** | **L** | **L** | **P** | **I** | **P** | **S** | **A** | **S** | **D** | **D** | **L** | **Y** | **I** | **K** | **D** | **I** | **D** | **K** | **E** | **I** | **T** | **K** | **I** | **K** | **E** | **K** | **M** | **I** | **E** | **V** | **S** | **S** | **E** | **L** | **Q** | **I** | **V** | **T** | **S** | **R** | **P** | **Q** | **Y** | **V** | **D** | **G** | **Q** | **T** | **K** | **E** | **E** | **I** | **L** | **E** | **L** | **E** | **R** | **H** | **R** | **L** | **A** | **R** | **E** | **L** | **H** | **D** | **S** | **V** | **S** | **Q** | **Q** | **L** | **F** | **A** | **A** | **M** | **M** | **M** | **M** | **S** | **A** | **L** | **T** | **E** | **Q** | **A** | **E** | **K** | **S** | **E** | **T** | **P** | **E** | **M** | **F** | **R** | **K** | **Q** | **L** | **K** | **M** | **V** | **A** | **E** | **I** | **I** | **N** | **A** | **S** | **Q** | **S** | **E** | **M** | **R** | **A** | **L** | **L** | **L** | **H** | **L** | **R** | **P** | **V** | **N** | **L** | **E** | **E** | **K** | **S** | **L** | **K** | **Q** | **G** | **I** | **E** | **Q** | **L** | **L** | **K** | **E** | **L** | **Q** | **N** | **K** | **I** | **Q** | **I** | **S** | **L** | **K** | **W** | **D** | **V** | **E** | **D** | **V** | **K** | **L** | **S** | **S** | **S** | **I** | **E** | **D** | **H** | **L** | **F** | **R** | **I** | **V** | **Q** | **E** | **L** | **L** | **S** | **N** | **T** | **L** | **R** | **H** | **A** | **K** | **A** | **N** | **E** | **L** | **E** | **V** | **Y** | **L** | **H** | **K** | **I** | **D** | **N** | **N** | **L** | **L** | **L** | **R** | **I** | **I** | **D** | **D** | **G** | **T** | **G** | **F** | **N** | **M** | **N** | **E** | **T** | **K** | **T** | **G** | **S** | **Y** | **G** | **L** | **N** | **N** | **I** | **K** | **E** | **R** | **V** | **A** | **G** | **I** | **G** | **G** | **T** | **V** | **K** | **I** | **I** | **S** | **F** | **K** | **G** | **Q** | **G** | **T** | **S** | **V** | **E** | **I** | **K** | **V** | **P** | **L** | **M** | **K** | **E** | **A** |
| **N54190** | **M** | **M** | **G** | **R** | **I** | **S** | **K** | **A** | **M** | **L** | **A** | **V** | **Y** | **S** | **G** | **I** | **A** | **A** | **F** | **L** | **I** | **I** | **L** | **F** | **S** | **L** | **F** | **T** | **Y** | **F** | **Y** | **A** | **S** | **N** | **Q** | **S** | **H** | **W** | **W** | **G** | **E** | **L** | **L** | **R** | **A** | **R** | **L** | **L** | **Y** | **V** | **P** | **L** | **I** | **F** | **H** | **L** | **M** | **A** | **I** | **S** | **L** | **G** | **V** | **G** | **L** | **I** | **V** | **F** | **L** | **L** | **L** | **S** | **L** | **I** | **Q** | **K** | **A** | **K** | **Y** | **G** | **K** | **I** | **E** | **E** | **K** | **L** | **R** | **A** | **L** | **S** | **S** | **G** | **N** | **Y** | **E** | **S** | **K** | **L** | **L** | **H** | **Q** | **P** | **I** | **P** | **S** | **A** | **S** | **D** | **D** | **L** | **Y** | **I** | **K** | **D** | **I** | **D** | **I** | **E** | **I** | **T** | **K** | **I** | **K** | **E** | **K** | **M** | **I** | **E** | **V** | **S** | **S** | **E** | **L** | **Q** | **I** | **M** | **T** | **S** | **R** | **P** | **Q** | **Y** | **V** | **D** | **G** | **Q** | **T** | **K** | **E** | **E** | **I** | **L** | **E** | **L** | **E** | **R** | **H** | **R** | **L** | **A** | **R** | **E** | **L** | **H** | **D** | **S** | **V** | **S** | **Q** | **Q** | **L** | **F** | **A** | **A** | **M** | **M** | **M** | **M** | **S** | **A** | **L** | **T** | **E** | **Q** | **A** | **E** | **K** | **S** | **E** | **T** | **P** | **E** | **M** | **F** | **R** | **K** | **Q** | **L** | **K** | **M** | **V** | **A** | **E** | **I** | **I** | **N** | **A** | **S** | **Q** | **S** | **E** | **M** | **R** | **A** | **L** | **L** | **L** | **H** | **L** | **R** | **P** | **V** | **N** | **L** | **E** | **E** | **K** | **S** | **L** | **K** | **Q** | **G** | **I** | **E** | **Q** | **L** | **L** | **K** | **E** | **L** | **Q** | **N** | **K** | **I** | **Q** | **I** | **S** | **L** | **K** | **W** | **D** | **V** | **E** | **D** | **V** | **K** | **L** | **T** | **S** | **S** | **I** | **E** | **D** | **H** | **L** | **F** | **R** | **I** | **V** | **Q** | **E** | **L** | **L** | **S** | **N** | **T** | **L** | **R** | **H** | **A** | **K** | **A** | **N** | **E** | **L** | **E** | **V** | **Y** | **L** | **H** | **K** | **I** | **D** | **N** | **N** | **L** | **L** | **L** | **R** | **I** | **I** | **D** | **D** | **G** | **T** | **G** | **F** | **N** | **M** | **N** | **E** | **T** | **K** | **T** | **G** | **S** | **Y** | **G** | **L** | **N** | **N** | **I** | **K** | **E** | **R** | **V** | **A** | **G** | **I** | **G** | **G** | **T** | **V** | **K** | **I** | **I** | **S** | **F** | **K** | **G** | **Q** | **G** | **T** | **S** | **V** | **E** | **I** | **K** | **V** | **P** | **L** | **M** | **K** | **E** | **A** |
| **N55290** | **M** | **M** | **G** | **K** | **I** | **S** | **R** | **A** | **M** | **L** | **A** | **V** | **Y** | **S** | **G** | **I** | **A** | **A** | **F** | **L** | **I** | **I** | **L** | **F** | **S** | **L** | **F** | **T** | **Y** | **F** | **Y** | **A** | **S** | **N** | **Q** | **S** | **H** | **W** | **W** | **G** | **E** | **L** | **L** | **R** | **A** | **R** | **L** | **L** | **Y** | **V** | **P** | **L** | **I** | **F** | **H** | **L** | **L** | **A** | **I** | **S** | **L** | **G** | **V** | **G** | **L** | **I** | **V** | **F** | **L** | **L** | **L** | **S** | **L** | **I** | **Q** | **K** | **T** | **K** | **Y** | **G** | **K** | **I** | **E** | **E** | **K** | **L** | **R** | **A** | **L** | **S** | **S** | **G** | **N** | **Y** | **E** | **S** | **K** | **L** | **L** | **L** | **L** | **P** | **I** | **P** | **S** | **A** | **S** | **D** | **D** | **L** | **Y** | **I** | **K** | **D** | **I** | **D** | **K** | **E** | **I** | **T** | **K** | **I** | **K** | **E** | **K** | **M** | **I** | **E** | **V** | **S** | **S** | **E** | **L** | **Q** | **I** | **V** | **T** | **S** | **R** | **P** | **Q** | **Y** | **V** | **D** | **G** | **Q** | **T** | **K** | **E** | **E** | **I** | **L** | **E** | **L** | **E** | **R** | **H** | **R** | **L** | **A** | **R** | **E** | **L** | **H** | **D** | **S** | **V** | **S** | **Q** | **Q** | **L** | **F** | **A** | **A** | **M** | **M** | **M** | **M** | **S** | **A** | **L** | **T** | **E** | **Q** | **A** | **E** | **K** | **S** | **E** | **T** | **P** | **E** | **M** | **F** | **R** | **K** | **Q** | **L** | **K** | **M** | **V** | **A** | **E** | **I** | **I** | **N** | **A** | **S** | **Q** | **S** | **E** | **M** | **R** | **A** | **L** | **L** | **L** | **H** | **L** | **R** | **P** | **V** | **N** | **L** | **E** | **E** | **K** | **S** | **L** | **K** | **Q** | **G** | **I** | **E** | **Q** | **L** | **L** | **K** | **E** | **L** | **Q** | **N** | **K** | **I** | **Q** | **I** | **S** | **L** | **K** | **W** | **D** | **V** | **E** | **D** | **V** | **K** | **L** | **S** | **S** | **S** | **I** | **E** | **D** | **H** | **L** | **F** | **R** | **I** | **V** | **Q** | **E** | **L** | **L** | **S** | **N** | **T** | **L** | **R** | **H** | **A** | **K** | **A** | **N** | **E** | **L** | **E** | **V** | **Y** | **L** | **H** | **K** | **I** | **D** | **N** | **N** | **L** | **L** | **L** | **R** | **I** | **I** | **D** | **D** | **G** | **T** | **G** | **F** | **N** | **M** | **N** | **E** | **T** | **K** | **T** | **G** | **S** | **Y** | **G** | **L** | **N** | **N** | **I** | **K** | **E** | **R** | **V** | **A** | **G** | **I** | **G** | **G** | **T** | **V** | **K** | **I** | **I** | **S** | **F** | **K** | **G** | **Q** | **G** | **T** | **S** | **V** | **E** | **I** | **K** | **V** | **P** | **L** | **M** | **K** | **E** | **A** |
| **N53655** | **M** | **M** | **G** | **K** | **I** | **S** | **R** | **A** | **M** | **L** | **A** | **V** | **Y** | **S** | **G** | **I** | **A** | **A** | **F** | **L** | **I** | **I** | **L** | **F** | **S** | **L** | **F** | **T** | **Y** | **F** | **Y** | **A** | **S** | **N** | **Q** | **S** | **H** | **W** | **W** | **G** | **E** | **L** | **L** | **R** | **A** | **R** | **L** | **L** | **Y** | **V** | **P** | **L** | **I** | **F** | **H** | **L** | **L** | **A** | **I** | **S** | **L** | **G** | **V** | **G** | **L** | **I** | **V** | **F** | **L** | **L** | **L** | **S** | **L** | **I** | **Q** | **K** | **T** | **K** | **Y** | **G** | **K** | **I** | **E** | **E** | **K** | **L** | **R** | **A** | **L** | **S** | **S** | **G** | **N** | **Y** | **E** | **S** | **K** | **L** | **L** | **L** | **L** | **P** | **I** | **P** | **S** | **A** | **S** | **D** | **D** | **L** | **Y** | **I** | **K** | **D** | **I** | **D** | **K** | **E** | **I** | **T** | **K** | **I** | **K** | **E** | **K** | **M** | **I** | **E** | **V** | **S** | **S** | **E** | **L** | **Q** | **I** | **V** | **T** | **S** | **R** | **P** | **Q** | **Y** | **V** | **D** | **G** | **Q** | **T** | **K** | **E** | **E** | **I** | **L** | **E** | **L** | **E** | **R** | **H** | **R** | **L** | **A** | **R** | **E** | **L** | **H** | **D** | **S** | **V** | **S** | **Q** | **Q** | **L** | **F** | **A** | **A** | **M** | **M** | **M** | **M** | **S** | **A** | **L** | **T** | **E** | **Q** | **A** | **E** | **K** | **S** | **E** | **T** | **P** | **E** | **M** | **F** | **R** | **K** | **Q** | **L** | **K** | **M** | **V** | **A** | **E** | **I** | **I** | **N** | **A** | **S** | **Q** | **S** | **E** | **M** | **R** | **A** | **L** | **L** | **L** | **H** | **L** | **R** | **P** | **V** | **N** | **L** | **E** | **E** | **K** | **S** | **L** | **K** | **Q** | **G** | **I** | **E** | **Q** | **L** | **L** | **K** | **E** | **L** | **Q** | **N** | **K** | **I** | **Q** | **I** | **S** | **L** | **K** | **W** | **D** | **V** | **E** | **D** | **V** | **K** | **L** | **S** | **S** | **S** | **I** | **E** | **D** | **H** | **L** | **F** | **R** | **I** | **V** | **Q** | **E** | **L** | **L** | **S** | **N** | **T** | **L** | **R** | **H** | **A** | **K** | **A** | **N** | **E** | **L** | **E** | **V** | **Y** | **L** | **H** | **K** | **I** | **D** | **N** | **N** | **L** | **L** | **L** | **R** | **I** | **I** | **D** | **D** | **G** | **T** | **G** | **F** | **N** | **M** | **N** | **E** | **T** | **K** | **T** | **G** | **S** | **Y** | **G** | **L** | **N** | **N** | **I** | **K** | **E** | **R** | **V** | **A** | **G** | **I** | **G** | **G** | **T** | **V** | **K** | **I** | **I** | **S** | **F** | **K** | **G** | **Q** | **G** | **T** | **S** | **V** | **E** | **I** | **K** | **V** | **P** | **L** | **M** | **K** | **E** | **A** |
| **N52777** | **M** | **M** | **G** | **R** | **I** | **S** | **K** | **A** | **M** | **L** | **A** | **V** | **Y** | **S** | **G** | **I** | **A** | **A** | **F** | **L** | **I** | **I** | **L** | **F** | **S** | **L** | **F** | **T** | **Y** | **F** | **Y** | **A** | **S** | **N** | **Q** | **S** | **H** | **W** | **W** | **G** | **E** | **L** | **L** | **R** | **A** | **R** | **L** | **L** | **Y** | **V** | **P** | **L** | **I** | **F** | **H** | **L** | **M** | **A** | **I** | **S** | **L** | **G** | **V** | **G** | **L** | **I** | **V** | **F** | **L** | **L** | **L** | **S** | **L** | **I** | **Q** | **K** | **A** | **K** | **Y** | **G** | **K** | **I** | **E** | **E** | **K** | **L** | **R** | **A** | **L** | **S** | **S** | **G** | **N** | **Y** | **E** | **S** | **K** | **L** | **L** | **H** | **Q** | **P** | **I** | **P** | **S** | **A** | **S** | **D** | **D** | **L** | **Y** | **I** | **K** | **D** | **I** | **D** | **M** | **E** | **I** | **T** | **K** | **I** | **K** | **E** | **K** | **M** | **I** | **E** | **V** | **S** | **S** | **E** | **L** | **Q** | **I** | **M** | **T** | **S** | **R** | **P** | **Q** | **Y** | **V** | **D** | **G** | **Q** | **T** | **K** | **E** | **E** | **I** | **L** | **E** | **L** | **E** | **R** | **H** | **R** | **L** | **A** | **R** | **E** | **L** | **H** | **D** | **S** | **V** | **S** | **Q** | **Q** | **L** | **F** | **A** | **A** | **M** | **M** | **M** | **M** | **S** | **A** | **L** | **T** | **E** | **Q** | **A** | **E** | **K** | **S** | **E** | **T** | **P** | **E** | **M** | **F** | **R** | **K** | **Q** | **L** | **K** | **M** | **V** | **A** | **E** | **I** | **I** | **N** | **A** | **S** | **Q** | **S** | **E** | **M** | **R** | **A** | **L** | **L** | **L** | **H** | **L** | **R** | **P** | **V** | **N** | **L** | **E** | **E** | **K** | **S** | **L** | **K** | **Q** | **G** | **I** | **E** | **Q** | **L** | **L** | **K** | **E** | **L** | **Q** | **N** | **K** | **I** | **Q** | **I** | **S** | **L** | **K** | **W** | **D** | **V** | **E** | **D** | **V** | **K** | **L** | **T** | **S** | **S** | **I** | **E** | **D** | **H** | **L** | **F** | **R** | **I** | **V** | **Q** | **E** | **L** | **L** | **S** | **N** | **T** | **L** | **R** | **H** | **A** | **K** | **A** | **N** | **E** | **L** | **E** | **V** | **Y** | **L** | **H** | **K** | **I** | **D** | **N** | **N** | **L** | **L** | **L** | **R** | **I** | **I** | **D** | **D** | **G** | **T** | **G** | **F** | **N** | **M** | **N** | **E** | **T** | **K** | **T** | **G** | **S** | **Y** | **G** | **L** | **N** | **N** | **I** | **K** | **E** | **R** | **V** | **A** | **G** | **I** | **G** | **G** | **T** | **V** | **K** | **I** | **I** | **S** | **F** | **K** | **G** | **Q** | **G** | **T** | **S** | **V** | **E** | **I** | **K** | **V** | **P** | **L** | **M** | **K** | **E** | **A** |
| **N59998F** | **M** | **M** | **G** | **R** | **I** | **S** | **K** | **A** | **M** | **L** | **A** | **V** | **Y** | **S** | **G** | **I** | **A** | **A** | **F** | **L** | **I** | **I** | **L** | **F** | **S** | **L** | **F** | **T** | **Y** | **F** | **Y** | **A** | **S** | **N** | **Q** | **S** | **H** | **W** | **W** | **G** | **E** | **L** | **L** | **R** | **A** | **R** | **L** | **L** | **Y** | **V** | **P** | **L** | **I** | **F** | **H** | **L** | **M** | **A** | **I** | **S** | **L** | **G** | **V** | **G** | **L** | **I** | **V** | **F** | **L** | **L** | **L** | **S** | **L** | **I** | **Q** | **K** | **T** | **K** | **Y** | **G** | **K** | **I** | **E** | **E** | **K** | **L** | **R** | **A** | **L** | **S** | **S** | **G** | **N** | **Y** | **E** | **S** | **K** | **L** | **L** | **H** | **Q** | **P** | **I** | **P** | **S** | **A** | **S** | **D** | **D** | **L** | **Y** | **I** | **K** | **D** | **I** | **D** | **M** | **E** | **I** | **T** | **K** | **I** | **K** | **E** | **K** | **M** | **I** | **E** | **V** | **S** | **S** | **E** | **L** | **Q** | **I** | **M** | **T** | **S** | **R** | **P** | **Q** | **Y** | **V** | **D** | **G** | **Q** | **T** | **K** | **E** | **E** | **I** | **L** | **E** | **L** | **E** | **R** | **H** | **R** | **L** | **A** | **R** | **E** | **L** | **H** | **D** | **S** | **V** | **S** | **Q** | **Q** | **L** | **F** | **A** | **A** | **M** | **M** | **M** | **M** | **S** | **A** | **L** | **T** | **E** | **Q** | **A** | **E** | **K** | **S** | **E** | **T** | **P** | **E** | **M** | **F** | **R** | **K** | **Q** | **L** | **K** | **M** | **V** | **A** | **E** | **I** | **I** | **N** | **A** | **S** | **Q** | **S** | **E** | **M** | **R** | **A** | **L** | **L** | **L** | **H** | **L** | **R** | **P** | **V** | **N** | **L** | **E** | **E** | **K** | **S** | **L** | **K** | **Q** | **G** | **I** | **E** | **Q** | **L** | **L** | **K** | **E** | **L** | **Q** | **N** | **K** | **I** | **Q** | **I** | **S** | **L** | **K** | **W** | **D** | **V** | **E** | **D** | **V** | **K** | **L** | **T** | **S** | **S** | **I** | **E** | **D** | **H** | **L** | **F** | **R** | **I** | **V** | **Q** | **E** | **L** | **L** | **S** | **N** | **T** | **L** | **R** | **H** | **A** | **K** | **A** | **N** | **E** | **L** | **E** | **V** | **Y** | **L** | **H** | **K** | **I** | **D** | **N** | **N** | **L** | **L** | **L** | **R** | **I** | **I** | **D** | **D** | **G** | **T** | **G** | **F** | **N** | **M** | **N** | **E** | **T** | **K** | **T** | **G** | **S** | **Y** | **G** | **L** | **N** | **N** | **I** | **K** | **E** | **R** | **V** | **A** | **G** | **I** | **G** | **G** | **T** | **V** | **K** | **I** | **I** | **S** | **F** | **K** | **G** | **Q** | **G** | **T** | **S** | **V** | **E** | **I** | **K** | **V** | **P** | **L** | **M** | **K** | **E** | **A** |
| **N60001F** | **M** | **M** | **G** | **K** | **I** | **S** | **R** | **A** | **M** | **L** | **A** | **V** | **Y** | **S** | **G** | **I** | **A** | **A** | **F** | **L** | **I** | **I** | **L** | **F** | **S** | **L** | **F** | **T** | **Y** | **F** | **Y** | **A** | **S** | **N** | **Q** | **S** | **H** | **W** | **W** | **G** | **E** | **L** | **L** | **R** | **A** | **R** | **L** | **L** | **Y** | **V** | **P** | **L** | **I** | **F** | **H** | **L** | **L** | **A** | **I** | **S** | **L** | **G** | **V** | **G** | **L** | **I** | **V** | **F** | **L** | **L** | **L** | **S** | **L** | **I** | **Q** | **K** | **T** | **K** | **Y** | **G** | **K** | **I** | **E** | **E** | **K** | **L** | **R** | **A** | **L** | **S** | **S** | **G** | **N** | **Y** | **E** | **S** | **K** | **L** | **L** | **L** | **L** | **P** | **I** | **P** | **S** | **A** | **S** | **D** | **D** | **L** | **Y** | **I** | **K** | **D** | **I** | **D** | **K** | **E** | **I** | **T** | **K** | **I** | **K** | **E** | **K** | **M** | **I** | **E** | **V** | **S** | **S** | **E** | **L** | **Q** | **I** | **V** | **T** | **S** | **R** | **P** | **Q** | **Y** | **V** | **D** | **G** | **Q** | **T** | **K** | **E** | **E** | **I** | **L** | **E** | **L** | **E** | **R** | **H** | **R** | **L** | **A** | **R** | **E** | **L** | **H** | **D** | **S** | **V** | **S** | **Q** | **Q** | **L** | **F** | **A** | **A** | **M** | **M** | **M** | **M** | **S** | **A** | **L** | **T** | **E** | **Q** | **A** | **E** | **K** | **S** | **E** | **T** | **P** | **E** | **M** | **F** | **R** | **K** | **Q** | **L** | **K** | **M** | **V** | **A** | **E** | **I** | **I** | **N** | **A** | **S** | **Q** | **S** | **E** | **M** | **R** | **A** | **L** | **L** | **L** | **H** | **L** | **R** | **P** | **V** | **N** | **L** | **E** | **E** | **K** | **S** | **L** | **K** | **Q** | **G** | **I** | **E** | **Q** | **L** | **L** | **K** | **E** | **L** | **Q** | **N** | **K** | **I** | **Q** | **I** | **S** | **L** | **K** | **W** | **D** | **V** | **E** | **D** | **V** | **K** | **L** | **S** | **S** | **S** | **I** | **E** | **D** | **H** | **L** | **F** | **R** | **I** | **V** | **Q** | **E** | **L** | **L** | **S** | **N** | **T** | **L** | **R** | **H** | **A** | **K** | **A** | **N** | **E** | **L** | **E** | **V** | **Y** | **L** | **H** | **K** | **I** | **D** | **N** | **N** | **L** | **L** | **L** | **R** | **I** | **I** | **D** | **D** | **G** | **T** | **G** | **F** | **N** | **M** | **N** | **E** | **T** | **K** | **T** | **G** | **S** | **Y** | **G** | **L** | **N** | **N** | **I** | **K** | **E** | **R** | **V** | **A** | **G** | **I** | **G** | **G** | **T** | **V** | **K** | **I** | **I** | **S** | **F** | **K** | **G** | **Q** | **G** | **T** | **S** | **V** | **E** | **I** | **K** | **V** | **P** | **L** | **M** | **K** | **E** | **A** |
| **N59947F** | **M** | **M** | **G** | **K** | **I** | **S** | **R** | **A** | **M** | **L** | **A** | **V** | **Y** | **S** | **G** | **I** | **A** | **A** | **F** | **L** | **I** | **I** | **L** | **F** | **S** | **L** | **F** | **T** | **Y** | **F** | **Y** | **A** | **S** | **N** | **Q** | **S** | **H** | **W** | **W** | **G** | **E** | **L** | **L** | **R** | **A** | **R** | **L** | **L** | **Y** | **V** | **P** | **L** | **I** | **F** | **H** | **L** | **L** | **A** | **I** | **S** | **L** | **G** | **V** | **G** | **L** | **I** | **V** | **F** | **L** | **L** | **L** | **S** | **L** | **I** | **Q** | **K** | **T** | **K** | **Y** | **G** | **K** | **I** | **E** | **E** | **K** | **L** | **R** | **A** | **L** | **S** | **S** | **G** | **N** | **Y** | **E** | **S** | **K** | **L** | **L** | **L** | **L** | **P** | **I** | **P** | **S** | **A** | **S** | **D** | **D** | **L** | **Y** | **I** | **K** | **D** | **I** | **D** | **K** | **E** | **I** | **T** | **K** | **I** | **K** | **E** | **K** | **M** | **I** | **E** | **V** | **S** | **S** | **E** | **L** | **Q** | **I** | **V** | **T** | **S** | **R** | **P** | **Q** | **Y** | **V** | **D** | **G** | **Q** | **T** | **K** | **E** | **E** | **I** | **L** | **E** | **L** | **E** | **R** | **H** | **R** | **L** | **A** | **R** | **E** | **L** | **H** | **D** | **S** | **V** | **S** | **Q** | **Q** | **L** | **F** | **A** | **A** | **M** | **M** | **M** | **M** | **S** | **A** | **L** | **T** | **E** | **Q** | **A** | **E** | **K** | **S** | **E** | **T** | **P** | **E** | **M** | **F** | **R** | **K** | **Q** | **L** | **K** | **M** | **V** | **A** | **E** | **I** | **I** | **N** | **A** | **S** | **Q** | **S** | **E** | **M** | **R** | **A** | **L** | **L** | **L** | **H** | **L** | **R** | **P** | **V** | **N** | **L** | **E** | **E** | **K** | **S** | **L** | **K** | **Q** | **G** | **I** | **E** | **Q** | **L** | **L** | **K** | **E** | **L** | **Q** | **N** | **K** | **I** | **Q** | **I** | **S** | **L** | **K** | **W** | **D** | **V** | **E** | **D** | **V** | **K** | **L** | **S** | **S** | **S** | **I** | **E** | **D** | **H** | **L** | **F** | **R** | **I** | **V** | **Q** | **E** | **L** | **L** | **S** | **N** | **T** | **L** | **R** | **H** | **A** | **K** | **A** | **N** | **E** | **L** | **E** | **V** | **Y** | **L** | **H** | **K** | **I** | **D** | **N** | **N** | **L** | **L** | **L** | **R** | **I** | **I** | **D** | **D** | **G** | **T** | **G** | **F** | **N** | **M** | **N** | **E** | **T** | **K** | **T** | **G** | **S** | **Y** | **G** | **L** | **N** | **N** | **I** | **K** | **E** | **R** | **V** | **A** | **G** | **I** | **G** | **G** | **T** | **V** | **K** | **I** | **I** | **S** | **F** | **K** | **G** | **Q** | **G** | **T** | **S** | **V** | **E** | **I** | **K** | **V** | **P** | **L** | **M** | **K** | **E** | **A** |
| **N59613F** | **M** | **M** | **G** | **K** | **I** | **S** | **R** | **A** | **M** | **L** | **A** | **V** | **Y** | **S** | **G** | **I** | **A** | **A** | **F** | **L** | **I** | **I** | **L** | **F** | **S** | **L** | **F** | **T** | **Y** | **F** | **Y** | **A** | **S** | **N** | **Q** | **S** | **H** | **W** | **W** | **G** | **E** | **L** | **L** | **R** | **A** | **R** | **L** | **L** | **Y** | **V** | **P** | **L** | **I** | **F** | **H** | **L** | **L** | **A** | **I** | **S** | **L** | **G** | **V** | **G** | **L** | **I** | **V** | **F** | **L** | **L** | **L** | **S** | **L** | **I** | **Q** | **K** | **T** | **K** | **Y** | **G** | **K** | **I** | **E** | **E** | **K** | **L** | **R** | **A** | **L** | **S** | **S** | **G** | **N** | **Y** | **E** | **S** | **K** | **L** | **L** | **L** | **L** | **P** | **I** | **P** | **S** | **A** | **S** | **D** | **D** | **L** | **Y** | **I** | **K** | **D** | **I** | **D** | **K** | **E** | **I** | **T** | **K** | **I** | **K** | **E** | **K** | **M** | **I** | **E** | **V** | **S** | **S** | **E** | **L** | **Q** | **I** | **V** | **T** | **S** | **R** | **P** | **Q** | **Y** | **V** | **D** | **G** | **Q** | **T** | **K** | **E** | **E** | **I** | **L** | **E** | **L** | **E** | **R** | **H** | **R** | **L** | **A** | **R** | **E** | **L** | **H** | **D** | **S** | **V** | **S** | **Q** | **Q** | **L** | **F** | **A** | **A** | **M** | **M** | **M** | **M** | **S** | **A** | **L** | **T** | **E** | **Q** | **A** | **E** | **K** | **S** | **E** | **T** | **P** | **E** | **M** | **F** | **R** | **K** | **Q** | **L** | **K** | **M** | **V** | **A** | **E** | **I** | **I** | **N** | **A** | **S** | **Q** | **S** | **E** | **M** | **R** | **A** | **L** | **L** | **L** | **H** | **L** | **R** | **P** | **V** | **N** | **L** | **E** | **E** | **K** | **S** | **L** | **K** | **Q** | **G** | **I** | **E** | **Q** | **L** | **L** | **K** | **E** | **L** | **Q** | **N** | **K** | **I** | **Q** | **I** | **S** | **L** | **K** | **W** | **D** | **V** | **E** | **D** | **V** | **K** | **L** | **S** | **S** | **S** | **I** | **E** | **D** | **H** | **L** | **F** | **R** | **I** | **V** | **Q** | **E** | **L** | **L** | **S** | **N** | **T** | **L** | **R** | **H** | **A** | **K** | **A** | **N** | **E** | **L** | **E** | **V** | **Y** | **L** | **H** | **K** | **I** | **D** | **N** | **N** | **L** | **L** | **L** | **R** | **I** | **I** | **D** | **D** | **G** | **T** | **G** | **F** | **N** | **M** | **N** | **E** | **T** | **K** | **T** | **G** | **S** | **Y** | **G** | **L** | **N** | **N** | **I** | **K** | **E** | **R** | **V** | **A** | **G** | **I** | **G** | **G** | **T** | **V** | **K** | **I** | **I** | **S** | **F** | **K** | **G** | **Q** | **G** | **T** | **S** | **V** | **E** | **I** | **K** | **V** | **P** | **L** | **M** | **K** | **E** | **A** |
| **N54538** | **M** | **M** | **G** | **K** | **I** | **S** | **R** | **A** | **M** | **L** | **A** | **V** | **Y** | **S** | **G** | **I** | **A** | **A** | **F** | **L** | **I** | **I** | **L** | **F** | **S** | **L** | **F** | **T** | **Y** | **F** | **Y** | **A** | **S** | **N** | **Q** | **S** | **H** | **W** | **W** | **G** | **E** | **L** | **L** | **R** | **A** | **R** | **L** | **L** | **Y** | **V** | **P** | **L** | **I** | **F** | **H** | **L** | **L** | **A** | **I** | **S** | **L** | **G** | **V** | **G** | **L** | **I** | **V** | **F** | **L** | **L** | **L** | **S** | **L** | **I** | **Q** | **K** | **T** | **K** | **Y** | **G** | **K** | **I** | **E** | **E** | **K** | **L** | **R** | **A** | **L** | **S** | **S** | **G** | **N** | **Y** | **E** | **S** | **K** | **L** | **L** | **L** | **L** | **P** | **I** | **P** | **S** | **A** | **S** | **D** | **D** | **L** | **Y** | **I** | **K** | **D** | **I** | **D** | **K** | **E** | **I** | **T** | **K** | **I** | **K** | **E** | **K** | **M** | **I** | **E** | **V** | **S** | **S** | **E** | **L** | **Q** | **I** | **V** | **T** | **S** | **R** | **P** | **Q** | **Y** | **V** | **D** | **G** | **Q** | **T** | **K** | **E** | **E** | **I** | **L** | **E** | **L** | **E** | **R** | **H** | **R** | **L** | **A** | **R** | **E** | **L** | **H** | **D** | **S** | **V** | **S** | **Q** | **Q** | **L** | **F** | **A** | **A** | **M** | **M** | **M** | **M** | **S** | **A** | **L** | **T** | **E** | **Q** | **A** | **E** | **K** | **S** | **E** | **T** | **P** | **E** | **M** | **F** | **R** | **K** | **Q** | **L** | **K** | **M** | **V** | **A** | **E** | **I** | **I** | **N** | **A** | **S** | **Q** | **S** | **E** | **M** | **R** | **A** | **L** | **L** | **L** | **H** | **L** | **R** | **P** | **V** | **N** | **L** | **E** | **E** | **K** | **S** | **L** | **K** | **Q** | **G** | **I** | **E** | **Q** | **L** | **L** | **K** | **E** | **L** | **Q** | **N** | **K** | **I** | **Q** | **I** | **S** | **L** | **K** | **W** | **D** | **V** | **E** | **D** | **V** | **K** | **L** | **S** | **S** | **S** | **I** | **E** | **D** | **H** | **L** | **F** | **R** | **I** | **V** | **Q** | **E** | **L** | **L** | **S** | **N** | **T** | **L** | **R** | **H** | **A** | **K** | **A** | **N** | **E** | **L** | **E** | **V** | **Y** | **L** | **H** | **K** | **I** | **D** | **N** | **N** | **L** | **L** | **L** | **R** | **I** | **I** | **D** | **D** | **G** | **T** | **G** | **F** | **N** | **M** | **N** | **E** | **T** | **K** | **T** | **G** | **S** | **Y** | **G** | **L** | **N** | **N** | **I** | **K** | **E** | **R** | **V** | **A** | **G** | **I** | **G** | **G** | **T** | **V** | **K** | **I** | **I** | **S** | **F** | **K** | **G** | **Q** | **G** | **T** | **S** | **V** | **E** | **I** | **K** | **V** | **P** | **L** | **M** | **K** | **E** | **A** |
| **N60061F** | **M** | **M** | **G** | **R** | **I** | **S** | **K** | **A** | **M** | **L** | **A** | **V** | **Y** | **S** | **G** | **I** | **A** | **A** | **F** | **L** | **I** | **I** | **L** | **F** | **S** | **L** | **F** | **T** | **Y** | **F** | **Y** | **A** | **S** | **N** | **Q** | **S** | **H** | **W** | **W** | **G** | **E** | **L** | **L** | **R** | **A** | **R** | **L** | **L** | **Y** | **V** | **P** | **L** | **I** | **F** | **H** | **L** | **M** | **A** | **I** | **S** | **L** | **G** | **V** | **G** | **L** | **I** | **V** | **F** | **L** | **L** | **L** | **S** | **L** | **I** | **Q** | **K** | **A** | **K** | **Y** | **G** | **K** | **I** | **E** | **E** | **K** | **L** | **R** | **A** | **L** | **S** | **S** | **G** | **N** | **Y** | **E** | **S** | **K** | **L** | **L** | **H** | **Q** | **P** | **I** | **P** | **S** | **T** | **S** | **D** | **D** | **L** | **Y** | **I** | **K** | **D** | **I** | **D** | **M** | **E** | **I** | **T** | **K** | **I** | **K** | **E** | **K** | **M** | **I** | **E** | **V** | **S** | **S** | **E** | **L** | **Q** | **I** | **M** | **T** | **S** | **R** | **P** | **Q** | **Y** | **V** | **D** | **G** | **Q** | **T** | **K** | **E** | **E** | **I** | **L** | **E** | **L** | **E** | **R** | **H** | **R** | **L** | **A** | **R** | **E** | **L** | **H** | **D** | **S** | **V** | **S** | **Q** | **Q** | **L** | **F** | **A** | **A** | **M** | **M** | **M** | **M** | **S** | **A** | **L** | **T** | **E** | **Q** | **A** | **E** | **K** | **S** | **E** | **T** | **P** | **E** | **M** | **F** | **R** | **K** | **Q** | **L** | **K** | **M** | **V** | **A** | **E** | **I** | **I** | **N** | **A** | **S** | **Q** | **S** | **E** | **M** | **R** | **A** | **L** | **L** | **L** | **H** | **L** | **R** | **P** | **V** | **N** | **L** | **E** | **E** | **K** | **S** | **L** | **K** | **Q** | **G** | **I** | **E** | **Q** | **L** | **L** | **K** | **E** | **L** | **Q** | **N** | **K** | **I** | **Q** | **I** | **S** | **L** | **K** | **W** | **D** | **V** | **E** | **D** | **V** | **K** | **L** | **T** | **S** | **S** | **I** | **E** | **D** | **H** | **L** | **F** | **R** | **I** | **V** | **Q** | **E** | **L** | **L** | **S** | **N** | **T** | **L** | **R** | **H** | **A** | **K** | **A** | **N** | **E** | **L** | **E** | **V** | **Y** | **L** | **H** | **K** | **I** | **D** | **N** | **N** | **L** | **L** | **L** | **R** | **I** | **I** | **D** | **D** | **G** | **T** | **G** | **F** | **N** | **M** | **N** | **E** | **T** | **K** | **T** | **G** | **S** | **Y** | **G** | **L** | **N** | **N** | **I** | **K** | **E** | **R** | **V** | **A** | **G** | **I** | **G** | **G** | **T** | **V** | **K** | **I** | **I** | **S** | **F** | **K** | **G** | **Q** | **G** | **T** | **S** | **V** | **E** | **I** | **K** | **V** | **P** | **L** | **M** | **K** | **E** | **A** |
| **N59439F** | **M** | **M** | **G** | **R** | **I** | **S** | **K** | **A** | **M** | **L** | **A** | **V** | **Y** | **S** | **G** | **I** | **A** | **A** | **F** | **L** | **I** | **I** | **L** | **F** | **S** | **L** | **F** | **T** | **Y** | **F** | **Y** | **A** | **S** | **N** | **Q** | **S** | **H** | **W** | **W** | **G** | **E** | **L** | **L** | **R** | **A** | **R** | **L** | **L** | **Y** | **V** | **P** | **L** | **I** | **F** | **H** | **L** | **M** | **A** | **I** | **S** | **L** | **G** | **V** | **G** | **L** | **I** | **V** | **F** | **L** | **L** | **L** | **S** | **L** | **I** | **Q** | **K** | **A** | **K** | **Y** | **G** | **K** | **I** | **E** | **E** | **K** | **L** | **R** | **A** | **L** | **S** | **S** | **G** | **N** | **Y** | **E** | **S** | **K** | **L** | **L** | **H** | **Q** | **P** | **I** | **P** | **S** | **A** | **S** | **D** | **D** | **L** | **Y** | **I** | **K** | **D** | **I** | **D** | **M** | **E** | **I** | **T** | **K** | **I** | **K** | **E** | **K** | **M** | **I** | **E** | **V** | **S** | **S** | **E** | **L** | **Q** | **I** | **M** | **T** | **S** | **R** | **P** | **Q** | **Y** | **V** | **D** | **G** | **Q** | **T** | **K** | **E** | **E** | **I** | **L** | **E** | **L** | **E** | **R** | **H** | **R** | **L** | **A** | **R** | **E** | **L** | **H** | **D** | **S** | **V** | **S** | **Q** | **Q** | **L** | **F** | **A** | **A** | **M** | **M** | **M** | **M** | **S** | **A** | **L** | **T** | **E** | **Q** | **A** | **E** | **K** | **S** | **E** | **T** | **P** | **E** | **M** | **F** | **R** | **K** | **Q** | **L** | **K** | **M** | **V** | **A** | **E** | **I** | **I** | **N** | **A** | **S** | **Q** | **S** | **E** | **M** | **R** | **A** | **L** | **L** | **L** | **H** | **L** | **R** | **P** | **V** | **N** | **L** | **E** | **E** | **K** | **S** | **L** | **K** | **Q** | **G** | **I** | **E** | **Q** | **L** | **L** | **K** | **E** | **L** | **Q** | **N** | **K** | **I** | **Q** | **I** | **S** | **L** | **K** | **W** | **D** | **V** | **E** | **D** | **V** | **K** | **L** | **T** | **S** | **S** | **I** | **E** | **D** | **H** | **L** | **F** | **R** | **I** | **V** | **Q** | **E** | **L** | **L** | **S** | **N** | **T** | **L** | **R** | **H** | **A** | **K** | **A** | **N** | **E** | **L** | **E** | **V** | **Y** | **L** | **H** | **K** | **I** | **D** | **N** | **N** | **L** | **L** | **L** | **R** | **I** | **I** | **D** | **D** | **G** | **T** | **G** | **F** | **N** | **M** | **N** | **E** | **T** | **K** | **T** | **G** | **S** | **Y** | **G** | **L** | **N** | **N** | **I** | **K** | **E** | **R** | **V** | **A** | **G** | **I** | **G** | **G** | **T** | **V** | **K** | **I** | **I** | **S** | **F** | **K** | **G** | **Q** | **G** | **T** | **S** | **V** | **E** | **I** | **K** | **V** | **P** | **L** | **M** | **K** | **E** | **A** |
| **N52636** | **M** | **M** | **G** | **R** | **I** | **S** | **K** | **A** | **M** | **L** | **A** | **V** | **Y** | **S** | **G** | **I** | **A** | **A** | **F** | **L** | **I** | **I** | **L** | **F** | **S** | **L** | **F** | **T** | **Y** | **F** | **Y** | **A** | **S** | **N** | **Q** | **S** | **H** | **W** | **W** | **G** | **E** | **L** | **L** | **R** | **A** | **R** | **L** | **L** | **Y** | **V** | **P** | **L** | **I** | **F** | **H** | **L** | **M** | **A** | **I** | **S** | **L** | **G** | **V** | **G** | **L** | **I** | **V** | **F** | **L** | **L** | **L** | **S** | **L** | **I** | **Q** | **K** | **A** | **K** | **Y** | **G** | **K** | **I** | **E** | **E** | **K** | **L** | **R** | **A** | **L** | **S** | **S** | **G** | **N** | **Y** | **E** | **S** | **K** | **L** | **L** | **H** | **Q** | **P** | **I** | **P** | **S** | **A** | **S** | **D** | **D** | **L** | **Y** | **I** | **K** | **D** | **I** | **D** | **M** | **E** | **I** | **T** | **K** | **I** | **K** | **E** | **K** | **M** | **I** | **E** | **V** | **S** | **S** | **E** | **L** | **Q** | **I** | **M** | **T** | **S** | **R** | **P** | **Q** | **Y** | **V** | **D** | **G** | **Q** | **T** | **K** | **E** | **E** | **I** | **L** | **E** | **L** | **E** | **R** | **H** | **R** | **L** | **A** | **R** | **E** | **L** | **H** | **D** | **S** | **V** | **S** | **Q** | **Q** | **L** | **F** | **A** | **A** | **M** | **M** | **M** | **M** | **S** | **A** | **L** | **T** | **E** | **Q** | **A** | **E** | **K** | **S** | **E** | **T** | **P** | **E** | **M** | **F** | **R** | **K** | **Q** | **L** | **K** | **M** | **V** | **A** | **E** | **I** | **I** | **N** | **A** | **S** | **Q** | **S** | **E** | **M** | **R** | **A** | **L** | **L** | **L** | **H** | **L** | **R** | **P** | **V** | **N** | **L** | **E** | **E** | **K** | **S** | **L** | **K** | **Q** | **G** | **I** | **E** | **Q** | **L** | **L** | **K** | **E** | **L** | **Q** | **N** | **K** | **I** | **Q** | **I** | **S** | **L** | **K** | **W** | **D** | **V** | **E** | **D** | **V** | **K** | **L** | **T** | **S** | **S** | **I** | **E** | **D** | **H** | **L** | **F** | **R** | **I** | **V** | **Q** | **E** | **L** | **L** | **S** | **N** | **T** | **L** | **R** | **H** | **A** | **K** | **A** | **N** | **E** | **L** | **E** | **V** | **Y** | **L** | **H** | **K** | **I** | **D** | **N** | **N** | **L** | **L** | **L** | **R** | **I** | **I** | **D** | **D** | **G** | **T** | **G** | **F** | **N** | **M** | **N** | **E** | **T** | **K** | **T** | **G** | **S** | **Y** | **G** | **L** | **N** | **N** | **I** | **K** | **E** | **R** | **V** | **A** | **G** | **I** | **G** | **G** | **T** | **V** | **K** | **I** | **I** | **S** | **F** | **K** | **G** | **Q** | **G** | **T** | **S** | **V** | **E** | **I** | **K** | **V** | **P** | **L** | **M** | **K** | **E** | **A** |
| **N54546** | **M** | **M** | **G** | **K** | **I** | **S** | **R** | **A** | **M** | **L** | **A** | **V** | **Y** | **S** | **G** | **I** | **A** | **A** | **F** | **L** | **I** | **I** | **L** | **F** | **S** | **L** | **F** | **T** | **Y** | **F** | **Y** | **A** | **S** | **N** | **Q** | **S** | **H** | **W** | **W** | **G** | **E** | **L** | **L** | **R** | **A** | **R** | **L** | **L** | **Y** | **V** | **P** | **L** | **I** | **F** | **H** | **L** | **L** | **A** | **I** | **S** | **L** | **G** | **V** | **G** | **L** | **I** | **V** | **F** | **L** | **L** | **L** | **S** | **L** | **I** | **Q** | **K** | **T** | **K** | **Y** | **G** | **K** | **I** | **E** | **E** | **K** | **L** | **R** | **A** | **L** | **S** | **S** | **G** | **N** | **Y** | **E** | **S** | **K** | **L** | **L** | **L** | **L** | **P** | **I** | **P** | **S** | **A** | **S** | **D** | **D** | **L** | **Y** | **I** | **K** | **D** | **I** | **D** | **K** | **E** | **I** | **T** | **K** | **I** | **K** | **E** | **K** | **M** | **I** | **E** | **V** | **S** | **S** | **E** | **L** | **Q** | **I** | **V** | **T** | **S** | **R** | **P** | **Q** | **Y** | **V** | **D** | **G** | **Q** | **T** | **K** | **E** | **E** | **I** | **L** | **E** | **L** | **E** | **R** | **H** | **R** | **L** | **A** | **R** | **E** | **L** | **H** | **D** | **S** | **V** | **S** | **Q** | **Q** | **L** | **F** | **A** | **A** | **M** | **M** | **M** | **M** | **S** | **A** | **L** | **T** | **E** | **Q** | **A** | **E** | **K** | **S** | **E** | **T** | **P** | **E** | **M** | **F** | **R** | **K** | **Q** | **L** | **K** | **M** | **V** | **A** | **E** | **I** | **I** | **N** | **A** | **S** | **Q** | **S** | **E** | **M** | **R** | **A** | **L** | **L** | **L** | **H** | **L** | **R** | **P** | **V** | **N** | **L** | **E** | **E** | **K** | **S** | **L** | **K** | **Q** | **G** | **I** | **E** | **Q** | **L** | **L** | **K** | **E** | **L** | **Q** | **N** | **K** | **I** | **Q** | **I** | **S** | **L** | **K** | **W** | **D** | **V** | **E** | **D** | **V** | **K** | **L** | **S** | **S** | **S** | **I** | **E** | **D** | **H** | **L** | **F** | **R** | **I** | **V** | **Q** | **E** | **L** | **L** | **S** | **N** | **T** | **L** | **R** | **H** | **A** | **K** | **A** | **N** | **E** | **L** | **E** | **V** | **Y** | **L** | **H** | **K** | **I** | **D** | **N** | **N** | **L** | **L** | **L** | **R** | **I** | **I** | **D** | **D** | **G** | **T** | **G** | **F** | **N** | **M** | **N** | **E** | **T** | **K** | **T** | **G** | **S** | **Y** | **G** | **L** | **N** | **N** | **I** | **K** | **E** | **R** | **V** | **A** | **G** | **I** | **G** | **G** | **T** | **V** | **K** | **I** | **I** | **S** | **F** | **K** | **G** | **Q** | **G** | **T** | **S** | **V** | **E** | **I** | **K** | **V** | **P** | **L** | **M** | **K** | **E** | **A** |
| **N60519F** | **M** | **M** | **G** | **R** | **I** | **S** | **K** | **A** | **M** | **L** | **A** | **V** | **Y** | **S** | **G** | **I** | **A** | **A** | **F** | **L** | **I** | **I** | **L** | **F** | **S** | **L** | **F** | **T** | **Y** | **F** | **Y** | **A** | **S** | **N** | **Q** | **S** | **H** | **W** | **W** | **G** | **E** | **L** | **L** | **R** | **A** | **R** | **L** | **L** | **Y** | **V** | **P** | **L** | **I** | **F** | **H** | **L** | **M** | **A** | **I** | **S** | **L** | **G** | **V** | **G** | **L** | **I** | **V** | **F** | **L** | **L** | **L** | **S** | **L** | **I** | **Q** | **K** | **A** | **K** | **Y** | **G** | **K** | **I** | **E** | **E** | **K** | **L** | **R** | **A** | **L** | **S** | **S** | **G** | **N** | **Y** | **E** | **S** | **K** | **L** | **L** | **H** | **Q** | **P** | **I** | **P** | **S** | **A** | **S** | **D** | **D** | **L** | **Y** | **I** | **K** | **D** | **I** | **D** | **M** | **E** | **I** | **T** | **K** | **I** | **K** | **E** | **K** | **M** | **I** | **E** | **V** | **S** | **S** | **E** | **L** | **Q** | **I** | **M** | **T** | **S** | **R** | **P** | **Q** | **Y** | **V** | **D** | **G** | **Q** | **T** | **K** | **E** | **E** | **I** | **L** | **E** | **L** | **E** | **R** | **H** | **R** | **L** | **A** | **R** | **E** | **L** | **H** | **D** | **S** | **V** | **S** | **Q** | **Q** | **L** | **F** | **A** | **A** | **M** | **M** | **M** | **M** | **S** | **A** | **L** | **T** | **E** | **Q** | **A** | **E** | **K** | **S** | **E** | **T** | **P** | **E** | **M** | **F** | **R** | **K** | **Q** | **L** | **K** | **M** | **V** | **A** | **E** | **I** | **I** | **N** | **A** | **S** | **Q** | **S** | **E** | **M** | **R** | **A** | **L** | **L** | **L** | **H** | **L** | **R** | **P** | **V** | **N** | **L** | **E** | **E** | **K** | **S** | **L** | **K** | **Q** | **G** | **I** | **E** | **Q** | **L** | **L** | **K** | **E** | **L** | **Q** | **N** | **K** | **I** | **Q** | **I** | **S** | **L** | **K** | **W** | **D** | **I** | **E** | **D** | **V** | **K** | **L** | **T** | **S** | **S** | **I** | **E** | **D** | **H** | **L** | **F** | **R** | **I** | **V** | **Q** | **E** | **L** | **L** | **S** | **N** | **T** | **L** | **R** | **H** | **A** | **K** | **A** | **N** | **E** | **L** | **E** | **V** | **Y** | **L** | **H** | **K** | **I** | **D** | **N** | **N** | **L** | **L** | **L** | **R** | **I** | **I** | **D** | **D** | **G** | **T** | **G** | **F** | **N** | **M** | **N** | **E** | **T** | **K** | **T** | **G** | **S** | **Y** | **G** | **L** | **N** | **N** | **I** | **K** | **E** | **R** | **V** | **A** | **G** | **I** | **G** | **G** | **T** | **V** | **K** | **I** | **I** | **S** | **F** | **K** | **G** | **Q** | **G** | **T** | **S** | **V** | **E** | **I** | **K** | **V** | **P** | **L** | **M** | **K** | **E** | **A** |
| **N53804** | **M** | **M** | **G** | **R** | **I** | **S** | **K** | **A** | **M** | **L** | **A** | **V** | **Y** | **S** | **G** | **I** | **A** | **A** | **F** | **L** | **I** | **I** | **L** | **F** | **S** | **L** | **F** | **T** | **Y** | **F** | **Y** | **A** | **S** | **N** | **Q** | **S** | **H** | **W** | **W** | **G** | **E** | **L** | **L** | **R** | **A** | **R** | **L** | **L** | **Y** | **V** | **P** | **L** | **I** | **F** | **H** | **L** | **M** | **A** | **I** | **S** | **L** | **G** | **V** | **G** | **L** | **I** | **V** | **F** | **L** | **L** | **L** | **S** | **L** | **I** | **Q** | **K** | **A** | **K** | **Y** | **G** | **K** | **I** | **E** | **E** | **K** | **L** | **R** | **A** | **L** | **S** | **S** | **G** | **N** | **Y** | **E** | **S** | **K** | **L** | **L** | **H** | **Q** | **P** | **I** | **P** | **S** | **A** | **S** | **D** | **D** | **L** | **Y** | **I** | **K** | **D** | **I** | **D** | **M** | **E** | **I** | **T** | **K** | **I** | **K** | **E** | **K** | **M** | **I** | **E** | **V** | **S** | **S** | **E** | **L** | **Q** | **I** | **M** | **T** | **S** | **R** | **P** | **Q** | **Y** | **V** | **D** | **G** | **Q** | **T** | **K** | **E** | **E** | **I** | **L** | **E** | **L** | **E** | **R** | **H** | **R** | **L** | **A** | **R** | **E** | **L** | **H** | **D** | **S** | **V** | **S** | **Q** | **Q** | **L** | **F** | **A** | **A** | **M** | **M** | **M** | **M** | **S** | **A** | **L** | **T** | **E** | **Q** | **A** | **E** | **K** | **S** | **E** | **T** | **P** | **E** | **M** | **F** | **R** | **K** | **Q** | **L** | **K** | **M** | **V** | **A** | **E** | **I** | **I** | **N** | **A** | **S** | **Q** | **S** | **E** | **M** | **R** | **A** | **L** | **L** | **L** | **H** | **L** | **R** | **P** | **V** | **N** | **L** | **E** | **E** | **K** | **S** | **L** | **K** | **Q** | **G** | **I** | **E** | **Q** | **L** | **L** | **K** | **E** | **L** | **Q** | **N** | **K** | **I** | **Q** | **I** | **S** | **L** | **K** | **W** | **D** | **V** | **E** | **D** | **V** | **K** | **L** | **T** | **S** | **S** | **I** | **E** | **D** | **H** | **L** | **F** | **R** | **I** | **V** | **Q** | **E** | **L** | **L** | **S** | **N** | **T** | **L** | **R** | **H** | **A** | **K** | **A** | **N** | **E** | **L** | **E** | **V** | **Y** | **L** | **H** | **K** | **I** | **D** | **N** | **N** | **L** | **L** | **L** | **R** | **I** | **I** | **D** | **D** | **G** | **T** | **G** | **F** | **N** | **M** | **N** | **E** | **T** | **K** | **T** | **G** | **S** | **Y** | **G** | **L** | **N** | **N** | **I** | **K** | **E** | **R** | **V** | **A** | **G** | **I** | **G** | **G** | **T** | **V** | **K** | **I** | **I** | **S** | **F** | **K** | **G** | **Q** | **G** | **T** | **S** | **V** | **E** | **I** | **K** | **V** | **P** | **L** | **M** | **K** | **E** | **A** |
| **N53595** | **M** | **M** | **G** | **K** | **I** | **S** | **R** | **A** | **M** | **L** | **A** | **V** | **Y** | **S** | **G** | **I** | **A** | **A** | **F** | **L** | **I** | **I** | **L** | **F** | **S** | **L** | **F** | **T** | **Y** | **F** | **Y** | **A** | **S** | **N** | **Q** | **S** | **H** | **W** | **W** | **G** | **E** | **L** | **L** | **R** | **A** | **R** | **L** | **L** | **Y** | **V** | **P** | **L** | **I** | **F** | **H** | **L** | **L** | **A** | **I** | **S** | **L** | **G** | **V** | **G** | **L** | **I** | **V** | **F** | **L** | **L** | **L** | **S** | **L** | **I** | **Q** | **K** | **T** | **K** | **Y** | **G** | **K** | **I** | **E** | **E** | **K** | **L** | **R** | **A** | **L** | **S** | **S** | **G** | **N** | **Y** | **E** | **S** | **K** | **L** | **L** | **L** | **L** | **P** | **I** | **P** | **S** | **A** | **S** | **D** | **D** | **L** | **Y** | **I** | **K** | **D** | **I** | **D** | **K** | **E** | **I** | **T** | **K** | **I** | **K** | **E** | **K** | **M** | **I** | **E** | **V** | **S** | **S** | **E** | **L** | **Q** | **I** | **V** | **T** | **S** | **R** | **P** | **Q** | **Y** | **V** | **D** | **G** | **Q** | **T** | **K** | **E** | **E** | **I** | **L** | **E** | **L** | **E** | **R** | **H** | **R** | **L** | **A** | **R** | **E** | **L** | **H** | **D** | **S** | **V** | **S** | **Q** | **Q** | **L** | **F** | **A** | **A** | **M** | **M** | **M** | **M** | **S** | **A** | **L** | **T** | **E** | **Q** | **A** | **E** | **K** | **S** | **E** | **T** | **P** | **E** | **M** | **F** | **R** | **K** | **Q** | **L** | **K** | **M** | **V** | **A** | **E** | **I** | **I** | **N** | **A** | **S** | **Q** | **S** | **E** | **M** | **R** | **A** | **L** | **L** | **L** | **H** | **L** | **R** | **P** | **V** | **N** | **L** | **E** | **E** | **K** | **S** | **L** | **K** | **Q** | **G** | **I** | **E** | **Q** | **L** | **L** | **K** | **E** | **L** | **Q** | **N** | **K** | **I** | **Q** | **I** | **S** | **L** | **K** | **W** | **D** | **V** | **E** | **D** | **V** | **K** | **L** | **S** | **S** | **S** | **I** | **E** | **D** | **H** | **L** | **F** | **R** | **I** | **V** | **Q** | **E** | **L** | **L** | **S** | **N** | **T** | **L** | **R** | **H** | **A** | **K** | **A** | **N** | **E** | **L** | **E** | **V** | **Y** | **L** | **H** | **K** | **I** | **D** | **N** | **N** | **L** | **L** | **L** | **R** | **I** | **I** | **D** | **D** | **G** | **T** | **G** | **F** | **N** | **M** | **N** | **E** | **T** | **K** | **T** | **G** | **S** | **Y** | **G** | **L** | **N** | **N** | **I** | **K** | **E** | **R** | **V** | **A** | **G** | **I** | **G** | **G** | **T** | **V** | **K** | **I** | **I** | **S** | **F** | **K** | **G** | **Q** | **G** | **T** | **S** | **V** | **E** | **I** | **K** | **V** | **P** | **L** | **M** | **K** | **E** | **A** |
| **N59589F** | **M** | **M** | **G** | **K** | **I** | **S** | **R** | **A** | **M** | **L** | **A** | **V** | **Y** | **S** | **G** | **I** | **A** | **A** | **F** | **L** | **I** | **I** | **L** | **F** | **S** | **L** | **F** | **T** | **Y** | **F** | **Y** | **A** | **S** | **N** | **Q** | **S** | **H** | **W** | **W** | **G** | **E** | **L** | **L** | **R** | **A** | **R** | **L** | **L** | **Y** | **V** | **P** | **L** | **I** | **F** | **H** | **L** | **L** | **A** | **I** | **S** | **L** | **G** | **V** | **G** | **L** | **I** | **V** | **F** | **L** | **L** | **L** | **S** | **L** | **I** | **Q** | **K** | **T** | **K** | **Y** | **G** | **K** | **I** | **E** | **E** | **K** | **L** | **R** | **A** | **L** | **S** | **S** | **G** | **N** | **Y** | **E** | **S** | **K** | **L** | **L** | **L** | **L** | **P** | **I** | **P** | **S** | **A** | **S** | **D** | **D** | **L** | **Y** | **I** | **K** | **D** | **I** | **D** | **K** | **E** | **I** | **T** | **K** | **I** | **K** | **E** | **K** | **M** | **I** | **E** | **V** | **S** | **S** | **E** | **L** | **Q** | **I** | **V** | **T** | **S** | **R** | **P** | **Q** | **Y** | **V** | **D** | **G** | **Q** | **T** | **K** | **E** | **E** | **I** | **L** | **E** | **L** | **E** | **R** | **H** | **R** | **L** | **A** | **R** | **E** | **L** | **H** | **D** | **S** | **V** | **S** | **Q** | **Q** | **L** | **F** | **A** | **A** | **M** | **M** | **M** | **M** | **S** | **A** | **L** | **T** | **E** | **Q** | **A** | **E** | **K** | **S** | **E** | **T** | **P** | **E** | **M** | **F** | **R** | **K** | **Q** | **L** | **K** | **M** | **V** | **A** | **E** | **I** | **I** | **N** | **A** | **S** | **Q** | **S** | **E** | **M** | **R** | **A** | **L** | **L** | **L** | **H** | **L** | **R** | **P** | **V** | **N** | **L** | **E** | **E** | **K** | **S** | **L** | **K** | **Q** | **G** | **I** | **E** | **Q** | **L** | **L** | **K** | **E** | **L** | **Q** | **N** | **K** | **I** | **Q** | **I** | **S** | **L** | **K** | **W** | **D** | **V** | **E** | **D** | **V** | **K** | **L** | **S** | **S** | **S** | **I** | **E** | **D** | **H** | **L** | **F** | **R** | **I** | **V** | **Q** | **E** | **L** | **L** | **S** | **N** | **T** | **L** | **R** | **H** | **A** | **K** | **A** | **N** | **E** | **L** | **E** | **V** | **Y** | **L** | **H** | **K** | **I** | **D** | **N** | **N** | **L** | **L** | **L** | **R** | **I** | **I** | **D** | **D** | **G** | **T** | **G** | **F** | **N** | **M** | **N** | **E** | **T** | **K** | **T** | **G** | **S** | **Y** | **G** | **L** | **N** | **N** | **I** | **K** | **E** | **R** | **V** | **A** | **G** | **I** | **G** | **G** | **T** | **V** | **K** | **I** | **I** | **S** | **F** | **K** | **G** | **Q** | **G** | **T** | **S** | **V** | **E** | **I** | **K** | **V** | **P** | **L** | **M** | **K** | **E** | **A** |
| **N59504F** | **M** | **M** | **G** | **R** | **I** | **S** | **K** | **A** | **M** | **L** | **A** | **V** | **Y** | **S** | **G** | **I** | **A** | **A** | **F** | **L** | **I** | **I** | **L** | **F** | **S** | **L** | **F** | **T** | **Y** | **F** | **Y** | **A** | **S** | **N** | **Q** | **S** | **H** | **W** | **W** | **G** | **E** | **L** | **L** | **R** | **A** | **R** | **L** | **L** | **Y** | **V** | **P** | **L** | **I** | **F** | **H** | **L** | **M** | **A** | **I** | **S** | **L** | **G** | **V** | **G** | **L** | **I** | **V** | **F** | **L** | **L** | **L** | **S** | **L** | **I** | **Q** | **K** | **A** | **K** | **Y** | **G** | **K** | **I** | **E** | **E** | **K** | **L** | **R** | **A** | **L** | **S** | **S** | **G** | **N** | **Y** | **E** | **S** | **K** | **L** | **L** | **H** | **Q** | **P** | **I** | **P** | **S** | **A** | **S** | **D** | **D** | **L** | **Y** | **I** | **K** | **D** | **I** | **D** | **M** | **E** | **I** | **T** | **K** | **I** | **K** | **E** | **K** | **M** | **I** | **E** | **V** | **S** | **S** | **E** | **L** | **Q** | **I** | **M** | **T** | **S** | **R** | **P** | **Q** | **Y** | **V** | **D** | **G** | **Q** | **T** | **K** | **E** | **E** | **I** | **L** | **E** | **L** | **E** | **R** | **H** | **R** | **L** | **A** | **R** | **E** | **L** | **H** | **D** | **S** | **V** | **S** | **Q** | **Q** | **L** | **F** | **A** | **A** | **M** | **M** | **M** | **M** | **S** | **A** | **L** | **T** | **E** | **Q** | **A** | **E** | **K** | **S** | **E** | **T** | **P** | **E** | **M** | **F** | **R** | **K** | **Q** | **L** | **K** | **M** | **V** | **A** | **E** | **I** | **I** | **N** | **A** | **S** | **Q** | **S** | **E** | **M** | **R** | **A** | **L** | **L** | **L** | **H** | **L** | **R** | **P** | **V** | **N** | **L** | **E** | **E** | **K** | **S** | **L** | **K** | **Q** | **G** | **I** | **E** | **Q** | **L** | **L** | **K** | **E** | **L** | **Q** | **N** | **K** | **I** | **Q** | **I** | **S** | **L** | **K** | **W** | **D** | **V** | **E** | **D** | **V** | **K** | **L** | **T** | **S** | **S** | **I** | **E** | **D** | **H** | **L** | **F** | **R** | **I** | **V** | **Q** | **E** | **L** | **L** | **S** | **N** | **T** | **L** | **R** | **H** | **A** | **K** | **A** | **N** | **E** | **L** | **E** | **V** | **Y** | **L** | **H** | **K** | **I** | **D** | **N** | **N** | **L** | **L** | **L** | **R** | **I** | **I** | **D** | **D** | **G** | **T** | **G** | **F** | **N** | **M** | **N** | **E** | **T** | **K** | **T** | **G** | **S** | **Y** | **G** | **L** | **N** | **N** | **I** | **K** | **E** | **R** | **V** | **A** | **G** | **I** | **G** | **G** | **T** | **V** | **K** | **I** | **I** | **S** | **F** | **K** | **G** | **Q** | **G** | **T** | **S** | **V** | **E** | **I** | **K** | **V** | **P** | **L** | **M** | **K** | **E** | **A** |
| **N52459** | **M** | **M** | **G** | **K** | **I** | **S** | **R** | **A** | **M** | **L** | **A** | **V** | **Y** | **S** | **G** | **I** | **A** | **A** | **F** | **L** | **I** | **I** | **L** | **F** | **S** | **L** | **F** | **T** | **Y** | **F** | **Y** | **A** | **S** | **N** | **Q** | **S** | **H** | **W** | **W** | **G** | **E** | **L** | **L** | **R** | **A** | **R** | **L** | **L** | **Y** | **V** | **P** | **L** | **I** | **F** | **H** | **L** | **L** | **A** | **I** | **S** | **L** | **G** | **V** | **G** | **L** | **I** | **V** | **F** | **L** | **L** | **L** | **S** | **L** | **I** | **Q** | **K** | **T** | **K** | **Y** | **G** | **K** | **I** | **E** | **E** | **K** | **L** | **R** | **A** | **L** | **S** | **S** | **G** | **N** | **Y** | **E** | **S** | **K** | **L** | **L** | **L** | **L** | **P** | **I** | **P** | **S** | **A** | **S** | **D** | **D** | **L** | **Y** | **I** | **K** | **D** | **I** | **D** | **K** | **E** | **I** | **T** | **K** | **I** | **K** | **E** | **K** | **M** | **I** | **E** | **V** | **S** | **S** | **E** | **L** | **Q** | **I** | **V** | **T** | **S** | **R** | **P** | **Q** | **Y** | **V** | **D** | **G** | **Q** | **T** | **K** | **E** | **E** | **I** | **L** | **E** | **L** | **E** | **R** | **H** | **R** | **L** | **A** | **R** | **E** | **L** | **H** | **D** | **S** | **V** | **S** | **Q** | **Q** | **L** | **F** | **A** | **A** | **M** | **M** | **M** | **M** | **S** | **A** | **L** | **T** | **E** | **Q** | **A** | **E** | **K** | **S** | **E** | **T** | **P** | **E** | **M** | **F** | **R** | **K** | **Q** | **L** | **K** | **M** | **V** | **A** | **E** | **I** | **I** | **N** | **A** | **S** | **Q** | **S** | **E** | **M** | **R** | **A** | **L** | **L** | **L** | **H** | **L** | **R** | **P** | **V** | **N** | **L** | **E** | **E** | **K** | **S** | **L** | **K** | **Q** | **G** | **I** | **E** | **Q** | **L** | **L** | **K** | **E** | **L** | **Q** | **N** | **K** | **I** | **Q** | **I** | **S** | **L** | **K** | **W** | **D** | **V** | **E** | **D** | **V** | **K** | **L** | **S** | **S** | **S** | **I** | **E** | **D** | **H** | **L** | **F** | **R** | **I** | **V** | **Q** | **E** | **L** | **L** | **S** | **N** | **T** | **L** | **R** | **H** | **A** | **K** | **A** | **N** | **E** | **L** | **E** | **V** | **Y** | **L** | **H** | **K** | **I** | **D** | **N** | **N** | **L** | **L** | **L** | **R** | **I** | **I** | **D** | **D** | **G** | **T** | **G** | **F** | **N** | **M** | **N** | **E** | **T** | **K** | **T** | **G** | **S** | **Y** | **G** | **L** | **N** | **N** | **I** | **K** | **E** | **R** | **V** | **A** | **G** | **I** | **G** | **G** | **T** | **V** | **K** | **I** | **I** | **S** | **F** | **K** | **G** | **Q** | **G** | **T** | **S** | **V** | **E** | **I** | **K** | **V** | **P** | **L** | **M** | **K** | **E** | **A** |
| **N60324F** | **M** | **M** | **G** | **R** | **I** | **S** | **K** | **A** | **M** | **L** | **A** | **V** | **Y** | **S** | **G** | **I** | **A** | **A** | **F** | **L** | **I** | **I** | **L** | **F** | **S** | **L** | **F** | **T** | **Y** | **F** | **Y** | **A** | **S** | **N** | **Q** | **S** | **H** | **W** | **W** | **G** | **E** | **L** | **L** | **R** | **A** | **R** | **L** | **L** | **Y** | **V** | **P** | **L** | **I** | **F** | **H** | **L** | **M** | **A** | **I** | **S** | **L** | **G** | **V** | **G** | **L** | **I** | **V** | **F** | **L** | **L** | **L** | **S** | **L** | **I** | **Q** | **K** | **A** | **K** | **Y** | **G** | **K** | **I** | **E** | **E** | **K** | **L** | **R** | **A** | **L** | **S** | **S** | **G** | **N** | **Y** | **E** | **S** | **K** | **L** | **L** | **H** | **Q** | **P** | **I** | **P** | **S** | **A** | **S** | **D** | **D** | **L** | **Y** | **I** | **K** | **D** | **I** | **D** | **M** | **E** | **I** | **T** | **K** | **I** | **K** | **E** | **K** | **M** | **I** | **E** | **V** | **S** | **S** | **E** | **L** | **Q** | **I** | **M** | **T** | **S** | **R** | **P** | **Q** | **Y** | **V** | **D** | **G** | **Q** | **T** | **K** | **E** | **E** | **I** | **L** | **E** | **L** | **E** | **R** | **H** | **R** | **L** | **A** | **R** | **E** | **L** | **H** | **D** | **S** | **V** | **S** | **Q** | **Q** | **L** | **F** | **A** | **A** | **M** | **M** | **M** | **M** | **S** | **A** | **L** | **T** | **E** | **Q** | **A** | **E** | **K** | **S** | **E** | **T** | **P** | **G** | **M** | **F** | **R** | **K** | **Q** | **L** | **K** | **M** | **V** | **A** | **E** | **I** | **I** | **N** | **A** | **S** | **Q** | **S** | **E** | **M** | **R** | **A** | **L** | **L** | **L** | **H** | **L** | **R** | **P** | **V** | **N** | **L** | **E** | **E** | **K** | **S** | **L** | **K** | **Q** | **G** | **I** | **E** | **Q** | **L** | **L** | **K** | **E** | **L** | **Q** | **N** | **K** | **I** | **Q** | **I** | **S** | **L** | **K** | **W** | **D** | **V** | **E** | **D** | **V** | **K** | **L** | **T** | **S** | **S** | **I** | **E** | **D** | **H** | **L** | **F** | **R** | **I** | **V** | **Q** | **E** | **L** | **L** | **S** | **N** | **T** | **L** | **R** | **H** | **A** | **K** | **A** | **N** | **E** | **L** | **E** | **V** | **Y** | **L** | **H** | **K** | **I** | **D** | **N** | **N** | **L** | **L** | **L** | **R** | **I** | **I** | **D** | **D** | **G** | **T** | **G** | **F** | **N** | **M** | **N** | **E** | **T** | **K** | **T** | **G** | **S** | **Y** | **G** | **L** | **N** | **N** | **I** | **K** | **E** | **R** | **V** | **A** | **G** | **I** | **G** | **G** | **T** | **V** | **K** | **I** | **I** | **S** | **F** | **K** | **G** | **Q** | **G** | **T** | **S** | **V** | **E** | **I** | **K** | **V** | **P** | **L** | **M** | **K** | **E** | **A** |
| **N59745F** | **M** | **M** | **G** | **K** | **I** | **S** | **R** | **A** | **M** | **L** | **A** | **V** | **Y** | **S** | **G** | **I** | **A** | **A** | **F** | **L** | **I** | **I** | **L** | **F** | **S** | **L** | **F** | **T** | **Y** | **F** | **Y** | **A** | **S** | **N** | **Q** | **S** | **H** | **W** | **W** | **G** | **E** | **L** | **L** | **R** | **A** | **R** | **L** | **L** | **Y** | **V** | **P** | **L** | **I** | **F** | **H** | **L** | **L** | **A** | **I** | **S** | **L** | **G** | **V** | **G** | **L** | **I** | **V** | **F** | **L** | **L** | **L** | **S** | **L** | **I** | **Q** | **K** | **T** | **K** | **Y** | **G** | **K** | **I** | **E** | **E** | **K** | **L** | **R** | **A** | **L** | **S** | **S** | **G** | **N** | **Y** | **E** | **S** | **K** | **L** | **L** | **L** | **L** | **P** | **I** | **P** | **S** | **A** | **S** | **D** | **D** | **L** | **Y** | **I** | **K** | **D** | **I** | **D** | **K** | **E** | **I** | **T** | **K** | **I** | **K** | **E** | **K** | **M** | **I** | **E** | **V** | **S** | **S** | **E** | **L** | **Q** | **I** | **V** | **T** | **S** | **R** | **P** | **Q** | **Y** | **V** | **D** | **G** | **Q** | **T** | **K** | **E** | **E** | **I** | **L** | **E** | **L** | **E** | **R** | **H** | **R** | **L** | **A** | **R** | **E** | **L** | **H** | **D** | **S** | **V** | **S** | **Q** | **Q** | **L** | **F** | **A** | **A** | **M** | **M** | **M** | **M** | **S** | **A** | **L** | **T** | **E** | **Q** | **A** | **E** | **K** | **S** | **E** | **T** | **P** | **E** | **M** | **F** | **R** | **K** | **Q** | **L** | **K** | **M** | **V** | **A** | **E** | **I** | **I** | **N** | **A** | **S** | **Q** | **S** | **E** | **M** | **R** | **A** | **L** | **L** | **L** | **H** | **L** | **R** | **P** | **V** | **N** | **L** | **E** | **E** | **K** | **S** | **L** | **K** | **Q** | **G** | **I** | **E** | **Q** | **L** | **L** | **K** | **E** | **L** | **Q** | **N** | **K** | **I** | **Q** | **I** | **S** | **L** | **K** | **W** | **D** | **V** | **E** | **D** | **V** | **K** | **L** | **S** | **S** | **S** | **I** | **E** | **D** | **H** | **L** | **F** | **R** | **I** | **V** | **Q** | **E** | **L** | **L** | **S** | **N** | **T** | **L** | **R** | **H** | **A** | **K** | **A** | **N** | **E** | **L** | **E** | **V** | **Y** | **L** | **H** | **K** | **I** | **D** | **N** | **N** | **L** | **L** | **L** | **R** | **I** | **I** | **D** | **D** | **G** | **T** | **G** | **F** | **N** | **M** | **N** | **E** | **T** | **K** | **T** | **G** | **S** | **Y** | **G** | **L** | **N** | **N** | **I** | **K** | **E** | **R** | **V** | **V** | **G** | **I** | **G** | **G** | **T** | **V** | **K** | **I** | **I** | **S** | **F** | **K** | **G** | **Q** | **G** | **T** | **S** | **V** | **E** | **I** | **K** | **V** | **P** | **L** | **M** | **K** | **E** | **A** |
| **N59513F** | **M** | **M** | **G** | **K** | **I** | **S** | **R** | **A** | **M** | **L** | **A** | **V** | **Y** | **S** | **G** | **I** | **A** | **A** | **F** | **L** | **I** | **I** | **L** | **F** | **S** | **L** | **F** | **T** | **Y** | **F** | **Y** | **A** | **S** | **N** | **Q** | **S** | **H** | **W** | **W** | **G** | **E** | **L** | **L** | **R** | **A** | **R** | **L** | **L** | **Y** | **V** | **P** | **L** | **I** | **F** | **H** | **L** | **L** | **A** | **I** | **S** | **L** | **G** | **V** | **G** | **L** | **I** | **V** | **F** | **L** | **L** | **L** | **S** | **L** | **I** | **Q** | **K** | **T** | **K** | **Y** | **G** | **K** | **I** | **E** | **E** | **K** | **L** | **R** | **A** | **L** | **S** | **S** | **G** | **N** | **Y** | **E** | **S** | **K** | **L** | **L** | **L** | **F** | **P** | **I** | **P** | **S** | **A** | **S** | **D** | **D** | **L** | **Y** | **I** | **K** | **D** | **I** | **D** | **K** | **E** | **I** | **T** | **K** | **I** | **K** | **E** | **K** | **M** | **I** | **E** | **V** | **S** | **S** | **E** | **L** | **Q** | **I** | **V** | **T** | **S** | **R** | **P** | **Q** | **Y** | **V** | **D** | **G** | **Q** | **T** | **K** | **E** | **E** | **I** | **L** | **E** | **L** | **E** | **R** | **H** | **R** | **L** | **A** | **R** | **E** | **L** | **H** | **D** | **S** | **V** | **S** | **Q** | **Q** | **L** | **F** | **A** | **A** | **M** | **M** | **M** | **M** | **S** | **A** | **L** | **T** | **E** | **Q** | **A** | **E** | **K** | **S** | **E** | **T** | **P** | **E** | **M** | **F** | **R** | **K** | **Q** | **L** | **K** | **M** | **V** | **A** | **E** | **I** | **I** | **N** | **A** | **S** | **Q** | **S** | **E** | **M** | **R** | **A** | **L** | **L** | **L** | **H** | **L** | **R** | **P** | **V** | **N** | **L** | **E** | **E** | **K** | **S** | **L** | **K** | **Q** | **G** | **I** | **E** | **Q** | **L** | **L** | **K** | **E** | **L** | **Q** | **N** | **K** | **I** | **Q** | **I** | **S** | **L** | **K** | **W** | **D** | **V** | **E** | **D** | **V** | **K** | **L** | **S** | **S** | **S** | **I** | **E** | **D** | **H** | **L** | **F** | **R** | **I** | **V** | **Q** | **E** | **L** | **L** | **S** | **N** | **T** | **L** | **R** | **H** | **A** | **K** | **A** | **N** | **E** | **L** | **E** | **V** | **Y** | **L** | **H** | **K** | **I** | **D** | **N** | **N** | **L** | **L** | **L** | **R** | **I** | **I** | **D** | **D** | **G** | **T** | **G** | **F** | **N** | **M** | **N** | **E** | **T** | **K** | **T** | **G** | **S** | **Y** | **G** | **L** | **N** | **N** | **I** | **K** | **E** | **R** | **V** | **A** | **G** | **I** | **G** | **G** | **T** | **V** | **K** | **I** | **I** | **S** | **F** | **K** | **G** | **Q** | **G** | **T** | **S** | **V** | **E** | **I** | **K** | **V** | **P** | **L** | **M** | **K** | **E** | **A** |
 **SNP position** | . | . | . | 4 | . | . | 7 | . | . | . | . | . | . | . | . | . | . | . | . | . | . | . | . | . | . | . | . | . | . | . | . | . | . | . | . | 36 | . | . | . | . | . | . | . | . | . | . | . | . | . | . | . | . | . | . | . | . | 57 | . | . | . | . | . | . | . | . | . | . | . | . | . | . | . | . | . | . | . | 77 | . | . | 80 | . | . | . | . | . | . | . | . | . | . | . | . | . | . | . | . | . | . | . | 100 | 101 | . | . | . | . | 106 | . | . | . | . | . | . | . | . | . | . | 117 | . | . | 120 | 121 | . | . | . | . | . | . | . | . | . | . | . | . | . | . | 136 | . | . | . | . | . | . | . | . | . | . | . | . | . | . | . | . | . | . | . | . | . | . | . | . | . | . | . | . | . | . | . | . | . | . | . | . | . | . | . | . | . | . | . | 180 | . | . | . | . | . | . | . | . | . | . | 191 | 192 | . | . | . | . | . | . | . | . | . | . | . | . | . | . | . | . | . | . | . | . | . | . | . | . | . | . | . | . | . | . | . | . | . | . | . | . | . | . | . | . | . | . | . | . | . | . | . | . | . | . | . | . | . | . | . | . | . | . | 251 | 252 | . | . | . | . | . | 258 | . | . | . | . | . | 264 | . | . | . | . | . | . | . | . | . | . | . | . | . | . | . | . | . | . | . | . | . | . | . | . | . | . | . | . | . | . | . | . | . | . | . | . | . | . | . | . | . | . | . | . | . | . | . | . | . | . | . | . | . | . | . | . | . | . | . | . | . | . | 327 | . | . | . | . | . | . | . | . | . | . | . | . | . | . | . | . | . | . | . | . | . | . | . | . | . | . | . | . |

|  |  |
| --- | --- |
|  |

|  |  |
| --- | --- |
|  |

|  |  |
| --- | --- |
| **Legend** | |
  | **Reference Sequence** |

  |
|  | **Validated SNPs** |
|  | **Non-validated SNPs** |
|  | **New SNPs** |
|  | **GAPs** |
|  |  |

  
  |
|  |  |
