# Supplementary material for: sraX: A Novel Comprehensive Resistome Analysis Tool
Source: Front Microbiol. 2020 Feb 5;11:52. doi: 10.3389/fmicb.2020.00052 (PMC7025521; doi:10.3389/fmicb.2020.00052)
Supplement: Supplementary file 4 [file Data_Sheet_3.ZIP › liaS/liaS_Efaecalis_dna.html]

# liaS

|  |
| --- |
|  |

|  |
| --- |
|  |

|  |  |
| --- | --- |
| **Definition:** | LiaS is a histidine kinase found in the liaFSR signal transduction pathway. Mutations confer daptomycin resistance. |

|  |
| --- |
|  |

|  |
| --- |
|  |

|  |  |  |
| --- | --- | --- |
| **Reference organism:** | *Enterococcus faecalis V583* | [NCBI, CARD] |

|  |
| --- |
|  |

|  |
| --- |
|  |

|  |  |
| --- | --- |
| **Summary Data** | || **Parameter** | **Value_(n)_** || Total number of genomes | 98 |
| MSA length | 1104 |
| Total number of SNPs | 66 |
| SNP density (# SNPs/MSA length) | 0.060 |

|  |
| --- |
|  |

|  |
| --- |
|  |

|  |
| --- |
|  |

|  |  |  |  |  |  |  |  |  |  |  |  |  |  |  |  |  |  |  |  |  |  |  |  |  |  |  |  |  |  |  |  |  |  |  |  |  |  |  |  |  |  |  |  |  |  |  |  |  |  |  |  |  |  |  |  |  |  |  |  |  |  |  |  |  |  |  |  |  |  |  |  |  |  |  |  |  |  |  |  |  |  |  |  |  |  |  |  |  |  |  |  |  |  |  |  |  |  |  |  |  |  |  |  |  |  |  |  |  |  |  |  |  |  |  |  |  |  |  |  |  |  |  |  |  |  |  |  |  |  |  |  |  |  |  |  |  |  |  |  |  |  |  |  |  |  |  |  |  |  |  |  |  |  |  |  |  |  |  |  |  |  |  |  |  |  |  |  |  |  |  |  |  |  |  |  |  |  |  |  |  |  |  |  |  |  |  |  |  |  |  |  |  |  |  |  |  |  |  |  |  |  |  |  |  |  |  |  |  |  |  |  |  |  |  |  |  |  |  |  |  |  |  |  |  |  |  |  |  |  |  |  |  |  |  |  |  |  |  |  |  |  |  |  |  |  |  |  |  |  |  |  |  |  |  |  |  |  |  |  |  |  |  |  |  |  |  |  |  |  |  |  |  |  |  |  |  |  |  |  |  |  |  |  |  |  |  |  |  |  |  |  |  |  |  |  |  |  |  |  |  |  |  |  |  |  |  |  |  |  |  |  |  |  |  |  |  |  |  |  |  |  |  |  |  |  |  |  |  |  |  |  |  |  |  |  |  |  |  |  |  |  |  |  |  |  |  |  |  |  |  |  |  |  |  |  |  |  |  |  |  |  |  |  |  |  |  |  |  |  |  |  |  |  |  |  |  |  |  |  |  |  |  |  |  |  |  |  |  |  |  |  |  |  |  |  |  |  |  |  |  |  |  |  |  |  |  |  |  |  |  |  |  |  |  |  |  |  |  |  |  |  |  |  |  |  |  |  |  |  |  |  |  |  |  |  |  |  |  |  |  |  |  |  |  |  |  |  |  |  |  |  |  |  |  |  |  |  |  |  |  |  |  |  |  |  |  |  |  |  |  |  |  |  |  |  |  |  |  |  |  |  |  |  |  |  |  |  |  |  |  |  |  |  |  |  |  |  |  |  |  |  |  |  |  |  |  |  |  |  |  |  |  |  |  |  |  |  |  |  |  |  |  |  |  |  |  |  |  |  |  |  |  |  |  |  |  |  |  |  |  |  |  |  |  |  |  |  |  |  |  |  |  |  |  |  |  |  |  |  |  |  |  |  |  |  |  |  |  |  |  |  |  |  |  |  |  |  |  |  |  |  |  |  |  |  |  |  |  |  |  |  |  |  |  |  |  |  |  |  |  |  |  |  |  |  |  |  |  |  |  |  |  |  |  |  |  |  |  |  |  |  |  |  |  |  |  |  |  |  |  |  |  |  |  |  |  |  |  |  |  |  |  |  |  |  |  |  |  |  |  |  |  |  |  |  |  |  |  |  |  |  |  |  |  |  |  |  |  |  |  |  |  |  |  |  |  |  |  |  |  |  |  |  |  |  |  |  |  |  |  |  |  |  |  |  |  |  |  |  |  |  |  |  |  |  |  |  |  |  |  |  |  |  |  |  |  |  |  |  |  |  |  |  |  |  |  |  |  |  |  |  |  |  |  |  |  |  |  |  |  |  |  |  |  |  |  |  |  |  |  |  |  |  |  |  |  |  |  |  |  |  |  |  |  |  |  |  |  |  |  |  |  |  |  |  |  |  |  |  |  |  |  |  |  |  |  |  |  |  |  |  |  |  |  |  |  |  |  |  |  |  |  |  |  |  |  |  |  |  |  |  |  |  |  |  |  |  |  |  |  |  |  |  |  |  |  |  |  |  |  |  |  |  |  |  |  |  |  |  |  |  |  |  |  |  |  |  |  |  |  |  |  |  |  |  |  |  |  |  |  |  |  |  |  |  |  |  |  |  |  |  |  |  |  |  |  |  |  |  |  |  |  |  |  |  |  |  |  |  |  |  |  |  |  |  |  |  |  |  |  |  |  |  |  |  |  |  |  |  |  |  |  |  |  |  |  |  |  |  |  |  |  |  |  |  |  |  |  |  |  |  |  |  |  |  |  |  |  |  |  |  |  |  |  |  |  |  |  |  |  |  |  |  |  |  |  |  |  |  |  |  |  |  |  |  |  |  |  |  |  |  |  |  |  |  |  |  |  |  |  |  |  |  |  |  |  |  |  |  |  |  |  |  |  |  |  |  |  |  |  |  |  |  |  |  |  |  |  |  |  |  |  |  |  |  |  |  |  |  |  |  |  |  |  |  |  |  |  |  |  |  |  |  |  |  |  |  |  |  |  |  |  |  |  |  |  |  |  |  |  |  |  |  |  |  |  |  |  |  |  |  |  |  |  |  |  |  |  |  |  |  |  |  |  |  |  |  |  |  |  |  |  |  |  |  |  |  |  |  |  |  |  |  |  |  |  |  |  |  |  |  |  |  |  |  |
| --- | --- | --- | --- | --- | --- | --- | --- | --- | --- | --- | --- | --- | --- | --- | --- | --- | --- | --- | --- | --- | --- | --- | --- | --- | --- | --- | --- | --- | --- | --- | --- | --- | --- | --- | --- | --- | --- | --- | --- | --- | --- | --- | --- | --- | --- | --- | --- | --- | --- | --- | --- | --- | --- | --- | --- | --- | --- | --- | --- | --- | --- | --- | --- | --- | --- | --- | --- | --- | --- | --- | --- | --- | --- | --- | --- | --- | --- | --- | --- | --- | --- | --- | --- | --- | --- | --- | --- | --- | --- | --- | --- | --- | --- | --- | --- | --- | --- | --- | --- | --- | --- | --- | --- | --- | --- | --- | --- | --- | --- | --- | --- | --- | --- | --- | --- | --- | --- | --- | --- | --- | --- | --- | --- | --- | --- | --- | --- | --- | --- | --- | --- | --- | --- | --- | --- | --- | --- | --- | --- | --- | --- | --- | --- | --- | --- | --- | --- | --- | --- | --- | --- | --- | --- | --- | --- | --- | --- | --- | --- | --- | --- | --- | --- | --- | --- | --- | --- | --- | --- | --- | --- | --- | --- | --- | --- | --- | --- | --- | --- | --- | --- | --- | --- | --- | --- | --- | --- | --- | --- | --- | --- | --- | --- | --- | --- | --- | --- | --- | --- | --- | --- | --- | --- | --- | --- | --- | --- | --- | --- | --- | --- | --- | --- | --- | --- | --- | --- | --- | --- | --- | --- | --- | --- | --- | --- | --- | --- | --- | --- | --- | --- | --- | --- | --- | --- | --- | --- | --- | --- | --- | --- | --- | --- | --- | --- | --- | --- | --- | --- | --- | --- | --- | --- | --- | --- | --- | --- | --- | --- | --- | --- | --- | --- | --- | --- | --- | --- | --- | --- | --- | --- | --- | --- | --- | --- | --- | --- | --- | --- | --- | --- | --- | --- | --- | --- | --- | --- | --- | --- | --- | --- | --- | --- | --- | --- | --- | --- | --- | --- | --- | --- | --- | --- | --- | --- | --- | --- | --- | --- | --- | --- | --- | --- | --- | --- | --- | --- | --- | --- | --- | --- | --- | --- | --- | --- | --- | --- | --- | --- | --- | --- | --- | --- | --- | --- | --- | --- | --- | --- | --- | --- | --- | --- | --- | --- | --- | --- | --- | --- | --- | --- | --- | --- | --- | --- | --- | --- | --- | --- | --- | --- | --- | --- | --- | --- | --- | --- | --- | --- | --- | --- | --- | --- | --- | --- | --- | --- | --- | --- | --- | --- | --- | --- | --- | --- | --- | --- | --- | --- | --- | --- | --- | --- | --- | --- | --- | --- | --- | --- | --- | --- | --- | --- | --- | --- | --- | --- | --- | --- | --- | --- | --- | --- | --- | --- | --- | --- | --- | --- | --- | --- | --- | --- | --- | --- | --- | --- | --- | --- | --- | --- | --- | --- | --- | --- | --- | --- | --- | --- | --- | --- | --- | --- | --- | --- | --- | --- | --- | --- | --- | --- | --- | --- | --- | --- | --- | --- | --- | --- | --- | --- | --- | --- | --- | --- | --- | --- | --- | --- | --- | --- | --- | --- | --- | --- | --- | --- | --- | --- | --- | --- | --- | --- | --- | --- | --- | --- | --- | --- | --- | --- | --- | --- | --- | --- | --- | --- | --- | --- | --- | --- | --- | --- | --- | --- | --- | --- | --- | --- | --- | --- | --- | --- | --- | --- | --- | --- | --- | --- | --- | --- | --- | --- | --- | --- | --- | --- | --- | --- | --- | --- | --- | --- | --- | --- | --- | --- | --- | --- | --- | --- | --- | --- | --- | --- | --- | --- | --- | --- | --- | --- | --- | --- | --- | --- | --- | --- | --- | --- | --- | --- | --- | --- | --- | --- | --- | --- | --- | --- | --- | --- | --- | --- | --- | --- | --- | --- | --- | --- | --- | --- | --- | --- | --- | --- | --- | --- | --- | --- | --- | --- | --- | --- | --- | --- | --- | --- | --- | --- | --- | --- | --- | --- | --- | --- | --- | --- | --- | --- | --- | --- | --- | --- | --- | --- | --- | --- | --- | --- | --- | --- | --- | --- | --- | --- | --- | --- | --- | --- | --- | --- | --- | --- | --- | --- | --- | --- | --- | --- | --- | --- | --- | --- | --- | --- | --- | --- | --- | --- | --- | --- | --- | --- | --- | --- | --- | --- | --- | --- | --- | --- | --- | --- | --- | --- | --- | --- | --- | --- | --- | --- | --- | --- | --- | --- | --- | --- | --- | --- | --- | --- | --- | --- | --- | --- | --- | --- | --- | --- | --- | --- | --- | --- | --- | --- | --- | --- | --- | --- | --- | --- | --- | --- | --- | --- | --- | --- | --- | --- | --- | --- | --- | --- | --- | --- | --- | --- | --- | --- | --- | --- | --- | --- | --- | --- | --- | --- | --- | --- | --- | --- | --- | --- | --- | --- | --- | --- | --- | --- | --- | --- | --- | --- | --- | --- | --- | --- | --- | --- | --- | --- | --- | --- | --- | --- | --- | --- | --- | --- | --- | --- | --- | --- | --- | --- | --- | --- | --- | --- | --- | --- | --- | --- | --- | --- | --- | --- | --- | --- | --- | --- | --- | --- | --- | --- | --- | --- | --- | --- | --- | --- | --- | --- | --- | --- | --- | --- | --- | --- | --- | --- | --- | --- | --- | --- | --- | --- | --- | --- | --- | --- | --- | --- | --- | --- | --- | --- | --- | --- | --- | --- | --- | --- | --- | --- | --- | --- | --- | --- | --- | --- | --- | --- | --- | --- | --- | --- | --- | --- | --- | --- | --- | --- | --- | --- | --- | --- | --- | --- | --- | --- | --- | --- | --- | --- | --- | --- | --- | --- | --- | --- | --- | --- | --- | --- | --- | --- | --- | --- | --- | --- | --- | --- | --- | --- | --- | --- | --- | --- | --- | --- | --- | --- | --- | --- | --- | --- | --- | --- | --- | --- | --- | --- | --- | --- | --- | --- | --- | --- | --- | --- | --- | --- | --- | --- | --- | --- | --- | --- | --- | --- | --- | --- | --- | --- | --- | --- | --- | --- | --- | --- | --- | --- | --- | --- | --- | --- | --- | --- | --- | --- | --- | --- | --- | --- | --- | --- | --- | --- | --- | --- | --- | --- | --- | --- | --- | --- | --- | --- | --- | --- | --- | --- | --- | --- | --- | --- | --- | --- | --- | --- | --- | --- | --- | --- | --- | --- | --- | --- | --- | --- | --- | --- | --- | --- | --- | --- | --- | --- | --- | --- | --- | --- | --- | --- | --- | --- | --- | --- | --- | --- | --- | --- | --- | --- | --- | --- | --- | --- | --- | --- | --- | --- | --- | --- | --- | --- | --- | --- | --- | --- | --- | --- | --- | --- | --- | --- | --- | --- | --- | --- | --- | --- | --- | --- | --- | --- | --- | --- | --- | --- | --- | --- | --- | --- | --- | --- | --- | --- | --- | --- | --- | --- | --- | --- | --- | --- | --- | --- | --- | --- | --- | --- | --- | --- | --- | --- | --- | --- | --- | --- | --- | --- | --- | --- | --- | --- | --- | --- | --- | --- | --- | --- | --- | --- | --- | --- | --- | --- | --- | --- | --- | --- | --- | --- | --- | --- | --- | --- | --- | --- | --- | --- | --- | --- | --- | --- | --- | --- | --- | --- | --- | --- | --- | --- |
|  || **Enterococcus faecalis V583 (AAO82600.1)** | **A** | **T** | **G** | **A** | **C** | **C** | **G** | **A** | **T** | **C** | **G** | **G** | **A** | **T** | **T** | **T** | **C** | **A** | **A** | **G** | **A** | **C** | **G** | **C** | **A** | **T** | **G** | **A** | **T** | **T** | **T** | **C** | **A** | **T** | **T** | **A** | **T** | **A** | **T** | **G** | **C** | **G** | **T** | **C** | **C** | **C** | **T** | **T** | **A** | **G** | **C** | **A** | **C** | **C** | **T** | **T** | **T** | **A** | **T** | **T** | **G** | **T** | **T** | **A** | **T** | **C** | **T** | **T** | **A** | **A** | **T** | **T** | **A** | **C** | **A** | **T** | **T** | **G** | **T** | **T** | **T** | **T** | **C** | **A** | **T** | **A** | **T** | **T** | **T** | **T** | **C** | **A** | **T** | **T** | **C** | **G** | **A** | **T** | **T** | **A** | **A** | **A** | **C** | **A** | **A** | **A** | **A** | **C** | **C** | **G** | **G** | **T** | **G** | **G** | **T** | **T** | **A** | **T** | **T** | **A** | **G** | **A** | **G** | **C** | **T** | **T** | **C** | **T** | **T** | **C** | **A** | **G** | **A** | **G** | **A** | **A** | **A** | **A** | **G** | **T** | **C** | **T** | **T** | **T** | **T** | **A** | **T** | **T** | **T** | **A** | **C** | **C** | **A** | **C** | **T** | **A** | **A** | **T** | **T** | **G** | **T** | **G** | **C** | **A** | **C** | **A** | **T** | **T** | **G** | **T** | **T** | **C** | **T** | **C** | **A** | **T** | **A** | **T** | **C** | **C** | **T** | **T** | **A** | **C** | **T** | **A** | **A** | **T** | **A** | **G** | **G** | **C** | **T** | **T** | **A** | **T** | **T** | **G** | **A** | **C** | **C** | **T** | **T** | **T** | **T** | **T** | **A** | **C** | **T** | **G** | **A** | **T** | **T** | **T** | **C** | **A** | **T** | **T** | **G** | **G** | **T** | **T** | **C** | **A** | **A** | **A** | **A** | **A** | **G** | **G** | **G** | **C** | **A** | **A** | **T** | **A** | **T** | **G** | **G** | **A** | **C** | **G** | **G** | **A** | **T** | **T** | **G** | **A** | **A** | **G** | **A** | **A** | **A** | **A** | **A** | **C** | **T** | **T** | **C** | **G** | **G** | **T** | **T** | **A** | **T** | **T** | **G** | **G** | **C** | **C** | **A** | **A** | **C** | **G** | **G** | **T** | **A** | **A** | **T** | **T** | **A** | **T** | **G** | **A** | **A** | **A** | **G** | **T** | **C** | **C** | **A** | **G** | **T** | **C** | **T** | **T** | **A** | **A** | **A** | **C** | **A** | **A** | **A** | **C** | **C** | **A** | **A** | **C** | **G** | **A** | **C** | **C** | **A** | **G** | **T** | **G** | **A** | **A** | **A** | **A** | **T** | **C** | **A** | **A** | **G** | **A** | **C** | **C** | **A** | **T** | **T** | **A** | **T** | **C** | **T** | **A** | **A** | **C** | **C** | **G** | **A** | **A** | **G** | **T** | **C** | **G** | **A** | **A** | **C** | **A** | **A** | **G** | **A** | **T** | **A** | **T** | **T** | **T** | **G** | **G** | **T** | **C** | **G** | **A** | **T** | **T** | **A** | **A** | **A** | **A** | **A** | **T** | **A** | **A** | **A** | **T** | **T** | **A** | **T** | **T** | **A** | **G** | **A** | **G** | **A** | **T** | **G** | **T** | **C** | **T** | **A** | **A** | **A** | **G** | **A** | **A** | **T** | **T** | **G** | **C** | **A** | **A** | **T** | **T** | **A** | **T** | **T** | **A** | **A** | **A** | **C** | **A** | **G** | **T** | **C** | **G** | **A** | **C** | **C** | **G** | **C** | **A** | **A** | **T** | **T** | **A** | **A** | **T** | **G** | **G** | **A** | **T** | **G** | **G** | **G** | **C** | **A** | **A** | **A** | **C** | **A** | **A** | **A** | **A** | **G** | **A** | **A** | **G** | **A** | **A** | **A** | **T** | **T** | **T** | **T** | **A** | **G** | **A** | **G** | **A** | **A** | **C** | **G** | **A** | **G** | **C** | **G** | **G** | **C** | **A** | **T** | **C** | **G** | **T** | **T** | **T** | **G** | **G** | **C** | **G** | **C** | **G** | **G** | **G** | **A** | **G** | **T** | **T** | **G** | **C** | **A** | **T** | **G** | **A** | **T** | **T** | **C** | **A** | **G** | **T** | **C** | **A** | **G** | **T** | **C** | **A** | **A** | **C** | **A** | **A** | **C** | **T** | **T** | **T** | **T** | **T** | **G** | **C** | **A** | **G** | **C** | **C** | **A** | **T** | **G** | **A** | **T** | **G** | **A** | **T** | **G** | **T** | **T** | **G** | **T** | **C** | **T** | **G** | **C** | **A** | **T** | **T** | **A** | **A** | **A** | **T** | **G** | **A** | **A** | **C** | **A** | **A** | **G** | **C** | **A** | **C** | **A** | **A** | **C** | **G** | **A** | **A** | **C** | **A** | **G** | **A** | **A** | **A** | **C** | **C** | **C** | **C** | **G** | **G** | **A** | **A** | **C** | **C** | **A** | **T** | **A** | **T** | **C** | **G** | **T** | **A** | **A** | **A** | **C** | **A** | **A** | **C** | **T** | **A** | **G** | **C** | **C** | **A** | **T** | **G** | **G** | **T** | **G** | **G** | **C** | **A** | **G** | **A** | **A** | **A** | **T** | **C** | **A** | **T** | **T** | **A** | **A** | **T** | **G** | **C** | **C** | **T** | **C** | **C** | **C** | **A** | **A** | **T** | **C** | **G** | **G** | **A** | **A** | **A** | **T** | **G** | **C** | **G** | **C** | **G** | **C** | **G** | **C** | **T** | **A** | **C** | **T** | **A** | **T** | **T** | **G** | **C** | **A** | **C** | **T** | **T** | **G** | **C** | **G** | **T** | **C** | **C** | **T** | **A** | **T** | **C** | **A** | **G** | **T** | **C** | **T** | **A** | **G** | **A** | **A** | **G** | **G** | **A** | **A** | **A** | **A** | **A** | **G** | **T** | **T** | **T** | **G** | **C** | **G** | **T** | **A** | **A** | **A** | **G** | **G** | **T** | **A** | **T** | **T** | **G** | **A** | **A** | **C** | **A** | **A** | **T** | **T** | **A** | **C** | **T** | **G** | **A** | **A** | **A** | **G** | **A** | **A** | **C** | **T** | **A** | **C** | **A** | **A** | **A** | **C** | **A** | **A** | **A** | **A** | **A** | **T** | **T** | **A** | **A** | **A** | **A** | **T** | **T** | **G** | **A** | **A** | **T** | **T** | **G** | **A** | **T** | **T** | **T** | **G** | **G** | **G** | **A** | **T** | **G** | **T** | **T** | **G** | **A** | **A** | **G** | **A** | **T** | **G** | **T** | **T** | **C** | **A** | **T** | **T** | **T** | **A** | **A** | **A** | **T** | **A** | **G** | **C** | **A** | **G** | **C** | **A** | **T** | **T** | **G** | **A** | **G** | **G** | **A** | **T** | **C** | **A** | **T** | **C** | **T** | **T** | **T** | **T** | **C** | **C** | **G** | **A** | **A** | **T** | **T** | **G** | **T** | **G** | **C** | **A** | **G** | **G** | **A** | **G** | **T** | **T** | **A** | **C** | **T** | **T** | **T** | **C** | **A** | **A** | **A** | **T** | **A** | **C** | **C** | **T** | **T** | **A** | **A** | **G** | **A** | **C** | **A** | **T** | **G** | **C** | **C** | **A** | **A** | **A** | **G** | **C** | **A** | **A** | **A** | **G** | **G** | **A** | **A** | **T** | **T** | **A** | **G** | **A** | **G** | **G** | **T** | **A** | **T** | **A** | **C** | **T** | **T** | **A** | **C** | **A** | **C** | **C** | **A** | **A** | **G** | **T** | **C** | **G** | **A** | **T** | **A** | **A** | **A** | **A** | **A** | **C** | **G** | **T** | **G** | **T** | **T** | **A** | **T** | **T** | **G** | **C** | **G** | **T** | **A** | **T** | **T** | **G** | **T** | **T** | **G** | **A** | **T** | **G** | **A** | **T** | **G** | **G** | **T** | **G** | **T** | **C** | **G** | **G** | **C** | **T** | **T** | **T** | **G** | **A** | **T** | **A** | **T** | **G** | **A** | **A** | **G** | **G** | **A** | **A** | **C** | **A** | **A** | **A** | **G** | **T** | **A** | **A** | **T** | **A** | **A** | **A** | **G** | **C** | **C** | **G** | **G** | **T** | **A** | **G** | **T** | **T** | **A** | **T** | **G** | **G** | **C** | **T** | **T** | **A** | **A** | **A** | **T** | **A** | **A** | **T** | **A** | **T** | **T** | **C** | **G** | **A** | **G** | **A** | **A** | **C** | **G** | **T** | **G** | **T** | **T** | **G** | **T** | **C** | **G** | **G** | **C** | **A** | **T** | **G** | **G** | **G** | **C** | **G** | **G** | **T** | **A** | **C** | **A** | **G** | **T** | **T** | **A** | **A** | **A** | **A** | **T** | **T** | **A** | **T** | **T** | **A** | **G** | **T** | **T** | **T** | **T** | **A** | **A** | **A** | **G** | **G** | **G** | **C** | **A** | **G** | **G** | **G** | **A** | **A** | **C** | **C** | **A** | **G** | **C** | **G** | **T** | **T** | **G** | **A** | **A** | **A** | **T** | **T** | **A** | **A** | **A** | **G** | **T** | **T** | **C** | **C** | **T** | **G** | **T** | **C** | **A** | **T** | **A** | **A** | **A** | **G** | **G** | **A** | **G** | **G** | **A** | **A** | **A** | **C** | **T** | **G** | **C** | **A** | **A** | **G** | **T** | **G** | **A** | **T** | **C** | **A** | **A** | **A** | **G** | **T** | **A** | **A** | **T** | **G** | **T** | **T** | **A** | **G** | **T** | **G** | **G** | **A** | **T** | **G** | **A** |
| **N52734** | **A** | **T** | **G** | **A** | **C** | **C** | **G** | **A** | **T** | **C** | **G** | **G** | **A** | **T** | **T** | **T** | **C** | **A** | **A** | **G** | **A** | **C** | **G** | **C** | **A** | **T** | **G** | **A** | **T** | **T** | **T** | **C** | **A** | **T** | **T** | **A** | **T** | **A** | **T** | **G** | **C** | **G** | **T** | **C** | **C** | **C** | **T** | **T** | **A** | **G** | **C** | **A** | **C** | **C** | **T** | **T** | **T** | **A** | **T** | **T** | **G** | **T** | **T** | **A** | **T** | **C** | **T** | **T** | **A** | **A** | **T** | **T** | **A** | **C** | **A** | **T** | **T** | **G** | **T** | **T** | **T** | **T** | **C** | **A** | **T** | **A** | **T** | **T** | **T** | **T** | **C** | **A** | **T** | **T** | **C** | **G** | **A** | **T** | **T** | **A** | **A** | **A** | **C** | **A** | **A** | **A** | **A** | **C** | **C** | **G** | **G** | **T** | **G** | **G** | **T** | **T** | **A** | **T** | **T** | **A** | **G** | **A** | **G** | **C** | **T** | **T** | **C** | **T** | **T** | **C** | **A** | **G** | **A** | **G** | **A** | **A** | **A** | **A** | **G** | **T** | **C** | **T** | **T** | **T** | **T** | **A** | **T** | **T** | **T** | **A** | **C** | **C** | **A** | **C** | **T** | **A** | **A** | **T** | **T** | **G** | **T** | **G** | **C** | **A** | **C** | **A** | **T** | **T** | **G** | **T** | **T** | **C** | **T** | **C** | **A** | **T** | **A** | **T** | **C** | **C** | **T** | **T** | **A** | **C** | **T** | **A** | **A** | **T** | **A** | **G** | **G** | **C** | **T** | **T** | **A** | **T** | **T** | **G** | **A** | **C** | **C** | **T** | **T** | **T** | **T** | **T** | **A** | **C** | **T** | **G** | **A** | **T** | **T** | **T** | **C** | **A** | **T** | **T** | **G** | **G** | **T** | **T** | **C** | **A** | **A** | **A** | **A** | **A** | **G** | **G** | **G** | **C** | **A** | **A** | **T** | **A** | **T** | **G** | **G** | **A** | **C** | **G** | **G** | **A** | **T** | **T** | **G** | **A** | **A** | **G** | **A** | **A** | **A** | **A** | **A** | **C** | **T** | **T** | **C** | **G** | **G** | **T** | **T** | **A** | **T** | **T** | **G** | **G** | **C** | **C** | **A** | **A** | **C** | **G** | **G** | **T** | **A** | **A** | **T** | **T** | **A** | **T** | **G** | **A** | **A** | **A** | **G** | **T** | **C** | **C** | **A** | **G** | **T** | **C** | **T** | **T** | **A** | **A** | **A** | **C** | **A** | **A** | **A** | **C** | **C** | **A** | **A** | **C** | **G** | **A** | **C** | **C** | **A** | **G** | **T** | **G** | **A** | **A** | **A** | **A** | **T** | **C** | **A** | **A** | **G** | **A** | **C** | **C** | **A** | **T** | **T** | **A** | **T** | **C** | **T** | **A** | **A** | **C** | **C** | **G** | **A** | **A** | **G** | **T** | **C** | **G** | **A** | **A** | **C** | **A** | **A** | **G** | **A** | **T** | **A** | **T** | **T** | **T** | **G** | **G** | **T** | **C** | **G** | **A** | **T** | **T** | **A** | **A** | **A** | **A** | **A** | **T** | **A** | **A** | **A** | **T** | **T** | **A** | **T** | **T** | **A** | **G** | **A** | **G** | **A** | **T** | **G** | **T** | **C** | **T** | **A** | **A** | **A** | **G** | **A** | **A** | **T** | **T** | **G** | **C** | **A** | **A** | **T** | **T** | **A** | **T** | **T** | **A** | **A** | **A** | **C** | **A** | **G** | **T** | **C** | **G** | **A** | **C** | **C** | **G** | **C** | **A** | **A** | **T** | **T** | **A** | **A** | **T** | **G** | **G** | **A** | **T** | **G** | **G** | **G** | **C** | **A** | **A** | **A** | **C** | **A** | **A** | **A** | **A** | **G** | **A** | **A** | **G** | **A** | **A** | **A** | **T** | **T** | **T** | **T** | **A** | **G** | **A** | **G** | **A** | **A** | **C** | **G** | **A** | **G** | **C** | **G** | **G** | **C** | **A** | **T** | **C** | **G** | **T** | **T** | **T** | **G** | **G** | **C** | **G** | **C** | **G** | **G** | **G** | **A** | **G** | **T** | **T** | **G** | **C** | **A** | **T** | **G** | **A** | **T** | **T** | **C** | **A** | **G** | **T** | **C** | **A** | **G** | **T** | **C** | **A** | **A** | **C** | **A** | **A** | **C** | **T** | **T** | **T** | **T** | **T** | **G** | **C** | **A** | **G** | **C** | **C** | **A** | **T** | **G** | **A** | **T** | **G** | **A** | **T** | **G** | **T** | **T** | **G** | **T** | **C** | **T** | **G** | **C** | **C** | **T** | **T** | **A** | **A** | **A** | **T** | **G** | **A** | **A** | **C** | **A** | **A** | **G** | **C** | **A** | **C** | **A** | **A** | **C** | **G** | **A** | **A** | **C** | **A** | **G** | **A** | **A** | **A** | **C** | **C** | **C** | **C** | **G** | **G** | **A** | **A** | **C** | **C** | **A** | **T** | **A** | **T** | **C** | **G** | **T** | **A** | **A** | **A** | **C** | **A** | **A** | **C** | **T** | **A** | **G** | **C** | **C** | **A** | **T** | **G** | **G** | **T** | **G** | **G** | **C** | **A** | **G** | **A** | **A** | **A** | **T** | **C** | **A** | **T** | **T** | **A** | **A** | **T** | **G** | **C** | **C** | **T** | **C** | **C** | **C** | **A** | **A** | **T** | **C** | **G** | **G** | **A** | **A** | **A** | **T** | **G** | **C** | **G** | **C** | **G** | **C** | **G** | **T** | **T** | **A** | **C** | **T** | **A** | **T** | **T** | **G** | **C** | **A** | **C** | **T** | **T** | **G** | **C** | **G** | **T** | **C** | **C** | **T** | **A** | **T** | **C** | **A** | **G** | **T** | **C** | **T** | **A** | **G** | **A** | **A** | **G** | **G** | **A** | **A** | **A** | **A** | **A** | **G** | **T** | **T** | **T** | **G** | **C** | **G** | **T** | **A** | **A** | **A** | **G** | **G** | **T** | **A** | **T** | **T** | **G** | **A** | **A** | **C** | **A** | **A** | **T** | **T** | **A** | **C** | **T** | **G** | **A** | **A** | **A** | **G** | **A** | **A** | **C** | **T** | **A** | **C** | **A** | **A** | **A** | **C** | **A** | **A** | **A** | **A** | **A** | **T** | **T** | **A** | **A** | **A** | **A** | **T** | **T** | **G** | **A** | **A** | **T** | **T** | **G** | **A** | **T** | **T** | **T** | **G** | **G** | **G** | **A** | **T** | **G** | **T** | **T** | **G** | **A** | **A** | **G** | **A** | **T** | **G** | **T** | **T** | **C** | **A** | **T** | **T** | **T** | **A** | **A** | **A** | **T** | **A** | **G** | **C** | **A** | **G** | **C** | **A** | **T** | **T** | **G** | **A** | **G** | **G** | **A** | **T** | **C** | **A** | **T** | **C** | **T** | **T** | **T** | **T** | **C** | **C** | **G** | **A** | **A** | **T** | **T** | **G** | **T** | **G** | **C** | **A** | **G** | **G** | **A** | **A** | **T** | **T** | **A** | **C** | **T** | **T** | **T** | **C** | **A** | **A** | **A** | **T** | **A** | **C** | **C** | **T** | **T** | **A** | **A** | **G** | **A** | **C** | **A** | **T** | **G** | **C** | **C** | **A** | **A** | **A** | **G** | **C** | **A** | **A** | **A** | **G** | **G** | **A** | **A** | **T** | **T** | **A** | **G** | **A** | **G** | **G** | **T** | **A** | **T** | **A** | **C** | **T** | **T** | **A** | **C** | **A** | **C** | **C** | **A** | **A** | **G** | **T** | **C** | **G** | **A** | **T** | **A** | **A** | **A** | **A** | **A** | **C** | **G** | **T** | **G** | **T** | **T** | **A** | **T** | **T** | **G** | **C** | **G** | **T** | **A** | **T** | **T** | **G** | **T** | **T** | **G** | **A** | **T** | **G** | **A** | **C** | **G** | **G** | **T** | **G** | **T** | **C** | **G** | **G** | **C** | **T** | **T** | **T** | **G** | **A** | **T** | **A** | **T** | **G** | **A** | **A** | **G** | **G** | **A** | **A** | **C** | **A** | **A** | **A** | **G** | **T** | **A** | **A** | **T** | **A** | **A** | **A** | **G** | **C** | **C** | **G** | **G** | **T** | **A** | **G** | **T** | **T** | **A** | **T** | **G** | **G** | **C** | **T** | **T** | **A** | **A** | **A** | **T** | **A** | **A** | **T** | **A** | **T** | **C** | **C** | **G** | **A** | **G** | **A** | **A** | **C** | **G** | **T** | **G** | **T** | **T** | **G** | **T** | **C** | **G** | **G** | **C** | **A** | **T** | **G** | **G** | **G** | **C** | **G** | **G** | **T** | **A** | **C** | **A** | **G** | **T** | **T** | **A** | **A** | **A** | **A** | **T** | **T** | **A** | **T** | **T** | **A** | **G** | **T** | **T** | **T** | **T** | **A** | **A** | **A** | **G** | **G** | **G** | **C** | **A** | **G** | **G** | **G** | **A** | **A** | **C** | **C** | **A** | **G** | **C** | **G** | **T** | **T** | **G** | **A** | **A** | **A** | **T** | **T** | **A** | **A** | **A** | **G** | **T** | **T** | **C** | **C** | **T** | **G** | **T** | **C** | **A** | **T** | **A** | **A** | **A** | **G** | **G** | **A** | **G** | **G** | **A** | **A** | **A** | **C** | **T** | **G** | **C** | **A** | **A** | **G** | **T** | **G** | **A** | **T** | **C** | **A** | **A** | **A** | **G** | **T** | **A** | **A** | **T** | **G** | **T** | **T** | **A** | **G** | **T** | **G** | **G** | **A** | **-** | **-** | **-** |
| **N52587** | **A** | **T** | **G** | **A** | **C** | **C** | **G** | **A** | **T** | **C** | **G** | **G** | **A** | **T** | **T** | **T** | **C** | **A** | **A** | **G** | **A** | **C** | **G** | **C** | **A** | **T** | **G** | **A** | **T** | **T** | **T** | **C** | **A** | **T** | **T** | **A** | **T** | **A** | **T** | **G** | **C** | **G** | **T** | **C** | **C** | **C** | **T** | **T** | **A** | **G** | **C** | **A** | **C** | **C** | **T** | **T** | **T** | **A** | **T** | **T** | **G** | **T** | **T** | **A** | **T** | **T** | **T** | **T** | **A** | **A** | **T** | **T** | **A** | **C** | **C** | **T** | **T** | **G** | **T** | **T** | **T** | **T** | **C** | **A** | **T** | **A** | **T** | **T** | **T** | **T** | **C** | **A** | **T** | **T** | **C** | **A** | **A** | **T** | **T** | **A** | **A** | **A** | **C** | **A** | **A** | **A** | **A** | **C** | **C** | **G** | **G** | **T** | **G** | **G** | **C** | **T** | **C** | **T** | **T** | **A** | **G** | **A** | **A** | **C** | **T** | **T** | **C** | **T** | **T** | **C** | **A** | **G** | **A** | **G** | **A** | **A** | **A** | **A** | **G** | **T** | **C** | **T** | **T** | **T** | **T** | **A** | **T** | **C** | **T** | **G** | **C** | **C** | **A** | **C** | **T** | **A** | **A** | **T** | **T** | **G** | **T** | **G** | **C** | **A** | **T** | **A** | **T** | **T** | **G** | **T** | **T** | **C** | **T** | **T** | **A** | **T** | **A** | **T** | **C** | **C** | **T** | **T** | **A** | **C** | **T** | **A** | **A** | **T** | **A** | **G** | **G** | **C** | **T** | **T** | **A** | **T** | **T** | **G** | **A** | **C** | **C** | **T** | **T** | **T** | **T** | **T** | **A** | **C** | **T** | **G** | **A** | **T** | **T** | **T** | **C** | **G** | **T** | **T** | **G** | **G** | **T** | **C** | **C** | **A** | **A** | **A** | **A** | **A** | **G** | **G** | **C** | **C** | **A** | **A** | **T** | **A** | **T** | **G** | **G** | **A** | **C** | **G** | **G** | **A** | **T** | **T** | **G** | **A** | **A** | **G** | **A** | **A** | **A** | **A** | **A** | **C** | **T** | **T** | **C** | **G** | **G** | **T** | **T** | **A** | **T** | **T** | **A** | **G** | **C** | **C** | **A** | **A** | **T** | **G** | **G** | **C** | **A** | **A** | **T** | **T** | **A** | **T** | **G** | **A** | **A** | **A** | **G** | **T** | **C** | **C** | **A** | **G** | **T** | **C** | **T** | **T** | **A** | **A** | **A** | **C** | **A** | **A** | **A** | **C** | **C** | **A** | **A** | **T** | **G** | **A** | **C** | **T** | **A** | **G** | **T** | **G** | **A** | **A** | **A** | **A** | **T** | **C** | **A** | **A** | **G** | **A** | **C** | **C** | **A** | **T** | **T** | **A** | **T** | **C** | **T** | **A** | **A** | **T** | **C** | **G** | **A** | **A** | **G** | **T** | **C** | **G** | **A** | **A** | **C** | **A** | **A** | **G** | **A** | **T** | **A** | **T** | **T** | **T** | **G** | **G** | **T** | **C** | **G** | **A** | **T** | **T** | **A** | **A** | **A** | **A** | **A** | **C** | **A** | **A** | **A** | **T** | **T** | **A** | **T** | **T** | **A** | **G** | **A** | **G** | **A** | **T** | **G** | **T** | **C** | **T** | **A** | **A** | **A** | **G** | **A** | **A** | **T** | **T** | **G** | **C** | **A** | **A** | **T** | **T** | **A** | **T** | **T** | **A** | **A** | **A** | **C** | **A** | **G** | **T** | **C** | **G** | **A** | **C** | **C** | **G** | **C** | **A** | **A** | **T** | **T** | **A** | **A** | **T** | **G** | **G** | **A** | **T** | **G** | **G** | **G** | **C** | **A** | **A** | **A** | **C** | **A** | **A** | **A** | **A** | **G** | **A** | **A** | **G** | **A** | **A** | **A** | **T** | **T** | **T** | **T** | **A** | **G** | **A** | **G** | **A** | **A** | **C** | **G** | **A** | **G** | **C** | **G** | **G** | **C** | **A** | **T** | **C** | **G** | **T** | **T** | **T** | **G** | **G** | **C** | **A** | **C** | **G** | **G** | **G** | **A** | **G** | **T** | **T** | **G** | **C** | **A** | **T** | **G** | **A** | **T** | **T** | **C** | **A** | **G** | **T** | **C** | **A** | **G** | **T** | **C** | **A** | **A** | **C** | **A** | **A** | **C** | **T** | **T** | **T** | **T** | **T** | **G** | **C** | **A** | **G** | **C** | **C** | **A** | **T** | **G** | **A** | **T** | **G** | **A** | **T** | **G** | **T** | **T** | **G** | **T** | **C** | **T** | **G** | **C** | **C** | **T** | **T** | **A** | **A** | **A** | **T** | **G** | **A** | **A** | **C** | **A** | **A** | **G** | **C** | **A** | **C** | **A** | **A** | **C** | **G** | **A** | **A** | **C** | **A** | **G** | **A** | **A** | **A** | **C** | **C** | **C** | **C** | **T** | **G** | **A** | **A** | **C** | **C** | **A** | **T** | **A** | **T** | **C** | **G** | **T** | **A** | **A** | **A** | **C** | **A** | **A** | **C** | **T** | **A** | **G** | **C** | **C** | **A** | **T** | **G** | **G** | **T** | **G** | **G** | **C** | **A** | **G** | **A** | **A** | **A** | **T** | **C** | **A** | **T** | **T** | **A** | **A** | **T** | **G** | **C** | **C** | **T** | **C** | **C** | **C** | **A** | **A** | **T** | **C** | **G** | **G** | **A** | **A** | **A** | **T** | **G** | **C** | **G** | **C** | **G** | **C** | **G** | **T** | **T** | **A** | **C** | **T** | **A** | **T** | **T** | **A** | **C** | **A** | **C** | **T** | **T** | **G** | **C** | **G** | **T** | **C** | **C** | **T** | **A** | **T** | **T** | **A** | **G** | **T** | **C** | **T** | **A** | **G** | **A** | **A** | **G** | **G** | **G** | **A** | **A** | **A** | **A** | **G** | **T** | **T** | **T** | **G** | **C** | **G** | **T** | **A** | **A** | **A** | **G** | **G** | **C** | **A** | **T** | **T** | **G** | **A** | **A** | **C** | **A** | **A** | **T** | **T** | **A** | **T** | **T** | **G** | **A** | **A** | **A** | **G** | **A** | **A** | **C** | **T** | **A** | **C** | **A** | **A** | **A** | **C** | **A** | **A** | **A** | **A** | **A** | **T** | **T** | **A** | **A** | **A** | **A** | **T** | **T** | **G** | **A** | **A** | **T** | **T** | **G** | **A** | **T** | **T** | **T** | **G** | **G** | **G** | **A** | **T** | **G** | **T** | **T** | **G** | **A** | **A** | **G** | **A** | **T** | **G** | **T** | **T** | **C** | **A** | **T** | **T** | **T** | **A** | **A** | **A** | **T** | **A** | **G** | **C** | **A** | **G** | **C** | **A** | **T** | **T** | **G** | **A** | **G** | **G** | **A** | **T** | **C** | **A** | **T** | **C** | **T** | **T** | **T** | **T** | **C** | **C** | **G** | **A** | **A** | **T** | **T** | **G** | **T** | **T** | **C** | **A** | **G** | **G** | **A** | **A** | **T** | **T** | **A** | **C** | **T** | **T** | **T** | **C** | **A** | **A** | **A** | **T** | **A** | **C** | **C** | **T** | **T** | **A** | **A** | **G** | **A** | **C** | **A** | **T** | **G** | **C** | **C** | **A** | **A** | **A** | **G** | **C** | **A** | **A** | **A** | **A** | **G** | **A** | **A** | **T** | **T** | **A** | **G** | **A** | **G** | **G** | **T** | **A** | **T** | **A** | **C** | **T** | **T** | **A** | **C** | **A** | **C** | **C** | **A** | **A** | **G** | **T** | **C** | **G** | **A** | **T** | **A** | **A** | **A** | **A** | **A** | **T** | **G** | **T** | **T** | **T** | **T** | **A** | **T** | **T** | **G** | **C** | **G** | **C** | **A** | **T** | **T** | **G** | **T** | **T** | **G** | **A** | **T** | **G** | **A** | **T** | **G** | **G** | **T** | **G** | **T** | **C** | **G** | **G** | **C** | **T** | **T** | **T** | **G** | **A** | **T** | **A** | **T** | **G** | **A** | **A** | **G** | **G** | **A** | **A** | **C** | **A** | **A** | **A** | **G** | **T** | **A** | **A** | **T** | **A** | **A** | **G** | **G** | **C** | **C** | **G** | **G** | **T** | **A** | **G** | **T** | **T** | **A** | **T** | **G** | **G** | **C** | **T** | **T** | **A** | **A** | **A** | **T** | **A** | **A** | **T** | **A** | **T** | **T** | **C** | **G** | **A** | **G** | **A** | **A** | **C** | **G** | **T** | **G** | **T** | **T** | **G** | **T** | **C** | **G** | **G** | **C** | **A** | **T** | **G** | **G** | **G** | **C** | **G** | **G** | **T** | **A** | **C** | **A** | **G** | **T** | **T** | **A** | **A** | **A** | **A** | **T** | **T** | **A** | **T** | **T** | **A** | **G** | **T** | **T** | **T** | **T** | **A** | **A** | **A** | **G** | **G** | **G** | **C** | **A** | **G** | **G** | **G** | **A** | **A** | **C** | **C** | **A** | **G** | **C** | **G** | **T** | **T** | **G** | **A** | **A** | **A** | **T** | **T** | **A** | **A** | **A** | **G** | **T** | **T** | **C** | **C** | **T** | **G** | **T** | **C** | **A** | **T** | **A** | **A** | **A** | **G** | **G** | **A** | **G** | **G** | **A** | **A** | **A** | **C** | **T** | **G** | **C** | **A** | **A** | **G** | **T** | **G** | **A** | **T** | **C** | **A** | **A** | **A** | **G** | **T** | **A** | **A** | **T** | **G** | **T** | **T** | **A** | **G** | **T** | **G** | **G** | **A** | **-** | **-** | **-** |
| **N53405** | **A** | **T** | **G** | **A** | **C** | **C** | **G** | **A** | **T** | **C** | **G** | **G** | **A** | **T** | **T** | **T** | **C** | **A** | **A** | **G** | **A** | **C** | **G** | **C** | **A** | **T** | **G** | **A** | **T** | **T** | **T** | **C** | **A** | **T** | **T** | **A** | **T** | **A** | **T** | **G** | **C** | **G** | **T** | **C** | **C** | **C** | **T** | **T** | **A** | **G** | **C** | **A** | **C** | **C** | **T** | **T** | **T** | **A** | **T** | **T** | **G** | **T** | **T** | **A** | **T** | **C** | **T** | **T** | **A** | **A** | **T** | **T** | **A** | **C** | **A** | **T** | **T** | **G** | **T** | **T** | **T** | **T** | **C** | **A** | **T** | **A** | **T** | **T** | **T** | **T** | **C** | **A** | **T** | **T** | **C** | **G** | **A** | **T** | **T** | **A** | **A** | **A** | **C** | **A** | **A** | **A** | **A** | **C** | **C** | **G** | **G** | **T** | **G** | **G** | **C** | **T** | **A** | **T** | **T** | **A** | **G** | **A** | **G** | **C** | **T** | **T** | **C** | **T** | **T** | **C** | **A** | **G** | **A** | **G** | **A** | **A** | **A** | **A** | **G** | **T** | **C** | **T** | **T** | **T** | **T** | **A** | **T** | **T** | **T** | **A** | **C** | **C** | **A** | **C** | **T** | **A** | **A** | **T** | **T** | **G** | **T** | **G** | **C** | **A** | **C** | **A** | **T** | **T** | **G** | **T** | **T** | **C** | **T** | **C** | **A** | **T** | **A** | **T** | **C** | **C** | **T** | **T** | **A** | **C** | **T** | **A** | **A** | **T** | **A** | **G** | **G** | **C** | **T** | **T** | **A** | **T** | **T** | **G** | **A** | **C** | **C** | **T** | **T** | **T** | **T** | **T** | **A** | **C** | **T** | **G** | **A** | **T** | **T** | **T** | **C** | **A** | **T** | **T** | **G** | **G** | **T** | **T** | **C** | **A** | **A** | **A** | **A** | **A** | **G** | **G** | **G** | **C** | **A** | **A** | **T** | **A** | **T** | **G** | **G** | **A** | **C** | **G** | **G** | **A** | **T** | **T** | **G** | **A** | **A** | **G** | **A** | **A** | **A** | **A** | **A** | **C** | **T** | **T** | **C** | **G** | **G** | **T** | **T** | **A** | **T** | **T** | **G** | **G** | **C** | **C** | **A** | **A** | **C** | **G** | **G** | **T** | **A** | **A** | **T** | **T** | **A** | **T** | **G** | **A** | **A** | **A** | **G** | **T** | **C** | **C** | **A** | **G** | **T** | **C** | **T** | **T** | **A** | **A** | **A** | **C** | **A** | **A** | **A** | **C** | **C** | **A** | **A** | **C** | **G** | **A** | **C** | **C** | **A** | **G** | **T** | **G** | **A** | **A** | **A** | **A** | **T** | **C** | **A** | **A** | **G** | **A** | **C** | **C** | **A** | **T** | **T** | **A** | **T** | **C** | **T** | **A** | **A** | **C** | **C** | **G** | **A** | **A** | **G** | **T** | **C** | **G** | **A** | **A** | **C** | **A** | **A** | **G** | **A** | **T** | **A** | **T** | **T** | **T** | **G** | **G** | **T** | **C** | **G** | **A** | **T** | **T** | **A** | **A** | **A** | **A** | **A** | **T** | **A** | **A** | **A** | **T** | **T** | **A** | **T** | **T** | **A** | **G** | **A** | **G** | **A** | **T** | **G** | **T** | **C** | **T** | **A** | **A** | **A** | **G** | **A** | **A** | **T** | **T** | **G** | **C** | **A** | **A** | **T** | **T** | **A** | **T** | **T** | **A** | **A** | **A** | **C** | **A** | **G** | **T** | **C** | **G** | **A** | **C** | **C** | **G** | **C** | **A** | **A** | **T** | **T** | **A** | **A** | **T** | **G** | **G** | **A** | **T** | **G** | **G** | **G** | **C** | **A** | **A** | **A** | **C** | **A** | **A** | **A** | **A** | **G** | **A** | **A** | **G** | **A** | **A** | **A** | **T** | **T** | **T** | **T** | **A** | **G** | **A** | **G** | **A** | **A** | **C** | **G** | **A** | **G** | **C** | **G** | **G** | **C** | **A** | **T** | **C** | **G** | **T** | **T** | **T** | **G** | **G** | **C** | **G** | **C** | **G** | **G** | **G** | **A** | **G** | **T** | **T** | **G** | **C** | **A** | **T** | **G** | **A** | **T** | **T** | **C** | **A** | **G** | **T** | **C** | **A** | **G** | **T** | **C** | **A** | **A** | **C** | **A** | **A** | **C** | **T** | **T** | **T** | **T** | **T** | **G** | **C** | **A** | **G** | **C** | **C** | **A** | **T** | **G** | **A** | **T** | **G** | **A** | **T** | **G** | **T** | **T** | **G** | **T** | **C** | **T** | **G** | **C** | **C** | **T** | **T** | **A** | **A** | **A** | **T** | **G** | **A** | **A** | **C** | **A** | **A** | **G** | **C** | **A** | **C** | **A** | **A** | **C** | **G** | **A** | **A** | **C** | **A** | **G** | **A** | **A** | **A** | **C** | **T** | **C** | **C** | **T** | **G** | **A** | **A** | **C** | **C** | **A** | **T** | **A** | **T** | **C** | **G** | **C** | **A** | **A** | **A** | **C** | **A** | **A** | **C** | **T** | **A** | **G** | **C** | **C** | **A** | **T** | **G** | **G** | **T** | **G** | **G** | **C** | **A** | **G** | **A** | **A** | **A** | **T** | **C** | **A** | **T** | **T** | **A** | **A** | **T** | **G** | **C** | **C** | **T** | **C** | **C** | **C** | **A** | **A** | **T** | **C** | **G** | **G** | **A** | **A** | **A** | **T** | **G** | **C** | **G** | **C** | **G** | **C** | **G** | **T** | **T** | **A** | **C** | **T** | **A** | **T** | **T** | **G** | **C** | **A** | **C** | **T** | **T** | **G** | **C** | **G** | **T** | **C** | **C** | **T** | **A** | **T** | **C** | **A** | **G** | **T** | **C** | **T** | **A** | **G** | **A** | **A** | **G** | **G** | **A** | **A** | **A** | **A** | **A** | **G** | **T** | **T** | **T** | **G** | **C** | **G** | **T** | **A** | **A** | **A** | **G** | **G** | **T** | **A** | **T** | **T** | **G** | **A** | **A** | **C** | **A** | **A** | **T** | **T** | **A** | **C** | **T** | **G** | **A** | **A** | **A** | **G** | **A** | **A** | **C** | **T** | **A** | **C** | **A** | **A** | **A** | **C** | **A** | **A** | **A** | **A** | **A** | **T** | **T** | **A** | **A** | **A** | **A** | **T** | **T** | **G** | **A** | **A** | **T** | **T** | **G** | **A** | **T** | **T** | **T** | **G** | **G** | **G** | **A** | **T** | **G** | **T** | **T** | **G** | **A** | **A** | **G** | **A** | **T** | **G** | **T** | **T** | **C** | **A** | **T** | **T** | **T** | **A** | **A** | **A** | **T** | **A** | **G** | **C** | **A** | **G** | **C** | **A** | **T** | **T** | **G** | **A** | **G** | **G** | **A** | **T** | **C** | **A** | **T** | **C** | **T** | **T** | **T** | **T** | **C** | **C** | **G** | **A** | **A** | **T** | **T** | **G** | **T** | **G** | **C** | **A** | **G** | **G** | **A** | **A** | **T** | **T** | **A** | **C** | **T** | **T** | **T** | **C** | **A** | **A** | **A** | **T** | **A** | **C** | **C** | **T** | **T** | **A** | **A** | **G** | **A** | **C** | **A** | **T** | **G** | **C** | **C** | **A** | **A** | **A** | **G** | **C** | **A** | **A** | **A** | **G** | **G** | **A** | **A** | **T** | **T** | **A** | **G** | **A** | **G** | **G** | **T** | **A** | **T** | **A** | **C** | **T** | **T** | **A** | **C** | **A** | **C** | **C** | **A** | **A** | **G** | **T** | **C** | **G** | **A** | **T** | **A** | **A** | **A** | **A** | **A** | **C** | **G** | **T** | **G** | **T** | **T** | **A** | **T** | **T** | **G** | **C** | **G** | **T** | **A** | **T** | **T** | **G** | **T** | **T** | **G** | **A** | **T** | **G** | **A** | **T** | **G** | **G** | **T** | **G** | **T** | **C** | **G** | **G** | **C** | **T** | **T** | **T** | **G** | **A** | **T** | **A** | **T** | **G** | **A** | **A** | **G** | **G** | **A** | **A** | **C** | **A** | **A** | **A** | **G** | **T** | **A** | **A** | **T** | **A** | **A** | **A** | **G** | **C** | **C** | **G** | **G** | **T** | **A** | **G** | **T** | **T** | **A** | **T** | **G** | **G** | **C** | **T** | **T** | **A** | **A** | **A** | **T** | **A** | **A** | **T** | **A** | **T** | **C** | **C** | **G** | **A** | **G** | **A** | **A** | **C** | **G** | **T** | **G** | **T** | **T** | **G** | **T** | **C** | **G** | **G** | **C** | **A** | **T** | **G** | **G** | **G** | **C** | **G** | **G** | **T** | **A** | **C** | **A** | **G** | **T** | **T** | **A** | **A** | **A** | **A** | **T** | **T** | **A** | **T** | **T** | **A** | **G** | **T** | **T** | **T** | **T** | **A** | **A** | **A** | **G** | **G** | **G** | **C** | **A** | **G** | **G** | **G** | **A** | **A** | **C** | **C** | **A** | **G** | **C** | **G** | **T** | **T** | **G** | **A** | **A** | **A** | **T** | **T** | **A** | **A** | **A** | **G** | **T** | **T** | **C** | **C** | **T** | **G** | **T** | **C** | **A** | **T** | **A** | **A** | **A** | **G** | **G** | **A** | **G** | **G** | **A** | **A** | **A** | **C** | **T** | **G** | **C** | **A** | **A** | **G** | **T** | **G** | **A** | **T** | **C** | **A** | **A** | **A** | **G** | **T** | **A** | **A** | **T** | **G** | **T** | **T** | **A** | **G** | **T** | **G** | **G** | **A** | **-** | **-** | **-** |
| **N59409F** | **A** | **T** | **G** | **A** | **C** | **C** | **G** | **A** | **T** | **C** | **G** | **G** | **A** | **T** | **T** | **T** | **C** | **A** | **A** | **G** | **A** | **C** | **G** | **C** | **A** | **T** | **G** | **A** | **T** | **T** | **T** | **C** | **A** | **T** | **T** | **A** | **T** | **A** | **T** | **G** | **C** | **G** | **T** | **C** | **C** | **C** | **T** | **T** | **A** | **G** | **C** | **A** | **C** | **C** | **T** | **T** | **T** | **A** | **T** | **T** | **G** | **T** | **T** | **A** | **T** | **C** | **T** | **T** | **A** | **A** | **T** | **T** | **A** | **C** | **A** | **T** | **T** | **G** | **T** | **T** | **T** | **T** | **C** | **A** | **T** | **A** | **T** | **T** | **T** | **T** | **C** | **A** | **T** | **T** | **C** | **G** | **A** | **T** | **T** | **A** | **A** | **A** | **C** | **A** | **A** | **A** | **A** | **C** | **C** | **G** | **G** | **T** | **G** | **G** | **T** | **T** | **A** | **T** | **T** | **A** | **G** | **A** | **G** | **C** | **T** | **T** | **C** | **T** | **T** | **C** | **A** | **G** | **A** | **G** | **A** | **A** | **A** | **A** | **G** | **T** | **C** | **T** | **T** | **T** | **T** | **A** | **T** | **T** | **T** | **A** | **C** | **C** | **A** | **C** | **T** | **A** | **A** | **T** | **T** | **G** | **T** | **G** | **C** | **A** | **C** | **A** | **T** | **T** | **G** | **T** | **T** | **C** | **T** | **C** | **A** | **T** | **A** | **T** | **C** | **C** | **T** | **T** | **A** | **C** | **T** | **A** | **A** | **T** | **A** | **G** | **G** | **C** | **T** | **T** | **A** | **T** | **T** | **G** | **A** | **C** | **C** | **T** | **T** | **T** | **T** | **T** | **A** | **C** | **T** | **G** | **A** | **T** | **T** | **T** | **C** | **A** | **T** | **T** | **G** | **G** | **T** | **T** | **C** | **A** | **A** | **A** | **A** | **A** | **G** | **G** | **G** | **C** | **A** | **A** | **T** | **A** | **T** | **G** | **G** | **A** | **C** | **G** | **G** | **A** | **T** | **T** | **G** | **A** | **A** | **G** | **A** | **A** | **A** | **A** | **A** | **C** | **T** | **T** | **C** | **G** | **G** | **T** | **T** | **A** | **T** | **T** | **G** | **G** | **C** | **C** | **A** | **A** | **C** | **G** | **G** | **T** | **A** | **A** | **T** | **T** | **A** | **C** | **G** | **A** | **A** | **A** | **G** | **T** | **C** | **C** | **A** | **G** | **T** | **C** | **T** | **T** | **A** | **A** | **A** | **C** | **A** | **A** | **A** | **C** | **C** | **A** | **A** | **C** | **G** | **A** | **C** | **C** | **A** | **G** | **T** | **G** | **A** | **A** | **A** | **A** | **T** | **C** | **A** | **A** | **G** | **A** | **C** | **C** | **A** | **T** | **T** | **A** | **T** | **C** | **T** | **A** | **A** | **C** | **C** | **G** | **A** | **A** | **G** | **T** | **C** | **G** | **A** | **A** | **C** | **A** | **A** | **G** | **A** | **T** | **A** | **T** | **T** | **T** | **G** | **G** | **T** | **C** | **G** | **A** | **T** | **T** | **A** | **A** | **A** | **A** | **A** | **T** | **A** | **A** | **A** | **T** | **T** | **A** | **T** | **T** | **A** | **G** | **A** | **G** | **A** | **T** | **G** | **T** | **C** | **T** | **A** | **A** | **A** | **G** | **A** | **A** | **T** | **T** | **G** | **C** | **A** | **A** | **T** | **T** | **A** | **T** | **T** | **A** | **A** | **A** | **C** | **A** | **G** | **T** | **C** | **G** | **A** | **C** | **C** | **G** | **C** | **A** | **A** | **T** | **T** | **A** | **A** | **T** | **G** | **G** | **A** | **T** | **G** | **G** | **G** | **C** | **A** | **A** | **A** | **C** | **A** | **A** | **A** | **A** | **G** | **A** | **A** | **G** | **A** | **A** | **A** | **T** | **T** | **T** | **T** | **A** | **G** | **A** | **G** | **A** | **A** | **C** | **G** | **A** | **G** | **C** | **G** | **G** | **C** | **A** | **T** | **C** | **G** | **T** | **T** | **T** | **G** | **G** | **C** | **G** | **C** | **G** | **G** | **G** | **A** | **G** | **T** | **T** | **G** | **C** | **A** | **T** | **G** | **A** | **T** | **T** | **C** | **A** | **G** | **T** | **C** | **A** | **G** | **T** | **C** | **A** | **A** | **C** | **A** | **A** | **C** | **T** | **T** | **T** | **T** | **T** | **G** | **C** | **A** | **G** | **C** | **C** | **A** | **T** | **G** | **A** | **T** | **G** | **A** | **T** | **G** | **T** | **T** | **G** | **T** | **C** | **T** | **G** | **C** | **C** | **T** | **T** | **A** | **A** | **A** | **T** | **G** | **A** | **A** | **C** | **A** | **A** | **G** | **C** | **A** | **C** | **A** | **A** | **C** | **G** | **A** | **A** | **C** | **A** | **G** | **A** | **A** | **A** | **C** | **C** | **C** | **C** | **G** | **G** | **A** | **A** | **C** | **C** | **A** | **T** | **A** | **T** | **C** | **G** | **T** | **A** | **A** | **A** | **C** | **A** | **A** | **C** | **T** | **A** | **G** | **C** | **C** | **A** | **T** | **G** | **G** | **T** | **G** | **G** | **C** | **A** | **G** | **A** | **A** | **A** | **T** | **C** | **A** | **T** | **T** | **A** | **A** | **T** | **G** | **C** | **C** | **T** | **C** | **C** | **C** | **A** | **A** | **T** | **C** | **G** | **G** | **A** | **A** | **A** | **T** | **G** | **C** | **G** | **C** | **G** | **C** | **G** | **C** | **T** | **A** | **C** | **T** | **A** | **T** | **T** | **G** | **C** | **A** | **C** | **T** | **T** | **G** | **C** | **G** | **T** | **C** | **C** | **T** | **A** | **T** | **C** | **A** | **G** | **T** | **C** | **T** | **A** | **G** | **A** | **A** | **G** | **G** | **A** | **A** | **A** | **A** | **A** | **G** | **T** | **T** | **T** | **G** | **C** | **G** | **T** | **A** | **A** | **A** | **G** | **G** | **T** | **A** | **T** | **T** | **G** | **A** | **A** | **C** | **A** | **A** | **T** | **T** | **A** | **C** | **T** | **G** | **A** | **A** | **A** | **G** | **A** | **A** | **C** | **T** | **A** | **C** | **A** | **A** | **A** | **C** | **A** | **A** | **A** | **A** | **A** | **T** | **T** | **A** | **A** | **A** | **A** | **T** | **T** | **G** | **A** | **A** | **T** | **T** | **G** | **A** | **T** | **T** | **T** | **G** | **G** | **G** | **A** | **T** | **G** | **T** | **T** | **G** | **A** | **A** | **G** | **A** | **T** | **G** | **T** | **T** | **C** | **A** | **T** | **T** | **T** | **A** | **A** | **A** | **T** | **A** | **G** | **C** | **A** | **G** | **C** | **A** | **T** | **T** | **G** | **A** | **G** | **G** | **A** | **T** | **C** | **A** | **T** | **C** | **T** | **T** | **T** | **T** | **C** | **C** | **G** | **A** | **A** | **T** | **T** | **G** | **T** | **G** | **C** | **A** | **G** | **G** | **A** | **G** | **T** | **T** | **A** | **C** | **T** | **T** | **T** | **C** | **A** | **A** | **A** | **T** | **A** | **C** | **C** | **T** | **T** | **A** | **A** | **G** | **A** | **C** | **A** | **T** | **G** | **C** | **C** | **A** | **A** | **A** | **G** | **C** | **A** | **A** | **A** | **G** | **G** | **A** | **A** | **T** | **T** | **A** | **G** | **A** | **G** | **G** | **T** | **A** | **T** | **A** | **C** | **T** | **T** | **A** | **C** | **A** | **C** | **C** | **A** | **A** | **G** | **T** | **C** | **G** | **A** | **T** | **A** | **A** | **A** | **A** | **A** | **C** | **G** | **T** | **G** | **T** | **T** | **A** | **T** | **T** | **G** | **C** | **G** | **T** | **A** | **T** | **T** | **G** | **T** | **T** | **G** | **A** | **T** | **G** | **A** | **T** | **G** | **G** | **T** | **G** | **T** | **C** | **G** | **G** | **C** | **T** | **T** | **T** | **G** | **A** | **T** | **A** | **T** | **G** | **A** | **A** | **G** | **G** | **A** | **A** | **C** | **A** | **A** | **A** | **G** | **T** | **A** | **A** | **T** | **A** | **A** | **A** | **G** | **C** | **C** | **G** | **G** | **T** | **A** | **G** | **T** | **T** | **A** | **T** | **G** | **G** | **C** | **T** | **T** | **A** | **A** | **A** | **T** | **A** | **A** | **T** | **A** | **T** | **T** | **C** | **G** | **A** | **G** | **A** | **A** | **C** | **G** | **T** | **G** | **T** | **T** | **G** | **T** | **C** | **G** | **G** | **C** | **A** | **T** | **G** | **G** | **G** | **C** | **G** | **G** | **T** | **A** | **C** | **A** | **G** | **T** | **T** | **A** | **A** | **A** | **A** | **T** | **T** | **A** | **T** | **T** | **A** | **G** | **T** | **T** | **T** | **T** | **A** | **A** | **A** | **G** | **G** | **G** | **C** | **A** | **G** | **G** | **G** | **A** | **A** | **C** | **C** | **A** | **G** | **C** | **G** | **T** | **T** | **G** | **A** | **A** | **A** | **T** | **T** | **A** | **A** | **A** | **G** | **T** | **T** | **C** | **C** | **T** | **G** | **T** | **C** | **A** | **T** | **A** | **A** | **A** | **G** | **G** | **A** | **G** | **G** | **A** | **A** | **A** | **C** | **T** | **G** | **C** | **A** | **A** | **G** | **T** | **G** | **A** | **T** | **C** | **A** | **A** | **A** | **G** | **T** | **A** | **A** | **T** | **G** | **T** | **T** | **A** | **G** | **T** | **G** | **G** | **A** | **-** | **-** | **-** |
| **N55725** | **A** | **T** | **G** | **A** | **C** | **C** | **G** | **A** | **T** | **C** | **G** | **G** | **A** | **T** | **T** | **T** | **C** | **A** | **A** | **G** | **A** | **C** | **G** | **C** | **A** | **T** | **G** | **A** | **T** | **T** | **T** | **C** | **A** | **T** | **T** | **A** | **T** | **A** | **T** | **G** | **C** | **G** | **T** | **C** | **C** | **C** | **T** | **T** | **A** | **G** | **C** | **A** | **C** | **C** | **T** | **T** | **T** | **A** | **T** | **T** | **G** | **T** | **T** | **A** | **T** | **C** | **T** | **T** | **A** | **A** | **T** | **T** | **A** | **C** | **A** | **T** | **T** | **G** | **T** | **T** | **T** | **T** | **C** | **A** | **T** | **A** | **T** | **T** | **T** | **T** | **C** | **A** | **T** | **T** | **C** | **G** | **A** | **T** | **T** | **A** | **A** | **A** | **C** | **A** | **A** | **A** | **A** | **C** | **C** | **G** | **G** | **T** | **G** | **G** | **T** | **T** | **A** | **T** | **T** | **A** | **G** | **A** | **G** | **C** | **T** | **C** | **C** | **T** | **T** | **C** | **A** | **G** | **A** | **G** | **A** | **A** | **A** | **A** | **G** | **T** | **C** | **T** | **T** | **T** | **T** | **A** | **T** | **C** | **T** | **A** | **C** | **C** | **A** | **C** | **T** | **A** | **A** | **T** | **T** | **G** | **T** | **G** | **C** | **A** | **C** | **A** | **T** | **T** | **G** | **T** | **T** | **C** | **T** | **C** | **A** | **T** | **A** | **T** | **C** | **C** | **T** | **T** | **A** | **C** | **T** | **A** | **A** | **T** | **A** | **G** | **G** | **C** | **T** | **T** | **A** | **T** | **T** | **G** | **A** | **C** | **C** | **T** | **T** | **T** | **T** | **T** | **A** | **C** | **T** | **G** | **A** | **T** | **T** | **T** | **C** | **A** | **T** | **T** | **G** | **G** | **T** | **T** | **C** | **A** | **A** | **A** | **A** | **A** | **G** | **G** | **G** | **C** | **A** | **A** | **T** | **A** | **T** | **G** | **G** | **A** | **C** | **G** | **G** | **A** | **T** | **T** | **G** | **A** | **A** | **G** | **A** | **A** | **A** | **A** | **A** | **C** | **T** | **T** | **C** | **G** | **G** | **T** | **T** | **A** | **T** | **T** | **G** | **G** | **C** | **C** | **A** | **A** | **C** | **G** | **G** | **T** | **A** | **A** | **T** | **T** | **A** | **T** | **G** | **A** | **A** | **A** | **G** | **T** | **C** | **C** | **A** | **G** | **T** | **C** | **T** | **T** | **A** | **A** | **A** | **C** | **A** | **A** | **A** | **C** | **C** | **A** | **A** | **C** | **G** | **A** | **C** | **C** | **A** | **G** | **T** | **G** | **A** | **A** | **A** | **A** | **T** | **C** | **A** | **A** | **G** | **A** | **C** | **C** | **A** | **T** | **T** | **A** | **T** | **C** | **T** | **A** | **A** | **C** | **C** | **G** | **A** | **A** | **G** | **T** | **C** | **G** | **A** | **A** | **C** | **A** | **A** | **G** | **A** | **T** | **A** | **T** | **T** | **T** | **G** | **G** | **T** | **C** | **G** | **A** | **T** | **T** | **A** | **A** | **A** | **A** | **A** | **T** | **A** | **A** | **A** | **T** | **T** | **A** | **T** | **T** | **A** | **G** | **A** | **G** | **A** | **T** | **G** | **T** | **C** | **T** | **A** | **A** | **A** | **G** | **A** | **A** | **T** | **T** | **G** | **C** | **A** | **A** | **T** | **T** | **A** | **T** | **T** | **A** | **A** | **A** | **C** | **A** | **G** | **T** | **C** | **G** | **A** | **C** | **C** | **G** | **C** | **A** | **A** | **T** | **T** | **A** | **A** | **T** | **G** | **G** | **A** | **T** | **G** | **G** | **G** | **C** | **A** | **A** | **A** | **C** | **A** | **A** | **A** | **A** | **G** | **A** | **A** | **G** | **A** | **A** | **A** | **T** | **T** | **T** | **T** | **A** | **G** | **A** | **G** | **A** | **A** | **C** | **G** | **A** | **G** | **C** | **G** | **G** | **C** | **A** | **T** | **C** | **G** | **T** | **T** | **T** | **G** | **G** | **C** | **G** | **C** | **G** | **G** | **G** | **A** | **G** | **T** | **T** | **G** | **C** | **A** | **T** | **G** | **A** | **T** | **T** | **C** | **A** | **G** | **T** | **C** | **A** | **G** | **T** | **C** | **A** | **A** | **C** | **A** | **A** | **C** | **T** | **T** | **T** | **T** | **T** | **G** | **C** | **A** | **G** | **C** | **C** | **A** | **T** | **G** | **A** | **T** | **G** | **A** | **T** | **G** | **T** | **T** | **G** | **T** | **C** | **T** | **G** | **C** | **C** | **T** | **T** | **A** | **A** | **A** | **T** | **G** | **A** | **A** | **C** | **A** | **A** | **G** | **C** | **A** | **C** | **A** | **A** | **C** | **G** | **A** | **A** | **C** | **A** | **G** | **A** | **A** | **A** | **C** | **C** | **C** | **C** | **G** | **G** | **A** | **A** | **C** | **C** | **A** | **T** | **A** | **T** | **C** | **G** | **T** | **A** | **A** | **A** | **C** | **A** | **A** | **C** | **T** | **A** | **G** | **C** | **C** | **A** | **T** | **G** | **G** | **T** | **G** | **G** | **C** | **A** | **G** | **A** | **A** | **A** | **T** | **C** | **A** | **T** | **T** | **A** | **A** | **T** | **G** | **C** | **C** | **T** | **C** | **C** | **C** | **A** | **A** | **T** | **C** | **G** | **G** | **A** | **A** | **A** | **T** | **G** | **C** | **G** | **C** | **G** | **C** | **G** | **T** | **T** | **A** | **C** | **T** | **A** | **T** | **T** | **G** | **C** | **A** | **C** | **T** | **T** | **G** | **C** | **G** | **T** | **C** | **C** | **T** | **A** | **T** | **C** | **A** | **G** | **T** | **C** | **T** | **A** | **G** | **A** | **A** | **G** | **G** | **A** | **A** | **A** | **A** | **A** | **G** | **T** | **T** | **T** | **G** | **C** | **G** | **T** | **A** | **A** | **A** | **G** | **G** | **T** | **A** | **T** | **T** | **G** | **A** | **A** | **C** | **A** | **A** | **T** | **T** | **A** | **C** | **T** | **G** | **A** | **A** | **A** | **G** | **A** | **A** | **C** | **T** | **A** | **C** | **A** | **A** | **A** | **C** | **A** | **A** | **A** | **A** | **A** | **T** | **T** | **A** | **A** | **A** | **A** | **T** | **T** | **G** | **A** | **A** | **T** | **T** | **G** | **A** | **T** | **T** | **T** | **G** | **G** | **G** | **A** | **T** | **G** | **T** | **T** | **G** | **A** | **A** | **G** | **A** | **T** | **G** | **T** | **T** | **C** | **A** | **T** | **T** | **T** | **A** | **A** | **A** | **T** | **A** | **G** | **C** | **A** | **G** | **C** | **A** | **T** | **T** | **G** | **A** | **G** | **G** | **A** | **T** | **C** | **A** | **T** | **C** | **T** | **T** | **T** | **T** | **C** | **C** | **G** | **A** | **A** | **T** | **T** | **G** | **T** | **G** | **C** | **A** | **G** | **G** | **A** | **A** | **T** | **T** | **A** | **C** | **T** | **T** | **T** | **C** | **A** | **A** | **A** | **T** | **A** | **C** | **C** | **T** | **T** | **A** | **A** | **G** | **A** | **C** | **A** | **T** | **G** | **C** | **C** | **A** | **A** | **A** | **G** | **C** | **A** | **A** | **A** | **G** | **G** | **A** | **A** | **T** | **T** | **A** | **G** | **A** | **G** | **G** | **T** | **A** | **T** | **A** | **C** | **T** | **T** | **A** | **C** | **A** | **C** | **C** | **A** | **A** | **G** | **T** | **C** | **G** | **A** | **T** | **A** | **A** | **A** | **A** | **A** | **C** | **G** | **T** | **G** | **T** | **T** | **A** | **T** | **T** | **G** | **C** | **G** | **T** | **A** | **T** | **T** | **G** | **T** | **T** | **G** | **A** | **T** | **G** | **A** | **T** | **G** | **G** | **T** | **G** | **T** | **C** | **G** | **G** | **C** | **T** | **T** | **T** | **G** | **A** | **T** | **A** | **T** | **G** | **A** | **A** | **G** | **G** | **A** | **A** | **C** | **A** | **A** | **A** | **G** | **T** | **A** | **A** | **T** | **A** | **A** | **A** | **G** | **C** | **C** | **G** | **G** | **T** | **A** | **G** | **T** | **T** | **A** | **T** | **G** | **G** | **C** | **T** | **T** | **A** | **A** | **A** | **T** | **A** | **A** | **T** | **A** | **T** | **T** | **C** | **G** | **A** | **G** | **A** | **A** | **C** | **G** | **T** | **G** | **T** | **T** | **G** | **T** | **C** | **G** | **G** | **C** | **A** | **T** | **G** | **G** | **G** | **C** | **G** | **G** | **T** | **A** | **C** | **A** | **G** | **T** | **T** | **A** | **A** | **A** | **A** | **T** | **T** | **A** | **T** | **T** | **A** | **G** | **T** | **T** | **T** | **T** | **A** | **A** | **A** | **G** | **G** | **G** | **C** | **A** | **G** | **G** | **G** | **A** | **A** | **C** | **C** | **A** | **G** | **C** | **G** | **T** | **T** | **G** | **A** | **A** | **A** | **T** | **T** | **A** | **A** | **A** | **G** | **T** | **T** | **C** | **C** | **T** | **G** | **T** | **C** | **A** | **T** | **A** | **A** | **A** | **G** | **G** | **A** | **G** | **G** | **A** | **A** | **A** | **C** | **T** | **G** | **C** | **A** | **A** | **G** | **T** | **G** | **A** | **T** | **C** | **A** | **A** | **A** | **G** | **T** | **A** | **A** | **T** | **G** | **T** | **T** | **A** | **G** | **T** | **G** | **G** | **A** | **-** | **-** | **-** |
| **N53661** | **A** | **T** | **G** | **A** | **C** | **C** | **G** | **A** | **T** | **C** | **G** | **G** | **A** | **T** | **T** | **T** | **C** | **A** | **A** | **G** | **A** | **C** | **G** | **C** | **A** | **T** | **G** | **A** | **T** | **T** | **T** | **C** | **A** | **T** | **T** | **A** | **T** | **A** | **T** | **G** | **C** | **G** | **T** | **C** | **C** | **C** | **T** | **T** | **A** | **G** | **C** | **A** | **C** | **C** | **T** | **T** | **T** | **A** | **T** | **T** | **G** | **T** | **T** | **A** | **T** | **C** | **T** | **T** | **A** | **A** | **T** | **T** | **A** | **C** | **A** | **T** | **T** | **G** | **T** | **T** | **T** | **T** | **C** | **A** | **T** | **A** | **T** | **T** | **T** | **T** | **C** | **A** | **T** | **T** | **C** | **G** | **A** | **T** | **T** | **A** | **A** | **A** | **C** | **A** | **A** | **A** | **A** | **C** | **C** | **G** | **G** | **T** | **G** | **G** | **T** | **T** | **A** | **T** | **T** | **A** | **G** | **A** | **G** | **C** | **T** | **T** | **C** | **T** | **T** | **C** | **A** | **G** | **A** | **G** | **A** | **A** | **A** | **A** | **G** | **T** | **C** | **T** | **T** | **T** | **T** | **A** | **T** | **T** | **T** | **A** | **C** | **C** | **A** | **C** | **T** | **A** | **A** | **T** | **T** | **G** | **T** | **G** | **C** | **A** | **C** | **A** | **T** | **T** | **G** | **T** | **T** | **C** | **T** | **C** | **A** | **T** | **A** | **T** | **C** | **C** | **T** | **T** | **A** | **C** | **T** | **A** | **A** | **T** | **A** | **G** | **G** | **C** | **T** | **T** | **A** | **T** | **T** | **G** | **A** | **C** | **C** | **T** | **T** | **T** | **T** | **T** | **A** | **C** | **T** | **G** | **A** | **T** | **T** | **T** | **C** | **A** | **T** | **T** | **G** | **G** | **T** | **T** | **C** | **A** | **A** | **A** | **A** | **A** | **G** | **G** | **G** | **C** | **A** | **A** | **T** | **A** | **T** | **G** | **G** | **A** | **C** | **G** | **G** | **A** | **T** | **T** | **G** | **A** | **A** | **G** | **A** | **A** | **A** | **A** | **A** | **C** | **T** | **T** | **C** | **G** | **G** | **T** | **T** | **A** | **T** | **T** | **G** | **G** | **C** | **C** | **A** | **A** | **C** | **G** | **G** | **T** | **A** | **A** | **T** | **T** | **A** | **T** | **G** | **A** | **A** | **A** | **G** | **T** | **C** | **C** | **A** | **G** | **T** | **C** | **T** | **T** | **A** | **A** | **A** | **C** | **A** | **A** | **A** | **C** | **C** | **A** | **A** | **C** | **G** | **A** | **C** | **C** | **A** | **G** | **T** | **G** | **A** | **A** | **A** | **A** | **T** | **C** | **A** | **A** | **G** | **A** | **C** | **C** | **A** | **T** | **T** | **A** | **T** | **C** | **T** | **A** | **A** | **C** | **C** | **G** | **A** | **A** | **G** | **T** | **C** | **G** | **A** | **A** | **C** | **A** | **A** | **G** | **A** | **T** | **A** | **T** | **T** | **T** | **G** | **G** | **T** | **C** | **G** | **A** | **T** | **T** | **A** | **A** | **A** | **A** | **A** | **T** | **A** | **A** | **A** | **T** | **T** | **A** | **T** | **T** | **A** | **G** | **A** | **G** | **A** | **T** | **G** | **T** | **C** | **T** | **A** | **A** | **A** | **G** | **A** | **A** | **T** | **T** | **G** | **C** | **A** | **A** | **T** | **T** | **A** | **T** | **T** | **A** | **A** | **A** | **C** | **A** | **G** | **T** | **C** | **G** | **A** | **C** | **C** | **G** | **C** | **A** | **A** | **T** | **T** | **A** | **A** | **T** | **G** | **G** | **A** | **T** | **G** | **G** | **G** | **C** | **A** | **A** | **A** | **C** | **A** | **A** | **A** | **A** | **G** | **A** | **A** | **G** | **A** | **A** | **A** | **T** | **T** | **T** | **T** | **A** | **G** | **A** | **G** | **A** | **A** | **C** | **G** | **A** | **G** | **C** | **G** | **G** | **C** | **A** | **T** | **C** | **G** | **T** | **T** | **T** | **G** | **G** | **C** | **G** | **C** | **G** | **G** | **G** | **A** | **G** | **T** | **T** | **G** | **C** | **A** | **T** | **G** | **A** | **T** | **T** | **C** | **A** | **G** | **T** | **T** | **A** | **G** | **T** | **C** | **A** | **A** | **C** | **A** | **A** | **C** | **T** | **T** | **T** | **T** | **T** | **G** | **C** | **A** | **G** | **C** | **C** | **A** | **T** | **G** | **A** | **T** | **G** | **A** | **T** | **G** | **T** | **T** | **G** | **T** | **C** | **T** | **G** | **C** | **C** | **T** | **T** | **A** | **A** | **A** | **T** | **G** | **A** | **A** | **C** | **A** | **A** | **G** | **C** | **A** | **C** | **A** | **A** | **C** | **G** | **A** | **A** | **C** | **A** | **G** | **A** | **A** | **A** | **C** | **C** | **C** | **C** | **G** | **G** | **A** | **A** | **C** | **C** | **A** | **T** | **A** | **T** | **C** | **G** | **T** | **A** | **A** | **A** | **C** | **A** | **A** | **C** | **T** | **A** | **G** | **C** | **C** | **A** | **T** | **G** | **G** | **T** | **G** | **G** | **C** | **A** | **G** | **A** | **A** | **A** | **T** | **C** | **A** | **T** | **T** | **A** | **A** | **T** | **G** | **C** | **C** | **T** | **C** | **C** | **C** | **A** | **A** | **T** | **C** | **G** | **G** | **A** | **A** | **A** | **T** | **G** | **C** | **G** | **C** | **G** | **C** | **G** | **T** | **T** | **A** | **C** | **T** | **A** | **T** | **T** | **G** | **C** | **A** | **C** | **T** | **T** | **G** | **C** | **G** | **T** | **C** | **C** | **T** | **A** | **T** | **C** | **A** | **G** | **T** | **C** | **T** | **A** | **G** | **A** | **A** | **G** | **G** | **A** | **A** | **A** | **A** | **A** | **G** | **T** | **T** | **T** | **G** | **C** | **G** | **T** | **A** | **A** | **A** | **G** | **G** | **T** | **A** | **T** | **T** | **G** | **A** | **A** | **C** | **A** | **A** | **T** | **T** | **A** | **C** | **T** | **G** | **A** | **A** | **A** | **G** | **A** | **A** | **C** | **T** | **A** | **C** | **A** | **A** | **A** | **C** | **A** | **A** | **A** | **A** | **A** | **T** | **T** | **A** | **A** | **A** | **A** | **T** | **T** | **G** | **A** | **A** | **T** | **T** | **G** | **A** | **T** | **T** | **T** | **G** | **G** | **G** | **A** | **T** | **G** | **T** | **T** | **G** | **A** | **A** | **G** | **A** | **T** | **G** | **T** | **T** | **C** | **A** | **T** | **T** | **T** | **A** | **A** | **A** | **T** | **A** | **G** | **C** | **A** | **G** | **C** | **A** | **T** | **T** | **G** | **A** | **G** | **G** | **A** | **T** | **C** | **A** | **T** | **C** | **T** | **T** | **T** | **T** | **C** | **C** | **G** | **A** | **A** | **T** | **T** | **G** | **T** | **G** | **C** | **A** | **G** | **G** | **A** | **G** | **T** | **T** | **A** | **C** | **T** | **T** | **T** | **C** | **A** | **A** | **A** | **T** | **A** | **C** | **C** | **T** | **T** | **A** | **A** | **G** | **A** | **C** | **A** | **T** | **G** | **C** | **C** | **A** | **A** | **A** | **G** | **C** | **A** | **A** | **A** | **G** | **G** | **A** | **A** | **T** | **T** | **A** | **G** | **A** | **G** | **G** | **T** | **A** | **T** | **A** | **C** | **T** | **T** | **A** | **C** | **A** | **C** | **C** | **A** | **A** | **G** | **T** | **C** | **G** | **A** | **T** | **A** | **A** | **A** | **A** | **A** | **C** | **G** | **T** | **G** | **T** | **T** | **A** | **T** | **T** | **G** | **C** | **G** | **T** | **A** | **T** | **T** | **G** | **T** | **T** | **G** | **A** | **T** | **G** | **A** | **C** | **G** | **G** | **T** | **G** | **T** | **C** | **G** | **G** | **C** | **T** | **T** | **T** | **G** | **A** | **T** | **A** | **T** | **G** | **A** | **A** | **G** | **G** | **A** | **A** | **C** | **A** | **A** | **A** | **G** | **T** | **A** | **A** | **T** | **A** | **A** | **A** | **G** | **C** | **C** | **G** | **G** | **T** | **A** | **G** | **T** | **T** | **A** | **T** | **G** | **G** | **C** | **T** | **T** | **A** | **A** | **A** | **T** | **A** | **A** | **T** | **A** | **T** | **C** | **C** | **G** | **A** | **G** | **A** | **A** | **C** | **G** | **T** | **G** | **T** | **T** | **G** | **T** | **C** | **G** | **G** | **C** | **A** | **T** | **G** | **G** | **G** | **C** | **G** | **G** | **T** | **A** | **C** | **A** | **G** | **T** | **T** | **A** | **A** | **A** | **A** | **T** | **T** | **A** | **T** | **T** | **A** | **G** | **T** | **T** | **T** | **T** | **A** | **A** | **A** | **G** | **G** | **G** | **C** | **A** | **G** | **G** | **G** | **A** | **A** | **C** | **C** | **A** | **G** | **C** | **G** | **T** | **T** | **G** | **A** | **A** | **A** | **T** | **T** | **A** | **A** | **A** | **G** | **T** | **T** | **C** | **C** | **T** | **G** | **T** | **C** | **A** | **T** | **A** | **A** | **A** | **G** | **G** | **A** | **G** | **G** | **A** | **A** | **A** | **C** | **T** | **G** | **C** | **A** | **A** | **G** | **T** | **G** | **A** | **T** | **C** | **A** | **A** | **A** | **G** | **T** | **A** | **A** | **T** | **G** | **T** | **T** | **A** | **G** | **T** | **G** | **G** | **A** | **-** | **-** | **-** |
| **N54574** | **A** | **T** | **G** | **A** | **C** | **C** | **G** | **A** | **T** | **C** | **G** | **G** | **A** | **T** | **T** | **T** | **C** | **A** | **A** | **G** | **A** | **C** | **G** | **C** | **A** | **T** | **G** | **A** | **T** | **T** | **T** | **C** | **A** | **T** | **T** | **A** | **T** | **A** | **T** | **G** | **C** | **G** | **T** | **C** | **C** | **C** | **T** | **T** | **A** | **G** | **C** | **A** | **C** | **C** | **T** | **T** | **T** | **A** | **T** | **T** | **G** | **T** | **T** | **A** | **T** | **C** | **T** | **T** | **A** | **A** | **T** | **T** | **A** | **C** | **A** | **T** | **T** | **G** | **T** | **T** | **T** | **T** | **C** | **A** | **T** | **A** | **T** | **T** | **T** | **T** | **C** | **A** | **T** | **T** | **C** | **G** | **A** | **T** | **T** | **A** | **A** | **A** | **C** | **A** | **A** | **A** | **A** | **C** | **C** | **G** | **G** | **T** | **G** | **G** | **C** | **T** | **A** | **T** | **T** | **A** | **G** | **A** | **G** | **C** | **T** | **T** | **C** | **T** | **T** | **C** | **A** | **G** | **A** | **G** | **A** | **A** | **A** | **A** | **G** | **T** | **C** | **T** | **T** | **T** | **T** | **A** | **T** | **T** | **T** | **A** | **C** | **C** | **A** | **C** | **T** | **A** | **A** | **T** | **T** | **G** | **T** | **G** | **C** | **A** | **C** | **A** | **T** | **T** | **G** | **T** | **T** | **C** | **T** | **C** | **A** | **T** | **A** | **T** | **C** | **C** | **T** | **T** | **A** | **C** | **T** | **A** | **A** | **T** | **A** | **G** | **G** | **C** | **T** | **T** | **A** | **T** | **T** | **G** | **A** | **C** | **C** | **T** | **T** | **T** | **T** | **T** | **A** | **C** | **T** | **G** | **A** | **T** | **T** | **T** | **C** | **A** | **T** | **T** | **G** | **G** | **T** | **T** | **C** | **A** | **A** | **A** | **A** | **A** | **G** | **G** | **G** | **C** | **A** | **A** | **T** | **A** | **T** | **G** | **G** | **A** | **C** | **G** | **G** | **A** | **T** | **T** | **G** | **A** | **A** | **G** | **A** | **A** | **A** | **A** | **A** | **C** | **T** | **T** | **C** | **G** | **G** | **T** | **T** | **A** | **T** | **T** | **G** | **G** | **C** | **C** | **A** | **A** | **C** | **G** | **G** | **T** | **A** | **A** | **T** | **T** | **A** | **T** | **G** | **A** | **A** | **A** | **G** | **T** | **C** | **C** | **A** | **G** | **T** | **C** | **T** | **T** | **A** | **A** | **A** | **C** | **A** | **A** | **A** | **C** | **C** | **A** | **A** | **C** | **G** | **A** | **C** | **C** | **A** | **G** | **T** | **G** | **A** | **A** | **A** | **A** | **T** | **C** | **A** | **A** | **G** | **A** | **C** | **C** | **A** | **T** | **T** | **A** | **T** | **C** | **T** | **A** | **A** | **C** | **C** | **G** | **A** | **A** | **G** | **T** | **C** | **G** | **A** | **A** | **C** | **A** | **A** | **G** | **A** | **T** | **A** | **T** | **T** | **T** | **G** | **G** | **T** | **C** | **G** | **A** | **T** | **T** | **A** | **A** | **A** | **A** | **A** | **T** | **A** | **A** | **A** | **T** | **T** | **A** | **T** | **T** | **A** | **G** | **A** | **G** | **A** | **T** | **G** | **T** | **C** | **T** | **A** | **A** | **A** | **G** | **A** | **A** | **T** | **T** | **G** | **C** | **A** | **A** | **T** | **T** | **A** | **T** | **T** | **A** | **A** | **A** | **C** | **A** | **G** | **T** | **C** | **G** | **A** | **C** | **C** | **G** | **C** | **A** | **A** | **T** | **T** | **A** | **A** | **T** | **G** | **G** | **A** | **T** | **G** | **G** | **G** | **C** | **A** | **A** | **A** | **C** | **A** | **A** | **A** | **A** | **G** | **A** | **A** | **G** | **A** | **A** | **A** | **T** | **T** | **T** | **T** | **A** | **G** | **A** | **G** | **A** | **A** | **C** | **G** | **A** | **G** | **C** | **G** | **G** | **C** | **A** | **T** | **C** | **G** | **T** | **T** | **T** | **G** | **G** | **C** | **G** | **C** | **G** | **G** | **G** | **A** | **G** | **T** | **T** | **A** | **C** | **A** | **T** | **G** | **A** | **T** | **T** | **C** | **A** | **G** | **T** | **C** | **A** | **G** | **T** | **C** | **A** | **A** | **C** | **A** | **A** | **C** | **T** | **T** | **T** | **T** | **T** | **G** | **C** | **A** | **G** | **C** | **C** | **A** | **T** | **G** | **A** | **T** | **G** | **A** | **T** | **G** | **T** | **T** | **G** | **T** | **C** | **T** | **G** | **C** | **A** | **T** | **T** | **A** | **A** | **A** | **T** | **G** | **A** | **A** | **C** | **A** | **A** | **G** | **C** | **A** | **C** | **A** | **A** | **C** | **G** | **A** | **A** | **C** | **A** | **G** | **A** | **A** | **A** | **C** | **C** | **C** | **C** | **G** | **G** | **A** | **A** | **C** | **C** | **A** | **T** | **A** | **T** | **C** | **G** | **T** | **A** | **A** | **A** | **C** | **A** | **A** | **C** | **T** | **A** | **G** | **C** | **C** | **A** | **T** | **G** | **G** | **T** | **G** | **G** | **C** | **A** | **G** | **A** | **A** | **A** | **T** | **C** | **A** | **T** | **T** | **A** | **A** | **T** | **G** | **C** | **C** | **T** | **C** | **C** | **C** | **A** | **A** | **T** | **C** | **G** | **G** | **A** | **A** | **A** | **T** | **G** | **C** | **G** | **C** | **G** | **C** | **G** | **C** | **T** | **A** | **C** | **T** | **A** | **T** | **T** | **G** | **C** | **A** | **C** | **T** | **T** | **G** | **C** | **G** | **T** | **C** | **C** | **T** | **A** | **T** | **C** | **A** | **G** | **T** | **C** | **T** | **A** | **G** | **A** | **A** | **G** | **G** | **A** | **A** | **A** | **A** | **A** | **G** | **T** | **T** | **T** | **G** | **C** | **G** | **T** | **A** | **A** | **A** | **G** | **G** | **T** | **A** | **T** | **T** | **G** | **A** | **A** | **C** | **A** | **A** | **T** | **T** | **A** | **C** | **T** | **G** | **A** | **A** | **A** | **G** | **A** | **A** | **C** | **T** | **A** | **C** | **A** | **A** | **A** | **C** | **A** | **A** | **A** | **A** | **A** | **T** | **T** | **A** | **A** | **A** | **A** | **T** | **T** | **G** | **A** | **A** | **T** | **T** | **G** | **A** | **T** | **T** | **T** | **G** | **G** | **G** | **A** | **T** | **G** | **T** | **T** | **G** | **A** | **A** | **G** | **A** | **T** | **G** | **T** | **T** | **C** | **A** | **T** | **T** | **T** | **A** | **A** | **A** | **T** | **A** | **G** | **C** | **A** | **G** | **C** | **A** | **T** | **T** | **G** | **A** | **G** | **G** | **A** | **T** | **C** | **A** | **T** | **C** | **T** | **T** | **T** | **T** | **C** | **C** | **G** | **A** | **A** | **T** | **T** | **G** | **T** | **G** | **C** | **A** | **G** | **G** | **A** | **G** | **T** | **T** | **A** | **C** | **T** | **T** | **T** | **C** | **A** | **A** | **A** | **T** | **A** | **C** | **C** | **T** | **T** | **A** | **A** | **G** | **A** | **C** | **A** | **T** | **G** | **C** | **C** | **A** | **A** | **A** | **G** | **C** | **A** | **A** | **A** | **G** | **G** | **A** | **A** | **T** | **T** | **A** | **G** | **A** | **G** | **G** | **T** | **A** | **T** | **A** | **C** | **T** | **T** | **A** | **C** | **A** | **C** | **C** | **A** | **A** | **G** | **T** | **C** | **G** | **A** | **T** | **A** | **A** | **A** | **A** | **A** | **C** | **G** | **T** | **G** | **T** | **T** | **A** | **T** | **T** | **G** | **C** | **G** | **T** | **A** | **T** | **T** | **G** | **T** | **T** | **G** | **A** | **T** | **G** | **A** | **T** | **G** | **G** | **T** | **G** | **T** | **C** | **G** | **G** | **C** | **T** | **T** | **T** | **G** | **A** | **T** | **A** | **T** | **G** | **A** | **A** | **G** | **G** | **A** | **A** | **C** | **A** | **A** | **A** | **G** | **T** | **A** | **A** | **T** | **A** | **A** | **A** | **G** | **C** | **C** | **G** | **G** | **T** | **A** | **G** | **T** | **T** | **A** | **T** | **G** | **G** | **C** | **T** | **T** | **A** | **A** | **A** | **T** | **A** | **A** | **T** | **A** | **T** | **T** | **C** | **G** | **A** | **G** | **A** | **A** | **C** | **G** | **T** | **G** | **T** | **T** | **G** | **T** | **C** | **G** | **G** | **C** | **A** | **T** | **G** | **G** | **G** | **C** | **G** | **G** | **T** | **A** | **C** | **A** | **G** | **T** | **T** | **A** | **A** | **A** | **A** | **T** | **T** | **A** | **T** | **T** | **A** | **G** | **T** | **T** | **T** | **T** | **A** | **A** | **A** | **G** | **G** | **G** | **C** | **A** | **G** | **G** | **G** | **A** | **A** | **C** | **C** | **A** | **G** | **C** | **G** | **T** | **T** | **G** | **A** | **A** | **A** | **T** | **T** | **A** | **A** | **A** | **G** | **T** | **T** | **C** | **C** | **T** | **G** | **T** | **C** | **A** | **T** | **A** | **A** | **A** | **G** | **G** | **A** | **G** | **G** | **A** | **A** | **A** | **C** | **T** | **G** | **C** | **A** | **A** | **G** | **T** | **G** | **A** | **T** | **C** | **A** | **A** | **A** | **G** | **T** | **A** | **A** | **T** | **G** | **T** | **T** | **A** | **G** | **T** | **G** | **G** | **A** | **-** | **-** | **-** |
| **N59491F** | **A** | **T** | **G** | **A** | **C** | **C** | **G** | **A** | **T** | **C** | **G** | **G** | **A** | **T** | **T** | **T** | **C** | **A** | **A** | **G** | **A** | **C** | **G** | **C** | **A** | **T** | **G** | **A** | **T** | **T** | **T** | **C** | **A** | **T** | **T** | **A** | **T** | **A** | **T** | **G** | **C** | **G** | **T** | **C** | **C** | **C** | **T** | **T** | **A** | **G** | **C** | **A** | **C** | **C** | **T** | **T** | **T** | **A** | **T** | **T** | **G** | **T** | **T** | **A** | **T** | **C** | **T** | **T** | **A** | **A** | **T** | **T** | **A** | **C** | **A** | **T** | **T** | **G** | **T** | **T** | **T** | **T** | **C** | **A** | **T** | **A** | **T** | **T** | **T** | **T** | **C** | **A** | **T** | **T** | **C** | **G** | **A** | **T** | **T** | **A** | **A** | **A** | **C** | **A** | **A** | **A** | **A** | **C** | **C** | **G** | **G** | **T** | **G** | **G** | **C** | **T** | **A** | **T** | **T** | **A** | **G** | **A** | **G** | **C** | **T** | **T** | **C** | **T** | **T** | **C** | **A** | **G** | **A** | **G** | **A** | **A** | **A** | **A** | **G** | **T** | **C** | **T** | **T** | **T** | **T** | **A** | **T** | **T** | **T** | **A** | **C** | **C** | **A** | **C** | **T** | **A** | **A** | **T** | **T** | **G** | **T** | **G** | **C** | **A** | **C** | **A** | **T** | **T** | **G** | **T** | **T** | **C** | **T** | **C** | **A** | **T** | **A** | **T** | **C** | **C** | **T** | **T** | **A** | **C** | **T** | **A** | **A** | **T** | **A** | **G** | **G** | **C** | **T** | **T** | **A** | **T** | **T** | **G** | **A** | **C** | **C** | **T** | **T** | **T** | **T** | **T** | **A** | **C** | **T** | **G** | **A** | **T** | **T** | **T** | **C** | **A** | **T** | **T** | **G** | **G** | **T** | **T** | **C** | **A** | **A** | **A** | **A** | **A** | **G** | **G** | **G** | **C** | **A** | **A** | **T** | **A** | **T** | **G** | **G** | **A** | **C** | **G** | **G** | **A** | **T** | **T** | **G** | **A** | **A** | **G** | **A** | **A** | **A** | **A** | **A** | **C** | **T** | **T** | **C** | **G** | **G** | **T** | **T** | **A** | **T** | **T** | **G** | **G** | **C** | **C** | **A** | **A** | **T** | **G** | **G** | **T** | **A** | **A** | **T** | **T** | **A** | **T** | **G** | **A** | **A** | **A** | **G** | **T** | **C** | **C** | **A** | **G** | **T** | **C** | **T** | **T** | **A** | **A** | **A** | **C** | **A** | **A** | **A** | **C** | **C** | **A** | **A** | **C** | **G** | **A** | **C** | **C** | **A** | **G** | **T** | **G** | **A** | **A** | **A** | **A** | **T** | **C** | **A** | **A** | **G** | **A** | **T** | **C** | **A** | **T** | **T** | **A** | **T** | **C** | **T** | **A** | **A** | **C** | **C** | **G** | **A** | **A** | **G** | **T** | **C** | **G** | **A** | **A** | **C** | **A** | **A** | **G** | **A** | **T** | **A** | **T** | **T** | **T** | **G** | **G** | **T** | **C** | **G** | **A** | **T** | **T** | **A** | **A** | **A** | **A** | **A** | **T** | **A** | **A** | **A** | **T** | **T** | **A** | **T** | **T** | **A** | **G** | **A** | **G** | **A** | **T** | **G** | **T** | **C** | **T** | **A** | **A** | **A** | **G** | **A** | **A** | **T** | **T** | **G** | **C** | **A** | **A** | **T** | **T** | **A** | **T** | **T** | **A** | **A** | **A** | **C** | **A** | **G** | **T** | **C** | **G** | **G** | **C** | **C** | **G** | **C** | **A** | **A** | **T** | **T** | **A** | **A** | **T** | **G** | **G** | **A** | **T** | **G** | **G** | **G** | **C** | **A** | **A** | **A** | **C** | **A** | **A** | **A** | **A** | **G** | **A** | **A** | **G** | **A** | **A** | **A** | **T** | **T** | **T** | **T** | **A** | **G** | **A** | **G** | **A** | **A** | **C** | **G** | **A** | **G** | **C** | **G** | **G** | **C** | **A** | **T** | **C** | **G** | **T** | **T** | **T** | **G** | **G** | **C** | **G** | **C** | **G** | **G** | **G** | **A** | **G** | **T** | **T** | **G** | **C** | **A** | **T** | **G** | **A** | **T** | **T** | **C** | **A** | **G** | **T** | **C** | **A** | **G** | **T** | **C** | **A** | **A** | **C** | **A** | **A** | **C** | **T** | **T** | **T** | **T** | **T** | **G** | **C** | **A** | **G** | **C** | **C** | **A** | **T** | **G** | **A** | **T** | **G** | **A** | **T** | **G** | **T** | **T** | **G** | **T** | **C** | **T** | **G** | **C** | **C** | **T** | **T** | **A** | **A** | **A** | **T** | **G** | **A** | **A** | **C** | **A** | **A** | **G** | **C** | **A** | **C** | **A** | **A** | **C** | **G** | **A** | **A** | **C** | **A** | **G** | **A** | **A** | **A** | **C** | **C** | **C** | **C** | **G** | **G** | **A** | **A** | **C** | **C** | **A** | **T** | **A** | **T** | **C** | **G** | **T** | **A** | **A** | **A** | **C** | **A** | **A** | **C** | **T** | **A** | **G** | **C** | **C** | **A** | **T** | **G** | **G** | **T** | **G** | **G** | **C** | **A** | **G** | **A** | **A** | **A** | **T** | **C** | **A** | **T** | **T** | **A** | **A** | **T** | **G** | **C** | **C** | **T** | **C** | **C** | **C** | **A** | **A** | **T** | **C** | **G** | **G** | **A** | **A** | **A** | **T** | **G** | **C** | **G** | **C** | **G** | **C** | **G** | **T** | **T** | **A** | **C** | **T** | **A** | **T** | **T** | **G** | **C** | **A** | **C** | **T** | **T** | **G** | **C** | **G** | **T** | **C** | **C** | **T** | **A** | **T** | **C** | **A** | **G** | **T** | **C** | **T** | **A** | **G** | **A** | **A** | **G** | **G** | **A** | **A** | **A** | **A** | **A** | **G** | **T** | **T** | **T** | **G** | **C** | **G** | **T** | **A** | **A** | **A** | **G** | **G** | **T** | **A** | **T** | **T** | **G** | **A** | **A** | **C** | **A** | **A** | **T** | **T** | **A** | **C** | **T** | **A** | **A** | **A** | **A** | **G** | **A** | **A** | **C** | **T** | **A** | **C** | **A** | **A** | **A** | **C** | **A** | **A** | **A** | **A** | **A** | **T** | **T** | **A** | **A** | **A** | **A** | **T** | **T** | **G** | **A** | **A** | **T** | **T** | **G** | **A** | **T** | **T** | **T** | **G** | **G** | **G** | **A** | **T** | **G** | **T** | **T** | **G** | **A** | **A** | **G** | **A** | **T** | **G** | **T** | **T** | **C** | **A** | **T** | **T** | **T** | **A** | **A** | **A** | **T** | **A** | **G** | **C** | **A** | **G** | **C** | **A** | **T** | **T** | **G** | **A** | **G** | **G** | **A** | **T** | **C** | **A** | **T** | **C** | **T** | **T** | **T** | **T** | **C** | **C** | **G** | **A** | **A** | **T** | **T** | **G** | **T** | **G** | **C** | **A** | **G** | **G** | **A** | **A** | **T** | **T** | **A** | **C** | **T** | **T** | **T** | **C** | **C** | **A** | **A** | **T** | **A** | **C** | **C** | **T** | **T** | **A** | **A** | **G** | **A** | **C** | **A** | **T** | **G** | **C** | **C** | **A** | **A** | **A** | **G** | **C** | **A** | **A** | **A** | **G** | **G** | **A** | **A** | **T** | **T** | **A** | **G** | **A** | **G** | **G** | **T** | **A** | **T** | **A** | **C** | **T** | **T** | **A** | **C** | **A** | **C** | **C** | **A** | **A** | **G** | **T** | **C** | **G** | **A** | **T** | **A** | **A** | **A** | **A** | **A** | **C** | **G** | **T** | **G** | **T** | **T** | **A** | **T** | **T** | **G** | **C** | **G** | **T** | **A** | **T** | **T** | **G** | **T** | **T** | **G** | **A** | **T** | **G** | **A** | **T** | **G** | **G** | **T** | **G** | **T** | **C** | **G** | **G** | **C** | **T** | **T** | **T** | **G** | **A** | **T** | **A** | **T** | **G** | **A** | **A** | **G** | **G** | **A** | **A** | **C** | **A** | **A** | **A** | **G** | **T** | **A** | **A** | **T** | **A** | **A** | **A** | **G** | **C** | **C** | **G** | **G** | **T** | **A** | **G** | **T** | **T** | **A** | **T** | **G** | **G** | **C** | **T** | **T** | **A** | **A** | **A** | **T** | **A** | **A** | **T** | **A** | **T** | **T** | **C** | **G** | **A** | **G** | **A** | **A** | **C** | **G** | **T** | **G** | **T** | **T** | **G** | **T** | **C** | **G** | **G** | **C** | **A** | **T** | **G** | **G** | **G** | **C** | **G** | **G** | **T** | **A** | **C** | **A** | **G** | **T** | **T** | **A** | **A** | **A** | **A** | **T** | **T** | **A** | **T** | **T** | **A** | **G** | **T** | **T** | **T** | **T** | **A** | **A** | **A** | **G** | **G** | **G** | **C** | **A** | **G** | **G** | **G** | **A** | **A** | **C** | **C** | **A** | **G** | **C** | **G** | **T** | **T** | **G** | **A** | **A** | **A** | **T** | **T** | **A** | **A** | **A** | **G** | **T** | **T** | **C** | **C** | **T** | **G** | **T** | **C** | **A** | **T** | **A** | **A** | **A** | **G** | **G** | **A** | **G** | **G** | **A** | **A** | **A** | **C** | **T** | **G** | **C** | **A** | **A** | **G** | **T** | **G** | **A** | **T** | **C** | **A** | **A** | **A** | **G** | **T** | **A** | **A** | **T** | **G** | **T** | **T** | **A** | **G** | **T** | **G** | **G** | **A** | **-** | **-** | **-** |
| **N52729** | **A** | **T** | **G** | **A** | **C** | **C** | **G** | **A** | **T** | **C** | **G** | **G** | **A** | **T** | **T** | **T** | **C** | **A** | **A** | **G** | **A** | **C** | **G** | **C** | **A** | **T** | **G** | **A** | **T** | **T** | **T** | **C** | **A** | **T** | **T** | **A** | **T** | **A** | **T** | **G** | **C** | **G** | **T** | **C** | **C** | **C** | **T** | **T** | **A** | **G** | **C** | **A** | **C** | **C** | **T** | **T** | **T** | **A** | **T** | **T** | **G** | **T** | **T** | **A** | **T** | **C** | **T** | **T** | **A** | **A** | **T** | **T** | **A** | **C** | **A** | **T** | **T** | **G** | **T** | **T** | **T** | **T** | **C** | **A** | **T** | **A** | **T** | **T** | **T** | **T** | **C** | **A** | **T** | **T** | **C** | **G** | **A** | **T** | **T** | **A** | **A** | **A** | **C** | **A** | **A** | **A** | **A** | **C** | **C** | **G** | **G** | **T** | **G** | **G** | **T** | **T** | **A** | **T** | **T** | **A** | **G** | **A** | **G** | **C** | **T** | **T** | **C** | **T** | **T** | **C** | **A** | **G** | **A** | **G** | **A** | **A** | **A** | **A** | **G** | **T** | **C** | **T** | **T** | **T** | **T** | **A** | **T** | **C** | **T** | **A** | **C** | **C** | **A** | **C** | **T** | **A** | **A** | **T** | **T** | **G** | **T** | **G** | **C** | **A** | **C** | **A** | **T** | **T** | **G** | **T** | **T** | **C** | **T** | **C** | **A** | **T** | **A** | **T** | **C** | **C** | **T** | **T** | **A** | **C** | **T** | **A** | **A** | **T** | **A** | **G** | **G** | **C** | **T** | **T** | **A** | **T** | **T** | **G** | **A** | **C** | **C** | **T** | **T** | **T** | **T** | **T** | **A** | **C** | **T** | **G** | **A** | **T** | **T** | **T** | **C** | **A** | **T** | **T** | **G** | **G** | **T** | **T** | **C** | **A** | **A** | **A** | **A** | **A** | **G** | **G** | **G** | **C** | **A** | **A** | **T** | **A** | **T** | **G** | **G** | **A** | **C** | **G** | **G** | **A** | **T** | **T** | **G** | **A** | **A** | **G** | **A** | **A** | **A** | **A** | **A** | **C** | **T** | **T** | **C** | **G** | **G** | **T** | **T** | **A** | **T** | **T** | **G** | **G** | **C** | **C** | **A** | **A** | **C** | **G** | **G** | **T** | **A** | **A** | **T** | **T** | **A** | **T** | **G** | **A** | **A** | **A** | **G** | **T** | **C** | **C** | **A** | **G** | **T** | **C** | **T** | **T** | **A** | **A** | **A** | **C** | **A** | **A** | **A** | **C** | **C** | **A** | **A** | **C** | **G** | **A** | **C** | **C** | **A** | **G** | **T** | **G** | **A** | **A** | **A** | **A** | **T** | **C** | **A** | **A** | **G** | **A** | **T** | **C** | **A** | **T** | **T** | **A** | **T** | **C** | **T** | **A** | **A** | **C** | **C** | **G** | **A** | **A** | **G** | **T** | **C** | **G** | **A** | **A** | **C** | **A** | **A** | **G** | **A** | **T** | **A** | **T** | **T** | **T** | **G** | **G** | **T** | **C** | **G** | **A** | **T** | **T** | **A** | **A** | **A** | **A** | **A** | **T** | **A** | **A** | **A** | **T** | **T** | **A** | **T** | **T** | **A** | **G** | **A** | **G** | **A** | **T** | **G** | **T** | **C** | **T** | **A** | **A** | **A** | **G** | **A** | **A** | **T** | **T** | **G** | **C** | **A** | **A** | **T** | **T** | **A** | **T** | **T** | **A** | **A** | **A** | **C** | **A** | **G** | **T** | **C** | **G** | **A** | **C** | **C** | **G** | **C** | **A** | **A** | **T** | **T** | **A** | **A** | **T** | **G** | **G** | **A** | **T** | **G** | **G** | **G** | **C** | **A** | **A** | **A** | **C** | **A** | **A** | **A** | **A** | **G** | **A** | **A** | **G** | **A** | **A** | **A** | **T** | **T** | **T** | **T** | **A** | **G** | **A** | **G** | **A** | **A** | **C** | **G** | **A** | **G** | **C** | **G** | **G** | **C** | **A** | **T** | **C** | **G** | **T** | **T** | **T** | **G** | **G** | **C** | **G** | **C** | **G** | **G** | **G** | **A** | **G** | **T** | **T** | **G** | **C** | **A** | **T** | **G** | **A** | **T** | **T** | **C** | **A** | **G** | **T** | **C** | **A** | **G** | **T** | **C** | **A** | **A** | **C** | **A** | **A** | **C** | **T** | **T** | **T** | **T** | **T** | **G** | **C** | **A** | **G** | **C** | **C** | **A** | **T** | **G** | **A** | **T** | **G** | **A** | **T** | **G** | **T** | **T** | **G** | **T** | **C** | **T** | **G** | **C** | **C** | **T** | **T** | **A** | **A** | **A** | **T** | **G** | **A** | **A** | **C** | **A** | **A** | **G** | **C** | **A** | **C** | **A** | **A** | **C** | **G** | **A** | **A** | **C** | **A** | **G** | **A** | **A** | **A** | **C** | **C** | **C** | **C** | **G** | **G** | **A** | **A** | **C** | **C** | **A** | **T** | **A** | **T** | **C** | **G** | **T** | **A** | **A** | **A** | **C** | **A** | **A** | **C** | **T** | **A** | **G** | **C** | **C** | **A** | **T** | **G** | **G** | **T** | **G** | **G** | **C** | **A** | **G** | **A** | **A** | **A** | **T** | **C** | **A** | **T** | **T** | **A** | **A** | **T** | **G** | **C** | **C** | **T** | **C** | **C** | **C** | **A** | **A** | **T** | **C** | **G** | **G** | **A** | **A** | **A** | **T** | **G** | **C** | **G** | **C** | **G** | **C** | **G** | **T** | **T** | **A** | **C** | **T** | **A** | **T** | **T** | **G** | **C** | **A** | **C** | **T** | **T** | **G** | **C** | **G** | **T** | **C** | **C** | **T** | **A** | **T** | **C** | **A** | **G** | **T** | **C** | **T** | **A** | **G** | **A** | **A** | **G** | **G** | **A** | **A** | **A** | **A** | **A** | **G** | **T** | **T** | **T** | **G** | **C** | **G** | **T** | **A** | **A** | **A** | **G** | **G** | **T** | **A** | **T** | **T** | **G** | **A** | **A** | **C** | **A** | **A** | **T** | **T** | **A** | **C** | **T** | **G** | **A** | **A** | **A** | **G** | **A** | **A** | **C** | **T** | **A** | **C** | **A** | **A** | **A** | **C** | **A** | **A** | **A** | **A** | **A** | **T** | **T** | **A** | **A** | **A** | **A** | **T** | **T** | **G** | **A** | **A** | **T** | **T** | **G** | **A** | **T** | **T** | **T** | **G** | **G** | **G** | **A** | **T** | **G** | **T** | **T** | **G** | **A** | **A** | **G** | **A** | **T** | **G** | **T** | **T** | **C** | **A** | **T** | **T** | **T** | **A** | **A** | **A** | **T** | **A** | **G** | **C** | **A** | **G** | **C** | **A** | **T** | **T** | **G** | **A** | **G** | **G** | **A** | **T** | **C** | **A** | **T** | **C** | **T** | **T** | **T** | **T** | **C** | **C** | **G** | **A** | **A** | **T** | **T** | **G** | **T** | **G** | **C** | **A** | **G** | **G** | **A** | **G** | **T** | **T** | **A** | **C** | **T** | **T** | **T** | **C** | **A** | **A** | **A** | **T** | **A** | **C** | **C** | **T** | **T** | **A** | **A** | **G** | **A** | **C** | **A** | **T** | **G** | **C** | **C** | **A** | **A** | **A** | **G** | **C** | **A** | **A** | **A** | **G** | **G** | **A** | **A** | **T** | **T** | **A** | **G** | **A** | **G** | **G** | **T** | **A** | **T** | **A** | **C** | **T** | **T** | **A** | **C** | **A** | **C** | **C** | **A** | **A** | **G** | **T** | **C** | **G** | **A** | **T** | **A** | **A** | **A** | **A** | **A** | **C** | **G** | **T** | **G** | **T** | **T** | **A** | **T** | **T** | **G** | **C** | **G** | **T** | **A** | **T** | **T** | **G** | **T** | **T** | **G** | **A** | **T** | **G** | **A** | **C** | **G** | **G** | **T** | **G** | **T** | **C** | **G** | **G** | **C** | **T** | **T** | **T** | **G** | **A** | **T** | **A** | **T** | **G** | **A** | **A** | **G** | **G** | **A** | **A** | **C** | **A** | **A** | **A** | **G** | **T** | **A** | **A** | **T** | **A** | **A** | **A** | **G** | **C** | **C** | **G** | **G** | **T** | **A** | **G** | **T** | **T** | **A** | **T** | **G** | **G** | **C** | **T** | **T** | **A** | **A** | **A** | **T** | **A** | **A** | **T** | **A** | **T** | **C** | **C** | **G** | **A** | **G** | **A** | **A** | **C** | **G** | **T** | **G** | **T** | **T** | **G** | **T** | **C** | **G** | **G** | **C** | **A** | **T** | **G** | **G** | **G** | **C** | **G** | **G** | **T** | **A** | **C** | **A** | **G** | **T** | **T** | **A** | **A** | **A** | **A** | **T** | **T** | **A** | **T** | **T** | **A** | **G** | **T** | **T** | **T** | **T** | **A** | **A** | **A** | **G** | **G** | **G** | **C** | **A** | **G** | **G** | **G** | **A** | **A** | **C** | **C** | **A** | **G** | **C** | **G** | **T** | **T** | **G** | **A** | **A** | **A** | **T** | **T** | **A** | **A** | **A** | **G** | **T** | **T** | **C** | **C** | **T** | **G** | **T** | **C** | **A** | **T** | **A** | **A** | **A** | **G** | **G** | **A** | **G** | **G** | **A** | **A** | **A** | **C** | **T** | **G** | **C** | **A** | **A** | **G** | **T** | **G** | **A** | **T** | **C** | **A** | **A** | **A** | **G** | **T** | **A** | **A** | **T** | **G** | **T** | **T** | **A** | **G** | **T** | **G** | **G** | **A** | **-** | **-** | **-** |
| **N59959F** | **A** | **T** | **G** | **A** | **C** | **C** | **G** | **A** | **T** | **C** | **G** | **G** | **A** | **T** | **T** | **T** | **C** | **A** | **A** | **G** | **A** | **C** | **G** | **C** | **A** | **T** | **G** | **A** | **T** | **T** | **T** | **C** | **A** | **T** | **T** | **A** | **T** | **A** | **T** | **G** | **C** | **G** | **T** | **C** | **C** | **C** | **T** | **T** | **A** | **G** | **C** | **A** | **C** | **C** | **T** | **T** | **T** | **A** | **T** | **T** | **G** | **T** | **T** | **A** | **T** | **C** | **T** | **T** | **A** | **A** | **T** | **T** | **A** | **C** | **A** | **T** | **T** | **G** | **T** | **T** | **T** | **T** | **C** | **A** | **T** | **A** | **T** | **T** | **T** | **T** | **C** | **A** | **T** | **T** | **C** | **G** | **A** | **T** | **T** | **A** | **A** | **A** | **C** | **A** | **A** | **A** | **A** | **C** | **C** | **G** | **G** | **T** | **G** | **G** | **C** | **T** | **A** | **T** | **T** | **A** | **G** | **A** | **G** | **C** | **T** | **T** | **C** | **T** | **T** | **C** | **A** | **G** | **A** | **G** | **A** | **A** | **A** | **A** | **G** | **T** | **C** | **T** | **T** | **T** | **T** | **A** | **T** | **T** | **T** | **A** | **C** | **C** | **A** | **C** | **T** | **A** | **A** | **T** | **T** | **G** | **T** | **G** | **C** | **A** | **C** | **A** | **T** | **T** | **G** | **T** | **T** | **C** | **T** | **C** | **A** | **T** | **A** | **T** | **C** | **C** | **T** | **T** | **A** | **C** | **T** | **A** | **A** | **T** | **A** | **G** | **G** | **C** | **T** | **T** | **A** | **T** | **T** | **G** | **A** | **C** | **C** | **T** | **T** | **T** | **T** | **T** | **A** | **C** | **T** | **G** | **A** | **T** | **T** | **T** | **C** | **A** | **T** | **T** | **G** | **G** | **T** | **T** | **C** | **A** | **A** | **A** | **A** | **A** | **G** | **G** | **G** | **C** | **A** | **A** | **T** | **A** | **T** | **G** | **G** | **A** | **C** | **G** | **G** | **A** | **T** | **T** | **G** | **A** | **A** | **G** | **A** | **A** | **A** | **A** | **A** | **C** | **T** | **T** | **C** | **G** | **G** | **T** | **T** | **A** | **T** | **T** | **G** | **G** | **C** | **C** | **A** | **A** | **T** | **G** | **G** | **T** | **A** | **A** | **T** | **T** | **A** | **T** | **G** | **A** | **A** | **A** | **G** | **T** | **C** | **C** | **A** | **G** | **T** | **C** | **T** | **T** | **A** | **A** | **A** | **C** | **A** | **A** | **A** | **C** | **C** | **A** | **A** | **C** | **G** | **A** | **C** | **C** | **A** | **G** | **T** | **G** | **A** | **A** | **A** | **A** | **T** | **C** | **A** | **A** | **G** | **A** | **C** | **C** | **A** | **T** | **T** | **A** | **T** | **C** | **T** | **A** | **A** | **C** | **C** | **G** | **A** | **A** | **G** | **T** | **C** | **G** | **A** | **A** | **C** | **A** | **A** | **G** | **A** | **T** | **A** | **T** | **T** | **T** | **G** | **G** | **T** | **C** | **G** | **A** | **T** | **T** | **A** | **A** | **A** | **A** | **A** | **T** | **A** | **A** | **A** | **T** | **T** | **A** | **T** | **T** | **A** | **G** | **A** | **G** | **A** | **T** | **G** | **T** | **C** | **T** | **A** | **A** | **A** | **G** | **A** | **A** | **T** | **T** | **G** | **C** | **A** | **A** | **T** | **T** | **A** | **T** | **T** | **A** | **A** | **A** | **C** | **A** | **G** | **T** | **C** | **G** | **A** | **C** | **C** | **G** | **C** | **A** | **A** | **T** | **T** | **A** | **A** | **T** | **G** | **G** | **A** | **T** | **G** | **G** | **G** | **C** | **A** | **A** | **A** | **C** | **A** | **A** | **A** | **A** | **G** | **A** | **A** | **G** | **A** | **A** | **A** | **T** | **T** | **T** | **T** | **A** | **G** | **A** | **G** | **A** | **A** | **C** | **G** | **A** | **G** | **C** | **G** | **G** | **C** | **A** | **T** | **C** | **G** | **T** | **T** | **T** | **G** | **G** | **C** | **G** | **C** | **G** | **G** | **G** | **A** | **G** | **T** | **T** | **G** | **C** | **A** | **T** | **G** | **A** | **T** | **T** | **C** | **A** | **G** | **T** | **C** | **A** | **G** | **T** | **C** | **A** | **A** | **C** | **A** | **A** | **C** | **T** | **T** | **T** | **T** | **T** | **G** | **C** | **A** | **G** | **C** | **C** | **A** | **T** | **G** | **A** | **T** | **G** | **A** | **T** | **G** | **T** | **T** | **G** | **T** | **C** | **T** | **G** | **C** | **C** | **T** | **T** | **A** | **A** | **A** | **T** | **G** | **A** | **A** | **C** | **A** | **A** | **G** | **C** | **A** | **C** | **A** | **A** | **C** | **G** | **A** | **A** | **C** | **A** | **G** | **A** | **A** | **A** | **C** | **C** | **C** | **C** | **T** | **G** | **A** | **A** | **C** | **C** | **A** | **T** | **A** | **T** | **C** | **G** | **C** | **A** | **A** | **A** | **C** | **A** | **A** | **C** | **T** | **A** | **G** | **C** | **C** | **A** | **T** | **G** | **G** | **T** | **G** | **G** | **C** | **A** | **G** | **A** | **A** | **A** | **T** | **C** | **A** | **T** | **T** | **A** | **A** | **T** | **G** | **C** | **C** | **T** | **C** | **C** | **C** | **A** | **A** | **T** | **C** | **G** | **G** | **A** | **A** | **A** | **T** | **G** | **C** | **G** | **C** | **G** | **C** | **G** | **T** | **T** | **A** | **C** | **T** | **A** | **T** | **T** | **G** | **C** | **A** | **C** | **T** | **T** | **G** | **C** | **G** | **T** | **C** | **C** | **T** | **A** | **T** | **C** | **A** | **G** | **T** | **C** | **T** | **A** | **G** | **A** | **A** | **G** | **G** | **A** | **A** | **A** | **A** | **A** | **G** | **T** | **T** | **T** | **G** | **C** | **G** | **T** | **A** | **A** | **A** | **G** | **G** | **T** | **A** | **T** | **T** | **G** | **A** | **A** | **C** | **A** | **A** | **T** | **T** | **A** | **C** | **T** | **G** | **A** | **A** | **A** | **G** | **A** | **A** | **C** | **T** | **A** | **C** | **A** | **A** | **A** | **C** | **A** | **A** | **A** | **A** | **A** | **T** | **T** | **A** | **A** | **A** | **A** | **T** | **T** | **G** | **A** | **A** | **T** | **T** | **G** | **A** | **T** | **T** | **T** | **G** | **G** | **G** | **A** | **T** | **G** | **T** | **T** | **G** | **A** | **A** | **G** | **A** | **T** | **G** | **T** | **T** | **C** | **A** | **T** | **T** | **T** | **A** | **A** | **A** | **T** | **A** | **G** | **C** | **A** | **G** | **C** | **A** | **T** | **T** | **G** | **A** | **G** | **G** | **A** | **T** | **C** | **A** | **T** | **C** | **T** | **T** | **T** | **T** | **C** | **C** | **G** | **A** | **A** | **T** | **T** | **G** | **T** | **G** | **C** | **A** | **G** | **G** | **A** | **G** | **T** | **T** | **A** | **C** | **T** | **T** | **T** | **C** | **A** | **A** | **A** | **T** | **A** | **C** | **C** | **T** | **T** | **A** | **A** | **G** | **A** | **C** | **A** | **T** | **G** | **C** | **C** | **A** | **A** | **A** | **G** | **C** | **A** | **A** | **A** | **G** | **G** | **A** | **A** | **T** | **T** | **A** | **G** | **A** | **G** | **G** | **T** | **A** | **T** | **A** | **C** | **T** | **T** | **A** | **C** | **A** | **C** | **C** | **A** | **A** | **G** | **T** | **C** | **G** | **A** | **T** | **A** | **A** | **A** | **A** | **A** | **C** | **G** | **T** | **G** | **T** | **T** | **A** | **T** | **T** | **G** | **C** | **G** | **T** | **A** | **T** | **T** | **G** | **T** | **T** | **G** | **A** | **T** | **G** | **A** | **T** | **G** | **G** | **T** | **G** | **T** | **C** | **G** | **G** | **C** | **T** | **T** | **T** | **G** | **A** | **T** | **A** | **T** | **G** | **A** | **A** | **G** | **G** | **A** | **A** | **C** | **A** | **A** | **A** | **G** | **T** | **A** | **A** | **T** | **A** | **A** | **A** | **G** | **C** | **T** | **G** | **G** | **T** | **A** | **G** | **T** | **T** | **A** | **T** | **G** | **G** | **C** | **T** | **T** | **A** | **A** | **A** | **T** | **A** | **A** | **T** | **A** | **T** | **T** | **C** | **G** | **A** | **G** | **A** | **A** | **C** | **G** | **T** | **G** | **T** | **T** | **G** | **T** | **C** | **G** | **G** | **C** | **A** | **T** | **G** | **G** | **G** | **C** | **G** | **G** | **T** | **A** | **C** | **A** | **G** | **T** | **T** | **A** | **A** | **A** | **A** | **T** | **T** | **A** | **T** | **T** | **A** | **G** | **T** | **T** | **T** | **T** | **A** | **A** | **A** | **G** | **G** | **G** | **C** | **A** | **G** | **G** | **G** | **A** | **A** | **C** | **C** | **A** | **G** | **C** | **G** | **T** | **T** | **G** | **A** | **A** | **A** | **T** | **T** | **A** | **A** | **A** | **G** | **T** | **T** | **C** | **C** | **T** | **G** | **T** | **C** | **A** | **T** | **A** | **A** | **A** | **G** | **G** | **A** | **G** | **G** | **A** | **A** | **A** | **C** | **T** | **G** | **C** | **A** | **A** | **G** | **T** | **G** | **A** | **T** | **C** | **A** | **A** | **A** | **G** | **T** | **A** | **A** | **T** | **G** | **C** | **T** | **A** | **G** | **T** | **G** | **G** | **A** | **-** | **-** | **-** |
| **N59599F** | **A** | **T** | **G** | **A** | **C** | **C** | **G** | **A** | **T** | **C** | **G** | **G** | **A** | **T** | **T** | **T** | **C** | **A** | **A** | **G** | **A** | **C** | **G** | **C** | **A** | **T** | **G** | **A** | **T** | **T** | **T** | **C** | **A** | **T** | **T** | **A** | **T** | **A** | **T** | **G** | **C** | **G** | **T** | **C** | **C** | **C** | **T** | **T** | **A** | **G** | **C** | **A** | **C** | **C** | **T** | **T** | **T** | **A** | **T** | **T** | **G** | **T** | **T** | **A** | **T** | **C** | **T** | **T** | **A** | **A** | **T** | **T** | **A** | **C** | **A** | **T** | **T** | **G** | **T** | **T** | **T** | **T** | **C** | **A** | **T** | **A** | **T** | **T** | **T** | **T** | **C** | **A** | **T** | **T** | **C** | **G** | **A** | **T** | **T** | **A** | **A** | **A** | **C** | **A** | **A** | **A** | **A** | **C** | **C** | **G** | **G** | **T** | **G** | **G** | **C** | **T** | **A** | **T** | **T** | **A** | **G** | **A** | **G** | **C** | **T** | **T** | **C** | **T** | **T** | **C** | **A** | **G** | **A** | **G** | **A** | **A** | **A** | **A** | **G** | **T** | **C** | **T** | **T** | **T** | **T** | **A** | **T** | **C** | **T** | **A** | **C** | **C** | **A** | **C** | **T** | **A** | **A** | **T** | **T** | **G** | **T** | **G** | **C** | **A** | **C** | **A** | **T** | **T** | **G** | **T** | **T** | **C** | **T** | **C** | **A** | **T** | **A** | **T** | **C** | **C** | **T** | **T** | **A** | **C** | **T** | **A** | **A** | **T** | **A** | **G** | **G** | **C** | **T** | **T** | **A** | **T** | **T** | **G** | **A** | **C** | **C** | **T** | **T** | **T** | **T** | **T** | **A** | **C** | **T** | **G** | **A** | **T** | **T** | **T** | **C** | **A** | **T** | **T** | **G** | **G** | **T** | **T** | **C** | **A** | **A** | **A** | **A** | **A** | **G** | **G** | **G** | **C** | **A** | **A** | **T** | **A** | **T** | **G** | **G** | **A** | **C** | **G** | **G** | **A** | **T** | **T** | **G** | **A** | **A** | **G** | **A** | **A** | **A** | **A** | **A** | **C** | **T** | **T** | **C** | **G** | **G** | **T** | **T** | **A** | **T** | **T** | **G** | **G** | **C** | **C** | **A** | **A** | **C** | **G** | **G** | **T** | **A** | **A** | **T** | **T** | **A** | **T** | **G** | **A** | **A** | **A** | **G** | **T** | **C** | **C** | **A** | **G** | **T** | **C** | **T** | **T** | **A** | **A** | **A** | **C** | **A** | **A** | **A** | **C** | **C** | **A** | **A** | **C** | **G** | **A** | **C** | **C** | **A** | **G** | **T** | **G** | **A** | **A** | **A** | **A** | **T** | **C** | **A** | **A** | **G** | **A** | **C** | **C** | **A** | **T** | **T** | **A** | **T** | **C** | **T** | **A** | **A** | **C** | **C** | **G** | **A** | **A** | **G** | **T** | **C** | **G** | **A** | **A** | **C** | **A** | **A** | **G** | **A** | **T** | **A** | **T** | **T** | **T** | **G** | **G** | **T** | **C** | **G** | **A** | **T** | **T** | **A** | **A** | **A** | **A** | **A** | **T** | **A** | **A** | **A** | **T** | **T** | **A** | **T** | **T** | **A** | **G** | **A** | **G** | **A** | **T** | **G** | **T** | **C** | **T** | **A** | **A** | **A** | **G** | **A** | **A** | **T** | **T** | **G** | **C** | **A** | **A** | **T** | **T** | **A** | **T** | **T** | **A** | **A** | **A** | **C** | **A** | **G** | **T** | **C** | **G** | **A** | **C** | **C** | **G** | **C** | **A** | **A** | **T** | **T** | **A** | **A** | **T** | **G** | **G** | **A** | **T** | **G** | **G** | **G** | **C** | **A** | **A** | **A** | **C** | **A** | **A** | **A** | **A** | **G** | **A** | **A** | **G** | **A** | **A** | **A** | **T** | **T** | **T** | **T** | **A** | **G** | **A** | **G** | **A** | **A** | **C** | **G** | **A** | **G** | **C** | **G** | **G** | **C** | **A** | **T** | **C** | **G** | **T** | **T** | **T** | **G** | **G** | **C** | **G** | **C** | **G** | **G** | **G** | **A** | **G** | **T** | **T** | **G** | **C** | **A** | **T** | **G** | **A** | **T** | **T** | **C** | **A** | **G** | **T** | **C** | **A** | **G** | **T** | **C** | **A** | **A** | **C** | **A** | **A** | **C** | **T** | **T** | **T** | **T** | **T** | **G** | **C** | **A** | **G** | **C** | **C** | **A** | **T** | **G** | **A** | **T** | **G** | **A** | **T** | **G** | **T** | **T** | **G** | **T** | **C** | **T** | **G** | **C** | **A** | **T** | **T** | **A** | **A** | **A** | **T** | **G** | **A** | **A** | **C** | **A** | **A** | **G** | **C** | **A** | **C** | **A** | **A** | **C** | **G** | **A** | **A** | **C** | **A** | **G** | **A** | **A** | **A** | **C** | **C** | **C** | **C** | **G** | **G** | **A** | **A** | **C** | **C** | **A** | **T** | **A** | **T** | **C** | **G** | **T** | **A** | **A** | **A** | **C** | **A** | **A** | **C** | **T** | **A** | **G** | **C** | **C** | **A** | **T** | **G** | **G** | **T** | **G** | **G** | **C** | **A** | **G** | **A** | **A** | **A** | **T** | **C** | **A** | **T** | **T** | **A** | **A** | **T** | **G** | **C** | **C** | **T** | **C** | **C** | **C** | **A** | **A** | **T** | **C** | **G** | **G** | **A** | **A** | **A** | **T** | **G** | **C** | **G** | **C** | **G** | **C** | **G** | **C** | **T** | **A** | **C** | **T** | **A** | **T** | **T** | **G** | **C** | **A** | **C** | **T** | **T** | **G** | **C** | **G** | **T** | **C** | **C** | **T** | **A** | **T** | **C** | **A** | **G** | **T** | **C** | **T** | **A** | **G** | **A** | **A** | **G** | **G** | **A** | **A** | **A** | **A** | **A** | **G** | **T** | **T** | **T** | **G** | **C** | **G** | **T** | **A** | **A** | **A** | **G** | **G** | **T** | **A** | **T** | **T** | **G** | **A** | **A** | **C** | **A** | **A** | **T** | **T** | **A** | **C** | **T** | **G** | **A** | **A** | **A** | **G** | **A** | **A** | **C** | **T** | **A** | **C** | **A** | **A** | **A** | **C** | **A** | **A** | **A** | **A** | **A** | **T** | **T** | **A** | **A** | **A** | **A** | **T** | **T** | **G** | **A** | **A** | **T** | **T** | **G** | **A** | **T** | **T** | **T** | **G** | **G** | **G** | **A** | **T** | **G** | **T** | **T** | **G** | **A** | **A** | **G** | **A** | **T** | **G** | **T** | **T** | **C** | **A** | **T** | **T** | **T** | **A** | **A** | **A** | **T** | **A** | **G** | **C** | **A** | **G** | **C** | **A** | **T** | **T** | **G** | **A** | **G** | **G** | **A** | **T** | **C** | **A** | **T** | **C** | **T** | **T** | **T** | **T** | **C** | **C** | **G** | **A** | **A** | **T** | **T** | **G** | **T** | **G** | **C** | **A** | **G** | **G** | **A** | **G** | **T** | **T** | **A** | **C** | **T** | **T** | **T** | **C** | **A** | **A** | **A** | **T** | **A** | **C** | **C** | **T** | **T** | **A** | **A** | **G** | **A** | **C** | **A** | **T** | **G** | **C** | **C** | **A** | **A** | **A** | **G** | **C** | **A** | **A** | **A** | **G** | **G** | **A** | **A** | **T** | **T** | **A** | **G** | **A** | **G** | **G** | **T** | **A** | **T** | **A** | **C** | **T** | **T** | **A** | **C** | **A** | **C** | **C** | **A** | **A** | **G** | **T** | **C** | **G** | **A** | **T** | **A** | **A** | **A** | **A** | **A** | **C** | **G** | **T** | **G** | **T** | **T** | **A** | **T** | **T** | **G** | **C** | **G** | **T** | **A** | **T** | **T** | **G** | **T** | **T** | **G** | **A** | **T** | **G** | **A** | **T** | **G** | **G** | **T** | **G** | **T** | **C** | **G** | **G** | **C** | **T** | **T** | **T** | **G** | **A** | **T** | **A** | **T** | **G** | **A** | **A** | **G** | **G** | **A** | **A** | **C** | **A** | **A** | **A** | **G** | **T** | **A** | **A** | **T** | **A** | **A** | **A** | **G** | **C** | **C** | **G** | **G** | **T** | **A** | **G** | **T** | **T** | **A** | **T** | **G** | **G** | **C** | **T** | **T** | **A** | **A** | **A** | **T** | **A** | **A** | **T** | **A** | **T** | **T** | **C** | **G** | **A** | **G** | **A** | **A** | **C** | **G** | **T** | **G** | **T** | **T** | **G** | **T** | **C** | **G** | **G** | **C** | **A** | **T** | **G** | **G** | **G** | **C** | **G** | **G** | **T** | **A** | **C** | **A** | **G** | **T** | **T** | **A** | **A** | **A** | **A** | **T** | **T** | **A** | **T** | **T** | **A** | **G** | **T** | **T** | **T** | **T** | **A** | **A** | **A** | **G** | **G** | **G** | **C** | **A** | **G** | **G** | **G** | **A** | **A** | **C** | **C** | **A** | **G** | **C** | **G** | **T** | **T** | **G** | **A** | **A** | **A** | **T** | **T** | **A** | **A** | **A** | **G** | **T** | **T** | **C** | **C** | **T** | **G** | **T** | **C** | **A** | **T** | **A** | **A** | **A** | **G** | **G** | **A** | **G** | **G** | **A** | **A** | **A** | **C** | **T** | **G** | **C** | **A** | **A** | **G** | **T** | **G** | **A** | **T** | **C** | **A** | **A** | **A** | **G** | **T** | **A** | **A** | **T** | **G** | **T** | **T** | **A** | **G** | **T** | **G** | **G** | **A** | **-** | **-** | **-** |
| **N55724** | **A** | **T** | **G** | **A** | **C** | **C** | **G** | **A** | **T** | **C** | **G** | **G** | **A** | **T** | **T** | **T** | **C** | **A** | **A** | **G** | **A** | **C** | **G** | **C** | **A** | **T** | **G** | **A** | **T** | **T** | **T** | **C** | **A** | **T** | **T** | **A** | **T** | **A** | **T** | **G** | **C** | **G** | **T** | **C** | **C** | **C** | **T** | **T** | **A** | **G** | **C** | **A** | **C** | **C** | **T** | **T** | **T** | **A** | **T** | **T** | **G** | **T** | **T** | **A** | **T** | **C** | **T** | **T** | **A** | **A** | **T** | **T** | **A** | **C** | **A** | **T** | **T** | **G** | **T** | **T** | **T** | **T** | **C** | **A** | **T** | **A** | **T** | **T** | **T** | **T** | **C** | **A** | **T** | **T** | **C** | **G** | **A** | **T** | **T** | **A** | **A** | **A** | **C** | **A** | **A** | **A** | **A** | **C** | **C** | **G** | **G** | **T** | **G** | **G** | **C** | **T** | **A** | **T** | **T** | **A** | **G** | **A** | **G** | **C** | **T** | **T** | **C** | **T** | **T** | **C** | **A** | **G** | **A** | **G** | **A** | **A** | **A** | **A** | **G** | **T** | **C** | **T** | **T** | **T** | **T** | **A** | **T** | **C** | **T** | **A** | **C** | **C** | **A** | **C** | **T** | **A** | **A** | **T** | **T** | **G** | **T** | **G** | **C** | **A** | **C** | **A** | **T** | **T** | **G** | **T** | **T** | **C** | **T** | **C** | **A** | **T** | **A** | **T** | **C** | **C** | **T** | **T** | **A** | **C** | **T** | **A** | **A** | **T** | **A** | **G** | **G** | **C** | **T** | **T** | **A** | **T** | **T** | **G** | **A** | **C** | **C** | **T** | **T** | **T** | **T** | **T** | **A** | **C** | **T** | **G** | **A** | **T** | **T** | **T** | **C** | **A** | **T** | **T** | **G** | **G** | **T** | **T** | **C** | **A** | **A** | **A** | **A** | **A** | **G** | **G** | **G** | **C** | **A** | **A** | **T** | **A** | **T** | **G** | **G** | **A** | **C** | **G** | **G** | **A** | **T** | **T** | **G** | **A** | **A** | **G** | **A** | **A** | **A** | **A** | **A** | **C** | **T** | **T** | **C** | **G** | **G** | **T** | **T** | **A** | **T** | **T** | **G** | **G** | **C** | **C** | **A** | **A** | **C** | **G** | **G** | **T** | **A** | **A** | **T** | **T** | **A** | **T** | **G** | **A** | **A** | **A** | **G** | **T** | **C** | **C** | **A** | **G** | **T** | **C** | **T** | **T** | **A** | **A** | **A** | **C** | **A** | **A** | **A** | **C** | **C** | **A** | **A** | **C** | **G** | **A** | **C** | **C** | **A** | **G** | **T** | **G** | **A** | **A** | **A** | **A** | **T** | **C** | **A** | **A** | **G** | **A** | **C** | **C** | **A** | **T** | **T** | **A** | **T** | **C** | **T** | **A** | **A** | **C** | **C** | **G** | **A** | **A** | **G** | **T** | **C** | **G** | **A** | **A** | **C** | **A** | **A** | **G** | **A** | **T** | **A** | **T** | **T** | **T** | **G** | **G** | **T** | **C** | **G** | **A** | **T** | **T** | **A** | **A** | **A** | **A** | **A** | **T** | **A** | **A** | **A** | **T** | **T** | **A** | **T** | **T** | **A** | **G** | **A** | **G** | **A** | **T** | **G** | **T** | **C** | **T** | **A** | **A** | **A** | **G** | **A** | **A** | **T** | **T** | **G** | **C** | **A** | **A** | **T** | **T** | **A** | **T** | **T** | **A** | **A** | **A** | **C** | **A** | **G** | **T** | **C** | **G** | **A** | **C** | **C** | **G** | **C** | **A** | **A** | **T** | **T** | **A** | **A** | **T** | **G** | **G** | **A** | **T** | **G** | **G** | **G** | **C** | **A** | **A** | **A** | **C** | **A** | **A** | **A** | **A** | **G** | **A** | **A** | **G** | **A** | **A** | **A** | **T** | **T** | **T** | **T** | **A** | **G** | **A** | **G** | **A** | **A** | **C** | **G** | **A** | **G** | **C** | **G** | **G** | **C** | **A** | **T** | **C** | **G** | **T** | **T** | **T** | **G** | **G** | **C** | **G** | **C** | **G** | **G** | **G** | **A** | **G** | **T** | **T** | **G** | **C** | **A** | **T** | **G** | **A** | **T** | **T** | **C** | **A** | **G** | **T** | **C** | **A** | **G** | **T** | **C** | **A** | **A** | **C** | **A** | **A** | **C** | **T** | **T** | **T** | **T** | **T** | **G** | **C** | **A** | **G** | **C** | **C** | **A** | **T** | **G** | **A** | **T** | **G** | **A** | **T** | **G** | **T** | **T** | **G** | **T** | **C** | **T** | **G** | **C** | **A** | **T** | **T** | **A** | **A** | **A** | **T** | **G** | **A** | **A** | **C** | **A** | **A** | **G** | **C** | **A** | **C** | **A** | **A** | **C** | **G** | **A** | **A** | **C** | **A** | **G** | **A** | **A** | **A** | **C** | **C** | **C** | **C** | **G** | **G** | **A** | **A** | **C** | **C** | **A** | **T** | **A** | **T** | **C** | **G** | **T** | **A** | **A** | **A** | **C** | **A** | **A** | **C** | **T** | **A** | **G** | **C** | **C** | **A** | **T** | **G** | **G** | **T** | **G** | **G** | **C** | **A** | **G** | **A** | **A** | **A** | **T** | **C** | **A** | **T** | **T** | **A** | **A** | **T** | **G** | **C** | **C** | **T** | **C** | **C** | **C** | **A** | **A** | **T** | **C** | **G** | **G** | **A** | **A** | **A** | **T** | **G** | **C** | **G** | **C** | **G** | **C** | **G** | **C** | **T** | **A** | **C** | **T** | **A** | **T** | **T** | **G** | **C** | **A** | **C** | **T** | **T** | **G** | **C** | **G** | **T** | **C** | **C** | **T** | **A** | **T** | **C** | **A** | **G** | **T** | **C** | **T** | **A** | **G** | **A** | **A** | **G** | **G** | **A** | **A** | **A** | **A** | **A** | **G** | **T** | **T** | **T** | **G** | **C** | **G** | **T** | **A** | **A** | **A** | **G** | **G** | **T** | **A** | **T** | **T** | **G** | **A** | **A** | **C** | **A** | **A** | **T** | **T** | **A** | **C** | **T** | **G** | **A** | **A** | **A** | **G** | **A** | **A** | **C** | **T** | **A** | **C** | **A** | **A** | **A** | **C** | **A** | **A** | **A** | **A** | **A** | **T** | **T** | **A** | **A** | **A** | **A** | **T** | **T** | **G** | **A** | **A** | **T** | **T** | **G** | **A** | **T** | **T** | **T** | **G** | **G** | **G** | **A** | **T** | **G** | **T** | **T** | **G** | **A** | **A** | **G** | **A** | **T** | **G** | **T** | **T** | **C** | **A** | **T** | **T** | **T** | **A** | **A** | **A** | **T** | **A** | **G** | **C** | **A** | **G** | **C** | **A** | **T** | **T** | **G** | **A** | **G** | **G** | **A** | **T** | **C** | **A** | **T** | **C** | **T** | **T** | **T** | **T** | **C** | **C** | **G** | **A** | **A** | **T** | **T** | **G** | **T** | **G** | **C** | **A** | **G** | **G** | **A** | **G** | **T** | **T** | **A** | **C** | **T** | **T** | **T** | **C** | **A** | **A** | **A** | **T** | **A** | **C** | **C** | **T** | **T** | **A** | **A** | **G** | **A** | **C** | **A** | **T** | **G** | **C** | **C** | **A** | **A** | **A** | **G** | **C** | **A** | **A** | **A** | **G** | **G** | **A** | **A** | **T** | **T** | **A** | **G** | **A** | **G** | **G** | **T** | **A** | **T** | **A** | **C** | **T** | **T** | **A** | **C** | **A** | **C** | **C** | **A** | **A** | **G** | **T** | **C** | **G** | **A** | **T** | **A** | **A** | **A** | **A** | **A** | **C** | **G** | **T** | **G** | **T** | **T** | **A** | **T** | **T** | **G** | **C** | **G** | **T** | **A** | **T** | **T** | **G** | **T** | **T** | **G** | **A** | **T** | **G** | **A** | **T** | **G** | **G** | **T** | **G** | **T** | **C** | **G** | **G** | **C** | **T** | **T** | **T** | **G** | **A** | **T** | **A** | **T** | **G** | **A** | **A** | **G** | **G** | **A** | **A** | **C** | **A** | **A** | **A** | **G** | **T** | **A** | **A** | **T** | **A** | **A** | **A** | **G** | **C** | **C** | **G** | **G** | **T** | **A** | **G** | **T** | **T** | **A** | **T** | **G** | **G** | **C** | **T** | **T** | **A** | **A** | **A** | **T** | **A** | **A** | **T** | **A** | **T** | **C** | **C** | **G** | **A** | **G** | **A** | **A** | **C** | **G** | **T** | **G** | **T** | **T** | **G** | **T** | **C** | **G** | **G** | **C** | **A** | **T** | **G** | **G** | **G** | **C** | **G** | **G** | **T** | **A** | **C** | **A** | **G** | **T** | **T** | **A** | **A** | **A** | **A** | **T** | **T** | **A** | **T** | **T** | **A** | **G** | **T** | **T** | **T** | **T** | **A** | **A** | **A** | **G** | **G** | **G** | **C** | **A** | **G** | **G** | **G** | **A** | **A** | **C** | **C** | **A** | **G** | **C** | **G** | **T** | **T** | **G** | **A** | **A** | **A** | **T** | **T** | **A** | **A** | **A** | **G** | **T** | **T** | **C** | **C** | **T** | **G** | **T** | **C** | **A** | **T** | **A** | **A** | **A** | **G** | **G** | **A** | **G** | **G** | **A** | **A** | **A** | **C** | **T** | **G** | **C** | **A** | **A** | **G** | **T** | **G** | **A** | **T** | **C** | **A** | **A** | **A** | **G** | **T** | **A** | **A** | **T** | **G** | **T** | **T** | **A** | **G** | **T** | **G** | **G** | **A** | **-** | **-** | **-** |
| **N52648** | **A** | **T** | **G** | **A** | **C** | **C** | **G** | **A** | **T** | **C** | **G** | **G** | **A** | **T** | **T** | **T** | **C** | **A** | **A** | **G** | **A** | **C** | **G** | **C** | **A** | **T** | **G** | **A** | **T** | **T** | **T** | **C** | **A** | **T** | **T** | **A** | **T** | **A** | **T** | **G** | **C** | **G** | **T** | **C** | **C** | **C** | **T** | **T** | **A** | **G** | **C** | **A** | **C** | **C** | **T** | **T** | **T** | **A** | **T** | **T** | **G** | **T** | **T** | **A** | **T** | **C** | **T** | **T** | **A** | **A** | **T** | **T** | **A** | **C** | **A** | **T** | **T** | **G** | **T** | **T** | **T** | **T** | **C** | **A** | **T** | **A** | **T** | **T** | **T** | **T** | **C** | **A** | **T** | **T** | **C** | **G** | **A** | **T** | **T** | **A** | **A** | **A** | **C** | **A** | **A** | **A** | **A** | **C** | **C** | **G** | **G** | **T** | **G** | **G** | **C** | **T** | **A** | **T** | **T** | **A** | **G** | **A** | **G** | **C** | **T** | **T** | **C** | **T** | **T** | **C** | **A** | **G** | **A** | **G** | **A** | **A** | **A** | **A** | **G** | **T** | **C** | **T** | **T** | **T** | **T** | **A** | **T** | **C** | **T** | **A** | **C** | **C** | **A** | **C** | **T** | **A** | **A** | **T** | **T** | **G** | **T** | **G** | **C** | **A** | **C** | **A** | **T** | **T** | **G** | **T** | **T** | **C** | **T** | **C** | **A** | **T** | **A** | **T** | **C** | **C** | **T** | **T** | **A** | **C** | **T** | **A** | **A** | **T** | **A** | **G** | **G** | **C** | **T** | **T** | **A** | **T** | **T** | **G** | **A** | **C** | **C** | **T** | **T** | **T** | **T** | **T** | **A** | **C** | **T** | **G** | **A** | **T** | **T** | **T** | **C** | **A** | **T** | **T** | **G** | **G** | **T** | **T** | **C** | **A** | **A** | **A** | **A** | **A** | **G** | **G** | **G** | **C** | **A** | **A** | **T** | **A** | **T** | **G** | **G** | **A** | **C** | **G** | **G** | **A** | **T** | **T** | **G** | **A** | **A** | **G** | **A** | **A** | **A** | **A** | **A** | **C** | **T** | **T** | **C** | **G** | **G** | **T** | **T** | **A** | **T** | **T** | **G** | **G** | **C** | **C** | **A** | **A** | **T** | **G** | **G** | **C** | **A** | **A** | **T** | **T** | **A** | **T** | **G** | **A** | **A** | **A** | **G** | **T** | **C** | **C** | **A** | **G** | **T** | **C** | **T** | **T** | **A** | **A** | **A** | **C** | **A** | **A** | **A** | **C** | **C** | **A** | **A** | **C** | **G** | **A** | **C** | **C** | **A** | **G** | **T** | **G** | **A** | **A** | **A** | **A** | **T** | **C** | **A** | **A** | **G** | **A** | **T** | **C** | **A** | **T** | **T** | **A** | **T** | **C** | **T** | **A** | **A** | **C** | **C** | **G** | **A** | **A** | **G** | **T** | **C** | **G** | **A** | **A** | **C** | **A** | **A** | **G** | **A** | **T** | **A** | **T** | **T** | **T** | **G** | **G** | **T** | **C** | **G** | **A** | **T** | **T** | **A** | **A** | **A** | **A** | **A** | **T** | **A** | **A** | **A** | **T** | **T** | **A** | **T** | **T** | **A** | **G** | **A** | **G** | **A** | **T** | **G** | **T** | **C** | **T** | **A** | **A** | **A** | **G** | **A** | **A** | **T** | **T** | **G** | **C** | **A** | **A** | **T** | **T** | **A** | **T** | **T** | **A** | **A** | **A** | **C** | **A** | **G** | **T** | **C** | **G** | **G** | **C** | **C** | **G** | **C** | **A** | **A** | **T** | **T** | **A** | **A** | **T** | **G** | **G** | **A** | **T** | **G** | **G** | **G** | **C** | **A** | **A** | **A** | **C** | **A** | **A** | **A** | **A** | **G** | **A** | **A** | **G** | **A** | **A** | **A** | **T** | **T** | **T** | **T** | **A** | **G** | **A** | **G** | **A** | **A** | **C** | **G** | **A** | **G** | **C** | **G** | **G** | **C** | **A** | **T** | **C** | **G** | **T** | **T** | **T** | **G** | **G** | **C** | **G** | **C** | **G** | **G** | **G** | **A** | **G** | **T** | **T** | **G** | **C** | **A** | **T** | **G** | **A** | **T** | **T** | **C** | **A** | **G** | **T** | **C** | **A** | **G** | **T** | **C** | **A** | **A** | **C** | **A** | **A** | **C** | **T** | **T** | **T** | **T** | **T** | **G** | **C** | **A** | **G** | **C** | **C** | **A** | **T** | **G** | **A** | **T** | **G** | **A** | **T** | **G** | **T** | **T** | **G** | **T** | **C** | **T** | **G** | **C** | **C** | **T** | **T** | **A** | **A** | **A** | **T** | **G** | **A** | **A** | **C** | **A** | **A** | **G** | **C** | **A** | **C** | **A** | **A** | **C** | **G** | **A** | **A** | **C** | **A** | **G** | **A** | **A** | **A** | **C** | **C** | **C** | **C** | **G** | **G** | **A** | **A** | **C** | **C** | **A** | **T** | **A** | **T** | **C** | **G** | **T** | **A** | **A** | **A** | **C** | **A** | **A** | **C** | **T** | **A** | **G** | **C** | **C** | **A** | **T** | **G** | **G** | **T** | **G** | **G** | **C** | **A** | **G** | **A** | **A** | **A** | **T** | **C** | **A** | **T** | **T** | **A** | **A** | **T** | **G** | **C** | **C** | **T** | **C** | **C** | **C** | **A** | **A** | **T** | **C** | **G** | **G** | **A** | **A** | **A** | **T** | **G** | **C** | **G** | **C** | **G** | **C** | **G** | **T** | **T** | **A** | **C** | **T** | **A** | **T** | **T** | **G** | **C** | **A** | **C** | **T** | **T** | **G** | **C** | **G** | **T** | **C** | **C** | **T** | **A** | **T** | **C** | **A** | **G** | **T** | **C** | **T** | **A** | **G** | **A** | **A** | **G** | **G** | **A** | **A** | **A** | **A** | **A** | **G** | **T** | **T** | **T** | **G** | **C** | **G** | **T** | **A** | **A** | **A** | **G** | **G** | **T** | **A** | **T** | **T** | **G** | **A** | **A** | **C** | **A** | **A** | **T** | **T** | **A** | **C** | **T** | **G** | **A** | **A** | **A** | **G** | **A** | **A** | **C** | **T** | **A** | **C** | **A** | **A** | **A** | **C** | **A** | **A** | **A** | **A** | **A** | **T** | **T** | **A** | **A** | **A** | **A** | **T** | **T** | **G** | **A** | **A** | **T** | **T** | **G** | **A** | **T** | **T** | **T** | **G** | **G** | **G** | **A** | **T** | **G** | **T** | **T** | **G** | **A** | **A** | **G** | **A** | **T** | **G** | **T** | **T** | **C** | **A** | **T** | **T** | **T** | **A** | **A** | **A** | **T** | **A** | **G** | **C** | **A** | **G** | **C** | **A** | **T** | **T** | **G** | **A** | **G** | **G** | **A** | **T** | **C** | **A** | **T** | **C** | **T** | **T** | **T** | **T** | **C** | **C** | **G** | **A** | **A** | **T** | **T** | **G** | **T** | **G** | **C** | **A** | **G** | **G** | **A** | **A** | **T** | **T** | **A** | **C** | **T** | **T** | **T** | **C** | **C** | **A** | **A** | **T** | **A** | **C** | **C** | **T** | **T** | **A** | **A** | **G** | **A** | **C** | **A** | **T** | **G** | **C** | **C** | **A** | **A** | **A** | **G** | **C** | **A** | **A** | **A** | **G** | **G** | **A** | **A** | **T** | **T** | **A** | **G** | **A** | **G** | **G** | **T** | **A** | **T** | **A** | **C** | **T** | **T** | **A** | **C** | **A** | **C** | **C** | **A** | **A** | **G** | **T** | **C** | **G** | **A** | **T** | **A** | **A** | **A** | **A** | **A** | **C** | **G** | **T** | **G** | **T** | **T** | **A** | **T** | **T** | **G** | **C** | **G** | **T** | **A** | **T** | **T** | **G** | **T** | **T** | **G** | **A** | **T** | **G** | **A** | **T** | **G** | **G** | **T** | **G** | **T** | **C** | **G** | **G** | **C** | **T** | **T** | **T** | **G** | **A** | **T** | **A** | **T** | **G** | **A** | **A** | **G** | **G** | **A** | **A** | **C** | **A** | **A** | **A** | **G** | **T** | **A** | **A** | **T** | **A** | **A** | **A** | **G** | **C** | **C** | **G** | **G** | **T** | **A** | **G** | **T** | **T** | **A** | **T** | **G** | **G** | **C** | **T** | **T** | **A** | **A** | **A** | **T** | **A** | **A** | **T** | **A** | **T** | **T** | **C** | **G** | **A** | **G** | **A** | **A** | **C** | **G** | **T** | **G** | **T** | **T** | **G** | **T** | **C** | **G** | **G** | **C** | **A** | **T** | **G** | **G** | **G** | **C** | **G** | **G** | **T** | **A** | **C** | **A** | **G** | **T** | **T** | **A** | **A** | **A** | **A** | **T** | **T** | **A** | **T** | **T** | **A** | **G** | **T** | **T** | **T** | **T** | **A** | **A** | **A** | **G** | **G** | **G** | **C** | **A** | **G** | **G** | **G** | **A** | **A** | **C** | **C** | **A** | **G** | **C** | **G** | **T** | **T** | **G** | **A** | **A** | **A** | **T** | **T** | **A** | **A** | **A** | **G** | **T** | **T** | **C** | **C** | **T** | **G** | **T** | **C** | **A** | **T** | **A** | **A** | **A** | **G** | **G** | **A** | **G** | **G** | **A** | **A** | **A** | **C** | **T** | **G** | **C** | **A** | **A** | **G** | **T** | **G** | **A** | **T** | **C** | **A** | **A** | **A** | **G** | **T** | **A** | **A** | **T** | **G** | **T** | **T** | **A** | **G** | **T** | **G** | **G** | **A** | **-** | **-** | **-** |
| **N55753** | **A** | **T** | **G** | **A** | **C** | **C** | **G** | **A** | **T** | **C** | **G** | **G** | **A** | **T** | **T** | **T** | **C** | **A** | **A** | **G** | **A** | **C** | **G** | **C** | **A** | **T** | **G** | **A** | **T** | **T** | **T** | **C** | **A** | **T** | **T** | **A** | **T** | **A** | **T** | **G** | **C** | **G** | **T** | **C** | **C** | **C** | **T** | **T** | **A** | **G** | **C** | **A** | **C** | **C** | **T** | **T** | **T** | **A** | **T** | **T** | **G** | **T** | **T** | **A** | **T** | **C** | **T** | **T** | **A** | **A** | **T** | **T** | **A** | **C** | **A** | **T** | **T** | **G** | **T** | **T** | **T** | **T** | **C** | **A** | **T** | **A** | **T** | **T** | **T** | **T** | **C** | **A** | **T** | **T** | **C** | **G** | **A** | **T** | **T** | **A** | **A** | **A** | **C** | **A** | **A** | **A** | **A** | **C** | **C** | **G** | **G** | **T** | **G** | **G** | **T** | **T** | **A** | **T** | **T** | **A** | **G** | **A** | **G** | **C** | **T** | **C** | **C** | **T** | **T** | **C** | **A** | **G** | **A** | **G** | **A** | **A** | **A** | **A** | **G** | **T** | **C** | **T** | **T** | **T** | **T** | **A** | **T** | **C** | **T** | **A** | **C** | **C** | **A** | **C** | **T** | **A** | **A** | **T** | **T** | **G** | **T** | **G** | **C** | **A** | **C** | **A** | **T** | **T** | **G** | **T** | **T** | **C** | **T** | **C** | **A** | **T** | **A** | **T** | **C** | **C** | **T** | **T** | **A** | **C** | **T** | **A** | **A** | **T** | **A** | **G** | **G** | **C** | **T** | **T** | **A** | **T** | **T** | **G** | **A** | **C** | **C** | **T** | **T** | **T** | **T** | **T** | **A** | **C** | **T** | **G** | **A** | **T** | **T** | **T** | **C** | **A** | **T** | **T** | **G** | **G** | **T** | **T** | **C** | **A** | **A** | **A** | **A** | **A** | **G** | **G** | **G** | **C** | **A** | **A** | **T** | **A** | **T** | **G** | **G** | **A** | **C** | **G** | **G** | **A** | **T** | **T** | **G** | **A** | **A** | **G** | **A** | **A** | **A** | **A** | **A** | **C** | **T** | **T** | **C** | **G** | **G** | **T** | **T** | **A** | **T** | **T** | **G** | **G** | **C** | **C** | **A** | **A** | **C** | **G** | **G** | **T** | **A** | **A** | **T** | **T** | **A** | **T** | **G** | **A** | **A** | **A** | **G** | **T** | **C** | **C** | **A** | **G** | **T** | **C** | **T** | **T** | **A** | **A** | **A** | **C** | **A** | **A** | **A** | **C** | **C** | **A** | **A** | **C** | **G** | **A** | **C** | **C** | **A** | **G** | **T** | **G** | **A** | **A** | **A** | **A** | **T** | **C** | **A** | **A** | **G** | **A** | **C** | **C** | **A** | **T** | **T** | **A** | **T** | **C** | **T** | **A** | **A** | **C** | **C** | **G** | **A** | **A** | **G** | **T** | **C** | **G** | **A** | **A** | **C** | **A** | **A** | **G** | **A** | **T** | **A** | **T** | **T** | **T** | **G** | **G** | **T** | **C** | **G** | **A** | **T** | **T** | **A** | **A** | **A** | **A** | **A** | **T** | **A** | **A** | **A** | **T** | **T** | **A** | **T** | **T** | **A** | **G** | **A** | **G** | **A** | **T** | **G** | **T** | **C** | **T** | **A** | **A** | **A** | **G** | **A** | **A** | **T** | **T** | **G** | **C** | **A** | **A** | **T** | **T** | **A** | **T** | **T** | **A** | **A** | **A** | **C** | **A** | **G** | **T** | **C** | **G** | **A** | **C** | **C** | **G** | **C** | **A** | **A** | **T** | **T** | **A** | **A** | **T** | **G** | **G** | **A** | **T** | **G** | **G** | **G** | **C** | **A** | **A** | **A** | **C** | **A** | **A** | **A** | **A** | **G** | **A** | **A** | **G** | **A** | **A** | **A** | **T** | **T** | **T** | **T** | **A** | **G** | **A** | **G** | **A** | **A** | **C** | **G** | **A** | **G** | **C** | **G** | **G** | **C** | **A** | **T** | **C** | **G** | **T** | **T** | **T** | **G** | **G** | **C** | **G** | **C** | **G** | **G** | **G** | **A** | **G** | **T** | **T** | **G** | **C** | **A** | **T** | **G** | **A** | **T** | **T** | **C** | **A** | **G** | **T** | **C** | **A** | **G** | **T** | **C** | **A** | **A** | **C** | **A** | **A** | **C** | **T** | **T** | **T** | **T** | **T** | **G** | **C** | **A** | **G** | **C** | **C** | **A** | **T** | **G** | **A** | **T** | **G** | **A** | **T** | **G** | **T** | **T** | **G** | **T** | **C** | **T** | **G** | **C** | **C** | **T** | **T** | **A** | **A** | **A** | **T** | **G** | **A** | **A** | **C** | **A** | **A** | **G** | **C** | **A** | **C** | **A** | **A** | **C** | **G** | **A** | **A** | **C** | **A** | **G** | **A** | **A** | **A** | **C** | **C** | **C** | **C** | **G** | **G** | **A** | **A** | **C** | **C** | **A** | **T** | **A** | **T** | **C** | **G** | **T** | **A** | **A** | **A** | **C** | **A** | **A** | **C** | **T** | **A** | **G** | **C** | **C** | **A** | **T** | **G** | **G** | **T** | **G** | **G** | **C** | **A** | **G** | **A** | **A** | **A** | **T** | **C** | **A** | **T** | **T** | **A** | **A** | **T** | **G** | **C** | **C** | **T** | **C** | **C** | **C** | **A** | **A** | **T** | **C** | **G** | **G** | **A** | **A** | **A** | **T** | **G** | **C** | **G** | **C** | **G** | **C** | **G** | **T** | **T** | **A** | **C** | **T** | **A** | **T** | **T** | **G** | **C** | **A** | **C** | **T** | **T** | **G** | **C** | **G** | **T** | **C** | **C** | **T** | **A** | **T** | **C** | **A** | **G** | **T** | **C** | **T** | **A** | **G** | **A** | **A** | **G** | **G** | **A** | **A** | **A** | **A** | **A** | **G** | **T** | **T** | **T** | **G** | **C** | **G** | **T** | **A** | **A** | **A** | **G** | **G** | **T** | **A** | **T** | **T** | **G** | **A** | **A** | **C** | **A** | **A** | **T** | **T** | **A** | **C** | **T** | **G** | **A** | **A** | **A** | **G** | **A** | **A** | **C** | **T** | **A** | **C** | **A** | **A** | **A** | **C** | **A** | **A** | **A** | **A** | **A** | **T** | **T** | **A** | **A** | **A** | **A** | **T** | **T** | **G** | **A** | **A** | **T** | **T** | **G** | **A** | **T** | **T** | **T** | **G** | **G** | **G** | **A** | **T** | **G** | **T** | **T** | **G** | **A** | **A** | **G** | **A** | **T** | **G** | **T** | **T** | **C** | **A** | **T** | **T** | **T** | **A** | **A** | **A** | **T** | **A** | **G** | **C** | **A** | **G** | **C** | **A** | **T** | **T** | **G** | **A** | **G** | **G** | **A** | **T** | **C** | **A** | **T** | **C** | **T** | **T** | **T** | **T** | **C** | **C** | **G** | **A** | **A** | **T** | **T** | **G** | **T** | **G** | **C** | **A** | **G** | **G** | **A** | **A** | **T** | **T** | **A** | **C** | **T** | **T** | **T** | **C** | **A** | **A** | **A** | **T** | **A** | **C** | **C** | **T** | **T** | **A** | **A** | **G** | **A** | **C** | **A** | **T** | **G** | **C** | **C** | **A** | **A** | **A** | **G** | **C** | **A** | **A** | **A** | **G** | **G** | **A** | **A** | **T** | **T** | **A** | **G** | **A** | **G** | **G** | **T** | **A** | **T** | **A** | **C** | **T** | **T** | **A** | **C** | **A** | **C** | **C** | **A** | **A** | **G** | **T** | **C** | **G** | **A** | **T** | **A** | **A** | **A** | **A** | **A** | **C** | **G** | **T** | **G** | **T** | **T** | **A** | **T** | **T** | **G** | **C** | **G** | **T** | **A** | **T** | **T** | **G** | **T** | **T** | **G** | **A** | **T** | **G** | **A** | **T** | **G** | **G** | **T** | **G** | **T** | **C** | **G** | **G** | **C** | **T** | **T** | **T** | **G** | **A** | **T** | **A** | **T** | **G** | **A** | **A** | **G** | **G** | **A** | **A** | **C** | **A** | **A** | **A** | **G** | **T** | **A** | **A** | **T** | **A** | **A** | **A** | **G** | **C** | **C** | **G** | **G** | **T** | **A** | **G** | **T** | **T** | **A** | **T** | **G** | **G** | **C** | **T** | **T** | **A** | **A** | **A** | **T** | **A** | **A** | **T** | **A** | **T** | **T** | **C** | **G** | **A** | **G** | **A** | **A** | **C** | **G** | **T** | **G** | **T** | **T** | **G** | **T** | **C** | **G** | **G** | **C** | **A** | **T** | **G** | **G** | **G** | **C** | **G** | **G** | **T** | **A** | **C** | **A** | **G** | **T** | **T** | **A** | **A** | **A** | **A** | **T** | **T** | **A** | **T** | **T** | **A** | **G** | **T** | **T** | **T** | **T** | **A** | **A** | **A** | **G** | **G** | **G** | **C** | **A** | **G** | **G** | **G** | **A** | **A** | **C** | **C** | **A** | **G** | **C** | **G** | **T** | **T** | **G** | **A** | **A** | **A** | **T** | **T** | **A** | **A** | **A** | **G** | **T** | **T** | **C** | **C** | **T** | **G** | **T** | **C** | **A** | **T** | **A** | **A** | **A** | **G** | **G** | **A** | **G** | **G** | **A** | **A** | **A** | **C** | **T** | **G** | **C** | **A** | **A** | **G** | **T** | **G** | **A** | **T** | **C** | **A** | **A** | **A** | **G** | **T** | **A** | **A** | **T** | **G** | **T** | **T** | **A** | **G** | **T** | **G** | **G** | **A** | **-** | **-** | **-** |
| **N59505F** | **A** | **T** | **G** | **A** | **C** | **C** | **G** | **A** | **T** | **C** | **G** | **G** | **A** | **T** | **T** | **T** | **C** | **A** | **A** | **G** | **A** | **C** | **G** | **C** | **A** | **T** | **G** | **A** | **T** | **T** | **T** | **C** | **A** | **T** | **T** | **A** | **T** | **A** | **T** | **G** | **C** | **G** | **T** | **C** | **C** | **C** | **T** | **T** | **A** | **G** | **C** | **A** | **C** | **C** | **T** | **T** | **T** | **A** | **T** | **T** | **G** | **T** | **T** | **A** | **T** | **C** | **T** | **T** | **A** | **A** | **T** | **T** | **A** | **C** | **A** | **T** | **T** | **G** | **T** | **T** | **T** | **T** | **C** | **A** | **T** | **A** | **T** | **T** | **T** | **T** | **C** | **A** | **T** | **T** | **C** | **G** | **A** | **T** | **T** | **A** | **A** | **A** | **C** | **A** | **A** | **A** | **A** | **C** | **C** | **G** | **G** | **T** | **G** | **G** | **C** | **T** | **A** | **T** | **T** | **A** | **G** | **A** | **G** | **C** | **T** | **T** | **C** | **T** | **T** | **C** | **A** | **G** | **A** | **G** | **A** | **A** | **A** | **A** | **G** | **T** | **C** | **T** | **T** | **T** | **T** | **A** | **T** | **C** | **T** | **A** | **C** | **C** | **A** | **C** | **T** | **A** | **A** | **T** | **T** | **G** | **T** | **G** | **C** | **A** | **C** | **A** | **T** | **T** | **G** | **T** | **T** | **C** | **T** | **C** | **A** | **T** | **A** | **T** | **C** | **C** | **T** | **T** | **A** | **C** | **T** | **A** | **A** | **T** | **A** | **G** | **G** | **C** | **T** | **T** | **A** | **T** | **T** | **G** | **A** | **C** | **C** | **T** | **T** | **T** | **T** | **T** | **A** | **C** | **T** | **G** | **A** | **T** | **T** | **T** | **C** | **A** | **T** | **T** | **G** | **G** | **T** | **T** | **C** | **A** | **A** | **A** | **A** | **A** | **G** | **G** | **G** | **C** | **A** | **A** | **T** | **A** | **T** | **G** | **G** | **A** | **C** | **G** | **G** | **A** | **T** | **T** | **G** | **A** | **A** | **G** | **A** | **A** | **A** | **A** | **A** | **C** | **T** | **T** | **C** | **G** | **G** | **T** | **T** | **A** | **T** | **T** | **G** | **G** | **C** | **C** | **A** | **A** | **T** | **G** | **G** | **C** | **A** | **A** | **T** | **T** | **A** | **T** | **G** | **A** | **A** | **A** | **G** | **T** | **C** | **C** | **A** | **G** | **T** | **C** | **T** | **T** | **A** | **A** | **A** | **C** | **A** | **A** | **A** | **C** | **C** | **A** | **A** | **C** | **G** | **A** | **C** | **C** | **A** | **G** | **T** | **G** | **A** | **A** | **A** | **A** | **T** | **C** | **A** | **A** | **G** | **A** | **T** | **C** | **A** | **T** | **T** | **A** | **T** | **C** | **T** | **A** | **A** | **C** | **C** | **G** | **A** | **A** | **G** | **T** | **C** | **G** | **A** | **A** | **C** | **A** | **A** | **G** | **A** | **T** | **A** | **T** | **T** | **T** | **G** | **G** | **T** | **C** | **G** | **A** | **T** | **T** | **A** | **A** | **A** | **A** | **A** | **T** | **A** | **A** | **A** | **T** | **T** | **A** | **T** | **T** | **A** | **G** | **A** | **G** | **A** | **T** | **G** | **T** | **C** | **T** | **A** | **A** | **A** | **G** | **A** | **A** | **T** | **T** | **G** | **C** | **A** | **A** | **T** | **T** | **A** | **T** | **T** | **A** | **A** | **A** | **C** | **A** | **G** | **T** | **C** | **G** | **G** | **C** | **C** | **G** | **C** | **A** | **A** | **T** | **T** | **A** | **A** | **T** | **G** | **G** | **A** | **T** | **G** | **G** | **G** | **C** | **A** | **A** | **A** | **C** | **A** | **A** | **A** | **A** | **G** | **A** | **A** | **G** | **A** | **A** | **A** | **T** | **T** | **T** | **T** | **A** | **G** | **A** | **G** | **A** | **A** | **C** | **G** | **A** | **G** | **C** | **G** | **G** | **C** | **A** | **T** | **C** | **G** | **T** | **T** | **T** | **G** | **G** | **C** | **G** | **C** | **G** | **G** | **G** | **A** | **G** | **T** | **T** | **G** | **C** | **A** | **T** | **G** | **A** | **T** | **T** | **C** | **A** | **G** | **T** | **C** | **A** | **G** | **T** | **C** | **A** | **A** | **C** | **A** | **A** | **C** | **T** | **T** | **T** | **T** | **T** | **G** | **C** | **A** | **G** | **C** | **C** | **A** | **T** | **G** | **A** | **T** | **G** | **A** | **T** | **G** | **T** | **T** | **G** | **T** | **C** | **T** | **G** | **C** | **C** | **T** | **T** | **A** | **A** | **A** | **T** | **G** | **A** | **A** | **C** | **A** | **A** | **G** | **C** | **A** | **C** | **A** | **A** | **C** | **G** | **A** | **A** | **C** | **A** | **G** | **A** | **A** | **A** | **C** | **C** | **C** | **C** | **G** | **G** | **A** | **A** | **C** | **C** | **A** | **T** | **A** | **T** | **C** | **G** | **T** | **A** | **A** | **A** | **C** | **A** | **A** | **C** | **T** | **A** | **G** | **C** | **C** | **A** | **T** | **G** | **G** | **T** | **G** | **G** | **C** | **A** | **G** | **A** | **A** | **A** | **T** | **C** | **A** | **T** | **T** | **A** | **A** | **T** | **G** | **C** | **C** | **T** | **C** | **C** | **C** | **A** | **A** | **T** | **C** | **G** | **G** | **A** | **A** | **A** | **T** | **G** | **C** | **G** | **C** | **G** | **C** | **G** | **T** | **T** | **A** | **C** | **T** | **A** | **T** | **T** | **G** | **C** | **A** | **C** | **T** | **T** | **G** | **C** | **G** | **T** | **C** | **C** | **T** | **A** | **T** | **C** | **A** | **G** | **T** | **C** | **T** | **A** | **G** | **A** | **A** | **G** | **G** | **A** | **A** | **A** | **A** | **A** | **G** | **T** | **T** | **T** | **G** | **C** | **G** | **T** | **A** | **A** | **A** | **G** | **G** | **T** | **A** | **T** | **T** | **G** | **A** | **A** | **C** | **A** | **A** | **T** | **T** | **A** | **C** | **T** | **G** | **A** | **A** | **A** | **G** | **A** | **A** | **C** | **T** | **A** | **C** | **A** | **A** | **A** | **C** | **A** | **A** | **A** | **A** | **A** | **T** | **T** | **A** | **A** | **A** | **A** | **T** | **T** | **G** | **A** | **A** | **T** | **T** | **G** | **A** | **T** | **T** | **T** | **G** | **G** | **G** | **A** | **T** | **G** | **T** | **T** | **G** | **A** | **A** | **G** | **A** | **T** | **G** | **T** | **T** | **C** | **A** | **T** | **T** | **T** | **A** | **A** | **A** | **T** | **A** | **G** | **C** | **A** | **G** | **C** | **A** | **T** | **T** | **G** | **A** | **G** | **G** | **A** | **T** | **C** | **A** | **T** | **C** | **T** | **T** | **T** | **T** | **C** | **C** | **G** | **A** | **A** | **T** | **T** | **G** | **T** | **G** | **C** | **A** | **G** | **G** | **A** | **G** | **T** | **T** | **A** | **C** | **T** | **T** | **T** | **C** | **A** | **A** | **A** | **T** | **A** | **C** | **C** | **T** | **T** | **A** | **A** | **G** | **A** | **C** | **A** | **T** | **G** | **C** | **T** | **A** | **A** | **A** | **G** | **C** | **A** | **A** | **A** | **G** | **G** | **A** | **A** | **T** | **T** | **A** | **G** | **A** | **G** | **G** | **T** | **A** | **T** | **A** | **T** | **T** | **T** | **A** | **C** | **A** | **C** | **C** | **A** | **A** | **G** | **T** | **T** | **G** | **A** | **T** | **A** | **A** | **A** | **A** | **A** | **C** | **G** | **T** | **G** | **T** | **T** | **A** | **T** | **T** | **G** | **C** | **G** | **T** | **A** | **T** | **T** | **G** | **T** | **T** | **G** | **A** | **T** | **G** | **A** | **T** | **G** | **G** | **T** | **G** | **T** | **C** | **G** | **G** | **C** | **T** | **T** | **T** | **G** | **A** | **T** | **A** | **T** | **G** | **A** | **A** | **G** | **G** | **A** | **A** | **C** | **A** | **A** | **A** | **G** | **T** | **A** | **A** | **T** | **A** | **A** | **A** | **G** | **C** | **C** | **G** | **G** | **T** | **A** | **G** | **T** | **T** | **A** | **T** | **G** | **G** | **C** | **T** | **T** | **A** | **A** | **A** | **T** | **A** | **A** | **T** | **A** | **T** | **T** | **C** | **G** | **A** | **G** | **A** | **A** | **C** | **G** | **T** | **G** | **T** | **T** | **G** | **T** | **C** | **G** | **G** | **C** | **A** | **T** | **G** | **G** | **G** | **C** | **G** | **G** | **T** | **A** | **C** | **A** | **G** | **T** | **T** | **A** | **A** | **A** | **A** | **T** | **T** | **A** | **T** | **T** | **A** | **G** | **T** | **T** | **T** | **T** | **A** | **A** | **A** | **G** | **G** | **G** | **C** | **A** | **G** | **G** | **G** | **A** | **A** | **C** | **C** | **A** | **G** | **C** | **G** | **T** | **T** | **G** | **A** | **A** | **A** | **T** | **T** | **A** | **A** | **A** | **G** | **T** | **T** | **C** | **C** | **T** | **G** | **T** | **C** | **A** | **T** | **A** | **A** | **A** | **G** | **G** | **A** | **G** | **G** | **A** | **A** | **A** | **C** | **T** | **G** | **C** | **A** | **A** | **G** | **T** | **G** | **A** | **T** | **C** | **A** | **A** | **A** | **G** | **T** | **A** | **A** | **T** | **G** | **T** | **T** | **A** | **G** | **T** | **G** | **G** | **A** | **-** | **-** | **-** |
| **N59910F** | **A** | **T** | **G** | **A** | **C** | **C** | **G** | **A** | **T** | **C** | **G** | **G** | **A** | **T** | **T** | **T** | **C** | **A** | **A** | **G** | **A** | **C** | **G** | **C** | **A** | **T** | **G** | **A** | **T** | **T** | **T** | **C** | **A** | **T** | **T** | **A** | **T** | **A** | **T** | **G** | **C** | **G** | **T** | **C** | **C** | **C** | **T** | **T** | **A** | **G** | **C** | **A** | **C** | **C** | **T** | **T** | **T** | **A** | **T** | **T** | **G** | **T** | **T** | **A** | **T** | **C** | **T** | **T** | **A** | **A** | **T** | **T** | **A** | **C** | **A** | **T** | **T** | **G** | **T** | **T** | **T** | **T** | **C** | **A** | **T** | **A** | **T** | **T** | **T** | **T** | **C** | **A** | **T** | **T** | **C** | **G** | **A** | **T** | **T** | **A** | **A** | **A** | **C** | **A** | **A** | **A** | **A** | **C** | **C** | **G** | **G** | **T** | **G** | **G** | **T** | **T** | **A** | **T** | **T** | **A** | **G** | **A** | **G** | **C** | **T** | **T** | **C** | **T** | **T** | **C** | **A** | **G** | **A** | **G** | **A** | **A** | **A** | **A** | **G** | **T** | **C** | **T** | **T** | **T** | **T** | **A** | **T** | **T** | **T** | **A** | **C** | **C** | **A** | **C** | **T** | **A** | **A** | **T** | **T** | **G** | **T** | **G** | **C** | **A** | **C** | **A** | **T** | **T** | **G** | **T** | **T** | **C** | **T** | **C** | **A** | **T** | **A** | **T** | **C** | **C** | **T** | **T** | **A** | **C** | **T** | **A** | **A** | **T** | **A** | **G** | **G** | **C** | **T** | **T** | **A** | **T** | **T** | **G** | **A** | **C** | **C** | **T** | **T** | **T** | **T** | **T** | **A** | **C** | **T** | **G** | **A** | **T** | **T** | **T** | **C** | **A** | **T** | **T** | **G** | **G** | **T** | **T** | **C** | **A** | **A** | **A** | **A** | **A** | **G** | **G** | **G** | **C** | **A** | **A** | **T** | **A** | **T** | **G** | **G** | **A** | **C** | **G** | **G** | **A** | **T** | **T** | **G** | **A** | **A** | **G** | **A** | **A** | **A** | **A** | **A** | **C** | **T** | **T** | **C** | **G** | **G** | **T** | **T** | **A** | **T** | **T** | **G** | **G** | **C** | **C** | **A** | **A** | **C** | **G** | **G** | **T** | **A** | **A** | **T** | **T** | **A** | **T** | **G** | **A** | **A** | **A** | **G** | **T** | **C** | **C** | **A** | **G** | **T** | **C** | **T** | **T** | **A** | **A** | **A** | **C** | **A** | **A** | **A** | **C** | **C** | **A** | **A** | **C** | **G** | **A** | **C** | **C** | **A** | **G** | **T** | **G** | **A** | **A** | **A** | **A** | **T** | **C** | **A** | **A** | **G** | **A** | **C** | **C** | **A** | **T** | **T** | **A** | **T** | **C** | **T** | **A** | **A** | **C** | **C** | **G** | **A** | **A** | **G** | **T** | **C** | **G** | **A** | **A** | **C** | **A** | **A** | **G** | **A** | **T** | **A** | **T** | **T** | **T** | **G** | **G** | **T** | **C** | **G** | **A** | **T** | **T** | **A** | **A** | **A** | **A** | **A** | **T** | **A** | **A** | **A** | **T** | **T** | **A** | **T** | **T** | **A** | **G** | **A** | **G** | **A** | **T** | **G** | **T** | **C** | **T** | **A** | **A** | **A** | **G** | **A** | **A** | **T** | **T** | **G** | **C** | **A** | **A** | **T** | **T** | **A** | **T** | **T** | **A** | **A** | **A** | **C** | **A** | **G** | **T** | **C** | **G** | **A** | **C** | **C** | **G** | **C** | **A** | **A** | **T** | **T** | **A** | **A** | **T** | **G** | **G** | **A** | **T** | **G** | **G** | **G** | **C** | **A** | **A** | **A** | **C** | **A** | **A** | **A** | **A** | **G** | **A** | **A** | **G** | **A** | **A** | **A** | **T** | **T** | **T** | **T** | **A** | **G** | **A** | **G** | **A** | **A** | **C** | **G** | **A** | **G** | **C** | **G** | **G** | **C** | **A** | **T** | **C** | **G** | **T** | **T** | **T** | **G** | **G** | **C** | **G** | **C** | **G** | **G** | **G** | **A** | **G** | **T** | **T** | **G** | **C** | **A** | **T** | **G** | **A** | **T** | **T** | **C** | **A** | **G** | **T** | **C** | **A** | **G** | **T** | **C** | **A** | **A** | **C** | **A** | **A** | **C** | **T** | **T** | **T** | **T** | **T** | **G** | **C** | **A** | **G** | **C** | **C** | **A** | **T** | **G** | **A** | **T** | **G** | **A** | **T** | **G** | **T** | **T** | **G** | **T** | **C** | **T** | **G** | **C** | **A** | **T** | **T** | **A** | **A** | **A** | **T** | **G** | **A** | **A** | **C** | **A** | **A** | **G** | **C** | **A** | **C** | **A** | **A** | **C** | **G** | **A** | **A** | **C** | **A** | **G** | **A** | **A** | **A** | **C** | **C** | **C** | **C** | **G** | **G** | **A** | **A** | **C** | **C** | **A** | **T** | **A** | **T** | **C** | **G** | **T** | **A** | **A** | **A** | **C** | **A** | **A** | **C** | **T** | **A** | **G** | **C** | **C** | **A** | **T** | **G** | **G** | **T** | **G** | **G** | **C** | **A** | **G** | **A** | **A** | **A** | **T** | **C** | **A** | **T** | **T** | **A** | **A** | **T** | **G** | **C** | **C** | **T** | **C** | **C** | **C** | **A** | **A** | **T** | **C** | **G** | **G** | **A** | **A** | **A** | **T** | **G** | **C** | **G** | **C** | **G** | **C** | **G** | **C** | **T** | **A** | **C** | **T** | **A** | **T** | **T** | **G** | **C** | **A** | **C** | **T** | **T** | **G** | **C** | **G** | **T** | **C** | **C** | **T** | **A** | **T** | **C** | **A** | **G** | **T** | **C** | **T** | **A** | **G** | **A** | **A** | **G** | **G** | **A** | **A** | **A** | **A** | **A** | **G** | **T** | **T** | **T** | **G** | **C** | **G** | **T** | **A** | **A** | **A** | **G** | **G** | **T** | **A** | **T** | **T** | **G** | **A** | **A** | **C** | **A** | **A** | **T** | **T** | **A** | **C** | **T** | **G** | **A** | **A** | **A** | **G** | **A** | **A** | **C** | **T** | **A** | **C** | **A** | **A** | **A** | **C** | **A** | **A** | **A** | **A** | **A** | **T** | **T** | **A** | **A** | **A** | **A** | **T** | **T** | **G** | **A** | **A** | **T** | **T** | **G** | **A** | **T** | **T** | **T** | **G** | **G** | **G** | **A** | **T** | **G** | **T** | **T** | **G** | **A** | **A** | **G** | **A** | **T** | **G** | **T** | **T** | **C** | **A** | **T** | **T** | **T** | **A** | **A** | **A** | **T** | **A** | **G** | **C** | **A** | **G** | **C** | **A** | **T** | **T** | **G** | **A** | **G** | **G** | **A** | **T** | **C** | **A** | **T** | **C** | **T** | **T** | **T** | **T** | **C** | **C** | **G** | **A** | **A** | **T** | **T** | **G** | **T** | **G** | **C** | **A** | **G** | **G** | **A** | **G** | **T** | **T** | **A** | **C** | **T** | **T** | **T** | **C** | **A** | **A** | **A** | **T** | **A** | **C** | **C** | **T** | **T** | **A** | **A** | **G** | **A** | **C** | **A** | **T** | **G** | **C** | **C** | **A** | **A** | **A** | **G** | **C** | **A** | **A** | **A** | **G** | **G** | **A** | **A** | **T** | **T** | **A** | **G** | **A** | **G** | **G** | **T** | **A** | **T** | **A** | **C** | **T** | **T** | **A** | **C** | **A** | **C** | **C** | **A** | **A** | **G** | **T** | **C** | **G** | **A** | **T** | **A** | **A** | **A** | **A** | **A** | **C** | **G** | **T** | **G** | **C** | **T** | **A** | **T** | **T** | **G** | **C** | **G** | **T** | **A** | **T** | **T** | **G** | **T** | **T** | **G** | **A** | **T** | **G** | **A** | **C** | **G** | **G** | **T** | **G** | **T** | **C** | **G** | **G** | **C** | **T** | **T** | **T** | **G** | **A** | **T** | **A** | **T** | **G** | **A** | **A** | **G** | **G** | **A** | **A** | **C** | **A** | **A** | **A** | **G** | **T** | **A** | **A** | **T** | **A** | **A** | **A** | **G** | **C** | **C** | **G** | **G** | **T** | **A** | **G** | **T** | **T** | **A** | **T** | **G** | **G** | **C** | **T** | **T** | **A** | **A** | **A** | **T** | **A** | **A** | **T** | **A** | **T** | **C** | **C** | **G** | **A** | **G** | **A** | **A** | **C** | **G** | **T** | **G** | **T** | **T** | **G** | **T** | **C** | **G** | **G** | **C** | **A** | **T** | **G** | **G** | **G** | **C** | **G** | **G** | **T** | **A** | **C** | **A** | **G** | **T** | **T** | **A** | **A** | **A** | **A** | **T** | **T** | **A** | **T** | **T** | **A** | **G** | **T** | **T** | **T** | **T** | **A** | **A** | **A** | **G** | **G** | **G** | **C** | **A** | **G** | **G** | **G** | **A** | **A** | **C** | **C** | **A** | **G** | **C** | **G** | **T** | **T** | **G** | **A** | **A** | **A** | **T** | **T** | **A** | **A** | **A** | **G** | **T** | **T** | **C** | **C** | **T** | **G** | **T** | **C** | **A** | **T** | **A** | **A** | **A** | **G** | **G** | **A** | **G** | **G** | **A** | **A** | **A** | **C** | **T** | **G** | **C** | **A** | **A** | **G** | **T** | **G** | **A** | **T** | **C** | **A** | **A** | **A** | **G** | **T** | **A** | **A** | **T** | **G** | **T** | **T** | **A** | **G** | **T** | **G** | **G** | **A** | **-** | **-** | **-** |
| **N55706** | **A** | **T** | **G** | **A** | **C** | **C** | **G** | **A** | **T** | **C** | **G** | **G** | **A** | **T** | **T** | **T** | **C** | **A** | **A** | **G** | **A** | **C** | **G** | **C** | **A** | **T** | **G** | **A** | **T** | **T** | **T** | **C** | **A** | **T** | **T** | **A** | **T** | **A** | **T** | **G** | **C** | **G** | **T** | **C** | **C** | **C** | **T** | **T** | **A** | **G** | **C** | **A** | **C** | **C** | **T** | **T** | **T** | **A** | **T** | **T** | **G** | **T** | **T** | **A** | **T** | **C** | **T** | **T** | **A** | **A** | **T** | **T** | **A** | **C** | **A** | **T** | **T** | **G** | **T** | **T** | **T** | **T** | **C** | **A** | **T** | **A** | **T** | **T** | **T** | **T** | **C** | **A** | **T** | **T** | **C** | **G** | **A** | **T** | **T** | **A** | **A** | **A** | **C** | **A** | **A** | **A** | **A** | **C** | **C** | **G** | **G** | **T** | **G** | **G** | **C** | **T** | **A** | **T** | **T** | **A** | **G** | **A** | **G** | **C** | **T** | **T** | **C** | **T** | **T** | **C** | **A** | **G** | **A** | **G** | **A** | **A** | **A** | **A** | **G** | **T** | **C** | **T** | **T** | **T** | **T** | **A** | **T** | **T** | **T** | **A** | **C** | **C** | **A** | **C** | **T** | **A** | **A** | **T** | **T** | **G** | **T** | **G** | **C** | **A** | **C** | **A** | **T** | **T** | **G** | **T** | **T** | **C** | **T** | **C** | **A** | **T** | **A** | **T** | **C** | **C** | **T** | **T** | **A** | **C** | **T** | **A** | **A** | **T** | **A** | **G** | **G** | **C** | **T** | **T** | **A** | **T** | **T** | **G** | **A** | **C** | **C** | **T** | **T** | **T** | **T** | **T** | **A** | **C** | **T** | **G** | **A** | **T** | **T** | **T** | **C** | **A** | **T** | **T** | **G** | **G** | **T** | **T** | **C** | **A** | **A** | **A** | **A** | **A** | **G** | **G** | **G** | **C** | **A** | **A** | **T** | **A** | **T** | **G** | **G** | **A** | **C** | **G** | **G** | **A** | **T** | **T** | **G** | **A** | **A** | **G** | **A** | **A** | **A** | **A** | **A** | **C** | **T** | **T** | **C** | **G** | **G** | **T** | **T** | **A** | **T** | **T** | **G** | **G** | **C** | **C** | **A** | **A** | **C** | **G** | **G** | **T** | **A** | **A** | **T** | **T** | **A** | **T** | **G** | **A** | **A** | **A** | **G** | **T** | **C** | **C** | **A** | **G** | **T** | **C** | **T** | **T** | **A** | **A** | **A** | **C** | **A** | **A** | **A** | **C** | **C** | **A** | **A** | **C** | **G** | **A** | **C** | **C** | **A** | **G** | **T** | **G** | **A** | **A** | **A** | **A** | **T** | **C** | **A** | **A** | **G** | **A** | **C** | **C** | **A** | **T** | **T** | **A** | **T** | **C** | **T** | **A** | **A** | **C** | **C** | **G** | **A** | **A** | **G** | **T** | **C** | **G** | **A** | **A** | **C** | **A** | **A** | **G** | **A** | **T** | **A** | **T** | **T** | **T** | **G** | **G** | **T** | **C** | **G** | **A** | **T** | **T** | **A** | **A** | **A** | **A** | **A** | **T** | **A** | **A** | **A** | **T** | **T** | **A** | **T** | **T** | **A** | **G** | **A** | **G** | **A** | **T** | **G** | **T** | **C** | **T** | **A** | **A** | **A** | **G** | **A** | **A** | **T** | **T** | **G** | **C** | **A** | **A** | **T** | **T** | **A** | **T** | **T** | **A** | **A** | **A** | **C** | **A** | **G** | **T** | **C** | **G** | **A** | **C** | **C** | **G** | **C** | **A** | **A** | **T** | **T** | **A** | **A** | **T** | **G** | **G** | **A** | **T** | **G** | **G** | **G** | **C** | **A** | **A** | **A** | **C** | **A** | **A** | **A** | **A** | **G** | **A** | **A** | **G** | **A** | **A** | **A** | **T** | **T** | **T** | **T** | **A** | **G** | **A** | **G** | **A** | **A** | **C** | **G** | **A** | **G** | **C** | **G** | **G** | **C** | **A** | **T** | **C** | **G** | **T** | **T** | **T** | **G** | **G** | **C** | **G** | **C** | **G** | **G** | **G** | **A** | **G** | **T** | **T** | **G** | **C** | **A** | **T** | **G** | **A** | **T** | **T** | **C** | **A** | **G** | **T** | **C** | **A** | **G** | **T** | **C** | **A** | **A** | **C** | **A** | **A** | **C** | **T** | **T** | **T** | **T** | **T** | **G** | **C** | **A** | **G** | **C** | **C** | **A** | **T** | **G** | **A** | **T** | **G** | **A** | **T** | **G** | **T** | **T** | **G** | **T** | **C** | **T** | **G** | **C** | **C** | **T** | **T** | **A** | **A** | **A** | **T** | **G** | **A** | **A** | **C** | **A** | **A** | **G** | **C** | **A** | **C** | **A** | **A** | **C** | **G** | **A** | **A** | **C** | **A** | **G** | **A** | **A** | **A** | **C** | **T** | **C** | **C** | **T** | **G** | **A** | **A** | **C** | **C** | **A** | **T** | **A** | **T** | **C** | **G** | **C** | **A** | **A** | **A** | **C** | **A** | **A** | **C** | **T** | **A** | **G** | **C** | **C** | **A** | **T** | **G** | **G** | **T** | **G** | **G** | **C** | **A** | **G** | **A** | **A** | **A** | **T** | **C** | **A** | **T** | **T** | **A** | **A** | **T** | **G** | **C** | **C** | **T** | **C** | **C** | **C** | **A** | **A** | **T** | **C** | **G** | **G** | **A** | **A** | **A** | **T** | **G** | **C** | **G** | **C** | **G** | **C** | **G** | **T** | **T** | **A** | **C** | **T** | **A** | **T** | **T** | **G** | **C** | **A** | **C** | **T** | **T** | **G** | **C** | **G** | **T** | **C** | **C** | **T** | **A** | **T** | **C** | **A** | **G** | **T** | **C** | **T** | **A** | **G** | **A** | **A** | **G** | **G** | **A** | **A** | **A** | **A** | **A** | **G** | **T** | **T** | **T** | **G** | **C** | **G** | **T** | **A** | **A** | **A** | **G** | **G** | **T** | **A** | **T** | **T** | **G** | **A** | **A** | **C** | **A** | **A** | **T** | **T** | **A** | **C** | **T** | **G** | **A** | **A** | **A** | **G** | **A** | **A** | **C** | **T** | **A** | **C** | **A** | **A** | **A** | **C** | **A** | **A** | **A** | **A** | **A** | **T** | **T** | **A** | **A** | **A** | **A** | **T** | **T** | **G** | **A** | **A** | **T** | **T** | **G** | **A** | **T** | **T** | **T** | **G** | **G** | **G** | **A** | **T** | **G** | **T** | **T** | **G** | **A** | **A** | **G** | **A** | **T** | **G** | **T** | **T** | **C** | **A** | **T** | **T** | **T** | **A** | **A** | **A** | **T** | **A** | **G** | **C** | **A** | **G** | **C** | **A** | **T** | **T** | **G** | **A** | **G** | **G** | **A** | **T** | **C** | **A** | **T** | **C** | **T** | **T** | **T** | **T** | **C** | **C** | **G** | **A** | **A** | **T** | **T** | **G** | **T** | **G** | **C** | **A** | **G** | **G** | **A** | **A** | **T** | **T** | **A** | **C** | **T** | **T** | **T** | **C** | **A** | **A** | **A** | **T** | **A** | **C** | **C** | **T** | **T** | **A** | **A** | **G** | **A** | **C** | **A** | **T** | **G** | **C** | **C** | **A** | **A** | **A** | **G** | **C** | **A** | **A** | **A** | **G** | **G** | **A** | **A** | **T** | **T** | **A** | **G** | **A** | **G** | **G** | **T** | **A** | **T** | **A** | **C** | **T** | **T** | **A** | **C** | **A** | **C** | **C** | **A** | **A** | **G** | **T** | **C** | **G** | **A** | **T** | **A** | **A** | **A** | **A** | **A** | **C** | **G** | **T** | **G** | **T** | **T** | **A** | **T** | **T** | **G** | **C** | **G** | **T** | **A** | **T** | **T** | **G** | **T** | **T** | **G** | **A** | **T** | **G** | **A** | **T** | **G** | **G** | **T** | **G** | **T** | **C** | **G** | **G** | **C** | **T** | **T** | **T** | **G** | **A** | **T** | **A** | **T** | **G** | **A** | **A** | **G** | **G** | **A** | **A** | **C** | **A** | **A** | **A** | **G** | **T** | **A** | **A** | **T** | **A** | **A** | **A** | **G** | **C** | **C** | **G** | **G** | **T** | **A** | **G** | **T** | **T** | **A** | **T** | **G** | **G** | **C** | **T** | **T** | **A** | **A** | **A** | **T** | **A** | **A** | **T** | **A** | **T** | **C** | **C** | **G** | **A** | **G** | **A** | **A** | **C** | **G** | **T** | **G** | **T** | **T** | **G** | **T** | **C** | **G** | **G** | **C** | **A** | **T** | **G** | **G** | **G** | **C** | **G** | **G** | **T** | **A** | **C** | **A** | **G** | **T** | **T** | **A** | **A** | **A** | **A** | **T** | **T** | **A** | **T** | **T** | **A** | **G** | **T** | **T** | **T** | **T** | **A** | **A** | **A** | **G** | **G** | **G** | **C** | **A** | **G** | **G** | **G** | **A** | **A** | **C** | **C** | **A** | **G** | **C** | **G** | **T** | **T** | **G** | **A** | **A** | **A** | **T** | **T** | **A** | **A** | **A** | **G** | **T** | **T** | **C** | **C** | **T** | **G** | **T** | **C** | **A** | **T** | **A** | **A** | **A** | **G** | **G** | **A** | **G** | **G** | **A** | **A** | **A** | **C** | **T** | **G** | **C** | **A** | **A** | **G** | **T** | **G** | **A** | **T** | **C** | **A** | **A** | **A** | **G** | **T** | **A** | **A** | **T** | **G** | **T** | **T** | **A** | **G** | **T** | **G** | **G** | **A** | **-** | **-** | **-** |
| **N59554F** | **A** | **T** | **G** | **A** | **C** | **C** | **G** | **A** | **T** | **C** | **G** | **G** | **A** | **T** | **T** | **T** | **C** | **A** | **A** | **G** | **A** | **C** | **G** | **C** | **A** | **T** | **G** | **A** | **T** | **T** | **T** | **C** | **A** | **T** | **T** | **A** | **T** | **A** | **T** | **G** | **C** | **G** | **T** | **C** | **C** | **C** | **T** | **T** | **A** | **G** | **C** | **A** | **C** | **C** | **T** | **T** | **T** | **A** | **T** | **T** | **G** | **T** | **T** | **A** | **T** | **C** | **T** | **T** | **A** | **A** | **T** | **T** | **A** | **C** | **A** | **T** | **T** | **G** | **T** | **T** | **T** | **T** | **C** | **A** | **T** | **A** | **T** | **T** | **T** | **T** | **C** | **A** | **T** | **T** | **C** | **G** | **A** | **T** | **T** | **A** | **A** | **A** | **C** | **A** | **A** | **A** | **A** | **C** | **C** | **G** | **G** | **T** | **G** | **G** | **T** | **T** | **A** | **T** | **T** | **A** | **G** | **A** | **G** | **C** | **T** | **T** | **C** | **T** | **T** | **C** | **A** | **G** | **A** | **G** | **A** | **A** | **A** | **A** | **G** | **T** | **C** | **T** | **T** | **T** | **T** | **A** | **T** | **T** | **T** | **A** | **C** | **C** | **A** | **C** | **T** | **A** | **A** | **T** | **T** | **G** | **T** | **G** | **C** | **A** | **C** | **A** | **T** | **T** | **G** | **T** | **T** | **C** | **T** | **C** | **A** | **T** | **A** | **T** | **C** | **C** | **T** | **T** | **A** | **C** | **T** | **A** | **A** | **T** | **A** | **G** | **G** | **C** | **T** | **T** | **A** | **T** | **T** | **G** | **A** | **C** | **C** | **T** | **T** | **T** | **T** | **T** | **A** | **C** | **T** | **G** | **A** | **T** | **T** | **T** | **C** | **A** | **T** | **T** | **G** | **G** | **T** | **T** | **C** | **A** | **A** | **A** | **A** | **A** | **G** | **G** | **G** | **C** | **A** | **A** | **T** | **A** | **T** | **G** | **G** | **A** | **C** | **G** | **G** | **A** | **T** | **T** | **G** | **A** | **A** | **G** | **A** | **A** | **A** | **A** | **A** | **C** | **T** | **T** | **C** | **G** | **G** | **T** | **T** | **A** | **T** | **T** | **G** | **G** | **C** | **C** | **A** | **A** | **C** | **G** | **G** | **T** | **A** | **A** | **T** | **T** | **A** | **T** | **G** | **A** | **A** | **A** | **G** | **T** | **C** | **C** | **A** | **G** | **T** | **C** | **T** | **T** | **A** | **A** | **A** | **C** | **A** | **A** | **A** | **C** | **C** | **A** | **A** | **C** | **G** | **A** | **C** | **C** | **A** | **G** | **T** | **G** | **A** | **A** | **A** | **A** | **T** | **C** | **A** | **A** | **G** | **A** | **C** | **C** | **A** | **T** | **T** | **A** | **T** | **C** | **T** | **A** | **A** | **C** | **C** | **G** | **A** | **A** | **G** | **T** | **C** | **G** | **A** | **A** | **C** | **A** | **A** | **G** | **A** | **T** | **A** | **T** | **T** | **T** | **G** | **G** | **T** | **C** | **G** | **A** | **T** | **T** | **A** | **A** | **A** | **A** | **A** | **T** | **A** | **A** | **A** | **T** | **T** | **A** | **T** | **T** | **A** | **G** | **A** | **G** | **A** | **T** | **G** | **T** | **C** | **T** | **A** | **A** | **A** | **G** | **A** | **A** | **T** | **T** | **G** | **C** | **A** | **A** | **T** | **T** | **A** | **T** | **T** | **A** | **A** | **A** | **C** | **A** | **G** | **T** | **C** | **G** | **A** | **C** | **C** | **G** | **C** | **A** | **A** | **T** | **T** | **A** | **A** | **T** | **G** | **G** | **A** | **T** | **G** | **G** | **G** | **C** | **A** | **A** | **A** | **C** | **A** | **A** | **A** | **A** | **G** | **A** | **A** | **G** | **A** | **A** | **A** | **T** | **T** | **T** | **T** | **A** | **G** | **A** | **G** | **A** | **A** | **C** | **G** | **A** | **G** | **C** | **G** | **G** | **C** | **A** | **T** | **C** | **G** | **T** | **T** | **T** | **G** | **G** | **C** | **G** | **C** | **G** | **G** | **G** | **A** | **G** | **T** | **T** | **G** | **C** | **A** | **T** | **G** | **A** | **T** | **T** | **C** | **A** | **G** | **T** | **C** | **A** | **G** | **T** | **C** | **A** | **A** | **C** | **A** | **A** | **C** | **T** | **T** | **T** | **T** | **T** | **G** | **C** | **A** | **G** | **C** | **C** | **A** | **T** | **G** | **A** | **T** | **G** | **A** | **T** | **G** | **T** | **T** | **G** | **T** | **C** | **T** | **G** | **C** | **C** | **T** | **T** | **A** | **A** | **A** | **T** | **G** | **A** | **A** | **C** | **A** | **A** | **G** | **C** | **A** | **C** | **A** | **A** | **C** | **G** | **A** | **A** | **C** | **A** | **G** | **A** | **A** | **A** | **C** | **C** | **C** | **C** | **G** | **G** | **A** | **A** | **C** | **C** | **A** | **T** | **A** | **T** | **C** | **G** | **T** | **A** | **A** | **A** | **C** | **A** | **A** | **C** | **T** | **A** | **G** | **C** | **C** | **A** | **T** | **G** | **G** | **T** | **G** | **G** | **C** | **A** | **G** | **A** | **A** | **A** | **T** | **C** | **A** | **T** | **T** | **A** | **A** | **T** | **G** | **C** | **C** | **T** | **C** | **C** | **C** | **A** | **A** | **T** | **C** | **G** | **G** | **A** | **A** | **A** | **T** | **G** | **C** | **G** | **C** | **G** | **C** | **G** | **T** | **T** | **A** | **C** | **T** | **A** | **T** | **T** | **G** | **C** | **A** | **C** | **T** | **T** | **G** | **C** | **G** | **T** | **C** | **C** | **T** | **A** | **T** | **C** | **A** | **G** | **T** | **C** | **T** | **A** | **G** | **A** | **A** | **G** | **G** | **A** | **A** | **A** | **A** | **A** | **G** | **T** | **T** | **T** | **G** | **C** | **G** | **T** | **A** | **A** | **A** | **G** | **G** | **T** | **A** | **T** | **T** | **G** | **A** | **A** | **C** | **A** | **A** | **T** | **T** | **A** | **C** | **T** | **G** | **A** | **A** | **A** | **G** | **A** | **A** | **C** | **T** | **A** | **C** | **A** | **A** | **A** | **C** | **A** | **A** | **A** | **A** | **A** | **T** | **T** | **A** | **A** | **A** | **A** | **T** | **T** | **G** | **A** | **A** | **T** | **T** | **G** | **A** | **T** | **T** | **T** | **G** | **G** | **G** | **A** | **T** | **G** | **T** | **T** | **G** | **A** | **A** | **G** | **A** | **T** | **G** | **T** | **T** | **C** | **A** | **T** | **T** | **T** | **A** | **A** | **A** | **T** | **A** | **G** | **C** | **A** | **G** | **C** | **A** | **T** | **T** | **G** | **A** | **G** | **G** | **A** | **T** | **C** | **A** | **T** | **C** | **T** | **T** | **T** | **T** | **C** | **C** | **G** | **A** | **A** | **T** | **T** | **G** | **T** | **G** | **C** | **A** | **G** | **G** | **A** | **A** | **T** | **T** | **A** | **C** | **T** | **T** | **T** | **C** | **A** | **A** | **A** | **T** | **A** | **C** | **C** | **T** | **T** | **A** | **A** | **G** | **A** | **C** | **A** | **T** | **G** | **C** | **C** | **A** | **A** | **A** | **G** | **C** | **A** | **A** | **A** | **G** | **G** | **A** | **A** | **T** | **T** | **A** | **G** | **A** | **G** | **G** | **T** | **A** | **T** | **A** | **C** | **T** | **T** | **A** | **C** | **A** | **C** | **C** | **A** | **A** | **G** | **T** | **C** | **G** | **A** | **T** | **A** | **A** | **A** | **A** | **A** | **C** | **G** | **T** | **G** | **T** | **T** | **A** | **T** | **T** | **G** | **C** | **G** | **T** | **A** | **T** | **T** | **G** | **T** | **T** | **G** | **A** | **T** | **G** | **A** | **C** | **G** | **G** | **T** | **G** | **T** | **C** | **G** | **G** | **C** | **T** | **T** | **T** | **G** | **A** | **T** | **A** | **T** | **G** | **A** | **A** | **G** | **G** | **A** | **A** | **C** | **A** | **A** | **A** | **G** | **T** | **A** | **A** | **T** | **A** | **A** | **A** | **G** | **C** | **C** | **G** | **G** | **T** | **A** | **G** | **T** | **T** | **A** | **T** | **G** | **G** | **C** | **T** | **T** | **A** | **A** | **A** | **T** | **A** | **A** | **T** | **A** | **T** | **C** | **C** | **G** | **A** | **G** | **A** | **A** | **C** | **G** | **T** | **G** | **T** | **T** | **G** | **T** | **C** | **G** | **G** | **C** | **A** | **T** | **G** | **G** | **G** | **C** | **G** | **G** | **T** | **A** | **C** | **A** | **G** | **T** | **T** | **A** | **A** | **A** | **A** | **T** | **T** | **A** | **T** | **T** | **A** | **G** | **T** | **T** | **T** | **T** | **A** | **A** | **A** | **G** | **G** | **G** | **C** | **A** | **G** | **G** | **G** | **A** | **A** | **C** | **C** | **A** | **G** | **C** | **G** | **T** | **T** | **G** | **A** | **A** | **A** | **T** | **T** | **A** | **A** | **A** | **G** | **T** | **T** | **C** | **C** | **T** | **G** | **T** | **C** | **A** | **T** | **A** | **A** | **A** | **G** | **G** | **A** | **G** | **G** | **A** | **A** | **A** | **C** | **T** | **G** | **C** | **A** | **A** | **G** | **T** | **G** | **A** | **T** | **C** | **A** | **A** | **A** | **G** | **T** | **A** | **A** | **T** | **G** | **T** | **T** | **A** | **G** | **T** | **G** | **G** | **A** | **-** | **-** | **-** |
| **N54595** | **A** | **T** | **G** | **A** | **C** | **C** | **G** | **A** | **T** | **C** | **G** | **G** | **A** | **T** | **T** | **T** | **C** | **A** | **A** | **G** | **A** | **C** | **G** | **C** | **A** | **T** | **G** | **A** | **T** | **T** | **T** | **C** | **A** | **T** | **T** | **A** | **T** | **A** | **T** | **G** | **C** | **G** | **T** | **C** | **C** | **C** | **T** | **T** | **A** | **G** | **C** | **A** | **C** | **C** | **T** | **T** | **T** | **A** | **T** | **T** | **G** | **T** | **T** | **A** | **T** | **C** | **T** | **T** | **A** | **A** | **T** | **T** | **A** | **C** | **A** | **T** | **T** | **G** | **T** | **T** | **T** | **T** | **C** | **A** | **T** | **A** | **T** | **T** | **T** | **T** | **C** | **A** | **T** | **T** | **C** | **G** | **A** | **T** | **T** | **A** | **A** | **A** | **C** | **A** | **A** | **A** | **A** | **C** | **C** | **G** | **G** | **T** | **G** | **G** | **C** | **T** | **A** | **T** | **T** | **A** | **G** | **A** | **G** | **C** | **T** | **T** | **C** | **T** | **T** | **C** | **A** | **G** | **A** | **G** | **A** | **A** | **A** | **A** | **G** | **T** | **C** | **T** | **T** | **T** | **T** | **A** | **T** | **T** | **T** | **A** | **C** | **C** | **A** | **C** | **T** | **A** | **A** | **T** | **T** | **G** | **T** | **G** | **C** | **A** | **C** | **A** | **T** | **T** | **G** | **T** | **T** | **C** | **T** | **C** | **A** | **T** | **A** | **T** | **C** | **C** | **T** | **T** | **A** | **C** | **T** | **A** | **A** | **T** | **A** | **G** | **G** | **C** | **T** | **T** | **A** | **T** | **T** | **G** | **A** | **C** | **C** | **T** | **T** | **T** | **T** | **T** | **A** | **C** | **T** | **G** | **A** | **T** | **T** | **T** | **C** | **A** | **T** | **T** | **G** | **G** | **T** | **T** | **C** | **A** | **A** | **A** | **A** | **A** | **G** | **G** | **G** | **C** | **A** | **A** | **T** | **A** | **T** | **G** | **G** | **A** | **C** | **G** | **G** | **A** | **T** | **T** | **G** | **A** | **A** | **G** | **A** | **A** | **A** | **A** | **A** | **C** | **T** | **T** | **C** | **G** | **G** | **T** | **T** | **A** | **T** | **T** | **G** | **G** | **C** | **C** | **A** | **A** | **T** | **G** | **G** | **T** | **A** | **A** | **T** | **T** | **A** | **T** | **G** | **A** | **A** | **A** | **G** | **T** | **C** | **C** | **A** | **G** | **T** | **C** | **T** | **T** | **A** | **A** | **A** | **C** | **A** | **A** | **A** | **C** | **C** | **A** | **A** | **C** | **G** | **A** | **C** | **C** | **A** | **G** | **T** | **G** | **A** | **A** | **A** | **A** | **T** | **C** | **A** | **A** | **G** | **A** | **C** | **C** | **A** | **T** | **T** | **A** | **T** | **C** | **T** | **A** | **A** | **C** | **C** | **G** | **A** | **A** | **G** | **T** | **C** | **G** | **A** | **A** | **C** | **A** | **A** | **G** | **A** | **T** | **A** | **T** | **T** | **T** | **G** | **G** | **T** | **C** | **G** | **A** | **T** | **T** | **A** | **A** | **A** | **A** | **A** | **T** | **A** | **A** | **A** | **T** | **T** | **A** | **T** | **T** | **A** | **G** | **A** | **G** | **A** | **T** | **G** | **T** | **C** | **T** | **A** | **A** | **A** | **G** | **A** | **A** | **T** | **T** | **G** | **C** | **A** | **A** | **T** | **T** | **A** | **T** | **T** | **A** | **A** | **A** | **C** | **A** | **G** | **T** | **C** | **G** | **A** | **C** | **C** | **G** | **C** | **A** | **A** | **T** | **T** | **A** | **A** | **T** | **G** | **G** | **A** | **T** | **G** | **G** | **G** | **C** | **A** | **A** | **A** | **C** | **A** | **A** | **A** | **A** | **G** | **A** | **A** | **G** | **A** | **A** | **A** | **T** | **T** | **T** | **T** | **A** | **G** | **A** | **G** | **A** | **A** | **C** | **G** | **A** | **G** | **C** | **G** | **G** | **C** | **A** | **T** | **C** | **G** | **T** | **T** | **T** | **G** | **G** | **C** | **G** | **C** | **G** | **G** | **G** | **A** | **G** | **T** | **T** | **G** | **C** | **A** | **T** | **G** | **A** | **T** | **T** | **C** | **A** | **G** | **T** | **C** | **A** | **G** | **T** | **C** | **A** | **A** | **C** | **A** | **A** | **C** | **T** | **T** | **T** | **T** | **T** | **G** | **C** | **A** | **G** | **C** | **C** | **A** | **T** | **G** | **A** | **T** | **G** | **A** | **T** | **G** | **T** | **T** | **G** | **T** | **C** | **T** | **G** | **C** | **C** | **T** | **T** | **A** | **A** | **A** | **T** | **G** | **A** | **A** | **C** | **A** | **A** | **G** | **C** | **A** | **C** | **A** | **A** | **C** | **G** | **A** | **A** | **C** | **A** | **G** | **A** | **A** | **A** | **C** | **C** | **C** | **C** | **T** | **G** | **A** | **A** | **C** | **C** | **A** | **T** | **A** | **T** | **C** | **G** | **C** | **A** | **A** | **A** | **C** | **A** | **A** | **C** | **T** | **A** | **G** | **C** | **C** | **A** | **T** | **G** | **G** | **T** | **G** | **G** | **C** | **A** | **G** | **A** | **A** | **A** | **T** | **C** | **A** | **T** | **T** | **A** | **A** | **T** | **G** | **C** | **C** | **T** | **C** | **C** | **C** | **A** | **A** | **T** | **C** | **G** | **G** | **A** | **A** | **A** | **T** | **G** | **C** | **G** | **C** | **G** | **C** | **G** | **T** | **T** | **A** | **C** | **T** | **A** | **T** | **T** | **G** | **C** | **A** | **C** | **T** | **T** | **G** | **C** | **G** | **T** | **C** | **C** | **T** | **A** | **T** | **C** | **A** | **G** | **T** | **C** | **T** | **A** | **G** | **A** | **A** | **G** | **G** | **A** | **A** | **A** | **A** | **A** | **G** | **T** | **T** | **T** | **G** | **C** | **G** | **T** | **A** | **A** | **A** | **G** | **G** | **T** | **A** | **T** | **T** | **G** | **A** | **A** | **C** | **A** | **A** | **T** | **T** | **A** | **C** | **T** | **G** | **A** | **A** | **A** | **G** | **A** | **A** | **C** | **T** | **A** | **C** | **A** | **A** | **A** | **C** | **A** | **A** | **A** | **A** | **A** | **T** | **T** | **A** | **A** | **A** | **A** | **T** | **T** | **G** | **A** | **A** | **T** | **T** | **G** | **A** | **T** | **T** | **T** | **G** | **G** | **G** | **A** | **T** | **G** | **T** | **T** | **G** | **A** | **A** | **G** | **A** | **T** | **G** | **T** | **T** | **C** | **A** | **T** | **T** | **T** | **A** | **A** | **A** | **T** | **A** | **G** | **C** | **A** | **G** | **C** | **A** | **T** | **T** | **G** | **A** | **G** | **G** | **A** | **T** | **C** | **A** | **T** | **C** | **T** | **T** | **T** | **T** | **C** | **C** | **G** | **A** | **A** | **T** | **T** | **G** | **T** | **G** | **C** | **A** | **G** | **G** | **A** | **G** | **T** | **T** | **A** | **C** | **T** | **T** | **T** | **C** | **A** | **A** | **A** | **T** | **A** | **C** | **C** | **T** | **T** | **A** | **A** | **G** | **A** | **C** | **A** | **T** | **G** | **C** | **C** | **A** | **A** | **A** | **G** | **C** | **A** | **A** | **A** | **G** | **G** | **A** | **A** | **T** | **T** | **A** | **G** | **A** | **G** | **G** | **T** | **A** | **T** | **A** | **C** | **T** | **T** | **A** | **C** | **A** | **C** | **C** | **A** | **A** | **G** | **T** | **C** | **G** | **A** | **T** | **A** | **A** | **A** | **A** | **A** | **C** | **G** | **T** | **G** | **T** | **T** | **A** | **T** | **T** | **G** | **C** | **G** | **T** | **A** | **T** | **T** | **G** | **T** | **T** | **G** | **A** | **T** | **G** | **A** | **T** | **G** | **G** | **T** | **G** | **T** | **C** | **G** | **G** | **C** | **T** | **T** | **T** | **G** | **A** | **T** | **A** | **T** | **G** | **A** | **A** | **G** | **G** | **A** | **A** | **C** | **A** | **A** | **A** | **G** | **T** | **A** | **A** | **T** | **A** | **A** | **A** | **G** | **C** | **C** | **G** | **G** | **T** | **A** | **G** | **T** | **T** | **A** | **T** | **G** | **G** | **C** | **T** | **T** | **A** | **A** | **A** | **T** | **A** | **A** | **T** | **A** | **T** | **C** | **C** | **G** | **A** | **G** | **A** | **A** | **C** | **G** | **T** | **G** | **T** | **T** | **G** | **T** | **C** | **G** | **G** | **C** | **A** | **T** | **G** | **G** | **G** | **C** | **G** | **G** | **T** | **A** | **C** | **A** | **G** | **T** | **T** | **A** | **A** | **A** | **A** | **T** | **T** | **A** | **T** | **T** | **A** | **G** | **T** | **T** | **T** | **T** | **A** | **A** | **A** | **G** | **G** | **G** | **C** | **A** | **G** | **G** | **G** | **A** | **A** | **C** | **C** | **A** | **G** | **C** | **G** | **T** | **T** | **G** | **A** | **A** | **A** | **T** | **T** | **A** | **A** | **A** | **G** | **T** | **T** | **C** | **C** | **T** | **G** | **T** | **C** | **A** | **T** | **A** | **A** | **A** | **G** | **G** | **A** | **G** | **G** | **A** | **A** | **A** | **C** | **T** | **G** | **C** | **A** | **A** | **G** | **T** | **G** | **A** | **T** | **C** | **A** | **A** | **A** | **G** | **T** | **A** | **A** | **T** | **G** | **T** | **T** | **A** | **G** | **T** | **G** | **G** | **A** | **-** | **-** | **-** |
| **N54564** | **A** | **T** | **G** | **A** | **C** | **C** | **G** | **A** | **T** | **C** | **G** | **G** | **A** | **T** | **T** | **T** | **C** | **A** | **A** | **G** | **A** | **C** | **G** | **C** | **A** | **T** | **G** | **A** | **T** | **T** | **T** | **C** | **A** | **T** | **T** | **A** | **T** | **A** | **T** | **G** | **C** | **G** | **T** | **C** | **C** | **C** | **T** | **T** | **A** | **G** | **C** | **A** | **C** | **C** | **T** | **T** | **T** | **A** | **T** | **T** | **G** | **T** | **T** | **A** | **T** | **C** | **T** | **T** | **A** | **A** | **T** | **T** | **A** | **C** | **A** | **T** | **T** | **G** | **T** | **T** | **T** | **T** | **C** | **A** | **T** | **A** | **T** | **T** | **T** | **T** | **C** | **A** | **T** | **T** | **C** | **G** | **A** | **T** | **T** | **A** | **A** | **A** | **C** | **A** | **A** | **A** | **A** | **C** | **C** | **G** | **G** | **T** | **G** | **G** | **C** | **T** | **A** | **T** | **T** | **A** | **G** | **A** | **G** | **C** | **T** | **T** | **C** | **T** | **T** | **C** | **A** | **G** | **A** | **G** | **A** | **A** | **A** | **A** | **G** | **T** | **C** | **T** | **T** | **T** | **T** | **A** | **T** | **C** | **T** | **A** | **C** | **C** | **A** | **C** | **T** | **A** | **A** | **T** | **T** | **G** | **T** | **G** | **C** | **A** | **C** | **A** | **T** | **T** | **G** | **T** | **T** | **C** | **T** | **C** | **A** | **T** | **A** | **T** | **C** | **C** | **T** | **T** | **A** | **C** | **T** | **A** | **A** | **T** | **A** | **G** | **G** | **C** | **T** | **T** | **A** | **T** | **T** | **G** | **A** | **C** | **C** | **T** | **T** | **T** | **T** | **T** | **A** | **C** | **T** | **G** | **A** | **T** | **T** | **T** | **C** | **C** | **T** | **T** | **G** | **G** | **T** | **T** | **C** | **A** | **A** | **A** | **A** | **A** | **G** | **G** | **A** | **C** | **A** | **A** | **T** | **A** | **T** | **G** | **G** | **A** | **C** | **G** | **G** | **A** | **T** | **T** | **G** | **A** | **A** | **G** | **A** | **A** | **A** | **A** | **A** | **C** | **T** | **T** | **C** | **G** | **G** | **T** | **T** | **A** | **T** | **T** | **G** | **G** | **C** | **C** | **A** | **A** | **C** | **G** | **G** | **T** | **A** | **A** | **T** | **T** | **A** | **T** | **G** | **A** | **A** | **A** | **G** | **T** | **C** | **C** | **A** | **G** | **T** | **C** | **T** | **T** | **A** | **A** | **A** | **C** | **A** | **A** | **A** | **C** | **C** | **A** | **A** | **C** | **G** | **A** | **C** | **C** | **A** | **G** | **T** | **G** | **A** | **A** | **A** | **A** | **T** | **C** | **A** | **A** | **G** | **A** | **C** | **C** | **A** | **T** | **T** | **A** | **T** | **C** | **T** | **A** | **A** | **C** | **C** | **G** | **A** | **A** | **G** | **T** | **C** | **G** | **A** | **A** | **C** | **A** | **A** | **G** | **A** | **T** | **A** | **T** | **T** | **T** | **G** | **G** | **T** | **C** | **G** | **A** | **T** | **T** | **A** | **A** | **A** | **A** | **A** | **T** | **A** | **A** | **A** | **T** | **T** | **A** | **T** | **T** | **A** | **G** | **A** | **G** | **A** | **T** | **G** | **T** | **C** | **T** | **A** | **A** | **A** | **G** | **A** | **A** | **T** | **T** | **G** | **C** | **A** | **A** | **T** | **T** | **A** | **T** | **T** | **A** | **A** | **A** | **C** | **A** | **G** | **T** | **C** | **G** | **A** | **C** | **C** | **G** | **C** | **A** | **A** | **T** | **T** | **A** | **A** | **T** | **G** | **G** | **A** | **T** | **G** | **G** | **G** | **C** | **A** | **A** | **A** | **C** | **A** | **A** | **A** | **A** | **G** | **A** | **A** | **G** | **A** | **A** | **A** | **T** | **T** | **T** | **T** | **A** | **G** | **A** | **G** | **A** | **A** | **C** | **G** | **A** | **G** | **C** | **G** | **G** | **C** | **A** | **T** | **C** | **G** | **T** | **T** | **T** | **G** | **G** | **C** | **G** | **C** | **G** | **G** | **G** | **A** | **G** | **T** | **T** | **G** | **C** | **A** | **T** | **G** | **A** | **T** | **T** | **C** | **A** | **G** | **T** | **C** | **A** | **G** | **T** | **C** | **A** | **A** | **C** | **A** | **A** | **C** | **T** | **T** | **T** | **T** | **T** | **G** | **C** | **A** | **G** | **C** | **C** | **A** | **T** | **G** | **A** | **T** | **G** | **A** | **T** | **G** | **T** | **T** | **G** | **T** | **C** | **T** | **G** | **C** | **C** | **T** | **T** | **A** | **A** | **A** | **T** | **G** | **A** | **A** | **C** | **A** | **A** | **G** | **C** | **A** | **C** | **A** | **A** | **C** | **G** | **A** | **A** | **C** | **A** | **G** | **A** | **A** | **A** | **C** | **C** | **C** | **C** | **G** | **G** | **A** | **A** | **C** | **C** | **A** | **T** | **A** | **T** | **C** | **G** | **T** | **A** | **A** | **A** | **C** | **A** | **A** | **C** | **T** | **A** | **G** | **C** | **C** | **A** | **T** | **G** | **G** | **T** | **G** | **G** | **C** | **A** | **G** | **A** | **A** | **A** | **T** | **C** | **A** | **T** | **T** | **A** | **A** | **T** | **G** | **C** | **C** | **T** | **C** | **C** | **C** | **A** | **A** | **T** | **C** | **G** | **G** | **A** | **A** | **A** | **T** | **G** | **C** | **G** | **T** | **G** | **C** | **G** | **T** | **T** | **A** | **C** | **T** | **A** | **T** | **T** | **G** | **C** | **A** | **C** | **T** | **T** | **G** | **C** | **G** | **T** | **C** | **C** | **T** | **A** | **T** | **C** | **A** | **G** | **T** | **C** | **T** | **A** | **G** | **A** | **A** | **G** | **G** | **A** | **A** | **A** | **A** | **A** | **G** | **T** | **T** | **T** | **G** | **C** | **G** | **T** | **A** | **A** | **A** | **G** | **G** | **T** | **A** | **T** | **T** | **G** | **A** | **A** | **C** | **A** | **A** | **T** | **T** | **A** | **C** | **T** | **G** | **A** | **A** | **A** | **G** | **A** | **A** | **C** | **T** | **A** | **C** | **A** | **A** | **A** | **C** | **A** | **A** | **A** | **A** | **A** | **T** | **T** | **A** | **A** | **A** | **A** | **T** | **T** | **G** | **A** | **A** | **T** | **T** | **G** | **A** | **T** | **T** | **T** | **G** | **G** | **G** | **A** | **T** | **G** | **T** | **T** | **G** | **A** | **A** | **G** | **A** | **T** | **G** | **T** | **T** | **C** | **A** | **T** | **T** | **T** | **A** | **A** | **A** | **T** | **A** | **G** | **C** | **A** | **G** | **C** | **A** | **T** | **T** | **G** | **A** | **G** | **G** | **A** | **T** | **C** | **A** | **T** | **C** | **T** | **T** | **T** | **T** | **C** | **C** | **G** | **A** | **A** | **T** | **T** | **G** | **T** | **G** | **C** | **A** | **G** | **G** | **A** | **G** | **T** | **T** | **A** | **C** | **T** | **T** | **T** | **C** | **A** | **A** | **A** | **T** | **A** | **C** | **C** | **T** | **T** | **A** | **A** | **G** | **A** | **C** | **A** | **T** | **G** | **C** | **C** | **A** | **A** | **A** | **G** | **C** | **A** | **A** | **A** | **G** | **G** | **A** | **A** | **T** | **T** | **A** | **G** | **A** | **G** | **G** | **T** | **A** | **T** | **A** | **C** | **T** | **T** | **A** | **C** | **A** | **C** | **C** | **A** | **A** | **G** | **T** | **C** | **G** | **A** | **T** | **A** | **A** | **A** | **A** | **A** | **C** | **G** | **T** | **G** | **T** | **T** | **A** | **T** | **T** | **G** | **C** | **G** | **T** | **A** | **T** | **T** | **G** | **T** | **T** | **G** | **A** | **T** | **G** | **A** | **C** | **G** | **G** | **T** | **G** | **T** | **C** | **G** | **G** | **C** | **T** | **T** | **T** | **G** | **A** | **T** | **A** | **T** | **G** | **A** | **A** | **G** | **G** | **A** | **A** | **C** | **A** | **A** | **A** | **G** | **T** | **A** | **A** | **T** | **A** | **A** | **A** | **G** | **C** | **C** | **G** | **G** | **T** | **A** | **G** | **T** | **T** | **A** | **T** | **G** | **G** | **C** | **T** | **T** | **A** | **A** | **A** | **T** | **A** | **A** | **T** | **A** | **T** | **C** | **C** | **G** | **A** | **G** | **A** | **A** | **C** | **G** | **T** | **G** | **T** | **T** | **G** | **T** | **C** | **G** | **G** | **C** | **A** | **T** | **G** | **G** | **G** | **C** | **G** | **G** | **T** | **A** | **C** | **A** | **G** | **T** | **T** | **A** | **A** | **A** | **A** | **T** | **T** | **A** | **T** | **T** | **A** | **G** | **T** | **T** | **T** | **T** | **A** | **A** | **A** | **G** | **G** | **G** | **C** | **A** | **G** | **G** | **G** | **A** | **A** | **C** | **C** | **A** | **G** | **C** | **G** | **T** | **T** | **G** | **A** | **A** | **A** | **T** | **T** | **A** | **A** | **A** | **G** | **T** | **T** | **C** | **C** | **T** | **G** | **T** | **C** | **A** | **T** | **A** | **A** | **A** | **G** | **G** | **A** | **G** | **G** | **A** | **A** | **A** | **C** | **T** | **G** | **C** | **A** | **A** | **G** | **T** | **G** | **A** | **T** | **C** | **A** | **A** | **A** | **G** | **T** | **A** | **A** | **T** | **G** | **T** | **T** | **A** | **G** | **T** | **G** | **G** | **A** | **-** | **-** | **-** |
| **N60280F** | **A** | **T** | **G** | **A** | **C** | **C** | **G** | **A** | **T** | **C** | **G** | **G** | **A** | **T** | **T** | **T** | **C** | **A** | **A** | **G** | **A** | **C** | **G** | **C** | **A** | **T** | **G** | **A** | **T** | **T** | **T** | **C** | **A** | **T** | **T** | **A** | **T** | **A** | **T** | **G** | **C** | **G** | **T** | **C** | **C** | **C** | **T** | **T** | **A** | **G** | **C** | **A** | **C** | **C** | **T** | **T** | **T** | **A** | **T** | **T** | **G** | **T** | **T** | **A** | **T** | **C** | **T** | **T** | **A** | **A** | **T** | **T** | **A** | **C** | **A** | **T** | **T** | **G** | **T** | **T** | **T** | **T** | **C** | **A** | **T** | **A** | **T** | **T** | **T** | **T** | **C** | **A** | **T** | **T** | **C** | **G** | **A** | **T** | **T** | **A** | **A** | **A** | **C** | **A** | **A** | **A** | **A** | **C** | **C** | **G** | **G** | **T** | **G** | **G** | **C** | **T** | **A** | **T** | **T** | **A** | **G** | **A** | **G** | **C** | **T** | **T** | **C** | **T** | **T** | **C** | **A** | **G** | **A** | **G** | **A** | **A** | **A** | **A** | **G** | **T** | **C** | **T** | **T** | **T** | **T** | **A** | **T** | **T** | **T** | **A** | **C** | **C** | **A** | **C** | **T** | **A** | **A** | **T** | **T** | **G** | **T** | **G** | **C** | **A** | **C** | **A** | **T** | **T** | **G** | **T** | **T** | **C** | **T** | **C** | **A** | **T** | **A** | **T** | **C** | **C** | **T** | **T** | **A** | **C** | **T** | **A** | **A** | **T** | **A** | **G** | **G** | **C** | **T** | **T** | **A** | **T** | **T** | **G** | **A** | **C** | **C** | **T** | **T** | **T** | **T** | **T** | **A** | **C** | **T** | **G** | **A** | **T** | **T** | **T** | **C** | **A** | **T** | **T** | **G** | **G** | **T** | **T** | **C** | **A** | **A** | **A** | **A** | **A** | **G** | **G** | **G** | **C** | **A** | **A** | **T** | **A** | **T** | **G** | **G** | **A** | **C** | **G** | **G** | **A** | **T** | **T** | **G** | **A** | **A** | **G** | **A** | **A** | **A** | **A** | **A** | **C** | **T** | **T** | **C** | **G** | **G** | **T** | **T** | **A** | **T** | **T** | **G** | **G** | **C** | **C** | **A** | **A** | **T** | **G** | **G** | **T** | **A** | **A** | **T** | **T** | **A** | **T** | **G** | **A** | **A** | **A** | **G** | **T** | **C** | **C** | **A** | **G** | **T** | **C** | **T** | **T** | **A** | **A** | **A** | **C** | **A** | **A** | **A** | **C** | **C** | **A** | **A** | **C** | **G** | **A** | **C** | **C** | **A** | **G** | **T** | **G** | **A** | **A** | **A** | **A** | **T** | **C** | **A** | **A** | **G** | **A** | **C** | **C** | **A** | **T** | **T** | **A** | **T** | **C** | **T** | **A** | **A** | **C** | **C** | **G** | **A** | **A** | **G** | **T** | **C** | **G** | **A** | **A** | **C** | **A** | **A** | **G** | **A** | **T** | **A** | **T** | **T** | **T** | **G** | **G** | **T** | **C** | **G** | **A** | **T** | **T** | **A** | **A** | **A** | **A** | **A** | **T** | **A** | **A** | **A** | **T** | **T** | **A** | **T** | **T** | **A** | **G** | **A** | **G** | **A** | **T** | **G** | **T** | **C** | **T** | **A** | **A** | **A** | **G** | **A** | **A** | **T** | **T** | **G** | **C** | **A** | **A** | **T** | **T** | **A** | **T** | **T** | **A** | **A** | **A** | **C** | **A** | **G** | **T** | **C** | **G** | **A** | **C** | **C** | **G** | **C** | **A** | **A** | **T** | **T** | **A** | **A** | **T** | **G** | **G** | **A** | **T** | **G** | **G** | **G** | **C** | **A** | **A** | **A** | **C** | **A** | **A** | **A** | **A** | **G** | **A** | **A** | **G** | **A** | **A** | **A** | **T** | **T** | **T** | **T** | **A** | **G** | **A** | **G** | **A** | **A** | **C** | **G** | **A** | **G** | **C** | **G** | **G** | **C** | **A** | **T** | **C** | **G** | **T** | **T** | **T** | **G** | **G** | **C** | **G** | **C** | **G** | **G** | **G** | **A** | **G** | **T** | **T** | **G** | **C** | **A** | **T** | **G** | **A** | **T** | **T** | **C** | **A** | **G** | **T** | **C** | **A** | **G** | **T** | **C** | **A** | **A** | **C** | **A** | **A** | **C** | **T** | **T** | **T** | **T** | **T** | **G** | **C** | **A** | **G** | **C** | **C** | **A** | **T** | **G** | **A** | **T** | **G** | **A** | **T** | **G** | **T** | **T** | **G** | **T** | **C** | **T** | **G** | **C** | **C** | **T** | **T** | **A** | **A** | **A** | **T** | **G** | **A** | **A** | **C** | **A** | **A** | **G** | **C** | **A** | **C** | **A** | **A** | **C** | **G** | **A** | **A** | **C** | **A** | **G** | **A** | **A** | **A** | **C** | **C** | **C** | **C** | **T** | **G** | **A** | **A** | **C** | **C** | **A** | **T** | **A** | **T** | **C** | **G** | **C** | **A** | **A** | **A** | **C** | **A** | **A** | **C** | **T** | **A** | **G** | **C** | **C** | **A** | **T** | **G** | **G** | **T** | **G** | **G** | **C** | **A** | **G** | **A** | **A** | **A** | **T** | **C** | **A** | **T** | **T** | **A** | **A** | **T** | **G** | **C** | **C** | **T** | **C** | **C** | **C** | **A** | **A** | **T** | **C** | **G** | **G** | **A** | **A** | **A** | **T** | **G** | **C** | **G** | **C** | **G** | **C** | **G** | **T** | **T** | **A** | **C** | **T** | **A** | **T** | **T** | **G** | **C** | **A** | **C** | **T** | **T** | **G** | **C** | **G** | **T** | **C** | **C** | **T** | **A** | **T** | **C** | **A** | **G** | **T** | **C** | **T** | **A** | **G** | **A** | **A** | **G** | **G** | **A** | **A** | **A** | **A** | **A** | **G** | **T** | **T** | **T** | **G** | **C** | **G** | **T** | **A** | **A** | **A** | **G** | **G** | **T** | **A** | **T** | **T** | **G** | **A** | **A** | **C** | **A** | **A** | **T** | **T** | **A** | **C** | **T** | **G** | **A** | **A** | **A** | **G** | **A** | **A** | **C** | **T** | **A** | **C** | **A** | **A** | **A** | **C** | **A** | **A** | **A** | **A** | **A** | **T** | **T** | **A** | **A** | **A** | **A** | **T** | **T** | **G** | **A** | **A** | **T** | **T** | **G** | **A** | **T** | **T** | **T** | **G** | **G** | **G** | **A** | **T** | **G** | **T** | **T** | **G** | **A** | **A** | **G** | **A** | **T** | **G** | **T** | **T** | **C** | **A** | **T** | **T** | **T** | **A** | **A** | **A** | **T** | **A** | **G** | **C** | **A** | **G** | **C** | **A** | **T** | **T** | **G** | **A** | **G** | **G** | **A** | **T** | **C** | **A** | **T** | **C** | **T** | **T** | **T** | **T** | **C** | **C** | **G** | **A** | **A** | **T** | **T** | **G** | **T** | **G** | **C** | **A** | **G** | **G** | **A** | **A** | **T** | **T** | **A** | **C** | **T** | **T** | **T** | **C** | **A** | **A** | **A** | **T** | **A** | **C** | **T** | **T** | **T** | **A** | **A** | **G** | **A** | **C** | **A** | **T** | **G** | **C** | **C** | **A** | **A** | **A** | **G** | **C** | **A** | **A** | **A** | **G** | **G** | **A** | **A** | **T** | **T** | **A** | **G** | **A** | **G** | **G** | **T** | **A** | **T** | **A** | **C** | **T** | **T** | **A** | **C** | **A** | **C** | **C** | **A** | **A** | **G** | **T** | **C** | **G** | **A** | **T** | **A** | **A** | **A** | **A** | **A** | **C** | **G** | **T** | **G** | **C** | **T** | **A** | **T** | **T** | **G** | **C** | **G** | **T** | **A** | **T** | **T** | **G** | **T** | **T** | **G** | **A** | **T** | **G** | **A** | **C** | **G** | **G** | **T** | **G** | **T** | **C** | **G** | **G** | **C** | **T** | **T** | **T** | **G** | **A** | **T** | **A** | **T** | **G** | **A** | **A** | **G** | **G** | **A** | **A** | **C** | **A** | **A** | **A** | **G** | **T** | **A** | **A** | **T** | **A** | **A** | **A** | **G** | **C** | **C** | **G** | **G** | **T** | **A** | **G** | **T** | **T** | **A** | **T** | **G** | **G** | **C** | **T** | **T** | **A** | **A** | **A** | **T** | **A** | **A** | **T** | **A** | **T** | **C** | **C** | **G** | **A** | **G** | **A** | **A** | **C** | **G** | **T** | **G** | **T** | **T** | **G** | **T** | **C** | **G** | **G** | **C** | **A** | **T** | **G** | **G** | **G** | **C** | **G** | **G** | **T** | **A** | **C** | **A** | **G** | **T** | **T** | **A** | **A** | **A** | **A** | **T** | **T** | **A** | **T** | **T** | **A** | **G** | **T** | **T** | **T** | **T** | **A** | **A** | **A** | **G** | **G** | **G** | **C** | **A** | **G** | **G** | **G** | **A** | **A** | **C** | **C** | **A** | **G** | **C** | **G** | **T** | **T** | **G** | **A** | **A** | **A** | **T** | **T** | **A** | **A** | **A** | **G** | **T** | **T** | **C** | **C** | **T** | **G** | **T** | **C** | **A** | **T** | **A** | **A** | **A** | **G** | **G** | **A** | **G** | **G** | **A** | **A** | **A** | **C** | **T** | **G** | **C** | **A** | **A** | **G** | **T** | **G** | **A** | **T** | **C** | **A** | **A** | **A** | **G** | **T** | **A** | **A** | **T** | **G** | **T** | **T** | **A** | **G** | **T** | **G** | **G** | **A** | **-** | **-** | **-** |
| **N60392F** | **A** | **T** | **G** | **A** | **C** | **C** | **G** | **A** | **T** | **C** | **G** | **G** | **A** | **T** | **T** | **T** | **C** | **A** | **A** | **G** | **A** | **C** | **G** | **C** | **A** | **T** | **G** | **A** | **T** | **T** | **T** | **C** | **A** | **T** | **T** | **A** | **T** | **A** | **T** | **G** | **C** | **G** | **T** | **C** | **C** | **C** | **T** | **T** | **A** | **G** | **C** | **A** | **C** | **C** | **T** | **T** | **T** | **A** | **T** | **T** | **G** | **T** | **T** | **A** | **T** | **C** | **T** | **T** | **A** | **A** | **T** | **T** | **A** | **C** | **A** | **T** | **T** | **G** | **T** | **T** | **T** | **T** | **C** | **A** | **T** | **A** | **T** | **T** | **T** | **T** | **C** | **A** | **T** | **T** | **C** | **G** | **A** | **T** | **T** | **A** | **A** | **A** | **C** | **A** | **A** | **A** | **A** | **C** | **C** | **G** | **G** | **T** | **G** | **G** | **C** | **T** | **A** | **T** | **T** | **A** | **G** | **A** | **G** | **C** | **T** | **T** | **C** | **T** | **T** | **C** | **A** | **G** | **A** | **G** | **A** | **A** | **A** | **A** | **G** | **T** | **C** | **T** | **T** | **T** | **T** | **A** | **T** | **C** | **T** | **A** | **C** | **C** | **A** | **C** | **T** | **A** | **A** | **T** | **T** | **G** | **T** | **G** | **C** | **A** | **C** | **A** | **T** | **T** | **G** | **T** | **T** | **C** | **T** | **C** | **A** | **T** | **A** | **T** | **C** | **C** | **T** | **T** | **A** | **C** | **T** | **A** | **A** | **T** | **A** | **G** | **G** | **C** | **T** | **T** | **A** | **T** | **T** | **G** | **A** | **C** | **C** | **T** | **T** | **T** | **T** | **T** | **A** | **C** | **T** | **G** | **A** | **T** | **T** | **T** | **C** | **C** | **T** | **T** | **G** | **G** | **T** | **T** | **C** | **A** | **A** | **A** | **A** | **A** | **G** | **G** | **A** | **C** | **A** | **A** | **T** | **A** | **T** | **G** | **G** | **A** | **C** | **G** | **G** | **A** | **T** | **T** | **G** | **A** | **A** | **G** | **A** | **A** | **A** | **A** | **A** | **C** | **T** | **T** | **C** | **G** | **G** | **T** | **T** | **A** | **T** | **T** | **G** | **G** | **C** | **C** | **A** | **A** | **C** | **G** | **G** | **T** | **A** | **A** | **T** | **T** | **A** | **T** | **G** | **A** | **A** | **A** | **G** | **T** | **C** | **C** | **A** | **G** | **T** | **C** | **T** | **T** | **A** | **A** | **A** | **C** | **A** | **A** | **A** | **C** | **C** | **A** | **A** | **C** | **G** | **A** | **C** | **C** | **A** | **G** | **T** | **G** | **A** | **A** | **A** | **A** | **T** | **C** | **A** | **A** | **G** | **A** | **C** | **C** | **A** | **T** | **T** | **A** | **T** | **C** | **T** | **A** | **A** | **C** | **C** | **G** | **A** | **A** | **G** | **T** | **C** | **G** | **A** | **A** | **C** | **A** | **A** | **G** | **A** | **T** | **A** | **T** | **T** | **T** | **G** | **G** | **T** | **C** | **G** | **A** | **T** | **T** | **A** | **A** | **A** | **A** | **A** | **T** | **A** | **A** | **A** | **T** | **T** | **A** | **T** | **T** | **A** | **G** | **A** | **G** | **A** | **T** | **G** | **T** | **C** | **T** | **A** | **A** | **A** | **G** | **A** | **A** | **T** | **T** | **G** | **C** | **A** | **A** | **T** | **T** | **A** | **T** | **T** | **A** | **A** | **A** | **C** | **A** | **G** | **T** | **C** | **G** | **A** | **C** | **C** | **G** | **C** | **A** | **A** | **T** | **T** | **A** | **A** | **T** | **G** | **G** | **A** | **T** | **G** | **G** | **G** | **C** | **A** | **A** | **A** | **C** | **A** | **A** | **A** | **A** | **G** | **A** | **A** | **G** | **A** | **A** | **A** | **T** | **T** | **T** | **T** | **A** | **G** | **A** | **G** | **A** | **A** | **C** | **G** | **A** | **G** | **C** | **G** | **G** | **C** | **A** | **T** | **C** | **G** | **T** | **T** | **T** | **G** | **G** | **C** | **G** | **C** | **G** | **G** | **G** | **A** | **G** | **T** | **T** | **G** | **C** | **A** | **T** | **G** | **A** | **T** | **T** | **C** | **A** | **G** | **T** | **C** | **A** | **G** | **T** | **C** | **A** | **A** | **C** | **A** | **A** | **C** | **T** | **T** | **T** | **T** | **T** | **G** | **C** | **A** | **G** | **C** | **C** | **A** | **T** | **G** | **A** | **T** | **G** | **A** | **T** | **G** | **T** | **T** | **G** | **T** | **C** | **T** | **G** | **C** | **C** | **T** | **T** | **A** | **A** | **A** | **T** | **G** | **A** | **A** | **C** | **A** | **A** | **G** | **C** | **A** | **C** | **A** | **A** | **C** | **G** | **A** | **A** | **C** | **A** | **G** | **A** | **A** | **A** | **C** | **C** | **C** | **C** | **G** | **G** | **A** | **A** | **C** | **C** | **A** | **T** | **A** | **T** | **C** | **G** | **T** | **A** | **A** | **A** | **C** | **A** | **A** | **C** | **T** | **A** | **G** | **C** | **C** | **A** | **T** | **G** | **G** | **T** | **G** | **G** | **C** | **A** | **G** | **A** | **A** | **A** | **T** | **C** | **A** | **T** | **T** | **A** | **A** | **T** | **G** | **C** | **C** | **T** | **C** | **C** | **C** | **A** | **A** | **T** | **C** | **G** | **G** | **A** | **A** | **A** | **T** | **G** | **C** | **G** | **T** | **G** | **C** | **G** | **T** | **T** | **A** | **C** | **T** | **A** | **T** | **T** | **G** | **C** | **A** | **C** | **T** | **T** | **G** | **C** | **G** | **T** | **C** | **C** | **T** | **A** | **T** | **C** | **A** | **G** | **T** | **C** | **T** | **A** | **G** | **A** | **A** | **G** | **G** | **A** | **A** | **A** | **A** | **A** | **G** | **T** | **T** | **T** | **G** | **C** | **G** | **T** | **A** | **A** | **A** | **G** | **G** | **T** | **A** | **T** | **T** | **G** | **A** | **A** | **C** | **A** | **A** | **T** | **T** | **A** | **C** | **T** | **G** | **A** | **A** | **A** | **G** | **A** | **A** | **C** | **T** | **A** | **C** | **A** | **A** | **A** | **C** | **A** | **A** | **A** | **A** | **A** | **T** | **T** | **A** | **A** | **A** | **A** | **T** | **T** | **G** | **A** | **A** | **T** | **T** | **G** | **A** | **T** | **T** | **T** | **G** | **G** | **G** | **A** | **T** | **G** | **T** | **T** | **G** | **A** | **A** | **G** | **A** | **T** | **G** | **T** | **T** | **C** | **A** | **T** | **T** | **T** | **A** | **A** | **A** | **T** | **A** | **G** | **C** | **A** | **G** | **C** | **A** | **T** | **T** | **G** | **A** | **G** | **G** | **A** | **T** | **C** | **A** | **T** | **C** | **T** | **T** | **T** | **T** | **C** | **C** | **G** | **A** | **A** | **T** | **T** | **G** | **T** | **G** | **C** | **A** | **G** | **G** | **A** | **G** | **T** | **T** | **A** | **C** | **T** | **T** | **T** | **C** | **A** | **A** | **A** | **T** | **A** | **C** | **C** | **T** | **T** | **A** | **A** | **G** | **A** | **C** | **A** | **T** | **G** | **C** | **C** | **A** | **A** | **A** | **G** | **C** | **A** | **A** | **A** | **G** | **G** | **A** | **A** | **T** | **T** | **A** | **G** | **A** | **G** | **G** | **T** | **A** | **T** | **A** | **C** | **T** | **T** | **A** | **C** | **A** | **C** | **C** | **A** | **A** | **G** | **T** | **C** | **G** | **A** | **T** | **A** | **A** | **A** | **A** | **A** | **C** | **G** | **T** | **G** | **T** | **T** | **A** | **T** | **T** | **G** | **C** | **G** | **T** | **A** | **T** | **T** | **G** | **T** | **T** | **G** | **A** | **T** | **G** | **A** | **C** | **G** | **G** | **T** | **G** | **T** | **C** | **G** | **G** | **C** | **T** | **T** | **T** | **G** | **A** | **T** | **A** | **T** | **G** | **A** | **A** | **G** | **G** | **A** | **A** | **C** | **A** | **A** | **A** | **G** | **T** | **A** | **A** | **T** | **A** | **A** | **A** | **G** | **C** | **C** | **G** | **G** | **T** | **A** | **G** | **T** | **T** | **A** | **T** | **G** | **G** | **C** | **T** | **T** | **A** | **A** | **A** | **T** | **A** | **A** | **T** | **A** | **T** | **C** | **C** | **G** | **A** | **G** | **A** | **A** | **C** | **G** | **T** | **G** | **T** | **T** | **G** | **T** | **C** | **G** | **G** | **C** | **A** | **T** | **G** | **G** | **G** | **C** | **G** | **G** | **T** | **A** | **C** | **A** | **G** | **T** | **T** | **A** | **A** | **A** | **A** | **T** | **T** | **A** | **T** | **T** | **A** | **G** | **T** | **T** | **T** | **T** | **A** | **A** | **A** | **G** | **G** | **G** | **C** | **A** | **G** | **G** | **G** | **A** | **A** | **C** | **C** | **A** | **G** | **C** | **G** | **T** | **T** | **G** | **A** | **A** | **A** | **T** | **T** | **A** | **A** | **A** | **G** | **T** | **T** | **C** | **C** | **T** | **G** | **T** | **C** | **A** | **T** | **A** | **A** | **A** | **G** | **G** | **A** | **G** | **G** | **A** | **A** | **A** | **C** | **T** | **G** | **C** | **A** | **A** | **G** | **T** | **G** | **A** | **T** | **C** | **A** | **A** | **A** | **G** | **T** | **A** | **A** | **T** | **G** | **T** | **T** | **A** | **G** | **T** | **G** | **G** | **A** | **-** | **-** | **-** |
| **N59936F** | **A** | **T** | **G** | **A** | **C** | **C** | **G** | **A** | **T** | **C** | **G** | **G** | **A** | **T** | **T** | **T** | **C** | **A** | **A** | **G** | **A** | **C** | **G** | **C** | **A** | **T** | **G** | **A** | **T** | **T** | **T** | **C** | **A** | **T** | **T** | **A** | **T** | **A** | **T** | **G** | **C** | **G** | **T** | **C** | **C** | **C** | **T** | **T** | **A** | **G** | **C** | **A** | **C** | **C** | **T** | **T** | **T** | **A** | **T** | **T** | **G** | **T** | **T** | **A** | **T** | **C** | **T** | **T** | **A** | **A** | **T** | **T** | **A** | **C** | **A** | **T** | **T** | **G** | **T** | **T** | **T** | **T** | **C** | **A** | **T** | **A** | **T** | **T** | **T** | **T** | **C** | **A** | **T** | **T** | **C** | **G** | **A** | **T** | **T** | **A** | **A** | **A** | **C** | **A** | **A** | **A** | **A** | **C** | **C** | **G** | **G** | **T** | **G** | **G** | **C** | **T** | **A** | **T** | **T** | **A** | **G** | **A** | **G** | **C** | **T** | **T** | **C** | **T** | **T** | **C** | **A** | **G** | **A** | **G** | **A** | **A** | **A** | **A** | **G** | **T** | **C** | **T** | **T** | **T** | **T** | **A** | **T** | **C** | **T** | **A** | **C** | **C** | **A** | **C** | **T** | **A** | **A** | **T** | **T** | **G** | **T** | **G** | **C** | **A** | **C** | **A** | **T** | **T** | **G** | **T** | **T** | **C** | **T** | **C** | **A** | **T** | **A** | **T** | **C** | **C** | **T** | **T** | **A** | **C** | **T** | **A** | **A** | **T** | **A** | **G** | **G** | **C** | **T** | **T** | **A** | **T** | **T** | **G** | **A** | **C** | **C** | **T** | **T** | **T** | **T** | **T** | **A** | **C** | **T** | **G** | **A** | **T** | **T** | **T** | **C** | **A** | **T** | **T** | **G** | **G** | **T** | **T** | **C** | **A** | **A** | **A** | **A** | **A** | **G** | **G** | **G** | **C** | **A** | **A** | **T** | **A** | **T** | **G** | **G** | **A** | **C** | **G** | **G** | **A** | **T** | **T** | **G** | **A** | **A** | **G** | **A** | **A** | **A** | **A** | **A** | **C** | **T** | **T** | **C** | **G** | **G** | **T** | **T** | **A** | **T** | **T** | **G** | **G** | **C** | **C** | **A** | **A** | **T** | **G** | **G** | **C** | **A** | **A** | **T** | **T** | **A** | **T** | **G** | **A** | **A** | **A** | **G** | **T** | **C** | **C** | **A** | **G** | **T** | **C** | **T** | **T** | **A** | **A** | **A** | **C** | **A** | **A** | **A** | **C** | **C** | **A** | **A** | **C** | **G** | **A** | **C** | **C** | **A** | **G** | **T** | **G** | **A** | **A** | **A** | **A** | **T** | **C** | **A** | **A** | **G** | **A** | **T** | **C** | **A** | **T** | **T** | **A** | **T** | **C** | **T** | **A** | **A** | **C** | **C** | **G** | **A** | **A** | **G** | **T** | **C** | **G** | **A** | **A** | **C** | **A** | **A** | **G** | **A** | **T** | **A** | **T** | **T** | **T** | **G** | **G** | **T** | **C** | **G** | **A** | **T** | **T** | **A** | **A** | **A** | **A** | **A** | **T** | **A** | **A** | **A** | **T** | **T** | **A** | **T** | **T** | **A** | **G** | **A** | **G** | **A** | **T** | **G** | **T** | **C** | **T** | **A** | **A** | **A** | **G** | **A** | **A** | **T** | **T** | **G** | **C** | **A** | **A** | **T** | **T** | **A** | **T** | **T** | **A** | **A** | **A** | **C** | **A** | **G** | **T** | **C** | **G** | **G** | **C** | **C** | **G** | **C** | **A** | **A** | **T** | **T** | **A** | **A** | **T** | **G** | **G** | **A** | **T** | **G** | **G** | **G** | **C** | **A** | **A** | **A** | **C** | **A** | **A** | **A** | **A** | **G** | **A** | **A** | **G** | **A** | **A** | **A** | **T** | **T** | **T** | **T** | **A** | **G** | **A** | **G** | **A** | **A** | **C** | **G** | **A** | **G** | **C** | **G** | **G** | **C** | **A** | **T** | **C** | **G** | **T** | **T** | **T** | **G** | **G** | **C** | **G** | **C** | **G** | **G** | **G** | **A** | **G** | **T** | **T** | **G** | **C** | **A** | **T** | **G** | **A** | **T** | **T** | **C** | **A** | **G** | **T** | **C** | **A** | **G** | **T** | **C** | **A** | **A** | **C** | **A** | **A** | **C** | **T** | **T** | **T** | **T** | **T** | **G** | **C** | **A** | **G** | **C** | **C** | **A** | **T** | **G** | **A** | **T** | **G** | **A** | **T** | **G** | **T** | **T** | **G** | **T** | **C** | **T** | **G** | **C** | **C** | **T** | **T** | **A** | **A** | **A** | **T** | **G** | **A** | **A** | **C** | **A** | **A** | **G** | **C** | **A** | **C** | **A** | **A** | **C** | **G** | **A** | **A** | **C** | **A** | **G** | **A** | **A** | **A** | **C** | **C** | **C** | **C** | **G** | **G** | **A** | **A** | **C** | **C** | **A** | **T** | **A** | **T** | **C** | **G** | **T** | **A** | **A** | **A** | **C** | **A** | **A** | **C** | **T** | **A** | **G** | **C** | **C** | **A** | **T** | **G** | **G** | **T** | **G** | **G** | **C** | **A** | **G** | **A** | **A** | **A** | **T** | **C** | **A** | **T** | **T** | **A** | **A** | **T** | **G** | **C** | **C** | **T** | **C** | **C** | **C** | **A** | **A** | **T** | **C** | **G** | **G** | **A** | **A** | **A** | **T** | **G** | **C** | **G** | **C** | **G** | **C** | **G** | **T** | **T** | **A** | **C** | **T** | **A** | **T** | **T** | **G** | **C** | **A** | **C** | **T** | **T** | **G** | **C** | **G** | **T** | **C** | **C** | **T** | **A** | **T** | **C** | **A** | **G** | **T** | **C** | **T** | **A** | **G** | **A** | **A** | **G** | **G** | **A** | **A** | **A** | **A** | **A** | **G** | **T** | **T** | **T** | **G** | **C** | **G** | **T** | **A** | **A** | **A** | **G** | **G** | **T** | **A** | **T** | **T** | **G** | **A** | **A** | **C** | **A** | **A** | **T** | **T** | **A** | **C** | **T** | **G** | **A** | **A** | **A** | **G** | **A** | **A** | **C** | **T** | **A** | **C** | **A** | **A** | **A** | **C** | **A** | **A** | **A** | **A** | **A** | **T** | **T** | **A** | **A** | **A** | **A** | **T** | **T** | **G** | **A** | **A** | **T** | **T** | **G** | **A** | **T** | **T** | **T** | **G** | **G** | **G** | **A** | **T** | **G** | **T** | **T** | **G** | **A** | **A** | **G** | **A** | **T** | **G** | **T** | **T** | **C** | **A** | **T** | **T** | **T** | **A** | **A** | **A** | **T** | **A** | **G** | **C** | **A** | **G** | **C** | **A** | **T** | **T** | **G** | **A** | **G** | **G** | **A** | **T** | **C** | **A** | **T** | **C** | **T** | **T** | **T** | **T** | **C** | **C** | **G** | **A** | **A** | **T** | **T** | **G** | **T** | **G** | **C** | **A** | **G** | **G** | **A** | **A** | **T** | **T** | **A** | **C** | **T** | **T** | **T** | **C** | **C** | **A** | **A** | **T** | **A** | **C** | **C** | **T** | **T** | **A** | **A** | **G** | **A** | **C** | **A** | **T** | **G** | **C** | **C** | **A** | **A** | **A** | **G** | **C** | **A** | **A** | **A** | **G** | **G** | **A** | **A** | **T** | **T** | **A** | **G** | **A** | **G** | **G** | **T** | **A** | **T** | **A** | **C** | **T** | **T** | **A** | **C** | **A** | **C** | **C** | **A** | **A** | **G** | **T** | **C** | **G** | **A** | **T** | **A** | **A** | **A** | **A** | **A** | **C** | **G** | **T** | **G** | **T** | **T** | **A** | **T** | **T** | **G** | **C** | **G** | **T** | **A** | **T** | **T** | **G** | **T** | **T** | **G** | **A** | **T** | **G** | **A** | **T** | **G** | **G** | **T** | **G** | **T** | **C** | **G** | **G** | **C** | **T** | **T** | **T** | **G** | **A** | **T** | **A** | **T** | **G** | **A** | **A** | **G** | **G** | **A** | **A** | **C** | **A** | **A** | **A** | **G** | **T** | **A** | **A** | **T** | **A** | **A** | **A** | **G** | **C** | **C** | **G** | **G** | **T** | **A** | **G** | **T** | **T** | **A** | **T** | **G** | **G** | **C** | **T** | **T** | **A** | **A** | **A** | **T** | **A** | **A** | **T** | **A** | **T** | **T** | **C** | **G** | **A** | **G** | **A** | **A** | **C** | **G** | **T** | **G** | **T** | **T** | **G** | **T** | **C** | **G** | **G** | **C** | **A** | **T** | **G** | **G** | **G** | **C** | **G** | **G** | **T** | **A** | **C** | **A** | **G** | **T** | **T** | **A** | **A** | **A** | **A** | **T** | **T** | **A** | **T** | **T** | **A** | **G** | **T** | **T** | **T** | **T** | **A** | **A** | **A** | **G** | **G** | **G** | **C** | **A** | **G** | **G** | **G** | **A** | **A** | **C** | **C** | **A** | **G** | **C** | **G** | **T** | **T** | **G** | **A** | **A** | **A** | **T** | **T** | **A** | **A** | **A** | **G** | **T** | **T** | **C** | **C** | **T** | **G** | **T** | **C** | **A** | **T** | **A** | **A** | **A** | **G** | **G** | **A** | **G** | **G** | **A** | **A** | **A** | **C** | **T** | **G** | **C** | **A** | **A** | **G** | **T** | **G** | **A** | **T** | **C** | **A** | **A** | **A** | **G** | **T** | **A** | **A** | **T** | **G** | **T** | **T** | **A** | **G** | **T** | **G** | **G** | **A** | **-** | **-** | **-** |
| **N55268** | **A** | **T** | **G** | **A** | **C** | **C** | **G** | **A** | **T** | **C** | **G** | **G** | **A** | **T** | **T** | **T** | **C** | **A** | **A** | **G** | **A** | **C** | **G** | **C** | **A** | **T** | **G** | **A** | **T** | **T** | **T** | **C** | **A** | **T** | **T** | **A** | **T** | **A** | **T** | **G** | **C** | **G** | **T** | **C** | **C** | **C** | **T** | **T** | **A** | **G** | **C** | **A** | **C** | **C** | **T** | **T** | **T** | **A** | **T** | **T** | **G** | **T** | **T** | **A** | **T** | **C** | **T** | **T** | **A** | **A** | **T** | **T** | **A** | **C** | **A** | **T** | **T** | **G** | **T** | **T** | **T** | **T** | **C** | **A** | **T** | **A** | **T** | **T** | **T** | **T** | **C** | **A** | **T** | **T** | **C** | **G** | **A** | **T** | **T** | **A** | **A** | **A** | **C** | **A** | **A** | **A** | **A** | **C** | **C** | **G** | **G** | **T** | **G** | **G** | **C** | **T** | **A** | **T** | **T** | **A** | **G** | **A** | **G** | **C** | **T** | **T** | **C** | **T** | **T** | **C** | **A** | **G** | **A** | **G** | **A** | **A** | **A** | **A** | **G** | **T** | **C** | **T** | **T** | **T** | **T** | **A** | **T** | **T** | **T** | **A** | **C** | **C** | **A** | **C** | **T** | **A** | **A** | **T** | **T** | **G** | **T** | **G** | **C** | **A** | **C** | **A** | **T** | **T** | **G** | **T** | **T** | **C** | **T** | **C** | **A** | **T** | **A** | **T** | **C** | **C** | **T** | **T** | **A** | **C** | **T** | **A** | **A** | **T** | **A** | **G** | **G** | **C** | **T** | **T** | **A** | **T** | **T** | **G** | **A** | **C** | **C** | **T** | **T** | **T** | **T** | **T** | **A** | **C** | **T** | **G** | **A** | **T** | **T** | **T** | **C** | **A** | **T** | **T** | **G** | **G** | **T** | **T** | **C** | **A** | **A** | **A** | **A** | **A** | **G** | **G** | **G** | **C** | **A** | **A** | **T** | **A** | **T** | **G** | **G** | **A** | **C** | **G** | **G** | **A** | **T** | **T** | **G** | **A** | **A** | **G** | **A** | **A** | **A** | **A** | **A** | **C** | **T** | **T** | **C** | **G** | **G** | **T** | **T** | **A** | **T** | **T** | **G** | **G** | **C** | **C** | **A** | **A** | **C** | **G** | **G** | **T** | **A** | **A** | **T** | **T** | **A** | **T** | **G** | **A** | **A** | **A** | **G** | **T** | **C** | **C** | **A** | **G** | **T** | **C** | **T** | **T** | **A** | **A** | **A** | **C** | **A** | **A** | **A** | **C** | **C** | **A** | **A** | **C** | **G** | **A** | **C** | **C** | **A** | **G** | **T** | **G** | **A** | **A** | **A** | **A** | **T** | **C** | **A** | **A** | **G** | **A** | **T** | **C** | **A** | **T** | **T** | **A** | **T** | **C** | **T** | **A** | **A** | **C** | **C** | **G** | **A** | **A** | **G** | **T** | **C** | **G** | **A** | **A** | **C** | **A** | **A** | **G** | **A** | **T** | **A** | **T** | **T** | **T** | **G** | **G** | **T** | **C** | **G** | **A** | **T** | **T** | **A** | **A** | **A** | **A** | **A** | **T** | **A** | **A** | **A** | **T** | **T** | **A** | **T** | **T** | **A** | **G** | **A** | **G** | **A** | **T** | **G** | **T** | **C** | **T** | **A** | **A** | **A** | **G** | **A** | **A** | **T** | **T** | **G** | **C** | **A** | **A** | **T** | **T** | **A** | **T** | **T** | **A** | **A** | **A** | **C** | **A** | **G** | **T** | **C** | **G** | **A** | **C** | **C** | **G** | **C** | **A** | **A** | **T** | **T** | **A** | **A** | **T** | **G** | **G** | **A** | **T** | **G** | **G** | **G** | **C** | **A** | **A** | **A** | **C** | **A** | **A** | **A** | **A** | **G** | **A** | **A** | **G** | **A** | **A** | **A** | **T** | **T** | **T** | **T** | **A** | **G** | **A** | **G** | **A** | **A** | **C** | **G** | **A** | **G** | **C** | **G** | **G** | **C** | **A** | **T** | **C** | **G** | **T** | **T** | **T** | **G** | **G** | **C** | **G** | **C** | **G** | **G** | **G** | **A** | **G** | **T** | **T** | **G** | **C** | **A** | **T** | **G** | **A** | **T** | **T** | **C** | **A** | **G** | **T** | **C** | **A** | **G** | **T** | **C** | **A** | **A** | **C** | **A** | **A** | **C** | **T** | **T** | **T** | **T** | **T** | **G** | **C** | **A** | **G** | **C** | **C** | **A** | **T** | **G** | **A** | **T** | **G** | **A** | **T** | **G** | **T** | **T** | **G** | **T** | **C** | **T** | **G** | **C** | **C** | **T** | **T** | **A** | **A** | **A** | **T** | **G** | **A** | **A** | **C** | **A** | **A** | **G** | **C** | **A** | **C** | **A** | **A** | **C** | **G** | **A** | **A** | **C** | **A** | **G** | **A** | **A** | **A** | **C** | **C** | **C** | **C** | **T** | **G** | **A** | **A** | **C** | **C** | **A** | **T** | **A** | **T** | **C** | **G** | **T** | **A** | **A** | **A** | **C** | **A** | **A** | **C** | **T** | **A** | **G** | **C** | **C** | **A** | **T** | **G** | **G** | **T** | **G** | **G** | **C** | **A** | **G** | **A** | **A** | **A** | **T** | **C** | **A** | **T** | **T** | **A** | **A** | **T** | **G** | **C** | **C** | **T** | **C** | **C** | **C** | **A** | **A** | **T** | **C** | **G** | **G** | **A** | **A** | **A** | **T** | **G** | **C** | **G** | **C** | **G** | **C** | **G** | **T** | **T** | **A** | **C** | **T** | **A** | **T** | **T** | **G** | **C** | **A** | **C** | **T** | **T** | **G** | **C** | **G** | **T** | **C** | **C** | **T** | **A** | **T** | **C** | **A** | **G** | **T** | **C** | **T** | **A** | **G** | **A** | **A** | **G** | **G** | **A** | **A** | **A** | **A** | **A** | **G** | **T** | **T** | **T** | **G** | **C** | **G** | **T** | **A** | **A** | **A** | **G** | **G** | **T** | **A** | **T** | **T** | **G** | **A** | **A** | **C** | **A** | **A** | **T** | **T** | **A** | **C** | **T** | **G** | **A** | **A** | **A** | **G** | **A** | **A** | **C** | **T** | **A** | **C** | **A** | **A** | **A** | **C** | **A** | **A** | **A** | **A** | **A** | **T** | **T** | **A** | **A** | **A** | **A** | **T** | **T** | **G** | **A** | **A** | **T** | **T** | **G** | **A** | **T** | **T** | **T** | **G** | **G** | **G** | **A** | **T** | **G** | **T** | **T** | **G** | **A** | **A** | **G** | **A** | **T** | **G** | **T** | **T** | **C** | **A** | **T** | **T** | **T** | **A** | **A** | **A** | **T** | **A** | **G** | **C** | **A** | **G** | **C** | **A** | **T** | **T** | **G** | **A** | **G** | **G** | **A** | **T** | **C** | **A** | **T** | **C** | **T** | **T** | **T** | **T** | **C** | **C** | **G** | **A** | **A** | **T** | **T** | **G** | **T** | **G** | **C** | **A** | **G** | **G** | **A** | **G** | **T** | **T** | **A** | **C** | **T** | **T** | **T** | **C** | **A** | **A** | **A** | **T** | **A** | **C** | **C** | **T** | **T** | **A** | **A** | **G** | **A** | **C** | **A** | **T** | **G** | **C** | **C** | **A** | **A** | **A** | **G** | **C** | **A** | **A** | **A** | **G** | **G** | **A** | **A** | **T** | **T** | **A** | **G** | **A** | **G** | **G** | **T** | **A** | **T** | **A** | **C** | **T** | **T** | **A** | **C** | **A** | **C** | **C** | **A** | **A** | **G** | **T** | **C** | **G** | **A** | **T** | **A** | **A** | **A** | **A** | **A** | **C** | **G** | **T** | **G** | **C** | **T** | **A** | **T** | **T** | **G** | **C** | **G** | **T** | **A** | **T** | **T** | **G** | **T** | **T** | **G** | **A** | **T** | **G** | **A** | **C** | **G** | **G** | **T** | **G** | **T** | **C** | **G** | **G** | **C** | **T** | **T** | **T** | **G** | **A** | **T** | **A** | **T** | **G** | **A** | **A** | **G** | **G** | **A** | **A** | **C** | **A** | **A** | **A** | **G** | **T** | **A** | **A** | **T** | **A** | **A** | **A** | **G** | **C** | **C** | **G** | **G** | **T** | **A** | **G** | **T** | **T** | **A** | **T** | **G** | **G** | **C** | **T** | **T** | **A** | **A** | **A** | **T** | **A** | **A** | **T** | **A** | **T** | **T** | **C** | **G** | **A** | **G** | **A** | **A** | **C** | **G** | **T** | **G** | **T** | **T** | **G** | **T** | **C** | **G** | **G** | **C** | **A** | **T** | **G** | **G** | **G** | **C** | **G** | **G** | **T** | **A** | **C** | **A** | **G** | **T** | **T** | **A** | **A** | **A** | **A** | **T** | **T** | **A** | **T** | **T** | **A** | **G** | **T** | **T** | **T** | **T** | **A** | **A** | **A** | **G** | **G** | **G** | **C** | **A** | **G** | **G** | **G** | **A** | **A** | **C** | **C** | **A** | **G** | **C** | **G** | **T** | **T** | **G** | **A** | **A** | **A** | **T** | **T** | **A** | **A** | **A** | **G** | **T** | **T** | **C** | **C** | **T** | **G** | **T** | **C** | **A** | **T** | **A** | **A** | **A** | **G** | **G** | **A** | **G** | **G** | **A** | **A** | **A** | **C** | **T** | **G** | **C** | **A** | **A** | **G** | **T** | **G** | **A** | **T** | **C** | **A** | **A** | **A** | **G** | **T** | **A** | **A** | **T** | **G** | **T** | **T** | **A** | **G** | **T** | **G** | **G** | **A** | **-** | **-** | **-** |
| **N59555F** | **A** | **T** | **G** | **A** | **C** | **C** | **G** | **A** | **T** | **C** | **G** | **G** | **A** | **T** | **T** | **T** | **C** | **A** | **A** | **G** | **A** | **C** | **G** | **C** | **A** | **T** | **G** | **A** | **T** | **T** | **T** | **C** | **A** | **T** | **T** | **A** | **T** | **A** | **T** | **G** | **C** | **G** | **T** | **C** | **C** | **C** | **T** | **T** | **A** | **G** | **C** | **A** | **C** | **C** | **T** | **T** | **T** | **A** | **T** | **T** | **G** | **T** | **T** | **A** | **T** | **C** | **T** | **T** | **A** | **A** | **T** | **T** | **A** | **C** | **A** | **T** | **T** | **G** | **T** | **T** | **T** | **T** | **C** | **A** | **T** | **A** | **T** | **T** | **T** | **T** | **C** | **A** | **T** | **T** | **C** | **G** | **A** | **T** | **T** | **A** | **A** | **A** | **C** | **A** | **A** | **A** | **A** | **C** | **C** | **G** | **G** | **T** | **G** | **G** | **C** | **T** | **A** | **T** | **T** | **A** | **G** | **A** | **G** | **C** | **T** | **T** | **C** | **T** | **T** | **C** | **A** | **G** | **A** | **G** | **A** | **A** | **A** | **A** | **G** | **T** | **C** | **T** | **T** | **T** | **T** | **A** | **T** | **C** | **T** | **A** | **C** | **C** | **A** | **C** | **T** | **A** | **A** | **T** | **T** | **G** | **T** | **G** | **C** | **A** | **C** | **A** | **T** | **T** | **G** | **T** | **T** | **C** | **T** | **C** | **A** | **T** | **A** | **T** | **C** | **C** | **T** | **T** | **A** | **C** | **T** | **A** | **A** | **T** | **A** | **G** | **G** | **C** | **T** | **T** | **A** | **T** | **T** | **G** | **A** | **C** | **C** | **T** | **T** | **T** | **T** | **T** | **A** | **C** | **T** | **G** | **A** | **T** | **T** | **T** | **C** | **A** | **T** | **T** | **G** | **G** | **T** | **T** | **C** | **A** | **A** | **A** | **A** | **A** | **G** | **G** | **G** | **C** | **A** | **A** | **T** | **A** | **T** | **G** | **G** | **A** | **C** | **G** | **G** | **A** | **T** | **T** | **G** | **A** | **A** | **G** | **A** | **A** | **A** | **A** | **A** | **C** | **T** | **T** | **C** | **G** | **G** | **T** | **T** | **A** | **T** | **T** | **G** | **G** | **C** | **C** | **A** | **A** | **T** | **G** | **G** | **C** | **A** | **A** | **T** | **T** | **A** | **T** | **G** | **A** | **A** | **A** | **G** | **T** | **C** | **C** | **A** | **G** | **T** | **C** | **T** | **T** | **A** | **A** | **A** | **C** | **A** | **A** | **A** | **C** | **C** | **A** | **A** | **C** | **G** | **A** | **C** | **C** | **A** | **G** | **T** | **G** | **A** | **A** | **A** | **A** | **T** | **C** | **A** | **A** | **G** | **A** | **T** | **C** | **A** | **T** | **T** | **A** | **T** | **C** | **T** | **A** | **A** | **C** | **C** | **G** | **A** | **A** | **G** | **T** | **C** | **G** | **A** | **A** | **C** | **A** | **A** | **G** | **A** | **T** | **A** | **T** | **T** | **T** | **G** | **G** | **T** | **C** | **G** | **A** | **T** | **T** | **A** | **A** | **A** | **A** | **A** | **T** | **A** | **A** | **A** | **T** | **T** | **A** | **T** | **T** | **A** | **G** | **A** | **G** | **A** | **T** | **G** | **T** | **C** | **T** | **A** | **A** | **A** | **G** | **A** | **A** | **T** | **T** | **G** | **C** | **A** | **A** | **T** | **T** | **A** | **T** | **T** | **A** | **A** | **A** | **C** | **A** | **G** | **T** | **C** | **G** | **G** | **C** | **C** | **G** | **C** | **A** | **A** | **T** | **T** | **A** | **A** | **T** | **G** | **G** | **A** | **T** | **G** | **G** | **G** | **C** | **A** | **A** | **A** | **C** | **A** | **A** | **A** | **A** | **G** | **A** | **A** | **G** | **A** | **A** | **A** | **T** | **T** | **T** | **T** | **A** | **G** | **A** | **G** | **A** | **A** | **C** | **G** | **A** | **G** | **C** | **G** | **G** | **C** | **A** | **T** | **C** | **G** | **T** | **T** | **T** | **G** | **G** | **C** | **G** | **C** | **G** | **G** | **G** | **A** | **G** | **T** | **T** | **G** | **C** | **A** | **T** | **G** | **A** | **T** | **T** | **C** | **A** | **G** | **T** | **C** | **A** | **G** | **T** | **C** | **A** | **A** | **C** | **A** | **A** | **C** | **T** | **T** | **T** | **T** | **T** | **G** | **C** | **A** | **G** | **C** | **C** | **A** | **T** | **G** | **A** | **T** | **G** | **A** | **T** | **G** | **T** | **T** | **G** | **T** | **C** | **T** | **G** | **C** | **C** | **T** | **T** | **A** | **A** | **A** | **T** | **G** | **A** | **A** | **C** | **A** | **A** | **G** | **C** | **A** | **C** | **A** | **A** | **C** | **G** | **A** | **A** | **C** | **A** | **G** | **A** | **A** | **A** | **C** | **C** | **C** | **C** | **G** | **G** | **A** | **A** | **C** | **C** | **A** | **T** | **A** | **T** | **C** | **G** | **T** | **A** | **A** | **A** | **C** | **A** | **A** | **C** | **T** | **A** | **G** | **C** | **C** | **A** | **T** | **G** | **G** | **T** | **G** | **G** | **C** | **A** | **G** | **A** | **A** | **A** | **T** | **C** | **A** | **T** | **T** | **A** | **A** | **T** | **G** | **C** | **C** | **T** | **C** | **C** | **C** | **A** | **A** | **T** | **C** | **G** | **G** | **A** | **A** | **A** | **T** | **G** | **C** | **G** | **C** | **G** | **C** | **G** | **T** | **T** | **A** | **C** | **T** | **A** | **T** | **T** | **G** | **C** | **A** | **C** | **T** | **T** | **G** | **C** | **G** | **T** | **C** | **C** | **T** | **A** | **T** | **C** | **A** | **G** | **T** | **C** | **T** | **A** | **G** | **A** | **A** | **G** | **G** | **A** | **A** | **A** | **A** | **A** | **G** | **T** | **T** | **T** | **G** | **C** | **G** | **T** | **A** | **A** | **A** | **G** | **G** | **T** | **A** | **T** | **T** | **G** | **A** | **A** | **C** | **A** | **A** | **T** | **T** | **A** | **C** | **T** | **G** | **A** | **A** | **A** | **G** | **A** | **A** | **C** | **T** | **A** | **C** | **A** | **A** | **A** | **C** | **A** | **A** | **A** | **A** | **A** | **T** | **T** | **A** | **A** | **A** | **A** | **T** | **T** | **G** | **A** | **A** | **T** | **T** | **G** | **A** | **T** | **T** | **T** | **G** | **G** | **G** | **A** | **T** | **G** | **T** | **T** | **G** | **A** | **A** | **G** | **A** | **T** | **G** | **T** | **T** | **C** | **A** | **T** | **T** | **T** | **A** | **A** | **A** | **T** | **A** | **G** | **C** | **A** | **G** | **C** | **A** | **T** | **T** | **G** | **A** | **G** | **G** | **A** | **T** | **C** | **A** | **T** | **C** | **T** | **T** | **T** | **T** | **C** | **C** | **G** | **A** | **A** | **T** | **T** | **G** | **T** | **G** | **C** | **A** | **G** | **G** | **A** | **G** | **T** | **T** | **A** | **C** | **T** | **T** | **T** | **C** | **A** | **A** | **A** | **T** | **A** | **C** | **C** | **T** | **T** | **A** | **A** | **G** | **A** | **C** | **A** | **T** | **G** | **C** | **T** | **A** | **A** | **A** | **G** | **C** | **A** | **A** | **A** | **G** | **G** | **A** | **A** | **T** | **T** | **A** | **G** | **A** | **G** | **G** | **T** | **A** | **T** | **A** | **T** | **T** | **T** | **A** | **C** | **A** | **C** | **C** | **A** | **A** | **G** | **T** | **T** | **G** | **A** | **T** | **A** | **A** | **A** | **A** | **A** | **C** | **G** | **T** | **G** | **T** | **T** | **A** | **T** | **T** | **G** | **C** | **G** | **T** | **A** | **T** | **T** | **G** | **T** | **T** | **G** | **A** | **T** | **G** | **A** | **T** | **G** | **G** | **T** | **G** | **T** | **C** | **G** | **G** | **C** | **T** | **T** | **T** | **G** | **A** | **T** | **A** | **T** | **G** | **A** | **A** | **G** | **G** | **A** | **A** | **C** | **A** | **A** | **A** | **G** | **T** | **A** | **A** | **T** | **A** | **A** | **A** | **G** | **C** | **C** | **G** | **G** | **T** | **A** | **G** | **T** | **T** | **A** | **T** | **G** | **G** | **C** | **T** | **T** | **A** | **A** | **A** | **T** | **A** | **A** | **T** | **A** | **T** | **T** | **C** | **G** | **A** | **G** | **A** | **A** | **C** | **G** | **T** | **G** | **T** | **T** | **G** | **T** | **C** | **G** | **G** | **C** | **A** | **T** | **G** | **G** | **G** | **C** | **G** | **G** | **T** | **A** | **C** | **A** | **G** | **T** | **T** | **A** | **A** | **A** | **A** | **T** | **T** | **A** | **T** | **T** | **A** | **G** | **T** | **T** | **T** | **T** | **A** | **A** | **A** | **G** | **G** | **G** | **C** | **A** | **G** | **G** | **G** | **A** | **A** | **C** | **C** | **A** | **G** | **C** | **G** | **T** | **T** | **G** | **A** | **A** | **A** | **T** | **T** | **A** | **A** | **A** | **G** | **T** | **T** | **C** | **C** | **T** | **G** | **T** | **C** | **A** | **T** | **A** | **A** | **A** | **G** | **G** | **A** | **G** | **G** | **A** | **A** | **A** | **C** | **T** | **G** | **C** | **A** | **A** | **G** | **T** | **G** | **A** | **T** | **C** | **A** | **A** | **A** | **G** | **T** | **A** | **A** | **T** | **G** | **T** | **T** | **A** | **G** | **T** | **G** | **G** | **A** | **-** | **-** | **-** |
| **N54121** | **A** | **T** | **G** | **A** | **C** | **C** | **G** | **A** | **T** | **C** | **G** | **G** | **A** | **T** | **T** | **T** | **C** | **A** | **A** | **G** | **A** | **C** | **G** | **C** | **A** | **T** | **G** | **A** | **T** | **T** | **T** | **C** | **A** | **T** | **T** | **A** | **T** | **A** | **T** | **G** | **C** | **G** | **T** | **C** | **C** | **C** | **T** | **T** | **A** | **G** | **C** | **A** | **C** | **C** | **T** | **T** | **T** | **A** | **T** | **T** | **G** | **T** | **T** | **A** | **T** | **C** | **T** | **T** | **A** | **A** | **T** | **T** | **A** | **C** | **A** | **T** | **T** | **G** | **T** | **T** | **T** | **T** | **C** | **A** | **T** | **A** | **T** | **T** | **T** | **T** | **C** | **A** | **T** | **T** | **C** | **G** | **A** | **T** | **T** | **A** | **A** | **A** | **C** | **A** | **A** | **A** | **A** | **C** | **C** | **G** | **G** | **T** | **G** | **G** | **T** | **T** | **A** | **T** | **T** | **A** | **G** | **A** | **G** | **C** | **T** | **T** | **C** | **T** | **T** | **C** | **A** | **G** | **A** | **G** | **A** | **A** | **A** | **A** | **G** | **T** | **C** | **T** | **T** | **T** | **T** | **A** | **T** | **C** | **T** | **A** | **C** | **C** | **A** | **C** | **T** | **A** | **A** | **T** | **T** | **G** | **T** | **G** | **C** | **A** | **C** | **A** | **T** | **T** | **G** | **T** | **T** | **C** | **T** | **C** | **A** | **T** | **A** | **T** | **C** | **C** | **T** | **T** | **A** | **C** | **T** | **A** | **A** | **T** | **A** | **G** | **G** | **C** | **T** | **T** | **A** | **T** | **T** | **G** | **A** | **C** | **C** | **T** | **T** | **T** | **T** | **T** | **A** | **C** | **T** | **G** | **A** | **T** | **T** | **T** | **C** | **A** | **T** | **T** | **G** | **G** | **T** | **T** | **C** | **A** | **A** | **A** | **A** | **A** | **G** | **G** | **G** | **C** | **A** | **A** | **T** | **A** | **T** | **G** | **G** | **A** | **C** | **G** | **G** | **A** | **T** | **T** | **G** | **A** | **A** | **G** | **A** | **A** | **A** | **A** | **A** | **C** | **T** | **T** | **C** | **G** | **G** | **T** | **T** | **A** | **T** | **T** | **G** | **G** | **C** | **C** | **A** | **A** | **C** | **G** | **G** | **T** | **A** | **A** | **T** | **T** | **A** | **T** | **G** | **A** | **A** | **A** | **G** | **T** | **C** | **C** | **A** | **G** | **T** | **C** | **T** | **T** | **A** | **A** | **A** | **C** | **A** | **A** | **A** | **C** | **C** | **A** | **A** | **C** | **G** | **A** | **C** | **C** | **A** | **G** | **T** | **G** | **A** | **A** | **A** | **A** | **T** | **C** | **A** | **A** | **G** | **A** | **T** | **C** | **A** | **T** | **T** | **A** | **T** | **C** | **T** | **A** | **A** | **C** | **C** | **G** | **A** | **A** | **G** | **T** | **C** | **G** | **A** | **A** | **C** | **A** | **A** | **G** | **A** | **T** | **A** | **T** | **T** | **T** | **G** | **G** | **T** | **C** | **G** | **A** | **T** | **T** | **A** | **A** | **A** | **A** | **A** | **T** | **A** | **A** | **A** | **T** | **T** | **A** | **T** | **T** | **A** | **G** | **A** | **G** | **A** | **T** | **G** | **T** | **C** | **T** | **A** | **A** | **A** | **G** | **A** | **A** | **T** | **T** | **G** | **C** | **A** | **A** | **T** | **T** | **A** | **T** | **T** | **A** | **A** | **A** | **C** | **A** | **G** | **T** | **C** | **G** | **A** | **C** | **C** | **G** | **C** | **A** | **A** | **T** | **T** | **A** | **A** | **T** | **G** | **G** | **A** | **T** | **G** | **G** | **G** | **C** | **A** | **A** | **A** | **C** | **A** | **A** | **A** | **A** | **G** | **A** | **A** | **G** | **A** | **A** | **A** | **T** | **T** | **T** | **T** | **A** | **G** | **A** | **G** | **A** | **A** | **C** | **G** | **A** | **G** | **C** | **G** | **G** | **C** | **A** | **T** | **C** | **G** | **T** | **T** | **T** | **G** | **G** | **C** | **G** | **C** | **G** | **G** | **G** | **A** | **G** | **T** | **T** | **G** | **C** | **A** | **T** | **G** | **A** | **T** | **T** | **C** | **A** | **G** | **T** | **C** | **A** | **G** | **T** | **C** | **A** | **A** | **C** | **A** | **A** | **C** | **T** | **T** | **T** | **T** | **T** | **G** | **C** | **A** | **G** | **C** | **C** | **A** | **T** | **G** | **A** | **T** | **G** | **A** | **T** | **G** | **T** | **T** | **G** | **T** | **C** | **T** | **G** | **C** | **C** | **T** | **T** | **A** | **A** | **A** | **T** | **G** | **A** | **A** | **C** | **A** | **A** | **G** | **C** | **A** | **C** | **A** | **A** | **C** | **G** | **A** | **A** | **C** | **A** | **G** | **A** | **A** | **A** | **C** | **C** | **C** | **C** | **G** | **G** | **A** | **A** | **C** | **C** | **A** | **T** | **A** | **T** | **C** | **G** | **T** | **A** | **A** | **A** | **C** | **A** | **A** | **C** | **T** | **A** | **G** | **C** | **C** | **A** | **T** | **G** | **G** | **T** | **G** | **G** | **C** | **A** | **G** | **A** | **A** | **A** | **T** | **C** | **A** | **T** | **T** | **A** | **A** | **T** | **G** | **C** | **C** | **T** | **C** | **C** | **C** | **A** | **A** | **T** | **C** | **G** | **G** | **A** | **A** | **A** | **T** | **G** | **C** | **G** | **C** | **G** | **C** | **G** | **T** | **T** | **A** | **C** | **T** | **A** | **T** | **T** | **G** | **C** | **A** | **C** | **T** | **T** | **G** | **C** | **G** | **T** | **C** | **C** | **T** | **A** | **T** | **C** | **A** | **G** | **T** | **C** | **T** | **A** | **G** | **A** | **A** | **G** | **G** | **A** | **A** | **A** | **A** | **A** | **G** | **T** | **T** | **T** | **G** | **C** | **G** | **T** | **A** | **A** | **A** | **G** | **G** | **T** | **A** | **T** | **T** | **G** | **A** | **A** | **C** | **A** | **A** | **T** | **T** | **A** | **C** | **T** | **G** | **A** | **A** | **A** | **G** | **A** | **A** | **C** | **T** | **A** | **C** | **A** | **A** | **A** | **C** | **A** | **A** | **A** | **A** | **A** | **T** | **T** | **A** | **A** | **A** | **A** | **T** | **T** | **G** | **A** | **A** | **T** | **T** | **G** | **A** | **T** | **T** | **T** | **G** | **G** | **G** | **A** | **T** | **G** | **T** | **T** | **G** | **A** | **A** | **G** | **A** | **T** | **G** | **T** | **T** | **C** | **A** | **T** | **T** | **T** | **A** | **A** | **A** | **T** | **A** | **G** | **C** | **A** | **G** | **C** | **A** | **T** | **T** | **G** | **A** | **G** | **G** | **A** | **T** | **C** | **A** | **T** | **C** | **T** | **T** | **T** | **T** | **C** | **C** | **G** | **A** | **A** | **T** | **T** | **G** | **T** | **G** | **C** | **A** | **G** | **G** | **A** | **G** | **T** | **T** | **A** | **C** | **T** | **T** | **T** | **C** | **A** | **A** | **A** | **T** | **A** | **C** | **C** | **T** | **T** | **A** | **A** | **G** | **A** | **C** | **A** | **T** | **G** | **C** | **C** | **A** | **A** | **A** | **G** | **C** | **A** | **A** | **A** | **G** | **G** | **A** | **A** | **T** | **T** | **A** | **G** | **A** | **G** | **G** | **T** | **A** | **T** | **A** | **C** | **T** | **T** | **A** | **C** | **A** | **C** | **C** | **A** | **A** | **G** | **T** | **C** | **G** | **A** | **T** | **A** | **A** | **A** | **A** | **A** | **C** | **G** | **T** | **G** | **T** | **T** | **A** | **T** | **T** | **G** | **C** | **G** | **T** | **A** | **T** | **T** | **G** | **T** | **T** | **G** | **A** | **T** | **G** | **A** | **C** | **G** | **G** | **T** | **G** | **T** | **C** | **G** | **G** | **C** | **T** | **T** | **T** | **G** | **A** | **T** | **A** | **T** | **G** | **A** | **A** | **G** | **G** | **A** | **A** | **C** | **A** | **A** | **A** | **G** | **T** | **A** | **A** | **T** | **A** | **A** | **A** | **G** | **C** | **C** | **G** | **G** | **T** | **A** | **G** | **T** | **T** | **A** | **T** | **G** | **G** | **C** | **T** | **T** | **A** | **A** | **A** | **T** | **A** | **A** | **T** | **A** | **T** | **C** | **C** | **G** | **A** | **G** | **A** | **A** | **C** | **G** | **T** | **G** | **T** | **T** | **G** | **T** | **C** | **G** | **G** | **C** | **A** | **T** | **G** | **G** | **G** | **C** | **G** | **G** | **T** | **A** | **C** | **A** | **G** | **T** | **T** | **A** | **A** | **A** | **A** | **T** | **T** | **A** | **T** | **T** | **A** | **G** | **T** | **T** | **T** | **T** | **A** | **A** | **A** | **G** | **G** | **G** | **C** | **A** | **G** | **G** | **G** | **A** | **A** | **C** | **C** | **A** | **G** | **C** | **G** | **T** | **T** | **G** | **A** | **A** | **A** | **T** | **T** | **A** | **A** | **A** | **G** | **T** | **T** | **C** | **C** | **T** | **G** | **T** | **C** | **A** | **T** | **A** | **A** | **A** | **G** | **G** | **A** | **G** | **G** | **A** | **A** | **A** | **C** | **T** | **G** | **C** | **A** | **A** | **G** | **T** | **G** | **A** | **T** | **C** | **A** | **A** | **A** | **G** | **T** | **A** | **A** | **T** | **G** | **T** | **T** | **A** | **G** | **T** | **G** | **G** | **A** | **-** | **-** | **-** |
| **N60440F** | **A** | **T** | **G** | **A** | **C** | **C** | **G** | **A** | **T** | **C** | **G** | **G** | **A** | **T** | **T** | **T** | **C** | **A** | **A** | **G** | **A** | **C** | **G** | **C** | **A** | **T** | **G** | **A** | **T** | **T** | **T** | **C** | **A** | **T** | **T** | **A** | **T** | **A** | **T** | **G** | **C** | **G** | **T** | **C** | **C** | **C** | **T** | **T** | **A** | **G** | **C** | **A** | **C** | **C** | **T** | **T** | **T** | **A** | **T** | **T** | **G** | **T** | **T** | **A** | **T** | **C** | **T** | **T** | **A** | **A** | **T** | **T** | **A** | **C** | **A** | **T** | **T** | **G** | **T** | **T** | **T** | **T** | **C** | **A** | **T** | **A** | **T** | **T** | **T** | **T** | **C** | **A** | **T** | **T** | **C** | **G** | **A** | **T** | **T** | **A** | **A** | **A** | **C** | **A** | **A** | **A** | **A** | **C** | **C** | **G** | **G** | **T** | **G** | **G** | **C** | **T** | **A** | **T** | **T** | **A** | **G** | **A** | **G** | **C** | **T** | **T** | **C** | **T** | **T** | **C** | **A** | **G** | **A** | **G** | **A** | **A** | **A** | **A** | **G** | **T** | **C** | **T** | **T** | **T** | **T** | **A** | **T** | **T** | **T** | **A** | **C** | **C** | **A** | **C** | **T** | **A** | **A** | **T** | **T** | **G** | **T** | **G** | **C** | **A** | **C** | **A** | **T** | **T** | **G** | **T** | **T** | **C** | **T** | **C** | **A** | **T** | **A** | **T** | **C** | **C** | **T** | **T** | **A** | **C** | **T** | **A** | **A** | **T** | **A** | **G** | **G** | **C** | **T** | **T** | **A** | **T** | **T** | **G** | **A** | **C** | **C** | **T** | **T** | **T** | **T** | **T** | **A** | **C** | **T** | **G** | **A** | **T** | **T** | **T** | **C** | **A** | **T** | **T** | **G** | **G** | **T** | **T** | **C** | **A** | **A** | **A** | **A** | **A** | **G** | **G** | **G** | **C** | **A** | **A** | **T** | **A** | **T** | **G** | **G** | **A** | **C** | **G** | **G** | **A** | **T** | **T** | **G** | **A** | **A** | **G** | **A** | **A** | **A** | **A** | **A** | **C** | **T** | **T** | **C** | **G** | **G** | **T** | **T** | **A** | **T** | **T** | **G** | **G** | **C** | **C** | **A** | **A** | **T** | **G** | **G** | **T** | **A** | **A** | **T** | **T** | **A** | **T** | **G** | **A** | **A** | **A** | **G** | **T** | **C** | **C** | **A** | **G** | **T** | **C** | **T** | **T** | **A** | **A** | **A** | **C** | **A** | **A** | **A** | **C** | **C** | **A** | **A** | **C** | **G** | **A** | **C** | **C** | **A** | **G** | **T** | **G** | **A** | **A** | **A** | **A** | **T** | **C** | **A** | **A** | **G** | **A** | **C** | **C** | **A** | **T** | **T** | **A** | **T** | **C** | **T** | **A** | **A** | **C** | **C** | **G** | **A** | **A** | **G** | **T** | **C** | **G** | **A** | **A** | **C** | **A** | **A** | **G** | **A** | **T** | **A** | **T** | **T** | **T** | **G** | **G** | **T** | **C** | **G** | **A** | **T** | **T** | **A** | **A** | **A** | **A** | **A** | **T** | **A** | **A** | **A** | **T** | **T** | **A** | **T** | **T** | **A** | **G** | **A** | **G** | **A** | **T** | **G** | **T** | **C** | **T** | **A** | **A** | **A** | **G** | **A** | **A** | **T** | **T** | **G** | **C** | **A** | **A** | **T** | **T** | **A** | **T** | **T** | **A** | **A** | **A** | **C** | **A** | **G** | **T** | **C** | **G** | **A** | **C** | **C** | **G** | **C** | **A** | **A** | **T** | **T** | **A** | **A** | **T** | **G** | **G** | **A** | **T** | **G** | **G** | **G** | **C** | **A** | **A** | **A** | **C** | **A** | **A** | **A** | **A** | **G** | **A** | **A** | **G** | **A** | **A** | **A** | **T** | **T** | **T** | **T** | **A** | **G** | **A** | **G** | **A** | **A** | **C** | **G** | **A** | **G** | **C** | **G** | **G** | **C** | **A** | **T** | **C** | **G** | **T** | **T** | **T** | **G** | **G** | **C** | **G** | **C** | **G** | **G** | **G** | **A** | **G** | **T** | **T** | **G** | **C** | **A** | **T** | **G** | **A** | **T** | **T** | **C** | **A** | **G** | **T** | **C** | **A** | **G** | **T** | **C** | **A** | **A** | **C** | **A** | **A** | **C** | **T** | **T** | **T** | **T** | **T** | **G** | **C** | **A** | **G** | **C** | **C** | **A** | **T** | **G** | **A** | **T** | **G** | **A** | **T** | **G** | **T** | **T** | **G** | **T** | **C** | **T** | **G** | **C** | **C** | **T** | **T** | **A** | **A** | **A** | **T** | **G** | **A** | **A** | **C** | **A** | **A** | **G** | **C** | **A** | **C** | **A** | **A** | **C** | **G** | **A** | **A** | **C** | **A** | **G** | **A** | **A** | **A** | **C** | **C** | **C** | **C** | **T** | **G** | **A** | **A** | **C** | **C** | **A** | **T** | **A** | **T** | **C** | **G** | **C** | **A** | **A** | **A** | **C** | **A** | **A** | **C** | **T** | **A** | **G** | **C** | **C** | **A** | **T** | **G** | **G** | **T** | **G** | **G** | **C** | **A** | **G** | **A** | **A** | **A** | **T** | **C** | **A** | **T** | **T** | **A** | **A** | **T** | **G** | **C** | **C** | **T** | **C** | **C** | **C** | **A** | **A** | **T** | **C** | **G** | **G** | **A** | **A** | **A** | **T** | **G** | **C** | **G** | **C** | **G** | **C** | **G** | **T** | **T** | **A** | **C** | **T** | **A** | **T** | **T** | **G** | **C** | **A** | **C** | **T** | **T** | **G** | **C** | **G** | **T** | **C** | **C** | **T** | **A** | **T** | **C** | **A** | **G** | **T** | **C** | **T** | **A** | **G** | **A** | **A** | **G** | **G** | **A** | **A** | **A** | **A** | **A** | **G** | **T** | **T** | **T** | **G** | **C** | **G** | **T** | **A** | **A** | **A** | **G** | **G** | **T** | **A** | **T** | **T** | **G** | **A** | **A** | **C** | **A** | **A** | **T** | **T** | **A** | **C** | **T** | **G** | **A** | **A** | **A** | **G** | **A** | **A** | **C** | **T** | **A** | **C** | **A** | **A** | **A** | **C** | **A** | **A** | **A** | **A** | **A** | **T** | **T** | **A** | **A** | **A** | **A** | **T** | **T** | **G** | **A** | **A** | **T** | **T** | **G** | **A** | **T** | **T** | **T** | **G** | **G** | **G** | **A** | **T** | **G** | **T** | **T** | **G** | **A** | **A** | **G** | **A** | **T** | **G** | **T** | **T** | **C** | **A** | **T** | **T** | **T** | **A** | **A** | **A** | **T** | **A** | **G** | **C** | **A** | **G** | **C** | **A** | **T** | **T** | **G** | **A** | **G** | **G** | **A** | **T** | **C** | **A** | **T** | **C** | **T** | **T** | **T** | **T** | **C** | **C** | **G** | **A** | **A** | **T** | **T** | **G** | **T** | **G** | **C** | **A** | **G** | **G** | **A** | **G** | **T** | **T** | **A** | **C** | **T** | **T** | **T** | **C** | **A** | **A** | **A** | **T** | **A** | **C** | **C** | **T** | **T** | **A** | **A** | **G** | **A** | **C** | **A** | **T** | **G** | **C** | **C** | **A** | **A** | **A** | **G** | **C** | **A** | **A** | **A** | **G** | **G** | **A** | **A** | **T** | **T** | **A** | **G** | **A** | **G** | **G** | **T** | **A** | **T** | **A** | **C** | **T** | **T** | **A** | **C** | **A** | **C** | **C** | **A** | **A** | **G** | **T** | **C** | **G** | **A** | **T** | **A** | **A** | **A** | **A** | **A** | **C** | **G** | **T** | **G** | **T** | **T** | **A** | **T** | **T** | **G** | **C** | **G** | **T** | **A** | **T** | **T** | **G** | **T** | **T** | **G** | **A** | **T** | **G** | **A** | **T** | **G** | **G** | **T** | **G** | **T** | **C** | **G** | **G** | **C** | **T** | **T** | **T** | **G** | **A** | **T** | **A** | **T** | **G** | **A** | **A** | **G** | **G** | **A** | **A** | **C** | **A** | **A** | **A** | **G** | **T** | **A** | **A** | **T** | **A** | **A** | **A** | **G** | **C** | **T** | **G** | **G** | **T** | **A** | **G** | **T** | **T** | **A** | **T** | **G** | **G** | **C** | **T** | **T** | **A** | **A** | **A** | **T** | **A** | **A** | **T** | **A** | **T** | **T** | **C** | **G** | **A** | **G** | **A** | **A** | **C** | **G** | **T** | **G** | **T** | **T** | **G** | **T** | **C** | **G** | **G** | **C** | **A** | **T** | **G** | **G** | **G** | **C** | **G** | **G** | **T** | **A** | **C** | **A** | **G** | **T** | **T** | **A** | **A** | **A** | **A** | **T** | **T** | **A** | **T** | **T** | **A** | **G** | **T** | **T** | **T** | **T** | **A** | **A** | **A** | **G** | **G** | **G** | **C** | **A** | **G** | **G** | **G** | **A** | **A** | **C** | **C** | **A** | **G** | **C** | **G** | **T** | **T** | **G** | **A** | **A** | **A** | **T** | **T** | **A** | **A** | **A** | **G** | **T** | **T** | **C** | **C** | **T** | **G** | **T** | **C** | **A** | **T** | **A** | **A** | **A** | **G** | **G** | **A** | **G** | **G** | **A** | **A** | **A** | **C** | **T** | **G** | **C** | **A** | **A** | **G** | **T** | **G** | **A** | **T** | **C** | **A** | **A** | **A** | **G** | **T** | **A** | **A** | **T** | **G** | **C** | **T** | **A** | **G** | **T** | **G** | **G** | **A** | **-** | **-** | **-** |
| **N55240** | **A** | **T** | **G** | **A** | **C** | **C** | **G** | **A** | **T** | **C** | **G** | **G** | **A** | **T** | **T** | **T** | **C** | **A** | **A** | **G** | **A** | **C** | **G** | **C** | **A** | **T** | **G** | **A** | **T** | **T** | **T** | **C** | **A** | **T** | **T** | **A** | **T** | **A** | **T** | **G** | **C** | **G** | **T** | **C** | **C** | **C** | **T** | **T** | **A** | **G** | **C** | **A** | **C** | **C** | **T** | **T** | **T** | **A** | **T** | **T** | **G** | **T** | **T** | **A** | **T** | **C** | **T** | **T** | **A** | **A** | **T** | **T** | **A** | **C** | **A** | **T** | **T** | **G** | **T** | **T** | **T** | **T** | **C** | **A** | **T** | **A** | **T** | **T** | **T** | **T** | **C** | **A** | **T** | **T** | **C** | **G** | **A** | **T** | **T** | **A** | **A** | **A** | **C** | **A** | **A** | **A** | **A** | **C** | **C** | **G** | **G** | **T** | **G** | **G** | **T** | **T** | **A** | **T** | **T** | **A** | **G** | **A** | **G** | **C** | **T** | **T** | **C** | **T** | **T** | **C** | **A** | **G** | **A** | **G** | **A** | **A** | **A** | **A** | **G** | **T** | **C** | **T** | **T** | **T** | **T** | **A** | **T** | **T** | **T** | **A** | **C** | **C** | **A** | **C** | **T** | **A** | **A** | **T** | **T** | **G** | **T** | **G** | **C** | **A** | **C** | **A** | **T** | **T** | **G** | **T** | **T** | **C** | **T** | **C** | **A** | **T** | **A** | **T** | **C** | **C** | **T** | **T** | **A** | **C** | **T** | **A** | **A** | **T** | **A** | **G** | **G** | **C** | **T** | **T** | **A** | **T** | **T** | **G** | **A** | **C** | **C** | **T** | **T** | **T** | **T** | **T** | **A** | **C** | **T** | **G** | **A** | **T** | **T** | **T** | **C** | **A** | **T** | **T** | **G** | **G** | **T** | **T** | **C** | **A** | **A** | **A** | **A** | **A** | **G** | **G** | **G** | **C** | **A** | **A** | **T** | **A** | **T** | **G** | **G** | **A** | **C** | **G** | **G** | **A** | **T** | **T** | **G** | **A** | **A** | **G** | **A** | **A** | **A** | **A** | **A** | **C** | **T** | **T** | **C** | **G** | **G** | **T** | **T** | **A** | **T** | **T** | **G** | **G** | **C** | **C** | **A** | **A** | **C** | **G** | **G** | **T** | **A** | **A** | **T** | **T** | **A** | **T** | **G** | **A** | **A** | **A** | **G** | **T** | **C** | **C** | **A** | **G** | **T** | **C** | **T** | **T** | **A** | **A** | **A** | **C** | **A** | **A** | **A** | **C** | **C** | **A** | **A** | **C** | **G** | **A** | **C** | **C** | **A** | **G** | **T** | **G** | **A** | **A** | **A** | **A** | **T** | **C** | **A** | **A** | **G** | **A** | **C** | **C** | **A** | **T** | **T** | **A** | **T** | **C** | **T** | **A** | **A** | **C** | **C** | **G** | **A** | **A** | **G** | **T** | **C** | **G** | **A** | **A** | **C** | **A** | **A** | **G** | **A** | **T** | **A** | **T** | **T** | **T** | **G** | **G** | **T** | **C** | **G** | **A** | **T** | **T** | **A** | **A** | **A** | **A** | **A** | **T** | **A** | **A** | **A** | **T** | **T** | **A** | **T** | **T** | **A** | **G** | **A** | **G** | **A** | **T** | **G** | **T** | **C** | **T** | **A** | **A** | **A** | **G** | **A** | **A** | **T** | **T** | **G** | **C** | **A** | **A** | **T** | **T** | **A** | **T** | **T** | **A** | **A** | **A** | **C** | **A** | **G** | **T** | **C** | **G** | **A** | **C** | **C** | **G** | **C** | **A** | **A** | **T** | **T** | **A** | **A** | **T** | **G** | **G** | **A** | **T** | **G** | **G** | **G** | **C** | **A** | **A** | **A** | **C** | **A** | **A** | **A** | **A** | **G** | **A** | **A** | **G** | **A** | **A** | **A** | **T** | **T** | **T** | **T** | **A** | **G** | **A** | **G** | **A** | **A** | **C** | **G** | **A** | **G** | **C** | **G** | **G** | **C** | **A** | **T** | **C** | **G** | **T** | **T** | **T** | **G** | **G** | **C** | **G** | **C** | **G** | **G** | **G** | **A** | **G** | **T** | **T** | **G** | **C** | **A** | **T** | **G** | **A** | **T** | **T** | **C** | **A** | **G** | **T** | **C** | **A** | **G** | **T** | **C** | **A** | **A** | **C** | **A** | **A** | **C** | **T** | **T** | **T** | **T** | **T** | **G** | **C** | **A** | **G** | **C** | **C** | **A** | **T** | **G** | **A** | **T** | **G** | **A** | **T** | **G** | **T** | **T** | **G** | **T** | **C** | **T** | **G** | **C** | **C** | **T** | **T** | **A** | **A** | **A** | **T** | **G** | **A** | **A** | **C** | **A** | **A** | **G** | **C** | **A** | **C** | **A** | **A** | **C** | **G** | **A** | **A** | **C** | **A** | **G** | **A** | **A** | **A** | **C** | **T** | **C** | **C** | **T** | **G** | **A** | **A** | **C** | **C** | **A** | **T** | **A** | **T** | **C** | **G** | **C** | **A** | **A** | **A** | **C** | **A** | **A** | **C** | **T** | **A** | **G** | **C** | **C** | **A** | **T** | **G** | **G** | **T** | **G** | **G** | **C** | **A** | **G** | **A** | **A** | **A** | **T** | **C** | **A** | **T** | **T** | **A** | **A** | **T** | **G** | **C** | **C** | **T** | **C** | **C** | **C** | **A** | **A** | **T** | **C** | **G** | **G** | **A** | **A** | **A** | **T** | **G** | **C** | **G** | **T** | **G** | **C** | **G** | **C** | **T** | **A** | **C** | **T** | **A** | **T** | **T** | **G** | **C** | **A** | **C** | **T** | **T** | **G** | **C** | **G** | **T** | **C** | **C** | **T** | **A** | **T** | **C** | **A** | **G** | **T** | **C** | **T** | **A** | **G** | **A** | **A** | **G** | **G** | **A** | **A** | **A** | **A** | **A** | **G** | **T** | **T** | **T** | **G** | **C** | **G** | **T** | **A** | **A** | **A** | **G** | **G** | **T** | **A** | **T** | **T** | **G** | **A** | **A** | **C** | **A** | **A** | **T** | **T** | **A** | **C** | **T** | **G** | **A** | **A** | **A** | **G** | **A** | **A** | **C** | **T** | **A** | **C** | **A** | **A** | **A** | **C** | **A** | **A** | **A** | **A** | **A** | **T** | **T** | **A** | **A** | **A** | **A** | **T** | **T** | **G** | **A** | **A** | **T** | **T** | **G** | **A** | **T** | **T** | **T** | **G** | **G** | **G** | **A** | **T** | **G** | **T** | **T** | **G** | **A** | **A** | **G** | **A** | **T** | **G** | **T** | **T** | **C** | **A** | **T** | **T** | **T** | **A** | **A** | **A** | **T** | **A** | **G** | **C** | **A** | **G** | **C** | **A** | **T** | **T** | **G** | **A** | **G** | **G** | **A** | **T** | **C** | **A** | **T** | **C** | **T** | **T** | **T** | **T** | **C** | **C** | **G** | **A** | **A** | **T** | **T** | **G** | **T** | **G** | **C** | **A** | **G** | **G** | **A** | **G** | **T** | **T** | **A** | **C** | **T** | **T** | **T** | **C** | **A** | **A** | **A** | **T** | **A** | **C** | **C** | **T** | **T** | **A** | **A** | **G** | **A** | **C** | **A** | **T** | **G** | **C** | **C** | **A** | **A** | **A** | **G** | **C** | **A** | **A** | **A** | **G** | **G** | **A** | **A** | **T** | **T** | **A** | **G** | **A** | **G** | **G** | **T** | **A** | **T** | **A** | **C** | **T** | **T** | **A** | **C** | **A** | **C** | **C** | **A** | **A** | **G** | **T** | **C** | **G** | **A** | **T** | **A** | **A** | **A** | **A** | **A** | **C** | **G** | **T** | **G** | **T** | **T** | **A** | **T** | **T** | **G** | **C** | **G** | **T** | **A** | **T** | **T** | **G** | **T** | **T** | **G** | **A** | **T** | **G** | **A** | **T** | **G** | **G** | **T** | **G** | **T** | **C** | **G** | **G** | **C** | **T** | **T** | **T** | **G** | **A** | **T** | **A** | **T** | **G** | **A** | **A** | **G** | **G** | **A** | **A** | **C** | **A** | **A** | **A** | **G** | **T** | **A** | **A** | **T** | **A** | **A** | **A** | **G** | **C** | **C** | **G** | **G** | **T** | **A** | **G** | **T** | **T** | **A** | **T** | **G** | **G** | **C** | **T** | **T** | **A** | **A** | **A** | **T** | **A** | **A** | **T** | **A** | **T** | **C** | **C** | **G** | **A** | **G** | **A** | **A** | **C** | **G** | **T** | **G** | **T** | **T** | **G** | **T** | **C** | **G** | **G** | **C** | **A** | **T** | **G** | **G** | **G** | **C** | **G** | **G** | **T** | **A** | **C** | **A** | **G** | **T** | **T** | **A** | **A** | **A** | **A** | **T** | **T** | **A** | **T** | **T** | **A** | **G** | **T** | **T** | **T** | **T** | **A** | **A** | **A** | **G** | **G** | **G** | **C** | **A** | **G** | **G** | **G** | **A** | **A** | **C** | **C** | **A** | **G** | **C** | **G** | **T** | **T** | **G** | **A** | **A** | **A** | **T** | **T** | **A** | **A** | **A** | **G** | **T** | **T** | **C** | **C** | **T** | **G** | **T** | **C** | **A** | **T** | **A** | **A** | **A** | **G** | **G** | **A** | **G** | **G** | **A** | **A** | **A** | **C** | **T** | **G** | **C** | **A** | **A** | **G** | **T** | **G** | **A** | **T** | **C** | **A** | **A** | **A** | **G** | **T** | **A** | **A** | **T** | **G** | **T** | **T** | **A** | **G** | **T** | **G** | **G** | **A** | **-** | **-** | **-** |
| **N55338** | **A** | **T** | **G** | **A** | **C** | **C** | **G** | **A** | **T** | **C** | **G** | **G** | **A** | **T** | **T** | **T** | **C** | **A** | **A** | **G** | **A** | **C** | **G** | **C** | **A** | **T** | **G** | **A** | **T** | **T** | **T** | **C** | **A** | **T** | **T** | **A** | **T** | **A** | **T** | **G** | **C** | **G** | **T** | **C** | **C** | **C** | **T** | **T** | **A** | **G** | **C** | **A** | **C** | **C** | **T** | **T** | **T** | **A** | **T** | **T** | **G** | **T** | **T** | **A** | **T** | **C** | **T** | **T** | **A** | **A** | **T** | **T** | **A** | **C** | **A** | **T** | **T** | **G** | **T** | **T** | **T** | **T** | **C** | **A** | **T** | **A** | **T** | **T** | **T** | **T** | **C** | **A** | **T** | **T** | **C** | **G** | **A** | **T** | **T** | **A** | **A** | **A** | **C** | **A** | **A** | **A** | **A** | **C** | **C** | **G** | **G** | **T** | **G** | **G** | **T** | **T** | **A** | **T** | **T** | **A** | **G** | **A** | **G** | **C** | **T** | **T** | **C** | **T** | **T** | **C** | **A** | **G** | **A** | **G** | **A** | **A** | **A** | **A** | **G** | **T** | **C** | **T** | **T** | **T** | **T** | **A** | **T** | **C** | **T** | **A** | **C** | **C** | **A** | **C** | **T** | **A** | **A** | **T** | **T** | **G** | **T** | **G** | **C** | **A** | **C** | **A** | **T** | **T** | **G** | **T** | **T** | **C** | **T** | **C** | **A** | **T** | **A** | **T** | **C** | **C** | **T** | **T** | **A** | **C** | **T** | **A** | **A** | **T** | **A** | **G** | **G** | **C** | **T** | **T** | **A** | **T** | **T** | **G** | **A** | **C** | **C** | **T** | **T** | **T** | **T** | **T** | **A** | **C** | **T** | **G** | **A** | **T** | **T** | **T** | **C** | **A** | **T** | **T** | **G** | **G** | **T** | **T** | **C** | **A** | **A** | **A** | **A** | **A** | **G** | **G** | **G** | **C** | **A** | **A** | **T** | **A** | **T** | **G** | **G** | **A** | **C** | **G** | **G** | **A** | **T** | **T** | **G** | **A** | **A** | **G** | **A** | **A** | **A** | **A** | **A** | **C** | **T** | **T** | **C** | **G** | **G** | **T** | **T** | **A** | **T** | **T** | **G** | **G** | **C** | **C** | **A** | **A** | **C** | **G** | **G** | **T** | **A** | **A** | **T** | **T** | **A** | **T** | **G** | **A** | **A** | **A** | **G** | **T** | **C** | **C** | **A** | **G** | **T** | **C** | **T** | **T** | **A** | **A** | **A** | **C** | **A** | **A** | **A** | **C** | **C** | **A** | **A** | **C** | **G** | **A** | **C** | **C** | **A** | **G** | **T** | **G** | **A** | **A** | **A** | **A** | **T** | **C** | **A** | **A** | **G** | **A** | **T** | **C** | **A** | **T** | **T** | **A** | **T** | **C** | **T** | **A** | **A** | **C** | **C** | **G** | **A** | **A** | **G** | **T** | **C** | **G** | **A** | **A** | **C** | **A** | **A** | **G** | **A** | **T** | **A** | **T** | **T** | **T** | **G** | **G** | **T** | **C** | **G** | **A** | **T** | **T** | **A** | **A** | **A** | **A** | **A** | **T** | **A** | **A** | **A** | **T** | **T** | **A** | **T** | **T** | **A** | **G** | **A** | **G** | **A** | **T** | **G** | **T** | **C** | **T** | **A** | **A** | **A** | **G** | **A** | **A** | **T** | **T** | **G** | **C** | **A** | **A** | **T** | **T** | **A** | **T** | **T** | **A** | **A** | **A** | **C** | **A** | **G** | **T** | **C** | **G** | **A** | **C** | **C** | **G** | **C** | **A** | **A** | **T** | **T** | **A** | **A** | **T** | **G** | **G** | **A** | **T** | **G** | **G** | **G** | **C** | **A** | **A** | **A** | **C** | **A** | **A** | **A** | **A** | **G** | **A** | **A** | **G** | **A** | **A** | **A** | **T** | **T** | **T** | **T** | **A** | **G** | **A** | **G** | **A** | **A** | **C** | **G** | **A** | **G** | **C** | **G** | **G** | **C** | **A** | **T** | **C** | **G** | **T** | **T** | **T** | **G** | **G** | **C** | **G** | **C** | **G** | **G** | **G** | **A** | **G** | **T** | **T** | **G** | **C** | **A** | **T** | **G** | **A** | **T** | **T** | **C** | **A** | **G** | **T** | **C** | **A** | **G** | **T** | **C** | **A** | **A** | **C** | **A** | **A** | **C** | **T** | **T** | **T** | **T** | **T** | **G** | **C** | **A** | **G** | **C** | **C** | **A** | **T** | **G** | **A** | **T** | **G** | **A** | **T** | **G** | **T** | **T** | **G** | **T** | **C** | **T** | **G** | **C** | **C** | **T** | **T** | **A** | **A** | **A** | **T** | **G** | **A** | **A** | **C** | **A** | **A** | **G** | **C** | **A** | **C** | **A** | **A** | **C** | **G** | **A** | **A** | **C** | **A** | **G** | **A** | **A** | **A** | **C** | **C** | **C** | **C** | **G** | **G** | **A** | **A** | **C** | **C** | **A** | **T** | **A** | **T** | **C** | **G** | **T** | **A** | **A** | **A** | **C** | **A** | **A** | **C** | **T** | **A** | **G** | **C** | **C** | **A** | **T** | **G** | **G** | **T** | **G** | **G** | **C** | **A** | **G** | **A** | **A** | **A** | **T** | **C** | **A** | **T** | **T** | **A** | **A** | **T** | **G** | **C** | **C** | **T** | **C** | **T** | **C** | **A** | **A** | **T** | **C** | **G** | **G** | **A** | **A** | **A** | **T** | **G** | **C** | **G** | **C** | **G** | **C** | **G** | **T** | **T** | **A** | **C** | **T** | **A** | **T** | **T** | **G** | **C** | **A** | **C** | **T** | **T** | **G** | **C** | **G** | **T** | **C** | **C** | **T** | **A** | **T** | **C** | **A** | **G** | **T** | **C** | **T** | **A** | **G** | **A** | **A** | **G** | **G** | **A** | **A** | **A** | **A** | **A** | **G** | **T** | **T** | **T** | **G** | **C** | **G** | **T** | **A** | **A** | **A** | **G** | **G** | **T** | **A** | **T** | **T** | **G** | **A** | **A** | **C** | **A** | **A** | **T** | **T** | **A** | **C** | **T** | **G** | **A** | **A** | **A** | **G** | **A** | **A** | **C** | **T** | **A** | **C** | **A** | **A** | **A** | **C** | **A** | **A** | **A** | **A** | **A** | **T** | **T** | **A** | **A** | **A** | **A** | **T** | **T** | **G** | **A** | **A** | **T** | **T** | **G** | **A** | **T** | **T** | **T** | **G** | **G** | **G** | **A** | **T** | **G** | **T** | **T** | **G** | **A** | **A** | **G** | **A** | **T** | **G** | **T** | **T** | **C** | **A** | **T** | **T** | **T** | **A** | **A** | **A** | **T** | **A** | **G** | **C** | **A** | **G** | **C** | **A** | **T** | **T** | **G** | **A** | **G** | **G** | **A** | **T** | **C** | **A** | **T** | **C** | **T** | **T** | **T** | **T** | **C** | **C** | **G** | **A** | **A** | **T** | **T** | **G** | **T** | **G** | **C** | **A** | **G** | **G** | **A** | **A** | **T** | **T** | **A** | **C** | **T** | **T** | **T** | **C** | **A** | **A** | **A** | **T** | **A** | **C** | **C** | **T** | **T** | **A** | **A** | **G** | **A** | **C** | **A** | **T** | **G** | **C** | **C** | **A** | **A** | **A** | **G** | **C** | **A** | **A** | **A** | **G** | **G** | **A** | **A** | **T** | **T** | **A** | **G** | **A** | **G** | **G** | **T** | **A** | **T** | **A** | **C** | **T** | **T** | **A** | **C** | **A** | **C** | **C** | **A** | **A** | **G** | **T** | **C** | **G** | **A** | **T** | **A** | **A** | **A** | **A** | **A** | **C** | **G** | **T** | **G** | **C** | **T** | **A** | **T** | **T** | **G** | **C** | **G** | **T** | **A** | **T** | **T** | **G** | **T** | **T** | **G** | **A** | **T** | **G** | **A** | **C** | **G** | **G** | **T** | **G** | **T** | **C** | **G** | **G** | **C** | **T** | **T** | **T** | **G** | **A** | **T** | **A** | **T** | **G** | **A** | **A** | **G** | **G** | **A** | **A** | **C** | **A** | **A** | **A** | **G** | **T** | **A** | **A** | **T** | **A** | **A** | **A** | **G** | **C** | **C** | **G** | **G** | **T** | **A** | **G** | **T** | **T** | **A** | **T** | **G** | **G** | **C** | **T** | **T** | **A** | **A** | **A** | **T** | **A** | **A** | **T** | **A** | **T** | **C** | **C** | **G** | **A** | **G** | **A** | **A** | **C** | **G** | **T** | **G** | **T** | **T** | **G** | **T** | **C** | **G** | **G** | **C** | **A** | **T** | **G** | **G** | **G** | **C** | **G** | **G** | **T** | **A** | **C** | **A** | **G** | **T** | **T** | **A** | **A** | **A** | **A** | **T** | **T** | **A** | **T** | **T** | **A** | **G** | **T** | **T** | **T** | **T** | **A** | **A** | **A** | **G** | **G** | **G** | **C** | **A** | **G** | **G** | **G** | **A** | **A** | **C** | **C** | **A** | **G** | **C** | **G** | **T** | **T** | **G** | **A** | **A** | **A** | **T** | **T** | **A** | **A** | **A** | **G** | **T** | **T** | **C** | **C** | **T** | **G** | **T** | **C** | **A** | **T** | **A** | **A** | **A** | **G** | **G** | **A** | **G** | **G** | **A** | **A** | **A** | **C** | **T** | **G** | **C** | **A** | **A** | **G** | **T** | **G** | **A** | **T** | **C** | **A** | **A** | **A** | **G** | **T** | **A** | **A** | **T** | **G** | **T** | **T** | **A** | **G** | **T** | **G** | **G** | **A** | **-** | **-** | **-** |
| **N59454F** | **A** | **T** | **G** | **A** | **C** | **C** | **G** | **A** | **T** | **C** | **G** | **G** | **A** | **T** | **T** | **T** | **C** | **A** | **A** | **G** | **A** | **C** | **G** | **C** | **A** | **T** | **G** | **A** | **T** | **T** | **T** | **C** | **A** | **T** | **T** | **A** | **T** | **A** | **T** | **G** | **C** | **G** | **T** | **C** | **C** | **C** | **T** | **T** | **A** | **G** | **C** | **A** | **C** | **C** | **T** | **T** | **T** | **A** | **T** | **T** | **G** | **T** | **T** | **A** | **T** | **C** | **T** | **T** | **A** | **A** | **T** | **T** | **A** | **C** | **A** | **T** | **T** | **G** | **T** | **T** | **T** | **T** | **C** | **A** | **T** | **A** | **T** | **T** | **T** | **T** | **C** | **A** | **T** | **T** | **C** | **G** | **A** | **T** | **T** | **A** | **A** | **A** | **C** | **A** | **A** | **A** | **A** | **C** | **C** | **G** | **G** | **T** | **G** | **G** | **C** | **T** | **A** | **T** | **T** | **A** | **G** | **A** | **G** | **C** | **T** | **T** | **C** | **T** | **T** | **C** | **A** | **G** | **A** | **G** | **A** | **A** | **A** | **A** | **G** | **T** | **C** | **T** | **T** | **T** | **T** | **A** | **T** | **T** | **T** | **A** | **C** | **C** | **A** | **C** | **T** | **A** | **A** | **T** | **T** | **G** | **T** | **G** | **C** | **A** | **C** | **A** | **T** | **T** | **G** | **T** | **T** | **C** | **T** | **C** | **A** | **T** | **A** | **T** | **C** | **C** | **T** | **T** | **A** | **C** | **T** | **A** | **A** | **T** | **A** | **G** | **G** | **C** | **T** | **T** | **A** | **T** | **T** | **G** | **A** | **C** | **C** | **T** | **T** | **T** | **T** | **T** | **A** | **C** | **T** | **G** | **A** | **T** | **T** | **T** | **C** | **A** | **T** | **T** | **G** | **G** | **T** | **T** | **C** | **A** | **A** | **A** | **A** | **A** | **G** | **G** | **G** | **C** | **A** | **A** | **T** | **A** | **T** | **G** | **G** | **A** | **C** | **G** | **G** | **A** | **T** | **T** | **G** | **A** | **A** | **G** | **A** | **A** | **A** | **A** | **A** | **C** | **T** | **T** | **C** | **G** | **G** | **T** | **T** | **A** | **T** | **T** | **G** | **G** | **C** | **C** | **A** | **A** | **C** | **G** | **G** | **T** | **A** | **A** | **T** | **T** | **A** | **T** | **G** | **A** | **A** | **A** | **G** | **T** | **C** | **C** | **A** | **G** | **T** | **C** | **T** | **T** | **A** | **A** | **A** | **C** | **A** | **A** | **A** | **C** | **C** | **A** | **A** | **C** | **G** | **A** | **C** | **C** | **A** | **G** | **T** | **G** | **A** | **A** | **A** | **A** | **T** | **C** | **A** | **A** | **G** | **A** | **C** | **C** | **A** | **T** | **T** | **A** | **T** | **C** | **T** | **A** | **A** | **C** | **C** | **G** | **A** | **A** | **G** | **T** | **C** | **G** | **A** | **A** | **C** | **A** | **A** | **G** | **A** | **T** | **A** | **T** | **T** | **T** | **G** | **G** | **T** | **C** | **G** | **A** | **T** | **T** | **A** | **A** | **A** | **A** | **A** | **T** | **A** | **A** | **A** | **T** | **T** | **A** | **T** | **T** | **A** | **G** | **A** | **G** | **A** | **T** | **G** | **T** | **C** | **T** | **A** | **A** | **A** | **G** | **A** | **A** | **T** | **T** | **G** | **C** | **A** | **A** | **T** | **T** | **A** | **T** | **T** | **A** | **A** | **A** | **C** | **A** | **G** | **T** | **C** | **G** | **A** | **C** | **C** | **G** | **C** | **A** | **A** | **T** | **T** | **A** | **A** | **T** | **G** | **G** | **A** | **T** | **G** | **G** | **G** | **C** | **A** | **A** | **A** | **C** | **A** | **A** | **A** | **A** | **G** | **A** | **A** | **G** | **A** | **A** | **A** | **T** | **T** | **T** | **T** | **A** | **G** | **A** | **G** | **A** | **A** | **C** | **G** | **A** | **G** | **C** | **G** | **G** | **C** | **A** | **T** | **C** | **G** | **T** | **T** | **T** | **G** | **G** | **C** | **G** | **C** | **G** | **G** | **G** | **A** | **G** | **T** | **T** | **G** | **C** | **A** | **T** | **G** | **A** | **T** | **T** | **C** | **A** | **G** | **T** | **C** | **A** | **G** | **T** | **C** | **A** | **A** | **C** | **A** | **A** | **C** | **T** | **T** | **T** | **T** | **T** | **G** | **C** | **A** | **G** | **C** | **C** | **A** | **T** | **G** | **A** | **T** | **G** | **A** | **T** | **G** | **T** | **T** | **G** | **T** | **C** | **T** | **G** | **C** | **C** | **T** | **T** | **A** | **A** | **A** | **T** | **G** | **A** | **A** | **C** | **A** | **A** | **G** | **C** | **A** | **C** | **A** | **A** | **C** | **G** | **A** | **A** | **C** | **A** | **G** | **A** | **A** | **A** | **C** | **T** | **C** | **C** | **T** | **G** | **A** | **A** | **C** | **C** | **A** | **T** | **A** | **T** | **C** | **G** | **C** | **A** | **A** | **A** | **C** | **A** | **A** | **C** | **T** | **A** | **G** | **C** | **C** | **A** | **T** | **G** | **G** | **T** | **G** | **G** | **C** | **A** | **G** | **A** | **A** | **A** | **T** | **C** | **A** | **T** | **T** | **A** | **A** | **T** | **G** | **C** | **C** | **T** | **C** | **C** | **C** | **A** | **A** | **T** | **C** | **G** | **G** | **A** | **A** | **A** | **T** | **G** | **C** | **G** | **C** | **G** | **C** | **G** | **T** | **T** | **A** | **C** | **T** | **A** | **T** | **T** | **G** | **C** | **A** | **C** | **T** | **T** | **G** | **C** | **G** | **T** | **C** | **C** | **T** | **A** | **T** | **C** | **A** | **G** | **T** | **C** | **T** | **A** | **G** | **A** | **A** | **G** | **G** | **A** | **A** | **A** | **A** | **A** | **G** | **T** | **T** | **T** | **G** | **C** | **G** | **T** | **A** | **A** | **A** | **G** | **G** | **T** | **A** | **T** | **T** | **G** | **A** | **A** | **C** | **A** | **A** | **T** | **T** | **A** | **C** | **T** | **G** | **A** | **A** | **A** | **G** | **A** | **A** | **C** | **T** | **A** | **C** | **A** | **A** | **A** | **C** | **A** | **A** | **A** | **A** | **A** | **T** | **T** | **A** | **A** | **A** | **A** | **T** | **T** | **G** | **A** | **A** | **T** | **T** | **G** | **A** | **T** | **T** | **T** | **G** | **G** | **G** | **A** | **T** | **G** | **T** | **T** | **G** | **A** | **A** | **G** | **A** | **T** | **G** | **T** | **T** | **C** | **A** | **T** | **T** | **T** | **A** | **A** | **A** | **T** | **A** | **G** | **C** | **A** | **G** | **C** | **A** | **T** | **T** | **G** | **A** | **G** | **G** | **A** | **T** | **C** | **A** | **T** | **C** | **T** | **T** | **T** | **T** | **C** | **C** | **G** | **A** | **A** | **T** | **T** | **G** | **T** | **G** | **C** | **A** | **G** | **G** | **A** | **A** | **T** | **T** | **A** | **C** | **T** | **T** | **T** | **C** | **A** | **A** | **A** | **T** | **A** | **C** | **C** | **T** | **T** | **A** | **A** | **G** | **A** | **C** | **A** | **T** | **G** | **C** | **C** | **A** | **A** | **A** | **G** | **C** | **A** | **A** | **A** | **G** | **G** | **A** | **A** | **T** | **T** | **A** | **G** | **A** | **G** | **G** | **T** | **A** | **T** | **A** | **C** | **T** | **T** | **A** | **C** | **A** | **C** | **C** | **A** | **A** | **G** | **T** | **C** | **G** | **A** | **T** | **A** | **A** | **A** | **A** | **A** | **C** | **G** | **T** | **G** | **T** | **T** | **A** | **T** | **T** | **G** | **C** | **G** | **T** | **A** | **T** | **T** | **G** | **T** | **T** | **G** | **A** | **T** | **G** | **A** | **T** | **G** | **G** | **T** | **G** | **T** | **C** | **G** | **G** | **C** | **T** | **T** | **T** | **G** | **A** | **T** | **A** | **T** | **G** | **A** | **A** | **G** | **G** | **A** | **A** | **C** | **A** | **A** | **A** | **G** | **T** | **A** | **A** | **T** | **A** | **A** | **A** | **G** | **C** | **C** | **G** | **G** | **T** | **A** | **G** | **T** | **T** | **A** | **T** | **G** | **G** | **C** | **T** | **T** | **A** | **A** | **A** | **T** | **A** | **A** | **T** | **A** | **T** | **C** | **C** | **G** | **A** | **G** | **A** | **A** | **C** | **G** | **T** | **G** | **T** | **T** | **G** | **T** | **C** | **G** | **G** | **C** | **A** | **T** | **G** | **G** | **G** | **C** | **G** | **G** | **T** | **A** | **C** | **A** | **G** | **T** | **T** | **A** | **A** | **A** | **A** | **T** | **T** | **A** | **T** | **T** | **A** | **G** | **T** | **T** | **T** | **T** | **A** | **A** | **A** | **G** | **G** | **G** | **C** | **A** | **G** | **G** | **G** | **A** | **A** | **C** | **C** | **A** | **G** | **C** | **G** | **T** | **T** | **G** | **A** | **A** | **A** | **T** | **T** | **A** | **A** | **A** | **G** | **T** | **T** | **C** | **C** | **T** | **G** | **T** | **C** | **A** | **T** | **A** | **A** | **A** | **G** | **G** | **A** | **G** | **G** | **A** | **A** | **A** | **C** | **T** | **G** | **C** | **A** | **A** | **G** | **T** | **G** | **A** | **T** | **C** | **A** | **A** | **A** | **G** | **T** | **A** | **A** | **T** | **G** | **T** | **T** | **A** | **G** | **T** | **G** | **G** | **A** | **-** | **-** | **-** |
| **N52773** | **A** | **T** | **G** | **A** | **C** | **C** | **G** | **A** | **T** | **C** | **G** | **G** | **A** | **T** | **T** | **T** | **C** | **A** | **A** | **G** | **A** | **C** | **G** | **C** | **A** | **T** | **G** | **A** | **T** | **T** | **T** | **C** | **A** | **T** | **T** | **A** | **T** | **A** | **T** | **G** | **C** | **G** | **T** | **C** | **C** | **C** | **T** | **T** | **A** | **G** | **C** | **A** | **C** | **C** | **T** | **T** | **T** | **A** | **T** | **T** | **G** | **T** | **T** | **A** | **T** | **C** | **T** | **T** | **A** | **A** | **T** | **T** | **A** | **C** | **A** | **T** | **T** | **G** | **T** | **T** | **T** | **T** | **C** | **A** | **T** | **A** | **T** | **T** | **T** | **T** | **C** | **A** | **T** | **T** | **C** | **G** | **A** | **T** | **T** | **A** | **A** | **A** | **C** | **A** | **A** | **A** | **A** | **C** | **C** | **G** | **G** | **T** | **G** | **G** | **T** | **T** | **A** | **T** | **T** | **A** | **G** | **A** | **G** | **C** | **T** | **T** | **C** | **T** | **T** | **C** | **A** | **G** | **A** | **G** | **A** | **A** | **A** | **A** | **G** | **T** | **C** | **T** | **T** | **T** | **T** | **A** | **T** | **C** | **T** | **A** | **C** | **C** | **A** | **C** | **T** | **A** | **A** | **T** | **T** | **G** | **T** | **G** | **C** | **A** | **C** | **A** | **T** | **T** | **G** | **T** | **T** | **C** | **T** | **C** | **A** | **T** | **A** | **T** | **C** | **C** | **T** | **T** | **A** | **C** | **T** | **A** | **A** | **T** | **A** | **G** | **G** | **C** | **T** | **T** | **A** | **T** | **T** | **G** | **A** | **C** | **C** | **T** | **T** | **T** | **T** | **T** | **A** | **C** | **T** | **G** | **A** | **T** | **T** | **T** | **C** | **A** | **T** | **T** | **G** | **G** | **T** | **T** | **C** | **A** | **A** | **A** | **A** | **A** | **G** | **G** | **G** | **C** | **A** | **A** | **T** | **A** | **T** | **G** | **G** | **A** | **C** | **G** | **G** | **A** | **T** | **T** | **G** | **A** | **A** | **G** | **A** | **A** | **A** | **A** | **A** | **C** | **T** | **T** | **C** | **G** | **G** | **T** | **T** | **A** | **T** | **T** | **G** | **G** | **C** | **C** | **A** | **A** | **C** | **G** | **G** | **T** | **A** | **A** | **T** | **T** | **A** | **T** | **G** | **A** | **A** | **A** | **G** | **T** | **C** | **C** | **A** | **G** | **T** | **C** | **T** | **T** | **A** | **A** | **A** | **C** | **A** | **A** | **A** | **C** | **C** | **A** | **A** | **C** | **G** | **A** | **C** | **C** | **A** | **G** | **T** | **G** | **A** | **A** | **A** | **A** | **T** | **C** | **A** | **A** | **G** | **A** | **C** | **C** | **A** | **T** | **T** | **A** | **T** | **C** | **T** | **A** | **A** | **C** | **C** | **G** | **A** | **A** | **G** | **T** | **C** | **G** | **A** | **A** | **C** | **A** | **A** | **G** | **A** | **T** | **A** | **T** | **T** | **T** | **G** | **G** | **T** | **C** | **G** | **A** | **T** | **T** | **A** | **A** | **A** | **A** | **A** | **T** | **A** | **A** | **A** | **T** | **T** | **A** | **T** | **T** | **A** | **G** | **A** | **G** | **A** | **T** | **G** | **T** | **C** | **T** | **A** | **A** | **A** | **G** | **A** | **A** | **T** | **T** | **G** | **C** | **A** | **A** | **T** | **T** | **A** | **T** | **T** | **A** | **A** | **A** | **C** | **A** | **G** | **T** | **C** | **G** | **A** | **C** | **C** | **G** | **C** | **A** | **A** | **T** | **T** | **A** | **A** | **T** | **G** | **G** | **A** | **T** | **G** | **G** | **G** | **C** | **A** | **A** | **A** | **C** | **A** | **A** | **A** | **A** | **G** | **A** | **A** | **G** | **A** | **A** | **A** | **T** | **T** | **T** | **T** | **A** | **G** | **A** | **G** | **A** | **A** | **C** | **G** | **A** | **G** | **C** | **G** | **G** | **C** | **A** | **T** | **C** | **G** | **T** | **T** | **T** | **G** | **G** | **C** | **G** | **C** | **G** | **G** | **G** | **A** | **G** | **T** | **T** | **G** | **C** | **A** | **T** | **G** | **A** | **T** | **T** | **C** | **A** | **G** | **T** | **C** | **A** | **G** | **T** | **C** | **A** | **A** | **C** | **A** | **A** | **C** | **T** | **T** | **T** | **T** | **T** | **G** | **C** | **A** | **G** | **C** | **C** | **A** | **T** | **G** | **A** | **T** | **G** | **A** | **T** | **G** | **T** | **T** | **G** | **T** | **C** | **T** | **G** | **C** | **A** | **T** | **T** | **A** | **A** | **A** | **T** | **G** | **A** | **A** | **C** | **A** | **A** | **G** | **C** | **A** | **C** | **A** | **A** | **C** | **G** | **A** | **A** | **C** | **A** | **G** | **A** | **A** | **A** | **C** | **C** | **C** | **C** | **G** | **G** | **A** | **A** | **C** | **C** | **A** | **T** | **A** | **T** | **C** | **G** | **T** | **A** | **A** | **A** | **C** | **A** | **A** | **C** | **T** | **A** | **G** | **C** | **C** | **A** | **T** | **G** | **G** | **T** | **G** | **G** | **C** | **A** | **G** | **A** | **A** | **A** | **T** | **C** | **A** | **T** | **T** | **A** | **A** | **T** | **G** | **C** | **C** | **T** | **C** | **C** | **C** | **A** | **A** | **T** | **C** | **G** | **G** | **A** | **A** | **A** | **T** | **G** | **C** | **G** | **C** | **G** | **C** | **G** | **C** | **T** | **A** | **C** | **T** | **A** | **T** | **T** | **G** | **C** | **A** | **C** | **T** | **T** | **G** | **C** | **G** | **T** | **C** | **C** | **T** | **A** | **T** | **C** | **A** | **G** | **T** | **C** | **T** | **A** | **G** | **A** | **A** | **G** | **G** | **A** | **A** | **A** | **A** | **A** | **G** | **T** | **T** | **T** | **G** | **C** | **G** | **T** | **A** | **A** | **A** | **G** | **G** | **T** | **A** | **T** | **T** | **G** | **A** | **A** | **C** | **A** | **A** | **T** | **T** | **A** | **C** | **T** | **G** | **A** | **A** | **A** | **G** | **A** | **A** | **C** | **T** | **A** | **C** | **A** | **A** | **A** | **C** | **A** | **A** | **A** | **A** | **A** | **T** | **T** | **A** | **A** | **A** | **A** | **T** | **T** | **G** | **A** | **A** | **T** | **T** | **G** | **A** | **T** | **T** | **T** | **G** | **G** | **G** | **A** | **T** | **G** | **T** | **T** | **G** | **A** | **A** | **G** | **A** | **T** | **G** | **T** | **T** | **C** | **A** | **T** | **T** | **T** | **A** | **A** | **A** | **T** | **A** | **G** | **C** | **A** | **G** | **C** | **A** | **T** | **T** | **G** | **A** | **G** | **G** | **A** | **T** | **C** | **A** | **T** | **C** | **T** | **T** | **T** | **T** | **C** | **C** | **G** | **A** | **A** | **T** | **T** | **G** | **T** | **G** | **C** | **A** | **G** | **G** | **A** | **G** | **T** | **T** | **A** | **C** | **T** | **T** | **T** | **C** | **A** | **A** | **A** | **T** | **A** | **C** | **C** | **T** | **T** | **A** | **A** | **G** | **A** | **C** | **A** | **T** | **G** | **C** | **C** | **A** | **A** | **A** | **G** | **C** | **A** | **A** | **A** | **G** | **G** | **A** | **A** | **T** | **T** | **A** | **G** | **A** | **G** | **G** | **T** | **A** | **T** | **A** | **C** | **T** | **T** | **A** | **C** | **A** | **C** | **C** | **A** | **A** | **G** | **T** | **C** | **G** | **A** | **T** | **A** | **A** | **A** | **A** | **A** | **C** | **G** | **T** | **G** | **T** | **T** | **A** | **T** | **T** | **G** | **C** | **G** | **T** | **A** | **T** | **T** | **G** | **T** | **T** | **G** | **A** | **T** | **G** | **A** | **T** | **G** | **G** | **T** | **G** | **T** | **C** | **G** | **G** | **C** | **T** | **T** | **T** | **G** | **A** | **T** | **A** | **T** | **G** | **A** | **A** | **G** | **G** | **A** | **A** | **C** | **A** | **A** | **A** | **G** | **T** | **A** | **A** | **T** | **A** | **A** | **A** | **G** | **C** | **C** | **G** | **G** | **T** | **A** | **G** | **T** | **T** | **A** | **T** | **G** | **G** | **C** | **T** | **T** | **A** | **A** | **A** | **T** | **A** | **A** | **T** | **A** | **T** | **C** | **C** | **G** | **A** | **G** | **A** | **A** | **C** | **G** | **T** | **G** | **T** | **T** | **G** | **T** | **C** | **G** | **G** | **C** | **A** | **T** | **G** | **G** | **G** | **C** | **G** | **G** | **T** | **A** | **C** | **A** | **G** | **T** | **T** | **A** | **A** | **A** | **A** | **T** | **T** | **A** | **T** | **T** | **A** | **G** | **T** | **T** | **T** | **T** | **A** | **A** | **A** | **G** | **G** | **G** | **C** | **A** | **G** | **G** | **G** | **A** | **A** | **C** | **C** | **A** | **G** | **C** | **G** | **T** | **T** | **G** | **A** | **A** | **A** | **T** | **T** | **A** | **A** | **A** | **G** | **T** | **T** | **C** | **C** | **T** | **G** | **T** | **C** | **A** | **T** | **A** | **A** | **A** | **G** | **G** | **A** | **G** | **G** | **A** | **A** | **A** | **C** | **T** | **G** | **C** | **A** | **A** | **G** | **T** | **G** | **A** | **T** | **C** | **A** | **A** | **A** | **G** | **T** | **A** | **A** | **T** | **G** | **T** | **T** | **A** | **G** | **T** | **G** | **G** | **A** | **-** | **-** | **-** |
| **N52623** | **A** | **T** | **G** | **A** | **C** | **C** | **G** | **A** | **T** | **C** | **G** | **G** | **A** | **T** | **T** | **T** | **C** | **A** | **A** | **G** | **A** | **C** | **G** | **C** | **A** | **T** | **G** | **A** | **T** | **T** | **T** | **C** | **A** | **T** | **T** | **A** | **T** | **A** | **T** | **G** | **C** | **G** | **T** | **C** | **C** | **C** | **T** | **T** | **A** | **G** | **C** | **A** | **C** | **C** | **T** | **T** | **T** | **A** | **T** | **T** | **G** | **T** | **T** | **A** | **T** | **C** | **T** | **T** | **A** | **A** | **T** | **T** | **A** | **C** | **A** | **T** | **T** | **G** | **T** | **T** | **T** | **T** | **C** | **A** | **T** | **A** | **T** | **T** | **T** | **T** | **C** | **A** | **T** | **T** | **C** | **G** | **A** | **T** | **T** | **A** | **A** | **A** | **C** | **A** | **A** | **A** | **A** | **C** | **C** | **G** | **G** | **T** | **G** | **G** | **T** | **T** | **A** | **T** | **T** | **A** | **G** | **A** | **G** | **C** | **T** | **T** | **C** | **T** | **T** | **C** | **A** | **G** | **A** | **G** | **A** | **A** | **A** | **A** | **G** | **T** | **C** | **T** | **T** | **T** | **T** | **A** | **T** | **T** | **T** | **A** | **C** | **C** | **A** | **C** | **T** | **A** | **A** | **T** | **T** | **G** | **T** | **G** | **C** | **A** | **C** | **A** | **T** | **T** | **G** | **T** | **T** | **C** | **T** | **C** | **A** | **T** | **A** | **T** | **C** | **C** | **T** | **T** | **A** | **C** | **T** | **A** | **A** | **T** | **A** | **G** | **G** | **C** | **T** | **T** | **A** | **T** | **T** | **G** | **A** | **C** | **C** | **T** | **T** | **T** | **T** | **T** | **A** | **C** | **T** | **G** | **A** | **T** | **T** | **T** | **C** | **A** | **T** | **T** | **G** | **G** | **T** | **T** | **C** | **A** | **A** | **A** | **A** | **A** | **G** | **G** | **G** | **C** | **A** | **A** | **T** | **A** | **T** | **G** | **G** | **A** | **C** | **G** | **G** | **A** | **T** | **T** | **G** | **A** | **A** | **G** | **A** | **A** | **A** | **A** | **A** | **C** | **T** | **T** | **C** | **G** | **G** | **T** | **T** | **A** | **T** | **T** | **G** | **G** | **C** | **C** | **A** | **A** | **C** | **G** | **G** | **T** | **A** | **A** | **T** | **T** | **A** | **T** | **G** | **A** | **A** | **A** | **G** | **T** | **C** | **C** | **A** | **G** | **T** | **C** | **T** | **T** | **A** | **A** | **A** | **C** | **A** | **A** | **A** | **C** | **C** | **A** | **A** | **C** | **G** | **A** | **C** | **C** | **A** | **G** | **T** | **G** | **A** | **A** | **A** | **A** | **T** | **C** | **A** | **A** | **G** | **A** | **C** | **C** | **A** | **T** | **T** | **A** | **T** | **C** | **T** | **A** | **A** | **C** | **C** | **G** | **A** | **A** | **G** | **T** | **C** | **G** | **A** | **A** | **C** | **A** | **A** | **G** | **A** | **T** | **A** | **T** | **T** | **T** | **G** | **G** | **T** | **C** | **G** | **A** | **T** | **T** | **A** | **A** | **A** | **A** | **A** | **T** | **A** | **A** | **A** | **T** | **T** | **A** | **T** | **T** | **A** | **G** | **A** | **G** | **A** | **T** | **G** | **T** | **C** | **T** | **A** | **A** | **A** | **G** | **A** | **A** | **T** | **T** | **G** | **C** | **A** | **A** | **T** | **T** | **A** | **T** | **T** | **A** | **A** | **A** | **C** | **A** | **G** | **T** | **C** | **G** | **A** | **C** | **C** | **G** | **C** | **A** | **A** | **T** | **T** | **A** | **A** | **T** | **G** | **G** | **A** | **T** | **G** | **G** | **G** | **C** | **A** | **A** | **A** | **C** | **A** | **A** | **A** | **A** | **G** | **A** | **A** | **G** | **A** | **A** | **A** | **T** | **T** | **T** | **T** | **A** | **G** | **A** | **G** | **A** | **A** | **C** | **G** | **A** | **G** | **C** | **G** | **G** | **C** | **A** | **T** | **C** | **G** | **T** | **T** | **T** | **G** | **G** | **C** | **G** | **C** | **G** | **G** | **G** | **A** | **G** | **T** | **T** | **G** | **C** | **A** | **T** | **G** | **A** | **T** | **T** | **C** | **A** | **G** | **T** | **C** | **A** | **G** | **T** | **C** | **A** | **A** | **C** | **A** | **A** | **C** | **T** | **T** | **T** | **T** | **T** | **G** | **C** | **A** | **G** | **C** | **C** | **A** | **T** | **G** | **A** | **T** | **G** | **A** | **T** | **G** | **T** | **T** | **G** | **T** | **C** | **T** | **G** | **C** | **A** | **T** | **T** | **A** | **A** | **A** | **T** | **G** | **A** | **A** | **C** | **A** | **A** | **G** | **C** | **A** | **C** | **A** | **A** | **C** | **G** | **A** | **A** | **C** | **A** | **G** | **A** | **A** | **A** | **C** | **C** | **C** | **C** | **G** | **G** | **A** | **A** | **C** | **C** | **A** | **T** | **A** | **T** | **C** | **G** | **T** | **A** | **A** | **A** | **C** | **A** | **A** | **C** | **T** | **A** | **G** | **C** | **C** | **A** | **T** | **G** | **G** | **T** | **G** | **G** | **C** | **A** | **G** | **A** | **A** | **A** | **T** | **C** | **A** | **T** | **T** | **A** | **A** | **T** | **G** | **C** | **C** | **T** | **C** | **C** | **C** | **A** | **A** | **T** | **C** | **G** | **G** | **A** | **A** | **A** | **T** | **G** | **C** | **G** | **C** | **G** | **C** | **G** | **C** | **T** | **A** | **C** | **T** | **A** | **T** | **T** | **G** | **C** | **A** | **C** | **T** | **T** | **G** | **C** | **G** | **T** | **C** | **C** | **T** | **A** | **T** | **C** | **A** | **G** | **T** | **C** | **T** | **A** | **G** | **A** | **A** | **G** | **G** | **A** | **A** | **A** | **A** | **A** | **G** | **T** | **T** | **T** | **G** | **C** | **G** | **T** | **A** | **A** | **A** | **G** | **G** | **T** | **A** | **T** | **T** | **G** | **A** | **A** | **C** | **A** | **A** | **T** | **T** | **A** | **C** | **T** | **G** | **A** | **A** | **A** | **G** | **A** | **A** | **C** | **T** | **A** | **C** | **A** | **A** | **A** | **C** | **A** | **A** | **A** | **A** | **A** | **T** | **T** | **A** | **A** | **A** | **A** | **T** | **T** | **G** | **A** | **A** | **T** | **T** | **G** | **A** | **T** | **T** | **T** | **G** | **G** | **G** | **A** | **T** | **G** | **T** | **T** | **G** | **A** | **A** | **G** | **A** | **T** | **G** | **T** | **T** | **C** | **A** | **T** | **T** | **T** | **A** | **A** | **A** | **T** | **A** | **G** | **C** | **A** | **G** | **C** | **A** | **T** | **T** | **G** | **A** | **G** | **G** | **A** | **T** | **C** | **A** | **T** | **C** | **T** | **T** | **T** | **T** | **C** | **C** | **G** | **A** | **A** | **T** | **T** | **G** | **T** | **G** | **C** | **A** | **G** | **G** | **A** | **G** | **T** | **T** | **A** | **C** | **T** | **T** | **T** | **C** | **A** | **A** | **A** | **T** | **A** | **C** | **C** | **T** | **T** | **A** | **A** | **G** | **A** | **C** | **A** | **T** | **G** | **C** | **C** | **A** | **A** | **A** | **G** | **C** | **A** | **A** | **A** | **G** | **G** | **A** | **A** | **T** | **T** | **A** | **G** | **A** | **G** | **G** | **T** | **A** | **T** | **A** | **C** | **T** | **T** | **A** | **C** | **A** | **C** | **C** | **A** | **A** | **G** | **T** | **C** | **G** | **A** | **T** | **A** | **A** | **A** | **A** | **A** | **C** | **G** | **T** | **G** | **T** | **T** | **A** | **T** | **T** | **G** | **C** | **G** | **T** | **A** | **T** | **T** | **G** | **T** | **T** | **G** | **A** | **T** | **G** | **A** | **T** | **G** | **G** | **T** | **G** | **T** | **C** | **G** | **G** | **C** | **T** | **T** | **T** | **G** | **A** | **T** | **A** | **T** | **G** | **A** | **A** | **G** | **G** | **A** | **A** | **C** | **A** | **A** | **A** | **G** | **T** | **A** | **A** | **T** | **A** | **A** | **A** | **G** | **C** | **C** | **G** | **G** | **T** | **A** | **G** | **T** | **T** | **A** | **T** | **G** | **G** | **C** | **T** | **T** | **A** | **A** | **A** | **T** | **A** | **A** | **T** | **A** | **T** | **T** | **C** | **G** | **A** | **G** | **A** | **A** | **C** | **G** | **T** | **G** | **T** | **T** | **G** | **T** | **C** | **G** | **G** | **C** | **A** | **T** | **G** | **G** | **G** | **C** | **G** | **G** | **T** | **A** | **C** | **A** | **G** | **T** | **T** | **A** | **A** | **A** | **A** | **T** | **T** | **A** | **T** | **T** | **A** | **G** | **T** | **T** | **T** | **T** | **A** | **A** | **A** | **G** | **G** | **G** | **C** | **A** | **G** | **G** | **G** | **A** | **A** | **C** | **C** | **A** | **G** | **C** | **G** | **T** | **T** | **G** | **A** | **A** | **A** | **T** | **T** | **A** | **A** | **A** | **G** | **T** | **T** | **C** | **C** | **T** | **G** | **T** | **C** | **A** | **T** | **A** | **A** | **A** | **G** | **G** | **A** | **G** | **G** | **A** | **A** | **A** | **C** | **T** | **G** | **C** | **A** | **A** | **G** | **T** | **G** | **A** | **T** | **C** | **A** | **A** | **A** | **G** | **T** | **A** | **A** | **T** | **G** | **T** | **T** | **A** | **G** | **T** | **G** | **G** | **A** | **-** | **-** | **-** |
| **N59455F** | **A** | **T** | **G** | **A** | **C** | **C** | **G** | **A** | **T** | **C** | **G** | **G** | **A** | **T** | **T** | **T** | **C** | **A** | **A** | **G** | **A** | **C** | **G** | **C** | **A** | **T** | **G** | **A** | **T** | **T** | **T** | **C** | **A** | **T** | **T** | **A** | **T** | **A** | **T** | **G** | **C** | **G** | **T** | **C** | **C** | **C** | **T** | **T** | **A** | **G** | **C** | **A** | **C** | **C** | **T** | **T** | **T** | **A** | **T** | **T** | **G** | **T** | **T** | **A** | **T** | **C** | **T** | **T** | **A** | **A** | **T** | **T** | **A** | **C** | **A** | **T** | **T** | **G** | **T** | **T** | **T** | **T** | **C** | **A** | **T** | **A** | **T** | **T** | **T** | **T** | **C** | **A** | **T** | **T** | **C** | **G** | **A** | **T** | **T** | **A** | **A** | **A** | **C** | **A** | **A** | **A** | **A** | **C** | **C** | **G** | **G** | **T** | **G** | **G** | **C** | **T** | **A** | **T** | **T** | **A** | **G** | **A** | **G** | **C** | **T** | **T** | **C** | **T** | **T** | **C** | **A** | **G** | **A** | **G** | **A** | **A** | **A** | **A** | **G** | **T** | **C** | **T** | **T** | **T** | **T** | **A** | **T** | **T** | **T** | **A** | **C** | **C** | **A** | **C** | **T** | **A** | **A** | **T** | **T** | **G** | **T** | **G** | **C** | **A** | **C** | **A** | **T** | **T** | **G** | **T** | **T** | **C** | **T** | **C** | **A** | **T** | **A** | **T** | **C** | **C** | **T** | **T** | **A** | **C** | **T** | **A** | **A** | **T** | **A** | **G** | **G** | **C** | **T** | **T** | **A** | **T** | **T** | **G** | **A** | **C** | **C** | **T** | **T** | **T** | **T** | **T** | **A** | **C** | **T** | **G** | **A** | **T** | **T** | **T** | **C** | **A** | **T** | **T** | **G** | **G** | **T** | **T** | **C** | **A** | **A** | **A** | **A** | **A** | **G** | **G** | **G** | **C** | **A** | **A** | **T** | **A** | **T** | **G** | **G** | **A** | **C** | **G** | **G** | **A** | **T** | **T** | **G** | **A** | **A** | **G** | **A** | **A** | **A** | **A** | **A** | **C** | **T** | **T** | **C** | **G** | **G** | **T** | **T** | **A** | **T** | **T** | **G** | **G** | **C** | **C** | **A** | **A** | **T** | **G** | **G** | **T** | **A** | **A** | **T** | **T** | **A** | **T** | **G** | **A** | **A** | **A** | **G** | **T** | **C** | **C** | **A** | **G** | **T** | **C** | **T** | **T** | **A** | **A** | **A** | **C** | **A** | **A** | **A** | **C** | **C** | **A** | **A** | **C** | **G** | **A** | **C** | **C** | **A** | **G** | **T** | **G** | **A** | **A** | **A** | **A** | **T** | **C** | **A** | **A** | **G** | **A** | **T** | **C** | **A** | **T** | **T** | **A** | **T** | **C** | **T** | **A** | **A** | **C** | **C** | **G** | **A** | **A** | **G** | **T** | **C** | **G** | **A** | **A** | **C** | **A** | **A** | **G** | **A** | **T** | **A** | **T** | **T** | **T** | **G** | **G** | **T** | **C** | **G** | **A** | **T** | **T** | **A** | **A** | **A** | **A** | **A** | **T** | **A** | **A** | **A** | **T** | **T** | **A** | **T** | **T** | **A** | **G** | **A** | **G** | **A** | **T** | **G** | **T** | **C** | **T** | **A** | **A** | **A** | **G** | **A** | **A** | **T** | **T** | **G** | **C** | **A** | **A** | **T** | **T** | **A** | **T** | **T** | **A** | **A** | **A** | **C** | **A** | **G** | **T** | **C** | **G** | **G** | **C** | **C** | **G** | **C** | **A** | **A** | **T** | **T** | **A** | **A** | **T** | **G** | **G** | **A** | **T** | **G** | **G** | **G** | **C** | **A** | **A** | **A** | **C** | **A** | **A** | **A** | **A** | **G** | **A** | **A** | **G** | **A** | **A** | **A** | **T** | **T** | **T** | **T** | **A** | **G** | **A** | **G** | **A** | **A** | **C** | **G** | **A** | **G** | **C** | **G** | **G** | **C** | **A** | **T** | **C** | **G** | **T** | **T** | **T** | **G** | **G** | **C** | **G** | **C** | **G** | **G** | **G** | **A** | **G** | **T** | **T** | **G** | **C** | **A** | **T** | **G** | **A** | **T** | **T** | **C** | **A** | **G** | **T** | **C** | **A** | **G** | **T** | **C** | **A** | **A** | **C** | **A** | **A** | **C** | **T** | **T** | **T** | **T** | **T** | **G** | **C** | **A** | **G** | **C** | **C** | **A** | **T** | **G** | **A** | **T** | **G** | **A** | **T** | **G** | **T** | **T** | **G** | **T** | **C** | **T** | **G** | **C** | **C** | **T** | **T** | **A** | **A** | **A** | **T** | **G** | **A** | **A** | **C** | **A** | **A** | **G** | **C** | **A** | **C** | **A** | **A** | **C** | **G** | **A** | **A** | **C** | **A** | **G** | **A** | **A** | **A** | **C** | **C** | **C** | **C** | **G** | **G** | **A** | **A** | **C** | **C** | **A** | **T** | **A** | **T** | **C** | **G** | **T** | **A** | **A** | **A** | **C** | **A** | **A** | **C** | **T** | **A** | **G** | **C** | **C** | **A** | **T** | **G** | **G** | **T** | **G** | **G** | **C** | **A** | **G** | **A** | **A** | **A** | **T** | **C** | **A** | **T** | **T** | **A** | **A** | **T** | **G** | **C** | **C** | **T** | **C** | **C** | **C** | **A** | **A** | **T** | **C** | **G** | **G** | **A** | **A** | **A** | **T** | **G** | **C** | **G** | **C** | **G** | **C** | **G** | **T** | **T** | **A** | **C** | **T** | **A** | **T** | **T** | **G** | **C** | **A** | **C** | **T** | **T** | **G** | **C** | **G** | **T** | **C** | **C** | **T** | **A** | **T** | **C** | **A** | **G** | **T** | **C** | **T** | **A** | **G** | **A** | **A** | **G** | **G** | **A** | **A** | **A** | **A** | **A** | **G** | **T** | **T** | **T** | **G** | **C** | **G** | **T** | **A** | **A** | **A** | **G** | **G** | **T** | **A** | **T** | **T** | **G** | **A** | **A** | **C** | **A** | **A** | **T** | **T** | **A** | **C** | **T** | **A** | **A** | **A** | **A** | **G** | **A** | **A** | **C** | **T** | **A** | **C** | **A** | **A** | **A** | **C** | **A** | **A** | **A** | **A** | **A** | **T** | **T** | **A** | **A** | **A** | **A** | **T** | **T** | **G** | **A** | **A** | **T** | **T** | **G** | **A** | **T** | **T** | **T** | **G** | **G** | **G** | **A** | **T** | **G** | **T** | **T** | **G** | **A** | **A** | **G** | **A** | **T** | **G** | **T** | **T** | **C** | **A** | **T** | **T** | **T** | **A** | **A** | **A** | **T** | **A** | **G** | **C** | **A** | **G** | **C** | **A** | **T** | **T** | **G** | **A** | **G** | **G** | **A** | **T** | **C** | **A** | **T** | **C** | **T** | **T** | **T** | **T** | **C** | **C** | **G** | **A** | **A** | **T** | **T** | **G** | **T** | **G** | **C** | **A** | **G** | **G** | **A** | **A** | **T** | **T** | **A** | **C** | **T** | **T** | **T** | **C** | **C** | **A** | **A** | **T** | **A** | **C** | **C** | **T** | **T** | **A** | **A** | **G** | **A** | **C** | **A** | **T** | **G** | **C** | **C** | **A** | **A** | **A** | **G** | **C** | **A** | **A** | **A** | **G** | **G** | **A** | **A** | **T** | **T** | **A** | **G** | **A** | **G** | **G** | **T** | **A** | **T** | **A** | **C** | **T** | **T** | **A** | **C** | **A** | **C** | **C** | **A** | **A** | **G** | **T** | **C** | **G** | **A** | **T** | **A** | **A** | **A** | **A** | **A** | **C** | **G** | **T** | **G** | **T** | **T** | **A** | **T** | **T** | **G** | **C** | **G** | **T** | **A** | **T** | **T** | **G** | **T** | **T** | **G** | **A** | **T** | **G** | **A** | **T** | **G** | **G** | **T** | **G** | **T** | **C** | **G** | **G** | **C** | **T** | **T** | **T** | **G** | **A** | **T** | **A** | **T** | **G** | **A** | **A** | **G** | **G** | **A** | **A** | **C** | **A** | **A** | **A** | **G** | **T** | **A** | **A** | **T** | **A** | **A** | **A** | **G** | **C** | **C** | **G** | **G** | **T** | **A** | **G** | **T** | **T** | **A** | **T** | **G** | **G** | **C** | **T** | **T** | **A** | **A** | **A** | **T** | **A** | **A** | **T** | **A** | **T** | **T** | **C** | **G** | **A** | **G** | **A** | **A** | **C** | **G** | **T** | **G** | **T** | **T** | **G** | **T** | **C** | **G** | **G** | **C** | **A** | **T** | **G** | **G** | **G** | **C** | **G** | **G** | **T** | **A** | **C** | **A** | **G** | **T** | **T** | **A** | **A** | **A** | **A** | **T** | **T** | **A** | **T** | **T** | **A** | **G** | **T** | **T** | **T** | **T** | **A** | **A** | **A** | **G** | **G** | **G** | **C** | **A** | **G** | **G** | **G** | **A** | **A** | **C** | **C** | **A** | **G** | **C** | **G** | **T** | **T** | **G** | **A** | **A** | **A** | **T** | **T** | **A** | **A** | **A** | **G** | **T** | **T** | **C** | **C** | **T** | **G** | **T** | **C** | **A** | **T** | **A** | **A** | **A** | **G** | **G** | **A** | **G** | **G** | **A** | **A** | **A** | **C** | **T** | **G** | **C** | **A** | **A** | **G** | **T** | **G** | **A** | **T** | **C** | **A** | **A** | **A** | **G** | **T** | **A** | **A** | **T** | **G** | **T** | **T** | **A** | **G** | **T** | **G** | **G** | **A** | **-** | **-** | **-** |
| **N59611F** | **A** | **T** | **G** | **A** | **C** | **C** | **G** | **A** | **T** | **C** | **G** | **G** | **A** | **T** | **T** | **T** | **C** | **A** | **A** | **G** | **A** | **C** | **G** | **C** | **A** | **T** | **G** | **A** | **T** | **T** | **T** | **C** | **A** | **T** | **T** | **A** | **T** | **A** | **T** | **G** | **C** | **G** | **T** | **C** | **C** | **C** | **T** | **T** | **A** | **G** | **C** | **A** | **C** | **C** | **T** | **T** | **T** | **A** | **T** | **T** | **G** | **T** | **T** | **A** | **T** | **C** | **T** | **T** | **A** | **A** | **T** | **T** | **A** | **C** | **A** | **T** | **T** | **G** | **T** | **T** | **T** | **T** | **C** | **A** | **T** | **A** | **T** | **T** | **T** | **T** | **C** | **A** | **T** | **T** | **C** | **G** | **A** | **T** | **T** | **A** | **A** | **A** | **C** | **A** | **A** | **A** | **A** | **C** | **C** | **G** | **G** | **T** | **G** | **G** | **C** | **T** | **A** | **T** | **T** | **A** | **G** | **A** | **G** | **C** | **T** | **T** | **C** | **T** | **T** | **C** | **A** | **G** | **A** | **G** | **A** | **A** | **A** | **A** | **G** | **T** | **C** | **T** | **T** | **T** | **T** | **A** | **T** | **T** | **T** | **A** | **C** | **C** | **A** | **C** | **T** | **A** | **A** | **T** | **T** | **G** | **T** | **G** | **C** | **A** | **C** | **A** | **T** | **T** | **G** | **T** | **T** | **C** | **T** | **C** | **A** | **T** | **A** | **T** | **C** | **C** | **T** | **T** | **A** | **C** | **T** | **A** | **A** | **T** | **A** | **G** | **G** | **C** | **T** | **T** | **A** | **T** | **T** | **G** | **A** | **C** | **C** | **T** | **T** | **T** | **T** | **T** | **A** | **C** | **T** | **G** | **A** | **T** | **T** | **T** | **C** | **A** | **T** | **T** | **G** | **G** | **T** | **T** | **C** | **A** | **A** | **A** | **A** | **A** | **G** | **G** | **G** | **C** | **A** | **A** | **T** | **A** | **T** | **G** | **G** | **A** | **C** | **G** | **G** | **A** | **T** | **T** | **G** | **A** | **A** | **G** | **A** | **A** | **A** | **A** | **A** | **C** | **T** | **T** | **C** | **G** | **G** | **T** | **T** | **A** | **T** | **T** | **G** | **G** | **C** | **C** | **A** | **A** | **C** | **G** | **G** | **T** | **A** | **A** | **T** | **T** | **A** | **T** | **G** | **A** | **A** | **A** | **G** | **T** | **C** | **C** | **A** | **G** | **T** | **C** | **T** | **T** | **A** | **A** | **A** | **C** | **A** | **A** | **A** | **C** | **C** | **A** | **A** | **C** | **G** | **A** | **C** | **C** | **A** | **G** | **T** | **G** | **A** | **A** | **A** | **A** | **T** | **C** | **A** | **A** | **G** | **A** | **C** | **C** | **A** | **T** | **T** | **A** | **T** | **C** | **T** | **A** | **A** | **C** | **C** | **G** | **A** | **A** | **G** | **T** | **C** | **G** | **A** | **A** | **C** | **A** | **A** | **G** | **A** | **T** | **A** | **T** | **T** | **T** | **G** | **G** | **T** | **C** | **G** | **A** | **T** | **T** | **A** | **A** | **A** | **A** | **A** | **T** | **A** | **A** | **A** | **T** | **T** | **A** | **T** | **T** | **A** | **G** | **A** | **G** | **A** | **T** | **G** | **T** | **C** | **T** | **A** | **A** | **A** | **G** | **A** | **A** | **T** | **T** | **G** | **C** | **A** | **A** | **T** | **T** | **A** | **T** | **T** | **A** | **A** | **A** | **C** | **A** | **G** | **T** | **C** | **G** | **A** | **C** | **C** | **G** | **C** | **A** | **A** | **T** | **T** | **A** | **A** | **T** | **G** | **G** | **A** | **T** | **G** | **G** | **G** | **C** | **A** | **A** | **A** | **C** | **A** | **A** | **A** | **A** | **G** | **A** | **A** | **G** | **A** | **A** | **A** | **T** | **T** | **T** | **T** | **A** | **G** | **A** | **G** | **A** | **A** | **C** | **G** | **A** | **G** | **C** | **G** | **G** | **C** | **A** | **T** | **C** | **G** | **T** | **T** | **T** | **G** | **G** | **C** | **G** | **C** | **G** | **G** | **G** | **A** | **G** | **T** | **T** | **G** | **C** | **A** | **T** | **G** | **A** | **T** | **T** | **C** | **A** | **G** | **T** | **C** | **A** | **G** | **T** | **C** | **A** | **A** | **C** | **A** | **A** | **C** | **T** | **T** | **T** | **T** | **T** | **G** | **C** | **A** | **G** | **C** | **C** | **A** | **T** | **G** | **A** | **T** | **G** | **A** | **T** | **G** | **T** | **T** | **G** | **T** | **C** | **T** | **G** | **C** | **C** | **T** | **T** | **A** | **A** | **A** | **T** | **G** | **A** | **A** | **C** | **A** | **A** | **G** | **C** | **A** | **C** | **A** | **A** | **C** | **G** | **A** | **A** | **C** | **A** | **G** | **A** | **A** | **A** | **C** | **T** | **C** | **C** | **T** | **G** | **A** | **A** | **C** | **C** | **A** | **T** | **A** | **T** | **C** | **G** | **C** | **A** | **A** | **A** | **C** | **A** | **A** | **C** | **T** | **A** | **G** | **C** | **C** | **A** | **T** | **G** | **G** | **T** | **G** | **G** | **C** | **A** | **G** | **A** | **A** | **A** | **T** | **C** | **A** | **T** | **T** | **A** | **A** | **T** | **G** | **C** | **C** | **T** | **C** | **C** | **C** | **A** | **A** | **T** | **C** | **G** | **G** | **A** | **A** | **A** | **T** | **G** | **C** | **G** | **C** | **G** | **C** | **G** | **T** | **T** | **A** | **C** | **T** | **A** | **T** | **T** | **G** | **C** | **A** | **C** | **T** | **T** | **G** | **C** | **G** | **T** | **C** | **C** | **T** | **A** | **T** | **C** | **A** | **G** | **T** | **C** | **T** | **A** | **G** | **A** | **A** | **G** | **G** | **A** | **A** | **A** | **A** | **A** | **G** | **T** | **T** | **T** | **G** | **C** | **G** | **T** | **A** | **A** | **A** | **G** | **G** | **T** | **A** | **T** | **T** | **G** | **A** | **A** | **C** | **A** | **A** | **T** | **T** | **A** | **C** | **T** | **G** | **A** | **A** | **A** | **G** | **A** | **A** | **C** | **T** | **A** | **C** | **A** | **A** | **A** | **C** | **A** | **A** | **A** | **A** | **A** | **T** | **T** | **A** | **A** | **A** | **A** | **T** | **T** | **G** | **A** | **A** | **T** | **T** | **G** | **A** | **T** | **T** | **T** | **G** | **G** | **G** | **A** | **T** | **G** | **T** | **T** | **G** | **A** | **A** | **G** | **A** | **T** | **G** | **T** | **T** | **C** | **A** | **T** | **T** | **T** | **A** | **A** | **A** | **T** | **A** | **G** | **C** | **A** | **G** | **C** | **A** | **T** | **T** | **G** | **A** | **G** | **G** | **A** | **T** | **C** | **A** | **T** | **C** | **T** | **T** | **T** | **T** | **C** | **C** | **G** | **A** | **A** | **T** | **T** | **G** | **T** | **G** | **C** | **A** | **G** | **G** | **A** | **A** | **T** | **T** | **A** | **C** | **T** | **T** | **T** | **C** | **A** | **A** | **A** | **T** | **A** | **C** | **C** | **T** | **T** | **A** | **A** | **G** | **A** | **C** | **A** | **T** | **G** | **C** | **C** | **A** | **A** | **A** | **G** | **C** | **A** | **A** | **A** | **G** | **G** | **A** | **A** | **T** | **T** | **A** | **G** | **A** | **G** | **G** | **T** | **A** | **T** | **A** | **C** | **T** | **T** | **A** | **C** | **A** | **C** | **C** | **A** | **A** | **G** | **T** | **C** | **G** | **A** | **T** | **A** | **A** | **A** | **A** | **A** | **C** | **G** | **T** | **G** | **T** | **T** | **A** | **T** | **T** | **G** | **C** | **G** | **T** | **A** | **T** | **T** | **G** | **T** | **T** | **G** | **A** | **T** | **G** | **A** | **T** | **G** | **G** | **T** | **G** | **T** | **C** | **G** | **G** | **C** | **T** | **T** | **T** | **G** | **A** | **T** | **A** | **T** | **G** | **A** | **A** | **G** | **G** | **A** | **A** | **C** | **A** | **A** | **A** | **G** | **T** | **A** | **A** | **T** | **A** | **A** | **A** | **G** | **C** | **C** | **G** | **G** | **T** | **A** | **G** | **T** | **T** | **A** | **T** | **G** | **G** | **C** | **T** | **T** | **A** | **A** | **A** | **T** | **A** | **A** | **T** | **A** | **T** | **C** | **C** | **G** | **A** | **G** | **A** | **A** | **C** | **G** | **T** | **G** | **T** | **T** | **G** | **T** | **C** | **G** | **G** | **C** | **A** | **T** | **G** | **G** | **G** | **C** | **G** | **G** | **T** | **A** | **C** | **A** | **G** | **T** | **T** | **A** | **A** | **A** | **A** | **T** | **T** | **A** | **T** | **T** | **A** | **G** | **T** | **T** | **T** | **T** | **A** | **A** | **A** | **G** | **G** | **G** | **C** | **A** | **G** | **G** | **G** | **A** | **A** | **C** | **C** | **A** | **G** | **C** | **G** | **T** | **T** | **G** | **A** | **A** | **A** | **T** | **T** | **A** | **A** | **A** | **G** | **T** | **T** | **C** | **C** | **T** | **G** | **T** | **C** | **A** | **T** | **A** | **A** | **A** | **G** | **G** | **A** | **G** | **G** | **A** | **A** | **A** | **C** | **T** | **G** | **C** | **A** | **A** | **G** | **T** | **G** | **A** | **T** | **C** | **A** | **A** | **A** | **G** | **T** | **A** | **A** | **T** | **G** | **T** | **T** | **A** | **G** | **T** | **G** | **G** | **A** | **-** | **-** | **-** |
| **N60027F** | **A** | **T** | **G** | **A** | **C** | **C** | **G** | **A** | **T** | **C** | **G** | **G** | **A** | **T** | **T** | **T** | **C** | **A** | **A** | **G** | **A** | **C** | **G** | **C** | **A** | **T** | **G** | **A** | **T** | **T** | **T** | **C** | **A** | **T** | **T** | **A** | **T** | **A** | **T** | **G** | **C** | **G** | **T** | **C** | **C** | **C** | **T** | **T** | **A** | **G** | **C** | **A** | **C** | **C** | **T** | **T** | **T** | **A** | **T** | **T** | **G** | **T** | **T** | **A** | **T** | **C** | **T** | **T** | **A** | **A** | **T** | **T** | **A** | **C** | **A** | **T** | **T** | **G** | **T** | **T** | **T** | **T** | **C** | **A** | **T** | **A** | **T** | **T** | **T** | **T** | **C** | **A** | **T** | **T** | **C** | **G** | **A** | **T** | **T** | **A** | **A** | **A** | **C** | **A** | **A** | **A** | **A** | **C** | **C** | **G** | **G** | **T** | **G** | **G** | **T** | **T** | **A** | **T** | **T** | **A** | **G** | **A** | **G** | **C** | **T** | **T** | **C** | **T** | **T** | **C** | **A** | **G** | **A** | **G** | **A** | **A** | **A** | **A** | **G** | **T** | **C** | **T** | **T** | **T** | **T** | **A** | **T** | **T** | **T** | **A** | **C** | **C** | **A** | **C** | **T** | **A** | **A** | **T** | **T** | **G** | **T** | **G** | **C** | **A** | **C** | **A** | **T** | **T** | **G** | **T** | **T** | **C** | **T** | **C** | **A** | **T** | **A** | **T** | **C** | **C** | **T** | **T** | **A** | **C** | **T** | **A** | **A** | **T** | **A** | **G** | **G** | **C** | **T** | **T** | **A** | **T** | **T** | **G** | **A** | **C** | **C** | **T** | **T** | **T** | **T** | **T** | **A** | **C** | **T** | **G** | **A** | **T** | **T** | **T** | **C** | **A** | **T** | **T** | **G** | **G** | **T** | **T** | **C** | **A** | **A** | **A** | **A** | **A** | **G** | **G** | **G** | **C** | **A** | **A** | **T** | **A** | **T** | **G** | **G** | **A** | **C** | **G** | **G** | **A** | **T** | **T** | **G** | **A** | **A** | **G** | **A** | **A** | **A** | **A** | **A** | **C** | **T** | **T** | **C** | **G** | **G** | **T** | **T** | **A** | **T** | **T** | **G** | **G** | **C** | **C** | **A** | **A** | **C** | **G** | **G** | **T** | **A** | **A** | **T** | **T** | **A** | **T** | **G** | **A** | **A** | **A** | **G** | **T** | **C** | **C** | **A** | **G** | **T** | **C** | **T** | **T** | **A** | **A** | **A** | **C** | **A** | **A** | **A** | **C** | **C** | **A** | **A** | **C** | **G** | **A** | **C** | **C** | **A** | **G** | **T** | **G** | **A** | **A** | **A** | **A** | **T** | **C** | **A** | **A** | **G** | **A** | **C** | **C** | **A** | **T** | **T** | **A** | **T** | **C** | **T** | **A** | **A** | **C** | **C** | **G** | **A** | **A** | **G** | **T** | **C** | **G** | **A** | **A** | **C** | **A** | **A** | **G** | **A** | **T** | **A** | **T** | **T** | **T** | **G** | **G** | **T** | **C** | **G** | **A** | **T** | **T** | **A** | **A** | **A** | **A** | **A** | **T** | **A** | **A** | **A** | **T** | **T** | **A** | **T** | **T** | **A** | **G** | **A** | **G** | **A** | **T** | **G** | **T** | **C** | **T** | **A** | **A** | **A** | **G** | **A** | **A** | **T** | **T** | **G** | **C** | **A** | **A** | **T** | **T** | **A** | **T** | **T** | **A** | **A** | **A** | **C** | **A** | **G** | **T** | **C** | **G** | **A** | **C** | **C** | **G** | **C** | **A** | **A** | **T** | **T** | **A** | **A** | **T** | **G** | **G** | **A** | **T** | **G** | **G** | **G** | **C** | **A** | **A** | **A** | **C** | **A** | **A** | **A** | **A** | **G** | **A** | **A** | **G** | **A** | **A** | **A** | **T** | **T** | **T** | **T** | **A** | **G** | **A** | **G** | **A** | **A** | **C** | **G** | **A** | **G** | **C** | **G** | **G** | **C** | **A** | **T** | **C** | **G** | **T** | **T** | **T** | **G** | **G** | **C** | **G** | **C** | **G** | **G** | **G** | **A** | **G** | **T** | **T** | **G** | **C** | **A** | **T** | **G** | **A** | **T** | **T** | **C** | **A** | **G** | **T** | **C** | **A** | **G** | **T** | **C** | **A** | **A** | **C** | **A** | **A** | **C** | **T** | **T** | **T** | **T** | **T** | **G** | **C** | **A** | **G** | **C** | **C** | **A** | **T** | **G** | **A** | **T** | **G** | **A** | **T** | **G** | **T** | **T** | **G** | **T** | **C** | **T** | **G** | **C** | **C** | **T** | **T** | **A** | **A** | **A** | **T** | **G** | **A** | **A** | **C** | **A** | **A** | **G** | **C** | **A** | **C** | **A** | **A** | **C** | **G** | **A** | **A** | **C** | **A** | **G** | **A** | **A** | **A** | **C** | **C** | **C** | **C** | **G** | **G** | **A** | **A** | **C** | **C** | **A** | **T** | **A** | **T** | **C** | **G** | **T** | **A** | **A** | **A** | **C** | **A** | **A** | **C** | **T** | **A** | **G** | **C** | **C** | **A** | **T** | **G** | **G** | **T** | **G** | **G** | **C** | **A** | **G** | **A** | **A** | **A** | **T** | **C** | **A** | **T** | **T** | **A** | **A** | **C** | **G** | **C** | **C** | **T** | **C** | **C** | **C** | **A** | **A** | **T** | **C** | **G** | **G** | **A** | **A** | **A** | **T** | **G** | **C** | **G** | **C** | **G** | **C** | **G** | **T** | **T** | **A** | **C** | **T** | **A** | **T** | **T** | **G** | **C** | **A** | **C** | **T** | **T** | **G** | **C** | **G** | **T** | **C** | **C** | **T** | **A** | **T** | **C** | **A** | **G** | **T** | **C** | **T** | **A** | **G** | **A** | **A** | **G** | **G** | **A** | **A** | **A** | **A** | **A** | **G** | **T** | **T** | **T** | **G** | **C** | **G** | **T** | **A** | **A** | **A** | **G** | **G** | **T** | **A** | **T** | **T** | **G** | **A** | **A** | **C** | **A** | **A** | **T** | **T** | **A** | **C** | **T** | **G** | **A** | **A** | **A** | **G** | **A** | **A** | **C** | **T** | **A** | **C** | **A** | **A** | **A** | **C** | **A** | **A** | **A** | **A** | **A** | **T** | **T** | **A** | **A** | **A** | **A** | **T** | **T** | **G** | **A** | **A** | **T** | **T** | **G** | **A** | **T** | **T** | **T** | **G** | **G** | **G** | **A** | **T** | **G** | **T** | **T** | **G** | **A** | **A** | **G** | **A** | **T** | **G** | **T** | **T** | **C** | **A** | **T** | **T** | **T** | **A** | **A** | **A** | **T** | **A** | **G** | **C** | **A** | **G** | **C** | **A** | **T** | **T** | **G** | **A** | **G** | **G** | **A** | **T** | **C** | **A** | **T** | **C** | **T** | **T** | **T** | **T** | **C** | **C** | **G** | **A** | **A** | **T** | **T** | **G** | **T** | **G** | **C** | **A** | **G** | **G** | **A** | **A** | **T** | **T** | **A** | **C** | **T** | **T** | **T** | **C** | **A** | **A** | **A** | **T** | **A** | **C** | **C** | **T** | **T** | **A** | **A** | **G** | **A** | **C** | **A** | **T** | **G** | **C** | **C** | **A** | **A** | **A** | **G** | **C** | **A** | **A** | **A** | **G** | **G** | **A** | **A** | **T** | **T** | **A** | **G** | **A** | **G** | **G** | **T** | **A** | **T** | **A** | **C** | **T** | **T** | **A** | **C** | **A** | **C** | **C** | **A** | **A** | **G** | **T** | **C** | **G** | **A** | **T** | **A** | **A** | **A** | **A** | **A** | **C** | **G** | **T** | **G** | **C** | **T** | **A** | **T** | **T** | **G** | **C** | **G** | **T** | **A** | **T** | **T** | **G** | **T** | **T** | **G** | **A** | **T** | **G** | **A** | **C** | **G** | **G** | **T** | **G** | **T** | **C** | **G** | **G** | **C** | **T** | **T** | **T** | **G** | **A** | **T** | **A** | **T** | **G** | **A** | **A** | **G** | **G** | **A** | **A** | **C** | **A** | **A** | **A** | **G** | **T** | **A** | **A** | **T** | **A** | **A** | **A** | **G** | **C** | **C** | **G** | **G** | **T** | **A** | **G** | **T** | **T** | **A** | **T** | **G** | **G** | **C** | **T** | **T** | **A** | **A** | **A** | **T** | **A** | **A** | **T** | **A** | **T** | **T** | **C** | **G** | **A** | **G** | **A** | **A** | **C** | **G** | **T** | **G** | **T** | **T** | **G** | **T** | **C** | **G** | **G** | **C** | **A** | **T** | **G** | **G** | **G** | **C** | **G** | **G** | **T** | **A** | **C** | **A** | **G** | **T** | **T** | **A** | **A** | **A** | **A** | **T** | **T** | **A** | **T** | **T** | **A** | **G** | **T** | **T** | **T** | **T** | **A** | **A** | **A** | **G** | **G** | **G** | **C** | **A** | **G** | **G** | **G** | **A** | **A** | **C** | **C** | **A** | **G** | **C** | **G** | **T** | **T** | **G** | **A** | **A** | **A** | **T** | **T** | **A** | **A** | **A** | **G** | **T** | **T** | **C** | **C** | **T** | **G** | **T** | **C** | **A** | **T** | **A** | **A** | **A** | **G** | **G** | **A** | **G** | **G** | **A** | **A** | **A** | **C** | **T** | **G** | **C** | **A** | **A** | **G** | **T** | **G** | **A** | **T** | **C** | **A** | **A** | **A** | **G** | **T** | **A** | **A** | **T** | **G** | **T** | **T** | **A** | **G** | **T** | **G** | **G** | **A** | **-** | **-** | **-** |
| **N60034F** | **A** | **T** | **G** | **A** | **C** | **C** | **G** | **A** | **T** | **C** | **G** | **G** | **A** | **T** | **T** | **T** | **C** | **A** | **A** | **G** | **A** | **C** | **G** | **C** | **A** | **T** | **G** | **A** | **T** | **T** | **T** | **C** | **A** | **T** | **T** | **A** | **T** | **A** | **T** | **G** | **C** | **G** | **T** | **C** | **C** | **C** | **T** | **T** | **A** | **G** | **C** | **A** | **C** | **C** | **T** | **T** | **T** | **A** | **T** | **T** | **G** | **T** | **T** | **A** | **T** | **C** | **T** | **T** | **A** | **A** | **T** | **T** | **A** | **C** | **A** | **T** | **T** | **G** | **T** | **T** | **T** | **T** | **C** | **A** | **T** | **A** | **T** | **T** | **T** | **T** | **C** | **A** | **T** | **T** | **C** | **G** | **A** | **T** | **T** | **A** | **A** | **A** | **C** | **A** | **A** | **A** | **A** | **C** | **C** | **G** | **G** | **T** | **G** | **G** | **T** | **T** | **A** | **T** | **T** | **A** | **G** | **A** | **G** | **C** | **T** | **T** | **C** | **T** | **T** | **C** | **A** | **G** | **A** | **G** | **A** | **A** | **A** | **A** | **G** | **T** | **C** | **T** | **T** | **T** | **T** | **A** | **T** | **T** | **T** | **A** | **C** | **C** | **A** | **C** | **T** | **A** | **A** | **T** | **T** | **G** | **T** | **G** | **C** | **A** | **C** | **A** | **T** | **T** | **G** | **T** | **T** | **C** | **T** | **C** | **A** | **T** | **A** | **T** | **C** | **C** | **T** | **T** | **A** | **C** | **T** | **A** | **A** | **T** | **A** | **G** | **G** | **C** | **T** | **T** | **A** | **T** | **T** | **G** | **A** | **C** | **C** | **T** | **T** | **T** | **T** | **T** | **A** | **C** | **T** | **G** | **A** | **T** | **T** | **T** | **C** | **A** | **T** | **T** | **G** | **G** | **T** | **T** | **C** | **A** | **A** | **A** | **A** | **A** | **G** | **G** | **G** | **C** | **A** | **A** | **T** | **A** | **T** | **G** | **G** | **A** | **C** | **G** | **G** | **A** | **T** | **T** | **G** | **A** | **A** | **G** | **A** | **A** | **A** | **A** | **A** | **C** | **T** | **T** | **C** | **G** | **G** | **T** | **T** | **A** | **T** | **T** | **G** | **G** | **C** | **C** | **A** | **A** | **C** | **G** | **G** | **T** | **A** | **A** | **T** | **T** | **A** | **T** | **G** | **A** | **A** | **A** | **G** | **T** | **C** | **C** | **A** | **G** | **T** | **C** | **T** | **T** | **A** | **A** | **A** | **C** | **A** | **A** | **A** | **C** | **C** | **A** | **A** | **C** | **G** | **A** | **C** | **C** | **A** | **G** | **T** | **G** | **A** | **A** | **A** | **A** | **T** | **C** | **A** | **A** | **G** | **A** | **C** | **C** | **A** | **T** | **T** | **A** | **T** | **C** | **T** | **A** | **A** | **C** | **C** | **G** | **A** | **A** | **G** | **T** | **C** | **G** | **A** | **A** | **C** | **A** | **A** | **G** | **A** | **T** | **A** | **T** | **T** | **T** | **G** | **G** | **T** | **C** | **G** | **A** | **T** | **T** | **A** | **A** | **A** | **A** | **T** | **T** | **A** | **A** | **A** | **T** | **T** | **A** | **T** | **T** | **A** | **G** | **A** | **G** | **A** | **T** | **G** | **T** | **C** | **T** | **A** | **A** | **A** | **G** | **A** | **A** | **T** | **T** | **G** | **C** | **A** | **A** | **T** | **T** | **A** | **T** | **T** | **A** | **A** | **A** | **C** | **A** | **G** | **T** | **C** | **G** | **A** | **C** | **C** | **G** | **C** | **A** | **A** | **T** | **T** | **A** | **A** | **T** | **G** | **G** | **A** | **T** | **G** | **G** | **G** | **C** | **A** | **A** | **A** | **C** | **A** | **A** | **A** | **A** | **G** | **A** | **A** | **G** | **A** | **A** | **A** | **T** | **T** | **T** | **T** | **A** | **G** | **A** | **G** | **A** | **A** | **C** | **G** | **A** | **G** | **C** | **G** | **G** | **C** | **A** | **T** | **C** | **G** | **T** | **T** | **T** | **G** | **G** | **C** | **G** | **C** | **G** | **G** | **G** | **A** | **G** | **T** | **T** | **G** | **C** | **A** | **T** | **G** | **A** | **T** | **T** | **C** | **A** | **G** | **T** | **C** | **A** | **G** | **T** | **C** | **A** | **A** | **C** | **A** | **A** | **C** | **T** | **T** | **T** | **T** | **T** | **G** | **C** | **A** | **G** | **C** | **C** | **A** | **T** | **G** | **A** | **T** | **G** | **A** | **T** | **G** | **T** | **T** | **G** | **T** | **C** | **T** | **G** | **C** | **C** | **T** | **T** | **A** | **A** | **A** | **T** | **G** | **A** | **A** | **C** | **A** | **A** | **G** | **C** | **A** | **C** | **A** | **A** | **C** | **G** | **A** | **A** | **C** | **A** | **G** | **A** | **A** | **A** | **C** | **C** | **C** | **C** | **G** | **G** | **A** | **A** | **C** | **C** | **A** | **T** | **A** | **T** | **C** | **G** | **T** | **A** | **A** | **A** | **C** | **A** | **A** | **C** | **T** | **A** | **G** | **C** | **C** | **A** | **T** | **G** | **G** | **T** | **G** | **G** | **C** | **A** | **G** | **A** | **A** | **A** | **T** | **C** | **A** | **T** | **T** | **A** | **A** | **T** | **G** | **C** | **C** | **T** | **C** | **C** | **C** | **A** | **A** | **T** | **C** | **G** | **G** | **A** | **A** | **A** | **T** | **G** | **C** | **G** | **C** | **G** | **C** | **G** | **T** | **T** | **A** | **C** | **T** | **A** | **T** | **T** | **G** | **C** | **A** | **C** | **T** | **T** | **G** | **C** | **G** | **T** | **C** | **C** | **T** | **A** | **T** | **C** | **A** | **G** | **T** | **C** | **T** | **A** | **G** | **A** | **A** | **G** | **G** | **A** | **A** | **A** | **A** | **A** | **G** | **T** | **T** | **T** | **G** | **C** | **G** | **T** | **A** | **A** | **A** | **G** | **G** | **T** | **A** | **T** | **T** | **G** | **A** | **A** | **C** | **A** | **A** | **T** | **T** | **A** | **C** | **T** | **G** | **A** | **A** | **A** | **G** | **A** | **A** | **C** | **T** | **A** | **C** | **A** | **A** | **A** | **C** | **A** | **A** | **A** | **A** | **A** | **T** | **T** | **A** | **A** | **A** | **A** | **T** | **T** | **G** | **A** | **A** | **T** | **T** | **G** | **A** | **T** | **T** | **T** | **G** | **G** | **G** | **A** | **T** | **G** | **T** | **T** | **G** | **A** | **A** | **G** | **A** | **T** | **G** | **T** | **T** | **C** | **A** | **T** | **T** | **T** | **A** | **A** | **A** | **T** | **A** | **G** | **C** | **A** | **G** | **C** | **A** | **T** | **T** | **G** | **A** | **G** | **G** | **A** | **T** | **C** | **A** | **T** | **C** | **T** | **T** | **T** | **T** | **C** | **C** | **G** | **A** | **A** | **T** | **T** | **G** | **T** | **G** | **C** | **A** | **G** | **G** | **A** | **A** | **T** | **T** | **A** | **C** | **T** | **T** | **T** | **C** | **A** | **A** | **A** | **T** | **A** | **C** | **C** | **T** | **T** | **A** | **A** | **G** | **A** | **C** | **A** | **T** | **G** | **C** | **C** | **A** | **A** | **A** | **G** | **C** | **A** | **A** | **A** | **G** | **G** | **A** | **A** | **T** | **T** | **A** | **G** | **A** | **G** | **G** | **T** | **A** | **T** | **A** | **C** | **T** | **T** | **A** | **C** | **A** | **C** | **C** | **A** | **A** | **G** | **T** | **C** | **G** | **A** | **T** | **A** | **A** | **A** | **A** | **A** | **C** | **G** | **T** | **G** | **T** | **T** | **A** | **T** | **T** | **G** | **C** | **G** | **T** | **A** | **T** | **T** | **G** | **T** | **T** | **G** | **A** | **T** | **G** | **A** | **C** | **G** | **G** | **T** | **G** | **T** | **C** | **G** | **G** | **C** | **T** | **T** | **T** | **G** | **A** | **T** | **A** | **T** | **G** | **A** | **A** | **G** | **G** | **A** | **A** | **C** | **A** | **A** | **A** | **G** | **T** | **A** | **A** | **T** | **A** | **A** | **A** | **G** | **C** | **C** | **G** | **G** | **T** | **A** | **G** | **T** | **T** | **A** | **T** | **G** | **G** | **C** | **T** | **T** | **A** | **A** | **A** | **T** | **A** | **A** | **T** | **A** | **T** | **C** | **C** | **G** | **A** | **G** | **A** | **A** | **C** | **G** | **T** | **G** | **T** | **T** | **G** | **T** | **C** | **G** | **G** | **C** | **A** | **T** | **G** | **G** | **G** | **C** | **G** | **G** | **T** | **A** | **C** | **A** | **G** | **T** | **T** | **A** | **A** | **A** | **A** | **T** | **T** | **A** | **T** | **T** | **A** | **G** | **T** | **T** | **T** | **T** | **A** | **A** | **A** | **G** | **G** | **G** | **C** | **A** | **G** | **G** | **G** | **A** | **A** | **C** | **C** | **A** | **G** | **C** | **G** | **T** | **T** | **G** | **A** | **A** | **A** | **T** | **T** | **A** | **A** | **A** | **G** | **T** | **T** | **C** | **C** | **T** | **G** | **T** | **C** | **A** | **T** | **A** | **A** | **A** | **G** | **G** | **A** | **G** | **G** | **A** | **A** | **A** | **C** | **T** | **G** | **C** | **A** | **A** | **G** | **T** | **G** | **A** | **T** | **C** | **A** | **A** | **A** | **G** | **T** | **A** | **A** | **T** | **G** | **T** | **T** | **A** | **G** | **T** | **G** | **G** | **A** | **-** | **-** | **-** |
| **N59420F** | **A** | **T** | **G** | **A** | **C** | **C** | **G** | **A** | **T** | **C** | **G** | **G** | **A** | **T** | **T** | **T** | **C** | **A** | **A** | **G** | **A** | **C** | **G** | **C** | **A** | **T** | **G** | **A** | **T** | **T** | **T** | **C** | **A** | **T** | **T** | **A** | **T** | **A** | **T** | **G** | **C** | **G** | **T** | **C** | **C** | **C** | **T** | **T** | **A** | **G** | **C** | **A** | **C** | **C** | **T** | **T** | **T** | **A** | **T** | **T** | **G** | **T** | **T** | **A** | **T** | **C** | **T** | **T** | **A** | **A** | **T** | **T** | **A** | **C** | **A** | **T** | **T** | **G** | **T** | **T** | **T** | **T** | **C** | **A** | **T** | **A** | **T** | **T** | **T** | **T** | **C** | **A** | **T** | **T** | **C** | **G** | **A** | **T** | **T** | **A** | **A** | **A** | **C** | **A** | **A** | **A** | **A** | **C** | **C** | **G** | **G** | **T** | **G** | **G** | **T** | **T** | **A** | **T** | **T** | **A** | **G** | **A** | **G** | **C** | **T** | **T** | **C** | **T** | **T** | **C** | **A** | **G** | **A** | **G** | **A** | **A** | **A** | **A** | **G** | **T** | **C** | **T** | **T** | **T** | **T** | **A** | **T** | **T** | **T** | **A** | **C** | **C** | **A** | **C** | **T** | **A** | **A** | **T** | **T** | **G** | **T** | **G** | **C** | **A** | **C** | **A** | **T** | **T** | **G** | **T** | **T** | **C** | **T** | **C** | **A** | **T** | **A** | **T** | **C** | **C** | **T** | **T** | **A** | **C** | **T** | **A** | **A** | **T** | **A** | **G** | **G** | **C** | **T** | **T** | **A** | **T** | **T** | **G** | **A** | **C** | **C** | **T** | **T** | **T** | **T** | **T** | **A** | **C** | **T** | **G** | **A** | **T** | **T** | **T** | **C** | **A** | **T** | **T** | **G** | **G** | **T** | **T** | **C** | **A** | **A** | **A** | **A** | **A** | **G** | **G** | **G** | **C** | **A** | **A** | **T** | **A** | **T** | **G** | **G** | **A** | **C** | **G** | **G** | **A** | **T** | **T** | **G** | **A** | **A** | **G** | **A** | **A** | **A** | **A** | **A** | **C** | **T** | **T** | **C** | **G** | **G** | **T** | **T** | **A** | **T** | **T** | **G** | **G** | **C** | **C** | **A** | **A** | **C** | **G** | **G** | **T** | **A** | **A** | **T** | **T** | **A** | **T** | **G** | **A** | **A** | **A** | **G** | **T** | **C** | **C** | **A** | **G** | **T** | **C** | **T** | **T** | **A** | **A** | **A** | **C** | **A** | **A** | **A** | **C** | **C** | **A** | **A** | **C** | **G** | **A** | **C** | **C** | **A** | **G** | **T** | **G** | **A** | **A** | **A** | **A** | **T** | **C** | **A** | **A** | **G** | **A** | **C** | **C** | **A** | **T** | **T** | **A** | **T** | **C** | **T** | **A** | **A** | **C** | **C** | **G** | **A** | **A** | **G** | **T** | **C** | **G** | **A** | **A** | **C** | **A** | **A** | **G** | **A** | **T** | **A** | **T** | **T** | **T** | **G** | **G** | **T** | **C** | **G** | **A** | **T** | **T** | **A** | **A** | **A** | **A** | **A** | **T** | **A** | **A** | **A** | **T** | **T** | **A** | **T** | **T** | **A** | **G** | **A** | **G** | **A** | **T** | **G** | **T** | **C** | **T** | **A** | **A** | **A** | **G** | **A** | **A** | **T** | **T** | **G** | **C** | **A** | **A** | **T** | **T** | **A** | **T** | **T** | **A** | **A** | **A** | **C** | **A** | **G** | **T** | **C** | **G** | **A** | **C** | **C** | **G** | **C** | **A** | **A** | **T** | **T** | **A** | **A** | **T** | **G** | **G** | **A** | **T** | **G** | **G** | **G** | **C** | **A** | **A** | **A** | **C** | **A** | **A** | **A** | **A** | **G** | **A** | **A** | **G** | **A** | **A** | **A** | **T** | **T** | **T** | **T** | **A** | **G** | **A** | **G** | **A** | **A** | **C** | **G** | **A** | **G** | **C** | **G** | **G** | **C** | **A** | **T** | **C** | **G** | **T** | **T** | **T** | **G** | **G** | **C** | **G** | **C** | **G** | **G** | **G** | **A** | **G** | **T** | **T** | **G** | **C** | **A** | **T** | **G** | **A** | **T** | **T** | **C** | **A** | **G** | **T** | **C** | **A** | **G** | **T** | **C** | **A** | **A** | **C** | **A** | **A** | **C** | **T** | **T** | **T** | **T** | **T** | **G** | **C** | **A** | **G** | **C** | **C** | **A** | **T** | **G** | **A** | **T** | **G** | **A** | **T** | **G** | **T** | **T** | **G** | **T** | **C** | **T** | **G** | **C** | **A** | **T** | **T** | **A** | **A** | **A** | **T** | **G** | **A** | **A** | **C** | **A** | **A** | **G** | **C** | **A** | **C** | **A** | **A** | **C** | **G** | **A** | **A** | **C** | **A** | **G** | **A** | **A** | **A** | **C** | **C** | **C** | **C** | **G** | **G** | **A** | **A** | **C** | **C** | **A** | **T** | **A** | **T** | **C** | **G** | **T** | **A** | **A** | **A** | **C** | **A** | **A** | **C** | **T** | **A** | **G** | **C** | **C** | **A** | **T** | **G** | **G** | **T** | **G** | **G** | **C** | **A** | **G** | **A** | **A** | **A** | **T** | **C** | **A** | **T** | **T** | **A** | **A** | **T** | **G** | **C** | **C** | **T** | **C** | **C** | **C** | **A** | **A** | **T** | **C** | **G** | **G** | **A** | **A** | **A** | **T** | **G** | **C** | **G** | **C** | **G** | **C** | **G** | **C** | **T** | **A** | **C** | **T** | **A** | **T** | **T** | **G** | **C** | **A** | **C** | **T** | **T** | **G** | **C** | **G** | **T** | **C** | **C** | **T** | **A** | **T** | **C** | **A** | **G** | **T** | **C** | **T** | **A** | **G** | **A** | **A** | **G** | **G** | **A** | **A** | **A** | **A** | **A** | **G** | **T** | **T** | **T** | **G** | **C** | **G** | **T** | **A** | **A** | **A** | **G** | **G** | **T** | **A** | **T** | **T** | **G** | **A** | **A** | **C** | **A** | **A** | **T** | **T** | **A** | **C** | **T** | **G** | **A** | **A** | **A** | **G** | **A** | **A** | **C** | **T** | **A** | **C** | **A** | **A** | **A** | **C** | **A** | **A** | **A** | **A** | **A** | **T** | **T** | **A** | **A** | **A** | **A** | **T** | **T** | **G** | **A** | **A** | **T** | **T** | **G** | **A** | **T** | **T** | **T** | **G** | **G** | **G** | **A** | **T** | **G** | **T** | **T** | **G** | **A** | **A** | **G** | **A** | **T** | **G** | **T** | **T** | **C** | **A** | **T** | **T** | **T** | **A** | **A** | **A** | **T** | **A** | **G** | **C** | **A** | **G** | **C** | **A** | **T** | **T** | **G** | **A** | **G** | **G** | **A** | **T** | **C** | **A** | **T** | **C** | **T** | **T** | **T** | **T** | **C** | **C** | **G** | **A** | **A** | **T** | **T** | **G** | **T** | **G** | **C** | **A** | **G** | **G** | **A** | **G** | **T** | **T** | **A** | **C** | **T** | **T** | **T** | **C** | **A** | **A** | **A** | **T** | **A** | **C** | **C** | **T** | **T** | **A** | **A** | **G** | **A** | **C** | **A** | **T** | **G** | **C** | **C** | **A** | **A** | **A** | **G** | **C** | **A** | **A** | **A** | **G** | **G** | **A** | **A** | **T** | **T** | **A** | **G** | **A** | **G** | **G** | **T** | **A** | **T** | **A** | **C** | **T** | **T** | **A** | **C** | **A** | **C** | **C** | **A** | **A** | **G** | **T** | **C** | **G** | **A** | **T** | **A** | **A** | **A** | **A** | **A** | **C** | **G** | **T** | **G** | **T** | **T** | **A** | **T** | **T** | **G** | **C** | **G** | **T** | **A** | **T** | **T** | **G** | **T** | **T** | **G** | **A** | **T** | **G** | **A** | **T** | **G** | **G** | **T** | **G** | **T** | **C** | **G** | **G** | **C** | **T** | **T** | **T** | **G** | **A** | **T** | **A** | **T** | **G** | **A** | **A** | **G** | **G** | **A** | **A** | **C** | **A** | **A** | **A** | **G** | **T** | **A** | **A** | **T** | **A** | **A** | **A** | **G** | **C** | **C** | **G** | **G** | **T** | **A** | **G** | **T** | **T** | **A** | **T** | **G** | **G** | **C** | **T** | **T** | **A** | **A** | **A** | **T** | **A** | **A** | **T** | **A** | **T** | **C** | **C** | **G** | **A** | **G** | **A** | **A** | **C** | **G** | **T** | **G** | **T** | **T** | **G** | **T** | **C** | **G** | **G** | **C** | **A** | **T** | **G** | **G** | **G** | **C** | **G** | **G** | **T** | **A** | **C** | **A** | **G** | **T** | **T** | **A** | **A** | **A** | **A** | **T** | **T** | **A** | **T** | **T** | **A** | **G** | **T** | **T** | **T** | **T** | **A** | **A** | **A** | **G** | **G** | **G** | **C** | **A** | **G** | **G** | **G** | **A** | **A** | **C** | **C** | **A** | **G** | **C** | **G** | **T** | **T** | **G** | **A** | **A** | **A** | **T** | **T** | **A** | **A** | **A** | **G** | **T** | **T** | **C** | **C** | **T** | **G** | **T** | **C** | **A** | **T** | **A** | **A** | **A** | **G** | **G** | **A** | **G** | **G** | **A** | **A** | **A** | **C** | **T** | **G** | **C** | **A** | **A** | **G** | **T** | **G** | **A** | **T** | **C** | **A** | **A** | **A** | **G** | **T** | **A** | **A** | **T** | **G** | **T** | **T** | **A** | **G** | **T** | **G** | **G** | **A** | **-** | **-** | **-** |
| **N55113** | **A** | **T** | **G** | **A** | **C** | **C** | **G** | **A** | **T** | **C** | **G** | **G** | **A** | **T** | **T** | **T** | **C** | **A** | **A** | **G** | **A** | **C** | **G** | **C** | **A** | **T** | **G** | **A** | **T** | **T** | **T** | **C** | **A** | **T** | **T** | **A** | **T** | **A** | **T** | **G** | **C** | **G** | **T** | **C** | **C** | **C** | **T** | **T** | **A** | **G** | **C** | **A** | **C** | **C** | **T** | **T** | **T** | **A** | **T** | **T** | **G** | **T** | **T** | **A** | **T** | **C** | **T** | **T** | **A** | **A** | **T** | **T** | **A** | **C** | **A** | **T** | **T** | **G** | **T** | **T** | **T** | **T** | **C** | **A** | **T** | **A** | **T** | **T** | **T** | **T** | **C** | **A** | **T** | **T** | **C** | **G** | **A** | **T** | **T** | **A** | **A** | **A** | **C** | **A** | **A** | **A** | **A** | **C** | **C** | **G** | **G** | **T** | **G** | **G** | **C** | **T** | **A** | **T** | **T** | **A** | **G** | **A** | **G** | **C** | **T** | **T** | **C** | **T** | **T** | **C** | **A** | **G** | **A** | **G** | **A** | **A** | **A** | **A** | **G** | **T** | **C** | **T** | **T** | **T** | **T** | **A** | **T** | **T** | **T** | **A** | **C** | **C** | **A** | **C** | **T** | **A** | **A** | **T** | **T** | **G** | **T** | **G** | **C** | **A** | **C** | **A** | **T** | **T** | **G** | **T** | **T** | **C** | **T** | **C** | **A** | **T** | **A** | **T** | **C** | **C** | **T** | **T** | **A** | **C** | **T** | **A** | **A** | **T** | **A** | **G** | **G** | **C** | **T** | **T** | **A** | **T** | **T** | **G** | **A** | **C** | **C** | **T** | **T** | **T** | **T** | **T** | **A** | **C** | **T** | **G** | **A** | **T** | **T** | **T** | **C** | **A** | **T** | **T** | **G** | **G** | **T** | **T** | **C** | **A** | **A** | **A** | **A** | **A** | **G** | **G** | **G** | **C** | **A** | **A** | **T** | **A** | **T** | **G** | **G** | **A** | **C** | **G** | **G** | **A** | **T** | **T** | **G** | **A** | **A** | **G** | **A** | **A** | **A** | **A** | **A** | **C** | **T** | **T** | **C** | **G** | **G** | **T** | **T** | **A** | **T** | **T** | **G** | **G** | **C** | **C** | **A** | **A** | **C** | **G** | **G** | **T** | **A** | **A** | **T** | **T** | **A** | **T** | **G** | **A** | **A** | **A** | **G** | **T** | **C** | **C** | **A** | **G** | **T** | **C** | **T** | **T** | **A** | **A** | **A** | **C** | **A** | **A** | **A** | **C** | **C** | **A** | **A** | **C** | **G** | **A** | **C** | **C** | **A** | **G** | **T** | **G** | **A** | **A** | **A** | **A** | **T** | **C** | **A** | **A** | **G** | **A** | **T** | **C** | **A** | **T** | **T** | **A** | **T** | **C** | **T** | **A** | **A** | **C** | **C** | **G** | **A** | **A** | **G** | **T** | **C** | **G** | **A** | **A** | **C** | **A** | **A** | **G** | **A** | **T** | **A** | **T** | **T** | **T** | **G** | **G** | **T** | **C** | **G** | **A** | **T** | **T** | **A** | **A** | **A** | **A** | **A** | **T** | **A** | **A** | **A** | **T** | **T** | **A** | **T** | **T** | **A** | **G** | **A** | **G** | **A** | **T** | **G** | **T** | **C** | **T** | **A** | **A** | **A** | **G** | **A** | **A** | **T** | **T** | **G** | **C** | **A** | **A** | **T** | **T** | **A** | **T** | **T** | **A** | **A** | **A** | **C** | **A** | **G** | **T** | **C** | **G** | **A** | **C** | **C** | **G** | **C** | **A** | **A** | **T** | **T** | **A** | **A** | **T** | **G** | **G** | **A** | **T** | **G** | **G** | **G** | **C** | **A** | **A** | **A** | **C** | **A** | **A** | **A** | **A** | **G** | **A** | **A** | **G** | **A** | **A** | **A** | **T** | **T** | **T** | **T** | **A** | **G** | **A** | **G** | **A** | **A** | **C** | **G** | **A** | **G** | **C** | **G** | **G** | **C** | **A** | **T** | **C** | **G** | **T** | **T** | **T** | **G** | **G** | **C** | **G** | **C** | **G** | **G** | **G** | **A** | **G** | **T** | **T** | **G** | **C** | **A** | **T** | **G** | **A** | **T** | **T** | **C** | **A** | **G** | **T** | **C** | **A** | **G** | **T** | **C** | **A** | **A** | **C** | **A** | **A** | **C** | **T** | **T** | **T** | **T** | **T** | **G** | **C** | **A** | **G** | **C** | **C** | **A** | **T** | **G** | **A** | **T** | **G** | **A** | **T** | **G** | **T** | **T** | **G** | **T** | **C** | **T** | **G** | **C** | **C** | **T** | **T** | **A** | **A** | **A** | **T** | **G** | **A** | **A** | **C** | **A** | **A** | **G** | **C** | **A** | **C** | **A** | **A** | **C** | **G** | **A** | **A** | **C** | **A** | **G** | **A** | **A** | **A** | **C** | **C** | **C** | **C** | **T** | **G** | **A** | **A** | **C** | **C** | **A** | **T** | **A** | **T** | **C** | **G** | **T** | **A** | **A** | **A** | **C** | **A** | **A** | **C** | **T** | **A** | **G** | **C** | **C** | **A** | **T** | **G** | **G** | **T** | **G** | **G** | **C** | **A** | **G** | **A** | **A** | **A** | **T** | **C** | **A** | **T** | **T** | **A** | **A** | **T** | **G** | **C** | **C** | **T** | **C** | **C** | **C** | **A** | **A** | **T** | **C** | **G** | **G** | **A** | **A** | **A** | **T** | **G** | **C** | **G** | **C** | **G** | **C** | **G** | **T** | **T** | **A** | **C** | **T** | **A** | **T** | **T** | **G** | **C** | **A** | **C** | **T** | **T** | **G** | **C** | **G** | **T** | **C** | **C** | **T** | **A** | **T** | **C** | **A** | **G** | **T** | **C** | **T** | **A** | **G** | **A** | **A** | **G** | **G** | **A** | **A** | **A** | **A** | **A** | **G** | **T** | **T** | **T** | **G** | **C** | **G** | **T** | **A** | **A** | **A** | **G** | **G** | **T** | **A** | **T** | **T** | **G** | **A** | **A** | **C** | **A** | **A** | **T** | **T** | **A** | **C** | **T** | **G** | **A** | **A** | **A** | **G** | **A** | **A** | **C** | **T** | **A** | **C** | **A** | **A** | **A** | **C** | **A** | **A** | **A** | **A** | **A** | **T** | **T** | **A** | **A** | **A** | **A** | **T** | **T** | **G** | **A** | **A** | **T** | **T** | **G** | **A** | **T** | **T** | **T** | **G** | **G** | **G** | **A** | **T** | **G** | **T** | **T** | **G** | **A** | **A** | **G** | **A** | **T** | **G** | **T** | **T** | **C** | **A** | **T** | **T** | **T** | **A** | **A** | **A** | **T** | **A** | **G** | **C** | **A** | **G** | **C** | **A** | **T** | **T** | **G** | **A** | **G** | **G** | **A** | **T** | **C** | **A** | **T** | **C** | **T** | **T** | **T** | **T** | **C** | **C** | **G** | **A** | **A** | **T** | **T** | **G** | **T** | **G** | **C** | **A** | **G** | **G** | **A** | **G** | **T** | **T** | **A** | **C** | **T** | **T** | **T** | **C** | **A** | **A** | **A** | **T** | **A** | **C** | **C** | **T** | **T** | **A** | **A** | **G** | **A** | **C** | **A** | **T** | **G** | **C** | **C** | **A** | **A** | **A** | **G** | **C** | **A** | **A** | **A** | **G** | **G** | **A** | **A** | **T** | **T** | **A** | **G** | **A** | **G** | **G** | **T** | **A** | **T** | **A** | **C** | **T** | **T** | **A** | **C** | **A** | **C** | **C** | **A** | **A** | **G** | **T** | **C** | **G** | **A** | **T** | **A** | **A** | **A** | **A** | **A** | **C** | **G** | **T** | **G** | **C** | **T** | **A** | **T** | **T** | **G** | **C** | **G** | **T** | **A** | **T** | **T** | **G** | **T** | **T** | **G** | **A** | **T** | **G** | **A** | **C** | **G** | **G** | **T** | **G** | **T** | **C** | **G** | **G** | **C** | **T** | **T** | **T** | **G** | **A** | **T** | **A** | **T** | **G** | **A** | **A** | **G** | **G** | **A** | **A** | **C** | **A** | **A** | **A** | **G** | **T** | **A** | **A** | **T** | **A** | **A** | **A** | **G** | **C** | **C** | **G** | **G** | **T** | **A** | **G** | **T** | **T** | **A** | **T** | **G** | **G** | **C** | **T** | **T** | **A** | **A** | **A** | **T** | **A** | **A** | **T** | **A** | **T** | **T** | **C** | **G** | **A** | **G** | **A** | **A** | **C** | **G** | **T** | **G** | **T** | **T** | **G** | **T** | **C** | **G** | **G** | **C** | **A** | **T** | **G** | **G** | **G** | **C** | **G** | **G** | **T** | **A** | **C** | **A** | **G** | **T** | **T** | **A** | **A** | **A** | **A** | **T** | **T** | **A** | **T** | **T** | **A** | **G** | **T** | **T** | **T** | **T** | **A** | **A** | **A** | **G** | **G** | **G** | **C** | **A** | **G** | **G** | **G** | **A** | **A** | **C** | **C** | **A** | **G** | **C** | **G** | **T** | **T** | **G** | **A** | **A** | **A** | **T** | **T** | **A** | **A** | **A** | **G** | **T** | **T** | **C** | **C** | **T** | **G** | **T** | **C** | **A** | **T** | **A** | **A** | **A** | **G** | **G** | **A** | **G** | **G** | **A** | **A** | **A** | **C** | **T** | **G** | **C** | **A** | **A** | **G** | **T** | **G** | **A** | **T** | **C** | **A** | **A** | **A** | **G** | **T** | **A** | **A** | **T** | **G** | **T** | **T** | **A** | **G** | **T** | **G** | **G** | **A** | **-** | **-** | **-** |
| **N59550F** | **A** | **T** | **G** | **A** | **C** | **C** | **G** | **A** | **T** | **C** | **G** | **G** | **A** | **T** | **T** | **T** | **C** | **A** | **A** | **G** | **A** | **C** | **G** | **C** | **A** | **T** | **G** | **A** | **T** | **T** | **T** | **C** | **A** | **T** | **T** | **A** | **T** | **A** | **T** | **G** | **C** | **G** | **T** | **C** | **C** | **C** | **T** | **T** | **A** | **G** | **C** | **A** | **C** | **C** | **T** | **T** | **T** | **A** | **T** | **T** | **G** | **T** | **T** | **A** | **T** | **C** | **T** | **T** | **A** | **A** | **T** | **T** | **A** | **C** | **A** | **T** | **T** | **G** | **T** | **T** | **T** | **T** | **C** | **A** | **T** | **A** | **T** | **T** | **T** | **T** | **C** | **A** | **T** | **T** | **C** | **G** | **A** | **T** | **T** | **A** | **A** | **A** | **C** | **A** | **A** | **A** | **A** | **C** | **C** | **G** | **G** | **T** | **G** | **G** | **T** | **T** | **A** | **T** | **T** | **A** | **G** | **A** | **G** | **C** | **T** | **T** | **C** | **T** | **T** | **C** | **A** | **G** | **A** | **G** | **A** | **A** | **A** | **A** | **G** | **T** | **C** | **T** | **T** | **T** | **T** | **A** | **T** | **T** | **T** | **A** | **C** | **C** | **A** | **C** | **T** | **A** | **A** | **T** | **T** | **G** | **T** | **G** | **C** | **A** | **C** | **A** | **T** | **T** | **G** | **T** | **T** | **C** | **T** | **C** | **A** | **T** | **A** | **T** | **C** | **C** | **T** | **T** | **A** | **C** | **T** | **A** | **A** | **T** | **A** | **G** | **G** | **C** | **T** | **T** | **A** | **T** | **T** | **G** | **A** | **C** | **C** | **T** | **T** | **T** | **T** | **T** | **A** | **C** | **T** | **G** | **A** | **T** | **T** | **T** | **C** | **A** | **T** | **T** | **G** | **G** | **T** | **T** | **C** | **A** | **A** | **A** | **A** | **A** | **G** | **G** | **G** | **C** | **A** | **A** | **T** | **A** | **T** | **G** | **G** | **A** | **C** | **G** | **G** | **A** | **T** | **T** | **G** | **A** | **A** | **G** | **A** | **A** | **A** | **A** | **A** | **C** | **T** | **T** | **C** | **G** | **G** | **T** | **T** | **A** | **T** | **T** | **G** | **G** | **C** | **C** | **A** | **A** | **C** | **G** | **G** | **T** | **A** | **A** | **T** | **T** | **A** | **T** | **G** | **A** | **A** | **A** | **G** | **T** | **C** | **C** | **A** | **G** | **T** | **C** | **T** | **T** | **A** | **A** | **A** | **C** | **A** | **A** | **A** | **C** | **C** | **A** | **A** | **C** | **G** | **A** | **C** | **C** | **A** | **G** | **T** | **G** | **A** | **A** | **A** | **A** | **T** | **C** | **A** | **A** | **G** | **A** | **C** | **C** | **A** | **T** | **T** | **A** | **T** | **C** | **T** | **A** | **A** | **C** | **C** | **G** | **A** | **A** | **G** | **T** | **C** | **G** | **A** | **A** | **C** | **A** | **A** | **G** | **A** | **T** | **A** | **T** | **T** | **T** | **G** | **G** | **T** | **C** | **G** | **A** | **T** | **T** | **A** | **A** | **A** | **A** | **A** | **T** | **A** | **A** | **A** | **T** | **T** | **A** | **T** | **T** | **A** | **G** | **A** | **G** | **A** | **T** | **G** | **T** | **C** | **T** | **A** | **A** | **A** | **G** | **A** | **A** | **T** | **T** | **G** | **C** | **A** | **A** | **T** | **T** | **A** | **T** | **T** | **A** | **A** | **A** | **C** | **A** | **G** | **T** | **C** | **G** | **A** | **C** | **C** | **G** | **C** | **A** | **A** | **T** | **T** | **A** | **A** | **T** | **G** | **G** | **A** | **T** | **G** | **G** | **G** | **C** | **A** | **A** | **A** | **C** | **A** | **A** | **A** | **A** | **G** | **A** | **A** | **G** | **A** | **A** | **A** | **T** | **T** | **T** | **T** | **A** | **G** | **A** | **G** | **A** | **A** | **C** | **G** | **A** | **G** | **C** | **G** | **G** | **C** | **A** | **T** | **C** | **G** | **T** | **T** | **T** | **G** | **G** | **C** | **G** | **C** | **G** | **G** | **G** | **A** | **G** | **T** | **T** | **G** | **C** | **A** | **T** | **G** | **A** | **T** | **T** | **C** | **A** | **G** | **T** | **C** | **A** | **G** | **T** | **C** | **A** | **A** | **C** | **A** | **A** | **C** | **T** | **T** | **T** | **T** | **T** | **G** | **C** | **A** | **G** | **C** | **C** | **A** | **T** | **G** | **A** | **T** | **G** | **A** | **T** | **G** | **T** | **T** | **G** | **T** | **C** | **T** | **G** | **C** | **A** | **T** | **T** | **A** | **A** | **A** | **T** | **G** | **A** | **A** | **C** | **A** | **A** | **G** | **C** | **A** | **C** | **A** | **A** | **C** | **G** | **A** | **A** | **C** | **A** | **G** | **A** | **A** | **A** | **C** | **C** | **C** | **C** | **G** | **G** | **A** | **A** | **C** | **C** | **A** | **T** | **A** | **T** | **C** | **G** | **T** | **A** | **A** | **A** | **C** | **A** | **A** | **C** | **T** | **A** | **G** | **C** | **C** | **A** | **T** | **G** | **G** | **T** | **G** | **G** | **C** | **A** | **G** | **A** | **A** | **A** | **T** | **C** | **A** | **T** | **T** | **A** | **A** | **T** | **G** | **C** | **C** | **T** | **C** | **C** | **C** | **A** | **A** | **T** | **C** | **G** | **G** | **A** | **A** | **A** | **T** | **G** | **C** | **G** | **C** | **G** | **C** | **G** | **C** | **T** | **A** | **C** | **T** | **A** | **T** | **T** | **G** | **C** | **A** | **C** | **T** | **T** | **G** | **C** | **G** | **T** | **C** | **C** | **T** | **A** | **T** | **C** | **A** | **G** | **T** | **C** | **T** | **A** | **G** | **A** | **A** | **G** | **G** | **A** | **A** | **A** | **A** | **A** | **G** | **T** | **T** | **T** | **G** | **C** | **G** | **T** | **A** | **A** | **A** | **G** | **G** | **T** | **A** | **T** | **T** | **G** | **A** | **A** | **C** | **A** | **A** | **T** | **T** | **A** | **C** | **T** | **G** | **A** | **A** | **A** | **G** | **A** | **A** | **C** | **T** | **A** | **C** | **A** | **A** | **A** | **C** | **A** | **A** | **A** | **A** | **A** | **T** | **T** | **A** | **A** | **A** | **A** | **T** | **T** | **G** | **A** | **A** | **T** | **T** | **G** | **A** | **T** | **T** | **T** | **G** | **G** | **G** | **A** | **T** | **G** | **T** | **T** | **G** | **A** | **A** | **G** | **A** | **T** | **G** | **T** | **T** | **C** | **A** | **T** | **T** | **T** | **A** | **A** | **A** | **T** | **A** | **G** | **C** | **A** | **G** | **C** | **A** | **T** | **T** | **G** | **A** | **G** | **G** | **A** | **T** | **C** | **A** | **T** | **C** | **T** | **T** | **T** | **T** | **C** | **C** | **G** | **A** | **A** | **T** | **T** | **G** | **T** | **G** | **C** | **A** | **G** | **G** | **A** | **G** | **T** | **T** | **A** | **C** | **T** | **T** | **T** | **C** | **A** | **A** | **A** | **T** | **A** | **C** | **C** | **T** | **T** | **A** | **A** | **G** | **A** | **C** | **A** | **T** | **G** | **C** | **C** | **A** | **A** | **A** | **G** | **C** | **A** | **A** | **A** | **G** | **G** | **A** | **A** | **T** | **T** | **A** | **G** | **A** | **G** | **G** | **T** | **A** | **T** | **A** | **C** | **T** | **T** | **A** | **C** | **A** | **C** | **C** | **A** | **A** | **G** | **T** | **C** | **G** | **A** | **T** | **A** | **A** | **A** | **A** | **A** | **C** | **G** | **T** | **G** | **C** | **T** | **A** | **T** | **T** | **G** | **C** | **G** | **T** | **A** | **T** | **T** | **G** | **T** | **T** | **G** | **A** | **T** | **G** | **A** | **C** | **G** | **G** | **T** | **G** | **T** | **C** | **G** | **G** | **C** | **T** | **T** | **T** | **G** | **A** | **T** | **A** | **T** | **G** | **A** | **A** | **G** | **G** | **A** | **A** | **C** | **A** | **A** | **A** | **G** | **T** | **A** | **A** | **T** | **A** | **A** | **A** | **G** | **C** | **C** | **G** | **G** | **T** | **A** | **G** | **T** | **T** | **A** | **T** | **G** | **G** | **C** | **T** | **T** | **A** | **A** | **A** | **T** | **A** | **A** | **T** | **A** | **T** | **C** | **C** | **G** | **A** | **G** | **A** | **A** | **C** | **G** | **T** | **G** | **T** | **T** | **G** | **T** | **C** | **G** | **G** | **C** | **A** | **T** | **G** | **G** | **G** | **C** | **G** | **G** | **T** | **A** | **C** | **A** | **G** | **T** | **T** | **A** | **A** | **A** | **A** | **T** | **T** | **A** | **T** | **T** | **A** | **G** | **T** | **T** | **T** | **T** | **A** | **A** | **A** | **G** | **G** | **G** | **C** | **A** | **G** | **G** | **G** | **A** | **A** | **C** | **C** | **A** | **G** | **C** | **G** | **T** | **T** | **G** | **A** | **A** | **A** | **T** | **T** | **A** | **A** | **A** | **G** | **T** | **T** | **C** | **C** | **T** | **G** | **T** | **C** | **A** | **T** | **A** | **A** | **A** | **G** | **G** | **A** | **G** | **G** | **A** | **A** | **A** | **C** | **T** | **G** | **C** | **A** | **A** | **G** | **T** | **G** | **A** | **T** | **C** | **A** | **A** | **A** | **G** | **T** | **A** | **A** | **T** | **G** | **T** | **T** | **A** | **G** | **T** | **G** | **G** | **A** | **-** | **-** | **-** |
| **N59862F** | **A** | **T** | **G** | **A** | **C** | **C** | **G** | **A** | **T** | **C** | **G** | **G** | **A** | **T** | **T** | **T** | **C** | **A** | **A** | **G** | **A** | **C** | **G** | **C** | **A** | **T** | **G** | **A** | **T** | **T** | **T** | **C** | **A** | **T** | **T** | **A** | **T** | **A** | **T** | **G** | **C** | **G** | **T** | **C** | **C** | **C** | **T** | **T** | **A** | **G** | **C** | **A** | **C** | **C** | **T** | **T** | **T** | **A** | **T** | **T** | **G** | **T** | **T** | **A** | **T** | **C** | **T** | **T** | **A** | **A** | **T** | **T** | **A** | **C** | **A** | **T** | **T** | **G** | **T** | **T** | **T** | **T** | **C** | **A** | **T** | **A** | **T** | **T** | **T** | **T** | **C** | **A** | **T** | **T** | **C** | **G** | **A** | **T** | **T** | **A** | **A** | **A** | **C** | **A** | **A** | **A** | **A** | **C** | **C** | **G** | **G** | **T** | **G** | **G** | **T** | **T** | **A** | **T** | **T** | **A** | **G** | **A** | **G** | **C** | **T** | **T** | **C** | **T** | **T** | **C** | **A** | **G** | **A** | **G** | **A** | **A** | **A** | **A** | **G** | **T** | **C** | **T** | **T** | **T** | **T** | **A** | **T** | **C** | **T** | **A** | **C** | **C** | **A** | **C** | **T** | **A** | **A** | **T** | **T** | **G** | **T** | **G** | **C** | **A** | **C** | **A** | **T** | **T** | **G** | **T** | **T** | **C** | **T** | **C** | **A** | **T** | **A** | **T** | **C** | **C** | **T** | **T** | **A** | **C** | **T** | **A** | **A** | **T** | **A** | **G** | **G** | **C** | **T** | **T** | **A** | **T** | **T** | **G** | **A** | **C** | **C** | **T** | **T** | **T** | **T** | **T** | **A** | **C** | **T** | **G** | **A** | **T** | **T** | **T** | **C** | **A** | **T** | **T** | **G** | **G** | **T** | **T** | **C** | **A** | **A** | **A** | **A** | **A** | **G** | **G** | **G** | **C** | **A** | **A** | **T** | **A** | **T** | **G** | **G** | **A** | **C** | **G** | **G** | **A** | **T** | **T** | **G** | **A** | **A** | **G** | **A** | **A** | **A** | **A** | **A** | **C** | **T** | **T** | **C** | **G** | **G** | **T** | **T** | **A** | **T** | **T** | **G** | **G** | **C** | **C** | **A** | **A** | **C** | **G** | **G** | **T** | **A** | **A** | **T** | **T** | **A** | **T** | **G** | **A** | **A** | **A** | **G** | **T** | **C** | **C** | **A** | **G** | **T** | **C** | **T** | **T** | **A** | **A** | **A** | **C** | **A** | **A** | **A** | **C** | **C** | **A** | **A** | **C** | **G** | **A** | **C** | **C** | **A** | **G** | **T** | **G** | **A** | **A** | **A** | **A** | **T** | **C** | **A** | **A** | **G** | **A** | **T** | **C** | **A** | **T** | **T** | **A** | **T** | **C** | **T** | **A** | **A** | **C** | **C** | **G** | **A** | **A** | **G** | **T** | **C** | **G** | **A** | **A** | **C** | **A** | **A** | **G** | **A** | **T** | **A** | **T** | **T** | **T** | **G** | **G** | **T** | **C** | **G** | **A** | **T** | **T** | **A** | **A** | **A** | **A** | **A** | **T** | **A** | **A** | **A** | **T** | **T** | **A** | **T** | **T** | **A** | **G** | **A** | **G** | **A** | **T** | **G** | **T** | **C** | **T** | **A** | **A** | **A** | **G** | **A** | **A** | **T** | **T** | **G** | **C** | **A** | **A** | **T** | **T** | **A** | **T** | **T** | **A** | **A** | **A** | **C** | **A** | **G** | **T** | **C** | **G** | **A** | **C** | **C** | **G** | **C** | **A** | **A** | **T** | **T** | **A** | **A** | **T** | **G** | **G** | **A** | **T** | **G** | **G** | **G** | **C** | **A** | **A** | **A** | **C** | **A** | **A** | **A** | **A** | **G** | **A** | **A** | **G** | **A** | **A** | **A** | **T** | **T** | **T** | **T** | **A** | **G** | **A** | **G** | **A** | **A** | **C** | **G** | **A** | **G** | **C** | **G** | **G** | **C** | **A** | **T** | **C** | **G** | **T** | **T** | **T** | **G** | **G** | **C** | **G** | **C** | **G** | **G** | **G** | **A** | **G** | **T** | **T** | **G** | **C** | **A** | **T** | **G** | **A** | **T** | **T** | **C** | **A** | **G** | **T** | **C** | **A** | **G** | **T** | **C** | **A** | **A** | **C** | **A** | **A** | **C** | **T** | **T** | **T** | **T** | **T** | **G** | **C** | **A** | **G** | **C** | **C** | **A** | **T** | **G** | **A** | **T** | **G** | **A** | **T** | **G** | **T** | **T** | **G** | **T** | **C** | **T** | **G** | **C** | **C** | **T** | **T** | **A** | **A** | **A** | **T** | **G** | **A** | **A** | **C** | **A** | **A** | **G** | **C** | **A** | **C** | **A** | **A** | **C** | **G** | **A** | **A** | **C** | **A** | **G** | **A** | **A** | **A** | **C** | **C** | **C** | **C** | **G** | **G** | **A** | **A** | **C** | **C** | **A** | **T** | **A** | **T** | **C** | **G** | **T** | **A** | **A** | **A** | **C** | **A** | **A** | **C** | **T** | **A** | **G** | **C** | **C** | **A** | **T** | **G** | **G** | **T** | **G** | **G** | **C** | **A** | **G** | **A** | **A** | **A** | **T** | **C** | **A** | **T** | **T** | **A** | **A** | **T** | **G** | **C** | **C** | **T** | **C** | **C** | **C** | **A** | **A** | **T** | **C** | **G** | **G** | **A** | **A** | **A** | **T** | **G** | **C** | **G** | **C** | **G** | **C** | **G** | **T** | **T** | **A** | **C** | **T** | **A** | **T** | **T** | **G** | **C** | **A** | **C** | **T** | **T** | **G** | **C** | **G** | **T** | **C** | **C** | **T** | **A** | **T** | **C** | **A** | **G** | **T** | **C** | **T** | **A** | **G** | **A** | **A** | **G** | **G** | **A** | **A** | **A** | **A** | **A** | **G** | **T** | **T** | **T** | **G** | **C** | **G** | **T** | **A** | **A** | **A** | **G** | **G** | **T** | **A** | **T** | **T** | **G** | **A** | **A** | **C** | **A** | **A** | **T** | **T** | **A** | **C** | **T** | **G** | **A** | **A** | **A** | **G** | **A** | **A** | **C** | **T** | **A** | **C** | **A** | **A** | **A** | **C** | **A** | **A** | **A** | **A** | **A** | **T** | **T** | **A** | **A** | **A** | **A** | **T** | **T** | **G** | **A** | **A** | **T** | **T** | **G** | **A** | **T** | **T** | **T** | **G** | **G** | **G** | **A** | **T** | **G** | **T** | **T** | **G** | **A** | **A** | **G** | **A** | **T** | **G** | **T** | **T** | **C** | **A** | **T** | **T** | **T** | **A** | **A** | **A** | **T** | **A** | **G** | **C** | **A** | **G** | **C** | **A** | **T** | **T** | **G** | **A** | **G** | **G** | **A** | **T** | **C** | **A** | **T** | **C** | **T** | **T** | **T** | **T** | **C** | **C** | **G** | **A** | **A** | **T** | **T** | **G** | **T** | **G** | **C** | **A** | **G** | **G** | **A** | **A** | **T** | **T** | **A** | **C** | **T** | **T** | **T** | **C** | **C** | **A** | **A** | **T** | **A** | **C** | **C** | **T** | **T** | **A** | **A** | **G** | **A** | **C** | **A** | **T** | **G** | **C** | **C** | **A** | **A** | **A** | **G** | **C** | **A** | **A** | **A** | **G** | **G** | **A** | **A** | **T** | **T** | **A** | **G** | **A** | **G** | **G** | **T** | **A** | **T** | **A** | **C** | **T** | **T** | **A** | **C** | **A** | **C** | **C** | **A** | **A** | **G** | **T** | **C** | **G** | **A** | **T** | **A** | **A** | **A** | **A** | **A** | **C** | **G** | **T** | **G** | **T** | **T** | **A** | **T** | **T** | **G** | **C** | **G** | **T** | **A** | **T** | **T** | **G** | **T** | **T** | **G** | **A** | **T** | **G** | **A** | **T** | **G** | **G** | **T** | **G** | **T** | **C** | **G** | **G** | **C** | **T** | **T** | **T** | **G** | **A** | **T** | **A** | **T** | **G** | **A** | **A** | **G** | **G** | **A** | **A** | **C** | **A** | **A** | **A** | **G** | **T** | **A** | **A** | **T** | **A** | **A** | **A** | **G** | **C** | **T** | **G** | **G** | **T** | **A** | **G** | **T** | **T** | **A** | **T** | **G** | **G** | **C** | **T** | **T** | **A** | **A** | **A** | **T** | **A** | **A** | **T** | **A** | **T** | **T** | **C** | **G** | **A** | **G** | **A** | **A** | **C** | **G** | **T** | **G** | **T** | **T** | **G** | **T** | **C** | **G** | **G** | **C** | **A** | **T** | **G** | **G** | **G** | **C** | **G** | **G** | **T** | **A** | **C** | **A** | **G** | **T** | **T** | **A** | **A** | **A** | **A** | **T** | **T** | **A** | **T** | **T** | **A** | **G** | **T** | **T** | **T** | **T** | **A** | **A** | **A** | **G** | **G** | **G** | **C** | **A** | **G** | **G** | **G** | **A** | **A** | **C** | **C** | **A** | **G** | **C** | **G** | **T** | **T** | **G** | **A** | **A** | **A** | **T** | **T** | **A** | **A** | **A** | **G** | **T** | **T** | **C** | **C** | **T** | **G** | **T** | **C** | **A** | **T** | **A** | **A** | **A** | **G** | **G** | **A** | **G** | **G** | **A** | **A** | **A** | **C** | **T** | **G** | **C** | **A** | **A** | **G** | **T** | **G** | **A** | **T** | **C** | **A** | **A** | **A** | **G** | **T** | **A** | **A** | **T** | **G** | **T** | **T** | **A** | **G** | **T** | **G** | **G** | **A** | **-** | **-** | **-** |
| **N53761** | **A** | **T** | **G** | **A** | **C** | **C** | **G** | **A** | **T** | **C** | **G** | **G** | **A** | **T** | **T** | **T** | **C** | **A** | **A** | **G** | **A** | **C** | **G** | **C** | **A** | **T** | **G** | **A** | **T** | **T** | **T** | **C** | **A** | **T** | **T** | **A** | **T** | **A** | **T** | **G** | **C** | **G** | **T** | **C** | **C** | **C** | **T** | **T** | **A** | **G** | **C** | **A** | **C** | **C** | **T** | **T** | **T** | **A** | **T** | **T** | **G** | **T** | **T** | **A** | **T** | **C** | **T** | **T** | **A** | **A** | **T** | **T** | **A** | **C** | **A** | **T** | **T** | **G** | **T** | **T** | **T** | **T** | **C** | **A** | **T** | **A** | **T** | **T** | **T** | **T** | **C** | **A** | **T** | **T** | **C** | **G** | **A** | **T** | **T** | **A** | **A** | **A** | **C** | **A** | **A** | **A** | **A** | **C** | **C** | **G** | **G** | **T** | **G** | **G** | **C** | **T** | **A** | **T** | **T** | **A** | **G** | **A** | **G** | **C** | **T** | **T** | **C** | **T** | **T** | **C** | **A** | **G** | **A** | **G** | **A** | **A** | **A** | **A** | **G** | **T** | **C** | **T** | **T** | **T** | **T** | **A** | **T** | **T** | **T** | **A** | **C** | **C** | **A** | **C** | **T** | **A** | **A** | **T** | **T** | **G** | **T** | **G** | **C** | **A** | **C** | **A** | **T** | **T** | **G** | **T** | **T** | **C** | **T** | **C** | **A** | **T** | **A** | **T** | **C** | **C** | **T** | **T** | **A** | **C** | **T** | **A** | **A** | **T** | **A** | **G** | **G** | **C** | **T** | **T** | **A** | **T** | **T** | **G** | **A** | **C** | **C** | **T** | **T** | **T** | **T** | **T** | **A** | **C** | **T** | **G** | **A** | **T** | **T** | **T** | **C** | **A** | **T** | **T** | **G** | **G** | **T** | **T** | **C** | **A** | **A** | **A** | **A** | **A** | **G** | **G** | **G** | **C** | **A** | **A** | **T** | **A** | **T** | **G** | **G** | **A** | **C** | **G** | **G** | **A** | **T** | **T** | **G** | **A** | **A** | **G** | **A** | **A** | **A** | **A** | **A** | **C** | **T** | **T** | **C** | **G** | **G** | **T** | **T** | **A** | **T** | **T** | **G** | **G** | **C** | **C** | **A** | **A** | **T** | **G** | **G** | **T** | **A** | **A** | **T** | **T** | **A** | **T** | **G** | **A** | **A** | **A** | **G** | **T** | **C** | **C** | **A** | **G** | **T** | **C** | **T** | **T** | **A** | **A** | **A** | **C** | **A** | **A** | **A** | **C** | **C** | **A** | **A** | **C** | **G** | **A** | **C** | **C** | **A** | **G** | **T** | **G** | **A** | **A** | **A** | **A** | **T** | **C** | **A** | **A** | **G** | **A** | **C** | **C** | **A** | **T** | **T** | **A** | **T** | **C** | **T** | **A** | **A** | **C** | **C** | **G** | **A** | **A** | **G** | **T** | **C** | **G** | **A** | **A** | **C** | **A** | **A** | **G** | **A** | **T** | **A** | **T** | **T** | **T** | **G** | **G** | **T** | **C** | **G** | **A** | **T** | **T** | **A** | **A** | **A** | **A** | **A** | **T** | **A** | **A** | **A** | **T** | **T** | **A** | **T** | **T** | **A** | **G** | **A** | **G** | **A** | **T** | **G** | **T** | **C** | **T** | **A** | **A** | **A** | **G** | **A** | **A** | **T** | **T** | **G** | **C** | **A** | **A** | **T** | **T** | **A** | **T** | **T** | **A** | **A** | **A** | **C** | **A** | **G** | **T** | **C** | **G** | **A** | **C** | **C** | **G** | **C** | **A** | **A** | **T** | **T** | **A** | **A** | **T** | **G** | **G** | **A** | **T** | **G** | **G** | **G** | **C** | **A** | **A** | **A** | **C** | **A** | **A** | **A** | **A** | **G** | **A** | **A** | **G** | **A** | **A** | **A** | **T** | **T** | **T** | **T** | **A** | **G** | **A** | **G** | **A** | **A** | **C** | **G** | **A** | **G** | **C** | **G** | **G** | **C** | **A** | **T** | **C** | **G** | **T** | **T** | **T** | **G** | **G** | **C** | **G** | **C** | **G** | **G** | **G** | **A** | **G** | **T** | **T** | **G** | **C** | **A** | **T** | **G** | **A** | **T** | **T** | **C** | **A** | **G** | **T** | **C** | **A** | **G** | **T** | **C** | **A** | **A** | **C** | **A** | **A** | **C** | **T** | **T** | **T** | **T** | **T** | **G** | **C** | **A** | **G** | **C** | **C** | **A** | **T** | **G** | **A** | **T** | **G** | **A** | **T** | **G** | **T** | **T** | **G** | **T** | **C** | **T** | **G** | **C** | **C** | **T** | **T** | **A** | **A** | **A** | **T** | **G** | **A** | **A** | **C** | **A** | **A** | **G** | **C** | **A** | **C** | **A** | **A** | **C** | **G** | **A** | **A** | **C** | **A** | **G** | **A** | **A** | **A** | **C** | **C** | **C** | **C** | **T** | **G** | **A** | **A** | **C** | **C** | **A** | **T** | **A** | **T** | **C** | **G** | **C** | **A** | **A** | **A** | **C** | **A** | **A** | **C** | **T** | **A** | **G** | **C** | **C** | **A** | **T** | **G** | **G** | **T** | **G** | **G** | **C** | **A** | **G** | **A** | **A** | **A** | **T** | **C** | **A** | **T** | **T** | **A** | **A** | **T** | **G** | **C** | **C** | **T** | **C** | **C** | **C** | **A** | **A** | **T** | **C** | **G** | **G** | **A** | **A** | **A** | **T** | **G** | **C** | **G** | **C** | **G** | **C** | **G** | **T** | **T** | **A** | **C** | **T** | **A** | **T** | **T** | **G** | **C** | **A** | **C** | **T** | **T** | **G** | **C** | **G** | **T** | **C** | **C** | **T** | **A** | **T** | **C** | **A** | **G** | **T** | **C** | **T** | **A** | **G** | **A** | **A** | **G** | **G** | **A** | **A** | **A** | **A** | **A** | **G** | **T** | **T** | **T** | **G** | **C** | **G** | **T** | **A** | **A** | **A** | **G** | **G** | **T** | **A** | **T** | **T** | **G** | **A** | **A** | **C** | **A** | **A** | **T** | **T** | **A** | **C** | **T** | **G** | **A** | **A** | **A** | **G** | **A** | **A** | **C** | **T** | **A** | **C** | **A** | **A** | **A** | **C** | **A** | **A** | **A** | **A** | **A** | **T** | **T** | **A** | **A** | **A** | **A** | **T** | **T** | **G** | **A** | **A** | **T** | **T** | **G** | **A** | **T** | **T** | **T** | **G** | **G** | **G** | **A** | **T** | **G** | **T** | **T** | **G** | **A** | **A** | **G** | **A** | **T** | **G** | **T** | **T** | **C** | **A** | **T** | **T** | **T** | **A** | **A** | **A** | **T** | **A** | **G** | **C** | **A** | **G** | **C** | **A** | **T** | **T** | **G** | **A** | **G** | **G** | **A** | **T** | **C** | **A** | **T** | **C** | **T** | **T** | **T** | **T** | **C** | **C** | **G** | **A** | **A** | **T** | **T** | **G** | **T** | **G** | **C** | **A** | **G** | **G** | **A** | **A** | **T** | **T** | **A** | **C** | **T** | **T** | **T** | **C** | **A** | **A** | **A** | **T** | **A** | **C** | **C** | **T** | **T** | **A** | **A** | **G** | **A** | **C** | **A** | **T** | **G** | **C** | **C** | **A** | **A** | **A** | **G** | **C** | **A** | **A** | **A** | **G** | **G** | **A** | **A** | **T** | **T** | **A** | **G** | **A** | **G** | **G** | **T** | **A** | **T** | **A** | **C** | **T** | **T** | **A** | **C** | **A** | **C** | **C** | **A** | **A** | **G** | **T** | **C** | **G** | **A** | **T** | **A** | **A** | **A** | **A** | **A** | **C** | **G** | **T** | **G** | **C** | **T** | **A** | **T** | **T** | **G** | **C** | **G** | **T** | **A** | **T** | **T** | **G** | **T** | **T** | **G** | **A** | **T** | **G** | **A** | **C** | **G** | **G** | **T** | **G** | **T** | **C** | **G** | **G** | **C** | **T** | **T** | **T** | **G** | **A** | **T** | **A** | **T** | **G** | **A** | **A** | **G** | **G** | **A** | **A** | **C** | **A** | **A** | **A** | **G** | **T** | **A** | **A** | **T** | **A** | **A** | **A** | **G** | **C** | **C** | **G** | **G** | **T** | **A** | **G** | **T** | **T** | **A** | **T** | **G** | **G** | **C** | **T** | **T** | **A** | **A** | **A** | **T** | **A** | **A** | **T** | **A** | **T** | **C** | **C** | **G** | **A** | **G** | **A** | **A** | **C** | **G** | **T** | **G** | **T** | **T** | **G** | **T** | **C** | **G** | **G** | **C** | **A** | **T** | **G** | **G** | **G** | **C** | **G** | **G** | **T** | **A** | **C** | **A** | **G** | **T** | **T** | **A** | **A** | **A** | **A** | **T** | **T** | **A** | **T** | **T** | **A** | **G** | **T** | **T** | **T** | **T** | **A** | **A** | **A** | **G** | **G** | **G** | **C** | **A** | **G** | **G** | **G** | **A** | **A** | **C** | **T** | **A** | **G** | **C** | **G** | **T** | **T** | **G** | **A** | **A** | **A** | **T** | **T** | **A** | **A** | **A** | **G** | **T** | **T** | **C** | **C** | **T** | **G** | **T** | **C** | **A** | **T** | **A** | **A** | **A** | **G** | **G** | **A** | **G** | **G** | **A** | **A** | **A** | **C** | **T** | **G** | **C** | **A** | **A** | **G** | **T** | **G** | **A** | **T** | **C** | **A** | **A** | **A** | **G** | **T** | **A** | **A** | **T** | **G** | **T** | **T** | **A** | **G** | **T** | **G** | **G** | **A** | **-** | **-** | **-** |
| **N54156** | **A** | **T** | **G** | **A** | **C** | **C** | **G** | **A** | **T** | **C** | **G** | **G** | **A** | **T** | **T** | **T** | **C** | **A** | **A** | **G** | **A** | **C** | **G** | **C** | **A** | **T** | **G** | **A** | **T** | **T** | **T** | **C** | **A** | **T** | **T** | **A** | **T** | **A** | **T** | **G** | **C** | **G** | **T** | **C** | **C** | **C** | **T** | **T** | **A** | **G** | **C** | **A** | **C** | **C** | **T** | **T** | **T** | **A** | **T** | **T** | **G** | **T** | **T** | **A** | **T** | **C** | **T** | **T** | **A** | **A** | **T** | **T** | **A** | **C** | **A** | **T** | **T** | **G** | **T** | **T** | **T** | **T** | **C** | **A** | **T** | **A** | **T** | **T** | **T** | **T** | **C** | **A** | **T** | **T** | **C** | **G** | **A** | **T** | **T** | **A** | **A** | **A** | **C** | **A** | **A** | **A** | **A** | **C** | **C** | **G** | **G** | **T** | **G** | **G** | **T** | **T** | **A** | **T** | **T** | **A** | **G** | **A** | **G** | **C** | **T** | **T** | **C** | **T** | **T** | **C** | **A** | **G** | **A** | **G** | **A** | **A** | **A** | **A** | **G** | **T** | **C** | **T** | **T** | **T** | **T** | **A** | **T** | **T** | **T** | **A** | **C** | **C** | **A** | **C** | **T** | **A** | **A** | **T** | **T** | **G** | **T** | **G** | **C** | **A** | **C** | **A** | **T** | **T** | **G** | **T** | **T** | **C** | **T** | **C** | **A** | **T** | **A** | **T** | **C** | **C** | **T** | **T** | **A** | **C** | **T** | **A** | **A** | **T** | **A** | **G** | **G** | **C** | **T** | **T** | **A** | **T** | **T** | **G** | **A** | **C** | **C** | **T** | **T** | **T** | **T** | **T** | **A** | **C** | **T** | **G** | **A** | **T** | **T** | **T** | **C** | **A** | **T** | **T** | **G** | **G** | **T** | **T** | **C** | **A** | **A** | **A** | **A** | **A** | **G** | **G** | **G** | **C** | **A** | **A** | **T** | **A** | **T** | **G** | **G** | **A** | **C** | **G** | **G** | **A** | **T** | **T** | **G** | **A** | **A** | **G** | **A** | **A** | **A** | **A** | **A** | **C** | **T** | **T** | **C** | **G** | **G** | **T** | **T** | **A** | **T** | **T** | **G** | **G** | **C** | **C** | **A** | **A** | **C** | **G** | **G** | **T** | **A** | **A** | **T** | **T** | **A** | **T** | **G** | **A** | **A** | **A** | **G** | **T** | **C** | **C** | **A** | **G** | **T** | **C** | **T** | **T** | **A** | **A** | **A** | **C** | **A** | **A** | **A** | **C** | **C** | **A** | **A** | **C** | **G** | **A** | **C** | **C** | **A** | **G** | **T** | **G** | **A** | **A** | **A** | **A** | **T** | **C** | **A** | **A** | **G** | **A** | **C** | **C** | **A** | **T** | **T** | **A** | **T** | **C** | **T** | **A** | **A** | **C** | **C** | **G** | **A** | **A** | **G** | **T** | **C** | **G** | **A** | **A** | **C** | **A** | **A** | **G** | **A** | **T** | **A** | **T** | **T** | **T** | **G** | **G** | **T** | **C** | **G** | **A** | **T** | **T** | **A** | **A** | **A** | **A** | **A** | **T** | **A** | **A** | **A** | **T** | **T** | **A** | **T** | **T** | **A** | **G** | **A** | **G** | **A** | **T** | **G** | **T** | **C** | **T** | **A** | **A** | **A** | **G** | **A** | **A** | **T** | **T** | **G** | **C** | **A** | **A** | **T** | **T** | **A** | **T** | **T** | **A** | **A** | **A** | **C** | **A** | **G** | **T** | **C** | **G** | **A** | **C** | **C** | **G** | **C** | **A** | **A** | **T** | **T** | **A** | **A** | **T** | **G** | **G** | **A** | **T** | **G** | **G** | **G** | **C** | **A** | **A** | **A** | **C** | **A** | **A** | **A** | **A** | **G** | **A** | **A** | **G** | **A** | **A** | **A** | **T** | **T** | **T** | **T** | **A** | **G** | **A** | **G** | **A** | **A** | **C** | **G** | **A** | **G** | **C** | **G** | **G** | **C** | **A** | **T** | **C** | **G** | **T** | **T** | **T** | **G** | **G** | **C** | **G** | **C** | **G** | **G** | **G** | **A** | **G** | **T** | **T** | **G** | **C** | **A** | **T** | **G** | **A** | **T** | **T** | **C** | **A** | **G** | **T** | **C** | **A** | **G** | **T** | **C** | **A** | **A** | **C** | **A** | **A** | **C** | **T** | **T** | **T** | **T** | **T** | **G** | **C** | **A** | **G** | **C** | **C** | **A** | **T** | **G** | **A** | **T** | **G** | **A** | **T** | **G** | **T** | **T** | **G** | **T** | **C** | **T** | **G** | **C** | **C** | **T** | **T** | **A** | **A** | **A** | **T** | **G** | **A** | **A** | **C** | **A** | **A** | **G** | **C** | **A** | **C** | **A** | **A** | **C** | **G** | **A** | **A** | **C** | **A** | **G** | **A** | **A** | **A** | **C** | **T** | **C** | **C** | **T** | **G** | **A** | **A** | **C** | **C** | **A** | **T** | **A** | **T** | **C** | **G** | **C** | **A** | **A** | **A** | **C** | **A** | **A** | **C** | **T** | **A** | **G** | **C** | **C** | **A** | **T** | **G** | **G** | **T** | **G** | **G** | **C** | **A** | **G** | **A** | **A** | **A** | **T** | **C** | **A** | **T** | **T** | **A** | **A** | **T** | **G** | **C** | **C** | **T** | **C** | **C** | **C** | **A** | **A** | **T** | **C** | **G** | **G** | **A** | **A** | **A** | **T** | **G** | **C** | **G** | **T** | **G** | **C** | **G** | **C** | **T** | **A** | **C** | **T** | **A** | **T** | **T** | **G** | **C** | **A** | **C** | **T** | **T** | **G** | **C** | **G** | **T** | **C** | **C** | **T** | **A** | **T** | **C** | **A** | **G** | **T** | **C** | **T** | **A** | **G** | **A** | **A** | **G** | **G** | **A** | **A** | **A** | **A** | **A** | **G** | **T** | **T** | **T** | **G** | **C** | **G** | **T** | **A** | **A** | **A** | **G** | **G** | **T** | **A** | **T** | **T** | **G** | **A** | **A** | **C** | **A** | **A** | **T** | **T** | **A** | **C** | **T** | **G** | **A** | **A** | **A** | **G** | **A** | **A** | **C** | **T** | **A** | **C** | **A** | **A** | **A** | **C** | **A** | **A** | **A** | **A** | **A** | **T** | **T** | **A** | **A** | **A** | **A** | **T** | **T** | **G** | **A** | **A** | **T** | **T** | **G** | **A** | **T** | **T** | **T** | **G** | **G** | **G** | **A** | **T** | **G** | **T** | **T** | **G** | **A** | **A** | **G** | **A** | **T** | **G** | **T** | **T** | **C** | **A** | **T** | **T** | **T** | **A** | **A** | **A** | **T** | **A** | **G** | **C** | **A** | **G** | **C** | **A** | **T** | **T** | **G** | **A** | **G** | **G** | **A** | **T** | **C** | **A** | **T** | **C** | **T** | **T** | **T** | **T** | **C** | **C** | **G** | **A** | **A** | **T** | **T** | **G** | **T** | **G** | **C** | **A** | **G** | **G** | **A** | **G** | **T** | **T** | **A** | **C** | **T** | **T** | **T** | **C** | **A** | **A** | **A** | **T** | **A** | **C** | **C** | **T** | **T** | **A** | **A** | **G** | **A** | **C** | **A** | **T** | **G** | **C** | **C** | **A** | **A** | **A** | **G** | **C** | **A** | **A** | **A** | **G** | **G** | **A** | **A** | **T** | **T** | **A** | **G** | **A** | **G** | **G** | **T** | **A** | **T** | **A** | **C** | **T** | **T** | **A** | **C** | **A** | **C** | **C** | **A** | **A** | **G** | **T** | **C** | **G** | **A** | **T** | **A** | **A** | **A** | **A** | **A** | **C** | **G** | **T** | **G** | **T** | **T** | **A** | **T** | **T** | **G** | **C** | **G** | **T** | **A** | **T** | **T** | **G** | **T** | **T** | **G** | **A** | **T** | **G** | **A** | **T** | **G** | **G** | **T** | **G** | **T** | **C** | **G** | **G** | **C** | **T** | **T** | **T** | **G** | **A** | **T** | **A** | **T** | **G** | **A** | **A** | **G** | **G** | **A** | **A** | **C** | **A** | **A** | **A** | **G** | **T** | **A** | **A** | **T** | **A** | **A** | **A** | **G** | **C** | **C** | **G** | **G** | **T** | **A** | **G** | **T** | **T** | **A** | **T** | **G** | **G** | **C** | **T** | **T** | **A** | **A** | **A** | **T** | **A** | **A** | **T** | **A** | **T** | **C** | **C** | **G** | **A** | **G** | **A** | **A** | **C** | **G** | **T** | **G** | **T** | **T** | **G** | **T** | **C** | **G** | **G** | **C** | **A** | **T** | **G** | **G** | **G** | **C** | **G** | **G** | **T** | **A** | **C** | **A** | **G** | **T** | **T** | **A** | **A** | **A** | **A** | **T** | **T** | **A** | **T** | **T** | **A** | **G** | **T** | **T** | **T** | **T** | **A** | **A** | **A** | **G** | **G** | **G** | **C** | **A** | **G** | **G** | **G** | **A** | **A** | **C** | **C** | **A** | **G** | **C** | **G** | **T** | **T** | **G** | **A** | **A** | **A** | **T** | **T** | **A** | **A** | **A** | **G** | **T** | **T** | **C** | **C** | **T** | **G** | **T** | **C** | **A** | **T** | **A** | **A** | **A** | **G** | **G** | **A** | **G** | **G** | **A** | **A** | **A** | **C** | **T** | **G** | **C** | **A** | **A** | **G** | **T** | **G** | **A** | **T** | **C** | **A** | **A** | **A** | **G** | **T** | **A** | **A** | **T** | **G** | **T** | **T** | **A** | **G** | **T** | **G** | **G** | **A** | **-** | **-** | **-** |
| **N59466F** | **A** | **T** | **G** | **A** | **C** | **C** | **G** | **A** | **T** | **C** | **G** | **G** | **A** | **T** | **T** | **T** | **C** | **A** | **A** | **G** | **A** | **C** | **G** | **C** | **A** | **T** | **G** | **A** | **T** | **T** | **T** | **C** | **A** | **T** | **T** | **A** | **T** | **A** | **T** | **G** | **C** | **G** | **T** | **C** | **C** | **C** | **T** | **T** | **A** | **G** | **C** | **A** | **C** | **C** | **T** | **T** | **T** | **A** | **T** | **T** | **G** | **T** | **T** | **A** | **T** | **C** | **T** | **T** | **A** | **A** | **T** | **T** | **A** | **C** | **A** | **T** | **T** | **G** | **T** | **T** | **T** | **T** | **C** | **A** | **T** | **A** | **T** | **T** | **T** | **T** | **C** | **A** | **T** | **T** | **C** | **G** | **A** | **T** | **T** | **A** | **A** | **A** | **C** | **A** | **A** | **A** | **A** | **C** | **C** | **G** | **G** | **T** | **G** | **G** | **T** | **T** | **A** | **T** | **T** | **A** | **G** | **A** | **G** | **C** | **T** | **T** | **C** | **T** | **T** | **C** | **A** | **G** | **A** | **G** | **A** | **A** | **A** | **A** | **G** | **T** | **C** | **T** | **T** | **T** | **T** | **A** | **T** | **T** | **T** | **A** | **C** | **C** | **A** | **C** | **T** | **A** | **A** | **T** | **T** | **G** | **T** | **G** | **C** | **A** | **C** | **A** | **T** | **T** | **G** | **T** | **T** | **C** | **T** | **C** | **A** | **T** | **A** | **T** | **C** | **C** | **T** | **T** | **A** | **C** | **T** | **A** | **A** | **T** | **A** | **G** | **G** | **C** | **T** | **T** | **A** | **T** | **T** | **G** | **A** | **C** | **C** | **T** | **T** | **T** | **T** | **T** | **A** | **C** | **T** | **G** | **A** | **T** | **T** | **T** | **C** | **A** | **T** | **T** | **G** | **G** | **T** | **T** | **C** | **A** | **A** | **A** | **A** | **A** | **G** | **G** | **G** | **C** | **A** | **A** | **T** | **A** | **T** | **G** | **G** | **A** | **C** | **G** | **G** | **A** | **T** | **T** | **G** | **A** | **A** | **G** | **A** | **A** | **A** | **A** | **A** | **C** | **T** | **T** | **C** | **G** | **G** | **T** | **T** | **A** | **T** | **T** | **G** | **G** | **C** | **C** | **A** | **A** | **C** | **G** | **G** | **T** | **A** | **A** | **T** | **T** | **A** | **T** | **G** | **A** | **A** | **A** | **G** | **T** | **C** | **C** | **A** | **G** | **T** | **C** | **T** | **T** | **A** | **A** | **A** | **C** | **A** | **A** | **A** | **C** | **C** | **A** | **A** | **C** | **G** | **A** | **C** | **C** | **A** | **G** | **T** | **G** | **A** | **A** | **A** | **A** | **T** | **C** | **A** | **A** | **G** | **A** | **C** | **C** | **A** | **T** | **T** | **A** | **T** | **C** | **T** | **A** | **A** | **C** | **C** | **G** | **A** | **A** | **G** | **T** | **C** | **G** | **A** | **A** | **C** | **A** | **A** | **G** | **A** | **T** | **A** | **T** | **T** | **T** | **G** | **G** | **T** | **C** | **G** | **A** | **T** | **T** | **A** | **A** | **A** | **A** | **A** | **T** | **A** | **A** | **A** | **T** | **T** | **A** | **T** | **T** | **A** | **G** | **A** | **G** | **A** | **T** | **G** | **T** | **C** | **T** | **A** | **A** | **A** | **G** | **A** | **A** | **T** | **T** | **G** | **C** | **A** | **A** | **T** | **T** | **A** | **T** | **T** | **A** | **A** | **A** | **C** | **A** | **G** | **T** | **C** | **G** | **A** | **C** | **C** | **G** | **C** | **A** | **A** | **T** | **T** | **A** | **A** | **T** | **G** | **G** | **A** | **T** | **G** | **G** | **G** | **C** | **A** | **A** | **A** | **C** | **A** | **A** | **A** | **A** | **G** | **A** | **A** | **G** | **A** | **A** | **A** | **T** | **T** | **T** | **T** | **A** | **G** | **A** | **G** | **A** | **A** | **C** | **G** | **A** | **G** | **C** | **G** | **G** | **C** | **A** | **T** | **C** | **G** | **T** | **T** | **T** | **G** | **G** | **C** | **G** | **C** | **G** | **G** | **G** | **A** | **G** | **T** | **T** | **G** | **C** | **A** | **T** | **G** | **A** | **T** | **T** | **C** | **A** | **G** | **T** | **C** | **A** | **G** | **T** | **C** | **A** | **A** | **C** | **A** | **A** | **C** | **T** | **T** | **T** | **T** | **T** | **G** | **C** | **A** | **G** | **C** | **C** | **A** | **T** | **G** | **A** | **T** | **G** | **A** | **T** | **G** | **T** | **T** | **G** | **T** | **C** | **T** | **G** | **C** | **A** | **T** | **T** | **A** | **A** | **A** | **T** | **G** | **A** | **A** | **C** | **A** | **A** | **G** | **C** | **A** | **C** | **A** | **A** | **C** | **G** | **A** | **A** | **C** | **A** | **G** | **A** | **A** | **A** | **C** | **C** | **C** | **C** | **G** | **G** | **A** | **A** | **C** | **C** | **A** | **T** | **A** | **T** | **C** | **G** | **T** | **A** | **A** | **A** | **C** | **A** | **A** | **C** | **T** | **A** | **G** | **C** | **C** | **A** | **T** | **G** | **G** | **T** | **G** | **G** | **C** | **A** | **G** | **A** | **A** | **A** | **T** | **C** | **A** | **T** | **T** | **A** | **A** | **T** | **G** | **C** | **C** | **T** | **C** | **C** | **C** | **A** | **A** | **T** | **C** | **G** | **G** | **A** | **A** | **A** | **T** | **G** | **C** | **G** | **C** | **G** | **C** | **G** | **C** | **T** | **A** | **C** | **T** | **A** | **T** | **T** | **G** | **C** | **A** | **C** | **T** | **T** | **G** | **C** | **G** | **T** | **C** | **C** | **T** | **A** | **T** | **C** | **A** | **G** | **T** | **C** | **T** | **A** | **G** | **A** | **A** | **G** | **G** | **A** | **A** | **A** | **A** | **A** | **G** | **T** | **T** | **T** | **G** | **C** | **G** | **T** | **A** | **A** | **A** | **G** | **G** | **T** | **A** | **T** | **T** | **G** | **A** | **A** | **C** | **A** | **A** | **T** | **T** | **A** | **C** | **T** | **G** | **A** | **A** | **A** | **G** | **A** | **A** | **C** | **T** | **A** | **C** | **A** | **A** | **A** | **C** | **A** | **A** | **A** | **A** | **A** | **T** | **T** | **A** | **A** | **A** | **A** | **T** | **T** | **G** | **A** | **A** | **T** | **T** | **G** | **A** | **T** | **T** | **T** | **G** | **G** | **G** | **A** | **T** | **G** | **T** | **T** | **G** | **A** | **A** | **G** | **A** | **T** | **G** | **T** | **T** | **C** | **A** | **T** | **T** | **T** | **A** | **A** | **A** | **T** | **A** | **G** | **C** | **A** | **G** | **C** | **A** | **T** | **T** | **G** | **A** | **G** | **G** | **A** | **T** | **C** | **A** | **T** | **C** | **T** | **T** | **T** | **T** | **C** | **C** | **G** | **A** | **A** | **T** | **T** | **G** | **T** | **G** | **C** | **A** | **G** | **G** | **A** | **G** | **T** | **T** | **A** | **C** | **T** | **T** | **T** | **C** | **A** | **A** | **A** | **T** | **A** | **C** | **C** | **T** | **T** | **A** | **A** | **G** | **A** | **C** | **A** | **T** | **G** | **C** | **C** | **A** | **A** | **A** | **G** | **C** | **A** | **A** | **A** | **G** | **G** | **A** | **A** | **T** | **T** | **A** | **G** | **A** | **G** | **G** | **T** | **A** | **T** | **A** | **C** | **T** | **T** | **A** | **C** | **A** | **C** | **C** | **A** | **A** | **G** | **T** | **C** | **G** | **A** | **T** | **A** | **A** | **A** | **A** | **A** | **C** | **G** | **T** | **G** | **T** | **T** | **A** | **T** | **T** | **G** | **C** | **G** | **T** | **A** | **T** | **T** | **G** | **T** | **T** | **G** | **A** | **T** | **G** | **A** | **T** | **G** | **G** | **T** | **G** | **T** | **C** | **G** | **G** | **C** | **T** | **T** | **T** | **G** | **A** | **T** | **A** | **T** | **G** | **A** | **A** | **G** | **G** | **A** | **A** | **C** | **A** | **A** | **A** | **G** | **T** | **A** | **A** | **T** | **A** | **A** | **A** | **G** | **C** | **C** | **G** | **G** | **T** | **A** | **G** | **T** | **T** | **A** | **T** | **G** | **G** | **C** | **T** | **T** | **A** | **A** | **A** | **T** | **A** | **A** | **T** | **A** | **T** | **T** | **C** | **G** | **A** | **G** | **A** | **A** | **C** | **G** | **T** | **G** | **T** | **T** | **G** | **T** | **C** | **G** | **G** | **C** | **A** | **T** | **G** | **G** | **G** | **C** | **G** | **G** | **T** | **A** | **C** | **A** | **G** | **T** | **T** | **A** | **A** | **A** | **A** | **T** | **T** | **A** | **T** | **T** | **A** | **G** | **T** | **T** | **T** | **T** | **A** | **A** | **A** | **G** | **G** | **G** | **C** | **A** | **G** | **G** | **G** | **A** | **A** | **C** | **C** | **A** | **G** | **C** | **G** | **T** | **T** | **G** | **A** | **A** | **A** | **T** | **T** | **A** | **A** | **A** | **G** | **T** | **T** | **C** | **C** | **T** | **G** | **T** | **C** | **A** | **T** | **A** | **A** | **A** | **G** | **G** | **A** | **G** | **G** | **A** | **A** | **A** | **C** | **T** | **G** | **C** | **A** | **A** | **G** | **T** | **G** | **A** | **T** | **C** | **A** | **A** | **A** | **G** | **T** | **A** | **A** | **T** | **G** | **T** | **T** | **A** | **G** | **T** | **G** | **G** | **A** | **-** | **-** | **-** |
| **N59609F** | **A** | **T** | **G** | **A** | **C** | **C** | **G** | **A** | **T** | **C** | **G** | **G** | **A** | **T** | **T** | **T** | **C** | **A** | **A** | **G** | **A** | **C** | **G** | **C** | **A** | **T** | **G** | **A** | **T** | **T** | **T** | **C** | **A** | **T** | **T** | **A** | **T** | **A** | **T** | **G** | **C** | **G** | **T** | **C** | **C** | **C** | **T** | **T** | **A** | **G** | **C** | **A** | **C** | **C** | **T** | **T** | **T** | **A** | **T** | **T** | **G** | **T** | **T** | **A** | **T** | **C** | **T** | **T** | **A** | **A** | **T** | **T** | **A** | **C** | **A** | **T** | **T** | **G** | **T** | **T** | **T** | **T** | **C** | **A** | **T** | **A** | **T** | **T** | **T** | **T** | **C** | **A** | **T** | **T** | **C** | **G** | **A** | **T** | **T** | **A** | **A** | **A** | **C** | **A** | **A** | **A** | **A** | **C** | **C** | **G** | **G** | **T** | **G** | **G** | **C** | **T** | **A** | **T** | **T** | **A** | **G** | **A** | **G** | **C** | **T** | **T** | **C** | **T** | **T** | **C** | **A** | **G** | **A** | **G** | **A** | **A** | **A** | **A** | **G** | **T** | **C** | **T** | **T** | **T** | **T** | **A** | **T** | **C** | **T** | **A** | **C** | **C** | **A** | **C** | **T** | **A** | **A** | **T** | **T** | **G** | **T** | **G** | **C** | **A** | **C** | **A** | **T** | **T** | **G** | **T** | **T** | **C** | **T** | **C** | **A** | **T** | **A** | **T** | **C** | **C** | **T** | **T** | **A** | **C** | **T** | **A** | **A** | **T** | **A** | **G** | **G** | **C** | **T** | **T** | **A** | **T** | **T** | **G** | **A** | **C** | **C** | **T** | **T** | **T** | **T** | **T** | **A** | **C** | **T** | **G** | **A** | **T** | **T** | **T** | **C** | **A** | **T** | **T** | **G** | **G** | **T** | **T** | **C** | **A** | **A** | **A** | **A** | **A** | **G** | **G** | **G** | **C** | **A** | **A** | **T** | **A** | **T** | **G** | **G** | **A** | **C** | **G** | **G** | **A** | **T** | **T** | **G** | **A** | **A** | **G** | **A** | **A** | **A** | **A** | **A** | **C** | **T** | **T** | **C** | **G** | **G** | **T** | **T** | **A** | **T** | **T** | **G** | **G** | **C** | **C** | **A** | **A** | **C** | **G** | **G** | **T** | **A** | **A** | **T** | **T** | **A** | **T** | **G** | **A** | **A** | **A** | **G** | **T** | **C** | **C** | **A** | **G** | **T** | **C** | **T** | **T** | **A** | **A** | **A** | **C** | **A** | **A** | **A** | **C** | **C** | **A** | **A** | **C** | **G** | **A** | **C** | **C** | **A** | **G** | **T** | **G** | **A** | **A** | **A** | **A** | **T** | **C** | **A** | **A** | **G** | **A** | **C** | **C** | **A** | **T** | **T** | **A** | **T** | **C** | **T** | **A** | **A** | **C** | **C** | **G** | **A** | **A** | **G** | **T** | **C** | **G** | **A** | **A** | **C** | **A** | **A** | **G** | **A** | **T** | **A** | **T** | **T** | **T** | **G** | **G** | **T** | **C** | **G** | **A** | **T** | **T** | **A** | **A** | **A** | **A** | **A** | **T** | **A** | **A** | **A** | **T** | **T** | **A** | **T** | **T** | **A** | **G** | **A** | **G** | **A** | **T** | **G** | **T** | **C** | **T** | **A** | **A** | **A** | **G** | **A** | **A** | **T** | **T** | **G** | **C** | **A** | **A** | **T** | **T** | **A** | **T** | **T** | **A** | **A** | **A** | **C** | **A** | **G** | **T** | **C** | **G** | **A** | **C** | **C** | **G** | **C** | **A** | **A** | **T** | **T** | **A** | **A** | **T** | **G** | **G** | **A** | **T** | **G** | **G** | **G** | **C** | **A** | **A** | **A** | **C** | **A** | **A** | **A** | **A** | **G** | **A** | **A** | **G** | **A** | **A** | **A** | **T** | **T** | **T** | **T** | **A** | **G** | **A** | **G** | **A** | **A** | **C** | **G** | **A** | **G** | **C** | **G** | **G** | **C** | **A** | **T** | **C** | **G** | **T** | **T** | **T** | **G** | **G** | **C** | **G** | **C** | **G** | **G** | **G** | **A** | **G** | **T** | **T** | **G** | **C** | **A** | **T** | **G** | **A** | **T** | **T** | **C** | **A** | **G** | **T** | **C** | **A** | **G** | **T** | **C** | **A** | **A** | **C** | **A** | **A** | **C** | **T** | **T** | **T** | **T** | **T** | **G** | **C** | **A** | **G** | **C** | **C** | **A** | **T** | **G** | **A** | **T** | **G** | **A** | **T** | **G** | **T** | **T** | **G** | **T** | **C** | **T** | **G** | **C** | **A** | **T** | **T** | **A** | **A** | **A** | **T** | **G** | **A** | **A** | **C** | **A** | **A** | **G** | **C** | **A** | **C** | **A** | **A** | **C** | **G** | **A** | **A** | **C** | **A** | **G** | **A** | **A** | **A** | **C** | **C** | **C** | **C** | **G** | **G** | **A** | **A** | **C** | **C** | **A** | **T** | **A** | **T** | **C** | **G** | **T** | **A** | **A** | **A** | **C** | **A** | **A** | **C** | **T** | **A** | **G** | **C** | **C** | **A** | **T** | **G** | **G** | **T** | **G** | **G** | **C** | **A** | **G** | **A** | **A** | **A** | **T** | **C** | **A** | **T** | **T** | **A** | **A** | **T** | **G** | **C** | **C** | **T** | **C** | **C** | **C** | **A** | **A** | **T** | **C** | **G** | **G** | **A** | **A** | **A** | **T** | **G** | **C** | **G** | **C** | **G** | **C** | **G** | **C** | **T** | **A** | **C** | **T** | **A** | **T** | **T** | **G** | **C** | **A** | **C** | **T** | **T** | **G** | **C** | **G** | **T** | **C** | **C** | **T** | **A** | **T** | **C** | **A** | **G** | **T** | **C** | **T** | **A** | **G** | **A** | **A** | **G** | **G** | **A** | **A** | **A** | **A** | **A** | **G** | **T** | **T** | **T** | **G** | **C** | **G** | **T** | **A** | **A** | **A** | **G** | **G** | **T** | **A** | **T** | **T** | **G** | **A** | **A** | **C** | **A** | **A** | **T** | **T** | **A** | **C** | **T** | **G** | **A** | **A** | **A** | **G** | **A** | **A** | **C** | **T** | **A** | **C** | **A** | **A** | **A** | **C** | **A** | **A** | **A** | **A** | **A** | **T** | **T** | **A** | **A** | **A** | **A** | **T** | **T** | **G** | **A** | **A** | **T** | **T** | **G** | **A** | **T** | **T** | **T** | **G** | **G** | **G** | **A** | **T** | **G** | **T** | **T** | **G** | **A** | **A** | **G** | **A** | **T** | **G** | **T** | **T** | **C** | **A** | **T** | **T** | **T** | **A** | **A** | **A** | **T** | **A** | **G** | **C** | **A** | **G** | **C** | **A** | **T** | **T** | **G** | **A** | **G** | **G** | **A** | **T** | **C** | **A** | **T** | **C** | **T** | **T** | **T** | **T** | **C** | **C** | **G** | **A** | **A** | **T** | **T** | **G** | **T** | **G** | **C** | **A** | **G** | **G** | **A** | **G** | **T** | **T** | **A** | **C** | **T** | **T** | **T** | **C** | **A** | **A** | **A** | **T** | **A** | **C** | **C** | **T** | **T** | **A** | **A** | **G** | **A** | **C** | **A** | **T** | **G** | **C** | **C** | **A** | **A** | **A** | **G** | **C** | **A** | **A** | **A** | **G** | **G** | **A** | **A** | **T** | **T** | **A** | **G** | **A** | **G** | **G** | **T** | **A** | **T** | **A** | **C** | **T** | **T** | **A** | **C** | **A** | **C** | **C** | **A** | **A** | **G** | **T** | **C** | **G** | **A** | **T** | **A** | **A** | **A** | **A** | **A** | **C** | **G** | **T** | **G** | **T** | **T** | **A** | **T** | **T** | **G** | **C** | **G** | **T** | **A** | **T** | **T** | **G** | **T** | **T** | **G** | **A** | **T** | **G** | **A** | **T** | **G** | **G** | **T** | **G** | **T** | **C** | **G** | **G** | **C** | **T** | **T** | **T** | **G** | **A** | **T** | **A** | **T** | **G** | **A** | **A** | **G** | **G** | **A** | **A** | **C** | **A** | **A** | **A** | **G** | **T** | **A** | **A** | **T** | **A** | **A** | **A** | **G** | **C** | **C** | **G** | **G** | **T** | **A** | **G** | **T** | **T** | **A** | **T** | **G** | **G** | **C** | **T** | **T** | **A** | **A** | **A** | **T** | **A** | **A** | **T** | **A** | **T** | **T** | **C** | **G** | **A** | **G** | **A** | **A** | **C** | **G** | **T** | **G** | **T** | **T** | **G** | **T** | **C** | **G** | **G** | **C** | **A** | **T** | **G** | **G** | **G** | **C** | **G** | **G** | **T** | **A** | **C** | **A** | **G** | **T** | **T** | **A** | **A** | **A** | **A** | **T** | **T** | **A** | **T** | **T** | **A** | **G** | **T** | **T** | **T** | **T** | **A** | **A** | **A** | **G** | **G** | **G** | **C** | **A** | **G** | **G** | **G** | **A** | **A** | **C** | **C** | **A** | **G** | **C** | **G** | **T** | **T** | **G** | **A** | **A** | **A** | **T** | **T** | **A** | **A** | **A** | **G** | **T** | **T** | **C** | **C** | **T** | **G** | **T** | **C** | **A** | **T** | **A** | **A** | **A** | **G** | **G** | **A** | **G** | **G** | **A** | **A** | **A** | **C** | **T** | **G** | **C** | **A** | **A** | **G** | **T** | **G** | **A** | **T** | **C** | **A** | **A** | **A** | **G** | **T** | **A** | **A** | **T** | **G** | **T** | **T** | **A** | **G** | **T** | **G** | **G** | **A** | **-** | **-** | **-** |
| **N60268F** | **A** | **T** | **G** | **A** | **C** | **C** | **G** | **A** | **T** | **C** | **G** | **G** | **A** | **T** | **T** | **T** | **C** | **A** | **A** | **G** | **A** | **C** | **G** | **C** | **A** | **T** | **G** | **A** | **T** | **T** | **T** | **C** | **A** | **T** | **T** | **A** | **T** | **A** | **T** | **G** | **C** | **G** | **T** | **C** | **C** | **C** | **T** | **T** | **A** | **G** | **C** | **A** | **C** | **C** | **T** | **T** | **T** | **A** | **T** | **T** | **G** | **T** | **T** | **A** | **T** | **C** | **T** | **T** | **A** | **A** | **T** | **T** | **A** | **C** | **A** | **T** | **T** | **G** | **T** | **T** | **T** | **T** | **C** | **A** | **T** | **A** | **T** | **T** | **T** | **T** | **C** | **A** | **T** | **T** | **C** | **G** | **A** | **T** | **T** | **A** | **A** | **A** | **C** | **A** | **A** | **A** | **A** | **C** | **C** | **G** | **G** | **T** | **G** | **G** | **T** | **T** | **A** | **T** | **T** | **A** | **G** | **A** | **G** | **C** | **T** | **T** | **C** | **T** | **T** | **C** | **A** | **G** | **A** | **G** | **A** | **A** | **A** | **A** | **G** | **T** | **C** | **T** | **T** | **T** | **T** | **A** | **T** | **T** | **T** | **A** | **C** | **C** | **A** | **C** | **T** | **A** | **A** | **T** | **T** | **G** | **T** | **G** | **C** | **A** | **C** | **A** | **T** | **T** | **G** | **T** | **T** | **C** | **T** | **C** | **A** | **T** | **A** | **T** | **C** | **C** | **T** | **T** | **A** | **C** | **T** | **A** | **A** | **T** | **A** | **G** | **G** | **C** | **T** | **T** | **A** | **T** | **T** | **G** | **A** | **C** | **C** | **T** | **T** | **T** | **T** | **T** | **A** | **C** | **T** | **G** | **A** | **T** | **T** | **T** | **C** | **A** | **T** | **T** | **G** | **G** | **T** | **T** | **C** | **A** | **A** | **A** | **A** | **A** | **G** | **G** | **G** | **C** | **A** | **A** | **T** | **A** | **T** | **G** | **G** | **A** | **C** | **G** | **G** | **A** | **T** | **T** | **G** | **A** | **A** | **G** | **A** | **A** | **A** | **A** | **A** | **C** | **T** | **T** | **C** | **G** | **G** | **T** | **T** | **A** | **T** | **T** | **G** | **G** | **C** | **C** | **A** | **A** | **C** | **G** | **G** | **T** | **A** | **A** | **T** | **T** | **A** | **T** | **G** | **A** | **A** | **A** | **G** | **T** | **C** | **C** | **A** | **G** | **T** | **C** | **T** | **T** | **A** | **A** | **A** | **C** | **A** | **A** | **A** | **C** | **C** | **A** | **A** | **C** | **G** | **A** | **C** | **C** | **A** | **G** | **T** | **G** | **A** | **A** | **A** | **A** | **T** | **C** | **A** | **A** | **G** | **A** | **C** | **C** | **A** | **T** | **T** | **A** | **T** | **C** | **T** | **A** | **A** | **C** | **C** | **G** | **A** | **A** | **G** | **T** | **C** | **G** | **A** | **A** | **C** | **A** | **A** | **G** | **A** | **T** | **A** | **T** | **T** | **T** | **G** | **G** | **T** | **C** | **G** | **A** | **T** | **T** | **A** | **A** | **A** | **A** | **A** | **T** | **A** | **A** | **A** | **T** | **T** | **A** | **T** | **T** | **A** | **G** | **A** | **G** | **A** | **T** | **G** | **T** | **C** | **T** | **A** | **A** | **A** | **G** | **A** | **A** | **T** | **T** | **G** | **C** | **A** | **A** | **T** | **T** | **A** | **T** | **T** | **A** | **A** | **A** | **C** | **A** | **G** | **T** | **C** | **G** | **A** | **C** | **C** | **G** | **C** | **A** | **A** | **T** | **T** | **A** | **A** | **T** | **G** | **G** | **A** | **T** | **G** | **G** | **G** | **C** | **A** | **A** | **A** | **C** | **A** | **A** | **A** | **A** | **G** | **A** | **A** | **G** | **A** | **A** | **A** | **T** | **T** | **T** | **T** | **A** | **G** | **A** | **G** | **A** | **A** | **C** | **G** | **A** | **G** | **C** | **G** | **G** | **C** | **A** | **T** | **C** | **G** | **T** | **T** | **T** | **G** | **G** | **C** | **G** | **C** | **G** | **G** | **G** | **A** | **G** | **T** | **T** | **G** | **C** | **A** | **T** | **G** | **A** | **T** | **T** | **C** | **A** | **G** | **T** | **C** | **A** | **G** | **T** | **C** | **A** | **A** | **C** | **A** | **A** | **C** | **T** | **T** | **T** | **T** | **T** | **G** | **C** | **A** | **G** | **C** | **C** | **A** | **T** | **G** | **A** | **T** | **G** | **A** | **T** | **G** | **T** | **T** | **G** | **T** | **C** | **T** | **G** | **C** | **A** | **T** | **T** | **A** | **A** | **A** | **T** | **G** | **A** | **A** | **C** | **A** | **A** | **G** | **C** | **A** | **C** | **A** | **A** | **C** | **G** | **A** | **A** | **C** | **A** | **G** | **A** | **A** | **A** | **C** | **C** | **C** | **C** | **G** | **G** | **A** | **A** | **C** | **C** | **A** | **T** | **A** | **T** | **C** | **G** | **T** | **A** | **A** | **A** | **C** | **A** | **A** | **C** | **T** | **A** | **G** | **C** | **C** | **A** | **T** | **G** | **G** | **T** | **G** | **G** | **C** | **A** | **G** | **A** | **A** | **A** | **T** | **C** | **A** | **T** | **T** | **A** | **A** | **T** | **G** | **C** | **C** | **T** | **C** | **C** | **C** | **A** | **A** | **T** | **C** | **G** | **G** | **A** | **A** | **A** | **T** | **G** | **C** | **G** | **C** | **G** | **C** | **G** | **C** | **T** | **A** | **C** | **T** | **A** | **T** | **T** | **G** | **C** | **A** | **C** | **T** | **T** | **G** | **C** | **G** | **T** | **C** | **C** | **T** | **A** | **T** | **C** | **A** | **G** | **T** | **C** | **T** | **A** | **G** | **A** | **A** | **G** | **G** | **A** | **A** | **A** | **A** | **A** | **G** | **T** | **T** | **T** | **G** | **C** | **G** | **T** | **A** | **A** | **A** | **G** | **G** | **T** | **A** | **T** | **T** | **G** | **A** | **A** | **C** | **A** | **A** | **T** | **T** | **A** | **C** | **T** | **G** | **A** | **A** | **A** | **G** | **A** | **A** | **C** | **T** | **A** | **C** | **A** | **A** | **A** | **C** | **A** | **A** | **A** | **A** | **A** | **T** | **T** | **A** | **A** | **A** | **A** | **T** | **T** | **G** | **A** | **A** | **T** | **T** | **G** | **A** | **T** | **T** | **T** | **G** | **G** | **G** | **A** | **T** | **G** | **T** | **T** | **G** | **A** | **A** | **G** | **A** | **T** | **G** | **T** | **T** | **C** | **A** | **T** | **T** | **T** | **A** | **A** | **A** | **T** | **A** | **G** | **C** | **A** | **G** | **C** | **A** | **T** | **T** | **G** | **A** | **G** | **G** | **A** | **T** | **C** | **A** | **T** | **C** | **T** | **T** | **T** | **T** | **C** | **C** | **G** | **A** | **A** | **T** | **T** | **G** | **T** | **G** | **C** | **A** | **G** | **G** | **A** | **G** | **T** | **T** | **A** | **C** | **T** | **T** | **T** | **C** | **A** | **A** | **A** | **T** | **A** | **C** | **C** | **T** | **T** | **A** | **A** | **G** | **A** | **C** | **A** | **T** | **G** | **C** | **C** | **A** | **A** | **A** | **G** | **C** | **A** | **A** | **A** | **G** | **G** | **A** | **A** | **T** | **T** | **A** | **G** | **A** | **G** | **G** | **T** | **A** | **T** | **A** | **C** | **T** | **T** | **A** | **C** | **A** | **C** | **C** | **A** | **A** | **G** | **T** | **C** | **G** | **A** | **T** | **A** | **A** | **A** | **A** | **A** | **C** | **G** | **T** | **G** | **C** | **T** | **A** | **T** | **T** | **G** | **C** | **G** | **T** | **A** | **T** | **T** | **G** | **T** | **T** | **G** | **A** | **T** | **G** | **A** | **C** | **G** | **G** | **T** | **G** | **T** | **C** | **G** | **G** | **C** | **T** | **T** | **T** | **G** | **A** | **T** | **A** | **T** | **G** | **A** | **A** | **G** | **G** | **A** | **A** | **C** | **A** | **A** | **A** | **G** | **T** | **A** | **A** | **T** | **A** | **A** | **A** | **G** | **C** | **C** | **G** | **G** | **T** | **A** | **G** | **T** | **T** | **A** | **T** | **G** | **G** | **C** | **T** | **T** | **A** | **A** | **A** | **T** | **A** | **A** | **T** | **A** | **T** | **C** | **C** | **G** | **A** | **G** | **A** | **A** | **C** | **G** | **T** | **G** | **T** | **T** | **G** | **T** | **C** | **G** | **G** | **C** | **A** | **T** | **G** | **G** | **G** | **C** | **G** | **G** | **T** | **A** | **C** | **A** | **G** | **T** | **T** | **A** | **A** | **A** | **A** | **T** | **T** | **A** | **T** | **T** | **A** | **G** | **T** | **T** | **T** | **T** | **A** | **A** | **A** | **G** | **G** | **G** | **C** | **A** | **G** | **G** | **G** | **A** | **A** | **C** | **C** | **A** | **G** | **C** | **G** | **T** | **T** | **G** | **A** | **A** | **A** | **T** | **T** | **A** | **A** | **A** | **G** | **T** | **T** | **C** | **C** | **T** | **G** | **T** | **C** | **A** | **T** | **A** | **A** | **A** | **G** | **G** | **A** | **G** | **G** | **A** | **A** | **A** | **C** | **T** | **G** | **C** | **A** | **A** | **G** | **T** | **G** | **A** | **T** | **C** | **A** | **A** | **A** | **G** | **T** | **A** | **A** | **T** | **G** | **T** | **T** | **A** | **G** | **T** | **G** | **G** | **A** | **-** | **-** | **-** |
| **N59896F** | **A** | **T** | **G** | **A** | **C** | **C** | **G** | **A** | **T** | **C** | **G** | **G** | **A** | **T** | **T** | **T** | **C** | **A** | **A** | **G** | **A** | **C** | **G** | **C** | **A** | **T** | **G** | **A** | **T** | **T** | **T** | **C** | **A** | **T** | **T** | **A** | **T** | **A** | **T** | **G** | **C** | **G** | **T** | **C** | **C** | **C** | **T** | **T** | **A** | **G** | **C** | **A** | **C** | **C** | **T** | **T** | **T** | **A** | **T** | **T** | **G** | **T** | **T** | **A** | **T** | **C** | **T** | **T** | **A** | **A** | **T** | **T** | **A** | **C** | **A** | **T** | **T** | **G** | **T** | **T** | **T** | **T** | **C** | **A** | **T** | **A** | **T** | **T** | **T** | **T** | **C** | **A** | **T** | **T** | **C** | **G** | **A** | **T** | **T** | **A** | **A** | **A** | **C** | **A** | **A** | **A** | **A** | **C** | **C** | **G** | **G** | **T** | **G** | **G** | **C** | **T** | **A** | **T** | **T** | **A** | **G** | **A** | **G** | **C** | **T** | **T** | **C** | **T** | **T** | **C** | **A** | **G** | **A** | **G** | **A** | **A** | **A** | **A** | **G** | **T** | **C** | **T** | **T** | **T** | **T** | **A** | **T** | **T** | **T** | **A** | **C** | **C** | **A** | **C** | **T** | **A** | **A** | **T** | **T** | **G** | **T** | **G** | **C** | **A** | **C** | **A** | **T** | **T** | **G** | **T** | **T** | **C** | **T** | **C** | **A** | **T** | **A** | **T** | **C** | **C** | **T** | **T** | **A** | **C** | **T** | **A** | **A** | **T** | **A** | **G** | **G** | **C** | **T** | **T** | **A** | **T** | **T** | **G** | **A** | **C** | **C** | **T** | **T** | **T** | **T** | **T** | **A** | **C** | **T** | **G** | **A** | **T** | **T** | **T** | **C** | **A** | **T** | **T** | **G** | **G** | **T** | **T** | **C** | **A** | **A** | **A** | **A** | **A** | **G** | **G** | **G** | **C** | **A** | **A** | **T** | **A** | **T** | **G** | **G** | **A** | **C** | **G** | **G** | **A** | **T** | **T** | **G** | **A** | **A** | **G** | **A** | **A** | **A** | **A** | **A** | **C** | **T** | **T** | **C** | **G** | **G** | **T** | **T** | **A** | **T** | **T** | **G** | **G** | **C** | **C** | **A** | **A** | **C** | **G** | **G** | **T** | **A** | **A** | **T** | **T** | **A** | **T** | **G** | **A** | **A** | **A** | **G** | **T** | **C** | **C** | **A** | **G** | **T** | **C** | **T** | **T** | **A** | **A** | **A** | **C** | **A** | **A** | **A** | **C** | **C** | **A** | **A** | **C** | **G** | **A** | **C** | **C** | **A** | **G** | **T** | **G** | **A** | **A** | **A** | **A** | **T** | **C** | **A** | **A** | **G** | **A** | **C** | **C** | **A** | **T** | **T** | **A** | **T** | **C** | **T** | **A** | **A** | **C** | **C** | **G** | **A** | **A** | **G** | **T** | **C** | **G** | **A** | **A** | **C** | **A** | **A** | **G** | **A** | **T** | **A** | **T** | **T** | **T** | **G** | **G** | **T** | **C** | **G** | **A** | **T** | **T** | **A** | **A** | **A** | **A** | **A** | **T** | **A** | **A** | **A** | **T** | **T** | **A** | **T** | **T** | **A** | **G** | **A** | **G** | **A** | **T** | **G** | **T** | **C** | **T** | **A** | **A** | **A** | **G** | **A** | **A** | **T** | **T** | **G** | **C** | **A** | **A** | **T** | **T** | **A** | **T** | **T** | **A** | **A** | **A** | **C** | **A** | **G** | **T** | **C** | **G** | **A** | **C** | **C** | **G** | **C** | **A** | **A** | **T** | **T** | **A** | **A** | **T** | **G** | **G** | **A** | **T** | **G** | **G** | **G** | **C** | **A** | **A** | **A** | **C** | **A** | **A** | **A** | **A** | **G** | **A** | **A** | **G** | **A** | **A** | **A** | **T** | **T** | **T** | **T** | **A** | **G** | **A** | **G** | **A** | **A** | **C** | **G** | **A** | **G** | **C** | **G** | **G** | **C** | **A** | **T** | **C** | **G** | **T** | **T** | **T** | **G** | **G** | **C** | **G** | **C** | **G** | **G** | **G** | **A** | **G** | **T** | **T** | **G** | **C** | **A** | **T** | **G** | **A** | **T** | **T** | **C** | **A** | **G** | **T** | **C** | **A** | **G** | **T** | **C** | **A** | **A** | **C** | **A** | **A** | **C** | **T** | **T** | **T** | **T** | **T** | **G** | **C** | **A** | **G** | **C** | **C** | **A** | **T** | **G** | **A** | **T** | **G** | **A** | **T** | **G** | **T** | **T** | **G** | **T** | **C** | **T** | **G** | **C** | **C** | **T** | **T** | **A** | **A** | **A** | **T** | **G** | **A** | **A** | **C** | **A** | **A** | **G** | **C** | **A** | **C** | **A** | **A** | **C** | **G** | **A** | **A** | **C** | **A** | **G** | **A** | **A** | **A** | **C** | **T** | **C** | **C** | **T** | **G** | **A** | **A** | **C** | **C** | **A** | **T** | **A** | **T** | **C** | **G** | **C** | **A** | **A** | **A** | **C** | **A** | **A** | **C** | **T** | **A** | **G** | **C** | **C** | **A** | **T** | **G** | **G** | **T** | **G** | **G** | **C** | **A** | **G** | **A** | **A** | **A** | **T** | **C** | **A** | **T** | **T** | **A** | **A** | **T** | **G** | **C** | **C** | **T** | **C** | **C** | **C** | **A** | **A** | **T** | **C** | **G** | **G** | **A** | **A** | **A** | **T** | **G** | **C** | **G** | **C** | **G** | **C** | **G** | **T** | **T** | **A** | **C** | **T** | **A** | **T** | **T** | **G** | **C** | **A** | **C** | **T** | **T** | **G** | **C** | **G** | **T** | **C** | **C** | **T** | **A** | **T** | **C** | **A** | **G** | **T** | **C** | **T** | **A** | **G** | **A** | **A** | **G** | **G** | **A** | **A** | **A** | **A** | **A** | **G** | **T** | **T** | **T** | **G** | **C** | **G** | **T** | **A** | **A** | **A** | **G** | **G** | **T** | **A** | **T** | **T** | **G** | **A** | **A** | **C** | **A** | **A** | **T** | **T** | **A** | **C** | **T** | **G** | **A** | **A** | **A** | **G** | **A** | **A** | **C** | **T** | **A** | **C** | **A** | **A** | **A** | **C** | **A** | **A** | **A** | **A** | **A** | **T** | **T** | **A** | **A** | **A** | **A** | **T** | **T** | **G** | **A** | **A** | **T** | **T** | **G** | **A** | **T** | **T** | **T** | **G** | **G** | **G** | **A** | **T** | **G** | **T** | **T** | **G** | **A** | **A** | **G** | **A** | **T** | **G** | **T** | **T** | **C** | **A** | **T** | **T** | **T** | **A** | **A** | **A** | **T** | **A** | **G** | **C** | **A** | **G** | **C** | **A** | **T** | **T** | **G** | **A** | **G** | **G** | **A** | **T** | **C** | **A** | **T** | **C** | **T** | **T** | **T** | **T** | **C** | **C** | **G** | **A** | **A** | **T** | **T** | **G** | **T** | **G** | **C** | **A** | **G** | **G** | **A** | **A** | **T** | **T** | **A** | **C** | **T** | **T** | **T** | **C** | **A** | **A** | **A** | **T** | **A** | **C** | **C** | **T** | **T** | **A** | **A** | **G** | **A** | **C** | **A** | **T** | **G** | **C** | **C** | **A** | **A** | **A** | **G** | **C** | **A** | **A** | **A** | **G** | **G** | **A** | **A** | **T** | **T** | **A** | **G** | **A** | **G** | **G** | **T** | **A** | **T** | **A** | **C** | **T** | **T** | **A** | **C** | **A** | **C** | **C** | **A** | **A** | **G** | **T** | **C** | **G** | **A** | **T** | **A** | **A** | **A** | **A** | **A** | **C** | **G** | **T** | **G** | **T** | **T** | **A** | **T** | **T** | **G** | **C** | **G** | **T** | **A** | **T** | **T** | **G** | **T** | **T** | **G** | **A** | **T** | **G** | **A** | **T** | **G** | **G** | **T** | **G** | **T** | **C** | **G** | **G** | **C** | **T** | **T** | **T** | **G** | **A** | **T** | **A** | **T** | **G** | **A** | **A** | **G** | **G** | **A** | **A** | **C** | **A** | **A** | **A** | **G** | **T** | **A** | **A** | **T** | **A** | **A** | **A** | **G** | **C** | **C** | **G** | **G** | **T** | **A** | **G** | **T** | **T** | **A** | **T** | **G** | **G** | **C** | **T** | **T** | **A** | **A** | **A** | **T** | **A** | **A** | **T** | **A** | **T** | **C** | **C** | **G** | **A** | **G** | **A** | **A** | **C** | **G** | **T** | **G** | **T** | **T** | **G** | **T** | **C** | **G** | **G** | **C** | **A** | **T** | **G** | **G** | **G** | **C** | **G** | **G** | **T** | **A** | **C** | **A** | **G** | **T** | **T** | **A** | **A** | **A** | **A** | **T** | **T** | **A** | **T** | **T** | **A** | **G** | **T** | **T** | **T** | **T** | **A** | **A** | **A** | **G** | **G** | **G** | **C** | **A** | **G** | **G** | **G** | **A** | **A** | **C** | **C** | **A** | **G** | **C** | **G** | **T** | **T** | **G** | **A** | **A** | **A** | **T** | **T** | **A** | **A** | **A** | **G** | **T** | **T** | **C** | **C** | **T** | **G** | **T** | **C** | **A** | **T** | **A** | **A** | **A** | **G** | **G** | **A** | **G** | **G** | **A** | **A** | **A** | **C** | **T** | **G** | **C** | **A** | **A** | **G** | **T** | **G** | **A** | **T** | **C** | **A** | **A** | **A** | **G** | **T** | **A** | **A** | **T** | **G** | **T** | **T** | **A** | **G** | **T** | **G** | **G** | **A** | **-** | **-** | **-** |
| **N54118** | **A** | **T** | **G** | **A** | **C** | **C** | **G** | **A** | **T** | **C** | **G** | **G** | **A** | **T** | **T** | **T** | **C** | **A** | **A** | **G** | **A** | **C** | **G** | **C** | **A** | **T** | **G** | **A** | **T** | **T** | **T** | **C** | **A** | **T** | **T** | **A** | **T** | **A** | **T** | **G** | **C** | **G** | **T** | **C** | **C** | **C** | **T** | **T** | **A** | **G** | **C** | **A** | **C** | **C** | **T** | **T** | **T** | **A** | **T** | **T** | **G** | **T** | **T** | **A** | **T** | **C** | **T** | **T** | **A** | **A** | **T** | **T** | **A** | **C** | **A** | **T** | **T** | **G** | **T** | **T** | **T** | **T** | **C** | **A** | **T** | **A** | **T** | **T** | **T** | **T** | **C** | **A** | **T** | **T** | **C** | **G** | **A** | **T** | **T** | **A** | **A** | **A** | **C** | **A** | **A** | **A** | **A** | **C** | **C** | **G** | **G** | **T** | **G** | **G** | **T** | **T** | **A** | **T** | **T** | **A** | **G** | **A** | **G** | **C** | **T** | **T** | **C** | **T** | **T** | **C** | **A** | **G** | **A** | **G** | **A** | **A** | **A** | **A** | **G** | **T** | **C** | **T** | **T** | **T** | **T** | **A** | **T** | **C** | **T** | **A** | **C** | **C** | **A** | **C** | **T** | **A** | **A** | **T** | **T** | **G** | **T** | **G** | **C** | **A** | **C** | **A** | **T** | **T** | **G** | **T** | **T** | **C** | **T** | **C** | **A** | **T** | **A** | **T** | **C** | **C** | **T** | **T** | **A** | **C** | **T** | **A** | **A** | **T** | **A** | **G** | **G** | **C** | **T** | **T** | **A** | **T** | **T** | **G** | **A** | **C** | **C** | **T** | **T** | **T** | **T** | **T** | **A** | **C** | **T** | **G** | **A** | **T** | **T** | **T** | **C** | **A** | **T** | **T** | **G** | **G** | **T** | **T** | **C** | **A** | **A** | **A** | **A** | **A** | **G** | **G** | **G** | **C** | **A** | **A** | **T** | **A** | **T** | **G** | **G** | **A** | **C** | **G** | **G** | **A** | **T** | **T** | **G** | **A** | **A** | **G** | **A** | **A** | **A** | **A** | **A** | **C** | **T** | **T** | **C** | **G** | **G** | **T** | **T** | **A** | **T** | **T** | **G** | **G** | **C** | **C** | **A** | **A** | **C** | **G** | **G** | **T** | **A** | **A** | **T** | **T** | **A** | **T** | **G** | **A** | **A** | **A** | **G** | **T** | **C** | **C** | **A** | **G** | **T** | **C** | **T** | **T** | **A** | **A** | **A** | **C** | **A** | **A** | **A** | **C** | **C** | **A** | **A** | **C** | **G** | **A** | **C** | **C** | **A** | **G** | **T** | **G** | **A** | **A** | **A** | **A** | **T** | **C** | **A** | **A** | **G** | **A** | **T** | **C** | **A** | **T** | **T** | **A** | **T** | **C** | **T** | **A** | **A** | **C** | **C** | **G** | **A** | **A** | **G** | **T** | **C** | **G** | **A** | **A** | **C** | **A** | **A** | **G** | **A** | **T** | **A** | **T** | **T** | **T** | **G** | **G** | **T** | **C** | **G** | **A** | **T** | **T** | **A** | **A** | **A** | **A** | **A** | **T** | **A** | **A** | **A** | **T** | **T** | **A** | **T** | **T** | **A** | **G** | **A** | **G** | **A** | **T** | **G** | **T** | **C** | **T** | **A** | **A** | **A** | **G** | **A** | **A** | **T** | **T** | **G** | **C** | **A** | **A** | **T** | **T** | **A** | **T** | **T** | **A** | **A** | **A** | **C** | **A** | **G** | **T** | **C** | **G** | **A** | **C** | **C** | **G** | **C** | **A** | **A** | **T** | **T** | **A** | **A** | **T** | **G** | **G** | **A** | **T** | **G** | **G** | **G** | **C** | **A** | **A** | **A** | **C** | **A** | **A** | **A** | **A** | **G** | **A** | **A** | **G** | **A** | **A** | **A** | **T** | **T** | **T** | **T** | **A** | **G** | **A** | **G** | **A** | **A** | **C** | **G** | **A** | **G** | **C** | **G** | **G** | **C** | **A** | **T** | **C** | **G** | **T** | **T** | **T** | **G** | **G** | **C** | **G** | **C** | **G** | **G** | **G** | **A** | **G** | **T** | **T** | **G** | **C** | **A** | **T** | **G** | **A** | **T** | **T** | **C** | **A** | **G** | **T** | **C** | **A** | **G** | **T** | **C** | **A** | **A** | **C** | **A** | **A** | **C** | **T** | **T** | **T** | **T** | **T** | **G** | **C** | **A** | **G** | **C** | **C** | **A** | **T** | **G** | **A** | **T** | **G** | **A** | **T** | **G** | **T** | **T** | **G** | **T** | **C** | **T** | **G** | **C** | **C** | **T** | **T** | **A** | **A** | **A** | **T** | **G** | **A** | **A** | **C** | **A** | **A** | **G** | **C** | **A** | **C** | **A** | **A** | **C** | **G** | **A** | **A** | **C** | **A** | **G** | **A** | **A** | **A** | **C** | **C** | **C** | **C** | **G** | **G** | **A** | **A** | **C** | **C** | **A** | **T** | **A** | **T** | **C** | **G** | **T** | **A** | **A** | **A** | **C** | **A** | **A** | **C** | **T** | **A** | **G** | **C** | **C** | **A** | **T** | **G** | **G** | **T** | **G** | **G** | **C** | **A** | **G** | **A** | **A** | **A** | **T** | **C** | **A** | **T** | **T** | **A** | **A** | **T** | **G** | **C** | **C** | **T** | **C** | **C** | **C** | **A** | **A** | **T** | **C** | **G** | **G** | **A** | **A** | **A** | **T** | **G** | **C** | **G** | **C** | **G** | **C** | **G** | **T** | **T** | **A** | **C** | **T** | **A** | **T** | **T** | **G** | **C** | **A** | **C** | **T** | **T** | **G** | **C** | **G** | **T** | **C** | **C** | **T** | **A** | **T** | **C** | **A** | **G** | **T** | **C** | **T** | **A** | **G** | **A** | **A** | **G** | **G** | **A** | **A** | **A** | **A** | **A** | **G** | **T** | **T** | **T** | **G** | **C** | **G** | **T** | **A** | **A** | **A** | **G** | **G** | **T** | **A** | **T** | **T** | **G** | **A** | **A** | **C** | **A** | **A** | **T** | **T** | **A** | **C** | **T** | **G** | **A** | **A** | **A** | **G** | **A** | **A** | **C** | **T** | **A** | **C** | **A** | **A** | **A** | **C** | **A** | **A** | **A** | **A** | **A** | **T** | **T** | **A** | **A** | **A** | **A** | **T** | **T** | **G** | **A** | **A** | **T** | **T** | **G** | **A** | **T** | **T** | **T** | **G** | **G** | **G** | **A** | **T** | **G** | **T** | **T** | **G** | **A** | **A** | **G** | **A** | **T** | **G** | **T** | **T** | **C** | **A** | **T** | **T** | **T** | **A** | **A** | **A** | **T** | **A** | **G** | **C** | **A** | **G** | **C** | **A** | **T** | **T** | **G** | **A** | **G** | **G** | **A** | **T** | **C** | **A** | **T** | **C** | **T** | **T** | **T** | **T** | **C** | **C** | **G** | **A** | **A** | **T** | **T** | **G** | **T** | **G** | **C** | **A** | **G** | **G** | **A** | **G** | **T** | **T** | **A** | **C** | **T** | **T** | **T** | **C** | **A** | **A** | **A** | **T** | **A** | **C** | **C** | **T** | **T** | **A** | **A** | **G** | **A** | **C** | **A** | **T** | **G** | **C** | **C** | **A** | **A** | **A** | **G** | **C** | **A** | **A** | **A** | **G** | **G** | **A** | **A** | **T** | **T** | **A** | **G** | **A** | **G** | **G** | **T** | **A** | **T** | **A** | **C** | **T** | **T** | **A** | **C** | **A** | **C** | **C** | **A** | **A** | **G** | **T** | **C** | **G** | **A** | **T** | **A** | **A** | **A** | **A** | **A** | **C** | **G** | **T** | **G** | **T** | **T** | **A** | **T** | **T** | **G** | **C** | **G** | **T** | **A** | **T** | **T** | **G** | **T** | **T** | **G** | **A** | **T** | **G** | **A** | **C** | **G** | **G** | **T** | **G** | **T** | **C** | **G** | **G** | **C** | **T** | **T** | **T** | **G** | **A** | **T** | **A** | **T** | **G** | **A** | **A** | **G** | **G** | **A** | **A** | **C** | **A** | **A** | **A** | **G** | **T** | **A** | **A** | **T** | **A** | **A** | **A** | **G** | **C** | **C** | **G** | **G** | **T** | **A** | **G** | **T** | **T** | **A** | **T** | **G** | **G** | **C** | **T** | **T** | **A** | **A** | **A** | **T** | **A** | **A** | **T** | **A** | **T** | **C** | **C** | **G** | **A** | **G** | **A** | **A** | **C** | **G** | **T** | **G** | **T** | **T** | **G** | **T** | **C** | **G** | **G** | **C** | **A** | **T** | **G** | **G** | **G** | **C** | **G** | **G** | **T** | **A** | **C** | **A** | **G** | **T** | **T** | **A** | **A** | **A** | **A** | **T** | **T** | **A** | **T** | **T** | **A** | **G** | **T** | **T** | **T** | **T** | **A** | **A** | **A** | **G** | **G** | **G** | **C** | **A** | **G** | **G** | **G** | **A** | **A** | **C** | **C** | **A** | **G** | **C** | **G** | **T** | **T** | **G** | **A** | **A** | **A** | **T** | **T** | **A** | **A** | **A** | **G** | **T** | **T** | **C** | **C** | **T** | **G** | **T** | **C** | **A** | **T** | **A** | **A** | **A** | **G** | **G** | **A** | **G** | **G** | **A** | **A** | **A** | **C** | **T** | **G** | **C** | **A** | **A** | **G** | **T** | **G** | **A** | **T** | **C** | **A** | **A** | **A** | **G** | **T** | **A** | **A** | **T** | **G** | **T** | **T** | **A** | **G** | **T** | **G** | **G** | **A** | **-** | **-** | **-** |
| **N59458F** | **A** | **T** | **G** | **A** | **C** | **C** | **G** | **A** | **T** | **C** | **G** | **G** | **A** | **T** | **T** | **T** | **C** | **A** | **A** | **G** | **A** | **C** | **G** | **C** | **A** | **T** | **G** | **A** | **T** | **T** | **T** | **C** | **A** | **T** | **T** | **A** | **T** | **A** | **T** | **G** | **C** | **G** | **T** | **C** | **C** | **C** | **T** | **T** | **A** | **G** | **C** | **A** | **C** | **C** | **T** | **T** | **T** | **A** | **T** | **T** | **G** | **T** | **T** | **A** | **T** | **C** | **T** | **T** | **A** | **A** | **T** | **T** | **A** | **C** | **A** | **T** | **T** | **G** | **T** | **T** | **T** | **T** | **C** | **A** | **T** | **A** | **T** | **T** | **T** | **T** | **C** | **A** | **T** | **T** | **C** | **G** | **A** | **T** | **T** | **A** | **A** | **A** | **C** | **A** | **A** | **A** | **A** | **C** | **C** | **G** | **G** | **T** | **G** | **G** | **C** | **T** | **A** | **T** | **T** | **A** | **G** | **A** | **G** | **C** | **T** | **T** | **C** | **T** | **T** | **C** | **A** | **G** | **A** | **G** | **A** | **A** | **A** | **A** | **G** | **T** | **C** | **T** | **T** | **T** | **T** | **A** | **T** | **T** | **T** | **A** | **C** | **C** | **A** | **C** | **T** | **A** | **A** | **T** | **T** | **G** | **T** | **G** | **C** | **A** | **C** | **A** | **T** | **T** | **G** | **T** | **T** | **C** | **T** | **C** | **A** | **T** | **A** | **T** | **C** | **C** | **T** | **T** | **A** | **C** | **T** | **A** | **A** | **T** | **A** | **G** | **G** | **C** | **T** | **T** | **A** | **T** | **T** | **G** | **A** | **C** | **C** | **T** | **T** | **T** | **T** | **T** | **A** | **C** | **T** | **G** | **A** | **T** | **T** | **T** | **C** | **A** | **T** | **T** | **G** | **G** | **T** | **T** | **C** | **A** | **A** | **A** | **A** | **A** | **G** | **G** | **G** | **C** | **A** | **A** | **T** | **A** | **T** | **G** | **G** | **A** | **C** | **G** | **G** | **A** | **T** | **T** | **G** | **A** | **A** | **G** | **A** | **A** | **A** | **A** | **A** | **C** | **T** | **T** | **C** | **G** | **G** | **T** | **T** | **A** | **T** | **T** | **G** | **G** | **C** | **C** | **A** | **A** | **C** | **G** | **G** | **T** | **A** | **A** | **T** | **T** | **A** | **T** | **G** | **A** | **A** | **A** | **G** | **T** | **C** | **C** | **A** | **G** | **T** | **C** | **T** | **T** | **A** | **A** | **A** | **C** | **A** | **A** | **A** | **C** | **C** | **A** | **A** | **C** | **G** | **A** | **C** | **C** | **A** | **G** | **T** | **G** | **A** | **A** | **A** | **A** | **T** | **C** | **A** | **A** | **G** | **A** | **C** | **C** | **A** | **T** | **T** | **A** | **T** | **C** | **T** | **A** | **A** | **C** | **C** | **G** | **A** | **A** | **G** | **T** | **C** | **G** | **A** | **A** | **C** | **A** | **A** | **G** | **A** | **T** | **A** | **T** | **T** | **T** | **G** | **G** | **T** | **C** | **G** | **A** | **T** | **T** | **A** | **A** | **A** | **A** | **A** | **T** | **A** | **A** | **A** | **T** | **T** | **A** | **T** | **T** | **A** | **G** | **A** | **G** | **A** | **T** | **G** | **T** | **C** | **T** | **A** | **A** | **A** | **G** | **A** | **A** | **T** | **T** | **G** | **C** | **A** | **A** | **T** | **T** | **A** | **T** | **T** | **A** | **A** | **A** | **C** | **A** | **G** | **T** | **C** | **G** | **A** | **C** | **C** | **G** | **C** | **A** | **A** | **T** | **T** | **A** | **A** | **T** | **G** | **G** | **A** | **T** | **G** | **G** | **G** | **C** | **A** | **A** | **A** | **C** | **A** | **A** | **A** | **A** | **G** | **A** | **A** | **G** | **A** | **A** | **A** | **T** | **T** | **T** | **T** | **A** | **G** | **A** | **G** | **A** | **A** | **C** | **G** | **A** | **G** | **C** | **G** | **G** | **C** | **A** | **T** | **C** | **G** | **T** | **T** | **T** | **G** | **G** | **C** | **G** | **C** | **G** | **G** | **G** | **A** | **G** | **T** | **T** | **G** | **C** | **A** | **T** | **G** | **A** | **T** | **T** | **C** | **A** | **G** | **T** | **C** | **A** | **G** | **T** | **C** | **A** | **A** | **C** | **A** | **A** | **C** | **T** | **T** | **T** | **T** | **T** | **G** | **C** | **A** | **G** | **C** | **C** | **A** | **T** | **G** | **A** | **T** | **G** | **A** | **T** | **G** | **T** | **T** | **G** | **T** | **C** | **T** | **G** | **C** | **C** | **T** | **T** | **A** | **A** | **A** | **T** | **G** | **A** | **A** | **C** | **A** | **A** | **G** | **C** | **A** | **C** | **A** | **A** | **C** | **G** | **A** | **A** | **C** | **A** | **G** | **A** | **A** | **A** | **C** | **T** | **C** | **C** | **T** | **G** | **A** | **A** | **C** | **C** | **A** | **T** | **A** | **T** | **C** | **G** | **C** | **A** | **A** | **A** | **C** | **A** | **A** | **C** | **T** | **A** | **G** | **C** | **C** | **A** | **T** | **G** | **G** | **T** | **G** | **G** | **C** | **A** | **G** | **A** | **A** | **A** | **T** | **C** | **A** | **T** | **T** | **A** | **A** | **T** | **G** | **C** | **C** | **T** | **C** | **C** | **C** | **A** | **A** | **T** | **C** | **G** | **G** | **A** | **A** | **A** | **T** | **G** | **C** | **G** | **C** | **G** | **C** | **G** | **T** | **T** | **A** | **C** | **T** | **A** | **T** | **T** | **G** | **C** | **A** | **C** | **T** | **T** | **G** | **C** | **G** | **T** | **C** | **C** | **T** | **A** | **T** | **C** | **A** | **G** | **T** | **C** | **T** | **A** | **G** | **A** | **A** | **G** | **G** | **A** | **A** | **A** | **A** | **A** | **G** | **T** | **T** | **T** | **G** | **C** | **G** | **T** | **A** | **A** | **A** | **G** | **G** | **T** | **A** | **T** | **T** | **G** | **A** | **A** | **C** | **A** | **A** | **T** | **T** | **A** | **C** | **T** | **G** | **A** | **A** | **A** | **G** | **A** | **A** | **C** | **T** | **A** | **C** | **A** | **A** | **A** | **C** | **A** | **A** | **A** | **A** | **A** | **T** | **T** | **A** | **A** | **A** | **A** | **T** | **T** | **G** | **A** | **A** | **T** | **T** | **G** | **A** | **T** | **T** | **T** | **G** | **G** | **G** | **A** | **T** | **G** | **T** | **T** | **G** | **A** | **A** | **G** | **A** | **T** | **G** | **T** | **T** | **C** | **A** | **T** | **T** | **T** | **A** | **A** | **A** | **T** | **A** | **G** | **C** | **A** | **G** | **C** | **A** | **T** | **T** | **G** | **A** | **G** | **G** | **A** | **T** | **C** | **A** | **T** | **C** | **T** | **T** | **T** | **T** | **C** | **C** | **G** | **A** | **A** | **T** | **T** | **G** | **T** | **G** | **C** | **A** | **G** | **G** | **A** | **A** | **T** | **T** | **A** | **C** | **T** | **T** | **T** | **C** | **A** | **A** | **A** | **T** | **A** | **C** | **C** | **T** | **T** | **A** | **A** | **G** | **A** | **C** | **A** | **T** | **G** | **C** | **C** | **A** | **A** | **A** | **G** | **C** | **A** | **A** | **A** | **G** | **G** | **A** | **A** | **T** | **T** | **A** | **G** | **A** | **G** | **G** | **T** | **A** | **T** | **A** | **C** | **T** | **T** | **A** | **C** | **A** | **C** | **C** | **A** | **A** | **G** | **T** | **C** | **G** | **A** | **T** | **A** | **A** | **A** | **A** | **A** | **C** | **G** | **T** | **G** | **T** | **T** | **A** | **T** | **T** | **G** | **C** | **G** | **T** | **A** | **T** | **T** | **G** | **T** | **T** | **G** | **A** | **T** | **G** | **A** | **T** | **G** | **G** | **T** | **G** | **T** | **C** | **G** | **G** | **C** | **T** | **T** | **T** | **G** | **A** | **T** | **A** | **T** | **G** | **A** | **A** | **G** | **G** | **A** | **A** | **C** | **A** | **A** | **A** | **G** | **T** | **A** | **A** | **T** | **A** | **A** | **A** | **G** | **C** | **C** | **G** | **G** | **T** | **A** | **G** | **T** | **T** | **A** | **T** | **G** | **G** | **C** | **T** | **T** | **A** | **A** | **A** | **T** | **A** | **A** | **T** | **A** | **T** | **C** | **C** | **G** | **A** | **G** | **A** | **A** | **C** | **G** | **T** | **G** | **T** | **T** | **G** | **T** | **C** | **G** | **G** | **C** | **A** | **T** | **G** | **G** | **G** | **C** | **G** | **G** | **T** | **A** | **C** | **A** | **G** | **T** | **T** | **A** | **A** | **A** | **A** | **T** | **T** | **A** | **T** | **T** | **A** | **G** | **T** | **T** | **T** | **T** | **A** | **A** | **A** | **G** | **G** | **G** | **C** | **A** | **G** | **G** | **G** | **A** | **A** | **C** | **C** | **A** | **G** | **C** | **G** | **T** | **T** | **G** | **A** | **A** | **A** | **T** | **T** | **A** | **A** | **A** | **G** | **T** | **T** | **C** | **C** | **T** | **G** | **T** | **C** | **A** | **T** | **A** | **A** | **A** | **G** | **G** | **A** | **G** | **G** | **A** | **A** | **A** | **C** | **T** | **G** | **C** | **A** | **A** | **G** | **T** | **G** | **A** | **T** | **C** | **A** | **A** | **A** | **G** | **T** | **A** | **A** | **T** | **G** | **T** | **T** | **A** | **G** | **T** | **G** | **G** | **A** | **-** | **-** | **-** |
| **N52454** | **A** | **T** | **G** | **A** | **C** | **C** | **G** | **A** | **T** | **C** | **G** | **G** | **A** | **T** | **T** | **T** | **C** | **A** | **A** | **G** | **A** | **C** | **G** | **C** | **A** | **T** | **G** | **A** | **T** | **T** | **T** | **C** | **A** | **T** | **T** | **A** | **T** | **A** | **T** | **G** | **C** | **G** | **T** | **C** | **C** | **C** | **T** | **T** | **A** | **G** | **C** | **A** | **C** | **C** | **T** | **T** | **T** | **A** | **T** | **T** | **G** | **T** | **T** | **A** | **T** | **C** | **T** | **T** | **A** | **A** | **T** | **T** | **A** | **C** | **A** | **T** | **T** | **G** | **T** | **T** | **T** | **T** | **C** | **A** | **T** | **A** | **T** | **T** | **T** | **T** | **C** | **A** | **T** | **T** | **C** | **G** | **A** | **T** | **T** | **A** | **A** | **A** | **C** | **A** | **A** | **A** | **A** | **C** | **C** | **G** | **G** | **T** | **G** | **G** | **T** | **T** | **A** | **T** | **T** | **A** | **G** | **A** | **G** | **C** | **T** | **T** | **C** | **T** | **T** | **C** | **A** | **G** | **A** | **G** | **A** | **A** | **A** | **A** | **G** | **T** | **C** | **T** | **T** | **T** | **T** | **A** | **T** | **T** | **T** | **A** | **C** | **C** | **A** | **C** | **T** | **A** | **A** | **T** | **T** | **G** | **T** | **G** | **C** | **A** | **C** | **A** | **T** | **T** | **G** | **T** | **T** | **C** | **T** | **C** | **A** | **T** | **A** | **T** | **C** | **C** | **T** | **T** | **A** | **C** | **T** | **A** | **A** | **T** | **A** | **G** | **G** | **C** | **T** | **T** | **A** | **T** | **T** | **G** | **A** | **C** | **C** | **T** | **T** | **T** | **T** | **T** | **A** | **C** | **T** | **G** | **A** | **T** | **T** | **T** | **C** | **A** | **T** | **T** | **G** | **G** | **T** | **T** | **C** | **A** | **A** | **A** | **A** | **A** | **G** | **G** | **G** | **C** | **A** | **A** | **T** | **A** | **T** | **G** | **G** | **A** | **C** | **G** | **G** | **A** | **T** | **T** | **G** | **A** | **A** | **G** | **A** | **A** | **A** | **A** | **A** | **C** | **T** | **T** | **C** | **G** | **G** | **T** | **T** | **A** | **T** | **T** | **G** | **G** | **C** | **C** | **A** | **A** | **C** | **G** | **G** | **T** | **A** | **A** | **T** | **T** | **A** | **T** | **G** | **A** | **A** | **A** | **G** | **T** | **C** | **C** | **A** | **G** | **T** | **C** | **T** | **T** | **A** | **A** | **A** | **C** | **A** | **A** | **A** | **C** | **C** | **A** | **A** | **C** | **G** | **A** | **C** | **C** | **A** | **G** | **T** | **G** | **A** | **A** | **A** | **A** | **T** | **C** | **A** | **A** | **G** | **A** | **C** | **C** | **A** | **T** | **T** | **A** | **T** | **C** | **T** | **A** | **A** | **C** | **C** | **G** | **A** | **A** | **G** | **T** | **C** | **G** | **A** | **A** | **C** | **A** | **A** | **G** | **A** | **T** | **A** | **T** | **T** | **T** | **G** | **G** | **T** | **C** | **G** | **A** | **T** | **T** | **A** | **A** | **A** | **A** | **A** | **T** | **A** | **A** | **A** | **T** | **T** | **A** | **T** | **T** | **A** | **G** | **A** | **G** | **A** | **T** | **G** | **T** | **C** | **T** | **A** | **A** | **A** | **G** | **A** | **A** | **T** | **T** | **G** | **C** | **A** | **A** | **T** | **T** | **A** | **T** | **T** | **A** | **A** | **A** | **C** | **A** | **G** | **T** | **C** | **G** | **A** | **C** | **C** | **G** | **C** | **A** | **A** | **T** | **T** | **A** | **A** | **T** | **G** | **G** | **A** | **T** | **G** | **G** | **G** | **C** | **A** | **A** | **A** | **C** | **A** | **A** | **A** | **A** | **G** | **A** | **A** | **G** | **A** | **A** | **A** | **T** | **T** | **T** | **T** | **A** | **G** | **A** | **G** | **A** | **A** | **C** | **G** | **A** | **G** | **C** | **G** | **G** | **C** | **A** | **T** | **C** | **G** | **T** | **T** | **T** | **G** | **G** | **C** | **G** | **C** | **G** | **G** | **G** | **A** | **G** | **T** | **T** | **G** | **C** | **A** | **T** | **G** | **A** | **T** | **T** | **C** | **A** | **G** | **T** | **C** | **A** | **G** | **T** | **C** | **A** | **A** | **C** | **A** | **A** | **C** | **T** | **T** | **T** | **T** | **T** | **G** | **C** | **A** | **G** | **C** | **C** | **A** | **T** | **G** | **A** | **T** | **G** | **A** | **T** | **G** | **T** | **T** | **G** | **T** | **C** | **T** | **G** | **C** | **C** | **T** | **T** | **A** | **A** | **A** | **T** | **G** | **A** | **A** | **C** | **A** | **A** | **G** | **C** | **A** | **C** | **A** | **A** | **C** | **G** | **A** | **A** | **C** | **A** | **G** | **A** | **A** | **A** | **C** | **T** | **C** | **C** | **T** | **G** | **A** | **A** | **C** | **C** | **A** | **T** | **A** | **T** | **C** | **G** | **C** | **A** | **A** | **A** | **C** | **A** | **A** | **C** | **T** | **A** | **G** | **C** | **C** | **A** | **T** | **G** | **G** | **T** | **G** | **G** | **C** | **A** | **G** | **A** | **A** | **A** | **T** | **C** | **A** | **T** | **T** | **A** | **A** | **T** | **G** | **C** | **C** | **T** | **C** | **C** | **C** | **A** | **A** | **T** | **C** | **G** | **G** | **A** | **A** | **A** | **T** | **G** | **C** | **G** | **T** | **G** | **C** | **G** | **C** | **T** | **A** | **C** | **T** | **A** | **T** | **T** | **G** | **C** | **A** | **C** | **T** | **T** | **G** | **C** | **G** | **T** | **C** | **C** | **T** | **A** | **T** | **C** | **A** | **G** | **T** | **C** | **T** | **A** | **G** | **A** | **A** | **G** | **G** | **A** | **A** | **A** | **A** | **A** | **G** | **T** | **T** | **T** | **G** | **C** | **G** | **T** | **A** | **A** | **A** | **G** | **G** | **T** | **A** | **T** | **T** | **G** | **A** | **A** | **C** | **A** | **A** | **T** | **T** | **A** | **C** | **T** | **G** | **A** | **A** | **A** | **G** | **A** | **A** | **C** | **T** | **A** | **C** | **A** | **A** | **A** | **C** | **A** | **A** | **A** | **A** | **A** | **T** | **T** | **A** | **A** | **A** | **A** | **T** | **T** | **G** | **A** | **A** | **T** | **T** | **G** | **A** | **T** | **T** | **T** | **G** | **G** | **G** | **A** | **T** | **G** | **T** | **T** | **G** | **A** | **A** | **G** | **A** | **T** | **G** | **T** | **T** | **C** | **A** | **T** | **T** | **T** | **A** | **A** | **A** | **T** | **A** | **G** | **C** | **A** | **G** | **C** | **A** | **T** | **T** | **G** | **A** | **G** | **G** | **A** | **T** | **C** | **A** | **T** | **C** | **T** | **T** | **T** | **T** | **C** | **C** | **G** | **A** | **A** | **T** | **T** | **G** | **T** | **G** | **C** | **A** | **G** | **G** | **A** | **G** | **T** | **T** | **A** | **C** | **T** | **T** | **T** | **C** | **A** | **A** | **A** | **T** | **A** | **C** | **C** | **T** | **T** | **A** | **A** | **G** | **A** | **C** | **A** | **T** | **G** | **C** | **C** | **A** | **A** | **A** | **G** | **C** | **A** | **A** | **A** | **G** | **G** | **A** | **A** | **T** | **T** | **A** | **G** | **A** | **G** | **G** | **T** | **A** | **T** | **A** | **C** | **T** | **T** | **A** | **C** | **A** | **C** | **C** | **A** | **A** | **G** | **T** | **C** | **G** | **A** | **T** | **A** | **A** | **A** | **A** | **A** | **C** | **G** | **T** | **G** | **T** | **T** | **A** | **T** | **T** | **G** | **C** | **G** | **T** | **A** | **T** | **T** | **G** | **T** | **T** | **G** | **A** | **T** | **G** | **A** | **T** | **G** | **G** | **T** | **G** | **T** | **C** | **G** | **G** | **C** | **T** | **T** | **T** | **G** | **A** | **T** | **A** | **T** | **G** | **A** | **A** | **G** | **G** | **A** | **A** | **C** | **A** | **A** | **A** | **G** | **T** | **A** | **A** | **T** | **A** | **A** | **A** | **G** | **C** | **C** | **G** | **G** | **T** | **A** | **G** | **T** | **T** | **A** | **T** | **G** | **G** | **C** | **T** | **T** | **A** | **A** | **A** | **T** | **A** | **A** | **T** | **A** | **T** | **C** | **C** | **G** | **A** | **G** | **A** | **A** | **C** | **G** | **T** | **G** | **T** | **T** | **G** | **T** | **C** | **G** | **G** | **C** | **A** | **T** | **G** | **G** | **G** | **C** | **G** | **G** | **T** | **A** | **C** | **A** | **G** | **T** | **T** | **A** | **A** | **A** | **A** | **T** | **T** | **A** | **T** | **T** | **A** | **G** | **T** | **T** | **T** | **T** | **A** | **A** | **A** | **G** | **G** | **G** | **C** | **A** | **G** | **G** | **G** | **A** | **A** | **C** | **C** | **A** | **G** | **C** | **G** | **T** | **T** | **G** | **A** | **A** | **A** | **T** | **T** | **A** | **A** | **A** | **G** | **T** | **T** | **C** | **C** | **T** | **G** | **T** | **C** | **A** | **T** | **A** | **A** | **A** | **G** | **G** | **A** | **G** | **G** | **A** | **A** | **A** | **C** | **T** | **G** | **C** | **A** | **A** | **G** | **T** | **G** | **A** | **T** | **C** | **A** | **A** | **A** | **G** | **T** | **A** | **A** | **T** | **G** | **T** | **T** | **A** | **G** | **T** | **G** | **G** | **A** | **-** | **-** | **-** |
| **N59457F** | **A** | **T** | **G** | **A** | **C** | **C** | **G** | **A** | **T** | **C** | **G** | **G** | **A** | **T** | **T** | **T** | **C** | **A** | **A** | **G** | **A** | **C** | **G** | **C** | **A** | **T** | **G** | **A** | **T** | **T** | **T** | **C** | **A** | **T** | **T** | **A** | **T** | **A** | **T** | **G** | **C** | **G** | **T** | **C** | **C** | **C** | **T** | **T** | **A** | **G** | **C** | **A** | **C** | **C** | **T** | **T** | **T** | **A** | **T** | **T** | **G** | **T** | **T** | **A** | **T** | **C** | **T** | **T** | **A** | **A** | **T** | **T** | **A** | **C** | **A** | **T** | **T** | **G** | **T** | **T** | **T** | **T** | **C** | **A** | **T** | **A** | **T** | **T** | **T** | **T** | **C** | **A** | **T** | **T** | **C** | **G** | **A** | **T** | **T** | **A** | **A** | **A** | **C** | **A** | **A** | **A** | **A** | **C** | **C** | **G** | **G** | **T** | **G** | **G** | **T** | **T** | **A** | **T** | **T** | **A** | **G** | **A** | **G** | **C** | **T** | **T** | **C** | **T** | **T** | **C** | **A** | **G** | **A** | **G** | **A** | **A** | **A** | **A** | **G** | **T** | **C** | **T** | **T** | **T** | **T** | **A** | **T** | **T** | **T** | **A** | **C** | **C** | **A** | **C** | **T** | **A** | **A** | **T** | **T** | **G** | **T** | **G** | **C** | **A** | **C** | **A** | **T** | **T** | **G** | **T** | **T** | **C** | **T** | **C** | **A** | **T** | **A** | **T** | **C** | **C** | **T** | **T** | **A** | **C** | **T** | **A** | **A** | **T** | **A** | **G** | **G** | **C** | **T** | **T** | **A** | **T** | **T** | **G** | **A** | **C** | **C** | **T** | **T** | **T** | **T** | **T** | **A** | **C** | **T** | **G** | **A** | **T** | **T** | **T** | **C** | **A** | **T** | **T** | **G** | **G** | **T** | **T** | **C** | **A** | **A** | **A** | **A** | **A** | **G** | **G** | **G** | **C** | **A** | **A** | **T** | **A** | **T** | **G** | **G** | **A** | **C** | **G** | **G** | **A** | **T** | **T** | **G** | **A** | **A** | **G** | **A** | **A** | **A** | **A** | **A** | **C** | **T** | **T** | **C** | **G** | **G** | **T** | **T** | **A** | **T** | **T** | **G** | **G** | **C** | **C** | **A** | **A** | **C** | **G** | **G** | **T** | **A** | **A** | **T** | **T** | **A** | **T** | **G** | **A** | **A** | **A** | **G** | **T** | **C** | **C** | **A** | **G** | **T** | **C** | **T** | **T** | **A** | **A** | **A** | **C** | **A** | **A** | **A** | **C** | **C** | **A** | **A** | **C** | **G** | **A** | **C** | **C** | **A** | **G** | **T** | **G** | **A** | **A** | **A** | **A** | **T** | **C** | **A** | **A** | **G** | **A** | **C** | **C** | **A** | **T** | **T** | **A** | **T** | **C** | **T** | **A** | **A** | **C** | **C** | **G** | **A** | **A** | **G** | **T** | **C** | **G** | **A** | **A** | **C** | **A** | **A** | **G** | **A** | **T** | **A** | **T** | **T** | **T** | **G** | **G** | **T** | **C** | **G** | **A** | **T** | **T** | **A** | **A** | **A** | **A** | **A** | **T** | **A** | **A** | **A** | **T** | **T** | **A** | **T** | **T** | **A** | **G** | **A** | **G** | **A** | **T** | **G** | **T** | **C** | **T** | **A** | **A** | **A** | **G** | **A** | **A** | **T** | **T** | **G** | **C** | **A** | **A** | **T** | **T** | **A** | **T** | **T** | **A** | **A** | **A** | **C** | **A** | **G** | **T** | **C** | **G** | **A** | **C** | **C** | **G** | **C** | **A** | **A** | **T** | **T** | **A** | **A** | **T** | **G** | **G** | **A** | **T** | **G** | **G** | **G** | **C** | **A** | **A** | **A** | **C** | **A** | **A** | **A** | **A** | **G** | **A** | **A** | **G** | **A** | **A** | **A** | **T** | **T** | **T** | **T** | **A** | **G** | **A** | **G** | **A** | **A** | **C** | **G** | **A** | **G** | **C** | **G** | **G** | **C** | **A** | **T** | **C** | **G** | **T** | **T** | **T** | **G** | **G** | **C** | **G** | **C** | **G** | **G** | **G** | **A** | **G** | **T** | **T** | **G** | **C** | **A** | **T** | **G** | **A** | **T** | **T** | **C** | **A** | **G** | **T** | **T** | **A** | **G** | **T** | **C** | **A** | **A** | **C** | **A** | **A** | **C** | **T** | **T** | **T** | **T** | **T** | **G** | **C** | **A** | **G** | **C** | **C** | **A** | **T** | **G** | **A** | **T** | **G** | **A** | **T** | **G** | **T** | **T** | **G** | **T** | **C** | **T** | **G** | **C** | **C** | **T** | **T** | **A** | **A** | **A** | **T** | **G** | **A** | **A** | **C** | **A** | **A** | **G** | **C** | **A** | **C** | **A** | **A** | **C** | **G** | **A** | **A** | **C** | **A** | **G** | **A** | **A** | **A** | **C** | **C** | **C** | **C** | **G** | **G** | **A** | **A** | **C** | **C** | **A** | **T** | **A** | **T** | **C** | **G** | **T** | **A** | **A** | **A** | **C** | **A** | **A** | **C** | **T** | **A** | **G** | **C** | **C** | **A** | **T** | **G** | **G** | **T** | **G** | **G** | **C** | **A** | **G** | **A** | **A** | **A** | **T** | **C** | **A** | **T** | **T** | **A** | **A** | **T** | **G** | **C** | **C** | **T** | **C** | **C** | **C** | **A** | **A** | **T** | **C** | **G** | **G** | **A** | **A** | **A** | **T** | **G** | **C** | **G** | **C** | **G** | **C** | **G** | **T** | **T** | **A** | **C** | **T** | **A** | **T** | **T** | **G** | **C** | **A** | **C** | **T** | **T** | **G** | **C** | **G** | **T** | **C** | **C** | **T** | **A** | **T** | **C** | **A** | **G** | **T** | **C** | **T** | **A** | **G** | **A** | **A** | **G** | **G** | **A** | **A** | **A** | **A** | **A** | **G** | **T** | **T** | **T** | **G** | **C** | **G** | **T** | **A** | **A** | **A** | **G** | **G** | **T** | **A** | **T** | **T** | **G** | **A** | **A** | **C** | **A** | **A** | **T** | **T** | **A** | **C** | **T** | **G** | **A** | **A** | **A** | **G** | **A** | **A** | **C** | **T** | **A** | **C** | **A** | **A** | **A** | **C** | **A** | **A** | **A** | **A** | **A** | **T** | **T** | **A** | **A** | **A** | **A** | **T** | **T** | **G** | **A** | **A** | **T** | **T** | **G** | **A** | **T** | **T** | **T** | **G** | **G** | **G** | **A** | **T** | **G** | **T** | **T** | **G** | **A** | **A** | **G** | **A** | **T** | **G** | **T** | **T** | **C** | **A** | **T** | **T** | **T** | **A** | **A** | **A** | **T** | **A** | **G** | **C** | **A** | **G** | **C** | **A** | **T** | **T** | **G** | **A** | **G** | **G** | **A** | **T** | **C** | **A** | **T** | **C** | **T** | **T** | **T** | **T** | **C** | **C** | **G** | **A** | **A** | **T** | **T** | **G** | **T** | **G** | **C** | **A** | **G** | **G** | **A** | **G** | **T** | **T** | **A** | **C** | **T** | **T** | **T** | **C** | **A** | **A** | **A** | **T** | **A** | **C** | **C** | **T** | **T** | **A** | **A** | **G** | **A** | **C** | **A** | **T** | **G** | **C** | **C** | **A** | **A** | **A** | **G** | **C** | **A** | **A** | **A** | **G** | **G** | **A** | **A** | **T** | **T** | **A** | **G** | **A** | **G** | **G** | **T** | **A** | **T** | **A** | **C** | **T** | **T** | **A** | **C** | **A** | **C** | **C** | **A** | **A** | **G** | **T** | **C** | **G** | **A** | **T** | **A** | **A** | **A** | **A** | **A** | **C** | **G** | **T** | **G** | **T** | **T** | **A** | **T** | **T** | **G** | **C** | **G** | **T** | **A** | **T** | **T** | **G** | **T** | **T** | **G** | **A** | **T** | **G** | **A** | **T** | **G** | **G** | **T** | **G** | **T** | **C** | **G** | **G** | **C** | **T** | **T** | **T** | **G** | **A** | **T** | **A** | **T** | **G** | **A** | **A** | **G** | **G** | **A** | **A** | **C** | **A** | **A** | **A** | **G** | **T** | **A** | **A** | **T** | **A** | **A** | **A** | **G** | **C** | **T** | **G** | **G** | **T** | **A** | **G** | **T** | **T** | **A** | **T** | **G** | **G** | **C** | **T** | **T** | **A** | **A** | **A** | **T** | **A** | **A** | **T** | **A** | **T** | **C** | **C** | **G** | **A** | **G** | **A** | **A** | **C** | **G** | **T** | **G** | **T** | **T** | **G** | **T** | **C** | **G** | **G** | **C** | **A** | **T** | **G** | **G** | **G** | **C** | **G** | **G** | **T** | **A** | **C** | **A** | **G** | **T** | **T** | **A** | **A** | **A** | **A** | **T** | **T** | **A** | **T** | **T** | **A** | **G** | **T** | **T** | **T** | **T** | **A** | **A** | **A** | **G** | **G** | **G** | **C** | **A** | **G** | **G** | **G** | **A** | **A** | **C** | **C** | **A** | **G** | **C** | **G** | **T** | **T** | **G** | **A** | **A** | **A** | **T** | **T** | **A** | **A** | **A** | **G** | **T** | **T** | **C** | **C** | **T** | **G** | **T** | **C** | **A** | **T** | **A** | **A** | **A** | **G** | **G** | **A** | **G** | **G** | **A** | **A** | **A** | **C** | **T** | **G** | **C** | **A** | **A** | **G** | **T** | **G** | **A** | **T** | **C** | **A** | **A** | **A** | **G** | **T** | **A** | **A** | **T** | **G** | **T** | **T** | **A** | **G** | **T** | **G** | **G** | **A** | **-** | **-** | **-** |
| **N60114F** | **A** | **T** | **G** | **A** | **C** | **C** | **G** | **A** | **T** | **C** | **G** | **G** | **A** | **T** | **T** | **T** | **C** | **A** | **A** | **G** | **A** | **C** | **G** | **C** | **A** | **T** | **G** | **A** | **T** | **T** | **T** | **C** | **A** | **T** | **T** | **A** | **T** | **A** | **T** | **G** | **C** | **G** | **T** | **C** | **C** | **C** | **T** | **T** | **A** | **G** | **C** | **A** | **C** | **C** | **T** | **T** | **T** | **A** | **T** | **T** | **G** | **T** | **T** | **A** | **T** | **C** | **T** | **T** | **A** | **A** | **T** | **T** | **A** | **C** | **A** | **T** | **T** | **G** | **T** | **T** | **T** | **T** | **C** | **A** | **T** | **A** | **T** | **T** | **T** | **T** | **C** | **A** | **T** | **T** | **C** | **G** | **A** | **T** | **T** | **A** | **A** | **A** | **C** | **A** | **A** | **A** | **A** | **C** | **C** | **G** | **G** | **T** | **G** | **G** | **C** | **T** | **A** | **T** | **T** | **A** | **G** | **A** | **G** | **C** | **T** | **T** | **C** | **T** | **T** | **C** | **A** | **G** | **A** | **G** | **A** | **A** | **A** | **A** | **G** | **T** | **C** | **T** | **T** | **T** | **T** | **A** | **T** | **T** | **T** | **A** | **C** | **C** | **A** | **C** | **T** | **A** | **A** | **T** | **T** | **G** | **T** | **G** | **C** | **A** | **C** | **A** | **T** | **T** | **G** | **T** | **T** | **C** | **T** | **C** | **A** | **T** | **A** | **T** | **C** | **C** | **T** | **T** | **A** | **C** | **T** | **A** | **A** | **T** | **A** | **G** | **G** | **C** | **T** | **T** | **A** | **T** | **T** | **G** | **A** | **C** | **C** | **T** | **T** | **T** | **T** | **T** | **A** | **C** | **T** | **G** | **A** | **T** | **T** | **T** | **C** | **A** | **T** | **T** | **G** | **G** | **T** | **T** | **C** | **A** | **A** | **A** | **A** | **A** | **G** | **G** | **G** | **C** | **A** | **A** | **T** | **A** | **T** | **G** | **G** | **A** | **C** | **G** | **G** | **A** | **T** | **T** | **G** | **A** | **A** | **G** | **A** | **A** | **A** | **A** | **A** | **C** | **T** | **T** | **C** | **G** | **G** | **T** | **T** | **A** | **T** | **T** | **G** | **G** | **C** | **C** | **A** | **A** | **C** | **G** | **G** | **T** | **A** | **A** | **T** | **T** | **A** | **T** | **G** | **A** | **A** | **A** | **G** | **T** | **C** | **C** | **A** | **G** | **T** | **C** | **T** | **T** | **A** | **A** | **A** | **C** | **A** | **A** | **A** | **C** | **C** | **A** | **A** | **C** | **G** | **A** | **C** | **C** | **A** | **G** | **T** | **G** | **A** | **A** | **A** | **A** | **T** | **C** | **A** | **A** | **G** | **A** | **C** | **C** | **A** | **T** | **T** | **A** | **T** | **C** | **T** | **A** | **A** | **C** | **C** | **G** | **A** | **A** | **G** | **T** | **C** | **G** | **A** | **A** | **C** | **A** | **A** | **G** | **A** | **T** | **A** | **T** | **T** | **T** | **G** | **G** | **T** | **C** | **G** | **A** | **T** | **T** | **A** | **A** | **A** | **A** | **A** | **T** | **A** | **A** | **A** | **T** | **T** | **A** | **T** | **T** | **A** | **G** | **A** | **G** | **A** | **T** | **G** | **T** | **C** | **T** | **A** | **A** | **A** | **G** | **A** | **A** | **T** | **T** | **G** | **C** | **A** | **A** | **T** | **T** | **A** | **T** | **T** | **A** | **A** | **A** | **C** | **A** | **G** | **T** | **C** | **G** | **A** | **C** | **C** | **G** | **C** | **A** | **A** | **T** | **T** | **A** | **A** | **T** | **G** | **G** | **A** | **T** | **G** | **G** | **G** | **C** | **A** | **A** | **A** | **C** | **A** | **A** | **A** | **A** | **G** | **A** | **A** | **G** | **A** | **A** | **A** | **T** | **T** | **T** | **T** | **A** | **G** | **A** | **G** | **A** | **A** | **C** | **G** | **A** | **G** | **C** | **G** | **G** | **C** | **A** | **T** | **C** | **G** | **T** | **T** | **T** | **G** | **G** | **C** | **G** | **C** | **G** | **G** | **G** | **A** | **G** | **T** | **T** | **G** | **C** | **A** | **T** | **G** | **A** | **T** | **T** | **C** | **A** | **G** | **T** | **C** | **A** | **G** | **T** | **C** | **A** | **A** | **C** | **A** | **A** | **C** | **T** | **T** | **T** | **T** | **T** | **G** | **C** | **A** | **G** | **C** | **C** | **A** | **T** | **G** | **A** | **T** | **G** | **A** | **T** | **G** | **T** | **T** | **G** | **T** | **C** | **T** | **G** | **C** | **C** | **T** | **T** | **A** | **A** | **A** | **T** | **G** | **A** | **A** | **C** | **A** | **A** | **G** | **C** | **A** | **C** | **A** | **A** | **C** | **G** | **A** | **A** | **C** | **A** | **G** | **A** | **A** | **A** | **C** | **T** | **C** | **C** | **T** | **G** | **A** | **A** | **C** | **C** | **A** | **T** | **A** | **T** | **C** | **G** | **C** | **A** | **A** | **A** | **C** | **A** | **A** | **C** | **T** | **A** | **G** | **C** | **C** | **A** | **T** | **G** | **G** | **T** | **G** | **G** | **C** | **A** | **G** | **A** | **A** | **A** | **T** | **C** | **A** | **T** | **T** | **A** | **A** | **T** | **G** | **C** | **C** | **T** | **C** | **C** | **C** | **A** | **A** | **T** | **C** | **G** | **G** | **A** | **A** | **A** | **T** | **G** | **C** | **G** | **C** | **G** | **C** | **G** | **T** | **T** | **A** | **C** | **T** | **A** | **T** | **T** | **G** | **C** | **A** | **C** | **T** | **T** | **G** | **C** | **G** | **T** | **C** | **C** | **T** | **A** | **T** | **C** | **A** | **G** | **T** | **C** | **T** | **A** | **G** | **A** | **A** | **G** | **G** | **A** | **A** | **A** | **A** | **A** | **G** | **T** | **T** | **T** | **G** | **C** | **G** | **T** | **A** | **A** | **A** | **G** | **G** | **T** | **A** | **T** | **T** | **G** | **A** | **A** | **C** | **A** | **A** | **T** | **T** | **A** | **C** | **T** | **G** | **A** | **A** | **A** | **G** | **A** | **A** | **C** | **T** | **A** | **C** | **A** | **A** | **A** | **C** | **A** | **A** | **A** | **A** | **A** | **T** | **T** | **A** | **A** | **A** | **A** | **T** | **T** | **G** | **A** | **A** | **T** | **T** | **G** | **A** | **T** | **T** | **T** | **G** | **G** | **G** | **A** | **T** | **G** | **T** | **T** | **G** | **A** | **A** | **G** | **A** | **T** | **G** | **T** | **T** | **C** | **A** | **T** | **T** | **T** | **A** | **A** | **A** | **T** | **A** | **G** | **C** | **A** | **G** | **C** | **A** | **T** | **T** | **G** | **A** | **G** | **G** | **A** | **T** | **C** | **A** | **T** | **C** | **T** | **T** | **T** | **T** | **C** | **C** | **G** | **A** | **A** | **T** | **T** | **G** | **T** | **G** | **C** | **A** | **G** | **G** | **A** | **A** | **T** | **T** | **A** | **C** | **T** | **T** | **T** | **C** | **A** | **A** | **A** | **T** | **A** | **C** | **C** | **T** | **T** | **A** | **A** | **G** | **A** | **C** | **A** | **T** | **G** | **C** | **C** | **A** | **A** | **A** | **G** | **C** | **A** | **A** | **A** | **G** | **G** | **A** | **A** | **T** | **T** | **A** | **G** | **A** | **G** | **G** | **T** | **A** | **T** | **A** | **C** | **T** | **T** | **A** | **C** | **A** | **C** | **C** | **A** | **A** | **G** | **T** | **C** | **G** | **A** | **T** | **A** | **A** | **A** | **A** | **A** | **C** | **G** | **T** | **G** | **T** | **T** | **A** | **T** | **T** | **G** | **C** | **G** | **T** | **A** | **T** | **T** | **G** | **T** | **T** | **G** | **A** | **T** | **G** | **A** | **T** | **G** | **G** | **T** | **G** | **T** | **C** | **G** | **G** | **C** | **T** | **T** | **T** | **G** | **A** | **T** | **A** | **T** | **G** | **A** | **A** | **G** | **G** | **A** | **A** | **C** | **A** | **A** | **A** | **G** | **T** | **A** | **A** | **T** | **A** | **A** | **A** | **G** | **C** | **C** | **G** | **G** | **T** | **A** | **G** | **T** | **T** | **A** | **T** | **G** | **G** | **C** | **T** | **T** | **A** | **A** | **A** | **T** | **A** | **A** | **T** | **A** | **T** | **C** | **C** | **G** | **A** | **G** | **A** | **A** | **C** | **G** | **T** | **G** | **T** | **T** | **G** | **T** | **C** | **G** | **G** | **C** | **A** | **T** | **G** | **G** | **G** | **C** | **G** | **G** | **T** | **A** | **C** | **A** | **G** | **T** | **T** | **A** | **A** | **A** | **A** | **T** | **T** | **A** | **T** | **T** | **A** | **G** | **T** | **T** | **T** | **T** | **A** | **A** | **A** | **G** | **G** | **G** | **C** | **A** | **G** | **G** | **G** | **A** | **A** | **C** | **C** | **A** | **G** | **C** | **G** | **T** | **T** | **G** | **A** | **A** | **A** | **T** | **T** | **A** | **A** | **A** | **G** | **T** | **T** | **C** | **C** | **T** | **G** | **T** | **C** | **A** | **T** | **A** | **A** | **A** | **G** | **G** | **A** | **G** | **G** | **A** | **A** | **A** | **C** | **T** | **G** | **C** | **A** | **A** | **G** | **T** | **G** | **A** | **T** | **C** | **A** | **A** | **A** | **G** | **T** | **A** | **A** | **T** | **G** | **T** | **T** | **A** | **G** | **T** | **G** | **G** | **A** | **-** | **-** | **-** |
| **N52724** | **A** | **T** | **G** | **A** | **C** | **C** | **G** | **A** | **T** | **C** | **G** | **G** | **A** | **T** | **T** | **T** | **C** | **A** | **A** | **G** | **A** | **C** | **G** | **C** | **A** | **T** | **G** | **A** | **T** | **T** | **T** | **C** | **A** | **T** | **T** | **A** | **T** | **A** | **T** | **G** | **C** | **G** | **T** | **C** | **C** | **C** | **T** | **T** | **A** | **G** | **C** | **A** | **C** | **C** | **T** | **T** | **T** | **A** | **T** | **T** | **G** | **T** | **T** | **A** | **T** | **C** | **T** | **T** | **A** | **A** | **T** | **T** | **A** | **C** | **A** | **T** | **T** | **G** | **T** | **T** | **T** | **T** | **C** | **A** | **T** | **A** | **T** | **T** | **T** | **T** | **C** | **A** | **T** | **T** | **C** | **G** | **A** | **T** | **T** | **A** | **A** | **A** | **C** | **A** | **A** | **A** | **A** | **C** | **C** | **G** | **G** | **T** | **G** | **G** | **C** | **T** | **A** | **T** | **T** | **A** | **G** | **A** | **G** | **C** | **T** | **T** | **C** | **T** | **T** | **C** | **A** | **G** | **A** | **G** | **A** | **A** | **A** | **A** | **G** | **T** | **C** | **T** | **T** | **T** | **T** | **A** | **T** | **T** | **T** | **A** | **C** | **C** | **A** | **C** | **T** | **A** | **A** | **T** | **T** | **G** | **T** | **G** | **C** | **A** | **C** | **A** | **T** | **T** | **G** | **T** | **T** | **C** | **T** | **C** | **A** | **T** | **A** | **T** | **C** | **C** | **T** | **T** | **A** | **C** | **T** | **A** | **A** | **T** | **A** | **G** | **G** | **C** | **T** | **T** | **A** | **T** | **T** | **G** | **A** | **C** | **C** | **T** | **T** | **T** | **T** | **T** | **A** | **C** | **T** | **G** | **A** | **T** | **T** | **T** | **C** | **A** | **T** | **T** | **G** | **G** | **T** | **T** | **C** | **A** | **A** | **A** | **A** | **A** | **G** | **G** | **G** | **C** | **A** | **A** | **T** | **A** | **T** | **G** | **G** | **A** | **C** | **G** | **G** | **A** | **T** | **T** | **G** | **A** | **A** | **G** | **A** | **A** | **A** | **A** | **A** | **C** | **T** | **T** | **C** | **G** | **G** | **T** | **T** | **A** | **T** | **T** | **G** | **G** | **C** | **C** | **A** | **A** | **T** | **G** | **G** | **T** | **A** | **A** | **T** | **T** | **A** | **T** | **G** | **A** | **A** | **A** | **G** | **T** | **C** | **C** | **A** | **G** | **T** | **C** | **T** | **T** | **A** | **A** | **A** | **C** | **A** | **A** | **A** | **C** | **C** | **A** | **A** | **C** | **G** | **A** | **C** | **C** | **A** | **G** | **T** | **G** | **A** | **G** | **A** | **A** | **T** | **C** | **A** | **A** | **G** | **A** | **C** | **C** | **A** | **T** | **T** | **A** | **T** | **C** | **T** | **A** | **A** | **C** | **C** | **G** | **A** | **A** | **G** | **T** | **C** | **G** | **A** | **A** | **C** | **A** | **A** | **G** | **A** | **T** | **A** | **T** | **T** | **T** | **G** | **G** | **T** | **C** | **G** | **A** | **T** | **T** | **A** | **A** | **A** | **A** | **A** | **T** | **A** | **A** | **A** | **T** | **T** | **A** | **T** | **T** | **A** | **G** | **A** | **G** | **A** | **T** | **G** | **T** | **C** | **T** | **A** | **A** | **A** | **G** | **A** | **A** | **T** | **T** | **G** | **C** | **A** | **A** | **T** | **T** | **A** | **T** | **T** | **A** | **A** | **A** | **C** | **A** | **G** | **T** | **C** | **G** | **G** | **C** | **C** | **G** | **C** | **A** | **A** | **T** | **T** | **A** | **A** | **T** | **G** | **G** | **A** | **T** | **G** | **G** | **G** | **C** | **A** | **A** | **A** | **C** | **A** | **A** | **A** | **A** | **G** | **A** | **A** | **G** | **A** | **A** | **A** | **T** | **T** | **T** | **T** | **A** | **G** | **A** | **G** | **A** | **A** | **C** | **G** | **A** | **G** | **C** | **G** | **G** | **C** | **A** | **T** | **C** | **G** | **T** | **T** | **T** | **G** | **G** | **C** | **G** | **C** | **G** | **G** | **G** | **A** | **G** | **T** | **T** | **G** | **C** | **A** | **T** | **G** | **A** | **T** | **T** | **C** | **A** | **G** | **T** | **C** | **A** | **G** | **T** | **C** | **A** | **A** | **C** | **A** | **A** | **C** | **T** | **T** | **T** | **T** | **T** | **G** | **C** | **A** | **G** | **C** | **C** | **A** | **T** | **G** | **A** | **T** | **G** | **A** | **T** | **G** | **T** | **T** | **G** | **T** | **C** | **T** | **G** | **C** | **C** | **T** | **T** | **A** | **A** | **A** | **T** | **G** | **A** | **A** | **C** | **A** | **A** | **G** | **C** | **A** | **C** | **A** | **A** | **C** | **G** | **A** | **A** | **C** | **A** | **G** | **A** | **A** | **A** | **C** | **C** | **C** | **C** | **G** | **G** | **A** | **A** | **C** | **C** | **A** | **T** | **A** | **T** | **C** | **G** | **T** | **A** | **A** | **A** | **C** | **A** | **A** | **C** | **T** | **A** | **G** | **C** | **C** | **A** | **T** | **G** | **G** | **T** | **G** | **G** | **C** | **A** | **G** | **A** | **A** | **A** | **T** | **C** | **A** | **T** | **T** | **A** | **A** | **T** | **G** | **C** | **C** | **T** | **C** | **C** | **C** | **A** | **A** | **T** | **C** | **G** | **G** | **A** | **A** | **A** | **T** | **G** | **C** | **G** | **C** | **G** | **C** | **G** | **T** | **T** | **A** | **C** | **T** | **A** | **T** | **T** | **G** | **C** | **A** | **C** | **T** | **T** | **G** | **C** | **G** | **T** | **C** | **C** | **T** | **A** | **T** | **C** | **A** | **G** | **T** | **C** | **T** | **A** | **G** | **A** | **A** | **G** | **G** | **A** | **A** | **A** | **A** | **A** | **G** | **T** | **T** | **T** | **G** | **C** | **G** | **T** | **A** | **A** | **A** | **G** | **G** | **T** | **A** | **T** | **T** | **G** | **A** | **A** | **C** | **A** | **A** | **T** | **T** | **A** | **C** | **T** | **G** | **A** | **A** | **A** | **G** | **A** | **A** | **C** | **T** | **A** | **C** | **A** | **A** | **A** | **C** | **A** | **A** | **A** | **A** | **A** | **T** | **T** | **A** | **A** | **A** | **A** | **T** | **T** | **G** | **A** | **A** | **T** | **T** | **G** | **A** | **T** | **T** | **T** | **G** | **G** | **G** | **A** | **T** | **G** | **T** | **T** | **G** | **A** | **A** | **G** | **A** | **T** | **G** | **T** | **T** | **C** | **A** | **T** | **T** | **T** | **A** | **A** | **A** | **T** | **A** | **G** | **C** | **A** | **G** | **C** | **A** | **T** | **T** | **G** | **A** | **G** | **G** | **A** | **T** | **C** | **A** | **T** | **C** | **T** | **T** | **T** | **T** | **C** | **C** | **G** | **A** | **A** | **T** | **T** | **G** | **T** | **G** | **C** | **A** | **G** | **G** | **A** | **A** | **T** | **T** | **A** | **C** | **T** | **T** | **T** | **C** | **C** | **A** | **A** | **T** | **A** | **C** | **C** | **T** | **T** | **A** | **A** | **G** | **A** | **C** | **A** | **T** | **G** | **C** | **C** | **A** | **A** | **A** | **G** | **C** | **A** | **A** | **A** | **G** | **G** | **A** | **A** | **T** | **T** | **A** | **G** | **A** | **G** | **G** | **T** | **A** | **T** | **A** | **C** | **T** | **T** | **A** | **C** | **A** | **C** | **C** | **A** | **A** | **G** | **T** | **C** | **G** | **A** | **T** | **A** | **A** | **A** | **A** | **A** | **C** | **G** | **T** | **G** | **T** | **T** | **A** | **T** | **T** | **G** | **C** | **G** | **T** | **A** | **T** | **T** | **G** | **T** | **T** | **G** | **A** | **T** | **G** | **A** | **T** | **G** | **G** | **T** | **G** | **T** | **C** | **G** | **G** | **C** | **T** | **T** | **T** | **G** | **A** | **T** | **A** | **T** | **G** | **A** | **A** | **G** | **G** | **A** | **A** | **C** | **A** | **A** | **A** | **G** | **T** | **A** | **A** | **T** | **A** | **A** | **A** | **G** | **C** | **C** | **G** | **G** | **T** | **A** | **G** | **T** | **T** | **A** | **T** | **G** | **G** | **C** | **T** | **T** | **A** | **A** | **A** | **T** | **A** | **A** | **T** | **A** | **T** | **T** | **C** | **G** | **A** | **G** | **A** | **A** | **C** | **G** | **T** | **G** | **T** | **T** | **G** | **T** | **C** | **G** | **G** | **C** | **A** | **T** | **G** | **G** | **G** | **C** | **G** | **G** | **T** | **A** | **C** | **A** | **G** | **T** | **T** | **A** | **A** | **A** | **A** | **T** | **T** | **A** | **T** | **T** | **A** | **G** | **T** | **T** | **T** | **T** | **A** | **A** | **A** | **G** | **G** | **G** | **C** | **A** | **G** | **G** | **G** | **A** | **A** | **C** | **C** | **A** | **G** | **C** | **G** | **T** | **T** | **G** | **A** | **A** | **A** | **T** | **T** | **A** | **A** | **A** | **G** | **T** | **T** | **C** | **C** | **T** | **G** | **T** | **C** | **A** | **T** | **A** | **A** | **A** | **G** | **G** | **A** | **G** | **G** | **A** | **A** | **A** | **C** | **T** | **G** | **C** | **A** | **A** | **G** | **T** | **G** | **A** | **T** | **C** | **A** | **A** | **A** | **G** | **T** | **A** | **A** | **T** | **G** | **T** | **T** | **A** | **G** | **T** | **G** | **G** | **A** | **-** | **-** | **-** |
| **N52564** | **A** | **T** | **G** | **A** | **C** | **C** | **G** | **A** | **T** | **C** | **G** | **G** | **A** | **T** | **T** | **T** | **C** | **A** | **A** | **G** | **A** | **C** | **G** | **C** | **A** | **T** | **G** | **A** | **T** | **T** | **T** | **C** | **A** | **T** | **T** | **A** | **T** | **A** | **T** | **G** | **C** | **G** | **T** | **C** | **C** | **C** | **T** | **T** | **A** | **G** | **C** | **A** | **C** | **C** | **T** | **T** | **T** | **A** | **T** | **T** | **G** | **T** | **T** | **A** | **T** | **C** | **T** | **T** | **A** | **A** | **T** | **T** | **A** | **C** | **A** | **T** | **T** | **G** | **T** | **T** | **T** | **T** | **C** | **A** | **T** | **A** | **T** | **T** | **T** | **T** | **C** | **A** | **T** | **T** | **C** | **G** | **A** | **T** | **T** | **A** | **A** | **A** | **C** | **A** | **A** | **A** | **A** | **C** | **C** | **G** | **G** | **T** | **G** | **G** | **T** | **T** | **A** | **T** | **T** | **A** | **G** | **A** | **G** | **C** | **T** | **T** | **C** | **T** | **T** | **C** | **A** | **G** | **A** | **G** | **A** | **A** | **A** | **A** | **G** | **T** | **C** | **T** | **T** | **T** | **T** | **A** | **T** | **T** | **T** | **A** | **C** | **C** | **A** | **C** | **T** | **A** | **A** | **T** | **T** | **G** | **T** | **G** | **C** | **A** | **C** | **A** | **T** | **T** | **G** | **T** | **T** | **C** | **T** | **C** | **A** | **T** | **A** | **T** | **C** | **C** | **T** | **T** | **A** | **C** | **T** | **A** | **A** | **T** | **A** | **G** | **G** | **C** | **T** | **T** | **A** | **T** | **T** | **G** | **A** | **C** | **C** | **T** | **T** | **T** | **T** | **T** | **A** | **C** | **T** | **G** | **A** | **T** | **T** | **T** | **C** | **A** | **T** | **T** | **G** | **G** | **T** | **T** | **C** | **A** | **A** | **A** | **A** | **A** | **G** | **G** | **G** | **C** | **A** | **A** | **T** | **A** | **T** | **G** | **G** | **A** | **C** | **G** | **G** | **A** | **T** | **T** | **G** | **A** | **A** | **G** | **A** | **A** | **A** | **A** | **A** | **C** | **T** | **T** | **C** | **G** | **G** | **T** | **T** | **A** | **T** | **T** | **G** | **G** | **C** | **C** | **A** | **A** | **C** | **G** | **G** | **T** | **A** | **A** | **T** | **T** | **A** | **T** | **G** | **A** | **A** | **A** | **G** | **T** | **C** | **C** | **A** | **G** | **T** | **C** | **T** | **T** | **A** | **A** | **A** | **C** | **A** | **A** | **A** | **C** | **C** | **A** | **A** | **C** | **G** | **A** | **C** | **C** | **A** | **G** | **T** | **G** | **A** | **A** | **A** | **A** | **T** | **C** | **A** | **A** | **G** | **A** | **C** | **C** | **A** | **T** | **T** | **A** | **T** | **C** | **T** | **A** | **A** | **C** | **C** | **G** | **A** | **A** | **G** | **T** | **C** | **G** | **A** | **A** | **C** | **A** | **A** | **G** | **A** | **T** | **A** | **T** | **T** | **T** | **G** | **G** | **T** | **C** | **G** | **A** | **T** | **T** | **A** | **A** | **A** | **A** | **A** | **T** | **A** | **A** | **A** | **T** | **T** | **A** | **T** | **T** | **A** | **G** | **A** | **G** | **A** | **T** | **G** | **T** | **C** | **T** | **A** | **A** | **A** | **G** | **A** | **A** | **T** | **T** | **G** | **C** | **A** | **A** | **T** | **T** | **A** | **T** | **T** | **A** | **A** | **A** | **C** | **A** | **G** | **T** | **C** | **G** | **A** | **C** | **C** | **G** | **C** | **A** | **A** | **T** | **T** | **A** | **A** | **T** | **G** | **G** | **A** | **T** | **G** | **G** | **G** | **C** | **A** | **A** | **A** | **C** | **A** | **A** | **A** | **A** | **G** | **A** | **A** | **G** | **A** | **A** | **A** | **T** | **T** | **T** | **T** | **A** | **G** | **A** | **G** | **A** | **A** | **C** | **G** | **A** | **G** | **C** | **G** | **G** | **C** | **A** | **T** | **C** | **G** | **T** | **T** | **T** | **G** | **G** | **C** | **G** | **C** | **G** | **G** | **G** | **A** | **G** | **T** | **T** | **G** | **C** | **A** | **T** | **G** | **A** | **T** | **T** | **C** | **A** | **G** | **T** | **C** | **A** | **G** | **T** | **C** | **A** | **A** | **C** | **A** | **A** | **C** | **T** | **T** | **T** | **T** | **T** | **G** | **C** | **A** | **G** | **C** | **C** | **A** | **T** | **G** | **A** | **T** | **G** | **A** | **T** | **G** | **T** | **T** | **G** | **T** | **C** | **T** | **G** | **C** | **C** | **T** | **T** | **A** | **A** | **A** | **T** | **G** | **A** | **A** | **C** | **A** | **A** | **G** | **C** | **A** | **C** | **A** | **A** | **C** | **G** | **A** | **A** | **C** | **A** | **G** | **A** | **A** | **A** | **C** | **T** | **C** | **C** | **T** | **G** | **A** | **A** | **C** | **C** | **A** | **T** | **A** | **T** | **C** | **G** | **C** | **A** | **A** | **A** | **C** | **A** | **A** | **C** | **T** | **A** | **G** | **C** | **C** | **A** | **T** | **G** | **G** | **T** | **G** | **G** | **C** | **A** | **G** | **A** | **A** | **A** | **T** | **C** | **A** | **T** | **T** | **A** | **A** | **T** | **G** | **C** | **C** | **T** | **C** | **C** | **C** | **A** | **A** | **T** | **C** | **G** | **G** | **A** | **A** | **A** | **T** | **G** | **C** | **G** | **T** | **G** | **C** | **G** | **C** | **T** | **A** | **C** | **T** | **A** | **T** | **T** | **G** | **C** | **A** | **C** | **T** | **T** | **G** | **C** | **G** | **T** | **C** | **C** | **T** | **A** | **T** | **C** | **A** | **G** | **T** | **C** | **T** | **A** | **G** | **A** | **A** | **G** | **G** | **A** | **A** | **A** | **A** | **A** | **G** | **T** | **T** | **T** | **G** | **C** | **G** | **T** | **A** | **A** | **A** | **G** | **G** | **T** | **A** | **T** | **T** | **G** | **A** | **A** | **C** | **A** | **A** | **T** | **T** | **A** | **C** | **T** | **G** | **A** | **A** | **A** | **G** | **A** | **A** | **C** | **T** | **A** | **C** | **A** | **A** | **A** | **C** | **A** | **A** | **A** | **A** | **A** | **T** | **T** | **A** | **A** | **A** | **A** | **T** | **T** | **G** | **A** | **A** | **T** | **T** | **G** | **A** | **T** | **T** | **T** | **G** | **G** | **G** | **A** | **T** | **G** | **T** | **T** | **G** | **A** | **A** | **G** | **A** | **T** | **G** | **T** | **T** | **C** | **A** | **T** | **T** | **T** | **A** | **A** | **A** | **T** | **A** | **G** | **C** | **A** | **G** | **C** | **A** | **T** | **T** | **G** | **A** | **G** | **G** | **A** | **T** | **C** | **A** | **T** | **C** | **T** | **T** | **T** | **T** | **C** | **C** | **G** | **A** | **A** | **T** | **T** | **G** | **T** | **G** | **C** | **A** | **G** | **G** | **A** | **G** | **T** | **T** | **A** | **C** | **T** | **T** | **T** | **C** | **A** | **A** | **A** | **T** | **A** | **C** | **C** | **T** | **T** | **A** | **A** | **G** | **A** | **C** | **A** | **T** | **G** | **C** | **C** | **A** | **A** | **A** | **G** | **C** | **A** | **A** | **A** | **G** | **G** | **A** | **A** | **T** | **T** | **A** | **G** | **A** | **G** | **G** | **T** | **A** | **T** | **A** | **C** | **T** | **T** | **A** | **C** | **A** | **C** | **C** | **A** | **A** | **G** | **T** | **C** | **G** | **A** | **T** | **A** | **A** | **A** | **A** | **A** | **C** | **G** | **T** | **G** | **T** | **T** | **A** | **T** | **T** | **G** | **C** | **G** | **T** | **A** | **T** | **T** | **G** | **T** | **T** | **G** | **A** | **T** | **G** | **A** | **T** | **G** | **G** | **T** | **G** | **T** | **C** | **G** | **G** | **C** | **T** | **T** | **T** | **G** | **A** | **T** | **A** | **T** | **G** | **A** | **A** | **G** | **G** | **A** | **A** | **C** | **A** | **A** | **A** | **G** | **T** | **A** | **A** | **T** | **A** | **A** | **A** | **G** | **C** | **C** | **G** | **G** | **T** | **A** | **G** | **T** | **T** | **A** | **T** | **G** | **G** | **C** | **T** | **T** | **A** | **A** | **A** | **T** | **A** | **A** | **T** | **A** | **T** | **C** | **C** | **G** | **A** | **G** | **A** | **A** | **C** | **G** | **T** | **G** | **T** | **T** | **G** | **T** | **C** | **G** | **G** | **C** | **A** | **T** | **G** | **G** | **G** | **C** | **G** | **G** | **T** | **A** | **C** | **A** | **G** | **T** | **T** | **A** | **A** | **A** | **A** | **T** | **T** | **A** | **T** | **T** | **A** | **G** | **T** | **T** | **T** | **T** | **A** | **A** | **A** | **G** | **G** | **G** | **C** | **A** | **G** | **G** | **G** | **A** | **A** | **C** | **C** | **A** | **G** | **C** | **G** | **T** | **T** | **G** | **A** | **A** | **A** | **T** | **T** | **A** | **A** | **A** | **G** | **T** | **T** | **C** | **C** | **T** | **G** | **T** | **C** | **A** | **T** | **A** | **A** | **A** | **G** | **G** | **A** | **G** | **G** | **A** | **A** | **A** | **C** | **T** | **G** | **C** | **A** | **A** | **G** | **T** | **G** | **A** | **T** | **C** | **A** | **A** | **A** | **G** | **T** | **A** | **A** | **T** | **G** | **T** | **T** | **A** | **G** | **T** | **G** | **G** | **A** | **-** | **-** | **-** |
| **N53453** | **A** | **T** | **G** | **A** | **C** | **C** | **G** | **A** | **T** | **C** | **G** | **G** | **A** | **T** | **T** | **T** | **C** | **A** | **A** | **G** | **A** | **C** | **G** | **C** | **A** | **T** | **G** | **A** | **T** | **T** | **T** | **C** | **A** | **T** | **T** | **A** | **T** | **A** | **T** | **G** | **C** | **G** | **T** | **C** | **C** | **C** | **T** | **T** | **A** | **G** | **C** | **A** | **C** | **C** | **T** | **T** | **T** | **A** | **T** | **T** | **G** | **T** | **T** | **A** | **T** | **C** | **T** | **T** | **A** | **A** | **T** | **T** | **A** | **C** | **A** | **T** | **T** | **G** | **T** | **T** | **T** | **T** | **C** | **A** | **T** | **A** | **T** | **T** | **T** | **T** | **C** | **A** | **T** | **T** | **C** | **G** | **A** | **T** | **T** | **A** | **A** | **A** | **C** | **A** | **A** | **A** | **A** | **C** | **C** | **G** | **G** | **T** | **G** | **G** | **C** | **T** | **A** | **T** | **T** | **A** | **G** | **A** | **G** | **C** | **T** | **T** | **C** | **T** | **T** | **C** | **A** | **G** | **A** | **G** | **A** | **A** | **A** | **A** | **G** | **T** | **C** | **T** | **T** | **T** | **T** | **A** | **T** | **T** | **T** | **A** | **C** | **C** | **A** | **C** | **T** | **A** | **A** | **T** | **T** | **G** | **T** | **G** | **C** | **A** | **C** | **A** | **T** | **T** | **G** | **T** | **T** | **C** | **T** | **C** | **A** | **T** | **A** | **T** | **C** | **C** | **T** | **T** | **A** | **C** | **T** | **A** | **A** | **T** | **A** | **G** | **G** | **C** | **T** | **T** | **A** | **T** | **T** | **G** | **A** | **C** | **C** | **T** | **T** | **T** | **T** | **T** | **A** | **C** | **T** | **G** | **A** | **T** | **T** | **T** | **C** | **A** | **T** | **T** | **G** | **G** | **T** | **T** | **C** | **A** | **A** | **A** | **A** | **A** | **G** | **G** | **G** | **C** | **A** | **A** | **T** | **A** | **T** | **G** | **G** | **A** | **C** | **G** | **G** | **A** | **T** | **T** | **G** | **A** | **A** | **G** | **A** | **A** | **A** | **A** | **A** | **C** | **T** | **T** | **C** | **G** | **G** | **T** | **T** | **A** | **T** | **T** | **G** | **G** | **C** | **C** | **A** | **A** | **T** | **G** | **G** | **T** | **A** | **A** | **T** | **T** | **A** | **T** | **G** | **A** | **A** | **A** | **G** | **T** | **C** | **C** | **A** | **G** | **T** | **C** | **T** | **T** | **A** | **A** | **A** | **C** | **A** | **A** | **A** | **C** | **C** | **A** | **A** | **C** | **G** | **A** | **C** | **C** | **A** | **G** | **T** | **G** | **A** | **A** | **A** | **A** | **T** | **C** | **A** | **A** | **G** | **A** | **C** | **C** | **A** | **T** | **T** | **A** | **T** | **C** | **T** | **A** | **A** | **C** | **C** | **G** | **A** | **A** | **G** | **T** | **C** | **G** | **A** | **A** | **C** | **A** | **A** | **G** | **A** | **T** | **A** | **T** | **T** | **T** | **G** | **G** | **T** | **C** | **G** | **A** | **T** | **T** | **A** | **A** | **A** | **A** | **A** | **T** | **A** | **A** | **A** | **T** | **T** | **A** | **T** | **T** | **A** | **G** | **A** | **G** | **A** | **T** | **G** | **T** | **C** | **T** | **A** | **A** | **A** | **G** | **A** | **A** | **T** | **T** | **G** | **C** | **A** | **A** | **T** | **T** | **A** | **T** | **T** | **A** | **A** | **A** | **C** | **A** | **G** | **T** | **C** | **G** | **A** | **C** | **C** | **G** | **C** | **A** | **A** | **T** | **T** | **A** | **A** | **T** | **G** | **G** | **A** | **T** | **G** | **G** | **G** | **C** | **A** | **A** | **A** | **C** | **A** | **A** | **A** | **A** | **G** | **A** | **A** | **G** | **A** | **A** | **A** | **T** | **T** | **T** | **T** | **A** | **G** | **A** | **G** | **A** | **A** | **C** | **G** | **A** | **G** | **C** | **G** | **G** | **C** | **A** | **T** | **C** | **G** | **T** | **T** | **T** | **G** | **G** | **C** | **G** | **C** | **G** | **G** | **G** | **A** | **G** | **T** | **T** | **G** | **C** | **A** | **T** | **G** | **A** | **T** | **T** | **C** | **A** | **G** | **T** | **C** | **A** | **G** | **T** | **C** | **A** | **A** | **C** | **A** | **A** | **C** | **T** | **T** | **T** | **T** | **T** | **G** | **C** | **A** | **G** | **C** | **C** | **A** | **T** | **G** | **A** | **T** | **G** | **A** | **T** | **G** | **T** | **T** | **G** | **T** | **C** | **T** | **G** | **C** | **C** | **T** | **T** | **A** | **A** | **A** | **T** | **G** | **A** | **A** | **C** | **A** | **A** | **G** | **C** | **A** | **C** | **A** | **A** | **C** | **G** | **A** | **A** | **C** | **A** | **G** | **A** | **A** | **A** | **C** | **C** | **C** | **C** | **T** | **G** | **A** | **A** | **C** | **C** | **A** | **T** | **A** | **T** | **C** | **G** | **C** | **A** | **A** | **A** | **C** | **A** | **A** | **C** | **T** | **A** | **G** | **C** | **C** | **A** | **T** | **G** | **G** | **T** | **G** | **G** | **C** | **A** | **G** | **A** | **A** | **A** | **T** | **C** | **A** | **T** | **T** | **A** | **A** | **T** | **G** | **C** | **C** | **T** | **C** | **C** | **C** | **A** | **A** | **T** | **C** | **G** | **G** | **A** | **A** | **A** | **T** | **G** | **C** | **G** | **C** | **G** | **C** | **G** | **T** | **T** | **A** | **C** | **T** | **A** | **T** | **T** | **G** | **C** | **A** | **C** | **T** | **T** | **G** | **C** | **G** | **T** | **C** | **C** | **T** | **A** | **T** | **C** | **A** | **G** | **T** | **C** | **T** | **A** | **G** | **A** | **A** | **G** | **G** | **A** | **A** | **A** | **A** | **A** | **G** | **T** | **T** | **T** | **G** | **C** | **G** | **T** | **A** | **A** | **A** | **G** | **G** | **T** | **A** | **T** | **T** | **G** | **A** | **A** | **C** | **A** | **A** | **T** | **T** | **A** | **C** | **T** | **G** | **A** | **A** | **A** | **G** | **A** | **A** | **C** | **T** | **A** | **C** | **A** | **A** | **A** | **C** | **A** | **A** | **A** | **A** | **A** | **T** | **T** | **A** | **A** | **A** | **A** | **T** | **T** | **G** | **A** | **A** | **T** | **T** | **G** | **A** | **T** | **T** | **T** | **G** | **G** | **G** | **A** | **T** | **G** | **T** | **T** | **G** | **A** | **A** | **G** | **A** | **T** | **G** | **T** | **T** | **C** | **A** | **T** | **T** | **T** | **A** | **A** | **A** | **T** | **A** | **G** | **C** | **A** | **G** | **C** | **A** | **T** | **T** | **G** | **A** | **G** | **G** | **A** | **T** | **C** | **A** | **T** | **C** | **T** | **T** | **T** | **T** | **C** | **C** | **G** | **A** | **A** | **T** | **T** | **G** | **T** | **G** | **C** | **A** | **G** | **G** | **A** | **G** | **T** | **T** | **A** | **C** | **T** | **T** | **T** | **C** | **A** | **A** | **A** | **T** | **A** | **C** | **C** | **T** | **T** | **A** | **A** | **G** | **A** | **C** | **A** | **T** | **G** | **C** | **C** | **A** | **A** | **A** | **G** | **C** | **A** | **A** | **A** | **G** | **G** | **A** | **A** | **T** | **T** | **A** | **G** | **A** | **G** | **G** | **T** | **A** | **T** | **A** | **C** | **T** | **T** | **A** | **C** | **A** | **C** | **C** | **A** | **A** | **G** | **T** | **C** | **G** | **A** | **T** | **A** | **A** | **A** | **A** | **A** | **C** | **G** | **T** | **G** | **T** | **T** | **A** | **T** | **T** | **G** | **C** | **G** | **T** | **A** | **T** | **T** | **G** | **T** | **T** | **G** | **A** | **T** | **G** | **A** | **T** | **G** | **G** | **T** | **G** | **T** | **C** | **G** | **G** | **C** | **T** | **T** | **T** | **G** | **A** | **T** | **A** | **T** | **G** | **A** | **A** | **G** | **G** | **A** | **A** | **C** | **A** | **A** | **A** | **G** | **T** | **A** | **A** | **T** | **A** | **A** | **A** | **G** | **C** | **C** | **G** | **G** | **T** | **A** | **G** | **T** | **T** | **A** | **T** | **G** | **G** | **C** | **T** | **T** | **A** | **A** | **A** | **T** | **A** | **A** | **T** | **A** | **T** | **C** | **C** | **G** | **A** | **G** | **A** | **A** | **C** | **G** | **T** | **G** | **T** | **T** | **G** | **T** | **C** | **G** | **G** | **C** | **A** | **T** | **G** | **G** | **G** | **C** | **G** | **G** | **T** | **A** | **C** | **A** | **G** | **T** | **T** | **A** | **A** | **A** | **A** | **T** | **T** | **A** | **T** | **T** | **A** | **G** | **T** | **T** | **T** | **T** | **A** | **A** | **A** | **G** | **G** | **G** | **C** | **A** | **G** | **G** | **G** | **A** | **A** | **C** | **C** | **A** | **G** | **C** | **G** | **T** | **T** | **G** | **A** | **A** | **A** | **T** | **T** | **A** | **A** | **A** | **G** | **T** | **T** | **C** | **C** | **T** | **G** | **T** | **C** | **A** | **T** | **A** | **A** | **A** | **G** | **G** | **A** | **G** | **G** | **A** | **A** | **A** | **C** | **T** | **G** | **C** | **A** | **A** | **G** | **T** | **G** | **A** | **T** | **C** | **A** | **A** | **A** | **G** | **T** | **A** | **A** | **T** | **G** | **T** | **T** | **A** | **G** | **T** | **G** | **G** | **A** | **-** | **-** | **-** |
| **N55185** | **A** | **T** | **G** | **A** | **C** | **C** | **G** | **A** | **T** | **C** | **G** | **G** | **A** | **T** | **T** | **T** | **C** | **A** | **A** | **G** | **A** | **C** | **G** | **C** | **A** | **T** | **G** | **A** | **T** | **T** | **T** | **C** | **A** | **T** | **T** | **A** | **T** | **A** | **T** | **G** | **C** | **G** | **T** | **C** | **C** | **C** | **T** | **T** | **A** | **G** | **C** | **A** | **C** | **C** | **T** | **T** | **T** | **A** | **T** | **T** | **G** | **T** | **T** | **A** | **T** | **C** | **T** | **T** | **A** | **A** | **T** | **T** | **A** | **C** | **A** | **T** | **T** | **G** | **T** | **T** | **T** | **T** | **C** | **A** | **T** | **A** | **T** | **T** | **T** | **T** | **C** | **A** | **T** | **T** | **C** | **G** | **A** | **T** | **T** | **A** | **A** | **A** | **C** | **A** | **A** | **A** | **A** | **C** | **C** | **G** | **G** | **T** | **G** | **G** | **C** | **T** | **A** | **T** | **T** | **A** | **G** | **A** | **G** | **C** | **T** | **T** | **C** | **T** | **T** | **C** | **A** | **G** | **A** | **G** | **A** | **A** | **A** | **A** | **G** | **T** | **C** | **T** | **T** | **T** | **T** | **A** | **T** | **T** | **T** | **A** | **C** | **C** | **A** | **C** | **T** | **A** | **A** | **T** | **T** | **G** | **T** | **G** | **C** | **A** | **C** | **A** | **T** | **T** | **G** | **T** | **T** | **C** | **T** | **C** | **A** | **T** | **A** | **T** | **C** | **C** | **T** | **T** | **A** | **C** | **T** | **A** | **A** | **T** | **A** | **G** | **G** | **C** | **T** | **T** | **A** | **T** | **T** | **G** | **A** | **C** | **C** | **T** | **T** | **T** | **T** | **T** | **A** | **C** | **T** | **G** | **A** | **T** | **T** | **T** | **C** | **A** | **T** | **T** | **G** | **G** | **T** | **T** | **C** | **A** | **A** | **A** | **A** | **A** | **G** | **G** | **G** | **C** | **A** | **A** | **T** | **A** | **T** | **G** | **G** | **A** | **C** | **G** | **G** | **A** | **T** | **T** | **G** | **A** | **A** | **G** | **A** | **A** | **A** | **A** | **A** | **C** | **T** | **T** | **C** | **G** | **G** | **T** | **T** | **A** | **T** | **T** | **G** | **G** | **C** | **C** | **A** | **A** | **T** | **G** | **G** | **T** | **A** | **A** | **T** | **T** | **A** | **T** | **G** | **A** | **A** | **A** | **G** | **T** | **C** | **C** | **A** | **G** | **T** | **C** | **T** | **T** | **A** | **A** | **A** | **C** | **A** | **A** | **A** | **C** | **C** | **A** | **A** | **C** | **G** | **A** | **C** | **C** | **A** | **G** | **T** | **G** | **A** | **G** | **A** | **A** | **T** | **C** | **A** | **A** | **G** | **A** | **C** | **C** | **A** | **T** | **T** | **A** | **T** | **C** | **T** | **A** | **A** | **C** | **C** | **G** | **A** | **A** | **G** | **T** | **C** | **G** | **A** | **A** | **C** | **A** | **A** | **G** | **A** | **T** | **A** | **T** | **T** | **T** | **G** | **G** | **T** | **C** | **G** | **A** | **T** | **T** | **A** | **A** | **A** | **A** | **A** | **T** | **A** | **A** | **A** | **T** | **T** | **A** | **T** | **T** | **A** | **G** | **A** | **G** | **A** | **T** | **G** | **T** | **C** | **T** | **A** | **A** | **A** | **G** | **A** | **A** | **T** | **T** | **G** | **C** | **A** | **A** | **T** | **T** | **A** | **T** | **T** | **A** | **A** | **A** | **C** | **A** | **G** | **T** | **C** | **G** | **G** | **C** | **C** | **G** | **C** | **A** | **A** | **T** | **T** | **A** | **A** | **T** | **G** | **G** | **A** | **T** | **G** | **G** | **G** | **C** | **A** | **A** | **A** | **C** | **A** | **A** | **A** | **A** | **G** | **A** | **A** | **G** | **A** | **A** | **A** | **T** | **T** | **T** | **T** | **A** | **G** | **A** | **G** | **A** | **A** | **C** | **G** | **A** | **G** | **C** | **G** | **G** | **C** | **A** | **T** | **C** | **G** | **T** | **T** | **T** | **G** | **G** | **C** | **G** | **C** | **G** | **G** | **G** | **A** | **G** | **T** | **T** | **G** | **C** | **A** | **T** | **G** | **A** | **T** | **T** | **C** | **A** | **G** | **T** | **C** | **A** | **G** | **T** | **C** | **A** | **A** | **C** | **A** | **A** | **C** | **T** | **T** | **T** | **T** | **T** | **G** | **C** | **A** | **G** | **C** | **C** | **A** | **T** | **G** | **A** | **T** | **G** | **A** | **T** | **G** | **T** | **T** | **G** | **T** | **C** | **T** | **G** | **C** | **C** | **T** | **T** | **A** | **A** | **A** | **T** | **G** | **A** | **A** | **C** | **A** | **A** | **G** | **C** | **A** | **C** | **A** | **A** | **C** | **G** | **A** | **A** | **C** | **A** | **G** | **A** | **A** | **A** | **C** | **C** | **C** | **C** | **G** | **G** | **A** | **A** | **C** | **C** | **A** | **T** | **A** | **T** | **C** | **G** | **T** | **A** | **A** | **A** | **C** | **A** | **A** | **C** | **T** | **A** | **G** | **C** | **C** | **A** | **T** | **G** | **G** | **T** | **G** | **G** | **C** | **A** | **G** | **A** | **A** | **A** | **T** | **C** | **A** | **T** | **T** | **A** | **A** | **T** | **G** | **C** | **C** | **T** | **C** | **C** | **C** | **A** | **A** | **T** | **C** | **G** | **G** | **A** | **A** | **A** | **T** | **G** | **C** | **G** | **C** | **G** | **C** | **G** | **T** | **T** | **A** | **C** | **T** | **A** | **T** | **T** | **G** | **C** | **A** | **C** | **T** | **T** | **G** | **C** | **G** | **T** | **C** | **C** | **T** | **A** | **T** | **C** | **A** | **G** | **T** | **C** | **T** | **A** | **G** | **A** | **A** | **G** | **G** | **A** | **A** | **A** | **A** | **A** | **G** | **T** | **T** | **T** | **G** | **C** | **G** | **T** | **A** | **A** | **A** | **G** | **G** | **T** | **A** | **T** | **T** | **G** | **A** | **A** | **C** | **A** | **A** | **T** | **T** | **A** | **C** | **T** | **G** | **A** | **A** | **A** | **G** | **A** | **A** | **C** | **T** | **A** | **C** | **A** | **A** | **A** | **C** | **A** | **A** | **A** | **A** | **A** | **T** | **T** | **A** | **A** | **A** | **A** | **T** | **T** | **G** | **A** | **A** | **T** | **T** | **G** | **A** | **T** | **T** | **T** | **G** | **G** | **G** | **A** | **T** | **G** | **T** | **T** | **G** | **A** | **A** | **G** | **A** | **T** | **G** | **T** | **T** | **C** | **A** | **T** | **T** | **T** | **A** | **A** | **A** | **T** | **A** | **G** | **C** | **A** | **G** | **C** | **A** | **T** | **T** | **G** | **A** | **G** | **G** | **A** | **T** | **C** | **A** | **T** | **C** | **T** | **T** | **T** | **T** | **C** | **C** | **G** | **A** | **A** | **T** | **T** | **G** | **T** | **G** | **C** | **A** | **G** | **G** | **A** | **A** | **T** | **T** | **A** | **C** | **T** | **T** | **T** | **C** | **C** | **A** | **A** | **T** | **A** | **C** | **C** | **T** | **T** | **A** | **A** | **G** | **A** | **C** | **A** | **T** | **G** | **C** | **C** | **A** | **A** | **A** | **G** | **C** | **A** | **A** | **A** | **G** | **G** | **A** | **A** | **T** | **T** | **A** | **G** | **A** | **G** | **G** | **T** | **A** | **T** | **A** | **C** | **T** | **T** | **A** | **C** | **A** | **C** | **C** | **A** | **A** | **G** | **T** | **C** | **G** | **A** | **T** | **A** | **A** | **A** | **A** | **A** | **C** | **G** | **T** | **G** | **T** | **T** | **A** | **T** | **T** | **G** | **C** | **G** | **T** | **A** | **T** | **T** | **G** | **T** | **T** | **G** | **A** | **T** | **G** | **A** | **T** | **G** | **G** | **T** | **G** | **T** | **C** | **G** | **G** | **C** | **T** | **T** | **T** | **G** | **A** | **T** | **A** | **T** | **G** | **A** | **A** | **G** | **G** | **A** | **A** | **C** | **A** | **A** | **A** | **G** | **T** | **A** | **A** | **T** | **A** | **A** | **A** | **G** | **C** | **C** | **G** | **G** | **T** | **A** | **G** | **T** | **T** | **A** | **T** | **G** | **G** | **C** | **T** | **T** | **A** | **A** | **A** | **T** | **A** | **A** | **T** | **A** | **T** | **T** | **C** | **G** | **A** | **G** | **A** | **A** | **C** | **G** | **T** | **G** | **T** | **T** | **G** | **T** | **C** | **G** | **G** | **C** | **A** | **T** | **G** | **G** | **G** | **C** | **G** | **G** | **T** | **A** | **C** | **A** | **G** | **T** | **T** | **A** | **A** | **A** | **A** | **T** | **T** | **A** | **T** | **T** | **A** | **G** | **T** | **T** | **T** | **T** | **A** | **A** | **A** | **G** | **G** | **G** | **C** | **A** | **G** | **G** | **G** | **A** | **A** | **C** | **C** | **A** | **G** | **C** | **G** | **T** | **T** | **G** | **A** | **A** | **A** | **T** | **T** | **A** | **A** | **A** | **G** | **T** | **T** | **C** | **C** | **T** | **G** | **T** | **C** | **A** | **T** | **A** | **A** | **A** | **G** | **G** | **A** | **G** | **G** | **A** | **A** | **A** | **C** | **T** | **G** | **C** | **A** | **A** | **G** | **T** | **G** | **A** | **T** | **C** | **A** | **A** | **A** | **G** | **T** | **A** | **A** | **T** | **G** | **T** | **T** | **A** | **G** | **T** | **G** | **G** | **A** | **-** | **-** | **-** |
| **N54548** | **A** | **T** | **G** | **A** | **C** | **C** | **G** | **A** | **T** | **C** | **G** | **G** | **A** | **T** | **T** | **T** | **C** | **A** | **A** | **G** | **A** | **C** | **G** | **C** | **A** | **T** | **G** | **A** | **T** | **T** | **T** | **C** | **A** | **T** | **T** | **A** | **T** | **A** | **T** | **G** | **C** | **G** | **T** | **C** | **C** | **C** | **T** | **T** | **A** | **G** | **C** | **A** | **C** | **C** | **T** | **T** | **T** | **A** | **T** | **T** | **G** | **T** | **T** | **A** | **T** | **T** | **T** | **T** | **A** | **A** | **T** | **T** | **A** | **C** | **C** | **T** | **T** | **G** | **T** | **T** | **T** | **T** | **C** | **A** | **T** | **A** | **T** | **T** | **T** | **T** | **C** | **A** | **T** | **T** | **C** | **A** | **A** | **T** | **T** | **A** | **A** | **A** | **C** | **A** | **A** | **A** | **A** | **C** | **C** | **G** | **G** | **T** | **G** | **G** | **C** | **T** | **C** | **T** | **T** | **A** | **G** | **A** | **A** | **C** | **T** | **T** | **C** | **T** | **T** | **C** | **A** | **G** | **A** | **G** | **A** | **A** | **A** | **A** | **G** | **T** | **C** | **T** | **T** | **T** | **T** | **A** | **T** | **C** | **T** | **G** | **C** | **C** | **A** | **C** | **T** | **A** | **A** | **T** | **T** | **G** | **T** | **G** | **C** | **A** | **T** | **A** | **T** | **T** | **G** | **T** | **T** | **C** | **T** | **T** | **A** | **T** | **A** | **T** | **C** | **C** | **T** | **T** | **A** | **C** | **T** | **A** | **A** | **T** | **A** | **G** | **G** | **C** | **T** | **T** | **A** | **T** | **T** | **G** | **A** | **C** | **C** | **T** | **T** | **T** | **T** | **T** | **A** | **C** | **T** | **G** | **A** | **T** | **T** | **T** | **C** | **G** | **T** | **T** | **G** | **G** | **T** | **C** | **C** | **A** | **A** | **A** | **A** | **A** | **G** | **G** | **C** | **C** | **A** | **A** | **T** | **A** | **T** | **G** | **G** | **A** | **C** | **G** | **G** | **A** | **T** | **T** | **G** | **A** | **A** | **G** | **A** | **A** | **A** | **A** | **A** | **C** | **T** | **T** | **C** | **G** | **G** | **T** | **T** | **A** | **T** | **T** | **A** | **G** | **C** | **C** | **A** | **A** | **T** | **G** | **G** | **C** | **A** | **A** | **T** | **T** | **A** | **T** | **G** | **A** | **A** | **A** | **G** | **T** | **C** | **C** | **A** | **G** | **T** | **C** | **T** | **T** | **A** | **A** | **A** | **C** | **A** | **A** | **A** | **C** | **C** | **A** | **A** | **T** | **G** | **A** | **C** | **T** | **A** | **G** | **T** | **G** | **A** | **A** | **A** | **A** | **T** | **C** | **A** | **A** | **G** | **A** | **C** | **C** | **A** | **T** | **T** | **A** | **T** | **C** | **T** | **A** | **A** | **T** | **C** | **G** | **A** | **A** | **G** | **T** | **C** | **G** | **A** | **A** | **C** | **A** | **A** | **G** | **A** | **T** | **A** | **T** | **T** | **T** | **G** | **G** | **T** | **C** | **G** | **A** | **T** | **T** | **A** | **A** | **A** | **A** | **A** | **C** | **A** | **A** | **A** | **T** | **T** | **A** | **T** | **T** | **A** | **G** | **A** | **G** | **A** | **T** | **G** | **T** | **C** | **T** | **A** | **A** | **A** | **G** | **A** | **A** | **T** | **T** | **G** | **C** | **A** | **A** | **T** | **T** | **A** | **T** | **T** | **A** | **A** | **A** | **C** | **A** | **G** | **T** | **C** | **G** | **A** | **C** | **C** | **G** | **C** | **A** | **A** | **T** | **T** | **A** | **A** | **T** | **G** | **G** | **A** | **T** | **G** | **G** | **G** | **C** | **A** | **A** | **A** | **C** | **A** | **A** | **A** | **A** | **G** | **A** | **A** | **G** | **A** | **A** | **A** | **T** | **T** | **T** | **T** | **A** | **G** | **A** | **G** | **A** | **A** | **C** | **G** | **A** | **G** | **C** | **G** | **G** | **C** | **A** | **T** | **C** | **G** | **T** | **T** | **T** | **G** | **G** | **C** | **A** | **C** | **G** | **G** | **G** | **A** | **G** | **T** | **T** | **G** | **C** | **A** | **T** | **G** | **A** | **T** | **T** | **C** | **A** | **G** | **T** | **C** | **A** | **G** | **T** | **C** | **A** | **A** | **C** | **A** | **A** | **C** | **T** | **T** | **T** | **T** | **T** | **G** | **C** | **A** | **G** | **C** | **C** | **A** | **T** | **G** | **A** | **T** | **G** | **A** | **T** | **G** | **T** | **T** | **G** | **T** | **C** | **T** | **G** | **C** | **C** | **T** | **T** | **A** | **A** | **A** | **T** | **G** | **A** | **A** | **C** | **A** | **A** | **G** | **C** | **A** | **C** | **A** | **A** | **C** | **G** | **A** | **A** | **C** | **A** | **G** | **A** | **A** | **A** | **C** | **C** | **C** | **C** | **T** | **G** | **A** | **A** | **C** | **C** | **A** | **T** | **A** | **T** | **C** | **G** | **T** | **A** | **A** | **A** | **C** | **A** | **A** | **C** | **T** | **A** | **G** | **C** | **C** | **A** | **T** | **G** | **G** | **T** | **G** | **G** | **C** | **A** | **G** | **A** | **A** | **A** | **T** | **C** | **A** | **T** | **T** | **A** | **A** | **T** | **G** | **C** | **C** | **T** | **C** | **C** | **C** | **A** | **A** | **T** | **C** | **G** | **G** | **A** | **A** | **A** | **T** | **G** | **C** | **G** | **C** | **G** | **C** | **G** | **T** | **T** | **A** | **C** | **T** | **A** | **T** | **T** | **A** | **C** | **A** | **C** | **T** | **T** | **G** | **C** | **G** | **T** | **C** | **C** | **T** | **A** | **T** | **T** | **A** | **G** | **T** | **C** | **T** | **A** | **G** | **A** | **A** | **G** | **G** | **G** | **A** | **A** | **A** | **A** | **G** | **T** | **T** | **T** | **G** | **C** | **G** | **T** | **A** | **A** | **A** | **G** | **G** | **C** | **A** | **T** | **T** | **G** | **A** | **A** | **C** | **A** | **A** | **T** | **T** | **A** | **T** | **T** | **G** | **A** | **A** | **A** | **G** | **A** | **A** | **C** | **T** | **A** | **C** | **A** | **A** | **A** | **C** | **A** | **A** | **A** | **A** | **A** | **T** | **T** | **A** | **A** | **A** | **A** | **T** | **T** | **G** | **A** | **A** | **T** | **T** | **G** | **A** | **T** | **T** | **T** | **G** | **G** | **G** | **A** | **T** | **G** | **T** | **T** | **G** | **A** | **A** | **G** | **A** | **T** | **G** | **T** | **T** | **C** | **A** | **T** | **T** | **T** | **A** | **A** | **A** | **T** | **A** | **G** | **C** | **A** | **G** | **C** | **A** | **T** | **T** | **G** | **A** | **G** | **G** | **A** | **T** | **C** | **A** | **T** | **C** | **T** | **T** | **T** | **T** | **C** | **C** | **G** | **A** | **A** | **T** | **T** | **G** | **T** | **T** | **C** | **A** | **G** | **G** | **A** | **A** | **T** | **T** | **A** | **C** | **T** | **T** | **T** | **C** | **A** | **A** | **A** | **T** | **A** | **C** | **C** | **T** | **T** | **A** | **A** | **G** | **A** | **C** | **A** | **T** | **G** | **C** | **C** | **A** | **A** | **A** | **G** | **C** | **A** | **A** | **A** | **A** | **G** | **A** | **A** | **T** | **T** | **A** | **G** | **A** | **G** | **G** | **T** | **A** | **T** | **A** | **C** | **T** | **T** | **A** | **C** | **A** | **C** | **C** | **A** | **A** | **G** | **T** | **C** | **G** | **A** | **T** | **A** | **A** | **A** | **A** | **A** | **T** | **G** | **T** | **T** | **T** | **T** | **A** | **T** | **T** | **G** | **C** | **G** | **C** | **A** | **T** | **T** | **G** | **T** | **T** | **G** | **A** | **T** | **G** | **A** | **T** | **G** | **G** | **T** | **G** | **T** | **C** | **G** | **G** | **C** | **T** | **T** | **T** | **G** | **A** | **T** | **A** | **T** | **G** | **A** | **A** | **G** | **G** | **A** | **A** | **C** | **A** | **A** | **A** | **G** | **T** | **A** | **A** | **T** | **A** | **A** | **G** | **G** | **C** | **C** | **G** | **G** | **T** | **A** | **G** | **T** | **T** | **A** | **T** | **G** | **G** | **C** | **T** | **T** | **A** | **A** | **A** | **T** | **A** | **A** | **T** | **A** | **T** | **T** | **C** | **G** | **A** | **G** | **A** | **A** | **C** | **G** | **T** | **G** | **T** | **T** | **G** | **T** | **C** | **G** | **G** | **C** | **A** | **T** | **G** | **G** | **G** | **C** | **G** | **G** | **T** | **A** | **C** | **A** | **G** | **T** | **T** | **A** | **A** | **A** | **A** | **T** | **T** | **A** | **T** | **T** | **A** | **G** | **T** | **T** | **T** | **T** | **A** | **A** | **A** | **G** | **G** | **G** | **C** | **A** | **G** | **G** | **G** | **A** | **A** | **C** | **C** | **A** | **G** | **C** | **G** | **T** | **T** | **G** | **A** | **A** | **A** | **T** | **T** | **A** | **A** | **A** | **G** | **T** | **T** | **C** | **C** | **T** | **G** | **T** | **C** | **A** | **T** | **A** | **A** | **A** | **G** | **G** | **A** | **G** | **G** | **A** | **A** | **A** | **C** | **T** | **G** | **C** | **A** | **A** | **G** | **T** | **G** | **A** | **T** | **C** | **A** | **A** | **A** | **G** | **T** | **A** | **A** | **T** | **G** | **T** | **T** | **A** | **G** | **T** | **G** | **G** | **A** | **-** | **-** | **-** |
| **N55119** | **A** | **T** | **G** | **A** | **C** | **C** | **G** | **A** | **T** | **C** | **G** | **G** | **A** | **T** | **T** | **T** | **C** | **A** | **A** | **G** | **A** | **C** | **G** | **C** | **A** | **T** | **G** | **A** | **T** | **T** | **T** | **C** | **A** | **T** | **T** | **A** | **T** | **A** | **T** | **G** | **C** | **G** | **T** | **C** | **C** | **C** | **T** | **T** | **A** | **G** | **C** | **A** | **C** | **C** | **T** | **T** | **T** | **A** | **T** | **T** | **G** | **T** | **T** | **A** | **T** | **C** | **T** | **T** | **A** | **A** | **T** | **T** | **A** | **C** | **A** | **T** | **T** | **G** | **T** | **T** | **T** | **T** | **C** | **A** | **T** | **A** | **T** | **T** | **T** | **T** | **C** | **A** | **T** | **T** | **C** | **G** | **A** | **T** | **T** | **A** | **A** | **A** | **C** | **A** | **A** | **A** | **A** | **C** | **C** | **G** | **G** | **T** | **G** | **G** | **T** | **T** | **A** | **T** | **T** | **A** | **G** | **A** | **G** | **C** | **T** | **T** | **C** | **T** | **T** | **C** | **A** | **G** | **A** | **G** | **A** | **A** | **A** | **A** | **G** | **T** | **C** | **T** | **T** | **T** | **T** | **A** | **T** | **C** | **T** | **A** | **C** | **C** | **A** | **C** | **T** | **A** | **A** | **T** | **T** | **G** | **T** | **G** | **C** | **A** | **C** | **A** | **T** | **T** | **G** | **T** | **T** | **C** | **T** | **C** | **A** | **T** | **A** | **T** | **C** | **C** | **T** | **T** | **A** | **C** | **T** | **A** | **A** | **T** | **A** | **G** | **G** | **C** | **T** | **T** | **A** | **T** | **T** | **G** | **A** | **C** | **C** | **T** | **T** | **T** | **T** | **T** | **A** | **C** | **T** | **G** | **A** | **T** | **T** | **T** | **C** | **A** | **T** | **T** | **G** | **G** | **T** | **T** | **C** | **A** | **A** | **A** | **A** | **A** | **G** | **G** | **G** | **C** | **A** | **A** | **T** | **A** | **T** | **G** | **G** | **A** | **C** | **G** | **G** | **A** | **T** | **T** | **G** | **A** | **A** | **G** | **A** | **A** | **A** | **A** | **A** | **C** | **T** | **T** | **C** | **G** | **G** | **T** | **T** | **A** | **T** | **T** | **G** | **G** | **C** | **C** | **A** | **A** | **C** | **G** | **G** | **T** | **A** | **A** | **T** | **T** | **A** | **T** | **G** | **A** | **A** | **A** | **G** | **T** | **C** | **C** | **A** | **G** | **T** | **C** | **T** | **T** | **A** | **A** | **A** | **C** | **A** | **A** | **A** | **C** | **C** | **A** | **A** | **C** | **G** | **A** | **C** | **C** | **A** | **G** | **T** | **G** | **A** | **A** | **A** | **A** | **T** | **C** | **A** | **A** | **G** | **A** | **T** | **C** | **A** | **T** | **T** | **A** | **T** | **C** | **T** | **A** | **A** | **C** | **C** | **G** | **A** | **A** | **G** | **T** | **C** | **G** | **A** | **A** | **C** | **A** | **A** | **G** | **A** | **T** | **A** | **T** | **T** | **T** | **G** | **G** | **T** | **C** | **G** | **A** | **T** | **T** | **A** | **A** | **A** | **A** | **A** | **T** | **A** | **A** | **A** | **T** | **T** | **A** | **T** | **T** | **A** | **G** | **A** | **G** | **A** | **T** | **G** | **T** | **C** | **T** | **A** | **A** | **A** | **G** | **A** | **A** | **T** | **T** | **G** | **C** | **A** | **A** | **T** | **T** | **A** | **T** | **T** | **A** | **A** | **A** | **C** | **A** | **G** | **T** | **C** | **G** | **A** | **C** | **C** | **G** | **C** | **A** | **A** | **T** | **T** | **A** | **A** | **T** | **G** | **G** | **A** | **T** | **G** | **G** | **G** | **C** | **A** | **A** | **A** | **C** | **A** | **A** | **A** | **A** | **G** | **A** | **A** | **G** | **A** | **A** | **A** | **T** | **T** | **T** | **T** | **A** | **G** | **A** | **G** | **A** | **A** | **C** | **G** | **A** | **G** | **C** | **G** | **G** | **C** | **A** | **T** | **C** | **G** | **T** | **T** | **T** | **G** | **G** | **C** | **G** | **C** | **G** | **G** | **G** | **A** | **G** | **T** | **T** | **G** | **C** | **A** | **T** | **G** | **A** | **T** | **T** | **C** | **A** | **G** | **T** | **C** | **A** | **G** | **T** | **C** | **A** | **A** | **C** | **A** | **A** | **C** | **T** | **T** | **T** | **T** | **T** | **G** | **C** | **A** | **G** | **C** | **C** | **A** | **T** | **G** | **A** | **T** | **G** | **A** | **T** | **G** | **T** | **T** | **G** | **T** | **C** | **T** | **G** | **C** | **C** | **T** | **T** | **A** | **A** | **A** | **T** | **G** | **A** | **A** | **C** | **A** | **A** | **G** | **C** | **A** | **C** | **A** | **A** | **C** | **G** | **A** | **A** | **C** | **A** | **G** | **A** | **A** | **A** | **C** | **C** | **C** | **C** | **G** | **G** | **A** | **A** | **C** | **C** | **A** | **T** | **A** | **T** | **C** | **G** | **T** | **A** | **A** | **A** | **C** | **A** | **A** | **C** | **T** | **A** | **G** | **C** | **C** | **A** | **T** | **G** | **G** | **T** | **G** | **G** | **C** | **A** | **G** | **A** | **A** | **A** | **T** | **C** | **A** | **T** | **T** | **A** | **A** | **T** | **G** | **C** | **C** | **T** | **C** | **C** | **C** | **A** | **A** | **T** | **C** | **G** | **G** | **A** | **A** | **A** | **T** | **G** | **C** | **G** | **C** | **G** | **C** | **G** | **T** | **T** | **A** | **C** | **T** | **A** | **T** | **T** | **G** | **C** | **A** | **C** | **T** | **T** | **G** | **C** | **G** | **T** | **C** | **C** | **T** | **A** | **T** | **C** | **A** | **G** | **T** | **C** | **T** | **A** | **G** | **A** | **A** | **G** | **G** | **A** | **A** | **A** | **A** | **A** | **G** | **T** | **T** | **T** | **G** | **C** | **G** | **T** | **A** | **A** | **A** | **G** | **G** | **T** | **A** | **T** | **T** | **G** | **A** | **A** | **C** | **A** | **A** | **T** | **T** | **A** | **C** | **T** | **G** | **A** | **A** | **A** | **G** | **A** | **A** | **C** | **T** | **A** | **C** | **A** | **A** | **A** | **C** | **A** | **A** | **A** | **A** | **A** | **T** | **T** | **A** | **A** | **A** | **A** | **T** | **T** | **G** | **A** | **A** | **T** | **T** | **G** | **A** | **T** | **T** | **T** | **G** | **G** | **G** | **A** | **T** | **G** | **T** | **T** | **G** | **A** | **A** | **G** | **A** | **T** | **G** | **T** | **T** | **C** | **A** | **T** | **T** | **T** | **A** | **A** | **A** | **T** | **A** | **G** | **C** | **A** | **G** | **C** | **A** | **T** | **T** | **G** | **A** | **G** | **G** | **A** | **T** | **C** | **A** | **T** | **C** | **T** | **T** | **T** | **T** | **C** | **C** | **G** | **A** | **A** | **T** | **T** | **G** | **T** | **G** | **C** | **A** | **G** | **G** | **A** | **G** | **T** | **T** | **A** | **C** | **T** | **T** | **T** | **C** | **A** | **A** | **A** | **T** | **A** | **C** | **C** | **T** | **T** | **A** | **A** | **G** | **A** | **C** | **A** | **T** | **G** | **C** | **C** | **A** | **A** | **A** | **G** | **C** | **A** | **A** | **A** | **G** | **G** | **A** | **A** | **T** | **T** | **A** | **G** | **A** | **G** | **G** | **T** | **A** | **T** | **A** | **C** | **T** | **T** | **A** | **C** | **A** | **C** | **C** | **A** | **A** | **G** | **T** | **C** | **G** | **A** | **T** | **A** | **A** | **A** | **A** | **A** | **C** | **G** | **T** | **G** | **T** | **T** | **A** | **T** | **T** | **G** | **C** | **G** | **T** | **A** | **T** | **T** | **G** | **T** | **T** | **G** | **A** | **T** | **G** | **A** | **C** | **G** | **G** | **T** | **G** | **T** | **C** | **G** | **G** | **C** | **T** | **T** | **T** | **G** | **A** | **T** | **A** | **T** | **G** | **A** | **A** | **G** | **G** | **A** | **A** | **C** | **A** | **A** | **A** | **G** | **T** | **A** | **A** | **T** | **A** | **A** | **A** | **G** | **C** | **C** | **G** | **G** | **T** | **A** | **G** | **T** | **T** | **A** | **T** | **G** | **G** | **C** | **T** | **T** | **A** | **A** | **A** | **T** | **A** | **A** | **T** | **A** | **T** | **C** | **C** | **G** | **A** | **G** | **A** | **A** | **C** | **G** | **T** | **G** | **T** | **T** | **G** | **T** | **C** | **G** | **G** | **C** | **A** | **T** | **G** | **G** | **G** | **C** | **G** | **G** | **T** | **A** | **C** | **A** | **G** | **T** | **T** | **A** | **A** | **A** | **A** | **T** | **T** | **A** | **T** | **T** | **A** | **G** | **T** | **T** | **T** | **T** | **A** | **A** | **A** | **G** | **G** | **G** | **C** | **A** | **G** | **G** | **G** | **A** | **A** | **C** | **C** | **A** | **G** | **C** | **G** | **T** | **T** | **G** | **A** | **A** | **A** | **T** | **T** | **A** | **A** | **A** | **G** | **T** | **T** | **C** | **C** | **T** | **G** | **T** | **C** | **A** | **T** | **A** | **A** | **A** | **G** | **G** | **A** | **G** | **G** | **A** | **A** | **A** | **C** | **T** | **G** | **C** | **A** | **A** | **G** | **T** | **G** | **A** | **T** | **C** | **A** | **A** | **A** | **G** | **T** | **A** | **A** | **T** | **G** | **T** | **T** | **A** | **G** | **T** | **G** | **G** | **A** | **-** | **-** | **-** |
| **N59963F** | **A** | **T** | **G** | **A** | **C** | **C** | **G** | **A** | **T** | **C** | **G** | **G** | **A** | **T** | **T** | **T** | **C** | **A** | **A** | **G** | **A** | **C** | **G** | **C** | **A** | **T** | **G** | **A** | **T** | **T** | **T** | **C** | **A** | **T** | **T** | **A** | **T** | **A** | **T** | **G** | **C** | **G** | **T** | **C** | **C** | **C** | **T** | **T** | **A** | **G** | **C** | **A** | **C** | **C** | **T** | **T** | **T** | **A** | **T** | **T** | **G** | **T** | **T** | **A** | **T** | **C** | **T** | **T** | **A** | **A** | **T** | **T** | **A** | **C** | **A** | **T** | **T** | **G** | **T** | **T** | **T** | **T** | **C** | **A** | **T** | **A** | **T** | **T** | **T** | **T** | **C** | **A** | **T** | **T** | **C** | **G** | **A** | **T** | **T** | **A** | **A** | **A** | **C** | **A** | **A** | **A** | **A** | **C** | **C** | **G** | **G** | **T** | **G** | **G** | **T** | **T** | **A** | **T** | **T** | **A** | **G** | **A** | **G** | **C** | **T** | **T** | **C** | **T** | **T** | **C** | **A** | **G** | **A** | **G** | **A** | **A** | **A** | **A** | **G** | **T** | **C** | **T** | **T** | **T** | **T** | **A** | **T** | **C** | **T** | **A** | **C** | **C** | **A** | **C** | **T** | **A** | **A** | **T** | **T** | **G** | **T** | **G** | **C** | **A** | **C** | **A** | **T** | **T** | **G** | **T** | **T** | **C** | **T** | **C** | **A** | **T** | **A** | **T** | **C** | **C** | **T** | **T** | **A** | **C** | **T** | **A** | **A** | **T** | **A** | **G** | **G** | **C** | **T** | **T** | **A** | **T** | **T** | **G** | **A** | **C** | **C** | **T** | **T** | **T** | **T** | **T** | **A** | **C** | **T** | **G** | **A** | **T** | **T** | **T** | **C** | **A** | **T** | **T** | **G** | **G** | **T** | **T** | **C** | **A** | **A** | **A** | **A** | **A** | **G** | **G** | **G** | **C** | **A** | **A** | **T** | **A** | **T** | **G** | **G** | **A** | **C** | **G** | **G** | **A** | **T** | **T** | **G** | **A** | **A** | **G** | **A** | **A** | **A** | **A** | **A** | **C** | **T** | **T** | **C** | **G** | **G** | **T** | **T** | **A** | **T** | **T** | **G** | **G** | **C** | **C** | **A** | **A** | **T** | **G** | **G** | **T** | **A** | **A** | **T** | **T** | **A** | **T** | **G** | **A** | **A** | **A** | **G** | **T** | **C** | **C** | **A** | **G** | **T** | **C** | **T** | **T** | **A** | **A** | **A** | **C** | **A** | **A** | **A** | **C** | **C** | **A** | **A** | **C** | **G** | **A** | **C** | **C** | **A** | **G** | **T** | **G** | **A** | **A** | **A** | **A** | **T** | **C** | **A** | **A** | **G** | **A** | **T** | **C** | **A** | **T** | **T** | **A** | **T** | **C** | **T** | **A** | **A** | **C** | **C** | **G** | **A** | **A** | **G** | **T** | **C** | **G** | **A** | **A** | **C** | **A** | **A** | **G** | **A** | **T** | **A** | **T** | **T** | **T** | **G** | **G** | **T** | **C** | **G** | **A** | **T** | **T** | **A** | **A** | **A** | **A** | **A** | **T** | **A** | **A** | **A** | **T** | **T** | **A** | **T** | **T** | **A** | **G** | **A** | **G** | **A** | **T** | **G** | **T** | **C** | **T** | **A** | **A** | **A** | **G** | **A** | **A** | **T** | **T** | **G** | **C** | **A** | **A** | **T** | **T** | **A** | **T** | **T** | **A** | **A** | **A** | **C** | **A** | **G** | **T** | **C** | **G** | **G** | **C** | **C** | **G** | **C** | **A** | **A** | **T** | **T** | **A** | **A** | **T** | **G** | **G** | **A** | **T** | **G** | **G** | **G** | **C** | **A** | **A** | **A** | **C** | **A** | **A** | **A** | **A** | **G** | **A** | **A** | **G** | **A** | **A** | **A** | **T** | **T** | **T** | **T** | **A** | **G** | **A** | **G** | **A** | **A** | **C** | **G** | **A** | **G** | **C** | **G** | **G** | **C** | **A** | **T** | **C** | **G** | **T** | **T** | **T** | **G** | **G** | **C** | **G** | **C** | **G** | **G** | **G** | **A** | **G** | **T** | **T** | **G** | **C** | **A** | **T** | **G** | **A** | **T** | **T** | **C** | **A** | **G** | **T** | **C** | **A** | **G** | **T** | **C** | **A** | **A** | **C** | **A** | **A** | **C** | **T** | **T** | **T** | **T** | **T** | **G** | **C** | **A** | **G** | **C** | **C** | **A** | **T** | **G** | **A** | **T** | **G** | **A** | **T** | **G** | **T** | **T** | **G** | **T** | **C** | **T** | **G** | **C** | **C** | **T** | **T** | **A** | **A** | **A** | **T** | **G** | **A** | **A** | **C** | **A** | **A** | **G** | **C** | **A** | **C** | **A** | **A** | **C** | **G** | **A** | **A** | **C** | **A** | **G** | **A** | **A** | **A** | **C** | **C** | **C** | **C** | **G** | **G** | **A** | **A** | **C** | **C** | **A** | **T** | **A** | **T** | **C** | **G** | **T** | **A** | **A** | **A** | **C** | **A** | **A** | **C** | **T** | **A** | **G** | **C** | **C** | **A** | **T** | **G** | **G** | **T** | **G** | **G** | **C** | **A** | **G** | **A** | **A** | **A** | **T** | **C** | **A** | **T** | **T** | **A** | **A** | **T** | **G** | **C** | **C** | **T** | **C** | **C** | **C** | **A** | **A** | **T** | **C** | **G** | **G** | **A** | **A** | **A** | **T** | **G** | **C** | **G** | **C** | **G** | **C** | **G** | **T** | **T** | **A** | **C** | **T** | **A** | **T** | **T** | **G** | **C** | **A** | **C** | **T** | **T** | **G** | **C** | **G** | **T** | **C** | **C** | **T** | **A** | **T** | **C** | **A** | **G** | **T** | **C** | **T** | **A** | **G** | **A** | **A** | **G** | **G** | **A** | **A** | **A** | **A** | **A** | **G** | **T** | **T** | **T** | **G** | **C** | **G** | **T** | **A** | **A** | **A** | **G** | **G** | **T** | **A** | **T** | **T** | **G** | **A** | **A** | **C** | **A** | **A** | **T** | **T** | **A** | **C** | **T** | **G** | **A** | **A** | **A** | **G** | **A** | **A** | **C** | **T** | **A** | **C** | **A** | **A** | **A** | **C** | **A** | **A** | **A** | **A** | **A** | **T** | **T** | **A** | **A** | **A** | **A** | **T** | **T** | **G** | **A** | **A** | **T** | **T** | **G** | **A** | **T** | **T** | **T** | **G** | **G** | **G** | **A** | **T** | **G** | **T** | **T** | **G** | **A** | **A** | **G** | **A** | **T** | **G** | **T** | **T** | **C** | **A** | **T** | **T** | **T** | **A** | **A** | **A** | **T** | **A** | **G** | **C** | **A** | **G** | **C** | **A** | **T** | **T** | **G** | **A** | **G** | **G** | **A** | **T** | **C** | **A** | **T** | **C** | **T** | **T** | **T** | **T** | **C** | **C** | **G** | **A** | **A** | **T** | **T** | **G** | **T** | **G** | **C** | **A** | **G** | **G** | **A** | **G** | **T** | **T** | **A** | **C** | **T** | **T** | **T** | **C** | **A** | **A** | **A** | **T** | **A** | **C** | **C** | **T** | **T** | **A** | **A** | **G** | **A** | **C** | **A** | **T** | **G** | **C** | **C** | **A** | **A** | **A** | **G** | **C** | **A** | **A** | **A** | **G** | **G** | **A** | **A** | **T** | **T** | **A** | **G** | **A** | **G** | **G** | **T** | **A** | **T** | **A** | **C** | **T** | **T** | **A** | **C** | **A** | **C** | **C** | **A** | **A** | **G** | **T** | **C** | **G** | **A** | **T** | **A** | **A** | **A** | **A** | **A** | **C** | **G** | **T** | **G** | **T** | **T** | **A** | **T** | **T** | **G** | **C** | **G** | **T** | **A** | **T** | **T** | **G** | **T** | **T** | **G** | **A** | **T** | **G** | **A** | **C** | **G** | **G** | **T** | **G** | **T** | **C** | **G** | **G** | **C** | **T** | **T** | **T** | **G** | **A** | **T** | **A** | **T** | **G** | **A** | **A** | **G** | **G** | **A** | **A** | **C** | **A** | **A** | **A** | **G** | **T** | **A** | **A** | **T** | **A** | **A** | **A** | **G** | **C** | **C** | **G** | **G** | **T** | **A** | **G** | **T** | **T** | **A** | **T** | **G** | **G** | **C** | **T** | **T** | **A** | **A** | **A** | **T** | **A** | **A** | **T** | **A** | **T** | **C** | **C** | **G** | **A** | **G** | **A** | **A** | **C** | **G** | **T** | **G** | **T** | **T** | **G** | **T** | **C** | **G** | **G** | **C** | **A** | **T** | **G** | **G** | **G** | **C** | **G** | **G** | **T** | **A** | **C** | **A** | **G** | **T** | **T** | **A** | **A** | **A** | **A** | **T** | **T** | **A** | **T** | **T** | **A** | **G** | **T** | **T** | **T** | **T** | **A** | **A** | **A** | **G** | **G** | **G** | **C** | **A** | **G** | **G** | **G** | **A** | **A** | **C** | **C** | **A** | **G** | **C** | **G** | **T** | **T** | **G** | **A** | **A** | **A** | **T** | **T** | **A** | **A** | **A** | **G** | **T** | **T** | **C** | **C** | **T** | **G** | **T** | **C** | **A** | **T** | **A** | **A** | **A** | **G** | **G** | **A** | **G** | **G** | **A** | **A** | **A** | **C** | **T** | **G** | **C** | **A** | **A** | **G** | **T** | **G** | **A** | **T** | **C** | **A** | **A** | **A** | **G** | **T** | **A** | **A** | **T** | **G** | **T** | **T** | **A** | **G** | **T** | **G** | **G** | **A** | **-** | **-** | **-** |
| **N59646F** | **A** | **T** | **G** | **A** | **C** | **C** | **G** | **A** | **T** | **C** | **G** | **G** | **A** | **T** | **T** | **T** | **C** | **A** | **A** | **G** | **A** | **C** | **G** | **C** | **A** | **T** | **G** | **A** | **T** | **T** | **T** | **C** | **A** | **T** | **T** | **A** | **T** | **A** | **T** | **G** | **C** | **G** | **T** | **C** | **C** | **C** | **T** | **T** | **A** | **G** | **C** | **A** | **C** | **C** | **T** | **T** | **T** | **A** | **T** | **T** | **G** | **T** | **T** | **A** | **T** | **C** | **T** | **T** | **A** | **A** | **T** | **T** | **A** | **C** | **A** | **T** | **T** | **G** | **T** | **T** | **T** | **T** | **C** | **A** | **T** | **A** | **T** | **T** | **T** | **T** | **C** | **A** | **T** | **T** | **C** | **G** | **A** | **T** | **T** | **A** | **A** | **A** | **C** | **A** | **A** | **A** | **A** | **C** | **C** | **G** | **G** | **T** | **G** | **G** | **T** | **T** | **A** | **T** | **T** | **A** | **G** | **A** | **G** | **C** | **T** | **T** | **C** | **T** | **T** | **C** | **A** | **G** | **A** | **G** | **A** | **A** | **A** | **A** | **G** | **T** | **C** | **T** | **T** | **T** | **T** | **A** | **T** | **T** | **T** | **A** | **C** | **C** | **A** | **C** | **T** | **A** | **A** | **T** | **T** | **G** | **T** | **G** | **C** | **A** | **C** | **A** | **T** | **T** | **G** | **T** | **T** | **C** | **T** | **C** | **A** | **T** | **A** | **T** | **C** | **C** | **T** | **T** | **A** | **C** | **T** | **A** | **A** | **T** | **A** | **G** | **G** | **C** | **T** | **T** | **A** | **T** | **T** | **G** | **A** | **C** | **C** | **T** | **T** | **T** | **T** | **T** | **A** | **C** | **T** | **G** | **A** | **T** | **T** | **T** | **C** | **A** | **T** | **T** | **G** | **G** | **T** | **T** | **C** | **A** | **A** | **A** | **A** | **A** | **G** | **G** | **G** | **C** | **A** | **A** | **T** | **A** | **T** | **G** | **G** | **A** | **C** | **G** | **G** | **A** | **T** | **T** | **G** | **A** | **A** | **G** | **A** | **A** | **A** | **A** | **A** | **C** | **T** | **T** | **C** | **G** | **G** | **T** | **T** | **A** | **T** | **T** | **G** | **G** | **C** | **C** | **A** | **A** | **C** | **G** | **G** | **T** | **A** | **A** | **T** | **T** | **A** | **T** | **G** | **A** | **A** | **A** | **G** | **T** | **C** | **C** | **A** | **G** | **T** | **C** | **T** | **T** | **A** | **A** | **A** | **C** | **A** | **A** | **A** | **C** | **C** | **A** | **A** | **C** | **G** | **A** | **C** | **C** | **A** | **G** | **T** | **G** | **A** | **A** | **A** | **A** | **T** | **C** | **A** | **A** | **G** | **A** | **C** | **C** | **A** | **T** | **T** | **A** | **T** | **C** | **T** | **A** | **A** | **C** | **C** | **G** | **A** | **A** | **G** | **T** | **C** | **G** | **A** | **A** | **C** | **A** | **A** | **G** | **A** | **T** | **A** | **T** | **T** | **T** | **G** | **G** | **T** | **C** | **G** | **A** | **T** | **T** | **A** | **A** | **A** | **A** | **A** | **T** | **A** | **A** | **A** | **T** | **T** | **A** | **T** | **T** | **A** | **G** | **A** | **G** | **A** | **T** | **G** | **T** | **C** | **T** | **A** | **A** | **A** | **G** | **A** | **A** | **T** | **T** | **G** | **C** | **A** | **A** | **T** | **T** | **A** | **T** | **T** | **A** | **A** | **A** | **C** | **A** | **G** | **T** | **C** | **G** | **A** | **C** | **C** | **G** | **C** | **A** | **A** | **T** | **T** | **A** | **A** | **T** | **G** | **G** | **A** | **T** | **G** | **G** | **G** | **C** | **A** | **A** | **A** | **C** | **A** | **A** | **A** | **A** | **G** | **A** | **A** | **G** | **A** | **A** | **A** | **T** | **T** | **T** | **T** | **A** | **G** | **A** | **G** | **A** | **A** | **C** | **G** | **A** | **G** | **C** | **G** | **G** | **C** | **A** | **T** | **C** | **G** | **T** | **T** | **T** | **G** | **G** | **C** | **G** | **C** | **G** | **G** | **G** | **A** | **G** | **T** | **T** | **G** | **C** | **A** | **T** | **G** | **A** | **T** | **T** | **C** | **A** | **G** | **T** | **C** | **A** | **G** | **T** | **C** | **A** | **A** | **C** | **A** | **A** | **C** | **T** | **T** | **T** | **T** | **T** | **G** | **C** | **A** | **G** | **C** | **C** | **A** | **T** | **G** | **A** | **T** | **G** | **A** | **T** | **G** | **T** | **T** | **G** | **T** | **C** | **T** | **G** | **C** | **C** | **T** | **T** | **A** | **A** | **A** | **T** | **G** | **A** | **A** | **C** | **A** | **A** | **G** | **C** | **A** | **C** | **A** | **A** | **C** | **G** | **A** | **A** | **C** | **A** | **G** | **A** | **A** | **A** | **C** | **C** | **C** | **C** | **G** | **G** | **A** | **A** | **C** | **C** | **A** | **T** | **A** | **T** | **C** | **G** | **T** | **A** | **A** | **A** | **C** | **A** | **A** | **C** | **T** | **A** | **G** | **C** | **C** | **A** | **T** | **G** | **G** | **T** | **G** | **G** | **C** | **A** | **G** | **A** | **A** | **A** | **T** | **C** | **A** | **T** | **T** | **A** | **A** | **T** | **G** | **C** | **C** | **T** | **C** | **C** | **C** | **A** | **A** | **T** | **C** | **G** | **G** | **A** | **A** | **A** | **T** | **G** | **C** | **G** | **C** | **G** | **C** | **G** | **T** | **T** | **A** | **C** | **T** | **A** | **T** | **T** | **G** | **C** | **A** | **C** | **T** | **T** | **G** | **C** | **G** | **T** | **C** | **C** | **T** | **A** | **T** | **C** | **A** | **G** | **T** | **C** | **T** | **A** | **G** | **A** | **A** | **G** | **G** | **A** | **A** | **A** | **A** | **A** | **G** | **T** | **T** | **T** | **G** | **C** | **G** | **T** | **A** | **A** | **A** | **G** | **G** | **T** | **A** | **T** | **T** | **G** | **A** | **A** | **C** | **A** | **A** | **T** | **T** | **A** | **C** | **T** | **G** | **A** | **A** | **A** | **G** | **A** | **A** | **C** | **T** | **A** | **C** | **A** | **A** | **A** | **C** | **A** | **A** | **A** | **A** | **A** | **T** | **T** | **A** | **A** | **A** | **A** | **T** | **T** | **G** | **A** | **A** | **T** | **T** | **G** | **A** | **T** | **T** | **T** | **G** | **G** | **G** | **A** | **T** | **G** | **T** | **T** | **G** | **A** | **A** | **G** | **A** | **T** | **G** | **T** | **T** | **C** | **A** | **T** | **T** | **T** | **A** | **A** | **A** | **T** | **A** | **G** | **C** | **A** | **G** | **C** | **A** | **T** | **T** | **G** | **A** | **G** | **G** | **A** | **T** | **C** | **A** | **T** | **C** | **T** | **T** | **T** | **T** | **C** | **C** | **G** | **A** | **A** | **T** | **T** | **G** | **T** | **G** | **C** | **A** | **G** | **G** | **A** | **A** | **T** | **T** | **A** | **C** | **T** | **T** | **T** | **C** | **A** | **A** | **A** | **T** | **A** | **C** | **C** | **T** | **T** | **A** | **A** | **G** | **A** | **C** | **A** | **T** | **G** | **C** | **C** | **A** | **A** | **A** | **G** | **C** | **A** | **A** | **A** | **G** | **G** | **A** | **A** | **T** | **T** | **A** | **G** | **A** | **G** | **G** | **T** | **A** | **T** | **A** | **C** | **T** | **T** | **A** | **C** | **A** | **C** | **C** | **A** | **A** | **G** | **T** | **C** | **G** | **A** | **T** | **A** | **A** | **A** | **A** | **A** | **C** | **G** | **T** | **G** | **T** | **T** | **A** | **T** | **T** | **G** | **C** | **G** | **T** | **A** | **T** | **T** | **G** | **T** | **T** | **G** | **A** | **T** | **G** | **A** | **C** | **G** | **G** | **T** | **G** | **T** | **C** | **G** | **G** | **C** | **T** | **T** | **T** | **G** | **A** | **T** | **A** | **T** | **G** | **A** | **A** | **G** | **G** | **A** | **A** | **C** | **A** | **A** | **A** | **G** | **T** | **A** | **A** | **T** | **A** | **A** | **A** | **G** | **C** | **C** | **G** | **G** | **T** | **A** | **G** | **T** | **T** | **A** | **T** | **G** | **G** | **C** | **T** | **T** | **A** | **A** | **A** | **T** | **A** | **A** | **T** | **A** | **T** | **C** | **C** | **G** | **A** | **G** | **A** | **A** | **C** | **G** | **T** | **G** | **T** | **T** | **G** | **T** | **C** | **G** | **G** | **C** | **A** | **T** | **G** | **G** | **G** | **C** | **G** | **G** | **T** | **A** | **C** | **A** | **G** | **T** | **T** | **A** | **A** | **A** | **A** | **T** | **T** | **A** | **T** | **T** | **A** | **G** | **T** | **T** | **T** | **T** | **A** | **A** | **A** | **G** | **G** | **G** | **C** | **A** | **G** | **G** | **G** | **A** | **A** | **C** | **C** | **A** | **G** | **C** | **G** | **T** | **T** | **G** | **A** | **A** | **A** | **T** | **T** | **A** | **A** | **A** | **G** | **T** | **T** | **C** | **C** | **T** | **G** | **T** | **C** | **A** | **T** | **A** | **A** | **A** | **G** | **G** | **A** | **G** | **G** | **A** | **A** | **A** | **C** | **T** | **G** | **C** | **A** | **A** | **G** | **T** | **G** | **A** | **T** | **C** | **A** | **A** | **A** | **G** | **T** | **A** | **A** | **T** | **G** | **T** | **T** | **A** | **G** | **T** | **G** | **G** | **A** | **-** | **-** | **-** |
| **N60100F** | **A** | **T** | **G** | **A** | **C** | **C** | **G** | **A** | **T** | **C** | **G** | **G** | **A** | **T** | **T** | **T** | **C** | **A** | **A** | **G** | **A** | **C** | **G** | **C** | **A** | **T** | **G** | **A** | **T** | **T** | **T** | **C** | **A** | **T** | **T** | **A** | **T** | **A** | **T** | **G** | **C** | **G** | **T** | **C** | **C** | **C** | **T** | **T** | **A** | **G** | **C** | **A** | **C** | **C** | **T** | **T** | **T** | **A** | **T** | **T** | **G** | **T** | **T** | **A** | **T** | **C** | **T** | **T** | **A** | **A** | **T** | **T** | **A** | **C** | **A** | **T** | **T** | **G** | **T** | **T** | **T** | **T** | **C** | **A** | **T** | **A** | **T** | **T** | **T** | **T** | **C** | **A** | **T** | **T** | **C** | **G** | **A** | **T** | **T** | **A** | **A** | **A** | **C** | **A** | **A** | **A** | **A** | **C** | **C** | **G** | **G** | **T** | **G** | **G** | **T** | **T** | **A** | **T** | **T** | **A** | **G** | **A** | **G** | **C** | **T** | **T** | **C** | **T** | **T** | **C** | **A** | **G** | **A** | **G** | **A** | **A** | **A** | **A** | **G** | **T** | **C** | **T** | **T** | **T** | **T** | **A** | **T** | **T** | **T** | **A** | **C** | **C** | **A** | **C** | **T** | **A** | **A** | **T** | **T** | **G** | **T** | **G** | **C** | **A** | **C** | **A** | **T** | **T** | **G** | **T** | **T** | **C** | **T** | **C** | **A** | **T** | **A** | **T** | **C** | **C** | **T** | **T** | **A** | **C** | **T** | **A** | **A** | **T** | **A** | **G** | **G** | **C** | **T** | **T** | **A** | **T** | **T** | **G** | **A** | **C** | **C** | **T** | **T** | **T** | **T** | **T** | **A** | **C** | **T** | **G** | **A** | **T** | **T** | **T** | **C** | **A** | **T** | **T** | **G** | **G** | **T** | **T** | **C** | **A** | **A** | **A** | **A** | **A** | **G** | **G** | **G** | **C** | **A** | **A** | **T** | **A** | **T** | **G** | **G** | **A** | **C** | **G** | **G** | **A** | **T** | **T** | **G** | **A** | **A** | **G** | **A** | **A** | **A** | **A** | **A** | **C** | **T** | **T** | **C** | **G** | **G** | **T** | **T** | **A** | **T** | **T** | **G** | **G** | **C** | **C** | **A** | **A** | **C** | **G** | **G** | **T** | **A** | **A** | **T** | **T** | **A** | **T** | **G** | **A** | **A** | **A** | **G** | **T** | **C** | **C** | **A** | **G** | **T** | **C** | **T** | **T** | **A** | **A** | **A** | **C** | **A** | **A** | **A** | **C** | **C** | **A** | **A** | **C** | **G** | **A** | **C** | **C** | **A** | **G** | **T** | **G** | **A** | **A** | **A** | **A** | **T** | **C** | **A** | **A** | **G** | **A** | **C** | **C** | **A** | **T** | **T** | **A** | **T** | **C** | **T** | **A** | **A** | **C** | **C** | **G** | **A** | **A** | **G** | **T** | **T** | **G** | **A** | **A** | **C** | **A** | **A** | **G** | **A** | **T** | **A** | **T** | **T** | **T** | **G** | **G** | **T** | **C** | **G** | **A** | **T** | **T** | **A** | **A** | **A** | **A** | **A** | **T** | **A** | **A** | **A** | **T** | **T** | **A** | **T** | **T** | **A** | **G** | **A** | **G** | **A** | **T** | **G** | **T** | **C** | **T** | **A** | **A** | **A** | **G** | **A** | **A** | **T** | **T** | **G** | **C** | **A** | **A** | **T** | **T** | **A** | **T** | **T** | **A** | **A** | **A** | **C** | **A** | **G** | **T** | **C** | **G** | **A** | **C** | **C** | **G** | **C** | **A** | **A** | **T** | **T** | **A** | **A** | **T** | **G** | **G** | **A** | **T** | **G** | **G** | **G** | **C** | **A** | **A** | **A** | **C** | **A** | **A** | **A** | **A** | **G** | **A** | **A** | **G** | **A** | **A** | **A** | **T** | **T** | **T** | **T** | **A** | **G** | **A** | **G** | **A** | **A** | **C** | **G** | **A** | **G** | **C** | **G** | **G** | **C** | **A** | **T** | **C** | **G** | **T** | **T** | **T** | **G** | **G** | **C** | **G** | **C** | **G** | **G** | **G** | **A** | **G** | **T** | **T** | **G** | **C** | **A** | **T** | **G** | **A** | **T** | **T** | **C** | **A** | **G** | **T** | **C** | **A** | **G** | **T** | **C** | **A** | **A** | **C** | **A** | **A** | **C** | **T** | **T** | **T** | **T** | **T** | **G** | **C** | **A** | **G** | **C** | **C** | **A** | **T** | **G** | **A** | **T** | **G** | **A** | **T** | **G** | **T** | **T** | **G** | **T** | **C** | **T** | **G** | **C** | **C** | **T** | **T** | **A** | **A** | **A** | **T** | **G** | **A** | **A** | **C** | **A** | **A** | **G** | **C** | **A** | **C** | **A** | **A** | **C** | **G** | **A** | **A** | **C** | **A** | **G** | **A** | **A** | **A** | **C** | **C** | **C** | **C** | **G** | **G** | **A** | **A** | **C** | **C** | **A** | **T** | **A** | **T** | **C** | **G** | **T** | **A** | **A** | **A** | **C** | **A** | **A** | **C** | **T** | **A** | **G** | **C** | **C** | **A** | **T** | **G** | **G** | **T** | **G** | **G** | **C** | **A** | **G** | **A** | **A** | **A** | **T** | **C** | **A** | **T** | **T** | **A** | **A** | **T** | **G** | **C** | **C** | **T** | **C** | **C** | **C** | **A** | **A** | **T** | **C** | **G** | **G** | **A** | **A** | **A** | **T** | **G** | **C** | **G** | **C** | **G** | **C** | **G** | **T** | **T** | **A** | **C** | **T** | **A** | **T** | **T** | **G** | **C** | **A** | **C** | **T** | **T** | **G** | **C** | **G** | **T** | **C** | **C** | **T** | **A** | **T** | **C** | **A** | **G** | **T** | **C** | **T** | **A** | **G** | **A** | **A** | **G** | **G** | **A** | **A** | **A** | **A** | **A** | **G** | **T** | **T** | **T** | **G** | **C** | **G** | **T** | **A** | **A** | **A** | **G** | **G** | **T** | **A** | **T** | **T** | **G** | **A** | **A** | **C** | **A** | **A** | **T** | **T** | **A** | **C** | **T** | **G** | **A** | **A** | **A** | **G** | **A** | **A** | **C** | **T** | **A** | **C** | **A** | **A** | **A** | **C** | **A** | **A** | **A** | **A** | **A** | **T** | **T** | **A** | **A** | **A** | **A** | **T** | **T** | **G** | **A** | **A** | **T** | **T** | **G** | **A** | **T** | **T** | **T** | **G** | **G** | **G** | **A** | **T** | **G** | **T** | **T** | **G** | **A** | **A** | **G** | **A** | **T** | **G** | **T** | **T** | **C** | **A** | **T** | **T** | **T** | **A** | **A** | **A** | **T** | **A** | **G** | **C** | **A** | **G** | **C** | **A** | **T** | **T** | **G** | **A** | **G** | **G** | **A** | **T** | **C** | **A** | **T** | **C** | **T** | **T** | **T** | **T** | **C** | **C** | **G** | **A** | **A** | **T** | **T** | **G** | **T** | **G** | **C** | **A** | **G** | **G** | **A** | **A** | **T** | **T** | **A** | **C** | **T** | **T** | **T** | **C** | **C** | **A** | **A** | **T** | **A** | **C** | **C** | **T** | **T** | **A** | **A** | **G** | **A** | **C** | **A** | **T** | **G** | **C** | **C** | **A** | **A** | **A** | **G** | **C** | **A** | **A** | **A** | **G** | **G** | **A** | **A** | **T** | **T** | **A** | **G** | **A** | **G** | **G** | **T** | **A** | **T** | **A** | **C** | **T** | **T** | **A** | **C** | **A** | **C** | **C** | **A** | **A** | **G** | **T** | **C** | **G** | **A** | **T** | **A** | **A** | **A** | **A** | **A** | **C** | **G** | **T** | **G** | **T** | **T** | **A** | **T** | **T** | **G** | **C** | **G** | **T** | **A** | **T** | **T** | **G** | **T** | **T** | **G** | **A** | **T** | **G** | **A** | **T** | **G** | **G** | **T** | **G** | **T** | **C** | **G** | **G** | **C** | **T** | **T** | **T** | **G** | **A** | **T** | **A** | **T** | **G** | **A** | **A** | **G** | **G** | **A** | **A** | **C** | **A** | **A** | **A** | **G** | **T** | **A** | **A** | **T** | **A** | **A** | **A** | **G** | **C** | **C** | **G** | **G** | **T** | **A** | **G** | **T** | **T** | **A** | **T** | **G** | **G** | **C** | **T** | **T** | **A** | **A** | **A** | **T** | **A** | **A** | **T** | **A** | **T** | **T** | **C** | **G** | **A** | **G** | **A** | **A** | **C** | **G** | **T** | **G** | **T** | **T** | **G** | **T** | **C** | **G** | **G** | **C** | **A** | **T** | **G** | **G** | **G** | **C** | **G** | **G** | **T** | **A** | **C** | **A** | **G** | **T** | **T** | **A** | **A** | **A** | **A** | **T** | **T** | **A** | **T** | **T** | **A** | **G** | **T** | **T** | **T** | **T** | **A** | **A** | **A** | **G** | **G** | **G** | **C** | **A** | **G** | **G** | **G** | **A** | **A** | **C** | **C** | **A** | **G** | **C** | **G** | **T** | **T** | **G** | **A** | **A** | **A** | **T** | **T** | **A** | **A** | **A** | **G** | **T** | **T** | **C** | **C** | **T** | **G** | **T** | **C** | **A** | **T** | **A** | **A** | **A** | **G** | **G** | **A** | **G** | **G** | **A** | **A** | **A** | **C** | **T** | **G** | **C** | **A** | **A** | **G** | **T** | **G** | **A** | **T** | **C** | **A** | **A** | **A** | **G** | **T** | **A** | **A** | **T** | **G** | **T** | **T** | **A** | **G** | **T** | **G** | **G** | **A** | **-** | **-** | **-** |
| **N59841F** | **A** | **T** | **G** | **A** | **C** | **C** | **G** | **A** | **T** | **C** | **G** | **G** | **A** | **T** | **T** | **T** | **C** | **A** | **A** | **G** | **A** | **C** | **G** | **C** | **A** | **T** | **G** | **A** | **T** | **T** | **T** | **C** | **A** | **T** | **T** | **A** | **T** | **A** | **T** | **G** | **C** | **G** | **T** | **C** | **C** | **C** | **T** | **T** | **A** | **G** | **C** | **A** | **C** | **C** | **T** | **T** | **T** | **A** | **T** | **T** | **G** | **T** | **T** | **A** | **T** | **C** | **T** | **T** | **A** | **A** | **T** | **T** | **A** | **C** | **A** | **T** | **T** | **G** | **T** | **T** | **T** | **T** | **C** | **A** | **T** | **A** | **T** | **T** | **T** | **T** | **C** | **A** | **T** | **T** | **C** | **G** | **A** | **T** | **T** | **A** | **A** | **A** | **C** | **A** | **A** | **A** | **A** | **C** | **C** | **G** | **G** | **T** | **G** | **G** | **C** | **T** | **A** | **T** | **T** | **A** | **G** | **A** | **G** | **C** | **T** | **T** | **C** | **T** | **T** | **C** | **A** | **G** | **A** | **G** | **A** | **A** | **A** | **A** | **G** | **T** | **C** | **T** | **T** | **T** | **T** | **A** | **T** | **C** | **T** | **A** | **C** | **C** | **A** | **C** | **T** | **A** | **A** | **T** | **T** | **G** | **T** | **G** | **C** | **A** | **C** | **A** | **T** | **T** | **G** | **T** | **T** | **C** | **T** | **C** | **A** | **T** | **A** | **T** | **C** | **C** | **T** | **T** | **A** | **C** | **T** | **A** | **A** | **T** | **A** | **G** | **G** | **C** | **T** | **T** | **A** | **T** | **T** | **G** | **A** | **C** | **C** | **T** | **T** | **T** | **T** | **T** | **A** | **C** | **T** | **G** | **A** | **T** | **T** | **T** | **C** | **A** | **T** | **T** | **G** | **G** | **T** | **T** | **C** | **A** | **A** | **A** | **A** | **A** | **G** | **G** | **G** | **C** | **A** | **A** | **T** | **A** | **T** | **G** | **G** | **A** | **C** | **G** | **G** | **A** | **T** | **T** | **G** | **A** | **A** | **G** | **A** | **A** | **A** | **A** | **A** | **C** | **T** | **T** | **C** | **G** | **G** | **T** | **T** | **A** | **T** | **T** | **G** | **G** | **C** | **C** | **A** | **A** | **T** | **G** | **G** | **C** | **A** | **A** | **T** | **T** | **A** | **T** | **G** | **A** | **A** | **A** | **G** | **T** | **C** | **C** | **A** | **G** | **T** | **C** | **T** | **T** | **A** | **A** | **A** | **C** | **A** | **A** | **A** | **C** | **C** | **A** | **A** | **C** | **G** | **A** | **C** | **C** | **A** | **G** | **T** | **G** | **A** | **A** | **A** | **A** | **T** | **C** | **A** | **A** | **G** | **A** | **T** | **C** | **A** | **T** | **T** | **A** | **T** | **C** | **T** | **A** | **A** | **C** | **C** | **G** | **A** | **A** | **G** | **T** | **C** | **G** | **A** | **A** | **C** | **A** | **A** | **G** | **A** | **T** | **A** | **T** | **T** | **T** | **G** | **G** | **T** | **C** | **G** | **A** | **T** | **T** | **A** | **A** | **A** | **A** | **A** | **T** | **A** | **A** | **A** | **T** | **T** | **A** | **T** | **T** | **A** | **G** | **A** | **G** | **A** | **T** | **G** | **T** | **C** | **T** | **A** | **A** | **A** | **G** | **A** | **A** | **T** | **T** | **G** | **C** | **A** | **A** | **T** | **T** | **A** | **T** | **T** | **A** | **A** | **A** | **C** | **A** | **G** | **T** | **C** | **G** | **G** | **C** | **C** | **G** | **C** | **A** | **A** | **T** | **T** | **A** | **A** | **T** | **G** | **G** | **A** | **T** | **G** | **G** | **G** | **C** | **A** | **A** | **A** | **C** | **A** | **A** | **A** | **A** | **G** | **A** | **A** | **G** | **A** | **A** | **A** | **T** | **T** | **T** | **T** | **A** | **G** | **A** | **G** | **A** | **A** | **C** | **G** | **A** | **G** | **C** | **G** | **G** | **C** | **A** | **T** | **C** | **G** | **T** | **T** | **T** | **G** | **G** | **C** | **G** | **C** | **G** | **G** | **G** | **A** | **G** | **T** | **T** | **G** | **C** | **A** | **T** | **G** | **A** | **T** | **T** | **C** | **A** | **G** | **T** | **C** | **A** | **G** | **T** | **C** | **A** | **A** | **C** | **A** | **A** | **C** | **T** | **T** | **T** | **T** | **T** | **G** | **C** | **A** | **G** | **C** | **C** | **A** | **T** | **G** | **A** | **T** | **G** | **A** | **T** | **G** | **T** | **T** | **G** | **T** | **C** | **T** | **G** | **C** | **C** | **T** | **T** | **A** | **A** | **A** | **T** | **G** | **A** | **A** | **C** | **A** | **A** | **G** | **C** | **A** | **C** | **A** | **A** | **C** | **G** | **A** | **A** | **C** | **A** | **G** | **A** | **A** | **A** | **C** | **C** | **C** | **C** | **G** | **G** | **A** | **A** | **C** | **C** | **A** | **T** | **A** | **T** | **C** | **G** | **T** | **A** | **A** | **A** | **C** | **A** | **A** | **C** | **T** | **A** | **G** | **C** | **C** | **A** | **T** | **G** | **G** | **T** | **G** | **G** | **C** | **A** | **G** | **A** | **A** | **A** | **T** | **C** | **A** | **T** | **T** | **A** | **A** | **T** | **G** | **C** | **C** | **T** | **C** | **C** | **C** | **A** | **A** | **T** | **C** | **G** | **G** | **A** | **A** | **A** | **T** | **G** | **C** | **G** | **C** | **G** | **C** | **G** | **T** | **T** | **A** | **C** | **T** | **A** | **T** | **T** | **G** | **C** | **A** | **C** | **T** | **T** | **G** | **C** | **G** | **T** | **C** | **C** | **T** | **A** | **T** | **C** | **A** | **G** | **T** | **C** | **T** | **A** | **G** | **A** | **A** | **G** | **G** | **A** | **A** | **A** | **A** | **A** | **G** | **T** | **T** | **T** | **G** | **C** | **G** | **T** | **A** | **A** | **A** | **G** | **G** | **T** | **A** | **T** | **T** | **G** | **A** | **A** | **C** | **A** | **A** | **T** | **T** | **A** | **C** | **T** | **G** | **A** | **A** | **A** | **G** | **A** | **A** | **C** | **T** | **A** | **C** | **A** | **A** | **A** | **C** | **A** | **A** | **A** | **A** | **A** | **T** | **T** | **A** | **A** | **A** | **A** | **T** | **T** | **G** | **A** | **A** | **T** | **T** | **G** | **A** | **T** | **T** | **T** | **G** | **G** | **G** | **A** | **T** | **G** | **T** | **T** | **G** | **A** | **A** | **G** | **A** | **T** | **G** | **T** | **T** | **C** | **A** | **T** | **T** | **T** | **A** | **A** | **A** | **T** | **A** | **G** | **C** | **A** | **G** | **C** | **A** | **T** | **T** | **G** | **A** | **G** | **G** | **A** | **T** | **C** | **A** | **T** | **C** | **T** | **T** | **T** | **T** | **C** | **C** | **G** | **A** | **A** | **T** | **T** | **G** | **T** | **G** | **C** | **A** | **G** | **G** | **A** | **G** | **T** | **T** | **A** | **C** | **T** | **T** | **T** | **C** | **A** | **A** | **A** | **T** | **A** | **C** | **C** | **T** | **T** | **A** | **A** | **G** | **A** | **C** | **A** | **T** | **G** | **C** | **T** | **A** | **A** | **A** | **G** | **C** | **A** | **A** | **A** | **G** | **G** | **A** | **A** | **T** | **T** | **A** | **G** | **A** | **G** | **G** | **T** | **A** | **T** | **A** | **T** | **T** | **T** | **A** | **C** | **A** | **C** | **C** | **A** | **A** | **G** | **T** | **T** | **G** | **A** | **T** | **A** | **A** | **A** | **A** | **A** | **C** | **G** | **T** | **G** | **T** | **T** | **A** | **T** | **T** | **G** | **C** | **G** | **T** | **A** | **T** | **T** | **G** | **T** | **T** | **G** | **A** | **T** | **G** | **A** | **T** | **G** | **G** | **T** | **G** | **T** | **C** | **G** | **G** | **C** | **T** | **T** | **T** | **G** | **A** | **T** | **A** | **T** | **G** | **A** | **A** | **G** | **G** | **A** | **A** | **C** | **A** | **A** | **A** | **G** | **T** | **A** | **A** | **T** | **A** | **A** | **A** | **G** | **C** | **C** | **G** | **G** | **T** | **A** | **G** | **T** | **T** | **A** | **T** | **G** | **G** | **C** | **T** | **T** | **A** | **A** | **A** | **T** | **A** | **A** | **T** | **A** | **T** | **T** | **C** | **G** | **A** | **G** | **A** | **A** | **C** | **G** | **T** | **G** | **T** | **T** | **G** | **T** | **C** | **G** | **G** | **C** | **A** | **T** | **G** | **G** | **G** | **C** | **G** | **G** | **T** | **A** | **C** | **A** | **G** | **T** | **T** | **A** | **A** | **A** | **A** | **T** | **T** | **A** | **T** | **T** | **A** | **G** | **T** | **T** | **T** | **T** | **A** | **A** | **A** | **G** | **G** | **G** | **C** | **A** | **G** | **G** | **G** | **A** | **A** | **C** | **C** | **A** | **G** | **C** | **G** | **T** | **T** | **G** | **A** | **A** | **A** | **T** | **T** | **A** | **A** | **A** | **G** | **T** | **T** | **C** | **C** | **T** | **G** | **T** | **C** | **A** | **T** | **A** | **A** | **A** | **G** | **G** | **A** | **G** | **G** | **A** | **A** | **A** | **C** | **T** | **G** | **C** | **A** | **A** | **G** | **T** | **G** | **A** | **T** | **C** | **A** | **A** | **A** | **G** | **T** | **A** | **A** | **T** | **G** | **T** | **T** | **A** | **G** | **T** | **G** | **G** | **A** | **-** | **-** | **-** |
| **N59544F** | **A** | **T** | **G** | **A** | **C** | **C** | **G** | **A** | **T** | **C** | **G** | **G** | **A** | **T** | **T** | **T** | **C** | **A** | **A** | **G** | **A** | **C** | **G** | **C** | **A** | **T** | **G** | **A** | **T** | **T** | **T** | **C** | **A** | **T** | **T** | **A** | **T** | **A** | **T** | **G** | **C** | **G** | **T** | **C** | **C** | **C** | **T** | **T** | **A** | **G** | **C** | **A** | **C** | **C** | **T** | **T** | **T** | **A** | **T** | **T** | **G** | **T** | **T** | **A** | **T** | **C** | **T** | **T** | **A** | **A** | **T** | **T** | **A** | **C** | **A** | **T** | **T** | **G** | **T** | **T** | **T** | **T** | **C** | **A** | **T** | **A** | **T** | **T** | **T** | **T** | **C** | **A** | **T** | **T** | **C** | **G** | **A** | **T** | **T** | **A** | **A** | **A** | **C** | **A** | **A** | **A** | **A** | **C** | **C** | **G** | **G** | **T** | **G** | **G** | **T** | **T** | **A** | **T** | **T** | **A** | **G** | **A** | **G** | **C** | **T** | **T** | **C** | **T** | **T** | **C** | **A** | **G** | **A** | **G** | **A** | **A** | **A** | **A** | **G** | **T** | **C** | **T** | **T** | **T** | **T** | **A** | **T** | **T** | **T** | **A** | **C** | **C** | **A** | **C** | **T** | **A** | **A** | **T** | **T** | **G** | **T** | **G** | **C** | **A** | **C** | **A** | **T** | **T** | **G** | **T** | **T** | **C** | **T** | **C** | **A** | **T** | **A** | **T** | **C** | **C** | **T** | **T** | **A** | **C** | **T** | **A** | **A** | **T** | **A** | **G** | **G** | **C** | **T** | **T** | **A** | **T** | **T** | **G** | **A** | **C** | **C** | **T** | **T** | **T** | **T** | **T** | **A** | **C** | **T** | **G** | **A** | **T** | **T** | **T** | **C** | **A** | **T** | **T** | **G** | **G** | **T** | **T** | **C** | **A** | **A** | **A** | **A** | **A** | **G** | **G** | **G** | **C** | **A** | **A** | **T** | **A** | **T** | **G** | **G** | **A** | **C** | **G** | **G** | **A** | **T** | **T** | **G** | **A** | **A** | **G** | **A** | **A** | **A** | **A** | **A** | **C** | **T** | **T** | **C** | **G** | **G** | **T** | **T** | **A** | **T** | **T** | **G** | **G** | **C** | **C** | **A** | **A** | **C** | **G** | **G** | **T** | **A** | **A** | **T** | **T** | **A** | **T** | **G** | **A** | **A** | **A** | **G** | **T** | **C** | **C** | **A** | **G** | **T** | **C** | **T** | **T** | **A** | **A** | **A** | **C** | **A** | **A** | **A** | **C** | **C** | **A** | **A** | **C** | **G** | **A** | **C** | **C** | **A** | **G** | **T** | **G** | **A** | **A** | **A** | **A** | **T** | **C** | **A** | **A** | **G** | **A** | **C** | **C** | **A** | **T** | **T** | **A** | **T** | **C** | **T** | **A** | **A** | **C** | **C** | **G** | **A** | **A** | **G** | **T** | **C** | **G** | **A** | **A** | **C** | **A** | **A** | **G** | **A** | **T** | **A** | **T** | **T** | **T** | **G** | **G** | **T** | **C** | **G** | **A** | **T** | **T** | **A** | **A** | **A** | **A** | **A** | **T** | **A** | **A** | **A** | **T** | **T** | **A** | **T** | **T** | **A** | **G** | **A** | **G** | **A** | **T** | **G** | **T** | **C** | **T** | **A** | **A** | **A** | **G** | **A** | **A** | **T** | **T** | **G** | **C** | **A** | **A** | **T** | **T** | **A** | **T** | **T** | **A** | **A** | **A** | **C** | **A** | **G** | **T** | **C** | **G** | **A** | **C** | **C** | **G** | **C** | **A** | **A** | **T** | **T** | **A** | **A** | **T** | **G** | **G** | **A** | **T** | **G** | **G** | **G** | **C** | **A** | **A** | **A** | **C** | **A** | **A** | **A** | **A** | **G** | **A** | **A** | **G** | **A** | **A** | **A** | **T** | **T** | **T** | **T** | **A** | **G** | **A** | **G** | **A** | **A** | **C** | **G** | **A** | **G** | **C** | **G** | **G** | **C** | **A** | **T** | **C** | **G** | **T** | **T** | **T** | **G** | **G** | **C** | **G** | **C** | **G** | **G** | **G** | **A** | **G** | **T** | **T** | **G** | **C** | **A** | **T** | **G** | **A** | **T** | **T** | **C** | **A** | **G** | **T** | **C** | **A** | **G** | **T** | **C** | **A** | **A** | **C** | **A** | **A** | **C** | **T** | **T** | **T** | **T** | **T** | **G** | **C** | **A** | **G** | **C** | **C** | **A** | **T** | **G** | **A** | **T** | **G** | **A** | **T** | **G** | **T** | **T** | **G** | **T** | **C** | **T** | **G** | **C** | **A** | **T** | **T** | **A** | **A** | **A** | **T** | **G** | **A** | **A** | **C** | **A** | **A** | **G** | **C** | **A** | **C** | **A** | **A** | **C** | **G** | **A** | **A** | **C** | **A** | **G** | **A** | **A** | **A** | **C** | **C** | **C** | **C** | **G** | **G** | **A** | **A** | **C** | **C** | **A** | **T** | **A** | **T** | **C** | **G** | **T** | **A** | **A** | **A** | **C** | **A** | **A** | **C** | **T** | **A** | **G** | **C** | **C** | **A** | **T** | **G** | **G** | **T** | **G** | **G** | **C** | **A** | **G** | **A** | **A** | **A** | **T** | **C** | **A** | **T** | **T** | **A** | **A** | **T** | **G** | **C** | **C** | **T** | **C** | **C** | **C** | **A** | **A** | **T** | **C** | **G** | **G** | **A** | **A** | **A** | **T** | **G** | **C** | **G** | **C** | **G** | **C** | **G** | **C** | **T** | **A** | **C** | **T** | **A** | **T** | **T** | **G** | **C** | **A** | **C** | **T** | **T** | **G** | **C** | **G** | **T** | **C** | **C** | **T** | **A** | **T** | **C** | **A** | **G** | **T** | **C** | **T** | **A** | **G** | **A** | **A** | **G** | **G** | **A** | **A** | **A** | **A** | **A** | **G** | **T** | **T** | **T** | **G** | **C** | **G** | **T** | **A** | **A** | **A** | **G** | **G** | **T** | **A** | **T** | **T** | **G** | **A** | **A** | **C** | **A** | **A** | **T** | **T** | **A** | **C** | **T** | **G** | **A** | **A** | **A** | **G** | **A** | **A** | **C** | **T** | **A** | **C** | **A** | **A** | **A** | **C** | **A** | **A** | **A** | **A** | **A** | **T** | **T** | **A** | **A** | **A** | **A** | **T** | **T** | **G** | **A** | **A** | **T** | **T** | **G** | **A** | **T** | **T** | **T** | **G** | **G** | **G** | **A** | **T** | **G** | **T** | **T** | **G** | **A** | **A** | **G** | **A** | **T** | **G** | **T** | **T** | **C** | **A** | **T** | **T** | **T** | **A** | **A** | **A** | **T** | **A** | **G** | **C** | **A** | **G** | **C** | **A** | **T** | **T** | **G** | **A** | **G** | **G** | **A** | **T** | **C** | **A** | **T** | **C** | **T** | **T** | **T** | **T** | **C** | **C** | **G** | **A** | **A** | **T** | **T** | **G** | **T** | **G** | **C** | **A** | **G** | **G** | **A** | **G** | **T** | **T** | **A** | **C** | **T** | **T** | **T** | **C** | **A** | **A** | **A** | **T** | **A** | **C** | **C** | **T** | **T** | **A** | **A** | **G** | **A** | **C** | **A** | **T** | **G** | **C** | **C** | **A** | **A** | **A** | **G** | **C** | **A** | **A** | **A** | **G** | **G** | **A** | **A** | **T** | **T** | **A** | **G** | **A** | **G** | **G** | **T** | **A** | **T** | **A** | **C** | **T** | **T** | **A** | **C** | **A** | **C** | **C** | **A** | **A** | **G** | **T** | **C** | **G** | **A** | **T** | **A** | **A** | **A** | **A** | **A** | **C** | **G** | **T** | **G** | **T** | **T** | **A** | **T** | **T** | **G** | **C** | **G** | **T** | **A** | **T** | **T** | **G** | **T** | **T** | **G** | **A** | **T** | **G** | **A** | **T** | **G** | **G** | **T** | **G** | **T** | **C** | **G** | **G** | **C** | **T** | **T** | **T** | **G** | **A** | **T** | **A** | **T** | **G** | **A** | **A** | **G** | **G** | **A** | **A** | **C** | **A** | **A** | **A** | **G** | **T** | **A** | **A** | **T** | **A** | **A** | **A** | **G** | **C** | **C** | **G** | **G** | **T** | **A** | **G** | **T** | **T** | **A** | **T** | **G** | **G** | **C** | **T** | **T** | **A** | **A** | **A** | **T** | **A** | **A** | **T** | **A** | **T** | **T** | **C** | **G** | **A** | **G** | **A** | **A** | **C** | **G** | **T** | **G** | **T** | **T** | **G** | **T** | **C** | **G** | **G** | **C** | **A** | **T** | **G** | **G** | **G** | **C** | **G** | **G** | **T** | **A** | **C** | **A** | **G** | **T** | **T** | **A** | **A** | **A** | **A** | **T** | **T** | **A** | **T** | **T** | **A** | **G** | **T** | **T** | **T** | **T** | **A** | **A** | **A** | **G** | **G** | **G** | **C** | **A** | **G** | **G** | **G** | **A** | **A** | **C** | **C** | **A** | **G** | **C** | **G** | **T** | **T** | **G** | **A** | **A** | **A** | **T** | **T** | **A** | **A** | **A** | **G** | **T** | **T** | **C** | **C** | **T** | **G** | **T** | **C** | **A** | **T** | **A** | **A** | **A** | **G** | **G** | **A** | **G** | **G** | **A** | **A** | **A** | **C** | **T** | **G** | **C** | **A** | **A** | **G** | **T** | **G** | **A** | **T** | **C** | **A** | **A** | **A** | **G** | **T** | **A** | **A** | **T** | **G** | **T** | **T** | **A** | **G** | **T** | **G** | **G** | **A** | **-** | **-** | **-** |
| **N55106** | **A** | **T** | **G** | **A** | **C** | **C** | **G** | **A** | **T** | **C** | **G** | **G** | **A** | **T** | **T** | **T** | **C** | **A** | **A** | **G** | **A** | **C** | **G** | **C** | **A** | **T** | **G** | **A** | **T** | **T** | **T** | **C** | **A** | **T** | **T** | **A** | **T** | **A** | **T** | **G** | **C** | **G** | **T** | **C** | **C** | **C** | **T** | **T** | **A** | **G** | **C** | **A** | **C** | **C** | **T** | **T** | **T** | **A** | **T** | **T** | **G** | **T** | **T** | **A** | **T** | **C** | **T** | **T** | **A** | **A** | **T** | **T** | **A** | **C** | **A** | **T** | **T** | **G** | **T** | **T** | **T** | **T** | **C** | **A** | **T** | **A** | **T** | **T** | **T** | **T** | **C** | **A** | **T** | **T** | **C** | **G** | **A** | **T** | **T** | **A** | **A** | **A** | **C** | **A** | **A** | **A** | **A** | **C** | **C** | **G** | **G** | **T** | **G** | **G** | **C** | **T** | **A** | **T** | **T** | **A** | **G** | **A** | **G** | **C** | **T** | **T** | **C** | **T** | **T** | **C** | **A** | **G** | **A** | **G** | **A** | **A** | **A** | **A** | **G** | **T** | **C** | **T** | **T** | **T** | **T** | **A** | **T** | **T** | **T** | **A** | **C** | **C** | **A** | **C** | **T** | **A** | **A** | **T** | **T** | **G** | **T** | **G** | **C** | **A** | **C** | **A** | **T** | **T** | **G** | **T** | **T** | **C** | **T** | **C** | **A** | **T** | **A** | **T** | **C** | **C** | **T** | **T** | **A** | **C** | **T** | **A** | **A** | **T** | **A** | **G** | **G** | **C** | **T** | **T** | **A** | **T** | **T** | **G** | **A** | **C** | **C** | **T** | **T** | **T** | **T** | **T** | **A** | **C** | **T** | **G** | **A** | **T** | **T** | **T** | **C** | **A** | **T** | **T** | **G** | **G** | **T** | **T** | **C** | **A** | **A** | **A** | **A** | **A** | **G** | **G** | **G** | **C** | **A** | **A** | **T** | **A** | **T** | **G** | **G** | **A** | **C** | **G** | **G** | **A** | **T** | **T** | **G** | **A** | **A** | **G** | **A** | **A** | **A** | **A** | **A** | **C** | **T** | **T** | **C** | **G** | **G** | **T** | **T** | **A** | **T** | **T** | **G** | **G** | **C** | **C** | **A** | **A** | **C** | **G** | **G** | **T** | **A** | **A** | **T** | **T** | **A** | **T** | **G** | **A** | **A** | **A** | **G** | **T** | **C** | **C** | **A** | **G** | **T** | **C** | **T** | **T** | **A** | **A** | **A** | **C** | **A** | **A** | **A** | **C** | **C** | **A** | **A** | **C** | **G** | **A** | **C** | **C** | **A** | **G** | **T** | **G** | **A** | **A** | **A** | **A** | **T** | **C** | **A** | **A** | **G** | **A** | **C** | **C** | **A** | **T** | **T** | **A** | **T** | **C** | **T** | **A** | **A** | **C** | **C** | **G** | **A** | **A** | **G** | **T** | **C** | **G** | **A** | **A** | **C** | **A** | **A** | **G** | **A** | **T** | **A** | **T** | **T** | **T** | **G** | **G** | **T** | **C** | **G** | **A** | **T** | **T** | **A** | **A** | **A** | **A** | **A** | **T** | **A** | **A** | **A** | **T** | **T** | **A** | **T** | **T** | **A** | **G** | **A** | **G** | **A** | **T** | **G** | **T** | **C** | **T** | **A** | **A** | **A** | **G** | **A** | **A** | **T** | **T** | **G** | **C** | **A** | **A** | **T** | **T** | **A** | **T** | **T** | **A** | **A** | **A** | **C** | **A** | **G** | **T** | **C** | **G** | **A** | **C** | **C** | **G** | **C** | **A** | **A** | **T** | **T** | **A** | **A** | **T** | **G** | **G** | **A** | **T** | **G** | **G** | **G** | **C** | **A** | **A** | **A** | **C** | **A** | **A** | **A** | **A** | **G** | **A** | **A** | **G** | **A** | **A** | **A** | **T** | **T** | **T** | **T** | **A** | **G** | **A** | **G** | **A** | **A** | **C** | **G** | **A** | **G** | **C** | **G** | **G** | **C** | **A** | **T** | **C** | **G** | **T** | **T** | **T** | **G** | **G** | **C** | **G** | **C** | **G** | **G** | **G** | **A** | **G** | **T** | **T** | **G** | **C** | **A** | **T** | **G** | **A** | **T** | **T** | **C** | **A** | **G** | **T** | **C** | **A** | **G** | **T** | **C** | **A** | **A** | **C** | **A** | **A** | **C** | **T** | **T** | **T** | **T** | **T** | **G** | **C** | **A** | **G** | **C** | **C** | **A** | **T** | **G** | **A** | **T** | **G** | **A** | **T** | **G** | **T** | **T** | **G** | **T** | **C** | **T** | **G** | **C** | **C** | **T** | **T** | **A** | **A** | **A** | **T** | **G** | **A** | **A** | **C** | **A** | **A** | **G** | **C** | **A** | **C** | **A** | **A** | **C** | **G** | **A** | **A** | **C** | **A** | **G** | **A** | **A** | **A** | **C** | **T** | **C** | **C** | **T** | **G** | **A** | **A** | **C** | **C** | **A** | **T** | **A** | **T** | **C** | **G** | **C** | **A** | **A** | **A** | **C** | **A** | **A** | **C** | **T** | **A** | **G** | **C** | **C** | **A** | **T** | **G** | **G** | **T** | **G** | **G** | **C** | **A** | **G** | **A** | **A** | **A** | **T** | **C** | **A** | **T** | **T** | **A** | **A** | **T** | **G** | **C** | **C** | **T** | **C** | **C** | **C** | **A** | **A** | **T** | **C** | **G** | **G** | **A** | **A** | **A** | **T** | **G** | **C** | **G** | **C** | **G** | **C** | **G** | **T** | **T** | **A** | **C** | **T** | **A** | **T** | **T** | **G** | **C** | **A** | **C** | **T** | **T** | **G** | **C** | **G** | **T** | **C** | **C** | **T** | **A** | **T** | **C** | **A** | **G** | **T** | **C** | **T** | **A** | **G** | **A** | **A** | **G** | **G** | **A** | **A** | **A** | **A** | **A** | **G** | **T** | **T** | **T** | **G** | **C** | **G** | **T** | **A** | **A** | **A** | **G** | **G** | **T** | **A** | **T** | **T** | **G** | **A** | **A** | **C** | **A** | **A** | **T** | **T** | **A** | **C** | **T** | **G** | **A** | **A** | **A** | **G** | **A** | **A** | **C** | **T** | **A** | **C** | **A** | **A** | **A** | **C** | **A** | **A** | **A** | **A** | **A** | **T** | **T** | **A** | **A** | **A** | **A** | **T** | **T** | **G** | **A** | **A** | **T** | **T** | **G** | **A** | **T** | **T** | **T** | **G** | **G** | **G** | **A** | **T** | **G** | **T** | **T** | **G** | **A** | **A** | **G** | **A** | **T** | **G** | **T** | **T** | **C** | **A** | **T** | **T** | **T** | **A** | **A** | **A** | **T** | **A** | **G** | **C** | **A** | **G** | **C** | **A** | **T** | **T** | **G** | **A** | **G** | **G** | **A** | **T** | **C** | **A** | **T** | **C** | **T** | **T** | **T** | **T** | **C** | **C** | **G** | **A** | **A** | **T** | **T** | **G** | **T** | **G** | **C** | **A** | **G** | **G** | **A** | **A** | **T** | **T** | **A** | **C** | **T** | **T** | **T** | **C** | **A** | **A** | **A** | **T** | **A** | **C** | **C** | **T** | **T** | **A** | **A** | **G** | **A** | **C** | **A** | **T** | **G** | **C** | **C** | **A** | **A** | **A** | **G** | **C** | **A** | **A** | **A** | **G** | **G** | **A** | **A** | **T** | **T** | **A** | **G** | **A** | **G** | **G** | **T** | **A** | **T** | **A** | **C** | **T** | **T** | **A** | **C** | **A** | **C** | **C** | **A** | **A** | **G** | **T** | **C** | **G** | **A** | **T** | **A** | **A** | **A** | **A** | **A** | **C** | **G** | **T** | **G** | **T** | **T** | **A** | **T** | **T** | **G** | **C** | **G** | **T** | **A** | **T** | **T** | **G** | **T** | **T** | **G** | **A** | **T** | **G** | **A** | **T** | **G** | **G** | **T** | **G** | **T** | **C** | **G** | **G** | **C** | **T** | **T** | **T** | **G** | **A** | **T** | **A** | **T** | **G** | **A** | **A** | **G** | **G** | **A** | **A** | **C** | **A** | **A** | **A** | **G** | **T** | **A** | **A** | **T** | **A** | **A** | **A** | **G** | **C** | **C** | **G** | **G** | **T** | **A** | **G** | **T** | **T** | **A** | **T** | **G** | **G** | **C** | **T** | **T** | **A** | **A** | **A** | **T** | **A** | **A** | **T** | **A** | **T** | **C** | **C** | **G** | **A** | **G** | **A** | **A** | **C** | **G** | **T** | **G** | **T** | **T** | **G** | **T** | **C** | **G** | **G** | **C** | **A** | **T** | **G** | **G** | **G** | **C** | **G** | **G** | **T** | **A** | **C** | **A** | **G** | **T** | **T** | **A** | **A** | **A** | **A** | **T** | **T** | **A** | **T** | **T** | **A** | **G** | **T** | **T** | **T** | **T** | **A** | **A** | **A** | **G** | **G** | **G** | **C** | **A** | **G** | **G** | **G** | **A** | **A** | **C** | **C** | **A** | **G** | **C** | **G** | **T** | **T** | **G** | **A** | **A** | **A** | **T** | **T** | **A** | **A** | **A** | **G** | **T** | **T** | **C** | **C** | **T** | **G** | **T** | **C** | **A** | **T** | **A** | **A** | **A** | **G** | **G** | **A** | **G** | **G** | **A** | **A** | **A** | **C** | **T** | **G** | **C** | **A** | **A** | **G** | **T** | **G** | **A** | **T** | **C** | **A** | **A** | **A** | **G** | **T** | **A** | **A** | **T** | **G** | **T** | **T** | **A** | **G** | **T** | **G** | **G** | **A** | **-** | **-** | **-** |
| **N54097** | **A** | **T** | **G** | **A** | **C** | **C** | **G** | **A** | **T** | **C** | **G** | **G** | **A** | **T** | **T** | **T** | **C** | **A** | **A** | **G** | **A** | **C** | **G** | **C** | **A** | **T** | **G** | **A** | **T** | **T** | **T** | **C** | **A** | **T** | **T** | **A** | **T** | **A** | **T** | **G** | **C** | **G** | **T** | **C** | **C** | **C** | **T** | **T** | **A** | **G** | **C** | **A** | **C** | **C** | **T** | **T** | **T** | **A** | **T** | **T** | **G** | **T** | **T** | **A** | **T** | **C** | **T** | **T** | **A** | **A** | **T** | **T** | **A** | **C** | **A** | **T** | **T** | **G** | **T** | **T** | **T** | **T** | **C** | **A** | **T** | **A** | **T** | **T** | **T** | **T** | **C** | **A** | **T** | **T** | **C** | **G** | **A** | **T** | **T** | **A** | **A** | **A** | **C** | **A** | **A** | **A** | **A** | **C** | **C** | **G** | **G** | **T** | **G** | **G** | **C** | **T** | **A** | **T** | **T** | **A** | **G** | **A** | **G** | **C** | **T** | **T** | **C** | **T** | **T** | **C** | **A** | **G** | **A** | **G** | **A** | **A** | **A** | **A** | **G** | **T** | **C** | **T** | **T** | **T** | **T** | **A** | **T** | **T** | **T** | **A** | **C** | **C** | **A** | **C** | **T** | **A** | **A** | **T** | **T** | **G** | **T** | **G** | **C** | **A** | **C** | **A** | **T** | **T** | **G** | **T** | **T** | **C** | **T** | **C** | **A** | **T** | **A** | **T** | **C** | **C** | **T** | **T** | **A** | **C** | **T** | **A** | **A** | **T** | **A** | **G** | **G** | **C** | **T** | **T** | **A** | **T** | **T** | **G** | **A** | **C** | **C** | **T** | **T** | **T** | **T** | **T** | **A** | **C** | **T** | **G** | **A** | **T** | **T** | **T** | **C** | **A** | **T** | **T** | **G** | **G** | **T** | **T** | **C** | **A** | **A** | **A** | **A** | **A** | **G** | **G** | **G** | **C** | **A** | **A** | **T** | **A** | **T** | **G** | **G** | **A** | **C** | **G** | **G** | **A** | **T** | **T** | **G** | **A** | **A** | **G** | **A** | **A** | **A** | **A** | **A** | **C** | **T** | **T** | **C** | **G** | **G** | **T** | **T** | **A** | **T** | **T** | **G** | **G** | **C** | **C** | **A** | **A** | **C** | **G** | **G** | **T** | **A** | **A** | **T** | **T** | **A** | **T** | **G** | **A** | **A** | **A** | **G** | **T** | **C** | **C** | **A** | **G** | **T** | **C** | **T** | **T** | **A** | **A** | **A** | **C** | **A** | **A** | **A** | **C** | **C** | **A** | **A** | **C** | **G** | **A** | **C** | **C** | **A** | **G** | **T** | **G** | **A** | **A** | **A** | **A** | **T** | **C** | **A** | **A** | **G** | **A** | **C** | **C** | **A** | **T** | **T** | **A** | **T** | **C** | **T** | **A** | **A** | **C** | **C** | **G** | **A** | **A** | **G** | **T** | **C** | **G** | **A** | **A** | **C** | **A** | **A** | **G** | **A** | **T** | **A** | **T** | **T** | **T** | **G** | **G** | **T** | **C** | **G** | **A** | **T** | **T** | **A** | **A** | **A** | **A** | **A** | **T** | **A** | **A** | **A** | **T** | **T** | **A** | **T** | **T** | **A** | **G** | **A** | **G** | **A** | **T** | **G** | **T** | **C** | **T** | **A** | **A** | **A** | **G** | **A** | **A** | **T** | **T** | **G** | **C** | **A** | **A** | **T** | **T** | **A** | **T** | **T** | **A** | **A** | **A** | **C** | **A** | **G** | **T** | **C** | **G** | **A** | **C** | **C** | **G** | **C** | **A** | **A** | **T** | **T** | **A** | **A** | **T** | **G** | **G** | **A** | **T** | **G** | **G** | **G** | **C** | **A** | **A** | **A** | **C** | **A** | **A** | **A** | **A** | **G** | **A** | **A** | **G** | **A** | **A** | **A** | **T** | **T** | **T** | **T** | **A** | **G** | **A** | **G** | **A** | **A** | **C** | **G** | **A** | **G** | **C** | **G** | **G** | **C** | **A** | **T** | **C** | **G** | **T** | **T** | **T** | **G** | **G** | **C** | **G** | **C** | **G** | **G** | **G** | **A** | **G** | **T** | **T** | **G** | **C** | **A** | **T** | **G** | **A** | **T** | **T** | **C** | **A** | **G** | **T** | **C** | **A** | **G** | **T** | **C** | **A** | **A** | **C** | **A** | **A** | **C** | **T** | **T** | **T** | **T** | **T** | **G** | **C** | **A** | **G** | **C** | **C** | **A** | **T** | **G** | **A** | **T** | **G** | **A** | **T** | **G** | **T** | **T** | **G** | **T** | **C** | **T** | **G** | **C** | **C** | **T** | **T** | **A** | **A** | **A** | **T** | **G** | **A** | **A** | **C** | **A** | **A** | **G** | **C** | **A** | **C** | **A** | **A** | **C** | **G** | **A** | **A** | **C** | **A** | **G** | **A** | **A** | **A** | **C** | **T** | **C** | **C** | **T** | **G** | **A** | **A** | **C** | **C** | **A** | **T** | **A** | **T** | **C** | **G** | **C** | **A** | **A** | **A** | **C** | **A** | **A** | **C** | **T** | **A** | **G** | **C** | **C** | **A** | **T** | **G** | **G** | **T** | **G** | **G** | **C** | **A** | **G** | **A** | **A** | **A** | **T** | **C** | **A** | **T** | **T** | **A** | **A** | **T** | **G** | **C** | **C** | **T** | **C** | **C** | **C** | **A** | **A** | **T** | **C** | **G** | **G** | **A** | **A** | **A** | **T** | **G** | **C** | **G** | **C** | **G** | **C** | **G** | **T** | **T** | **A** | **C** | **T** | **A** | **T** | **T** | **G** | **C** | **A** | **C** | **T** | **T** | **G** | **C** | **G** | **T** | **C** | **C** | **T** | **A** | **T** | **C** | **A** | **G** | **T** | **C** | **T** | **A** | **G** | **A** | **A** | **G** | **G** | **A** | **A** | **A** | **A** | **A** | **G** | **T** | **T** | **T** | **G** | **C** | **G** | **T** | **A** | **A** | **A** | **G** | **G** | **T** | **A** | **T** | **T** | **G** | **A** | **A** | **C** | **A** | **A** | **T** | **T** | **A** | **C** | **T** | **G** | **A** | **A** | **A** | **G** | **A** | **A** | **C** | **T** | **A** | **C** | **A** | **A** | **A** | **C** | **A** | **A** | **A** | **A** | **A** | **T** | **T** | **A** | **A** | **A** | **A** | **T** | **T** | **G** | **A** | **A** | **T** | **T** | **G** | **A** | **T** | **T** | **T** | **G** | **G** | **G** | **A** | **T** | **G** | **T** | **T** | **G** | **A** | **A** | **G** | **A** | **T** | **G** | **T** | **T** | **C** | **A** | **T** | **T** | **T** | **A** | **A** | **A** | **T** | **A** | **G** | **C** | **A** | **G** | **C** | **A** | **T** | **T** | **G** | **A** | **G** | **G** | **A** | **T** | **C** | **A** | **T** | **C** | **T** | **T** | **T** | **T** | **C** | **C** | **G** | **A** | **A** | **T** | **T** | **G** | **T** | **G** | **C** | **A** | **G** | **G** | **A** | **A** | **T** | **T** | **A** | **C** | **T** | **T** | **T** | **C** | **A** | **A** | **A** | **T** | **A** | **C** | **C** | **T** | **T** | **A** | **A** | **G** | **A** | **C** | **A** | **T** | **G** | **C** | **C** | **A** | **A** | **A** | **G** | **C** | **A** | **A** | **A** | **G** | **G** | **A** | **A** | **T** | **T** | **A** | **G** | **A** | **G** | **G** | **T** | **A** | **T** | **A** | **C** | **T** | **T** | **A** | **C** | **A** | **C** | **C** | **A** | **A** | **G** | **T** | **C** | **G** | **A** | **T** | **A** | **A** | **A** | **A** | **A** | **C** | **G** | **T** | **G** | **T** | **T** | **A** | **T** | **T** | **G** | **C** | **G** | **T** | **A** | **T** | **T** | **G** | **T** | **T** | **G** | **A** | **T** | **G** | **A** | **T** | **G** | **G** | **T** | **G** | **T** | **C** | **G** | **G** | **C** | **T** | **T** | **T** | **G** | **A** | **T** | **A** | **T** | **G** | **A** | **A** | **G** | **G** | **A** | **A** | **C** | **A** | **A** | **A** | **G** | **T** | **A** | **A** | **T** | **A** | **A** | **A** | **G** | **C** | **C** | **G** | **G** | **T** | **A** | **G** | **T** | **T** | **A** | **T** | **G** | **G** | **C** | **T** | **T** | **A** | **A** | **A** | **T** | **A** | **A** | **T** | **A** | **T** | **C** | **C** | **G** | **A** | **G** | **A** | **A** | **C** | **G** | **T** | **G** | **T** | **T** | **G** | **T** | **C** | **G** | **G** | **C** | **A** | **T** | **G** | **G** | **G** | **C** | **G** | **G** | **T** | **A** | **C** | **A** | **G** | **T** | **T** | **A** | **A** | **A** | **A** | **T** | **T** | **A** | **T** | **T** | **A** | **G** | **T** | **T** | **T** | **T** | **A** | **A** | **A** | **G** | **G** | **G** | **C** | **A** | **G** | **G** | **G** | **A** | **A** | **C** | **C** | **A** | **G** | **C** | **G** | **T** | **T** | **G** | **A** | **A** | **A** | **T** | **T** | **A** | **A** | **A** | **G** | **T** | **T** | **C** | **C** | **T** | **G** | **T** | **C** | **A** | **T** | **A** | **A** | **A** | **G** | **G** | **A** | **G** | **G** | **A** | **A** | **A** | **C** | **T** | **G** | **C** | **A** | **A** | **G** | **T** | **G** | **A** | **T** | **C** | **A** | **A** | **A** | **G** | **T** | **A** | **A** | **T** | **G** | **T** | **T** | **A** | **G** | **T** | **G** | **G** | **A** | **-** | **-** | **-** |
| **N52662** | **A** | **T** | **G** | **A** | **C** | **C** | **G** | **A** | **T** | **C** | **G** | **G** | **A** | **T** | **T** | **T** | **C** | **A** | **A** | **G** | **A** | **C** | **G** | **C** | **A** | **T** | **G** | **A** | **T** | **T** | **T** | **C** | **A** | **T** | **T** | **A** | **T** | **A** | **T** | **G** | **C** | **G** | **T** | **C** | **C** | **C** | **T** | **T** | **A** | **G** | **C** | **A** | **C** | **C** | **T** | **T** | **T** | **A** | **T** | **T** | **G** | **T** | **T** | **A** | **T** | **T** | **T** | **T** | **A** | **A** | **T** | **T** | **A** | **C** | **C** | **T** | **T** | **G** | **T** | **T** | **T** | **T** | **C** | **A** | **T** | **A** | **T** | **T** | **T** | **T** | **C** | **A** | **T** | **T** | **C** | **A** | **A** | **T** | **T** | **A** | **A** | **A** | **C** | **A** | **A** | **A** | **A** | **C** | **C** | **G** | **G** | **T** | **G** | **G** | **C** | **T** | **C** | **T** | **T** | **A** | **G** | **A** | **A** | **C** | **T** | **T** | **C** | **T** | **T** | **C** | **A** | **G** | **A** | **G** | **A** | **A** | **A** | **A** | **G** | **T** | **C** | **T** | **T** | **T** | **T** | **A** | **T** | **C** | **T** | **G** | **C** | **C** | **A** | **C** | **T** | **A** | **A** | **T** | **T** | **G** | **T** | **G** | **C** | **A** | **T** | **A** | **T** | **T** | **G** | **T** | **T** | **C** | **T** | **T** | **A** | **T** | **A** | **T** | **C** | **C** | **T** | **T** | **A** | **C** | **T** | **A** | **A** | **T** | **A** | **G** | **G** | **C** | **T** | **T** | **A** | **T** | **T** | **G** | **A** | **C** | **C** | **T** | **T** | **T** | **T** | **T** | **A** | **C** | **T** | **G** | **A** | **T** | **T** | **T** | **C** | **G** | **T** | **T** | **G** | **G** | **T** | **C** | **C** | **A** | **A** | **A** | **A** | **A** | **G** | **G** | **C** | **C** | **A** | **A** | **T** | **A** | **T** | **G** | **G** | **A** | **C** | **G** | **G** | **A** | **T** | **T** | **G** | **A** | **A** | **G** | **A** | **A** | **A** | **A** | **A** | **C** | **T** | **T** | **C** | **G** | **G** | **T** | **T** | **A** | **T** | **T** | **A** | **G** | **C** | **C** | **A** | **A** | **T** | **G** | **G** | **C** | **A** | **A** | **T** | **T** | **A** | **T** | **G** | **A** | **A** | **A** | **G** | **T** | **C** | **C** | **A** | **G** | **T** | **C** | **T** | **T** | **A** | **A** | **A** | **C** | **A** | **A** | **A** | **C** | **C** | **A** | **A** | **T** | **G** | **A** | **C** | **T** | **A** | **G** | **T** | **G** | **A** | **A** | **A** | **A** | **T** | **C** | **A** | **A** | **G** | **A** | **C** | **C** | **A** | **T** | **T** | **A** | **T** | **C** | **T** | **A** | **A** | **T** | **C** | **G** | **A** | **A** | **G** | **T** | **C** | **G** | **A** | **A** | **C** | **A** | **A** | **G** | **A** | **T** | **A** | **T** | **T** | **T** | **G** | **G** | **T** | **C** | **G** | **A** | **T** | **T** | **A** | **A** | **A** | **A** | **A** | **C** | **A** | **A** | **A** | **T** | **T** | **A** | **T** | **T** | **A** | **G** | **A** | **G** | **A** | **T** | **G** | **T** | **C** | **T** | **A** | **A** | **A** | **G** | **A** | **A** | **T** | **T** | **G** | **C** | **A** | **A** | **T** | **T** | **A** | **T** | **T** | **A** | **A** | **A** | **C** | **A** | **G** | **T** | **C** | **G** | **A** | **C** | **C** | **G** | **C** | **A** | **A** | **T** | **T** | **A** | **A** | **T** | **G** | **G** | **A** | **T** | **G** | **G** | **G** | **C** | **A** | **A** | **A** | **C** | **A** | **A** | **A** | **A** | **G** | **A** | **A** | **G** | **A** | **A** | **A** | **T** | **T** | **T** | **T** | **A** | **G** | **A** | **G** | **A** | **A** | **C** | **G** | **A** | **G** | **C** | **G** | **G** | **C** | **A** | **T** | **C** | **G** | **T** | **T** | **T** | **G** | **G** | **C** | **A** | **C** | **G** | **G** | **G** | **A** | **G** | **T** | **T** | **G** | **C** | **A** | **T** | **G** | **A** | **T** | **T** | **C** | **A** | **G** | **T** | **C** | **A** | **G** | **T** | **C** | **A** | **A** | **C** | **A** | **A** | **C** | **T** | **T** | **T** | **T** | **T** | **G** | **C** | **A** | **G** | **C** | **C** | **A** | **T** | **G** | **A** | **T** | **G** | **A** | **T** | **G** | **T** | **T** | **G** | **T** | **C** | **T** | **G** | **C** | **C** | **T** | **T** | **A** | **A** | **A** | **T** | **G** | **A** | **A** | **C** | **A** | **A** | **G** | **C** | **A** | **C** | **A** | **A** | **C** | **G** | **A** | **A** | **C** | **A** | **G** | **A** | **A** | **A** | **C** | **C** | **C** | **C** | **T** | **G** | **A** | **A** | **C** | **C** | **A** | **T** | **A** | **T** | **C** | **G** | **T** | **A** | **A** | **A** | **C** | **A** | **A** | **C** | **T** | **A** | **G** | **C** | **C** | **A** | **T** | **G** | **G** | **T** | **G** | **G** | **C** | **A** | **G** | **A** | **A** | **A** | **T** | **C** | **A** | **T** | **T** | **A** | **A** | **T** | **G** | **C** | **C** | **T** | **C** | **C** | **C** | **A** | **A** | **T** | **C** | **G** | **G** | **A** | **A** | **A** | **T** | **G** | **C** | **G** | **C** | **G** | **C** | **G** | **T** | **T** | **A** | **C** | **T** | **A** | **T** | **T** | **A** | **C** | **A** | **C** | **T** | **T** | **G** | **C** | **G** | **T** | **C** | **C** | **T** | **A** | **T** | **T** | **A** | **G** | **T** | **C** | **T** | **A** | **G** | **A** | **A** | **G** | **G** | **G** | **A** | **A** | **A** | **A** | **G** | **T** | **T** | **T** | **G** | **C** | **G** | **T** | **A** | **A** | **A** | **G** | **G** | **C** | **A** | **T** | **T** | **G** | **A** | **A** | **C** | **A** | **A** | **T** | **T** | **A** | **T** | **T** | **G** | **A** | **A** | **A** | **G** | **A** | **A** | **C** | **T** | **A** | **C** | **A** | **A** | **A** | **C** | **A** | **A** | **A** | **A** | **A** | **T** | **T** | **A** | **A** | **A** | **A** | **T** | **T** | **G** | **A** | **A** | **T** | **T** | **G** | **A** | **T** | **T** | **T** | **G** | **G** | **G** | **A** | **T** | **G** | **T** | **T** | **G** | **A** | **A** | **G** | **A** | **T** | **G** | **T** | **T** | **C** | **A** | **T** | **T** | **T** | **A** | **A** | **A** | **T** | **A** | **G** | **C** | **A** | **G** | **C** | **A** | **T** | **T** | **G** | **A** | **G** | **G** | **A** | **T** | **C** | **A** | **T** | **C** | **T** | **T** | **T** | **T** | **C** | **C** | **G** | **A** | **A** | **T** | **T** | **G** | **T** | **T** | **C** | **A** | **G** | **G** | **A** | **A** | **T** | **T** | **A** | **C** | **T** | **T** | **T** | **C** | **A** | **A** | **A** | **T** | **A** | **C** | **C** | **T** | **T** | **A** | **A** | **G** | **A** | **C** | **A** | **T** | **G** | **C** | **C** | **A** | **A** | **A** | **G** | **C** | **A** | **A** | **A** | **A** | **G** | **A** | **A** | **T** | **T** | **A** | **G** | **A** | **G** | **G** | **T** | **A** | **T** | **A** | **C** | **T** | **T** | **A** | **C** | **A** | **C** | **C** | **A** | **A** | **G** | **T** | **C** | **G** | **A** | **T** | **A** | **A** | **A** | **A** | **A** | **T** | **G** | **T** | **T** | **T** | **T** | **A** | **T** | **T** | **G** | **C** | **G** | **C** | **A** | **T** | **T** | **G** | **T** | **T** | **G** | **A** | **T** | **G** | **A** | **T** | **G** | **G** | **T** | **G** | **T** | **C** | **G** | **G** | **C** | **T** | **T** | **T** | **G** | **A** | **T** | **A** | **T** | **G** | **A** | **A** | **G** | **G** | **A** | **A** | **C** | **A** | **A** | **A** | **G** | **T** | **A** | **A** | **T** | **A** | **A** | **G** | **G** | **C** | **C** | **G** | **G** | **T** | **A** | **G** | **T** | **T** | **A** | **T** | **G** | **G** | **C** | **T** | **T** | **A** | **A** | **A** | **T** | **A** | **A** | **T** | **A** | **T** | **T** | **C** | **G** | **A** | **G** | **A** | **A** | **C** | **G** | **T** | **G** | **T** | **T** | **G** | **T** | **C** | **G** | **G** | **C** | **A** | **T** | **G** | **G** | **G** | **C** | **G** | **G** | **T** | **A** | **C** | **A** | **G** | **T** | **T** | **A** | **A** | **A** | **A** | **T** | **T** | **A** | **T** | **T** | **A** | **G** | **T** | **T** | **T** | **T** | **A** | **A** | **A** | **G** | **G** | **G** | **C** | **A** | **G** | **G** | **G** | **A** | **A** | **C** | **C** | **A** | **G** | **C** | **G** | **T** | **T** | **G** | **A** | **A** | **A** | **T** | **T** | **A** | **A** | **A** | **G** | **T** | **T** | **C** | **C** | **T** | **G** | **T** | **C** | **A** | **T** | **A** | **A** | **A** | **G** | **G** | **A** | **G** | **G** | **A** | **A** | **A** | **C** | **T** | **G** | **C** | **A** | **A** | **G** | **T** | **G** | **A** | **T** | **C** | **A** | **A** | **A** | **G** | **T** | **A** | **A** | **T** | **G** | **T** | **T** | **A** | **G** | **T** | **G** | **G** | **A** | **-** | **-** | **-** |
| **N59509F** | **A** | **T** | **G** | **A** | **C** | **C** | **G** | **A** | **T** | **C** | **G** | **G** | **A** | **T** | **T** | **T** | **C** | **A** | **A** | **G** | **A** | **C** | **G** | **C** | **A** | **T** | **G** | **A** | **T** | **T** | **T** | **C** | **A** | **T** | **T** | **A** | **T** | **A** | **T** | **G** | **C** | **G** | **T** | **C** | **C** | **C** | **T** | **T** | **A** | **G** | **C** | **A** | **C** | **C** | **T** | **T** | **T** | **A** | **T** | **T** | **G** | **T** | **T** | **A** | **T** | **C** | **T** | **T** | **A** | **A** | **T** | **T** | **A** | **C** | **A** | **T** | **T** | **G** | **T** | **T** | **T** | **T** | **C** | **A** | **T** | **A** | **T** | **T** | **T** | **T** | **C** | **A** | **T** | **T** | **C** | **G** | **A** | **T** | **T** | **A** | **A** | **A** | **C** | **A** | **A** | **A** | **A** | **C** | **C** | **G** | **G** | **T** | **G** | **G** | **T** | **T** | **A** | **T** | **T** | **A** | **G** | **A** | **G** | **C** | **T** | **T** | **C** | **T** | **T** | **C** | **A** | **G** | **A** | **G** | **A** | **A** | **A** | **A** | **G** | **T** | **C** | **T** | **T** | **T** | **T** | **A** | **T** | **T** | **T** | **A** | **C** | **C** | **A** | **C** | **T** | **A** | **A** | **T** | **T** | **G** | **T** | **G** | **C** | **A** | **C** | **A** | **T** | **T** | **G** | **T** | **T** | **C** | **T** | **C** | **A** | **T** | **A** | **T** | **C** | **C** | **T** | **T** | **A** | **C** | **T** | **A** | **A** | **T** | **A** | **G** | **G** | **C** | **T** | **T** | **A** | **T** | **T** | **G** | **A** | **C** | **C** | **T** | **T** | **T** | **T** | **T** | **A** | **C** | **T** | **G** | **A** | **T** | **T** | **T** | **C** | **A** | **T** | **T** | **G** | **G** | **T** | **T** | **C** | **A** | **A** | **A** | **A** | **A** | **G** | **G** | **G** | **C** | **A** | **A** | **T** | **A** | **T** | **G** | **G** | **A** | **C** | **G** | **G** | **A** | **T** | **T** | **G** | **A** | **A** | **G** | **A** | **A** | **A** | **A** | **A** | **C** | **T** | **T** | **C** | **G** | **G** | **T** | **T** | **A** | **T** | **T** | **G** | **G** | **C** | **C** | **A** | **A** | **C** | **G** | **G** | **T** | **A** | **A** | **T** | **T** | **A** | **T** | **G** | **A** | **A** | **A** | **G** | **T** | **C** | **C** | **A** | **G** | **T** | **C** | **T** | **T** | **A** | **A** | **A** | **C** | **A** | **A** | **A** | **C** | **C** | **A** | **A** | **C** | **G** | **A** | **C** | **C** | **A** | **G** | **T** | **G** | **A** | **A** | **A** | **A** | **T** | **C** | **A** | **A** | **G** | **A** | **C** | **C** | **A** | **T** | **T** | **A** | **T** | **C** | **T** | **A** | **A** | **C** | **C** | **G** | **A** | **A** | **G** | **T** | **C** | **G** | **A** | **A** | **C** | **A** | **A** | **G** | **A** | **T** | **A** | **T** | **T** | **T** | **G** | **G** | **T** | **C** | **G** | **A** | **T** | **T** | **A** | **A** | **A** | **A** | **A** | **T** | **A** | **A** | **A** | **T** | **T** | **A** | **T** | **T** | **A** | **G** | **A** | **G** | **A** | **T** | **G** | **T** | **C** | **T** | **A** | **A** | **A** | **G** | **A** | **A** | **T** | **T** | **G** | **C** | **A** | **A** | **T** | **T** | **A** | **T** | **T** | **A** | **A** | **A** | **C** | **A** | **G** | **T** | **C** | **G** | **A** | **C** | **C** | **G** | **C** | **A** | **A** | **T** | **T** | **A** | **A** | **T** | **G** | **G** | **A** | **T** | **G** | **G** | **G** | **C** | **A** | **A** | **A** | **C** | **A** | **A** | **A** | **A** | **G** | **A** | **A** | **G** | **A** | **A** | **A** | **T** | **T** | **T** | **T** | **A** | **G** | **A** | **G** | **A** | **A** | **C** | **G** | **A** | **G** | **C** | **G** | **G** | **C** | **A** | **T** | **C** | **G** | **T** | **T** | **T** | **G** | **G** | **C** | **G** | **C** | **G** | **G** | **G** | **A** | **G** | **T** | **T** | **G** | **C** | **A** | **T** | **G** | **A** | **T** | **T** | **C** | **A** | **G** | **T** | **C** | **A** | **G** | **T** | **C** | **A** | **A** | **C** | **A** | **A** | **C** | **T** | **T** | **T** | **T** | **T** | **G** | **C** | **A** | **G** | **C** | **C** | **A** | **T** | **G** | **A** | **T** | **G** | **A** | **T** | **G** | **T** | **T** | **G** | **T** | **C** | **T** | **G** | **C** | **A** | **T** | **T** | **A** | **A** | **A** | **T** | **G** | **A** | **A** | **C** | **A** | **A** | **G** | **C** | **A** | **C** | **A** | **A** | **C** | **G** | **A** | **A** | **C** | **A** | **G** | **A** | **A** | **A** | **C** | **C** | **C** | **C** | **G** | **G** | **A** | **A** | **C** | **C** | **A** | **T** | **A** | **T** | **C** | **G** | **T** | **A** | **A** | **A** | **C** | **A** | **A** | **C** | **T** | **A** | **G** | **C** | **C** | **A** | **T** | **G** | **G** | **T** | **G** | **G** | **C** | **A** | **G** | **A** | **A** | **A** | **T** | **C** | **A** | **T** | **T** | **A** | **A** | **T** | **G** | **C** | **C** | **T** | **C** | **C** | **C** | **A** | **A** | **T** | **C** | **G** | **G** | **A** | **A** | **A** | **T** | **G** | **C** | **G** | **C** | **G** | **C** | **G** | **C** | **T** | **A** | **C** | **T** | **A** | **T** | **T** | **G** | **C** | **A** | **C** | **T** | **T** | **G** | **C** | **G** | **T** | **C** | **C** | **T** | **A** | **T** | **C** | **A** | **G** | **T** | **C** | **T** | **A** | **G** | **A** | **A** | **G** | **G** | **A** | **A** | **A** | **A** | **A** | **G** | **T** | **T** | **T** | **G** | **C** | **G** | **T** | **A** | **A** | **A** | **G** | **G** | **T** | **A** | **T** | **T** | **G** | **A** | **A** | **C** | **A** | **A** | **T** | **T** | **A** | **C** | **T** | **G** | **A** | **A** | **A** | **G** | **A** | **A** | **C** | **T** | **A** | **C** | **A** | **A** | **A** | **C** | **A** | **A** | **A** | **A** | **A** | **T** | **T** | **A** | **A** | **A** | **A** | **T** | **T** | **G** | **A** | **A** | **T** | **T** | **G** | **A** | **T** | **T** | **T** | **G** | **G** | **G** | **A** | **T** | **G** | **T** | **T** | **G** | **A** | **A** | **G** | **A** | **T** | **G** | **T** | **T** | **C** | **A** | **T** | **T** | **T** | **A** | **A** | **A** | **T** | **A** | **G** | **C** | **A** | **G** | **C** | **A** | **T** | **T** | **G** | **A** | **G** | **G** | **A** | **T** | **C** | **A** | **T** | **C** | **T** | **T** | **T** | **T** | **C** | **C** | **G** | **A** | **A** | **T** | **T** | **G** | **T** | **G** | **C** | **A** | **G** | **G** | **A** | **G** | **T** | **T** | **A** | **C** | **T** | **T** | **T** | **C** | **A** | **A** | **A** | **T** | **A** | **C** | **C** | **T** | **T** | **A** | **A** | **G** | **A** | **C** | **A** | **T** | **G** | **C** | **C** | **A** | **A** | **A** | **G** | **C** | **A** | **A** | **A** | **G** | **G** | **A** | **A** | **T** | **T** | **A** | **G** | **A** | **G** | **G** | **T** | **A** | **T** | **A** | **C** | **T** | **T** | **A** | **C** | **A** | **C** | **C** | **A** | **A** | **G** | **T** | **C** | **G** | **A** | **T** | **A** | **A** | **A** | **A** | **A** | **C** | **G** | **T** | **G** | **C** | **T** | **A** | **T** | **T** | **G** | **C** | **G** | **T** | **A** | **T** | **T** | **G** | **T** | **T** | **G** | **A** | **T** | **G** | **A** | **C** | **G** | **G** | **T** | **G** | **T** | **C** | **G** | **G** | **C** | **T** | **T** | **T** | **G** | **A** | **T** | **A** | **T** | **G** | **A** | **A** | **G** | **G** | **A** | **A** | **C** | **A** | **A** | **A** | **G** | **T** | **A** | **A** | **T** | **A** | **A** | **A** | **G** | **C** | **C** | **G** | **G** | **T** | **A** | **G** | **T** | **T** | **A** | **T** | **G** | **G** | **C** | **T** | **T** | **A** | **A** | **A** | **T** | **A** | **A** | **T** | **A** | **T** | **C** | **C** | **G** | **A** | **G** | **A** | **A** | **C** | **G** | **T** | **G** | **T** | **T** | **G** | **T** | **C** | **G** | **G** | **C** | **A** | **T** | **G** | **G** | **G** | **C** | **G** | **G** | **T** | **A** | **C** | **A** | **G** | **T** | **T** | **A** | **A** | **A** | **A** | **T** | **T** | **A** | **T** | **T** | **A** | **G** | **T** | **T** | **T** | **T** | **A** | **A** | **A** | **G** | **G** | **G** | **C** | **A** | **G** | **G** | **G** | **A** | **A** | **C** | **C** | **A** | **G** | **C** | **G** | **T** | **T** | **G** | **A** | **A** | **A** | **T** | **T** | **A** | **A** | **A** | **G** | **T** | **T** | **C** | **C** | **T** | **G** | **T** | **C** | **A** | **T** | **A** | **A** | **A** | **G** | **G** | **A** | **G** | **G** | **A** | **A** | **A** | **C** | **T** | **G** | **C** | **A** | **A** | **G** | **T** | **G** | **A** | **T** | **C** | **A** | **A** | **A** | **G** | **T** | **A** | **A** | **T** | **G** | **T** | **T** | **A** | **G** | **T** | **G** | **G** | **A** | **-** | **-** | **-** |
| **N59591F** | **A** | **T** | **G** | **A** | **C** | **C** | **G** | **A** | **T** | **C** | **G** | **G** | **A** | **T** | **T** | **T** | **C** | **A** | **A** | **G** | **G** | **C** | **G** | **C** | **A** | **T** | **G** | **A** | **T** | **T** | **T** | **C** | **A** | **T** | **T** | **A** | **T** | **A** | **T** | **G** | **C** | **G** | **T** | **C** | **C** | **C** | **T** | **T** | **A** | **G** | **C** | **A** | **C** | **C** | **T** | **T** | **T** | **A** | **T** | **T** | **G** | **T** | **T** | **A** | **T** | **C** | **T** | **T** | **A** | **A** | **T** | **T** | **A** | **C** | **A** | **T** | **T** | **G** | **T** | **T** | **T** | **T** | **C** | **A** | **T** | **A** | **T** | **T** | **T** | **T** | **C** | **A** | **T** | **T** | **C** | **G** | **A** | **T** | **T** | **A** | **A** | **A** | **C** | **A** | **A** | **A** | **A** | **C** | **C** | **G** | **G** | **T** | **G** | **G** | **T** | **T** | **A** | **T** | **T** | **A** | **G** | **A** | **G** | **C** | **T** | **T** | **C** | **T** | **T** | **C** | **A** | **G** | **A** | **G** | **A** | **A** | **A** | **A** | **G** | **T** | **C** | **T** | **T** | **T** | **T** | **A** | **T** | **T** | **T** | **A** | **C** | **C** | **A** | **C** | **T** | **A** | **A** | **T** | **T** | **G** | **T** | **G** | **C** | **A** | **C** | **A** | **T** | **T** | **G** | **T** | **T** | **C** | **T** | **C** | **A** | **T** | **A** | **T** | **C** | **C** | **T** | **T** | **A** | **C** | **T** | **A** | **A** | **T** | **A** | **G** | **G** | **C** | **T** | **T** | **A** | **T** | **T** | **G** | **A** | **C** | **C** | **T** | **T** | **T** | **T** | **T** | **A** | **C** | **T** | **G** | **A** | **T** | **T** | **T** | **C** | **A** | **T** | **T** | **G** | **G** | **T** | **T** | **C** | **A** | **A** | **A** | **A** | **A** | **G** | **G** | **G** | **C** | **A** | **A** | **T** | **A** | **T** | **G** | **G** | **A** | **C** | **G** | **G** | **A** | **T** | **T** | **G** | **A** | **A** | **G** | **A** | **A** | **A** | **A** | **A** | **C** | **T** | **T** | **C** | **G** | **G** | **T** | **T** | **A** | **T** | **T** | **G** | **G** | **C** | **C** | **A** | **A** | **C** | **G** | **G** | **T** | **A** | **A** | **T** | **T** | **A** | **T** | **G** | **A** | **A** | **A** | **G** | **T** | **C** | **C** | **A** | **G** | **T** | **C** | **T** | **T** | **A** | **A** | **A** | **C** | **A** | **A** | **A** | **C** | **C** | **A** | **A** | **C** | **G** | **A** | **C** | **C** | **A** | **G** | **T** | **G** | **A** | **A** | **A** | **A** | **T** | **C** | **A** | **A** | **G** | **A** | **C** | **C** | **A** | **T** | **T** | **A** | **T** | **C** | **T** | **A** | **A** | **C** | **C** | **G** | **A** | **A** | **G** | **T** | **C** | **G** | **A** | **A** | **C** | **A** | **A** | **G** | **A** | **T** | **A** | **T** | **T** | **T** | **G** | **G** | **T** | **C** | **G** | **A** | **T** | **T** | **A** | **A** | **A** | **A** | **A** | **T** | **A** | **A** | **A** | **T** | **T** | **A** | **T** | **T** | **A** | **G** | **A** | **G** | **A** | **T** | **G** | **T** | **C** | **T** | **A** | **A** | **A** | **G** | **A** | **A** | **T** | **T** | **G** | **C** | **A** | **A** | **T** | **T** | **A** | **T** | **T** | **A** | **A** | **A** | **C** | **A** | **G** | **T** | **C** | **G** | **G** | **C** | **C** | **G** | **C** | **A** | **A** | **T** | **T** | **A** | **A** | **T** | **G** | **G** | **A** | **T** | **G** | **G** | **G** | **C** | **A** | **A** | **A** | **C** | **A** | **A** | **A** | **A** | **G** | **A** | **A** | **G** | **A** | **A** | **A** | **T** | **T** | **T** | **T** | **A** | **G** | **A** | **G** | **A** | **A** | **C** | **G** | **A** | **G** | **C** | **G** | **G** | **C** | **A** | **T** | **C** | **G** | **T** | **T** | **T** | **G** | **G** | **C** | **G** | **C** | **G** | **G** | **G** | **A** | **G** | **T** | **T** | **G** | **C** | **A** | **T** | **G** | **A** | **T** | **T** | **C** | **A** | **G** | **T** | **C** | **A** | **G** | **T** | **C** | **A** | **A** | **C** | **A** | **A** | **C** | **T** | **T** | **T** | **T** | **T** | **G** | **C** | **A** | **G** | **C** | **C** | **A** | **T** | **G** | **A** | **T** | **G** | **A** | **T** | **G** | **T** | **T** | **G** | **T** | **C** | **T** | **G** | **C** | **C** | **T** | **T** | **A** | **A** | **A** | **T** | **G** | **A** | **A** | **C** | **A** | **A** | **G** | **C** | **A** | **C** | **A** | **A** | **C** | **G** | **A** | **A** | **C** | **A** | **G** | **A** | **A** | **A** | **C** | **C** | **C** | **C** | **G** | **G** | **A** | **A** | **C** | **C** | **A** | **T** | **A** | **T** | **C** | **G** | **T** | **A** | **A** | **A** | **C** | **A** | **A** | **C** | **T** | **A** | **G** | **C** | **C** | **A** | **T** | **G** | **G** | **T** | **G** | **G** | **C** | **A** | **G** | **A** | **A** | **A** | **T** | **C** | **A** | **T** | **T** | **A** | **A** | **T** | **G** | **C** | **C** | **T** | **C** | **C** | **C** | **A** | **A** | **T** | **C** | **G** | **G** | **A** | **A** | **A** | **T** | **G** | **C** | **G** | **C** | **G** | **C** | **G** | **T** | **T** | **A** | **C** | **T** | **A** | **T** | **T** | **G** | **C** | **A** | **C** | **T** | **T** | **G** | **C** | **G** | **T** | **C** | **C** | **T** | **A** | **T** | **C** | **A** | **G** | **T** | **C** | **T** | **A** | **G** | **A** | **A** | **G** | **G** | **A** | **A** | **A** | **A** | **A** | **G** | **T** | **T** | **T** | **G** | **C** | **G** | **T** | **A** | **A** | **A** | **G** | **G** | **T** | **A** | **T** | **T** | **G** | **A** | **A** | **C** | **A** | **A** | **T** | **T** | **A** | **C** | **T** | **G** | **A** | **A** | **A** | **G** | **A** | **A** | **C** | **T** | **A** | **C** | **A** | **A** | **A** | **C** | **A** | **A** | **A** | **A** | **A** | **T** | **T** | **A** | **A** | **A** | **A** | **T** | **T** | **G** | **A** | **A** | **T** | **T** | **G** | **A** | **T** | **T** | **T** | **G** | **G** | **G** | **A** | **T** | **G** | **T** | **T** | **G** | **A** | **A** | **G** | **A** | **T** | **G** | **T** | **T** | **C** | **A** | **T** | **T** | **T** | **A** | **A** | **A** | **T** | **A** | **G** | **C** | **A** | **G** | **C** | **A** | **T** | **T** | **G** | **A** | **G** | **G** | **A** | **T** | **C** | **A** | **T** | **C** | **T** | **T** | **T** | **T** | **C** | **C** | **G** | **A** | **A** | **T** | **T** | **G** | **T** | **G** | **C** | **A** | **G** | **G** | **A** | **A** | **T** | **T** | **A** | **C** | **T** | **T** | **T** | **C** | **A** | **A** | **A** | **T** | **A** | **C** | **C** | **T** | **T** | **A** | **A** | **G** | **A** | **C** | **A** | **T** | **G** | **C** | **C** | **A** | **A** | **A** | **G** | **C** | **A** | **A** | **A** | **G** | **G** | **A** | **A** | **T** | **T** | **A** | **G** | **A** | **G** | **G** | **T** | **A** | **T** | **A** | **C** | **T** | **T** | **A** | **C** | **A** | **C** | **C** | **A** | **A** | **G** | **T** | **C** | **G** | **A** | **T** | **A** | **A** | **A** | **A** | **A** | **C** | **G** | **T** | **G** | **T** | **T** | **A** | **T** | **T** | **G** | **C** | **G** | **T** | **A** | **T** | **T** | **G** | **T** | **T** | **G** | **A** | **T** | **G** | **A** | **T** | **G** | **G** | **T** | **G** | **T** | **C** | **G** | **G** | **C** | **T** | **T** | **T** | **G** | **A** | **T** | **A** | **T** | **G** | **A** | **A** | **G** | **G** | **A** | **A** | **C** | **A** | **A** | **A** | **G** | **T** | **A** | **A** | **T** | **A** | **A** | **A** | **G** | **C** | **C** | **G** | **G** | **T** | **A** | **G** | **T** | **T** | **A** | **T** | **G** | **G** | **C** | **T** | **T** | **A** | **A** | **A** | **T** | **A** | **A** | **T** | **A** | **T** | **C** | **C** | **G** | **A** | **G** | **A** | **A** | **C** | **G** | **T** | **G** | **T** | **T** | **G** | **T** | **C** | **G** | **G** | **C** | **A** | **T** | **G** | **G** | **G** | **C** | **G** | **G** | **T** | **A** | **C** | **A** | **G** | **T** | **T** | **A** | **A** | **A** | **A** | **T** | **T** | **A** | **T** | **T** | **A** | **G** | **T** | **T** | **T** | **T** | **A** | **A** | **A** | **G** | **G** | **G** | **C** | **A** | **G** | **G** | **G** | **A** | **A** | **C** | **C** | **A** | **G** | **C** | **G** | **T** | **T** | **G** | **A** | **A** | **A** | **T** | **T** | **A** | **A** | **A** | **G** | **T** | **T** | **C** | **C** | **T** | **G** | **T** | **C** | **A** | **T** | **A** | **A** | **A** | **G** | **G** | **A** | **G** | **G** | **A** | **A** | **A** | **C** | **T** | **G** | **C** | **A** | **A** | **G** | **T** | **G** | **A** | **T** | **C** | **A** | **A** | **A** | **G** | **T** | **A** | **A** | **T** | **G** | **T** | **T** | **A** | **G** | **T** | **G** | **G** | **A** | **-** | **-** | **-** |
| **N59836F** | **A** | **T** | **G** | **A** | **C** | **C** | **G** | **A** | **T** | **C** | **G** | **G** | **A** | **T** | **T** | **T** | **C** | **A** | **A** | **G** | **A** | **C** | **G** | **C** | **A** | **T** | **G** | **A** | **T** | **T** | **T** | **C** | **A** | **T** | **T** | **A** | **T** | **A** | **T** | **G** | **C** | **G** | **T** | **C** | **C** | **C** | **T** | **T** | **A** | **G** | **C** | **A** | **C** | **C** | **T** | **T** | **T** | **A** | **T** | **T** | **G** | **T** | **T** | **A** | **T** | **C** | **T** | **T** | **A** | **A** | **T** | **T** | **A** | **C** | **A** | **T** | **T** | **G** | **T** | **T** | **T** | **T** | **C** | **A** | **T** | **A** | **T** | **T** | **T** | **T** | **C** | **A** | **T** | **T** | **C** | **G** | **A** | **T** | **T** | **A** | **A** | **A** | **C** | **A** | **A** | **A** | **A** | **C** | **C** | **G** | **G** | **T** | **G** | **G** | **T** | **T** | **A** | **T** | **T** | **A** | **G** | **A** | **G** | **C** | **T** | **T** | **C** | **T** | **T** | **C** | **A** | **G** | **A** | **G** | **A** | **A** | **A** | **A** | **G** | **T** | **C** | **T** | **T** | **T** | **T** | **A** | **T** | **T** | **T** | **A** | **C** | **C** | **A** | **C** | **T** | **A** | **A** | **T** | **T** | **G** | **T** | **G** | **C** | **A** | **C** | **A** | **T** | **T** | **G** | **T** | **T** | **C** | **T** | **C** | **A** | **T** | **A** | **T** | **C** | **C** | **T** | **T** | **A** | **C** | **T** | **A** | **A** | **T** | **A** | **G** | **G** | **C** | **T** | **T** | **A** | **T** | **T** | **G** | **A** | **C** | **C** | **T** | **T** | **T** | **T** | **T** | **A** | **C** | **T** | **G** | **A** | **T** | **T** | **T** | **C** | **A** | **T** | **T** | **G** | **G** | **T** | **T** | **C** | **A** | **A** | **A** | **A** | **A** | **G** | **G** | **G** | **C** | **A** | **A** | **T** | **A** | **T** | **G** | **G** | **A** | **C** | **G** | **G** | **A** | **T** | **T** | **G** | **A** | **A** | **G** | **A** | **A** | **A** | **A** | **A** | **C** | **T** | **T** | **C** | **G** | **G** | **T** | **T** | **A** | **T** | **T** | **G** | **G** | **C** | **C** | **A** | **A** | **C** | **G** | **G** | **T** | **A** | **A** | **T** | **T** | **A** | **T** | **G** | **A** | **A** | **A** | **G** | **T** | **C** | **C** | **A** | **G** | **T** | **C** | **T** | **T** | **A** | **A** | **A** | **C** | **A** | **A** | **A** | **C** | **C** | **A** | **A** | **C** | **G** | **A** | **C** | **C** | **A** | **G** | **T** | **G** | **A** | **A** | **A** | **A** | **T** | **C** | **A** | **A** | **G** | **A** | **C** | **C** | **A** | **T** | **T** | **A** | **T** | **C** | **T** | **A** | **A** | **C** | **C** | **G** | **A** | **A** | **G** | **T** | **C** | **G** | **A** | **A** | **C** | **A** | **A** | **G** | **A** | **T** | **A** | **T** | **T** | **T** | **G** | **G** | **T** | **C** | **G** | **A** | **T** | **T** | **A** | **A** | **A** | **A** | **A** | **T** | **A** | **A** | **A** | **T** | **T** | **A** | **T** | **T** | **A** | **G** | **A** | **G** | **A** | **T** | **G** | **T** | **C** | **T** | **A** | **A** | **A** | **G** | **A** | **A** | **T** | **T** | **G** | **C** | **A** | **A** | **T** | **T** | **A** | **T** | **T** | **A** | **A** | **A** | **C** | **A** | **G** | **T** | **C** | **G** | **A** | **C** | **C** | **G** | **C** | **A** | **A** | **T** | **T** | **A** | **A** | **T** | **G** | **G** | **A** | **T** | **G** | **G** | **G** | **C** | **A** | **A** | **A** | **C** | **A** | **A** | **A** | **A** | **G** | **A** | **A** | **G** | **A** | **A** | **A** | **T** | **T** | **T** | **T** | **A** | **G** | **A** | **G** | **A** | **A** | **C** | **G** | **A** | **G** | **C** | **G** | **G** | **C** | **A** | **T** | **C** | **G** | **T** | **T** | **T** | **G** | **G** | **C** | **G** | **C** | **G** | **G** | **G** | **A** | **G** | **T** | **T** | **G** | **C** | **A** | **T** | **G** | **A** | **T** | **T** | **C** | **A** | **G** | **T** | **C** | **A** | **G** | **T** | **C** | **A** | **A** | **C** | **A** | **A** | **C** | **T** | **T** | **T** | **T** | **T** | **G** | **C** | **A** | **G** | **C** | **C** | **A** | **T** | **G** | **A** | **T** | **G** | **A** | **T** | **G** | **T** | **T** | **G** | **T** | **C** | **T** | **G** | **C** | **C** | **T** | **T** | **A** | **A** | **A** | **T** | **G** | **A** | **A** | **C** | **A** | **A** | **G** | **C** | **A** | **C** | **A** | **A** | **C** | **G** | **A** | **A** | **C** | **A** | **G** | **A** | **A** | **A** | **C** | **T** | **C** | **C** | **T** | **G** | **A** | **A** | **C** | **C** | **A** | **T** | **A** | **T** | **C** | **G** | **C** | **A** | **A** | **A** | **C** | **A** | **A** | **C** | **T** | **A** | **G** | **C** | **C** | **A** | **T** | **G** | **G** | **T** | **G** | **G** | **C** | **A** | **G** | **A** | **A** | **A** | **T** | **C** | **A** | **T** | **T** | **A** | **A** | **T** | **G** | **C** | **C** | **T** | **C** | **C** | **C** | **A** | **A** | **T** | **C** | **G** | **G** | **A** | **A** | **A** | **T** | **G** | **C** | **G** | **T** | **G** | **C** | **G** | **C** | **T** | **A** | **C** | **T** | **A** | **T** | **T** | **G** | **C** | **A** | **C** | **T** | **T** | **G** | **C** | **G** | **T** | **C** | **C** | **T** | **A** | **T** | **C** | **A** | **G** | **T** | **C** | **T** | **A** | **G** | **A** | **A** | **G** | **G** | **A** | **A** | **A** | **A** | **A** | **G** | **T** | **T** | **T** | **G** | **C** | **G** | **T** | **A** | **A** | **A** | **G** | **G** | **T** | **A** | **T** | **T** | **G** | **A** | **A** | **C** | **A** | **A** | **T** | **T** | **A** | **C** | **T** | **G** | **A** | **A** | **A** | **G** | **A** | **A** | **C** | **T** | **A** | **C** | **A** | **A** | **A** | **C** | **A** | **A** | **A** | **A** | **A** | **T** | **T** | **A** | **A** | **A** | **A** | **T** | **T** | **G** | **A** | **A** | **T** | **T** | **G** | **A** | **T** | **T** | **T** | **G** | **G** | **G** | **A** | **T** | **G** | **T** | **T** | **G** | **A** | **A** | **G** | **A** | **T** | **G** | **T** | **T** | **C** | **A** | **T** | **T** | **T** | **A** | **A** | **A** | **T** | **A** | **G** | **C** | **A** | **G** | **C** | **A** | **T** | **T** | **G** | **A** | **G** | **G** | **A** | **T** | **C** | **A** | **T** | **C** | **T** | **T** | **T** | **T** | **C** | **C** | **G** | **A** | **A** | **T** | **T** | **G** | **T** | **G** | **C** | **A** | **G** | **G** | **A** | **G** | **T** | **T** | **A** | **C** | **T** | **T** | **T** | **C** | **A** | **A** | **A** | **T** | **A** | **C** | **C** | **T** | **T** | **A** | **A** | **G** | **A** | **C** | **A** | **T** | **G** | **C** | **C** | **A** | **A** | **A** | **G** | **C** | **A** | **A** | **A** | **G** | **G** | **A** | **A** | **T** | **T** | **A** | **G** | **A** | **G** | **G** | **T** | **A** | **T** | **A** | **C** | **T** | **T** | **A** | **C** | **A** | **C** | **C** | **A** | **A** | **G** | **T** | **C** | **G** | **A** | **T** | **A** | **A** | **A** | **A** | **A** | **C** | **G** | **T** | **G** | **T** | **T** | **A** | **T** | **T** | **G** | **C** | **G** | **T** | **A** | **T** | **T** | **G** | **T** | **T** | **G** | **A** | **T** | **G** | **A** | **T** | **G** | **G** | **T** | **G** | **T** | **C** | **G** | **G** | **C** | **T** | **T** | **T** | **G** | **A** | **T** | **A** | **T** | **G** | **A** | **A** | **G** | **G** | **A** | **A** | **C** | **A** | **A** | **A** | **G** | **T** | **A** | **A** | **T** | **A** | **A** | **A** | **G** | **C** | **C** | **G** | **G** | **T** | **A** | **G** | **T** | **T** | **A** | **T** | **G** | **G** | **C** | **T** | **T** | **A** | **A** | **A** | **T** | **A** | **A** | **T** | **A** | **T** | **C** | **C** | **G** | **A** | **G** | **A** | **A** | **C** | **G** | **T** | **G** | **T** | **T** | **G** | **T** | **C** | **G** | **G** | **C** | **A** | **T** | **G** | **G** | **G** | **C** | **G** | **G** | **T** | **A** | **C** | **A** | **G** | **T** | **T** | **A** | **A** | **A** | **A** | **T** | **T** | **A** | **T** | **T** | **A** | **G** | **T** | **T** | **T** | **T** | **A** | **A** | **A** | **G** | **G** | **G** | **C** | **A** | **G** | **G** | **G** | **A** | **A** | **C** | **C** | **A** | **G** | **C** | **G** | **T** | **T** | **G** | **A** | **A** | **A** | **T** | **T** | **A** | **A** | **A** | **G** | **T** | **T** | **C** | **C** | **T** | **G** | **T** | **C** | **A** | **T** | **A** | **A** | **A** | **G** | **G** | **A** | **G** | **G** | **A** | **A** | **A** | **C** | **T** | **G** | **C** | **A** | **A** | **G** | **T** | **G** | **A** | **T** | **C** | **A** | **A** | **A** | **G** | **T** | **A** | **A** | **T** | **G** | **T** | **T** | **A** | **G** | **T** | **G** | **G** | **A** | **-** | **-** | **-** |
| **N59592F** | **A** | **T** | **G** | **A** | **C** | **C** | **G** | **A** | **T** | **C** | **G** | **G** | **A** | **T** | **T** | **T** | **C** | **A** | **A** | **G** | **A** | **C** | **G** | **C** | **A** | **T** | **G** | **A** | **T** | **T** | **T** | **C** | **A** | **T** | **T** | **A** | **T** | **A** | **T** | **G** | **C** | **G** | **T** | **C** | **C** | **C** | **T** | **T** | **A** | **G** | **C** | **A** | **C** | **C** | **T** | **T** | **T** | **A** | **T** | **T** | **G** | **T** | **T** | **A** | **T** | **C** | **T** | **T** | **A** | **A** | **T** | **T** | **A** | **C** | **A** | **T** | **T** | **G** | **T** | **T** | **T** | **T** | **C** | **A** | **T** | **A** | **T** | **T** | **T** | **T** | **C** | **A** | **T** | **T** | **C** | **G** | **A** | **T** | **T** | **A** | **A** | **A** | **C** | **A** | **A** | **A** | **A** | **C** | **C** | **G** | **G** | **T** | **G** | **G** | **C** | **T** | **A** | **T** | **T** | **A** | **G** | **A** | **G** | **C** | **T** | **T** | **C** | **T** | **T** | **C** | **A** | **G** | **A** | **G** | **A** | **A** | **A** | **A** | **G** | **T** | **C** | **T** | **T** | **T** | **T** | **A** | **T** | **T** | **T** | **A** | **C** | **C** | **A** | **C** | **T** | **A** | **A** | **T** | **T** | **G** | **T** | **G** | **C** | **A** | **C** | **A** | **T** | **T** | **G** | **T** | **T** | **C** | **T** | **C** | **A** | **T** | **A** | **T** | **C** | **C** | **T** | **T** | **A** | **C** | **T** | **A** | **A** | **T** | **A** | **G** | **G** | **C** | **T** | **T** | **A** | **T** | **T** | **G** | **A** | **C** | **C** | **T** | **T** | **T** | **T** | **T** | **A** | **C** | **T** | **G** | **A** | **T** | **T** | **T** | **C** | **A** | **T** | **T** | **G** | **G** | **T** | **T** | **C** | **A** | **A** | **A** | **A** | **A** | **G** | **G** | **G** | **C** | **A** | **A** | **T** | **A** | **T** | **G** | **G** | **A** | **C** | **G** | **G** | **A** | **T** | **T** | **G** | **A** | **A** | **G** | **A** | **A** | **A** | **A** | **A** | **C** | **T** | **T** | **C** | **G** | **G** | **T** | **T** | **A** | **T** | **T** | **G** | **G** | **C** | **C** | **A** | **A** | **T** | **G** | **G** | **T** | **A** | **A** | **T** | **T** | **A** | **T** | **G** | **A** | **A** | **A** | **G** | **T** | **C** | **C** | **A** | **G** | **T** | **C** | **T** | **T** | **A** | **A** | **A** | **C** | **A** | **A** | **A** | **C** | **C** | **A** | **A** | **C** | **G** | **A** | **C** | **C** | **A** | **G** | **T** | **G** | **A** | **A** | **A** | **A** | **T** | **C** | **A** | **A** | **G** | **A** | **C** | **C** | **A** | **T** | **T** | **A** | **T** | **C** | **T** | **A** | **A** | **C** | **C** | **G** | **A** | **A** | **G** | **T** | **C** | **G** | **A** | **A** | **C** | **A** | **A** | **G** | **A** | **T** | **A** | **T** | **T** | **T** | **G** | **G** | **T** | **C** | **G** | **A** | **T** | **T** | **A** | **A** | **A** | **A** | **A** | **T** | **A** | **A** | **A** | **T** | **T** | **A** | **T** | **T** | **A** | **G** | **A** | **G** | **A** | **T** | **G** | **T** | **C** | **T** | **A** | **A** | **A** | **G** | **A** | **A** | **T** | **T** | **G** | **C** | **A** | **A** | **T** | **T** | **A** | **T** | **T** | **A** | **A** | **A** | **C** | **A** | **G** | **T** | **C** | **G** | **A** | **C** | **C** | **G** | **C** | **A** | **A** | **T** | **T** | **A** | **A** | **T** | **G** | **G** | **A** | **T** | **G** | **G** | **G** | **C** | **A** | **A** | **A** | **C** | **A** | **A** | **A** | **A** | **G** | **A** | **A** | **G** | **A** | **A** | **A** | **T** | **T** | **T** | **T** | **A** | **G** | **A** | **G** | **A** | **A** | **C** | **G** | **A** | **G** | **C** | **G** | **G** | **C** | **A** | **T** | **C** | **G** | **T** | **T** | **T** | **G** | **G** | **C** | **G** | **C** | **G** | **G** | **G** | **A** | **G** | **T** | **T** | **G** | **C** | **A** | **T** | **G** | **A** | **T** | **T** | **C** | **A** | **G** | **T** | **C** | **A** | **G** | **T** | **C** | **A** | **A** | **C** | **A** | **A** | **C** | **T** | **T** | **T** | **T** | **T** | **G** | **C** | **A** | **G** | **C** | **C** | **A** | **T** | **G** | **A** | **T** | **G** | **A** | **T** | **G** | **T** | **T** | **G** | **T** | **C** | **T** | **G** | **C** | **C** | **T** | **T** | **A** | **A** | **A** | **T** | **G** | **A** | **A** | **C** | **A** | **A** | **G** | **C** | **A** | **C** | **A** | **A** | **C** | **G** | **A** | **A** | **C** | **A** | **G** | **A** | **A** | **A** | **C** | **C** | **C** | **C** | **T** | **G** | **A** | **A** | **C** | **C** | **A** | **T** | **A** | **T** | **C** | **G** | **C** | **A** | **A** | **A** | **C** | **A** | **A** | **C** | **T** | **A** | **G** | **C** | **C** | **A** | **T** | **G** | **G** | **T** | **G** | **G** | **C** | **A** | **G** | **A** | **A** | **A** | **T** | **C** | **A** | **T** | **T** | **A** | **A** | **T** | **G** | **C** | **C** | **T** | **C** | **C** | **C** | **A** | **A** | **T** | **C** | **G** | **G** | **A** | **A** | **A** | **T** | **G** | **C** | **G** | **C** | **G** | **C** | **G** | **T** | **T** | **A** | **C** | **T** | **A** | **T** | **T** | **G** | **C** | **A** | **C** | **T** | **T** | **G** | **C** | **G** | **T** | **C** | **C** | **T** | **A** | **T** | **C** | **A** | **G** | **T** | **C** | **T** | **A** | **G** | **A** | **A** | **G** | **G** | **A** | **A** | **A** | **A** | **A** | **G** | **T** | **T** | **T** | **G** | **C** | **G** | **T** | **A** | **A** | **A** | **G** | **G** | **T** | **A** | **T** | **T** | **G** | **A** | **A** | **C** | **A** | **A** | **T** | **T** | **A** | **C** | **T** | **G** | **A** | **A** | **A** | **G** | **A** | **A** | **C** | **T** | **A** | **C** | **A** | **A** | **A** | **C** | **A** | **A** | **A** | **A** | **A** | **T** | **T** | **A** | **A** | **A** | **A** | **T** | **T** | **G** | **A** | **A** | **T** | **T** | **G** | **A** | **T** | **T** | **T** | **G** | **G** | **G** | **A** | **T** | **G** | **T** | **T** | **G** | **A** | **A** | **G** | **A** | **T** | **G** | **T** | **T** | **C** | **A** | **T** | **T** | **T** | **A** | **A** | **A** | **T** | **A** | **G** | **C** | **A** | **G** | **C** | **A** | **T** | **T** | **G** | **A** | **G** | **G** | **A** | **T** | **C** | **A** | **T** | **C** | **T** | **T** | **T** | **T** | **C** | **C** | **G** | **A** | **A** | **T** | **T** | **G** | **T** | **G** | **C** | **A** | **G** | **G** | **A** | **A** | **T** | **T** | **A** | **C** | **T** | **T** | **T** | **C** | **A** | **A** | **A** | **T** | **A** | **C** | **T** | **T** | **T** | **A** | **A** | **G** | **A** | **C** | **A** | **T** | **G** | **C** | **C** | **A** | **A** | **A** | **G** | **C** | **A** | **A** | **A** | **G** | **G** | **A** | **A** | **T** | **T** | **A** | **G** | **A** | **G** | **G** | **T** | **A** | **T** | **A** | **C** | **T** | **T** | **A** | **C** | **A** | **C** | **C** | **A** | **A** | **G** | **T** | **C** | **G** | **A** | **T** | **A** | **A** | **A** | **A** | **A** | **C** | **G** | **T** | **G** | **C** | **T** | **A** | **T** | **T** | **G** | **C** | **G** | **T** | **A** | **T** | **T** | **G** | **T** | **T** | **G** | **A** | **T** | **G** | **A** | **C** | **G** | **G** | **T** | **G** | **T** | **C** | **G** | **G** | **C** | **T** | **T** | **T** | **G** | **A** | **T** | **A** | **T** | **G** | **A** | **A** | **G** | **G** | **A** | **A** | **C** | **A** | **A** | **A** | **G** | **T** | **A** | **A** | **T** | **A** | **A** | **A** | **G** | **C** | **C** | **G** | **G** | **T** | **A** | **G** | **T** | **T** | **A** | **T** | **G** | **G** | **C** | **T** | **T** | **A** | **A** | **A** | **T** | **A** | **A** | **T** | **A** | **T** | **C** | **C** | **G** | **A** | **G** | **A** | **A** | **C** | **G** | **T** | **G** | **T** | **T** | **G** | **T** | **C** | **G** | **G** | **C** | **A** | **T** | **G** | **G** | **G** | **C** | **G** | **G** | **T** | **A** | **C** | **A** | **G** | **T** | **T** | **A** | **A** | **A** | **A** | **T** | **T** | **A** | **T** | **T** | **A** | **G** | **T** | **T** | **T** | **T** | **A** | **A** | **A** | **G** | **G** | **G** | **C** | **A** | **G** | **G** | **G** | **A** | **A** | **C** | **C** | **A** | **G** | **C** | **G** | **T** | **T** | **G** | **A** | **A** | **A** | **T** | **T** | **A** | **A** | **A** | **G** | **T** | **T** | **C** | **C** | **T** | **G** | **T** | **C** | **A** | **T** | **A** | **A** | **A** | **G** | **G** | **A** | **G** | **G** | **A** | **A** | **A** | **C** | **T** | **G** | **C** | **A** | **A** | **G** | **T** | **G** | **A** | **T** | **C** | **A** | **A** | **A** | **G** | **T** | **A** | **A** | **T** | **G** | **T** | **T** | **A** | **G** | **T** | **G** | **G** | **A** | **-** | **-** | **-** |
| **N60271F** | **A** | **T** | **G** | **A** | **C** | **C** | **G** | **A** | **T** | **C** | **G** | **G** | **A** | **T** | **T** | **T** | **C** | **A** | **A** | **G** | **A** | **C** | **G** | **C** | **A** | **T** | **G** | **A** | **T** | **T** | **T** | **C** | **A** | **T** | **T** | **A** | **T** | **A** | **T** | **G** | **C** | **G** | **T** | **C** | **C** | **C** | **T** | **T** | **A** | **G** | **C** | **A** | **C** | **C** | **T** | **T** | **T** | **A** | **T** | **T** | **G** | **T** | **T** | **A** | **T** | **C** | **T** | **T** | **A** | **A** | **T** | **T** | **A** | **C** | **A** | **T** | **T** | **G** | **T** | **T** | **T** | **T** | **C** | **A** | **T** | **A** | **T** | **T** | **T** | **T** | **C** | **A** | **T** | **T** | **C** | **G** | **A** | **T** | **T** | **A** | **A** | **A** | **C** | **A** | **A** | **A** | **A** | **C** | **C** | **G** | **G** | **T** | **G** | **G** | **T** | **T** | **A** | **T** | **T** | **A** | **G** | **A** | **G** | **C** | **T** | **T** | **C** | **T** | **T** | **C** | **A** | **G** | **A** | **G** | **A** | **A** | **A** | **A** | **G** | **T** | **C** | **T** | **T** | **T** | **T** | **A** | **T** | **T** | **T** | **A** | **C** | **C** | **A** | **C** | **T** | **A** | **A** | **T** | **T** | **G** | **T** | **G** | **C** | **A** | **C** | **A** | **T** | **T** | **G** | **T** | **T** | **C** | **T** | **C** | **A** | **T** | **A** | **T** | **C** | **C** | **T** | **T** | **A** | **C** | **T** | **A** | **A** | **T** | **A** | **G** | **G** | **C** | **T** | **T** | **A** | **T** | **T** | **G** | **A** | **C** | **C** | **T** | **T** | **T** | **T** | **T** | **A** | **C** | **T** | **G** | **A** | **T** | **T** | **T** | **C** | **A** | **T** | **T** | **G** | **G** | **T** | **T** | **C** | **A** | **A** | **A** | **A** | **A** | **G** | **G** | **G** | **C** | **A** | **A** | **T** | **A** | **T** | **G** | **G** | **A** | **C** | **G** | **G** | **A** | **T** | **T** | **G** | **A** | **A** | **G** | **A** | **A** | **A** | **A** | **A** | **C** | **T** | **T** | **C** | **G** | **G** | **T** | **T** | **A** | **T** | **T** | **G** | **G** | **C** | **C** | **A** | **A** | **C** | **G** | **G** | **T** | **A** | **A** | **T** | **T** | **A** | **T** | **G** | **A** | **A** | **A** | **G** | **T** | **C** | **C** | **A** | **G** | **T** | **C** | **T** | **T** | **A** | **A** | **A** | **C** | **A** | **A** | **A** | **C** | **C** | **A** | **A** | **C** | **G** | **A** | **C** | **C** | **A** | **G** | **T** | **G** | **A** | **A** | **A** | **A** | **T** | **C** | **A** | **A** | **G** | **A** | **C** | **C** | **A** | **T** | **T** | **A** | **T** | **C** | **T** | **A** | **A** | **C** | **C** | **G** | **A** | **A** | **G** | **T** | **C** | **G** | **A** | **A** | **C** | **A** | **A** | **G** | **A** | **T** | **A** | **T** | **T** | **T** | **G** | **G** | **T** | **C** | **G** | **A** | **T** | **T** | **A** | **A** | **A** | **A** | **A** | **T** | **A** | **A** | **A** | **T** | **T** | **A** | **T** | **T** | **A** | **G** | **A** | **G** | **A** | **T** | **G** | **T** | **C** | **T** | **A** | **A** | **A** | **G** | **A** | **A** | **T** | **T** | **G** | **C** | **A** | **A** | **T** | **T** | **A** | **T** | **T** | **A** | **A** | **A** | **C** | **A** | **G** | **T** | **C** | **G** | **A** | **C** | **C** | **G** | **C** | **A** | **A** | **T** | **T** | **A** | **A** | **T** | **G** | **G** | **A** | **T** | **G** | **G** | **G** | **C** | **A** | **A** | **A** | **C** | **A** | **A** | **A** | **A** | **G** | **A** | **A** | **G** | **A** | **A** | **A** | **T** | **T** | **T** | **T** | **A** | **G** | **A** | **G** | **A** | **A** | **C** | **G** | **A** | **G** | **C** | **G** | **G** | **C** | **A** | **T** | **C** | **G** | **T** | **T** | **T** | **G** | **G** | **C** | **G** | **C** | **G** | **G** | **G** | **A** | **G** | **T** | **T** | **G** | **C** | **A** | **T** | **G** | **A** | **T** | **T** | **C** | **A** | **G** | **T** | **C** | **A** | **G** | **T** | **C** | **A** | **A** | **C** | **A** | **A** | **C** | **T** | **T** | **T** | **T** | **T** | **G** | **C** | **A** | **G** | **C** | **C** | **A** | **T** | **G** | **A** | **T** | **G** | **A** | **T** | **G** | **T** | **T** | **G** | **T** | **C** | **T** | **G** | **C** | **C** | **T** | **T** | **A** | **A** | **A** | **T** | **G** | **A** | **A** | **C** | **A** | **A** | **G** | **C** | **A** | **C** | **A** | **A** | **C** | **G** | **A** | **A** | **C** | **A** | **G** | **A** | **A** | **A** | **C** | **C** | **C** | **C** | **G** | **G** | **A** | **A** | **C** | **C** | **A** | **T** | **A** | **T** | **C** | **G** | **T** | **A** | **A** | **A** | **C** | **A** | **A** | **C** | **T** | **A** | **G** | **C** | **C** | **A** | **T** | **G** | **G** | **T** | **G** | **G** | **C** | **A** | **G** | **A** | **A** | **A** | **T** | **C** | **A** | **T** | **T** | **A** | **A** | **C** | **G** | **C** | **C** | **T** | **C** | **C** | **C** | **A** | **A** | **T** | **C** | **G** | **G** | **A** | **A** | **A** | **T** | **G** | **C** | **G** | **C** | **G** | **C** | **G** | **T** | **T** | **A** | **C** | **T** | **A** | **T** | **T** | **G** | **C** | **A** | **C** | **T** | **T** | **G** | **C** | **G** | **T** | **C** | **C** | **T** | **A** | **T** | **C** | **A** | **G** | **T** | **C** | **T** | **A** | **G** | **A** | **A** | **G** | **G** | **A** | **A** | **A** | **A** | **A** | **G** | **T** | **T** | **T** | **G** | **C** | **G** | **T** | **A** | **A** | **A** | **G** | **G** | **T** | **A** | **T** | **T** | **G** | **A** | **A** | **C** | **A** | **A** | **T** | **T** | **A** | **C** | **T** | **G** | **A** | **A** | **A** | **G** | **A** | **A** | **C** | **T** | **A** | **C** | **A** | **A** | **A** | **C** | **A** | **A** | **A** | **A** | **A** | **T** | **T** | **A** | **A** | **A** | **A** | **T** | **T** | **G** | **A** | **A** | **T** | **T** | **G** | **A** | **T** | **T** | **T** | **G** | **G** | **G** | **A** | **T** | **G** | **T** | **T** | **G** | **A** | **A** | **G** | **A** | **T** | **G** | **T** | **T** | **C** | **A** | **T** | **T** | **T** | **A** | **A** | **A** | **T** | **A** | **G** | **C** | **A** | **G** | **C** | **A** | **T** | **T** | **G** | **A** | **G** | **G** | **A** | **T** | **C** | **A** | **T** | **C** | **T** | **T** | **T** | **T** | **C** | **C** | **G** | **A** | **A** | **T** | **T** | **G** | **T** | **G** | **C** | **A** | **G** | **G** | **A** | **A** | **T** | **T** | **A** | **C** | **T** | **T** | **T** | **C** | **A** | **A** | **A** | **T** | **A** | **C** | **C** | **T** | **T** | **A** | **A** | **G** | **A** | **C** | **A** | **T** | **G** | **C** | **C** | **A** | **A** | **A** | **G** | **C** | **A** | **A** | **A** | **G** | **G** | **A** | **A** | **T** | **T** | **A** | **G** | **A** | **G** | **G** | **T** | **A** | **T** | **A** | **C** | **T** | **T** | **A** | **C** | **A** | **C** | **C** | **A** | **A** | **G** | **T** | **C** | **G** | **A** | **T** | **A** | **A** | **A** | **A** | **A** | **C** | **G** | **T** | **G** | **C** | **T** | **A** | **T** | **T** | **G** | **C** | **G** | **T** | **A** | **T** | **T** | **G** | **T** | **T** | **G** | **A** | **T** | **G** | **A** | **C** | **G** | **G** | **T** | **G** | **T** | **C** | **G** | **G** | **C** | **T** | **T** | **T** | **G** | **A** | **T** | **A** | **T** | **G** | **A** | **A** | **G** | **G** | **A** | **A** | **C** | **A** | **A** | **A** | **G** | **T** | **A** | **A** | **T** | **A** | **A** | **A** | **G** | **C** | **C** | **G** | **G** | **T** | **A** | **G** | **T** | **T** | **A** | **T** | **G** | **G** | **C** | **T** | **T** | **A** | **A** | **A** | **T** | **A** | **A** | **T** | **A** | **T** | **T** | **C** | **G** | **A** | **G** | **A** | **A** | **C** | **G** | **T** | **G** | **T** | **T** | **G** | **T** | **C** | **G** | **G** | **C** | **A** | **T** | **G** | **G** | **G** | **C** | **G** | **G** | **T** | **A** | **C** | **A** | **G** | **T** | **T** | **A** | **A** | **A** | **A** | **T** | **T** | **A** | **T** | **T** | **A** | **G** | **T** | **T** | **T** | **T** | **A** | **A** | **A** | **G** | **G** | **G** | **C** | **A** | **G** | **G** | **G** | **A** | **A** | **C** | **C** | **A** | **G** | **C** | **G** | **T** | **T** | **G** | **A** | **A** | **A** | **T** | **T** | **A** | **A** | **A** | **G** | **T** | **T** | **C** | **C** | **T** | **G** | **T** | **C** | **A** | **T** | **A** | **A** | **A** | **G** | **G** | **A** | **G** | **G** | **A** | **A** | **A** | **C** | **T** | **G** | **C** | **A** | **A** | **G** | **T** | **G** | **A** | **T** | **C** | **A** | **A** | **A** | **G** | **T** | **A** | **A** | **T** | **G** | **T** | **T** | **A** | **G** | **T** | **G** | **G** | **A** | **-** | **-** | **-** |
| **N52469** | **A** | **T** | **G** | **A** | **C** | **C** | **G** | **A** | **T** | **C** | **G** | **G** | **A** | **T** | **T** | **T** | **C** | **A** | **A** | **G** | **A** | **C** | **G** | **C** | **A** | **T** | **G** | **A** | **T** | **T** | **T** | **C** | **A** | **T** | **T** | **A** | **T** | **A** | **T** | **G** | **C** | **G** | **T** | **C** | **C** | **C** | **T** | **T** | **A** | **G** | **C** | **A** | **C** | **C** | **T** | **T** | **T** | **A** | **T** | **T** | **G** | **T** | **T** | **A** | **T** | **C** | **T** | **T** | **A** | **A** | **T** | **T** | **A** | **C** | **A** | **T** | **T** | **G** | **T** | **T** | **T** | **T** | **C** | **A** | **T** | **A** | **T** | **T** | **T** | **T** | **C** | **A** | **T** | **T** | **C** | **G** | **A** | **T** | **T** | **A** | **A** | **A** | **C** | **A** | **A** | **A** | **A** | **C** | **C** | **G** | **G** | **T** | **G** | **G** | **C** | **T** | **A** | **T** | **T** | **A** | **G** | **A** | **G** | **C** | **T** | **T** | **C** | **T** | **T** | **C** | **A** | **G** | **A** | **G** | **A** | **A** | **A** | **A** | **G** | **T** | **C** | **T** | **T** | **T** | **T** | **A** | **T** | **T** | **T** | **A** | **C** | **C** | **A** | **C** | **T** | **A** | **A** | **T** | **T** | **G** | **T** | **G** | **C** | **A** | **C** | **A** | **T** | **T** | **G** | **T** | **T** | **C** | **T** | **C** | **A** | **T** | **A** | **T** | **C** | **C** | **T** | **T** | **A** | **C** | **T** | **A** | **A** | **T** | **A** | **G** | **G** | **C** | **T** | **T** | **A** | **T** | **T** | **G** | **A** | **C** | **C** | **T** | **T** | **T** | **T** | **T** | **A** | **C** | **T** | **G** | **A** | **T** | **T** | **T** | **C** | **A** | **T** | **T** | **G** | **G** | **T** | **T** | **C** | **A** | **A** | **A** | **A** | **A** | **G** | **G** | **G** | **C** | **A** | **A** | **T** | **A** | **T** | **G** | **G** | **A** | **C** | **G** | **G** | **A** | **T** | **T** | **G** | **A** | **A** | **G** | **A** | **A** | **A** | **A** | **A** | **C** | **T** | **T** | **C** | **G** | **G** | **T** | **T** | **A** | **T** | **T** | **G** | **G** | **C** | **C** | **A** | **A** | **T** | **G** | **G** | **T** | **A** | **A** | **T** | **T** | **A** | **T** | **G** | **A** | **A** | **A** | **G** | **T** | **C** | **C** | **A** | **G** | **T** | **C** | **T** | **T** | **A** | **A** | **A** | **C** | **A** | **A** | **A** | **C** | **C** | **A** | **A** | **C** | **G** | **A** | **C** | **C** | **A** | **G** | **T** | **G** | **A** | **A** | **A** | **A** | **T** | **C** | **A** | **A** | **G** | **A** | **C** | **C** | **A** | **T** | **T** | **A** | **T** | **C** | **T** | **A** | **A** | **C** | **C** | **G** | **A** | **A** | **G** | **T** | **C** | **G** | **A** | **A** | **C** | **A** | **A** | **G** | **A** | **T** | **A** | **T** | **T** | **T** | **G** | **G** | **T** | **C** | **G** | **A** | **T** | **T** | **A** | **A** | **A** | **A** | **A** | **T** | **A** | **A** | **A** | **T** | **T** | **A** | **T** | **T** | **A** | **G** | **A** | **G** | **A** | **T** | **G** | **T** | **C** | **T** | **A** | **A** | **A** | **G** | **A** | **A** | **T** | **T** | **G** | **C** | **A** | **A** | **T** | **T** | **A** | **T** | **T** | **A** | **A** | **A** | **C** | **A** | **G** | **T** | **C** | **G** | **A** | **C** | **C** | **G** | **C** | **A** | **A** | **T** | **T** | **A** | **A** | **T** | **G** | **G** | **A** | **T** | **G** | **G** | **G** | **C** | **A** | **A** | **A** | **C** | **A** | **A** | **A** | **A** | **G** | **A** | **A** | **G** | **A** | **A** | **A** | **T** | **T** | **T** | **T** | **A** | **G** | **A** | **G** | **A** | **A** | **C** | **G** | **A** | **G** | **C** | **G** | **G** | **C** | **A** | **T** | **C** | **G** | **T** | **T** | **T** | **G** | **G** | **C** | **G** | **C** | **G** | **G** | **G** | **A** | **G** | **T** | **T** | **G** | **C** | **A** | **T** | **G** | **A** | **T** | **T** | **C** | **A** | **G** | **T** | **C** | **A** | **G** | **T** | **C** | **A** | **A** | **C** | **A** | **A** | **C** | **T** | **T** | **T** | **T** | **T** | **G** | **C** | **A** | **G** | **C** | **C** | **A** | **T** | **G** | **A** | **T** | **G** | **A** | **T** | **G** | **T** | **T** | **G** | **T** | **C** | **T** | **G** | **C** | **C** | **T** | **T** | **A** | **A** | **A** | **T** | **G** | **A** | **A** | **C** | **A** | **A** | **G** | **C** | **A** | **C** | **A** | **A** | **C** | **G** | **A** | **A** | **C** | **A** | **G** | **A** | **A** | **A** | **C** | **C** | **C** | **C** | **T** | **G** | **A** | **A** | **C** | **C** | **A** | **T** | **A** | **T** | **C** | **G** | **C** | **A** | **A** | **A** | **C** | **A** | **A** | **C** | **T** | **A** | **G** | **C** | **C** | **A** | **T** | **G** | **G** | **T** | **G** | **G** | **C** | **A** | **G** | **A** | **A** | **A** | **T** | **C** | **A** | **T** | **T** | **A** | **A** | **T** | **G** | **C** | **C** | **T** | **C** | **C** | **C** | **A** | **A** | **T** | **C** | **G** | **G** | **A** | **A** | **A** | **T** | **G** | **C** | **G** | **C** | **G** | **C** | **G** | **T** | **T** | **A** | **C** | **T** | **A** | **T** | **T** | **G** | **C** | **A** | **C** | **T** | **T** | **G** | **C** | **G** | **T** | **C** | **C** | **T** | **A** | **T** | **C** | **A** | **G** | **T** | **C** | **T** | **A** | **G** | **A** | **A** | **G** | **G** | **A** | **A** | **A** | **A** | **A** | **G** | **T** | **T** | **T** | **G** | **C** | **G** | **T** | **A** | **A** | **A** | **G** | **G** | **T** | **A** | **T** | **T** | **G** | **A** | **A** | **C** | **A** | **A** | **T** | **T** | **A** | **C** | **T** | **G** | **A** | **A** | **A** | **G** | **A** | **A** | **C** | **T** | **A** | **C** | **A** | **A** | **A** | **C** | **A** | **A** | **A** | **A** | **A** | **T** | **T** | **A** | **A** | **A** | **A** | **T** | **T** | **G** | **A** | **A** | **T** | **T** | **G** | **A** | **T** | **T** | **T** | **G** | **G** | **G** | **A** | **T** | **G** | **T** | **T** | **G** | **A** | **A** | **G** | **A** | **T** | **G** | **T** | **T** | **C** | **A** | **T** | **T** | **T** | **A** | **A** | **A** | **T** | **A** | **G** | **C** | **A** | **G** | **C** | **A** | **T** | **T** | **G** | **A** | **G** | **G** | **A** | **T** | **C** | **A** | **T** | **C** | **T** | **T** | **T** | **T** | **C** | **C** | **G** | **A** | **A** | **T** | **T** | **G** | **T** | **G** | **C** | **A** | **G** | **G** | **A** | **G** | **T** | **T** | **A** | **C** | **T** | **T** | **T** | **C** | **A** | **A** | **A** | **T** | **A** | **C** | **C** | **T** | **T** | **A** | **A** | **G** | **A** | **C** | **A** | **T** | **G** | **C** | **C** | **A** | **A** | **A** | **G** | **C** | **A** | **A** | **A** | **G** | **G** | **A** | **A** | **T** | **T** | **A** | **G** | **A** | **G** | **G** | **T** | **A** | **T** | **A** | **C** | **T** | **T** | **A** | **C** | **A** | **C** | **C** | **A** | **A** | **G** | **T** | **C** | **G** | **A** | **T** | **A** | **A** | **A** | **A** | **A** | **C** | **G** | **T** | **G** | **T** | **T** | **A** | **T** | **T** | **G** | **C** | **G** | **T** | **A** | **T** | **T** | **G** | **T** | **T** | **G** | **A** | **T** | **G** | **A** | **T** | **G** | **G** | **T** | **G** | **T** | **C** | **G** | **G** | **C** | **T** | **T** | **T** | **G** | **A** | **T** | **A** | **T** | **G** | **A** | **A** | **G** | **G** | **A** | **A** | **C** | **A** | **A** | **A** | **G** | **T** | **A** | **A** | **T** | **A** | **A** | **A** | **G** | **C** | **C** | **G** | **G** | **T** | **A** | **G** | **T** | **T** | **A** | **T** | **G** | **G** | **C** | **T** | **T** | **A** | **A** | **A** | **T** | **A** | **A** | **T** | **A** | **T** | **C** | **C** | **G** | **A** | **G** | **A** | **A** | **C** | **G** | **T** | **G** | **T** | **T** | **G** | **T** | **C** | **G** | **G** | **C** | **A** | **T** | **G** | **G** | **G** | **C** | **G** | **G** | **T** | **A** | **C** | **A** | **G** | **T** | **T** | **A** | **A** | **A** | **A** | **T** | **T** | **A** | **T** | **T** | **A** | **G** | **T** | **T** | **T** | **T** | **A** | **A** | **A** | **G** | **G** | **G** | **C** | **A** | **G** | **G** | **G** | **A** | **A** | **C** | **C** | **A** | **G** | **C** | **G** | **T** | **T** | **G** | **A** | **A** | **A** | **T** | **T** | **A** | **A** | **A** | **G** | **T** | **T** | **C** | **C** | **T** | **G** | **T** | **C** | **A** | **T** | **A** | **A** | **A** | **G** | **G** | **A** | **G** | **G** | **A** | **A** | **A** | **C** | **T** | **G** | **C** | **A** | **A** | **G** | **T** | **G** | **A** | **T** | **C** | **A** | **A** | **A** | **G** | **T** | **A** | **A** | **T** | **G** | **T** | **T** | **A** | **G** | **T** | **G** | **G** | **A** | **-** | **-** | **-** |
| **N54553** | **A** | **T** | **G** | **A** | **C** | **C** | **G** | **A** | **T** | **C** | **G** | **G** | **A** | **T** | **T** | **T** | **C** | **A** | **A** | **G** | **A** | **C** | **G** | **C** | **A** | **T** | **G** | **A** | **T** | **T** | **T** | **C** | **A** | **T** | **T** | **A** | **T** | **A** | **T** | **G** | **C** | **G** | **T** | **C** | **C** | **C** | **T** | **T** | **A** | **G** | **C** | **A** | **C** | **C** | **T** | **T** | **T** | **A** | **T** | **T** | **G** | **T** | **T** | **A** | **T** | **C** | **T** | **T** | **A** | **A** | **T** | **T** | **A** | **C** | **A** | **T** | **T** | **G** | **T** | **T** | **T** | **T** | **C** | **A** | **T** | **A** | **T** | **T** | **T** | **T** | **C** | **A** | **T** | **T** | **C** | **G** | **A** | **T** | **T** | **A** | **A** | **A** | **C** | **A** | **A** | **A** | **A** | **C** | **C** | **G** | **G** | **T** | **G** | **G** | **T** | **T** | **A** | **T** | **T** | **A** | **G** | **A** | **G** | **C** | **T** | **T** | **C** | **T** | **T** | **C** | **A** | **G** | **A** | **G** | **A** | **A** | **A** | **A** | **G** | **T** | **C** | **T** | **T** | **T** | **T** | **A** | **T** | **C** | **T** | **A** | **C** | **C** | **A** | **C** | **T** | **A** | **A** | **T** | **T** | **G** | **T** | **G** | **C** | **A** | **C** | **A** | **T** | **T** | **G** | **T** | **T** | **C** | **T** | **C** | **A** | **T** | **A** | **T** | **C** | **C** | **T** | **T** | **A** | **C** | **T** | **A** | **A** | **T** | **A** | **G** | **G** | **C** | **T** | **T** | **A** | **T** | **T** | **G** | **A** | **C** | **C** | **T** | **T** | **T** | **T** | **T** | **A** | **C** | **T** | **G** | **A** | **T** | **T** | **T** | **C** | **A** | **T** | **T** | **G** | **G** | **T** | **T** | **C** | **A** | **A** | **A** | **A** | **A** | **G** | **G** | **G** | **C** | **A** | **A** | **T** | **A** | **T** | **G** | **G** | **A** | **C** | **G** | **G** | **A** | **T** | **T** | **G** | **A** | **A** | **G** | **A** | **A** | **A** | **A** | **A** | **C** | **T** | **T** | **C** | **G** | **G** | **T** | **T** | **A** | **T** | **T** | **G** | **G** | **C** | **C** | **A** | **A** | **T** | **G** | **G** | **T** | **A** | **A** | **T** | **T** | **A** | **T** | **G** | **A** | **A** | **A** | **G** | **T** | **C** | **C** | **A** | **G** | **T** | **C** | **T** | **T** | **A** | **A** | **A** | **C** | **A** | **A** | **A** | **C** | **C** | **A** | **A** | **C** | **G** | **A** | **C** | **C** | **A** | **G** | **T** | **G** | **A** | **A** | **A** | **A** | **T** | **C** | **A** | **A** | **G** | **A** | **T** | **C** | **A** | **T** | **T** | **A** | **T** | **C** | **T** | **A** | **A** | **C** | **C** | **G** | **A** | **A** | **G** | **T** | **C** | **G** | **A** | **A** | **C** | **A** | **A** | **G** | **A** | **T** | **A** | **T** | **T** | **T** | **G** | **G** | **T** | **C** | **G** | **A** | **T** | **T** | **A** | **A** | **A** | **A** | **A** | **T** | **A** | **A** | **A** | **T** | **T** | **A** | **T** | **T** | **A** | **G** | **A** | **G** | **A** | **T** | **G** | **T** | **C** | **T** | **A** | **A** | **A** | **G** | **A** | **A** | **T** | **T** | **G** | **C** | **A** | **A** | **T** | **T** | **A** | **T** | **T** | **A** | **A** | **A** | **C** | **A** | **G** | **T** | **C** | **G** | **G** | **C** | **C** | **G** | **C** | **A** | **A** | **T** | **T** | **A** | **A** | **T** | **G** | **G** | **A** | **T** | **G** | **G** | **G** | **C** | **A** | **A** | **A** | **C** | **A** | **A** | **A** | **A** | **G** | **A** | **A** | **G** | **A** | **A** | **A** | **T** | **T** | **T** | **T** | **A** | **G** | **A** | **G** | **A** | **A** | **C** | **G** | **A** | **G** | **C** | **G** | **G** | **C** | **A** | **T** | **C** | **G** | **T** | **T** | **T** | **G** | **G** | **C** | **G** | **C** | **G** | **G** | **G** | **A** | **G** | **T** | **T** | **G** | **C** | **A** | **T** | **G** | **A** | **T** | **T** | **C** | **A** | **G** | **T** | **C** | **A** | **G** | **T** | **C** | **A** | **A** | **C** | **A** | **A** | **C** | **T** | **T** | **T** | **T** | **T** | **G** | **C** | **A** | **G** | **C** | **C** | **A** | **T** | **G** | **A** | **T** | **G** | **A** | **T** | **G** | **T** | **T** | **G** | **T** | **C** | **T** | **G** | **C** | **C** | **T** | **T** | **A** | **A** | **A** | **T** | **G** | **A** | **A** | **C** | **A** | **A** | **G** | **C** | **A** | **C** | **A** | **A** | **C** | **G** | **A** | **A** | **C** | **A** | **G** | **A** | **A** | **A** | **C** | **C** | **C** | **C** | **G** | **G** | **A** | **A** | **C** | **C** | **A** | **T** | **A** | **T** | **C** | **G** | **T** | **A** | **A** | **A** | **C** | **A** | **A** | **C** | **T** | **A** | **G** | **C** | **C** | **A** | **T** | **G** | **G** | **T** | **G** | **G** | **C** | **A** | **G** | **A** | **A** | **A** | **T** | **C** | **A** | **T** | **T** | **A** | **A** | **T** | **G** | **C** | **C** | **T** | **C** | **C** | **C** | **A** | **A** | **T** | **C** | **G** | **G** | **A** | **A** | **A** | **T** | **G** | **C** | **G** | **C** | **G** | **C** | **G** | **T** | **T** | **A** | **C** | **T** | **A** | **T** | **T** | **G** | **C** | **A** | **C** | **T** | **T** | **G** | **C** | **G** | **T** | **C** | **C** | **T** | **A** | **T** | **C** | **A** | **G** | **T** | **C** | **T** | **A** | **G** | **A** | **A** | **G** | **G** | **A** | **A** | **A** | **A** | **A** | **G** | **T** | **T** | **T** | **G** | **C** | **G** | **T** | **A** | **A** | **A** | **G** | **G** | **T** | **A** | **T** | **T** | **G** | **A** | **A** | **C** | **A** | **A** | **T** | **T** | **A** | **C** | **T** | **G** | **A** | **A** | **A** | **G** | **A** | **A** | **C** | **T** | **A** | **C** | **A** | **A** | **A** | **C** | **A** | **A** | **A** | **A** | **A** | **T** | **T** | **A** | **A** | **A** | **A** | **T** | **T** | **G** | **A** | **A** | **T** | **T** | **G** | **A** | **T** | **T** | **T** | **G** | **G** | **G** | **A** | **T** | **G** | **T** | **T** | **G** | **A** | **A** | **G** | **A** | **T** | **G** | **T** | **T** | **C** | **A** | **T** | **T** | **T** | **A** | **A** | **A** | **T** | **A** | **G** | **C** | **A** | **G** | **C** | **A** | **T** | **T** | **G** | **A** | **G** | **G** | **A** | **T** | **C** | **A** | **T** | **C** | **T** | **T** | **T** | **T** | **C** | **C** | **G** | **A** | **A** | **T** | **T** | **G** | **T** | **G** | **C** | **A** | **G** | **G** | **A** | **G** | **T** | **T** | **A** | **C** | **T** | **T** | **T** | **C** | **A** | **A** | **A** | **T** | **A** | **C** | **C** | **T** | **T** | **A** | **A** | **G** | **A** | **C** | **A** | **T** | **G** | **C** | **C** | **A** | **A** | **A** | **G** | **C** | **A** | **A** | **A** | **G** | **G** | **A** | **A** | **T** | **T** | **A** | **G** | **A** | **G** | **G** | **T** | **A** | **T** | **A** | **C** | **T** | **T** | **A** | **C** | **A** | **C** | **C** | **A** | **A** | **G** | **T** | **C** | **G** | **A** | **T** | **A** | **A** | **A** | **A** | **A** | **C** | **G** | **T** | **G** | **T** | **T** | **A** | **T** | **T** | **G** | **C** | **G** | **T** | **A** | **T** | **T** | **G** | **T** | **T** | **G** | **A** | **T** | **G** | **A** | **C** | **G** | **G** | **T** | **G** | **T** | **C** | **G** | **G** | **C** | **T** | **T** | **T** | **G** | **A** | **T** | **A** | **T** | **G** | **A** | **A** | **G** | **G** | **A** | **A** | **C** | **A** | **A** | **A** | **G** | **T** | **A** | **A** | **T** | **A** | **A** | **A** | **G** | **C** | **C** | **G** | **G** | **T** | **A** | **G** | **T** | **T** | **A** | **T** | **G** | **G** | **C** | **T** | **T** | **A** | **A** | **A** | **T** | **A** | **A** | **T** | **A** | **T** | **C** | **C** | **G** | **A** | **G** | **A** | **A** | **C** | **G** | **T** | **G** | **T** | **T** | **G** | **T** | **C** | **G** | **G** | **C** | **A** | **T** | **G** | **G** | **G** | **C** | **G** | **G** | **T** | **A** | **C** | **A** | **G** | **T** | **T** | **A** | **A** | **A** | **A** | **T** | **T** | **A** | **T** | **T** | **A** | **G** | **T** | **T** | **T** | **T** | **A** | **A** | **A** | **G** | **G** | **G** | **C** | **A** | **G** | **G** | **G** | **A** | **A** | **C** | **C** | **A** | **G** | **C** | **G** | **T** | **T** | **G** | **A** | **A** | **A** | **T** | **T** | **A** | **A** | **A** | **G** | **T** | **T** | **C** | **C** | **T** | **G** | **T** | **C** | **A** | **T** | **A** | **A** | **A** | **G** | **G** | **A** | **G** | **G** | **A** | **A** | **A** | **C** | **T** | **G** | **C** | **A** | **A** | **G** | **T** | **G** | **A** | **T** | **C** | **A** | **A** | **A** | **G** | **T** | **A** | **A** | **T** | **G** | **T** | **T** | **A** | **G** | **T** | **G** | **G** | **A** | **-** | **-** | **-** |
| **N59847F** | **A** | **T** | **G** | **A** | **C** | **C** | **G** | **A** | **T** | **C** | **G** | **G** | **A** | **T** | **T** | **T** | **C** | **A** | **A** | **G** | **A** | **C** | **G** | **C** | **A** | **T** | **G** | **A** | **T** | **T** | **T** | **C** | **A** | **T** | **T** | **A** | **T** | **A** | **T** | **G** | **C** | **G** | **T** | **C** | **C** | **C** | **T** | **T** | **A** | **G** | **C** | **A** | **C** | **C** | **T** | **T** | **T** | **A** | **T** | **T** | **G** | **T** | **T** | **A** | **T** | **C** | **T** | **T** | **A** | **A** | **T** | **T** | **A** | **C** | **A** | **T** | **T** | **G** | **T** | **T** | **T** | **T** | **C** | **A** | **T** | **A** | **T** | **T** | **T** | **T** | **C** | **A** | **T** | **T** | **C** | **G** | **A** | **T** | **T** | **A** | **A** | **A** | **C** | **A** | **A** | **A** | **A** | **C** | **C** | **G** | **G** | **T** | **G** | **G** | **T** | **T** | **A** | **T** | **T** | **A** | **G** | **A** | **G** | **C** | **T** | **T** | **C** | **T** | **T** | **C** | **A** | **G** | **A** | **G** | **A** | **A** | **A** | **A** | **G** | **T** | **C** | **T** | **T** | **T** | **T** | **A** | **T** | **T** | **T** | **A** | **C** | **C** | **A** | **C** | **T** | **A** | **A** | **T** | **T** | **G** | **T** | **G** | **C** | **A** | **C** | **A** | **T** | **T** | **G** | **T** | **T** | **C** | **T** | **C** | **A** | **T** | **A** | **T** | **C** | **C** | **T** | **T** | **A** | **C** | **T** | **A** | **A** | **T** | **A** | **G** | **G** | **C** | **T** | **T** | **A** | **T** | **T** | **G** | **A** | **C** | **C** | **T** | **T** | **T** | **T** | **T** | **A** | **C** | **T** | **G** | **A** | **T** | **T** | **T** | **C** | **A** | **T** | **T** | **G** | **G** | **T** | **T** | **C** | **A** | **A** | **A** | **A** | **A** | **G** | **G** | **G** | **C** | **A** | **A** | **T** | **A** | **T** | **G** | **G** | **A** | **C** | **G** | **G** | **A** | **T** | **T** | **G** | **A** | **A** | **G** | **A** | **A** | **A** | **A** | **A** | **C** | **T** | **T** | **C** | **G** | **G** | **T** | **T** | **A** | **T** | **T** | **G** | **G** | **C** | **C** | **A** | **A** | **C** | **G** | **G** | **T** | **A** | **A** | **T** | **T** | **A** | **T** | **G** | **A** | **A** | **A** | **G** | **T** | **C** | **C** | **A** | **G** | **T** | **C** | **T** | **T** | **A** | **A** | **A** | **C** | **A** | **A** | **A** | **C** | **C** | **A** | **A** | **C** | **G** | **A** | **C** | **C** | **A** | **G** | **T** | **G** | **A** | **A** | **A** | **A** | **T** | **C** | **A** | **A** | **G** | **A** | **C** | **C** | **A** | **T** | **T** | **A** | **T** | **C** | **T** | **A** | **A** | **C** | **C** | **G** | **A** | **A** | **G** | **T** | **C** | **G** | **A** | **A** | **C** | **A** | **A** | **G** | **A** | **T** | **A** | **T** | **T** | **T** | **G** | **G** | **T** | **C** | **G** | **A** | **T** | **T** | **A** | **A** | **A** | **A** | **A** | **T** | **A** | **A** | **A** | **T** | **T** | **A** | **T** | **T** | **A** | **G** | **A** | **G** | **A** | **T** | **G** | **T** | **C** | **T** | **A** | **A** | **A** | **G** | **A** | **A** | **T** | **T** | **G** | **C** | **A** | **A** | **T** | **T** | **A** | **T** | **T** | **A** | **A** | **A** | **C** | **A** | **G** | **T** | **C** | **G** | **A** | **C** | **C** | **G** | **C** | **A** | **A** | **T** | **T** | **A** | **A** | **T** | **G** | **G** | **A** | **T** | **G** | **G** | **G** | **C** | **A** | **A** | **A** | **C** | **A** | **A** | **A** | **A** | **G** | **A** | **A** | **G** | **A** | **A** | **A** | **T** | **T** | **T** | **T** | **A** | **G** | **A** | **G** | **A** | **A** | **C** | **G** | **A** | **G** | **C** | **G** | **G** | **C** | **A** | **T** | **C** | **G** | **T** | **T** | **T** | **G** | **G** | **C** | **G** | **C** | **G** | **G** | **G** | **A** | **G** | **T** | **T** | **G** | **C** | **A** | **T** | **G** | **A** | **T** | **T** | **C** | **A** | **G** | **T** | **C** | **A** | **G** | **T** | **C** | **A** | **A** | **C** | **A** | **A** | **C** | **T** | **T** | **T** | **T** | **T** | **G** | **C** | **A** | **G** | **C** | **C** | **A** | **T** | **G** | **A** | **T** | **G** | **A** | **T** | **G** | **T** | **T** | **G** | **T** | **C** | **T** | **G** | **C** | **A** | **T** | **T** | **A** | **A** | **A** | **T** | **G** | **A** | **A** | **C** | **A** | **A** | **G** | **C** | **A** | **C** | **A** | **A** | **C** | **G** | **A** | **A** | **C** | **A** | **G** | **A** | **A** | **A** | **C** | **C** | **C** | **C** | **G** | **G** | **A** | **A** | **C** | **C** | **A** | **T** | **A** | **T** | **C** | **G** | **T** | **A** | **A** | **A** | **C** | **A** | **A** | **C** | **T** | **A** | **G** | **C** | **C** | **A** | **T** | **G** | **G** | **T** | **G** | **G** | **C** | **A** | **G** | **A** | **A** | **A** | **T** | **C** | **A** | **T** | **T** | **A** | **A** | **T** | **G** | **C** | **C** | **T** | **C** | **C** | **C** | **A** | **A** | **T** | **C** | **G** | **G** | **A** | **A** | **A** | **T** | **G** | **C** | **G** | **C** | **G** | **C** | **G** | **C** | **T** | **A** | **C** | **T** | **A** | **T** | **T** | **G** | **C** | **A** | **C** | **T** | **T** | **G** | **C** | **G** | **T** | **C** | **C** | **T** | **A** | **T** | **C** | **A** | **G** | **T** | **C** | **T** | **A** | **G** | **A** | **A** | **G** | **G** | **A** | **A** | **A** | **A** | **A** | **G** | **T** | **T** | **T** | **G** | **C** | **G** | **T** | **A** | **A** | **A** | **G** | **G** | **T** | **A** | **T** | **T** | **G** | **A** | **A** | **C** | **A** | **A** | **T** | **T** | **A** | **C** | **T** | **G** | **A** | **A** | **A** | **G** | **A** | **A** | **C** | **T** | **A** | **C** | **A** | **A** | **A** | **C** | **A** | **A** | **A** | **A** | **A** | **T** | **T** | **A** | **A** | **A** | **A** | **T** | **T** | **G** | **A** | **A** | **T** | **T** | **G** | **A** | **T** | **T** | **T** | **G** | **G** | **G** | **A** | **T** | **G** | **T** | **T** | **G** | **A** | **A** | **G** | **A** | **T** | **G** | **T** | **T** | **C** | **A** | **T** | **T** | **T** | **A** | **A** | **A** | **T** | **A** | **G** | **C** | **A** | **G** | **C** | **A** | **T** | **T** | **G** | **A** | **G** | **G** | **A** | **T** | **C** | **A** | **T** | **C** | **T** | **T** | **T** | **T** | **C** | **C** | **G** | **A** | **A** | **T** | **T** | **G** | **T** | **G** | **C** | **A** | **G** | **G** | **A** | **G** | **T** | **T** | **A** | **C** | **T** | **T** | **T** | **C** | **A** | **A** | **A** | **T** | **A** | **C** | **C** | **T** | **T** | **A** | **A** | **G** | **A** | **C** | **A** | **T** | **G** | **C** | **C** | **A** | **A** | **A** | **G** | **C** | **A** | **A** | **A** | **G** | **G** | **A** | **A** | **T** | **T** | **A** | **G** | **A** | **G** | **G** | **T** | **A** | **T** | **A** | **C** | **T** | **T** | **A** | **C** | **A** | **C** | **C** | **A** | **A** | **G** | **T** | **C** | **G** | **A** | **T** | **A** | **A** | **A** | **A** | **A** | **C** | **G** | **T** | **G** | **T** | **T** | **A** | **T** | **T** | **G** | **C** | **G** | **T** | **A** | **T** | **T** | **G** | **T** | **T** | **G** | **A** | **T** | **G** | **A** | **T** | **G** | **G** | **T** | **G** | **T** | **C** | **G** | **G** | **C** | **T** | **T** | **T** | **G** | **A** | **T** | **A** | **T** | **G** | **A** | **A** | **G** | **G** | **A** | **A** | **C** | **A** | **A** | **A** | **G** | **T** | **A** | **A** | **T** | **A** | **A** | **A** | **G** | **C** | **C** | **G** | **G** | **T** | **A** | **G** | **T** | **T** | **A** | **T** | **G** | **G** | **C** | **T** | **T** | **A** | **A** | **A** | **T** | **A** | **A** | **T** | **A** | **T** | **T** | **C** | **G** | **A** | **G** | **A** | **A** | **C** | **G** | **T** | **G** | **T** | **T** | **G** | **T** | **C** | **G** | **G** | **C** | **A** | **T** | **G** | **G** | **G** | **C** | **G** | **G** | **T** | **A** | **C** | **A** | **G** | **T** | **T** | **A** | **A** | **A** | **A** | **T** | **T** | **A** | **T** | **T** | **A** | **G** | **T** | **T** | **T** | **T** | **A** | **A** | **A** | **G** | **G** | **G** | **C** | **A** | **G** | **G** | **G** | **A** | **A** | **C** | **C** | **A** | **G** | **C** | **G** | **T** | **T** | **G** | **A** | **A** | **A** | **T** | **T** | **A** | **A** | **A** | **G** | **T** | **T** | **C** | **C** | **T** | **G** | **T** | **C** | **A** | **T** | **A** | **A** | **A** | **G** | **G** | **A** | **G** | **G** | **A** | **A** | **A** | **C** | **T** | **G** | **C** | **A** | **A** | **G** | **T** | **G** | **A** | **T** | **C** | **A** | **A** | **A** | **G** | **T** | **A** | **A** | **T** | **G** | **T** | **T** | **A** | **G** | **T** | **G** | **G** | **A** | **-** | **-** | **-** |
| **N54676** | **A** | **T** | **G** | **A** | **C** | **C** | **G** | **A** | **T** | **C** | **G** | **G** | **A** | **T** | **T** | **T** | **C** | **A** | **A** | **G** | **A** | **C** | **G** | **C** | **A** | **T** | **G** | **A** | **T** | **T** | **T** | **C** | **A** | **T** | **T** | **A** | **T** | **A** | **T** | **G** | **C** | **G** | **T** | **C** | **C** | **C** | **T** | **T** | **A** | **G** | **C** | **A** | **C** | **C** | **T** | **T** | **T** | **A** | **T** | **T** | **G** | **T** | **T** | **A** | **T** | **C** | **T** | **T** | **A** | **A** | **T** | **T** | **A** | **C** | **A** | **T** | **T** | **G** | **T** | **T** | **T** | **T** | **C** | **A** | **T** | **A** | **T** | **T** | **T** | **T** | **C** | **A** | **T** | **T** | **C** | **G** | **A** | **T** | **T** | **A** | **A** | **A** | **C** | **A** | **A** | **A** | **A** | **C** | **C** | **G** | **G** | **T** | **G** | **G** | **C** | **T** | **A** | **T** | **T** | **A** | **G** | **A** | **G** | **C** | **T** | **T** | **C** | **T** | **T** | **C** | **A** | **G** | **A** | **G** | **A** | **A** | **A** | **A** | **G** | **T** | **C** | **T** | **T** | **T** | **T** | **A** | **T** | **C** | **T** | **A** | **C** | **C** | **A** | **C** | **T** | **A** | **A** | **T** | **T** | **G** | **T** | **G** | **C** | **A** | **C** | **A** | **T** | **T** | **G** | **T** | **T** | **C** | **T** | **C** | **A** | **T** | **A** | **T** | **C** | **C** | **T** | **T** | **A** | **C** | **T** | **A** | **A** | **T** | **A** | **G** | **G** | **C** | **T** | **T** | **A** | **T** | **T** | **G** | **A** | **C** | **C** | **T** | **T** | **T** | **T** | **T** | **A** | **C** | **T** | **G** | **A** | **T** | **T** | **T** | **C** | **A** | **T** | **T** | **G** | **G** | **T** | **T** | **C** | **A** | **A** | **A** | **A** | **A** | **G** | **G** | **G** | **C** | **A** | **A** | **T** | **A** | **T** | **G** | **G** | **A** | **C** | **G** | **G** | **A** | **T** | **T** | **G** | **A** | **A** | **G** | **A** | **A** | **A** | **A** | **A** | **C** | **T** | **T** | **C** | **G** | **G** | **T** | **T** | **A** | **T** | **T** | **G** | **G** | **C** | **C** | **A** | **A** | **T** | **G** | **G** | **C** | **A** | **A** | **T** | **T** | **A** | **T** | **G** | **A** | **A** | **A** | **G** | **T** | **C** | **C** | **A** | **G** | **T** | **C** | **T** | **T** | **A** | **A** | **A** | **C** | **A** | **A** | **A** | **C** | **C** | **A** | **A** | **C** | **G** | **A** | **C** | **C** | **A** | **G** | **T** | **G** | **A** | **A** | **A** | **A** | **T** | **C** | **A** | **A** | **G** | **A** | **T** | **C** | **A** | **T** | **T** | **A** | **T** | **C** | **T** | **A** | **A** | **C** | **C** | **G** | **A** | **A** | **G** | **T** | **C** | **G** | **A** | **A** | **C** | **A** | **A** | **G** | **A** | **T** | **A** | **T** | **T** | **T** | **G** | **G** | **T** | **C** | **G** | **A** | **T** | **T** | **A** | **A** | **A** | **A** | **A** | **T** | **A** | **A** | **A** | **T** | **T** | **A** | **T** | **T** | **A** | **G** | **A** | **G** | **A** | **T** | **G** | **T** | **C** | **T** | **A** | **A** | **A** | **G** | **A** | **A** | **T** | **T** | **G** | **C** | **A** | **A** | **T** | **T** | **A** | **T** | **T** | **A** | **A** | **A** | **C** | **A** | **G** | **T** | **C** | **G** | **G** | **C** | **C** | **G** | **C** | **A** | **A** | **T** | **T** | **A** | **A** | **T** | **G** | **G** | **A** | **T** | **G** | **G** | **G** | **C** | **A** | **A** | **A** | **C** | **A** | **A** | **A** | **A** | **G** | **A** | **A** | **G** | **A** | **A** | **A** | **T** | **T** | **T** | **T** | **A** | **G** | **A** | **G** | **A** | **A** | **C** | **G** | **A** | **G** | **C** | **G** | **G** | **C** | **A** | **T** | **C** | **G** | **T** | **T** | **T** | **G** | **G** | **C** | **G** | **C** | **G** | **G** | **G** | **A** | **G** | **T** | **T** | **G** | **C** | **A** | **T** | **G** | **A** | **T** | **T** | **C** | **A** | **G** | **T** | **C** | **A** | **G** | **T** | **C** | **A** | **A** | **C** | **A** | **A** | **C** | **T** | **T** | **T** | **T** | **T** | **G** | **C** | **A** | **G** | **C** | **C** | **A** | **T** | **G** | **A** | **T** | **G** | **A** | **T** | **G** | **T** | **T** | **G** | **T** | **C** | **T** | **G** | **C** | **C** | **T** | **T** | **A** | **A** | **A** | **T** | **G** | **A** | **A** | **C** | **A** | **A** | **G** | **C** | **A** | **C** | **A** | **A** | **C** | **G** | **A** | **A** | **C** | **A** | **G** | **A** | **A** | **A** | **C** | **C** | **C** | **C** | **G** | **G** | **A** | **A** | **C** | **C** | **A** | **T** | **A** | **T** | **C** | **G** | **T** | **A** | **A** | **A** | **C** | **A** | **A** | **C** | **T** | **A** | **G** | **C** | **C** | **A** | **T** | **G** | **G** | **T** | **G** | **G** | **C** | **A** | **G** | **A** | **A** | **A** | **T** | **C** | **A** | **T** | **T** | **A** | **A** | **T** | **G** | **C** | **C** | **T** | **C** | **C** | **C** | **A** | **A** | **T** | **C** | **G** | **G** | **A** | **A** | **A** | **T** | **G** | **C** | **G** | **C** | **G** | **C** | **G** | **T** | **T** | **A** | **C** | **T** | **A** | **T** | **T** | **G** | **C** | **A** | **C** | **T** | **T** | **G** | **C** | **G** | **T** | **C** | **C** | **T** | **A** | **T** | **C** | **A** | **G** | **T** | **C** | **T** | **A** | **G** | **A** | **A** | **G** | **G** | **A** | **A** | **A** | **A** | **A** | **G** | **T** | **T** | **T** | **G** | **C** | **G** | **T** | **A** | **A** | **A** | **G** | **G** | **T** | **A** | **T** | **T** | **G** | **A** | **A** | **C** | **A** | **A** | **T** | **T** | **A** | **C** | **T** | **G** | **A** | **A** | **A** | **G** | **A** | **A** | **C** | **T** | **A** | **C** | **A** | **A** | **A** | **C** | **A** | **A** | **A** | **A** | **A** | **T** | **T** | **A** | **A** | **A** | **A** | **T** | **T** | **G** | **A** | **A** | **T** | **T** | **G** | **A** | **T** | **T** | **T** | **G** | **G** | **G** | **A** | **T** | **G** | **T** | **T** | **G** | **A** | **A** | **G** | **A** | **T** | **G** | **T** | **T** | **C** | **A** | **T** | **T** | **T** | **A** | **A** | **A** | **T** | **A** | **G** | **C** | **A** | **G** | **C** | **A** | **T** | **T** | **G** | **A** | **G** | **G** | **A** | **T** | **C** | **A** | **T** | **C** | **T** | **T** | **T** | **T** | **C** | **C** | **G** | **A** | **A** | **T** | **T** | **G** | **T** | **G** | **C** | **A** | **G** | **G** | **A** | **A** | **T** | **T** | **A** | **C** | **T** | **T** | **T** | **C** | **C** | **A** | **A** | **T** | **A** | **C** | **C** | **T** | **T** | **A** | **A** | **G** | **A** | **C** | **A** | **T** | **G** | **C** | **C** | **A** | **A** | **A** | **G** | **C** | **A** | **A** | **A** | **G** | **G** | **A** | **A** | **T** | **T** | **A** | **G** | **A** | **G** | **G** | **T** | **A** | **T** | **A** | **C** | **T** | **T** | **A** | **C** | **A** | **C** | **C** | **A** | **A** | **G** | **T** | **C** | **G** | **A** | **T** | **A** | **A** | **A** | **A** | **A** | **C** | **G** | **T** | **G** | **T** | **T** | **A** | **T** | **T** | **G** | **C** | **G** | **T** | **A** | **T** | **T** | **G** | **T** | **T** | **G** | **A** | **T** | **G** | **A** | **T** | **G** | **G** | **T** | **G** | **T** | **C** | **G** | **G** | **C** | **T** | **T** | **T** | **G** | **A** | **T** | **A** | **T** | **G** | **A** | **A** | **G** | **G** | **A** | **A** | **C** | **A** | **A** | **A** | **G** | **T** | **A** | **A** | **T** | **A** | **A** | **A** | **G** | **C** | **C** | **G** | **G** | **T** | **A** | **G** | **T** | **T** | **A** | **T** | **G** | **G** | **C** | **T** | **T** | **A** | **A** | **A** | **T** | **A** | **A** | **T** | **A** | **T** | **T** | **C** | **G** | **A** | **G** | **A** | **A** | **C** | **G** | **T** | **G** | **T** | **T** | **G** | **T** | **C** | **G** | **G** | **C** | **A** | **T** | **G** | **G** | **G** | **C** | **G** | **G** | **T** | **A** | **C** | **A** | **G** | **T** | **T** | **A** | **A** | **A** | **A** | **T** | **T** | **A** | **T** | **T** | **A** | **G** | **T** | **T** | **T** | **T** | **A** | **A** | **A** | **G** | **G** | **G** | **C** | **A** | **G** | **G** | **G** | **A** | **A** | **C** | **C** | **A** | **G** | **C** | **G** | **T** | **T** | **G** | **A** | **A** | **A** | **T** | **T** | **A** | **A** | **A** | **G** | **T** | **T** | **C** | **C** | **T** | **G** | **T** | **C** | **A** | **T** | **A** | **A** | **A** | **G** | **G** | **A** | **G** | **G** | **A** | **A** | **A** | **C** | **T** | **G** | **C** | **A** | **A** | **G** | **T** | **G** | **A** | **T** | **C** | **A** | **A** | **A** | **G** | **T** | **A** | **A** | **T** | **G** | **T** | **T** | **A** | **G** | **T** | **G** | **G** | **A** | **-** | **-** | **-** |
| **N59600F** | **A** | **T** | **G** | **A** | **C** | **C** | **G** | **A** | **T** | **C** | **G** | **G** | **A** | **T** | **T** | **T** | **C** | **A** | **A** | **G** | **A** | **C** | **G** | **C** | **A** | **T** | **G** | **A** | **T** | **T** | **T** | **C** | **A** | **T** | **T** | **A** | **T** | **A** | **T** | **G** | **C** | **G** | **T** | **C** | **C** | **C** | **T** | **T** | **A** | **G** | **C** | **A** | **C** | **C** | **T** | **T** | **T** | **A** | **T** | **T** | **G** | **T** | **T** | **A** | **T** | **C** | **T** | **T** | **A** | **A** | **T** | **T** | **A** | **C** | **A** | **T** | **T** | **G** | **T** | **T** | **T** | **T** | **C** | **A** | **T** | **A** | **T** | **T** | **T** | **T** | **C** | **A** | **T** | **T** | **C** | **G** | **A** | **T** | **T** | **A** | **A** | **A** | **C** | **A** | **A** | **A** | **A** | **C** | **C** | **G** | **G** | **T** | **G** | **G** | **T** | **T** | **A** | **T** | **T** | **A** | **G** | **A** | **G** | **C** | **T** | **T** | **C** | **T** | **T** | **C** | **A** | **G** | **A** | **G** | **A** | **A** | **A** | **A** | **G** | **T** | **C** | **T** | **T** | **T** | **T** | **A** | **T** | **C** | **T** | **A** | **C** | **C** | **A** | **C** | **T** | **A** | **A** | **T** | **T** | **G** | **T** | **G** | **C** | **A** | **C** | **A** | **T** | **T** | **G** | **T** | **T** | **C** | **T** | **C** | **A** | **T** | **A** | **T** | **C** | **C** | **T** | **T** | **A** | **C** | **T** | **A** | **A** | **T** | **A** | **G** | **G** | **C** | **T** | **T** | **A** | **T** | **T** | **G** | **A** | **C** | **C** | **T** | **T** | **T** | **T** | **T** | **A** | **C** | **T** | **G** | **A** | **T** | **T** | **T** | **C** | **A** | **T** | **T** | **G** | **G** | **T** | **T** | **C** | **A** | **A** | **A** | **A** | **A** | **G** | **G** | **G** | **C** | **A** | **A** | **T** | **A** | **T** | **G** | **G** | **A** | **C** | **G** | **G** | **A** | **T** | **T** | **G** | **A** | **A** | **G** | **A** | **A** | **A** | **A** | **A** | **C** | **T** | **T** | **C** | **G** | **G** | **T** | **T** | **A** | **T** | **T** | **G** | **G** | **C** | **C** | **A** | **A** | **C** | **G** | **G** | **T** | **A** | **A** | **T** | **T** | **A** | **T** | **G** | **A** | **A** | **A** | **G** | **T** | **C** | **C** | **A** | **G** | **T** | **C** | **T** | **T** | **A** | **A** | **A** | **C** | **A** | **A** | **A** | **C** | **C** | **A** | **A** | **C** | **G** | **A** | **C** | **C** | **A** | **G** | **T** | **G** | **A** | **A** | **A** | **A** | **T** | **C** | **A** | **A** | **G** | **A** | **T** | **C** | **A** | **T** | **T** | **A** | **T** | **C** | **T** | **A** | **A** | **C** | **C** | **G** | **A** | **A** | **G** | **T** | **C** | **G** | **A** | **A** | **C** | **A** | **A** | **G** | **A** | **T** | **A** | **T** | **T** | **T** | **G** | **G** | **T** | **C** | **G** | **A** | **T** | **T** | **A** | **A** | **A** | **A** | **A** | **T** | **A** | **A** | **A** | **T** | **T** | **A** | **T** | **T** | **A** | **G** | **A** | **G** | **A** | **T** | **G** | **T** | **C** | **T** | **A** | **A** | **A** | **G** | **A** | **A** | **T** | **T** | **G** | **C** | **A** | **A** | **T** | **T** | **A** | **T** | **T** | **A** | **A** | **A** | **C** | **A** | **G** | **T** | **C** | **G** | **A** | **C** | **C** | **G** | **C** | **A** | **A** | **T** | **T** | **A** | **A** | **T** | **G** | **G** | **A** | **T** | **G** | **G** | **G** | **C** | **A** | **A** | **A** | **C** | **A** | **A** | **A** | **A** | **G** | **A** | **A** | **G** | **A** | **A** | **A** | **T** | **T** | **T** | **T** | **A** | **G** | **A** | **G** | **A** | **A** | **C** | **G** | **A** | **G** | **C** | **G** | **G** | **C** | **A** | **T** | **C** | **G** | **T** | **T** | **T** | **G** | **G** | **C** | **G** | **C** | **G** | **G** | **G** | **A** | **G** | **T** | **T** | **G** | **C** | **A** | **T** | **G** | **A** | **T** | **T** | **C** | **A** | **G** | **T** | **C** | **A** | **G** | **T** | **C** | **A** | **A** | **C** | **A** | **A** | **C** | **T** | **T** | **T** | **T** | **T** | **G** | **C** | **A** | **G** | **C** | **C** | **A** | **T** | **G** | **A** | **T** | **G** | **A** | **T** | **G** | **T** | **T** | **G** | **T** | **C** | **T** | **G** | **C** | **C** | **T** | **T** | **A** | **A** | **A** | **T** | **G** | **A** | **A** | **C** | **A** | **A** | **G** | **C** | **A** | **C** | **A** | **A** | **C** | **G** | **A** | **A** | **C** | **A** | **G** | **A** | **A** | **A** | **C** | **C** | **C** | **C** | **G** | **G** | **A** | **A** | **C** | **C** | **A** | **T** | **A** | **T** | **C** | **G** | **T** | **A** | **A** | **A** | **C** | **A** | **A** | **C** | **T** | **A** | **G** | **C** | **C** | **A** | **T** | **G** | **G** | **T** | **G** | **G** | **C** | **A** | **G** | **A** | **A** | **A** | **T** | **C** | **A** | **T** | **T** | **A** | **A** | **T** | **G** | **C** | **C** | **T** | **C** | **C** | **C** | **A** | **A** | **T** | **C** | **G** | **G** | **A** | **A** | **A** | **T** | **G** | **C** | **G** | **C** | **G** | **C** | **G** | **T** | **T** | **A** | **C** | **T** | **A** | **T** | **T** | **G** | **C** | **A** | **C** | **T** | **T** | **G** | **C** | **G** | **T** | **C** | **C** | **T** | **A** | **T** | **C** | **A** | **G** | **T** | **C** | **T** | **A** | **G** | **A** | **A** | **G** | **G** | **A** | **A** | **A** | **A** | **A** | **G** | **T** | **T** | **T** | **G** | **C** | **G** | **T** | **A** | **A** | **A** | **G** | **G** | **T** | **A** | **T** | **T** | **G** | **A** | **A** | **C** | **A** | **A** | **T** | **T** | **A** | **C** | **T** | **G** | **A** | **A** | **A** | **G** | **A** | **A** | **C** | **T** | **A** | **C** | **A** | **A** | **A** | **C** | **A** | **A** | **A** | **A** | **A** | **T** | **T** | **A** | **A** | **A** | **A** | **T** | **T** | **G** | **A** | **A** | **T** | **T** | **G** | **A** | **T** | **T** | **T** | **G** | **G** | **G** | **A** | **T** | **G** | **T** | **T** | **G** | **A** | **A** | **G** | **A** | **T** | **G** | **T** | **T** | **C** | **A** | **T** | **T** | **T** | **A** | **A** | **A** | **T** | **A** | **G** | **C** | **A** | **G** | **C** | **A** | **T** | **T** | **G** | **A** | **G** | **G** | **A** | **T** | **C** | **A** | **T** | **C** | **T** | **T** | **T** | **T** | **C** | **C** | **G** | **A** | **A** | **T** | **T** | **G** | **T** | **G** | **C** | **A** | **G** | **G** | **A** | **G** | **T** | **T** | **A** | **C** | **T** | **T** | **T** | **C** | **A** | **A** | **A** | **T** | **A** | **C** | **C** | **T** | **T** | **A** | **A** | **G** | **A** | **C** | **A** | **T** | **G** | **C** | **C** | **A** | **A** | **A** | **G** | **C** | **A** | **A** | **A** | **G** | **G** | **A** | **A** | **T** | **T** | **A** | **G** | **A** | **G** | **G** | **T** | **A** | **T** | **A** | **C** | **T** | **T** | **A** | **C** | **A** | **C** | **C** | **A** | **A** | **G** | **T** | **C** | **G** | **A** | **T** | **A** | **A** | **A** | **A** | **A** | **C** | **G** | **T** | **G** | **T** | **T** | **A** | **T** | **T** | **G** | **C** | **G** | **T** | **A** | **T** | **T** | **G** | **T** | **T** | **G** | **A** | **T** | **G** | **A** | **C** | **G** | **G** | **T** | **G** | **T** | **C** | **G** | **G** | **C** | **T** | **T** | **T** | **G** | **A** | **T** | **A** | **T** | **G** | **A** | **A** | **G** | **G** | **A** | **A** | **C** | **A** | **A** | **A** | **G** | **T** | **A** | **A** | **T** | **A** | **A** | **A** | **G** | **C** | **C** | **G** | **G** | **T** | **A** | **G** | **T** | **T** | **A** | **T** | **G** | **G** | **C** | **T** | **T** | **A** | **A** | **A** | **T** | **A** | **A** | **T** | **A** | **T** | **C** | **C** | **G** | **A** | **G** | **A** | **A** | **C** | **G** | **T** | **G** | **T** | **T** | **G** | **T** | **C** | **G** | **G** | **C** | **A** | **T** | **G** | **G** | **G** | **C** | **G** | **G** | **T** | **A** | **C** | **A** | **G** | **T** | **T** | **A** | **A** | **A** | **A** | **T** | **T** | **A** | **T** | **T** | **A** | **G** | **T** | **T** | **T** | **T** | **A** | **A** | **A** | **G** | **G** | **G** | **C** | **A** | **G** | **G** | **G** | **A** | **A** | **C** | **C** | **A** | **G** | **C** | **G** | **T** | **T** | **G** | **A** | **A** | **A** | **T** | **T** | **A** | **A** | **A** | **G** | **T** | **T** | **C** | **C** | **T** | **G** | **T** | **C** | **A** | **T** | **A** | **A** | **A** | **G** | **G** | **A** | **G** | **G** | **A** | **A** | **A** | **C** | **T** | **G** | **C** | **A** | **A** | **G** | **T** | **G** | **A** | **T** | **C** | **A** | **A** | **A** | **G** | **T** | **A** | **A** | **T** | **G** | **T** | **T** | **A** | **G** | **T** | **G** | **G** | **A** | **-** | **-** | **-** |
| **N53519** | **A** | **T** | **G** | **A** | **C** | **C** | **G** | **A** | **T** | **C** | **G** | **G** | **A** | **T** | **T** | **T** | **C** | **A** | **A** | **G** | **A** | **C** | **G** | **C** | **A** | **T** | **G** | **A** | **T** | **T** | **T** | **C** | **A** | **T** | **T** | **A** | **T** | **A** | **T** | **G** | **C** | **G** | **T** | **C** | **C** | **C** | **T** | **T** | **A** | **G** | **C** | **A** | **C** | **C** | **T** | **T** | **T** | **A** | **T** | **T** | **G** | **T** | **T** | **A** | **T** | **C** | **T** | **T** | **A** | **A** | **T** | **T** | **A** | **C** | **A** | **T** | **T** | **G** | **T** | **T** | **T** | **T** | **C** | **A** | **T** | **A** | **T** | **T** | **T** | **T** | **C** | **A** | **T** | **T** | **C** | **G** | **A** | **T** | **T** | **A** | **A** | **A** | **C** | **A** | **A** | **A** | **A** | **C** | **C** | **G** | **G** | **T** | **G** | **G** | **T** | **T** | **A** | **T** | **T** | **A** | **G** | **A** | **G** | **C** | **T** | **T** | **C** | **T** | **T** | **C** | **A** | **G** | **A** | **G** | **A** | **A** | **A** | **A** | **G** | **T** | **C** | **T** | **T** | **T** | **T** | **A** | **T** | **T** | **T** | **A** | **C** | **C** | **A** | **C** | **T** | **A** | **A** | **T** | **T** | **G** | **T** | **G** | **C** | **A** | **C** | **A** | **T** | **T** | **G** | **T** | **T** | **C** | **T** | **C** | **A** | **T** | **A** | **T** | **C** | **C** | **T** | **T** | **A** | **C** | **T** | **A** | **A** | **T** | **A** | **G** | **G** | **C** | **T** | **T** | **A** | **T** | **T** | **G** | **A** | **C** | **C** | **T** | **T** | **T** | **T** | **T** | **A** | **C** | **T** | **G** | **A** | **T** | **T** | **T** | **C** | **A** | **T** | **T** | **G** | **G** | **T** | **T** | **C** | **A** | **A** | **A** | **A** | **A** | **G** | **G** | **G** | **C** | **A** | **A** | **T** | **A** | **T** | **G** | **G** | **A** | **C** | **G** | **G** | **A** | **T** | **T** | **G** | **A** | **A** | **G** | **A** | **A** | **A** | **A** | **A** | **C** | **T** | **T** | **C** | **G** | **G** | **T** | **T** | **A** | **T** | **T** | **G** | **G** | **C** | **C** | **A** | **A** | **C** | **G** | **G** | **T** | **A** | **A** | **T** | **T** | **A** | **T** | **G** | **A** | **A** | **A** | **G** | **T** | **C** | **C** | **A** | **G** | **T** | **C** | **T** | **T** | **A** | **A** | **A** | **C** | **A** | **A** | **A** | **C** | **C** | **A** | **A** | **C** | **G** | **A** | **C** | **C** | **A** | **G** | **T** | **G** | **A** | **A** | **A** | **A** | **T** | **C** | **A** | **A** | **G** | **A** | **C** | **C** | **A** | **T** | **T** | **A** | **T** | **C** | **T** | **A** | **A** | **C** | **C** | **G** | **A** | **A** | **G** | **T** | **C** | **G** | **A** | **A** | **C** | **A** | **A** | **G** | **A** | **T** | **A** | **T** | **T** | **T** | **G** | **G** | **T** | **C** | **G** | **A** | **T** | **T** | **A** | **A** | **A** | **A** | **A** | **T** | **A** | **A** | **A** | **T** | **T** | **A** | **T** | **T** | **A** | **G** | **A** | **G** | **A** | **T** | **G** | **T** | **C** | **T** | **A** | **A** | **A** | **G** | **A** | **A** | **T** | **T** | **G** | **C** | **A** | **A** | **T** | **T** | **A** | **T** | **T** | **A** | **A** | **A** | **C** | **A** | **G** | **T** | **C** | **G** | **A** | **C** | **C** | **G** | **C** | **A** | **A** | **T** | **T** | **A** | **A** | **T** | **G** | **G** | **A** | **T** | **G** | **G** | **G** | **C** | **A** | **A** | **A** | **C** | **A** | **A** | **A** | **A** | **G** | **A** | **A** | **G** | **A** | **A** | **A** | **T** | **T** | **T** | **T** | **A** | **G** | **A** | **G** | **A** | **A** | **C** | **G** | **A** | **G** | **C** | **G** | **G** | **C** | **A** | **T** | **C** | **G** | **T** | **T** | **T** | **G** | **G** | **C** | **G** | **C** | **G** | **G** | **G** | **A** | **G** | **T** | **T** | **G** | **C** | **A** | **T** | **G** | **A** | **T** | **T** | **C** | **A** | **G** | **T** | **C** | **A** | **G** | **T** | **C** | **A** | **A** | **C** | **A** | **A** | **C** | **T** | **T** | **T** | **T** | **T** | **G** | **C** | **A** | **G** | **C** | **C** | **A** | **T** | **G** | **A** | **T** | **G** | **A** | **T** | **G** | **T** | **T** | **G** | **T** | **C** | **T** | **G** | **C** | **C** | **T** | **T** | **A** | **A** | **A** | **T** | **G** | **A** | **A** | **C** | **A** | **A** | **G** | **C** | **A** | **C** | **A** | **A** | **C** | **G** | **A** | **A** | **C** | **A** | **G** | **A** | **A** | **A** | **C** | **C** | **C** | **C** | **G** | **G** | **A** | **A** | **C** | **C** | **A** | **T** | **A** | **T** | **C** | **G** | **T** | **A** | **A** | **A** | **C** | **A** | **A** | **C** | **T** | **A** | **G** | **C** | **C** | **A** | **T** | **G** | **G** | **T** | **G** | **G** | **C** | **A** | **G** | **A** | **A** | **A** | **T** | **C** | **A** | **T** | **T** | **A** | **A** | **T** | **G** | **C** | **C** | **T** | **C** | **C** | **C** | **A** | **A** | **T** | **C** | **G** | **G** | **A** | **A** | **A** | **T** | **G** | **C** | **G** | **C** | **G** | **C** | **G** | **T** | **T** | **A** | **C** | **T** | **A** | **T** | **T** | **G** | **C** | **A** | **C** | **T** | **T** | **G** | **C** | **G** | **T** | **C** | **C** | **T** | **A** | **T** | **C** | **A** | **G** | **T** | **C** | **T** | **A** | **G** | **A** | **A** | **G** | **G** | **A** | **A** | **A** | **A** | **A** | **G** | **T** | **T** | **T** | **G** | **C** | **G** | **T** | **A** | **A** | **A** | **G** | **G** | **T** | **A** | **T** | **T** | **G** | **A** | **A** | **C** | **A** | **A** | **T** | **T** | **A** | **C** | **T** | **G** | **A** | **A** | **A** | **G** | **A** | **A** | **C** | **T** | **A** | **C** | **A** | **A** | **A** | **C** | **A** | **A** | **A** | **A** | **A** | **T** | **T** | **A** | **A** | **A** | **A** | **T** | **T** | **G** | **A** | **A** | **T** | **T** | **G** | **A** | **T** | **T** | **T** | **G** | **G** | **G** | **A** | **T** | **G** | **T** | **T** | **G** | **A** | **A** | **G** | **A** | **T** | **G** | **T** | **T** | **C** | **A** | **T** | **T** | **T** | **A** | **A** | **A** | **T** | **A** | **G** | **C** | **A** | **G** | **C** | **A** | **T** | **T** | **G** | **A** | **G** | **G** | **A** | **T** | **C** | **A** | **T** | **C** | **T** | **T** | **T** | **T** | **C** | **C** | **G** | **A** | **A** | **T** | **T** | **G** | **T** | **G** | **C** | **A** | **G** | **G** | **A** | **A** | **T** | **T** | **A** | **C** | **T** | **T** | **T** | **C** | **A** | **A** | **A** | **T** | **A** | **C** | **C** | **T** | **T** | **A** | **A** | **G** | **A** | **C** | **A** | **T** | **G** | **C** | **C** | **A** | **A** | **A** | **G** | **C** | **A** | **A** | **A** | **G** | **G** | **A** | **A** | **T** | **T** | **A** | **G** | **A** | **G** | **G** | **T** | **A** | **T** | **A** | **C** | **T** | **T** | **A** | **C** | **A** | **C** | **C** | **A** | **A** | **G** | **T** | **C** | **G** | **A** | **T** | **A** | **A** | **A** | **A** | **A** | **C** | **G** | **T** | **G** | **T** | **T** | **A** | **T** | **T** | **G** | **C** | **G** | **T** | **A** | **T** | **T** | **G** | **T** | **T** | **G** | **A** | **T** | **G** | **A** | **C** | **G** | **G** | **T** | **G** | **T** | **C** | **G** | **G** | **C** | **T** | **T** | **T** | **G** | **A** | **T** | **A** | **T** | **G** | **A** | **A** | **G** | **G** | **A** | **A** | **C** | **A** | **A** | **A** | **G** | **T** | **A** | **A** | **T** | **A** | **A** | **A** | **G** | **C** | **C** | **G** | **G** | **T** | **A** | **G** | **T** | **T** | **A** | **T** | **G** | **G** | **C** | **T** | **T** | **A** | **A** | **A** | **T** | **A** | **A** | **T** | **A** | **T** | **C** | **C** | **G** | **A** | **G** | **A** | **A** | **C** | **G** | **T** | **G** | **T** | **T** | **G** | **T** | **C** | **G** | **G** | **C** | **A** | **T** | **G** | **G** | **G** | **C** | **G** | **G** | **T** | **A** | **C** | **A** | **G** | **T** | **T** | **A** | **A** | **A** | **A** | **T** | **T** | **A** | **T** | **T** | **A** | **G** | **T** | **T** | **T** | **T** | **A** | **A** | **A** | **G** | **G** | **G** | **C** | **A** | **G** | **G** | **G** | **A** | **A** | **C** | **C** | **A** | **G** | **C** | **G** | **T** | **T** | **G** | **A** | **A** | **A** | **T** | **T** | **A** | **A** | **A** | **G** | **T** | **T** | **C** | **C** | **T** | **G** | **T** | **C** | **A** | **T** | **A** | **A** | **A** | **G** | **G** | **A** | **G** | **G** | **A** | **A** | **A** | **C** | **T** | **G** | **C** | **A** | **A** | **G** | **T** | **G** | **A** | **T** | **C** | **A** | **A** | **A** | **G** | **T** | **A** | **A** | **T** | **G** | **T** | **T** | **A** | **G** | **T** | **G** | **G** | **A** | **-** | **-** | **-** |
| **N53766** | **A** | **T** | **G** | **A** | **C** | **C** | **G** | **A** | **T** | **C** | **G** | **G** | **A** | **T** | **T** | **T** | **C** | **A** | **A** | **G** | **A** | **C** | **G** | **C** | **A** | **T** | **G** | **A** | **T** | **T** | **T** | **C** | **A** | **T** | **T** | **A** | **T** | **A** | **T** | **G** | **C** | **G** | **T** | **C** | **C** | **C** | **T** | **T** | **A** | **G** | **C** | **A** | **C** | **C** | **T** | **T** | **T** | **A** | **T** | **T** | **G** | **T** | **T** | **A** | **T** | **C** | **T** | **T** | **A** | **A** | **T** | **T** | **A** | **C** | **A** | **T** | **T** | **G** | **T** | **T** | **T** | **T** | **C** | **A** | **T** | **A** | **T** | **T** | **T** | **T** | **C** | **A** | **T** | **T** | **C** | **G** | **A** | **T** | **T** | **A** | **A** | **A** | **C** | **A** | **A** | **A** | **A** | **C** | **C** | **G** | **G** | **T** | **G** | **G** | **T** | **T** | **A** | **T** | **T** | **A** | **G** | **A** | **G** | **C** | **T** | **T** | **C** | **T** | **T** | **C** | **A** | **G** | **A** | **G** | **A** | **A** | **A** | **A** | **G** | **T** | **C** | **T** | **T** | **T** | **T** | **A** | **T** | **T** | **T** | **A** | **C** | **C** | **A** | **C** | **T** | **A** | **A** | **T** | **T** | **G** | **T** | **G** | **C** | **A** | **C** | **A** | **T** | **T** | **G** | **T** | **T** | **C** | **T** | **C** | **A** | **T** | **A** | **T** | **C** | **C** | **T** | **T** | **A** | **C** | **T** | **A** | **A** | **T** | **A** | **G** | **G** | **C** | **T** | **T** | **A** | **T** | **T** | **G** | **A** | **C** | **C** | **T** | **T** | **T** | **T** | **T** | **A** | **C** | **T** | **G** | **A** | **T** | **T** | **T** | **C** | **A** | **T** | **T** | **G** | **G** | **T** | **T** | **C** | **A** | **A** | **A** | **A** | **A** | **G** | **G** | **G** | **C** | **A** | **A** | **T** | **A** | **T** | **G** | **G** | **A** | **C** | **G** | **G** | **A** | **T** | **T** | **G** | **A** | **A** | **G** | **A** | **A** | **A** | **A** | **A** | **C** | **T** | **T** | **C** | **G** | **G** | **T** | **T** | **A** | **T** | **T** | **G** | **G** | **C** | **C** | **A** | **A** | **C** | **G** | **G** | **T** | **A** | **A** | **T** | **T** | **A** | **T** | **G** | **A** | **A** | **A** | **G** | **T** | **C** | **C** | **A** | **G** | **T** | **C** | **T** | **T** | **A** | **A** | **A** | **C** | **A** | **A** | **A** | **C** | **C** | **A** | **A** | **C** | **G** | **A** | **C** | **C** | **A** | **G** | **T** | **G** | **A** | **A** | **A** | **A** | **T** | **C** | **A** | **A** | **G** | **A** | **C** | **C** | **A** | **T** | **T** | **A** | **T** | **C** | **T** | **A** | **A** | **C** | **C** | **G** | **A** | **A** | **G** | **T** | **C** | **G** | **A** | **A** | **C** | **A** | **A** | **G** | **A** | **T** | **A** | **T** | **T** | **T** | **G** | **G** | **T** | **C** | **G** | **A** | **T** | **T** | **A** | **A** | **A** | **A** | **A** | **T** | **A** | **A** | **A** | **T** | **T** | **A** | **T** | **T** | **A** | **G** | **A** | **G** | **A** | **T** | **G** | **T** | **C** | **T** | **A** | **A** | **A** | **G** | **A** | **A** | **T** | **T** | **G** | **C** | **A** | **A** | **T** | **T** | **A** | **T** | **T** | **A** | **A** | **A** | **C** | **A** | **G** | **T** | **C** | **G** | **A** | **C** | **C** | **G** | **C** | **A** | **A** | **T** | **T** | **A** | **A** | **T** | **G** | **G** | **A** | **T** | **G** | **G** | **G** | **C** | **A** | **A** | **A** | **C** | **A** | **A** | **A** | **A** | **G** | **A** | **A** | **G** | **A** | **A** | **A** | **T** | **T** | **T** | **T** | **A** | **G** | **A** | **G** | **A** | **A** | **C** | **G** | **A** | **G** | **C** | **G** | **G** | **C** | **A** | **T** | **C** | **G** | **T** | **T** | **T** | **G** | **G** | **C** | **G** | **C** | **G** | **G** | **G** | **A** | **G** | **T** | **T** | **G** | **C** | **A** | **T** | **G** | **A** | **T** | **T** | **C** | **A** | **G** | **T** | **C** | **A** | **G** | **T** | **C** | **A** | **A** | **C** | **A** | **A** | **C** | **T** | **T** | **T** | **T** | **T** | **G** | **C** | **A** | **G** | **C** | **C** | **A** | **T** | **G** | **A** | **T** | **G** | **A** | **T** | **G** | **T** | **T** | **G** | **T** | **C** | **T** | **G** | **C** | **C** | **T** | **T** | **A** | **A** | **A** | **T** | **G** | **A** | **A** | **C** | **A** | **A** | **G** | **C** | **A** | **C** | **A** | **A** | **C** | **G** | **A** | **A** | **C** | **A** | **G** | **A** | **A** | **A** | **C** | **C** | **C** | **C** | **G** | **G** | **A** | **A** | **C** | **C** | **A** | **T** | **A** | **T** | **C** | **G** | **T** | **A** | **A** | **A** | **C** | **A** | **A** | **C** | **T** | **A** | **G** | **C** | **C** | **A** | **T** | **G** | **G** | **T** | **G** | **G** | **C** | **A** | **G** | **A** | **A** | **A** | **T** | **C** | **A** | **T** | **T** | **A** | **A** | **T** | **G** | **C** | **C** | **T** | **C** | **C** | **C** | **A** | **A** | **T** | **C** | **G** | **G** | **A** | **A** | **A** | **T** | **G** | **C** | **G** | **C** | **G** | **C** | **G** | **T** | **T** | **A** | **C** | **T** | **A** | **T** | **T** | **G** | **C** | **A** | **C** | **T** | **T** | **G** | **C** | **G** | **T** | **C** | **C** | **T** | **A** | **T** | **C** | **A** | **G** | **T** | **C** | **T** | **A** | **G** | **A** | **A** | **G** | **G** | **A** | **A** | **A** | **A** | **A** | **G** | **T** | **T** | **T** | **G** | **C** | **G** | **T** | **A** | **A** | **A** | **G** | **G** | **T** | **A** | **T** | **T** | **G** | **A** | **A** | **C** | **A** | **A** | **T** | **T** | **A** | **C** | **T** | **G** | **A** | **A** | **A** | **G** | **A** | **A** | **C** | **T** | **A** | **C** | **A** | **A** | **A** | **C** | **A** | **A** | **A** | **A** | **A** | **T** | **T** | **A** | **A** | **A** | **A** | **T** | **T** | **G** | **A** | **A** | **T** | **T** | **G** | **A** | **T** | **T** | **T** | **G** | **G** | **G** | **A** | **T** | **G** | **T** | **T** | **G** | **A** | **A** | **G** | **A** | **T** | **G** | **T** | **T** | **C** | **A** | **T** | **T** | **T** | **A** | **A** | **A** | **T** | **A** | **G** | **C** | **A** | **G** | **C** | **A** | **T** | **T** | **G** | **A** | **G** | **G** | **A** | **T** | **C** | **A** | **T** | **C** | **T** | **T** | **T** | **T** | **C** | **C** | **G** | **A** | **A** | **T** | **T** | **G** | **T** | **G** | **C** | **A** | **G** | **G** | **A** | **A** | **T** | **T** | **A** | **C** | **T** | **T** | **T** | **C** | **A** | **A** | **A** | **T** | **A** | **C** | **C** | **T** | **T** | **A** | **A** | **G** | **A** | **C** | **A** | **T** | **G** | **C** | **C** | **A** | **A** | **A** | **G** | **C** | **A** | **A** | **A** | **G** | **G** | **A** | **A** | **T** | **T** | **A** | **G** | **A** | **G** | **G** | **T** | **A** | **T** | **A** | **C** | **T** | **T** | **A** | **C** | **A** | **C** | **C** | **A** | **A** | **G** | **T** | **C** | **G** | **A** | **T** | **A** | **A** | **A** | **A** | **A** | **C** | **G** | **T** | **G** | **T** | **T** | **A** | **T** | **T** | **G** | **C** | **G** | **T** | **A** | **T** | **T** | **G** | **T** | **T** | **G** | **A** | **T** | **G** | **A** | **C** | **G** | **G** | **T** | **G** | **T** | **C** | **G** | **G** | **C** | **T** | **T** | **T** | **G** | **A** | **T** | **A** | **T** | **G** | **A** | **A** | **G** | **G** | **A** | **A** | **C** | **A** | **A** | **A** | **G** | **T** | **A** | **A** | **T** | **A** | **A** | **A** | **G** | **C** | **C** | **G** | **G** | **T** | **A** | **G** | **T** | **T** | **A** | **T** | **G** | **G** | **C** | **T** | **T** | **A** | **A** | **A** | **T** | **A** | **A** | **T** | **A** | **T** | **C** | **C** | **G** | **A** | **G** | **A** | **A** | **C** | **G** | **T** | **G** | **T** | **T** | **G** | **T** | **C** | **G** | **G** | **C** | **A** | **T** | **G** | **G** | **G** | **C** | **G** | **G** | **T** | **A** | **C** | **A** | **G** | **T** | **T** | **A** | **A** | **A** | **A** | **T** | **T** | **A** | **T** | **T** | **A** | **G** | **T** | **T** | **T** | **T** | **A** | **A** | **A** | **G** | **G** | **G** | **C** | **A** | **G** | **G** | **G** | **A** | **A** | **C** | **C** | **A** | **G** | **C** | **G** | **T** | **T** | **G** | **A** | **A** | **A** | **T** | **T** | **A** | **A** | **A** | **G** | **T** | **T** | **C** | **C** | **T** | **G** | **T** | **C** | **A** | **T** | **A** | **A** | **A** | **G** | **G** | **A** | **G** | **G** | **A** | **A** | **A** | **C** | **T** | **G** | **C** | **A** | **A** | **G** | **T** | **G** | **A** | **T** | **C** | **A** | **A** | **A** | **G** | **T** | **A** | **A** | **T** | **G** | **T** | **T** | **A** | **G** | **T** | **G** | **G** | **A** | **-** | **-** | **-** |
| **N59584F** | **A** | **T** | **G** | **A** | **C** | **C** | **G** | **A** | **T** | **C** | **G** | **G** | **A** | **T** | **T** | **T** | **C** | **A** | **A** | **G** | **A** | **C** | **G** | **C** | **A** | **T** | **G** | **A** | **T** | **T** | **T** | **C** | **A** | **T** | **T** | **A** | **T** | **A** | **T** | **G** | **C** | **G** | **T** | **C** | **C** | **C** | **T** | **T** | **A** | **G** | **C** | **A** | **C** | **C** | **T** | **T** | **T** | **A** | **T** | **T** | **G** | **T** | **T** | **A** | **T** | **C** | **T** | **T** | **A** | **A** | **T** | **T** | **A** | **C** | **A** | **T** | **T** | **G** | **T** | **T** | **T** | **T** | **C** | **A** | **T** | **A** | **T** | **T** | **T** | **T** | **C** | **A** | **T** | **T** | **C** | **G** | **A** | **T** | **T** | **A** | **A** | **A** | **C** | **A** | **A** | **A** | **A** | **C** | **C** | **G** | **G** | **T** | **G** | **G** | **C** | **T** | **A** | **T** | **T** | **A** | **G** | **A** | **G** | **C** | **T** | **T** | **C** | **T** | **T** | **C** | **A** | **G** | **A** | **G** | **A** | **A** | **A** | **A** | **G** | **T** | **C** | **T** | **T** | **T** | **T** | **A** | **T** | **T** | **T** | **A** | **C** | **C** | **A** | **C** | **T** | **A** | **A** | **T** | **T** | **G** | **T** | **G** | **C** | **A** | **C** | **A** | **T** | **T** | **G** | **T** | **T** | **C** | **T** | **C** | **A** | **T** | **A** | **T** | **C** | **C** | **T** | **T** | **A** | **C** | **T** | **A** | **A** | **T** | **A** | **G** | **G** | **C** | **T** | **T** | **A** | **T** | **T** | **G** | **A** | **C** | **C** | **T** | **T** | **T** | **T** | **T** | **A** | **C** | **T** | **G** | **A** | **T** | **T** | **T** | **C** | **A** | **T** | **T** | **G** | **G** | **T** | **T** | **C** | **A** | **A** | **A** | **A** | **A** | **G** | **G** | **G** | **C** | **A** | **A** | **T** | **A** | **T** | **G** | **G** | **A** | **C** | **G** | **G** | **A** | **T** | **T** | **G** | **A** | **A** | **G** | **A** | **A** | **A** | **A** | **A** | **C** | **T** | **T** | **C** | **G** | **G** | **T** | **T** | **A** | **T** | **T** | **G** | **G** | **C** | **C** | **A** | **A** | **C** | **G** | **G** | **T** | **A** | **A** | **T** | **T** | **A** | **T** | **G** | **A** | **A** | **A** | **G** | **T** | **C** | **C** | **A** | **G** | **T** | **C** | **T** | **T** | **A** | **A** | **A** | **C** | **A** | **A** | **A** | **C** | **C** | **A** | **A** | **C** | **G** | **A** | **C** | **C** | **A** | **G** | **T** | **G** | **A** | **A** | **A** | **A** | **T** | **C** | **A** | **A** | **G** | **A** | **C** | **C** | **A** | **T** | **T** | **A** | **T** | **C** | **T** | **A** | **A** | **C** | **C** | **G** | **A** | **A** | **G** | **T** | **C** | **G** | **A** | **A** | **C** | **A** | **A** | **G** | **A** | **T** | **A** | **T** | **T** | **T** | **G** | **G** | **T** | **C** | **G** | **A** | **T** | **T** | **A** | **A** | **A** | **A** | **A** | **T** | **A** | **A** | **A** | **T** | **T** | **A** | **T** | **T** | **A** | **G** | **A** | **G** | **A** | **T** | **G** | **T** | **C** | **T** | **A** | **A** | **A** | **G** | **A** | **A** | **T** | **T** | **G** | **C** | **A** | **A** | **T** | **T** | **A** | **T** | **T** | **A** | **A** | **A** | **C** | **A** | **G** | **T** | **C** | **G** | **A** | **C** | **C** | **G** | **C** | **A** | **A** | **T** | **T** | **A** | **A** | **T** | **G** | **G** | **A** | **T** | **G** | **G** | **G** | **C** | **A** | **A** | **A** | **C** | **A** | **A** | **A** | **A** | **G** | **A** | **A** | **G** | **A** | **A** | **A** | **T** | **T** | **T** | **T** | **A** | **G** | **A** | **G** | **A** | **A** | **C** | **G** | **A** | **G** | **C** | **G** | **G** | **C** | **A** | **T** | **C** | **G** | **T** | **T** | **T** | **G** | **G** | **C** | **G** | **C** | **G** | **G** | **G** | **A** | **G** | **T** | **T** | **G** | **C** | **A** | **T** | **G** | **A** | **T** | **T** | **C** | **A** | **G** | **T** | **C** | **A** | **G** | **T** | **C** | **A** | **A** | **C** | **A** | **A** | **C** | **T** | **T** | **T** | **T** | **T** | **G** | **C** | **A** | **G** | **C** | **C** | **A** | **T** | **G** | **A** | **T** | **G** | **A** | **T** | **G** | **T** | **T** | **G** | **T** | **C** | **T** | **G** | **C** | **C** | **T** | **T** | **A** | **A** | **A** | **T** | **G** | **A** | **A** | **C** | **A** | **A** | **G** | **C** | **A** | **C** | **A** | **A** | **C** | **G** | **A** | **A** | **C** | **A** | **G** | **A** | **A** | **A** | **C** | **T** | **C** | **C** | **T** | **G** | **A** | **A** | **C** | **C** | **A** | **T** | **A** | **T** | **C** | **G** | **C** | **A** | **A** | **A** | **C** | **A** | **A** | **C** | **T** | **A** | **G** | **C** | **C** | **A** | **T** | **G** | **G** | **T** | **G** | **G** | **C** | **A** | **G** | **A** | **A** | **A** | **T** | **C** | **A** | **T** | **T** | **A** | **A** | **T** | **G** | **C** | **C** | **T** | **C** | **C** | **C** | **A** | **A** | **T** | **C** | **G** | **G** | **A** | **A** | **A** | **T** | **G** | **C** | **G** | **T** | **G** | **C** | **G** | **C** | **T** | **A** | **C** | **T** | **A** | **T** | **T** | **G** | **C** | **A** | **C** | **T** | **T** | **G** | **C** | **G** | **T** | **C** | **C** | **T** | **A** | **T** | **C** | **A** | **G** | **T** | **C** | **T** | **A** | **G** | **A** | **A** | **G** | **G** | **A** | **A** | **A** | **A** | **A** | **G** | **T** | **T** | **T** | **G** | **C** | **G** | **T** | **A** | **A** | **A** | **G** | **G** | **T** | **A** | **T** | **T** | **G** | **A** | **A** | **C** | **A** | **A** | **T** | **T** | **A** | **C** | **T** | **G** | **A** | **A** | **A** | **G** | **A** | **A** | **C** | **T** | **A** | **C** | **A** | **A** | **A** | **C** | **A** | **A** | **A** | **A** | **A** | **T** | **T** | **A** | **A** | **A** | **A** | **T** | **T** | **G** | **A** | **A** | **T** | **T** | **G** | **A** | **T** | **T** | **T** | **G** | **G** | **G** | **A** | **T** | **G** | **T** | **T** | **G** | **A** | **A** | **G** | **A** | **T** | **G** | **T** | **T** | **C** | **A** | **T** | **T** | **T** | **A** | **A** | **A** | **T** | **A** | **G** | **C** | **A** | **G** | **C** | **A** | **T** | **T** | **G** | **A** | **G** | **G** | **A** | **T** | **C** | **A** | **T** | **C** | **T** | **T** | **T** | **T** | **C** | **C** | **G** | **A** | **A** | **T** | **T** | **G** | **T** | **G** | **C** | **A** | **G** | **G** | **A** | **G** | **T** | **T** | **A** | **C** | **T** | **T** | **T** | **C** | **A** | **A** | **A** | **T** | **A** | **C** | **C** | **T** | **T** | **A** | **A** | **G** | **A** | **C** | **A** | **T** | **G** | **C** | **C** | **A** | **A** | **A** | **G** | **C** | **A** | **A** | **A** | **G** | **G** | **A** | **A** | **T** | **T** | **A** | **G** | **A** | **G** | **G** | **T** | **A** | **T** | **A** | **C** | **T** | **T** | **A** | **C** | **A** | **C** | **C** | **A** | **A** | **G** | **T** | **C** | **G** | **A** | **T** | **A** | **A** | **A** | **A** | **A** | **C** | **G** | **T** | **G** | **C** | **T** | **A** | **T** | **T** | **G** | **C** | **G** | **T** | **A** | **T** | **T** | **G** | **T** | **T** | **G** | **A** | **T** | **G** | **A** | **C** | **G** | **G** | **T** | **G** | **T** | **C** | **G** | **G** | **C** | **T** | **T** | **T** | **G** | **A** | **T** | **A** | **T** | **G** | **A** | **A** | **G** | **G** | **A** | **A** | **C** | **A** | **A** | **A** | **G** | **T** | **A** | **A** | **T** | **A** | **A** | **A** | **G** | **C** | **C** | **G** | **G** | **T** | **A** | **G** | **T** | **T** | **A** | **T** | **G** | **G** | **C** | **T** | **T** | **A** | **A** | **A** | **T** | **A** | **A** | **T** | **A** | **T** | **C** | **C** | **G** | **A** | **G** | **A** | **A** | **C** | **G** | **T** | **G** | **T** | **T** | **G** | **T** | **C** | **G** | **G** | **C** | **A** | **T** | **G** | **G** | **G** | **C** | **G** | **G** | **T** | **A** | **C** | **A** | **G** | **T** | **T** | **A** | **A** | **A** | **A** | **T** | **T** | **A** | **T** | **T** | **A** | **G** | **T** | **T** | **T** | **T** | **A** | **A** | **A** | **G** | **G** | **G** | **C** | **A** | **G** | **G** | **G** | **A** | **A** | **C** | **C** | **A** | **G** | **C** | **G** | **T** | **T** | **G** | **A** | **A** | **A** | **T** | **T** | **A** | **A** | **A** | **G** | **T** | **T** | **C** | **C** | **T** | **G** | **T** | **C** | **A** | **T** | **A** | **A** | **A** | **G** | **G** | **A** | **G** | **G** | **A** | **A** | **A** | **C** | **T** | **G** | **C** | **A** | **A** | **G** | **T** | **G** | **A** | **T** | **C** | **A** | **A** | **A** | **G** | **T** | **A** | **A** | **T** | **G** | **T** | **T** | **A** | **G** | **T** | **G** | **G** | **A** | **-** | **-** | **-** |
| **N53476** | **A** | **T** | **G** | **A** | **C** | **C** | **G** | **A** | **T** | **C** | **G** | **G** | **A** | **T** | **T** | **T** | **C** | **A** | **A** | **G** | **A** | **C** | **G** | **C** | **A** | **T** | **G** | **A** | **T** | **T** | **T** | **C** | **A** | **T** | **T** | **A** | **T** | **A** | **T** | **G** | **C** | **G** | **T** | **C** | **C** | **C** | **T** | **T** | **A** | **G** | **C** | **A** | **C** | **C** | **T** | **T** | **T** | **A** | **T** | **T** | **G** | **T** | **T** | **A** | **T** | **C** | **T** | **T** | **A** | **A** | **T** | **T** | **A** | **C** | **A** | **T** | **T** | **G** | **T** | **T** | **T** | **T** | **C** | **A** | **T** | **A** | **T** | **T** | **T** | **T** | **C** | **A** | **T** | **T** | **C** | **G** | **A** | **T** | **T** | **A** | **A** | **A** | **C** | **A** | **A** | **A** | **A** | **C** | **C** | **G** | **G** | **T** | **G** | **G** | **T** | **T** | **A** | **T** | **T** | **A** | **G** | **A** | **G** | **C** | **T** | **T** | **C** | **T** | **T** | **C** | **A** | **G** | **A** | **G** | **A** | **A** | **A** | **A** | **G** | **T** | **C** | **T** | **T** | **T** | **T** | **A** | **T** | **C** | **T** | **A** | **C** | **C** | **A** | **C** | **T** | **A** | **A** | **T** | **T** | **G** | **T** | **G** | **C** | **A** | **C** | **A** | **T** | **T** | **G** | **T** | **T** | **C** | **T** | **C** | **A** | **T** | **A** | **T** | **C** | **C** | **T** | **T** | **A** | **C** | **T** | **A** | **A** | **T** | **A** | **G** | **G** | **C** | **T** | **T** | **A** | **T** | **T** | **G** | **A** | **C** | **C** | **T** | **T** | **T** | **T** | **T** | **A** | **C** | **T** | **G** | **A** | **T** | **T** | **T** | **C** | **A** | **T** | **T** | **G** | **G** | **T** | **T** | **C** | **A** | **A** | **A** | **A** | **A** | **G** | **G** | **G** | **C** | **A** | **A** | **T** | **A** | **T** | **G** | **G** | **A** | **C** | **G** | **G** | **A** | **T** | **T** | **G** | **A** | **A** | **G** | **A** | **A** | **A** | **A** | **A** | **C** | **T** | **T** | **C** | **G** | **G** | **T** | **T** | **A** | **T** | **T** | **G** | **G** | **C** | **C** | **A** | **A** | **C** | **G** | **G** | **T** | **A** | **A** | **T** | **T** | **A** | **T** | **G** | **A** | **A** | **A** | **G** | **T** | **C** | **C** | **A** | **G** | **T** | **C** | **T** | **T** | **A** | **A** | **A** | **C** | **A** | **A** | **A** | **C** | **C** | **A** | **A** | **C** | **G** | **A** | **C** | **C** | **A** | **G** | **T** | **G** | **A** | **A** | **A** | **A** | **T** | **C** | **A** | **A** | **G** | **A** | **T** | **C** | **A** | **T** | **T** | **A** | **T** | **C** | **T** | **A** | **A** | **C** | **C** | **G** | **A** | **A** | **G** | **T** | **C** | **G** | **A** | **A** | **C** | **A** | **A** | **G** | **A** | **T** | **A** | **T** | **T** | **T** | **G** | **G** | **T** | **C** | **G** | **A** | **T** | **T** | **A** | **A** | **A** | **A** | **A** | **T** | **A** | **A** | **A** | **T** | **T** | **A** | **T** | **T** | **A** | **G** | **A** | **G** | **A** | **T** | **G** | **T** | **C** | **T** | **A** | **A** | **A** | **G** | **A** | **A** | **T** | **T** | **G** | **C** | **A** | **A** | **T** | **T** | **A** | **T** | **T** | **A** | **A** | **A** | **C** | **A** | **G** | **T** | **C** | **G** | **A** | **C** | **C** | **G** | **C** | **A** | **A** | **T** | **T** | **A** | **A** | **T** | **G** | **G** | **A** | **T** | **G** | **G** | **G** | **C** | **A** | **A** | **A** | **C** | **A** | **A** | **A** | **A** | **G** | **A** | **A** | **G** | **A** | **A** | **A** | **T** | **T** | **T** | **T** | **A** | **G** | **A** | **G** | **A** | **A** | **C** | **G** | **A** | **G** | **C** | **G** | **G** | **C** | **A** | **T** | **C** | **G** | **T** | **T** | **T** | **G** | **G** | **C** | **G** | **C** | **G** | **G** | **G** | **A** | **G** | **T** | **T** | **G** | **C** | **A** | **T** | **G** | **A** | **T** | **T** | **C** | **A** | **G** | **T** | **C** | **A** | **G** | **T** | **C** | **A** | **A** | **C** | **A** | **A** | **C** | **T** | **T** | **T** | **T** | **T** | **G** | **C** | **A** | **G** | **C** | **C** | **A** | **T** | **G** | **A** | **T** | **G** | **A** | **T** | **G** | **T** | **T** | **G** | **T** | **C** | **T** | **G** | **C** | **C** | **T** | **T** | **A** | **A** | **A** | **T** | **G** | **A** | **A** | **C** | **A** | **A** | **G** | **C** | **A** | **C** | **A** | **A** | **C** | **G** | **A** | **A** | **C** | **A** | **G** | **A** | **A** | **A** | **C** | **C** | **C** | **C** | **G** | **G** | **A** | **A** | **C** | **C** | **A** | **T** | **A** | **T** | **C** | **G** | **T** | **A** | **A** | **A** | **C** | **A** | **A** | **C** | **T** | **A** | **G** | **C** | **C** | **A** | **T** | **G** | **G** | **T** | **G** | **G** | **C** | **A** | **G** | **A** | **A** | **A** | **T** | **C** | **A** | **T** | **T** | **A** | **A** | **T** | **G** | **C** | **C** | **T** | **C** | **T** | **C** | **A** | **A** | **T** | **C** | **G** | **G** | **A** | **A** | **A** | **T** | **G** | **C** | **G** | **C** | **G** | **C** | **G** | **T** | **T** | **A** | **C** | **T** | **A** | **T** | **T** | **G** | **C** | **A** | **C** | **T** | **T** | **G** | **C** | **G** | **T** | **C** | **C** | **T** | **A** | **T** | **C** | **A** | **G** | **T** | **C** | **T** | **A** | **G** | **A** | **A** | **G** | **G** | **A** | **A** | **A** | **A** | **A** | **G** | **T** | **T** | **T** | **G** | **C** | **G** | **T** | **A** | **A** | **A** | **G** | **G** | **T** | **A** | **T** | **T** | **G** | **A** | **A** | **C** | **A** | **A** | **T** | **T** | **A** | **C** | **T** | **G** | **A** | **A** | **A** | **G** | **A** | **A** | **C** | **T** | **A** | **C** | **A** | **A** | **A** | **C** | **A** | **A** | **A** | **A** | **A** | **T** | **T** | **A** | **A** | **A** | **A** | **T** | **T** | **G** | **A** | **A** | **T** | **T** | **G** | **A** | **T** | **T** | **T** | **G** | **G** | **G** | **A** | **T** | **G** | **T** | **T** | **G** | **A** | **A** | **G** | **A** | **T** | **G** | **T** | **T** | **C** | **A** | **T** | **T** | **T** | **A** | **A** | **A** | **T** | **A** | **G** | **C** | **A** | **G** | **C** | **A** | **T** | **T** | **G** | **A** | **G** | **G** | **A** | **T** | **C** | **A** | **T** | **C** | **T** | **T** | **T** | **T** | **C** | **C** | **G** | **A** | **A** | **T** | **T** | **G** | **T** | **G** | **C** | **A** | **G** | **G** | **A** | **A** | **T** | **T** | **A** | **C** | **T** | **T** | **T** | **C** | **A** | **A** | **A** | **T** | **A** | **C** | **C** | **T** | **T** | **A** | **A** | **G** | **A** | **C** | **A** | **T** | **G** | **C** | **C** | **A** | **A** | **A** | **G** | **C** | **A** | **A** | **A** | **G** | **G** | **A** | **A** | **T** | **T** | **A** | **G** | **A** | **G** | **G** | **T** | **A** | **T** | **A** | **C** | **T** | **T** | **A** | **C** | **A** | **C** | **C** | **A** | **A** | **G** | **T** | **C** | **G** | **A** | **T** | **A** | **A** | **A** | **A** | **A** | **C** | **G** | **T** | **G** | **C** | **T** | **A** | **T** | **T** | **G** | **C** | **G** | **T** | **A** | **T** | **T** | **G** | **T** | **T** | **G** | **A** | **T** | **G** | **A** | **C** | **G** | **G** | **T** | **G** | **T** | **C** | **G** | **G** | **C** | **T** | **T** | **T** | **G** | **A** | **T** | **A** | **T** | **G** | **A** | **A** | **G** | **G** | **A** | **A** | **C** | **A** | **A** | **A** | **G** | **T** | **A** | **A** | **T** | **A** | **A** | **A** | **G** | **C** | **C** | **G** | **G** | **T** | **A** | **G** | **T** | **T** | **A** | **T** | **G** | **G** | **C** | **T** | **T** | **A** | **A** | **A** | **T** | **A** | **A** | **T** | **A** | **T** | **C** | **C** | **G** | **A** | **G** | **A** | **A** | **C** | **G** | **T** | **G** | **T** | **T** | **G** | **T** | **C** | **G** | **G** | **C** | **A** | **T** | **G** | **G** | **G** | **C** | **G** | **G** | **T** | **A** | **C** | **A** | **G** | **T** | **T** | **A** | **A** | **A** | **A** | **T** | **T** | **A** | **T** | **T** | **A** | **G** | **T** | **T** | **T** | **T** | **A** | **A** | **A** | **G** | **G** | **G** | **C** | **A** | **G** | **G** | **G** | **A** | **A** | **C** | **C** | **A** | **G** | **C** | **G** | **T** | **T** | **G** | **A** | **A** | **A** | **T** | **T** | **A** | **A** | **A** | **G** | **T** | **T** | **C** | **C** | **T** | **G** | **T** | **C** | **A** | **T** | **A** | **A** | **A** | **G** | **G** | **A** | **G** | **G** | **A** | **A** | **A** | **C** | **T** | **G** | **C** | **A** | **A** | **G** | **T** | **G** | **A** | **T** | **C** | **A** | **A** | **A** | **G** | **T** | **A** | **A** | **T** | **G** | **T** | **T** | **A** | **G** | **T** | **G** | **G** | **A** | **-** | **-** | **-** |
| **N52755** | **A** | **T** | **G** | **A** | **C** | **C** | **G** | **A** | **T** | **C** | **G** | **G** | **A** | **T** | **T** | **T** | **C** | **A** | **A** | **G** | **A** | **C** | **G** | **C** | **A** | **T** | **G** | **A** | **T** | **T** | **T** | **C** | **A** | **T** | **T** | **A** | **T** | **A** | **T** | **G** | **C** | **G** | **T** | **C** | **C** | **C** | **T** | **T** | **A** | **G** | **C** | **A** | **C** | **C** | **T** | **T** | **T** | **A** | **T** | **T** | **G** | **T** | **T** | **A** | **T** | **C** | **T** | **T** | **A** | **A** | **T** | **T** | **A** | **C** | **A** | **T** | **T** | **G** | **T** | **T** | **T** | **T** | **C** | **A** | **T** | **A** | **T** | **T** | **T** | **T** | **C** | **A** | **T** | **T** | **C** | **G** | **A** | **T** | **T** | **A** | **A** | **A** | **C** | **A** | **A** | **A** | **A** | **C** | **C** | **G** | **G** | **T** | **G** | **G** | **T** | **T** | **A** | **T** | **T** | **A** | **G** | **A** | **G** | **C** | **T** | **T** | **C** | **T** | **T** | **C** | **A** | **G** | **A** | **G** | **A** | **A** | **A** | **A** | **G** | **T** | **C** | **T** | **T** | **T** | **T** | **A** | **T** | **T** | **T** | **A** | **C** | **C** | **A** | **C** | **T** | **A** | **A** | **T** | **T** | **G** | **T** | **G** | **C** | **A** | **C** | **A** | **T** | **T** | **G** | **T** | **T** | **C** | **T** | **C** | **A** | **T** | **A** | **T** | **C** | **C** | **T** | **T** | **A** | **C** | **T** | **A** | **A** | **T** | **A** | **G** | **G** | **C** | **T** | **T** | **A** | **T** | **T** | **G** | **A** | **C** | **C** | **T** | **T** | **T** | **T** | **T** | **A** | **C** | **T** | **G** | **A** | **T** | **T** | **T** | **C** | **A** | **T** | **T** | **G** | **G** | **T** | **T** | **C** | **A** | **A** | **A** | **A** | **A** | **G** | **G** | **G** | **C** | **A** | **A** | **T** | **A** | **T** | **G** | **G** | **A** | **C** | **G** | **G** | **A** | **T** | **T** | **G** | **A** | **A** | **G** | **A** | **A** | **A** | **A** | **A** | **C** | **T** | **T** | **C** | **G** | **G** | **T** | **T** | **A** | **T** | **T** | **G** | **G** | **C** | **C** | **A** | **A** | **C** | **G** | **G** | **T** | **A** | **A** | **T** | **T** | **A** | **T** | **G** | **A** | **A** | **A** | **G** | **T** | **C** | **C** | **A** | **G** | **T** | **C** | **T** | **T** | **A** | **A** | **A** | **C** | **A** | **A** | **A** | **C** | **C** | **A** | **A** | **C** | **G** | **A** | **C** | **C** | **A** | **G** | **T** | **G** | **A** | **A** | **A** | **A** | **T** | **C** | **A** | **A** | **G** | **A** | **C** | **C** | **A** | **T** | **T** | **A** | **T** | **C** | **T** | **A** | **A** | **C** | **C** | **G** | **A** | **A** | **G** | **T** | **C** | **G** | **A** | **A** | **C** | **A** | **A** | **G** | **A** | **T** | **A** | **T** | **T** | **T** | **G** | **G** | **T** | **C** | **G** | **A** | **T** | **T** | **A** | **A** | **A** | **A** | **A** | **T** | **A** | **A** | **A** | **T** | **T** | **A** | **T** | **T** | **A** | **G** | **A** | **G** | **A** | **T** | **G** | **T** | **C** | **T** | **A** | **A** | **A** | **G** | **A** | **A** | **T** | **T** | **G** | **C** | **A** | **A** | **T** | **T** | **A** | **T** | **T** | **A** | **A** | **A** | **C** | **A** | **G** | **T** | **C** | **G** | **A** | **C** | **C** | **G** | **C** | **A** | **A** | **T** | **T** | **A** | **A** | **T** | **G** | **G** | **A** | **T** | **G** | **G** | **G** | **C** | **A** | **A** | **A** | **C** | **A** | **A** | **A** | **A** | **G** | **A** | **A** | **G** | **A** | **A** | **A** | **T** | **T** | **T** | **T** | **A** | **G** | **A** | **G** | **A** | **A** | **C** | **G** | **A** | **G** | **C** | **G** | **G** | **C** | **A** | **T** | **C** | **G** | **T** | **T** | **T** | **G** | **G** | **C** | **G** | **C** | **G** | **G** | **G** | **A** | **G** | **T** | **T** | **G** | **C** | **A** | **T** | **G** | **A** | **T** | **T** | **C** | **A** | **G** | **T** | **C** | **A** | **G** | **T** | **C** | **A** | **A** | **C** | **A** | **A** | **C** | **T** | **T** | **T** | **T** | **T** | **G** | **C** | **A** | **G** | **C** | **C** | **A** | **T** | **G** | **A** | **T** | **G** | **A** | **T** | **G** | **T** | **T** | **G** | **T** | **C** | **T** | **G** | **C** | **A** | **T** | **T** | **A** | **A** | **A** | **T** | **G** | **A** | **A** | **C** | **A** | **A** | **G** | **C** | **A** | **C** | **A** | **A** | **C** | **G** | **A** | **A** | **C** | **A** | **G** | **A** | **A** | **A** | **C** | **C** | **C** | **C** | **G** | **G** | **A** | **A** | **C** | **C** | **A** | **T** | **A** | **T** | **C** | **G** | **T** | **A** | **A** | **A** | **C** | **A** | **A** | **C** | **T** | **A** | **G** | **C** | **C** | **A** | **T** | **G** | **G** | **T** | **G** | **G** | **C** | **A** | **G** | **A** | **A** | **A** | **T** | **C** | **A** | **T** | **T** | **A** | **A** | **T** | **G** | **C** | **C** | **T** | **C** | **C** | **C** | **A** | **A** | **T** | **C** | **G** | **G** | **A** | **A** | **A** | **T** | **G** | **C** | **G** | **C** | **G** | **C** | **G** | **C** | **T** | **A** | **C** | **T** | **A** | **T** | **T** | **G** | **C** | **A** | **C** | **T** | **T** | **G** | **C** | **G** | **T** | **C** | **C** | **T** | **A** | **T** | **C** | **A** | **G** | **T** | **C** | **T** | **A** | **G** | **A** | **A** | **G** | **G** | **A** | **A** | **A** | **A** | **A** | **G** | **T** | **T** | **T** | **G** | **C** | **G** | **T** | **A** | **A** | **A** | **G** | **G** | **T** | **A** | **T** | **T** | **G** | **A** | **A** | **C** | **A** | **A** | **T** | **T** | **A** | **C** | **T** | **G** | **A** | **A** | **A** | **G** | **A** | **A** | **C** | **T** | **A** | **C** | **A** | **A** | **A** | **C** | **A** | **A** | **A** | **A** | **A** | **T** | **T** | **A** | **A** | **A** | **A** | **T** | **T** | **G** | **A** | **A** | **T** | **T** | **G** | **A** | **T** | **T** | **T** | **G** | **G** | **G** | **A** | **T** | **G** | **T** | **T** | **G** | **A** | **A** | **G** | **A** | **T** | **G** | **T** | **T** | **C** | **A** | **T** | **T** | **T** | **A** | **A** | **A** | **T** | **A** | **G** | **C** | **A** | **G** | **C** | **A** | **T** | **T** | **G** | **A** | **G** | **G** | **A** | **T** | **C** | **A** | **T** | **C** | **T** | **T** | **T** | **T** | **C** | **C** | **G** | **A** | **A** | **T** | **T** | **G** | **T** | **G** | **C** | **A** | **G** | **G** | **A** | **G** | **T** | **T** | **A** | **C** | **T** | **T** | **T** | **C** | **A** | **A** | **A** | **T** | **A** | **C** | **C** | **T** | **T** | **A** | **A** | **G** | **A** | **C** | **A** | **T** | **G** | **C** | **C** | **A** | **A** | **A** | **G** | **C** | **A** | **A** | **A** | **G** | **G** | **A** | **A** | **T** | **T** | **A** | **G** | **A** | **G** | **G** | **T** | **A** | **T** | **A** | **C** | **T** | **T** | **A** | **C** | **A** | **C** | **C** | **A** | **A** | **G** | **T** | **C** | **G** | **A** | **T** | **A** | **A** | **A** | **A** | **A** | **C** | **G** | **T** | **G** | **T** | **T** | **A** | **T** | **T** | **G** | **C** | **G** | **T** | **A** | **T** | **T** | **G** | **T** | **T** | **G** | **A** | **T** | **G** | **A** | **T** | **G** | **G** | **T** | **G** | **T** | **C** | **G** | **G** | **C** | **T** | **T** | **T** | **G** | **A** | **T** | **A** | **T** | **G** | **A** | **A** | **G** | **G** | **A** | **A** | **C** | **A** | **A** | **A** | **G** | **T** | **A** | **A** | **T** | **A** | **A** | **A** | **G** | **C** | **C** | **G** | **G** | **T** | **A** | **G** | **T** | **T** | **A** | **T** | **G** | **G** | **C** | **T** | **T** | **A** | **A** | **A** | **T** | **A** | **A** | **T** | **A** | **T** | **T** | **C** | **G** | **A** | **G** | **A** | **A** | **C** | **G** | **T** | **G** | **T** | **T** | **G** | **T** | **C** | **G** | **G** | **C** | **A** | **T** | **G** | **G** | **G** | **C** | **G** | **G** | **T** | **A** | **C** | **A** | **G** | **T** | **T** | **A** | **A** | **A** | **A** | **T** | **T** | **A** | **T** | **T** | **A** | **G** | **T** | **T** | **T** | **T** | **A** | **A** | **A** | **G** | **G** | **G** | **C** | **A** | **G** | **G** | **G** | **A** | **A** | **C** | **C** | **A** | **G** | **C** | **G** | **T** | **T** | **G** | **A** | **A** | **A** | **T** | **T** | **A** | **A** | **A** | **G** | **T** | **T** | **C** | **C** | **T** | **G** | **T** | **C** | **A** | **T** | **A** | **A** | **A** | **G** | **G** | **A** | **G** | **G** | **A** | **A** | **A** | **C** | **T** | **G** | **C** | **A** | **A** | **G** | **T** | **G** | **A** | **T** | **C** | **A** | **A** | **A** | **G** | **T** | **A** | **A** | **T** | **G** | **T** | **T** | **A** | **G** | **T** | **G** | **G** | **A** | **-** | **-** | **-** |
| **N52789** | **A** | **T** | **G** | **A** | **C** | **C** | **G** | **A** | **T** | **C** | **G** | **G** | **A** | **T** | **T** | **T** | **C** | **A** | **A** | **G** | **A** | **C** | **G** | **C** | **A** | **T** | **G** | **A** | **T** | **T** | **T** | **C** | **A** | **T** | **T** | **A** | **T** | **A** | **T** | **G** | **C** | **G** | **T** | **C** | **C** | **C** | **T** | **T** | **A** | **G** | **C** | **A** | **C** | **C** | **T** | **T** | **T** | **A** | **T** | **T** | **G** | **T** | **T** | **A** | **T** | **C** | **T** | **T** | **A** | **A** | **T** | **T** | **A** | **C** | **A** | **T** | **T** | **G** | **T** | **T** | **T** | **T** | **C** | **A** | **T** | **A** | **T** | **T** | **T** | **T** | **C** | **A** | **T** | **T** | **C** | **G** | **A** | **T** | **T** | **A** | **A** | **A** | **C** | **A** | **A** | **A** | **A** | **C** | **C** | **G** | **G** | **T** | **G** | **G** | **C** | **T** | **A** | **T** | **T** | **A** | **G** | **A** | **G** | **C** | **T** | **T** | **C** | **T** | **T** | **C** | **A** | **G** | **A** | **G** | **A** | **A** | **A** | **A** | **G** | **T** | **C** | **T** | **T** | **T** | **T** | **A** | **T** | **C** | **T** | **A** | **C** | **C** | **A** | **C** | **T** | **A** | **A** | **T** | **T** | **G** | **T** | **G** | **C** | **A** | **C** | **A** | **T** | **T** | **G** | **T** | **T** | **C** | **T** | **C** | **A** | **T** | **A** | **T** | **C** | **C** | **T** | **T** | **A** | **C** | **T** | **A** | **A** | **T** | **A** | **G** | **G** | **C** | **T** | **T** | **A** | **T** | **T** | **G** | **A** | **C** | **C** | **T** | **T** | **T** | **T** | **T** | **A** | **C** | **T** | **G** | **A** | **T** | **T** | **T** | **C** | **A** | **T** | **T** | **G** | **G** | **T** | **T** | **C** | **A** | **A** | **A** | **A** | **A** | **G** | **G** | **G** | **C** | **A** | **A** | **T** | **A** | **T** | **G** | **G** | **A** | **C** | **G** | **G** | **A** | **T** | **T** | **G** | **A** | **A** | **G** | **A** | **A** | **A** | **A** | **A** | **C** | **T** | **T** | **C** | **G** | **G** | **T** | **T** | **A** | **T** | **T** | **G** | **G** | **C** | **C** | **A** | **A** | **T** | **G** | **G** | **C** | **A** | **A** | **T** | **T** | **A** | **T** | **G** | **A** | **A** | **A** | **G** | **T** | **C** | **C** | **A** | **G** | **T** | **C** | **T** | **T** | **A** | **A** | **A** | **C** | **A** | **A** | **A** | **C** | **C** | **A** | **A** | **C** | **G** | **A** | **C** | **C** | **A** | **G** | **T** | **G** | **A** | **A** | **A** | **A** | **T** | **C** | **A** | **A** | **G** | **A** | **T** | **C** | **A** | **T** | **T** | **A** | **T** | **C** | **T** | **A** | **A** | **C** | **C** | **G** | **A** | **A** | **G** | **T** | **C** | **G** | **A** | **A** | **C** | **A** | **A** | **G** | **A** | **T** | **A** | **T** | **T** | **T** | **G** | **G** | **T** | **C** | **G** | **A** | **T** | **T** | **A** | **A** | **A** | **A** | **A** | **T** | **A** | **A** | **A** | **T** | **T** | **A** | **T** | **T** | **A** | **G** | **A** | **G** | **A** | **T** | **G** | **T** | **C** | **T** | **A** | **A** | **A** | **G** | **A** | **A** | **T** | **T** | **G** | **C** | **A** | **A** | **T** | **T** | **A** | **T** | **T** | **A** | **A** | **A** | **C** | **A** | **G** | **T** | **C** | **G** | **G** | **C** | **C** | **G** | **C** | **A** | **A** | **T** | **T** | **A** | **A** | **T** | **G** | **G** | **A** | **T** | **G** | **G** | **G** | **C** | **A** | **A** | **A** | **C** | **A** | **A** | **A** | **A** | **G** | **A** | **A** | **G** | **A** | **A** | **A** | **T** | **T** | **T** | **T** | **A** | **G** | **A** | **G** | **A** | **A** | **C** | **G** | **A** | **G** | **C** | **G** | **G** | **C** | **A** | **T** | **C** | **G** | **T** | **T** | **T** | **G** | **G** | **C** | **G** | **C** | **G** | **G** | **G** | **A** | **G** | **T** | **T** | **G** | **C** | **A** | **T** | **G** | **A** | **T** | **T** | **C** | **A** | **G** | **T** | **C** | **A** | **G** | **T** | **C** | **A** | **A** | **C** | **A** | **A** | **C** | **T** | **T** | **T** | **T** | **T** | **G** | **C** | **A** | **G** | **C** | **C** | **A** | **T** | **G** | **A** | **T** | **G** | **A** | **T** | **G** | **T** | **T** | **G** | **T** | **C** | **T** | **G** | **C** | **C** | **T** | **T** | **A** | **A** | **A** | **T** | **G** | **A** | **A** | **C** | **A** | **A** | **G** | **C** | **A** | **C** | **A** | **A** | **C** | **G** | **A** | **A** | **C** | **A** | **G** | **A** | **A** | **A** | **C** | **C** | **C** | **C** | **G** | **G** | **A** | **A** | **C** | **C** | **A** | **T** | **A** | **T** | **C** | **G** | **T** | **A** | **A** | **A** | **C** | **A** | **A** | **C** | **T** | **A** | **G** | **C** | **C** | **A** | **T** | **G** | **G** | **T** | **G** | **G** | **C** | **A** | **G** | **A** | **A** | **A** | **T** | **C** | **A** | **T** | **T** | **A** | **A** | **T** | **G** | **C** | **C** | **T** | **C** | **C** | **C** | **A** | **A** | **T** | **C** | **G** | **G** | **A** | **A** | **A** | **T** | **G** | **C** | **G** | **C** | **G** | **C** | **G** | **T** | **T** | **A** | **C** | **T** | **A** | **T** | **T** | **G** | **C** | **A** | **C** | **T** | **T** | **G** | **C** | **G** | **T** | **C** | **C** | **T** | **A** | **T** | **C** | **A** | **G** | **T** | **C** | **T** | **A** | **G** | **A** | **A** | **G** | **G** | **A** | **A** | **A** | **A** | **A** | **G** | **T** | **T** | **T** | **G** | **C** | **G** | **T** | **A** | **A** | **A** | **G** | **G** | **T** | **A** | **T** | **T** | **G** | **A** | **A** | **C** | **A** | **A** | **T** | **T** | **A** | **C** | **T** | **G** | **A** | **A** | **A** | **G** | **A** | **A** | **C** | **T** | **A** | **C** | **A** | **A** | **A** | **C** | **A** | **A** | **A** | **A** | **A** | **T** | **T** | **A** | **A** | **A** | **A** | **T** | **T** | **G** | **A** | **A** | **T** | **T** | **G** | **A** | **T** | **T** | **T** | **G** | **G** | **G** | **A** | **T** | **G** | **T** | **T** | **G** | **A** | **A** | **G** | **A** | **T** | **G** | **T** | **T** | **C** | **A** | **T** | **T** | **T** | **A** | **A** | **A** | **T** | **A** | **G** | **C** | **A** | **G** | **C** | **A** | **T** | **T** | **G** | **A** | **G** | **G** | **A** | **T** | **C** | **A** | **T** | **C** | **T** | **T** | **T** | **T** | **C** | **C** | **G** | **A** | **A** | **T** | **T** | **G** | **T** | **G** | **C** | **A** | **G** | **G** | **A** | **A** | **T** | **T** | **A** | **C** | **T** | **T** | **T** | **C** | **C** | **A** | **A** | **T** | **A** | **C** | **C** | **T** | **T** | **A** | **A** | **G** | **A** | **C** | **A** | **T** | **G** | **C** | **C** | **A** | **A** | **A** | **G** | **C** | **A** | **A** | **A** | **G** | **G** | **A** | **A** | **T** | **T** | **A** | **G** | **A** | **G** | **G** | **T** | **A** | **T** | **A** | **C** | **T** | **T** | **A** | **C** | **A** | **C** | **C** | **A** | **A** | **G** | **T** | **C** | **G** | **A** | **T** | **A** | **A** | **A** | **A** | **A** | **C** | **G** | **T** | **G** | **T** | **T** | **A** | **T** | **T** | **G** | **C** | **G** | **T** | **A** | **T** | **T** | **G** | **T** | **T** | **G** | **A** | **T** | **G** | **A** | **T** | **G** | **G** | **T** | **G** | **T** | **C** | **G** | **G** | **C** | **T** | **T** | **T** | **G** | **A** | **T** | **A** | **T** | **G** | **A** | **A** | **G** | **G** | **A** | **A** | **C** | **A** | **A** | **A** | **G** | **T** | **A** | **A** | **T** | **A** | **A** | **A** | **G** | **C** | **C** | **G** | **G** | **T** | **A** | **G** | **T** | **T** | **A** | **T** | **G** | **G** | **C** | **T** | **T** | **A** | **A** | **A** | **T** | **A** | **A** | **T** | **A** | **T** | **T** | **C** | **G** | **A** | **G** | **A** | **A** | **C** | **G** | **T** | **G** | **T** | **T** | **G** | **T** | **C** | **G** | **G** | **C** | **A** | **T** | **G** | **G** | **G** | **C** | **G** | **G** | **T** | **A** | **C** | **A** | **G** | **T** | **T** | **A** | **A** | **A** | **A** | **T** | **T** | **A** | **T** | **T** | **A** | **G** | **T** | **T** | **T** | **T** | **A** | **A** | **A** | **G** | **G** | **G** | **C** | **A** | **G** | **G** | **G** | **A** | **A** | **C** | **C** | **A** | **G** | **C** | **G** | **T** | **T** | **G** | **A** | **A** | **A** | **T** | **T** | **A** | **A** | **A** | **G** | **T** | **T** | **C** | **C** | **T** | **G** | **T** | **C** | **A** | **T** | **A** | **A** | **A** | **G** | **G** | **A** | **G** | **G** | **A** | **A** | **A** | **C** | **T** | **G** | **C** | **A** | **A** | **G** | **T** | **G** | **A** | **T** | **C** | **A** | **A** | **A** | **G** | **T** | **A** | **A** | **T** | **G** | **T** | **T** | **A** | **G** | **T** | **G** | **G** | **A** | **-** | **-** | **-** |
| **N59462F** | **A** | **T** | **G** | **A** | **C** | **C** | **G** | **A** | **T** | **C** | **G** | **G** | **A** | **T** | **T** | **T** | **C** | **A** | **A** | **G** | **A** | **C** | **G** | **C** | **A** | **T** | **G** | **A** | **T** | **T** | **T** | **C** | **A** | **T** | **T** | **A** | **T** | **A** | **T** | **G** | **C** | **G** | **T** | **C** | **C** | **C** | **T** | **T** | **A** | **G** | **C** | **A** | **C** | **C** | **T** | **T** | **T** | **A** | **T** | **T** | **G** | **T** | **T** | **A** | **T** | **C** | **T** | **T** | **A** | **A** | **T** | **T** | **A** | **C** | **A** | **T** | **T** | **G** | **T** | **T** | **T** | **T** | **C** | **A** | **T** | **A** | **T** | **T** | **T** | **T** | **C** | **A** | **T** | **T** | **C** | **G** | **A** | **T** | **T** | **A** | **A** | **A** | **C** | **A** | **A** | **A** | **A** | **C** | **C** | **G** | **G** | **T** | **G** | **G** | **C** | **T** | **A** | **T** | **T** | **A** | **G** | **A** | **G** | **C** | **T** | **T** | **C** | **T** | **T** | **C** | **A** | **G** | **A** | **G** | **A** | **A** | **A** | **A** | **G** | **T** | **C** | **T** | **T** | **T** | **T** | **A** | **T** | **C** | **T** | **A** | **C** | **C** | **A** | **C** | **T** | **A** | **A** | **T** | **T** | **G** | **T** | **G** | **C** | **A** | **C** | **A** | **T** | **T** | **G** | **T** | **T** | **C** | **T** | **C** | **A** | **T** | **A** | **T** | **C** | **C** | **T** | **T** | **A** | **C** | **T** | **A** | **A** | **T** | **A** | **G** | **G** | **C** | **T** | **T** | **A** | **T** | **T** | **G** | **A** | **C** | **C** | **T** | **T** | **T** | **T** | **T** | **A** | **C** | **T** | **G** | **A** | **T** | **T** | **T** | **C** | **A** | **T** | **T** | **G** | **G** | **T** | **T** | **C** | **A** | **A** | **A** | **A** | **A** | **G** | **G** | **G** | **C** | **A** | **A** | **T** | **A** | **T** | **G** | **G** | **A** | **C** | **G** | **G** | **A** | **T** | **T** | **G** | **A** | **A** | **G** | **A** | **A** | **A** | **A** | **A** | **C** | **T** | **T** | **C** | **G** | **G** | **T** | **T** | **A** | **T** | **T** | **G** | **G** | **C** | **C** | **A** | **A** | **C** | **G** | **G** | **T** | **A** | **A** | **T** | **T** | **A** | **T** | **G** | **A** | **A** | **A** | **G** | **T** | **C** | **C** | **A** | **G** | **T** | **C** | **T** | **T** | **A** | **A** | **A** | **C** | **A** | **A** | **A** | **C** | **C** | **A** | **A** | **C** | **G** | **A** | **C** | **C** | **A** | **G** | **T** | **G** | **A** | **A** | **A** | **A** | **T** | **C** | **A** | **A** | **G** | **A** | **C** | **C** | **A** | **T** | **T** | **A** | **T** | **C** | **T** | **A** | **A** | **C** | **C** | **G** | **A** | **A** | **G** | **T** | **C** | **G** | **A** | **A** | **C** | **A** | **A** | **G** | **A** | **T** | **A** | **T** | **T** | **T** | **G** | **G** | **T** | **C** | **G** | **A** | **T** | **T** | **A** | **A** | **A** | **A** | **A** | **T** | **A** | **A** | **A** | **T** | **T** | **A** | **T** | **T** | **A** | **G** | **A** | **G** | **A** | **T** | **G** | **T** | **C** | **T** | **A** | **A** | **A** | **G** | **A** | **A** | **T** | **T** | **G** | **C** | **A** | **A** | **T** | **T** | **A** | **T** | **T** | **A** | **A** | **A** | **C** | **A** | **G** | **T** | **C** | **G** | **A** | **C** | **C** | **G** | **C** | **A** | **A** | **T** | **T** | **A** | **A** | **T** | **G** | **G** | **A** | **T** | **G** | **G** | **G** | **C** | **A** | **A** | **A** | **C** | **A** | **A** | **A** | **A** | **G** | **A** | **A** | **G** | **A** | **A** | **A** | **T** | **T** | **T** | **T** | **A** | **G** | **A** | **G** | **A** | **A** | **C** | **G** | **A** | **G** | **C** | **G** | **G** | **C** | **A** | **T** | **C** | **G** | **T** | **T** | **T** | **G** | **G** | **C** | **G** | **C** | **G** | **G** | **G** | **A** | **G** | **T** | **T** | **G** | **C** | **A** | **T** | **G** | **A** | **T** | **T** | **C** | **A** | **G** | **T** | **C** | **A** | **G** | **T** | **C** | **A** | **A** | **C** | **A** | **A** | **C** | **T** | **T** | **T** | **T** | **T** | **G** | **C** | **A** | **G** | **C** | **C** | **A** | **T** | **G** | **A** | **T** | **G** | **A** | **T** | **G** | **T** | **T** | **G** | **T** | **C** | **T** | **G** | **C** | **A** | **T** | **T** | **A** | **A** | **A** | **T** | **G** | **A** | **A** | **C** | **A** | **A** | **G** | **C** | **A** | **C** | **A** | **A** | **C** | **G** | **A** | **A** | **C** | **A** | **G** | **A** | **A** | **A** | **C** | **C** | **C** | **C** | **G** | **G** | **A** | **A** | **C** | **C** | **A** | **T** | **A** | **T** | **C** | **G** | **T** | **A** | **A** | **A** | **C** | **A** | **A** | **C** | **T** | **A** | **G** | **C** | **C** | **A** | **T** | **G** | **G** | **T** | **G** | **G** | **C** | **A** | **G** | **A** | **A** | **A** | **T** | **C** | **A** | **T** | **T** | **A** | **A** | **T** | **G** | **C** | **C** | **T** | **C** | **C** | **C** | **A** | **A** | **T** | **C** | **G** | **G** | **A** | **A** | **A** | **T** | **G** | **C** | **G** | **C** | **G** | **C** | **G** | **C** | **T** | **A** | **C** | **T** | **A** | **T** | **T** | **G** | **C** | **A** | **C** | **T** | **T** | **G** | **C** | **G** | **T** | **C** | **C** | **T** | **A** | **T** | **C** | **A** | **G** | **T** | **C** | **T** | **A** | **G** | **A** | **A** | **G** | **G** | **A** | **A** | **A** | **A** | **A** | **G** | **T** | **T** | **T** | **G** | **C** | **G** | **T** | **A** | **A** | **A** | **G** | **G** | **T** | **A** | **T** | **T** | **G** | **A** | **A** | **C** | **A** | **A** | **T** | **T** | **A** | **C** | **T** | **G** | **A** | **A** | **A** | **G** | **A** | **A** | **C** | **T** | **A** | **C** | **A** | **A** | **A** | **C** | **A** | **A** | **A** | **A** | **A** | **T** | **T** | **A** | **A** | **A** | **A** | **T** | **T** | **G** | **A** | **A** | **T** | **T** | **G** | **A** | **T** | **T** | **T** | **G** | **G** | **G** | **A** | **T** | **G** | **T** | **T** | **G** | **A** | **A** | **G** | **A** | **T** | **G** | **T** | **T** | **C** | **A** | **T** | **T** | **T** | **A** | **A** | **A** | **T** | **A** | **G** | **C** | **A** | **G** | **C** | **A** | **T** | **T** | **G** | **A** | **G** | **G** | **A** | **T** | **C** | **A** | **T** | **C** | **T** | **T** | **T** | **T** | **C** | **C** | **G** | **A** | **A** | **T** | **T** | **G** | **T** | **G** | **C** | **A** | **G** | **G** | **A** | **G** | **T** | **T** | **A** | **C** | **T** | **T** | **T** | **C** | **A** | **A** | **A** | **T** | **A** | **C** | **C** | **T** | **T** | **A** | **A** | **G** | **A** | **C** | **A** | **T** | **G** | **C** | **C** | **A** | **A** | **A** | **G** | **C** | **A** | **A** | **A** | **G** | **G** | **A** | **A** | **T** | **T** | **A** | **G** | **A** | **G** | **G** | **T** | **A** | **T** | **A** | **C** | **T** | **T** | **A** | **C** | **A** | **C** | **C** | **A** | **A** | **G** | **T** | **C** | **G** | **A** | **T** | **A** | **A** | **A** | **A** | **A** | **C** | **G** | **T** | **G** | **T** | **T** | **A** | **T** | **T** | **G** | **C** | **G** | **T** | **A** | **T** | **T** | **G** | **T** | **T** | **G** | **A** | **T** | **G** | **A** | **T** | **G** | **G** | **T** | **G** | **T** | **C** | **G** | **G** | **C** | **T** | **T** | **T** | **G** | **A** | **T** | **A** | **T** | **G** | **A** | **A** | **G** | **G** | **A** | **A** | **C** | **A** | **A** | **A** | **G** | **T** | **A** | **A** | **T** | **A** | **A** | **A** | **G** | **C** | **C** | **G** | **G** | **T** | **A** | **G** | **T** | **T** | **A** | **T** | **G** | **G** | **C** | **T** | **T** | **A** | **A** | **A** | **T** | **A** | **A** | **T** | **A** | **T** | **C** | **C** | **G** | **A** | **G** | **A** | **A** | **C** | **G** | **T** | **G** | **T** | **T** | **G** | **T** | **C** | **G** | **G** | **C** | **A** | **T** | **G** | **G** | **G** | **C** | **G** | **G** | **T** | **A** | **C** | **A** | **G** | **T** | **T** | **A** | **A** | **A** | **A** | **T** | **T** | **A** | **T** | **T** | **A** | **G** | **T** | **T** | **T** | **T** | **A** | **A** | **A** | **G** | **G** | **G** | **C** | **A** | **G** | **G** | **G** | **A** | **A** | **C** | **C** | **A** | **G** | **C** | **G** | **T** | **T** | **G** | **A** | **A** | **A** | **T** | **T** | **A** | **A** | **A** | **G** | **T** | **T** | **C** | **C** | **T** | **G** | **T** | **C** | **A** | **T** | **A** | **A** | **A** | **G** | **G** | **A** | **G** | **G** | **A** | **A** | **A** | **C** | **T** | **G** | **C** | **A** | **A** | **G** | **T** | **G** | **A** | **T** | **C** | **A** | **A** | **A** | **G** | **T** | **A** | **A** | **T** | **G** | **T** | **T** | **A** | **G** | **T** | **G** | **G** | **A** | **-** | **-** | **-** |
| **N53420** | **A** | **T** | **G** | **A** | **C** | **C** | **G** | **A** | **T** | **C** | **G** | **G** | **A** | **T** | **T** | **T** | **C** | **A** | **A** | **G** | **A** | **C** | **G** | **C** | **A** | **T** | **G** | **A** | **T** | **T** | **T** | **C** | **A** | **T** | **T** | **A** | **T** | **A** | **T** | **G** | **C** | **G** | **T** | **C** | **C** | **C** | **T** | **T** | **A** | **G** | **C** | **A** | **C** | **C** | **T** | **T** | **T** | **A** | **T** | **T** | **G** | **T** | **T** | **A** | **T** | **T** | **T** | **T** | **A** | **A** | **T** | **T** | **A** | **C** | **C** | **T** | **T** | **G** | **T** | **T** | **T** | **T** | **C** | **A** | **T** | **A** | **T** | **T** | **T** | **T** | **C** | **A** | **T** | **T** | **C** | **A** | **A** | **T** | **T** | **A** | **A** | **A** | **C** | **A** | **A** | **A** | **A** | **C** | **C** | **G** | **G** | **T** | **G** | **G** | **C** | **T** | **C** | **T** | **T** | **A** | **G** | **A** | **A** | **C** | **T** | **T** | **C** | **T** | **T** | **C** | **A** | **G** | **A** | **G** | **A** | **A** | **A** | **A** | **G** | **T** | **C** | **T** | **T** | **T** | **T** | **A** | **T** | **C** | **T** | **G** | **C** | **C** | **A** | **C** | **T** | **A** | **A** | **T** | **T** | **G** | **T** | **G** | **C** | **A** | **T** | **A** | **T** | **T** | **G** | **T** | **T** | **C** | **T** | **T** | **A** | **T** | **A** | **T** | **C** | **C** | **T** | **T** | **A** | **C** | **T** | **A** | **A** | **T** | **A** | **G** | **G** | **C** | **T** | **T** | **A** | **T** | **T** | **G** | **A** | **C** | **C** | **T** | **T** | **T** | **T** | **T** | **A** | **C** | **T** | **G** | **A** | **T** | **T** | **T** | **C** | **G** | **T** | **T** | **G** | **G** | **T** | **C** | **C** | **A** | **A** | **A** | **A** | **A** | **G** | **G** | **C** | **C** | **A** | **A** | **T** | **A** | **T** | **G** | **G** | **A** | **C** | **G** | **G** | **A** | **T** | **T** | **G** | **A** | **A** | **G** | **A** | **A** | **A** | **A** | **A** | **C** | **T** | **T** | **C** | **G** | **G** | **T** | **T** | **A** | **T** | **T** | **A** | **G** | **C** | **C** | **A** | **A** | **T** | **G** | **G** | **C** | **A** | **A** | **T** | **T** | **A** | **T** | **G** | **A** | **A** | **A** | **G** | **T** | **C** | **C** | **A** | **G** | **T** | **C** | **T** | **T** | **A** | **A** | **A** | **C** | **A** | **A** | **A** | **C** | **C** | **A** | **A** | **T** | **G** | **A** | **C** | **T** | **A** | **G** | **T** | **G** | **A** | **A** | **A** | **A** | **T** | **C** | **A** | **A** | **G** | **A** | **C** | **C** | **A** | **T** | **T** | **A** | **T** | **C** | **T** | **A** | **A** | **T** | **C** | **G** | **A** | **A** | **G** | **T** | **C** | **G** | **A** | **A** | **C** | **A** | **A** | **G** | **A** | **T** | **A** | **T** | **T** | **T** | **G** | **G** | **T** | **C** | **G** | **A** | **T** | **T** | **A** | **A** | **A** | **A** | **A** | **C** | **A** | **A** | **A** | **T** | **T** | **A** | **T** | **T** | **A** | **G** | **A** | **G** | **A** | **T** | **G** | **T** | **C** | **T** | **A** | **A** | **A** | **G** | **A** | **A** | **T** | **T** | **G** | **C** | **A** | **A** | **T** | **T** | **A** | **T** | **T** | **A** | **A** | **A** | **C** | **A** | **G** | **T** | **C** | **G** | **A** | **C** | **C** | **G** | **C** | **A** | **A** | **T** | **T** | **A** | **A** | **T** | **G** | **G** | **A** | **T** | **G** | **G** | **G** | **C** | **A** | **A** | **A** | **C** | **A** | **A** | **A** | **A** | **G** | **A** | **A** | **G** | **A** | **A** | **A** | **T** | **T** | **T** | **T** | **A** | **G** | **A** | **G** | **A** | **A** | **C** | **G** | **A** | **G** | **C** | **G** | **G** | **C** | **A** | **T** | **C** | **G** | **T** | **T** | **T** | **G** | **G** | **C** | **A** | **C** | **G** | **G** | **G** | **A** | **G** | **T** | **T** | **G** | **C** | **A** | **T** | **G** | **A** | **T** | **T** | **C** | **A** | **G** | **T** | **C** | **A** | **G** | **T** | **C** | **A** | **A** | **C** | **A** | **A** | **C** | **T** | **T** | **T** | **T** | **T** | **G** | **C** | **A** | **G** | **C** | **C** | **A** | **T** | **G** | **A** | **T** | **G** | **A** | **T** | **G** | **T** | **T** | **G** | **T** | **C** | **T** | **G** | **C** | **C** | **T** | **T** | **A** | **A** | **A** | **T** | **G** | **A** | **A** | **C** | **A** | **A** | **G** | **C** | **A** | **C** | **A** | **A** | **C** | **G** | **A** | **A** | **C** | **A** | **G** | **A** | **A** | **A** | **C** | **C** | **C** | **C** | **T** | **G** | **A** | **A** | **C** | **C** | **A** | **T** | **A** | **T** | **C** | **G** | **T** | **A** | **A** | **A** | **C** | **A** | **A** | **C** | **T** | **A** | **G** | **C** | **C** | **A** | **T** | **G** | **G** | **T** | **G** | **G** | **C** | **A** | **G** | **A** | **A** | **A** | **T** | **C** | **A** | **T** | **T** | **A** | **A** | **T** | **G** | **C** | **C** | **T** | **C** | **C** | **C** | **A** | **A** | **T** | **C** | **G** | **G** | **A** | **A** | **A** | **T** | **G** | **C** | **G** | **C** | **G** | **C** | **G** | **T** | **T** | **A** | **C** | **T** | **A** | **T** | **T** | **A** | **C** | **A** | **C** | **T** | **T** | **G** | **C** | **G** | **T** | **C** | **C** | **T** | **A** | **T** | **T** | **A** | **G** | **T** | **C** | **T** | **A** | **G** | **A** | **A** | **G** | **G** | **G** | **A** | **A** | **A** | **A** | **G** | **T** | **T** | **T** | **G** | **C** | **G** | **T** | **A** | **A** | **A** | **G** | **G** | **C** | **A** | **T** | **T** | **G** | **A** | **A** | **C** | **A** | **A** | **T** | **T** | **A** | **T** | **T** | **G** | **A** | **A** | **A** | **G** | **A** | **A** | **C** | **T** | **A** | **C** | **A** | **A** | **A** | **C** | **A** | **A** | **A** | **A** | **A** | **T** | **T** | **A** | **A** | **A** | **A** | **T** | **T** | **G** | **A** | **A** | **T** | **T** | **G** | **A** | **T** | **T** | **T** | **G** | **G** | **G** | **A** | **T** | **G** | **T** | **T** | **G** | **A** | **A** | **G** | **A** | **T** | **G** | **T** | **T** | **C** | **A** | **T** | **T** | **T** | **A** | **A** | **A** | **T** | **A** | **G** | **C** | **A** | **G** | **C** | **A** | **T** | **T** | **G** | **A** | **G** | **G** | **A** | **T** | **C** | **A** | **T** | **C** | **T** | **T** | **T** | **T** | **C** | **C** | **G** | **A** | **A** | **T** | **T** | **G** | **T** | **T** | **C** | **A** | **G** | **G** | **A** | **A** | **T** | **T** | **A** | **C** | **T** | **T** | **T** | **C** | **A** | **A** | **A** | **T** | **A** | **C** | **C** | **T** | **T** | **A** | **A** | **G** | **A** | **C** | **A** | **T** | **G** | **C** | **C** | **A** | **A** | **A** | **G** | **C** | **A** | **A** | **A** | **A** | **G** | **A** | **A** | **T** | **T** | **A** | **G** | **A** | **G** | **G** | **T** | **A** | **T** | **A** | **C** | **T** | **T** | **A** | **C** | **A** | **C** | **C** | **A** | **A** | **G** | **T** | **C** | **G** | **A** | **T** | **A** | **A** | **A** | **A** | **A** | **T** | **G** | **T** | **T** | **T** | **T** | **A** | **T** | **T** | **G** | **C** | **G** | **C** | **A** | **T** | **T** | **G** | **T** | **T** | **G** | **A** | **T** | **G** | **A** | **T** | **G** | **G** | **T** | **G** | **T** | **C** | **G** | **G** | **C** | **T** | **T** | **T** | **G** | **A** | **T** | **A** | **T** | **G** | **A** | **A** | **G** | **G** | **A** | **A** | **C** | **A** | **A** | **A** | **G** | **T** | **A** | **A** | **T** | **A** | **A** | **G** | **G** | **C** | **C** | **G** | **G** | **T** | **A** | **G** | **T** | **T** | **A** | **T** | **G** | **G** | **C** | **T** | **T** | **A** | **A** | **A** | **T** | **A** | **A** | **T** | **A** | **T** | **T** | **C** | **G** | **A** | **G** | **A** | **A** | **C** | **G** | **T** | **G** | **T** | **T** | **G** | **T** | **C** | **G** | **G** | **C** | **A** | **T** | **G** | **G** | **G** | **C** | **G** | **G** | **T** | **A** | **C** | **A** | **G** | **T** | **T** | **A** | **A** | **A** | **A** | **T** | **T** | **A** | **T** | **T** | **A** | **G** | **T** | **T** | **T** | **T** | **A** | **A** | **A** | **G** | **G** | **G** | **C** | **A** | **G** | **G** | **G** | **A** | **A** | **C** | **C** | **A** | **G** | **C** | **G** | **T** | **T** | **G** | **A** | **A** | **A** | **T** | **T** | **A** | **A** | **A** | **G** | **T** | **T** | **C** | **C** | **T** | **G** | **T** | **C** | **A** | **T** | **A** | **A** | **A** | **G** | **G** | **A** | **G** | **G** | **A** | **A** | **A** | **C** | **T** | **G** | **C** | **A** | **A** | **G** | **T** | **G** | **A** | **T** | **C** | **A** | **A** | **A** | **G** | **T** | **A** | **A** | **T** | **G** | **T** | **T** | **A** | **G** | **T** | **G** | **G** | **A** | **-** | **-** | **-** |
| **N59606F** | **A** | **T** | **G** | **A** | **C** | **C** | **G** | **A** | **T** | **C** | **G** | **G** | **A** | **T** | **T** | **T** | **C** | **A** | **A** | **G** | **A** | **C** | **G** | **C** | **A** | **T** | **G** | **A** | **T** | **T** | **T** | **C** | **A** | **T** | **T** | **A** | **T** | **A** | **T** | **G** | **C** | **G** | **T** | **C** | **C** | **C** | **T** | **T** | **A** | **G** | **C** | **A** | **C** | **C** | **T** | **T** | **T** | **A** | **T** | **T** | **G** | **T** | **T** | **A** | **T** | **C** | **T** | **T** | **A** | **A** | **T** | **T** | **A** | **C** | **A** | **T** | **T** | **G** | **T** | **T** | **T** | **T** | **C** | **A** | **T** | **A** | **T** | **T** | **T** | **T** | **C** | **A** | **T** | **T** | **C** | **G** | **A** | **T** | **T** | **A** | **A** | **A** | **C** | **A** | **A** | **A** | **A** | **C** | **C** | **G** | **G** | **T** | **G** | **G** | **T** | **T** | **A** | **T** | **T** | **A** | **G** | **A** | **G** | **C** | **T** | **T** | **C** | **T** | **T** | **C** | **A** | **G** | **A** | **G** | **A** | **A** | **A** | **A** | **G** | **T** | **C** | **T** | **T** | **T** | **T** | **A** | **T** | **C** | **T** | **A** | **C** | **C** | **A** | **C** | **T** | **A** | **A** | **T** | **T** | **G** | **T** | **G** | **C** | **A** | **C** | **A** | **T** | **T** | **G** | **T** | **T** | **C** | **T** | **C** | **A** | **T** | **A** | **T** | **C** | **C** | **T** | **T** | **A** | **C** | **T** | **A** | **A** | **T** | **A** | **G** | **G** | **C** | **T** | **T** | **A** | **T** | **T** | **G** | **A** | **C** | **C** | **T** | **T** | **T** | **T** | **T** | **A** | **C** | **T** | **G** | **A** | **T** | **T** | **T** | **C** | **A** | **T** | **T** | **G** | **G** | **T** | **T** | **C** | **A** | **A** | **A** | **A** | **A** | **G** | **G** | **G** | **C** | **A** | **A** | **T** | **A** | **T** | **G** | **G** | **A** | **C** | **G** | **G** | **A** | **T** | **T** | **G** | **A** | **A** | **G** | **A** | **A** | **A** | **A** | **A** | **C** | **T** | **T** | **C** | **G** | **G** | **T** | **T** | **A** | **T** | **T** | **G** | **G** | **C** | **C** | **A** | **A** | **T** | **G** | **G** | **T** | **A** | **A** | **T** | **T** | **A** | **T** | **G** | **A** | **A** | **A** | **G** | **T** | **C** | **C** | **A** | **G** | **T** | **C** | **T** | **T** | **A** | **A** | **A** | **C** | **A** | **A** | **A** | **C** | **C** | **A** | **A** | **C** | **G** | **A** | **C** | **C** | **A** | **G** | **T** | **G** | **A** | **A** | **A** | **A** | **T** | **C** | **A** | **A** | **G** | **A** | **T** | **C** | **A** | **T** | **T** | **A** | **T** | **C** | **T** | **A** | **A** | **C** | **C** | **G** | **A** | **A** | **G** | **T** | **C** | **G** | **A** | **A** | **C** | **A** | **A** | **G** | **A** | **T** | **A** | **T** | **T** | **T** | **G** | **G** | **T** | **C** | **G** | **A** | **T** | **T** | **A** | **A** | **A** | **A** | **A** | **T** | **A** | **A** | **A** | **T** | **T** | **A** | **T** | **T** | **A** | **G** | **A** | **G** | **A** | **T** | **G** | **T** | **C** | **T** | **A** | **A** | **A** | **G** | **A** | **A** | **T** | **T** | **G** | **C** | **A** | **A** | **T** | **T** | **A** | **T** | **T** | **A** | **A** | **A** | **C** | **A** | **G** | **T** | **C** | **G** | **G** | **C** | **C** | **G** | **C** | **A** | **A** | **T** | **T** | **A** | **A** | **T** | **G** | **G** | **A** | **T** | **G** | **G** | **G** | **C** | **A** | **A** | **A** | **C** | **A** | **A** | **A** | **A** | **G** | **A** | **A** | **G** | **A** | **A** | **A** | **T** | **T** | **T** | **T** | **A** | **G** | **A** | **G** | **A** | **A** | **C** | **G** | **A** | **G** | **C** | **G** | **G** | **C** | **A** | **T** | **C** | **G** | **T** | **T** | **T** | **G** | **G** | **C** | **G** | **C** | **G** | **G** | **G** | **A** | **G** | **T** | **T** | **G** | **C** | **A** | **T** | **G** | **A** | **T** | **T** | **C** | **A** | **G** | **T** | **C** | **A** | **G** | **T** | **C** | **A** | **A** | **C** | **A** | **A** | **C** | **T** | **T** | **T** | **T** | **T** | **G** | **C** | **A** | **G** | **C** | **C** | **A** | **T** | **G** | **A** | **T** | **G** | **A** | **T** | **G** | **T** | **T** | **G** | **T** | **C** | **T** | **G** | **C** | **C** | **T** | **T** | **A** | **A** | **A** | **T** | **G** | **A** | **A** | **C** | **A** | **A** | **G** | **C** | **A** | **C** | **A** | **A** | **C** | **G** | **A** | **A** | **C** | **A** | **G** | **A** | **A** | **A** | **C** | **C** | **C** | **C** | **G** | **G** | **A** | **A** | **C** | **C** | **A** | **T** | **A** | **T** | **C** | **G** | **T** | **A** | **A** | **A** | **C** | **A** | **A** | **C** | **T** | **A** | **G** | **C** | **C** | **A** | **T** | **G** | **G** | **T** | **G** | **G** | **C** | **A** | **G** | **A** | **A** | **A** | **T** | **C** | **A** | **T** | **T** | **A** | **A** | **T** | **G** | **C** | **C** | **T** | **C** | **C** | **C** | **A** | **A** | **T** | **C** | **G** | **G** | **A** | **A** | **A** | **T** | **G** | **C** | **G** | **C** | **G** | **C** | **G** | **T** | **T** | **A** | **C** | **T** | **A** | **T** | **T** | **G** | **C** | **A** | **C** | **T** | **T** | **G** | **C** | **G** | **T** | **C** | **C** | **T** | **A** | **T** | **C** | **A** | **G** | **T** | **C** | **T** | **A** | **G** | **A** | **A** | **G** | **G** | **A** | **A** | **A** | **A** | **A** | **G** | **T** | **T** | **T** | **G** | **C** | **G** | **T** | **A** | **A** | **A** | **G** | **G** | **T** | **A** | **T** | **T** | **G** | **A** | **A** | **C** | **A** | **A** | **T** | **T** | **A** | **C** | **T** | **G** | **A** | **A** | **A** | **G** | **A** | **A** | **C** | **T** | **A** | **C** | **A** | **A** | **A** | **C** | **A** | **A** | **A** | **A** | **A** | **T** | **T** | **A** | **A** | **A** | **A** | **T** | **T** | **G** | **A** | **A** | **T** | **T** | **G** | **A** | **T** | **T** | **T** | **G** | **G** | **G** | **A** | **T** | **G** | **T** | **T** | **G** | **A** | **A** | **G** | **A** | **T** | **G** | **T** | **T** | **C** | **A** | **T** | **T** | **T** | **A** | **A** | **A** | **T** | **A** | **G** | **C** | **A** | **G** | **C** | **A** | **T** | **T** | **G** | **A** | **G** | **G** | **A** | **T** | **C** | **A** | **T** | **C** | **T** | **T** | **T** | **T** | **C** | **C** | **G** | **A** | **A** | **T** | **T** | **G** | **T** | **G** | **C** | **A** | **G** | **G** | **A** | **G** | **T** | **T** | **A** | **C** | **T** | **T** | **T** | **C** | **A** | **A** | **A** | **T** | **A** | **C** | **C** | **T** | **T** | **A** | **A** | **G** | **A** | **C** | **A** | **T** | **G** | **C** | **C** | **A** | **A** | **A** | **G** | **C** | **A** | **A** | **A** | **G** | **G** | **A** | **A** | **T** | **T** | **A** | **G** | **A** | **G** | **G** | **T** | **A** | **T** | **A** | **C** | **T** | **T** | **A** | **C** | **A** | **C** | **C** | **A** | **A** | **G** | **T** | **C** | **G** | **A** | **T** | **A** | **A** | **A** | **A** | **A** | **C** | **G** | **T** | **G** | **T** | **T** | **A** | **T** | **T** | **G** | **C** | **G** | **T** | **A** | **T** | **T** | **G** | **T** | **T** | **G** | **A** | **T** | **G** | **A** | **C** | **G** | **G** | **T** | **G** | **T** | **C** | **G** | **G** | **C** | **T** | **T** | **T** | **G** | **A** | **T** | **A** | **T** | **G** | **A** | **A** | **G** | **G** | **A** | **A** | **C** | **A** | **A** | **A** | **G** | **T** | **A** | **A** | **T** | **A** | **A** | **A** | **G** | **C** | **C** | **G** | **G** | **T** | **A** | **G** | **T** | **T** | **A** | **T** | **G** | **G** | **C** | **T** | **T** | **A** | **A** | **A** | **T** | **A** | **A** | **T** | **A** | **T** | **C** | **C** | **G** | **A** | **G** | **A** | **A** | **C** | **G** | **T** | **G** | **T** | **T** | **G** | **T** | **C** | **G** | **G** | **C** | **A** | **T** | **G** | **G** | **G** | **C** | **G** | **G** | **T** | **A** | **C** | **A** | **G** | **T** | **T** | **A** | **A** | **A** | **A** | **T** | **T** | **A** | **T** | **T** | **A** | **G** | **T** | **T** | **T** | **T** | **A** | **A** | **A** | **G** | **G** | **G** | **C** | **A** | **G** | **G** | **G** | **A** | **A** | **C** | **C** | **A** | **G** | **C** | **G** | **T** | **T** | **G** | **A** | **A** | **A** | **T** | **T** | **A** | **A** | **A** | **G** | **T** | **T** | **C** | **C** | **T** | **G** | **T** | **C** | **A** | **T** | **A** | **A** | **A** | **G** | **G** | **A** | **G** | **G** | **A** | **A** | **A** | **C** | **T** | **G** | **C** | **A** | **A** | **G** | **T** | **G** | **A** | **T** | **C** | **A** | **A** | **A** | **G** | **T** | **A** | **A** | **T** | **G** | **T** | **T** | **A** | **G** | **T** | **G** | **G** | **A** | **-** | **-** | **-** |
| **N55265** | **A** | **T** | **G** | **A** | **C** | **C** | **G** | **A** | **T** | **C** | **G** | **G** | **A** | **T** | **T** | **T** | **C** | **A** | **A** | **G** | **A** | **C** | **G** | **C** | **A** | **T** | **G** | **A** | **T** | **T** | **T** | **C** | **A** | **T** | **T** | **A** | **T** | **A** | **T** | **G** | **C** | **G** | **T** | **C** | **C** | **C** | **T** | **T** | **A** | **G** | **C** | **A** | **C** | **C** | **T** | **T** | **T** | **A** | **T** | **T** | **G** | **T** | **T** | **A** | **T** | **T** | **T** | **T** | **A** | **A** | **T** | **T** | **A** | **C** | **C** | **T** | **T** | **G** | **T** | **T** | **T** | **T** | **C** | **A** | **T** | **A** | **T** | **T** | **T** | **T** | **C** | **A** | **T** | **T** | **C** | **A** | **A** | **T** | **T** | **A** | **A** | **A** | **C** | **A** | **A** | **A** | **A** | **C** | **C** | **G** | **G** | **T** | **G** | **G** | **C** | **T** | **C** | **T** | **T** | **A** | **G** | **A** | **A** | **C** | **T** | **T** | **C** | **T** | **T** | **C** | **A** | **G** | **A** | **G** | **A** | **A** | **A** | **A** | **G** | **T** | **C** | **T** | **T** | **T** | **T** | **A** | **T** | **C** | **T** | **G** | **C** | **C** | **A** | **C** | **T** | **A** | **A** | **T** | **T** | **G** | **T** | **G** | **C** | **A** | **T** | **A** | **T** | **T** | **G** | **T** | **T** | **C** | **T** | **T** | **A** | **T** | **A** | **T** | **C** | **C** | **T** | **T** | **A** | **C** | **T** | **A** | **A** | **T** | **A** | **G** | **G** | **C** | **T** | **T** | **A** | **T** | **T** | **G** | **A** | **C** | **C** | **T** | **T** | **T** | **T** | **T** | **A** | **C** | **T** | **G** | **A** | **T** | **T** | **T** | **C** | **G** | **T** | **T** | **G** | **G** | **T** | **C** | **C** | **A** | **A** | **A** | **A** | **A** | **G** | **G** | **C** | **C** | **A** | **A** | **T** | **A** | **T** | **G** | **G** | **A** | **C** | **G** | **G** | **A** | **T** | **T** | **G** | **A** | **A** | **G** | **A** | **A** | **A** | **A** | **A** | **C** | **T** | **T** | **C** | **G** | **G** | **T** | **T** | **A** | **T** | **T** | **A** | **G** | **C** | **C** | **A** | **A** | **T** | **G** | **G** | **C** | **A** | **A** | **T** | **T** | **A** | **T** | **G** | **A** | **A** | **A** | **G** | **T** | **C** | **C** | **A** | **G** | **T** | **C** | **T** | **T** | **A** | **A** | **A** | **C** | **A** | **A** | **A** | **C** | **C** | **A** | **A** | **T** | **G** | **A** | **C** | **T** | **A** | **G** | **T** | **G** | **A** | **A** | **A** | **A** | **T** | **C** | **A** | **A** | **G** | **A** | **C** | **C** | **A** | **T** | **T** | **A** | **T** | **C** | **T** | **A** | **A** | **T** | **C** | **G** | **A** | **A** | **G** | **T** | **C** | **G** | **A** | **A** | **C** | **A** | **A** | **G** | **A** | **T** | **A** | **T** | **T** | **T** | **G** | **G** | **T** | **C** | **G** | **A** | **T** | **T** | **A** | **A** | **A** | **A** | **A** | **C** | **A** | **A** | **A** | **T** | **T** | **A** | **T** | **T** | **A** | **G** | **A** | **G** | **A** | **T** | **G** | **T** | **C** | **T** | **A** | **A** | **A** | **G** | **A** | **A** | **T** | **T** | **G** | **C** | **A** | **A** | **T** | **T** | **A** | **T** | **T** | **A** | **A** | **A** | **C** | **A** | **G** | **T** | **C** | **G** | **A** | **C** | **C** | **G** | **C** | **A** | **A** | **T** | **T** | **A** | **A** | **T** | **G** | **G** | **A** | **T** | **G** | **G** | **G** | **C** | **A** | **A** | **A** | **C** | **A** | **A** | **A** | **A** | **G** | **A** | **A** | **G** | **A** | **A** | **A** | **T** | **T** | **T** | **T** | **A** | **G** | **A** | **G** | **A** | **A** | **C** | **G** | **A** | **G** | **C** | **G** | **G** | **C** | **A** | **T** | **C** | **G** | **T** | **T** | **T** | **G** | **G** | **C** | **A** | **C** | **G** | **G** | **G** | **A** | **G** | **T** | **T** | **G** | **C** | **A** | **T** | **G** | **A** | **T** | **T** | **C** | **A** | **G** | **T** | **C** | **A** | **G** | **T** | **C** | **A** | **A** | **C** | **A** | **A** | **C** | **T** | **T** | **T** | **T** | **T** | **G** | **C** | **A** | **G** | **C** | **C** | **A** | **T** | **G** | **A** | **T** | **G** | **A** | **T** | **G** | **T** | **T** | **G** | **T** | **C** | **T** | **G** | **C** | **C** | **T** | **T** | **A** | **A** | **A** | **T** | **G** | **A** | **A** | **C** | **A** | **A** | **G** | **C** | **A** | **C** | **A** | **A** | **C** | **G** | **A** | **A** | **C** | **A** | **G** | **A** | **A** | **A** | **C** | **C** | **C** | **C** | **T** | **G** | **A** | **A** | **C** | **C** | **A** | **T** | **A** | **T** | **C** | **G** | **T** | **A** | **A** | **A** | **C** | **A** | **A** | **C** | **T** | **A** | **G** | **C** | **C** | **A** | **T** | **G** | **G** | **T** | **G** | **G** | **C** | **A** | **G** | **A** | **A** | **A** | **T** | **C** | **A** | **T** | **T** | **A** | **A** | **T** | **G** | **C** | **C** | **T** | **C** | **C** | **C** | **A** | **A** | **T** | **C** | **G** | **G** | **A** | **A** | **A** | **T** | **G** | **C** | **G** | **C** | **G** | **C** | **G** | **T** | **T** | **A** | **C** | **T** | **A** | **T** | **T** | **A** | **C** | **A** | **C** | **T** | **T** | **G** | **C** | **G** | **T** | **C** | **C** | **T** | **A** | **T** | **T** | **A** | **G** | **T** | **C** | **T** | **A** | **G** | **A** | **A** | **G** | **G** | **G** | **A** | **A** | **A** | **A** | **G** | **T** | **T** | **T** | **G** | **C** | **G** | **T** | **A** | **A** | **A** | **G** | **G** | **C** | **A** | **T** | **T** | **G** | **A** | **A** | **C** | **A** | **A** | **T** | **T** | **A** | **T** | **T** | **G** | **A** | **A** | **A** | **G** | **A** | **A** | **C** | **T** | **A** | **C** | **A** | **A** | **A** | **C** | **A** | **A** | **A** | **A** | **A** | **T** | **T** | **A** | **A** | **A** | **A** | **T** | **T** | **G** | **A** | **A** | **T** | **T** | **G** | **A** | **T** | **T** | **T** | **G** | **G** | **G** | **A** | **T** | **G** | **T** | **T** | **G** | **A** | **A** | **G** | **A** | **T** | **G** | **T** | **T** | **C** | **A** | **T** | **T** | **T** | **A** | **A** | **A** | **T** | **A** | **G** | **C** | **A** | **G** | **C** | **A** | **T** | **T** | **G** | **A** | **G** | **G** | **A** | **T** | **C** | **A** | **T** | **C** | **T** | **T** | **T** | **T** | **C** | **C** | **G** | **A** | **A** | **T** | **T** | **G** | **T** | **T** | **C** | **A** | **G** | **G** | **A** | **A** | **T** | **T** | **A** | **C** | **T** | **T** | **T** | **C** | **A** | **A** | **A** | **T** | **A** | **C** | **C** | **T** | **T** | **A** | **A** | **G** | **A** | **C** | **A** | **T** | **G** | **C** | **C** | **A** | **A** | **A** | **G** | **C** | **A** | **A** | **A** | **A** | **G** | **A** | **A** | **T** | **T** | **A** | **G** | **A** | **G** | **G** | **T** | **A** | **T** | **A** | **C** | **T** | **T** | **A** | **C** | **A** | **C** | **C** | **A** | **A** | **G** | **T** | **C** | **G** | **A** | **T** | **A** | **A** | **A** | **A** | **A** | **T** | **G** | **T** | **T** | **T** | **T** | **A** | **T** | **T** | **G** | **C** | **G** | **C** | **A** | **T** | **T** | **G** | **T** | **T** | **G** | **A** | **T** | **G** | **A** | **T** | **G** | **G** | **T** | **G** | **T** | **C** | **G** | **G** | **C** | **T** | **T** | **T** | **G** | **A** | **T** | **A** | **T** | **G** | **A** | **A** | **G** | **G** | **A** | **A** | **C** | **A** | **A** | **A** | **G** | **T** | **A** | **A** | **T** | **A** | **A** | **G** | **G** | **C** | **C** | **G** | **G** | **T** | **A** | **G** | **T** | **T** | **A** | **T** | **G** | **G** | **C** | **T** | **T** | **A** | **A** | **A** | **T** | **A** | **A** | **T** | **A** | **T** | **T** | **C** | **G** | **A** | **G** | **A** | **A** | **C** | **G** | **T** | **G** | **T** | **T** | **G** | **T** | **C** | **G** | **G** | **C** | **A** | **T** | **G** | **G** | **G** | **C** | **G** | **G** | **T** | **A** | **C** | **A** | **G** | **T** | **T** | **A** | **A** | **A** | **A** | **T** | **T** | **A** | **T** | **T** | **A** | **G** | **T** | **T** | **T** | **T** | **A** | **A** | **A** | **G** | **G** | **G** | **C** | **A** | **G** | **G** | **G** | **A** | **A** | **C** | **C** | **A** | **G** | **C** | **G** | **T** | **T** | **G** | **A** | **A** | **A** | **T** | **T** | **A** | **A** | **A** | **G** | **T** | **T** | **C** | **C** | **T** | **G** | **T** | **C** | **A** | **T** | **A** | **A** | **A** | **G** | **G** | **A** | **G** | **G** | **A** | **A** | **A** | **C** | **T** | **G** | **C** | **A** | **A** | **G** | **T** | **G** | **A** | **T** | **C** | **A** | **A** | **A** | **G** | **T** | **A** | **A** | **T** | **G** | **T** | **T** | **A** | **G** | **T** | **G** | **G** | **A** | **-** | **-** | **-** |
| **N53418** | **A** | **T** | **G** | **A** | **C** | **C** | **G** | **A** | **T** | **C** | **G** | **G** | **A** | **T** | **T** | **T** | **C** | **A** | **A** | **G** | **A** | **C** | **G** | **C** | **A** | **T** | **G** | **A** | **T** | **T** | **T** | **C** | **A** | **T** | **T** | **A** | **T** | **A** | **T** | **G** | **C** | **G** | **T** | **C** | **C** | **C** | **T** | **T** | **A** | **G** | **C** | **A** | **C** | **C** | **T** | **T** | **T** | **A** | **T** | **T** | **G** | **T** | **T** | **A** | **T** | **C** | **T** | **T** | **A** | **A** | **T** | **T** | **A** | **C** | **A** | **T** | **T** | **G** | **T** | **T** | **T** | **T** | **C** | **A** | **T** | **A** | **T** | **T** | **T** | **T** | **C** | **A** | **T** | **T** | **C** | **G** | **A** | **T** | **T** | **A** | **A** | **A** | **C** | **A** | **A** | **A** | **A** | **C** | **C** | **G** | **G** | **T** | **G** | **G** | **C** | **T** | **A** | **T** | **T** | **A** | **G** | **A** | **G** | **C** | **T** | **T** | **C** | **T** | **T** | **C** | **A** | **G** | **A** | **G** | **A** | **A** | **A** | **A** | **G** | **T** | **C** | **T** | **T** | **T** | **T** | **A** | **T** | **T** | **T** | **A** | **C** | **C** | **A** | **C** | **T** | **A** | **A** | **T** | **T** | **G** | **T** | **G** | **C** | **A** | **C** | **A** | **T** | **T** | **G** | **T** | **T** | **C** | **T** | **C** | **A** | **T** | **A** | **T** | **C** | **C** | **T** | **T** | **A** | **C** | **T** | **A** | **A** | **T** | **A** | **G** | **G** | **C** | **T** | **T** | **A** | **T** | **T** | **G** | **A** | **C** | **C** | **T** | **T** | **T** | **T** | **T** | **A** | **C** | **T** | **G** | **A** | **T** | **T** | **T** | **C** | **A** | **T** | **T** | **G** | **G** | **T** | **T** | **C** | **A** | **A** | **A** | **A** | **A** | **G** | **G** | **G** | **C** | **A** | **A** | **T** | **A** | **T** | **G** | **G** | **A** | **C** | **G** | **G** | **A** | **T** | **T** | **G** | **A** | **A** | **G** | **A** | **A** | **A** | **A** | **A** | **C** | **T** | **T** | **C** | **G** | **G** | **T** | **T** | **A** | **T** | **T** | **G** | **G** | **C** | **C** | **A** | **A** | **C** | **G** | **G** | **T** | **A** | **A** | **T** | **T** | **A** | **T** | **G** | **A** | **A** | **A** | **G** | **T** | **C** | **C** | **A** | **G** | **T** | **C** | **T** | **T** | **A** | **A** | **A** | **C** | **A** | **A** | **A** | **C** | **C** | **A** | **A** | **C** | **G** | **A** | **C** | **C** | **A** | **G** | **T** | **G** | **A** | **A** | **A** | **A** | **T** | **C** | **A** | **A** | **G** | **A** | **C** | **C** | **A** | **T** | **T** | **A** | **T** | **C** | **T** | **A** | **A** | **C** | **C** | **G** | **A** | **A** | **G** | **T** | **C** | **G** | **A** | **A** | **C** | **A** | **A** | **G** | **A** | **T** | **A** | **T** | **T** | **T** | **G** | **G** | **T** | **C** | **G** | **A** | **T** | **T** | **A** | **A** | **A** | **A** | **A** | **T** | **A** | **A** | **A** | **T** | **T** | **A** | **T** | **T** | **A** | **G** | **A** | **G** | **A** | **T** | **G** | **T** | **C** | **T** | **A** | **A** | **A** | **G** | **A** | **A** | **T** | **T** | **G** | **C** | **A** | **A** | **T** | **T** | **A** | **T** | **T** | **A** | **A** | **A** | **C** | **A** | **G** | **T** | **C** | **G** | **A** | **C** | **C** | **G** | **C** | **A** | **A** | **T** | **T** | **A** | **A** | **T** | **G** | **G** | **A** | **T** | **G** | **G** | **G** | **C** | **A** | **A** | **A** | **C** | **A** | **A** | **A** | **A** | **G** | **A** | **A** | **G** | **A** | **A** | **A** | **T** | **T** | **T** | **T** | **A** | **G** | **A** | **G** | **A** | **A** | **C** | **G** | **A** | **G** | **C** | **G** | **G** | **C** | **A** | **T** | **C** | **G** | **T** | **T** | **T** | **G** | **G** | **C** | **G** | **C** | **G** | **G** | **G** | **A** | **G** | **T** | **T** | **G** | **C** | **A** | **T** | **G** | **A** | **T** | **T** | **C** | **A** | **G** | **T** | **C** | **A** | **G** | **T** | **C** | **A** | **A** | **C** | **A** | **A** | **C** | **T** | **T** | **T** | **T** | **T** | **G** | **C** | **A** | **G** | **C** | **C** | **A** | **T** | **G** | **A** | **T** | **G** | **A** | **T** | **G** | **T** | **T** | **G** | **T** | **C** | **T** | **G** | **C** | **C** | **T** | **T** | **A** | **A** | **A** | **T** | **G** | **A** | **A** | **C** | **A** | **A** | **G** | **C** | **A** | **C** | **A** | **A** | **C** | **G** | **A** | **A** | **C** | **A** | **G** | **A** | **A** | **A** | **C** | **T** | **C** | **C** | **T** | **G** | **A** | **A** | **C** | **C** | **A** | **T** | **A** | **T** | **C** | **G** | **C** | **A** | **A** | **A** | **C** | **A** | **A** | **C** | **T** | **A** | **G** | **C** | **C** | **A** | **T** | **G** | **G** | **T** | **G** | **G** | **C** | **A** | **G** | **A** | **A** | **A** | **T** | **C** | **A** | **T** | **T** | **A** | **A** | **T** | **G** | **C** | **C** | **T** | **C** | **C** | **C** | **A** | **A** | **T** | **C** | **G** | **G** | **A** | **A** | **A** | **T** | **G** | **C** | **G** | **C** | **G** | **C** | **G** | **T** | **T** | **A** | **C** | **T** | **A** | **T** | **T** | **G** | **C** | **A** | **C** | **T** | **T** | **G** | **C** | **G** | **T** | **C** | **C** | **T** | **A** | **T** | **C** | **A** | **G** | **T** | **C** | **T** | **A** | **G** | **A** | **A** | **G** | **G** | **A** | **A** | **A** | **A** | **A** | **G** | **T** | **T** | **T** | **G** | **C** | **G** | **T** | **A** | **A** | **A** | **G** | **G** | **T** | **A** | **T** | **T** | **G** | **A** | **A** | **C** | **A** | **A** | **T** | **T** | **A** | **C** | **T** | **G** | **A** | **A** | **A** | **G** | **A** | **A** | **C** | **T** | **A** | **C** | **A** | **A** | **A** | **C** | **A** | **A** | **A** | **A** | **A** | **T** | **T** | **A** | **A** | **A** | **A** | **T** | **T** | **G** | **A** | **A** | **T** | **T** | **G** | **A** | **T** | **T** | **T** | **G** | **G** | **G** | **A** | **T** | **G** | **T** | **T** | **G** | **A** | **A** | **G** | **A** | **T** | **G** | **T** | **T** | **C** | **A** | **T** | **T** | **T** | **A** | **A** | **A** | **T** | **A** | **G** | **C** | **A** | **G** | **C** | **A** | **T** | **T** | **G** | **A** | **G** | **G** | **A** | **T** | **C** | **A** | **T** | **C** | **T** | **T** | **T** | **T** | **C** | **C** | **G** | **A** | **A** | **T** | **T** | **G** | **T** | **G** | **C** | **A** | **G** | **G** | **A** | **A** | **T** | **T** | **A** | **C** | **T** | **T** | **T** | **C** | **A** | **A** | **A** | **T** | **A** | **C** | **C** | **T** | **T** | **A** | **A** | **G** | **A** | **C** | **A** | **T** | **G** | **C** | **C** | **A** | **A** | **A** | **G** | **C** | **A** | **A** | **A** | **G** | **G** | **A** | **A** | **T** | **T** | **A** | **G** | **A** | **G** | **G** | **T** | **A** | **T** | **A** | **C** | **T** | **T** | **A** | **C** | **A** | **C** | **C** | **A** | **A** | **G** | **T** | **C** | **G** | **A** | **T** | **A** | **A** | **A** | **A** | **A** | **C** | **G** | **T** | **G** | **T** | **T** | **A** | **T** | **T** | **G** | **C** | **G** | **T** | **A** | **T** | **T** | **G** | **T** | **T** | **G** | **A** | **T** | **G** | **A** | **T** | **G** | **G** | **T** | **G** | **T** | **C** | **G** | **G** | **C** | **T** | **T** | **T** | **G** | **A** | **T** | **A** | **T** | **G** | **A** | **A** | **G** | **G** | **A** | **A** | **C** | **A** | **A** | **A** | **G** | **T** | **A** | **A** | **T** | **A** | **A** | **A** | **G** | **C** | **C** | **G** | **G** | **T** | **A** | **G** | **T** | **T** | **A** | **T** | **G** | **G** | **C** | **T** | **T** | **A** | **A** | **A** | **T** | **A** | **A** | **T** | **A** | **T** | **C** | **C** | **G** | **A** | **G** | **A** | **A** | **C** | **G** | **T** | **G** | **T** | **T** | **G** | **T** | **C** | **G** | **G** | **C** | **A** | **T** | **G** | **G** | **G** | **C** | **G** | **G** | **T** | **A** | **C** | **A** | **G** | **T** | **T** | **A** | **A** | **A** | **A** | **T** | **T** | **A** | **T** | **T** | **A** | **G** | **T** | **T** | **T** | **T** | **A** | **A** | **A** | **G** | **G** | **G** | **C** | **A** | **G** | **G** | **G** | **A** | **A** | **C** | **C** | **A** | **G** | **C** | **G** | **T** | **T** | **G** | **A** | **A** | **A** | **T** | **T** | **A** | **A** | **A** | **G** | **T** | **T** | **C** | **C** | **T** | **G** | **T** | **C** | **A** | **T** | **A** | **A** | **A** | **G** | **G** | **A** | **G** | **G** | **A** | **A** | **A** | **C** | **T** | **G** | **C** | **A** | **A** | **G** | **T** | **G** | **A** | **T** | **C** | **A** | **A** | **A** | **G** | **T** | **A** | **A** | **T** | **G** | **T** | **T** | **A** | **G** | **T** | **G** | **G** | **A** | **-** | **-** | **-** |
| **N60063F** | **A** | **T** | **G** | **A** | **C** | **C** | **G** | **A** | **T** | **C** | **G** | **G** | **A** | **T** | **T** | **T** | **C** | **A** | **A** | **G** | **A** | **C** | **G** | **C** | **A** | **T** | **G** | **A** | **T** | **T** | **T** | **C** | **A** | **T** | **T** | **A** | **T** | **A** | **T** | **G** | **C** | **G** | **T** | **C** | **C** | **C** | **T** | **T** | **A** | **G** | **C** | **A** | **C** | **C** | **T** | **T** | **T** | **A** | **T** | **T** | **G** | **T** | **T** | **A** | **T** | **C** | **T** | **T** | **A** | **A** | **T** | **T** | **A** | **C** | **A** | **T** | **T** | **G** | **T** | **T** | **T** | **T** | **C** | **A** | **T** | **A** | **T** | **T** | **T** | **T** | **C** | **A** | **T** | **T** | **C** | **G** | **A** | **T** | **T** | **A** | **A** | **A** | **C** | **A** | **A** | **A** | **A** | **C** | **C** | **G** | **G** | **T** | **G** | **G** | **T** | **T** | **A** | **T** | **T** | **A** | **G** | **A** | **G** | **C** | **T** | **T** | **C** | **T** | **T** | **C** | **A** | **G** | **A** | **G** | **A** | **A** | **A** | **A** | **G** | **T** | **C** | **T** | **T** | **T** | **T** | **A** | **T** | **T** | **T** | **A** | **C** | **C** | **A** | **C** | **T** | **A** | **A** | **T** | **T** | **G** | **T** | **G** | **C** | **A** | **C** | **A** | **T** | **T** | **G** | **T** | **T** | **C** | **T** | **C** | **A** | **T** | **A** | **T** | **C** | **C** | **T** | **T** | **A** | **C** | **T** | **A** | **A** | **T** | **A** | **G** | **G** | **C** | **T** | **T** | **A** | **T** | **T** | **G** | **A** | **C** | **C** | **T** | **T** | **T** | **T** | **T** | **A** | **C** | **T** | **G** | **A** | **T** | **T** | **T** | **C** | **A** | **T** | **T** | **G** | **G** | **T** | **T** | **C** | **A** | **A** | **A** | **A** | **A** | **G** | **G** | **G** | **C** | **A** | **A** | **T** | **A** | **T** | **G** | **G** | **A** | **C** | **G** | **G** | **A** | **T** | **T** | **G** | **A** | **A** | **G** | **A** | **A** | **A** | **A** | **A** | **C** | **T** | **T** | **C** | **G** | **G** | **T** | **T** | **A** | **T** | **T** | **G** | **G** | **C** | **C** | **A** | **A** | **C** | **G** | **G** | **T** | **A** | **A** | **T** | **T** | **A** | **T** | **G** | **A** | **A** | **A** | **G** | **T** | **C** | **C** | **A** | **G** | **T** | **C** | **T** | **T** | **A** | **A** | **A** | **C** | **A** | **A** | **A** | **C** | **C** | **A** | **A** | **C** | **G** | **A** | **C** | **C** | **A** | **G** | **T** | **G** | **A** | **A** | **A** | **A** | **T** | **C** | **A** | **A** | **G** | **A** | **C** | **C** | **A** | **T** | **T** | **A** | **T** | **C** | **T** | **A** | **A** | **C** | **C** | **G** | **A** | **A** | **G** | **T** | **C** | **G** | **A** | **A** | **C** | **A** | **A** | **G** | **A** | **T** | **A** | **T** | **T** | **T** | **G** | **G** | **T** | **C** | **G** | **A** | **T** | **T** | **A** | **A** | **A** | **A** | **A** | **T** | **A** | **A** | **A** | **T** | **T** | **A** | **T** | **T** | **A** | **G** | **A** | **G** | **A** | **T** | **G** | **T** | **C** | **T** | **A** | **A** | **A** | **G** | **A** | **A** | **T** | **T** | **G** | **C** | **A** | **A** | **T** | **T** | **A** | **T** | **T** | **A** | **A** | **A** | **C** | **A** | **G** | **T** | **C** | **G** | **A** | **C** | **C** | **G** | **C** | **A** | **A** | **T** | **T** | **A** | **A** | **T** | **G** | **G** | **A** | **T** | **G** | **G** | **G** | **C** | **A** | **A** | **A** | **C** | **A** | **A** | **A** | **A** | **G** | **A** | **A** | **G** | **A** | **A** | **A** | **T** | **T** | **T** | **T** | **A** | **G** | **A** | **G** | **A** | **A** | **C** | **G** | **A** | **G** | **C** | **G** | **G** | **C** | **A** | **T** | **C** | **G** | **T** | **T** | **T** | **G** | **G** | **C** | **G** | **C** | **G** | **G** | **G** | **A** | **G** | **T** | **T** | **G** | **C** | **A** | **T** | **G** | **A** | **T** | **T** | **C** | **A** | **G** | **T** | **C** | **A** | **G** | **T** | **C** | **A** | **A** | **C** | **A** | **A** | **C** | **T** | **T** | **T** | **T** | **T** | **G** | **C** | **A** | **G** | **C** | **C** | **A** | **T** | **G** | **A** | **T** | **G** | **A** | **T** | **G** | **T** | **T** | **G** | **T** | **C** | **T** | **G** | **C** | **C** | **T** | **T** | **A** | **A** | **A** | **T** | **G** | **A** | **A** | **C** | **A** | **A** | **G** | **C** | **A** | **C** | **A** | **A** | **C** | **G** | **A** | **A** | **C** | **A** | **G** | **A** | **A** | **A** | **C** | **C** | **C** | **C** | **G** | **G** | **A** | **A** | **C** | **C** | **A** | **T** | **A** | **T** | **C** | **G** | **T** | **A** | **A** | **A** | **C** | **A** | **A** | **C** | **T** | **A** | **G** | **C** | **C** | **A** | **T** | **G** | **G** | **T** | **G** | **G** | **C** | **A** | **G** | **A** | **A** | **A** | **T** | **C** | **A** | **T** | **T** | **A** | **A** | **T** | **G** | **C** | **C** | **T** | **C** | **C** | **C** | **A** | **A** | **T** | **C** | **G** | **G** | **A** | **A** | **A** | **T** | **G** | **C** | **G** | **C** | **G** | **C** | **G** | **T** | **T** | **A** | **C** | **T** | **A** | **T** | **T** | **G** | **C** | **A** | **C** | **T** | **T** | **G** | **C** | **G** | **T** | **C** | **C** | **T** | **A** | **T** | **C** | **A** | **G** | **T** | **C** | **T** | **A** | **G** | **A** | **A** | **G** | **G** | **A** | **A** | **A** | **A** | **A** | **G** | **T** | **T** | **T** | **G** | **C** | **G** | **T** | **A** | **A** | **A** | **G** | **G** | **T** | **A** | **T** | **T** | **G** | **A** | **A** | **C** | **A** | **A** | **T** | **T** | **A** | **C** | **T** | **G** | **A** | **A** | **A** | **G** | **A** | **A** | **C** | **T** | **A** | **C** | **A** | **A** | **A** | **C** | **A** | **A** | **A** | **A** | **A** | **T** | **T** | **A** | **A** | **A** | **A** | **T** | **T** | **G** | **A** | **A** | **T** | **T** | **G** | **A** | **T** | **T** | **T** | **G** | **G** | **G** | **A** | **T** | **G** | **T** | **T** | **G** | **A** | **A** | **G** | **A** | **T** | **G** | **T** | **T** | **C** | **A** | **T** | **T** | **T** | **A** | **A** | **A** | **T** | **A** | **G** | **C** | **A** | **G** | **C** | **A** | **T** | **T** | **G** | **A** | **G** | **G** | **A** | **T** | **C** | **A** | **T** | **C** | **T** | **T** | **T** | **T** | **C** | **C** | **G** | **A** | **A** | **T** | **T** | **G** | **T** | **G** | **C** | **A** | **G** | **G** | **A** | **A** | **T** | **T** | **A** | **C** | **T** | **T** | **T** | **C** | **A** | **A** | **A** | **T** | **A** | **C** | **C** | **T** | **T** | **A** | **A** | **G** | **A** | **C** | **A** | **T** | **G** | **C** | **C** | **A** | **A** | **A** | **G** | **C** | **A** | **A** | **A** | **G** | **G** | **A** | **A** | **T** | **T** | **A** | **G** | **A** | **G** | **G** | **T** | **A** | **T** | **A** | **C** | **T** | **T** | **A** | **C** | **A** | **C** | **C** | **A** | **A** | **G** | **T** | **C** | **G** | **A** | **T** | **A** | **A** | **A** | **A** | **A** | **C** | **G** | **T** | **G** | **T** | **T** | **A** | **T** | **T** | **G** | **C** | **G** | **T** | **A** | **T** | **T** | **G** | **T** | **T** | **G** | **A** | **T** | **G** | **A** | **C** | **G** | **G** | **T** | **G** | **T** | **C** | **G** | **G** | **C** | **T** | **T** | **T** | **G** | **A** | **T** | **A** | **T** | **G** | **A** | **A** | **G** | **G** | **A** | **A** | **C** | **A** | **A** | **A** | **G** | **T** | **A** | **A** | **T** | **A** | **A** | **A** | **G** | **C** | **C** | **G** | **G** | **T** | **A** | **G** | **T** | **T** | **A** | **T** | **G** | **G** | **C** | **T** | **T** | **A** | **A** | **A** | **T** | **A** | **A** | **T** | **A** | **T** | **C** | **C** | **G** | **A** | **G** | **A** | **A** | **C** | **G** | **T** | **G** | **T** | **T** | **G** | **T** | **C** | **G** | **G** | **C** | **A** | **T** | **G** | **G** | **G** | **C** | **G** | **G** | **T** | **A** | **C** | **A** | **G** | **T** | **T** | **A** | **A** | **A** | **A** | **T** | **T** | **A** | **T** | **T** | **A** | **G** | **T** | **T** | **T** | **T** | **A** | **A** | **A** | **G** | **G** | **G** | **C** | **A** | **G** | **G** | **G** | **A** | **A** | **C** | **C** | **A** | **G** | **C** | **G** | **T** | **T** | **G** | **A** | **A** | **A** | **T** | **T** | **A** | **A** | **A** | **G** | **T** | **T** | **C** | **C** | **T** | **G** | **T** | **C** | **A** | **T** | **A** | **A** | **A** | **G** | **G** | **A** | **G** | **G** | **A** | **A** | **A** | **C** | **T** | **G** | **C** | **A** | **A** | **G** | **T** | **G** | **A** | **T** | **C** | **A** | **A** | **A** | **G** | **T** | **A** | **A** | **T** | **G** | **T** | **T** | **A** | **G** | **T** | **G** | **G** | **A** | **-** | **-** | **-** |
| **N53434** | **A** | **T** | **G** | **A** | **C** | **C** | **G** | **A** | **T** | **C** | **G** | **G** | **A** | **T** | **T** | **T** | **C** | **A** | **A** | **G** | **A** | **C** | **G** | **C** | **A** | **T** | **G** | **A** | **T** | **T** | **T** | **C** | **A** | **T** | **T** | **A** | **T** | **A** | **T** | **G** | **C** | **G** | **T** | **C** | **C** | **C** | **T** | **T** | **A** | **G** | **C** | **A** | **C** | **C** | **T** | **T** | **T** | **A** | **T** | **T** | **G** | **T** | **T** | **A** | **T** | **C** | **T** | **T** | **A** | **A** | **T** | **T** | **A** | **C** | **A** | **T** | **T** | **G** | **T** | **T** | **T** | **T** | **C** | **A** | **T** | **A** | **T** | **T** | **T** | **T** | **C** | **A** | **T** | **T** | **C** | **G** | **A** | **T** | **T** | **A** | **A** | **A** | **C** | **A** | **A** | **A** | **A** | **C** | **C** | **G** | **G** | **T** | **G** | **G** | **T** | **T** | **A** | **T** | **T** | **A** | **G** | **A** | **G** | **C** | **T** | **T** | **C** | **T** | **T** | **C** | **A** | **G** | **A** | **G** | **A** | **A** | **A** | **A** | **G** | **T** | **C** | **T** | **T** | **T** | **T** | **A** | **T** | **T** | **T** | **A** | **C** | **C** | **A** | **C** | **T** | **A** | **A** | **T** | **T** | **G** | **T** | **G** | **C** | **A** | **C** | **A** | **T** | **T** | **G** | **T** | **T** | **C** | **T** | **C** | **A** | **T** | **A** | **T** | **C** | **C** | **T** | **T** | **A** | **C** | **T** | **A** | **A** | **T** | **A** | **G** | **G** | **C** | **T** | **T** | **A** | **T** | **T** | **G** | **A** | **C** | **C** | **T** | **T** | **T** | **T** | **T** | **A** | **C** | **T** | **G** | **A** | **T** | **T** | **T** | **C** | **A** | **T** | **T** | **G** | **G** | **T** | **T** | **C** | **A** | **A** | **A** | **A** | **A** | **G** | **G** | **G** | **C** | **A** | **A** | **T** | **A** | **T** | **G** | **G** | **A** | **C** | **G** | **G** | **A** | **T** | **T** | **G** | **A** | **A** | **G** | **A** | **A** | **A** | **A** | **A** | **C** | **T** | **T** | **C** | **G** | **G** | **T** | **T** | **A** | **T** | **T** | **G** | **G** | **C** | **C** | **A** | **A** | **C** | **G** | **G** | **T** | **A** | **A** | **T** | **T** | **A** | **T** | **G** | **A** | **A** | **A** | **G** | **T** | **C** | **C** | **A** | **G** | **T** | **C** | **T** | **T** | **A** | **A** | **A** | **C** | **A** | **A** | **A** | **C** | **C** | **A** | **A** | **C** | **G** | **A** | **C** | **C** | **A** | **G** | **T** | **G** | **A** | **A** | **A** | **A** | **T** | **C** | **A** | **A** | **G** | **A** | **C** | **C** | **A** | **T** | **T** | **A** | **T** | **C** | **T** | **A** | **A** | **C** | **C** | **G** | **A** | **A** | **G** | **T** | **C** | **G** | **A** | **A** | **C** | **A** | **A** | **G** | **A** | **T** | **A** | **T** | **T** | **T** | **G** | **G** | **T** | **C** | **G** | **A** | **T** | **T** | **A** | **A** | **A** | **A** | **A** | **T** | **A** | **A** | **A** | **T** | **T** | **A** | **T** | **T** | **A** | **G** | **A** | **G** | **A** | **T** | **G** | **T** | **C** | **T** | **A** | **A** | **A** | **G** | **A** | **A** | **T** | **T** | **G** | **C** | **A** | **A** | **T** | **T** | **A** | **T** | **T** | **A** | **A** | **A** | **C** | **A** | **G** | **T** | **C** | **G** | **A** | **C** | **C** | **G** | **C** | **A** | **A** | **T** | **T** | **A** | **A** | **T** | **G** | **G** | **A** | **T** | **G** | **G** | **G** | **C** | **A** | **A** | **A** | **C** | **A** | **A** | **A** | **A** | **G** | **A** | **A** | **G** | **A** | **A** | **A** | **T** | **T** | **T** | **T** | **A** | **G** | **A** | **G** | **A** | **A** | **C** | **G** | **A** | **G** | **C** | **G** | **G** | **C** | **A** | **T** | **C** | **G** | **T** | **T** | **T** | **G** | **G** | **C** | **G** | **C** | **G** | **G** | **G** | **A** | **G** | **T** | **T** | **G** | **C** | **A** | **T** | **G** | **A** | **T** | **T** | **C** | **A** | **G** | **T** | **C** | **A** | **G** | **T** | **C** | **A** | **A** | **C** | **A** | **A** | **C** | **T** | **T** | **T** | **T** | **T** | **G** | **C** | **A** | **G** | **C** | **C** | **A** | **T** | **G** | **A** | **T** | **G** | **A** | **T** | **G** | **T** | **T** | **G** | **T** | **C** | **T** | **G** | **C** | **C** | **T** | **T** | **A** | **A** | **A** | **T** | **G** | **A** | **A** | **C** | **A** | **A** | **G** | **C** | **A** | **C** | **A** | **A** | **C** | **G** | **A** | **A** | **C** | **A** | **G** | **A** | **A** | **A** | **C** | **T** | **C** | **C** | **T** | **G** | **A** | **A** | **C** | **C** | **A** | **T** | **A** | **T** | **C** | **G** | **C** | **A** | **A** | **A** | **C** | **A** | **A** | **C** | **T** | **A** | **G** | **C** | **C** | **A** | **T** | **G** | **G** | **T** | **G** | **G** | **C** | **A** | **G** | **A** | **A** | **A** | **T** | **C** | **A** | **T** | **T** | **A** | **A** | **T** | **G** | **C** | **C** | **T** | **C** | **C** | **C** | **A** | **A** | **T** | **C** | **G** | **G** | **A** | **A** | **A** | **T** | **G** | **C** | **G** | **T** | **G** | **C** | **G** | **C** | **T** | **A** | **C** | **T** | **A** | **T** | **T** | **G** | **C** | **A** | **C** | **T** | **T** | **G** | **C** | **G** | **T** | **C** | **C** | **T** | **A** | **T** | **C** | **A** | **G** | **T** | **C** | **T** | **A** | **G** | **A** | **A** | **G** | **G** | **A** | **A** | **A** | **A** | **A** | **G** | **T** | **T** | **T** | **G** | **C** | **G** | **T** | **A** | **A** | **A** | **G** | **G** | **T** | **A** | **T** | **T** | **G** | **A** | **A** | **C** | **A** | **A** | **T** | **T** | **A** | **C** | **T** | **G** | **A** | **A** | **A** | **G** | **A** | **A** | **C** | **T** | **A** | **C** | **A** | **A** | **A** | **C** | **A** | **A** | **A** | **A** | **A** | **T** | **T** | **A** | **A** | **A** | **A** | **T** | **T** | **G** | **A** | **A** | **T** | **T** | **G** | **A** | **T** | **T** | **T** | **G** | **G** | **G** | **A** | **T** | **G** | **T** | **T** | **G** | **A** | **A** | **G** | **A** | **T** | **G** | **T** | **T** | **C** | **A** | **T** | **T** | **T** | **A** | **A** | **A** | **T** | **A** | **G** | **C** | **A** | **G** | **C** | **A** | **T** | **T** | **G** | **A** | **G** | **G** | **A** | **T** | **C** | **A** | **T** | **C** | **T** | **T** | **T** | **T** | **C** | **C** | **G** | **A** | **A** | **T** | **T** | **G** | **T** | **G** | **C** | **A** | **G** | **G** | **A** | **G** | **T** | **T** | **A** | **C** | **T** | **T** | **T** | **C** | **A** | **A** | **A** | **T** | **A** | **C** | **C** | **T** | **T** | **A** | **A** | **G** | **A** | **C** | **A** | **T** | **G** | **C** | **C** | **A** | **A** | **A** | **G** | **C** | **A** | **A** | **A** | **G** | **G** | **A** | **A** | **T** | **T** | **A** | **G** | **A** | **G** | **G** | **T** | **A** | **T** | **A** | **C** | **T** | **T** | **A** | **C** | **A** | **C** | **C** | **A** | **A** | **G** | **T** | **C** | **G** | **A** | **T** | **A** | **A** | **A** | **A** | **A** | **C** | **G** | **T** | **G** | **T** | **T** | **A** | **T** | **T** | **G** | **C** | **G** | **T** | **A** | **T** | **T** | **G** | **T** | **T** | **G** | **A** | **T** | **G** | **A** | **T** | **G** | **G** | **T** | **G** | **T** | **C** | **G** | **G** | **C** | **T** | **T** | **T** | **G** | **A** | **T** | **A** | **T** | **G** | **A** | **A** | **G** | **G** | **A** | **A** | **C** | **A** | **A** | **A** | **G** | **T** | **A** | **A** | **T** | **A** | **A** | **A** | **G** | **C** | **C** | **G** | **G** | **T** | **A** | **G** | **T** | **T** | **A** | **T** | **G** | **G** | **C** | **T** | **T** | **A** | **A** | **A** | **T** | **A** | **A** | **T** | **A** | **T** | **C** | **C** | **G** | **A** | **G** | **A** | **A** | **C** | **G** | **T** | **G** | **T** | **T** | **G** | **T** | **C** | **G** | **G** | **C** | **A** | **T** | **G** | **G** | **G** | **C** | **G** | **G** | **T** | **A** | **C** | **A** | **G** | **T** | **T** | **A** | **A** | **A** | **A** | **T** | **T** | **A** | **T** | **T** | **A** | **G** | **T** | **T** | **T** | **T** | **A** | **A** | **A** | **G** | **G** | **G** | **C** | **A** | **G** | **G** | **G** | **A** | **A** | **C** | **C** | **A** | **G** | **C** | **G** | **T** | **T** | **G** | **A** | **A** | **A** | **T** | **T** | **A** | **A** | **A** | **G** | **T** | **T** | **C** | **C** | **T** | **G** | **T** | **C** | **A** | **T** | **A** | **A** | **A** | **G** | **G** | **A** | **G** | **G** | **A** | **A** | **A** | **C** | **T** | **G** | **C** | **A** | **A** | **G** | **T** | **G** | **A** | **T** | **C** | **A** | **A** | **A** | **G** | **T** | **A** | **A** | **T** | **G** | **T** | **T** | **A** | **G** | **T** | **G** | **G** | **A** | **-** | **-** | **-** |
| **N59845F** | **A** | **T** | **G** | **A** | **C** | **C** | **G** | **A** | **T** | **C** | **G** | **G** | **A** | **T** | **T** | **T** | **C** | **A** | **A** | **G** | **A** | **C** | **G** | **C** | **A** | **T** | **G** | **A** | **T** | **T** | **T** | **C** | **A** | **T** | **T** | **A** | **T** | **A** | **T** | **G** | **C** | **G** | **T** | **C** | **C** | **C** | **T** | **T** | **A** | **G** | **C** | **A** | **C** | **C** | **T** | **T** | **T** | **A** | **T** | **T** | **G** | **T** | **T** | **A** | **T** | **C** | **T** | **T** | **A** | **A** | **T** | **T** | **A** | **C** | **A** | **T** | **T** | **G** | **T** | **T** | **T** | **T** | **C** | **A** | **T** | **A** | **T** | **T** | **T** | **T** | **C** | **A** | **T** | **T** | **C** | **G** | **A** | **T** | **T** | **A** | **A** | **A** | **C** | **A** | **A** | **A** | **A** | **C** | **C** | **G** | **G** | **T** | **G** | **G** | **T** | **T** | **A** | **T** | **T** | **A** | **G** | **A** | **G** | **C** | **T** | **T** | **C** | **T** | **T** | **C** | **A** | **G** | **A** | **G** | **A** | **A** | **A** | **A** | **G** | **T** | **C** | **T** | **T** | **T** | **T** | **A** | **T** | **T** | **T** | **A** | **C** | **C** | **A** | **C** | **T** | **A** | **A** | **T** | **T** | **G** | **T** | **G** | **C** | **A** | **C** | **A** | **T** | **T** | **G** | **T** | **T** | **C** | **T** | **C** | **A** | **T** | **A** | **T** | **C** | **C** | **T** | **T** | **A** | **C** | **T** | **A** | **A** | **T** | **A** | **G** | **G** | **C** | **T** | **T** | **A** | **T** | **T** | **G** | **A** | **C** | **C** | **T** | **T** | **T** | **T** | **T** | **A** | **C** | **T** | **G** | **A** | **T** | **T** | **T** | **C** | **A** | **T** | **T** | **G** | **G** | **T** | **T** | **C** | **A** | **A** | **A** | **A** | **A** | **G** | **G** | **G** | **C** | **A** | **A** | **T** | **A** | **T** | **G** | **G** | **A** | **C** | **G** | **G** | **A** | **T** | **T** | **G** | **A** | **A** | **G** | **A** | **A** | **A** | **A** | **A** | **C** | **T** | **T** | **C** | **G** | **G** | **T** | **T** | **A** | **T** | **T** | **G** | **G** | **C** | **C** | **A** | **A** | **C** | **G** | **G** | **T** | **A** | **A** | **T** | **T** | **A** | **T** | **G** | **A** | **A** | **A** | **G** | **T** | **C** | **C** | **A** | **G** | **T** | **C** | **T** | **T** | **A** | **A** | **A** | **C** | **A** | **A** | **A** | **C** | **C** | **A** | **A** | **C** | **G** | **A** | **C** | **C** | **A** | **G** | **T** | **G** | **A** | **A** | **A** | **A** | **T** | **C** | **A** | **A** | **G** | **A** | **C** | **C** | **A** | **T** | **T** | **A** | **T** | **C** | **T** | **A** | **A** | **C** | **C** | **G** | **A** | **A** | **G** | **T** | **C** | **G** | **A** | **A** | **C** | **A** | **A** | **G** | **A** | **T** | **A** | **T** | **T** | **T** | **G** | **G** | **T** | **C** | **G** | **A** | **T** | **T** | **A** | **A** | **A** | **A** | **A** | **T** | **A** | **A** | **A** | **T** | **T** | **A** | **T** | **T** | **A** | **G** | **A** | **G** | **A** | **T** | **G** | **T** | **C** | **T** | **A** | **A** | **A** | **G** | **A** | **A** | **T** | **T** | **G** | **C** | **A** | **A** | **T** | **T** | **A** | **T** | **T** | **A** | **A** | **A** | **C** | **A** | **G** | **T** | **C** | **G** | **A** | **C** | **C** | **G** | **C** | **A** | **A** | **T** | **T** | **A** | **A** | **T** | **G** | **G** | **A** | **T** | **G** | **G** | **G** | **C** | **A** | **A** | **A** | **C** | **A** | **A** | **A** | **A** | **G** | **A** | **A** | **G** | **A** | **A** | **A** | **T** | **T** | **T** | **T** | **A** | **G** | **A** | **G** | **A** | **A** | **C** | **G** | **A** | **G** | **C** | **G** | **G** | **C** | **A** | **T** | **C** | **G** | **T** | **T** | **T** | **G** | **G** | **C** | **G** | **C** | **G** | **G** | **G** | **A** | **G** | **T** | **T** | **G** | **C** | **A** | **T** | **G** | **A** | **T** | **T** | **C** | **A** | **G** | **T** | **C** | **A** | **G** | **T** | **C** | **A** | **A** | **C** | **A** | **A** | **C** | **T** | **T** | **T** | **T** | **T** | **G** | **C** | **A** | **G** | **C** | **C** | **A** | **T** | **G** | **A** | **T** | **G** | **A** | **T** | **G** | **T** | **T** | **G** | **T** | **C** | **T** | **G** | **C** | **C** | **T** | **T** | **A** | **A** | **A** | **T** | **G** | **A** | **A** | **C** | **A** | **A** | **G** | **C** | **A** | **C** | **A** | **A** | **C** | **G** | **A** | **A** | **C** | **A** | **G** | **A** | **A** | **A** | **C** | **C** | **C** | **C** | **G** | **G** | **A** | **A** | **C** | **C** | **A** | **T** | **A** | **T** | **C** | **G** | **T** | **A** | **A** | **A** | **C** | **A** | **A** | **C** | **T** | **A** | **G** | **C** | **C** | **A** | **T** | **G** | **G** | **T** | **G** | **G** | **C** | **A** | **G** | **A** | **A** | **A** | **T** | **C** | **A** | **T** | **T** | **A** | **A** | **T** | **G** | **C** | **C** | **T** | **C** | **T** | **C** | **A** | **A** | **T** | **C** | **G** | **G** | **A** | **A** | **A** | **T** | **G** | **C** | **G** | **C** | **G** | **C** | **G** | **T** | **T** | **A** | **C** | **T** | **A** | **T** | **T** | **G** | **C** | **A** | **C** | **T** | **T** | **G** | **C** | **G** | **T** | **C** | **C** | **T** | **A** | **T** | **C** | **A** | **G** | **T** | **C** | **T** | **A** | **G** | **A** | **A** | **G** | **G** | **A** | **A** | **A** | **A** | **A** | **G** | **T** | **T** | **T** | **G** | **C** | **G** | **T** | **A** | **A** | **A** | **G** | **G** | **T** | **A** | **T** | **T** | **G** | **A** | **A** | **C** | **A** | **A** | **T** | **T** | **A** | **C** | **T** | **G** | **A** | **A** | **A** | **G** | **A** | **A** | **C** | **T** | **A** | **C** | **A** | **A** | **A** | **C** | **A** | **A** | **A** | **A** | **A** | **T** | **T** | **A** | **A** | **A** | **A** | **T** | **T** | **G** | **A** | **A** | **T** | **T** | **G** | **A** | **T** | **T** | **T** | **G** | **G** | **G** | **A** | **T** | **G** | **T** | **T** | **G** | **A** | **A** | **G** | **A** | **T** | **G** | **T** | **T** | **C** | **A** | **T** | **T** | **T** | **A** | **A** | **A** | **T** | **A** | **G** | **C** | **A** | **G** | **C** | **A** | **T** | **T** | **G** | **A** | **G** | **G** | **A** | **T** | **C** | **A** | **T** | **C** | **T** | **T** | **T** | **T** | **C** | **C** | **G** | **A** | **A** | **T** | **T** | **G** | **T** | **G** | **C** | **A** | **G** | **G** | **A** | **A** | **T** | **T** | **A** | **C** | **T** | **T** | **T** | **C** | **A** | **A** | **A** | **T** | **A** | **C** | **C** | **T** | **T** | **A** | **A** | **G** | **A** | **C** | **A** | **T** | **G** | **C** | **C** | **A** | **A** | **A** | **G** | **C** | **A** | **A** | **A** | **G** | **G** | **A** | **A** | **T** | **T** | **A** | **G** | **A** | **G** | **G** | **T** | **A** | **T** | **A** | **C** | **T** | **T** | **A** | **C** | **A** | **C** | **C** | **A** | **A** | **G** | **T** | **C** | **G** | **A** | **T** | **A** | **A** | **A** | **A** | **A** | **C** | **G** | **T** | **G** | **C** | **T** | **A** | **T** | **T** | **G** | **C** | **G** | **T** | **A** | **T** | **T** | **G** | **T** | **T** | **G** | **A** | **T** | **G** | **A** | **C** | **G** | **G** | **T** | **G** | **T** | **C** | **G** | **G** | **C** | **T** | **T** | **T** | **G** | **A** | **T** | **A** | **T** | **G** | **A** | **A** | **G** | **G** | **A** | **A** | **C** | **A** | **A** | **A** | **G** | **T** | **A** | **A** | **T** | **A** | **A** | **A** | **G** | **C** | **C** | **G** | **G** | **T** | **A** | **G** | **T** | **T** | **A** | **T** | **G** | **G** | **C** | **T** | **T** | **A** | **A** | **A** | **T** | **A** | **A** | **T** | **A** | **T** | **C** | **C** | **G** | **A** | **G** | **A** | **A** | **C** | **G** | **T** | **G** | **T** | **T** | **G** | **T** | **C** | **G** | **G** | **C** | **A** | **T** | **G** | **G** | **G** | **C** | **G** | **G** | **T** | **A** | **C** | **A** | **G** | **T** | **T** | **A** | **A** | **A** | **A** | **T** | **T** | **A** | **T** | **T** | **A** | **G** | **T** | **T** | **T** | **T** | **A** | **A** | **A** | **G** | **G** | **G** | **C** | **A** | **G** | **G** | **G** | **A** | **A** | **C** | **C** | **A** | **G** | **C** | **G** | **T** | **T** | **G** | **A** | **A** | **A** | **T** | **T** | **A** | **A** | **A** | **G** | **T** | **T** | **C** | **C** | **T** | **G** | **T** | **C** | **A** | **T** | **A** | **A** | **A** | **G** | **G** | **A** | **G** | **G** | **A** | **A** | **A** | **C** | **T** | **G** | **C** | **A** | **A** | **G** | **T** | **G** | **A** | **T** | **C** | **A** | **A** | **A** | **G** | **T** | **A** | **A** | **T** | **G** | **T** | **T** | **A** | **G** | **T** | **G** | **G** | **A** | **-** | **-** | **-** |
| **N59689F** | **A** | **T** | **G** | **A** | **C** | **C** | **G** | **A** | **T** | **C** | **G** | **G** | **A** | **T** | **T** | **T** | **C** | **A** | **A** | **G** | **A** | **C** | **G** | **C** | **A** | **T** | **G** | **A** | **T** | **T** | **T** | **C** | **A** | **T** | **T** | **A** | **T** | **A** | **T** | **G** | **C** | **G** | **T** | **C** | **C** | **C** | **T** | **T** | **A** | **G** | **C** | **A** | **C** | **C** | **T** | **T** | **T** | **A** | **T** | **T** | **G** | **T** | **T** | **A** | **T** | **C** | **T** | **T** | **A** | **A** | **T** | **T** | **A** | **C** | **A** | **T** | **T** | **G** | **T** | **T** | **T** | **T** | **C** | **A** | **T** | **A** | **T** | **T** | **T** | **T** | **C** | **A** | **T** | **T** | **C** | **G** | **A** | **T** | **T** | **A** | **A** | **A** | **C** | **A** | **A** | **A** | **A** | **C** | **C** | **G** | **G** | **T** | **G** | **G** | **C** | **T** | **A** | **T** | **T** | **A** | **G** | **A** | **G** | **C** | **T** | **T** | **C** | **T** | **T** | **C** | **A** | **G** | **A** | **G** | **A** | **A** | **A** | **A** | **G** | **T** | **C** | **T** | **T** | **T** | **T** | **A** | **T** | **T** | **T** | **A** | **C** | **C** | **A** | **C** | **T** | **A** | **A** | **T** | **T** | **G** | **T** | **G** | **C** | **A** | **C** | **A** | **T** | **T** | **G** | **T** | **T** | **C** | **T** | **C** | **A** | **T** | **A** | **T** | **C** | **C** | **T** | **T** | **A** | **C** | **T** | **A** | **A** | **T** | **A** | **G** | **G** | **C** | **T** | **T** | **A** | **T** | **T** | **G** | **A** | **C** | **C** | **T** | **T** | **T** | **T** | **T** | **A** | **C** | **T** | **G** | **A** | **T** | **T** | **T** | **C** | **A** | **T** | **T** | **G** | **G** | **T** | **T** | **C** | **A** | **A** | **A** | **A** | **A** | **G** | **G** | **G** | **C** | **A** | **A** | **T** | **A** | **T** | **G** | **G** | **A** | **C** | **G** | **G** | **A** | **T** | **T** | **G** | **A** | **A** | **G** | **A** | **A** | **A** | **A** | **A** | **C** | **T** | **T** | **C** | **G** | **G** | **T** | **T** | **A** | **T** | **T** | **G** | **G** | **C** | **C** | **A** | **A** | **C** | **G** | **G** | **T** | **A** | **A** | **T** | **T** | **A** | **T** | **G** | **A** | **A** | **A** | **G** | **T** | **C** | **C** | **A** | **G** | **T** | **C** | **T** | **T** | **A** | **A** | **A** | **C** | **A** | **A** | **A** | **C** | **C** | **A** | **A** | **C** | **G** | **A** | **C** | **C** | **A** | **G** | **T** | **G** | **A** | **A** | **A** | **A** | **T** | **C** | **A** | **A** | **G** | **A** | **C** | **C** | **A** | **T** | **T** | **A** | **T** | **C** | **T** | **A** | **A** | **C** | **C** | **G** | **A** | **A** | **G** | **T** | **C** | **G** | **A** | **A** | **C** | **A** | **A** | **G** | **A** | **T** | **A** | **T** | **T** | **T** | **G** | **G** | **T** | **C** | **G** | **A** | **T** | **T** | **A** | **A** | **A** | **A** | **A** | **T** | **A** | **A** | **A** | **T** | **T** | **A** | **T** | **T** | **A** | **G** | **A** | **G** | **A** | **T** | **G** | **T** | **C** | **T** | **A** | **A** | **A** | **G** | **A** | **A** | **T** | **T** | **G** | **C** | **A** | **A** | **T** | **T** | **A** | **T** | **T** | **A** | **A** | **A** | **C** | **A** | **G** | **T** | **C** | **G** | **A** | **C** | **C** | **G** | **C** | **A** | **A** | **T** | **T** | **A** | **A** | **T** | **G** | **G** | **A** | **T** | **G** | **G** | **G** | **C** | **A** | **A** | **A** | **C** | **A** | **A** | **A** | **A** | **G** | **A** | **A** | **G** | **A** | **A** | **A** | **T** | **T** | **T** | **T** | **A** | **G** | **A** | **G** | **A** | **A** | **C** | **G** | **A** | **G** | **C** | **G** | **G** | **C** | **A** | **T** | **C** | **G** | **T** | **T** | **T** | **G** | **G** | **C** | **G** | **C** | **G** | **G** | **G** | **A** | **G** | **T** | **T** | **G** | **C** | **A** | **T** | **G** | **A** | **T** | **T** | **C** | **A** | **G** | **T** | **C** | **A** | **G** | **T** | **C** | **A** | **A** | **C** | **A** | **A** | **C** | **T** | **T** | **T** | **T** | **T** | **G** | **C** | **A** | **G** | **C** | **C** | **A** | **T** | **G** | **A** | **T** | **G** | **A** | **T** | **G** | **T** | **T** | **G** | **T** | **C** | **T** | **G** | **C** | **C** | **T** | **T** | **A** | **A** | **A** | **T** | **G** | **A** | **A** | **C** | **A** | **A** | **G** | **C** | **A** | **C** | **A** | **A** | **C** | **G** | **A** | **A** | **C** | **A** | **G** | **A** | **A** | **A** | **C** | **T** | **C** | **C** | **T** | **G** | **A** | **A** | **C** | **C** | **A** | **T** | **A** | **T** | **C** | **G** | **C** | **A** | **A** | **A** | **C** | **A** | **A** | **C** | **T** | **A** | **G** | **C** | **C** | **A** | **T** | **G** | **G** | **T** | **G** | **G** | **C** | **A** | **G** | **A** | **A** | **A** | **T** | **C** | **A** | **T** | **T** | **A** | **A** | **T** | **G** | **C** | **C** | **T** | **C** | **C** | **C** | **A** | **A** | **T** | **C** | **G** | **G** | **A** | **A** | **A** | **T** | **G** | **C** | **G** | **C** | **G** | **C** | **G** | **T** | **T** | **A** | **C** | **T** | **A** | **T** | **T** | **G** | **C** | **A** | **C** | **T** | **T** | **G** | **C** | **G** | **T** | **C** | **C** | **T** | **A** | **T** | **C** | **A** | **G** | **T** | **C** | **T** | **A** | **G** | **A** | **A** | **G** | **G** | **A** | **A** | **A** | **A** | **A** | **G** | **T** | **T** | **T** | **G** | **C** | **G** | **T** | **A** | **A** | **A** | **G** | **G** | **T** | **A** | **T** | **T** | **G** | **A** | **A** | **C** | **A** | **A** | **T** | **T** | **A** | **C** | **T** | **G** | **A** | **A** | **A** | **G** | **A** | **A** | **C** | **T** | **A** | **C** | **A** | **A** | **A** | **C** | **A** | **A** | **A** | **A** | **A** | **T** | **T** | **A** | **A** | **A** | **A** | **T** | **T** | **G** | **A** | **A** | **T** | **T** | **G** | **A** | **T** | **T** | **T** | **G** | **G** | **G** | **A** | **T** | **G** | **T** | **T** | **G** | **A** | **A** | **G** | **A** | **T** | **G** | **T** | **T** | **C** | **A** | **T** | **T** | **T** | **A** | **A** | **A** | **T** | **A** | **G** | **C** | **A** | **G** | **C** | **A** | **T** | **T** | **G** | **A** | **G** | **G** | **A** | **T** | **C** | **A** | **T** | **C** | **T** | **T** | **T** | **T** | **C** | **C** | **G** | **A** | **A** | **T** | **T** | **G** | **T** | **G** | **C** | **A** | **G** | **G** | **A** | **A** | **T** | **T** | **A** | **C** | **T** | **T** | **T** | **C** | **A** | **A** | **A** | **T** | **A** | **C** | **C** | **T** | **T** | **A** | **A** | **G** | **A** | **C** | **A** | **T** | **G** | **C** | **C** | **A** | **A** | **A** | **G** | **C** | **A** | **A** | **A** | **G** | **G** | **A** | **A** | **T** | **T** | **A** | **G** | **A** | **G** | **G** | **T** | **A** | **T** | **A** | **C** | **T** | **T** | **A** | **C** | **A** | **C** | **C** | **A** | **A** | **G** | **T** | **C** | **G** | **A** | **T** | **A** | **A** | **A** | **A** | **A** | **C** | **G** | **T** | **G** | **T** | **T** | **A** | **T** | **T** | **G** | **C** | **G** | **T** | **A** | **T** | **T** | **G** | **T** | **T** | **G** | **A** | **T** | **G** | **A** | **T** | **G** | **G** | **T** | **G** | **T** | **C** | **G** | **G** | **C** | **T** | **T** | **T** | **G** | **A** | **T** | **A** | **T** | **G** | **A** | **A** | **G** | **G** | **A** | **A** | **C** | **A** | **A** | **A** | **G** | **T** | **A** | **A** | **T** | **A** | **A** | **A** | **G** | **C** | **C** | **G** | **G** | **T** | **A** | **G** | **T** | **T** | **A** | **T** | **G** | **G** | **C** | **T** | **T** | **A** | **A** | **A** | **T** | **A** | **A** | **T** | **A** | **T** | **C** | **C** | **G** | **A** | **G** | **A** | **A** | **C** | **G** | **T** | **G** | **T** | **T** | **G** | **T** | **C** | **G** | **G** | **C** | **A** | **T** | **G** | **G** | **G** | **C** | **G** | **G** | **T** | **A** | **C** | **A** | **G** | **T** | **T** | **A** | **A** | **A** | **A** | **T** | **T** | **A** | **T** | **T** | **A** | **G** | **T** | **T** | **T** | **T** | **A** | **A** | **A** | **G** | **G** | **G** | **C** | **A** | **G** | **G** | **G** | **A** | **A** | **C** | **C** | **A** | **G** | **C** | **G** | **T** | **T** | **G** | **A** | **A** | **A** | **T** | **T** | **A** | **A** | **A** | **G** | **T** | **T** | **C** | **C** | **T** | **G** | **T** | **C** | **A** | **T** | **A** | **A** | **A** | **G** | **G** | **A** | **G** | **G** | **A** | **A** | **A** | **C** | **T** | **G** | **C** | **A** | **A** | **G** | **T** | **G** | **A** | **T** | **C** | **A** | **A** | **A** | **G** | **T** | **A** | **A** | **T** | **G** | **T** | **T** | **A** | **G** | **T** | **G** | **G** | **A** | **-** | **-** | **-** |
| **N54588** | **A** | **T** | **G** | **A** | **C** | **C** | **G** | **A** | **T** | **C** | **G** | **G** | **A** | **T** | **T** | **T** | **C** | **A** | **A** | **G** | **A** | **C** | **G** | **C** | **A** | **T** | **G** | **A** | **T** | **T** | **T** | **C** | **A** | **T** | **T** | **A** | **T** | **A** | **T** | **G** | **C** | **G** | **T** | **C** | **C** | **C** | **T** | **T** | **A** | **G** | **C** | **A** | **C** | **C** | **T** | **T** | **T** | **A** | **T** | **T** | **G** | **T** | **T** | **A** | **T** | **C** | **T** | **T** | **A** | **A** | **T** | **T** | **A** | **C** | **A** | **T** | **T** | **G** | **T** | **T** | **T** | **T** | **C** | **A** | **T** | **A** | **T** | **T** | **T** | **T** | **C** | **A** | **T** | **T** | **C** | **G** | **A** | **T** | **T** | **A** | **A** | **A** | **C** | **A** | **A** | **A** | **A** | **C** | **C** | **G** | **G** | **T** | **G** | **G** | **C** | **T** | **A** | **T** | **T** | **A** | **G** | **A** | **G** | **C** | **T** | **T** | **C** | **T** | **T** | **C** | **A** | **G** | **A** | **G** | **A** | **A** | **A** | **A** | **G** | **T** | **C** | **T** | **T** | **T** | **T** | **A** | **T** | **T** | **T** | **A** | **C** | **C** | **A** | **C** | **T** | **A** | **A** | **T** | **T** | **G** | **T** | **G** | **C** | **A** | **C** | **A** | **T** | **T** | **G** | **T** | **T** | **C** | **T** | **C** | **A** | **T** | **A** | **T** | **C** | **C** | **T** | **T** | **A** | **C** | **T** | **A** | **A** | **T** | **A** | **G** | **G** | **C** | **T** | **T** | **A** | **T** | **T** | **G** | **A** | **C** | **C** | **T** | **T** | **T** | **T** | **T** | **A** | **C** | **T** | **G** | **A** | **T** | **T** | **T** | **C** | **A** | **T** | **T** | **G** | **G** | **T** | **T** | **C** | **A** | **A** | **A** | **A** | **A** | **G** | **G** | **G** | **C** | **A** | **A** | **T** | **A** | **T** | **G** | **G** | **A** | **C** | **G** | **G** | **A** | **T** | **T** | **G** | **A** | **A** | **G** | **A** | **A** | **A** | **A** | **A** | **C** | **T** | **T** | **C** | **G** | **G** | **T** | **T** | **A** | **T** | **T** | **G** | **G** | **C** | **C** | **A** | **A** | **C** | **G** | **G** | **T** | **A** | **A** | **T** | **T** | **A** | **T** | **G** | **A** | **A** | **A** | **G** | **T** | **C** | **C** | **A** | **G** | **T** | **C** | **T** | **T** | **A** | **A** | **A** | **C** | **A** | **A** | **A** | **C** | **C** | **A** | **A** | **C** | **G** | **A** | **C** | **C** | **A** | **G** | **T** | **G** | **A** | **A** | **A** | **A** | **T** | **C** | **A** | **A** | **G** | **A** | **C** | **C** | **A** | **T** | **T** | **A** | **T** | **C** | **T** | **A** | **A** | **C** | **C** | **G** | **A** | **A** | **G** | **T** | **C** | **G** | **A** | **A** | **C** | **A** | **A** | **G** | **A** | **T** | **A** | **T** | **T** | **T** | **G** | **G** | **T** | **C** | **G** | **A** | **T** | **T** | **A** | **A** | **A** | **A** | **A** | **T** | **A** | **A** | **A** | **T** | **T** | **A** | **T** | **T** | **A** | **G** | **A** | **G** | **A** | **T** | **G** | **T** | **C** | **T** | **A** | **A** | **A** | **G** | **A** | **A** | **T** | **T** | **G** | **C** | **A** | **A** | **T** | **T** | **A** | **T** | **T** | **A** | **A** | **A** | **C** | **A** | **G** | **T** | **C** | **G** | **A** | **C** | **C** | **G** | **C** | **A** | **A** | **T** | **T** | **A** | **A** | **T** | **G** | **G** | **A** | **T** | **G** | **G** | **G** | **C** | **A** | **A** | **A** | **C** | **A** | **A** | **A** | **A** | **G** | **A** | **A** | **G** | **A** | **A** | **A** | **T** | **T** | **T** | **T** | **A** | **G** | **A** | **G** | **A** | **A** | **C** | **G** | **A** | **G** | **C** | **G** | **G** | **C** | **A** | **T** | **C** | **G** | **T** | **T** | **T** | **G** | **G** | **C** | **G** | **C** | **G** | **G** | **G** | **A** | **G** | **T** | **T** | **G** | **C** | **A** | **T** | **G** | **A** | **T** | **T** | **C** | **A** | **G** | **T** | **C** | **A** | **G** | **T** | **C** | **A** | **A** | **C** | **A** | **A** | **T** | **T** | **T** | **T** | **T** | **T** | **G** | **C** | **A** | **G** | **C** | **C** | **A** | **T** | **G** | **A** | **T** | **G** | **A** | **T** | **G** | **T** | **T** | **G** | **T** | **C** | **T** | **G** | **C** | **C** | **T** | **T** | **A** | **A** | **A** | **T** | **G** | **A** | **A** | **C** | **A** | **A** | **G** | **C** | **A** | **C** | **A** | **A** | **C** | **G** | **A** | **A** | **C** | **A** | **G** | **A** | **A** | **A** | **C** | **T** | **C** | **C** | **T** | **G** | **A** | **A** | **C** | **C** | **A** | **T** | **A** | **T** | **C** | **G** | **C** | **A** | **A** | **A** | **C** | **A** | **A** | **C** | **T** | **A** | **G** | **C** | **C** | **A** | **T** | **G** | **G** | **T** | **G** | **G** | **C** | **A** | **G** | **A** | **A** | **A** | **T** | **C** | **A** | **T** | **T** | **A** | **A** | **T** | **G** | **C** | **C** | **T** | **C** | **C** | **C** | **A** | **A** | **T** | **C** | **G** | **G** | **A** | **A** | **A** | **T** | **G** | **C** | **G** | **T** | **G** | **C** | **G** | **C** | **T** | **A** | **C** | **T** | **A** | **T** | **T** | **G** | **C** | **A** | **C** | **T** | **T** | **G** | **C** | **G** | **T** | **C** | **C** | **T** | **A** | **T** | **C** | **A** | **G** | **T** | **C** | **T** | **A** | **G** | **A** | **A** | **G** | **G** | **A** | **A** | **A** | **A** | **A** | **G** | **T** | **T** | **T** | **G** | **C** | **G** | **T** | **A** | **A** | **A** | **G** | **G** | **T** | **A** | **T** | **T** | **G** | **A** | **A** | **C** | **A** | **A** | **T** | **T** | **A** | **C** | **T** | **G** | **A** | **A** | **A** | **G** | **A** | **A** | **C** | **T** | **A** | **C** | **A** | **A** | **A** | **C** | **A** | **A** | **A** | **A** | **A** | **T** | **T** | **A** | **A** | **A** | **A** | **T** | **T** | **G** | **A** | **A** | **T** | **T** | **G** | **A** | **T** | **T** | **T** | **G** | **G** | **G** | **A** | **T** | **G** | **T** | **T** | **G** | **A** | **A** | **G** | **A** | **T** | **G** | **T** | **T** | **C** | **A** | **T** | **T** | **T** | **A** | **A** | **A** | **T** | **A** | **G** | **C** | **A** | **G** | **C** | **A** | **T** | **T** | **G** | **A** | **G** | **G** | **A** | **T** | **C** | **A** | **T** | **C** | **T** | **T** | **T** | **T** | **C** | **C** | **G** | **A** | **A** | **T** | **T** | **G** | **T** | **G** | **C** | **A** | **G** | **G** | **A** | **G** | **T** | **T** | **A** | **C** | **T** | **T** | **T** | **C** | **A** | **A** | **A** | **T** | **A** | **C** | **C** | **T** | **T** | **A** | **A** | **G** | **A** | **C** | **A** | **T** | **G** | **C** | **C** | **A** | **A** | **A** | **G** | **C** | **A** | **A** | **A** | **G** | **G** | **A** | **A** | **T** | **T** | **A** | **G** | **A** | **G** | **G** | **T** | **A** | **T** | **A** | **C** | **T** | **T** | **A** | **C** | **A** | **C** | **C** | **A** | **A** | **G** | **T** | **C** | **G** | **A** | **T** | **A** | **A** | **A** | **A** | **A** | **C** | **G** | **T** | **G** | **T** | **T** | **A** | **T** | **T** | **G** | **C** | **G** | **T** | **A** | **T** | **T** | **G** | **T** | **T** | **G** | **A** | **T** | **G** | **A** | **T** | **G** | **G** | **T** | **G** | **T** | **C** | **G** | **G** | **C** | **T** | **T** | **T** | **G** | **A** | **T** | **A** | **T** | **G** | **A** | **A** | **G** | **G** | **A** | **A** | **C** | **A** | **A** | **A** | **G** | **T** | **A** | **A** | **T** | **A** | **A** | **A** | **G** | **C** | **C** | **G** | **G** | **T** | **A** | **G** | **T** | **T** | **A** | **T** | **G** | **G** | **C** | **T** | **T** | **A** | **A** | **A** | **T** | **A** | **A** | **T** | **A** | **T** | **C** | **C** | **G** | **A** | **G** | **A** | **A** | **C** | **G** | **T** | **G** | **T** | **T** | **G** | **T** | **C** | **G** | **G** | **C** | **A** | **T** | **G** | **G** | **G** | **C** | **G** | **G** | **T** | **A** | **C** | **A** | **G** | **T** | **T** | **A** | **A** | **A** | **A** | **T** | **T** | **A** | **T** | **T** | **A** | **G** | **T** | **T** | **T** | **T** | **A** | **A** | **A** | **G** | **G** | **G** | **C** | **A** | **G** | **G** | **G** | **A** | **A** | **C** | **C** | **A** | **G** | **C** | **G** | **T** | **T** | **G** | **A** | **A** | **A** | **T** | **T** | **A** | **A** | **A** | **G** | **T** | **T** | **C** | **C** | **T** | **G** | **T** | **C** | **A** | **T** | **A** | **A** | **A** | **G** | **G** | **A** | **G** | **G** | **A** | **A** | **A** | **C** | **T** | **G** | **C** | **A** | **A** | **G** | **T** | **G** | **A** | **T** | **C** | **A** | **A** | **A** | **G** | **T** | **A** | **A** | **T** | **G** | **T** | **T** | **A** | **G** | **T** | **G** | **G** | **A** | **-** | **-** | **-** |
| **N53457** | **A** | **T** | **G** | **A** | **C** | **C** | **G** | **A** | **T** | **C** | **G** | **G** | **A** | **T** | **T** | **T** | **C** | **A** | **A** | **G** | **A** | **C** | **G** | **C** | **A** | **T** | **G** | **A** | **T** | **T** | **T** | **C** | **A** | **T** | **T** | **A** | **T** | **A** | **T** | **G** | **C** | **G** | **T** | **C** | **C** | **C** | **T** | **T** | **A** | **G** | **C** | **A** | **C** | **C** | **T** | **T** | **T** | **A** | **T** | **T** | **G** | **T** | **T** | **A** | **T** | **C** | **T** | **T** | **A** | **A** | **T** | **T** | **A** | **C** | **A** | **T** | **T** | **G** | **T** | **T** | **T** | **T** | **C** | **A** | **T** | **A** | **T** | **T** | **T** | **T** | **C** | **A** | **T** | **T** | **C** | **G** | **A** | **T** | **T** | **A** | **A** | **A** | **C** | **A** | **A** | **A** | **A** | **C** | **C** | **G** | **G** | **T** | **G** | **G** | **C** | **T** | **A** | **T** | **T** | **A** | **G** | **A** | **G** | **C** | **T** | **T** | **C** | **T** | **T** | **C** | **A** | **G** | **A** | **G** | **A** | **A** | **A** | **A** | **G** | **T** | **C** | **T** | **T** | **T** | **T** | **A** | **T** | **T** | **T** | **A** | **C** | **C** | **A** | **C** | **T** | **A** | **A** | **T** | **T** | **G** | **T** | **G** | **C** | **A** | **C** | **A** | **T** | **T** | **G** | **T** | **T** | **C** | **T** | **C** | **A** | **T** | **A** | **T** | **C** | **C** | **T** | **T** | **A** | **C** | **T** | **A** | **A** | **T** | **A** | **G** | **G** | **C** | **T** | **T** | **A** | **T** | **T** | **G** | **A** | **C** | **C** | **T** | **T** | **T** | **T** | **T** | **A** | **C** | **T** | **G** | **A** | **T** | **T** | **T** | **C** | **A** | **T** | **T** | **G** | **G** | **T** | **T** | **C** | **A** | **A** | **A** | **A** | **A** | **G** | **G** | **G** | **C** | **A** | **A** | **T** | **A** | **T** | **G** | **G** | **A** | **C** | **G** | **G** | **A** | **T** | **T** | **G** | **A** | **A** | **G** | **A** | **A** | **A** | **A** | **A** | **C** | **T** | **T** | **C** | **G** | **G** | **T** | **T** | **A** | **T** | **T** | **G** | **G** | **C** | **C** | **A** | **A** | **T** | **G** | **G** | **T** | **A** | **A** | **T** | **T** | **A** | **T** | **G** | **A** | **A** | **A** | **G** | **T** | **C** | **C** | **A** | **G** | **T** | **C** | **T** | **T** | **A** | **A** | **A** | **C** | **A** | **A** | **A** | **C** | **C** | **A** | **A** | **C** | **G** | **A** | **C** | **C** | **A** | **G** | **T** | **G** | **A** | **A** | **A** | **A** | **T** | **C** | **A** | **A** | **G** | **A** | **C** | **C** | **A** | **T** | **T** | **A** | **T** | **C** | **T** | **A** | **A** | **C** | **C** | **G** | **A** | **A** | **G** | **T** | **C** | **G** | **A** | **A** | **C** | **A** | **A** | **G** | **A** | **T** | **A** | **T** | **T** | **T** | **G** | **G** | **T** | **C** | **G** | **A** | **T** | **T** | **A** | **A** | **A** | **A** | **A** | **T** | **A** | **A** | **A** | **T** | **T** | **A** | **T** | **T** | **A** | **G** | **A** | **G** | **A** | **T** | **G** | **T** | **C** | **T** | **A** | **A** | **A** | **G** | **A** | **A** | **T** | **T** | **G** | **C** | **A** | **A** | **T** | **T** | **A** | **T** | **T** | **A** | **A** | **A** | **C** | **A** | **G** | **T** | **C** | **G** | **A** | **C** | **C** | **G** | **C** | **A** | **A** | **T** | **T** | **A** | **A** | **T** | **G** | **G** | **A** | **T** | **G** | **G** | **G** | **C** | **A** | **A** | **A** | **C** | **A** | **A** | **A** | **A** | **G** | **A** | **A** | **G** | **A** | **A** | **A** | **T** | **T** | **T** | **T** | **A** | **G** | **A** | **G** | **A** | **A** | **C** | **G** | **A** | **G** | **C** | **G** | **G** | **C** | **A** | **T** | **C** | **G** | **T** | **T** | **T** | **G** | **G** | **C** | **G** | **C** | **G** | **G** | **G** | **A** | **G** | **T** | **T** | **G** | **C** | **A** | **T** | **G** | **A** | **T** | **T** | **C** | **A** | **G** | **T** | **C** | **A** | **G** | **T** | **C** | **A** | **A** | **C** | **A** | **A** | **C** | **T** | **T** | **T** | **T** | **T** | **G** | **C** | **A** | **G** | **C** | **C** | **A** | **T** | **G** | **A** | **T** | **G** | **A** | **T** | **G** | **T** | **T** | **G** | **T** | **C** | **T** | **G** | **C** | **C** | **T** | **T** | **A** | **A** | **A** | **T** | **G** | **A** | **A** | **C** | **A** | **A** | **G** | **C** | **A** | **C** | **A** | **A** | **C** | **G** | **A** | **A** | **C** | **A** | **G** | **A** | **A** | **A** | **C** | **C** | **C** | **C** | **T** | **G** | **A** | **A** | **C** | **C** | **A** | **T** | **A** | **T** | **C** | **G** | **C** | **A** | **A** | **A** | **C** | **A** | **A** | **C** | **T** | **A** | **G** | **C** | **C** | **A** | **T** | **G** | **G** | **T** | **G** | **G** | **C** | **A** | **G** | **A** | **A** | **A** | **T** | **C** | **A** | **T** | **T** | **A** | **A** | **T** | **G** | **C** | **C** | **T** | **C** | **C** | **C** | **A** | **A** | **T** | **C** | **G** | **G** | **A** | **A** | **A** | **T** | **G** | **C** | **G** | **C** | **G** | **C** | **G** | **T** | **T** | **A** | **C** | **T** | **A** | **T** | **T** | **G** | **C** | **A** | **C** | **T** | **T** | **G** | **C** | **G** | **T** | **C** | **C** | **T** | **A** | **T** | **C** | **A** | **G** | **T** | **C** | **T** | **A** | **G** | **A** | **A** | **G** | **G** | **A** | **A** | **A** | **A** | **A** | **G** | **T** | **T** | **T** | **G** | **C** | **G** | **T** | **A** | **A** | **A** | **G** | **G** | **T** | **A** | **T** | **T** | **G** | **A** | **A** | **C** | **A** | **A** | **T** | **T** | **A** | **C** | **T** | **G** | **A** | **A** | **A** | **G** | **A** | **A** | **C** | **T** | **A** | **C** | **A** | **A** | **A** | **C** | **A** | **A** | **A** | **A** | **A** | **T** | **T** | **A** | **A** | **A** | **A** | **T** | **T** | **G** | **A** | **A** | **T** | **T** | **G** | **A** | **T** | **T** | **T** | **G** | **G** | **G** | **A** | **T** | **G** | **T** | **T** | **G** | **A** | **A** | **G** | **A** | **T** | **G** | **T** | **T** | **C** | **A** | **T** | **T** | **T** | **A** | **A** | **A** | **T** | **A** | **G** | **C** | **A** | **G** | **C** | **A** | **T** | **T** | **G** | **A** | **G** | **G** | **A** | **T** | **C** | **A** | **T** | **C** | **T** | **T** | **T** | **T** | **C** | **C** | **G** | **A** | **A** | **T** | **T** | **G** | **T** | **G** | **C** | **A** | **G** | **G** | **A** | **G** | **T** | **T** | **A** | **C** | **T** | **T** | **T** | **C** | **A** | **A** | **A** | **T** | **A** | **C** | **C** | **T** | **T** | **A** | **A** | **G** | **A** | **C** | **A** | **T** | **G** | **C** | **C** | **A** | **A** | **A** | **G** | **C** | **A** | **A** | **A** | **G** | **G** | **A** | **A** | **T** | **T** | **A** | **G** | **A** | **G** | **G** | **T** | **A** | **T** | **A** | **C** | **T** | **T** | **A** | **C** | **A** | **C** | **C** | **A** | **A** | **G** | **T** | **C** | **G** | **A** | **T** | **A** | **A** | **A** | **A** | **A** | **C** | **G** | **T** | **G** | **T** | **T** | **A** | **T** | **T** | **G** | **C** | **G** | **T** | **A** | **T** | **T** | **G** | **T** | **T** | **G** | **A** | **T** | **G** | **A** | **T** | **G** | **G** | **T** | **G** | **T** | **C** | **G** | **G** | **C** | **T** | **T** | **T** | **G** | **A** | **T** | **A** | **T** | **G** | **A** | **A** | **G** | **G** | **A** | **A** | **C** | **A** | **A** | **A** | **G** | **T** | **A** | **A** | **T** | **A** | **A** | **A** | **G** | **C** | **T** | **G** | **G** | **T** | **A** | **G** | **T** | **T** | **A** | **T** | **G** | **G** | **C** | **T** | **T** | **A** | **A** | **A** | **T** | **A** | **A** | **T** | **A** | **T** | **T** | **C** | **G** | **A** | **G** | **A** | **A** | **C** | **G** | **T** | **G** | **T** | **T** | **G** | **T** | **C** | **G** | **G** | **C** | **A** | **T** | **G** | **G** | **G** | **C** | **G** | **G** | **T** | **A** | **C** | **A** | **G** | **T** | **T** | **A** | **A** | **A** | **A** | **T** | **T** | **A** | **T** | **T** | **A** | **G** | **T** | **T** | **T** | **T** | **A** | **A** | **A** | **G** | **G** | **G** | **C** | **A** | **G** | **G** | **G** | **A** | **A** | **C** | **C** | **A** | **G** | **C** | **G** | **T** | **T** | **G** | **A** | **A** | **A** | **T** | **T** | **A** | **A** | **A** | **G** | **T** | **T** | **C** | **C** | **T** | **G** | **T** | **C** | **A** | **T** | **A** | **A** | **A** | **G** | **G** | **A** | **G** | **G** | **A** | **A** | **A** | **C** | **T** | **G** | **C** | **A** | **A** | **G** | **T** | **G** | **A** | **T** | **C** | **A** | **A** | **A** | **G** | **T** | **A** | **A** | **T** | **G** | **C** | **T** | **A** | **G** | **T** | **G** | **G** | **A** | **-** | **-** | **-** |
| **N59508F** | **A** | **T** | **G** | **A** | **C** | **C** | **G** | **A** | **T** | **C** | **G** | **G** | **A** | **T** | **T** | **T** | **C** | **A** | **A** | **G** | **A** | **C** | **G** | **C** | **A** | **T** | **G** | **A** | **T** | **T** | **T** | **C** | **A** | **T** | **T** | **A** | **T** | **A** | **T** | **G** | **C** | **G** | **T** | **C** | **C** | **C** | **T** | **T** | **A** | **G** | **C** | **A** | **C** | **C** | **T** | **T** | **T** | **A** | **T** | **T** | **G** | **T** | **T** | **A** | **T** | **C** | **T** | **T** | **A** | **A** | **T** | **T** | **A** | **C** | **A** | **T** | **T** | **G** | **T** | **T** | **T** | **T** | **C** | **A** | **T** | **A** | **T** | **T** | **T** | **T** | **C** | **A** | **T** | **T** | **C** | **G** | **A** | **T** | **T** | **A** | **A** | **A** | **C** | **A** | **A** | **A** | **A** | **C** | **C** | **G** | **G** | **T** | **G** | **G** | **T** | **T** | **A** | **T** | **T** | **A** | **G** | **A** | **G** | **C** | **T** | **T** | **C** | **T** | **T** | **C** | **A** | **G** | **A** | **G** | **A** | **A** | **A** | **A** | **G** | **T** | **C** | **T** | **T** | **T** | **T** | **A** | **T** | **C** | **T** | **A** | **C** | **C** | **A** | **C** | **T** | **A** | **A** | **T** | **T** | **G** | **T** | **G** | **C** | **A** | **C** | **A** | **T** | **T** | **G** | **T** | **T** | **C** | **T** | **C** | **A** | **T** | **A** | **T** | **C** | **C** | **T** | **T** | **A** | **C** | **T** | **A** | **A** | **T** | **A** | **G** | **G** | **C** | **T** | **T** | **A** | **T** | **T** | **G** | **A** | **C** | **C** | **T** | **T** | **T** | **T** | **T** | **A** | **C** | **T** | **G** | **A** | **T** | **T** | **T** | **C** | **A** | **T** | **T** | **G** | **G** | **T** | **T** | **C** | **A** | **A** | **A** | **A** | **A** | **G** | **G** | **G** | **C** | **A** | **A** | **T** | **A** | **T** | **G** | **G** | **A** | **C** | **G** | **G** | **A** | **T** | **T** | **G** | **A** | **A** | **G** | **A** | **A** | **A** | **A** | **A** | **C** | **T** | **T** | **C** | **G** | **G** | **T** | **T** | **A** | **T** | **T** | **G** | **G** | **C** | **C** | **A** | **A** | **C** | **G** | **G** | **T** | **A** | **A** | **T** | **T** | **A** | **T** | **G** | **A** | **A** | **A** | **G** | **T** | **C** | **C** | **A** | **G** | **T** | **C** | **T** | **T** | **A** | **A** | **A** | **C** | **A** | **A** | **A** | **C** | **C** | **A** | **A** | **C** | **G** | **A** | **C** | **C** | **A** | **G** | **T** | **G** | **A** | **A** | **A** | **A** | **T** | **C** | **A** | **A** | **G** | **A** | **T** | **C** | **A** | **T** | **T** | **A** | **T** | **C** | **T** | **A** | **A** | **C** | **C** | **G** | **A** | **A** | **G** | **T** | **C** | **G** | **A** | **A** | **C** | **A** | **A** | **G** | **A** | **T** | **A** | **T** | **T** | **T** | **G** | **G** | **T** | **C** | **G** | **A** | **T** | **T** | **A** | **A** | **A** | **A** | **A** | **T** | **A** | **A** | **A** | **T** | **T** | **A** | **T** | **T** | **A** | **G** | **A** | **G** | **A** | **T** | **G** | **T** | **C** | **T** | **A** | **A** | **A** | **G** | **A** | **A** | **T** | **T** | **G** | **C** | **A** | **A** | **T** | **T** | **A** | **T** | **T** | **A** | **A** | **A** | **C** | **A** | **G** | **T** | **C** | **G** | **A** | **C** | **C** | **G** | **C** | **A** | **A** | **T** | **T** | **A** | **A** | **T** | **G** | **G** | **A** | **T** | **G** | **G** | **G** | **C** | **A** | **A** | **A** | **C** | **A** | **A** | **A** | **A** | **G** | **A** | **A** | **G** | **A** | **A** | **A** | **T** | **T** | **T** | **T** | **A** | **G** | **A** | **G** | **A** | **A** | **C** | **G** | **A** | **G** | **C** | **G** | **G** | **C** | **A** | **T** | **C** | **G** | **T** | **T** | **T** | **G** | **G** | **C** | **G** | **C** | **G** | **G** | **G** | **A** | **G** | **T** | **T** | **G** | **C** | **A** | **T** | **G** | **A** | **T** | **T** | **C** | **A** | **G** | **T** | **C** | **A** | **G** | **T** | **C** | **A** | **A** | **C** | **A** | **A** | **C** | **T** | **T** | **T** | **T** | **T** | **G** | **C** | **A** | **G** | **C** | **C** | **A** | **T** | **G** | **A** | **T** | **G** | **A** | **T** | **G** | **T** | **T** | **G** | **T** | **C** | **T** | **G** | **C** | **C** | **T** | **T** | **A** | **A** | **A** | **T** | **G** | **A** | **A** | **C** | **A** | **A** | **G** | **C** | **A** | **C** | **A** | **A** | **C** | **G** | **A** | **A** | **C** | **A** | **G** | **A** | **A** | **A** | **C** | **C** | **C** | **C** | **G** | **G** | **A** | **A** | **C** | **C** | **A** | **T** | **A** | **T** | **C** | **G** | **T** | **A** | **A** | **A** | **C** | **A** | **A** | **C** | **T** | **A** | **G** | **C** | **C** | **A** | **T** | **G** | **G** | **T** | **G** | **G** | **C** | **A** | **G** | **A** | **A** | **A** | **T** | **C** | **A** | **T** | **T** | **A** | **A** | **T** | **G** | **C** | **C** | **T** | **C** | **C** | **C** | **A** | **A** | **T** | **C** | **G** | **G** | **A** | **A** | **A** | **T** | **G** | **C** | **G** | **C** | **G** | **C** | **G** | **T** | **T** | **A** | **C** | **T** | **A** | **T** | **T** | **G** | **C** | **A** | **C** | **T** | **T** | **G** | **C** | **G** | **T** | **C** | **C** | **T** | **A** | **T** | **C** | **A** | **G** | **T** | **C** | **T** | **A** | **G** | **A** | **A** | **G** | **G** | **A** | **A** | **A** | **A** | **A** | **G** | **T** | **T** | **T** | **G** | **C** | **G** | **T** | **A** | **A** | **A** | **G** | **G** | **T** | **A** | **T** | **T** | **G** | **A** | **A** | **C** | **A** | **A** | **T** | **T** | **A** | **C** | **T** | **G** | **A** | **A** | **A** | **G** | **A** | **A** | **C** | **T** | **A** | **C** | **A** | **A** | **A** | **C** | **A** | **A** | **A** | **A** | **A** | **T** | **T** | **A** | **A** | **A** | **A** | **T** | **T** | **G** | **A** | **A** | **T** | **T** | **G** | **A** | **T** | **T** | **T** | **G** | **G** | **G** | **A** | **T** | **G** | **T** | **T** | **G** | **A** | **A** | **G** | **A** | **T** | **G** | **T** | **T** | **C** | **A** | **T** | **T** | **T** | **A** | **A** | **A** | **T** | **A** | **G** | **C** | **A** | **G** | **C** | **A** | **T** | **T** | **G** | **A** | **G** | **G** | **A** | **T** | **C** | **A** | **T** | **C** | **T** | **T** | **T** | **T** | **C** | **C** | **G** | **A** | **A** | **T** | **T** | **G** | **T** | **G** | **C** | **A** | **G** | **G** | **A** | **G** | **T** | **T** | **A** | **C** | **T** | **T** | **T** | **C** | **A** | **A** | **A** | **T** | **A** | **C** | **C** | **T** | **T** | **A** | **A** | **G** | **A** | **C** | **A** | **T** | **G** | **C** | **C** | **A** | **A** | **A** | **G** | **C** | **A** | **A** | **A** | **G** | **G** | **A** | **A** | **T** | **T** | **A** | **G** | **A** | **G** | **G** | **T** | **A** | **T** | **A** | **C** | **T** | **T** | **A** | **C** | **A** | **C** | **C** | **A** | **A** | **G** | **T** | **C** | **G** | **A** | **T** | **A** | **A** | **A** | **A** | **A** | **C** | **G** | **T** | **G** | **T** | **T** | **A** | **T** | **T** | **G** | **C** | **G** | **T** | **A** | **T** | **T** | **G** | **T** | **T** | **G** | **A** | **T** | **G** | **A** | **C** | **G** | **G** | **T** | **G** | **T** | **C** | **G** | **G** | **C** | **T** | **T** | **T** | **G** | **A** | **T** | **A** | **T** | **G** | **A** | **A** | **G** | **G** | **A** | **A** | **C** | **A** | **A** | **A** | **G** | **T** | **A** | **A** | **T** | **A** | **A** | **A** | **G** | **C** | **C** | **G** | **G** | **T** | **A** | **G** | **T** | **T** | **A** | **T** | **G** | **G** | **C** | **T** | **T** | **A** | **A** | **A** | **T** | **A** | **A** | **T** | **A** | **T** | **C** | **C** | **G** | **A** | **G** | **A** | **A** | **C** | **G** | **T** | **G** | **T** | **T** | **G** | **T** | **C** | **G** | **G** | **C** | **A** | **T** | **G** | **G** | **G** | **C** | **G** | **G** | **T** | **A** | **C** | **A** | **G** | **T** | **T** | **A** | **A** | **A** | **A** | **T** | **T** | **A** | **T** | **T** | **A** | **G** | **T** | **T** | **T** | **T** | **A** | **A** | **A** | **G** | **G** | **G** | **C** | **A** | **G** | **G** | **G** | **A** | **A** | **C** | **C** | **A** | **G** | **C** | **G** | **T** | **T** | **G** | **A** | **A** | **A** | **T** | **T** | **A** | **A** | **A** | **G** | **T** | **T** | **C** | **C** | **T** | **G** | **T** | **C** | **A** | **T** | **A** | **A** | **A** | **G** | **G** | **A** | **G** | **G** | **A** | **A** | **A** | **C** | **T** | **G** | **C** | **A** | **A** | **G** | **T** | **G** | **A** | **T** | **C** | **A** | **A** | **A** | **G** | **T** | **A** | **A** | **T** | **G** | **T** | **T** | **A** | **G** | **T** | **G** | **G** | **A** | **-** | **-** | **-** |
| **N59860F** | **A** | **T** | **G** | **A** | **C** | **C** | **G** | **A** | **T** | **C** | **G** | **G** | **A** | **T** | **T** | **T** | **C** | **A** | **A** | **G** | **A** | **C** | **G** | **C** | **A** | **T** | **G** | **A** | **T** | **T** | **T** | **C** | **A** | **T** | **T** | **A** | **T** | **A** | **T** | **G** | **C** | **G** | **T** | **C** | **C** | **C** | **T** | **T** | **A** | **G** | **C** | **A** | **C** | **C** | **T** | **T** | **T** | **A** | **T** | **T** | **G** | **T** | **T** | **A** | **T** | **C** | **T** | **T** | **A** | **A** | **T** | **T** | **A** | **C** | **A** | **T** | **T** | **G** | **T** | **T** | **T** | **T** | **C** | **A** | **T** | **A** | **T** | **T** | **T** | **T** | **C** | **A** | **T** | **T** | **C** | **G** | **A** | **T** | **T** | **A** | **A** | **A** | **C** | **A** | **A** | **A** | **A** | **C** | **C** | **G** | **G** | **T** | **G** | **G** | **T** | **T** | **A** | **T** | **T** | **A** | **G** | **A** | **G** | **C** | **T** | **T** | **C** | **T** | **T** | **C** | **A** | **G** | **A** | **G** | **A** | **A** | **A** | **A** | **G** | **T** | **C** | **T** | **T** | **T** | **T** | **A** | **T** | **T** | **T** | **A** | **C** | **C** | **A** | **C** | **T** | **A** | **A** | **T** | **T** | **G** | **T** | **G** | **C** | **A** | **C** | **A** | **T** | **T** | **G** | **T** | **T** | **C** | **T** | **C** | **A** | **T** | **A** | **T** | **C** | **C** | **T** | **T** | **A** | **C** | **T** | **A** | **A** | **T** | **A** | **G** | **G** | **C** | **T** | **T** | **A** | **T** | **T** | **G** | **A** | **C** | **C** | **T** | **T** | **T** | **T** | **T** | **A** | **C** | **T** | **G** | **A** | **T** | **T** | **T** | **C** | **A** | **T** | **T** | **G** | **G** | **T** | **T** | **C** | **A** | **A** | **A** | **A** | **A** | **G** | **G** | **G** | **C** | **A** | **A** | **T** | **A** | **T** | **G** | **G** | **A** | **C** | **G** | **G** | **A** | **T** | **T** | **G** | **A** | **A** | **G** | **A** | **A** | **A** | **A** | **A** | **C** | **T** | **T** | **C** | **G** | **G** | **T** | **T** | **A** | **T** | **T** | **G** | **G** | **C** | **C** | **A** | **A** | **C** | **G** | **G** | **T** | **A** | **A** | **T** | **T** | **A** | **T** | **G** | **A** | **A** | **A** | **G** | **T** | **C** | **C** | **A** | **G** | **T** | **C** | **T** | **T** | **A** | **A** | **A** | **C** | **A** | **A** | **A** | **C** | **C** | **A** | **A** | **C** | **G** | **A** | **C** | **C** | **A** | **G** | **T** | **G** | **A** | **A** | **A** | **A** | **T** | **C** | **A** | **A** | **G** | **A** | **C** | **C** | **A** | **T** | **T** | **A** | **T** | **C** | **T** | **A** | **A** | **C** | **C** | **G** | **A** | **A** | **G** | **T** | **C** | **G** | **A** | **A** | **C** | **A** | **A** | **G** | **A** | **T** | **A** | **T** | **T** | **T** | **G** | **G** | **T** | **C** | **G** | **A** | **T** | **T** | **A** | **A** | **A** | **A** | **A** | **T** | **A** | **A** | **A** | **T** | **T** | **A** | **T** | **T** | **A** | **G** | **A** | **G** | **A** | **T** | **G** | **T** | **C** | **T** | **A** | **A** | **A** | **G** | **A** | **A** | **T** | **T** | **G** | **C** | **A** | **A** | **T** | **T** | **A** | **T** | **T** | **A** | **A** | **A** | **C** | **A** | **G** | **T** | **C** | **G** | **A** | **C** | **C** | **G** | **C** | **A** | **A** | **T** | **T** | **A** | **A** | **T** | **G** | **G** | **A** | **T** | **G** | **G** | **G** | **C** | **A** | **A** | **A** | **C** | **A** | **A** | **A** | **A** | **G** | **A** | **A** | **G** | **A** | **A** | **A** | **T** | **T** | **T** | **T** | **A** | **G** | **A** | **G** | **A** | **A** | **C** | **G** | **A** | **G** | **C** | **G** | **G** | **C** | **A** | **T** | **C** | **G** | **T** | **T** | **T** | **G** | **G** | **C** | **G** | **C** | **G** | **G** | **G** | **A** | **G** | **T** | **T** | **G** | **C** | **A** | **T** | **G** | **A** | **T** | **T** | **C** | **A** | **G** | **T** | **C** | **A** | **G** | **T** | **C** | **A** | **A** | **C** | **A** | **A** | **C** | **T** | **T** | **T** | **T** | **T** | **G** | **C** | **A** | **G** | **C** | **C** | **A** | **T** | **G** | **A** | **T** | **G** | **A** | **T** | **G** | **T** | **T** | **G** | **T** | **C** | **T** | **G** | **C** | **C** | **T** | **T** | **A** | **A** | **A** | **T** | **G** | **A** | **A** | **C** | **A** | **A** | **G** | **C** | **A** | **C** | **A** | **A** | **C** | **G** | **A** | **A** | **C** | **A** | **G** | **A** | **A** | **A** | **C** | **T** | **C** | **C** | **T** | **G** | **A** | **A** | **C** | **C** | **A** | **T** | **A** | **T** | **C** | **G** | **C** | **A** | **A** | **A** | **C** | **A** | **A** | **C** | **T** | **A** | **G** | **C** | **C** | **A** | **T** | **G** | **G** | **T** | **G** | **G** | **C** | **A** | **G** | **A** | **A** | **A** | **T** | **C** | **A** | **T** | **T** | **A** | **A** | **T** | **G** | **C** | **C** | **T** | **C** | **C** | **C** | **A** | **A** | **T** | **C** | **G** | **G** | **A** | **A** | **A** | **T** | **G** | **C** | **G** | **T** | **G** | **C** | **G** | **C** | **T** | **A** | **C** | **T** | **A** | **T** | **T** | **G** | **C** | **A** | **C** | **T** | **T** | **G** | **C** | **G** | **T** | **C** | **C** | **T** | **A** | **T** | **C** | **A** | **G** | **T** | **C** | **T** | **A** | **G** | **A** | **A** | **G** | **G** | **A** | **A** | **A** | **A** | **A** | **G** | **T** | **T** | **T** | **G** | **C** | **G** | **T** | **A** | **A** | **A** | **G** | **G** | **T** | **A** | **T** | **T** | **G** | **A** | **A** | **C** | **A** | **A** | **T** | **T** | **A** | **C** | **T** | **G** | **A** | **A** | **A** | **G** | **A** | **A** | **C** | **T** | **A** | **C** | **A** | **A** | **A** | **C** | **A** | **A** | **A** | **A** | **A** | **T** | **T** | **A** | **A** | **A** | **A** | **T** | **T** | **G** | **A** | **A** | **T** | **T** | **G** | **A** | **T** | **T** | **T** | **G** | **G** | **G** | **A** | **T** | **G** | **T** | **T** | **G** | **A** | **A** | **G** | **A** | **T** | **G** | **T** | **T** | **C** | **A** | **T** | **T** | **T** | **A** | **A** | **A** | **T** | **A** | **G** | **C** | **A** | **G** | **C** | **A** | **T** | **T** | **G** | **A** | **G** | **G** | **A** | **T** | **C** | **A** | **T** | **C** | **T** | **T** | **T** | **T** | **C** | **C** | **G** | **A** | **A** | **T** | **T** | **G** | **T** | **G** | **C** | **A** | **G** | **G** | **A** | **G** | **T** | **T** | **A** | **C** | **T** | **T** | **T** | **C** | **A** | **A** | **A** | **T** | **A** | **C** | **C** | **T** | **T** | **A** | **A** | **G** | **A** | **C** | **A** | **T** | **G** | **C** | **C** | **A** | **A** | **A** | **G** | **C** | **A** | **A** | **A** | **G** | **G** | **A** | **A** | **T** | **T** | **A** | **G** | **A** | **G** | **G** | **T** | **A** | **T** | **A** | **C** | **T** | **T** | **A** | **C** | **A** | **C** | **C** | **A** | **A** | **G** | **T** | **C** | **G** | **A** | **T** | **A** | **A** | **A** | **A** | **A** | **C** | **G** | **T** | **G** | **T** | **T** | **A** | **T** | **T** | **G** | **C** | **G** | **T** | **A** | **T** | **T** | **G** | **T** | **T** | **G** | **A** | **T** | **G** | **A** | **T** | **G** | **G** | **T** | **G** | **T** | **C** | **G** | **G** | **C** | **T** | **T** | **T** | **G** | **A** | **T** | **A** | **T** | **G** | **A** | **A** | **G** | **G** | **A** | **A** | **C** | **A** | **A** | **A** | **G** | **T** | **A** | **A** | **T** | **A** | **A** | **A** | **G** | **C** | **C** | **G** | **G** | **T** | **A** | **G** | **T** | **T** | **A** | **T** | **G** | **G** | **C** | **T** | **T** | **A** | **A** | **A** | **T** | **A** | **A** | **T** | **A** | **T** | **C** | **C** | **G** | **A** | **G** | **A** | **A** | **C** | **G** | **T** | **G** | **T** | **T** | **G** | **T** | **C** | **G** | **G** | **C** | **A** | **T** | **G** | **G** | **G** | **C** | **G** | **G** | **T** | **A** | **C** | **A** | **G** | **T** | **T** | **A** | **A** | **A** | **A** | **T** | **T** | **A** | **T** | **T** | **A** | **G** | **T** | **T** | **T** | **T** | **A** | **A** | **A** | **G** | **G** | **G** | **C** | **A** | **G** | **G** | **G** | **A** | **A** | **C** | **C** | **A** | **G** | **C** | **G** | **T** | **T** | **G** | **A** | **A** | **A** | **T** | **T** | **A** | **A** | **A** | **G** | **T** | **T** | **C** | **C** | **T** | **G** | **T** | **C** | **A** | **T** | **A** | **A** | **A** | **G** | **G** | **A** | **G** | **G** | **A** | **A** | **A** | **C** | **T** | **G** | **C** | **A** | **A** | **G** | **T** | **G** | **A** | **T** | **C** | **A** | **A** | **A** | **G** | **T** | **A** | **A** | **T** | **G** | **T** | **T** | **A** | **G** | **T** | **G** | **G** | **A** | **-** | **-** | **-** |
| **N52467** | **A** | **T** | **G** | **A** | **C** | **C** | **G** | **A** | **T** | **C** | **G** | **G** | **A** | **T** | **T** | **T** | **C** | **A** | **A** | **G** | **A** | **C** | **G** | **C** | **A** | **T** | **G** | **A** | **T** | **T** | **T** | **C** | **A** | **T** | **T** | **A** | **T** | **A** | **T** | **G** | **C** | **G** | **T** | **C** | **C** | **C** | **T** | **T** | **A** | **G** | **C** | **A** | **C** | **C** | **T** | **T** | **T** | **A** | **T** | **T** | **G** | **T** | **T** | **A** | **T** | **T** | **T** | **T** | **A** | **A** | **T** | **T** | **A** | **C** | **C** | **T** | **T** | **G** | **T** | **T** | **T** | **T** | **C** | **A** | **T** | **A** | **T** | **T** | **T** | **T** | **C** | **A** | **T** | **T** | **C** | **A** | **A** | **T** | **T** | **A** | **A** | **A** | **C** | **A** | **A** | **A** | **A** | **C** | **C** | **G** | **G** | **T** | **G** | **G** | **C** | **T** | **C** | **T** | **T** | **A** | **G** | **A** | **A** | **C** | **T** | **T** | **C** | **T** | **T** | **C** | **A** | **G** | **A** | **G** | **A** | **A** | **A** | **A** | **G** | **T** | **C** | **T** | **T** | **T** | **T** | **A** | **T** | **C** | **T** | **G** | **C** | **C** | **A** | **C** | **T** | **A** | **A** | **T** | **T** | **G** | **T** | **G** | **C** | **A** | **T** | **A** | **T** | **T** | **G** | **T** | **T** | **C** | **T** | **T** | **A** | **T** | **A** | **T** | **C** | **C** | **T** | **T** | **A** | **C** | **T** | **A** | **A** | **T** | **A** | **G** | **G** | **C** | **T** | **T** | **A** | **T** | **T** | **G** | **A** | **C** | **C** | **T** | **T** | **T** | **T** | **T** | **A** | **C** | **T** | **G** | **A** | **T** | **T** | **T** | **C** | **G** | **T** | **T** | **G** | **G** | **T** | **C** | **C** | **A** | **A** | **A** | **A** | **A** | **G** | **G** | **C** | **C** | **A** | **A** | **T** | **A** | **T** | **G** | **G** | **A** | **C** | **G** | **G** | **A** | **T** | **T** | **G** | **A** | **A** | **G** | **A** | **A** | **A** | **A** | **A** | **C** | **T** | **T** | **C** | **G** | **G** | **T** | **T** | **A** | **T** | **T** | **A** | **G** | **C** | **C** | **A** | **A** | **T** | **G** | **G** | **C** | **A** | **A** | **T** | **T** | **A** | **T** | **G** | **A** | **A** | **A** | **G** | **T** | **C** | **C** | **A** | **G** | **T** | **C** | **T** | **T** | **A** | **A** | **A** | **C** | **A** | **A** | **A** | **C** | **C** | **A** | **A** | **T** | **G** | **A** | **C** | **T** | **A** | **G** | **T** | **G** | **A** | **A** | **A** | **A** | **T** | **C** | **A** | **A** | **G** | **A** | **C** | **C** | **A** | **T** | **T** | **A** | **T** | **C** | **T** | **A** | **A** | **T** | **C** | **G** | **A** | **A** | **G** | **T** | **C** | **G** | **A** | **A** | **C** | **A** | **A** | **G** | **A** | **T** | **A** | **T** | **T** | **T** | **G** | **G** | **T** | **C** | **G** | **A** | **T** | **T** | **A** | **A** | **A** | **A** | **A** | **C** | **A** | **A** | **A** | **T** | **T** | **A** | **T** | **T** | **A** | **G** | **A** | **G** | **A** | **T** | **G** | **T** | **C** | **T** | **A** | **A** | **A** | **G** | **A** | **A** | **T** | **T** | **G** | **C** | **A** | **A** | **T** | **T** | **A** | **T** | **T** | **A** | **A** | **A** | **C** | **A** | **G** | **T** | **C** | **G** | **A** | **C** | **C** | **G** | **C** | **A** | **A** | **T** | **T** | **A** | **A** | **T** | **G** | **G** | **A** | **T** | **G** | **G** | **G** | **C** | **A** | **A** | **A** | **C** | **A** | **A** | **A** | **A** | **G** | **A** | **A** | **G** | **A** | **A** | **A** | **T** | **T** | **T** | **T** | **A** | **G** | **A** | **G** | **A** | **A** | **C** | **G** | **A** | **G** | **C** | **G** | **G** | **C** | **A** | **T** | **C** | **G** | **T** | **T** | **T** | **G** | **G** | **C** | **A** | **C** | **G** | **G** | **G** | **A** | **G** | **T** | **T** | **G** | **C** | **A** | **T** | **G** | **A** | **T** | **T** | **C** | **A** | **G** | **T** | **C** | **A** | **G** | **T** | **C** | **A** | **A** | **C** | **A** | **A** | **C** | **T** | **T** | **T** | **T** | **T** | **G** | **C** | **A** | **G** | **C** | **C** | **A** | **T** | **G** | **A** | **T** | **G** | **A** | **T** | **G** | **T** | **T** | **G** | **T** | **C** | **T** | **G** | **C** | **C** | **T** | **T** | **A** | **A** | **A** | **T** | **G** | **A** | **A** | **C** | **A** | **A** | **G** | **C** | **A** | **C** | **A** | **A** | **C** | **G** | **A** | **A** | **C** | **A** | **G** | **A** | **A** | **A** | **C** | **C** | **C** | **C** | **T** | **G** | **A** | **A** | **C** | **C** | **A** | **T** | **A** | **T** | **C** | **G** | **T** | **A** | **A** | **A** | **C** | **A** | **A** | **C** | **T** | **A** | **G** | **C** | **C** | **A** | **T** | **G** | **G** | **T** | **G** | **G** | **C** | **A** | **G** | **A** | **A** | **A** | **T** | **C** | **A** | **T** | **T** | **A** | **A** | **T** | **G** | **C** | **C** | **T** | **C** | **C** | **C** | **A** | **A** | **T** | **C** | **G** | **G** | **A** | **A** | **A** | **T** | **G** | **C** | **G** | **C** | **G** | **C** | **G** | **T** | **T** | **A** | **C** | **T** | **A** | **T** | **T** | **A** | **C** | **A** | **C** | **T** | **T** | **G** | **C** | **G** | **T** | **C** | **C** | **T** | **A** | **T** | **T** | **A** | **G** | **T** | **C** | **T** | **A** | **G** | **A** | **A** | **G** | **G** | **G** | **A** | **A** | **A** | **A** | **G** | **T** | **T** | **T** | **G** | **C** | **G** | **T** | **A** | **A** | **A** | **G** | **G** | **C** | **A** | **T** | **T** | **G** | **A** | **A** | **C** | **A** | **A** | **T** | **T** | **A** | **T** | **T** | **G** | **A** | **A** | **A** | **G** | **A** | **A** | **C** | **T** | **A** | **C** | **A** | **A** | **A** | **C** | **A** | **A** | **A** | **A** | **A** | **T** | **T** | **A** | **A** | **A** | **A** | **T** | **T** | **G** | **A** | **A** | **T** | **T** | **G** | **A** | **T** | **T** | **T** | **G** | **G** | **G** | **A** | **T** | **G** | **T** | **T** | **G** | **A** | **A** | **G** | **A** | **T** | **G** | **T** | **T** | **C** | **A** | **T** | **T** | **T** | **A** | **A** | **A** | **T** | **A** | **G** | **C** | **A** | **G** | **C** | **A** | **T** | **T** | **G** | **A** | **G** | **G** | **A** | **T** | **C** | **A** | **T** | **C** | **T** | **T** | **T** | **T** | **C** | **C** | **G** | **A** | **A** | **T** | **T** | **G** | **T** | **T** | **C** | **A** | **G** | **G** | **A** | **A** | **T** | **T** | **A** | **C** | **T** | **T** | **T** | **C** | **A** | **A** | **A** | **T** | **A** | **C** | **C** | **T** | **T** | **A** | **A** | **G** | **A** | **C** | **A** | **T** | **G** | **C** | **C** | **A** | **A** | **A** | **G** | **C** | **A** | **A** | **A** | **A** | **G** | **A** | **A** | **T** | **T** | **A** | **G** | **A** | **G** | **G** | **T** | **A** | **T** | **A** | **C** | **T** | **T** | **A** | **C** | **A** | **C** | **C** | **A** | **A** | **G** | **T** | **C** | **G** | **A** | **T** | **A** | **A** | **A** | **A** | **A** | **T** | **G** | **T** | **T** | **T** | **T** | **A** | **T** | **T** | **G** | **C** | **G** | **C** | **A** | **T** | **T** | **G** | **T** | **T** | **G** | **A** | **T** | **G** | **A** | **T** | **G** | **G** | **T** | **G** | **T** | **C** | **G** | **G** | **C** | **T** | **T** | **T** | **G** | **A** | **T** | **A** | **T** | **G** | **A** | **A** | **G** | **G** | **A** | **A** | **C** | **A** | **A** | **A** | **G** | **T** | **A** | **A** | **T** | **A** | **A** | **G** | **G** | **C** | **C** | **G** | **G** | **T** | **A** | **G** | **T** | **T** | **A** | **T** | **G** | **G** | **C** | **T** | **T** | **A** | **A** | **A** | **T** | **A** | **A** | **T** | **A** | **T** | **T** | **C** | **G** | **A** | **G** | **A** | **A** | **C** | **G** | **T** | **G** | **T** | **T** | **G** | **T** | **C** | **G** | **G** | **C** | **A** | **T** | **G** | **G** | **G** | **C** | **G** | **G** | **T** | **A** | **C** | **A** | **G** | **T** | **T** | **A** | **A** | **A** | **A** | **T** | **T** | **A** | **T** | **T** | **A** | **G** | **T** | **T** | **T** | **T** | **A** | **A** | **A** | **G** | **G** | **G** | **C** | **A** | **G** | **G** | **G** | **A** | **A** | **C** | **C** | **A** | **G** | **C** | **G** | **T** | **T** | **G** | **A** | **A** | **A** | **T** | **T** | **A** | **A** | **A** | **G** | **T** | **T** | **C** | **C** | **T** | **G** | **T** | **C** | **A** | **T** | **A** | **A** | **A** | **G** | **G** | **A** | **G** | **G** | **A** | **A** | **A** | **C** | **T** | **G** | **C** | **A** | **A** | **G** | **T** | **G** | **A** | **T** | **C** | **A** | **A** | **A** | **G** | **T** | **A** | **A** | **T** | **G** | **T** | **T** | **A** | **G** | **T** | **G** | **G** | **A** | **-** | **-** | **-** |
| **N54576** | **A** | **T** | **G** | **A** | **C** | **C** | **G** | **A** | **T** | **C** | **G** | **G** | **A** | **T** | **T** | **T** | **C** | **A** | **A** | **G** | **A** | **C** | **G** | **C** | **A** | **T** | **G** | **A** | **T** | **T** | **T** | **C** | **A** | **T** | **T** | **A** | **T** | **A** | **T** | **G** | **C** | **G** | **T** | **C** | **C** | **C** | **T** | **T** | **A** | **G** | **C** | **A** | **C** | **C** | **T** | **T** | **T** | **A** | **T** | **T** | **G** | **T** | **T** | **A** | **T** | **C** | **T** | **T** | **A** | **A** | **T** | **T** | **A** | **C** | **A** | **T** | **T** | **G** | **T** | **T** | **T** | **T** | **C** | **A** | **T** | **A** | **T** | **T** | **T** | **T** | **C** | **A** | **T** | **T** | **C** | **G** | **A** | **T** | **T** | **A** | **A** | **A** | **C** | **A** | **A** | **A** | **A** | **C** | **C** | **G** | **G** | **T** | **G** | **G** | **T** | **T** | **A** | **T** | **T** | **A** | **G** | **A** | **G** | **C** | **T** | **T** | **C** | **T** | **T** | **C** | **A** | **G** | **A** | **G** | **A** | **A** | **A** | **A** | **G** | **T** | **C** | **T** | **T** | **T** | **T** | **A** | **T** | **T** | **T** | **A** | **C** | **C** | **A** | **C** | **T** | **A** | **A** | **T** | **T** | **G** | **T** | **G** | **C** | **A** | **C** | **A** | **T** | **T** | **G** | **T** | **T** | **C** | **T** | **C** | **A** | **T** | **A** | **T** | **C** | **C** | **T** | **T** | **A** | **C** | **T** | **A** | **A** | **T** | **A** | **G** | **G** | **C** | **T** | **T** | **A** | **T** | **T** | **G** | **A** | **C** | **C** | **T** | **T** | **T** | **T** | **T** | **A** | **C** | **T** | **G** | **A** | **T** | **T** | **T** | **C** | **A** | **T** | **T** | **G** | **G** | **T** | **T** | **C** | **A** | **A** | **A** | **A** | **A** | **G** | **G** | **G** | **C** | **A** | **A** | **T** | **A** | **T** | **G** | **G** | **A** | **C** | **G** | **G** | **A** | **T** | **T** | **G** | **A** | **A** | **G** | **A** | **A** | **A** | **A** | **A** | **C** | **T** | **T** | **C** | **G** | **G** | **T** | **T** | **A** | **T** | **T** | **G** | **G** | **C** | **C** | **A** | **A** | **C** | **G** | **G** | **T** | **A** | **A** | **T** | **T** | **A** | **T** | **G** | **A** | **A** | **A** | **G** | **T** | **C** | **C** | **A** | **G** | **T** | **C** | **T** | **T** | **A** | **A** | **A** | **C** | **A** | **A** | **A** | **C** | **C** | **A** | **A** | **C** | **G** | **A** | **C** | **C** | **A** | **G** | **T** | **G** | **A** | **A** | **A** | **A** | **T** | **C** | **A** | **A** | **G** | **A** | **C** | **C** | **A** | **T** | **T** | **A** | **T** | **C** | **T** | **A** | **A** | **C** | **C** | **G** | **A** | **A** | **G** | **T** | **C** | **G** | **A** | **A** | **C** | **A** | **A** | **G** | **A** | **T** | **A** | **T** | **T** | **T** | **G** | **G** | **T** | **C** | **G** | **A** | **T** | **T** | **A** | **A** | **A** | **A** | **A** | **T** | **A** | **A** | **A** | **T** | **T** | **A** | **T** | **T** | **A** | **G** | **A** | **G** | **A** | **T** | **G** | **T** | **C** | **T** | **A** | **A** | **A** | **G** | **A** | **A** | **T** | **T** | **G** | **C** | **A** | **A** | **T** | **T** | **A** | **T** | **T** | **A** | **A** | **A** | **C** | **A** | **G** | **T** | **C** | **G** | **A** | **C** | **C** | **G** | **C** | **A** | **A** | **T** | **T** | **A** | **A** | **T** | **G** | **G** | **A** | **T** | **G** | **G** | **G** | **C** | **A** | **A** | **A** | **C** | **A** | **A** | **A** | **A** | **G** | **A** | **A** | **G** | **A** | **A** | **A** | **T** | **T** | **T** | **T** | **A** | **G** | **A** | **G** | **A** | **A** | **C** | **G** | **A** | **G** | **C** | **G** | **G** | **C** | **A** | **T** | **C** | **G** | **T** | **T** | **T** | **G** | **G** | **C** | **G** | **C** | **G** | **G** | **G** | **A** | **G** | **T** | **T** | **G** | **C** | **A** | **T** | **G** | **A** | **T** | **T** | **C** | **A** | **G** | **T** | **T** | **A** | **G** | **T** | **C** | **A** | **A** | **C** | **A** | **A** | **C** | **T** | **T** | **T** | **T** | **T** | **G** | **C** | **A** | **G** | **C** | **C** | **A** | **T** | **G** | **A** | **T** | **G** | **A** | **T** | **G** | **T** | **T** | **G** | **T** | **C** | **T** | **G** | **C** | **C** | **T** | **T** | **A** | **A** | **A** | **T** | **G** | **A** | **A** | **C** | **A** | **A** | **G** | **C** | **A** | **C** | **A** | **A** | **C** | **G** | **A** | **A** | **C** | **A** | **G** | **A** | **A** | **A** | **C** | **C** | **C** | **C** | **G** | **G** | **A** | **A** | **C** | **C** | **A** | **T** | **A** | **T** | **C** | **G** | **T** | **A** | **A** | **A** | **C** | **A** | **A** | **C** | **T** | **A** | **G** | **C** | **C** | **A** | **T** | **G** | **G** | **T** | **G** | **G** | **C** | **A** | **G** | **A** | **A** | **A** | **T** | **C** | **A** | **T** | **T** | **A** | **A** | **T** | **G** | **C** | **C** | **T** | **C** | **C** | **C** | **A** | **A** | **T** | **C** | **G** | **G** | **A** | **A** | **A** | **T** | **G** | **C** | **G** | **C** | **G** | **C** | **G** | **T** | **T** | **A** | **C** | **T** | **A** | **T** | **T** | **G** | **C** | **A** | **C** | **T** | **T** | **G** | **C** | **G** | **T** | **C** | **C** | **T** | **A** | **T** | **C** | **A** | **G** | **T** | **C** | **T** | **A** | **G** | **A** | **A** | **G** | **G** | **A** | **A** | **A** | **A** | **A** | **G** | **T** | **T** | **T** | **G** | **C** | **G** | **T** | **A** | **A** | **A** | **G** | **G** | **T** | **A** | **T** | **T** | **G** | **A** | **A** | **C** | **A** | **A** | **T** | **T** | **A** | **C** | **T** | **G** | **A** | **A** | **A** | **G** | **A** | **A** | **C** | **T** | **A** | **C** | **A** | **A** | **A** | **C** | **A** | **A** | **A** | **A** | **A** | **T** | **T** | **A** | **A** | **A** | **A** | **T** | **T** | **G** | **A** | **A** | **T** | **T** | **G** | **A** | **T** | **T** | **T** | **G** | **G** | **G** | **A** | **T** | **G** | **T** | **T** | **G** | **A** | **A** | **G** | **A** | **T** | **G** | **T** | **T** | **C** | **A** | **T** | **T** | **T** | **A** | **A** | **A** | **T** | **A** | **G** | **C** | **A** | **G** | **C** | **A** | **T** | **T** | **G** | **A** | **G** | **G** | **A** | **T** | **C** | **A** | **T** | **C** | **T** | **T** | **T** | **T** | **C** | **C** | **G** | **A** | **A** | **T** | **T** | **G** | **T** | **G** | **C** | **A** | **G** | **G** | **A** | **G** | **T** | **T** | **A** | **C** | **T** | **T** | **T** | **C** | **A** | **A** | **A** | **T** | **A** | **C** | **C** | **T** | **T** | **A** | **A** | **G** | **A** | **C** | **A** | **T** | **G** | **C** | **C** | **A** | **A** | **A** | **G** | **C** | **A** | **A** | **A** | **G** | **G** | **A** | **A** | **T** | **T** | **A** | **G** | **A** | **G** | **G** | **T** | **A** | **T** | **A** | **C** | **T** | **T** | **A** | **C** | **A** | **C** | **C** | **A** | **A** | **G** | **T** | **C** | **G** | **A** | **T** | **A** | **A** | **A** | **A** | **A** | **C** | **G** | **T** | **G** | **T** | **T** | **A** | **T** | **T** | **G** | **C** | **G** | **T** | **A** | **T** | **T** | **G** | **T** | **T** | **G** | **A** | **T** | **G** | **A** | **C** | **G** | **G** | **T** | **G** | **T** | **C** | **G** | **G** | **C** | **T** | **T** | **T** | **G** | **A** | **T** | **A** | **T** | **G** | **A** | **A** | **G** | **G** | **A** | **A** | **C** | **A** | **A** | **A** | **G** | **T** | **A** | **A** | **T** | **A** | **A** | **A** | **G** | **C** | **C** | **G** | **G** | **T** | **A** | **G** | **T** | **T** | **A** | **T** | **G** | **G** | **C** | **T** | **T** | **A** | **A** | **A** | **T** | **A** | **A** | **T** | **A** | **T** | **C** | **C** | **G** | **A** | **G** | **A** | **A** | **C** | **G** | **T** | **G** | **T** | **T** | **G** | **T** | **C** | **G** | **G** | **C** | **A** | **T** | **G** | **G** | **G** | **C** | **G** | **G** | **T** | **A** | **C** | **A** | **G** | **T** | **T** | **A** | **A** | **A** | **A** | **T** | **T** | **A** | **T** | **T** | **A** | **G** | **T** | **T** | **T** | **T** | **A** | **A** | **A** | **G** | **G** | **G** | **C** | **A** | **G** | **G** | **G** | **A** | **A** | **C** | **C** | **A** | **G** | **C** | **G** | **T** | **T** | **G** | **A** | **A** | **A** | **T** | **T** | **A** | **A** | **A** | **G** | **T** | **T** | **C** | **C** | **T** | **G** | **T** | **C** | **A** | **T** | **A** | **A** | **A** | **G** | **G** | **A** | **G** | **G** | **A** | **A** | **A** | **C** | **T** | **G** | **C** | **A** | **A** | **G** | **T** | **G** | **A** | **T** | **C** | **A** | **A** | **A** | **G** | **T** | **A** | **A** | **T** | **G** | **T** | **T** | **A** | **G** | **T** | **G** | **G** | **A** | **-** | **-** | **-** |
| **N55181** | **A** | **T** | **G** | **A** | **C** | **C** | **G** | **A** | **T** | **C** | **G** | **G** | **A** | **T** | **T** | **T** | **C** | **A** | **A** | **G** | **A** | **C** | **G** | **C** | **A** | **T** | **G** | **A** | **T** | **T** | **T** | **C** | **A** | **T** | **T** | **A** | **T** | **A** | **T** | **G** | **C** | **G** | **T** | **C** | **C** | **C** | **T** | **T** | **A** | **G** | **C** | **A** | **C** | **C** | **T** | **T** | **T** | **A** | **T** | **T** | **G** | **T** | **T** | **A** | **T** | **C** | **T** | **T** | **A** | **A** | **T** | **T** | **A** | **C** | **A** | **T** | **T** | **G** | **T** | **T** | **T** | **T** | **C** | **A** | **T** | **A** | **T** | **T** | **T** | **T** | **C** | **A** | **T** | **T** | **C** | **G** | **A** | **T** | **T** | **A** | **A** | **A** | **C** | **A** | **A** | **A** | **A** | **C** | **C** | **G** | **G** | **T** | **G** | **G** | **C** | **T** | **A** | **T** | **T** | **A** | **G** | **A** | **G** | **C** | **T** | **T** | **C** | **T** | **T** | **C** | **A** | **G** | **A** | **G** | **A** | **A** | **A** | **A** | **G** | **T** | **C** | **T** | **T** | **T** | **T** | **A** | **T** | **C** | **T** | **A** | **C** | **C** | **A** | **C** | **T** | **A** | **A** | **T** | **T** | **G** | **T** | **G** | **C** | **A** | **C** | **A** | **T** | **T** | **G** | **T** | **T** | **C** | **T** | **C** | **A** | **T** | **A** | **T** | **C** | **C** | **T** | **T** | **A** | **C** | **T** | **A** | **A** | **T** | **A** | **G** | **G** | **C** | **T** | **T** | **A** | **T** | **T** | **G** | **A** | **C** | **C** | **T** | **T** | **T** | **T** | **T** | **A** | **C** | **T** | **G** | **A** | **T** | **T** | **T** | **C** | **A** | **T** | **T** | **G** | **G** | **T** | **T** | **C** | **A** | **A** | **A** | **A** | **A** | **G** | **G** | **G** | **C** | **A** | **A** | **T** | **A** | **T** | **G** | **G** | **A** | **C** | **G** | **G** | **A** | **T** | **T** | **G** | **A** | **A** | **G** | **A** | **A** | **A** | **A** | **A** | **C** | **T** | **T** | **C** | **G** | **G** | **T** | **T** | **A** | **T** | **T** | **G** | **G** | **C** | **C** | **A** | **A** | **T** | **G** | **G** | **C** | **A** | **A** | **T** | **T** | **A** | **T** | **G** | **A** | **A** | **A** | **G** | **T** | **C** | **C** | **A** | **G** | **T** | **C** | **T** | **T** | **A** | **A** | **A** | **C** | **A** | **A** | **A** | **C** | **C** | **A** | **A** | **C** | **G** | **A** | **C** | **C** | **A** | **G** | **T** | **G** | **A** | **A** | **A** | **A** | **T** | **C** | **A** | **A** | **G** | **A** | **T** | **C** | **A** | **T** | **T** | **A** | **T** | **C** | **T** | **A** | **A** | **C** | **C** | **G** | **A** | **A** | **G** | **T** | **C** | **G** | **A** | **A** | **C** | **A** | **A** | **G** | **A** | **T** | **A** | **T** | **T** | **T** | **G** | **G** | **T** | **C** | **G** | **A** | **T** | **T** | **A** | **A** | **A** | **A** | **A** | **T** | **A** | **A** | **A** | **T** | **T** | **A** | **T** | **T** | **A** | **G** | **A** | **G** | **A** | **T** | **G** | **T** | **C** | **T** | **A** | **A** | **A** | **G** | **A** | **A** | **T** | **T** | **G** | **C** | **A** | **A** | **T** | **T** | **A** | **T** | **T** | **A** | **A** | **A** | **C** | **A** | **G** | **T** | **C** | **G** | **G** | **C** | **C** | **G** | **C** | **A** | **A** | **T** | **T** | **A** | **A** | **T** | **G** | **G** | **A** | **T** | **G** | **G** | **G** | **C** | **A** | **A** | **A** | **C** | **A** | **A** | **A** | **A** | **G** | **A** | **A** | **G** | **A** | **A** | **A** | **T** | **T** | **T** | **T** | **A** | **G** | **A** | **G** | **A** | **A** | **C** | **G** | **A** | **G** | **C** | **G** | **G** | **C** | **A** | **T** | **C** | **G** | **T** | **T** | **T** | **G** | **G** | **C** | **G** | **C** | **G** | **G** | **G** | **A** | **G** | **T** | **T** | **G** | **C** | **A** | **T** | **G** | **A** | **T** | **T** | **C** | **A** | **G** | **T** | **C** | **A** | **G** | **T** | **C** | **A** | **A** | **C** | **A** | **A** | **C** | **T** | **T** | **T** | **T** | **T** | **G** | **C** | **A** | **G** | **C** | **C** | **A** | **T** | **G** | **A** | **T** | **G** | **A** | **T** | **G** | **T** | **T** | **G** | **T** | **C** | **T** | **G** | **C** | **C** | **T** | **T** | **A** | **A** | **A** | **T** | **G** | **A** | **A** | **C** | **A** | **A** | **G** | **C** | **A** | **C** | **A** | **A** | **C** | **G** | **A** | **A** | **C** | **A** | **G** | **A** | **A** | **A** | **C** | **C** | **C** | **C** | **G** | **G** | **A** | **A** | **C** | **C** | **A** | **T** | **A** | **T** | **C** | **G** | **T** | **A** | **A** | **A** | **C** | **A** | **A** | **C** | **T** | **A** | **G** | **C** | **C** | **A** | **T** | **G** | **G** | **T** | **G** | **G** | **C** | **A** | **G** | **A** | **A** | **A** | **T** | **C** | **A** | **T** | **T** | **A** | **A** | **T** | **G** | **C** | **C** | **T** | **C** | **C** | **C** | **A** | **A** | **T** | **C** | **G** | **G** | **A** | **A** | **A** | **T** | **G** | **C** | **G** | **C** | **G** | **C** | **G** | **T** | **T** | **A** | **C** | **T** | **A** | **T** | **T** | **G** | **C** | **A** | **C** | **T** | **T** | **G** | **C** | **G** | **T** | **C** | **C** | **T** | **A** | **T** | **C** | **A** | **G** | **T** | **C** | **T** | **A** | **G** | **A** | **A** | **G** | **G** | **A** | **A** | **A** | **A** | **A** | **G** | **T** | **T** | **T** | **G** | **C** | **G** | **T** | **A** | **A** | **A** | **G** | **G** | **T** | **A** | **T** | **T** | **G** | **A** | **A** | **C** | **A** | **A** | **T** | **T** | **A** | **C** | **T** | **G** | **A** | **A** | **A** | **G** | **A** | **A** | **C** | **T** | **A** | **C** | **A** | **A** | **A** | **C** | **A** | **A** | **A** | **A** | **A** | **T** | **T** | **A** | **A** | **A** | **A** | **T** | **T** | **G** | **A** | **A** | **T** | **T** | **G** | **A** | **T** | **T** | **T** | **G** | **G** | **G** | **A** | **T** | **G** | **T** | **T** | **G** | **A** | **A** | **G** | **A** | **T** | **G** | **T** | **T** | **C** | **A** | **T** | **T** | **T** | **A** | **A** | **A** | **T** | **A** | **G** | **C** | **A** | **G** | **C** | **A** | **T** | **T** | **G** | **A** | **G** | **G** | **A** | **T** | **C** | **A** | **T** | **C** | **T** | **T** | **T** | **T** | **C** | **C** | **G** | **A** | **A** | **T** | **T** | **G** | **T** | **G** | **C** | **A** | **G** | **G** | **A** | **A** | **T** | **T** | **A** | **C** | **T** | **T** | **T** | **C** | **C** | **A** | **A** | **T** | **A** | **C** | **C** | **T** | **T** | **A** | **A** | **G** | **A** | **C** | **A** | **T** | **G** | **C** | **C** | **A** | **A** | **A** | **G** | **C** | **A** | **A** | **A** | **G** | **G** | **A** | **A** | **T** | **T** | **A** | **G** | **A** | **G** | **G** | **T** | **A** | **T** | **A** | **C** | **T** | **T** | **A** | **C** | **A** | **C** | **C** | **A** | **A** | **G** | **T** | **C** | **G** | **A** | **T** | **A** | **A** | **A** | **A** | **A** | **C** | **G** | **T** | **G** | **T** | **T** | **A** | **T** | **T** | **G** | **C** | **G** | **T** | **A** | **T** | **T** | **G** | **T** | **T** | **G** | **A** | **T** | **G** | **A** | **T** | **G** | **G** | **T** | **G** | **T** | **C** | **G** | **G** | **C** | **T** | **T** | **T** | **G** | **A** | **T** | **A** | **T** | **G** | **A** | **A** | **G** | **G** | **A** | **A** | **C** | **A** | **A** | **A** | **G** | **T** | **A** | **A** | **T** | **A** | **A** | **A** | **G** | **C** | **C** | **G** | **G** | **T** | **A** | **G** | **T** | **T** | **A** | **T** | **G** | **G** | **C** | **T** | **T** | **A** | **A** | **A** | **T** | **A** | **A** | **T** | **A** | **T** | **T** | **C** | **G** | **A** | **G** | **A** | **A** | **C** | **G** | **T** | **G** | **T** | **T** | **G** | **T** | **C** | **G** | **G** | **C** | **A** | **T** | **G** | **G** | **G** | **C** | **G** | **G** | **T** | **A** | **C** | **A** | **G** | **T** | **T** | **A** | **A** | **A** | **A** | **T** | **T** | **A** | **T** | **T** | **A** | **G** | **T** | **T** | **T** | **T** | **A** | **A** | **A** | **G** | **G** | **G** | **C** | **A** | **G** | **G** | **G** | **A** | **A** | **C** | **C** | **A** | **G** | **C** | **G** | **T** | **T** | **G** | **A** | **A** | **A** | **T** | **T** | **A** | **A** | **A** | **G** | **T** | **T** | **C** | **C** | **T** | **G** | **T** | **C** | **A** | **T** | **A** | **A** | **A** | **G** | **G** | **A** | **G** | **G** | **A** | **A** | **A** | **C** | **T** | **G** | **C** | **A** | **A** | **G** | **T** | **G** | **A** | **T** | **C** | **A** | **A** | **A** | **G** | **T** | **A** | **A** | **T** | **G** | **T** | **T** | **A** | **G** | **T** | **G** | **G** | **A** | **-** | **-** | **-** |
 **SNP position** | . | . | . | . | . | . | . | . | . | . | . | . | . | . | . | . | . | . | . | . | 21 | . | . | . | . | . | . | . | . | . | . | . | . | . | . | . | . | . | . | . | . | . | . | . | . | . | . | . | . | . | . | . | . | . | . | . | . | . | . | . | . | . | . | . | . | 66 | . | . | . | . | . | . | . | . | 75 | . | . | . | . | . | . | . | . | . | . | . | . | . | . | . | . | . | . | . | . | 96 | . | . | . | . | . | . | . | . | . | . | . | . | . | . | . | . | . | . | 115 | . | 117 | . | . | . | . | . | 123 | . | . | 126 | . | . | . | . | . | . | . | . | . | . | . | . | . | . | . | . | . | . | . | . | . | 148 | . | 150 | . | . | . | . | . | . | . | . | . | . | . | . | . | . | 165 | . | . | . | . | . | . | . | . | 174 | . | . | . | . | . | . | . | . | . | . | . | . | . | . | . | . | . | . | . | . | . | . | . | . | . | . | . | . | . | . | . | . | . | . | . | . | . | . | . | . | . | 216 | . | . | . | . | . | 222 | . | . | . | . | . | . | . | . | 231 | . | . | . | . | . | . | . | . | . | . | . | . | . | . | . | . | . | . | . | . | . | . | . | . | . | . | . | . | . | . | . | . | . | . | . | 267 | . | . | . | . | . | 273 | . | . | 276 | . | . | . | . | . | 282 | . | . | . | . | . | . | . | . | . | . | . | . | . | . | . | . | . | . | . | . | . | . | . | . | . | 308 | . | . | . | 312 | . | . | . | . | . | 318 | . | . | . | . | . | . | . | . | 327 | . | . | . | . | . | . | . | . | . | . | 338 | . | . | . | . | . | . | 345 | . | . | . | . | . | . | . | . | . | . | . | . | . | . | . | . | . | . | . | . | . | . | . | . | . | 371 | 372 | . | . | . | . | . | . | . | . | . | . | . | . | . | . | . | . | . | . | . | . | . | . | . | . | . | . | . | . | . | . | . | . | . | . | . | . | . | . | . | . | . | . | . | . | 417 | . | . | . | . | . | . | . | . | . | . | . | . | . | . | . | . | . | . | . | . | . | . | . | . | . | . | . | . | . | . | . | . | . | . | . | . | . | . | . | . | . | . | . | . | . | . | . | . | . | . | . | . | . | . | . | . | . | . | . | . | . | . | 480 | . | . | . | . | . | . | . | . | 489 | . | . | . | . | . | . | . | . | . | . | . | 501 | . | . | . | . | . | . | . | . | . | 511 | . | . | . | . | . | . | . | . | . | . | . | . | . | . | . | . | . | . | . | . | . | . | . | . | . | . | . | . | 540 | . | . | . | . | . | . | . | . | . | . | . | . | . | . | . | . | . | . | . | . | . | . | . | . | . | . | . | . | . | 570 | . | . | 573 | . | . | . | . | . | . | . | . | . | . | . | 585 | . | . | . | . | . | . | . | . | . | . | . | . | . | . | . | . | . | . | . | . | . | . | . | . | . | . | . | . | . | . | . | . | 618 | . | . | . | . | . | 624 | . | . | . | . | . | . | . | . | . | . | . | . | . | . | 639 | . | . | . | 643 | . | . | . | . | . | . | . | 651 | . | . | . | . | . | . | . | . | . | . | . | . | . | . | 666 | . | . | . | . | . | . | . | . | . | . | . | 678 | . | . | . | . | . | . | . | . | . | . | . | . | . | . | . | . | . | 696 | . | . | . | . | . | . | . | . | . | . | . | . | 709 | . | 711 | . | . | . | . | . | . | . | . | . | . | . | . | . | . | . | . | . | . | . | . | . | . | . | . | . | . | . | . | . | . | . | . | . | . | . | . | . | . | . | . | . | . | . | . | . | . | . | . | . | . | . | . | . | . | . | . | . | . | . | . | . | . | . | . | . | . | . | . | . | . | . | . | . | . | . | . | . | . | . | . | . | . | . | . | . | . | . | . | . | . | . | . | . | . | . | 807 | . | . | . | . | . | 813 | . | . | . | . | . | . | . | . | 822 | . | . | . | . | . | 828 | . | . | . | . | . | . | . | . | . | . | . | 840 | . | . | . | . | . | . | . | . | 849 | . | . | . | . | . | . | . | . | . | . | . | . | . | . | 864 | . | . | . | . | . | . | . | . | . | . | . | 876 | . | . | . | . | . | . | . | . | 885 | . | . | 888 | 889 | . | . | . | . | . | . | . | 897 | . | . | . | . | . | . | . | . | . | . | . | 909 | . | . | . | . | . | . | . | . | . | . | . | . | . | . | . | . | . | . | . | . | . | . | . | . | . | . | . | . | . | . | . | . | . | . | . | 945 | . | . | 948 | . | . | . | . | . | . | . | . | . | . | . | . | . | . | . | . | . | . | . | . | . | . | . | 972 | . | . | . | . | . | . | . | . | . | . | . | . | . | . | . | . | . | . | . | . | . | . | . | . | . | . | . | . | . | . | . | . | . | . | . | . | . | . | . | . | . | . | . | . | . | . | . | . | . | . | . | . | . | . | . | . | . | . | . | . | . | . | 1035 | . | . | . | . | . | . | . | . | . | . | . | . | . | . | . | . | . | . | . | . | . | . | . | . | . | . | . | . | . | . | . | . | . | . | . | . | . | . | . | . | . | . | . | . | . | . | . | . | . | . | . | . | . | . | . | . | . | . | 1094 | . | . | . | . | . | . | . | . | . | . |

|  |
| --- |
|  |

|  |  |
| --- | --- |
|  |

|  |  |
| --- | --- |
| **Legend** | |
  | **Reference Sequence** |

  |
|  | **Detected SNPs** |
|  |  |
