# Supplementary material for: sraX: A Novel Comprehensive Resistome Analysis Tool
Source: Front Microbiol. 2020 Feb 5;11:52. doi: 10.3389/fmicb.2020.00052 (PMC7025521; doi:10.3389/fmicb.2020.00052)
Supplement: Supplementary file 4 [file Data_Sheet_3.ZIP › liaS/liaS_Efaecium_dna.html]

# liaS

|  |
| --- |
|  |

|  |
| --- |
|  |

|  |  |
| --- | --- |
| **Definition:** | LiaS is a histidine kinase found in the liaFSR signal transduction pathway. Mutations confer daptomycin resistance. |

|  |
| --- |
|  |

|  |
| --- |
|  |

|  |  |  |
| --- | --- | --- |
| **Reference organism:** | *Enterococcus faecium DO* | [NCBI, CARD] |

|  |
| --- |
|  |

|  |
| --- |
|  |

|  |  |
| --- | --- |
| **Summary Data** | || **Parameter** | **Value_(n)_** || Total number of genomes | 101 |
| MSA length | 1068 |
| Total number of SNPs | 106 |
| SNP density (# SNPs/MSA length) | 0.099 |

|  |
| --- |
|  |

|  |
| --- |
|  |

|  |
| --- |
|  |

|  |  |  |  |  |  |  |  |  |  |  |  |  |  |  |  |  |  |  |  |  |  |  |  |  |  |  |  |  |  |  |  |  |  |  |  |  |  |  |  |  |  |  |  |  |  |  |  |  |  |  |  |  |  |  |  |  |  |  |  |  |  |  |  |  |  |  |  |  |  |  |  |  |  |  |  |  |  |  |  |  |  |  |  |  |  |  |  |  |  |  |  |  |  |  |  |  |  |  |  |  |  |  |  |  |  |  |  |  |  |  |  |  |  |  |  |  |  |  |  |  |  |  |  |  |  |  |  |  |  |  |  |  |  |  |  |  |  |  |  |  |  |  |  |  |  |  |  |  |  |  |  |  |  |  |  |  |  |  |  |  |  |  |  |  |  |  |  |  |  |  |  |  |  |  |  |  |  |  |  |  |  |  |  |  |  |  |  |  |  |  |  |  |  |  |  |  |  |  |  |  |  |  |  |  |  |  |  |  |  |  |  |  |  |  |  |  |  |  |  |  |  |  |  |  |  |  |  |  |  |  |  |  |  |  |  |  |  |  |  |  |  |  |  |  |  |  |  |  |  |  |  |  |  |  |  |  |  |  |  |  |  |  |  |  |  |  |  |  |  |  |  |  |  |  |  |  |  |  |  |  |  |  |  |  |  |  |  |  |  |  |  |  |  |  |  |  |  |  |  |  |  |  |  |  |  |  |  |  |  |  |  |  |  |  |  |  |  |  |  |  |  |  |  |  |  |  |  |  |  |  |  |  |  |  |  |  |  |  |  |  |  |  |  |  |  |  |  |  |  |  |  |  |  |  |  |  |  |  |  |  |  |  |  |  |  |  |  |  |  |  |  |  |  |  |  |  |  |  |  |  |  |  |  |  |  |  |  |  |  |  |  |  |  |  |  |  |  |  |  |  |  |  |  |  |  |  |  |  |  |  |  |  |  |  |  |  |  |  |  |  |  |  |  |  |  |  |  |  |  |  |  |  |  |  |  |  |  |  |  |  |  |  |  |  |  |  |  |  |  |  |  |  |  |  |  |  |  |  |  |  |  |  |  |  |  |  |  |  |  |  |  |  |  |  |  |  |  |  |  |  |  |  |  |  |  |  |  |  |  |  |  |  |  |  |  |  |  |  |  |  |  |  |  |  |  |  |  |  |  |  |  |  |  |  |  |  |  |  |  |  |  |  |  |  |  |  |  |  |  |  |  |  |  |  |  |  |  |  |  |  |  |  |  |  |  |  |  |  |  |  |  |  |  |  |  |  |  |  |  |  |  |  |  |  |  |  |  |  |  |  |  |  |  |  |  |  |  |  |  |  |  |  |  |  |  |  |  |  |  |  |  |  |  |  |  |  |  |  |  |  |  |  |  |  |  |  |  |  |  |  |  |  |  |  |  |  |  |  |  |  |  |  |  |  |  |  |  |  |  |  |  |  |  |  |  |  |  |  |  |  |  |  |  |  |  |  |  |  |  |  |  |  |  |  |  |  |  |  |  |  |  |  |  |  |  |  |  |  |  |  |  |  |  |  |  |  |  |  |  |  |  |  |  |  |  |  |  |  |  |  |  |  |  |  |  |  |  |  |  |  |  |  |  |  |  |  |  |  |  |  |  |  |  |  |  |  |  |  |  |  |  |  |  |  |  |  |  |  |  |  |  |  |  |  |  |  |  |  |  |  |  |  |  |  |  |  |  |  |  |  |  |  |  |  |  |  |  |  |  |  |  |  |  |  |  |  |  |  |  |  |  |  |  |  |  |  |  |  |  |  |  |  |  |  |  |  |  |  |  |  |  |  |  |  |  |  |  |  |  |  |  |  |  |  |  |  |  |  |  |  |  |  |  |  |  |  |  |  |  |  |  |  |  |  |  |  |  |  |  |  |  |  |  |  |  |  |  |  |  |  |  |  |  |  |  |  |  |  |  |  |  |  |  |  |  |  |  |  |  |  |  |  |  |  |  |  |  |  |  |  |  |  |  |  |  |  |  |  |  |  |  |  |  |  |  |  |  |  |  |  |  |  |  |  |  |  |  |  |  |  |  |  |  |  |  |  |  |  |  |  |  |  |  |  |  |  |  |  |  |  |  |  |  |  |  |  |  |  |  |  |  |  |  |  |  |  |  |  |  |  |  |  |  |  |  |  |  |  |  |  |  |  |  |  |  |  |  |  |  |  |  |  |  |  |  |  |  |  |  |  |  |  |  |  |  |  |  |  |  |  |  |  |  |  |  |  |  |  |  |  |  |  |  |  |  |  |  |  |  |  |  |  |  |  |  |  |  |  |  |  |  |  |  |  |  |  |  |  |  |  |  |  |  |  |  |  |  |  |  |  |  |  |  |  |  |  |  |  |  |  |  |  |  |  |  |  |  |  |  |  |  |  |  |  |  |  |  |  |  |  |  |  |  |  |  |  |  |  |  |
| --- | --- | --- | --- | --- | --- | --- | --- | --- | --- | --- | --- | --- | --- | --- | --- | --- | --- | --- | --- | --- | --- | --- | --- | --- | --- | --- | --- | --- | --- | --- | --- | --- | --- | --- | --- | --- | --- | --- | --- | --- | --- | --- | --- | --- | --- | --- | --- | --- | --- | --- | --- | --- | --- | --- | --- | --- | --- | --- | --- | --- | --- | --- | --- | --- | --- | --- | --- | --- | --- | --- | --- | --- | --- | --- | --- | --- | --- | --- | --- | --- | --- | --- | --- | --- | --- | --- | --- | --- | --- | --- | --- | --- | --- | --- | --- | --- | --- | --- | --- | --- | --- | --- | --- | --- | --- | --- | --- | --- | --- | --- | --- | --- | --- | --- | --- | --- | --- | --- | --- | --- | --- | --- | --- | --- | --- | --- | --- | --- | --- | --- | --- | --- | --- | --- | --- | --- | --- | --- | --- | --- | --- | --- | --- | --- | --- | --- | --- | --- | --- | --- | --- | --- | --- | --- | --- | --- | --- | --- | --- | --- | --- | --- | --- | --- | --- | --- | --- | --- | --- | --- | --- | --- | --- | --- | --- | --- | --- | --- | --- | --- | --- | --- | --- | --- | --- | --- | --- | --- | --- | --- | --- | --- | --- | --- | --- | --- | --- | --- | --- | --- | --- | --- | --- | --- | --- | --- | --- | --- | --- | --- | --- | --- | --- | --- | --- | --- | --- | --- | --- | --- | --- | --- | --- | --- | --- | --- | --- | --- | --- | --- | --- | --- | --- | --- | --- | --- | --- | --- | --- | --- | --- | --- | --- | --- | --- | --- | --- | --- | --- | --- | --- | --- | --- | --- | --- | --- | --- | --- | --- | --- | --- | --- | --- | --- | --- | --- | --- | --- | --- | --- | --- | --- | --- | --- | --- | --- | --- | --- | --- | --- | --- | --- | --- | --- | --- | --- | --- | --- | --- | --- | --- | --- | --- | --- | --- | --- | --- | --- | --- | --- | --- | --- | --- | --- | --- | --- | --- | --- | --- | --- | --- | --- | --- | --- | --- | --- | --- | --- | --- | --- | --- | --- | --- | --- | --- | --- | --- | --- | --- | --- | --- | --- | --- | --- | --- | --- | --- | --- | --- | --- | --- | --- | --- | --- | --- | --- | --- | --- | --- | --- | --- | --- | --- | --- | --- | --- | --- | --- | --- | --- | --- | --- | --- | --- | --- | --- | --- | --- | --- | --- | --- | --- | --- | --- | --- | --- | --- | --- | --- | --- | --- | --- | --- | --- | --- | --- | --- | --- | --- | --- | --- | --- | --- | --- | --- | --- | --- | --- | --- | --- | --- | --- | --- | --- | --- | --- | --- | --- | --- | --- | --- | --- | --- | --- | --- | --- | --- | --- | --- | --- | --- | --- | --- | --- | --- | --- | --- | --- | --- | --- | --- | --- | --- | --- | --- | --- | --- | --- | --- | --- | --- | --- | --- | --- | --- | --- | --- | --- | --- | --- | --- | --- | --- | --- | --- | --- | --- | --- | --- | --- | --- | --- | --- | --- | --- | --- | --- | --- | --- | --- | --- | --- | --- | --- | --- | --- | --- | --- | --- | --- | --- | --- | --- | --- | --- | --- | --- | --- | --- | --- | --- | --- | --- | --- | --- | --- | --- | --- | --- | --- | --- | --- | --- | --- | --- | --- | --- | --- | --- | --- | --- | --- | --- | --- | --- | --- | --- | --- | --- | --- | --- | --- | --- | --- | --- | --- | --- | --- | --- | --- | --- | --- | --- | --- | --- | --- | --- | --- | --- | --- | --- | --- | --- | --- | --- | --- | --- | --- | --- | --- | --- | --- | --- | --- | --- | --- | --- | --- | --- | --- | --- | --- | --- | --- | --- | --- | --- | --- | --- | --- | --- | --- | --- | --- | --- | --- | --- | --- | --- | --- | --- | --- | --- | --- | --- | --- | --- | --- | --- | --- | --- | --- | --- | --- | --- | --- | --- | --- | --- | --- | --- | --- | --- | --- | --- | --- | --- | --- | --- | --- | --- | --- | --- | --- | --- | --- | --- | --- | --- | --- | --- | --- | --- | --- | --- | --- | --- | --- | --- | --- | --- | --- | --- | --- | --- | --- | --- | --- | --- | --- | --- | --- | --- | --- | --- | --- | --- | --- | --- | --- | --- | --- | --- | --- | --- | --- | --- | --- | --- | --- | --- | --- | --- | --- | --- | --- | --- | --- | --- | --- | --- | --- | --- | --- | --- | --- | --- | --- | --- | --- | --- | --- | --- | --- | --- | --- | --- | --- | --- | --- | --- | --- | --- | --- | --- | --- | --- | --- | --- | --- | --- | --- | --- | --- | --- | --- | --- | --- | --- | --- | --- | --- | --- | --- | --- | --- | --- | --- | --- | --- | --- | --- | --- | --- | --- | --- | --- | --- | --- | --- | --- | --- | --- | --- | --- | --- | --- | --- | --- | --- | --- | --- | --- | --- | --- | --- | --- | --- | --- | --- | --- | --- | --- | --- | --- | --- | --- | --- | --- | --- | --- | --- | --- | --- | --- | --- | --- | --- | --- | --- | --- | --- | --- | --- | --- | --- | --- | --- | --- | --- | --- | --- | --- | --- | --- | --- | --- | --- | --- | --- | --- | --- | --- | --- | --- | --- | --- | --- | --- | --- | --- | --- | --- | --- | --- | --- | --- | --- | --- | --- | --- | --- | --- | --- | --- | --- | --- | --- | --- | --- | --- | --- | --- | --- | --- | --- | --- | --- | --- | --- | --- | --- | --- | --- | --- | --- | --- | --- | --- | --- | --- | --- | --- | --- | --- | --- | --- | --- | --- | --- | --- | --- | --- | --- | --- | --- | --- | --- | --- | --- | --- | --- | --- | --- | --- | --- | --- | --- | --- | --- | --- | --- | --- | --- | --- | --- | --- | --- | --- | --- | --- | --- | --- | --- | --- | --- | --- | --- | --- | --- | --- | --- | --- | --- | --- | --- | --- | --- | --- | --- | --- | --- | --- | --- | --- | --- | --- | --- | --- | --- | --- | --- | --- | --- | --- | --- | --- | --- | --- | --- | --- | --- | --- | --- | --- | --- | --- | --- | --- | --- | --- | --- | --- | --- | --- | --- | --- | --- | --- | --- | --- | --- | --- | --- | --- | --- | --- | --- | --- | --- | --- | --- | --- | --- | --- | --- | --- | --- | --- | --- | --- | --- | --- | --- | --- | --- | --- | --- | --- | --- | --- | --- | --- | --- | --- | --- | --- | --- | --- | --- | --- | --- | --- | --- | --- | --- | --- | --- | --- | --- | --- | --- | --- | --- | --- | --- | --- | --- | --- | --- | --- | --- | --- | --- | --- | --- | --- | --- | --- | --- | --- | --- | --- | --- | --- | --- | --- | --- | --- | --- | --- | --- | --- | --- | --- | --- | --- | --- | --- | --- | --- | --- | --- | --- | --- | --- | --- | --- | --- | --- | --- | --- | --- | --- | --- | --- | --- | --- | --- | --- | --- | --- | --- | --- | --- | --- | --- | --- | --- | --- | --- | --- | --- | --- | --- | --- | --- | --- | --- |
|  || **Enterococcus faecium DO (AFK58561.1)** | **A** | **T** | **G** | **A** | **T** | **G** | **G** | **G** | **A** | **A** | **A** | **A** | **A** | **T** | **A** | **T** | **C** | **C** | **A** | **G** | **A** | **G** | **C** | **G** | **A** | **T** | **G** | **C** | **T** | **A** | **G** | **C** | **T** | **G** | **T** | **T** | **T** | **A** | **T** | **T** | **C** | **G** | **G** | **G** | **G** | **A** | **T** | **T** | **G** | **C** | **G** | **G** | **C** | **T** | **T** | **T** | **C** | **C** | **T** | **T** | **A** | **T** | **T** | **A** | **T** | **T** | **C** | **T** | **A** | **T** | **T** | **T** | **T** | **C** | **A** | **C** | **T** | **T** | **T** | **T** | **C** | **A** | **C** | **T** | **T** | **A** | **T** | **T** | **T** | **T** | **T** | **A** | **T** | **G** | **C** | **C** | **A** | **G** | **C** | **A** | **A** | **T** | **C** | **A** | **A** | **A** | **G** | **T** | **C** | **A** | **T** | **T** | **G** | **G** | **T** | **G** | **G** | **G** | **G** | **A** | **G** | **A** | **A** | **T** | **T** | **A** | **C** | **T** | **G** | **C** | **G** | **T** | **G** | **C** | **A** | **C** | **G** | **T** | **T** | **T** | **A** | **T** | **T** | **A** | **T** | **A** | **T** | **G** | **T** | **T** | **C** | **C** | **G** | **C** | **T** | **T** | **A** | **T** | **C** | **T** | **T** | **C** | **C** | **A** | **T** | **T** | **T** | **G** | **T** | **T** | **A** | **G** | **C** | **C** | **A** | **T** | **T** | **T** | **C** | **C** | **T** | **T** | **A** | **G** | **G** | **T** | **G** | **T** | **A** | **G** | **G** | **A** | **T** | **T** | **A** | **A** | **T** | **T** | **G** | **T** | **C** | **T** | **T** | **T** | **C** | **T** | **A** | **T** | **T** | **A** | **T** | **T** | **A** | **T** | **C** | **A** | **C** | **T** | **T** | **A** | **T** | **T** | **C** | **A** | **A** | **A** | **A** | **G** | **A** | **C** | **A** | **A** | **A** | **A** | **T** | **A** | **C** | **G** | **G** | **G** | **A** | **A** | **A** | **A** | **T** | **C** | **G** | **A** | **A** | **G** | **A** | **A** | **A** | **A** | **G** | **C** | **T** | **G** | **C** | **G** | **T** | **G** | **C** | **A** | **C** | **T** | **T** | **T** | **C** | **T** | **T** | **C** | **T** | **G** | **G** | **C** | **A** | **A** | **T** | **T** | **A** | **T** | **G** | **A** | **A** | **T** | **C** | **C** | **A** | **A** | **A** | **T** | **T** | **G** | **T** | **T** | **G** | **C** | **T** | **T** | **C** | **T** | **T** | **C** | **C** | **G** | **A** | **T** | **C** | **C** | **C** | **A** | **A** | **G** | **T** | **G** | **C** | **A** | **T** | **C** | **G** | **G** | **A** | **C** | **G** | **A** | **T** | **T** | **T** | **A** | **T** | **A** | **C** | **A** | **T** | **C** | **A** | **A** | **A** | **G** | **A** | **T** | **A** | **T** | **T** | **G** | **A** | **T** | **A** | **A** | **G** | **G** | **A** | **A** | **A** | **T** | **C** | **A** | **C** | **T** | **A** | **A** | **G** | **A** | **T** | **A** | **A** | **A** | **A** | **G** | **A** | **A** | **A** | **A** | **A** | **A** | **T** | **G** | **A** | **T** | **T** | **G** | **A** | **A** | **G** | **T** | **A** | **T** | **C** | **T** | **A** | **G** | **T** | **G** | **A** | **A** | **T** | **T** | **A** | **C** | **A** | **A** | **A** | **T** | **T** | **G** | **T** | **A** | **A** | **C** | **G** | **A** | **G** | **C** | **C** | **G** | **T** | **C** | **C** | **C** | **C** | **A** | **A** | **T** | **A** | **T** | **G** | **T** | **A** | **G** | **A** | **T** | **G** | **G** | **A** | **C** | **A** | **A** | **A** | **C** | **C** | **A** | **A** | **A** | **G** | **A** | **A** | **G** | **A** | **A** | **A** | **T** | **T** | **T** | **T** | **A** | **G** | **A** | **A** | **T** | **T** | **A** | **G** | **A** | **A** | **A** | **G** | **A** | **C** | **A** | **T** | **C** | **G** | **A** | **T** | **T** | **A** | **G** | **C** | **C** | **C** | **G** | **T** | **G** | **A** | **G** | **C** | **T** | **G** | **C** | **A** | **T** | **G** | **A** | **T** | **T** | **C** | **A** | **G** | **T** | **T** | **T** | **C** | **G** | **C** | **A** | **A** | **C** | **A** | **A** | **T** | **T** | **G** | **T** | **T** | **T** | **G** | **C** | **A** | **G** | **C** | **A** | **A** | **T** | **G** | **A** | **T** | **G** | **A** | **T** | **G** | **A** | **T** | **G** | **T** | **C** | **G** | **G** | **C** | **A** | **T** | **T** | **G** | **A** | **C** | **G** | **G** | **A** | **G** | **C** | **A** | **A** | **G** | **C** | **A** | **G** | **A** | **A** | **A** | **A** | **A** | **A** | **G** | **C** | **G** | **A** | **G** | **A** | **C** | **A** | **C** | **C** | **A** | **G** | **A** | **A** | **A** | **T** | **G** | **T** | **T** | **T** | **C** | **G** | **C** | **A** | **A** | **G** | **C** | **A** | **G** | **T** | **T** | **G** | **A** | **A** | **A** | **A** | **T** | **G** | **G** | **T** | **A** | **G** | **C** | **A** | **G** | **A** | **G** | **A** | **T** | **C** | **A** | **T** | **C** | **A** | **A** | **C** | **G** | **C** | **T** | **T** | **C** | **C** | **C** | **A** | **G** | **T** | **C** | **T** | **G** | **A** | **G** | **A** | **T** | **G** | **C** | **G** | **T** | **G** | **C** | **G** | **C** | **T** | **G** | **C** | **T** | **T** | **C** | **T** | **T** | **C** | **A** | **T** | **C** | **T** | **A** | **C** | **G** | **T** | **C** | **C** | **A** | **G** | **T** | **C** | **A** | **A** | **T** | **T** | **T** | **A** | **G** | **A** | **A** | **G** | **A** | **G** | **A** | **A** | **A** | **A** | **G** | **T** | **T** | **T** | **A** | **A** | **A** | **G** | **C** | **A** | **A** | **G** | **G** | **T** | **A** | **T** | **C** | **G** | **A** | **G** | **C** | **A** | **G** | **T** | **T** | **A** | **T** | **T** | **G** | **A** | **A** | **G** | **G** | **A** | **A** | **T** | **T** | **G** | **C** | **A** | **A** | **A** | **A** | **T** | **A** | **A** | **A** | **A** | **T** | **C** | **C** | **A** | **G** | **A** | **T** | **T** | **T** | **C** | **G** | **C** | **T** | **G** | **A** | **A** | **A** | **T** | **G** | **G** | **G** | **A** | **T** | **G** | **T** | **A** | **G** | **A** | **A** | **G** | **A** | **T** | **G** | **T** | **A** | **A** | **A** | **A** | **C** | **T** | **A** | **T** | **C** | **T** | **A** | **G** | **T** | **T** | **C** | **C** | **A** | **T** | **T** | **G** | **A** | **G** | **G** | **A** | **T** | **C** | **A** | **C** | **T** | **T** | **G** | **T** | **T** | **C** | **C** | **G** | **A** | **A** | **T** | **C** | **G** | **T** | **T** | **C** | **A** | **A** | **G** | **A** | **A** | **T** | **T** | **G** | **T** | **T** | **A** | **T** | **C** | **A** | **A** | **A** | **T** | **A** | **C** | **A** | **T** | **T** | **A** | **A** | **G** | **A** | **C** | **A** | **T** | **G** | **C** | **T** | **A** | **A** | **A** | **G** | **C** | **C** | **A** | **A** | **T** | **G** | **A** | **A** | **T** | **T** | **A** | **G** | **A** | **G** | **G** | **T** | **A** | **T** | **A** | **T** | **T** | **T** | **G** | **C** | **A** | **C** | **A** | **A** | **A** | **A** | **T** | **A** | **G** | **A** | **C** | **A** | **A** | **T** | **A** | **A** | **T** | **C** | **T** | **T** | **T** | **T** | **G** | **T** | **T** | **A** | **C** | **G** | **T** | **A** | **T** | **T** | **A** | **T** | **T** | **G** | **A** | **C** | **G** | **A** | **C** | **G** | **G** | **A** | **A** | **C** | **A** | **G** | **G** | **G** | **T** | **T** | **T** | **A** | **A** | **T** | **A** | **T** | **G** | **A** | **A** | **T** | **G** | **A** | **A** | **A** | **C** | **G** | **A** | **A** | **A** | **A** | **C** | **G** | **G** | **G** | **A** | **A** | **G** | **T** | **T** | **A** | **T** | **G** | **G** | **A** | **T** | **T** | **G** | **A** | **A** | **C** | **A** | **A** | **T** | **A** | **T** | **C** | **A** | **A** | **A** | **G** | **A** | **A** | **C** | **G** | **A** | **G** | **T** | **A** | **G** | **C** | **T** | **G** | **G** | **T** | **A** | **T** | **T** | **G** | **G** | **T** | **G** | **G** | **A** | **A** | **C** | **A** | **G** | **T** | **A** | **A** | **A** | **A** | **A** | **T** | **C** | **A** | **T** | **T** | **A** | **G** | **T** | **T** | **T** | **T** | **A** | **A** | **A** | **G** | **G** | **T** | **C** | **A** | **A** | **G** | **G** | **T** | **A** | **C** | **A** | **A** | **G** | **T** | **G** | **T** | **A** | **G** | **A** | **A** | **A** | **T** | **C** | **A** | **A** | **G** | **G** | **T** | **C** | **C** | **C** | **T** | **T** | **T** | **G** | **A** | **T** | **G** | **A** | **A** | **G** | **G** | **A** | **G** | **G** | **C** | **A** | **T** | **A** | **G** |
| **N53548** | **A** | **T** | **G** | **A** | **T** | **G** | **G** | **G** | **A** | **A** | **G** | **A** | **A** | **T** | **A** | **T** | **C** | **C** | **A** | **A** | **A** | **G** | **C** | **A** | **A** | **T** | **G** | **C** | **T** | **A** | **G** | **C** | **T** | **G** | **T** | **T** | **T** | **A** | **T** | **T** | **C** | **G** | **G** | **G** | **G** | **A** | **T** | **T** | **G** | **C** | **G** | **G** | **C** | **T** | **T** | **T** | **C** | **C** | **T** | **T** | **A** | **T** | **T** | **A** | **T** | **T** | **C** | **T** | **A** | **T** | **T** | **T** | **T** | **C** | **A** | **C** | **T** | **T** | **T** | **T** | **C** | **A** | **C** | **T** | **T** | **A** | **T** | **T** | **T** | **T** | **T** | **A** | **T** | **G** | **C** | **C** | **A** | **G** | **T** | **A** | **A** | **C** | **C** | **A** | **A** | **A** | **G** | **T** | **C** | **A** | **T** | **T** | **G** | **G** | **T** | **G** | **G** | **G** | **G** | **A** | **G** | **A** | **A** | **T** | **T** | **G** | **C** | **T** | **G** | **C** | **G** | **T** | **G** | **C** | **G** | **C** | **G** | **T** | **T** | **T** | **A** | **T** | **T** | **A** | **T** | **A** | **C** | **G** | **T** | **T** | **C** | **C** | **G** | **C** | **T** | **T** | **A** | **T** | **C** | **T** | **T** | **C** | **C** | **A** | **T** | **T** | **T** | **G** | **A** | **T** | **G** | **G** | **C** | **A** | **A** | **T** | **T** | **T** | **C** | **A** | **T** | **T** | **A** | **G** | **G** | **T** | **G** | **T** | **A** | **G** | **G** | **A** | **T** | **T** | **G** | **A** | **T** | **C** | **G** | **T** | **A** | **T** | **T** | **T** | **C** | **T** | **A** | **T** | **T** | **G** | **T** | **T** | **A** | **T** | **C** | **A** | **C** | **T** | **T** | **A** | **T** | **T** | **C** | **A** | **A** | **A** | **A** | **G** | **G** | **C** | **A** | **A** | **A** | **A** | **T** | **A** | **C** | **G** | **G** | **G** | **A** | **A** | **A** | **A** | **T** | **C** | **G** | **A** | **A** | **G** | **A** | **A** | **A** | **A** | **G** | **C** | **T** | **T** | **C** | **G** | **T** | **G** | **C** | **A** | **C** | **T** | **T** | **T** | **C** | **T** | **T** | **C** | **T** | **G** | **G** | **C** | **A** | **A** | **T** | **T** | **A** | **T** | **G** | **A** | **A** | **T** | **C** | **C** | **A** | **A** | **A** | **C** | **T** | **T** | **T** | **T** | **G** | **C** | **A** | **T** | **C** | **A** | **G** | **C** | **C** | **G** | **A** | **T** | **C** | **C** | **C** | **A** | **A** | **G** | **C** | **G** | **C** | **G** | **T** | **C** | **G** | **G** | **A** | **T** | **G** | **A** | **T** | **C** | **T** | **G** | **T** | **A** | **C** | **A** | **T** | **C** | **A** | **A** | **A** | **G** | **A** | **T** | **A** | **T** | **T** | **G** | **A** | **T** | **A** | **T** | **G** | **G** | **A** | **A** | **A** | **T** | **T** | **A** | **C** | **T** | **A** | **A** | **A** | **A** | **T** | **A** | **A** | **A** | **A** | **G** | **A** | **A** | **A** | **A** | **A** | **A** | **T** | **G** | **A** | **T** | **C** | **G** | **A** | **A** | **G** | **T** | **A** | **T** | **C** | **C** | **A** | **G** | **T** | **G** | **A** | **A** | **T** | **T** | **A** | **C** | **A** | **G** | **A** | **T** | **T** | **A** | **T** | **G** | **A** | **C** | **T** | **A** | **G** | **C** | **C** | **G** | **T** | **C** | **C** | **T** | **C** | **A** | **A** | **T** | **A** | **T** | **G** | **T** | **A** | **G** | **A** | **T** | **G** | **G** | **A** | **C** | **A** | **A** | **A** | **C** | **C** | **A** | **A** | **A** | **G** | **A** | **A** | **G** | **A** | **G** | **A** | **T** | **T** | **T** | **T** | **A** | **G** | **A** | **A** | **T** | **T** | **A** | **G** | **A** | **A** | **A** | **G** | **G** | **C** | **A** | **T** | **C** | **G** | **A** | **T** | **T** | **A** | **G** | **C** | **T** | **C** | **G** | **T** | **G** | **A** | **G** | **C** | **T** | **T** | **C** | **A** | **T** | **G** | **A** | **T** | **T** | **C** | **A** | **G** | **T** | **T** | **T** | **C** | **A** | **C** | **A** | **G** | **C** | **A** | **A** | **T** | **T** | **A** | **T** | **T** | **T** | **G** | **C** | **A** | **G** | **C** | **G** | **A** | **T** | **G** | **A** | **T** | **G** | **A** | **T** | **G** | **A** | **T** | **G** | **T** | **C** | **G** | **G** | **C** | **A** | **T** | **T** | **G** | **A** | **C** | **G** | **G** | **A** | **G** | **C** | **A** | **A** | **G** | **C** | **A** | **G** | **A** | **A** | **A** | **A** | **A** | **A** | **G** | **T** | **G** | **A** | **G** | **A** | **C** | **A** | **C** | **C** | **A** | **G** | **A** | **A** | **A** | **T** | **G** | **T** | **T** | **C** | **C** | **G** | **T** | **A** | **A** | **G** | **C** | **A** | **G** | **T** | **T** | **G** | **A** | **A** | **A** | **A** | **T** | **G** | **G** | **T** | **G** | **G** | **C** | **A** | **G** | **A** | **G** | **A** | **T** | **C** | **A** | **T** | **C** | **A** | **A** | **C** | **G** | **C** | **T** | **T** | **C** | **C** | **C** | **A** | **G** | **T** | **C** | **T** | **G** | **A** | **G** | **A** | **T** | **G** | **C** | **G** | **T** | **G** | **C** | **G** | **C** | **T** | **G** | **C** | **T** | **T** | **C** | **T** | **T** | **C** | **A** | **T** | **C** | **T** | **G** | **C** | **G** | **T** | **C** | **C** | **A** | **G** | **T** | **C** | **A** | **A** | **T** | **T** | **T** | **A** | **G** | **A** | **A** | **G** | **A** | **A** | **A** | **A** | **A** | **A** | **G** | **T** | **T** | **T** | **A** | **A** | **A** | **A** | **C** | **A** | **A** | **G** | **G** | **C** | **A** | **T** | **T** | **G** | **A** | **G** | **C** | **A** | **G** | **T** | **T** | **A** | **T** | **T** | **G** | **A** | **A** | **G** | **G** | **A** | **A** | **T** | **T** | **G** | **C** | **A** | **G** | **A** | **A** | **T** | **A** | **A** | **A** | **A** | **T** | **C** | **C** | **A** | **G** | **A** | **T** | **T** | **T** | **C** | **G** | **C** | **T** | **A** | **A** | **A** | **A** | **T** | **G** | **G** | **G** | **A** | **T** | **G** | **T** | **A** | **G** | **A** | **A** | **G** | **A** | **T** | **G** | **T** | **A** | **A** | **A** | **A** | **T** | **T** | **A** | **A** | **C** | **T** | **A** | **G** | **T** | **T** | **C** | **C** | **A** | **T** | **T** | **G** | **A** | **G** | **G** | **A** | **T** | **C** | **A** | **C** | **T** | **T** | **G** | **T** | **T** | **C** | **C** | **G** | **A** | **A** | **T** | **C** | **G** | **T** | **T** | **C** | **A** | **A** | **G** | **A** | **A** | **T** | **T** | **G** | **T** | **T** | **A** | **T** | **C** | **A** | **A** | **A** | **C** | **A** | **C** | **A** | **T** | **T** | **A** | **A** | **G** | **A** | **C** | **A** | **T** | **G** | **C** | **T** | **A** | **A** | **G** | **G** | **C** | **T** | **A** | **A** | **T** | **G** | **A** | **A** | **T** | **T** | **A** | **G** | **A** | **A** | **G** | **T** | **C** | **T** | **A** | **T** | **T** | **T** | **G** | **C** | **A** | **T** | **A** | **A** | **A** | **A** | **T** | **A** | **G** | **A** | **T** | **A** | **A** | **C** | **A** | **A** | **T** | **C** | **T** | **T** | **T** | **T** | **A** | **T** | **T** | **A** | **C** | **G** | **T** | **A** | **T** | **C** | **A** | **T** | **T** | **G** | **A** | **C** | **G** | **A** | **C** | **G** | **G** | **G** | **A** | **C** | **A** | **G** | **G** | **A** | **T** | **T** | **T** | **A** | **A** | **T** | **A** | **T** | **G** | **A** | **A** | **T** | **G** | **A** | **A** | **A** | **C** | **C** | **A** | **A** | **A** | **A** | **C** | **G** | **G** | **G** | **A** | **A** | **G** | **T** | **T** | **A** | **T** | **G** | **G** | **A** | **T** | **T** | **G** | **A** | **A** | **C** | **A** | **A** | **T** | **A** | **T** | **C** | **A** | **A** | **A** | **G** | **A** | **A** | **C** | **G** | **A** | **G** | **T** | **A** | **G** | **C** | **T** | **G** | **G** | **T** | **A** | **T** | **T** | **G** | **G** | **T** | **G** | **G** | **A** | **A** | **C** | **A** | **G** | **T** | **A** | **A** | **A** | **A** | **A** | **T** | **C** | **A** | **T** | **T** | **A** | **G** | **T** | **T** | **T** | **T** | **A** | **A** | **A** | **G** | **G** | **G** | **C** | **A** | **A** | **G** | **G** | **T** | **A** | **C** | **G** | **A** | **G** | **C** | **G** | **T** | **G** | **G** | **A** | **G** | **A** | **T** | **C** | **A** | **A** | **G** | **G** | **T** | **T** | **C** | **C** | **T** | **T** | **T** | **G** | **A** | **T** | **G** | **A** | **A** | **G** | **G** | **A** | **G** | **G** | **C** | **A** | **-** | **-** | **-** |
| **N53595** | **A** | **T** | **G** | **A** | **T** | **G** | **G** | **G** | **A** | **A** | **A** | **A** | **A** | **T** | **A** | **T** | **C** | **C** | **A** | **G** | **A** | **G** | **C** | **G** | **A** | **T** | **G** | **C** | **T** | **A** | **G** | **C** | **T** | **G** | **T** | **T** | **T** | **A** | **T** | **T** | **C** | **G** | **G** | **G** | **G** | **A** | **T** | **T** | **G** | **C** | **G** | **G** | **C** | **T** | **T** | **T** | **C** | **C** | **T** | **T** | **A** | **T** | **T** | **A** | **T** | **A** | **C** | **T** | **A** | **T** | **T** | **T** | **T** | **C** | **A** | **C** | **T** | **T** | **T** | **T** | **C** | **A** | **C** | **T** | **T** | **A** | **T** | **T** | **T** | **T** | **T** | **A** | **T** | **G** | **C** | **C** | **A** | **G** | **T** | **A** | **A** | **T** | **C** | **A** | **A** | **A** | **G** | **T** | **C** | **A** | **T** | **T** | **G** | **G** | **T** | **G** | **G** | **G** | **G** | **A** | **G** | **A** | **A** | **T** | **T** | **A** | **C** | **T** | **G** | **C** | **G** | **T** | **G** | **C** | **A** | **C** | **G** | **T** | **T** | **T** | **A** | **T** | **T** | **A** | **T** | **A** | **T** | **G** | **T** | **T** | **C** | **C** | **G** | **C** | **T** | **T** | **A** | **T** | **C** | **T** | **T** | **C** | **C** | **A** | **T** | **T** | **T** | **G** | **T** | **T** | **A** | **G** | **C** | **T** | **A** | **T** | **T** | **T** | **C** | **C** | **T** | **T** | **A** | **G** | **G** | **T** | **G** | **T** | **A** | **G** | **G** | **A** | **T** | **T** | **A** | **A** | **T** | **C** | **G** | **T** | **C** | **T** | **T** | **T** | **C** | **T** | **A** | **T** | **T** | **A** | **T** | **T** | **A** | **T** | **C** | **A** | **C** | **T** | **T** | **A** | **T** | **T** | **C** | **A** | **A** | **A** | **A** | **G** | **A** | **C** | **A** | **A** | **A** | **A** | **T** | **A** | **C** | **G** | **G** | **G** | **A** | **A** | **A** | **A** | **T** | **C** | **G** | **A** | **A** | **G** | **A** | **A** | **A** | **A** | **G** | **C** | **T** | **G** | **C** | **G** | **T** | **G** | **C** | **A** | **C** | **T** | **C** | **T** | **C** | **T** | **T** | **C** | **T** | **G** | **G** | **C** | **A** | **A** | **T** | **T** | **A** | **T** | **G** | **A** | **A** | **T** | **C** | **C** | **A** | **A** | **A** | **T** | **T** | **G** | **T** | **T** | **G** | **C** | **T** | **T** | **C** | **T** | **T** | **C** | **C** | **G** | **A** | **T** | **C** | **C** | **C** | **A** | **A** | **G** | **T** | **G** | **C** | **A** | **T** | **C** | **G** | **G** | **A** | **C** | **G** | **A** | **T** | **T** | **T** | **A** | **T** | **A** | **C** | **A** | **T** | **C** | **A** | **A** | **A** | **G** | **A** | **T** | **A** | **T** | **T** | **G** | **A** | **T** | **A** | **A** | **G** | **G** | **A** | **A** | **A** | **T** | **C** | **A** | **C** | **T** | **A** | **A** | **A** | **A** | **T** | **A** | **A** | **A** | **A** | **G** | **A** | **A** | **A** | **A** | **A** | **A** | **T** | **G** | **A** | **T** | **T** | **G** | **A** | **A** | **G** | **T** | **A** | **T** | **C** | **T** | **A** | **G** | **T** | **G** | **A** | **A** | **T** | **T** | **A** | **C** | **A** | **A** | **A** | **T** | **T** | **G** | **T** | **A** | **A** | **C** | **G** | **A** | **G** | **C** | **C** | **G** | **T** | **C** | **C** | **C** | **C** | **A** | **A** | **T** | **A** | **T** | **G** | **T** | **A** | **G** | **A** | **T** | **G** | **G** | **A** | **C** | **A** | **A** | **A** | **C** | **C** | **A** | **A** | **A** | **G** | **A** | **A** | **G** | **A** | **A** | **A** | **T** | **T** | **T** | **T** | **A** | **G** | **A** | **A** | **T** | **T** | **A** | **G** | **A** | **A** | **A** | **G** | **A** | **C** | **A** | **T** | **C** | **G** | **A** | **T** | **T** | **A** | **G** | **C** | **C** | **C** | **G** | **T** | **G** | **A** | **G** | **C** | **T** | **G** | **C** | **A** | **T** | **G** | **A** | **T** | **T** | **C** | **A** | **G** | **T** | **T** | **T** | **C** | **G** | **C** | **A** | **A** | **C** | **A** | **A** | **T** | **T** | **G** | **T** | **T** | **T** | **G** | **C** | **A** | **G** | **C** | **A** | **A** | **T** | **G** | **A** | **T** | **G** | **A** | **T** | **G** | **A** | **T** | **G** | **T** | **C** | **G** | **G** | **C** | **A** | **T** | **T** | **A** | **A** | **C** | **G** | **G** | **A** | **G** | **C** | **A** | **A** | **G** | **C** | **A** | **G** | **A** | **A** | **A** | **A** | **A** | **A** | **G** | **C** | **G** | **A** | **G** | **A** | **C** | **A** | **C** | **C** | **A** | **G** | **A** | **A** | **A** | **T** | **G** | **T** | **T** | **C** | **C** | **G** | **C** | **A** | **A** | **G** | **C** | **A** | **G** | **T** | **T** | **G** | **A** | **A** | **A** | **A** | **T** | **G** | **G** | **T** | **A** | **G** | **C** | **A** | **G** | **A** | **G** | **A** | **T** | **C** | **A** | **T** | **C** | **A** | **A** | **C** | **G** | **C** | **T** | **T** | **C** | **C** | **C** | **A** | **G** | **T** | **C** | **T** | **G** | **A** | **G** | **A** | **T** | **G** | **C** | **G** | **T** | **G** | **C** | **G** | **C** | **T** | **G** | **C** | **T** | **T** | **C** | **T** | **T** | **C** | **A** | **T** | **C** | **T** | **G** | **C** | **G** | **T** | **C** | **C** | **A** | **G** | **T** | **C** | **A** | **A** | **T** | **T** | **T** | **A** | **G** | **A** | **A** | **G** | **A** | **G** | **A** | **A** | **A** | **A** | **G** | **T** | **T** | **T** | **A** | **A** | **A** | **G** | **C** | **A** | **A** | **G** | **G** | **T** | **A** | **T** | **C** | **G** | **A** | **G** | **C** | **A** | **G** | **T** | **T** | **A** | **T** | **T** | **G** | **A** | **A** | **G** | **G** | **A** | **A** | **T** | **T** | **G** | **C** | **A** | **A** | **A** | **A** | **T** | **A** | **A** | **A** | **A** | **T** | **C** | **C** | **A** | **G** | **A** | **T** | **T** | **T** | **C** | **G** | **C** | **T** | **G** | **A** | **A** | **A** | **T** | **G** | **G** | **G** | **A** | **T** | **G** | **T** | **A** | **G** | **A** | **A** | **G** | **A** | **T** | **G** | **T** | **A** | **A** | **A** | **A** | **C** | **T** | **A** | **T** | **C** | **T** | **A** | **G** | **T** | **T** | **C** | **C** | **A** | **T** | **T** | **G** | **A** | **G** | **G** | **A** | **T** | **C** | **A** | **C** | **T** | **T** | **G** | **T** | **T** | **C** | **C** | **G** | **A** | **A** | **T** | **C** | **G** | **T** | **T** | **C** | **A** | **A** | **G** | **A** | **A** | **T** | **T** | **G** | **T** | **T** | **A** | **T** | **C** | **A** | **A** | **A** | **T** | **A** | **C** | **A** | **T** | **T** | **A** | **A** | **G** | **A** | **C** | **A** | **T** | **G** | **C** | **T** | **A** | **A** | **G** | **G** | **C** | **T** | **A** | **A** | **T** | **G** | **A** | **A** | **T** | **T** | **A** | **G** | **A** | **A** | **G** | **T** | **C** | **T** | **A** | **T** | **T** | **T** | **G** | **C** | **A** | **T** | **A** | **A** | **A** | **A** | **T** | **A** | **G** | **A** | **T** | **A** | **A** | **C** | **A** | **A** | **T** | **C** | **T** | **T** | **T** | **T** | **A** | **T** | **T** | **A** | **C** | **G** | **T** | **A** | **T** | **C** | **A** | **T** | **T** | **G** | **A** | **C** | **G** | **A** | **C** | **G** | **G** | **G** | **A** | **C** | **A** | **G** | **G** | **A** | **T** | **T** | **T** | **A** | **A** | **T** | **A** | **T** | **G** | **A** | **A** | **T** | **G** | **A** | **A** | **A** | **C** | **C** | **A** | **A** | **A** | **A** | **C** | **G** | **G** | **G** | **A** | **A** | **G** | **T** | **T** | **A** | **T** | **G** | **G** | **A** | **T** | **T** | **G** | **A** | **A** | **C** | **A** | **A** | **T** | **A** | **T** | **C** | **A** | **A** | **A** | **G** | **A** | **A** | **C** | **G** | **A** | **G** | **T** | **A** | **G** | **C** | **T** | **G** | **G** | **T** | **A** | **T** | **T** | **G** | **G** | **T** | **G** | **G** | **A** | **A** | **C** | **A** | **G** | **T** | **A** | **A** | **A** | **A** | **A** | **T** | **C** | **A** | **T** | **T** | **A** | **G** | **T** | **T** | **T** | **T** | **A** | **A** | **A** | **G** | **G** | **G** | **C** | **A** | **A** | **G** | **G** | **T** | **A** | **C** | **G** | **A** | **G** | **C** | **G** | **T** | **G** | **G** | **A** | **G** | **A** | **T** | **C** | **A** | **A** | **G** | **G** | **T** | **C** | **C** | **C** | **T** | **T** | **T** | **G** | **A** | **T** | **G** | **A** | **A** | **G** | **G** | **A** | **G** | **G** | **C** | **A** | **-** | **-** | **-** |
| **N60061F** | **A** | **T** | **G** | **A** | **T** | **G** | **G** | **G** | **A** | **A** | **G** | **A** | **A** | **T** | **A** | **T** | **C** | **C** | **A** | **A** | **A** | **G** | **C** | **A** | **A** | **T** | **G** | **C** | **T** | **A** | **G** | **C** | **T** | **G** | **T** | **T** | **T** | **A** | **T** | **T** | **C** | **G** | **G** | **G** | **G** | **A** | **T** | **T** | **G** | **C** | **G** | **G** | **C** | **T** | **T** | **T** | **C** | **C** | **T** | **T** | **A** | **T** | **T** | **A** | **T** | **T** | **C** | **T** | **A** | **T** | **T** | **T** | **T** | **C** | **A** | **C** | **T** | **T** | **T** | **T** | **C** | **A** | **C** | **T** | **T** | **A** | **T** | **T** | **T** | **T** | **T** | **A** | **T** | **G** | **C** | **C** | **A** | **G** | **T** | **A** | **A** | **C** | **C** | **A** | **A** | **A** | **G** | **T** | **C** | **A** | **T** | **T** | **G** | **G** | **T** | **G** | **G** | **G** | **G** | **A** | **G** | **A** | **A** | **T** | **T** | **G** | **C** | **T** | **G** | **C** | **G** | **T** | **G** | **C** | **G** | **C** | **G** | **T** | **T** | **T** | **A** | **T** | **T** | **A** | **T** | **A** | **C** | **G** | **T** | **T** | **C** | **C** | **G** | **C** | **T** | **T** | **A** | **T** | **C** | **T** | **T** | **C** | **C** | **A** | **T** | **T** | **T** | **G** | **A** | **T** | **G** | **G** | **C** | **A** | **A** | **T** | **T** | **T** | **C** | **A** | **T** | **T** | **A** | **G** | **G** | **T** | **G** | **T** | **A** | **G** | **G** | **A** | **T** | **T** | **G** | **A** | **T** | **C** | **G** | **T** | **A** | **T** | **T** | **T** | **C** | **T** | **A** | **T** | **T** | **G** | **T** | **T** | **A** | **T** | **C** | **A** | **C** | **T** | **T** | **A** | **T** | **T** | **C** | **A** | **A** | **A** | **A** | **G** | **G** | **C** | **A** | **A** | **A** | **A** | **T** | **A** | **C** | **G** | **G** | **G** | **A** | **A** | **A** | **A** | **T** | **C** | **G** | **A** | **A** | **G** | **A** | **A** | **A** | **A** | **G** | **C** | **T** | **T** | **C** | **G** | **T** | **G** | **C** | **A** | **C** | **T** | **T** | **T** | **C** | **T** | **T** | **C** | **T** | **G** | **G** | **C** | **A** | **A** | **T** | **T** | **A** | **T** | **G** | **A** | **A** | **T** | **C** | **C** | **A** | **A** | **A** | **C** | **T** | **T** | **T** | **T** | **G** | **C** | **A** | **T** | **C** | **A** | **G** | **C** | **C** | **G** | **A** | **T** | **C** | **C** | **C** | **A** | **A** | **G** | **C** | **A** | **C** | **G** | **T** | **C** | **G** | **G** | **A** | **T** | **G** | **A** | **T** | **C** | **T** | **G** | **T** | **A** | **C** | **A** | **T** | **C** | **A** | **A** | **A** | **G** | **A** | **T** | **A** | **T** | **T** | **G** | **A** | **T** | **A** | **T** | **G** | **G** | **A** | **A** | **A** | **T** | **T** | **A** | **C** | **T** | **A** | **A** | **A** | **A** | **T** | **A** | **A** | **A** | **A** | **G** | **A** | **A** | **A** | **A** | **A** | **A** | **T** | **G** | **A** | **T** | **C** | **G** | **A** | **A** | **G** | **T** | **A** | **T** | **C** | **C** | **A** | **G** | **T** | **G** | **A** | **A** | **T** | **T** | **A** | **C** | **A** | **G** | **A** | **T** | **T** | **A** | **T** | **G** | **A** | **C** | **T** | **A** | **G** | **C** | **C** | **G** | **T** | **C** | **C** | **T** | **C** | **A** | **A** | **T** | **A** | **T** | **G** | **T** | **A** | **G** | **A** | **T** | **G** | **G** | **A** | **C** | **A** | **A** | **A** | **C** | **C** | **A** | **A** | **A** | **G** | **A** | **A** | **G** | **A** | **G** | **A** | **T** | **T** | **T** | **T** | **A** | **G** | **A** | **A** | **T** | **T** | **A** | **G** | **A** | **A** | **A** | **G** | **G** | **C** | **A** | **T** | **C** | **G** | **A** | **T** | **T** | **A** | **G** | **C** | **T** | **C** | **G** | **T** | **G** | **A** | **G** | **C** | **T** | **T** | **C** | **A** | **T** | **G** | **A** | **T** | **T** | **C** | **A** | **G** | **T** | **T** | **T** | **C** | **A** | **C** | **A** | **G** | **C** | **A** | **A** | **T** | **T** | **A** | **T** | **T** | **T** | **G** | **C** | **A** | **G** | **C** | **G** | **A** | **T** | **G** | **A** | **T** | **G** | **A** | **T** | **G** | **A** | **T** | **G** | **T** | **C** | **G** | **G** | **C** | **A** | **T** | **T** | **G** | **A** | **C** | **G** | **G** | **A** | **G** | **C** | **A** | **A** | **G** | **C** | **A** | **G** | **A** | **A** | **A** | **A** | **A** | **A** | **G** | **T** | **G** | **A** | **G** | **A** | **C** | **A** | **C** | **C** | **A** | **G** | **A** | **A** | **A** | **T** | **G** | **T** | **T** | **C** | **C** | **G** | **T** | **A** | **A** | **G** | **C** | **A** | **G** | **T** | **T** | **G** | **A** | **A** | **A** | **A** | **T** | **G** | **G** | **T** | **G** | **G** | **C** | **A** | **G** | **A** | **G** | **A** | **T** | **C** | **A** | **T** | **C** | **A** | **A** | **C** | **G** | **C** | **T** | **T** | **C** | **C** | **C** | **A** | **G** | **T** | **C** | **T** | **G** | **A** | **G** | **A** | **T** | **G** | **C** | **G** | **T** | **G** | **C** | **G** | **C** | **T** | **G** | **C** | **T** | **T** | **C** | **T** | **T** | **C** | **A** | **T** | **C** | **T** | **G** | **C** | **G** | **T** | **C** | **C** | **A** | **G** | **T** | **C** | **A** | **A** | **T** | **T** | **T** | **A** | **G** | **A** | **A** | **G** | **A** | **A** | **A** | **A** | **A** | **A** | **G** | **T** | **T** | **T** | **A** | **A** | **A** | **A** | **C** | **A** | **A** | **G** | **G** | **C** | **A** | **T** | **T** | **G** | **A** | **G** | **C** | **A** | **G** | **T** | **T** | **A** | **T** | **T** | **G** | **A** | **A** | **G** | **G** | **A** | **A** | **T** | **T** | **G** | **C** | **A** | **G** | **A** | **A** | **T** | **A** | **A** | **A** | **A** | **T** | **C** | **C** | **A** | **G** | **A** | **T** | **T** | **T** | **C** | **G** | **C** | **T** | **A** | **A** | **A** | **A** | **T** | **G** | **G** | **G** | **A** | **T** | **G** | **T** | **A** | **G** | **A** | **A** | **G** | **A** | **T** | **G** | **T** | **A** | **A** | **A** | **A** | **T** | **T** | **A** | **A** | **C** | **T** | **A** | **G** | **T** | **T** | **C** | **C** | **A** | **T** | **T** | **G** | **A** | **G** | **G** | **A** | **T** | **C** | **A** | **C** | **T** | **T** | **G** | **T** | **T** | **C** | **C** | **G** | **A** | **A** | **T** | **C** | **G** | **T** | **T** | **C** | **A** | **A** | **G** | **A** | **A** | **T** | **T** | **G** | **T** | **T** | **A** | **T** | **C** | **A** | **A** | **A** | **C** | **A** | **C** | **A** | **T** | **T** | **A** | **A** | **G** | **A** | **C** | **A** | **T** | **G** | **C** | **T** | **A** | **A** | **G** | **G** | **C** | **T** | **A** | **A** | **T** | **G** | **A** | **A** | **T** | **T** | **A** | **G** | **A** | **A** | **G** | **T** | **C** | **T** | **A** | **T** | **T** | **T** | **G** | **C** | **A** | **T** | **A** | **A** | **A** | **A** | **T** | **A** | **G** | **A** | **T** | **A** | **A** | **C** | **A** | **A** | **T** | **C** | **T** | **T** | **T** | **T** | **A** | **T** | **T** | **A** | **C** | **G** | **T** | **A** | **T** | **C** | **A** | **T** | **T** | **G** | **A** | **C** | **G** | **A** | **C** | **G** | **G** | **G** | **A** | **C** | **A** | **G** | **G** | **A** | **T** | **T** | **T** | **A** | **A** | **T** | **A** | **T** | **G** | **A** | **A** | **T** | **G** | **A** | **A** | **A** | **C** | **C** | **A** | **A** | **A** | **A** | **C** | **G** | **G** | **G** | **A** | **A** | **G** | **T** | **T** | **A** | **T** | **G** | **G** | **A** | **T** | **T** | **G** | **A** | **A** | **C** | **A** | **A** | **T** | **A** | **T** | **C** | **A** | **A** | **A** | **G** | **A** | **A** | **C** | **G** | **A** | **G** | **T** | **A** | **G** | **C** | **T** | **G** | **G** | **T** | **A** | **T** | **T** | **G** | **G** | **T** | **G** | **G** | **A** | **A** | **C** | **A** | **G** | **T** | **A** | **A** | **A** | **A** | **A** | **T** | **C** | **A** | **T** | **T** | **A** | **G** | **T** | **T** | **T** | **T** | **A** | **A** | **A** | **G** | **G** | **G** | **C** | **A** | **A** | **G** | **G** | **T** | **A** | **C** | **G** | **A** | **G** | **C** | **G** | **T** | **G** | **G** | **A** | **G** | **A** | **T** | **C** | **A** | **A** | **G** | **G** | **T** | **C** | **C** | **C** | **T** | **T** | **T** | **G** | **A** | **T** | **G** | **A** | **A** | **G** | **G** | **A** | **G** | **G** | **C** | **A** | **-** | **-** | **-** |
| **N55077** | **A** | **T** | **G** | **A** | **T** | **G** | **G** | **G** | **A** | **A** | **G** | **A** | **A** | **T** | **A** | **T** | **C** | **C** | **A** | **A** | **A** | **G** | **C** | **A** | **A** | **T** | **G** | **C** | **T** | **A** | **G** | **C** | **T** | **G** | **T** | **T** | **T** | **A** | **T** | **T** | **C** | **G** | **G** | **G** | **G** | **A** | **T** | **T** | **G** | **C** | **G** | **G** | **C** | **T** | **T** | **T** | **C** | **C** | **T** | **T** | **A** | **T** | **T** | **A** | **T** | **T** | **C** | **T** | **A** | **T** | **T** | **T** | **T** | **C** | **A** | **C** | **T** | **T** | **T** | **T** | **C** | **A** | **C** | **T** | **T** | **A** | **T** | **T** | **T** | **T** | **T** | **A** | **T** | **G** | **C** | **C** | **A** | **G** | **T** | **A** | **A** | **C** | **C** | **A** | **A** | **A** | **G** | **T** | **C** | **A** | **T** | **T** | **G** | **G** | **T** | **G** | **G** | **G** | **G** | **A** | **G** | **A** | **A** | **T** | **T** | **G** | **C** | **T** | **G** | **C** | **G** | **T** | **G** | **C** | **G** | **C** | **G** | **T** | **T** | **T** | **A** | **T** | **T** | **A** | **T** | **A** | **C** | **G** | **T** | **T** | **C** | **C** | **G** | **C** | **T** | **T** | **A** | **T** | **C** | **T** | **T** | **C** | **C** | **A** | **T** | **T** | **T** | **G** | **A** | **T** | **G** | **G** | **C** | **A** | **A** | **T** | **T** | **T** | **C** | **A** | **T** | **T** | **A** | **G** | **G** | **T** | **G** | **T** | **A** | **G** | **G** | **A** | **T** | **T** | **G** | **A** | **T** | **C** | **G** | **T** | **A** | **T** | **T** | **T** | **C** | **T** | **A** | **T** | **T** | **G** | **T** | **T** | **A** | **T** | **C** | **A** | **C** | **T** | **T** | **A** | **T** | **T** | **C** | **A** | **A** | **A** | **A** | **G** | **G** | **C** | **A** | **A** | **A** | **A** | **T** | **A** | **C** | **G** | **G** | **G** | **A** | **A** | **A** | **A** | **T** | **C** | **G** | **A** | **A** | **G** | **A** | **A** | **A** | **A** | **G** | **C** | **T** | **T** | **C** | **G** | **T** | **G** | **C** | **A** | **C** | **T** | **T** | **T** | **C** | **T** | **T** | **C** | **T** | **G** | **G** | **C** | **A** | **A** | **T** | **T** | **A** | **T** | **G** | **A** | **A** | **T** | **C** | **C** | **A** | **A** | **A** | **C** | **T** | **T** | **T** | **T** | **G** | **C** | **A** | **T** | **C** | **A** | **G** | **C** | **C** | **G** | **A** | **T** | **C** | **C** | **C** | **A** | **A** | **G** | **C** | **G** | **C** | **G** | **T** | **C** | **G** | **G** | **A** | **T** | **G** | **A** | **T** | **C** | **T** | **G** | **T** | **A** | **C** | **A** | **T** | **C** | **A** | **A** | **A** | **G** | **A** | **T** | **A** | **T** | **T** | **G** | **A** | **T** | **A** | **T** | **G** | **G** | **A** | **A** | **A** | **T** | **T** | **A** | **C** | **T** | **A** | **A** | **A** | **A** | **T** | **A** | **A** | **A** | **A** | **G** | **A** | **A** | **A** | **A** | **A** | **A** | **T** | **G** | **A** | **T** | **C** | **G** | **A** | **A** | **G** | **T** | **A** | **T** | **C** | **C** | **A** | **G** | **T** | **G** | **A** | **A** | **T** | **T** | **A** | **C** | **A** | **G** | **A** | **T** | **T** | **A** | **T** | **G** | **A** | **C** | **T** | **A** | **G** | **C** | **C** | **G** | **T** | **C** | **C** | **T** | **C** | **A** | **A** | **T** | **A** | **T** | **G** | **T** | **A** | **G** | **A** | **T** | **G** | **G** | **A** | **C** | **A** | **A** | **A** | **C** | **C** | **A** | **A** | **A** | **G** | **A** | **A** | **G** | **A** | **G** | **A** | **T** | **T** | **T** | **T** | **A** | **G** | **A** | **A** | **T** | **T** | **A** | **G** | **A** | **A** | **A** | **G** | **G** | **C** | **A** | **T** | **C** | **G** | **A** | **T** | **T** | **A** | **G** | **C** | **T** | **C** | **G** | **T** | **G** | **A** | **G** | **C** | **T** | **T** | **C** | **A** | **T** | **G** | **A** | **T** | **T** | **C** | **A** | **G** | **T** | **T** | **T** | **C** | **A** | **C** | **A** | **G** | **C** | **A** | **A** | **T** | **T** | **A** | **T** | **T** | **T** | **G** | **C** | **A** | **G** | **C** | **G** | **A** | **T** | **G** | **A** | **T** | **G** | **A** | **T** | **G** | **A** | **T** | **G** | **T** | **C** | **G** | **G** | **C** | **A** | **T** | **T** | **G** | **A** | **C** | **G** | **G** | **A** | **G** | **C** | **A** | **A** | **G** | **C** | **A** | **G** | **A** | **A** | **A** | **A** | **A** | **A** | **G** | **T** | **G** | **A** | **G** | **A** | **C** | **A** | **C** | **C** | **A** | **G** | **A** | **A** | **A** | **T** | **G** | **T** | **T** | **C** | **C** | **G** | **T** | **A** | **A** | **G** | **C** | **A** | **G** | **T** | **T** | **G** | **A** | **A** | **A** | **A** | **T** | **G** | **G** | **T** | **G** | **G** | **C** | **A** | **G** | **A** | **G** | **A** | **T** | **C** | **A** | **T** | **C** | **A** | **A** | **C** | **G** | **C** | **T** | **T** | **C** | **C** | **C** | **A** | **G** | **T** | **C** | **T** | **G** | **A** | **G** | **A** | **T** | **G** | **C** | **G** | **T** | **G** | **C** | **G** | **C** | **T** | **G** | **C** | **T** | **T** | **C** | **T** | **T** | **C** | **A** | **T** | **C** | **T** | **G** | **C** | **G** | **T** | **C** | **C** | **A** | **G** | **T** | **C** | **A** | **A** | **T** | **T** | **T** | **A** | **G** | **A** | **A** | **G** | **A** | **A** | **A** | **A** | **A** | **A** | **G** | **T** | **T** | **T** | **A** | **A** | **A** | **A** | **C** | **A** | **A** | **G** | **G** | **C** | **A** | **T** | **T** | **G** | **A** | **G** | **C** | **A** | **G** | **T** | **T** | **A** | **T** | **T** | **G** | **A** | **A** | **G** | **G** | **A** | **A** | **T** | **T** | **G** | **C** | **A** | **G** | **A** | **A** | **T** | **A** | **A** | **A** | **A** | **T** | **C** | **C** | **A** | **G** | **A** | **T** | **T** | **T** | **C** | **G** | **C** | **T** | **A** | **A** | **A** | **A** | **T** | **G** | **G** | **G** | **A** | **T** | **G** | **T** | **A** | **G** | **A** | **A** | **G** | **A** | **T** | **G** | **T** | **A** | **A** | **A** | **A** | **T** | **T** | **A** | **A** | **C** | **T** | **A** | **G** | **T** | **T** | **C** | **C** | **A** | **T** | **T** | **G** | **A** | **G** | **G** | **A** | **T** | **C** | **A** | **C** | **T** | **T** | **G** | **T** | **T** | **C** | **C** | **G** | **A** | **A** | **T** | **C** | **G** | **T** | **T** | **C** | **A** | **A** | **G** | **A** | **A** | **T** | **T** | **G** | **T** | **T** | **A** | **T** | **C** | **A** | **A** | **A** | **C** | **A** | **C** | **A** | **T** | **T** | **A** | **A** | **G** | **A** | **C** | **A** | **T** | **G** | **C** | **T** | **A** | **A** | **G** | **G** | **C** | **T** | **A** | **A** | **T** | **G** | **A** | **A** | **T** | **T** | **A** | **G** | **A** | **A** | **G** | **T** | **C** | **T** | **A** | **T** | **T** | **T** | **G** | **C** | **A** | **T** | **A** | **A** | **A** | **A** | **T** | **A** | **G** | **A** | **T** | **A** | **A** | **C** | **A** | **A** | **T** | **C** | **T** | **T** | **T** | **T** | **A** | **T** | **T** | **A** | **C** | **G** | **T** | **A** | **T** | **C** | **A** | **T** | **T** | **G** | **A** | **C** | **G** | **A** | **C** | **G** | **G** | **G** | **A** | **C** | **A** | **G** | **G** | **A** | **T** | **T** | **T** | **A** | **A** | **T** | **A** | **T** | **G** | **A** | **A** | **T** | **G** | **A** | **A** | **A** | **C** | **C** | **A** | **A** | **A** | **A** | **C** | **G** | **G** | **G** | **A** | **A** | **G** | **T** | **T** | **A** | **T** | **G** | **G** | **A** | **T** | **T** | **G** | **A** | **A** | **C** | **A** | **A** | **T** | **A** | **T** | **C** | **A** | **A** | **A** | **G** | **A** | **A** | **C** | **G** | **A** | **G** | **T** | **A** | **G** | **C** | **T** | **G** | **G** | **T** | **A** | **T** | **T** | **G** | **G** | **T** | **G** | **G** | **A** | **A** | **C** | **A** | **G** | **T** | **A** | **A** | **A** | **A** | **A** | **T** | **C** | **A** | **T** | **T** | **A** | **G** | **T** | **T** | **T** | **T** | **A** | **A** | **A** | **G** | **G** | **G** | **C** | **A** | **A** | **G** | **G** | **T** | **A** | **C** | **G** | **A** | **G** | **C** | **G** | **T** | **G** | **G** | **A** | **G** | **A** | **T** | **C** | **A** | **A** | **G** | **G** | **T** | **T** | **C** | **C** | **T** | **T** | **T** | **G** | **A** | **T** | **G** | **A** | **A** | **G** | **G** | **A** | **G** | **G** | **C** | **A** | **-** | **-** | **-** |
| **N52777** | **A** | **T** | **G** | **A** | **T** | **G** | **G** | **G** | **A** | **A** | **G** | **A** | **A** | **T** | **A** | **T** | **C** | **C** | **A** | **A** | **A** | **G** | **C** | **A** | **A** | **T** | **G** | **C** | **T** | **A** | **G** | **C** | **T** | **G** | **T** | **T** | **T** | **A** | **T** | **T** | **C** | **G** | **G** | **G** | **G** | **A** | **T** | **T** | **G** | **C** | **G** | **G** | **C** | **T** | **T** | **T** | **C** | **C** | **T** | **T** | **A** | **T** | **T** | **A** | **T** | **T** | **C** | **T** | **A** | **T** | **T** | **T** | **T** | **C** | **A** | **C** | **T** | **T** | **T** | **T** | **C** | **A** | **C** | **T** | **T** | **A** | **T** | **T** | **T** | **T** | **T** | **A** | **T** | **G** | **C** | **C** | **A** | **G** | **T** | **A** | **A** | **C** | **C** | **A** | **A** | **A** | **G** | **T** | **C** | **A** | **T** | **T** | **G** | **G** | **T** | **G** | **G** | **G** | **G** | **A** | **G** | **A** | **A** | **T** | **T** | **G** | **C** | **T** | **G** | **C** | **G** | **T** | **G** | **C** | **G** | **C** | **G** | **T** | **T** | **T** | **A** | **T** | **T** | **A** | **T** | **A** | **C** | **G** | **T** | **T** | **C** | **C** | **G** | **C** | **T** | **T** | **A** | **T** | **C** | **T** | **T** | **C** | **C** | **A** | **T** | **T** | **T** | **G** | **A** | **T** | **G** | **G** | **C** | **A** | **A** | **T** | **T** | **T** | **C** | **A** | **T** | **T** | **A** | **G** | **G** | **T** | **G** | **T** | **A** | **G** | **G** | **A** | **T** | **T** | **G** | **A** | **T** | **C** | **G** | **T** | **A** | **T** | **T** | **T** | **C** | **T** | **A** | **T** | **T** | **G** | **T** | **T** | **A** | **T** | **C** | **A** | **C** | **T** | **T** | **A** | **T** | **T** | **C** | **A** | **A** | **A** | **A** | **G** | **G** | **C** | **A** | **A** | **A** | **A** | **T** | **A** | **C** | **G** | **G** | **G** | **A** | **A** | **A** | **A** | **T** | **C** | **G** | **A** | **A** | **G** | **A** | **A** | **A** | **A** | **G** | **C** | **T** | **T** | **C** | **G** | **T** | **G** | **C** | **A** | **C** | **T** | **T** | **T** | **C** | **T** | **T** | **C** | **T** | **G** | **G** | **C** | **A** | **A** | **T** | **T** | **A** | **T** | **G** | **A** | **A** | **T** | **C** | **C** | **A** | **A** | **A** | **C** | **T** | **T** | **T** | **T** | **G** | **C** | **A** | **T** | **C** | **A** | **G** | **C** | **C** | **G** | **A** | **T** | **C** | **C** | **C** | **A** | **A** | **G** | **C** | **G** | **C** | **G** | **T** | **C** | **G** | **G** | **A** | **T** | **G** | **A** | **T** | **C** | **T** | **G** | **T** | **A** | **C** | **A** | **T** | **C** | **A** | **A** | **A** | **G** | **A** | **T** | **A** | **T** | **T** | **G** | **A** | **T** | **A** | **T** | **G** | **G** | **A** | **A** | **A** | **T** | **T** | **A** | **C** | **T** | **A** | **A** | **A** | **A** | **T** | **A** | **A** | **A** | **A** | **G** | **A** | **A** | **A** | **A** | **A** | **A** | **T** | **G** | **A** | **T** | **C** | **G** | **A** | **A** | **G** | **T** | **A** | **T** | **C** | **C** | **A** | **G** | **T** | **G** | **A** | **A** | **T** | **T** | **A** | **C** | **A** | **G** | **A** | **T** | **T** | **A** | **T** | **G** | **A** | **C** | **T** | **A** | **G** | **C** | **C** | **G** | **T** | **C** | **C** | **T** | **C** | **A** | **A** | **T** | **A** | **T** | **G** | **T** | **A** | **G** | **A** | **T** | **G** | **G** | **A** | **C** | **A** | **A** | **A** | **C** | **C** | **A** | **A** | **A** | **G** | **A** | **A** | **G** | **A** | **G** | **A** | **T** | **T** | **T** | **T** | **A** | **G** | **A** | **A** | **T** | **T** | **A** | **G** | **A** | **A** | **A** | **G** | **G** | **C** | **A** | **T** | **C** | **G** | **A** | **T** | **T** | **A** | **G** | **C** | **T** | **C** | **G** | **T** | **G** | **A** | **G** | **C** | **T** | **T** | **C** | **A** | **T** | **G** | **A** | **T** | **T** | **C** | **A** | **G** | **T** | **T** | **T** | **C** | **A** | **C** | **A** | **G** | **C** | **A** | **A** | **T** | **T** | **A** | **T** | **T** | **T** | **G** | **C** | **A** | **G** | **C** | **G** | **A** | **T** | **G** | **A** | **T** | **G** | **A** | **T** | **G** | **A** | **T** | **G** | **T** | **C** | **G** | **G** | **C** | **A** | **T** | **T** | **G** | **A** | **C** | **G** | **G** | **A** | **G** | **C** | **A** | **A** | **G** | **C** | **A** | **G** | **A** | **A** | **A** | **A** | **A** | **A** | **G** | **T** | **G** | **A** | **G** | **A** | **C** | **A** | **C** | **C** | **A** | **G** | **A** | **A** | **A** | **T** | **G** | **T** | **T** | **C** | **C** | **G** | **T** | **A** | **A** | **G** | **C** | **A** | **G** | **T** | **T** | **G** | **A** | **A** | **A** | **A** | **T** | **G** | **G** | **T** | **G** | **G** | **C** | **A** | **G** | **A** | **G** | **A** | **T** | **C** | **A** | **T** | **C** | **A** | **A** | **C** | **G** | **C** | **T** | **T** | **C** | **C** | **C** | **A** | **G** | **T** | **C** | **T** | **G** | **A** | **G** | **A** | **T** | **G** | **C** | **G** | **T** | **G** | **C** | **G** | **C** | **T** | **G** | **C** | **T** | **T** | **C** | **T** | **T** | **C** | **A** | **T** | **C** | **T** | **G** | **C** | **G** | **T** | **C** | **C** | **A** | **G** | **T** | **C** | **A** | **A** | **T** | **T** | **T** | **A** | **G** | **A** | **A** | **G** | **A** | **A** | **A** | **A** | **A** | **A** | **G** | **T** | **T** | **T** | **A** | **A** | **A** | **A** | **C** | **A** | **A** | **G** | **G** | **C** | **A** | **T** | **T** | **G** | **A** | **G** | **C** | **A** | **G** | **T** | **T** | **A** | **T** | **T** | **G** | **A** | **A** | **G** | **G** | **A** | **A** | **T** | **T** | **G** | **C** | **A** | **G** | **A** | **A** | **T** | **A** | **A** | **A** | **A** | **T** | **C** | **C** | **A** | **G** | **A** | **T** | **T** | **T** | **C** | **G** | **C** | **T** | **A** | **A** | **A** | **A** | **T** | **G** | **G** | **G** | **A** | **T** | **G** | **T** | **A** | **G** | **A** | **A** | **G** | **A** | **T** | **G** | **T** | **A** | **A** | **A** | **A** | **T** | **T** | **A** | **A** | **C** | **T** | **A** | **G** | **T** | **T** | **C** | **C** | **A** | **T** | **T** | **G** | **A** | **G** | **G** | **A** | **T** | **C** | **A** | **C** | **T** | **T** | **G** | **T** | **T** | **C** | **C** | **G** | **A** | **A** | **T** | **C** | **G** | **T** | **T** | **C** | **A** | **A** | **G** | **A** | **A** | **T** | **T** | **G** | **T** | **T** | **A** | **T** | **C** | **A** | **A** | **A** | **C** | **A** | **C** | **A** | **T** | **T** | **A** | **A** | **G** | **A** | **C** | **A** | **T** | **G** | **C** | **T** | **A** | **A** | **G** | **G** | **C** | **T** | **A** | **A** | **T** | **G** | **A** | **A** | **T** | **T** | **A** | **G** | **A** | **A** | **G** | **T** | **C** | **T** | **A** | **T** | **T** | **T** | **G** | **C** | **A** | **T** | **A** | **A** | **A** | **A** | **T** | **A** | **G** | **A** | **T** | **A** | **A** | **C** | **A** | **A** | **T** | **C** | **T** | **T** | **T** | **T** | **A** | **T** | **T** | **A** | **C** | **G** | **T** | **A** | **T** | **C** | **A** | **T** | **T** | **G** | **A** | **C** | **G** | **A** | **C** | **G** | **G** | **G** | **A** | **C** | **A** | **G** | **G** | **A** | **T** | **T** | **T** | **A** | **A** | **T** | **A** | **T** | **G** | **A** | **A** | **T** | **G** | **A** | **A** | **A** | **C** | **C** | **A** | **A** | **A** | **A** | **C** | **G** | **G** | **G** | **A** | **A** | **G** | **T** | **T** | **A** | **T** | **G** | **G** | **A** | **T** | **T** | **G** | **A** | **A** | **C** | **A** | **A** | **T** | **A** | **T** | **C** | **A** | **A** | **A** | **G** | **A** | **A** | **C** | **G** | **A** | **G** | **T** | **A** | **G** | **C** | **T** | **G** | **G** | **T** | **A** | **T** | **T** | **G** | **G** | **T** | **G** | **G** | **A** | **A** | **C** | **A** | **G** | **T** | **A** | **A** | **A** | **A** | **A** | **T** | **C** | **A** | **T** | **T** | **A** | **G** | **T** | **T** | **T** | **T** | **A** | **A** | **A** | **G** | **G** | **G** | **C** | **A** | **A** | **G** | **G** | **T** | **A** | **C** | **G** | **A** | **G** | **C** | **G** | **T** | **G** | **G** | **A** | **G** | **A** | **T** | **C** | **A** | **A** | **G** | **G** | **T** | **C** | **C** | **C** | **T** | **T** | **T** | **G** | **A** | **T** | **G** | **A** | **A** | **G** | **G** | **A** | **G** | **G** | **C** | **A** | **-** | **-** | **-** |
| **N60284F** | **A** | **T** | **G** | **A** | **T** | **G** | **G** | **G** | **A** | **A** | **G** | **A** | **A** | **T** | **A** | **T** | **C** | **C** | **A** | **A** | **A** | **G** | **C** | **A** | **A** | **T** | **G** | **C** | **T** | **A** | **G** | **C** | **T** | **G** | **T** | **T** | **T** | **A** | **T** | **T** | **C** | **G** | **G** | **G** | **G** | **A** | **T** | **T** | **G** | **C** | **G** | **G** | **C** | **T** | **T** | **T** | **C** | **C** | **T** | **T** | **A** | **T** | **T** | **A** | **T** | **T** | **C** | **T** | **A** | **T** | **T** | **T** | **T** | **C** | **A** | **C** | **T** | **T** | **T** | **T** | **C** | **A** | **C** | **T** | **T** | **A** | **T** | **T** | **T** | **T** | **T** | **A** | **T** | **G** | **C** | **C** | **A** | **G** | **T** | **A** | **A** | **C** | **C** | **A** | **A** | **A** | **G** | **T** | **C** | **A** | **T** | **T** | **G** | **G** | **T** | **G** | **G** | **G** | **G** | **A** | **G** | **A** | **A** | **T** | **T** | **G** | **C** | **T** | **G** | **C** | **G** | **T** | **G** | **C** | **G** | **C** | **G** | **T** | **T** | **T** | **A** | **T** | **T** | **A** | **T** | **A** | **C** | **G** | **T** | **T** | **C** | **C** | **G** | **C** | **T** | **T** | **A** | **T** | **C** | **T** | **T** | **C** | **C** | **A** | **T** | **T** | **T** | **G** | **A** | **T** | **G** | **G** | **C** | **A** | **A** | **T** | **T** | **T** | **C** | **A** | **T** | **T** | **A** | **G** | **G** | **T** | **G** | **T** | **A** | **G** | **G** | **A** | **T** | **T** | **G** | **A** | **T** | **C** | **G** | **T** | **A** | **T** | **T** | **T** | **C** | **T** | **A** | **T** | **T** | **G** | **T** | **T** | **A** | **T** | **C** | **A** | **C** | **T** | **T** | **A** | **T** | **T** | **C** | **A** | **A** | **A** | **A** | **G** | **G** | **C** | **A** | **A** | **A** | **A** | **T** | **A** | **C** | **G** | **G** | **G** | **A** | **A** | **A** | **A** | **T** | **C** | **G** | **A** | **A** | **G** | **A** | **A** | **A** | **A** | **G** | **C** | **T** | **T** | **C** | **G** | **T** | **G** | **C** | **A** | **C** | **T** | **T** | **T** | **C** | **T** | **T** | **C** | **T** | **G** | **G** | **C** | **A** | **A** | **T** | **T** | **A** | **T** | **G** | **A** | **A** | **T** | **C** | **C** | **A** | **A** | **A** | **C** | **T** | **T** | **T** | **T** | **G** | **C** | **A** | **T** | **C** | **A** | **G** | **C** | **C** | **G** | **A** | **T** | **C** | **C** | **C** | **A** | **A** | **G** | **C** | **G** | **C** | **G** | **T** | **C** | **G** | **G** | **A** | **T** | **G** | **A** | **T** | **C** | **T** | **G** | **T** | **A** | **C** | **A** | **T** | **C** | **A** | **A** | **A** | **G** | **A** | **T** | **A** | **T** | **T** | **G** | **A** | **T** | **A** | **T** | **G** | **G** | **A** | **A** | **A** | **T** | **T** | **A** | **C** | **T** | **A** | **A** | **A** | **A** | **T** | **A** | **A** | **A** | **A** | **G** | **A** | **A** | **A** | **A** | **A** | **A** | **T** | **G** | **A** | **T** | **C** | **G** | **A** | **A** | **G** | **T** | **A** | **T** | **C** | **C** | **A** | **G** | **T** | **G** | **A** | **A** | **T** | **T** | **A** | **C** | **A** | **G** | **A** | **T** | **T** | **A** | **T** | **G** | **A** | **C** | **T** | **A** | **G** | **C** | **C** | **G** | **T** | **C** | **C** | **T** | **C** | **A** | **A** | **T** | **A** | **T** | **G** | **T** | **A** | **G** | **A** | **T** | **G** | **G** | **A** | **C** | **A** | **A** | **A** | **C** | **C** | **A** | **A** | **A** | **G** | **A** | **A** | **G** | **A** | **G** | **A** | **T** | **T** | **T** | **T** | **A** | **G** | **A** | **A** | **T** | **T** | **A** | **G** | **A** | **A** | **A** | **G** | **G** | **C** | **A** | **T** | **C** | **G** | **A** | **T** | **T** | **A** | **G** | **C** | **T** | **C** | **G** | **T** | **G** | **A** | **G** | **C** | **T** | **T** | **C** | **A** | **T** | **G** | **A** | **T** | **T** | **C** | **A** | **G** | **T** | **T** | **T** | **C** | **A** | **C** | **A** | **G** | **C** | **A** | **A** | **T** | **T** | **A** | **T** | **T** | **T** | **G** | **C** | **A** | **G** | **C** | **G** | **A** | **T** | **G** | **A** | **T** | **G** | **A** | **T** | **G** | **A** | **T** | **G** | **T** | **C** | **G** | **G** | **C** | **A** | **T** | **T** | **G** | **A** | **C** | **G** | **G** | **A** | **G** | **C** | **A** | **A** | **G** | **C** | **A** | **G** | **A** | **A** | **A** | **A** | **A** | **A** | **G** | **T** | **G** | **A** | **G** | **A** | **C** | **A** | **C** | **C** | **A** | **G** | **A** | **A** | **A** | **T** | **G** | **T** | **T** | **C** | **C** | **G** | **T** | **A** | **A** | **G** | **C** | **A** | **G** | **T** | **T** | **G** | **A** | **A** | **A** | **A** | **T** | **G** | **G** | **T** | **G** | **G** | **C** | **A** | **G** | **A** | **G** | **A** | **T** | **C** | **A** | **T** | **C** | **A** | **A** | **C** | **G** | **C** | **T** | **T** | **C** | **C** | **C** | **A** | **G** | **T** | **C** | **T** | **G** | **A** | **G** | **A** | **T** | **G** | **C** | **G** | **T** | **G** | **C** | **G** | **C** | **T** | **G** | **C** | **T** | **T** | **C** | **T** | **T** | **C** | **A** | **T** | **C** | **T** | **G** | **C** | **G** | **T** | **C** | **C** | **A** | **G** | **T** | **C** | **A** | **A** | **T** | **T** | **T** | **A** | **G** | **A** | **A** | **G** | **A** | **A** | **A** | **A** | **A** | **A** | **G** | **T** | **T** | **T** | **A** | **A** | **A** | **A** | **C** | **A** | **A** | **G** | **G** | **C** | **A** | **T** | **T** | **G** | **A** | **G** | **C** | **A** | **G** | **T** | **T** | **A** | **T** | **T** | **G** | **A** | **A** | **G** | **G** | **A** | **A** | **T** | **T** | **G** | **C** | **A** | **G** | **A** | **A** | **T** | **A** | **A** | **A** | **A** | **T** | **C** | **C** | **A** | **G** | **A** | **T** | **T** | **T** | **C** | **G** | **C** | **T** | **A** | **A** | **A** | **A** | **T** | **G** | **G** | **G** | **A** | **T** | **G** | **T** | **A** | **G** | **A** | **A** | **G** | **A** | **T** | **G** | **T** | **A** | **A** | **A** | **A** | **T** | **T** | **A** | **A** | **C** | **T** | **A** | **G** | **T** | **T** | **C** | **C** | **A** | **T** | **T** | **G** | **A** | **G** | **G** | **A** | **T** | **C** | **A** | **C** | **T** | **T** | **G** | **T** | **T** | **C** | **C** | **G** | **A** | **A** | **T** | **C** | **G** | **T** | **T** | **C** | **A** | **A** | **G** | **A** | **A** | **T** | **T** | **G** | **T** | **T** | **A** | **T** | **C** | **A** | **A** | **A** | **C** | **A** | **C** | **A** | **T** | **T** | **A** | **A** | **G** | **A** | **C** | **A** | **T** | **G** | **C** | **T** | **A** | **A** | **G** | **G** | **C** | **T** | **A** | **A** | **T** | **G** | **A** | **A** | **T** | **T** | **A** | **G** | **A** | **A** | **G** | **T** | **C** | **T** | **A** | **T** | **T** | **T** | **G** | **C** | **A** | **T** | **A** | **A** | **A** | **A** | **T** | **A** | **G** | **A** | **T** | **A** | **A** | **C** | **A** | **A** | **T** | **C** | **T** | **T** | **T** | **T** | **A** | **T** | **T** | **A** | **C** | **G** | **T** | **A** | **T** | **C** | **A** | **T** | **T** | **G** | **A** | **C** | **G** | **A** | **C** | **G** | **G** | **G** | **A** | **C** | **A** | **G** | **G** | **A** | **T** | **T** | **T** | **A** | **A** | **T** | **A** | **T** | **G** | **A** | **A** | **T** | **G** | **A** | **A** | **A** | **C** | **C** | **A** | **A** | **A** | **A** | **C** | **G** | **G** | **G** | **A** | **A** | **G** | **T** | **T** | **A** | **T** | **G** | **G** | **A** | **T** | **T** | **G** | **A** | **A** | **C** | **A** | **A** | **T** | **A** | **T** | **C** | **A** | **A** | **A** | **G** | **A** | **A** | **C** | **G** | **A** | **G** | **T** | **A** | **G** | **C** | **T** | **G** | **G** | **T** | **A** | **T** | **T** | **G** | **G** | **T** | **G** | **G** | **A** | **A** | **C** | **A** | **G** | **T** | **A** | **A** | **A** | **A** | **A** | **T** | **C** | **A** | **T** | **T** | **A** | **G** | **T** | **T** | **T** | **T** | **A** | **A** | **A** | **G** | **G** | **G** | **C** | **A** | **A** | **G** | **G** | **T** | **A** | **C** | **G** | **A** | **G** | **C** | **G** | **T** | **G** | **G** | **A** | **G** | **A** | **T** | **C** | **A** | **A** | **G** | **G** | **T** | **C** | **C** | **C** | **T** | **T** | **T** | **G** | **A** | **T** | **G** | **A** | **A** | **G** | **G** | **A** | **G** | **G** | **C** | **A** | **-** | **-** | **-** |
| **N60252F** | **A** | **T** | **G** | **A** | **T** | **G** | **G** | **G** | **A** | **A** | **G** | **A** | **A** | **T** | **A** | **T** | **C** | **C** | **A** | **A** | **A** | **G** | **C** | **A** | **A** | **T** | **G** | **C** | **T** | **A** | **G** | **C** | **T** | **G** | **T** | **T** | **T** | **A** | **T** | **T** | **C** | **G** | **G** | **G** | **G** | **A** | **T** | **T** | **G** | **C** | **G** | **G** | **C** | **T** | **T** | **T** | **C** | **C** | **T** | **T** | **A** | **T** | **T** | **A** | **T** | **T** | **C** | **T** | **A** | **T** | **T** | **T** | **T** | **C** | **A** | **C** | **T** | **T** | **T** | **T** | **C** | **A** | **C** | **T** | **T** | **A** | **T** | **T** | **T** | **T** | **T** | **A** | **T** | **G** | **C** | **C** | **A** | **G** | **T** | **A** | **A** | **C** | **C** | **A** | **A** | **A** | **G** | **T** | **C** | **A** | **T** | **T** | **G** | **G** | **T** | **G** | **G** | **G** | **G** | **A** | **G** | **A** | **A** | **T** | **T** | **G** | **C** | **T** | **G** | **C** | **G** | **T** | **G** | **C** | **G** | **C** | **G** | **T** | **T** | **T** | **A** | **T** | **T** | **A** | **T** | **A** | **C** | **G** | **T** | **T** | **C** | **C** | **G** | **C** | **T** | **T** | **A** | **T** | **C** | **T** | **T** | **C** | **C** | **A** | **T** | **T** | **T** | **G** | **A** | **T** | **G** | **G** | **C** | **A** | **A** | **T** | **T** | **T** | **C** | **A** | **T** | **T** | **A** | **G** | **G** | **T** | **G** | **T** | **A** | **G** | **G** | **A** | **T** | **T** | **G** | **A** | **T** | **C** | **G** | **T** | **A** | **T** | **T** | **T** | **C** | **T** | **A** | **T** | **T** | **G** | **T** | **T** | **A** | **T** | **C** | **A** | **C** | **T** | **T** | **A** | **T** | **T** | **C** | **A** | **A** | **A** | **A** | **G** | **G** | **C** | **A** | **A** | **A** | **A** | **T** | **A** | **C** | **G** | **G** | **G** | **A** | **A** | **A** | **A** | **T** | **C** | **G** | **A** | **A** | **G** | **A** | **A** | **A** | **A** | **G** | **C** | **T** | **T** | **C** | **G** | **T** | **G** | **C** | **A** | **C** | **T** | **T** | **T** | **C** | **T** | **T** | **C** | **T** | **G** | **G** | **C** | **A** | **A** | **T** | **T** | **A** | **T** | **G** | **A** | **A** | **T** | **C** | **C** | **A** | **A** | **A** | **C** | **T** | **T** | **T** | **T** | **G** | **C** | **A** | **T** | **C** | **A** | **G** | **C** | **C** | **G** | **A** | **T** | **C** | **C** | **C** | **A** | **A** | **G** | **C** | **G** | **C** | **G** | **T** | **C** | **G** | **G** | **A** | **T** | **G** | **A** | **T** | **C** | **T** | **G** | **T** | **A** | **C** | **A** | **T** | **C** | **A** | **A** | **A** | **G** | **A** | **T** | **A** | **T** | **T** | **G** | **A** | **T** | **A** | **T** | **G** | **G** | **A** | **A** | **A** | **T** | **T** | **A** | **C** | **T** | **A** | **A** | **A** | **A** | **T** | **A** | **A** | **A** | **A** | **G** | **A** | **A** | **A** | **A** | **A** | **A** | **T** | **G** | **A** | **T** | **C** | **G** | **A** | **A** | **G** | **T** | **A** | **T** | **C** | **C** | **A** | **G** | **T** | **G** | **A** | **A** | **T** | **T** | **A** | **C** | **A** | **G** | **A** | **T** | **T** | **A** | **T** | **G** | **A** | **C** | **T** | **A** | **G** | **C** | **C** | **G** | **T** | **C** | **C** | **T** | **C** | **A** | **A** | **T** | **A** | **T** | **G** | **T** | **A** | **G** | **A** | **T** | **G** | **G** | **A** | **C** | **A** | **A** | **A** | **C** | **C** | **A** | **A** | **A** | **G** | **A** | **A** | **G** | **A** | **G** | **A** | **T** | **T** | **T** | **T** | **A** | **G** | **A** | **A** | **T** | **T** | **A** | **G** | **A** | **A** | **A** | **G** | **G** | **C** | **A** | **T** | **C** | **G** | **A** | **T** | **T** | **A** | **G** | **C** | **T** | **C** | **G** | **T** | **G** | **A** | **G** | **C** | **T** | **T** | **C** | **A** | **T** | **G** | **A** | **T** | **T** | **C** | **A** | **G** | **T** | **T** | **T** | **C** | **A** | **C** | **A** | **G** | **C** | **A** | **A** | **T** | **T** | **A** | **T** | **T** | **T** | **G** | **C** | **A** | **G** | **C** | **G** | **A** | **T** | **G** | **A** | **T** | **G** | **A** | **T** | **G** | **A** | **T** | **G** | **T** | **C** | **G** | **G** | **C** | **A** | **T** | **T** | **G** | **A** | **C** | **G** | **G** | **A** | **G** | **C** | **A** | **A** | **G** | **C** | **A** | **G** | **A** | **A** | **A** | **A** | **A** | **A** | **G** | **T** | **G** | **A** | **G** | **A** | **C** | **A** | **C** | **C** | **A** | **G** | **A** | **A** | **A** | **T** | **G** | **T** | **T** | **C** | **C** | **G** | **T** | **A** | **A** | **G** | **C** | **A** | **G** | **T** | **T** | **G** | **A** | **A** | **A** | **A** | **T** | **G** | **G** | **T** | **G** | **G** | **C** | **A** | **G** | **A** | **G** | **A** | **T** | **C** | **A** | **T** | **C** | **A** | **A** | **C** | **G** | **C** | **T** | **T** | **C** | **C** | **C** | **A** | **G** | **T** | **C** | **T** | **G** | **A** | **G** | **A** | **T** | **G** | **C** | **G** | **T** | **G** | **C** | **G** | **C** | **T** | **G** | **C** | **T** | **T** | **C** | **T** | **T** | **C** | **A** | **T** | **C** | **T** | **G** | **C** | **G** | **T** | **C** | **C** | **A** | **G** | **T** | **C** | **A** | **A** | **T** | **T** | **T** | **A** | **G** | **A** | **A** | **G** | **A** | **A** | **A** | **A** | **A** | **A** | **G** | **T** | **T** | **T** | **A** | **A** | **A** | **A** | **C** | **A** | **A** | **G** | **G** | **C** | **A** | **T** | **T** | **G** | **A** | **G** | **C** | **A** | **G** | **T** | **T** | **A** | **T** | **T** | **G** | **A** | **A** | **G** | **G** | **A** | **A** | **T** | **T** | **G** | **C** | **A** | **G** | **A** | **A** | **T** | **A** | **A** | **A** | **A** | **T** | **C** | **C** | **A** | **G** | **A** | **T** | **T** | **T** | **C** | **G** | **C** | **T** | **A** | **A** | **A** | **A** | **T** | **G** | **G** | **G** | **A** | **T** | **G** | **T** | **A** | **G** | **A** | **A** | **G** | **A** | **T** | **G** | **T** | **A** | **A** | **A** | **A** | **T** | **T** | **A** | **A** | **C** | **T** | **A** | **G** | **T** | **T** | **C** | **C** | **A** | **T** | **T** | **G** | **A** | **G** | **G** | **A** | **T** | **C** | **A** | **C** | **T** | **T** | **G** | **T** | **T** | **C** | **C** | **G** | **A** | **A** | **T** | **C** | **G** | **T** | **T** | **C** | **A** | **A** | **G** | **A** | **A** | **T** | **T** | **G** | **T** | **T** | **A** | **T** | **C** | **A** | **A** | **A** | **C** | **A** | **C** | **A** | **T** | **T** | **A** | **A** | **G** | **A** | **C** | **A** | **T** | **G** | **C** | **T** | **A** | **A** | **G** | **G** | **C** | **T** | **A** | **A** | **T** | **G** | **A** | **A** | **T** | **T** | **A** | **G** | **A** | **A** | **G** | **T** | **C** | **T** | **A** | **T** | **T** | **T** | **G** | **C** | **A** | **T** | **A** | **A** | **A** | **A** | **T** | **A** | **G** | **A** | **T** | **A** | **A** | **C** | **A** | **A** | **T** | **C** | **T** | **T** | **T** | **T** | **A** | **T** | **T** | **A** | **C** | **G** | **T** | **A** | **T** | **C** | **A** | **T** | **T** | **G** | **A** | **C** | **G** | **A** | **C** | **G** | **G** | **G** | **A** | **C** | **A** | **G** | **G** | **A** | **T** | **T** | **T** | **A** | **A** | **T** | **A** | **T** | **G** | **A** | **A** | **T** | **G** | **A** | **A** | **A** | **C** | **C** | **A** | **A** | **A** | **A** | **C** | **G** | **G** | **G** | **A** | **A** | **G** | **T** | **T** | **A** | **T** | **G** | **G** | **A** | **T** | **T** | **G** | **A** | **A** | **C** | **A** | **A** | **T** | **A** | **T** | **C** | **A** | **A** | **A** | **G** | **A** | **A** | **C** | **G** | **A** | **G** | **T** | **A** | **G** | **C** | **T** | **G** | **G** | **T** | **A** | **T** | **T** | **G** | **G** | **T** | **G** | **G** | **A** | **A** | **C** | **A** | **G** | **T** | **A** | **A** | **A** | **A** | **A** | **T** | **C** | **A** | **T** | **T** | **A** | **G** | **T** | **T** | **T** | **T** | **A** | **A** | **A** | **G** | **G** | **G** | **C** | **A** | **A** | **G** | **G** | **T** | **A** | **C** | **G** | **A** | **G** | **C** | **G** | **T** | **G** | **G** | **A** | **G** | **A** | **T** | **C** | **A** | **A** | **G** | **G** | **T** | **C** | **C** | **C** | **T** | **T** | **T** | **G** | **A** | **T** | **G** | **A** | **A** | **G** | **G** | **A** | **G** | **G** | **C** | **A** | **-** | **-** | **-** |
| **N59653F** | **A** | **T** | **G** | **A** | **T** | **G** | **G** | **G** | **A** | **A** | **A** | **A** | **A** | **T** | **A** | **T** | **C** | **C** | **A** | **G** | **A** | **G** | **C** | **G** | **A** | **T** | **G** | **C** | **T** | **A** | **G** | **C** | **T** | **G** | **T** | **T** | **T** | **A** | **T** | **T** | **C** | **G** | **G** | **G** | **G** | **A** | **T** | **T** | **G** | **C** | **G** | **G** | **C** | **T** | **T** | **T** | **C** | **C** | **T** | **T** | **A** | **T** | **T** | **A** | **T** | **T** | **C** | **T** | **A** | **T** | **T** | **T** | **T** | **C** | **A** | **C** | **T** | **T** | **T** | **T** | **C** | **A** | **C** | **T** | **T** | **A** | **T** | **T** | **T** | **T** | **T** | **A** | **T** | **G** | **C** | **C** | **A** | **G** | **C** | **A** | **A** | **T** | **C** | **A** | **A** | **A** | **G** | **T** | **C** | **A** | **T** | **T** | **G** | **G** | **T** | **G** | **G** | **G** | **G** | **A** | **G** | **A** | **A** | **T** | **T** | **A** | **C** | **T** | **G** | **C** | **G** | **T** | **G** | **C** | **A** | **C** | **G** | **T** | **T** | **T** | **A** | **T** | **T** | **A** | **T** | **A** | **T** | **G** | **T** | **T** | **C** | **C** | **G** | **C** | **T** | **T** | **A** | **T** | **C** | **T** | **T** | **C** | **C** | **A** | **T** | **T** | **T** | **G** | **T** | **T** | **A** | **G** | **C** | **C** | **A** | **T** | **T** | **T** | **C** | **C** | **T** | **T** | **A** | **G** | **G** | **T** | **G** | **T** | **A** | **G** | **G** | **A** | **T** | **T** | **A** | **A** | **T** | **T** | **G** | **T** | **C** | **T** | **T** | **T** | **C** | **T** | **A** | **T** | **T** | **A** | **T** | **T** | **A** | **T** | **C** | **A** | **C** | **T** | **T** | **A** | **T** | **T** | **C** | **A** | **A** | **A** | **A** | **G** | **A** | **C** | **A** | **A** | **A** | **A** | **T** | **A** | **C** | **G** | **G** | **G** | **A** | **A** | **A** | **A** | **T** | **C** | **G** | **A** | **A** | **G** | **A** | **A** | **A** | **A** | **G** | **C** | **T** | **G** | **C** | **G** | **T** | **G** | **C** | **A** | **C** | **T** | **T** | **T** | **C** | **T** | **T** | **C** | **T** | **G** | **G** | **C** | **A** | **A** | **T** | **T** | **A** | **T** | **G** | **A** | **A** | **T** | **C** | **C** | **A** | **A** | **A** | **T** | **T** | **G** | **T** | **T** | **G** | **C** | **T** | **T** | **C** | **T** | **T** | **C** | **C** | **G** | **A** | **T** | **C** | **C** | **C** | **A** | **A** | **G** | **T** | **G** | **C** | **A** | **T** | **C** | **G** | **G** | **A** | **C** | **G** | **A** | **T** | **T** | **T** | **A** | **T** | **A** | **C** | **A** | **T** | **C** | **A** | **A** | **A** | **G** | **A** | **T** | **A** | **T** | **T** | **G** | **A** | **T** | **A** | **A** | **G** | **G** | **A** | **A** | **A** | **T** | **C** | **A** | **C** | **T** | **A** | **A** | **G** | **A** | **T** | **A** | **A** | **A** | **A** | **G** | **A** | **A** | **A** | **A** | **A** | **A** | **T** | **G** | **A** | **T** | **T** | **G** | **A** | **A** | **G** | **T** | **A** | **T** | **C** | **T** | **A** | **G** | **T** | **G** | **A** | **A** | **T** | **T** | **A** | **C** | **A** | **A** | **A** | **T** | **T** | **G** | **T** | **A** | **A** | **C** | **G** | **A** | **G** | **C** | **C** | **G** | **T** | **C** | **C** | **C** | **C** | **A** | **A** | **T** | **A** | **T** | **G** | **T** | **A** | **G** | **A** | **T** | **G** | **G** | **A** | **C** | **A** | **A** | **A** | **C** | **C** | **A** | **A** | **A** | **G** | **A** | **A** | **G** | **A** | **A** | **A** | **T** | **T** | **T** | **T** | **A** | **G** | **A** | **A** | **T** | **T** | **A** | **G** | **A** | **A** | **A** | **G** | **A** | **C** | **A** | **T** | **C** | **G** | **A** | **T** | **T** | **A** | **G** | **C** | **C** | **C** | **G** | **T** | **G** | **A** | **G** | **C** | **T** | **G** | **C** | **A** | **T** | **G** | **A** | **T** | **T** | **C** | **A** | **G** | **T** | **T** | **T** | **C** | **G** | **C** | **A** | **A** | **C** | **A** | **A** | **T** | **T** | **G** | **T** | **T** | **T** | **G** | **C** | **A** | **G** | **C** | **A** | **A** | **T** | **G** | **A** | **T** | **G** | **A** | **T** | **G** | **A** | **T** | **G** | **T** | **C** | **G** | **G** | **C** | **A** | **T** | **T** | **G** | **A** | **C** | **G** | **G** | **A** | **G** | **C** | **A** | **A** | **G** | **C** | **A** | **G** | **A** | **A** | **A** | **A** | **A** | **A** | **G** | **C** | **G** | **A** | **G** | **A** | **C** | **A** | **C** | **C** | **A** | **G** | **A** | **A** | **A** | **T** | **G** | **T** | **T** | **C** | **C** | **G** | **C** | **A** | **A** | **G** | **C** | **A** | **G** | **T** | **T** | **G** | **A** | **A** | **A** | **A** | **T** | **G** | **G** | **T** | **A** | **G** | **C** | **A** | **G** | **A** | **G** | **A** | **T** | **C** | **A** | **T** | **C** | **A** | **A** | **C** | **G** | **C** | **T** | **T** | **C** | **C** | **C** | **A** | **G** | **T** | **C** | **T** | **G** | **A** | **G** | **A** | **T** | **G** | **C** | **G** | **T** | **G** | **C** | **G** | **C** | **T** | **G** | **C** | **T** | **T** | **C** | **T** | **T** | **C** | **A** | **T** | **C** | **T** | **A** | **C** | **G** | **T** | **C** | **C** | **A** | **G** | **T** | **C** | **A** | **A** | **T** | **T** | **T** | **A** | **G** | **A** | **A** | **G** | **A** | **G** | **A** | **A** | **A** | **A** | **G** | **T** | **T** | **T** | **A** | **A** | **A** | **G** | **C** | **A** | **A** | **G** | **G** | **T** | **A** | **T** | **C** | **G** | **A** | **G** | **C** | **A** | **G** | **T** | **T** | **A** | **T** | **T** | **G** | **A** | **A** | **G** | **G** | **A** | **A** | **T** | **T** | **G** | **C** | **A** | **A** | **A** | **A** | **T** | **A** | **A** | **A** | **A** | **T** | **C** | **C** | **A** | **G** | **A** | **T** | **T** | **T** | **C** | **G** | **C** | **T** | **G** | **A** | **A** | **A** | **T** | **G** | **G** | **G** | **A** | **T** | **G** | **T** | **A** | **G** | **A** | **A** | **G** | **A** | **T** | **G** | **T** | **A** | **A** | **A** | **A** | **C** | **T** | **A** | **T** | **C** | **T** | **A** | **G** | **T** | **T** | **C** | **C** | **A** | **T** | **T** | **G** | **A** | **G** | **G** | **A** | **T** | **C** | **A** | **C** | **T** | **T** | **G** | **T** | **T** | **C** | **C** | **G** | **A** | **A** | **T** | **C** | **G** | **T** | **T** | **C** | **A** | **A** | **G** | **A** | **A** | **T** | **T** | **G** | **T** | **T** | **A** | **T** | **C** | **A** | **A** | **A** | **T** | **A** | **C** | **A** | **T** | **T** | **A** | **A** | **G** | **A** | **C** | **A** | **T** | **G** | **C** | **C** | **A** | **A** | **A** | **G** | **C** | **C** | **A** | **A** | **T** | **G** | **A** | **A** | **T** | **T** | **A** | **G** | **A** | **G** | **G** | **T** | **A** | **T** | **A** | **T** | **T** | **T** | **G** | **C** | **A** | **C** | **A** | **A** | **A** | **A** | **T** | **A** | **G** | **A** | **C** | **A** | **A** | **T** | **A** | **A** | **T** | **C** | **T** | **T** | **T** | **T** | **G** | **T** | **T** | **A** | **C** | **G** | **T** | **A** | **T** | **T** | **A** | **T** | **T** | **G** | **A** | **C** | **G** | **A** | **C** | **G** | **G** | **A** | **A** | **C** | **A** | **G** | **G** | **G** | **T** | **T** | **T** | **A** | **A** | **T** | **A** | **T** | **G** | **A** | **A** | **T** | **G** | **A** | **A** | **A** | **C** | **G** | **A** | **A** | **A** | **A** | **C** | **G** | **G** | **G** | **A** | **A** | **G** | **T** | **T** | **A** | **T** | **G** | **G** | **A** | **T** | **T** | **G** | **A** | **A** | **C** | **A** | **A** | **T** | **A** | **T** | **C** | **A** | **A** | **A** | **G** | **A** | **A** | **C** | **G** | **A** | **G** | **T** | **A** | **G** | **C** | **T** | **G** | **G** | **T** | **A** | **T** | **T** | **G** | **G** | **T** | **G** | **G** | **A** | **A** | **C** | **A** | **G** | **T** | **A** | **A** | **A** | **A** | **A** | **T** | **C** | **A** | **T** | **T** | **A** | **G** | **T** | **T** | **T** | **T** | **A** | **A** | **A** | **G** | **G** | **G** | **C** | **A** | **A** | **G** | **G** | **T** | **A** | **C** | **A** | **A** | **G** | **T** | **G** | **T** | **A** | **G** | **A** | **A** | **A** | **T** | **C** | **A** | **A** | **G** | **G** | **T** | **C** | **C** | **C** | **T** | **T** | **T** | **G** | **A** | **T** | **G** | **A** | **A** | **G** | **G** | **A** | **G** | **G** | **C** | **A** | **-** | **-** | **-** |
| **N54150** | **A** | **T** | **G** | **A** | **T** | **G** | **G** | **G** | **A** | **A** | **G** | **A** | **A** | **T** | **A** | **T** | **C** | **C** | **A** | **A** | **A** | **G** | **C** | **A** | **A** | **T** | **G** | **C** | **T** | **A** | **G** | **C** | **T** | **G** | **T** | **T** | **T** | **A** | **T** | **T** | **C** | **G** | **G** | **G** | **G** | **A** | **T** | **T** | **G** | **C** | **G** | **G** | **C** | **T** | **T** | **T** | **C** | **C** | **T** | **T** | **A** | **T** | **T** | **A** | **T** | **T** | **C** | **T** | **A** | **T** | **T** | **T** | **T** | **C** | **A** | **C** | **T** | **T** | **T** | **T** | **C** | **A** | **C** | **T** | **T** | **A** | **T** | **T** | **T** | **T** | **T** | **A** | **T** | **G** | **C** | **C** | **A** | **G** | **T** | **A** | **A** | **C** | **C** | **A** | **A** | **A** | **G** | **T** | **C** | **A** | **T** | **T** | **G** | **G** | **T** | **G** | **G** | **G** | **G** | **A** | **G** | **A** | **A** | **T** | **T** | **G** | **C** | **T** | **G** | **C** | **G** | **T** | **G** | **C** | **G** | **C** | **G** | **T** | **T** | **T** | **A** | **T** | **T** | **A** | **T** | **A** | **C** | **G** | **T** | **T** | **C** | **C** | **G** | **C** | **T** | **T** | **A** | **T** | **C** | **T** | **T** | **C** | **C** | **A** | **T** | **T** | **T** | **G** | **A** | **T** | **G** | **G** | **C** | **A** | **A** | **T** | **T** | **T** | **C** | **A** | **T** | **T** | **A** | **G** | **G** | **T** | **G** | **T** | **A** | **G** | **G** | **A** | **T** | **T** | **G** | **A** | **T** | **C** | **G** | **T** | **A** | **T** | **T** | **T** | **C** | **T** | **A** | **T** | **T** | **G** | **T** | **T** | **A** | **T** | **C** | **A** | **C** | **T** | **T** | **A** | **T** | **T** | **C** | **A** | **A** | **A** | **A** | **G** | **G** | **C** | **A** | **A** | **A** | **A** | **T** | **A** | **C** | **G** | **G** | **G** | **A** | **A** | **A** | **A** | **T** | **C** | **G** | **A** | **A** | **G** | **A** | **A** | **A** | **A** | **G** | **C** | **T** | **T** | **C** | **G** | **T** | **G** | **C** | **A** | **C** | **T** | **T** | **T** | **C** | **T** | **T** | **C** | **T** | **G** | **G** | **C** | **A** | **A** | **T** | **T** | **A** | **T** | **G** | **A** | **A** | **T** | **C** | **C** | **A** | **A** | **A** | **C** | **T** | **T** | **T** | **T** | **G** | **C** | **A** | **T** | **C** | **A** | **G** | **C** | **C** | **G** | **A** | **T** | **C** | **C** | **C** | **A** | **A** | **G** | **C** | **G** | **C** | **G** | **T** | **C** | **G** | **G** | **A** | **T** | **G** | **A** | **T** | **C** | **T** | **G** | **T** | **A** | **C** | **A** | **T** | **C** | **A** | **A** | **A** | **G** | **A** | **T** | **A** | **T** | **T** | **G** | **A** | **T** | **A** | **T** | **G** | **G** | **A** | **A** | **A** | **T** | **T** | **A** | **C** | **T** | **A** | **A** | **A** | **A** | **T** | **A** | **A** | **A** | **A** | **G** | **A** | **A** | **A** | **A** | **A** | **A** | **T** | **G** | **A** | **T** | **C** | **G** | **A** | **A** | **G** | **T** | **A** | **T** | **C** | **C** | **A** | **G** | **T** | **G** | **A** | **A** | **T** | **T** | **A** | **C** | **A** | **G** | **A** | **T** | **T** | **A** | **T** | **G** | **A** | **C** | **T** | **A** | **G** | **C** | **C** | **G** | **T** | **C** | **C** | **T** | **C** | **A** | **A** | **T** | **A** | **T** | **G** | **T** | **A** | **G** | **A** | **T** | **G** | **G** | **A** | **C** | **A** | **A** | **A** | **C** | **C** | **A** | **A** | **A** | **G** | **A** | **A** | **G** | **A** | **G** | **A** | **T** | **T** | **T** | **T** | **A** | **G** | **A** | **A** | **T** | **T** | **A** | **G** | **A** | **A** | **A** | **G** | **G** | **C** | **A** | **T** | **C** | **G** | **A** | **T** | **T** | **A** | **G** | **C** | **T** | **C** | **G** | **T** | **G** | **A** | **G** | **C** | **T** | **T** | **C** | **A** | **T** | **G** | **A** | **T** | **T** | **C** | **A** | **G** | **T** | **T** | **T** | **C** | **A** | **C** | **A** | **G** | **C** | **A** | **A** | **T** | **T** | **A** | **T** | **T** | **T** | **G** | **C** | **A** | **G** | **C** | **G** | **A** | **T** | **G** | **A** | **T** | **G** | **A** | **T** | **G** | **A** | **T** | **G** | **T** | **C** | **G** | **G** | **C** | **A** | **T** | **T** | **G** | **A** | **C** | **G** | **G** | **A** | **G** | **C** | **A** | **A** | **G** | **C** | **A** | **G** | **A** | **A** | **A** | **A** | **A** | **A** | **G** | **T** | **G** | **A** | **G** | **A** | **C** | **A** | **C** | **C** | **A** | **G** | **A** | **A** | **A** | **T** | **G** | **T** | **T** | **C** | **C** | **G** | **T** | **A** | **A** | **G** | **C** | **A** | **G** | **T** | **T** | **G** | **A** | **A** | **A** | **A** | **T** | **G** | **G** | **T** | **G** | **G** | **C** | **A** | **G** | **A** | **G** | **A** | **T** | **C** | **A** | **T** | **C** | **A** | **A** | **C** | **G** | **C** | **T** | **T** | **C** | **C** | **C** | **A** | **G** | **T** | **C** | **T** | **G** | **A** | **G** | **A** | **T** | **G** | **C** | **G** | **T** | **G** | **C** | **G** | **C** | **T** | **G** | **C** | **T** | **T** | **C** | **T** | **T** | **C** | **A** | **T** | **C** | **T** | **G** | **C** | **G** | **T** | **C** | **C** | **A** | **G** | **T** | **C** | **A** | **A** | **T** | **T** | **T** | **A** | **G** | **A** | **A** | **G** | **A** | **A** | **A** | **A** | **A** | **A** | **G** | **T** | **T** | **T** | **A** | **A** | **A** | **A** | **C** | **A** | **A** | **G** | **G** | **C** | **A** | **T** | **T** | **G** | **A** | **G** | **C** | **A** | **G** | **T** | **T** | **A** | **T** | **T** | **G** | **A** | **A** | **G** | **G** | **A** | **A** | **T** | **T** | **G** | **C** | **A** | **G** | **A** | **A** | **T** | **A** | **A** | **A** | **A** | **T** | **C** | **C** | **A** | **G** | **A** | **T** | **T** | **T** | **C** | **G** | **C** | **T** | **A** | **A** | **A** | **A** | **T** | **G** | **G** | **G** | **A** | **T** | **G** | **T** | **A** | **G** | **A** | **A** | **G** | **A** | **T** | **G** | **T** | **A** | **A** | **A** | **A** | **T** | **T** | **A** | **A** | **C** | **T** | **A** | **G** | **T** | **T** | **C** | **C** | **A** | **T** | **T** | **G** | **A** | **G** | **G** | **A** | **T** | **C** | **A** | **C** | **T** | **T** | **G** | **T** | **T** | **C** | **C** | **G** | **A** | **A** | **T** | **C** | **G** | **T** | **T** | **C** | **A** | **A** | **G** | **A** | **A** | **T** | **T** | **G** | **T** | **T** | **A** | **T** | **C** | **A** | **A** | **A** | **C** | **A** | **C** | **A** | **T** | **T** | **A** | **A** | **G** | **A** | **C** | **A** | **T** | **G** | **C** | **T** | **A** | **A** | **G** | **G** | **C** | **T** | **A** | **A** | **T** | **G** | **A** | **A** | **T** | **T** | **A** | **G** | **A** | **A** | **G** | **T** | **C** | **T** | **A** | **T** | **T** | **T** | **G** | **C** | **A** | **T** | **A** | **A** | **A** | **A** | **T** | **A** | **G** | **A** | **T** | **A** | **A** | **C** | **A** | **A** | **T** | **C** | **T** | **T** | **T** | **T** | **A** | **T** | **T** | **A** | **C** | **G** | **T** | **A** | **T** | **C** | **A** | **T** | **T** | **G** | **A** | **C** | **G** | **A** | **C** | **G** | **G** | **G** | **A** | **C** | **A** | **G** | **G** | **A** | **T** | **T** | **T** | **A** | **A** | **T** | **A** | **T** | **G** | **A** | **A** | **T** | **G** | **A** | **A** | **A** | **C** | **C** | **A** | **A** | **A** | **A** | **C** | **G** | **G** | **G** | **A** | **A** | **G** | **T** | **T** | **A** | **T** | **G** | **G** | **A** | **T** | **T** | **G** | **A** | **A** | **C** | **A** | **A** | **T** | **A** | **T** | **C** | **A** | **A** | **A** | **G** | **A** | **A** | **C** | **G** | **A** | **G** | **T** | **A** | **G** | **C** | **T** | **G** | **G** | **T** | **A** | **T** | **T** | **G** | **G** | **T** | **G** | **G** | **A** | **A** | **C** | **A** | **G** | **T** | **A** | **A** | **A** | **A** | **A** | **T** | **C** | **A** | **T** | **T** | **A** | **G** | **T** | **T** | **T** | **T** | **A** | **A** | **A** | **G** | **G** | **G** | **C** | **A** | **A** | **G** | **G** | **T** | **A** | **C** | **G** | **A** | **G** | **C** | **G** | **T** | **G** | **G** | **A** | **G** | **A** | **T** | **C** | **A** | **A** | **G** | **G** | **T** | **C** | **C** | **C** | **T** | **T** | **T** | **G** | **A** | **T** | **G** | **A** | **A** | **G** | **G** | **A** | **G** | **G** | **C** | **A** | **-** | **-** | **-** |
| **N54035** | **A** | **T** | **G** | **A** | **T** | **G** | **G** | **G** | **A** | **A** | **G** | **A** | **A** | **T** | **A** | **T** | **C** | **C** | **A** | **A** | **A** | **G** | **C** | **A** | **A** | **T** | **G** | **C** | **T** | **A** | **G** | **C** | **T** | **G** | **T** | **T** | **T** | **A** | **T** | **T** | **C** | **G** | **G** | **G** | **G** | **A** | **T** | **T** | **G** | **C** | **G** | **G** | **C** | **T** | **T** | **T** | **C** | **C** | **T** | **T** | **A** | **T** | **T** | **A** | **T** | **T** | **C** | **T** | **A** | **T** | **T** | **T** | **T** | **C** | **A** | **C** | **T** | **T** | **T** | **T** | **C** | **A** | **C** | **T** | **T** | **A** | **T** | **T** | **T** | **T** | **T** | **A** | **T** | **G** | **C** | **C** | **A** | **G** | **T** | **A** | **A** | **C** | **C** | **A** | **A** | **A** | **G** | **T** | **C** | **A** | **T** | **T** | **G** | **G** | **T** | **G** | **G** | **G** | **G** | **A** | **G** | **A** | **A** | **T** | **T** | **G** | **C** | **T** | **G** | **C** | **G** | **T** | **G** | **C** | **G** | **C** | **G** | **T** | **T** | **T** | **A** | **T** | **T** | **A** | **T** | **A** | **C** | **G** | **T** | **T** | **C** | **C** | **G** | **C** | **T** | **T** | **A** | **T** | **C** | **T** | **T** | **C** | **C** | **A** | **T** | **T** | **T** | **G** | **A** | **T** | **G** | **G** | **C** | **A** | **A** | **T** | **T** | **T** | **C** | **A** | **T** | **T** | **A** | **G** | **G** | **T** | **G** | **T** | **A** | **G** | **G** | **A** | **T** | **T** | **G** | **A** | **T** | **C** | **G** | **T** | **A** | **T** | **T** | **T** | **C** | **T** | **A** | **T** | **T** | **G** | **T** | **T** | **A** | **T** | **C** | **A** | **C** | **T** | **T** | **A** | **T** | **T** | **C** | **A** | **A** | **A** | **A** | **G** | **G** | **C** | **A** | **A** | **A** | **A** | **T** | **A** | **C** | **G** | **G** | **G** | **A** | **A** | **A** | **A** | **T** | **C** | **G** | **A** | **A** | **G** | **A** | **A** | **A** | **A** | **G** | **C** | **T** | **T** | **C** | **G** | **T** | **G** | **C** | **A** | **C** | **T** | **T** | **T** | **C** | **T** | **T** | **C** | **T** | **G** | **G** | **C** | **A** | **A** | **T** | **T** | **A** | **T** | **G** | **A** | **A** | **T** | **C** | **C** | **A** | **A** | **A** | **C** | **T** | **T** | **T** | **T** | **G** | **C** | **A** | **T** | **C** | **A** | **G** | **C** | **C** | **G** | **A** | **T** | **C** | **C** | **C** | **A** | **A** | **G** | **C** | **A** | **C** | **G** | **T** | **C** | **G** | **G** | **A** | **T** | **G** | **A** | **T** | **C** | **T** | **G** | **T** | **A** | **C** | **A** | **T** | **C** | **A** | **A** | **A** | **G** | **A** | **T** | **A** | **T** | **T** | **G** | **A** | **T** | **A** | **T** | **G** | **G** | **A** | **A** | **A** | **T** | **T** | **A** | **C** | **T** | **A** | **A** | **A** | **A** | **T** | **A** | **A** | **A** | **A** | **G** | **A** | **A** | **A** | **A** | **A** | **A** | **T** | **G** | **A** | **T** | **C** | **G** | **A** | **A** | **G** | **T** | **A** | **T** | **C** | **C** | **A** | **G** | **T** | **G** | **A** | **A** | **T** | **T** | **A** | **C** | **A** | **G** | **A** | **T** | **T** | **A** | **T** | **G** | **A** | **C** | **T** | **A** | **G** | **C** | **C** | **G** | **T** | **C** | **C** | **T** | **C** | **A** | **A** | **T** | **A** | **T** | **G** | **T** | **A** | **G** | **A** | **T** | **G** | **G** | **A** | **C** | **A** | **A** | **A** | **C** | **C** | **A** | **A** | **A** | **G** | **A** | **A** | **G** | **A** | **G** | **A** | **T** | **T** | **T** | **T** | **A** | **G** | **A** | **A** | **T** | **T** | **A** | **G** | **A** | **A** | **A** | **G** | **G** | **C** | **A** | **T** | **C** | **G** | **A** | **T** | **T** | **A** | **G** | **C** | **T** | **C** | **G** | **T** | **G** | **A** | **G** | **C** | **T** | **T** | **C** | **A** | **T** | **G** | **A** | **T** | **T** | **C** | **A** | **G** | **T** | **T** | **T** | **C** | **A** | **C** | **A** | **G** | **C** | **A** | **A** | **T** | **T** | **A** | **T** | **T** | **T** | **G** | **C** | **A** | **G** | **C** | **G** | **A** | **T** | **G** | **A** | **T** | **G** | **A** | **T** | **G** | **A** | **T** | **G** | **T** | **C** | **G** | **G** | **C** | **A** | **T** | **T** | **G** | **A** | **C** | **G** | **G** | **A** | **G** | **C** | **A** | **A** | **G** | **C** | **A** | **G** | **A** | **A** | **A** | **A** | **A** | **A** | **G** | **T** | **G** | **A** | **G** | **A** | **C** | **A** | **C** | **C** | **A** | **G** | **A** | **A** | **A** | **T** | **G** | **T** | **T** | **C** | **C** | **G** | **T** | **A** | **A** | **G** | **C** | **A** | **G** | **T** | **T** | **G** | **A** | **A** | **A** | **A** | **T** | **G** | **G** | **T** | **G** | **G** | **C** | **A** | **G** | **A** | **G** | **A** | **T** | **C** | **A** | **T** | **C** | **A** | **A** | **C** | **G** | **C** | **T** | **T** | **C** | **C** | **C** | **A** | **G** | **T** | **C** | **T** | **G** | **A** | **G** | **A** | **T** | **G** | **C** | **G** | **T** | **G** | **C** | **G** | **C** | **T** | **G** | **C** | **T** | **T** | **C** | **T** | **T** | **C** | **A** | **T** | **C** | **T** | **G** | **C** | **G** | **T** | **C** | **C** | **A** | **G** | **T** | **C** | **A** | **A** | **T** | **T** | **T** | **A** | **G** | **A** | **A** | **G** | **A** | **A** | **A** | **A** | **A** | **A** | **G** | **T** | **T** | **T** | **A** | **A** | **A** | **A** | **C** | **A** | **A** | **G** | **G** | **C** | **A** | **T** | **T** | **G** | **A** | **G** | **C** | **A** | **G** | **T** | **T** | **A** | **T** | **T** | **G** | **A** | **A** | **G** | **G** | **A** | **A** | **T** | **T** | **G** | **C** | **A** | **G** | **A** | **A** | **T** | **A** | **A** | **A** | **A** | **T** | **C** | **C** | **A** | **G** | **A** | **T** | **T** | **T** | **C** | **G** | **C** | **T** | **A** | **A** | **A** | **A** | **T** | **G** | **G** | **G** | **A** | **T** | **G** | **T** | **A** | **G** | **A** | **A** | **G** | **A** | **T** | **G** | **T** | **A** | **A** | **A** | **A** | **T** | **T** | **A** | **A** | **C** | **T** | **A** | **G** | **T** | **T** | **C** | **C** | **A** | **T** | **T** | **G** | **A** | **G** | **G** | **A** | **T** | **C** | **A** | **C** | **T** | **T** | **G** | **T** | **T** | **C** | **C** | **G** | **A** | **A** | **T** | **C** | **G** | **T** | **T** | **C** | **A** | **A** | **G** | **A** | **A** | **T** | **T** | **G** | **T** | **T** | **A** | **T** | **C** | **A** | **A** | **A** | **C** | **A** | **C** | **A** | **T** | **T** | **A** | **A** | **G** | **A** | **C** | **A** | **T** | **G** | **C** | **T** | **A** | **A** | **G** | **G** | **C** | **T** | **A** | **A** | **T** | **G** | **A** | **A** | **T** | **T** | **A** | **G** | **A** | **A** | **G** | **T** | **C** | **T** | **A** | **T** | **T** | **T** | **G** | **C** | **A** | **T** | **A** | **A** | **A** | **A** | **T** | **A** | **G** | **A** | **T** | **A** | **A** | **C** | **A** | **A** | **T** | **C** | **T** | **T** | **T** | **T** | **A** | **T** | **T** | **A** | **C** | **G** | **T** | **A** | **T** | **C** | **A** | **T** | **T** | **G** | **A** | **C** | **G** | **A** | **C** | **G** | **G** | **G** | **A** | **C** | **A** | **G** | **G** | **A** | **T** | **T** | **T** | **A** | **A** | **T** | **A** | **T** | **G** | **A** | **A** | **T** | **G** | **A** | **A** | **A** | **C** | **C** | **A** | **A** | **A** | **A** | **C** | **G** | **G** | **G** | **A** | **A** | **G** | **T** | **T** | **A** | **T** | **G** | **G** | **A** | **T** | **T** | **G** | **A** | **A** | **C** | **A** | **A** | **T** | **A** | **T** | **C** | **A** | **A** | **A** | **G** | **A** | **A** | **C** | **G** | **A** | **G** | **T** | **A** | **G** | **C** | **T** | **G** | **G** | **T** | **A** | **T** | **T** | **G** | **G** | **T** | **G** | **G** | **A** | **A** | **C** | **A** | **G** | **T** | **A** | **A** | **A** | **A** | **A** | **T** | **C** | **A** | **T** | **T** | **A** | **G** | **T** | **T** | **T** | **T** | **A** | **A** | **A** | **G** | **G** | **G** | **C** | **A** | **A** | **G** | **G** | **T** | **A** | **C** | **G** | **A** | **G** | **C** | **G** | **T** | **G** | **G** | **A** | **G** | **A** | **T** | **C** | **A** | **A** | **G** | **G** | **T** | **C** | **C** | **C** | **T** | **T** | **T** | **G** | **A** | **T** | **G** | **A** | **A** | **G** | **G** | **A** | **G** | **G** | **C** | **A** | **-** | **-** | **-** |
| **N55072** | **A** | **T** | **G** | **A** | **T** | **G** | **G** | **G** | **A** | **A** | **G** | **A** | **A** | **T** | **A** | **T** | **C** | **C** | **A** | **A** | **A** | **G** | **C** | **A** | **A** | **T** | **G** | **C** | **T** | **A** | **G** | **C** | **T** | **G** | **T** | **T** | **T** | **A** | **T** | **T** | **C** | **G** | **G** | **G** | **G** | **A** | **T** | **T** | **G** | **C** | **G** | **G** | **C** | **T** | **T** | **T** | **C** | **C** | **T** | **T** | **A** | **T** | **T** | **A** | **T** | **T** | **C** | **T** | **A** | **T** | **T** | **T** | **T** | **C** | **A** | **C** | **T** | **T** | **T** | **T** | **C** | **A** | **C** | **T** | **T** | **A** | **T** | **T** | **T** | **T** | **T** | **A** | **T** | **G** | **C** | **C** | **A** | **G** | **T** | **A** | **A** | **C** | **C** | **A** | **A** | **A** | **G** | **T** | **C** | **A** | **T** | **T** | **G** | **G** | **T** | **G** | **G** | **G** | **G** | **A** | **G** | **A** | **A** | **T** | **T** | **G** | **C** | **T** | **G** | **C** | **G** | **T** | **G** | **C** | **G** | **C** | **G** | **T** | **T** | **T** | **A** | **T** | **T** | **A** | **T** | **A** | **C** | **G** | **T** | **T** | **C** | **C** | **G** | **C** | **T** | **T** | **A** | **T** | **C** | **T** | **T** | **C** | **C** | **A** | **T** | **T** | **T** | **G** | **A** | **T** | **G** | **G** | **C** | **A** | **A** | **T** | **T** | **T** | **C** | **A** | **T** | **T** | **A** | **G** | **G** | **T** | **G** | **T** | **A** | **G** | **G** | **A** | **T** | **T** | **G** | **A** | **T** | **C** | **G** | **T** | **A** | **T** | **T** | **T** | **C** | **T** | **A** | **T** | **T** | **G** | **T** | **T** | **A** | **T** | **C** | **A** | **C** | **T** | **T** | **A** | **T** | **T** | **C** | **A** | **A** | **A** | **A** | **G** | **G** | **C** | **A** | **A** | **A** | **A** | **T** | **A** | **C** | **G** | **G** | **G** | **A** | **A** | **A** | **A** | **T** | **C** | **G** | **A** | **A** | **G** | **A** | **A** | **A** | **A** | **G** | **C** | **T** | **T** | **C** | **G** | **T** | **G** | **C** | **A** | **C** | **T** | **T** | **T** | **C** | **T** | **T** | **C** | **T** | **G** | **G** | **C** | **A** | **A** | **T** | **T** | **A** | **T** | **G** | **A** | **A** | **T** | **C** | **C** | **A** | **A** | **A** | **C** | **T** | **T** | **T** | **T** | **G** | **C** | **A** | **T** | **C** | **A** | **G** | **C** | **C** | **G** | **A** | **T** | **C** | **C** | **C** | **A** | **A** | **G** | **C** | **G** | **C** | **G** | **T** | **C** | **G** | **G** | **A** | **T** | **G** | **A** | **T** | **C** | **T** | **G** | **T** | **A** | **C** | **A** | **T** | **C** | **A** | **A** | **A** | **G** | **A** | **T** | **A** | **T** | **T** | **G** | **A** | **T** | **A** | **T** | **G** | **G** | **A** | **A** | **A** | **T** | **T** | **A** | **C** | **T** | **A** | **A** | **A** | **A** | **T** | **A** | **A** | **A** | **A** | **G** | **A** | **A** | **A** | **A** | **A** | **A** | **T** | **G** | **A** | **T** | **C** | **G** | **A** | **A** | **G** | **T** | **A** | **T** | **C** | **C** | **A** | **G** | **T** | **G** | **A** | **A** | **T** | **T** | **A** | **C** | **A** | **G** | **A** | **T** | **T** | **A** | **T** | **G** | **A** | **C** | **T** | **A** | **G** | **C** | **C** | **G** | **T** | **C** | **C** | **T** | **C** | **A** | **A** | **T** | **A** | **T** | **G** | **T** | **A** | **G** | **A** | **T** | **G** | **G** | **A** | **C** | **A** | **A** | **A** | **C** | **C** | **A** | **A** | **A** | **G** | **A** | **A** | **G** | **A** | **G** | **A** | **T** | **T** | **T** | **T** | **A** | **G** | **A** | **A** | **T** | **T** | **A** | **G** | **A** | **A** | **A** | **G** | **G** | **C** | **A** | **T** | **C** | **G** | **A** | **T** | **T** | **A** | **G** | **C** | **T** | **C** | **G** | **T** | **G** | **A** | **G** | **C** | **T** | **T** | **C** | **A** | **T** | **G** | **A** | **T** | **T** | **C** | **A** | **G** | **T** | **T** | **T** | **C** | **A** | **C** | **A** | **G** | **C** | **A** | **A** | **T** | **T** | **A** | **T** | **T** | **T** | **G** | **C** | **A** | **G** | **C** | **G** | **A** | **T** | **G** | **A** | **T** | **G** | **A** | **T** | **G** | **A** | **T** | **G** | **T** | **C** | **G** | **G** | **C** | **A** | **T** | **T** | **G** | **A** | **C** | **G** | **G** | **A** | **G** | **C** | **A** | **A** | **G** | **C** | **A** | **G** | **A** | **A** | **A** | **A** | **A** | **A** | **G** | **T** | **G** | **A** | **G** | **A** | **C** | **A** | **C** | **C** | **A** | **G** | **A** | **A** | **A** | **T** | **G** | **T** | **T** | **C** | **C** | **G** | **T** | **A** | **A** | **G** | **C** | **A** | **G** | **T** | **T** | **G** | **A** | **A** | **A** | **A** | **T** | **G** | **G** | **T** | **G** | **G** | **C** | **A** | **G** | **A** | **G** | **A** | **T** | **C** | **A** | **T** | **C** | **A** | **A** | **C** | **G** | **C** | **T** | **T** | **C** | **C** | **C** | **A** | **G** | **T** | **C** | **T** | **G** | **A** | **G** | **A** | **T** | **G** | **C** | **G** | **T** | **G** | **C** | **G** | **C** | **T** | **G** | **C** | **T** | **T** | **C** | **T** | **T** | **C** | **A** | **T** | **C** | **T** | **G** | **C** | **G** | **T** | **C** | **C** | **A** | **G** | **T** | **C** | **A** | **A** | **T** | **T** | **T** | **A** | **G** | **A** | **A** | **G** | **A** | **A** | **A** | **A** | **A** | **A** | **G** | **T** | **T** | **T** | **A** | **A** | **A** | **A** | **C** | **A** | **A** | **G** | **G** | **C** | **A** | **T** | **T** | **G** | **A** | **G** | **C** | **A** | **G** | **T** | **T** | **A** | **T** | **T** | **G** | **A** | **A** | **G** | **G** | **A** | **A** | **T** | **T** | **G** | **C** | **A** | **G** | **A** | **A** | **T** | **A** | **A** | **A** | **A** | **T** | **C** | **C** | **A** | **G** | **A** | **T** | **T** | **T** | **C** | **G** | **C** | **T** | **A** | **A** | **A** | **A** | **T** | **G** | **G** | **G** | **A** | **T** | **G** | **T** | **A** | **G** | **A** | **A** | **G** | **A** | **T** | **G** | **T** | **A** | **A** | **A** | **A** | **T** | **T** | **A** | **A** | **C** | **T** | **A** | **G** | **T** | **T** | **C** | **C** | **A** | **T** | **T** | **G** | **A** | **G** | **G** | **A** | **T** | **C** | **A** | **C** | **T** | **T** | **G** | **T** | **T** | **C** | **C** | **G** | **A** | **A** | **T** | **C** | **G** | **T** | **T** | **C** | **A** | **A** | **G** | **A** | **A** | **T** | **T** | **G** | **T** | **T** | **A** | **T** | **C** | **A** | **A** | **A** | **C** | **A** | **C** | **A** | **T** | **T** | **A** | **A** | **G** | **A** | **C** | **A** | **T** | **G** | **C** | **T** | **A** | **A** | **G** | **G** | **C** | **T** | **A** | **A** | **T** | **G** | **A** | **A** | **T** | **T** | **A** | **G** | **A** | **A** | **G** | **T** | **C** | **T** | **A** | **T** | **T** | **T** | **G** | **C** | **A** | **T** | **A** | **A** | **A** | **A** | **T** | **A** | **G** | **A** | **T** | **A** | **A** | **C** | **A** | **A** | **T** | **C** | **T** | **T** | **T** | **T** | **A** | **T** | **T** | **A** | **C** | **G** | **T** | **A** | **T** | **C** | **A** | **T** | **T** | **G** | **A** | **C** | **G** | **A** | **C** | **G** | **G** | **G** | **A** | **C** | **A** | **G** | **G** | **A** | **T** | **T** | **T** | **A** | **A** | **T** | **A** | **T** | **G** | **A** | **A** | **T** | **G** | **A** | **A** | **A** | **C** | **C** | **A** | **A** | **A** | **A** | **C** | **G** | **G** | **G** | **A** | **A** | **G** | **T** | **T** | **A** | **T** | **G** | **G** | **A** | **T** | **T** | **G** | **A** | **A** | **C** | **A** | **A** | **T** | **A** | **T** | **C** | **A** | **A** | **A** | **G** | **A** | **A** | **C** | **G** | **A** | **G** | **T** | **A** | **G** | **C** | **T** | **G** | **G** | **T** | **A** | **T** | **T** | **G** | **G** | **T** | **G** | **G** | **A** | **A** | **C** | **A** | **G** | **T** | **A** | **A** | **A** | **A** | **A** | **T** | **C** | **A** | **T** | **T** | **A** | **G** | **T** | **T** | **T** | **T** | **A** | **A** | **A** | **G** | **G** | **G** | **C** | **A** | **A** | **G** | **G** | **T** | **A** | **C** | **G** | **A** | **G** | **C** | **G** | **T** | **G** | **G** | **A** | **G** | **A** | **T** | **C** | **A** | **A** | **G** | **G** | **T** | **C** | **C** | **C** | **T** | **T** | **T** | **G** | **A** | **T** | **G** | **A** | **A** | **G** | **G** | **A** | **G** | **G** | **C** | **A** | **-** | **-** | **-** |
| **N59998F** | **A** | **T** | **G** | **A** | **T** | **G** | **G** | **G** | **A** | **A** | **G** | **A** | **A** | **T** | **A** | **T** | **C** | **C** | **A** | **A** | **A** | **G** | **C** | **A** | **A** | **T** | **G** | **C** | **T** | **A** | **G** | **C** | **T** | **G** | **T** | **T** | **T** | **A** | **T** | **T** | **C** | **G** | **G** | **G** | **G** | **A** | **T** | **T** | **G** | **C** | **G** | **G** | **C** | **T** | **T** | **T** | **C** | **C** | **T** | **T** | **A** | **T** | **T** | **A** | **T** | **T** | **C** | **T** | **A** | **T** | **T** | **T** | **T** | **C** | **A** | **C** | **T** | **T** | **T** | **T** | **C** | **A** | **C** | **T** | **T** | **A** | **T** | **T** | **T** | **T** | **T** | **A** | **T** | **G** | **C** | **C** | **A** | **G** | **T** | **A** | **A** | **C** | **C** | **A** | **A** | **A** | **G** | **T** | **C** | **A** | **T** | **T** | **G** | **G** | **T** | **G** | **G** | **G** | **G** | **A** | **G** | **A** | **A** | **T** | **T** | **G** | **C** | **T** | **G** | **C** | **G** | **T** | **G** | **C** | **G** | **C** | **G** | **T** | **T** | **T** | **A** | **T** | **T** | **A** | **T** | **A** | **C** | **G** | **T** | **T** | **C** | **C** | **G** | **C** | **T** | **T** | **A** | **T** | **C** | **T** | **T** | **C** | **C** | **A** | **T** | **T** | **T** | **G** | **A** | **T** | **G** | **G** | **C** | **A** | **A** | **T** | **T** | **T** | **C** | **A** | **T** | **T** | **A** | **G** | **G** | **T** | **G** | **T** | **A** | **G** | **G** | **A** | **T** | **T** | **G** | **A** | **T** | **C** | **G** | **T** | **A** | **T** | **T** | **T** | **C** | **T** | **A** | **T** | **T** | **G** | **T** | **T** | **A** | **T** | **C** | **A** | **C** | **T** | **T** | **A** | **T** | **T** | **C** | **A** | **A** | **A** | **A** | **G** | **A** | **C** | **A** | **A** | **A** | **A** | **T** | **A** | **C** | **G** | **G** | **G** | **A** | **A** | **A** | **A** | **T** | **C** | **G** | **A** | **A** | **G** | **A** | **A** | **A** | **A** | **G** | **C** | **T** | **T** | **C** | **G** | **T** | **G** | **C** | **A** | **C** | **T** | **T** | **T** | **C** | **T** | **T** | **C** | **T** | **G** | **G** | **C** | **A** | **A** | **T** | **T** | **A** | **T** | **G** | **A** | **A** | **T** | **C** | **C** | **A** | **A** | **A** | **C** | **T** | **T** | **T** | **T** | **G** | **C** | **A** | **T** | **C** | **A** | **G** | **C** | **C** | **G** | **A** | **T** | **C** | **C** | **C** | **A** | **A** | **G** | **C** | **G** | **C** | **G** | **T** | **C** | **G** | **G** | **A** | **T** | **G** | **A** | **T** | **C** | **T** | **G** | **T** | **A** | **C** | **A** | **T** | **C** | **A** | **A** | **A** | **G** | **A** | **T** | **A** | **T** | **T** | **G** | **A** | **T** | **A** | **T** | **G** | **G** | **A** | **A** | **A** | **T** | **T** | **A** | **C** | **T** | **A** | **A** | **A** | **A** | **T** | **A** | **A** | **A** | **A** | **G** | **A** | **A** | **A** | **A** | **A** | **A** | **T** | **G** | **A** | **T** | **C** | **G** | **A** | **A** | **G** | **T** | **A** | **T** | **C** | **C** | **A** | **G** | **T** | **G** | **A** | **A** | **T** | **T** | **A** | **C** | **A** | **G** | **A** | **T** | **T** | **A** | **T** | **G** | **A** | **C** | **T** | **A** | **G** | **C** | **C** | **G** | **T** | **C** | **C** | **T** | **C** | **A** | **A** | **T** | **A** | **T** | **G** | **T** | **A** | **G** | **A** | **T** | **G** | **G** | **A** | **C** | **A** | **A** | **A** | **C** | **C** | **A** | **A** | **A** | **G** | **A** | **A** | **G** | **A** | **G** | **A** | **T** | **T** | **T** | **T** | **A** | **G** | **A** | **A** | **T** | **T** | **A** | **G** | **A** | **A** | **A** | **G** | **G** | **C** | **A** | **T** | **C** | **G** | **A** | **T** | **T** | **A** | **G** | **C** | **T** | **C** | **G** | **T** | **G** | **A** | **G** | **C** | **T** | **T** | **C** | **A** | **T** | **G** | **A** | **T** | **T** | **C** | **A** | **G** | **T** | **T** | **T** | **C** | **A** | **C** | **A** | **G** | **C** | **A** | **A** | **T** | **T** | **A** | **T** | **T** | **T** | **G** | **C** | **A** | **G** | **C** | **G** | **A** | **T** | **G** | **A** | **T** | **G** | **A** | **T** | **G** | **A** | **T** | **G** | **T** | **C** | **G** | **G** | **C** | **A** | **T** | **T** | **G** | **A** | **C** | **G** | **G** | **A** | **G** | **C** | **A** | **A** | **G** | **C** | **A** | **G** | **A** | **A** | **A** | **A** | **A** | **A** | **G** | **T** | **G** | **A** | **G** | **A** | **C** | **A** | **C** | **C** | **A** | **G** | **A** | **A** | **A** | **T** | **G** | **T** | **T** | **C** | **C** | **G** | **T** | **A** | **A** | **G** | **C** | **A** | **G** | **T** | **T** | **G** | **A** | **A** | **A** | **A** | **T** | **G** | **G** | **T** | **G** | **G** | **C** | **A** | **G** | **A** | **G** | **A** | **T** | **C** | **A** | **T** | **C** | **A** | **A** | **C** | **G** | **C** | **T** | **T** | **C** | **C** | **C** | **A** | **G** | **T** | **C** | **T** | **G** | **A** | **G** | **A** | **T** | **G** | **C** | **G** | **T** | **G** | **C** | **G** | **C** | **T** | **G** | **C** | **T** | **T** | **C** | **T** | **T** | **C** | **A** | **T** | **C** | **T** | **G** | **C** | **G** | **T** | **C** | **C** | **A** | **G** | **T** | **C** | **A** | **A** | **T** | **T** | **T** | **A** | **G** | **A** | **A** | **G** | **A** | **A** | **A** | **A** | **A** | **A** | **G** | **T** | **T** | **T** | **A** | **A** | **A** | **A** | **C** | **A** | **A** | **G** | **G** | **C** | **A** | **T** | **T** | **G** | **A** | **G** | **C** | **A** | **G** | **T** | **T** | **A** | **T** | **T** | **G** | **A** | **A** | **G** | **G** | **A** | **A** | **T** | **T** | **G** | **C** | **A** | **G** | **A** | **A** | **T** | **A** | **A** | **A** | **A** | **T** | **C** | **C** | **A** | **G** | **A** | **T** | **T** | **T** | **C** | **G** | **C** | **T** | **A** | **A** | **A** | **A** | **T** | **G** | **G** | **G** | **A** | **T** | **G** | **T** | **A** | **G** | **A** | **A** | **G** | **A** | **T** | **G** | **T** | **A** | **A** | **A** | **A** | **T** | **T** | **A** | **A** | **C** | **T** | **A** | **G** | **T** | **T** | **C** | **C** | **A** | **T** | **T** | **G** | **A** | **G** | **G** | **A** | **T** | **C** | **A** | **C** | **T** | **T** | **G** | **T** | **T** | **C** | **C** | **G** | **A** | **A** | **T** | **C** | **G** | **T** | **T** | **C** | **A** | **A** | **G** | **A** | **A** | **T** | **T** | **G** | **T** | **T** | **A** | **T** | **C** | **A** | **A** | **A** | **C** | **A** | **C** | **A** | **T** | **T** | **A** | **A** | **G** | **A** | **C** | **A** | **T** | **G** | **C** | **T** | **A** | **A** | **G** | **G** | **C** | **T** | **A** | **A** | **T** | **G** | **A** | **A** | **T** | **T** | **A** | **G** | **A** | **A** | **G** | **T** | **C** | **T** | **A** | **T** | **T** | **T** | **G** | **C** | **A** | **T** | **A** | **A** | **A** | **A** | **T** | **A** | **G** | **A** | **T** | **A** | **A** | **C** | **A** | **A** | **T** | **C** | **T** | **T** | **T** | **T** | **A** | **T** | **T** | **A** | **C** | **G** | **T** | **A** | **T** | **C** | **A** | **T** | **T** | **G** | **A** | **C** | **G** | **A** | **C** | **G** | **G** | **G** | **A** | **C** | **A** | **G** | **G** | **A** | **T** | **T** | **T** | **A** | **A** | **T** | **A** | **T** | **G** | **A** | **A** | **T** | **G** | **A** | **A** | **A** | **C** | **C** | **A** | **A** | **A** | **A** | **C** | **G** | **G** | **G** | **A** | **A** | **G** | **T** | **T** | **A** | **T** | **G** | **G** | **A** | **T** | **T** | **G** | **A** | **A** | **C** | **A** | **A** | **T** | **A** | **T** | **C** | **A** | **A** | **A** | **G** | **A** | **A** | **C** | **G** | **A** | **G** | **T** | **A** | **G** | **C** | **T** | **G** | **G** | **T** | **A** | **T** | **T** | **G** | **G** | **T** | **G** | **G** | **A** | **A** | **C** | **A** | **G** | **T** | **A** | **A** | **A** | **A** | **A** | **T** | **C** | **A** | **T** | **T** | **A** | **G** | **T** | **T** | **T** | **T** | **A** | **A** | **A** | **G** | **G** | **G** | **C** | **A** | **A** | **G** | **G** | **T** | **A** | **C** | **G** | **A** | **G** | **C** | **G** | **T** | **G** | **G** | **A** | **G** | **A** | **T** | **C** | **A** | **A** | **G** | **G** | **T** | **C** | **C** | **C** | **T** | **T** | **T** | **G** | **A** | **T** | **G** | **A** | **A** | **G** | **G** | **A** | **G** | **G** | **C** | **A** | **-** | **-** | **-** |
| **N60200F** | **A** | **T** | **G** | **A** | **T** | **G** | **G** | **G** | **A** | **A** | **G** | **A** | **A** | **T** | **A** | **T** | **C** | **C** | **A** | **A** | **A** | **G** | **C** | **A** | **A** | **T** | **G** | **C** | **T** | **A** | **G** | **C** | **T** | **G** | **T** | **T** | **T** | **A** | **T** | **T** | **C** | **G** | **G** | **G** | **G** | **A** | **T** | **T** | **G** | **C** | **G** | **G** | **C** | **T** | **T** | **T** | **C** | **C** | **T** | **T** | **A** | **T** | **T** | **A** | **T** | **T** | **C** | **T** | **A** | **T** | **T** | **T** | **T** | **C** | **A** | **C** | **T** | **T** | **T** | **T** | **C** | **A** | **C** | **T** | **T** | **A** | **T** | **T** | **T** | **T** | **T** | **A** | **T** | **G** | **C** | **C** | **A** | **G** | **T** | **A** | **A** | **C** | **C** | **A** | **A** | **A** | **G** | **T** | **C** | **A** | **T** | **T** | **G** | **G** | **T** | **G** | **G** | **G** | **G** | **A** | **G** | **A** | **A** | **T** | **T** | **G** | **C** | **T** | **G** | **C** | **G** | **T** | **G** | **C** | **G** | **C** | **G** | **T** | **T** | **T** | **A** | **T** | **T** | **A** | **T** | **A** | **C** | **G** | **T** | **T** | **C** | **C** | **G** | **C** | **T** | **T** | **A** | **T** | **C** | **T** | **T** | **C** | **C** | **A** | **T** | **T** | **T** | **G** | **A** | **T** | **G** | **G** | **C** | **A** | **A** | **T** | **T** | **T** | **C** | **A** | **T** | **T** | **A** | **G** | **G** | **T** | **G** | **T** | **A** | **G** | **G** | **A** | **T** | **T** | **G** | **A** | **T** | **C** | **G** | **T** | **A** | **T** | **T** | **T** | **C** | **T** | **A** | **T** | **T** | **G** | **T** | **T** | **A** | **T** | **C** | **A** | **C** | **T** | **T** | **A** | **T** | **T** | **C** | **A** | **A** | **A** | **A** | **G** | **G** | **C** | **A** | **A** | **A** | **A** | **T** | **A** | **C** | **G** | **G** | **G** | **A** | **A** | **A** | **A** | **T** | **C** | **G** | **A** | **A** | **G** | **A** | **A** | **A** | **A** | **G** | **C** | **T** | **T** | **C** | **G** | **T** | **G** | **C** | **A** | **C** | **T** | **T** | **T** | **C** | **T** | **T** | **C** | **T** | **G** | **G** | **C** | **A** | **A** | **T** | **T** | **A** | **T** | **G** | **A** | **A** | **T** | **C** | **C** | **A** | **A** | **A** | **C** | **T** | **T** | **T** | **T** | **G** | **C** | **A** | **T** | **C** | **A** | **G** | **C** | **C** | **G** | **A** | **T** | **C** | **C** | **C** | **A** | **A** | **G** | **C** | **G** | **C** | **G** | **T** | **C** | **G** | **G** | **A** | **T** | **G** | **A** | **T** | **C** | **T** | **G** | **T** | **A** | **C** | **A** | **T** | **C** | **A** | **A** | **A** | **G** | **A** | **T** | **A** | **T** | **T** | **G** | **A** | **T** | **A** | **T** | **G** | **G** | **A** | **A** | **A** | **T** | **T** | **A** | **C** | **T** | **A** | **A** | **A** | **A** | **T** | **A** | **A** | **A** | **A** | **G** | **A** | **A** | **A** | **A** | **A** | **A** | **T** | **G** | **A** | **T** | **C** | **G** | **A** | **A** | **G** | **T** | **A** | **T** | **C** | **C** | **A** | **G** | **T** | **G** | **A** | **A** | **T** | **T** | **A** | **C** | **A** | **G** | **A** | **T** | **T** | **A** | **T** | **G** | **A** | **C** | **T** | **A** | **G** | **C** | **C** | **G** | **T** | **C** | **C** | **T** | **C** | **A** | **A** | **T** | **A** | **T** | **G** | **T** | **A** | **G** | **A** | **T** | **G** | **G** | **A** | **C** | **A** | **A** | **A** | **C** | **C** | **A** | **A** | **A** | **G** | **A** | **A** | **G** | **A** | **G** | **A** | **T** | **T** | **T** | **T** | **A** | **G** | **A** | **A** | **T** | **T** | **A** | **G** | **A** | **A** | **A** | **G** | **G** | **C** | **A** | **T** | **C** | **G** | **A** | **T** | **T** | **A** | **G** | **C** | **T** | **C** | **G** | **T** | **G** | **A** | **G** | **C** | **T** | **T** | **C** | **A** | **T** | **G** | **A** | **T** | **T** | **C** | **A** | **G** | **T** | **T** | **T** | **C** | **A** | **C** | **A** | **G** | **C** | **A** | **A** | **T** | **T** | **A** | **T** | **T** | **T** | **G** | **C** | **A** | **G** | **C** | **G** | **A** | **T** | **G** | **A** | **T** | **G** | **A** | **T** | **G** | **A** | **T** | **G** | **T** | **C** | **G** | **G** | **C** | **A** | **T** | **T** | **G** | **A** | **C** | **G** | **G** | **A** | **G** | **C** | **A** | **A** | **G** | **C** | **A** | **G** | **A** | **A** | **A** | **A** | **A** | **A** | **G** | **T** | **G** | **A** | **G** | **A** | **C** | **A** | **C** | **C** | **A** | **G** | **A** | **A** | **A** | **T** | **G** | **T** | **T** | **C** | **C** | **G** | **T** | **A** | **A** | **G** | **C** | **A** | **G** | **T** | **T** | **G** | **A** | **A** | **A** | **A** | **T** | **G** | **G** | **T** | **G** | **G** | **C** | **A** | **G** | **A** | **G** | **A** | **T** | **C** | **A** | **T** | **C** | **A** | **A** | **C** | **G** | **C** | **T** | **T** | **C** | **C** | **C** | **A** | **G** | **T** | **C** | **T** | **G** | **A** | **G** | **A** | **T** | **G** | **C** | **G** | **T** | **G** | **C** | **G** | **C** | **T** | **G** | **C** | **T** | **T** | **C** | **T** | **T** | **C** | **A** | **T** | **C** | **T** | **G** | **C** | **G** | **T** | **C** | **C** | **A** | **G** | **T** | **C** | **A** | **A** | **T** | **T** | **T** | **A** | **G** | **A** | **A** | **G** | **A** | **A** | **A** | **A** | **A** | **A** | **G** | **T** | **T** | **T** | **A** | **A** | **A** | **A** | **C** | **A** | **A** | **G** | **G** | **C** | **A** | **T** | **T** | **G** | **A** | **G** | **C** | **A** | **G** | **T** | **T** | **A** | **T** | **T** | **G** | **A** | **A** | **G** | **G** | **A** | **A** | **T** | **T** | **G** | **C** | **A** | **G** | **A** | **A** | **T** | **A** | **A** | **A** | **A** | **T** | **C** | **C** | **A** | **G** | **A** | **T** | **T** | **T** | **C** | **G** | **C** | **T** | **A** | **A** | **A** | **A** | **T** | **G** | **G** | **G** | **A** | **T** | **G** | **T** | **A** | **G** | **A** | **A** | **G** | **A** | **T** | **G** | **T** | **A** | **A** | **A** | **A** | **T** | **T** | **A** | **A** | **C** | **T** | **A** | **G** | **T** | **T** | **C** | **C** | **A** | **T** | **T** | **G** | **A** | **G** | **G** | **A** | **T** | **C** | **A** | **C** | **T** | **T** | **G** | **T** | **T** | **C** | **C** | **G** | **A** | **A** | **T** | **C** | **G** | **T** | **T** | **C** | **A** | **A** | **G** | **A** | **A** | **T** | **T** | **G** | **T** | **T** | **A** | **T** | **C** | **A** | **A** | **A** | **C** | **A** | **C** | **A** | **T** | **T** | **A** | **A** | **G** | **A** | **C** | **A** | **T** | **G** | **C** | **T** | **A** | **A** | **G** | **G** | **C** | **T** | **A** | **A** | **T** | **G** | **A** | **A** | **T** | **T** | **A** | **G** | **A** | **A** | **G** | **T** | **C** | **T** | **A** | **T** | **T** | **T** | **G** | **C** | **A** | **T** | **A** | **A** | **A** | **A** | **T** | **A** | **G** | **A** | **T** | **A** | **A** | **C** | **A** | **A** | **T** | **C** | **T** | **T** | **T** | **T** | **A** | **T** | **T** | **A** | **C** | **G** | **T** | **A** | **T** | **C** | **A** | **T** | **T** | **G** | **A** | **C** | **G** | **A** | **C** | **G** | **G** | **G** | **A** | **C** | **A** | **G** | **G** | **A** | **T** | **T** | **T** | **A** | **A** | **T** | **A** | **T** | **G** | **A** | **A** | **T** | **G** | **A** | **A** | **A** | **C** | **C** | **A** | **A** | **A** | **A** | **C** | **G** | **G** | **G** | **A** | **A** | **G** | **T** | **T** | **A** | **T** | **G** | **G** | **A** | **T** | **T** | **G** | **A** | **A** | **C** | **A** | **A** | **T** | **A** | **T** | **C** | **A** | **A** | **A** | **G** | **A** | **A** | **C** | **G** | **A** | **G** | **T** | **A** | **G** | **C** | **T** | **G** | **G** | **T** | **A** | **T** | **T** | **G** | **G** | **T** | **G** | **G** | **A** | **A** | **C** | **A** | **G** | **T** | **A** | **A** | **A** | **A** | **A** | **T** | **C** | **A** | **T** | **T** | **A** | **G** | **T** | **T** | **T** | **T** | **A** | **A** | **A** | **G** | **G** | **G** | **C** | **A** | **A** | **G** | **G** | **T** | **A** | **C** | **G** | **A** | **G** | **C** | **G** | **T** | **G** | **G** | **A** | **G** | **A** | **T** | **C** | **A** | **A** | **G** | **G** | **T** | **C** | **C** | **C** | **T** | **T** | **T** | **G** | **A** | **T** | **G** | **A** | **A** | **G** | **G** | **A** | **G** | **G** | **C** | **A** | **-** | **-** | **-** |
| **N54155** | **A** | **T** | **G** | **A** | **T** | **G** | **G** | **G** | **A** | **A** | **A** | **A** | **A** | **T** | **A** | **T** | **C** | **C** | **A** | **G** | **A** | **G** | **C** | **G** | **A** | **T** | **G** | **C** | **T** | **A** | **G** | **C** | **T** | **G** | **T** | **T** | **T** | **A** | **T** | **T** | **C** | **G** | **G** | **G** | **G** | **A** | **T** | **T** | **G** | **C** | **G** | **G** | **C** | **T** | **T** | **T** | **C** | **C** | **T** | **T** | **A** | **T** | **T** | **A** | **T** | **A** | **C** | **T** | **A** | **T** | **T** | **T** | **T** | **C** | **A** | **C** | **T** | **T** | **T** | **T** | **C** | **A** | **C** | **T** | **T** | **A** | **T** | **T** | **T** | **T** | **T** | **A** | **T** | **G** | **C** | **C** | **A** | **G** | **T** | **A** | **A** | **T** | **C** | **A** | **A** | **A** | **G** | **T** | **C** | **A** | **T** | **T** | **G** | **G** | **T** | **G** | **G** | **G** | **G** | **A** | **G** | **A** | **A** | **T** | **T** | **A** | **C** | **T** | **G** | **C** | **G** | **T** | **G** | **C** | **A** | **C** | **G** | **T** | **T** | **T** | **A** | **T** | **T** | **A** | **T** | **A** | **T** | **G** | **T** | **T** | **C** | **C** | **G** | **C** | **T** | **T** | **A** | **T** | **C** | **T** | **T** | **C** | **C** | **A** | **T** | **T** | **T** | **G** | **T** | **T** | **A** | **G** | **C** | **T** | **A** | **T** | **T** | **T** | **C** | **C** | **T** | **T** | **A** | **G** | **G** | **T** | **G** | **T** | **A** | **G** | **G** | **A** | **T** | **T** | **A** | **A** | **T** | **C** | **G** | **T** | **C** | **T** | **T** | **T** | **C** | **T** | **A** | **T** | **T** | **A** | **T** | **T** | **A** | **T** | **C** | **A** | **C** | **T** | **T** | **A** | **T** | **T** | **C** | **A** | **A** | **A** | **A** | **G** | **A** | **C** | **A** | **A** | **A** | **A** | **T** | **A** | **C** | **G** | **G** | **G** | **A** | **A** | **A** | **A** | **T** | **C** | **G** | **A** | **A** | **G** | **A** | **A** | **A** | **A** | **G** | **C** | **T** | **G** | **C** | **G** | **T** | **G** | **C** | **A** | **C** | **T** | **C** | **T** | **C** | **T** | **T** | **C** | **T** | **G** | **G** | **C** | **A** | **A** | **T** | **T** | **A** | **T** | **G** | **A** | **A** | **T** | **C** | **C** | **A** | **A** | **A** | **T** | **T** | **G** | **T** | **T** | **G** | **C** | **T** | **T** | **C** | **T** | **T** | **C** | **C** | **G** | **A** | **T** | **C** | **C** | **C** | **A** | **A** | **G** | **T** | **G** | **C** | **A** | **T** | **C** | **G** | **G** | **A** | **C** | **G** | **A** | **T** | **T** | **T** | **A** | **T** | **A** | **C** | **A** | **T** | **C** | **A** | **A** | **A** | **G** | **A** | **T** | **A** | **T** | **T** | **G** | **A** | **T** | **A** | **A** | **G** | **G** | **A** | **A** | **A** | **T** | **C** | **A** | **C** | **T** | **A** | **A** | **A** | **A** | **T** | **A** | **A** | **A** | **A** | **G** | **A** | **A** | **A** | **A** | **A** | **A** | **T** | **G** | **A** | **T** | **T** | **G** | **A** | **A** | **G** | **T** | **A** | **T** | **C** | **T** | **A** | **G** | **T** | **G** | **A** | **A** | **T** | **T** | **A** | **C** | **A** | **A** | **A** | **T** | **T** | **G** | **T** | **A** | **A** | **C** | **G** | **A** | **G** | **C** | **C** | **G** | **T** | **C** | **C** | **C** | **C** | **A** | **A** | **T** | **A** | **T** | **G** | **T** | **A** | **G** | **A** | **T** | **G** | **G** | **A** | **C** | **A** | **A** | **A** | **C** | **C** | **A** | **A** | **A** | **G** | **A** | **A** | **G** | **A** | **A** | **A** | **T** | **T** | **T** | **T** | **A** | **G** | **A** | **A** | **T** | **T** | **A** | **G** | **A** | **A** | **A** | **G** | **A** | **C** | **A** | **T** | **C** | **G** | **A** | **T** | **T** | **A** | **G** | **C** | **C** | **C** | **G** | **T** | **G** | **A** | **G** | **C** | **T** | **G** | **C** | **A** | **T** | **G** | **A** | **T** | **T** | **C** | **A** | **G** | **T** | **T** | **T** | **C** | **G** | **C** | **A** | **A** | **C** | **A** | **A** | **T** | **T** | **G** | **T** | **T** | **T** | **G** | **C** | **A** | **G** | **C** | **A** | **A** | **T** | **G** | **A** | **T** | **G** | **A** | **T** | **G** | **A** | **T** | **G** | **T** | **C** | **G** | **G** | **C** | **A** | **T** | **T** | **A** | **A** | **C** | **G** | **G** | **A** | **G** | **C** | **A** | **A** | **G** | **C** | **A** | **G** | **A** | **A** | **A** | **A** | **A** | **A** | **G** | **C** | **G** | **A** | **G** | **A** | **C** | **A** | **C** | **C** | **A** | **G** | **A** | **A** | **A** | **T** | **G** | **T** | **T** | **C** | **C** | **G** | **C** | **A** | **A** | **G** | **C** | **A** | **G** | **T** | **T** | **G** | **A** | **A** | **A** | **A** | **T** | **G** | **G** | **T** | **A** | **G** | **C** | **A** | **G** | **A** | **G** | **A** | **T** | **C** | **A** | **T** | **C** | **A** | **A** | **C** | **G** | **C** | **T** | **T** | **C** | **C** | **C** | **A** | **G** | **T** | **C** | **T** | **G** | **A** | **G** | **A** | **T** | **G** | **C** | **G** | **T** | **G** | **C** | **G** | **C** | **T** | **G** | **C** | **T** | **T** | **C** | **T** | **T** | **C** | **A** | **T** | **C** | **T** | **G** | **C** | **G** | **T** | **C** | **C** | **A** | **G** | **T** | **C** | **A** | **A** | **T** | **T** | **T** | **A** | **G** | **A** | **A** | **G** | **A** | **G** | **A** | **A** | **A** | **A** | **G** | **T** | **T** | **T** | **A** | **A** | **A** | **G** | **C** | **A** | **A** | **G** | **G** | **T** | **A** | **T** | **C** | **G** | **A** | **G** | **C** | **A** | **G** | **T** | **T** | **A** | **T** | **T** | **G** | **A** | **A** | **G** | **G** | **A** | **A** | **T** | **T** | **G** | **C** | **A** | **A** | **A** | **A** | **T** | **A** | **A** | **A** | **A** | **T** | **C** | **C** | **A** | **G** | **A** | **T** | **T** | **T** | **C** | **G** | **C** | **T** | **G** | **A** | **A** | **A** | **T** | **G** | **G** | **G** | **A** | **T** | **G** | **T** | **A** | **G** | **A** | **A** | **G** | **A** | **T** | **G** | **T** | **A** | **A** | **A** | **A** | **C** | **T** | **A** | **T** | **C** | **T** | **A** | **G** | **T** | **T** | **C** | **C** | **A** | **T** | **T** | **G** | **A** | **G** | **G** | **A** | **T** | **C** | **A** | **C** | **T** | **T** | **G** | **T** | **T** | **C** | **C** | **G** | **A** | **A** | **T** | **C** | **G** | **T** | **T** | **C** | **A** | **A** | **G** | **A** | **A** | **T** | **T** | **G** | **T** | **T** | **A** | **T** | **C** | **A** | **A** | **A** | **T** | **A** | **C** | **A** | **T** | **T** | **A** | **A** | **G** | **A** | **C** | **A** | **T** | **G** | **C** | **T** | **A** | **A** | **G** | **G** | **C** | **T** | **A** | **A** | **T** | **G** | **A** | **A** | **T** | **T** | **A** | **G** | **A** | **A** | **G** | **T** | **C** | **T** | **A** | **T** | **T** | **T** | **G** | **C** | **A** | **T** | **A** | **A** | **A** | **A** | **T** | **A** | **G** | **A** | **T** | **A** | **A** | **C** | **A** | **A** | **T** | **C** | **T** | **T** | **T** | **T** | **A** | **T** | **T** | **A** | **C** | **G** | **T** | **A** | **T** | **C** | **A** | **T** | **T** | **G** | **A** | **C** | **G** | **A** | **C** | **G** | **G** | **G** | **A** | **C** | **A** | **G** | **G** | **A** | **T** | **T** | **T** | **A** | **A** | **T** | **A** | **T** | **G** | **A** | **A** | **T** | **G** | **A** | **A** | **A** | **C** | **C** | **A** | **A** | **A** | **A** | **C** | **G** | **G** | **G** | **A** | **A** | **G** | **T** | **T** | **A** | **T** | **G** | **G** | **A** | **T** | **T** | **G** | **A** | **A** | **C** | **A** | **A** | **T** | **A** | **T** | **C** | **A** | **A** | **A** | **G** | **A** | **A** | **C** | **G** | **A** | **G** | **T** | **A** | **G** | **C** | **T** | **G** | **G** | **T** | **A** | **T** | **T** | **G** | **G** | **T** | **G** | **G** | **A** | **A** | **C** | **A** | **G** | **T** | **A** | **A** | **A** | **A** | **A** | **T** | **C** | **A** | **T** | **T** | **A** | **G** | **T** | **T** | **T** | **T** | **A** | **A** | **A** | **G** | **G** | **G** | **C** | **A** | **A** | **G** | **G** | **T** | **A** | **C** | **G** | **A** | **G** | **C** | **G** | **T** | **G** | **G** | **A** | **G** | **A** | **T** | **C** | **A** | **A** | **G** | **G** | **T** | **C** | **C** | **C** | **T** | **T** | **T** | **G** | **A** | **T** | **G** | **A** | **A** | **G** | **G** | **A** | **G** | **G** | **C** | **A** | **-** | **-** | **-** |
| **N53655** | **A** | **T** | **G** | **A** | **T** | **G** | **G** | **G** | **A** | **A** | **A** | **A** | **A** | **T** | **A** | **T** | **C** | **C** | **A** | **G** | **A** | **G** | **C** | **G** | **A** | **T** | **G** | **C** | **T** | **A** | **G** | **C** | **T** | **G** | **T** | **T** | **T** | **A** | **T** | **T** | **C** | **G** | **G** | **G** | **G** | **A** | **T** | **T** | **G** | **C** | **G** | **G** | **C** | **T** | **T** | **T** | **C** | **C** | **T** | **T** | **A** | **T** | **T** | **A** | **T** | **A** | **C** | **T** | **A** | **T** | **T** | **T** | **T** | **C** | **A** | **C** | **T** | **T** | **T** | **T** | **C** | **A** | **C** | **T** | **T** | **A** | **T** | **T** | **T** | **T** | **T** | **A** | **T** | **G** | **C** | **C** | **A** | **G** | **T** | **A** | **A** | **T** | **C** | **A** | **A** | **A** | **G** | **T** | **C** | **A** | **T** | **T** | **G** | **G** | **T** | **G** | **G** | **G** | **G** | **A** | **G** | **A** | **A** | **T** | **T** | **A** | **C** | **T** | **G** | **C** | **G** | **T** | **G** | **C** | **A** | **C** | **G** | **T** | **T** | **T** | **A** | **T** | **T** | **A** | **T** | **A** | **T** | **G** | **T** | **T** | **C** | **C** | **G** | **C** | **T** | **T** | **A** | **T** | **C** | **T** | **T** | **C** | **C** | **A** | **T** | **T** | **T** | **G** | **T** | **T** | **A** | **G** | **C** | **T** | **A** | **T** | **T** | **T** | **C** | **C** | **T** | **T** | **A** | **G** | **G** | **T** | **G** | **T** | **A** | **G** | **G** | **A** | **T** | **T** | **A** | **A** | **T** | **C** | **G** | **T** | **C** | **T** | **T** | **T** | **C** | **T** | **A** | **T** | **T** | **A** | **T** | **T** | **A** | **T** | **C** | **A** | **C** | **T** | **T** | **A** | **T** | **T** | **C** | **A** | **A** | **A** | **A** | **G** | **A** | **C** | **A** | **A** | **A** | **A** | **T** | **A** | **C** | **G** | **G** | **G** | **A** | **A** | **A** | **A** | **T** | **C** | **G** | **A** | **A** | **G** | **A** | **A** | **A** | **A** | **G** | **C** | **T** | **G** | **C** | **G** | **T** | **G** | **C** | **A** | **C** | **T** | **C** | **T** | **C** | **T** | **T** | **C** | **T** | **G** | **G** | **C** | **A** | **A** | **T** | **T** | **A** | **T** | **G** | **A** | **A** | **T** | **C** | **C** | **A** | **A** | **A** | **T** | **T** | **G** | **T** | **T** | **G** | **C** | **T** | **T** | **C** | **T** | **T** | **C** | **C** | **G** | **A** | **T** | **C** | **C** | **C** | **A** | **A** | **G** | **T** | **G** | **C** | **A** | **T** | **C** | **G** | **G** | **A** | **C** | **G** | **A** | **T** | **T** | **T** | **A** | **T** | **A** | **C** | **A** | **T** | **C** | **A** | **A** | **A** | **G** | **A** | **T** | **A** | **T** | **T** | **G** | **A** | **T** | **A** | **A** | **G** | **G** | **A** | **A** | **A** | **T** | **C** | **A** | **C** | **T** | **A** | **A** | **A** | **A** | **T** | **A** | **A** | **A** | **A** | **G** | **A** | **A** | **A** | **A** | **A** | **A** | **T** | **G** | **A** | **T** | **T** | **G** | **A** | **A** | **G** | **T** | **A** | **T** | **C** | **T** | **A** | **G** | **T** | **G** | **A** | **A** | **T** | **T** | **A** | **C** | **A** | **A** | **A** | **T** | **T** | **G** | **T** | **A** | **A** | **C** | **G** | **A** | **G** | **C** | **C** | **G** | **T** | **C** | **C** | **C** | **C** | **A** | **A** | **T** | **A** | **T** | **G** | **T** | **A** | **G** | **A** | **T** | **G** | **G** | **A** | **C** | **A** | **A** | **A** | **C** | **C** | **A** | **A** | **A** | **G** | **A** | **A** | **G** | **A** | **A** | **A** | **T** | **T** | **T** | **T** | **A** | **G** | **A** | **A** | **T** | **T** | **A** | **G** | **A** | **A** | **A** | **G** | **A** | **C** | **A** | **T** | **C** | **G** | **A** | **T** | **T** | **A** | **G** | **C** | **C** | **C** | **G** | **T** | **G** | **A** | **G** | **C** | **T** | **G** | **C** | **A** | **T** | **G** | **A** | **T** | **T** | **C** | **A** | **G** | **T** | **T** | **T** | **C** | **G** | **C** | **A** | **A** | **C** | **A** | **A** | **T** | **T** | **G** | **T** | **T** | **T** | **G** | **C** | **A** | **G** | **C** | **A** | **A** | **T** | **G** | **A** | **T** | **G** | **A** | **T** | **G** | **A** | **T** | **G** | **T** | **C** | **G** | **G** | **C** | **A** | **T** | **T** | **A** | **A** | **C** | **G** | **G** | **A** | **G** | **C** | **A** | **A** | **G** | **C** | **A** | **G** | **A** | **A** | **A** | **A** | **A** | **A** | **G** | **C** | **G** | **A** | **G** | **A** | **C** | **A** | **C** | **C** | **A** | **G** | **A** | **A** | **A** | **T** | **G** | **T** | **T** | **C** | **C** | **G** | **C** | **A** | **A** | **G** | **C** | **A** | **G** | **T** | **T** | **G** | **A** | **A** | **A** | **A** | **T** | **G** | **G** | **T** | **A** | **G** | **C** | **A** | **G** | **A** | **G** | **A** | **T** | **C** | **A** | **T** | **C** | **A** | **A** | **C** | **G** | **C** | **T** | **T** | **C** | **C** | **C** | **A** | **G** | **T** | **C** | **T** | **G** | **A** | **G** | **A** | **T** | **G** | **C** | **G** | **T** | **G** | **C** | **G** | **C** | **T** | **G** | **C** | **T** | **T** | **C** | **T** | **T** | **C** | **A** | **T** | **C** | **T** | **G** | **C** | **G** | **T** | **C** | **C** | **A** | **G** | **T** | **C** | **A** | **A** | **T** | **T** | **T** | **A** | **G** | **A** | **A** | **G** | **A** | **G** | **A** | **A** | **A** | **A** | **G** | **T** | **T** | **T** | **A** | **A** | **A** | **G** | **C** | **A** | **A** | **G** | **G** | **T** | **A** | **T** | **C** | **G** | **A** | **G** | **C** | **A** | **G** | **T** | **T** | **A** | **T** | **T** | **G** | **A** | **A** | **G** | **G** | **A** | **A** | **T** | **T** | **G** | **C** | **A** | **A** | **A** | **A** | **T** | **A** | **A** | **A** | **A** | **T** | **C** | **C** | **A** | **G** | **A** | **T** | **T** | **T** | **C** | **G** | **C** | **T** | **G** | **A** | **A** | **A** | **T** | **G** | **G** | **G** | **A** | **T** | **G** | **T** | **A** | **G** | **A** | **A** | **G** | **A** | **T** | **G** | **T** | **A** | **A** | **A** | **A** | **C** | **T** | **A** | **T** | **C** | **T** | **A** | **G** | **T** | **T** | **C** | **C** | **A** | **T** | **T** | **G** | **A** | **G** | **G** | **A** | **T** | **C** | **A** | **C** | **T** | **T** | **G** | **T** | **T** | **C** | **C** | **G** | **A** | **A** | **T** | **C** | **G** | **T** | **T** | **C** | **A** | **A** | **G** | **A** | **A** | **T** | **T** | **G** | **T** | **T** | **A** | **T** | **C** | **A** | **A** | **A** | **T** | **A** | **C** | **A** | **T** | **T** | **A** | **A** | **G** | **A** | **C** | **A** | **T** | **G** | **C** | **T** | **A** | **A** | **G** | **G** | **C** | **T** | **A** | **A** | **T** | **G** | **A** | **A** | **T** | **T** | **A** | **G** | **A** | **A** | **G** | **T** | **C** | **T** | **A** | **T** | **T** | **T** | **G** | **C** | **A** | **T** | **A** | **A** | **A** | **A** | **T** | **A** | **G** | **A** | **T** | **A** | **A** | **C** | **A** | **A** | **T** | **C** | **T** | **T** | **T** | **T** | **A** | **T** | **T** | **A** | **C** | **G** | **T** | **A** | **T** | **C** | **A** | **T** | **T** | **G** | **A** | **C** | **G** | **A** | **C** | **G** | **G** | **G** | **A** | **C** | **A** | **G** | **G** | **A** | **T** | **T** | **T** | **A** | **A** | **T** | **A** | **T** | **G** | **A** | **A** | **T** | **G** | **A** | **A** | **A** | **C** | **C** | **A** | **A** | **A** | **A** | **C** | **G** | **G** | **G** | **A** | **A** | **G** | **T** | **T** | **A** | **T** | **G** | **G** | **A** | **T** | **T** | **G** | **A** | **A** | **C** | **A** | **A** | **T** | **A** | **T** | **C** | **A** | **A** | **A** | **G** | **A** | **A** | **C** | **G** | **A** | **G** | **T** | **A** | **G** | **C** | **T** | **G** | **G** | **T** | **A** | **T** | **T** | **G** | **G** | **T** | **G** | **G** | **A** | **A** | **C** | **A** | **G** | **T** | **A** | **A** | **A** | **A** | **A** | **T** | **C** | **A** | **T** | **T** | **A** | **G** | **T** | **T** | **T** | **T** | **A** | **A** | **A** | **G** | **G** | **G** | **C** | **A** | **A** | **G** | **G** | **T** | **A** | **C** | **G** | **A** | **G** | **C** | **G** | **T** | **G** | **G** | **A** | **G** | **A** | **T** | **C** | **A** | **A** | **G** | **G** | **T** | **C** | **C** | **C** | **T** | **T** | **T** | **G** | **A** | **T** | **G** | **A** | **A** | **G** | **G** | **A** | **G** | **G** | **C** | **A** | **-** | **-** | **-** |
| **N54152** | **A** | **T** | **G** | **A** | **T** | **G** | **G** | **G** | **A** | **A** | **G** | **A** | **A** | **T** | **A** | **T** | **C** | **C** | **A** | **A** | **A** | **G** | **C** | **A** | **A** | **T** | **G** | **C** | **T** | **A** | **G** | **C** | **T** | **G** | **T** | **T** | **T** | **A** | **T** | **T** | **C** | **G** | **G** | **G** | **G** | **A** | **T** | **T** | **G** | **C** | **G** | **G** | **C** | **T** | **T** | **T** | **C** | **C** | **T** | **T** | **A** | **T** | **T** | **A** | **T** | **T** | **C** | **T** | **A** | **T** | **T** | **T** | **T** | **C** | **A** | **C** | **T** | **T** | **T** | **T** | **C** | **A** | **C** | **T** | **T** | **A** | **T** | **T** | **T** | **T** | **T** | **A** | **T** | **G** | **C** | **C** | **A** | **G** | **T** | **A** | **A** | **C** | **C** | **A** | **A** | **A** | **G** | **T** | **C** | **A** | **T** | **T** | **G** | **G** | **T** | **G** | **G** | **G** | **G** | **A** | **G** | **A** | **A** | **T** | **T** | **G** | **C** | **T** | **G** | **C** | **G** | **T** | **G** | **C** | **G** | **C** | **G** | **T** | **T** | **T** | **A** | **T** | **T** | **A** | **T** | **A** | **C** | **G** | **T** | **T** | **C** | **C** | **G** | **C** | **T** | **T** | **A** | **T** | **C** | **T** | **T** | **C** | **C** | **A** | **T** | **T** | **T** | **G** | **A** | **T** | **G** | **G** | **C** | **A** | **A** | **T** | **T** | **T** | **C** | **A** | **T** | **T** | **A** | **G** | **G** | **T** | **G** | **T** | **A** | **G** | **G** | **A** | **T** | **T** | **G** | **A** | **T** | **C** | **G** | **T** | **A** | **T** | **T** | **T** | **C** | **T** | **A** | **T** | **T** | **G** | **T** | **T** | **A** | **T** | **C** | **A** | **C** | **T** | **T** | **A** | **T** | **T** | **C** | **A** | **A** | **A** | **A** | **G** | **G** | **C** | **A** | **A** | **A** | **A** | **T** | **A** | **C** | **G** | **G** | **G** | **A** | **A** | **A** | **A** | **T** | **C** | **G** | **A** | **A** | **G** | **A** | **A** | **A** | **A** | **G** | **C** | **T** | **T** | **C** | **G** | **T** | **G** | **C** | **A** | **C** | **T** | **T** | **T** | **C** | **T** | **T** | **C** | **T** | **G** | **G** | **C** | **A** | **A** | **T** | **T** | **A** | **T** | **G** | **A** | **A** | **T** | **C** | **C** | **A** | **A** | **A** | **C** | **T** | **T** | **T** | **T** | **G** | **C** | **A** | **T** | **C** | **A** | **G** | **C** | **C** | **G** | **A** | **T** | **C** | **C** | **C** | **A** | **A** | **G** | **C** | **G** | **C** | **G** | **T** | **C** | **G** | **G** | **A** | **T** | **G** | **A** | **T** | **C** | **T** | **G** | **T** | **A** | **C** | **A** | **T** | **C** | **A** | **A** | **A** | **G** | **A** | **T** | **A** | **T** | **T** | **G** | **A** | **T** | **A** | **T** | **G** | **G** | **A** | **A** | **A** | **T** | **T** | **A** | **C** | **T** | **A** | **A** | **A** | **A** | **T** | **A** | **A** | **A** | **A** | **G** | **A** | **A** | **A** | **A** | **A** | **A** | **T** | **G** | **A** | **T** | **C** | **G** | **A** | **A** | **G** | **T** | **A** | **T** | **C** | **C** | **A** | **G** | **T** | **G** | **A** | **A** | **T** | **T** | **A** | **C** | **A** | **G** | **A** | **T** | **T** | **A** | **T** | **G** | **A** | **C** | **T** | **A** | **G** | **C** | **C** | **G** | **T** | **C** | **C** | **T** | **C** | **A** | **A** | **T** | **A** | **T** | **G** | **T** | **A** | **G** | **A** | **T** | **G** | **G** | **A** | **C** | **A** | **A** | **A** | **C** | **C** | **A** | **A** | **A** | **G** | **A** | **A** | **G** | **A** | **G** | **A** | **T** | **T** | **T** | **T** | **A** | **G** | **A** | **A** | **T** | **T** | **A** | **G** | **A** | **A** | **A** | **G** | **G** | **C** | **A** | **T** | **C** | **G** | **A** | **T** | **T** | **A** | **G** | **C** | **T** | **C** | **G** | **T** | **G** | **A** | **G** | **C** | **T** | **T** | **C** | **A** | **T** | **G** | **A** | **T** | **T** | **C** | **A** | **G** | **T** | **T** | **T** | **C** | **A** | **C** | **A** | **G** | **C** | **A** | **A** | **T** | **T** | **A** | **T** | **T** | **T** | **G** | **C** | **A** | **G** | **C** | **G** | **A** | **T** | **G** | **A** | **T** | **G** | **A** | **T** | **G** | **A** | **T** | **G** | **T** | **C** | **G** | **G** | **C** | **A** | **T** | **T** | **G** | **A** | **C** | **G** | **G** | **A** | **G** | **C** | **A** | **A** | **G** | **C** | **A** | **G** | **A** | **A** | **A** | **A** | **A** | **A** | **G** | **T** | **G** | **A** | **G** | **A** | **C** | **A** | **C** | **C** | **A** | **G** | **A** | **A** | **A** | **T** | **G** | **T** | **T** | **C** | **C** | **G** | **T** | **A** | **A** | **G** | **C** | **A** | **G** | **T** | **T** | **G** | **A** | **A** | **A** | **A** | **T** | **G** | **G** | **T** | **G** | **G** | **C** | **A** | **G** | **A** | **G** | **A** | **T** | **C** | **A** | **T** | **C** | **A** | **A** | **C** | **G** | **C** | **T** | **T** | **C** | **C** | **C** | **A** | **G** | **T** | **C** | **T** | **G** | **A** | **G** | **A** | **T** | **G** | **C** | **G** | **T** | **G** | **C** | **G** | **C** | **T** | **G** | **C** | **T** | **T** | **C** | **T** | **T** | **C** | **A** | **T** | **C** | **T** | **G** | **C** | **G** | **T** | **C** | **C** | **A** | **G** | **T** | **C** | **A** | **A** | **T** | **T** | **T** | **A** | **G** | **A** | **A** | **G** | **A** | **A** | **A** | **A** | **A** | **A** | **G** | **T** | **T** | **T** | **A** | **A** | **A** | **A** | **C** | **A** | **A** | **G** | **G** | **C** | **A** | **T** | **T** | **G** | **A** | **G** | **C** | **A** | **G** | **T** | **T** | **A** | **T** | **T** | **G** | **A** | **A** | **G** | **G** | **A** | **A** | **T** | **T** | **G** | **C** | **A** | **G** | **A** | **A** | **T** | **A** | **A** | **A** | **A** | **T** | **C** | **C** | **A** | **G** | **A** | **T** | **T** | **T** | **C** | **G** | **C** | **T** | **A** | **A** | **A** | **A** | **T** | **G** | **G** | **G** | **A** | **T** | **G** | **T** | **A** | **G** | **A** | **A** | **G** | **A** | **T** | **G** | **T** | **A** | **A** | **A** | **A** | **T** | **T** | **A** | **A** | **C** | **T** | **A** | **G** | **T** | **T** | **C** | **C** | **A** | **T** | **T** | **G** | **A** | **G** | **G** | **A** | **T** | **C** | **A** | **C** | **T** | **T** | **G** | **T** | **T** | **C** | **C** | **G** | **A** | **A** | **T** | **C** | **G** | **T** | **T** | **C** | **A** | **A** | **G** | **A** | **A** | **T** | **T** | **G** | **T** | **T** | **A** | **T** | **C** | **A** | **A** | **A** | **C** | **A** | **C** | **A** | **T** | **T** | **A** | **A** | **G** | **A** | **C** | **A** | **T** | **G** | **C** | **T** | **A** | **A** | **G** | **G** | **C** | **T** | **A** | **A** | **T** | **G** | **A** | **A** | **T** | **T** | **A** | **G** | **A** | **A** | **G** | **T** | **C** | **T** | **A** | **T** | **T** | **T** | **G** | **C** | **A** | **T** | **A** | **A** | **A** | **A** | **T** | **A** | **G** | **A** | **T** | **A** | **A** | **C** | **A** | **A** | **T** | **C** | **T** | **T** | **T** | **T** | **A** | **T** | **T** | **A** | **C** | **G** | **T** | **A** | **T** | **C** | **A** | **T** | **T** | **G** | **A** | **C** | **G** | **A** | **C** | **G** | **G** | **G** | **A** | **C** | **A** | **G** | **G** | **A** | **T** | **T** | **T** | **A** | **A** | **T** | **A** | **T** | **G** | **A** | **A** | **T** | **G** | **A** | **A** | **A** | **C** | **C** | **A** | **A** | **A** | **A** | **C** | **G** | **G** | **G** | **A** | **A** | **G** | **T** | **T** | **A** | **T** | **G** | **G** | **A** | **T** | **T** | **G** | **A** | **A** | **C** | **A** | **A** | **T** | **A** | **T** | **C** | **A** | **A** | **A** | **G** | **A** | **A** | **C** | **G** | **A** | **G** | **T** | **A** | **G** | **C** | **T** | **G** | **G** | **T** | **A** | **T** | **T** | **G** | **G** | **T** | **G** | **G** | **A** | **A** | **C** | **A** | **G** | **T** | **A** | **A** | **A** | **A** | **A** | **T** | **C** | **A** | **T** | **T** | **A** | **G** | **T** | **T** | **T** | **T** | **A** | **A** | **A** | **G** | **G** | **G** | **C** | **A** | **A** | **G** | **G** | **T** | **A** | **C** | **G** | **A** | **G** | **C** | **G** | **T** | **G** | **G** | **A** | **G** | **A** | **T** | **C** | **A** | **A** | **G** | **G** | **T** | **C** | **C** | **C** | **T** | **T** | **T** | **G** | **A** | **T** | **G** | **A** | **A** | **G** | **G** | **A** | **G** | **G** | **C** | **A** | **-** | **-** | **-** |
| **N59503F** | **A** | **T** | **G** | **A** | **T** | **G** | **G** | **G** | **A** | **A** | **G** | **A** | **A** | **T** | **A** | **T** | **C** | **C** | **A** | **A** | **A** | **G** | **C** | **A** | **A** | **T** | **G** | **C** | **T** | **A** | **G** | **C** | **T** | **G** | **T** | **T** | **T** | **A** | **T** | **T** | **C** | **G** | **G** | **G** | **G** | **A** | **T** | **T** | **G** | **C** | **G** | **G** | **C** | **T** | **T** | **T** | **C** | **C** | **T** | **T** | **A** | **T** | **T** | **A** | **T** | **T** | **C** | **T** | **A** | **T** | **T** | **T** | **T** | **C** | **A** | **C** | **T** | **T** | **T** | **T** | **C** | **A** | **C** | **T** | **T** | **A** | **T** | **T** | **T** | **T** | **T** | **A** | **T** | **G** | **C** | **C** | **A** | **G** | **T** | **A** | **A** | **C** | **C** | **A** | **A** | **A** | **G** | **T** | **C** | **A** | **T** | **T** | **G** | **G** | **T** | **G** | **G** | **G** | **G** | **A** | **G** | **A** | **A** | **T** | **T** | **G** | **C** | **T** | **G** | **C** | **G** | **T** | **G** | **C** | **G** | **C** | **G** | **T** | **T** | **T** | **A** | **T** | **T** | **A** | **T** | **A** | **C** | **G** | **T** | **T** | **C** | **C** | **G** | **C** | **T** | **T** | **A** | **T** | **C** | **T** | **T** | **C** | **C** | **A** | **T** | **T** | **T** | **G** | **A** | **T** | **G** | **G** | **C** | **A** | **A** | **T** | **T** | **T** | **C** | **A** | **T** | **T** | **A** | **G** | **G** | **T** | **G** | **T** | **A** | **G** | **G** | **A** | **T** | **T** | **G** | **A** | **T** | **C** | **G** | **T** | **A** | **T** | **T** | **T** | **C** | **T** | **A** | **T** | **T** | **G** | **T** | **T** | **A** | **T** | **C** | **A** | **C** | **T** | **T** | **A** | **T** | **T** | **C** | **A** | **A** | **A** | **A** | **G** | **A** | **C** | **A** | **A** | **A** | **A** | **T** | **A** | **C** | **G** | **G** | **G** | **A** | **A** | **A** | **A** | **T** | **C** | **G** | **A** | **A** | **G** | **A** | **A** | **A** | **A** | **G** | **C** | **T** | **T** | **C** | **G** | **T** | **G** | **C** | **A** | **C** | **T** | **T** | **T** | **C** | **T** | **T** | **C** | **T** | **G** | **G** | **C** | **A** | **A** | **T** | **T** | **A** | **T** | **G** | **A** | **A** | **T** | **C** | **C** | **A** | **A** | **A** | **C** | **T** | **T** | **T** | **T** | **G** | **C** | **A** | **T** | **C** | **A** | **G** | **C** | **C** | **G** | **A** | **T** | **C** | **C** | **C** | **A** | **A** | **G** | **C** | **G** | **C** | **G** | **T** | **C** | **G** | **G** | **A** | **T** | **G** | **A** | **T** | **C** | **T** | **G** | **T** | **A** | **C** | **A** | **T** | **C** | **A** | **A** | **A** | **G** | **A** | **T** | **A** | **T** | **T** | **G** | **A** | **T** | **A** | **T** | **G** | **G** | **A** | **A** | **A** | **T** | **T** | **A** | **C** | **T** | **A** | **A** | **A** | **A** | **T** | **A** | **A** | **A** | **A** | **G** | **A** | **A** | **A** | **A** | **A** | **A** | **T** | **G** | **A** | **T** | **C** | **G** | **A** | **A** | **G** | **T** | **A** | **T** | **C** | **C** | **A** | **G** | **T** | **G** | **A** | **A** | **T** | **T** | **A** | **C** | **A** | **G** | **A** | **T** | **T** | **A** | **T** | **G** | **A** | **C** | **T** | **A** | **G** | **C** | **C** | **G** | **T** | **C** | **C** | **T** | **C** | **A** | **A** | **T** | **A** | **T** | **G** | **T** | **A** | **G** | **A** | **T** | **G** | **G** | **A** | **C** | **A** | **A** | **A** | **C** | **C** | **A** | **A** | **A** | **G** | **A** | **A** | **G** | **A** | **G** | **A** | **T** | **T** | **T** | **T** | **A** | **G** | **A** | **A** | **T** | **T** | **A** | **G** | **A** | **A** | **A** | **G** | **G** | **C** | **A** | **T** | **C** | **G** | **A** | **T** | **T** | **A** | **G** | **C** | **T** | **C** | **G** | **T** | **G** | **A** | **G** | **C** | **T** | **T** | **C** | **A** | **T** | **G** | **A** | **T** | **T** | **C** | **A** | **G** | **T** | **T** | **T** | **C** | **A** | **C** | **A** | **G** | **C** | **A** | **A** | **T** | **T** | **A** | **T** | **T** | **T** | **G** | **C** | **A** | **G** | **C** | **G** | **A** | **T** | **G** | **A** | **T** | **G** | **A** | **T** | **G** | **A** | **T** | **G** | **T** | **C** | **G** | **G** | **C** | **A** | **T** | **T** | **G** | **A** | **C** | **G** | **G** | **A** | **G** | **C** | **A** | **A** | **G** | **C** | **A** | **G** | **A** | **A** | **A** | **A** | **A** | **A** | **G** | **T** | **G** | **A** | **G** | **A** | **C** | **A** | **C** | **C** | **A** | **G** | **A** | **A** | **A** | **T** | **G** | **T** | **T** | **C** | **C** | **G** | **T** | **A** | **A** | **G** | **C** | **A** | **G** | **T** | **T** | **G** | **A** | **A** | **A** | **A** | **T** | **G** | **G** | **T** | **G** | **G** | **C** | **A** | **G** | **A** | **G** | **A** | **T** | **C** | **A** | **T** | **C** | **A** | **A** | **C** | **G** | **C** | **T** | **T** | **C** | **C** | **C** | **A** | **G** | **T** | **C** | **T** | **G** | **A** | **G** | **A** | **T** | **G** | **C** | **G** | **T** | **G** | **C** | **G** | **C** | **T** | **G** | **C** | **T** | **T** | **C** | **T** | **T** | **C** | **A** | **T** | **C** | **T** | **G** | **C** | **G** | **T** | **C** | **C** | **A** | **G** | **T** | **C** | **A** | **A** | **T** | **T** | **T** | **A** | **G** | **A** | **A** | **G** | **A** | **A** | **A** | **A** | **A** | **A** | **G** | **T** | **T** | **T** | **A** | **A** | **A** | **A** | **C** | **A** | **A** | **G** | **G** | **C** | **A** | **T** | **T** | **G** | **A** | **G** | **C** | **A** | **G** | **T** | **T** | **A** | **T** | **T** | **G** | **A** | **A** | **G** | **G** | **A** | **A** | **T** | **T** | **G** | **C** | **A** | **G** | **A** | **A** | **T** | **A** | **A** | **A** | **A** | **T** | **C** | **C** | **A** | **G** | **A** | **T** | **T** | **T** | **C** | **G** | **C** | **T** | **A** | **A** | **A** | **A** | **T** | **G** | **G** | **G** | **A** | **T** | **G** | **T** | **A** | **G** | **A** | **A** | **G** | **A** | **T** | **G** | **T** | **A** | **A** | **A** | **A** | **T** | **T** | **A** | **A** | **C** | **T** | **A** | **G** | **T** | **T** | **C** | **C** | **A** | **T** | **T** | **G** | **A** | **G** | **G** | **A** | **T** | **C** | **A** | **C** | **T** | **T** | **G** | **T** | **T** | **C** | **C** | **G** | **A** | **A** | **T** | **C** | **G** | **T** | **T** | **C** | **A** | **A** | **G** | **A** | **A** | **T** | **T** | **G** | **T** | **T** | **A** | **T** | **C** | **A** | **A** | **A** | **C** | **A** | **C** | **A** | **T** | **T** | **A** | **A** | **G** | **A** | **C** | **A** | **T** | **G** | **C** | **T** | **A** | **A** | **G** | **G** | **C** | **T** | **A** | **A** | **T** | **G** | **A** | **A** | **T** | **T** | **A** | **G** | **A** | **A** | **G** | **T** | **C** | **T** | **A** | **T** | **T** | **T** | **G** | **C** | **A** | **T** | **A** | **A** | **A** | **A** | **T** | **A** | **G** | **A** | **T** | **A** | **A** | **C** | **A** | **A** | **T** | **C** | **T** | **T** | **T** | **T** | **A** | **T** | **T** | **A** | **C** | **G** | **T** | **A** | **T** | **C** | **A** | **T** | **T** | **G** | **A** | **C** | **G** | **A** | **C** | **G** | **G** | **G** | **A** | **C** | **A** | **G** | **G** | **A** | **T** | **T** | **T** | **A** | **A** | **T** | **A** | **T** | **G** | **A** | **A** | **T** | **G** | **A** | **A** | **A** | **C** | **C** | **A** | **A** | **A** | **A** | **C** | **G** | **G** | **G** | **A** | **A** | **G** | **T** | **T** | **A** | **T** | **G** | **G** | **A** | **T** | **T** | **G** | **A** | **A** | **C** | **A** | **A** | **T** | **A** | **T** | **C** | **A** | **A** | **A** | **G** | **A** | **A** | **C** | **G** | **A** | **G** | **T** | **A** | **G** | **C** | **T** | **G** | **G** | **T** | **A** | **T** | **T** | **G** | **G** | **T** | **G** | **G** | **A** | **A** | **C** | **A** | **G** | **T** | **A** | **A** | **A** | **A** | **A** | **T** | **C** | **A** | **T** | **T** | **A** | **G** | **T** | **T** | **T** | **T** | **A** | **A** | **A** | **G** | **G** | **G** | **C** | **A** | **A** | **G** | **G** | **T** | **A** | **C** | **G** | **A** | **G** | **C** | **G** | **T** | **G** | **G** | **A** | **G** | **A** | **T** | **C** | **A** | **A** | **G** | **G** | **T** | **C** | **C** | **C** | **T** | **T** | **T** | **G** | **A** | **T** | **G** | **A** | **A** | **G** | **G** | **A** | **G** | **G** | **C** | **A** | **-** | **-** | **-** |
| **N59788F** | **A** | **T** | **G** | **A** | **T** | **G** | **G** | **G** | **A** | **A** | **G** | **A** | **A** | **T** | **A** | **T** | **C** | **C** | **A** | **A** | **A** | **G** | **C** | **A** | **A** | **T** | **G** | **C** | **T** | **A** | **G** | **C** | **T** | **G** | **T** | **T** | **T** | **A** | **T** | **T** | **C** | **G** | **G** | **G** | **G** | **A** | **T** | **T** | **G** | **C** | **G** | **G** | **C** | **T** | **T** | **T** | **C** | **C** | **T** | **T** | **A** | **T** | **T** | **A** | **T** | **T** | **C** | **T** | **A** | **T** | **T** | **T** | **T** | **C** | **A** | **C** | **T** | **T** | **T** | **T** | **C** | **A** | **C** | **T** | **T** | **A** | **T** | **T** | **T** | **T** | **T** | **A** | **T** | **G** | **C** | **C** | **A** | **G** | **T** | **A** | **A** | **C** | **C** | **A** | **A** | **A** | **G** | **T** | **C** | **A** | **T** | **T** | **G** | **G** | **T** | **G** | **G** | **G** | **G** | **A** | **G** | **A** | **A** | **T** | **T** | **G** | **C** | **T** | **G** | **C** | **G** | **T** | **G** | **C** | **G** | **C** | **G** | **T** | **T** | **T** | **A** | **T** | **T** | **A** | **T** | **A** | **C** | **G** | **T** | **T** | **C** | **C** | **G** | **C** | **T** | **T** | **A** | **T** | **C** | **T** | **T** | **C** | **C** | **A** | **T** | **T** | **T** | **G** | **A** | **T** | **G** | **G** | **C** | **A** | **A** | **T** | **T** | **T** | **C** | **A** | **T** | **T** | **A** | **G** | **G** | **T** | **G** | **T** | **A** | **G** | **G** | **A** | **T** | **T** | **G** | **A** | **T** | **C** | **G** | **T** | **A** | **T** | **T** | **T** | **C** | **T** | **A** | **T** | **T** | **G** | **T** | **T** | **A** | **T** | **C** | **A** | **C** | **T** | **T** | **A** | **T** | **T** | **C** | **A** | **A** | **A** | **A** | **G** | **G** | **C** | **A** | **A** | **A** | **A** | **T** | **A** | **C** | **G** | **G** | **G** | **A** | **A** | **A** | **A** | **T** | **C** | **G** | **A** | **A** | **G** | **A** | **A** | **A** | **A** | **G** | **C** | **T** | **T** | **C** | **G** | **T** | **G** | **C** | **A** | **C** | **T** | **T** | **T** | **C** | **T** | **T** | **C** | **T** | **G** | **G** | **C** | **A** | **A** | **T** | **T** | **A** | **T** | **G** | **A** | **A** | **T** | **C** | **C** | **A** | **A** | **A** | **C** | **T** | **T** | **T** | **T** | **G** | **C** | **A** | **T** | **C** | **A** | **G** | **C** | **C** | **G** | **A** | **T** | **C** | **C** | **C** | **A** | **A** | **G** | **C** | **G** | **C** | **G** | **T** | **C** | **G** | **G** | **A** | **T** | **G** | **A** | **T** | **C** | **T** | **G** | **T** | **A** | **C** | **A** | **T** | **C** | **A** | **A** | **A** | **G** | **A** | **T** | **A** | **T** | **T** | **G** | **A** | **T** | **A** | **T** | **G** | **G** | **A** | **A** | **A** | **T** | **T** | **A** | **C** | **T** | **A** | **A** | **A** | **A** | **T** | **A** | **A** | **A** | **A** | **G** | **A** | **A** | **A** | **A** | **A** | **A** | **T** | **G** | **A** | **T** | **C** | **G** | **A** | **A** | **G** | **T** | **A** | **T** | **C** | **C** | **A** | **G** | **T** | **G** | **A** | **A** | **T** | **T** | **A** | **C** | **A** | **G** | **A** | **T** | **T** | **A** | **T** | **G** | **A** | **C** | **T** | **A** | **G** | **C** | **C** | **G** | **T** | **C** | **C** | **T** | **C** | **A** | **A** | **T** | **A** | **T** | **G** | **T** | **A** | **G** | **A** | **T** | **G** | **G** | **A** | **C** | **A** | **A** | **A** | **C** | **C** | **A** | **A** | **A** | **G** | **A** | **A** | **G** | **A** | **G** | **A** | **T** | **T** | **T** | **T** | **A** | **G** | **A** | **A** | **T** | **T** | **A** | **G** | **A** | **A** | **A** | **G** | **G** | **C** | **A** | **T** | **C** | **G** | **A** | **T** | **T** | **A** | **G** | **C** | **T** | **C** | **G** | **T** | **G** | **A** | **G** | **C** | **T** | **T** | **C** | **A** | **T** | **G** | **A** | **T** | **T** | **C** | **A** | **G** | **T** | **T** | **T** | **C** | **A** | **C** | **A** | **G** | **C** | **A** | **A** | **T** | **T** | **A** | **T** | **T** | **T** | **G** | **C** | **A** | **G** | **C** | **G** | **A** | **T** | **G** | **A** | **T** | **G** | **A** | **T** | **G** | **A** | **T** | **G** | **T** | **C** | **G** | **G** | **C** | **A** | **T** | **T** | **G** | **A** | **C** | **G** | **G** | **A** | **G** | **C** | **A** | **A** | **G** | **C** | **A** | **G** | **A** | **A** | **A** | **A** | **A** | **A** | **G** | **T** | **G** | **A** | **G** | **A** | **C** | **A** | **C** | **C** | **A** | **G** | **A** | **A** | **A** | **T** | **G** | **T** | **T** | **C** | **C** | **G** | **T** | **A** | **A** | **G** | **C** | **A** | **G** | **T** | **T** | **G** | **A** | **A** | **A** | **A** | **T** | **G** | **G** | **T** | **G** | **G** | **C** | **A** | **G** | **A** | **G** | **A** | **T** | **C** | **A** | **T** | **C** | **A** | **A** | **C** | **G** | **C** | **T** | **T** | **C** | **C** | **C** | **A** | **G** | **T** | **C** | **T** | **G** | **A** | **G** | **A** | **T** | **G** | **C** | **G** | **T** | **G** | **C** | **G** | **C** | **T** | **G** | **C** | **T** | **T** | **C** | **T** | **T** | **C** | **A** | **T** | **C** | **T** | **G** | **C** | **G** | **T** | **C** | **C** | **A** | **G** | **T** | **C** | **A** | **A** | **T** | **T** | **T** | **A** | **G** | **A** | **A** | **G** | **A** | **A** | **A** | **A** | **A** | **A** | **G** | **T** | **T** | **T** | **A** | **A** | **A** | **A** | **C** | **A** | **A** | **G** | **G** | **C** | **A** | **T** | **T** | **G** | **A** | **G** | **C** | **A** | **G** | **T** | **T** | **A** | **T** | **T** | **G** | **A** | **A** | **G** | **G** | **A** | **A** | **T** | **T** | **G** | **C** | **A** | **G** | **A** | **A** | **T** | **A** | **A** | **A** | **A** | **T** | **C** | **C** | **A** | **G** | **A** | **T** | **T** | **T** | **C** | **G** | **C** | **T** | **A** | **A** | **A** | **A** | **T** | **G** | **G** | **G** | **A** | **T** | **G** | **T** | **A** | **G** | **A** | **A** | **G** | **A** | **T** | **G** | **T** | **A** | **A** | **A** | **A** | **T** | **T** | **A** | **A** | **C** | **T** | **A** | **G** | **T** | **T** | **C** | **C** | **A** | **T** | **T** | **G** | **A** | **G** | **G** | **A** | **T** | **C** | **A** | **C** | **T** | **T** | **G** | **T** | **T** | **C** | **C** | **G** | **A** | **A** | **T** | **C** | **G** | **T** | **T** | **C** | **A** | **A** | **G** | **A** | **A** | **T** | **T** | **G** | **T** | **T** | **A** | **T** | **C** | **A** | **A** | **A** | **C** | **A** | **C** | **A** | **T** | **T** | **A** | **A** | **G** | **A** | **C** | **A** | **T** | **G** | **C** | **T** | **A** | **A** | **G** | **G** | **C** | **T** | **A** | **A** | **T** | **G** | **A** | **A** | **T** | **T** | **A** | **G** | **A** | **A** | **G** | **T** | **C** | **T** | **A** | **T** | **T** | **T** | **G** | **C** | **A** | **T** | **A** | **A** | **A** | **A** | **T** | **A** | **G** | **A** | **T** | **A** | **A** | **C** | **A** | **A** | **T** | **C** | **T** | **T** | **T** | **T** | **A** | **T** | **T** | **A** | **C** | **G** | **T** | **A** | **T** | **C** | **A** | **T** | **T** | **G** | **A** | **C** | **G** | **A** | **C** | **G** | **G** | **G** | **A** | **C** | **A** | **G** | **G** | **A** | **T** | **T** | **T** | **A** | **A** | **T** | **A** | **T** | **G** | **A** | **A** | **T** | **G** | **A** | **A** | **A** | **C** | **C** | **A** | **A** | **A** | **A** | **C** | **G** | **G** | **G** | **A** | **A** | **G** | **T** | **T** | **A** | **T** | **G** | **G** | **A** | **T** | **T** | **G** | **A** | **A** | **C** | **A** | **A** | **T** | **A** | **T** | **C** | **A** | **A** | **A** | **G** | **A** | **A** | **C** | **G** | **A** | **G** | **T** | **A** | **G** | **C** | **T** | **G** | **G** | **T** | **A** | **T** | **T** | **G** | **G** | **T** | **G** | **G** | **A** | **A** | **C** | **A** | **G** | **T** | **A** | **A** | **A** | **A** | **A** | **T** | **C** | **A** | **T** | **T** | **A** | **G** | **T** | **T** | **T** | **T** | **A** | **A** | **A** | **G** | **G** | **G** | **C** | **A** | **A** | **G** | **G** | **T** | **A** | **C** | **G** | **A** | **G** | **C** | **G** | **T** | **G** | **G** | **A** | **G** | **A** | **T** | **C** | **A** | **A** | **G** | **G** | **T** | **C** | **C** | **C** | **T** | **T** | **T** | **G** | **A** | **T** | **G** | **A** | **A** | **G** | **G** | **A** | **G** | **G** | **C** | **A** | **-** | **-** | **-** |
| **N52459** | **A** | **T** | **G** | **A** | **T** | **G** | **G** | **G** | **A** | **A** | **A** | **A** | **A** | **T** | **A** | **T** | **C** | **C** | **A** | **G** | **A** | **G** | **C** | **G** | **A** | **T** | **G** | **C** | **T** | **A** | **G** | **C** | **T** | **G** | **T** | **T** | **T** | **A** | **T** | **T** | **C** | **G** | **G** | **G** | **G** | **A** | **T** | **T** | **G** | **C** | **G** | **G** | **C** | **T** | **T** | **T** | **C** | **C** | **T** | **T** | **A** | **T** | **T** | **A** | **T** | **T** | **C** | **T** | **A** | **T** | **T** | **T** | **T** | **C** | **A** | **C** | **T** | **T** | **T** | **T** | **C** | **A** | **C** | **T** | **T** | **A** | **T** | **T** | **T** | **T** | **T** | **A** | **T** | **G** | **C** | **C** | **A** | **G** | **T** | **A** | **A** | **T** | **C** | **A** | **A** | **A** | **G** | **T** | **C** | **A** | **T** | **T** | **G** | **G** | **T** | **G** | **G** | **G** | **G** | **A** | **G** | **A** | **A** | **T** | **T** | **A** | **C** | **T** | **G** | **C** | **G** | **T** | **G** | **C** | **A** | **C** | **G** | **T** | **T** | **T** | **A** | **T** | **T** | **A** | **T** | **A** | **T** | **G** | **T** | **T** | **C** | **C** | **G** | **C** | **T** | **T** | **A** | **T** | **C** | **T** | **T** | **C** | **C** | **A** | **T** | **T** | **T** | **G** | **T** | **T** | **A** | **G** | **C** | **T** | **A** | **T** | **T** | **T** | **C** | **C** | **T** | **T** | **A** | **G** | **G** | **T** | **G** | **T** | **A** | **G** | **G** | **A** | **T** | **T** | **A** | **A** | **T** | **C** | **G** | **T** | **C** | **T** | **T** | **T** | **C** | **T** | **A** | **T** | **T** | **A** | **T** | **T** | **A** | **T** | **C** | **A** | **C** | **T** | **T** | **A** | **T** | **T** | **C** | **A** | **A** | **A** | **A** | **G** | **A** | **C** | **A** | **A** | **A** | **A** | **T** | **A** | **C** | **G** | **G** | **G** | **A** | **A** | **A** | **A** | **T** | **C** | **G** | **A** | **A** | **G** | **A** | **A** | **A** | **A** | **G** | **C** | **T** | **G** | **C** | **G** | **T** | **G** | **C** | **A** | **C** | **T** | **C** | **T** | **C** | **T** | **T** | **C** | **T** | **G** | **G** | **C** | **A** | **A** | **T** | **T** | **A** | **T** | **G** | **A** | **A** | **T** | **C** | **C** | **A** | **A** | **A** | **T** | **T** | **G** | **T** | **T** | **G** | **C** | **T** | **T** | **C** | **T** | **T** | **C** | **C** | **G** | **A** | **T** | **C** | **C** | **C** | **A** | **A** | **G** | **T** | **G** | **C** | **A** | **T** | **C** | **G** | **G** | **A** | **C** | **G** | **A** | **T** | **T** | **T** | **A** | **T** | **A** | **C** | **A** | **T** | **C** | **A** | **A** | **A** | **G** | **A** | **T** | **A** | **T** | **T** | **G** | **A** | **T** | **A** | **A** | **G** | **G** | **A** | **A** | **A** | **T** | **C** | **A** | **C** | **T** | **A** | **A** | **A** | **A** | **T** | **A** | **A** | **A** | **A** | **G** | **A** | **A** | **A** | **A** | **A** | **A** | **T** | **G** | **A** | **T** | **T** | **G** | **A** | **A** | **G** | **T** | **A** | **T** | **C** | **T** | **A** | **G** | **T** | **G** | **A** | **A** | **T** | **T** | **A** | **C** | **A** | **A** | **A** | **T** | **T** | **G** | **T** | **A** | **A** | **C** | **G** | **A** | **G** | **C** | **C** | **G** | **T** | **C** | **C** | **C** | **C** | **A** | **A** | **T** | **A** | **T** | **G** | **T** | **A** | **G** | **A** | **T** | **G** | **G** | **A** | **C** | **A** | **A** | **A** | **C** | **C** | **A** | **A** | **A** | **G** | **A** | **A** | **G** | **A** | **A** | **A** | **T** | **T** | **T** | **T** | **A** | **G** | **A** | **A** | **T** | **T** | **A** | **G** | **A** | **A** | **A** | **G** | **A** | **C** | **A** | **T** | **C** | **G** | **A** | **T** | **T** | **A** | **G** | **C** | **C** | **C** | **G** | **T** | **G** | **A** | **G** | **C** | **T** | **G** | **C** | **A** | **T** | **G** | **A** | **T** | **T** | **C** | **A** | **G** | **T** | **T** | **T** | **C** | **G** | **C** | **A** | **A** | **C** | **A** | **A** | **T** | **T** | **G** | **T** | **T** | **T** | **G** | **C** | **A** | **G** | **C** | **A** | **A** | **T** | **G** | **A** | **T** | **G** | **A** | **T** | **G** | **A** | **T** | **G** | **T** | **C** | **G** | **G** | **C** | **A** | **T** | **T** | **A** | **A** | **C** | **G** | **G** | **A** | **G** | **C** | **A** | **A** | **G** | **C** | **A** | **G** | **A** | **A** | **A** | **A** | **A** | **A** | **G** | **C** | **G** | **A** | **G** | **A** | **C** | **A** | **C** | **C** | **A** | **G** | **A** | **A** | **A** | **T** | **G** | **T** | **T** | **C** | **C** | **G** | **C** | **A** | **A** | **G** | **C** | **A** | **G** | **T** | **T** | **G** | **A** | **A** | **A** | **A** | **T** | **G** | **G** | **T** | **A** | **G** | **C** | **A** | **G** | **A** | **G** | **A** | **T** | **C** | **A** | **T** | **C** | **A** | **A** | **C** | **G** | **C** | **T** | **T** | **C** | **C** | **C** | **A** | **G** | **T** | **C** | **T** | **G** | **A** | **G** | **A** | **T** | **G** | **C** | **G** | **T** | **G** | **C** | **G** | **C** | **T** | **G** | **C** | **T** | **T** | **C** | **T** | **T** | **C** | **A** | **T** | **C** | **T** | **G** | **C** | **G** | **T** | **C** | **C** | **A** | **G** | **T** | **C** | **A** | **A** | **T** | **T** | **T** | **A** | **G** | **A** | **A** | **G** | **A** | **G** | **A** | **A** | **A** | **A** | **G** | **T** | **T** | **T** | **A** | **A** | **A** | **G** | **C** | **A** | **A** | **G** | **G** | **T** | **A** | **T** | **C** | **G** | **A** | **G** | **C** | **A** | **G** | **T** | **T** | **A** | **T** | **T** | **G** | **A** | **A** | **G** | **G** | **A** | **A** | **T** | **T** | **G** | **C** | **A** | **A** | **A** | **A** | **T** | **A** | **A** | **A** | **A** | **T** | **C** | **C** | **A** | **G** | **A** | **T** | **T** | **T** | **C** | **G** | **C** | **T** | **G** | **A** | **A** | **A** | **T** | **G** | **G** | **G** | **A** | **T** | **G** | **T** | **A** | **G** | **A** | **A** | **G** | **A** | **T** | **G** | **T** | **A** | **A** | **A** | **A** | **C** | **T** | **A** | **T** | **C** | **T** | **A** | **G** | **T** | **T** | **C** | **C** | **A** | **T** | **T** | **G** | **A** | **G** | **G** | **A** | **T** | **C** | **A** | **C** | **T** | **T** | **G** | **T** | **T** | **C** | **C** | **G** | **A** | **A** | **T** | **C** | **G** | **T** | **T** | **C** | **A** | **A** | **G** | **A** | **A** | **T** | **T** | **G** | **T** | **T** | **A** | **T** | **C** | **A** | **A** | **A** | **T** | **A** | **C** | **A** | **T** | **T** | **A** | **A** | **G** | **A** | **C** | **A** | **T** | **G** | **C** | **C** | **A** | **A** | **A** | **G** | **C** | **C** | **A** | **A** | **T** | **G** | **A** | **A** | **T** | **T** | **A** | **G** | **A** | **G** | **G** | **T** | **A** | **T** | **A** | **T** | **T** | **T** | **G** | **C** | **A** | **C** | **A** | **A** | **A** | **A** | **T** | **A** | **G** | **A** | **C** | **A** | **A** | **T** | **A** | **A** | **T** | **C** | **T** | **T** | **T** | **T** | **G** | **T** | **T** | **A** | **C** | **G** | **T** | **A** | **T** | **T** | **A** | **T** | **T** | **G** | **A** | **C** | **G** | **A** | **C** | **G** | **G** | **A** | **A** | **C** | **A** | **G** | **G** | **G** | **T** | **T** | **T** | **A** | **A** | **T** | **A** | **T** | **G** | **A** | **A** | **T** | **G** | **A** | **A** | **A** | **C** | **G** | **A** | **A** | **A** | **A** | **C** | **G** | **G** | **G** | **A** | **A** | **G** | **T** | **T** | **A** | **T** | **G** | **G** | **A** | **T** | **T** | **G** | **A** | **A** | **C** | **A** | **A** | **T** | **A** | **T** | **C** | **A** | **A** | **A** | **G** | **A** | **A** | **C** | **G** | **A** | **G** | **T** | **A** | **G** | **C** | **T** | **G** | **G** | **T** | **A** | **T** | **T** | **G** | **G** | **T** | **G** | **G** | **A** | **A** | **C** | **A** | **G** | **T** | **A** | **A** | **A** | **A** | **A** | **T** | **C** | **A** | **T** | **T** | **A** | **G** | **T** | **T** | **T** | **T** | **A** | **A** | **A** | **G** | **G** | **G** | **C** | **A** | **A** | **G** | **G** | **T** | **A** | **C** | **A** | **A** | **G** | **T** | **G** | **T** | **A** | **G** | **A** | **A** | **A** | **T** | **C** | **A** | **A** | **G** | **G** | **T** | **C** | **C** | **C** | **T** | **T** | **T** | **G** | **A** | **T** | **G** | **A** | **A** | **G** | **G** | **A** | **G** | **G** | **C** | **A** | **-** | **-** | **-** |
| **N54567** | **A** | **T** | **G** | **A** | **T** | **G** | **G** | **G** | **A** | **A** | **A** | **A** | **A** | **T** | **A** | **T** | **C** | **C** | **A** | **G** | **A** | **G** | **C** | **G** | **A** | **T** | **G** | **C** | **T** | **A** | **G** | **C** | **T** | **G** | **T** | **T** | **T** | **A** | **T** | **T** | **C** | **G** | **G** | **G** | **G** | **A** | **T** | **T** | **G** | **C** | **G** | **G** | **C** | **T** | **T** | **T** | **C** | **C** | **T** | **T** | **A** | **T** | **T** | **A** | **T** | **A** | **C** | **T** | **A** | **T** | **T** | **T** | **T** | **C** | **A** | **C** | **T** | **T** | **T** | **T** | **C** | **A** | **C** | **T** | **T** | **A** | **T** | **T** | **T** | **T** | **T** | **A** | **T** | **G** | **C** | **C** | **A** | **G** | **T** | **A** | **A** | **T** | **C** | **A** | **A** | **A** | **G** | **T** | **C** | **A** | **T** | **T** | **G** | **G** | **T** | **G** | **G** | **G** | **G** | **A** | **G** | **A** | **A** | **T** | **T** | **A** | **C** | **T** | **G** | **C** | **G** | **T** | **G** | **C** | **A** | **C** | **G** | **T** | **T** | **T** | **A** | **T** | **T** | **A** | **T** | **A** | **T** | **G** | **T** | **T** | **C** | **C** | **G** | **C** | **T** | **T** | **A** | **T** | **C** | **T** | **T** | **C** | **C** | **A** | **T** | **T** | **T** | **G** | **T** | **T** | **A** | **G** | **C** | **T** | **A** | **T** | **T** | **T** | **C** | **C** | **T** | **T** | **A** | **G** | **G** | **T** | **G** | **T** | **A** | **G** | **G** | **A** | **T** | **T** | **A** | **A** | **T** | **C** | **G** | **T** | **C** | **T** | **T** | **T** | **C** | **T** | **A** | **T** | **T** | **A** | **T** | **T** | **A** | **T** | **C** | **A** | **C** | **T** | **T** | **A** | **T** | **T** | **C** | **A** | **A** | **A** | **A** | **G** | **A** | **C** | **A** | **A** | **A** | **A** | **T** | **A** | **C** | **G** | **G** | **G** | **A** | **A** | **A** | **A** | **T** | **C** | **G** | **A** | **A** | **G** | **A** | **A** | **A** | **A** | **G** | **C** | **T** | **G** | **C** | **G** | **T** | **G** | **C** | **A** | **C** | **T** | **C** | **T** | **C** | **T** | **T** | **C** | **T** | **G** | **G** | **C** | **A** | **A** | **T** | **T** | **A** | **T** | **G** | **A** | **A** | **T** | **C** | **C** | **A** | **A** | **A** | **T** | **T** | **G** | **T** | **T** | **G** | **C** | **T** | **T** | **C** | **T** | **T** | **C** | **C** | **G** | **A** | **T** | **C** | **C** | **C** | **A** | **A** | **G** | **T** | **G** | **C** | **A** | **T** | **C** | **G** | **G** | **A** | **C** | **G** | **A** | **T** | **T** | **T** | **A** | **T** | **A** | **C** | **A** | **T** | **C** | **A** | **A** | **A** | **G** | **A** | **T** | **A** | **T** | **T** | **G** | **A** | **T** | **A** | **A** | **G** | **G** | **A** | **A** | **A** | **T** | **C** | **A** | **C** | **T** | **A** | **A** | **A** | **A** | **T** | **A** | **A** | **A** | **A** | **G** | **A** | **A** | **A** | **A** | **A** | **A** | **T** | **G** | **A** | **T** | **T** | **G** | **A** | **A** | **G** | **T** | **A** | **T** | **C** | **T** | **A** | **G** | **T** | **G** | **A** | **A** | **T** | **T** | **A** | **C** | **A** | **A** | **A** | **T** | **T** | **G** | **T** | **A** | **A** | **C** | **G** | **A** | **G** | **C** | **C** | **G** | **T** | **C** | **C** | **C** | **C** | **A** | **A** | **T** | **A** | **T** | **G** | **T** | **A** | **G** | **A** | **T** | **G** | **G** | **A** | **C** | **A** | **A** | **A** | **C** | **C** | **A** | **A** | **A** | **G** | **A** | **A** | **G** | **A** | **A** | **A** | **T** | **T** | **T** | **T** | **A** | **G** | **A** | **A** | **T** | **T** | **A** | **G** | **A** | **A** | **A** | **G** | **A** | **C** | **A** | **T** | **C** | **G** | **A** | **T** | **T** | **A** | **G** | **C** | **C** | **C** | **G** | **T** | **G** | **A** | **G** | **C** | **T** | **G** | **C** | **A** | **T** | **G** | **A** | **T** | **T** | **C** | **A** | **G** | **T** | **T** | **T** | **C** | **G** | **C** | **A** | **A** | **C** | **A** | **A** | **T** | **T** | **G** | **T** | **T** | **T** | **G** | **C** | **A** | **G** | **C** | **A** | **A** | **T** | **G** | **A** | **T** | **G** | **A** | **T** | **G** | **A** | **T** | **G** | **T** | **C** | **G** | **G** | **C** | **A** | **T** | **T** | **A** | **A** | **C** | **G** | **G** | **A** | **G** | **C** | **A** | **A** | **G** | **C** | **A** | **G** | **A** | **A** | **A** | **A** | **A** | **A** | **G** | **C** | **G** | **A** | **G** | **A** | **C** | **A** | **C** | **C** | **A** | **G** | **A** | **A** | **A** | **T** | **G** | **T** | **T** | **C** | **C** | **G** | **C** | **A** | **A** | **G** | **C** | **A** | **G** | **T** | **T** | **G** | **A** | **A** | **A** | **A** | **T** | **G** | **G** | **T** | **A** | **G** | **C** | **A** | **G** | **A** | **G** | **A** | **T** | **C** | **A** | **T** | **C** | **A** | **A** | **C** | **G** | **C** | **T** | **T** | **C** | **C** | **C** | **A** | **G** | **T** | **C** | **T** | **G** | **A** | **G** | **A** | **T** | **G** | **C** | **G** | **T** | **G** | **C** | **G** | **C** | **T** | **G** | **C** | **T** | **T** | **C** | **T** | **T** | **C** | **A** | **T** | **C** | **T** | **G** | **C** | **G** | **T** | **C** | **C** | **A** | **G** | **T** | **C** | **A** | **A** | **T** | **T** | **T** | **A** | **G** | **A** | **A** | **G** | **A** | **G** | **A** | **A** | **A** | **A** | **G** | **T** | **T** | **T** | **A** | **A** | **A** | **G** | **C** | **A** | **A** | **G** | **G** | **T** | **A** | **T** | **C** | **G** | **A** | **G** | **C** | **A** | **G** | **T** | **T** | **A** | **T** | **T** | **G** | **A** | **A** | **G** | **G** | **A** | **A** | **T** | **T** | **G** | **C** | **A** | **A** | **A** | **A** | **T** | **A** | **A** | **A** | **A** | **T** | **C** | **C** | **A** | **G** | **A** | **T** | **T** | **T** | **C** | **G** | **C** | **T** | **G** | **A** | **A** | **A** | **T** | **G** | **G** | **G** | **A** | **T** | **G** | **T** | **A** | **G** | **A** | **A** | **G** | **A** | **T** | **G** | **T** | **A** | **A** | **A** | **A** | **C** | **T** | **A** | **T** | **C** | **T** | **A** | **G** | **T** | **T** | **C** | **C** | **A** | **T** | **T** | **G** | **A** | **G** | **G** | **A** | **T** | **C** | **A** | **C** | **T** | **T** | **G** | **T** | **T** | **C** | **C** | **G** | **A** | **A** | **T** | **C** | **G** | **T** | **T** | **C** | **A** | **A** | **G** | **A** | **A** | **T** | **T** | **G** | **T** | **T** | **A** | **T** | **C** | **A** | **A** | **A** | **T** | **A** | **C** | **A** | **T** | **T** | **A** | **A** | **G** | **A** | **C** | **A** | **T** | **G** | **C** | **T** | **A** | **A** | **G** | **G** | **C** | **T** | **A** | **A** | **T** | **G** | **A** | **A** | **T** | **T** | **A** | **G** | **A** | **A** | **G** | **T** | **C** | **T** | **A** | **T** | **T** | **T** | **G** | **C** | **A** | **T** | **A** | **A** | **A** | **A** | **T** | **A** | **G** | **A** | **T** | **A** | **A** | **C** | **A** | **A** | **T** | **C** | **T** | **T** | **T** | **T** | **A** | **T** | **T** | **A** | **C** | **G** | **T** | **A** | **T** | **C** | **A** | **T** | **T** | **G** | **A** | **C** | **G** | **A** | **C** | **G** | **G** | **G** | **A** | **C** | **A** | **G** | **G** | **A** | **T** | **T** | **T** | **A** | **A** | **T** | **A** | **T** | **G** | **A** | **A** | **T** | **G** | **A** | **A** | **A** | **C** | **C** | **A** | **A** | **A** | **A** | **C** | **G** | **G** | **G** | **A** | **A** | **G** | **T** | **T** | **A** | **T** | **G** | **G** | **A** | **T** | **T** | **G** | **A** | **A** | **C** | **A** | **A** | **T** | **A** | **T** | **C** | **A** | **A** | **A** | **G** | **A** | **A** | **C** | **G** | **A** | **G** | **T** | **A** | **G** | **C** | **T** | **G** | **G** | **T** | **A** | **T** | **T** | **G** | **G** | **T** | **G** | **G** | **A** | **A** | **C** | **A** | **G** | **T** | **A** | **A** | **A** | **A** | **A** | **T** | **C** | **A** | **T** | **T** | **A** | **G** | **T** | **T** | **T** | **T** | **A** | **A** | **A** | **G** | **G** | **G** | **C** | **A** | **A** | **G** | **G** | **T** | **A** | **C** | **G** | **A** | **G** | **C** | **G** | **T** | **G** | **G** | **A** | **G** | **A** | **T** | **C** | **A** | **A** | **G** | **G** | **T** | **C** | **C** | **C** | **T** | **T** | **T** | **G** | **A** | **T** | **G** | **A** | **A** | **G** | **G** | **A** | **G** | **G** | **C** | **A** | **-** | **-** | **-** |
| **N59435F** | **A** | **T** | **G** | **A** | **T** | **G** | **G** | **G** | **A** | **A** | **G** | **A** | **A** | **T** | **A** | **T** | **C** | **C** | **A** | **A** | **A** | **G** | **C** | **A** | **A** | **T** | **G** | **C** | **T** | **A** | **G** | **C** | **T** | **G** | **T** | **T** | **T** | **A** | **T** | **T** | **C** | **G** | **G** | **G** | **G** | **A** | **T** | **T** | **G** | **C** | **G** | **G** | **C** | **T** | **T** | **T** | **C** | **C** | **T** | **T** | **A** | **T** | **T** | **A** | **T** | **T** | **C** | **T** | **A** | **T** | **T** | **T** | **T** | **C** | **A** | **C** | **T** | **T** | **T** | **T** | **C** | **A** | **C** | **T** | **T** | **A** | **T** | **T** | **T** | **T** | **T** | **A** | **T** | **G** | **C** | **C** | **A** | **G** | **T** | **A** | **A** | **C** | **C** | **A** | **A** | **A** | **G** | **T** | **C** | **A** | **T** | **T** | **G** | **G** | **T** | **G** | **G** | **G** | **G** | **A** | **G** | **A** | **A** | **T** | **T** | **G** | **C** | **T** | **G** | **C** | **G** | **T** | **G** | **C** | **G** | **C** | **G** | **T** | **T** | **T** | **A** | **T** | **T** | **A** | **T** | **A** | **C** | **G** | **T** | **T** | **C** | **C** | **G** | **C** | **T** | **T** | **A** | **T** | **C** | **T** | **T** | **C** | **C** | **A** | **T** | **T** | **T** | **G** | **A** | **T** | **G** | **G** | **C** | **A** | **A** | **T** | **T** | **T** | **C** | **A** | **T** | **T** | **A** | **G** | **G** | **T** | **G** | **T** | **A** | **G** | **G** | **A** | **T** | **T** | **G** | **A** | **T** | **C** | **G** | **T** | **A** | **T** | **T** | **T** | **C** | **T** | **A** | **T** | **T** | **G** | **T** | **T** | **A** | **T** | **C** | **A** | **C** | **T** | **T** | **A** | **T** | **T** | **C** | **A** | **A** | **A** | **A** | **G** | **A** | **C** | **A** | **A** | **A** | **A** | **T** | **A** | **C** | **G** | **G** | **G** | **A** | **A** | **A** | **A** | **T** | **C** | **G** | **A** | **A** | **G** | **A** | **A** | **A** | **A** | **G** | **C** | **T** | **T** | **C** | **G** | **T** | **G** | **C** | **A** | **C** | **T** | **T** | **T** | **C** | **T** | **T** | **C** | **T** | **G** | **G** | **C** | **A** | **A** | **T** | **T** | **A** | **T** | **G** | **A** | **A** | **T** | **C** | **C** | **A** | **A** | **A** | **C** | **T** | **T** | **T** | **T** | **G** | **C** | **A** | **T** | **C** | **A** | **G** | **C** | **C** | **G** | **A** | **T** | **C** | **C** | **C** | **A** | **A** | **G** | **C** | **G** | **C** | **G** | **T** | **C** | **G** | **G** | **A** | **T** | **G** | **A** | **T** | **C** | **T** | **G** | **T** | **A** | **C** | **A** | **T** | **C** | **A** | **A** | **A** | **G** | **A** | **T** | **A** | **T** | **T** | **G** | **A** | **T** | **A** | **T** | **G** | **G** | **A** | **A** | **A** | **T** | **T** | **A** | **C** | **T** | **A** | **A** | **A** | **A** | **T** | **A** | **A** | **A** | **A** | **G** | **A** | **A** | **A** | **A** | **A** | **A** | **T** | **G** | **A** | **T** | **C** | **G** | **A** | **A** | **G** | **T** | **A** | **T** | **C** | **C** | **A** | **G** | **T** | **G** | **A** | **A** | **T** | **T** | **A** | **C** | **A** | **G** | **A** | **T** | **T** | **A** | **T** | **G** | **A** | **C** | **T** | **A** | **G** | **C** | **C** | **G** | **T** | **C** | **C** | **T** | **C** | **A** | **A** | **T** | **A** | **T** | **G** | **T** | **A** | **G** | **A** | **T** | **G** | **G** | **A** | **C** | **A** | **A** | **A** | **C** | **C** | **A** | **A** | **A** | **G** | **A** | **A** | **G** | **A** | **G** | **A** | **T** | **T** | **T** | **T** | **A** | **G** | **A** | **A** | **T** | **T** | **A** | **G** | **A** | **A** | **A** | **G** | **G** | **C** | **A** | **T** | **C** | **G** | **A** | **T** | **T** | **A** | **G** | **C** | **T** | **C** | **G** | **T** | **G** | **A** | **G** | **C** | **T** | **T** | **C** | **A** | **T** | **G** | **A** | **T** | **T** | **C** | **A** | **G** | **T** | **T** | **T** | **C** | **A** | **C** | **A** | **G** | **C** | **A** | **A** | **T** | **T** | **A** | **T** | **T** | **T** | **G** | **C** | **A** | **G** | **C** | **G** | **A** | **T** | **G** | **A** | **T** | **G** | **A** | **T** | **G** | **A** | **T** | **G** | **T** | **C** | **G** | **G** | **C** | **A** | **T** | **T** | **G** | **A** | **C** | **G** | **G** | **A** | **G** | **C** | **A** | **A** | **G** | **C** | **A** | **G** | **A** | **A** | **A** | **A** | **A** | **A** | **G** | **T** | **G** | **A** | **G** | **A** | **C** | **A** | **C** | **C** | **A** | **G** | **A** | **A** | **A** | **T** | **G** | **T** | **T** | **C** | **C** | **G** | **T** | **A** | **A** | **G** | **C** | **A** | **G** | **T** | **T** | **G** | **A** | **A** | **A** | **A** | **T** | **G** | **G** | **T** | **G** | **G** | **C** | **A** | **G** | **A** | **G** | **A** | **T** | **C** | **A** | **T** | **C** | **A** | **A** | **C** | **G** | **C** | **T** | **T** | **C** | **C** | **C** | **A** | **G** | **T** | **C** | **T** | **G** | **A** | **G** | **A** | **T** | **G** | **C** | **G** | **T** | **G** | **C** | **G** | **C** | **T** | **G** | **C** | **T** | **T** | **C** | **T** | **T** | **C** | **A** | **T** | **C** | **T** | **G** | **C** | **G** | **T** | **C** | **C** | **A** | **G** | **T** | **C** | **A** | **A** | **T** | **T** | **T** | **A** | **G** | **A** | **A** | **G** | **A** | **A** | **A** | **A** | **A** | **A** | **G** | **T** | **T** | **T** | **A** | **A** | **A** | **A** | **C** | **A** | **A** | **G** | **G** | **C** | **A** | **T** | **T** | **G** | **A** | **G** | **C** | **A** | **G** | **T** | **T** | **A** | **T** | **T** | **G** | **A** | **A** | **G** | **G** | **A** | **A** | **T** | **T** | **G** | **C** | **A** | **G** | **A** | **A** | **T** | **A** | **A** | **A** | **A** | **T** | **C** | **C** | **A** | **G** | **A** | **T** | **T** | **T** | **C** | **G** | **C** | **T** | **A** | **A** | **A** | **A** | **T** | **G** | **G** | **G** | **A** | **T** | **G** | **T** | **A** | **G** | **A** | **A** | **G** | **A** | **T** | **G** | **T** | **A** | **A** | **A** | **A** | **T** | **T** | **A** | **A** | **C** | **T** | **A** | **G** | **T** | **T** | **C** | **C** | **A** | **T** | **T** | **G** | **A** | **G** | **G** | **A** | **T** | **C** | **A** | **C** | **T** | **T** | **G** | **T** | **T** | **C** | **C** | **G** | **A** | **A** | **T** | **C** | **G** | **T** | **T** | **C** | **A** | **A** | **G** | **A** | **A** | **T** | **T** | **G** | **T** | **T** | **A** | **T** | **C** | **A** | **A** | **A** | **C** | **A** | **C** | **A** | **T** | **T** | **A** | **A** | **G** | **A** | **C** | **A** | **T** | **G** | **C** | **T** | **A** | **A** | **G** | **G** | **C** | **T** | **A** | **A** | **T** | **G** | **A** | **A** | **T** | **T** | **A** | **G** | **A** | **A** | **G** | **T** | **C** | **T** | **A** | **T** | **T** | **T** | **G** | **C** | **A** | **T** | **A** | **A** | **A** | **A** | **T** | **A** | **G** | **A** | **T** | **A** | **A** | **C** | **A** | **A** | **T** | **C** | **T** | **T** | **T** | **T** | **A** | **T** | **T** | **A** | **C** | **G** | **T** | **A** | **T** | **C** | **A** | **T** | **T** | **G** | **A** | **C** | **G** | **A** | **C** | **G** | **G** | **G** | **A** | **C** | **A** | **G** | **G** | **A** | **T** | **T** | **T** | **A** | **A** | **T** | **A** | **T** | **G** | **A** | **A** | **T** | **G** | **A** | **A** | **A** | **C** | **C** | **A** | **A** | **A** | **A** | **C** | **G** | **G** | **G** | **A** | **A** | **G** | **T** | **T** | **A** | **T** | **G** | **G** | **A** | **T** | **T** | **G** | **A** | **A** | **C** | **A** | **A** | **T** | **A** | **T** | **C** | **A** | **A** | **A** | **G** | **A** | **A** | **C** | **G** | **A** | **G** | **T** | **A** | **G** | **C** | **T** | **G** | **G** | **T** | **A** | **T** | **T** | **G** | **G** | **T** | **G** | **G** | **A** | **A** | **C** | **A** | **G** | **T** | **A** | **A** | **A** | **A** | **A** | **T** | **C** | **A** | **T** | **T** | **A** | **G** | **T** | **T** | **T** | **T** | **A** | **A** | **A** | **G** | **G** | **G** | **C** | **A** | **A** | **G** | **G** | **T** | **A** | **C** | **G** | **A** | **G** | **C** | **G** | **T** | **G** | **G** | **A** | **G** | **A** | **T** | **C** | **A** | **A** | **G** | **G** | **T** | **C** | **C** | **C** | **T** | **T** | **T** | **G** | **A** | **T** | **G** | **A** | **A** | **G** | **G** | **A** | **G** | **G** | **C** | **A** | **-** | **-** | **-** |
| **N59779F** | **A** | **T** | **G** | **A** | **T** | **G** | **G** | **G** | **A** | **A** | **G** | **A** | **A** | **T** | **A** | **T** | **C** | **C** | **A** | **A** | **A** | **G** | **C** | **A** | **A** | **T** | **G** | **C** | **T** | **A** | **G** | **C** | **T** | **G** | **T** | **T** | **T** | **A** | **T** | **T** | **C** | **G** | **G** | **G** | **G** | **A** | **T** | **T** | **G** | **C** | **G** | **G** | **C** | **T** | **T** | **T** | **C** | **C** | **T** | **T** | **A** | **T** | **T** | **A** | **T** | **T** | **C** | **T** | **A** | **T** | **T** | **T** | **T** | **C** | **A** | **C** | **T** | **T** | **T** | **T** | **C** | **A** | **C** | **T** | **T** | **A** | **T** | **T** | **T** | **T** | **T** | **A** | **T** | **G** | **C** | **C** | **A** | **G** | **T** | **A** | **A** | **C** | **C** | **A** | **A** | **A** | **G** | **T** | **C** | **A** | **T** | **T** | **G** | **G** | **T** | **G** | **G** | **G** | **G** | **A** | **G** | **A** | **A** | **T** | **T** | **G** | **C** | **T** | **G** | **C** | **G** | **T** | **G** | **C** | **G** | **C** | **G** | **T** | **T** | **T** | **A** | **T** | **T** | **A** | **T** | **A** | **C** | **G** | **T** | **T** | **C** | **C** | **G** | **C** | **T** | **T** | **A** | **T** | **C** | **T** | **T** | **C** | **C** | **A** | **T** | **T** | **T** | **G** | **A** | **T** | **G** | **G** | **C** | **A** | **A** | **T** | **T** | **T** | **C** | **A** | **T** | **T** | **A** | **G** | **G** | **T** | **G** | **T** | **A** | **G** | **G** | **A** | **T** | **T** | **G** | **A** | **T** | **C** | **G** | **T** | **A** | **T** | **T** | **T** | **C** | **T** | **A** | **T** | **T** | **G** | **T** | **T** | **A** | **T** | **C** | **A** | **C** | **T** | **T** | **A** | **T** | **T** | **C** | **A** | **A** | **A** | **A** | **G** | **G** | **C** | **A** | **A** | **A** | **A** | **T** | **A** | **C** | **G** | **G** | **G** | **A** | **A** | **A** | **A** | **T** | **C** | **G** | **A** | **A** | **G** | **A** | **A** | **A** | **A** | **G** | **C** | **T** | **T** | **C** | **G** | **T** | **G** | **C** | **A** | **C** | **T** | **T** | **T** | **C** | **T** | **T** | **C** | **T** | **G** | **G** | **C** | **A** | **A** | **T** | **T** | **A** | **T** | **G** | **A** | **A** | **T** | **C** | **C** | **A** | **A** | **A** | **C** | **T** | **T** | **T** | **T** | **G** | **C** | **A** | **T** | **C** | **A** | **G** | **C** | **C** | **G** | **A** | **T** | **C** | **C** | **C** | **A** | **A** | **G** | **C** | **G** | **C** | **G** | **T** | **C** | **G** | **G** | **A** | **T** | **G** | **A** | **T** | **C** | **T** | **G** | **T** | **A** | **C** | **A** | **T** | **C** | **A** | **A** | **A** | **G** | **A** | **T** | **A** | **T** | **T** | **G** | **A** | **T** | **A** | **T** | **G** | **G** | **A** | **A** | **A** | **T** | **T** | **A** | **C** | **T** | **A** | **A** | **A** | **A** | **T** | **A** | **A** | **A** | **A** | **G** | **A** | **A** | **A** | **A** | **A** | **A** | **T** | **G** | **A** | **T** | **C** | **G** | **A** | **A** | **G** | **T** | **A** | **T** | **C** | **C** | **A** | **G** | **T** | **G** | **A** | **A** | **T** | **T** | **A** | **C** | **A** | **G** | **A** | **T** | **T** | **A** | **T** | **G** | **A** | **C** | **T** | **A** | **G** | **C** | **C** | **G** | **T** | **C** | **C** | **T** | **C** | **A** | **A** | **T** | **A** | **T** | **G** | **T** | **A** | **G** | **A** | **T** | **G** | **G** | **A** | **C** | **A** | **A** | **A** | **C** | **C** | **A** | **A** | **A** | **G** | **A** | **A** | **G** | **A** | **G** | **A** | **T** | **T** | **T** | **T** | **A** | **G** | **A** | **A** | **T** | **T** | **A** | **G** | **A** | **A** | **A** | **G** | **G** | **C** | **A** | **T** | **C** | **G** | **A** | **T** | **T** | **A** | **G** | **C** | **T** | **C** | **G** | **T** | **G** | **A** | **G** | **C** | **T** | **T** | **C** | **A** | **T** | **G** | **A** | **T** | **T** | **C** | **A** | **G** | **T** | **T** | **T** | **C** | **A** | **C** | **A** | **G** | **C** | **A** | **A** | **T** | **T** | **A** | **T** | **T** | **T** | **G** | **C** | **A** | **G** | **C** | **G** | **A** | **T** | **G** | **A** | **T** | **G** | **A** | **T** | **G** | **A** | **T** | **G** | **T** | **C** | **G** | **G** | **C** | **A** | **T** | **T** | **G** | **A** | **C** | **G** | **G** | **A** | **G** | **C** | **A** | **A** | **G** | **C** | **A** | **G** | **A** | **A** | **A** | **A** | **A** | **A** | **G** | **T** | **G** | **A** | **G** | **A** | **C** | **A** | **C** | **C** | **A** | **G** | **A** | **A** | **A** | **T** | **G** | **T** | **T** | **C** | **C** | **G** | **T** | **A** | **A** | **G** | **C** | **A** | **G** | **T** | **T** | **G** | **A** | **A** | **A** | **A** | **T** | **G** | **G** | **T** | **G** | **G** | **C** | **A** | **G** | **A** | **G** | **A** | **T** | **C** | **A** | **T** | **C** | **A** | **A** | **C** | **G** | **C** | **T** | **T** | **C** | **C** | **C** | **A** | **G** | **T** | **C** | **T** | **G** | **A** | **G** | **A** | **T** | **G** | **C** | **G** | **T** | **G** | **C** | **G** | **C** | **T** | **G** | **C** | **T** | **T** | **C** | **T** | **T** | **C** | **A** | **T** | **C** | **T** | **G** | **C** | **G** | **T** | **C** | **C** | **A** | **G** | **T** | **C** | **A** | **A** | **T** | **T** | **T** | **A** | **G** | **A** | **A** | **G** | **A** | **A** | **A** | **A** | **A** | **A** | **G** | **T** | **T** | **T** | **A** | **A** | **A** | **A** | **C** | **A** | **A** | **G** | **G** | **C** | **A** | **T** | **T** | **G** | **A** | **G** | **C** | **A** | **G** | **T** | **T** | **A** | **T** | **T** | **G** | **A** | **A** | **G** | **G** | **A** | **A** | **T** | **T** | **G** | **C** | **A** | **G** | **A** | **A** | **T** | **A** | **A** | **A** | **A** | **T** | **C** | **C** | **A** | **G** | **A** | **T** | **T** | **T** | **C** | **G** | **C** | **T** | **A** | **A** | **A** | **A** | **T** | **G** | **G** | **G** | **A** | **T** | **G** | **T** | **A** | **G** | **A** | **A** | **G** | **A** | **T** | **G** | **T** | **A** | **A** | **A** | **A** | **T** | **T** | **A** | **A** | **C** | **T** | **A** | **G** | **T** | **T** | **C** | **C** | **A** | **T** | **T** | **G** | **A** | **G** | **G** | **A** | **T** | **C** | **A** | **C** | **T** | **T** | **G** | **T** | **T** | **C** | **C** | **G** | **A** | **A** | **T** | **C** | **G** | **T** | **T** | **C** | **A** | **A** | **G** | **A** | **A** | **T** | **T** | **G** | **T** | **T** | **A** | **T** | **C** | **A** | **A** | **A** | **C** | **A** | **C** | **A** | **T** | **T** | **A** | **A** | **G** | **A** | **C** | **A** | **T** | **G** | **C** | **T** | **A** | **A** | **G** | **G** | **C** | **T** | **A** | **A** | **T** | **G** | **A** | **A** | **T** | **T** | **A** | **G** | **A** | **A** | **G** | **T** | **C** | **T** | **A** | **T** | **T** | **T** | **G** | **C** | **A** | **T** | **A** | **A** | **A** | **A** | **T** | **A** | **G** | **A** | **T** | **A** | **A** | **C** | **A** | **A** | **T** | **C** | **T** | **T** | **T** | **T** | **A** | **T** | **T** | **A** | **C** | **G** | **T** | **A** | **T** | **C** | **A** | **T** | **T** | **G** | **A** | **C** | **G** | **A** | **C** | **G** | **G** | **G** | **A** | **C** | **A** | **G** | **G** | **A** | **T** | **T** | **T** | **A** | **A** | **T** | **A** | **T** | **G** | **A** | **A** | **T** | **G** | **A** | **A** | **A** | **C** | **C** | **A** | **A** | **A** | **A** | **C** | **G** | **G** | **G** | **A** | **A** | **G** | **T** | **T** | **A** | **T** | **G** | **G** | **A** | **T** | **T** | **G** | **A** | **A** | **C** | **A** | **A** | **T** | **A** | **T** | **C** | **A** | **A** | **A** | **G** | **A** | **A** | **C** | **G** | **A** | **G** | **T** | **A** | **G** | **C** | **T** | **G** | **G** | **T** | **A** | **T** | **T** | **G** | **G** | **T** | **G** | **G** | **A** | **A** | **C** | **A** | **G** | **T** | **A** | **A** | **A** | **A** | **A** | **T** | **C** | **A** | **T** | **T** | **A** | **G** | **T** | **T** | **T** | **T** | **A** | **A** | **A** | **G** | **G** | **G** | **C** | **A** | **A** | **G** | **G** | **T** | **A** | **C** | **G** | **A** | **G** | **C** | **G** | **T** | **G** | **G** | **A** | **G** | **A** | **T** | **C** | **A** | **A** | **G** | **G** | **T** | **C** | **C** | **C** | **T** | **T** | **T** | **G** | **A** | **T** | **G** | **A** | **A** | **G** | **G** | **A** | **G** | **G** | **C** | **A** | **-** | **-** | **-** |
| **N54070** | **A** | **T** | **G** | **A** | **T** | **G** | **G** | **G** | **A** | **A** | **G** | **A** | **A** | **T** | **T** | **T** | **C** | **C** | **A** | **A** | **A** | **G** | **C** | **A** | **A** | **T** | **G** | **C** | **T** | **A** | **G** | **C** | **T** | **G** | **T** | **T** | **T** | **A** | **T** | **T** | **C** | **G** | **G** | **G** | **G** | **A** | **T** | **T** | **G** | **C** | **G** | **G** | **C** | **T** | **T** | **T** | **C** | **C** | **T** | **T** | **A** | **T** | **T** | **A** | **T** | **T** | **C** | **T** | **A** | **T** | **T** | **T** | **T** | **C** | **A** | **C** | **T** | **T** | **T** | **T** | **C** | **A** | **C** | **T** | **T** | **A** | **T** | **T** | **T** | **T** | **T** | **A** | **T** | **G** | **C** | **C** | **A** | **G** | **T** | **A** | **A** | **C** | **C** | **A** | **A** | **A** | **G** | **T** | **C** | **A** | **T** | **T** | **G** | **G** | **T** | **G** | **G** | **G** | **G** | **A** | **G** | **A** | **A** | **T** | **T** | **G** | **C** | **T** | **G** | **C** | **G** | **T** | **G** | **C** | **G** | **C** | **G** | **T** | **T** | **T** | **A** | **T** | **T** | **A** | **T** | **A** | **C** | **G** | **T** | **T** | **C** | **C** | **G** | **C** | **T** | **T** | **A** | **T** | **C** | **T** | **T** | **C** | **C** | **A** | **T** | **T** | **T** | **G** | **A** | **T** | **G** | **G** | **C** | **C** | **A** | **T** | **T** | **T** | **C** | **A** | **T** | **T** | **A** | **G** | **G** | **T** | **G** | **T** | **A** | **G** | **G** | **A** | **T** | **T** | **G** | **A** | **T** | **C** | **G** | **T** | **A** | **T** | **T** | **T** | **C** | **T** | **A** | **T** | **T** | **G** | **T** | **T** | **A** | **T** | **C** | **A** | **C** | **T** | **T** | **A** | **T** | **T** | **C** | **A** | **A** | **A** | **A** | **G** | **G** | **C** | **A** | **A** | **A** | **A** | **T** | **A** | **C** | **G** | **G** | **G** | **A** | **A** | **A** | **A** | **T** | **C** | **G** | **A** | **A** | **G** | **A** | **A** | **A** | **A** | **G** | **C** | **T** | **T** | **C** | **G** | **T** | **G** | **C** | **A** | **C** | **T** | **T** | **T** | **C** | **T** | **T** | **C** | **T** | **G** | **G** | **C** | **A** | **A** | **T** | **T** | **A** | **T** | **G** | **A** | **A** | **T** | **C** | **C** | **A** | **A** | **A** | **C** | **T** | **T** | **T** | **T** | **G** | **C** | **A** | **T** | **C** | **A** | **G** | **C** | **C** | **G** | **A** | **T** | **C** | **C** | **C** | **A** | **A** | **G** | **C** | **G** | **C** | **G** | **T** | **C** | **G** | **G** | **A** | **T** | **G** | **A** | **T** | **C** | **T** | **G** | **T** | **A** | **C** | **A** | **T** | **C** | **A** | **A** | **A** | **G** | **A** | **T** | **A** | **T** | **T** | **G** | **A** | **T** | **A** | **T** | **A** | **G** | **A** | **A** | **A** | **T** | **T** | **A** | **C** | **T** | **A** | **A** | **A** | **A** | **T** | **A** | **A** | **A** | **A** | **G** | **A** | **A** | **A** | **A** | **A** | **A** | **T** | **G** | **A** | **T** | **C** | **G** | **A** | **A** | **G** | **T** | **A** | **T** | **C** | **C** | **A** | **G** | **T** | **G** | **A** | **A** | **T** | **T** | **A** | **C** | **A** | **G** | **A** | **T** | **T** | **A** | **T** | **G** | **A** | **C** | **T** | **A** | **G** | **C** | **C** | **G** | **T** | **C** | **C** | **T** | **C** | **A** | **A** | **T** | **A** | **T** | **G** | **T** | **A** | **G** | **A** | **T** | **G** | **G** | **A** | **C** | **A** | **A** | **A** | **C** | **C** | **A** | **A** | **A** | **G** | **A** | **A** | **G** | **A** | **G** | **A** | **T** | **T** | **T** | **T** | **A** | **G** | **A** | **A** | **T** | **T** | **A** | **G** | **A** | **A** | **A** | **G** | **G** | **C** | **A** | **T** | **C** | **G** | **A** | **T** | **T** | **A** | **G** | **C** | **T** | **C** | **G** | **T** | **G** | **A** | **G** | **C** | **T** | **T** | **C** | **A** | **T** | **G** | **A** | **T** | **T** | **C** | **A** | **G** | **T** | **T** | **T** | **C** | **A** | **C** | **A** | **G** | **C** | **A** | **A** | **T** | **T** | **A** | **T** | **T** | **T** | **G** | **C** | **A** | **G** | **C** | **G** | **A** | **T** | **G** | **A** | **T** | **G** | **A** | **T** | **G** | **A** | **T** | **G** | **T** | **C** | **G** | **G** | **C** | **A** | **T** | **T** | **G** | **A** | **C** | **G** | **G** | **A** | **G** | **C** | **A** | **A** | **G** | **C** | **A** | **G** | **A** | **A** | **A** | **A** | **A** | **A** | **G** | **T** | **G** | **A** | **G** | **A** | **C** | **A** | **C** | **C** | **A** | **G** | **A** | **A** | **A** | **T** | **G** | **T** | **T** | **C** | **C** | **G** | **T** | **A** | **A** | **G** | **C** | **A** | **G** | **T** | **T** | **G** | **A** | **A** | **A** | **A** | **T** | **G** | **G** | **T** | **G** | **G** | **C** | **A** | **G** | **A** | **G** | **A** | **T** | **C** | **A** | **T** | **C** | **A** | **A** | **C** | **G** | **C** | **T** | **T** | **C** | **C** | **C** | **A** | **G** | **T** | **C** | **T** | **G** | **A** | **G** | **A** | **T** | **G** | **C** | **G** | **T** | **G** | **C** | **G** | **C** | **T** | **G** | **C** | **T** | **T** | **C** | **T** | **T** | **C** | **A** | **T** | **C** | **T** | **G** | **C** | **G** | **T** | **C** | **C** | **A** | **G** | **T** | **C** | **A** | **A** | **T** | **T** | **T** | **A** | **G** | **A** | **A** | **G** | **A** | **A** | **A** | **A** | **A** | **A** | **G** | **T** | **T** | **T** | **A** | **A** | **A** | **A** | **C** | **A** | **A** | **G** | **G** | **C** | **A** | **T** | **T** | **G** | **A** | **G** | **C** | **A** | **G** | **T** | **T** | **A** | **T** | **T** | **G** | **A** | **A** | **G** | **G** | **A** | **A** | **T** | **T** | **G** | **C** | **A** | **G** | **A** | **A** | **T** | **A** | **A** | **A** | **A** | **T** | **C** | **C** | **A** | **G** | **A** | **T** | **T** | **T** | **C** | **G** | **C** | **T** | **A** | **A** | **A** | **A** | **T** | **G** | **G** | **G** | **A** | **T** | **G** | **T** | **A** | **G** | **A** | **A** | **G** | **A** | **T** | **G** | **T** | **A** | **A** | **A** | **A** | **T** | **T** | **A** | **A** | **C** | **T** | **A** | **G** | **T** | **T** | **C** | **C** | **A** | **T** | **T** | **G** | **A** | **G** | **G** | **A** | **T** | **C** | **A** | **C** | **T** | **T** | **G** | **T** | **T** | **C** | **C** | **G** | **A** | **A** | **T** | **C** | **G** | **T** | **T** | **C** | **A** | **A** | **G** | **A** | **A** | **T** | **T** | **G** | **T** | **T** | **A** | **T** | **C** | **A** | **A** | **A** | **C** | **A** | **C** | **A** | **T** | **T** | **A** | **A** | **G** | **A** | **C** | **A** | **T** | **G** | **C** | **T** | **A** | **A** | **G** | **G** | **C** | **T** | **A** | **A** | **T** | **G** | **A** | **A** | **T** | **T** | **A** | **G** | **A** | **A** | **G** | **T** | **C** | **T** | **A** | **T** | **T** | **T** | **G** | **C** | **A** | **T** | **A** | **A** | **A** | **A** | **T** | **A** | **G** | **A** | **T** | **A** | **A** | **C** | **A** | **A** | **T** | **C** | **T** | **T** | **T** | **T** | **A** | **T** | **T** | **A** | **C** | **G** | **T** | **A** | **T** | **C** | **A** | **T** | **T** | **G** | **A** | **C** | **G** | **A** | **C** | **G** | **G** | **G** | **A** | **C** | **A** | **G** | **G** | **A** | **T** | **T** | **T** | **A** | **A** | **T** | **A** | **T** | **G** | **A** | **A** | **T** | **G** | **A** | **A** | **A** | **C** | **C** | **A** | **A** | **A** | **A** | **C** | **G** | **G** | **G** | **A** | **A** | **G** | **T** | **T** | **A** | **T** | **G** | **G** | **A** | **T** | **T** | **G** | **A** | **A** | **C** | **A** | **A** | **T** | **A** | **T** | **C** | **A** | **A** | **A** | **G** | **A** | **A** | **C** | **G** | **A** | **G** | **T** | **A** | **G** | **C** | **T** | **G** | **G** | **T** | **A** | **T** | **T** | **G** | **G** | **T** | **G** | **G** | **A** | **A** | **C** | **A** | **G** | **T** | **A** | **A** | **A** | **A** | **A** | **T** | **C** | **A** | **T** | **T** | **A** | **G** | **T** | **T** | **T** | **T** | **A** | **A** | **A** | **G** | **G** | **G** | **C** | **A** | **A** | **G** | **G** | **T** | **A** | **C** | **G** | **A** | **G** | **C** | **G** | **T** | **G** | **G** | **A** | **G** | **A** | **T** | **C** | **A** | **A** | **G** | **G** | **T** | **C** | **C** | **C** | **T** | **T** | **T** | **G** | **A** | **T** | **G** | **A** | **A** | **G** | **G** | **A** | **G** | **G** | **C** | **A** | **-** | **-** | **-** |
| **N59943F** | **A** | **T** | **G** | **A** | **T** | **G** | **G** | **G** | **A** | **A** | **A** | **A** | **A** | **T** | **A** | **T** | **C** | **C** | **A** | **G** | **A** | **G** | **C** | **G** | **A** | **T** | **G** | **C** | **T** | **A** | **G** | **C** | **T** | **G** | **T** | **T** | **T** | **A** | **T** | **T** | **C** | **G** | **G** | **G** | **G** | **A** | **T** | **T** | **G** | **C** | **G** | **G** | **C** | **T** | **T** | **T** | **C** | **C** | **T** | **T** | **A** | **T** | **T** | **A** | **T** | **T** | **C** | **T** | **A** | **T** | **T** | **T** | **T** | **C** | **A** | **C** | **T** | **T** | **T** | **T** | **C** | **A** | **C** | **T** | **T** | **A** | **T** | **T** | **T** | **T** | **T** | **A** | **T** | **G** | **C** | **C** | **A** | **G** | **T** | **A** | **A** | **T** | **C** | **A** | **A** | **A** | **G** | **T** | **C** | **A** | **T** | **T** | **G** | **G** | **T** | **G** | **G** | **G** | **G** | **A** | **G** | **A** | **A** | **T** | **T** | **A** | **C** | **T** | **G** | **C** | **G** | **T** | **G** | **C** | **A** | **C** | **G** | **T** | **T** | **T** | **A** | **T** | **T** | **A** | **T** | **A** | **T** | **G** | **T** | **T** | **C** | **C** | **G** | **C** | **T** | **T** | **A** | **T** | **C** | **T** | **T** | **C** | **C** | **A** | **T** | **T** | **T** | **G** | **T** | **T** | **A** | **G** | **C** | **T** | **A** | **T** | **T** | **T** | **C** | **C** | **T** | **T** | **A** | **G** | **G** | **T** | **G** | **T** | **A** | **G** | **G** | **A** | **T** | **T** | **A** | **A** | **T** | **C** | **G** | **T** | **C** | **T** | **T** | **T** | **C** | **T** | **A** | **T** | **T** | **A** | **T** | **T** | **A** | **T** | **C** | **A** | **C** | **T** | **T** | **A** | **T** | **T** | **C** | **A** | **A** | **A** | **A** | **G** | **A** | **C** | **A** | **A** | **A** | **A** | **T** | **A** | **C** | **G** | **G** | **G** | **A** | **A** | **A** | **A** | **T** | **C** | **G** | **A** | **A** | **G** | **A** | **A** | **A** | **A** | **G** | **C** | **T** | **G** | **C** | **G** | **T** | **G** | **C** | **A** | **C** | **T** | **C** | **T** | **C** | **T** | **T** | **C** | **T** | **G** | **G** | **C** | **A** | **A** | **T** | **T** | **A** | **T** | **G** | **A** | **A** | **T** | **C** | **C** | **A** | **A** | **A** | **T** | **T** | **G** | **T** | **T** | **G** | **C** | **T** | **T** | **C** | **T** | **T** | **C** | **C** | **G** | **A** | **T** | **C** | **C** | **C** | **A** | **A** | **G** | **T** | **G** | **C** | **A** | **T** | **C** | **G** | **G** | **A** | **C** | **G** | **A** | **T** | **T** | **T** | **A** | **T** | **A** | **C** | **A** | **T** | **C** | **A** | **A** | **A** | **G** | **A** | **T** | **A** | **T** | **T** | **G** | **A** | **T** | **A** | **A** | **G** | **G** | **A** | **A** | **A** | **T** | **C** | **A** | **C** | **T** | **A** | **A** | **A** | **A** | **T** | **A** | **A** | **A** | **A** | **G** | **A** | **A** | **A** | **A** | **A** | **A** | **T** | **G** | **A** | **T** | **T** | **G** | **A** | **A** | **G** | **T** | **A** | **T** | **C** | **T** | **A** | **G** | **T** | **G** | **A** | **A** | **T** | **T** | **A** | **C** | **A** | **A** | **A** | **T** | **T** | **G** | **T** | **A** | **A** | **C** | **G** | **A** | **G** | **C** | **C** | **G** | **T** | **C** | **C** | **C** | **C** | **A** | **A** | **T** | **A** | **T** | **G** | **T** | **A** | **G** | **A** | **T** | **G** | **G** | **A** | **C** | **A** | **A** | **A** | **C** | **C** | **A** | **A** | **A** | **G** | **A** | **A** | **G** | **A** | **A** | **A** | **T** | **T** | **T** | **T** | **A** | **G** | **A** | **A** | **T** | **T** | **A** | **G** | **A** | **A** | **A** | **G** | **A** | **C** | **A** | **T** | **C** | **G** | **A** | **T** | **T** | **A** | **G** | **C** | **C** | **C** | **G** | **T** | **G** | **A** | **G** | **C** | **T** | **G** | **C** | **A** | **T** | **G** | **A** | **T** | **T** | **C** | **A** | **G** | **T** | **T** | **T** | **C** | **G** | **C** | **A** | **A** | **C** | **A** | **A** | **T** | **T** | **G** | **T** | **T** | **T** | **G** | **C** | **A** | **G** | **C** | **A** | **A** | **T** | **G** | **A** | **T** | **G** | **A** | **T** | **G** | **A** | **T** | **G** | **T** | **C** | **G** | **G** | **C** | **A** | **T** | **T** | **A** | **A** | **C** | **G** | **G** | **A** | **G** | **C** | **A** | **A** | **G** | **C** | **A** | **G** | **A** | **A** | **A** | **A** | **A** | **A** | **G** | **C** | **G** | **A** | **G** | **A** | **C** | **A** | **C** | **C** | **A** | **G** | **A** | **A** | **A** | **T** | **G** | **T** | **T** | **C** | **C** | **G** | **C** | **A** | **A** | **G** | **C** | **A** | **G** | **T** | **T** | **G** | **A** | **A** | **A** | **A** | **T** | **G** | **G** | **T** | **A** | **G** | **C** | **A** | **G** | **A** | **G** | **A** | **T** | **C** | **A** | **T** | **C** | **A** | **A** | **C** | **G** | **C** | **T** | **T** | **C** | **C** | **C** | **A** | **G** | **T** | **C** | **T** | **G** | **A** | **G** | **A** | **T** | **G** | **C** | **G** | **T** | **G** | **C** | **G** | **C** | **T** | **G** | **C** | **T** | **T** | **C** | **T** | **T** | **C** | **A** | **T** | **C** | **T** | **G** | **C** | **G** | **T** | **C** | **C** | **A** | **G** | **T** | **C** | **A** | **A** | **T** | **T** | **T** | **A** | **G** | **A** | **A** | **G** | **A** | **G** | **A** | **A** | **A** | **A** | **G** | **T** | **T** | **T** | **A** | **A** | **A** | **G** | **C** | **A** | **A** | **G** | **G** | **T** | **A** | **T** | **C** | **G** | **A** | **G** | **C** | **A** | **G** | **T** | **T** | **A** | **T** | **T** | **G** | **A** | **A** | **G** | **G** | **A** | **A** | **T** | **T** | **G** | **C** | **A** | **A** | **A** | **A** | **T** | **A** | **A** | **A** | **A** | **T** | **T** | **C** | **A** | **G** | **A** | **T** | **T** | **T** | **C** | **G** | **C** | **T** | **G** | **A** | **A** | **A** | **T** | **G** | **G** | **G** | **A** | **T** | **G** | **T** | **A** | **G** | **A** | **A** | **G** | **A** | **T** | **G** | **T** | **A** | **A** | **A** | **A** | **C** | **T** | **A** | **T** | **C** | **T** | **A** | **G** | **C** | **T** | **C** | **C** | **A** | **T** | **T** | **G** | **A** | **G** | **G** | **A** | **T** | **C** | **A** | **C** | **T** | **T** | **G** | **T** | **T** | **C** | **C** | **G** | **A** | **A** | **T** | **C** | **G** | **T** | **T** | **C** | **A** | **A** | **G** | **A** | **A** | **T** | **T** | **G** | **T** | **T** | **A** | **T** | **C** | **A** | **A** | **A** | **T** | **A** | **C** | **A** | **T** | **T** | **A** | **A** | **G** | **A** | **C** | **A** | **T** | **G** | **C** | **C** | **A** | **A** | **A** | **G** | **C** | **C** | **A** | **A** | **T** | **G** | **A** | **A** | **T** | **T** | **A** | **G** | **A** | **G** | **G** | **T** | **A** | **T** | **A** | **T** | **T** | **T** | **G** | **C** | **A** | **T** | **A** | **A** | **A** | **A** | **T** | **A** | **G** | **A** | **C** | **A** | **A** | **T** | **A** | **A** | **T** | **C** | **T** | **T** | **T** | **T** | **A** | **T** | **T** | **A** | **C** | **G** | **T** | **A** | **T** | **T** | **A** | **T** | **T** | **G** | **A** | **C** | **G** | **A** | **T** | **G** | **G** | **A** | **A** | **C** | **A** | **G** | **G** | **G** | **T** | **T** | **T** | **A** | **A** | **T** | **A** | **T** | **G** | **A** | **A** | **T** | **G** | **A** | **A** | **A** | **C** | **G** | **A** | **A** | **A** | **A** | **C** | **G** | **G** | **G** | **A** | **A** | **G** | **T** | **T** | **A** | **T** | **G** | **G** | **A** | **T** | **T** | **G** | **A** | **A** | **C** | **A** | **A** | **T** | **A** | **T** | **C** | **A** | **A** | **A** | **G** | **A** | **A** | **C** | **G** | **A** | **G** | **T** | **A** | **G** | **C** | **T** | **G** | **G** | **T** | **A** | **T** | **T** | **G** | **G** | **T** | **G** | **G** | **A** | **A** | **C** | **A** | **G** | **T** | **A** | **A** | **A** | **A** | **A** | **T** | **C** | **A** | **T** | **T** | **A** | **G** | **T** | **T** | **T** | **T** | **A** | **A** | **A** | **G** | **G** | **G** | **C** | **A** | **A** | **G** | **G** | **T** | **A** | **C** | **A** | **A** | **G** | **T** | **G** | **T** | **A** | **G** | **A** | **A** | **A** | **T** | **C** | **A** | **A** | **G** | **G** | **T** | **C** | **C** | **C** | **T** | **T** | **T** | **G** | **A** | **T** | **G** | **A** | **A** | **G** | **G** | **A** | **G** | **G** | **C** | **A** | **-** | **-** | **-** |
| **N52769** | **A** | **T** | **G** | **A** | **T** | **G** | **G** | **G** | **A** | **A** | **A** | **A** | **A** | **T** | **A** | **T** | **C** | **C** | **A** | **G** | **A** | **G** | **C** | **G** | **A** | **T** | **G** | **C** | **T** | **A** | **G** | **C** | **T** | **G** | **T** | **T** | **T** | **A** | **T** | **T** | **C** | **G** | **G** | **G** | **G** | **A** | **T** | **T** | **G** | **C** | **G** | **G** | **C** | **T** | **T** | **T** | **C** | **C** | **T** | **T** | **A** | **T** | **T** | **A** | **T** | **T** | **C** | **T** | **A** | **T** | **T** | **T** | **T** | **C** | **A** | **C** | **T** | **T** | **T** | **T** | **C** | **A** | **C** | **T** | **T** | **A** | **T** | **T** | **T** | **T** | **T** | **A** | **T** | **G** | **C** | **C** | **A** | **G** | **T** | **A** | **A** | **T** | **C** | **A** | **A** | **A** | **G** | **T** | **C** | **A** | **T** | **T** | **G** | **G** | **T** | **G** | **G** | **G** | **G** | **A** | **G** | **A** | **A** | **T** | **T** | **A** | **C** | **T** | **G** | **C** | **G** | **T** | **G** | **C** | **A** | **C** | **G** | **T** | **T** | **T** | **A** | **T** | **T** | **A** | **T** | **A** | **T** | **G** | **T** | **T** | **C** | **C** | **G** | **C** | **T** | **T** | **A** | **T** | **C** | **T** | **T** | **C** | **C** | **A** | **T** | **T** | **T** | **G** | **T** | **T** | **A** | **G** | **C** | **T** | **A** | **T** | **T** | **T** | **C** | **C** | **T** | **T** | **A** | **G** | **G** | **T** | **G** | **T** | **A** | **G** | **G** | **A** | **T** | **T** | **A** | **A** | **T** | **C** | **G** | **T** | **C** | **T** | **T** | **T** | **C** | **T** | **A** | **T** | **T** | **A** | **T** | **T** | **A** | **T** | **C** | **A** | **C** | **T** | **T** | **A** | **T** | **T** | **C** | **A** | **A** | **A** | **A** | **G** | **A** | **C** | **A** | **A** | **A** | **A** | **T** | **A** | **C** | **G** | **G** | **G** | **A** | **A** | **A** | **A** | **T** | **C** | **G** | **A** | **A** | **G** | **A** | **A** | **A** | **A** | **G** | **C** | **T** | **G** | **C** | **G** | **T** | **G** | **C** | **A** | **C** | **T** | **C** | **T** | **C** | **T** | **T** | **C** | **T** | **G** | **G** | **C** | **A** | **A** | **T** | **T** | **A** | **T** | **G** | **A** | **A** | **T** | **C** | **C** | **A** | **A** | **A** | **T** | **T** | **G** | **T** | **T** | **G** | **C** | **T** | **T** | **C** | **T** | **T** | **C** | **C** | **G** | **A** | **T** | **C** | **C** | **C** | **A** | **A** | **G** | **T** | **G** | **C** | **A** | **T** | **C** | **G** | **G** | **A** | **C** | **G** | **A** | **T** | **T** | **T** | **A** | **T** | **A** | **C** | **A** | **T** | **C** | **A** | **A** | **A** | **G** | **A** | **T** | **A** | **T** | **T** | **G** | **A** | **T** | **A** | **A** | **G** | **G** | **A** | **A** | **A** | **T** | **C** | **A** | **C** | **T** | **A** | **A** | **A** | **A** | **T** | **A** | **A** | **A** | **A** | **G** | **A** | **A** | **A** | **A** | **A** | **A** | **T** | **G** | **A** | **T** | **T** | **G** | **A** | **A** | **G** | **T** | **A** | **T** | **C** | **T** | **A** | **G** | **T** | **G** | **A** | **A** | **T** | **T** | **A** | **C** | **A** | **A** | **A** | **T** | **T** | **G** | **T** | **A** | **A** | **C** | **G** | **A** | **G** | **C** | **C** | **G** | **T** | **C** | **C** | **C** | **C** | **A** | **A** | **T** | **A** | **T** | **G** | **T** | **A** | **G** | **A** | **T** | **G** | **G** | **A** | **C** | **A** | **A** | **A** | **C** | **C** | **A** | **A** | **A** | **G** | **A** | **A** | **G** | **A** | **A** | **A** | **T** | **T** | **T** | **T** | **A** | **G** | **A** | **A** | **T** | **T** | **A** | **G** | **A** | **A** | **A** | **G** | **A** | **C** | **A** | **T** | **C** | **G** | **A** | **T** | **T** | **A** | **G** | **C** | **C** | **C** | **G** | **T** | **G** | **A** | **G** | **C** | **T** | **G** | **C** | **A** | **T** | **G** | **A** | **T** | **T** | **C** | **A** | **G** | **T** | **T** | **T** | **C** | **G** | **C** | **A** | **A** | **C** | **A** | **A** | **T** | **T** | **A** | **T** | **T** | **T** | **G** | **C** | **A** | **G** | **C** | **A** | **A** | **T** | **G** | **A** | **T** | **G** | **A** | **T** | **G** | **A** | **T** | **G** | **T** | **C** | **G** | **G** | **C** | **A** | **T** | **T** | **G** | **A** | **C** | **G** | **G** | **A** | **G** | **C** | **A** | **A** | **G** | **C** | **A** | **G** | **A** | **A** | **A** | **A** | **A** | **A** | **G** | **C** | **G** | **A** | **G** | **A** | **C** | **A** | **C** | **C** | **A** | **G** | **A** | **A** | **A** | **T** | **G** | **T** | **T** | **C** | **C** | **G** | **C** | **A** | **A** | **G** | **C** | **A** | **G** | **T** | **T** | **G** | **A** | **A** | **A** | **A** | **T** | **G** | **G** | **T** | **A** | **G** | **C** | **A** | **G** | **A** | **G** | **A** | **T** | **C** | **A** | **T** | **C** | **A** | **A** | **C** | **G** | **C** | **T** | **T** | **C** | **C** | **C** | **A** | **G** | **T** | **C** | **T** | **G** | **A** | **G** | **A** | **T** | **G** | **C** | **G** | **T** | **G** | **C** | **G** | **C** | **T** | **G** | **C** | **T** | **T** | **C** | **T** | **T** | **C** | **A** | **T** | **C** | **T** | **G** | **C** | **G** | **T** | **C** | **C** | **A** | **G** | **T** | **C** | **A** | **A** | **T** | **T** | **T** | **A** | **G** | **A** | **A** | **G** | **A** | **G** | **A** | **A** | **A** | **A** | **G** | **T** | **T** | **T** | **A** | **A** | **A** | **G** | **C** | **A** | **A** | **G** | **G** | **T** | **A** | **T** | **C** | **G** | **A** | **G** | **C** | **A** | **G** | **T** | **T** | **A** | **T** | **T** | **G** | **A** | **A** | **G** | **G** | **A** | **A** | **T** | **T** | **G** | **C** | **A** | **A** | **A** | **A** | **T** | **A** | **A** | **A** | **A** | **T** | **T** | **C** | **A** | **G** | **A** | **T** | **T** | **T** | **C** | **G** | **C** | **T** | **G** | **A** | **A** | **A** | **T** | **G** | **G** | **G** | **A** | **T** | **G** | **T** | **A** | **G** | **A** | **A** | **G** | **A** | **T** | **G** | **T** | **A** | **A** | **A** | **A** | **C** | **T** | **A** | **T** | **C** | **T** | **A** | **G** | **C** | **T** | **C** | **C** | **A** | **T** | **T** | **G** | **A** | **G** | **G** | **A** | **T** | **C** | **A** | **C** | **T** | **T** | **G** | **T** | **T** | **C** | **C** | **G** | **A** | **A** | **T** | **C** | **G** | **T** | **T** | **C** | **A** | **A** | **G** | **A** | **A** | **T** | **T** | **G** | **T** | **T** | **A** | **T** | **C** | **A** | **A** | **A** | **T** | **A** | **C** | **A** | **T** | **T** | **A** | **A** | **G** | **A** | **C** | **A** | **T** | **G** | **C** | **C** | **A** | **A** | **A** | **G** | **C** | **C** | **A** | **A** | **T** | **G** | **A** | **A** | **T** | **T** | **A** | **G** | **A** | **G** | **G** | **T** | **A** | **T** | **A** | **T** | **T** | **T** | **G** | **C** | **A** | **T** | **A** | **A** | **A** | **A** | **T** | **A** | **G** | **A** | **C** | **A** | **A** | **T** | **A** | **A** | **T** | **C** | **T** | **T** | **T** | **T** | **A** | **T** | **T** | **A** | **C** | **G** | **T** | **A** | **T** | **T** | **A** | **T** | **T** | **G** | **A** | **C** | **G** | **A** | **T** | **G** | **G** | **A** | **A** | **C** | **A** | **G** | **G** | **G** | **T** | **T** | **T** | **A** | **A** | **T** | **A** | **T** | **G** | **A** | **A** | **T** | **G** | **A** | **A** | **A** | **C** | **G** | **A** | **A** | **A** | **A** | **C** | **G** | **G** | **G** | **A** | **A** | **G** | **T** | **T** | **A** | **T** | **G** | **G** | **A** | **T** | **T** | **G** | **A** | **A** | **C** | **A** | **A** | **T** | **A** | **T** | **C** | **A** | **A** | **A** | **G** | **A** | **A** | **C** | **G** | **A** | **G** | **T** | **A** | **G** | **C** | **T** | **G** | **G** | **T** | **A** | **T** | **T** | **G** | **G** | **T** | **G** | **G** | **A** | **A** | **C** | **A** | **G** | **T** | **A** | **A** | **A** | **A** | **A** | **T** | **C** | **A** | **T** | **T** | **A** | **G** | **T** | **T** | **T** | **T** | **A** | **A** | **A** | **G** | **G** | **G** | **C** | **A** | **A** | **G** | **G** | **T** | **A** | **C** | **A** | **A** | **G** | **T** | **G** | **T** | **A** | **G** | **A** | **A** | **A** | **T** | **C** | **A** | **A** | **G** | **G** | **T** | **C** | **C** | **C** | **T** | **T** | **T** | **G** | **A** | **T** | **G** | **A** | **A** | **G** | **G** | **A** | **G** | **G** | **C** | **A** | **-** | **-** | **-** |
| **N59504F** | **A** | **T** | **G** | **A** | **T** | **G** | **G** | **G** | **A** | **A** | **G** | **A** | **A** | **T** | **A** | **T** | **C** | **C** | **A** | **A** | **A** | **G** | **C** | **A** | **A** | **T** | **G** | **C** | **T** | **A** | **G** | **C** | **T** | **G** | **T** | **T** | **T** | **A** | **T** | **T** | **C** | **G** | **G** | **G** | **G** | **A** | **T** | **T** | **G** | **C** | **G** | **G** | **C** | **T** | **T** | **T** | **C** | **C** | **T** | **T** | **A** | **T** | **T** | **A** | **T** | **T** | **C** | **T** | **A** | **T** | **T** | **T** | **T** | **C** | **A** | **C** | **T** | **T** | **T** | **T** | **C** | **A** | **C** | **T** | **T** | **A** | **T** | **T** | **T** | **T** | **T** | **A** | **T** | **G** | **C** | **C** | **A** | **G** | **T** | **A** | **A** | **C** | **C** | **A** | **A** | **A** | **G** | **T** | **C** | **A** | **T** | **T** | **G** | **G** | **T** | **G** | **G** | **G** | **G** | **A** | **G** | **A** | **A** | **T** | **T** | **G** | **C** | **T** | **G** | **C** | **G** | **T** | **G** | **C** | **G** | **C** | **G** | **T** | **T** | **T** | **A** | **T** | **T** | **A** | **T** | **A** | **C** | **G** | **T** | **T** | **C** | **C** | **G** | **C** | **T** | **T** | **A** | **T** | **C** | **T** | **T** | **C** | **C** | **A** | **T** | **T** | **T** | **G** | **A** | **T** | **G** | **G** | **C** | **A** | **A** | **T** | **T** | **T** | **C** | **A** | **T** | **T** | **A** | **G** | **G** | **T** | **G** | **T** | **A** | **G** | **G** | **A** | **T** | **T** | **G** | **A** | **T** | **C** | **G** | **T** | **A** | **T** | **T** | **T** | **C** | **T** | **A** | **T** | **T** | **G** | **T** | **T** | **A** | **T** | **C** | **A** | **C** | **T** | **T** | **A** | **T** | **T** | **C** | **A** | **A** | **A** | **A** | **G** | **G** | **C** | **A** | **A** | **A** | **A** | **T** | **A** | **C** | **G** | **G** | **G** | **A** | **A** | **A** | **A** | **T** | **C** | **G** | **A** | **A** | **G** | **A** | **A** | **A** | **A** | **G** | **C** | **T** | **T** | **C** | **G** | **T** | **G** | **C** | **A** | **C** | **T** | **T** | **T** | **C** | **T** | **T** | **C** | **T** | **G** | **G** | **C** | **A** | **A** | **T** | **T** | **A** | **T** | **G** | **A** | **A** | **T** | **C** | **C** | **A** | **A** | **A** | **C** | **T** | **T** | **T** | **T** | **G** | **C** | **A** | **T** | **C** | **A** | **G** | **C** | **C** | **G** | **A** | **T** | **C** | **C** | **C** | **A** | **A** | **G** | **C** | **G** | **C** | **G** | **T** | **C** | **G** | **G** | **A** | **T** | **G** | **A** | **T** | **C** | **T** | **G** | **T** | **A** | **C** | **A** | **T** | **C** | **A** | **A** | **A** | **G** | **A** | **T** | **A** | **T** | **T** | **G** | **A** | **T** | **A** | **T** | **G** | **G** | **A** | **A** | **A** | **T** | **T** | **A** | **C** | **T** | **A** | **A** | **A** | **A** | **T** | **A** | **A** | **A** | **A** | **G** | **A** | **A** | **A** | **A** | **A** | **A** | **T** | **G** | **A** | **T** | **C** | **G** | **A** | **A** | **G** | **T** | **A** | **T** | **C** | **C** | **A** | **G** | **T** | **G** | **A** | **A** | **T** | **T** | **A** | **C** | **A** | **G** | **A** | **T** | **T** | **A** | **T** | **G** | **A** | **C** | **T** | **A** | **G** | **C** | **C** | **G** | **T** | **C** | **C** | **T** | **C** | **A** | **A** | **T** | **A** | **T** | **G** | **T** | **A** | **G** | **A** | **T** | **G** | **G** | **A** | **C** | **A** | **A** | **A** | **C** | **C** | **A** | **A** | **A** | **G** | **A** | **A** | **G** | **A** | **G** | **A** | **T** | **T** | **T** | **T** | **A** | **G** | **A** | **A** | **T** | **T** | **A** | **G** | **A** | **A** | **A** | **G** | **G** | **C** | **A** | **T** | **C** | **G** | **A** | **T** | **T** | **A** | **G** | **C** | **T** | **C** | **G** | **T** | **G** | **A** | **G** | **C** | **T** | **T** | **C** | **A** | **T** | **G** | **A** | **T** | **T** | **C** | **A** | **G** | **T** | **T** | **T** | **C** | **A** | **C** | **A** | **G** | **C** | **A** | **A** | **T** | **T** | **A** | **T** | **T** | **T** | **G** | **C** | **A** | **G** | **C** | **G** | **A** | **T** | **G** | **A** | **T** | **G** | **A** | **T** | **G** | **A** | **T** | **G** | **T** | **C** | **G** | **G** | **C** | **A** | **T** | **T** | **G** | **A** | **C** | **G** | **G** | **A** | **G** | **C** | **A** | **A** | **G** | **C** | **A** | **G** | **A** | **A** | **A** | **A** | **A** | **A** | **G** | **T** | **G** | **A** | **G** | **A** | **C** | **A** | **C** | **C** | **A** | **G** | **A** | **A** | **A** | **T** | **G** | **T** | **T** | **C** | **C** | **G** | **T** | **A** | **A** | **G** | **C** | **A** | **G** | **T** | **T** | **G** | **A** | **A** | **A** | **A** | **T** | **G** | **G** | **T** | **G** | **G** | **C** | **A** | **G** | **A** | **G** | **A** | **T** | **C** | **A** | **T** | **C** | **A** | **A** | **C** | **G** | **C** | **T** | **T** | **C** | **C** | **C** | **A** | **G** | **T** | **C** | **T** | **G** | **A** | **G** | **A** | **T** | **G** | **C** | **G** | **T** | **G** | **C** | **G** | **C** | **T** | **G** | **C** | **T** | **T** | **C** | **T** | **T** | **C** | **A** | **T** | **C** | **T** | **G** | **C** | **G** | **T** | **C** | **C** | **A** | **G** | **T** | **C** | **A** | **A** | **T** | **T** | **T** | **A** | **G** | **A** | **A** | **G** | **A** | **A** | **A** | **A** | **A** | **A** | **G** | **T** | **T** | **T** | **A** | **A** | **A** | **A** | **C** | **A** | **A** | **G** | **G** | **C** | **A** | **T** | **T** | **G** | **A** | **G** | **C** | **A** | **G** | **T** | **T** | **A** | **T** | **T** | **G** | **A** | **A** | **G** | **G** | **A** | **A** | **T** | **T** | **G** | **C** | **A** | **G** | **A** | **A** | **T** | **A** | **A** | **A** | **A** | **T** | **C** | **C** | **A** | **G** | **A** | **T** | **T** | **T** | **C** | **G** | **C** | **T** | **A** | **A** | **A** | **A** | **T** | **G** | **G** | **G** | **A** | **T** | **G** | **T** | **A** | **G** | **A** | **A** | **G** | **A** | **T** | **G** | **T** | **A** | **A** | **A** | **A** | **T** | **T** | **A** | **A** | **C** | **T** | **A** | **G** | **T** | **T** | **C** | **C** | **A** | **T** | **T** | **G** | **A** | **G** | **G** | **A** | **T** | **C** | **A** | **C** | **T** | **T** | **G** | **T** | **T** | **C** | **C** | **G** | **A** | **A** | **T** | **C** | **G** | **T** | **T** | **C** | **A** | **A** | **G** | **A** | **A** | **T** | **T** | **G** | **T** | **T** | **A** | **T** | **C** | **A** | **A** | **A** | **C** | **A** | **C** | **A** | **T** | **T** | **A** | **A** | **G** | **A** | **C** | **A** | **T** | **G** | **C** | **T** | **A** | **A** | **G** | **G** | **C** | **T** | **A** | **A** | **T** | **G** | **A** | **A** | **T** | **T** | **A** | **G** | **A** | **A** | **G** | **T** | **C** | **T** | **A** | **T** | **T** | **T** | **G** | **C** | **A** | **T** | **A** | **A** | **A** | **A** | **T** | **A** | **G** | **A** | **T** | **A** | **A** | **C** | **A** | **A** | **T** | **C** | **T** | **T** | **T** | **T** | **A** | **T** | **T** | **A** | **C** | **G** | **T** | **A** | **T** | **C** | **A** | **T** | **T** | **G** | **A** | **C** | **G** | **A** | **C** | **G** | **G** | **G** | **A** | **C** | **A** | **G** | **G** | **A** | **T** | **T** | **T** | **A** | **A** | **T** | **A** | **T** | **G** | **A** | **A** | **T** | **G** | **A** | **A** | **A** | **C** | **C** | **A** | **A** | **A** | **A** | **C** | **G** | **G** | **G** | **A** | **A** | **G** | **T** | **T** | **A** | **T** | **G** | **G** | **A** | **T** | **T** | **G** | **A** | **A** | **C** | **A** | **A** | **T** | **A** | **T** | **C** | **A** | **A** | **A** | **G** | **A** | **A** | **C** | **G** | **A** | **G** | **T** | **A** | **G** | **C** | **T** | **G** | **G** | **T** | **A** | **T** | **T** | **G** | **G** | **T** | **G** | **G** | **A** | **A** | **C** | **A** | **G** | **T** | **A** | **A** | **A** | **A** | **A** | **T** | **C** | **A** | **T** | **T** | **A** | **G** | **T** | **T** | **T** | **T** | **A** | **A** | **A** | **G** | **G** | **G** | **C** | **A** | **A** | **G** | **G** | **T** | **A** | **C** | **G** | **A** | **G** | **C** | **G** | **T** | **G** | **G** | **A** | **G** | **A** | **T** | **C** | **A** | **A** | **G** | **G** | **T** | **C** | **C** | **C** | **T** | **T** | **T** | **G** | **A** | **T** | **G** | **A** | **A** | **G** | **G** | **A** | **G** | **G** | **C** | **A** | **-** | **-** | **-** |
| **N53559** | **A** | **T** | **G** | **A** | **T** | **G** | **G** | **G** | **A** | **A** | **G** | **A** | **A** | **T** | **T** | **T** | **C** | **C** | **A** | **A** | **A** | **G** | **C** | **A** | **A** | **T** | **G** | **C** | **T** | **A** | **G** | **C** | **T** | **G** | **T** | **T** | **T** | **A** | **T** | **T** | **C** | **G** | **G** | **G** | **G** | **A** | **T** | **T** | **G** | **C** | **G** | **G** | **C** | **T** | **T** | **T** | **C** | **C** | **T** | **T** | **A** | **T** | **T** | **A** | **T** | **T** | **C** | **T** | **A** | **T** | **T** | **T** | **T** | **C** | **A** | **C** | **T** | **T** | **T** | **T** | **C** | **A** | **C** | **T** | **T** | **A** | **T** | **T** | **T** | **T** | **T** | **A** | **T** | **G** | **C** | **C** | **A** | **G** | **T** | **A** | **A** | **C** | **C** | **A** | **A** | **A** | **G** | **T** | **C** | **A** | **T** | **T** | **G** | **G** | **T** | **G** | **G** | **G** | **G** | **A** | **G** | **A** | **A** | **T** | **T** | **G** | **C** | **T** | **G** | **C** | **G** | **T** | **G** | **C** | **G** | **C** | **G** | **T** | **T** | **T** | **A** | **T** | **T** | **A** | **T** | **A** | **C** | **G** | **T** | **T** | **C** | **C** | **G** | **C** | **T** | **T** | **A** | **T** | **C** | **T** | **T** | **C** | **C** | **A** | **T** | **T** | **T** | **G** | **A** | **T** | **G** | **G** | **C** | **C** | **A** | **T** | **T** | **T** | **C** | **A** | **T** | **T** | **A** | **G** | **G** | **T** | **G** | **T** | **A** | **G** | **G** | **A** | **T** | **T** | **G** | **A** | **T** | **C** | **G** | **T** | **A** | **T** | **T** | **T** | **C** | **T** | **A** | **T** | **T** | **G** | **T** | **T** | **A** | **T** | **C** | **A** | **C** | **T** | **T** | **A** | **T** | **T** | **C** | **A** | **A** | **A** | **A** | **G** | **G** | **C** | **A** | **A** | **A** | **A** | **T** | **A** | **C** | **G** | **G** | **G** | **A** | **A** | **A** | **A** | **T** | **C** | **G** | **A** | **A** | **G** | **A** | **A** | **A** | **A** | **G** | **C** | **T** | **T** | **C** | **G** | **T** | **G** | **C** | **A** | **C** | **T** | **T** | **T** | **C** | **T** | **T** | **C** | **T** | **G** | **G** | **C** | **A** | **A** | **T** | **T** | **A** | **T** | **G** | **A** | **A** | **T** | **C** | **C** | **A** | **A** | **A** | **C** | **T** | **T** | **T** | **T** | **G** | **C** | **A** | **T** | **C** | **A** | **G** | **C** | **C** | **G** | **A** | **T** | **C** | **C** | **C** | **A** | **A** | **G** | **C** | **G** | **C** | **G** | **T** | **C** | **G** | **G** | **A** | **T** | **G** | **A** | **T** | **C** | **T** | **G** | **T** | **A** | **C** | **A** | **T** | **C** | **A** | **A** | **A** | **G** | **A** | **T** | **A** | **T** | **T** | **G** | **A** | **T** | **A** | **T** | **A** | **G** | **A** | **A** | **A** | **T** | **T** | **A** | **C** | **T** | **A** | **A** | **A** | **A** | **T** | **A** | **A** | **A** | **A** | **G** | **A** | **A** | **A** | **A** | **A** | **A** | **T** | **G** | **A** | **T** | **C** | **G** | **A** | **A** | **G** | **T** | **A** | **T** | **C** | **C** | **A** | **G** | **T** | **G** | **A** | **A** | **T** | **T** | **A** | **C** | **A** | **G** | **A** | **T** | **T** | **A** | **T** | **G** | **A** | **C** | **T** | **A** | **G** | **C** | **C** | **G** | **T** | **C** | **C** | **T** | **C** | **A** | **A** | **T** | **A** | **T** | **G** | **T** | **A** | **G** | **A** | **T** | **G** | **G** | **A** | **C** | **A** | **A** | **A** | **C** | **C** | **A** | **A** | **A** | **G** | **A** | **A** | **G** | **A** | **G** | **A** | **T** | **T** | **T** | **T** | **A** | **G** | **A** | **A** | **T** | **T** | **A** | **G** | **A** | **A** | **A** | **G** | **G** | **C** | **A** | **T** | **C** | **G** | **A** | **T** | **T** | **A** | **G** | **C** | **T** | **C** | **G** | **T** | **G** | **A** | **G** | **C** | **T** | **T** | **C** | **A** | **T** | **G** | **A** | **T** | **T** | **C** | **A** | **G** | **T** | **T** | **T** | **C** | **A** | **C** | **A** | **G** | **C** | **A** | **A** | **T** | **T** | **A** | **T** | **T** | **T** | **G** | **C** | **A** | **G** | **C** | **G** | **A** | **T** | **G** | **A** | **T** | **G** | **A** | **T** | **G** | **A** | **T** | **G** | **T** | **C** | **G** | **G** | **C** | **A** | **T** | **T** | **G** | **A** | **C** | **G** | **G** | **A** | **G** | **C** | **A** | **A** | **G** | **C** | **A** | **G** | **A** | **A** | **A** | **A** | **A** | **A** | **G** | **T** | **G** | **A** | **G** | **A** | **C** | **A** | **C** | **C** | **A** | **G** | **A** | **A** | **A** | **T** | **G** | **T** | **T** | **C** | **C** | **G** | **T** | **A** | **A** | **G** | **C** | **A** | **G** | **T** | **T** | **G** | **A** | **A** | **A** | **A** | **T** | **G** | **G** | **T** | **G** | **G** | **C** | **A** | **G** | **A** | **G** | **A** | **T** | **C** | **A** | **T** | **C** | **A** | **A** | **C** | **G** | **C** | **T** | **T** | **C** | **C** | **C** | **A** | **G** | **T** | **C** | **T** | **G** | **A** | **G** | **A** | **T** | **G** | **C** | **G** | **T** | **G** | **C** | **G** | **C** | **T** | **G** | **C** | **T** | **T** | **C** | **T** | **T** | **C** | **A** | **T** | **C** | **T** | **G** | **C** | **G** | **T** | **C** | **C** | **A** | **G** | **T** | **C** | **A** | **A** | **T** | **T** | **T** | **A** | **G** | **A** | **A** | **G** | **A** | **A** | **A** | **A** | **A** | **A** | **G** | **T** | **T** | **T** | **A** | **A** | **A** | **A** | **C** | **A** | **A** | **G** | **G** | **C** | **A** | **T** | **T** | **G** | **A** | **G** | **C** | **A** | **G** | **T** | **T** | **A** | **T** | **T** | **G** | **A** | **A** | **G** | **G** | **A** | **A** | **T** | **T** | **G** | **C** | **A** | **G** | **A** | **A** | **T** | **A** | **A** | **A** | **A** | **T** | **C** | **C** | **A** | **G** | **A** | **T** | **T** | **T** | **C** | **G** | **C** | **T** | **A** | **A** | **A** | **A** | **T** | **G** | **G** | **G** | **A** | **T** | **G** | **T** | **A** | **G** | **A** | **A** | **G** | **A** | **T** | **G** | **T** | **A** | **A** | **A** | **A** | **T** | **T** | **A** | **A** | **C** | **T** | **A** | **G** | **T** | **T** | **C** | **C** | **A** | **T** | **T** | **G** | **A** | **G** | **G** | **A** | **T** | **C** | **A** | **C** | **T** | **T** | **G** | **T** | **T** | **C** | **C** | **G** | **A** | **A** | **T** | **C** | **G** | **T** | **T** | **C** | **A** | **A** | **G** | **A** | **A** | **T** | **T** | **G** | **T** | **T** | **A** | **T** | **C** | **A** | **A** | **A** | **C** | **A** | **C** | **A** | **T** | **T** | **A** | **A** | **G** | **A** | **C** | **A** | **T** | **G** | **C** | **T** | **A** | **A** | **G** | **G** | **C** | **T** | **A** | **A** | **T** | **G** | **A** | **A** | **T** | **T** | **A** | **G** | **A** | **A** | **G** | **T** | **C** | **T** | **A** | **T** | **T** | **T** | **G** | **C** | **A** | **T** | **A** | **A** | **A** | **A** | **T** | **A** | **G** | **A** | **T** | **A** | **A** | **C** | **A** | **A** | **T** | **C** | **T** | **T** | **T** | **T** | **A** | **T** | **T** | **A** | **C** | **G** | **T** | **A** | **T** | **C** | **A** | **T** | **T** | **G** | **A** | **C** | **G** | **A** | **C** | **G** | **G** | **G** | **A** | **C** | **A** | **G** | **G** | **A** | **T** | **T** | **T** | **A** | **A** | **T** | **A** | **T** | **G** | **A** | **A** | **T** | **G** | **A** | **A** | **A** | **C** | **C** | **A** | **A** | **A** | **A** | **C** | **G** | **G** | **G** | **A** | **A** | **G** | **T** | **T** | **A** | **T** | **G** | **G** | **A** | **T** | **T** | **G** | **A** | **A** | **C** | **A** | **A** | **T** | **A** | **T** | **C** | **A** | **A** | **A** | **G** | **A** | **A** | **C** | **G** | **A** | **G** | **T** | **A** | **G** | **C** | **T** | **G** | **G** | **T** | **A** | **T** | **T** | **G** | **G** | **T** | **G** | **G** | **A** | **A** | **C** | **A** | **G** | **T** | **A** | **A** | **A** | **A** | **A** | **T** | **C** | **A** | **T** | **T** | **A** | **G** | **T** | **T** | **T** | **T** | **A** | **A** | **A** | **G** | **G** | **G** | **C** | **A** | **A** | **G** | **G** | **T** | **A** | **C** | **G** | **A** | **G** | **C** | **G** | **T** | **G** | **G** | **A** | **G** | **A** | **T** | **C** | **A** | **A** | **G** | **G** | **T** | **C** | **C** | **C** | **T** | **T** | **T** | **G** | **A** | **T** | **G** | **A** | **A** | **G** | **G** | **A** | **G** | **G** | **C** | **A** | **-** | **-** | **-** |
| **N55290** | **A** | **T** | **G** | **A** | **T** | **G** | **G** | **G** | **A** | **A** | **A** | **A** | **A** | **T** | **A** | **T** | **C** | **C** | **A** | **G** | **A** | **G** | **C** | **G** | **A** | **T** | **G** | **C** | **T** | **A** | **G** | **C** | **T** | **G** | **T** | **T** | **T** | **A** | **T** | **T** | **C** | **G** | **G** | **G** | **G** | **A** | **T** | **T** | **G** | **C** | **G** | **G** | **C** | **T** | **T** | **T** | **C** | **C** | **T** | **T** | **A** | **T** | **T** | **A** | **T** | **T** | **C** | **T** | **A** | **T** | **T** | **T** | **T** | **C** | **A** | **C** | **T** | **T** | **T** | **T** | **C** | **A** | **C** | **T** | **T** | **A** | **T** | **T** | **T** | **T** | **T** | **A** | **T** | **G** | **C** | **C** | **A** | **G** | **T** | **A** | **A** | **T** | **C** | **A** | **A** | **A** | **G** | **T** | **C** | **A** | **T** | **T** | **G** | **G** | **T** | **G** | **G** | **G** | **G** | **A** | **G** | **A** | **A** | **T** | **T** | **A** | **C** | **T** | **G** | **C** | **G** | **T** | **G** | **C** | **A** | **C** | **G** | **T** | **T** | **T** | **A** | **T** | **T** | **A** | **T** | **A** | **T** | **G** | **T** | **T** | **C** | **C** | **G** | **C** | **T** | **T** | **A** | **T** | **C** | **T** | **T** | **C** | **C** | **A** | **T** | **T** | **T** | **G** | **T** | **T** | **A** | **G** | **C** | **T** | **A** | **T** | **T** | **T** | **C** | **C** | **T** | **T** | **A** | **G** | **G** | **T** | **G** | **T** | **A** | **G** | **G** | **A** | **T** | **T** | **A** | **A** | **T** | **C** | **G** | **T** | **C** | **T** | **T** | **T** | **C** | **T** | **A** | **T** | **T** | **A** | **T** | **T** | **A** | **T** | **C** | **A** | **C** | **T** | **T** | **A** | **T** | **T** | **C** | **A** | **A** | **A** | **A** | **G** | **A** | **C** | **A** | **A** | **A** | **A** | **T** | **A** | **C** | **G** | **G** | **G** | **A** | **A** | **A** | **A** | **T** | **C** | **G** | **A** | **A** | **G** | **A** | **A** | **A** | **A** | **G** | **C** | **T** | **G** | **C** | **G** | **T** | **G** | **C** | **A** | **C** | **T** | **C** | **T** | **C** | **T** | **T** | **C** | **T** | **G** | **G** | **C** | **A** | **A** | **T** | **T** | **A** | **T** | **G** | **A** | **A** | **T** | **C** | **C** | **A** | **A** | **A** | **T** | **T** | **G** | **T** | **T** | **G** | **C** | **T** | **T** | **C** | **T** | **T** | **C** | **C** | **G** | **A** | **T** | **C** | **C** | **C** | **A** | **A** | **G** | **T** | **G** | **C** | **A** | **T** | **C** | **G** | **G** | **A** | **C** | **G** | **A** | **T** | **T** | **T** | **A** | **T** | **A** | **C** | **A** | **T** | **C** | **A** | **A** | **A** | **G** | **A** | **T** | **A** | **T** | **T** | **G** | **A** | **T** | **A** | **A** | **G** | **G** | **A** | **A** | **A** | **T** | **C** | **A** | **C** | **T** | **A** | **A** | **A** | **A** | **T** | **A** | **A** | **A** | **A** | **G** | **A** | **A** | **A** | **A** | **A** | **A** | **T** | **G** | **A** | **T** | **T** | **G** | **A** | **A** | **G** | **T** | **A** | **T** | **C** | **T** | **A** | **G** | **T** | **G** | **A** | **A** | **T** | **T** | **A** | **C** | **A** | **A** | **A** | **T** | **T** | **G** | **T** | **A** | **A** | **C** | **G** | **A** | **G** | **C** | **C** | **G** | **T** | **C** | **C** | **C** | **C** | **A** | **A** | **T** | **A** | **T** | **G** | **T** | **A** | **G** | **A** | **T** | **G** | **G** | **A** | **C** | **A** | **A** | **A** | **C** | **C** | **A** | **A** | **A** | **G** | **A** | **A** | **G** | **A** | **A** | **A** | **T** | **T** | **T** | **T** | **A** | **G** | **A** | **A** | **T** | **T** | **A** | **G** | **A** | **A** | **A** | **G** | **A** | **C** | **A** | **T** | **C** | **G** | **A** | **T** | **T** | **A** | **G** | **C** | **C** | **C** | **G** | **T** | **G** | **A** | **G** | **C** | **T** | **G** | **C** | **A** | **T** | **G** | **A** | **T** | **T** | **C** | **A** | **G** | **T** | **T** | **T** | **C** | **G** | **C** | **A** | **A** | **C** | **A** | **A** | **T** | **T** | **A** | **T** | **T** | **T** | **G** | **C** | **A** | **G** | **C** | **A** | **A** | **T** | **G** | **A** | **T** | **G** | **A** | **T** | **G** | **A** | **T** | **G** | **T** | **C** | **G** | **G** | **C** | **A** | **T** | **T** | **G** | **A** | **C** | **G** | **G** | **A** | **G** | **C** | **A** | **A** | **G** | **C** | **A** | **G** | **A** | **A** | **A** | **A** | **A** | **A** | **G** | **C** | **G** | **A** | **G** | **A** | **C** | **A** | **C** | **C** | **A** | **G** | **A** | **A** | **A** | **T** | **G** | **T** | **T** | **C** | **C** | **G** | **C** | **A** | **A** | **G** | **C** | **A** | **G** | **T** | **T** | **G** | **A** | **A** | **A** | **A** | **T** | **G** | **G** | **T** | **A** | **G** | **C** | **A** | **G** | **A** | **G** | **A** | **T** | **C** | **A** | **T** | **C** | **A** | **A** | **C** | **G** | **C** | **T** | **T** | **C** | **C** | **C** | **A** | **G** | **T** | **C** | **T** | **G** | **A** | **G** | **A** | **T** | **G** | **C** | **G** | **T** | **G** | **C** | **G** | **C** | **T** | **G** | **C** | **T** | **T** | **C** | **T** | **T** | **C** | **A** | **T** | **C** | **T** | **G** | **C** | **G** | **T** | **C** | **C** | **A** | **G** | **T** | **C** | **A** | **A** | **T** | **T** | **T** | **A** | **G** | **A** | **A** | **G** | **A** | **G** | **A** | **A** | **A** | **A** | **G** | **T** | **T** | **T** | **A** | **A** | **A** | **G** | **C** | **A** | **A** | **G** | **G** | **T** | **A** | **T** | **C** | **G** | **A** | **G** | **C** | **A** | **G** | **T** | **T** | **A** | **T** | **T** | **G** | **A** | **A** | **G** | **G** | **A** | **A** | **T** | **T** | **G** | **C** | **A** | **A** | **A** | **A** | **T** | **A** | **A** | **A** | **A** | **T** | **T** | **C** | **A** | **G** | **A** | **T** | **T** | **T** | **C** | **G** | **C** | **T** | **G** | **A** | **A** | **A** | **T** | **G** | **G** | **G** | **A** | **T** | **G** | **T** | **A** | **G** | **A** | **A** | **G** | **A** | **T** | **G** | **T** | **A** | **A** | **A** | **A** | **C** | **T** | **A** | **T** | **C** | **T** | **A** | **G** | **C** | **T** | **C** | **C** | **A** | **T** | **T** | **G** | **A** | **G** | **G** | **A** | **T** | **C** | **A** | **C** | **T** | **T** | **G** | **T** | **T** | **C** | **C** | **G** | **A** | **A** | **T** | **C** | **G** | **T** | **T** | **C** | **A** | **A** | **G** | **A** | **A** | **T** | **T** | **G** | **T** | **T** | **A** | **T** | **C** | **A** | **A** | **A** | **T** | **A** | **C** | **A** | **T** | **T** | **A** | **A** | **G** | **A** | **C** | **A** | **T** | **G** | **C** | **C** | **A** | **A** | **A** | **G** | **C** | **C** | **A** | **A** | **T** | **G** | **A** | **A** | **T** | **T** | **A** | **G** | **A** | **G** | **G** | **T** | **A** | **T** | **A** | **T** | **T** | **T** | **G** | **C** | **A** | **T** | **A** | **A** | **A** | **A** | **T** | **A** | **G** | **A** | **C** | **A** | **A** | **T** | **A** | **A** | **T** | **C** | **T** | **T** | **T** | **T** | **A** | **T** | **T** | **A** | **C** | **G** | **T** | **A** | **T** | **T** | **A** | **T** | **T** | **G** | **A** | **C** | **G** | **A** | **T** | **G** | **G** | **A** | **A** | **C** | **A** | **G** | **G** | **G** | **T** | **T** | **T** | **A** | **A** | **T** | **A** | **T** | **G** | **A** | **A** | **T** | **G** | **A** | **A** | **A** | **C** | **G** | **A** | **A** | **A** | **A** | **C** | **G** | **G** | **G** | **A** | **A** | **G** | **T** | **T** | **A** | **T** | **G** | **G** | **A** | **T** | **T** | **G** | **A** | **A** | **C** | **A** | **A** | **T** | **A** | **T** | **C** | **A** | **A** | **A** | **G** | **A** | **A** | **C** | **G** | **A** | **G** | **T** | **A** | **G** | **C** | **T** | **G** | **G** | **T** | **A** | **T** | **T** | **G** | **G** | **T** | **G** | **G** | **A** | **A** | **C** | **A** | **G** | **T** | **A** | **A** | **A** | **A** | **A** | **T** | **C** | **A** | **T** | **T** | **A** | **G** | **T** | **T** | **T** | **T** | **A** | **A** | **A** | **G** | **G** | **G** | **C** | **A** | **A** | **G** | **G** | **T** | **A** | **C** | **A** | **A** | **G** | **T** | **G** | **T** | **A** | **G** | **A** | **A** | **A** | **T** | **C** | **A** | **A** | **G** | **G** | **T** | **C** | **C** | **C** | **T** | **T** | **T** | **G** | **A** | **T** | **G** | **A** | **A** | **G** | **G** | **A** | **G** | **G** | **C** | **A** | **-** | **-** | **-** |
| **N53533** | **A** | **T** | **G** | **A** | **T** | **G** | **G** | **G** | **A** | **A** | **G** | **A** | **A** | **T** | **A** | **T** | **C** | **C** | **A** | **A** | **A** | **G** | **C** | **A** | **A** | **T** | **G** | **C** | **T** | **A** | **G** | **C** | **T** | **G** | **T** | **T** | **T** | **A** | **T** | **T** | **C** | **G** | **G** | **G** | **G** | **A** | **T** | **T** | **G** | **C** | **G** | **G** | **C** | **T** | **T** | **T** | **C** | **C** | **T** | **T** | **A** | **T** | **T** | **A** | **T** | **T** | **C** | **T** | **A** | **T** | **T** | **T** | **T** | **C** | **A** | **C** | **T** | **T** | **T** | **T** | **C** | **A** | **C** | **T** | **T** | **A** | **T** | **T** | **T** | **T** | **T** | **A** | **T** | **G** | **C** | **C** | **A** | **G** | **T** | **A** | **A** | **C** | **C** | **A** | **A** | **A** | **G** | **T** | **C** | **A** | **T** | **T** | **G** | **G** | **T** | **G** | **G** | **G** | **G** | **A** | **G** | **A** | **A** | **T** | **T** | **G** | **C** | **T** | **G** | **C** | **G** | **T** | **G** | **C** | **G** | **C** | **G** | **T** | **T** | **T** | **A** | **T** | **T** | **A** | **T** | **A** | **C** | **G** | **T** | **T** | **C** | **C** | **G** | **C** | **T** | **T** | **A** | **T** | **C** | **T** | **T** | **C** | **C** | **A** | **T** | **T** | **T** | **G** | **A** | **T** | **G** | **G** | **C** | **A** | **A** | **T** | **T** | **T** | **C** | **A** | **T** | **T** | **A** | **G** | **G** | **T** | **G** | **T** | **A** | **G** | **G** | **A** | **T** | **T** | **G** | **A** | **T** | **C** | **G** | **T** | **A** | **T** | **T** | **T** | **C** | **T** | **A** | **T** | **T** | **G** | **T** | **T** | **A** | **T** | **C** | **A** | **C** | **T** | **T** | **A** | **T** | **T** | **C** | **A** | **A** | **A** | **A** | **G** | **G** | **C** | **A** | **A** | **A** | **A** | **T** | **A** | **C** | **G** | **G** | **G** | **A** | **A** | **A** | **A** | **T** | **C** | **G** | **A** | **A** | **G** | **A** | **A** | **A** | **A** | **G** | **C** | **T** | **T** | **C** | **G** | **T** | **G** | **C** | **A** | **C** | **T** | **T** | **T** | **C** | **T** | **T** | **C** | **T** | **G** | **G** | **C** | **A** | **A** | **T** | **T** | **A** | **T** | **G** | **A** | **A** | **T** | **C** | **C** | **A** | **A** | **A** | **C** | **T** | **T** | **T** | **T** | **G** | **C** | **A** | **T** | **C** | **A** | **G** | **C** | **C** | **G** | **A** | **T** | **C** | **C** | **C** | **A** | **A** | **G** | **C** | **G** | **C** | **G** | **T** | **C** | **G** | **G** | **A** | **T** | **G** | **A** | **T** | **C** | **T** | **G** | **T** | **A** | **C** | **A** | **T** | **C** | **A** | **A** | **A** | **G** | **A** | **T** | **A** | **T** | **T** | **G** | **A** | **T** | **A** | **T** | **G** | **G** | **A** | **A** | **A** | **T** | **T** | **A** | **C** | **T** | **A** | **A** | **A** | **A** | **T** | **A** | **A** | **A** | **A** | **G** | **A** | **A** | **A** | **A** | **A** | **A** | **T** | **G** | **A** | **T** | **C** | **G** | **A** | **A** | **G** | **T** | **A** | **T** | **C** | **C** | **A** | **G** | **T** | **G** | **A** | **A** | **T** | **T** | **A** | **C** | **A** | **G** | **A** | **T** | **T** | **A** | **T** | **G** | **A** | **C** | **T** | **A** | **G** | **C** | **C** | **G** | **T** | **C** | **C** | **T** | **C** | **A** | **A** | **T** | **A** | **T** | **G** | **T** | **A** | **G** | **A** | **T** | **G** | **G** | **A** | **C** | **A** | **A** | **A** | **C** | **C** | **A** | **A** | **A** | **G** | **A** | **A** | **G** | **A** | **G** | **A** | **T** | **T** | **T** | **T** | **A** | **G** | **A** | **A** | **T** | **T** | **A** | **G** | **A** | **A** | **A** | **G** | **G** | **C** | **A** | **T** | **C** | **G** | **A** | **T** | **T** | **A** | **G** | **C** | **T** | **C** | **G** | **T** | **G** | **A** | **G** | **C** | **T** | **T** | **C** | **A** | **T** | **G** | **A** | **T** | **T** | **C** | **A** | **G** | **T** | **T** | **T** | **C** | **A** | **C** | **A** | **G** | **C** | **A** | **A** | **T** | **T** | **A** | **T** | **T** | **T** | **G** | **C** | **A** | **G** | **C** | **G** | **A** | **T** | **G** | **A** | **T** | **G** | **A** | **T** | **G** | **A** | **T** | **G** | **T** | **C** | **G** | **G** | **C** | **A** | **T** | **T** | **G** | **A** | **C** | **G** | **G** | **A** | **G** | **C** | **A** | **A** | **G** | **C** | **A** | **G** | **A** | **A** | **A** | **A** | **A** | **A** | **G** | **T** | **G** | **A** | **G** | **A** | **C** | **A** | **C** | **C** | **A** | **G** | **A** | **A** | **A** | **T** | **G** | **T** | **T** | **C** | **C** | **G** | **T** | **A** | **A** | **G** | **C** | **A** | **G** | **T** | **T** | **G** | **A** | **A** | **A** | **A** | **T** | **G** | **G** | **T** | **G** | **G** | **C** | **A** | **G** | **A** | **G** | **A** | **T** | **C** | **A** | **T** | **C** | **A** | **A** | **C** | **G** | **C** | **T** | **T** | **C** | **C** | **C** | **A** | **G** | **T** | **C** | **T** | **G** | **A** | **G** | **A** | **T** | **G** | **C** | **G** | **T** | **G** | **C** | **G** | **C** | **T** | **G** | **C** | **T** | **T** | **C** | **T** | **T** | **C** | **A** | **T** | **C** | **T** | **G** | **C** | **G** | **T** | **C** | **C** | **A** | **G** | **T** | **C** | **A** | **A** | **T** | **T** | **T** | **A** | **G** | **A** | **A** | **G** | **A** | **A** | **A** | **A** | **A** | **A** | **G** | **T** | **T** | **T** | **A** | **A** | **A** | **A** | **C** | **A** | **A** | **G** | **G** | **C** | **A** | **T** | **T** | **G** | **A** | **G** | **C** | **A** | **G** | **T** | **T** | **A** | **T** | **T** | **G** | **A** | **A** | **G** | **G** | **A** | **A** | **T** | **T** | **G** | **C** | **A** | **G** | **A** | **A** | **T** | **A** | **A** | **A** | **A** | **T** | **C** | **C** | **A** | **G** | **A** | **T** | **T** | **T** | **C** | **G** | **C** | **T** | **A** | **A** | **A** | **A** | **T** | **G** | **G** | **G** | **A** | **T** | **G** | **T** | **A** | **G** | **A** | **A** | **G** | **A** | **T** | **G** | **T** | **A** | **A** | **A** | **A** | **T** | **T** | **A** | **A** | **C** | **T** | **A** | **G** | **T** | **T** | **C** | **C** | **A** | **T** | **T** | **G** | **A** | **G** | **G** | **A** | **T** | **C** | **A** | **C** | **T** | **T** | **G** | **T** | **T** | **C** | **C** | **G** | **A** | **A** | **T** | **C** | **G** | **T** | **T** | **C** | **A** | **A** | **G** | **A** | **A** | **T** | **T** | **G** | **T** | **T** | **A** | **T** | **C** | **A** | **A** | **A** | **C** | **A** | **C** | **A** | **T** | **T** | **A** | **A** | **G** | **A** | **C** | **A** | **T** | **G** | **C** | **T** | **A** | **A** | **G** | **G** | **C** | **T** | **A** | **A** | **T** | **G** | **A** | **A** | **T** | **T** | **A** | **G** | **A** | **A** | **G** | **T** | **C** | **T** | **A** | **T** | **T** | **T** | **G** | **C** | **A** | **T** | **A** | **A** | **A** | **A** | **T** | **A** | **G** | **A** | **T** | **A** | **A** | **C** | **A** | **A** | **T** | **C** | **T** | **T** | **T** | **T** | **A** | **T** | **T** | **A** | **C** | **G** | **T** | **A** | **T** | **C** | **A** | **T** | **T** | **G** | **A** | **C** | **G** | **A** | **C** | **G** | **G** | **G** | **A** | **C** | **A** | **G** | **G** | **A** | **T** | **T** | **T** | **A** | **A** | **T** | **A** | **T** | **G** | **A** | **A** | **T** | **G** | **A** | **A** | **A** | **C** | **C** | **A** | **A** | **A** | **A** | **C** | **G** | **G** | **G** | **A** | **A** | **G** | **T** | **T** | **A** | **T** | **G** | **G** | **A** | **T** | **T** | **G** | **A** | **A** | **C** | **A** | **A** | **T** | **A** | **T** | **C** | **A** | **A** | **A** | **G** | **A** | **A** | **C** | **G** | **A** | **G** | **T** | **A** | **G** | **C** | **T** | **G** | **G** | **T** | **A** | **T** | **T** | **G** | **G** | **T** | **G** | **G** | **A** | **A** | **C** | **A** | **G** | **T** | **A** | **A** | **A** | **A** | **A** | **T** | **C** | **A** | **T** | **T** | **A** | **G** | **T** | **T** | **T** | **T** | **A** | **A** | **A** | **G** | **G** | **G** | **C** | **A** | **A** | **G** | **G** | **T** | **A** | **C** | **G** | **A** | **G** | **C** | **G** | **T** | **G** | **G** | **A** | **G** | **A** | **T** | **C** | **A** | **A** | **G** | **G** | **T** | **C** | **C** | **C** | **T** | **T** | **T** | **G** | **A** | **T** | **G** | **A** | **A** | **G** | **G** | **A** | **G** | **G** | **C** | **A** | **-** | **-** | **-** |
| **N52629** | **A** | **T** | **G** | **A** | **T** | **G** | **G** | **G** | **A** | **A** | **G** | **A** | **A** | **T** | **A** | **T** | **C** | **C** | **A** | **A** | **A** | **G** | **C** | **A** | **A** | **T** | **G** | **C** | **T** | **A** | **G** | **C** | **T** | **G** | **T** | **T** | **T** | **A** | **T** | **T** | **C** | **G** | **G** | **G** | **G** | **A** | **T** | **T** | **G** | **C** | **G** | **G** | **C** | **T** | **T** | **T** | **C** | **C** | **T** | **T** | **A** | **T** | **T** | **A** | **T** | **T** | **C** | **T** | **A** | **T** | **T** | **T** | **T** | **C** | **A** | **C** | **T** | **T** | **T** | **T** | **C** | **A** | **C** | **T** | **T** | **A** | **T** | **T** | **T** | **T** | **T** | **A** | **T** | **G** | **C** | **C** | **A** | **G** | **T** | **A** | **A** | **C** | **C** | **A** | **A** | **A** | **G** | **T** | **C** | **A** | **T** | **T** | **G** | **G** | **T** | **G** | **G** | **G** | **G** | **A** | **G** | **A** | **A** | **T** | **T** | **G** | **C** | **T** | **G** | **C** | **G** | **T** | **G** | **C** | **G** | **C** | **G** | **T** | **T** | **T** | **A** | **T** | **T** | **A** | **T** | **A** | **C** | **G** | **T** | **T** | **C** | **C** | **G** | **C** | **T** | **T** | **A** | **T** | **C** | **T** | **T** | **C** | **C** | **A** | **T** | **T** | **T** | **G** | **A** | **T** | **G** | **G** | **C** | **A** | **A** | **T** | **T** | **T** | **C** | **A** | **T** | **T** | **A** | **G** | **G** | **T** | **G** | **T** | **A** | **G** | **G** | **A** | **T** | **T** | **G** | **A** | **T** | **C** | **G** | **T** | **A** | **T** | **T** | **T** | **C** | **T** | **A** | **T** | **T** | **G** | **T** | **T** | **A** | **T** | **C** | **A** | **C** | **T** | **T** | **A** | **T** | **T** | **C** | **A** | **A** | **A** | **A** | **G** | **G** | **C** | **A** | **A** | **A** | **A** | **T** | **A** | **C** | **G** | **G** | **G** | **A** | **A** | **A** | **A** | **T** | **C** | **G** | **A** | **A** | **G** | **A** | **A** | **A** | **A** | **G** | **C** | **T** | **T** | **C** | **G** | **T** | **G** | **C** | **A** | **C** | **T** | **T** | **T** | **C** | **T** | **T** | **C** | **T** | **G** | **G** | **C** | **A** | **A** | **T** | **T** | **A** | **T** | **G** | **A** | **A** | **T** | **C** | **C** | **A** | **A** | **A** | **C** | **T** | **T** | **T** | **T** | **G** | **C** | **A** | **T** | **C** | **A** | **G** | **C** | **C** | **G** | **A** | **T** | **C** | **C** | **C** | **A** | **A** | **G** | **C** | **G** | **C** | **G** | **T** | **C** | **G** | **G** | **A** | **T** | **G** | **A** | **T** | **C** | **T** | **G** | **T** | **A** | **C** | **A** | **T** | **C** | **A** | **A** | **A** | **G** | **A** | **T** | **A** | **T** | **T** | **G** | **A** | **T** | **A** | **T** | **G** | **G** | **A** | **A** | **A** | **T** | **T** | **A** | **C** | **T** | **A** | **A** | **A** | **A** | **T** | **A** | **A** | **A** | **A** | **G** | **A** | **A** | **A** | **A** | **A** | **A** | **T** | **G** | **A** | **T** | **C** | **G** | **A** | **A** | **G** | **T** | **A** | **T** | **C** | **C** | **A** | **G** | **T** | **G** | **A** | **A** | **T** | **T** | **A** | **C** | **A** | **G** | **A** | **T** | **T** | **A** | **T** | **G** | **A** | **C** | **T** | **A** | **G** | **C** | **C** | **G** | **T** | **C** | **C** | **T** | **C** | **A** | **A** | **T** | **A** | **T** | **G** | **T** | **A** | **G** | **A** | **T** | **G** | **G** | **A** | **C** | **A** | **A** | **A** | **C** | **C** | **A** | **A** | **A** | **G** | **A** | **A** | **G** | **A** | **G** | **A** | **T** | **T** | **T** | **T** | **A** | **G** | **A** | **A** | **T** | **T** | **A** | **G** | **A** | **A** | **A** | **G** | **G** | **C** | **A** | **T** | **C** | **G** | **A** | **T** | **T** | **A** | **G** | **C** | **T** | **C** | **G** | **T** | **G** | **A** | **G** | **C** | **T** | **T** | **C** | **A** | **T** | **G** | **A** | **T** | **T** | **C** | **A** | **G** | **T** | **T** | **T** | **C** | **A** | **C** | **A** | **G** | **C** | **A** | **A** | **T** | **T** | **A** | **T** | **T** | **T** | **G** | **C** | **A** | **G** | **C** | **G** | **A** | **T** | **G** | **A** | **T** | **G** | **A** | **T** | **G** | **A** | **T** | **G** | **T** | **C** | **G** | **G** | **C** | **A** | **T** | **T** | **G** | **A** | **C** | **G** | **G** | **A** | **G** | **C** | **A** | **A** | **G** | **C** | **A** | **G** | **A** | **A** | **A** | **A** | **A** | **A** | **G** | **T** | **G** | **A** | **G** | **A** | **C** | **A** | **C** | **C** | **A** | **G** | **A** | **A** | **A** | **T** | **G** | **T** | **T** | **C** | **C** | **G** | **T** | **A** | **A** | **G** | **C** | **A** | **G** | **T** | **T** | **G** | **A** | **A** | **A** | **A** | **T** | **G** | **G** | **T** | **G** | **G** | **C** | **A** | **G** | **A** | **G** | **A** | **T** | **C** | **A** | **T** | **C** | **A** | **A** | **C** | **G** | **C** | **T** | **T** | **C** | **C** | **C** | **A** | **G** | **T** | **C** | **T** | **G** | **A** | **G** | **A** | **T** | **G** | **C** | **G** | **T** | **G** | **C** | **G** | **C** | **T** | **G** | **C** | **T** | **T** | **C** | **T** | **T** | **C** | **A** | **T** | **C** | **T** | **G** | **C** | **G** | **T** | **C** | **C** | **A** | **G** | **T** | **C** | **A** | **A** | **T** | **T** | **T** | **A** | **G** | **A** | **A** | **G** | **A** | **A** | **A** | **A** | **A** | **A** | **G** | **T** | **T** | **T** | **A** | **A** | **A** | **A** | **C** | **A** | **A** | **G** | **G** | **C** | **A** | **T** | **T** | **G** | **A** | **G** | **C** | **A** | **G** | **T** | **T** | **A** | **T** | **T** | **G** | **A** | **A** | **G** | **G** | **A** | **A** | **T** | **T** | **G** | **C** | **A** | **G** | **A** | **A** | **T** | **A** | **A** | **A** | **A** | **T** | **C** | **C** | **A** | **G** | **A** | **T** | **T** | **T** | **C** | **G** | **C** | **T** | **A** | **A** | **A** | **A** | **T** | **G** | **G** | **G** | **A** | **T** | **G** | **T** | **A** | **G** | **A** | **A** | **G** | **A** | **T** | **G** | **T** | **A** | **A** | **A** | **A** | **T** | **T** | **A** | **A** | **C** | **T** | **A** | **G** | **T** | **T** | **C** | **C** | **A** | **T** | **T** | **G** | **A** | **G** | **G** | **A** | **T** | **C** | **A** | **C** | **T** | **T** | **G** | **T** | **T** | **C** | **C** | **G** | **A** | **A** | **T** | **C** | **G** | **T** | **T** | **C** | **A** | **A** | **G** | **A** | **A** | **T** | **T** | **G** | **T** | **T** | **A** | **T** | **C** | **A** | **A** | **A** | **C** | **A** | **C** | **A** | **T** | **T** | **A** | **A** | **G** | **A** | **C** | **A** | **T** | **G** | **C** | **T** | **A** | **A** | **G** | **G** | **C** | **T** | **A** | **A** | **T** | **G** | **A** | **A** | **T** | **T** | **A** | **G** | **A** | **A** | **G** | **T** | **C** | **T** | **A** | **T** | **T** | **T** | **G** | **C** | **A** | **T** | **A** | **A** | **A** | **A** | **T** | **A** | **G** | **A** | **T** | **A** | **A** | **C** | **A** | **A** | **T** | **C** | **T** | **T** | **T** | **T** | **A** | **T** | **T** | **A** | **C** | **G** | **T** | **A** | **T** | **C** | **A** | **T** | **T** | **G** | **A** | **C** | **G** | **A** | **C** | **G** | **G** | **G** | **A** | **C** | **A** | **G** | **G** | **A** | **T** | **T** | **T** | **A** | **A** | **T** | **A** | **T** | **G** | **A** | **A** | **T** | **G** | **A** | **A** | **A** | **C** | **C** | **A** | **A** | **A** | **A** | **C** | **G** | **G** | **G** | **A** | **A** | **G** | **T** | **T** | **A** | **T** | **G** | **G** | **A** | **T** | **T** | **G** | **A** | **A** | **C** | **A** | **A** | **T** | **A** | **T** | **C** | **A** | **A** | **A** | **G** | **A** | **A** | **C** | **G** | **A** | **G** | **T** | **A** | **G** | **C** | **T** | **G** | **G** | **T** | **A** | **T** | **T** | **G** | **G** | **T** | **G** | **G** | **A** | **A** | **C** | **A** | **G** | **T** | **A** | **A** | **A** | **A** | **A** | **T** | **C** | **A** | **T** | **T** | **A** | **G** | **T** | **T** | **T** | **T** | **A** | **A** | **A** | **G** | **G** | **G** | **C** | **A** | **A** | **G** | **G** | **T** | **A** | **C** | **G** | **A** | **G** | **C** | **G** | **T** | **G** | **G** | **A** | **G** | **A** | **T** | **C** | **A** | **A** | **G** | **G** | **T** | **C** | **C** | **C** | **T** | **T** | **T** | **G** | **A** | **T** | **G** | **A** | **A** | **G** | **G** | **A** | **G** | **G** | **C** | **A** | **-** | **-** | **-** |
| **N54538** | **A** | **T** | **G** | **A** | **T** | **G** | **G** | **G** | **A** | **A** | **A** | **A** | **A** | **T** | **A** | **T** | **C** | **C** | **A** | **G** | **A** | **G** | **C** | **G** | **A** | **T** | **G** | **C** | **T** | **A** | **G** | **C** | **T** | **G** | **T** | **T** | **T** | **A** | **T** | **T** | **C** | **G** | **G** | **G** | **G** | **A** | **T** | **T** | **G** | **C** | **G** | **G** | **C** | **T** | **T** | **T** | **C** | **C** | **T** | **T** | **A** | **T** | **T** | **A** | **T** | **T** | **C** | **T** | **A** | **T** | **T** | **T** | **T** | **C** | **A** | **C** | **T** | **T** | **T** | **T** | **C** | **A** | **C** | **T** | **T** | **A** | **T** | **T** | **T** | **T** | **T** | **A** | **T** | **G** | **C** | **C** | **A** | **G** | **T** | **A** | **A** | **T** | **C** | **A** | **A** | **A** | **G** | **T** | **C** | **A** | **T** | **T** | **G** | **G** | **T** | **G** | **G** | **G** | **G** | **A** | **G** | **A** | **A** | **T** | **T** | **A** | **C** | **T** | **G** | **C** | **G** | **T** | **G** | **C** | **A** | **C** | **G** | **T** | **T** | **T** | **A** | **T** | **T** | **A** | **T** | **A** | **T** | **G** | **T** | **T** | **C** | **C** | **G** | **C** | **T** | **T** | **A** | **T** | **C** | **T** | **T** | **C** | **C** | **A** | **T** | **T** | **T** | **G** | **T** | **T** | **A** | **G** | **C** | **T** | **A** | **T** | **T** | **T** | **C** | **C** | **T** | **T** | **A** | **G** | **G** | **T** | **G** | **T** | **A** | **G** | **G** | **A** | **T** | **T** | **A** | **A** | **T** | **C** | **G** | **T** | **C** | **T** | **T** | **T** | **C** | **T** | **A** | **T** | **T** | **A** | **T** | **T** | **A** | **T** | **C** | **A** | **C** | **T** | **T** | **A** | **T** | **T** | **C** | **A** | **A** | **A** | **A** | **G** | **A** | **C** | **A** | **A** | **A** | **A** | **T** | **A** | **C** | **G** | **G** | **G** | **A** | **A** | **A** | **A** | **T** | **C** | **G** | **A** | **A** | **G** | **A** | **A** | **A** | **A** | **G** | **C** | **T** | **G** | **C** | **G** | **T** | **G** | **C** | **A** | **C** | **T** | **C** | **T** | **C** | **T** | **T** | **C** | **T** | **G** | **G** | **C** | **A** | **A** | **T** | **T** | **A** | **T** | **G** | **A** | **A** | **T** | **C** | **C** | **A** | **A** | **A** | **T** | **T** | **G** | **T** | **T** | **G** | **C** | **T** | **T** | **C** | **T** | **T** | **C** | **C** | **G** | **A** | **T** | **C** | **C** | **C** | **A** | **A** | **G** | **T** | **G** | **C** | **A** | **T** | **C** | **G** | **G** | **A** | **C** | **G** | **A** | **T** | **T** | **T** | **A** | **T** | **A** | **C** | **A** | **T** | **C** | **A** | **A** | **A** | **G** | **A** | **T** | **A** | **T** | **T** | **G** | **A** | **T** | **A** | **A** | **G** | **G** | **A** | **A** | **A** | **T** | **C** | **A** | **C** | **T** | **A** | **A** | **A** | **A** | **T** | **A** | **A** | **A** | **A** | **G** | **A** | **A** | **A** | **A** | **A** | **A** | **T** | **G** | **A** | **T** | **T** | **G** | **A** | **A** | **G** | **T** | **A** | **T** | **C** | **T** | **A** | **G** | **T** | **G** | **A** | **A** | **T** | **T** | **A** | **C** | **A** | **A** | **A** | **T** | **T** | **G** | **T** | **A** | **A** | **C** | **G** | **A** | **G** | **C** | **C** | **G** | **T** | **C** | **C** | **C** | **C** | **A** | **A** | **T** | **A** | **T** | **G** | **T** | **A** | **G** | **A** | **T** | **G** | **G** | **A** | **C** | **A** | **A** | **A** | **C** | **C** | **A** | **A** | **A** | **G** | **A** | **A** | **G** | **A** | **A** | **A** | **T** | **T** | **T** | **T** | **A** | **G** | **A** | **A** | **T** | **T** | **A** | **G** | **A** | **A** | **A** | **G** | **A** | **C** | **A** | **T** | **C** | **G** | **A** | **T** | **T** | **A** | **G** | **C** | **C** | **C** | **G** | **T** | **G** | **A** | **G** | **C** | **T** | **G** | **C** | **A** | **T** | **G** | **A** | **T** | **T** | **C** | **A** | **G** | **T** | **T** | **T** | **C** | **G** | **C** | **A** | **A** | **C** | **A** | **A** | **T** | **T** | **G** | **T** | **T** | **T** | **G** | **C** | **A** | **G** | **C** | **A** | **A** | **T** | **G** | **A** | **T** | **G** | **A** | **T** | **G** | **A** | **T** | **G** | **T** | **C** | **G** | **G** | **C** | **A** | **T** | **T** | **A** | **A** | **C** | **G** | **G** | **A** | **G** | **C** | **A** | **A** | **G** | **C** | **A** | **G** | **A** | **A** | **A** | **A** | **A** | **A** | **G** | **C** | **G** | **A** | **G** | **A** | **C** | **A** | **C** | **C** | **A** | **G** | **A** | **A** | **A** | **T** | **G** | **T** | **T** | **C** | **C** | **G** | **C** | **A** | **A** | **G** | **C** | **A** | **G** | **T** | **T** | **G** | **A** | **A** | **A** | **A** | **T** | **G** | **G** | **T** | **A** | **G** | **C** | **A** | **G** | **A** | **G** | **A** | **T** | **C** | **A** | **T** | **C** | **A** | **A** | **C** | **G** | **C** | **T** | **T** | **C** | **C** | **C** | **A** | **G** | **T** | **C** | **T** | **G** | **A** | **G** | **A** | **T** | **G** | **C** | **G** | **T** | **G** | **C** | **G** | **C** | **T** | **G** | **C** | **T** | **T** | **C** | **T** | **T** | **C** | **A** | **T** | **C** | **T** | **G** | **C** | **G** | **T** | **C** | **C** | **A** | **G** | **T** | **C** | **A** | **A** | **T** | **T** | **T** | **A** | **G** | **A** | **A** | **G** | **A** | **G** | **A** | **A** | **A** | **A** | **G** | **T** | **T** | **T** | **A** | **A** | **A** | **G** | **C** | **A** | **A** | **G** | **G** | **T** | **A** | **T** | **C** | **G** | **A** | **G** | **C** | **A** | **G** | **T** | **T** | **A** | **T** | **T** | **G** | **A** | **A** | **G** | **G** | **A** | **A** | **T** | **T** | **G** | **C** | **A** | **A** | **A** | **A** | **T** | **A** | **A** | **A** | **A** | **T** | **C** | **C** | **A** | **G** | **A** | **T** | **T** | **T** | **C** | **G** | **C** | **T** | **G** | **A** | **A** | **A** | **T** | **G** | **G** | **G** | **A** | **T** | **G** | **T** | **A** | **G** | **A** | **A** | **G** | **A** | **T** | **G** | **T** | **A** | **A** | **A** | **A** | **C** | **T** | **A** | **T** | **C** | **T** | **A** | **G** | **T** | **T** | **C** | **C** | **A** | **T** | **T** | **G** | **A** | **G** | **G** | **A** | **T** | **C** | **A** | **C** | **T** | **T** | **G** | **T** | **T** | **C** | **C** | **G** | **A** | **A** | **T** | **C** | **G** | **T** | **T** | **C** | **A** | **A** | **G** | **A** | **A** | **T** | **T** | **G** | **T** | **T** | **A** | **T** | **C** | **A** | **A** | **A** | **T** | **A** | **C** | **A** | **T** | **T** | **A** | **A** | **G** | **A** | **C** | **A** | **T** | **G** | **C** | **C** | **A** | **A** | **A** | **G** | **C** | **C** | **A** | **A** | **T** | **G** | **A** | **A** | **T** | **T** | **A** | **G** | **A** | **G** | **G** | **T** | **A** | **T** | **A** | **T** | **T** | **T** | **G** | **C** | **A** | **C** | **A** | **A** | **A** | **A** | **T** | **A** | **G** | **A** | **C** | **A** | **A** | **T** | **A** | **A** | **T** | **C** | **T** | **T** | **T** | **T** | **G** | **T** | **T** | **A** | **C** | **G** | **T** | **A** | **T** | **T** | **A** | **T** | **T** | **G** | **A** | **C** | **G** | **A** | **C** | **G** | **G** | **A** | **A** | **C** | **A** | **G** | **G** | **G** | **T** | **T** | **T** | **A** | **A** | **T** | **A** | **T** | **G** | **A** | **A** | **T** | **G** | **A** | **A** | **A** | **C** | **G** | **A** | **A** | **A** | **A** | **C** | **G** | **G** | **G** | **A** | **A** | **G** | **T** | **T** | **A** | **T** | **G** | **G** | **A** | **T** | **T** | **G** | **A** | **A** | **C** | **A** | **A** | **T** | **A** | **T** | **C** | **A** | **A** | **A** | **G** | **A** | **A** | **C** | **G** | **A** | **G** | **T** | **A** | **G** | **C** | **T** | **G** | **G** | **T** | **A** | **T** | **T** | **G** | **G** | **T** | **G** | **G** | **A** | **A** | **C** | **A** | **G** | **T** | **A** | **A** | **A** | **A** | **A** | **T** | **C** | **A** | **T** | **T** | **A** | **G** | **T** | **T** | **T** | **T** | **A** | **A** | **A** | **G** | **G** | **G** | **C** | **A** | **A** | **G** | **G** | **T** | **A** | **C** | **A** | **A** | **G** | **T** | **G** | **T** | **A** | **G** | **A** | **A** | **A** | **T** | **C** | **A** | **A** | **G** | **G** | **T** | **C** | **C** | **C** | **T** | **T** | **T** | **G** | **A** | **T** | **G** | **A** | **A** | **G** | **G** | **A** | **G** | **G** | **C** | **A** | **-** | **-** | **-** |
| **N59469F** | **A** | **T** | **G** | **A** | **T** | **G** | **G** | **G** | **A** | **A** | **G** | **A** | **A** | **T** | **A** | **T** | **C** | **C** | **A** | **A** | **A** | **G** | **C** | **A** | **A** | **T** | **G** | **C** | **T** | **A** | **G** | **C** | **T** | **G** | **T** | **T** | **T** | **A** | **T** | **T** | **C** | **G** | **G** | **G** | **G** | **A** | **T** | **T** | **G** | **C** | **G** | **G** | **C** | **T** | **T** | **T** | **C** | **C** | **T** | **T** | **A** | **T** | **T** | **A** | **T** | **T** | **C** | **T** | **A** | **T** | **T** | **T** | **T** | **C** | **A** | **C** | **T** | **T** | **T** | **T** | **C** | **A** | **C** | **T** | **T** | **A** | **T** | **T** | **T** | **T** | **T** | **A** | **T** | **G** | **C** | **C** | **A** | **G** | **T** | **A** | **A** | **C** | **C** | **A** | **A** | **A** | **G** | **T** | **C** | **A** | **T** | **T** | **G** | **G** | **T** | **G** | **G** | **G** | **G** | **A** | **G** | **A** | **A** | **T** | **T** | **G** | **C** | **T** | **G** | **C** | **G** | **T** | **G** | **C** | **G** | **C** | **G** | **T** | **T** | **T** | **A** | **T** | **T** | **A** | **T** | **A** | **C** | **G** | **T** | **T** | **C** | **C** | **G** | **C** | **T** | **T** | **A** | **T** | **C** | **T** | **T** | **C** | **C** | **A** | **T** | **T** | **T** | **G** | **A** | **T** | **G** | **G** | **C** | **A** | **A** | **T** | **T** | **T** | **C** | **A** | **T** | **T** | **A** | **G** | **G** | **T** | **G** | **T** | **A** | **G** | **G** | **A** | **T** | **T** | **G** | **A** | **T** | **C** | **G** | **T** | **A** | **T** | **T** | **T** | **C** | **T** | **A** | **T** | **T** | **G** | **T** | **T** | **A** | **T** | **C** | **A** | **C** | **T** | **T** | **A** | **T** | **T** | **C** | **A** | **A** | **A** | **A** | **G** | **G** | **C** | **A** | **A** | **A** | **A** | **T** | **A** | **C** | **G** | **G** | **G** | **A** | **A** | **A** | **A** | **T** | **C** | **G** | **A** | **A** | **G** | **A** | **A** | **A** | **A** | **G** | **C** | **T** | **T** | **C** | **G** | **T** | **G** | **C** | **A** | **C** | **T** | **T** | **T** | **C** | **T** | **T** | **C** | **T** | **G** | **G** | **C** | **A** | **A** | **T** | **T** | **A** | **T** | **G** | **A** | **A** | **T** | **C** | **C** | **A** | **A** | **A** | **C** | **T** | **T** | **T** | **T** | **G** | **C** | **A** | **T** | **C** | **A** | **G** | **C** | **C** | **G** | **A** | **T** | **C** | **C** | **C** | **A** | **A** | **G** | **C** | **A** | **C** | **G** | **T** | **C** | **G** | **G** | **A** | **T** | **G** | **A** | **T** | **C** | **T** | **G** | **T** | **A** | **C** | **A** | **T** | **C** | **A** | **A** | **A** | **G** | **A** | **T** | **A** | **T** | **T** | **G** | **A** | **T** | **A** | **T** | **G** | **G** | **A** | **A** | **A** | **T** | **T** | **A** | **C** | **T** | **A** | **A** | **A** | **A** | **T** | **A** | **A** | **A** | **A** | **G** | **A** | **A** | **A** | **A** | **A** | **A** | **T** | **G** | **A** | **T** | **C** | **G** | **A** | **A** | **G** | **T** | **A** | **T** | **C** | **C** | **A** | **G** | **T** | **G** | **A** | **A** | **T** | **T** | **A** | **C** | **A** | **G** | **A** | **T** | **T** | **A** | **T** | **G** | **A** | **C** | **T** | **A** | **G** | **C** | **C** | **G** | **T** | **C** | **C** | **T** | **C** | **A** | **A** | **T** | **A** | **T** | **G** | **T** | **A** | **G** | **A** | **T** | **G** | **G** | **A** | **C** | **A** | **A** | **A** | **C** | **C** | **A** | **A** | **A** | **G** | **A** | **A** | **G** | **A** | **G** | **A** | **T** | **T** | **T** | **T** | **A** | **G** | **A** | **A** | **T** | **T** | **A** | **G** | **A** | **A** | **A** | **G** | **G** | **C** | **A** | **T** | **C** | **G** | **A** | **T** | **T** | **A** | **G** | **C** | **T** | **C** | **G** | **T** | **G** | **A** | **G** | **C** | **T** | **T** | **C** | **A** | **T** | **G** | **A** | **T** | **T** | **C** | **A** | **G** | **T** | **T** | **T** | **C** | **A** | **C** | **A** | **G** | **C** | **A** | **A** | **T** | **T** | **A** | **T** | **T** | **T** | **G** | **C** | **A** | **G** | **C** | **G** | **A** | **T** | **G** | **A** | **T** | **G** | **A** | **T** | **G** | **A** | **T** | **G** | **T** | **C** | **G** | **G** | **C** | **A** | **T** | **T** | **G** | **A** | **C** | **G** | **G** | **A** | **G** | **C** | **A** | **A** | **G** | **C** | **A** | **G** | **A** | **A** | **A** | **A** | **A** | **A** | **G** | **T** | **G** | **A** | **G** | **A** | **C** | **A** | **C** | **C** | **A** | **G** | **A** | **A** | **A** | **T** | **G** | **T** | **T** | **C** | **C** | **G** | **T** | **A** | **A** | **G** | **C** | **A** | **G** | **T** | **T** | **G** | **A** | **A** | **A** | **A** | **T** | **G** | **G** | **T** | **G** | **G** | **C** | **A** | **G** | **A** | **G** | **A** | **T** | **C** | **A** | **T** | **C** | **A** | **A** | **C** | **G** | **C** | **T** | **T** | **C** | **C** | **C** | **A** | **G** | **T** | **C** | **T** | **G** | **A** | **G** | **A** | **T** | **G** | **C** | **G** | **T** | **G** | **C** | **G** | **C** | **T** | **G** | **C** | **T** | **T** | **C** | **T** | **T** | **C** | **A** | **T** | **C** | **T** | **G** | **C** | **G** | **T** | **C** | **C** | **A** | **G** | **T** | **C** | **A** | **A** | **T** | **T** | **T** | **A** | **G** | **A** | **A** | **G** | **A** | **A** | **A** | **A** | **A** | **A** | **G** | **T** | **T** | **T** | **A** | **A** | **A** | **A** | **C** | **A** | **A** | **G** | **G** | **C** | **A** | **T** | **T** | **G** | **A** | **G** | **C** | **A** | **G** | **T** | **T** | **A** | **T** | **T** | **G** | **A** | **A** | **G** | **G** | **A** | **A** | **T** | **T** | **G** | **C** | **A** | **G** | **A** | **A** | **T** | **A** | **A** | **A** | **A** | **T** | **C** | **C** | **A** | **G** | **A** | **T** | **T** | **T** | **C** | **G** | **C** | **T** | **A** | **A** | **A** | **A** | **T** | **G** | **G** | **G** | **A** | **T** | **G** | **T** | **A** | **G** | **A** | **A** | **G** | **A** | **T** | **G** | **T** | **A** | **A** | **A** | **A** | **T** | **T** | **A** | **A** | **C** | **T** | **A** | **G** | **T** | **T** | **C** | **C** | **A** | **T** | **T** | **G** | **A** | **G** | **G** | **A** | **T** | **C** | **A** | **C** | **T** | **T** | **G** | **T** | **T** | **C** | **C** | **G** | **A** | **A** | **T** | **C** | **G** | **T** | **T** | **C** | **A** | **A** | **G** | **A** | **A** | **T** | **T** | **G** | **T** | **T** | **A** | **T** | **C** | **A** | **A** | **A** | **C** | **A** | **C** | **A** | **T** | **T** | **A** | **A** | **G** | **A** | **C** | **A** | **T** | **G** | **C** | **T** | **A** | **A** | **G** | **G** | **C** | **T** | **A** | **A** | **T** | **G** | **A** | **A** | **T** | **T** | **A** | **G** | **A** | **A** | **G** | **T** | **C** | **T** | **A** | **T** | **T** | **T** | **G** | **C** | **A** | **T** | **A** | **A** | **A** | **A** | **T** | **A** | **G** | **A** | **T** | **A** | **A** | **C** | **A** | **A** | **T** | **C** | **T** | **T** | **T** | **T** | **A** | **T** | **T** | **A** | **C** | **G** | **T** | **A** | **T** | **C** | **A** | **T** | **T** | **G** | **A** | **C** | **G** | **A** | **C** | **G** | **G** | **G** | **A** | **C** | **A** | **G** | **G** | **A** | **T** | **T** | **T** | **A** | **A** | **T** | **A** | **T** | **G** | **A** | **A** | **T** | **G** | **A** | **A** | **A** | **C** | **C** | **A** | **A** | **A** | **A** | **C** | **G** | **G** | **G** | **A** | **A** | **G** | **T** | **T** | **A** | **T** | **G** | **G** | **A** | **T** | **T** | **G** | **A** | **A** | **C** | **A** | **A** | **T** | **A** | **T** | **C** | **A** | **A** | **A** | **G** | **A** | **A** | **C** | **G** | **A** | **G** | **T** | **A** | **G** | **C** | **T** | **G** | **G** | **T** | **A** | **T** | **T** | **G** | **G** | **T** | **G** | **G** | **A** | **A** | **C** | **A** | **G** | **T** | **A** | **A** | **A** | **A** | **A** | **T** | **C** | **A** | **T** | **T** | **A** | **G** | **T** | **T** | **T** | **T** | **A** | **A** | **A** | **G** | **G** | **G** | **C** | **A** | **A** | **G** | **G** | **T** | **A** | **C** | **G** | **A** | **G** | **C** | **G** | **T** | **G** | **G** | **A** | **G** | **A** | **T** | **C** | **A** | **A** | **G** | **G** | **T** | **C** | **C** | **C** | **T** | **T** | **T** | **G** | **A** | **T** | **G** | **A** | **A** | **G** | **G** | **A** | **G** | **G** | **C** | **A** | **-** | **-** | **-** |
| **N55229** | **A** | **T** | **G** | **A** | **T** | **G** | **G** | **G** | **A** | **A** | **G** | **A** | **A** | **T** | **A** | **T** | **C** | **C** | **A** | **A** | **A** | **G** | **C** | **A** | **A** | **T** | **G** | **C** | **T** | **A** | **G** | **C** | **T** | **G** | **T** | **T** | **T** | **A** | **T** | **T** | **C** | **G** | **G** | **G** | **G** | **A** | **T** | **T** | **G** | **C** | **G** | **G** | **C** | **T** | **T** | **T** | **C** | **C** | **T** | **T** | **A** | **T** | **T** | **A** | **T** | **T** | **C** | **T** | **A** | **T** | **T** | **T** | **T** | **C** | **A** | **C** | **T** | **T** | **T** | **T** | **C** | **A** | **C** | **T** | **T** | **A** | **T** | **T** | **T** | **T** | **T** | **A** | **T** | **G** | **C** | **C** | **A** | **G** | **T** | **A** | **A** | **C** | **C** | **A** | **A** | **A** | **G** | **T** | **C** | **A** | **T** | **T** | **G** | **G** | **T** | **G** | **G** | **G** | **G** | **A** | **G** | **A** | **A** | **T** | **T** | **G** | **C** | **T** | **G** | **C** | **G** | **T** | **G** | **C** | **G** | **C** | **G** | **T** | **T** | **T** | **A** | **T** | **T** | **A** | **T** | **A** | **C** | **G** | **T** | **T** | **C** | **C** | **G** | **C** | **T** | **T** | **A** | **T** | **C** | **T** | **T** | **C** | **C** | **A** | **T** | **T** | **T** | **G** | **A** | **T** | **G** | **G** | **C** | **A** | **A** | **T** | **T** | **T** | **C** | **A** | **T** | **T** | **A** | **G** | **G** | **T** | **G** | **T** | **A** | **G** | **G** | **A** | **T** | **T** | **G** | **A** | **T** | **C** | **G** | **T** | **A** | **T** | **T** | **T** | **C** | **T** | **A** | **T** | **T** | **G** | **T** | **T** | **A** | **T** | **C** | **A** | **C** | **T** | **T** | **A** | **T** | **T** | **C** | **A** | **A** | **A** | **A** | **G** | **G** | **C** | **A** | **A** | **A** | **A** | **T** | **A** | **C** | **G** | **A** | **G** | **A** | **A** | **A** | **A** | **T** | **C** | **G** | **A** | **A** | **G** | **A** | **A** | **A** | **A** | **G** | **C** | **T** | **T** | **C** | **G** | **T** | **G** | **C** | **A** | **C** | **T** | **T** | **T** | **C** | **T** | **T** | **C** | **T** | **G** | **G** | **C** | **A** | **A** | **T** | **T** | **A** | **T** | **G** | **A** | **A** | **T** | **C** | **C** | **A** | **A** | **A** | **C** | **T** | **T** | **T** | **T** | **G** | **C** | **A** | **T** | **C** | **A** | **G** | **C** | **C** | **G** | **A** | **T** | **C** | **C** | **C** | **A** | **A** | **G** | **C** | **G** | **C** | **G** | **T** | **C** | **G** | **G** | **A** | **T** | **G** | **A** | **T** | **C** | **T** | **G** | **T** | **A** | **C** | **A** | **T** | **C** | **A** | **A** | **A** | **G** | **A** | **T** | **A** | **T** | **T** | **G** | **A** | **T** | **A** | **T** | **G** | **G** | **A** | **A** | **A** | **T** | **T** | **A** | **C** | **T** | **A** | **A** | **A** | **A** | **T** | **A** | **A** | **A** | **A** | **G** | **A** | **A** | **A** | **A** | **A** | **A** | **T** | **G** | **A** | **T** | **C** | **G** | **A** | **A** | **G** | **T** | **A** | **T** | **C** | **C** | **A** | **G** | **T** | **G** | **A** | **A** | **T** | **T** | **A** | **C** | **A** | **G** | **A** | **T** | **T** | **A** | **T** | **G** | **A** | **C** | **T** | **A** | **G** | **C** | **C** | **G** | **T** | **C** | **C** | **T** | **C** | **A** | **A** | **T** | **A** | **T** | **G** | **T** | **A** | **G** | **A** | **T** | **G** | **G** | **A** | **C** | **A** | **A** | **A** | **C** | **C** | **A** | **A** | **A** | **G** | **A** | **A** | **G** | **A** | **G** | **A** | **T** | **T** | **T** | **T** | **A** | **G** | **A** | **A** | **T** | **T** | **A** | **G** | **A** | **A** | **A** | **G** | **G** | **C** | **A** | **T** | **C** | **G** | **A** | **T** | **T** | **A** | **G** | **C** | **T** | **C** | **G** | **T** | **G** | **A** | **G** | **C** | **T** | **T** | **C** | **A** | **T** | **G** | **A** | **T** | **T** | **C** | **A** | **G** | **T** | **T** | **T** | **C** | **A** | **C** | **A** | **G** | **C** | **A** | **A** | **T** | **T** | **A** | **T** | **T** | **T** | **G** | **C** | **A** | **G** | **C** | **G** | **A** | **T** | **G** | **A** | **T** | **G** | **A** | **T** | **G** | **A** | **T** | **G** | **T** | **C** | **G** | **G** | **C** | **A** | **T** | **T** | **G** | **A** | **C** | **G** | **G** | **A** | **G** | **C** | **A** | **A** | **G** | **C** | **A** | **G** | **A** | **A** | **A** | **A** | **A** | **A** | **G** | **T** | **G** | **A** | **G** | **A** | **C** | **A** | **A** | **C** | **A** | **G** | **A** | **A** | **A** | **T** | **G** | **T** | **T** | **C** | **C** | **G** | **T** | **A** | **A** | **G** | **C** | **A** | **G** | **T** | **T** | **G** | **A** | **A** | **A** | **A** | **T** | **G** | **G** | **T** | **G** | **G** | **C** | **A** | **G** | **A** | **G** | **A** | **T** | **C** | **A** | **T** | **C** | **A** | **A** | **C** | **G** | **C** | **T** | **T** | **C** | **C** | **C** | **A** | **G** | **T** | **C** | **T** | **G** | **A** | **G** | **A** | **T** | **G** | **C** | **G** | **T** | **G** | **C** | **G** | **C** | **T** | **G** | **C** | **T** | **T** | **C** | **T** | **T** | **C** | **A** | **T** | **C** | **T** | **G** | **C** | **G** | **T** | **C** | **C** | **A** | **G** | **T** | **C** | **A** | **A** | **T** | **T** | **T** | **A** | **G** | **A** | **A** | **G** | **A** | **A** | **A** | **A** | **A** | **A** | **G** | **T** | **T** | **T** | **A** | **A** | **A** | **A** | **C** | **A** | **A** | **G** | **G** | **C** | **A** | **T** | **T** | **G** | **A** | **G** | **C** | **A** | **G** | **T** | **T** | **A** | **T** | **T** | **G** | **A** | **A** | **G** | **G** | **A** | **A** | **T** | **T** | **G** | **C** | **A** | **G** | **A** | **A** | **T** | **A** | **A** | **A** | **A** | **T** | **C** | **C** | **A** | **G** | **A** | **T** | **T** | **T** | **C** | **G** | **C** | **T** | **A** | **A** | **A** | **A** | **T** | **G** | **G** | **G** | **A** | **T** | **G** | **T** | **A** | **G** | **A** | **A** | **G** | **A** | **T** | **G** | **T** | **A** | **A** | **A** | **A** | **T** | **T** | **A** | **A** | **C** | **T** | **A** | **G** | **T** | **T** | **C** | **C** | **A** | **T** | **T** | **G** | **A** | **G** | **G** | **A** | **T** | **C** | **A** | **C** | **T** | **T** | **G** | **T** | **T** | **C** | **C** | **G** | **A** | **A** | **T** | **C** | **G** | **T** | **T** | **C** | **A** | **A** | **G** | **A** | **A** | **T** | **T** | **G** | **T** | **T** | **A** | **T** | **C** | **A** | **A** | **A** | **C** | **A** | **C** | **A** | **T** | **T** | **A** | **A** | **G** | **A** | **C** | **A** | **T** | **G** | **C** | **T** | **A** | **A** | **G** | **G** | **C** | **T** | **A** | **A** | **T** | **G** | **A** | **A** | **T** | **T** | **A** | **G** | **A** | **A** | **G** | **T** | **C** | **T** | **A** | **T** | **T** | **T** | **G** | **C** | **A** | **T** | **A** | **A** | **A** | **A** | **T** | **A** | **G** | **A** | **T** | **A** | **A** | **C** | **A** | **A** | **T** | **C** | **T** | **T** | **T** | **T** | **A** | **T** | **T** | **A** | **C** | **G** | **T** | **A** | **T** | **T** | **A** | **T** | **T** | **G** | **A** | **C** | **G** | **A** | **C** | **G** | **G** | **G** | **A** | **C** | **A** | **G** | **G** | **A** | **T** | **T** | **T** | **A** | **A** | **T** | **A** | **T** | **G** | **A** | **A** | **T** | **G** | **A** | **A** | **A** | **C** | **C** | **A** | **A** | **A** | **A** | **C** | **G** | **G** | **G** | **A** | **A** | **G** | **T** | **T** | **A** | **T** | **G** | **G** | **A** | **T** | **T** | **G** | **A** | **A** | **C** | **A** | **A** | **T** | **A** | **T** | **C** | **A** | **A** | **A** | **G** | **A** | **A** | **C** | **G** | **A** | **G** | **T** | **A** | **G** | **C** | **T** | **G** | **G** | **T** | **A** | **T** | **T** | **G** | **G** | **T** | **G** | **G** | **A** | **A** | **C** | **A** | **G** | **T** | **A** | **A** | **A** | **A** | **A** | **T** | **C** | **A** | **T** | **T** | **A** | **G** | **T** | **T** | **T** | **T** | **A** | **A** | **A** | **G** | **G** | **G** | **C** | **A** | **A** | **G** | **G** | **T** | **A** | **C** | **G** | **A** | **G** | **C** | **G** | **T** | **G** | **G** | **A** | **G** | **A** | **T** | **C** | **A** | **A** | **G** | **G** | **T** | **C** | **C** | **C** | **T** | **T** | **T** | **G** | **A** | **T** | **G** | **A** | **A** | **G** | **G** | **A** | **G** | **G** | **C** | **A** | **-** | **-** | **-** |
| **N59513F** | **A** | **T** | **G** | **A** | **T** | **G** | **G** | **G** | **A** | **A** | **A** | **A** | **A** | **T** | **A** | **T** | **C** | **C** | **A** | **G** | **A** | **G** | **C** | **G** | **A** | **T** | **G** | **C** | **T** | **A** | **G** | **C** | **T** | **G** | **T** | **T** | **T** | **A** | **T** | **T** | **C** | **G** | **G** | **G** | **G** | **A** | **T** | **T** | **G** | **C** | **G** | **G** | **C** | **T** | **T** | **T** | **C** | **C** | **T** | **T** | **A** | **T** | **T** | **A** | **T** | **T** | **C** | **T** | **A** | **T** | **T** | **T** | **T** | **C** | **A** | **C** | **T** | **T** | **T** | **T** | **C** | **A** | **C** | **T** | **T** | **A** | **T** | **T** | **T** | **T** | **T** | **A** | **T** | **G** | **C** | **C** | **A** | **G** | **C** | **A** | **A** | **T** | **C** | **A** | **A** | **A** | **G** | **T** | **C** | **A** | **T** | **T** | **G** | **G** | **T** | **G** | **G** | **G** | **G** | **A** | **G** | **A** | **A** | **T** | **T** | **A** | **C** | **T** | **G** | **C** | **G** | **T** | **G** | **C** | **A** | **C** | **G** | **T** | **T** | **T** | **A** | **T** | **T** | **A** | **T** | **A** | **T** | **G** | **T** | **T** | **C** | **C** | **G** | **C** | **T** | **T** | **A** | **T** | **C** | **T** | **T** | **C** | **C** | **A** | **T** | **T** | **T** | **G** | **T** | **T** | **A** | **G** | **C** | **C** | **A** | **T** | **T** | **T** | **C** | **C** | **T** | **T** | **A** | **G** | **G** | **T** | **G** | **T** | **A** | **G** | **G** | **A** | **T** | **T** | **A** | **A** | **T** | **T** | **G** | **T** | **C** | **T** | **T** | **T** | **C** | **T** | **A** | **T** | **T** | **A** | **T** | **T** | **A** | **T** | **C** | **A** | **C** | **T** | **T** | **A** | **T** | **T** | **C** | **A** | **A** | **A** | **A** | **G** | **A** | **C** | **A** | **A** | **A** | **A** | **T** | **A** | **C** | **G** | **G** | **G** | **A** | **A** | **A** | **A** | **T** | **C** | **G** | **A** | **A** | **G** | **A** | **A** | **A** | **A** | **G** | **C** | **T** | **G** | **C** | **G** | **T** | **G** | **C** | **A** | **C** | **T** | **T** | **T** | **C** | **T** | **T** | **C** | **T** | **G** | **G** | **C** | **A** | **A** | **T** | **T** | **A** | **T** | **G** | **A** | **A** | **T** | **C** | **C** | **A** | **A** | **A** | **T** | **T** | **G** | **T** | **T** | **G** | **C** | **T** | **T** | **T** | **T** | **T** | **C** | **C** | **G** | **A** | **T** | **C** | **C** | **C** | **A** | **A** | **G** | **T** | **G** | **C** | **A** | **T** | **C** | **G** | **G** | **A** | **C** | **G** | **A** | **T** | **T** | **T** | **A** | **T** | **A** | **C** | **A** | **T** | **C** | **A** | **A** | **A** | **G** | **A** | **T** | **A** | **T** | **T** | **G** | **A** | **T** | **A** | **A** | **G** | **G** | **A** | **A** | **A** | **T** | **C** | **A** | **C** | **T** | **A** | **A** | **G** | **A** | **T** | **A** | **A** | **A** | **A** | **G** | **A** | **A** | **A** | **A** | **A** | **A** | **T** | **G** | **A** | **T** | **T** | **G** | **A** | **A** | **G** | **T** | **A** | **T** | **C** | **T** | **A** | **G** | **T** | **G** | **A** | **A** | **T** | **T** | **A** | **C** | **A** | **A** | **A** | **T** | **T** | **G** | **T** | **A** | **A** | **C** | **G** | **A** | **G** | **C** | **C** | **G** | **T** | **C** | **C** | **C** | **C** | **A** | **A** | **T** | **A** | **T** | **G** | **T** | **A** | **G** | **A** | **T** | **G** | **G** | **A** | **C** | **A** | **A** | **A** | **C** | **C** | **A** | **A** | **A** | **G** | **A** | **A** | **G** | **A** | **A** | **A** | **T** | **T** | **T** | **T** | **A** | **G** | **A** | **A** | **T** | **T** | **A** | **G** | **A** | **A** | **A** | **G** | **A** | **C** | **A** | **T** | **C** | **G** | **A** | **T** | **T** | **A** | **G** | **C** | **C** | **C** | **G** | **T** | **G** | **A** | **G** | **C** | **T** | **G** | **C** | **A** | **T** | **G** | **A** | **T** | **T** | **C** | **A** | **G** | **T** | **T** | **T** | **C** | **G** | **C** | **A** | **A** | **C** | **A** | **A** | **T** | **T** | **G** | **T** | **T** | **T** | **G** | **C** | **A** | **G** | **C** | **A** | **A** | **T** | **G** | **A** | **T** | **G** | **A** | **T** | **G** | **A** | **T** | **G** | **T** | **C** | **G** | **G** | **C** | **A** | **T** | **T** | **G** | **A** | **C** | **G** | **G** | **A** | **G** | **C** | **A** | **A** | **G** | **C** | **A** | **G** | **A** | **A** | **A** | **A** | **A** | **A** | **G** | **C** | **G** | **A** | **G** | **A** | **C** | **A** | **C** | **C** | **A** | **G** | **A** | **A** | **A** | **T** | **G** | **T** | **T** | **C** | **C** | **G** | **C** | **A** | **A** | **G** | **C** | **A** | **G** | **T** | **T** | **G** | **A** | **A** | **A** | **A** | **T** | **G** | **G** | **T** | **A** | **G** | **C** | **A** | **G** | **A** | **G** | **A** | **T** | **C** | **A** | **T** | **C** | **A** | **A** | **C** | **G** | **C** | **T** | **T** | **C** | **C** | **C** | **A** | **G** | **T** | **C** | **T** | **G** | **A** | **G** | **A** | **T** | **G** | **C** | **G** | **T** | **G** | **C** | **G** | **C** | **T** | **G** | **C** | **T** | **T** | **C** | **T** | **T** | **C** | **A** | **T** | **C** | **T** | **A** | **C** | **G** | **T** | **C** | **C** | **A** | **G** | **T** | **C** | **A** | **A** | **T** | **T** | **T** | **A** | **G** | **A** | **A** | **G** | **A** | **G** | **A** | **A** | **A** | **A** | **G** | **T** | **T** | **T** | **A** | **A** | **A** | **G** | **C** | **A** | **A** | **G** | **G** | **T** | **A** | **T** | **C** | **G** | **A** | **G** | **C** | **A** | **G** | **T** | **T** | **A** | **T** | **T** | **G** | **A** | **A** | **G** | **G** | **A** | **A** | **T** | **T** | **G** | **C** | **A** | **A** | **A** | **A** | **T** | **A** | **A** | **A** | **A** | **T** | **C** | **C** | **A** | **G** | **A** | **T** | **T** | **T** | **C** | **G** | **C** | **T** | **G** | **A** | **A** | **A** | **T** | **G** | **G** | **G** | **A** | **T** | **G** | **T** | **A** | **G** | **A** | **A** | **G** | **A** | **T** | **G** | **T** | **A** | **A** | **A** | **A** | **C** | **T** | **A** | **T** | **C** | **T** | **A** | **G** | **T** | **T** | **C** | **C** | **A** | **T** | **T** | **G** | **A** | **G** | **G** | **A** | **T** | **C** | **A** | **C** | **T** | **T** | **G** | **T** | **T** | **C** | **C** | **G** | **A** | **A** | **T** | **C** | **G** | **T** | **T** | **C** | **A** | **A** | **G** | **A** | **A** | **T** | **T** | **G** | **T** | **T** | **A** | **T** | **C** | **A** | **A** | **A** | **T** | **A** | **C** | **A** | **T** | **T** | **A** | **A** | **G** | **A** | **C** | **A** | **T** | **G** | **C** | **C** | **A** | **A** | **A** | **G** | **C** | **C** | **A** | **A** | **T** | **G** | **A** | **A** | **T** | **T** | **A** | **G** | **A** | **G** | **G** | **T** | **A** | **T** | **A** | **T** | **T** | **T** | **G** | **C** | **A** | **C** | **A** | **A** | **A** | **A** | **T** | **A** | **G** | **A** | **C** | **A** | **A** | **T** | **A** | **A** | **T** | **C** | **T** | **T** | **T** | **T** | **G** | **T** | **T** | **A** | **C** | **G** | **T** | **A** | **T** | **T** | **A** | **T** | **T** | **G** | **A** | **C** | **G** | **A** | **C** | **G** | **G** | **A** | **A** | **C** | **A** | **G** | **G** | **G** | **T** | **T** | **T** | **A** | **A** | **T** | **A** | **T** | **G** | **A** | **A** | **T** | **G** | **A** | **A** | **A** | **C** | **G** | **A** | **A** | **A** | **A** | **C** | **G** | **G** | **G** | **A** | **A** | **G** | **T** | **T** | **A** | **T** | **G** | **G** | **A** | **T** | **T** | **G** | **A** | **A** | **C** | **A** | **A** | **T** | **A** | **T** | **C** | **A** | **A** | **A** | **G** | **A** | **A** | **C** | **G** | **A** | **G** | **T** | **A** | **G** | **C** | **T** | **G** | **G** | **T** | **A** | **T** | **T** | **G** | **G** | **T** | **G** | **G** | **A** | **A** | **C** | **A** | **G** | **T** | **A** | **A** | **A** | **A** | **A** | **T** | **C** | **A** | **T** | **T** | **A** | **G** | **T** | **T** | **T** | **T** | **A** | **A** | **A** | **G** | **G** | **G** | **C** | **A** | **A** | **G** | **G** | **T** | **A** | **C** | **A** | **A** | **G** | **T** | **G** | **T** | **A** | **G** | **A** | **A** | **A** | **T** | **C** | **A** | **A** | **G** | **G** | **T** | **C** | **C** | **C** | **T** | **T** | **T** | **G** | **A** | **T** | **G** | **A** | **A** | **G** | **G** | **A** | **G** | **G** | **C** | **A** | **-** | **-** | **-** |
| **N60123F** | **A** | **T** | **G** | **A** | **T** | **G** | **G** | **G** | **A** | **A** | **G** | **A** | **A** | **T** | **A** | **T** | **C** | **C** | **A** | **A** | **A** | **G** | **C** | **A** | **A** | **T** | **G** | **C** | **T** | **A** | **G** | **C** | **T** | **G** | **T** | **T** | **T** | **A** | **T** | **T** | **C** | **G** | **G** | **G** | **G** | **A** | **T** | **T** | **G** | **C** | **G** | **G** | **C** | **T** | **T** | **T** | **C** | **C** | **T** | **T** | **A** | **T** | **T** | **A** | **T** | **T** | **C** | **T** | **A** | **T** | **T** | **T** | **T** | **C** | **A** | **C** | **T** | **T** | **T** | **T** | **C** | **A** | **C** | **T** | **T** | **A** | **T** | **T** | **T** | **T** | **T** | **A** | **T** | **G** | **C** | **C** | **A** | **G** | **T** | **A** | **A** | **C** | **C** | **A** | **A** | **A** | **G** | **T** | **C** | **A** | **T** | **T** | **G** | **G** | **T** | **G** | **G** | **G** | **G** | **A** | **G** | **A** | **A** | **T** | **T** | **G** | **C** | **T** | **G** | **C** | **G** | **T** | **G** | **C** | **G** | **C** | **G** | **T** | **T** | **T** | **A** | **T** | **T** | **A** | **T** | **A** | **C** | **G** | **T** | **T** | **C** | **C** | **G** | **C** | **T** | **T** | **A** | **T** | **C** | **T** | **T** | **C** | **C** | **A** | **T** | **T** | **T** | **G** | **A** | **T** | **G** | **G** | **C** | **A** | **A** | **T** | **T** | **T** | **C** | **A** | **T** | **T** | **A** | **G** | **G** | **T** | **G** | **T** | **A** | **G** | **G** | **A** | **T** | **T** | **G** | **A** | **T** | **C** | **G** | **T** | **A** | **T** | **T** | **T** | **C** | **T** | **A** | **T** | **T** | **G** | **T** | **T** | **A** | **T** | **C** | **A** | **C** | **T** | **T** | **A** | **T** | **T** | **C** | **A** | **A** | **A** | **A** | **G** | **A** | **C** | **A** | **A** | **A** | **A** | **T** | **A** | **C** | **G** | **G** | **G** | **A** | **A** | **A** | **A** | **T** | **C** | **G** | **A** | **A** | **G** | **A** | **A** | **A** | **A** | **G** | **C** | **T** | **T** | **C** | **G** | **T** | **G** | **C** | **A** | **C** | **T** | **T** | **T** | **C** | **T** | **T** | **C** | **T** | **G** | **G** | **C** | **A** | **A** | **T** | **T** | **A** | **T** | **G** | **A** | **A** | **T** | **C** | **C** | **A** | **A** | **A** | **C** | **T** | **T** | **T** | **T** | **G** | **C** | **A** | **T** | **C** | **A** | **G** | **C** | **C** | **G** | **A** | **T** | **C** | **C** | **C** | **A** | **A** | **G** | **C** | **G** | **C** | **G** | **T** | **C** | **G** | **G** | **A** | **T** | **G** | **A** | **T** | **C** | **T** | **G** | **T** | **A** | **C** | **A** | **T** | **C** | **A** | **A** | **A** | **G** | **A** | **T** | **A** | **T** | **T** | **G** | **A** | **T** | **A** | **T** | **G** | **G** | **A** | **A** | **A** | **T** | **T** | **A** | **C** | **T** | **A** | **A** | **A** | **A** | **T** | **A** | **A** | **A** | **A** | **G** | **A** | **A** | **A** | **A** | **A** | **A** | **T** | **G** | **A** | **T** | **C** | **G** | **A** | **A** | **G** | **T** | **A** | **T** | **C** | **C** | **A** | **G** | **T** | **G** | **A** | **A** | **T** | **T** | **A** | **C** | **A** | **G** | **A** | **T** | **T** | **A** | **T** | **G** | **A** | **C** | **T** | **A** | **G** | **C** | **C** | **G** | **T** | **C** | **C** | **T** | **C** | **A** | **A** | **T** | **A** | **T** | **G** | **T** | **A** | **G** | **A** | **T** | **G** | **G** | **A** | **C** | **A** | **A** | **A** | **C** | **C** | **A** | **A** | **A** | **G** | **A** | **A** | **G** | **A** | **G** | **A** | **T** | **T** | **T** | **T** | **A** | **G** | **A** | **A** | **T** | **T** | **A** | **G** | **A** | **A** | **A** | **G** | **G** | **C** | **A** | **T** | **C** | **G** | **A** | **T** | **T** | **A** | **G** | **C** | **T** | **C** | **G** | **T** | **G** | **A** | **G** | **C** | **T** | **T** | **C** | **A** | **T** | **G** | **A** | **T** | **T** | **C** | **A** | **G** | **T** | **T** | **T** | **C** | **A** | **C** | **A** | **G** | **C** | **A** | **A** | **T** | **T** | **A** | **T** | **T** | **T** | **G** | **C** | **A** | **G** | **C** | **G** | **A** | **T** | **G** | **A** | **T** | **G** | **A** | **T** | **G** | **A** | **T** | **G** | **T** | **C** | **G** | **G** | **C** | **A** | **T** | **T** | **G** | **A** | **C** | **G** | **G** | **A** | **G** | **C** | **A** | **A** | **G** | **C** | **A** | **G** | **A** | **A** | **A** | **A** | **A** | **A** | **G** | **T** | **G** | **A** | **G** | **A** | **C** | **A** | **C** | **C** | **A** | **G** | **A** | **A** | **A** | **T** | **G** | **T** | **T** | **C** | **C** | **G** | **T** | **A** | **A** | **G** | **C** | **A** | **G** | **T** | **T** | **G** | **A** | **A** | **A** | **A** | **T** | **G** | **G** | **T** | **G** | **G** | **C** | **A** | **G** | **A** | **G** | **A** | **T** | **C** | **A** | **T** | **C** | **A** | **A** | **C** | **G** | **C** | **T** | **T** | **C** | **C** | **C** | **A** | **G** | **T** | **C** | **T** | **G** | **A** | **G** | **A** | **T** | **G** | **C** | **G** | **T** | **G** | **C** | **G** | **C** | **T** | **G** | **C** | **T** | **T** | **C** | **T** | **T** | **C** | **A** | **T** | **C** | **T** | **G** | **C** | **G** | **T** | **C** | **C** | **A** | **G** | **T** | **C** | **A** | **A** | **T** | **T** | **T** | **A** | **G** | **A** | **A** | **G** | **A** | **A** | **A** | **A** | **A** | **A** | **G** | **T** | **T** | **T** | **A** | **A** | **A** | **A** | **C** | **A** | **A** | **G** | **G** | **C** | **A** | **T** | **T** | **G** | **A** | **G** | **C** | **A** | **G** | **T** | **T** | **A** | **T** | **T** | **G** | **A** | **A** | **G** | **G** | **A** | **A** | **T** | **T** | **G** | **C** | **A** | **G** | **A** | **A** | **T** | **A** | **A** | **A** | **A** | **T** | **C** | **C** | **A** | **G** | **A** | **T** | **T** | **T** | **C** | **G** | **C** | **T** | **A** | **A** | **A** | **A** | **T** | **G** | **G** | **G** | **A** | **T** | **G** | **T** | **A** | **G** | **A** | **A** | **G** | **A** | **T** | **G** | **T** | **A** | **A** | **A** | **A** | **T** | **T** | **A** | **A** | **C** | **T** | **A** | **G** | **T** | **T** | **C** | **C** | **A** | **T** | **T** | **G** | **A** | **G** | **G** | **A** | **T** | **C** | **A** | **C** | **T** | **T** | **G** | **T** | **T** | **C** | **C** | **G** | **A** | **A** | **T** | **C** | **G** | **T** | **T** | **C** | **A** | **A** | **G** | **A** | **A** | **T** | **T** | **G** | **T** | **T** | **A** | **T** | **C** | **A** | **A** | **A** | **C** | **A** | **C** | **A** | **T** | **T** | **A** | **A** | **G** | **A** | **C** | **A** | **T** | **G** | **C** | **T** | **A** | **A** | **G** | **G** | **C** | **T** | **A** | **A** | **T** | **G** | **A** | **A** | **T** | **T** | **A** | **G** | **A** | **A** | **G** | **T** | **C** | **T** | **A** | **T** | **T** | **T** | **G** | **C** | **A** | **T** | **A** | **A** | **A** | **A** | **T** | **A** | **G** | **A** | **T** | **A** | **A** | **C** | **A** | **A** | **T** | **C** | **T** | **T** | **T** | **T** | **A** | **T** | **T** | **A** | **C** | **G** | **T** | **A** | **T** | **C** | **A** | **T** | **T** | **G** | **A** | **C** | **G** | **A** | **C** | **G** | **G** | **G** | **A** | **C** | **A** | **G** | **G** | **A** | **T** | **T** | **T** | **A** | **A** | **T** | **A** | **T** | **G** | **A** | **A** | **T** | **G** | **A** | **A** | **A** | **C** | **C** | **A** | **A** | **A** | **A** | **C** | **G** | **G** | **G** | **A** | **A** | **G** | **T** | **T** | **A** | **T** | **G** | **G** | **A** | **T** | **T** | **G** | **A** | **A** | **C** | **A** | **A** | **T** | **A** | **T** | **C** | **A** | **A** | **A** | **G** | **A** | **A** | **C** | **G** | **A** | **G** | **T** | **A** | **G** | **C** | **T** | **G** | **G** | **T** | **A** | **T** | **T** | **G** | **G** | **T** | **G** | **G** | **A** | **A** | **C** | **A** | **G** | **T** | **A** | **A** | **A** | **A** | **A** | **T** | **C** | **A** | **T** | **T** | **A** | **G** | **T** | **T** | **T** | **T** | **A** | **A** | **A** | **G** | **G** | **G** | **C** | **A** | **A** | **G** | **G** | **T** | **A** | **C** | **G** | **A** | **G** | **C** | **G** | **T** | **G** | **G** | **A** | **G** | **A** | **T** | **C** | **A** | **A** | **G** | **G** | **T** | **C** | **C** | **C** | **T** | **T** | **T** | **G** | **A** | **T** | **G** | **A** | **A** | **G** | **G** | **A** | **G** | **G** | **C** | **A** | **-** | **-** | **-** |
| **N59891F** | **A** | **T** | **G** | **A** | **T** | **G** | **G** | **G** | **A** | **A** | **G** | **A** | **A** | **T** | **A** | **T** | **C** | **C** | **A** | **A** | **A** | **G** | **C** | **A** | **A** | **T** | **G** | **C** | **T** | **A** | **G** | **C** | **T** | **G** | **T** | **T** | **T** | **A** | **T** | **T** | **C** | **G** | **G** | **G** | **G** | **A** | **T** | **T** | **G** | **C** | **G** | **G** | **C** | **T** | **T** | **T** | **C** | **C** | **T** | **T** | **A** | **T** | **T** | **A** | **T** | **T** | **C** | **T** | **A** | **T** | **T** | **T** | **T** | **C** | **A** | **C** | **T** | **T** | **T** | **T** | **C** | **A** | **C** | **T** | **T** | **A** | **T** | **T** | **T** | **T** | **T** | **A** | **T** | **G** | **C** | **C** | **A** | **G** | **T** | **A** | **A** | **C** | **C** | **A** | **A** | **A** | **G** | **T** | **C** | **A** | **T** | **T** | **G** | **G** | **T** | **G** | **G** | **G** | **G** | **A** | **G** | **A** | **A** | **T** | **T** | **G** | **C** | **T** | **G** | **C** | **G** | **T** | **G** | **C** | **G** | **C** | **G** | **T** | **T** | **T** | **A** | **T** | **T** | **A** | **T** | **A** | **C** | **G** | **T** | **T** | **C** | **C** | **G** | **C** | **T** | **T** | **A** | **T** | **C** | **T** | **T** | **C** | **C** | **A** | **T** | **T** | **T** | **G** | **A** | **T** | **G** | **G** | **C** | **A** | **A** | **T** | **T** | **T** | **C** | **A** | **T** | **T** | **A** | **G** | **G** | **T** | **G** | **T** | **A** | **G** | **G** | **A** | **T** | **T** | **G** | **A** | **T** | **C** | **G** | **T** | **A** | **T** | **T** | **T** | **C** | **T** | **A** | **T** | **T** | **G** | **T** | **T** | **A** | **T** | **C** | **A** | **C** | **T** | **T** | **A** | **T** | **T** | **C** | **A** | **A** | **A** | **A** | **G** | **G** | **C** | **A** | **A** | **A** | **A** | **T** | **A** | **C** | **G** | **G** | **G** | **A** | **A** | **A** | **A** | **T** | **C** | **G** | **A** | **A** | **G** | **A** | **A** | **A** | **A** | **G** | **C** | **T** | **T** | **C** | **G** | **T** | **G** | **C** | **A** | **C** | **T** | **T** | **T** | **C** | **T** | **T** | **C** | **T** | **G** | **G** | **C** | **A** | **A** | **T** | **T** | **A** | **T** | **G** | **A** | **A** | **T** | **C** | **C** | **A** | **A** | **A** | **C** | **T** | **T** | **T** | **T** | **G** | **C** | **A** | **T** | **C** | **A** | **G** | **C** | **C** | **G** | **A** | **T** | **C** | **C** | **C** | **A** | **A** | **G** | **C** | **G** | **C** | **G** | **T** | **C** | **G** | **G** | **A** | **T** | **G** | **A** | **T** | **C** | **T** | **G** | **T** | **A** | **C** | **A** | **T** | **C** | **A** | **A** | **A** | **G** | **A** | **T** | **A** | **T** | **T** | **G** | **A** | **T** | **A** | **T** | **G** | **G** | **A** | **A** | **A** | **T** | **T** | **A** | **C** | **T** | **A** | **A** | **A** | **A** | **T** | **A** | **A** | **A** | **A** | **G** | **A** | **A** | **A** | **A** | **A** | **A** | **T** | **G** | **A** | **T** | **C** | **G** | **A** | **A** | **G** | **T** | **A** | **T** | **C** | **C** | **A** | **G** | **T** | **G** | **A** | **A** | **T** | **T** | **A** | **C** | **A** | **G** | **A** | **T** | **T** | **A** | **T** | **G** | **A** | **C** | **T** | **A** | **G** | **C** | **C** | **G** | **T** | **C** | **C** | **T** | **C** | **A** | **A** | **T** | **A** | **T** | **G** | **T** | **A** | **G** | **A** | **T** | **G** | **G** | **A** | **C** | **A** | **A** | **A** | **C** | **C** | **A** | **A** | **A** | **G** | **A** | **A** | **G** | **A** | **G** | **A** | **T** | **T** | **T** | **T** | **A** | **G** | **A** | **A** | **T** | **T** | **A** | **G** | **A** | **A** | **A** | **G** | **G** | **C** | **A** | **T** | **C** | **G** | **A** | **T** | **T** | **A** | **G** | **C** | **T** | **C** | **G** | **T** | **G** | **A** | **G** | **C** | **T** | **T** | **C** | **A** | **T** | **G** | **A** | **T** | **T** | **C** | **A** | **G** | **T** | **T** | **T** | **C** | **A** | **C** | **A** | **G** | **C** | **A** | **A** | **T** | **T** | **A** | **T** | **T** | **T** | **G** | **C** | **A** | **G** | **C** | **G** | **A** | **T** | **G** | **A** | **T** | **G** | **A** | **T** | **G** | **A** | **T** | **G** | **T** | **C** | **G** | **G** | **C** | **A** | **T** | **T** | **G** | **A** | **C** | **G** | **G** | **A** | **G** | **C** | **A** | **A** | **G** | **C** | **A** | **G** | **A** | **A** | **A** | **A** | **A** | **A** | **G** | **T** | **G** | **A** | **G** | **A** | **C** | **A** | **C** | **C** | **A** | **G** | **A** | **A** | **A** | **T** | **G** | **T** | **T** | **C** | **C** | **G** | **T** | **A** | **A** | **G** | **C** | **A** | **G** | **T** | **T** | **G** | **A** | **A** | **A** | **A** | **T** | **G** | **G** | **T** | **G** | **G** | **C** | **A** | **G** | **A** | **G** | **A** | **T** | **C** | **A** | **T** | **C** | **A** | **A** | **C** | **G** | **C** | **T** | **T** | **C** | **C** | **C** | **A** | **G** | **T** | **C** | **T** | **G** | **A** | **G** | **A** | **T** | **G** | **C** | **G** | **T** | **G** | **C** | **G** | **C** | **T** | **G** | **C** | **T** | **T** | **C** | **T** | **T** | **C** | **A** | **T** | **C** | **T** | **G** | **C** | **G** | **T** | **C** | **C** | **A** | **G** | **T** | **C** | **A** | **A** | **T** | **T** | **T** | **A** | **G** | **A** | **A** | **G** | **A** | **A** | **A** | **A** | **A** | **A** | **G** | **T** | **T** | **T** | **A** | **A** | **A** | **A** | **C** | **A** | **A** | **G** | **G** | **C** | **A** | **T** | **T** | **G** | **A** | **G** | **C** | **A** | **G** | **T** | **T** | **A** | **T** | **T** | **G** | **A** | **A** | **G** | **G** | **A** | **A** | **T** | **T** | **G** | **C** | **A** | **G** | **A** | **A** | **T** | **A** | **A** | **A** | **A** | **T** | **C** | **C** | **A** | **G** | **A** | **T** | **T** | **T** | **C** | **G** | **C** | **T** | **A** | **A** | **A** | **A** | **T** | **G** | **G** | **G** | **A** | **T** | **G** | **T** | **A** | **G** | **A** | **A** | **G** | **A** | **T** | **G** | **T** | **A** | **A** | **A** | **A** | **T** | **T** | **A** | **A** | **C** | **T** | **A** | **G** | **T** | **T** | **C** | **C** | **A** | **T** | **T** | **G** | **A** | **G** | **G** | **A** | **T** | **C** | **A** | **C** | **T** | **T** | **G** | **T** | **T** | **C** | **C** | **G** | **A** | **A** | **T** | **C** | **G** | **T** | **T** | **C** | **A** | **A** | **G** | **A** | **A** | **T** | **T** | **G** | **T** | **T** | **A** | **T** | **C** | **A** | **A** | **A** | **C** | **A** | **C** | **A** | **T** | **T** | **A** | **A** | **G** | **A** | **C** | **A** | **T** | **G** | **C** | **T** | **A** | **A** | **G** | **G** | **C** | **T** | **A** | **A** | **T** | **G** | **A** | **A** | **T** | **T** | **A** | **G** | **A** | **A** | **G** | **T** | **C** | **T** | **A** | **T** | **T** | **T** | **G** | **C** | **A** | **T** | **A** | **A** | **A** | **A** | **T** | **A** | **G** | **A** | **T** | **A** | **A** | **C** | **A** | **A** | **T** | **C** | **T** | **T** | **T** | **T** | **A** | **T** | **T** | **A** | **C** | **G** | **T** | **A** | **T** | **C** | **A** | **T** | **T** | **G** | **A** | **C** | **G** | **A** | **C** | **G** | **G** | **G** | **A** | **C** | **A** | **G** | **G** | **A** | **T** | **T** | **T** | **A** | **A** | **T** | **A** | **T** | **G** | **A** | **A** | **T** | **G** | **A** | **A** | **A** | **C** | **C** | **A** | **A** | **A** | **A** | **C** | **G** | **G** | **G** | **A** | **A** | **G** | **T** | **T** | **A** | **T** | **G** | **G** | **A** | **T** | **T** | **G** | **A** | **A** | **C** | **A** | **A** | **T** | **A** | **T** | **C** | **A** | **A** | **A** | **G** | **A** | **A** | **C** | **G** | **A** | **G** | **T** | **A** | **G** | **C** | **T** | **G** | **G** | **T** | **A** | **T** | **T** | **G** | **G** | **T** | **G** | **G** | **A** | **A** | **C** | **A** | **G** | **T** | **A** | **A** | **A** | **A** | **A** | **T** | **C** | **A** | **T** | **T** | **A** | **G** | **T** | **T** | **T** | **T** | **A** | **A** | **A** | **G** | **G** | **G** | **C** | **A** | **A** | **G** | **G** | **T** | **A** | **C** | **G** | **A** | **G** | **C** | **G** | **T** | **G** | **G** | **A** | **G** | **A** | **T** | **C** | **A** | **A** | **G** | **G** | **T** | **C** | **C** | **C** | **T** | **T** | **T** | **G** | **A** | **T** | **G** | **A** | **A** | **G** | **G** | **A** | **G** | **G** | **C** | **A** | **-** | **-** | **-** |
| **N54546** | **A** | **T** | **G** | **A** | **T** | **G** | **G** | **G** | **A** | **A** | **A** | **A** | **A** | **T** | **A** | **T** | **C** | **C** | **A** | **G** | **A** | **G** | **C** | **G** | **A** | **T** | **G** | **C** | **T** | **A** | **G** | **C** | **T** | **G** | **T** | **T** | **T** | **A** | **T** | **T** | **C** | **G** | **G** | **G** | **G** | **A** | **T** | **T** | **G** | **C** | **G** | **G** | **C** | **T** | **T** | **T** | **C** | **C** | **T** | **T** | **A** | **T** | **T** | **A** | **T** | **T** | **C** | **T** | **A** | **T** | **T** | **T** | **T** | **C** | **A** | **C** | **T** | **T** | **T** | **T** | **C** | **A** | **C** | **T** | **T** | **A** | **T** | **T** | **T** | **T** | **T** | **A** | **T** | **G** | **C** | **C** | **A** | **G** | **T** | **A** | **A** | **T** | **C** | **A** | **A** | **A** | **G** | **T** | **C** | **A** | **T** | **T** | **G** | **G** | **T** | **G** | **G** | **G** | **G** | **A** | **G** | **A** | **A** | **T** | **T** | **A** | **C** | **T** | **G** | **C** | **G** | **T** | **G** | **C** | **A** | **C** | **G** | **T** | **T** | **T** | **A** | **T** | **T** | **A** | **T** | **A** | **T** | **G** | **T** | **T** | **C** | **C** | **G** | **C** | **T** | **T** | **A** | **T** | **C** | **T** | **T** | **C** | **C** | **A** | **T** | **T** | **T** | **G** | **T** | **T** | **A** | **G** | **C** | **T** | **A** | **T** | **T** | **T** | **C** | **C** | **T** | **T** | **A** | **G** | **G** | **T** | **G** | **T** | **A** | **G** | **G** | **A** | **T** | **T** | **A** | **A** | **T** | **C** | **G** | **T** | **C** | **T** | **T** | **T** | **C** | **T** | **A** | **T** | **T** | **A** | **T** | **T** | **A** | **T** | **C** | **A** | **C** | **T** | **T** | **A** | **T** | **T** | **C** | **A** | **A** | **A** | **A** | **G** | **A** | **C** | **A** | **A** | **A** | **A** | **T** | **A** | **C** | **G** | **G** | **G** | **A** | **A** | **A** | **A** | **T** | **C** | **G** | **A** | **A** | **G** | **A** | **A** | **A** | **A** | **G** | **C** | **T** | **G** | **C** | **G** | **T** | **G** | **C** | **A** | **C** | **T** | **C** | **T** | **C** | **T** | **T** | **C** | **T** | **G** | **G** | **C** | **A** | **A** | **T** | **T** | **A** | **T** | **G** | **A** | **A** | **T** | **C** | **C** | **A** | **A** | **A** | **T** | **T** | **G** | **T** | **T** | **G** | **C** | **T** | **T** | **C** | **T** | **T** | **C** | **C** | **G** | **A** | **T** | **C** | **C** | **C** | **A** | **A** | **G** | **T** | **G** | **C** | **A** | **T** | **C** | **G** | **G** | **A** | **C** | **G** | **A** | **T** | **T** | **T** | **A** | **T** | **A** | **C** | **A** | **T** | **C** | **A** | **A** | **A** | **G** | **A** | **T** | **A** | **T** | **T** | **G** | **A** | **T** | **A** | **A** | **G** | **G** | **A** | **A** | **A** | **T** | **C** | **A** | **C** | **T** | **A** | **A** | **A** | **A** | **T** | **A** | **A** | **A** | **A** | **G** | **A** | **A** | **A** | **A** | **A** | **A** | **T** | **G** | **A** | **T** | **T** | **G** | **A** | **A** | **G** | **T** | **A** | **T** | **C** | **T** | **A** | **G** | **T** | **G** | **A** | **A** | **T** | **T** | **A** | **C** | **A** | **A** | **A** | **T** | **T** | **G** | **T** | **A** | **A** | **C** | **G** | **A** | **G** | **C** | **C** | **G** | **T** | **C** | **C** | **C** | **C** | **A** | **A** | **T** | **A** | **T** | **G** | **T** | **A** | **G** | **A** | **T** | **G** | **G** | **A** | **C** | **A** | **A** | **A** | **C** | **C** | **A** | **A** | **A** | **G** | **A** | **A** | **G** | **A** | **A** | **A** | **T** | **T** | **T** | **T** | **A** | **G** | **A** | **A** | **T** | **T** | **A** | **G** | **A** | **A** | **A** | **G** | **A** | **C** | **A** | **T** | **C** | **G** | **A** | **T** | **T** | **A** | **G** | **C** | **C** | **C** | **G** | **T** | **G** | **A** | **G** | **C** | **T** | **G** | **C** | **A** | **T** | **G** | **A** | **T** | **T** | **C** | **A** | **G** | **T** | **T** | **T** | **C** | **G** | **C** | **A** | **A** | **C** | **A** | **A** | **T** | **T** | **G** | **T** | **T** | **T** | **G** | **C** | **A** | **G** | **C** | **A** | **A** | **T** | **G** | **A** | **T** | **G** | **A** | **T** | **G** | **A** | **T** | **G** | **T** | **C** | **G** | **G** | **C** | **A** | **T** | **T** | **A** | **A** | **C** | **G** | **G** | **A** | **G** | **C** | **A** | **A** | **G** | **C** | **A** | **G** | **A** | **A** | **A** | **A** | **A** | **A** | **G** | **C** | **G** | **A** | **G** | **A** | **C** | **A** | **C** | **C** | **A** | **G** | **A** | **A** | **A** | **T** | **G** | **T** | **T** | **C** | **C** | **G** | **C** | **A** | **A** | **G** | **C** | **A** | **G** | **T** | **T** | **G** | **A** | **A** | **A** | **A** | **T** | **G** | **G** | **T** | **A** | **G** | **C** | **A** | **G** | **A** | **G** | **A** | **T** | **C** | **A** | **T** | **C** | **A** | **A** | **C** | **G** | **C** | **T** | **T** | **C** | **C** | **C** | **A** | **G** | **T** | **C** | **T** | **G** | **A** | **G** | **A** | **T** | **G** | **C** | **G** | **T** | **G** | **C** | **G** | **C** | **T** | **G** | **C** | **T** | **T** | **C** | **T** | **T** | **C** | **A** | **T** | **C** | **T** | **G** | **C** | **G** | **T** | **C** | **C** | **A** | **G** | **T** | **C** | **A** | **A** | **T** | **T** | **T** | **A** | **G** | **A** | **A** | **G** | **A** | **G** | **A** | **A** | **A** | **A** | **G** | **T** | **T** | **T** | **A** | **A** | **A** | **G** | **C** | **A** | **A** | **G** | **G** | **T** | **A** | **T** | **C** | **G** | **A** | **G** | **C** | **A** | **G** | **T** | **T** | **A** | **T** | **T** | **G** | **A** | **A** | **G** | **G** | **A** | **A** | **T** | **T** | **G** | **C** | **A** | **A** | **A** | **A** | **T** | **A** | **A** | **A** | **A** | **T** | **C** | **C** | **A** | **G** | **A** | **T** | **T** | **T** | **C** | **G** | **C** | **T** | **G** | **A** | **A** | **A** | **T** | **G** | **G** | **G** | **A** | **T** | **G** | **T** | **A** | **G** | **A** | **A** | **G** | **A** | **T** | **G** | **T** | **A** | **A** | **A** | **A** | **C** | **T** | **A** | **T** | **C** | **T** | **A** | **G** | **T** | **T** | **C** | **C** | **A** | **T** | **T** | **G** | **A** | **G** | **G** | **A** | **T** | **C** | **A** | **C** | **T** | **T** | **G** | **T** | **T** | **C** | **C** | **G** | **A** | **A** | **T** | **C** | **G** | **T** | **T** | **C** | **A** | **A** | **G** | **A** | **A** | **T** | **T** | **G** | **T** | **T** | **A** | **T** | **C** | **A** | **A** | **A** | **T** | **A** | **C** | **A** | **T** | **T** | **A** | **A** | **G** | **A** | **C** | **A** | **T** | **G** | **C** | **C** | **A** | **A** | **A** | **G** | **C** | **C** | **A** | **A** | **T** | **G** | **A** | **A** | **T** | **T** | **A** | **G** | **A** | **G** | **G** | **T** | **A** | **T** | **A** | **T** | **T** | **T** | **G** | **C** | **A** | **C** | **A** | **A** | **A** | **A** | **T** | **A** | **G** | **A** | **C** | **A** | **A** | **T** | **A** | **A** | **T** | **C** | **T** | **T** | **T** | **T** | **G** | **T** | **T** | **A** | **C** | **G** | **T** | **A** | **T** | **T** | **A** | **T** | **T** | **G** | **A** | **C** | **G** | **A** | **C** | **G** | **G** | **A** | **A** | **C** | **A** | **G** | **G** | **G** | **T** | **T** | **T** | **A** | **A** | **T** | **A** | **T** | **G** | **A** | **A** | **T** | **G** | **A** | **A** | **A** | **C** | **G** | **A** | **A** | **A** | **A** | **C** | **G** | **G** | **G** | **A** | **A** | **G** | **T** | **T** | **A** | **T** | **G** | **G** | **A** | **T** | **T** | **G** | **A** | **A** | **C** | **A** | **A** | **T** | **A** | **T** | **C** | **A** | **A** | **A** | **G** | **A** | **A** | **C** | **G** | **A** | **G** | **T** | **A** | **G** | **C** | **T** | **G** | **G** | **T** | **A** | **T** | **T** | **G** | **G** | **T** | **G** | **G** | **A** | **A** | **C** | **A** | **G** | **T** | **A** | **A** | **A** | **A** | **A** | **T** | **C** | **A** | **T** | **T** | **A** | **G** | **T** | **T** | **T** | **T** | **A** | **A** | **A** | **G** | **G** | **G** | **C** | **A** | **A** | **G** | **G** | **T** | **A** | **C** | **A** | **A** | **G** | **T** | **G** | **T** | **A** | **G** | **A** | **A** | **A** | **T** | **C** | **A** | **A** | **G** | **G** | **T** | **C** | **C** | **C** | **T** | **T** | **T** | **G** | **A** | **T** | **G** | **A** | **A** | **G** | **G** | **A** | **G** | **G** | **C** | **A** | **-** | **-** | **-** |
| **N52824** | **A** | **T** | **G** | **A** | **T** | **G** | **G** | **G** | **A** | **A** | **A** | **A** | **A** | **T** | **A** | **T** | **C** | **C** | **A** | **G** | **A** | **G** | **C** | **G** | **A** | **T** | **G** | **C** | **T** | **A** | **G** | **C** | **T** | **G** | **T** | **T** | **T** | **A** | **T** | **T** | **C** | **G** | **G** | **G** | **G** | **A** | **T** | **T** | **G** | **C** | **G** | **G** | **C** | **T** | **T** | **T** | **C** | **C** | **T** | **T** | **A** | **T** | **T** | **A** | **T** | **A** | **C** | **T** | **A** | **T** | **T** | **T** | **T** | **C** | **A** | **C** | **T** | **T** | **T** | **T** | **C** | **A** | **C** | **T** | **T** | **A** | **T** | **T** | **T** | **T** | **T** | **A** | **T** | **G** | **C** | **C** | **A** | **G** | **T** | **A** | **A** | **T** | **C** | **A** | **A** | **A** | **G** | **T** | **C** | **A** | **T** | **T** | **G** | **G** | **T** | **G** | **G** | **G** | **G** | **A** | **G** | **A** | **A** | **T** | **T** | **A** | **C** | **T** | **G** | **C** | **G** | **T** | **G** | **C** | **A** | **C** | **G** | **T** | **T** | **T** | **A** | **T** | **T** | **A** | **T** | **A** | **T** | **G** | **T** | **T** | **C** | **C** | **G** | **C** | **T** | **T** | **A** | **T** | **C** | **T** | **T** | **C** | **C** | **A** | **T** | **T** | **T** | **G** | **T** | **T** | **A** | **G** | **C** | **T** | **A** | **T** | **T** | **T** | **C** | **C** | **T** | **T** | **A** | **G** | **G** | **T** | **G** | **T** | **A** | **G** | **G** | **A** | **T** | **T** | **A** | **A** | **T** | **C** | **G** | **T** | **C** | **T** | **T** | **T** | **C** | **T** | **A** | **T** | **T** | **A** | **T** | **T** | **A** | **T** | **C** | **A** | **C** | **T** | **T** | **A** | **T** | **T** | **C** | **A** | **A** | **A** | **A** | **G** | **A** | **C** | **A** | **A** | **A** | **A** | **T** | **A** | **C** | **G** | **G** | **G** | **A** | **A** | **A** | **A** | **T** | **C** | **G** | **A** | **A** | **G** | **A** | **A** | **A** | **A** | **G** | **C** | **T** | **G** | **C** | **G** | **T** | **G** | **C** | **A** | **C** | **T** | **C** | **T** | **C** | **T** | **T** | **C** | **T** | **G** | **G** | **C** | **A** | **A** | **T** | **T** | **A** | **T** | **G** | **A** | **A** | **T** | **C** | **C** | **A** | **A** | **A** | **T** | **T** | **G** | **T** | **T** | **G** | **C** | **T** | **T** | **C** | **T** | **T** | **C** | **C** | **G** | **A** | **T** | **C** | **C** | **C** | **A** | **A** | **G** | **T** | **G** | **C** | **A** | **T** | **C** | **G** | **G** | **A** | **C** | **G** | **A** | **T** | **T** | **T** | **A** | **T** | **A** | **C** | **A** | **T** | **C** | **A** | **A** | **A** | **G** | **A** | **T** | **A** | **T** | **T** | **G** | **A** | **T** | **A** | **A** | **G** | **G** | **A** | **A** | **A** | **T** | **C** | **A** | **C** | **T** | **A** | **A** | **A** | **A** | **T** | **A** | **A** | **A** | **A** | **G** | **A** | **A** | **A** | **A** | **A** | **A** | **T** | **G** | **A** | **T** | **T** | **G** | **A** | **A** | **G** | **T** | **A** | **T** | **C** | **T** | **A** | **G** | **T** | **G** | **A** | **A** | **T** | **T** | **A** | **C** | **A** | **A** | **A** | **T** | **T** | **G** | **T** | **A** | **A** | **C** | **G** | **A** | **G** | **C** | **C** | **G** | **T** | **C** | **C** | **C** | **C** | **A** | **A** | **T** | **A** | **T** | **G** | **T** | **A** | **G** | **A** | **T** | **G** | **G** | **A** | **C** | **A** | **A** | **A** | **C** | **C** | **A** | **A** | **A** | **G** | **A** | **A** | **G** | **A** | **A** | **A** | **T** | **T** | **T** | **T** | **A** | **G** | **A** | **A** | **T** | **T** | **A** | **G** | **A** | **A** | **A** | **G** | **A** | **C** | **A** | **T** | **C** | **G** | **A** | **T** | **T** | **A** | **G** | **C** | **C** | **C** | **G** | **T** | **G** | **A** | **G** | **C** | **T** | **G** | **C** | **A** | **T** | **G** | **A** | **T** | **T** | **C** | **A** | **G** | **T** | **T** | **T** | **C** | **G** | **C** | **A** | **A** | **C** | **A** | **A** | **T** | **T** | **G** | **T** | **T** | **T** | **G** | **C** | **A** | **G** | **C** | **A** | **A** | **T** | **G** | **A** | **T** | **G** | **A** | **T** | **G** | **A** | **T** | **G** | **T** | **C** | **G** | **G** | **C** | **A** | **T** | **T** | **A** | **A** | **C** | **G** | **G** | **A** | **G** | **C** | **A** | **A** | **G** | **C** | **A** | **G** | **A** | **A** | **A** | **A** | **A** | **A** | **G** | **C** | **G** | **A** | **G** | **A** | **C** | **A** | **C** | **C** | **A** | **G** | **A** | **A** | **A** | **T** | **G** | **T** | **T** | **C** | **C** | **G** | **C** | **A** | **A** | **G** | **C** | **A** | **G** | **T** | **T** | **G** | **A** | **A** | **A** | **A** | **T** | **G** | **G** | **T** | **A** | **G** | **C** | **A** | **G** | **A** | **G** | **A** | **T** | **C** | **A** | **T** | **C** | **A** | **A** | **C** | **G** | **C** | **T** | **T** | **C** | **C** | **C** | **A** | **G** | **T** | **C** | **T** | **G** | **A** | **G** | **A** | **T** | **G** | **C** | **G** | **T** | **G** | **C** | **G** | **C** | **T** | **G** | **C** | **T** | **T** | **C** | **T** | **T** | **C** | **A** | **T** | **C** | **T** | **G** | **C** | **G** | **T** | **C** | **C** | **A** | **G** | **T** | **C** | **A** | **A** | **T** | **T** | **T** | **A** | **G** | **A** | **A** | **G** | **A** | **G** | **A** | **A** | **A** | **A** | **G** | **T** | **T** | **T** | **A** | **A** | **A** | **G** | **C** | **A** | **A** | **G** | **G** | **T** | **A** | **T** | **C** | **G** | **A** | **G** | **C** | **A** | **G** | **T** | **T** | **A** | **T** | **T** | **G** | **A** | **A** | **G** | **G** | **A** | **A** | **T** | **T** | **G** | **C** | **A** | **A** | **A** | **A** | **T** | **A** | **A** | **A** | **A** | **T** | **C** | **C** | **A** | **G** | **A** | **T** | **T** | **T** | **C** | **G** | **C** | **T** | **G** | **A** | **A** | **A** | **T** | **G** | **G** | **G** | **A** | **T** | **G** | **T** | **A** | **G** | **A** | **A** | **G** | **A** | **T** | **G** | **T** | **A** | **A** | **A** | **A** | **C** | **T** | **A** | **T** | **C** | **T** | **A** | **G** | **T** | **T** | **C** | **C** | **A** | **T** | **T** | **G** | **A** | **G** | **G** | **A** | **T** | **C** | **A** | **C** | **T** | **T** | **G** | **T** | **T** | **C** | **C** | **G** | **A** | **A** | **T** | **C** | **G** | **T** | **T** | **C** | **A** | **A** | **G** | **A** | **A** | **T** | **T** | **G** | **T** | **T** | **A** | **T** | **C** | **A** | **A** | **A** | **T** | **A** | **C** | **A** | **T** | **T** | **A** | **A** | **G** | **A** | **C** | **A** | **T** | **G** | **C** | **T** | **A** | **A** | **G** | **G** | **C** | **T** | **A** | **A** | **T** | **G** | **A** | **A** | **T** | **T** | **A** | **G** | **A** | **A** | **G** | **T** | **C** | **T** | **A** | **T** | **T** | **T** | **G** | **C** | **A** | **T** | **A** | **A** | **A** | **A** | **T** | **A** | **G** | **A** | **T** | **A** | **A** | **C** | **A** | **A** | **T** | **C** | **T** | **T** | **T** | **T** | **A** | **T** | **T** | **A** | **C** | **G** | **T** | **A** | **T** | **C** | **A** | **T** | **T** | **G** | **A** | **C** | **G** | **A** | **C** | **G** | **G** | **G** | **A** | **C** | **A** | **G** | **G** | **A** | **T** | **T** | **T** | **A** | **A** | **T** | **A** | **T** | **G** | **A** | **A** | **T** | **G** | **A** | **A** | **A** | **C** | **C** | **A** | **A** | **A** | **A** | **C** | **G** | **G** | **G** | **A** | **A** | **G** | **T** | **T** | **A** | **T** | **G** | **G** | **A** | **T** | **T** | **G** | **A** | **A** | **C** | **A** | **A** | **T** | **A** | **T** | **C** | **A** | **A** | **A** | **G** | **A** | **A** | **C** | **G** | **A** | **G** | **T** | **A** | **G** | **C** | **T** | **G** | **G** | **T** | **A** | **T** | **T** | **G** | **G** | **T** | **G** | **G** | **A** | **A** | **C** | **A** | **G** | **T** | **A** | **A** | **A** | **A** | **A** | **T** | **C** | **A** | **T** | **T** | **A** | **G** | **T** | **T** | **T** | **T** | **A** | **A** | **A** | **G** | **G** | **G** | **C** | **A** | **A** | **G** | **G** | **T** | **A** | **C** | **G** | **A** | **G** | **C** | **G** | **T** | **G** | **G** | **A** | **G** | **A** | **T** | **C** | **A** | **A** | **G** | **G** | **T** | **C** | **C** | **C** | **T** | **T** | **T** | **G** | **A** | **T** | **G** | **A** | **A** | **G** | **G** | **A** | **G** | **G** | **C** | **A** | **-** | **-** | **-** |
| **N59745F** | **A** | **T** | **G** | **A** | **T** | **G** | **G** | **G** | **A** | **A** | **A** | **A** | **A** | **T** | **A** | **T** | **C** | **C** | **A** | **G** | **A** | **G** | **C** | **G** | **A** | **T** | **G** | **C** | **T** | **A** | **G** | **C** | **T** | **G** | **T** | **T** | **T** | **A** | **T** | **T** | **C** | **G** | **G** | **G** | **G** | **A** | **T** | **T** | **G** | **C** | **G** | **G** | **C** | **T** | **T** | **T** | **C** | **C** | **T** | **T** | **A** | **T** | **T** | **A** | **T** | **T** | **C** | **T** | **A** | **T** | **T** | **T** | **T** | **C** | **A** | **C** | **T** | **T** | **T** | **T** | **C** | **A** | **C** | **T** | **T** | **A** | **T** | **T** | **T** | **T** | **T** | **A** | **T** | **G** | **C** | **C** | **A** | **G** | **C** | **A** | **A** | **T** | **C** | **A** | **A** | **A** | **G** | **T** | **C** | **A** | **T** | **T** | **G** | **G** | **T** | **G** | **G** | **G** | **G** | **A** | **G** | **A** | **A** | **T** | **T** | **A** | **C** | **T** | **G** | **C** | **G** | **T** | **G** | **C** | **A** | **C** | **G** | **T** | **T** | **T** | **A** | **T** | **T** | **A** | **T** | **A** | **T** | **G** | **T** | **T** | **C** | **C** | **G** | **C** | **T** | **T** | **A** | **T** | **C** | **T** | **T** | **C** | **C** | **A** | **T** | **T** | **T** | **G** | **T** | **T** | **A** | **G** | **C** | **C** | **A** | **T** | **T** | **T** | **C** | **C** | **T** | **T** | **A** | **G** | **G** | **T** | **G** | **T** | **A** | **G** | **G** | **A** | **T** | **T** | **A** | **A** | **T** | **T** | **G** | **T** | **C** | **T** | **T** | **T** | **C** | **T** | **A** | **T** | **T** | **A** | **T** | **T** | **A** | **T** | **C** | **A** | **C** | **T** | **T** | **A** | **T** | **T** | **C** | **A** | **A** | **A** | **A** | **G** | **A** | **C** | **A** | **A** | **A** | **A** | **T** | **A** | **C** | **G** | **G** | **G** | **A** | **A** | **A** | **A** | **T** | **C** | **G** | **A** | **A** | **G** | **A** | **A** | **A** | **A** | **G** | **C** | **T** | **G** | **C** | **G** | **T** | **G** | **C** | **A** | **C** | **T** | **T** | **T** | **C** | **T** | **T** | **C** | **T** | **G** | **G** | **C** | **A** | **A** | **T** | **T** | **A** | **T** | **G** | **A** | **A** | **T** | **C** | **C** | **A** | **A** | **A** | **T** | **T** | **G** | **T** | **T** | **G** | **C** | **T** | **T** | **C** | **T** | **T** | **C** | **C** | **G** | **A** | **T** | **C** | **C** | **C** | **A** | **A** | **G** | **T** | **G** | **C** | **A** | **T** | **C** | **G** | **G** | **A** | **C** | **G** | **A** | **T** | **T** | **T** | **A** | **T** | **A** | **C** | **A** | **T** | **C** | **A** | **A** | **A** | **G** | **A** | **T** | **A** | **T** | **T** | **G** | **A** | **T** | **A** | **A** | **G** | **G** | **A** | **A** | **A** | **T** | **C** | **A** | **C** | **T** | **A** | **A** | **G** | **A** | **T** | **A** | **A** | **A** | **A** | **G** | **A** | **A** | **A** | **A** | **A** | **A** | **T** | **G** | **A** | **T** | **T** | **G** | **A** | **A** | **G** | **T** | **A** | **T** | **C** | **T** | **A** | **G** | **T** | **G** | **A** | **A** | **T** | **T** | **A** | **C** | **A** | **A** | **A** | **T** | **T** | **G** | **T** | **A** | **A** | **C** | **G** | **A** | **G** | **C** | **C** | **G** | **T** | **C** | **C** | **C** | **C** | **A** | **A** | **T** | **A** | **T** | **G** | **T** | **A** | **G** | **A** | **T** | **G** | **G** | **A** | **C** | **A** | **A** | **A** | **C** | **C** | **A** | **A** | **A** | **G** | **A** | **A** | **G** | **A** | **A** | **A** | **T** | **T** | **T** | **T** | **A** | **G** | **A** | **A** | **T** | **T** | **A** | **G** | **A** | **A** | **A** | **G** | **A** | **C** | **A** | **T** | **C** | **G** | **A** | **T** | **T** | **A** | **G** | **C** | **C** | **C** | **G** | **T** | **G** | **A** | **G** | **C** | **T** | **G** | **C** | **A** | **T** | **G** | **A** | **T** | **T** | **C** | **A** | **G** | **T** | **T** | **T** | **C** | **G** | **C** | **A** | **A** | **C** | **A** | **A** | **T** | **T** | **G** | **T** | **T** | **T** | **G** | **C** | **A** | **G** | **C** | **A** | **A** | **T** | **G** | **A** | **T** | **G** | **A** | **T** | **G** | **A** | **T** | **G** | **T** | **C** | **G** | **G** | **C** | **A** | **T** | **T** | **G** | **A** | **C** | **G** | **G** | **A** | **G** | **C** | **A** | **A** | **G** | **C** | **A** | **G** | **A** | **A** | **A** | **A** | **A** | **A** | **G** | **C** | **G** | **A** | **G** | **A** | **C** | **A** | **C** | **C** | **A** | **G** | **A** | **A** | **A** | **T** | **G** | **T** | **T** | **C** | **C** | **G** | **C** | **A** | **A** | **G** | **C** | **A** | **G** | **T** | **T** | **G** | **A** | **A** | **A** | **A** | **T** | **G** | **G** | **T** | **A** | **G** | **C** | **A** | **G** | **A** | **G** | **A** | **T** | **C** | **A** | **T** | **C** | **A** | **A** | **C** | **G** | **C** | **T** | **T** | **C** | **C** | **C** | **A** | **G** | **T** | **C** | **T** | **G** | **A** | **G** | **A** | **T** | **G** | **C** | **G** | **T** | **G** | **C** | **G** | **C** | **T** | **G** | **C** | **T** | **T** | **C** | **T** | **T** | **C** | **A** | **T** | **C** | **T** | **A** | **C** | **G** | **T** | **C** | **C** | **A** | **G** | **T** | **C** | **A** | **A** | **T** | **T** | **T** | **A** | **G** | **A** | **A** | **G** | **A** | **G** | **A** | **A** | **A** | **A** | **G** | **T** | **T** | **T** | **A** | **A** | **A** | **G** | **C** | **A** | **A** | **G** | **G** | **T** | **A** | **T** | **C** | **G** | **A** | **G** | **C** | **A** | **G** | **T** | **T** | **A** | **T** | **T** | **G** | **A** | **A** | **G** | **G** | **A** | **A** | **T** | **T** | **G** | **C** | **A** | **A** | **A** | **A** | **T** | **A** | **A** | **A** | **A** | **T** | **C** | **C** | **A** | **G** | **A** | **T** | **T** | **T** | **C** | **G** | **C** | **T** | **G** | **A** | **A** | **A** | **T** | **G** | **G** | **G** | **A** | **T** | **G** | **T** | **A** | **G** | **A** | **A** | **G** | **A** | **T** | **G** | **T** | **A** | **A** | **A** | **A** | **C** | **T** | **A** | **T** | **C** | **T** | **A** | **G** | **T** | **T** | **C** | **C** | **A** | **T** | **T** | **G** | **A** | **G** | **G** | **A** | **T** | **C** | **A** | **C** | **T** | **T** | **G** | **T** | **T** | **C** | **C** | **G** | **A** | **A** | **T** | **C** | **G** | **T** | **T** | **C** | **A** | **A** | **G** | **A** | **A** | **T** | **T** | **G** | **T** | **T** | **A** | **T** | **C** | **A** | **A** | **A** | **T** | **A** | **C** | **A** | **T** | **T** | **A** | **A** | **G** | **A** | **C** | **A** | **T** | **G** | **C** | **C** | **A** | **A** | **A** | **G** | **C** | **C** | **A** | **A** | **T** | **G** | **A** | **A** | **T** | **T** | **A** | **G** | **A** | **G** | **G** | **T** | **A** | **T** | **A** | **T** | **T** | **T** | **G** | **C** | **A** | **C** | **A** | **A** | **A** | **A** | **T** | **A** | **G** | **A** | **C** | **A** | **A** | **T** | **A** | **A** | **T** | **C** | **T** | **T** | **T** | **T** | **G** | **T** | **T** | **A** | **C** | **G** | **T** | **A** | **T** | **T** | **A** | **T** | **T** | **G** | **A** | **C** | **G** | **A** | **C** | **G** | **G** | **A** | **A** | **C** | **A** | **G** | **G** | **G** | **T** | **T** | **T** | **A** | **A** | **T** | **A** | **T** | **G** | **A** | **A** | **T** | **G** | **A** | **A** | **A** | **C** | **G** | **A** | **A** | **A** | **A** | **C** | **G** | **G** | **G** | **A** | **A** | **G** | **T** | **T** | **A** | **T** | **G** | **G** | **A** | **T** | **T** | **G** | **A** | **A** | **C** | **A** | **A** | **T** | **A** | **T** | **C** | **A** | **A** | **A** | **G** | **A** | **A** | **C** | **G** | **A** | **G** | **T** | **A** | **G** | **T** | **T** | **G** | **G** | **T** | **A** | **T** | **T** | **G** | **G** | **T** | **G** | **G** | **A** | **A** | **C** | **A** | **G** | **T** | **A** | **A** | **A** | **A** | **A** | **T** | **C** | **A** | **T** | **T** | **A** | **G** | **T** | **T** | **T** | **T** | **A** | **A** | **A** | **G** | **G** | **G** | **C** | **A** | **A** | **G** | **G** | **T** | **A** | **C** | **A** | **A** | **G** | **T** | **G** | **T** | **A** | **G** | **A** | **A** | **A** | **T** | **C** | **A** | **A** | **G** | **G** | **T** | **C** | **C** | **C** | **T** | **T** | **T** | **G** | **A** | **T** | **G** | **A** | **A** | **G** | **G** | **A** | **G** | **G** | **C** | **A** | **-** | **-** | **-** |
| **N59542F** | **A** | **T** | **G** | **A** | **T** | **G** | **G** | **G** | **A** | **A** | **G** | **A** | **A** | **T** | **A** | **T** | **C** | **C** | **A** | **A** | **A** | **G** | **C** | **A** | **A** | **T** | **G** | **C** | **T** | **A** | **G** | **C** | **T** | **G** | **T** | **T** | **T** | **A** | **T** | **T** | **C** | **G** | **G** | **G** | **G** | **A** | **T** | **T** | **G** | **C** | **G** | **G** | **C** | **T** | **T** | **T** | **C** | **C** | **T** | **T** | **A** | **T** | **T** | **A** | **T** | **T** | **C** | **T** | **A** | **T** | **T** | **T** | **T** | **C** | **A** | **C** | **T** | **T** | **T** | **T** | **C** | **A** | **C** | **T** | **T** | **A** | **T** | **T** | **T** | **T** | **T** | **A** | **T** | **G** | **C** | **C** | **A** | **G** | **T** | **A** | **A** | **C** | **C** | **A** | **A** | **A** | **G** | **T** | **C** | **A** | **T** | **T** | **G** | **G** | **T** | **G** | **G** | **G** | **G** | **A** | **G** | **A** | **A** | **T** | **T** | **G** | **C** | **T** | **G** | **C** | **G** | **T** | **G** | **C** | **G** | **C** | **G** | **T** | **T** | **T** | **A** | **T** | **T** | **A** | **T** | **A** | **C** | **G** | **T** | **T** | **C** | **C** | **G** | **C** | **T** | **T** | **A** | **T** | **C** | **T** | **T** | **C** | **C** | **A** | **T** | **T** | **T** | **G** | **A** | **T** | **G** | **G** | **C** | **A** | **A** | **T** | **T** | **T** | **C** | **A** | **T** | **T** | **A** | **G** | **G** | **T** | **G** | **T** | **A** | **G** | **G** | **A** | **T** | **T** | **G** | **A** | **T** | **C** | **G** | **T** | **A** | **T** | **T** | **T** | **C** | **T** | **A** | **T** | **T** | **G** | **T** | **T** | **A** | **T** | **C** | **A** | **C** | **T** | **T** | **A** | **T** | **T** | **C** | **A** | **A** | **A** | **A** | **G** | **G** | **C** | **A** | **A** | **A** | **A** | **T** | **A** | **C** | **G** | **G** | **G** | **A** | **A** | **A** | **A** | **T** | **C** | **G** | **A** | **A** | **G** | **A** | **A** | **A** | **A** | **G** | **C** | **T** | **T** | **C** | **G** | **T** | **G** | **C** | **A** | **C** | **T** | **T** | **T** | **C** | **T** | **T** | **C** | **T** | **G** | **G** | **C** | **A** | **A** | **T** | **T** | **A** | **T** | **G** | **A** | **A** | **T** | **C** | **C** | **A** | **A** | **A** | **C** | **T** | **T** | **T** | **T** | **G** | **C** | **A** | **T** | **C** | **A** | **G** | **C** | **C** | **G** | **A** | **T** | **C** | **C** | **C** | **A** | **A** | **G** | **C** | **G** | **C** | **G** | **T** | **C** | **G** | **G** | **A** | **T** | **G** | **A** | **T** | **C** | **T** | **G** | **T** | **A** | **C** | **A** | **T** | **C** | **A** | **A** | **A** | **G** | **A** | **T** | **A** | **T** | **T** | **G** | **A** | **T** | **A** | **T** | **G** | **G** | **A** | **A** | **A** | **T** | **T** | **A** | **C** | **T** | **A** | **A** | **A** | **A** | **T** | **A** | **A** | **A** | **A** | **G** | **A** | **A** | **A** | **A** | **A** | **A** | **T** | **G** | **A** | **T** | **C** | **G** | **A** | **A** | **G** | **T** | **A** | **T** | **C** | **C** | **A** | **G** | **T** | **G** | **A** | **A** | **T** | **T** | **A** | **C** | **A** | **G** | **A** | **T** | **T** | **A** | **T** | **G** | **A** | **C** | **T** | **A** | **G** | **C** | **C** | **G** | **T** | **C** | **C** | **T** | **C** | **A** | **A** | **T** | **A** | **T** | **G** | **T** | **A** | **G** | **A** | **T** | **G** | **G** | **A** | **C** | **A** | **A** | **A** | **C** | **C** | **A** | **A** | **A** | **G** | **A** | **A** | **G** | **A** | **G** | **A** | **T** | **T** | **T** | **T** | **A** | **G** | **A** | **A** | **T** | **T** | **A** | **G** | **A** | **A** | **A** | **G** | **G** | **C** | **A** | **T** | **C** | **G** | **A** | **T** | **T** | **A** | **G** | **C** | **T** | **C** | **G** | **T** | **G** | **A** | **G** | **C** | **T** | **T** | **C** | **A** | **T** | **G** | **A** | **T** | **T** | **C** | **A** | **G** | **T** | **T** | **T** | **C** | **A** | **C** | **A** | **G** | **C** | **A** | **A** | **T** | **T** | **A** | **T** | **T** | **T** | **G** | **C** | **A** | **G** | **C** | **G** | **A** | **T** | **G** | **A** | **T** | **G** | **A** | **T** | **G** | **A** | **T** | **G** | **T** | **C** | **G** | **G** | **C** | **A** | **T** | **T** | **G** | **A** | **C** | **G** | **G** | **A** | **G** | **C** | **A** | **A** | **G** | **C** | **A** | **G** | **A** | **A** | **A** | **A** | **A** | **A** | **G** | **T** | **G** | **A** | **G** | **A** | **C** | **A** | **C** | **C** | **A** | **G** | **A** | **A** | **A** | **T** | **G** | **T** | **T** | **C** | **C** | **G** | **T** | **A** | **A** | **G** | **C** | **A** | **G** | **T** | **T** | **G** | **A** | **A** | **A** | **A** | **T** | **G** | **G** | **T** | **G** | **G** | **C** | **A** | **G** | **A** | **G** | **A** | **T** | **C** | **A** | **T** | **C** | **A** | **A** | **C** | **G** | **C** | **T** | **T** | **C** | **C** | **C** | **A** | **G** | **T** | **C** | **T** | **G** | **A** | **G** | **A** | **T** | **G** | **C** | **G** | **T** | **G** | **C** | **G** | **C** | **T** | **G** | **C** | **T** | **T** | **C** | **T** | **T** | **C** | **A** | **T** | **C** | **T** | **G** | **C** | **G** | **T** | **C** | **C** | **A** | **G** | **T** | **C** | **A** | **A** | **T** | **T** | **T** | **A** | **G** | **A** | **A** | **G** | **A** | **A** | **A** | **A** | **A** | **A** | **G** | **T** | **T** | **T** | **A** | **A** | **A** | **A** | **C** | **A** | **A** | **G** | **G** | **C** | **A** | **T** | **T** | **G** | **A** | **G** | **C** | **A** | **G** | **T** | **T** | **A** | **T** | **T** | **G** | **A** | **A** | **G** | **G** | **A** | **A** | **T** | **T** | **G** | **C** | **A** | **G** | **A** | **A** | **T** | **A** | **A** | **A** | **A** | **T** | **C** | **C** | **A** | **G** | **A** | **T** | **T** | **T** | **C** | **G** | **C** | **T** | **A** | **A** | **A** | **A** | **T** | **G** | **G** | **G** | **A** | **T** | **G** | **T** | **A** | **G** | **A** | **A** | **G** | **A** | **T** | **G** | **T** | **A** | **A** | **A** | **A** | **T** | **T** | **A** | **A** | **C** | **T** | **A** | **G** | **T** | **T** | **C** | **C** | **A** | **T** | **T** | **G** | **A** | **G** | **G** | **A** | **T** | **C** | **A** | **C** | **T** | **T** | **G** | **T** | **T** | **C** | **C** | **G** | **A** | **A** | **T** | **C** | **G** | **T** | **T** | **C** | **A** | **A** | **G** | **A** | **A** | **T** | **T** | **G** | **T** | **T** | **A** | **T** | **C** | **A** | **A** | **A** | **C** | **A** | **C** | **A** | **T** | **T** | **A** | **A** | **G** | **A** | **C** | **A** | **T** | **G** | **C** | **T** | **A** | **A** | **G** | **G** | **C** | **T** | **A** | **A** | **T** | **G** | **A** | **A** | **T** | **T** | **A** | **G** | **A** | **A** | **G** | **T** | **C** | **T** | **A** | **T** | **T** | **T** | **G** | **C** | **A** | **T** | **A** | **A** | **A** | **A** | **T** | **A** | **G** | **A** | **T** | **A** | **A** | **C** | **A** | **A** | **T** | **C** | **T** | **T** | **T** | **T** | **A** | **T** | **T** | **A** | **C** | **G** | **T** | **A** | **T** | **C** | **A** | **T** | **T** | **G** | **A** | **C** | **G** | **A** | **C** | **G** | **G** | **G** | **A** | **C** | **A** | **G** | **G** | **A** | **T** | **T** | **T** | **A** | **A** | **T** | **A** | **T** | **G** | **A** | **A** | **T** | **G** | **A** | **A** | **A** | **C** | **C** | **A** | **A** | **A** | **A** | **C** | **G** | **G** | **G** | **A** | **A** | **G** | **T** | **T** | **A** | **T** | **G** | **G** | **A** | **T** | **T** | **G** | **A** | **A** | **C** | **A** | **A** | **T** | **A** | **T** | **C** | **A** | **A** | **A** | **G** | **A** | **A** | **C** | **G** | **A** | **G** | **T** | **A** | **G** | **C** | **T** | **G** | **G** | **T** | **A** | **T** | **T** | **G** | **G** | **T** | **G** | **G** | **A** | **A** | **C** | **A** | **G** | **T** | **A** | **A** | **A** | **A** | **A** | **T** | **C** | **A** | **T** | **T** | **A** | **G** | **T** | **T** | **T** | **T** | **A** | **A** | **A** | **G** | **G** | **G** | **C** | **A** | **A** | **G** | **G** | **T** | **A** | **C** | **G** | **A** | **G** | **C** | **G** | **T** | **G** | **G** | **A** | **G** | **A** | **T** | **C** | **A** | **A** | **G** | **G** | **T** | **C** | **C** | **C** | **T** | **T** | **T** | **G** | **A** | **T** | **G** | **A** | **A** | **G** | **G** | **A** | **G** | **G** | **C** | **A** | **-** | **-** | **-** |
| **N60365F** | **A** | **T** | **G** | **A** | **T** | **G** | **G** | **G** | **A** | **A** | **G** | **A** | **A** | **T** | **A** | **T** | **C** | **C** | **A** | **A** | **A** | **G** | **C** | **A** | **A** | **T** | **G** | **C** | **T** | **A** | **G** | **C** | **T** | **G** | **T** | **T** | **T** | **A** | **T** | **T** | **C** | **G** | **G** | **G** | **G** | **A** | **T** | **T** | **G** | **C** | **G** | **G** | **C** | **T** | **T** | **T** | **C** | **C** | **T** | **T** | **A** | **T** | **T** | **A** | **T** | **T** | **C** | **T** | **A** | **T** | **T** | **T** | **T** | **C** | **A** | **C** | **T** | **T** | **T** | **T** | **C** | **A** | **C** | **T** | **T** | **A** | **T** | **T** | **T** | **T** | **T** | **A** | **T** | **G** | **C** | **C** | **A** | **G** | **T** | **A** | **A** | **C** | **C** | **A** | **A** | **A** | **G** | **T** | **C** | **A** | **T** | **T** | **G** | **G** | **T** | **G** | **G** | **G** | **G** | **A** | **G** | **A** | **A** | **T** | **T** | **G** | **C** | **T** | **G** | **C** | **G** | **T** | **G** | **C** | **G** | **C** | **G** | **T** | **T** | **T** | **A** | **T** | **T** | **A** | **T** | **A** | **C** | **G** | **T** | **T** | **C** | **C** | **G** | **C** | **T** | **T** | **A** | **T** | **C** | **T** | **T** | **C** | **C** | **A** | **T** | **T** | **T** | **G** | **A** | **T** | **G** | **G** | **C** | **A** | **A** | **T** | **T** | **T** | **C** | **A** | **T** | **T** | **A** | **G** | **G** | **T** | **G** | **T** | **A** | **G** | **G** | **A** | **T** | **T** | **G** | **A** | **T** | **C** | **G** | **T** | **A** | **T** | **T** | **T** | **C** | **T** | **A** | **T** | **T** | **G** | **T** | **T** | **A** | **T** | **C** | **A** | **C** | **T** | **T** | **A** | **T** | **T** | **C** | **A** | **A** | **A** | **A** | **G** | **G** | **C** | **A** | **A** | **A** | **A** | **T** | **A** | **C** | **G** | **G** | **G** | **A** | **A** | **A** | **A** | **T** | **C** | **G** | **A** | **A** | **G** | **A** | **A** | **A** | **A** | **G** | **C** | **T** | **T** | **C** | **G** | **T** | **G** | **C** | **A** | **C** | **T** | **T** | **T** | **C** | **T** | **T** | **C** | **T** | **G** | **G** | **C** | **A** | **A** | **T** | **T** | **A** | **T** | **G** | **A** | **A** | **T** | **C** | **C** | **A** | **A** | **A** | **C** | **T** | **T** | **T** | **T** | **G** | **C** | **A** | **T** | **C** | **A** | **G** | **C** | **C** | **G** | **A** | **T** | **C** | **C** | **C** | **A** | **A** | **G** | **C** | **G** | **C** | **G** | **T** | **C** | **G** | **G** | **A** | **T** | **G** | **A** | **T** | **C** | **T** | **G** | **T** | **A** | **C** | **A** | **T** | **C** | **A** | **A** | **A** | **G** | **A** | **T** | **A** | **T** | **T** | **G** | **A** | **T** | **A** | **T** | **G** | **G** | **A** | **A** | **A** | **T** | **T** | **A** | **C** | **T** | **A** | **A** | **A** | **A** | **T** | **A** | **A** | **A** | **A** | **G** | **A** | **A** | **A** | **A** | **A** | **A** | **T** | **G** | **A** | **T** | **C** | **G** | **A** | **A** | **G** | **T** | **A** | **T** | **C** | **C** | **A** | **G** | **T** | **G** | **A** | **A** | **T** | **T** | **A** | **C** | **A** | **G** | **A** | **T** | **T** | **A** | **T** | **G** | **A** | **C** | **T** | **A** | **G** | **C** | **C** | **G** | **T** | **C** | **C** | **T** | **C** | **A** | **A** | **T** | **A** | **T** | **G** | **T** | **A** | **G** | **A** | **T** | **G** | **G** | **A** | **C** | **A** | **A** | **A** | **C** | **C** | **A** | **A** | **A** | **G** | **A** | **A** | **G** | **A** | **G** | **A** | **T** | **T** | **T** | **T** | **A** | **G** | **A** | **A** | **T** | **T** | **A** | **G** | **A** | **A** | **A** | **G** | **G** | **C** | **A** | **T** | **C** | **G** | **A** | **T** | **T** | **A** | **G** | **C** | **T** | **C** | **G** | **T** | **G** | **A** | **G** | **C** | **T** | **T** | **C** | **A** | **T** | **G** | **A** | **T** | **T** | **C** | **A** | **G** | **T** | **T** | **T** | **C** | **A** | **C** | **A** | **G** | **C** | **A** | **A** | **T** | **T** | **A** | **T** | **T** | **T** | **G** | **C** | **A** | **G** | **C** | **G** | **A** | **T** | **G** | **A** | **T** | **G** | **A** | **T** | **G** | **A** | **T** | **G** | **T** | **C** | **G** | **G** | **C** | **A** | **T** | **T** | **G** | **A** | **C** | **G** | **G** | **A** | **G** | **C** | **A** | **A** | **G** | **C** | **A** | **G** | **A** | **A** | **A** | **A** | **A** | **A** | **G** | **T** | **G** | **A** | **G** | **A** | **C** | **A** | **C** | **C** | **A** | **G** | **A** | **A** | **A** | **T** | **G** | **T** | **T** | **C** | **C** | **G** | **T** | **A** | **A** | **G** | **C** | **A** | **G** | **T** | **T** | **G** | **A** | **A** | **A** | **A** | **T** | **G** | **G** | **T** | **G** | **G** | **C** | **A** | **G** | **A** | **G** | **A** | **T** | **C** | **A** | **T** | **C** | **A** | **A** | **C** | **G** | **C** | **T** | **T** | **C** | **C** | **C** | **A** | **G** | **T** | **C** | **T** | **G** | **A** | **G** | **A** | **T** | **G** | **C** | **G** | **T** | **G** | **C** | **G** | **C** | **T** | **G** | **C** | **T** | **T** | **C** | **T** | **T** | **C** | **A** | **T** | **C** | **T** | **G** | **C** | **G** | **T** | **C** | **C** | **A** | **G** | **T** | **C** | **A** | **A** | **T** | **T** | **T** | **A** | **G** | **A** | **A** | **G** | **A** | **A** | **A** | **A** | **A** | **A** | **G** | **T** | **T** | **T** | **A** | **A** | **A** | **A** | **C** | **A** | **A** | **G** | **G** | **C** | **A** | **T** | **T** | **G** | **A** | **G** | **C** | **A** | **G** | **T** | **T** | **A** | **T** | **T** | **G** | **A** | **A** | **G** | **G** | **A** | **A** | **T** | **T** | **G** | **C** | **A** | **G** | **A** | **A** | **T** | **A** | **A** | **A** | **A** | **T** | **C** | **C** | **A** | **G** | **A** | **T** | **T** | **T** | **C** | **G** | **C** | **T** | **A** | **A** | **A** | **A** | **T** | **G** | **G** | **G** | **A** | **T** | **G** | **T** | **A** | **G** | **A** | **A** | **G** | **A** | **T** | **G** | **T** | **A** | **A** | **A** | **A** | **T** | **T** | **A** | **A** | **C** | **T** | **A** | **G** | **T** | **T** | **C** | **C** | **A** | **T** | **T** | **G** | **A** | **G** | **G** | **A** | **T** | **C** | **A** | **C** | **T** | **T** | **G** | **T** | **T** | **C** | **C** | **G** | **A** | **A** | **T** | **C** | **G** | **T** | **T** | **C** | **A** | **A** | **G** | **A** | **A** | **T** | **T** | **G** | **T** | **T** | **A** | **T** | **C** | **A** | **A** | **A** | **C** | **A** | **C** | **A** | **T** | **T** | **A** | **A** | **G** | **A** | **C** | **A** | **T** | **G** | **C** | **T** | **A** | **A** | **G** | **G** | **C** | **T** | **A** | **A** | **T** | **G** | **A** | **A** | **T** | **T** | **A** | **G** | **A** | **A** | **G** | **T** | **C** | **T** | **A** | **T** | **T** | **T** | **G** | **C** | **A** | **T** | **A** | **A** | **A** | **A** | **T** | **A** | **G** | **A** | **T** | **A** | **A** | **C** | **A** | **A** | **T** | **C** | **T** | **T** | **T** | **T** | **A** | **T** | **T** | **A** | **C** | **G** | **T** | **A** | **T** | **C** | **A** | **T** | **T** | **G** | **A** | **C** | **G** | **A** | **C** | **G** | **G** | **G** | **A** | **C** | **A** | **G** | **G** | **A** | **T** | **T** | **T** | **A** | **A** | **T** | **A** | **T** | **G** | **A** | **A** | **T** | **G** | **A** | **A** | **A** | **C** | **C** | **A** | **A** | **A** | **A** | **C** | **G** | **G** | **G** | **A** | **A** | **G** | **T** | **T** | **A** | **T** | **G** | **G** | **A** | **T** | **T** | **G** | **A** | **A** | **C** | **A** | **A** | **T** | **A** | **T** | **C** | **A** | **A** | **A** | **G** | **A** | **A** | **C** | **G** | **A** | **G** | **T** | **A** | **G** | **C** | **T** | **G** | **G** | **T** | **A** | **T** | **T** | **G** | **G** | **T** | **G** | **G** | **A** | **A** | **C** | **A** | **G** | **T** | **A** | **A** | **A** | **A** | **A** | **T** | **C** | **A** | **T** | **T** | **A** | **G** | **T** | **T** | **T** | **T** | **A** | **A** | **A** | **G** | **G** | **G** | **C** | **A** | **A** | **G** | **G** | **T** | **A** | **C** | **G** | **A** | **G** | **C** | **G** | **T** | **G** | **G** | **A** | **G** | **A** | **T** | **C** | **A** | **A** | **G** | **G** | **T** | **C** | **C** | **C** | **T** | **T** | **T** | **G** | **A** | **T** | **G** | **A** | **A** | **G** | **G** | **A** | **G** | **G** | **C** | **A** | **-** | **-** | **-** |
| **N59484F** | **A** | **T** | **G** | **A** | **T** | **G** | **G** | **G** | **A** | **A** | **G** | **A** | **A** | **T** | **A** | **T** | **C** | **C** | **A** | **A** | **A** | **G** | **C** | **A** | **A** | **T** | **G** | **C** | **T** | **A** | **G** | **C** | **T** | **G** | **T** | **T** | **T** | **A** | **T** | **T** | **C** | **G** | **G** | **G** | **G** | **A** | **T** | **T** | **G** | **C** | **G** | **G** | **C** | **T** | **T** | **T** | **C** | **C** | **T** | **T** | **A** | **T** | **T** | **A** | **T** | **T** | **C** | **T** | **A** | **T** | **T** | **T** | **T** | **C** | **A** | **C** | **T** | **T** | **T** | **T** | **C** | **A** | **C** | **T** | **T** | **A** | **T** | **T** | **T** | **T** | **T** | **A** | **T** | **G** | **C** | **C** | **A** | **G** | **T** | **A** | **A** | **C** | **C** | **A** | **A** | **A** | **G** | **T** | **C** | **A** | **T** | **T** | **G** | **G** | **T** | **G** | **G** | **G** | **G** | **A** | **G** | **A** | **A** | **T** | **T** | **G** | **C** | **T** | **G** | **C** | **G** | **T** | **G** | **C** | **G** | **C** | **G** | **T** | **T** | **T** | **A** | **T** | **T** | **A** | **T** | **A** | **C** | **G** | **T** | **T** | **C** | **C** | **G** | **C** | **T** | **T** | **A** | **T** | **C** | **T** | **T** | **C** | **C** | **A** | **T** | **T** | **T** | **G** | **A** | **T** | **G** | **G** | **C** | **A** | **A** | **T** | **T** | **T** | **C** | **A** | **T** | **T** | **A** | **G** | **G** | **T** | **G** | **T** | **A** | **G** | **G** | **A** | **T** | **T** | **G** | **A** | **T** | **C** | **G** | **T** | **A** | **T** | **T** | **T** | **C** | **T** | **A** | **T** | **T** | **G** | **T** | **T** | **A** | **T** | **C** | **A** | **C** | **T** | **T** | **A** | **T** | **T** | **C** | **A** | **A** | **A** | **A** | **G** | **A** | **C** | **A** | **A** | **A** | **A** | **T** | **A** | **C** | **G** | **G** | **G** | **A** | **A** | **A** | **A** | **T** | **C** | **G** | **A** | **A** | **G** | **A** | **A** | **A** | **A** | **G** | **C** | **T** | **T** | **C** | **G** | **T** | **G** | **C** | **A** | **C** | **T** | **T** | **T** | **C** | **T** | **T** | **C** | **T** | **G** | **G** | **C** | **A** | **A** | **T** | **T** | **A** | **T** | **G** | **A** | **A** | **T** | **C** | **C** | **A** | **A** | **A** | **C** | **T** | **T** | **T** | **T** | **G** | **C** | **A** | **T** | **C** | **A** | **G** | **C** | **C** | **G** | **A** | **T** | **C** | **C** | **C** | **A** | **A** | **G** | **C** | **G** | **C** | **G** | **T** | **C** | **G** | **G** | **A** | **T** | **G** | **A** | **T** | **C** | **T** | **G** | **T** | **A** | **C** | **A** | **T** | **C** | **A** | **A** | **A** | **G** | **A** | **T** | **A** | **T** | **T** | **G** | **A** | **T** | **A** | **T** | **G** | **G** | **A** | **A** | **A** | **T** | **T** | **A** | **C** | **T** | **A** | **A** | **A** | **A** | **T** | **A** | **A** | **A** | **A** | **G** | **A** | **A** | **A** | **A** | **A** | **A** | **T** | **G** | **A** | **T** | **C** | **G** | **A** | **A** | **G** | **T** | **A** | **T** | **C** | **C** | **A** | **G** | **T** | **G** | **A** | **A** | **T** | **T** | **A** | **C** | **A** | **G** | **A** | **T** | **T** | **A** | **T** | **G** | **A** | **C** | **T** | **A** | **G** | **C** | **C** | **G** | **T** | **C** | **C** | **T** | **C** | **A** | **A** | **T** | **A** | **T** | **G** | **T** | **A** | **G** | **A** | **T** | **G** | **G** | **A** | **C** | **A** | **A** | **A** | **C** | **C** | **A** | **A** | **A** | **G** | **A** | **A** | **G** | **A** | **G** | **A** | **T** | **T** | **T** | **T** | **A** | **G** | **A** | **A** | **T** | **T** | **A** | **G** | **A** | **A** | **A** | **G** | **G** | **C** | **A** | **T** | **C** | **G** | **A** | **T** | **T** | **A** | **G** | **C** | **T** | **C** | **G** | **T** | **G** | **A** | **G** | **C** | **T** | **T** | **C** | **A** | **T** | **G** | **A** | **T** | **T** | **C** | **A** | **G** | **T** | **T** | **T** | **C** | **A** | **C** | **A** | **G** | **C** | **A** | **A** | **T** | **T** | **A** | **T** | **T** | **T** | **G** | **C** | **A** | **G** | **C** | **G** | **A** | **T** | **G** | **A** | **T** | **G** | **A** | **T** | **G** | **A** | **T** | **G** | **T** | **C** | **G** | **G** | **C** | **A** | **T** | **T** | **G** | **A** | **C** | **G** | **G** | **A** | **G** | **C** | **A** | **A** | **G** | **C** | **A** | **G** | **A** | **A** | **A** | **A** | **A** | **A** | **G** | **T** | **G** | **A** | **G** | **A** | **C** | **A** | **C** | **C** | **A** | **G** | **A** | **A** | **A** | **T** | **G** | **T** | **T** | **C** | **C** | **G** | **T** | **A** | **A** | **G** | **C** | **A** | **G** | **T** | **T** | **G** | **A** | **A** | **A** | **A** | **T** | **G** | **G** | **T** | **G** | **G** | **C** | **A** | **G** | **A** | **G** | **A** | **T** | **C** | **A** | **T** | **C** | **A** | **A** | **C** | **G** | **C** | **T** | **T** | **C** | **C** | **C** | **A** | **G** | **T** | **C** | **T** | **G** | **A** | **G** | **A** | **T** | **G** | **C** | **G** | **T** | **G** | **C** | **G** | **C** | **T** | **G** | **C** | **T** | **T** | **C** | **T** | **T** | **C** | **A** | **T** | **C** | **T** | **G** | **C** | **G** | **T** | **C** | **C** | **A** | **G** | **T** | **C** | **A** | **A** | **T** | **T** | **T** | **A** | **G** | **A** | **A** | **G** | **A** | **A** | **A** | **A** | **A** | **A** | **G** | **T** | **T** | **T** | **A** | **A** | **A** | **A** | **C** | **A** | **A** | **G** | **G** | **C** | **A** | **T** | **T** | **G** | **A** | **G** | **C** | **A** | **G** | **T** | **T** | **A** | **T** | **T** | **G** | **A** | **A** | **G** | **G** | **A** | **A** | **T** | **T** | **G** | **C** | **A** | **G** | **A** | **A** | **T** | **A** | **A** | **A** | **A** | **T** | **C** | **C** | **A** | **G** | **A** | **T** | **T** | **T** | **C** | **G** | **C** | **T** | **A** | **A** | **A** | **A** | **T** | **G** | **G** | **G** | **A** | **T** | **G** | **T** | **A** | **G** | **A** | **A** | **G** | **A** | **T** | **G** | **T** | **A** | **A** | **A** | **A** | **T** | **T** | **A** | **A** | **C** | **T** | **A** | **G** | **T** | **T** | **C** | **C** | **A** | **T** | **T** | **G** | **A** | **G** | **G** | **A** | **T** | **C** | **A** | **C** | **T** | **T** | **G** | **T** | **T** | **C** | **C** | **G** | **A** | **A** | **T** | **C** | **G** | **T** | **T** | **C** | **A** | **A** | **G** | **A** | **A** | **T** | **T** | **G** | **T** | **T** | **A** | **T** | **C** | **A** | **A** | **A** | **C** | **A** | **C** | **A** | **T** | **T** | **A** | **A** | **G** | **A** | **C** | **A** | **T** | **G** | **C** | **T** | **A** | **A** | **G** | **G** | **C** | **T** | **A** | **A** | **T** | **G** | **A** | **A** | **T** | **T** | **A** | **G** | **A** | **A** | **G** | **T** | **C** | **T** | **A** | **T** | **T** | **T** | **G** | **C** | **A** | **T** | **A** | **A** | **A** | **A** | **T** | **A** | **G** | **A** | **T** | **A** | **A** | **C** | **A** | **A** | **T** | **C** | **T** | **T** | **T** | **T** | **A** | **T** | **T** | **A** | **C** | **G** | **T** | **A** | **T** | **C** | **A** | **T** | **T** | **G** | **A** | **C** | **G** | **A** | **C** | **G** | **G** | **G** | **A** | **C** | **A** | **G** | **G** | **A** | **T** | **T** | **T** | **A** | **A** | **T** | **A** | **T** | **G** | **A** | **A** | **T** | **G** | **A** | **A** | **A** | **C** | **C** | **A** | **A** | **A** | **A** | **C** | **G** | **G** | **G** | **A** | **A** | **G** | **T** | **T** | **A** | **T** | **G** | **G** | **A** | **T** | **T** | **G** | **A** | **A** | **C** | **A** | **A** | **T** | **A** | **T** | **C** | **A** | **A** | **A** | **G** | **A** | **A** | **C** | **G** | **A** | **G** | **T** | **A** | **G** | **C** | **T** | **G** | **G** | **T** | **A** | **T** | **T** | **G** | **G** | **T** | **G** | **G** | **A** | **A** | **C** | **A** | **G** | **T** | **A** | **A** | **A** | **A** | **A** | **T** | **C** | **A** | **T** | **T** | **A** | **G** | **T** | **T** | **T** | **T** | **A** | **A** | **A** | **G** | **G** | **G** | **C** | **A** | **A** | **G** | **G** | **T** | **A** | **C** | **G** | **A** | **G** | **C** | **G** | **T** | **G** | **G** | **A** | **G** | **A** | **T** | **C** | **A** | **A** | **G** | **G** | **T** | **C** | **C** | **C** | **T** | **T** | **T** | **G** | **A** | **T** | **G** | **A** | **A** | **G** | **G** | **A** | **G** | **G** | **C** | **A** | **-** | **-** | **-** |
| **N60449F** | **A** | **T** | **G** | **A** | **T** | **G** | **G** | **G** | **A** | **A** | **G** | **A** | **A** | **T** | **A** | **T** | **C** | **C** | **A** | **A** | **A** | **G** | **C** | **A** | **A** | **T** | **G** | **C** | **T** | **A** | **G** | **C** | **T** | **G** | **T** | **T** | **T** | **A** | **T** | **T** | **C** | **G** | **G** | **G** | **G** | **A** | **T** | **T** | **G** | **C** | **G** | **G** | **C** | **T** | **T** | **T** | **C** | **C** | **T** | **T** | **A** | **T** | **T** | **A** | **T** | **T** | **C** | **T** | **A** | **T** | **T** | **T** | **T** | **C** | **A** | **C** | **T** | **T** | **T** | **T** | **C** | **A** | **C** | **T** | **T** | **A** | **T** | **T** | **T** | **T** | **T** | **A** | **T** | **G** | **C** | **C** | **A** | **G** | **T** | **A** | **A** | **C** | **C** | **A** | **A** | **A** | **G** | **T** | **C** | **A** | **T** | **T** | **G** | **G** | **T** | **G** | **G** | **G** | **G** | **A** | **G** | **A** | **A** | **T** | **T** | **G** | **C** | **T** | **G** | **C** | **G** | **T** | **G** | **C** | **G** | **C** | **G** | **T** | **T** | **T** | **A** | **T** | **T** | **A** | **T** | **A** | **C** | **G** | **T** | **T** | **C** | **C** | **G** | **C** | **T** | **T** | **A** | **T** | **C** | **T** | **T** | **C** | **C** | **A** | **T** | **T** | **T** | **G** | **A** | **T** | **G** | **G** | **C** | **A** | **A** | **T** | **T** | **T** | **C** | **A** | **T** | **T** | **A** | **G** | **G** | **T** | **G** | **T** | **A** | **G** | **G** | **A** | **T** | **T** | **G** | **A** | **T** | **C** | **G** | **T** | **A** | **T** | **T** | **T** | **C** | **T** | **A** | **T** | **T** | **G** | **T** | **T** | **A** | **T** | **C** | **A** | **C** | **T** | **T** | **A** | **T** | **T** | **C** | **A** | **A** | **A** | **A** | **G** | **G** | **C** | **A** | **A** | **A** | **A** | **T** | **A** | **C** | **G** | **G** | **G** | **A** | **A** | **A** | **A** | **T** | **C** | **G** | **A** | **A** | **G** | **A** | **A** | **A** | **A** | **G** | **C** | **T** | **T** | **C** | **G** | **T** | **G** | **C** | **A** | **C** | **T** | **T** | **T** | **C** | **T** | **T** | **C** | **T** | **G** | **G** | **C** | **A** | **A** | **T** | **T** | **A** | **T** | **G** | **A** | **A** | **T** | **C** | **C** | **A** | **A** | **A** | **C** | **T** | **T** | **T** | **T** | **G** | **C** | **A** | **T** | **C** | **A** | **G** | **C** | **C** | **G** | **A** | **T** | **C** | **C** | **C** | **A** | **A** | **G** | **C** | **G** | **C** | **G** | **T** | **C** | **G** | **G** | **A** | **T** | **G** | **A** | **T** | **C** | **T** | **G** | **T** | **A** | **C** | **A** | **T** | **C** | **A** | **A** | **A** | **G** | **A** | **T** | **A** | **T** | **T** | **G** | **A** | **T** | **A** | **T** | **G** | **G** | **A** | **A** | **A** | **T** | **T** | **A** | **C** | **T** | **A** | **A** | **A** | **A** | **T** | **A** | **A** | **A** | **A** | **G** | **A** | **A** | **A** | **A** | **A** | **A** | **T** | **G** | **A** | **T** | **C** | **G** | **A** | **A** | **G** | **T** | **A** | **T** | **C** | **C** | **A** | **G** | **T** | **G** | **A** | **A** | **T** | **T** | **A** | **C** | **A** | **G** | **A** | **T** | **T** | **A** | **T** | **G** | **A** | **C** | **T** | **A** | **G** | **C** | **C** | **G** | **T** | **C** | **C** | **T** | **C** | **A** | **A** | **T** | **A** | **T** | **G** | **T** | **A** | **G** | **A** | **T** | **G** | **G** | **A** | **C** | **A** | **A** | **A** | **C** | **C** | **A** | **A** | **A** | **G** | **A** | **A** | **G** | **A** | **G** | **A** | **T** | **T** | **T** | **T** | **A** | **G** | **A** | **A** | **T** | **T** | **A** | **G** | **A** | **A** | **A** | **G** | **G** | **C** | **A** | **T** | **C** | **G** | **A** | **T** | **T** | **A** | **G** | **C** | **T** | **C** | **G** | **T** | **G** | **A** | **G** | **C** | **T** | **T** | **C** | **A** | **T** | **G** | **A** | **T** | **T** | **C** | **A** | **G** | **T** | **T** | **T** | **C** | **A** | **C** | **A** | **G** | **C** | **A** | **A** | **T** | **T** | **A** | **T** | **T** | **T** | **G** | **C** | **A** | **G** | **C** | **G** | **A** | **T** | **G** | **A** | **T** | **G** | **A** | **T** | **G** | **A** | **T** | **G** | **T** | **C** | **G** | **G** | **C** | **A** | **T** | **T** | **G** | **A** | **C** | **G** | **G** | **A** | **G** | **C** | **A** | **A** | **G** | **C** | **A** | **G** | **A** | **A** | **A** | **A** | **A** | **A** | **G** | **T** | **G** | **A** | **G** | **A** | **C** | **A** | **C** | **C** | **A** | **G** | **A** | **A** | **A** | **T** | **G** | **T** | **T** | **C** | **C** | **G** | **T** | **A** | **A** | **G** | **C** | **A** | **G** | **T** | **T** | **G** | **A** | **A** | **A** | **A** | **T** | **G** | **G** | **T** | **G** | **G** | **C** | **A** | **G** | **A** | **G** | **A** | **T** | **C** | **A** | **T** | **C** | **A** | **A** | **C** | **G** | **C** | **T** | **T** | **C** | **C** | **C** | **A** | **G** | **T** | **C** | **T** | **G** | **A** | **G** | **A** | **T** | **G** | **C** | **G** | **T** | **G** | **C** | **G** | **C** | **T** | **G** | **C** | **T** | **T** | **C** | **T** | **T** | **C** | **A** | **T** | **C** | **T** | **G** | **C** | **G** | **T** | **C** | **C** | **A** | **G** | **T** | **C** | **A** | **A** | **T** | **T** | **T** | **A** | **G** | **A** | **A** | **G** | **A** | **A** | **A** | **A** | **A** | **A** | **G** | **T** | **T** | **T** | **A** | **A** | **A** | **A** | **C** | **A** | **A** | **G** | **G** | **C** | **A** | **T** | **T** | **G** | **A** | **G** | **C** | **A** | **G** | **T** | **T** | **A** | **T** | **T** | **G** | **A** | **A** | **G** | **G** | **A** | **A** | **T** | **T** | **G** | **C** | **A** | **G** | **A** | **A** | **T** | **A** | **A** | **A** | **A** | **T** | **C** | **C** | **A** | **G** | **A** | **T** | **T** | **T** | **C** | **G** | **C** | **T** | **A** | **A** | **A** | **A** | **T** | **G** | **G** | **G** | **A** | **T** | **G** | **T** | **A** | **G** | **A** | **A** | **G** | **A** | **T** | **G** | **T** | **A** | **A** | **A** | **A** | **T** | **T** | **A** | **A** | **C** | **T** | **A** | **G** | **T** | **T** | **C** | **C** | **A** | **T** | **T** | **G** | **A** | **G** | **G** | **A** | **T** | **C** | **A** | **C** | **T** | **T** | **G** | **T** | **T** | **C** | **C** | **G** | **A** | **A** | **T** | **C** | **G** | **T** | **T** | **C** | **A** | **A** | **G** | **A** | **A** | **T** | **T** | **G** | **T** | **T** | **A** | **T** | **C** | **A** | **A** | **A** | **C** | **A** | **C** | **A** | **T** | **T** | **A** | **A** | **G** | **A** | **C** | **A** | **T** | **G** | **C** | **T** | **A** | **A** | **G** | **G** | **C** | **T** | **A** | **A** | **T** | **G** | **A** | **A** | **T** | **T** | **A** | **G** | **A** | **A** | **G** | **T** | **C** | **T** | **A** | **T** | **T** | **T** | **G** | **C** | **A** | **T** | **A** | **A** | **A** | **A** | **T** | **A** | **G** | **A** | **T** | **A** | **A** | **C** | **A** | **A** | **T** | **C** | **T** | **T** | **T** | **T** | **A** | **T** | **T** | **A** | **C** | **G** | **T** | **A** | **T** | **C** | **A** | **T** | **T** | **G** | **A** | **C** | **G** | **A** | **C** | **G** | **G** | **G** | **A** | **C** | **A** | **G** | **G** | **A** | **T** | **T** | **T** | **A** | **A** | **T** | **A** | **T** | **G** | **A** | **A** | **T** | **G** | **A** | **A** | **A** | **C** | **C** | **A** | **A** | **A** | **A** | **C** | **G** | **G** | **G** | **A** | **A** | **G** | **T** | **T** | **A** | **T** | **G** | **G** | **A** | **T** | **T** | **G** | **A** | **A** | **C** | **A** | **A** | **T** | **A** | **T** | **C** | **A** | **A** | **A** | **G** | **A** | **A** | **C** | **G** | **A** | **G** | **T** | **A** | **G** | **C** | **T** | **G** | **G** | **T** | **A** | **T** | **T** | **G** | **G** | **T** | **G** | **G** | **A** | **A** | **C** | **A** | **G** | **T** | **A** | **A** | **A** | **A** | **A** | **T** | **C** | **A** | **T** | **T** | **A** | **G** | **T** | **T** | **T** | **T** | **A** | **A** | **A** | **G** | **G** | **G** | **C** | **A** | **A** | **G** | **G** | **T** | **A** | **C** | **G** | **A** | **G** | **C** | **G** | **T** | **G** | **G** | **A** | **G** | **A** | **T** | **C** | **A** | **A** | **G** | **G** | **T** | **C** | **C** | **C** | **T** | **T** | **T** | **G** | **A** | **T** | **G** | **A** | **A** | **G** | **G** | **A** | **G** | **G** | **C** | **A** | **-** | **-** | **-** |
| **N60001F** | **A** | **T** | **G** | **A** | **T** | **G** | **G** | **G** | **A** | **A** | **A** | **A** | **A** | **T** | **A** | **T** | **C** | **C** | **A** | **G** | **A** | **G** | **C** | **G** | **A** | **T** | **G** | **C** | **T** | **A** | **G** | **C** | **T** | **G** | **T** | **T** | **T** | **A** | **T** | **T** | **C** | **G** | **G** | **G** | **G** | **A** | **T** | **T** | **G** | **C** | **G** | **G** | **C** | **T** | **T** | **T** | **C** | **C** | **T** | **T** | **A** | **T** | **T** | **A** | **T** | **T** | **C** | **T** | **A** | **T** | **T** | **T** | **T** | **C** | **A** | **C** | **T** | **T** | **T** | **T** | **C** | **A** | **C** | **T** | **T** | **A** | **T** | **T** | **T** | **T** | **T** | **A** | **T** | **G** | **C** | **C** | **A** | **G** | **T** | **A** | **A** | **T** | **C** | **A** | **A** | **A** | **G** | **T** | **C** | **A** | **T** | **T** | **G** | **G** | **T** | **G** | **G** | **G** | **G** | **A** | **G** | **A** | **A** | **T** | **T** | **A** | **C** | **T** | **G** | **C** | **G** | **T** | **G** | **C** | **A** | **C** | **G** | **T** | **T** | **T** | **A** | **T** | **T** | **A** | **T** | **A** | **T** | **G** | **T** | **T** | **C** | **C** | **G** | **C** | **T** | **T** | **A** | **T** | **C** | **T** | **T** | **C** | **C** | **A** | **T** | **T** | **T** | **G** | **T** | **T** | **A** | **G** | **C** | **C** | **A** | **T** | **T** | **T** | **C** | **C** | **T** | **T** | **A** | **G** | **G** | **T** | **G** | **T** | **A** | **G** | **G** | **A** | **T** | **T** | **A** | **A** | **T** | **C** | **G** | **T** | **C** | **T** | **T** | **T** | **C** | **T** | **A** | **T** | **T** | **A** | **T** | **T** | **A** | **T** | **C** | **A** | **C** | **T** | **T** | **A** | **T** | **T** | **C** | **A** | **A** | **A** | **A** | **G** | **A** | **C** | **A** | **A** | **A** | **A** | **T** | **A** | **C** | **G** | **G** | **G** | **A** | **A** | **A** | **A** | **T** | **C** | **G** | **A** | **A** | **G** | **A** | **A** | **A** | **A** | **G** | **C** | **T** | **G** | **C** | **G** | **T** | **G** | **C** | **A** | **C** | **T** | **C** | **T** | **C** | **T** | **T** | **C** | **T** | **G** | **G** | **C** | **A** | **A** | **T** | **T** | **A** | **T** | **G** | **A** | **A** | **T** | **C** | **C** | **A** | **A** | **A** | **T** | **T** | **G** | **T** | **T** | **G** | **C** | **T** | **T** | **C** | **T** | **T** | **C** | **C** | **G** | **A** | **T** | **C** | **C** | **C** | **A** | **A** | **G** | **T** | **G** | **C** | **A** | **T** | **C** | **G** | **G** | **A** | **C** | **G** | **A** | **T** | **T** | **T** | **A** | **T** | **A** | **C** | **A** | **T** | **C** | **A** | **A** | **A** | **G** | **A** | **T** | **A** | **T** | **T** | **G** | **A** | **T** | **A** | **A** | **G** | **G** | **A** | **A** | **A** | **T** | **C** | **A** | **C** | **T** | **A** | **A** | **G** | **A** | **T** | **A** | **A** | **A** | **A** | **G** | **A** | **A** | **A** | **A** | **A** | **A** | **T** | **G** | **A** | **T** | **T** | **G** | **A** | **A** | **G** | **T** | **A** | **T** | **C** | **T** | **A** | **G** | **T** | **G** | **A** | **A** | **T** | **T** | **A** | **C** | **A** | **A** | **A** | **T** | **T** | **G** | **T** | **A** | **A** | **C** | **G** | **A** | **G** | **C** | **C** | **G** | **T** | **C** | **C** | **C** | **C** | **A** | **A** | **T** | **A** | **T** | **G** | **T** | **A** | **G** | **A** | **T** | **G** | **G** | **A** | **C** | **A** | **A** | **A** | **C** | **C** | **A** | **A** | **A** | **G** | **A** | **A** | **G** | **A** | **A** | **A** | **T** | **T** | **T** | **T** | **A** | **G** | **A** | **A** | **T** | **T** | **A** | **G** | **A** | **A** | **A** | **G** | **A** | **C** | **A** | **T** | **C** | **G** | **A** | **T** | **T** | **A** | **G** | **C** | **C** | **C** | **G** | **T** | **G** | **A** | **G** | **C** | **T** | **G** | **C** | **A** | **T** | **G** | **A** | **T** | **T** | **C** | **A** | **G** | **T** | **T** | **T** | **C** | **G** | **C** | **A** | **A** | **C** | **A** | **A** | **T** | **T** | **G** | **T** | **T** | **T** | **G** | **C** | **A** | **G** | **C** | **A** | **A** | **T** | **G** | **A** | **T** | **G** | **A** | **T** | **G** | **A** | **T** | **G** | **T** | **C** | **G** | **G** | **C** | **A** | **T** | **T** | **G** | **A** | **C** | **G** | **G** | **A** | **G** | **C** | **A** | **A** | **G** | **C** | **A** | **G** | **A** | **A** | **A** | **A** | **A** | **A** | **G** | **C** | **G** | **A** | **G** | **A** | **C** | **A** | **C** | **C** | **A** | **G** | **A** | **A** | **A** | **T** | **G** | **T** | **T** | **C** | **C** | **G** | **C** | **A** | **A** | **G** | **C** | **A** | **G** | **T** | **T** | **G** | **A** | **A** | **A** | **A** | **T** | **G** | **G** | **T** | **A** | **G** | **C** | **A** | **G** | **A** | **G** | **A** | **T** | **C** | **A** | **T** | **C** | **A** | **A** | **C** | **G** | **C** | **T** | **T** | **C** | **C** | **C** | **A** | **G** | **T** | **C** | **T** | **G** | **A** | **G** | **A** | **T** | **G** | **C** | **G** | **T** | **G** | **C** | **G** | **C** | **T** | **G** | **C** | **T** | **T** | **C** | **T** | **T** | **C** | **A** | **T** | **C** | **T** | **G** | **C** | **G** | **T** | **C** | **C** | **A** | **G** | **T** | **C** | **A** | **A** | **T** | **T** | **T** | **G** | **G** | **A** | **A** | **G** | **A** | **G** | **A** | **A** | **A** | **A** | **G** | **T** | **T** | **T** | **A** | **A** | **A** | **G** | **C** | **A** | **A** | **G** | **G** | **T** | **A** | **T** | **T** | **G** | **A** | **G** | **C** | **A** | **G** | **T** | **T** | **A** | **T** | **T** | **G** | **A** | **A** | **G** | **G** | **A** | **A** | **T** | **T** | **G** | **C** | **A** | **A** | **A** | **A** | **T** | **A** | **A** | **A** | **A** | **T** | **C** | **C** | **A** | **G** | **A** | **T** | **T** | **T** | **C** | **G** | **C** | **T** | **G** | **A** | **A** | **A** | **T** | **G** | **G** | **G** | **A** | **T** | **G** | **T** | **A** | **G** | **A** | **A** | **G** | **A** | **T** | **G** | **T** | **A** | **A** | **A** | **A** | **C** | **T** | **A** | **T** | **C** | **T** | **A** | **G** | **T** | **T** | **C** | **C** | **A** | **T** | **T** | **G** | **A** | **G** | **G** | **A** | **T** | **C** | **A** | **C** | **T** | **T** | **G** | **T** | **T** | **C** | **C** | **G** | **A** | **A** | **T** | **C** | **G** | **T** | **T** | **C** | **A** | **A** | **G** | **A** | **A** | **T** | **T** | **G** | **T** | **T** | **A** | **T** | **C** | **A** | **A** | **A** | **T** | **A** | **C** | **A** | **T** | **T** | **A** | **A** | **G** | **A** | **C** | **A** | **T** | **G** | **C** | **C** | **A** | **A** | **A** | **G** | **C** | **C** | **A** | **A** | **T** | **G** | **A** | **A** | **T** | **T** | **A** | **G** | **A** | **G** | **G** | **T** | **A** | **T** | **A** | **T** | **T** | **T** | **G** | **C** | **A** | **C** | **A** | **A** | **A** | **A** | **T** | **A** | **G** | **A** | **C** | **A** | **A** | **T** | **A** | **A** | **T** | **C** | **T** | **T** | **T** | **T** | **G** | **T** | **T** | **A** | **C** | **G** | **T** | **A** | **T** | **T** | **A** | **T** | **T** | **G** | **A** | **C** | **G** | **A** | **C** | **G** | **G** | **A** | **A** | **C** | **A** | **G** | **G** | **G** | **T** | **T** | **T** | **A** | **A** | **T** | **A** | **T** | **G** | **A** | **A** | **T** | **G** | **A** | **A** | **A** | **C** | **G** | **A** | **A** | **A** | **A** | **C** | **G** | **G** | **G** | **A** | **A** | **G** | **T** | **T** | **A** | **T** | **G** | **G** | **A** | **T** | **T** | **G** | **A** | **A** | **C** | **A** | **A** | **T** | **A** | **T** | **C** | **A** | **A** | **A** | **G** | **A** | **A** | **C** | **G** | **A** | **G** | **T** | **A** | **G** | **C** | **T** | **G** | **G** | **T** | **A** | **T** | **T** | **G** | **G** | **T** | **G** | **G** | **A** | **A** | **C** | **A** | **G** | **T** | **A** | **A** | **A** | **A** | **A** | **T** | **C** | **A** | **T** | **T** | **A** | **G** | **T** | **T** | **T** | **T** | **A** | **A** | **A** | **G** | **G** | **G** | **C** | **A** | **A** | **G** | **G** | **T** | **A** | **C** | **A** | **A** | **G** | **T** | **G** | **T** | **A** | **G** | **A** | **A** | **A** | **T** | **C** | **A** | **A** | **G** | **G** | **T** | **C** | **C** | **C** | **T** | **T** | **T** | **G** | **A** | **T** | **G** | **A** | **A** | **G** | **G** | **A** | **G** | **G** | **C** | **A** | **-** | **-** | **-** |
| **N54551** | **A** | **T** | **G** | **A** | **T** | **G** | **G** | **G** | **A** | **A** | **G** | **A** | **A** | **T** | **T** | **T** | **C** | **C** | **A** | **A** | **A** | **G** | **C** | **A** | **A** | **T** | **G** | **C** | **T** | **A** | **G** | **C** | **T** | **G** | **T** | **T** | **T** | **A** | **T** | **T** | **C** | **G** | **G** | **G** | **G** | **A** | **T** | **T** | **G** | **C** | **G** | **G** | **C** | **T** | **T** | **T** | **C** | **C** | **T** | **T** | **A** | **T** | **T** | **A** | **T** | **T** | **C** | **T** | **A** | **T** | **T** | **T** | **T** | **C** | **A** | **C** | **T** | **T** | **T** | **T** | **C** | **A** | **C** | **T** | **T** | **A** | **T** | **T** | **T** | **T** | **T** | **A** | **T** | **G** | **C** | **C** | **A** | **G** | **T** | **A** | **A** | **C** | **C** | **A** | **A** | **A** | **G** | **T** | **C** | **A** | **T** | **T** | **G** | **G** | **T** | **G** | **G** | **G** | **G** | **A** | **G** | **A** | **A** | **T** | **T** | **G** | **C** | **T** | **G** | **C** | **G** | **T** | **G** | **C** | **G** | **C** | **G** | **T** | **T** | **T** | **A** | **T** | **T** | **A** | **T** | **A** | **C** | **G** | **T** | **T** | **C** | **C** | **G** | **C** | **T** | **T** | **A** | **T** | **C** | **T** | **T** | **C** | **C** | **A** | **T** | **T** | **T** | **G** | **A** | **T** | **G** | **G** | **C** | **C** | **A** | **T** | **T** | **T** | **C** | **A** | **T** | **T** | **A** | **G** | **G** | **T** | **G** | **T** | **A** | **G** | **G** | **A** | **T** | **T** | **G** | **A** | **T** | **C** | **G** | **T** | **A** | **T** | **T** | **T** | **C** | **T** | **A** | **T** | **T** | **G** | **T** | **T** | **A** | **T** | **C** | **A** | **C** | **T** | **T** | **A** | **T** | **T** | **C** | **A** | **A** | **A** | **A** | **G** | **G** | **C** | **A** | **A** | **A** | **A** | **T** | **A** | **C** | **G** | **G** | **G** | **A** | **A** | **A** | **A** | **T** | **C** | **G** | **A** | **A** | **G** | **A** | **A** | **A** | **A** | **G** | **C** | **T** | **T** | **C** | **G** | **T** | **G** | **C** | **A** | **C** | **T** | **T** | **T** | **C** | **T** | **T** | **C** | **T** | **G** | **G** | **C** | **A** | **A** | **T** | **T** | **A** | **T** | **G** | **A** | **A** | **T** | **C** | **C** | **A** | **A** | **A** | **C** | **T** | **T** | **T** | **T** | **G** | **C** | **A** | **T** | **C** | **A** | **G** | **C** | **C** | **G** | **A** | **T** | **C** | **C** | **C** | **A** | **A** | **G** | **C** | **G** | **C** | **G** | **T** | **C** | **G** | **G** | **A** | **T** | **G** | **A** | **T** | **C** | **T** | **G** | **T** | **A** | **C** | **A** | **T** | **C** | **A** | **A** | **A** | **G** | **A** | **T** | **A** | **T** | **T** | **G** | **A** | **T** | **A** | **T** | **A** | **G** | **A** | **A** | **A** | **T** | **T** | **A** | **C** | **T** | **A** | **A** | **A** | **A** | **T** | **A** | **A** | **A** | **A** | **G** | **A** | **A** | **A** | **A** | **A** | **A** | **T** | **G** | **A** | **T** | **C** | **G** | **A** | **A** | **G** | **T** | **A** | **T** | **C** | **C** | **A** | **G** | **T** | **G** | **A** | **A** | **T** | **T** | **A** | **C** | **A** | **G** | **A** | **T** | **T** | **A** | **T** | **G** | **A** | **C** | **T** | **A** | **G** | **C** | **C** | **G** | **T** | **C** | **C** | **T** | **C** | **A** | **A** | **T** | **A** | **T** | **G** | **T** | **A** | **G** | **A** | **T** | **G** | **G** | **A** | **C** | **A** | **A** | **A** | **C** | **C** | **A** | **A** | **A** | **G** | **A** | **A** | **G** | **A** | **G** | **A** | **T** | **T** | **T** | **T** | **A** | **G** | **A** | **A** | **T** | **T** | **A** | **G** | **A** | **A** | **A** | **G** | **G** | **C** | **A** | **T** | **C** | **G** | **A** | **T** | **T** | **A** | **G** | **C** | **T** | **C** | **G** | **T** | **G** | **A** | **G** | **C** | **T** | **T** | **C** | **A** | **T** | **G** | **A** | **T** | **T** | **C** | **A** | **G** | **T** | **T** | **T** | **C** | **A** | **C** | **A** | **G** | **C** | **A** | **A** | **T** | **T** | **A** | **T** | **T** | **T** | **G** | **C** | **A** | **G** | **C** | **G** | **A** | **T** | **G** | **A** | **T** | **G** | **A** | **T** | **G** | **A** | **T** | **G** | **T** | **C** | **G** | **G** | **C** | **A** | **T** | **T** | **G** | **A** | **C** | **G** | **G** | **A** | **G** | **C** | **A** | **A** | **G** | **C** | **A** | **G** | **A** | **A** | **A** | **A** | **A** | **A** | **G** | **T** | **G** | **A** | **G** | **A** | **C** | **A** | **C** | **C** | **A** | **G** | **A** | **A** | **A** | **T** | **G** | **T** | **T** | **C** | **C** | **G** | **T** | **A** | **A** | **G** | **C** | **A** | **G** | **T** | **T** | **G** | **A** | **A** | **A** | **A** | **T** | **G** | **G** | **T** | **G** | **G** | **C** | **A** | **G** | **A** | **G** | **A** | **T** | **C** | **A** | **T** | **C** | **A** | **A** | **C** | **G** | **C** | **T** | **T** | **C** | **C** | **C** | **A** | **G** | **T** | **C** | **T** | **G** | **A** | **G** | **A** | **T** | **G** | **C** | **G** | **T** | **G** | **C** | **G** | **C** | **T** | **G** | **C** | **T** | **T** | **C** | **T** | **T** | **C** | **A** | **T** | **C** | **T** | **G** | **C** | **G** | **T** | **C** | **C** | **A** | **G** | **T** | **C** | **A** | **A** | **T** | **T** | **T** | **A** | **G** | **A** | **A** | **G** | **A** | **A** | **A** | **A** | **A** | **A** | **G** | **T** | **T** | **T** | **A** | **A** | **A** | **A** | **C** | **A** | **A** | **G** | **G** | **C** | **A** | **T** | **T** | **G** | **A** | **G** | **C** | **A** | **G** | **T** | **T** | **A** | **T** | **T** | **G** | **A** | **A** | **G** | **G** | **A** | **A** | **T** | **T** | **G** | **C** | **A** | **G** | **A** | **A** | **T** | **A** | **A** | **A** | **A** | **T** | **C** | **C** | **A** | **G** | **A** | **T** | **T** | **T** | **C** | **G** | **C** | **T** | **A** | **A** | **A** | **A** | **T** | **G** | **G** | **G** | **A** | **T** | **G** | **T** | **A** | **G** | **A** | **A** | **G** | **A** | **T** | **G** | **T** | **A** | **A** | **A** | **A** | **T** | **T** | **A** | **A** | **C** | **T** | **A** | **G** | **T** | **T** | **C** | **C** | **A** | **T** | **T** | **G** | **A** | **G** | **G** | **A** | **T** | **C** | **A** | **C** | **T** | **T** | **G** | **T** | **T** | **C** | **C** | **G** | **A** | **A** | **T** | **C** | **G** | **T** | **T** | **C** | **A** | **A** | **G** | **A** | **A** | **T** | **T** | **G** | **T** | **T** | **A** | **T** | **C** | **A** | **A** | **A** | **C** | **A** | **C** | **A** | **T** | **T** | **A** | **A** | **G** | **A** | **C** | **A** | **T** | **G** | **C** | **T** | **A** | **A** | **G** | **G** | **C** | **T** | **A** | **A** | **T** | **G** | **A** | **A** | **T** | **T** | **A** | **G** | **A** | **A** | **G** | **T** | **C** | **T** | **A** | **T** | **T** | **T** | **G** | **C** | **A** | **T** | **A** | **A** | **A** | **A** | **T** | **A** | **G** | **A** | **T** | **A** | **A** | **C** | **A** | **A** | **T** | **C** | **T** | **T** | **T** | **T** | **A** | **T** | **T** | **A** | **C** | **G** | **T** | **A** | **T** | **C** | **A** | **T** | **T** | **G** | **A** | **C** | **G** | **A** | **C** | **G** | **G** | **G** | **A** | **C** | **A** | **G** | **G** | **A** | **T** | **T** | **T** | **A** | **A** | **T** | **A** | **T** | **G** | **A** | **A** | **T** | **G** | **A** | **A** | **A** | **C** | **C** | **A** | **A** | **A** | **A** | **C** | **G** | **G** | **G** | **A** | **A** | **G** | **T** | **T** | **A** | **T** | **G** | **G** | **A** | **T** | **T** | **G** | **A** | **A** | **C** | **A** | **A** | **T** | **A** | **T** | **C** | **A** | **A** | **A** | **G** | **A** | **A** | **C** | **G** | **A** | **G** | **T** | **A** | **G** | **C** | **T** | **G** | **G** | **T** | **A** | **T** | **T** | **G** | **G** | **T** | **G** | **G** | **A** | **A** | **C** | **A** | **G** | **T** | **A** | **A** | **A** | **A** | **A** | **T** | **C** | **A** | **T** | **T** | **A** | **G** | **T** | **T** | **T** | **T** | **A** | **A** | **A** | **G** | **G** | **G** | **C** | **A** | **A** | **G** | **G** | **T** | **A** | **C** | **G** | **A** | **G** | **C** | **G** | **T** | **G** | **G** | **A** | **G** | **A** | **T** | **C** | **A** | **A** | **G** | **G** | **T** | **C** | **C** | **C** | **T** | **T** | **T** | **G** | **A** | **T** | **G** | **A** | **A** | **G** | **G** | **A** | **G** | **G** | **C** | **A** | **-** | **-** | **-** |
| **N59559F** | **A** | **T** | **G** | **A** | **T** | **G** | **G** | **G** | **A** | **A** | **A** | **A** | **A** | **T** | **A** | **T** | **C** | **C** | **A** | **G** | **A** | **G** | **C** | **G** | **A** | **T** | **G** | **C** | **T** | **A** | **G** | **C** | **T** | **G** | **T** | **T** | **T** | **A** | **T** | **T** | **C** | **G** | **G** | **G** | **G** | **A** | **T** | **T** | **G** | **C** | **G** | **G** | **C** | **T** | **T** | **T** | **C** | **C** | **T** | **T** | **A** | **T** | **T** | **A** | **T** | **T** | **C** | **T** | **A** | **T** | **T** | **T** | **T** | **C** | **A** | **C** | **T** | **T** | **T** | **T** | **C** | **A** | **C** | **T** | **T** | **A** | **T** | **T** | **T** | **T** | **T** | **A** | **T** | **G** | **C** | **C** | **A** | **G** | **T** | **A** | **A** | **T** | **C** | **A** | **A** | **A** | **G** | **T** | **C** | **A** | **T** | **T** | **G** | **G** | **T** | **G** | **G** | **G** | **G** | **A** | **G** | **A** | **A** | **T** | **T** | **A** | **C** | **T** | **G** | **C** | **G** | **T** | **G** | **C** | **A** | **C** | **G** | **T** | **T** | **T** | **A** | **T** | **T** | **A** | **T** | **A** | **T** | **G** | **T** | **T** | **C** | **C** | **G** | **C** | **T** | **T** | **A** | **T** | **C** | **T** | **T** | **C** | **C** | **A** | **T** | **T** | **T** | **G** | **T** | **T** | **A** | **G** | **C** | **T** | **A** | **T** | **T** | **T** | **C** | **C** | **T** | **T** | **A** | **G** | **G** | **T** | **G** | **T** | **A** | **G** | **G** | **A** | **T** | **T** | **A** | **A** | **T** | **C** | **G** | **T** | **C** | **T** | **T** | **T** | **C** | **T** | **A** | **T** | **T** | **A** | **T** | **T** | **A** | **T** | **C** | **A** | **C** | **T** | **T** | **A** | **T** | **T** | **C** | **A** | **A** | **A** | **A** | **G** | **A** | **C** | **A** | **A** | **A** | **A** | **T** | **A** | **C** | **G** | **G** | **G** | **A** | **A** | **A** | **A** | **T** | **C** | **G** | **A** | **A** | **G** | **A** | **A** | **A** | **A** | **G** | **C** | **T** | **G** | **C** | **G** | **T** | **G** | **C** | **A** | **C** | **T** | **C** | **T** | **C** | **T** | **T** | **C** | **T** | **G** | **G** | **C** | **A** | **A** | **T** | **T** | **A** | **T** | **G** | **A** | **A** | **T** | **C** | **C** | **A** | **A** | **A** | **T** | **T** | **G** | **T** | **T** | **G** | **C** | **T** | **T** | **C** | **T** | **T** | **C** | **C** | **G** | **A** | **T** | **C** | **C** | **C** | **A** | **A** | **G** | **T** | **G** | **C** | **A** | **T** | **C** | **G** | **G** | **A** | **C** | **G** | **A** | **T** | **T** | **T** | **A** | **T** | **A** | **C** | **A** | **T** | **C** | **A** | **A** | **A** | **G** | **A** | **T** | **A** | **T** | **T** | **G** | **A** | **T** | **A** | **A** | **G** | **G** | **A** | **A** | **A** | **T** | **C** | **A** | **C** | **T** | **A** | **A** | **A** | **A** | **T** | **A** | **A** | **A** | **A** | **G** | **A** | **A** | **A** | **A** | **A** | **A** | **T** | **G** | **A** | **T** | **T** | **G** | **A** | **A** | **G** | **T** | **A** | **T** | **C** | **T** | **A** | **G** | **T** | **G** | **A** | **A** | **T** | **T** | **A** | **C** | **A** | **A** | **A** | **T** | **T** | **G** | **T** | **A** | **A** | **C** | **G** | **A** | **G** | **C** | **C** | **G** | **T** | **C** | **C** | **C** | **C** | **A** | **A** | **T** | **A** | **T** | **G** | **T** | **A** | **G** | **A** | **T** | **G** | **G** | **A** | **C** | **A** | **A** | **A** | **C** | **C** | **A** | **A** | **A** | **G** | **A** | **A** | **G** | **A** | **A** | **A** | **T** | **T** | **T** | **T** | **A** | **G** | **A** | **A** | **T** | **T** | **A** | **G** | **A** | **A** | **A** | **G** | **A** | **C** | **A** | **T** | **C** | **G** | **A** | **T** | **T** | **A** | **G** | **C** | **C** | **C** | **G** | **T** | **G** | **A** | **G** | **C** | **T** | **G** | **C** | **A** | **T** | **G** | **A** | **T** | **T** | **C** | **A** | **G** | **T** | **T** | **T** | **C** | **G** | **C** | **A** | **A** | **C** | **A** | **A** | **T** | **T** | **A** | **T** | **T** | **T** | **G** | **C** | **A** | **G** | **C** | **A** | **A** | **T** | **G** | **A** | **T** | **G** | **A** | **T** | **G** | **A** | **T** | **G** | **T** | **C** | **G** | **G** | **C** | **A** | **T** | **T** | **G** | **A** | **C** | **G** | **G** | **A** | **G** | **C** | **A** | **A** | **G** | **C** | **A** | **G** | **A** | **A** | **A** | **A** | **A** | **A** | **G** | **C** | **G** | **A** | **G** | **A** | **C** | **A** | **C** | **C** | **A** | **G** | **A** | **A** | **A** | **T** | **G** | **T** | **T** | **C** | **C** | **G** | **C** | **A** | **A** | **G** | **C** | **A** | **G** | **T** | **T** | **G** | **A** | **A** | **A** | **A** | **T** | **G** | **G** | **T** | **A** | **G** | **C** | **A** | **G** | **A** | **G** | **A** | **T** | **C** | **A** | **T** | **C** | **A** | **A** | **C** | **G** | **C** | **T** | **T** | **C** | **C** | **C** | **A** | **G** | **T** | **C** | **T** | **G** | **A** | **G** | **A** | **T** | **G** | **C** | **G** | **T** | **G** | **C** | **G** | **C** | **T** | **G** | **C** | **T** | **T** | **C** | **T** | **T** | **C** | **A** | **T** | **C** | **T** | **G** | **C** | **G** | **T** | **C** | **C** | **A** | **G** | **T** | **C** | **A** | **A** | **T** | **T** | **T** | **A** | **G** | **A** | **A** | **G** | **A** | **G** | **A** | **A** | **A** | **A** | **G** | **T** | **T** | **T** | **A** | **A** | **A** | **G** | **C** | **A** | **A** | **G** | **G** | **T** | **A** | **T** | **C** | **G** | **A** | **G** | **C** | **A** | **G** | **T** | **T** | **A** | **T** | **T** | **G** | **A** | **A** | **G** | **G** | **A** | **A** | **T** | **T** | **G** | **C** | **A** | **A** | **A** | **A** | **T** | **A** | **A** | **A** | **A** | **T** | **T** | **C** | **A** | **G** | **A** | **T** | **T** | **T** | **C** | **G** | **C** | **T** | **G** | **A** | **A** | **A** | **T** | **G** | **G** | **G** | **A** | **T** | **G** | **T** | **A** | **G** | **A** | **A** | **G** | **A** | **T** | **G** | **T** | **A** | **A** | **A** | **A** | **C** | **T** | **A** | **T** | **C** | **T** | **A** | **G** | **C** | **T** | **C** | **C** | **A** | **T** | **T** | **G** | **A** | **G** | **G** | **A** | **T** | **C** | **A** | **C** | **T** | **T** | **G** | **T** | **T** | **C** | **C** | **G** | **A** | **A** | **T** | **C** | **G** | **T** | **T** | **C** | **A** | **A** | **G** | **A** | **A** | **T** | **T** | **G** | **T** | **T** | **A** | **T** | **C** | **A** | **A** | **A** | **T** | **A** | **C** | **A** | **T** | **T** | **A** | **A** | **G** | **A** | **C** | **A** | **T** | **G** | **C** | **C** | **A** | **A** | **A** | **G** | **C** | **C** | **A** | **A** | **T** | **G** | **A** | **A** | **T** | **T** | **A** | **G** | **A** | **G** | **G** | **T** | **A** | **T** | **A** | **T** | **T** | **T** | **G** | **C** | **A** | **T** | **A** | **A** | **A** | **A** | **T** | **A** | **G** | **A** | **C** | **A** | **A** | **T** | **A** | **A** | **T** | **C** | **T** | **T** | **T** | **T** | **A** | **T** | **T** | **A** | **C** | **G** | **T** | **A** | **T** | **T** | **A** | **T** | **T** | **G** | **A** | **C** | **G** | **A** | **T** | **G** | **G** | **A** | **A** | **C** | **A** | **G** | **G** | **G** | **T** | **T** | **T** | **A** | **A** | **T** | **A** | **T** | **G** | **A** | **A** | **T** | **G** | **A** | **A** | **A** | **C** | **G** | **A** | **A** | **A** | **A** | **C** | **G** | **G** | **G** | **A** | **A** | **G** | **T** | **T** | **A** | **T** | **G** | **G** | **A** | **T** | **T** | **G** | **A** | **A** | **C** | **A** | **A** | **T** | **A** | **T** | **C** | **A** | **A** | **A** | **G** | **A** | **A** | **C** | **G** | **A** | **G** | **T** | **A** | **G** | **C** | **T** | **G** | **G** | **T** | **A** | **T** | **T** | **G** | **G** | **T** | **G** | **G** | **A** | **A** | **C** | **A** | **G** | **T** | **A** | **A** | **A** | **A** | **A** | **T** | **C** | **A** | **T** | **T** | **A** | **G** | **T** | **T** | **T** | **T** | **A** | **A** | **A** | **G** | **G** | **G** | **C** | **A** | **A** | **G** | **G** | **T** | **A** | **C** | **A** | **A** | **G** | **T** | **G** | **T** | **A** | **G** | **A** | **A** | **A** | **T** | **C** | **A** | **A** | **G** | **G** | **T** | **C** | **C** | **C** | **T** | **T** | **T** | **G** | **A** | **T** | **G** | **A** | **A** | **G** | **G** | **A** | **G** | **G** | **C** | **A** | **-** | **-** | **-** |
| **N59517F** | **A** | **T** | **G** | **A** | **T** | **G** | **G** | **G** | **A** | **A** | **G** | **A** | **A** | **T** | **A** | **T** | **C** | **C** | **A** | **A** | **A** | **G** | **C** | **A** | **A** | **T** | **G** | **C** | **T** | **A** | **G** | **C** | **T** | **G** | **T** | **T** | **T** | **A** | **T** | **T** | **C** | **G** | **G** | **G** | **G** | **A** | **T** | **T** | **G** | **C** | **G** | **G** | **C** | **T** | **T** | **T** | **C** | **C** | **T** | **T** | **A** | **T** | **T** | **A** | **T** | **T** | **C** | **T** | **A** | **T** | **T** | **T** | **T** | **C** | **A** | **C** | **T** | **T** | **T** | **T** | **C** | **A** | **C** | **T** | **T** | **A** | **T** | **T** | **T** | **T** | **T** | **A** | **T** | **G** | **C** | **C** | **A** | **G** | **T** | **A** | **A** | **C** | **C** | **A** | **A** | **A** | **G** | **T** | **C** | **A** | **T** | **T** | **G** | **G** | **T** | **G** | **G** | **G** | **G** | **A** | **G** | **A** | **A** | **T** | **T** | **G** | **C** | **T** | **G** | **C** | **G** | **T** | **G** | **C** | **G** | **C** | **G** | **T** | **T** | **T** | **A** | **T** | **T** | **A** | **T** | **A** | **C** | **G** | **T** | **T** | **C** | **C** | **G** | **C** | **T** | **T** | **A** | **T** | **C** | **T** | **T** | **C** | **C** | **A** | **T** | **T** | **T** | **G** | **A** | **T** | **G** | **G** | **C** | **A** | **A** | **T** | **T** | **T** | **C** | **A** | **T** | **T** | **A** | **G** | **G** | **T** | **G** | **T** | **A** | **G** | **G** | **A** | **T** | **T** | **G** | **A** | **T** | **C** | **G** | **T** | **A** | **T** | **T** | **T** | **C** | **T** | **A** | **T** | **T** | **G** | **T** | **T** | **A** | **T** | **C** | **A** | **C** | **T** | **T** | **A** | **T** | **T** | **C** | **A** | **A** | **A** | **A** | **G** | **G** | **C** | **A** | **A** | **A** | **A** | **T** | **A** | **C** | **G** | **G** | **G** | **A** | **A** | **A** | **A** | **T** | **C** | **G** | **A** | **A** | **G** | **A** | **A** | **A** | **A** | **G** | **C** | **T** | **T** | **C** | **G** | **T** | **G** | **C** | **A** | **C** | **T** | **T** | **T** | **C** | **T** | **T** | **C** | **T** | **G** | **G** | **C** | **A** | **A** | **T** | **T** | **A** | **T** | **G** | **A** | **A** | **T** | **C** | **C** | **A** | **A** | **A** | **C** | **T** | **T** | **T** | **T** | **G** | **C** | **A** | **T** | **C** | **A** | **G** | **C** | **C** | **G** | **A** | **T** | **C** | **C** | **C** | **A** | **A** | **G** | **C** | **G** | **C** | **G** | **T** | **C** | **G** | **G** | **A** | **T** | **G** | **A** | **T** | **C** | **T** | **G** | **T** | **A** | **C** | **A** | **T** | **C** | **A** | **A** | **A** | **G** | **A** | **T** | **A** | **T** | **T** | **G** | **A** | **T** | **A** | **T** | **G** | **G** | **A** | **A** | **A** | **T** | **T** | **A** | **C** | **T** | **A** | **A** | **A** | **A** | **T** | **A** | **A** | **A** | **A** | **G** | **A** | **A** | **A** | **A** | **A** | **A** | **T** | **G** | **A** | **T** | **C** | **G** | **A** | **A** | **G** | **T** | **A** | **T** | **C** | **C** | **A** | **G** | **T** | **G** | **A** | **A** | **T** | **T** | **A** | **C** | **A** | **G** | **A** | **T** | **T** | **A** | **T** | **G** | **A** | **C** | **T** | **A** | **G** | **C** | **C** | **G** | **T** | **C** | **C** | **T** | **C** | **A** | **A** | **T** | **A** | **T** | **G** | **T** | **A** | **G** | **A** | **T** | **G** | **G** | **A** | **C** | **A** | **A** | **A** | **C** | **C** | **A** | **A** | **A** | **G** | **A** | **A** | **G** | **A** | **G** | **A** | **T** | **T** | **T** | **T** | **A** | **G** | **A** | **A** | **T** | **T** | **A** | **G** | **A** | **A** | **A** | **G** | **G** | **C** | **A** | **T** | **C** | **G** | **A** | **T** | **T** | **A** | **G** | **C** | **T** | **C** | **G** | **T** | **G** | **A** | **G** | **C** | **T** | **T** | **C** | **A** | **T** | **G** | **A** | **T** | **T** | **C** | **A** | **G** | **T** | **T** | **T** | **C** | **A** | **C** | **A** | **G** | **C** | **A** | **A** | **T** | **T** | **A** | **T** | **T** | **T** | **G** | **C** | **A** | **G** | **C** | **G** | **A** | **T** | **G** | **A** | **T** | **G** | **A** | **T** | **G** | **A** | **T** | **G** | **T** | **C** | **G** | **G** | **C** | **A** | **T** | **T** | **G** | **A** | **C** | **G** | **G** | **A** | **G** | **C** | **A** | **A** | **G** | **C** | **A** | **G** | **A** | **A** | **A** | **A** | **A** | **A** | **G** | **T** | **G** | **A** | **G** | **A** | **C** | **A** | **C** | **C** | **A** | **G** | **A** | **A** | **A** | **T** | **G** | **T** | **T** | **C** | **C** | **G** | **T** | **A** | **A** | **G** | **C** | **A** | **G** | **T** | **T** | **G** | **A** | **A** | **A** | **A** | **T** | **G** | **G** | **T** | **G** | **G** | **C** | **A** | **G** | **A** | **G** | **A** | **T** | **C** | **A** | **T** | **C** | **A** | **A** | **C** | **G** | **C** | **T** | **T** | **C** | **C** | **C** | **A** | **G** | **T** | **C** | **T** | **G** | **A** | **G** | **A** | **T** | **G** | **C** | **G** | **T** | **G** | **C** | **G** | **C** | **T** | **G** | **C** | **T** | **T** | **C** | **T** | **T** | **C** | **A** | **T** | **C** | **T** | **G** | **C** | **G** | **T** | **C** | **C** | **A** | **G** | **T** | **C** | **A** | **A** | **T** | **T** | **T** | **A** | **G** | **A** | **A** | **G** | **A** | **A** | **A** | **A** | **A** | **A** | **G** | **T** | **T** | **T** | **A** | **A** | **A** | **A** | **C** | **A** | **A** | **G** | **G** | **C** | **A** | **T** | **T** | **G** | **A** | **G** | **C** | **A** | **G** | **T** | **T** | **A** | **T** | **T** | **G** | **A** | **A** | **G** | **G** | **A** | **A** | **T** | **T** | **G** | **C** | **A** | **G** | **A** | **A** | **T** | **A** | **A** | **A** | **A** | **T** | **C** | **C** | **A** | **G** | **A** | **T** | **T** | **T** | **C** | **G** | **C** | **T** | **A** | **A** | **A** | **A** | **T** | **G** | **G** | **G** | **A** | **T** | **G** | **T** | **A** | **G** | **A** | **A** | **G** | **A** | **T** | **G** | **T** | **A** | **A** | **A** | **A** | **T** | **T** | **A** | **A** | **C** | **T** | **A** | **G** | **T** | **T** | **C** | **C** | **A** | **T** | **T** | **G** | **A** | **G** | **G** | **A** | **T** | **C** | **A** | **C** | **T** | **T** | **G** | **T** | **T** | **C** | **C** | **G** | **A** | **A** | **T** | **C** | **G** | **T** | **T** | **C** | **A** | **A** | **G** | **A** | **A** | **T** | **T** | **G** | **T** | **T** | **A** | **T** | **C** | **A** | **A** | **A** | **C** | **A** | **C** | **A** | **T** | **T** | **A** | **A** | **G** | **A** | **C** | **A** | **T** | **G** | **C** | **T** | **A** | **A** | **G** | **G** | **C** | **T** | **A** | **A** | **T** | **G** | **A** | **A** | **T** | **T** | **A** | **G** | **A** | **A** | **G** | **T** | **C** | **T** | **A** | **T** | **T** | **T** | **G** | **C** | **A** | **T** | **A** | **A** | **A** | **A** | **T** | **A** | **G** | **A** | **T** | **A** | **A** | **C** | **A** | **A** | **T** | **C** | **T** | **T** | **T** | **T** | **A** | **T** | **T** | **A** | **C** | **G** | **T** | **A** | **T** | **C** | **A** | **T** | **T** | **G** | **A** | **C** | **G** | **A** | **C** | **G** | **G** | **G** | **A** | **C** | **A** | **G** | **G** | **A** | **T** | **T** | **T** | **A** | **A** | **T** | **A** | **T** | **G** | **A** | **A** | **T** | **G** | **A** | **A** | **A** | **C** | **C** | **A** | **A** | **A** | **A** | **C** | **G** | **G** | **G** | **A** | **A** | **G** | **T** | **T** | **A** | **T** | **G** | **G** | **A** | **T** | **T** | **G** | **A** | **A** | **C** | **A** | **A** | **T** | **A** | **T** | **C** | **A** | **A** | **A** | **G** | **A** | **A** | **C** | **G** | **A** | **G** | **T** | **A** | **G** | **C** | **T** | **G** | **G** | **T** | **A** | **T** | **T** | **G** | **G** | **T** | **G** | **G** | **A** | **A** | **C** | **A** | **G** | **T** | **A** | **A** | **A** | **A** | **A** | **T** | **C** | **A** | **T** | **T** | **A** | **G** | **T** | **T** | **T** | **T** | **A** | **A** | **A** | **G** | **G** | **G** | **C** | **A** | **A** | **G** | **G** | **T** | **A** | **C** | **G** | **A** | **G** | **C** | **G** | **T** | **G** | **G** | **A** | **G** | **A** | **T** | **C** | **A** | **A** | **G** | **G** | **T** | **C** | **C** | **C** | **T** | **T** | **T** | **G** | **A** | **T** | **G** | **A** | **A** | **G** | **G** | **A** | **G** | **G** | **C** | **A** | **-** | **-** | **-** |
| **N54190** | **A** | **T** | **G** | **A** | **T** | **G** | **G** | **G** | **A** | **A** | **G** | **A** | **A** | **T** | **T** | **T** | **C** | **C** | **A** | **A** | **A** | **G** | **C** | **A** | **A** | **T** | **G** | **C** | **T** | **A** | **G** | **C** | **T** | **G** | **T** | **T** | **T** | **A** | **T** | **T** | **C** | **G** | **G** | **G** | **G** | **A** | **T** | **T** | **G** | **C** | **G** | **G** | **C** | **T** | **T** | **T** | **C** | **C** | **T** | **T** | **A** | **T** | **T** | **A** | **T** | **T** | **C** | **T** | **A** | **T** | **T** | **T** | **T** | **C** | **A** | **C** | **T** | **T** | **T** | **T** | **C** | **A** | **C** | **T** | **T** | **A** | **T** | **T** | **T** | **T** | **T** | **A** | **T** | **G** | **C** | **C** | **A** | **G** | **T** | **A** | **A** | **C** | **C** | **A** | **A** | **A** | **G** | **T** | **C** | **A** | **T** | **T** | **G** | **G** | **T** | **G** | **G** | **G** | **G** | **A** | **G** | **A** | **A** | **T** | **T** | **G** | **C** | **T** | **G** | **C** | **G** | **T** | **G** | **C** | **G** | **C** | **G** | **T** | **T** | **T** | **A** | **T** | **T** | **A** | **T** | **A** | **C** | **G** | **T** | **T** | **C** | **C** | **G** | **C** | **T** | **T** | **A** | **T** | **C** | **T** | **T** | **C** | **C** | **A** | **T** | **T** | **T** | **G** | **A** | **T** | **G** | **G** | **C** | **C** | **A** | **T** | **T** | **T** | **C** | **A** | **T** | **T** | **A** | **G** | **G** | **T** | **G** | **T** | **A** | **G** | **G** | **A** | **T** | **T** | **G** | **A** | **T** | **C** | **G** | **T** | **A** | **T** | **T** | **T** | **C** | **T** | **A** | **T** | **T** | **G** | **T** | **T** | **A** | **T** | **C** | **A** | **C** | **T** | **T** | **A** | **T** | **T** | **C** | **A** | **A** | **A** | **A** | **G** | **G** | **C** | **A** | **A** | **A** | **A** | **T** | **A** | **C** | **G** | **G** | **G** | **A** | **A** | **A** | **A** | **T** | **C** | **G** | **A** | **A** | **G** | **A** | **A** | **A** | **A** | **G** | **C** | **T** | **T** | **C** | **G** | **T** | **G** | **C** | **A** | **C** | **T** | **T** | **T** | **C** | **T** | **T** | **C** | **T** | **G** | **G** | **C** | **A** | **A** | **T** | **T** | **A** | **T** | **G** | **A** | **A** | **T** | **C** | **C** | **A** | **A** | **A** | **C** | **T** | **T** | **T** | **T** | **G** | **C** | **A** | **T** | **C** | **A** | **G** | **C** | **C** | **G** | **A** | **T** | **C** | **C** | **C** | **A** | **A** | **G** | **C** | **G** | **C** | **G** | **T** | **C** | **G** | **G** | **A** | **T** | **G** | **A** | **T** | **C** | **T** | **G** | **T** | **A** | **C** | **A** | **T** | **C** | **A** | **A** | **A** | **G** | **A** | **T** | **A** | **T** | **T** | **G** | **A** | **T** | **A** | **T** | **A** | **G** | **A** | **A** | **A** | **T** | **T** | **A** | **C** | **T** | **A** | **A** | **A** | **A** | **T** | **A** | **A** | **A** | **A** | **G** | **A** | **A** | **A** | **A** | **A** | **A** | **T** | **G** | **A** | **T** | **C** | **G** | **A** | **A** | **G** | **T** | **A** | **T** | **C** | **C** | **A** | **G** | **T** | **G** | **A** | **A** | **T** | **T** | **A** | **C** | **A** | **G** | **A** | **T** | **T** | **A** | **T** | **G** | **A** | **C** | **T** | **A** | **G** | **C** | **C** | **G** | **T** | **C** | **C** | **T** | **C** | **A** | **A** | **T** | **A** | **T** | **G** | **T** | **A** | **G** | **A** | **T** | **G** | **G** | **A** | **C** | **A** | **A** | **A** | **C** | **C** | **A** | **A** | **A** | **G** | **A** | **A** | **G** | **A** | **G** | **A** | **T** | **T** | **T** | **T** | **A** | **G** | **A** | **A** | **T** | **T** | **A** | **G** | **A** | **A** | **A** | **G** | **G** | **C** | **A** | **T** | **C** | **G** | **A** | **T** | **T** | **A** | **G** | **C** | **T** | **C** | **G** | **T** | **G** | **A** | **G** | **C** | **T** | **T** | **C** | **A** | **T** | **G** | **A** | **T** | **T** | **C** | **A** | **G** | **T** | **T** | **T** | **C** | **A** | **C** | **A** | **G** | **C** | **A** | **A** | **T** | **T** | **A** | **T** | **T** | **T** | **G** | **C** | **A** | **G** | **C** | **G** | **A** | **T** | **G** | **A** | **T** | **G** | **A** | **T** | **G** | **A** | **T** | **G** | **T** | **C** | **G** | **G** | **C** | **A** | **T** | **T** | **G** | **A** | **C** | **G** | **G** | **A** | **G** | **C** | **A** | **A** | **G** | **C** | **A** | **G** | **A** | **A** | **A** | **A** | **A** | **A** | **G** | **T** | **G** | **A** | **G** | **A** | **C** | **A** | **C** | **C** | **A** | **G** | **A** | **A** | **A** | **T** | **G** | **T** | **T** | **C** | **C** | **G** | **T** | **A** | **A** | **G** | **C** | **A** | **G** | **T** | **T** | **G** | **A** | **A** | **A** | **A** | **T** | **G** | **G** | **T** | **G** | **G** | **C** | **A** | **G** | **A** | **G** | **A** | **T** | **C** | **A** | **T** | **C** | **A** | **A** | **C** | **G** | **C** | **T** | **T** | **C** | **C** | **C** | **A** | **G** | **T** | **C** | **T** | **G** | **A** | **G** | **A** | **T** | **G** | **C** | **G** | **T** | **G** | **C** | **G** | **C** | **T** | **G** | **C** | **T** | **T** | **C** | **T** | **T** | **C** | **A** | **T** | **C** | **T** | **G** | **C** | **G** | **T** | **C** | **C** | **A** | **G** | **T** | **C** | **A** | **A** | **T** | **T** | **T** | **A** | **G** | **A** | **A** | **G** | **A** | **A** | **A** | **A** | **A** | **A** | **G** | **T** | **T** | **T** | **A** | **A** | **A** | **A** | **C** | **A** | **A** | **G** | **G** | **C** | **A** | **T** | **T** | **G** | **A** | **G** | **C** | **A** | **G** | **T** | **T** | **A** | **T** | **T** | **G** | **A** | **A** | **G** | **G** | **A** | **A** | **T** | **T** | **G** | **C** | **A** | **G** | **A** | **A** | **T** | **A** | **A** | **A** | **A** | **T** | **C** | **C** | **A** | **G** | **A** | **T** | **T** | **T** | **C** | **G** | **C** | **T** | **A** | **A** | **A** | **A** | **T** | **G** | **G** | **G** | **A** | **T** | **G** | **T** | **A** | **G** | **A** | **A** | **G** | **A** | **T** | **G** | **T** | **A** | **A** | **A** | **A** | **T** | **T** | **A** | **A** | **C** | **T** | **A** | **G** | **T** | **T** | **C** | **C** | **A** | **T** | **T** | **G** | **A** | **G** | **G** | **A** | **T** | **C** | **A** | **C** | **T** | **T** | **G** | **T** | **T** | **C** | **C** | **G** | **A** | **A** | **T** | **C** | **G** | **T** | **T** | **C** | **A** | **A** | **G** | **A** | **A** | **T** | **T** | **G** | **T** | **T** | **A** | **T** | **C** | **A** | **A** | **A** | **C** | **A** | **C** | **A** | **T** | **T** | **A** | **A** | **G** | **A** | **C** | **A** | **T** | **G** | **C** | **T** | **A** | **A** | **G** | **G** | **C** | **T** | **A** | **A** | **T** | **G** | **A** | **A** | **T** | **T** | **A** | **G** | **A** | **A** | **G** | **T** | **C** | **T** | **A** | **T** | **T** | **T** | **G** | **C** | **A** | **T** | **A** | **A** | **A** | **A** | **T** | **A** | **G** | **A** | **T** | **A** | **A** | **C** | **A** | **A** | **T** | **C** | **T** | **T** | **T** | **T** | **A** | **T** | **T** | **A** | **C** | **G** | **T** | **A** | **T** | **C** | **A** | **T** | **T** | **G** | **A** | **C** | **G** | **A** | **C** | **G** | **G** | **G** | **A** | **C** | **A** | **G** | **G** | **A** | **T** | **T** | **T** | **A** | **A** | **T** | **A** | **T** | **G** | **A** | **A** | **T** | **G** | **A** | **A** | **A** | **C** | **C** | **A** | **A** | **A** | **A** | **C** | **G** | **G** | **G** | **A** | **A** | **G** | **T** | **T** | **A** | **T** | **G** | **G** | **A** | **T** | **T** | **G** | **A** | **A** | **C** | **A** | **A** | **T** | **A** | **T** | **C** | **A** | **A** | **A** | **G** | **A** | **A** | **C** | **G** | **A** | **G** | **T** | **A** | **G** | **C** | **T** | **G** | **G** | **T** | **A** | **T** | **T** | **G** | **G** | **T** | **G** | **G** | **A** | **A** | **C** | **A** | **G** | **T** | **A** | **A** | **A** | **A** | **A** | **T** | **C** | **A** | **T** | **T** | **A** | **G** | **T** | **T** | **T** | **T** | **A** | **A** | **A** | **G** | **G** | **G** | **C** | **A** | **A** | **G** | **G** | **T** | **A** | **C** | **G** | **A** | **G** | **C** | **G** | **T** | **G** | **G** | **A** | **G** | **A** | **T** | **C** | **A** | **A** | **G** | **G** | **T** | **C** | **C** | **C** | **T** | **T** | **T** | **G** | **A** | **T** | **G** | **A** | **A** | **G** | **G** | **A** | **G** | **G** | **C** | **A** | **-** | **-** | **-** |
| **N60190F** | **A** | **T** | **G** | **A** | **T** | **G** | **G** | **G** | **A** | **A** | **A** | **A** | **A** | **T** | **A** | **T** | **C** | **C** | **A** | **G** | **A** | **G** | **C** | **G** | **A** | **T** | **G** | **C** | **T** | **A** | **G** | **C** | **T** | **G** | **T** | **T** | **T** | **A** | **T** | **T** | **C** | **G** | **G** | **G** | **G** | **A** | **T** | **T** | **G** | **C** | **G** | **G** | **C** | **T** | **T** | **T** | **C** | **C** | **T** | **T** | **A** | **T** | **T** | **A** | **T** | **T** | **C** | **T** | **A** | **T** | **T** | **T** | **T** | **C** | **A** | **C** | **T** | **T** | **T** | **T** | **C** | **A** | **C** | **T** | **T** | **A** | **T** | **T** | **T** | **T** | **T** | **A** | **T** | **G** | **C** | **C** | **A** | **G** | **C** | **A** | **A** | **T** | **C** | **A** | **A** | **A** | **G** | **T** | **C** | **A** | **T** | **T** | **G** | **G** | **T** | **G** | **G** | **G** | **G** | **A** | **G** | **A** | **A** | **T** | **T** | **A** | **C** | **T** | **G** | **C** | **G** | **T** | **G** | **C** | **A** | **C** | **G** | **T** | **T** | **T** | **A** | **T** | **T** | **A** | **T** | **A** | **T** | **G** | **T** | **T** | **C** | **C** | **G** | **C** | **T** | **T** | **A** | **T** | **C** | **T** | **T** | **C** | **C** | **A** | **T** | **T** | **T** | **G** | **T** | **T** | **A** | **G** | **C** | **C** | **A** | **T** | **T** | **T** | **C** | **C** | **T** | **T** | **A** | **G** | **G** | **T** | **G** | **T** | **A** | **G** | **G** | **A** | **T** | **T** | **A** | **A** | **T** | **T** | **G** | **T** | **C** | **T** | **T** | **T** | **C** | **T** | **A** | **T** | **T** | **A** | **T** | **T** | **A** | **T** | **C** | **A** | **C** | **T** | **T** | **A** | **T** | **T** | **C** | **A** | **A** | **A** | **A** | **G** | **A** | **C** | **A** | **A** | **A** | **A** | **T** | **A** | **C** | **G** | **G** | **G** | **A** | **A** | **A** | **A** | **T** | **C** | **G** | **A** | **A** | **G** | **A** | **A** | **A** | **A** | **G** | **C** | **T** | **G** | **C** | **G** | **T** | **G** | **C** | **A** | **C** | **T** | **T** | **T** | **C** | **T** | **T** | **C** | **T** | **G** | **G** | **C** | **A** | **A** | **T** | **T** | **A** | **T** | **G** | **A** | **A** | **T** | **C** | **C** | **A** | **A** | **A** | **T** | **T** | **G** | **T** | **T** | **G** | **C** | **T** | **T** | **C** | **T** | **T** | **C** | **C** | **G** | **A** | **T** | **C** | **C** | **C** | **A** | **A** | **G** | **T** | **G** | **C** | **A** | **T** | **C** | **G** | **G** | **A** | **C** | **G** | **A** | **T** | **T** | **T** | **A** | **T** | **A** | **C** | **A** | **T** | **C** | **A** | **A** | **A** | **G** | **A** | **T** | **A** | **T** | **T** | **G** | **A** | **T** | **A** | **A** | **G** | **G** | **A** | **A** | **A** | **T** | **C** | **A** | **C** | **T** | **A** | **A** | **G** | **A** | **T** | **A** | **A** | **A** | **A** | **G** | **A** | **A** | **A** | **A** | **A** | **A** | **T** | **G** | **A** | **T** | **T** | **G** | **A** | **A** | **G** | **T** | **A** | **T** | **C** | **T** | **A** | **G** | **T** | **G** | **A** | **A** | **T** | **T** | **A** | **C** | **A** | **A** | **A** | **T** | **T** | **G** | **T** | **A** | **A** | **C** | **G** | **A** | **G** | **C** | **C** | **G** | **T** | **C** | **C** | **C** | **C** | **A** | **A** | **T** | **A** | **T** | **G** | **T** | **A** | **G** | **A** | **T** | **G** | **G** | **A** | **C** | **A** | **A** | **A** | **C** | **C** | **A** | **A** | **A** | **G** | **A** | **A** | **G** | **A** | **A** | **A** | **T** | **T** | **T** | **T** | **A** | **G** | **A** | **A** | **T** | **T** | **A** | **G** | **A** | **A** | **A** | **G** | **A** | **C** | **A** | **T** | **C** | **G** | **A** | **T** | **T** | **A** | **G** | **C** | **C** | **C** | **G** | **T** | **G** | **A** | **G** | **C** | **T** | **G** | **C** | **A** | **T** | **G** | **A** | **T** | **T** | **C** | **A** | **G** | **T** | **T** | **T** | **C** | **G** | **C** | **A** | **A** | **C** | **A** | **A** | **T** | **T** | **G** | **T** | **T** | **T** | **G** | **C** | **A** | **G** | **C** | **A** | **A** | **T** | **G** | **A** | **T** | **G** | **A** | **T** | **G** | **A** | **T** | **G** | **T** | **C** | **G** | **G** | **C** | **A** | **T** | **T** | **G** | **A** | **C** | **G** | **G** | **A** | **G** | **C** | **A** | **A** | **G** | **C** | **A** | **G** | **A** | **A** | **A** | **A** | **A** | **A** | **G** | **C** | **G** | **A** | **G** | **A** | **C** | **A** | **C** | **C** | **A** | **G** | **A** | **A** | **A** | **T** | **G** | **T** | **T** | **C** | **C** | **G** | **C** | **A** | **A** | **G** | **C** | **A** | **G** | **T** | **T** | **G** | **A** | **A** | **A** | **A** | **T** | **G** | **G** | **T** | **A** | **G** | **C** | **A** | **G** | **A** | **G** | **A** | **T** | **C** | **A** | **T** | **C** | **A** | **A** | **C** | **G** | **C** | **T** | **T** | **C** | **C** | **C** | **A** | **G** | **T** | **C** | **T** | **G** | **A** | **G** | **A** | **T** | **G** | **C** | **G** | **T** | **G** | **C** | **G** | **C** | **T** | **G** | **C** | **T** | **T** | **C** | **T** | **T** | **C** | **A** | **T** | **C** | **T** | **A** | **C** | **G** | **T** | **C** | **C** | **A** | **G** | **T** | **C** | **A** | **A** | **T** | **T** | **T** | **A** | **G** | **A** | **A** | **G** | **A** | **G** | **A** | **A** | **A** | **A** | **G** | **T** | **T** | **T** | **A** | **A** | **A** | **G** | **C** | **A** | **A** | **G** | **G** | **T** | **A** | **T** | **C** | **G** | **A** | **G** | **C** | **A** | **G** | **T** | **T** | **A** | **T** | **T** | **G** | **A** | **A** | **G** | **G** | **A** | **A** | **T** | **T** | **G** | **C** | **A** | **A** | **A** | **A** | **T** | **A** | **A** | **A** | **A** | **T** | **C** | **C** | **A** | **G** | **A** | **T** | **T** | **T** | **C** | **G** | **C** | **T** | **G** | **A** | **A** | **A** | **T** | **G** | **G** | **G** | **A** | **T** | **G** | **T** | **A** | **G** | **A** | **A** | **G** | **A** | **T** | **G** | **T** | **A** | **A** | **A** | **A** | **C** | **T** | **A** | **T** | **C** | **T** | **A** | **G** | **T** | **T** | **C** | **C** | **A** | **T** | **T** | **G** | **A** | **G** | **G** | **A** | **T** | **C** | **A** | **C** | **T** | **T** | **G** | **T** | **T** | **C** | **C** | **G** | **A** | **A** | **T** | **C** | **G** | **T** | **T** | **C** | **A** | **A** | **G** | **A** | **A** | **T** | **T** | **G** | **T** | **T** | **A** | **T** | **C** | **A** | **A** | **A** | **T** | **A** | **C** | **A** | **T** | **T** | **A** | **A** | **G** | **A** | **C** | **A** | **T** | **G** | **C** | **C** | **A** | **A** | **A** | **G** | **C** | **C** | **A** | **A** | **T** | **G** | **A** | **A** | **T** | **T** | **A** | **G** | **A** | **G** | **G** | **T** | **A** | **T** | **A** | **T** | **T** | **T** | **G** | **C** | **A** | **C** | **A** | **A** | **A** | **A** | **T** | **A** | **G** | **A** | **C** | **A** | **A** | **T** | **A** | **A** | **T** | **C** | **T** | **T** | **T** | **T** | **G** | **T** | **T** | **A** | **C** | **G** | **T** | **A** | **T** | **T** | **A** | **T** | **T** | **G** | **A** | **C** | **G** | **A** | **C** | **G** | **G** | **A** | **A** | **C** | **A** | **G** | **G** | **G** | **T** | **T** | **T** | **A** | **A** | **T** | **A** | **T** | **G** | **A** | **A** | **T** | **G** | **A** | **A** | **A** | **C** | **G** | **A** | **A** | **A** | **A** | **C** | **G** | **G** | **G** | **A** | **A** | **G** | **T** | **T** | **A** | **T** | **G** | **G** | **A** | **T** | **T** | **G** | **A** | **A** | **C** | **A** | **A** | **T** | **A** | **T** | **C** | **A** | **A** | **A** | **G** | **A** | **A** | **C** | **G** | **A** | **G** | **T** | **A** | **G** | **T** | **T** | **G** | **G** | **T** | **A** | **T** | **T** | **G** | **G** | **T** | **G** | **G** | **A** | **A** | **C** | **A** | **G** | **T** | **A** | **A** | **A** | **A** | **A** | **T** | **C** | **A** | **T** | **T** | **A** | **G** | **T** | **T** | **T** | **T** | **A** | **A** | **A** | **G** | **G** | **G** | **C** | **A** | **A** | **G** | **G** | **T** | **A** | **C** | **A** | **A** | **G** | **T** | **G** | **T** | **A** | **G** | **A** | **A** | **A** | **T** | **C** | **A** | **A** | **G** | **G** | **T** | **C** | **C** | **C** | **T** | **T** | **T** | **G** | **A** | **T** | **G** | **A** | **A** | **G** | **G** | **A** | **G** | **G** | **C** | **A** | **-** | **-** | **-** |
| **N52656** | **A** | **T** | **G** | **A** | **T** | **G** | **G** | **G** | **A** | **A** | **A** | **A** | **A** | **T** | **A** | **T** | **C** | **C** | **A** | **G** | **A** | **G** | **C** | **G** | **A** | **T** | **G** | **C** | **T** | **A** | **G** | **C** | **T** | **G** | **T** | **T** | **T** | **A** | **T** | **T** | **C** | **G** | **G** | **G** | **G** | **A** | **T** | **T** | **G** | **C** | **G** | **G** | **C** | **T** | **T** | **T** | **C** | **C** | **T** | **T** | **A** | **T** | **T** | **A** | **T** | **T** | **C** | **T** | **A** | **T** | **T** | **T** | **T** | **C** | **A** | **C** | **T** | **T** | **T** | **T** | **C** | **A** | **C** | **T** | **T** | **A** | **T** | **T** | **T** | **T** | **T** | **A** | **T** | **G** | **C** | **C** | **A** | **G** | **T** | **A** | **A** | **T** | **C** | **A** | **A** | **A** | **G** | **T** | **C** | **A** | **T** | **T** | **G** | **G** | **T** | **G** | **G** | **G** | **G** | **A** | **G** | **A** | **A** | **T** | **T** | **A** | **C** | **T** | **G** | **C** | **G** | **T** | **G** | **C** | **A** | **C** | **G** | **T** | **T** | **T** | **A** | **T** | **T** | **A** | **T** | **A** | **T** | **G** | **T** | **T** | **C** | **C** | **G** | **C** | **T** | **T** | **A** | **T** | **C** | **T** | **T** | **C** | **C** | **A** | **T** | **T** | **T** | **G** | **T** | **T** | **A** | **G** | **C** | **T** | **A** | **T** | **T** | **T** | **C** | **C** | **T** | **T** | **A** | **G** | **G** | **T** | **G** | **T** | **A** | **G** | **G** | **A** | **T** | **T** | **A** | **A** | **T** | **C** | **G** | **T** | **C** | **T** | **T** | **T** | **C** | **T** | **A** | **T** | **T** | **A** | **T** | **T** | **A** | **T** | **C** | **A** | **C** | **T** | **T** | **A** | **T** | **T** | **C** | **A** | **A** | **A** | **A** | **G** | **A** | **C** | **A** | **A** | **A** | **A** | **T** | **A** | **C** | **G** | **G** | **G** | **A** | **A** | **A** | **A** | **T** | **C** | **G** | **A** | **A** | **G** | **A** | **A** | **A** | **A** | **G** | **C** | **T** | **G** | **C** | **G** | **T** | **G** | **C** | **A** | **C** | **T** | **C** | **T** | **C** | **T** | **T** | **C** | **T** | **G** | **G** | **C** | **A** | **A** | **T** | **T** | **A** | **T** | **G** | **A** | **A** | **T** | **C** | **C** | **A** | **A** | **A** | **T** | **T** | **G** | **T** | **T** | **G** | **C** | **T** | **T** | **C** | **T** | **T** | **C** | **C** | **G** | **A** | **T** | **C** | **C** | **C** | **A** | **A** | **G** | **T** | **G** | **C** | **A** | **T** | **C** | **G** | **G** | **A** | **C** | **G** | **A** | **T** | **T** | **T** | **A** | **T** | **A** | **C** | **A** | **T** | **C** | **A** | **A** | **A** | **G** | **A** | **T** | **A** | **T** | **T** | **G** | **A** | **T** | **A** | **A** | **G** | **G** | **A** | **A** | **A** | **T** | **C** | **A** | **C** | **T** | **A** | **A** | **A** | **A** | **T** | **A** | **A** | **A** | **A** | **G** | **A** | **A** | **A** | **A** | **A** | **A** | **T** | **G** | **A** | **T** | **T** | **G** | **A** | **A** | **G** | **T** | **A** | **T** | **C** | **T** | **A** | **G** | **T** | **G** | **A** | **A** | **T** | **T** | **A** | **C** | **A** | **A** | **A** | **T** | **T** | **G** | **T** | **A** | **A** | **C** | **G** | **A** | **G** | **C** | **C** | **G** | **T** | **C** | **C** | **C** | **C** | **A** | **A** | **T** | **A** | **T** | **G** | **T** | **A** | **G** | **A** | **T** | **G** | **G** | **A** | **C** | **A** | **A** | **A** | **C** | **C** | **A** | **A** | **A** | **G** | **A** | **A** | **G** | **A** | **A** | **A** | **T** | **T** | **T** | **T** | **A** | **G** | **A** | **A** | **T** | **T** | **A** | **G** | **A** | **A** | **A** | **G** | **A** | **C** | **A** | **T** | **C** | **G** | **A** | **T** | **T** | **A** | **G** | **C** | **C** | **C** | **G** | **T** | **G** | **A** | **G** | **C** | **T** | **G** | **C** | **A** | **T** | **G** | **A** | **T** | **T** | **C** | **A** | **G** | **T** | **T** | **T** | **C** | **G** | **C** | **A** | **A** | **C** | **A** | **A** | **T** | **T** | **G** | **T** | **T** | **T** | **G** | **C** | **A** | **G** | **C** | **A** | **A** | **T** | **G** | **A** | **T** | **G** | **A** | **T** | **G** | **A** | **T** | **G** | **T** | **C** | **G** | **G** | **C** | **A** | **T** | **T** | **A** | **A** | **C** | **G** | **G** | **A** | **G** | **C** | **A** | **A** | **G** | **C** | **A** | **G** | **A** | **A** | **A** | **A** | **A** | **A** | **G** | **C** | **G** | **A** | **G** | **A** | **C** | **A** | **C** | **C** | **A** | **G** | **A** | **A** | **A** | **T** | **G** | **T** | **T** | **C** | **C** | **G** | **C** | **A** | **A** | **G** | **C** | **A** | **G** | **T** | **T** | **G** | **A** | **A** | **A** | **A** | **T** | **G** | **G** | **T** | **A** | **G** | **C** | **A** | **G** | **A** | **G** | **A** | **T** | **C** | **A** | **T** | **C** | **A** | **A** | **C** | **G** | **C** | **T** | **T** | **C** | **C** | **C** | **A** | **G** | **T** | **C** | **T** | **G** | **A** | **G** | **A** | **T** | **G** | **C** | **G** | **T** | **G** | **C** | **G** | **C** | **T** | **G** | **C** | **T** | **T** | **C** | **T** | **T** | **C** | **A** | **T** | **C** | **T** | **G** | **C** | **G** | **T** | **C** | **C** | **A** | **G** | **T** | **C** | **A** | **A** | **T** | **T** | **T** | **A** | **G** | **A** | **A** | **G** | **A** | **G** | **A** | **A** | **A** | **A** | **G** | **T** | **T** | **T** | **A** | **A** | **A** | **G** | **C** | **A** | **A** | **G** | **G** | **T** | **A** | **T** | **C** | **G** | **A** | **G** | **C** | **A** | **G** | **T** | **T** | **A** | **T** | **T** | **G** | **A** | **A** | **G** | **G** | **A** | **A** | **T** | **T** | **G** | **C** | **A** | **A** | **A** | **A** | **T** | **A** | **A** | **A** | **A** | **T** | **C** | **C** | **A** | **G** | **A** | **T** | **T** | **T** | **C** | **G** | **C** | **T** | **G** | **A** | **A** | **A** | **T** | **G** | **G** | **G** | **A** | **T** | **G** | **T** | **A** | **G** | **A** | **A** | **G** | **A** | **T** | **G** | **T** | **A** | **A** | **A** | **A** | **C** | **T** | **A** | **T** | **C** | **T** | **A** | **G** | **T** | **T** | **C** | **C** | **A** | **T** | **T** | **G** | **A** | **G** | **G** | **A** | **T** | **C** | **A** | **C** | **T** | **T** | **G** | **T** | **T** | **C** | **C** | **G** | **A** | **A** | **T** | **C** | **G** | **T** | **T** | **C** | **A** | **A** | **G** | **A** | **A** | **T** | **T** | **G** | **T** | **T** | **A** | **T** | **C** | **A** | **A** | **A** | **T** | **A** | **C** | **A** | **T** | **T** | **A** | **A** | **G** | **A** | **C** | **A** | **T** | **G** | **C** | **C** | **A** | **A** | **A** | **G** | **C** | **C** | **A** | **A** | **T** | **G** | **A** | **A** | **T** | **T** | **A** | **G** | **A** | **G** | **G** | **T** | **A** | **T** | **A** | **T** | **T** | **T** | **G** | **C** | **A** | **C** | **A** | **A** | **A** | **A** | **T** | **A** | **G** | **A** | **C** | **A** | **A** | **T** | **A** | **A** | **T** | **C** | **T** | **T** | **T** | **T** | **G** | **T** | **T** | **A** | **C** | **G** | **T** | **A** | **T** | **T** | **A** | **T** | **T** | **G** | **A** | **C** | **G** | **A** | **C** | **G** | **G** | **A** | **A** | **C** | **A** | **G** | **G** | **G** | **T** | **T** | **T** | **A** | **A** | **T** | **A** | **T** | **G** | **A** | **A** | **T** | **G** | **A** | **A** | **A** | **C** | **G** | **A** | **A** | **A** | **A** | **C** | **G** | **G** | **G** | **A** | **A** | **G** | **T** | **T** | **A** | **T** | **G** | **G** | **A** | **T** | **T** | **G** | **A** | **A** | **C** | **A** | **A** | **T** | **A** | **T** | **C** | **A** | **A** | **A** | **G** | **A** | **A** | **C** | **G** | **A** | **G** | **T** | **A** | **G** | **C** | **T** | **G** | **G** | **T** | **A** | **T** | **T** | **G** | **G** | **T** | **G** | **G** | **A** | **A** | **C** | **A** | **G** | **T** | **A** | **A** | **A** | **A** | **A** | **T** | **C** | **A** | **T** | **T** | **A** | **G** | **T** | **T** | **T** | **T** | **A** | **A** | **A** | **G** | **G** | **G** | **C** | **A** | **A** | **G** | **G** | **T** | **A** | **C** | **A** | **A** | **G** | **T** | **G** | **T** | **A** | **G** | **A** | **A** | **A** | **T** | **C** | **A** | **A** | **G** | **G** | **T** | **C** | **C** | **C** | **T** | **T** | **T** | **G** | **A** | **T** | **G** | **A** | **A** | **G** | **G** | **A** | **G** | **G** | **C** | **A** | **-** | **-** | **-** |
| **N53804** | **A** | **T** | **G** | **A** | **T** | **G** | **G** | **G** | **A** | **A** | **G** | **A** | **A** | **T** | **A** | **T** | **C** | **C** | **A** | **A** | **A** | **G** | **C** | **A** | **A** | **T** | **G** | **C** | **T** | **A** | **G** | **C** | **T** | **G** | **T** | **T** | **T** | **A** | **T** | **T** | **C** | **G** | **G** | **G** | **G** | **A** | **T** | **T** | **G** | **C** | **G** | **G** | **C** | **T** | **T** | **T** | **C** | **C** | **T** | **T** | **A** | **T** | **T** | **A** | **T** | **T** | **C** | **T** | **A** | **T** | **T** | **T** | **T** | **C** | **A** | **C** | **T** | **T** | **T** | **T** | **C** | **A** | **C** | **T** | **T** | **A** | **T** | **T** | **T** | **T** | **T** | **A** | **T** | **G** | **C** | **C** | **A** | **G** | **T** | **A** | **A** | **C** | **C** | **A** | **A** | **A** | **G** | **T** | **C** | **A** | **T** | **T** | **G** | **G** | **T** | **G** | **G** | **G** | **G** | **A** | **G** | **A** | **A** | **T** | **T** | **G** | **C** | **T** | **G** | **C** | **G** | **T** | **G** | **C** | **G** | **C** | **G** | **T** | **T** | **T** | **A** | **T** | **T** | **A** | **T** | **A** | **C** | **G** | **T** | **T** | **C** | **C** | **G** | **C** | **T** | **T** | **A** | **T** | **C** | **T** | **T** | **C** | **C** | **A** | **T** | **T** | **T** | **G** | **A** | **T** | **G** | **G** | **C** | **A** | **A** | **T** | **T** | **T** | **C** | **A** | **T** | **T** | **A** | **G** | **G** | **T** | **G** | **T** | **A** | **G** | **G** | **A** | **T** | **T** | **G** | **A** | **T** | **C** | **G** | **T** | **A** | **T** | **T** | **T** | **C** | **T** | **A** | **T** | **T** | **G** | **T** | **T** | **A** | **T** | **C** | **A** | **C** | **T** | **T** | **A** | **T** | **T** | **C** | **A** | **A** | **A** | **A** | **G** | **G** | **C** | **A** | **A** | **A** | **A** | **T** | **A** | **C** | **G** | **G** | **G** | **A** | **A** | **A** | **A** | **T** | **C** | **G** | **A** | **A** | **G** | **A** | **A** | **A** | **A** | **G** | **C** | **T** | **T** | **C** | **G** | **T** | **G** | **C** | **A** | **C** | **T** | **T** | **T** | **C** | **T** | **T** | **C** | **T** | **G** | **G** | **C** | **A** | **A** | **T** | **T** | **A** | **T** | **G** | **A** | **A** | **T** | **C** | **C** | **A** | **A** | **A** | **C** | **T** | **T** | **T** | **T** | **G** | **C** | **A** | **T** | **C** | **A** | **G** | **C** | **C** | **G** | **A** | **T** | **C** | **C** | **C** | **A** | **A** | **G** | **C** | **G** | **C** | **G** | **T** | **C** | **G** | **G** | **A** | **T** | **G** | **A** | **T** | **C** | **T** | **G** | **T** | **A** | **C** | **A** | **T** | **C** | **A** | **A** | **A** | **G** | **A** | **T** | **A** | **T** | **T** | **G** | **A** | **T** | **A** | **T** | **G** | **G** | **A** | **A** | **A** | **T** | **T** | **A** | **C** | **T** | **A** | **A** | **A** | **A** | **T** | **A** | **A** | **A** | **A** | **G** | **A** | **A** | **A** | **A** | **A** | **A** | **T** | **G** | **A** | **T** | **C** | **G** | **A** | **A** | **G** | **T** | **A** | **T** | **C** | **C** | **A** | **G** | **T** | **G** | **A** | **A** | **T** | **T** | **A** | **C** | **A** | **G** | **A** | **T** | **T** | **A** | **T** | **G** | **A** | **C** | **T** | **A** | **G** | **C** | **C** | **G** | **T** | **C** | **C** | **T** | **C** | **A** | **A** | **T** | **A** | **T** | **G** | **T** | **A** | **G** | **A** | **T** | **G** | **G** | **A** | **C** | **A** | **A** | **A** | **C** | **C** | **A** | **A** | **A** | **G** | **A** | **A** | **G** | **A** | **G** | **A** | **T** | **T** | **T** | **T** | **A** | **G** | **A** | **A** | **T** | **T** | **A** | **G** | **A** | **A** | **A** | **G** | **G** | **C** | **A** | **T** | **C** | **G** | **A** | **T** | **T** | **A** | **G** | **C** | **T** | **C** | **G** | **T** | **G** | **A** | **G** | **C** | **T** | **T** | **C** | **A** | **T** | **G** | **A** | **T** | **T** | **C** | **A** | **G** | **T** | **T** | **T** | **C** | **A** | **C** | **A** | **G** | **C** | **A** | **A** | **T** | **T** | **A** | **T** | **T** | **T** | **G** | **C** | **A** | **G** | **C** | **G** | **A** | **T** | **G** | **A** | **T** | **G** | **A** | **T** | **G** | **A** | **T** | **G** | **T** | **C** | **G** | **G** | **C** | **A** | **T** | **T** | **G** | **A** | **C** | **G** | **G** | **A** | **G** | **C** | **A** | **A** | **G** | **C** | **A** | **G** | **A** | **A** | **A** | **A** | **A** | **A** | **G** | **T** | **G** | **A** | **G** | **A** | **C** | **A** | **C** | **C** | **A** | **G** | **A** | **A** | **A** | **T** | **G** | **T** | **T** | **C** | **C** | **G** | **T** | **A** | **A** | **G** | **C** | **A** | **G** | **T** | **T** | **G** | **A** | **A** | **A** | **A** | **T** | **G** | **G** | **T** | **G** | **G** | **C** | **A** | **G** | **A** | **G** | **A** | **T** | **C** | **A** | **T** | **C** | **A** | **A** | **C** | **G** | **C** | **T** | **T** | **C** | **C** | **C** | **A** | **G** | **T** | **C** | **T** | **G** | **A** | **G** | **A** | **T** | **G** | **C** | **G** | **T** | **G** | **C** | **G** | **C** | **T** | **G** | **C** | **T** | **T** | **C** | **T** | **T** | **C** | **A** | **T** | **C** | **T** | **G** | **C** | **G** | **T** | **C** | **C** | **A** | **G** | **T** | **C** | **A** | **A** | **T** | **T** | **T** | **A** | **G** | **A** | **A** | **G** | **A** | **A** | **A** | **A** | **A** | **A** | **G** | **T** | **T** | **T** | **A** | **A** | **A** | **A** | **C** | **A** | **A** | **G** | **G** | **C** | **A** | **T** | **T** | **G** | **A** | **G** | **C** | **A** | **G** | **T** | **T** | **A** | **T** | **T** | **G** | **A** | **A** | **G** | **G** | **A** | **A** | **T** | **T** | **G** | **C** | **A** | **G** | **A** | **A** | **T** | **A** | **A** | **A** | **A** | **T** | **C** | **C** | **A** | **G** | **A** | **T** | **T** | **T** | **C** | **G** | **C** | **T** | **A** | **A** | **A** | **A** | **T** | **G** | **G** | **G** | **A** | **T** | **G** | **T** | **A** | **G** | **A** | **A** | **G** | **A** | **T** | **G** | **T** | **A** | **A** | **A** | **A** | **T** | **T** | **A** | **A** | **C** | **T** | **A** | **G** | **T** | **T** | **C** | **C** | **A** | **T** | **T** | **G** | **A** | **G** | **G** | **A** | **T** | **C** | **A** | **C** | **T** | **T** | **G** | **T** | **T** | **C** | **C** | **G** | **A** | **A** | **T** | **C** | **G** | **T** | **T** | **C** | **A** | **A** | **G** | **A** | **A** | **T** | **T** | **G** | **T** | **T** | **A** | **T** | **C** | **A** | **A** | **A** | **C** | **A** | **C** | **A** | **T** | **T** | **A** | **A** | **G** | **A** | **C** | **A** | **T** | **G** | **C** | **T** | **A** | **A** | **G** | **G** | **C** | **T** | **A** | **A** | **T** | **G** | **A** | **A** | **T** | **T** | **A** | **G** | **A** | **A** | **G** | **T** | **C** | **T** | **A** | **T** | **T** | **T** | **G** | **C** | **A** | **T** | **A** | **A** | **A** | **A** | **T** | **A** | **G** | **A** | **T** | **A** | **A** | **C** | **A** | **A** | **T** | **C** | **T** | **T** | **T** | **T** | **A** | **T** | **T** | **A** | **C** | **G** | **T** | **A** | **T** | **C** | **A** | **T** | **T** | **G** | **A** | **C** | **G** | **A** | **C** | **G** | **G** | **G** | **A** | **C** | **A** | **G** | **G** | **A** | **T** | **T** | **T** | **A** | **A** | **T** | **A** | **T** | **G** | **A** | **A** | **T** | **G** | **A** | **A** | **A** | **C** | **C** | **A** | **A** | **A** | **A** | **C** | **G** | **G** | **G** | **A** | **A** | **G** | **T** | **T** | **A** | **T** | **G** | **G** | **A** | **T** | **T** | **G** | **A** | **A** | **C** | **A** | **A** | **T** | **A** | **T** | **C** | **A** | **A** | **A** | **G** | **A** | **A** | **C** | **G** | **A** | **G** | **T** | **A** | **G** | **C** | **T** | **G** | **G** | **T** | **A** | **T** | **T** | **G** | **G** | **T** | **G** | **G** | **A** | **A** | **C** | **A** | **G** | **T** | **A** | **A** | **A** | **A** | **A** | **T** | **C** | **A** | **T** | **T** | **A** | **G** | **T** | **T** | **T** | **T** | **A** | **A** | **A** | **G** | **G** | **G** | **C** | **A** | **A** | **G** | **G** | **T** | **A** | **C** | **G** | **A** | **G** | **C** | **G** | **T** | **G** | **G** | **A** | **G** | **A** | **T** | **C** | **A** | **A** | **G** | **G** | **T** | **C** | **C** | **C** | **T** | **T** | **T** | **G** | **A** | **T** | **G** | **A** | **A** | **G** | **G** | **A** | **G** | **G** | **C** | **A** | **-** | **-** | **-** |
| **N52829** | **A** | **T** | **G** | **A** | **T** | **G** | **G** | **G** | **A** | **A** | **G** | **A** | **A** | **T** | **T** | **T** | **C** | **C** | **A** | **A** | **A** | **G** | **C** | **A** | **A** | **T** | **G** | **C** | **T** | **A** | **G** | **C** | **T** | **G** | **T** | **T** | **T** | **A** | **T** | **T** | **C** | **G** | **G** | **G** | **G** | **A** | **T** | **T** | **G** | **C** | **G** | **G** | **C** | **T** | **T** | **T** | **C** | **C** | **T** | **T** | **A** | **T** | **T** | **A** | **T** | **T** | **C** | **T** | **A** | **T** | **T** | **T** | **T** | **C** | **A** | **C** | **T** | **T** | **T** | **T** | **C** | **A** | **C** | **T** | **T** | **A** | **T** | **T** | **T** | **T** | **T** | **A** | **T** | **G** | **C** | **C** | **A** | **G** | **T** | **A** | **A** | **C** | **C** | **A** | **A** | **A** | **G** | **T** | **C** | **A** | **T** | **T** | **G** | **G** | **T** | **G** | **G** | **G** | **G** | **A** | **G** | **A** | **A** | **T** | **T** | **G** | **C** | **T** | **G** | **C** | **G** | **T** | **G** | **C** | **G** | **C** | **G** | **T** | **T** | **T** | **A** | **T** | **T** | **A** | **T** | **A** | **C** | **G** | **T** | **T** | **C** | **C** | **G** | **C** | **T** | **T** | **A** | **T** | **C** | **T** | **T** | **C** | **C** | **A** | **T** | **T** | **T** | **G** | **A** | **T** | **G** | **G** | **C** | **C** | **A** | **T** | **T** | **T** | **C** | **A** | **T** | **T** | **A** | **G** | **G** | **T** | **G** | **T** | **A** | **G** | **G** | **A** | **T** | **T** | **G** | **A** | **T** | **C** | **G** | **T** | **A** | **T** | **T** | **T** | **C** | **T** | **A** | **T** | **T** | **G** | **T** | **T** | **A** | **T** | **C** | **A** | **C** | **T** | **T** | **A** | **T** | **T** | **C** | **A** | **A** | **A** | **A** | **G** | **G** | **C** | **A** | **A** | **A** | **A** | **T** | **A** | **C** | **G** | **G** | **G** | **A** | **A** | **A** | **A** | **T** | **C** | **G** | **A** | **A** | **G** | **A** | **A** | **A** | **A** | **G** | **C** | **T** | **T** | **C** | **G** | **T** | **G** | **C** | **A** | **C** | **T** | **T** | **T** | **C** | **T** | **T** | **C** | **T** | **G** | **G** | **C** | **A** | **A** | **T** | **T** | **A** | **T** | **G** | **A** | **A** | **T** | **C** | **C** | **A** | **A** | **A** | **C** | **T** | **T** | **T** | **T** | **G** | **C** | **A** | **T** | **C** | **A** | **G** | **C** | **C** | **G** | **A** | **T** | **C** | **C** | **C** | **A** | **A** | **G** | **C** | **G** | **C** | **G** | **T** | **C** | **G** | **G** | **A** | **T** | **G** | **A** | **T** | **C** | **T** | **G** | **T** | **A** | **C** | **A** | **T** | **C** | **A** | **A** | **A** | **G** | **A** | **T** | **A** | **T** | **T** | **G** | **A** | **T** | **A** | **T** | **A** | **G** | **A** | **A** | **A** | **T** | **T** | **A** | **C** | **T** | **A** | **A** | **A** | **A** | **T** | **A** | **A** | **A** | **A** | **G** | **A** | **A** | **A** | **A** | **A** | **A** | **T** | **G** | **A** | **T** | **C** | **G** | **A** | **A** | **G** | **T** | **A** | **T** | **C** | **C** | **A** | **G** | **T** | **G** | **A** | **A** | **T** | **T** | **A** | **C** | **A** | **G** | **A** | **T** | **T** | **A** | **T** | **G** | **A** | **C** | **T** | **A** | **G** | **C** | **C** | **G** | **T** | **C** | **C** | **T** | **C** | **A** | **A** | **T** | **A** | **T** | **G** | **T** | **A** | **G** | **A** | **T** | **G** | **G** | **A** | **C** | **A** | **A** | **A** | **C** | **C** | **A** | **A** | **A** | **G** | **A** | **A** | **G** | **A** | **G** | **A** | **T** | **T** | **T** | **T** | **A** | **G** | **A** | **A** | **T** | **T** | **A** | **G** | **A** | **A** | **A** | **G** | **G** | **C** | **A** | **T** | **C** | **G** | **A** | **T** | **T** | **A** | **G** | **C** | **T** | **C** | **G** | **T** | **G** | **A** | **G** | **C** | **T** | **T** | **C** | **A** | **T** | **G** | **A** | **T** | **T** | **C** | **A** | **G** | **T** | **T** | **T** | **C** | **A** | **C** | **A** | **G** | **C** | **A** | **A** | **T** | **T** | **A** | **T** | **T** | **T** | **G** | **C** | **A** | **G** | **C** | **G** | **A** | **T** | **G** | **A** | **T** | **G** | **A** | **T** | **G** | **A** | **T** | **G** | **T** | **C** | **G** | **G** | **C** | **A** | **T** | **T** | **G** | **A** | **C** | **G** | **G** | **A** | **G** | **C** | **A** | **A** | **G** | **C** | **A** | **G** | **A** | **A** | **A** | **A** | **A** | **A** | **G** | **T** | **G** | **A** | **G** | **A** | **C** | **A** | **C** | **C** | **A** | **G** | **A** | **A** | **A** | **T** | **G** | **T** | **T** | **C** | **C** | **G** | **T** | **A** | **A** | **G** | **C** | **A** | **G** | **T** | **T** | **G** | **A** | **A** | **A** | **A** | **T** | **G** | **G** | **T** | **G** | **G** | **C** | **A** | **G** | **A** | **G** | **A** | **T** | **C** | **A** | **T** | **C** | **A** | **A** | **C** | **G** | **C** | **T** | **T** | **C** | **C** | **C** | **A** | **G** | **T** | **C** | **T** | **G** | **A** | **G** | **A** | **T** | **G** | **C** | **G** | **T** | **G** | **C** | **G** | **C** | **T** | **G** | **C** | **T** | **T** | **C** | **T** | **T** | **C** | **A** | **T** | **C** | **T** | **G** | **C** | **G** | **T** | **C** | **C** | **A** | **G** | **T** | **C** | **A** | **A** | **T** | **T** | **T** | **A** | **G** | **A** | **A** | **G** | **A** | **A** | **A** | **A** | **A** | **A** | **G** | **T** | **T** | **T** | **A** | **A** | **A** | **A** | **C** | **A** | **A** | **G** | **G** | **C** | **A** | **T** | **T** | **G** | **A** | **G** | **C** | **A** | **G** | **T** | **T** | **A** | **T** | **T** | **G** | **A** | **A** | **G** | **G** | **A** | **A** | **T** | **T** | **G** | **C** | **A** | **G** | **A** | **A** | **T** | **A** | **A** | **A** | **A** | **T** | **C** | **C** | **A** | **G** | **A** | **T** | **T** | **T** | **C** | **G** | **C** | **T** | **A** | **A** | **A** | **A** | **T** | **G** | **G** | **G** | **A** | **T** | **G** | **T** | **A** | **G** | **A** | **A** | **G** | **A** | **T** | **G** | **T** | **A** | **A** | **A** | **A** | **T** | **T** | **A** | **A** | **C** | **T** | **A** | **G** | **T** | **T** | **C** | **C** | **A** | **T** | **T** | **G** | **A** | **G** | **G** | **A** | **T** | **C** | **A** | **C** | **T** | **T** | **G** | **T** | **T** | **C** | **C** | **G** | **A** | **A** | **T** | **C** | **G** | **T** | **T** | **C** | **A** | **A** | **G** | **A** | **A** | **T** | **T** | **G** | **T** | **T** | **A** | **T** | **C** | **A** | **A** | **A** | **C** | **A** | **C** | **A** | **T** | **T** | **A** | **A** | **G** | **A** | **C** | **A** | **T** | **G** | **C** | **T** | **A** | **A** | **G** | **G** | **C** | **T** | **A** | **A** | **T** | **G** | **A** | **A** | **T** | **T** | **A** | **G** | **A** | **A** | **G** | **T** | **C** | **T** | **A** | **T** | **T** | **T** | **G** | **C** | **A** | **T** | **A** | **A** | **A** | **A** | **T** | **A** | **G** | **A** | **T** | **A** | **A** | **C** | **A** | **A** | **T** | **C** | **T** | **T** | **T** | **T** | **A** | **T** | **T** | **A** | **C** | **G** | **T** | **A** | **T** | **C** | **A** | **T** | **T** | **G** | **A** | **C** | **G** | **A** | **C** | **G** | **G** | **G** | **A** | **C** | **A** | **G** | **G** | **A** | **T** | **T** | **T** | **A** | **A** | **T** | **A** | **T** | **G** | **A** | **A** | **T** | **G** | **A** | **A** | **A** | **C** | **C** | **A** | **A** | **A** | **A** | **C** | **G** | **G** | **G** | **A** | **A** | **G** | **T** | **T** | **A** | **T** | **G** | **G** | **A** | **T** | **T** | **G** | **A** | **A** | **C** | **A** | **A** | **T** | **A** | **T** | **C** | **A** | **A** | **A** | **G** | **A** | **A** | **C** | **G** | **A** | **G** | **T** | **A** | **G** | **C** | **T** | **G** | **G** | **T** | **A** | **T** | **T** | **G** | **G** | **T** | **G** | **G** | **A** | **A** | **C** | **A** | **G** | **T** | **A** | **A** | **A** | **A** | **A** | **T** | **C** | **A** | **T** | **T** | **A** | **G** | **T** | **T** | **T** | **T** | **A** | **A** | **A** | **G** | **G** | **G** | **C** | **A** | **A** | **G** | **G** | **T** | **A** | **C** | **G** | **A** | **G** | **C** | **G** | **T** | **G** | **G** | **A** | **G** | **A** | **T** | **C** | **A** | **A** | **G** | **G** | **T** | **C** | **C** | **C** | **T** | **T** | **T** | **G** | **A** | **T** | **G** | **A** | **A** | **G** | **G** | **A** | **G** | **G** | **C** | **A** | **-** | **-** | **-** |
| **N60048F** | **A** | **T** | **G** | **A** | **T** | **G** | **G** | **G** | **A** | **A** | **G** | **A** | **A** | **T** | **A** | **T** | **C** | **C** | **A** | **A** | **A** | **G** | **C** | **A** | **A** | **T** | **G** | **C** | **T** | **A** | **G** | **C** | **T** | **G** | **T** | **T** | **T** | **A** | **T** | **T** | **C** | **G** | **G** | **G** | **G** | **A** | **T** | **T** | **G** | **C** | **G** | **G** | **C** | **T** | **T** | **T** | **C** | **C** | **T** | **T** | **A** | **T** | **T** | **A** | **T** | **T** | **C** | **T** | **A** | **T** | **T** | **T** | **T** | **C** | **A** | **C** | **T** | **T** | **T** | **T** | **C** | **A** | **C** | **T** | **T** | **A** | **T** | **T** | **T** | **T** | **T** | **A** | **T** | **G** | **C** | **C** | **A** | **G** | **T** | **A** | **A** | **C** | **C** | **A** | **A** | **A** | **G** | **T** | **C** | **A** | **T** | **T** | **G** | **G** | **T** | **G** | **G** | **G** | **G** | **A** | **G** | **A** | **A** | **T** | **T** | **G** | **C** | **T** | **G** | **C** | **G** | **T** | **G** | **C** | **G** | **C** | **G** | **T** | **T** | **T** | **A** | **T** | **T** | **A** | **T** | **A** | **C** | **G** | **T** | **T** | **C** | **C** | **G** | **C** | **T** | **T** | **A** | **T** | **C** | **T** | **T** | **C** | **C** | **A** | **T** | **T** | **T** | **G** | **A** | **T** | **G** | **G** | **C** | **A** | **A** | **T** | **T** | **T** | **C** | **A** | **T** | **T** | **A** | **G** | **G** | **T** | **G** | **T** | **A** | **G** | **G** | **A** | **T** | **T** | **G** | **A** | **T** | **C** | **G** | **T** | **A** | **T** | **T** | **T** | **C** | **T** | **A** | **T** | **T** | **G** | **T** | **T** | **A** | **T** | **C** | **A** | **C** | **T** | **T** | **A** | **T** | **T** | **C** | **A** | **A** | **A** | **A** | **G** | **G** | **C** | **A** | **A** | **A** | **A** | **T** | **A** | **C** | **G** | **G** | **G** | **A** | **A** | **A** | **A** | **T** | **C** | **G** | **A** | **A** | **G** | **A** | **A** | **A** | **A** | **G** | **C** | **T** | **T** | **C** | **G** | **T** | **G** | **C** | **A** | **C** | **T** | **T** | **T** | **C** | **T** | **T** | **C** | **T** | **G** | **G** | **C** | **A** | **A** | **T** | **T** | **A** | **T** | **G** | **A** | **A** | **T** | **C** | **C** | **A** | **A** | **A** | **C** | **T** | **T** | **T** | **T** | **G** | **C** | **A** | **T** | **C** | **A** | **G** | **C** | **C** | **G** | **A** | **T** | **C** | **C** | **C** | **A** | **A** | **G** | **C** | **A** | **C** | **G** | **T** | **C** | **G** | **G** | **A** | **T** | **G** | **A** | **T** | **C** | **T** | **G** | **T** | **A** | **C** | **A** | **T** | **C** | **A** | **A** | **A** | **G** | **A** | **T** | **A** | **T** | **T** | **G** | **A** | **T** | **A** | **T** | **G** | **G** | **A** | **A** | **A** | **T** | **T** | **A** | **C** | **T** | **A** | **A** | **A** | **A** | **T** | **A** | **A** | **A** | **A** | **G** | **A** | **A** | **A** | **A** | **A** | **A** | **T** | **G** | **A** | **T** | **C** | **G** | **A** | **A** | **G** | **T** | **A** | **T** | **C** | **C** | **A** | **G** | **T** | **G** | **A** | **A** | **T** | **T** | **A** | **C** | **A** | **G** | **A** | **T** | **T** | **A** | **T** | **G** | **A** | **C** | **T** | **A** | **G** | **C** | **C** | **G** | **T** | **C** | **C** | **T** | **C** | **A** | **A** | **T** | **A** | **T** | **G** | **T** | **A** | **G** | **A** | **T** | **G** | **G** | **A** | **C** | **A** | **A** | **A** | **C** | **C** | **A** | **A** | **A** | **G** | **A** | **A** | **G** | **A** | **G** | **A** | **T** | **T** | **T** | **T** | **A** | **G** | **A** | **A** | **T** | **T** | **A** | **G** | **A** | **A** | **A** | **G** | **G** | **C** | **A** | **T** | **C** | **G** | **A** | **T** | **T** | **A** | **G** | **C** | **T** | **C** | **G** | **T** | **G** | **A** | **G** | **C** | **T** | **T** | **C** | **A** | **T** | **G** | **A** | **T** | **T** | **C** | **A** | **G** | **T** | **T** | **T** | **C** | **A** | **C** | **A** | **G** | **C** | **A** | **A** | **T** | **T** | **A** | **T** | **T** | **T** | **G** | **C** | **A** | **G** | **C** | **G** | **A** | **T** | **G** | **A** | **T** | **G** | **A** | **T** | **G** | **A** | **T** | **G** | **T** | **C** | **G** | **G** | **C** | **A** | **T** | **T** | **G** | **A** | **C** | **G** | **G** | **A** | **G** | **C** | **A** | **A** | **G** | **C** | **A** | **G** | **A** | **A** | **A** | **A** | **A** | **A** | **G** | **T** | **G** | **A** | **G** | **A** | **C** | **A** | **C** | **C** | **A** | **G** | **A** | **A** | **A** | **T** | **G** | **T** | **T** | **C** | **C** | **G** | **T** | **A** | **A** | **G** | **C** | **A** | **G** | **T** | **T** | **G** | **A** | **A** | **A** | **A** | **T** | **G** | **G** | **T** | **G** | **G** | **C** | **A** | **G** | **A** | **G** | **A** | **T** | **C** | **A** | **T** | **C** | **A** | **A** | **C** | **G** | **C** | **T** | **T** | **C** | **C** | **C** | **A** | **G** | **T** | **C** | **T** | **G** | **A** | **G** | **A** | **T** | **G** | **C** | **G** | **T** | **G** | **C** | **G** | **C** | **T** | **G** | **C** | **T** | **T** | **C** | **T** | **T** | **C** | **A** | **T** | **C** | **T** | **G** | **C** | **G** | **T** | **C** | **C** | **A** | **G** | **T** | **C** | **A** | **A** | **T** | **T** | **T** | **A** | **G** | **A** | **A** | **G** | **A** | **A** | **A** | **A** | **A** | **A** | **G** | **T** | **T** | **T** | **A** | **A** | **A** | **A** | **C** | **A** | **A** | **G** | **G** | **C** | **A** | **T** | **T** | **G** | **A** | **G** | **C** | **A** | **G** | **T** | **T** | **A** | **T** | **T** | **G** | **A** | **A** | **G** | **G** | **A** | **A** | **T** | **T** | **G** | **C** | **A** | **G** | **A** | **A** | **T** | **A** | **A** | **A** | **A** | **T** | **C** | **C** | **A** | **G** | **A** | **T** | **T** | **T** | **C** | **G** | **C** | **T** | **A** | **A** | **A** | **A** | **T** | **G** | **G** | **G** | **A** | **T** | **G** | **T** | **A** | **G** | **A** | **A** | **G** | **A** | **T** | **G** | **T** | **A** | **A** | **A** | **A** | **T** | **T** | **A** | **A** | **C** | **T** | **A** | **G** | **T** | **T** | **C** | **C** | **A** | **T** | **T** | **G** | **A** | **G** | **G** | **A** | **T** | **C** | **A** | **C** | **T** | **T** | **G** | **T** | **T** | **C** | **C** | **G** | **A** | **A** | **T** | **C** | **G** | **T** | **T** | **C** | **A** | **A** | **G** | **A** | **A** | **T** | **T** | **G** | **T** | **T** | **A** | **T** | **C** | **A** | **A** | **A** | **C** | **A** | **C** | **A** | **T** | **T** | **A** | **A** | **G** | **A** | **C** | **A** | **T** | **G** | **C** | **T** | **A** | **A** | **G** | **G** | **C** | **T** | **A** | **A** | **T** | **G** | **A** | **A** | **T** | **T** | **A** | **G** | **A** | **A** | **G** | **T** | **C** | **T** | **A** | **T** | **T** | **T** | **G** | **C** | **A** | **T** | **A** | **A** | **A** | **A** | **T** | **A** | **G** | **A** | **T** | **A** | **A** | **C** | **A** | **A** | **T** | **C** | **T** | **T** | **T** | **T** | **A** | **T** | **T** | **A** | **C** | **G** | **T** | **A** | **T** | **C** | **A** | **T** | **T** | **G** | **A** | **C** | **G** | **A** | **C** | **G** | **G** | **G** | **A** | **C** | **A** | **G** | **G** | **A** | **T** | **T** | **T** | **A** | **A** | **T** | **A** | **T** | **G** | **A** | **A** | **T** | **G** | **A** | **A** | **A** | **C** | **C** | **A** | **A** | **A** | **A** | **C** | **G** | **G** | **G** | **A** | **A** | **G** | **T** | **T** | **A** | **T** | **G** | **G** | **A** | **T** | **T** | **G** | **A** | **A** | **C** | **A** | **A** | **T** | **A** | **T** | **C** | **A** | **A** | **A** | **G** | **A** | **A** | **C** | **G** | **A** | **G** | **T** | **A** | **G** | **C** | **T** | **G** | **G** | **T** | **A** | **T** | **T** | **G** | **G** | **T** | **G** | **G** | **A** | **A** | **C** | **A** | **G** | **T** | **A** | **A** | **A** | **A** | **A** | **T** | **C** | **A** | **T** | **T** | **A** | **G** | **T** | **T** | **T** | **T** | **A** | **A** | **A** | **G** | **G** | **G** | **C** | **A** | **A** | **G** | **G** | **T** | **A** | **C** | **G** | **A** | **G** | **C** | **G** | **T** | **G** | **G** | **A** | **G** | **A** | **T** | **C** | **A** | **A** | **G** | **G** | **T** | **C** | **C** | **C** | **T** | **T** | **T** | **G** | **A** | **T** | **G** | **A** | **A** | **G** | **G** | **A** | **G** | **G** | **C** | **A** | **-** | **-** | **-** |
| **N52702** | **A** | **T** | **G** | **A** | **T** | **G** | **G** | **G** | **A** | **A** | **G** | **A** | **A** | **T** | **A** | **T** | **C** | **C** | **A** | **A** | **A** | **G** | **C** | **A** | **A** | **T** | **G** | **C** | **T** | **A** | **G** | **C** | **T** | **G** | **T** | **T** | **T** | **A** | **T** | **T** | **C** | **G** | **G** | **G** | **G** | **A** | **T** | **T** | **G** | **C** | **G** | **G** | **C** | **T** | **T** | **T** | **C** | **C** | **T** | **T** | **A** | **T** | **T** | **A** | **T** | **T** | **C** | **T** | **A** | **T** | **T** | **T** | **T** | **C** | **A** | **C** | **T** | **T** | **T** | **T** | **C** | **A** | **C** | **T** | **T** | **A** | **T** | **T** | **T** | **T** | **T** | **A** | **T** | **G** | **C** | **C** | **A** | **G** | **T** | **A** | **A** | **C** | **C** | **A** | **A** | **A** | **G** | **T** | **C** | **A** | **T** | **T** | **G** | **G** | **T** | **G** | **G** | **G** | **G** | **A** | **G** | **A** | **A** | **T** | **T** | **G** | **C** | **T** | **G** | **C** | **G** | **T** | **G** | **C** | **G** | **C** | **G** | **T** | **T** | **T** | **A** | **T** | **T** | **A** | **T** | **A** | **C** | **G** | **T** | **T** | **C** | **C** | **G** | **C** | **T** | **T** | **A** | **T** | **C** | **T** | **T** | **C** | **C** | **A** | **T** | **T** | **T** | **G** | **A** | **T** | **G** | **G** | **C** | **A** | **A** | **T** | **T** | **T** | **C** | **A** | **T** | **T** | **A** | **G** | **G** | **T** | **G** | **T** | **A** | **G** | **G** | **A** | **T** | **T** | **G** | **A** | **T** | **C** | **G** | **T** | **A** | **T** | **T** | **T** | **C** | **T** | **A** | **T** | **T** | **G** | **T** | **T** | **A** | **T** | **C** | **A** | **C** | **T** | **T** | **A** | **T** | **T** | **C** | **A** | **A** | **A** | **A** | **G** | **G** | **C** | **A** | **A** | **A** | **A** | **T** | **A** | **C** | **G** | **G** | **G** | **A** | **A** | **A** | **A** | **T** | **C** | **G** | **A** | **A** | **G** | **A** | **A** | **A** | **A** | **G** | **C** | **T** | **T** | **C** | **G** | **T** | **G** | **C** | **A** | **C** | **T** | **T** | **T** | **C** | **T** | **T** | **C** | **T** | **G** | **G** | **C** | **A** | **A** | **T** | **T** | **A** | **T** | **G** | **A** | **A** | **T** | **C** | **C** | **A** | **A** | **A** | **C** | **T** | **T** | **T** | **T** | **G** | **C** | **A** | **T** | **C** | **A** | **G** | **C** | **C** | **G** | **A** | **T** | **C** | **C** | **C** | **A** | **A** | **G** | **C** | **G** | **C** | **G** | **T** | **C** | **G** | **G** | **A** | **T** | **G** | **A** | **T** | **C** | **T** | **G** | **T** | **A** | **C** | **A** | **T** | **C** | **A** | **A** | **A** | **G** | **A** | **T** | **A** | **T** | **T** | **G** | **A** | **T** | **A** | **T** | **G** | **G** | **A** | **A** | **A** | **T** | **T** | **A** | **C** | **T** | **A** | **A** | **A** | **A** | **T** | **A** | **A** | **A** | **A** | **G** | **A** | **A** | **A** | **A** | **A** | **A** | **T** | **G** | **A** | **T** | **C** | **G** | **A** | **A** | **G** | **T** | **A** | **T** | **C** | **C** | **A** | **G** | **T** | **G** | **A** | **A** | **T** | **T** | **A** | **C** | **A** | **G** | **A** | **T** | **T** | **A** | **T** | **G** | **A** | **C** | **T** | **A** | **G** | **C** | **C** | **G** | **T** | **C** | **C** | **T** | **C** | **A** | **A** | **T** | **A** | **T** | **G** | **T** | **A** | **G** | **A** | **T** | **G** | **G** | **C** | **C** | **A** | **A** | **A** | **C** | **C** | **A** | **A** | **A** | **G** | **A** | **A** | **G** | **A** | **G** | **A** | **T** | **T** | **T** | **T** | **A** | **G** | **A** | **A** | **T** | **T** | **A** | **G** | **A** | **A** | **A** | **G** | **G** | **C** | **A** | **T** | **C** | **G** | **A** | **T** | **T** | **A** | **G** | **C** | **T** | **C** | **G** | **T** | **G** | **A** | **G** | **C** | **T** | **T** | **C** | **A** | **T** | **G** | **A** | **T** | **T** | **C** | **A** | **G** | **T** | **T** | **T** | **C** | **A** | **C** | **A** | **G** | **C** | **A** | **A** | **T** | **T** | **A** | **T** | **T** | **T** | **G** | **C** | **A** | **G** | **C** | **G** | **A** | **T** | **G** | **A** | **T** | **G** | **A** | **T** | **G** | **A** | **T** | **G** | **T** | **C** | **G** | **G** | **C** | **A** | **T** | **T** | **G** | **A** | **C** | **G** | **G** | **A** | **G** | **C** | **A** | **A** | **G** | **C** | **A** | **G** | **A** | **A** | **A** | **A** | **A** | **A** | **G** | **T** | **G** | **A** | **G** | **A** | **C** | **A** | **C** | **C** | **A** | **G** | **A** | **A** | **A** | **T** | **G** | **T** | **T** | **C** | **C** | **G** | **T** | **A** | **A** | **G** | **C** | **A** | **G** | **T** | **T** | **G** | **A** | **A** | **A** | **A** | **T** | **G** | **G** | **T** | **G** | **G** | **C** | **A** | **G** | **A** | **G** | **A** | **T** | **C** | **A** | **T** | **C** | **A** | **A** | **C** | **G** | **C** | **T** | **T** | **C** | **C** | **C** | **A** | **G** | **T** | **C** | **T** | **G** | **A** | **G** | **A** | **T** | **G** | **C** | **G** | **T** | **G** | **C** | **G** | **C** | **T** | **G** | **C** | **T** | **T** | **C** | **T** | **T** | **C** | **A** | **T** | **C** | **T** | **G** | **C** | **G** | **T** | **C** | **C** | **A** | **G** | **T** | **C** | **A** | **A** | **T** | **T** | **T** | **A** | **G** | **A** | **A** | **G** | **A** | **A** | **A** | **A** | **A** | **A** | **G** | **T** | **T** | **T** | **A** | **A** | **A** | **A** | **C** | **A** | **A** | **G** | **G** | **C** | **A** | **T** | **T** | **G** | **A** | **G** | **C** | **A** | **G** | **T** | **T** | **A** | **T** | **T** | **G** | **A** | **A** | **G** | **G** | **A** | **A** | **T** | **T** | **G** | **C** | **A** | **G** | **A** | **A** | **T** | **A** | **A** | **A** | **A** | **T** | **C** | **C** | **A** | **G** | **A** | **T** | **T** | **T** | **C** | **G** | **C** | **T** | **A** | **A** | **A** | **A** | **T** | **G** | **G** | **G** | **A** | **T** | **G** | **T** | **A** | **G** | **A** | **A** | **G** | **A** | **T** | **G** | **T** | **A** | **A** | **A** | **A** | **T** | **T** | **A** | **A** | **C** | **T** | **A** | **G** | **T** | **T** | **C** | **C** | **A** | **T** | **T** | **G** | **A** | **G** | **G** | **A** | **T** | **C** | **A** | **C** | **T** | **T** | **G** | **T** | **T** | **C** | **C** | **G** | **A** | **A** | **T** | **C** | **G** | **T** | **T** | **C** | **A** | **A** | **G** | **A** | **A** | **T** | **T** | **G** | **T** | **T** | **A** | **T** | **C** | **A** | **A** | **A** | **C** | **A** | **C** | **A** | **T** | **T** | **A** | **A** | **G** | **A** | **C** | **A** | **T** | **G** | **C** | **T** | **A** | **A** | **G** | **G** | **C** | **T** | **A** | **A** | **T** | **G** | **A** | **A** | **T** | **T** | **A** | **G** | **A** | **A** | **G** | **T** | **C** | **T** | **A** | **T** | **T** | **T** | **G** | **C** | **A** | **T** | **A** | **A** | **A** | **A** | **T** | **A** | **G** | **A** | **T** | **A** | **A** | **C** | **A** | **A** | **T** | **C** | **T** | **T** | **T** | **T** | **A** | **T** | **T** | **A** | **C** | **G** | **T** | **A** | **T** | **C** | **A** | **T** | **T** | **G** | **A** | **C** | **G** | **A** | **C** | **G** | **G** | **G** | **A** | **C** | **A** | **G** | **G** | **C** | **T** | **T** | **T** | **A** | **A** | **T** | **A** | **T** | **G** | **A** | **A** | **T** | **G** | **A** | **A** | **A** | **C** | **C** | **A** | **A** | **A** | **A** | **C** | **G** | **G** | **G** | **A** | **A** | **G** | **T** | **T** | **A** | **T** | **G** | **G** | **A** | **T** | **T** | **G** | **A** | **A** | **C** | **A** | **A** | **T** | **A** | **T** | **C** | **A** | **A** | **A** | **G** | **A** | **A** | **C** | **G** | **A** | **G** | **T** | **A** | **G** | **C** | **T** | **G** | **G** | **T** | **A** | **T** | **T** | **G** | **G** | **T** | **G** | **G** | **A** | **A** | **C** | **A** | **G** | **T** | **A** | **A** | **A** | **A** | **A** | **T** | **C** | **A** | **T** | **T** | **A** | **G** | **T** | **T** | **T** | **T** | **A** | **A** | **A** | **G** | **G** | **G** | **C** | **A** | **A** | **G** | **G** | **T** | **A** | **C** | **G** | **A** | **G** | **C** | **G** | **T** | **G** | **G** | **A** | **G** | **A** | **T** | **C** | **A** | **A** | **G** | **G** | **T** | **C** | **C** | **C** | **T** | **T** | **T** | **G** | **A** | **T** | **G** | **A** | **A** | **G** | **G** | **A** | **G** | **G** | **C** | **A** | **-** | **-** | **-** |
| **N55299** | **A** | **T** | **G** | **A** | **T** | **G** | **G** | **G** | **A** | **A** | **G** | **A** | **A** | **T** | **A** | **T** | **C** | **C** | **A** | **A** | **A** | **G** | **C** | **A** | **A** | **T** | **G** | **C** | **T** | **A** | **G** | **C** | **T** | **G** | **T** | **T** | **T** | **A** | **T** | **T** | **C** | **G** | **G** | **G** | **G** | **A** | **T** | **T** | **G** | **C** | **G** | **G** | **C** | **T** | **T** | **T** | **C** | **C** | **T** | **T** | **A** | **T** | **T** | **A** | **T** | **T** | **C** | **T** | **A** | **T** | **T** | **T** | **T** | **C** | **A** | **C** | **T** | **T** | **T** | **T** | **C** | **A** | **C** | **T** | **T** | **A** | **T** | **T** | **T** | **T** | **T** | **A** | **T** | **G** | **C** | **C** | **A** | **G** | **T** | **A** | **A** | **C** | **C** | **A** | **A** | **A** | **G** | **T** | **C** | **A** | **T** | **T** | **G** | **G** | **T** | **G** | **G** | **G** | **G** | **A** | **G** | **A** | **A** | **T** | **T** | **G** | **C** | **T** | **G** | **C** | **G** | **T** | **G** | **C** | **G** | **C** | **G** | **T** | **T** | **T** | **A** | **T** | **T** | **A** | **T** | **A** | **C** | **G** | **T** | **T** | **C** | **C** | **G** | **C** | **T** | **T** | **A** | **T** | **C** | **T** | **T** | **C** | **C** | **A** | **T** | **T** | **T** | **G** | **A** | **T** | **G** | **G** | **C** | **A** | **A** | **T** | **T** | **T** | **C** | **A** | **T** | **T** | **A** | **G** | **G** | **T** | **G** | **T** | **A** | **G** | **G** | **A** | **T** | **T** | **G** | **A** | **T** | **C** | **G** | **T** | **A** | **T** | **T** | **T** | **C** | **T** | **A** | **T** | **T** | **G** | **T** | **T** | **A** | **T** | **C** | **A** | **C** | **T** | **T** | **A** | **T** | **T** | **C** | **A** | **A** | **A** | **A** | **G** | **G** | **C** | **A** | **A** | **A** | **A** | **T** | **A** | **C** | **G** | **G** | **G** | **A** | **A** | **A** | **A** | **T** | **C** | **G** | **A** | **A** | **G** | **A** | **A** | **A** | **A** | **G** | **C** | **T** | **T** | **C** | **G** | **T** | **G** | **C** | **A** | **C** | **T** | **T** | **T** | **C** | **T** | **T** | **C** | **T** | **G** | **G** | **C** | **A** | **A** | **T** | **T** | **A** | **T** | **G** | **A** | **A** | **T** | **C** | **C** | **A** | **A** | **A** | **C** | **T** | **T** | **T** | **T** | **G** | **C** | **A** | **T** | **C** | **A** | **G** | **C** | **C** | **G** | **A** | **T** | **C** | **C** | **C** | **A** | **A** | **G** | **C** | **G** | **C** | **G** | **T** | **C** | **G** | **G** | **A** | **T** | **G** | **A** | **T** | **C** | **T** | **G** | **T** | **A** | **C** | **A** | **T** | **C** | **A** | **A** | **A** | **G** | **A** | **T** | **A** | **T** | **T** | **G** | **A** | **T** | **A** | **T** | **G** | **G** | **A** | **A** | **A** | **T** | **T** | **A** | **C** | **T** | **A** | **A** | **A** | **A** | **T** | **A** | **A** | **A** | **A** | **G** | **A** | **A** | **A** | **A** | **A** | **A** | **T** | **G** | **A** | **T** | **C** | **G** | **A** | **A** | **G** | **T** | **A** | **T** | **C** | **C** | **A** | **G** | **T** | **G** | **A** | **A** | **T** | **T** | **A** | **C** | **A** | **G** | **A** | **T** | **T** | **A** | **T** | **G** | **A** | **C** | **T** | **A** | **G** | **C** | **C** | **G** | **T** | **C** | **C** | **T** | **C** | **A** | **A** | **T** | **A** | **T** | **G** | **T** | **A** | **G** | **A** | **T** | **G** | **G** | **A** | **C** | **A** | **A** | **A** | **C** | **C** | **A** | **A** | **A** | **G** | **A** | **A** | **G** | **A** | **G** | **A** | **T** | **T** | **T** | **T** | **A** | **G** | **A** | **A** | **T** | **T** | **A** | **G** | **A** | **A** | **A** | **G** | **G** | **C** | **A** | **T** | **C** | **G** | **A** | **T** | **T** | **A** | **G** | **C** | **T** | **C** | **G** | **T** | **G** | **A** | **G** | **C** | **T** | **T** | **C** | **A** | **T** | **G** | **A** | **T** | **T** | **C** | **A** | **G** | **T** | **T** | **T** | **C** | **A** | **C** | **A** | **G** | **C** | **A** | **A** | **T** | **T** | **A** | **T** | **T** | **T** | **G** | **C** | **A** | **G** | **C** | **G** | **A** | **T** | **G** | **A** | **T** | **G** | **A** | **T** | **G** | **A** | **T** | **G** | **T** | **C** | **G** | **G** | **C** | **A** | **T** | **T** | **G** | **A** | **C** | **G** | **G** | **A** | **G** | **C** | **A** | **A** | **G** | **C** | **A** | **G** | **A** | **A** | **A** | **A** | **A** | **A** | **G** | **T** | **G** | **A** | **G** | **A** | **C** | **A** | **C** | **C** | **A** | **G** | **A** | **A** | **A** | **T** | **G** | **T** | **T** | **C** | **C** | **G** | **T** | **A** | **A** | **G** | **C** | **A** | **G** | **T** | **T** | **G** | **A** | **A** | **A** | **A** | **T** | **G** | **G** | **T** | **G** | **G** | **C** | **A** | **G** | **A** | **G** | **A** | **T** | **C** | **A** | **T** | **C** | **A** | **A** | **C** | **G** | **C** | **T** | **T** | **C** | **C** | **C** | **A** | **G** | **T** | **C** | **T** | **G** | **A** | **G** | **A** | **T** | **G** | **C** | **G** | **T** | **G** | **C** | **G** | **C** | **T** | **G** | **C** | **T** | **T** | **C** | **T** | **T** | **C** | **A** | **T** | **C** | **T** | **G** | **C** | **G** | **T** | **C** | **C** | **A** | **G** | **T** | **C** | **A** | **A** | **T** | **T** | **T** | **A** | **G** | **A** | **A** | **G** | **A** | **A** | **A** | **A** | **A** | **A** | **G** | **T** | **T** | **T** | **A** | **A** | **A** | **A** | **C** | **A** | **A** | **G** | **G** | **C** | **A** | **T** | **T** | **G** | **A** | **G** | **C** | **A** | **G** | **T** | **T** | **A** | **T** | **T** | **G** | **A** | **A** | **G** | **G** | **A** | **A** | **T** | **T** | **G** | **C** | **A** | **G** | **A** | **A** | **T** | **A** | **A** | **A** | **A** | **T** | **C** | **C** | **A** | **G** | **A** | **T** | **T** | **T** | **C** | **G** | **C** | **T** | **A** | **A** | **A** | **A** | **T** | **G** | **G** | **G** | **A** | **T** | **G** | **T** | **A** | **G** | **A** | **A** | **G** | **A** | **T** | **G** | **T** | **A** | **A** | **A** | **A** | **T** | **T** | **A** | **A** | **C** | **T** | **A** | **G** | **T** | **T** | **C** | **C** | **A** | **T** | **T** | **G** | **A** | **G** | **G** | **A** | **T** | **C** | **A** | **C** | **T** | **T** | **G** | **T** | **T** | **C** | **C** | **G** | **A** | **A** | **T** | **C** | **G** | **T** | **T** | **C** | **A** | **A** | **G** | **A** | **A** | **T** | **T** | **G** | **T** | **T** | **A** | **T** | **C** | **A** | **A** | **A** | **C** | **A** | **C** | **A** | **T** | **T** | **A** | **A** | **G** | **A** | **C** | **A** | **T** | **G** | **C** | **T** | **A** | **A** | **G** | **G** | **C** | **T** | **A** | **A** | **T** | **G** | **A** | **A** | **T** | **T** | **A** | **G** | **A** | **A** | **G** | **T** | **C** | **T** | **A** | **T** | **T** | **T** | **G** | **C** | **A** | **T** | **A** | **A** | **A** | **A** | **T** | **A** | **G** | **A** | **T** | **A** | **A** | **C** | **A** | **A** | **T** | **C** | **T** | **T** | **T** | **T** | **A** | **T** | **T** | **A** | **C** | **G** | **T** | **A** | **T** | **C** | **A** | **T** | **T** | **G** | **A** | **C** | **G** | **A** | **C** | **G** | **G** | **G** | **A** | **C** | **A** | **G** | **G** | **A** | **T** | **T** | **T** | **A** | **A** | **T** | **A** | **T** | **G** | **A** | **A** | **T** | **G** | **A** | **A** | **A** | **C** | **C** | **A** | **A** | **A** | **A** | **C** | **G** | **G** | **G** | **A** | **A** | **G** | **T** | **T** | **A** | **T** | **G** | **G** | **A** | **T** | **T** | **G** | **A** | **A** | **C** | **A** | **A** | **T** | **A** | **T** | **C** | **A** | **A** | **A** | **G** | **A** | **A** | **C** | **G** | **A** | **G** | **T** | **A** | **G** | **C** | **T** | **G** | **G** | **T** | **A** | **T** | **T** | **G** | **G** | **T** | **G** | **G** | **A** | **A** | **C** | **A** | **G** | **T** | **A** | **A** | **A** | **A** | **A** | **T** | **C** | **A** | **T** | **T** | **A** | **G** | **T** | **T** | **T** | **T** | **A** | **A** | **A** | **G** | **G** | **G** | **C** | **A** | **A** | **G** | **G** | **T** | **A** | **C** | **G** | **A** | **G** | **C** | **G** | **T** | **G** | **G** | **A** | **G** | **A** | **T** | **C** | **A** | **A** | **G** | **G** | **T** | **C** | **C** | **C** | **T** | **T** | **T** | **G** | **A** | **T** | **G** | **A** | **A** | **G** | **G** | **A** | **G** | **G** | **C** | **A** | **-** | **-** | **-** |
| **N59492F** | **A** | **T** | **G** | **A** | **T** | **G** | **G** | **G** | **A** | **A** | **G** | **A** | **A** | **T** | **A** | **T** | **C** | **C** | **A** | **A** | **A** | **G** | **C** | **A** | **A** | **T** | **G** | **C** | **T** | **A** | **G** | **C** | **T** | **G** | **T** | **T** | **T** | **A** | **T** | **T** | **C** | **G** | **G** | **G** | **G** | **A** | **T** | **T** | **G** | **C** | **G** | **G** | **C** | **T** | **T** | **T** | **C** | **C** | **T** | **T** | **A** | **T** | **T** | **A** | **T** | **T** | **C** | **T** | **A** | **T** | **T** | **T** | **T** | **C** | **A** | **C** | **T** | **T** | **T** | **T** | **C** | **A** | **C** | **T** | **T** | **A** | **T** | **T** | **T** | **T** | **T** | **A** | **T** | **G** | **C** | **C** | **A** | **G** | **T** | **A** | **A** | **C** | **C** | **A** | **A** | **A** | **G** | **T** | **C** | **A** | **T** | **T** | **G** | **G** | **T** | **G** | **G** | **G** | **G** | **A** | **G** | **A** | **A** | **T** | **T** | **G** | **C** | **T** | **G** | **C** | **G** | **T** | **G** | **C** | **G** | **C** | **G** | **T** | **T** | **T** | **A** | **T** | **T** | **A** | **T** | **A** | **C** | **G** | **T** | **T** | **C** | **C** | **G** | **C** | **T** | **T** | **A** | **T** | **C** | **T** | **T** | **C** | **C** | **A** | **T** | **T** | **T** | **G** | **A** | **T** | **G** | **G** | **C** | **A** | **A** | **T** | **T** | **T** | **C** | **A** | **T** | **T** | **A** | **G** | **G** | **T** | **G** | **T** | **A** | **G** | **G** | **A** | **T** | **T** | **G** | **A** | **T** | **C** | **G** | **T** | **A** | **T** | **T** | **T** | **C** | **T** | **A** | **T** | **T** | **G** | **T** | **T** | **A** | **T** | **C** | **A** | **C** | **T** | **T** | **A** | **T** | **T** | **C** | **A** | **A** | **A** | **A** | **G** | **A** | **C** | **A** | **A** | **A** | **A** | **T** | **A** | **C** | **G** | **G** | **G** | **A** | **A** | **A** | **A** | **T** | **C** | **G** | **A** | **A** | **G** | **A** | **A** | **A** | **A** | **G** | **C** | **T** | **T** | **C** | **G** | **T** | **G** | **C** | **A** | **C** | **T** | **T** | **T** | **C** | **T** | **T** | **C** | **T** | **G** | **G** | **C** | **A** | **A** | **T** | **T** | **A** | **T** | **G** | **A** | **A** | **T** | **C** | **C** | **A** | **A** | **A** | **C** | **T** | **T** | **T** | **T** | **G** | **C** | **A** | **T** | **C** | **A** | **G** | **C** | **C** | **G** | **A** | **T** | **C** | **C** | **C** | **A** | **A** | **G** | **C** | **G** | **C** | **G** | **T** | **C** | **G** | **G** | **A** | **T** | **G** | **A** | **T** | **C** | **T** | **G** | **T** | **A** | **C** | **A** | **T** | **C** | **A** | **A** | **A** | **G** | **A** | **T** | **A** | **T** | **T** | **G** | **A** | **T** | **A** | **T** | **G** | **G** | **A** | **A** | **A** | **T** | **T** | **A** | **C** | **T** | **A** | **A** | **A** | **A** | **T** | **A** | **A** | **A** | **A** | **G** | **A** | **A** | **A** | **A** | **A** | **A** | **T** | **G** | **A** | **T** | **C** | **G** | **A** | **A** | **G** | **T** | **A** | **T** | **C** | **C** | **A** | **G** | **T** | **G** | **A** | **A** | **T** | **T** | **A** | **C** | **A** | **G** | **A** | **T** | **T** | **A** | **T** | **G** | **A** | **C** | **T** | **A** | **G** | **C** | **C** | **G** | **T** | **C** | **C** | **T** | **C** | **A** | **A** | **T** | **A** | **T** | **G** | **T** | **A** | **G** | **A** | **T** | **G** | **G** | **A** | **C** | **A** | **A** | **A** | **C** | **C** | **A** | **A** | **A** | **G** | **A** | **A** | **G** | **A** | **G** | **A** | **T** | **T** | **T** | **T** | **A** | **G** | **A** | **A** | **T** | **T** | **A** | **G** | **A** | **A** | **A** | **G** | **G** | **C** | **A** | **T** | **C** | **G** | **A** | **T** | **T** | **A** | **G** | **C** | **T** | **C** | **G** | **T** | **G** | **A** | **G** | **C** | **T** | **T** | **C** | **A** | **T** | **G** | **A** | **T** | **T** | **C** | **A** | **G** | **T** | **T** | **T** | **C** | **A** | **C** | **A** | **G** | **C** | **A** | **A** | **T** | **T** | **A** | **T** | **T** | **T** | **G** | **C** | **A** | **G** | **C** | **G** | **A** | **T** | **G** | **A** | **T** | **G** | **A** | **T** | **G** | **A** | **T** | **G** | **T** | **C** | **G** | **G** | **C** | **A** | **T** | **T** | **G** | **A** | **C** | **G** | **G** | **A** | **G** | **C** | **A** | **A** | **G** | **C** | **A** | **G** | **A** | **A** | **A** | **A** | **A** | **A** | **G** | **T** | **G** | **A** | **G** | **A** | **C** | **A** | **C** | **C** | **A** | **G** | **A** | **A** | **A** | **T** | **G** | **T** | **T** | **C** | **C** | **G** | **T** | **A** | **A** | **G** | **C** | **A** | **G** | **T** | **T** | **G** | **A** | **A** | **A** | **A** | **T** | **G** | **G** | **T** | **G** | **G** | **C** | **A** | **G** | **A** | **G** | **A** | **T** | **C** | **A** | **T** | **C** | **A** | **A** | **C** | **G** | **C** | **T** | **T** | **C** | **C** | **C** | **A** | **G** | **T** | **C** | **T** | **G** | **A** | **G** | **A** | **T** | **G** | **C** | **G** | **T** | **G** | **C** | **G** | **C** | **T** | **G** | **C** | **T** | **T** | **C** | **T** | **T** | **C** | **A** | **T** | **C** | **T** | **G** | **C** | **G** | **T** | **C** | **C** | **A** | **G** | **T** | **C** | **A** | **A** | **T** | **T** | **T** | **A** | **G** | **A** | **A** | **G** | **A** | **A** | **A** | **A** | **A** | **A** | **G** | **T** | **T** | **T** | **A** | **A** | **A** | **A** | **C** | **A** | **A** | **G** | **G** | **C** | **A** | **T** | **T** | **G** | **A** | **G** | **C** | **A** | **G** | **T** | **T** | **A** | **T** | **T** | **G** | **A** | **A** | **G** | **G** | **A** | **A** | **T** | **T** | **G** | **C** | **A** | **G** | **A** | **A** | **T** | **A** | **A** | **A** | **A** | **T** | **C** | **C** | **A** | **G** | **A** | **T** | **T** | **T** | **C** | **G** | **C** | **T** | **A** | **A** | **A** | **A** | **T** | **G** | **G** | **G** | **A** | **T** | **G** | **T** | **A** | **G** | **A** | **A** | **G** | **A** | **T** | **G** | **T** | **A** | **A** | **A** | **A** | **T** | **T** | **A** | **A** | **C** | **T** | **A** | **G** | **T** | **T** | **C** | **C** | **A** | **T** | **T** | **G** | **A** | **G** | **G** | **A** | **T** | **C** | **A** | **C** | **T** | **T** | **G** | **T** | **T** | **C** | **C** | **G** | **A** | **A** | **T** | **C** | **G** | **T** | **T** | **C** | **A** | **A** | **G** | **A** | **A** | **T** | **T** | **G** | **T** | **T** | **A** | **T** | **C** | **A** | **A** | **A** | **C** | **A** | **C** | **A** | **T** | **T** | **A** | **A** | **G** | **A** | **C** | **A** | **T** | **G** | **C** | **T** | **A** | **A** | **G** | **G** | **C** | **T** | **A** | **A** | **T** | **G** | **A** | **A** | **T** | **T** | **A** | **G** | **A** | **A** | **G** | **T** | **C** | **T** | **A** | **T** | **T** | **T** | **G** | **C** | **A** | **T** | **A** | **A** | **A** | **A** | **T** | **A** | **G** | **A** | **T** | **A** | **A** | **C** | **A** | **A** | **T** | **C** | **T** | **T** | **T** | **T** | **A** | **T** | **T** | **A** | **C** | **G** | **T** | **A** | **T** | **C** | **A** | **T** | **T** | **G** | **A** | **C** | **G** | **A** | **C** | **G** | **G** | **G** | **A** | **C** | **A** | **G** | **G** | **A** | **T** | **T** | **T** | **A** | **A** | **T** | **A** | **T** | **G** | **A** | **A** | **T** | **G** | **A** | **A** | **A** | **C** | **C** | **A** | **A** | **A** | **A** | **C** | **G** | **G** | **G** | **A** | **A** | **G** | **T** | **T** | **A** | **T** | **G** | **G** | **A** | **T** | **T** | **G** | **A** | **A** | **C** | **A** | **A** | **T** | **A** | **T** | **C** | **A** | **A** | **A** | **G** | **A** | **A** | **C** | **G** | **A** | **G** | **T** | **A** | **G** | **C** | **T** | **G** | **G** | **T** | **A** | **T** | **T** | **G** | **G** | **T** | **G** | **G** | **A** | **A** | **C** | **A** | **G** | **T** | **A** | **A** | **A** | **A** | **A** | **T** | **C** | **A** | **T** | **T** | **A** | **G** | **T** | **T** | **T** | **T** | **A** | **A** | **A** | **G** | **G** | **G** | **C** | **A** | **A** | **G** | **G** | **T** | **A** | **C** | **G** | **A** | **G** | **C** | **G** | **T** | **G** | **G** | **A** | **G** | **A** | **T** | **C** | **A** | **A** | **G** | **G** | **T** | **C** | **C** | **C** | **T** | **T** | **T** | **G** | **A** | **T** | **G** | **A** | **A** | **G** | **G** | **A** | **G** | **G** | **C** | **A** | **-** | **-** | **-** |
| **N53439** | **A** | **T** | **G** | **A** | **T** | **G** | **G** | **G** | **A** | **A** | **G** | **A** | **A** | **T** | **A** | **T** | **C** | **C** | **A** | **A** | **A** | **G** | **C** | **A** | **A** | **T** | **G** | **C** | **T** | **A** | **G** | **C** | **T** | **G** | **T** | **T** | **T** | **A** | **T** | **T** | **C** | **G** | **G** | **G** | **G** | **A** | **T** | **T** | **G** | **C** | **G** | **G** | **C** | **T** | **T** | **T** | **C** | **C** | **T** | **T** | **A** | **T** | **T** | **A** | **T** | **T** | **C** | **T** | **A** | **T** | **T** | **T** | **T** | **C** | **A** | **C** | **T** | **T** | **T** | **T** | **C** | **A** | **C** | **T** | **T** | **A** | **T** | **T** | **T** | **T** | **T** | **A** | **T** | **G** | **C** | **C** | **A** | **G** | **T** | **A** | **A** | **C** | **C** | **A** | **A** | **A** | **G** | **T** | **C** | **A** | **T** | **T** | **G** | **G** | **T** | **G** | **G** | **G** | **G** | **A** | **G** | **A** | **A** | **T** | **T** | **G** | **C** | **T** | **G** | **C** | **G** | **T** | **G** | **C** | **G** | **C** | **G** | **T** | **T** | **T** | **A** | **T** | **T** | **A** | **T** | **A** | **C** | **G** | **T** | **T** | **C** | **C** | **G** | **C** | **T** | **T** | **A** | **T** | **C** | **T** | **T** | **C** | **C** | **A** | **T** | **T** | **T** | **G** | **A** | **T** | **G** | **G** | **C** | **A** | **A** | **T** | **T** | **T** | **C** | **A** | **T** | **T** | **A** | **G** | **G** | **T** | **G** | **T** | **A** | **G** | **G** | **A** | **T** | **T** | **G** | **A** | **T** | **C** | **G** | **T** | **A** | **T** | **T** | **T** | **C** | **T** | **A** | **T** | **T** | **G** | **T** | **T** | **A** | **T** | **C** | **A** | **C** | **T** | **T** | **A** | **T** | **T** | **C** | **A** | **A** | **A** | **A** | **G** | **G** | **C** | **A** | **A** | **A** | **A** | **T** | **A** | **C** | **G** | **G** | **G** | **A** | **A** | **A** | **A** | **T** | **C** | **G** | **A** | **A** | **G** | **A** | **A** | **A** | **A** | **G** | **C** | **T** | **T** | **C** | **G** | **T** | **G** | **C** | **A** | **C** | **T** | **T** | **T** | **C** | **T** | **T** | **C** | **T** | **G** | **G** | **C** | **A** | **A** | **T** | **T** | **A** | **T** | **G** | **A** | **A** | **T** | **C** | **C** | **A** | **A** | **A** | **C** | **T** | **T** | **T** | **T** | **G** | **C** | **A** | **T** | **C** | **A** | **G** | **C** | **C** | **G** | **A** | **T** | **C** | **C** | **C** | **A** | **A** | **G** | **C** | **G** | **C** | **G** | **T** | **C** | **G** | **G** | **A** | **T** | **G** | **A** | **T** | **C** | **T** | **G** | **T** | **A** | **C** | **A** | **T** | **C** | **A** | **A** | **A** | **G** | **A** | **T** | **A** | **T** | **T** | **G** | **A** | **T** | **A** | **T** | **G** | **G** | **A** | **A** | **A** | **T** | **T** | **A** | **C** | **T** | **A** | **A** | **A** | **A** | **T** | **A** | **A** | **A** | **A** | **G** | **A** | **A** | **A** | **A** | **A** | **A** | **T** | **G** | **A** | **T** | **C** | **G** | **A** | **A** | **G** | **T** | **A** | **T** | **C** | **C** | **A** | **G** | **T** | **G** | **A** | **A** | **T** | **T** | **A** | **C** | **A** | **G** | **A** | **T** | **T** | **A** | **T** | **G** | **A** | **C** | **T** | **A** | **G** | **C** | **C** | **G** | **T** | **C** | **C** | **T** | **C** | **A** | **A** | **T** | **A** | **T** | **G** | **T** | **A** | **G** | **A** | **T** | **G** | **G** | **A** | **C** | **A** | **A** | **A** | **C** | **C** | **A** | **A** | **A** | **G** | **A** | **A** | **G** | **A** | **G** | **A** | **T** | **T** | **T** | **T** | **A** | **G** | **A** | **A** | **T** | **T** | **A** | **G** | **A** | **A** | **A** | **G** | **G** | **C** | **A** | **T** | **C** | **G** | **A** | **T** | **T** | **A** | **G** | **C** | **T** | **C** | **G** | **T** | **G** | **A** | **G** | **C** | **T** | **T** | **C** | **A** | **T** | **G** | **A** | **T** | **T** | **C** | **A** | **G** | **T** | **T** | **T** | **C** | **A** | **C** | **A** | **G** | **C** | **A** | **A** | **T** | **T** | **A** | **T** | **T** | **T** | **G** | **C** | **A** | **G** | **C** | **G** | **A** | **T** | **G** | **A** | **T** | **G** | **A** | **T** | **G** | **A** | **T** | **G** | **T** | **C** | **G** | **G** | **C** | **A** | **T** | **T** | **G** | **A** | **C** | **G** | **G** | **A** | **G** | **C** | **A** | **A** | **G** | **C** | **A** | **G** | **A** | **A** | **A** | **A** | **A** | **A** | **G** | **T** | **G** | **A** | **G** | **A** | **C** | **A** | **C** | **C** | **A** | **G** | **G** | **A** | **A** | **T** | **G** | **T** | **T** | **C** | **C** | **G** | **T** | **A** | **A** | **G** | **C** | **A** | **G** | **T** | **T** | **G** | **A** | **A** | **A** | **A** | **T** | **G** | **G** | **T** | **G** | **G** | **C** | **A** | **G** | **A** | **G** | **A** | **T** | **C** | **A** | **T** | **C** | **A** | **A** | **C** | **G** | **C** | **T** | **T** | **C** | **C** | **C** | **A** | **G** | **T** | **C** | **T** | **G** | **A** | **G** | **A** | **T** | **G** | **C** | **G** | **T** | **G** | **C** | **G** | **C** | **T** | **G** | **C** | **T** | **T** | **C** | **T** | **T** | **C** | **A** | **T** | **C** | **T** | **G** | **C** | **G** | **T** | **C** | **C** | **A** | **G** | **T** | **C** | **A** | **A** | **T** | **T** | **T** | **A** | **G** | **A** | **A** | **G** | **A** | **A** | **A** | **A** | **A** | **A** | **G** | **T** | **T** | **T** | **A** | **A** | **A** | **A** | **C** | **A** | **A** | **G** | **G** | **C** | **A** | **T** | **T** | **G** | **A** | **G** | **C** | **A** | **G** | **T** | **T** | **A** | **T** | **T** | **G** | **A** | **A** | **G** | **G** | **A** | **A** | **T** | **T** | **G** | **C** | **A** | **G** | **A** | **A** | **T** | **A** | **A** | **A** | **A** | **T** | **C** | **C** | **A** | **G** | **A** | **T** | **T** | **T** | **C** | **G** | **C** | **T** | **A** | **A** | **A** | **A** | **T** | **G** | **G** | **G** | **A** | **T** | **G** | **T** | **A** | **G** | **A** | **A** | **G** | **A** | **T** | **G** | **T** | **A** | **A** | **A** | **A** | **T** | **T** | **A** | **A** | **C** | **T** | **A** | **G** | **T** | **T** | **C** | **C** | **A** | **T** | **T** | **G** | **A** | **G** | **G** | **A** | **T** | **C** | **A** | **C** | **T** | **T** | **G** | **T** | **T** | **C** | **C** | **G** | **A** | **A** | **T** | **C** | **G** | **T** | **T** | **C** | **A** | **A** | **G** | **A** | **A** | **T** | **T** | **G** | **T** | **T** | **A** | **T** | **C** | **A** | **A** | **A** | **C** | **A** | **C** | **A** | **T** | **T** | **A** | **A** | **G** | **A** | **C** | **A** | **T** | **G** | **C** | **T** | **A** | **A** | **G** | **G** | **C** | **T** | **A** | **A** | **T** | **G** | **A** | **A** | **T** | **T** | **A** | **G** | **A** | **A** | **G** | **T** | **C** | **T** | **A** | **T** | **T** | **T** | **G** | **C** | **A** | **T** | **A** | **A** | **A** | **A** | **T** | **A** | **G** | **A** | **T** | **A** | **A** | **C** | **A** | **A** | **T** | **C** | **T** | **T** | **T** | **T** | **A** | **T** | **T** | **A** | **C** | **G** | **T** | **A** | **T** | **C** | **A** | **T** | **T** | **G** | **A** | **C** | **G** | **A** | **C** | **G** | **G** | **G** | **A** | **C** | **A** | **G** | **G** | **C** | **T** | **T** | **T** | **A** | **A** | **T** | **A** | **T** | **G** | **A** | **A** | **T** | **G** | **A** | **A** | **A** | **C** | **C** | **A** | **A** | **A** | **A** | **C** | **G** | **G** | **G** | **A** | **A** | **G** | **T** | **T** | **A** | **T** | **G** | **G** | **A** | **T** | **T** | **G** | **A** | **A** | **C** | **A** | **A** | **T** | **A** | **T** | **C** | **A** | **A** | **A** | **G** | **A** | **A** | **C** | **G** | **A** | **G** | **T** | **A** | **G** | **C** | **T** | **G** | **G** | **T** | **A** | **T** | **T** | **G** | **G** | **T** | **G** | **G** | **A** | **A** | **C** | **A** | **G** | **T** | **A** | **A** | **A** | **A** | **A** | **T** | **C** | **A** | **T** | **T** | **A** | **G** | **T** | **T** | **T** | **T** | **A** | **A** | **A** | **G** | **G** | **G** | **C** | **A** | **A** | **G** | **G** | **T** | **A** | **C** | **G** | **A** | **G** | **C** | **G** | **T** | **G** | **G** | **A** | **G** | **A** | **T** | **C** | **A** | **A** | **G** | **G** | **T** | **C** | **C** | **C** | **T** | **T** | **T** | **G** | **A** | **T** | **G** | **A** | **A** | **G** | **G** | **A** | **G** | **G** | **C** | **A** | **-** | **-** | **-** |
| **N60425F** | **A** | **T** | **G** | **A** | **T** | **G** | **G** | **G** | **A** | **A** | **G** | **A** | **A** | **T** | **A** | **T** | **C** | **C** | **A** | **A** | **A** | **G** | **C** | **A** | **A** | **T** | **G** | **C** | **T** | **A** | **G** | **C** | **T** | **G** | **T** | **T** | **T** | **A** | **T** | **T** | **C** | **G** | **G** | **G** | **G** | **A** | **T** | **T** | **G** | **C** | **G** | **G** | **C** | **T** | **T** | **T** | **C** | **C** | **T** | **T** | **A** | **T** | **T** | **A** | **T** | **T** | **C** | **T** | **A** | **T** | **T** | **T** | **T** | **C** | **A** | **C** | **T** | **T** | **T** | **T** | **C** | **A** | **C** | **T** | **T** | **A** | **T** | **T** | **T** | **T** | **T** | **A** | **T** | **G** | **C** | **C** | **A** | **G** | **T** | **A** | **A** | **C** | **C** | **A** | **A** | **A** | **G** | **T** | **C** | **A** | **T** | **T** | **G** | **G** | **T** | **G** | **G** | **G** | **G** | **A** | **G** | **A** | **A** | **T** | **T** | **G** | **C** | **T** | **G** | **C** | **G** | **T** | **G** | **C** | **G** | **C** | **G** | **T** | **T** | **T** | **A** | **T** | **T** | **A** | **T** | **A** | **C** | **G** | **T** | **T** | **C** | **C** | **G** | **C** | **T** | **T** | **A** | **T** | **C** | **T** | **T** | **C** | **C** | **A** | **T** | **T** | **T** | **G** | **A** | **T** | **G** | **G** | **C** | **A** | **A** | **T** | **T** | **T** | **C** | **A** | **T** | **T** | **A** | **G** | **G** | **T** | **G** | **T** | **A** | **G** | **G** | **A** | **T** | **T** | **G** | **A** | **T** | **C** | **G** | **T** | **A** | **T** | **T** | **T** | **C** | **T** | **A** | **T** | **T** | **G** | **T** | **T** | **A** | **T** | **C** | **A** | **C** | **T** | **T** | **A** | **T** | **T** | **C** | **A** | **A** | **A** | **A** | **G** | **G** | **C** | **A** | **A** | **A** | **A** | **T** | **A** | **C** | **G** | **G** | **G** | **A** | **A** | **A** | **A** | **T** | **C** | **G** | **A** | **A** | **G** | **A** | **A** | **A** | **A** | **G** | **C** | **T** | **T** | **C** | **G** | **T** | **G** | **C** | **A** | **C** | **T** | **T** | **T** | **C** | **T** | **T** | **C** | **T** | **G** | **G** | **C** | **A** | **A** | **T** | **T** | **A** | **T** | **G** | **A** | **A** | **T** | **C** | **C** | **A** | **A** | **A** | **C** | **T** | **T** | **T** | **T** | **G** | **C** | **A** | **T** | **C** | **A** | **G** | **C** | **C** | **G** | **A** | **T** | **C** | **C** | **C** | **A** | **A** | **G** | **C** | **A** | **C** | **G** | **T** | **C** | **G** | **G** | **A** | **T** | **G** | **A** | **T** | **C** | **T** | **G** | **T** | **A** | **C** | **A** | **T** | **C** | **A** | **A** | **A** | **G** | **A** | **T** | **A** | **T** | **T** | **G** | **A** | **T** | **A** | **T** | **G** | **G** | **A** | **A** | **A** | **T** | **T** | **A** | **C** | **T** | **A** | **A** | **A** | **A** | **T** | **A** | **A** | **A** | **A** | **G** | **A** | **A** | **A** | **A** | **A** | **A** | **T** | **G** | **A** | **T** | **C** | **G** | **A** | **A** | **G** | **T** | **A** | **T** | **C** | **C** | **A** | **G** | **T** | **G** | **A** | **A** | **T** | **T** | **A** | **C** | **A** | **G** | **A** | **T** | **T** | **A** | **T** | **G** | **A** | **C** | **T** | **A** | **G** | **C** | **C** | **G** | **T** | **C** | **C** | **T** | **C** | **A** | **A** | **T** | **A** | **T** | **G** | **T** | **A** | **G** | **A** | **T** | **G** | **G** | **A** | **C** | **A** | **A** | **A** | **C** | **C** | **A** | **A** | **A** | **G** | **A** | **A** | **G** | **A** | **G** | **A** | **T** | **T** | **T** | **T** | **A** | **G** | **A** | **A** | **T** | **T** | **A** | **G** | **A** | **A** | **A** | **G** | **G** | **C** | **A** | **T** | **C** | **G** | **A** | **T** | **T** | **A** | **G** | **C** | **T** | **C** | **G** | **T** | **G** | **A** | **G** | **C** | **T** | **T** | **C** | **A** | **T** | **G** | **A** | **T** | **T** | **C** | **A** | **G** | **T** | **T** | **T** | **C** | **A** | **C** | **A** | **G** | **C** | **A** | **A** | **T** | **T** | **A** | **T** | **T** | **T** | **G** | **C** | **A** | **G** | **C** | **G** | **A** | **T** | **G** | **A** | **T** | **G** | **A** | **T** | **G** | **A** | **T** | **G** | **T** | **C** | **G** | **G** | **C** | **A** | **T** | **T** | **G** | **A** | **C** | **G** | **G** | **A** | **G** | **C** | **A** | **A** | **G** | **C** | **A** | **G** | **A** | **A** | **A** | **A** | **A** | **A** | **G** | **T** | **G** | **A** | **G** | **A** | **C** | **A** | **C** | **C** | **A** | **G** | **A** | **A** | **A** | **T** | **G** | **T** | **T** | **C** | **C** | **G** | **T** | **A** | **A** | **G** | **C** | **A** | **G** | **T** | **T** | **G** | **A** | **A** | **A** | **A** | **T** | **G** | **G** | **T** | **G** | **G** | **C** | **A** | **G** | **A** | **G** | **A** | **T** | **C** | **A** | **T** | **C** | **A** | **A** | **C** | **G** | **C** | **T** | **T** | **C** | **C** | **C** | **A** | **G** | **T** | **C** | **T** | **G** | **A** | **G** | **A** | **T** | **G** | **C** | **G** | **T** | **G** | **C** | **G** | **C** | **T** | **G** | **C** | **T** | **T** | **C** | **T** | **T** | **C** | **A** | **T** | **C** | **T** | **G** | **C** | **G** | **T** | **C** | **C** | **A** | **G** | **T** | **C** | **A** | **A** | **T** | **T** | **T** | **A** | **G** | **A** | **A** | **G** | **A** | **A** | **A** | **A** | **A** | **A** | **G** | **T** | **T** | **T** | **A** | **A** | **A** | **A** | **C** | **A** | **A** | **G** | **G** | **C** | **A** | **T** | **T** | **G** | **A** | **G** | **C** | **A** | **G** | **T** | **T** | **A** | **T** | **T** | **G** | **A** | **A** | **G** | **G** | **A** | **A** | **T** | **T** | **G** | **C** | **A** | **G** | **A** | **A** | **T** | **A** | **A** | **A** | **A** | **T** | **C** | **C** | **A** | **G** | **A** | **T** | **T** | **T** | **C** | **G** | **C** | **T** | **A** | **A** | **A** | **A** | **T** | **G** | **G** | **G** | **A** | **T** | **G** | **T** | **A** | **G** | **A** | **A** | **G** | **A** | **T** | **G** | **T** | **A** | **A** | **A** | **A** | **T** | **T** | **A** | **A** | **C** | **T** | **A** | **G** | **T** | **T** | **C** | **C** | **A** | **T** | **T** | **G** | **A** | **G** | **G** | **A** | **T** | **C** | **A** | **C** | **T** | **T** | **G** | **T** | **T** | **C** | **C** | **G** | **A** | **A** | **T** | **C** | **G** | **T** | **T** | **C** | **A** | **A** | **G** | **A** | **A** | **T** | **T** | **G** | **T** | **T** | **A** | **T** | **C** | **A** | **A** | **A** | **C** | **A** | **C** | **A** | **T** | **T** | **A** | **A** | **G** | **A** | **C** | **A** | **T** | **G** | **C** | **T** | **A** | **A** | **G** | **G** | **C** | **T** | **A** | **A** | **T** | **G** | **A** | **A** | **T** | **T** | **A** | **G** | **A** | **A** | **G** | **T** | **C** | **T** | **A** | **T** | **T** | **T** | **G** | **C** | **A** | **T** | **A** | **A** | **A** | **A** | **T** | **A** | **G** | **A** | **T** | **A** | **A** | **C** | **A** | **A** | **T** | **C** | **T** | **T** | **T** | **T** | **A** | **T** | **T** | **A** | **C** | **G** | **T** | **A** | **T** | **C** | **A** | **T** | **T** | **G** | **A** | **C** | **G** | **A** | **C** | **G** | **G** | **G** | **A** | **C** | **A** | **G** | **G** | **A** | **T** | **T** | **T** | **A** | **A** | **T** | **A** | **T** | **G** | **A** | **A** | **T** | **G** | **A** | **A** | **A** | **C** | **C** | **A** | **A** | **A** | **A** | **C** | **G** | **G** | **G** | **A** | **A** | **G** | **T** | **T** | **A** | **T** | **G** | **G** | **A** | **T** | **T** | **G** | **A** | **A** | **C** | **A** | **A** | **T** | **A** | **T** | **C** | **A** | **A** | **A** | **G** | **A** | **A** | **C** | **G** | **A** | **G** | **T** | **A** | **G** | **C** | **T** | **G** | **G** | **T** | **A** | **T** | **T** | **G** | **G** | **T** | **G** | **G** | **A** | **A** | **C** | **A** | **G** | **T** | **A** | **A** | **A** | **A** | **A** | **T** | **C** | **A** | **T** | **T** | **A** | **G** | **T** | **T** | **T** | **T** | **A** | **A** | **A** | **G** | **G** | **G** | **C** | **A** | **A** | **G** | **G** | **T** | **A** | **C** | **G** | **A** | **G** | **C** | **G** | **T** | **G** | **G** | **A** | **G** | **A** | **T** | **C** | **A** | **A** | **G** | **G** | **T** | **C** | **C** | **C** | **T** | **T** | **T** | **G** | **A** | **T** | **G** | **A** | **A** | **G** | **G** | **A** | **G** | **G** | **C** | **A** | **-** | **-** | **-** |
| **N60299F** | **A** | **T** | **G** | **A** | **T** | **G** | **G** | **G** | **A** | **A** | **G** | **A** | **A** | **T** | **A** | **T** | **C** | **C** | **A** | **A** | **A** | **G** | **C** | **A** | **A** | **T** | **G** | **C** | **T** | **A** | **G** | **C** | **T** | **G** | **T** | **T** | **T** | **A** | **T** | **T** | **C** | **G** | **G** | **G** | **G** | **A** | **T** | **T** | **G** | **C** | **G** | **G** | **C** | **T** | **T** | **T** | **C** | **C** | **T** | **T** | **A** | **T** | **T** | **A** | **T** | **T** | **C** | **T** | **A** | **T** | **T** | **T** | **T** | **C** | **A** | **C** | **T** | **T** | **T** | **T** | **C** | **A** | **C** | **T** | **T** | **A** | **T** | **T** | **T** | **T** | **T** | **A** | **T** | **G** | **C** | **C** | **A** | **G** | **T** | **A** | **A** | **C** | **C** | **A** | **A** | **A** | **G** | **T** | **C** | **A** | **T** | **T** | **G** | **G** | **T** | **G** | **G** | **G** | **G** | **A** | **G** | **A** | **A** | **T** | **T** | **G** | **C** | **T** | **G** | **C** | **G** | **T** | **G** | **C** | **G** | **C** | **G** | **T** | **T** | **T** | **A** | **T** | **T** | **A** | **T** | **A** | **C** | **G** | **T** | **T** | **C** | **C** | **G** | **C** | **T** | **T** | **A** | **T** | **C** | **T** | **T** | **C** | **C** | **A** | **T** | **T** | **T** | **G** | **A** | **T** | **G** | **G** | **C** | **A** | **A** | **T** | **T** | **T** | **C** | **A** | **T** | **T** | **A** | **G** | **G** | **T** | **G** | **T** | **A** | **G** | **G** | **A** | **T** | **T** | **G** | **A** | **T** | **C** | **G** | **T** | **A** | **T** | **T** | **T** | **C** | **T** | **A** | **T** | **T** | **G** | **T** | **T** | **A** | **T** | **C** | **A** | **C** | **T** | **T** | **A** | **T** | **T** | **C** | **A** | **A** | **A** | **A** | **G** | **G** | **C** | **A** | **A** | **A** | **A** | **T** | **A** | **C** | **G** | **G** | **G** | **A** | **A** | **A** | **A** | **T** | **C** | **G** | **A** | **A** | **G** | **A** | **A** | **A** | **A** | **G** | **C** | **T** | **T** | **C** | **G** | **T** | **G** | **C** | **A** | **C** | **T** | **T** | **T** | **C** | **T** | **T** | **C** | **T** | **G** | **G** | **C** | **A** | **A** | **T** | **T** | **A** | **T** | **G** | **A** | **A** | **T** | **C** | **C** | **A** | **A** | **A** | **C** | **T** | **T** | **T** | **T** | **G** | **C** | **A** | **T** | **C** | **A** | **G** | **C** | **C** | **G** | **A** | **T** | **C** | **C** | **C** | **A** | **A** | **G** | **C** | **G** | **C** | **G** | **T** | **C** | **G** | **G** | **A** | **T** | **G** | **A** | **T** | **C** | **T** | **G** | **T** | **A** | **C** | **A** | **T** | **C** | **A** | **A** | **A** | **G** | **A** | **T** | **A** | **T** | **T** | **G** | **A** | **T** | **A** | **T** | **G** | **G** | **A** | **A** | **A** | **T** | **T** | **A** | **C** | **T** | **A** | **A** | **A** | **A** | **T** | **A** | **A** | **A** | **A** | **G** | **A** | **A** | **A** | **A** | **A** | **A** | **T** | **G** | **A** | **T** | **C** | **G** | **A** | **A** | **G** | **T** | **A** | **T** | **C** | **C** | **A** | **G** | **T** | **G** | **A** | **A** | **T** | **T** | **A** | **C** | **A** | **G** | **A** | **T** | **T** | **A** | **T** | **G** | **A** | **C** | **T** | **A** | **G** | **C** | **C** | **G** | **T** | **C** | **C** | **T** | **C** | **A** | **A** | **T** | **A** | **T** | **G** | **T** | **A** | **G** | **A** | **T** | **G** | **G** | **A** | **C** | **A** | **A** | **A** | **C** | **C** | **A** | **A** | **A** | **G** | **A** | **A** | **G** | **A** | **G** | **A** | **T** | **T** | **T** | **T** | **A** | **G** | **A** | **A** | **T** | **T** | **A** | **G** | **A** | **A** | **A** | **G** | **G** | **C** | **A** | **T** | **C** | **G** | **A** | **T** | **T** | **A** | **G** | **C** | **T** | **C** | **G** | **T** | **G** | **A** | **G** | **C** | **T** | **T** | **C** | **A** | **T** | **G** | **A** | **T** | **T** | **C** | **A** | **G** | **T** | **T** | **T** | **C** | **A** | **C** | **A** | **G** | **C** | **A** | **A** | **T** | **T** | **A** | **T** | **T** | **T** | **G** | **C** | **A** | **G** | **C** | **G** | **A** | **T** | **G** | **A** | **T** | **G** | **A** | **T** | **G** | **A** | **T** | **G** | **T** | **C** | **G** | **G** | **C** | **A** | **T** | **T** | **G** | **A** | **C** | **G** | **G** | **A** | **G** | **C** | **A** | **A** | **G** | **C** | **A** | **G** | **A** | **A** | **A** | **A** | **A** | **A** | **G** | **T** | **G** | **A** | **G** | **A** | **C** | **A** | **C** | **C** | **A** | **G** | **G** | **A** | **A** | **T** | **G** | **T** | **T** | **C** | **C** | **G** | **T** | **A** | **A** | **G** | **C** | **A** | **G** | **T** | **T** | **G** | **A** | **A** | **A** | **A** | **T** | **G** | **G** | **T** | **G** | **G** | **C** | **A** | **G** | **A** | **G** | **A** | **T** | **C** | **A** | **T** | **C** | **A** | **A** | **C** | **G** | **C** | **T** | **T** | **C** | **C** | **C** | **A** | **G** | **T** | **C** | **T** | **G** | **A** | **G** | **A** | **T** | **G** | **C** | **G** | **T** | **G** | **C** | **G** | **C** | **T** | **G** | **C** | **T** | **T** | **C** | **T** | **T** | **C** | **A** | **T** | **C** | **T** | **G** | **C** | **G** | **T** | **C** | **C** | **A** | **G** | **T** | **C** | **A** | **A** | **T** | **T** | **T** | **A** | **G** | **A** | **A** | **G** | **A** | **A** | **A** | **A** | **A** | **A** | **G** | **T** | **T** | **T** | **A** | **A** | **A** | **A** | **C** | **A** | **A** | **G** | **G** | **C** | **A** | **T** | **T** | **G** | **A** | **G** | **C** | **A** | **G** | **T** | **T** | **A** | **T** | **T** | **G** | **A** | **A** | **G** | **G** | **A** | **A** | **T** | **T** | **G** | **C** | **A** | **G** | **A** | **A** | **T** | **A** | **A** | **A** | **A** | **T** | **C** | **C** | **A** | **G** | **A** | **T** | **T** | **T** | **C** | **G** | **C** | **T** | **A** | **A** | **A** | **A** | **T** | **G** | **G** | **G** | **A** | **T** | **G** | **T** | **A** | **G** | **A** | **A** | **G** | **A** | **T** | **G** | **T** | **A** | **A** | **A** | **A** | **T** | **T** | **A** | **A** | **C** | **T** | **A** | **G** | **T** | **T** | **C** | **C** | **A** | **T** | **T** | **G** | **A** | **G** | **G** | **A** | **T** | **C** | **A** | **C** | **T** | **T** | **G** | **T** | **T** | **C** | **C** | **G** | **A** | **A** | **T** | **C** | **G** | **T** | **T** | **C** | **A** | **A** | **G** | **A** | **A** | **T** | **T** | **G** | **T** | **T** | **A** | **T** | **C** | **A** | **A** | **A** | **C** | **A** | **C** | **A** | **T** | **T** | **A** | **A** | **G** | **A** | **C** | **A** | **T** | **G** | **C** | **T** | **A** | **A** | **G** | **G** | **C** | **T** | **A** | **A** | **T** | **G** | **A** | **A** | **T** | **T** | **A** | **G** | **A** | **A** | **G** | **T** | **C** | **T** | **A** | **T** | **T** | **T** | **G** | **C** | **A** | **T** | **A** | **A** | **A** | **A** | **T** | **A** | **G** | **A** | **T** | **A** | **A** | **C** | **A** | **A** | **T** | **C** | **T** | **T** | **T** | **T** | **A** | **T** | **T** | **A** | **C** | **G** | **T** | **A** | **T** | **C** | **A** | **T** | **T** | **G** | **A** | **C** | **G** | **A** | **C** | **G** | **G** | **G** | **A** | **C** | **A** | **G** | **G** | **C** | **T** | **T** | **T** | **A** | **A** | **T** | **A** | **T** | **G** | **A** | **A** | **T** | **G** | **A** | **A** | **A** | **C** | **C** | **A** | **A** | **A** | **A** | **C** | **G** | **G** | **G** | **A** | **A** | **G** | **T** | **T** | **A** | **T** | **G** | **G** | **A** | **T** | **T** | **G** | **A** | **A** | **C** | **A** | **A** | **T** | **A** | **T** | **C** | **A** | **A** | **A** | **G** | **A** | **A** | **C** | **G** | **A** | **G** | **T** | **A** | **G** | **C** | **T** | **G** | **G** | **T** | **A** | **T** | **T** | **G** | **G** | **T** | **G** | **G** | **A** | **A** | **C** | **A** | **G** | **T** | **A** | **A** | **A** | **A** | **A** | **T** | **C** | **A** | **T** | **T** | **A** | **G** | **T** | **T** | **T** | **T** | **A** | **A** | **A** | **G** | **G** | **G** | **C** | **A** | **A** | **G** | **G** | **T** | **A** | **C** | **G** | **A** | **G** | **C** | **G** | **T** | **G** | **G** | **A** | **G** | **A** | **T** | **C** | **A** | **A** | **G** | **G** | **T** | **C** | **C** | **C** | **T** | **T** | **T** | **G** | **A** | **T** | **G** | **A** | **A** | **G** | **G** | **A** | **G** | **G** | **C** | **A** | **-** | **-** | **-** |
| **N60417F** | **A** | **T** | **G** | **A** | **T** | **G** | **G** | **G** | **A** | **A** | **A** | **A** | **A** | **T** | **A** | **T** | **C** | **C** | **A** | **G** | **A** | **G** | **C** | **G** | **A** | **T** | **G** | **C** | **T** | **A** | **G** | **C** | **T** | **G** | **T** | **T** | **T** | **A** | **T** | **T** | **C** | **G** | **G** | **G** | **G** | **A** | **T** | **T** | **G** | **C** | **G** | **G** | **C** | **T** | **T** | **T** | **C** | **C** | **T** | **T** | **A** | **T** | **T** | **A** | **T** | **T** | **C** | **T** | **A** | **T** | **T** | **T** | **T** | **C** | **A** | **C** | **T** | **T** | **T** | **T** | **C** | **A** | **C** | **T** | **T** | **A** | **T** | **T** | **T** | **T** | **T** | **A** | **T** | **G** | **C** | **C** | **A** | **G** | **T** | **A** | **A** | **T** | **C** | **A** | **A** | **A** | **G** | **T** | **C** | **A** | **T** | **T** | **G** | **G** | **T** | **G** | **G** | **G** | **G** | **A** | **G** | **A** | **A** | **T** | **T** | **A** | **C** | **T** | **G** | **C** | **G** | **T** | **G** | **C** | **A** | **C** | **G** | **T** | **T** | **T** | **A** | **T** | **T** | **A** | **T** | **A** | **T** | **G** | **T** | **T** | **C** | **C** | **G** | **C** | **T** | **T** | **A** | **T** | **C** | **T** | **T** | **C** | **C** | **A** | **T** | **T** | **T** | **G** | **T** | **T** | **A** | **G** | **C** | **T** | **A** | **T** | **T** | **T** | **C** | **C** | **T** | **T** | **A** | **G** | **G** | **T** | **G** | **T** | **A** | **G** | **G** | **A** | **T** | **T** | **A** | **A** | **T** | **C** | **G** | **T** | **C** | **T** | **T** | **T** | **C** | **T** | **A** | **T** | **T** | **A** | **T** | **T** | **A** | **T** | **C** | **A** | **C** | **T** | **T** | **A** | **T** | **T** | **C** | **A** | **A** | **A** | **A** | **G** | **A** | **C** | **A** | **A** | **A** | **A** | **T** | **A** | **C** | **G** | **G** | **G** | **A** | **A** | **A** | **A** | **T** | **C** | **G** | **A** | **A** | **G** | **A** | **A** | **A** | **A** | **G** | **C** | **T** | **G** | **C** | **G** | **T** | **G** | **C** | **A** | **C** | **T** | **C** | **T** | **C** | **T** | **T** | **C** | **T** | **G** | **G** | **C** | **A** | **A** | **T** | **T** | **A** | **T** | **G** | **A** | **A** | **T** | **C** | **C** | **A** | **A** | **A** | **T** | **T** | **G** | **T** | **T** | **G** | **C** | **T** | **T** | **C** | **T** | **T** | **C** | **C** | **G** | **A** | **T** | **C** | **C** | **C** | **A** | **A** | **G** | **T** | **G** | **C** | **A** | **T** | **C** | **G** | **G** | **A** | **C** | **G** | **A** | **T** | **T** | **T** | **A** | **T** | **A** | **C** | **A** | **T** | **C** | **A** | **A** | **A** | **G** | **A** | **T** | **A** | **T** | **T** | **G** | **A** | **T** | **A** | **A** | **G** | **G** | **A** | **A** | **A** | **T** | **C** | **A** | **C** | **T** | **A** | **A** | **A** | **A** | **T** | **A** | **A** | **A** | **A** | **G** | **A** | **A** | **A** | **A** | **A** | **A** | **T** | **G** | **A** | **T** | **T** | **G** | **A** | **A** | **G** | **T** | **A** | **T** | **C** | **T** | **A** | **G** | **T** | **G** | **A** | **A** | **T** | **T** | **A** | **C** | **A** | **A** | **A** | **T** | **T** | **G** | **T** | **A** | **A** | **C** | **G** | **A** | **G** | **C** | **C** | **G** | **T** | **C** | **C** | **C** | **C** | **A** | **A** | **T** | **A** | **T** | **G** | **T** | **A** | **G** | **A** | **T** | **G** | **G** | **A** | **C** | **A** | **A** | **A** | **C** | **C** | **A** | **A** | **A** | **G** | **A** | **A** | **G** | **A** | **A** | **A** | **T** | **T** | **T** | **T** | **A** | **G** | **A** | **A** | **T** | **T** | **A** | **G** | **A** | **A** | **A** | **G** | **A** | **C** | **A** | **T** | **C** | **G** | **A** | **T** | **T** | **A** | **G** | **C** | **C** | **C** | **G** | **T** | **G** | **A** | **G** | **C** | **T** | **G** | **C** | **A** | **T** | **G** | **A** | **T** | **T** | **C** | **A** | **G** | **T** | **T** | **T** | **C** | **G** | **C** | **A** | **A** | **C** | **A** | **A** | **T** | **T** | **A** | **T** | **T** | **T** | **G** | **C** | **A** | **G** | **C** | **A** | **A** | **T** | **G** | **A** | **T** | **G** | **A** | **T** | **G** | **A** | **T** | **G** | **T** | **C** | **G** | **G** | **C** | **A** | **T** | **T** | **G** | **A** | **C** | **G** | **G** | **A** | **G** | **C** | **A** | **A** | **G** | **C** | **A** | **G** | **A** | **A** | **A** | **A** | **A** | **A** | **G** | **C** | **G** | **A** | **G** | **A** | **C** | **A** | **C** | **C** | **A** | **G** | **A** | **A** | **A** | **T** | **G** | **T** | **T** | **C** | **C** | **G** | **C** | **A** | **A** | **G** | **C** | **A** | **G** | **T** | **T** | **G** | **A** | **A** | **A** | **A** | **T** | **G** | **G** | **T** | **A** | **G** | **C** | **A** | **G** | **A** | **G** | **A** | **T** | **C** | **A** | **T** | **C** | **A** | **A** | **C** | **G** | **C** | **T** | **T** | **C** | **C** | **C** | **A** | **G** | **T** | **C** | **T** | **G** | **A** | **G** | **A** | **T** | **G** | **C** | **G** | **T** | **G** | **C** | **G** | **C** | **T** | **G** | **C** | **T** | **T** | **C** | **T** | **T** | **C** | **A** | **T** | **C** | **T** | **G** | **C** | **G** | **T** | **C** | **C** | **A** | **G** | **T** | **C** | **A** | **A** | **T** | **T** | **T** | **A** | **G** | **A** | **A** | **G** | **A** | **G** | **A** | **A** | **A** | **A** | **G** | **T** | **T** | **T** | **A** | **A** | **A** | **G** | **C** | **A** | **A** | **G** | **G** | **T** | **A** | **T** | **C** | **G** | **A** | **G** | **C** | **A** | **G** | **T** | **T** | **A** | **T** | **T** | **G** | **A** | **A** | **G** | **G** | **A** | **A** | **T** | **T** | **G** | **C** | **A** | **A** | **A** | **A** | **T** | **A** | **A** | **A** | **A** | **T** | **T** | **C** | **A** | **G** | **A** | **T** | **T** | **T** | **C** | **G** | **C** | **T** | **G** | **A** | **A** | **A** | **T** | **G** | **G** | **G** | **A** | **T** | **G** | **T** | **A** | **G** | **A** | **A** | **G** | **A** | **T** | **G** | **T** | **A** | **A** | **A** | **A** | **C** | **T** | **A** | **T** | **C** | **T** | **A** | **G** | **C** | **T** | **C** | **C** | **A** | **T** | **T** | **G** | **A** | **G** | **G** | **A** | **T** | **C** | **A** | **C** | **T** | **T** | **G** | **T** | **T** | **C** | **C** | **G** | **A** | **A** | **T** | **C** | **G** | **T** | **T** | **C** | **A** | **A** | **G** | **A** | **A** | **T** | **T** | **G** | **T** | **T** | **A** | **T** | **C** | **A** | **A** | **A** | **T** | **A** | **C** | **A** | **T** | **T** | **A** | **A** | **G** | **A** | **C** | **A** | **T** | **G** | **C** | **C** | **A** | **A** | **A** | **G** | **C** | **C** | **A** | **A** | **T** | **G** | **A** | **A** | **T** | **T** | **A** | **G** | **A** | **G** | **G** | **T** | **A** | **T** | **A** | **T** | **T** | **T** | **G** | **C** | **A** | **T** | **A** | **A** | **A** | **A** | **T** | **A** | **G** | **A** | **C** | **A** | **A** | **T** | **A** | **A** | **T** | **C** | **T** | **T** | **T** | **T** | **A** | **T** | **T** | **A** | **C** | **G** | **T** | **A** | **T** | **T** | **A** | **T** | **T** | **G** | **A** | **C** | **G** | **A** | **T** | **G** | **G** | **A** | **A** | **C** | **A** | **G** | **G** | **G** | **T** | **T** | **T** | **A** | **A** | **T** | **A** | **T** | **G** | **A** | **A** | **T** | **G** | **A** | **A** | **A** | **C** | **G** | **A** | **A** | **A** | **A** | **C** | **G** | **G** | **G** | **A** | **A** | **G** | **T** | **T** | **A** | **T** | **G** | **G** | **A** | **T** | **T** | **G** | **A** | **A** | **C** | **A** | **A** | **T** | **A** | **T** | **C** | **A** | **A** | **A** | **G** | **A** | **A** | **C** | **G** | **A** | **G** | **T** | **A** | **G** | **C** | **T** | **G** | **G** | **T** | **A** | **T** | **T** | **G** | **G** | **T** | **G** | **G** | **A** | **A** | **C** | **A** | **G** | **T** | **A** | **A** | **A** | **A** | **A** | **T** | **C** | **A** | **T** | **T** | **A** | **G** | **T** | **T** | **T** | **T** | **A** | **A** | **A** | **G** | **G** | **G** | **C** | **A** | **A** | **G** | **G** | **T** | **A** | **C** | **A** | **A** | **G** | **T** | **G** | **T** | **A** | **G** | **A** | **A** | **A** | **T** | **C** | **A** | **A** | **G** | **G** | **T** | **C** | **C** | **C** | **T** | **T** | **T** | **G** | **A** | **T** | **G** | **A** | **A** | **G** | **G** | **A** | **G** | **G** | **C** | **A** | **-** | **-** | **-** |
| **N59947F** | **A** | **T** | **G** | **A** | **T** | **G** | **G** | **G** | **A** | **A** | **A** | **A** | **A** | **T** | **A** | **T** | **C** | **C** | **A** | **G** | **A** | **G** | **C** | **G** | **A** | **T** | **G** | **C** | **T** | **A** | **G** | **C** | **T** | **G** | **T** | **T** | **T** | **A** | **T** | **T** | **C** | **G** | **G** | **G** | **G** | **A** | **T** | **T** | **G** | **C** | **G** | **G** | **C** | **T** | **T** | **T** | **C** | **C** | **T** | **T** | **A** | **T** | **T** | **A** | **T** | **T** | **C** | **T** | **A** | **T** | **T** | **T** | **T** | **C** | **A** | **C** | **T** | **T** | **T** | **T** | **C** | **A** | **C** | **T** | **T** | **A** | **T** | **T** | **T** | **T** | **T** | **A** | **T** | **G** | **C** | **C** | **A** | **G** | **T** | **A** | **A** | **T** | **C** | **A** | **A** | **A** | **G** | **T** | **C** | **A** | **T** | **T** | **G** | **G** | **T** | **G** | **G** | **G** | **G** | **A** | **G** | **A** | **A** | **T** | **T** | **A** | **C** | **T** | **G** | **C** | **G** | **T** | **G** | **C** | **A** | **C** | **G** | **T** | **T** | **T** | **A** | **T** | **T** | **A** | **T** | **A** | **T** | **G** | **T** | **T** | **C** | **C** | **G** | **C** | **T** | **T** | **A** | **T** | **C** | **T** | **T** | **C** | **C** | **A** | **T** | **T** | **T** | **G** | **T** | **T** | **A** | **G** | **C** | **T** | **A** | **T** | **T** | **T** | **C** | **C** | **T** | **T** | **A** | **G** | **G** | **T** | **G** | **T** | **A** | **G** | **G** | **A** | **T** | **T** | **A** | **A** | **T** | **C** | **G** | **T** | **C** | **T** | **T** | **T** | **C** | **T** | **A** | **T** | **T** | **A** | **T** | **T** | **A** | **T** | **C** | **A** | **C** | **T** | **T** | **A** | **T** | **T** | **C** | **A** | **A** | **A** | **A** | **G** | **A** | **C** | **A** | **A** | **A** | **A** | **T** | **A** | **C** | **G** | **G** | **G** | **A** | **A** | **A** | **A** | **T** | **C** | **G** | **A** | **A** | **G** | **A** | **A** | **A** | **A** | **G** | **C** | **T** | **G** | **C** | **G** | **T** | **G** | **C** | **A** | **C** | **T** | **C** | **T** | **C** | **T** | **T** | **C** | **T** | **G** | **G** | **C** | **A** | **A** | **T** | **T** | **A** | **T** | **G** | **A** | **A** | **T** | **C** | **C** | **A** | **A** | **A** | **T** | **T** | **G** | **T** | **T** | **G** | **C** | **T** | **T** | **C** | **T** | **T** | **C** | **C** | **G** | **A** | **T** | **C** | **C** | **C** | **A** | **A** | **G** | **T** | **G** | **C** | **A** | **T** | **C** | **G** | **G** | **A** | **C** | **G** | **A** | **T** | **T** | **T** | **A** | **T** | **A** | **C** | **A** | **T** | **C** | **A** | **A** | **A** | **G** | **A** | **T** | **A** | **T** | **T** | **G** | **A** | **T** | **A** | **A** | **G** | **G** | **A** | **A** | **A** | **T** | **C** | **A** | **C** | **T** | **A** | **A** | **A** | **A** | **T** | **A** | **A** | **A** | **A** | **G** | **A** | **A** | **A** | **A** | **A** | **A** | **T** | **G** | **A** | **T** | **T** | **G** | **A** | **A** | **G** | **T** | **A** | **T** | **C** | **T** | **A** | **G** | **T** | **G** | **A** | **A** | **T** | **T** | **A** | **C** | **A** | **A** | **A** | **T** | **T** | **G** | **T** | **A** | **A** | **C** | **G** | **A** | **G** | **C** | **C** | **G** | **T** | **C** | **C** | **C** | **C** | **A** | **A** | **T** | **A** | **T** | **G** | **T** | **A** | **G** | **A** | **T** | **G** | **G** | **A** | **C** | **A** | **A** | **A** | **C** | **C** | **A** | **A** | **A** | **G** | **A** | **A** | **G** | **A** | **A** | **A** | **T** | **T** | **T** | **T** | **A** | **G** | **A** | **A** | **T** | **T** | **A** | **G** | **A** | **A** | **A** | **G** | **A** | **C** | **A** | **T** | **C** | **G** | **A** | **T** | **T** | **A** | **G** | **C** | **C** | **C** | **G** | **T** | **G** | **A** | **G** | **C** | **T** | **G** | **C** | **A** | **T** | **G** | **A** | **T** | **T** | **C** | **A** | **G** | **T** | **T** | **T** | **C** | **G** | **C** | **A** | **A** | **C** | **A** | **A** | **T** | **T** | **G** | **T** | **T** | **T** | **G** | **C** | **A** | **G** | **C** | **A** | **A** | **T** | **G** | **A** | **T** | **G** | **A** | **T** | **G** | **A** | **T** | **G** | **T** | **C** | **G** | **G** | **C** | **A** | **T** | **T** | **A** | **A** | **C** | **G** | **G** | **A** | **G** | **C** | **A** | **A** | **G** | **C** | **A** | **G** | **A** | **A** | **A** | **A** | **A** | **A** | **G** | **C** | **G** | **A** | **G** | **A** | **C** | **A** | **C** | **C** | **A** | **G** | **A** | **A** | **A** | **T** | **G** | **T** | **T** | **C** | **C** | **G** | **C** | **A** | **A** | **G** | **C** | **A** | **G** | **T** | **T** | **G** | **A** | **A** | **A** | **A** | **T** | **G** | **G** | **T** | **A** | **G** | **C** | **A** | **G** | **A** | **G** | **A** | **T** | **C** | **A** | **T** | **C** | **A** | **A** | **C** | **G** | **C** | **T** | **T** | **C** | **C** | **C** | **A** | **G** | **T** | **C** | **T** | **G** | **A** | **G** | **A** | **T** | **G** | **C** | **G** | **T** | **G** | **C** | **G** | **C** | **T** | **G** | **C** | **T** | **T** | **C** | **T** | **T** | **C** | **A** | **T** | **C** | **T** | **G** | **C** | **G** | **T** | **C** | **C** | **A** | **G** | **T** | **C** | **A** | **A** | **T** | **T** | **T** | **A** | **G** | **A** | **A** | **G** | **A** | **G** | **A** | **A** | **A** | **A** | **G** | **T** | **T** | **T** | **A** | **A** | **A** | **G** | **C** | **A** | **A** | **G** | **G** | **T** | **A** | **T** | **C** | **G** | **A** | **G** | **C** | **A** | **G** | **T** | **T** | **A** | **T** | **T** | **G** | **A** | **A** | **G** | **G** | **A** | **A** | **T** | **T** | **G** | **C** | **A** | **A** | **A** | **A** | **T** | **A** | **A** | **A** | **A** | **T** | **T** | **C** | **A** | **G** | **A** | **T** | **T** | **T** | **C** | **G** | **C** | **T** | **G** | **A** | **A** | **A** | **T** | **G** | **G** | **G** | **A** | **T** | **G** | **T** | **A** | **G** | **A** | **A** | **G** | **A** | **T** | **G** | **T** | **A** | **A** | **A** | **A** | **C** | **T** | **A** | **T** | **C** | **T** | **A** | **G** | **C** | **T** | **C** | **C** | **A** | **T** | **T** | **G** | **A** | **G** | **G** | **A** | **T** | **C** | **A** | **C** | **T** | **T** | **G** | **T** | **T** | **C** | **C** | **G** | **A** | **A** | **T** | **C** | **G** | **T** | **T** | **C** | **A** | **A** | **G** | **A** | **A** | **T** | **T** | **G** | **T** | **T** | **A** | **T** | **C** | **A** | **A** | **A** | **T** | **A** | **C** | **A** | **T** | **T** | **A** | **A** | **G** | **A** | **C** | **A** | **T** | **G** | **C** | **C** | **A** | **A** | **A** | **G** | **C** | **C** | **A** | **A** | **T** | **G** | **A** | **A** | **T** | **T** | **A** | **G** | **A** | **G** | **G** | **T** | **A** | **T** | **A** | **T** | **T** | **T** | **G** | **C** | **A** | **T** | **A** | **A** | **A** | **A** | **T** | **A** | **G** | **A** | **C** | **A** | **A** | **T** | **A** | **A** | **T** | **C** | **T** | **T** | **T** | **T** | **A** | **T** | **T** | **A** | **C** | **G** | **T** | **A** | **T** | **T** | **A** | **T** | **T** | **G** | **A** | **C** | **G** | **A** | **T** | **G** | **G** | **A** | **A** | **C** | **A** | **G** | **G** | **G** | **T** | **T** | **T** | **A** | **A** | **T** | **A** | **T** | **G** | **A** | **A** | **T** | **G** | **A** | **A** | **A** | **C** | **G** | **A** | **A** | **A** | **A** | **C** | **G** | **G** | **G** | **A** | **A** | **G** | **T** | **T** | **A** | **T** | **G** | **G** | **A** | **T** | **T** | **G** | **A** | **A** | **C** | **A** | **A** | **T** | **A** | **T** | **C** | **A** | **A** | **A** | **G** | **A** | **A** | **C** | **G** | **A** | **G** | **T** | **A** | **G** | **C** | **T** | **G** | **G** | **T** | **A** | **T** | **T** | **G** | **G** | **T** | **G** | **G** | **A** | **A** | **C** | **A** | **G** | **T** | **A** | **A** | **A** | **A** | **A** | **T** | **C** | **A** | **T** | **T** | **A** | **G** | **T** | **T** | **T** | **T** | **A** | **A** | **A** | **G** | **G** | **G** | **C** | **A** | **A** | **G** | **G** | **T** | **A** | **C** | **A** | **A** | **G** | **T** | **G** | **T** | **A** | **G** | **A** | **A** | **A** | **T** | **C** | **A** | **A** | **G** | **G** | **T** | **C** | **C** | **C** | **T** | **T** | **T** | **G** | **A** | **T** | **G** | **A** | **A** | **G** | **G** | **A** | **G** | **G** | **C** | **A** | **-** | **-** | **-** |
| **N59951F** | **A** | **T** | **G** | **A** | **T** | **G** | **G** | **G** | **A** | **A** | **G** | **A** | **A** | **T** | **A** | **T** | **C** | **C** | **A** | **A** | **A** | **G** | **C** | **A** | **A** | **T** | **G** | **C** | **T** | **A** | **G** | **C** | **T** | **G** | **T** | **T** | **T** | **A** | **T** | **T** | **C** | **G** | **G** | **G** | **G** | **A** | **T** | **T** | **G** | **C** | **G** | **G** | **C** | **T** | **T** | **T** | **C** | **C** | **T** | **T** | **A** | **T** | **T** | **A** | **T** | **T** | **C** | **T** | **A** | **T** | **T** | **T** | **T** | **C** | **A** | **C** | **T** | **T** | **T** | **T** | **C** | **A** | **C** | **T** | **T** | **A** | **T** | **T** | **T** | **T** | **T** | **A** | **T** | **G** | **C** | **C** | **A** | **G** | **T** | **A** | **A** | **C** | **C** | **A** | **A** | **A** | **G** | **T** | **C** | **A** | **T** | **T** | **G** | **G** | **T** | **G** | **G** | **G** | **G** | **A** | **G** | **A** | **A** | **T** | **T** | **G** | **C** | **T** | **G** | **C** | **G** | **T** | **G** | **C** | **G** | **C** | **G** | **T** | **T** | **T** | **A** | **T** | **T** | **A** | **T** | **A** | **C** | **G** | **T** | **T** | **C** | **C** | **G** | **C** | **T** | **T** | **A** | **T** | **C** | **T** | **T** | **C** | **C** | **A** | **T** | **T** | **T** | **G** | **A** | **T** | **G** | **G** | **C** | **A** | **A** | **T** | **T** | **T** | **C** | **A** | **T** | **T** | **A** | **G** | **G** | **T** | **G** | **T** | **A** | **G** | **G** | **A** | **T** | **T** | **G** | **A** | **T** | **C** | **G** | **T** | **A** | **T** | **T** | **T** | **C** | **T** | **A** | **T** | **T** | **G** | **T** | **T** | **A** | **T** | **C** | **A** | **C** | **T** | **T** | **A** | **T** | **T** | **C** | **A** | **A** | **A** | **A** | **G** | **G** | **C** | **A** | **A** | **A** | **A** | **T** | **A** | **C** | **G** | **G** | **G** | **A** | **A** | **A** | **A** | **T** | **C** | **G** | **A** | **A** | **G** | **A** | **A** | **A** | **A** | **G** | **C** | **T** | **T** | **C** | **G** | **T** | **G** | **C** | **A** | **C** | **T** | **T** | **T** | **C** | **T** | **T** | **C** | **T** | **G** | **G** | **C** | **A** | **A** | **T** | **T** | **A** | **T** | **G** | **A** | **A** | **T** | **C** | **C** | **A** | **A** | **A** | **C** | **T** | **T** | **T** | **T** | **G** | **C** | **A** | **T** | **C** | **A** | **G** | **C** | **C** | **G** | **A** | **T** | **C** | **C** | **C** | **A** | **A** | **G** | **C** | **A** | **C** | **G** | **T** | **C** | **G** | **G** | **A** | **T** | **G** | **A** | **T** | **C** | **T** | **G** | **T** | **A** | **C** | **A** | **T** | **C** | **A** | **A** | **A** | **G** | **A** | **T** | **A** | **T** | **T** | **G** | **A** | **T** | **A** | **T** | **G** | **G** | **A** | **A** | **A** | **T** | **T** | **A** | **C** | **T** | **A** | **A** | **A** | **A** | **T** | **A** | **A** | **A** | **A** | **G** | **A** | **A** | **A** | **A** | **A** | **A** | **T** | **G** | **A** | **T** | **C** | **G** | **A** | **A** | **G** | **T** | **A** | **T** | **C** | **C** | **A** | **G** | **T** | **G** | **A** | **A** | **T** | **T** | **A** | **C** | **A** | **G** | **A** | **T** | **T** | **A** | **T** | **G** | **A** | **C** | **T** | **A** | **G** | **C** | **C** | **G** | **T** | **C** | **C** | **T** | **C** | **A** | **A** | **T** | **A** | **T** | **G** | **T** | **A** | **G** | **A** | **T** | **G** | **G** | **A** | **C** | **A** | **A** | **A** | **C** | **C** | **A** | **A** | **A** | **G** | **A** | **A** | **G** | **A** | **G** | **A** | **T** | **T** | **T** | **T** | **A** | **G** | **A** | **A** | **T** | **T** | **A** | **G** | **A** | **A** | **A** | **G** | **G** | **C** | **A** | **T** | **C** | **G** | **A** | **T** | **T** | **A** | **G** | **C** | **T** | **C** | **G** | **T** | **G** | **A** | **G** | **C** | **T** | **T** | **C** | **A** | **T** | **G** | **A** | **T** | **T** | **C** | **A** | **G** | **T** | **T** | **T** | **C** | **A** | **C** | **A** | **G** | **C** | **A** | **A** | **T** | **T** | **A** | **T** | **T** | **T** | **G** | **C** | **A** | **G** | **C** | **G** | **A** | **T** | **G** | **A** | **T** | **G** | **A** | **T** | **G** | **A** | **T** | **G** | **T** | **C** | **G** | **G** | **C** | **A** | **T** | **T** | **G** | **A** | **C** | **G** | **G** | **A** | **G** | **C** | **A** | **A** | **G** | **C** | **A** | **G** | **A** | **A** | **A** | **A** | **A** | **A** | **G** | **T** | **G** | **A** | **G** | **A** | **C** | **A** | **C** | **C** | **A** | **G** | **A** | **A** | **A** | **T** | **G** | **T** | **T** | **C** | **C** | **G** | **T** | **A** | **A** | **G** | **C** | **A** | **G** | **T** | **T** | **G** | **A** | **A** | **A** | **A** | **T** | **G** | **G** | **T** | **G** | **G** | **C** | **A** | **G** | **A** | **G** | **A** | **T** | **C** | **A** | **T** | **C** | **A** | **A** | **C** | **G** | **C** | **T** | **T** | **C** | **C** | **C** | **A** | **G** | **T** | **C** | **T** | **G** | **A** | **G** | **A** | **T** | **G** | **C** | **G** | **T** | **G** | **C** | **G** | **C** | **T** | **G** | **C** | **T** | **T** | **C** | **T** | **T** | **C** | **A** | **T** | **C** | **T** | **G** | **C** | **G** | **T** | **C** | **C** | **A** | **G** | **T** | **C** | **A** | **A** | **T** | **T** | **T** | **A** | **G** | **A** | **A** | **G** | **A** | **A** | **A** | **A** | **A** | **A** | **G** | **T** | **T** | **T** | **A** | **A** | **A** | **A** | **C** | **A** | **A** | **G** | **G** | **C** | **A** | **T** | **T** | **G** | **A** | **G** | **C** | **A** | **G** | **T** | **T** | **A** | **T** | **T** | **G** | **A** | **A** | **G** | **G** | **A** | **A** | **T** | **T** | **G** | **C** | **A** | **G** | **A** | **A** | **T** | **A** | **A** | **A** | **A** | **T** | **C** | **C** | **A** | **G** | **A** | **T** | **T** | **T** | **C** | **G** | **C** | **T** | **A** | **A** | **A** | **A** | **T** | **G** | **G** | **G** | **A** | **T** | **G** | **T** | **A** | **G** | **A** | **A** | **G** | **A** | **T** | **G** | **T** | **A** | **A** | **A** | **A** | **T** | **T** | **A** | **A** | **C** | **T** | **A** | **G** | **T** | **T** | **C** | **C** | **A** | **T** | **T** | **G** | **A** | **G** | **G** | **A** | **T** | **C** | **A** | **C** | **T** | **T** | **G** | **T** | **T** | **C** | **C** | **G** | **A** | **A** | **T** | **C** | **G** | **T** | **T** | **C** | **A** | **A** | **G** | **A** | **A** | **T** | **T** | **G** | **T** | **T** | **A** | **T** | **C** | **A** | **A** | **A** | **C** | **A** | **C** | **A** | **T** | **T** | **A** | **A** | **G** | **A** | **C** | **A** | **T** | **G** | **C** | **T** | **A** | **A** | **G** | **G** | **C** | **T** | **A** | **A** | **T** | **G** | **A** | **A** | **T** | **T** | **A** | **G** | **A** | **A** | **G** | **T** | **C** | **T** | **A** | **T** | **T** | **T** | **G** | **C** | **A** | **T** | **A** | **A** | **A** | **A** | **T** | **A** | **G** | **A** | **T** | **A** | **A** | **C** | **A** | **A** | **T** | **C** | **T** | **T** | **T** | **T** | **A** | **T** | **T** | **A** | **C** | **G** | **T** | **A** | **T** | **C** | **A** | **T** | **T** | **G** | **A** | **C** | **G** | **A** | **C** | **G** | **G** | **G** | **A** | **C** | **A** | **G** | **G** | **A** | **T** | **T** | **T** | **A** | **A** | **T** | **A** | **T** | **G** | **A** | **A** | **T** | **G** | **A** | **A** | **A** | **C** | **C** | **A** | **A** | **A** | **A** | **C** | **G** | **G** | **G** | **A** | **A** | **G** | **T** | **T** | **A** | **T** | **G** | **G** | **A** | **T** | **T** | **G** | **A** | **A** | **C** | **A** | **A** | **T** | **A** | **T** | **C** | **A** | **A** | **A** | **G** | **A** | **A** | **C** | **G** | **A** | **G** | **T** | **A** | **G** | **C** | **T** | **G** | **G** | **T** | **A** | **T** | **T** | **G** | **G** | **T** | **G** | **G** | **A** | **A** | **C** | **A** | **G** | **T** | **A** | **A** | **A** | **A** | **A** | **T** | **C** | **A** | **T** | **T** | **A** | **G** | **T** | **T** | **T** | **T** | **A** | **A** | **A** | **G** | **G** | **G** | **C** | **A** | **A** | **G** | **G** | **T** | **A** | **C** | **G** | **A** | **G** | **C** | **G** | **T** | **G** | **G** | **A** | **G** | **A** | **T** | **C** | **A** | **A** | **G** | **G** | **T** | **C** | **C** | **C** | **T** | **T** | **T** | **G** | **A** | **T** | **G** | **A** | **A** | **G** | **G** | **A** | **G** | **G** | **C** | **A** | **-** | **-** | **-** |
| **N59439F** | **A** | **T** | **G** | **A** | **T** | **G** | **G** | **G** | **A** | **A** | **G** | **A** | **A** | **T** | **A** | **T** | **C** | **C** | **A** | **A** | **A** | **G** | **C** | **A** | **A** | **T** | **G** | **C** | **T** | **A** | **G** | **C** | **T** | **G** | **T** | **T** | **T** | **A** | **T** | **T** | **C** | **G** | **G** | **G** | **G** | **A** | **T** | **T** | **G** | **C** | **G** | **G** | **C** | **T** | **T** | **T** | **C** | **C** | **T** | **T** | **A** | **T** | **T** | **A** | **T** | **T** | **C** | **T** | **A** | **T** | **T** | **T** | **T** | **C** | **A** | **C** | **T** | **T** | **T** | **T** | **C** | **A** | **C** | **T** | **T** | **A** | **T** | **T** | **T** | **T** | **T** | **A** | **T** | **G** | **C** | **C** | **A** | **G** | **T** | **A** | **A** | **C** | **C** | **A** | **A** | **A** | **G** | **T** | **C** | **A** | **T** | **T** | **G** | **G** | **T** | **G** | **G** | **G** | **G** | **A** | **G** | **A** | **A** | **T** | **T** | **G** | **C** | **T** | **G** | **C** | **G** | **T** | **G** | **C** | **G** | **C** | **G** | **T** | **T** | **T** | **A** | **T** | **T** | **A** | **T** | **A** | **C** | **G** | **T** | **T** | **C** | **C** | **G** | **C** | **T** | **T** | **A** | **T** | **C** | **T** | **T** | **C** | **C** | **A** | **T** | **T** | **T** | **G** | **A** | **T** | **G** | **G** | **C** | **A** | **A** | **T** | **T** | **T** | **C** | **A** | **T** | **T** | **A** | **G** | **G** | **T** | **G** | **T** | **A** | **G** | **G** | **A** | **T** | **T** | **G** | **A** | **T** | **C** | **G** | **T** | **A** | **T** | **T** | **T** | **C** | **T** | **A** | **T** | **T** | **G** | **T** | **T** | **A** | **T** | **C** | **A** | **C** | **T** | **T** | **A** | **T** | **T** | **C** | **A** | **A** | **A** | **A** | **G** | **G** | **C** | **A** | **A** | **A** | **A** | **T** | **A** | **C** | **G** | **G** | **G** | **A** | **A** | **A** | **A** | **T** | **C** | **G** | **A** | **A** | **G** | **A** | **A** | **A** | **A** | **G** | **C** | **T** | **T** | **C** | **G** | **T** | **G** | **C** | **A** | **C** | **T** | **T** | **T** | **C** | **T** | **T** | **C** | **T** | **G** | **G** | **C** | **A** | **A** | **T** | **T** | **A** | **T** | **G** | **A** | **A** | **T** | **C** | **C** | **A** | **A** | **A** | **C** | **T** | **T** | **T** | **T** | **G** | **C** | **A** | **T** | **C** | **A** | **G** | **C** | **C** | **G** | **A** | **T** | **C** | **C** | **C** | **A** | **A** | **G** | **C** | **G** | **C** | **G** | **T** | **C** | **G** | **G** | **A** | **T** | **G** | **A** | **T** | **C** | **T** | **G** | **T** | **A** | **C** | **A** | **T** | **C** | **A** | **A** | **A** | **G** | **A** | **T** | **A** | **T** | **T** | **G** | **A** | **T** | **A** | **T** | **G** | **G** | **A** | **A** | **A** | **T** | **T** | **A** | **C** | **T** | **A** | **A** | **A** | **A** | **T** | **A** | **A** | **A** | **A** | **G** | **A** | **A** | **A** | **A** | **A** | **A** | **T** | **G** | **A** | **T** | **C** | **G** | **A** | **A** | **G** | **T** | **A** | **T** | **C** | **C** | **A** | **G** | **T** | **G** | **A** | **A** | **T** | **T** | **A** | **C** | **A** | **G** | **A** | **T** | **T** | **A** | **T** | **G** | **A** | **C** | **T** | **A** | **G** | **C** | **C** | **G** | **T** | **C** | **C** | **T** | **C** | **A** | **A** | **T** | **A** | **T** | **G** | **T** | **A** | **G** | **A** | **T** | **G** | **G** | **A** | **C** | **A** | **A** | **A** | **C** | **C** | **A** | **A** | **A** | **G** | **A** | **A** | **G** | **A** | **G** | **A** | **T** | **T** | **T** | **T** | **A** | **G** | **A** | **A** | **T** | **T** | **A** | **G** | **A** | **A** | **A** | **G** | **G** | **C** | **A** | **T** | **C** | **G** | **A** | **T** | **T** | **A** | **G** | **C** | **T** | **C** | **G** | **T** | **G** | **A** | **G** | **C** | **T** | **T** | **C** | **A** | **T** | **G** | **A** | **T** | **T** | **C** | **A** | **G** | **T** | **T** | **T** | **C** | **A** | **C** | **A** | **G** | **C** | **A** | **A** | **T** | **T** | **A** | **T** | **T** | **T** | **G** | **C** | **A** | **G** | **C** | **G** | **A** | **T** | **G** | **A** | **T** | **G** | **A** | **T** | **G** | **A** | **T** | **G** | **T** | **C** | **G** | **G** | **C** | **A** | **T** | **T** | **G** | **A** | **C** | **G** | **G** | **A** | **G** | **C** | **A** | **A** | **G** | **C** | **A** | **G** | **A** | **A** | **A** | **A** | **A** | **A** | **G** | **T** | **G** | **A** | **G** | **A** | **C** | **A** | **C** | **C** | **A** | **G** | **A** | **A** | **A** | **T** | **G** | **T** | **T** | **C** | **C** | **G** | **T** | **A** | **A** | **G** | **C** | **A** | **G** | **T** | **T** | **G** | **A** | **A** | **A** | **A** | **T** | **G** | **G** | **T** | **G** | **G** | **C** | **A** | **G** | **A** | **G** | **A** | **T** | **C** | **A** | **T** | **C** | **A** | **A** | **C** | **G** | **C** | **T** | **T** | **C** | **C** | **C** | **A** | **G** | **T** | **C** | **T** | **G** | **A** | **G** | **A** | **T** | **G** | **C** | **G** | **T** | **G** | **C** | **G** | **C** | **T** | **G** | **C** | **T** | **T** | **C** | **T** | **T** | **C** | **A** | **T** | **C** | **T** | **G** | **C** | **G** | **T** | **C** | **C** | **A** | **G** | **T** | **C** | **A** | **A** | **T** | **T** | **T** | **A** | **G** | **A** | **A** | **G** | **A** | **A** | **A** | **A** | **A** | **A** | **G** | **T** | **T** | **T** | **A** | **A** | **A** | **A** | **C** | **A** | **A** | **G** | **G** | **C** | **A** | **T** | **T** | **G** | **A** | **G** | **C** | **A** | **G** | **T** | **T** | **A** | **T** | **T** | **G** | **A** | **A** | **G** | **G** | **A** | **A** | **T** | **T** | **G** | **C** | **A** | **G** | **A** | **A** | **T** | **A** | **A** | **A** | **A** | **T** | **C** | **C** | **A** | **G** | **A** | **T** | **T** | **T** | **C** | **G** | **C** | **T** | **A** | **A** | **A** | **A** | **T** | **G** | **G** | **G** | **A** | **T** | **G** | **T** | **A** | **G** | **A** | **A** | **G** | **A** | **T** | **G** | **T** | **A** | **A** | **A** | **A** | **T** | **T** | **A** | **A** | **C** | **T** | **A** | **G** | **T** | **T** | **C** | **C** | **A** | **T** | **T** | **G** | **A** | **G** | **G** | **A** | **T** | **C** | **A** | **C** | **T** | **T** | **G** | **T** | **T** | **C** | **C** | **G** | **A** | **A** | **T** | **C** | **G** | **T** | **T** | **C** | **A** | **A** | **G** | **A** | **A** | **T** | **T** | **G** | **T** | **T** | **A** | **T** | **C** | **A** | **A** | **A** | **C** | **A** | **C** | **A** | **T** | **T** | **A** | **A** | **G** | **A** | **C** | **A** | **T** | **G** | **C** | **T** | **A** | **A** | **G** | **G** | **C** | **T** | **A** | **A** | **T** | **G** | **A** | **A** | **T** | **T** | **A** | **G** | **A** | **A** | **G** | **T** | **C** | **T** | **A** | **T** | **T** | **T** | **G** | **C** | **A** | **T** | **A** | **A** | **A** | **A** | **T** | **A** | **G** | **A** | **T** | **A** | **A** | **C** | **A** | **A** | **T** | **C** | **T** | **T** | **T** | **T** | **A** | **T** | **T** | **A** | **C** | **G** | **T** | **A** | **T** | **C** | **A** | **T** | **T** | **G** | **A** | **C** | **G** | **A** | **C** | **G** | **G** | **G** | **A** | **C** | **A** | **G** | **G** | **A** | **T** | **T** | **T** | **A** | **A** | **T** | **A** | **T** | **G** | **A** | **A** | **T** | **G** | **A** | **A** | **A** | **C** | **C** | **A** | **A** | **A** | **A** | **C** | **G** | **G** | **G** | **A** | **A** | **G** | **T** | **T** | **A** | **T** | **G** | **G** | **A** | **T** | **T** | **G** | **A** | **A** | **C** | **A** | **A** | **T** | **A** | **T** | **C** | **A** | **A** | **A** | **G** | **A** | **A** | **C** | **G** | **A** | **G** | **T** | **A** | **G** | **C** | **T** | **G** | **G** | **T** | **A** | **T** | **T** | **G** | **G** | **T** | **G** | **G** | **A** | **A** | **C** | **A** | **G** | **T** | **A** | **A** | **A** | **A** | **A** | **T** | **C** | **A** | **T** | **T** | **A** | **G** | **T** | **T** | **T** | **T** | **A** | **A** | **A** | **G** | **G** | **G** | **C** | **A** | **A** | **G** | **G** | **T** | **A** | **C** | **G** | **A** | **G** | **C** | **G** | **T** | **G** | **G** | **A** | **G** | **A** | **T** | **C** | **A** | **A** | **G** | **G** | **T** | **C** | **C** | **C** | **T** | **T** | **T** | **G** | **A** | **T** | **G** | **A** | **A** | **G** | **G** | **A** | **G** | **G** | **C** | **A** | **-** | **-** | **-** |
| **N55655** | **A** | **T** | **G** | **A** | **T** | **G** | **G** | **G** | **A** | **A** | **G** | **A** | **A** | **T** | **A** | **T** | **C** | **C** | **A** | **A** | **A** | **G** | **C** | **A** | **A** | **T** | **G** | **C** | **T** | **A** | **G** | **C** | **T** | **G** | **T** | **T** | **T** | **A** | **T** | **T** | **C** | **G** | **G** | **G** | **G** | **A** | **T** | **T** | **G** | **C** | **G** | **G** | **C** | **T** | **T** | **T** | **C** | **C** | **T** | **T** | **A** | **T** | **T** | **A** | **T** | **T** | **C** | **T** | **A** | **T** | **T** | **T** | **T** | **C** | **A** | **C** | **T** | **T** | **T** | **T** | **C** | **A** | **C** | **T** | **T** | **A** | **T** | **T** | **T** | **T** | **T** | **A** | **T** | **G** | **C** | **C** | **A** | **G** | **T** | **A** | **A** | **C** | **C** | **A** | **A** | **A** | **G** | **G** | **C** | **A** | **T** | **T** | **G** | **G** | **T** | **G** | **G** | **G** | **G** | **A** | **G** | **A** | **A** | **T** | **T** | **G** | **C** | **T** | **G** | **C** | **G** | **T** | **G** | **C** | **G** | **C** | **G** | **T** | **T** | **T** | **A** | **T** | **T** | **A** | **T** | **A** | **C** | **G** | **T** | **T** | **C** | **C** | **G** | **C** | **T** | **T** | **A** | **T** | **C** | **T** | **T** | **C** | **C** | **A** | **T** | **T** | **T** | **G** | **A** | **T** | **G** | **G** | **C** | **A** | **A** | **T** | **T** | **T** | **C** | **A** | **T** | **T** | **A** | **G** | **G** | **T** | **G** | **T** | **A** | **G** | **G** | **A** | **T** | **T** | **G** | **A** | **T** | **C** | **G** | **T** | **A** | **T** | **T** | **T** | **C** | **T** | **A** | **T** | **T** | **G** | **T** | **T** | **A** | **T** | **C** | **A** | **C** | **T** | **T** | **A** | **T** | **T** | **C** | **A** | **A** | **A** | **A** | **G** | **G** | **C** | **A** | **A** | **A** | **A** | **T** | **A** | **C** | **G** | **G** | **G** | **A** | **A** | **A** | **A** | **T** | **C** | **G** | **A** | **A** | **G** | **A** | **A** | **A** | **A** | **G** | **C** | **T** | **T** | **C** | **G** | **T** | **G** | **C** | **A** | **C** | **T** | **T** | **T** | **C** | **T** | **T** | **C** | **T** | **G** | **G** | **C** | **A** | **A** | **T** | **T** | **A** | **T** | **G** | **A** | **A** | **T** | **C** | **C** | **A** | **A** | **A** | **C** | **T** | **T** | **T** | **T** | **G** | **C** | **A** | **T** | **C** | **A** | **G** | **C** | **C** | **G** | **A** | **T** | **C** | **C** | **C** | **A** | **A** | **G** | **C** | **G** | **C** | **G** | **T** | **C** | **G** | **G** | **A** | **T** | **G** | **A** | **T** | **C** | **T** | **G** | **T** | **A** | **C** | **A** | **T** | **C** | **A** | **A** | **A** | **G** | **A** | **T** | **A** | **T** | **T** | **G** | **A** | **T** | **A** | **T** | **G** | **G** | **A** | **A** | **A** | **T** | **T** | **A** | **C** | **T** | **A** | **A** | **A** | **A** | **T** | **A** | **A** | **A** | **A** | **G** | **A** | **A** | **A** | **A** | **A** | **A** | **T** | **G** | **A** | **T** | **C** | **G** | **A** | **A** | **G** | **T** | **A** | **T** | **C** | **C** | **A** | **G** | **T** | **G** | **A** | **A** | **T** | **T** | **A** | **C** | **A** | **G** | **A** | **T** | **T** | **A** | **T** | **G** | **A** | **C** | **T** | **A** | **G** | **C** | **C** | **G** | **T** | **C** | **C** | **T** | **C** | **A** | **A** | **T** | **A** | **T** | **G** | **T** | **A** | **G** | **A** | **T** | **G** | **G** | **A** | **C** | **A** | **A** | **A** | **C** | **C** | **A** | **A** | **A** | **G** | **A** | **A** | **G** | **A** | **G** | **A** | **T** | **T** | **T** | **T** | **A** | **G** | **A** | **A** | **T** | **T** | **A** | **G** | **A** | **A** | **A** | **G** | **G** | **C** | **A** | **T** | **C** | **G** | **A** | **T** | **T** | **A** | **G** | **C** | **T** | **C** | **G** | **T** | **G** | **A** | **G** | **C** | **T** | **T** | **C** | **A** | **T** | **G** | **A** | **T** | **T** | **C** | **A** | **G** | **T** | **T** | **T** | **C** | **A** | **C** | **A** | **G** | **C** | **A** | **A** | **T** | **T** | **A** | **T** | **T** | **T** | **G** | **C** | **A** | **G** | **C** | **G** | **A** | **T** | **G** | **A** | **T** | **G** | **A** | **T** | **G** | **A** | **T** | **G** | **T** | **C** | **G** | **G** | **C** | **A** | **T** | **T** | **G** | **A** | **C** | **G** | **G** | **A** | **G** | **C** | **A** | **A** | **G** | **C** | **A** | **G** | **A** | **A** | **A** | **A** | **A** | **A** | **G** | **T** | **G** | **A** | **G** | **A** | **C** | **A** | **C** | **C** | **A** | **G** | **A** | **A** | **A** | **T** | **G** | **T** | **T** | **C** | **C** | **G** | **T** | **A** | **A** | **G** | **C** | **A** | **G** | **T** | **T** | **G** | **A** | **A** | **A** | **A** | **T** | **G** | **G** | **T** | **G** | **G** | **C** | **A** | **G** | **A** | **G** | **A** | **T** | **C** | **A** | **T** | **C** | **A** | **A** | **C** | **G** | **C** | **T** | **T** | **C** | **C** | **C** | **A** | **G** | **T** | **C** | **T** | **G** | **A** | **G** | **A** | **T** | **G** | **C** | **G** | **T** | **G** | **C** | **G** | **C** | **T** | **G** | **C** | **T** | **T** | **C** | **T** | **T** | **C** | **A** | **T** | **C** | **T** | **G** | **C** | **G** | **T** | **C** | **C** | **A** | **G** | **T** | **C** | **A** | **A** | **T** | **T** | **T** | **A** | **G** | **A** | **A** | **G** | **A** | **A** | **A** | **A** | **A** | **A** | **G** | **T** | **T** | **T** | **A** | **A** | **A** | **A** | **C** | **A** | **A** | **G** | **G** | **C** | **A** | **T** | **T** | **G** | **A** | **G** | **C** | **A** | **G** | **T** | **T** | **A** | **T** | **T** | **G** | **A** | **A** | **G** | **G** | **A** | **A** | **T** | **T** | **G** | **C** | **A** | **G** | **A** | **A** | **T** | **A** | **A** | **A** | **A** | **T** | **C** | **C** | **A** | **G** | **A** | **T** | **T** | **T** | **C** | **G** | **C** | **T** | **A** | **A** | **A** | **A** | **T** | **G** | **G** | **G** | **A** | **T** | **G** | **T** | **A** | **G** | **A** | **A** | **G** | **A** | **T** | **G** | **T** | **A** | **A** | **A** | **A** | **T** | **T** | **A** | **A** | **C** | **T** | **A** | **G** | **T** | **T** | **C** | **C** | **A** | **T** | **T** | **G** | **A** | **G** | **G** | **A** | **T** | **C** | **A** | **C** | **T** | **T** | **G** | **T** | **T** | **C** | **C** | **G** | **A** | **A** | **T** | **C** | **G** | **T** | **T** | **C** | **A** | **A** | **G** | **A** | **A** | **T** | **T** | **G** | **T** | **T** | **A** | **T** | **C** | **A** | **A** | **A** | **C** | **A** | **C** | **A** | **T** | **T** | **A** | **A** | **G** | **G** | **C** | **A** | **T** | **G** | **C** | **T** | **A** | **A** | **G** | **G** | **C** | **T** | **A** | **A** | **T** | **G** | **A** | **A** | **T** | **T** | **A** | **G** | **A** | **A** | **G** | **T** | **C** | **T** | **A** | **T** | **T** | **T** | **G** | **C** | **A** | **T** | **A** | **A** | **A** | **A** | **T** | **A** | **G** | **A** | **T** | **A** | **A** | **C** | **A** | **A** | **T** | **C** | **T** | **T** | **T** | **T** | **A** | **T** | **T** | **A** | **C** | **G** | **T** | **A** | **T** | **C** | **A** | **T** | **T** | **G** | **A** | **C** | **G** | **A** | **C** | **G** | **G** | **G** | **A** | **C** | **A** | **G** | **G** | **A** | **T** | **T** | **T** | **A** | **A** | **T** | **A** | **T** | **G** | **A** | **A** | **T** | **G** | **A** | **A** | **A** | **C** | **C** | **A** | **A** | **A** | **A** | **C** | **G** | **G** | **G** | **A** | **A** | **G** | **T** | **T** | **A** | **T** | **G** | **G** | **A** | **T** | **T** | **G** | **A** | **A** | **C** | **A** | **A** | **T** | **A** | **T** | **C** | **A** | **A** | **A** | **G** | **A** | **A** | **C** | **G** | **A** | **G** | **T** | **A** | **G** | **C** | **T** | **G** | **G** | **T** | **A** | **T** | **T** | **G** | **G** | **T** | **G** | **G** | **A** | **A** | **C** | **A** | **G** | **T** | **A** | **A** | **A** | **A** | **A** | **T** | **C** | **A** | **T** | **T** | **A** | **G** | **T** | **T** | **T** | **T** | **A** | **A** | **A** | **G** | **G** | **G** | **C** | **A** | **A** | **G** | **G** | **T** | **A** | **C** | **G** | **A** | **G** | **C** | **G** | **T** | **G** | **G** | **A** | **G** | **A** | **T** | **C** | **A** | **A** | **G** | **G** | **T** | **C** | **C** | **C** | **T** | **T** | **T** | **G** | **A** | **T** | **G** | **A** | **A** | **G** | **G** | **A** | **G** | **G** | **C** | **A** | **-** | **-** | **-** |
| **N53479** | **A** | **T** | **G** | **A** | **T** | **G** | **G** | **G** | **A** | **A** | **G** | **A** | **A** | **T** | **A** | **T** | **C** | **C** | **A** | **A** | **A** | **G** | **C** | **A** | **A** | **T** | **G** | **C** | **T** | **A** | **G** | **C** | **T** | **G** | **T** | **T** | **T** | **A** | **T** | **T** | **C** | **G** | **G** | **G** | **G** | **A** | **T** | **T** | **G** | **C** | **G** | **G** | **C** | **T** | **T** | **T** | **C** | **C** | **T** | **T** | **A** | **T** | **T** | **A** | **T** | **T** | **C** | **T** | **A** | **T** | **T** | **T** | **T** | **C** | **A** | **C** | **T** | **T** | **T** | **T** | **C** | **A** | **C** | **T** | **T** | **A** | **T** | **T** | **T** | **T** | **T** | **A** | **T** | **G** | **C** | **C** | **A** | **G** | **T** | **A** | **A** | **C** | **C** | **A** | **A** | **A** | **G** | **T** | **C** | **A** | **T** | **T** | **G** | **G** | **T** | **G** | **G** | **G** | **G** | **A** | **G** | **A** | **A** | **T** | **T** | **G** | **C** | **T** | **G** | **C** | **G** | **T** | **G** | **C** | **G** | **C** | **G** | **T** | **T** | **T** | **A** | **T** | **T** | **A** | **T** | **A** | **C** | **G** | **T** | **T** | **C** | **C** | **G** | **C** | **T** | **T** | **A** | **T** | **C** | **T** | **T** | **C** | **C** | **A** | **T** | **T** | **T** | **G** | **A** | **T** | **G** | **G** | **C** | **A** | **A** | **T** | **T** | **T** | **C** | **A** | **T** | **T** | **A** | **G** | **G** | **T** | **G** | **T** | **A** | **G** | **G** | **A** | **T** | **T** | **G** | **A** | **T** | **C** | **G** | **T** | **A** | **T** | **T** | **T** | **C** | **T** | **A** | **T** | **T** | **G** | **T** | **T** | **A** | **T** | **C** | **A** | **C** | **T** | **T** | **A** | **T** | **T** | **C** | **A** | **A** | **A** | **A** | **G** | **G** | **C** | **A** | **A** | **A** | **A** | **T** | **A** | **C** | **G** | **G** | **G** | **A** | **A** | **A** | **A** | **T** | **C** | **G** | **A** | **A** | **G** | **A** | **A** | **A** | **A** | **G** | **C** | **T** | **T** | **C** | **G** | **T** | **G** | **C** | **A** | **C** | **T** | **T** | **T** | **C** | **T** | **T** | **C** | **T** | **G** | **G** | **C** | **A** | **A** | **T** | **T** | **A** | **T** | **G** | **A** | **A** | **T** | **C** | **C** | **A** | **A** | **A** | **C** | **T** | **T** | **T** | **T** | **G** | **C** | **A** | **T** | **C** | **A** | **G** | **C** | **C** | **G** | **A** | **T** | **C** | **C** | **C** | **A** | **A** | **G** | **C** | **G** | **C** | **G** | **T** | **C** | **G** | **G** | **A** | **T** | **G** | **A** | **T** | **C** | **T** | **G** | **T** | **A** | **C** | **A** | **T** | **C** | **A** | **A** | **A** | **G** | **A** | **T** | **A** | **T** | **T** | **G** | **A** | **T** | **A** | **T** | **G** | **G** | **A** | **A** | **A** | **T** | **T** | **A** | **C** | **T** | **A** | **A** | **A** | **A** | **T** | **A** | **A** | **A** | **A** | **G** | **A** | **A** | **A** | **A** | **A** | **A** | **T** | **G** | **A** | **T** | **C** | **G** | **A** | **A** | **G** | **T** | **A** | **T** | **C** | **C** | **A** | **G** | **T** | **G** | **A** | **A** | **T** | **T** | **A** | **C** | **A** | **G** | **A** | **T** | **T** | **A** | **T** | **G** | **A** | **C** | **T** | **A** | **G** | **C** | **C** | **G** | **T** | **C** | **C** | **T** | **C** | **A** | **A** | **T** | **A** | **T** | **G** | **T** | **A** | **G** | **A** | **T** | **G** | **G** | **A** | **C** | **A** | **A** | **A** | **C** | **C** | **A** | **A** | **A** | **G** | **A** | **A** | **G** | **A** | **G** | **A** | **T** | **T** | **T** | **T** | **A** | **G** | **A** | **A** | **T** | **T** | **A** | **G** | **A** | **A** | **A** | **G** | **G** | **C** | **A** | **T** | **C** | **G** | **A** | **T** | **T** | **A** | **G** | **C** | **T** | **C** | **G** | **T** | **G** | **A** | **G** | **C** | **T** | **T** | **C** | **A** | **T** | **G** | **A** | **T** | **T** | **C** | **A** | **G** | **T** | **T** | **T** | **C** | **A** | **C** | **A** | **G** | **C** | **A** | **A** | **T** | **T** | **A** | **T** | **T** | **T** | **G** | **C** | **A** | **G** | **C** | **G** | **A** | **T** | **G** | **A** | **T** | **G** | **A** | **T** | **G** | **A** | **T** | **G** | **T** | **C** | **G** | **G** | **C** | **A** | **T** | **T** | **G** | **A** | **C** | **G** | **G** | **A** | **G** | **C** | **A** | **A** | **G** | **C** | **A** | **G** | **A** | **A** | **A** | **A** | **A** | **A** | **G** | **T** | **G** | **A** | **G** | **A** | **C** | **A** | **C** | **C** | **A** | **G** | **A** | **A** | **A** | **T** | **G** | **T** | **T** | **C** | **C** | **G** | **T** | **A** | **A** | **G** | **C** | **A** | **G** | **T** | **T** | **G** | **A** | **A** | **A** | **A** | **T** | **G** | **G** | **T** | **G** | **G** | **C** | **A** | **G** | **A** | **G** | **A** | **T** | **C** | **A** | **T** | **C** | **A** | **A** | **C** | **G** | **C** | **T** | **T** | **C** | **C** | **C** | **A** | **G** | **T** | **C** | **T** | **G** | **A** | **G** | **A** | **T** | **G** | **C** | **G** | **T** | **G** | **C** | **G** | **C** | **T** | **G** | **C** | **T** | **T** | **C** | **T** | **T** | **C** | **A** | **T** | **C** | **T** | **G** | **C** | **G** | **T** | **C** | **C** | **A** | **G** | **T** | **C** | **A** | **A** | **T** | **T** | **T** | **A** | **G** | **A** | **A** | **G** | **A** | **A** | **A** | **A** | **A** | **A** | **G** | **T** | **T** | **T** | **A** | **A** | **A** | **A** | **C** | **A** | **A** | **G** | **G** | **C** | **A** | **T** | **T** | **G** | **A** | **G** | **C** | **A** | **G** | **T** | **T** | **A** | **T** | **T** | **G** | **A** | **A** | **G** | **G** | **A** | **A** | **T** | **T** | **G** | **C** | **A** | **G** | **A** | **A** | **T** | **A** | **A** | **A** | **A** | **T** | **C** | **C** | **A** | **G** | **A** | **T** | **T** | **T** | **C** | **G** | **C** | **T** | **A** | **A** | **A** | **A** | **T** | **G** | **G** | **G** | **A** | **T** | **G** | **T** | **A** | **G** | **A** | **A** | **G** | **A** | **T** | **G** | **T** | **A** | **A** | **A** | **A** | **T** | **T** | **A** | **A** | **C** | **T** | **A** | **G** | **T** | **T** | **C** | **C** | **A** | **T** | **T** | **G** | **A** | **G** | **G** | **A** | **T** | **C** | **A** | **C** | **T** | **T** | **G** | **T** | **T** | **C** | **C** | **G** | **A** | **A** | **T** | **C** | **G** | **T** | **T** | **C** | **A** | **A** | **G** | **A** | **A** | **T** | **T** | **G** | **T** | **T** | **A** | **T** | **C** | **A** | **A** | **A** | **C** | **A** | **C** | **A** | **T** | **T** | **A** | **A** | **G** | **A** | **C** | **A** | **T** | **G** | **C** | **T** | **A** | **A** | **G** | **G** | **C** | **T** | **A** | **A** | **T** | **G** | **A** | **A** | **T** | **T** | **A** | **G** | **A** | **A** | **G** | **T** | **C** | **T** | **A** | **T** | **T** | **T** | **G** | **C** | **A** | **T** | **A** | **A** | **A** | **A** | **T** | **A** | **G** | **A** | **T** | **A** | **A** | **C** | **A** | **A** | **T** | **C** | **T** | **T** | **T** | **T** | **A** | **T** | **T** | **A** | **C** | **G** | **T** | **A** | **T** | **C** | **A** | **T** | **T** | **G** | **A** | **C** | **G** | **A** | **C** | **G** | **G** | **G** | **A** | **C** | **A** | **G** | **G** | **A** | **T** | **T** | **T** | **A** | **A** | **T** | **A** | **T** | **G** | **A** | **A** | **T** | **G** | **A** | **A** | **A** | **C** | **C** | **A** | **A** | **A** | **A** | **C** | **G** | **G** | **G** | **A** | **A** | **G** | **T** | **T** | **A** | **T** | **G** | **G** | **A** | **T** | **T** | **G** | **A** | **A** | **C** | **A** | **A** | **T** | **A** | **T** | **C** | **A** | **A** | **A** | **G** | **A** | **A** | **C** | **G** | **A** | **G** | **T** | **A** | **G** | **C** | **T** | **G** | **G** | **T** | **A** | **T** | **T** | **G** | **G** | **T** | **G** | **G** | **A** | **A** | **C** | **A** | **G** | **T** | **A** | **A** | **A** | **A** | **A** | **T** | **C** | **A** | **T** | **T** | **A** | **G** | **T** | **T** | **T** | **T** | **A** | **A** | **A** | **G** | **G** | **G** | **C** | **A** | **A** | **G** | **G** | **T** | **A** | **C** | **G** | **A** | **G** | **C** | **G** | **T** | **G** | **G** | **A** | **G** | **A** | **T** | **C** | **A** | **A** | **G** | **G** | **T** | **C** | **C** | **C** | **T** | **T** | **T** | **G** | **A** | **T** | **G** | **A** | **A** | **G** | **G** | **A** | **G** | **G** | **C** | **A** | **-** | **-** | **-** |
| **N53805** | **A** | **T** | **G** | **A** | **T** | **G** | **G** | **G** | **A** | **A** | **G** | **A** | **A** | **T** | **A** | **T** | **C** | **C** | **A** | **A** | **A** | **G** | **C** | **A** | **A** | **T** | **G** | **C** | **T** | **A** | **G** | **C** | **T** | **G** | **T** | **T** | **T** | **A** | **T** | **T** | **C** | **G** | **G** | **G** | **G** | **A** | **T** | **T** | **G** | **C** | **G** | **G** | **C** | **T** | **T** | **T** | **C** | **C** | **T** | **T** | **A** | **T** | **T** | **A** | **T** | **T** | **C** | **T** | **A** | **T** | **T** | **T** | **T** | **C** | **A** | **C** | **T** | **T** | **T** | **T** | **C** | **A** | **C** | **T** | **T** | **A** | **T** | **T** | **T** | **T** | **T** | **A** | **T** | **G** | **C** | **C** | **A** | **G** | **T** | **A** | **A** | **C** | **C** | **A** | **A** | **A** | **G** | **G** | **C** | **A** | **T** | **T** | **G** | **G** | **T** | **G** | **G** | **G** | **G** | **A** | **G** | **A** | **A** | **T** | **T** | **G** | **C** | **T** | **G** | **C** | **G** | **T** | **G** | **C** | **G** | **C** | **G** | **T** | **T** | **T** | **A** | **T** | **T** | **A** | **T** | **A** | **C** | **G** | **T** | **T** | **C** | **C** | **G** | **C** | **T** | **T** | **A** | **T** | **C** | **T** | **T** | **C** | **C** | **A** | **T** | **T** | **T** | **G** | **A** | **T** | **G** | **G** | **C** | **A** | **A** | **T** | **T** | **T** | **C** | **A** | **T** | **T** | **A** | **G** | **G** | **T** | **G** | **T** | **A** | **G** | **G** | **A** | **T** | **T** | **G** | **A** | **T** | **C** | **G** | **T** | **A** | **T** | **T** | **T** | **C** | **T** | **A** | **T** | **T** | **G** | **T** | **T** | **A** | **T** | **C** | **A** | **C** | **T** | **T** | **A** | **T** | **T** | **C** | **A** | **A** | **A** | **A** | **G** | **G** | **C** | **A** | **A** | **A** | **A** | **T** | **A** | **C** | **G** | **G** | **G** | **A** | **A** | **A** | **A** | **T** | **C** | **G** | **A** | **A** | **G** | **A** | **A** | **A** | **A** | **G** | **C** | **T** | **T** | **C** | **G** | **T** | **G** | **C** | **A** | **C** | **T** | **T** | **T** | **C** | **T** | **T** | **C** | **T** | **G** | **G** | **C** | **A** | **A** | **T** | **T** | **A** | **T** | **G** | **A** | **A** | **T** | **C** | **C** | **A** | **A** | **A** | **C** | **T** | **T** | **T** | **T** | **G** | **C** | **A** | **T** | **C** | **A** | **G** | **C** | **C** | **G** | **A** | **T** | **C** | **C** | **C** | **A** | **A** | **G** | **C** | **G** | **C** | **G** | **T** | **C** | **G** | **G** | **A** | **T** | **G** | **A** | **T** | **C** | **T** | **G** | **T** | **A** | **C** | **A** | **T** | **C** | **A** | **A** | **A** | **G** | **A** | **T** | **A** | **T** | **T** | **G** | **A** | **T** | **A** | **T** | **G** | **G** | **A** | **A** | **A** | **T** | **T** | **A** | **C** | **T** | **A** | **A** | **A** | **A** | **T** | **A** | **A** | **A** | **A** | **G** | **A** | **A** | **A** | **A** | **A** | **A** | **T** | **G** | **A** | **T** | **C** | **G** | **A** | **A** | **G** | **T** | **A** | **T** | **C** | **C** | **A** | **G** | **T** | **G** | **A** | **A** | **T** | **T** | **A** | **C** | **A** | **G** | **A** | **T** | **T** | **A** | **T** | **G** | **A** | **C** | **T** | **A** | **G** | **C** | **C** | **G** | **T** | **C** | **C** | **T** | **C** | **A** | **A** | **T** | **A** | **T** | **G** | **T** | **A** | **G** | **A** | **T** | **G** | **G** | **A** | **C** | **A** | **A** | **A** | **C** | **C** | **A** | **A** | **A** | **G** | **A** | **A** | **G** | **A** | **G** | **A** | **T** | **T** | **T** | **T** | **A** | **G** | **A** | **A** | **T** | **T** | **A** | **G** | **A** | **A** | **A** | **G** | **G** | **C** | **A** | **T** | **C** | **G** | **A** | **T** | **T** | **A** | **G** | **C** | **T** | **C** | **G** | **T** | **G** | **A** | **G** | **C** | **T** | **T** | **C** | **A** | **T** | **G** | **A** | **T** | **T** | **C** | **A** | **G** | **T** | **T** | **T** | **C** | **A** | **C** | **A** | **G** | **C** | **A** | **A** | **T** | **T** | **A** | **T** | **T** | **T** | **G** | **C** | **A** | **G** | **C** | **G** | **A** | **T** | **G** | **A** | **T** | **G** | **A** | **T** | **G** | **A** | **T** | **G** | **T** | **C** | **G** | **G** | **C** | **A** | **T** | **T** | **G** | **A** | **C** | **G** | **G** | **A** | **G** | **C** | **A** | **A** | **G** | **C** | **A** | **G** | **A** | **A** | **A** | **A** | **A** | **A** | **G** | **T** | **G** | **A** | **G** | **A** | **C** | **A** | **C** | **C** | **A** | **G** | **A** | **A** | **A** | **T** | **G** | **T** | **T** | **C** | **C** | **G** | **T** | **A** | **A** | **G** | **C** | **A** | **G** | **T** | **T** | **G** | **A** | **A** | **A** | **A** | **T** | **G** | **G** | **T** | **G** | **G** | **C** | **A** | **G** | **A** | **G** | **A** | **T** | **C** | **A** | **T** | **C** | **A** | **A** | **C** | **G** | **C** | **T** | **T** | **C** | **C** | **C** | **A** | **G** | **T** | **C** | **T** | **G** | **A** | **G** | **A** | **T** | **G** | **C** | **G** | **T** | **G** | **C** | **G** | **C** | **T** | **G** | **C** | **T** | **T** | **C** | **T** | **T** | **C** | **A** | **T** | **C** | **T** | **G** | **C** | **G** | **T** | **C** | **C** | **A** | **G** | **T** | **C** | **A** | **A** | **T** | **T** | **T** | **A** | **G** | **A** | **A** | **G** | **A** | **A** | **A** | **A** | **A** | **A** | **G** | **T** | **T** | **T** | **A** | **A** | **A** | **A** | **C** | **A** | **A** | **G** | **G** | **C** | **A** | **T** | **T** | **G** | **A** | **G** | **C** | **A** | **G** | **T** | **T** | **A** | **T** | **T** | **G** | **A** | **A** | **G** | **G** | **A** | **A** | **T** | **T** | **G** | **C** | **A** | **G** | **A** | **A** | **T** | **A** | **A** | **A** | **A** | **T** | **C** | **C** | **A** | **G** | **A** | **T** | **T** | **T** | **C** | **G** | **C** | **T** | **A** | **A** | **A** | **A** | **T** | **G** | **G** | **G** | **A** | **T** | **G** | **T** | **A** | **G** | **A** | **A** | **G** | **A** | **T** | **G** | **T** | **A** | **A** | **A** | **A** | **T** | **T** | **A** | **A** | **C** | **T** | **A** | **G** | **T** | **T** | **C** | **C** | **A** | **T** | **T** | **G** | **A** | **G** | **G** | **A** | **T** | **C** | **A** | **C** | **T** | **T** | **G** | **T** | **T** | **C** | **C** | **G** | **A** | **A** | **T** | **C** | **G** | **T** | **T** | **C** | **A** | **A** | **G** | **A** | **A** | **T** | **T** | **G** | **T** | **T** | **A** | **T** | **C** | **A** | **A** | **A** | **C** | **A** | **C** | **A** | **T** | **T** | **A** | **A** | **G** | **G** | **C** | **A** | **T** | **G** | **C** | **T** | **A** | **A** | **G** | **G** | **C** | **T** | **A** | **A** | **T** | **G** | **A** | **A** | **T** | **T** | **A** | **G** | **A** | **A** | **G** | **T** | **C** | **T** | **A** | **T** | **T** | **T** | **G** | **C** | **A** | **T** | **A** | **A** | **A** | **A** | **T** | **A** | **G** | **A** | **T** | **A** | **A** | **C** | **A** | **A** | **T** | **C** | **T** | **T** | **T** | **T** | **A** | **T** | **T** | **A** | **C** | **G** | **T** | **A** | **T** | **C** | **A** | **T** | **T** | **G** | **A** | **C** | **G** | **A** | **C** | **G** | **G** | **G** | **A** | **C** | **A** | **G** | **G** | **A** | **T** | **T** | **T** | **A** | **A** | **T** | **A** | **T** | **G** | **A** | **A** | **T** | **G** | **A** | **A** | **A** | **C** | **C** | **A** | **A** | **A** | **A** | **C** | **G** | **G** | **G** | **A** | **A** | **G** | **T** | **T** | **A** | **T** | **G** | **G** | **A** | **T** | **T** | **G** | **A** | **A** | **C** | **A** | **A** | **T** | **A** | **T** | **C** | **A** | **A** | **A** | **G** | **A** | **A** | **C** | **G** | **A** | **G** | **T** | **A** | **G** | **C** | **T** | **G** | **G** | **T** | **A** | **T** | **T** | **G** | **G** | **T** | **G** | **G** | **A** | **A** | **C** | **A** | **G** | **T** | **A** | **A** | **A** | **A** | **A** | **T** | **C** | **A** | **T** | **T** | **A** | **G** | **T** | **T** | **T** | **T** | **A** | **A** | **A** | **G** | **G** | **G** | **C** | **A** | **A** | **G** | **G** | **T** | **A** | **C** | **G** | **A** | **G** | **C** | **G** | **T** | **G** | **G** | **A** | **G** | **A** | **T** | **C** | **A** | **A** | **G** | **G** | **T** | **C** | **C** | **C** | **T** | **T** | **T** | **G** | **A** | **T** | **G** | **A** | **A** | **G** | **G** | **A** | **G** | **G** | **C** | **A** | **-** | **-** | **-** |
| **N55317** | **A** | **T** | **G** | **A** | **T** | **G** | **G** | **G** | **A** | **A** | **A** | **A** | **A** | **T** | **A** | **T** | **C** | **C** | **A** | **G** | **A** | **G** | **C** | **G** | **A** | **T** | **G** | **C** | **T** | **A** | **G** | **C** | **T** | **G** | **T** | **T** | **T** | **A** | **T** | **T** | **C** | **G** | **G** | **G** | **G** | **A** | **T** | **T** | **G** | **C** | **G** | **G** | **C** | **T** | **T** | **T** | **C** | **C** | **T** | **T** | **A** | **T** | **T** | **A** | **T** | **T** | **C** | **T** | **A** | **T** | **T** | **T** | **T** | **C** | **A** | **C** | **T** | **T** | **T** | **T** | **C** | **A** | **C** | **T** | **T** | **A** | **T** | **T** | **T** | **T** | **T** | **A** | **T** | **G** | **C** | **C** | **A** | **G** | **T** | **A** | **A** | **T** | **C** | **A** | **A** | **A** | **G** | **T** | **C** | **A** | **T** | **T** | **G** | **G** | **T** | **G** | **G** | **G** | **G** | **A** | **G** | **A** | **A** | **T** | **T** | **A** | **C** | **T** | **G** | **C** | **G** | **T** | **G** | **C** | **A** | **C** | **G** | **T** | **T** | **T** | **A** | **T** | **T** | **A** | **T** | **A** | **T** | **G** | **T** | **T** | **C** | **C** | **G** | **C** | **T** | **T** | **A** | **T** | **C** | **T** | **T** | **C** | **C** | **A** | **T** | **T** | **T** | **G** | **T** | **T** | **A** | **G** | **C** | **T** | **A** | **T** | **T** | **T** | **C** | **C** | **T** | **T** | **A** | **G** | **G** | **T** | **G** | **T** | **A** | **G** | **G** | **A** | **T** | **T** | **A** | **A** | **T** | **C** | **G** | **T** | **C** | **T** | **T** | **T** | **C** | **T** | **A** | **T** | **T** | **A** | **T** | **T** | **A** | **T** | **C** | **A** | **C** | **T** | **T** | **A** | **T** | **T** | **C** | **A** | **A** | **A** | **A** | **G** | **A** | **C** | **A** | **A** | **A** | **A** | **T** | **A** | **C** | **G** | **G** | **G** | **A** | **A** | **A** | **A** | **T** | **C** | **G** | **A** | **A** | **G** | **A** | **A** | **A** | **A** | **G** | **C** | **T** | **G** | **C** | **G** | **T** | **G** | **C** | **A** | **C** | **T** | **C** | **T** | **C** | **T** | **T** | **C** | **T** | **G** | **G** | **C** | **A** | **A** | **T** | **T** | **A** | **T** | **G** | **A** | **A** | **T** | **C** | **C** | **A** | **A** | **A** | **T** | **T** | **G** | **T** | **T** | **G** | **C** | **T** | **T** | **C** | **T** | **T** | **C** | **C** | **G** | **A** | **T** | **C** | **C** | **C** | **A** | **A** | **G** | **T** | **G** | **C** | **A** | **T** | **C** | **G** | **G** | **A** | **C** | **G** | **A** | **T** | **T** | **T** | **A** | **T** | **A** | **C** | **A** | **T** | **C** | **A** | **A** | **A** | **G** | **A** | **T** | **A** | **T** | **T** | **G** | **A** | **T** | **A** | **A** | **G** | **G** | **A** | **A** | **A** | **T** | **C** | **A** | **C** | **T** | **A** | **A** | **A** | **A** | **T** | **A** | **A** | **A** | **A** | **G** | **A** | **A** | **A** | **A** | **A** | **A** | **T** | **G** | **A** | **T** | **T** | **G** | **A** | **A** | **G** | **T** | **A** | **T** | **C** | **T** | **A** | **G** | **T** | **G** | **A** | **A** | **T** | **T** | **A** | **C** | **A** | **A** | **A** | **T** | **T** | **G** | **T** | **A** | **A** | **C** | **G** | **A** | **G** | **C** | **C** | **G** | **T** | **C** | **C** | **C** | **C** | **A** | **A** | **T** | **A** | **T** | **G** | **T** | **A** | **G** | **A** | **T** | **G** | **G** | **A** | **C** | **A** | **A** | **A** | **C** | **C** | **A** | **A** | **A** | **G** | **A** | **A** | **G** | **A** | **A** | **A** | **T** | **T** | **T** | **T** | **A** | **G** | **A** | **A** | **T** | **T** | **A** | **G** | **A** | **A** | **A** | **G** | **A** | **C** | **A** | **T** | **C** | **G** | **A** | **T** | **T** | **A** | **G** | **C** | **C** | **C** | **G** | **T** | **G** | **A** | **G** | **C** | **T** | **G** | **C** | **A** | **T** | **G** | **A** | **T** | **T** | **C** | **A** | **G** | **T** | **T** | **T** | **C** | **G** | **C** | **A** | **A** | **C** | **A** | **A** | **T** | **T** | **A** | **T** | **T** | **T** | **G** | **C** | **A** | **G** | **C** | **A** | **A** | **T** | **G** | **A** | **T** | **G** | **A** | **T** | **G** | **A** | **T** | **G** | **T** | **C** | **G** | **G** | **C** | **A** | **T** | **T** | **G** | **A** | **C** | **G** | **G** | **A** | **G** | **C** | **A** | **A** | **G** | **C** | **A** | **G** | **A** | **A** | **A** | **A** | **A** | **A** | **G** | **C** | **G** | **A** | **G** | **A** | **C** | **A** | **C** | **C** | **A** | **G** | **A** | **A** | **A** | **T** | **G** | **T** | **T** | **C** | **C** | **G** | **C** | **A** | **A** | **G** | **C** | **A** | **G** | **T** | **T** | **G** | **A** | **A** | **A** | **A** | **T** | **G** | **G** | **T** | **A** | **G** | **C** | **A** | **G** | **A** | **G** | **A** | **T** | **C** | **A** | **T** | **C** | **A** | **A** | **C** | **G** | **C** | **T** | **T** | **C** | **C** | **C** | **A** | **G** | **T** | **C** | **T** | **G** | **A** | **G** | **A** | **T** | **G** | **C** | **G** | **T** | **G** | **C** | **G** | **C** | **T** | **G** | **C** | **T** | **T** | **C** | **T** | **T** | **C** | **A** | **T** | **C** | **T** | **G** | **C** | **G** | **T** | **C** | **C** | **A** | **G** | **T** | **C** | **A** | **A** | **T** | **T** | **T** | **A** | **G** | **A** | **A** | **G** | **A** | **G** | **A** | **A** | **A** | **A** | **G** | **T** | **T** | **T** | **A** | **A** | **A** | **G** | **C** | **A** | **A** | **G** | **G** | **T** | **A** | **T** | **C** | **G** | **A** | **G** | **C** | **A** | **G** | **T** | **T** | **A** | **T** | **T** | **G** | **A** | **A** | **G** | **G** | **A** | **A** | **T** | **T** | **G** | **C** | **A** | **A** | **A** | **A** | **T** | **A** | **A** | **A** | **A** | **T** | **T** | **C** | **A** | **G** | **A** | **T** | **T** | **T** | **C** | **G** | **C** | **T** | **G** | **A** | **A** | **A** | **T** | **G** | **G** | **G** | **A** | **T** | **G** | **T** | **A** | **G** | **A** | **A** | **G** | **A** | **T** | **G** | **T** | **A** | **A** | **A** | **A** | **C** | **T** | **A** | **T** | **C** | **T** | **A** | **G** | **C** | **T** | **C** | **C** | **A** | **T** | **T** | **G** | **A** | **G** | **G** | **A** | **T** | **C** | **A** | **C** | **T** | **T** | **G** | **T** | **T** | **C** | **C** | **G** | **A** | **A** | **T** | **C** | **G** | **T** | **T** | **C** | **A** | **A** | **G** | **A** | **A** | **T** | **T** | **G** | **T** | **T** | **A** | **T** | **C** | **A** | **A** | **A** | **T** | **A** | **C** | **A** | **T** | **T** | **A** | **A** | **G** | **A** | **C** | **A** | **T** | **G** | **C** | **C** | **A** | **A** | **A** | **G** | **C** | **C** | **A** | **A** | **T** | **G** | **A** | **A** | **T** | **T** | **A** | **G** | **A** | **G** | **G** | **T** | **A** | **T** | **A** | **T** | **T** | **T** | **G** | **C** | **A** | **T** | **A** | **A** | **A** | **A** | **T** | **A** | **G** | **A** | **C** | **A** | **A** | **T** | **A** | **A** | **T** | **C** | **T** | **T** | **T** | **T** | **A** | **T** | **T** | **A** | **C** | **G** | **T** | **A** | **T** | **T** | **A** | **T** | **T** | **G** | **A** | **C** | **G** | **A** | **T** | **G** | **G** | **A** | **A** | **C** | **A** | **G** | **G** | **G** | **T** | **T** | **T** | **A** | **A** | **T** | **A** | **T** | **G** | **A** | **A** | **T** | **G** | **A** | **A** | **A** | **C** | **G** | **A** | **A** | **A** | **A** | **C** | **G** | **G** | **G** | **A** | **A** | **G** | **T** | **T** | **A** | **T** | **G** | **G** | **A** | **T** | **T** | **G** | **A** | **A** | **C** | **A** | **A** | **T** | **A** | **T** | **C** | **A** | **A** | **A** | **G** | **A** | **A** | **C** | **G** | **A** | **G** | **T** | **A** | **G** | **C** | **T** | **G** | **G** | **T** | **A** | **T** | **T** | **G** | **G** | **T** | **G** | **G** | **A** | **A** | **C** | **A** | **G** | **T** | **A** | **A** | **A** | **A** | **A** | **T** | **C** | **A** | **T** | **T** | **A** | **G** | **T** | **T** | **T** | **T** | **A** | **A** | **A** | **G** | **G** | **G** | **C** | **A** | **A** | **G** | **G** | **T** | **A** | **C** | **A** | **A** | **G** | **T** | **G** | **T** | **A** | **G** | **A** | **A** | **A** | **T** | **C** | **A** | **A** | **G** | **G** | **T** | **C** | **C** | **C** | **T** | **T** | **T** | **G** | **A** | **T** | **G** | **A** | **A** | **G** | **G** | **A** | **G** | **G** | **C** | **A** | **-** | **-** | **-** |
| **N59711F** | **A** | **T** | **G** | **A** | **T** | **G** | **G** | **G** | **A** | **A** | **A** | **A** | **A** | **T** | **A** | **T** | **C** | **C** | **A** | **G** | **A** | **G** | **C** | **G** | **A** | **T** | **G** | **C** | **T** | **A** | **G** | **C** | **T** | **G** | **T** | **T** | **T** | **A** | **T** | **T** | **C** | **G** | **G** | **G** | **G** | **A** | **T** | **T** | **G** | **C** | **G** | **G** | **C** | **T** | **T** | **T** | **C** | **C** | **T** | **T** | **A** | **T** | **T** | **A** | **T** | **T** | **C** | **T** | **A** | **T** | **T** | **T** | **T** | **C** | **A** | **C** | **T** | **T** | **T** | **T** | **C** | **A** | **C** | **T** | **T** | **A** | **T** | **T** | **T** | **T** | **T** | **A** | **T** | **G** | **C** | **C** | **A** | **G** | **C** | **A** | **A** | **T** | **C** | **A** | **A** | **A** | **G** | **T** | **C** | **A** | **T** | **T** | **G** | **G** | **T** | **G** | **G** | **G** | **G** | **A** | **G** | **A** | **A** | **T** | **T** | **A** | **C** | **T** | **G** | **C** | **G** | **T** | **G** | **C** | **A** | **C** | **G** | **T** | **T** | **T** | **A** | **T** | **T** | **A** | **T** | **A** | **T** | **G** | **T** | **T** | **C** | **C** | **G** | **C** | **T** | **T** | **A** | **T** | **C** | **T** | **T** | **C** | **C** | **A** | **T** | **T** | **T** | **G** | **T** | **T** | **A** | **G** | **C** | **C** | **A** | **T** | **T** | **T** | **C** | **C** | **T** | **T** | **A** | **G** | **G** | **T** | **G** | **T** | **A** | **G** | **G** | **A** | **T** | **T** | **A** | **A** | **T** | **T** | **G** | **T** | **C** | **T** | **T** | **T** | **C** | **T** | **A** | **T** | **T** | **A** | **T** | **T** | **A** | **T** | **C** | **A** | **C** | **T** | **T** | **A** | **T** | **T** | **C** | **A** | **A** | **A** | **A** | **G** | **A** | **C** | **A** | **A** | **A** | **A** | **T** | **A** | **C** | **G** | **G** | **G** | **A** | **A** | **A** | **A** | **T** | **C** | **G** | **A** | **A** | **G** | **A** | **A** | **A** | **A** | **G** | **C** | **T** | **G** | **C** | **G** | **T** | **G** | **C** | **A** | **C** | **T** | **T** | **T** | **C** | **T** | **T** | **C** | **T** | **G** | **G** | **C** | **A** | **A** | **T** | **T** | **A** | **T** | **G** | **A** | **A** | **T** | **C** | **C** | **A** | **A** | **A** | **T** | **T** | **G** | **T** | **T** | **G** | **C** | **T** | **T** | **C** | **T** | **T** | **C** | **C** | **G** | **A** | **T** | **C** | **C** | **C** | **A** | **A** | **G** | **T** | **G** | **C** | **A** | **T** | **C** | **G** | **G** | **A** | **C** | **G** | **A** | **T** | **T** | **T** | **A** | **T** | **A** | **C** | **A** | **T** | **C** | **A** | **A** | **A** | **G** | **A** | **T** | **A** | **T** | **T** | **G** | **A** | **T** | **A** | **A** | **G** | **G** | **A** | **A** | **A** | **T** | **C** | **A** | **C** | **T** | **A** | **A** | **G** | **A** | **T** | **A** | **A** | **A** | **A** | **G** | **A** | **A** | **A** | **A** | **A** | **A** | **T** | **G** | **A** | **T** | **T** | **G** | **A** | **A** | **G** | **T** | **A** | **T** | **C** | **T** | **A** | **G** | **T** | **G** | **A** | **A** | **T** | **T** | **A** | **C** | **A** | **A** | **A** | **T** | **T** | **G** | **T** | **A** | **A** | **C** | **G** | **A** | **G** | **C** | **C** | **G** | **T** | **C** | **C** | **C** | **C** | **A** | **A** | **T** | **A** | **T** | **G** | **T** | **A** | **G** | **A** | **T** | **G** | **G** | **A** | **C** | **A** | **A** | **A** | **C** | **C** | **A** | **A** | **A** | **G** | **A** | **A** | **G** | **A** | **A** | **A** | **T** | **T** | **T** | **T** | **A** | **G** | **A** | **A** | **T** | **T** | **A** | **G** | **A** | **A** | **A** | **G** | **A** | **C** | **A** | **T** | **C** | **G** | **A** | **T** | **T** | **A** | **G** | **C** | **C** | **C** | **G** | **T** | **G** | **A** | **G** | **C** | **T** | **G** | **C** | **A** | **T** | **G** | **A** | **T** | **T** | **C** | **A** | **G** | **T** | **T** | **T** | **C** | **G** | **C** | **A** | **A** | **C** | **A** | **A** | **T** | **T** | **G** | **T** | **T** | **T** | **G** | **C** | **A** | **G** | **C** | **A** | **A** | **T** | **G** | **A** | **T** | **G** | **A** | **T** | **G** | **A** | **T** | **G** | **T** | **C** | **G** | **G** | **C** | **A** | **T** | **T** | **G** | **A** | **C** | **G** | **G** | **A** | **G** | **C** | **A** | **A** | **G** | **C** | **A** | **G** | **A** | **A** | **A** | **A** | **A** | **A** | **G** | **C** | **G** | **A** | **G** | **A** | **C** | **A** | **C** | **C** | **A** | **G** | **A** | **A** | **A** | **T** | **G** | **T** | **T** | **C** | **C** | **G** | **C** | **A** | **A** | **G** | **C** | **A** | **G** | **T** | **T** | **G** | **A** | **A** | **A** | **A** | **T** | **G** | **G** | **T** | **A** | **G** | **C** | **A** | **G** | **A** | **G** | **A** | **T** | **C** | **A** | **T** | **C** | **A** | **A** | **C** | **G** | **C** | **T** | **T** | **C** | **C** | **C** | **A** | **G** | **T** | **C** | **T** | **G** | **A** | **G** | **A** | **T** | **G** | **C** | **G** | **T** | **G** | **C** | **G** | **C** | **T** | **G** | **C** | **T** | **T** | **C** | **T** | **T** | **C** | **A** | **T** | **C** | **T** | **A** | **C** | **G** | **T** | **C** | **C** | **A** | **G** | **T** | **C** | **A** | **A** | **T** | **T** | **T** | **A** | **G** | **A** | **A** | **G** | **A** | **G** | **A** | **A** | **A** | **A** | **G** | **T** | **T** | **T** | **A** | **A** | **A** | **G** | **C** | **A** | **A** | **G** | **G** | **T** | **A** | **T** | **C** | **G** | **A** | **G** | **C** | **A** | **G** | **T** | **T** | **A** | **T** | **T** | **G** | **A** | **A** | **G** | **G** | **A** | **A** | **T** | **T** | **G** | **C** | **A** | **A** | **A** | **A** | **T** | **A** | **A** | **A** | **A** | **T** | **C** | **C** | **A** | **G** | **A** | **T** | **T** | **T** | **C** | **G** | **C** | **T** | **G** | **A** | **A** | **A** | **T** | **G** | **G** | **G** | **A** | **T** | **G** | **T** | **A** | **G** | **A** | **A** | **G** | **A** | **T** | **G** | **T** | **A** | **A** | **A** | **A** | **C** | **T** | **A** | **T** | **C** | **T** | **A** | **G** | **T** | **T** | **C** | **C** | **A** | **T** | **T** | **G** | **A** | **G** | **G** | **A** | **T** | **C** | **A** | **C** | **T** | **T** | **G** | **T** | **T** | **C** | **C** | **G** | **A** | **A** | **T** | **C** | **G** | **T** | **T** | **C** | **A** | **A** | **G** | **A** | **A** | **T** | **T** | **G** | **T** | **T** | **A** | **T** | **C** | **A** | **A** | **A** | **T** | **A** | **C** | **A** | **T** | **T** | **A** | **A** | **G** | **A** | **C** | **A** | **T** | **G** | **C** | **T** | **A** | **A** | **A** | **G** | **C** | **C** | **A** | **A** | **T** | **G** | **A** | **A** | **T** | **T** | **A** | **G** | **A** | **G** | **G** | **T** | **A** | **T** | **A** | **T** | **T** | **T** | **G** | **C** | **A** | **C** | **A** | **A** | **A** | **A** | **T** | **A** | **G** | **A** | **C** | **A** | **A** | **T** | **A** | **A** | **T** | **C** | **T** | **T** | **T** | **T** | **G** | **T** | **T** | **A** | **C** | **G** | **T** | **A** | **T** | **T** | **A** | **T** | **T** | **G** | **A** | **C** | **G** | **A** | **C** | **G** | **G** | **A** | **A** | **C** | **A** | **G** | **G** | **G** | **T** | **T** | **T** | **A** | **A** | **T** | **A** | **T** | **G** | **A** | **A** | **T** | **G** | **A** | **A** | **A** | **C** | **G** | **A** | **A** | **A** | **A** | **C** | **G** | **G** | **G** | **A** | **A** | **G** | **T** | **T** | **A** | **T** | **G** | **G** | **A** | **T** | **T** | **G** | **A** | **A** | **C** | **A** | **A** | **T** | **A** | **T** | **C** | **A** | **A** | **A** | **G** | **A** | **A** | **C** | **G** | **A** | **G** | **T** | **A** | **G** | **C** | **T** | **G** | **G** | **T** | **A** | **T** | **T** | **G** | **G** | **T** | **G** | **G** | **A** | **A** | **C** | **A** | **G** | **T** | **A** | **A** | **A** | **A** | **A** | **T** | **C** | **A** | **T** | **T** | **A** | **G** | **T** | **T** | **T** | **T** | **A** | **A** | **A** | **G** | **G** | **T** | **C** | **A** | **A** | **G** | **G** | **T** | **A** | **C** | **A** | **A** | **G** | **T** | **G** | **T** | **A** | **G** | **A** | **A** | **A** | **T** | **C** | **A** | **A** | **G** | **G** | **T** | **C** | **C** | **C** | **T** | **T** | **T** | **G** | **A** | **T** | **G** | **A** | **A** | **G** | **G** | **A** | **G** | **G** | **C** | **A** | **-** | **-** | **-** |
| **N52751** | **A** | **T** | **G** | **A** | **T** | **G** | **G** | **G** | **A** | **A** | **G** | **A** | **A** | **T** | **A** | **T** | **C** | **C** | **A** | **A** | **A** | **G** | **C** | **A** | **A** | **T** | **G** | **C** | **T** | **A** | **G** | **C** | **T** | **G** | **T** | **T** | **T** | **A** | **T** | **T** | **C** | **G** | **G** | **G** | **G** | **A** | **T** | **T** | **G** | **C** | **G** | **G** | **C** | **T** | **T** | **T** | **C** | **C** | **T** | **T** | **A** | **T** | **T** | **A** | **T** | **T** | **C** | **T** | **A** | **T** | **T** | **T** | **T** | **C** | **A** | **C** | **T** | **T** | **T** | **T** | **C** | **A** | **C** | **T** | **T** | **A** | **T** | **T** | **T** | **T** | **T** | **A** | **T** | **G** | **C** | **C** | **A** | **G** | **T** | **A** | **A** | **C** | **C** | **A** | **A** | **A** | **G** | **T** | **C** | **A** | **T** | **T** | **G** | **G** | **T** | **G** | **G** | **G** | **G** | **A** | **G** | **A** | **A** | **T** | **T** | **G** | **C** | **T** | **G** | **C** | **G** | **T** | **G** | **C** | **G** | **C** | **G** | **T** | **T** | **T** | **A** | **T** | **T** | **A** | **T** | **A** | **C** | **G** | **T** | **T** | **C** | **C** | **G** | **C** | **T** | **T** | **A** | **T** | **C** | **T** | **T** | **C** | **C** | **A** | **T** | **T** | **T** | **G** | **A** | **T** | **G** | **G** | **C** | **A** | **A** | **T** | **T** | **T** | **C** | **A** | **T** | **T** | **A** | **G** | **G** | **T** | **G** | **T** | **A** | **G** | **G** | **A** | **T** | **T** | **G** | **A** | **T** | **C** | **G** | **T** | **A** | **T** | **T** | **T** | **C** | **T** | **A** | **T** | **T** | **G** | **T** | **T** | **A** | **T** | **C** | **A** | **C** | **T** | **T** | **A** | **T** | **T** | **C** | **A** | **A** | **A** | **A** | **G** | **G** | **C** | **A** | **A** | **A** | **A** | **T** | **A** | **C** | **G** | **G** | **G** | **A** | **A** | **A** | **A** | **T** | **C** | **G** | **A** | **A** | **G** | **A** | **A** | **A** | **A** | **G** | **C** | **T** | **T** | **C** | **G** | **T** | **G** | **C** | **A** | **C** | **T** | **T** | **T** | **C** | **T** | **T** | **C** | **T** | **G** | **G** | **C** | **A** | **A** | **T** | **T** | **A** | **T** | **G** | **A** | **A** | **T** | **C** | **C** | **A** | **A** | **A** | **C** | **T** | **T** | **T** | **T** | **G** | **C** | **A** | **T** | **C** | **A** | **G** | **C** | **C** | **G** | **A** | **T** | **C** | **C** | **C** | **A** | **A** | **G** | **C** | **G** | **C** | **G** | **T** | **C** | **G** | **G** | **A** | **T** | **G** | **A** | **T** | **C** | **T** | **G** | **T** | **A** | **C** | **A** | **T** | **C** | **A** | **A** | **A** | **G** | **A** | **T** | **A** | **T** | **T** | **G** | **A** | **T** | **A** | **T** | **G** | **G** | **A** | **A** | **A** | **T** | **T** | **A** | **C** | **T** | **A** | **A** | **A** | **A** | **T** | **A** | **A** | **A** | **A** | **G** | **A** | **A** | **A** | **A** | **A** | **A** | **T** | **G** | **A** | **T** | **C** | **G** | **A** | **A** | **G** | **T** | **A** | **T** | **C** | **C** | **A** | **G** | **T** | **G** | **A** | **A** | **T** | **T** | **A** | **C** | **A** | **G** | **A** | **T** | **T** | **A** | **T** | **G** | **A** | **C** | **T** | **A** | **G** | **C** | **C** | **G** | **T** | **C** | **C** | **T** | **C** | **A** | **A** | **T** | **A** | **T** | **G** | **T** | **A** | **G** | **A** | **T** | **G** | **G** | **C** | **C** | **A** | **A** | **A** | **C** | **C** | **A** | **A** | **A** | **G** | **A** | **A** | **G** | **A** | **G** | **A** | **T** | **T** | **T** | **T** | **A** | **G** | **A** | **A** | **T** | **T** | **A** | **G** | **A** | **A** | **A** | **G** | **G** | **C** | **A** | **T** | **C** | **G** | **A** | **T** | **T** | **A** | **G** | **C** | **T** | **C** | **G** | **T** | **G** | **A** | **G** | **C** | **T** | **T** | **C** | **A** | **T** | **G** | **A** | **T** | **T** | **C** | **A** | **G** | **T** | **T** | **T** | **C** | **A** | **C** | **A** | **G** | **C** | **A** | **A** | **T** | **T** | **A** | **T** | **T** | **T** | **G** | **C** | **A** | **G** | **C** | **G** | **A** | **T** | **G** | **A** | **T** | **G** | **A** | **T** | **G** | **A** | **T** | **G** | **T** | **C** | **G** | **G** | **C** | **A** | **T** | **T** | **G** | **A** | **C** | **G** | **G** | **A** | **G** | **C** | **A** | **A** | **G** | **C** | **A** | **G** | **A** | **A** | **A** | **A** | **A** | **A** | **G** | **T** | **G** | **A** | **G** | **A** | **C** | **A** | **C** | **C** | **A** | **G** | **A** | **A** | **A** | **T** | **G** | **T** | **T** | **C** | **C** | **G** | **T** | **A** | **A** | **G** | **C** | **A** | **G** | **T** | **T** | **G** | **A** | **A** | **A** | **A** | **T** | **G** | **G** | **T** | **G** | **G** | **C** | **A** | **G** | **A** | **G** | **A** | **T** | **C** | **A** | **T** | **C** | **A** | **A** | **C** | **G** | **C** | **T** | **T** | **C** | **C** | **C** | **A** | **G** | **T** | **C** | **T** | **G** | **A** | **G** | **A** | **T** | **G** | **C** | **G** | **T** | **G** | **C** | **G** | **C** | **T** | **G** | **C** | **T** | **T** | **C** | **T** | **T** | **C** | **A** | **T** | **C** | **T** | **G** | **C** | **G** | **T** | **C** | **C** | **A** | **G** | **T** | **C** | **A** | **A** | **T** | **T** | **T** | **A** | **G** | **A** | **A** | **G** | **A** | **A** | **A** | **A** | **A** | **A** | **G** | **T** | **T** | **T** | **A** | **A** | **A** | **A** | **C** | **A** | **A** | **G** | **G** | **C** | **A** | **T** | **T** | **G** | **A** | **G** | **C** | **A** | **G** | **T** | **T** | **A** | **T** | **T** | **G** | **A** | **A** | **G** | **G** | **A** | **A** | **T** | **T** | **G** | **C** | **A** | **G** | **A** | **A** | **T** | **A** | **A** | **A** | **A** | **T** | **C** | **C** | **A** | **G** | **A** | **T** | **T** | **T** | **C** | **G** | **C** | **T** | **A** | **A** | **A** | **A** | **T** | **G** | **G** | **G** | **A** | **T** | **G** | **T** | **A** | **G** | **A** | **A** | **G** | **A** | **T** | **G** | **T** | **A** | **A** | **A** | **A** | **T** | **T** | **A** | **A** | **C** | **T** | **A** | **G** | **T** | **T** | **C** | **C** | **A** | **T** | **T** | **G** | **A** | **G** | **G** | **A** | **T** | **C** | **A** | **C** | **T** | **T** | **G** | **T** | **T** | **C** | **C** | **G** | **A** | **A** | **T** | **C** | **G** | **T** | **T** | **C** | **A** | **A** | **G** | **A** | **A** | **T** | **T** | **G** | **T** | **T** | **A** | **T** | **C** | **A** | **A** | **A** | **C** | **A** | **C** | **A** | **T** | **T** | **A** | **A** | **G** | **A** | **C** | **A** | **T** | **G** | **C** | **T** | **A** | **A** | **G** | **G** | **C** | **T** | **A** | **A** | **T** | **G** | **A** | **A** | **T** | **T** | **A** | **G** | **A** | **A** | **G** | **T** | **C** | **T** | **A** | **T** | **T** | **T** | **G** | **C** | **A** | **T** | **A** | **A** | **A** | **A** | **T** | **A** | **G** | **A** | **T** | **A** | **A** | **C** | **A** | **A** | **T** | **C** | **T** | **T** | **T** | **T** | **A** | **T** | **T** | **A** | **C** | **G** | **T** | **A** | **T** | **C** | **A** | **T** | **T** | **G** | **A** | **C** | **G** | **A** | **C** | **G** | **G** | **G** | **A** | **C** | **A** | **G** | **G** | **C** | **T** | **T** | **T** | **A** | **A** | **T** | **A** | **T** | **G** | **A** | **A** | **T** | **G** | **A** | **A** | **A** | **C** | **C** | **A** | **A** | **A** | **A** | **C** | **G** | **G** | **G** | **A** | **A** | **G** | **T** | **T** | **A** | **T** | **G** | **G** | **A** | **T** | **T** | **G** | **A** | **A** | **C** | **A** | **A** | **T** | **A** | **T** | **C** | **A** | **A** | **A** | **G** | **A** | **A** | **C** | **G** | **A** | **G** | **T** | **A** | **G** | **C** | **T** | **G** | **G** | **T** | **A** | **T** | **T** | **G** | **G** | **T** | **G** | **G** | **A** | **A** | **C** | **A** | **G** | **T** | **A** | **A** | **A** | **A** | **A** | **T** | **C** | **A** | **T** | **T** | **A** | **G** | **T** | **T** | **T** | **T** | **A** | **A** | **A** | **G** | **G** | **G** | **C** | **A** | **A** | **G** | **G** | **T** | **A** | **C** | **G** | **A** | **G** | **C** | **G** | **T** | **G** | **G** | **A** | **G** | **A** | **T** | **C** | **A** | **A** | **G** | **G** | **T** | **C** | **C** | **C** | **T** | **T** | **T** | **G** | **A** | **T** | **G** | **A** | **A** | **G** | **G** | **A** | **G** | **G** | **C** | **A** | **-** | **-** | **-** |
| **N60490F** | **A** | **T** | **G** | **A** | **T** | **G** | **G** | **G** | **A** | **A** | **G** | **A** | **A** | **T** | **A** | **T** | **C** | **C** | **A** | **A** | **A** | **G** | **C** | **A** | **A** | **T** | **G** | **C** | **T** | **A** | **G** | **C** | **T** | **G** | **T** | **T** | **T** | **A** | **T** | **T** | **C** | **G** | **G** | **G** | **G** | **A** | **T** | **T** | **G** | **C** | **G** | **G** | **C** | **T** | **T** | **T** | **C** | **C** | **T** | **T** | **A** | **T** | **T** | **A** | **T** | **T** | **C** | **T** | **A** | **T** | **T** | **T** | **T** | **C** | **A** | **C** | **T** | **T** | **T** | **T** | **C** | **A** | **C** | **T** | **T** | **A** | **T** | **T** | **T** | **T** | **T** | **A** | **T** | **G** | **C** | **C** | **A** | **G** | **T** | **A** | **A** | **C** | **C** | **A** | **A** | **A** | **G** | **T** | **C** | **A** | **T** | **T** | **G** | **G** | **T** | **G** | **G** | **G** | **G** | **A** | **G** | **A** | **A** | **T** | **T** | **G** | **C** | **T** | **G** | **C** | **G** | **T** | **G** | **C** | **G** | **C** | **G** | **T** | **T** | **T** | **A** | **T** | **T** | **A** | **T** | **A** | **C** | **G** | **T** | **T** | **C** | **C** | **G** | **C** | **T** | **T** | **A** | **T** | **C** | **T** | **T** | **C** | **C** | **A** | **T** | **T** | **T** | **G** | **A** | **T** | **G** | **G** | **C** | **A** | **A** | **T** | **T** | **T** | **C** | **A** | **T** | **T** | **A** | **G** | **G** | **T** | **G** | **T** | **A** | **G** | **G** | **A** | **T** | **T** | **G** | **A** | **T** | **C** | **G** | **T** | **A** | **T** | **T** | **T** | **C** | **T** | **A** | **T** | **T** | **G** | **T** | **T** | **A** | **T** | **C** | **A** | **C** | **T** | **T** | **A** | **T** | **T** | **C** | **A** | **A** | **A** | **A** | **G** | **A** | **C** | **A** | **A** | **A** | **A** | **T** | **A** | **C** | **G** | **G** | **G** | **A** | **A** | **A** | **A** | **T** | **C** | **G** | **A** | **A** | **G** | **A** | **A** | **A** | **A** | **G** | **C** | **T** | **T** | **C** | **G** | **T** | **G** | **C** | **A** | **C** | **T** | **T** | **T** | **C** | **T** | **T** | **C** | **T** | **G** | **G** | **C** | **A** | **A** | **T** | **T** | **A** | **T** | **G** | **A** | **A** | **T** | **C** | **C** | **A** | **A** | **A** | **C** | **T** | **T** | **T** | **T** | **G** | **C** | **A** | **T** | **C** | **A** | **G** | **C** | **C** | **G** | **A** | **T** | **C** | **C** | **C** | **A** | **A** | **G** | **C** | **G** | **C** | **G** | **T** | **C** | **G** | **G** | **A** | **T** | **G** | **A** | **T** | **C** | **T** | **G** | **T** | **A** | **C** | **A** | **T** | **C** | **A** | **A** | **A** | **G** | **A** | **T** | **A** | **T** | **T** | **G** | **A** | **T** | **A** | **T** | **G** | **G** | **A** | **A** | **A** | **T** | **T** | **A** | **C** | **T** | **A** | **A** | **A** | **A** | **T** | **A** | **A** | **A** | **A** | **G** | **A** | **A** | **A** | **A** | **A** | **A** | **T** | **G** | **A** | **T** | **C** | **G** | **A** | **A** | **G** | **T** | **A** | **T** | **C** | **C** | **A** | **G** | **T** | **G** | **A** | **A** | **T** | **T** | **A** | **C** | **A** | **G** | **A** | **T** | **T** | **A** | **T** | **G** | **A** | **C** | **T** | **A** | **G** | **C** | **C** | **G** | **T** | **C** | **C** | **T** | **C** | **A** | **A** | **T** | **A** | **T** | **G** | **T** | **A** | **G** | **A** | **T** | **G** | **G** | **A** | **C** | **A** | **A** | **A** | **C** | **C** | **A** | **A** | **A** | **G** | **A** | **A** | **G** | **A** | **G** | **A** | **T** | **T** | **T** | **T** | **A** | **G** | **A** | **A** | **T** | **T** | **A** | **G** | **A** | **A** | **A** | **G** | **G** | **C** | **A** | **T** | **C** | **G** | **A** | **T** | **T** | **A** | **G** | **C** | **T** | **C** | **G** | **T** | **G** | **A** | **G** | **C** | **T** | **T** | **C** | **A** | **T** | **G** | **A** | **T** | **T** | **C** | **A** | **G** | **T** | **T** | **T** | **C** | **A** | **C** | **A** | **G** | **C** | **A** | **A** | **T** | **T** | **A** | **T** | **T** | **T** | **G** | **C** | **A** | **G** | **C** | **G** | **A** | **T** | **G** | **A** | **T** | **G** | **A** | **T** | **G** | **A** | **T** | **G** | **T** | **C** | **G** | **G** | **C** | **A** | **T** | **T** | **G** | **A** | **C** | **G** | **G** | **A** | **G** | **C** | **A** | **A** | **G** | **C** | **A** | **G** | **A** | **A** | **A** | **A** | **A** | **A** | **G** | **T** | **G** | **A** | **G** | **A** | **C** | **A** | **C** | **C** | **A** | **G** | **A** | **A** | **A** | **T** | **G** | **T** | **T** | **C** | **C** | **G** | **T** | **A** | **A** | **G** | **C** | **A** | **G** | **T** | **T** | **G** | **A** | **A** | **A** | **A** | **T** | **G** | **G** | **T** | **G** | **G** | **C** | **A** | **G** | **A** | **G** | **A** | **T** | **C** | **A** | **T** | **C** | **A** | **A** | **C** | **G** | **C** | **T** | **T** | **C** | **C** | **C** | **A** | **G** | **T** | **C** | **T** | **G** | **A** | **G** | **A** | **T** | **G** | **C** | **G** | **T** | **G** | **C** | **G** | **C** | **T** | **G** | **C** | **T** | **T** | **C** | **T** | **T** | **C** | **A** | **T** | **C** | **T** | **G** | **C** | **G** | **T** | **C** | **C** | **A** | **G** | **T** | **C** | **A** | **A** | **T** | **T** | **T** | **A** | **G** | **A** | **A** | **G** | **A** | **A** | **A** | **A** | **A** | **A** | **G** | **T** | **T** | **T** | **A** | **A** | **A** | **A** | **C** | **A** | **A** | **G** | **G** | **C** | **A** | **T** | **T** | **G** | **A** | **G** | **C** | **A** | **G** | **T** | **T** | **A** | **T** | **T** | **G** | **A** | **A** | **G** | **G** | **A** | **A** | **T** | **T** | **G** | **C** | **A** | **G** | **A** | **A** | **T** | **A** | **A** | **A** | **A** | **T** | **C** | **C** | **A** | **G** | **A** | **T** | **T** | **T** | **C** | **G** | **C** | **T** | **A** | **A** | **A** | **A** | **T** | **G** | **G** | **G** | **A** | **T** | **G** | **T** | **A** | **G** | **A** | **A** | **G** | **A** | **T** | **G** | **T** | **A** | **A** | **A** | **A** | **T** | **T** | **A** | **A** | **C** | **T** | **A** | **G** | **T** | **T** | **C** | **C** | **A** | **T** | **T** | **G** | **A** | **G** | **G** | **A** | **T** | **C** | **A** | **C** | **T** | **T** | **G** | **T** | **T** | **C** | **C** | **G** | **A** | **A** | **T** | **C** | **G** | **T** | **T** | **C** | **A** | **A** | **G** | **A** | **A** | **T** | **T** | **G** | **T** | **T** | **A** | **T** | **C** | **A** | **A** | **A** | **C** | **A** | **C** | **A** | **T** | **T** | **A** | **A** | **G** | **A** | **C** | **A** | **T** | **G** | **C** | **T** | **A** | **A** | **G** | **G** | **C** | **T** | **A** | **A** | **T** | **G** | **A** | **A** | **T** | **T** | **A** | **G** | **A** | **A** | **G** | **T** | **C** | **T** | **A** | **T** | **T** | **T** | **G** | **C** | **A** | **T** | **A** | **A** | **A** | **A** | **T** | **A** | **G** | **A** | **T** | **A** | **A** | **C** | **A** | **A** | **T** | **C** | **T** | **T** | **T** | **T** | **A** | **T** | **T** | **A** | **C** | **G** | **T** | **A** | **T** | **C** | **A** | **T** | **T** | **G** | **A** | **C** | **G** | **A** | **C** | **G** | **G** | **G** | **A** | **C** | **A** | **G** | **G** | **A** | **T** | **T** | **T** | **A** | **A** | **T** | **A** | **T** | **G** | **A** | **A** | **T** | **G** | **A** | **A** | **A** | **C** | **C** | **A** | **A** | **A** | **A** | **C** | **G** | **G** | **G** | **A** | **A** | **G** | **T** | **T** | **A** | **T** | **G** | **G** | **A** | **T** | **T** | **G** | **A** | **A** | **C** | **A** | **A** | **T** | **A** | **T** | **C** | **A** | **A** | **A** | **G** | **A** | **A** | **C** | **G** | **A** | **G** | **T** | **A** | **G** | **C** | **T** | **G** | **G** | **T** | **A** | **T** | **T** | **G** | **G** | **T** | **G** | **G** | **A** | **A** | **C** | **A** | **G** | **T** | **A** | **A** | **A** | **A** | **A** | **T** | **C** | **A** | **T** | **T** | **A** | **G** | **T** | **T** | **T** | **T** | **A** | **A** | **A** | **G** | **G** | **G** | **C** | **A** | **A** | **G** | **G** | **T** | **A** | **C** | **G** | **A** | **G** | **C** | **G** | **T** | **G** | **G** | **A** | **G** | **A** | **T** | **C** | **A** | **A** | **G** | **G** | **T** | **C** | **C** | **C** | **T** | **T** | **T** | **G** | **A** | **T** | **G** | **A** | **A** | **G** | **G** | **A** | **G** | **G** | **C** | **A** | **-** | **-** | **-** |
| **N59907F** | **A** | **T** | **G** | **A** | **T** | **G** | **G** | **G** | **A** | **A** | **G** | **A** | **A** | **T** | **A** | **T** | **C** | **C** | **A** | **A** | **A** | **G** | **C** | **A** | **A** | **T** | **G** | **C** | **T** | **A** | **G** | **C** | **T** | **G** | **T** | **T** | **T** | **A** | **T** | **T** | **C** | **G** | **G** | **G** | **G** | **A** | **T** | **T** | **G** | **C** | **G** | **G** | **C** | **T** | **T** | **T** | **C** | **C** | **T** | **T** | **A** | **T** | **T** | **A** | **T** | **T** | **C** | **T** | **A** | **T** | **T** | **T** | **T** | **C** | **A** | **C** | **T** | **T** | **T** | **T** | **C** | **A** | **C** | **T** | **T** | **A** | **T** | **T** | **T** | **T** | **T** | **A** | **T** | **G** | **C** | **C** | **A** | **G** | **T** | **A** | **A** | **C** | **C** | **A** | **A** | **A** | **G** | **T** | **C** | **A** | **T** | **T** | **G** | **G** | **T** | **G** | **G** | **G** | **G** | **A** | **G** | **A** | **A** | **T** | **T** | **G** | **C** | **T** | **G** | **C** | **G** | **T** | **G** | **C** | **G** | **C** | **G** | **T** | **T** | **T** | **A** | **T** | **T** | **A** | **T** | **A** | **C** | **G** | **T** | **T** | **C** | **C** | **G** | **C** | **T** | **T** | **A** | **T** | **C** | **T** | **T** | **C** | **C** | **A** | **T** | **T** | **T** | **G** | **A** | **T** | **G** | **G** | **C** | **A** | **A** | **T** | **T** | **T** | **C** | **A** | **T** | **T** | **A** | **G** | **G** | **T** | **G** | **T** | **A** | **G** | **G** | **A** | **T** | **T** | **G** | **A** | **T** | **C** | **G** | **T** | **A** | **T** | **T** | **T** | **C** | **T** | **A** | **T** | **T** | **G** | **T** | **T** | **A** | **T** | **C** | **A** | **C** | **T** | **T** | **A** | **T** | **T** | **C** | **A** | **A** | **A** | **A** | **G** | **G** | **C** | **A** | **A** | **A** | **A** | **T** | **A** | **C** | **G** | **G** | **G** | **A** | **A** | **A** | **A** | **T** | **C** | **G** | **A** | **A** | **G** | **A** | **A** | **A** | **A** | **G** | **C** | **T** | **T** | **C** | **G** | **T** | **G** | **C** | **A** | **C** | **T** | **T** | **T** | **C** | **T** | **T** | **C** | **T** | **G** | **G** | **C** | **A** | **A** | **T** | **T** | **A** | **T** | **G** | **A** | **A** | **T** | **C** | **C** | **A** | **A** | **A** | **C** | **T** | **T** | **T** | **T** | **G** | **C** | **A** | **T** | **C** | **A** | **G** | **C** | **C** | **G** | **A** | **T** | **C** | **C** | **C** | **A** | **A** | **G** | **C** | **A** | **C** | **G** | **T** | **C** | **G** | **G** | **A** | **T** | **G** | **A** | **T** | **C** | **T** | **G** | **T** | **A** | **C** | **A** | **T** | **C** | **A** | **A** | **A** | **G** | **A** | **T** | **A** | **T** | **T** | **G** | **A** | **T** | **A** | **T** | **G** | **G** | **A** | **A** | **A** | **T** | **T** | **A** | **C** | **T** | **A** | **A** | **A** | **A** | **T** | **A** | **A** | **A** | **A** | **G** | **A** | **A** | **A** | **A** | **A** | **A** | **T** | **G** | **A** | **T** | **C** | **G** | **A** | **A** | **G** | **T** | **A** | **T** | **C** | **C** | **A** | **G** | **T** | **G** | **A** | **A** | **T** | **T** | **A** | **C** | **A** | **G** | **A** | **T** | **T** | **A** | **T** | **G** | **A** | **C** | **T** | **A** | **G** | **C** | **C** | **G** | **T** | **C** | **C** | **T** | **C** | **A** | **A** | **T** | **A** | **T** | **G** | **T** | **A** | **G** | **A** | **T** | **G** | **G** | **A** | **C** | **A** | **A** | **A** | **C** | **C** | **A** | **A** | **A** | **G** | **A** | **A** | **G** | **A** | **G** | **A** | **T** | **T** | **T** | **T** | **A** | **G** | **A** | **A** | **T** | **T** | **A** | **G** | **A** | **A** | **A** | **G** | **G** | **C** | **A** | **T** | **C** | **G** | **A** | **T** | **T** | **A** | **G** | **C** | **T** | **C** | **G** | **T** | **G** | **A** | **G** | **C** | **T** | **T** | **C** | **A** | **T** | **G** | **A** | **T** | **T** | **C** | **A** | **G** | **T** | **T** | **T** | **C** | **A** | **C** | **A** | **G** | **C** | **A** | **A** | **T** | **T** | **A** | **T** | **T** | **T** | **G** | **C** | **A** | **G** | **C** | **G** | **A** | **T** | **G** | **A** | **T** | **G** | **A** | **T** | **G** | **A** | **T** | **G** | **T** | **C** | **G** | **G** | **C** | **A** | **T** | **T** | **G** | **A** | **C** | **G** | **G** | **A** | **G** | **C** | **A** | **A** | **G** | **C** | **A** | **G** | **A** | **A** | **A** | **A** | **A** | **A** | **G** | **T** | **G** | **A** | **G** | **A** | **C** | **A** | **C** | **C** | **A** | **G** | **A** | **A** | **A** | **T** | **G** | **T** | **T** | **C** | **C** | **G** | **T** | **A** | **A** | **G** | **C** | **A** | **G** | **T** | **T** | **G** | **A** | **A** | **A** | **A** | **T** | **G** | **G** | **T** | **G** | **G** | **C** | **A** | **G** | **A** | **G** | **A** | **T** | **C** | **A** | **T** | **C** | **A** | **A** | **C** | **G** | **C** | **T** | **T** | **C** | **C** | **C** | **A** | **G** | **T** | **C** | **T** | **G** | **A** | **G** | **A** | **T** | **G** | **C** | **G** | **T** | **G** | **C** | **G** | **C** | **T** | **G** | **C** | **T** | **T** | **C** | **T** | **T** | **C** | **A** | **T** | **C** | **T** | **G** | **C** | **G** | **T** | **C** | **C** | **A** | **G** | **T** | **C** | **A** | **A** | **T** | **T** | **T** | **A** | **G** | **A** | **A** | **G** | **A** | **A** | **A** | **A** | **A** | **A** | **G** | **T** | **T** | **T** | **A** | **A** | **A** | **A** | **C** | **A** | **A** | **G** | **G** | **C** | **A** | **T** | **T** | **G** | **A** | **G** | **C** | **A** | **G** | **T** | **T** | **A** | **T** | **T** | **G** | **A** | **A** | **G** | **G** | **A** | **A** | **T** | **T** | **G** | **C** | **A** | **G** | **A** | **A** | **T** | **A** | **A** | **A** | **A** | **T** | **C** | **C** | **A** | **G** | **A** | **T** | **T** | **T** | **C** | **G** | **C** | **T** | **A** | **A** | **A** | **A** | **T** | **G** | **G** | **A** | **A** | **T** | **G** | **T** | **A** | **G** | **A** | **A** | **G** | **A** | **T** | **G** | **T** | **A** | **A** | **A** | **A** | **T** | **T** | **A** | **A** | **C** | **T** | **A** | **G** | **T** | **T** | **C** | **C** | **A** | **T** | **T** | **G** | **A** | **G** | **G** | **A** | **T** | **C** | **A** | **C** | **T** | **T** | **G** | **T** | **T** | **C** | **C** | **G** | **A** | **A** | **T** | **C** | **G** | **T** | **T** | **C** | **A** | **A** | **G** | **A** | **A** | **T** | **T** | **G** | **T** | **T** | **A** | **T** | **C** | **A** | **A** | **A** | **C** | **A** | **C** | **A** | **T** | **T** | **A** | **A** | **G** | **A** | **C** | **A** | **T** | **G** | **C** | **T** | **A** | **A** | **G** | **G** | **C** | **T** | **A** | **A** | **T** | **G** | **A** | **A** | **T** | **T** | **A** | **G** | **A** | **A** | **G** | **T** | **C** | **T** | **A** | **T** | **T** | **T** | **G** | **C** | **A** | **T** | **A** | **A** | **A** | **A** | **T** | **A** | **G** | **A** | **T** | **A** | **A** | **C** | **A** | **A** | **T** | **C** | **T** | **T** | **T** | **T** | **A** | **T** | **T** | **A** | **C** | **G** | **T** | **A** | **T** | **C** | **A** | **T** | **T** | **G** | **A** | **C** | **G** | **A** | **C** | **G** | **G** | **G** | **A** | **C** | **A** | **G** | **G** | **A** | **T** | **T** | **T** | **A** | **A** | **T** | **A** | **T** | **G** | **A** | **A** | **T** | **G** | **A** | **A** | **A** | **C** | **C** | **A** | **A** | **A** | **A** | **C** | **G** | **G** | **G** | **A** | **A** | **G** | **T** | **T** | **A** | **T** | **G** | **G** | **A** | **T** | **T** | **G** | **A** | **A** | **C** | **A** | **A** | **T** | **A** | **T** | **C** | **A** | **A** | **A** | **G** | **A** | **A** | **C** | **G** | **A** | **G** | **T** | **A** | **G** | **C** | **T** | **G** | **G** | **T** | **A** | **T** | **T** | **G** | **G** | **T** | **G** | **G** | **A** | **A** | **C** | **A** | **G** | **T** | **A** | **A** | **A** | **A** | **A** | **T** | **C** | **A** | **T** | **T** | **A** | **G** | **T** | **T** | **T** | **T** | **A** | **A** | **A** | **G** | **G** | **G** | **C** | **A** | **A** | **G** | **G** | **T** | **A** | **C** | **G** | **A** | **G** | **C** | **G** | **T** | **G** | **G** | **A** | **G** | **A** | **T** | **C** | **A** | **A** | **G** | **G** | **T** | **C** | **C** | **C** | **T** | **T** | **T** | **G** | **A** | **T** | **G** | **A** | **A** | **G** | **G** | **A** | **G** | **G** | **C** | **A** | **-** | **-** | **-** |
| **N60519F** | **A** | **T** | **G** | **A** | **T** | **G** | **G** | **G** | **A** | **A** | **G** | **A** | **A** | **T** | **A** | **T** | **C** | **C** | **A** | **A** | **A** | **G** | **C** | **A** | **A** | **T** | **G** | **C** | **T** | **A** | **G** | **C** | **T** | **G** | **T** | **T** | **T** | **A** | **T** | **T** | **C** | **G** | **G** | **G** | **G** | **A** | **T** | **T** | **G** | **C** | **G** | **G** | **C** | **T** | **T** | **T** | **C** | **C** | **T** | **T** | **A** | **T** | **T** | **A** | **T** | **T** | **C** | **T** | **A** | **T** | **T** | **T** | **T** | **C** | **A** | **C** | **T** | **T** | **T** | **T** | **C** | **A** | **C** | **T** | **T** | **A** | **T** | **T** | **T** | **T** | **T** | **A** | **T** | **G** | **C** | **C** | **A** | **G** | **T** | **A** | **A** | **C** | **C** | **A** | **A** | **A** | **G** | **T** | **C** | **A** | **T** | **T** | **G** | **G** | **T** | **G** | **G** | **G** | **G** | **A** | **G** | **A** | **A** | **T** | **T** | **G** | **C** | **T** | **G** | **C** | **G** | **T** | **G** | **C** | **G** | **C** | **G** | **T** | **T** | **T** | **A** | **T** | **T** | **A** | **T** | **A** | **C** | **G** | **T** | **T** | **C** | **C** | **G** | **C** | **T** | **T** | **A** | **T** | **C** | **T** | **T** | **C** | **C** | **A** | **T** | **T** | **T** | **G** | **A** | **T** | **G** | **G** | **C** | **A** | **A** | **T** | **T** | **T** | **C** | **A** | **T** | **T** | **A** | **G** | **G** | **T** | **G** | **T** | **A** | **G** | **G** | **A** | **T** | **T** | **G** | **A** | **T** | **C** | **G** | **T** | **A** | **T** | **T** | **T** | **C** | **T** | **A** | **T** | **T** | **G** | **T** | **T** | **A** | **T** | **C** | **A** | **C** | **T** | **T** | **A** | **T** | **T** | **C** | **A** | **A** | **A** | **A** | **G** | **G** | **C** | **A** | **A** | **A** | **A** | **T** | **A** | **C** | **G** | **G** | **G** | **A** | **A** | **A** | **A** | **T** | **C** | **G** | **A** | **A** | **G** | **A** | **A** | **A** | **A** | **G** | **C** | **T** | **T** | **C** | **G** | **T** | **G** | **C** | **A** | **C** | **T** | **T** | **T** | **C** | **T** | **T** | **C** | **T** | **G** | **G** | **C** | **A** | **A** | **T** | **T** | **A** | **T** | **G** | **A** | **A** | **T** | **C** | **C** | **A** | **A** | **A** | **C** | **T** | **T** | **T** | **T** | **G** | **C** | **A** | **T** | **C** | **A** | **G** | **C** | **C** | **G** | **A** | **T** | **C** | **C** | **C** | **A** | **A** | **G** | **C** | **G** | **C** | **G** | **T** | **C** | **G** | **G** | **A** | **T** | **G** | **A** | **T** | **C** | **T** | **G** | **T** | **A** | **C** | **A** | **T** | **C** | **A** | **A** | **A** | **G** | **A** | **T** | **A** | **T** | **T** | **G** | **A** | **T** | **A** | **T** | **G** | **G** | **A** | **A** | **A** | **T** | **T** | **A** | **C** | **T** | **A** | **A** | **A** | **A** | **T** | **A** | **A** | **A** | **A** | **G** | **A** | **A** | **A** | **A** | **A** | **A** | **T** | **G** | **A** | **T** | **C** | **G** | **A** | **A** | **G** | **T** | **A** | **T** | **C** | **C** | **A** | **G** | **T** | **G** | **A** | **A** | **T** | **T** | **A** | **C** | **A** | **G** | **A** | **T** | **T** | **A** | **T** | **G** | **A** | **C** | **T** | **A** | **G** | **C** | **C** | **G** | **T** | **C** | **C** | **T** | **C** | **A** | **A** | **T** | **A** | **T** | **G** | **T** | **A** | **G** | **A** | **T** | **G** | **G** | **A** | **C** | **A** | **A** | **A** | **C** | **C** | **A** | **A** | **A** | **G** | **A** | **A** | **G** | **A** | **G** | **A** | **T** | **T** | **T** | **T** | **A** | **G** | **A** | **A** | **T** | **T** | **A** | **G** | **A** | **A** | **A** | **G** | **G** | **C** | **A** | **T** | **C** | **G** | **A** | **T** | **T** | **A** | **G** | **C** | **T** | **C** | **G** | **T** | **G** | **A** | **G** | **C** | **T** | **T** | **C** | **A** | **T** | **G** | **A** | **T** | **T** | **C** | **A** | **G** | **T** | **T** | **T** | **C** | **A** | **C** | **A** | **G** | **C** | **A** | **A** | **T** | **T** | **A** | **T** | **T** | **T** | **G** | **C** | **A** | **G** | **C** | **G** | **A** | **T** | **G** | **A** | **T** | **G** | **A** | **T** | **G** | **A** | **T** | **G** | **T** | **C** | **G** | **G** | **C** | **A** | **T** | **T** | **G** | **A** | **C** | **G** | **G** | **A** | **G** | **C** | **A** | **A** | **G** | **C** | **A** | **G** | **A** | **A** | **A** | **A** | **A** | **A** | **G** | **T** | **G** | **A** | **G** | **A** | **C** | **A** | **C** | **C** | **A** | **G** | **A** | **A** | **A** | **T** | **G** | **T** | **T** | **C** | **C** | **G** | **T** | **A** | **A** | **G** | **C** | **A** | **G** | **T** | **T** | **G** | **A** | **A** | **A** | **A** | **T** | **G** | **G** | **T** | **G** | **G** | **C** | **A** | **G** | **A** | **G** | **A** | **T** | **C** | **A** | **T** | **C** | **A** | **A** | **C** | **G** | **C** | **T** | **T** | **C** | **C** | **C** | **A** | **G** | **T** | **C** | **T** | **G** | **A** | **G** | **A** | **T** | **G** | **C** | **G** | **T** | **G** | **C** | **G** | **C** | **T** | **G** | **C** | **T** | **T** | **C** | **T** | **T** | **C** | **A** | **T** | **C** | **T** | **G** | **C** | **G** | **T** | **C** | **C** | **A** | **G** | **T** | **C** | **A** | **A** | **T** | **T** | **T** | **A** | **G** | **A** | **A** | **G** | **A** | **A** | **A** | **A** | **A** | **A** | **G** | **T** | **T** | **T** | **A** | **A** | **A** | **A** | **C** | **A** | **A** | **G** | **G** | **C** | **A** | **T** | **T** | **G** | **A** | **G** | **C** | **A** | **G** | **T** | **T** | **A** | **T** | **T** | **G** | **A** | **A** | **G** | **G** | **A** | **A** | **T** | **T** | **G** | **C** | **A** | **G** | **A** | **A** | **T** | **A** | **A** | **A** | **A** | **T** | **C** | **C** | **A** | **G** | **A** | **T** | **T** | **T** | **C** | **G** | **C** | **T** | **A** | **A** | **A** | **A** | **T** | **G** | **G** | **G** | **A** | **T** | **A** | **T** | **A** | **G** | **A** | **A** | **G** | **A** | **T** | **G** | **T** | **A** | **A** | **A** | **A** | **T** | **T** | **A** | **A** | **C** | **T** | **A** | **G** | **T** | **T** | **C** | **C** | **A** | **T** | **T** | **G** | **A** | **G** | **G** | **A** | **T** | **C** | **A** | **C** | **T** | **T** | **G** | **T** | **T** | **C** | **C** | **G** | **A** | **A** | **T** | **C** | **G** | **T** | **T** | **C** | **A** | **A** | **G** | **A** | **A** | **T** | **T** | **G** | **T** | **T** | **A** | **T** | **C** | **A** | **A** | **A** | **C** | **A** | **C** | **A** | **T** | **T** | **A** | **A** | **G** | **A** | **C** | **A** | **T** | **G** | **C** | **T** | **A** | **A** | **G** | **G** | **C** | **T** | **A** | **A** | **T** | **G** | **A** | **A** | **T** | **T** | **A** | **G** | **A** | **A** | **G** | **T** | **C** | **T** | **A** | **T** | **T** | **T** | **G** | **C** | **A** | **T** | **A** | **A** | **A** | **A** | **T** | **A** | **G** | **A** | **T** | **A** | **A** | **C** | **A** | **A** | **T** | **C** | **T** | **T** | **T** | **T** | **A** | **T** | **T** | **A** | **C** | **G** | **T** | **A** | **T** | **C** | **A** | **T** | **T** | **G** | **A** | **C** | **G** | **A** | **C** | **G** | **G** | **G** | **A** | **C** | **A** | **G** | **G** | **A** | **T** | **T** | **T** | **A** | **A** | **T** | **A** | **T** | **G** | **A** | **A** | **T** | **G** | **A** | **A** | **A** | **C** | **C** | **A** | **A** | **A** | **A** | **C** | **G** | **G** | **G** | **A** | **A** | **G** | **T** | **T** | **A** | **T** | **G** | **G** | **A** | **T** | **T** | **G** | **A** | **A** | **C** | **A** | **A** | **T** | **A** | **T** | **C** | **A** | **A** | **A** | **G** | **A** | **A** | **C** | **G** | **A** | **G** | **T** | **A** | **G** | **C** | **T** | **G** | **G** | **T** | **A** | **T** | **T** | **G** | **G** | **T** | **G** | **G** | **A** | **A** | **C** | **A** | **G** | **T** | **A** | **A** | **A** | **A** | **A** | **T** | **C** | **A** | **T** | **T** | **A** | **G** | **T** | **T** | **T** | **T** | **A** | **A** | **A** | **G** | **G** | **G** | **C** | **A** | **A** | **G** | **G** | **T** | **A** | **C** | **G** | **A** | **G** | **C** | **G** | **T** | **G** | **G** | **A** | **G** | **A** | **T** | **C** | **A** | **A** | **G** | **G** | **T** | **C** | **C** | **C** | **T** | **T** | **T** | **G** | **A** | **T** | **G** | **A** | **A** | **G** | **G** | **A** | **G** | **G** | **C** | **A** | **-** | **-** | **-** |
| **N52636** | **A** | **T** | **G** | **A** | **T** | **G** | **G** | **G** | **A** | **A** | **G** | **A** | **A** | **T** | **A** | **T** | **C** | **C** | **A** | **A** | **A** | **G** | **C** | **A** | **A** | **T** | **G** | **C** | **T** | **A** | **G** | **C** | **T** | **G** | **T** | **T** | **T** | **A** | **T** | **T** | **C** | **G** | **G** | **G** | **G** | **A** | **T** | **T** | **G** | **C** | **G** | **G** | **C** | **T** | **T** | **T** | **C** | **C** | **T** | **T** | **A** | **T** | **T** | **A** | **T** | **T** | **C** | **T** | **A** | **T** | **T** | **T** | **T** | **C** | **A** | **C** | **T** | **T** | **T** | **T** | **C** | **A** | **C** | **T** | **T** | **A** | **T** | **T** | **T** | **T** | **T** | **A** | **T** | **G** | **C** | **C** | **A** | **G** | **T** | **A** | **A** | **C** | **C** | **A** | **A** | **A** | **G** | **T** | **C** | **A** | **T** | **T** | **G** | **G** | **T** | **G** | **G** | **G** | **G** | **A** | **G** | **A** | **A** | **T** | **T** | **G** | **C** | **T** | **G** | **C** | **G** | **T** | **G** | **C** | **G** | **C** | **G** | **T** | **T** | **T** | **A** | **T** | **T** | **A** | **T** | **A** | **C** | **G** | **T** | **T** | **C** | **C** | **G** | **C** | **T** | **T** | **A** | **T** | **C** | **T** | **T** | **C** | **C** | **A** | **T** | **T** | **T** | **G** | **A** | **T** | **G** | **G** | **C** | **A** | **A** | **T** | **T** | **T** | **C** | **A** | **T** | **T** | **A** | **G** | **G** | **T** | **G** | **T** | **A** | **G** | **G** | **A** | **T** | **T** | **G** | **A** | **T** | **C** | **G** | **T** | **A** | **T** | **T** | **T** | **C** | **T** | **A** | **T** | **T** | **G** | **T** | **T** | **A** | **T** | **C** | **A** | **C** | **T** | **T** | **A** | **T** | **T** | **C** | **A** | **A** | **A** | **A** | **G** | **G** | **C** | **A** | **A** | **A** | **A** | **T** | **A** | **C** | **G** | **G** | **G** | **A** | **A** | **A** | **A** | **T** | **C** | **G** | **A** | **A** | **G** | **A** | **A** | **A** | **A** | **G** | **C** | **T** | **T** | **C** | **G** | **T** | **G** | **C** | **A** | **C** | **T** | **T** | **T** | **C** | **T** | **T** | **C** | **T** | **G** | **G** | **C** | **A** | **A** | **T** | **T** | **A** | **T** | **G** | **A** | **A** | **T** | **C** | **C** | **A** | **A** | **A** | **C** | **T** | **T** | **T** | **T** | **G** | **C** | **A** | **T** | **C** | **A** | **G** | **C** | **C** | **G** | **A** | **T** | **C** | **C** | **C** | **A** | **A** | **G** | **C** | **G** | **C** | **G** | **T** | **C** | **G** | **G** | **A** | **T** | **G** | **A** | **T** | **C** | **T** | **G** | **T** | **A** | **C** | **A** | **T** | **C** | **A** | **A** | **A** | **G** | **A** | **T** | **A** | **T** | **T** | **G** | **A** | **T** | **A** | **T** | **G** | **G** | **A** | **A** | **A** | **T** | **T** | **A** | **C** | **T** | **A** | **A** | **A** | **A** | **T** | **A** | **A** | **A** | **A** | **G** | **A** | **A** | **A** | **A** | **A** | **A** | **T** | **G** | **A** | **T** | **C** | **G** | **A** | **A** | **G** | **T** | **A** | **T** | **C** | **C** | **A** | **G** | **T** | **G** | **A** | **A** | **T** | **T** | **A** | **C** | **A** | **G** | **A** | **T** | **T** | **A** | **T** | **G** | **A** | **C** | **T** | **A** | **G** | **C** | **C** | **G** | **T** | **C** | **C** | **T** | **C** | **A** | **A** | **T** | **A** | **T** | **G** | **T** | **A** | **G** | **A** | **T** | **G** | **G** | **A** | **C** | **A** | **A** | **A** | **C** | **C** | **A** | **A** | **A** | **G** | **A** | **A** | **G** | **A** | **G** | **A** | **T** | **T** | **T** | **T** | **A** | **G** | **A** | **A** | **T** | **T** | **A** | **G** | **A** | **A** | **A** | **G** | **G** | **C** | **A** | **T** | **C** | **G** | **A** | **T** | **T** | **A** | **G** | **C** | **T** | **C** | **G** | **T** | **G** | **A** | **G** | **C** | **T** | **T** | **C** | **A** | **T** | **G** | **A** | **T** | **T** | **C** | **A** | **G** | **T** | **T** | **T** | **C** | **A** | **C** | **A** | **G** | **C** | **A** | **A** | **T** | **T** | **A** | **T** | **T** | **T** | **G** | **C** | **A** | **G** | **C** | **G** | **A** | **T** | **G** | **A** | **T** | **G** | **A** | **T** | **G** | **A** | **T** | **G** | **T** | **C** | **G** | **G** | **C** | **A** | **T** | **T** | **G** | **A** | **C** | **G** | **G** | **A** | **G** | **C** | **A** | **A** | **G** | **C** | **A** | **G** | **A** | **A** | **A** | **A** | **A** | **A** | **G** | **T** | **G** | **A** | **G** | **A** | **C** | **A** | **C** | **C** | **A** | **G** | **A** | **A** | **A** | **T** | **G** | **T** | **T** | **C** | **C** | **G** | **T** | **A** | **A** | **G** | **C** | **A** | **G** | **T** | **T** | **G** | **A** | **A** | **A** | **A** | **T** | **G** | **G** | **T** | **G** | **G** | **C** | **A** | **G** | **A** | **G** | **A** | **T** | **C** | **A** | **T** | **C** | **A** | **A** | **C** | **G** | **C** | **T** | **T** | **C** | **C** | **C** | **A** | **G** | **T** | **C** | **T** | **G** | **A** | **G** | **A** | **T** | **G** | **C** | **G** | **T** | **G** | **C** | **G** | **C** | **T** | **G** | **C** | **T** | **T** | **C** | **T** | **T** | **C** | **A** | **T** | **C** | **T** | **G** | **C** | **G** | **T** | **C** | **C** | **A** | **G** | **T** | **C** | **A** | **A** | **T** | **T** | **T** | **A** | **G** | **A** | **A** | **G** | **A** | **A** | **A** | **A** | **A** | **A** | **G** | **T** | **T** | **T** | **A** | **A** | **A** | **A** | **C** | **A** | **A** | **G** | **G** | **C** | **A** | **T** | **T** | **G** | **A** | **G** | **C** | **A** | **G** | **T** | **T** | **A** | **T** | **T** | **G** | **A** | **A** | **G** | **G** | **A** | **A** | **T** | **T** | **G** | **C** | **A** | **G** | **A** | **A** | **T** | **A** | **A** | **A** | **A** | **T** | **C** | **C** | **A** | **G** | **A** | **T** | **T** | **T** | **C** | **G** | **C** | **T** | **A** | **A** | **A** | **A** | **T** | **G** | **G** | **G** | **A** | **T** | **G** | **T** | **A** | **G** | **A** | **A** | **G** | **A** | **T** | **G** | **T** | **A** | **A** | **A** | **A** | **T** | **T** | **A** | **A** | **C** | **T** | **A** | **G** | **T** | **T** | **C** | **C** | **A** | **T** | **T** | **G** | **A** | **G** | **G** | **A** | **T** | **C** | **A** | **C** | **T** | **T** | **G** | **T** | **T** | **C** | **C** | **G** | **A** | **A** | **T** | **C** | **G** | **T** | **T** | **C** | **A** | **A** | **G** | **A** | **A** | **T** | **T** | **G** | **T** | **T** | **A** | **T** | **C** | **A** | **A** | **A** | **C** | **A** | **C** | **A** | **T** | **T** | **A** | **A** | **G** | **A** | **C** | **A** | **T** | **G** | **C** | **T** | **A** | **A** | **G** | **G** | **C** | **T** | **A** | **A** | **T** | **G** | **A** | **A** | **T** | **T** | **A** | **G** | **A** | **A** | **G** | **T** | **C** | **T** | **A** | **T** | **T** | **T** | **G** | **C** | **A** | **T** | **A** | **A** | **A** | **A** | **T** | **A** | **G** | **A** | **T** | **A** | **A** | **C** | **A** | **A** | **T** | **C** | **T** | **T** | **T** | **T** | **A** | **T** | **T** | **A** | **C** | **G** | **T** | **A** | **T** | **C** | **A** | **T** | **T** | **G** | **A** | **C** | **G** | **A** | **C** | **G** | **G** | **G** | **A** | **C** | **A** | **G** | **G** | **A** | **T** | **T** | **T** | **A** | **A** | **T** | **A** | **T** | **G** | **A** | **A** | **T** | **G** | **A** | **A** | **A** | **C** | **C** | **A** | **A** | **A** | **A** | **C** | **G** | **G** | **G** | **A** | **A** | **G** | **T** | **T** | **A** | **T** | **G** | **G** | **A** | **T** | **T** | **G** | **A** | **A** | **C** | **A** | **A** | **T** | **A** | **T** | **C** | **A** | **A** | **A** | **G** | **A** | **A** | **C** | **G** | **A** | **G** | **T** | **A** | **G** | **C** | **T** | **G** | **G** | **T** | **A** | **T** | **T** | **G** | **G** | **T** | **G** | **G** | **A** | **A** | **C** | **A** | **G** | **T** | **A** | **A** | **A** | **A** | **A** | **T** | **C** | **A** | **T** | **T** | **A** | **G** | **T** | **T** | **T** | **T** | **A** | **A** | **A** | **G** | **G** | **G** | **C** | **A** | **A** | **G** | **G** | **T** | **A** | **C** | **G** | **A** | **G** | **C** | **G** | **T** | **G** | **G** | **A** | **G** | **A** | **T** | **C** | **A** | **A** | **G** | **G** | **T** | **C** | **C** | **C** | **T** | **T** | **T** | **G** | **A** | **T** | **G** | **A** | **A** | **G** | **G** | **A** | **G** | **G** | **C** | **A** | **-** | **-** | **-** |
| **N60045F** | **A** | **T** | **G** | **A** | **T** | **G** | **G** | **G** | **A** | **A** | **G** | **A** | **A** | **T** | **A** | **T** | **C** | **C** | **A** | **A** | **A** | **G** | **C** | **A** | **A** | **T** | **G** | **C** | **T** | **A** | **G** | **C** | **T** | **G** | **T** | **T** | **T** | **A** | **T** | **T** | **C** | **G** | **G** | **G** | **G** | **A** | **T** | **T** | **G** | **C** | **G** | **G** | **C** | **T** | **T** | **T** | **C** | **C** | **T** | **T** | **A** | **T** | **T** | **A** | **T** | **T** | **C** | **T** | **A** | **T** | **T** | **T** | **T** | **C** | **A** | **C** | **T** | **T** | **T** | **T** | **C** | **A** | **C** | **T** | **T** | **A** | **T** | **T** | **T** | **T** | **T** | **A** | **T** | **G** | **C** | **C** | **A** | **G** | **T** | **A** | **A** | **C** | **C** | **A** | **A** | **A** | **G** | **T** | **C** | **A** | **T** | **T** | **G** | **G** | **T** | **G** | **G** | **G** | **G** | **A** | **G** | **A** | **A** | **T** | **T** | **G** | **C** | **T** | **G** | **C** | **G** | **T** | **G** | **C** | **G** | **C** | **G** | **T** | **T** | **T** | **A** | **T** | **T** | **A** | **T** | **A** | **C** | **G** | **T** | **T** | **C** | **C** | **G** | **C** | **T** | **T** | **A** | **T** | **C** | **T** | **T** | **C** | **C** | **A** | **T** | **T** | **T** | **G** | **A** | **T** | **G** | **G** | **C** | **A** | **A** | **T** | **T** | **T** | **C** | **A** | **T** | **T** | **A** | **G** | **G** | **T** | **G** | **T** | **A** | **G** | **G** | **A** | **T** | **T** | **G** | **A** | **T** | **C** | **G** | **T** | **A** | **T** | **T** | **T** | **C** | **T** | **A** | **T** | **T** | **G** | **T** | **T** | **A** | **T** | **C** | **A** | **C** | **T** | **T** | **A** | **T** | **T** | **C** | **A** | **A** | **A** | **A** | **G** | **G** | **C** | **A** | **A** | **A** | **A** | **T** | **A** | **C** | **G** | **G** | **G** | **A** | **A** | **A** | **A** | **T** | **C** | **G** | **A** | **A** | **G** | **A** | **A** | **A** | **A** | **G** | **C** | **T** | **T** | **C** | **G** | **T** | **G** | **C** | **A** | **C** | **T** | **T** | **T** | **C** | **T** | **T** | **C** | **T** | **G** | **G** | **C** | **A** | **A** | **T** | **T** | **A** | **T** | **G** | **A** | **A** | **T** | **C** | **C** | **A** | **A** | **A** | **C** | **T** | **T** | **T** | **T** | **G** | **C** | **A** | **T** | **C** | **A** | **G** | **C** | **C** | **G** | **A** | **T** | **C** | **C** | **C** | **A** | **A** | **G** | **C** | **A** | **C** | **G** | **T** | **C** | **G** | **G** | **A** | **T** | **G** | **A** | **T** | **C** | **T** | **G** | **T** | **A** | **C** | **A** | **T** | **C** | **A** | **A** | **A** | **G** | **A** | **T** | **A** | **T** | **T** | **G** | **A** | **T** | **A** | **T** | **G** | **G** | **A** | **A** | **A** | **T** | **T** | **A** | **C** | **T** | **A** | **A** | **A** | **A** | **T** | **A** | **A** | **A** | **A** | **G** | **A** | **A** | **A** | **A** | **A** | **A** | **T** | **G** | **A** | **T** | **C** | **G** | **A** | **A** | **G** | **T** | **A** | **T** | **C** | **C** | **A** | **G** | **T** | **G** | **A** | **A** | **T** | **T** | **A** | **C** | **A** | **G** | **A** | **T** | **T** | **A** | **T** | **G** | **A** | **C** | **T** | **A** | **G** | **C** | **C** | **G** | **T** | **C** | **C** | **T** | **C** | **A** | **A** | **T** | **A** | **T** | **G** | **T** | **A** | **G** | **A** | **T** | **G** | **G** | **A** | **C** | **A** | **A** | **A** | **C** | **C** | **A** | **A** | **A** | **G** | **A** | **A** | **G** | **A** | **G** | **A** | **T** | **T** | **T** | **T** | **A** | **G** | **A** | **A** | **T** | **T** | **A** | **G** | **A** | **A** | **A** | **G** | **G** | **C** | **A** | **T** | **C** | **G** | **A** | **T** | **T** | **A** | **G** | **C** | **T** | **C** | **G** | **T** | **G** | **A** | **G** | **C** | **T** | **T** | **C** | **A** | **T** | **G** | **A** | **T** | **T** | **C** | **A** | **G** | **T** | **T** | **T** | **C** | **A** | **C** | **A** | **G** | **C** | **A** | **A** | **T** | **T** | **A** | **T** | **T** | **T** | **G** | **C** | **A** | **G** | **C** | **G** | **A** | **T** | **G** | **A** | **T** | **G** | **A** | **T** | **G** | **A** | **T** | **G** | **T** | **C** | **G** | **G** | **C** | **A** | **T** | **T** | **G** | **A** | **C** | **G** | **G** | **A** | **G** | **C** | **A** | **A** | **G** | **C** | **A** | **G** | **A** | **A** | **A** | **A** | **A** | **A** | **G** | **T** | **G** | **A** | **G** | **A** | **C** | **A** | **C** | **C** | **A** | **G** | **A** | **A** | **A** | **T** | **G** | **T** | **T** | **C** | **C** | **G** | **T** | **A** | **A** | **G** | **C** | **A** | **G** | **T** | **T** | **G** | **A** | **A** | **A** | **A** | **T** | **G** | **G** | **T** | **G** | **G** | **C** | **A** | **G** | **A** | **G** | **A** | **T** | **C** | **A** | **T** | **C** | **A** | **A** | **C** | **G** | **C** | **T** | **T** | **C** | **C** | **C** | **A** | **G** | **T** | **C** | **T** | **G** | **A** | **G** | **A** | **T** | **G** | **C** | **G** | **T** | **G** | **C** | **G** | **C** | **T** | **G** | **C** | **T** | **T** | **C** | **T** | **T** | **C** | **A** | **T** | **C** | **T** | **G** | **C** | **G** | **T** | **C** | **C** | **A** | **G** | **T** | **C** | **A** | **A** | **T** | **T** | **T** | **A** | **G** | **A** | **A** | **G** | **A** | **A** | **A** | **A** | **A** | **A** | **G** | **T** | **T** | **T** | **A** | **A** | **A** | **A** | **C** | **A** | **A** | **G** | **G** | **C** | **A** | **T** | **T** | **G** | **A** | **G** | **C** | **A** | **G** | **T** | **T** | **A** | **T** | **T** | **G** | **A** | **A** | **G** | **G** | **A** | **A** | **T** | **T** | **G** | **C** | **A** | **G** | **A** | **A** | **T** | **A** | **A** | **A** | **A** | **T** | **C** | **C** | **A** | **G** | **A** | **T** | **T** | **T** | **C** | **G** | **C** | **T** | **A** | **A** | **A** | **A** | **T** | **G** | **G** | **A** | **A** | **T** | **G** | **T** | **A** | **G** | **A** | **A** | **G** | **A** | **T** | **G** | **T** | **A** | **A** | **A** | **A** | **T** | **T** | **A** | **A** | **C** | **T** | **A** | **G** | **T** | **T** | **C** | **C** | **A** | **T** | **T** | **G** | **A** | **G** | **G** | **A** | **T** | **C** | **A** | **C** | **T** | **T** | **G** | **T** | **T** | **C** | **C** | **G** | **A** | **A** | **T** | **C** | **G** | **T** | **T** | **C** | **A** | **A** | **G** | **A** | **A** | **T** | **T** | **G** | **T** | **T** | **A** | **T** | **C** | **A** | **A** | **A** | **C** | **A** | **C** | **A** | **T** | **T** | **A** | **A** | **G** | **A** | **C** | **A** | **T** | **G** | **C** | **T** | **A** | **A** | **G** | **G** | **C** | **T** | **A** | **A** | **T** | **G** | **A** | **A** | **T** | **T** | **A** | **G** | **A** | **A** | **G** | **T** | **C** | **T** | **A** | **T** | **T** | **T** | **G** | **C** | **A** | **T** | **A** | **A** | **A** | **A** | **T** | **A** | **G** | **A** | **T** | **A** | **A** | **C** | **A** | **A** | **T** | **C** | **T** | **T** | **T** | **T** | **A** | **T** | **T** | **A** | **C** | **G** | **T** | **A** | **T** | **C** | **A** | **T** | **T** | **G** | **A** | **C** | **G** | **A** | **C** | **G** | **G** | **G** | **A** | **C** | **A** | **G** | **G** | **A** | **T** | **T** | **T** | **A** | **A** | **T** | **A** | **T** | **G** | **A** | **A** | **T** | **G** | **A** | **A** | **A** | **C** | **C** | **A** | **A** | **A** | **A** | **C** | **G** | **G** | **G** | **A** | **A** | **G** | **T** | **T** | **A** | **T** | **G** | **G** | **A** | **T** | **T** | **G** | **A** | **A** | **C** | **A** | **A** | **T** | **A** | **T** | **C** | **A** | **A** | **A** | **G** | **A** | **A** | **C** | **G** | **A** | **G** | **T** | **A** | **G** | **C** | **T** | **G** | **G** | **T** | **A** | **T** | **T** | **G** | **G** | **T** | **G** | **G** | **A** | **A** | **C** | **A** | **G** | **T** | **A** | **A** | **A** | **A** | **A** | **T** | **C** | **A** | **T** | **T** | **A** | **G** | **T** | **T** | **T** | **T** | **A** | **A** | **A** | **G** | **G** | **G** | **C** | **A** | **A** | **G** | **G** | **T** | **A** | **C** | **G** | **A** | **G** | **C** | **G** | **T** | **G** | **G** | **A** | **G** | **A** | **T** | **C** | **A** | **A** | **G** | **G** | **T** | **C** | **C** | **C** | **T** | **T** | **T** | **G** | **A** | **T** | **G** | **A** | **A** | **G** | **G** | **A** | **G** | **G** | **C** | **A** | **-** | **-** | **-** |
| **N54107** | **A** | **T** | **G** | **A** | **T** | **G** | **G** | **G** | **A** | **A** | **G** | **A** | **A** | **T** | **A** | **T** | **C** | **C** | **A** | **A** | **A** | **G** | **C** | **A** | **A** | **T** | **G** | **C** | **T** | **A** | **G** | **C** | **T** | **G** | **T** | **T** | **T** | **A** | **T** | **T** | **C** | **G** | **G** | **G** | **G** | **A** | **T** | **T** | **G** | **C** | **G** | **G** | **C** | **T** | **T** | **T** | **C** | **C** | **T** | **T** | **A** | **T** | **T** | **A** | **T** | **T** | **C** | **T** | **A** | **T** | **T** | **T** | **T** | **C** | **A** | **C** | **T** | **T** | **T** | **T** | **C** | **A** | **C** | **T** | **T** | **A** | **T** | **T** | **T** | **T** | **T** | **A** | **T** | **G** | **C** | **C** | **A** | **G** | **T** | **A** | **A** | **C** | **C** | **A** | **A** | **A** | **G** | **T** | **C** | **A** | **T** | **T** | **G** | **G** | **T** | **G** | **G** | **G** | **G** | **A** | **G** | **A** | **A** | **T** | **T** | **G** | **C** | **T** | **G** | **C** | **G** | **T** | **G** | **C** | **G** | **C** | **G** | **T** | **T** | **T** | **A** | **T** | **T** | **A** | **T** | **A** | **C** | **G** | **T** | **T** | **C** | **C** | **G** | **C** | **T** | **T** | **A** | **T** | **C** | **T** | **T** | **C** | **C** | **A** | **T** | **T** | **T** | **G** | **A** | **T** | **G** | **G** | **C** | **A** | **A** | **T** | **T** | **T** | **C** | **A** | **T** | **T** | **A** | **G** | **G** | **T** | **G** | **T** | **A** | **G** | **G** | **A** | **T** | **T** | **G** | **A** | **T** | **C** | **G** | **T** | **A** | **T** | **T** | **T** | **C** | **T** | **A** | **T** | **T** | **G** | **T** | **T** | **A** | **T** | **C** | **A** | **C** | **T** | **T** | **A** | **T** | **T** | **C** | **A** | **A** | **A** | **A** | **G** | **G** | **C** | **A** | **A** | **A** | **A** | **T** | **A** | **C** | **G** | **G** | **G** | **A** | **A** | **A** | **A** | **T** | **C** | **G** | **A** | **A** | **G** | **A** | **A** | **A** | **A** | **G** | **C** | **T** | **T** | **C** | **G** | **T** | **G** | **C** | **A** | **C** | **T** | **T** | **T** | **C** | **T** | **T** | **C** | **T** | **G** | **G** | **C** | **A** | **A** | **T** | **T** | **A** | **T** | **G** | **A** | **A** | **T** | **C** | **C** | **A** | **A** | **A** | **C** | **T** | **T** | **T** | **T** | **G** | **C** | **A** | **T** | **C** | **A** | **G** | **C** | **C** | **G** | **A** | **T** | **C** | **C** | **C** | **A** | **A** | **G** | **C** | **G** | **C** | **G** | **T** | **C** | **G** | **G** | **A** | **T** | **G** | **A** | **T** | **C** | **T** | **G** | **T** | **A** | **C** | **A** | **T** | **C** | **A** | **A** | **A** | **G** | **A** | **T** | **A** | **T** | **T** | **G** | **A** | **T** | **A** | **T** | **G** | **G** | **A** | **A** | **A** | **T** | **T** | **A** | **C** | **T** | **A** | **A** | **A** | **A** | **T** | **A** | **A** | **A** | **A** | **G** | **A** | **A** | **A** | **A** | **A** | **A** | **T** | **G** | **A** | **T** | **C** | **G** | **A** | **A** | **G** | **T** | **A** | **T** | **C** | **C** | **A** | **G** | **T** | **G** | **A** | **A** | **T** | **T** | **A** | **C** | **A** | **G** | **A** | **T** | **T** | **A** | **T** | **G** | **A** | **C** | **T** | **A** | **G** | **C** | **C** | **G** | **T** | **C** | **C** | **T** | **C** | **A** | **A** | **T** | **A** | **T** | **G** | **T** | **A** | **G** | **A** | **T** | **G** | **G** | **C** | **C** | **A** | **A** | **A** | **C** | **C** | **A** | **A** | **A** | **G** | **A** | **A** | **G** | **A** | **G** | **A** | **T** | **T** | **T** | **T** | **A** | **G** | **A** | **A** | **T** | **T** | **A** | **G** | **A** | **A** | **A** | **G** | **G** | **C** | **A** | **T** | **C** | **G** | **A** | **T** | **T** | **A** | **G** | **C** | **T** | **C** | **G** | **T** | **G** | **A** | **G** | **C** | **T** | **T** | **C** | **A** | **T** | **G** | **A** | **T** | **T** | **C** | **A** | **G** | **T** | **T** | **T** | **C** | **A** | **C** | **A** | **G** | **C** | **A** | **A** | **T** | **T** | **A** | **T** | **T** | **T** | **G** | **C** | **A** | **G** | **C** | **G** | **A** | **T** | **G** | **A** | **T** | **G** | **A** | **T** | **G** | **A** | **T** | **G** | **T** | **C** | **G** | **G** | **C** | **A** | **T** | **T** | **G** | **A** | **C** | **G** | **G** | **A** | **G** | **C** | **A** | **A** | **G** | **C** | **A** | **G** | **A** | **A** | **A** | **A** | **A** | **A** | **G** | **T** | **G** | **A** | **G** | **A** | **C** | **A** | **C** | **C** | **A** | **G** | **A** | **A** | **A** | **T** | **G** | **T** | **T** | **C** | **C** | **G** | **T** | **A** | **A** | **G** | **C** | **A** | **G** | **T** | **T** | **G** | **A** | **A** | **A** | **A** | **T** | **G** | **G** | **T** | **G** | **G** | **C** | **A** | **G** | **A** | **G** | **A** | **T** | **C** | **A** | **T** | **C** | **A** | **A** | **C** | **G** | **C** | **T** | **T** | **C** | **C** | **C** | **A** | **G** | **T** | **C** | **T** | **G** | **A** | **G** | **A** | **T** | **G** | **C** | **G** | **T** | **G** | **C** | **G** | **C** | **T** | **G** | **C** | **T** | **T** | **C** | **T** | **T** | **C** | **A** | **T** | **C** | **T** | **G** | **C** | **G** | **T** | **C** | **C** | **A** | **G** | **T** | **C** | **A** | **A** | **T** | **T** | **T** | **A** | **G** | **A** | **A** | **G** | **A** | **A** | **A** | **A** | **A** | **A** | **G** | **T** | **T** | **T** | **A** | **A** | **A** | **A** | **C** | **A** | **A** | **G** | **G** | **C** | **A** | **T** | **T** | **G** | **A** | **G** | **C** | **A** | **G** | **T** | **T** | **A** | **T** | **T** | **G** | **A** | **A** | **G** | **G** | **A** | **A** | **T** | **T** | **G** | **C** | **A** | **G** | **A** | **A** | **T** | **A** | **A** | **A** | **A** | **T** | **C** | **C** | **A** | **G** | **A** | **T** | **T** | **T** | **C** | **G** | **C** | **T** | **A** | **A** | **A** | **A** | **T** | **G** | **G** | **G** | **A** | **T** | **G** | **T** | **A** | **G** | **A** | **A** | **G** | **A** | **T** | **G** | **T** | **A** | **A** | **A** | **A** | **T** | **T** | **A** | **A** | **C** | **T** | **A** | **G** | **T** | **T** | **C** | **C** | **A** | **T** | **T** | **G** | **A** | **G** | **G** | **A** | **T** | **C** | **A** | **C** | **T** | **T** | **G** | **T** | **T** | **C** | **C** | **G** | **A** | **A** | **T** | **C** | **G** | **T** | **T** | **C** | **A** | **A** | **G** | **A** | **A** | **T** | **T** | **G** | **T** | **T** | **A** | **T** | **C** | **A** | **A** | **A** | **C** | **A** | **C** | **A** | **T** | **T** | **A** | **A** | **G** | **A** | **C** | **A** | **T** | **G** | **C** | **T** | **A** | **A** | **G** | **G** | **C** | **T** | **A** | **A** | **T** | **G** | **A** | **A** | **T** | **T** | **A** | **G** | **A** | **A** | **G** | **T** | **C** | **T** | **A** | **T** | **T** | **T** | **G** | **C** | **A** | **T** | **A** | **A** | **A** | **A** | **T** | **A** | **G** | **A** | **T** | **A** | **A** | **C** | **A** | **A** | **T** | **C** | **T** | **T** | **T** | **T** | **A** | **T** | **T** | **A** | **C** | **G** | **T** | **A** | **T** | **C** | **A** | **T** | **T** | **G** | **A** | **C** | **G** | **A** | **C** | **G** | **G** | **G** | **A** | **C** | **A** | **G** | **G** | **C** | **T** | **T** | **T** | **A** | **A** | **T** | **A** | **T** | **G** | **A** | **A** | **T** | **G** | **A** | **A** | **A** | **C** | **C** | **A** | **A** | **A** | **A** | **C** | **G** | **G** | **G** | **A** | **A** | **G** | **T** | **T** | **A** | **T** | **G** | **G** | **A** | **T** | **T** | **G** | **A** | **A** | **C** | **A** | **A** | **T** | **A** | **T** | **C** | **A** | **A** | **A** | **G** | **A** | **A** | **C** | **G** | **A** | **G** | **T** | **A** | **G** | **C** | **T** | **G** | **G** | **T** | **A** | **T** | **T** | **G** | **G** | **T** | **G** | **G** | **A** | **A** | **C** | **A** | **G** | **T** | **A** | **A** | **A** | **A** | **A** | **T** | **C** | **A** | **T** | **T** | **A** | **G** | **T** | **T** | **T** | **T** | **A** | **A** | **A** | **G** | **G** | **G** | **C** | **A** | **A** | **G** | **G** | **T** | **A** | **C** | **G** | **A** | **G** | **C** | **G** | **T** | **G** | **G** | **A** | **G** | **A** | **T** | **C** | **A** | **A** | **G** | **G** | **T** | **C** | **C** | **C** | **T** | **T** | **T** | **G** | **A** | **T** | **G** | **A** | **A** | **G** | **G** | **A** | **G** | **G** | **C** | **A** | **-** | **-** | **-** |
| **N54519** | **A** | **T** | **G** | **A** | **T** | **G** | **G** | **G** | **A** | **A** | **A** | **A** | **A** | **T** | **A** | **T** | **C** | **C** | **A** | **G** | **A** | **G** | **C** | **G** | **A** | **T** | **G** | **C** | **T** | **A** | **G** | **C** | **T** | **G** | **T** | **T** | **T** | **A** | **T** | **T** | **C** | **G** | **G** | **G** | **G** | **A** | **T** | **T** | **G** | **C** | **G** | **G** | **C** | **T** | **T** | **T** | **C** | **C** | **T** | **T** | **A** | **T** | **T** | **A** | **T** | **T** | **C** | **T** | **A** | **T** | **T** | **T** | **T** | **C** | **A** | **C** | **T** | **T** | **T** | **T** | **C** | **A** | **C** | **T** | **T** | **A** | **T** | **T** | **T** | **T** | **T** | **A** | **T** | **G** | **C** | **C** | **A** | **G** | **T** | **A** | **A** | **T** | **C** | **A** | **A** | **A** | **G** | **T** | **C** | **A** | **T** | **T** | **G** | **G** | **T** | **G** | **G** | **G** | **G** | **A** | **G** | **A** | **A** | **T** | **T** | **A** | **C** | **T** | **G** | **C** | **G** | **T** | **G** | **C** | **A** | **C** | **G** | **T** | **T** | **T** | **A** | **T** | **T** | **A** | **T** | **A** | **T** | **G** | **T** | **T** | **C** | **C** | **G** | **C** | **T** | **T** | **A** | **T** | **C** | **T** | **T** | **C** | **C** | **A** | **T** | **T** | **T** | **G** | **T** | **T** | **A** | **G** | **C** | **T** | **A** | **T** | **T** | **T** | **C** | **C** | **T** | **T** | **A** | **G** | **G** | **T** | **G** | **T** | **A** | **G** | **G** | **A** | **T** | **T** | **A** | **A** | **T** | **C** | **G** | **T** | **C** | **T** | **T** | **T** | **C** | **T** | **A** | **T** | **T** | **A** | **T** | **T** | **A** | **T** | **C** | **A** | **C** | **T** | **T** | **A** | **T** | **T** | **C** | **A** | **A** | **A** | **A** | **G** | **A** | **C** | **A** | **A** | **A** | **A** | **T** | **A** | **C** | **G** | **G** | **G** | **A** | **A** | **A** | **A** | **T** | **C** | **G** | **A** | **A** | **G** | **A** | **A** | **A** | **A** | **G** | **C** | **T** | **G** | **C** | **G** | **T** | **G** | **C** | **A** | **C** | **T** | **C** | **T** | **C** | **T** | **T** | **C** | **T** | **G** | **G** | **C** | **A** | **A** | **T** | **T** | **A** | **T** | **G** | **A** | **A** | **T** | **C** | **C** | **A** | **A** | **A** | **T** | **T** | **G** | **T** | **T** | **G** | **C** | **T** | **T** | **C** | **T** | **T** | **C** | **C** | **G** | **A** | **T** | **C** | **C** | **C** | **A** | **A** | **G** | **T** | **G** | **C** | **A** | **T** | **C** | **G** | **G** | **A** | **C** | **G** | **A** | **T** | **T** | **T** | **A** | **T** | **A** | **C** | **A** | **T** | **C** | **A** | **A** | **A** | **G** | **A** | **T** | **A** | **T** | **T** | **G** | **A** | **T** | **A** | **A** | **G** | **G** | **A** | **A** | **A** | **T** | **C** | **A** | **C** | **T** | **A** | **A** | **A** | **A** | **T** | **A** | **A** | **A** | **A** | **G** | **A** | **A** | **A** | **A** | **A** | **A** | **T** | **G** | **A** | **T** | **T** | **G** | **A** | **A** | **G** | **T** | **A** | **T** | **C** | **T** | **A** | **G** | **T** | **G** | **A** | **A** | **T** | **T** | **A** | **C** | **A** | **A** | **A** | **T** | **T** | **G** | **T** | **A** | **A** | **C** | **G** | **A** | **G** | **C** | **C** | **G** | **T** | **C** | **C** | **C** | **C** | **A** | **A** | **T** | **A** | **T** | **G** | **T** | **A** | **G** | **A** | **T** | **G** | **G** | **A** | **C** | **A** | **A** | **A** | **C** | **C** | **A** | **A** | **A** | **G** | **A** | **A** | **G** | **A** | **A** | **A** | **T** | **T** | **T** | **T** | **A** | **G** | **A** | **A** | **T** | **T** | **A** | **G** | **A** | **A** | **A** | **G** | **A** | **C** | **A** | **T** | **C** | **G** | **A** | **T** | **T** | **A** | **G** | **C** | **C** | **C** | **G** | **T** | **G** | **A** | **G** | **C** | **T** | **G** | **C** | **A** | **T** | **G** | **A** | **T** | **T** | **C** | **A** | **G** | **T** | **T** | **T** | **C** | **G** | **C** | **A** | **A** | **C** | **A** | **A** | **T** | **T** | **A** | **T** | **T** | **T** | **G** | **C** | **A** | **G** | **C** | **A** | **A** | **T** | **G** | **A** | **T** | **G** | **A** | **T** | **G** | **A** | **T** | **G** | **T** | **C** | **G** | **G** | **C** | **A** | **T** | **T** | **G** | **A** | **C** | **G** | **G** | **A** | **G** | **C** | **A** | **A** | **G** | **C** | **A** | **G** | **A** | **A** | **A** | **A** | **A** | **A** | **G** | **C** | **G** | **A** | **G** | **A** | **C** | **A** | **C** | **C** | **A** | **G** | **A** | **A** | **A** | **T** | **G** | **T** | **T** | **C** | **C** | **G** | **C** | **A** | **A** | **G** | **C** | **A** | **G** | **T** | **T** | **G** | **A** | **A** | **A** | **A** | **T** | **G** | **G** | **T** | **A** | **G** | **C** | **A** | **G** | **A** | **G** | **A** | **T** | **C** | **A** | **T** | **C** | **A** | **A** | **C** | **G** | **C** | **T** | **T** | **C** | **C** | **C** | **A** | **G** | **T** | **C** | **T** | **G** | **A** | **G** | **A** | **T** | **G** | **C** | **G** | **T** | **G** | **C** | **G** | **C** | **T** | **G** | **C** | **T** | **T** | **C** | **T** | **T** | **C** | **A** | **T** | **C** | **T** | **G** | **C** | **G** | **T** | **C** | **C** | **A** | **G** | **T** | **C** | **A** | **A** | **T** | **T** | **T** | **A** | **G** | **A** | **A** | **G** | **A** | **G** | **A** | **A** | **A** | **A** | **G** | **T** | **T** | **T** | **A** | **A** | **A** | **G** | **C** | **A** | **A** | **G** | **G** | **T** | **A** | **T** | **C** | **G** | **A** | **G** | **C** | **A** | **G** | **T** | **T** | **A** | **T** | **T** | **G** | **A** | **A** | **G** | **G** | **A** | **A** | **T** | **T** | **G** | **C** | **A** | **A** | **A** | **A** | **T** | **A** | **A** | **A** | **A** | **T** | **T** | **C** | **A** | **G** | **A** | **T** | **T** | **T** | **C** | **G** | **C** | **T** | **G** | **A** | **A** | **A** | **T** | **G** | **G** | **G** | **A** | **T** | **G** | **T** | **A** | **G** | **A** | **A** | **G** | **A** | **T** | **G** | **T** | **A** | **A** | **A** | **A** | **C** | **T** | **A** | **T** | **C** | **T** | **A** | **G** | **C** | **T** | **C** | **C** | **A** | **T** | **T** | **G** | **A** | **G** | **G** | **A** | **T** | **C** | **A** | **C** | **T** | **T** | **G** | **T** | **T** | **C** | **C** | **G** | **A** | **A** | **T** | **C** | **G** | **T** | **T** | **C** | **A** | **A** | **G** | **A** | **A** | **T** | **T** | **G** | **T** | **T** | **A** | **T** | **C** | **A** | **A** | **A** | **T** | **A** | **C** | **A** | **T** | **T** | **A** | **A** | **G** | **A** | **C** | **A** | **T** | **G** | **C** | **C** | **A** | **A** | **A** | **G** | **C** | **C** | **A** | **A** | **T** | **G** | **A** | **A** | **T** | **T** | **A** | **G** | **A** | **G** | **G** | **T** | **A** | **T** | **A** | **T** | **T** | **T** | **G** | **C** | **A** | **T** | **A** | **A** | **A** | **A** | **T** | **A** | **G** | **A** | **C** | **A** | **A** | **T** | **A** | **A** | **T** | **C** | **T** | **T** | **T** | **T** | **A** | **T** | **T** | **A** | **C** | **G** | **T** | **A** | **T** | **T** | **A** | **T** | **T** | **G** | **A** | **C** | **G** | **A** | **T** | **G** | **G** | **A** | **A** | **C** | **A** | **G** | **G** | **G** | **T** | **T** | **T** | **A** | **A** | **T** | **A** | **T** | **G** | **A** | **A** | **T** | **G** | **A** | **A** | **A** | **C** | **G** | **A** | **A** | **A** | **A** | **C** | **G** | **G** | **G** | **A** | **A** | **G** | **T** | **T** | **A** | **T** | **G** | **G** | **A** | **T** | **T** | **G** | **A** | **A** | **C** | **A** | **A** | **T** | **A** | **T** | **C** | **A** | **A** | **A** | **G** | **A** | **A** | **C** | **G** | **A** | **G** | **T** | **A** | **G** | **C** | **T** | **G** | **G** | **T** | **A** | **T** | **T** | **G** | **G** | **T** | **G** | **G** | **A** | **A** | **C** | **A** | **G** | **T** | **A** | **A** | **A** | **A** | **A** | **T** | **C** | **A** | **T** | **T** | **A** | **G** | **T** | **T** | **T** | **T** | **A** | **A** | **A** | **G** | **G** | **G** | **C** | **A** | **A** | **G** | **G** | **T** | **A** | **C** | **A** | **A** | **G** | **T** | **G** | **T** | **A** | **G** | **A** | **A** | **A** | **T** | **C** | **A** | **A** | **G** | **G** | **T** | **C** | **C** | **C** | **T** | **T** | **T** | **G** | **A** | **T** | **G** | **A** | **A** | **G** | **G** | **A** | **G** | **G** | **C** | **A** | **-** | **-** | **-** |
| **N55337** | **A** | **T** | **G** | **A** | **T** | **G** | **G** | **G** | **A** | **A** | **G** | **A** | **A** | **T** | **A** | **T** | **C** | **C** | **A** | **A** | **A** | **G** | **C** | **A** | **A** | **T** | **G** | **C** | **T** | **A** | **G** | **C** | **T** | **G** | **T** | **T** | **T** | **A** | **T** | **T** | **C** | **G** | **G** | **G** | **G** | **A** | **T** | **T** | **G** | **C** | **G** | **G** | **C** | **T** | **T** | **T** | **C** | **C** | **T** | **T** | **A** | **T** | **T** | **A** | **T** | **T** | **C** | **T** | **A** | **T** | **T** | **T** | **T** | **C** | **A** | **C** | **T** | **T** | **T** | **T** | **C** | **A** | **C** | **T** | **T** | **A** | **T** | **T** | **T** | **T** | **T** | **A** | **T** | **G** | **C** | **C** | **A** | **G** | **T** | **A** | **A** | **C** | **C** | **A** | **A** | **A** | **G** | **G** | **C** | **A** | **T** | **T** | **G** | **G** | **T** | **G** | **G** | **G** | **G** | **A** | **G** | **A** | **A** | **T** | **T** | **G** | **C** | **T** | **G** | **C** | **G** | **T** | **G** | **C** | **G** | **C** | **G** | **T** | **T** | **T** | **A** | **T** | **T** | **A** | **T** | **A** | **C** | **G** | **T** | **T** | **C** | **C** | **G** | **C** | **T** | **T** | **A** | **T** | **C** | **T** | **T** | **C** | **C** | **A** | **T** | **T** | **T** | **G** | **A** | **T** | **G** | **G** | **C** | **A** | **A** | **T** | **T** | **T** | **C** | **A** | **T** | **T** | **A** | **G** | **G** | **T** | **G** | **T** | **A** | **G** | **G** | **A** | **T** | **T** | **G** | **A** | **T** | **C** | **G** | **T** | **A** | **T** | **T** | **T** | **C** | **T** | **A** | **T** | **T** | **G** | **T** | **T** | **A** | **T** | **C** | **A** | **C** | **T** | **T** | **A** | **T** | **T** | **C** | **A** | **A** | **A** | **A** | **G** | **G** | **C** | **A** | **A** | **A** | **A** | **T** | **A** | **C** | **G** | **G** | **G** | **A** | **A** | **A** | **A** | **T** | **C** | **G** | **A** | **A** | **G** | **A** | **A** | **A** | **A** | **G** | **C** | **T** | **T** | **C** | **G** | **T** | **G** | **C** | **A** | **C** | **T** | **T** | **T** | **C** | **T** | **T** | **C** | **T** | **G** | **G** | **C** | **A** | **A** | **T** | **T** | **A** | **T** | **G** | **A** | **A** | **T** | **C** | **C** | **A** | **A** | **A** | **C** | **T** | **T** | **T** | **T** | **G** | **C** | **A** | **T** | **C** | **A** | **G** | **C** | **C** | **G** | **A** | **T** | **C** | **C** | **C** | **A** | **A** | **G** | **C** | **G** | **C** | **G** | **T** | **C** | **G** | **G** | **A** | **T** | **G** | **A** | **T** | **C** | **T** | **G** | **T** | **A** | **C** | **A** | **T** | **C** | **A** | **A** | **A** | **G** | **A** | **T** | **A** | **T** | **T** | **G** | **A** | **T** | **A** | **T** | **G** | **G** | **A** | **A** | **A** | **T** | **T** | **A** | **C** | **T** | **A** | **A** | **A** | **A** | **T** | **A** | **A** | **A** | **A** | **G** | **A** | **A** | **A** | **A** | **A** | **A** | **T** | **G** | **A** | **T** | **C** | **G** | **A** | **A** | **G** | **T** | **A** | **T** | **C** | **C** | **A** | **G** | **T** | **G** | **A** | **A** | **T** | **T** | **A** | **C** | **A** | **G** | **A** | **T** | **T** | **A** | **T** | **G** | **A** | **C** | **T** | **A** | **G** | **C** | **C** | **G** | **T** | **C** | **C** | **T** | **C** | **A** | **A** | **T** | **A** | **T** | **G** | **T** | **A** | **G** | **A** | **T** | **G** | **G** | **A** | **C** | **A** | **A** | **A** | **C** | **C** | **A** | **A** | **A** | **G** | **A** | **A** | **G** | **A** | **G** | **A** | **T** | **T** | **T** | **T** | **A** | **G** | **A** | **A** | **T** | **T** | **A** | **G** | **A** | **A** | **A** | **G** | **G** | **C** | **A** | **T** | **C** | **G** | **A** | **T** | **T** | **A** | **G** | **C** | **T** | **C** | **G** | **T** | **G** | **A** | **G** | **C** | **T** | **T** | **C** | **A** | **T** | **G** | **A** | **T** | **T** | **C** | **A** | **G** | **T** | **T** | **T** | **C** | **A** | **C** | **A** | **G** | **C** | **A** | **A** | **T** | **T** | **A** | **T** | **T** | **T** | **G** | **C** | **A** | **G** | **C** | **G** | **A** | **T** | **G** | **A** | **T** | **G** | **A** | **T** | **G** | **A** | **T** | **G** | **T** | **C** | **G** | **G** | **C** | **A** | **T** | **T** | **G** | **A** | **C** | **G** | **G** | **A** | **G** | **C** | **A** | **A** | **G** | **C** | **A** | **G** | **A** | **A** | **A** | **A** | **A** | **A** | **G** | **T** | **G** | **A** | **G** | **A** | **C** | **A** | **C** | **C** | **A** | **G** | **A** | **A** | **A** | **T** | **G** | **T** | **T** | **C** | **C** | **G** | **T** | **A** | **A** | **G** | **C** | **A** | **G** | **T** | **T** | **G** | **A** | **A** | **A** | **A** | **T** | **G** | **G** | **T** | **G** | **G** | **C** | **A** | **G** | **A** | **G** | **A** | **T** | **C** | **A** | **T** | **C** | **A** | **A** | **C** | **G** | **C** | **T** | **T** | **C** | **C** | **C** | **A** | **G** | **T** | **C** | **T** | **G** | **A** | **G** | **A** | **T** | **G** | **C** | **G** | **T** | **G** | **C** | **G** | **C** | **T** | **G** | **C** | **T** | **T** | **C** | **T** | **T** | **C** | **A** | **T** | **C** | **T** | **G** | **C** | **G** | **T** | **C** | **C** | **A** | **G** | **T** | **C** | **A** | **A** | **T** | **T** | **T** | **A** | **G** | **A** | **A** | **G** | **A** | **A** | **A** | **A** | **A** | **A** | **G** | **T** | **T** | **T** | **A** | **A** | **A** | **A** | **C** | **A** | **A** | **G** | **G** | **C** | **A** | **T** | **T** | **G** | **A** | **G** | **C** | **A** | **G** | **T** | **T** | **A** | **T** | **T** | **G** | **A** | **A** | **G** | **G** | **A** | **A** | **T** | **T** | **G** | **C** | **A** | **G** | **A** | **A** | **T** | **A** | **A** | **A** | **A** | **T** | **C** | **C** | **A** | **G** | **A** | **T** | **T** | **T** | **C** | **G** | **C** | **T** | **A** | **A** | **A** | **A** | **T** | **G** | **G** | **G** | **A** | **T** | **G** | **T** | **A** | **G** | **A** | **A** | **G** | **A** | **T** | **G** | **T** | **A** | **A** | **A** | **A** | **T** | **T** | **A** | **A** | **C** | **T** | **A** | **G** | **T** | **T** | **C** | **C** | **A** | **T** | **T** | **G** | **A** | **G** | **G** | **A** | **T** | **C** | **A** | **C** | **T** | **T** | **G** | **T** | **T** | **C** | **C** | **G** | **A** | **A** | **T** | **C** | **G** | **T** | **T** | **C** | **A** | **A** | **G** | **A** | **A** | **T** | **T** | **G** | **T** | **T** | **A** | **T** | **C** | **A** | **A** | **A** | **C** | **A** | **C** | **A** | **T** | **T** | **A** | **A** | **G** | **G** | **C** | **A** | **T** | **G** | **C** | **T** | **A** | **A** | **G** | **G** | **C** | **T** | **A** | **A** | **T** | **G** | **A** | **A** | **T** | **T** | **A** | **G** | **A** | **A** | **G** | **T** | **C** | **T** | **A** | **T** | **T** | **T** | **G** | **C** | **A** | **T** | **A** | **A** | **A** | **A** | **T** | **A** | **G** | **A** | **T** | **A** | **A** | **C** | **A** | **A** | **T** | **C** | **T** | **T** | **T** | **T** | **A** | **T** | **T** | **A** | **C** | **G** | **T** | **A** | **T** | **C** | **A** | **T** | **T** | **G** | **A** | **C** | **G** | **A** | **C** | **G** | **G** | **G** | **A** | **C** | **A** | **G** | **G** | **A** | **T** | **T** | **T** | **A** | **A** | **T** | **A** | **T** | **G** | **A** | **A** | **T** | **G** | **A** | **A** | **A** | **C** | **C** | **A** | **A** | **A** | **A** | **C** | **G** | **G** | **G** | **A** | **A** | **G** | **T** | **T** | **A** | **T** | **G** | **G** | **A** | **T** | **T** | **G** | **A** | **A** | **C** | **A** | **A** | **T** | **A** | **T** | **C** | **A** | **A** | **A** | **G** | **A** | **A** | **C** | **G** | **A** | **G** | **T** | **A** | **G** | **C** | **T** | **G** | **G** | **T** | **A** | **T** | **T** | **G** | **G** | **T** | **G** | **G** | **A** | **A** | **C** | **A** | **G** | **T** | **A** | **A** | **A** | **A** | **A** | **T** | **C** | **A** | **T** | **T** | **A** | **G** | **T** | **T** | **T** | **T** | **A** | **A** | **A** | **G** | **G** | **G** | **C** | **A** | **A** | **G** | **G** | **T** | **A** | **C** | **G** | **A** | **G** | **C** | **G** | **T** | **G** | **G** | **A** | **G** | **A** | **T** | **C** | **A** | **A** | **G** | **G** | **T** | **C** | **C** | **C** | **T** | **T** | **T** | **G** | **A** | **T** | **G** | **A** | **A** | **G** | **G** | **A** | **G** | **G** | **C** | **A** | **-** | **-** | **-** |
| **N52643** | **A** | **T** | **G** | **A** | **T** | **G** | **G** | **G** | **A** | **A** | **G** | **A** | **A** | **T** | **T** | **T** | **C** | **C** | **A** | **A** | **A** | **G** | **C** | **A** | **A** | **T** | **G** | **C** | **T** | **A** | **G** | **C** | **T** | **G** | **T** | **T** | **T** | **A** | **T** | **T** | **C** | **G** | **G** | **G** | **G** | **A** | **T** | **T** | **G** | **C** | **G** | **G** | **C** | **T** | **T** | **T** | **C** | **C** | **T** | **T** | **A** | **T** | **T** | **A** | **T** | **T** | **C** | **T** | **A** | **T** | **T** | **T** | **T** | **C** | **A** | **C** | **T** | **T** | **T** | **T** | **C** | **A** | **C** | **T** | **T** | **A** | **T** | **T** | **T** | **T** | **T** | **A** | **T** | **G** | **C** | **C** | **A** | **G** | **T** | **A** | **A** | **C** | **C** | **A** | **A** | **A** | **G** | **T** | **C** | **A** | **T** | **T** | **G** | **G** | **T** | **G** | **G** | **G** | **G** | **A** | **G** | **A** | **A** | **T** | **T** | **G** | **C** | **T** | **G** | **C** | **G** | **T** | **G** | **C** | **G** | **C** | **G** | **T** | **T** | **T** | **A** | **T** | **T** | **A** | **T** | **A** | **C** | **G** | **T** | **T** | **C** | **C** | **G** | **C** | **T** | **T** | **A** | **T** | **C** | **T** | **T** | **C** | **C** | **A** | **T** | **T** | **T** | **G** | **A** | **T** | **G** | **G** | **C** | **C** | **A** | **T** | **T** | **T** | **C** | **A** | **T** | **T** | **A** | **G** | **G** | **T** | **G** | **T** | **A** | **G** | **G** | **A** | **T** | **T** | **G** | **A** | **T** | **C** | **G** | **T** | **A** | **T** | **T** | **T** | **C** | **T** | **A** | **T** | **T** | **G** | **T** | **T** | **A** | **T** | **C** | **A** | **C** | **T** | **T** | **A** | **T** | **T** | **C** | **A** | **A** | **A** | **A** | **G** | **G** | **C** | **A** | **A** | **A** | **A** | **T** | **A** | **C** | **G** | **G** | **G** | **A** | **A** | **A** | **A** | **T** | **C** | **G** | **A** | **A** | **G** | **A** | **A** | **A** | **A** | **G** | **C** | **T** | **T** | **C** | **G** | **T** | **G** | **C** | **A** | **C** | **T** | **T** | **T** | **C** | **T** | **T** | **C** | **T** | **G** | **G** | **C** | **A** | **A** | **T** | **T** | **A** | **T** | **G** | **A** | **A** | **T** | **C** | **C** | **A** | **A** | **A** | **C** | **T** | **T** | **T** | **T** | **G** | **C** | **A** | **T** | **C** | **A** | **G** | **C** | **C** | **G** | **A** | **T** | **C** | **C** | **C** | **A** | **A** | **G** | **C** | **G** | **C** | **G** | **T** | **C** | **G** | **G** | **A** | **T** | **G** | **A** | **T** | **C** | **T** | **G** | **T** | **A** | **C** | **A** | **T** | **C** | **A** | **A** | **A** | **G** | **A** | **T** | **A** | **T** | **T** | **G** | **A** | **T** | **A** | **T** | **A** | **G** | **A** | **A** | **A** | **T** | **T** | **A** | **C** | **T** | **A** | **A** | **A** | **A** | **T** | **A** | **A** | **A** | **A** | **G** | **A** | **A** | **A** | **A** | **A** | **A** | **T** | **G** | **A** | **T** | **C** | **G** | **A** | **A** | **G** | **T** | **A** | **T** | **C** | **C** | **A** | **G** | **T** | **G** | **A** | **A** | **T** | **T** | **A** | **C** | **A** | **G** | **A** | **T** | **T** | **A** | **T** | **G** | **A** | **C** | **T** | **A** | **G** | **C** | **C** | **G** | **T** | **C** | **C** | **T** | **C** | **A** | **A** | **T** | **A** | **T** | **G** | **T** | **A** | **G** | **A** | **T** | **G** | **G** | **A** | **C** | **A** | **A** | **A** | **C** | **C** | **A** | **A** | **A** | **G** | **A** | **A** | **G** | **A** | **G** | **A** | **T** | **T** | **T** | **T** | **A** | **G** | **A** | **A** | **T** | **T** | **A** | **G** | **A** | **A** | **A** | **G** | **G** | **C** | **A** | **T** | **C** | **G** | **A** | **T** | **T** | **A** | **G** | **C** | **T** | **C** | **G** | **T** | **G** | **A** | **G** | **C** | **T** | **T** | **C** | **A** | **T** | **G** | **A** | **T** | **T** | **C** | **A** | **G** | **T** | **T** | **T** | **C** | **A** | **C** | **A** | **G** | **C** | **A** | **A** | **T** | **T** | **A** | **T** | **T** | **T** | **G** | **C** | **A** | **G** | **C** | **G** | **A** | **T** | **G** | **A** | **T** | **G** | **A** | **T** | **G** | **A** | **T** | **G** | **T** | **C** | **G** | **G** | **C** | **A** | **T** | **T** | **G** | **A** | **C** | **G** | **G** | **A** | **G** | **C** | **A** | **A** | **G** | **C** | **A** | **G** | **A** | **A** | **A** | **A** | **A** | **A** | **G** | **T** | **G** | **A** | **G** | **A** | **C** | **A** | **C** | **C** | **A** | **G** | **A** | **A** | **A** | **T** | **G** | **T** | **T** | **C** | **C** | **G** | **T** | **A** | **A** | **G** | **C** | **A** | **G** | **T** | **T** | **G** | **A** | **A** | **A** | **A** | **T** | **G** | **G** | **T** | **G** | **G** | **C** | **A** | **G** | **A** | **G** | **A** | **T** | **C** | **A** | **T** | **C** | **A** | **A** | **C** | **G** | **C** | **T** | **T** | **C** | **C** | **C** | **A** | **G** | **T** | **C** | **T** | **G** | **A** | **G** | **A** | **T** | **G** | **C** | **G** | **T** | **G** | **C** | **G** | **C** | **T** | **G** | **C** | **T** | **T** | **C** | **T** | **T** | **C** | **A** | **T** | **C** | **T** | **G** | **C** | **G** | **T** | **C** | **C** | **A** | **G** | **T** | **C** | **A** | **A** | **T** | **T** | **T** | **A** | **G** | **A** | **A** | **G** | **A** | **A** | **A** | **A** | **A** | **A** | **G** | **T** | **T** | **T** | **A** | **A** | **A** | **A** | **C** | **A** | **A** | **G** | **G** | **C** | **A** | **T** | **T** | **G** | **A** | **G** | **C** | **A** | **G** | **T** | **T** | **A** | **T** | **T** | **G** | **A** | **A** | **G** | **G** | **A** | **A** | **T** | **T** | **G** | **C** | **A** | **G** | **A** | **A** | **T** | **A** | **A** | **A** | **A** | **T** | **C** | **C** | **A** | **G** | **A** | **T** | **T** | **T** | **C** | **G** | **C** | **T** | **A** | **A** | **A** | **A** | **T** | **G** | **G** | **G** | **A** | **T** | **G** | **T** | **A** | **G** | **A** | **A** | **G** | **A** | **T** | **G** | **T** | **A** | **A** | **A** | **A** | **T** | **T** | **A** | **A** | **C** | **T** | **A** | **G** | **T** | **T** | **C** | **C** | **A** | **T** | **T** | **G** | **A** | **G** | **G** | **A** | **T** | **C** | **A** | **C** | **T** | **T** | **G** | **T** | **T** | **C** | **C** | **G** | **A** | **A** | **T** | **C** | **G** | **T** | **T** | **C** | **A** | **A** | **G** | **A** | **A** | **T** | **T** | **G** | **T** | **T** | **A** | **T** | **C** | **A** | **A** | **A** | **C** | **A** | **C** | **A** | **T** | **T** | **A** | **A** | **G** | **A** | **C** | **A** | **T** | **G** | **C** | **T** | **A** | **A** | **G** | **G** | **C** | **T** | **A** | **A** | **T** | **G** | **A** | **A** | **T** | **T** | **A** | **G** | **A** | **A** | **G** | **T** | **C** | **T** | **A** | **T** | **T** | **T** | **G** | **C** | **A** | **T** | **A** | **A** | **A** | **A** | **T** | **A** | **G** | **A** | **T** | **A** | **A** | **C** | **A** | **A** | **T** | **C** | **T** | **T** | **T** | **T** | **A** | **T** | **T** | **A** | **C** | **G** | **T** | **A** | **T** | **C** | **A** | **T** | **T** | **G** | **A** | **C** | **G** | **A** | **C** | **G** | **G** | **G** | **A** | **C** | **A** | **G** | **G** | **A** | **T** | **T** | **T** | **A** | **A** | **T** | **A** | **T** | **G** | **A** | **A** | **T** | **G** | **A** | **A** | **A** | **C** | **C** | **A** | **A** | **A** | **A** | **C** | **G** | **G** | **G** | **A** | **A** | **G** | **T** | **T** | **A** | **T** | **G** | **G** | **A** | **T** | **T** | **G** | **A** | **A** | **C** | **A** | **A** | **T** | **A** | **T** | **C** | **A** | **A** | **A** | **G** | **A** | **A** | **C** | **G** | **A** | **G** | **T** | **A** | **G** | **C** | **T** | **G** | **G** | **T** | **A** | **T** | **T** | **G** | **G** | **T** | **G** | **G** | **A** | **A** | **C** | **A** | **G** | **T** | **A** | **A** | **A** | **A** | **A** | **T** | **C** | **A** | **T** | **T** | **A** | **G** | **T** | **T** | **T** | **T** | **A** | **A** | **A** | **G** | **G** | **G** | **C** | **A** | **A** | **G** | **G** | **T** | **A** | **C** | **G** | **A** | **G** | **C** | **G** | **T** | **G** | **G** | **A** | **G** | **A** | **T** | **C** | **A** | **A** | **G** | **G** | **T** | **C** | **C** | **C** | **T** | **T** | **T** | **G** | **A** | **T** | **G** | **A** | **A** | **G** | **G** | **A** | **G** | **G** | **C** | **A** | **-** | **-** | **-** |
| **N59613F** | **A** | **T** | **G** | **A** | **T** | **G** | **G** | **G** | **A** | **A** | **A** | **A** | **A** | **T** | **A** | **T** | **C** | **C** | **A** | **G** | **A** | **G** | **C** | **G** | **A** | **T** | **G** | **C** | **T** | **A** | **G** | **C** | **T** | **G** | **T** | **T** | **T** | **A** | **T** | **T** | **C** | **G** | **G** | **G** | **G** | **A** | **T** | **T** | **G** | **C** | **G** | **G** | **C** | **T** | **T** | **T** | **C** | **C** | **T** | **T** | **A** | **T** | **T** | **A** | **T** | **T** | **C** | **T** | **A** | **T** | **T** | **T** | **T** | **C** | **A** | **C** | **T** | **T** | **T** | **T** | **C** | **A** | **C** | **T** | **T** | **A** | **T** | **T** | **T** | **T** | **T** | **A** | **T** | **G** | **C** | **C** | **A** | **G** | **T** | **A** | **A** | **T** | **C** | **A** | **A** | **A** | **G** | **T** | **C** | **A** | **T** | **T** | **G** | **G** | **T** | **G** | **G** | **G** | **G** | **A** | **G** | **A** | **A** | **T** | **T** | **A** | **C** | **T** | **G** | **C** | **G** | **T** | **G** | **C** | **A** | **C** | **G** | **T** | **T** | **T** | **A** | **T** | **T** | **A** | **T** | **A** | **T** | **G** | **T** | **T** | **C** | **C** | **G** | **C** | **T** | **T** | **A** | **T** | **C** | **T** | **T** | **C** | **C** | **A** | **T** | **T** | **T** | **G** | **T** | **T** | **A** | **G** | **C** | **T** | **A** | **T** | **T** | **T** | **C** | **C** | **T** | **T** | **A** | **G** | **G** | **T** | **G** | **T** | **A** | **G** | **G** | **A** | **T** | **T** | **A** | **A** | **T** | **C** | **G** | **T** | **C** | **T** | **T** | **T** | **C** | **T** | **A** | **T** | **T** | **A** | **T** | **T** | **A** | **T** | **C** | **A** | **C** | **T** | **T** | **A** | **T** | **T** | **C** | **A** | **A** | **A** | **A** | **G** | **A** | **C** | **A** | **A** | **A** | **A** | **T** | **A** | **C** | **G** | **G** | **G** | **A** | **A** | **A** | **A** | **T** | **C** | **G** | **A** | **A** | **G** | **A** | **A** | **A** | **A** | **G** | **C** | **T** | **G** | **C** | **G** | **T** | **G** | **C** | **A** | **C** | **T** | **C** | **T** | **C** | **T** | **T** | **C** | **T** | **G** | **G** | **C** | **A** | **A** | **T** | **T** | **A** | **T** | **G** | **A** | **A** | **T** | **C** | **C** | **A** | **A** | **A** | **T** | **T** | **G** | **T** | **T** | **G** | **C** | **T** | **T** | **C** | **T** | **T** | **C** | **C** | **G** | **A** | **T** | **C** | **C** | **C** | **A** | **A** | **G** | **T** | **G** | **C** | **A** | **T** | **C** | **G** | **G** | **A** | **C** | **G** | **A** | **T** | **T** | **T** | **A** | **T** | **A** | **C** | **A** | **T** | **C** | **A** | **A** | **A** | **G** | **A** | **T** | **A** | **T** | **T** | **G** | **A** | **T** | **A** | **A** | **G** | **G** | **A** | **A** | **A** | **T** | **C** | **A** | **C** | **T** | **A** | **A** | **A** | **A** | **T** | **A** | **A** | **A** | **A** | **G** | **A** | **A** | **A** | **A** | **A** | **A** | **T** | **G** | **A** | **T** | **T** | **G** | **A** | **A** | **G** | **T** | **A** | **T** | **C** | **T** | **A** | **G** | **T** | **G** | **A** | **A** | **T** | **T** | **A** | **C** | **A** | **A** | **A** | **T** | **T** | **G** | **T** | **A** | **A** | **C** | **G** | **A** | **G** | **C** | **C** | **G** | **T** | **C** | **C** | **C** | **C** | **A** | **A** | **T** | **A** | **T** | **G** | **T** | **A** | **G** | **A** | **T** | **G** | **G** | **A** | **C** | **A** | **A** | **A** | **C** | **C** | **A** | **A** | **A** | **G** | **A** | **A** | **G** | **A** | **A** | **A** | **T** | **T** | **T** | **T** | **A** | **G** | **A** | **A** | **T** | **T** | **A** | **G** | **A** | **A** | **A** | **G** | **A** | **C** | **A** | **T** | **C** | **G** | **A** | **T** | **T** | **A** | **G** | **C** | **C** | **C** | **G** | **T** | **G** | **A** | **G** | **C** | **T** | **G** | **C** | **A** | **T** | **G** | **A** | **T** | **T** | **C** | **A** | **G** | **T** | **T** | **T** | **C** | **G** | **C** | **A** | **A** | **C** | **A** | **A** | **T** | **T** | **G** | **T** | **T** | **T** | **G** | **C** | **A** | **G** | **C** | **A** | **A** | **T** | **G** | **A** | **T** | **G** | **A** | **T** | **G** | **A** | **T** | **G** | **T** | **C** | **G** | **G** | **C** | **A** | **T** | **T** | **A** | **A** | **C** | **G** | **G** | **A** | **G** | **C** | **A** | **A** | **G** | **C** | **A** | **G** | **A** | **A** | **A** | **A** | **A** | **A** | **G** | **C** | **G** | **A** | **G** | **A** | **C** | **A** | **C** | **C** | **A** | **G** | **A** | **A** | **A** | **T** | **G** | **T** | **T** | **C** | **C** | **G** | **C** | **A** | **A** | **G** | **C** | **A** | **G** | **T** | **T** | **G** | **A** | **A** | **A** | **A** | **T** | **G** | **G** | **T** | **A** | **G** | **C** | **A** | **G** | **A** | **G** | **A** | **T** | **C** | **A** | **T** | **C** | **A** | **A** | **C** | **G** | **C** | **T** | **T** | **C** | **C** | **C** | **A** | **G** | **T** | **C** | **T** | **G** | **A** | **G** | **A** | **T** | **G** | **C** | **G** | **T** | **G** | **C** | **G** | **C** | **T** | **G** | **C** | **T** | **T** | **C** | **T** | **T** | **C** | **A** | **T** | **C** | **T** | **G** | **C** | **G** | **T** | **C** | **C** | **A** | **G** | **T** | **C** | **A** | **A** | **T** | **T** | **T** | **A** | **G** | **A** | **A** | **G** | **A** | **G** | **A** | **A** | **A** | **A** | **G** | **T** | **T** | **T** | **A** | **A** | **A** | **G** | **C** | **A** | **A** | **G** | **G** | **T** | **A** | **T** | **C** | **G** | **A** | **G** | **C** | **A** | **G** | **T** | **T** | **A** | **T** | **T** | **G** | **A** | **A** | **G** | **G** | **A** | **A** | **T** | **T** | **G** | **C** | **A** | **A** | **A** | **A** | **T** | **A** | **A** | **A** | **A** | **T** | **T** | **C** | **A** | **G** | **A** | **T** | **T** | **T** | **C** | **G** | **C** | **T** | **G** | **A** | **A** | **A** | **T** | **G** | **G** | **G** | **A** | **T** | **G** | **T** | **A** | **G** | **A** | **A** | **G** | **A** | **T** | **G** | **T** | **A** | **A** | **A** | **A** | **C** | **T** | **A** | **T** | **C** | **T** | **A** | **G** | **C** | **T** | **C** | **C** | **A** | **T** | **T** | **G** | **A** | **G** | **G** | **A** | **T** | **C** | **A** | **C** | **T** | **T** | **G** | **T** | **T** | **C** | **C** | **G** | **A** | **A** | **T** | **C** | **G** | **T** | **T** | **C** | **A** | **A** | **G** | **A** | **A** | **T** | **T** | **G** | **T** | **T** | **A** | **T** | **C** | **A** | **A** | **A** | **T** | **A** | **C** | **A** | **T** | **T** | **A** | **A** | **G** | **A** | **C** | **A** | **T** | **G** | **C** | **C** | **A** | **A** | **A** | **G** | **C** | **C** | **A** | **A** | **T** | **G** | **A** | **A** | **T** | **T** | **A** | **G** | **A** | **G** | **G** | **T** | **A** | **T** | **A** | **T** | **T** | **T** | **G** | **C** | **A** | **T** | **A** | **A** | **A** | **A** | **T** | **A** | **G** | **A** | **C** | **A** | **A** | **T** | **A** | **A** | **T** | **C** | **T** | **T** | **T** | **T** | **A** | **T** | **T** | **A** | **C** | **G** | **T** | **A** | **T** | **T** | **A** | **T** | **T** | **G** | **A** | **C** | **G** | **A** | **T** | **G** | **G** | **A** | **A** | **C** | **A** | **G** | **G** | **G** | **T** | **T** | **T** | **A** | **A** | **T** | **A** | **T** | **G** | **A** | **A** | **T** | **G** | **A** | **A** | **A** | **C** | **G** | **A** | **A** | **A** | **A** | **C** | **G** | **G** | **G** | **A** | **A** | **G** | **T** | **T** | **A** | **T** | **G** | **G** | **A** | **T** | **T** | **G** | **A** | **A** | **C** | **A** | **A** | **T** | **A** | **T** | **C** | **A** | **A** | **A** | **G** | **A** | **A** | **C** | **G** | **A** | **G** | **T** | **A** | **G** | **C** | **T** | **G** | **G** | **T** | **A** | **T** | **T** | **G** | **G** | **T** | **G** | **G** | **A** | **A** | **C** | **A** | **G** | **T** | **A** | **A** | **A** | **A** | **A** | **T** | **C** | **A** | **T** | **T** | **A** | **G** | **T** | **T** | **T** | **T** | **A** | **A** | **A** | **G** | **G** | **G** | **C** | **A** | **A** | **G** | **G** | **T** | **A** | **C** | **A** | **A** | **G** | **T** | **G** | **T** | **A** | **G** | **A** | **A** | **A** | **T** | **C** | **A** | **A** | **G** | **G** | **T** | **C** | **C** | **C** | **T** | **T** | **T** | **G** | **A** | **T** | **G** | **A** | **A** | **G** | **G** | **A** | **G** | **G** | **C** | **A** | **-** | **-** | **-** |
| **N54550** | **A** | **T** | **G** | **A** | **T** | **G** | **G** | **G** | **A** | **A** | **G** | **A** | **A** | **T** | **A** | **T** | **C** | **C** | **A** | **A** | **A** | **G** | **C** | **A** | **A** | **T** | **G** | **C** | **T** | **A** | **G** | **C** | **T** | **G** | **T** | **T** | **T** | **A** | **T** | **T** | **C** | **G** | **G** | **G** | **G** | **A** | **T** | **T** | **G** | **C** | **G** | **G** | **C** | **T** | **T** | **T** | **C** | **C** | **T** | **T** | **A** | **T** | **T** | **A** | **T** | **T** | **C** | **T** | **A** | **T** | **T** | **T** | **T** | **C** | **A** | **C** | **T** | **T** | **T** | **T** | **C** | **A** | **C** | **T** | **T** | **A** | **T** | **T** | **T** | **T** | **T** | **A** | **T** | **G** | **C** | **C** | **A** | **G** | **T** | **A** | **A** | **C** | **C** | **A** | **A** | **A** | **G** | **T** | **C** | **A** | **T** | **T** | **G** | **G** | **T** | **G** | **G** | **G** | **G** | **A** | **G** | **A** | **A** | **T** | **T** | **G** | **C** | **T** | **G** | **C** | **G** | **T** | **G** | **C** | **G** | **C** | **G** | **T** | **T** | **T** | **A** | **T** | **T** | **A** | **T** | **A** | **C** | **G** | **T** | **T** | **C** | **C** | **G** | **C** | **T** | **T** | **A** | **T** | **C** | **T** | **T** | **C** | **C** | **A** | **T** | **T** | **T** | **G** | **A** | **T** | **G** | **G** | **C** | **A** | **A** | **T** | **T** | **T** | **C** | **A** | **T** | **T** | **A** | **G** | **G** | **T** | **G** | **T** | **A** | **G** | **G** | **A** | **T** | **T** | **G** | **A** | **T** | **C** | **G** | **T** | **A** | **T** | **T** | **T** | **C** | **T** | **A** | **T** | **T** | **G** | **T** | **T** | **A** | **T** | **C** | **A** | **C** | **T** | **T** | **A** | **T** | **T** | **C** | **A** | **A** | **A** | **A** | **G** | **A** | **C** | **A** | **A** | **A** | **A** | **T** | **A** | **C** | **G** | **G** | **G** | **A** | **A** | **A** | **A** | **T** | **C** | **G** | **A** | **A** | **G** | **A** | **A** | **A** | **A** | **G** | **C** | **T** | **T** | **C** | **G** | **T** | **G** | **C** | **A** | **C** | **T** | **T** | **T** | **C** | **T** | **T** | **C** | **T** | **G** | **G** | **C** | **A** | **A** | **T** | **T** | **A** | **T** | **G** | **A** | **A** | **T** | **C** | **C** | **A** | **A** | **A** | **C** | **T** | **T** | **T** | **T** | **G** | **C** | **A** | **T** | **C** | **A** | **G** | **C** | **C** | **G** | **A** | **T** | **C** | **C** | **C** | **A** | **A** | **G** | **C** | **G** | **C** | **G** | **T** | **C** | **G** | **G** | **A** | **T** | **G** | **A** | **T** | **C** | **T** | **G** | **T** | **A** | **C** | **A** | **T** | **C** | **A** | **A** | **A** | **G** | **A** | **T** | **A** | **T** | **T** | **G** | **A** | **T** | **A** | **T** | **G** | **G** | **A** | **A** | **A** | **T** | **T** | **A** | **C** | **T** | **A** | **A** | **A** | **A** | **T** | **A** | **A** | **A** | **A** | **G** | **A** | **A** | **A** | **A** | **A** | **A** | **T** | **G** | **A** | **T** | **C** | **G** | **A** | **A** | **G** | **T** | **A** | **T** | **C** | **C** | **A** | **G** | **T** | **G** | **A** | **A** | **T** | **T** | **A** | **C** | **A** | **G** | **A** | **T** | **T** | **A** | **T** | **G** | **A** | **C** | **T** | **A** | **G** | **C** | **C** | **G** | **T** | **C** | **C** | **T** | **C** | **A** | **A** | **T** | **A** | **T** | **G** | **T** | **A** | **G** | **A** | **T** | **G** | **G** | **A** | **C** | **A** | **A** | **A** | **C** | **C** | **A** | **A** | **A** | **G** | **A** | **A** | **G** | **A** | **G** | **A** | **T** | **T** | **T** | **T** | **A** | **G** | **A** | **A** | **T** | **T** | **A** | **G** | **A** | **A** | **A** | **G** | **G** | **C** | **A** | **T** | **C** | **G** | **A** | **T** | **T** | **A** | **G** | **C** | **T** | **C** | **G** | **T** | **G** | **A** | **G** | **C** | **T** | **T** | **C** | **A** | **T** | **G** | **A** | **T** | **T** | **C** | **A** | **G** | **T** | **T** | **T** | **C** | **A** | **C** | **A** | **G** | **C** | **A** | **A** | **T** | **T** | **A** | **T** | **T** | **T** | **G** | **C** | **A** | **G** | **C** | **G** | **A** | **T** | **G** | **A** | **T** | **G** | **A** | **T** | **G** | **A** | **T** | **G** | **T** | **C** | **G** | **G** | **C** | **A** | **T** | **T** | **G** | **A** | **C** | **G** | **G** | **A** | **G** | **C** | **A** | **A** | **G** | **C** | **A** | **G** | **A** | **A** | **A** | **A** | **A** | **A** | **G** | **T** | **G** | **A** | **G** | **A** | **C** | **A** | **C** | **C** | **A** | **G** | **A** | **A** | **A** | **T** | **G** | **T** | **T** | **C** | **C** | **G** | **T** | **A** | **A** | **G** | **C** | **A** | **G** | **T** | **T** | **G** | **A** | **A** | **A** | **A** | **T** | **G** | **G** | **T** | **G** | **G** | **C** | **A** | **G** | **A** | **G** | **A** | **T** | **C** | **A** | **T** | **C** | **A** | **A** | **C** | **G** | **C** | **T** | **T** | **C** | **C** | **C** | **A** | **G** | **T** | **C** | **T** | **G** | **A** | **G** | **A** | **T** | **G** | **C** | **G** | **T** | **G** | **C** | **G** | **C** | **T** | **G** | **C** | **T** | **T** | **C** | **T** | **T** | **C** | **A** | **T** | **C** | **T** | **G** | **C** | **G** | **T** | **C** | **C** | **A** | **G** | **T** | **C** | **A** | **A** | **T** | **T** | **T** | **A** | **G** | **A** | **A** | **G** | **A** | **A** | **A** | **A** | **A** | **A** | **G** | **T** | **T** | **T** | **A** | **A** | **A** | **A** | **C** | **A** | **A** | **G** | **G** | **C** | **A** | **T** | **T** | **G** | **A** | **G** | **C** | **A** | **G** | **T** | **T** | **A** | **T** | **T** | **G** | **A** | **A** | **G** | **G** | **A** | **A** | **T** | **T** | **G** | **C** | **A** | **G** | **A** | **A** | **T** | **A** | **A** | **A** | **A** | **T** | **C** | **C** | **A** | **G** | **A** | **T** | **T** | **T** | **C** | **G** | **C** | **T** | **A** | **A** | **A** | **A** | **T** | **G** | **G** | **G** | **A** | **T** | **G** | **T** | **A** | **G** | **A** | **A** | **G** | **A** | **T** | **G** | **T** | **A** | **A** | **A** | **A** | **T** | **T** | **A** | **A** | **C** | **T** | **A** | **G** | **T** | **T** | **C** | **C** | **A** | **T** | **T** | **G** | **A** | **G** | **G** | **A** | **T** | **C** | **A** | **C** | **T** | **T** | **G** | **T** | **T** | **C** | **C** | **G** | **A** | **A** | **T** | **C** | **G** | **T** | **T** | **C** | **A** | **A** | **G** | **A** | **A** | **T** | **T** | **G** | **T** | **T** | **A** | **T** | **C** | **A** | **A** | **A** | **C** | **A** | **C** | **A** | **T** | **T** | **A** | **A** | **G** | **A** | **C** | **A** | **T** | **G** | **C** | **T** | **A** | **A** | **G** | **G** | **C** | **T** | **A** | **A** | **T** | **G** | **A** | **A** | **T** | **T** | **A** | **G** | **A** | **A** | **G** | **T** | **C** | **T** | **A** | **T** | **T** | **T** | **G** | **C** | **A** | **T** | **A** | **A** | **A** | **A** | **T** | **A** | **G** | **A** | **T** | **A** | **A** | **C** | **A** | **A** | **T** | **C** | **T** | **T** | **T** | **T** | **A** | **T** | **T** | **A** | **C** | **G** | **T** | **A** | **T** | **C** | **A** | **T** | **T** | **G** | **A** | **C** | **G** | **A** | **C** | **G** | **G** | **G** | **A** | **C** | **A** | **G** | **G** | **A** | **T** | **T** | **T** | **A** | **A** | **T** | **A** | **T** | **G** | **A** | **A** | **T** | **G** | **A** | **A** | **A** | **C** | **C** | **A** | **A** | **A** | **A** | **C** | **G** | **G** | **G** | **A** | **A** | **G** | **T** | **T** | **A** | **T** | **G** | **G** | **A** | **T** | **T** | **G** | **A** | **A** | **C** | **A** | **A** | **T** | **A** | **T** | **C** | **A** | **A** | **A** | **G** | **A** | **A** | **C** | **G** | **A** | **G** | **T** | **A** | **G** | **C** | **T** | **G** | **G** | **T** | **A** | **T** | **T** | **G** | **G** | **T** | **G** | **G** | **A** | **A** | **C** | **A** | **G** | **T** | **A** | **A** | **A** | **A** | **A** | **T** | **C** | **A** | **T** | **T** | **A** | **G** | **T** | **T** | **T** | **T** | **A** | **A** | **A** | **G** | **G** | **G** | **C** | **A** | **A** | **G** | **G** | **T** | **A** | **C** | **G** | **A** | **G** | **C** | **G** | **T** | **G** | **G** | **A** | **G** | **A** | **T** | **C** | **A** | **A** | **G** | **G** | **T** | **C** | **C** | **C** | **T** | **T** | **T** | **G** | **A** | **T** | **G** | **A** | **A** | **G** | **G** | **A** | **G** | **G** | **C** | **A** | **-** | **-** | **-** |
| **N53702** | **A** | **T** | **G** | **A** | **T** | **G** | **G** | **G** | **A** | **A** | **A** | **A** | **A** | **T** | **A** | **T** | **C** | **C** | **A** | **G** | **A** | **G** | **C** | **G** | **A** | **T** | **G** | **C** | **T** | **A** | **G** | **C** | **T** | **G** | **T** | **T** | **T** | **A** | **T** | **T** | **C** | **G** | **G** | **G** | **G** | **A** | **T** | **T** | **G** | **C** | **G** | **G** | **C** | **T** | **T** | **T** | **C** | **C** | **T** | **T** | **A** | **T** | **T** | **A** | **T** | **A** | **C** | **T** | **A** | **T** | **T** | **T** | **T** | **C** | **A** | **C** | **T** | **T** | **T** | **T** | **C** | **A** | **C** | **T** | **T** | **A** | **T** | **T** | **T** | **T** | **T** | **A** | **T** | **G** | **C** | **C** | **A** | **G** | **T** | **A** | **A** | **T** | **C** | **A** | **A** | **A** | **G** | **T** | **C** | **A** | **T** | **T** | **G** | **G** | **T** | **G** | **G** | **G** | **G** | **A** | **G** | **A** | **A** | **T** | **T** | **A** | **C** | **T** | **G** | **C** | **G** | **T** | **G** | **C** | **A** | **C** | **G** | **T** | **T** | **T** | **A** | **T** | **T** | **A** | **T** | **A** | **T** | **G** | **T** | **T** | **C** | **C** | **G** | **C** | **T** | **T** | **A** | **T** | **C** | **T** | **T** | **C** | **C** | **A** | **T** | **T** | **T** | **G** | **T** | **T** | **A** | **G** | **C** | **T** | **A** | **T** | **T** | **T** | **C** | **C** | **T** | **T** | **A** | **G** | **G** | **T** | **G** | **T** | **A** | **G** | **G** | **A** | **T** | **T** | **A** | **A** | **T** | **C** | **G** | **T** | **C** | **T** | **T** | **T** | **C** | **T** | **A** | **T** | **T** | **A** | **T** | **T** | **A** | **T** | **C** | **A** | **C** | **T** | **T** | **A** | **T** | **T** | **C** | **A** | **A** | **A** | **A** | **G** | **A** | **C** | **A** | **A** | **A** | **A** | **T** | **A** | **C** | **G** | **G** | **G** | **A** | **A** | **A** | **A** | **T** | **C** | **G** | **A** | **A** | **G** | **A** | **A** | **A** | **A** | **G** | **C** | **T** | **G** | **C** | **G** | **T** | **G** | **C** | **A** | **C** | **T** | **C** | **T** | **C** | **T** | **T** | **C** | **T** | **G** | **G** | **C** | **A** | **A** | **T** | **T** | **A** | **T** | **G** | **A** | **A** | **T** | **C** | **C** | **A** | **A** | **A** | **T** | **T** | **G** | **T** | **T** | **G** | **C** | **T** | **T** | **C** | **T** | **T** | **C** | **C** | **G** | **A** | **T** | **C** | **C** | **C** | **A** | **A** | **G** | **T** | **G** | **C** | **A** | **T** | **C** | **G** | **G** | **A** | **C** | **G** | **A** | **T** | **T** | **T** | **A** | **T** | **A** | **C** | **A** | **T** | **C** | **A** | **A** | **A** | **G** | **A** | **T** | **A** | **T** | **T** | **G** | **A** | **T** | **A** | **A** | **G** | **G** | **A** | **A** | **A** | **T** | **C** | **A** | **C** | **T** | **A** | **A** | **A** | **A** | **T** | **A** | **A** | **A** | **A** | **G** | **A** | **A** | **A** | **A** | **A** | **A** | **T** | **G** | **A** | **T** | **T** | **G** | **A** | **A** | **G** | **T** | **A** | **T** | **C** | **T** | **A** | **G** | **T** | **G** | **A** | **A** | **T** | **T** | **A** | **C** | **A** | **A** | **A** | **T** | **T** | **G** | **T** | **A** | **A** | **C** | **G** | **A** | **G** | **C** | **C** | **G** | **T** | **C** | **C** | **C** | **C** | **A** | **A** | **T** | **A** | **T** | **G** | **T** | **A** | **G** | **A** | **T** | **G** | **G** | **A** | **C** | **A** | **A** | **A** | **C** | **C** | **A** | **A** | **A** | **G** | **A** | **A** | **G** | **A** | **A** | **A** | **T** | **T** | **T** | **T** | **A** | **G** | **A** | **A** | **T** | **T** | **A** | **G** | **A** | **A** | **A** | **G** | **A** | **C** | **A** | **T** | **C** | **G** | **A** | **T** | **T** | **A** | **G** | **C** | **C** | **C** | **G** | **T** | **G** | **A** | **G** | **C** | **T** | **G** | **C** | **A** | **T** | **G** | **A** | **T** | **T** | **C** | **A** | **G** | **T** | **T** | **T** | **C** | **G** | **C** | **A** | **A** | **C** | **A** | **A** | **T** | **T** | **G** | **T** | **T** | **T** | **G** | **C** | **A** | **G** | **C** | **A** | **A** | **T** | **G** | **A** | **T** | **G** | **A** | **T** | **G** | **A** | **T** | **G** | **T** | **C** | **G** | **G** | **C** | **A** | **T** | **T** | **A** | **A** | **C** | **G** | **G** | **A** | **G** | **C** | **A** | **A** | **G** | **C** | **A** | **G** | **A** | **A** | **A** | **A** | **A** | **A** | **G** | **C** | **G** | **A** | **G** | **A** | **C** | **A** | **C** | **C** | **A** | **G** | **A** | **A** | **A** | **T** | **G** | **T** | **T** | **C** | **C** | **G** | **C** | **A** | **A** | **G** | **C** | **A** | **G** | **T** | **T** | **G** | **A** | **A** | **A** | **A** | **T** | **G** | **G** | **T** | **A** | **G** | **C** | **A** | **G** | **A** | **G** | **A** | **T** | **C** | **A** | **T** | **C** | **A** | **A** | **C** | **G** | **C** | **T** | **T** | **C** | **C** | **C** | **A** | **G** | **T** | **C** | **T** | **G** | **A** | **G** | **A** | **T** | **G** | **C** | **G** | **T** | **G** | **C** | **G** | **C** | **T** | **G** | **C** | **T** | **T** | **C** | **T** | **T** | **C** | **A** | **T** | **C** | **T** | **G** | **C** | **G** | **T** | **C** | **C** | **A** | **G** | **T** | **C** | **A** | **A** | **T** | **T** | **T** | **A** | **G** | **A** | **A** | **G** | **A** | **G** | **A** | **A** | **A** | **A** | **G** | **T** | **T** | **T** | **A** | **A** | **A** | **G** | **C** | **A** | **A** | **G** | **G** | **T** | **A** | **T** | **C** | **G** | **A** | **G** | **C** | **A** | **G** | **T** | **T** | **A** | **T** | **T** | **G** | **A** | **A** | **G** | **G** | **A** | **A** | **T** | **T** | **G** | **C** | **A** | **A** | **A** | **A** | **T** | **A** | **A** | **A** | **A** | **T** | **C** | **C** | **A** | **G** | **A** | **T** | **T** | **T** | **C** | **G** | **C** | **T** | **G** | **A** | **A** | **A** | **T** | **G** | **G** | **G** | **A** | **T** | **G** | **T** | **A** | **G** | **A** | **A** | **G** | **A** | **T** | **G** | **T** | **A** | **A** | **A** | **A** | **C** | **T** | **A** | **T** | **C** | **T** | **A** | **G** | **T** | **T** | **C** | **C** | **A** | **T** | **T** | **G** | **A** | **G** | **G** | **A** | **T** | **C** | **A** | **C** | **T** | **T** | **G** | **T** | **T** | **C** | **C** | **G** | **A** | **A** | **T** | **C** | **G** | **T** | **T** | **C** | **A** | **A** | **G** | **A** | **A** | **T** | **T** | **G** | **T** | **T** | **A** | **T** | **C** | **A** | **A** | **A** | **T** | **A** | **C** | **A** | **T** | **T** | **A** | **A** | **G** | **A** | **C** | **A** | **T** | **G** | **C** | **T** | **A** | **A** | **G** | **G** | **C** | **T** | **A** | **A** | **T** | **G** | **A** | **A** | **T** | **T** | **A** | **G** | **A** | **A** | **G** | **T** | **C** | **T** | **A** | **T** | **T** | **T** | **G** | **C** | **A** | **T** | **A** | **A** | **A** | **A** | **T** | **A** | **G** | **A** | **T** | **A** | **A** | **C** | **A** | **A** | **T** | **C** | **T** | **T** | **T** | **T** | **A** | **T** | **T** | **A** | **C** | **G** | **T** | **A** | **T** | **C** | **A** | **T** | **T** | **G** | **A** | **C** | **G** | **A** | **C** | **G** | **G** | **G** | **A** | **C** | **A** | **G** | **G** | **A** | **T** | **T** | **T** | **A** | **A** | **T** | **A** | **T** | **G** | **A** | **A** | **T** | **G** | **A** | **A** | **A** | **C** | **C** | **A** | **A** | **A** | **A** | **C** | **G** | **G** | **G** | **A** | **A** | **G** | **T** | **T** | **A** | **T** | **G** | **G** | **A** | **T** | **T** | **G** | **A** | **A** | **C** | **A** | **A** | **T** | **A** | **T** | **C** | **A** | **A** | **A** | **G** | **A** | **A** | **C** | **G** | **A** | **G** | **T** | **A** | **G** | **C** | **T** | **G** | **G** | **T** | **A** | **T** | **T** | **G** | **G** | **T** | **G** | **G** | **A** | **A** | **C** | **A** | **G** | **T** | **A** | **A** | **A** | **A** | **A** | **T** | **C** | **A** | **T** | **T** | **A** | **G** | **T** | **T** | **T** | **T** | **A** | **A** | **A** | **G** | **G** | **G** | **C** | **A** | **A** | **G** | **G** | **T** | **A** | **C** | **G** | **A** | **G** | **C** | **G** | **T** | **G** | **G** | **A** | **G** | **A** | **T** | **C** | **A** | **A** | **G** | **G** | **T** | **C** | **C** | **C** | **T** | **T** | **T** | **G** | **A** | **T** | **G** | **A** | **A** | **G** | **G** | **A** | **G** | **G** | **C** | **A** | **-** | **-** | **-** |
| **N59567F** | **A** | **T** | **G** | **A** | **T** | **G** | **G** | **G** | **A** | **A** | **G** | **A** | **A** | **T** | **A** | **T** | **C** | **C** | **A** | **A** | **A** | **G** | **C** | **A** | **A** | **T** | **G** | **C** | **T** | **A** | **G** | **C** | **T** | **G** | **T** | **T** | **T** | **A** | **T** | **T** | **C** | **G** | **G** | **G** | **G** | **A** | **T** | **T** | **G** | **C** | **G** | **G** | **C** | **T** | **T** | **T** | **C** | **C** | **T** | **T** | **A** | **T** | **T** | **A** | **T** | **T** | **C** | **T** | **A** | **T** | **T** | **T** | **T** | **C** | **A** | **C** | **T** | **T** | **T** | **T** | **C** | **A** | **C** | **T** | **T** | **A** | **T** | **T** | **T** | **T** | **T** | **A** | **T** | **G** | **C** | **C** | **A** | **G** | **T** | **A** | **A** | **C** | **C** | **A** | **A** | **A** | **G** | **T** | **C** | **A** | **T** | **T** | **G** | **G** | **T** | **G** | **G** | **G** | **G** | **A** | **G** | **A** | **A** | **T** | **T** | **G** | **C** | **T** | **G** | **C** | **G** | **T** | **G** | **C** | **G** | **C** | **G** | **T** | **T** | **T** | **A** | **T** | **T** | **A** | **T** | **A** | **C** | **G** | **T** | **T** | **C** | **C** | **G** | **C** | **T** | **T** | **A** | **T** | **C** | **T** | **T** | **C** | **C** | **A** | **T** | **T** | **T** | **G** | **A** | **T** | **G** | **G** | **C** | **A** | **A** | **T** | **T** | **T** | **C** | **A** | **T** | **T** | **A** | **G** | **G** | **T** | **G** | **T** | **A** | **G** | **G** | **A** | **T** | **T** | **G** | **A** | **T** | **C** | **G** | **T** | **A** | **T** | **T** | **T** | **C** | **T** | **A** | **T** | **T** | **G** | **T** | **T** | **A** | **T** | **C** | **A** | **C** | **T** | **T** | **A** | **T** | **T** | **C** | **A** | **A** | **A** | **A** | **G** | **A** | **C** | **A** | **A** | **A** | **A** | **T** | **A** | **C** | **G** | **G** | **G** | **A** | **A** | **A** | **A** | **T** | **C** | **G** | **A** | **A** | **G** | **A** | **A** | **A** | **A** | **G** | **C** | **T** | **T** | **C** | **G** | **T** | **G** | **C** | **A** | **C** | **T** | **T** | **T** | **C** | **T** | **T** | **C** | **T** | **G** | **G** | **C** | **A** | **A** | **T** | **T** | **A** | **T** | **G** | **A** | **A** | **T** | **C** | **C** | **A** | **A** | **A** | **C** | **T** | **T** | **T** | **T** | **G** | **C** | **A** | **T** | **C** | **A** | **G** | **C** | **C** | **G** | **A** | **T** | **C** | **C** | **C** | **A** | **A** | **G** | **C** | **G** | **C** | **G** | **T** | **C** | **G** | **G** | **A** | **T** | **G** | **A** | **T** | **C** | **T** | **G** | **T** | **A** | **C** | **A** | **T** | **C** | **A** | **A** | **A** | **G** | **A** | **T** | **A** | **T** | **T** | **G** | **A** | **T** | **A** | **T** | **G** | **G** | **A** | **A** | **A** | **T** | **T** | **A** | **C** | **T** | **A** | **A** | **A** | **A** | **T** | **A** | **A** | **A** | **A** | **G** | **A** | **A** | **A** | **A** | **A** | **A** | **T** | **G** | **A** | **T** | **C** | **G** | **A** | **A** | **G** | **T** | **A** | **T** | **C** | **C** | **A** | **G** | **T** | **G** | **A** | **A** | **T** | **T** | **A** | **C** | **A** | **G** | **A** | **T** | **T** | **A** | **T** | **G** | **A** | **C** | **T** | **A** | **G** | **C** | **C** | **G** | **T** | **C** | **C** | **T** | **C** | **A** | **A** | **T** | **A** | **T** | **G** | **T** | **A** | **G** | **A** | **T** | **G** | **G** | **A** | **C** | **A** | **A** | **A** | **C** | **C** | **A** | **A** | **A** | **G** | **A** | **A** | **G** | **A** | **G** | **A** | **T** | **T** | **T** | **T** | **A** | **G** | **A** | **A** | **T** | **T** | **A** | **G** | **A** | **A** | **A** | **G** | **G** | **C** | **A** | **T** | **C** | **G** | **A** | **T** | **T** | **A** | **G** | **C** | **T** | **C** | **G** | **T** | **G** | **A** | **G** | **C** | **T** | **T** | **C** | **A** | **T** | **G** | **A** | **T** | **T** | **C** | **A** | **G** | **T** | **T** | **T** | **C** | **A** | **C** | **A** | **G** | **C** | **A** | **A** | **C** | **T** | **A** | **T** | **T** | **T** | **G** | **C** | **A** | **G** | **C** | **G** | **A** | **T** | **G** | **A** | **T** | **G** | **A** | **T** | **G** | **A** | **T** | **G** | **T** | **C** | **G** | **G** | **C** | **A** | **T** | **T** | **G** | **A** | **C** | **G** | **G** | **A** | **G** | **C** | **A** | **A** | **G** | **C** | **A** | **G** | **A** | **A** | **A** | **A** | **A** | **A** | **G** | **T** | **G** | **A** | **G** | **A** | **C** | **A** | **C** | **C** | **A** | **G** | **A** | **A** | **A** | **T** | **G** | **T** | **T** | **C** | **C** | **G** | **T** | **A** | **A** | **G** | **C** | **A** | **G** | **T** | **T** | **G** | **A** | **A** | **A** | **A** | **T** | **G** | **G** | **T** | **G** | **G** | **C** | **A** | **G** | **A** | **G** | **A** | **T** | **C** | **A** | **T** | **C** | **A** | **A** | **C** | **G** | **C** | **T** | **T** | **C** | **C** | **C** | **A** | **G** | **T** | **C** | **T** | **G** | **A** | **G** | **A** | **T** | **G** | **C** | **G** | **T** | **G** | **C** | **G** | **C** | **T** | **G** | **C** | **T** | **T** | **C** | **T** | **T** | **C** | **A** | **T** | **C** | **T** | **G** | **C** | **G** | **T** | **C** | **C** | **A** | **G** | **T** | **C** | **A** | **A** | **T** | **T** | **T** | **A** | **G** | **A** | **A** | **G** | **A** | **A** | **A** | **A** | **A** | **A** | **G** | **T** | **T** | **T** | **A** | **A** | **A** | **A** | **C** | **A** | **A** | **G** | **G** | **C** | **A** | **T** | **T** | **G** | **A** | **G** | **C** | **A** | **G** | **T** | **T** | **A** | **T** | **T** | **G** | **A** | **A** | **G** | **G** | **A** | **A** | **T** | **T** | **G** | **C** | **A** | **G** | **A** | **A** | **T** | **A** | **A** | **A** | **A** | **T** | **C** | **C** | **A** | **G** | **A** | **T** | **T** | **T** | **C** | **G** | **C** | **T** | **A** | **A** | **A** | **A** | **T** | **G** | **G** | **G** | **A** | **T** | **G** | **T** | **A** | **G** | **A** | **A** | **G** | **A** | **T** | **G** | **T** | **A** | **A** | **A** | **A** | **T** | **T** | **A** | **A** | **C** | **T** | **A** | **G** | **T** | **T** | **C** | **C** | **A** | **T** | **T** | **G** | **A** | **G** | **G** | **A** | **T** | **C** | **A** | **C** | **T** | **T** | **G** | **T** | **T** | **C** | **C** | **G** | **A** | **A** | **T** | **C** | **G** | **T** | **T** | **C** | **A** | **A** | **G** | **A** | **A** | **T** | **T** | **G** | **T** | **T** | **A** | **T** | **C** | **A** | **A** | **A** | **C** | **A** | **C** | **A** | **T** | **T** | **A** | **A** | **G** | **A** | **C** | **A** | **T** | **G** | **C** | **T** | **A** | **A** | **G** | **G** | **C** | **T** | **A** | **A** | **T** | **G** | **A** | **A** | **T** | **T** | **A** | **G** | **A** | **A** | **G** | **T** | **C** | **T** | **A** | **T** | **T** | **T** | **G** | **C** | **A** | **T** | **A** | **A** | **A** | **A** | **T** | **A** | **G** | **A** | **T** | **A** | **A** | **C** | **A** | **A** | **T** | **C** | **T** | **T** | **T** | **T** | **A** | **T** | **T** | **A** | **C** | **G** | **T** | **A** | **T** | **C** | **A** | **T** | **T** | **G** | **A** | **C** | **G** | **A** | **C** | **G** | **G** | **G** | **A** | **C** | **A** | **G** | **G** | **A** | **T** | **T** | **T** | **A** | **A** | **T** | **A** | **T** | **G** | **A** | **A** | **T** | **G** | **A** | **A** | **A** | **C** | **C** | **A** | **A** | **A** | **A** | **C** | **G** | **G** | **G** | **A** | **A** | **G** | **T** | **T** | **A** | **T** | **G** | **G** | **A** | **T** | **T** | **G** | **A** | **A** | **C** | **A** | **A** | **T** | **A** | **T** | **C** | **A** | **A** | **A** | **G** | **A** | **A** | **C** | **G** | **A** | **G** | **T** | **A** | **G** | **C** | **T** | **G** | **G** | **T** | **A** | **T** | **T** | **G** | **G** | **T** | **G** | **G** | **A** | **A** | **C** | **A** | **G** | **T** | **A** | **A** | **A** | **A** | **A** | **T** | **C** | **A** | **T** | **T** | **A** | **G** | **T** | **T** | **T** | **T** | **A** | **A** | **A** | **G** | **G** | **G** | **C** | **A** | **A** | **G** | **G** | **T** | **A** | **C** | **G** | **A** | **G** | **C** | **G** | **T** | **G** | **G** | **A** | **G** | **A** | **T** | **C** | **A** | **A** | **G** | **G** | **T** | **C** | **C** | **C** | **T** | **T** | **T** | **G** | **A** | **T** | **G** | **A** | **A** | **G** | **G** | **A** | **G** | **G** | **C** | **A** | **-** | **-** | **-** |
| **N54560** | **A** | **T** | **G** | **A** | **T** | **G** | **G** | **G** | **A** | **A** | **G** | **A** | **A** | **T** | **A** | **T** | **C** | **C** | **A** | **A** | **A** | **G** | **C** | **A** | **A** | **T** | **G** | **C** | **T** | **A** | **G** | **C** | **T** | **G** | **T** | **T** | **T** | **A** | **T** | **T** | **C** | **G** | **G** | **G** | **G** | **A** | **T** | **T** | **G** | **C** | **G** | **G** | **C** | **T** | **T** | **T** | **C** | **C** | **T** | **T** | **A** | **T** | **T** | **A** | **T** | **T** | **C** | **T** | **A** | **T** | **T** | **T** | **T** | **C** | **A** | **C** | **T** | **T** | **T** | **T** | **C** | **A** | **C** | **T** | **T** | **A** | **T** | **T** | **T** | **T** | **T** | **A** | **T** | **G** | **C** | **C** | **A** | **G** | **T** | **A** | **A** | **C** | **C** | **A** | **A** | **A** | **G** | **T** | **C** | **A** | **T** | **T** | **G** | **G** | **T** | **G** | **G** | **G** | **G** | **A** | **G** | **A** | **A** | **T** | **T** | **G** | **C** | **T** | **G** | **C** | **G** | **T** | **G** | **C** | **G** | **C** | **G** | **T** | **T** | **T** | **A** | **T** | **T** | **A** | **T** | **A** | **C** | **G** | **T** | **T** | **C** | **C** | **G** | **C** | **T** | **T** | **A** | **T** | **C** | **T** | **T** | **C** | **C** | **A** | **T** | **T** | **T** | **G** | **A** | **T** | **G** | **G** | **C** | **A** | **A** | **T** | **T** | **T** | **C** | **A** | **T** | **T** | **A** | **G** | **G** | **T** | **G** | **T** | **A** | **G** | **G** | **A** | **T** | **T** | **G** | **A** | **T** | **C** | **G** | **T** | **A** | **T** | **T** | **T** | **C** | **T** | **A** | **T** | **T** | **G** | **T** | **T** | **A** | **T** | **C** | **A** | **C** | **T** | **T** | **A** | **T** | **T** | **C** | **A** | **A** | **A** | **A** | **G** | **G** | **C** | **A** | **A** | **A** | **A** | **T** | **A** | **C** | **G** | **G** | **G** | **A** | **A** | **A** | **A** | **T** | **C** | **G** | **A** | **A** | **G** | **A** | **A** | **A** | **A** | **G** | **C** | **T** | **T** | **C** | **G** | **T** | **G** | **C** | **A** | **C** | **T** | **T** | **T** | **C** | **T** | **T** | **C** | **T** | **G** | **G** | **C** | **A** | **A** | **T** | **T** | **A** | **T** | **G** | **A** | **A** | **T** | **C** | **C** | **A** | **A** | **A** | **C** | **T** | **T** | **T** | **T** | **G** | **C** | **A** | **T** | **C** | **A** | **G** | **C** | **C** | **G** | **A** | **T** | **C** | **C** | **C** | **A** | **A** | **G** | **C** | **A** | **C** | **G** | **T** | **C** | **G** | **G** | **A** | **T** | **G** | **A** | **T** | **C** | **T** | **G** | **T** | **A** | **C** | **A** | **T** | **C** | **A** | **A** | **A** | **G** | **A** | **T** | **A** | **T** | **T** | **G** | **A** | **T** | **A** | **T** | **G** | **G** | **A** | **A** | **A** | **T** | **T** | **A** | **C** | **T** | **A** | **A** | **A** | **A** | **T** | **A** | **A** | **A** | **A** | **G** | **A** | **A** | **A** | **A** | **A** | **A** | **T** | **G** | **A** | **T** | **C** | **G** | **A** | **A** | **G** | **T** | **A** | **T** | **C** | **C** | **A** | **G** | **T** | **G** | **A** | **A** | **T** | **T** | **A** | **C** | **A** | **G** | **A** | **T** | **T** | **A** | **T** | **G** | **A** | **C** | **T** | **A** | **G** | **C** | **C** | **G** | **T** | **C** | **C** | **T** | **C** | **A** | **A** | **T** | **A** | **T** | **G** | **T** | **A** | **G** | **A** | **T** | **G** | **G** | **A** | **C** | **A** | **A** | **A** | **C** | **C** | **A** | **A** | **A** | **G** | **A** | **A** | **G** | **A** | **G** | **A** | **T** | **T** | **T** | **T** | **A** | **G** | **A** | **A** | **T** | **T** | **A** | **G** | **A** | **A** | **A** | **G** | **G** | **C** | **A** | **T** | **C** | **G** | **A** | **T** | **T** | **A** | **G** | **C** | **T** | **C** | **G** | **T** | **G** | **A** | **G** | **C** | **T** | **T** | **C** | **A** | **T** | **G** | **A** | **T** | **T** | **C** | **A** | **G** | **T** | **T** | **T** | **C** | **A** | **C** | **A** | **G** | **C** | **A** | **A** | **T** | **T** | **A** | **T** | **T** | **T** | **G** | **C** | **A** | **G** | **C** | **G** | **A** | **T** | **G** | **A** | **T** | **G** | **A** | **T** | **G** | **A** | **T** | **G** | **T** | **C** | **G** | **G** | **C** | **A** | **T** | **T** | **G** | **A** | **C** | **G** | **G** | **A** | **G** | **C** | **A** | **A** | **G** | **C** | **A** | **G** | **A** | **A** | **A** | **A** | **A** | **A** | **G** | **T** | **G** | **A** | **G** | **A** | **C** | **A** | **C** | **C** | **A** | **G** | **A** | **A** | **A** | **T** | **G** | **T** | **T** | **C** | **C** | **G** | **T** | **A** | **A** | **G** | **C** | **A** | **G** | **T** | **T** | **G** | **A** | **A** | **A** | **A** | **T** | **G** | **G** | **T** | **G** | **G** | **C** | **A** | **G** | **A** | **G** | **A** | **T** | **C** | **A** | **T** | **C** | **A** | **A** | **C** | **G** | **C** | **T** | **T** | **C** | **C** | **C** | **A** | **G** | **T** | **C** | **T** | **G** | **A** | **G** | **A** | **T** | **G** | **C** | **G** | **T** | **G** | **C** | **G** | **C** | **T** | **G** | **C** | **T** | **T** | **C** | **T** | **T** | **C** | **A** | **T** | **C** | **T** | **G** | **C** | **G** | **T** | **C** | **C** | **A** | **G** | **T** | **C** | **A** | **A** | **T** | **T** | **T** | **A** | **G** | **A** | **A** | **G** | **A** | **A** | **A** | **A** | **A** | **A** | **G** | **T** | **T** | **T** | **A** | **A** | **A** | **A** | **C** | **A** | **A** | **G** | **G** | **C** | **A** | **T** | **T** | **G** | **A** | **G** | **C** | **A** | **G** | **T** | **T** | **A** | **T** | **T** | **G** | **A** | **A** | **G** | **G** | **A** | **A** | **T** | **T** | **G** | **C** | **A** | **G** | **A** | **A** | **T** | **A** | **A** | **A** | **A** | **T** | **C** | **C** | **A** | **G** | **A** | **T** | **T** | **T** | **C** | **G** | **C** | **T** | **A** | **A** | **A** | **A** | **T** | **G** | **G** | **G** | **A** | **T** | **G** | **T** | **A** | **G** | **A** | **A** | **G** | **A** | **T** | **G** | **T** | **A** | **A** | **A** | **A** | **T** | **T** | **A** | **A** | **C** | **T** | **A** | **G** | **T** | **T** | **C** | **C** | **A** | **T** | **T** | **G** | **A** | **G** | **G** | **A** | **T** | **C** | **A** | **C** | **T** | **T** | **G** | **T** | **T** | **C** | **C** | **G** | **A** | **A** | **T** | **C** | **G** | **T** | **T** | **C** | **A** | **A** | **G** | **A** | **A** | **T** | **T** | **G** | **T** | **T** | **A** | **T** | **C** | **A** | **A** | **A** | **C** | **A** | **C** | **A** | **T** | **T** | **A** | **A** | **G** | **A** | **C** | **A** | **T** | **G** | **C** | **T** | **A** | **A** | **G** | **G** | **C** | **T** | **A** | **A** | **T** | **G** | **A** | **A** | **T** | **T** | **A** | **G** | **A** | **A** | **G** | **T** | **C** | **T** | **A** | **T** | **T** | **T** | **G** | **C** | **A** | **T** | **A** | **A** | **A** | **A** | **T** | **A** | **G** | **A** | **T** | **A** | **A** | **C** | **A** | **A** | **T** | **C** | **T** | **T** | **T** | **T** | **A** | **T** | **T** | **A** | **C** | **G** | **T** | **A** | **T** | **C** | **A** | **T** | **T** | **G** | **A** | **C** | **G** | **A** | **C** | **G** | **G** | **G** | **A** | **C** | **A** | **G** | **G** | **A** | **T** | **T** | **T** | **A** | **A** | **T** | **A** | **T** | **G** | **A** | **A** | **T** | **G** | **A** | **A** | **A** | **C** | **C** | **A** | **A** | **A** | **A** | **C** | **G** | **G** | **G** | **A** | **A** | **G** | **T** | **T** | **A** | **T** | **G** | **G** | **A** | **T** | **T** | **G** | **A** | **A** | **C** | **A** | **A** | **T** | **A** | **T** | **C** | **A** | **A** | **A** | **G** | **A** | **A** | **C** | **G** | **A** | **G** | **T** | **A** | **G** | **C** | **T** | **G** | **G** | **T** | **A** | **T** | **T** | **G** | **G** | **T** | **G** | **G** | **A** | **A** | **C** | **A** | **G** | **T** | **A** | **A** | **A** | **A** | **A** | **T** | **C** | **A** | **T** | **T** | **A** | **G** | **T** | **T** | **T** | **T** | **A** | **A** | **A** | **G** | **G** | **G** | **C** | **A** | **A** | **G** | **G** | **T** | **A** | **C** | **G** | **A** | **G** | **C** | **G** | **T** | **G** | **G** | **A** | **G** | **A** | **T** | **C** | **A** | **A** | **G** | **G** | **T** | **C** | **C** | **C** | **T** | **T** | **T** | **G** | **A** | **T** | **G** | **A** | **A** | **G** | **G** | **A** | **G** | **G** | **C** | **A** | **-** | **-** | **-** |
| **N59754F** | **A** | **T** | **G** | **A** | **T** | **G** | **G** | **G** | **A** | **A** | **G** | **A** | **A** | **T** | **A** | **T** | **C** | **C** | **A** | **A** | **A** | **G** | **C** | **A** | **A** | **T** | **G** | **C** | **T** | **A** | **G** | **C** | **T** | **G** | **T** | **T** | **T** | **A** | **T** | **T** | **C** | **G** | **G** | **G** | **G** | **A** | **T** | **T** | **G** | **C** | **G** | **G** | **C** | **T** | **T** | **T** | **C** | **C** | **T** | **T** | **A** | **T** | **T** | **A** | **T** | **T** | **C** | **T** | **A** | **T** | **T** | **T** | **T** | **C** | **A** | **C** | **T** | **T** | **T** | **T** | **C** | **A** | **C** | **T** | **T** | **A** | **T** | **T** | **T** | **T** | **T** | **A** | **T** | **G** | **C** | **C** | **A** | **G** | **T** | **A** | **A** | **C** | **C** | **A** | **A** | **A** | **G** | **T** | **C** | **A** | **T** | **T** | **G** | **G** | **T** | **G** | **G** | **G** | **G** | **A** | **G** | **A** | **A** | **T** | **T** | **G** | **C** | **T** | **G** | **C** | **G** | **T** | **G** | **C** | **G** | **C** | **G** | **T** | **T** | **T** | **A** | **T** | **T** | **A** | **T** | **A** | **C** | **G** | **T** | **T** | **C** | **C** | **G** | **C** | **T** | **T** | **A** | **T** | **C** | **T** | **T** | **C** | **C** | **A** | **T** | **T** | **T** | **G** | **A** | **T** | **G** | **G** | **C** | **A** | **A** | **T** | **T** | **T** | **C** | **A** | **T** | **T** | **A** | **G** | **G** | **T** | **G** | **T** | **A** | **G** | **G** | **A** | **T** | **T** | **G** | **A** | **T** | **C** | **G** | **T** | **A** | **T** | **T** | **T** | **C** | **T** | **A** | **T** | **T** | **G** | **T** | **T** | **A** | **T** | **C** | **A** | **C** | **T** | **T** | **A** | **T** | **T** | **C** | **A** | **A** | **A** | **A** | **G** | **G** | **C** | **A** | **A** | **A** | **A** | **T** | **A** | **C** | **G** | **G** | **G** | **A** | **A** | **A** | **A** | **T** | **C** | **G** | **A** | **A** | **G** | **A** | **A** | **A** | **A** | **G** | **C** | **T** | **T** | **C** | **G** | **T** | **G** | **C** | **A** | **C** | **T** | **T** | **T** | **C** | **T** | **T** | **C** | **T** | **G** | **G** | **C** | **A** | **A** | **T** | **T** | **A** | **T** | **G** | **A** | **A** | **T** | **C** | **C** | **A** | **A** | **A** | **C** | **T** | **T** | **T** | **T** | **G** | **C** | **A** | **T** | **C** | **A** | **G** | **C** | **C** | **G** | **A** | **T** | **C** | **C** | **C** | **A** | **A** | **G** | **C** | **G** | **C** | **G** | **T** | **C** | **G** | **G** | **A** | **T** | **G** | **A** | **T** | **C** | **T** | **G** | **T** | **A** | **C** | **A** | **T** | **C** | **A** | **A** | **A** | **G** | **A** | **T** | **A** | **T** | **T** | **G** | **A** | **T** | **A** | **T** | **G** | **G** | **A** | **A** | **A** | **T** | **T** | **A** | **C** | **T** | **A** | **A** | **A** | **A** | **T** | **A** | **A** | **A** | **A** | **G** | **A** | **A** | **A** | **A** | **A** | **A** | **T** | **G** | **A** | **T** | **C** | **G** | **A** | **A** | **G** | **T** | **A** | **T** | **C** | **C** | **A** | **G** | **T** | **G** | **A** | **A** | **T** | **T** | **A** | **C** | **A** | **G** | **A** | **T** | **T** | **A** | **T** | **G** | **A** | **C** | **T** | **A** | **G** | **C** | **C** | **G** | **T** | **C** | **C** | **T** | **C** | **A** | **A** | **T** | **A** | **T** | **G** | **T** | **A** | **G** | **A** | **T** | **G** | **G** | **A** | **C** | **A** | **A** | **A** | **C** | **C** | **A** | **A** | **A** | **G** | **A** | **A** | **G** | **A** | **G** | **A** | **T** | **T** | **T** | **T** | **A** | **G** | **A** | **A** | **T** | **T** | **A** | **G** | **A** | **A** | **A** | **G** | **G** | **C** | **A** | **T** | **C** | **G** | **A** | **T** | **T** | **A** | **G** | **C** | **T** | **C** | **G** | **T** | **G** | **A** | **G** | **C** | **T** | **T** | **C** | **A** | **T** | **G** | **A** | **T** | **T** | **C** | **A** | **G** | **T** | **T** | **T** | **C** | **A** | **C** | **A** | **G** | **C** | **A** | **A** | **T** | **T** | **A** | **T** | **T** | **T** | **G** | **C** | **A** | **G** | **C** | **G** | **A** | **T** | **G** | **A** | **T** | **G** | **A** | **T** | **G** | **A** | **T** | **G** | **T** | **C** | **G** | **G** | **C** | **A** | **T** | **T** | **G** | **A** | **C** | **G** | **G** | **A** | **G** | **C** | **A** | **A** | **G** | **C** | **A** | **G** | **A** | **A** | **A** | **A** | **A** | **A** | **G** | **T** | **G** | **A** | **G** | **A** | **C** | **A** | **C** | **C** | **A** | **G** | **G** | **A** | **A** | **T** | **G** | **T** | **T** | **C** | **C** | **G** | **T** | **A** | **A** | **G** | **C** | **A** | **G** | **T** | **T** | **G** | **A** | **A** | **A** | **A** | **T** | **G** | **G** | **T** | **G** | **G** | **C** | **A** | **G** | **A** | **G** | **A** | **T** | **C** | **A** | **T** | **C** | **A** | **A** | **C** | **G** | **C** | **T** | **T** | **C** | **C** | **C** | **A** | **G** | **T** | **C** | **T** | **G** | **A** | **G** | **A** | **T** | **G** | **C** | **G** | **T** | **G** | **C** | **G** | **C** | **T** | **G** | **C** | **T** | **T** | **C** | **T** | **T** | **C** | **A** | **T** | **C** | **T** | **G** | **C** | **G** | **T** | **C** | **C** | **A** | **G** | **T** | **C** | **A** | **A** | **T** | **T** | **T** | **A** | **G** | **A** | **A** | **G** | **A** | **A** | **A** | **A** | **A** | **A** | **G** | **T** | **T** | **T** | **A** | **A** | **A** | **A** | **C** | **A** | **A** | **G** | **G** | **C** | **A** | **T** | **T** | **G** | **A** | **G** | **C** | **A** | **G** | **T** | **T** | **A** | **T** | **T** | **G** | **A** | **A** | **G** | **G** | **A** | **A** | **T** | **T** | **G** | **C** | **A** | **G** | **A** | **A** | **T** | **A** | **A** | **A** | **A** | **T** | **C** | **C** | **A** | **G** | **A** | **T** | **T** | **T** | **C** | **G** | **C** | **T** | **A** | **A** | **A** | **A** | **T** | **G** | **G** | **G** | **A** | **T** | **G** | **T** | **A** | **G** | **A** | **A** | **G** | **A** | **T** | **G** | **T** | **A** | **A** | **A** | **A** | **T** | **T** | **A** | **A** | **C** | **T** | **A** | **G** | **T** | **T** | **C** | **C** | **A** | **T** | **T** | **G** | **A** | **G** | **G** | **A** | **T** | **C** | **A** | **C** | **T** | **T** | **G** | **T** | **T** | **C** | **C** | **G** | **A** | **A** | **T** | **C** | **G** | **T** | **T** | **C** | **A** | **A** | **G** | **A** | **A** | **T** | **T** | **G** | **T** | **T** | **A** | **T** | **C** | **A** | **A** | **A** | **C** | **A** | **C** | **A** | **T** | **T** | **A** | **A** | **G** | **A** | **C** | **A** | **T** | **G** | **C** | **T** | **A** | **A** | **G** | **G** | **C** | **T** | **A** | **A** | **T** | **G** | **A** | **A** | **T** | **T** | **A** | **G** | **A** | **A** | **G** | **T** | **C** | **T** | **A** | **T** | **T** | **T** | **G** | **C** | **A** | **T** | **A** | **A** | **A** | **A** | **T** | **A** | **G** | **A** | **T** | **A** | **A** | **C** | **A** | **A** | **T** | **C** | **T** | **T** | **T** | **T** | **A** | **T** | **T** | **A** | **C** | **G** | **T** | **A** | **T** | **C** | **A** | **T** | **T** | **G** | **A** | **C** | **G** | **A** | **C** | **G** | **G** | **G** | **A** | **C** | **A** | **G** | **G** | **C** | **T** | **T** | **T** | **A** | **A** | **T** | **A** | **T** | **G** | **A** | **A** | **T** | **G** | **A** | **A** | **A** | **C** | **C** | **A** | **A** | **A** | **A** | **C** | **G** | **G** | **G** | **A** | **A** | **G** | **T** | **T** | **A** | **T** | **G** | **G** | **A** | **T** | **T** | **G** | **A** | **A** | **C** | **A** | **A** | **T** | **A** | **T** | **C** | **A** | **A** | **A** | **G** | **A** | **A** | **C** | **G** | **A** | **G** | **T** | **A** | **G** | **C** | **T** | **G** | **G** | **T** | **A** | **T** | **T** | **G** | **G** | **T** | **G** | **G** | **A** | **A** | **C** | **A** | **G** | **T** | **A** | **A** | **A** | **A** | **A** | **T** | **C** | **A** | **T** | **T** | **A** | **G** | **T** | **T** | **T** | **T** | **A** | **A** | **A** | **G** | **G** | **G** | **C** | **A** | **A** | **G** | **G** | **T** | **A** | **C** | **G** | **A** | **G** | **C** | **G** | **T** | **G** | **G** | **A** | **G** | **A** | **T** | **C** | **A** | **A** | **G** | **G** | **T** | **C** | **C** | **C** | **T** | **T** | **T** | **G** | **A** | **T** | **G** | **A** | **A** | **G** | **G** | **A** | **G** | **G** | **C** | **A** | **-** | **-** | **-** |
| **N60260F** | **A** | **T** | **G** | **A** | **T** | **G** | **G** | **G** | **A** | **A** | **G** | **A** | **A** | **T** | **A** | **T** | **C** | **C** | **A** | **A** | **A** | **G** | **C** | **A** | **A** | **T** | **G** | **C** | **T** | **A** | **G** | **C** | **T** | **G** | **T** | **T** | **T** | **A** | **T** | **T** | **C** | **G** | **G** | **G** | **G** | **A** | **T** | **T** | **G** | **C** | **G** | **G** | **C** | **T** | **T** | **T** | **C** | **C** | **T** | **T** | **A** | **T** | **T** | **A** | **T** | **T** | **C** | **T** | **A** | **T** | **T** | **T** | **T** | **C** | **A** | **C** | **T** | **T** | **T** | **T** | **C** | **A** | **C** | **T** | **T** | **A** | **T** | **T** | **T** | **T** | **T** | **A** | **T** | **G** | **C** | **C** | **A** | **G** | **T** | **A** | **A** | **C** | **C** | **A** | **A** | **A** | **G** | **T** | **C** | **A** | **T** | **T** | **G** | **G** | **T** | **G** | **G** | **G** | **G** | **A** | **G** | **A** | **A** | **T** | **T** | **G** | **C** | **T** | **G** | **C** | **G** | **T** | **G** | **C** | **G** | **C** | **G** | **T** | **T** | **T** | **A** | **T** | **T** | **A** | **T** | **A** | **C** | **G** | **T** | **T** | **C** | **C** | **G** | **C** | **T** | **T** | **A** | **T** | **C** | **T** | **T** | **C** | **C** | **A** | **T** | **T** | **T** | **G** | **A** | **T** | **G** | **G** | **C** | **A** | **A** | **T** | **T** | **T** | **C** | **A** | **T** | **T** | **A** | **G** | **G** | **T** | **G** | **T** | **A** | **G** | **G** | **A** | **T** | **T** | **G** | **A** | **T** | **C** | **G** | **T** | **A** | **T** | **T** | **T** | **C** | **T** | **A** | **T** | **T** | **G** | **T** | **T** | **A** | **T** | **C** | **A** | **C** | **T** | **T** | **A** | **T** | **T** | **C** | **A** | **A** | **A** | **A** | **G** | **G** | **C** | **A** | **A** | **A** | **A** | **T** | **A** | **C** | **G** | **G** | **G** | **A** | **A** | **A** | **A** | **T** | **C** | **G** | **A** | **A** | **G** | **A** | **A** | **A** | **A** | **G** | **C** | **T** | **T** | **C** | **G** | **T** | **G** | **C** | **A** | **C** | **T** | **T** | **T** | **C** | **T** | **T** | **C** | **T** | **G** | **G** | **C** | **A** | **A** | **T** | **T** | **A** | **T** | **G** | **A** | **A** | **T** | **C** | **C** | **A** | **A** | **A** | **C** | **T** | **T** | **T** | **T** | **G** | **C** | **A** | **T** | **C** | **A** | **G** | **C** | **C** | **G** | **A** | **T** | **C** | **C** | **C** | **A** | **A** | **G** | **C** | **G** | **C** | **G** | **T** | **C** | **G** | **G** | **A** | **T** | **G** | **A** | **T** | **C** | **T** | **G** | **T** | **A** | **C** | **A** | **T** | **C** | **A** | **A** | **A** | **G** | **A** | **T** | **A** | **T** | **T** | **G** | **A** | **T** | **A** | **T** | **G** | **G** | **A** | **A** | **A** | **T** | **T** | **A** | **C** | **T** | **A** | **A** | **A** | **A** | **T** | **A** | **A** | **A** | **A** | **G** | **A** | **A** | **A** | **A** | **A** | **A** | **T** | **G** | **A** | **T** | **C** | **G** | **A** | **A** | **G** | **T** | **A** | **T** | **C** | **C** | **A** | **G** | **T** | **G** | **A** | **A** | **T** | **T** | **A** | **C** | **A** | **G** | **A** | **T** | **T** | **A** | **T** | **G** | **A** | **C** | **T** | **A** | **G** | **C** | **C** | **G** | **T** | **C** | **C** | **T** | **C** | **A** | **A** | **T** | **A** | **T** | **G** | **T** | **A** | **G** | **A** | **T** | **G** | **G** | **A** | **C** | **A** | **A** | **A** | **C** | **C** | **A** | **A** | **A** | **G** | **A** | **A** | **G** | **A** | **G** | **A** | **T** | **T** | **T** | **T** | **A** | **G** | **A** | **A** | **T** | **T** | **A** | **G** | **A** | **A** | **A** | **G** | **G** | **C** | **A** | **T** | **C** | **G** | **A** | **T** | **T** | **A** | **G** | **C** | **T** | **C** | **G** | **T** | **G** | **A** | **G** | **C** | **T** | **T** | **C** | **A** | **T** | **G** | **A** | **T** | **T** | **C** | **A** | **G** | **T** | **T** | **T** | **C** | **A** | **C** | **A** | **G** | **C** | **A** | **A** | **T** | **T** | **A** | **T** | **T** | **T** | **G** | **C** | **A** | **G** | **C** | **G** | **A** | **T** | **G** | **A** | **T** | **G** | **A** | **T** | **G** | **A** | **T** | **G** | **T** | **C** | **G** | **G** | **C** | **A** | **T** | **T** | **G** | **A** | **C** | **G** | **G** | **A** | **G** | **C** | **A** | **A** | **G** | **C** | **A** | **G** | **A** | **A** | **A** | **A** | **A** | **A** | **G** | **T** | **G** | **A** | **G** | **A** | **C** | **A** | **C** | **C** | **A** | **G** | **A** | **A** | **A** | **T** | **G** | **T** | **T** | **C** | **C** | **G** | **T** | **A** | **A** | **G** | **C** | **A** | **G** | **T** | **T** | **G** | **A** | **A** | **A** | **A** | **T** | **G** | **G** | **T** | **G** | **G** | **C** | **A** | **G** | **A** | **G** | **A** | **T** | **C** | **A** | **T** | **C** | **A** | **A** | **C** | **G** | **C** | **T** | **T** | **C** | **C** | **C** | **A** | **G** | **T** | **C** | **T** | **G** | **A** | **G** | **A** | **T** | **G** | **C** | **G** | **T** | **G** | **C** | **G** | **C** | **T** | **G** | **C** | **T** | **T** | **C** | **T** | **T** | **C** | **A** | **T** | **C** | **T** | **G** | **C** | **G** | **T** | **C** | **C** | **A** | **G** | **T** | **C** | **A** | **A** | **T** | **T** | **T** | **A** | **G** | **A** | **A** | **G** | **A** | **A** | **A** | **A** | **A** | **A** | **G** | **T** | **T** | **T** | **A** | **A** | **A** | **A** | **C** | **A** | **A** | **G** | **G** | **C** | **A** | **T** | **T** | **G** | **A** | **G** | **C** | **A** | **G** | **T** | **T** | **A** | **T** | **T** | **G** | **A** | **A** | **G** | **G** | **A** | **A** | **T** | **T** | **G** | **C** | **A** | **G** | **A** | **A** | **T** | **A** | **A** | **A** | **A** | **T** | **C** | **C** | **A** | **G** | **A** | **T** | **T** | **T** | **C** | **G** | **C** | **T** | **A** | **A** | **A** | **A** | **T** | **G** | **G** | **G** | **A** | **T** | **G** | **T** | **A** | **G** | **A** | **A** | **G** | **A** | **T** | **G** | **T** | **A** | **A** | **A** | **A** | **T** | **T** | **A** | **A** | **C** | **T** | **A** | **G** | **T** | **T** | **C** | **C** | **A** | **T** | **T** | **G** | **A** | **G** | **G** | **A** | **T** | **C** | **A** | **C** | **T** | **T** | **G** | **T** | **T** | **C** | **C** | **G** | **A** | **A** | **T** | **C** | **G** | **T** | **T** | **C** | **A** | **A** | **G** | **A** | **A** | **T** | **T** | **G** | **T** | **T** | **A** | **T** | **C** | **A** | **A** | **A** | **C** | **A** | **C** | **A** | **T** | **T** | **A** | **A** | **G** | **A** | **C** | **A** | **T** | **G** | **C** | **T** | **A** | **A** | **G** | **G** | **C** | **T** | **A** | **A** | **T** | **G** | **A** | **A** | **T** | **T** | **A** | **G** | **A** | **A** | **G** | **T** | **C** | **T** | **A** | **T** | **T** | **T** | **G** | **C** | **A** | **T** | **A** | **A** | **A** | **A** | **T** | **A** | **G** | **A** | **T** | **A** | **A** | **C** | **A** | **A** | **T** | **C** | **T** | **T** | **T** | **T** | **A** | **T** | **T** | **A** | **C** | **G** | **T** | **A** | **T** | **C** | **A** | **T** | **T** | **G** | **A** | **C** | **G** | **A** | **C** | **G** | **G** | **G** | **A** | **C** | **A** | **G** | **G** | **A** | **T** | **T** | **T** | **A** | **A** | **T** | **A** | **T** | **G** | **A** | **A** | **T** | **G** | **A** | **A** | **A** | **C** | **C** | **A** | **A** | **A** | **A** | **C** | **G** | **G** | **G** | **A** | **A** | **G** | **T** | **T** | **A** | **T** | **G** | **G** | **A** | **T** | **T** | **G** | **A** | **A** | **C** | **A** | **A** | **T** | **A** | **T** | **C** | **A** | **A** | **A** | **G** | **A** | **A** | **C** | **G** | **A** | **G** | **T** | **A** | **G** | **C** | **T** | **G** | **G** | **T** | **A** | **T** | **T** | **G** | **G** | **T** | **G** | **G** | **A** | **A** | **C** | **A** | **G** | **T** | **A** | **A** | **A** | **A** | **A** | **T** | **C** | **A** | **T** | **T** | **A** | **G** | **T** | **T** | **T** | **T** | **A** | **A** | **A** | **G** | **G** | **G** | **C** | **A** | **A** | **G** | **G** | **T** | **A** | **C** | **G** | **A** | **G** | **C** | **G** | **T** | **G** | **G** | **A** | **G** | **A** | **T** | **C** | **A** | **A** | **G** | **G** | **T** | **C** | **C** | **C** | **T** | **T** | **T** | **G** | **A** | **T** | **G** | **A** | **A** | **G** | **G** | **A** | **G** | **G** | **C** | **A** | **-** | **-** | **-** |
| **N52690** | **A** | **T** | **G** | **A** | **T** | **G** | **G** | **G** | **A** | **A** | **G** | **A** | **A** | **T** | **A** | **T** | **C** | **C** | **A** | **A** | **A** | **G** | **C** | **A** | **A** | **T** | **G** | **C** | **T** | **A** | **G** | **C** | **T** | **G** | **T** | **T** | **T** | **A** | **T** | **T** | **C** | **G** | **G** | **G** | **G** | **A** | **T** | **T** | **G** | **C** | **G** | **G** | **C** | **T** | **T** | **T** | **C** | **C** | **T** | **T** | **A** | **T** | **T** | **A** | **T** | **T** | **C** | **T** | **A** | **T** | **T** | **T** | **T** | **C** | **A** | **C** | **T** | **T** | **T** | **T** | **C** | **A** | **C** | **T** | **T** | **A** | **T** | **T** | **T** | **T** | **T** | **A** | **T** | **G** | **C** | **C** | **A** | **G** | **T** | **A** | **A** | **C** | **C** | **A** | **A** | **A** | **G** | **T** | **C** | **A** | **T** | **T** | **G** | **G** | **T** | **G** | **G** | **G** | **G** | **A** | **G** | **A** | **A** | **T** | **T** | **G** | **C** | **T** | **G** | **C** | **G** | **T** | **G** | **C** | **G** | **C** | **G** | **T** | **T** | **T** | **A** | **T** | **T** | **A** | **T** | **A** | **C** | **G** | **T** | **T** | **C** | **C** | **G** | **C** | **T** | **T** | **A** | **T** | **C** | **T** | **T** | **C** | **C** | **A** | **T** | **T** | **T** | **G** | **A** | **T** | **G** | **G** | **C** | **A** | **A** | **T** | **T** | **T** | **C** | **A** | **T** | **T** | **A** | **G** | **G** | **T** | **G** | **T** | **A** | **G** | **G** | **A** | **T** | **T** | **G** | **A** | **T** | **C** | **G** | **T** | **A** | **T** | **T** | **T** | **C** | **T** | **A** | **T** | **T** | **G** | **T** | **T** | **A** | **T** | **C** | **A** | **C** | **T** | **T** | **A** | **T** | **T** | **C** | **A** | **A** | **A** | **A** | **G** | **G** | **C** | **A** | **A** | **A** | **A** | **T** | **A** | **C** | **G** | **G** | **G** | **A** | **A** | **A** | **A** | **T** | **C** | **G** | **A** | **A** | **G** | **A** | **A** | **A** | **A** | **G** | **C** | **T** | **T** | **C** | **G** | **T** | **G** | **C** | **A** | **C** | **T** | **T** | **T** | **C** | **T** | **T** | **C** | **T** | **G** | **G** | **C** | **A** | **A** | **T** | **T** | **A** | **T** | **G** | **A** | **A** | **T** | **C** | **C** | **A** | **A** | **A** | **C** | **T** | **T** | **T** | **T** | **G** | **C** | **A** | **T** | **C** | **A** | **G** | **C** | **C** | **G** | **A** | **T** | **C** | **C** | **C** | **A** | **A** | **G** | **C** | **G** | **C** | **G** | **T** | **C** | **G** | **G** | **A** | **T** | **G** | **A** | **T** | **C** | **T** | **G** | **T** | **A** | **C** | **A** | **T** | **C** | **A** | **A** | **A** | **G** | **A** | **T** | **A** | **T** | **T** | **G** | **A** | **T** | **A** | **T** | **G** | **G** | **A** | **A** | **A** | **T** | **T** | **A** | **C** | **T** | **A** | **A** | **A** | **A** | **T** | **A** | **A** | **A** | **A** | **G** | **A** | **A** | **A** | **A** | **A** | **A** | **T** | **G** | **A** | **T** | **C** | **G** | **A** | **A** | **G** | **T** | **A** | **T** | **C** | **C** | **A** | **G** | **T** | **G** | **A** | **A** | **T** | **T** | **A** | **C** | **A** | **G** | **A** | **T** | **T** | **A** | **T** | **G** | **A** | **C** | **T** | **A** | **G** | **C** | **C** | **G** | **T** | **C** | **C** | **T** | **C** | **A** | **A** | **T** | **A** | **T** | **G** | **T** | **A** | **G** | **A** | **T** | **G** | **G** | **A** | **C** | **A** | **A** | **A** | **C** | **C** | **A** | **A** | **A** | **G** | **A** | **A** | **G** | **A** | **G** | **A** | **T** | **T** | **T** | **T** | **A** | **G** | **A** | **A** | **T** | **T** | **A** | **G** | **A** | **A** | **A** | **G** | **G** | **C** | **A** | **T** | **C** | **G** | **A** | **T** | **T** | **A** | **G** | **C** | **T** | **C** | **G** | **T** | **G** | **A** | **G** | **C** | **T** | **T** | **C** | **A** | **T** | **G** | **A** | **T** | **T** | **C** | **A** | **G** | **T** | **T** | **T** | **C** | **A** | **C** | **A** | **G** | **C** | **A** | **A** | **T** | **T** | **A** | **T** | **T** | **T** | **G** | **C** | **A** | **G** | **C** | **G** | **A** | **T** | **G** | **A** | **T** | **G** | **A** | **T** | **G** | **A** | **T** | **G** | **T** | **C** | **G** | **G** | **C** | **A** | **T** | **T** | **G** | **A** | **C** | **G** | **G** | **A** | **G** | **C** | **A** | **A** | **G** | **C** | **A** | **G** | **A** | **A** | **A** | **A** | **A** | **A** | **G** | **T** | **G** | **A** | **G** | **A** | **C** | **A** | **C** | **C** | **A** | **G** | **A** | **A** | **A** | **T** | **G** | **T** | **T** | **C** | **C** | **G** | **T** | **A** | **A** | **G** | **C** | **A** | **G** | **T** | **T** | **G** | **A** | **A** | **A** | **A** | **T** | **G** | **G** | **T** | **G** | **G** | **C** | **A** | **G** | **A** | **G** | **A** | **T** | **C** | **A** | **T** | **C** | **A** | **A** | **C** | **G** | **C** | **T** | **T** | **C** | **C** | **C** | **A** | **G** | **T** | **C** | **T** | **G** | **A** | **G** | **A** | **T** | **G** | **C** | **G** | **T** | **G** | **C** | **G** | **C** | **T** | **G** | **C** | **T** | **T** | **C** | **T** | **T** | **C** | **A** | **T** | **C** | **T** | **G** | **C** | **G** | **T** | **C** | **C** | **A** | **G** | **T** | **C** | **A** | **A** | **T** | **T** | **T** | **A** | **G** | **A** | **A** | **G** | **A** | **A** | **A** | **A** | **A** | **A** | **G** | **T** | **T** | **T** | **A** | **A** | **A** | **A** | **C** | **A** | **A** | **G** | **G** | **C** | **A** | **T** | **T** | **G** | **A** | **G** | **C** | **A** | **G** | **T** | **T** | **A** | **T** | **T** | **G** | **A** | **A** | **G** | **G** | **A** | **A** | **T** | **T** | **G** | **C** | **A** | **G** | **A** | **A** | **T** | **A** | **A** | **A** | **A** | **T** | **C** | **C** | **A** | **G** | **A** | **T** | **T** | **T** | **C** | **G** | **C** | **T** | **A** | **A** | **A** | **A** | **T** | **G** | **G** | **G** | **A** | **T** | **G** | **T** | **A** | **G** | **A** | **A** | **G** | **A** | **T** | **G** | **T** | **A** | **A** | **A** | **A** | **T** | **T** | **A** | **A** | **C** | **T** | **A** | **G** | **T** | **T** | **C** | **C** | **A** | **T** | **T** | **G** | **A** | **G** | **G** | **A** | **T** | **C** | **A** | **C** | **T** | **T** | **G** | **T** | **T** | **C** | **C** | **G** | **A** | **A** | **T** | **C** | **G** | **T** | **T** | **C** | **A** | **A** | **G** | **A** | **A** | **T** | **T** | **G** | **T** | **T** | **A** | **T** | **C** | **A** | **A** | **A** | **C** | **A** | **C** | **A** | **T** | **T** | **A** | **A** | **G** | **A** | **C** | **A** | **T** | **G** | **C** | **T** | **A** | **A** | **G** | **G** | **C** | **T** | **A** | **A** | **T** | **G** | **A** | **A** | **T** | **T** | **A** | **G** | **A** | **A** | **G** | **T** | **C** | **T** | **A** | **T** | **T** | **T** | **G** | **C** | **A** | **T** | **A** | **A** | **A** | **A** | **T** | **A** | **G** | **A** | **T** | **A** | **A** | **C** | **A** | **A** | **T** | **C** | **T** | **T** | **T** | **T** | **A** | **T** | **T** | **A** | **C** | **G** | **T** | **A** | **T** | **C** | **A** | **T** | **T** | **G** | **A** | **C** | **G** | **A** | **C** | **G** | **G** | **G** | **A** | **C** | **A** | **G** | **G** | **A** | **T** | **T** | **T** | **A** | **A** | **T** | **A** | **T** | **G** | **A** | **A** | **T** | **G** | **A** | **A** | **A** | **C** | **C** | **A** | **A** | **A** | **A** | **C** | **G** | **G** | **G** | **A** | **A** | **G** | **T** | **T** | **A** | **T** | **G** | **G** | **A** | **T** | **T** | **G** | **A** | **A** | **C** | **A** | **A** | **T** | **A** | **T** | **C** | **A** | **A** | **A** | **G** | **A** | **A** | **C** | **G** | **A** | **G** | **T** | **A** | **G** | **C** | **T** | **G** | **G** | **T** | **A** | **T** | **T** | **G** | **G** | **T** | **G** | **G** | **A** | **A** | **C** | **A** | **G** | **T** | **A** | **A** | **A** | **A** | **A** | **T** | **C** | **A** | **T** | **T** | **A** | **G** | **T** | **T** | **T** | **T** | **A** | **A** | **A** | **G** | **G** | **G** | **C** | **A** | **A** | **G** | **G** | **T** | **A** | **C** | **G** | **A** | **G** | **C** | **G** | **T** | **G** | **G** | **A** | **G** | **A** | **T** | **C** | **A** | **A** | **G** | **G** | **T** | **C** | **C** | **C** | **T** | **T** | **T** | **G** | **A** | **T** | **G** | **A** | **A** | **G** | **G** | **A** | **G** | **G** | **C** | **A** | **-** | **-** | **-** |
| **N60463F** | **A** | **T** | **G** | **A** | **T** | **G** | **G** | **G** | **A** | **A** | **G** | **A** | **A** | **T** | **A** | **T** | **C** | **C** | **A** | **A** | **A** | **G** | **C** | **A** | **A** | **T** | **G** | **C** | **T** | **A** | **G** | **C** | **T** | **G** | **T** | **T** | **T** | **A** | **T** | **T** | **C** | **G** | **G** | **G** | **G** | **A** | **T** | **T** | **G** | **C** | **G** | **G** | **C** | **T** | **T** | **T** | **C** | **C** | **T** | **T** | **A** | **T** | **T** | **A** | **T** | **T** | **C** | **T** | **A** | **T** | **T** | **T** | **T** | **C** | **A** | **C** | **T** | **T** | **T** | **T** | **C** | **A** | **C** | **T** | **T** | **A** | **T** | **T** | **T** | **T** | **T** | **A** | **T** | **G** | **C** | **C** | **A** | **G** | **T** | **A** | **A** | **C** | **C** | **A** | **A** | **A** | **G** | **T** | **C** | **A** | **T** | **T** | **G** | **G** | **T** | **G** | **G** | **G** | **G** | **A** | **G** | **A** | **A** | **T** | **T** | **G** | **C** | **T** | **G** | **C** | **G** | **T** | **G** | **C** | **G** | **C** | **G** | **T** | **T** | **T** | **A** | **T** | **T** | **A** | **T** | **A** | **C** | **G** | **T** | **T** | **C** | **C** | **G** | **C** | **T** | **T** | **A** | **T** | **C** | **T** | **T** | **C** | **C** | **A** | **T** | **T** | **T** | **G** | **A** | **T** | **G** | **G** | **C** | **A** | **A** | **T** | **T** | **T** | **C** | **A** | **T** | **T** | **A** | **G** | **G** | **T** | **G** | **T** | **A** | **G** | **G** | **A** | **T** | **T** | **G** | **A** | **T** | **C** | **G** | **T** | **A** | **T** | **T** | **T** | **C** | **T** | **A** | **T** | **T** | **G** | **T** | **T** | **A** | **T** | **C** | **A** | **C** | **T** | **T** | **A** | **T** | **T** | **C** | **A** | **A** | **A** | **A** | **G** | **A** | **C** | **A** | **A** | **A** | **A** | **T** | **A** | **C** | **G** | **G** | **G** | **A** | **A** | **A** | **A** | **T** | **C** | **G** | **A** | **A** | **G** | **A** | **A** | **A** | **A** | **G** | **C** | **T** | **T** | **C** | **G** | **T** | **G** | **C** | **A** | **C** | **T** | **T** | **T** | **C** | **T** | **T** | **C** | **T** | **G** | **G** | **C** | **A** | **A** | **T** | **T** | **A** | **T** | **G** | **A** | **A** | **T** | **C** | **C** | **A** | **A** | **A** | **C** | **T** | **T** | **T** | **T** | **G** | **C** | **A** | **T** | **C** | **A** | **G** | **C** | **C** | **G** | **A** | **T** | **C** | **C** | **C** | **A** | **A** | **G** | **C** | **G** | **C** | **G** | **T** | **C** | **G** | **G** | **A** | **T** | **G** | **A** | **T** | **C** | **T** | **G** | **T** | **A** | **C** | **A** | **T** | **C** | **A** | **A** | **A** | **G** | **A** | **T** | **A** | **T** | **T** | **G** | **A** | **T** | **A** | **T** | **G** | **G** | **A** | **A** | **A** | **T** | **T** | **A** | **C** | **T** | **A** | **A** | **A** | **A** | **T** | **A** | **A** | **A** | **A** | **G** | **A** | **A** | **A** | **A** | **A** | **A** | **T** | **G** | **A** | **T** | **C** | **G** | **A** | **A** | **G** | **T** | **A** | **T** | **C** | **C** | **A** | **G** | **T** | **G** | **A** | **A** | **T** | **T** | **A** | **C** | **A** | **G** | **A** | **T** | **T** | **A** | **T** | **G** | **A** | **C** | **T** | **A** | **G** | **C** | **C** | **G** | **T** | **C** | **C** | **T** | **C** | **A** | **A** | **T** | **A** | **T** | **G** | **T** | **A** | **G** | **A** | **T** | **G** | **G** | **A** | **C** | **A** | **A** | **A** | **C** | **C** | **A** | **A** | **A** | **G** | **A** | **A** | **G** | **A** | **G** | **A** | **T** | **T** | **T** | **T** | **A** | **G** | **A** | **A** | **T** | **T** | **A** | **G** | **A** | **A** | **A** | **G** | **G** | **C** | **A** | **T** | **C** | **G** | **A** | **T** | **T** | **A** | **G** | **C** | **T** | **C** | **G** | **T** | **G** | **A** | **G** | **C** | **T** | **T** | **C** | **A** | **T** | **G** | **A** | **T** | **T** | **C** | **A** | **G** | **T** | **T** | **T** | **C** | **A** | **C** | **A** | **G** | **C** | **A** | **A** | **T** | **T** | **A** | **T** | **T** | **T** | **G** | **C** | **A** | **G** | **C** | **G** | **A** | **T** | **G** | **A** | **T** | **G** | **A** | **T** | **G** | **A** | **T** | **G** | **T** | **C** | **G** | **G** | **C** | **A** | **T** | **T** | **G** | **A** | **C** | **G** | **G** | **A** | **G** | **C** | **A** | **A** | **G** | **C** | **A** | **G** | **A** | **A** | **A** | **A** | **A** | **A** | **G** | **T** | **G** | **A** | **G** | **A** | **C** | **A** | **C** | **C** | **A** | **G** | **A** | **A** | **A** | **T** | **G** | **T** | **T** | **C** | **C** | **G** | **T** | **A** | **A** | **G** | **C** | **A** | **G** | **T** | **T** | **G** | **A** | **A** | **A** | **A** | **T** | **G** | **G** | **T** | **G** | **G** | **C** | **A** | **G** | **A** | **G** | **A** | **T** | **C** | **A** | **T** | **C** | **A** | **A** | **C** | **G** | **C** | **T** | **T** | **C** | **C** | **C** | **A** | **G** | **T** | **C** | **T** | **G** | **A** | **G** | **A** | **T** | **G** | **C** | **G** | **T** | **G** | **C** | **G** | **C** | **T** | **G** | **C** | **T** | **T** | **C** | **T** | **T** | **C** | **A** | **T** | **C** | **T** | **G** | **C** | **G** | **T** | **C** | **C** | **A** | **G** | **T** | **C** | **A** | **A** | **T** | **T** | **T** | **A** | **G** | **A** | **A** | **G** | **A** | **A** | **A** | **A** | **A** | **A** | **G** | **T** | **T** | **T** | **A** | **A** | **A** | **A** | **C** | **A** | **A** | **G** | **G** | **C** | **A** | **T** | **T** | **G** | **A** | **G** | **C** | **A** | **G** | **T** | **T** | **A** | **T** | **T** | **G** | **A** | **A** | **G** | **G** | **A** | **A** | **T** | **T** | **G** | **C** | **A** | **G** | **A** | **A** | **T** | **A** | **A** | **A** | **A** | **T** | **C** | **C** | **A** | **G** | **A** | **T** | **T** | **T** | **C** | **G** | **C** | **T** | **A** | **A** | **A** | **A** | **T** | **G** | **G** | **G** | **A** | **T** | **G** | **T** | **A** | **G** | **A** | **A** | **G** | **A** | **T** | **G** | **T** | **A** | **A** | **A** | **A** | **T** | **T** | **A** | **A** | **C** | **T** | **A** | **G** | **T** | **T** | **C** | **C** | **A** | **T** | **T** | **G** | **A** | **G** | **G** | **A** | **T** | **C** | **A** | **C** | **T** | **T** | **G** | **T** | **T** | **C** | **C** | **G** | **A** | **A** | **T** | **C** | **G** | **T** | **T** | **C** | **A** | **A** | **G** | **A** | **A** | **T** | **T** | **G** | **T** | **T** | **A** | **T** | **C** | **A** | **A** | **A** | **C** | **A** | **C** | **A** | **T** | **T** | **A** | **A** | **G** | **A** | **C** | **A** | **T** | **G** | **C** | **T** | **A** | **A** | **G** | **G** | **C** | **T** | **A** | **A** | **T** | **G** | **A** | **A** | **T** | **T** | **A** | **G** | **A** | **A** | **G** | **T** | **C** | **T** | **A** | **T** | **T** | **T** | **G** | **C** | **A** | **T** | **A** | **A** | **A** | **A** | **T** | **A** | **G** | **A** | **T** | **A** | **A** | **C** | **A** | **A** | **T** | **C** | **T** | **T** | **T** | **T** | **A** | **T** | **T** | **A** | **C** | **G** | **T** | **A** | **T** | **C** | **A** | **T** | **T** | **G** | **A** | **C** | **G** | **A** | **C** | **G** | **G** | **G** | **A** | **C** | **A** | **G** | **G** | **A** | **T** | **T** | **T** | **A** | **A** | **T** | **A** | **T** | **G** | **A** | **A** | **T** | **G** | **A** | **A** | **A** | **C** | **C** | **A** | **A** | **A** | **A** | **C** | **G** | **G** | **G** | **A** | **A** | **G** | **T** | **T** | **A** | **T** | **G** | **G** | **A** | **T** | **T** | **G** | **A** | **A** | **C** | **A** | **A** | **T** | **A** | **T** | **C** | **A** | **A** | **A** | **G** | **A** | **A** | **C** | **G** | **A** | **G** | **T** | **A** | **G** | **C** | **T** | **G** | **G** | **T** | **A** | **T** | **T** | **G** | **G** | **T** | **G** | **G** | **A** | **A** | **C** | **A** | **G** | **T** | **A** | **A** | **A** | **A** | **A** | **T** | **C** | **A** | **T** | **T** | **A** | **G** | **T** | **T** | **T** | **T** | **A** | **A** | **A** | **G** | **G** | **G** | **C** | **A** | **A** | **G** | **G** | **T** | **A** | **C** | **G** | **A** | **G** | **C** | **G** | **T** | **G** | **G** | **A** | **G** | **A** | **T** | **C** | **A** | **A** | **G** | **G** | **T** | **C** | **C** | **C** | **T** | **T** | **T** | **G** | **A** | **T** | **G** | **A** | **A** | **G** | **G** | **A** | **G** | **G** | **C** | **A** | **-** | **-** | **-** |
| **N59589F** | **A** | **T** | **G** | **A** | **T** | **G** | **G** | **G** | **A** | **A** | **A** | **A** | **A** | **T** | **A** | **T** | **C** | **C** | **A** | **G** | **A** | **G** | **C** | **G** | **A** | **T** | **G** | **C** | **T** | **A** | **G** | **C** | **T** | **G** | **T** | **T** | **T** | **A** | **T** | **T** | **C** | **G** | **G** | **G** | **G** | **A** | **T** | **T** | **G** | **C** | **G** | **G** | **C** | **T** | **T** | **T** | **C** | **C** | **T** | **T** | **A** | **T** | **T** | **A** | **T** | **T** | **C** | **T** | **A** | **T** | **T** | **T** | **T** | **C** | **A** | **C** | **T** | **T** | **T** | **T** | **C** | **A** | **C** | **T** | **T** | **A** | **T** | **T** | **T** | **T** | **T** | **A** | **T** | **G** | **C** | **C** | **A** | **G** | **C** | **A** | **A** | **T** | **C** | **A** | **A** | **A** | **G** | **T** | **C** | **A** | **T** | **T** | **G** | **G** | **T** | **G** | **G** | **G** | **G** | **A** | **G** | **A** | **A** | **T** | **T** | **A** | **C** | **T** | **G** | **C** | **G** | **T** | **G** | **C** | **A** | **C** | **G** | **T** | **T** | **T** | **A** | **T** | **T** | **A** | **T** | **A** | **T** | **G** | **T** | **T** | **C** | **C** | **G** | **C** | **T** | **T** | **A** | **T** | **C** | **T** | **T** | **C** | **C** | **A** | **T** | **T** | **T** | **G** | **T** | **T** | **A** | **G** | **C** | **C** | **A** | **T** | **T** | **T** | **C** | **C** | **T** | **T** | **A** | **G** | **G** | **T** | **G** | **T** | **A** | **G** | **G** | **A** | **T** | **T** | **A** | **A** | **T** | **T** | **G** | **T** | **C** | **T** | **T** | **T** | **C** | **T** | **A** | **T** | **T** | **A** | **T** | **T** | **A** | **T** | **C** | **A** | **C** | **T** | **T** | **A** | **T** | **T** | **C** | **A** | **A** | **A** | **A** | **G** | **A** | **C** | **A** | **A** | **A** | **A** | **T** | **A** | **C** | **G** | **G** | **G** | **A** | **A** | **A** | **A** | **T** | **C** | **G** | **A** | **A** | **G** | **A** | **A** | **A** | **A** | **G** | **C** | **T** | **G** | **C** | **G** | **T** | **G** | **C** | **A** | **C** | **T** | **T** | **T** | **C** | **T** | **T** | **C** | **T** | **G** | **G** | **C** | **A** | **A** | **T** | **T** | **A** | **T** | **G** | **A** | **A** | **T** | **C** | **C** | **A** | **A** | **A** | **T** | **T** | **G** | **T** | **T** | **G** | **C** | **T** | **T** | **C** | **T** | **T** | **C** | **C** | **G** | **A** | **T** | **C** | **C** | **C** | **A** | **A** | **G** | **T** | **G** | **C** | **A** | **T** | **C** | **G** | **G** | **A** | **C** | **G** | **A** | **T** | **T** | **T** | **A** | **T** | **A** | **C** | **A** | **T** | **C** | **A** | **A** | **A** | **G** | **A** | **T** | **A** | **T** | **T** | **G** | **A** | **T** | **A** | **A** | **G** | **G** | **A** | **A** | **A** | **T** | **C** | **A** | **C** | **T** | **A** | **A** | **G** | **A** | **T** | **A** | **A** | **A** | **A** | **G** | **A** | **A** | **A** | **A** | **A** | **A** | **T** | **G** | **A** | **T** | **T** | **G** | **A** | **A** | **G** | **T** | **A** | **T** | **C** | **T** | **A** | **G** | **T** | **G** | **A** | **A** | **T** | **T** | **A** | **C** | **A** | **A** | **A** | **T** | **T** | **G** | **T** | **A** | **A** | **C** | **G** | **A** | **G** | **C** | **C** | **G** | **T** | **C** | **C** | **C** | **C** | **A** | **A** | **T** | **A** | **T** | **G** | **T** | **A** | **G** | **A** | **T** | **G** | **G** | **A** | **C** | **A** | **A** | **A** | **C** | **C** | **A** | **A** | **A** | **G** | **A** | **A** | **G** | **A** | **A** | **A** | **T** | **T** | **T** | **T** | **A** | **G** | **A** | **A** | **T** | **T** | **A** | **G** | **A** | **A** | **A** | **G** | **A** | **C** | **A** | **T** | **C** | **G** | **A** | **T** | **T** | **A** | **G** | **C** | **C** | **C** | **G** | **T** | **G** | **A** | **G** | **C** | **T** | **G** | **C** | **A** | **T** | **G** | **A** | **T** | **T** | **C** | **A** | **G** | **T** | **T** | **T** | **C** | **G** | **C** | **A** | **A** | **C** | **A** | **A** | **T** | **T** | **G** | **T** | **T** | **T** | **G** | **C** | **A** | **G** | **C** | **A** | **A** | **T** | **G** | **A** | **T** | **G** | **A** | **T** | **G** | **A** | **T** | **G** | **T** | **C** | **G** | **G** | **C** | **A** | **T** | **T** | **G** | **A** | **C** | **G** | **G** | **A** | **G** | **C** | **A** | **A** | **G** | **C** | **A** | **G** | **A** | **A** | **A** | **A** | **A** | **A** | **G** | **C** | **G** | **A** | **G** | **A** | **C** | **A** | **C** | **C** | **A** | **G** | **A** | **A** | **A** | **T** | **G** | **T** | **T** | **C** | **C** | **G** | **C** | **A** | **A** | **G** | **C** | **A** | **G** | **T** | **T** | **G** | **A** | **A** | **A** | **A** | **T** | **G** | **G** | **T** | **A** | **G** | **C** | **A** | **G** | **A** | **G** | **A** | **T** | **C** | **A** | **T** | **C** | **A** | **A** | **C** | **G** | **C** | **T** | **T** | **C** | **C** | **C** | **A** | **G** | **T** | **C** | **T** | **G** | **A** | **G** | **A** | **T** | **G** | **C** | **G** | **T** | **G** | **C** | **G** | **C** | **T** | **G** | **C** | **T** | **T** | **C** | **T** | **T** | **C** | **A** | **T** | **C** | **T** | **A** | **C** | **G** | **T** | **C** | **C** | **A** | **G** | **T** | **C** | **A** | **A** | **T** | **T** | **T** | **A** | **G** | **A** | **A** | **G** | **A** | **G** | **A** | **A** | **A** | **A** | **G** | **T** | **T** | **T** | **A** | **A** | **A** | **G** | **C** | **A** | **A** | **G** | **G** | **T** | **A** | **T** | **C** | **G** | **A** | **G** | **C** | **A** | **G** | **T** | **T** | **A** | **T** | **T** | **G** | **A** | **A** | **G** | **G** | **A** | **A** | **T** | **T** | **G** | **C** | **A** | **A** | **A** | **A** | **T** | **A** | **A** | **A** | **A** | **T** | **C** | **C** | **A** | **G** | **A** | **T** | **T** | **T** | **C** | **G** | **C** | **T** | **G** | **A** | **A** | **A** | **T** | **G** | **G** | **G** | **A** | **T** | **G** | **T** | **A** | **G** | **A** | **A** | **G** | **A** | **T** | **G** | **T** | **A** | **A** | **A** | **A** | **C** | **T** | **A** | **T** | **C** | **T** | **A** | **G** | **T** | **T** | **C** | **C** | **A** | **T** | **T** | **G** | **A** | **G** | **G** | **A** | **T** | **C** | **A** | **C** | **T** | **T** | **G** | **T** | **T** | **C** | **C** | **G** | **A** | **A** | **T** | **C** | **G** | **T** | **T** | **C** | **A** | **A** | **G** | **A** | **A** | **T** | **T** | **G** | **T** | **T** | **A** | **T** | **C** | **A** | **A** | **A** | **T** | **A** | **C** | **A** | **T** | **T** | **A** | **A** | **G** | **A** | **C** | **A** | **T** | **G** | **C** | **C** | **A** | **A** | **A** | **G** | **C** | **C** | **A** | **A** | **T** | **G** | **A** | **A** | **T** | **T** | **A** | **G** | **A** | **G** | **G** | **T** | **A** | **T** | **A** | **T** | **T** | **T** | **G** | **C** | **A** | **C** | **A** | **A** | **A** | **A** | **T** | **A** | **G** | **A** | **C** | **A** | **A** | **T** | **A** | **A** | **T** | **C** | **T** | **T** | **T** | **T** | **G** | **T** | **T** | **A** | **C** | **G** | **T** | **A** | **T** | **T** | **A** | **T** | **T** | **G** | **A** | **C** | **G** | **A** | **C** | **G** | **G** | **A** | **A** | **C** | **A** | **G** | **G** | **G** | **T** | **T** | **T** | **A** | **A** | **T** | **A** | **T** | **G** | **A** | **A** | **T** | **G** | **A** | **A** | **A** | **C** | **G** | **A** | **A** | **A** | **A** | **C** | **G** | **G** | **G** | **A** | **A** | **G** | **T** | **T** | **A** | **T** | **G** | **G** | **A** | **T** | **T** | **G** | **A** | **A** | **C** | **A** | **A** | **T** | **A** | **T** | **C** | **A** | **A** | **A** | **G** | **A** | **A** | **C** | **G** | **A** | **G** | **T** | **A** | **G** | **C** | **T** | **G** | **G** | **T** | **A** | **T** | **T** | **G** | **G** | **T** | **G** | **G** | **A** | **A** | **C** | **A** | **G** | **T** | **A** | **A** | **A** | **A** | **A** | **T** | **C** | **A** | **T** | **T** | **A** | **G** | **T** | **T** | **T** | **T** | **A** | **A** | **A** | **G** | **G** | **G** | **C** | **A** | **A** | **G** | **G** | **T** | **A** | **C** | **A** | **A** | **G** | **T** | **G** | **T** | **A** | **G** | **A** | **A** | **A** | **T** | **C** | **A** | **A** | **G** | **G** | **T** | **C** | **C** | **C** | **T** | **T** | **T** | **G** | **A** | **T** | **G** | **A** | **A** | **G** | **G** | **A** | **G** | **G** | **C** | **A** | **-** | **-** | **-** |
| **N59531F** | **A** | **T** | **G** | **A** | **T** | **G** | **G** | **G** | **A** | **A** | **A** | **A** | **A** | **T** | **A** | **T** | **C** | **C** | **A** | **G** | **A** | **G** | **C** | **G** | **A** | **T** | **G** | **C** | **T** | **A** | **G** | **C** | **T** | **G** | **T** | **T** | **T** | **A** | **T** | **T** | **C** | **G** | **G** | **G** | **G** | **A** | **T** | **T** | **G** | **C** | **G** | **G** | **C** | **T** | **T** | **T** | **C** | **C** | **T** | **T** | **A** | **T** | **T** | **A** | **T** | **T** | **C** | **T** | **A** | **T** | **T** | **T** | **T** | **C** | **A** | **C** | **T** | **T** | **T** | **T** | **C** | **A** | **C** | **T** | **T** | **A** | **T** | **T** | **T** | **T** | **T** | **A** | **T** | **G** | **C** | **C** | **A** | **G** | **T** | **A** | **A** | **T** | **C** | **A** | **A** | **A** | **G** | **T** | **C** | **A** | **T** | **T** | **G** | **G** | **T** | **G** | **G** | **G** | **G** | **A** | **G** | **A** | **A** | **T** | **T** | **A** | **C** | **T** | **G** | **C** | **G** | **T** | **G** | **C** | **A** | **C** | **G** | **T** | **T** | **T** | **A** | **T** | **T** | **A** | **T** | **A** | **T** | **G** | **T** | **T** | **C** | **C** | **G** | **C** | **T** | **T** | **A** | **T** | **C** | **T** | **T** | **C** | **C** | **A** | **T** | **T** | **T** | **G** | **T** | **T** | **A** | **G** | **C** | **T** | **A** | **T** | **T** | **T** | **C** | **C** | **T** | **T** | **A** | **G** | **G** | **T** | **G** | **T** | **A** | **G** | **G** | **A** | **T** | **T** | **A** | **A** | **T** | **C** | **G** | **T** | **C** | **T** | **T** | **T** | **C** | **T** | **A** | **T** | **T** | **A** | **T** | **T** | **A** | **T** | **C** | **A** | **C** | **T** | **T** | **A** | **T** | **T** | **C** | **A** | **A** | **A** | **A** | **G** | **A** | **C** | **A** | **A** | **A** | **A** | **T** | **A** | **C** | **G** | **G** | **G** | **A** | **A** | **A** | **A** | **T** | **C** | **G** | **A** | **A** | **G** | **A** | **A** | **A** | **A** | **G** | **C** | **T** | **G** | **C** | **G** | **T** | **G** | **C** | **A** | **C** | **T** | **C** | **T** | **C** | **T** | **T** | **C** | **T** | **G** | **G** | **C** | **A** | **A** | **T** | **T** | **A** | **T** | **G** | **A** | **A** | **T** | **C** | **C** | **A** | **A** | **A** | **T** | **T** | **G** | **T** | **T** | **G** | **C** | **T** | **T** | **C** | **T** | **T** | **C** | **C** | **G** | **A** | **T** | **C** | **C** | **C** | **A** | **A** | **G** | **T** | **G** | **C** | **A** | **T** | **C** | **G** | **G** | **A** | **C** | **G** | **A** | **T** | **T** | **T** | **A** | **T** | **A** | **C** | **A** | **T** | **C** | **A** | **A** | **A** | **G** | **A** | **T** | **A** | **T** | **T** | **G** | **A** | **T** | **A** | **A** | **G** | **G** | **A** | **A** | **A** | **T** | **C** | **A** | **C** | **T** | **A** | **G** | **A** | **A** | **T** | **A** | **A** | **A** | **A** | **G** | **A** | **A** | **A** | **A** | **A** | **A** | **T** | **G** | **A** | **T** | **T** | **G** | **A** | **A** | **G** | **T** | **A** | **T** | **C** | **T** | **A** | **G** | **T** | **G** | **A** | **A** | **T** | **T** | **A** | **C** | **A** | **A** | **A** | **T** | **T** | **G** | **T** | **A** | **A** | **C** | **G** | **A** | **G** | **C** | **C** | **G** | **T** | **C** | **C** | **C** | **C** | **A** | **A** | **T** | **A** | **T** | **G** | **T** | **A** | **G** | **A** | **T** | **G** | **G** | **A** | **C** | **A** | **A** | **A** | **C** | **C** | **A** | **A** | **A** | **G** | **A** | **A** | **G** | **A** | **A** | **A** | **T** | **T** | **T** | **T** | **A** | **G** | **A** | **A** | **T** | **T** | **A** | **G** | **A** | **A** | **A** | **G** | **A** | **C** | **A** | **T** | **C** | **G** | **A** | **T** | **T** | **A** | **G** | **C** | **C** | **C** | **G** | **T** | **G** | **A** | **G** | **C** | **T** | **A** | **C** | **A** | **T** | **G** | **A** | **T** | **T** | **C** | **A** | **G** | **T** | **T** | **T** | **C** | **G** | **C** | **A** | **A** | **C** | **A** | **A** | **T** | **T** | **A** | **T** | **T** | **T** | **G** | **C** | **A** | **G** | **C** | **A** | **A** | **T** | **G** | **A** | **T** | **G** | **A** | **T** | **G** | **A** | **T** | **G** | **T** | **C** | **G** | **G** | **C** | **A** | **T** | **T** | **G** | **A** | **C** | **G** | **G** | **A** | **G** | **C** | **A** | **A** | **G** | **C** | **A** | **G** | **A** | **A** | **A** | **A** | **A** | **A** | **G** | **C** | **G** | **A** | **G** | **A** | **C** | **A** | **C** | **C** | **A** | **G** | **A** | **A** | **A** | **T** | **G** | **T** | **T** | **C** | **C** | **G** | **C** | **A** | **A** | **G** | **C** | **A** | **G** | **T** | **T** | **G** | **A** | **A** | **A** | **A** | **T** | **G** | **G** | **T** | **A** | **G** | **C** | **A** | **G** | **A** | **G** | **A** | **T** | **C** | **A** | **T** | **C** | **A** | **A** | **C** | **G** | **C** | **T** | **T** | **C** | **C** | **C** | **A** | **G** | **T** | **C** | **T** | **G** | **A** | **G** | **A** | **T** | **G** | **C** | **G** | **T** | **G** | **C** | **G** | **C** | **T** | **G** | **C** | **T** | **T** | **C** | **T** | **T** | **C** | **A** | **T** | **C** | **T** | **G** | **C** | **G** | **T** | **C** | **C** | **A** | **G** | **T** | **C** | **A** | **A** | **T** | **T** | **T** | **A** | **G** | **A** | **A** | **G** | **A** | **G** | **A** | **A** | **A** | **A** | **G** | **T** | **T** | **T** | **A** | **A** | **A** | **G** | **C** | **A** | **A** | **G** | **G** | **T** | **A** | **T** | **C** | **G** | **A** | **G** | **C** | **A** | **G** | **T** | **T** | **A** | **T** | **T** | **G** | **A** | **A** | **G** | **G** | **A** | **A** | **T** | **T** | **G** | **C** | **A** | **A** | **A** | **A** | **T** | **A** | **A** | **A** | **A** | **T** | **C** | **C** | **A** | **G** | **A** | **T** | **T** | **T** | **C** | **G** | **C** | **T** | **G** | **A** | **A** | **A** | **T** | **G** | **G** | **G** | **A** | **T** | **G** | **T** | **A** | **G** | **A** | **A** | **G** | **A** | **T** | **G** | **T** | **A** | **A** | **A** | **A** | **C** | **T** | **A** | **T** | **C** | **T** | **A** | **G** | **T** | **T** | **C** | **C** | **A** | **T** | **T** | **G** | **A** | **G** | **G** | **A** | **T** | **C** | **A** | **C** | **T** | **T** | **G** | **T** | **T** | **C** | **C** | **G** | **A** | **A** | **T** | **C** | **G** | **T** | **T** | **C** | **A** | **A** | **G** | **A** | **A** | **T** | **T** | **G** | **T** | **T** | **A** | **T** | **C** | **A** | **A** | **A** | **T** | **A** | **C** | **A** | **T** | **T** | **A** | **A** | **G** | **A** | **C** | **A** | **T** | **G** | **C** | **C** | **A** | **A** | **A** | **G** | **C** | **C** | **A** | **A** | **T** | **G** | **A** | **A** | **T** | **T** | **A** | **G** | **A** | **G** | **G** | **T** | **A** | **T** | **A** | **T** | **T** | **T** | **G** | **C** | **A** | **T** | **A** | **A** | **A** | **A** | **T** | **A** | **G** | **A** | **C** | **A** | **A** | **T** | **A** | **A** | **T** | **C** | **T** | **T** | **T** | **T** | **A** | **T** | **T** | **G** | **C** | **G** | **T** | **A** | **T** | **T** | **A** | **T** | **T** | **G** | **A** | **C** | **G** | **A** | **C** | **G** | **G** | **A** | **A** | **C** | **A** | **G** | **G** | **G** | **T** | **T** | **T** | **A** | **A** | **T** | **A** | **T** | **G** | **A** | **A** | **T** | **G** | **A** | **A** | **A** | **C** | **G** | **A** | **A** | **A** | **A** | **C** | **G** | **G** | **G** | **A** | **A** | **G** | **T** | **T** | **A** | **T** | **G** | **G** | **A** | **T** | **T** | **G** | **A** | **A** | **C** | **A** | **A** | **T** | **A** | **T** | **C** | **A** | **A** | **A** | **G** | **A** | **A** | **C** | **G** | **A** | **G** | **T** | **A** | **G** | **C** | **T** | **G** | **G** | **T** | **A** | **T** | **T** | **G** | **G** | **T** | **G** | **G** | **A** | **A** | **C** | **A** | **G** | **T** | **A** | **A** | **A** | **A** | **A** | **T** | **C** | **A** | **T** | **T** | **A** | **G** | **T** | **T** | **T** | **T** | **A** | **A** | **A** | **G** | **G** | **G** | **C** | **A** | **A** | **G** | **G** | **T** | **A** | **C** | **A** | **A** | **G** | **T** | **G** | **T** | **A** | **G** | **A** | **A** | **A** | **T** | **C** | **A** | **A** | **G** | **G** | **T** | **C** | **C** | **C** | **T** | **T** | **T** | **G** | **A** | **T** | **G** | **A** | **A** | **G** | **G** | **A** | **G** | **G** | **C** | **A** | **-** | **-** | **-** |
| **N54599** | **A** | **T** | **G** | **A** | **T** | **G** | **G** | **G** | **A** | **A** | **A** | **A** | **A** | **T** | **A** | **T** | **C** | **C** | **A** | **G** | **A** | **G** | **C** | **G** | **A** | **T** | **G** | **C** | **T** | **A** | **G** | **C** | **T** | **G** | **T** | **T** | **T** | **A** | **T** | **T** | **C** | **G** | **G** | **G** | **G** | **A** | **T** | **T** | **G** | **C** | **G** | **G** | **C** | **T** | **T** | **T** | **C** | **C** | **T** | **T** | **A** | **T** | **T** | **A** | **T** | **T** | **C** | **T** | **A** | **T** | **T** | **T** | **T** | **C** | **A** | **C** | **T** | **T** | **T** | **T** | **C** | **A** | **C** | **T** | **T** | **A** | **T** | **T** | **T** | **T** | **T** | **A** | **T** | **G** | **C** | **C** | **A** | **G** | **T** | **A** | **A** | **T** | **C** | **A** | **A** | **A** | **G** | **T** | **C** | **A** | **T** | **T** | **G** | **G** | **T** | **G** | **G** | **G** | **G** | **A** | **G** | **A** | **A** | **T** | **T** | **A** | **C** | **T** | **G** | **C** | **G** | **T** | **G** | **C** | **A** | **C** | **G** | **T** | **T** | **T** | **A** | **T** | **T** | **A** | **T** | **A** | **T** | **G** | **T** | **T** | **C** | **C** | **G** | **C** | **T** | **T** | **A** | **T** | **C** | **T** | **T** | **C** | **C** | **A** | **T** | **T** | **T** | **G** | **T** | **T** | **A** | **G** | **C** | **T** | **A** | **T** | **T** | **T** | **C** | **C** | **T** | **T** | **A** | **G** | **G** | **T** | **G** | **T** | **A** | **G** | **G** | **A** | **T** | **T** | **A** | **A** | **T** | **C** | **G** | **T** | **C** | **T** | **T** | **T** | **C** | **T** | **A** | **T** | **T** | **A** | **T** | **T** | **A** | **T** | **C** | **A** | **C** | **T** | **T** | **A** | **T** | **T** | **C** | **A** | **A** | **A** | **A** | **G** | **A** | **C** | **A** | **A** | **A** | **A** | **T** | **A** | **C** | **G** | **G** | **G** | **A** | **A** | **A** | **A** | **T** | **C** | **G** | **A** | **A** | **G** | **A** | **A** | **A** | **A** | **G** | **C** | **T** | **G** | **C** | **G** | **T** | **G** | **C** | **A** | **C** | **T** | **C** | **T** | **C** | **T** | **T** | **C** | **T** | **G** | **G** | **C** | **A** | **A** | **T** | **T** | **A** | **T** | **G** | **A** | **A** | **T** | **C** | **C** | **A** | **A** | **A** | **T** | **T** | **G** | **T** | **T** | **G** | **C** | **T** | **T** | **C** | **T** | **T** | **C** | **C** | **G** | **A** | **T** | **C** | **C** | **C** | **A** | **A** | **G** | **T** | **G** | **C** | **A** | **T** | **C** | **G** | **G** | **A** | **C** | **G** | **A** | **T** | **T** | **T** | **A** | **T** | **A** | **C** | **A** | **T** | **C** | **A** | **A** | **A** | **G** | **A** | **T** | **A** | **T** | **T** | **G** | **A** | **T** | **A** | **A** | **G** | **G** | **A** | **A** | **A** | **T** | **C** | **A** | **C** | **T** | **A** | **A** | **A** | **A** | **T** | **A** | **A** | **A** | **A** | **G** | **A** | **A** | **A** | **A** | **A** | **A** | **T** | **G** | **A** | **T** | **T** | **G** | **A** | **A** | **G** | **T** | **A** | **T** | **C** | **T** | **A** | **G** | **T** | **G** | **A** | **A** | **T** | **T** | **A** | **C** | **A** | **A** | **A** | **T** | **T** | **G** | **T** | **A** | **A** | **C** | **G** | **A** | **G** | **C** | **C** | **G** | **T** | **C** | **C** | **C** | **C** | **A** | **A** | **T** | **A** | **T** | **G** | **T** | **A** | **G** | **A** | **T** | **G** | **G** | **A** | **C** | **A** | **A** | **A** | **C** | **C** | **A** | **A** | **A** | **G** | **A** | **A** | **G** | **A** | **A** | **A** | **T** | **T** | **T** | **T** | **A** | **G** | **A** | **A** | **T** | **T** | **A** | **G** | **A** | **A** | **A** | **G** | **A** | **C** | **A** | **T** | **C** | **G** | **A** | **T** | **T** | **A** | **G** | **C** | **C** | **C** | **G** | **T** | **G** | **A** | **G** | **C** | **T** | **G** | **C** | **A** | **T** | **G** | **A** | **T** | **T** | **C** | **A** | **G** | **T** | **T** | **T** | **C** | **G** | **C** | **A** | **A** | **C** | **A** | **A** | **T** | **T** | **G** | **T** | **T** | **T** | **G** | **C** | **A** | **G** | **C** | **A** | **A** | **T** | **G** | **A** | **T** | **G** | **A** | **T** | **G** | **A** | **T** | **G** | **T** | **C** | **G** | **G** | **C** | **A** | **T** | **T** | **A** | **A** | **C** | **G** | **G** | **A** | **G** | **C** | **A** | **A** | **G** | **C** | **A** | **G** | **A** | **A** | **A** | **A** | **A** | **A** | **G** | **C** | **G** | **A** | **G** | **A** | **C** | **A** | **C** | **C** | **A** | **G** | **A** | **A** | **A** | **T** | **G** | **T** | **T** | **C** | **C** | **G** | **C** | **A** | **A** | **G** | **C** | **A** | **G** | **T** | **T** | **G** | **A** | **A** | **A** | **A** | **T** | **G** | **G** | **T** | **A** | **G** | **C** | **A** | **G** | **A** | **G** | **A** | **T** | **C** | **A** | **T** | **C** | **A** | **A** | **C** | **G** | **C** | **T** | **T** | **C** | **C** | **C** | **A** | **G** | **T** | **C** | **T** | **G** | **A** | **G** | **A** | **T** | **G** | **C** | **G** | **T** | **G** | **C** | **G** | **C** | **T** | **G** | **C** | **T** | **T** | **C** | **T** | **T** | **C** | **A** | **T** | **C** | **T** | **G** | **C** | **G** | **T** | **C** | **C** | **A** | **G** | **T** | **C** | **A** | **A** | **T** | **T** | **T** | **A** | **G** | **A** | **A** | **G** | **A** | **G** | **A** | **A** | **A** | **A** | **G** | **T** | **T** | **T** | **A** | **A** | **A** | **G** | **C** | **A** | **A** | **G** | **G** | **T** | **A** | **T** | **C** | **G** | **A** | **G** | **C** | **A** | **A** | **T** | **T** | **A** | **T** | **T** | **G** | **A** | **A** | **G** | **G** | **A** | **A** | **T** | **T** | **G** | **C** | **A** | **A** | **A** | **A** | **T** | **A** | **A** | **A** | **A** | **T** | **C** | **C** | **A** | **G** | **A** | **T** | **T** | **T** | **C** | **G** | **C** | **T** | **G** | **A** | **A** | **A** | **T** | **G** | **G** | **G** | **A** | **T** | **G** | **T** | **A** | **G** | **A** | **A** | **G** | **A** | **T** | **G** | **T** | **A** | **A** | **A** | **A** | **C** | **T** | **A** | **T** | **C** | **T** | **A** | **G** | **T** | **T** | **C** | **C** | **A** | **T** | **T** | **G** | **A** | **G** | **G** | **A** | **T** | **C** | **A** | **C** | **T** | **T** | **G** | **T** | **T** | **C** | **C** | **G** | **A** | **A** | **T** | **C** | **G** | **T** | **T** | **C** | **A** | **A** | **G** | **A** | **A** | **T** | **T** | **G** | **T** | **T** | **A** | **T** | **C** | **A** | **A** | **A** | **T** | **A** | **C** | **A** | **T** | **T** | **A** | **A** | **G** | **A** | **C** | **A** | **T** | **G** | **C** | **C** | **A** | **A** | **A** | **G** | **C** | **C** | **A** | **A** | **T** | **G** | **A** | **A** | **T** | **T** | **A** | **G** | **A** | **G** | **G** | **T** | **A** | **T** | **A** | **T** | **T** | **T** | **G** | **C** | **A** | **C** | **A** | **A** | **A** | **A** | **T** | **A** | **G** | **A** | **C** | **A** | **A** | **T** | **A** | **A** | **T** | **C** | **T** | **T** | **T** | **T** | **G** | **T** | **T** | **A** | **C** | **G** | **T** | **A** | **T** | **T** | **A** | **T** | **T** | **G** | **A** | **C** | **G** | **A** | **C** | **G** | **G** | **A** | **A** | **C** | **A** | **G** | **G** | **G** | **T** | **T** | **T** | **A** | **A** | **T** | **A** | **T** | **G** | **A** | **A** | **T** | **G** | **A** | **A** | **A** | **C** | **G** | **A** | **A** | **A** | **A** | **C** | **G** | **G** | **G** | **A** | **A** | **G** | **T** | **T** | **A** | **T** | **G** | **G** | **A** | **T** | **T** | **G** | **A** | **A** | **C** | **A** | **A** | **T** | **A** | **T** | **C** | **A** | **A** | **A** | **G** | **A** | **A** | **C** | **G** | **A** | **G** | **T** | **A** | **G** | **C** | **T** | **G** | **G** | **T** | **A** | **T** | **T** | **G** | **G** | **T** | **G** | **G** | **A** | **A** | **C** | **A** | **G** | **T** | **A** | **A** | **A** | **A** | **A** | **T** | **C** | **A** | **T** | **T** | **A** | **G** | **T** | **T** | **T** | **T** | **A** | **A** | **A** | **G** | **G** | **G** | **C** | **A** | **A** | **G** | **G** | **T** | **A** | **C** | **A** | **A** | **G** | **T** | **G** | **T** | **A** | **G** | **A** | **A** | **A** | **T** | **C** | **A** | **A** | **G** | **G** | **T** | **C** | **C** | **C** | **T** | **T** | **T** | **G** | **A** | **T** | **G** | **A** | **A** | **G** | **G** | **A** | **G** | **G** | **C** | **A** | **-** | **-** | **-** |
| **N60324F** | **A** | **T** | **G** | **A** | **T** | **G** | **G** | **G** | **A** | **A** | **G** | **A** | **A** | **T** | **A** | **T** | **C** | **C** | **A** | **A** | **A** | **G** | **C** | **A** | **A** | **T** | **G** | **C** | **T** | **A** | **G** | **C** | **T** | **G** | **T** | **T** | **T** | **A** | **T** | **T** | **C** | **G** | **G** | **G** | **G** | **A** | **T** | **T** | **G** | **C** | **G** | **G** | **C** | **T** | **T** | **T** | **C** | **C** | **T** | **T** | **A** | **T** | **T** | **A** | **T** | **T** | **C** | **T** | **A** | **T** | **T** | **T** | **T** | **C** | **A** | **C** | **T** | **T** | **T** | **T** | **C** | **A** | **C** | **T** | **T** | **A** | **T** | **T** | **T** | **T** | **T** | **A** | **T** | **G** | **C** | **C** | **A** | **G** | **T** | **A** | **A** | **C** | **C** | **A** | **A** | **A** | **G** | **T** | **C** | **A** | **T** | **T** | **G** | **G** | **T** | **G** | **G** | **G** | **G** | **A** | **G** | **A** | **A** | **T** | **T** | **G** | **C** | **T** | **G** | **C** | **G** | **T** | **G** | **C** | **G** | **C** | **G** | **T** | **T** | **T** | **A** | **T** | **T** | **A** | **T** | **A** | **C** | **G** | **T** | **T** | **C** | **C** | **G** | **C** | **T** | **T** | **A** | **T** | **C** | **T** | **T** | **C** | **C** | **A** | **T** | **T** | **T** | **G** | **A** | **T** | **G** | **G** | **C** | **A** | **A** | **T** | **T** | **T** | **C** | **A** | **T** | **T** | **A** | **G** | **G** | **T** | **G** | **T** | **A** | **G** | **G** | **A** | **T** | **T** | **G** | **A** | **T** | **C** | **G** | **T** | **A** | **T** | **T** | **T** | **C** | **T** | **A** | **T** | **T** | **G** | **T** | **T** | **A** | **T** | **C** | **A** | **C** | **T** | **T** | **A** | **T** | **T** | **C** | **A** | **A** | **A** | **A** | **G** | **G** | **C** | **A** | **A** | **A** | **A** | **T** | **A** | **C** | **G** | **G** | **G** | **A** | **A** | **A** | **A** | **T** | **C** | **G** | **A** | **A** | **G** | **A** | **A** | **A** | **A** | **G** | **C** | **T** | **T** | **C** | **G** | **T** | **G** | **C** | **A** | **C** | **T** | **T** | **T** | **C** | **T** | **T** | **C** | **T** | **G** | **G** | **C** | **A** | **A** | **T** | **T** | **A** | **T** | **G** | **A** | **A** | **T** | **C** | **C** | **A** | **A** | **A** | **C** | **T** | **T** | **T** | **T** | **G** | **C** | **A** | **T** | **C** | **A** | **G** | **C** | **C** | **G** | **A** | **T** | **C** | **C** | **C** | **A** | **A** | **G** | **C** | **G** | **C** | **G** | **T** | **C** | **G** | **G** | **A** | **T** | **G** | **A** | **T** | **C** | **T** | **G** | **T** | **A** | **C** | **A** | **T** | **C** | **A** | **A** | **A** | **G** | **A** | **T** | **A** | **T** | **T** | **G** | **A** | **T** | **A** | **T** | **G** | **G** | **A** | **A** | **A** | **T** | **T** | **A** | **C** | **T** | **A** | **A** | **A** | **A** | **T** | **A** | **A** | **A** | **A** | **G** | **A** | **A** | **A** | **A** | **A** | **A** | **T** | **G** | **A** | **T** | **C** | **G** | **A** | **A** | **G** | **T** | **A** | **T** | **C** | **C** | **A** | **G** | **T** | **G** | **A** | **A** | **T** | **T** | **A** | **C** | **A** | **G** | **A** | **T** | **T** | **A** | **T** | **G** | **A** | **C** | **T** | **A** | **G** | **C** | **C** | **G** | **T** | **C** | **C** | **T** | **C** | **A** | **A** | **T** | **A** | **T** | **G** | **T** | **A** | **G** | **A** | **T** | **G** | **G** | **A** | **C** | **A** | **A** | **A** | **C** | **C** | **A** | **A** | **A** | **G** | **A** | **A** | **G** | **A** | **G** | **A** | **T** | **T** | **T** | **T** | **A** | **G** | **A** | **A** | **T** | **T** | **A** | **G** | **A** | **A** | **A** | **G** | **G** | **C** | **A** | **T** | **C** | **G** | **A** | **T** | **T** | **A** | **G** | **C** | **T** | **C** | **G** | **T** | **G** | **A** | **G** | **C** | **T** | **T** | **C** | **A** | **T** | **G** | **A** | **T** | **T** | **C** | **A** | **G** | **T** | **T** | **T** | **C** | **A** | **C** | **A** | **G** | **C** | **A** | **A** | **T** | **T** | **A** | **T** | **T** | **T** | **G** | **C** | **A** | **G** | **C** | **G** | **A** | **T** | **G** | **A** | **T** | **G** | **A** | **T** | **G** | **A** | **T** | **G** | **T** | **C** | **G** | **G** | **C** | **A** | **T** | **T** | **G** | **A** | **C** | **G** | **G** | **A** | **G** | **C** | **A** | **A** | **G** | **C** | **A** | **G** | **A** | **A** | **A** | **A** | **A** | **A** | **G** | **T** | **G** | **A** | **G** | **A** | **C** | **A** | **C** | **C** | **A** | **G** | **G** | **A** | **A** | **T** | **G** | **T** | **T** | **C** | **C** | **G** | **T** | **A** | **A** | **G** | **C** | **A** | **G** | **T** | **T** | **G** | **A** | **A** | **A** | **A** | **T** | **G** | **G** | **T** | **G** | **G** | **C** | **A** | **G** | **A** | **G** | **A** | **T** | **C** | **A** | **T** | **C** | **A** | **A** | **C** | **G** | **C** | **T** | **T** | **C** | **C** | **C** | **A** | **G** | **T** | **C** | **T** | **G** | **A** | **G** | **A** | **T** | **G** | **C** | **G** | **T** | **G** | **C** | **G** | **C** | **T** | **G** | **C** | **T** | **T** | **C** | **T** | **T** | **C** | **A** | **T** | **C** | **T** | **G** | **C** | **G** | **T** | **C** | **C** | **A** | **G** | **T** | **C** | **A** | **A** | **T** | **T** | **T** | **A** | **G** | **A** | **A** | **G** | **A** | **A** | **A** | **A** | **A** | **A** | **G** | **T** | **T** | **T** | **A** | **A** | **A** | **A** | **C** | **A** | **A** | **G** | **G** | **C** | **A** | **T** | **T** | **G** | **A** | **G** | **C** | **A** | **G** | **T** | **T** | **A** | **T** | **T** | **G** | **A** | **A** | **G** | **G** | **A** | **A** | **T** | **T** | **G** | **C** | **A** | **G** | **A** | **A** | **T** | **A** | **A** | **A** | **A** | **T** | **C** | **C** | **A** | **G** | **A** | **T** | **T** | **T** | **C** | **G** | **C** | **T** | **A** | **A** | **A** | **A** | **T** | **G** | **G** | **G** | **A** | **T** | **G** | **T** | **A** | **G** | **A** | **A** | **G** | **A** | **T** | **G** | **T** | **A** | **A** | **A** | **A** | **T** | **T** | **A** | **A** | **C** | **T** | **A** | **G** | **T** | **T** | **C** | **C** | **A** | **T** | **T** | **G** | **A** | **G** | **G** | **A** | **T** | **C** | **A** | **C** | **T** | **T** | **G** | **T** | **T** | **C** | **C** | **G** | **A** | **A** | **T** | **C** | **G** | **T** | **T** | **C** | **A** | **A** | **G** | **A** | **A** | **T** | **T** | **G** | **T** | **T** | **A** | **T** | **C** | **A** | **A** | **A** | **C** | **A** | **C** | **A** | **T** | **T** | **A** | **A** | **G** | **A** | **C** | **A** | **T** | **G** | **C** | **T** | **A** | **A** | **G** | **G** | **C** | **T** | **A** | **A** | **T** | **G** | **A** | **A** | **T** | **T** | **A** | **G** | **A** | **A** | **G** | **T** | **C** | **T** | **A** | **T** | **T** | **T** | **G** | **C** | **A** | **T** | **A** | **A** | **A** | **A** | **T** | **A** | **G** | **A** | **T** | **A** | **A** | **C** | **A** | **A** | **T** | **C** | **T** | **T** | **T** | **T** | **A** | **T** | **T** | **A** | **C** | **G** | **T** | **A** | **T** | **C** | **A** | **T** | **T** | **G** | **A** | **C** | **G** | **A** | **C** | **G** | **G** | **G** | **A** | **C** | **A** | **G** | **G** | **C** | **T** | **T** | **T** | **A** | **A** | **T** | **A** | **T** | **G** | **A** | **A** | **T** | **G** | **A** | **A** | **A** | **C** | **C** | **A** | **A** | **A** | **A** | **C** | **G** | **G** | **G** | **A** | **A** | **G** | **T** | **T** | **A** | **T** | **G** | **G** | **A** | **T** | **T** | **G** | **A** | **A** | **C** | **A** | **A** | **T** | **A** | **T** | **C** | **A** | **A** | **A** | **G** | **A** | **A** | **C** | **G** | **A** | **G** | **T** | **A** | **G** | **C** | **T** | **G** | **G** | **T** | **A** | **T** | **T** | **G** | **G** | **T** | **G** | **G** | **A** | **A** | **C** | **A** | **G** | **T** | **A** | **A** | **A** | **A** | **A** | **T** | **C** | **A** | **T** | **T** | **A** | **G** | **T** | **T** | **T** | **T** | **A** | **A** | **A** | **G** | **G** | **G** | **C** | **A** | **A** | **G** | **G** | **T** | **A** | **C** | **G** | **A** | **G** | **C** | **G** | **T** | **G** | **G** | **A** | **G** | **A** | **T** | **C** | **A** | **A** | **G** | **G** | **T** | **C** | **C** | **C** | **T** | **T** | **T** | **G** | **A** | **T** | **G** | **A** | **A** | **G** | **G** | **A** | **G** | **G** | **C** | **A** | **-** | **-** | **-** |
| **N59624F** | **A** | **T** | **G** | **A** | **T** | **G** | **G** | **G** | **A** | **A** | **A** | **A** | **A** | **T** | **A** | **T** | **C** | **C** | **A** | **G** | **A** | **G** | **C** | **G** | **A** | **T** | **G** | **C** | **T** | **A** | **G** | **C** | **T** | **G** | **T** | **T** | **T** | **A** | **T** | **T** | **C** | **G** | **G** | **G** | **G** | **A** | **T** | **T** | **G** | **C** | **G** | **G** | **C** | **T** | **T** | **T** | **C** | **C** | **T** | **T** | **A** | **T** | **T** | **A** | **T** | **T** | **C** | **T** | **A** | **T** | **T** | **T** | **T** | **C** | **A** | **C** | **T** | **T** | **T** | **T** | **C** | **A** | **C** | **T** | **T** | **A** | **T** | **T** | **T** | **T** | **T** | **A** | **T** | **G** | **C** | **C** | **A** | **G** | **C** | **A** | **A** | **T** | **C** | **A** | **A** | **A** | **G** | **T** | **C** | **A** | **T** | **T** | **G** | **G** | **T** | **G** | **G** | **G** | **G** | **A** | **G** | **A** | **A** | **T** | **T** | **A** | **C** | **T** | **G** | **C** | **G** | **T** | **G** | **C** | **A** | **C** | **G** | **T** | **T** | **T** | **A** | **T** | **T** | **A** | **T** | **A** | **T** | **G** | **T** | **T** | **C** | **C** | **G** | **C** | **T** | **T** | **A** | **T** | **C** | **T** | **T** | **C** | **C** | **A** | **T** | **T** | **T** | **G** | **T** | **T** | **A** | **G** | **C** | **C** | **A** | **T** | **T** | **T** | **C** | **C** | **T** | **T** | **A** | **G** | **G** | **T** | **G** | **T** | **A** | **G** | **G** | **A** | **T** | **T** | **A** | **A** | **T** | **T** | **G** | **T** | **C** | **T** | **T** | **T** | **C** | **T** | **A** | **T** | **T** | **A** | **T** | **T** | **A** | **T** | **C** | **A** | **C** | **T** | **T** | **A** | **T** | **T** | **C** | **A** | **A** | **A** | **A** | **G** | **A** | **C** | **A** | **A** | **A** | **A** | **T** | **A** | **C** | **G** | **G** | **G** | **A** | **A** | **A** | **A** | **T** | **C** | **G** | **A** | **A** | **G** | **A** | **A** | **A** | **A** | **G** | **C** | **T** | **G** | **C** | **G** | **T** | **G** | **C** | **A** | **C** | **T** | **T** | **T** | **C** | **T** | **T** | **C** | **T** | **G** | **G** | **C** | **A** | **A** | **T** | **T** | **A** | **T** | **G** | **A** | **A** | **T** | **C** | **C** | **A** | **A** | **A** | **T** | **T** | **G** | **T** | **T** | **G** | **C** | **T** | **T** | **C** | **T** | **T** | **C** | **C** | **G** | **A** | **T** | **C** | **C** | **C** | **A** | **A** | **G** | **T** | **G** | **C** | **A** | **T** | **C** | **G** | **G** | **A** | **C** | **G** | **A** | **T** | **T** | **T** | **A** | **T** | **A** | **C** | **A** | **T** | **C** | **A** | **A** | **A** | **G** | **A** | **T** | **A** | **T** | **T** | **G** | **A** | **T** | **A** | **A** | **G** | **G** | **A** | **A** | **A** | **T** | **C** | **A** | **C** | **T** | **A** | **A** | **G** | **A** | **T** | **A** | **A** | **A** | **A** | **G** | **A** | **A** | **A** | **A** | **A** | **A** | **T** | **G** | **A** | **T** | **T** | **G** | **A** | **A** | **G** | **T** | **A** | **T** | **C** | **T** | **A** | **G** | **T** | **G** | **A** | **A** | **T** | **T** | **A** | **C** | **A** | **A** | **A** | **T** | **T** | **G** | **T** | **A** | **A** | **C** | **G** | **A** | **G** | **C** | **C** | **G** | **T** | **C** | **C** | **C** | **C** | **A** | **A** | **T** | **A** | **T** | **G** | **T** | **A** | **G** | **A** | **T** | **G** | **G** | **A** | **C** | **A** | **A** | **A** | **C** | **C** | **A** | **A** | **A** | **G** | **A** | **A** | **G** | **A** | **A** | **A** | **T** | **T** | **T** | **T** | **A** | **G** | **A** | **A** | **T** | **T** | **A** | **G** | **A** | **A** | **A** | **G** | **A** | **C** | **A** | **T** | **C** | **G** | **A** | **T** | **T** | **A** | **G** | **C** | **C** | **C** | **G** | **T** | **G** | **A** | **G** | **C** | **T** | **G** | **C** | **A** | **T** | **G** | **A** | **T** | **T** | **C** | **A** | **G** | **T** | **T** | **T** | **C** | **G** | **C** | **A** | **A** | **C** | **A** | **A** | **T** | **T** | **G** | **T** | **T** | **T** | **G** | **C** | **A** | **G** | **C** | **A** | **A** | **T** | **G** | **A** | **T** | **G** | **A** | **T** | **G** | **A** | **T** | **G** | **T** | **C** | **G** | **G** | **C** | **A** | **T** | **T** | **G** | **A** | **C** | **G** | **G** | **A** | **G** | **C** | **A** | **A** | **G** | **C** | **A** | **G** | **A** | **A** | **A** | **A** | **A** | **A** | **G** | **C** | **G** | **A** | **G** | **A** | **C** | **A** | **C** | **C** | **A** | **G** | **A** | **A** | **A** | **T** | **G** | **T** | **T** | **C** | **C** | **G** | **C** | **A** | **A** | **G** | **C** | **A** | **G** | **T** | **T** | **G** | **A** | **A** | **A** | **A** | **T** | **G** | **G** | **T** | **A** | **G** | **C** | **A** | **G** | **A** | **G** | **A** | **T** | **C** | **A** | **T** | **C** | **A** | **A** | **C** | **G** | **C** | **T** | **T** | **C** | **C** | **C** | **A** | **G** | **T** | **C** | **T** | **G** | **A** | **G** | **A** | **T** | **G** | **C** | **G** | **T** | **G** | **C** | **G** | **C** | **T** | **G** | **C** | **T** | **T** | **C** | **T** | **T** | **C** | **A** | **T** | **C** | **T** | **A** | **C** | **G** | **T** | **C** | **C** | **A** | **G** | **T** | **C** | **A** | **A** | **T** | **T** | **T** | **A** | **G** | **A** | **A** | **G** | **A** | **G** | **A** | **A** | **A** | **A** | **G** | **T** | **T** | **T** | **A** | **A** | **A** | **G** | **C** | **A** | **A** | **G** | **G** | **T** | **A** | **T** | **C** | **G** | **A** | **G** | **C** | **A** | **G** | **T** | **T** | **A** | **T** | **T** | **G** | **A** | **A** | **G** | **G** | **A** | **A** | **T** | **T** | **G** | **C** | **A** | **A** | **A** | **A** | **T** | **A** | **A** | **A** | **A** | **T** | **C** | **C** | **A** | **G** | **A** | **T** | **T** | **T** | **C** | **G** | **C** | **T** | **G** | **A** | **A** | **A** | **T** | **G** | **G** | **G** | **A** | **T** | **G** | **T** | **A** | **G** | **A** | **A** | **G** | **A** | **T** | **G** | **T** | **A** | **A** | **A** | **A** | **C** | **T** | **A** | **T** | **C** | **T** | **A** | **G** | **T** | **T** | **C** | **C** | **A** | **T** | **T** | **G** | **A** | **G** | **G** | **A** | **T** | **C** | **A** | **C** | **T** | **T** | **G** | **T** | **T** | **C** | **C** | **G** | **A** | **A** | **T** | **C** | **G** | **T** | **T** | **C** | **A** | **A** | **G** | **A** | **A** | **T** | **T** | **G** | **T** | **T** | **A** | **T** | **C** | **A** | **A** | **A** | **T** | **A** | **C** | **A** | **T** | **T** | **A** | **A** | **G** | **A** | **C** | **A** | **T** | **G** | **C** | **C** | **A** | **A** | **A** | **G** | **C** | **C** | **A** | **A** | **T** | **G** | **A** | **A** | **T** | **T** | **A** | **G** | **A** | **G** | **G** | **T** | **A** | **T** | **A** | **T** | **T** | **T** | **G** | **C** | **A** | **C** | **A** | **A** | **A** | **A** | **T** | **A** | **G** | **A** | **C** | **A** | **A** | **T** | **A** | **A** | **T** | **C** | **T** | **T** | **T** | **T** | **G** | **T** | **T** | **A** | **C** | **G** | **T** | **A** | **T** | **T** | **A** | **T** | **T** | **G** | **A** | **C** | **G** | **A** | **C** | **G** | **G** | **A** | **A** | **C** | **A** | **G** | **G** | **G** | **T** | **T** | **T** | **A** | **A** | **T** | **A** | **T** | **G** | **A** | **A** | **T** | **G** | **A** | **A** | **A** | **C** | **G** | **A** | **A** | **A** | **A** | **C** | **G** | **G** | **G** | **A** | **A** | **G** | **T** | **T** | **A** | **T** | **G** | **G** | **A** | **T** | **T** | **G** | **A** | **A** | **C** | **A** | **A** | **T** | **A** | **T** | **C** | **A** | **A** | **A** | **G** | **A** | **A** | **C** | **G** | **A** | **G** | **T** | **A** | **G** | **C** | **T** | **G** | **G** | **T** | **A** | **T** | **T** | **G** | **G** | **T** | **G** | **G** | **A** | **A** | **C** | **A** | **G** | **T** | **A** | **A** | **A** | **A** | **A** | **T** | **C** | **A** | **T** | **T** | **A** | **G** | **T** | **T** | **T** | **T** | **A** | **A** | **A** | **G** | **G** | **G** | **C** | **A** | **A** | **G** | **G** | **T** | **A** | **C** | **A** | **A** | **G** | **T** | **G** | **T** | **A** | **G** | **A** | **A** | **A** | **T** | **C** | **A** | **A** | **G** | **G** | **T** | **C** | **C** | **C** | **T** | **T** | **T** | **G** | **A** | **T** | **G** | **A** | **A** | **G** | **G** | **A** | **G** | **G** | **C** | **A** | **-** | **-** | **-** |
| **N52732** | **A** | **T** | **G** | **A** | **T** | **G** | **G** | **G** | **A** | **A** | **A** | **A** | **A** | **T** | **A** | **T** | **C** | **C** | **A** | **G** | **A** | **G** | **C** | **G** | **A** | **T** | **G** | **C** | **T** | **A** | **G** | **C** | **T** | **G** | **T** | **T** | **T** | **A** | **T** | **T** | **C** | **G** | **G** | **G** | **G** | **A** | **T** | **T** | **G** | **C** | **G** | **G** | **C** | **T** | **T** | **T** | **C** | **C** | **T** | **T** | **A** | **T** | **T** | **A** | **T** | **T** | **C** | **T** | **A** | **T** | **T** | **T** | **T** | **C** | **A** | **C** | **T** | **T** | **T** | **T** | **C** | **A** | **C** | **T** | **T** | **A** | **T** | **T** | **T** | **T** | **T** | **A** | **T** | **G** | **C** | **C** | **A** | **G** | **T** | **A** | **A** | **T** | **C** | **A** | **A** | **A** | **G** | **T** | **C** | **A** | **T** | **T** | **G** | **G** | **T** | **G** | **G** | **G** | **G** | **A** | **G** | **A** | **A** | **T** | **T** | **A** | **C** | **T** | **G** | **C** | **G** | **T** | **G** | **C** | **A** | **C** | **G** | **T** | **T** | **T** | **A** | **T** | **T** | **A** | **T** | **A** | **T** | **G** | **T** | **T** | **C** | **C** | **G** | **C** | **T** | **T** | **A** | **T** | **C** | **T** | **T** | **C** | **C** | **A** | **T** | **T** | **T** | **G** | **T** | **T** | **A** | **G** | **C** | **T** | **A** | **T** | **T** | **T** | **C** | **C** | **T** | **T** | **A** | **G** | **G** | **T** | **G** | **T** | **A** | **G** | **G** | **A** | **T** | **T** | **A** | **A** | **T** | **C** | **G** | **T** | **C** | **T** | **T** | **T** | **C** | **T** | **A** | **T** | **T** | **A** | **T** | **T** | **A** | **T** | **C** | **A** | **C** | **T** | **T** | **A** | **T** | **T** | **C** | **A** | **A** | **A** | **A** | **G** | **A** | **C** | **A** | **A** | **A** | **A** | **T** | **A** | **C** | **G** | **G** | **G** | **A** | **A** | **A** | **A** | **T** | **C** | **G** | **A** | **A** | **G** | **A** | **A** | **A** | **A** | **G** | **C** | **T** | **G** | **C** | **G** | **T** | **G** | **C** | **A** | **C** | **T** | **C** | **T** | **C** | **T** | **T** | **C** | **T** | **G** | **G** | **C** | **A** | **A** | **T** | **T** | **A** | **T** | **G** | **A** | **A** | **T** | **C** | **C** | **A** | **A** | **A** | **T** | **T** | **G** | **T** | **T** | **G** | **C** | **T** | **T** | **C** | **T** | **T** | **C** | **C** | **G** | **A** | **T** | **C** | **C** | **C** | **A** | **A** | **G** | **T** | **G** | **C** | **A** | **T** | **C** | **G** | **G** | **A** | **C** | **G** | **A** | **T** | **T** | **T** | **A** | **T** | **A** | **C** | **A** | **T** | **C** | **A** | **A** | **A** | **G** | **A** | **T** | **A** | **T** | **T** | **G** | **A** | **T** | **A** | **A** | **G** | **G** | **A** | **A** | **A** | **T** | **C** | **A** | **C** | **T** | **A** | **A** | **A** | **A** | **T** | **A** | **A** | **A** | **A** | **G** | **A** | **A** | **A** | **A** | **A** | **A** | **T** | **G** | **A** | **T** | **T** | **G** | **A** | **A** | **G** | **T** | **A** | **T** | **C** | **T** | **A** | **G** | **T** | **G** | **A** | **A** | **T** | **T** | **A** | **C** | **A** | **A** | **A** | **T** | **T** | **G** | **T** | **A** | **A** | **C** | **G** | **A** | **G** | **C** | **C** | **G** | **T** | **C** | **C** | **C** | **C** | **A** | **A** | **T** | **A** | **T** | **G** | **T** | **A** | **G** | **A** | **T** | **G** | **G** | **A** | **C** | **A** | **A** | **A** | **C** | **C** | **A** | **A** | **A** | **G** | **A** | **A** | **G** | **A** | **A** | **A** | **T** | **T** | **T** | **T** | **A** | **G** | **A** | **A** | **T** | **T** | **A** | **G** | **A** | **A** | **A** | **G** | **A** | **C** | **A** | **T** | **C** | **G** | **A** | **T** | **T** | **A** | **G** | **C** | **C** | **C** | **G** | **T** | **G** | **A** | **G** | **C** | **T** | **G** | **C** | **A** | **T** | **G** | **A** | **T** | **T** | **C** | **A** | **G** | **T** | **T** | **T** | **C** | **G** | **C** | **A** | **A** | **C** | **A** | **A** | **T** | **T** | **A** | **T** | **T** | **T** | **G** | **C** | **A** | **G** | **C** | **A** | **A** | **T** | **G** | **A** | **T** | **G** | **A** | **T** | **G** | **A** | **T** | **G** | **T** | **C** | **G** | **G** | **C** | **A** | **T** | **T** | **G** | **A** | **C** | **G** | **G** | **A** | **G** | **C** | **A** | **A** | **G** | **C** | **A** | **G** | **A** | **A** | **A** | **A** | **A** | **A** | **G** | **C** | **G** | **A** | **G** | **A** | **C** | **A** | **C** | **C** | **A** | **G** | **A** | **A** | **A** | **T** | **G** | **T** | **T** | **C** | **C** | **G** | **C** | **A** | **A** | **G** | **C** | **A** | **G** | **T** | **T** | **G** | **A** | **A** | **A** | **A** | **T** | **G** | **G** | **T** | **A** | **G** | **C** | **A** | **G** | **A** | **G** | **A** | **T** | **C** | **A** | **T** | **C** | **A** | **A** | **C** | **G** | **C** | **T** | **T** | **C** | **C** | **C** | **A** | **G** | **T** | **C** | **T** | **G** | **A** | **G** | **A** | **T** | **G** | **C** | **G** | **T** | **G** | **C** | **G** | **C** | **T** | **G** | **C** | **T** | **T** | **C** | **T** | **T** | **C** | **A** | **T** | **C** | **T** | **G** | **C** | **G** | **T** | **C** | **C** | **A** | **G** | **T** | **C** | **A** | **A** | **T** | **T** | **T** | **A** | **G** | **A** | **A** | **G** | **A** | **G** | **A** | **A** | **A** | **A** | **G** | **T** | **T** | **T** | **A** | **A** | **A** | **G** | **C** | **A** | **A** | **G** | **G** | **T** | **A** | **T** | **C** | **G** | **A** | **G** | **C** | **A** | **G** | **T** | **T** | **A** | **T** | **T** | **G** | **A** | **A** | **G** | **G** | **A** | **A** | **T** | **T** | **G** | **C** | **A** | **A** | **A** | **A** | **T** | **A** | **A** | **A** | **A** | **T** | **T** | **C** | **A** | **G** | **A** | **T** | **T** | **T** | **C** | **G** | **C** | **T** | **G** | **A** | **A** | **A** | **T** | **G** | **G** | **G** | **A** | **T** | **G** | **T** | **A** | **G** | **A** | **A** | **G** | **A** | **T** | **G** | **T** | **A** | **A** | **A** | **A** | **C** | **T** | **A** | **T** | **C** | **T** | **A** | **G** | **C** | **T** | **C** | **C** | **A** | **T** | **T** | **G** | **A** | **G** | **G** | **A** | **T** | **C** | **A** | **C** | **T** | **T** | **G** | **T** | **T** | **C** | **C** | **G** | **A** | **A** | **T** | **C** | **G** | **T** | **T** | **C** | **A** | **A** | **G** | **A** | **A** | **T** | **T** | **G** | **T** | **T** | **A** | **T** | **C** | **A** | **A** | **A** | **T** | **A** | **C** | **A** | **T** | **T** | **A** | **A** | **G** | **A** | **C** | **A** | **T** | **G** | **C** | **C** | **A** | **A** | **A** | **G** | **C** | **C** | **A** | **A** | **T** | **G** | **A** | **A** | **T** | **T** | **A** | **G** | **A** | **G** | **G** | **T** | **A** | **T** | **A** | **T** | **T** | **T** | **G** | **C** | **A** | **T** | **A** | **A** | **A** | **A** | **T** | **A** | **G** | **A** | **C** | **A** | **A** | **T** | **A** | **A** | **T** | **C** | **T** | **T** | **T** | **T** | **A** | **T** | **T** | **A** | **C** | **G** | **T** | **A** | **T** | **T** | **A** | **T** | **T** | **G** | **A** | **C** | **G** | **A** | **T** | **G** | **G** | **A** | **A** | **C** | **A** | **G** | **G** | **G** | **T** | **T** | **T** | **A** | **A** | **T** | **A** | **T** | **G** | **A** | **A** | **T** | **G** | **A** | **A** | **A** | **C** | **G** | **A** | **A** | **A** | **A** | **C** | **G** | **G** | **G** | **A** | **A** | **G** | **T** | **T** | **A** | **T** | **G** | **G** | **A** | **T** | **T** | **G** | **A** | **A** | **C** | **A** | **A** | **T** | **A** | **T** | **C** | **A** | **A** | **A** | **G** | **A** | **A** | **C** | **G** | **A** | **G** | **T** | **A** | **G** | **C** | **T** | **G** | **G** | **T** | **A** | **T** | **T** | **G** | **G** | **T** | **G** | **G** | **A** | **A** | **C** | **A** | **G** | **T** | **A** | **A** | **A** | **A** | **A** | **T** | **C** | **A** | **T** | **T** | **A** | **G** | **T** | **T** | **T** | **T** | **A** | **A** | **A** | **G** | **G** | **G** | **C** | **A** | **A** | **G** | **G** | **T** | **A** | **C** | **A** | **A** | **G** | **T** | **G** | **T** | **A** | **G** | **A** | **A** | **A** | **T** | **C** | **A** | **A** | **G** | **G** | **T** | **C** | **C** | **C** | **T** | **T** | **T** | **G** | **A** | **T** | **G** | **A** | **A** | **G** | **G** | **A** | **G** | **G** | **C** | **A** | **-** | **-** | **-** |
| **N60336F** | **A** | **T** | **G** | **A** | **T** | **G** | **G** | **G** | **A** | **A** | **G** | **A** | **A** | **T** | **A** | **T** | **C** | **C** | **A** | **A** | **A** | **G** | **C** | **A** | **A** | **T** | **G** | **C** | **T** | **A** | **G** | **C** | **T** | **G** | **T** | **T** | **T** | **A** | **T** | **T** | **C** | **G** | **G** | **G** | **G** | **A** | **T** | **T** | **G** | **C** | **G** | **G** | **C** | **T** | **T** | **T** | **C** | **C** | **T** | **T** | **A** | **T** | **T** | **A** | **T** | **T** | **C** | **T** | **A** | **T** | **T** | **T** | **T** | **C** | **A** | **C** | **T** | **T** | **T** | **T** | **C** | **A** | **C** | **T** | **T** | **A** | **T** | **T** | **T** | **T** | **T** | **A** | **T** | **G** | **C** | **C** | **A** | **G** | **T** | **A** | **A** | **C** | **C** | **A** | **A** | **A** | **G** | **T** | **C** | **A** | **T** | **T** | **G** | **G** | **T** | **G** | **G** | **G** | **G** | **A** | **G** | **A** | **A** | **T** | **T** | **G** | **C** | **T** | **G** | **C** | **G** | **T** | **G** | **C** | **G** | **C** | **G** | **T** | **T** | **T** | **A** | **T** | **T** | **A** | **T** | **A** | **C** | **G** | **T** | **T** | **C** | **C** | **G** | **C** | **T** | **T** | **A** | **T** | **C** | **T** | **T** | **C** | **C** | **A** | **T** | **T** | **T** | **G** | **A** | **T** | **G** | **G** | **C** | **A** | **A** | **T** | **T** | **T** | **C** | **A** | **T** | **T** | **A** | **G** | **G** | **T** | **G** | **T** | **A** | **G** | **G** | **A** | **T** | **T** | **G** | **A** | **T** | **C** | **G** | **T** | **A** | **T** | **T** | **T** | **C** | **T** | **A** | **T** | **T** | **G** | **T** | **T** | **A** | **T** | **C** | **A** | **C** | **T** | **T** | **A** | **T** | **T** | **C** | **A** | **A** | **A** | **A** | **G** | **G** | **C** | **A** | **A** | **A** | **A** | **T** | **A** | **C** | **G** | **G** | **G** | **A** | **A** | **A** | **A** | **T** | **C** | **G** | **A** | **A** | **G** | **A** | **A** | **A** | **A** | **G** | **C** | **T** | **T** | **C** | **G** | **T** | **G** | **C** | **A** | **C** | **T** | **T** | **T** | **C** | **T** | **T** | **C** | **T** | **G** | **G** | **C** | **A** | **A** | **T** | **T** | **A** | **T** | **G** | **A** | **A** | **T** | **C** | **C** | **A** | **A** | **A** | **C** | **T** | **T** | **T** | **T** | **G** | **C** | **A** | **T** | **C** | **A** | **G** | **C** | **C** | **G** | **A** | **T** | **C** | **C** | **C** | **A** | **A** | **G** | **C** | **G** | **C** | **G** | **T** | **C** | **G** | **G** | **A** | **T** | **G** | **A** | **T** | **C** | **T** | **G** | **T** | **A** | **C** | **A** | **T** | **C** | **A** | **A** | **A** | **G** | **A** | **T** | **A** | **T** | **T** | **G** | **A** | **T** | **A** | **T** | **G** | **G** | **A** | **A** | **A** | **T** | **T** | **A** | **C** | **T** | **A** | **A** | **A** | **A** | **T** | **A** | **A** | **A** | **A** | **G** | **A** | **A** | **A** | **A** | **A** | **A** | **T** | **G** | **A** | **T** | **C** | **G** | **A** | **A** | **G** | **T** | **A** | **T** | **C** | **C** | **A** | **G** | **T** | **G** | **A** | **A** | **T** | **T** | **A** | **C** | **A** | **G** | **A** | **T** | **T** | **A** | **T** | **G** | **A** | **C** | **T** | **A** | **G** | **C** | **C** | **G** | **T** | **C** | **C** | **T** | **C** | **A** | **A** | **T** | **A** | **T** | **G** | **T** | **A** | **G** | **A** | **T** | **G** | **G** | **A** | **C** | **A** | **A** | **A** | **C** | **C** | **A** | **A** | **A** | **G** | **A** | **A** | **G** | **A** | **G** | **A** | **T** | **T** | **T** | **T** | **A** | **G** | **A** | **A** | **T** | **T** | **A** | **G** | **A** | **A** | **A** | **G** | **G** | **C** | **A** | **T** | **C** | **G** | **A** | **T** | **T** | **A** | **G** | **C** | **T** | **C** | **G** | **T** | **G** | **A** | **G** | **C** | **T** | **T** | **C** | **A** | **T** | **G** | **A** | **T** | **T** | **C** | **A** | **G** | **T** | **T** | **T** | **C** | **A** | **C** | **A** | **G** | **C** | **A** | **A** | **T** | **T** | **A** | **T** | **T** | **T** | **G** | **C** | **A** | **G** | **C** | **G** | **A** | **T** | **G** | **A** | **T** | **G** | **A** | **T** | **G** | **A** | **T** | **G** | **T** | **C** | **G** | **G** | **C** | **A** | **T** | **T** | **G** | **A** | **C** | **G** | **G** | **A** | **G** | **C** | **A** | **A** | **G** | **C** | **A** | **G** | **A** | **A** | **A** | **A** | **A** | **A** | **G** | **T** | **G** | **A** | **G** | **A** | **C** | **A** | **C** | **C** | **A** | **G** | **A** | **A** | **A** | **T** | **G** | **T** | **T** | **C** | **C** | **G** | **T** | **A** | **A** | **G** | **C** | **A** | **G** | **T** | **T** | **G** | **A** | **A** | **A** | **A** | **T** | **G** | **G** | **T** | **G** | **G** | **C** | **A** | **G** | **A** | **G** | **A** | **T** | **C** | **A** | **T** | **C** | **A** | **A** | **C** | **G** | **C** | **T** | **T** | **C** | **C** | **C** | **A** | **G** | **T** | **C** | **T** | **G** | **A** | **G** | **A** | **T** | **G** | **C** | **G** | **T** | **G** | **C** | **G** | **C** | **T** | **G** | **C** | **T** | **T** | **C** | **T** | **T** | **C** | **A** | **T** | **C** | **T** | **G** | **C** | **G** | **T** | **C** | **C** | **A** | **G** | **T** | **C** | **A** | **A** | **T** | **T** | **T** | **A** | **G** | **A** | **A** | **G** | **A** | **A** | **A** | **A** | **A** | **A** | **G** | **T** | **T** | **T** | **A** | **A** | **A** | **A** | **C** | **A** | **A** | **G** | **G** | **C** | **A** | **T** | **T** | **G** | **A** | **G** | **C** | **A** | **G** | **T** | **T** | **A** | **T** | **T** | **G** | **A** | **A** | **G** | **G** | **A** | **A** | **T** | **T** | **G** | **C** | **A** | **G** | **A** | **A** | **T** | **A** | **A** | **A** | **A** | **T** | **C** | **C** | **A** | **G** | **A** | **T** | **T** | **T** | **C** | **G** | **C** | **T** | **A** | **A** | **A** | **A** | **T** | **G** | **G** | **G** | **A** | **T** | **G** | **T** | **A** | **G** | **A** | **A** | **G** | **A** | **T** | **G** | **T** | **A** | **A** | **A** | **A** | **T** | **T** | **A** | **A** | **C** | **T** | **A** | **G** | **T** | **T** | **C** | **C** | **A** | **T** | **T** | **G** | **A** | **G** | **G** | **A** | **T** | **C** | **A** | **C** | **T** | **T** | **G** | **T** | **T** | **C** | **C** | **G** | **A** | **A** | **T** | **C** | **G** | **T** | **T** | **C** | **A** | **A** | **G** | **A** | **A** | **T** | **T** | **G** | **T** | **T** | **A** | **T** | **C** | **A** | **A** | **A** | **C** | **A** | **C** | **A** | **T** | **T** | **A** | **A** | **G** | **A** | **C** | **A** | **T** | **G** | **C** | **T** | **A** | **A** | **G** | **G** | **C** | **T** | **A** | **A** | **T** | **G** | **A** | **A** | **T** | **T** | **A** | **G** | **A** | **A** | **G** | **T** | **C** | **T** | **A** | **T** | **T** | **T** | **G** | **C** | **A** | **T** | **A** | **A** | **A** | **A** | **T** | **A** | **G** | **A** | **T** | **A** | **A** | **C** | **A** | **A** | **T** | **C** | **T** | **T** | **T** | **T** | **A** | **T** | **T** | **A** | **C** | **G** | **T** | **A** | **T** | **C** | **A** | **T** | **T** | **G** | **A** | **C** | **G** | **A** | **C** | **G** | **G** | **G** | **A** | **C** | **A** | **G** | **G** | **A** | **T** | **T** | **T** | **A** | **A** | **T** | **A** | **T** | **G** | **A** | **A** | **T** | **G** | **A** | **A** | **A** | **C** | **C** | **A** | **A** | **A** | **A** | **C** | **G** | **G** | **G** | **A** | **A** | **G** | **T** | **T** | **A** | **T** | **G** | **G** | **A** | **T** | **T** | **G** | **A** | **A** | **C** | **A** | **A** | **T** | **A** | **T** | **C** | **A** | **A** | **A** | **G** | **A** | **A** | **C** | **G** | **A** | **G** | **T** | **A** | **G** | **C** | **T** | **G** | **G** | **T** | **A** | **T** | **T** | **G** | **G** | **T** | **G** | **G** | **A** | **A** | **C** | **A** | **G** | **T** | **A** | **A** | **A** | **A** | **A** | **T** | **C** | **A** | **T** | **T** | **A** | **G** | **T** | **T** | **T** | **T** | **A** | **A** | **A** | **G** | **G** | **G** | **C** | **A** | **A** | **G** | **G** | **T** | **A** | **C** | **G** | **A** | **G** | **C** | **G** | **T** | **G** | **G** | **A** | **G** | **A** | **T** | **C** | **A** | **A** | **G** | **G** | **T** | **C** | **C** | **C** | **T** | **T** | **T** | **G** | **A** | **T** | **G** | **A** | **A** | **G** | **G** | **A** | **G** | **G** | **C** | **A** | **-** | **-** | **-** |
| **N54671** | **A** | **T** | **G** | **A** | **T** | **G** | **G** | **G** | **A** | **A** | **A** | **A** | **A** | **T** | **A** | **T** | **C** | **C** | **A** | **G** | **A** | **G** | **C** | **G** | **A** | **T** | **G** | **C** | **T** | **A** | **G** | **C** | **T** | **G** | **T** | **T** | **T** | **A** | **T** | **T** | **C** | **G** | **G** | **G** | **G** | **A** | **T** | **T** | **G** | **C** | **G** | **G** | **C** | **T** | **T** | **T** | **C** | **C** | **T** | **T** | **A** | **T** | **T** | **A** | **T** | **A** | **C** | **T** | **A** | **T** | **T** | **T** | **T** | **C** | **A** | **C** | **T** | **T** | **T** | **T** | **C** | **A** | **C** | **T** | **T** | **A** | **T** | **T** | **T** | **T** | **T** | **A** | **T** | **G** | **C** | **C** | **A** | **G** | **T** | **A** | **A** | **T** | **C** | **A** | **A** | **A** | **G** | **T** | **C** | **A** | **T** | **T** | **G** | **G** | **T** | **G** | **G** | **G** | **G** | **A** | **G** | **A** | **A** | **T** | **T** | **A** | **C** | **T** | **G** | **C** | **G** | **T** | **G** | **C** | **A** | **C** | **G** | **T** | **T** | **T** | **A** | **T** | **T** | **A** | **T** | **A** | **T** | **G** | **T** | **T** | **C** | **C** | **G** | **C** | **T** | **T** | **A** | **T** | **C** | **T** | **T** | **C** | **C** | **A** | **T** | **T** | **T** | **G** | **T** | **T** | **A** | **G** | **C** | **T** | **A** | **T** | **T** | **T** | **C** | **C** | **T** | **T** | **A** | **G** | **G** | **T** | **G** | **T** | **A** | **G** | **G** | **A** | **T** | **T** | **A** | **A** | **T** | **C** | **G** | **T** | **C** | **T** | **T** | **T** | **C** | **T** | **A** | **T** | **T** | **A** | **T** | **T** | **A** | **T** | **C** | **A** | **C** | **T** | **T** | **A** | **T** | **T** | **C** | **A** | **A** | **A** | **A** | **G** | **A** | **C** | **A** | **A** | **A** | **A** | **T** | **A** | **C** | **G** | **G** | **G** | **A** | **A** | **A** | **A** | **T** | **C** | **G** | **A** | **A** | **G** | **A** | **A** | **A** | **A** | **G** | **C** | **T** | **G** | **C** | **G** | **T** | **G** | **C** | **A** | **C** | **T** | **C** | **T** | **C** | **T** | **T** | **C** | **T** | **G** | **G** | **C** | **A** | **A** | **T** | **T** | **A** | **T** | **G** | **A** | **A** | **T** | **C** | **C** | **A** | **A** | **A** | **T** | **T** | **G** | **T** | **T** | **G** | **C** | **T** | **T** | **C** | **T** | **T** | **C** | **C** | **G** | **A** | **T** | **C** | **C** | **C** | **A** | **A** | **G** | **T** | **G** | **C** | **A** | **T** | **C** | **G** | **G** | **A** | **C** | **G** | **A** | **T** | **T** | **T** | **A** | **T** | **A** | **C** | **A** | **T** | **C** | **A** | **A** | **A** | **G** | **A** | **T** | **A** | **T** | **T** | **G** | **A** | **T** | **A** | **A** | **G** | **G** | **A** | **A** | **A** | **T** | **C** | **A** | **C** | **T** | **A** | **A** | **A** | **A** | **T** | **A** | **A** | **A** | **A** | **G** | **A** | **A** | **A** | **A** | **A** | **A** | **T** | **G** | **A** | **T** | **T** | **G** | **A** | **A** | **G** | **T** | **A** | **T** | **C** | **T** | **A** | **G** | **T** | **G** | **A** | **A** | **T** | **T** | **A** | **C** | **A** | **A** | **A** | **T** | **T** | **G** | **T** | **A** | **A** | **C** | **G** | **A** | **G** | **C** | **C** | **G** | **T** | **C** | **C** | **C** | **C** | **A** | **A** | **T** | **A** | **T** | **G** | **T** | **A** | **G** | **A** | **T** | **G** | **G** | **A** | **C** | **A** | **A** | **A** | **C** | **C** | **A** | **A** | **A** | **G** | **A** | **A** | **G** | **A** | **A** | **A** | **T** | **T** | **T** | **T** | **A** | **G** | **A** | **A** | **T** | **T** | **A** | **G** | **A** | **A** | **A** | **G** | **A** | **C** | **A** | **T** | **C** | **G** | **A** | **T** | **T** | **A** | **G** | **C** | **C** | **C** | **G** | **T** | **G** | **A** | **G** | **C** | **T** | **G** | **C** | **A** | **T** | **G** | **A** | **T** | **T** | **C** | **A** | **G** | **T** | **T** | **T** | **C** | **G** | **C** | **A** | **A** | **C** | **A** | **A** | **T** | **T** | **G** | **T** | **T** | **T** | **G** | **C** | **A** | **G** | **C** | **A** | **A** | **T** | **G** | **A** | **T** | **G** | **A** | **T** | **G** | **A** | **T** | **G** | **T** | **C** | **G** | **G** | **C** | **A** | **T** | **T** | **A** | **A** | **C** | **G** | **G** | **A** | **G** | **C** | **A** | **A** | **G** | **C** | **A** | **G** | **A** | **A** | **A** | **A** | **A** | **A** | **G** | **C** | **G** | **A** | **G** | **A** | **C** | **A** | **C** | **C** | **A** | **G** | **A** | **A** | **A** | **T** | **G** | **T** | **T** | **C** | **C** | **G** | **C** | **A** | **A** | **G** | **C** | **A** | **G** | **T** | **T** | **G** | **A** | **A** | **A** | **A** | **T** | **G** | **G** | **T** | **A** | **G** | **C** | **A** | **G** | **A** | **G** | **A** | **T** | **C** | **A** | **T** | **C** | **A** | **A** | **C** | **G** | **C** | **T** | **T** | **C** | **C** | **C** | **A** | **G** | **T** | **C** | **T** | **G** | **A** | **G** | **A** | **T** | **G** | **C** | **G** | **T** | **G** | **C** | **G** | **C** | **T** | **G** | **C** | **T** | **T** | **C** | **T** | **T** | **C** | **A** | **T** | **C** | **T** | **G** | **C** | **G** | **T** | **C** | **C** | **A** | **G** | **T** | **C** | **A** | **A** | **T** | **T** | **T** | **A** | **G** | **A** | **A** | **G** | **A** | **G** | **A** | **A** | **A** | **A** | **G** | **T** | **T** | **T** | **A** | **A** | **A** | **G** | **C** | **A** | **A** | **G** | **G** | **T** | **A** | **T** | **C** | **G** | **A** | **G** | **C** | **A** | **G** | **T** | **T** | **A** | **T** | **T** | **G** | **A** | **A** | **G** | **G** | **A** | **A** | **T** | **T** | **G** | **C** | **A** | **A** | **A** | **A** | **T** | **A** | **A** | **A** | **A** | **T** | **C** | **C** | **A** | **G** | **A** | **T** | **T** | **T** | **C** | **G** | **C** | **T** | **G** | **A** | **A** | **A** | **T** | **G** | **G** | **G** | **A** | **T** | **G** | **T** | **A** | **G** | **A** | **A** | **G** | **A** | **T** | **G** | **T** | **A** | **A** | **A** | **A** | **C** | **T** | **A** | **T** | **C** | **T** | **A** | **G** | **T** | **T** | **C** | **C** | **A** | **T** | **T** | **G** | **A** | **G** | **G** | **A** | **T** | **C** | **A** | **C** | **T** | **T** | **G** | **T** | **T** | **C** | **C** | **G** | **A** | **A** | **T** | **C** | **G** | **T** | **T** | **C** | **A** | **A** | **G** | **A** | **A** | **T** | **T** | **G** | **T** | **T** | **A** | **T** | **C** | **A** | **A** | **A** | **T** | **A** | **C** | **A** | **T** | **T** | **A** | **A** | **G** | **A** | **C** | **A** | **T** | **G** | **C** | **T** | **A** | **A** | **G** | **G** | **C** | **T** | **A** | **A** | **T** | **G** | **A** | **A** | **T** | **T** | **A** | **G** | **A** | **A** | **G** | **T** | **C** | **T** | **A** | **T** | **T** | **T** | **G** | **C** | **A** | **T** | **A** | **A** | **A** | **A** | **T** | **A** | **G** | **A** | **T** | **A** | **A** | **C** | **A** | **A** | **T** | **C** | **T** | **T** | **T** | **T** | **A** | **T** | **T** | **A** | **C** | **G** | **T** | **A** | **T** | **C** | **A** | **T** | **T** | **G** | **A** | **C** | **G** | **A** | **C** | **G** | **G** | **G** | **A** | **C** | **A** | **G** | **G** | **A** | **T** | **T** | **T** | **A** | **A** | **T** | **A** | **T** | **G** | **A** | **A** | **T** | **G** | **A** | **A** | **A** | **C** | **C** | **A** | **A** | **A** | **A** | **C** | **G** | **G** | **G** | **A** | **A** | **G** | **T** | **T** | **A** | **T** | **G** | **G** | **A** | **T** | **T** | **G** | **A** | **A** | **C** | **A** | **A** | **T** | **A** | **T** | **C** | **A** | **A** | **A** | **G** | **A** | **A** | **C** | **G** | **A** | **G** | **T** | **A** | **G** | **C** | **T** | **G** | **G** | **T** | **A** | **T** | **T** | **G** | **G** | **T** | **G** | **G** | **A** | **A** | **C** | **A** | **G** | **T** | **A** | **A** | **A** | **A** | **A** | **T** | **C** | **A** | **T** | **T** | **A** | **G** | **T** | **T** | **T** | **T** | **A** | **A** | **A** | **G** | **G** | **G** | **C** | **A** | **A** | **G** | **G** | **T** | **A** | **C** | **G** | **A** | **G** | **C** | **G** | **T** | **G** | **G** | **A** | **G** | **A** | **T** | **C** | **A** | **A** | **G** | **G** | **T** | **C** | **C** | **C** | **T** | **T** | **T** | **G** | **A** | **T** | **G** | **A** | **A** | **G** | **G** | **A** | **G** | **G** | **C** | **A** | **-** | **-** | **-** |
| **N60357F** | **A** | **T** | **G** | **A** | **T** | **G** | **G** | **G** | **A** | **A** | **G** | **A** | **A** | **T** | **A** | **T** | **C** | **C** | **A** | **A** | **A** | **G** | **C** | **A** | **A** | **T** | **G** | **C** | **T** | **A** | **G** | **C** | **T** | **G** | **T** | **T** | **T** | **A** | **T** | **T** | **C** | **G** | **G** | **G** | **G** | **A** | **T** | **T** | **G** | **C** | **G** | **G** | **C** | **T** | **T** | **T** | **C** | **C** | **T** | **T** | **A** | **T** | **T** | **A** | **T** | **T** | **C** | **T** | **A** | **T** | **T** | **T** | **T** | **C** | **A** | **C** | **T** | **T** | **T** | **T** | **C** | **A** | **C** | **T** | **T** | **A** | **T** | **T** | **T** | **T** | **T** | **A** | **T** | **G** | **C** | **C** | **A** | **G** | **T** | **A** | **A** | **C** | **C** | **A** | **A** | **A** | **G** | **T** | **C** | **A** | **T** | **T** | **G** | **G** | **T** | **G** | **G** | **G** | **G** | **A** | **G** | **A** | **A** | **T** | **T** | **G** | **C** | **T** | **G** | **C** | **G** | **T** | **G** | **C** | **G** | **C** | **G** | **T** | **T** | **T** | **A** | **T** | **T** | **A** | **T** | **A** | **C** | **G** | **T** | **T** | **C** | **C** | **G** | **C** | **T** | **T** | **A** | **T** | **C** | **T** | **T** | **C** | **C** | **A** | **T** | **T** | **T** | **G** | **A** | **T** | **G** | **G** | **C** | **A** | **A** | **T** | **T** | **T** | **C** | **A** | **T** | **T** | **A** | **G** | **G** | **T** | **G** | **T** | **A** | **G** | **G** | **A** | **T** | **T** | **G** | **A** | **T** | **C** | **G** | **T** | **A** | **T** | **T** | **T** | **C** | **T** | **A** | **T** | **T** | **G** | **T** | **T** | **A** | **T** | **C** | **A** | **C** | **T** | **T** | **A** | **T** | **T** | **C** | **A** | **A** | **A** | **A** | **G** | **G** | **C** | **A** | **A** | **A** | **A** | **T** | **A** | **C** | **G** | **G** | **G** | **A** | **A** | **A** | **A** | **T** | **C** | **G** | **A** | **A** | **G** | **A** | **A** | **A** | **A** | **G** | **C** | **T** | **T** | **C** | **G** | **T** | **G** | **C** | **A** | **C** | **T** | **T** | **T** | **C** | **T** | **T** | **C** | **T** | **G** | **G** | **C** | **A** | **A** | **T** | **T** | **A** | **T** | **G** | **A** | **A** | **T** | **C** | **C** | **A** | **A** | **A** | **C** | **T** | **T** | **T** | **T** | **G** | **C** | **A** | **T** | **C** | **A** | **G** | **C** | **C** | **G** | **A** | **T** | **C** | **C** | **C** | **A** | **A** | **G** | **C** | **G** | **C** | **G** | **T** | **C** | **G** | **G** | **A** | **T** | **G** | **A** | **T** | **C** | **T** | **G** | **T** | **A** | **C** | **A** | **T** | **C** | **A** | **A** | **A** | **G** | **A** | **T** | **A** | **T** | **T** | **G** | **A** | **T** | **A** | **T** | **G** | **G** | **A** | **A** | **A** | **T** | **T** | **A** | **C** | **T** | **A** | **A** | **A** | **A** | **T** | **A** | **A** | **A** | **A** | **G** | **A** | **A** | **A** | **A** | **A** | **A** | **T** | **G** | **A** | **T** | **C** | **G** | **A** | **A** | **G** | **T** | **A** | **T** | **C** | **C** | **A** | **G** | **T** | **G** | **A** | **A** | **T** | **T** | **A** | **C** | **A** | **G** | **A** | **T** | **T** | **A** | **T** | **G** | **A** | **C** | **T** | **A** | **G** | **C** | **C** | **G** | **T** | **C** | **C** | **T** | **C** | **A** | **A** | **T** | **A** | **T** | **G** | **T** | **A** | **G** | **A** | **T** | **G** | **G** | **A** | **C** | **A** | **A** | **A** | **C** | **C** | **A** | **A** | **A** | **G** | **A** | **A** | **G** | **A** | **G** | **A** | **T** | **T** | **T** | **T** | **A** | **G** | **A** | **A** | **T** | **T** | **A** | **G** | **A** | **A** | **A** | **G** | **G** | **C** | **A** | **T** | **C** | **G** | **A** | **T** | **T** | **A** | **G** | **C** | **T** | **C** | **G** | **T** | **G** | **A** | **G** | **C** | **T** | **T** | **C** | **A** | **T** | **G** | **A** | **T** | **T** | **C** | **A** | **G** | **T** | **T** | **T** | **C** | **A** | **C** | **A** | **G** | **C** | **A** | **A** | **T** | **T** | **A** | **T** | **T** | **T** | **G** | **C** | **A** | **G** | **C** | **G** | **A** | **T** | **G** | **A** | **T** | **G** | **A** | **T** | **G** | **A** | **T** | **G** | **T** | **C** | **G** | **G** | **C** | **A** | **T** | **T** | **G** | **A** | **C** | **G** | **G** | **A** | **G** | **C** | **A** | **A** | **G** | **C** | **A** | **G** | **A** | **A** | **A** | **A** | **A** | **A** | **G** | **T** | **G** | **A** | **G** | **A** | **C** | **A** | **C** | **C** | **A** | **G** | **G** | **A** | **A** | **T** | **G** | **T** | **T** | **C** | **C** | **G** | **T** | **A** | **A** | **G** | **C** | **A** | **G** | **T** | **T** | **G** | **A** | **A** | **A** | **A** | **T** | **G** | **G** | **T** | **G** | **G** | **C** | **A** | **G** | **A** | **G** | **A** | **T** | **C** | **A** | **T** | **C** | **A** | **A** | **C** | **G** | **C** | **T** | **T** | **C** | **C** | **C** | **A** | **G** | **T** | **C** | **T** | **G** | **A** | **G** | **A** | **T** | **G** | **C** | **G** | **T** | **G** | **C** | **G** | **C** | **T** | **G** | **C** | **T** | **T** | **C** | **T** | **T** | **C** | **A** | **T** | **C** | **T** | **G** | **C** | **G** | **T** | **C** | **C** | **A** | **G** | **T** | **C** | **A** | **A** | **T** | **T** | **T** | **A** | **G** | **A** | **A** | **G** | **A** | **A** | **A** | **A** | **A** | **A** | **G** | **T** | **T** | **T** | **A** | **A** | **A** | **A** | **C** | **A** | **A** | **G** | **G** | **C** | **A** | **T** | **T** | **G** | **A** | **G** | **C** | **A** | **G** | **T** | **T** | **A** | **T** | **T** | **G** | **A** | **A** | **G** | **G** | **A** | **A** | **T** | **T** | **G** | **C** | **A** | **G** | **A** | **A** | **T** | **A** | **A** | **A** | **A** | **T** | **C** | **C** | **A** | **G** | **A** | **T** | **T** | **T** | **C** | **G** | **C** | **T** | **A** | **A** | **A** | **A** | **T** | **G** | **G** | **G** | **A** | **T** | **G** | **T** | **A** | **G** | **A** | **A** | **G** | **A** | **T** | **G** | **T** | **A** | **A** | **A** | **A** | **T** | **T** | **A** | **A** | **C** | **T** | **A** | **G** | **T** | **T** | **C** | **C** | **A** | **T** | **T** | **G** | **A** | **G** | **G** | **A** | **T** | **C** | **A** | **C** | **T** | **T** | **G** | **T** | **T** | **C** | **C** | **G** | **A** | **A** | **T** | **C** | **G** | **T** | **T** | **C** | **A** | **A** | **G** | **A** | **A** | **T** | **T** | **G** | **T** | **T** | **A** | **T** | **C** | **A** | **A** | **A** | **C** | **A** | **C** | **A** | **T** | **T** | **A** | **A** | **G** | **A** | **C** | **A** | **T** | **G** | **C** | **T** | **A** | **A** | **G** | **G** | **C** | **T** | **A** | **A** | **T** | **G** | **A** | **A** | **T** | **T** | **A** | **G** | **A** | **A** | **G** | **T** | **C** | **T** | **A** | **T** | **T** | **T** | **G** | **C** | **A** | **T** | **A** | **A** | **A** | **A** | **T** | **A** | **G** | **A** | **T** | **A** | **A** | **C** | **A** | **A** | **T** | **C** | **T** | **T** | **T** | **T** | **A** | **T** | **T** | **A** | **C** | **G** | **T** | **A** | **T** | **C** | **A** | **T** | **T** | **G** | **A** | **C** | **G** | **A** | **C** | **G** | **G** | **G** | **A** | **C** | **A** | **G** | **G** | **C** | **T** | **T** | **T** | **A** | **A** | **T** | **A** | **T** | **G** | **A** | **A** | **T** | **G** | **A** | **A** | **A** | **C** | **C** | **A** | **A** | **A** | **A** | **C** | **G** | **G** | **G** | **A** | **A** | **G** | **T** | **T** | **A** | **T** | **G** | **G** | **A** | **T** | **T** | **G** | **A** | **A** | **C** | **A** | **A** | **T** | **A** | **T** | **C** | **A** | **A** | **A** | **G** | **A** | **A** | **C** | **G** | **A** | **G** | **T** | **A** | **G** | **C** | **T** | **G** | **G** | **T** | **A** | **T** | **T** | **G** | **G** | **T** | **G** | **G** | **A** | **A** | **C** | **A** | **G** | **T** | **A** | **A** | **A** | **A** | **A** | **T** | **C** | **A** | **T** | **T** | **A** | **G** | **T** | **T** | **T** | **T** | **A** | **A** | **A** | **G** | **G** | **G** | **C** | **A** | **A** | **G** | **G** | **T** | **A** | **C** | **G** | **A** | **G** | **C** | **G** | **T** | **G** | **G** | **A** | **G** | **A** | **T** | **C** | **A** | **A** | **G** | **G** | **T** | **C** | **C** | **C** | **T** | **T** | **T** | **G** | **A** | **T** | **G** | **A** | **A** | **G** | **G** | **A** | **G** | **G** | **C** | **A** | **-** | **-** | **-** |
| **N52649** | **A** | **T** | **G** | **A** | **T** | **G** | **G** | **G** | **A** | **A** | **G** | **A** | **A** | **T** | **A** | **T** | **C** | **C** | **A** | **A** | **A** | **G** | **C** | **A** | **A** | **T** | **G** | **C** | **T** | **A** | **G** | **C** | **T** | **G** | **T** | **T** | **T** | **A** | **T** | **T** | **C** | **G** | **G** | **G** | **G** | **A** | **T** | **T** | **G** | **C** | **G** | **G** | **C** | **T** | **T** | **T** | **C** | **C** | **T** | **T** | **A** | **T** | **T** | **A** | **T** | **T** | **C** | **T** | **A** | **T** | **T** | **T** | **T** | **C** | **A** | **C** | **T** | **T** | **T** | **T** | **C** | **A** | **C** | **T** | **T** | **A** | **T** | **T** | **T** | **T** | **T** | **A** | **T** | **G** | **C** | **C** | **A** | **G** | **T** | **A** | **A** | **C** | **C** | **A** | **A** | **A** | **G** | **T** | **C** | **A** | **T** | **T** | **G** | **G** | **T** | **G** | **G** | **G** | **G** | **A** | **G** | **A** | **A** | **T** | **T** | **G** | **C** | **T** | **G** | **C** | **G** | **T** | **G** | **C** | **G** | **C** | **G** | **T** | **T** | **T** | **A** | **T** | **T** | **A** | **T** | **A** | **C** | **G** | **T** | **T** | **C** | **C** | **G** | **C** | **T** | **T** | **A** | **T** | **C** | **T** | **T** | **C** | **C** | **A** | **T** | **T** | **T** | **G** | **A** | **T** | **G** | **G** | **C** | **A** | **A** | **T** | **T** | **T** | **C** | **A** | **T** | **T** | **A** | **G** | **G** | **T** | **G** | **T** | **A** | **G** | **G** | **A** | **T** | **T** | **G** | **A** | **T** | **C** | **G** | **T** | **A** | **T** | **T** | **T** | **C** | **T** | **A** | **T** | **T** | **G** | **T** | **T** | **A** | **T** | **C** | **A** | **C** | **T** | **T** | **A** | **T** | **T** | **C** | **A** | **A** | **A** | **A** | **G** | **G** | **C** | **A** | **A** | **A** | **A** | **T** | **A** | **C** | **G** | **G** | **G** | **A** | **A** | **A** | **A** | **T** | **C** | **G** | **A** | **A** | **G** | **A** | **A** | **A** | **A** | **G** | **C** | **T** | **T** | **C** | **G** | **T** | **G** | **C** | **A** | **C** | **T** | **T** | **T** | **C** | **T** | **T** | **C** | **T** | **G** | **G** | **C** | **A** | **A** | **T** | **T** | **A** | **T** | **G** | **A** | **A** | **T** | **C** | **C** | **A** | **A** | **A** | **C** | **T** | **T** | **T** | **T** | **G** | **C** | **A** | **T** | **C** | **A** | **G** | **C** | **C** | **G** | **A** | **T** | **C** | **C** | **C** | **A** | **A** | **G** | **C** | **G** | **C** | **G** | **T** | **C** | **G** | **G** | **A** | **T** | **G** | **A** | **T** | **C** | **T** | **G** | **T** | **A** | **C** | **A** | **T** | **C** | **A** | **A** | **A** | **G** | **A** | **T** | **A** | **T** | **T** | **G** | **A** | **T** | **A** | **T** | **G** | **G** | **A** | **A** | **A** | **T** | **T** | **A** | **C** | **T** | **A** | **A** | **A** | **A** | **T** | **A** | **A** | **A** | **A** | **G** | **A** | **A** | **A** | **A** | **A** | **A** | **T** | **G** | **A** | **T** | **C** | **G** | **A** | **A** | **G** | **T** | **A** | **T** | **C** | **C** | **A** | **G** | **T** | **G** | **A** | **A** | **T** | **T** | **A** | **C** | **A** | **G** | **A** | **T** | **T** | **A** | **T** | **G** | **A** | **C** | **T** | **A** | **G** | **C** | **C** | **G** | **T** | **C** | **C** | **T** | **C** | **A** | **A** | **T** | **A** | **T** | **G** | **T** | **A** | **G** | **A** | **T** | **G** | **G** | **A** | **C** | **A** | **A** | **A** | **C** | **C** | **A** | **A** | **A** | **G** | **A** | **A** | **G** | **A** | **G** | **A** | **T** | **T** | **T** | **T** | **A** | **G** | **A** | **A** | **T** | **T** | **A** | **G** | **A** | **A** | **A** | **G** | **G** | **C** | **A** | **T** | **C** | **G** | **A** | **T** | **T** | **A** | **G** | **C** | **T** | **C** | **G** | **T** | **G** | **A** | **G** | **C** | **T** | **T** | **C** | **A** | **T** | **G** | **A** | **T** | **T** | **C** | **A** | **G** | **T** | **T** | **T** | **C** | **A** | **C** | **A** | **G** | **C** | **A** | **A** | **T** | **T** | **A** | **T** | **T** | **T** | **G** | **C** | **A** | **G** | **C** | **G** | **A** | **T** | **G** | **A** | **T** | **G** | **A** | **T** | **G** | **A** | **T** | **G** | **T** | **C** | **G** | **G** | **C** | **A** | **T** | **T** | **G** | **A** | **C** | **G** | **G** | **A** | **G** | **C** | **A** | **A** | **G** | **C** | **A** | **G** | **A** | **A** | **A** | **A** | **A** | **A** | **G** | **T** | **G** | **A** | **G** | **A** | **C** | **A** | **C** | **C** | **A** | **G** | **A** | **A** | **A** | **T** | **G** | **T** | **T** | **C** | **C** | **G** | **T** | **A** | **A** | **G** | **C** | **A** | **G** | **T** | **T** | **G** | **A** | **A** | **A** | **A** | **T** | **G** | **G** | **T** | **G** | **G** | **C** | **A** | **G** | **A** | **G** | **A** | **T** | **C** | **A** | **T** | **C** | **A** | **A** | **C** | **G** | **C** | **T** | **T** | **C** | **C** | **C** | **A** | **G** | **T** | **C** | **T** | **G** | **A** | **G** | **A** | **T** | **G** | **C** | **G** | **T** | **G** | **C** | **G** | **C** | **T** | **G** | **C** | **T** | **T** | **C** | **T** | **T** | **C** | **A** | **T** | **C** | **T** | **G** | **C** | **G** | **T** | **C** | **C** | **A** | **G** | **T** | **C** | **A** | **A** | **T** | **T** | **T** | **A** | **G** | **A** | **A** | **G** | **A** | **A** | **A** | **A** | **A** | **A** | **G** | **T** | **T** | **T** | **A** | **A** | **A** | **A** | **C** | **A** | **A** | **G** | **G** | **C** | **A** | **T** | **T** | **G** | **A** | **G** | **C** | **A** | **G** | **T** | **T** | **A** | **T** | **T** | **G** | **A** | **A** | **G** | **G** | **A** | **A** | **T** | **T** | **G** | **C** | **A** | **G** | **A** | **A** | **T** | **A** | **A** | **A** | **A** | **T** | **C** | **C** | **A** | **G** | **A** | **T** | **T** | **T** | **C** | **G** | **C** | **T** | **A** | **A** | **A** | **A** | **T** | **G** | **G** | **G** | **A** | **T** | **G** | **T** | **A** | **G** | **A** | **A** | **G** | **A** | **T** | **G** | **T** | **A** | **A** | **A** | **A** | **T** | **T** | **A** | **A** | **C** | **T** | **A** | **G** | **T** | **T** | **C** | **C** | **A** | **T** | **T** | **G** | **A** | **G** | **G** | **A** | **T** | **C** | **A** | **C** | **T** | **T** | **G** | **T** | **T** | **C** | **C** | **G** | **A** | **A** | **T** | **C** | **G** | **T** | **T** | **C** | **A** | **A** | **G** | **A** | **A** | **T** | **T** | **G** | **T** | **T** | **A** | **T** | **C** | **A** | **A** | **A** | **C** | **A** | **C** | **A** | **T** | **T** | **A** | **A** | **G** | **A** | **C** | **A** | **T** | **G** | **C** | **T** | **A** | **A** | **G** | **G** | **C** | **T** | **A** | **A** | **T** | **G** | **A** | **A** | **T** | **T** | **A** | **G** | **A** | **A** | **G** | **T** | **C** | **T** | **A** | **T** | **T** | **T** | **G** | **C** | **A** | **T** | **A** | **A** | **A** | **A** | **T** | **A** | **G** | **A** | **T** | **A** | **A** | **C** | **A** | **A** | **T** | **C** | **T** | **T** | **T** | **T** | **A** | **T** | **T** | **A** | **C** | **G** | **T** | **A** | **T** | **C** | **A** | **T** | **T** | **G** | **A** | **C** | **G** | **A** | **C** | **G** | **G** | **G** | **A** | **C** | **A** | **G** | **G** | **A** | **T** | **T** | **T** | **A** | **A** | **T** | **A** | **T** | **G** | **A** | **A** | **T** | **G** | **A** | **A** | **A** | **C** | **C** | **A** | **A** | **A** | **A** | **C** | **G** | **G** | **G** | **A** | **A** | **G** | **T** | **T** | **A** | **T** | **G** | **G** | **A** | **T** | **T** | **G** | **A** | **A** | **C** | **A** | **A** | **T** | **A** | **T** | **C** | **A** | **A** | **A** | **G** | **A** | **A** | **C** | **G** | **A** | **G** | **T** | **A** | **G** | **C** | **T** | **G** | **G** | **T** | **A** | **T** | **T** | **G** | **G** | **T** | **G** | **G** | **A** | **A** | **C** | **A** | **G** | **T** | **A** | **A** | **A** | **A** | **A** | **T** | **C** | **A** | **T** | **T** | **A** | **G** | **T** | **T** | **T** | **T** | **A** | **A** | **A** | **G** | **G** | **G** | **C** | **A** | **A** | **G** | **G** | **T** | **A** | **C** | **G** | **A** | **G** | **C** | **G** | **T** | **G** | **G** | **A** | **G** | **A** | **T** | **C** | **A** | **A** | **G** | **G** | **T** | **C** | **C** | **C** | **T** | **T** | **T** | **G** | **A** | **T** | **G** | **A** | **A** | **G** | **G** | **A** | **G** | **G** | **C** | **A** | **-** | **-** | **-** |
| **N60363F** | **A** | **T** | **G** | **A** | **T** | **G** | **G** | **G** | **A** | **A** | **G** | **A** | **A** | **T** | **A** | **T** | **C** | **C** | **A** | **A** | **A** | **G** | **C** | **A** | **A** | **T** | **G** | **C** | **T** | **A** | **G** | **C** | **T** | **G** | **T** | **T** | **T** | **A** | **T** | **T** | **C** | **G** | **G** | **G** | **G** | **A** | **T** | **T** | **G** | **C** | **G** | **G** | **C** | **T** | **T** | **T** | **C** | **C** | **T** | **T** | **A** | **T** | **T** | **A** | **T** | **T** | **C** | **T** | **A** | **T** | **T** | **T** | **T** | **C** | **A** | **C** | **T** | **T** | **T** | **T** | **C** | **A** | **C** | **T** | **T** | **A** | **T** | **T** | **T** | **T** | **T** | **A** | **T** | **G** | **C** | **C** | **A** | **G** | **T** | **A** | **A** | **C** | **C** | **A** | **A** | **A** | **G** | **T** | **C** | **A** | **T** | **T** | **G** | **G** | **T** | **G** | **G** | **G** | **G** | **A** | **G** | **A** | **A** | **T** | **T** | **G** | **C** | **T** | **G** | **C** | **G** | **T** | **G** | **C** | **G** | **C** | **G** | **T** | **T** | **T** | **A** | **T** | **T** | **A** | **T** | **A** | **C** | **G** | **T** | **T** | **C** | **C** | **G** | **C** | **T** | **T** | **A** | **T** | **C** | **T** | **T** | **C** | **C** | **A** | **T** | **T** | **T** | **G** | **A** | **T** | **G** | **G** | **C** | **A** | **A** | **T** | **T** | **T** | **C** | **A** | **T** | **T** | **A** | **G** | **G** | **T** | **G** | **T** | **A** | **G** | **G** | **A** | **T** | **T** | **G** | **A** | **T** | **C** | **G** | **T** | **A** | **T** | **T** | **T** | **C** | **T** | **A** | **T** | **T** | **G** | **T** | **T** | **A** | **T** | **C** | **A** | **C** | **T** | **T** | **A** | **T** | **T** | **C** | **A** | **A** | **A** | **A** | **G** | **A** | **C** | **A** | **A** | **A** | **A** | **T** | **A** | **C** | **G** | **G** | **G** | **A** | **A** | **A** | **A** | **T** | **C** | **G** | **A** | **A** | **G** | **A** | **A** | **A** | **A** | **G** | **C** | **T** | **T** | **C** | **G** | **T** | **G** | **C** | **A** | **C** | **T** | **T** | **T** | **C** | **T** | **T** | **C** | **T** | **G** | **G** | **C** | **A** | **A** | **T** | **T** | **A** | **T** | **G** | **A** | **A** | **T** | **C** | **C** | **A** | **A** | **A** | **C** | **T** | **T** | **T** | **T** | **G** | **C** | **A** | **T** | **C** | **A** | **G** | **C** | **C** | **G** | **A** | **T** | **C** | **C** | **C** | **A** | **A** | **G** | **C** | **G** | **C** | **G** | **T** | **C** | **G** | **G** | **A** | **T** | **G** | **A** | **T** | **C** | **T** | **G** | **T** | **A** | **C** | **A** | **T** | **C** | **A** | **A** | **A** | **G** | **A** | **T** | **A** | **T** | **T** | **G** | **A** | **T** | **A** | **T** | **G** | **G** | **A** | **A** | **A** | **T** | **T** | **A** | **C** | **T** | **A** | **A** | **A** | **A** | **T** | **A** | **A** | **A** | **A** | **G** | **A** | **A** | **A** | **A** | **A** | **A** | **T** | **G** | **A** | **T** | **C** | **G** | **A** | **A** | **G** | **T** | **A** | **T** | **C** | **C** | **A** | **G** | **T** | **G** | **A** | **A** | **T** | **T** | **A** | **C** | **A** | **G** | **A** | **T** | **T** | **A** | **T** | **G** | **A** | **C** | **T** | **A** | **G** | **C** | **C** | **G** | **T** | **C** | **C** | **T** | **C** | **A** | **A** | **T** | **A** | **T** | **G** | **T** | **A** | **G** | **A** | **T** | **G** | **G** | **A** | **C** | **A** | **A** | **A** | **C** | **C** | **A** | **A** | **A** | **G** | **A** | **A** | **G** | **A** | **G** | **A** | **T** | **T** | **T** | **T** | **A** | **G** | **A** | **A** | **T** | **T** | **A** | **G** | **A** | **A** | **A** | **G** | **G** | **C** | **A** | **T** | **C** | **G** | **A** | **T** | **T** | **A** | **G** | **C** | **T** | **C** | **G** | **T** | **G** | **A** | **G** | **C** | **T** | **T** | **C** | **A** | **T** | **G** | **A** | **T** | **T** | **C** | **A** | **G** | **T** | **T** | **T** | **C** | **A** | **C** | **A** | **G** | **C** | **A** | **A** | **T** | **T** | **A** | **T** | **T** | **T** | **G** | **C** | **A** | **G** | **C** | **G** | **A** | **T** | **G** | **A** | **T** | **G** | **A** | **T** | **G** | **A** | **T** | **G** | **T** | **C** | **G** | **G** | **C** | **A** | **T** | **T** | **G** | **A** | **C** | **G** | **G** | **A** | **G** | **C** | **A** | **A** | **G** | **C** | **A** | **G** | **A** | **A** | **A** | **A** | **A** | **A** | **G** | **T** | **G** | **A** | **G** | **A** | **C** | **A** | **C** | **C** | **A** | **G** | **A** | **A** | **A** | **T** | **G** | **T** | **T** | **C** | **C** | **G** | **T** | **A** | **A** | **G** | **C** | **A** | **G** | **T** | **T** | **G** | **A** | **A** | **A** | **A** | **T** | **G** | **G** | **T** | **G** | **G** | **C** | **A** | **G** | **A** | **G** | **A** | **T** | **C** | **A** | **T** | **C** | **A** | **A** | **C** | **G** | **C** | **T** | **T** | **C** | **C** | **C** | **A** | **G** | **T** | **C** | **T** | **G** | **A** | **G** | **A** | **T** | **G** | **C** | **G** | **T** | **G** | **C** | **G** | **C** | **T** | **G** | **C** | **T** | **T** | **C** | **T** | **T** | **C** | **A** | **T** | **C** | **T** | **G** | **C** | **G** | **T** | **C** | **C** | **A** | **G** | **T** | **C** | **A** | **A** | **T** | **T** | **T** | **A** | **G** | **A** | **A** | **G** | **A** | **A** | **A** | **A** | **A** | **A** | **G** | **T** | **T** | **T** | **A** | **A** | **A** | **A** | **C** | **A** | **A** | **G** | **G** | **C** | **A** | **T** | **T** | **G** | **A** | **G** | **C** | **A** | **G** | **T** | **T** | **A** | **T** | **T** | **G** | **A** | **A** | **G** | **G** | **A** | **A** | **T** | **T** | **G** | **C** | **A** | **G** | **A** | **A** | **T** | **A** | **A** | **A** | **A** | **T** | **C** | **C** | **A** | **G** | **A** | **T** | **T** | **T** | **C** | **G** | **C** | **T** | **A** | **A** | **A** | **A** | **T** | **G** | **G** | **G** | **A** | **T** | **G** | **T** | **A** | **G** | **A** | **A** | **G** | **A** | **T** | **G** | **T** | **A** | **A** | **A** | **A** | **T** | **T** | **A** | **A** | **C** | **T** | **A** | **G** | **T** | **T** | **C** | **C** | **A** | **T** | **T** | **G** | **A** | **G** | **G** | **A** | **T** | **C** | **A** | **C** | **T** | **T** | **G** | **T** | **T** | **C** | **C** | **G** | **A** | **A** | **T** | **C** | **G** | **T** | **T** | **C** | **A** | **A** | **G** | **A** | **A** | **T** | **T** | **G** | **T** | **T** | **A** | **T** | **C** | **A** | **A** | **A** | **C** | **A** | **C** | **A** | **T** | **T** | **A** | **A** | **G** | **A** | **C** | **A** | **T** | **G** | **C** | **T** | **A** | **A** | **G** | **G** | **C** | **T** | **A** | **A** | **T** | **G** | **A** | **A** | **T** | **T** | **A** | **G** | **A** | **A** | **G** | **T** | **C** | **T** | **A** | **T** | **T** | **T** | **G** | **C** | **A** | **T** | **A** | **A** | **A** | **A** | **T** | **A** | **G** | **A** | **T** | **A** | **A** | **C** | **A** | **A** | **T** | **C** | **T** | **T** | **T** | **T** | **A** | **T** | **T** | **A** | **C** | **G** | **T** | **A** | **T** | **C** | **A** | **T** | **T** | **G** | **A** | **C** | **G** | **A** | **C** | **G** | **G** | **G** | **A** | **C** | **A** | **G** | **G** | **A** | **T** | **T** | **T** | **A** | **A** | **T** | **A** | **T** | **G** | **A** | **A** | **T** | **G** | **A** | **A** | **A** | **C** | **C** | **A** | **A** | **A** | **A** | **C** | **G** | **G** | **G** | **A** | **A** | **G** | **T** | **T** | **A** | **T** | **G** | **G** | **A** | **T** | **T** | **G** | **A** | **A** | **C** | **A** | **A** | **T** | **A** | **T** | **C** | **A** | **A** | **A** | **G** | **A** | **A** | **C** | **G** | **A** | **G** | **T** | **A** | **G** | **C** | **T** | **G** | **G** | **T** | **A** | **T** | **T** | **G** | **G** | **T** | **G** | **G** | **A** | **A** | **C** | **A** | **G** | **T** | **A** | **A** | **A** | **A** | **A** | **T** | **C** | **A** | **T** | **T** | **A** | **G** | **T** | **T** | **T** | **T** | **A** | **A** | **A** | **G** | **G** | **G** | **C** | **A** | **A** | **G** | **G** | **T** | **A** | **C** | **G** | **A** | **G** | **C** | **G** | **T** | **G** | **G** | **A** | **G** | **A** | **T** | **C** | **A** | **A** | **G** | **G** | **T** | **C** | **C** | **C** | **T** | **T** | **T** | **G** | **A** | **T** | **G** | **A** | **A** | **G** | **G** | **A** | **G** | **G** | **C** | **A** | **-** | **-** | **-** |
| **N55279** | **A** | **T** | **G** | **A** | **T** | **G** | **G** | **G** | **A** | **A** | **A** | **A** | **A** | **T** | **A** | **T** | **C** | **C** | **A** | **G** | **A** | **G** | **C** | **G** | **A** | **T** | **G** | **C** | **T** | **A** | **G** | **C** | **T** | **G** | **T** | **T** | **T** | **A** | **T** | **T** | **C** | **G** | **G** | **G** | **G** | **A** | **T** | **T** | **G** | **C** | **G** | **G** | **C** | **T** | **T** | **T** | **C** | **C** | **T** | **T** | **A** | **T** | **T** | **A** | **T** | **T** | **C** | **T** | **A** | **T** | **T** | **T** | **T** | **C** | **A** | **C** | **T** | **T** | **T** | **T** | **C** | **A** | **C** | **T** | **T** | **A** | **T** | **T** | **T** | **T** | **T** | **A** | **T** | **G** | **C** | **C** | **A** | **G** | **C** | **A** | **A** | **T** | **C** | **A** | **A** | **A** | **G** | **T** | **C** | **A** | **T** | **T** | **G** | **G** | **T** | **G** | **G** | **G** | **G** | **A** | **G** | **A** | **A** | **T** | **T** | **A** | **C** | **T** | **G** | **C** | **G** | **T** | **G** | **C** | **A** | **C** | **G** | **T** | **T** | **T** | **A** | **T** | **T** | **A** | **T** | **A** | **T** | **G** | **T** | **T** | **C** | **C** | **G** | **C** | **T** | **T** | **A** | **T** | **C** | **T** | **T** | **C** | **C** | **A** | **T** | **T** | **T** | **G** | **T** | **T** | **A** | **G** | **C** | **C** | **A** | **T** | **T** | **T** | **C** | **C** | **T** | **T** | **A** | **G** | **G** | **T** | **G** | **T** | **A** | **G** | **G** | **A** | **T** | **T** | **A** | **A** | **T** | **T** | **G** | **T** | **C** | **T** | **T** | **T** | **C** | **T** | **A** | **T** | **T** | **A** | **T** | **T** | **A** | **T** | **C** | **A** | **C** | **T** | **T** | **A** | **T** | **T** | **C** | **A** | **A** | **A** | **A** | **G** | **A** | **C** | **A** | **A** | **A** | **A** | **T** | **A** | **C** | **G** | **G** | **G** | **A** | **A** | **A** | **A** | **T** | **C** | **G** | **A** | **A** | **G** | **A** | **A** | **A** | **A** | **G** | **C** | **T** | **G** | **C** | **G** | **T** | **G** | **C** | **A** | **C** | **T** | **T** | **T** | **C** | **T** | **T** | **C** | **T** | **G** | **G** | **C** | **A** | **A** | **T** | **T** | **A** | **T** | **G** | **A** | **A** | **T** | **C** | **C** | **A** | **A** | **A** | **T** | **T** | **G** | **T** | **T** | **G** | **C** | **T** | **T** | **C** | **T** | **T** | **C** | **C** | **G** | **A** | **T** | **C** | **C** | **C** | **A** | **A** | **G** | **T** | **G** | **C** | **A** | **T** | **C** | **G** | **G** | **A** | **C** | **G** | **A** | **T** | **T** | **T** | **A** | **T** | **A** | **C** | **A** | **T** | **C** | **A** | **A** | **A** | **G** | **A** | **T** | **A** | **T** | **T** | **G** | **A** | **T** | **A** | **A** | **G** | **G** | **A** | **A** | **A** | **T** | **C** | **A** | **C** | **T** | **A** | **A** | **G** | **A** | **T** | **A** | **A** | **A** | **A** | **G** | **A** | **A** | **A** | **A** | **A** | **A** | **T** | **G** | **A** | **T** | **T** | **G** | **A** | **A** | **G** | **T** | **A** | **T** | **C** | **T** | **A** | **G** | **T** | **G** | **A** | **A** | **T** | **T** | **A** | **C** | **A** | **A** | **A** | **T** | **T** | **G** | **T** | **A** | **A** | **C** | **G** | **A** | **G** | **C** | **C** | **G** | **T** | **C** | **C** | **C** | **C** | **A** | **A** | **T** | **A** | **T** | **G** | **T** | **A** | **G** | **A** | **T** | **G** | **G** | **A** | **C** | **A** | **A** | **A** | **C** | **C** | **A** | **A** | **A** | **G** | **A** | **A** | **G** | **A** | **A** | **A** | **T** | **T** | **T** | **T** | **A** | **G** | **A** | **A** | **T** | **T** | **A** | **G** | **A** | **A** | **A** | **G** | **A** | **C** | **A** | **T** | **C** | **G** | **A** | **T** | **T** | **A** | **G** | **C** | **C** | **C** | **G** | **T** | **G** | **A** | **G** | **C** | **T** | **G** | **C** | **A** | **T** | **G** | **A** | **T** | **T** | **C** | **A** | **G** | **T** | **T** | **T** | **C** | **G** | **C** | **A** | **A** | **C** | **A** | **A** | **T** | **T** | **G** | **T** | **T** | **T** | **G** | **C** | **A** | **G** | **C** | **A** | **A** | **T** | **G** | **A** | **T** | **G** | **A** | **T** | **G** | **A** | **T** | **G** | **T** | **C** | **G** | **G** | **C** | **A** | **T** | **T** | **G** | **A** | **C** | **G** | **G** | **A** | **G** | **C** | **A** | **A** | **G** | **C** | **A** | **G** | **A** | **A** | **A** | **A** | **A** | **A** | **G** | **C** | **G** | **A** | **G** | **A** | **C** | **A** | **C** | **C** | **A** | **G** | **A** | **A** | **A** | **T** | **G** | **T** | **T** | **C** | **C** | **G** | **C** | **A** | **A** | **G** | **C** | **A** | **G** | **T** | **T** | **G** | **A** | **A** | **A** | **A** | **T** | **G** | **G** | **T** | **A** | **G** | **C** | **A** | **G** | **A** | **G** | **A** | **T** | **C** | **A** | **T** | **C** | **A** | **A** | **C** | **G** | **C** | **T** | **T** | **C** | **C** | **C** | **A** | **G** | **T** | **C** | **T** | **G** | **A** | **G** | **A** | **T** | **G** | **C** | **G** | **T** | **G** | **C** | **G** | **C** | **T** | **G** | **C** | **T** | **T** | **C** | **T** | **T** | **C** | **A** | **T** | **C** | **T** | **A** | **C** | **G** | **T** | **C** | **C** | **A** | **G** | **T** | **C** | **A** | **A** | **T** | **T** | **T** | **A** | **G** | **A** | **A** | **G** | **A** | **G** | **A** | **A** | **A** | **A** | **G** | **T** | **T** | **T** | **A** | **A** | **A** | **G** | **C** | **A** | **A** | **G** | **G** | **T** | **A** | **T** | **C** | **G** | **A** | **G** | **C** | **A** | **G** | **T** | **T** | **A** | **T** | **T** | **G** | **A** | **A** | **G** | **G** | **A** | **A** | **T** | **T** | **G** | **C** | **A** | **A** | **A** | **A** | **T** | **A** | **A** | **A** | **A** | **T** | **C** | **C** | **A** | **G** | **A** | **T** | **T** | **T** | **C** | **G** | **C** | **T** | **G** | **A** | **A** | **A** | **T** | **G** | **G** | **G** | **A** | **T** | **G** | **T** | **A** | **G** | **A** | **A** | **G** | **A** | **T** | **G** | **T** | **A** | **A** | **A** | **A** | **C** | **T** | **A** | **T** | **C** | **T** | **A** | **G** | **T** | **T** | **C** | **C** | **A** | **T** | **T** | **G** | **A** | **G** | **G** | **A** | **T** | **C** | **A** | **C** | **T** | **T** | **G** | **T** | **T** | **C** | **C** | **G** | **A** | **A** | **T** | **C** | **G** | **T** | **T** | **C** | **A** | **A** | **G** | **A** | **A** | **T** | **T** | **G** | **T** | **T** | **A** | **T** | **C** | **A** | **A** | **A** | **T** | **A** | **C** | **A** | **T** | **T** | **A** | **A** | **G** | **A** | **C** | **A** | **T** | **G** | **C** | **T** | **A** | **A** | **A** | **G** | **C** | **C** | **A** | **A** | **T** | **G** | **A** | **A** | **T** | **T** | **A** | **G** | **A** | **G** | **G** | **T** | **A** | **T** | **A** | **T** | **T** | **T** | **G** | **C** | **A** | **C** | **A** | **A** | **A** | **A** | **T** | **A** | **G** | **A** | **C** | **A** | **A** | **T** | **A** | **A** | **T** | **C** | **T** | **T** | **T** | **T** | **G** | **T** | **T** | **A** | **C** | **G** | **T** | **A** | **T** | **T** | **A** | **T** | **T** | **G** | **A** | **C** | **G** | **A** | **C** | **G** | **G** | **A** | **A** | **C** | **A** | **G** | **G** | **G** | **T** | **T** | **T** | **A** | **A** | **T** | **A** | **T** | **G** | **A** | **A** | **T** | **G** | **A** | **A** | **A** | **C** | **G** | **A** | **A** | **A** | **A** | **C** | **G** | **G** | **G** | **A** | **A** | **G** | **T** | **T** | **A** | **T** | **G** | **G** | **A** | **T** | **T** | **G** | **A** | **A** | **C** | **A** | **A** | **T** | **A** | **T** | **C** | **A** | **A** | **A** | **G** | **A** | **A** | **C** | **G** | **A** | **G** | **T** | **A** | **G** | **C** | **T** | **G** | **G** | **T** | **A** | **T** | **T** | **G** | **G** | **T** | **G** | **G** | **A** | **A** | **C** | **A** | **G** | **T** | **A** | **A** | **A** | **A** | **A** | **T** | **C** | **A** | **T** | **T** | **A** | **G** | **T** | **T** | **T** | **T** | **A** | **A** | **A** | **G** | **G** | **T** | **C** | **A** | **A** | **G** | **G** | **T** | **A** | **C** | **A** | **A** | **G** | **T** | **G** | **T** | **A** | **G** | **A** | **A** | **A** | **T** | **C** | **A** | **A** | **G** | **G** | **T** | **C** | **C** | **C** | **T** | **T** | **T** | **G** | **A** | **T** | **G** | **A** | **A** | **G** | **G** | **A** | **G** | **G** | **C** | **A** | **-** | **-** | **-** |
| **N55728** | **A** | **T** | **G** | **A** | **T** | **G** | **G** | **G** | **A** | **A** | **A** | **A** | **A** | **T** | **A** | **T** | **C** | **C** | **A** | **G** | **A** | **G** | **C** | **G** | **A** | **T** | **G** | **C** | **T** | **A** | **G** | **C** | **T** | **G** | **T** | **T** | **T** | **A** | **T** | **T** | **C** | **G** | **G** | **G** | **G** | **A** | **T** | **T** | **G** | **C** | **G** | **G** | **C** | **T** | **T** | **T** | **C** | **C** | **T** | **T** | **A** | **T** | **T** | **A** | **T** | **A** | **C** | **T** | **A** | **T** | **T** | **T** | **T** | **C** | **A** | **C** | **T** | **T** | **T** | **T** | **C** | **A** | **C** | **T** | **T** | **A** | **T** | **T** | **T** | **T** | **T** | **A** | **T** | **G** | **C** | **C** | **A** | **G** | **T** | **A** | **A** | **T** | **C** | **A** | **A** | **A** | **G** | **T** | **C** | **A** | **T** | **T** | **G** | **G** | **T** | **G** | **G** | **G** | **G** | **A** | **G** | **A** | **A** | **T** | **T** | **A** | **C** | **T** | **G** | **C** | **G** | **T** | **G** | **C** | **A** | **C** | **G** | **T** | **T** | **T** | **A** | **T** | **T** | **A** | **T** | **A** | **T** | **G** | **T** | **T** | **C** | **C** | **G** | **C** | **T** | **T** | **A** | **T** | **C** | **T** | **T** | **C** | **C** | **A** | **T** | **T** | **T** | **G** | **T** | **T** | **A** | **G** | **C** | **T** | **A** | **T** | **T** | **T** | **C** | **C** | **T** | **T** | **A** | **G** | **G** | **T** | **G** | **T** | **A** | **G** | **G** | **A** | **T** | **T** | **A** | **A** | **T** | **C** | **G** | **T** | **C** | **T** | **T** | **T** | **C** | **T** | **A** | **T** | **T** | **A** | **T** | **T** | **A** | **T** | **C** | **A** | **C** | **T** | **T** | **A** | **T** | **T** | **C** | **A** | **A** | **A** | **A** | **G** | **A** | **C** | **A** | **A** | **A** | **A** | **T** | **A** | **C** | **G** | **G** | **G** | **A** | **A** | **A** | **A** | **T** | **C** | **G** | **A** | **A** | **G** | **A** | **A** | **A** | **A** | **G** | **C** | **T** | **G** | **C** | **G** | **T** | **G** | **C** | **A** | **C** | **T** | **C** | **T** | **C** | **T** | **T** | **C** | **T** | **G** | **G** | **C** | **A** | **A** | **T** | **T** | **A** | **T** | **G** | **A** | **A** | **T** | **C** | **C** | **A** | **A** | **A** | **T** | **T** | **G** | **T** | **T** | **G** | **C** | **T** | **T** | **C** | **T** | **T** | **C** | **C** | **G** | **A** | **T** | **C** | **C** | **C** | **A** | **A** | **G** | **T** | **G** | **C** | **A** | **T** | **C** | **G** | **G** | **A** | **C** | **G** | **A** | **T** | **T** | **T** | **A** | **T** | **A** | **C** | **A** | **T** | **C** | **A** | **A** | **A** | **G** | **A** | **T** | **A** | **T** | **T** | **G** | **A** | **T** | **A** | **A** | **G** | **G** | **A** | **A** | **A** | **T** | **C** | **A** | **C** | **T** | **A** | **A** | **A** | **A** | **T** | **A** | **A** | **A** | **A** | **G** | **A** | **A** | **A** | **A** | **A** | **A** | **T** | **G** | **A** | **T** | **T** | **G** | **A** | **A** | **G** | **T** | **A** | **T** | **C** | **T** | **A** | **G** | **T** | **G** | **A** | **A** | **T** | **T** | **A** | **C** | **A** | **A** | **A** | **T** | **T** | **G** | **T** | **A** | **A** | **C** | **G** | **A** | **G** | **C** | **C** | **G** | **T** | **C** | **C** | **C** | **C** | **A** | **A** | **T** | **A** | **T** | **G** | **T** | **A** | **G** | **A** | **T** | **G** | **G** | **A** | **C** | **A** | **A** | **A** | **C** | **C** | **A** | **A** | **A** | **G** | **A** | **A** | **G** | **A** | **A** | **A** | **T** | **T** | **T** | **T** | **A** | **G** | **A** | **A** | **T** | **T** | **A** | **G** | **A** | **A** | **A** | **G** | **A** | **C** | **A** | **T** | **C** | **G** | **A** | **T** | **T** | **A** | **G** | **C** | **C** | **C** | **G** | **T** | **G** | **A** | **G** | **C** | **T** | **G** | **C** | **A** | **T** | **G** | **A** | **T** | **T** | **C** | **A** | **G** | **T** | **T** | **T** | **C** | **G** | **C** | **A** | **A** | **C** | **A** | **A** | **T** | **T** | **G** | **T** | **T** | **T** | **G** | **C** | **A** | **G** | **C** | **A** | **A** | **T** | **G** | **A** | **T** | **G** | **A** | **T** | **G** | **A** | **T** | **G** | **T** | **C** | **G** | **G** | **C** | **A** | **T** | **T** | **A** | **A** | **C** | **G** | **G** | **A** | **G** | **C** | **A** | **A** | **G** | **C** | **A** | **G** | **A** | **A** | **A** | **A** | **A** | **A** | **G** | **C** | **G** | **A** | **G** | **A** | **C** | **A** | **C** | **C** | **A** | **G** | **A** | **A** | **A** | **T** | **G** | **T** | **T** | **C** | **C** | **G** | **C** | **A** | **A** | **G** | **C** | **A** | **G** | **T** | **T** | **G** | **A** | **A** | **A** | **A** | **T** | **G** | **G** | **T** | **A** | **G** | **C** | **A** | **G** | **A** | **G** | **A** | **T** | **C** | **A** | **T** | **C** | **A** | **A** | **C** | **G** | **C** | **T** | **T** | **C** | **C** | **C** | **A** | **G** | **T** | **C** | **T** | **G** | **A** | **G** | **A** | **T** | **G** | **C** | **G** | **T** | **G** | **C** | **G** | **C** | **T** | **G** | **C** | **T** | **T** | **C** | **T** | **T** | **C** | **A** | **T** | **C** | **T** | **G** | **C** | **G** | **T** | **C** | **C** | **A** | **G** | **T** | **C** | **A** | **A** | **T** | **T** | **T** | **A** | **G** | **A** | **A** | **G** | **A** | **G** | **A** | **A** | **A** | **A** | **G** | **T** | **T** | **T** | **A** | **A** | **A** | **G** | **C** | **A** | **A** | **G** | **G** | **T** | **A** | **T** | **C** | **G** | **A** | **G** | **C** | **A** | **G** | **T** | **T** | **A** | **T** | **T** | **G** | **A** | **A** | **G** | **G** | **A** | **A** | **T** | **T** | **G** | **C** | **A** | **A** | **A** | **A** | **T** | **A** | **A** | **A** | **A** | **T** | **C** | **C** | **A** | **G** | **A** | **T** | **T** | **T** | **C** | **G** | **C** | **T** | **G** | **A** | **A** | **A** | **T** | **G** | **G** | **G** | **A** | **T** | **G** | **T** | **A** | **G** | **A** | **A** | **G** | **A** | **T** | **G** | **T** | **A** | **A** | **A** | **A** | **C** | **T** | **A** | **T** | **C** | **T** | **A** | **G** | **T** | **T** | **C** | **C** | **A** | **T** | **T** | **G** | **A** | **G** | **G** | **A** | **T** | **C** | **A** | **C** | **T** | **T** | **G** | **T** | **T** | **C** | **C** | **G** | **A** | **A** | **T** | **C** | **G** | **T** | **T** | **C** | **A** | **A** | **G** | **A** | **A** | **T** | **T** | **G** | **T** | **T** | **A** | **T** | **C** | **A** | **A** | **A** | **T** | **A** | **C** | **A** | **T** | **T** | **A** | **A** | **G** | **A** | **C** | **A** | **T** | **G** | **C** | **T** | **A** | **A** | **G** | **G** | **C** | **T** | **A** | **A** | **T** | **G** | **A** | **A** | **T** | **T** | **A** | **G** | **A** | **A** | **G** | **T** | **C** | **T** | **A** | **T** | **T** | **T** | **G** | **C** | **A** | **T** | **A** | **A** | **A** | **A** | **T** | **A** | **G** | **A** | **T** | **A** | **A** | **C** | **A** | **A** | **T** | **C** | **T** | **T** | **T** | **T** | **A** | **T** | **T** | **A** | **C** | **G** | **T** | **A** | **T** | **C** | **A** | **T** | **T** | **G** | **A** | **C** | **G** | **A** | **C** | **G** | **G** | **G** | **A** | **C** | **A** | **G** | **G** | **A** | **T** | **T** | **T** | **A** | **A** | **T** | **A** | **T** | **G** | **A** | **A** | **T** | **G** | **A** | **A** | **A** | **C** | **C** | **A** | **A** | **A** | **A** | **C** | **G** | **G** | **G** | **A** | **A** | **G** | **T** | **T** | **A** | **T** | **G** | **G** | **A** | **T** | **T** | **G** | **A** | **A** | **C** | **A** | **A** | **T** | **A** | **T** | **C** | **A** | **A** | **A** | **G** | **A** | **A** | **C** | **G** | **A** | **G** | **T** | **A** | **G** | **C** | **T** | **G** | **G** | **T** | **A** | **T** | **T** | **G** | **G** | **T** | **G** | **G** | **A** | **A** | **C** | **A** | **G** | **T** | **A** | **A** | **A** | **A** | **A** | **T** | **C** | **A** | **T** | **T** | **A** | **G** | **T** | **T** | **T** | **T** | **A** | **A** | **A** | **G** | **G** | **G** | **C** | **A** | **A** | **G** | **G** | **T** | **A** | **C** | **G** | **A** | **G** | **C** | **G** | **T** | **G** | **G** | **A** | **G** | **A** | **T** | **C** | **A** | **A** | **G** | **G** | **T** | **C** | **C** | **C** | **T** | **T** | **T** | **G** | **A** | **T** | **G** | **A** | **A** | **G** | **G** | **A** | **G** | **G** | **C** | **A** | **-** | **-** | **-** |
 **SNP position** | . | . | . | . | . | . | . | . | . | . | 11 | . | . | . | 15 | . | . | . | . | 20 | . | . | . | 24 | . | . | . | . | . | . | . | . | . | . | . | . | . | . | . | . | . | . | . | . | . | . | . | . | . | . | . | . | . | . | . | . | . | . | . | . | . | . | . | . | . | 66 | . | . | . | . | . | . | . | . | . | . | . | . | . | . | . | . | . | . | . | . | . | . | . | . | . | . | . | . | . | . | . | . | 99 | . | . | 102 | . | . | . | . | . | 108 | . | . | . | . | . | . | . | . | . | . | . | . | . | . | . | . | . | 126 | . | . | . | . | . | . | . | . | 135 | . | . | . | . | . | . | . | . | . | . | . | 147 | . | . | . | . | . | . | . | . | . | . | . | . | . | . | . | . | . | . | . | . | . | 169 | . | 171 | . | . | 174 | . | . | . | . | . | 180 | . | . | . | . | . | . | . | . | . | . | . | . | . | . | 195 | . | . | 198 | . | . | 201 | . | . | . | . | . | . | . | . | 210 | . | . | . | . | . | . | . | . | . | . | . | . | . | . | . | . | . | . | 229 | . | . | . | . | . | . | . | . | . | 239 | . | . | . | . | . | . | . | . | . | . | . | . | . | . | . | . | . | . | 258 | . | . | . | . | . | . | . | . | 267 | . | . | . | . | . | . | . | . | . | . | . | . | . | . | . | . | . | . | . | . | . | . | . | . | 292 | . | 294 | . | . | . | . | 299 | . | 301 | 302 | 303 | . | . | . | . | . | . | . | . | . | . | . | 315 | 316 | . | 318 | . | . | . | . | . | 324 | . | . | . | 328 | . | 330 | . | . | . | . | . | . | . | . | . | . | . | . | . | . | . | . | . | . | . | 350 | 351 | . | . | . | . | . | 357 | . | . | . | . | 362 | 363 | . | . | . | . | . | . | . | . | . | . | . | . | . | . | . | . | . | 381 | . | . | . | . | . | . | . | . | 390 | . | . | . | . | . | . | . | . | . | . | . | 402 | . | . | . | 406 | . | 408 | . | . | 411 | . | . | . | . | . | . | . | . | 420 | . | . | . | . | . | . | . | . | . | . | . | . | . | . | 435 | . | . | . | . | . | . | . | . | . | . | . | . | . | . | 450 | . | . | . | . | . | . | . | . | . | . | . | . | . | . | . | . | . | 468 | . | . | . | . | . | . | . | . | . | . | . | 480 | . | . | . | . | . | . | . | . | 489 | . | . | . | . | . | . | . | . | . | . | . | . | . | . | 504 | . | . | 507 | . | . | . | 511 | . | 513 | . | . | . | . | . | . | . | . | 522 | . | . | . | . | . | . | . | . | . | . | . | . | . | . | . | . | . | . | . | . | 543 | . | . | . | . | . | . | . | . | . | . | . | . | . | . | . | . | . | . | . | . | 564 | . | . | . | . | . | . | 571 | . | . | . | 575 | . | . | . | . | . | . | 582 | . | . | 585 | . | . | . | . | . | . | . | . | . | . | . | . | . | . | . | . | . | 603 | . | . | . | . | . | . | . | . | . | . | . | . | . | . | . | . | . | . | . | . | . | . | . | . | . | . | . | . | . | . | . | . | . | . | . | . | . | . | . | . | . | . | . | . | . | . | . | . | . | . | . | . | . | 657 | . | . | . | . | . | . | . | . | . | . | . | . | . | . | 672 | . | . | . | . | . | 678 | . | . | . | . | . | . | . | . | . | . | . | 690 | . | . | . | . | . | 696 | . | . | 699 | . | . | . | . | . | 705 | . | . | . | . | . | . | . | . | . | . | . | . | . | . | . | . | . | 723 | . | . | . | . | . | . | . | . | 732 | . | . | . | . | . | . | . | . | . | . | . | 744 | . | . | . | . | . | . | 751 | . | . | 754 | . | . | . | . | . | . | . | . | . | . | . | . | . | . | 769 | . | . | 772 | . | . | . | . | 777 | . | . | . | . | . | . | . | . | . | . | . | . | . | . | . | . | . | . | . | . | . | . | . | . | . | . | . | . | . | . | . | . | . | . | . | . | . | . | . | . | . | . | . | . | . | . | . | 825 | . | . | . | . | . | . | . | . | 834 | . | . | . | . | . | 840 | . | . | 843 | . | . | 846 | . | . | . | . | . | . | . | . | . | . | . | 858 | . | . | 861 | . | . | . | . | . | . | . | . | 870 | . | . | . | . | . | . | . | . | 879 | . | . | 882 | . | . | . | . | . | . | . | . | 891 | . | . | 894 | . | . | . | . | . | 900 | . | . | . | . | . | . | . | . | 909 | . | . | 912 | . | . | . | . | . | 918 | . | . | . | . | . | . | . | . | . | . | . | . | . | . | . | . | . | 936 | . | . | . | . | . | . | . | . | . | . | . | . | . | . | . | . | . | . | . | . | . | . | . | . | . | . | . | . | . | . | . | . | . | . | . | . | . | . | . | . | . | . | . | 980 | . | . | . | . | . | . | . | . | . | . | . | . | . | . | . | . | . | . | . | . | . | . | . | . | . | . | . | . | . | . | . | . | . | . | . | . | . | . | . | 1020 | . | . | . | . | . | . | . | . | 1029 | . | . | 1032 | . | . | 1035 | . | . | 1038 | . | . | . | . | . | . | . | . | 1047 | . | . | . | . | . | . | . | . | . | . | . | . | . | . | . | . | . | . | . | . | . |

|  |
| --- |
|  |

|  |  |
| --- | --- |
|  |

|  |  |
| --- | --- |
| **Legend** | |
  | **Reference Sequence** |

  |
|  | **Detected SNPs** |
|  |  |
