# Supplementary material for: sraX: A Novel Comprehensive Resistome Analysis Tool
Source: Front Microbiol. 2020 Feb 5;11:52. doi: 10.3389/fmicb.2020.00052 (PMC7025521; doi:10.3389/fmicb.2020.00052)
Supplement: Supplementary file 4 [file Data_Sheet_3.ZIP › liaR/liaR_dna.html]

# liaR

|  |
| --- |
|  |

|  |
| --- |
|  |

|  |  |
| --- | --- |
| **Definition:** | LiaR is a response regulator found in the liaFSR signal transduction pathway. Mutations confer daptomycin resistance. |

|  |
| --- |
|  |

|  |
| --- |
|  |

|  |  |  |
| --- | --- | --- |
| **Reference organism:** | *Enterococcus faecalis V583* | [NCBI, CARD] |

|  |
| --- |
|  |

|  |
| --- |
|  |

|  |  |
| --- | --- |
| **Summary Data** | || **Parameter** | **Value_(n)_** || Total number of genomes | 198 |
| MSA length | 633 |
| Total number of SNPs | 205 |
| SNP density (# SNPs/MSA length) | 0.324 |

|  |
| --- |
|  |

|  |
| --- |
|  |

|  |
| --- |
|  |

|  |  |  |  |  |  |  |  |  |  |  |  |  |  |  |  |  |  |  |  |  |  |  |  |  |  |  |  |  |  |  |  |  |  |  |  |  |  |  |  |  |  |  |  |  |  |  |  |  |  |  |  |  |  |  |  |  |  |  |  |  |  |  |  |  |  |  |  |  |  |  |  |  |  |  |  |  |  |  |  |  |  |  |  |  |  |  |  |  |  |  |  |  |  |  |  |  |  |  |  |  |  |  |  |  |  |  |  |  |  |  |  |  |  |  |  |  |  |  |  |  |  |  |  |  |  |  |  |  |  |  |  |  |  |  |  |  |  |  |  |  |  |  |  |  |  |  |  |  |  |  |  |  |  |  |  |  |  |  |  |  |  |  |  |  |  |  |  |  |  |  |  |  |  |  |  |  |  |  |  |  |  |  |  |  |  |  |  |  |  |  |  |  |  |  |  |  |  |  |  |  |  |  |  |  |  |  |  |  |  |  |  |  |  |  |  |  |  |  |  |  |  |  |  |  |  |  |  |  |  |  |  |  |  |  |  |  |  |  |  |  |  |  |  |  |  |  |  |  |  |  |  |  |  |  |  |  |  |  |  |  |  |  |  |  |  |  |  |  |  |  |  |  |  |  |  |  |  |  |  |  |  |  |  |  |  |  |  |  |  |  |  |  |  |  |  |  |  |  |  |  |  |  |  |  |  |  |  |  |  |  |  |  |  |  |  |  |  |  |  |  |  |  |  |  |  |  |  |  |  |  |  |  |  |  |  |  |  |  |  |  |  |  |  |  |  |  |  |  |  |  |  |  |  |  |  |  |  |  |  |  |  |  |  |  |  |  |  |  |  |  |  |  |  |  |  |  |  |  |  |  |  |  |  |  |  |  |  |  |  |  |  |  |  |  |  |  |  |  |  |  |  |  |  |  |  |  |  |  |  |  |  |  |  |  |  |  |  |  |  |  |  |  |  |  |  |  |  |  |  |  |  |  |  |  |  |  |  |  |  |  |  |  |  |  |  |  |  |  |  |  |  |  |  |  |  |  |  |  |  |  |  |  |  |  |  |  |  |  |  |  |  |  |  |  |  |  |  |  |  |  |  |  |  |  |  |  |  |  |  |  |  |  |  |  |  |  |  |  |  |  |  |  |  |  |  |  |  |  |  |  |  |  |  |  |  |  |  |  |  |  |  |  |  |  |  |  |  |  |  |  |  |  |  |  |  |  |  |  |  |  |  |  |  |  |  |  |  |  |  |  |  |  |  |  |  |  |  |  |  |  |  |  |  |  |  |  |  |  |  |  |  |  |  |  |  |  |  |  |  |  |  |  |  |  |  |  |  |  |  |  |  |  |  |  |  |  |  |  |  |  |  |  |  |  |  |  |  |  |  |  |  |  |  |  |  |  |  |  |  |  |  |  |  |  |  |  |  |  |  |  |  |  |  |  |
| --- | --- | --- | --- | --- | --- | --- | --- | --- | --- | --- | --- | --- | --- | --- | --- | --- | --- | --- | --- | --- | --- | --- | --- | --- | --- | --- | --- | --- | --- | --- | --- | --- | --- | --- | --- | --- | --- | --- | --- | --- | --- | --- | --- | --- | --- | --- | --- | --- | --- | --- | --- | --- | --- | --- | --- | --- | --- | --- | --- | --- | --- | --- | --- | --- | --- | --- | --- | --- | --- | --- | --- | --- | --- | --- | --- | --- | --- | --- | --- | --- | --- | --- | --- | --- | --- | --- | --- | --- | --- | --- | --- | --- | --- | --- | --- | --- | --- | --- | --- | --- | --- | --- | --- | --- | --- | --- | --- | --- | --- | --- | --- | --- | --- | --- | --- | --- | --- | --- | --- | --- | --- | --- | --- | --- | --- | --- | --- | --- | --- | --- | --- | --- | --- | --- | --- | --- | --- | --- | --- | --- | --- | --- | --- | --- | --- | --- | --- | --- | --- | --- | --- | --- | --- | --- | --- | --- | --- | --- | --- | --- | --- | --- | --- | --- | --- | --- | --- | --- | --- | --- | --- | --- | --- | --- | --- | --- | --- | --- | --- | --- | --- | --- | --- | --- | --- | --- | --- | --- | --- | --- | --- | --- | --- | --- | --- | --- | --- | --- | --- | --- | --- | --- | --- | --- | --- | --- | --- | --- | --- | --- | --- | --- | --- | --- | --- | --- | --- | --- | --- | --- | --- | --- | --- | --- | --- | --- | --- | --- | --- | --- | --- | --- | --- | --- | --- | --- | --- | --- | --- | --- | --- | --- | --- | --- | --- | --- | --- | --- | --- | --- | --- | --- | --- | --- | --- | --- | --- | --- | --- | --- | --- | --- | --- | --- | --- | --- | --- | --- | --- | --- | --- | --- | --- | --- | --- | --- | --- | --- | --- | --- | --- | --- | --- | --- | --- | --- | --- | --- | --- | --- | --- | --- | --- | --- | --- | --- | --- | --- | --- | --- | --- | --- | --- | --- | --- | --- | --- | --- | --- | --- | --- | --- | --- | --- | --- | --- | --- | --- | --- | --- | --- | --- | --- | --- | --- | --- | --- | --- | --- | --- | --- | --- | --- | --- | --- | --- | --- | --- | --- | --- | --- | --- | --- | --- | --- | --- | --- | --- | --- | --- | --- | --- | --- | --- | --- | --- | --- | --- | --- | --- | --- | --- | --- | --- | --- | --- | --- | --- | --- | --- | --- | --- | --- | --- | --- | --- | --- | --- | --- | --- | --- | --- | --- | --- | --- | --- | --- | --- | --- | --- | --- | --- | --- | --- | --- | --- | --- | --- | --- | --- | --- | --- | --- | --- | --- | --- | --- | --- | --- | --- | --- | --- | --- | --- | --- | --- | --- | --- | --- | --- | --- | --- | --- | --- | --- | --- | --- | --- | --- | --- | --- | --- | --- | --- | --- | --- | --- | --- | --- | --- | --- | --- | --- | --- | --- | --- | --- | --- | --- | --- | --- | --- | --- | --- | --- | --- | --- | --- | --- | --- | --- | --- | --- | --- | --- | --- | --- | --- | --- | --- | --- | --- | --- | --- | --- | --- | --- | --- | --- | --- | --- | --- | --- | --- | --- | --- | --- | --- | --- | --- | --- | --- | --- | --- | --- | --- | --- | --- | --- | --- | --- | --- | --- | --- | --- | --- | --- | --- | --- | --- | --- | --- | --- | --- | --- | --- | --- | --- | --- | --- | --- | --- | --- | --- | --- | --- | --- | --- | --- | --- | --- | --- | --- | --- | --- | --- | --- | --- | --- | --- | --- | --- | --- | --- | --- | --- | --- | --- | --- | --- | --- | --- | --- | --- | --- | --- | --- | --- | --- | --- | --- | --- | --- | --- | --- | --- | --- | --- | --- | --- | --- | --- | --- | --- | --- | --- | --- | --- | --- | --- | --- | --- | --- | --- | --- | --- | --- | --- | --- | --- | --- | --- | --- | --- | --- | --- | --- | --- | --- | --- | --- | --- | --- | --- | --- | --- | --- | --- | --- | --- | --- | --- | --- | --- | --- | --- | --- | --- | --- | --- | --- | --- | --- | --- | --- | --- | --- | --- | --- | --- | --- | --- | --- | --- |
|  || **Enterococcus faecalis V583 (AAO82599.1)** | **G** | **T** | **G** | **A** | **T** | **C** | **A** | **A** | **A** | **G** | **T** | **A** | **A** | **T** | **G** | **T** | **T** | **A** | **G** | **T** | **G** | **G** | **A** | **T** | **G** | **A** | **C** | **C** | **A** | **T** | **G** | **A** | **A** | **A** | **T** | **G** | **G** | **T** | **C** | **C** | **G** | **T** | **T** | **T** | **A** | **G** | **G** | **C** | **G** | **T** | **T** | **T** | **C** | **A** | **T** | **C** | **A** | **T** | **A** | **T** | **T** | **T** | **A** | **T** | **C** | **T** | **A** | **T** | **T** | **C** | **A** | **A** | **G** | **A** | **G** | **G** | **A** | **T** | **A** | **T** | **A** | **G** | **A** | **A** | **G** | **T** | **C** | **G** | **T** | **A** | **G** | **G** | **C** | **G** | **A** | **A** | **G** | **C** | **A** | **G** | **A** | **A** | **A** | **A** | **C** | **G** | **G** | **T** | **A** | **A** | **G** | **A** | **T** | **T** | **G** | **G** | **C** | **T** | **A** | **T** | **G** | **A** | **A** | **A** | **A** | **A** | **G** | **C** | **A** | **T** | **T** | **G** | **G** | **A** | **A** | **C** | **T** | **A** | **C** | **G** | **T** | **C** | **C** | **A** | **G** | **A** | **T** | **G** | **T** | **T** | **A** | **T** | **T** | **T** | **T** | **G** | **A** | **T** | **G** | **G** | **A** | **T** | **T** | **T** | **G** | **G** | **T** | **A** | **A** | **T** | **G** | **G** | **A** | **A** | **G** | **A** | **A** | **A** | **T** | **G** | **G** | **A** | **C** | **G** | **G** | **C** | **A** | **T** | **T** | **G** | **A** | **T** | **T** | **C** | **A** | **A** | **C** | **A** | **A** | **A** | **A** | **G** | **C** | **G** | **A** | **T** | **C** | **T** | **T** | **G** | **A** | **A** | **A** | **G** | **A** | **T** | **T** | **G** | **G** | **C** | **C** | **A** | **G** | **A** | **A** | **G** | **C** | **C** | **A** | **A** | **G** | **A** | **T** | **T** | **A** | **T** | **T** | **A** | **T** | **T** | **G** | **T** | **G** | **A** | **C** | **G** | **A** | **G** | **T** | **T** | **T** | **T** | **A** | **T** | **T** | **G** | **A** | **T** | **G** | **A** | **T** | **G** | **A** | **A** | **A** | **A** | **A** | **G** | **T** | **G** | **T** | **A** | **T** | **C** | **C** | **G** | **G** | **C** | **G** | **A** | **T** | **T** | **G** | **A** | **A** | **G** | **C** | **T** | **G** | **G** | **T** | **G** | **C** | **A** | **G** | **C** | **G** | **G** | **G** | **C** | **T** | **A** | **C** | **C** | **T** | **A** | **T** | **T** | **A** | **A** | **A** | **G** | **A** | **C** | **A** | **T** | **C** | **A** | **A** | **C** | **A** | **G** | **C** | **A** | **C** | **A** | **T** | **G** | **A** | **G** | **A** | **T** | **T** | **G** | **C** | **T** | **G** | **A** | **T** | **G** | **C** | **A** | **A** | **T** | **T** | **C** | **G** | **G** | **G** | **C** | **G** | **A** | **C** | **T** | **T** | **A** | **T** | **C** | **G** | **C** | **G** | **G** | **A** | **G** | **A** | **G** | **C** | **G** | **T** | **G** | **T** | **G** | **T** | **T** | **G** | **G** | **A** | **A** | **C** | **C** | **T** | **G** | **A** | **A** | **G** | **T** | **G** | **A** | **C** | **G** | **C** | **A** | **T** | **A** | **A** | **G** | **A** | **T** | **G** | **A** | **T** | **G** | **G** | **A** | **A** | **C** | **G** | **G** | **T** | **T** | **A** | **A** | **C** | **A** | **A** | **A** | **A** | **A** | **A** | **A** | **C** | **A** | **A** | **G** | **A** | **G** | **C** | **C** | **G** | **G** | **T** | **G** | **T** | **T** | **G** | **C** | **A** | **C** | **G** | **A** | **A** | **G** | **A** | **T** | **T** | **T** | **G** | **A** | **C** | **A** | **A** | **A** | **C** | **C** | **G** | **G** | **G** | **A** | **A** | **C** | **A** | **C** | **G** | **A** | **A** | **A** | **T** | **T** | **T** | **T** | **A** | **A** | **T** | **G** | **T** | **T** | **G** | **A** | **T** | **T** | **G** | **C** | **A** | **C** | **A** | **A** | **G** | **G** | **T** | **A** | **A** | **A** | **A** | **G** | **T** | **A** | **A** | **T** | **C** | **A** | **G** | **G** | **A** | **A** | **A** | **T** | **A** | **G** | **C** | **T** | **G** | **A** | **T** | **G** | **A** | **A** | **C** | **T** | **C** | **T** | **T** | **T** | **A** | **T** | **C** | **A** | **C** | **T** | **T** | **T** | **G** | **A** | **A** | **A** | **A** | **C** | **A** | **G** | **T** | **T** | **A** | **A** | **A** | **A** | **C** | **A** | **C** | **A** | **T** | **G** | **T** | **T** | **T** | **C** | **A** | **A** | **A** | **C** | **A** | **T** | **T** | **T** | **T** | **A** | **G** | **C** | **A** | **A** | **A** | **A** | **C** | **T** | **A** | **G** | **A** | **T** | **G** | **T** | **G** | **G** | **A** | **T** | **G** | **A** | **T** | **C** | **G** | **G** | **A** | **C** | **C** | **C** | **A** | **A** | **G** | **C** | **G** | **G** | **C** | **G** | **A** | **T** | **T** | **T** | **A** | **T** | **G** | **C** | **T** | **T** | **T** | **T** | **C** | **A** | **A** | **C** | **A** | **T** | **G** | **G** | **T** | **T** | **T** | **A** | **G** | **C** | **C** | **A** | **A** | **A** | **T** | **A** | **A** |
| **N59862F** | **G** | **T** | **G** | **A** | **T** | **C** | **A** | **A** | **A** | **G** | **T** | **A** | **A** | **T** | **G** | **T** | **T** | **A** | **G** | **T** | **G** | **G** | **A** | **T** | **G** | **A** | **C** | **C** | **A** | **T** | **G** | **A** | **A** | **A** | **T** | **G** | **G** | **T** | **C** | **C** | **G** | **T** | **T** | **T** | **A** | **G** | **G** | **C** | **G** | **T** | **T** | **T** | **C** | **A** | **T** | **C** | **A** | **T** | **A** | **T** | **T** | **T** | **A** | **T** | **C** | **T** | **A** | **T** | **T** | **C** | **A** | **A** | **G** | **A** | **G** | **G** | **A** | **T** | **A** | **T** | **A** | **G** | **A** | **A** | **G** | **T** | **C** | **G** | **T** | **A** | **G** | **G** | **C** | **G** | **A** | **A** | **G** | **C** | **A** | **G** | **A** | **A** | **A** | **A** | **C** | **G** | **G** | **T** | **A** | **A** | **G** | **A** | **T** | **T** | **G** | **G** | **C** | **T** | **A** | **T** | **G** | **A** | **A** | **A** | **A** | **A** | **G** | **C** | **A** | **T** | **T** | **G** | **G** | **A** | **A** | **C** | **T** | **A** | **C** | **G** | **T** | **C** | **C** | **A** | **G** | **A** | **T** | **G** | **T** | **T** | **A** | **T** | **T** | **T** | **T** | **G** | **A** | **T** | **G** | **G** | **A** | **T** | **T** | **T** | **G** | **G** | **T** | **A** | **A** | **T** | **G** | **G** | **A** | **A** | **G** | **A** | **A** | **A** | **T** | **G** | **G** | **A** | **T** | **G** | **G** | **C** | **A** | **T** | **T** | **G** | **A** | **T** | **T** | **C** | **A** | **A** | **C** | **C** | **A** | **A** | **A** | **G** | **C** | **G** | **A** | **T** | **C** | **T** | **T** | **G** | **A** | **A** | **A** | **G** | **A** | **T** | **T** | **G** | **G** | **C** | **C** | **A** | **G** | **A** | **A** | **G** | **C** | **C** | **A** | **A** | **G** | **A** | **T** | **T** | **A** | **T** | **T** | **A** | **T** | **T** | **G** | **T** | **G** | **A** | **C** | **G** | **A** | **G** | **T** | **T** | **T** | **T** | **A** | **T** | **T** | **G** | **A** | **T** | **G** | **A** | **T** | **G** | **A** | **A** | **A** | **A** | **A** | **G** | **T** | **G** | **T** | **A** | **T** | **C** | **C** | **G** | **G** | **C** | **G** | **A** | **T** | **T** | **G** | **A** | **A** | **G** | **C** | **T** | **G** | **G** | **T** | **G** | **C** | **A** | **G** | **C** | **G** | **G** | **G** | **C** | **T** | **A** | **C** | **C** | **T** | **A** | **T** | **T** | **A** | **A** | **A** | **A** | **A** | **C** | **A** | **T** | **C** | **A** | **A** | **C** | **A** | **G** | **C** | **A** | **C** | **A** | **T** | **G** | **A** | **G** | **A** | **T** | **T** | **G** | **C** | **T** | **G** | **A** | **T** | **G** | **C** | **A** | **A** | **T** | **T** | **C** | **G** | **G** | **G** | **C** | **G** | **A** | **C** | **T** | **T** | **A** | **T** | **C** | **G** | **C** | **G** | **G** | **A** | **G** | **A** | **G** | **C** | **G** | **T** | **G** | **T** | **G** | **T** | **T** | **G** | **G** | **A** | **G** | **C** | **C** | **T** | **G** | **A** | **A** | **G** | **T** | **G** | **A** | **C** | **G** | **C** | **A** | **T** | **A** | **A** | **G** | **A** | **T** | **G** | **A** | **T** | **G** | **G** | **A** | **A** | **C** | **G** | **G** | **T** | **T** | **A** | **A** | **C** | **A** | **A** | **A** | **A** | **A** | **A** | **A** | **C** | **A** | **A** | **G** | **A** | **G** | **C** | **C** | **G** | **G** | **T** | **G** | **T** | **T** | **G** | **C** | **A** | **C** | **G** | **A** | **A** | **G** | **A** | **T** | **T** | **T** | **G** | **A** | **C** | **A** | **A** | **A** | **C** | **C** | **G** | **G** | **G** | **A** | **A** | **C** | **A** | **C** | **G** | **A** | **A** | **A** | **T** | **T** | **T** | **T** | **A** | **A** | **T** | **G** | **T** | **T** | **G** | **A** | **T** | **T** | **G** | **C** | **A** | **C** | **A** | **A** | **G** | **G** | **T** | **A** | **A** | **A** | **A** | **G** | **T** | **A** | **A** | **T** | **C** | **A** | **G** | **G** | **A** | **A** | **A** | **T** | **A** | **G** | **C** | **T** | **G** | **A** | **T** | **G** | **A** | **A** | **C** | **T** | **C** | **T** | **T** | **T** | **A** | **T** | **C** | **A** | **C** | **T** | **T** | **T** | **G** | **A** | **A** | **A** | **A** | **C** | **A** | **G** | **T** | **T** | **A** | **A** | **A** | **A** | **C** | **A** | **C** | **A** | **T** | **G** | **T** | **T** | **T** | **C** | **A** | **A** | **A** | **C** | **A** | **T** | **T** | **T** | **T** | **A** | **G** | **C** | **A** | **A** | **A** | **A** | **C** | **T** | **A** | **G** | **A** | **T** | **G** | **T** | **G** | **G** | **A** | **T** | **G** | **A** | **T** | **C** | **G** | **G** | **A** | **C** | **C** | **C** | **A** | **A** | **G** | **C** | **G** | **G** | **C** | **G** | **A** | **T** | **T** | **T** | **A** | **T** | **G** | **C** | **T** | **T** | **T** | **T** | **C** | **A** | **A** | **C** | **A** | **T** | **G** | **G** | **T** | **T** | **T** | **A** | **G** | **C** | **C** | **A** | **A** | **A** | **-** | **-** | **-** |
| **N55724** | **G** | **T** | **G** | **A** | **T** | **C** | **A** | **A** | **A** | **G** | **T** | **A** | **A** | **T** | **G** | **T** | **T** | **A** | **G** | **T** | **G** | **G** | **A** | **T** | **G** | **A** | **C** | **C** | **A** | **T** | **G** | **A** | **A** | **A** | **T** | **G** | **G** | **T** | **C** | **C** | **G** | **T** | **T** | **T** | **A** | **G** | **G** | **C** | **G** | **T** | **T** | **T** | **C** | **A** | **T** | **C** | **A** | **T** | **A** | **T** | **T** | **T** | **A** | **T** | **C** | **T** | **A** | **T** | **T** | **C** | **A** | **A** | **G** | **A** | **G** | **G** | **A** | **T** | **A** | **T** | **A** | **G** | **A** | **A** | **G** | **T** | **C** | **G** | **T** | **A** | **G** | **G** | **C** | **G** | **A** | **A** | **G** | **C** | **A** | **G** | **A** | **A** | **A** | **A** | **C** | **G** | **G** | **T** | **A** | **A** | **G** | **A** | **T** | **T** | **G** | **G** | **C** | **T** | **A** | **T** | **G** | **A** | **A** | **A** | **A** | **A** | **G** | **C** | **A** | **T** | **T** | **G** | **G** | **A** | **A** | **C** | **T** | **A** | **C** | **G** | **T** | **C** | **C** | **A** | **G** | **A** | **T** | **G** | **T** | **T** | **A** | **T** | **T** | **T** | **T** | **G** | **A** | **T** | **G** | **G** | **A** | **T** | **T** | **T** | **G** | **G** | **T** | **A** | **A** | **T** | **G** | **G** | **A** | **A** | **G** | **A** | **A** | **A** | **T** | **G** | **G** | **A** | **C** | **G** | **G** | **C** | **A** | **T** | **T** | **G** | **A** | **T** | **T** | **C** | **A** | **A** | **C** | **A** | **A** | **A** | **A** | **G** | **C** | **G** | **A** | **T** | **C** | **T** | **T** | **G** | **A** | **A** | **A** | **G** | **A** | **T** | **T** | **G** | **G** | **C** | **C** | **A** | **G** | **A** | **A** | **G** | **C** | **C** | **A** | **A** | **G** | **A** | **T** | **T** | **A** | **T** | **T** | **A** | **T** | **T** | **G** | **T** | **G** | **A** | **C** | **G** | **A** | **G** | **T** | **T** | **T** | **T** | **A** | **T** | **T** | **G** | **A** | **T** | **G** | **A** | **T** | **G** | **A** | **A** | **A** | **A** | **A** | **G** | **T** | **G** | **T** | **A** | **T** | **C** | **C** | **G** | **G** | **C** | **G** | **A** | **T** | **T** | **G** | **A** | **A** | **G** | **C** | **T** | **G** | **G** | **T** | **G** | **C** | **A** | **G** | **C** | **G** | **G** | **G** | **C** | **T** | **A** | **C** | **C** | **T** | **A** | **T** | **T** | **A** | **A** | **A** | **A** | **A** | **C** | **A** | **T** | **C** | **A** | **A** | **C** | **A** | **G** | **C** | **A** | **C** | **A** | **T** | **G** | **A** | **G** | **A** | **T** | **T** | **G** | **C** | **T** | **G** | **A** | **T** | **G** | **C** | **A** | **A** | **T** | **T** | **C** | **G** | **G** | **G** | **C** | **G** | **A** | **C** | **T** | **T** | **A** | **T** | **C** | **G** | **C** | **G** | **G** | **A** | **G** | **A** | **G** | **C** | **G** | **T** | **G** | **T** | **G** | **T** | **T** | **G** | **G** | **A** | **A** | **C** | **C** | **T** | **G** | **A** | **A** | **G** | **T** | **G** | **A** | **C** | **G** | **C** | **A** | **T** | **A** | **A** | **G** | **A** | **T** | **G** | **A** | **T** | **G** | **G** | **A** | **A** | **C** | **G** | **G** | **T** | **T** | **A** | **A** | **C** | **A** | **A** | **A** | **A** | **A** | **A** | **A** | **C** | **A** | **A** | **G** | **A** | **G** | **C** | **C** | **G** | **G** | **T** | **G** | **T** | **T** | **G** | **C** | **A** | **C** | **G** | **A** | **A** | **G** | **A** | **T** | **T** | **T** | **G** | **A** | **C** | **A** | **A** | **A** | **C** | **C** | **G** | **G** | **G** | **A** | **A** | **C** | **A** | **C** | **G** | **A** | **A** | **A** | **T** | **T** | **T** | **T** | **A** | **A** | **T** | **G** | **T** | **T** | **G** | **A** | **T** | **T** | **G** | **C** | **A** | **C** | **A** | **A** | **G** | **G** | **T** | **A** | **A** | **A** | **A** | **G** | **T** | **A** | **A** | **T** | **C** | **A** | **G** | **G** | **A** | **A** | **A** | **T** | **A** | **G** | **C** | **T** | **G** | **A** | **T** | **G** | **A** | **A** | **C** | **T** | **C** | **T** | **T** | **T** | **A** | **T** | **C** | **A** | **C** | **T** | **T** | **T** | **G** | **A** | **A** | **A** | **A** | **C** | **A** | **G** | **T** | **T** | **A** | **A** | **A** | **A** | **C** | **A** | **C** | **A** | **T** | **G** | **T** | **T** | **T** | **C** | **A** | **A** | **A** | **C** | **A** | **T** | **T** | **T** | **T** | **A** | **G** | **C** | **A** | **A** | **A** | **A** | **C** | **T** | **A** | **G** | **A** | **T** | **G** | **T** | **G** | **G** | **A** | **T** | **G** | **A** | **T** | **C** | **G** | **G** | **A** | **C** | **C** | **C** | **A** | **A** | **G** | **C** | **G** | **G** | **C** | **G** | **A** | **T** | **T** | **T** | **A** | **T** | **G** | **C** | **T** | **T** | **T** | **T** | **C** | **A** | **A** | **C** | **A** | **T** | **G** | **G** | **T** | **T** | **T** | **A** | **G** | **C** | **C** | **A** | **A** | **A** | **-** | **-** | **-** |
| **N60336F** | **A** | **T** | **G** | **A** | **T** | **A** | **A** | **A** | **A** | **G** | **T** | **T** | **T** | **T** | **A** | **T** | **T** | **A** | **G** | **T** | **A** | **G** | **A** | **T** | **G** | **A** | **C** | **C** | **A** | **T** | **G** | **A** | **A** | **A** | **T** | **G** | **G** | **T** | **A** | **C** | **G** | **C** | **T** | **T** | **A** | **G** | **G** | **C** | **G** | **T** | **C** | **T** | **C** | **T** | **T** | **C** | **T** | **T** | **A** | **T** | **T** | **T** | **A** | **T** | **C** | **T** | **A** | **T** | **C** | **C** | **A** | **A** | **G** | **A** | **A** | **G** | **A** | **C** | **A** | **T** | **T** | **G** | **A** | **A** | **G** | **T** | **G** | **A** | **T** | **T** | **G** | **G** | **A** | **G** | **A** | **A** | **G** | **C** | **T** | **G** | **A** | **A** | **A** | **A** | **T** | **G** | **G** | **A** | **C** | **G** | **T** | **C** | **A** | **A** | **G** | **G** | **C** | **T** | **A** | **T** | **G** | **A** | **A** | **A** | **A** | **G** | **G** | **C** | **G** | **A** | **T** | **G** | **G** | **C** | **A** | **C** | **T** | **T** | **C** | **G** | **A** | **C** | **C** | **A** | **G** | **A** | **T** | **G** | **T** | **C** | **A** | **T** | **T** | **T** | **T** | **A** | **A** | **T** | **G** | **G** | **A** | **T** | **C** | **T** | **A** | **G** | **T** | **C** | **A** | **T** | **G** | **G** | **A** | **A** | **G** | **A** | **A** | **A** | **T** | **G** | **G** | **A** | **T** | **G** | **G** | **C** | **A** | **T** | **C** | **G** | **A** | **A** | **T** | **C** | **A** | **A** | **C** | **A** | **A** | **A** | **A** | **G** | **C** | **A** | **A** | **T** | **C** | **T** | **T** | **A** | **A** | **A** | **A** | **G** | **A** | **T** | **T** | **G** | **G** | **C** | **C** | **A** | **A** | **A** | **A** | **G** | **C** | **C** | **A** | **A** | **A** | **A** | **T** | **C** | **A** | **T** | **T** | **A** | **T** | **C** | **G** | **T** | **C** | **A** | **C** | **A** | **A** | **G** | **T** | **T** | **T** | **T** | **A** | **T** | **T** | **G** | **A** | **T** | **G** | **A** | **T** | **G** | **A** | **A** | **A** | **A** | **A** | **G** | **T** | **T** | **T** | **A** | **T** | **C** | **C** | **T** | **G** | **C** | **A** | **A** | **T** | **T** | **G** | **A** | **A** | **G** | **C** | **A** | **G** | **G** | **A** | **G** | **C** | **A** | **G** | **C** | **A** | **G** | **G** | **A** | **T** | **A** | **T** | **T** | **T** | **G** | **T** | **T** | **G** | **A** | **A** | **G** | **A** | **C** | **G** | **T** | **C** | **T** | **A** | **C** | **C** | **G** | **C** | **A** | **C** | **A** | **T** | **G** | **A** | **A** | **A** | **T** | **A** | **G** | **C** | **T** | **G** | **A** | **T** | **G** | **C** | **T** | **A** | **T** | **A** | **A** | **G** | **G** | **G** | **C** | **T** | **A** | **C** | **G** | **C** | **A** | **A** | **C** | **G** | **C** | **G** | **G** | **C** | **G** | **A** | **G** | **C** | **G** | **A** | **G** | **T** | **A** | **T** | **T** | **G** | **G** | **A** | **A** | **C** | **C** | **T** | **G** | **A** | **A** | **G** | **T** | **G** | **A** | **C** | **A** | **A** | **C** | **A** | **A** | **A** | **A** | **A** | **T** | **G** | **A** | **T** | **G** | **G** | **A** | **A** | **A** | **A** | **A** | **A** | **T** | **G** | **A** | **G** | **T** | **C** | **G** | **G** | **C** | **G** | **A** | **A** | **A** | **T** | **G** | **A** | **T** | **C** | **C** | **A** | **G** | **T** | **A** | **T** | **T** | **G** | **C** | **A** | **T** | **G** | **A** | **A** | **G** | **A** | **A** | **T** | **T** | **A** | **A** | **C** | **G** | **A** | **A** | **T** | **C** | **G** | **A** | **G** | **A** | **A** | **A** | **A** | **T** | **G** | **A** | **A** | **A** | **T** | **T** | **T** | **T** | **G** | **A** | **T** | **G** | **C** | **T** | **G** | **A** | **T** | **T** | **T** | **C** | **T** | **G** | **A** | **A** | **G** | **G** | **G** | **A** | **A** | **A** | **A** | **G** | **T** | **A** | **A** | **T** | **C** | **A** | **G** | **G** | **A** | **A** | **A** | **T** | **C** | **G** | **C** | **G** | **G** | **A** | **T** | **G** | **A** | **A** | **T** | **T** | **G** | **T** | **T** | **T** | **A** | **T** | **C** | **A** | **C** | **A** | **C** | **T** | **G** | **A** | **A** | **A** | **A** | **C** | **A** | **G** | **T** | **T** | **A** | **A** | **A** | **A** | **C** | **G** | **C** | **A** | **T** | **G** | **T** | **G** | **T** | **C** | **G** | **A** | **A** | **T** | **A** | **T** | **A** | **T** | **T** | **A** | **G** | **C** | **T** | **A** | **A** | **G** | **T** | **T** | **A** | **G** | **A** | **G** | **G** | **T** | **A** | **G** | **A** | **A** | **G** | **A** | **C** | **C** | **G** | **A** | **A** | **C** | **T** | **C** | **A** | **A** | **G** | **C** | **A** | **G** | **C** | **T** | **A** | **T** | **C** | **T** | **A** | **T** | **G** | **C** | **A** | **T** | **T** | **T** | **A** | **A** | **A** | **C** | **A** | **T** | **G** | **G** | **T** | **T** | **T** | **G** | **G** | **T** | **G** | **A** | **A** | **A** | **-** | **-** | **-** |
| **N55279** | **A** | **T** | **G** | **A** | **T** | **A** | **A** | **A** | **A** | **G** | **T** | **T** | **T** | **T** | **A** | **T** | **T** | **A** | **G** | **T** | **A** | **G** | **A** | **T** | **G** | **A** | **C** | **C** | **A** | **T** | **G** | **A** | **A** | **A** | **T** | **G** | **G** | **T** | **G** | **C** | **G** | **C** | **T** | **T** | **A** | **G** | **G** | **C** | **G** | **T** | **C** | **T** | **C** | **T** | **T** | **C** | **T** | **T** | **A** | **T** | **T** | **T** | **A** | **T** | **C** | **T** | **A** | **T** | **T** | **C** | **A** | **A** | **G** | **A** | **A** | **G** | **A** | **C** | **A** | **T** | **T** | **G** | **A** | **A** | **G** | **T** | **A** | **A** | **T** | **T** | **G** | **G** | **A** | **G** | **A** | **A** | **G** | **C** | **T** | **G** | **A** | **A** | **A** | **A** | **T** | **G** | **G** | **A** | **C** | **G** | **A** | **C** | **A** | **A** | **G** | **G** | **C** | **T** | **A** | **T** | **G** | **A** | **G** | **A** | **A** | **G** | **G** | **C** | **G** | **A** | **T** | **G** | **A** | **C** | **A** | **C** | **T** | **T** | **C** | **G** | **A** | **C** | **C** | **G** | **G** | **A** | **C** | **G** | **T** | **C** | **A** | **T** | **T** | **T** | **T** | **A** | **A** | **T** | **G** | **G** | **A** | **C** | **T** | **T** | **A** | **G** | **T** | **C** | **A** | **T** | **G** | **G** | **A** | **A** | **G** | **A** | **A** | **A** | **T** | **G** | **G** | **A** | **T** | **G** | **G** | **T** | **A** | **T** | **C** | **G** | **A** | **A** | **T** | **C** | **A** | **A** | **C** | **A** | **A** | **A** | **A** | **G** | **C** | **A** | **A** | **T** | **C** | **T** | **T** | **A** | **A** | **A** | **A** | **G** | **A** | **T** | **T** | **G** | **G** | **C** | **C** | **A** | **G** | **A** | **A** | **G** | **C** | **T** | **A** | **A** | **A** | **A** | **T** | **C** | **A** | **T** | **T** | **A** | **T** | **C** | **G** | **T** | **A** | **A** | **C** | **C** | **A** | **G** | **T** | **T** | **T** | **T** | **A** | **T** | **T** | **G** | **A** | **T** | **G** | **A** | **C** | **G** | **A** | **A** | **A** | **A** | **A** | **G** | **T** | **T** | **T** | **A** | **T** | **C** | **C** | **T** | **G** | **C** | **C** | **A** | **T** | **T** | **G** | **A** | **A** | **G** | **C** | **A** | **G** | **G** | **T** | **G** | **C** | **A** | **G** | **C** | **A** | **G** | **G** | **G** | **T** | **A** | **T** | **T** | **T** | **G** | **T** | **T** | **G** | **A** | **A** | **G** | **A** | **C** | **C** | **T** | **C** | **T** | **A** | **C** | **C** | **G** | **C** | **A** | **C** | **A** | **T** | **G** | **A** | **A** | **A** | **T** | **T** | **G** | **C** | **T** | **G** | **A** | **T** | **G** | **C** | **T** | **A** | **T** | **A** | **A** | **G** | **G** | **G** | **C** | **A** | **A** | **C** | **G** | **C** | **A** | **A** | **C** | **G** | **T** | **G** | **G** | **C** | **G** | **A** | **G** | **C** | **G** | **A** | **G** | **T** | **A** | **T** | **T** | **A** | **G** | **A** | **A** | **C** | **C** | **A** | **G** | **A** | **A** | **G** | **T** | **G** | **A** | **C** | **A** | **A** | **C** | **A** | **A** | **A** | **A** | **A** | **T** | **G** | **A** | **T** | **G** | **G** | **A** | **A** | **A** | **A** | **A** | **A** | **T** | **G** | **A** | **G** | **T** | **C** | **G** | **G** | **C** | **G** | **A** | **A** | **A** | **T** | **G** | **A** | **G** | **C** | **C** | **T** | **G** | **T** | **A** | **T** | **T** | **A** | **C** | **A** | **T** | **G** | **A** | **A** | **G** | **A** | **A** | **C** | **T** | **A** | **A** | **C** | **G** | **A** | **A** | **T** | **C** | **G** | **G** | **G** | **A** | **A** | **A** | **A** | **T** | **G** | **A** | **A** | **A** | **T** | **T** | **T** | **T** | **A** | **A** | **T** | **G** | **C** | **T** | **G** | **A** | **T** | **T** | **T** | **C** | **T** | **G** | **A** | **A** | **G** | **G** | **A** | **A** | **A** | **A** | **A** | **G** | **T** | **A** | **A** | **T** | **C** | **A** | **G** | **G** | **A** | **A** | **A** | **T** | **A** | **G** | **C** | **G** | **G** | **A** | **C** | **G** | **A** | **A** | **T** | **T** | **G** | **T** | **T** | **T** | **A** | **T** | **T** | **A** | **C** | **A** | **C** | **T** | **G** | **A** | **A** | **A** | **A** | **C** | **A** | **G** | **T** | **A** | **A** | **A** | **A** | **A** | **C** | **A** | **C** | **A** | **T** | **G** | **T** | **T** | **T** | **C** | **C** | **A** | **A** | **T** | **A** | **T** | **A** | **T** | **T** | **G** | **G** | **C** | **T** | **A** | **A** | **G** | **C** | **T** | **A** | **G** | **A** | **A** | **G** | **T** | **G** | **G** | **A** | **A** | **G** | **A** | **C** | **C** | **G** | **T** | **A** | **C** | **A** | **C** | **A** | **A** | **G** | **C** | **C** | **G** | **C** | **T** | **A** | **T** | **C** | **T** | **A** | **T** | **G** | **C** | **A** | **T** | **T** | **C** | **A** | **A** | **A** | **C** | **A** | **T** | **G** | **G** | **T** | **T** | **T** | **G** | **G** | **T** | **G** | **A** | **A** | **A** | **-** | **-** | **-** |
| **N55337** | **A** | **T** | **G** | **A** | **T** | **A** | **A** | **A** | **A** | **G** | **T** | **T** | **T** | **T** | **A** | **T** | **T** | **A** | **G** | **T** | **A** | **G** | **A** | **T** | **G** | **A** | **C** | **C** | **A** | **T** | **G** | **A** | **A** | **A** | **T** | **G** | **G** | **T** | **A** | **C** | **G** | **C** | **T** | **T** | **A** | **G** | **G** | **C** | **G** | **T** | **C** | **T** | **C** | **T** | **T** | **C** | **T** | **T** | **A** | **T** | **T** | **T** | **A** | **T** | **C** | **T** | **A** | **T** | **C** | **C** | **A** | **A** | **G** | **A** | **A** | **G** | **A** | **C** | **A** | **T** | **T** | **G** | **A** | **A** | **G** | **T** | **G** | **A** | **T** | **T** | **G** | **G** | **A** | **G** | **A** | **A** | **G** | **C** | **T** | **G** | **A** | **A** | **A** | **A** | **T** | **G** | **G** | **A** | **C** | **G** | **T** | **C** | **A** | **A** | **G** | **G** | **C** | **T** | **A** | **T** | **G** | **A** | **A** | **A** | **A** | **G** | **G** | **C** | **G** | **A** | **T** | **G** | **G** | **C** | **A** | **C** | **T** | **T** | **C** | **G** | **A** | **C** | **C** | **A** | **G** | **A** | **T** | **G** | **T** | **C** | **A** | **T** | **T** | **T** | **T** | **A** | **A** | **T** | **G** | **G** | **A** | **T** | **C** | **T** | **A** | **G** | **T** | **C** | **A** | **T** | **G** | **G** | **A** | **A** | **G** | **A** | **A** | **A** | **T** | **G** | **G** | **A** | **T** | **G** | **G** | **C** | **A** | **T** | **C** | **G** | **A** | **A** | **T** | **C** | **A** | **A** | **C** | **A** | **A** | **A** | **A** | **G** | **C** | **A** | **A** | **T** | **C** | **T** | **T** | **A** | **A** | **A** | **A** | **G** | **A** | **T** | **T** | **G** | **G** | **C** | **C** | **A** | **A** | **A** | **A** | **G** | **C** | **C** | **A** | **A** | **A** | **A** | **T** | **C** | **A** | **T** | **T** | **A** | **T** | **C** | **G** | **T** | **C** | **A** | **C** | **A** | **A** | **G** | **T** | **T** | **T** | **T** | **A** | **T** | **T** | **G** | **A** | **T** | **G** | **A** | **T** | **G** | **A** | **A** | **A** | **A** | **A** | **G** | **T** | **T** | **T** | **A** | **T** | **C** | **C** | **T** | **G** | **C** | **A** | **A** | **T** | **T** | **G** | **A** | **A** | **G** | **C** | **A** | **G** | **G** | **A** | **G** | **C** | **A** | **G** | **C** | **A** | **G** | **G** | **A** | **T** | **A** | **T** | **T** | **T** | **A** | **T** | **T** | **G** | **A** | **A** | **G** | **A** | **C** | **G** | **T** | **C** | **T** | **A** | **C** | **C** | **G** | **C** | **A** | **C** | **A** | **T** | **G** | **A** | **A** | **A** | **T** | **A** | **G** | **C** | **T** | **G** | **A** | **T** | **G** | **C** | **T** | **A** | **T** | **A** | **A** | **G** | **G** | **G** | **C** | **T** | **A** | **C** | **G** | **C** | **A** | **A** | **C** | **G** | **C** | **G** | **G** | **C** | **G** | **A** | **G** | **C** | **G** | **A** | **G** | **T** | **A** | **T** | **T** | **G** | **G** | **A** | **A** | **C** | **C** | **T** | **G** | **A** | **A** | **G** | **T** | **G** | **A** | **C** | **A** | **A** | **C** | **A** | **A** | **A** | **A** | **A** | **T** | **G** | **A** | **T** | **G** | **G** | **A** | **A** | **A** | **A** | **A** | **A** | **T** | **G** | **A** | **G** | **T** | **C** | **G** | **G** | **C** | **G** | **A** | **A** | **A** | **T** | **G** | **A** | **T** | **C** | **C** | **A** | **G** | **T** | **A** | **T** | **T** | **G** | **C** | **A** | **T** | **G** | **A** | **A** | **G** | **A** | **A** | **T** | **T** | **A** | **A** | **C** | **G** | **A** | **A** | **T** | **C** | **G** | **A** | **G** | **A** | **A** | **A** | **A** | **T** | **G** | **A** | **A** | **A** | **T** | **T** | **T** | **T** | **G** | **A** | **T** | **G** | **C** | **T** | **G** | **A** | **T** | **T** | **T** | **C** | **T** | **G** | **A** | **A** | **G** | **G** | **G** | **A** | **A** | **A** | **A** | **G** | **T** | **A** | **A** | **T** | **C** | **A** | **G** | **G** | **A** | **A** | **A** | **T** | **C** | **G** | **C** | **G** | **G** | **A** | **T** | **G** | **A** | **A** | **T** | **T** | **G** | **T** | **T** | **T** | **A** | **T** | **C** | **A** | **C** | **A** | **C** | **T** | **G** | **A** | **A** | **A** | **A** | **C** | **A** | **G** | **T** | **T** | **A** | **A** | **A** | **A** | **C** | **G** | **C** | **A** | **T** | **G** | **T** | **G** | **T** | **C** | **G** | **A** | **A** | **T** | **A** | **T** | **A** | **T** | **T** | **A** | **G** | **C** | **T** | **A** | **A** | **G** | **T** | **T** | **A** | **G** | **A** | **G** | **G** | **T** | **A** | **G** | **A** | **A** | **G** | **A** | **C** | **C** | **G** | **A** | **A** | **C** | **T** | **C** | **A** | **A** | **G** | **C** | **A** | **G** | **C** | **T** | **A** | **T** | **C** | **T** | **A** | **T** | **G** | **C** | **G** | **T** | **T** | **T** | **A** | **A** | **A** | **C** | **A** | **T** | **G** | **G** | **T** | **T** | **T** | **G** | **G** | **T** | **T** | **A** | **A** | **A** | **-** | **-** | **-** |
| **N59505F** | **G** | **T** | **G** | **A** | **T** | **C** | **A** | **A** | **A** | **G** | **T** | **A** | **A** | **T** | **G** | **T** | **T** | **A** | **G** | **T** | **G** | **G** | **A** | **T** | **G** | **A** | **C** | **C** | **A** | **T** | **G** | **A** | **A** | **A** | **T** | **G** | **G** | **T** | **C** | **C** | **G** | **T** | **T** | **T** | **A** | **G** | **G** | **C** | **G** | **T** | **T** | **T** | **C** | **A** | **T** | **C** | **A** | **T** | **A** | **T** | **T** | **T** | **A** | **T** | **C** | **T** | **A** | **T** | **T** | **C** | **A** | **A** | **G** | **A** | **G** | **G** | **A** | **T** | **A** | **T** | **A** | **G** | **A** | **A** | **G** | **T** | **C** | **G** | **T** | **A** | **G** | **G** | **C** | **G** | **A** | **A** | **G** | **C** | **A** | **G** | **A** | **A** | **A** | **A** | **C** | **G** | **G** | **T** | **A** | **A** | **G** | **A** | **T** | **T** | **G** | **G** | **C** | **T** | **A** | **T** | **G** | **A** | **A** | **A** | **A** | **A** | **G** | **C** | **A** | **T** | **T** | **G** | **G** | **A** | **A** | **C** | **T** | **A** | **C** | **G** | **T** | **C** | **C** | **A** | **G** | **A** | **T** | **G** | **T** | **T** | **A** | **T** | **T** | **T** | **T** | **G** | **A** | **T** | **G** | **G** | **A** | **T** | **T** | **T** | **G** | **G** | **T** | **A** | **A** | **T** | **G** | **G** | **A** | **A** | **G** | **A** | **A** | **A** | **T** | **G** | **G** | **A** | **C** | **G** | **G** | **C** | **A** | **T** | **T** | **G** | **A** | **T** | **T** | **C** | **A** | **A** | **C** | **A** | **A** | **A** | **A** | **G** | **C** | **G** | **A** | **T** | **C** | **T** | **T** | **G** | **A** | **A** | **A** | **G** | **A** | **T** | **T** | **G** | **G** | **C** | **C** | **A** | **G** | **A** | **A** | **G** | **C** | **C** | **A** | **A** | **G** | **A** | **T** | **T** | **A** | **T** | **T** | **A** | **T** | **T** | **G** | **T** | **G** | **A** | **C** | **G** | **A** | **G** | **T** | **T** | **T** | **T** | **A** | **T** | **T** | **G** | **A** | **T** | **G** | **A** | **T** | **G** | **A** | **G** | **A** | **A** | **A** | **G** | **T** | **G** | **T** | **A** | **T** | **C** | **C** | **G** | **G** | **C** | **G** | **A** | **T** | **T** | **G** | **A** | **A** | **G** | **C** | **T** | **G** | **G** | **T** | **G** | **C** | **A** | **G** | **C** | **G** | **G** | **G** | **C** | **T** | **A** | **C** | **C** | **T** | **A** | **T** | **T** | **A** | **A** | **A** | **A** | **A** | **C** | **A** | **T** | **C** | **A** | **A** | **C** | **A** | **G** | **C** | **A** | **C** | **A** | **T** | **G** | **A** | **G** | **A** | **T** | **T** | **G** | **C** | **T** | **G** | **A** | **T** | **G** | **C** | **A** | **A** | **T** | **T** | **C** | **G** | **G** | **G** | **C** | **G** | **A** | **C** | **T** | **T** | **A** | **T** | **C** | **G** | **C** | **G** | **G** | **A** | **G** | **A** | **G** | **C** | **G** | **T** | **G** | **T** | **G** | **T** | **T** | **G** | **G** | **A** | **A** | **C** | **C** | **T** | **G** | **A** | **A** | **G** | **T** | **G** | **A** | **C** | **G** | **C** | **A** | **T** | **A** | **A** | **G** | **A** | **T** | **G** | **A** | **T** | **G** | **G** | **A** | **A** | **C** | **G** | **G** | **T** | **T** | **A** | **A** | **C** | **A** | **A** | **A** | **A** | **A** | **A** | **A** | **C** | **A** | **A** | **G** | **A** | **G** | **C** | **C** | **G** | **G** | **T** | **G** | **T** | **T** | **G** | **C** | **A** | **T** | **G** | **A** | **A** | **G** | **A** | **T** | **T** | **T** | **G** | **A** | **C** | **A** | **A** | **A** | **T** | **C** | **G** | **A** | **G** | **A** | **A** | **C** | **A** | **C** | **G** | **A** | **A** | **A** | **T** | **T** | **T** | **T** | **A** | **A** | **T** | **G** | **T** | **T** | **G** | **A** | **T** | **T** | **G** | **C** | **A** | **C** | **A** | **A** | **G** | **G** | **T** | **A** | **A** | **A** | **A** | **G** | **T** | **A** | **A** | **T** | **C** | **A** | **G** | **G** | **A** | **A** | **A** | **T** | **A** | **G** | **C** | **T** | **G** | **A** | **T** | **G** | **A** | **A** | **C** | **T** | **C** | **T** | **T** | **T** | **A** | **T** | **C** | **A** | **C** | **T** | **T** | **T** | **G** | **A** | **A** | **A** | **A** | **C** | **A** | **G** | **T** | **T** | **A** | **A** | **A** | **A** | **C** | **A** | **C** | **A** | **T** | **G** | **T** | **T** | **T** | **C** | **A** | **A** | **A** | **C** | **A** | **T** | **T** | **T** | **T** | **A** | **G** | **C** | **A** | **A** | **A** | **A** | **C** | **T** | **A** | **G** | **A** | **T** | **G** | **T** | **G** | **G** | **A** | **T** | **G** | **A** | **T** | **C** | **G** | **G** | **A** | **C** | **C** | **C** | **A** | **A** | **G** | **C** | **G** | **G** | **C** | **G** | **A** | **T** | **T** | **T** | **A** | **T** | **G** | **C** | **T** | **T** | **T** | **T** | **C** | **A** | **A** | **C** | **A** | **T** | **G** | **G** | **T** | **T** | **T** | **A** | **G** | **C** | **C** | **A** | **A** | **A** | **-** | **-** | **-** |
| **N53655** | **A** | **T** | **G** | **A** | **T** | **A** | **A** | **A** | **A** | **G** | **T** | **T** | **T** | **T** | **A** | **T** | **T** | **A** | **G** | **T** | **A** | **G** | **A** | **T** | **G** | **A** | **C** | **C** | **A** | **T** | **G** | **A** | **A** | **A** | **T** | **G** | **G** | **T** | **A** | **C** | **G** | **C** | **T** | **T** | **A** | **G** | **G** | **C** | **G** | **T** | **C** | **T** | **C** | **T** | **T** | **C** | **T** | **T** | **A** | **T** | **T** | **T** | **A** | **T** | **C** | **T** | **A** | **T** | **C** | **C** | **A** | **A** | **G** | **A** | **A** | **G** | **A** | **C** | **A** | **T** | **T** | **G** | **A** | **A** | **G** | **T** | **G** | **A** | **T** | **T** | **G** | **G** | **A** | **G** | **A** | **A** | **G** | **C** | **T** | **G** | **A** | **A** | **A** | **A** | **T** | **G** | **G** | **A** | **C** | **G** | **T** | **C** | **A** | **A** | **G** | **G** | **C** | **T** | **A** | **T** | **G** | **A** | **A** | **A** | **A** | **G** | **G** | **C** | **G** | **A** | **T** | **G** | **G** | **C** | **A** | **C** | **T** | **T** | **C** | **G** | **A** | **C** | **C** | **G** | **G** | **A** | **T** | **G** | **T** | **C** | **A** | **T** | **T** | **T** | **T** | **A** | **A** | **T** | **G** | **G** | **A** | **T** | **C** | **T** | **A** | **G** | **T** | **C** | **A** | **T** | **G** | **G** | **A** | **A** | **G** | **A** | **A** | **A** | **T** | **G** | **G** | **A** | **T** | **G** | **G** | **C** | **A** | **T** | **C** | **G** | **A** | **A** | **T** | **C** | **A** | **A** | **C** | **A** | **A** | **A** | **A** | **G** | **C** | **A** | **A** | **T** | **C** | **T** | **T** | **A** | **A** | **A** | **A** | **G** | **A** | **T** | **T** | **G** | **G** | **C** | **C** | **A** | **A** | **A** | **A** | **G** | **C** | **C** | **A** | **A** | **A** | **A** | **T** | **C** | **A** | **T** | **T** | **A** | **T** | **C** | **G** | **T** | **C** | **A** | **C** | **A** | **A** | **G** | **T** | **T** | **T** | **T** | **A** | **T** | **T** | **G** | **A** | **T** | **G** | **A** | **T** | **G** | **A** | **A** | **A** | **A** | **A** | **G** | **T** | **T** | **T** | **A** | **T** | **C** | **C** | **T** | **G** | **C** | **A** | **A** | **T** | **T** | **G** | **A** | **A** | **G** | **C** | **A** | **G** | **G** | **A** | **G** | **C** | **A** | **G** | **C** | **A** | **G** | **G** | **A** | **T** | **A** | **T** | **T** | **T** | **G** | **T** | **T** | **G** | **A** | **A** | **G** | **A** | **C** | **G** | **T** | **C** | **T** | **A** | **C** | **C** | **G** | **C** | **A** | **C** | **A** | **T** | **G** | **A** | **A** | **A** | **T** | **A** | **G** | **C** | **T** | **G** | **A** | **T** | **G** | **C** | **T** | **A** | **T** | **A** | **A** | **G** | **G** | **G** | **C** | **T** | **A** | **C** | **G** | **C** | **A** | **A** | **C** | **G** | **C** | **G** | **G** | **C** | **G** | **A** | **G** | **C** | **G** | **A** | **G** | **T** | **A** | **T** | **T** | **G** | **G** | **A** | **A** | **C** | **C** | **T** | **G** | **A** | **A** | **G** | **T** | **G** | **A** | **C** | **A** | **A** | **C** | **A** | **A** | **A** | **A** | **A** | **T** | **G** | **A** | **T** | **G** | **G** | **A** | **A** | **A** | **A** | **A** | **A** | **T** | **G** | **A** | **G** | **T** | **C** | **G** | **G** | **C** | **G** | **A** | **A** | **A** | **T** | **G** | **A** | **T** | **C** | **C** | **A** | **G** | **T** | **A** | **T** | **T** | **G** | **C** | **A** | **T** | **G** | **A** | **A** | **G** | **A** | **A** | **T** | **T** | **A** | **A** | **C** | **G** | **A** | **A** | **T** | **C** | **G** | **A** | **G** | **A** | **A** | **A** | **A** | **T** | **G** | **A** | **A** | **A** | **T** | **T** | **T** | **T** | **G** | **A** | **T** | **G** | **C** | **T** | **G** | **A** | **T** | **T** | **T** | **C** | **T** | **G** | **A** | **A** | **G** | **G** | **G** | **A** | **A** | **A** | **A** | **G** | **T** | **A** | **A** | **T** | **C** | **A** | **G** | **G** | **A** | **A** | **A** | **T** | **C** | **G** | **C** | **G** | **G** | **A** | **T** | **G** | **A** | **A** | **T** | **T** | **G** | **T** | **T** | **T** | **A** | **T** | **C** | **A** | **C** | **A** | **C** | **T** | **G** | **A** | **A** | **A** | **A** | **C** | **A** | **G** | **T** | **C** | **A** | **A** | **A** | **A** | **C** | **G** | **C** | **A** | **T** | **G** | **T** | **G** | **T** | **C** | **G** | **A** | **A** | **T** | **A** | **T** | **A** | **T** | **T** | **A** | **G** | **C** | **T** | **A** | **A** | **G** | **T** | **T** | **A** | **G** | **A** | **G** | **G** | **T** | **A** | **G** | **A** | **A** | **G** | **A** | **C** | **C** | **G** | **A** | **A** | **C** | **T** | **C** | **A** | **A** | **G** | **C** | **A** | **G** | **C** | **T** | **A** | **T** | **C** | **T** | **A** | **T** | **G** | **C** | **G** | **T** | **T** | **T** | **A** | **A** | **A** | **C** | **A** | **T** | **G** | **G** | **T** | **T** | **T** | **G** | **G** | **T** | **G** | **A** | **A** | **A** | **-** | **-** | **-** |
| **N52649** | **A** | **T** | **G** | **A** | **T** | **A** | **A** | **A** | **A** | **G** | **T** | **T** | **T** | **T** | **A** | **T** | **T** | **A** | **G** | **T** | **A** | **G** | **A** | **T** | **G** | **A** | **C** | **C** | **A** | **T** | **G** | **A** | **A** | **A** | **T** | **G** | **G** | **T** | **A** | **C** | **G** | **C** | **T** | **T** | **A** | **G** | **G** | **C** | **G** | **T** | **C** | **T** | **C** | **T** | **T** | **C** | **T** | **T** | **A** | **T** | **T** | **T** | **A** | **T** | **C** | **T** | **A** | **T** | **C** | **C** | **A** | **A** | **G** | **A** | **A** | **G** | **A** | **C** | **A** | **T** | **T** | **G** | **A** | **A** | **G** | **T** | **G** | **A** | **T** | **T** | **G** | **G** | **A** | **G** | **A** | **A** | **G** | **C** | **T** | **G** | **A** | **A** | **A** | **A** | **T** | **G** | **G** | **A** | **C** | **G** | **T** | **C** | **A** | **A** | **G** | **G** | **C** | **T** | **A** | **T** | **G** | **A** | **A** | **A** | **A** | **G** | **G** | **C** | **G** | **A** | **T** | **G** | **G** | **C** | **A** | **C** | **T** | **T** | **C** | **G** | **A** | **C** | **C** | **A** | **G** | **A** | **T** | **G** | **T** | **C** | **A** | **T** | **T** | **T** | **T** | **A** | **A** | **T** | **G** | **G** | **A** | **T** | **C** | **T** | **A** | **G** | **T** | **C** | **A** | **T** | **G** | **G** | **A** | **A** | **G** | **A** | **A** | **A** | **T** | **G** | **G** | **A** | **T** | **G** | **G** | **C** | **A** | **T** | **C** | **G** | **A** | **A** | **T** | **C** | **A** | **A** | **C** | **A** | **A** | **A** | **A** | **G** | **C** | **A** | **A** | **T** | **C** | **T** | **T** | **A** | **A** | **A** | **A** | **G** | **A** | **T** | **T** | **G** | **G** | **C** | **C** | **A** | **A** | **A** | **A** | **G** | **C** | **C** | **A** | **A** | **A** | **A** | **T** | **C** | **A** | **T** | **T** | **A** | **T** | **T** | **G** | **T** | **C** | **A** | **C** | **A** | **A** | **G** | **T** | **T** | **T** | **T** | **A** | **T** | **T** | **G** | **A** | **T** | **G** | **A** | **T** | **G** | **A** | **A** | **A** | **A** | **A** | **G** | **T** | **T** | **T** | **A** | **T** | **C** | **C** | **T** | **G** | **C** | **A** | **A** | **T** | **T** | **G** | **A** | **A** | **G** | **C** | **A** | **G** | **G** | **A** | **G** | **C** | **A** | **G** | **C** | **A** | **G** | **G** | **A** | **T** | **A** | **T** | **T** | **T** | **A** | **T** | **T** | **G** | **A** | **A** | **G** | **A** | **C** | **G** | **T** | **C** | **T** | **A** | **C** | **C** | **G** | **C** | **A** | **C** | **A** | **T** | **G** | **A** | **A** | **A** | **T** | **A** | **G** | **C** | **T** | **G** | **A** | **T** | **G** | **C** | **T** | **A** | **T** | **A** | **A** | **G** | **G** | **G** | **C** | **T** | **A** | **C** | **G** | **C** | **A** | **A** | **C** | **G** | **C** | **G** | **G** | **C** | **G** | **A** | **G** | **C** | **G** | **A** | **G** | **T** | **A** | **C** | **T** | **A** | **G** | **A** | **A** | **C** | **C** | **A** | **G** | **A** | **A** | **G** | **T** | **G** | **A** | **C** | **A** | **A** | **C** | **A** | **A** | **A** | **A** | **A** | **T** | **G** | **A** | **T** | **G** | **G** | **A** | **A** | **A** | **A** | **A** | **A** | **T** | **G** | **A** | **G** | **T** | **C** | **G** | **G** | **C** | **G** | **A** | **A** | **A** | **T** | **G** | **A** | **T** | **C** | **C** | **A** | **G** | **T** | **A** | **T** | **T** | **G** | **C** | **A** | **T** | **G** | **A** | **A** | **G** | **A** | **A** | **T** | **T** | **A** | **A** | **C** | **G** | **A** | **A** | **T** | **C** | **G** | **A** | **G** | **A** | **A** | **A** | **A** | **T** | **G** | **A** | **A** | **A** | **T** | **T** | **T** | **T** | **G** | **A** | **T** | **G** | **C** | **T** | **G** | **A** | **T** | **T** | **T** | **C** | **T** | **G** | **A** | **A** | **G** | **G** | **G** | **A** | **A** | **A** | **A** | **G** | **T** | **A** | **A** | **T** | **C** | **A** | **G** | **G** | **A** | **A** | **A** | **T** | **C** | **G** | **C** | **G** | **G** | **A** | **T** | **G** | **A** | **A** | **T** | **T** | **G** | **T** | **T** | **T** | **A** | **T** | **C** | **A** | **C** | **A** | **C** | **T** | **G** | **A** | **A** | **A** | **A** | **C** | **A** | **G** | **T** | **T** | **A** | **A** | **A** | **A** | **C** | **G** | **C** | **A** | **T** | **G** | **T** | **G** | **T** | **C** | **G** | **A** | **A** | **T** | **A** | **T** | **A** | **T** | **T** | **A** | **G** | **C** | **T** | **A** | **A** | **G** | **T** | **T** | **A** | **G** | **A** | **G** | **G** | **T** | **A** | **G** | **A** | **A** | **G** | **A** | **C** | **C** | **G** | **A** | **A** | **C** | **T** | **C** | **A** | **A** | **G** | **C** | **A** | **G** | **C** | **T** | **A** | **T** | **C** | **T** | **A** | **T** | **G** | **C** | **G** | **T** | **T** | **T** | **A** | **A** | **A** | **C** | **A** | **T** | **G** | **G** | **T** | **T** | **T** | **G** | **G** | **T** | **G** | **A** | **A** | **A** | **-** | **-** | **-** |
| **N59592F** | **G** | **T** | **G** | **A** | **T** | **C** | **A** | **A** | **A** | **G** | **T** | **A** | **A** | **T** | **G** | **T** | **T** | **A** | **G** | **T** | **G** | **G** | **A** | **T** | **G** | **A** | **C** | **C** | **A** | **T** | **G** | **A** | **A** | **A** | **T** | **G** | **G** | **T** | **C** | **C** | **G** | **T** | **T** | **T** | **A** | **G** | **G** | **C** | **G** | **T** | **T** | **T** | **C** | **A** | **T** | **C** | **A** | **T** | **A** | **T** | **T** | **T** | **A** | **T** | **C** | **T** | **A** | **T** | **T** | **C** | **A** | **A** | **G** | **A** | **G** | **G** | **A** | **T** | **A** | **T** | **A** | **G** | **A** | **A** | **G** | **T** | **C** | **G** | **T** | **A** | **G** | **G** | **C** | **G** | **A** | **A** | **G** | **C** | **A** | **G** | **A** | **A** | **A** | **A** | **C** | **G** | **G** | **T** | **A** | **A** | **G** | **A** | **T** | **T** | **G** | **G** | **C** | **T** | **A** | **T** | **G** | **A** | **A** | **A** | **A** | **A** | **G** | **C** | **A** | **T** | **T** | **G** | **G** | **A** | **A** | **C** | **T** | **A** | **C** | **G** | **T** | **C** | **C** | **A** | **G** | **A** | **T** | **G** | **T** | **T** | **A** | **T** | **T** | **T** | **T** | **G** | **A** | **T** | **G** | **G** | **A** | **T** | **T** | **T** | **G** | **G** | **T** | **A** | **A** | **T** | **G** | **G** | **A** | **A** | **G** | **A** | **A** | **A** | **T** | **G** | **G** | **A** | **T** | **G** | **G** | **C** | **A** | **T** | **T** | **G** | **A** | **T** | **T** | **C** | **A** | **A** | **C** | **C** | **A** | **A** | **A** | **G** | **C** | **G** | **A** | **T** | **C** | **T** | **T** | **G** | **A** | **A** | **A** | **G** | **A** | **T** | **T** | **G** | **G** | **C** | **C** | **A** | **G** | **A** | **A** | **G** | **C** | **C** | **A** | **A** | **G** | **A** | **T** | **T** | **A** | **T** | **T** | **A** | **T** | **T** | **G** | **T** | **G** | **A** | **C** | **G** | **A** | **G** | **T** | **T** | **T** | **T** | **A** | **T** | **T** | **G** | **A** | **T** | **G** | **A** | **T** | **G** | **A** | **A** | **A** | **A** | **A** | **G** | **T** | **G** | **T** | **A** | **T** | **C** | **C** | **G** | **G** | **C** | **G** | **A** | **T** | **T** | **G** | **A** | **A** | **G** | **C** | **T** | **G** | **G** | **T** | **G** | **C** | **A** | **G** | **C** | **A** | **G** | **G** | **C** | **T** | **A** | **C** | **C** | **T** | **A** | **T** | **T** | **A** | **A** | **A** | **A** | **A** | **C** | **A** | **T** | **C** | **A** | **A** | **C** | **A** | **G** | **C** | **A** | **C** | **A** | **T** | **G** | **A** | **G** | **A** | **T** | **T** | **G** | **C** | **T** | **G** | **A** | **T** | **G** | **C** | **A** | **A** | **T** | **T** | **C** | **G** | **G** | **G** | **C** | **G** | **A** | **C** | **T** | **T** | **A** | **T** | **C** | **G** | **C** | **G** | **G** | **A** | **G** | **A** | **G** | **C** | **G** | **T** | **G** | **T** | **G** | **T** | **T** | **G** | **G** | **A** | **A** | **C** | **C** | **T** | **G** | **A** | **A** | **G** | **T** | **G** | **A** | **C** | **G** | **C** | **A** | **T** | **A** | **A** | **G** | **A** | **T** | **G** | **A** | **T** | **G** | **G** | **A** | **A** | **C** | **G** | **G** | **T** | **T** | **A** | **A** | **C** | **A** | **A** | **A** | **A** | **A** | **A** | **A** | **C** | **A** | **A** | **G** | **A** | **G** | **C** | **C** | **G** | **G** | **T** | **G** | **T** | **T** | **G** | **C** | **A** | **C** | **G** | **A** | **A** | **G** | **A** | **T** | **T** | **T** | **G** | **A** | **C** | **A** | **A** | **A** | **C** | **C** | **G** | **G** | **G** | **A** | **A** | **C** | **A** | **C** | **G** | **A** | **A** | **A** | **T** | **T** | **T** | **T** | **A** | **A** | **T** | **G** | **T** | **T** | **G** | **A** | **T** | **T** | **G** | **C** | **A** | **C** | **A** | **A** | **G** | **G** | **T** | **A** | **A** | **A** | **A** | **G** | **T** | **A** | **A** | **T** | **C** | **A** | **G** | **G** | **A** | **A** | **A** | **T** | **T** | **G** | **C** | **T** | **G** | **A** | **T** | **G** | **A** | **A** | **C** | **T** | **C** | **T** | **T** | **T** | **A** | **T** | **C** | **A** | **C** | **T** | **T** | **T** | **G** | **A** | **A** | **A** | **A** | **C** | **A** | **G** | **T** | **T** | **A** | **A** | **A** | **A** | **C** | **A** | **C** | **A** | **T** | **G** | **T** | **T** | **T** | **C** | **A** | **A** | **A** | **C** | **A** | **T** | **T** | **T** | **T** | **A** | **G** | **C** | **A** | **A** | **A** | **A** | **C** | **T** | **A** | **G** | **A** | **T** | **G** | **T** | **G** | **G** | **A** | **T** | **G** | **A** | **T** | **C** | **G** | **G** | **A** | **C** | **C** | **C** | **A** | **A** | **G** | **C** | **G** | **G** | **C** | **G** | **A** | **T** | **T** | **T** | **A** | **T** | **G** | **C** | **T** | **T** | **T** | **T** | **C** | **A** | **A** | **C** | **A** | **T** | **G** | **G** | **T** | **T** | **T** | **A** | **G** | **C** | **C** | **A** | **A** | **A** | **-** | **-** | **-** |
| **N60034F** | **G** | **T** | **G** | **A** | **T** | **C** | **A** | **A** | **A** | **G** | **T** | **A** | **A** | **T** | **G** | **T** | **T** | **A** | **G** | **T** | **G** | **G** | **A** | **T** | **G** | **A** | **C** | **C** | **A** | **T** | **G** | **A** | **A** | **A** | **T** | **G** | **G** | **T** | **C** | **C** | **G** | **T** | **T** | **T** | **A** | **G** | **G** | **C** | **G** | **T** | **T** | **T** | **C** | **A** | **T** | **C** | **A** | **T** | **A** | **T** | **T** | **T** | **A** | **T** | **C** | **T** | **A** | **T** | **T** | **C** | **A** | **A** | **G** | **A** | **G** | **G** | **A** | **T** | **A** | **T** | **A** | **G** | **A** | **A** | **G** | **T** | **C** | **G** | **T** | **A** | **G** | **G** | **C** | **G** | **A** | **A** | **G** | **C** | **A** | **G** | **A** | **A** | **A** | **A** | **C** | **G** | **G** | **T** | **A** | **A** | **G** | **A** | **T** | **T** | **G** | **G** | **C** | **T** | **A** | **T** | **G** | **A** | **A** | **A** | **A** | **A** | **G** | **C** | **A** | **T** | **T** | **G** | **G** | **A** | **A** | **C** | **T** | **A** | **C** | **G** | **T** | **C** | **C** | **A** | **G** | **A** | **T** | **G** | **T** | **T** | **A** | **T** | **T** | **T** | **T** | **G** | **A** | **T** | **G** | **G** | **A** | **T** | **T** | **T** | **G** | **G** | **T** | **A** | **A** | **T** | **G** | **G** | **A** | **A** | **G** | **A** | **A** | **A** | **T** | **G** | **G** | **A** | **C** | **G** | **G** | **C** | **A** | **T** | **T** | **G** | **A** | **T** | **T** | **C** | **A** | **A** | **C** | **A** | **A** | **A** | **A** | **G** | **C** | **G** | **A** | **T** | **C** | **T** | **T** | **G** | **A** | **A** | **A** | **G** | **A** | **T** | **T** | **G** | **G** | **C** | **C** | **A** | **G** | **A** | **A** | **G** | **C** | **C** | **A** | **A** | **G** | **A** | **T** | **T** | **A** | **T** | **T** | **A** | **T** | **T** | **G** | **T** | **G** | **A** | **C** | **G** | **A** | **G** | **T** | **T** | **T** | **T** | **A** | **T** | **T** | **G** | **A** | **T** | **G** | **A** | **T** | **G** | **A** | **A** | **A** | **A** | **A** | **G** | **T** | **G** | **T** | **A** | **T** | **C** | **C** | **G** | **G** | **C** | **G** | **A** | **T** | **T** | **G** | **A** | **A** | **G** | **C** | **T** | **G** | **G** | **T** | **G** | **C** | **A** | **G** | **C** | **G** | **G** | **G** | **C** | **T** | **A** | **C** | **C** | **T** | **A** | **T** | **T** | **A** | **A** | **A** | **A** | **A** | **C** | **A** | **T** | **C** | **A** | **A** | **C** | **A** | **G** | **C** | **A** | **C** | **A** | **T** | **G** | **A** | **G** | **A** | **T** | **T** | **G** | **C** | **T** | **G** | **A** | **T** | **G** | **C** | **A** | **A** | **T** | **T** | **C** | **G** | **G** | **G** | **C** | **G** | **A** | **C** | **T** | **T** | **A** | **T** | **C** | **G** | **C** | **G** | **G** | **A** | **G** | **A** | **G** | **C** | **G** | **T** | **G** | **T** | **G** | **T** | **T** | **G** | **G** | **A** | **A** | **C** | **C** | **T** | **G** | **A** | **A** | **G** | **T** | **G** | **A** | **C** | **G** | **C** | **A** | **T** | **A** | **A** | **G** | **A** | **T** | **G** | **A** | **T** | **G** | **G** | **A** | **A** | **C** | **G** | **G** | **T** | **T** | **A** | **A** | **C** | **A** | **A** | **A** | **A** | **A** | **A** | **A** | **C** | **A** | **A** | **G** | **A** | **G** | **C** | **C** | **G** | **G** | **T** | **G** | **T** | **T** | **G** | **C** | **A** | **C** | **G** | **A** | **A** | **G** | **A** | **T** | **T** | **T** | **G** | **A** | **C** | **A** | **A** | **A** | **C** | **C** | **G** | **A** | **G** | **A** | **A** | **C** | **A** | **C** | **G** | **A** | **A** | **A** | **T** | **T** | **T** | **T** | **A** | **A** | **T** | **G** | **T** | **T** | **G** | **A** | **T** | **T** | **G** | **C** | **A** | **C** | **A** | **A** | **G** | **G** | **T** | **A** | **A** | **A** | **A** | **G** | **T** | **A** | **A** | **T** | **C** | **A** | **G** | **G** | **A** | **A** | **A** | **T** | **T** | **G** | **C** | **T** | **G** | **A** | **T** | **G** | **A** | **A** | **C** | **T** | **C** | **T** | **T** | **T** | **A** | **T** | **C** | **A** | **C** | **T** | **T** | **T** | **G** | **A** | **A** | **A** | **A** | **C** | **A** | **G** | **T** | **T** | **A** | **A** | **A** | **A** | **C** | **A** | **C** | **A** | **T** | **G** | **T** | **T** | **T** | **C** | **A** | **A** | **A** | **C** | **A** | **T** | **T** | **T** | **T** | **A** | **G** | **C** | **A** | **A** | **A** | **A** | **C** | **T** | **A** | **G** | **A** | **T** | **G** | **T** | **G** | **G** | **A** | **T** | **G** | **A** | **T** | **C** | **G** | **G** | **A** | **C** | **C** | **C** | **A** | **A** | **G** | **C** | **G** | **G** | **C** | **G** | **A** | **T** | **T** | **T** | **A** | **T** | **G** | **C** | **T** | **T** | **T** | **T** | **C** | **A** | **A** | **C** | **A** | **T** | **G** | **G** | **T** | **T** | **T** | **A** | **G** | **C** | **C** | **A** | **A** | **A** | **-** | **-** | **-** |
| **N60100F** | **G** | **T** | **G** | **A** | **T** | **C** | **A** | **A** | **A** | **G** | **T** | **A** | **A** | **T** | **G** | **T** | **T** | **A** | **G** | **T** | **G** | **G** | **A** | **T** | **G** | **A** | **C** | **C** | **A** | **T** | **G** | **A** | **A** | **A** | **T** | **G** | **G** | **T** | **C** | **C** | **G** | **T** | **T** | **T** | **A** | **G** | **G** | **C** | **G** | **T** | **T** | **T** | **C** | **A** | **T** | **C** | **A** | **T** | **A** | **T** | **T** | **T** | **A** | **T** | **C** | **T** | **A** | **T** | **T** | **C** | **A** | **A** | **G** | **A** | **G** | **G** | **A** | **T** | **A** | **T** | **A** | **G** | **A** | **A** | **G** | **T** | **C** | **G** | **T** | **A** | **G** | **G** | **C** | **G** | **A** | **A** | **G** | **C** | **A** | **G** | **A** | **A** | **A** | **A** | **C** | **G** | **G** | **T** | **A** | **A** | **G** | **A** | **T** | **T** | **G** | **G** | **C** | **T** | **A** | **T** | **G** | **A** | **A** | **A** | **A** | **A** | **G** | **C** | **A** | **T** | **T** | **G** | **G** | **A** | **A** | **C** | **T** | **A** | **C** | **G** | **T** | **C** | **C** | **A** | **G** | **A** | **T** | **G** | **T** | **T** | **A** | **T** | **T** | **T** | **T** | **G** | **A** | **T** | **G** | **G** | **A** | **T** | **T** | **T** | **G** | **G** | **T** | **A** | **A** | **T** | **G** | **G** | **A** | **A** | **G** | **A** | **A** | **A** | **T** | **G** | **G** | **A** | **C** | **G** | **G** | **C** | **A** | **T** | **T** | **G** | **A** | **T** | **T** | **C** | **A** | **A** | **C** | **A** | **A** | **A** | **A** | **G** | **C** | **G** | **A** | **T** | **C** | **T** | **T** | **G** | **A** | **A** | **A** | **G** | **A** | **T** | **T** | **G** | **G** | **C** | **C** | **A** | **G** | **A** | **A** | **G** | **C** | **C** | **A** | **A** | **G** | **A** | **T** | **T** | **A** | **T** | **T** | **A** | **T** | **T** | **G** | **T** | **G** | **A** | **C** | **G** | **A** | **G** | **T** | **T** | **T** | **T** | **A** | **T** | **T** | **G** | **A** | **T** | **G** | **A** | **T** | **G** | **A** | **A** | **A** | **A** | **A** | **G** | **T** | **G** | **T** | **A** | **T** | **C** | **C** | **G** | **G** | **C** | **G** | **A** | **T** | **T** | **G** | **A** | **A** | **G** | **C** | **T** | **G** | **G** | **T** | **G** | **C** | **A** | **G** | **C** | **G** | **G** | **G** | **C** | **T** | **A** | **C** | **C** | **T** | **A** | **T** | **T** | **A** | **A** | **A** | **A** | **A** | **C** | **A** | **T** | **C** | **A** | **A** | **C** | **A** | **G** | **C** | **A** | **C** | **A** | **T** | **G** | **A** | **G** | **A** | **T** | **T** | **G** | **C** | **T** | **G** | **A** | **T** | **G** | **C** | **A** | **A** | **T** | **T** | **C** | **G** | **G** | **G** | **C** | **G** | **A** | **C** | **T** | **T** | **A** | **T** | **C** | **G** | **C** | **G** | **G** | **A** | **G** | **A** | **G** | **C** | **G** | **T** | **G** | **T** | **G** | **T** | **T** | **G** | **G** | **A** | **A** | **C** | **C** | **T** | **G** | **A** | **A** | **G** | **T** | **G** | **A** | **C** | **G** | **C** | **A** | **T** | **A** | **A** | **G** | **A** | **T** | **G** | **A** | **T** | **G** | **G** | **A** | **A** | **C** | **G** | **G** | **T** | **T** | **A** | **A** | **C** | **A** | **A** | **A** | **A** | **A** | **A** | **A** | **C** | **A** | **A** | **G** | **A** | **G** | **C** | **C** | **G** | **G** | **T** | **G** | **T** | **T** | **G** | **C** | **A** | **C** | **G** | **A** | **A** | **G** | **A** | **T** | **T** | **T** | **G** | **A** | **C** | **A** | **A** | **A** | **C** | **C** | **G** | **G** | **G** | **A** | **A** | **C** | **A** | **C** | **G** | **A** | **A** | **A** | **T** | **T** | **T** | **T** | **A** | **A** | **T** | **G** | **T** | **T** | **G** | **A** | **T** | **T** | **G** | **C** | **A** | **C** | **A** | **A** | **G** | **G** | **T** | **A** | **A** | **A** | **A** | **G** | **T** | **A** | **A** | **T** | **C** | **A** | **G** | **G** | **A** | **A** | **A** | **T** | **A** | **G** | **C** | **T** | **G** | **A** | **T** | **G** | **A** | **A** | **C** | **T** | **C** | **T** | **T** | **T** | **A** | **T** | **C** | **A** | **C** | **T** | **T** | **T** | **G** | **A** | **A** | **A** | **A** | **C** | **A** | **G** | **T** | **T** | **A** | **A** | **A** | **A** | **C** | **A** | **C** | **A** | **T** | **G** | **T** | **T** | **T** | **C** | **A** | **A** | **A** | **C** | **A** | **T** | **T** | **T** | **T** | **A** | **G** | **C** | **A** | **A** | **A** | **A** | **C** | **T** | **A** | **G** | **A** | **T** | **G** | **T** | **G** | **G** | **A** | **T** | **G** | **A** | **T** | **C** | **G** | **G** | **A** | **C** | **C** | **C** | **A** | **A** | **G** | **C** | **G** | **G** | **C** | **G** | **A** | **T** | **T** | **T** | **A** | **T** | **G** | **C** | **T** | **T** | **T** | **T** | **C** | **A** | **A** | **C** | **A** | **T** | **G** | **G** | **T** | **T** | **T** | **A** | **G** | **C** | **C** | **A** | **A** | **A** | **-** | **-** | **-** |
| **N59503F** | **A** | **T** | **G** | **A** | **T** | **A** | **A** | **A** | **A** | **G** | **T** | **T** | **T** | **T** | **A** | **T** | **T** | **A** | **G** | **T** | **A** | **G** | **A** | **T** | **G** | **A** | **C** | **C** | **A** | **T** | **G** | **A** | **A** | **A** | **T** | **G** | **G** | **T** | **A** | **C** | **G** | **C** | **T** | **T** | **A** | **G** | **G** | **C** | **G** | **T** | **C** | **T** | **C** | **T** | **T** | **C** | **T** | **T** | **A** | **T** | **T** | **T** | **A** | **T** | **C** | **T** | **A** | **T** | **C** | **C** | **A** | **A** | **G** | **A** | **A** | **G** | **A** | **C** | **A** | **T** | **T** | **G** | **A** | **A** | **G** | **T** | **G** | **A** | **T** | **T** | **G** | **G** | **A** | **G** | **A** | **A** | **G** | **C** | **T** | **G** | **A** | **A** | **A** | **A** | **T** | **G** | **G** | **A** | **C** | **G** | **T** | **C** | **A** | **A** | **G** | **G** | **C** | **T** | **A** | **T** | **G** | **A** | **A** | **A** | **A** | **G** | **G** | **C** | **G** | **A** | **T** | **G** | **G** | **C** | **A** | **C** | **T** | **T** | **C** | **G** | **A** | **C** | **C** | **A** | **G** | **A** | **T** | **G** | **T** | **C** | **A** | **T** | **T** | **T** | **T** | **A** | **A** | **T** | **G** | **G** | **A** | **T** | **C** | **T** | **A** | **G** | **T** | **C** | **A** | **T** | **G** | **G** | **A** | **A** | **G** | **A** | **A** | **A** | **T** | **G** | **G** | **A** | **T** | **G** | **G** | **C** | **A** | **T** | **C** | **G** | **A** | **A** | **T** | **C** | **A** | **A** | **C** | **A** | **A** | **A** | **A** | **G** | **C** | **A** | **A** | **T** | **C** | **T** | **T** | **A** | **A** | **A** | **A** | **G** | **A** | **T** | **T** | **G** | **G** | **C** | **C** | **A** | **A** | **A** | **A** | **G** | **C** | **C** | **A** | **A** | **A** | **A** | **T** | **C** | **A** | **T** | **T** | **A** | **T** | **C** | **G** | **T** | **C** | **A** | **C** | **A** | **A** | **G** | **T** | **T** | **T** | **T** | **A** | **T** | **T** | **G** | **A** | **T** | **G** | **A** | **T** | **G** | **A** | **A** | **A** | **A** | **A** | **G** | **T** | **T** | **T** | **A** | **T** | **C** | **C** | **T** | **G** | **C** | **A** | **A** | **T** | **T** | **G** | **A** | **A** | **G** | **C** | **A** | **G** | **G** | **A** | **G** | **C** | **A** | **G** | **C** | **A** | **G** | **G** | **A** | **T** | **A** | **T** | **T** | **T** | **G** | **T** | **T** | **G** | **A** | **A** | **G** | **A** | **C** | **G** | **T** | **C** | **T** | **A** | **C** | **C** | **G** | **C** | **A** | **C** | **A** | **T** | **G** | **A** | **A** | **A** | **T** | **A** | **G** | **C** | **T** | **G** | **A** | **T** | **G** | **C** | **T** | **A** | **T** | **A** | **A** | **G** | **G** | **G** | **C** | **T** | **A** | **C** | **G** | **C** | **A** | **A** | **C** | **G** | **C** | **G** | **G** | **C** | **G** | **A** | **G** | **C** | **G** | **A** | **G** | **T** | **A** | **T** | **T** | **G** | **G** | **A** | **A** | **C** | **C** | **T** | **G** | **A** | **A** | **G** | **T** | **G** | **A** | **C** | **A** | **A** | **C** | **A** | **A** | **A** | **A** | **A** | **T** | **G** | **A** | **T** | **G** | **G** | **A** | **A** | **A** | **A** | **A** | **A** | **T** | **G** | **A** | **G** | **T** | **C** | **G** | **G** | **C** | **G** | **A** | **T** | **A** | **T** | **G** | **A** | **T** | **C** | **C** | **A** | **G** | **T** | **A** | **T** | **T** | **G** | **C** | **A** | **T** | **G** | **A** | **A** | **G** | **A** | **A** | **T** | **T** | **A** | **A** | **C** | **G** | **A** | **A** | **T** | **C** | **G** | **A** | **G** | **A** | **A** | **A** | **A** | **T** | **G** | **A** | **A** | **A** | **T** | **T** | **T** | **T** | **G** | **A** | **T** | **G** | **C** | **T** | **G** | **A** | **T** | **T** | **T** | **C** | **T** | **G** | **A** | **A** | **G** | **G** | **G** | **A** | **A** | **A** | **A** | **G** | **T** | **A** | **A** | **T** | **C** | **A** | **G** | **G** | **A** | **A** | **A** | **T** | **C** | **G** | **C** | **G** | **G** | **A** | **T** | **G** | **A** | **A** | **T** | **T** | **G** | **T** | **T** | **T** | **A** | **T** | **C** | **A** | **C** | **A** | **C** | **T** | **G** | **A** | **A** | **A** | **A** | **C** | **A** | **G** | **T** | **T** | **A** | **A** | **A** | **A** | **C** | **G** | **C** | **A** | **T** | **G** | **T** | **G** | **T** | **C** | **G** | **A** | **A** | **T** | **A** | **T** | **A** | **T** | **T** | **A** | **G** | **C** | **T** | **A** | **A** | **G** | **T** | **T** | **A** | **G** | **A** | **G** | **G** | **T** | **A** | **G** | **A** | **A** | **G** | **A** | **C** | **C** | **G** | **A** | **A** | **C** | **T** | **C** | **A** | **A** | **G** | **C** | **A** | **G** | **C** | **T** | **A** | **T** | **C** | **T** | **A** | **T** | **G** | **C** | **A** | **T** | **T** | **T** | **A** | **A** | **A** | **C** | **A** | **T** | **G** | **G** | **T** | **T** | **T** | **G** | **G** | **T** | **G** | **A** | **A** | **A** | **-** | **-** | **-** |
| **N59611F** | **G** | **T** | **G** | **A** | **T** | **C** | **A** | **A** | **A** | **G** | **T** | **A** | **A** | **T** | **G** | **T** | **T** | **A** | **G** | **T** | **G** | **G** | **A** | **T** | **G** | **A** | **C** | **C** | **A** | **T** | **G** | **A** | **A** | **A** | **T** | **G** | **G** | **T** | **C** | **C** | **G** | **T** | **T** | **T** | **A** | **G** | **G** | **C** | **G** | **T** | **T** | **T** | **C** | **A** | **T** | **C** | **A** | **T** | **A** | **T** | **T** | **T** | **A** | **T** | **C** | **T** | **A** | **T** | **T** | **C** | **A** | **A** | **G** | **A** | **G** | **G** | **A** | **T** | **A** | **T** | **A** | **G** | **A** | **A** | **G** | **T** | **C** | **G** | **T** | **A** | **G** | **G** | **C** | **G** | **A** | **A** | **G** | **C** | **A** | **G** | **A** | **A** | **A** | **A** | **C** | **G** | **G** | **T** | **A** | **A** | **G** | **A** | **T** | **T** | **G** | **G** | **C** | **T** | **A** | **T** | **G** | **A** | **A** | **A** | **A** | **A** | **G** | **C** | **A** | **T** | **T** | **G** | **G** | **A** | **A** | **C** | **T** | **A** | **C** | **G** | **T** | **C** | **C** | **A** | **G** | **A** | **T** | **G** | **T** | **T** | **A** | **T** | **T** | **T** | **T** | **G** | **A** | **T** | **G** | **G** | **A** | **T** | **T** | **T** | **G** | **G** | **T** | **A** | **A** | **T** | **G** | **G** | **A** | **A** | **G** | **A** | **A** | **A** | **T** | **G** | **G** | **A** | **C** | **G** | **G** | **C** | **A** | **T** | **T** | **G** | **A** | **T** | **T** | **C** | **A** | **A** | **C** | **C** | **A** | **A** | **A** | **G** | **C** | **G** | **A** | **T** | **C** | **T** | **T** | **G** | **A** | **A** | **A** | **G** | **A** | **T** | **T** | **G** | **G** | **C** | **C** | **A** | **G** | **A** | **A** | **G** | **C** | **C** | **A** | **A** | **G** | **A** | **T** | **T** | **A** | **T** | **T** | **A** | **T** | **T** | **G** | **T** | **G** | **A** | **C** | **G** | **A** | **G** | **T** | **T** | **T** | **T** | **A** | **T** | **T** | **G** | **A** | **T** | **G** | **A** | **T** | **G** | **A** | **A** | **A** | **A** | **A** | **G** | **T** | **G** | **T** | **A** | **T** | **C** | **C** | **G** | **G** | **C** | **G** | **A** | **T** | **T** | **G** | **A** | **A** | **G** | **C** | **T** | **G** | **G** | **T** | **G** | **C** | **A** | **G** | **C** | **G** | **G** | **G** | **C** | **T** | **A** | **C** | **C** | **T** | **A** | **T** | **T** | **A** | **A** | **A** | **A** | **A** | **C** | **A** | **T** | **C** | **A** | **A** | **C** | **A** | **G** | **C** | **A** | **C** | **A** | **T** | **G** | **A** | **G** | **A** | **T** | **T** | **G** | **C** | **T** | **G** | **A** | **T** | **G** | **C** | **A** | **A** | **T** | **T** | **C** | **G** | **G** | **G** | **C** | **G** | **A** | **C** | **T** | **T** | **A** | **T** | **C** | **G** | **C** | **G** | **G** | **A** | **G** | **A** | **G** | **C** | **G** | **T** | **G** | **T** | **G** | **T** | **T** | **G** | **G** | **A** | **A** | **C** | **C** | **T** | **G** | **A** | **A** | **G** | **T** | **G** | **A** | **C** | **G** | **C** | **A** | **T** | **A** | **A** | **G** | **A** | **T** | **G** | **A** | **T** | **G** | **G** | **A** | **A** | **C** | **G** | **G** | **T** | **T** | **A** | **A** | **C** | **A** | **A** | **A** | **A** | **A** | **A** | **A** | **C** | **A** | **A** | **G** | **A** | **G** | **C** | **C** | **G** | **G** | **T** | **G** | **T** | **T** | **G** | **C** | **A** | **C** | **G** | **A** | **A** | **G** | **A** | **T** | **T** | **T** | **G** | **A** | **C** | **A** | **A** | **A** | **C** | **C** | **G** | **G** | **G** | **A** | **A** | **C** | **A** | **C** | **G** | **A** | **A** | **A** | **T** | **T** | **T** | **T** | **A** | **A** | **T** | **G** | **T** | **T** | **G** | **A** | **T** | **T** | **G** | **C** | **A** | **C** | **A** | **A** | **G** | **G** | **T** | **A** | **A** | **A** | **A** | **G** | **T** | **A** | **A** | **T** | **C** | **A** | **G** | **G** | **A** | **A** | **A** | **T** | **A** | **G** | **C** | **T** | **G** | **A** | **T** | **G** | **A** | **A** | **C** | **T** | **C** | **T** | **T** | **T** | **A** | **T** | **C** | **A** | **C** | **T** | **T** | **T** | **G** | **A** | **A** | **A** | **A** | **C** | **A** | **G** | **T** | **T** | **A** | **A** | **A** | **A** | **C** | **A** | **C** | **A** | **T** | **G** | **T** | **T** | **T** | **C** | **A** | **A** | **A** | **C** | **A** | **T** | **T** | **T** | **T** | **A** | **G** | **C** | **A** | **A** | **A** | **A** | **C** | **T** | **A** | **G** | **A** | **T** | **G** | **T** | **G** | **G** | **A** | **T** | **G** | **A** | **T** | **C** | **G** | **G** | **A** | **C** | **C** | **C** | **A** | **A** | **G** | **C** | **G** | **G** | **C** | **G** | **A** | **T** | **T** | **T** | **A** | **T** | **G** | **C** | **T** | **T** | **T** | **T** | **C** | **A** | **A** | **C** | **A** | **T** | **G** | **G** | **T** | **T** | **T** | **A** | **G** | **C** | **C** | **A** | **A** | **A** | **-** | **-** | **-** |
| **N60045F** | **A** | **T** | **G** | **A** | **T** | **A** | **A** | **A** | **A** | **G** | **T** | **T** | **T** | **T** | **A** | **T** | **T** | **A** | **G** | **T** | **A** | **G** | **A** | **T** | **G** | **A** | **C** | **C** | **A** | **T** | **G** | **A** | **A** | **A** | **T** | **G** | **G** | **T** | **A** | **C** | **G** | **C** | **T** | **T** | **A** | **G** | **G** | **C** | **G** | **T** | **C** | **T** | **C** | **T** | **T** | **C** | **T** | **T** | **A** | **T** | **T** | **T** | **A** | **T** | **C** | **T** | **A** | **T** | **C** | **C** | **A** | **A** | **G** | **A** | **A** | **G** | **A** | **C** | **A** | **T** | **T** | **G** | **A** | **A** | **G** | **T** | **G** | **A** | **T** | **T** | **G** | **G** | **A** | **G** | **A** | **A** | **G** | **C** | **T** | **G** | **A** | **A** | **A** | **A** | **T** | **G** | **G** | **A** | **C** | **G** | **T** | **C** | **A** | **A** | **G** | **G** | **C** | **T** | **A** | **T** | **G** | **A** | **A** | **A** | **A** | **G** | **G** | **C** | **G** | **A** | **T** | **G** | **G** | **C** | **A** | **C** | **T** | **T** | **C** | **G** | **A** | **C** | **C** | **A** | **G** | **A** | **T** | **G** | **T** | **C** | **A** | **T** | **T** | **T** | **T** | **A** | **A** | **T** | **G** | **G** | **A** | **T** | **C** | **T** | **A** | **G** | **T** | **C** | **A** | **T** | **G** | **G** | **A** | **A** | **G** | **A** | **A** | **A** | **T** | **G** | **G** | **A** | **T** | **G** | **G** | **C** | **A** | **T** | **C** | **G** | **A** | **A** | **T** | **C** | **A** | **A** | **C** | **A** | **A** | **A** | **A** | **G** | **C** | **A** | **A** | **T** | **C** | **T** | **T** | **A** | **A** | **A** | **A** | **G** | **A** | **T** | **T** | **G** | **G** | **C** | **C** | **A** | **A** | **A** | **A** | **G** | **C** | **C** | **A** | **A** | **A** | **A** | **T** | **C** | **A** | **T** | **T** | **A** | **T** | **C** | **G** | **T** | **C** | **A** | **C** | **A** | **A** | **G** | **T** | **T** | **T** | **T** | **A** | **T** | **T** | **G** | **A** | **T** | **G** | **A** | **T** | **G** | **A** | **A** | **A** | **A** | **A** | **G** | **T** | **T** | **T** | **A** | **T** | **C** | **C** | **T** | **G** | **C** | **A** | **A** | **T** | **T** | **G** | **A** | **A** | **G** | **C** | **A** | **G** | **G** | **A** | **G** | **C** | **A** | **G** | **C** | **A** | **G** | **G** | **A** | **T** | **A** | **T** | **T** | **T** | **G** | **T** | **T** | **G** | **A** | **A** | **G** | **A** | **C** | **G** | **T** | **C** | **T** | **A** | **C** | **C** | **G** | **C** | **A** | **C** | **A** | **T** | **G** | **A** | **A** | **A** | **T** | **A** | **G** | **C** | **T** | **G** | **A** | **T** | **G** | **C** | **T** | **A** | **T** | **A** | **A** | **G** | **G** | **G** | **C** | **T** | **A** | **C** | **G** | **C** | **A** | **A** | **C** | **G** | **C** | **G** | **G** | **C** | **G** | **A** | **G** | **C** | **G** | **A** | **G** | **T** | **A** | **T** | **T** | **G** | **G** | **A** | **A** | **C** | **C** | **T** | **G** | **A** | **A** | **G** | **T** | **G** | **A** | **C** | **A** | **A** | **C** | **A** | **A** | **A** | **A** | **A** | **T** | **G** | **A** | **T** | **G** | **G** | **A** | **A** | **A** | **A** | **A** | **A** | **T** | **G** | **A** | **G** | **T** | **C** | **G** | **G** | **C** | **G** | **A** | **A** | **A** | **T** | **G** | **A** | **T** | **C** | **C** | **A** | **G** | **T** | **A** | **T** | **T** | **G** | **C** | **A** | **T** | **G** | **A** | **A** | **G** | **A** | **A** | **T** | **T** | **A** | **A** | **C** | **G** | **A** | **A** | **T** | **C** | **G** | **A** | **G** | **A** | **A** | **A** | **A** | **T** | **G** | **A** | **A** | **A** | **T** | **T** | **T** | **T** | **G** | **A** | **T** | **G** | **C** | **T** | **G** | **A** | **T** | **T** | **T** | **C** | **T** | **G** | **A** | **A** | **G** | **G** | **G** | **A** | **A** | **A** | **A** | **G** | **T** | **A** | **A** | **T** | **C** | **A** | **G** | **G** | **A** | **A** | **A** | **T** | **C** | **G** | **C** | **G** | **G** | **A** | **T** | **G** | **A** | **A** | **T** | **T** | **G** | **T** | **T** | **T** | **A** | **T** | **C** | **A** | **C** | **A** | **C** | **T** | **G** | **A** | **A** | **A** | **A** | **C** | **A** | **G** | **T** | **T** | **A** | **A** | **A** | **A** | **C** | **G** | **C** | **A** | **T** | **G** | **T** | **G** | **T** | **C** | **G** | **A** | **A** | **T** | **A** | **T** | **A** | **T** | **T** | **A** | **G** | **C** | **T** | **A** | **A** | **G** | **T** | **T** | **A** | **G** | **A** | **G** | **G** | **T** | **A** | **G** | **A** | **A** | **G** | **A** | **C** | **C** | **G** | **A** | **A** | **C** | **T** | **C** | **A** | **A** | **G** | **C** | **A** | **G** | **C** | **T** | **A** | **T** | **C** | **T** | **A** | **T** | **G** | **C** | **G** | **T** | **T** | **T** | **A** | **A** | **A** | **C** | **A** | **T** | **G** | **G** | **T** | **T** | **T** | **G** | **G** | **T** | **G** | **A** | **A** | **A** | **-** | **-** | **-** |
| **N59555F** | **G** | **T** | **G** | **A** | **T** | **C** | **A** | **A** | **A** | **G** | **T** | **A** | **A** | **T** | **G** | **T** | **T** | **A** | **G** | **T** | **G** | **G** | **A** | **T** | **G** | **A** | **C** | **C** | **A** | **T** | **G** | **A** | **A** | **A** | **T** | **G** | **G** | **T** | **C** | **C** | **G** | **T** | **T** | **T** | **A** | **G** | **G** | **C** | **G** | **T** | **T** | **T** | **C** | **A** | **T** | **C** | **A** | **T** | **A** | **T** | **T** | **T** | **A** | **T** | **C** | **T** | **A** | **T** | **T** | **C** | **A** | **A** | **G** | **A** | **G** | **G** | **A** | **T** | **A** | **T** | **A** | **G** | **A** | **A** | **G** | **T** | **C** | **G** | **T** | **A** | **G** | **G** | **C** | **G** | **A** | **A** | **G** | **C** | **A** | **G** | **A** | **A** | **A** | **A** | **C** | **G** | **G** | **T** | **A** | **A** | **G** | **A** | **T** | **T** | **G** | **G** | **C** | **T** | **A** | **T** | **G** | **A** | **A** | **A** | **A** | **A** | **G** | **C** | **A** | **T** | **T** | **G** | **G** | **A** | **A** | **C** | **T** | **A** | **C** | **G** | **T** | **C** | **C** | **A** | **G** | **A** | **T** | **G** | **T** | **T** | **A** | **T** | **T** | **T** | **T** | **G** | **A** | **T** | **G** | **G** | **A** | **T** | **T** | **T** | **G** | **G** | **T** | **A** | **A** | **T** | **G** | **G** | **A** | **A** | **G** | **A** | **A** | **A** | **T** | **G** | **G** | **A** | **C** | **G** | **G** | **C** | **A** | **T** | **T** | **G** | **A** | **T** | **T** | **C** | **A** | **A** | **C** | **A** | **A** | **A** | **A** | **G** | **C** | **G** | **A** | **T** | **C** | **T** | **T** | **G** | **A** | **A** | **A** | **G** | **A** | **T** | **T** | **G** | **G** | **C** | **C** | **A** | **G** | **A** | **A** | **G** | **C** | **C** | **A** | **A** | **G** | **A** | **T** | **T** | **A** | **T** | **T** | **A** | **T** | **T** | **G** | **T** | **G** | **A** | **C** | **G** | **A** | **G** | **T** | **T** | **T** | **T** | **A** | **T** | **T** | **G** | **A** | **T** | **G** | **A** | **T** | **G** | **A** | **G** | **A** | **A** | **A** | **G** | **T** | **G** | **T** | **A** | **T** | **C** | **C** | **G** | **G** | **C** | **G** | **A** | **T** | **T** | **G** | **A** | **A** | **G** | **C** | **T** | **G** | **G** | **T** | **G** | **C** | **A** | **G** | **C** | **G** | **G** | **G** | **C** | **T** | **A** | **C** | **C** | **T** | **A** | **T** | **T** | **A** | **A** | **A** | **A** | **A** | **C** | **A** | **T** | **C** | **A** | **A** | **C** | **A** | **G** | **C** | **A** | **C** | **A** | **T** | **G** | **A** | **G** | **A** | **T** | **T** | **G** | **C** | **T** | **G** | **A** | **T** | **G** | **C** | **A** | **A** | **T** | **T** | **C** | **G** | **G** | **G** | **C** | **G** | **A** | **C** | **T** | **T** | **A** | **T** | **C** | **G** | **C** | **G** | **G** | **A** | **G** | **A** | **G** | **C** | **G** | **T** | **G** | **T** | **G** | **T** | **T** | **G** | **G** | **A** | **A** | **C** | **C** | **T** | **G** | **A** | **A** | **G** | **T** | **G** | **A** | **C** | **G** | **C** | **A** | **T** | **A** | **A** | **G** | **A** | **T** | **G** | **A** | **T** | **G** | **G** | **A** | **A** | **C** | **G** | **G** | **T** | **T** | **A** | **A** | **C** | **A** | **A** | **A** | **A** | **A** | **A** | **A** | **C** | **A** | **A** | **G** | **A** | **G** | **C** | **C** | **G** | **G** | **T** | **G** | **T** | **T** | **G** | **C** | **A** | **T** | **G** | **A** | **A** | **G** | **A** | **T** | **T** | **T** | **G** | **A** | **C** | **A** | **A** | **A** | **T** | **C** | **G** | **A** | **G** | **A** | **A** | **C** | **A** | **C** | **G** | **A** | **A** | **A** | **T** | **T** | **T** | **T** | **A** | **A** | **T** | **G** | **T** | **T** | **G** | **A** | **T** | **T** | **G** | **C** | **A** | **C** | **A** | **A** | **G** | **G** | **T** | **A** | **A** | **A** | **A** | **G** | **T** | **A** | **A** | **T** | **C** | **A** | **G** | **G** | **A** | **A** | **A** | **T** | **A** | **G** | **C** | **T** | **G** | **A** | **T** | **G** | **A** | **A** | **C** | **T** | **C** | **T** | **T** | **T** | **A** | **T** | **C** | **A** | **C** | **T** | **T** | **T** | **G** | **A** | **A** | **A** | **A** | **C** | **A** | **G** | **T** | **T** | **A** | **A** | **A** | **A** | **C** | **A** | **C** | **A** | **T** | **G** | **T** | **T** | **T** | **C** | **A** | **A** | **A** | **C** | **A** | **T** | **T** | **T** | **T** | **A** | **G** | **C** | **A** | **A** | **A** | **A** | **C** | **T** | **A** | **G** | **A** | **T** | **G** | **T** | **G** | **G** | **A** | **T** | **G** | **A** | **T** | **C** | **G** | **G** | **A** | **C** | **C** | **C** | **A** | **A** | **G** | **C** | **G** | **G** | **C** | **G** | **A** | **T** | **T** | **T** | **A** | **T** | **G** | **C** | **T** | **T** | **T** | **T** | **C** | **A** | **A** | **C** | **A** | **T** | **G** | **G** | **T** | **T** | **T** | **A** | **G** | **C** | **C** | **A** | **A** | **A** | **-** | **-** | **-** |
| **N59457F** | **G** | **T** | **G** | **A** | **T** | **C** | **A** | **A** | **A** | **G** | **T** | **A** | **A** | **T** | **G** | **T** | **T** | **A** | **G** | **T** | **G** | **G** | **A** | **T** | **G** | **A** | **C** | **C** | **A** | **T** | **G** | **A** | **A** | **A** | **T** | **G** | **G** | **T** | **C** | **C** | **G** | **T** | **T** | **T** | **A** | **G** | **G** | **C** | **G** | **T** | **T** | **T** | **C** | **A** | **T** | **C** | **A** | **T** | **A** | **T** | **T** | **T** | **A** | **T** | **C** | **T** | **A** | **T** | **T** | **C** | **A** | **A** | **G** | **A** | **G** | **G** | **A** | **T** | **A** | **T** | **A** | **G** | **A** | **A** | **G** | **T** | **C** | **G** | **T** | **A** | **G** | **G** | **C** | **G** | **A** | **A** | **G** | **C** | **A** | **G** | **A** | **A** | **A** | **A** | **C** | **G** | **G** | **T** | **A** | **A** | **G** | **A** | **T** | **T** | **G** | **G** | **C** | **T** | **A** | **T** | **G** | **A** | **A** | **A** | **A** | **A** | **G** | **C** | **A** | **T** | **T** | **G** | **G** | **A** | **A** | **C** | **T** | **A** | **C** | **G** | **T** | **C** | **C** | **A** | **G** | **A** | **T** | **G** | **T** | **T** | **A** | **T** | **T** | **T** | **T** | **G** | **A** | **T** | **G** | **G** | **A** | **T** | **T** | **T** | **G** | **G** | **T** | **A** | **A** | **T** | **G** | **G** | **A** | **A** | **G** | **A** | **A** | **A** | **T** | **G** | **G** | **A** | **C** | **G** | **G** | **C** | **A** | **T** | **T** | **G** | **A** | **T** | **T** | **C** | **A** | **A** | **C** | **A** | **A** | **A** | **A** | **G** | **C** | **G** | **A** | **T** | **C** | **T** | **T** | **G** | **A** | **A** | **A** | **G** | **A** | **T** | **T** | **G** | **G** | **C** | **C** | **A** | **G** | **A** | **A** | **G** | **C** | **C** | **A** | **A** | **G** | **A** | **T** | **T** | **A** | **T** | **T** | **A** | **T** | **T** | **G** | **T** | **G** | **A** | **C** | **G** | **A** | **G** | **T** | **T** | **T** | **T** | **A** | **T** | **T** | **G** | **A** | **T** | **G** | **A** | **T** | **G** | **A** | **A** | **A** | **A** | **A** | **G** | **T** | **G** | **T** | **A** | **T** | **C** | **C** | **G** | **G** | **C** | **G** | **A** | **T** | **T** | **G** | **A** | **A** | **G** | **C** | **T** | **G** | **G** | **T** | **G** | **C** | **A** | **G** | **C** | **G** | **G** | **G** | **C** | **T** | **A** | **C** | **C** | **T** | **A** | **T** | **T** | **A** | **A** | **A** | **A** | **A** | **C** | **A** | **T** | **C** | **A** | **A** | **C** | **A** | **G** | **C** | **A** | **C** | **A** | **T** | **G** | **A** | **G** | **A** | **T** | **T** | **G** | **C** | **T** | **G** | **A** | **T** | **G** | **C** | **A** | **A** | **T** | **T** | **C** | **G** | **G** | **G** | **C** | **G** | **A** | **C** | **T** | **T** | **A** | **T** | **C** | **G** | **C** | **G** | **G** | **A** | **G** | **A** | **G** | **C** | **G** | **T** | **G** | **T** | **G** | **T** | **T** | **G** | **G** | **A** | **A** | **C** | **C** | **T** | **G** | **A** | **A** | **G** | **T** | **G** | **A** | **C** | **G** | **C** | **A** | **T** | **A** | **A** | **G** | **A** | **T** | **G** | **A** | **T** | **G** | **G** | **A** | **A** | **C** | **G** | **G** | **T** | **T** | **A** | **A** | **C** | **A** | **A** | **A** | **A** | **A** | **A** | **A** | **C** | **A** | **A** | **G** | **A** | **G** | **C** | **C** | **G** | **G** | **T** | **G** | **T** | **T** | **G** | **C** | **A** | **C** | **G** | **A** | **A** | **G** | **A** | **T** | **T** | **T** | **G** | **A** | **C** | **A** | **A** | **A** | **C** | **C** | **G** | **A** | **G** | **A** | **A** | **C** | **A** | **C** | **G** | **A** | **A** | **A** | **T** | **T** | **T** | **T** | **A** | **A** | **T** | **G** | **T** | **T** | **G** | **A** | **T** | **T** | **G** | **C** | **A** | **C** | **A** | **A** | **G** | **G** | **T** | **A** | **A** | **A** | **A** | **G** | **T** | **A** | **A** | **T** | **C** | **A** | **G** | **G** | **A** | **A** | **A** | **T** | **T** | **G** | **C** | **T** | **G** | **A** | **T** | **G** | **A** | **A** | **C** | **T** | **C** | **T** | **T** | **T** | **A** | **T** | **C** | **A** | **C** | **T** | **T** | **T** | **G** | **A** | **A** | **A** | **A** | **C** | **A** | **G** | **T** | **T** | **A** | **A** | **A** | **A** | **C** | **A** | **C** | **A** | **T** | **G** | **T** | **T** | **T** | **C** | **A** | **A** | **A** | **C** | **A** | **T** | **T** | **T** | **T** | **A** | **G** | **C** | **A** | **A** | **A** | **A** | **C** | **T** | **A** | **G** | **A** | **T** | **G** | **T** | **G** | **G** | **A** | **T** | **G** | **A** | **T** | **C** | **G** | **G** | **A** | **C** | **C** | **C** | **A** | **A** | **G** | **C** | **G** | **G** | **C** | **G** | **A** | **T** | **T** | **T** | **A** | **T** | **G** | **C** | **T** | **T** | **T** | **T** | **C** | **A** | **A** | **C** | **A** | **T** | **G** | **G** | **T** | **T** | **T** | **A** | **G** | **C** | **C** | **A** | **A** | **A** | **-** | **-** | **-** |
| **N52656** | **A** | **T** | **G** | **A** | **T** | **A** | **A** | **A** | **A** | **G** | **T** | **T** | **T** | **T** | **A** | **T** | **T** | **A** | **G** | **T** | **A** | **G** | **A** | **T** | **G** | **A** | **C** | **C** | **A** | **T** | **G** | **A** | **A** | **A** | **T** | **G** | **G** | **T** | **G** | **C** | **G** | **C** | **T** | **T** | **A** | **G** | **G** | **T** | **G** | **T** | **C** | **T** | **C** | **T** | **T** | **C** | **T** | **T** | **A** | **T** | **T** | **T** | **A** | **T** | **C** | **T** | **A** | **T** | **T** | **C** | **A** | **A** | **G** | **A** | **A** | **G** | **A** | **C** | **A** | **T** | **T** | **G** | **A** | **A** | **G** | **T** | **A** | **A** | **T** | **T** | **G** | **G** | **A** | **G** | **A** | **A** | **G** | **C** | **T** | **G** | **A** | **A** | **A** | **A** | **T** | **G** | **G** | **A** | **C** | **G** | **A** | **C** | **A** | **A** | **G** | **G** | **C** | **T** | **A** | **T** | **G** | **A** | **G** | **A** | **A** | **G** | **G** | **C** | **G** | **A** | **T** | **G** | **A** | **C** | **A** | **C** | **T** | **T** | **C** | **G** | **A** | **C** | **C** | **G** | **G** | **A** | **C** | **G** | **T** | **C** | **A** | **T** | **T** | **T** | **T** | **A** | **A** | **T** | **G** | **G** | **A** | **C** | **T** | **T** | **A** | **G** | **T** | **C** | **A** | **T** | **G** | **G** | **A** | **A** | **G** | **A** | **A** | **A** | **T** | **G** | **G** | **A** | **T** | **G** | **G** | **T** | **A** | **T** | **C** | **G** | **A** | **A** | **T** | **C** | **A** | **A** | **C** | **A** | **A** | **A** | **A** | **G** | **C** | **A** | **A** | **T** | **C** | **T** | **T** | **A** | **A** | **A** | **A** | **G** | **A** | **T** | **T** | **G** | **G** | **C** | **C** | **A** | **G** | **A** | **A** | **G** | **C** | **T** | **A** | **A** | **A** | **A** | **T** | **C** | **A** | **T** | **T** | **A** | **T** | **C** | **G** | **T** | **A** | **A** | **C** | **C** | **A** | **G** | **T** | **T** | **T** | **T** | **A** | **T** | **T** | **G** | **A** | **T** | **G** | **A** | **C** | **G** | **A** | **A** | **A** | **A** | **A** | **G** | **T** | **T** | **T** | **A** | **T** | **C** | **C** | **T** | **G** | **C** | **C** | **A** | **T** | **T** | **G** | **A** | **A** | **G** | **C** | **A** | **G** | **G** | **T** | **G** | **C** | **A** | **G** | **C** | **A** | **G** | **G** | **G** | **T** | **A** | **T** | **T** | **T** | **G** | **T** | **T** | **G** | **A** | **A** | **G** | **A** | **C** | **C** | **T** | **C** | **T** | **A** | **C** | **C** | **G** | **C** | **A** | **C** | **A** | **T** | **G** | **A** | **A** | **A** | **T** | **T** | **G** | **C** | **T** | **G** | **A** | **T** | **G** | **C** | **T** | **A** | **T** | **A** | **A** | **G** | **G** | **G** | **C** | **A** | **A** | **C** | **G** | **C** | **A** | **A** | **C** | **G** | **C** | **G** | **G** | **C** | **G** | **A** | **G** | **C** | **G** | **A** | **G** | **T** | **A** | **T** | **T** | **A** | **G** | **A** | **A** | **C** | **C** | **A** | **G** | **A** | **A** | **G** | **T** | **G** | **A** | **C** | **A** | **A** | **C** | **A** | **A** | **A** | **A** | **A** | **T** | **G** | **A** | **T** | **G** | **G** | **A** | **A** | **A** | **A** | **A** | **A** | **T** | **G** | **A** | **G** | **T** | **C** | **G** | **G** | **C** | **G** | **A** | **A** | **A** | **T** | **G** | **A** | **G** | **C** | **C** | **T** | **G** | **T** | **A** | **T** | **T** | **A** | **C** | **A** | **T** | **G** | **A** | **A** | **G** | **A** | **A** | **T** | **T** | **A** | **A** | **C** | **G** | **A** | **A** | **T** | **C** | **G** | **G** | **G** | **A** | **A** | **A** | **A** | **T** | **G** | **A** | **A** | **A** | **T** | **T** | **T** | **T** | **A** | **A** | **T** | **G** | **C** | **T** | **G** | **A** | **T** | **T** | **T** | **C** | **T** | **G** | **A** | **A** | **G** | **G** | **A** | **A** | **A** | **A** | **A** | **G** | **T** | **A** | **A** | **T** | **C** | **A** | **G** | **G** | **A** | **A** | **A** | **T** | **A** | **G** | **C** | **G** | **G** | **A** | **C** | **G** | **A** | **A** | **T** | **T** | **G** | **T** | **T** | **C** | **A** | **T** | **T** | **A** | **C** | **A** | **C** | **T** | **G** | **A** | **A** | **A** | **A** | **C** | **A** | **G** | **T** | **A** | **A** | **A** | **A** | **A** | **C** | **A** | **C** | **A** | **T** | **G** | **T** | **T** | **T** | **C** | **C** | **A** | **A** | **T** | **A** | **T** | **A** | **T** | **T** | **G** | **G** | **C** | **T** | **A** | **A** | **G** | **C** | **T** | **A** | **G** | **A** | **A** | **G** | **T** | **G** | **G** | **A** | **A** | **G** | **A** | **C** | **C** | **G** | **T** | **A** | **C** | **A** | **C** | **A** | **A** | **G** | **C** | **C** | **G** | **C** | **T** | **A** | **T** | **C** | **T** | **A** | **T** | **G** | **C** | **A** | **T** | **T** | **C** | **A** | **A** | **A** | **C** | **A** | **T** | **G** | **G** | **T** | **T** | **T** | **G** | **G** | **T** | **G** | **-** | **-** | **-** | **-** | **-** | **-** |
| **N53559** | **A** | **T** | **G** | **A** | **T** | **A** | **A** | **A** | **A** | **G** | **T** | **T** | **T** | **T** | **A** | **T** | **T** | **A** | **G** | **T** | **A** | **G** | **A** | **T** | **G** | **A** | **C** | **C** | **A** | **T** | **G** | **A** | **A** | **A** | **T** | **G** | **G** | **T** | **A** | **C** | **G** | **C** | **T** | **T** | **A** | **G** | **G** | **C** | **G** | **T** | **C** | **T** | **C** | **T** | **T** | **C** | **T** | **T** | **A** | **T** | **T** | **T** | **A** | **T** | **C** | **T** | **A** | **T** | **C** | **C** | **A** | **A** | **G** | **A** | **A** | **G** | **A** | **C** | **A** | **T** | **T** | **G** | **A** | **A** | **G** | **T** | **G** | **A** | **T** | **T** | **G** | **G** | **A** | **G** | **A** | **A** | **G** | **C** | **T** | **G** | **A** | **A** | **A** | **A** | **T** | **G** | **G** | **A** | **C** | **G** | **T** | **C** | **A** | **A** | **G** | **G** | **C** | **T** | **A** | **T** | **G** | **A** | **A** | **A** | **A** | **G** | **G** | **C** | **G** | **A** | **T** | **G** | **G** | **C** | **A** | **C** | **T** | **T** | **C** | **G** | **A** | **C** | **C** | **G** | **G** | **A** | **T** | **G** | **T** | **C** | **A** | **T** | **T** | **T** | **T** | **A** | **A** | **T** | **G** | **G** | **A** | **T** | **C** | **T** | **A** | **G** | **T** | **C** | **A** | **T** | **G** | **G** | **A** | **A** | **G** | **A** | **A** | **A** | **T** | **G** | **G** | **A** | **T** | **G** | **G** | **C** | **A** | **T** | **C** | **G** | **A** | **A** | **T** | **C** | **A** | **A** | **C** | **A** | **A** | **A** | **A** | **G** | **C** | **A** | **A** | **T** | **C** | **T** | **T** | **A** | **A** | **A** | **A** | **G** | **A** | **T** | **T** | **G** | **G** | **C** | **C** | **A** | **A** | **A** | **A** | **G** | **C** | **C** | **A** | **A** | **A** | **A** | **T** | **C** | **A** | **T** | **T** | **A** | **T** | **C** | **G** | **T** | **C** | **A** | **C** | **A** | **A** | **G** | **T** | **T** | **T** | **T** | **A** | **T** | **T** | **G** | **A** | **T** | **G** | **A** | **T** | **G** | **A** | **A** | **A** | **A** | **A** | **G** | **T** | **T** | **T** | **A** | **T** | **C** | **C** | **T** | **G** | **C** | **A** | **A** | **T** | **T** | **G** | **A** | **A** | **G** | **C** | **A** | **G** | **G** | **A** | **G** | **C** | **A** | **G** | **C** | **A** | **G** | **G** | **A** | **T** | **A** | **T** | **T** | **T** | **G** | **T** | **T** | **G** | **A** | **A** | **G** | **A** | **C** | **G** | **T** | **C** | **T** | **A** | **C** | **C** | **G** | **C** | **A** | **C** | **A** | **T** | **G** | **A** | **A** | **A** | **T** | **A** | **G** | **C** | **T** | **G** | **A** | **T** | **G** | **C** | **T** | **A** | **T** | **A** | **A** | **G** | **G** | **G** | **C** | **T** | **A** | **C** | **G** | **C** | **A** | **A** | **C** | **G** | **C** | **G** | **G** | **C** | **G** | **A** | **G** | **C** | **G** | **A** | **G** | **T** | **A** | **T** | **T** | **G** | **G** | **A** | **A** | **C** | **C** | **T** | **G** | **A** | **A** | **G** | **T** | **G** | **A** | **C** | **A** | **A** | **C** | **A** | **A** | **A** | **A** | **A** | **T** | **G** | **A** | **T** | **G** | **G** | **A** | **A** | **A** | **A** | **A** | **A** | **T** | **G** | **A** | **G** | **T** | **C** | **G** | **G** | **C** | **G** | **A** | **A** | **A** | **T** | **G** | **A** | **T** | **C** | **C** | **A** | **G** | **T** | **A** | **T** | **T** | **G** | **C** | **A** | **T** | **G** | **A** | **A** | **G** | **A** | **A** | **T** | **T** | **A** | **A** | **C** | **G** | **A** | **A** | **T** | **C** | **G** | **A** | **G** | **A** | **A** | **A** | **A** | **T** | **G** | **A** | **A** | **A** | **T** | **T** | **T** | **T** | **G** | **A** | **T** | **G** | **C** | **T** | **G** | **A** | **T** | **T** | **T** | **C** | **T** | **G** | **A** | **A** | **G** | **G** | **G** | **A** | **A** | **A** | **A** | **G** | **T** | **A** | **A** | **T** | **C** | **A** | **G** | **G** | **A** | **A** | **A** | **T** | **C** | **G** | **C** | **G** | **G** | **A** | **T** | **G** | **A** | **A** | **T** | **T** | **G** | **T** | **T** | **T** | **A** | **T** | **C** | **A** | **C** | **A** | **C** | **T** | **G** | **A** | **A** | **A** | **A** | **C** | **A** | **G** | **T** | **C** | **A** | **A** | **A** | **A** | **C** | **G** | **C** | **A** | **T** | **G** | **T** | **G** | **T** | **C** | **G** | **A** | **A** | **T** | **A** | **T** | **A** | **T** | **T** | **A** | **G** | **C** | **T** | **A** | **A** | **G** | **T** | **T** | **A** | **G** | **A** | **G** | **G** | **T** | **A** | **G** | **A** | **A** | **G** | **A** | **C** | **C** | **G** | **A** | **A** | **C** | **T** | **C** | **A** | **A** | **G** | **C** | **A** | **G** | **C** | **T** | **A** | **T** | **C** | **T** | **A** | **T** | **G** | **C** | **G** | **T** | **T** | **T** | **A** | **A** | **A** | **C** | **A** | **T** | **G** | **G** | **T** | **T** | **T** | **G** | **G** | **T** | **G** | **A** | **A** | **A** | **-** | **-** | **-** |
| **N52824** | **A** | **T** | **G** | **A** | **T** | **A** | **A** | **A** | **A** | **G** | **T** | **T** | **T** | **T** | **A** | **T** | **T** | **A** | **G** | **T** | **A** | **G** | **A** | **T** | **G** | **A** | **C** | **C** | **A** | **T** | **G** | **A** | **A** | **A** | **T** | **G** | **G** | **T** | **A** | **C** | **G** | **C** | **T** | **T** | **A** | **G** | **G** | **C** | **G** | **T** | **C** | **T** | **C** | **T** | **T** | **C** | **T** | **T** | **A** | **T** | **T** | **T** | **A** | **T** | **C** | **T** | **A** | **T** | **C** | **C** | **A** | **A** | **G** | **A** | **A** | **G** | **A** | **C** | **A** | **T** | **T** | **G** | **A** | **A** | **G** | **T** | **G** | **A** | **T** | **T** | **G** | **G** | **A** | **G** | **A** | **A** | **G** | **C** | **T** | **G** | **A** | **A** | **A** | **A** | **T** | **G** | **G** | **A** | **C** | **G** | **T** | **C** | **A** | **A** | **G** | **G** | **C** | **T** | **A** | **T** | **G** | **A** | **A** | **A** | **A** | **G** | **G** | **C** | **G** | **A** | **T** | **G** | **G** | **C** | **A** | **C** | **T** | **T** | **C** | **G** | **A** | **C** | **C** | **G** | **G** | **A** | **T** | **G** | **T** | **C** | **A** | **T** | **T** | **T** | **T** | **A** | **A** | **T** | **G** | **G** | **A** | **T** | **C** | **T** | **A** | **G** | **T** | **C** | **A** | **T** | **G** | **G** | **A** | **A** | **G** | **A** | **A** | **A** | **T** | **G** | **G** | **A** | **T** | **G** | **G** | **C** | **A** | **T** | **C** | **G** | **A** | **A** | **T** | **C** | **A** | **A** | **C** | **A** | **A** | **A** | **A** | **G** | **C** | **A** | **A** | **T** | **C** | **T** | **T** | **A** | **A** | **A** | **A** | **G** | **A** | **T** | **T** | **G** | **G** | **C** | **C** | **A** | **A** | **A** | **A** | **G** | **C** | **C** | **A** | **A** | **A** | **A** | **T** | **C** | **A** | **T** | **T** | **A** | **T** | **C** | **G** | **T** | **C** | **A** | **C** | **A** | **A** | **G** | **T** | **T** | **T** | **T** | **A** | **T** | **T** | **G** | **A** | **T** | **G** | **A** | **T** | **G** | **A** | **A** | **A** | **A** | **A** | **G** | **T** | **T** | **T** | **A** | **T** | **C** | **C** | **T** | **G** | **C** | **A** | **A** | **T** | **T** | **G** | **A** | **A** | **G** | **C** | **A** | **G** | **G** | **A** | **G** | **C** | **A** | **G** | **C** | **A** | **G** | **G** | **A** | **T** | **A** | **T** | **T** | **T** | **G** | **T** | **T** | **G** | **A** | **A** | **G** | **A** | **C** | **G** | **T** | **C** | **T** | **A** | **C** | **C** | **G** | **C** | **A** | **C** | **A** | **T** | **G** | **A** | **A** | **A** | **T** | **A** | **G** | **C** | **T** | **G** | **A** | **T** | **G** | **C** | **T** | **A** | **T** | **A** | **A** | **G** | **G** | **G** | **C** | **T** | **A** | **C** | **G** | **C** | **A** | **A** | **C** | **G** | **C** | **G** | **G** | **C** | **G** | **A** | **G** | **C** | **G** | **A** | **G** | **T** | **A** | **T** | **T** | **G** | **G** | **A** | **A** | **C** | **C** | **T** | **G** | **A** | **A** | **G** | **T** | **G** | **A** | **C** | **A** | **A** | **C** | **A** | **A** | **A** | **A** | **A** | **T** | **G** | **A** | **T** | **G** | **G** | **A** | **A** | **A** | **A** | **A** | **A** | **T** | **G** | **A** | **G** | **T** | **C** | **G** | **G** | **C** | **G** | **A** | **A** | **A** | **T** | **G** | **A** | **T** | **C** | **C** | **A** | **G** | **T** | **A** | **T** | **T** | **G** | **C** | **A** | **T** | **G** | **A** | **A** | **G** | **A** | **A** | **T** | **T** | **A** | **A** | **C** | **G** | **A** | **A** | **T** | **C** | **G** | **A** | **G** | **A** | **A** | **A** | **A** | **T** | **G** | **A** | **A** | **A** | **T** | **T** | **T** | **T** | **G** | **A** | **T** | **G** | **C** | **T** | **G** | **A** | **T** | **T** | **T** | **C** | **T** | **G** | **A** | **A** | **G** | **G** | **G** | **A** | **A** | **A** | **A** | **G** | **T** | **A** | **A** | **T** | **C** | **A** | **G** | **G** | **A** | **A** | **A** | **T** | **C** | **G** | **C** | **G** | **G** | **A** | **T** | **G** | **A** | **A** | **T** | **T** | **G** | **T** | **T** | **T** | **A** | **T** | **C** | **A** | **C** | **A** | **C** | **T** | **G** | **A** | **A** | **A** | **A** | **C** | **A** | **G** | **T** | **C** | **A** | **A** | **A** | **A** | **C** | **G** | **C** | **A** | **T** | **G** | **T** | **G** | **T** | **C** | **G** | **A** | **A** | **T** | **A** | **T** | **A** | **T** | **T** | **A** | **G** | **C** | **T** | **A** | **A** | **G** | **T** | **T** | **A** | **G** | **A** | **G** | **G** | **T** | **A** | **G** | **A** | **A** | **G** | **A** | **C** | **C** | **G** | **A** | **A** | **C** | **T** | **C** | **A** | **A** | **G** | **C** | **A** | **G** | **C** | **T** | **A** | **T** | **C** | **T** | **A** | **T** | **G** | **C** | **G** | **T** | **T** | **T** | **A** | **A** | **A** | **C** | **A** | **T** | **G** | **G** | **T** | **T** | **T** | **G** | **G** | **T** | **G** | **A** | **A** | **A** | **-** | **-** | **-** |
| **N59589F** | **A** | **T** | **G** | **A** | **T** | **A** | **A** | **A** | **A** | **G** | **T** | **T** | **T** | **T** | **A** | **T** | **T** | **A** | **G** | **T** | **A** | **G** | **A** | **T** | **G** | **A** | **C** | **C** | **A** | **T** | **G** | **A** | **A** | **A** | **T** | **G** | **G** | **T** | **G** | **C** | **G** | **C** | **T** | **T** | **A** | **G** | **G** | **C** | **G** | **T** | **C** | **T** | **C** | **T** | **T** | **C** | **T** | **T** | **A** | **T** | **T** | **T** | **A** | **T** | **C** | **T** | **A** | **T** | **T** | **C** | **A** | **A** | **G** | **A** | **A** | **G** | **A** | **C** | **A** | **T** | **T** | **G** | **A** | **A** | **G** | **T** | **A** | **A** | **T** | **T** | **G** | **G** | **A** | **G** | **A** | **A** | **G** | **C** | **T** | **G** | **A** | **A** | **A** | **A** | **T** | **G** | **G** | **A** | **C** | **G** | **A** | **C** | **A** | **A** | **G** | **G** | **C** | **T** | **A** | **T** | **G** | **A** | **G** | **A** | **A** | **G** | **G** | **C** | **G** | **A** | **T** | **G** | **A** | **C** | **A** | **C** | **T** | **T** | **C** | **G** | **A** | **C** | **C** | **G** | **G** | **A** | **C** | **G** | **T** | **C** | **A** | **T** | **T** | **T** | **T** | **A** | **A** | **T** | **G** | **G** | **A** | **C** | **T** | **T** | **A** | **G** | **T** | **C** | **A** | **T** | **G** | **G** | **A** | **A** | **G** | **A** | **A** | **A** | **T** | **G** | **G** | **A** | **T** | **G** | **G** | **T** | **A** | **T** | **C** | **G** | **A** | **A** | **T** | **C** | **A** | **A** | **C** | **A** | **A** | **A** | **A** | **G** | **C** | **A** | **A** | **T** | **C** | **T** | **T** | **A** | **A** | **A** | **A** | **G** | **A** | **T** | **T** | **G** | **G** | **C** | **C** | **A** | **G** | **A** | **A** | **G** | **C** | **T** | **A** | **A** | **A** | **A** | **T** | **C** | **A** | **T** | **T** | **A** | **T** | **C** | **G** | **T** | **A** | **A** | **C** | **C** | **A** | **G** | **T** | **T** | **T** | **T** | **A** | **T** | **T** | **G** | **A** | **T** | **G** | **A** | **C** | **G** | **A** | **A** | **A** | **A** | **A** | **G** | **T** | **T** | **T** | **A** | **T** | **C** | **C** | **T** | **G** | **C** | **C** | **A** | **T** | **T** | **G** | **A** | **A** | **G** | **C** | **A** | **G** | **G** | **T** | **G** | **C** | **A** | **G** | **C** | **A** | **G** | **G** | **G** | **T** | **A** | **T** | **T** | **T** | **G** | **T** | **T** | **G** | **A** | **A** | **G** | **A** | **C** | **C** | **T** | **C** | **T** | **A** | **C** | **C** | **G** | **C** | **A** | **C** | **A** | **T** | **G** | **A** | **A** | **A** | **T** | **T** | **G** | **C** | **T** | **G** | **A** | **T** | **G** | **C** | **T** | **A** | **T** | **A** | **A** | **G** | **G** | **G** | **C** | **A** | **A** | **C** | **G** | **C** | **A** | **A** | **C** | **G** | **T** | **G** | **G** | **C** | **G** | **A** | **G** | **C** | **G** | **A** | **G** | **T** | **A** | **T** | **T** | **A** | **G** | **A** | **A** | **C** | **C** | **A** | **G** | **A** | **A** | **G** | **T** | **G** | **A** | **C** | **A** | **A** | **C** | **A** | **A** | **A** | **A** | **A** | **T** | **G** | **A** | **T** | **G** | **G** | **A** | **A** | **A** | **A** | **A** | **A** | **T** | **G** | **A** | **G** | **T** | **C** | **G** | **G** | **C** | **G** | **A** | **A** | **A** | **T** | **G** | **A** | **G** | **C** | **C** | **T** | **G** | **T** | **A** | **T** | **T** | **A** | **C** | **A** | **T** | **G** | **A** | **A** | **G** | **A** | **A** | **C** | **T** | **A** | **A** | **C** | **G** | **A** | **A** | **T** | **C** | **G** | **G** | **G** | **A** | **A** | **A** | **A** | **T** | **G** | **A** | **A** | **A** | **T** | **T** | **T** | **T** | **A** | **A** | **T** | **G** | **C** | **T** | **G** | **A** | **T** | **T** | **T** | **C** | **T** | **G** | **A** | **A** | **G** | **G** | **A** | **A** | **A** | **A** | **A** | **G** | **T** | **A** | **A** | **T** | **C** | **A** | **G** | **G** | **A** | **A** | **A** | **T** | **A** | **G** | **C** | **G** | **G** | **A** | **C** | **G** | **A** | **A** | **T** | **T** | **G** | **T** | **T** | **T** | **A** | **T** | **T** | **A** | **C** | **A** | **C** | **T** | **G** | **A** | **A** | **A** | **A** | **C** | **A** | **G** | **T** | **A** | **A** | **A** | **A** | **A** | **C** | **A** | **C** | **A** | **T** | **G** | **T** | **T** | **T** | **C** | **C** | **A** | **A** | **T** | **A** | **T** | **A** | **T** | **T** | **G** | **G** | **C** | **T** | **A** | **A** | **G** | **C** | **T** | **A** | **G** | **A** | **A** | **G** | **T** | **G** | **G** | **A** | **A** | **G** | **A** | **C** | **C** | **G** | **T** | **A** | **C** | **A** | **C** | **A** | **A** | **G** | **C** | **C** | **G** | **C** | **T** | **A** | **T** | **C** | **T** | **A** | **T** | **G** | **C** | **A** | **T** | **T** | **C** | **A** | **A** | **A** | **C** | **A** | **T** | **G** | **G** | **T** | **T** | **T** | **G** | **G** | **T** | **G** | **A** | **A** | **A** | **-** | **-** | **-** |
| **N59492F** | **A** | **T** | **G** | **A** | **T** | **A** | **A** | **A** | **A** | **G** | **T** | **T** | **T** | **T** | **A** | **T** | **T** | **A** | **G** | **T** | **A** | **G** | **A** | **T** | **G** | **A** | **C** | **C** | **A** | **T** | **G** | **A** | **A** | **A** | **T** | **G** | **G** | **T** | **A** | **C** | **G** | **C** | **T** | **T** | **A** | **G** | **G** | **C** | **G** | **T** | **C** | **T** | **C** | **T** | **T** | **C** | **T** | **T** | **A** | **T** | **T** | **T** | **A** | **T** | **C** | **T** | **A** | **T** | **C** | **C** | **A** | **A** | **G** | **A** | **A** | **G** | **A** | **C** | **A** | **T** | **T** | **G** | **A** | **A** | **G** | **T** | **G** | **A** | **T** | **T** | **G** | **G** | **A** | **G** | **A** | **A** | **G** | **C** | **T** | **G** | **A** | **A** | **A** | **A** | **T** | **G** | **G** | **A** | **C** | **G** | **T** | **C** | **A** | **A** | **G** | **G** | **C** | **T** | **A** | **T** | **G** | **A** | **A** | **A** | **A** | **G** | **G** | **C** | **G** | **A** | **T** | **G** | **G** | **C** | **A** | **C** | **T** | **T** | **C** | **G** | **A** | **C** | **C** | **A** | **G** | **A** | **T** | **G** | **T** | **C** | **A** | **T** | **T** | **T** | **T** | **A** | **A** | **T** | **G** | **G** | **A** | **T** | **C** | **T** | **A** | **G** | **T** | **C** | **A** | **T** | **G** | **G** | **A** | **A** | **G** | **A** | **A** | **A** | **T** | **G** | **G** | **A** | **T** | **G** | **G** | **C** | **A** | **T** | **C** | **G** | **A** | **A** | **T** | **C** | **A** | **A** | **C** | **A** | **A** | **A** | **A** | **G** | **C** | **A** | **A** | **T** | **C** | **T** | **T** | **A** | **A** | **A** | **A** | **G** | **A** | **T** | **T** | **G** | **G** | **C** | **C** | **A** | **A** | **A** | **A** | **G** | **C** | **C** | **A** | **A** | **A** | **A** | **T** | **C** | **A** | **T** | **T** | **A** | **T** | **C** | **G** | **T** | **C** | **A** | **C** | **A** | **A** | **G** | **T** | **T** | **T** | **T** | **A** | **T** | **T** | **G** | **A** | **T** | **G** | **A** | **T** | **G** | **A** | **A** | **A** | **A** | **A** | **G** | **T** | **T** | **T** | **A** | **T** | **C** | **C** | **T** | **G** | **C** | **A** | **A** | **T** | **T** | **G** | **A** | **A** | **G** | **C** | **A** | **G** | **G** | **A** | **G** | **C** | **A** | **G** | **C** | **A** | **G** | **G** | **A** | **T** | **A** | **T** | **T** | **T** | **G** | **T** | **T** | **G** | **A** | **A** | **G** | **A** | **C** | **G** | **T** | **C** | **T** | **A** | **C** | **C** | **G** | **C** | **A** | **C** | **A** | **T** | **G** | **A** | **A** | **A** | **T** | **A** | **G** | **C** | **T** | **G** | **A** | **T** | **G** | **C** | **T** | **A** | **T** | **A** | **A** | **G** | **G** | **G** | **C** | **T** | **A** | **C** | **G** | **C** | **A** | **A** | **C** | **G** | **C** | **G** | **G** | **C** | **G** | **A** | **G** | **C** | **G** | **A** | **G** | **T** | **A** | **T** | **T** | **G** | **G** | **A** | **A** | **C** | **C** | **T** | **G** | **A** | **A** | **G** | **T** | **G** | **A** | **C** | **A** | **A** | **C** | **A** | **A** | **A** | **A** | **A** | **T** | **G** | **A** | **T** | **G** | **G** | **A** | **A** | **A** | **A** | **A** | **A** | **T** | **G** | **A** | **G** | **T** | **C** | **G** | **G** | **C** | **G** | **A** | **T** | **A** | **T** | **G** | **A** | **T** | **C** | **C** | **A** | **G** | **T** | **A** | **T** | **T** | **G** | **C** | **A** | **T** | **G** | **A** | **A** | **G** | **A** | **A** | **T** | **T** | **A** | **A** | **C** | **G** | **A** | **A** | **T** | **C** | **G** | **A** | **G** | **A** | **A** | **A** | **A** | **T** | **G** | **A** | **A** | **A** | **T** | **T** | **T** | **T** | **G** | **A** | **T** | **G** | **C** | **T** | **G** | **A** | **T** | **T** | **T** | **C** | **T** | **G** | **A** | **A** | **G** | **G** | **G** | **A** | **A** | **A** | **A** | **G** | **T** | **A** | **A** | **T** | **C** | **A** | **G** | **G** | **A** | **A** | **A** | **T** | **C** | **G** | **C** | **G** | **G** | **A** | **T** | **G** | **A** | **A** | **T** | **T** | **G** | **T** | **T** | **T** | **A** | **T** | **C** | **A** | **C** | **A** | **C** | **T** | **G** | **A** | **A** | **A** | **A** | **C** | **A** | **G** | **T** | **T** | **A** | **A** | **A** | **A** | **C** | **G** | **C** | **A** | **T** | **G** | **T** | **G** | **T** | **C** | **G** | **A** | **A** | **T** | **A** | **T** | **A** | **T** | **T** | **A** | **G** | **C** | **T** | **A** | **A** | **G** | **T** | **T** | **A** | **G** | **A** | **G** | **G** | **T** | **A** | **G** | **A** | **A** | **G** | **A** | **C** | **C** | **G** | **A** | **A** | **C** | **T** | **C** | **A** | **A** | **G** | **C** | **A** | **G** | **C** | **T** | **A** | **T** | **C** | **T** | **A** | **T** | **G** | **C** | **A** | **T** | **T** | **T** | **A** | **A** | **A** | **C** | **A** | **T** | **G** | **G** | **T** | **T** | **T** | **G** | **G** | **T** | **G** | **A** | **A** | **A** | **-** | **-** | **-** |
| **N60280F** | **G** | **T** | **G** | **A** | **T** | **C** | **A** | **A** | **A** | **G** | **T** | **A** | **A** | **T** | **G** | **T** | **T** | **A** | **G** | **T** | **G** | **G** | **A** | **T** | **G** | **A** | **C** | **C** | **A** | **T** | **G** | **A** | **A** | **A** | **T** | **G** | **G** | **T** | **C** | **C** | **G** | **T** | **T** | **T** | **A** | **G** | **G** | **C** | **G** | **T** | **T** | **T** | **C** | **A** | **T** | **C** | **A** | **T** | **A** | **T** | **T** | **T** | **A** | **T** | **C** | **T** | **A** | **T** | **T** | **C** | **A** | **A** | **G** | **A** | **G** | **G** | **A** | **T** | **A** | **T** | **A** | **G** | **A** | **A** | **G** | **T** | **C** | **G** | **T** | **A** | **G** | **G** | **C** | **G** | **A** | **A** | **G** | **C** | **A** | **G** | **A** | **A** | **A** | **A** | **C** | **G** | **G** | **T** | **A** | **A** | **G** | **A** | **T** | **T** | **G** | **G** | **C** | **T** | **A** | **T** | **G** | **A** | **A** | **A** | **A** | **A** | **G** | **C** | **A** | **T** | **T** | **G** | **G** | **A** | **A** | **C** | **T** | **A** | **C** | **G** | **T** | **C** | **C** | **A** | **G** | **A** | **T** | **G** | **T** | **T** | **A** | **T** | **T** | **T** | **T** | **G** | **A** | **T** | **G** | **G** | **A** | **T** | **T** | **T** | **G** | **G** | **T** | **A** | **A** | **T** | **G** | **G** | **A** | **A** | **G** | **A** | **A** | **A** | **T** | **G** | **G** | **A** | **T** | **G** | **G** | **C** | **A** | **T** | **T** | **G** | **A** | **T** | **T** | **C** | **A** | **A** | **C** | **C** | **A** | **A** | **A** | **G** | **C** | **G** | **A** | **T** | **C** | **T** | **T** | **G** | **A** | **A** | **A** | **G** | **A** | **T** | **T** | **G** | **G** | **C** | **C** | **A** | **G** | **A** | **A** | **G** | **C** | **C** | **A** | **A** | **G** | **A** | **T** | **T** | **A** | **T** | **T** | **A** | **T** | **T** | **G** | **T** | **G** | **A** | **C** | **G** | **A** | **G** | **T** | **T** | **T** | **T** | **A** | **T** | **T** | **G** | **A** | **T** | **G** | **A** | **T** | **G** | **A** | **A** | **A** | **A** | **A** | **G** | **T** | **G** | **T** | **A** | **T** | **C** | **C** | **G** | **G** | **C** | **G** | **A** | **T** | **T** | **G** | **A** | **A** | **G** | **C** | **T** | **G** | **G** | **T** | **G** | **C** | **A** | **G** | **C** | **A** | **G** | **G** | **C** | **T** | **A** | **C** | **C** | **T** | **A** | **T** | **T** | **A** | **A** | **A** | **A** | **A** | **C** | **A** | **T** | **C** | **A** | **A** | **C** | **A** | **G** | **C** | **A** | **C** | **A** | **T** | **G** | **A** | **G** | **A** | **T** | **T** | **G** | **C** | **T** | **G** | **A** | **T** | **G** | **C** | **A** | **A** | **T** | **T** | **C** | **G** | **G** | **G** | **C** | **G** | **A** | **C** | **T** | **T** | **A** | **T** | **C** | **G** | **C** | **G** | **G** | **A** | **G** | **A** | **G** | **C** | **G** | **T** | **G** | **T** | **G** | **T** | **T** | **G** | **G** | **A** | **A** | **C** | **C** | **T** | **G** | **A** | **A** | **G** | **T** | **G** | **A** | **C** | **G** | **C** | **A** | **T** | **A** | **A** | **G** | **A** | **T** | **G** | **A** | **T** | **G** | **G** | **A** | **A** | **C** | **G** | **G** | **T** | **T** | **A** | **A** | **C** | **A** | **A** | **A** | **A** | **A** | **A** | **A** | **C** | **A** | **A** | **G** | **A** | **G** | **C** | **C** | **G** | **G** | **T** | **G** | **T** | **T** | **G** | **C** | **A** | **C** | **G** | **A** | **A** | **G** | **A** | **T** | **T** | **T** | **G** | **A** | **C** | **A** | **A** | **A** | **C** | **C** | **G** | **G** | **G** | **A** | **A** | **C** | **A** | **C** | **G** | **A** | **A** | **A** | **T** | **T** | **T** | **T** | **A** | **A** | **T** | **G** | **T** | **T** | **G** | **A** | **T** | **T** | **G** | **C** | **A** | **C** | **A** | **A** | **G** | **G** | **T** | **A** | **A** | **A** | **A** | **G** | **T** | **A** | **A** | **T** | **C** | **A** | **G** | **G** | **A** | **A** | **A** | **T** | **T** | **G** | **C** | **T** | **G** | **A** | **T** | **G** | **A** | **A** | **C** | **T** | **C** | **T** | **T** | **T** | **A** | **T** | **C** | **A** | **C** | **T** | **T** | **T** | **G** | **A** | **A** | **A** | **A** | **C** | **A** | **G** | **T** | **T** | **A** | **A** | **A** | **A** | **C** | **A** | **C** | **A** | **T** | **G** | **T** | **T** | **T** | **C** | **A** | **A** | **A** | **C** | **A** | **T** | **T** | **T** | **T** | **A** | **G** | **C** | **A** | **A** | **A** | **A** | **C** | **T** | **A** | **G** | **A** | **T** | **G** | **T** | **G** | **G** | **A** | **T** | **G** | **A** | **T** | **C** | **G** | **G** | **A** | **C** | **C** | **C** | **A** | **A** | **G** | **C** | **G** | **G** | **C** | **G** | **A** | **T** | **T** | **T** | **A** | **T** | **G** | **C** | **T** | **T** | **T** | **T** | **C** | **A** | **A** | **C** | **A** | **T** | **G** | **G** | **T** | **T** | **T** | **A** | **G** | **C** | **C** | **A** | **A** | **A** | **-** | **-** | **-** |
| **N53595** | **A** | **T** | **G** | **A** | **T** | **A** | **A** | **A** | **A** | **G** | **T** | **T** | **T** | **T** | **A** | **T** | **T** | **A** | **G** | **T** | **A** | **G** | **A** | **T** | **G** | **A** | **C** | **C** | **A** | **T** | **G** | **A** | **A** | **A** | **T** | **G** | **G** | **T** | **A** | **C** | **G** | **C** | **T** | **T** | **A** | **G** | **G** | **C** | **G** | **T** | **C** | **T** | **C** | **T** | **T** | **C** | **T** | **T** | **A** | **T** | **T** | **T** | **A** | **T** | **C** | **T** | **A** | **T** | **C** | **C** | **A** | **A** | **G** | **A** | **A** | **G** | **A** | **C** | **A** | **T** | **T** | **G** | **A** | **A** | **G** | **T** | **G** | **A** | **T** | **T** | **G** | **G** | **A** | **G** | **A** | **A** | **G** | **C** | **T** | **G** | **A** | **A** | **A** | **A** | **T** | **G** | **G** | **A** | **C** | **G** | **T** | **C** | **A** | **A** | **G** | **G** | **C** | **T** | **A** | **T** | **G** | **A** | **A** | **A** | **A** | **G** | **G** | **C** | **G** | **A** | **T** | **G** | **G** | **C** | **A** | **C** | **T** | **T** | **C** | **G** | **A** | **C** | **C** | **G** | **G** | **A** | **T** | **G** | **T** | **C** | **A** | **T** | **T** | **T** | **T** | **A** | **A** | **T** | **G** | **G** | **A** | **T** | **C** | **T** | **A** | **G** | **T** | **C** | **A** | **T** | **G** | **G** | **A** | **A** | **G** | **A** | **A** | **A** | **T** | **G** | **G** | **A** | **T** | **G** | **G** | **C** | **A** | **T** | **C** | **G** | **A** | **A** | **T** | **C** | **A** | **A** | **C** | **A** | **A** | **A** | **A** | **G** | **C** | **A** | **A** | **T** | **C** | **T** | **T** | **A** | **A** | **A** | **A** | **G** | **A** | **T** | **T** | **G** | **G** | **C** | **C** | **A** | **A** | **A** | **A** | **G** | **C** | **C** | **A** | **A** | **A** | **A** | **T** | **C** | **A** | **T** | **T** | **A** | **T** | **C** | **G** | **T** | **C** | **A** | **C** | **A** | **A** | **G** | **T** | **T** | **T** | **T** | **A** | **T** | **T** | **G** | **A** | **T** | **G** | **A** | **T** | **G** | **A** | **A** | **A** | **A** | **A** | **G** | **T** | **T** | **T** | **A** | **T** | **C** | **C** | **T** | **G** | **C** | **A** | **A** | **T** | **T** | **G** | **A** | **A** | **G** | **C** | **A** | **G** | **G** | **A** | **G** | **C** | **A** | **G** | **C** | **A** | **G** | **G** | **A** | **T** | **A** | **T** | **T** | **T** | **G** | **T** | **T** | **G** | **A** | **A** | **G** | **A** | **C** | **G** | **T** | **C** | **T** | **A** | **C** | **C** | **G** | **C** | **A** | **C** | **A** | **T** | **G** | **A** | **A** | **A** | **T** | **A** | **G** | **C** | **T** | **G** | **A** | **T** | **G** | **C** | **T** | **A** | **T** | **A** | **A** | **G** | **G** | **G** | **C** | **T** | **A** | **C** | **G** | **C** | **A** | **A** | **C** | **G** | **C** | **G** | **G** | **C** | **G** | **A** | **G** | **C** | **G** | **A** | **G** | **T** | **A** | **T** | **T** | **G** | **G** | **A** | **A** | **C** | **C** | **T** | **G** | **A** | **A** | **G** | **T** | **G** | **A** | **C** | **A** | **A** | **C** | **A** | **A** | **A** | **A** | **A** | **T** | **G** | **A** | **T** | **G** | **G** | **A** | **A** | **A** | **A** | **A** | **A** | **T** | **G** | **A** | **G** | **T** | **C** | **G** | **G** | **C** | **G** | **A** | **A** | **A** | **T** | **G** | **A** | **T** | **C** | **C** | **A** | **G** | **T** | **A** | **T** | **T** | **G** | **C** | **A** | **T** | **G** | **A** | **A** | **G** | **A** | **A** | **T** | **T** | **A** | **A** | **C** | **G** | **A** | **A** | **T** | **C** | **G** | **A** | **G** | **A** | **A** | **A** | **A** | **T** | **G** | **A** | **A** | **A** | **T** | **T** | **T** | **T** | **G** | **A** | **T** | **G** | **C** | **T** | **G** | **A** | **T** | **T** | **T** | **C** | **T** | **G** | **A** | **A** | **G** | **G** | **G** | **A** | **A** | **A** | **A** | **G** | **T** | **A** | **A** | **T** | **C** | **A** | **G** | **G** | **A** | **A** | **A** | **T** | **C** | **G** | **C** | **G** | **G** | **A** | **T** | **G** | **A** | **A** | **T** | **T** | **G** | **T** | **T** | **T** | **A** | **T** | **C** | **A** | **C** | **A** | **C** | **T** | **G** | **A** | **A** | **A** | **A** | **C** | **A** | **G** | **T** | **C** | **A** | **A** | **A** | **A** | **C** | **G** | **C** | **A** | **T** | **G** | **T** | **G** | **T** | **C** | **G** | **A** | **A** | **T** | **A** | **T** | **A** | **T** | **T** | **A** | **G** | **C** | **T** | **A** | **A** | **G** | **T** | **T** | **A** | **G** | **A** | **G** | **G** | **T** | **A** | **G** | **A** | **A** | **G** | **A** | **C** | **C** | **G** | **A** | **A** | **C** | **T** | **C** | **A** | **A** | **G** | **C** | **A** | **G** | **C** | **T** | **A** | **T** | **C** | **T** | **A** | **T** | **G** | **C** | **G** | **T** | **T** | **T** | **A** | **A** | **A** | **C** | **A** | **T** | **G** | **G** | **T** | **T** | **T** | **G** | **G** | **T** | **G** | **A** | **A** | **A** | **-** | **-** | **-** |
| **N54595** | **G** | **T** | **G** | **A** | **T** | **C** | **A** | **A** | **A** | **G** | **T** | **A** | **A** | **T** | **G** | **T** | **T** | **A** | **G** | **T** | **G** | **G** | **A** | **T** | **G** | **A** | **C** | **C** | **A** | **T** | **G** | **A** | **A** | **A** | **T** | **G** | **G** | **T** | **C** | **C** | **G** | **T** | **T** | **T** | **A** | **G** | **G** | **C** | **G** | **T** | **T** | **T** | **C** | **A** | **T** | **C** | **A** | **T** | **A** | **T** | **T** | **T** | **A** | **T** | **C** | **T** | **A** | **T** | **T** | **C** | **A** | **A** | **G** | **A** | **G** | **G** | **A** | **T** | **A** | **T** | **A** | **G** | **A** | **A** | **G** | **T** | **C** | **G** | **T** | **A** | **G** | **G** | **C** | **G** | **A** | **A** | **G** | **C** | **A** | **G** | **A** | **A** | **A** | **A** | **C** | **G** | **G** | **T** | **A** | **A** | **G** | **A** | **T** | **T** | **G** | **G** | **C** | **T** | **A** | **T** | **G** | **A** | **A** | **A** | **A** | **A** | **G** | **C** | **A** | **T** | **T** | **G** | **G** | **A** | **A** | **C** | **T** | **A** | **C** | **G** | **T** | **C** | **C** | **A** | **G** | **A** | **T** | **G** | **T** | **T** | **A** | **T** | **T** | **T** | **T** | **G** | **A** | **T** | **G** | **G** | **A** | **T** | **T** | **T** | **G** | **G** | **T** | **A** | **A** | **T** | **G** | **G** | **A** | **A** | **G** | **A** | **A** | **A** | **T** | **G** | **G** | **A** | **C** | **G** | **G** | **C** | **A** | **T** | **T** | **G** | **A** | **T** | **T** | **C** | **A** | **A** | **C** | **A** | **A** | **A** | **A** | **G** | **C** | **G** | **A** | **T** | **C** | **T** | **T** | **G** | **A** | **A** | **A** | **G** | **A** | **T** | **T** | **G** | **G** | **C** | **C** | **A** | **G** | **A** | **A** | **G** | **C** | **C** | **A** | **A** | **G** | **A** | **T** | **T** | **A** | **T** | **T** | **A** | **T** | **T** | **G** | **T** | **G** | **A** | **C** | **G** | **A** | **G** | **T** | **T** | **T** | **T** | **A** | **T** | **T** | **G** | **A** | **T** | **G** | **A** | **T** | **G** | **A** | **A** | **A** | **A** | **A** | **G** | **T** | **G** | **T** | **A** | **T** | **C** | **C** | **G** | **G** | **C** | **G** | **A** | **T** | **T** | **G** | **A** | **A** | **G** | **C** | **T** | **G** | **G** | **T** | **G** | **C** | **A** | **G** | **C** | **G** | **G** | **G** | **C** | **T** | **A** | **C** | **C** | **T** | **A** | **T** | **T** | **A** | **A** | **A** | **A** | **A** | **C** | **A** | **T** | **C** | **A** | **A** | **C** | **A** | **G** | **C** | **A** | **C** | **A** | **T** | **G** | **A** | **G** | **A** | **T** | **T** | **G** | **C** | **T** | **G** | **A** | **T** | **G** | **C** | **A** | **A** | **T** | **T** | **C** | **G** | **G** | **G** | **C** | **G** | **A** | **C** | **T** | **T** | **A** | **T** | **C** | **G** | **C** | **G** | **G** | **A** | **G** | **A** | **G** | **C** | **G** | **T** | **G** | **T** | **G** | **T** | **T** | **G** | **G** | **A** | **A** | **C** | **C** | **T** | **G** | **A** | **A** | **G** | **T** | **G** | **A** | **C** | **G** | **C** | **A** | **T** | **A** | **A** | **G** | **A** | **T** | **G** | **A** | **T** | **G** | **G** | **A** | **A** | **C** | **G** | **G** | **T** | **T** | **A** | **A** | **C** | **A** | **A** | **A** | **A** | **A** | **A** | **A** | **C** | **A** | **A** | **G** | **A** | **G** | **C** | **C** | **G** | **G** | **T** | **G** | **C** | **T** | **A** | **C** | **A** | **T** | **G** | **A** | **A** | **G** | **A** | **T** | **T** | **T** | **G** | **A** | **C** | **A** | **A** | **A** | **C** | **C** | **G** | **A** | **G** | **A** | **A** | **C** | **A** | **C** | **G** | **A** | **A** | **A** | **T** | **T** | **T** | **T** | **A** | **A** | **T** | **G** | **T** | **T** | **G** | **A** | **T** | **T** | **G** | **C** | **A** | **C** | **A** | **A** | **G** | **G** | **T** | **A** | **A** | **A** | **A** | **G** | **T** | **A** | **A** | **T** | **C** | **A** | **G** | **G** | **A** | **A** | **A** | **T** | **A** | **G** | **C** | **T** | **G** | **A** | **T** | **G** | **A** | **A** | **C** | **T** | **C** | **T** | **T** | **T** | **A** | **T** | **C** | **A** | **C** | **T** | **T** | **T** | **G** | **A** | **A** | **A** | **A** | **C** | **A** | **G** | **T** | **T** | **A** | **A** | **A** | **A** | **C** | **A** | **C** | **A** | **T** | **G** | **T** | **T** | **T** | **C** | **A** | **A** | **A** | **C** | **A** | **T** | **T** | **T** | **T** | **A** | **G** | **C** | **A** | **A** | **A** | **A** | **C** | **T** | **A** | **G** | **A** | **T** | **G** | **T** | **G** | **G** | **A** | **T** | **G** | **A** | **T** | **C** | **G** | **G** | **A** | **C** | **C** | **C** | **A** | **A** | **G** | **C** | **G** | **G** | **C** | **G** | **A** | **T** | **T** | **T** | **A** | **T** | **G** | **C** | **T** | **T** | **T** | **T** | **C** | **A** | **A** | **C** | **A** | **T** | **G** | **G** | **T** | **T** | **T** | **A** | **G** | **C** | **C** | **A** | **A** | **A** | **-** | **-** | **-** |
| **N55706** | **G** | **T** | **G** | **A** | **T** | **C** | **A** | **A** | **A** | **G** | **T** | **A** | **A** | **T** | **G** | **T** | **T** | **A** | **G** | **T** | **G** | **G** | **A** | **T** | **G** | **A** | **C** | **C** | **A** | **T** | **G** | **A** | **A** | **A** | **T** | **G** | **G** | **T** | **C** | **C** | **G** | **T** | **T** | **T** | **A** | **G** | **G** | **C** | **G** | **T** | **T** | **T** | **C** | **A** | **T** | **C** | **A** | **T** | **A** | **T** | **T** | **T** | **A** | **T** | **C** | **T** | **A** | **T** | **T** | **C** | **A** | **A** | **G** | **A** | **G** | **G** | **A** | **T** | **A** | **T** | **A** | **G** | **A** | **A** | **G** | **T** | **C** | **G** | **T** | **A** | **G** | **G** | **C** | **G** | **A** | **A** | **G** | **C** | **A** | **G** | **A** | **A** | **A** | **A** | **C** | **G** | **G** | **T** | **A** | **A** | **G** | **A** | **T** | **T** | **G** | **G** | **C** | **T** | **A** | **T** | **G** | **A** | **A** | **A** | **A** | **A** | **G** | **C** | **A** | **T** | **T** | **G** | **G** | **A** | **A** | **C** | **T** | **A** | **C** | **G** | **T** | **C** | **C** | **A** | **G** | **A** | **T** | **G** | **T** | **T** | **A** | **T** | **T** | **T** | **T** | **G** | **A** | **T** | **G** | **G** | **A** | **T** | **T** | **T** | **G** | **G** | **T** | **A** | **A** | **T** | **G** | **G** | **A** | **A** | **G** | **A** | **A** | **A** | **T** | **G** | **G** | **A** | **C** | **G** | **G** | **C** | **A** | **T** | **T** | **G** | **A** | **T** | **T** | **C** | **A** | **A** | **C** | **C** | **A** | **A** | **A** | **G** | **C** | **G** | **A** | **T** | **C** | **T** | **T** | **G** | **A** | **A** | **A** | **G** | **A** | **T** | **T** | **G** | **G** | **C** | **C** | **A** | **G** | **A** | **A** | **G** | **C** | **C** | **A** | **A** | **G** | **A** | **T** | **T** | **A** | **T** | **T** | **A** | **T** | **T** | **G** | **T** | **G** | **A** | **C** | **G** | **A** | **G** | **T** | **T** | **T** | **T** | **A** | **T** | **T** | **G** | **A** | **T** | **G** | **A** | **T** | **G** | **A** | **A** | **A** | **A** | **A** | **G** | **T** | **G** | **T** | **A** | **T** | **C** | **C** | **G** | **G** | **C** | **G** | **A** | **T** | **T** | **G** | **A** | **A** | **G** | **C** | **T** | **G** | **G** | **T** | **G** | **C** | **A** | **G** | **C** | **G** | **G** | **G** | **C** | **T** | **A** | **C** | **C** | **T** | **A** | **T** | **T** | **A** | **A** | **A** | **A** | **A** | **C** | **A** | **T** | **C** | **A** | **A** | **C** | **A** | **G** | **C** | **A** | **C** | **A** | **T** | **G** | **A** | **G** | **A** | **T** | **T** | **G** | **C** | **T** | **G** | **A** | **T** | **G** | **C** | **A** | **A** | **T** | **T** | **C** | **G** | **G** | **G** | **C** | **G** | **A** | **C** | **T** | **T** | **A** | **T** | **C** | **G** | **C** | **G** | **G** | **A** | **G** | **A** | **G** | **C** | **G** | **T** | **G** | **T** | **G** | **T** | **T** | **G** | **G** | **A** | **A** | **C** | **C** | **T** | **G** | **A** | **A** | **G** | **T** | **G** | **A** | **C** | **G** | **C** | **A** | **T** | **A** | **A** | **G** | **A** | **T** | **G** | **A** | **T** | **G** | **G** | **A** | **A** | **C** | **G** | **G** | **T** | **T** | **A** | **A** | **C** | **A** | **A** | **A** | **A** | **A** | **A** | **A** | **C** | **A** | **A** | **G** | **A** | **G** | **C** | **C** | **G** | **G** | **T** | **G** | **T** | **T** | **G** | **C** | **A** | **C** | **G** | **A** | **A** | **G** | **A** | **T** | **T** | **T** | **G** | **A** | **C** | **A** | **A** | **A** | **C** | **C** | **G** | **G** | **G** | **A** | **A** | **C** | **A** | **C** | **G** | **A** | **A** | **A** | **T** | **T** | **T** | **T** | **A** | **A** | **T** | **G** | **T** | **T** | **G** | **A** | **T** | **T** | **G** | **C** | **A** | **C** | **A** | **A** | **G** | **G** | **T** | **A** | **A** | **A** | **A** | **G** | **T** | **A** | **A** | **T** | **C** | **A** | **G** | **G** | **A** | **A** | **A** | **T** | **A** | **G** | **C** | **T** | **G** | **A** | **T** | **G** | **A** | **A** | **C** | **T** | **C** | **T** | **T** | **T** | **A** | **T** | **C** | **A** | **C** | **T** | **T** | **T** | **G** | **A** | **A** | **A** | **A** | **C** | **A** | **G** | **T** | **T** | **A** | **A** | **A** | **A** | **C** | **A** | **C** | **A** | **T** | **G** | **T** | **T** | **T** | **C** | **A** | **A** | **A** | **C** | **A** | **T** | **T** | **T** | **T** | **A** | **G** | **C** | **A** | **A** | **A** | **A** | **C** | **T** | **A** | **G** | **A** | **T** | **G** | **T** | **G** | **G** | **A** | **T** | **G** | **A** | **T** | **C** | **G** | **G** | **A** | **C** | **C** | **C** | **A** | **A** | **G** | **C** | **G** | **G** | **C** | **G** | **A** | **T** | **T** | **T** | **A** | **T** | **G** | **C** | **T** | **T** | **T** | **T** | **C** | **A** | **A** | **C** | **A** | **T** | **G** | **G** | **T** | **T** | **T** | **A** | **G** | **C** | **C** | **A** | **A** | **A** | **-** | **-** | **-** |
| **N60001F** | **A** | **T** | **G** | **A** | **T** | **A** | **A** | **A** | **A** | **G** | **T** | **T** | **T** | **T** | **A** | **T** | **T** | **A** | **G** | **T** | **A** | **G** | **A** | **T** | **G** | **A** | **C** | **C** | **A** | **T** | **G** | **A** | **A** | **A** | **T** | **G** | **G** | **T** | **G** | **C** | **G** | **C** | **T** | **T** | **A** | **G** | **G** | **T** | **G** | **T** | **C** | **T** | **C** | **T** | **T** | **C** | **T** | **T** | **A** | **T** | **T** | **T** | **A** | **T** | **C** | **T** | **A** | **T** | **T** | **C** | **A** | **A** | **G** | **A** | **A** | **G** | **A** | **C** | **A** | **T** | **T** | **G** | **A** | **A** | **G** | **T** | **A** | **A** | **T** | **T** | **G** | **G** | **A** | **G** | **A** | **A** | **G** | **C** | **T** | **A** | **A** | **A** | **A** | **A** | **T** | **G** | **G** | **A** | **C** | **G** | **A** | **C** | **A** | **A** | **G** | **G** | **C** | **T** | **A** | **T** | **G** | **A** | **G** | **A** | **A** | **G** | **G** | **C** | **G** | **A** | **T** | **G** | **A** | **C** | **A** | **C** | **T** | **T** | **C** | **G** | **A** | **C** | **C** | **G** | **G** | **A** | **C** | **G** | **T** | **C** | **A** | **T** | **T** | **T** | **T** | **A** | **A** | **T** | **G** | **G** | **A** | **C** | **T** | **T** | **A** | **G** | **T** | **C** | **A** | **T** | **G** | **G** | **A** | **A** | **G** | **A** | **A** | **A** | **T** | **G** | **G** | **A** | **T** | **G** | **G** | **T** | **A** | **T** | **C** | **G** | **A** | **A** | **T** | **C** | **A** | **A** | **C** | **A** | **A** | **A** | **A** | **G** | **C** | **A** | **A** | **T** | **C** | **T** | **T** | **A** | **A** | **A** | **A** | **G** | **A** | **T** | **T** | **G** | **G** | **C** | **C** | **A** | **G** | **A** | **A** | **G** | **C** | **T** | **A** | **A** | **A** | **A** | **T** | **C** | **A** | **T** | **T** | **A** | **T** | **C** | **G** | **T** | **A** | **A** | **C** | **C** | **A** | **G** | **T** | **T** | **T** | **T** | **A** | **T** | **T** | **G** | **A** | **T** | **G** | **A** | **C** | **G** | **A** | **A** | **A** | **A** | **A** | **G** | **T** | **T** | **T** | **A** | **T** | **C** | **C** | **T** | **G** | **C** | **C** | **A** | **T** | **T** | **G** | **A** | **A** | **G** | **C** | **A** | **G** | **G** | **T** | **G** | **C** | **A** | **G** | **C** | **A** | **G** | **G** | **G** | **T** | **A** | **T** | **T** | **T** | **G** | **T** | **T** | **G** | **A** | **A** | **G** | **A** | **C** | **C** | **T** | **C** | **T** | **A** | **C** | **C** | **G** | **C** | **A** | **C** | **A** | **T** | **G** | **A** | **A** | **A** | **T** | **T** | **G** | **C** | **T** | **G** | **A** | **T** | **G** | **C** | **T** | **A** | **T** | **A** | **A** | **G** | **G** | **G** | **C** | **A** | **A** | **C** | **G** | **C** | **A** | **A** | **C** | **G** | **C** | **G** | **G** | **C** | **G** | **A** | **G** | **C** | **G** | **A** | **G** | **T** | **A** | **T** | **T** | **A** | **G** | **A** | **A** | **C** | **C** | **A** | **G** | **A** | **A** | **G** | **T** | **G** | **A** | **C** | **A** | **A** | **C** | **A** | **A** | **A** | **A** | **A** | **T** | **G** | **A** | **T** | **G** | **G** | **A** | **A** | **A** | **A** | **A** | **A** | **T** | **G** | **A** | **G** | **T** | **C** | **G** | **G** | **C** | **G** | **A** | **A** | **A** | **T** | **G** | **A** | **G** | **C** | **C** | **T** | **G** | **T** | **A** | **T** | **T** | **A** | **C** | **A** | **T** | **G** | **A** | **A** | **G** | **A** | **A** | **C** | **T** | **A** | **A** | **C** | **G** | **A** | **A** | **T** | **C** | **G** | **G** | **G** | **A** | **A** | **A** | **A** | **T** | **G** | **A** | **A** | **A** | **T** | **T** | **T** | **T** | **A** | **A** | **T** | **G** | **C** | **T** | **G** | **A** | **T** | **T** | **T** | **C** | **T** | **G** | **A** | **A** | **G** | **G** | **A** | **A** | **A** | **A** | **A** | **G** | **T** | **A** | **A** | **T** | **C** | **A** | **G** | **G** | **A** | **A** | **A** | **T** | **A** | **G** | **C** | **G** | **G** | **A** | **C** | **G** | **A** | **A** | **T** | **T** | **G** | **T** | **T** | **T** | **A** | **T** | **T** | **A** | **C** | **A** | **C** | **T** | **G** | **A** | **A** | **A** | **A** | **C** | **A** | **G** | **T** | **A** | **A** | **A** | **A** | **A** | **C** | **A** | **C** | **A** | **T** | **G** | **T** | **T** | **T** | **C** | **C** | **A** | **A** | **T** | **A** | **T** | **A** | **T** | **T** | **G** | **G** | **C** | **T** | **A** | **A** | **G** | **C** | **T** | **A** | **G** | **A** | **A** | **G** | **T** | **G** | **G** | **A** | **A** | **G** | **A** | **C** | **C** | **G** | **T** | **A** | **C** | **A** | **C** | **A** | **A** | **G** | **C** | **C** | **G** | **C** | **T** | **A** | **T** | **C** | **T** | **A** | **T** | **G** | **C** | **A** | **T** | **T** | **C** | **A** | **A** | **A** | **C** | **A** | **T** | **G** | **G** | **T** | **T** | **T** | **G** | **G** | **T** | **G** | **A** | **A** | **A** | **-** | **-** | **-** |
| **N59653F** | **A** | **T** | **G** | **A** | **T** | **A** | **A** | **A** | **A** | **G** | **T** | **T** | **T** | **T** | **A** | **T** | **T** | **A** | **G** | **T** | **A** | **G** | **A** | **T** | **G** | **A** | **C** | **C** | **A** | **T** | **G** | **A** | **A** | **A** | **T** | **G** | **G** | **T** | **G** | **C** | **G** | **C** | **T** | **T** | **A** | **G** | **G** | **C** | **G** | **T** | **C** | **T** | **C** | **T** | **T** | **C** | **T** | **T** | **A** | **T** | **T** | **T** | **A** | **T** | **C** | **T** | **A** | **T** | **T** | **C** | **A** | **A** | **G** | **A** | **A** | **G** | **A** | **C** | **A** | **T** | **T** | **G** | **A** | **A** | **G** | **T** | **A** | **A** | **T** | **T** | **G** | **G** | **A** | **G** | **A** | **A** | **G** | **C** | **T** | **G** | **A** | **A** | **A** | **A** | **T** | **G** | **G** | **A** | **C** | **G** | **A** | **C** | **A** | **A** | **G** | **G** | **C** | **T** | **A** | **T** | **G** | **A** | **G** | **A** | **A** | **G** | **G** | **C** | **G** | **A** | **T** | **G** | **A** | **C** | **A** | **C** | **T** | **T** | **C** | **G** | **A** | **C** | **C** | **G** | **G** | **A** | **C** | **G** | **T** | **C** | **A** | **T** | **T** | **T** | **T** | **A** | **A** | **T** | **G** | **G** | **A** | **C** | **T** | **T** | **A** | **G** | **T** | **C** | **A** | **T** | **G** | **G** | **A** | **A** | **G** | **A** | **A** | **A** | **T** | **G** | **G** | **A** | **T** | **G** | **G** | **T** | **A** | **T** | **C** | **G** | **A** | **A** | **T** | **C** | **A** | **A** | **C** | **A** | **A** | **A** | **A** | **G** | **C** | **A** | **A** | **T** | **C** | **T** | **T** | **A** | **A** | **A** | **A** | **G** | **A** | **T** | **T** | **G** | **G** | **C** | **C** | **A** | **G** | **A** | **A** | **G** | **C** | **T** | **A** | **A** | **A** | **A** | **T** | **C** | **A** | **T** | **T** | **A** | **T** | **C** | **G** | **T** | **A** | **A** | **C** | **C** | **A** | **G** | **T** | **T** | **T** | **T** | **A** | **T** | **T** | **G** | **A** | **T** | **G** | **A** | **C** | **G** | **A** | **A** | **A** | **A** | **A** | **G** | **T** | **T** | **T** | **A** | **T** | **C** | **C** | **T** | **G** | **C** | **C** | **A** | **T** | **T** | **G** | **A** | **A** | **G** | **C** | **A** | **G** | **G** | **T** | **G** | **C** | **A** | **G** | **C** | **A** | **G** | **G** | **G** | **T** | **A** | **T** | **T** | **T** | **G** | **T** | **T** | **G** | **A** | **A** | **G** | **A** | **C** | **C** | **T** | **C** | **T** | **A** | **C** | **C** | **G** | **C** | **A** | **C** | **A** | **T** | **G** | **A** | **A** | **A** | **T** | **T** | **G** | **C** | **T** | **G** | **A** | **T** | **G** | **C** | **T** | **A** | **T** | **A** | **A** | **G** | **G** | **G** | **C** | **A** | **A** | **C** | **G** | **C** | **A** | **A** | **C** | **G** | **T** | **G** | **G** | **C** | **G** | **A** | **G** | **C** | **G** | **A** | **G** | **T** | **A** | **T** | **T** | **A** | **G** | **A** | **A** | **C** | **C** | **A** | **G** | **A** | **A** | **G** | **T** | **G** | **A** | **C** | **A** | **A** | **C** | **A** | **A** | **A** | **A** | **A** | **T** | **G** | **A** | **T** | **G** | **G** | **A** | **A** | **A** | **A** | **A** | **A** | **T** | **G** | **A** | **G** | **T** | **C** | **G** | **G** | **C** | **G** | **A** | **A** | **A** | **T** | **G** | **A** | **G** | **C** | **C** | **T** | **G** | **T** | **A** | **T** | **T** | **A** | **C** | **A** | **T** | **G** | **A** | **A** | **G** | **A** | **A** | **C** | **T** | **A** | **A** | **C** | **G** | **A** | **A** | **T** | **C** | **G** | **G** | **G** | **A** | **A** | **A** | **A** | **T** | **G** | **A** | **A** | **A** | **T** | **T** | **T** | **T** | **A** | **A** | **T** | **G** | **C** | **T** | **G** | **A** | **T** | **T** | **T** | **C** | **T** | **G** | **A** | **A** | **G** | **G** | **A** | **A** | **A** | **A** | **A** | **G** | **T** | **A** | **A** | **T** | **C** | **A** | **G** | **G** | **A** | **A** | **A** | **T** | **A** | **G** | **C** | **G** | **G** | **A** | **C** | **G** | **A** | **A** | **T** | **T** | **G** | **T** | **T** | **T** | **A** | **T** | **T** | **A** | **C** | **A** | **C** | **T** | **G** | **A** | **A** | **A** | **A** | **C** | **A** | **G** | **T** | **A** | **A** | **A** | **A** | **A** | **C** | **A** | **C** | **A** | **T** | **G** | **T** | **T** | **T** | **C** | **C** | **A** | **A** | **T** | **A** | **T** | **A** | **T** | **T** | **G** | **G** | **C** | **T** | **A** | **A** | **G** | **C** | **T** | **A** | **G** | **A** | **A** | **G** | **T** | **G** | **G** | **A** | **A** | **G** | **A** | **C** | **C** | **G** | **T** | **A** | **C** | **A** | **C** | **A** | **A** | **G** | **C** | **C** | **G** | **C** | **T** | **A** | **T** | **C** | **T** | **A** | **T** | **G** | **C** | **A** | **T** | **T** | **C** | **A** | **A** | **A** | **C** | **A** | **T** | **G** | **G** | **T** | **T** | **T** | **G** | **G** | **T** | **G** | **A** | **A** | **A** | **-** | **-** | **-** |
| **N60048F** | **A** | **T** | **G** | **A** | **T** | **A** | **A** | **A** | **A** | **G** | **T** | **T** | **T** | **T** | **A** | **T** | **T** | **A** | **G** | **T** | **A** | **G** | **A** | **T** | **G** | **A** | **C** | **C** | **A** | **T** | **G** | **A** | **A** | **A** | **T** | **G** | **G** | **T** | **A** | **C** | **G** | **C** | **T** | **T** | **A** | **G** | **G** | **C** | **G** | **T** | **C** | **T** | **C** | **T** | **T** | **C** | **T** | **T** | **A** | **T** | **T** | **T** | **A** | **T** | **C** | **T** | **A** | **T** | **C** | **C** | **A** | **A** | **G** | **A** | **A** | **G** | **A** | **C** | **A** | **T** | **T** | **G** | **A** | **A** | **G** | **T** | **G** | **A** | **T** | **T** | **G** | **G** | **A** | **G** | **A** | **A** | **G** | **C** | **T** | **G** | **A** | **A** | **A** | **A** | **T** | **G** | **G** | **A** | **C** | **G** | **T** | **C** | **A** | **A** | **G** | **G** | **C** | **T** | **A** | **T** | **G** | **A** | **A** | **A** | **A** | **G** | **G** | **C** | **G** | **A** | **T** | **G** | **G** | **C** | **A** | **C** | **T** | **T** | **C** | **G** | **A** | **C** | **C** | **A** | **G** | **A** | **T** | **G** | **T** | **C** | **A** | **T** | **T** | **T** | **T** | **A** | **A** | **T** | **G** | **G** | **A** | **T** | **C** | **T** | **A** | **G** | **T** | **C** | **A** | **T** | **G** | **G** | **A** | **A** | **G** | **A** | **A** | **A** | **T** | **G** | **G** | **A** | **T** | **G** | **G** | **C** | **A** | **T** | **C** | **G** | **A** | **A** | **T** | **C** | **A** | **A** | **C** | **A** | **A** | **A** | **A** | **G** | **C** | **A** | **A** | **T** | **C** | **T** | **T** | **A** | **A** | **A** | **A** | **G** | **A** | **T** | **T** | **G** | **G** | **C** | **C** | **A** | **A** | **A** | **A** | **G** | **C** | **C** | **A** | **A** | **A** | **A** | **T** | **C** | **A** | **T** | **T** | **A** | **T** | **C** | **G** | **T** | **C** | **A** | **C** | **A** | **A** | **G** | **T** | **T** | **T** | **T** | **A** | **T** | **T** | **G** | **A** | **T** | **G** | **A** | **T** | **G** | **A** | **A** | **A** | **A** | **A** | **G** | **T** | **T** | **T** | **A** | **T** | **C** | **C** | **T** | **G** | **C** | **A** | **A** | **T** | **T** | **G** | **A** | **A** | **G** | **C** | **A** | **G** | **G** | **A** | **G** | **C** | **A** | **G** | **C** | **A** | **G** | **G** | **A** | **T** | **A** | **T** | **T** | **T** | **G** | **T** | **T** | **G** | **A** | **A** | **G** | **A** | **C** | **G** | **T** | **C** | **T** | **A** | **C** | **C** | **G** | **C** | **A** | **C** | **A** | **T** | **G** | **A** | **A** | **A** | **T** | **A** | **G** | **C** | **T** | **G** | **A** | **T** | **G** | **C** | **T** | **A** | **T** | **A** | **A** | **G** | **G** | **G** | **C** | **T** | **A** | **C** | **G** | **C** | **A** | **A** | **C** | **G** | **C** | **G** | **G** | **C** | **G** | **A** | **G** | **C** | **G** | **A** | **G** | **T** | **A** | **T** | **T** | **G** | **G** | **A** | **A** | **C** | **C** | **T** | **G** | **A** | **A** | **G** | **T** | **G** | **A** | **C** | **A** | **A** | **C** | **A** | **A** | **A** | **A** | **A** | **T** | **G** | **A** | **T** | **G** | **G** | **A** | **A** | **A** | **A** | **A** | **A** | **T** | **G** | **A** | **G** | **T** | **C** | **G** | **G** | **C** | **G** | **A** | **A** | **A** | **T** | **G** | **A** | **T** | **C** | **C** | **A** | **G** | **T** | **A** | **T** | **T** | **G** | **C** | **A** | **T** | **G** | **A** | **A** | **G** | **A** | **A** | **T** | **T** | **A** | **A** | **C** | **G** | **A** | **A** | **T** | **C** | **G** | **A** | **G** | **A** | **A** | **A** | **A** | **T** | **G** | **A** | **A** | **A** | **T** | **T** | **T** | **T** | **G** | **A** | **T** | **G** | **C** | **T** | **G** | **A** | **T** | **T** | **T** | **C** | **T** | **G** | **A** | **A** | **G** | **G** | **G** | **A** | **A** | **A** | **A** | **G** | **T** | **A** | **A** | **T** | **C** | **A** | **G** | **G** | **A** | **A** | **A** | **T** | **C** | **G** | **C** | **G** | **G** | **A** | **T** | **G** | **A** | **A** | **T** | **T** | **G** | **T** | **T** | **T** | **A** | **T** | **C** | **A** | **C** | **A** | **C** | **T** | **G** | **A** | **A** | **A** | **A** | **C** | **A** | **G** | **T** | **T** | **A** | **A** | **A** | **A** | **C** | **G** | **C** | **A** | **T** | **G** | **T** | **G** | **T** | **C** | **G** | **A** | **A** | **T** | **A** | **T** | **A** | **T** | **T** | **A** | **G** | **C** | **T** | **A** | **A** | **G** | **T** | **T** | **A** | **G** | **A** | **G** | **G** | **T** | **A** | **G** | **A** | **A** | **G** | **A** | **C** | **C** | **G** | **A** | **A** | **C** | **T** | **C** | **A** | **A** | **G** | **C** | **A** | **G** | **C** | **T** | **A** | **T** | **C** | **T** | **A** | **T** | **G** | **C** | **G** | **T** | **T** | **T** | **A** | **A** | **A** | **C** | **A** | **T** | **G** | **G** | **T** | **T** | **T** | **G** | **G** | **T** | **G** | **A** | **A** | **A** | **-** | **-** | **-** |
| **N59420F** | **G** | **T** | **G** | **A** | **T** | **C** | **A** | **A** | **A** | **G** | **T** | **A** | **A** | **T** | **G** | **T** | **T** | **A** | **G** | **T** | **G** | **G** | **A** | **T** | **G** | **A** | **C** | **C** | **A** | **T** | **G** | **A** | **A** | **A** | **T** | **G** | **G** | **T** | **C** | **C** | **G** | **T** | **T** | **T** | **A** | **G** | **G** | **C** | **G** | **T** | **T** | **T** | **C** | **A** | **T** | **C** | **A** | **T** | **A** | **T** | **T** | **T** | **A** | **T** | **C** | **T** | **A** | **T** | **T** | **C** | **A** | **A** | **G** | **A** | **G** | **G** | **A** | **T** | **A** | **T** | **A** | **G** | **A** | **A** | **G** | **T** | **C** | **G** | **T** | **A** | **G** | **G** | **C** | **G** | **A** | **A** | **G** | **C** | **A** | **G** | **A** | **A** | **A** | **A** | **T** | **G** | **G** | **T** | **A** | **A** | **G** | **A** | **T** | **T** | **G** | **G** | **C** | **T** | **A** | **T** | **G** | **A** | **A** | **A** | **A** | **A** | **G** | **C** | **A** | **T** | **T** | **G** | **G** | **A** | **A** | **C** | **T** | **A** | **C** | **G** | **T** | **C** | **C** | **A** | **G** | **A** | **T** | **G** | **T** | **T** | **A** | **T** | **T** | **T** | **T** | **G** | **A** | **T** | **G** | **G** | **A** | **T** | **T** | **T** | **G** | **G** | **T** | **A** | **A** | **T** | **G** | **G** | **A** | **A** | **G** | **A** | **A** | **A** | **T** | **G** | **G** | **A** | **C** | **G** | **G** | **C** | **A** | **T** | **T** | **G** | **A** | **T** | **T** | **C** | **A** | **A** | **C** | **A** | **A** | **A** | **A** | **G** | **C** | **G** | **A** | **T** | **C** | **T** | **T** | **G** | **A** | **A** | **A** | **G** | **A** | **T** | **T** | **G** | **G** | **C** | **C** | **A** | **G** | **A** | **A** | **G** | **C** | **C** | **A** | **A** | **G** | **A** | **T** | **T** | **A** | **T** | **T** | **A** | **T** | **T** | **G** | **T** | **G** | **A** | **C** | **G** | **A** | **G** | **T** | **T** | **T** | **T** | **A** | **T** | **T** | **G** | **A** | **T** | **G** | **A** | **T** | **G** | **A** | **A** | **A** | **A** | **A** | **G** | **T** | **G** | **T** | **A** | **T** | **C** | **C** | **G** | **G** | **C** | **G** | **A** | **T** | **T** | **G** | **A** | **A** | **G** | **C** | **T** | **G** | **G** | **T** | **G** | **C** | **A** | **G** | **C** | **G** | **G** | **G** | **C** | **T** | **A** | **C** | **C** | **T** | **A** | **T** | **T** | **A** | **A** | **A** | **A** | **A** | **C** | **A** | **T** | **C** | **A** | **A** | **C** | **A** | **G** | **C** | **A** | **C** | **A** | **T** | **G** | **A** | **G** | **A** | **T** | **T** | **G** | **C** | **T** | **G** | **A** | **T** | **G** | **C** | **A** | **A** | **T** | **T** | **C** | **G** | **G** | **G** | **C** | **G** | **A** | **C** | **T** | **T** | **A** | **T** | **C** | **G** | **C** | **G** | **G** | **A** | **G** | **A** | **G** | **C** | **G** | **T** | **G** | **T** | **G** | **T** | **T** | **G** | **G** | **A** | **A** | **C** | **C** | **T** | **G** | **A** | **A** | **G** | **T** | **G** | **A** | **C** | **G** | **C** | **A** | **T** | **A** | **A** | **G** | **A** | **T** | **G** | **A** | **T** | **G** | **G** | **A** | **A** | **C** | **G** | **G** | **T** | **T** | **A** | **A** | **C** | **A** | **A** | **A** | **A** | **A** | **A** | **A** | **C** | **A** | **A** | **G** | **A** | **G** | **C** | **C** | **G** | **G** | **T** | **G** | **T** | **T** | **G** | **C** | **A** | **C** | **G** | **A** | **A** | **G** | **A** | **T** | **T** | **T** | **G** | **A** | **C** | **A** | **A** | **A** | **C** | **C** | **G** | **A** | **G** | **A** | **A** | **C** | **A** | **C** | **G** | **A** | **A** | **A** | **T** | **T** | **T** | **T** | **A** | **A** | **T** | **G** | **T** | **T** | **G** | **A** | **T** | **T** | **G** | **C** | **A** | **C** | **A** | **A** | **G** | **G** | **T** | **A** | **A** | **A** | **A** | **G** | **T** | **A** | **A** | **T** | **C** | **A** | **G** | **G** | **A** | **A** | **A** | **T** | **T** | **G** | **C** | **T** | **G** | **A** | **T** | **G** | **A** | **A** | **C** | **T** | **C** | **T** | **T** | **T** | **A** | **T** | **C** | **A** | **C** | **T** | **T** | **T** | **G** | **A** | **A** | **A** | **A** | **C** | **A** | **G** | **T** | **T** | **A** | **A** | **A** | **A** | **C** | **A** | **C** | **A** | **T** | **G** | **T** | **T** | **T** | **C** | **A** | **A** | **A** | **C** | **A** | **T** | **T** | **T** | **T** | **A** | **G** | **C** | **A** | **A** | **A** | **A** | **C** | **T** | **A** | **G** | **A** | **T** | **G** | **T** | **G** | **G** | **A** | **T** | **G** | **A** | **T** | **C** | **G** | **G** | **A** | **C** | **C** | **C** | **A** | **A** | **G** | **C** | **G** | **G** | **C** | **G** | **A** | **T** | **T** | **T** | **A** | **T** | **G** | **C** | **T** | **T** | **T** | **T** | **C** | **A** | **A** | **C** | **A** | **T** | **G** | **G** | **T** | **T** | **T** | **A** | **G** | **C** | **C** | **A** | **A** | **A** | **-** | **-** | **-** |
| **N52467** | **G** | **T** | **G** | **A** | **T** | **C** | **A** | **A** | **A** | **G** | **T** | **A** | **A** | **T** | **G** | **T** | **T** | **A** | **G** | **T** | **G** | **G** | **A** | **T** | **G** | **A** | **C** | **C** | **A** | **T** | **G** | **A** | **A** | **A** | **T** | **G** | **G** | **T** | **C** | **C** | **G** | **T** | **T** | **T** | **A** | **G** | **G** | **C** | **G** | **T** | **T** | **T** | **C** | **A** | **T** | **C** | **A** | **T** | **A** | **T** | **T** | **T** | **A** | **T** | **C** | **T** | **A** | **T** | **T** | **C** | **A** | **A** | **G** | **A** | **G** | **G** | **A** | **T** | **A** | **T** | **A** | **G** | **A** | **A** | **G** | **T** | **C** | **G** | **T** | **A** | **G** | **G** | **C** | **G** | **A** | **A** | **G** | **C** | **A** | **G** | **A** | **A** | **A** | **A** | **C** | **G** | **G** | **T** | **A** | **A** | **G** | **A** | **T** | **T** | **G** | **G** | **C** | **T** | **A** | **T** | **G** | **A** | **A** | **A** | **A** | **A** | **G** | **C** | **A** | **T** | **T** | **G** | **G** | **A** | **A** | **C** | **T** | **A** | **C** | **G** | **T** | **C** | **C** | **A** | **G** | **A** | **T** | **G** | **T** | **T** | **A** | **T** | **T** | **T** | **T** | **G** | **A** | **T** | **G** | **G** | **A** | **C** | **T** | **T** | **G** | **G** | **T** | **A** | **A** | **T** | **G** | **G** | **A** | **A** | **G** | **A** | **A** | **A** | **T** | **G** | **G** | **A** | **C** | **G** | **G** | **C** | **A** | **T** | **T** | **G** | **A** | **T** | **T** | **C** | **A** | **A** | **C** | **A** | **A** | **A** | **A** | **G** | **C** | **G** | **A** | **T** | **T** | **T** | **T** | **G** | **A** | **A** | **A** | **G** | **A** | **T** | **T** | **G** | **G** | **C** | **C** | **A** | **G** | **A** | **A** | **G** | **C** | **C** | **A** | **A** | **G** | **A** | **T** | **T** | **A** | **T** | **T** | **A** | **T** | **T** | **G** | **T** | **G** | **A** | **C** | **G** | **A** | **G** | **T** | **T** | **T** | **T** | **A** | **T** | **T** | **G** | **A** | **T** | **G** | **A** | **T** | **G** | **A** | **A** | **A** | **A** | **A** | **G** | **T** | **G** | **T** | **A** | **T** | **C** | **C** | **G** | **G** | **C** | **A** | **A** | **T** | **T** | **G** | **A** | **A** | **G** | **C** | **T** | **G** | **G** | **T** | **G** | **C** | **A** | **G** | **C** | **G** | **G** | **G** | **C** | **T** | **A** | **C** | **C** | **T** | **A** | **T** | **T** | **A** | **A** | **A** | **A** | **A** | **C** | **A** | **T** | **C** | **A** | **A** | **C** | **A** | **G** | **C** | **A** | **C** | **A** | **T** | **G** | **A** | **G** | **A** | **T** | **T** | **G** | **C** | **T** | **G** | **A** | **T** | **G** | **C** | **G** | **A** | **T** | **T** | **C** | **G** | **G** | **G** | **C** | **G** | **A** | **C** | **T** | **T** | **A** | **T** | **C** | **G** | **C** | **G** | **G** | **A** | **G** | **A** | **G** | **C** | **G** | **T** | **G** | **T** | **G** | **T** | **T** | **G** | **G** | **A** | **A** | **C** | **C** | **T** | **G** | **A** | **A** | **G** | **T** | **G** | **A** | **C** | **G** | **C** | **A** | **T** | **A** | **A** | **G** | **A** | **T** | **G** | **A** | **T** | **G** | **G** | **A** | **A** | **C** | **G** | **G** | **T** | **T** | **A** | **A** | **C** | **A** | **A** | **A** | **A** | **A** | **A** | **A** | **C** | **A** | **A** | **G** | **A** | **G** | **C** | **C** | **G** | **G** | **T** | **G** | **C** | **T** | **A** | **C** | **A** | **C** | **G** | **A** | **A** | **G** | **A** | **T** | **T** | **T** | **G** | **A** | **C** | **A** | **A** | **A** | **C** | **C** | **G** | **A** | **G** | **A** | **A** | **C** | **A** | **C** | **G** | **A** | **A** | **A** | **T** | **T** | **T** | **T** | **A** | **A** | **T** | **G** | **C** | **T** | **G** | **A** | **T** | **T** | **G** | **C** | **A** | **C** | **A** | **A** | **G** | **G** | **T** | **A** | **A** | **A** | **A** | **G** | **T** | **A** | **A** | **T** | **C** | **A** | **G** | **G** | **A** | **A** | **A** | **T** | **T** | **G** | **C** | **T** | **G** | **A** | **T** | **G** | **A** | **A** | **C** | **T** | **C** | **T** | **T** | **T** | **A** | **T** | **C** | **A** | **C** | **T** | **T** | **T** | **G** | **A** | **A** | **A** | **A** | **C** | **A** | **G** | **T** | **T** | **A** | **A** | **A** | **A** | **C** | **A** | **C** | **A** | **T** | **G** | **T** | **T** | **T** | **C** | **A** | **A** | **A** | **C** | **A** | **T** | **T** | **T** | **T** | **A** | **G** | **C** | **A** | **A** | **A** | **A** | **C** | **T** | **A** | **G** | **A** | **T** | **G** | **T** | **G** | **G** | **A** | **T** | **G** | **A** | **T** | **C** | **G** | **G** | **A** | **C** | **C** | **C** | **A** | **A** | **G** | **C** | **G** | **G** | **C** | **G** | **A** | **T** | **T** | **T** | **A** | **T** | **G** | **C** | **A** | **T** | **T** | **T** | **C** | **A** | **A** | **C** | **A** | **C** | **G** | **G** | **T** | **T** | **T** | **A** | **G** | **C** | **C** | **A** | **A** | **A** | **-** | **-** | **-** |
| **N60027F** | **G** | **T** | **G** | **A** | **T** | **C** | **A** | **A** | **A** | **G** | **T** | **A** | **A** | **T** | **G** | **T** | **T** | **A** | **G** | **T** | **G** | **G** | **A** | **T** | **G** | **A** | **C** | **C** | **A** | **T** | **G** | **A** | **A** | **A** | **T** | **G** | **G** | **T** | **C** | **C** | **G** | **T** | **T** | **T** | **A** | **G** | **G** | **C** | **G** | **T** | **T** | **T** | **C** | **A** | **T** | **C** | **A** | **T** | **A** | **T** | **T** | **T** | **A** | **T** | **C** | **T** | **A** | **T** | **T** | **C** | **A** | **A** | **G** | **A** | **G** | **G** | **A** | **T** | **A** | **T** | **A** | **G** | **A** | **A** | **G** | **T** | **C** | **G** | **T** | **A** | **G** | **G** | **C** | **G** | **A** | **A** | **G** | **C** | **A** | **G** | **A** | **A** | **A** | **A** | **C** | **G** | **G** | **T** | **A** | **A** | **G** | **A** | **T** | **T** | **G** | **G** | **C** | **T** | **A** | **T** | **G** | **A** | **A** | **A** | **A** | **A** | **G** | **C** | **A** | **T** | **T** | **G** | **G** | **A** | **A** | **C** | **T** | **A** | **C** | **G** | **T** | **C** | **C** | **A** | **G** | **A** | **T** | **G** | **T** | **T** | **A** | **T** | **T** | **T** | **T** | **G** | **A** | **T** | **G** | **G** | **A** | **T** | **T** | **T** | **G** | **G** | **T** | **A** | **A** | **T** | **G** | **G** | **A** | **A** | **G** | **A** | **A** | **A** | **T** | **G** | **G** | **A** | **C** | **G** | **G** | **C** | **A** | **T** | **T** | **G** | **A** | **T** | **T** | **C** | **A** | **A** | **C** | **A** | **A** | **A** | **A** | **G** | **C** | **G** | **A** | **T** | **C** | **T** | **T** | **G** | **A** | **A** | **A** | **G** | **A** | **T** | **T** | **G** | **G** | **C** | **C** | **A** | **G** | **A** | **A** | **G** | **C** | **C** | **A** | **A** | **G** | **A** | **T** | **T** | **A** | **T** | **T** | **A** | **T** | **T** | **G** | **T** | **G** | **A** | **C** | **G** | **A** | **G** | **T** | **T** | **T** | **T** | **A** | **T** | **T** | **G** | **A** | **T** | **G** | **A** | **T** | **G** | **A** | **A** | **A** | **A** | **A** | **G** | **T** | **G** | **T** | **A** | **T** | **C** | **C** | **G** | **G** | **C** | **G** | **A** | **T** | **T** | **G** | **A** | **A** | **G** | **C** | **T** | **G** | **G** | **T** | **G** | **C** | **A** | **G** | **C** | **G** | **G** | **G** | **C** | **T** | **A** | **C** | **C** | **T** | **A** | **T** | **T** | **A** | **A** | **A** | **A** | **A** | **C** | **A** | **T** | **C** | **A** | **A** | **C** | **A** | **G** | **C** | **A** | **C** | **A** | **T** | **G** | **A** | **G** | **A** | **T** | **T** | **G** | **C** | **T** | **G** | **A** | **T** | **G** | **C** | **A** | **A** | **T** | **T** | **C** | **G** | **G** | **G** | **C** | **G** | **A** | **C** | **T** | **T** | **A** | **T** | **C** | **G** | **C** | **G** | **G** | **A** | **G** | **A** | **G** | **C** | **G** | **T** | **G** | **T** | **G** | **T** | **T** | **G** | **G** | **A** | **A** | **C** | **C** | **T** | **G** | **A** | **A** | **G** | **T** | **G** | **A** | **C** | **A** | **C** | **A** | **T** | **A** | **A** | **G** | **A** | **T** | **G** | **A** | **T** | **G** | **G** | **A** | **A** | **C** | **G** | **G** | **T** | **T** | **A** | **A** | **C** | **A** | **A** | **A** | **A** | **A** | **A** | **A** | **C** | **A** | **A** | **G** | **A** | **G** | **C** | **C** | **G** | **G** | **T** | **G** | **T** | **T** | **G** | **C** | **A** | **C** | **G** | **A** | **A** | **G** | **A** | **T** | **T** | **T** | **G** | **A** | **C** | **A** | **A** | **A** | **C** | **C** | **G** | **G** | **G** | **A** | **A** | **C** | **A** | **C** | **G** | **A** | **A** | **A** | **T** | **T** | **T** | **T** | **A** | **A** | **T** | **G** | **T** | **T** | **G** | **A** | **T** | **T** | **G** | **C** | **A** | **C** | **A** | **A** | **G** | **G** | **T** | **A** | **A** | **A** | **A** | **G** | **T** | **A** | **A** | **T** | **C** | **A** | **G** | **G** | **A** | **A** | **A** | **T** | **A** | **G** | **C** | **T** | **G** | **A** | **T** | **G** | **A** | **A** | **C** | **T** | **C** | **T** | **T** | **T** | **A** | **T** | **C** | **A** | **C** | **T** | **T** | **T** | **G** | **A** | **A** | **A** | **A** | **C** | **A** | **G** | **T** | **T** | **A** | **A** | **A** | **A** | **C** | **A** | **C** | **A** | **T** | **G** | **T** | **T** | **T** | **C** | **A** | **A** | **A** | **C** | **A** | **T** | **T** | **T** | **T** | **A** | **G** | **C** | **A** | **A** | **A** | **A** | **C** | **T** | **A** | **G** | **A** | **T** | **G** | **T** | **G** | **G** | **A** | **T** | **G** | **A** | **T** | **C** | **G** | **G** | **A** | **C** | **C** | **C** | **A** | **A** | **G** | **C** | **G** | **G** | **C** | **G** | **A** | **T** | **T** | **T** | **A** | **T** | **G** | **C** | **T** | **T** | **T** | **T** | **C** | **A** | **A** | **C** | **A** | **T** | **G** | **G** | **T** | **T** | **T** | **A** | **G** | **C** | **C** | **A** | **A** | **A** | **-** | **-** | **-** |
| **N52769** | **A** | **T** | **G** | **A** | **T** | **A** | **A** | **A** | **A** | **G** | **T** | **T** | **T** | **T** | **A** | **T** | **T** | **A** | **G** | **T** | **A** | **G** | **A** | **T** | **G** | **A** | **C** | **C** | **A** | **T** | **G** | **A** | **A** | **A** | **T** | **G** | **G** | **T** | **G** | **C** | **G** | **C** | **T** | **T** | **A** | **G** | **G** | **C** | **G** | **T** | **C** | **T** | **C** | **T** | **T** | **C** | **T** | **T** | **A** | **T** | **T** | **T** | **A** | **T** | **C** | **T** | **A** | **T** | **C** | **C** | **A** | **A** | **G** | **A** | **A** | **G** | **A** | **C** | **A** | **T** | **T** | **G** | **A** | **A** | **G** | **T** | **A** | **A** | **T** | **T** | **G** | **G** | **A** | **G** | **A** | **A** | **G** | **C** | **T** | **G** | **A** | **A** | **A** | **A** | **T** | **G** | **G** | **A** | **C** | **G** | **A** | **C** | **A** | **A** | **G** | **G** | **C** | **T** | **A** | **T** | **G** | **A** | **G** | **A** | **A** | **G** | **G** | **C** | **G** | **A** | **T** | **G** | **A** | **C** | **A** | **C** | **T** | **T** | **C** | **G** | **A** | **C** | **C** | **G** | **G** | **A** | **C** | **G** | **T** | **C** | **A** | **T** | **T** | **T** | **T** | **A** | **A** | **T** | **G** | **G** | **A** | **C** | **T** | **T** | **A** | **G** | **T** | **C** | **A** | **T** | **G** | **G** | **A** | **A** | **G** | **A** | **A** | **A** | **T** | **G** | **G** | **A** | **T** | **G** | **G** | **T** | **A** | **T** | **C** | **G** | **A** | **A** | **T** | **C** | **A** | **A** | **C** | **A** | **A** | **A** | **A** | **G** | **C** | **A** | **A** | **T** | **C** | **T** | **T** | **A** | **A** | **A** | **A** | **G** | **A** | **T** | **T** | **G** | **G** | **C** | **C** | **A** | **G** | **A** | **A** | **G** | **C** | **T** | **A** | **A** | **A** | **A** | **T** | **C** | **A** | **T** | **T** | **A** | **T** | **C** | **G** | **T** | **A** | **A** | **C** | **C** | **A** | **G** | **T** | **T** | **T** | **T** | **A** | **T** | **T** | **G** | **A** | **T** | **G** | **A** | **C** | **G** | **A** | **A** | **A** | **A** | **A** | **G** | **T** | **T** | **T** | **A** | **T** | **C** | **C** | **T** | **G** | **C** | **C** | **A** | **T** | **T** | **G** | **A** | **A** | **G** | **C** | **A** | **G** | **G** | **C** | **G** | **C** | **A** | **G** | **C** | **A** | **G** | **G** | **G** | **T** | **A** | **T** | **T** | **T** | **G** | **T** | **T** | **G** | **A** | **A** | **G** | **A** | **C** | **T** | **T** | **C** | **T** | **A** | **C** | **C** | **G** | **C** | **A** | **C** | **A** | **T** | **G** | **A** | **A** | **A** | **T** | **T** | **G** | **C** | **T** | **G** | **A** | **T** | **G** | **C** | **T** | **A** | **T** | **A** | **A** | **G** | **G** | **G** | **C** | **A** | **A** | **C** | **G** | **C** | **A** | **A** | **C** | **G** | **C** | **G** | **G** | **C** | **G** | **A** | **G** | **C** | **G** | **A** | **G** | **T** | **A** | **C** | **T** | **A** | **G** | **A** | **A** | **C** | **C** | **A** | **G** | **A** | **A** | **G** | **T** | **G** | **A** | **C** | **A** | **A** | **C** | **A** | **A** | **A** | **A** | **A** | **T** | **G** | **A** | **T** | **G** | **G** | **A** | **A** | **A** | **A** | **A** | **A** | **T** | **G** | **A** | **G** | **T** | **C** | **G** | **G** | **C** | **G** | **A** | **A** | **A** | **T** | **G** | **A** | **G** | **C** | **C** | **T** | **G** | **T** | **A** | **T** | **T** | **A** | **C** | **A** | **T** | **G** | **A** | **A** | **G** | **A** | **A** | **C** | **T** | **A** | **A** | **C** | **G** | **A** | **A** | **T** | **C** | **G** | **G** | **G** | **A** | **A** | **A** | **A** | **T** | **G** | **A** | **A** | **A** | **T** | **T** | **T** | **T** | **A** | **A** | **T** | **G** | **C** | **T** | **G** | **A** | **T** | **T** | **T** | **C** | **T** | **G** | **A** | **A** | **G** | **G** | **G** | **A** | **A** | **A** | **A** | **G** | **T** | **A** | **A** | **T** | **C** | **A** | **G** | **G** | **A** | **A** | **A** | **T** | **A** | **G** | **C** | **G** | **G** | **A** | **C** | **G** | **A** | **A** | **T** | **T** | **G** | **T** | **T** | **T** | **A** | **T** | **T** | **A** | **C** | **A** | **C** | **T** | **G** | **A** | **A** | **A** | **A** | **C** | **A** | **G** | **T** | **A** | **A** | **A** | **A** | **A** | **C** | **A** | **C** | **A** | **T** | **G** | **T** | **T** | **T** | **C** | **C** | **A** | **A** | **T** | **A** | **T** | **A** | **T** | **T** | **G** | **G** | **C** | **T** | **A** | **A** | **G** | **C** | **T** | **A** | **G** | **A** | **A** | **G** | **T** | **G** | **G** | **A** | **A** | **G** | **A** | **C** | **C** | **G** | **T** | **A** | **C** | **A** | **C** | **A** | **A** | **G** | **C** | **C** | **G** | **C** | **T** | **A** | **T** | **C** | **T** | **A** | **T** | **G** | **C** | **A** | **T** | **T** | **C** | **A** | **A** | **A** | **C** | **A** | **T** | **G** | **G** | **T** | **T** | **T** | **G** | **G** | **T** | **G** | **A** | **A** | **A** | **-** | **-** | **-** |
| **N54676** | **G** | **T** | **G** | **A** | **T** | **C** | **A** | **A** | **A** | **G** | **T** | **A** | **A** | **T** | **G** | **T** | **T** | **A** | **G** | **T** | **G** | **G** | **A** | **T** | **G** | **A** | **C** | **C** | **A** | **T** | **G** | **A** | **A** | **A** | **T** | **G** | **G** | **T** | **C** | **C** | **G** | **T** | **T** | **T** | **A** | **G** | **G** | **C** | **G** | **T** | **T** | **T** | **C** | **A** | **T** | **C** | **A** | **T** | **A** | **T** | **T** | **T** | **A** | **T** | **C** | **T** | **A** | **T** | **T** | **C** | **A** | **A** | **G** | **A** | **G** | **G** | **A** | **T** | **A** | **T** | **A** | **G** | **A** | **A** | **G** | **T** | **C** | **G** | **T** | **A** | **G** | **G** | **C** | **G** | **A** | **A** | **G** | **C** | **A** | **G** | **A** | **A** | **A** | **A** | **C** | **G** | **G** | **T** | **A** | **A** | **G** | **A** | **T** | **T** | **G** | **G** | **C** | **T** | **A** | **T** | **G** | **A** | **A** | **A** | **A** | **A** | **G** | **C** | **A** | **T** | **T** | **G** | **G** | **A** | **A** | **C** | **T** | **A** | **C** | **G** | **T** | **C** | **C** | **A** | **G** | **A** | **T** | **G** | **T** | **T** | **A** | **T** | **T** | **T** | **T** | **G** | **A** | **T** | **G** | **G** | **A** | **T** | **T** | **T** | **G** | **G** | **T** | **A** | **A** | **T** | **G** | **G** | **A** | **A** | **G** | **A** | **A** | **A** | **T** | **G** | **G** | **A** | **C** | **G** | **G** | **C** | **A** | **T** | **T** | **G** | **A** | **T** | **T** | **C** | **A** | **A** | **C** | **A** | **A** | **A** | **A** | **G** | **C** | **A** | **A** | **T** | **C** | **T** | **T** | **G** | **A** | **A** | **A** | **G** | **A** | **T** | **T** | **G** | **G** | **C** | **C** | **A** | **G** | **A** | **A** | **G** | **C** | **C** | **A** | **A** | **G** | **A** | **T** | **T** | **A** | **T** | **T** | **A** | **T** | **T** | **G** | **T** | **G** | **A** | **C** | **G** | **A** | **G** | **T** | **T** | **T** | **T** | **A** | **T** | **T** | **G** | **A** | **T** | **G** | **A** | **T** | **G** | **A** | **G** | **A** | **A** | **A** | **G** | **T** | **G** | **T** | **A** | **T** | **C** | **C** | **G** | **G** | **C** | **G** | **A** | **T** | **T** | **G** | **A** | **A** | **G** | **C** | **T** | **G** | **G** | **T** | **G** | **C** | **A** | **G** | **C** | **G** | **G** | **G** | **C** | **T** | **A** | **C** | **C** | **T** | **A** | **T** | **T** | **A** | **A** | **A** | **A** | **A** | **C** | **A** | **T** | **C** | **A** | **A** | **C** | **A** | **G** | **C** | **A** | **C** | **A** | **T** | **G** | **A** | **G** | **A** | **T** | **T** | **G** | **C** | **T** | **G** | **A** | **T** | **G** | **C** | **A** | **A** | **T** | **T** | **C** | **G** | **G** | **G** | **C** | **G** | **A** | **C** | **T** | **T** | **A** | **T** | **C** | **G** | **C** | **G** | **G** | **A** | **G** | **A** | **G** | **C** | **G** | **T** | **G** | **T** | **G** | **T** | **T** | **G** | **G** | **A** | **A** | **C** | **C** | **T** | **G** | **A** | **A** | **G** | **T** | **G** | **A** | **C** | **G** | **C** | **A** | **T** | **A** | **A** | **G** | **A** | **T** | **G** | **A** | **T** | **G** | **G** | **A** | **A** | **C** | **G** | **G** | **T** | **T** | **A** | **A** | **C** | **A** | **A** | **A** | **A** | **A** | **A** | **A** | **C** | **A** | **A** | **G** | **A** | **G** | **C** | **C** | **G** | **G** | **T** | **G** | **T** | **T** | **G** | **C** | **A** | **T** | **G** | **A** | **A** | **G** | **A** | **T** | **T** | **T** | **G** | **A** | **C** | **A** | **A** | **A** | **T** | **C** | **G** | **A** | **G** | **A** | **A** | **C** | **A** | **C** | **G** | **A** | **A** | **A** | **T** | **T** | **T** | **T** | **A** | **A** | **T** | **G** | **T** | **T** | **G** | **A** | **T** | **T** | **G** | **C** | **A** | **C** | **A** | **A** | **G** | **G** | **T** | **A** | **A** | **A** | **A** | **G** | **T** | **A** | **A** | **T** | **C** | **A** | **G** | **G** | **A** | **A** | **A** | **T** | **A** | **G** | **C** | **T** | **G** | **A** | **T** | **G** | **A** | **A** | **C** | **T** | **C** | **T** | **T** | **T** | **A** | **T** | **C** | **A** | **C** | **T** | **T** | **T** | **G** | **A** | **A** | **A** | **A** | **C** | **A** | **G** | **T** | **T** | **A** | **A** | **A** | **A** | **C** | **A** | **C** | **A** | **T** | **G** | **T** | **T** | **T** | **C** | **A** | **A** | **A** | **C** | **A** | **T** | **T** | **T** | **T** | **A** | **G** | **C** | **A** | **A** | **A** | **A** | **C** | **T** | **A** | **G** | **A** | **T** | **G** | **T** | **G** | **G** | **A** | **T** | **G** | **A** | **T** | **C** | **G** | **G** | **A** | **C** | **C** | **C** | **A** | **A** | **G** | **C** | **G** | **G** | **C** | **G** | **A** | **T** | **T** | **T** | **A** | **T** | **G** | **C** | **T** | **T** | **T** | **T** | **C** | **A** | **A** | **C** | **A** | **T** | **G** | **G** | **T** | **T** | **T** | **A** | **G** | **C** | **C** | **A** | **A** | **A** | **-** | **-** | **-** |
| **N52454** | **G** | **T** | **G** | **A** | **T** | **C** | **A** | **A** | **A** | **G** | **T** | **A** | **A** | **T** | **G** | **T** | **T** | **A** | **G** | **T** | **G** | **G** | **A** | **T** | **G** | **A** | **C** | **C** | **A** | **T** | **G** | **A** | **A** | **A** | **T** | **G** | **G** | **T** | **C** | **C** | **G** | **T** | **T** | **T** | **A** | **G** | **G** | **C** | **G** | **T** | **T** | **T** | **C** | **A** | **T** | **C** | **A** | **T** | **A** | **T** | **T** | **T** | **A** | **T** | **C** | **T** | **A** | **T** | **T** | **C** | **A** | **A** | **G** | **A** | **G** | **G** | **A** | **T** | **A** | **T** | **A** | **G** | **A** | **A** | **G** | **T** | **C** | **G** | **T** | **A** | **G** | **G** | **C** | **G** | **A** | **A** | **G** | **C** | **A** | **G** | **A** | **A** | **A** | **A** | **C** | **G** | **G** | **T** | **A** | **A** | **G** | **A** | **T** | **T** | **G** | **G** | **C** | **T** | **A** | **T** | **G** | **A** | **A** | **A** | **A** | **A** | **G** | **C** | **A** | **T** | **T** | **G** | **G** | **A** | **A** | **C** | **T** | **A** | **C** | **G** | **T** | **C** | **C** | **A** | **G** | **A** | **T** | **G** | **T** | **T** | **A** | **T** | **T** | **T** | **T** | **G** | **A** | **T** | **G** | **G** | **A** | **T** | **T** | **T** | **G** | **G** | **T** | **A** | **A** | **T** | **G** | **G** | **A** | **A** | **G** | **A** | **A** | **A** | **T** | **G** | **G** | **A** | **C** | **G** | **G** | **C** | **A** | **T** | **T** | **G** | **A** | **T** | **T** | **C** | **A** | **A** | **C** | **A** | **A** | **A** | **A** | **G** | **C** | **G** | **A** | **T** | **C** | **T** | **T** | **G** | **A** | **A** | **A** | **G** | **A** | **T** | **T** | **G** | **G** | **C** | **C** | **A** | **G** | **A** | **A** | **G** | **C** | **C** | **A** | **A** | **G** | **A** | **T** | **T** | **A** | **T** | **T** | **A** | **T** | **T** | **G** | **T** | **G** | **A** | **C** | **G** | **A** | **G** | **T** | **T** | **T** | **T** | **A** | **T** | **T** | **G** | **A** | **T** | **G** | **A** | **T** | **G** | **A** | **A** | **A** | **A** | **A** | **G** | **T** | **G** | **T** | **A** | **T** | **C** | **C** | **G** | **G** | **C** | **G** | **A** | **T** | **T** | **G** | **A** | **A** | **G** | **C** | **T** | **G** | **G** | **T** | **G** | **C** | **A** | **G** | **C** | **G** | **G** | **G** | **C** | **T** | **A** | **C** | **C** | **T** | **A** | **T** | **T** | **A** | **A** | **A** | **A** | **A** | **C** | **A** | **T** | **C** | **A** | **A** | **C** | **A** | **G** | **C** | **A** | **C** | **A** | **T** | **G** | **A** | **G** | **A** | **T** | **T** | **G** | **C** | **T** | **G** | **A** | **T** | **G** | **C** | **A** | **A** | **T** | **T** | **C** | **G** | **G** | **G** | **C** | **G** | **A** | **C** | **T** | **T** | **A** | **T** | **C** | **G** | **C** | **G** | **G** | **A** | **G** | **A** | **G** | **C** | **G** | **T** | **G** | **T** | **G** | **T** | **T** | **G** | **G** | **A** | **A** | **C** | **C** | **T** | **G** | **A** | **A** | **G** | **T** | **G** | **A** | **C** | **G** | **C** | **A** | **T** | **A** | **A** | **G** | **A** | **T** | **G** | **A** | **T** | **G** | **G** | **A** | **A** | **C** | **G** | **G** | **T** | **T** | **A** | **A** | **C** | **A** | **A** | **A** | **A** | **A** | **A** | **A** | **C** | **A** | **A** | **G** | **A** | **G** | **C** | **C** | **G** | **G** | **T** | **G** | **T** | **T** | **G** | **C** | **A** | **C** | **G** | **A** | **A** | **G** | **A** | **T** | **T** | **T** | **G** | **A** | **C** | **A** | **A** | **A** | **C** | **C** | **G** | **G** | **G** | **A** | **A** | **C** | **A** | **C** | **G** | **A** | **A** | **A** | **T** | **T** | **T** | **T** | **A** | **A** | **T** | **G** | **T** | **T** | **G** | **A** | **T** | **T** | **G** | **C** | **A** | **C** | **A** | **A** | **G** | **G** | **T** | **A** | **A** | **A** | **A** | **G** | **T** | **A** | **A** | **T** | **C** | **A** | **G** | **G** | **A** | **A** | **A** | **T** | **A** | **G** | **C** | **T** | **G** | **A** | **T** | **G** | **A** | **A** | **C** | **T** | **C** | **T** | **T** | **T** | **A** | **T** | **C** | **A** | **C** | **T** | **T** | **T** | **G** | **A** | **A** | **A** | **A** | **C** | **A** | **G** | **T** | **T** | **A** | **A** | **A** | **A** | **C** | **A** | **C** | **A** | **T** | **G** | **T** | **T** | **T** | **C** | **A** | **A** | **A** | **C** | **A** | **T** | **T** | **T** | **T** | **A** | **G** | **C** | **A** | **A** | **A** | **A** | **C** | **T** | **A** | **G** | **A** | **T** | **G** | **T** | **G** | **G** | **A** | **T** | **G** | **A** | **T** | **C** | **G** | **G** | **A** | **C** | **C** | **C** | **A** | **A** | **G** | **C** | **G** | **G** | **C** | **G** | **A** | **T** | **T** | **T** | **A** | **T** | **G** | **C** | **T** | **T** | **T** | **T** | **C** | **A** | **A** | **C** | **A** | **T** | **G** | **G** | **T** | **T** | **T** | **A** | **G** | **C** | **C** | **A** | **A** | **A** | **-** | **-** | **-** |
| **N59469F** | **A** | **T** | **G** | **A** | **T** | **A** | **A** | **A** | **A** | **G** | **T** | **T** | **T** | **T** | **A** | **T** | **T** | **A** | **G** | **T** | **A** | **G** | **A** | **T** | **G** | **A** | **C** | **C** | **A** | **T** | **G** | **A** | **A** | **A** | **T** | **G** | **G** | **T** | **A** | **C** | **G** | **C** | **T** | **T** | **A** | **G** | **G** | **C** | **G** | **T** | **C** | **T** | **C** | **T** | **T** | **C** | **T** | **T** | **A** | **T** | **T** | **T** | **A** | **T** | **C** | **T** | **A** | **T** | **C** | **C** | **A** | **A** | **G** | **A** | **A** | **G** | **A** | **C** | **A** | **T** | **T** | **G** | **A** | **A** | **G** | **T** | **G** | **A** | **T** | **T** | **G** | **G** | **A** | **G** | **A** | **A** | **G** | **C** | **T** | **G** | **A** | **A** | **A** | **A** | **T** | **G** | **G** | **A** | **C** | **G** | **T** | **C** | **A** | **A** | **G** | **G** | **C** | **T** | **A** | **T** | **G** | **A** | **A** | **A** | **A** | **G** | **G** | **C** | **G** | **A** | **T** | **G** | **G** | **C** | **A** | **C** | **T** | **T** | **C** | **G** | **A** | **C** | **C** | **A** | **G** | **A** | **T** | **G** | **T** | **C** | **A** | **T** | **T** | **T** | **T** | **A** | **A** | **T** | **G** | **G** | **A** | **T** | **C** | **T** | **A** | **G** | **T** | **C** | **A** | **T** | **G** | **G** | **A** | **A** | **G** | **A** | **A** | **A** | **T** | **G** | **G** | **A** | **T** | **G** | **G** | **C** | **A** | **T** | **C** | **G** | **A** | **A** | **T** | **C** | **A** | **A** | **C** | **A** | **A** | **A** | **A** | **G** | **C** | **A** | **A** | **T** | **C** | **T** | **T** | **A** | **A** | **A** | **A** | **G** | **A** | **T** | **T** | **G** | **G** | **C** | **C** | **A** | **A** | **A** | **A** | **G** | **C** | **C** | **A** | **A** | **A** | **A** | **T** | **C** | **A** | **T** | **T** | **A** | **T** | **C** | **G** | **T** | **C** | **A** | **C** | **A** | **A** | **G** | **T** | **T** | **T** | **T** | **A** | **T** | **T** | **G** | **A** | **T** | **G** | **A** | **T** | **G** | **A** | **A** | **A** | **A** | **A** | **G** | **T** | **T** | **T** | **A** | **T** | **C** | **C** | **T** | **G** | **C** | **A** | **A** | **T** | **T** | **G** | **A** | **A** | **G** | **C** | **A** | **G** | **G** | **A** | **G** | **C** | **A** | **G** | **C** | **A** | **G** | **G** | **A** | **T** | **A** | **T** | **T** | **T** | **G** | **T** | **T** | **G** | **A** | **A** | **G** | **A** | **C** | **G** | **T** | **C** | **T** | **A** | **C** | **C** | **G** | **C** | **A** | **C** | **A** | **T** | **G** | **A** | **A** | **A** | **T** | **A** | **G** | **C** | **T** | **G** | **A** | **T** | **G** | **C** | **T** | **A** | **T** | **A** | **A** | **G** | **G** | **G** | **C** | **T** | **A** | **C** | **G** | **C** | **A** | **A** | **C** | **G** | **C** | **G** | **G** | **C** | **G** | **A** | **G** | **C** | **G** | **A** | **G** | **T** | **A** | **T** | **T** | **G** | **G** | **A** | **A** | **C** | **C** | **T** | **G** | **A** | **A** | **G** | **T** | **G** | **A** | **C** | **A** | **A** | **C** | **A** | **A** | **A** | **A** | **A** | **T** | **G** | **A** | **T** | **G** | **G** | **A** | **A** | **A** | **A** | **A** | **A** | **T** | **G** | **A** | **G** | **T** | **C** | **G** | **G** | **C** | **G** | **A** | **A** | **A** | **T** | **G** | **A** | **T** | **C** | **C** | **A** | **G** | **T** | **A** | **T** | **T** | **G** | **C** | **A** | **T** | **G** | **A** | **A** | **G** | **A** | **A** | **T** | **T** | **A** | **A** | **C** | **G** | **A** | **A** | **T** | **C** | **G** | **A** | **G** | **A** | **A** | **A** | **A** | **T** | **G** | **A** | **A** | **A** | **T** | **T** | **T** | **T** | **G** | **A** | **T** | **G** | **C** | **T** | **G** | **A** | **T** | **T** | **T** | **C** | **T** | **G** | **A** | **A** | **G** | **G** | **G** | **A** | **A** | **A** | **A** | **G** | **T** | **A** | **A** | **T** | **C** | **A** | **G** | **G** | **A** | **A** | **A** | **T** | **C** | **G** | **C** | **G** | **G** | **A** | **T** | **G** | **A** | **A** | **T** | **T** | **G** | **T** | **T** | **T** | **A** | **T** | **C** | **A** | **C** | **A** | **C** | **T** | **G** | **A** | **A** | **A** | **A** | **C** | **A** | **G** | **T** | **T** | **A** | **A** | **A** | **A** | **C** | **G** | **C** | **A** | **T** | **G** | **T** | **G** | **T** | **C** | **G** | **A** | **A** | **T** | **A** | **T** | **A** | **T** | **T** | **A** | **G** | **C** | **T** | **A** | **A** | **G** | **T** | **T** | **A** | **G** | **A** | **G** | **G** | **T** | **A** | **G** | **A** | **A** | **G** | **A** | **C** | **C** | **G** | **A** | **A** | **C** | **T** | **C** | **A** | **A** | **G** | **C** | **A** | **G** | **C** | **T** | **A** | **T** | **C** | **T** | **A** | **T** | **G** | **C** | **G** | **T** | **T** | **T** | **A** | **A** | **A** | **C** | **A** | **T** | **G** | **G** | **T** | **T** | **T** | **G** | **G** | **T** | **G** | **A** | **A** | **A** | **-** | **-** | **-** |
| **N59745F** | **A** | **T** | **G** | **A** | **T** | **A** | **A** | **A** | **A** | **G** | **T** | **T** | **T** | **T** | **A** | **T** | **T** | **A** | **G** | **T** | **A** | **G** | **A** | **T** | **G** | **A** | **C** | **C** | **A** | **T** | **G** | **A** | **A** | **A** | **T** | **G** | **G** | **T** | **G** | **C** | **G** | **C** | **T** | **T** | **A** | **G** | **G** | **C** | **G** | **T** | **C** | **T** | **C** | **T** | **T** | **C** | **T** | **T** | **A** | **T** | **T** | **T** | **A** | **T** | **C** | **T** | **A** | **T** | **T** | **C** | **A** | **A** | **G** | **A** | **A** | **G** | **A** | **C** | **A** | **T** | **T** | **G** | **A** | **A** | **G** | **T** | **A** | **A** | **T** | **T** | **G** | **G** | **A** | **G** | **A** | **A** | **G** | **C** | **T** | **G** | **A** | **A** | **A** | **A** | **T** | **G** | **G** | **A** | **C** | **G** | **A** | **C** | **A** | **A** | **G** | **G** | **C** | **T** | **A** | **T** | **G** | **A** | **G** | **A** | **A** | **G** | **G** | **C** | **G** | **A** | **T** | **G** | **A** | **C** | **A** | **C** | **T** | **T** | **C** | **G** | **A** | **C** | **C** | **G** | **G** | **A** | **C** | **G** | **T** | **C** | **A** | **T** | **T** | **T** | **T** | **A** | **A** | **T** | **G** | **G** | **A** | **C** | **T** | **T** | **A** | **G** | **T** | **C** | **A** | **T** | **G** | **G** | **A** | **A** | **G** | **A** | **A** | **A** | **T** | **G** | **G** | **A** | **T** | **G** | **G** | **T** | **A** | **T** | **C** | **G** | **A** | **A** | **T** | **C** | **A** | **A** | **C** | **A** | **A** | **A** | **A** | **G** | **C** | **A** | **A** | **T** | **C** | **T** | **T** | **A** | **A** | **A** | **A** | **G** | **A** | **T** | **T** | **G** | **G** | **C** | **C** | **A** | **G** | **A** | **A** | **G** | **C** | **T** | **A** | **A** | **A** | **A** | **T** | **C** | **A** | **T** | **T** | **A** | **T** | **C** | **G** | **T** | **A** | **A** | **C** | **C** | **A** | **G** | **T** | **T** | **T** | **T** | **A** | **T** | **T** | **G** | **A** | **T** | **G** | **A** | **C** | **G** | **A** | **A** | **A** | **A** | **A** | **G** | **T** | **T** | **T** | **A** | **T** | **C** | **C** | **T** | **G** | **C** | **C** | **A** | **T** | **T** | **G** | **A** | **G** | **G** | **C** | **A** | **G** | **G** | **T** | **G** | **C** | **A** | **G** | **C** | **A** | **G** | **G** | **G** | **T** | **A** | **T** | **T** | **T** | **G** | **T** | **T** | **G** | **A** | **A** | **G** | **A** | **C** | **C** | **T** | **C** | **T** | **A** | **C** | **C** | **G** | **C** | **A** | **C** | **A** | **T** | **G** | **A** | **A** | **A** | **T** | **T** | **G** | **C** | **T** | **G** | **A** | **T** | **G** | **C** | **T** | **A** | **T** | **A** | **A** | **G** | **G** | **G** | **C** | **A** | **A** | **C** | **G** | **C** | **A** | **A** | **C** | **G** | **T** | **G** | **G** | **C** | **G** | **A** | **G** | **C** | **G** | **A** | **G** | **T** | **A** | **T** | **T** | **A** | **G** | **A** | **A** | **C** | **C** | **A** | **G** | **A** | **A** | **G** | **T** | **G** | **A** | **C** | **A** | **A** | **C** | **A** | **A** | **A** | **A** | **A** | **T** | **G** | **A** | **T** | **G** | **G** | **A** | **A** | **A** | **A** | **A** | **A** | **T** | **G** | **A** | **G** | **T** | **C** | **G** | **G** | **C** | **G** | **A** | **A** | **A** | **T** | **G** | **A** | **G** | **C** | **C** | **T** | **G** | **T** | **A** | **T** | **T** | **A** | **C** | **A** | **T** | **G** | **A** | **A** | **G** | **A** | **A** | **C** | **T** | **A** | **A** | **C** | **G** | **A** | **A** | **T** | **C** | **G** | **G** | **G** | **A** | **A** | **A** | **A** | **T** | **G** | **A** | **A** | **A** | **T** | **T** | **T** | **T** | **A** | **A** | **T** | **G** | **C** | **T** | **G** | **A** | **T** | **T** | **T** | **C** | **T** | **G** | **A** | **A** | **G** | **G** | **A** | **A** | **A** | **A** | **A** | **G** | **T** | **A** | **A** | **T** | **C** | **A** | **G** | **G** | **A** | **A** | **A** | **T** | **A** | **G** | **C** | **G** | **G** | **A** | **C** | **G** | **A** | **A** | **T** | **T** | **G** | **T** | **T** | **T** | **A** | **T** | **T** | **A** | **C** | **A** | **C** | **T** | **G** | **A** | **A** | **A** | **A** | **C** | **A** | **G** | **T** | **A** | **A** | **A** | **A** | **A** | **C** | **A** | **C** | **A** | **T** | **G** | **T** | **T** | **T** | **C** | **C** | **A** | **A** | **T** | **A** | **T** | **A** | **T** | **T** | **G** | **G** | **C** | **T** | **A** | **A** | **G** | **C** | **T** | **A** | **G** | **A** | **A** | **G** | **T** | **G** | **G** | **A** | **A** | **G** | **A** | **C** | **C** | **G** | **T** | **A** | **C** | **A** | **C** | **A** | **A** | **G** | **C** | **C** | **G** | **C** | **T** | **A** | **T** | **C** | **T** | **A** | **T** | **G** | **C** | **A** | **T** | **T** | **C** | **A** | **A** | **A** | **C** | **A** | **T** | **G** | **G** | **T** | **T** | **T** | **G** | **G** | **T** | **G** | **A** | **A** | **A** | **-** | **-** | **-** |
| **N59466F** | **G** | **T** | **G** | **A** | **T** | **C** | **A** | **A** | **A** | **G** | **T** | **A** | **A** | **T** | **G** | **T** | **T** | **A** | **G** | **T** | **G** | **G** | **A** | **T** | **G** | **A** | **C** | **C** | **A** | **T** | **G** | **A** | **A** | **A** | **T** | **G** | **G** | **T** | **C** | **C** | **G** | **T** | **T** | **T** | **A** | **G** | **G** | **C** | **G** | **T** | **T** | **T** | **C** | **A** | **T** | **C** | **A** | **T** | **A** | **T** | **T** | **T** | **A** | **T** | **C** | **T** | **A** | **T** | **T** | **C** | **A** | **A** | **G** | **A** | **G** | **G** | **A** | **T** | **A** | **T** | **A** | **G** | **A** | **A** | **G** | **T** | **C** | **G** | **T** | **A** | **G** | **G** | **C** | **G** | **A** | **A** | **G** | **C** | **A** | **G** | **A** | **A** | **A** | **A** | **C** | **G** | **G** | **T** | **A** | **A** | **G** | **A** | **T** | **T** | **G** | **G** | **C** | **T** | **A** | **T** | **G** | **A** | **A** | **A** | **A** | **A** | **G** | **C** | **A** | **T** | **T** | **G** | **G** | **A** | **A** | **C** | **T** | **A** | **C** | **G** | **T** | **C** | **C** | **A** | **G** | **A** | **T** | **G** | **T** | **T** | **A** | **T** | **T** | **T** | **T** | **G** | **A** | **T** | **G** | **G** | **A** | **T** | **T** | **T** | **G** | **G** | **T** | **A** | **A** | **T** | **G** | **G** | **A** | **A** | **G** | **A** | **A** | **A** | **T** | **G** | **G** | **A** | **C** | **G** | **G** | **C** | **A** | **T** | **T** | **G** | **A** | **T** | **T** | **C** | **A** | **A** | **C** | **A** | **A** | **A** | **A** | **G** | **C** | **G** | **A** | **T** | **C** | **T** | **T** | **G** | **A** | **A** | **A** | **G** | **A** | **T** | **T** | **G** | **G** | **C** | **C** | **A** | **G** | **A** | **A** | **G** | **C** | **C** | **A** | **A** | **G** | **A** | **T** | **T** | **A** | **T** | **T** | **A** | **T** | **T** | **G** | **T** | **G** | **A** | **C** | **G** | **A** | **G** | **T** | **T** | **T** | **T** | **A** | **T** | **T** | **G** | **A** | **T** | **G** | **A** | **T** | **G** | **A** | **A** | **A** | **A** | **A** | **G** | **T** | **G** | **T** | **A** | **T** | **C** | **C** | **G** | **G** | **C** | **G** | **A** | **T** | **T** | **G** | **A** | **A** | **G** | **C** | **T** | **G** | **G** | **T** | **G** | **C** | **A** | **G** | **C** | **G** | **G** | **G** | **C** | **T** | **A** | **C** | **C** | **T** | **A** | **T** | **T** | **A** | **A** | **A** | **A** | **A** | **C** | **A** | **T** | **C** | **A** | **A** | **C** | **A** | **G** | **C** | **A** | **C** | **A** | **T** | **G** | **A** | **G** | **A** | **T** | **T** | **G** | **C** | **T** | **G** | **A** | **T** | **G** | **C** | **A** | **A** | **T** | **T** | **C** | **G** | **G** | **G** | **C** | **G** | **A** | **C** | **T** | **T** | **A** | **T** | **C** | **G** | **C** | **G** | **G** | **A** | **G** | **A** | **G** | **C** | **G** | **T** | **G** | **T** | **G** | **T** | **T** | **G** | **G** | **A** | **A** | **C** | **C** | **T** | **G** | **A** | **A** | **G** | **T** | **G** | **A** | **C** | **G** | **C** | **A** | **T** | **A** | **A** | **G** | **A** | **T** | **G** | **A** | **T** | **G** | **G** | **A** | **A** | **C** | **G** | **G** | **T** | **T** | **A** | **A** | **C** | **A** | **A** | **A** | **A** | **A** | **A** | **A** | **C** | **A** | **A** | **G** | **A** | **G** | **C** | **C** | **G** | **G** | **T** | **G** | **T** | **T** | **G** | **C** | **A** | **C** | **G** | **A** | **A** | **G** | **A** | **T** | **T** | **T** | **G** | **A** | **C** | **A** | **A** | **A** | **C** | **C** | **G** | **G** | **G** | **A** | **A** | **C** | **A** | **C** | **G** | **A** | **A** | **A** | **T** | **T** | **T** | **T** | **A** | **A** | **T** | **G** | **T** | **T** | **G** | **A** | **T** | **T** | **G** | **C** | **A** | **C** | **A** | **A** | **G** | **G** | **T** | **A** | **A** | **A** | **A** | **G** | **T** | **A** | **A** | **T** | **C** | **A** | **G** | **G** | **A** | **A** | **A** | **T** | **A** | **G** | **C** | **T** | **G** | **A** | **T** | **G** | **A** | **A** | **C** | **T** | **C** | **T** | **T** | **T** | **A** | **T** | **C** | **A** | **C** | **T** | **T** | **T** | **G** | **A** | **A** | **A** | **A** | **C** | **A** | **G** | **T** | **T** | **A** | **A** | **A** | **A** | **C** | **A** | **C** | **A** | **T** | **G** | **T** | **T** | **T** | **C** | **A** | **A** | **A** | **C** | **A** | **T** | **T** | **T** | **T** | **A** | **G** | **C** | **A** | **A** | **A** | **A** | **C** | **T** | **A** | **G** | **A** | **T** | **G** | **T** | **G** | **G** | **A** | **T** | **G** | **A** | **T** | **C** | **G** | **G** | **A** | **C** | **C** | **C** | **A** | **A** | **G** | **C** | **G** | **G** | **C** | **G** | **A** | **T** | **T** | **T** | **A** | **T** | **G** | **C** | **T** | **T** | **T** | **T** | **C** | **A** | **A** | **C** | **A** | **T** | **G** | **G** | **T** | **T** | **T** | **A** | **G** | **C** | **C** | **A** | **A** | **A** | **-** | **-** | **-** |
| **N54550** | **A** | **T** | **G** | **A** | **T** | **A** | **A** | **A** | **A** | **G** | **T** | **T** | **T** | **T** | **A** | **T** | **T** | **A** | **G** | **T** | **A** | **G** | **A** | **T** | **G** | **A** | **C** | **C** | **A** | **T** | **G** | **A** | **A** | **A** | **T** | **G** | **G** | **T** | **A** | **C** | **G** | **C** | **T** | **T** | **A** | **G** | **G** | **C** | **G** | **T** | **C** | **T** | **C** | **T** | **T** | **C** | **T** | **T** | **A** | **T** | **T** | **T** | **A** | **T** | **C** | **T** | **A** | **T** | **C** | **C** | **A** | **A** | **G** | **A** | **A** | **G** | **A** | **C** | **A** | **T** | **T** | **G** | **A** | **A** | **G** | **T** | **G** | **A** | **T** | **T** | **G** | **G** | **A** | **G** | **A** | **A** | **G** | **C** | **T** | **G** | **A** | **A** | **A** | **A** | **T** | **G** | **G** | **A** | **C** | **G** | **T** | **C** | **A** | **A** | **G** | **G** | **C** | **T** | **A** | **T** | **G** | **A** | **A** | **A** | **A** | **G** | **G** | **C** | **G** | **A** | **T** | **G** | **G** | **C** | **A** | **C** | **T** | **T** | **C** | **G** | **A** | **C** | **C** | **A** | **G** | **A** | **T** | **G** | **T** | **C** | **A** | **T** | **T** | **T** | **T** | **A** | **A** | **T** | **G** | **G** | **A** | **T** | **C** | **T** | **A** | **G** | **T** | **C** | **A** | **T** | **G** | **G** | **A** | **A** | **G** | **A** | **A** | **A** | **T** | **G** | **G** | **A** | **T** | **G** | **G** | **C** | **A** | **T** | **C** | **G** | **A** | **A** | **T** | **C** | **A** | **A** | **C** | **A** | **A** | **A** | **A** | **G** | **C** | **A** | **A** | **T** | **C** | **T** | **T** | **A** | **A** | **A** | **A** | **G** | **A** | **T** | **T** | **G** | **G** | **C** | **C** | **A** | **A** | **A** | **A** | **G** | **C** | **C** | **A** | **A** | **A** | **A** | **T** | **C** | **A** | **T** | **T** | **A** | **T** | **C** | **G** | **T** | **C** | **A** | **C** | **A** | **A** | **G** | **T** | **T** | **T** | **T** | **A** | **T** | **T** | **G** | **A** | **T** | **G** | **A** | **T** | **G** | **A** | **A** | **A** | **A** | **A** | **G** | **T** | **T** | **T** | **A** | **T** | **C** | **C** | **T** | **G** | **C** | **A** | **A** | **T** | **T** | **G** | **A** | **A** | **G** | **C** | **A** | **G** | **G** | **A** | **G** | **C** | **A** | **G** | **C** | **A** | **G** | **G** | **A** | **T** | **A** | **T** | **T** | **T** | **G** | **T** | **T** | **G** | **A** | **A** | **G** | **A** | **C** | **G** | **T** | **C** | **T** | **A** | **C** | **C** | **G** | **C** | **A** | **C** | **A** | **T** | **G** | **A** | **A** | **A** | **T** | **A** | **G** | **C** | **T** | **G** | **A** | **T** | **G** | **C** | **T** | **A** | **T** | **A** | **A** | **G** | **G** | **G** | **C** | **T** | **A** | **C** | **G** | **C** | **A** | **A** | **C** | **G** | **C** | **G** | **G** | **C** | **G** | **A** | **G** | **C** | **G** | **A** | **G** | **T** | **A** | **T** | **T** | **G** | **G** | **A** | **A** | **C** | **C** | **T** | **G** | **A** | **A** | **G** | **T** | **G** | **A** | **C** | **A** | **A** | **C** | **A** | **A** | **A** | **A** | **A** | **T** | **G** | **A** | **T** | **G** | **G** | **A** | **A** | **A** | **A** | **A** | **A** | **T** | **G** | **A** | **G** | **T** | **C** | **G** | **G** | **C** | **G** | **A** | **T** | **A** | **T** | **G** | **A** | **T** | **C** | **C** | **A** | **G** | **T** | **A** | **T** | **T** | **G** | **C** | **A** | **T** | **G** | **A** | **A** | **G** | **A** | **A** | **T** | **T** | **A** | **A** | **C** | **G** | **A** | **A** | **T** | **C** | **G** | **A** | **G** | **A** | **A** | **A** | **A** | **T** | **G** | **A** | **A** | **A** | **T** | **T** | **T** | **T** | **G** | **A** | **T** | **G** | **C** | **T** | **G** | **A** | **T** | **T** | **T** | **C** | **T** | **G** | **A** | **A** | **G** | **G** | **G** | **A** | **A** | **A** | **A** | **G** | **T** | **A** | **A** | **T** | **C** | **A** | **G** | **G** | **A** | **A** | **A** | **T** | **C** | **G** | **C** | **G** | **G** | **A** | **T** | **G** | **A** | **A** | **T** | **T** | **G** | **T** | **T** | **T** | **A** | **T** | **C** | **A** | **C** | **A** | **C** | **T** | **G** | **A** | **A** | **A** | **A** | **C** | **A** | **G** | **T** | **T** | **A** | **A** | **A** | **A** | **C** | **G** | **C** | **A** | **T** | **G** | **T** | **G** | **T** | **C** | **G** | **A** | **A** | **T** | **A** | **T** | **A** | **T** | **T** | **A** | **G** | **C** | **T** | **A** | **A** | **G** | **T** | **T** | **A** | **G** | **A** | **G** | **G** | **T** | **A** | **G** | **A** | **A** | **G** | **A** | **C** | **C** | **G** | **A** | **A** | **C** | **T** | **C** | **A** | **A** | **G** | **C** | **A** | **G** | **C** | **T** | **A** | **T** | **C** | **T** | **A** | **T** | **G** | **C** | **A** | **T** | **T** | **T** | **A** | **A** | **A** | **C** | **A** | **T** | **G** | **G** | **T** | **T** | **T** | **G** | **G** | **T** | **G** | **A** | **A** | **A** | **-** | **-** | **-** |
| **N52734** | **G** | **T** | **G** | **A** | **T** | **C** | **A** | **A** | **A** | **G** | **T** | **A** | **A** | **T** | **G** | **T** | **T** | **A** | **G** | **T** | **G** | **G** | **A** | **T** | **G** | **A** | **C** | **C** | **A** | **T** | **G** | **A** | **A** | **A** | **T** | **G** | **G** | **T** | **C** | **C** | **G** | **T** | **T** | **T** | **A** | **G** | **G** | **C** | **G** | **T** | **T** | **T** | **C** | **A** | **T** | **C** | **A** | **T** | **A** | **T** | **T** | **T** | **A** | **T** | **C** | **T** | **A** | **T** | **T** | **C** | **A** | **A** | **G** | **A** | **G** | **G** | **A** | **T** | **A** | **T** | **A** | **G** | **A** | **A** | **G** | **T** | **C** | **G** | **T** | **A** | **G** | **G** | **C** | **G** | **A** | **A** | **G** | **C** | **A** | **G** | **A** | **A** | **A** | **A** | **T** | **G** | **G** | **T** | **A** | **A** | **G** | **A** | **T** | **T** | **G** | **G** | **C** | **T** | **A** | **T** | **G** | **A** | **A** | **A** | **A** | **A** | **G** | **C** | **A** | **T** | **T** | **G** | **G** | **A** | **A** | **C** | **T** | **A** | **C** | **G** | **T** | **C** | **C** | **A** | **G** | **A** | **T** | **G** | **T** | **T** | **A** | **T** | **T** | **T** | **T** | **G** | **A** | **T** | **G** | **G** | **A** | **T** | **T** | **T** | **G** | **G** | **T** | **A** | **A** | **T** | **G** | **G** | **A** | **A** | **G** | **A** | **A** | **A** | **T** | **G** | **G** | **A** | **C** | **G** | **G** | **C** | **A** | **T** | **T** | **G** | **A** | **T** | **T** | **C** | **A** | **A** | **C** | **A** | **A** | **A** | **A** | **G** | **C** | **G** | **A** | **T** | **C** | **T** | **T** | **G** | **A** | **A** | **A** | **G** | **A** | **T** | **T** | **G** | **G** | **C** | **C** | **A** | **G** | **A** | **A** | **G** | **C** | **C** | **A** | **A** | **G** | **A** | **T** | **T** | **A** | **T** | **T** | **A** | **T** | **T** | **G** | **T** | **G** | **A** | **C** | **G** | **A** | **G** | **T** | **T** | **T** | **T** | **A** | **T** | **T** | **G** | **A** | **T** | **G** | **A** | **T** | **G** | **A** | **A** | **A** | **A** | **A** | **G** | **T** | **G** | **T** | **A** | **T** | **C** | **C** | **G** | **G** | **C** | **G** | **A** | **T** | **T** | **G** | **A** | **A** | **G** | **C** | **T** | **G** | **G** | **T** | **G** | **C** | **A** | **G** | **C** | **G** | **G** | **G** | **C** | **T** | **A** | **C** | **C** | **T** | **A** | **T** | **T** | **A** | **A** | **A** | **A** | **A** | **C** | **A** | **T** | **C** | **A** | **A** | **C** | **A** | **G** | **C** | **A** | **C** | **A** | **T** | **G** | **A** | **G** | **A** | **T** | **T** | **G** | **C** | **T** | **G** | **A** | **T** | **G** | **C** | **A** | **A** | **T** | **T** | **C** | **G** | **G** | **G** | **C** | **G** | **A** | **C** | **T** | **T** | **A** | **T** | **C** | **G** | **C** | **G** | **G** | **A** | **G** | **A** | **G** | **C** | **G** | **T** | **G** | **T** | **G** | **T** | **T** | **G** | **G** | **A** | **A** | **C** | **C** | **T** | **G** | **A** | **A** | **G** | **T** | **G** | **A** | **C** | **A** | **C** | **A** | **T** | **A** | **A** | **G** | **A** | **T** | **G** | **A** | **T** | **G** | **G** | **A** | **A** | **C** | **G** | **G** | **T** | **T** | **A** | **A** | **C** | **A** | **A** | **A** | **A** | **A** | **A** | **A** | **C** | **A** | **A** | **G** | **A** | **G** | **C** | **C** | **G** | **G** | **T** | **G** | **T** | **T** | **G** | **C** | **A** | **C** | **G** | **A** | **A** | **G** | **A** | **T** | **T** | **T** | **G** | **A** | **C** | **A** | **A** | **A** | **C** | **C** | **G** | **G** | **G** | **A** | **A** | **C** | **A** | **C** | **G** | **A** | **A** | **A** | **T** | **T** | **T** | **T** | **A** | **A** | **T** | **G** | **T** | **T** | **G** | **A** | **T** | **T** | **G** | **C** | **A** | **C** | **A** | **A** | **G** | **G** | **T** | **A** | **A** | **A** | **A** | **G** | **T** | **A** | **A** | **T** | **C** | **A** | **G** | **G** | **A** | **A** | **A** | **T** | **A** | **G** | **C** | **T** | **G** | **A** | **T** | **G** | **A** | **A** | **C** | **T** | **C** | **T** | **T** | **T** | **A** | **T** | **C** | **A** | **C** | **T** | **T** | **T** | **G** | **A** | **A** | **A** | **A** | **C** | **A** | **G** | **T** | **T** | **A** | **A** | **A** | **A** | **C** | **A** | **C** | **A** | **T** | **G** | **T** | **T** | **T** | **C** | **A** | **A** | **A** | **C** | **A** | **T** | **T** | **T** | **T** | **A** | **G** | **C** | **A** | **A** | **A** | **A** | **C** | **T** | **A** | **G** | **A** | **T** | **G** | **T** | **G** | **G** | **A** | **T** | **G** | **A** | **T** | **C** | **G** | **G** | **A** | **C** | **C** | **C** | **A** | **A** | **G** | **C** | **G** | **G** | **C** | **G** | **A** | **T** | **T** | **T** | **A** | **T** | **G** | **C** | **T** | **T** | **T** | **T** | **C** | **A** | **A** | **C** | **A** | **T** | **G** | **G** | **T** | **T** | **T** | **A** | **G** | **C** | **C** | **A** | **A** | **A** | **-** | **-** | **-** |
| **N59998F** | **A** | **T** | **G** | **A** | **T** | **A** | **A** | **A** | **A** | **G** | **T** | **T** | **T** | **T** | **A** | **T** | **T** | **A** | **G** | **T** | **A** | **G** | **A** | **T** | **G** | **A** | **C** | **C** | **A** | **T** | **G** | **A** | **A** | **A** | **T** | **G** | **G** | **T** | **A** | **C** | **G** | **C** | **T** | **T** | **A** | **G** | **G** | **C** | **G** | **T** | **C** | **T** | **C** | **T** | **T** | **C** | **T** | **T** | **A** | **T** | **T** | **T** | **A** | **T** | **C** | **T** | **A** | **T** | **C** | **C** | **A** | **A** | **G** | **A** | **A** | **G** | **A** | **C** | **A** | **T** | **T** | **G** | **A** | **A** | **G** | **T** | **G** | **A** | **T** | **T** | **G** | **G** | **A** | **G** | **A** | **A** | **G** | **C** | **T** | **G** | **A** | **A** | **A** | **A** | **T** | **G** | **G** | **A** | **C** | **G** | **T** | **C** | **A** | **A** | **G** | **G** | **C** | **T** | **A** | **T** | **G** | **A** | **A** | **A** | **A** | **G** | **G** | **C** | **G** | **A** | **T** | **G** | **G** | **C** | **A** | **C** | **T** | **T** | **C** | **G** | **A** | **C** | **C** | **A** | **G** | **A** | **T** | **G** | **T** | **C** | **A** | **T** | **T** | **T** | **T** | **A** | **A** | **T** | **G** | **G** | **A** | **T** | **C** | **T** | **A** | **G** | **T** | **C** | **A** | **T** | **G** | **G** | **A** | **A** | **G** | **A** | **A** | **A** | **T** | **G** | **G** | **A** | **T** | **G** | **G** | **C** | **A** | **T** | **C** | **G** | **A** | **A** | **T** | **C** | **A** | **A** | **C** | **A** | **A** | **A** | **A** | **G** | **C** | **A** | **A** | **T** | **C** | **T** | **T** | **A** | **A** | **A** | **A** | **G** | **A** | **T** | **T** | **G** | **G** | **C** | **C** | **A** | **A** | **A** | **A** | **G** | **C** | **C** | **A** | **A** | **A** | **A** | **T** | **C** | **A** | **T** | **T** | **A** | **T** | **C** | **G** | **T** | **C** | **A** | **C** | **A** | **A** | **G** | **T** | **T** | **T** | **T** | **A** | **T** | **T** | **G** | **A** | **T** | **G** | **A** | **T** | **G** | **A** | **A** | **A** | **A** | **A** | **G** | **T** | **T** | **T** | **A** | **T** | **C** | **C** | **T** | **G** | **C** | **A** | **A** | **T** | **T** | **G** | **A** | **A** | **G** | **C** | **A** | **G** | **G** | **A** | **G** | **C** | **A** | **G** | **C** | **A** | **G** | **G** | **A** | **T** | **A** | **T** | **T** | **T** | **G** | **T** | **T** | **G** | **A** | **A** | **G** | **A** | **C** | **G** | **T** | **C** | **T** | **A** | **C** | **C** | **G** | **C** | **A** | **C** | **A** | **T** | **G** | **A** | **A** | **A** | **T** | **A** | **G** | **C** | **T** | **G** | **A** | **T** | **G** | **C** | **T** | **A** | **T** | **A** | **A** | **G** | **G** | **G** | **C** | **T** | **A** | **C** | **G** | **C** | **A** | **A** | **C** | **G** | **C** | **G** | **G** | **C** | **G** | **A** | **G** | **C** | **G** | **A** | **G** | **T** | **A** | **T** | **T** | **G** | **G** | **A** | **A** | **C** | **C** | **T** | **G** | **A** | **A** | **G** | **T** | **G** | **A** | **C** | **A** | **A** | **C** | **A** | **A** | **A** | **A** | **A** | **T** | **G** | **A** | **T** | **G** | **G** | **A** | **A** | **A** | **A** | **A** | **A** | **T** | **G** | **A** | **G** | **T** | **C** | **G** | **G** | **C** | **G** | **A** | **T** | **A** | **T** | **G** | **A** | **T** | **C** | **C** | **A** | **G** | **T** | **A** | **T** | **T** | **G** | **C** | **A** | **T** | **G** | **A** | **A** | **G** | **A** | **A** | **T** | **T** | **A** | **A** | **C** | **G** | **A** | **A** | **T** | **C** | **G** | **A** | **G** | **A** | **A** | **A** | **A** | **T** | **G** | **A** | **A** | **A** | **T** | **T** | **T** | **T** | **G** | **A** | **T** | **G** | **C** | **T** | **G** | **A** | **T** | **T** | **T** | **C** | **T** | **G** | **A** | **A** | **G** | **G** | **G** | **A** | **A** | **A** | **A** | **G** | **T** | **A** | **A** | **T** | **C** | **A** | **G** | **G** | **A** | **A** | **A** | **T** | **C** | **G** | **C** | **G** | **G** | **A** | **T** | **G** | **A** | **A** | **T** | **T** | **G** | **T** | **T** | **T** | **A** | **T** | **C** | **A** | **C** | **A** | **C** | **T** | **G** | **A** | **A** | **A** | **A** | **C** | **A** | **G** | **T** | **T** | **A** | **A** | **A** | **A** | **C** | **G** | **C** | **A** | **T** | **G** | **T** | **G** | **T** | **C** | **G** | **A** | **A** | **T** | **A** | **T** | **A** | **T** | **T** | **A** | **G** | **C** | **T** | **A** | **A** | **G** | **T** | **T** | **A** | **G** | **A** | **G** | **G** | **T** | **A** | **G** | **A** | **A** | **G** | **A** | **C** | **C** | **G** | **A** | **A** | **C** | **T** | **C** | **A** | **A** | **G** | **C** | **A** | **G** | **C** | **T** | **A** | **T** | **C** | **T** | **A** | **T** | **G** | **C** | **A** | **T** | **T** | **T** | **A** | **A** | **A** | **C** | **A** | **T** | **G** | **G** | **T** | **T** | **T** | **G** | **G** | **T** | **G** | **A** | **A** | **A** | **-** | **-** | **-** |
| **N52789** | **G** | **T** | **G** | **A** | **T** | **C** | **A** | **A** | **A** | **G** | **T** | **A** | **A** | **T** | **G** | **T** | **T** | **A** | **G** | **T** | **G** | **G** | **A** | **T** | **G** | **A** | **C** | **C** | **A** | **T** | **G** | **A** | **A** | **A** | **T** | **G** | **G** | **T** | **C** | **C** | **G** | **T** | **T** | **T** | **A** | **G** | **G** | **C** | **G** | **T** | **T** | **T** | **C** | **A** | **T** | **C** | **A** | **T** | **A** | **T** | **T** | **T** | **A** | **T** | **C** | **T** | **A** | **T** | **T** | **C** | **A** | **A** | **G** | **A** | **G** | **G** | **A** | **T** | **A** | **T** | **A** | **G** | **A** | **A** | **G** | **T** | **C** | **G** | **T** | **A** | **G** | **G** | **C** | **G** | **A** | **A** | **G** | **C** | **A** | **G** | **A** | **A** | **A** | **A** | **C** | **G** | **G** | **T** | **A** | **A** | **G** | **A** | **T** | **T** | **G** | **G** | **C** | **T** | **A** | **T** | **G** | **A** | **A** | **A** | **A** | **A** | **G** | **C** | **A** | **T** | **T** | **G** | **G** | **A** | **A** | **C** | **T** | **A** | **C** | **G** | **T** | **C** | **C** | **A** | **G** | **A** | **T** | **G** | **T** | **T** | **A** | **T** | **T** | **T** | **T** | **G** | **A** | **T** | **G** | **G** | **A** | **T** | **T** | **T** | **G** | **G** | **T** | **A** | **A** | **T** | **G** | **G** | **A** | **A** | **G** | **A** | **A** | **A** | **T** | **G** | **G** | **A** | **C** | **G** | **G** | **C** | **A** | **T** | **T** | **G** | **A** | **T** | **T** | **C** | **A** | **A** | **C** | **A** | **A** | **A** | **A** | **G** | **C** | **A** | **A** | **T** | **C** | **T** | **T** | **G** | **A** | **A** | **A** | **G** | **A** | **T** | **T** | **G** | **G** | **C** | **C** | **A** | **G** | **A** | **A** | **G** | **C** | **C** | **A** | **A** | **G** | **A** | **T** | **T** | **A** | **T** | **T** | **A** | **T** | **T** | **G** | **T** | **G** | **A** | **C** | **G** | **A** | **G** | **T** | **T** | **T** | **T** | **A** | **T** | **T** | **G** | **A** | **T** | **G** | **A** | **T** | **G** | **A** | **G** | **A** | **A** | **A** | **G** | **T** | **G** | **T** | **A** | **T** | **C** | **C** | **G** | **G** | **C** | **G** | **A** | **T** | **T** | **G** | **A** | **A** | **G** | **C** | **T** | **G** | **G** | **T** | **G** | **C** | **A** | **G** | **C** | **G** | **G** | **G** | **C** | **T** | **A** | **C** | **C** | **T** | **A** | **T** | **T** | **A** | **A** | **A** | **A** | **A** | **C** | **A** | **T** | **C** | **A** | **A** | **C** | **A** | **G** | **C** | **A** | **C** | **A** | **T** | **G** | **A** | **G** | **A** | **T** | **T** | **G** | **C** | **T** | **G** | **A** | **T** | **G** | **C** | **A** | **A** | **T** | **T** | **C** | **G** | **G** | **G** | **C** | **G** | **A** | **C** | **T** | **T** | **A** | **T** | **C** | **G** | **C** | **G** | **G** | **A** | **G** | **A** | **G** | **C** | **G** | **T** | **G** | **T** | **G** | **T** | **T** | **G** | **G** | **A** | **A** | **C** | **C** | **T** | **G** | **A** | **A** | **G** | **T** | **G** | **A** | **C** | **G** | **C** | **A** | **T** | **A** | **A** | **G** | **A** | **T** | **G** | **A** | **T** | **G** | **G** | **A** | **A** | **C** | **G** | **G** | **T** | **T** | **A** | **A** | **C** | **A** | **A** | **A** | **A** | **A** | **A** | **A** | **C** | **A** | **A** | **G** | **A** | **G** | **C** | **C** | **G** | **G** | **T** | **G** | **T** | **T** | **G** | **C** | **A** | **T** | **G** | **A** | **A** | **G** | **A** | **T** | **T** | **T** | **G** | **A** | **C** | **A** | **A** | **A** | **T** | **C** | **G** | **A** | **G** | **A** | **A** | **C** | **A** | **C** | **G** | **A** | **A** | **A** | **T** | **T** | **T** | **T** | **A** | **A** | **T** | **G** | **T** | **T** | **G** | **A** | **T** | **T** | **G** | **C** | **A** | **C** | **A** | **A** | **G** | **G** | **T** | **A** | **A** | **A** | **A** | **G** | **T** | **A** | **A** | **T** | **C** | **A** | **G** | **G** | **A** | **A** | **A** | **T** | **A** | **G** | **C** | **T** | **G** | **A** | **T** | **G** | **A** | **A** | **C** | **T** | **C** | **T** | **T** | **T** | **A** | **T** | **C** | **A** | **C** | **T** | **T** | **T** | **G** | **A** | **A** | **A** | **A** | **C** | **A** | **G** | **T** | **T** | **A** | **A** | **A** | **A** | **C** | **A** | **C** | **A** | **T** | **G** | **T** | **T** | **T** | **C** | **A** | **A** | **A** | **C** | **A** | **T** | **T** | **T** | **T** | **A** | **G** | **C** | **A** | **A** | **A** | **A** | **C** | **T** | **A** | **G** | **A** | **T** | **G** | **T** | **G** | **G** | **A** | **T** | **G** | **A** | **T** | **C** | **G** | **G** | **A** | **C** | **C** | **C** | **A** | **A** | **G** | **C** | **G** | **G** | **C** | **G** | **A** | **T** | **T** | **T** | **A** | **T** | **G** | **C** | **T** | **T** | **T** | **T** | **C** | **A** | **A** | **C** | **A** | **T** | **G** | **G** | **T** | **T** | **T** | **A** | **G** | **C** | **C** | **A** | **A** | **A** | **-** | **-** | **-** |
| **N52751** | **A** | **T** | **G** | **A** | **T** | **A** | **A** | **A** | **A** | **G** | **T** | **T** | **T** | **T** | **A** | **T** | **T** | **A** | **G** | **T** | **A** | **G** | **A** | **T** | **G** | **A** | **C** | **C** | **A** | **T** | **G** | **A** | **A** | **A** | **T** | **G** | **G** | **T** | **A** | **C** | **G** | **C** | **T** | **T** | **A** | **G** | **G** | **C** | **G** | **T** | **C** | **T** | **C** | **T** | **T** | **C** | **T** | **T** | **A** | **T** | **T** | **T** | **A** | **T** | **C** | **T** | **A** | **T** | **C** | **C** | **A** | **A** | **G** | **A** | **A** | **G** | **A** | **C** | **A** | **T** | **T** | **G** | **A** | **A** | **G** | **T** | **G** | **A** | **T** | **T** | **G** | **G** | **A** | **G** | **A** | **A** | **G** | **C** | **T** | **G** | **A** | **A** | **A** | **A** | **T** | **G** | **G** | **A** | **C** | **G** | **T** | **C** | **A** | **A** | **G** | **G** | **C** | **T** | **A** | **T** | **G** | **A** | **A** | **A** | **A** | **G** | **G** | **C** | **G** | **A** | **T** | **G** | **G** | **C** | **A** | **C** | **T** | **T** | **C** | **G** | **A** | **C** | **C** | **A** | **G** | **A** | **T** | **G** | **T** | **C** | **A** | **T** | **T** | **T** | **T** | **A** | **A** | **T** | **G** | **G** | **A** | **T** | **C** | **T** | **A** | **G** | **T** | **C** | **A** | **T** | **G** | **G** | **A** | **A** | **G** | **A** | **A** | **A** | **T** | **G** | **G** | **A** | **T** | **G** | **G** | **C** | **A** | **T** | **C** | **G** | **A** | **A** | **T** | **C** | **A** | **A** | **C** | **A** | **A** | **A** | **A** | **G** | **C** | **A** | **A** | **T** | **C** | **T** | **T** | **A** | **A** | **A** | **A** | **G** | **A** | **T** | **T** | **G** | **G** | **C** | **C** | **A** | **A** | **A** | **A** | **G** | **C** | **C** | **A** | **A** | **A** | **A** | **T** | **C** | **A** | **T** | **T** | **A** | **T** | **C** | **G** | **T** | **C** | **A** | **C** | **A** | **A** | **G** | **T** | **T** | **T** | **T** | **A** | **T** | **T** | **G** | **A** | **T** | **G** | **A** | **T** | **G** | **A** | **A** | **A** | **A** | **A** | **G** | **T** | **T** | **T** | **A** | **T** | **C** | **C** | **T** | **G** | **C** | **A** | **A** | **T** | **T** | **G** | **A** | **A** | **G** | **C** | **A** | **G** | **G** | **A** | **G** | **C** | **A** | **G** | **C** | **A** | **G** | **G** | **A** | **T** | **A** | **T** | **T** | **T** | **A** | **T** | **T** | **G** | **A** | **A** | **G** | **A** | **C** | **G** | **T** | **C** | **T** | **A** | **C** | **C** | **G** | **C** | **A** | **C** | **A** | **T** | **G** | **A** | **A** | **A** | **T** | **A** | **G** | **C** | **T** | **G** | **A** | **T** | **G** | **C** | **T** | **A** | **T** | **A** | **A** | **G** | **G** | **G** | **C** | **T** | **A** | **C** | **G** | **C** | **A** | **A** | **C** | **G** | **C** | **G** | **G** | **C** | **G** | **A** | **G** | **C** | **G** | **A** | **G** | **T** | **A** | **C** | **T** | **A** | **G** | **A** | **A** | **C** | **C** | **A** | **G** | **A** | **A** | **G** | **T** | **G** | **A** | **C** | **A** | **A** | **C** | **A** | **A** | **A** | **A** | **A** | **T** | **G** | **A** | **T** | **G** | **G** | **A** | **A** | **A** | **A** | **A** | **A** | **T** | **G** | **A** | **G** | **T** | **C** | **G** | **G** | **C** | **G** | **A** | **A** | **A** | **T** | **G** | **A** | **T** | **C** | **C** | **A** | **G** | **T** | **A** | **T** | **T** | **G** | **C** | **A** | **T** | **G** | **A** | **A** | **G** | **A** | **A** | **T** | **T** | **A** | **A** | **C** | **G** | **A** | **A** | **T** | **C** | **G** | **A** | **G** | **A** | **A** | **A** | **A** | **T** | **G** | **A** | **A** | **A** | **T** | **T** | **T** | **T** | **G** | **A** | **T** | **G** | **C** | **T** | **G** | **A** | **T** | **T** | **T** | **C** | **T** | **G** | **A** | **A** | **G** | **G** | **G** | **A** | **A** | **A** | **A** | **G** | **T** | **A** | **A** | **T** | **C** | **A** | **G** | **G** | **A** | **A** | **A** | **T** | **C** | **G** | **C** | **G** | **G** | **A** | **T** | **G** | **A** | **A** | **T** | **T** | **G** | **T** | **T** | **T** | **A** | **T** | **C** | **A** | **C** | **A** | **C** | **T** | **G** | **A** | **A** | **A** | **A** | **C** | **A** | **G** | **T** | **T** | **A** | **A** | **A** | **A** | **C** | **G** | **C** | **A** | **T** | **G** | **T** | **G** | **T** | **C** | **G** | **A** | **A** | **T** | **A** | **T** | **A** | **T** | **T** | **A** | **G** | **C** | **T** | **A** | **A** | **G** | **T** | **T** | **A** | **G** | **A** | **G** | **G** | **T** | **A** | **G** | **A** | **A** | **G** | **A** | **C** | **C** | **G** | **A** | **A** | **C** | **T** | **C** | **A** | **A** | **G** | **C** | **A** | **G** | **C** | **T** | **A** | **T** | **C** | **T** | **A** | **T** | **G** | **C** | **G** | **T** | **T** | **T** | **A** | **A** | **A** | **C** | **A** | **T** | **G** | **G** | **T** | **T** | **T** | **G** | **G** | **T** | **G** | **A** | **A** | **A** | **-** | **-** | **-** |
| **N52648** | **G** | **T** | **G** | **A** | **T** | **C** | **A** | **A** | **A** | **G** | **T** | **A** | **A** | **T** | **G** | **T** | **T** | **A** | **G** | **T** | **G** | **G** | **A** | **T** | **G** | **A** | **C** | **C** | **A** | **T** | **G** | **A** | **A** | **A** | **T** | **G** | **G** | **T** | **C** | **C** | **G** | **T** | **T** | **T** | **A** | **G** | **G** | **C** | **G** | **T** | **T** | **T** | **C** | **A** | **T** | **C** | **A** | **T** | **A** | **T** | **T** | **T** | **A** | **T** | **C** | **T** | **A** | **T** | **T** | **C** | **A** | **A** | **G** | **A** | **G** | **G** | **A** | **T** | **A** | **T** | **A** | **G** | **A** | **A** | **G** | **T** | **C** | **G** | **T** | **A** | **G** | **G** | **C** | **G** | **A** | **A** | **G** | **C** | **A** | **G** | **A** | **A** | **A** | **A** | **C** | **G** | **G** | **T** | **A** | **A** | **G** | **A** | **T** | **T** | **G** | **G** | **C** | **T** | **A** | **T** | **G** | **A** | **A** | **A** | **A** | **A** | **G** | **C** | **A** | **T** | **T** | **G** | **G** | **A** | **A** | **C** | **T** | **A** | **C** | **G** | **T** | **C** | **C** | **A** | **G** | **A** | **T** | **G** | **T** | **T** | **A** | **T** | **T** | **T** | **T** | **G** | **A** | **T** | **G** | **G** | **A** | **T** | **T** | **T** | **G** | **G** | **T** | **A** | **A** | **T** | **G** | **G** | **A** | **A** | **G** | **A** | **A** | **A** | **T** | **G** | **G** | **A** | **C** | **G** | **G** | **C** | **A** | **T** | **T** | **G** | **A** | **T** | **T** | **C** | **A** | **A** | **C** | **A** | **A** | **A** | **A** | **G** | **C** | **A** | **A** | **T** | **C** | **T** | **T** | **G** | **A** | **A** | **A** | **G** | **A** | **T** | **T** | **G** | **G** | **C** | **C** | **A** | **G** | **A** | **A** | **G** | **C** | **C** | **A** | **A** | **G** | **A** | **T** | **T** | **A** | **T** | **T** | **A** | **T** | **T** | **G** | **T** | **G** | **A** | **C** | **G** | **A** | **G** | **T** | **T** | **T** | **T** | **A** | **T** | **T** | **G** | **A** | **T** | **G** | **A** | **T** | **G** | **A** | **G** | **A** | **A** | **A** | **G** | **T** | **G** | **T** | **A** | **T** | **C** | **C** | **G** | **G** | **C** | **G** | **A** | **T** | **T** | **G** | **A** | **A** | **G** | **C** | **T** | **G** | **G** | **T** | **G** | **C** | **A** | **G** | **C** | **G** | **G** | **G** | **C** | **T** | **A** | **C** | **C** | **T** | **A** | **T** | **T** | **A** | **A** | **A** | **A** | **A** | **C** | **A** | **T** | **C** | **A** | **A** | **C** | **A** | **G** | **C** | **A** | **C** | **A** | **T** | **G** | **A** | **G** | **A** | **T** | **T** | **G** | **C** | **T** | **G** | **A** | **T** | **G** | **C** | **A** | **A** | **T** | **T** | **C** | **G** | **G** | **G** | **C** | **G** | **A** | **C** | **T** | **T** | **A** | **T** | **C** | **G** | **C** | **G** | **G** | **A** | **G** | **A** | **G** | **C** | **G** | **T** | **G** | **T** | **G** | **T** | **T** | **G** | **G** | **A** | **A** | **C** | **C** | **T** | **G** | **A** | **A** | **G** | **T** | **G** | **A** | **C** | **G** | **C** | **A** | **T** | **A** | **A** | **G** | **A** | **T** | **G** | **A** | **T** | **G** | **G** | **A** | **A** | **C** | **G** | **G** | **T** | **T** | **A** | **A** | **C** | **A** | **A** | **A** | **A** | **A** | **A** | **A** | **C** | **A** | **A** | **G** | **A** | **G** | **C** | **C** | **G** | **G** | **T** | **G** | **T** | **T** | **G** | **C** | **A** | **T** | **G** | **A** | **A** | **G** | **A** | **T** | **T** | **T** | **G** | **A** | **C** | **A** | **A** | **A** | **T** | **C** | **G** | **A** | **G** | **A** | **A** | **C** | **A** | **C** | **G** | **A** | **A** | **A** | **T** | **T** | **T** | **T** | **A** | **A** | **T** | **G** | **T** | **T** | **G** | **A** | **T** | **T** | **G** | **C** | **A** | **C** | **A** | **A** | **G** | **G** | **T** | **A** | **A** | **A** | **A** | **G** | **T** | **A** | **A** | **T** | **C** | **A** | **G** | **G** | **A** | **A** | **A** | **T** | **A** | **G** | **C** | **T** | **G** | **A** | **T** | **G** | **A** | **A** | **C** | **T** | **C** | **T** | **T** | **T** | **A** | **T** | **C** | **A** | **C** | **T** | **T** | **T** | **G** | **A** | **A** | **A** | **A** | **C** | **A** | **G** | **T** | **T** | **A** | **A** | **A** | **A** | **C** | **A** | **C** | **A** | **T** | **G** | **T** | **T** | **T** | **C** | **A** | **A** | **A** | **C** | **A** | **T** | **T** | **T** | **T** | **A** | **G** | **C** | **A** | **A** | **A** | **A** | **C** | **T** | **A** | **G** | **A** | **T** | **G** | **T** | **G** | **G** | **A** | **T** | **G** | **A** | **T** | **C** | **G** | **G** | **A** | **C** | **C** | **C** | **A** | **A** | **G** | **C** | **G** | **G** | **C** | **G** | **A** | **T** | **T** | **T** | **A** | **T** | **G** | **C** | **T** | **T** | **T** | **T** | **C** | **A** | **A** | **C** | **A** | **T** | **G** | **G** | **T** | **T** | **T** | **A** | **G** | **C** | **C** | **A** | **A** | **A** | **-** | **-** | **-** |
| **N52459** | **A** | **T** | **G** | **A** | **T** | **A** | **A** | **A** | **A** | **G** | **T** | **T** | **T** | **T** | **A** | **T** | **T** | **A** | **G** | **T** | **A** | **G** | **A** | **T** | **G** | **A** | **C** | **C** | **A** | **T** | **G** | **A** | **A** | **A** | **T** | **G** | **G** | **T** | **G** | **C** | **G** | **C** | **T** | **T** | **A** | **G** | **G** | **T** | **G** | **T** | **C** | **T** | **C** | **T** | **T** | **C** | **T** | **T** | **A** | **T** | **T** | **T** | **A** | **T** | **C** | **T** | **A** | **T** | **T** | **C** | **A** | **A** | **G** | **A** | **A** | **G** | **A** | **C** | **A** | **T** | **T** | **G** | **A** | **A** | **G** | **T** | **A** | **A** | **T** | **T** | **G** | **G** | **A** | **G** | **A** | **A** | **G** | **C** | **T** | **G** | **A** | **A** | **A** | **A** | **T** | **G** | **G** | **A** | **C** | **G** | **A** | **C** | **A** | **A** | **G** | **G** | **C** | **T** | **A** | **T** | **G** | **A** | **G** | **A** | **A** | **G** | **G** | **C** | **G** | **A** | **T** | **G** | **A** | **C** | **A** | **C** | **T** | **T** | **C** | **G** | **A** | **C** | **C** | **G** | **G** | **A** | **C** | **G** | **T** | **C** | **A** | **T** | **T** | **T** | **T** | **A** | **A** | **T** | **G** | **G** | **A** | **C** | **T** | **T** | **A** | **G** | **T** | **C** | **A** | **T** | **G** | **G** | **A** | **A** | **G** | **A** | **A** | **A** | **T** | **G** | **G** | **A** | **T** | **G** | **G** | **T** | **A** | **T** | **C** | **G** | **A** | **A** | **T** | **C** | **A** | **A** | **C** | **A** | **A** | **A** | **A** | **G** | **C** | **A** | **A** | **T** | **C** | **T** | **T** | **A** | **A** | **A** | **A** | **G** | **A** | **T** | **T** | **G** | **G** | **C** | **C** | **A** | **G** | **A** | **A** | **G** | **C** | **T** | **A** | **A** | **A** | **A** | **T** | **C** | **A** | **T** | **T** | **A** | **T** | **C** | **G** | **T** | **A** | **A** | **C** | **C** | **A** | **G** | **T** | **T** | **T** | **T** | **A** | **T** | **T** | **G** | **A** | **T** | **G** | **A** | **C** | **G** | **A** | **A** | **A** | **A** | **A** | **G** | **T** | **T** | **T** | **A** | **T** | **C** | **C** | **T** | **G** | **C** | **C** | **A** | **T** | **T** | **G** | **A** | **A** | **G** | **C** | **A** | **G** | **G** | **T** | **G** | **C** | **A** | **G** | **C** | **A** | **G** | **G** | **G** | **T** | **A** | **T** | **T** | **T** | **G** | **T** | **T** | **G** | **A** | **A** | **G** | **A** | **C** | **C** | **T** | **C** | **T** | **A** | **C** | **C** | **G** | **C** | **A** | **C** | **A** | **T** | **G** | **A** | **A** | **A** | **T** | **T** | **G** | **C** | **T** | **G** | **A** | **T** | **G** | **C** | **T** | **A** | **T** | **A** | **A** | **G** | **G** | **G** | **C** | **A** | **A** | **C** | **G** | **C** | **A** | **A** | **C** | **G** | **C** | **G** | **G** | **C** | **G** | **A** | **G** | **C** | **G** | **A** | **G** | **T** | **A** | **T** | **T** | **A** | **G** | **A** | **A** | **C** | **C** | **A** | **G** | **A** | **A** | **G** | **T** | **G** | **A** | **C** | **A** | **A** | **C** | **A** | **A** | **A** | **A** | **A** | **T** | **G** | **A** | **T** | **G** | **G** | **A** | **A** | **A** | **A** | **A** | **A** | **T** | **G** | **A** | **G** | **T** | **C** | **G** | **G** | **C** | **G** | **A** | **A** | **A** | **T** | **G** | **A** | **G** | **C** | **C** | **T** | **G** | **T** | **A** | **T** | **T** | **A** | **C** | **A** | **T** | **G** | **A** | **A** | **G** | **A** | **A** | **C** | **T** | **A** | **A** | **C** | **G** | **A** | **A** | **T** | **C** | **G** | **G** | **G** | **A** | **A** | **A** | **A** | **T** | **G** | **A** | **A** | **A** | **T** | **T** | **T** | **T** | **A** | **A** | **T** | **G** | **C** | **T** | **G** | **A** | **T** | **T** | **T** | **C** | **T** | **G** | **A** | **A** | **G** | **G** | **A** | **A** | **A** | **A** | **A** | **G** | **T** | **A** | **A** | **T** | **C** | **A** | **G** | **G** | **A** | **A** | **A** | **T** | **A** | **G** | **C** | **G** | **G** | **A** | **C** | **G** | **A** | **A** | **T** | **T** | **G** | **T** | **T** | **C** | **A** | **T** | **T** | **A** | **C** | **A** | **C** | **T** | **G** | **A** | **A** | **A** | **A** | **C** | **A** | **G** | **T** | **A** | **A** | **A** | **A** | **A** | **C** | **A** | **C** | **A** | **T** | **G** | **T** | **T** | **T** | **C** | **C** | **A** | **A** | **T** | **A** | **T** | **A** | **T** | **T** | **G** | **G** | **C** | **T** | **A** | **A** | **G** | **C** | **T** | **A** | **G** | **A** | **A** | **G** | **T** | **G** | **G** | **A** | **A** | **G** | **A** | **C** | **C** | **G** | **T** | **A** | **C** | **A** | **C** | **A** | **A** | **G** | **C** | **C** | **G** | **C** | **T** | **A** | **T** | **C** | **T** | **A** | **T** | **G** | **C** | **A** | **T** | **T** | **C** | **A** | **A** | **A** | **C** | **A** | **T** | **G** | **G** | **T** | **T** | **T** | **G** | **G** | **T** | **G** | **A** | **A** | **A** | **-** | **-** | **-** |
| **N52469** | **G** | **T** | **G** | **A** | **T** | **C** | **A** | **A** | **A** | **G** | **T** | **A** | **A** | **T** | **G** | **T** | **T** | **A** | **G** | **T** | **G** | **G** | **A** | **T** | **G** | **A** | **C** | **C** | **A** | **T** | **G** | **A** | **A** | **A** | **T** | **G** | **G** | **T** | **C** | **C** | **G** | **T** | **T** | **T** | **A** | **G** | **G** | **C** | **G** | **T** | **T** | **T** | **C** | **A** | **T** | **C** | **A** | **T** | **A** | **T** | **T** | **T** | **A** | **T** | **C** | **T** | **A** | **T** | **T** | **C** | **A** | **A** | **G** | **A** | **G** | **G** | **A** | **T** | **A** | **T** | **A** | **G** | **A** | **A** | **G** | **T** | **C** | **G** | **T** | **A** | **G** | **G** | **C** | **G** | **A** | **A** | **G** | **C** | **A** | **G** | **A** | **A** | **A** | **A** | **C** | **G** | **G** | **T** | **A** | **A** | **G** | **A** | **T** | **T** | **G** | **G** | **C** | **T** | **A** | **T** | **G** | **A** | **A** | **A** | **A** | **A** | **G** | **C** | **A** | **T** | **T** | **G** | **G** | **A** | **A** | **C** | **T** | **A** | **C** | **G** | **T** | **C** | **C** | **A** | **G** | **A** | **T** | **G** | **T** | **T** | **A** | **T** | **T** | **T** | **T** | **G** | **A** | **T** | **G** | **G** | **A** | **T** | **T** | **T** | **G** | **G** | **T** | **A** | **A** | **T** | **G** | **G** | **A** | **A** | **G** | **A** | **A** | **A** | **T** | **G** | **G** | **A** | **C** | **G** | **G** | **C** | **A** | **T** | **T** | **G** | **A** | **T** | **T** | **C** | **A** | **A** | **C** | **A** | **A** | **A** | **A** | **G** | **C** | **G** | **A** | **T** | **C** | **T** | **T** | **G** | **A** | **A** | **A** | **G** | **A** | **T** | **T** | **G** | **G** | **C** | **C** | **A** | **G** | **A** | **A** | **G** | **C** | **C** | **A** | **A** | **G** | **A** | **T** | **T** | **A** | **T** | **T** | **A** | **T** | **T** | **G** | **T** | **G** | **A** | **C** | **G** | **A** | **G** | **T** | **T** | **T** | **T** | **A** | **T** | **T** | **G** | **A** | **T** | **G** | **A** | **T** | **G** | **A** | **A** | **A** | **A** | **A** | **G** | **T** | **G** | **T** | **A** | **T** | **C** | **C** | **G** | **G** | **C** | **G** | **A** | **T** | **T** | **G** | **A** | **A** | **G** | **C** | **T** | **G** | **G** | **T** | **G** | **C** | **A** | **G** | **C** | **G** | **G** | **G** | **C** | **T** | **A** | **C** | **C** | **T** | **A** | **T** | **T** | **A** | **A** | **A** | **A** | **A** | **C** | **A** | **T** | **C** | **A** | **A** | **C** | **A** | **G** | **C** | **A** | **C** | **A** | **T** | **G** | **A** | **G** | **A** | **T** | **T** | **G** | **C** | **T** | **G** | **A** | **T** | **G** | **C** | **A** | **A** | **T** | **T** | **C** | **G** | **G** | **G** | **C** | **G** | **A** | **C** | **T** | **T** | **A** | **T** | **C** | **G** | **C** | **G** | **G** | **A** | **G** | **A** | **G** | **C** | **G** | **T** | **G** | **T** | **G** | **T** | **T** | **G** | **G** | **A** | **A** | **C** | **C** | **T** | **G** | **A** | **A** | **G** | **T** | **G** | **A** | **C** | **G** | **C** | **A** | **T** | **A** | **A** | **G** | **A** | **T** | **G** | **A** | **T** | **G** | **G** | **A** | **A** | **C** | **G** | **G** | **T** | **T** | **A** | **A** | **C** | **A** | **A** | **A** | **A** | **A** | **A** | **A** | **C** | **A** | **A** | **G** | **A** | **G** | **C** | **C** | **G** | **G** | **T** | **G** | **C** | **T** | **A** | **C** | **A** | **T** | **G** | **A** | **A** | **G** | **A** | **T** | **T** | **T** | **G** | **A** | **C** | **A** | **A** | **A** | **C** | **C** | **G** | **A** | **G** | **A** | **A** | **C** | **A** | **C** | **G** | **A** | **A** | **A** | **T** | **T** | **T** | **T** | **A** | **A** | **T** | **G** | **T** | **T** | **G** | **A** | **T** | **T** | **G** | **C** | **A** | **C** | **A** | **A** | **G** | **G** | **T** | **A** | **A** | **A** | **A** | **G** | **T** | **A** | **A** | **T** | **C** | **A** | **G** | **G** | **A** | **A** | **A** | **T** | **A** | **G** | **C** | **T** | **G** | **A** | **T** | **G** | **A** | **A** | **C** | **T** | **C** | **T** | **T** | **T** | **A** | **T** | **C** | **A** | **C** | **T** | **T** | **T** | **G** | **A** | **A** | **A** | **A** | **C** | **A** | **G** | **T** | **T** | **A** | **A** | **A** | **A** | **C** | **A** | **C** | **A** | **T** | **G** | **T** | **T** | **T** | **C** | **A** | **A** | **A** | **C** | **A** | **T** | **T** | **T** | **T** | **A** | **G** | **C** | **A** | **A** | **A** | **A** | **C** | **T** | **A** | **G** | **A** | **T** | **G** | **T** | **G** | **G** | **A** | **T** | **G** | **A** | **T** | **C** | **G** | **G** | **A** | **C** | **C** | **C** | **A** | **A** | **G** | **C** | **G** | **G** | **C** | **G** | **A** | **T** | **T** | **T** | **A** | **T** | **G** | **C** | **T** | **T** | **T** | **T** | **C** | **A** | **A** | **C** | **A** | **T** | **G** | **G** | **T** | **T** | **T** | **A** | **G** | **C** | **C** | **A** | **A** | **A** | **-** | **-** | **-** |
| **N54588** | **G** | **T** | **G** | **A** | **T** | **C** | **A** | **A** | **A** | **G** | **T** | **A** | **A** | **T** | **G** | **T** | **T** | **A** | **G** | **T** | **G** | **G** | **A** | **T** | **G** | **A** | **C** | **C** | **A** | **T** | **G** | **A** | **A** | **A** | **T** | **G** | **G** | **T** | **C** | **C** | **G** | **T** | **T** | **T** | **A** | **G** | **G** | **C** | **G** | **T** | **T** | **T** | **C** | **A** | **T** | **C** | **A** | **T** | **A** | **T** | **T** | **T** | **A** | **T** | **C** | **T** | **A** | **T** | **T** | **C** | **A** | **A** | **G** | **A** | **G** | **G** | **A** | **T** | **A** | **T** | **A** | **G** | **A** | **A** | **G** | **T** | **C** | **G** | **T** | **A** | **G** | **G** | **C** | **G** | **A** | **A** | **G** | **C** | **A** | **G** | **A** | **A** | **A** | **A** | **C** | **G** | **G** | **T** | **A** | **A** | **G** | **A** | **T** | **T** | **G** | **G** | **C** | **T** | **A** | **T** | **G** | **A** | **A** | **A** | **A** | **A** | **G** | **C** | **A** | **T** | **T** | **G** | **G** | **A** | **A** | **C** | **T** | **A** | **C** | **G** | **T** | **C** | **C** | **A** | **G** | **A** | **T** | **G** | **T** | **T** | **A** | **T** | **T** | **T** | **T** | **G** | **A** | **T** | **G** | **G** | **A** | **T** | **T** | **T** | **G** | **G** | **T** | **A** | **A** | **T** | **G** | **G** | **A** | **A** | **G** | **A** | **A** | **A** | **T** | **G** | **G** | **A** | **C** | **G** | **G** | **C** | **A** | **T** | **T** | **G** | **A** | **T** | **T** | **C** | **A** | **A** | **C** | **A** | **A** | **A** | **A** | **G** | **C** | **G** | **A** | **T** | **C** | **T** | **T** | **G** | **A** | **A** | **A** | **G** | **A** | **T** | **T** | **G** | **G** | **C** | **C** | **A** | **G** | **A** | **A** | **G** | **C** | **C** | **A** | **A** | **G** | **A** | **T** | **T** | **A** | **T** | **T** | **A** | **T** | **T** | **G** | **T** | **G** | **A** | **C** | **G** | **A** | **G** | **T** | **T** | **T** | **T** | **A** | **T** | **T** | **G** | **A** | **T** | **G** | **A** | **T** | **G** | **A** | **A** | **A** | **A** | **A** | **G** | **T** | **G** | **T** | **A** | **T** | **C** | **C** | **G** | **G** | **C** | **G** | **A** | **T** | **T** | **G** | **A** | **A** | **G** | **C** | **T** | **G** | **G** | **T** | **G** | **C** | **A** | **G** | **C** | **G** | **G** | **G** | **C** | **T** | **A** | **C** | **C** | **T** | **A** | **T** | **T** | **A** | **A** | **A** | **A** | **A** | **C** | **A** | **T** | **C** | **A** | **A** | **C** | **A** | **G** | **C** | **A** | **C** | **A** | **T** | **G** | **A** | **G** | **A** | **T** | **T** | **G** | **C** | **T** | **G** | **A** | **T** | **G** | **C** | **A** | **A** | **T** | **T** | **C** | **G** | **G** | **G** | **C** | **G** | **A** | **C** | **T** | **T** | **A** | **T** | **C** | **G** | **C** | **G** | **G** | **A** | **G** | **A** | **G** | **C** | **G** | **T** | **G** | **T** | **G** | **T** | **T** | **G** | **G** | **A** | **A** | **C** | **C** | **T** | **G** | **A** | **A** | **G** | **T** | **G** | **A** | **C** | **G** | **C** | **A** | **T** | **A** | **A** | **G** | **A** | **T** | **G** | **A** | **T** | **G** | **G** | **A** | **A** | **C** | **G** | **G** | **T** | **T** | **A** | **A** | **C** | **A** | **A** | **A** | **A** | **A** | **A** | **A** | **C** | **A** | **A** | **G** | **A** | **G** | **C** | **C** | **G** | **G** | **T** | **G** | **T** | **T** | **G** | **C** | **A** | **C** | **G** | **A** | **A** | **G** | **A** | **T** | **T** | **T** | **G** | **A** | **C** | **A** | **A** | **A** | **C** | **C** | **G** | **G** | **G** | **A** | **A** | **C** | **A** | **C** | **G** | **A** | **A** | **A** | **T** | **T** | **T** | **T** | **A** | **A** | **T** | **G** | **T** | **T** | **G** | **A** | **T** | **T** | **G** | **C** | **A** | **C** | **A** | **A** | **G** | **G** | **T** | **A** | **A** | **A** | **A** | **G** | **T** | **A** | **A** | **T** | **C** | **A** | **G** | **G** | **A** | **A** | **A** | **T** | **A** | **G** | **C** | **T** | **G** | **A** | **T** | **G** | **A** | **A** | **C** | **T** | **C** | **T** | **T** | **T** | **A** | **T** | **C** | **A** | **C** | **T** | **T** | **T** | **G** | **A** | **A** | **A** | **A** | **C** | **A** | **G** | **T** | **T** | **A** | **A** | **A** | **A** | **C** | **A** | **C** | **A** | **T** | **G** | **T** | **T** | **T** | **C** | **A** | **A** | **A** | **C** | **A** | **T** | **T** | **T** | **T** | **A** | **G** | **C** | **A** | **A** | **A** | **A** | **C** | **T** | **A** | **G** | **A** | **T** | **G** | **T** | **G** | **G** | **A** | **T** | **G** | **A** | **T** | **C** | **G** | **G** | **A** | **C** | **C** | **C** | **A** | **A** | **G** | **C** | **G** | **G** | **C** | **G** | **A** | **T** | **T** | **T** | **A** | **T** | **G** | **C** | **T** | **T** | **T** | **T** | **C** | **A** | **A** | **C** | **A** | **T** | **G** | **G** | **T** | **T** | **T** | **A** | **G** | **C** | **C** | **A** | **A** | **A** | **-** | **-** | **-** |
| **N54097** | **G** | **T** | **G** | **A** | **T** | **C** | **A** | **A** | **A** | **G** | **T** | **A** | **A** | **T** | **G** | **T** | **T** | **A** | **G** | **T** | **G** | **G** | **A** | **T** | **G** | **A** | **C** | **C** | **A** | **T** | **G** | **A** | **A** | **A** | **T** | **G** | **G** | **T** | **C** | **C** | **G** | **T** | **T** | **T** | **A** | **G** | **G** | **C** | **G** | **T** | **T** | **T** | **C** | **A** | **T** | **C** | **A** | **T** | **A** | **T** | **T** | **T** | **A** | **T** | **C** | **T** | **A** | **T** | **T** | **C** | **A** | **A** | **G** | **A** | **G** | **G** | **A** | **T** | **A** | **T** | **A** | **G** | **A** | **A** | **G** | **T** | **C** | **G** | **T** | **A** | **G** | **G** | **C** | **G** | **A** | **A** | **G** | **C** | **A** | **G** | **A** | **A** | **A** | **A** | **C** | **G** | **G** | **T** | **A** | **A** | **G** | **A** | **T** | **T** | **G** | **G** | **C** | **T** | **A** | **T** | **G** | **A** | **A** | **A** | **A** | **A** | **G** | **C** | **A** | **T** | **T** | **G** | **G** | **A** | **A** | **C** | **T** | **A** | **C** | **G** | **T** | **C** | **C** | **A** | **G** | **A** | **T** | **G** | **T** | **T** | **A** | **T** | **T** | **T** | **T** | **G** | **A** | **T** | **G** | **G** | **A** | **T** | **T** | **T** | **G** | **G** | **T** | **A** | **A** | **T** | **G** | **G** | **A** | **A** | **G** | **A** | **A** | **A** | **T** | **G** | **G** | **A** | **C** | **G** | **G** | **C** | **A** | **T** | **T** | **G** | **A** | **T** | **T** | **C** | **A** | **A** | **C** | **C** | **A** | **A** | **A** | **G** | **C** | **G** | **A** | **T** | **C** | **T** | **T** | **G** | **A** | **A** | **A** | **G** | **A** | **T** | **T** | **G** | **G** | **C** | **C** | **A** | **G** | **A** | **A** | **G** | **C** | **C** | **A** | **A** | **G** | **A** | **T** | **T** | **A** | **T** | **T** | **A** | **T** | **T** | **G** | **T** | **G** | **A** | **C** | **G** | **A** | **G** | **T** | **T** | **T** | **T** | **A** | **T** | **T** | **G** | **A** | **T** | **G** | **A** | **T** | **G** | **A** | **A** | **A** | **A** | **A** | **G** | **T** | **G** | **T** | **A** | **T** | **C** | **C** | **G** | **G** | **C** | **G** | **A** | **T** | **T** | **G** | **A** | **A** | **G** | **C** | **T** | **G** | **G** | **T** | **G** | **C** | **A** | **G** | **C** | **G** | **G** | **G** | **C** | **T** | **A** | **C** | **C** | **T** | **A** | **T** | **T** | **A** | **A** | **A** | **A** | **A** | **C** | **A** | **T** | **C** | **A** | **A** | **C** | **A** | **G** | **C** | **A** | **C** | **A** | **T** | **G** | **A** | **G** | **A** | **T** | **T** | **G** | **C** | **T** | **G** | **A** | **T** | **G** | **C** | **A** | **A** | **T** | **T** | **C** | **G** | **G** | **G** | **C** | **G** | **A** | **C** | **T** | **T** | **A** | **T** | **C** | **G** | **C** | **G** | **G** | **A** | **G** | **A** | **G** | **C** | **G** | **T** | **G** | **T** | **G** | **T** | **T** | **G** | **G** | **A** | **A** | **C** | **C** | **T** | **G** | **A** | **A** | **G** | **T** | **G** | **A** | **C** | **G** | **C** | **A** | **T** | **A** | **A** | **G** | **A** | **T** | **G** | **A** | **T** | **G** | **G** | **A** | **A** | **C** | **G** | **G** | **T** | **T** | **A** | **A** | **C** | **A** | **A** | **A** | **A** | **A** | **A** | **A** | **C** | **A** | **A** | **G** | **A** | **G** | **C** | **C** | **G** | **G** | **T** | **G** | **T** | **T** | **G** | **C** | **A** | **C** | **G** | **A** | **A** | **G** | **A** | **T** | **T** | **T** | **G** | **A** | **C** | **A** | **A** | **A** | **C** | **C** | **G** | **G** | **G** | **A** | **A** | **C** | **A** | **C** | **G** | **A** | **A** | **A** | **T** | **T** | **T** | **T** | **A** | **A** | **T** | **G** | **T** | **T** | **G** | **A** | **T** | **T** | **G** | **C** | **A** | **C** | **A** | **A** | **G** | **G** | **T** | **A** | **A** | **A** | **A** | **G** | **T** | **A** | **A** | **T** | **C** | **A** | **G** | **G** | **A** | **A** | **A** | **T** | **A** | **G** | **C** | **T** | **G** | **A** | **T** | **G** | **A** | **A** | **C** | **T** | **C** | **T** | **T** | **T** | **A** | **T** | **C** | **A** | **C** | **T** | **T** | **T** | **G** | **A** | **A** | **A** | **A** | **C** | **A** | **G** | **T** | **T** | **A** | **A** | **A** | **A** | **C** | **A** | **C** | **A** | **T** | **G** | **T** | **T** | **T** | **C** | **A** | **A** | **A** | **C** | **A** | **T** | **T** | **T** | **T** | **A** | **G** | **C** | **A** | **A** | **A** | **A** | **C** | **T** | **A** | **G** | **A** | **T** | **G** | **T** | **G** | **G** | **A** | **T** | **G** | **A** | **T** | **C** | **G** | **G** | **A** | **C** | **C** | **C** | **A** | **A** | **G** | **C** | **G** | **G** | **C** | **G** | **A** | **T** | **T** | **T** | **A** | **T** | **G** | **C** | **T** | **T** | **T** | **T** | **C** | **A** | **A** | **C** | **A** | **T** | **G** | **G** | **T** | **T** | **T** | **A** | **G** | **C** | **C** | **A** | **A** | **A** | **-** | **-** | **-** |
| **N59963F** | **G** | **T** | **G** | **A** | **T** | **C** | **A** | **A** | **A** | **G** | **T** | **A** | **A** | **T** | **G** | **T** | **T** | **A** | **G** | **T** | **G** | **G** | **A** | **T** | **G** | **A** | **C** | **C** | **A** | **T** | **G** | **A** | **A** | **A** | **T** | **G** | **G** | **T** | **C** | **C** | **G** | **T** | **T** | **T** | **A** | **G** | **G** | **C** | **G** | **T** | **T** | **T** | **C** | **A** | **T** | **C** | **A** | **T** | **A** | **T** | **T** | **T** | **A** | **T** | **C** | **T** | **A** | **T** | **T** | **C** | **A** | **A** | **G** | **A** | **G** | **G** | **A** | **T** | **A** | **T** | **A** | **G** | **A** | **A** | **G** | **T** | **C** | **G** | **T** | **A** | **G** | **G** | **C** | **G** | **A** | **A** | **G** | **C** | **A** | **G** | **A** | **A** | **A** | **A** | **T** | **G** | **G** | **T** | **A** | **A** | **G** | **A** | **T** | **T** | **G** | **G** | **C** | **T** | **A** | **T** | **G** | **A** | **A** | **A** | **A** | **A** | **G** | **C** | **A** | **T** | **T** | **G** | **G** | **A** | **A** | **C** | **T** | **A** | **C** | **G** | **T** | **C** | **C** | **A** | **G** | **A** | **T** | **G** | **T** | **T** | **A** | **T** | **T** | **T** | **T** | **G** | **A** | **T** | **G** | **G** | **A** | **T** | **T** | **T** | **G** | **G** | **T** | **A** | **A** | **T** | **G** | **G** | **A** | **A** | **G** | **A** | **A** | **A** | **T** | **G** | **G** | **A** | **C** | **G** | **G** | **C** | **A** | **T** | **T** | **G** | **A** | **T** | **T** | **C** | **A** | **A** | **C** | **A** | **A** | **A** | **A** | **G** | **C** | **G** | **A** | **T** | **C** | **T** | **T** | **G** | **A** | **A** | **A** | **G** | **A** | **T** | **T** | **G** | **G** | **C** | **C** | **A** | **G** | **A** | **A** | **G** | **C** | **C** | **A** | **A** | **G** | **A** | **T** | **T** | **A** | **T** | **T** | **A** | **T** | **T** | **G** | **T** | **G** | **A** | **C** | **G** | **A** | **G** | **T** | **T** | **T** | **T** | **A** | **T** | **T** | **G** | **A** | **T** | **G** | **A** | **T** | **G** | **A** | **A** | **A** | **A** | **A** | **G** | **T** | **G** | **T** | **A** | **T** | **C** | **C** | **G** | **G** | **C** | **G** | **A** | **T** | **T** | **G** | **A** | **A** | **G** | **C** | **T** | **G** | **G** | **T** | **G** | **C** | **A** | **G** | **C** | **G** | **G** | **G** | **C** | **T** | **A** | **C** | **C** | **T** | **A** | **T** | **T** | **A** | **A** | **A** | **A** | **A** | **C** | **A** | **T** | **C** | **A** | **A** | **C** | **A** | **G** | **C** | **A** | **C** | **A** | **T** | **G** | **A** | **G** | **A** | **T** | **T** | **G** | **C** | **T** | **G** | **A** | **T** | **G** | **C** | **A** | **A** | **T** | **T** | **C** | **G** | **G** | **G** | **C** | **G** | **A** | **C** | **T** | **T** | **A** | **T** | **C** | **G** | **C** | **G** | **G** | **A** | **G** | **A** | **G** | **C** | **G** | **T** | **G** | **T** | **G** | **T** | **T** | **G** | **G** | **A** | **A** | **C** | **C** | **T** | **G** | **A** | **A** | **G** | **T** | **G** | **A** | **C** | **G** | **C** | **A** | **T** | **A** | **A** | **G** | **A** | **T** | **G** | **A** | **T** | **G** | **G** | **A** | **A** | **C** | **G** | **G** | **T** | **T** | **A** | **A** | **C** | **A** | **A** | **A** | **A** | **A** | **A** | **A** | **C** | **A** | **A** | **G** | **A** | **G** | **C** | **C** | **G** | **G** | **T** | **G** | **T** | **T** | **G** | **C** | **A** | **T** | **G** | **A** | **A** | **G** | **A** | **T** | **T** | **T** | **G** | **A** | **C** | **A** | **A** | **A** | **T** | **C** | **G** | **A** | **G** | **A** | **A** | **C** | **A** | **C** | **G** | **A** | **A** | **A** | **T** | **T** | **T** | **T** | **A** | **A** | **T** | **G** | **T** | **T** | **G** | **A** | **T** | **T** | **G** | **C** | **A** | **C** | **A** | **A** | **G** | **G** | **T** | **A** | **A** | **A** | **A** | **G** | **T** | **A** | **A** | **T** | **C** | **A** | **G** | **G** | **A** | **A** | **A** | **T** | **A** | **G** | **C** | **T** | **G** | **A** | **T** | **G** | **A** | **A** | **C** | **T** | **C** | **T** | **T** | **T** | **A** | **T** | **C** | **A** | **C** | **T** | **T** | **T** | **G** | **A** | **A** | **A** | **A** | **C** | **A** | **G** | **T** | **T** | **A** | **A** | **A** | **A** | **C** | **A** | **C** | **A** | **T** | **G** | **T** | **T** | **T** | **C** | **A** | **A** | **A** | **C** | **A** | **T** | **T** | **T** | **T** | **A** | **G** | **C** | **A** | **A** | **A** | **A** | **C** | **T** | **A** | **G** | **A** | **T** | **G** | **T** | **G** | **G** | **A** | **T** | **G** | **A** | **T** | **C** | **G** | **G** | **A** | **C** | **C** | **C** | **A** | **A** | **G** | **C** | **G** | **G** | **C** | **G** | **A** | **T** | **T** | **T** | **A** | **T** | **G** | **C** | **T** | **T** | **T** | **T** | **C** | **A** | **A** | **C** | **A** | **T** | **G** | **G** | **T** | **T** | **T** | **A** | **G** | **C** | **C** | **A** | **A** | **A** | **-** | **-** | **-** |
| **N59754F** | **A** | **T** | **G** | **A** | **T** | **A** | **A** | **A** | **A** | **G** | **T** | **T** | **T** | **T** | **A** | **T** | **T** | **A** | **G** | **T** | **A** | **G** | **A** | **T** | **G** | **A** | **C** | **C** | **A** | **T** | **G** | **A** | **A** | **A** | **T** | **G** | **G** | **T** | **A** | **C** | **G** | **C** | **T** | **T** | **A** | **G** | **G** | **C** | **G** | **T** | **C** | **T** | **C** | **T** | **T** | **C** | **T** | **T** | **A** | **T** | **T** | **T** | **A** | **T** | **C** | **T** | **A** | **T** | **C** | **C** | **A** | **A** | **G** | **A** | **A** | **G** | **A** | **C** | **A** | **T** | **T** | **G** | **A** | **A** | **G** | **T** | **G** | **A** | **T** | **T** | **G** | **G** | **A** | **G** | **A** | **A** | **G** | **C** | **T** | **G** | **A** | **A** | **A** | **A** | **T** | **G** | **G** | **A** | **C** | **G** | **T** | **C** | **A** | **A** | **G** | **G** | **C** | **T** | **A** | **T** | **G** | **A** | **A** | **A** | **A** | **G** | **G** | **C** | **G** | **A** | **T** | **G** | **G** | **C** | **A** | **C** | **T** | **T** | **C** | **G** | **A** | **C** | **C** | **A** | **G** | **A** | **T** | **G** | **T** | **C** | **A** | **T** | **T** | **T** | **T** | **A** | **A** | **T** | **G** | **G** | **A** | **T** | **C** | **T** | **A** | **G** | **T** | **C** | **A** | **T** | **G** | **G** | **A** | **A** | **G** | **A** | **A** | **A** | **T** | **G** | **G** | **A** | **T** | **G** | **G** | **C** | **A** | **T** | **C** | **G** | **A** | **A** | **T** | **C** | **A** | **A** | **C** | **A** | **A** | **A** | **A** | **G** | **C** | **A** | **A** | **T** | **C** | **T** | **T** | **A** | **A** | **A** | **A** | **G** | **A** | **T** | **T** | **G** | **G** | **C** | **C** | **A** | **A** | **A** | **A** | **G** | **C** | **C** | **A** | **A** | **A** | **A** | **T** | **C** | **A** | **T** | **T** | **A** | **T** | **C** | **G** | **T** | **C** | **A** | **C** | **A** | **A** | **G** | **T** | **T** | **T** | **T** | **A** | **T** | **T** | **G** | **A** | **T** | **G** | **A** | **T** | **G** | **A** | **A** | **A** | **A** | **A** | **G** | **T** | **T** | **T** | **A** | **T** | **C** | **C** | **T** | **G** | **C** | **A** | **A** | **T** | **T** | **G** | **A** | **A** | **G** | **C** | **A** | **G** | **G** | **A** | **G** | **C** | **A** | **G** | **C** | **A** | **G** | **G** | **A** | **T** | **A** | **T** | **T** | **T** | **A** | **T** | **T** | **G** | **A** | **A** | **G** | **A** | **C** | **G** | **T** | **C** | **T** | **A** | **C** | **C** | **G** | **C** | **A** | **C** | **A** | **T** | **G** | **A** | **A** | **A** | **T** | **A** | **G** | **C** | **T** | **G** | **A** | **T** | **G** | **C** | **T** | **A** | **T** | **A** | **A** | **G** | **G** | **G** | **C** | **T** | **A** | **C** | **G** | **C** | **A** | **A** | **C** | **G** | **C** | **G** | **G** | **C** | **G** | **A** | **G** | **C** | **G** | **A** | **G** | **T** | **A** | **T** | **T** | **G** | **G** | **A** | **A** | **C** | **C** | **T** | **G** | **A** | **A** | **G** | **T** | **G** | **A** | **C** | **A** | **A** | **C** | **A** | **A** | **A** | **A** | **A** | **T** | **G** | **A** | **T** | **G** | **G** | **A** | **A** | **A** | **A** | **A** | **A** | **T** | **G** | **A** | **G** | **T** | **C** | **G** | **G** | **C** | **G** | **A** | **A** | **A** | **T** | **G** | **A** | **T** | **C** | **C** | **A** | **G** | **T** | **A** | **T** | **T** | **G** | **C** | **A** | **T** | **G** | **A** | **A** | **G** | **A** | **A** | **T** | **T** | **A** | **A** | **C** | **G** | **A** | **A** | **T** | **C** | **G** | **A** | **G** | **A** | **A** | **A** | **A** | **T** | **G** | **A** | **A** | **A** | **T** | **T** | **T** | **T** | **G** | **A** | **T** | **G** | **C** | **T** | **G** | **A** | **T** | **T** | **T** | **C** | **T** | **G** | **A** | **A** | **G** | **G** | **G** | **A** | **A** | **A** | **A** | **G** | **T** | **A** | **A** | **T** | **C** | **A** | **G** | **G** | **A** | **A** | **A** | **T** | **C** | **G** | **C** | **G** | **G** | **A** | **T** | **G** | **A** | **A** | **T** | **T** | **G** | **T** | **T** | **T** | **A** | **T** | **C** | **A** | **C** | **A** | **C** | **T** | **G** | **A** | **A** | **A** | **A** | **C** | **A** | **G** | **T** | **T** | **A** | **A** | **A** | **A** | **C** | **G** | **C** | **A** | **T** | **G** | **T** | **G** | **T** | **C** | **G** | **A** | **A** | **T** | **A** | **T** | **A** | **T** | **T** | **A** | **G** | **C** | **T** | **A** | **A** | **G** | **T** | **T** | **A** | **G** | **A** | **G** | **G** | **T** | **A** | **G** | **A** | **A** | **G** | **A** | **C** | **C** | **G** | **A** | **A** | **C** | **T** | **C** | **A** | **A** | **G** | **C** | **A** | **G** | **C** | **T** | **A** | **T** | **C** | **T** | **A** | **T** | **G** | **C** | **G** | **T** | **T** | **T** | **A** | **A** | **A** | **C** | **A** | **T** | **G** | **G** | **T** | **T** | **T** | **G** | **G** | **T** | **G** | **A** | **A** | **A** | **-** | **-** | **-** |
| **N54548** | **G** | **T** | **G** | **A** | **T** | **C** | **A** | **A** | **A** | **G** | **T** | **A** | **A** | **T** | **G** | **T** | **T** | **A** | **G** | **T** | **G** | **G** | **A** | **T** | **G** | **A** | **C** | **C** | **A** | **T** | **G** | **A** | **A** | **A** | **T** | **G** | **G** | **T** | **C** | **C** | **G** | **T** | **T** | **T** | **A** | **G** | **G** | **C** | **G** | **T** | **T** | **T** | **C** | **A** | **T** | **C** | **A** | **T** | **A** | **T** | **T** | **T** | **A** | **T** | **C** | **T** | **A** | **T** | **T** | **C** | **A** | **A** | **G** | **A** | **G** | **G** | **A** | **T** | **A** | **T** | **A** | **G** | **A** | **A** | **G** | **T** | **C** | **G** | **T** | **A** | **G** | **G** | **C** | **G** | **A** | **A** | **G** | **C** | **A** | **G** | **A** | **A** | **A** | **A** | **C** | **G** | **G** | **T** | **A** | **A** | **G** | **A** | **T** | **T** | **G** | **G** | **C** | **T** | **A** | **T** | **G** | **A** | **A** | **A** | **A** | **A** | **G** | **C** | **A** | **T** | **T** | **G** | **G** | **A** | **A** | **C** | **T** | **A** | **C** | **G** | **T** | **C** | **C** | **A** | **G** | **A** | **T** | **G** | **T** | **T** | **A** | **T** | **T** | **T** | **T** | **G** | **A** | **T** | **G** | **G** | **A** | **C** | **T** | **T** | **G** | **G** | **T** | **A** | **A** | **T** | **G** | **G** | **A** | **A** | **G** | **A** | **A** | **A** | **T** | **G** | **G** | **A** | **C** | **G** | **G** | **C** | **A** | **T** | **T** | **G** | **A** | **T** | **T** | **C** | **A** | **A** | **C** | **A** | **A** | **A** | **A** | **G** | **C** | **G** | **A** | **T** | **T** | **T** | **T** | **G** | **A** | **A** | **A** | **G** | **A** | **T** | **T** | **G** | **G** | **C** | **C** | **A** | **G** | **A** | **A** | **G** | **C** | **C** | **A** | **A** | **G** | **A** | **T** | **T** | **A** | **T** | **T** | **A** | **T** | **T** | **G** | **T** | **G** | **A** | **C** | **G** | **A** | **G** | **T** | **T** | **T** | **T** | **A** | **T** | **T** | **G** | **A** | **T** | **G** | **A** | **T** | **G** | **A** | **A** | **A** | **A** | **A** | **G** | **T** | **G** | **T** | **A** | **T** | **C** | **C** | **G** | **G** | **C** | **A** | **A** | **T** | **T** | **G** | **A** | **A** | **G** | **C** | **T** | **G** | **G** | **T** | **G** | **C** | **A** | **G** | **C** | **G** | **G** | **G** | **C** | **T** | **A** | **C** | **C** | **T** | **A** | **T** | **T** | **A** | **A** | **A** | **A** | **A** | **C** | **A** | **T** | **C** | **A** | **A** | **C** | **A** | **G** | **C** | **A** | **C** | **A** | **T** | **G** | **A** | **G** | **A** | **T** | **T** | **G** | **C** | **T** | **G** | **A** | **T** | **G** | **C** | **G** | **A** | **T** | **T** | **C** | **G** | **G** | **G** | **C** | **G** | **A** | **C** | **T** | **T** | **A** | **T** | **C** | **G** | **C** | **G** | **G** | **A** | **G** | **A** | **G** | **C** | **G** | **T** | **G** | **T** | **G** | **T** | **T** | **G** | **G** | **A** | **A** | **C** | **C** | **T** | **G** | **A** | **A** | **G** | **T** | **G** | **A** | **C** | **G** | **C** | **A** | **T** | **A** | **A** | **G** | **A** | **T** | **G** | **A** | **T** | **G** | **G** | **A** | **A** | **C** | **G** | **G** | **T** | **T** | **A** | **A** | **C** | **A** | **A** | **A** | **A** | **A** | **A** | **A** | **C** | **A** | **A** | **G** | **A** | **G** | **C** | **C** | **G** | **G** | **T** | **G** | **C** | **T** | **A** | **C** | **A** | **C** | **G** | **A** | **A** | **G** | **A** | **T** | **T** | **T** | **G** | **A** | **C** | **A** | **A** | **A** | **C** | **C** | **G** | **A** | **G** | **A** | **A** | **C** | **A** | **C** | **G** | **A** | **A** | **A** | **T** | **T** | **T** | **T** | **A** | **A** | **T** | **G** | **C** | **T** | **G** | **A** | **T** | **T** | **G** | **C** | **A** | **C** | **A** | **A** | **G** | **G** | **T** | **A** | **A** | **A** | **A** | **G** | **T** | **A** | **A** | **T** | **C** | **A** | **G** | **G** | **A** | **A** | **A** | **T** | **T** | **G** | **C** | **T** | **G** | **A** | **T** | **G** | **A** | **A** | **C** | **T** | **C** | **T** | **T** | **T** | **A** | **T** | **C** | **A** | **C** | **T** | **T** | **T** | **G** | **A** | **A** | **A** | **A** | **C** | **A** | **G** | **T** | **T** | **A** | **A** | **A** | **A** | **C** | **A** | **C** | **A** | **T** | **G** | **T** | **T** | **T** | **C** | **A** | **A** | **A** | **C** | **A** | **T** | **T** | **T** | **T** | **A** | **G** | **C** | **A** | **A** | **A** | **A** | **C** | **T** | **A** | **G** | **A** | **T** | **G** | **T** | **G** | **G** | **A** | **T** | **G** | **A** | **T** | **C** | **G** | **G** | **A** | **C** | **C** | **C** | **A** | **A** | **G** | **C** | **G** | **G** | **C** | **G** | **A** | **T** | **T** | **T** | **A** | **T** | **G** | **C** | **A** | **T** | **T** | **T** | **C** | **A** | **A** | **C** | **A** | **C** | **G** | **G** | **T** | **T** | **T** | **A** | **G** | **C** | **C** | **A** | **A** | **A** | **-** | **-** | **-** |
| **N55113** | **G** | **T** | **G** | **A** | **T** | **C** | **A** | **A** | **A** | **G** | **T** | **A** | **A** | **T** | **G** | **T** | **T** | **A** | **G** | **T** | **G** | **G** | **A** | **T** | **G** | **A** | **C** | **C** | **A** | **T** | **G** | **A** | **A** | **A** | **T** | **G** | **G** | **T** | **C** | **C** | **G** | **T** | **T** | **T** | **A** | **G** | **G** | **C** | **G** | **T** | **T** | **T** | **C** | **A** | **T** | **C** | **A** | **T** | **A** | **T** | **T** | **T** | **A** | **T** | **C** | **T** | **A** | **T** | **T** | **C** | **A** | **A** | **G** | **A** | **G** | **G** | **A** | **T** | **A** | **T** | **A** | **G** | **A** | **A** | **G** | **T** | **C** | **G** | **T** | **A** | **G** | **G** | **C** | **G** | **A** | **A** | **G** | **C** | **A** | **G** | **A** | **A** | **A** | **A** | **C** | **G** | **G** | **T** | **A** | **A** | **G** | **A** | **T** | **T** | **G** | **G** | **C** | **T** | **A** | **T** | **G** | **A** | **A** | **A** | **A** | **A** | **G** | **C** | **A** | **T** | **T** | **G** | **G** | **A** | **A** | **C** | **T** | **A** | **C** | **G** | **T** | **C** | **C** | **A** | **G** | **A** | **T** | **G** | **T** | **T** | **A** | **T** | **T** | **T** | **T** | **G** | **A** | **T** | **G** | **G** | **A** | **T** | **T** | **T** | **G** | **G** | **T** | **A** | **A** | **T** | **G** | **G** | **A** | **A** | **G** | **A** | **A** | **A** | **T** | **G** | **G** | **A** | **C** | **G** | **G** | **C** | **A** | **T** | **T** | **G** | **A** | **T** | **T** | **C** | **A** | **A** | **C** | **A** | **A** | **A** | **A** | **G** | **C** | **G** | **A** | **T** | **C** | **T** | **T** | **G** | **A** | **A** | **A** | **G** | **A** | **T** | **T** | **G** | **G** | **C** | **C** | **A** | **G** | **A** | **A** | **G** | **C** | **C** | **A** | **A** | **G** | **A** | **T** | **T** | **A** | **T** | **T** | **A** | **T** | **T** | **G** | **T** | **G** | **A** | **C** | **G** | **A** | **G** | **T** | **T** | **T** | **T** | **A** | **T** | **T** | **G** | **A** | **T** | **G** | **A** | **T** | **G** | **A** | **A** | **A** | **A** | **A** | **G** | **T** | **G** | **T** | **A** | **T** | **C** | **C** | **G** | **G** | **C** | **G** | **A** | **T** | **T** | **G** | **A** | **A** | **G** | **C** | **T** | **G** | **G** | **T** | **G** | **C** | **A** | **G** | **C** | **G** | **G** | **G** | **C** | **T** | **A** | **C** | **C** | **T** | **A** | **T** | **T** | **A** | **A** | **A** | **A** | **A** | **C** | **A** | **T** | **C** | **A** | **A** | **C** | **A** | **G** | **C** | **A** | **C** | **A** | **T** | **G** | **A** | **G** | **A** | **T** | **T** | **G** | **C** | **T** | **G** | **A** | **T** | **G** | **C** | **A** | **A** | **T** | **T** | **C** | **G** | **G** | **G** | **C** | **G** | **A** | **C** | **T** | **T** | **A** | **T** | **C** | **G** | **C** | **G** | **G** | **A** | **G** | **A** | **G** | **C** | **G** | **T** | **G** | **T** | **G** | **T** | **T** | **G** | **G** | **A** | **A** | **C** | **C** | **T** | **G** | **A** | **A** | **G** | **T** | **G** | **A** | **C** | **G** | **C** | **A** | **T** | **A** | **A** | **G** | **A** | **T** | **G** | **A** | **T** | **G** | **G** | **A** | **A** | **C** | **G** | **G** | **T** | **T** | **A** | **A** | **C** | **A** | **A** | **A** | **A** | **A** | **A** | **A** | **C** | **A** | **A** | **G** | **A** | **G** | **C** | **C** | **G** | **G** | **T** | **G** | **C** | **T** | **A** | **C** | **A** | **T** | **G** | **A** | **A** | **G** | **A** | **T** | **T** | **T** | **G** | **A** | **C** | **A** | **A** | **A** | **C** | **C** | **G** | **A** | **G** | **A** | **A** | **C** | **A** | **C** | **G** | **A** | **A** | **A** | **T** | **T** | **T** | **T** | **A** | **A** | **T** | **G** | **T** | **T** | **G** | **A** | **T** | **T** | **G** | **C** | **A** | **C** | **A** | **A** | **G** | **G** | **T** | **A** | **A** | **A** | **A** | **G** | **T** | **A** | **A** | **T** | **C** | **A** | **G** | **G** | **A** | **A** | **A** | **T** | **A** | **G** | **C** | **T** | **G** | **A** | **T** | **G** | **A** | **A** | **C** | **T** | **C** | **T** | **T** | **T** | **A** | **T** | **C** | **A** | **C** | **T** | **T** | **T** | **G** | **A** | **A** | **A** | **A** | **C** | **A** | **G** | **T** | **T** | **A** | **A** | **A** | **A** | **C** | **A** | **C** | **A** | **T** | **G** | **T** | **T** | **T** | **C** | **A** | **A** | **A** | **C** | **A** | **T** | **T** | **T** | **T** | **A** | **G** | **C** | **A** | **A** | **A** | **A** | **C** | **T** | **A** | **G** | **A** | **T** | **G** | **T** | **G** | **G** | **A** | **T** | **G** | **A** | **T** | **C** | **G** | **G** | **A** | **C** | **C** | **C** | **A** | **A** | **G** | **C** | **G** | **G** | **C** | **G** | **A** | **T** | **T** | **T** | **A** | **T** | **G** | **C** | **T** | **T** | **T** | **T** | **C** | **A** | **A** | **C** | **A** | **T** | **G** | **G** | **T** | **T** | **T** | **A** | **G** | **C** | **C** | **A** | **A** | **A** | **-** | **-** | **-** |
| **N59458F** | **G** | **T** | **G** | **A** | **T** | **C** | **A** | **A** | **A** | **G** | **T** | **A** | **A** | **T** | **G** | **T** | **T** | **A** | **G** | **T** | **G** | **G** | **A** | **T** | **G** | **A** | **C** | **C** | **A** | **T** | **G** | **A** | **A** | **A** | **T** | **G** | **G** | **T** | **C** | **C** | **G** | **T** | **T** | **T** | **A** | **G** | **G** | **C** | **G** | **T** | **T** | **T** | **C** | **A** | **T** | **C** | **A** | **T** | **A** | **T** | **T** | **T** | **A** | **T** | **C** | **T** | **A** | **T** | **T** | **C** | **A** | **A** | **G** | **A** | **G** | **G** | **A** | **T** | **A** | **T** | **A** | **G** | **A** | **A** | **G** | **T** | **C** | **G** | **T** | **A** | **G** | **G** | **C** | **G** | **A** | **A** | **G** | **C** | **A** | **G** | **A** | **A** | **A** | **A** | **C** | **G** | **G** | **T** | **A** | **A** | **G** | **A** | **T** | **T** | **G** | **G** | **C** | **T** | **A** | **T** | **G** | **A** | **A** | **A** | **A** | **A** | **G** | **C** | **A** | **T** | **T** | **G** | **G** | **A** | **A** | **C** | **T** | **A** | **C** | **G** | **T** | **C** | **C** | **A** | **G** | **A** | **T** | **G** | **T** | **T** | **A** | **T** | **T** | **T** | **T** | **G** | **A** | **T** | **G** | **G** | **A** | **T** | **T** | **T** | **G** | **G** | **T** | **A** | **A** | **T** | **G** | **G** | **A** | **A** | **G** | **A** | **A** | **A** | **T** | **G** | **G** | **A** | **C** | **G** | **G** | **C** | **A** | **T** | **T** | **G** | **A** | **T** | **T** | **C** | **A** | **A** | **C** | **C** | **A** | **A** | **A** | **G** | **C** | **G** | **A** | **T** | **C** | **T** | **T** | **G** | **A** | **A** | **A** | **G** | **A** | **T** | **T** | **G** | **G** | **C** | **C** | **A** | **G** | **A** | **A** | **G** | **C** | **C** | **A** | **A** | **G** | **A** | **T** | **T** | **A** | **T** | **T** | **A** | **T** | **T** | **G** | **T** | **G** | **A** | **C** | **G** | **A** | **G** | **T** | **T** | **T** | **T** | **A** | **T** | **T** | **G** | **A** | **T** | **G** | **A** | **T** | **G** | **A** | **A** | **A** | **A** | **A** | **G** | **T** | **G** | **T** | **A** | **T** | **C** | **C** | **G** | **G** | **C** | **G** | **A** | **T** | **T** | **G** | **A** | **A** | **G** | **C** | **T** | **G** | **G** | **T** | **G** | **C** | **A** | **G** | **C** | **G** | **G** | **G** | **C** | **T** | **A** | **C** | **C** | **T** | **A** | **T** | **T** | **A** | **A** | **A** | **A** | **A** | **C** | **A** | **T** | **C** | **A** | **A** | **C** | **A** | **G** | **C** | **A** | **C** | **A** | **T** | **G** | **A** | **G** | **A** | **T** | **T** | **G** | **C** | **T** | **G** | **A** | **T** | **G** | **C** | **A** | **A** | **T** | **T** | **C** | **G** | **G** | **G** | **C** | **G** | **A** | **C** | **T** | **T** | **A** | **T** | **C** | **G** | **C** | **G** | **G** | **A** | **G** | **A** | **G** | **C** | **G** | **T** | **G** | **T** | **G** | **T** | **T** | **G** | **G** | **A** | **A** | **C** | **C** | **T** | **G** | **A** | **A** | **G** | **T** | **G** | **A** | **C** | **G** | **C** | **A** | **T** | **A** | **A** | **G** | **A** | **T** | **G** | **A** | **T** | **G** | **G** | **A** | **A** | **C** | **G** | **G** | **T** | **T** | **A** | **A** | **C** | **A** | **A** | **A** | **A** | **A** | **A** | **A** | **C** | **A** | **A** | **G** | **A** | **G** | **C** | **C** | **G** | **G** | **T** | **G** | **T** | **T** | **G** | **C** | **A** | **C** | **G** | **A** | **A** | **G** | **A** | **T** | **T** | **T** | **G** | **A** | **C** | **A** | **A** | **A** | **C** | **C** | **G** | **G** | **G** | **A** | **A** | **C** | **A** | **C** | **G** | **A** | **A** | **A** | **T** | **T** | **T** | **T** | **A** | **A** | **T** | **G** | **T** | **T** | **G** | **A** | **T** | **T** | **G** | **C** | **A** | **C** | **A** | **A** | **G** | **G** | **T** | **A** | **A** | **A** | **A** | **G** | **T** | **A** | **A** | **T** | **C** | **A** | **G** | **G** | **A** | **A** | **A** | **T** | **A** | **G** | **C** | **T** | **G** | **A** | **T** | **G** | **A** | **A** | **C** | **T** | **C** | **T** | **T** | **T** | **A** | **T** | **C** | **A** | **C** | **T** | **T** | **T** | **G** | **A** | **A** | **A** | **A** | **C** | **A** | **G** | **T** | **T** | **A** | **A** | **A** | **A** | **C** | **A** | **C** | **A** | **T** | **G** | **T** | **T** | **T** | **C** | **A** | **A** | **A** | **C** | **A** | **T** | **T** | **T** | **T** | **A** | **G** | **C** | **A** | **A** | **A** | **A** | **C** | **T** | **A** | **G** | **A** | **T** | **G** | **T** | **G** | **G** | **A** | **T** | **G** | **A** | **T** | **C** | **G** | **G** | **A** | **C** | **C** | **C** | **A** | **A** | **G** | **C** | **G** | **G** | **C** | **G** | **A** | **T** | **T** | **T** | **A** | **T** | **G** | **C** | **T** | **T** | **T** | **T** | **C** | **A** | **A** | **C** | **A** | **T** | **G** | **G** | **T** | **T** | **T** | **A** | **G** | **C** | **C** | **A** | **A** | **A** | **-** | **-** | **-** |
| **N53533** | **A** | **T** | **G** | **A** | **T** | **A** | **A** | **A** | **A** | **G** | **T** | **T** | **T** | **T** | **A** | **T** | **T** | **A** | **G** | **T** | **A** | **G** | **A** | **T** | **G** | **A** | **C** | **C** | **A** | **T** | **G** | **A** | **A** | **A** | **T** | **G** | **G** | **T** | **A** | **C** | **G** | **C** | **T** | **T** | **A** | **G** | **G** | **C** | **G** | **T** | **C** | **T** | **C** | **T** | **T** | **C** | **T** | **T** | **A** | **T** | **T** | **T** | **A** | **T** | **C** | **T** | **A** | **T** | **C** | **C** | **A** | **A** | **G** | **A** | **A** | **G** | **A** | **C** | **A** | **T** | **T** | **G** | **A** | **A** | **G** | **T** | **G** | **A** | **T** | **T** | **G** | **G** | **A** | **G** | **A** | **A** | **G** | **C** | **T** | **G** | **A** | **A** | **A** | **A** | **T** | **G** | **G** | **A** | **C** | **G** | **T** | **C** | **A** | **A** | **G** | **G** | **C** | **T** | **A** | **T** | **G** | **A** | **A** | **A** | **A** | **G** | **G** | **C** | **G** | **A** | **T** | **G** | **G** | **C** | **A** | **C** | **T** | **T** | **C** | **G** | **A** | **C** | **C** | **A** | **G** | **A** | **T** | **G** | **T** | **C** | **A** | **T** | **T** | **T** | **T** | **A** | **A** | **T** | **G** | **G** | **A** | **T** | **C** | **T** | **A** | **G** | **T** | **C** | **A** | **T** | **G** | **G** | **A** | **A** | **G** | **A** | **A** | **A** | **T** | **G** | **G** | **A** | **T** | **G** | **G** | **C** | **A** | **T** | **C** | **G** | **A** | **A** | **T** | **C** | **A** | **A** | **C** | **A** | **A** | **A** | **A** | **G** | **C** | **A** | **A** | **T** | **C** | **T** | **T** | **A** | **A** | **A** | **A** | **G** | **A** | **T** | **T** | **G** | **G** | **C** | **C** | **A** | **A** | **A** | **A** | **G** | **C** | **C** | **A** | **A** | **A** | **A** | **T** | **C** | **A** | **T** | **T** | **A** | **T** | **C** | **G** | **T** | **C** | **A** | **C** | **A** | **A** | **G** | **T** | **T** | **T** | **T** | **A** | **T** | **T** | **G** | **A** | **T** | **G** | **A** | **T** | **G** | **A** | **A** | **A** | **A** | **A** | **G** | **T** | **T** | **T** | **A** | **T** | **C** | **C** | **T** | **G** | **C** | **A** | **A** | **T** | **T** | **G** | **A** | **A** | **G** | **C** | **A** | **G** | **G** | **A** | **G** | **C** | **A** | **G** | **C** | **A** | **G** | **G** | **A** | **T** | **A** | **T** | **T** | **T** | **A** | **T** | **T** | **G** | **A** | **A** | **G** | **A** | **C** | **G** | **T** | **C** | **T** | **A** | **C** | **C** | **G** | **C** | **A** | **C** | **A** | **T** | **G** | **A** | **A** | **A** | **T** | **A** | **G** | **C** | **T** | **G** | **A** | **T** | **G** | **C** | **T** | **A** | **T** | **A** | **A** | **G** | **G** | **G** | **C** | **T** | **A** | **C** | **G** | **C** | **A** | **A** | **C** | **G** | **C** | **G** | **G** | **C** | **G** | **A** | **G** | **C** | **G** | **A** | **G** | **T** | **A** | **T** | **T** | **G** | **G** | **A** | **A** | **C** | **C** | **T** | **G** | **A** | **A** | **G** | **T** | **G** | **A** | **C** | **A** | **A** | **C** | **A** | **A** | **A** | **A** | **A** | **T** | **G** | **A** | **T** | **G** | **G** | **A** | **A** | **A** | **A** | **A** | **A** | **T** | **G** | **A** | **G** | **T** | **C** | **G** | **G** | **C** | **G** | **A** | **A** | **A** | **T** | **G** | **A** | **T** | **C** | **C** | **A** | **G** | **T** | **A** | **T** | **T** | **G** | **C** | **A** | **T** | **G** | **A** | **A** | **G** | **A** | **A** | **T** | **T** | **A** | **A** | **C** | **G** | **A** | **A** | **T** | **C** | **G** | **A** | **G** | **A** | **A** | **A** | **A** | **T** | **G** | **A** | **A** | **A** | **T** | **T** | **T** | **T** | **G** | **A** | **T** | **G** | **C** | **T** | **G** | **A** | **T** | **T** | **T** | **C** | **T** | **G** | **A** | **A** | **G** | **G** | **G** | **A** | **A** | **A** | **A** | **G** | **T** | **A** | **A** | **T** | **C** | **A** | **G** | **G** | **A** | **A** | **A** | **T** | **C** | **G** | **C** | **G** | **G** | **A** | **T** | **G** | **A** | **A** | **T** | **T** | **G** | **T** | **T** | **T** | **A** | **T** | **C** | **A** | **C** | **A** | **C** | **T** | **G** | **A** | **A** | **A** | **A** | **C** | **A** | **G** | **T** | **T** | **A** | **A** | **A** | **A** | **C** | **G** | **C** | **A** | **T** | **G** | **T** | **G** | **T** | **C** | **G** | **A** | **A** | **T** | **A** | **T** | **A** | **T** | **T** | **A** | **G** | **C** | **T** | **A** | **A** | **G** | **T** | **T** | **A** | **G** | **A** | **G** | **G** | **T** | **A** | **G** | **A** | **A** | **G** | **A** | **C** | **C** | **G** | **A** | **A** | **C** | **T** | **C** | **A** | **A** | **G** | **C** | **A** | **G** | **C** | **T** | **A** | **T** | **C** | **T** | **A** | **T** | **G** | **C** | **G** | **T** | **T** | **T** | **A** | **A** | **A** | **C** | **A** | **T** | **G** | **G** | **T** | **T** | **T** | **G** | **G** | **T** | **G** | **A** | **A** | **A** | **-** | **-** | **-** |
| **N53548** | **A** | **T** | **G** | **A** | **T** | **A** | **A** | **A** | **A** | **G** | **T** | **T** | **T** | **T** | **A** | **T** | **T** | **A** | **G** | **T** | **A** | **G** | **A** | **T** | **G** | **A** | **C** | **C** | **A** | **T** | **G** | **A** | **A** | **A** | **T** | **G** | **G** | **T** | **A** | **C** | **G** | **C** | **T** | **T** | **A** | **G** | **G** | **C** | **G** | **T** | **C** | **T** | **C** | **T** | **T** | **C** | **T** | **T** | **A** | **T** | **T** | **T** | **A** | **T** | **C** | **T** | **A** | **T** | **C** | **C** | **A** | **A** | **G** | **A** | **A** | **G** | **A** | **C** | **A** | **T** | **T** | **G** | **A** | **A** | **G** | **T** | **G** | **A** | **T** | **T** | **G** | **G** | **A** | **G** | **A** | **A** | **G** | **C** | **T** | **G** | **A** | **A** | **A** | **A** | **T** | **G** | **G** | **A** | **C** | **G** | **T** | **C** | **A** | **A** | **G** | **G** | **C** | **T** | **A** | **T** | **G** | **A** | **A** | **A** | **A** | **G** | **G** | **C** | **G** | **A** | **T** | **G** | **G** | **C** | **A** | **C** | **T** | **T** | **C** | **G** | **A** | **C** | **C** | **A** | **G** | **A** | **T** | **G** | **T** | **C** | **A** | **T** | **T** | **T** | **T** | **A** | **A** | **T** | **G** | **G** | **A** | **T** | **C** | **T** | **A** | **G** | **T** | **C** | **A** | **T** | **G** | **G** | **A** | **A** | **G** | **A** | **A** | **A** | **T** | **G** | **G** | **A** | **T** | **G** | **G** | **C** | **A** | **T** | **C** | **G** | **A** | **A** | **T** | **C** | **A** | **A** | **C** | **A** | **A** | **A** | **A** | **G** | **C** | **A** | **A** | **T** | **C** | **T** | **T** | **A** | **A** | **A** | **A** | **G** | **A** | **T** | **T** | **G** | **G** | **A** | **C** | **A** | **A** | **A** | **A** | **G** | **C** | **C** | **A** | **A** | **A** | **A** | **T** | **C** | **A** | **T** | **T** | **A** | **T** | **C** | **G** | **T** | **C** | **A** | **C** | **A** | **A** | **G** | **T** | **T** | **T** | **T** | **A** | **T** | **T** | **G** | **A** | **T** | **G** | **A** | **T** | **G** | **A** | **A** | **A** | **A** | **A** | **G** | **T** | **T** | **T** | **A** | **T** | **C** | **C** | **T** | **G** | **C** | **A** | **A** | **T** | **T** | **G** | **A** | **A** | **G** | **C** | **A** | **G** | **G** | **A** | **G** | **C** | **A** | **G** | **C** | **A** | **G** | **G** | **A** | **T** | **A** | **T** | **T** | **T** | **A** | **T** | **T** | **G** | **A** | **A** | **G** | **A** | **C** | **G** | **T** | **C** | **T** | **A** | **C** | **C** | **G** | **C** | **A** | **C** | **A** | **T** | **G** | **A** | **A** | **A** | **T** | **A** | **G** | **C** | **T** | **G** | **A** | **T** | **G** | **C** | **T** | **A** | **T** | **A** | **A** | **G** | **G** | **G** | **C** | **T** | **A** | **C** | **G** | **C** | **A** | **A** | **C** | **G** | **C** | **G** | **G** | **C** | **G** | **A** | **G** | **C** | **G** | **A** | **G** | **T** | **A** | **T** | **T** | **G** | **G** | **A** | **A** | **C** | **C** | **T** | **G** | **A** | **A** | **G** | **T** | **G** | **A** | **C** | **A** | **A** | **C** | **A** | **A** | **A** | **A** | **A** | **T** | **G** | **A** | **T** | **G** | **G** | **A** | **A** | **A** | **A** | **A** | **A** | **T** | **G** | **A** | **G** | **T** | **C** | **G** | **G** | **C** | **G** | **A** | **A** | **A** | **T** | **G** | **A** | **T** | **C** | **C** | **A** | **G** | **T** | **A** | **T** | **T** | **G** | **C** | **A** | **T** | **G** | **A** | **A** | **G** | **A** | **A** | **T** | **T** | **A** | **A** | **C** | **G** | **A** | **A** | **T** | **C** | **G** | **A** | **G** | **A** | **A** | **A** | **A** | **T** | **G** | **A** | **A** | **A** | **T** | **T** | **T** | **T** | **G** | **A** | **T** | **G** | **C** | **T** | **G** | **A** | **T** | **T** | **T** | **C** | **T** | **G** | **A** | **A** | **G** | **G** | **G** | **A** | **A** | **A** | **A** | **G** | **T** | **A** | **A** | **T** | **C** | **A** | **G** | **G** | **A** | **A** | **A** | **T** | **C** | **G** | **C** | **G** | **G** | **A** | **T** | **G** | **A** | **A** | **T** | **T** | **G** | **T** | **T** | **T** | **A** | **T** | **C** | **A** | **C** | **A** | **C** | **T** | **G** | **A** | **A** | **A** | **A** | **C** | **A** | **G** | **T** | **T** | **A** | **A** | **A** | **A** | **C** | **G** | **C** | **A** | **T** | **G** | **T** | **G** | **T** | **C** | **G** | **A** | **A** | **T** | **A** | **T** | **A** | **T** | **T** | **A** | **G** | **C** | **T** | **A** | **A** | **G** | **T** | **T** | **A** | **G** | **A** | **G** | **G** | **T** | **A** | **G** | **A** | **A** | **G** | **A** | **C** | **C** | **G** | **A** | **A** | **C** | **T** | **C** | **A** | **A** | **G** | **C** | **A** | **G** | **C** | **T** | **A** | **T** | **C** | **T** | **A** | **T** | **G** | **C** | **G** | **T** | **T** | **T** | **A** | **A** | **A** | **C** | **A** | **T** | **G** | **G** | **T** | **T** | **T** | **G** | **G** | **T** | **G** | **A** | **A** | **A** | **-** | **-** | **-** |
| **N52829** | **A** | **T** | **G** | **A** | **T** | **A** | **A** | **A** | **A** | **G** | **T** | **T** | **T** | **T** | **A** | **T** | **T** | **A** | **G** | **T** | **A** | **G** | **A** | **T** | **G** | **A** | **C** | **C** | **A** | **T** | **G** | **A** | **A** | **A** | **T** | **G** | **G** | **T** | **A** | **C** | **G** | **C** | **T** | **T** | **A** | **G** | **G** | **C** | **G** | **T** | **C** | **T** | **C** | **T** | **T** | **C** | **T** | **T** | **A** | **T** | **T** | **T** | **A** | **T** | **C** | **T** | **A** | **T** | **C** | **C** | **A** | **A** | **G** | **A** | **A** | **G** | **A** | **C** | **A** | **T** | **T** | **G** | **A** | **A** | **G** | **T** | **G** | **A** | **T** | **T** | **G** | **G** | **A** | **G** | **A** | **A** | **G** | **C** | **T** | **G** | **A** | **A** | **A** | **A** | **T** | **G** | **G** | **A** | **C** | **G** | **T** | **C** | **A** | **A** | **G** | **G** | **C** | **T** | **A** | **T** | **G** | **A** | **A** | **A** | **A** | **G** | **G** | **C** | **G** | **A** | **T** | **G** | **G** | **C** | **A** | **C** | **T** | **T** | **C** | **G** | **A** | **C** | **C** | **G** | **G** | **A** | **T** | **G** | **T** | **C** | **A** | **T** | **T** | **T** | **T** | **A** | **A** | **T** | **G** | **G** | **A** | **T** | **C** | **T** | **A** | **G** | **T** | **C** | **A** | **T** | **G** | **G** | **A** | **A** | **G** | **A** | **A** | **A** | **T** | **G** | **G** | **A** | **T** | **G** | **G** | **C** | **A** | **T** | **C** | **G** | **A** | **A** | **T** | **C** | **A** | **A** | **C** | **A** | **A** | **A** | **A** | **G** | **C** | **A** | **A** | **T** | **C** | **T** | **T** | **A** | **A** | **A** | **A** | **G** | **A** | **T** | **T** | **G** | **G** | **C** | **C** | **A** | **A** | **A** | **A** | **G** | **C** | **C** | **A** | **A** | **A** | **A** | **T** | **C** | **A** | **T** | **T** | **A** | **T** | **C** | **G** | **T** | **C** | **A** | **C** | **A** | **A** | **G** | **T** | **T** | **T** | **T** | **A** | **T** | **T** | **G** | **A** | **T** | **G** | **A** | **T** | **G** | **A** | **A** | **A** | **A** | **A** | **G** | **T** | **T** | **T** | **A** | **T** | **C** | **C** | **T** | **G** | **C** | **A** | **A** | **T** | **T** | **G** | **A** | **A** | **G** | **C** | **A** | **G** | **G** | **A** | **G** | **C** | **A** | **G** | **C** | **A** | **G** | **G** | **A** | **T** | **A** | **T** | **T** | **T** | **G** | **T** | **T** | **G** | **A** | **A** | **G** | **A** | **C** | **G** | **T** | **C** | **T** | **A** | **C** | **C** | **G** | **C** | **A** | **C** | **A** | **T** | **G** | **A** | **A** | **A** | **T** | **A** | **G** | **C** | **T** | **G** | **A** | **T** | **G** | **C** | **T** | **A** | **T** | **A** | **A** | **G** | **G** | **G** | **C** | **T** | **A** | **C** | **G** | **C** | **A** | **A** | **C** | **G** | **C** | **G** | **G** | **C** | **G** | **A** | **G** | **C** | **G** | **A** | **G** | **T** | **A** | **T** | **T** | **G** | **G** | **A** | **A** | **C** | **C** | **T** | **G** | **A** | **A** | **G** | **T** | **G** | **A** | **C** | **A** | **A** | **C** | **A** | **A** | **A** | **A** | **A** | **T** | **G** | **A** | **T** | **G** | **G** | **A** | **A** | **A** | **A** | **A** | **A** | **T** | **G** | **A** | **G** | **T** | **C** | **G** | **G** | **C** | **G** | **A** | **A** | **A** | **T** | **G** | **A** | **T** | **C** | **C** | **A** | **G** | **T** | **A** | **T** | **T** | **G** | **C** | **A** | **T** | **G** | **A** | **A** | **G** | **A** | **A** | **T** | **T** | **A** | **A** | **C** | **G** | **A** | **A** | **T** | **C** | **G** | **A** | **G** | **A** | **A** | **A** | **A** | **T** | **G** | **A** | **A** | **A** | **T** | **T** | **T** | **T** | **G** | **A** | **T** | **G** | **C** | **T** | **G** | **A** | **T** | **T** | **T** | **C** | **T** | **G** | **A** | **A** | **G** | **G** | **G** | **A** | **A** | **A** | **A** | **G** | **T** | **A** | **A** | **T** | **C** | **A** | **G** | **G** | **A** | **A** | **A** | **T** | **C** | **G** | **C** | **G** | **G** | **A** | **T** | **G** | **A** | **A** | **T** | **T** | **G** | **T** | **T** | **T** | **A** | **T** | **C** | **A** | **C** | **A** | **C** | **T** | **G** | **A** | **A** | **A** | **A** | **C** | **A** | **G** | **T** | **C** | **A** | **A** | **A** | **A** | **C** | **G** | **C** | **A** | **T** | **G** | **T** | **G** | **T** | **C** | **G** | **A** | **A** | **T** | **A** | **T** | **A** | **T** | **T** | **A** | **G** | **C** | **T** | **A** | **A** | **G** | **T** | **T** | **A** | **G** | **A** | **G** | **G** | **T** | **A** | **G** | **A** | **A** | **G** | **A** | **C** | **C** | **G** | **A** | **A** | **C** | **T** | **C** | **A** | **A** | **G** | **C** | **A** | **G** | **C** | **T** | **A** | **T** | **C** | **T** | **A** | **T** | **G** | **C** | **G** | **T** | **T** | **T** | **A** | **A** | **A** | **C** | **A** | **T** | **G** | **G** | **T** | **T** | **T** | **G** | **G** | **T** | **G** | **A** | **A** | **A** | **-** | **-** | **-** |
| **N53418** | **G** | **T** | **G** | **A** | **T** | **C** | **A** | **A** | **A** | **G** | **T** | **A** | **A** | **T** | **G** | **T** | **T** | **A** | **G** | **T** | **G** | **G** | **A** | **T** | **G** | **A** | **C** | **C** | **A** | **T** | **G** | **A** | **A** | **A** | **T** | **G** | **G** | **T** | **C** | **C** | **G** | **T** | **T** | **T** | **A** | **G** | **G** | **C** | **G** | **T** | **T** | **T** | **C** | **A** | **T** | **C** | **A** | **T** | **A** | **T** | **T** | **T** | **A** | **T** | **C** | **T** | **A** | **T** | **T** | **C** | **A** | **A** | **G** | **A** | **G** | **G** | **A** | **T** | **A** | **T** | **A** | **G** | **A** | **A** | **G** | **T** | **C** | **G** | **T** | **A** | **G** | **G** | **C** | **G** | **A** | **A** | **G** | **C** | **A** | **G** | **A** | **A** | **A** | **A** | **C** | **G** | **G** | **T** | **A** | **A** | **G** | **A** | **T** | **T** | **G** | **G** | **C** | **T** | **A** | **T** | **G** | **A** | **A** | **A** | **A** | **A** | **G** | **C** | **A** | **T** | **T** | **G** | **G** | **A** | **A** | **C** | **T** | **A** | **C** | **G** | **T** | **C** | **C** | **A** | **G** | **A** | **T** | **G** | **T** | **T** | **A** | **T** | **T** | **T** | **T** | **G** | **A** | **T** | **G** | **G** | **A** | **T** | **T** | **T** | **G** | **G** | **T** | **A** | **A** | **T** | **G** | **G** | **A** | **A** | **G** | **A** | **A** | **A** | **T** | **G** | **G** | **A** | **C** | **G** | **G** | **C** | **A** | **T** | **T** | **G** | **A** | **T** | **T** | **C** | **A** | **A** | **C** | **C** | **A** | **A** | **A** | **G** | **C** | **G** | **A** | **T** | **C** | **T** | **T** | **G** | **A** | **A** | **A** | **G** | **A** | **T** | **T** | **G** | **G** | **C** | **C** | **A** | **G** | **A** | **A** | **G** | **C** | **C** | **A** | **A** | **G** | **A** | **T** | **T** | **A** | **T** | **T** | **A** | **T** | **T** | **G** | **T** | **G** | **A** | **C** | **G** | **A** | **G** | **T** | **T** | **T** | **T** | **A** | **T** | **T** | **G** | **A** | **T** | **G** | **A** | **T** | **G** | **A** | **A** | **A** | **A** | **A** | **G** | **T** | **G** | **T** | **A** | **T** | **C** | **C** | **G** | **G** | **C** | **G** | **A** | **T** | **T** | **G** | **A** | **A** | **G** | **C** | **T** | **G** | **G** | **T** | **G** | **C** | **A** | **G** | **C** | **G** | **G** | **G** | **C** | **T** | **A** | **C** | **C** | **T** | **A** | **T** | **T** | **A** | **A** | **A** | **A** | **A** | **C** | **A** | **T** | **C** | **A** | **A** | **C** | **A** | **G** | **C** | **A** | **C** | **A** | **T** | **G** | **A** | **G** | **A** | **T** | **T** | **G** | **C** | **T** | **G** | **A** | **T** | **G** | **C** | **A** | **A** | **T** | **T** | **C** | **G** | **G** | **G** | **C** | **G** | **A** | **C** | **T** | **T** | **A** | **T** | **C** | **G** | **C** | **G** | **G** | **A** | **G** | **A** | **G** | **C** | **G** | **T** | **G** | **T** | **G** | **T** | **T** | **G** | **G** | **A** | **A** | **C** | **C** | **T** | **G** | **A** | **A** | **G** | **T** | **G** | **A** | **C** | **G** | **C** | **A** | **T** | **A** | **A** | **G** | **A** | **T** | **G** | **A** | **T** | **G** | **G** | **A** | **A** | **C** | **G** | **G** | **T** | **T** | **A** | **A** | **C** | **A** | **A** | **A** | **A** | **A** | **A** | **A** | **C** | **A** | **A** | **G** | **A** | **G** | **C** | **C** | **G** | **G** | **T** | **G** | **T** | **T** | **G** | **C** | **A** | **C** | **G** | **A** | **A** | **G** | **A** | **T** | **T** | **T** | **G** | **A** | **C** | **A** | **A** | **A** | **C** | **C** | **G** | **G** | **G** | **A** | **A** | **C** | **A** | **C** | **G** | **A** | **A** | **A** | **T** | **T** | **T** | **T** | **A** | **A** | **T** | **G** | **T** | **T** | **G** | **A** | **T** | **T** | **G** | **C** | **A** | **C** | **A** | **A** | **G** | **G** | **T** | **A** | **A** | **A** | **A** | **G** | **T** | **A** | **A** | **T** | **C** | **A** | **G** | **G** | **A** | **A** | **A** | **T** | **A** | **G** | **C** | **T** | **G** | **A** | **T** | **G** | **A** | **A** | **C** | **T** | **C** | **T** | **T** | **T** | **A** | **T** | **C** | **A** | **C** | **T** | **T** | **T** | **G** | **A** | **A** | **A** | **A** | **C** | **A** | **G** | **T** | **T** | **A** | **A** | **A** | **A** | **C** | **A** | **C** | **A** | **T** | **G** | **T** | **T** | **T** | **C** | **A** | **A** | **A** | **C** | **A** | **T** | **T** | **T** | **T** | **A** | **G** | **C** | **A** | **A** | **A** | **A** | **C** | **T** | **A** | **G** | **A** | **T** | **G** | **T** | **G** | **G** | **A** | **T** | **G** | **A** | **T** | **C** | **G** | **G** | **A** | **C** | **C** | **C** | **A** | **A** | **G** | **C** | **G** | **G** | **C** | **G** | **A** | **T** | **T** | **T** | **A** | **T** | **G** | **C** | **T** | **T** | **T** | **T** | **C** | **A** | **A** | **C** | **A** | **T** | **G** | **G** | **T** | **T** | **T** | **A** | **G** | **C** | **C** | **A** | **A** | **A** | **-** | **-** | **-** |
| **N54671** | **A** | **T** | **G** | **A** | **T** | **A** | **A** | **A** | **A** | **G** | **T** | **T** | **T** | **T** | **A** | **T** | **T** | **A** | **G** | **T** | **A** | **G** | **A** | **T** | **G** | **A** | **C** | **C** | **A** | **T** | **G** | **A** | **A** | **A** | **T** | **G** | **G** | **T** | **A** | **C** | **G** | **C** | **T** | **T** | **A** | **G** | **G** | **C** | **G** | **T** | **C** | **T** | **C** | **T** | **T** | **C** | **T** | **T** | **A** | **T** | **T** | **T** | **A** | **T** | **C** | **T** | **A** | **T** | **C** | **C** | **A** | **A** | **G** | **A** | **A** | **G** | **A** | **C** | **A** | **T** | **T** | **G** | **A** | **A** | **G** | **T** | **G** | **A** | **T** | **T** | **G** | **G** | **A** | **G** | **A** | **A** | **G** | **C** | **T** | **G** | **A** | **A** | **A** | **A** | **T** | **G** | **G** | **A** | **C** | **G** | **T** | **C** | **A** | **A** | **G** | **G** | **C** | **T** | **A** | **T** | **G** | **A** | **A** | **A** | **A** | **G** | **G** | **C** | **G** | **A** | **T** | **G** | **G** | **C** | **A** | **C** | **T** | **T** | **C** | **G** | **A** | **C** | **C** | **G** | **G** | **A** | **T** | **G** | **T** | **C** | **A** | **T** | **T** | **T** | **T** | **A** | **A** | **T** | **G** | **G** | **A** | **T** | **C** | **T** | **A** | **G** | **T** | **C** | **A** | **T** | **G** | **G** | **A** | **A** | **G** | **A** | **A** | **A** | **T** | **G** | **G** | **A** | **T** | **G** | **G** | **C** | **A** | **T** | **C** | **G** | **A** | **A** | **T** | **C** | **A** | **A** | **C** | **A** | **A** | **A** | **A** | **G** | **C** | **A** | **A** | **T** | **C** | **T** | **T** | **A** | **A** | **A** | **A** | **G** | **A** | **T** | **T** | **G** | **G** | **C** | **C** | **A** | **A** | **A** | **A** | **G** | **C** | **C** | **A** | **A** | **A** | **A** | **T** | **C** | **A** | **T** | **T** | **A** | **T** | **C** | **G** | **T** | **C** | **A** | **C** | **A** | **A** | **G** | **T** | **T** | **T** | **T** | **A** | **T** | **T** | **G** | **A** | **T** | **G** | **A** | **T** | **G** | **A** | **A** | **A** | **A** | **A** | **G** | **T** | **T** | **T** | **A** | **T** | **C** | **C** | **T** | **G** | **C** | **A** | **A** | **T** | **T** | **G** | **A** | **A** | **G** | **C** | **A** | **G** | **G** | **A** | **G** | **C** | **A** | **G** | **C** | **A** | **G** | **G** | **A** | **T** | **A** | **T** | **T** | **T** | **G** | **T** | **T** | **G** | **A** | **A** | **G** | **A** | **C** | **G** | **T** | **C** | **T** | **A** | **C** | **C** | **G** | **C** | **A** | **C** | **A** | **T** | **G** | **A** | **A** | **A** | **T** | **A** | **G** | **C** | **T** | **G** | **A** | **T** | **G** | **C** | **T** | **A** | **T** | **A** | **A** | **G** | **G** | **G** | **C** | **T** | **A** | **C** | **G** | **C** | **A** | **A** | **C** | **G** | **C** | **G** | **G** | **C** | **G** | **A** | **G** | **C** | **G** | **A** | **G** | **T** | **A** | **T** | **T** | **G** | **G** | **A** | **A** | **C** | **C** | **T** | **G** | **A** | **A** | **G** | **T** | **G** | **A** | **C** | **A** | **A** | **C** | **A** | **A** | **A** | **A** | **A** | **T** | **G** | **A** | **T** | **G** | **G** | **A** | **A** | **A** | **A** | **A** | **A** | **T** | **G** | **A** | **G** | **T** | **C** | **G** | **G** | **C** | **G** | **A** | **A** | **A** | **T** | **G** | **A** | **T** | **C** | **C** | **A** | **G** | **T** | **A** | **T** | **T** | **G** | **C** | **A** | **T** | **G** | **A** | **A** | **G** | **A** | **A** | **T** | **T** | **A** | **A** | **C** | **G** | **A** | **A** | **T** | **C** | **G** | **A** | **G** | **A** | **A** | **A** | **A** | **T** | **G** | **A** | **A** | **A** | **T** | **T** | **T** | **T** | **G** | **A** | **T** | **G** | **C** | **T** | **G** | **A** | **T** | **T** | **T** | **C** | **T** | **G** | **A** | **A** | **G** | **G** | **G** | **A** | **A** | **A** | **A** | **G** | **T** | **A** | **A** | **T** | **C** | **A** | **G** | **G** | **A** | **A** | **A** | **T** | **C** | **G** | **C** | **G** | **G** | **A** | **T** | **G** | **A** | **A** | **T** | **T** | **G** | **T** | **T** | **T** | **A** | **T** | **C** | **A** | **C** | **A** | **C** | **T** | **G** | **A** | **A** | **A** | **A** | **C** | **A** | **G** | **T** | **C** | **A** | **A** | **A** | **A** | **C** | **G** | **C** | **A** | **T** | **G** | **T** | **G** | **T** | **C** | **G** | **A** | **A** | **T** | **A** | **T** | **A** | **T** | **T** | **A** | **G** | **C** | **T** | **A** | **A** | **G** | **T** | **T** | **A** | **G** | **A** | **G** | **G** | **T** | **A** | **G** | **A** | **A** | **G** | **A** | **C** | **C** | **G** | **A** | **A** | **C** | **T** | **C** | **A** | **A** | **G** | **C** | **A** | **G** | **C** | **T** | **A** | **T** | **C** | **T** | **A** | **T** | **G** | **C** | **G** | **T** | **T** | **T** | **A** | **A** | **A** | **C** | **A** | **T** | **G** | **G** | **T** | **T** | **T** | **G** | **G** | **T** | **G** | **A** | **A** | **A** | **-** | **-** | **-** |
| **N54150** | **A** | **T** | **G** | **A** | **T** | **A** | **A** | **A** | **A** | **G** | **T** | **T** | **T** | **T** | **A** | **T** | **T** | **A** | **G** | **T** | **A** | **G** | **A** | **T** | **G** | **A** | **C** | **C** | **A** | **T** | **G** | **A** | **A** | **A** | **T** | **G** | **G** | **T** | **A** | **C** | **G** | **C** | **T** | **T** | **A** | **G** | **G** | **C** | **G** | **T** | **C** | **T** | **C** | **T** | **T** | **C** | **T** | **T** | **A** | **T** | **T** | **T** | **A** | **T** | **C** | **T** | **A** | **T** | **C** | **C** | **A** | **A** | **G** | **A** | **A** | **G** | **A** | **C** | **A** | **T** | **T** | **G** | **A** | **A** | **G** | **T** | **G** | **A** | **T** | **T** | **G** | **G** | **A** | **G** | **A** | **A** | **G** | **C** | **T** | **G** | **A** | **A** | **A** | **A** | **T** | **G** | **G** | **A** | **C** | **G** | **T** | **C** | **A** | **A** | **G** | **G** | **C** | **T** | **A** | **T** | **G** | **A** | **A** | **A** | **A** | **G** | **G** | **C** | **G** | **A** | **T** | **G** | **G** | **C** | **A** | **C** | **T** | **T** | **C** | **G** | **A** | **C** | **C** | **A** | **G** | **A** | **T** | **G** | **T** | **C** | **A** | **T** | **T** | **T** | **T** | **A** | **A** | **T** | **G** | **G** | **A** | **T** | **C** | **T** | **A** | **G** | **T** | **C** | **A** | **T** | **G** | **G** | **A** | **A** | **G** | **A** | **A** | **A** | **T** | **G** | **G** | **A** | **T** | **G** | **G** | **C** | **A** | **T** | **C** | **G** | **A** | **A** | **T** | **C** | **A** | **A** | **C** | **A** | **A** | **A** | **A** | **G** | **C** | **A** | **A** | **T** | **C** | **T** | **T** | **A** | **A** | **A** | **A** | **G** | **A** | **T** | **T** | **G** | **G** | **C** | **C** | **A** | **A** | **A** | **A** | **G** | **C** | **C** | **A** | **A** | **A** | **A** | **T** | **C** | **A** | **T** | **T** | **A** | **T** | **C** | **G** | **T** | **C** | **A** | **C** | **A** | **A** | **G** | **T** | **T** | **T** | **T** | **A** | **T** | **T** | **G** | **A** | **T** | **G** | **A** | **T** | **G** | **A** | **A** | **A** | **A** | **A** | **G** | **T** | **T** | **T** | **A** | **T** | **C** | **C** | **T** | **G** | **C** | **A** | **A** | **T** | **T** | **G** | **A** | **A** | **G** | **C** | **A** | **G** | **G** | **A** | **G** | **C** | **A** | **G** | **C** | **A** | **G** | **G** | **A** | **T** | **A** | **T** | **T** | **T** | **G** | **T** | **T** | **G** | **A** | **A** | **G** | **A** | **C** | **G** | **T** | **C** | **T** | **A** | **C** | **C** | **G** | **C** | **A** | **C** | **A** | **T** | **G** | **A** | **A** | **A** | **T** | **A** | **G** | **C** | **T** | **G** | **A** | **T** | **G** | **C** | **T** | **A** | **T** | **A** | **A** | **G** | **G** | **G** | **C** | **T** | **A** | **C** | **G** | **C** | **A** | **A** | **C** | **G** | **C** | **G** | **G** | **C** | **G** | **A** | **G** | **C** | **G** | **A** | **G** | **T** | **A** | **T** | **T** | **G** | **G** | **A** | **A** | **C** | **C** | **T** | **G** | **A** | **A** | **G** | **T** | **G** | **A** | **C** | **A** | **A** | **C** | **A** | **A** | **A** | **A** | **A** | **T** | **G** | **A** | **T** | **G** | **G** | **A** | **A** | **A** | **A** | **A** | **A** | **T** | **G** | **A** | **G** | **T** | **C** | **G** | **G** | **C** | **G** | **A** | **A** | **A** | **T** | **G** | **A** | **T** | **C** | **C** | **A** | **G** | **T** | **A** | **T** | **T** | **G** | **C** | **A** | **T** | **G** | **A** | **A** | **G** | **A** | **A** | **T** | **T** | **A** | **A** | **C** | **G** | **A** | **A** | **T** | **C** | **G** | **A** | **G** | **A** | **A** | **A** | **A** | **T** | **G** | **A** | **A** | **A** | **T** | **T** | **T** | **T** | **G** | **A** | **T** | **G** | **C** | **T** | **G** | **A** | **T** | **T** | **T** | **C** | **T** | **G** | **A** | **A** | **G** | **G** | **G** | **A** | **A** | **A** | **A** | **G** | **T** | **A** | **A** | **T** | **C** | **A** | **G** | **G** | **A** | **A** | **A** | **T** | **C** | **G** | **C** | **G** | **G** | **A** | **T** | **G** | **A** | **A** | **T** | **T** | **G** | **T** | **T** | **T** | **A** | **T** | **C** | **A** | **C** | **A** | **C** | **T** | **G** | **A** | **A** | **A** | **A** | **C** | **A** | **G** | **T** | **T** | **A** | **A** | **A** | **A** | **C** | **G** | **C** | **A** | **T** | **G** | **T** | **G** | **T** | **C** | **G** | **A** | **A** | **T** | **A** | **T** | **A** | **T** | **T** | **A** | **G** | **C** | **T** | **A** | **A** | **G** | **T** | **T** | **A** | **G** | **A** | **G** | **G** | **T** | **A** | **G** | **A** | **A** | **G** | **A** | **C** | **C** | **G** | **A** | **A** | **C** | **T** | **C** | **A** | **A** | **G** | **C** | **A** | **G** | **C** | **T** | **A** | **T** | **C** | **T** | **A** | **T** | **G** | **C** | **G** | **T** | **T** | **T** | **A** | **A** | **A** | **C** | **A** | **T** | **G** | **G** | **T** | **T** | **T** | **G** | **G** | **T** | **G** | **A** | **A** | **A** | **-** | **-** | **-** |
| **N54574** | **G** | **T** | **G** | **A** | **T** | **C** | **A** | **A** | **A** | **G** | **T** | **A** | **A** | **T** | **G** | **T** | **T** | **A** | **G** | **T** | **G** | **G** | **A** | **T** | **G** | **A** | **C** | **C** | **A** | **T** | **G** | **A** | **A** | **A** | **T** | **G** | **G** | **T** | **C** | **C** | **G** | **T** | **T** | **T** | **A** | **G** | **G** | **C** | **G** | **T** | **T** | **T** | **C** | **A** | **T** | **C** | **A** | **T** | **A** | **T** | **T** | **T** | **A** | **T** | **C** | **T** | **A** | **T** | **T** | **C** | **A** | **A** | **G** | **A** | **G** | **G** | **A** | **T** | **A** | **T** | **A** | **G** | **A** | **A** | **G** | **T** | **C** | **G** | **T** | **A** | **G** | **G** | **C** | **G** | **A** | **A** | **G** | **C** | **A** | **G** | **A** | **A** | **A** | **A** | **C** | **G** | **G** | **T** | **A** | **A** | **G** | **A** | **T** | **T** | **G** | **G** | **C** | **T** | **A** | **T** | **G** | **A** | **A** | **A** | **A** | **A** | **G** | **C** | **A** | **T** | **T** | **G** | **G** | **A** | **A** | **C** | **T** | **A** | **C** | **G** | **T** | **C** | **C** | **A** | **G** | **A** | **T** | **G** | **T** | **T** | **A** | **T** | **T** | **T** | **T** | **G** | **A** | **T** | **G** | **G** | **A** | **T** | **T** | **T** | **G** | **G** | **T** | **A** | **A** | **T** | **G** | **G** | **A** | **A** | **G** | **A** | **A** | **A** | **T** | **G** | **G** | **A** | **C** | **G** | **G** | **C** | **A** | **T** | **T** | **G** | **A** | **T** | **T** | **C** | **A** | **A** | **C** | **A** | **A** | **A** | **A** | **G** | **C** | **G** | **A** | **T** | **C** | **T** | **T** | **G** | **A** | **A** | **A** | **G** | **A** | **T** | **T** | **G** | **G** | **C** | **C** | **A** | **G** | **A** | **A** | **G** | **C** | **C** | **A** | **A** | **G** | **A** | **T** | **T** | **A** | **T** | **T** | **A** | **T** | **T** | **G** | **T** | **G** | **A** | **C** | **G** | **A** | **G** | **T** | **T** | **T** | **T** | **A** | **T** | **T** | **G** | **A** | **T** | **G** | **A** | **T** | **G** | **A** | **A** | **A** | **A** | **A** | **G** | **T** | **G** | **T** | **A** | **T** | **C** | **C** | **G** | **G** | **C** | **G** | **A** | **T** | **T** | **G** | **A** | **A** | **G** | **C** | **T** | **G** | **G** | **T** | **G** | **C** | **A** | **G** | **C** | **G** | **G** | **G** | **C** | **T** | **A** | **C** | **C** | **T** | **A** | **T** | **T** | **A** | **A** | **A** | **A** | **A** | **C** | **A** | **T** | **C** | **A** | **A** | **C** | **A** | **G** | **C** | **A** | **C** | **A** | **T** | **G** | **A** | **G** | **A** | **T** | **T** | **G** | **C** | **T** | **G** | **A** | **T** | **G** | **C** | **A** | **A** | **T** | **T** | **C** | **G** | **G** | **G** | **C** | **G** | **A** | **C** | **T** | **T** | **A** | **T** | **C** | **G** | **C** | **G** | **G** | **A** | **G** | **A** | **G** | **C** | **G** | **T** | **G** | **T** | **G** | **T** | **T** | **G** | **G** | **A** | **A** | **C** | **C** | **T** | **G** | **A** | **A** | **G** | **T** | **G** | **A** | **C** | **G** | **C** | **A** | **T** | **A** | **A** | **G** | **A** | **T** | **G** | **A** | **T** | **G** | **G** | **A** | **A** | **C** | **G** | **G** | **T** | **T** | **A** | **A** | **C** | **A** | **A** | **A** | **A** | **A** | **A** | **A** | **C** | **A** | **A** | **G** | **A** | **G** | **C** | **C** | **G** | **G** | **T** | **G** | **T** | **T** | **G** | **C** | **A** | **C** | **G** | **A** | **A** | **G** | **A** | **T** | **T** | **T** | **G** | **A** | **C** | **A** | **A** | **A** | **C** | **C** | **G** | **G** | **G** | **A** | **A** | **C** | **A** | **C** | **G** | **A** | **A** | **A** | **T** | **T** | **T** | **T** | **A** | **A** | **T** | **G** | **T** | **T** | **G** | **A** | **T** | **T** | **G** | **C** | **A** | **C** | **A** | **A** | **G** | **G** | **T** | **A** | **A** | **A** | **A** | **G** | **T** | **A** | **A** | **T** | **C** | **A** | **G** | **G** | **A** | **A** | **A** | **T** | **A** | **G** | **C** | **T** | **G** | **A** | **T** | **G** | **A** | **A** | **C** | **T** | **C** | **T** | **T** | **T** | **A** | **T** | **C** | **A** | **C** | **T** | **T** | **T** | **G** | **A** | **A** | **A** | **A** | **C** | **A** | **G** | **T** | **T** | **A** | **A** | **A** | **A** | **C** | **A** | **C** | **A** | **T** | **G** | **T** | **T** | **T** | **C** | **A** | **A** | **A** | **C** | **A** | **T** | **T** | **T** | **T** | **A** | **G** | **C** | **A** | **A** | **A** | **A** | **C** | **T** | **A** | **G** | **A** | **T** | **G** | **T** | **G** | **G** | **A** | **T** | **G** | **A** | **T** | **C** | **G** | **G** | **A** | **C** | **C** | **C** | **A** | **A** | **G** | **C** | **G** | **G** | **C** | **G** | **A** | **T** | **T** | **T** | **A** | **T** | **A** | **C** | **T** | **T** | **T** | **T** | **C** | **A** | **A** | **C** | **A** | **T** | **G** | **G** | **T** | **T** | **T** | **A** | **G** | **C** | **C** | **A** | **A** | **A** | **-** | **-** | **-** |
| **N55655** | **A** | **T** | **G** | **A** | **T** | **A** | **A** | **A** | **A** | **G** | **T** | **T** | **T** | **T** | **A** | **T** | **T** | **A** | **G** | **T** | **A** | **G** | **A** | **T** | **G** | **A** | **C** | **C** | **A** | **T** | **G** | **A** | **A** | **A** | **T** | **G** | **G** | **T** | **A** | **C** | **G** | **C** | **T** | **T** | **A** | **G** | **G** | **C** | **G** | **T** | **C** | **T** | **C** | **T** | **T** | **C** | **T** | **T** | **A** | **T** | **T** | **T** | **A** | **T** | **C** | **T** | **A** | **T** | **C** | **C** | **A** | **A** | **G** | **A** | **A** | **G** | **A** | **C** | **A** | **T** | **T** | **G** | **A** | **A** | **G** | **T** | **G** | **A** | **T** | **T** | **G** | **G** | **A** | **G** | **A** | **A** | **G** | **C** | **T** | **G** | **A** | **A** | **A** | **A** | **T** | **G** | **G** | **A** | **C** | **G** | **T** | **C** | **A** | **A** | **G** | **G** | **C** | **T** | **A** | **T** | **G** | **A** | **A** | **A** | **A** | **G** | **G** | **C** | **G** | **A** | **T** | **G** | **G** | **C** | **A** | **C** | **T** | **T** | **C** | **G** | **A** | **C** | **C** | **A** | **G** | **A** | **T** | **G** | **T** | **C** | **A** | **T** | **T** | **T** | **T** | **A** | **A** | **T** | **G** | **G** | **A** | **T** | **C** | **T** | **A** | **G** | **T** | **C** | **A** | **T** | **G** | **G** | **A** | **A** | **G** | **A** | **A** | **A** | **T** | **G** | **G** | **A** | **T** | **G** | **G** | **C** | **A** | **T** | **C** | **G** | **A** | **A** | **T** | **C** | **A** | **A** | **C** | **A** | **A** | **A** | **A** | **G** | **C** | **A** | **A** | **T** | **C** | **T** | **T** | **A** | **A** | **A** | **A** | **G** | **A** | **T** | **T** | **G** | **G** | **C** | **C** | **A** | **A** | **A** | **A** | **G** | **C** | **C** | **A** | **A** | **A** | **A** | **T** | **C** | **A** | **T** | **T** | **A** | **T** | **C** | **G** | **T** | **C** | **A** | **C** | **A** | **A** | **G** | **T** | **T** | **T** | **T** | **A** | **T** | **T** | **G** | **A** | **T** | **G** | **A** | **T** | **G** | **A** | **A** | **A** | **A** | **A** | **G** | **T** | **T** | **T** | **A** | **T** | **C** | **C** | **T** | **G** | **C** | **A** | **A** | **T** | **T** | **G** | **A** | **A** | **G** | **C** | **A** | **G** | **G** | **A** | **G** | **C** | **A** | **G** | **C** | **A** | **G** | **G** | **A** | **T** | **A** | **T** | **T** | **T** | **A** | **T** | **T** | **G** | **A** | **A** | **G** | **A** | **C** | **G** | **T** | **C** | **T** | **A** | **C** | **C** | **G** | **C** | **A** | **C** | **A** | **T** | **G** | **A** | **A** | **A** | **T** | **A** | **G** | **C** | **T** | **G** | **A** | **T** | **G** | **C** | **T** | **A** | **T** | **A** | **A** | **G** | **G** | **G** | **C** | **T** | **A** | **C** | **G** | **C** | **A** | **A** | **C** | **G** | **C** | **G** | **G** | **C** | **G** | **A** | **G** | **C** | **G** | **A** | **G** | **T** | **A** | **T** | **T** | **G** | **G** | **A** | **A** | **C** | **C** | **T** | **G** | **A** | **A** | **G** | **T** | **G** | **A** | **C** | **A** | **A** | **C** | **A** | **A** | **A** | **A** | **A** | **T** | **G** | **A** | **T** | **G** | **G** | **A** | **A** | **A** | **A** | **A** | **A** | **T** | **G** | **A** | **G** | **T** | **C** | **G** | **G** | **C** | **G** | **A** | **A** | **A** | **T** | **G** | **A** | **T** | **C** | **C** | **A** | **G** | **T** | **A** | **T** | **T** | **G** | **C** | **A** | **T** | **G** | **A** | **A** | **G** | **A** | **A** | **T** | **T** | **A** | **A** | **C** | **G** | **A** | **A** | **T** | **C** | **G** | **A** | **G** | **A** | **A** | **A** | **A** | **T** | **G** | **A** | **A** | **A** | **T** | **T** | **T** | **T** | **G** | **A** | **T** | **G** | **C** | **T** | **G** | **A** | **T** | **T** | **T** | **C** | **T** | **G** | **A** | **A** | **G** | **G** | **G** | **A** | **A** | **A** | **A** | **G** | **T** | **A** | **A** | **T** | **C** | **A** | **G** | **G** | **A** | **A** | **A** | **T** | **C** | **G** | **C** | **G** | **G** | **A** | **T** | **G** | **A** | **A** | **T** | **T** | **G** | **T** | **T** | **T** | **A** | **T** | **C** | **A** | **C** | **A** | **C** | **T** | **G** | **A** | **A** | **A** | **A** | **C** | **A** | **G** | **T** | **T** | **A** | **A** | **A** | **A** | **C** | **G** | **C** | **A** | **T** | **G** | **T** | **G** | **T** | **C** | **G** | **A** | **A** | **T** | **A** | **T** | **A** | **T** | **T** | **A** | **G** | **C** | **T** | **A** | **A** | **G** | **T** | **T** | **A** | **G** | **A** | **G** | **G** | **T** | **A** | **G** | **A** | **A** | **G** | **A** | **C** | **C** | **G** | **A** | **A** | **C** | **T** | **C** | **A** | **A** | **G** | **C** | **A** | **G** | **C** | **T** | **A** | **T** | **C** | **T** | **A** | **T** | **G** | **C** | **G** | **T** | **T** | **T** | **A** | **A** | **A** | **C** | **A** | **T** | **G** | **G** | **T** | **T** | **T** | **G** | **G** | **T** | **T** | **A** | **A** | **A** | **-** | **-** | **-** |
| **N60284F** | **A** | **T** | **G** | **A** | **T** | **A** | **A** | **A** | **A** | **G** | **T** | **T** | **T** | **T** | **A** | **T** | **T** | **A** | **G** | **T** | **A** | **G** | **A** | **T** | **G** | **A** | **C** | **C** | **A** | **T** | **G** | **A** | **A** | **A** | **T** | **G** | **G** | **T** | **A** | **C** | **G** | **C** | **T** | **T** | **A** | **G** | **G** | **C** | **G** | **T** | **C** | **T** | **C** | **T** | **T** | **C** | **T** | **T** | **A** | **T** | **T** | **T** | **A** | **T** | **C** | **T** | **A** | **T** | **C** | **C** | **A** | **A** | **G** | **A** | **A** | **G** | **A** | **C** | **A** | **T** | **T** | **G** | **A** | **A** | **G** | **T** | **G** | **A** | **T** | **T** | **G** | **G** | **A** | **G** | **A** | **A** | **G** | **C** | **T** | **G** | **A** | **A** | **A** | **A** | **T** | **G** | **G** | **A** | **C** | **G** | **T** | **C** | **A** | **A** | **G** | **G** | **C** | **T** | **A** | **T** | **G** | **A** | **A** | **A** | **A** | **G** | **G** | **C** | **G** | **A** | **T** | **G** | **G** | **C** | **A** | **C** | **T** | **T** | **C** | **G** | **A** | **C** | **C** | **A** | **G** | **A** | **T** | **G** | **T** | **C** | **A** | **T** | **T** | **T** | **T** | **A** | **A** | **T** | **G** | **G** | **A** | **T** | **C** | **T** | **A** | **G** | **T** | **C** | **A** | **T** | **G** | **G** | **A** | **A** | **G** | **A** | **A** | **A** | **T** | **G** | **G** | **A** | **T** | **G** | **G** | **C** | **A** | **T** | **C** | **G** | **A** | **A** | **T** | **C** | **A** | **A** | **C** | **A** | **A** | **A** | **A** | **G** | **C** | **A** | **A** | **T** | **C** | **T** | **T** | **A** | **A** | **A** | **A** | **G** | **A** | **T** | **T** | **G** | **G** | **C** | **C** | **A** | **A** | **A** | **A** | **G** | **C** | **C** | **A** | **A** | **A** | **A** | **T** | **C** | **A** | **T** | **T** | **A** | **T** | **C** | **G** | **T** | **C** | **A** | **C** | **A** | **A** | **G** | **T** | **T** | **T** | **T** | **A** | **T** | **T** | **G** | **A** | **T** | **G** | **A** | **T** | **G** | **A** | **A** | **A** | **A** | **A** | **G** | **T** | **T** | **T** | **A** | **T** | **C** | **C** | **T** | **G** | **C** | **A** | **A** | **T** | **T** | **G** | **A** | **A** | **G** | **C** | **A** | **G** | **G** | **A** | **G** | **C** | **A** | **G** | **C** | **A** | **G** | **G** | **A** | **T** | **A** | **T** | **T** | **T** | **A** | **T** | **T** | **G** | **A** | **A** | **G** | **A** | **C** | **G** | **T** | **C** | **T** | **A** | **C** | **C** | **G** | **C** | **A** | **C** | **A** | **T** | **G** | **A** | **A** | **A** | **T** | **A** | **G** | **C** | **T** | **G** | **A** | **T** | **G** | **C** | **T** | **A** | **T** | **A** | **A** | **G** | **G** | **G** | **C** | **T** | **A** | **C** | **G** | **C** | **A** | **A** | **C** | **G** | **C** | **G** | **G** | **C** | **G** | **A** | **G** | **C** | **G** | **A** | **G** | **T** | **A** | **T** | **T** | **G** | **G** | **A** | **A** | **C** | **C** | **T** | **G** | **A** | **A** | **G** | **T** | **G** | **A** | **C** | **A** | **A** | **C** | **A** | **A** | **A** | **A** | **A** | **T** | **G** | **A** | **T** | **G** | **G** | **A** | **A** | **A** | **A** | **A** | **A** | **T** | **G** | **A** | **G** | **T** | **C** | **G** | **G** | **C** | **G** | **A** | **A** | **A** | **T** | **G** | **A** | **T** | **C** | **C** | **A** | **G** | **T** | **A** | **T** | **T** | **G** | **C** | **A** | **T** | **G** | **A** | **A** | **G** | **A** | **A** | **T** | **T** | **A** | **A** | **C** | **G** | **A** | **A** | **T** | **C** | **G** | **A** | **G** | **A** | **A** | **A** | **A** | **T** | **G** | **A** | **A** | **A** | **T** | **T** | **T** | **T** | **G** | **A** | **T** | **G** | **C** | **T** | **G** | **A** | **T** | **T** | **T** | **C** | **T** | **G** | **A** | **A** | **G** | **G** | **G** | **A** | **A** | **A** | **A** | **G** | **T** | **A** | **A** | **T** | **C** | **A** | **G** | **G** | **A** | **A** | **A** | **T** | **C** | **G** | **C** | **G** | **G** | **A** | **T** | **G** | **A** | **A** | **T** | **T** | **G** | **T** | **T** | **T** | **A** | **T** | **C** | **A** | **C** | **A** | **C** | **T** | **G** | **A** | **A** | **A** | **A** | **C** | **A** | **G** | **T** | **T** | **A** | **A** | **A** | **A** | **C** | **G** | **C** | **A** | **T** | **G** | **T** | **G** | **T** | **C** | **G** | **A** | **A** | **T** | **A** | **T** | **A** | **T** | **T** | **A** | **G** | **C** | **T** | **A** | **A** | **G** | **T** | **T** | **A** | **G** | **A** | **G** | **G** | **T** | **A** | **G** | **A** | **A** | **G** | **A** | **C** | **C** | **G** | **A** | **A** | **C** | **T** | **C** | **A** | **A** | **G** | **C** | **A** | **G** | **C** | **T** | **A** | **T** | **C** | **T** | **A** | **T** | **G** | **C** | **G** | **T** | **T** | **T** | **A** | **A** | **A** | **C** | **A** | **T** | **G** | **G** | **T** | **T** | **T** | **G** | **G** | **T** | **G** | **A** | **A** | **A** | **-** | **-** | **-** |
| **N53476** | **G** | **T** | **G** | **A** | **T** | **C** | **A** | **A** | **A** | **G** | **T** | **A** | **A** | **T** | **G** | **T** | **T** | **A** | **G** | **T** | **G** | **G** | **A** | **T** | **G** | **A** | **C** | **C** | **A** | **T** | **G** | **A** | **A** | **A** | **T** | **G** | **G** | **T** | **C** | **C** | **G** | **T** | **T** | **T** | **A** | **G** | **G** | **C** | **G** | **T** | **T** | **T** | **C** | **A** | **T** | **C** | **A** | **T** | **A** | **T** | **T** | **T** | **A** | **T** | **C** | **T** | **A** | **T** | **T** | **C** | **A** | **A** | **G** | **A** | **G** | **G** | **A** | **T** | **A** | **T** | **A** | **G** | **A** | **A** | **G** | **T** | **C** | **G** | **T** | **A** | **G** | **G** | **C** | **G** | **A** | **A** | **G** | **C** | **A** | **G** | **A** | **A** | **A** | **A** | **T** | **G** | **G** | **T** | **A** | **A** | **G** | **A** | **T** | **T** | **G** | **G** | **C** | **T** | **A** | **T** | **G** | **A** | **A** | **A** | **A** | **A** | **G** | **C** | **A** | **T** | **T** | **G** | **G** | **A** | **A** | **C** | **T** | **A** | **C** | **G** | **T** | **C** | **C** | **A** | **G** | **A** | **T** | **G** | **T** | **T** | **A** | **T** | **T** | **T** | **T** | **G** | **A** | **T** | **G** | **G** | **A** | **T** | **T** | **T** | **G** | **G** | **T** | **A** | **A** | **T** | **G** | **G** | **A** | **A** | **G** | **A** | **A** | **A** | **T** | **G** | **G** | **A** | **C** | **G** | **G** | **C** | **A** | **T** | **T** | **G** | **A** | **T** | **T** | **C** | **A** | **A** | **C** | **A** | **A** | **A** | **A** | **G** | **C** | **G** | **A** | **T** | **C** | **T** | **T** | **G** | **A** | **A** | **A** | **G** | **A** | **T** | **T** | **G** | **G** | **C** | **C** | **A** | **G** | **A** | **A** | **G** | **C** | **C** | **A** | **A** | **G** | **A** | **T** | **T** | **A** | **T** | **T** | **A** | **T** | **T** | **G** | **T** | **G** | **A** | **C** | **G** | **A** | **G** | **T** | **T** | **T** | **T** | **A** | **T** | **T** | **G** | **A** | **T** | **G** | **A** | **T** | **G** | **A** | **A** | **A** | **A** | **A** | **G** | **T** | **G** | **T** | **A** | **T** | **C** | **C** | **G** | **G** | **C** | **G** | **A** | **T** | **T** | **G** | **A** | **A** | **G** | **C** | **T** | **G** | **G** | **T** | **G** | **C** | **A** | **G** | **C** | **G** | **G** | **G** | **C** | **T** | **A** | **C** | **C** | **T** | **A** | **T** | **T** | **A** | **A** | **A** | **A** | **A** | **C** | **A** | **T** | **C** | **A** | **A** | **C** | **A** | **G** | **C** | **A** | **C** | **A** | **T** | **G** | **A** | **G** | **A** | **T** | **T** | **G** | **C** | **T** | **G** | **A** | **T** | **G** | **C** | **A** | **A** | **T** | **T** | **C** | **G** | **G** | **G** | **C** | **G** | **A** | **C** | **T** | **T** | **A** | **T** | **C** | **G** | **C** | **G** | **G** | **A** | **G** | **A** | **G** | **C** | **G** | **T** | **G** | **T** | **G** | **T** | **T** | **G** | **G** | **A** | **A** | **C** | **C** | **T** | **G** | **A** | **A** | **G** | **T** | **G** | **A** | **C** | **G** | **C** | **A** | **T** | **A** | **A** | **G** | **A** | **T** | **G** | **A** | **T** | **G** | **G** | **A** | **A** | **C** | **G** | **G** | **T** | **T** | **A** | **A** | **C** | **A** | **A** | **A** | **A** | **A** | **A** | **A** | **C** | **A** | **A** | **G** | **A** | **G** | **C** | **C** | **G** | **G** | **T** | **G** | **T** | **T** | **G** | **C** | **A** | **C** | **G** | **A** | **A** | **G** | **A** | **T** | **T** | **T** | **G** | **A** | **C** | **A** | **A** | **A** | **C** | **C** | **G** | **A** | **G** | **A** | **A** | **C** | **A** | **C** | **G** | **A** | **A** | **A** | **T** | **T** | **T** | **T** | **A** | **A** | **T** | **G** | **T** | **T** | **G** | **A** | **T** | **T** | **G** | **C** | **A** | **C** | **A** | **A** | **G** | **G** | **T** | **A** | **A** | **A** | **A** | **G** | **T** | **A** | **A** | **T** | **C** | **A** | **G** | **G** | **A** | **A** | **A** | **T** | **A** | **G** | **C** | **T** | **G** | **A** | **T** | **G** | **A** | **A** | **C** | **T** | **C** | **T** | **T** | **T** | **A** | **T** | **C** | **A** | **C** | **T** | **T** | **T** | **G** | **A** | **A** | **A** | **A** | **C** | **A** | **G** | **T** | **T** | **A** | **A** | **A** | **A** | **C** | **A** | **C** | **A** | **T** | **G** | **T** | **T** | **T** | **C** | **A** | **A** | **A** | **C** | **A** | **T** | **T** | **T** | **T** | **A** | **G** | **C** | **A** | **A** | **A** | **A** | **C** | **T** | **A** | **G** | **A** | **T** | **G** | **T** | **G** | **G** | **A** | **T** | **G** | **A** | **T** | **C** | **G** | **G** | **A** | **C** | **C** | **C** | **A** | **A** | **G** | **C** | **G** | **G** | **C** | **G** | **A** | **T** | **T** | **T** | **A** | **T** | **G** | **C** | **T** | **T** | **T** | **T** | **C** | **A** | **A** | **C** | **A** | **T** | **G** | **G** | **T** | **T** | **T** | **A** | **G** | **C** | **C** | **A** | **A** | **A** | **-** | **-** | **-** |
| **N59508F** | **G** | **T** | **G** | **A** | **T** | **C** | **A** | **A** | **A** | **G** | **T** | **A** | **A** | **T** | **G** | **T** | **T** | **A** | **G** | **T** | **G** | **G** | **A** | **T** | **G** | **A** | **C** | **C** | **A** | **T** | **G** | **A** | **A** | **A** | **T** | **G** | **G** | **T** | **C** | **C** | **G** | **T** | **T** | **T** | **A** | **G** | **G** | **C** | **G** | **T** | **T** | **T** | **C** | **A** | **T** | **C** | **A** | **T** | **A** | **T** | **T** | **T** | **A** | **T** | **C** | **T** | **A** | **T** | **T** | **C** | **A** | **A** | **G** | **A** | **G** | **G** | **A** | **T** | **A** | **T** | **A** | **G** | **A** | **A** | **G** | **T** | **C** | **G** | **T** | **A** | **G** | **G** | **C** | **G** | **A** | **A** | **G** | **C** | **A** | **G** | **A** | **A** | **A** | **A** | **T** | **G** | **G** | **T** | **A** | **A** | **G** | **A** | **T** | **T** | **G** | **G** | **C** | **T** | **A** | **T** | **G** | **A** | **A** | **A** | **A** | **A** | **G** | **C** | **A** | **T** | **T** | **G** | **G** | **A** | **A** | **C** | **T** | **A** | **C** | **G** | **T** | **C** | **C** | **A** | **G** | **A** | **T** | **G** | **T** | **T** | **A** | **T** | **T** | **T** | **T** | **G** | **A** | **T** | **G** | **G** | **A** | **T** | **T** | **T** | **G** | **G** | **T** | **A** | **A** | **T** | **G** | **G** | **A** | **A** | **G** | **A** | **A** | **A** | **T** | **G** | **G** | **A** | **C** | **G** | **G** | **C** | **A** | **T** | **T** | **G** | **A** | **T** | **T** | **C** | **A** | **A** | **C** | **A** | **A** | **A** | **A** | **G** | **C** | **G** | **A** | **T** | **C** | **T** | **T** | **G** | **A** | **A** | **A** | **G** | **A** | **T** | **T** | **G** | **G** | **C** | **C** | **A** | **G** | **A** | **A** | **G** | **C** | **C** | **A** | **A** | **G** | **A** | **T** | **T** | **A** | **T** | **T** | **A** | **T** | **T** | **G** | **T** | **G** | **A** | **C** | **G** | **A** | **G** | **T** | **T** | **T** | **T** | **A** | **T** | **T** | **G** | **A** | **T** | **G** | **A** | **T** | **G** | **A** | **A** | **A** | **A** | **A** | **G** | **T** | **G** | **T** | **A** | **T** | **C** | **C** | **G** | **G** | **C** | **G** | **A** | **T** | **T** | **G** | **A** | **A** | **G** | **C** | **T** | **G** | **G** | **T** | **G** | **C** | **A** | **G** | **C** | **G** | **G** | **G** | **C** | **T** | **A** | **C** | **C** | **T** | **A** | **T** | **T** | **A** | **A** | **A** | **A** | **A** | **C** | **A** | **T** | **C** | **A** | **A** | **C** | **A** | **G** | **C** | **A** | **C** | **A** | **T** | **G** | **A** | **G** | **A** | **T** | **T** | **G** | **C** | **T** | **G** | **A** | **T** | **G** | **C** | **A** | **A** | **T** | **T** | **C** | **G** | **G** | **G** | **C** | **G** | **A** | **C** | **T** | **T** | **A** | **T** | **C** | **G** | **C** | **G** | **G** | **A** | **G** | **A** | **G** | **C** | **G** | **T** | **G** | **T** | **G** | **T** | **T** | **G** | **G** | **A** | **A** | **C** | **C** | **T** | **G** | **A** | **A** | **G** | **T** | **G** | **A** | **C** | **G** | **C** | **A** | **T** | **A** | **A** | **G** | **A** | **T** | **G** | **A** | **T** | **G** | **G** | **A** | **A** | **C** | **G** | **G** | **T** | **T** | **A** | **A** | **C** | **A** | **A** | **A** | **A** | **A** | **A** | **A** | **C** | **A** | **A** | **G** | **A** | **G** | **C** | **C** | **G** | **G** | **T** | **G** | **T** | **T** | **G** | **C** | **A** | **T** | **G** | **A** | **A** | **G** | **A** | **T** | **T** | **T** | **G** | **A** | **C** | **A** | **A** | **A** | **T** | **C** | **G** | **A** | **G** | **A** | **A** | **C** | **A** | **C** | **G** | **A** | **A** | **A** | **T** | **T** | **T** | **T** | **A** | **A** | **T** | **G** | **T** | **T** | **G** | **A** | **T** | **T** | **G** | **C** | **A** | **C** | **A** | **A** | **G** | **G** | **T** | **A** | **A** | **A** | **A** | **G** | **T** | **A** | **A** | **T** | **C** | **A** | **G** | **G** | **A** | **A** | **A** | **T** | **A** | **G** | **C** | **T** | **G** | **A** | **T** | **G** | **A** | **A** | **C** | **T** | **C** | **T** | **T** | **T** | **A** | **T** | **C** | **A** | **C** | **T** | **T** | **T** | **G** | **A** | **A** | **A** | **A** | **C** | **A** | **G** | **T** | **T** | **A** | **A** | **A** | **A** | **C** | **A** | **C** | **A** | **T** | **G** | **T** | **T** | **T** | **C** | **A** | **A** | **A** | **C** | **A** | **T** | **T** | **T** | **T** | **A** | **G** | **C** | **A** | **A** | **A** | **A** | **C** | **T** | **A** | **G** | **A** | **T** | **G** | **T** | **G** | **G** | **A** | **T** | **G** | **A** | **T** | **C** | **G** | **G** | **A** | **C** | **C** | **C** | **A** | **A** | **G** | **C** | **G** | **G** | **C** | **G** | **A** | **T** | **T** | **T** | **A** | **T** | **G** | **C** | **T** | **T** | **T** | **T** | **C** | **A** | **A** | **C** | **A** | **T** | **G** | **G** | **T** | **T** | **T** | **A** | **G** | **C** | **C** | **A** | **A** | **A** | **-** | **-** | **-** |
| **N59567F** | **A** | **T** | **G** | **A** | **T** | **A** | **A** | **A** | **A** | **G** | **T** | **T** | **T** | **T** | **A** | **T** | **T** | **A** | **G** | **T** | **A** | **G** | **A** | **T** | **G** | **A** | **C** | **C** | **A** | **T** | **G** | **A** | **A** | **A** | **T** | **G** | **G** | **T** | **A** | **C** | **G** | **C** | **T** | **T** | **A** | **G** | **G** | **C** | **G** | **T** | **C** | **T** | **C** | **T** | **T** | **C** | **T** | **T** | **A** | **T** | **T** | **T** | **A** | **T** | **C** | **T** | **A** | **T** | **C** | **C** | **A** | **A** | **G** | **A** | **A** | **G** | **A** | **C** | **A** | **T** | **T** | **G** | **A** | **A** | **G** | **T** | **G** | **A** | **T** | **T** | **G** | **G** | **A** | **G** | **A** | **A** | **G** | **C** | **T** | **G** | **A** | **A** | **A** | **A** | **T** | **G** | **G** | **A** | **C** | **G** | **T** | **C** | **A** | **A** | **G** | **G** | **C** | **T** | **A** | **T** | **G** | **A** | **A** | **A** | **A** | **G** | **G** | **C** | **G** | **A** | **T** | **G** | **G** | **C** | **A** | **C** | **T** | **T** | **C** | **G** | **A** | **C** | **C** | **A** | **G** | **A** | **T** | **G** | **T** | **C** | **A** | **T** | **T** | **T** | **T** | **A** | **A** | **T** | **G** | **G** | **A** | **T** | **C** | **T** | **A** | **G** | **T** | **C** | **A** | **T** | **G** | **G** | **A** | **A** | **G** | **A** | **A** | **A** | **T** | **G** | **G** | **A** | **T** | **G** | **G** | **C** | **A** | **T** | **C** | **G** | **A** | **A** | **T** | **C** | **A** | **A** | **C** | **A** | **A** | **A** | **A** | **G** | **C** | **A** | **A** | **T** | **C** | **T** | **T** | **A** | **A** | **A** | **A** | **G** | **A** | **T** | **T** | **G** | **G** | **C** | **C** | **A** | **A** | **A** | **A** | **G** | **C** | **C** | **A** | **A** | **A** | **A** | **T** | **C** | **A** | **T** | **T** | **A** | **T** | **C** | **G** | **T** | **C** | **A** | **C** | **A** | **A** | **G** | **T** | **T** | **T** | **T** | **A** | **T** | **T** | **G** | **A** | **T** | **G** | **A** | **T** | **G** | **A** | **A** | **A** | **A** | **A** | **G** | **T** | **T** | **T** | **A** | **T** | **C** | **C** | **T** | **G** | **C** | **A** | **A** | **T** | **T** | **G** | **A** | **A** | **G** | **C** | **A** | **G** | **G** | **A** | **G** | **C** | **A** | **G** | **C** | **A** | **G** | **G** | **A** | **T** | **A** | **T** | **T** | **T** | **G** | **T** | **T** | **G** | **A** | **A** | **G** | **A** | **C** | **G** | **T** | **C** | **T** | **A** | **C** | **C** | **G** | **C** | **A** | **C** | **A** | **T** | **G** | **A** | **A** | **A** | **T** | **A** | **G** | **C** | **T** | **G** | **A** | **T** | **G** | **C** | **T** | **A** | **T** | **A** | **A** | **G** | **G** | **G** | **C** | **T** | **A** | **C** | **G** | **C** | **A** | **A** | **C** | **G** | **C** | **G** | **G** | **C** | **G** | **A** | **G** | **C** | **G** | **A** | **G** | **T** | **A** | **T** | **T** | **G** | **G** | **A** | **A** | **C** | **C** | **T** | **G** | **A** | **A** | **G** | **T** | **G** | **A** | **C** | **A** | **A** | **C** | **A** | **A** | **A** | **A** | **A** | **T** | **G** | **A** | **T** | **G** | **G** | **A** | **A** | **A** | **A** | **A** | **A** | **T** | **G** | **A** | **G** | **T** | **C** | **G** | **G** | **C** | **G** | **A** | **T** | **A** | **T** | **G** | **A** | **T** | **C** | **C** | **A** | **G** | **T** | **A** | **T** | **T** | **G** | **C** | **A** | **T** | **G** | **A** | **A** | **G** | **A** | **A** | **T** | **T** | **A** | **A** | **C** | **G** | **A** | **A** | **T** | **C** | **G** | **A** | **G** | **A** | **A** | **A** | **A** | **T** | **G** | **A** | **A** | **A** | **T** | **T** | **T** | **T** | **G** | **A** | **T** | **G** | **C** | **T** | **G** | **A** | **T** | **T** | **T** | **C** | **T** | **G** | **A** | **A** | **G** | **G** | **G** | **A** | **A** | **A** | **A** | **G** | **T** | **A** | **A** | **T** | **C** | **A** | **G** | **G** | **A** | **A** | **A** | **T** | **C** | **G** | **C** | **G** | **G** | **A** | **T** | **G** | **A** | **A** | **T** | **T** | **G** | **T** | **T** | **T** | **A** | **T** | **C** | **A** | **C** | **A** | **C** | **T** | **G** | **A** | **A** | **A** | **A** | **C** | **A** | **G** | **T** | **T** | **A** | **A** | **A** | **A** | **C** | **G** | **C** | **A** | **T** | **G** | **T** | **G** | **T** | **C** | **G** | **A** | **A** | **T** | **A** | **T** | **A** | **T** | **T** | **A** | **G** | **C** | **T** | **A** | **A** | **G** | **T** | **T** | **A** | **G** | **A** | **G** | **G** | **T** | **A** | **G** | **A** | **A** | **G** | **A** | **C** | **C** | **G** | **A** | **A** | **C** | **T** | **C** | **A** | **A** | **G** | **C** | **A** | **G** | **C** | **T** | **A** | **T** | **C** | **T** | **A** | **T** | **G** | **C** | **A** | **T** | **T** | **T** | **A** | **A** | **A** | **C** | **A** | **T** | **G** | **G** | **T** | **T** | **T** | **G** | **G** | **T** | **G** | **A** | **A** | **A** | **-** | **-** | **-** |
| **N54519** | **A** | **T** | **G** | **A** | **T** | **A** | **A** | **A** | **A** | **G** | **T** | **T** | **T** | **T** | **A** | **T** | **T** | **A** | **G** | **T** | **A** | **G** | **A** | **T** | **G** | **A** | **C** | **C** | **A** | **T** | **G** | **A** | **A** | **A** | **T** | **G** | **G** | **T** | **G** | **C** | **G** | **C** | **T** | **T** | **A** | **G** | **G** | **C** | **G** | **T** | **C** | **T** | **C** | **T** | **T** | **C** | **T** | **T** | **A** | **T** | **T** | **T** | **A** | **T** | **C** | **T** | **A** | **T** | **C** | **C** | **A** | **A** | **G** | **A** | **A** | **G** | **A** | **C** | **A** | **T** | **T** | **G** | **A** | **A** | **G** | **T** | **A** | **A** | **T** | **T** | **G** | **G** | **A** | **G** | **A** | **A** | **G** | **C** | **T** | **G** | **A** | **A** | **A** | **A** | **T** | **G** | **G** | **A** | **C** | **G** | **A** | **C** | **A** | **A** | **G** | **G** | **C** | **T** | **A** | **T** | **G** | **A** | **G** | **A** | **A** | **G** | **G** | **C** | **G** | **A** | **T** | **G** | **A** | **C** | **A** | **C** | **T** | **T** | **C** | **G** | **A** | **C** | **C** | **G** | **G** | **A** | **C** | **G** | **T** | **C** | **A** | **T** | **T** | **T** | **T** | **A** | **A** | **T** | **G** | **G** | **A** | **C** | **T** | **T** | **A** | **G** | **T** | **C** | **A** | **T** | **G** | **G** | **A** | **A** | **G** | **A** | **A** | **A** | **T** | **G** | **G** | **A** | **T** | **G** | **G** | **T** | **A** | **T** | **C** | **G** | **A** | **A** | **T** | **C** | **A** | **A** | **C** | **A** | **A** | **A** | **A** | **G** | **C** | **A** | **A** | **T** | **C** | **T** | **T** | **A** | **A** | **A** | **A** | **G** | **A** | **T** | **T** | **G** | **G** | **C** | **C** | **A** | **G** | **A** | **A** | **G** | **C** | **T** | **A** | **A** | **A** | **A** | **T** | **C** | **A** | **T** | **T** | **A** | **T** | **C** | **G** | **T** | **A** | **A** | **C** | **C** | **A** | **G** | **T** | **T** | **T** | **T** | **A** | **T** | **T** | **G** | **A** | **T** | **G** | **A** | **C** | **G** | **A** | **A** | **A** | **A** | **A** | **G** | **T** | **T** | **T** | **A** | **T** | **C** | **C** | **T** | **G** | **C** | **C** | **A** | **T** | **T** | **G** | **A** | **A** | **G** | **C** | **A** | **G** | **G** | **C** | **G** | **C** | **A** | **G** | **C** | **A** | **G** | **G** | **G** | **T** | **A** | **T** | **T** | **T** | **G** | **T** | **T** | **G** | **A** | **A** | **G** | **A** | **C** | **T** | **T** | **C** | **T** | **A** | **C** | **C** | **G** | **C** | **A** | **C** | **A** | **T** | **G** | **A** | **A** | **A** | **T** | **T** | **G** | **C** | **T** | **G** | **A** | **T** | **G** | **C** | **T** | **A** | **T** | **A** | **A** | **G** | **G** | **G** | **C** | **A** | **A** | **C** | **G** | **C** | **A** | **A** | **C** | **G** | **C** | **G** | **G** | **C** | **G** | **A** | **G** | **C** | **G** | **A** | **G** | **T** | **A** | **C** | **T** | **A** | **G** | **A** | **A** | **C** | **C** | **A** | **G** | **A** | **A** | **G** | **T** | **G** | **A** | **C** | **A** | **A** | **C** | **A** | **A** | **A** | **A** | **A** | **T** | **G** | **A** | **T** | **G** | **G** | **A** | **A** | **A** | **A** | **A** | **A** | **T** | **G** | **A** | **G** | **T** | **C** | **G** | **G** | **C** | **G** | **A** | **A** | **A** | **T** | **G** | **A** | **G** | **C** | **C** | **T** | **G** | **T** | **A** | **T** | **T** | **A** | **C** | **A** | **T** | **G** | **A** | **A** | **G** | **A** | **A** | **C** | **T** | **A** | **A** | **C** | **A** | **A** | **A** | **T** | **C** | **G** | **G** | **G** | **A** | **A** | **A** | **A** | **T** | **G** | **A** | **A** | **A** | **T** | **T** | **T** | **T** | **A** | **A** | **T** | **G** | **C** | **T** | **G** | **A** | **T** | **T** | **T** | **C** | **T** | **G** | **A** | **A** | **G** | **G** | **G** | **A** | **A** | **A** | **A** | **G** | **T** | **A** | **A** | **T** | **C** | **A** | **G** | **G** | **A** | **A** | **A** | **T** | **A** | **G** | **C** | **G** | **G** | **A** | **C** | **G** | **A** | **A** | **T** | **T** | **G** | **T** | **T** | **T** | **A** | **T** | **T** | **A** | **C** | **A** | **C** | **T** | **G** | **A** | **A** | **A** | **A** | **C** | **A** | **G** | **T** | **A** | **A** | **A** | **A** | **A** | **C** | **A** | **C** | **A** | **T** | **G** | **T** | **T** | **T** | **C** | **C** | **A** | **A** | **T** | **A** | **T** | **A** | **T** | **T** | **G** | **G** | **C** | **T** | **A** | **A** | **G** | **C** | **T** | **A** | **G** | **A** | **A** | **G** | **T** | **G** | **G** | **A** | **A** | **G** | **A** | **C** | **C** | **G** | **T** | **A** | **C** | **A** | **C** | **A** | **A** | **G** | **C** | **C** | **G** | **C** | **T** | **A** | **T** | **C** | **T** | **A** | **T** | **G** | **C** | **A** | **T** | **T** | **C** | **A** | **A** | **A** | **C** | **A** | **T** | **G** | **G** | **T** | **T** | **T** | **G** | **G** | **T** | **G** | **A** | **A** | **A** | **-** | **-** | **-** |
| **N59613F** | **A** | **T** | **G** | **A** | **T** | **A** | **A** | **A** | **A** | **G** | **T** | **T** | **T** | **T** | **A** | **T** | **T** | **A** | **G** | **T** | **A** | **G** | **A** | **T** | **G** | **A** | **C** | **C** | **A** | **T** | **G** | **A** | **A** | **A** | **T** | **G** | **G** | **T** | **G** | **C** | **G** | **C** | **T** | **T** | **A** | **G** | **G** | **C** | **G** | **T** | **C** | **T** | **C** | **T** | **T** | **C** | **T** | **T** | **A** | **T** | **T** | **T** | **A** | **T** | **C** | **T** | **A** | **T** | **C** | **C** | **A** | **A** | **G** | **A** | **A** | **G** | **A** | **C** | **A** | **T** | **T** | **G** | **A** | **A** | **G** | **T** | **A** | **A** | **T** | **T** | **G** | **G** | **A** | **G** | **A** | **A** | **G** | **C** | **T** | **G** | **A** | **A** | **A** | **A** | **T** | **G** | **G** | **A** | **C** | **G** | **A** | **C** | **A** | **A** | **G** | **G** | **C** | **T** | **A** | **T** | **G** | **A** | **G** | **A** | **A** | **G** | **G** | **C** | **G** | **A** | **T** | **G** | **A** | **C** | **A** | **C** | **T** | **T** | **C** | **G** | **A** | **C** | **C** | **G** | **G** | **A** | **C** | **G** | **T** | **C** | **A** | **T** | **T** | **T** | **T** | **A** | **A** | **T** | **G** | **G** | **A** | **C** | **T** | **T** | **A** | **G** | **T** | **C** | **A** | **T** | **G** | **G** | **A** | **A** | **G** | **A** | **A** | **A** | **T** | **G** | **G** | **A** | **T** | **G** | **G** | **T** | **A** | **T** | **C** | **G** | **A** | **A** | **T** | **C** | **A** | **A** | **C** | **A** | **A** | **A** | **A** | **G** | **C** | **A** | **A** | **T** | **C** | **T** | **T** | **A** | **A** | **A** | **A** | **G** | **A** | **T** | **T** | **G** | **G** | **C** | **C** | **A** | **G** | **A** | **A** | **G** | **C** | **T** | **A** | **A** | **A** | **A** | **T** | **C** | **A** | **T** | **T** | **A** | **T** | **C** | **G** | **T** | **A** | **A** | **C** | **C** | **A** | **G** | **T** | **T** | **T** | **T** | **A** | **T** | **T** | **G** | **A** | **T** | **G** | **A** | **C** | **G** | **A** | **A** | **A** | **A** | **A** | **G** | **T** | **T** | **T** | **A** | **T** | **C** | **C** | **T** | **G** | **C** | **C** | **A** | **T** | **T** | **G** | **A** | **A** | **G** | **C** | **A** | **G** | **G** | **C** | **G** | **C** | **A** | **G** | **C** | **A** | **G** | **G** | **G** | **T** | **A** | **T** | **T** | **T** | **G** | **T** | **T** | **G** | **A** | **A** | **G** | **A** | **C** | **T** | **T** | **C** | **T** | **A** | **C** | **C** | **G** | **C** | **A** | **C** | **A** | **T** | **G** | **A** | **A** | **A** | **T** | **T** | **G** | **C** | **T** | **G** | **A** | **T** | **G** | **C** | **T** | **A** | **T** | **A** | **A** | **G** | **G** | **G** | **C** | **A** | **A** | **C** | **G** | **C** | **A** | **A** | **C** | **G** | **C** | **G** | **G** | **C** | **G** | **A** | **G** | **C** | **G** | **A** | **G** | **T** | **A** | **C** | **T** | **A** | **G** | **A** | **A** | **C** | **C** | **A** | **G** | **A** | **A** | **G** | **T** | **G** | **A** | **C** | **A** | **A** | **C** | **A** | **A** | **A** | **A** | **A** | **T** | **G** | **A** | **T** | **G** | **G** | **A** | **A** | **A** | **A** | **A** | **A** | **T** | **G** | **A** | **G** | **T** | **C** | **G** | **G** | **C** | **G** | **A** | **A** | **A** | **T** | **G** | **A** | **G** | **C** | **C** | **T** | **G** | **T** | **A** | **T** | **T** | **A** | **C** | **A** | **T** | **G** | **A** | **A** | **G** | **A** | **A** | **C** | **T** | **A** | **A** | **C** | **G** | **A** | **A** | **T** | **C** | **G** | **G** | **G** | **A** | **A** | **A** | **A** | **T** | **G** | **A** | **A** | **A** | **T** | **T** | **T** | **T** | **A** | **A** | **T** | **G** | **C** | **T** | **G** | **A** | **T** | **T** | **T** | **C** | **T** | **G** | **A** | **A** | **G** | **G** | **G** | **A** | **A** | **A** | **A** | **G** | **T** | **A** | **A** | **T** | **C** | **A** | **G** | **G** | **A** | **A** | **A** | **T** | **A** | **G** | **C** | **G** | **G** | **A** | **C** | **G** | **A** | **A** | **T** | **T** | **G** | **T** | **T** | **T** | **A** | **T** | **T** | **A** | **C** | **A** | **C** | **T** | **G** | **A** | **A** | **A** | **A** | **C** | **A** | **G** | **T** | **A** | **A** | **A** | **A** | **A** | **C** | **A** | **C** | **A** | **T** | **G** | **T** | **T** | **T** | **C** | **C** | **A** | **A** | **T** | **A** | **T** | **A** | **T** | **T** | **G** | **G** | **C** | **T** | **A** | **A** | **G** | **C** | **T** | **A** | **G** | **A** | **A** | **G** | **T** | **G** | **G** | **A** | **A** | **G** | **A** | **C** | **C** | **G** | **T** | **A** | **C** | **A** | **C** | **A** | **A** | **G** | **C** | **C** | **G** | **C** | **T** | **A** | **T** | **C** | **T** | **A** | **T** | **G** | **C** | **A** | **T** | **T** | **C** | **A** | **A** | **A** | **C** | **A** | **T** | **G** | **G** | **T** | **T** | **T** | **G** | **G** | **T** | **G** | **A** | **A** | **A** | **-** | **-** | **-** |
| **N54546** | **A** | **T** | **G** | **A** | **T** | **A** | **A** | **A** | **A** | **G** | **T** | **T** | **T** | **T** | **A** | **T** | **T** | **A** | **G** | **T** | **A** | **G** | **A** | **T** | **G** | **A** | **C** | **C** | **A** | **T** | **G** | **A** | **A** | **A** | **T** | **G** | **G** | **T** | **G** | **C** | **G** | **C** | **T** | **T** | **A** | **G** | **G** | **T** | **G** | **T** | **C** | **T** | **C** | **T** | **T** | **C** | **T** | **T** | **A** | **T** | **T** | **T** | **A** | **T** | **C** | **T** | **A** | **T** | **T** | **C** | **A** | **A** | **G** | **A** | **A** | **G** | **A** | **C** | **A** | **T** | **T** | **G** | **A** | **A** | **G** | **T** | **A** | **A** | **T** | **T** | **G** | **G** | **A** | **G** | **A** | **A** | **G** | **C** | **T** | **G** | **A** | **A** | **A** | **A** | **T** | **G** | **G** | **A** | **C** | **G** | **A** | **C** | **A** | **A** | **G** | **G** | **C** | **T** | **A** | **T** | **G** | **A** | **G** | **A** | **A** | **G** | **G** | **C** | **G** | **A** | **T** | **G** | **A** | **C** | **A** | **C** | **T** | **T** | **C** | **G** | **A** | **C** | **C** | **G** | **G** | **A** | **C** | **G** | **T** | **C** | **A** | **T** | **T** | **T** | **T** | **A** | **A** | **T** | **G** | **G** | **A** | **C** | **T** | **T** | **A** | **G** | **T** | **C** | **A** | **T** | **G** | **G** | **A** | **A** | **G** | **A** | **A** | **A** | **T** | **G** | **G** | **A** | **T** | **G** | **G** | **T** | **A** | **T** | **C** | **G** | **A** | **A** | **T** | **C** | **A** | **A** | **C** | **A** | **A** | **A** | **A** | **G** | **C** | **A** | **A** | **T** | **C** | **T** | **T** | **A** | **A** | **A** | **A** | **G** | **A** | **T** | **T** | **G** | **G** | **C** | **C** | **A** | **G** | **A** | **A** | **G** | **C** | **T** | **A** | **A** | **A** | **A** | **T** | **C** | **A** | **T** | **T** | **A** | **T** | **C** | **G** | **T** | **A** | **A** | **C** | **C** | **A** | **G** | **T** | **T** | **T** | **T** | **A** | **T** | **T** | **G** | **A** | **T** | **G** | **A** | **C** | **G** | **A** | **A** | **A** | **A** | **A** | **G** | **T** | **T** | **T** | **A** | **T** | **C** | **C** | **T** | **G** | **C** | **C** | **A** | **T** | **T** | **G** | **A** | **A** | **G** | **C** | **A** | **G** | **G** | **T** | **G** | **C** | **A** | **G** | **C** | **A** | **G** | **G** | **G** | **T** | **A** | **T** | **T** | **T** | **G** | **T** | **T** | **G** | **A** | **A** | **G** | **A** | **C** | **C** | **T** | **C** | **T** | **A** | **C** | **C** | **G** | **C** | **A** | **C** | **A** | **T** | **G** | **A** | **A** | **A** | **T** | **T** | **G** | **C** | **T** | **G** | **A** | **T** | **G** | **C** | **T** | **A** | **T** | **A** | **A** | **G** | **G** | **G** | **C** | **A** | **A** | **C** | **G** | **C** | **A** | **A** | **C** | **G** | **C** | **G** | **G** | **C** | **G** | **A** | **G** | **C** | **G** | **A** | **G** | **T** | **A** | **T** | **T** | **A** | **G** | **A** | **A** | **C** | **C** | **A** | **G** | **A** | **A** | **G** | **T** | **G** | **A** | **C** | **A** | **A** | **C** | **A** | **A** | **A** | **A** | **A** | **T** | **G** | **A** | **T** | **G** | **G** | **A** | **A** | **A** | **A** | **A** | **A** | **T** | **G** | **A** | **G** | **T** | **C** | **G** | **G** | **C** | **G** | **A** | **A** | **A** | **T** | **G** | **A** | **G** | **C** | **C** | **T** | **G** | **T** | **A** | **T** | **T** | **A** | **C** | **A** | **T** | **G** | **A** | **A** | **G** | **A** | **A** | **C** | **T** | **A** | **A** | **C** | **G** | **A** | **A** | **T** | **C** | **G** | **G** | **G** | **A** | **A** | **A** | **A** | **T** | **G** | **A** | **A** | **A** | **T** | **T** | **T** | **T** | **A** | **A** | **T** | **G** | **C** | **T** | **G** | **A** | **T** | **T** | **T** | **C** | **T** | **G** | **A** | **A** | **G** | **G** | **A** | **A** | **A** | **A** | **A** | **G** | **T** | **A** | **A** | **T** | **C** | **A** | **G** | **G** | **A** | **A** | **A** | **T** | **A** | **G** | **C** | **G** | **G** | **A** | **C** | **G** | **A** | **A** | **T** | **T** | **G** | **T** | **T** | **C** | **A** | **T** | **T** | **A** | **C** | **A** | **C** | **T** | **G** | **A** | **A** | **A** | **A** | **C** | **A** | **G** | **T** | **A** | **A** | **A** | **A** | **A** | **C** | **A** | **C** | **A** | **T** | **G** | **T** | **T** | **T** | **C** | **C** | **A** | **A** | **T** | **A** | **T** | **A** | **T** | **T** | **G** | **G** | **C** | **T** | **A** | **A** | **G** | **C** | **T** | **A** | **G** | **A** | **A** | **G** | **T** | **G** | **G** | **A** | **A** | **G** | **A** | **C** | **C** | **G** | **T** | **A** | **C** | **A** | **C** | **A** | **A** | **G** | **C** | **C** | **G** | **C** | **T** | **A** | **T** | **C** | **T** | **A** | **T** | **G** | **C** | **A** | **T** | **T** | **C** | **A** | **A** | **A** | **C** | **A** | **T** | **G** | **G** | **T** | **T** | **T** | **G** | **G** | **T** | **G** | **A** | **A** | **A** | **-** | **-** | **-** |
| **N59606F** | **G** | **T** | **G** | **A** | **T** | **C** | **A** | **A** | **A** | **G** | **T** | **A** | **A** | **T** | **G** | **T** | **T** | **A** | **G** | **T** | **G** | **G** | **A** | **T** | **G** | **A** | **C** | **C** | **A** | **T** | **G** | **A** | **A** | **A** | **T** | **G** | **G** | **T** | **C** | **C** | **G** | **T** | **T** | **T** | **A** | **G** | **G** | **C** | **G** | **T** | **T** | **T** | **C** | **A** | **T** | **C** | **A** | **T** | **A** | **T** | **T** | **T** | **A** | **T** | **C** | **T** | **A** | **T** | **T** | **C** | **A** | **A** | **G** | **A** | **G** | **G** | **A** | **T** | **A** | **T** | **A** | **G** | **A** | **A** | **G** | **T** | **C** | **G** | **T** | **A** | **G** | **G** | **C** | **G** | **A** | **A** | **G** | **C** | **A** | **G** | **A** | **A** | **A** | **A** | **T** | **G** | **G** | **T** | **A** | **A** | **G** | **A** | **T** | **T** | **G** | **G** | **C** | **T** | **A** | **T** | **G** | **A** | **A** | **A** | **A** | **A** | **G** | **C** | **A** | **T** | **T** | **G** | **G** | **A** | **A** | **C** | **T** | **A** | **C** | **G** | **T** | **C** | **C** | **A** | **G** | **A** | **T** | **G** | **T** | **T** | **A** | **T** | **T** | **T** | **T** | **G** | **A** | **T** | **G** | **G** | **A** | **T** | **T** | **T** | **G** | **G** | **T** | **A** | **A** | **T** | **G** | **G** | **A** | **A** | **G** | **A** | **A** | **A** | **T** | **G** | **G** | **A** | **C** | **G** | **G** | **C** | **A** | **T** | **T** | **G** | **A** | **T** | **T** | **C** | **A** | **A** | **C** | **A** | **A** | **A** | **A** | **G** | **C** | **G** | **A** | **T** | **C** | **T** | **T** | **G** | **A** | **A** | **A** | **G** | **A** | **T** | **T** | **G** | **G** | **C** | **C** | **A** | **G** | **A** | **A** | **G** | **C** | **C** | **A** | **A** | **G** | **A** | **T** | **T** | **A** | **T** | **T** | **A** | **T** | **T** | **G** | **T** | **G** | **A** | **C** | **G** | **A** | **G** | **T** | **T** | **T** | **T** | **A** | **T** | **T** | **G** | **A** | **T** | **G** | **A** | **T** | **G** | **A** | **A** | **A** | **A** | **A** | **G** | **T** | **G** | **T** | **A** | **T** | **C** | **C** | **G** | **G** | **C** | **G** | **A** | **T** | **T** | **G** | **A** | **A** | **G** | **C** | **T** | **G** | **G** | **T** | **G** | **C** | **A** | **G** | **C** | **G** | **G** | **G** | **C** | **T** | **A** | **C** | **C** | **T** | **A** | **T** | **T** | **A** | **A** | **A** | **A** | **A** | **C** | **A** | **T** | **C** | **A** | **A** | **C** | **A** | **G** | **C** | **A** | **C** | **A** | **T** | **G** | **A** | **G** | **A** | **T** | **T** | **G** | **C** | **T** | **G** | **A** | **T** | **G** | **C** | **A** | **A** | **T** | **T** | **C** | **G** | **G** | **G** | **C** | **G** | **A** | **C** | **T** | **T** | **A** | **T** | **C** | **G** | **C** | **G** | **G** | **A** | **G** | **A** | **G** | **C** | **G** | **T** | **G** | **T** | **G** | **T** | **T** | **G** | **G** | **A** | **A** | **C** | **C** | **T** | **G** | **A** | **A** | **G** | **T** | **G** | **A** | **C** | **G** | **C** | **A** | **T** | **A** | **A** | **G** | **A** | **T** | **G** | **A** | **T** | **G** | **G** | **A** | **A** | **C** | **G** | **G** | **T** | **T** | **A** | **A** | **C** | **A** | **A** | **A** | **A** | **A** | **A** | **A** | **C** | **A** | **A** | **G** | **A** | **G** | **C** | **C** | **G** | **G** | **T** | **G** | **T** | **T** | **G** | **C** | **A** | **T** | **G** | **A** | **A** | **G** | **A** | **T** | **T** | **T** | **G** | **A** | **C** | **A** | **A** | **A** | **T** | **C** | **G** | **A** | **G** | **A** | **A** | **C** | **A** | **C** | **G** | **A** | **A** | **A** | **T** | **T** | **T** | **T** | **A** | **A** | **T** | **G** | **T** | **T** | **G** | **A** | **T** | **T** | **G** | **C** | **A** | **C** | **A** | **A** | **G** | **G** | **T** | **A** | **A** | **A** | **A** | **G** | **T** | **A** | **A** | **T** | **C** | **A** | **G** | **G** | **A** | **A** | **A** | **T** | **A** | **G** | **C** | **T** | **G** | **A** | **T** | **G** | **A** | **A** | **C** | **T** | **C** | **T** | **T** | **T** | **A** | **T** | **C** | **A** | **C** | **T** | **T** | **T** | **G** | **A** | **A** | **A** | **A** | **C** | **A** | **G** | **T** | **T** | **A** | **A** | **A** | **A** | **C** | **A** | **C** | **A** | **T** | **G** | **T** | **T** | **T** | **C** | **A** | **A** | **A** | **C** | **A** | **T** | **T** | **T** | **T** | **A** | **G** | **C** | **A** | **A** | **A** | **A** | **C** | **T** | **A** | **G** | **A** | **T** | **G** | **T** | **G** | **G** | **A** | **T** | **G** | **A** | **T** | **C** | **G** | **G** | **A** | **C** | **C** | **C** | **A** | **A** | **G** | **C** | **G** | **G** | **C** | **G** | **A** | **T** | **T** | **T** | **A** | **T** | **G** | **C** | **T** | **T** | **T** | **T** | **C** | **A** | **A** | **C** | **A** | **T** | **G** | **G** | **T** | **T** | **T** | **A** | **G** | **C** | **C** | **A** | **A** | **A** | **-** | **-** | **-** |
| **N52564** | **G** | **T** | **G** | **A** | **T** | **C** | **A** | **A** | **A** | **G** | **T** | **A** | **A** | **T** | **G** | **T** | **T** | **A** | **G** | **T** | **G** | **G** | **A** | **T** | **G** | **A** | **C** | **C** | **A** | **T** | **G** | **A** | **A** | **A** | **T** | **G** | **G** | **T** | **C** | **C** | **G** | **T** | **T** | **T** | **A** | **G** | **G** | **C** | **G** | **T** | **T** | **T** | **C** | **A** | **T** | **C** | **A** | **T** | **A** | **T** | **T** | **T** | **A** | **T** | **C** | **T** | **A** | **T** | **T** | **C** | **A** | **A** | **G** | **A** | **G** | **G** | **A** | **T** | **A** | **T** | **A** | **G** | **A** | **A** | **G** | **T** | **C** | **G** | **T** | **A** | **G** | **G** | **C** | **G** | **A** | **A** | **G** | **C** | **A** | **G** | **A** | **A** | **A** | **A** | **C** | **G** | **G** | **T** | **A** | **A** | **G** | **A** | **T** | **T** | **G** | **G** | **C** | **T** | **A** | **T** | **G** | **A** | **A** | **A** | **A** | **A** | **G** | **C** | **A** | **T** | **T** | **G** | **G** | **A** | **A** | **C** | **T** | **A** | **C** | **G** | **T** | **C** | **C** | **A** | **G** | **A** | **T** | **G** | **T** | **T** | **A** | **T** | **T** | **T** | **T** | **G** | **A** | **T** | **G** | **G** | **A** | **T** | **T** | **T** | **G** | **G** | **T** | **A** | **A** | **T** | **G** | **G** | **A** | **A** | **G** | **A** | **A** | **A** | **T** | **G** | **G** | **A** | **C** | **G** | **G** | **C** | **A** | **T** | **T** | **G** | **A** | **T** | **T** | **C** | **A** | **A** | **C** | **A** | **A** | **A** | **A** | **G** | **C** | **G** | **A** | **T** | **C** | **T** | **T** | **G** | **A** | **A** | **A** | **G** | **A** | **T** | **T** | **G** | **G** | **C** | **C** | **A** | **G** | **A** | **A** | **G** | **C** | **C** | **A** | **A** | **G** | **A** | **T** | **T** | **A** | **T** | **T** | **A** | **T** | **T** | **G** | **T** | **G** | **A** | **C** | **G** | **A** | **G** | **T** | **T** | **T** | **T** | **A** | **T** | **T** | **G** | **A** | **T** | **G** | **A** | **T** | **G** | **A** | **A** | **A** | **A** | **A** | **G** | **T** | **G** | **T** | **A** | **T** | **C** | **C** | **G** | **G** | **C** | **G** | **A** | **T** | **T** | **G** | **A** | **A** | **G** | **C** | **T** | **G** | **G** | **T** | **G** | **C** | **A** | **G** | **C** | **G** | **G** | **G** | **C** | **T** | **A** | **C** | **C** | **T** | **A** | **T** | **T** | **A** | **A** | **A** | **A** | **A** | **C** | **A** | **T** | **C** | **A** | **A** | **C** | **A** | **G** | **C** | **A** | **C** | **A** | **T** | **G** | **A** | **G** | **A** | **T** | **T** | **G** | **C** | **T** | **G** | **A** | **T** | **G** | **C** | **A** | **A** | **T** | **T** | **C** | **G** | **G** | **G** | **C** | **G** | **A** | **C** | **T** | **T** | **A** | **T** | **C** | **G** | **C** | **G** | **G** | **A** | **G** | **A** | **G** | **C** | **G** | **T** | **G** | **T** | **G** | **T** | **T** | **G** | **G** | **A** | **A** | **C** | **C** | **T** | **G** | **A** | **A** | **G** | **T** | **G** | **A** | **C** | **G** | **C** | **A** | **T** | **A** | **A** | **G** | **A** | **T** | **G** | **A** | **T** | **G** | **G** | **A** | **A** | **C** | **G** | **G** | **T** | **T** | **A** | **A** | **C** | **A** | **A** | **A** | **A** | **A** | **A** | **A** | **C** | **A** | **A** | **G** | **A** | **G** | **C** | **C** | **G** | **G** | **T** | **G** | **T** | **T** | **G** | **C** | **A** | **C** | **G** | **A** | **A** | **G** | **A** | **T** | **T** | **T** | **G** | **A** | **C** | **A** | **A** | **A** | **C** | **C** | **G** | **G** | **G** | **A** | **A** | **C** | **A** | **C** | **G** | **A** | **A** | **A** | **T** | **T** | **T** | **T** | **A** | **A** | **T** | **G** | **T** | **T** | **G** | **A** | **T** | **T** | **G** | **C** | **A** | **C** | **A** | **A** | **G** | **G** | **T** | **A** | **A** | **A** | **A** | **G** | **T** | **A** | **A** | **T** | **C** | **A** | **G** | **G** | **A** | **A** | **A** | **T** | **A** | **G** | **C** | **T** | **G** | **A** | **T** | **G** | **A** | **A** | **C** | **T** | **C** | **T** | **T** | **T** | **A** | **T** | **C** | **A** | **C** | **T** | **T** | **T** | **G** | **A** | **A** | **A** | **A** | **C** | **A** | **G** | **T** | **T** | **A** | **A** | **A** | **A** | **C** | **A** | **C** | **A** | **T** | **G** | **T** | **T** | **T** | **C** | **A** | **A** | **A** | **C** | **A** | **T** | **T** | **T** | **T** | **A** | **G** | **C** | **A** | **A** | **A** | **A** | **C** | **T** | **A** | **G** | **A** | **T** | **G** | **T** | **G** | **G** | **A** | **T** | **G** | **A** | **T** | **C** | **G** | **G** | **A** | **C** | **C** | **C** | **A** | **A** | **G** | **C** | **G** | **G** | **C** | **G** | **A** | **T** | **T** | **T** | **A** | **T** | **G** | **C** | **T** | **T** | **T** | **T** | **C** | **A** | **A** | **C** | **A** | **T** | **G** | **G** | **T** | **T** | **T** | **A** | **G** | **C** | **C** | **A** | **A** | **A** | **-** | **-** | **-** |
| **N59891F** | **A** | **T** | **G** | **A** | **T** | **A** | **A** | **A** | **A** | **G** | **T** | **T** | **T** | **T** | **A** | **T** | **T** | **A** | **G** | **T** | **A** | **G** | **A** | **T** | **G** | **A** | **C** | **C** | **A** | **T** | **G** | **A** | **A** | **A** | **T** | **G** | **G** | **T** | **A** | **C** | **G** | **C** | **T** | **T** | **A** | **G** | **G** | **C** | **G** | **T** | **C** | **T** | **C** | **T** | **T** | **C** | **T** | **T** | **A** | **T** | **T** | **T** | **A** | **T** | **C** | **T** | **A** | **T** | **C** | **C** | **A** | **A** | **G** | **A** | **A** | **G** | **A** | **C** | **A** | **T** | **T** | **G** | **A** | **A** | **G** | **T** | **G** | **A** | **T** | **T** | **G** | **G** | **A** | **G** | **A** | **A** | **G** | **C** | **T** | **G** | **A** | **A** | **A** | **A** | **T** | **G** | **G** | **A** | **C** | **G** | **T** | **C** | **A** | **A** | **G** | **G** | **C** | **T** | **A** | **T** | **G** | **A** | **A** | **A** | **A** | **G** | **G** | **C** | **G** | **A** | **T** | **G** | **G** | **C** | **A** | **C** | **T** | **T** | **C** | **G** | **A** | **C** | **C** | **A** | **G** | **A** | **T** | **G** | **T** | **C** | **A** | **T** | **T** | **T** | **T** | **A** | **A** | **T** | **G** | **G** | **A** | **T** | **C** | **T** | **A** | **G** | **T** | **C** | **A** | **T** | **G** | **G** | **A** | **A** | **G** | **A** | **A** | **A** | **T** | **G** | **G** | **A** | **T** | **G** | **G** | **C** | **A** | **T** | **C** | **G** | **A** | **A** | **T** | **C** | **A** | **A** | **C** | **A** | **A** | **A** | **A** | **G** | **C** | **A** | **A** | **T** | **C** | **T** | **T** | **A** | **A** | **A** | **A** | **G** | **A** | **T** | **T** | **G** | **G** | **C** | **C** | **A** | **A** | **A** | **A** | **G** | **C** | **C** | **A** | **A** | **A** | **A** | **T** | **C** | **A** | **T** | **T** | **A** | **T** | **C** | **G** | **T** | **C** | **A** | **C** | **A** | **A** | **G** | **T** | **T** | **T** | **T** | **A** | **T** | **T** | **G** | **A** | **T** | **G** | **A** | **T** | **G** | **A** | **A** | **A** | **A** | **A** | **G** | **T** | **T** | **T** | **A** | **T** | **C** | **C** | **T** | **G** | **C** | **A** | **A** | **T** | **T** | **G** | **A** | **A** | **G** | **C** | **A** | **G** | **G** | **A** | **G** | **C** | **A** | **G** | **C** | **A** | **G** | **G** | **A** | **T** | **A** | **T** | **T** | **T** | **A** | **T** | **T** | **G** | **A** | **A** | **G** | **A** | **C** | **G** | **T** | **C** | **T** | **A** | **C** | **C** | **G** | **C** | **A** | **C** | **A** | **T** | **G** | **A** | **A** | **A** | **T** | **A** | **G** | **C** | **T** | **G** | **A** | **T** | **G** | **C** | **T** | **A** | **T** | **A** | **A** | **G** | **G** | **G** | **C** | **T** | **A** | **C** | **G** | **C** | **A** | **A** | **C** | **G** | **C** | **G** | **G** | **C** | **G** | **A** | **G** | **C** | **G** | **A** | **G** | **T** | **A** | **T** | **T** | **G** | **G** | **A** | **A** | **C** | **C** | **T** | **G** | **A** | **A** | **G** | **T** | **G** | **A** | **C** | **A** | **A** | **C** | **A** | **A** | **A** | **A** | **A** | **T** | **G** | **A** | **T** | **G** | **G** | **A** | **A** | **A** | **A** | **A** | **A** | **T** | **G** | **A** | **G** | **T** | **C** | **G** | **G** | **C** | **G** | **A** | **A** | **A** | **T** | **G** | **A** | **T** | **C** | **C** | **A** | **G** | **T** | **A** | **T** | **T** | **G** | **C** | **A** | **T** | **G** | **A** | **A** | **G** | **A** | **A** | **T** | **T** | **A** | **A** | **C** | **G** | **A** | **A** | **T** | **C** | **G** | **A** | **G** | **A** | **A** | **A** | **A** | **T** | **G** | **A** | **A** | **A** | **T** | **T** | **T** | **T** | **G** | **A** | **T** | **G** | **C** | **T** | **G** | **A** | **T** | **T** | **T** | **C** | **T** | **G** | **A** | **A** | **G** | **G** | **G** | **A** | **A** | **A** | **A** | **G** | **T** | **A** | **A** | **T** | **C** | **A** | **G** | **G** | **A** | **A** | **A** | **T** | **C** | **G** | **C** | **G** | **G** | **A** | **T** | **G** | **A** | **A** | **T** | **T** | **G** | **T** | **T** | **T** | **A** | **T** | **C** | **A** | **C** | **A** | **C** | **T** | **G** | **A** | **A** | **A** | **A** | **C** | **A** | **G** | **T** | **T** | **A** | **A** | **A** | **A** | **C** | **G** | **C** | **A** | **T** | **G** | **T** | **G** | **T** | **C** | **G** | **A** | **A** | **T** | **A** | **T** | **A** | **T** | **T** | **A** | **G** | **C** | **T** | **A** | **A** | **G** | **T** | **T** | **A** | **G** | **A** | **G** | **G** | **T** | **A** | **G** | **A** | **A** | **G** | **A** | **C** | **C** | **G** | **A** | **A** | **C** | **T** | **C** | **A** | **A** | **G** | **C** | **A** | **G** | **C** | **T** | **A** | **T** | **C** | **T** | **A** | **T** | **G** | **C** | **G** | **T** | **T** | **T** | **A** | **A** | **A** | **C** | **A** | **T** | **G** | **G** | **T** | **T** | **T** | **G** | **G** | **T** | **G** | **A** | **A** | **A** | **-** | **-** | **-** |
| **N60268F** | **G** | **T** | **G** | **A** | **T** | **C** | **A** | **A** | **A** | **G** | **T** | **A** | **A** | **T** | **G** | **T** | **T** | **A** | **G** | **T** | **G** | **G** | **A** | **T** | **G** | **A** | **C** | **C** | **A** | **T** | **G** | **A** | **A** | **A** | **T** | **G** | **G** | **T** | **C** | **C** | **G** | **T** | **T** | **T** | **A** | **G** | **G** | **C** | **G** | **T** | **T** | **T** | **C** | **A** | **T** | **C** | **A** | **T** | **A** | **T** | **T** | **T** | **A** | **T** | **C** | **T** | **A** | **T** | **T** | **C** | **A** | **A** | **G** | **A** | **G** | **G** | **A** | **T** | **A** | **T** | **A** | **G** | **A** | **A** | **G** | **T** | **C** | **G** | **T** | **A** | **G** | **G** | **C** | **G** | **A** | **A** | **G** | **C** | **A** | **G** | **A** | **A** | **A** | **A** | **C** | **G** | **G** | **T** | **A** | **A** | **G** | **A** | **T** | **T** | **G** | **G** | **C** | **T** | **A** | **T** | **G** | **A** | **A** | **A** | **A** | **A** | **G** | **C** | **A** | **T** | **T** | **G** | **G** | **A** | **A** | **C** | **T** | **A** | **C** | **G** | **T** | **C** | **C** | **A** | **G** | **A** | **T** | **G** | **T** | **T** | **A** | **T** | **T** | **T** | **T** | **G** | **A** | **T** | **G** | **G** | **A** | **T** | **T** | **T** | **G** | **G** | **T** | **A** | **A** | **T** | **G** | **G** | **A** | **A** | **G** | **A** | **A** | **A** | **T** | **G** | **G** | **A** | **C** | **G** | **G** | **C** | **A** | **T** | **T** | **G** | **A** | **T** | **T** | **C** | **A** | **A** | **C** | **A** | **A** | **A** | **A** | **G** | **C** | **G** | **A** | **T** | **C** | **T** | **T** | **G** | **A** | **A** | **A** | **G** | **A** | **T** | **T** | **G** | **G** | **C** | **C** | **A** | **G** | **A** | **A** | **G** | **C** | **C** | **A** | **A** | **G** | **A** | **T** | **T** | **A** | **T** | **T** | **A** | **T** | **T** | **G** | **T** | **G** | **A** | **C** | **G** | **A** | **G** | **T** | **T** | **T** | **T** | **A** | **T** | **T** | **G** | **A** | **T** | **G** | **A** | **T** | **G** | **A** | **A** | **A** | **A** | **A** | **G** | **T** | **G** | **T** | **A** | **T** | **C** | **C** | **G** | **G** | **C** | **G** | **A** | **T** | **T** | **G** | **A** | **A** | **G** | **C** | **T** | **G** | **G** | **T** | **G** | **C** | **A** | **G** | **C** | **G** | **G** | **G** | **C** | **T** | **A** | **C** | **C** | **T** | **A** | **T** | **T** | **A** | **A** | **A** | **A** | **A** | **C** | **A** | **T** | **C** | **A** | **A** | **C** | **A** | **G** | **C** | **A** | **C** | **A** | **T** | **G** | **A** | **G** | **A** | **T** | **T** | **G** | **C** | **T** | **G** | **A** | **T** | **G** | **C** | **A** | **A** | **T** | **T** | **C** | **G** | **G** | **G** | **C** | **G** | **A** | **C** | **T** | **T** | **A** | **T** | **C** | **G** | **C** | **G** | **G** | **A** | **G** | **A** | **G** | **C** | **G** | **T** | **G** | **T** | **G** | **T** | **T** | **G** | **G** | **A** | **A** | **C** | **C** | **T** | **G** | **A** | **A** | **G** | **T** | **G** | **A** | **C** | **G** | **C** | **A** | **T** | **A** | **A** | **G** | **A** | **T** | **G** | **A** | **T** | **G** | **G** | **A** | **A** | **C** | **G** | **G** | **T** | **T** | **A** | **A** | **C** | **A** | **A** | **A** | **A** | **A** | **A** | **A** | **C** | **A** | **A** | **G** | **A** | **G** | **C** | **C** | **G** | **G** | **T** | **G** | **T** | **T** | **G** | **C** | **A** | **C** | **G** | **A** | **A** | **G** | **A** | **T** | **T** | **T** | **G** | **A** | **C** | **A** | **A** | **A** | **C** | **C** | **G** | **A** | **G** | **A** | **A** | **C** | **A** | **C** | **G** | **A** | **A** | **A** | **T** | **T** | **T** | **T** | **A** | **A** | **T** | **G** | **T** | **T** | **G** | **A** | **T** | **T** | **G** | **C** | **A** | **C** | **A** | **A** | **G** | **G** | **T** | **A** | **A** | **A** | **A** | **G** | **T** | **A** | **A** | **T** | **C** | **A** | **G** | **G** | **A** | **A** | **A** | **T** | **T** | **G** | **C** | **T** | **G** | **A** | **T** | **G** | **A** | **A** | **C** | **T** | **C** | **T** | **T** | **T** | **A** | **T** | **C** | **A** | **C** | **T** | **T** | **T** | **G** | **A** | **A** | **A** | **A** | **C** | **A** | **G** | **T** | **T** | **A** | **A** | **A** | **A** | **C** | **A** | **C** | **A** | **T** | **G** | **T** | **T** | **T** | **C** | **A** | **A** | **A** | **C** | **A** | **T** | **T** | **T** | **T** | **A** | **G** | **C** | **A** | **A** | **A** | **A** | **C** | **T** | **A** | **G** | **A** | **T** | **G** | **T** | **G** | **G** | **A** | **T** | **G** | **A** | **T** | **C** | **G** | **G** | **A** | **C** | **C** | **C** | **A** | **A** | **G** | **C** | **G** | **G** | **C** | **G** | **A** | **T** | **T** | **T** | **A** | **T** | **G** | **C** | **T** | **T** | **T** | **T** | **C** | **A** | **A** | **C** | **A** | **T** | **G** | **G** | **T** | **T** | **T** | **A** | **G** | **C** | **C** | **A** | **A** | **A** | **-** | **-** | **-** |
| **N53766** | **G** | **T** | **G** | **A** | **T** | **C** | **A** | **A** | **A** | **G** | **T** | **A** | **A** | **T** | **G** | **T** | **T** | **A** | **G** | **T** | **G** | **G** | **A** | **T** | **G** | **A** | **C** | **C** | **A** | **T** | **G** | **A** | **A** | **A** | **T** | **G** | **G** | **T** | **C** | **C** | **G** | **T** | **T** | **T** | **A** | **G** | **G** | **C** | **G** | **T** | **T** | **T** | **C** | **A** | **T** | **C** | **A** | **T** | **A** | **T** | **T** | **T** | **A** | **T** | **C** | **T** | **A** | **T** | **T** | **C** | **A** | **A** | **G** | **A** | **G** | **G** | **A** | **T** | **A** | **T** | **A** | **G** | **A** | **A** | **G** | **T** | **C** | **G** | **T** | **A** | **G** | **G** | **C** | **G** | **A** | **A** | **G** | **C** | **A** | **G** | **A** | **A** | **A** | **A** | **T** | **G** | **G** | **T** | **A** | **A** | **G** | **A** | **T** | **T** | **G** | **G** | **C** | **T** | **A** | **T** | **G** | **A** | **A** | **A** | **A** | **A** | **G** | **C** | **A** | **T** | **T** | **G** | **G** | **A** | **A** | **C** | **T** | **A** | **C** | **G** | **T** | **C** | **C** | **A** | **G** | **A** | **T** | **G** | **T** | **T** | **A** | **T** | **T** | **T** | **T** | **G** | **A** | **T** | **G** | **G** | **A** | **T** | **T** | **T** | **G** | **G** | **T** | **A** | **A** | **T** | **G** | **G** | **A** | **A** | **G** | **A** | **A** | **A** | **T** | **G** | **G** | **A** | **C** | **G** | **G** | **C** | **A** | **T** | **T** | **G** | **A** | **T** | **T** | **C** | **A** | **A** | **C** | **A** | **A** | **A** | **A** | **G** | **C** | **G** | **A** | **T** | **C** | **T** | **T** | **G** | **A** | **A** | **A** | **G** | **A** | **T** | **T** | **G** | **G** | **C** | **C** | **A** | **G** | **A** | **A** | **G** | **C** | **C** | **A** | **A** | **G** | **A** | **T** | **T** | **A** | **T** | **T** | **A** | **T** | **T** | **G** | **T** | **G** | **A** | **C** | **G** | **A** | **G** | **T** | **T** | **T** | **T** | **A** | **T** | **T** | **G** | **A** | **T** | **G** | **A** | **T** | **G** | **A** | **A** | **A** | **A** | **A** | **G** | **T** | **G** | **T** | **A** | **T** | **C** | **C** | **G** | **G** | **C** | **G** | **A** | **T** | **T** | **G** | **A** | **A** | **G** | **C** | **T** | **G** | **G** | **T** | **G** | **C** | **A** | **G** | **C** | **G** | **G** | **G** | **C** | **T** | **A** | **C** | **C** | **T** | **A** | **T** | **T** | **A** | **A** | **A** | **A** | **A** | **C** | **A** | **T** | **C** | **A** | **A** | **C** | **A** | **G** | **C** | **A** | **C** | **A** | **T** | **G** | **A** | **G** | **A** | **T** | **T** | **G** | **C** | **T** | **G** | **A** | **T** | **G** | **C** | **A** | **A** | **T** | **T** | **C** | **G** | **G** | **G** | **C** | **G** | **A** | **C** | **T** | **T** | **A** | **T** | **C** | **G** | **C** | **G** | **G** | **A** | **G** | **A** | **G** | **C** | **G** | **T** | **G** | **T** | **G** | **T** | **T** | **G** | **G** | **A** | **A** | **C** | **C** | **T** | **G** | **A** | **A** | **G** | **T** | **G** | **A** | **C** | **G** | **C** | **A** | **T** | **A** | **A** | **G** | **A** | **T** | **G** | **A** | **T** | **G** | **G** | **A** | **A** | **C** | **G** | **G** | **T** | **T** | **A** | **A** | **C** | **A** | **A** | **A** | **A** | **A** | **A** | **A** | **C** | **A** | **A** | **G** | **A** | **G** | **C** | **C** | **G** | **G** | **T** | **G** | **T** | **T** | **G** | **C** | **A** | **C** | **G** | **A** | **A** | **G** | **A** | **T** | **T** | **T** | **G** | **A** | **C** | **A** | **A** | **A** | **C** | **C** | **G** | **G** | **G** | **A** | **A** | **C** | **A** | **C** | **G** | **A** | **A** | **A** | **T** | **T** | **T** | **T** | **A** | **A** | **T** | **G** | **T** | **T** | **G** | **A** | **T** | **T** | **G** | **C** | **A** | **C** | **A** | **A** | **G** | **G** | **T** | **A** | **A** | **A** | **A** | **G** | **T** | **A** | **A** | **T** | **C** | **A** | **G** | **G** | **A** | **A** | **A** | **T** | **A** | **G** | **C** | **T** | **G** | **A** | **T** | **G** | **A** | **A** | **C** | **T** | **C** | **T** | **T** | **T** | **A** | **T** | **C** | **A** | **C** | **T** | **T** | **T** | **G** | **A** | **A** | **A** | **A** | **C** | **A** | **G** | **T** | **T** | **A** | **A** | **A** | **A** | **C** | **A** | **C** | **A** | **T** | **G** | **T** | **T** | **T** | **C** | **A** | **A** | **A** | **C** | **A** | **T** | **T** | **T** | **T** | **A** | **G** | **C** | **A** | **A** | **A** | **A** | **C** | **T** | **A** | **G** | **A** | **T** | **G** | **T** | **G** | **G** | **A** | **T** | **G** | **A** | **T** | **C** | **G** | **G** | **A** | **C** | **C** | **C** | **A** | **A** | **G** | **C** | **G** | **G** | **C** | **G** | **A** | **T** | **T** | **T** | **A** | **T** | **G** | **C** | **T** | **T** | **T** | **T** | **C** | **A** | **A** | **C** | **A** | **T** | **G** | **G** | **T** | **T** | **T** | **A** | **G** | **C** | **C** | **A** | **A** | **A** | **-** | **-** | **-** |
| **N52629** | **A** | **T** | **G** | **A** | **T** | **A** | **A** | **A** | **A** | **G** | **T** | **T** | **T** | **T** | **A** | **T** | **T** | **A** | **G** | **T** | **A** | **G** | **A** | **T** | **G** | **A** | **C** | **C** | **A** | **T** | **G** | **A** | **A** | **A** | **T** | **G** | **G** | **T** | **A** | **C** | **G** | **C** | **T** | **T** | **A** | **G** | **G** | **C** | **G** | **T** | **C** | **T** | **C** | **T** | **T** | **C** | **T** | **T** | **A** | **T** | **T** | **T** | **A** | **T** | **C** | **T** | **A** | **T** | **C** | **C** | **A** | **A** | **G** | **A** | **A** | **G** | **A** | **C** | **A** | **T** | **T** | **G** | **A** | **A** | **G** | **T** | **G** | **A** | **T** | **T** | **G** | **G** | **A** | **G** | **A** | **A** | **G** | **C** | **T** | **G** | **A** | **A** | **A** | **A** | **T** | **G** | **G** | **A** | **C** | **G** | **T** | **C** | **A** | **A** | **G** | **G** | **C** | **T** | **A** | **T** | **G** | **A** | **A** | **A** | **A** | **G** | **G** | **C** | **G** | **A** | **T** | **G** | **G** | **C** | **A** | **C** | **T** | **T** | **C** | **G** | **A** | **C** | **C** | **A** | **G** | **A** | **T** | **G** | **T** | **C** | **A** | **T** | **T** | **T** | **T** | **A** | **A** | **T** | **G** | **G** | **A** | **T** | **C** | **T** | **A** | **G** | **T** | **C** | **A** | **T** | **G** | **G** | **A** | **A** | **G** | **A** | **A** | **A** | **T** | **G** | **G** | **A** | **T** | **G** | **G** | **C** | **A** | **T** | **C** | **G** | **A** | **A** | **T** | **C** | **A** | **A** | **C** | **A** | **A** | **A** | **A** | **G** | **C** | **A** | **A** | **T** | **C** | **T** | **T** | **A** | **A** | **A** | **A** | **G** | **A** | **T** | **T** | **G** | **G** | **C** | **C** | **A** | **A** | **A** | **A** | **G** | **C** | **C** | **A** | **A** | **A** | **A** | **T** | **C** | **A** | **T** | **T** | **A** | **T** | **C** | **G** | **T** | **C** | **A** | **C** | **A** | **A** | **G** | **T** | **T** | **T** | **T** | **A** | **T** | **T** | **G** | **A** | **T** | **G** | **A** | **T** | **G** | **A** | **A** | **A** | **A** | **A** | **G** | **T** | **T** | **T** | **A** | **T** | **C** | **C** | **T** | **G** | **C** | **A** | **A** | **T** | **T** | **G** | **A** | **A** | **G** | **C** | **A** | **G** | **G** | **A** | **G** | **C** | **A** | **G** | **C** | **A** | **G** | **G** | **A** | **T** | **A** | **T** | **T** | **T** | **G** | **T** | **T** | **G** | **A** | **A** | **G** | **A** | **C** | **G** | **T** | **C** | **T** | **A** | **C** | **C** | **G** | **C** | **A** | **C** | **A** | **T** | **G** | **A** | **A** | **A** | **T** | **A** | **G** | **C** | **T** | **G** | **A** | **T** | **G** | **C** | **T** | **A** | **T** | **A** | **A** | **G** | **G** | **G** | **C** | **T** | **A** | **C** | **G** | **C** | **A** | **A** | **C** | **G** | **C** | **G** | **G** | **C** | **G** | **A** | **G** | **C** | **G** | **A** | **G** | **T** | **A** | **T** | **T** | **G** | **G** | **A** | **A** | **C** | **C** | **T** | **G** | **A** | **A** | **G** | **T** | **G** | **A** | **C** | **A** | **A** | **C** | **A** | **A** | **A** | **A** | **A** | **T** | **G** | **A** | **T** | **G** | **G** | **A** | **A** | **A** | **A** | **A** | **A** | **T** | **G** | **A** | **G** | **T** | **C** | **G** | **G** | **C** | **G** | **A** | **A** | **A** | **T** | **G** | **A** | **T** | **C** | **C** | **A** | **G** | **T** | **A** | **T** | **T** | **G** | **C** | **A** | **T** | **G** | **A** | **A** | **G** | **A** | **A** | **T** | **T** | **A** | **A** | **C** | **G** | **A** | **A** | **T** | **C** | **G** | **A** | **G** | **A** | **A** | **A** | **A** | **T** | **G** | **A** | **A** | **A** | **T** | **T** | **T** | **T** | **G** | **A** | **T** | **G** | **C** | **T** | **G** | **A** | **T** | **T** | **T** | **C** | **T** | **G** | **A** | **A** | **G** | **G** | **G** | **A** | **A** | **A** | **A** | **G** | **T** | **A** | **A** | **T** | **C** | **A** | **G** | **G** | **A** | **A** | **A** | **T** | **C** | **G** | **C** | **G** | **G** | **A** | **T** | **G** | **A** | **A** | **T** | **T** | **G** | **T** | **T** | **T** | **A** | **T** | **C** | **A** | **C** | **A** | **C** | **T** | **G** | **A** | **A** | **A** | **A** | **C** | **A** | **G** | **T** | **T** | **A** | **A** | **A** | **A** | **C** | **G** | **C** | **A** | **T** | **G** | **T** | **G** | **T** | **C** | **G** | **A** | **A** | **T** | **A** | **T** | **A** | **T** | **T** | **A** | **G** | **C** | **T** | **A** | **A** | **G** | **T** | **T** | **A** | **G** | **A** | **G** | **G** | **T** | **A** | **G** | **A** | **A** | **G** | **A** | **C** | **C** | **G** | **A** | **A** | **C** | **T** | **C** | **A** | **A** | **G** | **C** | **A** | **G** | **C** | **T** | **A** | **T** | **C** | **T** | **A** | **T** | **G** | **C** | **G** | **T** | **T** | **T** | **A** | **A** | **A** | **C** | **A** | **T** | **G** | **G** | **T** | **T** | **T** | **G** | **G** | **T** | **G** | **A** | **A** | **A** | **-** | **-** | **-** |
| **N54118** | **G** | **T** | **G** | **A** | **T** | **C** | **A** | **A** | **A** | **G** | **T** | **A** | **A** | **T** | **G** | **T** | **T** | **A** | **G** | **T** | **G** | **G** | **A** | **T** | **G** | **A** | **C** | **C** | **A** | **T** | **G** | **A** | **A** | **A** | **T** | **G** | **G** | **T** | **C** | **C** | **G** | **T** | **T** | **T** | **A** | **G** | **G** | **C** | **G** | **T** | **T** | **T** | **C** | **A** | **T** | **C** | **A** | **T** | **A** | **T** | **T** | **T** | **A** | **T** | **C** | **T** | **A** | **T** | **T** | **C** | **A** | **A** | **G** | **A** | **G** | **G** | **A** | **T** | **A** | **T** | **A** | **G** | **A** | **A** | **G** | **T** | **C** | **G** | **T** | **A** | **G** | **G** | **C** | **G** | **A** | **A** | **G** | **C** | **A** | **G** | **A** | **A** | **A** | **A** | **T** | **G** | **G** | **T** | **A** | **A** | **G** | **A** | **T** | **T** | **G** | **G** | **C** | **T** | **A** | **T** | **G** | **A** | **A** | **A** | **A** | **A** | **G** | **C** | **A** | **T** | **T** | **G** | **G** | **A** | **A** | **C** | **T** | **A** | **C** | **G** | **T** | **C** | **C** | **A** | **G** | **A** | **T** | **G** | **T** | **T** | **A** | **T** | **T** | **T** | **T** | **G** | **A** | **T** | **G** | **G** | **A** | **T** | **T** | **T** | **G** | **G** | **T** | **A** | **A** | **T** | **G** | **G** | **A** | **A** | **G** | **A** | **A** | **A** | **T** | **G** | **G** | **A** | **C** | **G** | **G** | **C** | **A** | **T** | **T** | **G** | **A** | **T** | **T** | **C** | **A** | **A** | **C** | **A** | **A** | **A** | **A** | **G** | **C** | **G** | **A** | **T** | **C** | **T** | **T** | **G** | **A** | **A** | **A** | **G** | **A** | **T** | **T** | **G** | **G** | **C** | **C** | **A** | **G** | **A** | **A** | **G** | **C** | **C** | **A** | **A** | **G** | **A** | **T** | **T** | **A** | **T** | **T** | **A** | **T** | **T** | **G** | **T** | **G** | **A** | **C** | **G** | **A** | **G** | **T** | **T** | **T** | **T** | **A** | **T** | **T** | **G** | **A** | **T** | **G** | **A** | **T** | **G** | **A** | **A** | **A** | **A** | **A** | **G** | **T** | **G** | **T** | **A** | **T** | **C** | **C** | **G** | **G** | **C** | **G** | **A** | **T** | **T** | **G** | **A** | **A** | **G** | **C** | **T** | **G** | **G** | **T** | **G** | **C** | **A** | **G** | **C** | **G** | **G** | **G** | **C** | **T** | **A** | **C** | **C** | **T** | **A** | **T** | **T** | **A** | **A** | **A** | **A** | **A** | **C** | **A** | **T** | **C** | **A** | **A** | **C** | **A** | **G** | **C** | **A** | **C** | **A** | **T** | **G** | **A** | **G** | **A** | **T** | **T** | **G** | **C** | **T** | **G** | **A** | **T** | **G** | **C** | **A** | **A** | **T** | **T** | **C** | **G** | **G** | **G** | **C** | **G** | **A** | **C** | **T** | **T** | **A** | **T** | **C** | **G** | **C** | **G** | **G** | **A** | **G** | **A** | **G** | **C** | **G** | **T** | **G** | **T** | **G** | **T** | **T** | **G** | **G** | **A** | **A** | **C** | **C** | **T** | **G** | **A** | **A** | **G** | **T** | **G** | **A** | **C** | **G** | **C** | **A** | **T** | **A** | **A** | **G** | **A** | **T** | **G** | **A** | **T** | **G** | **G** | **A** | **A** | **C** | **G** | **G** | **T** | **T** | **A** | **A** | **C** | **A** | **A** | **A** | **A** | **A** | **A** | **A** | **C** | **A** | **A** | **G** | **A** | **G** | **C** | **C** | **G** | **G** | **T** | **G** | **T** | **T** | **G** | **C** | **A** | **T** | **G** | **A** | **A** | **G** | **A** | **T** | **T** | **T** | **G** | **A** | **C** | **A** | **A** | **A** | **T** | **C** | **G** | **A** | **G** | **A** | **A** | **C** | **A** | **C** | **G** | **A** | **A** | **A** | **T** | **T** | **T** | **T** | **A** | **A** | **T** | **G** | **T** | **T** | **G** | **A** | **T** | **T** | **G** | **C** | **A** | **C** | **A** | **A** | **G** | **G** | **T** | **A** | **A** | **A** | **A** | **G** | **T** | **A** | **A** | **T** | **C** | **A** | **G** | **G** | **A** | **A** | **A** | **T** | **A** | **G** | **C** | **T** | **G** | **A** | **T** | **G** | **A** | **A** | **C** | **T** | **C** | **T** | **T** | **T** | **A** | **T** | **C** | **A** | **C** | **T** | **T** | **T** | **G** | **A** | **A** | **A** | **A** | **C** | **A** | **G** | **T** | **T** | **A** | **A** | **A** | **A** | **C** | **A** | **C** | **A** | **T** | **G** | **T** | **T** | **T** | **C** | **A** | **A** | **A** | **C** | **A** | **T** | **T** | **T** | **T** | **A** | **G** | **C** | **A** | **A** | **A** | **A** | **C** | **T** | **A** | **G** | **A** | **T** | **G** | **T** | **G** | **G** | **A** | **T** | **G** | **A** | **T** | **C** | **G** | **G** | **A** | **C** | **C** | **C** | **A** | **A** | **G** | **C** | **G** | **G** | **C** | **G** | **A** | **T** | **T** | **T** | **A** | **T** | **G** | **C** | **T** | **T** | **T** | **T** | **C** | **A** | **A** | **C** | **A** | **T** | **G** | **G** | **T** | **T** | **T** | **A** | **G** | **C** | **C** | **A** | **A** | **A** | **-** | **-** | **-** |
| **N53405** | **G** | **T** | **G** | **A** | **T** | **C** | **A** | **A** | **A** | **G** | **T** | **A** | **A** | **T** | **G** | **T** | **T** | **A** | **G** | **T** | **G** | **G** | **A** | **T** | **G** | **A** | **C** | **C** | **A** | **T** | **G** | **A** | **A** | **A** | **T** | **G** | **G** | **T** | **C** | **C** | **G** | **T** | **T** | **T** | **A** | **G** | **G** | **C** | **G** | **T** | **T** | **T** | **C** | **A** | **T** | **C** | **A** | **T** | **A** | **T** | **T** | **T** | **A** | **T** | **C** | **T** | **A** | **T** | **T** | **C** | **A** | **A** | **G** | **A** | **G** | **G** | **A** | **T** | **A** | **T** | **A** | **G** | **A** | **A** | **G** | **T** | **C** | **G** | **T** | **A** | **G** | **G** | **C** | **G** | **A** | **A** | **G** | **C** | **A** | **G** | **A** | **A** | **A** | **A** | **C** | **G** | **G** | **T** | **A** | **A** | **G** | **A** | **T** | **T** | **G** | **G** | **C** | **T** | **A** | **T** | **G** | **A** | **A** | **A** | **A** | **A** | **G** | **C** | **A** | **T** | **T** | **G** | **G** | **A** | **A** | **C** | **T** | **A** | **C** | **G** | **T** | **C** | **C** | **A** | **G** | **A** | **T** | **G** | **T** | **T** | **A** | **T** | **T** | **T** | **T** | **G** | **A** | **T** | **G** | **G** | **A** | **T** | **T** | **T** | **G** | **G** | **T** | **A** | **A** | **T** | **G** | **G** | **A** | **A** | **G** | **A** | **A** | **A** | **T** | **G** | **G** | **A** | **C** | **G** | **G** | **C** | **A** | **T** | **T** | **G** | **A** | **T** | **T** | **C** | **A** | **A** | **C** | **C** | **A** | **A** | **A** | **G** | **C** | **G** | **A** | **T** | **C** | **T** | **T** | **G** | **A** | **A** | **A** | **G** | **A** | **T** | **T** | **G** | **G** | **C** | **C** | **A** | **G** | **A** | **A** | **G** | **C** | **C** | **A** | **A** | **G** | **A** | **T** | **T** | **A** | **T** | **T** | **A** | **T** | **T** | **G** | **T** | **G** | **A** | **C** | **G** | **A** | **G** | **T** | **T** | **T** | **T** | **A** | **T** | **T** | **G** | **A** | **T** | **G** | **A** | **T** | **G** | **A** | **A** | **A** | **A** | **A** | **G** | **T** | **G** | **T** | **A** | **T** | **C** | **C** | **G** | **G** | **C** | **G** | **A** | **T** | **T** | **G** | **A** | **A** | **G** | **C** | **T** | **G** | **G** | **T** | **G** | **C** | **A** | **G** | **C** | **G** | **G** | **G** | **C** | **T** | **A** | **C** | **C** | **T** | **A** | **T** | **T** | **A** | **A** | **A** | **A** | **A** | **C** | **A** | **T** | **C** | **A** | **A** | **C** | **A** | **G** | **C** | **A** | **C** | **A** | **T** | **G** | **A** | **G** | **A** | **T** | **T** | **G** | **C** | **T** | **G** | **A** | **T** | **G** | **C** | **A** | **A** | **T** | **T** | **C** | **G** | **G** | **G** | **C** | **G** | **A** | **C** | **T** | **T** | **A** | **T** | **C** | **G** | **C** | **G** | **G** | **A** | **G** | **A** | **G** | **C** | **G** | **T** | **G** | **T** | **G** | **T** | **T** | **G** | **G** | **A** | **A** | **C** | **C** | **T** | **G** | **A** | **A** | **G** | **T** | **G** | **A** | **C** | **G** | **C** | **A** | **T** | **A** | **A** | **G** | **A** | **T** | **G** | **A** | **T** | **G** | **G** | **A** | **A** | **C** | **G** | **G** | **T** | **T** | **A** | **A** | **C** | **A** | **A** | **A** | **A** | **A** | **A** | **A** | **C** | **A** | **A** | **G** | **A** | **G** | **C** | **C** | **G** | **G** | **T** | **G** | **T** | **T** | **G** | **C** | **A** | **C** | **G** | **A** | **A** | **G** | **A** | **T** | **T** | **T** | **G** | **A** | **C** | **A** | **A** | **A** | **C** | **C** | **G** | **G** | **G** | **A** | **A** | **C** | **A** | **C** | **G** | **A** | **A** | **A** | **T** | **T** | **T** | **T** | **A** | **A** | **T** | **G** | **T** | **T** | **G** | **A** | **T** | **T** | **G** | **C** | **A** | **C** | **A** | **A** | **G** | **G** | **T** | **A** | **A** | **A** | **A** | **G** | **T** | **A** | **A** | **T** | **C** | **A** | **G** | **G** | **A** | **A** | **A** | **T** | **A** | **G** | **C** | **T** | **G** | **A** | **T** | **G** | **A** | **A** | **C** | **T** | **C** | **T** | **T** | **T** | **A** | **T** | **C** | **A** | **C** | **T** | **T** | **T** | **G** | **A** | **A** | **A** | **A** | **C** | **A** | **G** | **T** | **T** | **A** | **A** | **A** | **A** | **C** | **A** | **C** | **A** | **T** | **G** | **T** | **T** | **T** | **C** | **A** | **A** | **A** | **C** | **A** | **T** | **T** | **T** | **T** | **A** | **G** | **C** | **A** | **A** | **A** | **A** | **C** | **T** | **A** | **G** | **A** | **T** | **G** | **T** | **G** | **G** | **A** | **T** | **G** | **A** | **T** | **C** | **G** | **G** | **A** | **C** | **C** | **C** | **A** | **A** | **G** | **C** | **G** | **G** | **C** | **G** | **A** | **T** | **T** | **T** | **A** | **T** | **G** | **C** | **T** | **T** | **T** | **T** | **C** | **A** | **A** | **C** | **A** | **T** | **G** | **G** | **T** | **T** | **T** | **A** | **G** | **C** | **C** | **A** | **A** | **A** | **-** | **-** | **-** |
| **N60252F** | **A** | **T** | **G** | **A** | **T** | **A** | **A** | **A** | **A** | **G** | **T** | **T** | **T** | **T** | **A** | **T** | **T** | **A** | **G** | **T** | **A** | **G** | **A** | **T** | **G** | **A** | **C** | **C** | **A** | **T** | **G** | **A** | **A** | **A** | **T** | **G** | **G** | **T** | **A** | **C** | **G** | **C** | **T** | **T** | **A** | **G** | **G** | **C** | **G** | **T** | **C** | **T** | **C** | **T** | **T** | **C** | **T** | **T** | **A** | **T** | **T** | **T** | **A** | **T** | **C** | **T** | **A** | **T** | **C** | **C** | **A** | **A** | **G** | **A** | **A** | **G** | **A** | **C** | **A** | **T** | **T** | **G** | **A** | **A** | **G** | **T** | **G** | **A** | **T** | **T** | **G** | **G** | **A** | **G** | **A** | **A** | **G** | **C** | **T** | **G** | **A** | **A** | **A** | **A** | **T** | **G** | **G** | **A** | **C** | **G** | **T** | **C** | **A** | **A** | **G** | **G** | **C** | **T** | **A** | **T** | **G** | **A** | **A** | **A** | **A** | **G** | **G** | **C** | **G** | **A** | **T** | **G** | **G** | **C** | **A** | **C** | **T** | **T** | **C** | **G** | **A** | **C** | **C** | **A** | **G** | **A** | **T** | **G** | **T** | **C** | **A** | **T** | **T** | **T** | **T** | **A** | **A** | **T** | **G** | **G** | **A** | **T** | **C** | **T** | **A** | **G** | **T** | **C** | **A** | **T** | **G** | **G** | **A** | **A** | **G** | **A** | **A** | **A** | **T** | **G** | **G** | **A** | **T** | **G** | **G** | **C** | **A** | **T** | **C** | **G** | **A** | **A** | **T** | **C** | **A** | **A** | **C** | **A** | **A** | **A** | **A** | **G** | **C** | **A** | **A** | **T** | **C** | **T** | **T** | **A** | **A** | **A** | **A** | **G** | **A** | **T** | **T** | **G** | **G** | **C** | **C** | **A** | **A** | **A** | **A** | **G** | **C** | **C** | **A** | **A** | **A** | **A** | **T** | **C** | **A** | **T** | **T** | **A** | **T** | **C** | **G** | **T** | **C** | **A** | **C** | **A** | **A** | **G** | **T** | **T** | **T** | **T** | **A** | **T** | **T** | **G** | **A** | **T** | **G** | **A** | **T** | **G** | **A** | **A** | **A** | **A** | **A** | **G** | **T** | **T** | **T** | **A** | **T** | **C** | **C** | **T** | **G** | **C** | **A** | **A** | **T** | **T** | **G** | **A** | **A** | **G** | **C** | **A** | **G** | **G** | **A** | **G** | **C** | **A** | **G** | **C** | **A** | **G** | **G** | **A** | **T** | **A** | **T** | **T** | **T** | **A** | **T** | **T** | **G** | **A** | **A** | **G** | **A** | **C** | **G** | **T** | **C** | **T** | **A** | **C** | **C** | **G** | **C** | **A** | **C** | **A** | **T** | **G** | **A** | **A** | **A** | **T** | **A** | **G** | **C** | **T** | **G** | **A** | **T** | **G** | **C** | **T** | **A** | **T** | **A** | **A** | **G** | **G** | **G** | **C** | **T** | **A** | **C** | **G** | **C** | **A** | **A** | **C** | **G** | **C** | **G** | **G** | **C** | **G** | **A** | **G** | **C** | **G** | **A** | **G** | **T** | **A** | **T** | **T** | **G** | **G** | **A** | **A** | **C** | **C** | **T** | **G** | **A** | **A** | **G** | **T** | **G** | **A** | **C** | **A** | **A** | **C** | **A** | **A** | **A** | **A** | **A** | **T** | **G** | **A** | **T** | **G** | **G** | **A** | **A** | **A** | **A** | **A** | **A** | **T** | **G** | **A** | **G** | **T** | **C** | **G** | **G** | **C** | **G** | **A** | **A** | **A** | **T** | **G** | **A** | **T** | **C** | **C** | **A** | **G** | **T** | **A** | **T** | **T** | **G** | **C** | **A** | **T** | **G** | **A** | **A** | **G** | **A** | **A** | **T** | **T** | **A** | **A** | **C** | **G** | **A** | **A** | **T** | **C** | **G** | **A** | **G** | **A** | **A** | **A** | **A** | **T** | **G** | **A** | **A** | **A** | **T** | **T** | **T** | **T** | **G** | **A** | **T** | **G** | **C** | **T** | **G** | **A** | **T** | **T** | **T** | **C** | **T** | **G** | **A** | **A** | **G** | **G** | **G** | **A** | **A** | **A** | **A** | **G** | **T** | **A** | **A** | **T** | **C** | **A** | **G** | **G** | **A** | **A** | **A** | **T** | **C** | **G** | **C** | **G** | **G** | **A** | **T** | **G** | **A** | **A** | **T** | **T** | **G** | **T** | **T** | **T** | **A** | **T** | **C** | **A** | **C** | **A** | **C** | **T** | **G** | **A** | **A** | **A** | **A** | **C** | **A** | **G** | **T** | **T** | **A** | **A** | **A** | **A** | **C** | **G** | **C** | **A** | **T** | **G** | **T** | **G** | **T** | **C** | **G** | **A** | **A** | **T** | **A** | **T** | **A** | **T** | **T** | **A** | **G** | **C** | **T** | **A** | **A** | **G** | **T** | **T** | **A** | **G** | **A** | **G** | **G** | **T** | **A** | **G** | **A** | **A** | **G** | **A** | **C** | **C** | **G** | **A** | **A** | **C** | **T** | **C** | **A** | **A** | **G** | **C** | **A** | **G** | **C** | **T** | **A** | **T** | **C** | **T** | **A** | **T** | **G** | **C** | **G** | **T** | **T** | **T** | **A** | **A** | **A** | **C** | **A** | **T** | **G** | **G** | **T** | **T** | **T** | **G** | **G** | **T** | **G** | **A** | **A** | **A** | **-** | **-** | **-** |
| **N60425F** | **A** | **T** | **G** | **A** | **T** | **A** | **A** | **A** | **A** | **G** | **T** | **T** | **T** | **T** | **A** | **T** | **T** | **A** | **G** | **T** | **A** | **G** | **A** | **T** | **G** | **A** | **C** | **C** | **A** | **T** | **G** | **A** | **A** | **A** | **T** | **G** | **G** | **T** | **A** | **C** | **G** | **C** | **T** | **T** | **A** | **G** | **G** | **C** | **G** | **T** | **C** | **T** | **C** | **T** | **T** | **C** | **T** | **T** | **A** | **T** | **T** | **T** | **A** | **T** | **C** | **T** | **A** | **T** | **C** | **C** | **A** | **A** | **G** | **A** | **A** | **G** | **A** | **C** | **A** | **T** | **T** | **G** | **A** | **A** | **G** | **T** | **G** | **A** | **T** | **T** | **G** | **G** | **A** | **G** | **A** | **A** | **G** | **C** | **T** | **G** | **A** | **A** | **A** | **A** | **T** | **G** | **G** | **A** | **C** | **G** | **T** | **C** | **A** | **A** | **G** | **G** | **C** | **T** | **A** | **T** | **G** | **A** | **A** | **A** | **A** | **G** | **G** | **C** | **G** | **A** | **T** | **G** | **G** | **C** | **A** | **C** | **T** | **T** | **C** | **G** | **A** | **C** | **C** | **A** | **G** | **A** | **T** | **G** | **T** | **C** | **A** | **T** | **T** | **T** | **T** | **A** | **A** | **T** | **G** | **G** | **A** | **T** | **C** | **T** | **A** | **G** | **T** | **C** | **A** | **T** | **G** | **G** | **A** | **A** | **G** | **A** | **A** | **A** | **T** | **G** | **G** | **A** | **T** | **G** | **G** | **C** | **A** | **T** | **C** | **G** | **A** | **A** | **T** | **C** | **A** | **A** | **C** | **A** | **A** | **A** | **A** | **G** | **C** | **A** | **A** | **T** | **C** | **T** | **T** | **A** | **A** | **A** | **A** | **G** | **A** | **T** | **T** | **G** | **G** | **C** | **C** | **A** | **A** | **A** | **A** | **G** | **C** | **C** | **A** | **A** | **A** | **A** | **T** | **C** | **A** | **T** | **T** | **A** | **T** | **C** | **G** | **T** | **C** | **A** | **C** | **A** | **A** | **G** | **T** | **T** | **T** | **T** | **A** | **T** | **T** | **G** | **A** | **T** | **G** | **A** | **T** | **G** | **A** | **A** | **A** | **A** | **A** | **G** | **T** | **T** | **T** | **A** | **T** | **C** | **C** | **T** | **G** | **C** | **A** | **A** | **T** | **T** | **G** | **A** | **A** | **G** | **C** | **A** | **G** | **G** | **A** | **G** | **C** | **A** | **G** | **C** | **A** | **G** | **G** | **A** | **T** | **A** | **T** | **T** | **T** | **G** | **T** | **T** | **G** | **A** | **A** | **G** | **A** | **C** | **G** | **T** | **C** | **T** | **A** | **C** | **C** | **G** | **C** | **A** | **C** | **A** | **T** | **G** | **A** | **A** | **A** | **T** | **A** | **G** | **C** | **T** | **G** | **A** | **T** | **G** | **C** | **T** | **A** | **T** | **A** | **A** | **G** | **G** | **G** | **C** | **T** | **A** | **C** | **G** | **C** | **A** | **A** | **C** | **G** | **C** | **G** | **G** | **C** | **G** | **A** | **G** | **C** | **G** | **A** | **G** | **T** | **A** | **T** | **T** | **G** | **G** | **A** | **A** | **C** | **C** | **T** | **G** | **A** | **A** | **G** | **T** | **G** | **A** | **C** | **A** | **A** | **C** | **A** | **A** | **A** | **A** | **A** | **T** | **G** | **A** | **T** | **G** | **G** | **A** | **A** | **A** | **A** | **A** | **A** | **T** | **G** | **A** | **G** | **T** | **C** | **G** | **G** | **C** | **G** | **A** | **A** | **A** | **T** | **G** | **A** | **T** | **C** | **C** | **A** | **G** | **T** | **A** | **T** | **T** | **G** | **C** | **A** | **T** | **G** | **A** | **A** | **G** | **A** | **A** | **T** | **T** | **A** | **A** | **C** | **G** | **A** | **A** | **T** | **C** | **G** | **A** | **G** | **A** | **A** | **A** | **A** | **T** | **G** | **A** | **A** | **A** | **T** | **T** | **T** | **T** | **G** | **A** | **T** | **G** | **C** | **T** | **G** | **A** | **T** | **T** | **T** | **C** | **T** | **G** | **A** | **A** | **G** | **G** | **G** | **A** | **A** | **A** | **A** | **G** | **T** | **A** | **A** | **T** | **C** | **A** | **G** | **G** | **A** | **A** | **A** | **T** | **C** | **G** | **C** | **G** | **G** | **A** | **T** | **G** | **A** | **A** | **T** | **T** | **G** | **T** | **T** | **T** | **A** | **T** | **C** | **A** | **C** | **A** | **C** | **T** | **G** | **A** | **A** | **A** | **A** | **C** | **A** | **G** | **T** | **T** | **A** | **A** | **A** | **A** | **C** | **G** | **C** | **A** | **T** | **G** | **T** | **G** | **T** | **C** | **G** | **A** | **A** | **T** | **A** | **T** | **A** | **T** | **T** | **A** | **G** | **C** | **T** | **A** | **A** | **G** | **T** | **T** | **A** | **G** | **A** | **G** | **G** | **T** | **A** | **G** | **A** | **A** | **G** | **A** | **C** | **C** | **G** | **A** | **A** | **C** | **T** | **C** | **A** | **A** | **G** | **C** | **A** | **G** | **C** | **T** | **A** | **T** | **C** | **T** | **A** | **T** | **G** | **C** | **G** | **T** | **T** | **T** | **A** | **A** | **A** | **C** | **A** | **T** | **G** | **G** | **T** | **T** | **T** | **G** | **G** | **T** | **G** | **A** | **A** | **A** | **-** | **-** | **-** |
| **N54152** | **A** | **T** | **G** | **A** | **T** | **A** | **A** | **A** | **A** | **G** | **T** | **T** | **T** | **T** | **A** | **T** | **T** | **A** | **G** | **T** | **A** | **G** | **A** | **T** | **G** | **A** | **C** | **C** | **A** | **T** | **G** | **A** | **A** | **A** | **T** | **G** | **G** | **T** | **A** | **C** | **G** | **C** | **T** | **T** | **A** | **G** | **G** | **C** | **G** | **T** | **C** | **T** | **C** | **T** | **T** | **C** | **T** | **T** | **A** | **T** | **T** | **T** | **A** | **T** | **C** | **T** | **A** | **T** | **C** | **C** | **A** | **A** | **G** | **A** | **A** | **G** | **A** | **C** | **A** | **T** | **T** | **G** | **A** | **A** | **G** | **T** | **G** | **A** | **T** | **T** | **G** | **G** | **A** | **G** | **A** | **A** | **G** | **C** | **T** | **G** | **A** | **A** | **A** | **A** | **T** | **G** | **G** | **A** | **C** | **G** | **T** | **C** | **A** | **A** | **G** | **G** | **C** | **T** | **A** | **T** | **G** | **A** | **A** | **A** | **A** | **G** | **G** | **C** | **G** | **A** | **T** | **G** | **G** | **C** | **A** | **C** | **T** | **T** | **C** | **G** | **A** | **C** | **C** | **A** | **G** | **A** | **T** | **G** | **T** | **C** | **A** | **T** | **T** | **T** | **T** | **A** | **A** | **T** | **G** | **G** | **A** | **T** | **C** | **T** | **A** | **G** | **T** | **C** | **A** | **T** | **G** | **G** | **A** | **A** | **G** | **A** | **A** | **A** | **T** | **G** | **G** | **A** | **T** | **G** | **G** | **C** | **A** | **T** | **C** | **G** | **A** | **A** | **T** | **C** | **A** | **A** | **C** | **A** | **A** | **A** | **A** | **G** | **C** | **A** | **A** | **T** | **C** | **T** | **T** | **A** | **A** | **A** | **A** | **G** | **A** | **T** | **T** | **G** | **G** | **C** | **C** | **A** | **A** | **A** | **A** | **G** | **C** | **C** | **A** | **A** | **A** | **A** | **T** | **C** | **A** | **T** | **T** | **A** | **T** | **C** | **G** | **T** | **C** | **A** | **C** | **A** | **A** | **G** | **T** | **T** | **T** | **T** | **A** | **T** | **T** | **G** | **A** | **T** | **G** | **A** | **T** | **G** | **A** | **A** | **A** | **A** | **A** | **G** | **T** | **T** | **T** | **A** | **T** | **C** | **C** | **T** | **G** | **C** | **A** | **A** | **T** | **T** | **G** | **A** | **A** | **G** | **C** | **A** | **G** | **G** | **A** | **G** | **C** | **A** | **G** | **C** | **A** | **G** | **G** | **A** | **T** | **A** | **T** | **T** | **T** | **G** | **T** | **T** | **G** | **A** | **A** | **G** | **A** | **C** | **G** | **T** | **C** | **T** | **A** | **C** | **C** | **G** | **C** | **A** | **C** | **A** | **T** | **G** | **A** | **A** | **A** | **T** | **A** | **G** | **C** | **T** | **G** | **A** | **T** | **G** | **C** | **T** | **A** | **T** | **A** | **A** | **G** | **G** | **G** | **C** | **T** | **A** | **C** | **G** | **C** | **A** | **A** | **C** | **G** | **C** | **G** | **G** | **C** | **G** | **A** | **G** | **C** | **G** | **A** | **G** | **T** | **A** | **T** | **T** | **G** | **G** | **A** | **A** | **C** | **C** | **T** | **G** | **A** | **A** | **G** | **T** | **G** | **A** | **C** | **A** | **A** | **C** | **A** | **A** | **A** | **A** | **A** | **T** | **G** | **A** | **T** | **G** | **G** | **A** | **A** | **A** | **A** | **A** | **A** | **T** | **G** | **A** | **G** | **T** | **C** | **G** | **G** | **C** | **G** | **A** | **A** | **A** | **T** | **G** | **A** | **T** | **C** | **C** | **A** | **G** | **T** | **A** | **T** | **T** | **G** | **C** | **A** | **T** | **G** | **A** | **A** | **G** | **A** | **A** | **T** | **T** | **A** | **A** | **C** | **G** | **A** | **A** | **T** | **C** | **G** | **A** | **G** | **A** | **A** | **A** | **A** | **T** | **G** | **A** | **A** | **A** | **T** | **T** | **T** | **T** | **G** | **A** | **T** | **G** | **C** | **T** | **G** | **A** | **T** | **T** | **T** | **C** | **T** | **G** | **A** | **A** | **G** | **G** | **G** | **A** | **A** | **A** | **A** | **G** | **T** | **A** | **A** | **T** | **C** | **A** | **G** | **G** | **A** | **A** | **A** | **T** | **C** | **G** | **C** | **G** | **G** | **A** | **T** | **G** | **A** | **A** | **T** | **T** | **G** | **T** | **T** | **T** | **A** | **T** | **C** | **A** | **C** | **A** | **C** | **T** | **G** | **A** | **A** | **A** | **A** | **C** | **A** | **G** | **T** | **T** | **A** | **A** | **A** | **A** | **C** | **G** | **C** | **A** | **T** | **G** | **T** | **G** | **T** | **C** | **G** | **A** | **A** | **T** | **A** | **T** | **A** | **T** | **T** | **A** | **G** | **C** | **T** | **A** | **A** | **G** | **T** | **T** | **A** | **G** | **A** | **G** | **G** | **T** | **A** | **G** | **A** | **A** | **G** | **A** | **C** | **C** | **G** | **A** | **A** | **C** | **T** | **C** | **A** | **A** | **G** | **C** | **A** | **G** | **C** | **T** | **A** | **T** | **C** | **T** | **A** | **T** | **G** | **C** | **G** | **T** | **T** | **T** | **A** | **A** | **A** | **C** | **A** | **T** | **G** | **G** | **T** | **T** | **T** | **G** | **G** | **T** | **G** | **A** | **A** | **A** | **-** | **-** | **-** |
| **N60123F** | **A** | **T** | **G** | **A** | **T** | **A** | **A** | **A** | **A** | **G** | **T** | **T** | **T** | **T** | **A** | **T** | **T** | **A** | **G** | **T** | **A** | **G** | **A** | **T** | **G** | **A** | **C** | **C** | **A** | **T** | **G** | **A** | **A** | **A** | **T** | **G** | **G** | **T** | **A** | **C** | **G** | **C** | **T** | **T** | **A** | **G** | **G** | **C** | **G** | **T** | **C** | **T** | **C** | **T** | **T** | **C** | **T** | **T** | **A** | **T** | **T** | **T** | **A** | **T** | **C** | **T** | **A** | **T** | **C** | **C** | **A** | **A** | **G** | **A** | **A** | **G** | **A** | **C** | **A** | **T** | **T** | **G** | **A** | **A** | **G** | **T** | **G** | **A** | **T** | **T** | **G** | **G** | **A** | **G** | **A** | **A** | **G** | **C** | **T** | **G** | **A** | **A** | **A** | **A** | **T** | **G** | **G** | **A** | **C** | **G** | **T** | **C** | **A** | **A** | **G** | **G** | **C** | **T** | **A** | **T** | **G** | **A** | **A** | **A** | **A** | **G** | **G** | **C** | **G** | **A** | **T** | **G** | **G** | **C** | **A** | **C** | **T** | **T** | **C** | **G** | **A** | **C** | **C** | **A** | **G** | **A** | **T** | **G** | **T** | **C** | **A** | **T** | **T** | **T** | **T** | **A** | **A** | **T** | **G** | **G** | **A** | **T** | **C** | **T** | **A** | **G** | **T** | **C** | **A** | **T** | **G** | **G** | **A** | **A** | **G** | **A** | **A** | **A** | **T** | **G** | **G** | **A** | **T** | **G** | **G** | **C** | **A** | **T** | **C** | **G** | **A** | **A** | **T** | **C** | **A** | **A** | **C** | **A** | **A** | **A** | **A** | **G** | **C** | **A** | **A** | **T** | **C** | **T** | **T** | **A** | **A** | **A** | **A** | **G** | **A** | **T** | **T** | **G** | **G** | **C** | **C** | **A** | **A** | **A** | **A** | **G** | **C** | **C** | **A** | **A** | **A** | **A** | **T** | **C** | **A** | **T** | **T** | **A** | **T** | **C** | **G** | **T** | **C** | **A** | **C** | **A** | **A** | **G** | **T** | **T** | **T** | **T** | **A** | **T** | **T** | **G** | **A** | **T** | **G** | **A** | **T** | **G** | **A** | **A** | **A** | **A** | **A** | **G** | **T** | **T** | **T** | **A** | **T** | **C** | **C** | **T** | **G** | **C** | **A** | **A** | **T** | **T** | **G** | **A** | **A** | **G** | **C** | **A** | **G** | **G** | **A** | **G** | **C** | **A** | **G** | **C** | **A** | **G** | **G** | **A** | **T** | **A** | **T** | **T** | **T** | **G** | **T** | **T** | **G** | **A** | **A** | **G** | **A** | **C** | **G** | **T** | **C** | **T** | **A** | **C** | **C** | **G** | **C** | **A** | **C** | **A** | **T** | **G** | **A** | **A** | **A** | **T** | **A** | **G** | **C** | **T** | **G** | **A** | **T** | **G** | **C** | **T** | **A** | **T** | **A** | **A** | **G** | **G** | **G** | **C** | **T** | **A** | **C** | **G** | **C** | **A** | **A** | **C** | **G** | **C** | **G** | **G** | **C** | **G** | **A** | **G** | **C** | **G** | **A** | **G** | **T** | **A** | **T** | **T** | **G** | **G** | **A** | **A** | **C** | **C** | **T** | **G** | **A** | **A** | **G** | **T** | **G** | **A** | **C** | **A** | **A** | **C** | **A** | **A** | **A** | **A** | **A** | **T** | **G** | **A** | **T** | **G** | **G** | **A** | **A** | **A** | **A** | **A** | **A** | **T** | **G** | **A** | **G** | **T** | **C** | **G** | **G** | **C** | **G** | **A** | **T** | **A** | **T** | **G** | **A** | **T** | **C** | **C** | **A** | **G** | **T** | **A** | **T** | **T** | **G** | **C** | **A** | **T** | **G** | **A** | **A** | **G** | **A** | **A** | **T** | **T** | **A** | **A** | **C** | **G** | **A** | **A** | **T** | **C** | **G** | **A** | **G** | **A** | **A** | **A** | **A** | **T** | **G** | **A** | **A** | **A** | **T** | **T** | **T** | **T** | **G** | **A** | **T** | **G** | **C** | **T** | **G** | **A** | **T** | **T** | **T** | **C** | **T** | **G** | **A** | **A** | **G** | **G** | **G** | **A** | **A** | **A** | **A** | **G** | **T** | **A** | **A** | **T** | **C** | **A** | **G** | **G** | **A** | **A** | **A** | **T** | **C** | **G** | **C** | **G** | **G** | **A** | **T** | **G** | **A** | **A** | **T** | **T** | **G** | **T** | **T** | **T** | **A** | **T** | **C** | **A** | **C** | **A** | **C** | **T** | **G** | **A** | **A** | **A** | **A** | **C** | **A** | **G** | **T** | **T** | **A** | **A** | **A** | **A** | **C** | **G** | **C** | **A** | **T** | **G** | **T** | **G** | **T** | **C** | **G** | **A** | **A** | **T** | **A** | **T** | **A** | **T** | **T** | **A** | **G** | **C** | **T** | **A** | **A** | **G** | **T** | **T** | **A** | **G** | **A** | **G** | **G** | **T** | **A** | **G** | **A** | **A** | **G** | **A** | **C** | **C** | **G** | **A** | **A** | **C** | **T** | **C** | **A** | **A** | **G** | **C** | **A** | **G** | **C** | **T** | **A** | **T** | **C** | **T** | **A** | **T** | **G** | **C** | **A** | **T** | **T** | **T** | **A** | **A** | **A** | **C** | **A** | **T** | **G** | **G** | **T** | **T** | **T** | **G** | **G** | **T** | **G** | **A** | **A** | **A** | **-** | **-** | **-** |
| **N59531F** | **A** | **T** | **G** | **A** | **T** | **A** | **A** | **A** | **A** | **G** | **T** | **T** | **T** | **T** | **A** | **T** | **T** | **A** | **G** | **T** | **A** | **G** | **A** | **T** | **G** | **A** | **C** | **C** | **A** | **T** | **G** | **A** | **A** | **A** | **T** | **G** | **G** | **T** | **G** | **C** | **G** | **C** | **T** | **T** | **A** | **G** | **G** | **C** | **G** | **T** | **C** | **T** | **C** | **T** | **T** | **C** | **T** | **T** | **A** | **T** | **T** | **T** | **A** | **T** | **C** | **T** | **A** | **T** | **T** | **C** | **A** | **A** | **G** | **A** | **A** | **G** | **A** | **C** | **A** | **T** | **T** | **G** | **A** | **A** | **G** | **T** | **A** | **A** | **T** | **T** | **G** | **G** | **A** | **G** | **A** | **A** | **G** | **C** | **T** | **G** | **A** | **A** | **A** | **A** | **T** | **G** | **G** | **A** | **C** | **G** | **A** | **C** | **A** | **A** | **G** | **G** | **C** | **T** | **A** | **T** | **G** | **A** | **G** | **A** | **A** | **G** | **G** | **C** | **G** | **A** | **T** | **G** | **A** | **C** | **A** | **C** | **T** | **T** | **C** | **G** | **A** | **C** | **C** | **G** | **G** | **A** | **C** | **G** | **T** | **C** | **A** | **T** | **T** | **T** | **T** | **A** | **A** | **T** | **G** | **G** | **A** | **C** | **T** | **T** | **A** | **G** | **T** | **C** | **A** | **T** | **G** | **G** | **A** | **A** | **G** | **A** | **A** | **A** | **T** | **G** | **G** | **A** | **T** | **G** | **G** | **T** | **A** | **T** | **C** | **G** | **A** | **A** | **T** | **C** | **A** | **A** | **C** | **A** | **A** | **A** | **A** | **G** | **C** | **A** | **A** | **T** | **C** | **T** | **T** | **A** | **A** | **A** | **A** | **G** | **A** | **T** | **T** | **G** | **G** | **C** | **C** | **A** | **G** | **A** | **A** | **G** | **C** | **T** | **A** | **A** | **A** | **A** | **T** | **C** | **A** | **T** | **T** | **A** | **T** | **C** | **G** | **T** | **A** | **A** | **C** | **C** | **A** | **G** | **T** | **T** | **T** | **T** | **A** | **T** | **T** | **G** | **A** | **T** | **G** | **A** | **C** | **G** | **A** | **A** | **A** | **A** | **A** | **G** | **T** | **T** | **T** | **A** | **T** | **C** | **C** | **T** | **G** | **C** | **C** | **A** | **T** | **T** | **G** | **A** | **A** | **G** | **C** | **A** | **G** | **G** | **T** | **G** | **C** | **A** | **G** | **C** | **A** | **G** | **G** | **G** | **T** | **A** | **T** | **T** | **T** | **G** | **T** | **T** | **G** | **A** | **A** | **G** | **A** | **C** | **T** | **T** | **C** | **T** | **A** | **C** | **C** | **G** | **C** | **A** | **C** | **A** | **T** | **G** | **A** | **A** | **A** | **T** | **T** | **G** | **C** | **T** | **G** | **A** | **T** | **G** | **C** | **T** | **A** | **T** | **A** | **A** | **G** | **G** | **G** | **C** | **A** | **A** | **C** | **G** | **C** | **A** | **A** | **C** | **G** | **C** | **G** | **G** | **C** | **G** | **A** | **G** | **C** | **G** | **A** | **G** | **T** | **A** | **T** | **T** | **A** | **G** | **A** | **A** | **C** | **C** | **A** | **G** | **A** | **A** | **G** | **T** | **G** | **A** | **C** | **A** | **A** | **C** | **A** | **A** | **A** | **A** | **A** | **T** | **G** | **A** | **T** | **G** | **G** | **A** | **A** | **A** | **A** | **A** | **A** | **T** | **G** | **A** | **G** | **T** | **C** | **G** | **A** | **C** | **A** | **A** | **A** | **A** | **T** | **G** | **A** | **G** | **C** | **C** | **T** | **G** | **T** | **A** | **T** | **T** | **A** | **C** | **A** | **T** | **G** | **A** | **A** | **G** | **A** | **A** | **C** | **T** | **A** | **A** | **C** | **G** | **A** | **A** | **T** | **C** | **G** | **G** | **G** | **A** | **A** | **A** | **A** | **T** | **G** | **A** | **A** | **A** | **T** | **T** | **T** | **T** | **A** | **A** | **T** | **G** | **C** | **T** | **G** | **A** | **T** | **T** | **T** | **C** | **T** | **G** | **A** | **A** | **G** | **G** | **G** | **A** | **A** | **A** | **A** | **G** | **T** | **A** | **A** | **T** | **C** | **A** | **G** | **G** | **A** | **A** | **A** | **T** | **A** | **G** | **C** | **G** | **G** | **A** | **C** | **G** | **A** | **A** | **T** | **T** | **G** | **T** | **T** | **C** | **A** | **T** | **T** | **A** | **C** | **A** | **C** | **T** | **G** | **A** | **A** | **A** | **A** | **C** | **A** | **G** | **T** | **A** | **A** | **A** | **A** | **A** | **C** | **A** | **C** | **A** | **T** | **G** | **T** | **T** | **T** | **C** | **C** | **A** | **A** | **T** | **A** | **T** | **A** | **T** | **T** | **G** | **G** | **C** | **T** | **A** | **A** | **G** | **C** | **T** | **A** | **G** | **A** | **A** | **G** | **T** | **G** | **G** | **A** | **A** | **G** | **A** | **C** | **C** | **G** | **T** | **A** | **C** | **A** | **C** | **A** | **A** | **G** | **C** | **C** | **G** | **C** | **T** | **A** | **T** | **C** | **T** | **A** | **T** | **G** | **C** | **A** | **T** | **T** | **C** | **A** | **A** | **A** | **C** | **A** | **T** | **G** | **G** | **T** | **T** | **T** | **G** | **G** | **T** | **G** | **A** | **A** | **A** | **-** | **-** | **-** |
| **N55265** | **G** | **T** | **G** | **A** | **T** | **C** | **A** | **A** | **A** | **G** | **T** | **A** | **A** | **T** | **G** | **T** | **T** | **A** | **G** | **T** | **G** | **G** | **A** | **T** | **G** | **A** | **C** | **C** | **A** | **T** | **G** | **A** | **A** | **A** | **T** | **G** | **G** | **T** | **C** | **C** | **G** | **T** | **T** | **T** | **A** | **G** | **G** | **C** | **G** | **T** | **T** | **T** | **C** | **A** | **T** | **C** | **A** | **T** | **A** | **T** | **T** | **T** | **A** | **T** | **C** | **T** | **A** | **T** | **T** | **C** | **A** | **A** | **G** | **A** | **G** | **G** | **A** | **T** | **A** | **T** | **A** | **G** | **A** | **A** | **G** | **T** | **C** | **G** | **T** | **A** | **G** | **G** | **C** | **G** | **A** | **A** | **G** | **C** | **A** | **G** | **A** | **A** | **A** | **A** | **C** | **G** | **G** | **T** | **A** | **A** | **G** | **A** | **T** | **T** | **G** | **G** | **C** | **T** | **A** | **T** | **G** | **A** | **A** | **A** | **A** | **A** | **G** | **C** | **A** | **T** | **T** | **G** | **G** | **A** | **A** | **C** | **T** | **A** | **C** | **G** | **T** | **C** | **C** | **A** | **G** | **A** | **T** | **G** | **T** | **T** | **A** | **T** | **T** | **T** | **T** | **G** | **A** | **T** | **G** | **G** | **A** | **C** | **T** | **T** | **G** | **G** | **T** | **A** | **A** | **T** | **G** | **G** | **A** | **A** | **G** | **A** | **A** | **A** | **T** | **G** | **G** | **A** | **C** | **G** | **G** | **C** | **A** | **T** | **T** | **G** | **A** | **T** | **T** | **C** | **A** | **A** | **C** | **A** | **A** | **A** | **A** | **G** | **C** | **G** | **A** | **T** | **T** | **T** | **T** | **G** | **A** | **A** | **A** | **G** | **A** | **T** | **T** | **G** | **G** | **C** | **C** | **A** | **G** | **A** | **A** | **G** | **C** | **C** | **A** | **A** | **G** | **A** | **T** | **T** | **A** | **T** | **T** | **A** | **T** | **T** | **G** | **T** | **G** | **A** | **C** | **G** | **A** | **G** | **T** | **T** | **T** | **T** | **A** | **T** | **T** | **G** | **A** | **T** | **G** | **A** | **T** | **G** | **A** | **A** | **A** | **A** | **A** | **G** | **T** | **G** | **T** | **A** | **T** | **C** | **C** | **G** | **G** | **C** | **A** | **A** | **T** | **T** | **G** | **A** | **A** | **G** | **C** | **T** | **G** | **G** | **T** | **G** | **C** | **A** | **G** | **C** | **G** | **G** | **G** | **C** | **T** | **A** | **C** | **C** | **T** | **A** | **T** | **T** | **A** | **A** | **A** | **A** | **A** | **C** | **A** | **T** | **C** | **A** | **A** | **C** | **A** | **G** | **C** | **A** | **C** | **A** | **T** | **G** | **A** | **G** | **A** | **T** | **T** | **G** | **C** | **T** | **G** | **A** | **T** | **G** | **C** | **G** | **A** | **T** | **T** | **C** | **G** | **G** | **G** | **C** | **G** | **A** | **C** | **T** | **T** | **A** | **T** | **C** | **G** | **C** | **G** | **G** | **A** | **G** | **A** | **G** | **C** | **G** | **T** | **G** | **T** | **G** | **T** | **T** | **G** | **G** | **A** | **A** | **C** | **C** | **T** | **G** | **A** | **A** | **G** | **T** | **G** | **A** | **C** | **G** | **C** | **A** | **T** | **A** | **A** | **G** | **A** | **T** | **G** | **A** | **T** | **G** | **G** | **A** | **A** | **C** | **G** | **G** | **T** | **T** | **A** | **A** | **C** | **A** | **A** | **A** | **A** | **A** | **A** | **A** | **C** | **A** | **A** | **G** | **A** | **G** | **C** | **C** | **G** | **G** | **T** | **G** | **C** | **T** | **A** | **C** | **A** | **C** | **G** | **A** | **A** | **G** | **A** | **T** | **T** | **T** | **G** | **A** | **C** | **A** | **A** | **A** | **C** | **C** | **G** | **A** | **G** | **A** | **A** | **C** | **A** | **C** | **G** | **A** | **A** | **A** | **T** | **T** | **T** | **T** | **A** | **A** | **T** | **G** | **C** | **T** | **G** | **A** | **T** | **T** | **G** | **C** | **A** | **C** | **A** | **A** | **G** | **G** | **T** | **A** | **A** | **A** | **A** | **G** | **T** | **A** | **A** | **T** | **C** | **A** | **G** | **G** | **A** | **A** | **A** | **T** | **T** | **G** | **C** | **T** | **G** | **A** | **T** | **G** | **A** | **A** | **C** | **T** | **C** | **T** | **T** | **T** | **A** | **T** | **C** | **A** | **C** | **T** | **T** | **T** | **G** | **A** | **A** | **A** | **A** | **C** | **A** | **G** | **T** | **T** | **A** | **A** | **A** | **A** | **C** | **A** | **C** | **A** | **T** | **G** | **T** | **T** | **T** | **C** | **A** | **A** | **A** | **C** | **A** | **T** | **T** | **T** | **T** | **A** | **G** | **C** | **A** | **A** | **A** | **A** | **C** | **T** | **A** | **G** | **A** | **T** | **G** | **T** | **G** | **G** | **A** | **T** | **G** | **A** | **T** | **C** | **G** | **G** | **A** | **C** | **C** | **C** | **A** | **A** | **G** | **C** | **G** | **G** | **C** | **G** | **A** | **T** | **T** | **T** | **A** | **T** | **G** | **C** | **A** | **T** | **T** | **T** | **C** | **A** | **A** | **C** | **A** | **C** | **G** | **G** | **T** | **T** | **T** | **A** | **G** | **C** | **C** | **A** | **A** | **A** | **-** | **-** | **-** |
| **N52777** | **A** | **T** | **G** | **A** | **T** | **A** | **A** | **A** | **A** | **G** | **T** | **T** | **T** | **T** | **A** | **T** | **T** | **A** | **G** | **T** | **A** | **G** | **A** | **T** | **G** | **A** | **C** | **C** | **A** | **T** | **G** | **A** | **A** | **A** | **T** | **G** | **G** | **T** | **A** | **C** | **G** | **C** | **T** | **T** | **A** | **G** | **G** | **C** | **G** | **T** | **C** | **T** | **C** | **T** | **T** | **C** | **T** | **T** | **A** | **T** | **T** | **T** | **A** | **T** | **C** | **T** | **A** | **T** | **C** | **C** | **A** | **A** | **G** | **A** | **A** | **G** | **A** | **C** | **A** | **T** | **T** | **G** | **A** | **A** | **G** | **T** | **G** | **A** | **T** | **T** | **G** | **G** | **A** | **G** | **A** | **A** | **G** | **C** | **T** | **G** | **A** | **A** | **A** | **A** | **T** | **G** | **G** | **A** | **C** | **G** | **T** | **C** | **A** | **A** | **G** | **G** | **C** | **T** | **A** | **T** | **G** | **A** | **A** | **A** | **A** | **G** | **G** | **C** | **G** | **A** | **T** | **G** | **G** | **C** | **A** | **C** | **T** | **T** | **C** | **G** | **A** | **C** | **C** | **A** | **G** | **A** | **T** | **G** | **T** | **C** | **A** | **T** | **T** | **T** | **T** | **A** | **A** | **T** | **G** | **G** | **A** | **T** | **C** | **T** | **A** | **G** | **T** | **C** | **A** | **T** | **G** | **G** | **A** | **A** | **G** | **A** | **A** | **A** | **T** | **G** | **G** | **A** | **T** | **G** | **G** | **C** | **A** | **T** | **C** | **G** | **A** | **A** | **T** | **C** | **A** | **A** | **C** | **A** | **A** | **A** | **A** | **G** | **C** | **A** | **A** | **T** | **C** | **T** | **T** | **A** | **A** | **A** | **A** | **G** | **A** | **T** | **T** | **G** | **G** | **C** | **C** | **A** | **A** | **A** | **A** | **G** | **C** | **C** | **A** | **A** | **A** | **A** | **T** | **C** | **A** | **T** | **T** | **A** | **T** | **C** | **G** | **T** | **C** | **A** | **C** | **A** | **A** | **G** | **T** | **T** | **T** | **T** | **A** | **T** | **T** | **G** | **A** | **T** | **G** | **A** | **T** | **G** | **A** | **A** | **A** | **A** | **A** | **G** | **T** | **T** | **T** | **A** | **T** | **C** | **C** | **T** | **G** | **C** | **A** | **A** | **T** | **T** | **G** | **A** | **A** | **G** | **C** | **A** | **G** | **G** | **A** | **G** | **C** | **A** | **G** | **C** | **A** | **G** | **G** | **A** | **T** | **A** | **T** | **T** | **T** | **G** | **T** | **T** | **G** | **A** | **A** | **G** | **A** | **C** | **G** | **T** | **C** | **T** | **A** | **C** | **C** | **G** | **C** | **A** | **C** | **A** | **T** | **G** | **A** | **A** | **A** | **T** | **A** | **G** | **C** | **T** | **G** | **A** | **T** | **G** | **C** | **T** | **A** | **T** | **A** | **A** | **G** | **G** | **G** | **C** | **T** | **A** | **C** | **G** | **C** | **A** | **A** | **C** | **G** | **C** | **G** | **G** | **C** | **G** | **A** | **G** | **C** | **G** | **A** | **G** | **T** | **A** | **T** | **T** | **G** | **G** | **A** | **A** | **C** | **C** | **T** | **G** | **A** | **A** | **G** | **T** | **G** | **A** | **C** | **A** | **A** | **C** | **A** | **A** | **A** | **A** | **A** | **T** | **G** | **A** | **T** | **G** | **G** | **A** | **A** | **A** | **A** | **A** | **A** | **T** | **G** | **A** | **G** | **T** | **C** | **G** | **G** | **C** | **G** | **A** | **A** | **A** | **T** | **G** | **A** | **T** | **C** | **C** | **A** | **G** | **T** | **A** | **T** | **T** | **G** | **C** | **A** | **T** | **G** | **A** | **A** | **G** | **A** | **A** | **T** | **T** | **A** | **A** | **C** | **G** | **A** | **A** | **T** | **C** | **G** | **A** | **G** | **A** | **A** | **A** | **A** | **T** | **G** | **A** | **A** | **A** | **T** | **T** | **T** | **T** | **G** | **A** | **T** | **G** | **C** | **T** | **G** | **A** | **T** | **T** | **T** | **C** | **T** | **G** | **A** | **A** | **G** | **G** | **G** | **A** | **A** | **A** | **A** | **G** | **T** | **A** | **A** | **T** | **C** | **A** | **G** | **G** | **A** | **A** | **A** | **T** | **C** | **G** | **C** | **G** | **G** | **A** | **T** | **G** | **A** | **A** | **T** | **T** | **G** | **T** | **T** | **T** | **A** | **T** | **C** | **A** | **C** | **A** | **C** | **T** | **G** | **A** | **A** | **A** | **A** | **C** | **A** | **G** | **T** | **T** | **A** | **A** | **A** | **A** | **C** | **G** | **C** | **A** | **T** | **G** | **T** | **G** | **T** | **C** | **G** | **A** | **A** | **T** | **A** | **T** | **A** | **T** | **T** | **A** | **G** | **C** | **T** | **A** | **A** | **G** | **T** | **T** | **A** | **G** | **A** | **G** | **G** | **T** | **A** | **G** | **A** | **A** | **G** | **A** | **C** | **C** | **G** | **A** | **A** | **C** | **T** | **C** | **A** | **A** | **G** | **C** | **A** | **G** | **C** | **T** | **A** | **T** | **C** | **T** | **A** | **T** | **G** | **C** | **G** | **T** | **T** | **T** | **A** | **A** | **A** | **C** | **A** | **T** | **G** | **G** | **T** | **T** | **T** | **G** | **G** | **T** | **G** | **A** | **A** | **A** | **-** | **-** | **-** |
| **N59517F** | **A** | **T** | **G** | **A** | **T** | **A** | **A** | **A** | **A** | **G** | **T** | **T** | **T** | **T** | **A** | **T** | **T** | **A** | **G** | **T** | **A** | **G** | **A** | **T** | **G** | **A** | **C** | **C** | **A** | **T** | **G** | **A** | **A** | **A** | **T** | **G** | **G** | **T** | **A** | **C** | **G** | **C** | **T** | **T** | **A** | **G** | **G** | **C** | **G** | **T** | **C** | **T** | **C** | **T** | **T** | **C** | **T** | **T** | **A** | **T** | **T** | **T** | **A** | **T** | **C** | **T** | **A** | **T** | **C** | **C** | **A** | **A** | **G** | **A** | **A** | **G** | **A** | **C** | **A** | **T** | **T** | **G** | **A** | **A** | **G** | **T** | **G** | **A** | **T** | **T** | **G** | **G** | **A** | **G** | **A** | **A** | **G** | **C** | **T** | **G** | **A** | **A** | **A** | **A** | **T** | **G** | **G** | **A** | **C** | **G** | **T** | **C** | **A** | **A** | **G** | **G** | **C** | **T** | **A** | **T** | **G** | **A** | **A** | **A** | **A** | **G** | **G** | **C** | **G** | **A** | **T** | **G** | **G** | **C** | **A** | **C** | **T** | **T** | **C** | **G** | **A** | **C** | **C** | **A** | **G** | **A** | **T** | **G** | **T** | **C** | **A** | **T** | **T** | **T** | **T** | **A** | **A** | **T** | **G** | **G** | **A** | **T** | **C** | **T** | **A** | **G** | **T** | **C** | **A** | **T** | **G** | **G** | **A** | **A** | **G** | **A** | **A** | **A** | **T** | **G** | **G** | **A** | **T** | **G** | **G** | **C** | **A** | **T** | **C** | **G** | **A** | **A** | **T** | **C** | **A** | **A** | **C** | **A** | **A** | **A** | **A** | **G** | **C** | **A** | **A** | **T** | **C** | **T** | **T** | **A** | **A** | **A** | **A** | **G** | **A** | **T** | **T** | **G** | **G** | **C** | **C** | **A** | **A** | **A** | **A** | **G** | **C** | **C** | **A** | **A** | **A** | **A** | **T** | **C** | **A** | **T** | **T** | **A** | **T** | **C** | **G** | **T** | **C** | **A** | **C** | **A** | **A** | **G** | **T** | **T** | **T** | **T** | **A** | **T** | **T** | **G** | **A** | **T** | **G** | **A** | **T** | **G** | **A** | **A** | **A** | **A** | **A** | **G** | **T** | **T** | **T** | **A** | **T** | **C** | **C** | **T** | **G** | **C** | **A** | **A** | **T** | **T** | **G** | **A** | **A** | **G** | **C** | **A** | **G** | **G** | **A** | **G** | **C** | **A** | **G** | **C** | **A** | **G** | **G** | **A** | **T** | **A** | **T** | **T** | **T** | **G** | **T** | **T** | **G** | **A** | **A** | **G** | **A** | **C** | **G** | **T** | **C** | **T** | **A** | **C** | **C** | **G** | **C** | **A** | **C** | **A** | **T** | **G** | **A** | **A** | **A** | **T** | **A** | **G** | **C** | **T** | **G** | **A** | **T** | **G** | **C** | **T** | **A** | **T** | **A** | **A** | **G** | **G** | **G** | **C** | **T** | **A** | **C** | **G** | **C** | **A** | **A** | **C** | **G** | **C** | **G** | **G** | **C** | **G** | **A** | **G** | **C** | **G** | **A** | **G** | **T** | **A** | **T** | **T** | **G** | **G** | **A** | **A** | **C** | **C** | **T** | **G** | **A** | **A** | **G** | **T** | **G** | **A** | **C** | **A** | **A** | **C** | **A** | **A** | **A** | **A** | **A** | **T** | **G** | **A** | **T** | **G** | **G** | **A** | **A** | **A** | **A** | **A** | **A** | **T** | **G** | **A** | **G** | **T** | **C** | **G** | **G** | **C** | **G** | **A** | **A** | **A** | **T** | **G** | **A** | **T** | **C** | **C** | **A** | **G** | **T** | **A** | **T** | **T** | **G** | **C** | **A** | **T** | **G** | **A** | **A** | **G** | **A** | **A** | **T** | **T** | **A** | **A** | **C** | **G** | **A** | **A** | **T** | **C** | **G** | **A** | **G** | **A** | **A** | **A** | **A** | **T** | **G** | **A** | **A** | **A** | **T** | **T** | **T** | **T** | **G** | **A** | **T** | **G** | **C** | **T** | **G** | **A** | **T** | **T** | **T** | **C** | **T** | **G** | **A** | **A** | **G** | **G** | **G** | **A** | **A** | **A** | **A** | **G** | **T** | **A** | **A** | **T** | **C** | **A** | **G** | **G** | **A** | **A** | **A** | **T** | **C** | **G** | **C** | **G** | **G** | **A** | **T** | **G** | **A** | **A** | **T** | **T** | **G** | **T** | **T** | **T** | **A** | **T** | **C** | **A** | **C** | **A** | **C** | **T** | **G** | **A** | **A** | **A** | **A** | **C** | **A** | **G** | **T** | **T** | **A** | **A** | **A** | **A** | **C** | **G** | **C** | **A** | **T** | **G** | **T** | **G** | **T** | **C** | **G** | **A** | **A** | **T** | **A** | **T** | **A** | **T** | **T** | **A** | **G** | **C** | **T** | **A** | **A** | **G** | **T** | **T** | **A** | **G** | **A** | **G** | **G** | **T** | **A** | **G** | **A** | **A** | **G** | **A** | **C** | **C** | **G** | **A** | **A** | **C** | **T** | **C** | **A** | **A** | **G** | **C** | **A** | **G** | **C** | **T** | **A** | **T** | **C** | **T** | **A** | **T** | **G** | **C** | **G** | **T** | **T** | **T** | **A** | **A** | **A** | **C** | **A** | **T** | **G** | **G** | **T** | **T** | **T** | **G** | **G** | **T** | **G** | **A** | **A** | **A** | **-** | **-** | **-** |
| **N59609F** | **G** | **T** | **G** | **A** | **T** | **C** | **A** | **A** | **A** | **G** | **T** | **A** | **A** | **T** | **G** | **T** | **T** | **A** | **G** | **T** | **G** | **G** | **A** | **T** | **G** | **A** | **C** | **C** | **A** | **T** | **G** | **A** | **A** | **A** | **T** | **G** | **G** | **T** | **C** | **C** | **G** | **T** | **T** | **T** | **A** | **G** | **G** | **C** | **G** | **T** | **T** | **T** | **C** | **A** | **T** | **C** | **A** | **T** | **A** | **T** | **T** | **T** | **A** | **T** | **C** | **T** | **A** | **T** | **T** | **C** | **A** | **A** | **G** | **A** | **G** | **G** | **A** | **T** | **A** | **T** | **A** | **G** | **A** | **A** | **G** | **T** | **C** | **G** | **T** | **A** | **G** | **G** | **C** | **G** | **A** | **A** | **G** | **C** | **A** | **G** | **A** | **A** | **A** | **A** | **T** | **G** | **G** | **T** | **A** | **A** | **G** | **A** | **T** | **T** | **G** | **G** | **C** | **T** | **A** | **T** | **G** | **A** | **A** | **A** | **A** | **A** | **G** | **C** | **A** | **T** | **T** | **G** | **G** | **A** | **A** | **C** | **T** | **A** | **C** | **G** | **T** | **C** | **C** | **A** | **G** | **A** | **T** | **G** | **T** | **T** | **A** | **T** | **T** | **T** | **T** | **G** | **A** | **T** | **G** | **G** | **A** | **T** | **T** | **T** | **G** | **G** | **T** | **A** | **A** | **T** | **G** | **G** | **A** | **A** | **G** | **A** | **A** | **A** | **T** | **G** | **G** | **A** | **C** | **G** | **G** | **C** | **A** | **T** | **T** | **G** | **A** | **T** | **T** | **C** | **A** | **A** | **C** | **A** | **A** | **A** | **A** | **G** | **C** | **G** | **A** | **T** | **C** | **T** | **T** | **G** | **A** | **A** | **A** | **G** | **A** | **T** | **T** | **G** | **G** | **C** | **C** | **A** | **G** | **A** | **A** | **G** | **C** | **C** | **A** | **A** | **G** | **A** | **T** | **T** | **A** | **T** | **T** | **A** | **T** | **T** | **G** | **T** | **G** | **A** | **C** | **G** | **A** | **G** | **T** | **T** | **T** | **T** | **A** | **T** | **T** | **G** | **A** | **T** | **G** | **A** | **T** | **G** | **A** | **A** | **A** | **A** | **A** | **G** | **T** | **G** | **T** | **A** | **T** | **C** | **C** | **G** | **G** | **C** | **G** | **A** | **T** | **T** | **G** | **A** | **A** | **G** | **C** | **T** | **G** | **G** | **T** | **G** | **C** | **A** | **G** | **C** | **A** | **G** | **G** | **C** | **T** | **A** | **C** | **C** | **T** | **A** | **T** | **T** | **A** | **A** | **A** | **A** | **A** | **C** | **A** | **T** | **C** | **A** | **A** | **C** | **A** | **G** | **C** | **T** | **C** | **A** | **T** | **G** | **A** | **G** | **A** | **T** | **T** | **G** | **C** | **T** | **G** | **A** | **T** | **G** | **C** | **A** | **A** | **T** | **T** | **C** | **G** | **G** | **G** | **C** | **G** | **A** | **C** | **T** | **T** | **A** | **T** | **C** | **G** | **C** | **G** | **G** | **A** | **G** | **A** | **G** | **C** | **G** | **T** | **G** | **T** | **G** | **T** | **T** | **G** | **G** | **A** | **A** | **C** | **C** | **T** | **G** | **A** | **A** | **G** | **T** | **G** | **A** | **C** | **G** | **C** | **A** | **T** | **A** | **A** | **G** | **A** | **T** | **G** | **A** | **T** | **G** | **G** | **A** | **A** | **C** | **G** | **G** | **T** | **T** | **A** | **A** | **C** | **A** | **A** | **A** | **A** | **A** | **A** | **A** | **C** | **A** | **A** | **G** | **A** | **G** | **C** | **C** | **G** | **G** | **T** | **G** | **T** | **T** | **G** | **C** | **A** | **C** | **G** | **A** | **A** | **G** | **A** | **T** | **T** | **T** | **G** | **A** | **C** | **A** | **A** | **A** | **C** | **C** | **G** | **A** | **G** | **A** | **A** | **C** | **A** | **C** | **G** | **A** | **A** | **A** | **T** | **T** | **T** | **T** | **A** | **A** | **T** | **G** | **T** | **T** | **G** | **A** | **T** | **T** | **G** | **C** | **A** | **C** | **A** | **A** | **G** | **G** | **T** | **A** | **A** | **A** | **A** | **G** | **T** | **A** | **A** | **T** | **C** | **A** | **G** | **G** | **A** | **A** | **A** | **T** | **A** | **G** | **C** | **T** | **G** | **A** | **T** | **G** | **A** | **A** | **C** | **T** | **C** | **T** | **T** | **T** | **A** | **T** | **C** | **A** | **C** | **T** | **T** | **T** | **G** | **A** | **A** | **A** | **A** | **C** | **A** | **G** | **T** | **T** | **A** | **A** | **A** | **A** | **C** | **A** | **C** | **A** | **T** | **G** | **T** | **T** | **T** | **C** | **A** | **A** | **A** | **C** | **A** | **T** | **T** | **T** | **T** | **A** | **G** | **C** | **A** | **A** | **A** | **A** | **C** | **T** | **A** | **G** | **A** | **T** | **G** | **T** | **G** | **G** | **A** | **T** | **G** | **A** | **T** | **C** | **G** | **G** | **A** | **C** | **C** | **C** | **A** | **A** | **G** | **C** | **G** | **G** | **C** | **G** | **A** | **T** | **T** | **T** | **A** | **T** | **G** | **C** | **T** | **T** | **T** | **T** | **C** | **A** | **A** | **C** | **A** | **T** | **G** | **G** | **T** | **T** | **T** | **A** | **G** | **C** | **C** | **A** | **A** | **A** | **-** | **-** | **-** |
| **N55338** | **G** | **T** | **G** | **A** | **T** | **C** | **A** | **A** | **A** | **G** | **T** | **A** | **A** | **T** | **G** | **T** | **T** | **A** | **G** | **T** | **G** | **G** | **A** | **T** | **G** | **A** | **C** | **C** | **A** | **T** | **G** | **A** | **A** | **A** | **T** | **G** | **G** | **T** | **C** | **C** | **G** | **T** | **T** | **T** | **A** | **G** | **G** | **C** | **G** | **T** | **T** | **T** | **C** | **A** | **T** | **C** | **A** | **T** | **A** | **T** | **T** | **T** | **A** | **T** | **C** | **T** | **A** | **T** | **T** | **C** | **A** | **A** | **G** | **A** | **G** | **G** | **A** | **T** | **A** | **T** | **A** | **G** | **A** | **A** | **G** | **T** | **C** | **G** | **T** | **A** | **G** | **G** | **C** | **G** | **A** | **A** | **G** | **C** | **A** | **G** | **A** | **A** | **A** | **A** | **T** | **G** | **G** | **T** | **A** | **A** | **G** | **A** | **T** | **T** | **G** | **G** | **C** | **T** | **A** | **T** | **G** | **A** | **A** | **A** | **A** | **A** | **G** | **C** | **A** | **T** | **T** | **G** | **G** | **A** | **A** | **C** | **T** | **A** | **C** | **G** | **T** | **C** | **C** | **A** | **G** | **A** | **T** | **G** | **T** | **T** | **A** | **T** | **T** | **T** | **T** | **G** | **A** | **T** | **G** | **G** | **A** | **T** | **T** | **T** | **G** | **G** | **T** | **A** | **A** | **T** | **G** | **G** | **A** | **A** | **G** | **A** | **A** | **A** | **T** | **G** | **G** | **A** | **C** | **G** | **G** | **C** | **A** | **T** | **T** | **G** | **A** | **T** | **T** | **C** | **A** | **A** | **C** | **A** | **A** | **A** | **A** | **G** | **C** | **G** | **A** | **T** | **C** | **T** | **T** | **G** | **A** | **A** | **A** | **G** | **A** | **T** | **T** | **G** | **G** | **C** | **C** | **A** | **G** | **A** | **A** | **G** | **C** | **C** | **A** | **A** | **G** | **A** | **T** | **T** | **A** | **T** | **T** | **A** | **T** | **T** | **G** | **T** | **G** | **A** | **C** | **G** | **A** | **G** | **T** | **T** | **T** | **T** | **A** | **T** | **T** | **G** | **A** | **T** | **G** | **A** | **T** | **G** | **A** | **A** | **A** | **A** | **A** | **G** | **T** | **G** | **T** | **A** | **T** | **C** | **C** | **G** | **G** | **C** | **G** | **A** | **T** | **T** | **G** | **A** | **A** | **G** | **C** | **T** | **G** | **G** | **T** | **G** | **C** | **A** | **G** | **C** | **G** | **G** | **G** | **C** | **T** | **A** | **C** | **C** | **T** | **A** | **T** | **T** | **A** | **A** | **A** | **A** | **A** | **C** | **A** | **T** | **C** | **A** | **A** | **C** | **A** | **G** | **C** | **A** | **C** | **A** | **T** | **G** | **A** | **G** | **A** | **T** | **T** | **G** | **C** | **T** | **G** | **A** | **T** | **G** | **C** | **A** | **A** | **T** | **T** | **C** | **G** | **G** | **G** | **C** | **G** | **A** | **C** | **T** | **T** | **A** | **T** | **C** | **G** | **C** | **G** | **G** | **A** | **G** | **A** | **G** | **C** | **G** | **T** | **G** | **T** | **G** | **T** | **T** | **G** | **G** | **A** | **A** | **C** | **C** | **T** | **G** | **A** | **A** | **G** | **T** | **G** | **A** | **C** | **G** | **C** | **A** | **T** | **A** | **A** | **G** | **A** | **T** | **G** | **A** | **T** | **G** | **G** | **A** | **A** | **C** | **G** | **G** | **T** | **T** | **A** | **A** | **C** | **A** | **A** | **A** | **A** | **A** | **A** | **A** | **C** | **A** | **A** | **G** | **A** | **G** | **C** | **C** | **G** | **G** | **T** | **G** | **T** | **T** | **G** | **C** | **A** | **C** | **G** | **A** | **A** | **G** | **A** | **T** | **T** | **T** | **G** | **A** | **C** | **A** | **A** | **A** | **C** | **C** | **G** | **A** | **G** | **A** | **A** | **C** | **A** | **C** | **G** | **A** | **A** | **A** | **T** | **T** | **T** | **T** | **A** | **A** | **T** | **G** | **T** | **T** | **G** | **A** | **T** | **T** | **G** | **C** | **A** | **C** | **A** | **A** | **G** | **G** | **T** | **A** | **A** | **A** | **A** | **G** | **T** | **A** | **A** | **T** | **C** | **A** | **G** | **G** | **A** | **A** | **A** | **T** | **A** | **G** | **C** | **T** | **G** | **A** | **T** | **G** | **A** | **A** | **C** | **T** | **C** | **T** | **T** | **T** | **A** | **T** | **C** | **A** | **C** | **T** | **T** | **T** | **G** | **A** | **A** | **A** | **A** | **C** | **A** | **G** | **T** | **T** | **A** | **A** | **A** | **A** | **C** | **A** | **C** | **A** | **T** | **G** | **T** | **T** | **T** | **C** | **A** | **A** | **A** | **C** | **A** | **T** | **T** | **T** | **T** | **A** | **G** | **C** | **A** | **A** | **A** | **A** | **C** | **T** | **A** | **G** | **A** | **T** | **G** | **T** | **G** | **G** | **A** | **T** | **G** | **A** | **T** | **C** | **G** | **G** | **A** | **C** | **C** | **C** | **A** | **A** | **G** | **C** | **G** | **G** | **C** | **G** | **A** | **T** | **T** | **T** | **A** | **T** | **G** | **C** | **T** | **T** | **T** | **T** | **C** | **A** | **A** | **C** | **A** | **T** | **G** | **G** | **T** | **T** | **T** | **A** | **G** | **C** | **C** | **A** | **A** | **A** | **-** | **-** | **-** |
| **N60200F** | **A** | **T** | **G** | **A** | **T** | **A** | **A** | **A** | **A** | **G** | **T** | **T** | **T** | **T** | **A** | **T** | **T** | **A** | **G** | **T** | **A** | **G** | **A** | **T** | **G** | **A** | **C** | **C** | **A** | **T** | **G** | **A** | **A** | **A** | **T** | **G** | **G** | **T** | **A** | **C** | **G** | **C** | **T** | **T** | **A** | **G** | **G** | **C** | **G** | **T** | **C** | **T** | **C** | **T** | **T** | **C** | **T** | **T** | **A** | **T** | **T** | **T** | **A** | **T** | **C** | **T** | **A** | **T** | **C** | **C** | **A** | **A** | **G** | **A** | **A** | **G** | **A** | **C** | **A** | **T** | **T** | **G** | **A** | **A** | **G** | **T** | **G** | **A** | **T** | **T** | **G** | **G** | **A** | **G** | **A** | **A** | **G** | **C** | **T** | **G** | **A** | **A** | **A** | **A** | **T** | **G** | **G** | **A** | **C** | **G** | **T** | **C** | **A** | **A** | **G** | **G** | **C** | **T** | **A** | **T** | **G** | **A** | **A** | **A** | **A** | **G** | **G** | **C** | **G** | **A** | **T** | **G** | **G** | **C** | **A** | **C** | **T** | **T** | **C** | **G** | **A** | **C** | **C** | **A** | **G** | **A** | **T** | **G** | **T** | **C** | **A** | **T** | **T** | **T** | **T** | **A** | **A** | **T** | **G** | **G** | **A** | **T** | **C** | **T** | **A** | **G** | **T** | **C** | **A** | **T** | **G** | **G** | **A** | **A** | **G** | **A** | **A** | **A** | **T** | **G** | **G** | **A** | **T** | **G** | **G** | **C** | **A** | **T** | **C** | **G** | **A** | **A** | **T** | **C** | **A** | **A** | **C** | **A** | **A** | **A** | **A** | **G** | **C** | **A** | **A** | **T** | **C** | **T** | **T** | **A** | **A** | **A** | **A** | **G** | **A** | **T** | **T** | **G** | **G** | **C** | **C** | **A** | **A** | **A** | **A** | **G** | **C** | **C** | **A** | **A** | **A** | **A** | **T** | **C** | **A** | **T** | **T** | **A** | **T** | **C** | **G** | **T** | **C** | **A** | **C** | **A** | **A** | **G** | **T** | **T** | **T** | **T** | **A** | **T** | **T** | **G** | **A** | **T** | **G** | **A** | **T** | **G** | **A** | **A** | **A** | **A** | **A** | **G** | **T** | **T** | **T** | **A** | **T** | **C** | **C** | **T** | **G** | **C** | **A** | **A** | **T** | **T** | **G** | **A** | **A** | **G** | **C** | **A** | **G** | **G** | **A** | **G** | **C** | **A** | **G** | **C** | **A** | **G** | **G** | **A** | **T** | **A** | **T** | **T** | **T** | **G** | **T** | **T** | **G** | **A** | **A** | **G** | **A** | **C** | **G** | **T** | **C** | **T** | **A** | **C** | **C** | **G** | **C** | **A** | **C** | **A** | **T** | **G** | **A** | **A** | **A** | **T** | **A** | **G** | **C** | **T** | **G** | **A** | **T** | **G** | **C** | **T** | **A** | **T** | **A** | **A** | **G** | **G** | **G** | **C** | **T** | **A** | **C** | **G** | **C** | **A** | **A** | **C** | **G** | **C** | **G** | **G** | **C** | **G** | **A** | **G** | **C** | **G** | **A** | **G** | **T** | **A** | **T** | **T** | **G** | **G** | **A** | **A** | **C** | **C** | **T** | **G** | **A** | **A** | **G** | **T** | **G** | **A** | **C** | **A** | **A** | **C** | **A** | **A** | **A** | **A** | **A** | **T** | **G** | **A** | **T** | **G** | **G** | **A** | **A** | **A** | **A** | **A** | **A** | **T** | **G** | **A** | **G** | **T** | **C** | **G** | **G** | **C** | **G** | **A** | **A** | **A** | **T** | **G** | **A** | **T** | **C** | **C** | **A** | **G** | **T** | **A** | **T** | **T** | **G** | **C** | **A** | **T** | **G** | **A** | **A** | **G** | **A** | **A** | **T** | **T** | **A** | **A** | **C** | **G** | **A** | **A** | **T** | **C** | **G** | **A** | **G** | **A** | **A** | **A** | **A** | **T** | **G** | **A** | **A** | **A** | **T** | **T** | **T** | **T** | **G** | **A** | **T** | **G** | **C** | **T** | **G** | **A** | **T** | **T** | **T** | **C** | **T** | **G** | **A** | **A** | **G** | **G** | **G** | **A** | **A** | **A** | **A** | **G** | **T** | **A** | **A** | **T** | **C** | **A** | **G** | **G** | **A** | **A** | **A** | **T** | **C** | **G** | **C** | **G** | **G** | **A** | **T** | **G** | **A** | **A** | **T** | **T** | **G** | **T** | **T** | **T** | **A** | **T** | **C** | **A** | **C** | **A** | **C** | **T** | **G** | **A** | **A** | **A** | **A** | **C** | **A** | **G** | **T** | **T** | **A** | **A** | **A** | **A** | **C** | **G** | **C** | **A** | **T** | **G** | **T** | **G** | **T** | **C** | **G** | **A** | **A** | **T** | **A** | **T** | **A** | **T** | **T** | **A** | **G** | **C** | **T** | **A** | **A** | **G** | **T** | **T** | **A** | **G** | **A** | **G** | **G** | **T** | **A** | **G** | **A** | **A** | **G** | **A** | **C** | **C** | **G** | **A** | **A** | **C** | **T** | **C** | **A** | **A** | **G** | **C** | **A** | **G** | **C** | **T** | **A** | **T** | **C** | **T** | **A** | **T** | **G** | **C** | **G** | **T** | **T** | **T** | **A** | **A** | **A** | **C** | **A** | **T** | **G** | **G** | **T** | **T** | **T** | **G** | **G** | **T** | **G** | **A** | **A** | **A** | **-** | **-** | **-** |
| **N60299F** | **A** | **T** | **G** | **A** | **T** | **A** | **A** | **A** | **A** | **G** | **T** | **T** | **T** | **T** | **A** | **T** | **T** | **A** | **G** | **T** | **A** | **G** | **A** | **T** | **G** | **A** | **C** | **C** | **A** | **T** | **G** | **A** | **A** | **A** | **T** | **G** | **G** | **T** | **A** | **C** | **G** | **C** | **T** | **T** | **A** | **G** | **G** | **C** | **G** | **T** | **C** | **T** | **C** | **T** | **T** | **C** | **T** | **T** | **A** | **T** | **T** | **T** | **A** | **T** | **C** | **T** | **A** | **T** | **C** | **C** | **A** | **A** | **G** | **A** | **A** | **G** | **A** | **C** | **A** | **T** | **T** | **G** | **A** | **A** | **G** | **T** | **G** | **A** | **T** | **T** | **G** | **G** | **A** | **G** | **A** | **A** | **G** | **C** | **T** | **G** | **A** | **A** | **A** | **A** | **T** | **G** | **G** | **A** | **C** | **G** | **T** | **C** | **A** | **A** | **G** | **G** | **C** | **T** | **A** | **T** | **G** | **A** | **A** | **A** | **A** | **G** | **G** | **C** | **G** | **A** | **T** | **G** | **G** | **C** | **A** | **C** | **T** | **T** | **C** | **G** | **A** | **C** | **C** | **A** | **G** | **A** | **T** | **G** | **T** | **C** | **A** | **T** | **T** | **T** | **T** | **A** | **A** | **T** | **G** | **G** | **A** | **T** | **C** | **T** | **A** | **G** | **T** | **C** | **A** | **T** | **G** | **G** | **A** | **A** | **G** | **A** | **A** | **A** | **T** | **G** | **G** | **A** | **T** | **G** | **G** | **C** | **A** | **T** | **C** | **G** | **A** | **A** | **T** | **C** | **A** | **A** | **C** | **A** | **A** | **A** | **A** | **G** | **C** | **A** | **A** | **T** | **C** | **T** | **T** | **A** | **A** | **A** | **A** | **G** | **A** | **T** | **T** | **G** | **G** | **C** | **C** | **A** | **A** | **A** | **A** | **G** | **C** | **C** | **A** | **A** | **A** | **A** | **T** | **C** | **A** | **T** | **T** | **A** | **T** | **C** | **G** | **T** | **C** | **A** | **C** | **A** | **A** | **G** | **T** | **T** | **T** | **T** | **A** | **T** | **T** | **G** | **A** | **T** | **G** | **A** | **T** | **G** | **A** | **A** | **A** | **A** | **A** | **G** | **T** | **T** | **T** | **A** | **T** | **C** | **C** | **T** | **G** | **C** | **A** | **A** | **T** | **T** | **G** | **A** | **A** | **G** | **C** | **A** | **G** | **G** | **A** | **G** | **C** | **A** | **G** | **C** | **A** | **G** | **G** | **A** | **T** | **A** | **T** | **T** | **T** | **A** | **T** | **T** | **G** | **A** | **A** | **G** | **A** | **C** | **G** | **T** | **C** | **T** | **A** | **C** | **C** | **G** | **C** | **A** | **C** | **A** | **T** | **G** | **A** | **A** | **A** | **T** | **A** | **G** | **C** | **T** | **G** | **A** | **T** | **G** | **C** | **T** | **A** | **T** | **A** | **A** | **G** | **G** | **G** | **C** | **T** | **A** | **C** | **G** | **C** | **A** | **A** | **C** | **G** | **C** | **G** | **G** | **C** | **G** | **A** | **G** | **C** | **G** | **A** | **G** | **T** | **A** | **T** | **T** | **G** | **G** | **A** | **A** | **C** | **C** | **T** | **G** | **A** | **A** | **G** | **T** | **G** | **A** | **C** | **A** | **A** | **C** | **A** | **A** | **A** | **A** | **A** | **T** | **G** | **A** | **T** | **G** | **G** | **A** | **A** | **A** | **A** | **A** | **A** | **T** | **G** | **A** | **G** | **T** | **C** | **G** | **G** | **C** | **G** | **A** | **A** | **A** | **T** | **G** | **A** | **T** | **C** | **C** | **A** | **G** | **T** | **A** | **T** | **T** | **G** | **C** | **A** | **T** | **G** | **A** | **A** | **G** | **A** | **A** | **T** | **T** | **A** | **A** | **C** | **G** | **A** | **A** | **T** | **C** | **G** | **A** | **G** | **A** | **A** | **A** | **A** | **T** | **G** | **A** | **A** | **A** | **T** | **T** | **T** | **T** | **G** | **A** | **T** | **G** | **C** | **T** | **G** | **A** | **T** | **T** | **T** | **C** | **T** | **G** | **A** | **A** | **G** | **G** | **G** | **A** | **A** | **A** | **A** | **G** | **T** | **A** | **A** | **T** | **C** | **A** | **G** | **G** | **A** | **A** | **A** | **T** | **C** | **G** | **C** | **G** | **G** | **A** | **T** | **G** | **A** | **A** | **T** | **T** | **G** | **T** | **T** | **T** | **A** | **T** | **C** | **A** | **C** | **A** | **C** | **T** | **G** | **A** | **A** | **A** | **A** | **C** | **A** | **G** | **T** | **T** | **A** | **A** | **A** | **A** | **C** | **G** | **C** | **A** | **T** | **G** | **T** | **G** | **T** | **C** | **G** | **A** | **A** | **T** | **A** | **T** | **A** | **T** | **T** | **A** | **G** | **C** | **T** | **A** | **A** | **G** | **T** | **T** | **A** | **G** | **A** | **G** | **G** | **T** | **A** | **G** | **A** | **A** | **G** | **A** | **C** | **C** | **G** | **A** | **A** | **C** | **T** | **C** | **A** | **A** | **G** | **C** | **A** | **G** | **C** | **T** | **A** | **T** | **C** | **T** | **A** | **T** | **G** | **C** | **G** | **T** | **T** | **T** | **A** | **A** | **A** | **C** | **A** | **T** | **G** | **G** | **T** | **T** | **T** | **G** | **G** | **T** | **G** | **A** | **A** | **A** | **-** | **-** | **-** |
| **N59591F** | **G** | **T** | **G** | **A** | **T** | **C** | **A** | **A** | **A** | **G** | **T** | **A** | **A** | **T** | **G** | **T** | **T** | **A** | **G** | **T** | **G** | **G** | **A** | **T** | **G** | **A** | **C** | **C** | **A** | **T** | **G** | **A** | **A** | **A** | **T** | **G** | **G** | **T** | **C** | **C** | **G** | **T** | **T** | **T** | **A** | **G** | **G** | **C** | **G** | **T** | **T** | **T** | **C** | **A** | **T** | **C** | **A** | **T** | **A** | **T** | **T** | **T** | **A** | **T** | **C** | **T** | **A** | **T** | **T** | **C** | **A** | **A** | **G** | **A** | **G** | **G** | **A** | **T** | **A** | **T** | **A** | **G** | **A** | **A** | **G** | **T** | **C** | **G** | **T** | **A** | **G** | **G** | **C** | **G** | **A** | **A** | **G** | **C** | **A** | **G** | **A** | **A** | **A** | **A** | **C** | **G** | **G** | **T** | **A** | **A** | **G** | **A** | **T** | **T** | **G** | **G** | **C** | **T** | **A** | **T** | **G** | **A** | **A** | **A** | **A** | **A** | **G** | **C** | **A** | **T** | **T** | **G** | **G** | **A** | **A** | **C** | **T** | **A** | **C** | **G** | **T** | **C** | **C** | **A** | **G** | **A** | **T** | **G** | **T** | **T** | **A** | **T** | **T** | **T** | **T** | **G** | **A** | **T** | **G** | **G** | **A** | **T** | **T** | **T** | **G** | **G** | **T** | **A** | **A** | **T** | **G** | **G** | **A** | **A** | **G** | **A** | **A** | **A** | **T** | **G** | **G** | **A** | **T** | **G** | **G** | **C** | **A** | **T** | **T** | **G** | **A** | **T** | **T** | **C** | **A** | **A** | **C** | **C** | **A** | **A** | **A** | **G** | **C** | **G** | **A** | **T** | **C** | **T** | **T** | **G** | **A** | **A** | **A** | **G** | **A** | **T** | **T** | **G** | **G** | **C** | **C** | **A** | **G** | **A** | **A** | **G** | **C** | **C** | **A** | **A** | **G** | **A** | **T** | **T** | **A** | **T** | **T** | **A** | **T** | **T** | **G** | **T** | **G** | **A** | **C** | **G** | **A** | **G** | **T** | **T** | **T** | **T** | **A** | **T** | **T** | **G** | **A** | **T** | **G** | **A** | **T** | **G** | **A** | **A** | **A** | **A** | **A** | **G** | **T** | **G** | **T** | **A** | **T** | **C** | **C** | **G** | **G** | **C** | **G** | **A** | **T** | **T** | **G** | **A** | **A** | **G** | **C** | **T** | **G** | **G** | **T** | **G** | **C** | **A** | **G** | **C** | **G** | **G** | **G** | **C** | **T** | **A** | **C** | **C** | **T** | **A** | **T** | **T** | **A** | **A** | **A** | **A** | **A** | **C** | **A** | **T** | **C** | **A** | **A** | **C** | **A** | **G** | **C** | **A** | **C** | **A** | **T** | **G** | **A** | **G** | **A** | **T** | **T** | **G** | **C** | **T** | **G** | **A** | **T** | **G** | **C** | **A** | **A** | **T** | **T** | **C** | **G** | **G** | **G** | **C** | **G** | **A** | **C** | **T** | **T** | **A** | **T** | **C** | **G** | **C** | **G** | **G** | **A** | **G** | **A** | **G** | **C** | **G** | **T** | **G** | **T** | **G** | **T** | **T** | **G** | **G** | **A** | **G** | **C** | **C** | **T** | **G** | **A** | **A** | **G** | **T** | **G** | **A** | **C** | **G** | **C** | **A** | **T** | **A** | **A** | **G** | **A** | **T** | **G** | **A** | **T** | **G** | **G** | **A** | **A** | **C** | **G** | **G** | **T** | **T** | **A** | **A** | **C** | **A** | **A** | **A** | **A** | **A** | **A** | **A** | **C** | **A** | **A** | **G** | **A** | **G** | **C** | **C** | **G** | **G** | **T** | **G** | **T** | **T** | **G** | **C** | **A** | **T** | **G** | **A** | **A** | **G** | **A** | **T** | **T** | **T** | **G** | **A** | **C** | **A** | **A** | **A** | **T** | **C** | **G** | **A** | **G** | **A** | **A** | **C** | **A** | **C** | **G** | **A** | **A** | **A** | **T** | **T** | **T** | **T** | **A** | **A** | **T** | **G** | **T** | **T** | **G** | **A** | **T** | **T** | **G** | **C** | **A** | **C** | **A** | **A** | **G** | **G** | **T** | **A** | **A** | **A** | **A** | **G** | **T** | **A** | **A** | **T** | **C** | **A** | **G** | **G** | **A** | **A** | **A** | **T** | **A** | **G** | **C** | **T** | **G** | **A** | **T** | **G** | **A** | **A** | **C** | **T** | **C** | **T** | **T** | **T** | **A** | **T** | **C** | **A** | **C** | **T** | **T** | **T** | **G** | **A** | **A** | **A** | **A** | **C** | **A** | **G** | **T** | **T** | **A** | **A** | **A** | **A** | **C** | **A** | **C** | **A** | **T** | **G** | **T** | **T** | **T** | **C** | **A** | **A** | **A** | **C** | **A** | **T** | **T** | **T** | **T** | **A** | **G** | **C** | **A** | **A** | **A** | **A** | **C** | **T** | **A** | **G** | **A** | **T** | **G** | **T** | **G** | **G** | **A** | **T** | **G** | **A** | **T** | **C** | **G** | **G** | **A** | **C** | **C** | **C** | **A** | **A** | **G** | **C** | **G** | **G** | **C** | **G** | **A** | **T** | **T** | **T** | **A** | **T** | **G** | **C** | **T** | **T** | **T** | **T** | **C** | **A** | **A** | **C** | **A** | **T** | **G** | **G** | **T** | **T** | **T** | **A** | **G** | **C** | **C** | **A** | **A** | **A** | **-** | **-** | **-** |
| **N52773** | **G** | **T** | **G** | **A** | **T** | **C** | **A** | **A** | **A** | **G** | **T** | **A** | **A** | **T** | **G** | **T** | **T** | **A** | **G** | **T** | **G** | **G** | **A** | **T** | **G** | **A** | **C** | **C** | **A** | **T** | **G** | **A** | **A** | **A** | **T** | **G** | **G** | **T** | **C** | **C** | **G** | **T** | **T** | **T** | **A** | **G** | **G** | **C** | **G** | **T** | **T** | **T** | **C** | **A** | **T** | **C** | **A** | **T** | **A** | **T** | **T** | **T** | **A** | **T** | **C** | **T** | **A** | **T** | **T** | **C** | **A** | **A** | **G** | **A** | **G** | **G** | **A** | **T** | **A** | **T** | **A** | **G** | **A** | **A** | **G** | **T** | **C** | **G** | **T** | **A** | **G** | **G** | **C** | **G** | **A** | **A** | **G** | **C** | **A** | **G** | **A** | **A** | **A** | **A** | **T** | **G** | **G** | **T** | **A** | **A** | **G** | **A** | **T** | **T** | **G** | **G** | **C** | **T** | **A** | **T** | **G** | **A** | **A** | **A** | **A** | **A** | **G** | **C** | **A** | **T** | **T** | **G** | **G** | **A** | **A** | **C** | **T** | **A** | **C** | **G** | **T** | **C** | **C** | **A** | **G** | **A** | **T** | **G** | **T** | **T** | **A** | **T** | **T** | **T** | **T** | **G** | **A** | **T** | **G** | **G** | **A** | **T** | **T** | **T** | **G** | **G** | **T** | **A** | **A** | **T** | **G** | **G** | **A** | **A** | **G** | **A** | **A** | **A** | **T** | **G** | **G** | **A** | **C** | **G** | **G** | **C** | **A** | **T** | **T** | **G** | **A** | **T** | **T** | **C** | **A** | **A** | **C** | **A** | **A** | **A** | **A** | **G** | **C** | **G** | **A** | **T** | **C** | **T** | **T** | **G** | **A** | **A** | **A** | **G** | **A** | **T** | **T** | **G** | **G** | **C** | **C** | **A** | **G** | **A** | **A** | **G** | **C** | **C** | **A** | **A** | **G** | **A** | **T** | **T** | **A** | **T** | **T** | **A** | **T** | **T** | **G** | **T** | **G** | **A** | **C** | **G** | **A** | **G** | **T** | **T** | **T** | **T** | **A** | **T** | **T** | **G** | **A** | **T** | **G** | **A** | **T** | **G** | **A** | **A** | **A** | **A** | **A** | **G** | **T** | **G** | **T** | **A** | **T** | **C** | **C** | **G** | **G** | **C** | **G** | **A** | **T** | **T** | **G** | **A** | **A** | **G** | **C** | **T** | **G** | **G** | **T** | **G** | **C** | **A** | **G** | **C** | **G** | **G** | **G** | **C** | **T** | **A** | **C** | **C** | **T** | **A** | **T** | **T** | **A** | **A** | **A** | **A** | **A** | **C** | **A** | **T** | **C** | **A** | **A** | **C** | **A** | **G** | **C** | **A** | **C** | **A** | **T** | **G** | **A** | **G** | **A** | **T** | **T** | **G** | **C** | **T** | **G** | **A** | **T** | **G** | **C** | **A** | **A** | **T** | **T** | **C** | **G** | **G** | **G** | **C** | **G** | **A** | **C** | **T** | **T** | **A** | **T** | **C** | **G** | **C** | **G** | **G** | **A** | **G** | **A** | **G** | **C** | **G** | **T** | **G** | **T** | **G** | **T** | **T** | **G** | **G** | **A** | **A** | **C** | **C** | **T** | **G** | **A** | **A** | **G** | **T** | **G** | **A** | **C** | **A** | **C** | **A** | **T** | **A** | **A** | **G** | **A** | **T** | **G** | **A** | **T** | **G** | **G** | **A** | **A** | **C** | **G** | **G** | **T** | **T** | **A** | **A** | **C** | **A** | **A** | **A** | **A** | **A** | **A** | **A** | **C** | **A** | **A** | **G** | **A** | **G** | **C** | **C** | **G** | **G** | **T** | **G** | **T** | **T** | **G** | **C** | **A** | **C** | **G** | **A** | **A** | **G** | **A** | **T** | **T** | **T** | **G** | **A** | **C** | **A** | **A** | **A** | **C** | **C** | **G** | **G** | **G** | **A** | **A** | **C** | **A** | **C** | **G** | **A** | **A** | **A** | **T** | **T** | **T** | **T** | **A** | **A** | **T** | **G** | **T** | **T** | **G** | **A** | **T** | **T** | **G** | **C** | **A** | **C** | **A** | **A** | **G** | **G** | **T** | **A** | **A** | **A** | **A** | **G** | **T** | **A** | **A** | **T** | **C** | **A** | **G** | **G** | **A** | **A** | **A** | **T** | **A** | **G** | **C** | **T** | **G** | **A** | **T** | **G** | **A** | **A** | **C** | **T** | **C** | **T** | **T** | **T** | **A** | **T** | **C** | **A** | **C** | **T** | **T** | **T** | **G** | **A** | **A** | **A** | **A** | **C** | **A** | **G** | **T** | **T** | **A** | **A** | **A** | **A** | **C** | **A** | **C** | **A** | **T** | **G** | **T** | **T** | **T** | **C** | **A** | **A** | **A** | **C** | **A** | **T** | **T** | **T** | **T** | **A** | **G** | **C** | **A** | **A** | **A** | **A** | **C** | **T** | **A** | **G** | **A** | **T** | **G** | **T** | **G** | **G** | **A** | **T** | **G** | **A** | **T** | **C** | **G** | **G** | **A** | **C** | **C** | **C** | **A** | **A** | **G** | **C** | **G** | **G** | **C** | **G** | **A** | **T** | **T** | **T** | **A** | **T** | **G** | **C** | **T** | **T** | **T** | **T** | **C** | **A** | **A** | **C** | **A** | **T** | **G** | **G** | **T** | **T** | **T** | **A** | **G** | **C** | **C** | **A** | **A** | **A** | **-** | **-** | **-** |
| **N60357F** | **A** | **T** | **G** | **A** | **T** | **A** | **A** | **A** | **A** | **G** | **T** | **T** | **T** | **T** | **A** | **T** | **T** | **A** | **G** | **T** | **A** | **G** | **A** | **T** | **G** | **A** | **C** | **C** | **A** | **T** | **G** | **A** | **A** | **A** | **T** | **G** | **G** | **T** | **A** | **C** | **G** | **C** | **T** | **T** | **A** | **G** | **G** | **C** | **G** | **T** | **C** | **T** | **C** | **T** | **T** | **C** | **T** | **T** | **A** | **T** | **T** | **T** | **A** | **T** | **C** | **T** | **A** | **T** | **C** | **C** | **A** | **A** | **G** | **A** | **A** | **G** | **A** | **C** | **A** | **T** | **T** | **G** | **A** | **A** | **G** | **T** | **G** | **A** | **T** | **T** | **G** | **G** | **A** | **G** | **A** | **A** | **G** | **C** | **T** | **G** | **A** | **A** | **A** | **A** | **T** | **G** | **G** | **A** | **C** | **G** | **T** | **C** | **A** | **A** | **G** | **G** | **C** | **T** | **A** | **T** | **G** | **A** | **A** | **A** | **A** | **G** | **G** | **C** | **G** | **A** | **T** | **G** | **G** | **C** | **A** | **C** | **T** | **T** | **C** | **G** | **A** | **C** | **C** | **A** | **G** | **A** | **T** | **G** | **T** | **C** | **A** | **T** | **T** | **T** | **T** | **A** | **A** | **T** | **G** | **G** | **A** | **T** | **C** | **T** | **A** | **G** | **T** | **C** | **A** | **T** | **G** | **G** | **A** | **A** | **G** | **A** | **A** | **A** | **T** | **G** | **G** | **A** | **T** | **G** | **G** | **C** | **A** | **T** | **C** | **G** | **A** | **A** | **T** | **C** | **A** | **A** | **C** | **A** | **A** | **A** | **A** | **G** | **C** | **A** | **A** | **T** | **C** | **T** | **T** | **A** | **A** | **A** | **A** | **G** | **A** | **T** | **T** | **G** | **G** | **C** | **C** | **A** | **A** | **A** | **A** | **G** | **C** | **C** | **A** | **A** | **A** | **A** | **T** | **C** | **A** | **T** | **T** | **A** | **T** | **C** | **G** | **T** | **C** | **A** | **C** | **A** | **A** | **G** | **T** | **T** | **T** | **T** | **A** | **T** | **T** | **G** | **A** | **T** | **G** | **A** | **T** | **G** | **A** | **A** | **A** | **A** | **A** | **G** | **T** | **T** | **T** | **A** | **T** | **C** | **C** | **T** | **G** | **C** | **A** | **A** | **T** | **T** | **G** | **A** | **A** | **G** | **C** | **A** | **G** | **G** | **A** | **G** | **C** | **A** | **G** | **C** | **A** | **G** | **G** | **A** | **T** | **A** | **T** | **T** | **T** | **A** | **T** | **T** | **G** | **A** | **A** | **G** | **A** | **C** | **G** | **T** | **C** | **T** | **A** | **C** | **C** | **G** | **C** | **A** | **C** | **A** | **T** | **G** | **A** | **A** | **A** | **T** | **A** | **G** | **C** | **T** | **G** | **A** | **T** | **G** | **C** | **T** | **A** | **T** | **A** | **A** | **G** | **G** | **G** | **C** | **T** | **A** | **C** | **G** | **C** | **A** | **A** | **C** | **G** | **C** | **G** | **G** | **C** | **G** | **A** | **G** | **C** | **G** | **A** | **G** | **T** | **A** | **T** | **T** | **G** | **G** | **A** | **A** | **C** | **C** | **T** | **G** | **A** | **A** | **G** | **T** | **G** | **A** | **C** | **A** | **A** | **C** | **A** | **A** | **A** | **A** | **A** | **T** | **G** | **A** | **T** | **G** | **G** | **A** | **A** | **A** | **A** | **A** | **A** | **T** | **G** | **A** | **G** | **T** | **C** | **G** | **G** | **C** | **G** | **A** | **A** | **A** | **T** | **G** | **A** | **T** | **C** | **C** | **A** | **G** | **T** | **A** | **T** | **T** | **G** | **C** | **A** | **T** | **G** | **A** | **A** | **G** | **A** | **A** | **T** | **T** | **A** | **A** | **C** | **G** | **A** | **A** | **T** | **C** | **G** | **A** | **G** | **A** | **A** | **A** | **A** | **T** | **G** | **A** | **A** | **A** | **T** | **T** | **T** | **T** | **G** | **A** | **T** | **G** | **C** | **T** | **G** | **A** | **T** | **T** | **T** | **C** | **T** | **G** | **A** | **A** | **G** | **G** | **G** | **A** | **A** | **A** | **A** | **G** | **T** | **A** | **A** | **T** | **C** | **A** | **G** | **G** | **A** | **A** | **A** | **T** | **C** | **G** | **C** | **G** | **G** | **A** | **T** | **G** | **A** | **A** | **T** | **T** | **G** | **T** | **T** | **T** | **A** | **T** | **C** | **A** | **C** | **A** | **C** | **T** | **G** | **A** | **A** | **A** | **A** | **C** | **A** | **G** | **T** | **T** | **A** | **A** | **A** | **A** | **C** | **G** | **C** | **A** | **T** | **G** | **T** | **G** | **T** | **C** | **G** | **A** | **A** | **T** | **A** | **T** | **A** | **T** | **T** | **A** | **G** | **C** | **T** | **A** | **A** | **G** | **T** | **T** | **A** | **G** | **A** | **G** | **G** | **T** | **A** | **G** | **A** | **A** | **G** | **A** | **C** | **C** | **G** | **A** | **A** | **C** | **T** | **C** | **A** | **A** | **G** | **C** | **A** | **G** | **C** | **T** | **A** | **T** | **C** | **T** | **A** | **T** | **G** | **C** | **G** | **T** | **T** | **T** | **A** | **A** | **A** | **C** | **A** | **T** | **G** | **G** | **T** | **T** | **T** | **G** | **G** | **T** | **G** | **A** | **A** | **A** | **-** | **-** | **-** |
| **N59509F** | **G** | **T** | **G** | **A** | **T** | **C** | **A** | **A** | **A** | **G** | **T** | **A** | **A** | **T** | **G** | **T** | **T** | **A** | **G** | **T** | **G** | **G** | **A** | **T** | **G** | **A** | **C** | **C** | **A** | **T** | **G** | **A** | **A** | **A** | **T** | **G** | **G** | **T** | **C** | **C** | **G** | **T** | **T** | **T** | **A** | **G** | **G** | **C** | **G** | **T** | **T** | **T** | **C** | **A** | **T** | **C** | **A** | **T** | **A** | **T** | **T** | **T** | **A** | **T** | **C** | **T** | **A** | **T** | **T** | **C** | **A** | **A** | **G** | **A** | **G** | **G** | **A** | **T** | **A** | **T** | **A** | **G** | **A** | **A** | **G** | **T** | **C** | **G** | **T** | **A** | **G** | **G** | **C** | **G** | **A** | **A** | **G** | **C** | **A** | **G** | **A** | **A** | **A** | **A** | **C** | **G** | **G** | **T** | **A** | **A** | **G** | **A** | **T** | **T** | **G** | **G** | **C** | **T** | **A** | **T** | **G** | **A** | **A** | **A** | **A** | **A** | **G** | **C** | **A** | **T** | **T** | **G** | **G** | **A** | **A** | **C** | **T** | **A** | **C** | **G** | **T** | **C** | **C** | **A** | **G** | **A** | **T** | **G** | **T** | **T** | **A** | **T** | **T** | **T** | **T** | **G** | **A** | **T** | **G** | **G** | **A** | **T** | **T** | **T** | **G** | **G** | **T** | **A** | **A** | **T** | **G** | **G** | **A** | **A** | **G** | **A** | **A** | **A** | **T** | **G** | **G** | **A** | **C** | **G** | **G** | **C** | **A** | **T** | **T** | **G** | **A** | **T** | **T** | **C** | **A** | **A** | **C** | **A** | **A** | **A** | **A** | **G** | **C** | **G** | **A** | **T** | **C** | **T** | **T** | **G** | **A** | **A** | **A** | **G** | **A** | **T** | **T** | **G** | **G** | **C** | **C** | **A** | **G** | **A** | **A** | **G** | **C** | **C** | **A** | **A** | **G** | **A** | **T** | **T** | **A** | **T** | **T** | **A** | **T** | **T** | **G** | **T** | **G** | **A** | **C** | **G** | **A** | **G** | **T** | **T** | **T** | **T** | **A** | **T** | **T** | **G** | **A** | **T** | **G** | **A** | **T** | **G** | **A** | **A** | **A** | **A** | **A** | **G** | **T** | **G** | **T** | **A** | **T** | **C** | **C** | **G** | **G** | **C** | **G** | **A** | **T** | **T** | **G** | **A** | **A** | **G** | **C** | **T** | **G** | **G** | **T** | **G** | **C** | **A** | **G** | **C** | **G** | **G** | **G** | **C** | **T** | **A** | **C** | **C** | **T** | **A** | **T** | **T** | **A** | **A** | **A** | **A** | **A** | **C** | **A** | **T** | **C** | **A** | **A** | **C** | **A** | **G** | **C** | **A** | **C** | **A** | **T** | **G** | **A** | **G** | **A** | **T** | **T** | **G** | **C** | **T** | **G** | **A** | **T** | **G** | **C** | **A** | **A** | **T** | **T** | **C** | **G** | **G** | **G** | **C** | **G** | **A** | **C** | **T** | **T** | **A** | **T** | **C** | **G** | **C** | **G** | **G** | **A** | **G** | **A** | **G** | **C** | **G** | **T** | **G** | **T** | **G** | **T** | **T** | **G** | **G** | **A** | **A** | **C** | **C** | **T** | **G** | **A** | **A** | **G** | **T** | **G** | **A** | **C** | **G** | **C** | **A** | **T** | **A** | **A** | **G** | **A** | **T** | **G** | **A** | **T** | **G** | **G** | **A** | **A** | **C** | **G** | **G** | **T** | **T** | **A** | **A** | **C** | **A** | **A** | **A** | **A** | **A** | **A** | **A** | **C** | **A** | **A** | **G** | **A** | **G** | **C** | **C** | **G** | **G** | **T** | **G** | **T** | **T** | **G** | **C** | **A** | **C** | **G** | **A** | **A** | **G** | **A** | **T** | **T** | **T** | **G** | **A** | **C** | **A** | **A** | **A** | **C** | **C** | **G** | **A** | **G** | **A** | **A** | **C** | **A** | **C** | **G** | **A** | **A** | **A** | **T** | **T** | **T** | **T** | **A** | **A** | **T** | **G** | **T** | **T** | **G** | **A** | **T** | **T** | **G** | **C** | **A** | **C** | **A** | **A** | **G** | **G** | **T** | **A** | **A** | **A** | **A** | **G** | **T** | **A** | **A** | **T** | **C** | **A** | **G** | **G** | **A** | **A** | **A** | **T** | **T** | **G** | **C** | **T** | **G** | **A** | **T** | **G** | **A** | **A** | **C** | **T** | **C** | **T** | **T** | **T** | **A** | **T** | **C** | **A** | **C** | **T** | **T** | **T** | **G** | **A** | **A** | **A** | **A** | **C** | **A** | **G** | **T** | **T** | **A** | **A** | **A** | **A** | **C** | **A** | **C** | **A** | **T** | **G** | **T** | **T** | **T** | **C** | **A** | **A** | **A** | **C** | **A** | **T** | **T** | **T** | **T** | **A** | **G** | **C** | **A** | **A** | **A** | **A** | **C** | **T** | **A** | **G** | **A** | **T** | **G** | **T** | **G** | **G** | **A** | **T** | **G** | **A** | **T** | **C** | **G** | **G** | **A** | **C** | **C** | **C** | **A** | **A** | **G** | **C** | **G** | **G** | **C** | **G** | **A** | **T** | **T** | **T** | **A** | **T** | **G** | **C** | **T** | **T** | **T** | **T** | **C** | **A** | **A** | **C** | **A** | **T** | **G** | **G** | **T** | **T** | **T** | **A** | **G** | **C** | **C** | **A** | **A** | **A** | **-** | **-** | **-** |
| **N55753** | **G** | **T** | **G** | **A** | **T** | **C** | **A** | **A** | **A** | **G** | **T** | **A** | **A** | **T** | **G** | **T** | **T** | **A** | **G** | **T** | **G** | **G** | **A** | **T** | **G** | **A** | **C** | **C** | **A** | **T** | **G** | **A** | **A** | **A** | **T** | **G** | **G** | **T** | **C** | **C** | **G** | **T** | **T** | **T** | **A** | **G** | **G** | **C** | **G** | **T** | **T** | **T** | **C** | **A** | **T** | **C** | **A** | **T** | **A** | **T** | **T** | **T** | **A** | **T** | **C** | **T** | **A** | **T** | **T** | **C** | **A** | **A** | **G** | **A** | **G** | **G** | **A** | **T** | **A** | **T** | **A** | **G** | **A** | **A** | **G** | **T** | **C** | **G** | **T** | **A** | **G** | **G** | **C** | **G** | **A** | **A** | **G** | **C** | **A** | **G** | **A** | **A** | **A** | **A** | **C** | **G** | **G** | **T** | **A** | **A** | **G** | **A** | **T** | **T** | **G** | **G** | **C** | **T** | **A** | **T** | **G** | **A** | **A** | **A** | **A** | **A** | **G** | **C** | **A** | **T** | **T** | **G** | **G** | **A** | **A** | **C** | **T** | **A** | **C** | **G** | **T** | **C** | **C** | **A** | **G** | **A** | **T** | **G** | **T** | **T** | **A** | **T** | **T** | **T** | **T** | **G** | **A** | **T** | **G** | **G** | **A** | **T** | **T** | **T** | **G** | **G** | **T** | **A** | **A** | **T** | **G** | **G** | **A** | **A** | **G** | **A** | **A** | **A** | **T** | **G** | **G** | **A** | **C** | **G** | **G** | **C** | **A** | **T** | **T** | **G** | **A** | **T** | **T** | **C** | **A** | **A** | **C** | **A** | **A** | **A** | **A** | **G** | **C** | **G** | **A** | **T** | **C** | **T** | **T** | **G** | **A** | **A** | **A** | **G** | **A** | **T** | **T** | **G** | **G** | **C** | **C** | **A** | **G** | **A** | **A** | **G** | **C** | **C** | **A** | **A** | **G** | **A** | **T** | **T** | **A** | **T** | **T** | **A** | **T** | **T** | **G** | **T** | **G** | **A** | **C** | **G** | **A** | **G** | **T** | **T** | **T** | **T** | **A** | **T** | **T** | **G** | **A** | **T** | **G** | **A** | **T** | **G** | **A** | **A** | **A** | **A** | **A** | **G** | **T** | **G** | **T** | **A** | **T** | **C** | **C** | **G** | **G** | **C** | **G** | **A** | **T** | **T** | **G** | **A** | **A** | **G** | **C** | **T** | **G** | **G** | **T** | **G** | **C** | **A** | **G** | **C** | **G** | **G** | **G** | **C** | **T** | **A** | **C** | **C** | **T** | **A** | **T** | **T** | **A** | **A** | **A** | **A** | **A** | **C** | **A** | **T** | **C** | **A** | **A** | **C** | **A** | **G** | **C** | **A** | **C** | **A** | **T** | **G** | **A** | **G** | **A** | **T** | **T** | **G** | **C** | **T** | **G** | **A** | **T** | **G** | **C** | **A** | **A** | **T** | **T** | **C** | **G** | **G** | **G** | **C** | **G** | **A** | **C** | **T** | **T** | **A** | **T** | **C** | **G** | **C** | **G** | **G** | **A** | **G** | **A** | **G** | **C** | **G** | **T** | **G** | **T** | **G** | **T** | **T** | **G** | **G** | **A** | **A** | **C** | **C** | **T** | **G** | **A** | **A** | **G** | **T** | **G** | **A** | **C** | **G** | **C** | **A** | **T** | **A** | **A** | **G** | **A** | **T** | **G** | **A** | **T** | **G** | **G** | **A** | **A** | **C** | **G** | **G** | **T** | **T** | **A** | **A** | **C** | **A** | **A** | **A** | **A** | **A** | **A** | **A** | **C** | **A** | **A** | **G** | **A** | **G** | **C** | **C** | **G** | **G** | **T** | **G** | **T** | **T** | **G** | **C** | **A** | **T** | **G** | **A** | **A** | **G** | **A** | **T** | **T** | **T** | **G** | **A** | **C** | **A** | **A** | **A** | **T** | **C** | **G** | **A** | **G** | **A** | **A** | **C** | **A** | **C** | **G** | **A** | **A** | **A** | **T** | **T** | **T** | **T** | **A** | **A** | **T** | **G** | **T** | **T** | **G** | **A** | **T** | **T** | **G** | **C** | **A** | **C** | **A** | **A** | **G** | **G** | **T** | **A** | **A** | **A** | **A** | **G** | **T** | **A** | **A** | **T** | **C** | **A** | **G** | **G** | **A** | **A** | **A** | **T** | **A** | **G** | **C** | **T** | **G** | **A** | **T** | **G** | **A** | **A** | **C** | **T** | **C** | **T** | **T** | **T** | **A** | **T** | **C** | **A** | **C** | **T** | **T** | **T** | **G** | **A** | **A** | **A** | **A** | **C** | **A** | **G** | **T** | **T** | **A** | **A** | **A** | **A** | **C** | **A** | **C** | **A** | **T** | **G** | **T** | **T** | **T** | **C** | **A** | **A** | **A** | **C** | **A** | **T** | **T** | **T** | **T** | **A** | **G** | **C** | **A** | **A** | **A** | **A** | **C** | **T** | **A** | **G** | **A** | **T** | **G** | **T** | **G** | **G** | **A** | **T** | **G** | **A** | **T** | **C** | **G** | **G** | **A** | **C** | **C** | **C** | **A** | **A** | **G** | **C** | **G** | **G** | **C** | **G** | **A** | **T** | **T** | **T** | **A** | **T** | **G** | **C** | **T** | **T** | **T** | **T** | **C** | **A** | **A** | **C** | **A** | **T** | **G** | **G** | **T** | **T** | **T** | **A** | **G** | **C** | **C** | **A** | **A** | **A** | **-** | **-** | **-** |
| **N59559F** | **A** | **T** | **G** | **A** | **T** | **A** | **A** | **A** | **A** | **G** | **T** | **T** | **T** | **T** | **A** | **T** | **T** | **A** | **G** | **T** | **A** | **G** | **A** | **T** | **G** | **A** | **C** | **C** | **A** | **T** | **G** | **A** | **A** | **A** | **T** | **G** | **G** | **T** | **G** | **C** | **G** | **C** | **T** | **T** | **A** | **G** | **G** | **C** | **G** | **T** | **C** | **T** | **C** | **T** | **T** | **C** | **T** | **T** | **A** | **T** | **T** | **T** | **A** | **T** | **C** | **T** | **A** | **T** | **C** | **C** | **A** | **A** | **G** | **A** | **A** | **G** | **A** | **C** | **A** | **T** | **T** | **G** | **A** | **A** | **G** | **T** | **A** | **A** | **T** | **T** | **G** | **G** | **A** | **G** | **A** | **A** | **G** | **C** | **T** | **G** | **A** | **A** | **A** | **A** | **T** | **G** | **G** | **A** | **C** | **G** | **A** | **C** | **A** | **A** | **G** | **G** | **C** | **T** | **A** | **T** | **G** | **A** | **G** | **A** | **A** | **G** | **G** | **C** | **G** | **A** | **T** | **G** | **A** | **C** | **A** | **C** | **T** | **T** | **C** | **G** | **A** | **C** | **C** | **G** | **G** | **A** | **C** | **G** | **T** | **C** | **A** | **T** | **T** | **T** | **T** | **A** | **A** | **T** | **G** | **G** | **A** | **C** | **T** | **T** | **A** | **C** | **T** | **C** | **A** | **T** | **G** | **G** | **A** | **A** | **G** | **A** | **A** | **A** | **T** | **G** | **G** | **A** | **T** | **G** | **G** | **T** | **A** | **T** | **C** | **G** | **A** | **A** | **T** | **C** | **A** | **A** | **C** | **A** | **A** | **A** | **A** | **G** | **C** | **A** | **A** | **T** | **C** | **T** | **T** | **A** | **A** | **A** | **A** | **G** | **A** | **T** | **T** | **G** | **G** | **C** | **C** | **A** | **G** | **A** | **A** | **G** | **C** | **T** | **A** | **A** | **A** | **A** | **T** | **C** | **A** | **T** | **T** | **A** | **T** | **C** | **G** | **T** | **A** | **A** | **C** | **C** | **A** | **G** | **T** | **T** | **T** | **T** | **A** | **T** | **T** | **G** | **A** | **T** | **G** | **A** | **C** | **G** | **A** | **A** | **A** | **A** | **A** | **G** | **T** | **T** | **T** | **A** | **T** | **C** | **C** | **T** | **G** | **C** | **C** | **A** | **T** | **T** | **G** | **A** | **A** | **G** | **C** | **A** | **G** | **G** | **C** | **G** | **C** | **A** | **G** | **C** | **A** | **G** | **G** | **G** | **T** | **A** | **T** | **T** | **T** | **G** | **T** | **T** | **G** | **A** | **A** | **G** | **A** | **C** | **T** | **T** | **C** | **T** | **A** | **C** | **C** | **G** | **C** | **A** | **C** | **A** | **T** | **G** | **A** | **A** | **A** | **T** | **T** | **G** | **C** | **T** | **G** | **A** | **T** | **G** | **C** | **T** | **A** | **T** | **A** | **A** | **G** | **G** | **G** | **C** | **A** | **A** | **C** | **G** | **C** | **A** | **A** | **C** | **G** | **C** | **G** | **G** | **C** | **G** | **A** | **G** | **C** | **G** | **A** | **G** | **T** | **A** | **C** | **T** | **A** | **G** | **A** | **A** | **C** | **C** | **A** | **G** | **A** | **A** | **G** | **T** | **G** | **A** | **C** | **A** | **A** | **C** | **A** | **A** | **A** | **A** | **A** | **T** | **G** | **A** | **T** | **G** | **G** | **A** | **A** | **A** | **A** | **A** | **A** | **T** | **G** | **A** | **G** | **T** | **C** | **G** | **G** | **C** | **G** | **A** | **A** | **A** | **T** | **G** | **A** | **G** | **C** | **C** | **T** | **G** | **T** | **A** | **T** | **T** | **A** | **C** | **A** | **T** | **G** | **A** | **A** | **G** | **A** | **A** | **C** | **T** | **A** | **A** | **C** | **G** | **A** | **A** | **T** | **C** | **G** | **G** | **G** | **A** | **A** | **A** | **A** | **T** | **G** | **A** | **A** | **A** | **T** | **T** | **T** | **T** | **A** | **A** | **T** | **G** | **C** | **T** | **G** | **A** | **T** | **T** | **T** | **C** | **T** | **G** | **A** | **A** | **G** | **G** | **G** | **A** | **A** | **A** | **A** | **G** | **T** | **A** | **A** | **T** | **C** | **A** | **G** | **G** | **A** | **A** | **A** | **T** | **A** | **G** | **C** | **G** | **G** | **A** | **C** | **G** | **A** | **A** | **T** | **T** | **G** | **T** | **T** | **T** | **A** | **T** | **T** | **A** | **C** | **A** | **C** | **T** | **G** | **A** | **A** | **A** | **A** | **C** | **A** | **G** | **T** | **A** | **A** | **A** | **A** | **A** | **C** | **A** | **C** | **A** | **T** | **G** | **T** | **T** | **T** | **C** | **C** | **A** | **A** | **T** | **A** | **T** | **A** | **T** | **T** | **G** | **G** | **C** | **T** | **A** | **A** | **G** | **C** | **T** | **A** | **G** | **A** | **A** | **G** | **T** | **G** | **G** | **A** | **A** | **G** | **A** | **C** | **C** | **G** | **T** | **A** | **C** | **A** | **C** | **A** | **A** | **G** | **C** | **C** | **G** | **C** | **T** | **A** | **T** | **C** | **T** | **A** | **T** | **G** | **C** | **A** | **T** | **T** | **C** | **A** | **A** | **A** | **C** | **A** | **T** | **G** | **G** | **T** | **T** | **T** | **G** | **G** | **T** | **G** | **A** | **A** | **A** | **-** | **-** | **-** |
| **N59600F** | **G** | **T** | **G** | **A** | **T** | **C** | **A** | **A** | **A** | **G** | **T** | **A** | **A** | **T** | **G** | **T** | **T** | **A** | **G** | **T** | **G** | **G** | **A** | **T** | **G** | **A** | **C** | **C** | **A** | **T** | **G** | **A** | **A** | **A** | **T** | **G** | **G** | **T** | **C** | **C** | **G** | **T** | **T** | **T** | **A** | **G** | **G** | **C** | **G** | **T** | **T** | **T** | **C** | **A** | **T** | **C** | **A** | **T** | **A** | **T** | **T** | **T** | **A** | **T** | **C** | **T** | **A** | **T** | **T** | **C** | **A** | **A** | **G** | **A** | **G** | **G** | **A** | **T** | **A** | **T** | **A** | **G** | **A** | **A** | **G** | **T** | **C** | **G** | **T** | **A** | **G** | **G** | **C** | **G** | **A** | **A** | **G** | **C** | **A** | **G** | **A** | **A** | **A** | **A** | **T** | **G** | **G** | **T** | **A** | **A** | **G** | **A** | **T** | **T** | **G** | **G** | **C** | **T** | **A** | **T** | **G** | **A** | **A** | **A** | **A** | **A** | **G** | **C** | **A** | **T** | **T** | **G** | **G** | **A** | **A** | **C** | **T** | **A** | **C** | **G** | **T** | **C** | **C** | **A** | **G** | **A** | **T** | **G** | **T** | **T** | **A** | **T** | **T** | **T** | **T** | **G** | **A** | **T** | **G** | **G** | **A** | **T** | **T** | **T** | **G** | **G** | **T** | **A** | **A** | **T** | **G** | **G** | **A** | **A** | **G** | **A** | **A** | **A** | **T** | **G** | **G** | **A** | **C** | **G** | **G** | **C** | **A** | **T** | **T** | **G** | **A** | **T** | **T** | **C** | **A** | **A** | **C** | **A** | **A** | **A** | **A** | **G** | **C** | **G** | **A** | **T** | **C** | **T** | **T** | **G** | **A** | **A** | **A** | **G** | **A** | **T** | **T** | **G** | **G** | **C** | **C** | **A** | **G** | **A** | **A** | **G** | **C** | **C** | **A** | **A** | **G** | **A** | **T** | **T** | **A** | **T** | **T** | **A** | **T** | **T** | **G** | **T** | **G** | **A** | **C** | **G** | **A** | **G** | **T** | **T** | **T** | **T** | **A** | **T** | **T** | **G** | **A** | **T** | **G** | **A** | **T** | **G** | **A** | **A** | **A** | **A** | **A** | **G** | **T** | **G** | **T** | **A** | **T** | **C** | **C** | **G** | **G** | **C** | **G** | **A** | **T** | **T** | **G** | **A** | **A** | **G** | **C** | **T** | **G** | **G** | **T** | **G** | **C** | **A** | **G** | **C** | **G** | **G** | **G** | **C** | **T** | **A** | **C** | **C** | **T** | **A** | **T** | **T** | **A** | **A** | **A** | **A** | **A** | **C** | **A** | **T** | **C** | **A** | **A** | **C** | **A** | **G** | **C** | **A** | **C** | **A** | **T** | **G** | **A** | **G** | **A** | **T** | **T** | **G** | **C** | **T** | **G** | **A** | **T** | **G** | **C** | **A** | **A** | **T** | **T** | **C** | **G** | **G** | **G** | **C** | **G** | **A** | **C** | **T** | **T** | **A** | **T** | **C** | **G** | **C** | **G** | **G** | **A** | **G** | **A** | **G** | **C** | **G** | **T** | **G** | **T** | **G** | **T** | **T** | **G** | **G** | **A** | **A** | **C** | **C** | **T** | **G** | **A** | **A** | **G** | **T** | **G** | **A** | **C** | **G** | **C** | **A** | **T** | **A** | **A** | **G** | **A** | **T** | **G** | **A** | **T** | **G** | **G** | **A** | **A** | **C** | **G** | **G** | **T** | **T** | **A** | **A** | **C** | **A** | **A** | **A** | **A** | **A** | **A** | **A** | **C** | **A** | **A** | **G** | **A** | **G** | **C** | **C** | **G** | **G** | **T** | **G** | **T** | **T** | **G** | **C** | **A** | **T** | **G** | **A** | **A** | **G** | **A** | **T** | **T** | **T** | **G** | **A** | **C** | **A** | **A** | **A** | **T** | **C** | **G** | **A** | **G** | **A** | **A** | **C** | **A** | **C** | **G** | **A** | **A** | **A** | **T** | **T** | **T** | **T** | **A** | **A** | **T** | **G** | **T** | **T** | **G** | **A** | **T** | **T** | **G** | **C** | **A** | **C** | **A** | **A** | **G** | **G** | **T** | **A** | **A** | **A** | **A** | **G** | **T** | **A** | **A** | **T** | **C** | **A** | **G** | **G** | **A** | **A** | **A** | **T** | **A** | **G** | **C** | **T** | **G** | **A** | **T** | **G** | **A** | **A** | **C** | **T** | **C** | **T** | **T** | **T** | **A** | **T** | **C** | **A** | **C** | **T** | **T** | **T** | **G** | **A** | **A** | **A** | **A** | **C** | **A** | **G** | **T** | **T** | **A** | **A** | **A** | **A** | **C** | **A** | **C** | **A** | **T** | **G** | **T** | **T** | **T** | **C** | **A** | **A** | **A** | **C** | **A** | **T** | **T** | **T** | **T** | **A** | **G** | **C** | **A** | **A** | **A** | **A** | **C** | **T** | **A** | **G** | **A** | **T** | **G** | **T** | **G** | **G** | **A** | **T** | **G** | **A** | **T** | **C** | **G** | **G** | **A** | **C** | **C** | **C** | **A** | **A** | **G** | **C** | **G** | **G** | **C** | **G** | **A** | **T** | **T** | **T** | **A** | **T** | **G** | **C** | **T** | **T** | **T** | **T** | **C** | **A** | **A** | **C** | **A** | **T** | **G** | **G** | **T** | **T** | **T** | **A** | **G** | **C** | **C** | **A** | **A** | **A** | **-** | **-** | **-** |
| **N55317** | **A** | **T** | **G** | **A** | **T** | **A** | **A** | **A** | **A** | **G** | **T** | **T** | **T** | **T** | **A** | **T** | **T** | **A** | **G** | **T** | **A** | **G** | **A** | **T** | **G** | **A** | **C** | **C** | **A** | **T** | **G** | **A** | **A** | **A** | **T** | **G** | **G** | **T** | **G** | **C** | **G** | **C** | **T** | **T** | **A** | **G** | **G** | **C** | **G** | **T** | **C** | **T** | **C** | **T** | **T** | **C** | **T** | **T** | **A** | **T** | **T** | **T** | **A** | **T** | **C** | **T** | **A** | **T** | **C** | **C** | **A** | **A** | **G** | **A** | **A** | **G** | **A** | **C** | **A** | **T** | **T** | **G** | **A** | **A** | **G** | **T** | **A** | **A** | **T** | **T** | **G** | **G** | **A** | **G** | **A** | **A** | **G** | **C** | **T** | **G** | **A** | **A** | **A** | **A** | **T** | **G** | **G** | **A** | **C** | **G** | **A** | **C** | **A** | **A** | **G** | **G** | **C** | **T** | **A** | **T** | **G** | **A** | **G** | **A** | **A** | **G** | **G** | **C** | **G** | **A** | **T** | **G** | **A** | **C** | **A** | **C** | **T** | **T** | **C** | **G** | **A** | **C** | **C** | **G** | **G** | **A** | **C** | **G** | **T** | **C** | **A** | **T** | **T** | **T** | **T** | **A** | **A** | **T** | **G** | **G** | **A** | **C** | **T** | **T** | **A** | **G** | **T** | **C** | **A** | **T** | **G** | **G** | **A** | **A** | **G** | **A** | **A** | **A** | **T** | **G** | **G** | **A** | **T** | **G** | **G** | **T** | **A** | **T** | **C** | **G** | **A** | **A** | **T** | **C** | **A** | **A** | **C** | **A** | **A** | **A** | **A** | **G** | **C** | **A** | **A** | **T** | **C** | **T** | **T** | **A** | **A** | **A** | **A** | **G** | **A** | **T** | **T** | **G** | **G** | **C** | **C** | **A** | **G** | **A** | **A** | **G** | **C** | **T** | **A** | **A** | **A** | **A** | **T** | **C** | **A** | **T** | **T** | **A** | **T** | **C** | **G** | **T** | **A** | **A** | **C** | **C** | **A** | **G** | **T** | **T** | **T** | **T** | **A** | **T** | **T** | **G** | **A** | **T** | **G** | **A** | **C** | **G** | **A** | **A** | **A** | **A** | **A** | **G** | **T** | **T** | **T** | **A** | **T** | **C** | **C** | **T** | **G** | **C** | **C** | **A** | **T** | **T** | **G** | **A** | **A** | **G** | **C** | **A** | **G** | **G** | **C** | **G** | **C** | **A** | **G** | **C** | **A** | **G** | **G** | **G** | **T** | **A** | **T** | **T** | **T** | **G** | **T** | **T** | **G** | **A** | **A** | **G** | **A** | **C** | **T** | **T** | **C** | **T** | **A** | **C** | **C** | **G** | **C** | **A** | **C** | **A** | **T** | **G** | **A** | **A** | **A** | **T** | **T** | **G** | **C** | **T** | **G** | **A** | **T** | **G** | **C** | **T** | **A** | **T** | **A** | **A** | **G** | **G** | **G** | **C** | **A** | **A** | **C** | **G** | **C** | **A** | **A** | **C** | **G** | **C** | **G** | **G** | **C** | **G** | **A** | **G** | **C** | **G** | **A** | **G** | **T** | **A** | **C** | **T** | **A** | **G** | **A** | **A** | **C** | **C** | **A** | **G** | **A** | **A** | **G** | **T** | **G** | **A** | **C** | **A** | **A** | **C** | **A** | **A** | **A** | **A** | **A** | **T** | **G** | **A** | **T** | **G** | **G** | **A** | **A** | **A** | **A** | **A** | **A** | **T** | **G** | **A** | **G** | **T** | **C** | **G** | **G** | **C** | **G** | **A** | **A** | **A** | **T** | **G** | **A** | **G** | **C** | **C** | **T** | **G** | **T** | **A** | **T** | **T** | **A** | **C** | **A** | **T** | **G** | **A** | **A** | **G** | **A** | **A** | **C** | **T** | **A** | **A** | **C** | **G** | **A** | **A** | **T** | **C** | **G** | **G** | **G** | **A** | **A** | **A** | **A** | **T** | **G** | **A** | **A** | **A** | **T** | **T** | **T** | **T** | **A** | **A** | **T** | **G** | **C** | **T** | **G** | **A** | **T** | **T** | **T** | **C** | **T** | **G** | **A** | **A** | **G** | **G** | **G** | **A** | **A** | **A** | **A** | **G** | **T** | **A** | **A** | **T** | **C** | **A** | **G** | **G** | **A** | **A** | **A** | **T** | **A** | **G** | **C** | **G** | **G** | **A** | **C** | **G** | **A** | **A** | **T** | **T** | **G** | **T** | **T** | **T** | **A** | **T** | **T** | **A** | **C** | **A** | **C** | **T** | **G** | **A** | **A** | **A** | **A** | **C** | **A** | **G** | **T** | **A** | **A** | **A** | **A** | **A** | **C** | **A** | **C** | **A** | **T** | **G** | **T** | **T** | **T** | **C** | **C** | **A** | **A** | **T** | **A** | **T** | **A** | **T** | **T** | **G** | **G** | **C** | **T** | **A** | **A** | **G** | **C** | **T** | **A** | **G** | **A** | **A** | **G** | **T** | **G** | **G** | **A** | **A** | **G** | **A** | **C** | **C** | **G** | **T** | **A** | **C** | **A** | **C** | **A** | **A** | **G** | **C** | **C** | **G** | **C** | **T** | **A** | **T** | **C** | **T** | **A** | **T** | **G** | **C** | **A** | **T** | **T** | **C** | **A** | **A** | **A** | **C** | **A** | **T** | **G** | **G** | **T** | **T** | **T** | **G** | **G** | **T** | **G** | **A** | **A** | **A** | **-** | **-** | **-** |
| **N54599** | **A** | **T** | **G** | **A** | **T** | **A** | **A** | **A** | **A** | **G** | **T** | **T** | **T** | **T** | **A** | **T** | **T** | **A** | **G** | **T** | **A** | **G** | **A** | **T** | **G** | **A** | **C** | **C** | **A** | **T** | **G** | **A** | **A** | **A** | **T** | **G** | **G** | **T** | **G** | **C** | **G** | **C** | **T** | **T** | **A** | **G** | **G** | **T** | **G** | **T** | **C** | **T** | **C** | **T** | **T** | **C** | **T** | **T** | **A** | **T** | **T** | **T** | **A** | **T** | **C** | **T** | **A** | **T** | **T** | **C** | **A** | **A** | **G** | **A** | **A** | **G** | **A** | **C** | **A** | **T** | **T** | **G** | **A** | **A** | **G** | **T** | **A** | **A** | **T** | **T** | **G** | **G** | **A** | **G** | **A** | **A** | **G** | **C** | **T** | **G** | **A** | **A** | **A** | **A** | **T** | **G** | **G** | **A** | **C** | **G** | **A** | **C** | **A** | **A** | **G** | **G** | **C** | **T** | **A** | **T** | **G** | **A** | **G** | **A** | **A** | **G** | **G** | **C** | **G** | **A** | **T** | **G** | **A** | **C** | **A** | **C** | **T** | **T** | **C** | **G** | **A** | **C** | **C** | **G** | **G** | **A** | **C** | **G** | **T** | **C** | **A** | **T** | **T** | **T** | **T** | **A** | **A** | **T** | **G** | **G** | **A** | **C** | **T** | **T** | **A** | **G** | **T** | **C** | **A** | **T** | **G** | **G** | **A** | **A** | **G** | **A** | **A** | **A** | **T** | **G** | **G** | **A** | **T** | **G** | **G** | **T** | **A** | **T** | **C** | **G** | **A** | **A** | **T** | **C** | **A** | **A** | **C** | **A** | **A** | **A** | **A** | **G** | **C** | **A** | **A** | **T** | **C** | **T** | **T** | **A** | **A** | **A** | **A** | **G** | **A** | **T** | **T** | **G** | **G** | **C** | **T** | **A** | **G** | **A** | **A** | **G** | **C** | **T** | **A** | **A** | **A** | **A** | **T** | **C** | **A** | **T** | **T** | **A** | **T** | **C** | **G** | **T** | **A** | **A** | **C** | **C** | **A** | **G** | **T** | **T** | **T** | **T** | **A** | **T** | **T** | **G** | **A** | **T** | **G** | **A** | **C** | **G** | **A** | **A** | **A** | **A** | **A** | **G** | **T** | **T** | **T** | **A** | **T** | **C** | **C** | **T** | **G** | **C** | **C** | **A** | **T** | **T** | **G** | **A** | **A** | **G** | **C** | **A** | **G** | **G** | **T** | **G** | **C** | **A** | **G** | **C** | **A** | **G** | **G** | **G** | **T** | **A** | **T** | **T** | **T** | **G** | **T** | **T** | **G** | **A** | **A** | **G** | **A** | **C** | **C** | **T** | **C** | **T** | **A** | **C** | **C** | **G** | **C** | **A** | **C** | **A** | **T** | **G** | **A** | **A** | **A** | **T** | **T** | **G** | **C** | **T** | **G** | **A** | **T** | **G** | **C** | **T** | **A** | **T** | **A** | **A** | **G** | **G** | **G** | **C** | **A** | **A** | **C** | **G** | **C** | **A** | **A** | **C** | **G** | **C** | **G** | **G** | **C** | **G** | **A** | **G** | **C** | **G** | **A** | **G** | **T** | **A** | **T** | **T** | **A** | **G** | **A** | **A** | **C** | **C** | **A** | **G** | **A** | **A** | **G** | **T** | **G** | **A** | **C** | **A** | **A** | **C** | **A** | **A** | **A** | **A** | **A** | **T** | **G** | **A** | **T** | **G** | **G** | **A** | **A** | **A** | **A** | **A** | **A** | **T** | **G** | **A** | **G** | **T** | **C** | **G** | **G** | **C** | **G** | **A** | **A** | **A** | **T** | **G** | **A** | **G** | **C** | **C** | **T** | **G** | **T** | **A** | **T** | **T** | **A** | **C** | **A** | **T** | **G** | **A** | **A** | **G** | **A** | **A** | **C** | **T** | **A** | **A** | **C** | **G** | **A** | **A** | **T** | **C** | **G** | **G** | **G** | **A** | **A** | **A** | **A** | **T** | **G** | **A** | **A** | **A** | **T** | **T** | **T** | **T** | **A** | **A** | **T** | **G** | **C** | **T** | **G** | **A** | **T** | **T** | **T** | **C** | **T** | **G** | **A** | **A** | **G** | **G** | **A** | **A** | **A** | **A** | **A** | **G** | **T** | **A** | **A** | **T** | **C** | **A** | **G** | **G** | **A** | **A** | **A** | **T** | **A** | **G** | **C** | **G** | **G** | **A** | **C** | **G** | **A** | **A** | **T** | **T** | **G** | **T** | **T** | **C** | **A** | **T** | **T** | **A** | **C** | **A** | **C** | **T** | **G** | **A** | **A** | **A** | **A** | **C** | **A** | **G** | **T** | **A** | **A** | **A** | **A** | **A** | **C** | **A** | **C** | **A** | **T** | **G** | **T** | **T** | **T** | **C** | **C** | **A** | **A** | **T** | **A** | **T** | **A** | **T** | **T** | **G** | **G** | **C** | **T** | **A** | **A** | **G** | **C** | **T** | **A** | **G** | **A** | **A** | **G** | **T** | **G** | **G** | **A** | **A** | **G** | **A** | **C** | **C** | **G** | **T** | **A** | **C** | **A** | **C** | **A** | **A** | **G** | **C** | **C** | **G** | **C** | **T** | **A** | **T** | **C** | **T** | **A** | **T** | **G** | **C** | **A** | **T** | **T** | **C** | **A** | **A** | **A** | **C** | **A** | **T** | **G** | **G** | **T** | **T** | **T** | **G** | **G** | **T** | **G** | **A** | **A** | **A** | **-** | **-** | **-** |
| **N59788F** | **A** | **T** | **G** | **A** | **T** | **A** | **A** | **A** | **A** | **G** | **T** | **T** | **T** | **T** | **A** | **T** | **T** | **A** | **G** | **T** | **A** | **G** | **A** | **T** | **G** | **A** | **C** | **C** | **A** | **T** | **G** | **A** | **A** | **A** | **T** | **G** | **G** | **T** | **A** | **C** | **G** | **C** | **T** | **T** | **A** | **G** | **G** | **C** | **G** | **T** | **C** | **T** | **C** | **T** | **T** | **C** | **T** | **T** | **A** | **T** | **T** | **T** | **A** | **T** | **C** | **T** | **A** | **T** | **C** | **C** | **A** | **A** | **G** | **A** | **A** | **G** | **A** | **C** | **A** | **T** | **T** | **G** | **A** | **A** | **G** | **T** | **G** | **A** | **T** | **T** | **G** | **G** | **A** | **G** | **A** | **A** | **G** | **C** | **T** | **G** | **A** | **A** | **A** | **A** | **T** | **G** | **G** | **A** | **C** | **G** | **T** | **C** | **A** | **A** | **G** | **G** | **C** | **T** | **A** | **T** | **G** | **A** | **A** | **A** | **A** | **G** | **G** | **C** | **G** | **A** | **T** | **G** | **G** | **C** | **A** | **C** | **T** | **T** | **C** | **G** | **A** | **C** | **C** | **A** | **G** | **A** | **T** | **G** | **T** | **C** | **A** | **T** | **T** | **T** | **T** | **A** | **A** | **T** | **G** | **G** | **A** | **T** | **C** | **T** | **A** | **G** | **T** | **C** | **A** | **T** | **G** | **G** | **A** | **A** | **G** | **A** | **A** | **A** | **T** | **G** | **G** | **A** | **T** | **G** | **G** | **C** | **A** | **T** | **C** | **G** | **A** | **A** | **T** | **C** | **A** | **A** | **C** | **A** | **A** | **A** | **A** | **G** | **C** | **A** | **A** | **T** | **C** | **T** | **T** | **A** | **A** | **A** | **A** | **G** | **A** | **T** | **T** | **G** | **G** | **C** | **C** | **A** | **A** | **A** | **A** | **G** | **C** | **C** | **A** | **A** | **A** | **A** | **T** | **C** | **A** | **T** | **T** | **A** | **T** | **C** | **G** | **T** | **C** | **A** | **C** | **A** | **A** | **G** | **T** | **T** | **T** | **T** | **A** | **T** | **T** | **G** | **A** | **T** | **G** | **A** | **T** | **G** | **A** | **A** | **A** | **A** | **A** | **G** | **T** | **T** | **T** | **A** | **T** | **C** | **C** | **T** | **G** | **C** | **A** | **A** | **T** | **T** | **G** | **A** | **A** | **G** | **C** | **A** | **G** | **G** | **A** | **G** | **C** | **A** | **G** | **C** | **A** | **G** | **G** | **A** | **T** | **A** | **T** | **T** | **T** | **A** | **T** | **T** | **G** | **A** | **A** | **G** | **A** | **C** | **G** | **T** | **C** | **T** | **A** | **C** | **C** | **G** | **C** | **A** | **C** | **A** | **T** | **G** | **A** | **A** | **A** | **T** | **A** | **G** | **C** | **T** | **G** | **A** | **T** | **G** | **C** | **T** | **A** | **T** | **A** | **A** | **G** | **G** | **G** | **C** | **T** | **A** | **C** | **G** | **C** | **A** | **A** | **C** | **G** | **C** | **G** | **G** | **C** | **G** | **A** | **G** | **C** | **G** | **A** | **G** | **T** | **A** | **T** | **T** | **G** | **G** | **A** | **A** | **C** | **C** | **T** | **G** | **A** | **A** | **G** | **T** | **G** | **A** | **C** | **A** | **A** | **C** | **A** | **A** | **A** | **A** | **A** | **T** | **G** | **A** | **T** | **G** | **G** | **A** | **A** | **A** | **A** | **A** | **A** | **T** | **G** | **A** | **G** | **T** | **C** | **G** | **G** | **C** | **G** | **A** | **A** | **A** | **T** | **G** | **A** | **T** | **C** | **C** | **A** | **G** | **T** | **A** | **T** | **T** | **G** | **C** | **A** | **T** | **G** | **A** | **A** | **G** | **A** | **A** | **T** | **T** | **A** | **A** | **C** | **G** | **A** | **A** | **T** | **C** | **G** | **A** | **G** | **A** | **A** | **A** | **A** | **T** | **G** | **A** | **A** | **A** | **T** | **T** | **T** | **T** | **G** | **A** | **T** | **G** | **C** | **T** | **G** | **A** | **T** | **T** | **T** | **C** | **T** | **G** | **A** | **A** | **G** | **G** | **G** | **A** | **A** | **A** | **A** | **G** | **T** | **A** | **A** | **T** | **C** | **A** | **G** | **G** | **A** | **A** | **A** | **T** | **C** | **G** | **C** | **G** | **G** | **A** | **T** | **G** | **A** | **A** | **T** | **T** | **G** | **T** | **T** | **T** | **A** | **T** | **C** | **A** | **C** | **A** | **C** | **T** | **G** | **A** | **A** | **A** | **A** | **C** | **A** | **G** | **T** | **T** | **A** | **A** | **A** | **A** | **C** | **G** | **C** | **A** | **T** | **G** | **T** | **G** | **T** | **C** | **G** | **A** | **A** | **T** | **A** | **T** | **A** | **T** | **T** | **A** | **G** | **C** | **T** | **A** | **A** | **G** | **T** | **T** | **A** | **G** | **A** | **G** | **G** | **T** | **A** | **G** | **A** | **A** | **G** | **A** | **C** | **C** | **G** | **A** | **A** | **C** | **T** | **C** | **A** | **A** | **G** | **C** | **A** | **G** | **C** | **T** | **A** | **T** | **C** | **T** | **A** | **T** | **G** | **C** | **G** | **T** | **T** | **T** | **A** | **A** | **A** | **C** | **A** | **T** | **G** | **G** | **T** | **T** | **T** | **G** | **G** | **T** | **G** | **A** | **A** | **A** | **-** | **-** | **-** |
| **N60440F** | **G** | **T** | **G** | **A** | **T** | **C** | **A** | **A** | **A** | **G** | **T** | **A** | **A** | **T** | **G** | **C** | **T** | **A** | **G** | **T** | **G** | **G** | **A** | **T** | **G** | **A** | **C** | **C** | **A** | **T** | **G** | **A** | **A** | **A** | **T** | **G** | **G** | **T** | **C** | **C** | **G** | **T** | **T** | **T** | **A** | **G** | **G** | **C** | **G** | **T** | **T** | **T** | **C** | **A** | **T** | **C** | **A** | **T** | **A** | **T** | **T** | **T** | **A** | **T** | **C** | **T** | **A** | **T** | **T** | **C** | **A** | **A** | **G** | **A** | **G** | **G** | **A** | **T** | **A** | **T** | **A** | **G** | **A** | **A** | **G** | **T** | **C** | **G** | **T** | **A** | **G** | **G** | **C** | **G** | **A** | **A** | **G** | **C** | **A** | **G** | **A** | **A** | **A** | **A** | **C** | **G** | **G** | **T** | **A** | **A** | **G** | **A** | **T** | **T** | **G** | **G** | **C** | **T** | **A** | **T** | **G** | **A** | **A** | **A** | **A** | **A** | **G** | **C** | **A** | **T** | **T** | **G** | **G** | **A** | **A** | **C** | **T** | **A** | **C** | **G** | **T** | **C** | **C** | **A** | **G** | **A** | **T** | **G** | **T** | **T** | **A** | **T** | **T** | **T** | **T** | **G** | **A** | **T** | **G** | **G** | **A** | **T** | **T** | **T** | **G** | **G** | **T** | **A** | **A** | **T** | **G** | **G** | **A** | **A** | **G** | **A** | **A** | **A** | **T** | **G** | **G** | **A** | **C** | **G** | **G** | **C** | **A** | **T** | **T** | **G** | **A** | **T** | **T** | **C** | **A** | **A** | **C** | **A** | **A** | **A** | **A** | **G** | **C** | **G** | **A** | **T** | **C** | **T** | **T** | **G** | **A** | **A** | **A** | **G** | **A** | **T** | **T** | **G** | **G** | **C** | **C** | **A** | **G** | **A** | **A** | **G** | **C** | **C** | **A** | **A** | **G** | **A** | **T** | **T** | **A** | **T** | **T** | **A** | **T** | **T** | **G** | **T** | **G** | **A** | **C** | **G** | **A** | **G** | **T** | **T** | **T** | **T** | **A** | **T** | **T** | **G** | **A** | **T** | **G** | **A** | **T** | **G** | **A** | **A** | **A** | **A** | **A** | **G** | **T** | **G** | **T** | **A** | **T** | **C** | **C** | **G** | **G** | **C** | **G** | **A** | **T** | **T** | **G** | **A** | **A** | **G** | **C** | **T** | **G** | **G** | **T** | **G** | **C** | **A** | **G** | **C** | **A** | **G** | **G** | **C** | **T** | **A** | **C** | **C** | **T** | **A** | **T** | **T** | **A** | **A** | **A** | **A** | **A** | **C** | **A** | **T** | **C** | **A** | **A** | **C** | **A** | **G** | **C** | **T** | **C** | **A** | **T** | **G** | **A** | **G** | **A** | **T** | **T** | **G** | **C** | **T** | **G** | **A** | **T** | **G** | **C** | **A** | **A** | **T** | **T** | **C** | **G** | **G** | **G** | **C** | **G** | **A** | **C** | **T** | **T** | **A** | **T** | **C** | **G** | **C** | **G** | **G** | **A** | **G** | **A** | **G** | **C** | **G** | **T** | **G** | **T** | **G** | **T** | **T** | **G** | **G** | **A** | **A** | **C** | **C** | **T** | **G** | **A** | **A** | **G** | **T** | **G** | **A** | **C** | **G** | **C** | **A** | **T** | **A** | **A** | **G** | **A** | **T** | **G** | **A** | **T** | **G** | **G** | **A** | **A** | **C** | **G** | **G** | **T** | **T** | **A** | **A** | **C** | **A** | **A** | **A** | **A** | **A** | **A** | **A** | **C** | **A** | **A** | **G** | **A** | **G** | **C** | **C** | **G** | **G** | **T** | **G** | **T** | **T** | **G** | **C** | **A** | **C** | **G** | **A** | **A** | **G** | **A** | **T** | **T** | **T** | **G** | **A** | **C** | **A** | **A** | **A** | **C** | **C** | **G** | **A** | **G** | **A** | **A** | **C** | **A** | **C** | **G** | **A** | **A** | **A** | **T** | **T** | **T** | **T** | **A** | **A** | **T** | **G** | **T** | **T** | **G** | **A** | **T** | **T** | **G** | **C** | **A** | **C** | **A** | **A** | **G** | **G** | **T** | **A** | **A** | **A** | **A** | **G** | **T** | **A** | **A** | **T** | **C** | **A** | **G** | **G** | **A** | **A** | **A** | **T** | **A** | **G** | **C** | **T** | **G** | **A** | **T** | **G** | **A** | **A** | **C** | **T** | **C** | **T** | **T** | **T** | **A** | **T** | **C** | **A** | **C** | **T** | **T** | **T** | **G** | **A** | **A** | **A** | **A** | **C** | **A** | **G** | **T** | **T** | **A** | **A** | **A** | **A** | **C** | **A** | **C** | **A** | **T** | **G** | **T** | **T** | **T** | **C** | **A** | **A** | **A** | **C** | **A** | **T** | **T** | **T** | **T** | **A** | **G** | **C** | **A** | **A** | **A** | **A** | **C** | **T** | **A** | **G** | **A** | **T** | **G** | **T** | **G** | **G** | **A** | **T** | **G** | **A** | **T** | **C** | **G** | **G** | **A** | **C** | **C** | **C** | **A** | **A** | **G** | **C** | **G** | **G** | **C** | **G** | **A** | **T** | **T** | **T** | **A** | **T** | **G** | **C** | **T** | **T** | **T** | **T** | **C** | **A** | **A** | **C** | **A** | **T** | **G** | **G** | **T** | **T** | **T** | **A** | **G** | **C** | **C** | **A** | **A** | **A** | **-** | **-** | **-** |
| **N59599F** | **G** | **T** | **G** | **A** | **T** | **C** | **A** | **A** | **A** | **G** | **T** | **A** | **A** | **T** | **G** | **T** | **T** | **A** | **G** | **T** | **G** | **G** | **A** | **T** | **G** | **A** | **C** | **C** | **A** | **T** | **G** | **A** | **A** | **A** | **T** | **G** | **G** | **T** | **C** | **C** | **G** | **T** | **T** | **T** | **A** | **G** | **G** | **C** | **G** | **T** | **T** | **T** | **C** | **A** | **T** | **C** | **A** | **T** | **A** | **T** | **T** | **T** | **A** | **T** | **C** | **T** | **A** | **T** | **T** | **C** | **A** | **A** | **G** | **A** | **G** | **G** | **A** | **T** | **A** | **T** | **A** | **G** | **A** | **A** | **G** | **T** | **C** | **G** | **T** | **A** | **G** | **G** | **C** | **G** | **A** | **A** | **G** | **C** | **A** | **G** | **A** | **A** | **A** | **A** | **T** | **G** | **G** | **T** | **A** | **A** | **G** | **A** | **T** | **T** | **G** | **G** | **C** | **T** | **A** | **T** | **G** | **A** | **A** | **A** | **A** | **A** | **G** | **C** | **A** | **T** | **T** | **G** | **G** | **A** | **A** | **C** | **T** | **A** | **C** | **G** | **T** | **C** | **C** | **A** | **G** | **A** | **T** | **G** | **T** | **T** | **A** | **T** | **T** | **T** | **T** | **G** | **A** | **T** | **G** | **G** | **A** | **T** | **T** | **T** | **G** | **G** | **T** | **A** | **A** | **T** | **G** | **G** | **A** | **A** | **G** | **A** | **A** | **A** | **T** | **G** | **G** | **A** | **C** | **G** | **G** | **C** | **A** | **T** | **T** | **G** | **A** | **T** | **T** | **C** | **A** | **A** | **C** | **A** | **A** | **A** | **A** | **G** | **C** | **G** | **A** | **T** | **C** | **T** | **T** | **G** | **A** | **A** | **A** | **G** | **A** | **T** | **T** | **G** | **G** | **C** | **C** | **A** | **G** | **A** | **A** | **G** | **C** | **C** | **A** | **A** | **G** | **A** | **T** | **T** | **A** | **T** | **T** | **A** | **T** | **T** | **G** | **T** | **G** | **A** | **C** | **G** | **A** | **G** | **T** | **T** | **T** | **T** | **A** | **T** | **T** | **G** | **A** | **T** | **G** | **A** | **T** | **G** | **A** | **A** | **A** | **A** | **A** | **G** | **T** | **G** | **T** | **A** | **T** | **C** | **C** | **G** | **G** | **C** | **G** | **A** | **T** | **T** | **G** | **A** | **A** | **G** | **C** | **T** | **G** | **G** | **T** | **G** | **C** | **A** | **G** | **C** | **A** | **G** | **G** | **C** | **T** | **A** | **C** | **C** | **T** | **A** | **T** | **T** | **A** | **A** | **A** | **A** | **A** | **C** | **A** | **T** | **C** | **A** | **A** | **C** | **A** | **G** | **C** | **T** | **C** | **A** | **T** | **G** | **A** | **G** | **A** | **T** | **T** | **G** | **C** | **T** | **G** | **A** | **T** | **G** | **C** | **A** | **A** | **T** | **T** | **C** | **G** | **G** | **G** | **C** | **G** | **A** | **C** | **T** | **T** | **A** | **T** | **C** | **G** | **C** | **G** | **G** | **A** | **G** | **A** | **G** | **C** | **G** | **T** | **G** | **T** | **G** | **T** | **T** | **G** | **G** | **A** | **A** | **C** | **C** | **T** | **G** | **A** | **A** | **G** | **T** | **G** | **A** | **C** | **G** | **C** | **A** | **T** | **A** | **A** | **G** | **A** | **T** | **G** | **A** | **T** | **G** | **G** | **A** | **A** | **C** | **G** | **G** | **T** | **T** | **A** | **A** | **C** | **A** | **A** | **A** | **A** | **A** | **A** | **A** | **C** | **A** | **A** | **G** | **A** | **G** | **C** | **C** | **G** | **G** | **T** | **G** | **T** | **T** | **G** | **C** | **A** | **C** | **G** | **A** | **A** | **G** | **A** | **T** | **T** | **T** | **G** | **A** | **C** | **A** | **A** | **A** | **C** | **C** | **G** | **A** | **G** | **A** | **A** | **C** | **A** | **C** | **G** | **A** | **A** | **A** | **T** | **T** | **T** | **T** | **A** | **A** | **T** | **G** | **T** | **T** | **G** | **A** | **T** | **T** | **G** | **C** | **A** | **C** | **A** | **A** | **G** | **G** | **T** | **A** | **A** | **A** | **A** | **G** | **T** | **A** | **A** | **T** | **C** | **A** | **G** | **G** | **A** | **A** | **A** | **T** | **A** | **G** | **C** | **T** | **G** | **A** | **T** | **G** | **A** | **A** | **C** | **T** | **C** | **T** | **T** | **T** | **A** | **T** | **C** | **A** | **C** | **T** | **T** | **T** | **G** | **A** | **A** | **A** | **A** | **C** | **A** | **G** | **T** | **T** | **A** | **A** | **A** | **A** | **C** | **A** | **C** | **A** | **T** | **G** | **T** | **T** | **T** | **C** | **A** | **A** | **A** | **C** | **A** | **T** | **T** | **T** | **T** | **A** | **G** | **C** | **A** | **A** | **A** | **A** | **C** | **T** | **A** | **G** | **A** | **T** | **G** | **T** | **G** | **G** | **A** | **T** | **G** | **A** | **T** | **C** | **G** | **G** | **A** | **C** | **C** | **C** | **A** | **A** | **G** | **C** | **G** | **G** | **C** | **G** | **A** | **T** | **T** | **T** | **A** | **T** | **G** | **C** | **T** | **T** | **T** | **T** | **C** | **A** | **A** | **C** | **A** | **T** | **G** | **G** | **T** | **T** | **T** | **A** | **G** | **C** | **C** | **A** | **A** | **A** | **-** | **-** | **-** |
| **N59947F** | **A** | **T** | **G** | **A** | **T** | **A** | **A** | **A** | **A** | **G** | **T** | **T** | **T** | **T** | **A** | **T** | **T** | **A** | **G** | **T** | **A** | **G** | **A** | **T** | **G** | **A** | **C** | **C** | **A** | **T** | **G** | **A** | **A** | **A** | **T** | **G** | **G** | **T** | **G** | **C** | **G** | **C** | **T** | **T** | **A** | **G** | **G** | **C** | **G** | **T** | **C** | **T** | **C** | **T** | **T** | **C** | **T** | **T** | **A** | **T** | **T** | **T** | **A** | **T** | **C** | **T** | **A** | **T** | **C** | **C** | **A** | **A** | **G** | **A** | **A** | **G** | **A** | **C** | **A** | **T** | **T** | **G** | **A** | **A** | **G** | **T** | **A** | **A** | **T** | **T** | **G** | **G** | **A** | **G** | **A** | **A** | **G** | **C** | **T** | **G** | **A** | **A** | **A** | **A** | **T** | **G** | **G** | **A** | **C** | **G** | **A** | **C** | **A** | **A** | **G** | **G** | **C** | **T** | **A** | **T** | **G** | **A** | **G** | **A** | **A** | **G** | **G** | **C** | **G** | **A** | **T** | **G** | **A** | **C** | **A** | **C** | **T** | **T** | **C** | **G** | **A** | **C** | **C** | **G** | **G** | **A** | **C** | **G** | **T** | **C** | **A** | **T** | **T** | **T** | **T** | **A** | **A** | **T** | **G** | **G** | **A** | **C** | **T** | **T** | **A** | **G** | **T** | **C** | **A** | **T** | **G** | **G** | **A** | **A** | **G** | **A** | **A** | **A** | **T** | **G** | **G** | **A** | **T** | **G** | **G** | **T** | **A** | **T** | **C** | **G** | **A** | **A** | **T** | **C** | **A** | **A** | **C** | **A** | **A** | **A** | **A** | **G** | **C** | **A** | **A** | **T** | **C** | **T** | **T** | **A** | **A** | **A** | **A** | **G** | **A** | **T** | **T** | **G** | **G** | **C** | **C** | **A** | **G** | **A** | **A** | **G** | **C** | **T** | **A** | **A** | **A** | **A** | **T** | **C** | **A** | **T** | **T** | **A** | **T** | **C** | **G** | **T** | **A** | **A** | **C** | **C** | **A** | **G** | **T** | **T** | **T** | **T** | **A** | **T** | **T** | **G** | **A** | **T** | **G** | **A** | **C** | **G** | **A** | **A** | **A** | **A** | **A** | **G** | **T** | **T** | **T** | **A** | **T** | **C** | **C** | **T** | **G** | **C** | **C** | **A** | **T** | **T** | **G** | **A** | **A** | **G** | **C** | **A** | **G** | **G** | **C** | **G** | **C** | **A** | **G** | **C** | **A** | **G** | **G** | **G** | **T** | **A** | **T** | **T** | **T** | **G** | **T** | **T** | **G** | **A** | **A** | **G** | **A** | **C** | **T** | **T** | **C** | **T** | **A** | **C** | **C** | **G** | **C** | **A** | **C** | **A** | **T** | **G** | **A** | **A** | **A** | **T** | **T** | **G** | **C** | **T** | **G** | **A** | **T** | **G** | **C** | **T** | **A** | **T** | **A** | **A** | **G** | **G** | **G** | **C** | **A** | **A** | **C** | **G** | **C** | **A** | **A** | **C** | **G** | **C** | **G** | **G** | **C** | **G** | **A** | **G** | **C** | **G** | **A** | **G** | **T** | **A** | **C** | **T** | **A** | **G** | **A** | **A** | **C** | **C** | **A** | **G** | **A** | **A** | **G** | **T** | **G** | **A** | **C** | **A** | **A** | **C** | **A** | **A** | **A** | **A** | **A** | **T** | **G** | **A** | **T** | **G** | **G** | **A** | **A** | **A** | **A** | **A** | **A** | **T** | **G** | **A** | **G** | **T** | **C** | **G** | **G** | **C** | **G** | **A** | **A** | **A** | **T** | **G** | **A** | **G** | **C** | **C** | **T** | **G** | **T** | **A** | **T** | **T** | **A** | **C** | **A** | **T** | **G** | **A** | **A** | **G** | **A** | **A** | **C** | **T** | **A** | **A** | **C** | **G** | **A** | **A** | **T** | **C** | **G** | **G** | **G** | **A** | **A** | **A** | **A** | **T** | **G** | **A** | **A** | **A** | **T** | **T** | **T** | **T** | **A** | **A** | **T** | **G** | **C** | **T** | **G** | **A** | **T** | **T** | **T** | **C** | **T** | **G** | **A** | **A** | **G** | **G** | **G** | **A** | **A** | **A** | **A** | **G** | **T** | **A** | **A** | **T** | **C** | **A** | **G** | **G** | **A** | **A** | **A** | **T** | **A** | **G** | **C** | **G** | **G** | **A** | **C** | **G** | **A** | **A** | **T** | **T** | **G** | **T** | **T** | **T** | **A** | **T** | **T** | **A** | **C** | **A** | **C** | **T** | **G** | **A** | **A** | **A** | **A** | **C** | **A** | **G** | **T** | **A** | **A** | **A** | **A** | **A** | **C** | **A** | **C** | **A** | **T** | **G** | **T** | **T** | **T** | **C** | **C** | **A** | **A** | **T** | **A** | **T** | **A** | **T** | **T** | **G** | **G** | **C** | **T** | **A** | **A** | **G** | **C** | **T** | **A** | **G** | **A** | **A** | **G** | **T** | **G** | **G** | **A** | **A** | **G** | **A** | **C** | **C** | **G** | **T** | **A** | **C** | **A** | **C** | **A** | **A** | **G** | **C** | **C** | **G** | **C** | **T** | **A** | **T** | **C** | **T** | **A** | **T** | **G** | **C** | **A** | **T** | **T** | **C** | **A** | **A** | **A** | **C** | **A** | **T** | **G** | **G** | **T** | **T** | **T** | **G** | **G** | **T** | **G** | **A** | **A** | **A** | **-** | **-** | **-** |
| **N53519** | **G** | **T** | **G** | **A** | **T** | **C** | **A** | **A** | **A** | **G** | **T** | **A** | **A** | **T** | **G** | **T** | **T** | **A** | **G** | **T** | **G** | **G** | **A** | **T** | **G** | **A** | **C** | **C** | **A** | **T** | **G** | **A** | **A** | **A** | **T** | **G** | **G** | **T** | **C** | **C** | **G** | **T** | **T** | **T** | **A** | **G** | **G** | **C** | **G** | **T** | **T** | **T** | **C** | **A** | **T** | **C** | **A** | **T** | **A** | **T** | **T** | **T** | **A** | **T** | **C** | **T** | **A** | **T** | **T** | **C** | **A** | **A** | **G** | **A** | **G** | **G** | **A** | **T** | **A** | **T** | **A** | **G** | **A** | **A** | **G** | **T** | **C** | **G** | **T** | **A** | **G** | **G** | **C** | **G** | **A** | **A** | **G** | **C** | **A** | **G** | **A** | **A** | **A** | **A** | **T** | **G** | **G** | **T** | **A** | **A** | **G** | **A** | **T** | **T** | **G** | **G** | **C** | **T** | **A** | **T** | **G** | **A** | **A** | **A** | **A** | **A** | **G** | **C** | **A** | **T** | **T** | **G** | **G** | **A** | **A** | **C** | **T** | **A** | **C** | **G** | **T** | **C** | **C** | **A** | **G** | **A** | **T** | **G** | **T** | **T** | **A** | **T** | **T** | **T** | **T** | **G** | **A** | **T** | **G** | **G** | **A** | **T** | **T** | **T** | **G** | **G** | **T** | **A** | **A** | **T** | **G** | **G** | **A** | **A** | **G** | **A** | **A** | **A** | **T** | **G** | **G** | **A** | **C** | **G** | **G** | **C** | **A** | **T** | **T** | **G** | **A** | **T** | **T** | **C** | **A** | **A** | **C** | **A** | **A** | **A** | **A** | **G** | **C** | **G** | **A** | **T** | **C** | **T** | **T** | **G** | **A** | **A** | **A** | **G** | **A** | **T** | **T** | **G** | **G** | **C** | **C** | **A** | **G** | **A** | **A** | **G** | **C** | **C** | **A** | **A** | **G** | **A** | **T** | **T** | **A** | **T** | **T** | **A** | **T** | **T** | **G** | **T** | **G** | **A** | **C** | **G** | **A** | **G** | **T** | **T** | **T** | **T** | **A** | **T** | **T** | **G** | **A** | **T** | **G** | **A** | **T** | **G** | **A** | **A** | **A** | **A** | **A** | **G** | **T** | **G** | **T** | **A** | **T** | **C** | **C** | **G** | **G** | **C** | **G** | **A** | **T** | **T** | **G** | **A** | **A** | **G** | **C** | **T** | **G** | **G** | **T** | **G** | **C** | **A** | **G** | **C** | **G** | **G** | **G** | **C** | **T** | **A** | **C** | **C** | **T** | **A** | **T** | **T** | **A** | **A** | **A** | **A** | **A** | **C** | **A** | **T** | **C** | **A** | **A** | **C** | **A** | **G** | **C** | **A** | **C** | **A** | **T** | **G** | **A** | **G** | **A** | **T** | **T** | **G** | **C** | **T** | **G** | **A** | **T** | **G** | **C** | **A** | **A** | **T** | **T** | **C** | **G** | **G** | **G** | **C** | **G** | **A** | **C** | **T** | **T** | **A** | **T** | **C** | **G** | **C** | **G** | **G** | **A** | **G** | **A** | **G** | **C** | **G** | **T** | **G** | **T** | **G** | **T** | **T** | **G** | **G** | **A** | **A** | **C** | **C** | **T** | **G** | **A** | **A** | **G** | **T** | **G** | **A** | **C** | **A** | **C** | **A** | **T** | **A** | **A** | **G** | **A** | **T** | **G** | **A** | **T** | **G** | **G** | **A** | **A** | **C** | **G** | **G** | **T** | **T** | **A** | **A** | **C** | **A** | **A** | **A** | **A** | **A** | **A** | **A** | **C** | **A** | **A** | **G** | **A** | **G** | **C** | **C** | **G** | **G** | **T** | **G** | **T** | **T** | **G** | **C** | **A** | **C** | **G** | **A** | **A** | **G** | **A** | **T** | **T** | **T** | **G** | **A** | **C** | **A** | **A** | **A** | **C** | **C** | **G** | **G** | **G** | **A** | **A** | **C** | **A** | **C** | **G** | **A** | **A** | **A** | **T** | **T** | **T** | **T** | **A** | **A** | **T** | **G** | **T** | **T** | **G** | **A** | **T** | **T** | **G** | **C** | **A** | **C** | **A** | **A** | **G** | **G** | **T** | **A** | **A** | **A** | **A** | **G** | **T** | **A** | **A** | **T** | **C** | **A** | **G** | **G** | **A** | **A** | **A** | **T** | **A** | **G** | **C** | **T** | **G** | **A** | **T** | **G** | **A** | **A** | **C** | **T** | **C** | **T** | **T** | **T** | **A** | **T** | **C** | **A** | **C** | **T** | **T** | **T** | **G** | **A** | **A** | **A** | **A** | **C** | **A** | **G** | **T** | **T** | **A** | **A** | **A** | **A** | **C** | **A** | **C** | **A** | **T** | **G** | **T** | **T** | **T** | **C** | **A** | **A** | **A** | **C** | **A** | **T** | **T** | **T** | **T** | **A** | **G** | **C** | **A** | **A** | **A** | **A** | **C** | **T** | **A** | **G** | **A** | **T** | **G** | **T** | **G** | **G** | **A** | **T** | **G** | **A** | **T** | **C** | **G** | **G** | **A** | **C** | **C** | **C** | **A** | **A** | **G** | **C** | **G** | **G** | **C** | **G** | **A** | **T** | **T** | **T** | **A** | **T** | **G** | **C** | **T** | **T** | **T** | **T** | **C** | **A** | **A** | **C** | **A** | **T** | **G** | **G** | **T** | **T** | **T** | **A** | **G** | **C** | **C** | **A** | **A** | **A** | **-** | **-** | **-** |
| **N53805** | **A** | **T** | **G** | **A** | **T** | **A** | **A** | **A** | **A** | **G** | **T** | **T** | **T** | **T** | **A** | **T** | **T** | **A** | **G** | **T** | **A** | **G** | **A** | **T** | **G** | **A** | **C** | **C** | **A** | **T** | **G** | **A** | **A** | **A** | **T** | **G** | **G** | **T** | **A** | **C** | **G** | **C** | **T** | **T** | **A** | **G** | **G** | **C** | **G** | **T** | **C** | **T** | **C** | **T** | **T** | **C** | **T** | **T** | **A** | **T** | **T** | **T** | **A** | **T** | **C** | **T** | **A** | **T** | **C** | **C** | **A** | **A** | **G** | **A** | **A** | **G** | **A** | **C** | **A** | **T** | **T** | **G** | **A** | **A** | **G** | **T** | **G** | **A** | **T** | **T** | **G** | **G** | **A** | **G** | **A** | **A** | **G** | **C** | **T** | **G** | **A** | **A** | **A** | **A** | **T** | **G** | **G** | **A** | **C** | **G** | **T** | **C** | **A** | **A** | **G** | **G** | **C** | **T** | **A** | **T** | **G** | **A** | **A** | **A** | **A** | **G** | **G** | **C** | **G** | **A** | **T** | **G** | **G** | **C** | **A** | **C** | **T** | **T** | **C** | **G** | **A** | **C** | **C** | **A** | **G** | **A** | **T** | **G** | **T** | **C** | **A** | **T** | **T** | **T** | **T** | **A** | **A** | **T** | **G** | **G** | **A** | **T** | **C** | **T** | **A** | **G** | **T** | **C** | **A** | **T** | **G** | **G** | **A** | **A** | **G** | **A** | **A** | **A** | **T** | **G** | **G** | **A** | **T** | **G** | **G** | **C** | **A** | **T** | **C** | **G** | **A** | **A** | **T** | **C** | **A** | **A** | **C** | **A** | **A** | **A** | **A** | **G** | **C** | **A** | **A** | **T** | **C** | **T** | **T** | **A** | **A** | **A** | **A** | **G** | **A** | **T** | **T** | **G** | **G** | **C** | **C** | **A** | **A** | **A** | **A** | **G** | **C** | **C** | **A** | **A** | **A** | **A** | **T** | **C** | **A** | **T** | **T** | **A** | **T** | **C** | **G** | **T** | **C** | **A** | **C** | **A** | **A** | **G** | **T** | **T** | **T** | **T** | **A** | **T** | **T** | **G** | **A** | **T** | **G** | **A** | **T** | **G** | **A** | **A** | **A** | **A** | **A** | **G** | **T** | **T** | **T** | **A** | **T** | **C** | **C** | **T** | **G** | **C** | **A** | **A** | **T** | **T** | **G** | **A** | **A** | **G** | **C** | **A** | **G** | **G** | **A** | **G** | **C** | **A** | **G** | **C** | **A** | **G** | **G** | **A** | **T** | **A** | **T** | **T** | **T** | **A** | **T** | **T** | **G** | **A** | **A** | **G** | **A** | **C** | **G** | **T** | **C** | **T** | **A** | **C** | **C** | **G** | **C** | **A** | **C** | **A** | **T** | **G** | **A** | **A** | **A** | **T** | **A** | **G** | **C** | **T** | **G** | **A** | **T** | **G** | **C** | **T** | **A** | **T** | **A** | **A** | **G** | **G** | **G** | **C** | **T** | **A** | **C** | **G** | **C** | **A** | **A** | **C** | **G** | **C** | **G** | **G** | **C** | **G** | **A** | **G** | **C** | **G** | **A** | **G** | **T** | **A** | **T** | **T** | **G** | **G** | **A** | **A** | **C** | **C** | **T** | **G** | **A** | **A** | **G** | **T** | **G** | **A** | **C** | **A** | **A** | **C** | **A** | **A** | **A** | **A** | **A** | **T** | **G** | **A** | **T** | **G** | **G** | **A** | **A** | **A** | **A** | **A** | **A** | **T** | **G** | **A** | **G** | **T** | **C** | **G** | **G** | **C** | **G** | **A** | **A** | **A** | **T** | **G** | **A** | **T** | **C** | **C** | **A** | **G** | **T** | **A** | **T** | **T** | **G** | **C** | **A** | **T** | **G** | **A** | **A** | **G** | **A** | **A** | **T** | **T** | **A** | **A** | **C** | **G** | **A** | **A** | **T** | **C** | **G** | **A** | **G** | **A** | **A** | **A** | **A** | **T** | **G** | **A** | **A** | **A** | **T** | **T** | **T** | **T** | **G** | **A** | **T** | **G** | **C** | **T** | **G** | **A** | **T** | **T** | **T** | **C** | **T** | **G** | **A** | **A** | **G** | **G** | **G** | **A** | **A** | **A** | **A** | **G** | **T** | **A** | **A** | **T** | **C** | **A** | **G** | **G** | **A** | **A** | **A** | **T** | **C** | **G** | **C** | **G** | **G** | **A** | **T** | **G** | **A** | **A** | **T** | **T** | **G** | **T** | **T** | **T** | **A** | **T** | **C** | **A** | **C** | **A** | **C** | **T** | **G** | **A** | **A** | **A** | **A** | **C** | **A** | **G** | **T** | **T** | **A** | **A** | **A** | **A** | **C** | **G** | **C** | **A** | **T** | **G** | **T** | **G** | **T** | **C** | **G** | **A** | **A** | **T** | **A** | **T** | **A** | **T** | **T** | **A** | **G** | **C** | **T** | **A** | **A** | **G** | **T** | **T** | **A** | **G** | **A** | **G** | **G** | **T** | **A** | **G** | **A** | **A** | **G** | **A** | **C** | **C** | **G** | **A** | **A** | **C** | **T** | **C** | **A** | **A** | **G** | **C** | **A** | **G** | **C** | **T** | **A** | **T** | **C** | **T** | **A** | **T** | **G** | **C** | **G** | **T** | **T** | **T** | **A** | **A** | **A** | **C** | **A** | **T** | **G** | **G** | **T** | **T** | **T** | **G** | **G** | **T** | **T** | **A** | **A** | **A** | **-** | **-** | **-** |
| **N55725** | **G** | **T** | **G** | **A** | **T** | **C** | **A** | **A** | **A** | **G** | **T** | **A** | **A** | **T** | **G** | **T** | **T** | **A** | **G** | **T** | **G** | **G** | **A** | **T** | **G** | **A** | **C** | **C** | **A** | **T** | **G** | **A** | **A** | **A** | **T** | **G** | **G** | **T** | **C** | **C** | **G** | **T** | **T** | **T** | **A** | **G** | **G** | **C** | **G** | **T** | **T** | **T** | **C** | **A** | **T** | **C** | **A** | **T** | **A** | **T** | **T** | **T** | **A** | **T** | **C** | **T** | **A** | **T** | **T** | **C** | **A** | **A** | **G** | **A** | **G** | **G** | **A** | **T** | **A** | **T** | **A** | **G** | **A** | **A** | **G** | **T** | **C** | **G** | **T** | **A** | **G** | **G** | **C** | **G** | **A** | **A** | **G** | **C** | **A** | **G** | **A** | **A** | **A** | **A** | **C** | **G** | **G** | **T** | **A** | **A** | **G** | **A** | **T** | **T** | **G** | **G** | **C** | **T** | **A** | **T** | **G** | **A** | **A** | **A** | **A** | **A** | **G** | **C** | **A** | **T** | **T** | **G** | **G** | **A** | **A** | **C** | **T** | **A** | **C** | **G** | **T** | **C** | **C** | **A** | **G** | **A** | **T** | **G** | **T** | **T** | **A** | **T** | **T** | **T** | **T** | **G** | **A** | **T** | **G** | **G** | **A** | **T** | **T** | **T** | **G** | **G** | **T** | **A** | **A** | **T** | **G** | **G** | **A** | **A** | **G** | **A** | **A** | **A** | **T** | **G** | **G** | **A** | **C** | **G** | **G** | **C** | **A** | **T** | **T** | **G** | **A** | **T** | **T** | **C** | **A** | **A** | **C** | **A** | **A** | **A** | **A** | **G** | **C** | **G** | **A** | **T** | **C** | **T** | **T** | **G** | **A** | **A** | **A** | **G** | **A** | **T** | **T** | **G** | **G** | **C** | **C** | **A** | **G** | **A** | **A** | **G** | **C** | **C** | **A** | **A** | **G** | **A** | **T** | **T** | **A** | **T** | **T** | **A** | **T** | **T** | **G** | **T** | **G** | **A** | **C** | **G** | **A** | **G** | **T** | **T** | **T** | **T** | **A** | **T** | **T** | **G** | **A** | **T** | **G** | **A** | **T** | **G** | **A** | **A** | **A** | **A** | **A** | **G** | **T** | **G** | **T** | **A** | **T** | **C** | **C** | **G** | **G** | **C** | **G** | **A** | **T** | **T** | **G** | **A** | **A** | **G** | **C** | **T** | **G** | **G** | **T** | **G** | **C** | **A** | **G** | **C** | **G** | **G** | **G** | **C** | **T** | **A** | **C** | **C** | **T** | **A** | **T** | **T** | **A** | **A** | **A** | **A** | **A** | **C** | **A** | **T** | **C** | **A** | **A** | **C** | **A** | **G** | **C** | **A** | **C** | **A** | **T** | **G** | **A** | **G** | **A** | **T** | **T** | **G** | **C** | **T** | **G** | **A** | **T** | **G** | **C** | **A** | **A** | **T** | **T** | **C** | **G** | **G** | **G** | **C** | **G** | **A** | **C** | **T** | **T** | **A** | **T** | **C** | **G** | **C** | **G** | **G** | **A** | **G** | **A** | **G** | **C** | **G** | **T** | **G** | **T** | **G** | **T** | **T** | **G** | **G** | **A** | **A** | **C** | **C** | **T** | **G** | **A** | **A** | **G** | **T** | **G** | **A** | **C** | **G** | **C** | **A** | **T** | **A** | **A** | **G** | **A** | **T** | **G** | **A** | **T** | **G** | **G** | **A** | **A** | **C** | **G** | **G** | **T** | **T** | **A** | **A** | **C** | **A** | **A** | **A** | **A** | **A** | **A** | **A** | **C** | **A** | **A** | **G** | **A** | **G** | **C** | **C** | **G** | **G** | **T** | **G** | **T** | **T** | **G** | **C** | **A** | **T** | **G** | **A** | **A** | **G** | **A** | **T** | **T** | **T** | **G** | **A** | **C** | **A** | **A** | **A** | **T** | **C** | **G** | **A** | **G** | **A** | **A** | **C** | **A** | **C** | **G** | **A** | **A** | **A** | **T** | **T** | **T** | **T** | **A** | **A** | **T** | **G** | **T** | **T** | **G** | **A** | **T** | **T** | **G** | **C** | **A** | **C** | **A** | **A** | **G** | **G** | **T** | **A** | **A** | **A** | **A** | **G** | **T** | **A** | **A** | **T** | **C** | **A** | **G** | **G** | **A** | **A** | **A** | **T** | **A** | **G** | **C** | **T** | **G** | **A** | **T** | **G** | **A** | **A** | **C** | **T** | **C** | **T** | **T** | **T** | **A** | **T** | **C** | **A** | **C** | **T** | **T** | **T** | **G** | **A** | **A** | **A** | **A** | **C** | **A** | **G** | **T** | **T** | **A** | **A** | **A** | **A** | **C** | **A** | **C** | **A** | **T** | **G** | **T** | **T** | **T** | **C** | **A** | **A** | **A** | **C** | **A** | **T** | **T** | **T** | **T** | **A** | **G** | **C** | **A** | **A** | **A** | **A** | **C** | **T** | **A** | **G** | **A** | **T** | **G** | **T** | **G** | **G** | **A** | **T** | **G** | **A** | **T** | **C** | **G** | **G** | **A** | **C** | **C** | **C** | **A** | **A** | **G** | **C** | **G** | **G** | **C** | **G** | **A** | **T** | **T** | **T** | **A** | **T** | **G** | **C** | **T** | **T** | **T** | **T** | **C** | **A** | **A** | **C** | **A** | **T** | **G** | **G** | **T** | **T** | **T** | **A** | **G** | **C** | **C** | **A** | **A** | **A** | **-** | **-** | **-** |
| **N54155** | **A** | **T** | **G** | **A** | **T** | **A** | **A** | **A** | **A** | **G** | **T** | **T** | **T** | **T** | **A** | **T** | **T** | **A** | **G** | **T** | **A** | **G** | **A** | **T** | **G** | **A** | **C** | **C** | **A** | **T** | **G** | **A** | **A** | **A** | **T** | **G** | **G** | **T** | **A** | **C** | **G** | **C** | **T** | **T** | **A** | **G** | **G** | **C** | **G** | **T** | **C** | **T** | **C** | **T** | **T** | **C** | **T** | **T** | **A** | **T** | **T** | **T** | **A** | **T** | **C** | **T** | **A** | **T** | **C** | **C** | **A** | **A** | **G** | **A** | **A** | **G** | **A** | **C** | **A** | **T** | **T** | **G** | **A** | **A** | **G** | **T** | **G** | **A** | **T** | **T** | **G** | **G** | **A** | **G** | **A** | **A** | **G** | **C** | **T** | **G** | **A** | **A** | **A** | **A** | **T** | **G** | **G** | **A** | **C** | **G** | **T** | **C** | **A** | **A** | **G** | **G** | **C** | **T** | **A** | **T** | **G** | **A** | **A** | **A** | **A** | **G** | **G** | **C** | **G** | **A** | **T** | **G** | **G** | **C** | **A** | **C** | **T** | **T** | **C** | **G** | **A** | **C** | **C** | **G** | **G** | **A** | **T** | **G** | **T** | **C** | **A** | **T** | **T** | **T** | **T** | **A** | **A** | **T** | **G** | **G** | **A** | **T** | **C** | **T** | **A** | **G** | **T** | **C** | **A** | **T** | **G** | **G** | **A** | **A** | **G** | **A** | **A** | **A** | **T** | **G** | **G** | **A** | **T** | **G** | **G** | **C** | **A** | **T** | **C** | **G** | **A** | **A** | **T** | **C** | **A** | **A** | **C** | **A** | **A** | **A** | **A** | **G** | **C** | **A** | **A** | **T** | **C** | **T** | **T** | **A** | **A** | **A** | **A** | **G** | **A** | **T** | **T** | **G** | **G** | **C** | **C** | **A** | **A** | **A** | **A** | **G** | **C** | **C** | **A** | **A** | **A** | **A** | **T** | **C** | **A** | **T** | **T** | **A** | **T** | **C** | **G** | **T** | **C** | **A** | **C** | **A** | **A** | **G** | **T** | **T** | **T** | **T** | **A** | **T** | **T** | **G** | **A** | **T** | **G** | **A** | **T** | **G** | **A** | **A** | **A** | **A** | **A** | **G** | **T** | **T** | **T** | **A** | **T** | **C** | **C** | **T** | **G** | **C** | **A** | **A** | **T** | **T** | **G** | **A** | **A** | **G** | **C** | **A** | **G** | **G** | **A** | **G** | **C** | **A** | **G** | **C** | **A** | **G** | **G** | **A** | **T** | **A** | **T** | **T** | **T** | **G** | **T** | **T** | **G** | **A** | **A** | **G** | **A** | **C** | **G** | **T** | **C** | **T** | **A** | **C** | **C** | **G** | **C** | **A** | **C** | **A** | **T** | **G** | **A** | **A** | **A** | **T** | **A** | **G** | **C** | **T** | **G** | **A** | **T** | **G** | **C** | **T** | **A** | **T** | **A** | **A** | **G** | **G** | **G** | **C** | **T** | **A** | **C** | **G** | **C** | **A** | **A** | **C** | **G** | **C** | **G** | **G** | **C** | **G** | **A** | **G** | **C** | **G** | **A** | **G** | **T** | **A** | **T** | **T** | **G** | **G** | **A** | **A** | **C** | **C** | **T** | **G** | **A** | **A** | **G** | **T** | **G** | **A** | **C** | **A** | **A** | **C** | **A** | **A** | **A** | **A** | **A** | **T** | **G** | **A** | **T** | **G** | **G** | **A** | **A** | **A** | **A** | **A** | **A** | **T** | **G** | **A** | **G** | **T** | **C** | **G** | **G** | **C** | **G** | **A** | **A** | **A** | **T** | **G** | **A** | **T** | **C** | **C** | **A** | **G** | **T** | **A** | **T** | **T** | **G** | **C** | **A** | **T** | **G** | **A** | **A** | **G** | **A** | **A** | **T** | **T** | **A** | **A** | **C** | **G** | **A** | **A** | **T** | **C** | **G** | **A** | **G** | **A** | **A** | **A** | **A** | **T** | **G** | **A** | **A** | **A** | **T** | **T** | **T** | **T** | **G** | **A** | **T** | **G** | **C** | **T** | **G** | **A** | **T** | **T** | **T** | **C** | **T** | **G** | **A** | **A** | **G** | **G** | **G** | **A** | **A** | **A** | **A** | **G** | **T** | **A** | **A** | **T** | **C** | **A** | **G** | **G** | **A** | **A** | **A** | **T** | **C** | **G** | **C** | **G** | **G** | **A** | **T** | **G** | **A** | **A** | **T** | **T** | **G** | **T** | **T** | **T** | **A** | **T** | **C** | **A** | **C** | **A** | **C** | **T** | **G** | **A** | **A** | **A** | **A** | **C** | **A** | **G** | **T** | **C** | **A** | **A** | **A** | **A** | **C** | **G** | **C** | **A** | **T** | **G** | **T** | **G** | **T** | **C** | **G** | **A** | **A** | **T** | **A** | **T** | **A** | **T** | **T** | **A** | **G** | **C** | **T** | **A** | **A** | **G** | **T** | **T** | **A** | **G** | **A** | **G** | **G** | **T** | **A** | **G** | **A** | **A** | **G** | **A** | **C** | **C** | **G** | **A** | **A** | **C** | **T** | **C** | **A** | **A** | **G** | **C** | **A** | **G** | **C** | **T** | **A** | **T** | **C** | **T** | **A** | **T** | **G** | **C** | **G** | **T** | **T** | **T** | **A** | **A** | **A** | **C** | **A** | **T** | **G** | **G** | **T** | **T** | **T** | **G** | **G** | **T** | **G** | **A** | **A** | **A** | **-** | **-** | **-** |
| **N55119** | **G** | **T** | **G** | **A** | **T** | **C** | **A** | **A** | **A** | **G** | **T** | **A** | **A** | **T** | **G** | **T** | **T** | **A** | **G** | **T** | **G** | **G** | **A** | **T** | **G** | **A** | **C** | **C** | **A** | **T** | **G** | **A** | **A** | **A** | **T** | **G** | **G** | **T** | **C** | **C** | **G** | **T** | **T** | **T** | **A** | **G** | **G** | **C** | **G** | **T** | **T** | **T** | **C** | **A** | **T** | **C** | **A** | **T** | **A** | **T** | **T** | **T** | **A** | **T** | **C** | **T** | **A** | **T** | **T** | **C** | **A** | **A** | **G** | **A** | **G** | **G** | **A** | **T** | **A** | **T** | **A** | **G** | **A** | **A** | **G** | **T** | **C** | **G** | **T** | **A** | **G** | **G** | **C** | **G** | **A** | **A** | **G** | **C** | **A** | **G** | **A** | **A** | **A** | **A** | **T** | **G** | **G** | **T** | **A** | **A** | **G** | **A** | **T** | **T** | **G** | **G** | **C** | **T** | **A** | **T** | **G** | **A** | **A** | **A** | **A** | **A** | **G** | **C** | **A** | **T** | **T** | **G** | **G** | **A** | **A** | **C** | **T** | **A** | **C** | **G** | **T** | **C** | **C** | **A** | **G** | **A** | **T** | **G** | **T** | **T** | **A** | **T** | **T** | **T** | **T** | **G** | **A** | **T** | **G** | **G** | **A** | **T** | **T** | **T** | **G** | **G** | **T** | **A** | **A** | **T** | **G** | **G** | **A** | **A** | **G** | **A** | **A** | **A** | **T** | **G** | **G** | **A** | **C** | **G** | **G** | **C** | **A** | **T** | **T** | **G** | **A** | **T** | **T** | **C** | **A** | **A** | **C** | **A** | **A** | **A** | **A** | **G** | **C** | **G** | **A** | **T** | **C** | **T** | **T** | **G** | **A** | **A** | **A** | **G** | **A** | **T** | **T** | **G** | **G** | **C** | **C** | **A** | **G** | **A** | **A** | **G** | **C** | **C** | **A** | **A** | **G** | **A** | **T** | **T** | **A** | **T** | **T** | **A** | **T** | **T** | **G** | **T** | **G** | **A** | **C** | **G** | **A** | **G** | **T** | **T** | **T** | **T** | **A** | **T** | **T** | **G** | **A** | **T** | **G** | **A** | **T** | **G** | **A** | **A** | **A** | **A** | **A** | **G** | **T** | **G** | **T** | **A** | **T** | **C** | **C** | **G** | **G** | **C** | **G** | **A** | **T** | **T** | **G** | **A** | **A** | **G** | **C** | **T** | **G** | **G** | **T** | **G** | **C** | **A** | **G** | **C** | **G** | **G** | **G** | **C** | **T** | **A** | **C** | **C** | **T** | **A** | **T** | **T** | **A** | **A** | **A** | **A** | **A** | **C** | **A** | **T** | **C** | **A** | **A** | **C** | **A** | **G** | **C** | **A** | **C** | **A** | **T** | **G** | **A** | **G** | **A** | **T** | **T** | **G** | **C** | **T** | **G** | **A** | **T** | **G** | **C** | **A** | **A** | **T** | **T** | **C** | **G** | **G** | **G** | **C** | **G** | **A** | **C** | **T** | **T** | **A** | **T** | **C** | **G** | **C** | **G** | **G** | **A** | **G** | **A** | **G** | **C** | **G** | **T** | **G** | **T** | **G** | **T** | **T** | **G** | **G** | **A** | **A** | **C** | **C** | **T** | **G** | **A** | **A** | **G** | **T** | **G** | **A** | **C** | **G** | **C** | **A** | **T** | **A** | **A** | **G** | **A** | **T** | **G** | **A** | **T** | **G** | **G** | **A** | **A** | **C** | **G** | **G** | **T** | **T** | **A** | **A** | **C** | **A** | **A** | **A** | **A** | **A** | **A** | **A** | **C** | **A** | **A** | **G** | **A** | **G** | **C** | **C** | **G** | **G** | **T** | **G** | **T** | **T** | **G** | **C** | **A** | **T** | **G** | **A** | **A** | **G** | **A** | **T** | **T** | **T** | **G** | **A** | **C** | **A** | **A** | **A** | **T** | **C** | **G** | **A** | **G** | **A** | **A** | **C** | **A** | **C** | **G** | **A** | **A** | **A** | **T** | **T** | **T** | **T** | **A** | **A** | **T** | **G** | **T** | **T** | **G** | **A** | **T** | **T** | **G** | **C** | **A** | **C** | **A** | **A** | **G** | **G** | **T** | **A** | **A** | **A** | **A** | **G** | **T** | **A** | **A** | **T** | **C** | **A** | **G** | **G** | **A** | **A** | **A** | **T** | **A** | **G** | **C** | **T** | **G** | **A** | **T** | **G** | **A** | **A** | **C** | **T** | **C** | **T** | **T** | **T** | **A** | **T** | **C** | **A** | **C** | **T** | **T** | **T** | **G** | **A** | **A** | **A** | **A** | **C** | **A** | **G** | **T** | **T** | **A** | **A** | **A** | **A** | **C** | **A** | **C** | **A** | **T** | **G** | **T** | **T** | **T** | **C** | **A** | **A** | **A** | **C** | **A** | **T** | **T** | **T** | **T** | **A** | **G** | **C** | **A** | **A** | **A** | **A** | **C** | **T** | **A** | **G** | **A** | **T** | **G** | **T** | **G** | **G** | **A** | **T** | **G** | **A** | **T** | **C** | **G** | **G** | **A** | **C** | **C** | **C** | **A** | **A** | **G** | **C** | **G** | **G** | **C** | **G** | **A** | **T** | **T** | **T** | **A** | **T** | **G** | **C** | **T** | **T** | **T** | **T** | **C** | **A** | **A** | **C** | **A** | **T** | **G** | **G** | **T** | **T** | **T** | **A** | **G** | **C** | **C** | **A** | **A** | **A** | **-** | **-** | **-** |
| **N59711F** | **A** | **T** | **G** | **A** | **T** | **A** | **A** | **A** | **A** | **G** | **T** | **T** | **T** | **T** | **A** | **T** | **T** | **A** | **G** | **T** | **A** | **G** | **A** | **T** | **G** | **A** | **C** | **C** | **A** | **T** | **G** | **A** | **A** | **A** | **T** | **G** | **G** | **T** | **G** | **C** | **G** | **C** | **T** | **T** | **A** | **G** | **G** | **C** | **G** | **T** | **C** | **T** | **C** | **T** | **T** | **C** | **T** | **T** | **A** | **T** | **T** | **T** | **A** | **T** | **C** | **T** | **A** | **T** | **T** | **C** | **A** | **A** | **G** | **A** | **A** | **G** | **A** | **C** | **A** | **T** | **T** | **G** | **A** | **A** | **G** | **T** | **A** | **A** | **T** | **T** | **G** | **G** | **A** | **G** | **A** | **A** | **G** | **C** | **T** | **G** | **A** | **A** | **A** | **A** | **T** | **G** | **G** | **A** | **C** | **G** | **A** | **C** | **A** | **A** | **G** | **G** | **C** | **T** | **A** | **T** | **G** | **A** | **G** | **A** | **A** | **G** | **G** | **C** | **G** | **A** | **T** | **G** | **A** | **C** | **A** | **C** | **T** | **T** | **C** | **G** | **A** | **C** | **C** | **G** | **G** | **A** | **C** | **G** | **T** | **C** | **A** | **T** | **T** | **T** | **T** | **A** | **A** | **T** | **G** | **G** | **A** | **C** | **T** | **T** | **A** | **G** | **T** | **C** | **A** | **T** | **G** | **G** | **A** | **A** | **G** | **A** | **A** | **A** | **T** | **G** | **G** | **A** | **T** | **G** | **G** | **T** | **A** | **T** | **C** | **G** | **A** | **A** | **T** | **C** | **A** | **A** | **C** | **A** | **A** | **A** | **A** | **G** | **C** | **A** | **A** | **T** | **C** | **T** | **T** | **A** | **A** | **A** | **A** | **G** | **A** | **T** | **T** | **G** | **G** | **C** | **C** | **A** | **G** | **A** | **A** | **G** | **C** | **T** | **A** | **A** | **A** | **A** | **T** | **C** | **A** | **T** | **T** | **A** | **T** | **C** | **G** | **T** | **A** | **A** | **C** | **C** | **A** | **G** | **T** | **T** | **T** | **T** | **A** | **T** | **T** | **G** | **A** | **T** | **G** | **A** | **C** | **G** | **A** | **A** | **A** | **A** | **A** | **G** | **T** | **T** | **T** | **A** | **T** | **C** | **C** | **T** | **G** | **C** | **C** | **A** | **T** | **T** | **G** | **A** | **A** | **G** | **C** | **A** | **G** | **G** | **T** | **G** | **C** | **A** | **G** | **C** | **A** | **G** | **G** | **G** | **T** | **A** | **T** | **T** | **T** | **G** | **T** | **T** | **G** | **A** | **A** | **G** | **A** | **C** | **C** | **T** | **C** | **T** | **A** | **C** | **C** | **G** | **C** | **A** | **C** | **A** | **T** | **G** | **A** | **A** | **A** | **T** | **T** | **G** | **C** | **T** | **G** | **A** | **T** | **G** | **C** | **T** | **A** | **T** | **A** | **A** | **G** | **G** | **G** | **C** | **A** | **A** | **C** | **G** | **C** | **A** | **A** | **C** | **G** | **T** | **G** | **G** | **C** | **G** | **A** | **G** | **C** | **G** | **A** | **G** | **T** | **A** | **T** | **T** | **A** | **G** | **A** | **A** | **C** | **C** | **A** | **G** | **A** | **A** | **G** | **T** | **G** | **A** | **C** | **A** | **A** | **C** | **A** | **A** | **A** | **A** | **A** | **T** | **G** | **A** | **T** | **G** | **G** | **A** | **A** | **A** | **A** | **A** | **A** | **T** | **G** | **A** | **G** | **T** | **C** | **G** | **G** | **C** | **G** | **A** | **A** | **A** | **T** | **G** | **A** | **G** | **C** | **C** | **T** | **G** | **T** | **A** | **T** | **T** | **A** | **C** | **A** | **T** | **G** | **A** | **A** | **G** | **A** | **A** | **C** | **T** | **A** | **A** | **C** | **G** | **A** | **A** | **T** | **C** | **G** | **G** | **G** | **A** | **A** | **A** | **A** | **T** | **G** | **A** | **A** | **A** | **T** | **T** | **T** | **T** | **A** | **A** | **T** | **G** | **C** | **T** | **G** | **A** | **T** | **T** | **T** | **C** | **T** | **G** | **A** | **A** | **G** | **G** | **A** | **A** | **A** | **A** | **A** | **G** | **T** | **A** | **A** | **T** | **C** | **A** | **G** | **G** | **A** | **A** | **A** | **T** | **A** | **G** | **C** | **G** | **G** | **A** | **C** | **G** | **A** | **A** | **T** | **T** | **G** | **T** | **T** | **T** | **A** | **T** | **T** | **A** | **C** | **A** | **C** | **T** | **G** | **A** | **A** | **A** | **A** | **C** | **A** | **G** | **T** | **A** | **A** | **A** | **A** | **A** | **C** | **A** | **C** | **A** | **T** | **G** | **T** | **T** | **T** | **C** | **C** | **A** | **A** | **T** | **A** | **T** | **A** | **T** | **T** | **G** | **G** | **C** | **T** | **A** | **A** | **G** | **C** | **T** | **A** | **G** | **A** | **A** | **G** | **T** | **G** | **G** | **A** | **A** | **G** | **A** | **C** | **C** | **G** | **T** | **A** | **C** | **A** | **C** | **A** | **A** | **G** | **C** | **C** | **G** | **C** | **T** | **A** | **T** | **C** | **T** | **A** | **T** | **G** | **C** | **A** | **T** | **T** | **C** | **A** | **A** | **A** | **C** | **A** | **T** | **G** | **G** | **T** | **T** | **T** | **G** | **G** | **T** | **G** | **A** | **A** | **A** | **-** | **-** | **-** |
| **N52587** | **G** | **T** | **G** | **A** | **T** | **C** | **A** | **A** | **A** | **G** | **T** | **A** | **A** | **T** | **G** | **T** | **T** | **A** | **G** | **T** | **G** | **G** | **A** | **T** | **G** | **A** | **C** | **C** | **A** | **T** | **G** | **A** | **A** | **A** | **T** | **G** | **G** | **T** | **C** | **C** | **G** | **T** | **T** | **T** | **A** | **G** | **G** | **C** | **G** | **T** | **T** | **T** | **C** | **A** | **T** | **C** | **A** | **T** | **A** | **T** | **T** | **T** | **A** | **T** | **C** | **T** | **A** | **T** | **T** | **C** | **A** | **A** | **G** | **A** | **G** | **G** | **A** | **T** | **A** | **T** | **A** | **G** | **A** | **A** | **G** | **T** | **C** | **G** | **T** | **A** | **G** | **G** | **C** | **G** | **A** | **A** | **G** | **C** | **A** | **G** | **A** | **A** | **A** | **A** | **C** | **G** | **G** | **T** | **A** | **A** | **G** | **A** | **T** | **T** | **G** | **G** | **C** | **T** | **A** | **T** | **G** | **A** | **A** | **A** | **A** | **A** | **G** | **C** | **A** | **T** | **T** | **G** | **G** | **A** | **A** | **C** | **T** | **A** | **C** | **G** | **T** | **C** | **C** | **A** | **G** | **A** | **T** | **G** | **T** | **T** | **A** | **T** | **T** | **T** | **T** | **G** | **A** | **T** | **G** | **G** | **A** | **C** | **T** | **T** | **G** | **G** | **T** | **A** | **A** | **T** | **G** | **G** | **A** | **A** | **G** | **A** | **A** | **A** | **T** | **G** | **G** | **A** | **C** | **G** | **G** | **C** | **A** | **T** | **T** | **G** | **A** | **T** | **T** | **C** | **A** | **A** | **C** | **A** | **A** | **A** | **A** | **G** | **C** | **G** | **A** | **T** | **T** | **T** | **T** | **G** | **A** | **A** | **A** | **G** | **A** | **T** | **T** | **G** | **G** | **C** | **C** | **A** | **G** | **A** | **A** | **G** | **C** | **C** | **A** | **A** | **G** | **A** | **T** | **T** | **A** | **T** | **T** | **A** | **T** | **T** | **G** | **T** | **G** | **A** | **C** | **G** | **A** | **G** | **T** | **T** | **T** | **T** | **A** | **T** | **T** | **G** | **A** | **T** | **G** | **A** | **T** | **G** | **A** | **A** | **A** | **A** | **A** | **G** | **T** | **G** | **T** | **A** | **T** | **C** | **C** | **G** | **G** | **C** | **A** | **A** | **T** | **T** | **G** | **A** | **A** | **G** | **C** | **T** | **G** | **G** | **T** | **G** | **C** | **A** | **G** | **C** | **G** | **G** | **G** | **C** | **T** | **A** | **C** | **C** | **T** | **A** | **T** | **T** | **A** | **A** | **A** | **A** | **A** | **C** | **A** | **T** | **C** | **A** | **A** | **C** | **A** | **G** | **C** | **A** | **C** | **A** | **T** | **G** | **A** | **G** | **A** | **T** | **T** | **G** | **C** | **T** | **G** | **A** | **T** | **G** | **C** | **G** | **A** | **T** | **T** | **C** | **G** | **G** | **G** | **C** | **G** | **A** | **C** | **T** | **T** | **A** | **T** | **C** | **G** | **C** | **G** | **G** | **A** | **G** | **A** | **G** | **C** | **G** | **T** | **G** | **T** | **G** | **T** | **T** | **G** | **G** | **A** | **A** | **C** | **C** | **T** | **G** | **A** | **A** | **G** | **T** | **G** | **A** | **C** | **G** | **C** | **A** | **T** | **A** | **A** | **G** | **A** | **T** | **G** | **A** | **T** | **G** | **G** | **A** | **A** | **C** | **G** | **G** | **T** | **T** | **A** | **A** | **C** | **A** | **A** | **A** | **A** | **A** | **A** | **A** | **C** | **A** | **A** | **G** | **A** | **G** | **C** | **C** | **G** | **G** | **T** | **G** | **C** | **T** | **A** | **C** | **A** | **C** | **G** | **A** | **A** | **G** | **A** | **T** | **T** | **T** | **G** | **A** | **C** | **A** | **A** | **A** | **C** | **C** | **G** | **A** | **G** | **A** | **A** | **C** | **A** | **C** | **G** | **A** | **A** | **A** | **T** | **T** | **T** | **T** | **A** | **A** | **T** | **G** | **C** | **T** | **G** | **A** | **T** | **T** | **G** | **C** | **A** | **C** | **A** | **A** | **G** | **G** | **T** | **A** | **A** | **A** | **A** | **G** | **T** | **A** | **A** | **T** | **C** | **A** | **G** | **G** | **A** | **A** | **A** | **T** | **T** | **G** | **C** | **T** | **G** | **A** | **T** | **G** | **A** | **A** | **C** | **T** | **C** | **T** | **T** | **T** | **A** | **T** | **C** | **A** | **C** | **T** | **T** | **T** | **G** | **A** | **A** | **A** | **A** | **C** | **A** | **G** | **T** | **T** | **A** | **A** | **A** | **A** | **C** | **A** | **C** | **A** | **T** | **G** | **T** | **T** | **T** | **C** | **A** | **A** | **A** | **C** | **A** | **T** | **T** | **T** | **T** | **A** | **G** | **C** | **A** | **A** | **A** | **A** | **C** | **T** | **A** | **G** | **A** | **T** | **G** | **T** | **G** | **G** | **A** | **T** | **G** | **A** | **T** | **C** | **G** | **G** | **A** | **C** | **C** | **C** | **A** | **A** | **G** | **C** | **G** | **G** | **C** | **G** | **A** | **T** | **T** | **T** | **A** | **T** | **G** | **C** | **A** | **T** | **T** | **T** | **C** | **A** | **A** | **C** | **A** | **C** | **G** | **G** | **T** | **T** | **T** | **A** | **G** | **C** | **C** | **A** | **A** | **A** | **-** | **-** | **-** |
| **N60260F** | **A** | **T** | **G** | **A** | **T** | **A** | **A** | **A** | **A** | **G** | **T** | **T** | **T** | **T** | **A** | **T** | **T** | **A** | **G** | **T** | **A** | **G** | **A** | **T** | **G** | **A** | **C** | **C** | **A** | **T** | **G** | **A** | **A** | **A** | **T** | **G** | **G** | **T** | **A** | **C** | **G** | **C** | **T** | **T** | **A** | **G** | **G** | **C** | **G** | **T** | **C** | **T** | **C** | **T** | **T** | **C** | **T** | **T** | **A** | **T** | **T** | **T** | **A** | **T** | **C** | **T** | **A** | **T** | **C** | **C** | **A** | **A** | **G** | **A** | **A** | **G** | **A** | **C** | **A** | **T** | **T** | **G** | **A** | **A** | **G** | **T** | **G** | **A** | **T** | **T** | **G** | **G** | **A** | **G** | **A** | **A** | **G** | **C** | **T** | **G** | **A** | **A** | **A** | **A** | **T** | **G** | **G** | **A** | **C** | **G** | **T** | **C** | **A** | **A** | **G** | **G** | **C** | **T** | **A** | **T** | **G** | **A** | **A** | **A** | **A** | **G** | **G** | **C** | **G** | **A** | **T** | **G** | **G** | **C** | **A** | **C** | **T** | **T** | **C** | **G** | **A** | **C** | **C** | **A** | **G** | **A** | **T** | **G** | **T** | **C** | **A** | **T** | **T** | **T** | **T** | **A** | **A** | **T** | **G** | **G** | **A** | **T** | **C** | **T** | **A** | **G** | **T** | **C** | **A** | **T** | **G** | **G** | **A** | **A** | **G** | **A** | **A** | **A** | **T** | **G** | **G** | **A** | **T** | **G** | **G** | **C** | **A** | **T** | **C** | **G** | **A** | **A** | **T** | **C** | **A** | **A** | **C** | **A** | **A** | **A** | **A** | **G** | **C** | **A** | **A** | **T** | **C** | **T** | **T** | **A** | **A** | **A** | **A** | **G** | **A** | **T** | **T** | **G** | **G** | **C** | **C** | **A** | **A** | **A** | **A** | **G** | **C** | **C** | **A** | **A** | **A** | **A** | **T** | **C** | **A** | **T** | **T** | **A** | **T** | **C** | **G** | **T** | **C** | **A** | **C** | **A** | **A** | **G** | **T** | **T** | **T** | **T** | **A** | **T** | **T** | **G** | **A** | **T** | **G** | **A** | **T** | **G** | **A** | **A** | **A** | **A** | **A** | **G** | **T** | **T** | **T** | **A** | **T** | **C** | **C** | **T** | **G** | **C** | **A** | **A** | **T** | **T** | **G** | **A** | **A** | **G** | **C** | **A** | **G** | **G** | **A** | **G** | **C** | **A** | **G** | **C** | **A** | **G** | **G** | **A** | **T** | **A** | **T** | **T** | **T** | **A** | **T** | **T** | **G** | **A** | **A** | **G** | **A** | **C** | **G** | **T** | **C** | **T** | **A** | **C** | **C** | **G** | **C** | **A** | **C** | **A** | **T** | **G** | **A** | **A** | **A** | **T** | **A** | **G** | **C** | **T** | **G** | **A** | **T** | **G** | **C** | **T** | **A** | **T** | **A** | **A** | **G** | **G** | **G** | **C** | **T** | **A** | **C** | **G** | **C** | **A** | **A** | **C** | **G** | **C** | **G** | **G** | **C** | **G** | **A** | **G** | **C** | **G** | **A** | **G** | **T** | **A** | **T** | **T** | **G** | **G** | **A** | **A** | **C** | **C** | **T** | **G** | **A** | **A** | **G** | **T** | **G** | **A** | **C** | **A** | **A** | **C** | **A** | **A** | **A** | **A** | **A** | **T** | **G** | **A** | **T** | **G** | **G** | **A** | **A** | **A** | **A** | **A** | **A** | **T** | **G** | **A** | **G** | **T** | **C** | **G** | **G** | **C** | **G** | **A** | **A** | **A** | **T** | **G** | **A** | **T** | **C** | **C** | **A** | **G** | **T** | **A** | **T** | **T** | **G** | **C** | **A** | **T** | **G** | **A** | **A** | **G** | **A** | **A** | **T** | **T** | **A** | **A** | **C** | **G** | **A** | **A** | **T** | **C** | **G** | **A** | **G** | **A** | **A** | **A** | **A** | **T** | **G** | **A** | **A** | **A** | **T** | **T** | **T** | **T** | **G** | **A** | **T** | **G** | **C** | **T** | **G** | **A** | **T** | **T** | **T** | **C** | **T** | **G** | **A** | **A** | **G** | **G** | **G** | **A** | **A** | **A** | **A** | **G** | **T** | **A** | **A** | **T** | **C** | **A** | **G** | **G** | **A** | **A** | **A** | **T** | **C** | **G** | **C** | **G** | **G** | **A** | **T** | **G** | **A** | **A** | **T** | **T** | **G** | **T** | **T** | **T** | **A** | **T** | **C** | **A** | **C** | **A** | **C** | **T** | **G** | **A** | **A** | **A** | **A** | **C** | **A** | **G** | **T** | **T** | **A** | **A** | **A** | **A** | **C** | **G** | **C** | **A** | **T** | **G** | **T** | **G** | **T** | **C** | **G** | **A** | **A** | **T** | **A** | **T** | **A** | **T** | **T** | **A** | **G** | **C** | **T** | **A** | **A** | **G** | **T** | **T** | **A** | **G** | **A** | **G** | **G** | **T** | **A** | **G** | **A** | **A** | **G** | **A** | **C** | **C** | **G** | **A** | **A** | **C** | **T** | **C** | **A** | **A** | **G** | **C** | **A** | **G** | **C** | **T** | **A** | **T** | **C** | **T** | **A** | **T** | **G** | **C** | **G** | **T** | **T** | **T** | **A** | **A** | **A** | **C** | **A** | **T** | **G** | **G** | **T** | **T** | **T** | **G** | **G** | **T** | **G** | **A** | **A** | **A** | **-** | **-** | **-** |
| **N59860F** | **G** | **T** | **G** | **A** | **T** | **C** | **A** | **A** | **A** | **G** | **T** | **A** | **A** | **T** | **G** | **T** | **T** | **A** | **G** | **T** | **G** | **G** | **A** | **T** | **G** | **A** | **C** | **C** | **A** | **T** | **G** | **A** | **A** | **A** | **T** | **G** | **G** | **T** | **C** | **C** | **G** | **T** | **T** | **T** | **A** | **G** | **G** | **C** | **G** | **T** | **T** | **T** | **C** | **A** | **T** | **C** | **A** | **T** | **A** | **T** | **T** | **T** | **A** | **T** | **C** | **T** | **A** | **T** | **T** | **C** | **A** | **A** | **G** | **A** | **G** | **G** | **A** | **T** | **A** | **T** | **A** | **G** | **A** | **A** | **G** | **T** | **C** | **G** | **T** | **A** | **G** | **G** | **C** | **G** | **A** | **A** | **G** | **C** | **A** | **G** | **A** | **A** | **A** | **A** | **C** | **G** | **G** | **T** | **A** | **A** | **G** | **A** | **T** | **T** | **G** | **G** | **C** | **T** | **A** | **T** | **G** | **A** | **A** | **A** | **A** | **A** | **G** | **C** | **A** | **T** | **T** | **G** | **G** | **A** | **A** | **C** | **T** | **A** | **C** | **G** | **T** | **C** | **C** | **A** | **G** | **A** | **T** | **G** | **T** | **T** | **A** | **T** | **T** | **T** | **T** | **G** | **A** | **T** | **G** | **G** | **A** | **T** | **T** | **T** | **G** | **G** | **T** | **A** | **A** | **T** | **G** | **G** | **A** | **A** | **G** | **A** | **A** | **A** | **T** | **G** | **G** | **A** | **C** | **G** | **G** | **C** | **A** | **T** | **T** | **G** | **A** | **T** | **T** | **C** | **A** | **A** | **C** | **A** | **A** | **A** | **A** | **G** | **C** | **G** | **A** | **T** | **C** | **T** | **T** | **G** | **A** | **A** | **A** | **G** | **A** | **T** | **T** | **G** | **G** | **C** | **C** | **A** | **G** | **A** | **A** | **G** | **C** | **C** | **A** | **A** | **G** | **A** | **T** | **T** | **A** | **T** | **T** | **A** | **T** | **T** | **G** | **T** | **G** | **A** | **C** | **G** | **A** | **G** | **T** | **T** | **T** | **T** | **A** | **T** | **T** | **G** | **A** | **T** | **G** | **A** | **T** | **G** | **A** | **A** | **A** | **A** | **A** | **G** | **T** | **G** | **T** | **A** | **T** | **C** | **C** | **G** | **G** | **C** | **G** | **A** | **T** | **T** | **G** | **A** | **A** | **G** | **C** | **T** | **G** | **G** | **T** | **G** | **C** | **A** | **G** | **C** | **G** | **G** | **G** | **C** | **T** | **A** | **C** | **C** | **T** | **A** | **T** | **T** | **A** | **A** | **A** | **A** | **A** | **C** | **A** | **T** | **C** | **A** | **A** | **C** | **A** | **G** | **C** | **A** | **C** | **A** | **T** | **G** | **A** | **G** | **A** | **T** | **T** | **G** | **C** | **T** | **G** | **A** | **T** | **G** | **C** | **A** | **A** | **T** | **T** | **C** | **G** | **G** | **G** | **C** | **G** | **A** | **C** | **T** | **T** | **A** | **T** | **C** | **G** | **C** | **G** | **G** | **A** | **G** | **A** | **G** | **C** | **G** | **T** | **G** | **T** | **G** | **T** | **T** | **G** | **G** | **A** | **A** | **C** | **C** | **T** | **G** | **A** | **A** | **G** | **T** | **G** | **A** | **C** | **G** | **C** | **A** | **T** | **A** | **A** | **G** | **A** | **T** | **G** | **A** | **T** | **G** | **G** | **A** | **A** | **C** | **G** | **G** | **T** | **T** | **A** | **A** | **C** | **A** | **A** | **A** | **A** | **A** | **A** | **A** | **C** | **A** | **A** | **G** | **A** | **G** | **C** | **C** | **G** | **G** | **T** | **G** | **T** | **T** | **G** | **C** | **A** | **C** | **G** | **A** | **A** | **G** | **A** | **T** | **T** | **T** | **G** | **A** | **C** | **A** | **A** | **A** | **C** | **C** | **G** | **G** | **G** | **A** | **A** | **C** | **A** | **C** | **G** | **A** | **A** | **A** | **T** | **T** | **T** | **T** | **A** | **A** | **T** | **G** | **T** | **T** | **G** | **A** | **T** | **T** | **G** | **C** | **A** | **C** | **A** | **A** | **G** | **G** | **T** | **A** | **A** | **A** | **A** | **G** | **T** | **A** | **A** | **T** | **C** | **A** | **G** | **G** | **A** | **A** | **A** | **T** | **A** | **G** | **C** | **T** | **G** | **A** | **T** | **G** | **A** | **A** | **C** | **T** | **C** | **T** | **T** | **T** | **A** | **T** | **C** | **A** | **C** | **T** | **T** | **T** | **G** | **A** | **A** | **A** | **A** | **C** | **A** | **G** | **T** | **T** | **A** | **A** | **A** | **A** | **C** | **A** | **C** | **A** | **T** | **G** | **T** | **T** | **T** | **C** | **A** | **A** | **A** | **C** | **A** | **T** | **T** | **T** | **T** | **A** | **G** | **C** | **A** | **A** | **A** | **A** | **C** | **T** | **A** | **G** | **A** | **T** | **G** | **T** | **G** | **G** | **A** | **T** | **G** | **A** | **T** | **C** | **G** | **G** | **A** | **C** | **C** | **C** | **A** | **A** | **G** | **C** | **G** | **G** | **C** | **G** | **A** | **T** | **T** | **T** | **A** | **T** | **G** | **C** | **T** | **T** | **T** | **T** | **C** | **A** | **A** | **C** | **A** | **T** | **G** | **G** | **T** | **T** | **T** | **A** | **G** | **C** | **C** | **A** | **A** | **A** | **-** | **-** | **-** |
| **N59841F** | **G** | **T** | **G** | **A** | **T** | **C** | **A** | **A** | **A** | **G** | **T** | **A** | **A** | **T** | **G** | **T** | **T** | **A** | **G** | **T** | **G** | **G** | **A** | **T** | **G** | **A** | **C** | **C** | **A** | **T** | **G** | **A** | **A** | **A** | **T** | **G** | **G** | **T** | **C** | **C** | **G** | **T** | **T** | **T** | **A** | **G** | **G** | **C** | **G** | **T** | **T** | **T** | **C** | **A** | **T** | **C** | **A** | **T** | **A** | **T** | **T** | **T** | **A** | **T** | **C** | **T** | **A** | **T** | **T** | **C** | **A** | **A** | **G** | **A** | **G** | **G** | **A** | **T** | **A** | **T** | **A** | **G** | **A** | **A** | **G** | **T** | **C** | **G** | **T** | **A** | **G** | **G** | **C** | **G** | **A** | **A** | **G** | **C** | **A** | **G** | **A** | **A** | **A** | **A** | **C** | **G** | **G** | **T** | **A** | **A** | **G** | **A** | **T** | **T** | **G** | **G** | **C** | **T** | **A** | **T** | **G** | **A** | **A** | **A** | **A** | **A** | **G** | **C** | **A** | **T** | **T** | **G** | **G** | **A** | **A** | **C** | **T** | **A** | **C** | **G** | **T** | **C** | **C** | **A** | **G** | **A** | **T** | **G** | **T** | **T** | **A** | **T** | **T** | **T** | **T** | **G** | **A** | **T** | **G** | **G** | **A** | **T** | **T** | **T** | **G** | **G** | **T** | **A** | **A** | **T** | **G** | **G** | **A** | **A** | **G** | **A** | **A** | **A** | **T** | **G** | **G** | **A** | **C** | **G** | **G** | **C** | **A** | **T** | **T** | **G** | **A** | **T** | **T** | **C** | **A** | **A** | **C** | **A** | **A** | **A** | **A** | **G** | **C** | **G** | **A** | **T** | **C** | **T** | **T** | **G** | **A** | **A** | **A** | **G** | **A** | **T** | **T** | **G** | **G** | **C** | **C** | **A** | **G** | **A** | **A** | **G** | **C** | **C** | **A** | **A** | **G** | **A** | **T** | **T** | **A** | **T** | **T** | **A** | **T** | **T** | **G** | **T** | **G** | **A** | **C** | **G** | **A** | **G** | **T** | **T** | **T** | **T** | **A** | **T** | **T** | **G** | **A** | **T** | **G** | **A** | **T** | **G** | **A** | **G** | **A** | **A** | **A** | **G** | **T** | **G** | **T** | **A** | **T** | **C** | **C** | **G** | **G** | **C** | **G** | **A** | **T** | **T** | **G** | **A** | **A** | **G** | **C** | **T** | **G** | **G** | **T** | **G** | **C** | **A** | **G** | **C** | **G** | **G** | **G** | **C** | **T** | **A** | **C** | **C** | **T** | **A** | **T** | **T** | **A** | **A** | **A** | **A** | **A** | **C** | **A** | **T** | **C** | **A** | **A** | **C** | **A** | **G** | **C** | **A** | **C** | **A** | **T** | **G** | **A** | **G** | **A** | **T** | **T** | **G** | **C** | **T** | **G** | **A** | **T** | **G** | **C** | **A** | **A** | **T** | **T** | **C** | **G** | **G** | **G** | **C** | **G** | **A** | **C** | **T** | **T** | **A** | **T** | **C** | **G** | **C** | **G** | **G** | **A** | **G** | **A** | **G** | **C** | **G** | **T** | **G** | **T** | **G** | **T** | **T** | **G** | **G** | **A** | **A** | **C** | **C** | **T** | **G** | **A** | **A** | **G** | **T** | **G** | **A** | **C** | **G** | **C** | **A** | **T** | **A** | **A** | **G** | **A** | **T** | **G** | **A** | **T** | **G** | **G** | **A** | **A** | **C** | **G** | **G** | **T** | **T** | **A** | **A** | **C** | **A** | **A** | **A** | **A** | **A** | **A** | **A** | **C** | **A** | **A** | **G** | **A** | **G** | **C** | **C** | **G** | **G** | **T** | **G** | **T** | **T** | **G** | **C** | **A** | **T** | **G** | **A** | **A** | **G** | **A** | **T** | **T** | **T** | **G** | **A** | **C** | **A** | **A** | **A** | **T** | **C** | **G** | **A** | **G** | **A** | **A** | **C** | **A** | **C** | **G** | **A** | **A** | **A** | **T** | **T** | **T** | **T** | **A** | **A** | **T** | **G** | **T** | **T** | **G** | **A** | **T** | **T** | **G** | **C** | **A** | **C** | **A** | **A** | **G** | **G** | **T** | **A** | **A** | **A** | **A** | **G** | **T** | **A** | **A** | **T** | **C** | **A** | **G** | **G** | **A** | **A** | **A** | **T** | **A** | **G** | **C** | **T** | **G** | **A** | **T** | **G** | **A** | **A** | **C** | **T** | **C** | **T** | **T** | **T** | **A** | **T** | **C** | **A** | **C** | **T** | **T** | **T** | **G** | **A** | **A** | **A** | **A** | **C** | **A** | **G** | **T** | **T** | **A** | **A** | **A** | **A** | **C** | **A** | **C** | **A** | **T** | **G** | **T** | **T** | **T** | **C** | **A** | **A** | **A** | **C** | **A** | **T** | **T** | **T** | **T** | **A** | **G** | **C** | **A** | **A** | **A** | **A** | **C** | **T** | **A** | **G** | **A** | **T** | **G** | **T** | **G** | **G** | **A** | **T** | **G** | **A** | **T** | **C** | **G** | **G** | **A** | **C** | **C** | **C** | **A** | **A** | **G** | **C** | **G** | **G** | **C** | **G** | **A** | **T** | **T** | **T** | **A** | **T** | **G** | **C** | **T** | **T** | **T** | **T** | **C** | **A** | **A** | **C** | **A** | **T** | **G** | **G** | **T** | **T** | **T** | **A** | **G** | **C** | **C** | **A** | **A** | **A** | **-** | **-** | **-** |
| **N52623** | **G** | **T** | **G** | **A** | **T** | **C** | **A** | **A** | **A** | **G** | **T** | **A** | **A** | **T** | **G** | **T** | **T** | **A** | **G** | **T** | **G** | **G** | **A** | **T** | **G** | **A** | **C** | **C** | **A** | **T** | **G** | **A** | **A** | **A** | **T** | **G** | **G** | **T** | **C** | **C** | **G** | **T** | **T** | **T** | **A** | **G** | **G** | **C** | **G** | **T** | **T** | **T** | **C** | **A** | **T** | **C** | **A** | **T** | **A** | **T** | **T** | **T** | **A** | **T** | **C** | **T** | **A** | **T** | **T** | **C** | **A** | **A** | **G** | **A** | **G** | **G** | **A** | **T** | **A** | **T** | **A** | **G** | **A** | **A** | **G** | **T** | **C** | **G** | **T** | **A** | **G** | **G** | **C** | **G** | **A** | **A** | **G** | **C** | **A** | **G** | **A** | **A** | **A** | **A** | **T** | **G** | **G** | **T** | **A** | **A** | **G** | **A** | **T** | **T** | **G** | **G** | **C** | **T** | **A** | **T** | **G** | **A** | **A** | **A** | **A** | **A** | **G** | **C** | **A** | **T** | **T** | **G** | **G** | **A** | **A** | **C** | **T** | **A** | **C** | **G** | **T** | **C** | **C** | **A** | **G** | **A** | **T** | **G** | **T** | **T** | **A** | **T** | **T** | **T** | **T** | **G** | **A** | **T** | **G** | **G** | **A** | **T** | **T** | **T** | **G** | **G** | **T** | **A** | **A** | **T** | **G** | **G** | **A** | **A** | **G** | **A** | **A** | **A** | **T** | **G** | **G** | **A** | **C** | **G** | **G** | **C** | **A** | **T** | **T** | **G** | **A** | **T** | **T** | **C** | **A** | **A** | **C** | **A** | **A** | **A** | **A** | **G** | **C** | **G** | **A** | **T** | **C** | **T** | **T** | **G** | **A** | **A** | **A** | **G** | **A** | **T** | **T** | **G** | **G** | **C** | **C** | **A** | **G** | **A** | **A** | **G** | **C** | **C** | **A** | **A** | **G** | **A** | **T** | **T** | **A** | **T** | **T** | **A** | **T** | **T** | **G** | **T** | **G** | **A** | **C** | **G** | **A** | **G** | **T** | **T** | **T** | **T** | **A** | **T** | **T** | **G** | **A** | **T** | **G** | **A** | **T** | **G** | **A** | **A** | **A** | **A** | **A** | **G** | **T** | **G** | **T** | **A** | **T** | **C** | **C** | **G** | **G** | **C** | **G** | **A** | **T** | **T** | **G** | **A** | **A** | **G** | **C** | **T** | **G** | **G** | **T** | **G** | **C** | **A** | **G** | **C** | **G** | **G** | **G** | **C** | **T** | **A** | **C** | **C** | **T** | **A** | **T** | **T** | **A** | **A** | **A** | **A** | **A** | **C** | **A** | **T** | **C** | **A** | **A** | **C** | **A** | **G** | **C** | **A** | **C** | **A** | **T** | **G** | **A** | **G** | **A** | **T** | **T** | **G** | **C** | **T** | **G** | **A** | **T** | **G** | **C** | **A** | **A** | **T** | **T** | **C** | **G** | **G** | **G** | **C** | **G** | **A** | **C** | **T** | **T** | **A** | **T** | **C** | **G** | **C** | **G** | **G** | **A** | **G** | **A** | **G** | **C** | **G** | **T** | **G** | **T** | **G** | **T** | **T** | **G** | **G** | **A** | **A** | **C** | **C** | **T** | **G** | **A** | **A** | **G** | **T** | **G** | **A** | **C** | **A** | **C** | **A** | **T** | **A** | **A** | **G** | **A** | **T** | **G** | **A** | **T** | **G** | **G** | **A** | **A** | **C** | **G** | **G** | **T** | **T** | **A** | **A** | **C** | **A** | **A** | **A** | **A** | **A** | **A** | **A** | **C** | **A** | **A** | **G** | **A** | **G** | **C** | **C** | **G** | **G** | **T** | **G** | **T** | **T** | **G** | **C** | **A** | **C** | **G** | **A** | **A** | **G** | **A** | **T** | **T** | **T** | **G** | **A** | **C** | **A** | **A** | **A** | **C** | **C** | **G** | **G** | **G** | **A** | **A** | **C** | **A** | **C** | **G** | **A** | **A** | **A** | **T** | **T** | **T** | **T** | **A** | **A** | **T** | **G** | **T** | **T** | **G** | **A** | **T** | **T** | **G** | **C** | **A** | **C** | **A** | **A** | **G** | **G** | **T** | **A** | **A** | **A** | **A** | **G** | **T** | **A** | **A** | **T** | **C** | **A** | **G** | **G** | **A** | **A** | **A** | **T** | **A** | **G** | **C** | **T** | **G** | **A** | **T** | **G** | **A** | **A** | **C** | **T** | **C** | **T** | **T** | **T** | **A** | **T** | **C** | **A** | **C** | **T** | **T** | **T** | **G** | **A** | **A** | **A** | **A** | **C** | **A** | **G** | **T** | **T** | **A** | **A** | **A** | **A** | **C** | **A** | **C** | **A** | **T** | **G** | **T** | **T** | **T** | **C** | **A** | **A** | **A** | **C** | **A** | **T** | **T** | **T** | **T** | **A** | **G** | **C** | **A** | **A** | **A** | **A** | **C** | **T** | **A** | **G** | **A** | **T** | **G** | **T** | **G** | **G** | **A** | **T** | **G** | **A** | **T** | **C** | **G** | **G** | **A** | **C** | **C** | **C** | **A** | **A** | **G** | **C** | **G** | **G** | **C** | **G** | **A** | **T** | **T** | **T** | **A** | **T** | **G** | **C** | **T** | **T** | **T** | **T** | **C** | **A** | **A** | **C** | **A** | **T** | **G** | **G** | **T** | **T** | **T** | **A** | **G** | **C** | **C** | **A** | **A** | **A** | **-** | **-** | **-** |
| **N60190F** | **A** | **T** | **G** | **A** | **T** | **A** | **A** | **A** | **A** | **G** | **T** | **T** | **T** | **T** | **A** | **T** | **T** | **A** | **G** | **T** | **A** | **G** | **A** | **T** | **G** | **A** | **C** | **C** | **A** | **T** | **G** | **A** | **A** | **A** | **T** | **G** | **G** | **T** | **G** | **C** | **G** | **C** | **T** | **T** | **A** | **G** | **G** | **C** | **G** | **T** | **C** | **T** | **C** | **T** | **T** | **C** | **T** | **T** | **A** | **T** | **T** | **T** | **A** | **T** | **C** | **T** | **A** | **T** | **T** | **C** | **A** | **A** | **G** | **A** | **A** | **G** | **A** | **C** | **A** | **T** | **T** | **G** | **A** | **A** | **G** | **T** | **A** | **A** | **T** | **T** | **G** | **G** | **A** | **G** | **A** | **A** | **G** | **C** | **T** | **G** | **A** | **A** | **A** | **A** | **T** | **G** | **G** | **A** | **C** | **G** | **A** | **C** | **A** | **A** | **G** | **G** | **C** | **T** | **A** | **T** | **G** | **A** | **G** | **A** | **A** | **G** | **G** | **C** | **G** | **A** | **T** | **G** | **A** | **C** | **A** | **C** | **T** | **T** | **C** | **G** | **A** | **C** | **C** | **G** | **G** | **A** | **C** | **G** | **T** | **C** | **A** | **T** | **T** | **T** | **T** | **A** | **A** | **T** | **G** | **G** | **A** | **C** | **T** | **T** | **A** | **G** | **T** | **C** | **A** | **T** | **G** | **G** | **A** | **A** | **G** | **A** | **A** | **A** | **T** | **G** | **G** | **A** | **T** | **G** | **G** | **T** | **A** | **T** | **C** | **G** | **A** | **A** | **T** | **C** | **A** | **A** | **C** | **A** | **A** | **A** | **A** | **G** | **C** | **A** | **A** | **T** | **C** | **T** | **T** | **A** | **A** | **A** | **A** | **G** | **A** | **T** | **T** | **G** | **G** | **C** | **C** | **A** | **G** | **A** | **A** | **G** | **C** | **T** | **A** | **A** | **A** | **A** | **T** | **C** | **A** | **T** | **T** | **A** | **T** | **C** | **G** | **T** | **A** | **A** | **C** | **C** | **A** | **G** | **T** | **T** | **T** | **T** | **A** | **T** | **T** | **G** | **A** | **T** | **G** | **A** | **C** | **G** | **A** | **A** | **A** | **A** | **A** | **G** | **T** | **T** | **T** | **A** | **T** | **C** | **C** | **T** | **G** | **C** | **C** | **A** | **T** | **T** | **G** | **A** | **A** | **G** | **C** | **A** | **G** | **G** | **T** | **G** | **C** | **A** | **G** | **C** | **A** | **G** | **G** | **G** | **T** | **A** | **T** | **T** | **T** | **G** | **T** | **T** | **G** | **A** | **A** | **G** | **A** | **C** | **C** | **T** | **C** | **T** | **A** | **C** | **C** | **G** | **C** | **A** | **C** | **A** | **T** | **G** | **A** | **A** | **A** | **T** | **T** | **G** | **C** | **T** | **G** | **A** | **T** | **G** | **C** | **T** | **A** | **T** | **A** | **A** | **G** | **G** | **G** | **C** | **A** | **A** | **C** | **G** | **C** | **A** | **A** | **C** | **G** | **T** | **G** | **G** | **C** | **G** | **A** | **G** | **C** | **G** | **A** | **G** | **T** | **A** | **T** | **T** | **A** | **G** | **A** | **A** | **C** | **C** | **A** | **G** | **A** | **A** | **G** | **T** | **G** | **A** | **C** | **A** | **A** | **C** | **A** | **A** | **A** | **A** | **A** | **T** | **G** | **A** | **T** | **G** | **G** | **A** | **A** | **A** | **A** | **A** | **A** | **T** | **G** | **A** | **G** | **T** | **C** | **G** | **G** | **C** | **G** | **A** | **A** | **A** | **T** | **G** | **A** | **G** | **C** | **C** | **T** | **G** | **T** | **A** | **T** | **T** | **A** | **C** | **A** | **T** | **G** | **A** | **A** | **G** | **A** | **A** | **C** | **T** | **A** | **A** | **C** | **G** | **A** | **A** | **T** | **C** | **G** | **G** | **G** | **A** | **A** | **A** | **A** | **T** | **G** | **A** | **A** | **A** | **T** | **T** | **T** | **T** | **A** | **A** | **T** | **G** | **C** | **T** | **G** | **A** | **T** | **T** | **T** | **C** | **T** | **G** | **A** | **A** | **G** | **G** | **A** | **A** | **A** | **A** | **A** | **G** | **T** | **A** | **A** | **T** | **C** | **A** | **G** | **G** | **A** | **A** | **A** | **T** | **A** | **G** | **C** | **G** | **G** | **A** | **C** | **G** | **A** | **A** | **T** | **T** | **G** | **T** | **T** | **T** | **A** | **T** | **T** | **A** | **C** | **A** | **C** | **T** | **G** | **A** | **A** | **A** | **A** | **C** | **A** | **G** | **T** | **A** | **A** | **A** | **A** | **A** | **C** | **A** | **C** | **A** | **T** | **G** | **T** | **T** | **T** | **C** | **C** | **A** | **A** | **T** | **A** | **T** | **A** | **T** | **T** | **G** | **G** | **C** | **T** | **A** | **A** | **G** | **C** | **T** | **A** | **G** | **A** | **A** | **G** | **T** | **G** | **G** | **A** | **A** | **G** | **A** | **C** | **C** | **G** | **T** | **A** | **C** | **A** | **C** | **A** | **A** | **G** | **C** | **C** | **G** | **C** | **T** | **A** | **T** | **C** | **T** | **A** | **T** | **G** | **C** | **A** | **T** | **T** | **C** | **A** | **A** | **A** | **C** | **A** | **T** | **G** | **G** | **T** | **T** | **T** | **G** | **G** | **T** | **G** | **A** | **A** | **A** | **-** | **-** | **-** |
| **N54538** | **A** | **T** | **G** | **A** | **T** | **A** | **A** | **A** | **A** | **G** | **T** | **T** | **T** | **T** | **A** | **T** | **T** | **A** | **G** | **T** | **A** | **G** | **A** | **T** | **G** | **A** | **C** | **C** | **A** | **T** | **G** | **A** | **A** | **A** | **T** | **G** | **G** | **T** | **G** | **C** | **G** | **C** | **T** | **T** | **A** | **G** | **G** | **T** | **G** | **T** | **C** | **T** | **C** | **T** | **T** | **C** | **T** | **T** | **A** | **T** | **T** | **T** | **A** | **T** | **C** | **T** | **A** | **T** | **T** | **C** | **A** | **A** | **G** | **A** | **A** | **G** | **A** | **C** | **A** | **T** | **T** | **G** | **A** | **A** | **G** | **T** | **A** | **A** | **T** | **T** | **G** | **G** | **A** | **G** | **A** | **A** | **G** | **C** | **T** | **G** | **A** | **A** | **A** | **A** | **T** | **G** | **G** | **A** | **C** | **G** | **A** | **C** | **A** | **A** | **G** | **G** | **C** | **T** | **A** | **T** | **G** | **A** | **G** | **A** | **A** | **G** | **G** | **C** | **G** | **A** | **T** | **G** | **A** | **C** | **A** | **C** | **T** | **T** | **C** | **G** | **A** | **C** | **C** | **G** | **G** | **A** | **C** | **G** | **T** | **C** | **A** | **T** | **T** | **T** | **T** | **A** | **A** | **T** | **G** | **G** | **A** | **C** | **T** | **T** | **A** | **G** | **T** | **C** | **A** | **T** | **G** | **G** | **A** | **A** | **G** | **A** | **A** | **A** | **T** | **G** | **G** | **A** | **T** | **G** | **G** | **T** | **A** | **T** | **C** | **G** | **A** | **A** | **T** | **C** | **A** | **A** | **C** | **A** | **A** | **A** | **A** | **G** | **C** | **A** | **A** | **T** | **C** | **T** | **T** | **A** | **A** | **A** | **A** | **G** | **A** | **T** | **T** | **G** | **G** | **C** | **C** | **A** | **G** | **A** | **A** | **G** | **C** | **T** | **A** | **A** | **A** | **A** | **T** | **C** | **A** | **T** | **T** | **A** | **T** | **C** | **G** | **T** | **A** | **A** | **C** | **C** | **A** | **G** | **T** | **T** | **T** | **T** | **A** | **T** | **T** | **G** | **A** | **T** | **G** | **A** | **C** | **G** | **A** | **A** | **A** | **A** | **A** | **G** | **T** | **T** | **T** | **A** | **T** | **C** | **C** | **T** | **G** | **C** | **C** | **A** | **T** | **T** | **G** | **A** | **A** | **G** | **C** | **A** | **G** | **G** | **T** | **G** | **C** | **A** | **G** | **C** | **A** | **G** | **G** | **G** | **T** | **A** | **T** | **T** | **T** | **G** | **T** | **T** | **G** | **A** | **A** | **G** | **A** | **C** | **C** | **T** | **C** | **T** | **A** | **C** | **C** | **G** | **C** | **A** | **C** | **A** | **T** | **G** | **A** | **A** | **A** | **T** | **T** | **G** | **C** | **T** | **G** | **A** | **T** | **G** | **C** | **T** | **A** | **T** | **A** | **A** | **G** | **G** | **G** | **C** | **A** | **A** | **C** | **G** | **C** | **A** | **A** | **C** | **G** | **C** | **G** | **G** | **C** | **G** | **A** | **G** | **C** | **G** | **A** | **G** | **T** | **A** | **T** | **T** | **A** | **G** | **A** | **A** | **C** | **C** | **A** | **G** | **A** | **A** | **G** | **T** | **G** | **A** | **C** | **A** | **A** | **C** | **A** | **A** | **A** | **A** | **A** | **T** | **G** | **A** | **T** | **G** | **G** | **A** | **A** | **A** | **A** | **A** | **A** | **T** | **G** | **A** | **G** | **T** | **C** | **G** | **G** | **C** | **G** | **A** | **A** | **A** | **T** | **G** | **A** | **G** | **C** | **C** | **T** | **G** | **T** | **A** | **T** | **T** | **A** | **C** | **A** | **T** | **G** | **A** | **A** | **G** | **A** | **A** | **C** | **T** | **A** | **A** | **C** | **G** | **A** | **A** | **T** | **C** | **G** | **G** | **G** | **A** | **A** | **A** | **A** | **T** | **G** | **A** | **A** | **A** | **T** | **T** | **T** | **T** | **A** | **A** | **T** | **G** | **C** | **T** | **G** | **A** | **T** | **T** | **T** | **C** | **T** | **G** | **A** | **A** | **G** | **G** | **A** | **A** | **A** | **A** | **A** | **G** | **T** | **A** | **A** | **T** | **C** | **A** | **G** | **G** | **A** | **A** | **A** | **T** | **A** | **G** | **C** | **G** | **G** | **A** | **C** | **G** | **A** | **A** | **T** | **T** | **G** | **T** | **T** | **C** | **A** | **T** | **T** | **A** | **C** | **A** | **C** | **T** | **G** | **A** | **A** | **A** | **A** | **C** | **A** | **G** | **T** | **A** | **A** | **A** | **A** | **A** | **C** | **A** | **C** | **A** | **T** | **G** | **T** | **T** | **T** | **C** | **C** | **A** | **A** | **T** | **A** | **T** | **A** | **T** | **T** | **G** | **G** | **C** | **T** | **A** | **A** | **G** | **C** | **T** | **A** | **G** | **A** | **A** | **G** | **T** | **G** | **G** | **A** | **A** | **G** | **A** | **C** | **C** | **G** | **T** | **A** | **C** | **A** | **C** | **A** | **A** | **G** | **C** | **C** | **G** | **C** | **T** | **A** | **T** | **C** | **T** | **A** | **T** | **G** | **C** | **A** | **T** | **T** | **C** | **A** | **A** | **A** | **C** | **A** | **T** | **G** | **G** | **T** | **T** | **T** | **G** | **G** | **T** | **G** | **A** | **A** | **A** | **-** | **-** | **-** |
| **N52690** | **A** | **T** | **G** | **A** | **T** | **A** | **A** | **A** | **A** | **G** | **T** | **T** | **T** | **T** | **A** | **T** | **T** | **A** | **G** | **T** | **A** | **G** | **A** | **T** | **G** | **A** | **C** | **C** | **A** | **T** | **G** | **A** | **A** | **A** | **T** | **G** | **G** | **T** | **A** | **C** | **G** | **C** | **T** | **T** | **A** | **G** | **G** | **C** | **G** | **T** | **C** | **T** | **C** | **T** | **T** | **C** | **T** | **T** | **A** | **T** | **T** | **T** | **A** | **T** | **C** | **T** | **A** | **T** | **C** | **C** | **A** | **A** | **G** | **A** | **A** | **G** | **A** | **C** | **A** | **T** | **T** | **G** | **A** | **A** | **G** | **T** | **G** | **A** | **T** | **T** | **G** | **G** | **A** | **G** | **A** | **A** | **G** | **C** | **T** | **G** | **A** | **A** | **A** | **A** | **T** | **G** | **G** | **A** | **C** | **G** | **T** | **C** | **A** | **A** | **G** | **G** | **C** | **T** | **A** | **T** | **G** | **A** | **A** | **A** | **A** | **G** | **G** | **C** | **G** | **A** | **T** | **G** | **G** | **C** | **A** | **C** | **T** | **T** | **C** | **G** | **A** | **C** | **C** | **A** | **G** | **A** | **T** | **G** | **T** | **C** | **A** | **T** | **T** | **T** | **T** | **A** | **A** | **T** | **G** | **G** | **A** | **T** | **C** | **T** | **A** | **G** | **T** | **C** | **A** | **T** | **G** | **G** | **A** | **A** | **G** | **A** | **A** | **A** | **T** | **G** | **G** | **A** | **T** | **G** | **G** | **C** | **A** | **T** | **C** | **G** | **A** | **A** | **T** | **C** | **A** | **A** | **C** | **A** | **A** | **A** | **A** | **G** | **C** | **A** | **A** | **T** | **C** | **T** | **T** | **A** | **A** | **A** | **A** | **G** | **A** | **T** | **T** | **G** | **G** | **C** | **C** | **A** | **A** | **A** | **A** | **G** | **C** | **C** | **A** | **A** | **A** | **A** | **T** | **C** | **A** | **T** | **T** | **A** | **T** | **C** | **G** | **T** | **T** | **A** | **C** | **A** | **A** | **G** | **T** | **T** | **T** | **T** | **A** | **T** | **T** | **G** | **A** | **T** | **G** | **A** | **T** | **G** | **A** | **A** | **A** | **A** | **A** | **G** | **T** | **T** | **T** | **A** | **T** | **C** | **C** | **T** | **G** | **C** | **A** | **A** | **T** | **T** | **G** | **A** | **A** | **G** | **C** | **A** | **G** | **G** | **A** | **G** | **C** | **A** | **G** | **C** | **A** | **G** | **G** | **A** | **T** | **A** | **T** | **T** | **T** | **G** | **T** | **T** | **G** | **A** | **A** | **G** | **A** | **C** | **G** | **T** | **C** | **T** | **A** | **C** | **C** | **G** | **C** | **A** | **C** | **A** | **T** | **G** | **A** | **A** | **A** | **T** | **A** | **G** | **C** | **T** | **G** | **A** | **T** | **G** | **C** | **T** | **A** | **T** | **A** | **A** | **G** | **G** | **G** | **C** | **T** | **A** | **C** | **G** | **C** | **A** | **A** | **C** | **G** | **C** | **G** | **G** | **C** | **G** | **A** | **G** | **C** | **G** | **A** | **G** | **T** | **A** | **C** | **T** | **A** | **G** | **A** | **A** | **C** | **C** | **A** | **G** | **A** | **A** | **G** | **T** | **G** | **A** | **C** | **A** | **A** | **C** | **A** | **A** | **A** | **A** | **A** | **T** | **G** | **A** | **T** | **G** | **G** | **A** | **A** | **A** | **A** | **A** | **A** | **T** | **G** | **A** | **G** | **T** | **C** | **G** | **G** | **C** | **G** | **A** | **A** | **A** | **T** | **G** | **A** | **T** | **C** | **C** | **A** | **G** | **T** | **A** | **T** | **T** | **G** | **C** | **A** | **T** | **G** | **A** | **A** | **G** | **A** | **A** | **T** | **T** | **A** | **A** | **C** | **G** | **A** | **A** | **T** | **C** | **G** | **A** | **G** | **A** | **A** | **A** | **A** | **T** | **G** | **A** | **A** | **A** | **T** | **T** | **T** | **T** | **G** | **A** | **T** | **G** | **C** | **T** | **G** | **A** | **T** | **T** | **T** | **C** | **T** | **G** | **A** | **A** | **G** | **G** | **G** | **A** | **A** | **A** | **A** | **G** | **T** | **A** | **A** | **T** | **C** | **A** | **G** | **G** | **A** | **A** | **A** | **T** | **C** | **G** | **C** | **G** | **G** | **A** | **T** | **G** | **A** | **A** | **T** | **T** | **G** | **T** | **T** | **T** | **A** | **T** | **C** | **A** | **C** | **A** | **C** | **T** | **G** | **A** | **A** | **A** | **A** | **C** | **A** | **G** | **T** | **T** | **A** | **A** | **A** | **A** | **C** | **G** | **C** | **A** | **T** | **G** | **T** | **G** | **T** | **C** | **G** | **A** | **A** | **T** | **A** | **T** | **A** | **T** | **T** | **A** | **G** | **C** | **T** | **A** | **A** | **G** | **T** | **T** | **A** | **G** | **A** | **G** | **G** | **T** | **A** | **G** | **A** | **A** | **G** | **A** | **C** | **C** | **G** | **A** | **A** | **C** | **T** | **C** | **A** | **A** | **G** | **C** | **A** | **G** | **C** | **T** | **A** | **T** | **C** | **T** | **A** | **T** | **G** | **C** | **G** | **T** | **T** | **T** | **A** | **A** | **A** | **C** | **A** | **T** | **G** | **G** | **T** | **T** | **T** | **G** | **G** | **T** | **G** | **A** | **A** | **A** | **-** | **-** | **-** |
| **N55185** | **G** | **T** | **G** | **A** | **T** | **C** | **A** | **A** | **A** | **G** | **T** | **A** | **A** | **T** | **G** | **T** | **T** | **A** | **G** | **T** | **G** | **G** | **A** | **T** | **G** | **A** | **C** | **C** | **A** | **T** | **G** | **A** | **A** | **A** | **T** | **G** | **G** | **T** | **C** | **C** | **G** | **T** | **T** | **T** | **A** | **G** | **G** | **C** | **G** | **T** | **T** | **T** | **C** | **A** | **T** | **C** | **A** | **T** | **A** | **T** | **T** | **T** | **A** | **T** | **C** | **T** | **A** | **T** | **T** | **C** | **A** | **A** | **G** | **A** | **G** | **G** | **A** | **T** | **A** | **T** | **A** | **G** | **A** | **A** | **G** | **T** | **C** | **G** | **T** | **A** | **G** | **G** | **C** | **G** | **A** | **A** | **G** | **C** | **A** | **G** | **A** | **A** | **A** | **A** | **C** | **G** | **G** | **T** | **A** | **A** | **G** | **A** | **T** | **T** | **G** | **G** | **C** | **T** | **A** | **T** | **G** | **A** | **A** | **A** | **A** | **A** | **G** | **C** | **A** | **T** | **T** | **G** | **G** | **A** | **A** | **C** | **T** | **A** | **C** | **G** | **T** | **C** | **C** | **A** | **G** | **A** | **T** | **G** | **T** | **T** | **A** | **T** | **T** | **T** | **T** | **G** | **A** | **T** | **G** | **G** | **A** | **T** | **T** | **T** | **G** | **G** | **T** | **A** | **A** | **T** | **G** | **G** | **A** | **A** | **G** | **A** | **A** | **A** | **T** | **G** | **G** | **A** | **C** | **G** | **G** | **C** | **A** | **T** | **T** | **G** | **A** | **T** | **T** | **C** | **A** | **A** | **C** | **A** | **A** | **A** | **A** | **G** | **C** | **G** | **A** | **T** | **C** | **T** | **T** | **G** | **A** | **A** | **A** | **G** | **A** | **T** | **T** | **G** | **G** | **C** | **C** | **A** | **G** | **A** | **A** | **G** | **C** | **C** | **A** | **A** | **G** | **A** | **T** | **T** | **A** | **T** | **T** | **A** | **T** | **T** | **G** | **T** | **G** | **A** | **C** | **G** | **A** | **G** | **T** | **T** | **T** | **T** | **A** | **T** | **T** | **G** | **A** | **T** | **G** | **A** | **T** | **G** | **A** | **A** | **A** | **A** | **A** | **G** | **T** | **A** | **T** | **A** | **T** | **C** | **C** | **G** | **G** | **C** | **G** | **A** | **T** | **T** | **G** | **A** | **A** | **G** | **C** | **T** | **G** | **G** | **T** | **G** | **C** | **A** | **G** | **C** | **G** | **G** | **G** | **C** | **T** | **A** | **C** | **C** | **T** | **A** | **T** | **T** | **A** | **A** | **A** | **A** | **A** | **C** | **A** | **T** | **C** | **A** | **A** | **C** | **A** | **G** | **C** | **A** | **C** | **A** | **T** | **G** | **A** | **G** | **A** | **T** | **T** | **G** | **C** | **T** | **G** | **A** | **T** | **G** | **C** | **A** | **A** | **T** | **T** | **C** | **G** | **G** | **G** | **C** | **G** | **A** | **C** | **T** | **T** | **A** | **T** | **C** | **G** | **C** | **G** | **G** | **A** | **G** | **A** | **G** | **C** | **G** | **T** | **G** | **T** | **G** | **T** | **T** | **G** | **G** | **A** | **A** | **C** | **C** | **T** | **G** | **A** | **A** | **G** | **T** | **G** | **A** | **C** | **G** | **C** | **A** | **T** | **A** | **A** | **G** | **A** | **T** | **G** | **A** | **T** | **G** | **G** | **A** | **A** | **C** | **G** | **G** | **T** | **T** | **A** | **A** | **C** | **A** | **A** | **A** | **A** | **A** | **A** | **A** | **C** | **A** | **A** | **G** | **A** | **G** | **C** | **C** | **G** | **G** | **T** | **G** | **T** | **T** | **G** | **C** | **A** | **T** | **G** | **A** | **A** | **G** | **A** | **T** | **T** | **T** | **G** | **A** | **C** | **A** | **A** | **A** | **T** | **C** | **G** | **A** | **G** | **A** | **A** | **C** | **A** | **C** | **G** | **A** | **A** | **A** | **T** | **T** | **T** | **T** | **A** | **A** | **T** | **G** | **T** | **T** | **G** | **A** | **T** | **T** | **G** | **C** | **A** | **C** | **A** | **A** | **G** | **G** | **T** | **A** | **A** | **A** | **A** | **G** | **T** | **A** | **A** | **T** | **C** | **A** | **G** | **G** | **A** | **A** | **A** | **T** | **A** | **G** | **C** | **T** | **G** | **A** | **T** | **G** | **A** | **A** | **C** | **T** | **C** | **T** | **T** | **T** | **A** | **T** | **C** | **A** | **C** | **T** | **T** | **T** | **G** | **A** | **A** | **A** | **A** | **C** | **A** | **G** | **T** | **T** | **A** | **A** | **A** | **A** | **C** | **A** | **C** | **A** | **T** | **G** | **T** | **T** | **T** | **C** | **A** | **A** | **A** | **C** | **A** | **T** | **T** | **T** | **T** | **A** | **G** | **C** | **A** | **A** | **A** | **A** | **C** | **T** | **A** | **G** | **A** | **T** | **G** | **T** | **G** | **G** | **A** | **T** | **G** | **A** | **T** | **C** | **G** | **G** | **A** | **C** | **C** | **C** | **A** | **A** | **G** | **C** | **A** | **G** | **C** | **G** | **A** | **T** | **T** | **T** | **A** | **T** | **G** | **C** | **T** | **T** | **T** | **T** | **C** | **A** | **A** | **C** | **A** | **T** | **G** | **G** | **T** | **T** | **T** | **A** | **G** | **C** | **C** | **A** | **A** | **A** | **-** | **-** | **-** |
| **N54070** | **A** | **T** | **G** | **A** | **T** | **A** | **A** | **A** | **A** | **G** | **T** | **T** | **T** | **T** | **A** | **T** | **T** | **A** | **G** | **T** | **A** | **G** | **A** | **T** | **G** | **A** | **C** | **C** | **A** | **T** | **G** | **A** | **A** | **A** | **T** | **G** | **G** | **T** | **A** | **C** | **G** | **C** | **T** | **T** | **A** | **G** | **G** | **C** | **G** | **T** | **C** | **T** | **C** | **T** | **T** | **C** | **T** | **T** | **A** | **T** | **T** | **T** | **A** | **T** | **C** | **T** | **A** | **T** | **C** | **C** | **A** | **A** | **G** | **A** | **A** | **G** | **A** | **C** | **A** | **T** | **T** | **G** | **A** | **A** | **G** | **T** | **G** | **A** | **T** | **T** | **G** | **G** | **A** | **G** | **A** | **A** | **G** | **C** | **T** | **G** | **A** | **A** | **A** | **A** | **T** | **G** | **G** | **A** | **C** | **G** | **T** | **C** | **A** | **A** | **G** | **G** | **C** | **T** | **A** | **T** | **G** | **A** | **A** | **A** | **A** | **G** | **G** | **C** | **G** | **A** | **T** | **G** | **G** | **C** | **A** | **C** | **T** | **T** | **C** | **G** | **A** | **C** | **C** | **G** | **G** | **A** | **T** | **G** | **T** | **C** | **A** | **T** | **T** | **T** | **T** | **A** | **A** | **T** | **G** | **G** | **A** | **T** | **C** | **T** | **A** | **G** | **T** | **C** | **A** | **T** | **G** | **G** | **A** | **A** | **G** | **A** | **A** | **A** | **T** | **G** | **G** | **A** | **T** | **G** | **G** | **C** | **A** | **T** | **C** | **G** | **A** | **A** | **T** | **C** | **A** | **A** | **C** | **A** | **A** | **A** | **A** | **G** | **C** | **A** | **A** | **T** | **C** | **T** | **T** | **A** | **A** | **A** | **A** | **G** | **A** | **T** | **T** | **G** | **G** | **C** | **C** | **A** | **A** | **A** | **A** | **G** | **C** | **C** | **A** | **A** | **A** | **A** | **T** | **C** | **A** | **T** | **T** | **A** | **T** | **C** | **G** | **T** | **C** | **A** | **C** | **A** | **A** | **G** | **T** | **T** | **T** | **T** | **A** | **T** | **T** | **G** | **A** | **T** | **G** | **A** | **T** | **G** | **A** | **A** | **A** | **A** | **A** | **G** | **T** | **T** | **T** | **A** | **T** | **C** | **C** | **T** | **G** | **C** | **A** | **A** | **T** | **T** | **G** | **A** | **A** | **G** | **C** | **A** | **G** | **G** | **A** | **G** | **C** | **A** | **G** | **C** | **A** | **G** | **G** | **A** | **T** | **A** | **T** | **T** | **T** | **G** | **T** | **T** | **G** | **A** | **A** | **G** | **A** | **C** | **G** | **T** | **C** | **T** | **A** | **C** | **C** | **G** | **C** | **A** | **C** | **A** | **T** | **G** | **A** | **A** | **A** | **T** | **A** | **G** | **C** | **T** | **G** | **A** | **T** | **G** | **C** | **T** | **A** | **T** | **A** | **A** | **G** | **G** | **G** | **C** | **T** | **A** | **C** | **G** | **C** | **A** | **A** | **C** | **G** | **C** | **G** | **G** | **C** | **G** | **A** | **G** | **C** | **G** | **A** | **G** | **T** | **A** | **T** | **T** | **G** | **G** | **A** | **A** | **C** | **C** | **T** | **G** | **A** | **A** | **G** | **T** | **G** | **A** | **C** | **A** | **A** | **C** | **A** | **A** | **A** | **A** | **A** | **T** | **G** | **A** | **T** | **G** | **G** | **A** | **A** | **A** | **A** | **A** | **A** | **T** | **G** | **A** | **G** | **T** | **C** | **G** | **G** | **C** | **G** | **A** | **A** | **A** | **T** | **G** | **A** | **T** | **C** | **C** | **A** | **G** | **T** | **A** | **T** | **T** | **G** | **C** | **A** | **T** | **G** | **A** | **A** | **G** | **A** | **A** | **T** | **T** | **A** | **A** | **C** | **G** | **A** | **A** | **T** | **C** | **G** | **A** | **G** | **A** | **A** | **A** | **A** | **T** | **G** | **A** | **A** | **A** | **T** | **T** | **T** | **T** | **G** | **A** | **T** | **G** | **C** | **T** | **G** | **A** | **T** | **T** | **T** | **C** | **T** | **G** | **A** | **A** | **G** | **G** | **G** | **A** | **A** | **A** | **A** | **G** | **T** | **A** | **A** | **T** | **C** | **A** | **G** | **G** | **A** | **A** | **A** | **T** | **C** | **G** | **C** | **G** | **G** | **A** | **T** | **G** | **A** | **A** | **T** | **T** | **G** | **T** | **T** | **T** | **A** | **T** | **C** | **A** | **C** | **A** | **C** | **T** | **G** | **A** | **A** | **A** | **A** | **C** | **A** | **G** | **T** | **C** | **A** | **A** | **A** | **A** | **C** | **G** | **C** | **A** | **T** | **G** | **T** | **G** | **T** | **C** | **G** | **A** | **A** | **T** | **A** | **T** | **A** | **T** | **T** | **A** | **G** | **C** | **T** | **A** | **A** | **G** | **T** | **T** | **A** | **G** | **A** | **G** | **G** | **T** | **A** | **G** | **A** | **A** | **G** | **A** | **C** | **C** | **G** | **A** | **A** | **C** | **T** | **C** | **A** | **A** | **G** | **C** | **A** | **G** | **C** | **T** | **A** | **T** | **C** | **T** | **A** | **T** | **G** | **C** | **G** | **T** | **T** | **T** | **A** | **A** | **A** | **C** | **A** | **T** | **G** | **G** | **T** | **T** | **T** | **G** | **G** | **T** | **G** | **A** | **A** | **A** | **-** | **-** | **-** |
| **N55728** | **A** | **T** | **G** | **A** | **T** | **A** | **A** | **A** | **A** | **G** | **T** | **T** | **T** | **T** | **A** | **T** | **T** | **A** | **G** | **T** | **A** | **G** | **A** | **T** | **G** | **A** | **C** | **C** | **A** | **T** | **G** | **A** | **A** | **A** | **T** | **G** | **G** | **T** | **A** | **C** | **G** | **C** | **T** | **T** | **A** | **G** | **G** | **C** | **G** | **T** | **C** | **T** | **C** | **T** | **T** | **C** | **T** | **T** | **A** | **T** | **T** | **T** | **A** | **T** | **C** | **T** | **A** | **T** | **C** | **C** | **A** | **A** | **G** | **A** | **A** | **G** | **A** | **C** | **A** | **T** | **T** | **G** | **A** | **A** | **G** | **T** | **G** | **A** | **T** | **T** | **G** | **G** | **A** | **G** | **A** | **A** | **G** | **C** | **T** | **G** | **A** | **A** | **A** | **A** | **T** | **G** | **G** | **A** | **C** | **G** | **T** | **C** | **A** | **A** | **G** | **G** | **C** | **T** | **A** | **T** | **G** | **A** | **A** | **A** | **A** | **G** | **G** | **C** | **G** | **A** | **T** | **G** | **G** | **C** | **A** | **C** | **T** | **T** | **C** | **G** | **A** | **C** | **C** | **G** | **G** | **A** | **T** | **G** | **T** | **C** | **A** | **T** | **T** | **T** | **T** | **A** | **A** | **T** | **G** | **G** | **A** | **T** | **C** | **T** | **A** | **G** | **T** | **C** | **A** | **T** | **G** | **G** | **A** | **A** | **G** | **A** | **A** | **A** | **T** | **G** | **G** | **A** | **T** | **G** | **G** | **C** | **A** | **T** | **C** | **G** | **A** | **A** | **T** | **C** | **A** | **A** | **C** | **A** | **A** | **A** | **A** | **G** | **C** | **A** | **A** | **T** | **C** | **T** | **T** | **A** | **A** | **A** | **A** | **G** | **A** | **T** | **T** | **G** | **G** | **C** | **C** | **A** | **A** | **A** | **A** | **G** | **C** | **C** | **A** | **A** | **A** | **A** | **T** | **C** | **A** | **T** | **T** | **A** | **T** | **C** | **G** | **T** | **C** | **A** | **C** | **A** | **A** | **G** | **T** | **T** | **T** | **T** | **A** | **T** | **T** | **G** | **A** | **T** | **G** | **A** | **T** | **G** | **A** | **A** | **A** | **A** | **A** | **G** | **T** | **T** | **T** | **A** | **T** | **C** | **C** | **T** | **G** | **C** | **A** | **A** | **T** | **T** | **G** | **A** | **A** | **G** | **C** | **A** | **G** | **G** | **A** | **G** | **C** | **A** | **G** | **C** | **A** | **G** | **G** | **A** | **T** | **A** | **T** | **T** | **T** | **G** | **T** | **T** | **G** | **A** | **A** | **G** | **A** | **C** | **G** | **T** | **C** | **T** | **A** | **C** | **C** | **G** | **C** | **A** | **C** | **A** | **T** | **G** | **A** | **A** | **A** | **T** | **A** | **G** | **C** | **T** | **G** | **A** | **T** | **G** | **C** | **T** | **A** | **T** | **A** | **A** | **G** | **G** | **G** | **C** | **T** | **A** | **C** | **G** | **C** | **A** | **A** | **C** | **G** | **C** | **G** | **G** | **C** | **G** | **A** | **G** | **C** | **G** | **A** | **G** | **T** | **A** | **T** | **T** | **G** | **G** | **A** | **A** | **C** | **C** | **T** | **G** | **A** | **A** | **G** | **T** | **G** | **A** | **C** | **A** | **A** | **C** | **A** | **A** | **A** | **A** | **A** | **T** | **G** | **A** | **T** | **G** | **G** | **A** | **A** | **A** | **A** | **A** | **A** | **T** | **G** | **A** | **G** | **T** | **C** | **G** | **G** | **C** | **G** | **A** | **A** | **A** | **T** | **G** | **A** | **T** | **C** | **C** | **A** | **G** | **T** | **A** | **T** | **T** | **G** | **C** | **A** | **T** | **G** | **A** | **A** | **G** | **A** | **A** | **T** | **T** | **A** | **A** | **C** | **G** | **A** | **A** | **T** | **C** | **G** | **A** | **G** | **A** | **A** | **A** | **A** | **T** | **G** | **A** | **A** | **A** | **T** | **T** | **T** | **T** | **G** | **A** | **T** | **G** | **C** | **T** | **G** | **A** | **T** | **T** | **T** | **C** | **T** | **G** | **A** | **A** | **G** | **G** | **G** | **A** | **A** | **A** | **A** | **G** | **T** | **A** | **A** | **T** | **C** | **A** | **G** | **G** | **A** | **A** | **A** | **T** | **C** | **G** | **C** | **G** | **G** | **A** | **T** | **G** | **A** | **A** | **T** | **T** | **G** | **T** | **T** | **T** | **A** | **T** | **C** | **A** | **C** | **A** | **C** | **T** | **G** | **A** | **A** | **A** | **A** | **C** | **A** | **G** | **T** | **C** | **A** | **A** | **A** | **A** | **C** | **G** | **C** | **A** | **T** | **G** | **T** | **G** | **T** | **C** | **G** | **A** | **A** | **T** | **A** | **T** | **A** | **T** | **T** | **A** | **G** | **C** | **T** | **A** | **A** | **G** | **T** | **T** | **A** | **G** | **A** | **G** | **G** | **T** | **A** | **G** | **A** | **A** | **G** | **A** | **C** | **C** | **G** | **A** | **A** | **C** | **T** | **C** | **A** | **A** | **G** | **C** | **A** | **G** | **C** | **T** | **A** | **T** | **C** | **T** | **A** | **T** | **G** | **C** | **G** | **T** | **T** | **T** | **A** | **A** | **A** | **C** | **A** | **T** | **G** | **G** | **T** | **T** | **T** | **G** | **G** | **T** | **G** | **A** | **A** | **A** | **-** | **-** | **-** |
| **N59550F** | **G** | **T** | **G** | **A** | **T** | **C** | **A** | **A** | **A** | **G** | **T** | **A** | **A** | **T** | **G** | **T** | **T** | **A** | **G** | **T** | **G** | **G** | **A** | **T** | **G** | **A** | **C** | **C** | **A** | **T** | **G** | **A** | **A** | **A** | **T** | **G** | **G** | **T** | **C** | **C** | **G** | **T** | **T** | **T** | **A** | **G** | **G** | **C** | **G** | **T** | **T** | **T** | **C** | **A** | **T** | **C** | **A** | **T** | **A** | **T** | **T** | **T** | **A** | **T** | **C** | **T** | **A** | **T** | **T** | **C** | **A** | **A** | **G** | **A** | **G** | **G** | **A** | **T** | **A** | **T** | **A** | **G** | **A** | **A** | **G** | **T** | **C** | **G** | **T** | **A** | **G** | **G** | **C** | **G** | **A** | **A** | **G** | **C** | **A** | **G** | **A** | **A** | **A** | **A** | **C** | **G** | **G** | **T** | **A** | **A** | **G** | **A** | **T** | **T** | **G** | **G** | **C** | **T** | **A** | **T** | **G** | **A** | **A** | **A** | **A** | **A** | **G** | **C** | **A** | **T** | **T** | **G** | **G** | **A** | **A** | **C** | **T** | **A** | **C** | **G** | **T** | **C** | **C** | **A** | **G** | **A** | **T** | **G** | **T** | **T** | **A** | **T** | **T** | **T** | **T** | **G** | **A** | **T** | **G** | **G** | **A** | **T** | **T** | **T** | **G** | **G** | **T** | **A** | **A** | **T** | **G** | **G** | **A** | **A** | **G** | **A** | **A** | **A** | **T** | **G** | **G** | **A** | **C** | **G** | **G** | **C** | **A** | **T** | **T** | **G** | **A** | **T** | **T** | **C** | **A** | **A** | **C** | **A** | **A** | **A** | **A** | **G** | **C** | **G** | **A** | **T** | **C** | **T** | **T** | **G** | **A** | **A** | **A** | **G** | **A** | **T** | **T** | **G** | **G** | **C** | **C** | **A** | **G** | **A** | **A** | **G** | **C** | **C** | **A** | **A** | **G** | **A** | **T** | **T** | **A** | **T** | **T** | **A** | **T** | **T** | **G** | **T** | **G** | **A** | **C** | **G** | **A** | **G** | **T** | **T** | **T** | **T** | **A** | **T** | **T** | **G** | **A** | **T** | **G** | **A** | **T** | **G** | **A** | **A** | **A** | **A** | **A** | **G** | **T** | **G** | **T** | **A** | **T** | **C** | **C** | **G** | **G** | **C** | **G** | **A** | **T** | **T** | **G** | **A** | **A** | **G** | **C** | **T** | **G** | **G** | **T** | **G** | **C** | **A** | **G** | **C** | **G** | **G** | **G** | **C** | **T** | **A** | **C** | **C** | **T** | **A** | **T** | **T** | **A** | **A** | **A** | **A** | **A** | **C** | **A** | **T** | **C** | **A** | **A** | **C** | **A** | **G** | **C** | **A** | **C** | **A** | **T** | **G** | **A** | **G** | **A** | **T** | **T** | **G** | **C** | **T** | **G** | **A** | **T** | **G** | **C** | **A** | **A** | **T** | **T** | **C** | **G** | **G** | **G** | **C** | **G** | **A** | **C** | **T** | **T** | **A** | **T** | **C** | **G** | **C** | **G** | **G** | **A** | **G** | **A** | **G** | **C** | **G** | **T** | **G** | **T** | **G** | **T** | **T** | **G** | **G** | **A** | **A** | **C** | **C** | **T** | **G** | **A** | **A** | **G** | **T** | **G** | **A** | **C** | **G** | **C** | **A** | **T** | **A** | **A** | **G** | **A** | **T** | **G** | **A** | **T** | **G** | **G** | **A** | **A** | **C** | **G** | **G** | **T** | **T** | **A** | **A** | **C** | **A** | **A** | **A** | **A** | **A** | **A** | **A** | **C** | **A** | **A** | **G** | **A** | **G** | **C** | **C** | **G** | **G** | **T** | **G** | **T** | **T** | **G** | **C** | **A** | **C** | **G** | **A** | **A** | **G** | **A** | **T** | **T** | **T** | **G** | **A** | **C** | **A** | **A** | **A** | **C** | **C** | **G** | **A** | **G** | **A** | **A** | **C** | **A** | **C** | **G** | **A** | **A** | **A** | **T** | **T** | **T** | **T** | **A** | **A** | **T** | **G** | **T** | **T** | **G** | **A** | **T** | **T** | **G** | **C** | **A** | **C** | **A** | **A** | **G** | **G** | **T** | **A** | **A** | **A** | **A** | **G** | **T** | **A** | **A** | **T** | **C** | **A** | **G** | **G** | **A** | **A** | **A** | **T** | **T** | **G** | **C** | **T** | **G** | **A** | **T** | **G** | **A** | **A** | **C** | **T** | **C** | **T** | **T** | **T** | **A** | **T** | **C** | **A** | **C** | **T** | **T** | **T** | **G** | **A** | **A** | **A** | **A** | **C** | **A** | **G** | **T** | **T** | **A** | **A** | **A** | **A** | **C** | **A** | **C** | **A** | **T** | **G** | **T** | **T** | **T** | **C** | **A** | **A** | **A** | **C** | **A** | **T** | **T** | **T** | **T** | **A** | **G** | **C** | **A** | **A** | **A** | **A** | **C** | **T** | **A** | **G** | **A** | **T** | **G** | **T** | **G** | **G** | **A** | **T** | **G** | **A** | **T** | **C** | **G** | **G** | **A** | **C** | **C** | **C** | **A** | **A** | **G** | **C** | **G** | **G** | **C** | **G** | **A** | **T** | **T** | **T** | **A** | **T** | **G** | **C** | **T** | **T** | **T** | **T** | **C** | **A** | **A** | **C** | **A** | **T** | **G** | **G** | **T** | **T** | **T** | **A** | **G** | **C** | **C** | **A** | **A** | **A** | **-** | **-** | **-** |
| **N52643** | **A** | **T** | **G** | **A** | **T** | **A** | **A** | **A** | **A** | **G** | **T** | **T** | **T** | **T** | **A** | **T** | **T** | **A** | **G** | **T** | **A** | **G** | **A** | **T** | **G** | **A** | **C** | **C** | **A** | **T** | **G** | **A** | **A** | **A** | **T** | **G** | **G** | **T** | **A** | **C** | **G** | **C** | **T** | **T** | **A** | **G** | **G** | **C** | **G** | **T** | **C** | **T** | **C** | **T** | **T** | **C** | **T** | **T** | **A** | **T** | **T** | **T** | **A** | **T** | **C** | **T** | **A** | **T** | **C** | **C** | **A** | **A** | **G** | **A** | **A** | **G** | **A** | **C** | **A** | **T** | **T** | **G** | **A** | **A** | **G** | **T** | **G** | **A** | **T** | **T** | **G** | **G** | **A** | **G** | **A** | **A** | **G** | **C** | **T** | **G** | **A** | **A** | **A** | **A** | **T** | **G** | **G** | **A** | **C** | **G** | **T** | **C** | **A** | **A** | **G** | **G** | **C** | **T** | **A** | **T** | **G** | **A** | **A** | **A** | **A** | **G** | **G** | **C** | **G** | **A** | **T** | **G** | **G** | **C** | **A** | **C** | **T** | **T** | **C** | **G** | **A** | **C** | **C** | **G** | **G** | **A** | **T** | **G** | **T** | **C** | **A** | **T** | **T** | **T** | **T** | **A** | **A** | **T** | **G** | **G** | **A** | **T** | **C** | **T** | **A** | **G** | **T** | **C** | **A** | **T** | **G** | **G** | **A** | **A** | **G** | **A** | **A** | **A** | **T** | **G** | **G** | **A** | **T** | **G** | **G** | **C** | **A** | **T** | **C** | **G** | **A** | **A** | **T** | **C** | **A** | **A** | **C** | **A** | **A** | **A** | **A** | **G** | **C** | **A** | **A** | **T** | **C** | **T** | **T** | **A** | **A** | **A** | **A** | **G** | **A** | **T** | **T** | **G** | **G** | **C** | **C** | **A** | **A** | **A** | **A** | **G** | **C** | **C** | **A** | **A** | **A** | **A** | **T** | **C** | **A** | **T** | **T** | **A** | **T** | **C** | **G** | **T** | **C** | **A** | **C** | **A** | **A** | **G** | **T** | **T** | **T** | **T** | **A** | **T** | **T** | **G** | **A** | **T** | **G** | **A** | **T** | **G** | **A** | **A** | **A** | **A** | **A** | **G** | **T** | **T** | **T** | **A** | **T** | **C** | **C** | **T** | **G** | **C** | **A** | **A** | **T** | **T** | **G** | **A** | **A** | **G** | **C** | **A** | **G** | **G** | **A** | **G** | **C** | **A** | **G** | **C** | **A** | **G** | **G** | **A** | **T** | **A** | **T** | **T** | **T** | **G** | **T** | **T** | **G** | **A** | **A** | **G** | **A** | **C** | **G** | **T** | **C** | **T** | **A** | **C** | **C** | **G** | **C** | **A** | **C** | **A** | **T** | **G** | **A** | **A** | **A** | **T** | **A** | **G** | **C** | **T** | **G** | **A** | **T** | **G** | **C** | **T** | **A** | **T** | **A** | **A** | **G** | **G** | **G** | **C** | **T** | **A** | **C** | **G** | **C** | **A** | **A** | **C** | **G** | **C** | **G** | **G** | **C** | **G** | **A** | **G** | **C** | **G** | **A** | **G** | **T** | **A** | **T** | **T** | **G** | **G** | **A** | **A** | **C** | **C** | **T** | **G** | **A** | **A** | **G** | **T** | **G** | **A** | **C** | **A** | **A** | **C** | **A** | **A** | **A** | **A** | **A** | **T** | **G** | **A** | **T** | **G** | **G** | **A** | **A** | **A** | **A** | **A** | **A** | **T** | **G** | **A** | **G** | **T** | **C** | **G** | **G** | **C** | **G** | **A** | **A** | **A** | **T** | **G** | **A** | **T** | **C** | **C** | **A** | **G** | **T** | **A** | **T** | **T** | **G** | **C** | **A** | **T** | **G** | **A** | **A** | **G** | **A** | **A** | **T** | **T** | **A** | **A** | **C** | **G** | **A** | **A** | **T** | **C** | **G** | **A** | **G** | **A** | **A** | **A** | **A** | **T** | **G** | **A** | **A** | **A** | **T** | **T** | **T** | **T** | **G** | **A** | **T** | **G** | **C** | **T** | **G** | **A** | **T** | **T** | **T** | **C** | **T** | **G** | **A** | **A** | **G** | **G** | **G** | **A** | **A** | **A** | **A** | **G** | **T** | **A** | **A** | **T** | **C** | **A** | **G** | **G** | **A** | **A** | **A** | **T** | **C** | **G** | **C** | **G** | **G** | **A** | **T** | **G** | **A** | **A** | **T** | **T** | **G** | **T** | **T** | **T** | **A** | **T** | **C** | **A** | **C** | **A** | **C** | **T** | **G** | **A** | **A** | **A** | **A** | **C** | **A** | **G** | **T** | **C** | **A** | **A** | **A** | **A** | **C** | **G** | **C** | **A** | **T** | **G** | **T** | **G** | **T** | **C** | **G** | **A** | **A** | **T** | **A** | **T** | **A** | **T** | **T** | **A** | **G** | **C** | **T** | **A** | **A** | **G** | **T** | **T** | **A** | **G** | **A** | **G** | **G** | **T** | **A** | **G** | **A** | **A** | **G** | **A** | **C** | **C** | **G** | **A** | **A** | **C** | **T** | **C** | **A** | **A** | **G** | **C** | **A** | **G** | **C** | **T** | **A** | **T** | **C** | **T** | **A** | **T** | **G** | **C** | **G** | **T** | **T** | **T** | **A** | **A** | **A** | **C** | **A** | **T** | **G** | **G** | **T** | **T** | **T** | **G** | **G** | **T** | **G** | **A** | **A** | **A** | **-** | **-** | **-** |
| **N59491F** | **G** | **T** | **G** | **A** | **T** | **C** | **A** | **A** | **A** | **G** | **T** | **A** | **A** | **T** | **G** | **T** | **T** | **A** | **G** | **T** | **G** | **G** | **A** | **T** | **G** | **A** | **C** | **C** | **A** | **T** | **G** | **A** | **A** | **A** | **T** | **G** | **G** | **T** | **C** | **C** | **G** | **T** | **T** | **T** | **A** | **G** | **G** | **C** | **G** | **T** | **T** | **T** | **C** | **A** | **T** | **C** | **A** | **T** | **A** | **T** | **T** | **T** | **A** | **T** | **C** | **T** | **A** | **T** | **T** | **C** | **A** | **A** | **G** | **A** | **G** | **G** | **A** | **T** | **A** | **T** | **A** | **G** | **A** | **A** | **G** | **T** | **C** | **G** | **T** | **A** | **G** | **G** | **C** | **G** | **A** | **A** | **G** | **C** | **A** | **G** | **A** | **A** | **A** | **A** | **C** | **G** | **G** | **T** | **A** | **A** | **G** | **A** | **T** | **T** | **G** | **G** | **C** | **T** | **A** | **T** | **G** | **A** | **A** | **A** | **A** | **A** | **G** | **C** | **A** | **T** | **T** | **G** | **G** | **A** | **A** | **C** | **T** | **A** | **C** | **G** | **T** | **C** | **C** | **A** | **G** | **A** | **T** | **G** | **T** | **T** | **A** | **T** | **T** | **T** | **T** | **G** | **A** | **T** | **G** | **G** | **A** | **T** | **T** | **T** | **G** | **G** | **T** | **A** | **A** | **T** | **G** | **G** | **A** | **A** | **G** | **A** | **A** | **A** | **T** | **G** | **G** | **A** | **C** | **G** | **G** | **C** | **A** | **T** | **T** | **G** | **A** | **T** | **T** | **C** | **A** | **A** | **C** | **A** | **A** | **A** | **A** | **G** | **C** | **G** | **A** | **T** | **C** | **T** | **T** | **G** | **A** | **A** | **A** | **G** | **A** | **T** | **T** | **G** | **G** | **C** | **C** | **A** | **G** | **A** | **A** | **G** | **C** | **C** | **A** | **A** | **G** | **A** | **T** | **T** | **A** | **T** | **T** | **A** | **T** | **T** | **G** | **T** | **G** | **A** | **C** | **G** | **A** | **G** | **T** | **T** | **T** | **T** | **A** | **T** | **T** | **G** | **A** | **T** | **G** | **A** | **T** | **G** | **A** | **G** | **A** | **A** | **A** | **G** | **T** | **G** | **T** | **A** | **T** | **C** | **C** | **G** | **G** | **C** | **G** | **A** | **T** | **T** | **G** | **A** | **A** | **G** | **C** | **T** | **G** | **G** | **T** | **G** | **C** | **A** | **G** | **C** | **G** | **G** | **G** | **C** | **T** | **A** | **C** | **C** | **T** | **A** | **T** | **T** | **A** | **A** | **A** | **A** | **A** | **C** | **A** | **T** | **C** | **A** | **A** | **C** | **A** | **G** | **C** | **A** | **C** | **A** | **T** | **G** | **A** | **G** | **A** | **T** | **T** | **G** | **C** | **T** | **G** | **A** | **T** | **G** | **C** | **A** | **A** | **T** | **T** | **C** | **G** | **G** | **G** | **C** | **G** | **A** | **C** | **T** | **T** | **A** | **T** | **C** | **G** | **T** | **G** | **G** | **A** | **G** | **A** | **G** | **C** | **G** | **T** | **G** | **T** | **G** | **T** | **T** | **G** | **G** | **A** | **A** | **C** | **C** | **T** | **G** | **A** | **A** | **G** | **T** | **G** | **A** | **C** | **G** | **C** | **A** | **T** | **A** | **A** | **G** | **A** | **T** | **G** | **A** | **T** | **G** | **G** | **A** | **A** | **C** | **G** | **G** | **T** | **T** | **A** | **A** | **C** | **A** | **A** | **A** | **A** | **A** | **A** | **A** | **C** | **A** | **A** | **G** | **A** | **G** | **C** | **C** | **G** | **G** | **T** | **G** | **T** | **T** | **G** | **C** | **A** | **C** | **G** | **A** | **A** | **G** | **A** | **T** | **T** | **T** | **G** | **A** | **C** | **C** | **A** | **A** | **C** | **C** | **G** | **A** | **G** | **A** | **A** | **C** | **A** | **C** | **G** | **A** | **A** | **A** | **T** | **T** | **T** | **T** | **A** | **A** | **T** | **G** | **T** | **T** | **G** | **A** | **T** | **T** | **G** | **C** | **A** | **C** | **A** | **A** | **G** | **G** | **T** | **A** | **A** | **A** | **A** | **G** | **T** | **A** | **A** | **T** | **C** | **A** | **G** | **G** | **A** | **A** | **A** | **T** | **T** | **G** | **C** | **T** | **G** | **A** | **T** | **G** | **A** | **A** | **C** | **T** | **C** | **T** | **T** | **T** | **A** | **T** | **C** | **A** | **C** | **T** | **T** | **T** | **G** | **A** | **A** | **A** | **A** | **C** | **A** | **G** | **T** | **T** | **A** | **A** | **A** | **A** | **C** | **A** | **C** | **A** | **T** | **G** | **T** | **T** | **T** | **C** | **A** | **A** | **A** | **C** | **A** | **T** | **T** | **T** | **T** | **A** | **G** | **C** | **A** | **A** | **A** | **A** | **C** | **T** | **A** | **G** | **A** | **T** | **G** | **T** | **G** | **G** | **A** | **T** | **G** | **A** | **T** | **C** | **G** | **G** | **A** | **C** | **C** | **C** | **A** | **A** | **G** | **C** | **G** | **G** | **C** | **G** | **A** | **T** | **T** | **T** | **A** | **T** | **G** | **C** | **T** | **T** | **T** | **T** | **C** | **A** | **A** | **C** | **A** | **T** | **G** | **G** | **T** | **T** | **T** | **A** | **G** | **C** | **C** | **A** | **A** | **A** | **-** | **-** | **-** |
| **N53661** | **G** | **T** | **G** | **A** | **T** | **C** | **A** | **A** | **A** | **G** | **T** | **A** | **A** | **T** | **G** | **T** | **T** | **A** | **G** | **T** | **G** | **G** | **A** | **T** | **G** | **A** | **C** | **C** | **A** | **T** | **G** | **A** | **A** | **A** | **T** | **G** | **G** | **T** | **C** | **C** | **G** | **T** | **T** | **T** | **A** | **G** | **G** | **C** | **G** | **T** | **T** | **T** | **C** | **A** | **T** | **C** | **A** | **T** | **A** | **T** | **T** | **T** | **A** | **T** | **C** | **T** | **A** | **T** | **T** | **C** | **A** | **A** | **G** | **A** | **G** | **G** | **A** | **T** | **A** | **T** | **A** | **G** | **A** | **A** | **G** | **T** | **C** | **G** | **T** | **A** | **G** | **G** | **C** | **G** | **A** | **A** | **G** | **C** | **A** | **G** | **A** | **A** | **A** | **A** | **T** | **G** | **G** | **T** | **A** | **A** | **G** | **A** | **T** | **T** | **G** | **G** | **C** | **T** | **A** | **T** | **G** | **A** | **A** | **A** | **A** | **A** | **G** | **C** | **A** | **T** | **T** | **G** | **G** | **A** | **A** | **C** | **T** | **A** | **C** | **G** | **T** | **C** | **C** | **A** | **G** | **A** | **T** | **G** | **T** | **T** | **A** | **T** | **T** | **T** | **T** | **G** | **A** | **T** | **G** | **G** | **A** | **T** | **T** | **T** | **G** | **G** | **T** | **A** | **A** | **T** | **G** | **G** | **A** | **A** | **G** | **A** | **A** | **A** | **T** | **G** | **G** | **A** | **C** | **G** | **G** | **C** | **A** | **T** | **T** | **G** | **A** | **T** | **T** | **C** | **A** | **A** | **C** | **A** | **A** | **A** | **A** | **G** | **C** | **G** | **A** | **T** | **C** | **T** | **T** | **G** | **A** | **A** | **A** | **G** | **A** | **T** | **T** | **G** | **G** | **C** | **C** | **A** | **G** | **A** | **A** | **G** | **C** | **C** | **A** | **A** | **G** | **A** | **T** | **T** | **A** | **T** | **T** | **A** | **T** | **T** | **G** | **T** | **G** | **A** | **C** | **G** | **A** | **G** | **T** | **T** | **T** | **T** | **A** | **T** | **T** | **G** | **A** | **T** | **G** | **A** | **T** | **G** | **A** | **A** | **A** | **A** | **A** | **G** | **T** | **G** | **T** | **A** | **T** | **C** | **C** | **G** | **G** | **C** | **G** | **A** | **T** | **T** | **G** | **A** | **A** | **G** | **C** | **T** | **G** | **G** | **T** | **G** | **C** | **A** | **G** | **C** | **G** | **G** | **G** | **C** | **T** | **A** | **C** | **C** | **T** | **A** | **T** | **T** | **A** | **A** | **A** | **A** | **A** | **C** | **A** | **T** | **C** | **A** | **A** | **C** | **A** | **G** | **C** | **A** | **C** | **A** | **T** | **G** | **A** | **G** | **A** | **T** | **T** | **G** | **C** | **T** | **G** | **A** | **T** | **G** | **C** | **A** | **A** | **T** | **T** | **C** | **G** | **G** | **G** | **C** | **G** | **A** | **C** | **T** | **T** | **A** | **T** | **C** | **G** | **C** | **G** | **G** | **A** | **G** | **A** | **G** | **C** | **G** | **T** | **G** | **T** | **G** | **T** | **T** | **G** | **G** | **A** | **A** | **C** | **C** | **T** | **G** | **A** | **A** | **G** | **T** | **G** | **A** | **C** | **G** | **C** | **A** | **T** | **A** | **A** | **G** | **A** | **T** | **G** | **A** | **T** | **G** | **G** | **A** | **A** | **C** | **G** | **G** | **T** | **T** | **A** | **A** | **C** | **A** | **A** | **A** | **A** | **A** | **A** | **A** | **C** | **A** | **A** | **G** | **A** | **G** | **C** | **C** | **G** | **G** | **T** | **G** | **T** | **T** | **G** | **C** | **A** | **T** | **G** | **A** | **A** | **G** | **A** | **T** | **T** | **T** | **G** | **A** | **C** | **A** | **A** | **A** | **T** | **C** | **G** | **A** | **G** | **A** | **A** | **C** | **A** | **C** | **G** | **A** | **A** | **A** | **T** | **T** | **T** | **T** | **A** | **A** | **T** | **G** | **T** | **T** | **G** | **A** | **T** | **T** | **G** | **C** | **A** | **C** | **A** | **A** | **G** | **G** | **T** | **A** | **A** | **A** | **A** | **G** | **T** | **A** | **A** | **T** | **C** | **A** | **G** | **G** | **A** | **A** | **A** | **T** | **A** | **G** | **C** | **T** | **G** | **A** | **T** | **G** | **A** | **A** | **C** | **T** | **C** | **T** | **T** | **T** | **A** | **T** | **C** | **A** | **C** | **T** | **T** | **T** | **G** | **A** | **A** | **A** | **A** | **C** | **A** | **G** | **T** | **T** | **A** | **A** | **A** | **A** | **C** | **A** | **C** | **A** | **T** | **G** | **T** | **T** | **T** | **C** | **A** | **A** | **A** | **C** | **A** | **T** | **T** | **T** | **T** | **A** | **G** | **C** | **A** | **A** | **A** | **A** | **C** | **T** | **A** | **G** | **A** | **T** | **G** | **T** | **G** | **G** | **A** | **T** | **G** | **A** | **T** | **C** | **G** | **G** | **A** | **C** | **C** | **C** | **A** | **A** | **G** | **C** | **G** | **G** | **C** | **G** | **A** | **T** | **T** | **T** | **A** | **T** | **G** | **C** | **T** | **T** | **T** | **T** | **C** | **A** | **A** | **C** | **A** | **T** | **G** | **G** | **T** | **T** | **T** | **A** | **G** | **C** | **C** | **A** | **A** | **A** | **-** | **-** | **-** |
| **N59504F** | **A** | **T** | **G** | **A** | **T** | **A** | **A** | **A** | **A** | **G** | **T** | **T** | **T** | **T** | **A** | **T** | **T** | **A** | **G** | **T** | **A** | **G** | **A** | **T** | **G** | **A** | **C** | **C** | **A** | **T** | **G** | **A** | **A** | **A** | **T** | **G** | **G** | **T** | **A** | **C** | **G** | **C** | **T** | **T** | **A** | **G** | **G** | **C** | **G** | **T** | **C** | **T** | **C** | **T** | **T** | **C** | **T** | **T** | **A** | **T** | **T** | **T** | **A** | **T** | **C** | **T** | **A** | **T** | **C** | **C** | **A** | **A** | **G** | **A** | **A** | **G** | **A** | **C** | **A** | **T** | **T** | **G** | **A** | **A** | **G** | **T** | **G** | **A** | **T** | **T** | **G** | **G** | **A** | **G** | **A** | **A** | **G** | **C** | **T** | **G** | **A** | **A** | **A** | **A** | **T** | **G** | **G** | **A** | **C** | **G** | **T** | **C** | **A** | **A** | **G** | **G** | **C** | **T** | **A** | **T** | **G** | **A** | **A** | **A** | **A** | **G** | **G** | **C** | **G** | **A** | **T** | **G** | **G** | **C** | **A** | **C** | **T** | **T** | **C** | **G** | **A** | **C** | **C** | **A** | **G** | **A** | **T** | **G** | **T** | **C** | **A** | **T** | **T** | **T** | **T** | **A** | **A** | **T** | **G** | **G** | **A** | **T** | **C** | **T** | **A** | **G** | **T** | **C** | **A** | **T** | **G** | **G** | **A** | **A** | **G** | **A** | **A** | **A** | **T** | **G** | **G** | **A** | **T** | **G** | **G** | **C** | **A** | **T** | **C** | **G** | **A** | **A** | **T** | **C** | **A** | **A** | **C** | **A** | **A** | **A** | **A** | **G** | **C** | **A** | **A** | **T** | **C** | **T** | **T** | **A** | **A** | **A** | **A** | **G** | **A** | **T** | **T** | **G** | **G** | **C** | **C** | **A** | **A** | **A** | **A** | **G** | **C** | **C** | **A** | **A** | **A** | **A** | **T** | **C** | **A** | **T** | **T** | **A** | **T** | **C** | **G** | **T** | **C** | **A** | **C** | **A** | **A** | **G** | **T** | **T** | **T** | **T** | **A** | **T** | **T** | **G** | **A** | **T** | **G** | **A** | **T** | **G** | **A** | **A** | **A** | **A** | **A** | **G** | **T** | **T** | **T** | **A** | **T** | **C** | **C** | **T** | **G** | **C** | **A** | **A** | **T** | **T** | **G** | **A** | **A** | **G** | **C** | **A** | **G** | **G** | **A** | **G** | **C** | **A** | **G** | **C** | **A** | **G** | **G** | **A** | **T** | **A** | **T** | **T** | **T** | **A** | **T** | **T** | **G** | **A** | **A** | **G** | **A** | **C** | **G** | **T** | **C** | **T** | **A** | **C** | **C** | **G** | **C** | **A** | **C** | **A** | **T** | **G** | **A** | **A** | **A** | **T** | **A** | **G** | **C** | **T** | **G** | **A** | **T** | **G** | **C** | **T** | **A** | **T** | **A** | **A** | **G** | **G** | **G** | **C** | **T** | **A** | **C** | **G** | **C** | **A** | **A** | **C** | **G** | **C** | **G** | **G** | **C** | **G** | **A** | **G** | **C** | **G** | **A** | **G** | **T** | **A** | **T** | **T** | **G** | **G** | **A** | **A** | **C** | **C** | **T** | **G** | **A** | **A** | **G** | **T** | **G** | **A** | **C** | **A** | **A** | **C** | **A** | **A** | **A** | **A** | **A** | **T** | **G** | **A** | **T** | **G** | **G** | **A** | **A** | **A** | **A** | **A** | **A** | **T** | **G** | **A** | **G** | **T** | **C** | **G** | **G** | **C** | **G** | **A** | **A** | **A** | **T** | **G** | **A** | **T** | **C** | **C** | **A** | **G** | **T** | **A** | **T** | **T** | **G** | **C** | **A** | **T** | **G** | **A** | **A** | **G** | **A** | **A** | **T** | **T** | **A** | **A** | **C** | **G** | **A** | **A** | **T** | **C** | **G** | **A** | **G** | **A** | **A** | **A** | **A** | **T** | **G** | **A** | **A** | **A** | **T** | **T** | **T** | **T** | **G** | **A** | **T** | **G** | **C** | **T** | **G** | **A** | **T** | **T** | **T** | **C** | **T** | **G** | **A** | **A** | **G** | **G** | **G** | **A** | **A** | **A** | **A** | **G** | **T** | **A** | **A** | **T** | **C** | **A** | **G** | **G** | **A** | **A** | **A** | **T** | **C** | **G** | **C** | **G** | **G** | **A** | **T** | **G** | **A** | **A** | **T** | **T** | **G** | **T** | **T** | **T** | **A** | **T** | **C** | **A** | **C** | **A** | **C** | **T** | **G** | **A** | **A** | **A** | **A** | **C** | **A** | **G** | **T** | **T** | **A** | **A** | **A** | **A** | **C** | **G** | **C** | **A** | **T** | **G** | **T** | **G** | **T** | **C** | **G** | **A** | **A** | **T** | **A** | **T** | **A** | **T** | **T** | **A** | **G** | **C** | **T** | **A** | **A** | **G** | **T** | **T** | **A** | **G** | **A** | **G** | **G** | **T** | **A** | **G** | **A** | **A** | **G** | **A** | **C** | **C** | **G** | **A** | **A** | **C** | **T** | **C** | **A** | **A** | **G** | **C** | **A** | **G** | **C** | **T** | **A** | **T** | **C** | **T** | **A** | **T** | **G** | **C** | **G** | **T** | **T** | **T** | **A** | **A** | **A** | **C** | **A** | **T** | **G** | **G** | **T** | **T** | **T** | **G** | **G** | **T** | **G** | **A** | **A** | **A** | **-** | **-** | **-** |
| **N59907F** | **A** | **T** | **G** | **A** | **T** | **A** | **A** | **A** | **A** | **G** | **T** | **T** | **T** | **T** | **A** | **T** | **T** | **A** | **G** | **T** | **A** | **G** | **A** | **T** | **G** | **A** | **C** | **C** | **A** | **T** | **G** | **A** | **A** | **A** | **T** | **G** | **G** | **T** | **A** | **C** | **G** | **C** | **T** | **T** | **A** | **G** | **G** | **C** | **G** | **T** | **C** | **T** | **C** | **T** | **T** | **C** | **T** | **T** | **A** | **T** | **T** | **T** | **A** | **T** | **C** | **T** | **A** | **T** | **C** | **C** | **A** | **A** | **G** | **A** | **A** | **G** | **A** | **C** | **A** | **T** | **T** | **G** | **A** | **A** | **G** | **T** | **G** | **A** | **T** | **T** | **G** | **G** | **A** | **G** | **A** | **A** | **G** | **C** | **T** | **G** | **A** | **A** | **A** | **A** | **T** | **G** | **G** | **A** | **C** | **G** | **T** | **C** | **A** | **A** | **G** | **G** | **C** | **T** | **A** | **T** | **G** | **A** | **A** | **A** | **A** | **G** | **G** | **C** | **G** | **A** | **T** | **G** | **G** | **C** | **A** | **C** | **T** | **T** | **C** | **G** | **A** | **C** | **C** | **A** | **G** | **A** | **T** | **G** | **T** | **C** | **A** | **T** | **T** | **T** | **T** | **A** | **A** | **T** | **G** | **G** | **A** | **T** | **C** | **T** | **A** | **G** | **T** | **C** | **A** | **T** | **G** | **G** | **A** | **A** | **G** | **A** | **A** | **A** | **T** | **G** | **G** | **A** | **T** | **G** | **G** | **C** | **A** | **T** | **C** | **G** | **A** | **A** | **T** | **C** | **A** | **A** | **C** | **A** | **A** | **A** | **A** | **G** | **C** | **A** | **A** | **T** | **C** | **T** | **T** | **A** | **A** | **A** | **A** | **G** | **A** | **T** | **T** | **G** | **G** | **C** | **C** | **A** | **A** | **A** | **A** | **G** | **C** | **C** | **A** | **A** | **A** | **A** | **T** | **C** | **A** | **T** | **T** | **A** | **T** | **C** | **G** | **T** | **C** | **A** | **C** | **A** | **A** | **G** | **T** | **T** | **T** | **T** | **A** | **T** | **T** | **G** | **A** | **T** | **G** | **A** | **T** | **G** | **A** | **A** | **A** | **A** | **A** | **G** | **T** | **T** | **T** | **A** | **T** | **C** | **C** | **T** | **G** | **C** | **A** | **A** | **T** | **T** | **G** | **A** | **A** | **G** | **C** | **A** | **G** | **G** | **A** | **G** | **C** | **A** | **G** | **C** | **A** | **G** | **G** | **A** | **T** | **A** | **T** | **T** | **T** | **G** | **T** | **T** | **G** | **A** | **A** | **G** | **A** | **C** | **G** | **T** | **C** | **T** | **A** | **C** | **C** | **G** | **C** | **A** | **C** | **A** | **T** | **G** | **A** | **A** | **A** | **T** | **A** | **G** | **C** | **T** | **G** | **A** | **T** | **G** | **C** | **T** | **A** | **T** | **A** | **A** | **G** | **G** | **G** | **C** | **T** | **A** | **C** | **G** | **C** | **A** | **A** | **C** | **G** | **C** | **G** | **G** | **C** | **G** | **A** | **G** | **C** | **G** | **A** | **G** | **T** | **A** | **T** | **T** | **G** | **G** | **A** | **A** | **C** | **C** | **T** | **G** | **A** | **A** | **G** | **T** | **G** | **A** | **C** | **A** | **A** | **C** | **A** | **A** | **A** | **A** | **A** | **T** | **G** | **A** | **T** | **G** | **G** | **A** | **A** | **A** | **A** | **A** | **A** | **T** | **G** | **A** | **G** | **T** | **C** | **G** | **G** | **C** | **G** | **A** | **A** | **A** | **T** | **G** | **A** | **T** | **C** | **C** | **A** | **G** | **T** | **A** | **T** | **T** | **G** | **C** | **A** | **T** | **G** | **A** | **A** | **G** | **A** | **A** | **T** | **T** | **A** | **A** | **C** | **G** | **A** | **A** | **T** | **C** | **G** | **A** | **G** | **A** | **A** | **A** | **A** | **T** | **G** | **A** | **A** | **A** | **T** | **T** | **T** | **T** | **G** | **A** | **T** | **G** | **C** | **T** | **G** | **A** | **T** | **T** | **T** | **C** | **T** | **G** | **A** | **A** | **G** | **G** | **G** | **A** | **A** | **A** | **A** | **G** | **T** | **A** | **A** | **T** | **C** | **A** | **G** | **G** | **A** | **A** | **A** | **T** | **C** | **G** | **C** | **G** | **G** | **A** | **T** | **G** | **A** | **A** | **T** | **T** | **G** | **T** | **T** | **T** | **A** | **T** | **C** | **A** | **C** | **A** | **C** | **T** | **G** | **A** | **A** | **A** | **A** | **C** | **A** | **G** | **T** | **T** | **A** | **A** | **A** | **A** | **C** | **G** | **C** | **A** | **T** | **G** | **T** | **G** | **T** | **C** | **G** | **A** | **A** | **T** | **A** | **T** | **A** | **T** | **T** | **A** | **G** | **C** | **T** | **A** | **A** | **G** | **T** | **T** | **A** | **G** | **A** | **G** | **G** | **T** | **A** | **G** | **A** | **A** | **G** | **A** | **C** | **C** | **G** | **A** | **A** | **C** | **T** | **C** | **A** | **A** | **G** | **C** | **A** | **G** | **C** | **T** | **A** | **T** | **C** | **T** | **A** | **T** | **G** | **C** | **G** | **T** | **T** | **T** | **A** | **A** | **A** | **C** | **A** | **T** | **G** | **G** | **T** | **T** | **T** | **G** | **G** | **T** | **G** | **A** | **A** | **A** | **-** | **-** | **-** |
| **N60365F** | **A** | **T** | **G** | **A** | **T** | **A** | **A** | **A** | **A** | **G** | **T** | **T** | **T** | **T** | **A** | **T** | **T** | **A** | **G** | **T** | **A** | **G** | **A** | **T** | **G** | **A** | **C** | **C** | **A** | **T** | **G** | **A** | **A** | **A** | **T** | **G** | **G** | **T** | **A** | **C** | **G** | **C** | **T** | **T** | **A** | **G** | **G** | **C** | **G** | **T** | **C** | **T** | **C** | **T** | **T** | **C** | **T** | **T** | **A** | **T** | **T** | **T** | **A** | **T** | **C** | **T** | **A** | **T** | **C** | **C** | **A** | **A** | **G** | **A** | **A** | **G** | **A** | **C** | **A** | **T** | **T** | **G** | **A** | **A** | **G** | **T** | **G** | **A** | **T** | **T** | **G** | **G** | **A** | **G** | **A** | **A** | **G** | **C** | **T** | **G** | **A** | **A** | **A** | **A** | **T** | **G** | **G** | **A** | **C** | **G** | **T** | **C** | **A** | **A** | **G** | **G** | **C** | **T** | **A** | **T** | **G** | **A** | **A** | **A** | **A** | **G** | **G** | **C** | **G** | **A** | **T** | **G** | **G** | **C** | **A** | **C** | **T** | **T** | **C** | **G** | **A** | **C** | **C** | **A** | **G** | **A** | **T** | **G** | **T** | **C** | **A** | **T** | **T** | **T** | **T** | **A** | **A** | **T** | **G** | **G** | **A** | **T** | **C** | **T** | **A** | **G** | **T** | **C** | **A** | **T** | **G** | **G** | **A** | **A** | **G** | **A** | **A** | **A** | **T** | **G** | **G** | **A** | **T** | **G** | **G** | **C** | **A** | **T** | **C** | **G** | **A** | **A** | **T** | **C** | **A** | **A** | **C** | **A** | **A** | **A** | **A** | **G** | **C** | **A** | **A** | **T** | **C** | **T** | **T** | **A** | **A** | **A** | **A** | **G** | **A** | **T** | **T** | **G** | **G** | **C** | **C** | **A** | **A** | **A** | **A** | **G** | **C** | **C** | **A** | **A** | **A** | **A** | **T** | **C** | **A** | **T** | **T** | **A** | **T** | **C** | **G** | **T** | **C** | **A** | **C** | **A** | **A** | **G** | **T** | **T** | **T** | **T** | **A** | **T** | **T** | **G** | **A** | **T** | **G** | **A** | **T** | **G** | **A** | **A** | **A** | **A** | **A** | **G** | **T** | **T** | **T** | **A** | **T** | **C** | **C** | **T** | **G** | **C** | **A** | **A** | **T** | **T** | **G** | **A** | **A** | **G** | **C** | **A** | **G** | **G** | **A** | **G** | **C** | **A** | **G** | **C** | **A** | **G** | **G** | **A** | **T** | **A** | **T** | **T** | **T** | **A** | **T** | **T** | **G** | **A** | **A** | **G** | **A** | **C** | **G** | **T** | **C** | **T** | **A** | **C** | **C** | **G** | **C** | **A** | **C** | **A** | **T** | **G** | **A** | **A** | **A** | **T** | **A** | **G** | **C** | **T** | **G** | **A** | **T** | **G** | **C** | **T** | **A** | **T** | **A** | **A** | **G** | **G** | **G** | **C** | **T** | **A** | **C** | **G** | **C** | **A** | **A** | **C** | **G** | **C** | **G** | **G** | **C** | **G** | **A** | **G** | **C** | **G** | **A** | **G** | **T** | **A** | **T** | **T** | **G** | **G** | **A** | **A** | **C** | **C** | **T** | **G** | **A** | **A** | **G** | **T** | **G** | **A** | **C** | **A** | **A** | **C** | **A** | **A** | **A** | **A** | **A** | **T** | **G** | **A** | **T** | **G** | **G** | **A** | **A** | **A** | **A** | **A** | **A** | **T** | **G** | **A** | **G** | **T** | **C** | **G** | **G** | **C** | **G** | **A** | **A** | **A** | **T** | **G** | **A** | **T** | **C** | **C** | **A** | **G** | **T** | **A** | **T** | **T** | **G** | **C** | **A** | **T** | **G** | **A** | **A** | **G** | **A** | **A** | **T** | **T** | **A** | **A** | **C** | **G** | **A** | **A** | **T** | **C** | **G** | **A** | **G** | **A** | **A** | **A** | **A** | **T** | **G** | **A** | **A** | **A** | **T** | **T** | **T** | **T** | **G** | **A** | **T** | **G** | **C** | **T** | **G** | **A** | **T** | **T** | **T** | **C** | **T** | **G** | **A** | **A** | **G** | **G** | **G** | **A** | **A** | **A** | **A** | **G** | **T** | **A** | **A** | **T** | **C** | **A** | **G** | **G** | **A** | **A** | **A** | **T** | **C** | **G** | **C** | **G** | **G** | **A** | **T** | **G** | **A** | **A** | **T** | **T** | **G** | **T** | **T** | **T** | **A** | **T** | **C** | **A** | **C** | **A** | **C** | **T** | **G** | **A** | **A** | **A** | **A** | **C** | **A** | **G** | **T** | **T** | **A** | **A** | **A** | **A** | **C** | **G** | **C** | **A** | **T** | **G** | **T** | **G** | **T** | **C** | **G** | **A** | **A** | **T** | **A** | **T** | **A** | **T** | **T** | **A** | **G** | **C** | **T** | **A** | **A** | **G** | **T** | **T** | **A** | **G** | **A** | **G** | **G** | **T** | **A** | **G** | **A** | **A** | **G** | **A** | **C** | **C** | **G** | **A** | **A** | **C** | **T** | **C** | **A** | **A** | **G** | **C** | **A** | **G** | **C** | **T** | **A** | **T** | **C** | **T** | **A** | **T** | **G** | **C** | **G** | **T** | **T** | **T** | **A** | **A** | **A** | **C** | **A** | **T** | **G** | **G** | **T** | **T** | **T** | **G** | **G** | **T** | **G** | **A** | **A** | **A** | **-** | **-** | **-** |
| **N59542F** | **A** | **T** | **G** | **A** | **T** | **A** | **A** | **A** | **A** | **G** | **T** | **T** | **T** | **T** | **A** | **T** | **T** | **A** | **G** | **T** | **A** | **G** | **A** | **T** | **G** | **A** | **C** | **C** | **A** | **T** | **G** | **A** | **A** | **A** | **T** | **G** | **G** | **T** | **A** | **C** | **G** | **C** | **T** | **T** | **A** | **G** | **G** | **C** | **G** | **T** | **C** | **T** | **C** | **T** | **T** | **C** | **T** | **T** | **A** | **T** | **T** | **T** | **A** | **T** | **C** | **T** | **A** | **T** | **C** | **C** | **A** | **A** | **G** | **A** | **A** | **G** | **A** | **C** | **A** | **T** | **T** | **G** | **A** | **A** | **G** | **T** | **G** | **A** | **T** | **T** | **G** | **G** | **A** | **G** | **A** | **A** | **G** | **C** | **T** | **G** | **A** | **A** | **A** | **A** | **T** | **G** | **G** | **A** | **C** | **G** | **T** | **C** | **A** | **A** | **G** | **G** | **C** | **T** | **A** | **T** | **G** | **A** | **A** | **A** | **A** | **G** | **G** | **C** | **G** | **A** | **T** | **G** | **G** | **C** | **A** | **C** | **T** | **T** | **C** | **G** | **A** | **C** | **C** | **A** | **G** | **A** | **T** | **G** | **T** | **C** | **A** | **T** | **T** | **T** | **T** | **A** | **A** | **T** | **G** | **G** | **A** | **T** | **C** | **T** | **A** | **G** | **T** | **C** | **A** | **T** | **G** | **G** | **A** | **A** | **G** | **A** | **A** | **A** | **T** | **G** | **G** | **A** | **T** | **G** | **G** | **C** | **A** | **T** | **C** | **G** | **A** | **A** | **T** | **C** | **A** | **A** | **C** | **A** | **A** | **A** | **A** | **G** | **C** | **A** | **A** | **T** | **C** | **T** | **T** | **A** | **A** | **A** | **A** | **G** | **A** | **T** | **T** | **G** | **G** | **C** | **C** | **A** | **A** | **A** | **A** | **G** | **C** | **C** | **A** | **A** | **A** | **A** | **T** | **C** | **A** | **T** | **T** | **A** | **T** | **C** | **G** | **T** | **C** | **A** | **C** | **A** | **A** | **G** | **T** | **T** | **T** | **T** | **A** | **T** | **T** | **G** | **A** | **T** | **G** | **A** | **T** | **G** | **A** | **A** | **A** | **A** | **A** | **G** | **T** | **T** | **T** | **A** | **T** | **C** | **C** | **T** | **G** | **C** | **A** | **A** | **T** | **T** | **G** | **A** | **A** | **G** | **C** | **A** | **G** | **G** | **A** | **G** | **C** | **A** | **G** | **C** | **A** | **G** | **G** | **A** | **T** | **A** | **T** | **T** | **T** | **A** | **T** | **T** | **G** | **A** | **A** | **G** | **A** | **C** | **G** | **T** | **C** | **T** | **A** | **C** | **C** | **G** | **C** | **A** | **C** | **A** | **T** | **G** | **A** | **A** | **A** | **T** | **A** | **G** | **C** | **T** | **G** | **A** | **T** | **G** | **C** | **T** | **A** | **T** | **A** | **A** | **G** | **G** | **G** | **C** | **T** | **A** | **C** | **G** | **C** | **A** | **A** | **C** | **G** | **C** | **G** | **G** | **C** | **G** | **A** | **G** | **C** | **G** | **A** | **G** | **T** | **A** | **T** | **T** | **G** | **G** | **A** | **A** | **C** | **C** | **T** | **G** | **A** | **A** | **G** | **T** | **G** | **A** | **C** | **A** | **A** | **C** | **A** | **A** | **A** | **A** | **A** | **T** | **G** | **A** | **T** | **G** | **G** | **A** | **A** | **A** | **A** | **A** | **A** | **T** | **G** | **A** | **G** | **T** | **C** | **G** | **G** | **C** | **G** | **A** | **A** | **A** | **T** | **G** | **A** | **T** | **C** | **C** | **A** | **G** | **T** | **A** | **T** | **T** | **G** | **C** | **A** | **T** | **G** | **A** | **A** | **G** | **A** | **A** | **T** | **T** | **A** | **A** | **C** | **G** | **A** | **A** | **T** | **C** | **G** | **A** | **G** | **A** | **A** | **A** | **A** | **T** | **G** | **A** | **A** | **A** | **T** | **T** | **T** | **T** | **G** | **A** | **T** | **G** | **C** | **T** | **G** | **A** | **T** | **T** | **T** | **C** | **T** | **G** | **A** | **A** | **G** | **G** | **G** | **A** | **A** | **A** | **A** | **G** | **T** | **A** | **A** | **T** | **C** | **A** | **G** | **G** | **A** | **A** | **A** | **T** | **C** | **G** | **C** | **G** | **G** | **A** | **T** | **G** | **A** | **A** | **T** | **T** | **G** | **T** | **T** | **T** | **A** | **T** | **C** | **A** | **C** | **A** | **C** | **T** | **G** | **A** | **A** | **A** | **A** | **C** | **A** | **G** | **T** | **T** | **A** | **A** | **A** | **A** | **C** | **G** | **C** | **A** | **T** | **G** | **T** | **G** | **T** | **C** | **G** | **A** | **A** | **T** | **A** | **T** | **A** | **T** | **T** | **A** | **G** | **C** | **T** | **A** | **A** | **G** | **T** | **T** | **A** | **G** | **A** | **G** | **G** | **T** | **A** | **G** | **A** | **A** | **G** | **A** | **C** | **C** | **G** | **A** | **A** | **C** | **T** | **C** | **A** | **A** | **G** | **C** | **A** | **G** | **C** | **T** | **A** | **T** | **C** | **T** | **A** | **T** | **G** | **C** | **G** | **T** | **T** | **T** | **A** | **A** | **A** | **C** | **A** | **T** | **G** | **G** | **T** | **T** | **T** | **G** | **G** | **T** | **G** | **A** | **A** | **A** | **-** | **-** | **-** |
| **N59484F** | **A** | **T** | **G** | **A** | **T** | **A** | **A** | **A** | **A** | **G** | **T** | **T** | **T** | **T** | **A** | **T** | **T** | **A** | **G** | **T** | **A** | **G** | **A** | **T** | **G** | **A** | **C** | **C** | **A** | **T** | **G** | **A** | **A** | **A** | **T** | **G** | **G** | **T** | **A** | **C** | **G** | **C** | **T** | **T** | **A** | **G** | **G** | **C** | **G** | **T** | **C** | **T** | **C** | **T** | **T** | **C** | **T** | **T** | **A** | **T** | **T** | **T** | **A** | **T** | **C** | **T** | **A** | **T** | **C** | **C** | **A** | **A** | **G** | **A** | **A** | **G** | **A** | **C** | **A** | **T** | **T** | **G** | **A** | **A** | **G** | **T** | **G** | **A** | **T** | **T** | **G** | **G** | **A** | **G** | **A** | **A** | **G** | **C** | **T** | **G** | **A** | **A** | **A** | **A** | **T** | **G** | **G** | **A** | **C** | **G** | **T** | **C** | **A** | **A** | **G** | **G** | **C** | **T** | **A** | **T** | **G** | **A** | **A** | **A** | **A** | **G** | **G** | **C** | **G** | **A** | **T** | **G** | **G** | **C** | **A** | **C** | **T** | **T** | **C** | **G** | **A** | **C** | **C** | **A** | **G** | **A** | **T** | **G** | **T** | **C** | **A** | **T** | **T** | **T** | **T** | **A** | **A** | **T** | **G** | **G** | **A** | **T** | **C** | **T** | **A** | **G** | **T** | **C** | **A** | **T** | **G** | **G** | **A** | **A** | **G** | **A** | **A** | **A** | **T** | **G** | **G** | **A** | **T** | **G** | **G** | **C** | **A** | **T** | **C** | **G** | **A** | **A** | **T** | **C** | **A** | **A** | **C** | **A** | **A** | **A** | **A** | **G** | **C** | **A** | **A** | **T** | **C** | **T** | **T** | **A** | **A** | **A** | **A** | **G** | **A** | **T** | **T** | **G** | **G** | **C** | **C** | **A** | **A** | **A** | **A** | **G** | **C** | **C** | **A** | **A** | **A** | **A** | **T** | **C** | **A** | **T** | **T** | **A** | **T** | **C** | **G** | **T** | **C** | **A** | **C** | **A** | **A** | **G** | **T** | **T** | **T** | **T** | **A** | **T** | **T** | **G** | **A** | **T** | **G** | **A** | **T** | **G** | **A** | **A** | **A** | **A** | **A** | **G** | **T** | **T** | **T** | **A** | **T** | **C** | **C** | **T** | **G** | **C** | **A** | **A** | **T** | **T** | **G** | **A** | **A** | **G** | **C** | **A** | **G** | **G** | **A** | **G** | **C** | **A** | **G** | **C** | **A** | **G** | **G** | **A** | **T** | **A** | **T** | **T** | **T** | **G** | **T** | **T** | **G** | **A** | **A** | **G** | **A** | **C** | **G** | **T** | **C** | **T** | **A** | **C** | **C** | **G** | **C** | **A** | **C** | **A** | **T** | **G** | **A** | **A** | **A** | **T** | **A** | **G** | **C** | **T** | **G** | **A** | **T** | **G** | **C** | **T** | **A** | **T** | **A** | **A** | **G** | **G** | **G** | **C** | **T** | **A** | **C** | **G** | **C** | **A** | **A** | **C** | **G** | **C** | **G** | **G** | **C** | **G** | **A** | **G** | **C** | **G** | **A** | **G** | **T** | **A** | **T** | **T** | **G** | **G** | **A** | **A** | **C** | **C** | **T** | **G** | **A** | **A** | **G** | **T** | **G** | **A** | **C** | **A** | **A** | **C** | **A** | **A** | **A** | **A** | **A** | **T** | **G** | **A** | **T** | **G** | **G** | **A** | **A** | **A** | **A** | **A** | **A** | **T** | **G** | **A** | **G** | **T** | **C** | **G** | **G** | **C** | **G** | **A** | **T** | **A** | **T** | **G** | **A** | **T** | **C** | **C** | **A** | **G** | **T** | **A** | **T** | **T** | **G** | **C** | **A** | **T** | **G** | **A** | **A** | **G** | **A** | **A** | **T** | **T** | **A** | **A** | **C** | **G** | **A** | **A** | **T** | **C** | **G** | **A** | **G** | **A** | **A** | **A** | **A** | **T** | **G** | **A** | **A** | **A** | **T** | **T** | **T** | **T** | **G** | **A** | **T** | **G** | **C** | **T** | **G** | **A** | **T** | **T** | **T** | **C** | **T** | **G** | **A** | **A** | **G** | **G** | **G** | **A** | **A** | **A** | **A** | **G** | **T** | **A** | **A** | **T** | **C** | **A** | **G** | **G** | **A** | **A** | **A** | **T** | **C** | **G** | **C** | **G** | **G** | **A** | **T** | **G** | **A** | **A** | **T** | **T** | **G** | **T** | **T** | **T** | **A** | **T** | **C** | **A** | **C** | **A** | **C** | **T** | **G** | **A** | **A** | **A** | **A** | **C** | **A** | **G** | **T** | **T** | **A** | **A** | **A** | **A** | **C** | **G** | **C** | **A** | **T** | **G** | **T** | **G** | **T** | **C** | **G** | **A** | **A** | **T** | **A** | **T** | **A** | **T** | **T** | **A** | **G** | **C** | **T** | **A** | **A** | **G** | **T** | **T** | **A** | **G** | **A** | **G** | **G** | **T** | **A** | **G** | **A** | **A** | **G** | **A** | **C** | **C** | **G** | **A** | **A** | **C** | **T** | **C** | **A** | **A** | **G** | **C** | **A** | **G** | **C** | **T** | **A** | **T** | **C** | **T** | **A** | **T** | **G** | **C** | **A** | **T** | **T** | **T** | **A** | **A** | **A** | **C** | **A** | **T** | **G** | **G** | **T** | **T** | **T** | **G** | **G** | **T** | **G** | **A** | **A** | **A** | **-** | **-** | **-** |
| **N52702** | **A** | **T** | **G** | **A** | **T** | **A** | **A** | **A** | **A** | **G** | **T** | **T** | **T** | **T** | **A** | **T** | **T** | **A** | **G** | **T** | **A** | **G** | **A** | **T** | **G** | **A** | **C** | **C** | **A** | **T** | **G** | **A** | **A** | **A** | **T** | **G** | **G** | **T** | **A** | **C** | **G** | **C** | **T** | **T** | **A** | **G** | **G** | **C** | **G** | **T** | **C** | **T** | **C** | **T** | **T** | **C** | **T** | **T** | **A** | **T** | **T** | **T** | **A** | **T** | **C** | **T** | **A** | **T** | **C** | **C** | **A** | **A** | **G** | **A** | **A** | **G** | **A** | **C** | **A** | **T** | **T** | **G** | **A** | **A** | **G** | **T** | **G** | **A** | **T** | **T** | **G** | **G** | **A** | **G** | **A** | **A** | **G** | **C** | **T** | **G** | **A** | **A** | **A** | **A** | **T** | **G** | **G** | **A** | **C** | **G** | **T** | **C** | **A** | **A** | **G** | **G** | **C** | **T** | **A** | **T** | **G** | **A** | **A** | **A** | **A** | **G** | **G** | **C** | **G** | **A** | **T** | **G** | **G** | **C** | **A** | **C** | **T** | **T** | **C** | **G** | **A** | **C** | **C** | **A** | **G** | **A** | **T** | **G** | **T** | **C** | **A** | **T** | **T** | **T** | **T** | **A** | **A** | **T** | **G** | **G** | **A** | **T** | **C** | **T** | **A** | **G** | **T** | **C** | **A** | **T** | **G** | **G** | **A** | **A** | **G** | **A** | **A** | **A** | **T** | **G** | **G** | **A** | **T** | **G** | **G** | **C** | **A** | **T** | **C** | **G** | **A** | **A** | **T** | **C** | **A** | **A** | **C** | **A** | **A** | **A** | **A** | **G** | **C** | **A** | **A** | **T** | **C** | **T** | **T** | **A** | **A** | **A** | **A** | **G** | **A** | **T** | **T** | **G** | **G** | **C** | **C** | **A** | **A** | **A** | **A** | **G** | **C** | **C** | **A** | **A** | **A** | **A** | **T** | **C** | **A** | **T** | **T** | **A** | **T** | **C** | **G** | **T** | **C** | **A** | **C** | **A** | **A** | **G** | **T** | **T** | **T** | **T** | **A** | **T** | **T** | **G** | **A** | **T** | **G** | **A** | **T** | **G** | **A** | **A** | **A** | **A** | **A** | **G** | **T** | **T** | **T** | **A** | **T** | **C** | **C** | **T** | **G** | **C** | **A** | **A** | **T** | **T** | **G** | **A** | **A** | **G** | **C** | **A** | **G** | **G** | **A** | **G** | **C** | **A** | **G** | **C** | **A** | **G** | **G** | **A** | **T** | **A** | **T** | **T** | **T** | **A** | **T** | **T** | **G** | **A** | **A** | **G** | **A** | **C** | **G** | **T** | **C** | **T** | **A** | **C** | **C** | **G** | **C** | **A** | **C** | **A** | **T** | **G** | **A** | **A** | **A** | **T** | **A** | **G** | **C** | **T** | **G** | **A** | **T** | **G** | **C** | **T** | **A** | **T** | **A** | **A** | **G** | **G** | **G** | **C** | **T** | **A** | **C** | **G** | **C** | **A** | **A** | **C** | **G** | **C** | **G** | **G** | **C** | **G** | **A** | **G** | **C** | **G** | **A** | **G** | **T** | **A** | **C** | **T** | **A** | **G** | **A** | **A** | **C** | **C** | **A** | **G** | **A** | **A** | **G** | **T** | **G** | **A** | **C** | **A** | **A** | **C** | **A** | **A** | **A** | **A** | **A** | **T** | **G** | **A** | **T** | **G** | **G** | **A** | **A** | **A** | **A** | **A** | **A** | **T** | **G** | **A** | **G** | **T** | **C** | **G** | **G** | **C** | **G** | **A** | **A** | **A** | **T** | **G** | **A** | **T** | **C** | **C** | **A** | **G** | **T** | **A** | **T** | **T** | **G** | **C** | **A** | **T** | **G** | **A** | **A** | **G** | **A** | **A** | **T** | **T** | **A** | **A** | **C** | **G** | **A** | **A** | **T** | **C** | **G** | **A** | **G** | **A** | **A** | **A** | **A** | **T** | **G** | **A** | **A** | **A** | **T** | **T** | **T** | **T** | **G** | **A** | **T** | **G** | **C** | **T** | **G** | **A** | **T** | **T** | **T** | **C** | **T** | **G** | **A** | **A** | **G** | **G** | **G** | **A** | **A** | **A** | **A** | **G** | **T** | **A** | **A** | **T** | **C** | **A** | **G** | **G** | **A** | **A** | **A** | **T** | **C** | **G** | **C** | **G** | **G** | **A** | **T** | **G** | **A** | **A** | **T** | **T** | **G** | **T** | **T** | **T** | **A** | **T** | **C** | **A** | **C** | **A** | **C** | **T** | **G** | **A** | **A** | **A** | **A** | **C** | **A** | **G** | **T** | **T** | **A** | **A** | **A** | **A** | **C** | **G** | **C** | **A** | **T** | **G** | **T** | **G** | **T** | **C** | **G** | **A** | **A** | **T** | **A** | **T** | **A** | **T** | **T** | **A** | **G** | **C** | **T** | **A** | **A** | **G** | **T** | **T** | **A** | **G** | **A** | **G** | **G** | **T** | **A** | **G** | **A** | **A** | **G** | **A** | **C** | **C** | **G** | **A** | **A** | **C** | **T** | **C** | **A** | **A** | **G** | **C** | **A** | **G** | **C** | **T** | **A** | **T** | **C** | **T** | **A** | **T** | **G** | **C** | **G** | **T** | **T** | **T** | **A** | **A** | **A** | **C** | **A** | **T** | **G** | **G** | **T** | **T** | **T** | **G** | **G** | **T** | **G** | **A** | **A** | **A** | **-** | **-** | **-** |
| **N53702** | **A** | **T** | **G** | **A** | **T** | **A** | **A** | **A** | **A** | **G** | **T** | **T** | **T** | **T** | **A** | **T** | **T** | **A** | **G** | **T** | **A** | **G** | **A** | **T** | **G** | **A** | **C** | **C** | **A** | **T** | **G** | **A** | **A** | **A** | **T** | **G** | **G** | **T** | **A** | **C** | **G** | **C** | **T** | **T** | **A** | **G** | **G** | **C** | **G** | **T** | **C** | **T** | **C** | **T** | **T** | **C** | **T** | **T** | **A** | **T** | **T** | **T** | **A** | **T** | **C** | **T** | **A** | **T** | **C** | **C** | **A** | **A** | **G** | **A** | **A** | **G** | **A** | **C** | **A** | **T** | **T** | **G** | **A** | **A** | **G** | **T** | **G** | **A** | **T** | **T** | **G** | **G** | **A** | **G** | **A** | **A** | **G** | **C** | **T** | **G** | **A** | **A** | **A** | **A** | **T** | **G** | **G** | **A** | **C** | **G** | **T** | **C** | **A** | **A** | **G** | **G** | **C** | **T** | **A** | **T** | **G** | **A** | **A** | **A** | **A** | **G** | **G** | **C** | **G** | **A** | **T** | **G** | **G** | **C** | **A** | **C** | **T** | **T** | **C** | **G** | **A** | **C** | **C** | **G** | **G** | **A** | **T** | **G** | **T** | **C** | **A** | **T** | **T** | **T** | **T** | **A** | **A** | **T** | **G** | **G** | **A** | **T** | **C** | **T** | **A** | **G** | **T** | **C** | **A** | **T** | **G** | **G** | **A** | **A** | **G** | **A** | **A** | **A** | **T** | **G** | **G** | **A** | **T** | **G** | **G** | **C** | **A** | **T** | **C** | **G** | **A** | **A** | **T** | **C** | **A** | **A** | **C** | **A** | **A** | **A** | **A** | **G** | **C** | **A** | **A** | **T** | **C** | **T** | **T** | **A** | **A** | **A** | **A** | **G** | **A** | **T** | **T** | **G** | **G** | **C** | **C** | **A** | **A** | **A** | **A** | **G** | **C** | **C** | **A** | **A** | **A** | **A** | **T** | **C** | **A** | **T** | **T** | **A** | **T** | **C** | **G** | **T** | **C** | **A** | **C** | **A** | **A** | **G** | **T** | **T** | **T** | **T** | **A** | **T** | **T** | **G** | **A** | **T** | **G** | **A** | **T** | **G** | **A** | **A** | **A** | **A** | **A** | **G** | **T** | **T** | **T** | **A** | **T** | **C** | **C** | **T** | **G** | **C** | **A** | **A** | **T** | **T** | **G** | **A** | **A** | **G** | **C** | **A** | **G** | **G** | **A** | **G** | **C** | **A** | **G** | **C** | **A** | **G** | **G** | **A** | **T** | **A** | **T** | **T** | **T** | **G** | **T** | **T** | **G** | **A** | **A** | **G** | **A** | **C** | **G** | **T** | **C** | **T** | **A** | **C** | **C** | **G** | **C** | **A** | **C** | **A** | **T** | **G** | **A** | **A** | **A** | **T** | **A** | **G** | **C** | **T** | **G** | **A** | **T** | **G** | **C** | **T** | **A** | **T** | **A** | **A** | **G** | **G** | **G** | **C** | **T** | **A** | **C** | **G** | **C** | **A** | **A** | **C** | **G** | **C** | **G** | **G** | **C** | **G** | **A** | **G** | **C** | **G** | **A** | **G** | **T** | **A** | **T** | **T** | **G** | **G** | **A** | **A** | **C** | **C** | **T** | **G** | **A** | **A** | **G** | **T** | **G** | **A** | **C** | **A** | **A** | **C** | **A** | **A** | **A** | **A** | **A** | **T** | **G** | **A** | **T** | **G** | **G** | **A** | **A** | **A** | **A** | **A** | **A** | **T** | **G** | **A** | **G** | **T** | **C** | **G** | **G** | **C** | **G** | **A** | **A** | **A** | **T** | **G** | **A** | **T** | **C** | **C** | **A** | **G** | **T** | **A** | **T** | **T** | **G** | **C** | **A** | **T** | **G** | **A** | **A** | **G** | **A** | **A** | **T** | **T** | **A** | **A** | **C** | **G** | **A** | **A** | **T** | **C** | **G** | **A** | **G** | **A** | **A** | **A** | **A** | **T** | **G** | **A** | **A** | **A** | **T** | **T** | **T** | **T** | **G** | **A** | **T** | **G** | **C** | **T** | **G** | **A** | **T** | **T** | **T** | **C** | **T** | **G** | **A** | **A** | **G** | **G** | **G** | **A** | **A** | **A** | **A** | **G** | **T** | **A** | **A** | **T** | **C** | **A** | **G** | **G** | **A** | **A** | **A** | **T** | **C** | **G** | **C** | **G** | **G** | **A** | **T** | **G** | **A** | **A** | **T** | **T** | **G** | **T** | **T** | **T** | **A** | **T** | **C** | **A** | **C** | **A** | **C** | **T** | **G** | **A** | **A** | **A** | **A** | **C** | **A** | **G** | **T** | **C** | **A** | **A** | **A** | **A** | **C** | **G** | **C** | **A** | **T** | **G** | **T** | **G** | **T** | **C** | **G** | **A** | **A** | **T** | **A** | **T** | **A** | **T** | **T** | **A** | **G** | **C** | **T** | **A** | **A** | **G** | **T** | **T** | **A** | **G** | **A** | **G** | **G** | **T** | **A** | **G** | **A** | **A** | **G** | **A** | **C** | **C** | **G** | **A** | **A** | **C** | **T** | **C** | **A** | **A** | **G** | **C** | **A** | **G** | **C** | **T** | **A** | **T** | **C** | **T** | **A** | **T** | **G** | **C** | **G** | **T** | **T** | **T** | **A** | **A** | **A** | **C** | **A** | **T** | **G** | **G** | **T** | **T** | **T** | **G** | **G** | **T** | **G** | **A** | **A** | **A** | **-** | **-** | **-** |
| **N53439** | **A** | **T** | **G** | **A** | **T** | **A** | **A** | **A** | **A** | **G** | **T** | **T** | **T** | **T** | **A** | **T** | **T** | **A** | **G** | **T** | **A** | **G** | **A** | **T** | **G** | **A** | **C** | **C** | **A** | **T** | **G** | **A** | **A** | **A** | **T** | **G** | **G** | **T** | **A** | **C** | **G** | **C** | **T** | **T** | **A** | **G** | **G** | **C** | **G** | **T** | **C** | **T** | **C** | **T** | **T** | **C** | **T** | **T** | **A** | **T** | **T** | **T** | **A** | **T** | **C** | **T** | **A** | **T** | **C** | **C** | **A** | **A** | **G** | **A** | **A** | **G** | **A** | **C** | **A** | **T** | **T** | **G** | **A** | **A** | **G** | **T** | **G** | **A** | **T** | **T** | **G** | **G** | **A** | **G** | **A** | **A** | **G** | **C** | **T** | **G** | **A** | **A** | **A** | **A** | **T** | **G** | **G** | **A** | **C** | **G** | **T** | **C** | **A** | **A** | **G** | **G** | **C** | **T** | **A** | **T** | **G** | **A** | **A** | **A** | **A** | **G** | **G** | **C** | **G** | **A** | **T** | **G** | **G** | **C** | **A** | **C** | **T** | **T** | **C** | **G** | **A** | **C** | **C** | **A** | **G** | **A** | **T** | **G** | **T** | **C** | **A** | **T** | **T** | **T** | **T** | **A** | **A** | **T** | **G** | **G** | **A** | **T** | **C** | **T** | **A** | **G** | **T** | **C** | **A** | **T** | **G** | **G** | **A** | **A** | **G** | **A** | **A** | **A** | **T** | **G** | **G** | **A** | **T** | **G** | **G** | **C** | **A** | **T** | **C** | **G** | **A** | **A** | **T** | **C** | **A** | **A** | **C** | **A** | **A** | **A** | **A** | **G** | **C** | **A** | **A** | **T** | **C** | **T** | **T** | **A** | **A** | **A** | **A** | **G** | **A** | **T** | **T** | **G** | **G** | **C** | **C** | **A** | **A** | **A** | **A** | **G** | **C** | **C** | **A** | **A** | **A** | **A** | **T** | **C** | **A** | **T** | **T** | **A** | **T** | **C** | **G** | **T** | **C** | **A** | **C** | **A** | **A** | **G** | **T** | **T** | **T** | **T** | **A** | **T** | **T** | **G** | **A** | **T** | **G** | **A** | **T** | **G** | **A** | **A** | **A** | **A** | **A** | **G** | **T** | **T** | **T** | **A** | **T** | **C** | **C** | **T** | **G** | **C** | **A** | **A** | **T** | **T** | **G** | **A** | **A** | **G** | **C** | **A** | **G** | **G** | **A** | **G** | **C** | **A** | **G** | **C** | **A** | **G** | **G** | **A** | **T** | **A** | **T** | **T** | **T** | **A** | **T** | **T** | **G** | **A** | **A** | **G** | **A** | **C** | **G** | **T** | **C** | **T** | **A** | **C** | **C** | **G** | **C** | **A** | **C** | **A** | **T** | **G** | **A** | **A** | **A** | **T** | **A** | **G** | **C** | **T** | **G** | **A** | **T** | **G** | **C** | **T** | **A** | **T** | **A** | **A** | **G** | **G** | **G** | **C** | **T** | **A** | **C** | **G** | **C** | **A** | **A** | **C** | **G** | **C** | **G** | **G** | **C** | **G** | **A** | **G** | **C** | **G** | **A** | **G** | **T** | **A** | **T** | **T** | **G** | **G** | **A** | **A** | **C** | **C** | **T** | **G** | **A** | **A** | **G** | **T** | **G** | **A** | **C** | **A** | **A** | **C** | **A** | **A** | **A** | **A** | **A** | **T** | **G** | **A** | **T** | **G** | **G** | **A** | **A** | **A** | **A** | **A** | **A** | **T** | **G** | **A** | **G** | **T** | **C** | **G** | **G** | **C** | **G** | **A** | **A** | **A** | **T** | **G** | **A** | **T** | **C** | **C** | **A** | **G** | **T** | **A** | **T** | **T** | **G** | **C** | **A** | **T** | **G** | **A** | **A** | **G** | **A** | **A** | **T** | **T** | **A** | **A** | **C** | **G** | **A** | **A** | **T** | **C** | **G** | **A** | **G** | **A** | **A** | **A** | **A** | **T** | **G** | **A** | **A** | **A** | **T** | **T** | **T** | **T** | **G** | **A** | **T** | **G** | **C** | **T** | **G** | **A** | **T** | **T** | **T** | **C** | **T** | **G** | **A** | **A** | **G** | **G** | **G** | **A** | **A** | **A** | **A** | **G** | **T** | **A** | **A** | **T** | **C** | **A** | **G** | **G** | **A** | **A** | **A** | **T** | **C** | **G** | **C** | **G** | **G** | **A** | **T** | **G** | **A** | **A** | **T** | **T** | **G** | **T** | **T** | **T** | **A** | **T** | **C** | **A** | **C** | **A** | **C** | **T** | **G** | **A** | **A** | **A** | **A** | **C** | **A** | **G** | **T** | **T** | **A** | **A** | **A** | **A** | **C** | **G** | **C** | **A** | **T** | **G** | **T** | **G** | **T** | **C** | **G** | **A** | **A** | **T** | **A** | **T** | **A** | **T** | **T** | **A** | **G** | **C** | **T** | **A** | **A** | **G** | **T** | **T** | **A** | **G** | **A** | **G** | **G** | **T** | **A** | **G** | **A** | **A** | **G** | **A** | **C** | **C** | **G** | **A** | **A** | **C** | **T** | **C** | **A** | **A** | **G** | **C** | **A** | **G** | **C** | **T** | **A** | **T** | **C** | **T** | **A** | **T** | **G** | **C** | **G** | **T** | **T** | **T** | **A** | **A** | **A** | **C** | **A** | **T** | **G** | **G** | **T** | **T** | **T** | **G** | **G** | **T** | **G** | **A** | **A** | **A** | **-** | **-** | **-** |
| **N59845F** | **G** | **T** | **G** | **A** | **T** | **C** | **A** | **A** | **A** | **G** | **T** | **A** | **A** | **T** | **G** | **T** | **T** | **A** | **G** | **T** | **G** | **G** | **A** | **T** | **G** | **A** | **C** | **C** | **A** | **T** | **G** | **A** | **A** | **A** | **T** | **G** | **G** | **T** | **C** | **C** | **G** | **T** | **T** | **T** | **A** | **G** | **G** | **C** | **G** | **T** | **T** | **T** | **C** | **A** | **T** | **C** | **A** | **T** | **A** | **T** | **T** | **T** | **A** | **T** | **C** | **T** | **A** | **T** | **T** | **C** | **A** | **A** | **G** | **A** | **G** | **G** | **A** | **T** | **A** | **T** | **A** | **G** | **A** | **A** | **G** | **T** | **C** | **G** | **T** | **A** | **G** | **G** | **C** | **G** | **A** | **A** | **G** | **C** | **A** | **G** | **A** | **A** | **A** | **A** | **T** | **G** | **G** | **T** | **A** | **A** | **G** | **A** | **T** | **T** | **G** | **G** | **C** | **T** | **A** | **T** | **G** | **A** | **A** | **A** | **A** | **A** | **G** | **C** | **A** | **T** | **T** | **G** | **G** | **A** | **A** | **C** | **T** | **A** | **C** | **G** | **T** | **C** | **C** | **A** | **G** | **A** | **T** | **G** | **T** | **T** | **A** | **T** | **T** | **T** | **T** | **G** | **A** | **T** | **G** | **G** | **A** | **T** | **T** | **T** | **G** | **G** | **T** | **A** | **A** | **T** | **G** | **G** | **A** | **A** | **G** | **A** | **A** | **A** | **T** | **G** | **G** | **A** | **C** | **G** | **G** | **C** | **A** | **T** | **T** | **G** | **A** | **T** | **T** | **C** | **A** | **A** | **C** | **A** | **A** | **A** | **A** | **G** | **C** | **G** | **A** | **T** | **C** | **T** | **T** | **G** | **A** | **A** | **A** | **G** | **A** | **T** | **T** | **G** | **G** | **C** | **C** | **A** | **G** | **A** | **A** | **G** | **C** | **C** | **A** | **A** | **G** | **A** | **T** | **T** | **A** | **T** | **T** | **A** | **T** | **T** | **G** | **T** | **G** | **A** | **C** | **G** | **A** | **G** | **T** | **T** | **T** | **T** | **A** | **T** | **T** | **G** | **A** | **T** | **G** | **A** | **T** | **G** | **A** | **A** | **A** | **A** | **A** | **G** | **T** | **G** | **T** | **A** | **T** | **C** | **C** | **G** | **G** | **C** | **G** | **A** | **T** | **T** | **G** | **A** | **A** | **G** | **C** | **T** | **G** | **G** | **T** | **G** | **C** | **A** | **G** | **C** | **G** | **G** | **G** | **C** | **T** | **A** | **C** | **C** | **T** | **A** | **T** | **T** | **A** | **A** | **A** | **A** | **A** | **C** | **A** | **T** | **C** | **A** | **A** | **C** | **A** | **G** | **C** | **A** | **C** | **A** | **T** | **G** | **A** | **G** | **A** | **T** | **T** | **G** | **C** | **T** | **G** | **A** | **T** | **G** | **C** | **A** | **A** | **T** | **T** | **C** | **G** | **G** | **G** | **C** | **G** | **A** | **C** | **T** | **T** | **A** | **T** | **C** | **G** | **C** | **G** | **G** | **A** | **G** | **A** | **G** | **C** | **G** | **T** | **G** | **T** | **G** | **T** | **T** | **G** | **G** | **A** | **A** | **C** | **C** | **T** | **G** | **A** | **A** | **G** | **T** | **G** | **A** | **C** | **A** | **C** | **A** | **T** | **A** | **A** | **G** | **A** | **T** | **G** | **A** | **T** | **G** | **G** | **A** | **A** | **C** | **G** | **G** | **T** | **T** | **A** | **A** | **C** | **A** | **A** | **A** | **A** | **A** | **A** | **A** | **C** | **A** | **A** | **G** | **A** | **G** | **C** | **C** | **G** | **G** | **T** | **G** | **T** | **T** | **G** | **C** | **A** | **C** | **G** | **A** | **A** | **G** | **A** | **T** | **T** | **T** | **G** | **A** | **C** | **A** | **A** | **A** | **C** | **C** | **G** | **G** | **G** | **A** | **A** | **C** | **A** | **C** | **G** | **A** | **A** | **A** | **T** | **T** | **T** | **T** | **A** | **A** | **T** | **G** | **T** | **T** | **G** | **A** | **T** | **T** | **G** | **C** | **A** | **C** | **A** | **A** | **G** | **G** | **T** | **A** | **A** | **A** | **A** | **G** | **T** | **A** | **A** | **T** | **C** | **A** | **G** | **G** | **A** | **A** | **A** | **T** | **A** | **G** | **C** | **T** | **G** | **A** | **T** | **G** | **A** | **A** | **C** | **T** | **C** | **T** | **T** | **T** | **A** | **T** | **C** | **A** | **C** | **T** | **T** | **T** | **G** | **A** | **A** | **A** | **A** | **C** | **A** | **G** | **T** | **T** | **A** | **A** | **A** | **A** | **C** | **A** | **C** | **A** | **T** | **G** | **T** | **T** | **T** | **C** | **A** | **A** | **A** | **C** | **A** | **T** | **T** | **T** | **T** | **A** | **G** | **C** | **A** | **A** | **A** | **A** | **C** | **T** | **A** | **G** | **A** | **T** | **G** | **T** | **G** | **G** | **A** | **T** | **G** | **A** | **T** | **C** | **G** | **G** | **A** | **C** | **C** | **C** | **A** | **A** | **G** | **C** | **G** | **G** | **C** | **G** | **A** | **T** | **T** | **T** | **A** | **T** | **G** | **C** | **T** | **T** | **T** | **T** | **C** | **A** | **A** | **C** | **A** | **T** | **G** | **G** | **T** | **T** | **T** | **A** | **G** | **C** | **C** | **A** | **A** | **A** | **-** | **-** | **-** |
| **N60417F** | **A** | **T** | **G** | **A** | **T** | **A** | **A** | **A** | **A** | **G** | **T** | **T** | **T** | **T** | **A** | **T** | **T** | **A** | **G** | **T** | **A** | **G** | **A** | **T** | **G** | **A** | **C** | **C** | **A** | **T** | **G** | **A** | **A** | **A** | **T** | **G** | **G** | **T** | **G** | **C** | **G** | **C** | **T** | **T** | **A** | **G** | **G** | **C** | **G** | **T** | **C** | **T** | **C** | **T** | **T** | **C** | **T** | **T** | **A** | **T** | **T** | **T** | **A** | **T** | **C** | **T** | **A** | **T** | **C** | **C** | **A** | **A** | **G** | **A** | **A** | **G** | **A** | **C** | **A** | **T** | **T** | **G** | **A** | **A** | **G** | **T** | **A** | **A** | **T** | **T** | **G** | **G** | **A** | **G** | **A** | **A** | **G** | **C** | **T** | **G** | **A** | **A** | **A** | **A** | **T** | **G** | **G** | **A** | **C** | **G** | **A** | **C** | **A** | **A** | **G** | **G** | **C** | **T** | **A** | **T** | **G** | **A** | **G** | **A** | **A** | **G** | **G** | **C** | **G** | **A** | **T** | **G** | **A** | **C** | **A** | **C** | **T** | **T** | **C** | **G** | **A** | **C** | **C** | **G** | **G** | **A** | **C** | **G** | **T** | **C** | **A** | **T** | **T** | **T** | **T** | **A** | **A** | **T** | **G** | **G** | **A** | **C** | **T** | **T** | **A** | **G** | **T** | **C** | **A** | **T** | **G** | **G** | **A** | **A** | **G** | **A** | **A** | **A** | **T** | **G** | **G** | **A** | **T** | **G** | **G** | **T** | **A** | **T** | **C** | **G** | **A** | **A** | **T** | **C** | **A** | **A** | **C** | **A** | **A** | **A** | **A** | **G** | **C** | **A** | **A** | **T** | **C** | **T** | **T** | **A** | **A** | **A** | **A** | **G** | **A** | **T** | **T** | **G** | **G** | **C** | **C** | **A** | **G** | **A** | **A** | **G** | **C** | **T** | **A** | **A** | **A** | **A** | **T** | **C** | **A** | **T** | **T** | **A** | **T** | **C** | **G** | **T** | **A** | **A** | **C** | **C** | **A** | **G** | **T** | **T** | **T** | **T** | **A** | **T** | **T** | **G** | **A** | **T** | **G** | **A** | **C** | **G** | **A** | **A** | **A** | **A** | **A** | **G** | **T** | **T** | **T** | **A** | **T** | **C** | **C** | **T** | **G** | **C** | **C** | **A** | **T** | **T** | **G** | **A** | **A** | **G** | **C** | **A** | **G** | **G** | **C** | **G** | **C** | **A** | **G** | **C** | **A** | **G** | **G** | **G** | **T** | **A** | **T** | **T** | **T** | **G** | **T** | **T** | **G** | **A** | **A** | **G** | **A** | **C** | **T** | **T** | **C** | **T** | **A** | **C** | **C** | **G** | **C** | **A** | **C** | **A** | **T** | **G** | **A** | **A** | **A** | **T** | **T** | **G** | **C** | **T** | **G** | **A** | **T** | **G** | **C** | **T** | **A** | **T** | **A** | **A** | **G** | **G** | **G** | **C** | **A** | **A** | **C** | **G** | **C** | **A** | **A** | **C** | **G** | **C** | **G** | **G** | **C** | **G** | **A** | **G** | **C** | **G** | **A** | **G** | **T** | **A** | **C** | **T** | **A** | **G** | **A** | **A** | **C** | **C** | **A** | **G** | **A** | **A** | **G** | **T** | **G** | **A** | **C** | **A** | **A** | **C** | **A** | **A** | **A** | **A** | **A** | **T** | **G** | **A** | **T** | **G** | **G** | **A** | **A** | **A** | **A** | **A** | **A** | **T** | **G** | **A** | **G** | **T** | **C** | **G** | **G** | **C** | **G** | **A** | **A** | **A** | **T** | **G** | **A** | **G** | **C** | **C** | **T** | **G** | **T** | **A** | **T** | **T** | **A** | **C** | **A** | **T** | **G** | **A** | **A** | **G** | **A** | **A** | **C** | **T** | **A** | **A** | **C** | **G** | **A** | **A** | **T** | **C** | **G** | **G** | **G** | **A** | **A** | **A** | **A** | **T** | **G** | **A** | **A** | **A** | **T** | **T** | **T** | **T** | **A** | **A** | **T** | **G** | **C** | **T** | **G** | **A** | **T** | **T** | **T** | **C** | **T** | **G** | **A** | **A** | **G** | **G** | **G** | **A** | **A** | **A** | **A** | **G** | **T** | **A** | **A** | **T** | **C** | **A** | **G** | **G** | **A** | **A** | **A** | **T** | **A** | **G** | **C** | **G** | **G** | **A** | **C** | **G** | **A** | **A** | **T** | **T** | **G** | **T** | **T** | **T** | **A** | **T** | **T** | **A** | **C** | **A** | **C** | **T** | **G** | **A** | **A** | **A** | **A** | **C** | **A** | **G** | **T** | **A** | **A** | **A** | **A** | **A** | **C** | **A** | **C** | **A** | **T** | **G** | **T** | **T** | **T** | **C** | **C** | **A** | **A** | **T** | **A** | **T** | **A** | **T** | **T** | **G** | **G** | **C** | **T** | **A** | **A** | **G** | **C** | **T** | **A** | **G** | **A** | **A** | **G** | **T** | **G** | **G** | **A** | **A** | **G** | **A** | **C** | **C** | **G** | **T** | **A** | **C** | **A** | **C** | **A** | **A** | **G** | **C** | **C** | **G** | **C** | **T** | **A** | **T** | **C** | **T** | **A** | **T** | **G** | **C** | **A** | **T** | **T** | **C** | **A** | **A** | **A** | **C** | **A** | **T** | **G** | **G** | **T** | **T** | **T** | **G** | **G** | **T** | **G** | **A** | **A** | **A** | **-** | **-** | **-** |
| **N54553** | **G** | **T** | **G** | **A** | **T** | **C** | **A** | **A** | **A** | **G** | **T** | **A** | **A** | **T** | **G** | **T** | **T** | **A** | **G** | **T** | **G** | **G** | **A** | **T** | **G** | **A** | **C** | **C** | **A** | **T** | **G** | **A** | **A** | **A** | **T** | **G** | **G** | **T** | **C** | **C** | **G** | **T** | **T** | **T** | **A** | **G** | **G** | **C** | **G** | **T** | **T** | **T** | **C** | **A** | **T** | **C** | **A** | **T** | **A** | **T** | **T** | **T** | **A** | **T** | **C** | **T** | **A** | **T** | **T** | **C** | **A** | **A** | **G** | **A** | **G** | **G** | **A** | **T** | **A** | **T** | **A** | **G** | **A** | **A** | **G** | **T** | **C** | **G** | **T** | **A** | **G** | **G** | **C** | **G** | **A** | **A** | **G** | **C** | **A** | **G** | **A** | **A** | **A** | **A** | **T** | **G** | **G** | **T** | **A** | **A** | **G** | **A** | **T** | **T** | **G** | **G** | **C** | **T** | **A** | **T** | **G** | **A** | **A** | **A** | **A** | **A** | **G** | **C** | **A** | **T** | **T** | **G** | **G** | **A** | **A** | **C** | **T** | **A** | **C** | **G** | **T** | **C** | **C** | **A** | **G** | **A** | **T** | **G** | **T** | **T** | **A** | **T** | **T** | **T** | **T** | **G** | **A** | **T** | **G** | **G** | **A** | **T** | **T** | **T** | **G** | **G** | **T** | **A** | **A** | **T** | **G** | **G** | **A** | **A** | **G** | **A** | **A** | **A** | **T** | **G** | **G** | **A** | **C** | **G** | **G** | **C** | **A** | **T** | **T** | **G** | **A** | **T** | **T** | **C** | **A** | **A** | **C** | **A** | **A** | **A** | **A** | **G** | **C** | **G** | **A** | **T** | **C** | **T** | **T** | **G** | **A** | **A** | **A** | **G** | **A** | **T** | **T** | **G** | **G** | **C** | **C** | **A** | **G** | **A** | **A** | **G** | **C** | **C** | **A** | **A** | **G** | **A** | **T** | **T** | **A** | **T** | **T** | **A** | **T** | **T** | **G** | **T** | **G** | **A** | **C** | **G** | **A** | **G** | **T** | **T** | **T** | **T** | **A** | **T** | **T** | **G** | **A** | **T** | **G** | **A** | **T** | **G** | **A** | **A** | **A** | **A** | **A** | **G** | **T** | **G** | **T** | **A** | **T** | **C** | **C** | **G** | **G** | **C** | **G** | **A** | **T** | **T** | **G** | **A** | **A** | **G** | **C** | **T** | **G** | **G** | **T** | **G** | **C** | **A** | **G** | **C** | **G** | **G** | **G** | **C** | **T** | **A** | **C** | **C** | **T** | **A** | **T** | **T** | **A** | **A** | **A** | **A** | **A** | **C** | **A** | **T** | **C** | **A** | **A** | **C** | **A** | **G** | **C** | **A** | **C** | **A** | **T** | **G** | **A** | **G** | **A** | **T** | **T** | **G** | **C** | **T** | **G** | **A** | **T** | **G** | **C** | **A** | **A** | **T** | **T** | **C** | **G** | **G** | **G** | **C** | **G** | **A** | **C** | **T** | **T** | **A** | **T** | **C** | **G** | **C** | **G** | **G** | **A** | **G** | **A** | **G** | **C** | **G** | **T** | **G** | **T** | **G** | **T** | **T** | **G** | **G** | **A** | **A** | **C** | **C** | **T** | **G** | **A** | **A** | **G** | **T** | **G** | **A** | **C** | **G** | **C** | **A** | **T** | **A** | **A** | **G** | **A** | **T** | **G** | **A** | **T** | **G** | **G** | **A** | **A** | **C** | **G** | **G** | **T** | **T** | **A** | **A** | **C** | **A** | **A** | **A** | **A** | **A** | **A** | **A** | **C** | **A** | **A** | **G** | **A** | **G** | **C** | **C** | **G** | **G** | **T** | **G** | **T** | **T** | **G** | **C** | **A** | **T** | **G** | **A** | **A** | **G** | **A** | **T** | **T** | **T** | **G** | **A** | **C** | **A** | **A** | **A** | **T** | **C** | **G** | **A** | **G** | **A** | **A** | **C** | **A** | **C** | **G** | **A** | **A** | **A** | **T** | **T** | **T** | **T** | **A** | **A** | **T** | **G** | **T** | **T** | **G** | **A** | **T** | **T** | **G** | **C** | **A** | **C** | **A** | **A** | **G** | **G** | **T** | **A** | **A** | **A** | **A** | **G** | **T** | **A** | **A** | **T** | **C** | **A** | **G** | **G** | **A** | **A** | **A** | **T** | **A** | **G** | **C** | **T** | **G** | **A** | **T** | **G** | **A** | **A** | **C** | **T** | **C** | **T** | **T** | **T** | **A** | **T** | **C** | **A** | **C** | **T** | **T** | **T** | **G** | **A** | **A** | **A** | **A** | **C** | **A** | **G** | **T** | **T** | **A** | **A** | **A** | **A** | **C** | **A** | **C** | **A** | **T** | **G** | **T** | **T** | **T** | **C** | **A** | **A** | **A** | **C** | **A** | **T** | **T** | **T** | **T** | **A** | **G** | **C** | **A** | **A** | **A** | **A** | **C** | **T** | **A** | **G** | **A** | **T** | **G** | **T** | **G** | **G** | **A** | **T** | **G** | **A** | **T** | **C** | **G** | **G** | **A** | **C** | **C** | **C** | **A** | **A** | **G** | **C** | **G** | **G** | **C** | **G** | **A** | **T** | **T** | **T** | **A** | **T** | **G** | **C** | **T** | **T** | **T** | **T** | **C** | **A** | **A** | **C** | **A** | **T** | **G** | **G** | **T** | **T** | **T** | **A** | **G** | **C** | **C** | **A** | **A** | **A** | **-** | **-** | **-** |
| **N53761** | **G** | **T** | **G** | **A** | **T** | **C** | **A** | **A** | **A** | **G** | **T** | **A** | **A** | **T** | **G** | **T** | **T** | **A** | **G** | **T** | **G** | **G** | **A** | **T** | **G** | **A** | **C** | **C** | **A** | **T** | **G** | **A** | **A** | **A** | **T** | **G** | **G** | **T** | **C** | **C** | **G** | **T** | **T** | **T** | **A** | **G** | **G** | **C** | **G** | **T** | **T** | **T** | **C** | **A** | **T** | **C** | **A** | **T** | **A** | **T** | **T** | **T** | **A** | **T** | **C** | **T** | **A** | **T** | **T** | **C** | **A** | **A** | **G** | **A** | **G** | **G** | **A** | **T** | **A** | **T** | **A** | **G** | **A** | **A** | **G** | **T** | **C** | **G** | **T** | **A** | **G** | **G** | **C** | **G** | **A** | **A** | **G** | **C** | **A** | **G** | **A** | **A** | **A** | **A** | **T** | **G** | **G** | **T** | **A** | **A** | **G** | **A** | **T** | **T** | **G** | **G** | **C** | **T** | **A** | **T** | **G** | **A** | **A** | **A** | **A** | **A** | **G** | **C** | **A** | **T** | **T** | **G** | **G** | **A** | **A** | **C** | **T** | **A** | **C** | **G** | **T** | **C** | **C** | **A** | **G** | **A** | **T** | **G** | **T** | **T** | **A** | **T** | **T** | **T** | **T** | **G** | **A** | **T** | **G** | **G** | **A** | **T** | **T** | **T** | **G** | **G** | **T** | **A** | **A** | **T** | **G** | **G** | **A** | **A** | **G** | **A** | **A** | **A** | **T** | **G** | **G** | **A** | **C** | **G** | **G** | **C** | **A** | **T** | **T** | **G** | **A** | **T** | **T** | **C** | **A** | **A** | **C** | **A** | **A** | **A** | **A** | **G** | **C** | **G** | **A** | **T** | **C** | **T** | **T** | **G** | **A** | **A** | **A** | **G** | **A** | **T** | **T** | **G** | **G** | **C** | **C** | **A** | **G** | **A** | **A** | **G** | **C** | **C** | **A** | **A** | **G** | **A** | **T** | **T** | **A** | **T** | **T** | **A** | **T** | **T** | **G** | **T** | **G** | **A** | **C** | **G** | **A** | **G** | **T** | **T** | **T** | **T** | **A** | **T** | **T** | **G** | **A** | **T** | **G** | **A** | **T** | **G** | **A** | **A** | **A** | **A** | **A** | **G** | **T** | **G** | **T** | **A** | **T** | **C** | **C** | **G** | **G** | **C** | **G** | **A** | **T** | **T** | **G** | **A** | **A** | **G** | **C** | **T** | **G** | **G** | **T** | **G** | **C** | **A** | **G** | **C** | **G** | **G** | **G** | **C** | **T** | **A** | **C** | **C** | **T** | **A** | **T** | **T** | **A** | **A** | **A** | **A** | **A** | **C** | **A** | **T** | **C** | **A** | **A** | **C** | **A** | **G** | **C** | **A** | **C** | **A** | **T** | **G** | **A** | **G** | **A** | **T** | **T** | **G** | **C** | **T** | **G** | **A** | **T** | **G** | **C** | **A** | **A** | **T** | **T** | **C** | **G** | **G** | **G** | **C** | **G** | **A** | **C** | **T** | **T** | **A** | **T** | **C** | **A** | **C** | **G** | **G** | **A** | **G** | **A** | **G** | **C** | **G** | **T** | **G** | **T** | **G** | **T** | **T** | **G** | **G** | **A** | **A** | **C** | **C** | **T** | **G** | **A** | **A** | **G** | **T** | **G** | **A** | **C** | **G** | **C** | **A** | **T** | **A** | **A** | **G** | **A** | **T** | **G** | **A** | **T** | **G** | **G** | **A** | **A** | **C** | **G** | **G** | **T** | **T** | **A** | **A** | **C** | **A** | **A** | **A** | **A** | **A** | **A** | **A** | **C** | **A** | **A** | **G** | **A** | **G** | **C** | **C** | **G** | **G** | **T** | **G** | **T** | **T** | **G** | **C** | **A** | **C** | **G** | **A** | **A** | **G** | **A** | **T** | **T** | **T** | **G** | **A** | **C** | **A** | **A** | **A** | **C** | **C** | **G** | **G** | **G** | **A** | **A** | **C** | **A** | **C** | **G** | **A** | **A** | **A** | **T** | **T** | **T** | **T** | **A** | **A** | **T** | **G** | **T** | **T** | **G** | **A** | **T** | **T** | **G** | **C** | **A** | **C** | **A** | **A** | **G** | **G** | **T** | **A** | **A** | **A** | **A** | **G** | **T** | **A** | **A** | **T** | **C** | **A** | **G** | **G** | **A** | **A** | **A** | **T** | **A** | **G** | **C** | **T** | **G** | **A** | **T** | **G** | **A** | **A** | **C** | **T** | **C** | **T** | **T** | **T** | **A** | **T** | **C** | **A** | **C** | **T** | **T** | **T** | **G** | **A** | **A** | **A** | **A** | **C** | **A** | **G** | **T** | **T** | **A** | **A** | **A** | **A** | **C** | **A** | **C** | **A** | **T** | **G** | **T** | **T** | **T** | **C** | **A** | **A** | **A** | **C** | **A** | **T** | **T** | **T** | **T** | **A** | **G** | **C** | **A** | **A** | **A** | **A** | **C** | **T** | **A** | **G** | **A** | **T** | **G** | **T** | **G** | **G** | **A** | **T** | **G** | **A** | **T** | **C** | **G** | **G** | **A** | **C** | **C** | **C** | **A** | **A** | **G** | **C** | **G** | **G** | **C** | **G** | **A** | **T** | **T** | **T** | **A** | **T** | **G** | **C** | **T** | **T** | **T** | **T** | **C** | **A** | **A** | **C** | **A** | **T** | **G** | **G** | **T** | **T** | **T** | **A** | **G** | **C** | **C** | **A** | **A** | **A** | **-** | **-** | **-** |
| **N59624F** | **A** | **T** | **G** | **A** | **T** | **A** | **A** | **A** | **A** | **G** | **T** | **T** | **T** | **T** | **A** | **T** | **T** | **A** | **G** | **T** | **A** | **G** | **A** | **T** | **G** | **A** | **C** | **C** | **A** | **T** | **G** | **A** | **A** | **A** | **T** | **G** | **G** | **T** | **G** | **C** | **G** | **C** | **T** | **T** | **A** | **G** | **G** | **C** | **G** | **T** | **C** | **T** | **C** | **T** | **T** | **C** | **T** | **T** | **A** | **T** | **T** | **T** | **A** | **T** | **C** | **T** | **A** | **T** | **T** | **C** | **A** | **A** | **G** | **A** | **A** | **G** | **A** | **C** | **A** | **T** | **T** | **G** | **A** | **A** | **G** | **T** | **A** | **A** | **T** | **T** | **G** | **G** | **A** | **G** | **A** | **A** | **G** | **C** | **T** | **G** | **A** | **A** | **A** | **A** | **T** | **G** | **G** | **A** | **C** | **G** | **A** | **C** | **A** | **A** | **G** | **G** | **C** | **T** | **A** | **T** | **G** | **A** | **G** | **A** | **A** | **G** | **G** | **C** | **G** | **A** | **T** | **G** | **A** | **C** | **A** | **C** | **T** | **T** | **C** | **G** | **A** | **C** | **C** | **G** | **G** | **A** | **C** | **G** | **T** | **C** | **A** | **T** | **T** | **T** | **T** | **A** | **A** | **T** | **G** | **G** | **A** | **C** | **T** | **T** | **A** | **G** | **T** | **C** | **A** | **T** | **G** | **G** | **A** | **A** | **G** | **A** | **A** | **A** | **T** | **G** | **G** | **A** | **T** | **G** | **G** | **T** | **A** | **T** | **C** | **G** | **A** | **A** | **T** | **C** | **A** | **A** | **C** | **A** | **A** | **A** | **A** | **G** | **C** | **A** | **A** | **T** | **C** | **T** | **T** | **A** | **A** | **A** | **A** | **G** | **A** | **T** | **T** | **G** | **G** | **C** | **C** | **A** | **G** | **A** | **A** | **G** | **C** | **T** | **A** | **A** | **A** | **A** | **T** | **C** | **A** | **T** | **T** | **A** | **T** | **C** | **G** | **T** | **A** | **A** | **C** | **C** | **A** | **G** | **T** | **T** | **T** | **T** | **A** | **T** | **T** | **G** | **A** | **T** | **G** | **A** | **C** | **G** | **A** | **A** | **A** | **A** | **A** | **G** | **T** | **T** | **T** | **A** | **T** | **C** | **C** | **T** | **G** | **C** | **C** | **A** | **T** | **T** | **G** | **A** | **A** | **G** | **C** | **A** | **G** | **G** | **T** | **G** | **C** | **A** | **G** | **C** | **A** | **G** | **G** | **G** | **T** | **A** | **T** | **T** | **T** | **G** | **T** | **T** | **G** | **A** | **A** | **G** | **A** | **C** | **C** | **T** | **C** | **T** | **A** | **C** | **C** | **G** | **C** | **A** | **C** | **A** | **T** | **G** | **A** | **A** | **A** | **T** | **T** | **G** | **C** | **T** | **G** | **A** | **T** | **G** | **C** | **T** | **A** | **T** | **A** | **A** | **G** | **G** | **G** | **C** | **A** | **A** | **C** | **G** | **C** | **A** | **A** | **C** | **G** | **T** | **G** | **G** | **C** | **G** | **A** | **G** | **C** | **G** | **A** | **G** | **T** | **A** | **T** | **T** | **A** | **G** | **A** | **A** | **C** | **C** | **A** | **G** | **A** | **A** | **G** | **T** | **G** | **A** | **C** | **A** | **A** | **C** | **A** | **A** | **A** | **A** | **A** | **T** | **G** | **A** | **T** | **G** | **G** | **A** | **A** | **A** | **A** | **A** | **A** | **T** | **G** | **A** | **G** | **T** | **C** | **G** | **G** | **C** | **G** | **A** | **A** | **A** | **T** | **G** | **A** | **G** | **C** | **C** | **T** | **G** | **T** | **A** | **T** | **T** | **A** | **C** | **A** | **T** | **G** | **A** | **A** | **G** | **A** | **A** | **C** | **T** | **A** | **A** | **C** | **G** | **A** | **A** | **T** | **C** | **G** | **G** | **G** | **A** | **A** | **A** | **A** | **T** | **G** | **A** | **A** | **A** | **T** | **T** | **T** | **T** | **A** | **A** | **T** | **G** | **C** | **T** | **G** | **A** | **T** | **T** | **T** | **C** | **T** | **G** | **A** | **A** | **G** | **G** | **A** | **A** | **A** | **A** | **A** | **G** | **T** | **A** | **A** | **T** | **C** | **A** | **G** | **G** | **A** | **A** | **A** | **T** | **A** | **G** | **C** | **G** | **G** | **A** | **C** | **G** | **A** | **A** | **T** | **T** | **G** | **T** | **T** | **T** | **A** | **T** | **T** | **A** | **C** | **A** | **C** | **T** | **G** | **A** | **A** | **A** | **A** | **C** | **A** | **G** | **T** | **A** | **A** | **A** | **A** | **A** | **C** | **A** | **C** | **A** | **T** | **G** | **T** | **T** | **T** | **C** | **C** | **A** | **A** | **T** | **A** | **T** | **A** | **T** | **T** | **G** | **G** | **C** | **T** | **A** | **A** | **G** | **C** | **T** | **A** | **G** | **A** | **A** | **G** | **T** | **G** | **G** | **A** | **A** | **G** | **A** | **C** | **C** | **G** | **T** | **A** | **C** | **A** | **C** | **A** | **A** | **G** | **C** | **C** | **G** | **C** | **T** | **A** | **T** | **C** | **T** | **A** | **T** | **G** | **C** | **A** | **T** | **T** | **C** | **A** | **A** | **A** | **C** | **A** | **T** | **G** | **G** | **T** | **T** | **T** | **G** | **G** | **T** | **G** | **A** | **A** | **A** | **-** | **-** | **-** |
| **N59584F** | **G** | **T** | **G** | **A** | **T** | **C** | **A** | **A** | **A** | **G** | **T** | **A** | **A** | **T** | **G** | **T** | **T** | **A** | **G** | **T** | **G** | **G** | **A** | **T** | **G** | **A** | **C** | **C** | **A** | **T** | **G** | **A** | **A** | **A** | **T** | **G** | **G** | **T** | **C** | **C** | **G** | **T** | **T** | **T** | **A** | **G** | **G** | **C** | **G** | **T** | **T** | **T** | **C** | **A** | **T** | **C** | **A** | **T** | **A** | **T** | **T** | **T** | **A** | **T** | **C** | **T** | **A** | **T** | **T** | **C** | **A** | **A** | **G** | **A** | **G** | **G** | **A** | **T** | **A** | **T** | **A** | **G** | **A** | **A** | **G** | **T** | **C** | **G** | **T** | **A** | **G** | **G** | **C** | **G** | **A** | **A** | **G** | **C** | **A** | **G** | **A** | **A** | **A** | **A** | **T** | **G** | **G** | **T** | **A** | **A** | **G** | **A** | **T** | **T** | **G** | **G** | **C** | **T** | **A** | **T** | **G** | **A** | **A** | **A** | **A** | **A** | **G** | **C** | **A** | **T** | **T** | **G** | **G** | **A** | **A** | **C** | **T** | **A** | **C** | **G** | **T** | **C** | **C** | **A** | **G** | **A** | **T** | **G** | **T** | **T** | **A** | **T** | **T** | **T** | **T** | **G** | **A** | **T** | **G** | **G** | **A** | **T** | **T** | **T** | **G** | **G** | **T** | **A** | **A** | **T** | **G** | **G** | **A** | **A** | **G** | **A** | **A** | **A** | **T** | **G** | **G** | **A** | **C** | **G** | **G** | **C** | **A** | **T** | **T** | **G** | **A** | **T** | **T** | **C** | **A** | **A** | **C** | **A** | **A** | **A** | **A** | **G** | **C** | **G** | **A** | **T** | **C** | **T** | **T** | **G** | **A** | **A** | **A** | **G** | **A** | **T** | **T** | **G** | **G** | **C** | **C** | **A** | **G** | **A** | **A** | **G** | **C** | **C** | **A** | **A** | **G** | **A** | **T** | **T** | **A** | **T** | **T** | **A** | **T** | **T** | **G** | **T** | **G** | **A** | **C** | **G** | **A** | **G** | **T** | **T** | **T** | **T** | **A** | **T** | **T** | **G** | **A** | **T** | **G** | **A** | **T** | **G** | **A** | **A** | **A** | **A** | **A** | **G** | **T** | **G** | **T** | **A** | **T** | **C** | **C** | **G** | **G** | **C** | **G** | **A** | **T** | **T** | **G** | **A** | **A** | **G** | **C** | **T** | **G** | **G** | **T** | **G** | **C** | **A** | **G** | **C** | **G** | **G** | **G** | **C** | **T** | **A** | **C** | **C** | **T** | **A** | **T** | **T** | **A** | **A** | **A** | **A** | **A** | **C** | **A** | **T** | **C** | **A** | **A** | **C** | **A** | **G** | **C** | **A** | **C** | **A** | **T** | **G** | **A** | **G** | **A** | **T** | **T** | **G** | **C** | **T** | **G** | **A** | **T** | **G** | **C** | **A** | **A** | **T** | **T** | **C** | **G** | **G** | **G** | **C** | **G** | **A** | **C** | **T** | **T** | **A** | **T** | **C** | **G** | **C** | **G** | **G** | **A** | **G** | **A** | **G** | **C** | **G** | **T** | **G** | **T** | **G** | **T** | **T** | **G** | **G** | **A** | **A** | **C** | **C** | **T** | **G** | **A** | **A** | **G** | **T** | **G** | **A** | **C** | **G** | **C** | **A** | **T** | **A** | **A** | **G** | **A** | **T** | **G** | **A** | **T** | **G** | **G** | **A** | **A** | **C** | **G** | **G** | **T** | **T** | **A** | **A** | **C** | **A** | **A** | **A** | **A** | **A** | **A** | **A** | **C** | **A** | **A** | **G** | **A** | **G** | **C** | **C** | **G** | **G** | **T** | **G** | **T** | **T** | **G** | **C** | **A** | **C** | **G** | **A** | **A** | **G** | **A** | **T** | **T** | **T** | **G** | **A** | **C** | **A** | **A** | **A** | **C** | **C** | **G** | **G** | **G** | **A** | **A** | **C** | **A** | **C** | **G** | **A** | **A** | **A** | **T** | **T** | **T** | **T** | **A** | **A** | **T** | **G** | **T** | **T** | **G** | **A** | **T** | **T** | **G** | **C** | **A** | **C** | **A** | **A** | **G** | **G** | **T** | **A** | **A** | **A** | **A** | **G** | **T** | **A** | **A** | **T** | **C** | **A** | **G** | **G** | **A** | **A** | **A** | **T** | **A** | **G** | **C** | **T** | **G** | **A** | **T** | **G** | **A** | **A** | **C** | **T** | **C** | **T** | **T** | **T** | **A** | **T** | **C** | **A** | **C** | **T** | **T** | **T** | **G** | **A** | **A** | **A** | **A** | **C** | **A** | **G** | **T** | **T** | **A** | **A** | **A** | **A** | **C** | **A** | **C** | **A** | **T** | **G** | **T** | **T** | **T** | **C** | **A** | **A** | **A** | **C** | **A** | **T** | **T** | **T** | **T** | **A** | **G** | **C** | **A** | **A** | **A** | **A** | **C** | **T** | **A** | **G** | **A** | **T** | **G** | **T** | **G** | **G** | **A** | **T** | **G** | **A** | **T** | **C** | **G** | **G** | **A** | **C** | **C** | **C** | **A** | **A** | **G** | **C** | **G** | **G** | **C** | **G** | **A** | **T** | **T** | **T** | **A** | **T** | **G** | **C** | **T** | **T** | **T** | **T** | **C** | **A** | **A** | **C** | **A** | **T** | **G** | **G** | **T** | **T** | **T** | **A** | **G** | **C** | **C** | **A** | **A** | **A** | **-** | **-** | **-** |
| **N59554F** | **G** | **T** | **G** | **A** | **T** | **C** | **A** | **A** | **A** | **G** | **T** | **A** | **A** | **T** | **G** | **T** | **T** | **A** | **G** | **T** | **G** | **G** | **A** | **T** | **G** | **A** | **C** | **C** | **A** | **T** | **G** | **A** | **A** | **A** | **T** | **G** | **G** | **T** | **C** | **C** | **G** | **T** | **T** | **T** | **A** | **G** | **G** | **C** | **G** | **T** | **T** | **T** | **C** | **A** | **T** | **C** | **A** | **T** | **A** | **T** | **T** | **T** | **A** | **T** | **C** | **T** | **A** | **T** | **T** | **C** | **A** | **A** | **G** | **A** | **G** | **G** | **A** | **T** | **A** | **T** | **A** | **G** | **A** | **A** | **G** | **T** | **C** | **G** | **T** | **A** | **G** | **G** | **C** | **G** | **A** | **A** | **G** | **C** | **A** | **G** | **A** | **A** | **A** | **A** | **T** | **G** | **G** | **T** | **A** | **A** | **G** | **A** | **T** | **T** | **G** | **G** | **C** | **T** | **A** | **T** | **G** | **A** | **A** | **A** | **A** | **A** | **G** | **C** | **A** | **T** | **T** | **G** | **G** | **A** | **A** | **C** | **T** | **A** | **C** | **G** | **T** | **C** | **C** | **A** | **G** | **A** | **T** | **G** | **T** | **T** | **A** | **T** | **T** | **T** | **T** | **G** | **A** | **T** | **G** | **G** | **A** | **T** | **T** | **T** | **G** | **G** | **T** | **A** | **A** | **T** | **G** | **G** | **A** | **A** | **G** | **A** | **A** | **A** | **T** | **G** | **G** | **A** | **C** | **G** | **G** | **C** | **A** | **T** | **T** | **G** | **A** | **T** | **T** | **C** | **A** | **A** | **C** | **A** | **A** | **A** | **A** | **G** | **C** | **G** | **A** | **T** | **C** | **T** | **T** | **G** | **A** | **A** | **A** | **G** | **A** | **T** | **T** | **G** | **G** | **C** | **C** | **A** | **G** | **A** | **A** | **G** | **C** | **C** | **A** | **A** | **G** | **A** | **T** | **T** | **A** | **T** | **T** | **A** | **T** | **T** | **G** | **T** | **G** | **A** | **C** | **G** | **A** | **G** | **T** | **T** | **T** | **T** | **A** | **T** | **T** | **G** | **A** | **T** | **G** | **A** | **T** | **G** | **A** | **A** | **A** | **A** | **A** | **G** | **T** | **G** | **T** | **A** | **T** | **C** | **C** | **G** | **G** | **C** | **G** | **A** | **T** | **T** | **G** | **A** | **A** | **G** | **C** | **T** | **G** | **G** | **T** | **G** | **C** | **A** | **G** | **C** | **G** | **G** | **G** | **C** | **T** | **A** | **C** | **C** | **T** | **A** | **T** | **T** | **A** | **A** | **A** | **A** | **A** | **C** | **A** | **T** | **C** | **A** | **A** | **C** | **A** | **G** | **C** | **A** | **C** | **A** | **T** | **G** | **A** | **G** | **A** | **T** | **T** | **G** | **C** | **T** | **G** | **A** | **T** | **G** | **C** | **A** | **A** | **T** | **T** | **C** | **G** | **G** | **G** | **C** | **G** | **A** | **C** | **T** | **T** | **A** | **T** | **C** | **G** | **C** | **G** | **G** | **A** | **G** | **A** | **G** | **C** | **G** | **T** | **G** | **T** | **G** | **T** | **T** | **G** | **G** | **A** | **A** | **C** | **C** | **T** | **G** | **A** | **A** | **G** | **T** | **G** | **A** | **C** | **G** | **C** | **A** | **T** | **A** | **A** | **G** | **A** | **T** | **G** | **A** | **T** | **G** | **G** | **A** | **A** | **C** | **G** | **G** | **T** | **T** | **A** | **A** | **C** | **A** | **A** | **A** | **A** | **A** | **A** | **A** | **C** | **A** | **A** | **G** | **A** | **G** | **C** | **C** | **G** | **G** | **T** | **G** | **T** | **T** | **G** | **C** | **A** | **C** | **G** | **A** | **A** | **G** | **A** | **T** | **T** | **T** | **G** | **A** | **C** | **A** | **A** | **A** | **C** | **C** | **G** | **G** | **G** | **A** | **A** | **C** | **A** | **C** | **G** | **A** | **A** | **A** | **T** | **T** | **T** | **T** | **A** | **A** | **T** | **G** | **T** | **T** | **G** | **A** | **T** | **T** | **G** | **C** | **A** | **C** | **A** | **A** | **G** | **G** | **T** | **A** | **A** | **A** | **A** | **G** | **T** | **A** | **A** | **T** | **C** | **A** | **G** | **G** | **A** | **A** | **A** | **T** | **A** | **G** | **C** | **T** | **G** | **A** | **T** | **G** | **A** | **A** | **C** | **T** | **C** | **T** | **T** | **T** | **A** | **T** | **C** | **A** | **C** | **T** | **T** | **T** | **G** | **A** | **A** | **A** | **A** | **C** | **A** | **G** | **T** | **T** | **A** | **A** | **A** | **A** | **C** | **A** | **C** | **A** | **T** | **G** | **T** | **T** | **T** | **C** | **A** | **A** | **A** | **C** | **A** | **T** | **T** | **T** | **T** | **A** | **G** | **C** | **A** | **A** | **A** | **A** | **C** | **T** | **A** | **G** | **A** | **T** | **G** | **T** | **G** | **G** | **A** | **T** | **G** | **A** | **T** | **C** | **G** | **G** | **A** | **C** | **C** | **C** | **A** | **A** | **G** | **C** | **G** | **G** | **C** | **G** | **A** | **T** | **T** | **T** | **A** | **T** | **G** | **C** | **T** | **T** | **T** | **T** | **C** | **A** | **A** | **C** | **A** | **T** | **G** | **G** | **T** | **T** | **T** | **A** | **G** | **C** | **C** | **A** | **A** | **A** | **-** | **-** | **-** |
| **N55106** | **G** | **T** | **G** | **A** | **T** | **C** | **A** | **A** | **A** | **G** | **T** | **A** | **A** | **T** | **G** | **T** | **T** | **A** | **G** | **T** | **G** | **G** | **A** | **T** | **G** | **A** | **C** | **C** | **A** | **T** | **G** | **A** | **A** | **A** | **T** | **G** | **G** | **T** | **C** | **C** | **G** | **T** | **T** | **T** | **A** | **G** | **G** | **C** | **G** | **T** | **T** | **T** | **C** | **A** | **T** | **C** | **A** | **T** | **A** | **T** | **T** | **T** | **A** | **T** | **C** | **T** | **A** | **T** | **T** | **C** | **A** | **A** | **G** | **A** | **G** | **G** | **A** | **T** | **A** | **T** | **A** | **G** | **A** | **A** | **G** | **T** | **C** | **G** | **T** | **A** | **G** | **G** | **C** | **G** | **A** | **A** | **G** | **C** | **A** | **G** | **A** | **A** | **A** | **A** | **C** | **G** | **G** | **T** | **A** | **A** | **G** | **A** | **T** | **T** | **G** | **G** | **C** | **T** | **A** | **T** | **G** | **A** | **A** | **A** | **A** | **A** | **G** | **C** | **A** | **T** | **T** | **G** | **G** | **A** | **A** | **C** | **T** | **A** | **C** | **G** | **T** | **C** | **C** | **A** | **G** | **A** | **T** | **G** | **T** | **T** | **A** | **T** | **T** | **T** | **T** | **G** | **A** | **T** | **G** | **G** | **A** | **T** | **T** | **T** | **G** | **G** | **T** | **A** | **A** | **T** | **G** | **G** | **A** | **A** | **G** | **A** | **A** | **A** | **T** | **G** | **G** | **A** | **C** | **G** | **G** | **C** | **A** | **T** | **T** | **G** | **A** | **T** | **T** | **C** | **A** | **A** | **C** | **C** | **A** | **A** | **A** | **G** | **C** | **G** | **A** | **T** | **C** | **T** | **T** | **G** | **A** | **A** | **A** | **G** | **A** | **T** | **T** | **G** | **G** | **C** | **C** | **A** | **G** | **A** | **A** | **G** | **C** | **C** | **A** | **A** | **G** | **A** | **T** | **T** | **A** | **T** | **T** | **A** | **T** | **T** | **G** | **T** | **G** | **A** | **C** | **G** | **A** | **G** | **T** | **T** | **T** | **T** | **A** | **T** | **T** | **G** | **A** | **T** | **G** | **A** | **T** | **G** | **A** | **A** | **A** | **A** | **A** | **G** | **T** | **G** | **T** | **A** | **T** | **C** | **C** | **G** | **G** | **C** | **G** | **A** | **T** | **T** | **G** | **A** | **A** | **G** | **C** | **T** | **G** | **G** | **T** | **G** | **C** | **A** | **G** | **C** | **G** | **G** | **G** | **C** | **T** | **A** | **C** | **C** | **T** | **A** | **T** | **T** | **A** | **A** | **A** | **A** | **A** | **C** | **A** | **T** | **C** | **A** | **A** | **C** | **A** | **G** | **C** | **A** | **C** | **A** | **T** | **G** | **A** | **G** | **A** | **T** | **T** | **G** | **C** | **T** | **G** | **A** | **T** | **G** | **C** | **A** | **A** | **T** | **T** | **C** | **G** | **G** | **G** | **C** | **G** | **A** | **C** | **T** | **T** | **A** | **T** | **C** | **G** | **C** | **G** | **G** | **A** | **G** | **A** | **G** | **C** | **G** | **T** | **G** | **T** | **G** | **T** | **T** | **G** | **G** | **A** | **A** | **C** | **C** | **T** | **G** | **A** | **A** | **G** | **T** | **G** | **A** | **C** | **G** | **C** | **A** | **T** | **A** | **A** | **G** | **A** | **T** | **G** | **A** | **T** | **G** | **G** | **A** | **A** | **C** | **G** | **G** | **T** | **T** | **A** | **A** | **C** | **A** | **A** | **A** | **A** | **A** | **A** | **A** | **C** | **A** | **A** | **G** | **A** | **G** | **C** | **C** | **G** | **G** | **T** | **G** | **T** | **T** | **G** | **C** | **A** | **C** | **G** | **A** | **A** | **G** | **A** | **T** | **T** | **T** | **G** | **A** | **C** | **A** | **A** | **A** | **C** | **C** | **G** | **G** | **G** | **A** | **A** | **C** | **A** | **C** | **G** | **A** | **A** | **A** | **T** | **T** | **T** | **T** | **A** | **A** | **T** | **G** | **T** | **T** | **G** | **A** | **T** | **T** | **G** | **C** | **A** | **C** | **A** | **A** | **G** | **G** | **T** | **A** | **A** | **A** | **A** | **G** | **T** | **A** | **A** | **T** | **C** | **A** | **G** | **G** | **A** | **A** | **A** | **T** | **A** | **G** | **C** | **T** | **G** | **A** | **T** | **G** | **A** | **A** | **C** | **T** | **C** | **T** | **T** | **T** | **A** | **T** | **C** | **A** | **C** | **T** | **T** | **T** | **G** | **A** | **A** | **A** | **A** | **C** | **A** | **G** | **T** | **T** | **A** | **A** | **A** | **A** | **C** | **A** | **C** | **A** | **T** | **G** | **T** | **T** | **T** | **C** | **A** | **A** | **A** | **C** | **A** | **T** | **T** | **T** | **T** | **A** | **G** | **C** | **A** | **A** | **A** | **A** | **C** | **T** | **A** | **G** | **A** | **T** | **G** | **T** | **G** | **G** | **A** | **T** | **G** | **A** | **T** | **C** | **G** | **G** | **A** | **C** | **C** | **C** | **A** | **A** | **G** | **C** | **G** | **G** | **C** | **G** | **A** | **T** | **T** | **T** | **A** | **T** | **G** | **C** | **T** | **T** | **T** | **T** | **C** | **A** | **A** | **C** | **A** | **T** | **G** | **G** | **T** | **T** | **T** | **A** | **G** | **C** | **C** | **A** | **A** | **A** | **-** | **-** | **-** |
| **N59943F** | **A** | **T** | **G** | **A** | **T** | **A** | **A** | **A** | **A** | **G** | **T** | **T** | **T** | **T** | **A** | **T** | **T** | **A** | **G** | **T** | **A** | **G** | **A** | **T** | **G** | **A** | **C** | **C** | **A** | **T** | **G** | **A** | **A** | **A** | **T** | **G** | **G** | **T** | **G** | **C** | **G** | **C** | **T** | **T** | **A** | **G** | **G** | **C** | **G** | **T** | **C** | **T** | **C** | **T** | **T** | **C** | **T** | **T** | **A** | **T** | **T** | **T** | **A** | **T** | **C** | **T** | **A** | **T** | **C** | **C** | **A** | **A** | **G** | **A** | **A** | **G** | **A** | **C** | **A** | **T** | **T** | **G** | **A** | **A** | **G** | **T** | **A** | **A** | **T** | **T** | **G** | **G** | **A** | **G** | **A** | **A** | **G** | **C** | **T** | **G** | **A** | **A** | **A** | **A** | **T** | **G** | **G** | **A** | **C** | **G** | **A** | **C** | **A** | **A** | **G** | **G** | **C** | **T** | **A** | **T** | **G** | **A** | **G** | **A** | **A** | **G** | **G** | **C** | **G** | **A** | **T** | **G** | **A** | **C** | **A** | **C** | **T** | **T** | **C** | **G** | **A** | **C** | **C** | **G** | **G** | **A** | **C** | **G** | **T** | **C** | **A** | **T** | **T** | **T** | **T** | **A** | **A** | **T** | **G** | **G** | **A** | **C** | **T** | **T** | **A** | **G** | **T** | **C** | **A** | **T** | **G** | **G** | **A** | **A** | **G** | **A** | **A** | **A** | **T** | **G** | **G** | **A** | **T** | **G** | **G** | **T** | **A** | **T** | **C** | **G** | **A** | **A** | **T** | **C** | **A** | **A** | **C** | **A** | **A** | **A** | **A** | **G** | **C** | **A** | **A** | **T** | **C** | **T** | **T** | **A** | **A** | **A** | **A** | **G** | **A** | **T** | **T** | **G** | **G** | **C** | **C** | **A** | **G** | **A** | **A** | **G** | **C** | **T** | **A** | **A** | **A** | **A** | **T** | **C** | **A** | **T** | **T** | **A** | **T** | **C** | **G** | **T** | **A** | **A** | **C** | **C** | **A** | **G** | **T** | **T** | **T** | **T** | **A** | **T** | **T** | **G** | **A** | **T** | **G** | **A** | **C** | **G** | **A** | **A** | **A** | **A** | **A** | **G** | **T** | **T** | **T** | **A** | **T** | **C** | **C** | **T** | **G** | **C** | **C** | **A** | **T** | **T** | **G** | **A** | **A** | **G** | **C** | **A** | **G** | **G** | **C** | **G** | **C** | **A** | **G** | **C** | **A** | **G** | **G** | **G** | **T** | **A** | **T** | **T** | **T** | **G** | **T** | **T** | **G** | **A** | **A** | **G** | **A** | **C** | **T** | **T** | **C** | **T** | **A** | **C** | **C** | **G** | **C** | **A** | **C** | **A** | **T** | **G** | **A** | **A** | **A** | **T** | **T** | **G** | **C** | **T** | **G** | **A** | **T** | **G** | **C** | **T** | **A** | **T** | **A** | **A** | **G** | **G** | **G** | **C** | **A** | **A** | **C** | **G** | **C** | **A** | **A** | **C** | **G** | **C** | **G** | **G** | **C** | **G** | **A** | **G** | **C** | **G** | **A** | **G** | **T** | **A** | **C** | **T** | **A** | **G** | **A** | **A** | **C** | **C** | **A** | **G** | **A** | **A** | **G** | **T** | **G** | **A** | **C** | **A** | **A** | **C** | **A** | **A** | **A** | **A** | **A** | **T** | **G** | **A** | **T** | **G** | **G** | **A** | **A** | **A** | **A** | **A** | **A** | **T** | **G** | **A** | **G** | **T** | **C** | **G** | **G** | **C** | **G** | **A** | **A** | **A** | **T** | **G** | **A** | **G** | **C** | **C** | **T** | **G** | **T** | **A** | **T** | **T** | **A** | **C** | **A** | **T** | **G** | **A** | **A** | **G** | **A** | **A** | **C** | **T** | **A** | **A** | **C** | **G** | **A** | **A** | **T** | **C** | **G** | **G** | **G** | **A** | **A** | **A** | **A** | **T** | **G** | **A** | **A** | **A** | **T** | **T** | **T** | **T** | **A** | **A** | **T** | **G** | **C** | **T** | **G** | **A** | **T** | **T** | **T** | **C** | **T** | **G** | **A** | **A** | **G** | **G** | **G** | **A** | **A** | **A** | **A** | **G** | **T** | **A** | **A** | **T** | **C** | **A** | **G** | **G** | **A** | **A** | **A** | **T** | **A** | **G** | **C** | **G** | **G** | **A** | **C** | **G** | **A** | **A** | **T** | **T** | **G** | **T** | **T** | **T** | **A** | **T** | **T** | **A** | **C** | **A** | **C** | **T** | **G** | **A** | **A** | **A** | **A** | **C** | **A** | **G** | **T** | **A** | **A** | **A** | **A** | **A** | **C** | **A** | **C** | **A** | **T** | **G** | **T** | **T** | **T** | **C** | **C** | **A** | **A** | **T** | **A** | **T** | **A** | **T** | **T** | **G** | **G** | **C** | **T** | **A** | **A** | **G** | **C** | **T** | **A** | **G** | **A** | **A** | **G** | **T** | **G** | **G** | **A** | **A** | **G** | **A** | **C** | **C** | **G** | **T** | **A** | **C** | **A** | **C** | **A** | **A** | **G** | **C** | **C** | **G** | **C** | **T** | **A** | **T** | **C** | **T** | **A** | **T** | **G** | **C** | **A** | **T** | **T** | **C** | **A** | **A** | **A** | **C** | **A** | **T** | **G** | **G** | **T** | **T** | **T** | **G** | **G** | **T** | **G** | **A** | **A** | **A** | **-** | **-** | **-** |
| **N55229** | **A** | **T** | **G** | **A** | **T** | **A** | **A** | **A** | **A** | **G** | **T** | **T** | **T** | **T** | **A** | **T** | **T** | **A** | **G** | **T** | **A** | **G** | **A** | **T** | **G** | **A** | **C** | **C** | **A** | **T** | **G** | **A** | **A** | **A** | **T** | **G** | **G** | **T** | **A** | **C** | **G** | **C** | **T** | **T** | **A** | **G** | **G** | **C** | **G** | **T** | **C** | **T** | **C** | **T** | **T** | **C** | **T** | **T** | **A** | **T** | **T** | **T** | **A** | **T** | **C** | **T** | **A** | **T** | **C** | **C** | **A** | **A** | **G** | **A** | **A** | **G** | **A** | **C** | **A** | **T** | **T** | **G** | **A** | **A** | **G** | **T** | **G** | **A** | **T** | **T** | **G** | **G** | **A** | **G** | **A** | **A** | **G** | **C** | **T** | **G** | **A** | **A** | **A** | **A** | **T** | **G** | **G** | **A** | **C** | **G** | **T** | **C** | **A** | **A** | **G** | **G** | **C** | **T** | **A** | **T** | **G** | **A** | **A** | **A** | **A** | **G** | **G** | **C** | **G** | **A** | **T** | **G** | **G** | **C** | **A** | **C** | **T** | **T** | **C** | **G** | **A** | **C** | **C** | **A** | **G** | **A** | **T** | **G** | **T** | **C** | **A** | **T** | **T** | **T** | **T** | **A** | **A** | **T** | **G** | **G** | **A** | **T** | **C** | **T** | **A** | **G** | **T** | **C** | **A** | **T** | **G** | **G** | **A** | **A** | **G** | **A** | **A** | **A** | **T** | **G** | **G** | **A** | **T** | **G** | **G** | **C** | **A** | **T** | **C** | **G** | **A** | **A** | **T** | **C** | **A** | **A** | **C** | **A** | **A** | **A** | **A** | **G** | **C** | **A** | **A** | **T** | **C** | **T** | **T** | **A** | **A** | **A** | **A** | **G** | **A** | **T** | **T** | **G** | **G** | **C** | **C** | **A** | **A** | **A** | **A** | **G** | **C** | **C** | **A** | **A** | **A** | **A** | **T** | **C** | **A** | **T** | **T** | **A** | **T** | **C** | **G** | **T** | **C** | **A** | **C** | **A** | **A** | **G** | **T** | **T** | **T** | **T** | **A** | **T** | **T** | **G** | **A** | **T** | **G** | **A** | **T** | **G** | **A** | **A** | **A** | **A** | **A** | **G** | **T** | **T** | **T** | **A** | **T** | **C** | **C** | **T** | **G** | **C** | **A** | **A** | **T** | **T** | **G** | **A** | **A** | **G** | **C** | **A** | **G** | **G** | **A** | **G** | **C** | **A** | **G** | **C** | **A** | **G** | **G** | **A** | **T** | **A** | **T** | **T** | **T** | **A** | **T** | **T** | **G** | **A** | **A** | **G** | **A** | **C** | **G** | **T** | **C** | **T** | **A** | **C** | **C** | **G** | **C** | **A** | **C** | **A** | **T** | **G** | **A** | **A** | **A** | **T** | **A** | **G** | **C** | **T** | **G** | **A** | **T** | **G** | **C** | **T** | **A** | **T** | **A** | **A** | **G** | **G** | **G** | **C** | **T** | **A** | **C** | **G** | **C** | **A** | **A** | **C** | **G** | **C** | **G** | **G** | **C** | **G** | **A** | **G** | **C** | **G** | **A** | **G** | **T** | **A** | **T** | **T** | **G** | **G** | **A** | **A** | **C** | **C** | **T** | **G** | **A** | **A** | **G** | **T** | **G** | **A** | **C** | **A** | **A** | **C** | **A** | **A** | **A** | **A** | **A** | **T** | **G** | **A** | **T** | **G** | **G** | **A** | **A** | **A** | **A** | **A** | **A** | **T** | **G** | **A** | **G** | **T** | **C** | **G** | **G** | **C** | **G** | **A** | **A** | **A** | **T** | **G** | **A** | **T** | **C** | **C** | **A** | **G** | **T** | **A** | **T** | **T** | **G** | **C** | **A** | **T** | **G** | **A** | **A** | **G** | **A** | **A** | **T** | **T** | **A** | **A** | **C** | **G** | **A** | **A** | **T** | **C** | **G** | **A** | **G** | **A** | **A** | **A** | **A** | **T** | **G** | **A** | **A** | **A** | **T** | **T** | **T** | **T** | **G** | **A** | **T** | **G** | **C** | **T** | **G** | **A** | **T** | **T** | **T** | **C** | **T** | **G** | **A** | **A** | **G** | **G** | **G** | **A** | **A** | **A** | **A** | **G** | **T** | **A** | **A** | **T** | **C** | **A** | **G** | **G** | **A** | **A** | **A** | **T** | **C** | **G** | **C** | **G** | **G** | **A** | **T** | **G** | **A** | **A** | **T** | **T** | **G** | **T** | **T** | **T** | **A** | **T** | **C** | **A** | **C** | **A** | **C** | **T** | **G** | **A** | **A** | **A** | **A** | **C** | **A** | **G** | **T** | **T** | **A** | **A** | **A** | **A** | **C** | **G** | **C** | **A** | **T** | **G** | **T** | **G** | **T** | **C** | **G** | **A** | **A** | **T** | **A** | **T** | **A** | **T** | **T** | **A** | **G** | **C** | **T** | **A** | **A** | **G** | **T** | **T** | **A** | **G** | **A** | **G** | **G** | **T** | **A** | **G** | **A** | **A** | **G** | **A** | **C** | **C** | **G** | **A** | **A** | **C** | **T** | **C** | **A** | **A** | **G** | **C** | **A** | **G** | **C** | **T** | **A** | **T** | **C** | **T** | **A** | **T** | **G** | **C** | **G** | **T** | **T** | **T** | **A** | **A** | **A** | **C** | **A** | **T** | **G** | **G** | **T** | **T** | **T** | **G** | **G** | **T** | **T** | **A** | **A** | **A** | **-** | **-** | **-** |
| **N53457** | **G** | **T** | **G** | **A** | **T** | **C** | **A** | **A** | **A** | **G** | **T** | **A** | **A** | **T** | **G** | **C** | **T** | **A** | **G** | **T** | **G** | **G** | **A** | **T** | **G** | **A** | **C** | **C** | **A** | **T** | **G** | **A** | **A** | **A** | **T** | **G** | **G** | **T** | **C** | **C** | **G** | **T** | **T** | **T** | **A** | **G** | **G** | **C** | **G** | **T** | **T** | **T** | **C** | **A** | **T** | **C** | **A** | **T** | **A** | **T** | **T** | **T** | **A** | **T** | **C** | **T** | **A** | **T** | **T** | **C** | **A** | **A** | **G** | **A** | **G** | **G** | **A** | **T** | **A** | **T** | **A** | **G** | **A** | **A** | **G** | **T** | **C** | **G** | **T** | **A** | **G** | **G** | **C** | **G** | **A** | **A** | **G** | **C** | **A** | **G** | **A** | **A** | **A** | **A** | **C** | **G** | **G** | **T** | **A** | **A** | **G** | **A** | **T** | **T** | **G** | **G** | **C** | **T** | **A** | **T** | **G** | **A** | **A** | **A** | **A** | **A** | **G** | **C** | **A** | **T** | **T** | **G** | **G** | **A** | **A** | **C** | **T** | **A** | **C** | **G** | **T** | **C** | **C** | **A** | **G** | **A** | **T** | **G** | **T** | **T** | **A** | **T** | **T** | **T** | **T** | **G** | **A** | **T** | **G** | **G** | **A** | **T** | **T** | **T** | **G** | **G** | **T** | **A** | **A** | **T** | **G** | **G** | **A** | **A** | **G** | **A** | **A** | **A** | **T** | **G** | **G** | **A** | **C** | **G** | **G** | **C** | **A** | **T** | **T** | **G** | **A** | **T** | **T** | **C** | **A** | **A** | **C** | **A** | **A** | **A** | **A** | **G** | **C** | **G** | **A** | **T** | **C** | **T** | **T** | **G** | **A** | **A** | **A** | **G** | **A** | **T** | **T** | **G** | **G** | **C** | **C** | **A** | **G** | **A** | **A** | **G** | **C** | **C** | **A** | **A** | **G** | **A** | **T** | **T** | **A** | **T** | **T** | **A** | **T** | **T** | **G** | **T** | **G** | **A** | **C** | **G** | **A** | **G** | **T** | **T** | **T** | **T** | **A** | **T** | **T** | **G** | **A** | **T** | **G** | **A** | **T** | **G** | **A** | **A** | **A** | **A** | **A** | **G** | **T** | **G** | **T** | **A** | **T** | **C** | **C** | **G** | **G** | **C** | **G** | **A** | **T** | **T** | **G** | **A** | **A** | **G** | **C** | **T** | **G** | **G** | **T** | **G** | **C** | **A** | **G** | **C** | **A** | **G** | **G** | **C** | **T** | **A** | **C** | **C** | **T** | **A** | **T** | **T** | **A** | **A** | **A** | **A** | **A** | **C** | **A** | **T** | **C** | **A** | **A** | **C** | **A** | **G** | **C** | **T** | **C** | **A** | **T** | **G** | **A** | **G** | **A** | **T** | **T** | **G** | **C** | **T** | **G** | **A** | **T** | **G** | **C** | **A** | **A** | **T** | **T** | **C** | **G** | **G** | **A** | **C** | **G** | **A** | **C** | **T** | **T** | **A** | **T** | **C** | **G** | **C** | **G** | **G** | **A** | **G** | **A** | **G** | **C** | **G** | **T** | **G** | **T** | **G** | **T** | **T** | **G** | **G** | **A** | **A** | **C** | **C** | **T** | **G** | **A** | **A** | **G** | **T** | **G** | **A** | **C** | **G** | **C** | **A** | **T** | **A** | **A** | **G** | **A** | **T** | **G** | **A** | **T** | **G** | **G** | **A** | **A** | **C** | **G** | **G** | **T** | **T** | **A** | **A** | **C** | **A** | **A** | **A** | **A** | **A** | **A** | **A** | **C** | **A** | **A** | **G** | **A** | **G** | **C** | **C** | **G** | **G** | **T** | **G** | **T** | **T** | **G** | **C** | **A** | **C** | **G** | **A** | **A** | **G** | **A** | **T** | **T** | **T** | **G** | **A** | **C** | **A** | **A** | **A** | **C** | **C** | **G** | **A** | **G** | **A** | **A** | **C** | **A** | **C** | **G** | **A** | **A** | **A** | **T** | **T** | **T** | **T** | **A** | **A** | **T** | **G** | **T** | **T** | **G** | **A** | **T** | **T** | **G** | **C** | **A** | **C** | **A** | **A** | **G** | **G** | **T** | **A** | **A** | **A** | **A** | **G** | **T** | **A** | **A** | **T** | **C** | **A** | **G** | **G** | **A** | **A** | **A** | **T** | **A** | **G** | **C** | **T** | **G** | **A** | **T** | **G** | **A** | **A** | **C** | **T** | **C** | **T** | **T** | **T** | **A** | **T** | **C** | **A** | **C** | **T** | **T** | **T** | **G** | **A** | **A** | **A** | **A** | **C** | **A** | **G** | **T** | **T** | **A** | **A** | **A** | **A** | **C** | **A** | **C** | **A** | **T** | **G** | **T** | **T** | **T** | **C** | **A** | **A** | **A** | **C** | **A** | **T** | **T** | **T** | **T** | **A** | **G** | **C** | **A** | **A** | **A** | **A** | **C** | **T** | **A** | **G** | **A** | **T** | **G** | **T** | **G** | **G** | **A** | **T** | **G** | **A** | **T** | **C** | **G** | **G** | **A** | **C** | **C** | **C** | **A** | **A** | **G** | **C** | **G** | **G** | **C** | **G** | **A** | **T** | **T** | **T** | **A** | **T** | **G** | **C** | **T** | **T** | **T** | **T** | **C** | **A** | **A** | **C** | **A** | **T** | **G** | **G** | **T** | **T** | **T** | **A** | **G** | **C** | **C** | **A** | **A** | **A** | **-** | **-** | **-** |
| **N52729** | **G** | **T** | **G** | **A** | **T** | **C** | **A** | **A** | **A** | **G** | **T** | **A** | **A** | **T** | **G** | **T** | **T** | **A** | **G** | **T** | **G** | **G** | **A** | **T** | **G** | **A** | **C** | **C** | **A** | **T** | **G** | **A** | **A** | **A** | **T** | **G** | **G** | **T** | **C** | **C** | **G** | **T** | **T** | **T** | **A** | **G** | **G** | **C** | **G** | **T** | **T** | **T** | **C** | **A** | **T** | **C** | **A** | **T** | **A** | **T** | **T** | **T** | **A** | **T** | **C** | **T** | **A** | **T** | **T** | **C** | **A** | **A** | **G** | **A** | **G** | **G** | **A** | **T** | **A** | **T** | **A** | **G** | **A** | **A** | **G** | **T** | **C** | **G** | **T** | **A** | **G** | **G** | **C** | **G** | **A** | **A** | **G** | **C** | **A** | **G** | **A** | **A** | **A** | **A** | **T** | **G** | **G** | **T** | **A** | **A** | **G** | **A** | **T** | **T** | **G** | **G** | **C** | **T** | **A** | **T** | **G** | **A** | **A** | **A** | **A** | **A** | **G** | **C** | **A** | **T** | **T** | **G** | **G** | **A** | **A** | **C** | **T** | **A** | **C** | **G** | **T** | **C** | **C** | **A** | **G** | **A** | **T** | **G** | **T** | **T** | **A** | **T** | **T** | **T** | **T** | **G** | **A** | **T** | **G** | **G** | **A** | **T** | **T** | **T** | **G** | **G** | **T** | **A** | **A** | **T** | **G** | **G** | **A** | **A** | **G** | **A** | **A** | **A** | **T** | **G** | **G** | **A** | **C** | **G** | **G** | **C** | **A** | **T** | **T** | **G** | **A** | **T** | **T** | **C** | **A** | **A** | **C** | **A** | **A** | **A** | **A** | **G** | **C** | **G** | **A** | **T** | **C** | **T** | **T** | **G** | **A** | **A** | **A** | **G** | **A** | **T** | **T** | **G** | **G** | **C** | **C** | **A** | **G** | **A** | **A** | **G** | **C** | **C** | **A** | **A** | **G** | **A** | **T** | **T** | **A** | **T** | **T** | **A** | **T** | **T** | **G** | **T** | **G** | **A** | **C** | **G** | **A** | **G** | **T** | **T** | **T** | **T** | **A** | **T** | **T** | **G** | **A** | **T** | **G** | **A** | **T** | **G** | **A** | **A** | **A** | **A** | **A** | **G** | **T** | **G** | **T** | **A** | **T** | **C** | **C** | **G** | **G** | **C** | **G** | **A** | **T** | **T** | **G** | **A** | **A** | **G** | **C** | **T** | **G** | **G** | **T** | **G** | **C** | **A** | **G** | **C** | **G** | **G** | **G** | **C** | **T** | **A** | **C** | **C** | **T** | **A** | **T** | **T** | **A** | **A** | **A** | **A** | **A** | **C** | **A** | **T** | **C** | **A** | **A** | **C** | **A** | **G** | **C** | **A** | **C** | **A** | **T** | **G** | **A** | **G** | **A** | **T** | **T** | **G** | **C** | **T** | **G** | **A** | **T** | **G** | **C** | **A** | **A** | **T** | **T** | **C** | **G** | **G** | **G** | **C** | **G** | **A** | **C** | **T** | **T** | **A** | **T** | **C** | **G** | **C** | **G** | **G** | **A** | **G** | **A** | **G** | **C** | **G** | **T** | **G** | **T** | **G** | **T** | **T** | **G** | **G** | **A** | **A** | **C** | **C** | **T** | **G** | **A** | **A** | **G** | **T** | **G** | **A** | **C** | **G** | **C** | **A** | **T** | **A** | **A** | **G** | **A** | **T** | **G** | **A** | **T** | **G** | **G** | **A** | **A** | **C** | **G** | **G** | **T** | **T** | **A** | **A** | **C** | **A** | **A** | **A** | **A** | **A** | **A** | **A** | **C** | **A** | **A** | **G** | **A** | **G** | **C** | **C** | **G** | **G** | **T** | **G** | **T** | **T** | **G** | **C** | **A** | **T** | **G** | **A** | **A** | **G** | **A** | **T** | **T** | **T** | **G** | **A** | **C** | **A** | **A** | **A** | **T** | **C** | **G** | **A** | **G** | **A** | **A** | **C** | **A** | **C** | **G** | **A** | **A** | **A** | **T** | **T** | **T** | **T** | **A** | **A** | **T** | **G** | **T** | **T** | **G** | **A** | **T** | **T** | **G** | **C** | **A** | **C** | **A** | **A** | **G** | **G** | **T** | **A** | **A** | **A** | **A** | **G** | **T** | **A** | **A** | **T** | **C** | **A** | **G** | **G** | **A** | **A** | **A** | **T** | **A** | **G** | **C** | **T** | **G** | **A** | **T** | **G** | **A** | **A** | **C** | **T** | **C** | **T** | **T** | **T** | **A** | **T** | **C** | **A** | **C** | **T** | **T** | **T** | **G** | **A** | **A** | **A** | **A** | **C** | **A** | **G** | **T** | **T** | **A** | **A** | **A** | **A** | **C** | **A** | **C** | **A** | **T** | **G** | **T** | **T** | **T** | **C** | **A** | **A** | **A** | **C** | **A** | **T** | **T** | **T** | **T** | **A** | **G** | **C** | **A** | **A** | **A** | **A** | **C** | **T** | **A** | **G** | **A** | **T** | **G** | **T** | **G** | **G** | **A** | **T** | **G** | **A** | **T** | **C** | **G** | **G** | **A** | **C** | **C** | **C** | **A** | **A** | **G** | **C** | **G** | **G** | **C** | **G** | **A** | **T** | **T** | **T** | **A** | **T** | **G** | **C** | **T** | **T** | **T** | **T** | **C** | **A** | **A** | **C** | **A** | **T** | **G** | **G** | **T** | **T** | **T** | **A** | **G** | **C** | **C** | **A** | **A** | **A** | **-** | **-** | **-** |
| **N52636** | **A** | **T** | **G** | **A** | **T** | **A** | **A** | **A** | **A** | **G** | **T** | **T** | **T** | **T** | **A** | **T** | **T** | **A** | **G** | **T** | **A** | **G** | **A** | **T** | **G** | **A** | **C** | **C** | **A** | **T** | **G** | **A** | **A** | **A** | **T** | **G** | **G** | **T** | **A** | **C** | **G** | **C** | **T** | **T** | **A** | **G** | **G** | **C** | **G** | **T** | **C** | **T** | **C** | **T** | **T** | **C** | **T** | **T** | **A** | **T** | **T** | **T** | **A** | **T** | **C** | **T** | **A** | **T** | **C** | **C** | **A** | **A** | **G** | **A** | **A** | **G** | **A** | **C** | **A** | **T** | **T** | **G** | **A** | **A** | **G** | **T** | **G** | **A** | **T** | **T** | **G** | **G** | **A** | **G** | **A** | **A** | **G** | **C** | **T** | **G** | **A** | **A** | **A** | **A** | **T** | **G** | **G** | **A** | **C** | **G** | **T** | **C** | **A** | **A** | **G** | **G** | **C** | **T** | **A** | **T** | **G** | **A** | **A** | **A** | **A** | **G** | **G** | **C** | **G** | **A** | **T** | **G** | **G** | **C** | **A** | **C** | **T** | **T** | **C** | **G** | **A** | **C** | **C** | **A** | **G** | **A** | **T** | **G** | **T** | **C** | **A** | **T** | **T** | **T** | **T** | **A** | **A** | **T** | **G** | **G** | **A** | **T** | **C** | **T** | **A** | **G** | **T** | **C** | **A** | **T** | **G** | **G** | **A** | **A** | **G** | **A** | **A** | **A** | **T** | **G** | **G** | **A** | **T** | **G** | **G** | **C** | **A** | **T** | **C** | **G** | **A** | **A** | **T** | **C** | **A** | **A** | **C** | **A** | **A** | **A** | **A** | **G** | **C** | **A** | **A** | **T** | **C** | **T** | **T** | **A** | **A** | **A** | **A** | **G** | **A** | **T** | **T** | **G** | **G** | **C** | **C** | **A** | **A** | **A** | **A** | **G** | **C** | **C** | **A** | **A** | **A** | **A** | **T** | **C** | **A** | **T** | **T** | **A** | **T** | **C** | **G** | **T** | **C** | **A** | **C** | **A** | **A** | **G** | **T** | **T** | **T** | **T** | **A** | **T** | **T** | **G** | **A** | **T** | **G** | **A** | **T** | **G** | **A** | **A** | **A** | **A** | **A** | **G** | **T** | **T** | **T** | **A** | **T** | **C** | **C** | **T** | **G** | **C** | **A** | **A** | **T** | **T** | **G** | **A** | **A** | **G** | **C** | **A** | **G** | **G** | **A** | **G** | **C** | **A** | **G** | **C** | **A** | **G** | **G** | **A** | **T** | **A** | **T** | **T** | **T** | **G** | **T** | **T** | **G** | **A** | **A** | **G** | **A** | **C** | **G** | **T** | **C** | **T** | **A** | **C** | **C** | **G** | **C** | **A** | **C** | **A** | **T** | **G** | **A** | **A** | **A** | **T** | **A** | **G** | **C** | **T** | **G** | **A** | **T** | **G** | **C** | **T** | **A** | **T** | **A** | **A** | **G** | **G** | **G** | **C** | **T** | **A** | **C** | **G** | **C** | **A** | **A** | **C** | **G** | **C** | **G** | **G** | **C** | **G** | **A** | **G** | **C** | **G** | **A** | **G** | **T** | **A** | **T** | **T** | **G** | **G** | **A** | **A** | **C** | **C** | **T** | **G** | **A** | **A** | **G** | **T** | **G** | **A** | **C** | **A** | **A** | **C** | **A** | **A** | **A** | **A** | **A** | **T** | **G** | **A** | **T** | **G** | **G** | **A** | **A** | **A** | **A** | **A** | **A** | **T** | **G** | **A** | **G** | **T** | **C** | **G** | **G** | **C** | **G** | **A** | **A** | **A** | **T** | **G** | **A** | **T** | **C** | **C** | **A** | **G** | **T** | **A** | **T** | **T** | **G** | **C** | **A** | **T** | **G** | **A** | **A** | **G** | **A** | **A** | **T** | **T** | **A** | **A** | **C** | **G** | **A** | **A** | **T** | **C** | **G** | **A** | **G** | **A** | **A** | **A** | **A** | **T** | **G** | **A** | **A** | **A** | **T** | **T** | **T** | **T** | **G** | **A** | **T** | **G** | **C** | **T** | **G** | **A** | **T** | **T** | **T** | **C** | **T** | **G** | **A** | **A** | **G** | **G** | **G** | **A** | **A** | **A** | **A** | **G** | **T** | **A** | **A** | **T** | **C** | **A** | **G** | **G** | **A** | **A** | **A** | **T** | **C** | **G** | **C** | **G** | **G** | **A** | **T** | **G** | **A** | **A** | **T** | **T** | **G** | **T** | **T** | **T** | **A** | **T** | **C** | **A** | **C** | **A** | **C** | **T** | **G** | **A** | **A** | **A** | **A** | **C** | **A** | **G** | **T** | **T** | **A** | **A** | **A** | **A** | **C** | **G** | **C** | **A** | **T** | **G** | **T** | **G** | **T** | **C** | **G** | **A** | **A** | **T** | **A** | **T** | **A** | **T** | **T** | **A** | **G** | **C** | **T** | **A** | **A** | **G** | **T** | **T** | **A** | **G** | **A** | **G** | **G** | **T** | **A** | **G** | **A** | **A** | **G** | **A** | **C** | **C** | **G** | **A** | **A** | **C** | **T** | **C** | **A** | **A** | **G** | **C** | **A** | **G** | **C** | **T** | **A** | **T** | **C** | **T** | **A** | **T** | **G** | **C** | **G** | **T** | **T** | **T** | **A** | **A** | **A** | **C** | **A** | **T** | **G** | **G** | **T** | **T** | **T** | **G** | **G** | **T** | **G** | **A** | **A** | **A** | **-** | **-** | **-** |
| **N60463F** | **A** | **T** | **G** | **A** | **T** | **A** | **A** | **A** | **A** | **G** | **T** | **T** | **T** | **T** | **A** | **T** | **T** | **A** | **G** | **T** | **A** | **G** | **A** | **T** | **G** | **A** | **C** | **C** | **A** | **T** | **G** | **A** | **A** | **A** | **T** | **G** | **G** | **T** | **A** | **C** | **G** | **C** | **T** | **T** | **A** | **G** | **G** | **C** | **G** | **T** | **C** | **T** | **C** | **T** | **T** | **C** | **T** | **T** | **A** | **T** | **T** | **T** | **A** | **T** | **C** | **T** | **A** | **T** | **C** | **C** | **A** | **A** | **G** | **A** | **A** | **G** | **A** | **C** | **A** | **T** | **T** | **G** | **A** | **A** | **G** | **T** | **G** | **A** | **T** | **T** | **G** | **G** | **A** | **G** | **A** | **A** | **G** | **C** | **T** | **G** | **A** | **A** | **A** | **A** | **T** | **G** | **G** | **A** | **C** | **G** | **T** | **C** | **A** | **A** | **G** | **G** | **C** | **T** | **A** | **T** | **G** | **A** | **A** | **A** | **A** | **G** | **G** | **C** | **G** | **A** | **T** | **G** | **G** | **C** | **A** | **C** | **T** | **T** | **C** | **G** | **A** | **C** | **C** | **A** | **G** | **A** | **T** | **G** | **T** | **C** | **A** | **T** | **T** | **T** | **T** | **A** | **A** | **T** | **G** | **G** | **A** | **T** | **C** | **T** | **A** | **G** | **T** | **C** | **A** | **T** | **G** | **G** | **A** | **A** | **G** | **A** | **A** | **A** | **T** | **G** | **G** | **A** | **T** | **G** | **G** | **C** | **A** | **T** | **C** | **G** | **A** | **A** | **T** | **C** | **A** | **A** | **C** | **A** | **A** | **A** | **A** | **G** | **C** | **A** | **A** | **T** | **C** | **T** | **T** | **A** | **A** | **A** | **A** | **G** | **A** | **T** | **T** | **G** | **G** | **C** | **C** | **A** | **A** | **A** | **A** | **G** | **C** | **C** | **A** | **A** | **A** | **A** | **T** | **C** | **A** | **T** | **T** | **A** | **T** | **C** | **G** | **T** | **C** | **A** | **C** | **A** | **A** | **G** | **T** | **T** | **T** | **T** | **A** | **T** | **T** | **G** | **A** | **T** | **G** | **A** | **T** | **G** | **A** | **A** | **A** | **A** | **A** | **G** | **T** | **T** | **T** | **A** | **T** | **C** | **C** | **T** | **G** | **C** | **A** | **A** | **T** | **T** | **G** | **A** | **A** | **G** | **C** | **A** | **G** | **G** | **A** | **G** | **C** | **A** | **G** | **C** | **A** | **G** | **G** | **A** | **T** | **A** | **T** | **T** | **T** | **G** | **T** | **T** | **G** | **A** | **A** | **G** | **A** | **C** | **G** | **T** | **C** | **T** | **A** | **C** | **C** | **G** | **C** | **A** | **C** | **A** | **T** | **G** | **A** | **A** | **A** | **T** | **A** | **G** | **C** | **T** | **G** | **A** | **T** | **G** | **C** | **T** | **A** | **T** | **A** | **A** | **G** | **G** | **G** | **C** | **T** | **A** | **C** | **G** | **C** | **A** | **A** | **C** | **G** | **C** | **G** | **G** | **C** | **G** | **A** | **G** | **C** | **G** | **A** | **G** | **T** | **A** | **T** | **T** | **G** | **G** | **A** | **A** | **C** | **C** | **T** | **G** | **A** | **A** | **G** | **T** | **G** | **A** | **C** | **A** | **A** | **C** | **A** | **A** | **A** | **A** | **A** | **T** | **G** | **A** | **T** | **G** | **G** | **A** | **A** | **A** | **A** | **A** | **A** | **T** | **G** | **A** | **G** | **T** | **C** | **G** | **G** | **C** | **G** | **A** | **T** | **A** | **T** | **G** | **A** | **T** | **C** | **C** | **A** | **G** | **T** | **A** | **T** | **T** | **G** | **C** | **A** | **T** | **G** | **A** | **A** | **G** | **A** | **A** | **T** | **T** | **A** | **A** | **C** | **G** | **A** | **A** | **T** | **C** | **G** | **A** | **G** | **A** | **A** | **A** | **A** | **T** | **G** | **A** | **A** | **A** | **T** | **T** | **T** | **T** | **G** | **A** | **T** | **G** | **C** | **T** | **G** | **A** | **T** | **T** | **T** | **C** | **T** | **G** | **A** | **A** | **G** | **G** | **G** | **A** | **A** | **A** | **A** | **G** | **T** | **A** | **A** | **T** | **C** | **A** | **G** | **G** | **A** | **A** | **A** | **T** | **C** | **G** | **C** | **G** | **G** | **A** | **T** | **G** | **A** | **A** | **T** | **T** | **G** | **T** | **T** | **T** | **A** | **T** | **C** | **A** | **C** | **A** | **C** | **T** | **G** | **A** | **A** | **A** | **A** | **C** | **A** | **G** | **T** | **T** | **A** | **A** | **A** | **A** | **C** | **G** | **C** | **A** | **T** | **G** | **T** | **G** | **T** | **C** | **G** | **A** | **A** | **T** | **A** | **T** | **A** | **T** | **T** | **A** | **G** | **C** | **T** | **A** | **A** | **G** | **T** | **T** | **A** | **G** | **A** | **G** | **G** | **T** | **A** | **G** | **A** | **A** | **G** | **A** | **C** | **C** | **G** | **A** | **A** | **C** | **T** | **C** | **A** | **A** | **G** | **C** | **A** | **G** | **C** | **T** | **A** | **T** | **C** | **T** | **A** | **T** | **G** | **C** | **A** | **T** | **T** | **T** | **A** | **A** | **A** | **C** | **A** | **T** | **G** | **G** | **T** | **T** | **T** | **G** | **G** | **T** | **G** | **A** | **A** | **A** | **-** | **-** | **-** |
| **N59646F** | **G** | **T** | **G** | **A** | **T** | **C** | **A** | **A** | **A** | **G** | **T** | **A** | **A** | **T** | **G** | **T** | **T** | **A** | **G** | **T** | **G** | **G** | **A** | **T** | **G** | **A** | **C** | **C** | **A** | **T** | **G** | **A** | **A** | **A** | **T** | **G** | **G** | **T** | **C** | **C** | **G** | **T** | **T** | **T** | **A** | **G** | **G** | **C** | **G** | **T** | **T** | **T** | **C** | **A** | **T** | **C** | **A** | **T** | **A** | **T** | **T** | **T** | **A** | **T** | **C** | **T** | **A** | **T** | **T** | **C** | **A** | **A** | **G** | **A** | **G** | **G** | **A** | **T** | **A** | **T** | **A** | **G** | **A** | **A** | **G** | **T** | **C** | **G** | **T** | **A** | **G** | **G** | **C** | **G** | **A** | **A** | **G** | **C** | **A** | **G** | **A** | **A** | **A** | **A** | **T** | **G** | **G** | **T** | **A** | **A** | **G** | **A** | **T** | **T** | **G** | **G** | **C** | **T** | **A** | **T** | **G** | **A** | **A** | **A** | **A** | **A** | **G** | **C** | **A** | **T** | **T** | **G** | **G** | **A** | **A** | **C** | **T** | **A** | **C** | **G** | **T** | **C** | **C** | **A** | **G** | **A** | **T** | **G** | **T** | **T** | **A** | **T** | **T** | **T** | **T** | **G** | **A** | **T** | **G** | **G** | **A** | **T** | **T** | **T** | **G** | **G** | **T** | **A** | **A** | **T** | **G** | **G** | **A** | **A** | **G** | **A** | **A** | **A** | **T** | **G** | **G** | **A** | **C** | **G** | **G** | **C** | **A** | **T** | **T** | **G** | **A** | **T** | **T** | **C** | **A** | **A** | **C** | **A** | **A** | **A** | **A** | **G** | **C** | **G** | **A** | **T** | **C** | **T** | **T** | **G** | **A** | **A** | **A** | **G** | **A** | **T** | **T** | **G** | **G** | **C** | **C** | **A** | **G** | **A** | **A** | **G** | **C** | **C** | **A** | **A** | **G** | **A** | **T** | **T** | **A** | **T** | **T** | **A** | **T** | **T** | **G** | **T** | **G** | **A** | **C** | **G** | **A** | **G** | **T** | **T** | **T** | **T** | **A** | **T** | **T** | **G** | **A** | **T** | **G** | **A** | **T** | **G** | **A** | **A** | **A** | **A** | **A** | **G** | **T** | **G** | **T** | **A** | **T** | **C** | **C** | **G** | **G** | **C** | **G** | **A** | **T** | **T** | **G** | **A** | **A** | **G** | **C** | **T** | **G** | **G** | **T** | **G** | **C** | **A** | **G** | **C** | **G** | **G** | **G** | **C** | **T** | **A** | **C** | **C** | **T** | **A** | **T** | **T** | **A** | **A** | **A** | **A** | **A** | **C** | **A** | **T** | **C** | **A** | **A** | **C** | **A** | **G** | **C** | **A** | **C** | **A** | **T** | **G** | **A** | **G** | **A** | **T** | **T** | **G** | **C** | **T** | **G** | **A** | **T** | **G** | **C** | **A** | **A** | **T** | **T** | **C** | **G** | **G** | **G** | **C** | **G** | **A** | **C** | **T** | **T** | **A** | **T** | **C** | **G** | **C** | **G** | **G** | **A** | **G** | **A** | **G** | **C** | **G** | **T** | **G** | **T** | **G** | **T** | **T** | **G** | **G** | **A** | **A** | **C** | **C** | **T** | **G** | **A** | **A** | **G** | **T** | **G** | **A** | **C** | **G** | **C** | **A** | **T** | **A** | **A** | **G** | **A** | **T** | **G** | **A** | **T** | **G** | **G** | **A** | **A** | **C** | **G** | **G** | **T** | **T** | **A** | **A** | **C** | **A** | **A** | **A** | **A** | **A** | **A** | **A** | **C** | **A** | **A** | **G** | **A** | **G** | **C** | **C** | **G** | **G** | **T** | **G** | **T** | **T** | **G** | **C** | **A** | **C** | **G** | **A** | **A** | **G** | **A** | **T** | **T** | **T** | **G** | **A** | **C** | **A** | **A** | **A** | **C** | **C** | **G** | **G** | **G** | **A** | **A** | **C** | **A** | **C** | **G** | **A** | **A** | **A** | **T** | **T** | **T** | **T** | **A** | **A** | **T** | **G** | **T** | **T** | **G** | **A** | **T** | **T** | **G** | **C** | **A** | **C** | **A** | **A** | **G** | **G** | **T** | **A** | **A** | **A** | **A** | **G** | **T** | **A** | **A** | **T** | **C** | **A** | **G** | **G** | **A** | **A** | **A** | **T** | **A** | **G** | **C** | **T** | **G** | **A** | **T** | **G** | **A** | **A** | **C** | **T** | **C** | **T** | **T** | **T** | **A** | **T** | **C** | **A** | **C** | **T** | **T** | **T** | **G** | **A** | **A** | **A** | **A** | **C** | **A** | **G** | **T** | **T** | **A** | **A** | **A** | **A** | **C** | **A** | **C** | **A** | **T** | **G** | **T** | **T** | **T** | **C** | **A** | **A** | **A** | **C** | **A** | **T** | **T** | **T** | **T** | **A** | **G** | **C** | **A** | **A** | **A** | **A** | **C** | **T** | **A** | **G** | **A** | **T** | **G** | **T** | **G** | **G** | **A** | **T** | **G** | **A** | **T** | **C** | **G** | **G** | **A** | **C** | **C** | **C** | **A** | **A** | **G** | **C** | **G** | **G** | **C** | **G** | **A** | **T** | **T** | **T** | **A** | **T** | **G** | **C** | **T** | **T** | **T** | **T** | **C** | **A** | **A** | **C** | **A** | **T** | **G** | **G** | **T** | **T** | **T** | **A** | **G** | **C** | **C** | **A** | **A** | **A** | **-** | **-** | **-** |
| **N53453** | **G** | **T** | **G** | **A** | **T** | **C** | **A** | **A** | **A** | **G** | **T** | **A** | **A** | **T** | **G** | **T** | **T** | **A** | **G** | **T** | **G** | **G** | **A** | **T** | **G** | **A** | **C** | **C** | **A** | **T** | **G** | **A** | **A** | **A** | **T** | **G** | **G** | **T** | **C** | **C** | **G** | **T** | **T** | **T** | **A** | **G** | **G** | **C** | **G** | **T** | **T** | **T** | **C** | **A** | **T** | **C** | **A** | **T** | **A** | **T** | **T** | **T** | **A** | **T** | **C** | **T** | **A** | **T** | **T** | **C** | **A** | **A** | **G** | **A** | **G** | **G** | **A** | **T** | **A** | **T** | **A** | **G** | **A** | **A** | **G** | **T** | **C** | **G** | **T** | **A** | **G** | **G** | **C** | **G** | **A** | **A** | **G** | **C** | **A** | **G** | **A** | **A** | **A** | **A** | **C** | **G** | **G** | **T** | **A** | **A** | **G** | **A** | **T** | **T** | **G** | **G** | **C** | **T** | **A** | **T** | **G** | **A** | **A** | **A** | **A** | **A** | **G** | **C** | **A** | **T** | **T** | **G** | **G** | **A** | **A** | **C** | **T** | **A** | **C** | **G** | **T** | **C** | **C** | **A** | **G** | **A** | **T** | **G** | **T** | **T** | **A** | **T** | **T** | **T** | **T** | **G** | **A** | **T** | **G** | **G** | **A** | **T** | **T** | **T** | **G** | **G** | **T** | **A** | **A** | **T** | **G** | **G** | **A** | **A** | **G** | **A** | **A** | **A** | **T** | **G** | **G** | **A** | **C** | **G** | **G** | **C** | **A** | **T** | **T** | **G** | **A** | **T** | **T** | **C** | **A** | **A** | **C** | **A** | **A** | **A** | **A** | **G** | **C** | **G** | **A** | **T** | **C** | **T** | **T** | **G** | **A** | **A** | **A** | **G** | **A** | **T** | **T** | **G** | **G** | **C** | **C** | **A** | **G** | **A** | **A** | **G** | **C** | **C** | **A** | **A** | **G** | **A** | **T** | **T** | **A** | **T** | **T** | **A** | **T** | **T** | **G** | **T** | **G** | **A** | **C** | **G** | **A** | **G** | **T** | **T** | **T** | **T** | **A** | **T** | **T** | **G** | **A** | **T** | **G** | **A** | **T** | **G** | **A** | **A** | **A** | **A** | **A** | **G** | **T** | **G** | **T** | **A** | **T** | **C** | **C** | **G** | **G** | **C** | **G** | **A** | **T** | **T** | **G** | **A** | **A** | **G** | **C** | **T** | **G** | **G** | **T** | **G** | **C** | **A** | **G** | **C** | **G** | **G** | **G** | **C** | **T** | **A** | **C** | **C** | **T** | **A** | **T** | **T** | **A** | **A** | **A** | **A** | **A** | **C** | **A** | **T** | **C** | **A** | **A** | **C** | **A** | **G** | **C** | **A** | **C** | **A** | **T** | **G** | **A** | **G** | **A** | **T** | **T** | **G** | **C** | **T** | **G** | **A** | **T** | **G** | **C** | **A** | **A** | **T** | **T** | **C** | **G** | **G** | **G** | **C** | **G** | **A** | **C** | **T** | **T** | **A** | **T** | **C** | **G** | **C** | **G** | **G** | **A** | **G** | **A** | **G** | **C** | **G** | **T** | **G** | **T** | **G** | **T** | **T** | **G** | **G** | **A** | **A** | **C** | **C** | **T** | **G** | **A** | **A** | **G** | **T** | **G** | **A** | **C** | **G** | **C** | **A** | **T** | **A** | **A** | **G** | **A** | **T** | **G** | **A** | **T** | **G** | **G** | **A** | **A** | **C** | **G** | **G** | **T** | **T** | **A** | **A** | **C** | **A** | **A** | **A** | **A** | **A** | **A** | **A** | **C** | **A** | **A** | **G** | **A** | **G** | **C** | **C** | **G** | **G** | **T** | **G** | **C** | **T** | **A** | **C** | **A** | **T** | **G** | **A** | **A** | **G** | **A** | **T** | **T** | **T** | **G** | **A** | **C** | **A** | **A** | **A** | **C** | **C** | **G** | **A** | **G** | **A** | **A** | **C** | **A** | **C** | **G** | **A** | **A** | **A** | **T** | **T** | **T** | **T** | **A** | **A** | **T** | **G** | **T** | **T** | **G** | **A** | **T** | **T** | **G** | **C** | **A** | **C** | **A** | **A** | **G** | **G** | **T** | **A** | **A** | **A** | **A** | **G** | **T** | **A** | **A** | **T** | **C** | **A** | **G** | **G** | **A** | **A** | **A** | **T** | **A** | **G** | **C** | **T** | **G** | **A** | **T** | **G** | **A** | **A** | **C** | **T** | **C** | **T** | **T** | **T** | **A** | **T** | **C** | **A** | **C** | **T** | **T** | **T** | **G** | **A** | **A** | **A** | **A** | **C** | **A** | **G** | **T** | **T** | **A** | **A** | **A** | **A** | **C** | **A** | **C** | **A** | **T** | **G** | **T** | **T** | **T** | **C** | **A** | **A** | **A** | **C** | **A** | **T** | **T** | **T** | **T** | **A** | **G** | **C** | **A** | **A** | **A** | **A** | **C** | **T** | **A** | **G** | **A** | **T** | **G** | **T** | **G** | **G** | **A** | **T** | **G** | **A** | **T** | **C** | **G** | **G** | **A** | **C** | **C** | **C** | **A** | **A** | **G** | **C** | **G** | **G** | **C** | **G** | **A** | **T** | **T** | **T** | **A** | **T** | **G** | **C** | **T** | **T** | **T** | **T** | **C** | **A** | **A** | **C** | **A** | **T** | **G** | **G** | **T** | **T** | **T** | **A** | **G** | **C** | **C** | **A** | **A** | **A** | **-** | **-** | **-** |
| **N52724** | **G** | **T** | **G** | **A** | **T** | **C** | **A** | **A** | **A** | **G** | **T** | **A** | **A** | **T** | **G** | **T** | **T** | **A** | **G** | **T** | **G** | **G** | **A** | **T** | **G** | **A** | **C** | **C** | **A** | **T** | **G** | **A** | **A** | **A** | **T** | **G** | **G** | **T** | **C** | **C** | **G** | **T** | **T** | **T** | **A** | **G** | **G** | **C** | **G** | **T** | **T** | **T** | **C** | **A** | **T** | **C** | **A** | **T** | **A** | **T** | **T** | **T** | **A** | **T** | **C** | **T** | **A** | **T** | **T** | **C** | **A** | **A** | **G** | **A** | **G** | **G** | **A** | **T** | **A** | **T** | **A** | **G** | **A** | **A** | **G** | **T** | **C** | **G** | **T** | **A** | **G** | **G** | **C** | **G** | **A** | **A** | **G** | **C** | **A** | **G** | **A** | **A** | **A** | **A** | **C** | **G** | **G** | **T** | **A** | **A** | **G** | **A** | **T** | **T** | **G** | **G** | **C** | **T** | **A** | **T** | **G** | **A** | **A** | **A** | **A** | **A** | **G** | **C** | **A** | **T** | **T** | **G** | **G** | **A** | **A** | **C** | **T** | **A** | **C** | **G** | **T** | **C** | **C** | **A** | **G** | **A** | **T** | **G** | **T** | **T** | **A** | **T** | **T** | **T** | **T** | **G** | **A** | **T** | **G** | **G** | **A** | **T** | **T** | **T** | **G** | **G** | **T** | **A** | **A** | **T** | **G** | **G** | **A** | **A** | **G** | **A** | **A** | **A** | **T** | **G** | **G** | **A** | **C** | **G** | **G** | **C** | **A** | **T** | **T** | **G** | **A** | **T** | **T** | **C** | **A** | **A** | **C** | **A** | **A** | **A** | **A** | **G** | **C** | **G** | **A** | **T** | **C** | **T** | **T** | **G** | **A** | **A** | **A** | **G** | **A** | **T** | **T** | **G** | **G** | **C** | **C** | **A** | **G** | **A** | **A** | **G** | **C** | **C** | **A** | **A** | **G** | **A** | **T** | **T** | **A** | **T** | **T** | **A** | **T** | **T** | **G** | **T** | **G** | **A** | **C** | **G** | **A** | **G** | **T** | **T** | **T** | **T** | **A** | **T** | **T** | **G** | **A** | **T** | **G** | **A** | **T** | **G** | **A** | **A** | **A** | **A** | **A** | **G** | **T** | **A** | **T** | **A** | **T** | **C** | **C** | **G** | **G** | **C** | **G** | **A** | **T** | **T** | **G** | **A** | **A** | **G** | **C** | **T** | **G** | **G** | **T** | **G** | **C** | **A** | **G** | **C** | **G** | **G** | **G** | **C** | **T** | **A** | **C** | **C** | **T** | **A** | **T** | **T** | **A** | **A** | **A** | **A** | **A** | **C** | **A** | **T** | **C** | **A** | **A** | **C** | **A** | **G** | **C** | **A** | **C** | **A** | **T** | **G** | **A** | **G** | **A** | **T** | **T** | **G** | **C** | **T** | **G** | **A** | **T** | **G** | **C** | **A** | **A** | **T** | **T** | **C** | **G** | **G** | **G** | **C** | **G** | **A** | **C** | **T** | **T** | **A** | **T** | **C** | **G** | **C** | **G** | **G** | **A** | **G** | **A** | **G** | **C** | **G** | **T** | **G** | **T** | **G** | **T** | **T** | **G** | **G** | **A** | **A** | **C** | **C** | **T** | **G** | **A** | **A** | **G** | **T** | **G** | **A** | **C** | **G** | **C** | **A** | **T** | **A** | **A** | **G** | **A** | **T** | **G** | **A** | **T** | **G** | **G** | **A** | **A** | **C** | **G** | **G** | **T** | **T** | **A** | **A** | **C** | **A** | **A** | **A** | **A** | **A** | **A** | **A** | **C** | **A** | **A** | **G** | **A** | **G** | **C** | **C** | **G** | **G** | **T** | **G** | **T** | **T** | **G** | **C** | **A** | **T** | **G** | **A** | **A** | **G** | **A** | **T** | **T** | **T** | **G** | **A** | **C** | **A** | **A** | **A** | **T** | **C** | **G** | **A** | **G** | **A** | **A** | **C** | **A** | **C** | **G** | **A** | **A** | **A** | **T** | **T** | **T** | **T** | **A** | **A** | **T** | **G** | **T** | **T** | **G** | **A** | **T** | **T** | **G** | **C** | **A** | **C** | **A** | **A** | **G** | **G** | **T** | **A** | **A** | **A** | **A** | **G** | **T** | **A** | **A** | **T** | **C** | **A** | **G** | **G** | **A** | **A** | **A** | **T** | **A** | **G** | **C** | **T** | **G** | **A** | **T** | **G** | **A** | **A** | **C** | **T** | **C** | **T** | **T** | **T** | **A** | **T** | **C** | **A** | **C** | **T** | **T** | **T** | **G** | **A** | **A** | **A** | **A** | **C** | **A** | **G** | **T** | **T** | **A** | **A** | **A** | **A** | **C** | **A** | **C** | **A** | **T** | **G** | **T** | **T** | **T** | **C** | **A** | **A** | **A** | **C** | **A** | **T** | **T** | **T** | **T** | **A** | **G** | **C** | **A** | **A** | **A** | **A** | **C** | **T** | **A** | **G** | **A** | **T** | **G** | **T** | **G** | **G** | **A** | **T** | **G** | **A** | **T** | **C** | **G** | **G** | **A** | **C** | **C** | **C** | **A** | **A** | **G** | **C** | **A** | **G** | **C** | **G** | **A** | **T** | **T** | **T** | **A** | **T** | **G** | **C** | **T** | **T** | **T** | **T** | **C** | **A** | **A** | **C** | **A** | **T** | **G** | **G** | **T** | **T** | **T** | **A** | **G** | **C** | **C** | **A** | **A** | **A** | **-** | **-** | **-** |
| **N54035** | **A** | **T** | **G** | **A** | **T** | **A** | **A** | **A** | **A** | **G** | **T** | **T** | **T** | **T** | **A** | **T** | **T** | **A** | **G** | **T** | **A** | **G** | **A** | **T** | **G** | **A** | **C** | **C** | **A** | **T** | **G** | **A** | **A** | **A** | **T** | **G** | **G** | **T** | **A** | **C** | **G** | **C** | **T** | **T** | **A** | **G** | **G** | **C** | **G** | **T** | **C** | **T** | **C** | **T** | **T** | **C** | **T** | **T** | **A** | **T** | **T** | **T** | **A** | **T** | **C** | **T** | **A** | **T** | **C** | **C** | **A** | **A** | **G** | **A** | **A** | **G** | **A** | **C** | **A** | **T** | **T** | **G** | **A** | **A** | **G** | **T** | **G** | **A** | **T** | **T** | **G** | **G** | **A** | **G** | **A** | **A** | **G** | **C** | **T** | **G** | **A** | **A** | **A** | **A** | **T** | **G** | **G** | **A** | **C** | **G** | **T** | **C** | **A** | **A** | **G** | **G** | **C** | **T** | **A** | **T** | **G** | **A** | **A** | **A** | **A** | **G** | **G** | **C** | **G** | **A** | **T** | **G** | **G** | **C** | **A** | **C** | **T** | **T** | **C** | **G** | **A** | **C** | **C** | **A** | **G** | **A** | **T** | **G** | **T** | **C** | **A** | **T** | **T** | **T** | **T** | **A** | **A** | **T** | **G** | **G** | **A** | **T** | **C** | **T** | **A** | **G** | **T** | **C** | **A** | **T** | **G** | **G** | **A** | **A** | **G** | **A** | **A** | **A** | **T** | **G** | **G** | **A** | **T** | **G** | **G** | **C** | **A** | **T** | **C** | **G** | **A** | **A** | **T** | **C** | **A** | **A** | **C** | **A** | **A** | **A** | **A** | **G** | **C** | **A** | **A** | **T** | **C** | **T** | **T** | **A** | **A** | **A** | **A** | **G** | **A** | **T** | **T** | **G** | **G** | **C** | **C** | **A** | **A** | **A** | **A** | **G** | **C** | **C** | **A** | **A** | **A** | **A** | **T** | **C** | **A** | **T** | **T** | **A** | **T** | **C** | **G** | **T** | **C** | **A** | **C** | **A** | **A** | **G** | **T** | **T** | **T** | **T** | **A** | **T** | **T** | **G** | **A** | **T** | **G** | **A** | **T** | **G** | **A** | **A** | **A** | **A** | **A** | **G** | **T** | **T** | **T** | **A** | **T** | **C** | **C** | **T** | **G** | **C** | **A** | **A** | **T** | **T** | **G** | **A** | **A** | **G** | **C** | **A** | **G** | **G** | **A** | **G** | **C** | **A** | **G** | **C** | **A** | **G** | **G** | **A** | **T** | **A** | **T** | **T** | **T** | **G** | **T** | **T** | **G** | **A** | **A** | **G** | **A** | **C** | **G** | **T** | **C** | **T** | **A** | **C** | **C** | **G** | **C** | **A** | **C** | **A** | **T** | **G** | **A** | **A** | **A** | **T** | **A** | **G** | **C** | **T** | **G** | **A** | **T** | **G** | **C** | **T** | **A** | **T** | **A** | **A** | **G** | **G** | **G** | **C** | **T** | **A** | **C** | **G** | **C** | **A** | **A** | **C** | **G** | **C** | **G** | **G** | **C** | **G** | **A** | **G** | **C** | **G** | **A** | **G** | **T** | **A** | **T** | **T** | **G** | **G** | **A** | **A** | **C** | **C** | **T** | **G** | **A** | **A** | **G** | **T** | **G** | **A** | **C** | **A** | **A** | **C** | **A** | **A** | **A** | **A** | **A** | **T** | **G** | **A** | **T** | **G** | **G** | **A** | **A** | **A** | **A** | **A** | **A** | **T** | **G** | **A** | **G** | **T** | **C** | **G** | **G** | **C** | **G** | **A** | **A** | **A** | **T** | **G** | **A** | **T** | **C** | **C** | **A** | **G** | **T** | **A** | **T** | **T** | **G** | **C** | **A** | **T** | **G** | **A** | **A** | **G** | **A** | **A** | **T** | **T** | **A** | **A** | **C** | **G** | **A** | **A** | **T** | **C** | **G** | **A** | **G** | **A** | **A** | **A** | **A** | **T** | **G** | **A** | **A** | **A** | **T** | **T** | **T** | **T** | **G** | **A** | **T** | **G** | **C** | **T** | **G** | **A** | **T** | **T** | **T** | **C** | **T** | **G** | **A** | **A** | **G** | **G** | **G** | **A** | **A** | **A** | **A** | **G** | **T** | **A** | **A** | **T** | **C** | **A** | **G** | **G** | **A** | **A** | **A** | **T** | **C** | **G** | **C** | **G** | **G** | **A** | **T** | **G** | **A** | **A** | **T** | **T** | **G** | **T** | **T** | **T** | **A** | **T** | **C** | **A** | **C** | **A** | **C** | **T** | **G** | **A** | **A** | **A** | **A** | **C** | **A** | **G** | **T** | **T** | **A** | **A** | **A** | **A** | **C** | **G** | **C** | **A** | **T** | **G** | **T** | **G** | **T** | **C** | **G** | **A** | **A** | **T** | **A** | **T** | **A** | **T** | **T** | **A** | **G** | **C** | **T** | **A** | **A** | **G** | **T** | **T** | **A** | **G** | **A** | **G** | **G** | **T** | **A** | **G** | **A** | **A** | **G** | **A** | **C** | **C** | **G** | **A** | **A** | **C** | **T** | **C** | **A** | **A** | **G** | **C** | **A** | **G** | **C** | **T** | **A** | **T** | **C** | **T** | **A** | **T** | **G** | **C** | **G** | **T** | **T** | **T** | **A** | **A** | **A** | **C** | **A** | **T** | **G** | **G** | **T** | **T** | **T** | **G** | **G** | **T** | **G** | **A** | **A** | **A** | **-** | **-** | **-** |
| **N59910F** | **G** | **T** | **G** | **A** | **T** | **C** | **A** | **A** | **A** | **G** | **T** | **A** | **A** | **T** | **G** | **T** | **T** | **A** | **G** | **T** | **G** | **G** | **A** | **T** | **G** | **A** | **C** | **C** | **A** | **T** | **G** | **A** | **A** | **A** | **T** | **G** | **G** | **T** | **C** | **C** | **G** | **T** | **T** | **T** | **A** | **G** | **G** | **C** | **G** | **T** | **T** | **T** | **C** | **A** | **T** | **C** | **A** | **T** | **A** | **T** | **T** | **T** | **A** | **T** | **C** | **T** | **A** | **T** | **T** | **C** | **A** | **A** | **G** | **A** | **G** | **G** | **A** | **T** | **A** | **T** | **A** | **G** | **A** | **A** | **G** | **T** | **C** | **G** | **T** | **A** | **G** | **G** | **C** | **G** | **A** | **A** | **G** | **C** | **A** | **G** | **A** | **A** | **A** | **A** | **C** | **G** | **G** | **T** | **A** | **A** | **G** | **A** | **T** | **T** | **G** | **G** | **C** | **T** | **A** | **T** | **G** | **A** | **A** | **A** | **A** | **A** | **G** | **C** | **A** | **T** | **T** | **G** | **G** | **A** | **A** | **C** | **T** | **A** | **C** | **G** | **T** | **C** | **C** | **A** | **G** | **A** | **T** | **G** | **T** | **T** | **A** | **T** | **T** | **T** | **T** | **G** | **A** | **T** | **G** | **G** | **A** | **T** | **T** | **T** | **G** | **G** | **T** | **A** | **A** | **T** | **G** | **G** | **A** | **A** | **G** | **A** | **A** | **A** | **T** | **G** | **G** | **A** | **C** | **G** | **G** | **C** | **A** | **T** | **T** | **G** | **A** | **T** | **T** | **C** | **A** | **A** | **C** | **A** | **A** | **A** | **A** | **G** | **C** | **G** | **A** | **T** | **C** | **T** | **T** | **G** | **A** | **A** | **A** | **G** | **A** | **T** | **T** | **G** | **G** | **C** | **C** | **A** | **G** | **A** | **A** | **G** | **C** | **C** | **A** | **A** | **G** | **A** | **T** | **T** | **A** | **T** | **T** | **A** | **T** | **T** | **G** | **T** | **G** | **A** | **C** | **G** | **A** | **G** | **T** | **T** | **T** | **T** | **A** | **T** | **T** | **G** | **A** | **T** | **G** | **A** | **T** | **G** | **A** | **A** | **A** | **A** | **A** | **G** | **T** | **G** | **T** | **A** | **T** | **C** | **C** | **G** | **G** | **C** | **G** | **A** | **T** | **T** | **G** | **A** | **A** | **G** | **C** | **T** | **G** | **G** | **T** | **G** | **C** | **A** | **G** | **C** | **G** | **G** | **G** | **C** | **T** | **A** | **C** | **C** | **T** | **A** | **T** | **T** | **A** | **A** | **A** | **A** | **A** | **C** | **A** | **T** | **C** | **A** | **A** | **C** | **A** | **G** | **C** | **A** | **C** | **A** | **T** | **G** | **A** | **G** | **A** | **T** | **T** | **G** | **C** | **T** | **G** | **A** | **T** | **G** | **C** | **A** | **A** | **T** | **T** | **C** | **G** | **G** | **G** | **C** | **G** | **A** | **C** | **T** | **T** | **A** | **T** | **C** | **G** | **C** | **G** | **G** | **A** | **G** | **A** | **G** | **C** | **G** | **T** | **G** | **T** | **G** | **T** | **T** | **G** | **G** | **A** | **A** | **C** | **C** | **T** | **G** | **A** | **A** | **G** | **T** | **G** | **A** | **C** | **G** | **C** | **A** | **T** | **A** | **A** | **G** | **A** | **T** | **G** | **A** | **T** | **G** | **G** | **A** | **A** | **C** | **G** | **G** | **T** | **T** | **A** | **A** | **C** | **A** | **A** | **A** | **A** | **A** | **A** | **A** | **C** | **A** | **A** | **G** | **A** | **G** | **C** | **C** | **G** | **G** | **T** | **G** | **T** | **T** | **G** | **C** | **A** | **C** | **G** | **A** | **A** | **G** | **A** | **T** | **T** | **T** | **G** | **A** | **C** | **A** | **A** | **A** | **C** | **C** | **G** | **A** | **G** | **A** | **A** | **C** | **A** | **C** | **G** | **A** | **A** | **A** | **T** | **T** | **T** | **T** | **A** | **A** | **T** | **G** | **T** | **T** | **G** | **A** | **T** | **T** | **G** | **C** | **A** | **C** | **A** | **A** | **G** | **G** | **T** | **A** | **A** | **A** | **A** | **G** | **T** | **A** | **A** | **T** | **C** | **A** | **G** | **G** | **A** | **A** | **A** | **T** | **T** | **G** | **C** | **T** | **G** | **A** | **T** | **G** | **A** | **A** | **C** | **T** | **C** | **T** | **T** | **T** | **A** | **T** | **C** | **A** | **C** | **T** | **T** | **T** | **G** | **A** | **A** | **A** | **A** | **C** | **A** | **G** | **T** | **T** | **A** | **A** | **A** | **A** | **C** | **A** | **C** | **A** | **T** | **G** | **T** | **T** | **T** | **C** | **A** | **A** | **A** | **C** | **A** | **T** | **T** | **T** | **T** | **A** | **G** | **C** | **A** | **A** | **A** | **A** | **C** | **T** | **A** | **G** | **A** | **T** | **G** | **T** | **G** | **G** | **A** | **T** | **G** | **A** | **T** | **C** | **G** | **G** | **A** | **C** | **C** | **C** | **A** | **A** | **G** | **C** | **G** | **G** | **C** | **G** | **A** | **T** | **T** | **T** | **A** | **T** | **G** | **C** | **T** | **T** | **T** | **T** | **C** | **A** | **A** | **C** | **A** | **T** | **G** | **G** | **T** | **T** | **T** | **A** | **G** | **C** | **C** | **A** | **A** | **A** | **-** | **-** | **-** |
| **N55268** | **G** | **T** | **G** | **A** | **T** | **C** | **A** | **A** | **A** | **G** | **T** | **A** | **A** | **T** | **G** | **T** | **T** | **A** | **G** | **T** | **G** | **G** | **A** | **T** | **G** | **A** | **C** | **C** | **A** | **T** | **G** | **A** | **A** | **A** | **T** | **G** | **G** | **T** | **C** | **C** | **G** | **T** | **T** | **T** | **A** | **G** | **G** | **C** | **G** | **T** | **T** | **T** | **C** | **A** | **T** | **C** | **A** | **T** | **A** | **T** | **T** | **T** | **A** | **T** | **C** | **T** | **A** | **T** | **T** | **C** | **A** | **A** | **G** | **A** | **G** | **G** | **A** | **T** | **A** | **T** | **A** | **G** | **A** | **A** | **G** | **T** | **C** | **G** | **T** | **A** | **G** | **G** | **C** | **G** | **A** | **A** | **G** | **C** | **A** | **G** | **A** | **A** | **A** | **A** | **C** | **G** | **G** | **T** | **A** | **A** | **G** | **A** | **T** | **T** | **G** | **G** | **C** | **T** | **A** | **T** | **G** | **A** | **A** | **A** | **A** | **A** | **G** | **C** | **A** | **T** | **T** | **G** | **G** | **A** | **A** | **C** | **T** | **A** | **C** | **G** | **T** | **C** | **C** | **A** | **G** | **A** | **T** | **G** | **T** | **T** | **A** | **T** | **T** | **T** | **T** | **G** | **A** | **T** | **G** | **G** | **A** | **T** | **T** | **T** | **G** | **G** | **T** | **A** | **A** | **T** | **G** | **G** | **A** | **A** | **G** | **A** | **A** | **A** | **T** | **G** | **G** | **A** | **C** | **G** | **G** | **C** | **A** | **T** | **T** | **G** | **A** | **T** | **T** | **C** | **A** | **A** | **C** | **A** | **A** | **A** | **A** | **G** | **C** | **G** | **A** | **T** | **C** | **T** | **T** | **G** | **A** | **A** | **A** | **G** | **A** | **T** | **T** | **G** | **G** | **C** | **C** | **A** | **G** | **A** | **A** | **G** | **C** | **C** | **A** | **A** | **G** | **A** | **T** | **T** | **A** | **T** | **T** | **A** | **T** | **T** | **G** | **T** | **G** | **A** | **C** | **G** | **A** | **G** | **T** | **T** | **T** | **T** | **A** | **T** | **T** | **G** | **A** | **T** | **G** | **A** | **T** | **G** | **A** | **A** | **A** | **A** | **A** | **G** | **T** | **G** | **T** | **A** | **T** | **C** | **C** | **G** | **G** | **C** | **G** | **A** | **T** | **T** | **G** | **A** | **A** | **G** | **C** | **T** | **G** | **G** | **T** | **G** | **C** | **A** | **G** | **C** | **G** | **G** | **G** | **C** | **T** | **A** | **C** | **C** | **T** | **A** | **T** | **T** | **A** | **A** | **A** | **A** | **A** | **C** | **A** | **T** | **C** | **A** | **A** | **C** | **A** | **G** | **C** | **A** | **C** | **A** | **T** | **G** | **A** | **G** | **A** | **T** | **T** | **G** | **C** | **T** | **G** | **A** | **T** | **G** | **C** | **A** | **A** | **T** | **T** | **C** | **G** | **G** | **G** | **C** | **G** | **A** | **C** | **T** | **T** | **A** | **T** | **C** | **G** | **C** | **G** | **G** | **A** | **G** | **A** | **G** | **C** | **G** | **T** | **G** | **T** | **G** | **T** | **T** | **G** | **G** | **A** | **A** | **C** | **C** | **T** | **G** | **A** | **A** | **G** | **T** | **G** | **A** | **C** | **G** | **C** | **A** | **T** | **A** | **A** | **G** | **A** | **T** | **G** | **A** | **T** | **G** | **G** | **A** | **A** | **C** | **G** | **G** | **T** | **T** | **A** | **A** | **C** | **A** | **A** | **A** | **A** | **A** | **A** | **A** | **C** | **A** | **A** | **G** | **A** | **G** | **C** | **C** | **G** | **G** | **T** | **G** | **C** | **T** | **A** | **C** | **A** | **T** | **G** | **A** | **A** | **G** | **A** | **T** | **T** | **T** | **G** | **A** | **C** | **A** | **A** | **A** | **C** | **C** | **G** | **A** | **G** | **A** | **A** | **C** | **A** | **C** | **G** | **A** | **A** | **A** | **T** | **T** | **T** | **T** | **A** | **A** | **T** | **G** | **T** | **T** | **G** | **A** | **T** | **T** | **G** | **C** | **A** | **C** | **A** | **A** | **G** | **G** | **T** | **A** | **A** | **A** | **A** | **G** | **T** | **A** | **A** | **T** | **C** | **A** | **G** | **G** | **A** | **A** | **A** | **T** | **A** | **G** | **C** | **T** | **G** | **A** | **T** | **G** | **A** | **A** | **C** | **T** | **C** | **T** | **T** | **T** | **A** | **T** | **C** | **A** | **C** | **T** | **T** | **T** | **G** | **A** | **A** | **A** | **A** | **C** | **A** | **G** | **T** | **T** | **A** | **A** | **A** | **A** | **C** | **A** | **C** | **A** | **T** | **G** | **T** | **T** | **T** | **C** | **A** | **A** | **A** | **C** | **A** | **T** | **T** | **T** | **T** | **A** | **G** | **C** | **A** | **A** | **A** | **A** | **C** | **T** | **A** | **G** | **A** | **T** | **G** | **T** | **G** | **G** | **A** | **T** | **G** | **A** | **T** | **C** | **G** | **G** | **A** | **C** | **C** | **C** | **A** | **A** | **G** | **C** | **G** | **G** | **C** | **G** | **A** | **T** | **T** | **T** | **A** | **T** | **G** | **C** | **T** | **T** | **T** | **T** | **C** | **A** | **A** | **C** | **A** | **T** | **G** | **G** | **T** | **T** | **T** | **A** | **G** | **C** | **C** | **A** | **A** | **A** | **-** | **-** | **-** |
| **N55181** | **G** | **T** | **G** | **A** | **T** | **C** | **A** | **A** | **A** | **G** | **T** | **A** | **A** | **T** | **G** | **T** | **T** | **A** | **G** | **T** | **G** | **G** | **A** | **T** | **G** | **A** | **C** | **C** | **A** | **T** | **G** | **A** | **A** | **A** | **T** | **G** | **G** | **T** | **C** | **C** | **G** | **T** | **T** | **T** | **A** | **G** | **G** | **C** | **G** | **T** | **T** | **T** | **C** | **A** | **T** | **C** | **A** | **T** | **A** | **T** | **T** | **T** | **A** | **T** | **C** | **T** | **A** | **T** | **T** | **C** | **A** | **A** | **G** | **A** | **G** | **G** | **A** | **T** | **A** | **T** | **A** | **G** | **A** | **A** | **G** | **T** | **C** | **G** | **T** | **A** | **G** | **G** | **C** | **G** | **A** | **A** | **G** | **C** | **A** | **G** | **A** | **A** | **A** | **A** | **C** | **G** | **G** | **T** | **A** | **A** | **G** | **A** | **T** | **T** | **G** | **G** | **C** | **T** | **A** | **T** | **G** | **A** | **A** | **A** | **A** | **A** | **G** | **C** | **A** | **T** | **T** | **G** | **G** | **A** | **A** | **C** | **T** | **A** | **C** | **G** | **T** | **C** | **C** | **A** | **G** | **A** | **T** | **G** | **T** | **T** | **A** | **T** | **T** | **T** | **T** | **G** | **A** | **T** | **G** | **G** | **A** | **T** | **T** | **T** | **G** | **G** | **T** | **A** | **A** | **T** | **G** | **G** | **A** | **A** | **G** | **A** | **A** | **A** | **T** | **G** | **G** | **A** | **C** | **G** | **G** | **C** | **A** | **T** | **T** | **G** | **A** | **T** | **T** | **C** | **A** | **A** | **C** | **A** | **A** | **A** | **A** | **G** | **C** | **A** | **A** | **T** | **C** | **T** | **T** | **G** | **A** | **A** | **A** | **G** | **A** | **T** | **T** | **G** | **G** | **C** | **C** | **A** | **G** | **A** | **A** | **G** | **C** | **C** | **A** | **A** | **G** | **A** | **T** | **T** | **A** | **T** | **T** | **A** | **T** | **T** | **G** | **T** | **G** | **A** | **C** | **G** | **A** | **G** | **T** | **T** | **T** | **T** | **A** | **T** | **T** | **G** | **A** | **T** | **G** | **A** | **T** | **G** | **A** | **G** | **A** | **A** | **A** | **G** | **T** | **G** | **T** | **A** | **T** | **C** | **C** | **G** | **G** | **C** | **G** | **A** | **T** | **T** | **G** | **A** | **A** | **G** | **C** | **T** | **G** | **G** | **T** | **G** | **C** | **A** | **G** | **C** | **G** | **G** | **G** | **C** | **T** | **A** | **C** | **C** | **T** | **A** | **T** | **T** | **A** | **A** | **A** | **A** | **A** | **C** | **A** | **T** | **C** | **A** | **A** | **C** | **A** | **G** | **C** | **A** | **C** | **A** | **T** | **G** | **A** | **G** | **A** | **T** | **T** | **G** | **C** | **T** | **G** | **A** | **T** | **G** | **C** | **A** | **A** | **T** | **T** | **C** | **G** | **G** | **G** | **C** | **G** | **A** | **C** | **T** | **T** | **A** | **T** | **C** | **G** | **C** | **G** | **G** | **A** | **G** | **A** | **G** | **C** | **G** | **T** | **G** | **T** | **G** | **T** | **T** | **G** | **G** | **A** | **A** | **C** | **C** | **T** | **G** | **A** | **A** | **G** | **T** | **G** | **A** | **C** | **G** | **C** | **A** | **T** | **A** | **A** | **G** | **A** | **T** | **G** | **A** | **T** | **G** | **G** | **A** | **A** | **C** | **G** | **G** | **T** | **T** | **A** | **A** | **C** | **A** | **A** | **A** | **A** | **A** | **A** | **A** | **C** | **A** | **A** | **G** | **A** | **G** | **C** | **C** | **G** | **G** | **T** | **G** | **T** | **T** | **G** | **C** | **A** | **T** | **G** | **A** | **A** | **G** | **A** | **T** | **T** | **T** | **G** | **A** | **C** | **A** | **A** | **A** | **T** | **C** | **G** | **A** | **G** | **A** | **A** | **C** | **A** | **C** | **G** | **A** | **A** | **A** | **T** | **T** | **T** | **T** | **A** | **A** | **T** | **G** | **T** | **T** | **G** | **A** | **T** | **T** | **G** | **C** | **A** | **C** | **A** | **A** | **G** | **G** | **T** | **A** | **A** | **A** | **A** | **G** | **T** | **A** | **A** | **T** | **C** | **A** | **G** | **G** | **A** | **A** | **A** | **T** | **A** | **G** | **C** | **T** | **G** | **A** | **T** | **G** | **A** | **A** | **C** | **T** | **C** | **T** | **T** | **T** | **A** | **T** | **C** | **A** | **C** | **T** | **T** | **T** | **G** | **A** | **A** | **A** | **A** | **C** | **A** | **G** | **T** | **T** | **A** | **A** | **A** | **A** | **C** | **A** | **C** | **A** | **T** | **G** | **T** | **T** | **T** | **C** | **A** | **A** | **A** | **C** | **A** | **T** | **T** | **T** | **T** | **A** | **G** | **C** | **A** | **A** | **A** | **A** | **C** | **T** | **A** | **G** | **A** | **T** | **G** | **T** | **G** | **G** | **A** | **T** | **G** | **A** | **T** | **C** | **G** | **G** | **A** | **C** | **C** | **C** | **A** | **A** | **G** | **C** | **G** | **G** | **C** | **G** | **A** | **T** | **T** | **T** | **A** | **T** | **G** | **C** | **T** | **T** | **T** | **T** | **C** | **A** | **A** | **C** | **A** | **T** | **G** | **G** | **T** | **T** | **T** | **A** | **G** | **C** | **C** | **A** | **A** | **A** | **-** | **-** | **-** |
| **N60519F** | **A** | **T** | **G** | **A** | **T** | **A** | **A** | **A** | **A** | **G** | **T** | **T** | **T** | **T** | **A** | **T** | **T** | **A** | **G** | **T** | **A** | **G** | **A** | **T** | **G** | **A** | **C** | **C** | **A** | **T** | **G** | **A** | **A** | **A** | **T** | **G** | **G** | **T** | **A** | **C** | **G** | **C** | **T** | **T** | **A** | **G** | **G** | **C** | **G** | **T** | **C** | **T** | **C** | **T** | **T** | **C** | **T** | **T** | **A** | **T** | **T** | **T** | **A** | **T** | **C** | **T** | **A** | **T** | **C** | **C** | **A** | **A** | **G** | **A** | **A** | **G** | **A** | **C** | **A** | **T** | **T** | **G** | **A** | **A** | **G** | **T** | **G** | **A** | **T** | **T** | **G** | **G** | **A** | **G** | **A** | **A** | **G** | **C** | **T** | **G** | **A** | **A** | **A** | **A** | **T** | **G** | **G** | **A** | **C** | **G** | **T** | **C** | **A** | **A** | **G** | **G** | **C** | **T** | **A** | **T** | **G** | **A** | **A** | **A** | **A** | **G** | **G** | **C** | **G** | **A** | **T** | **G** | **G** | **C** | **A** | **C** | **T** | **T** | **C** | **G** | **A** | **C** | **C** | **A** | **G** | **A** | **T** | **G** | **T** | **C** | **A** | **T** | **T** | **T** | **T** | **A** | **A** | **T** | **G** | **G** | **A** | **T** | **C** | **T** | **A** | **G** | **T** | **C** | **A** | **T** | **G** | **G** | **A** | **A** | **G** | **A** | **A** | **A** | **T** | **G** | **G** | **A** | **T** | **G** | **G** | **C** | **A** | **T** | **C** | **G** | **A** | **A** | **T** | **C** | **A** | **A** | **C** | **A** | **A** | **A** | **A** | **G** | **C** | **A** | **A** | **T** | **C** | **T** | **T** | **A** | **A** | **A** | **A** | **G** | **A** | **T** | **T** | **G** | **G** | **C** | **C** | **A** | **A** | **A** | **A** | **G** | **C** | **C** | **A** | **A** | **A** | **A** | **T** | **C** | **A** | **T** | **T** | **A** | **T** | **C** | **G** | **T** | **C** | **A** | **C** | **A** | **A** | **G** | **T** | **T** | **T** | **T** | **A** | **T** | **T** | **G** | **A** | **T** | **G** | **A** | **T** | **G** | **A** | **A** | **A** | **A** | **A** | **G** | **T** | **T** | **T** | **A** | **T** | **C** | **C** | **T** | **G** | **C** | **A** | **A** | **T** | **T** | **G** | **A** | **A** | **G** | **C** | **A** | **G** | **G** | **A** | **G** | **C** | **A** | **G** | **C** | **A** | **G** | **G** | **A** | **T** | **A** | **T** | **T** | **T** | **A** | **T** | **T** | **G** | **A** | **A** | **G** | **A** | **C** | **G** | **T** | **C** | **T** | **A** | **C** | **C** | **G** | **C** | **A** | **C** | **A** | **T** | **G** | **A** | **A** | **A** | **T** | **A** | **G** | **C** | **T** | **G** | **A** | **T** | **G** | **C** | **T** | **A** | **T** | **A** | **A** | **G** | **G** | **G** | **C** | **T** | **A** | **C** | **G** | **C** | **A** | **A** | **C** | **G** | **C** | **G** | **G** | **C** | **G** | **A** | **G** | **C** | **G** | **A** | **G** | **T** | **A** | **T** | **T** | **G** | **G** | **A** | **A** | **C** | **C** | **T** | **G** | **A** | **A** | **G** | **T** | **G** | **A** | **C** | **A** | **A** | **C** | **A** | **A** | **A** | **A** | **A** | **T** | **G** | **A** | **T** | **G** | **G** | **A** | **A** | **A** | **A** | **A** | **A** | **T** | **G** | **A** | **G** | **T** | **C** | **G** | **G** | **C** | **G** | **A** | **A** | **A** | **T** | **G** | **A** | **T** | **C** | **C** | **A** | **G** | **T** | **A** | **T** | **T** | **G** | **C** | **A** | **T** | **G** | **A** | **A** | **G** | **A** | **A** | **T** | **T** | **A** | **A** | **C** | **G** | **A** | **A** | **T** | **C** | **G** | **A** | **G** | **A** | **A** | **A** | **A** | **T** | **G** | **A** | **A** | **A** | **T** | **T** | **T** | **T** | **G** | **A** | **T** | **G** | **C** | **T** | **G** | **A** | **T** | **T** | **T** | **C** | **T** | **G** | **A** | **A** | **G** | **G** | **G** | **A** | **A** | **A** | **A** | **G** | **T** | **A** | **A** | **T** | **C** | **A** | **G** | **G** | **A** | **A** | **A** | **T** | **C** | **G** | **C** | **G** | **G** | **A** | **T** | **G** | **A** | **A** | **T** | **T** | **G** | **T** | **T** | **T** | **A** | **T** | **C** | **A** | **C** | **A** | **C** | **T** | **G** | **A** | **A** | **A** | **A** | **C** | **A** | **G** | **T** | **T** | **A** | **A** | **A** | **A** | **C** | **G** | **C** | **A** | **T** | **G** | **T** | **G** | **T** | **C** | **G** | **A** | **A** | **T** | **A** | **T** | **A** | **T** | **T** | **A** | **G** | **C** | **T** | **A** | **A** | **G** | **T** | **T** | **A** | **G** | **A** | **G** | **G** | **T** | **A** | **G** | **A** | **A** | **G** | **A** | **C** | **C** | **G** | **A** | **A** | **C** | **T** | **C** | **A** | **A** | **G** | **C** | **A** | **G** | **C** | **T** | **A** | **T** | **C** | **T** | **A** | **T** | **G** | **C** | **G** | **T** | **T** | **T** | **A** | **A** | **A** | **C** | **A** | **T** | **G** | **G** | **T** | **T** | **T** | **G** | **G** | **T** | **G** | **A** | **A** | **A** | **-** | **-** | **-** |
| **N60063F** | **G** | **T** | **G** | **A** | **T** | **C** | **A** | **A** | **A** | **G** | **T** | **A** | **A** | **T** | **G** | **T** | **T** | **A** | **G** | **T** | **G** | **G** | **A** | **T** | **G** | **A** | **C** | **C** | **A** | **T** | **G** | **A** | **A** | **A** | **T** | **G** | **G** | **T** | **C** | **C** | **G** | **T** | **T** | **T** | **A** | **G** | **G** | **C** | **G** | **T** | **T** | **T** | **C** | **A** | **T** | **C** | **A** | **T** | **A** | **T** | **T** | **T** | **A** | **T** | **C** | **T** | **A** | **T** | **T** | **C** | **A** | **A** | **G** | **A** | **G** | **G** | **A** | **T** | **A** | **T** | **A** | **G** | **A** | **A** | **G** | **T** | **C** | **G** | **T** | **A** | **G** | **G** | **C** | **G** | **A** | **A** | **G** | **C** | **A** | **G** | **A** | **A** | **A** | **A** | **T** | **G** | **G** | **T** | **A** | **A** | **G** | **A** | **T** | **T** | **G** | **G** | **C** | **T** | **A** | **T** | **G** | **A** | **A** | **A** | **A** | **A** | **G** | **C** | **A** | **T** | **T** | **G** | **G** | **A** | **A** | **C** | **T** | **A** | **C** | **G** | **T** | **C** | **C** | **A** | **G** | **A** | **T** | **G** | **T** | **T** | **A** | **T** | **T** | **T** | **T** | **G** | **A** | **T** | **G** | **G** | **A** | **T** | **T** | **T** | **G** | **G** | **T** | **A** | **A** | **T** | **G** | **G** | **A** | **A** | **G** | **A** | **A** | **A** | **T** | **G** | **G** | **A** | **C** | **G** | **G** | **C** | **A** | **T** | **T** | **G** | **A** | **T** | **T** | **C** | **A** | **A** | **C** | **A** | **A** | **A** | **A** | **G** | **C** | **G** | **A** | **T** | **C** | **T** | **T** | **G** | **A** | **A** | **A** | **G** | **A** | **T** | **T** | **G** | **G** | **C** | **C** | **A** | **G** | **A** | **A** | **G** | **C** | **C** | **A** | **A** | **G** | **A** | **T** | **T** | **A** | **T** | **T** | **A** | **T** | **T** | **G** | **T** | **G** | **A** | **C** | **G** | **A** | **G** | **T** | **T** | **T** | **T** | **A** | **T** | **T** | **G** | **A** | **T** | **G** | **A** | **T** | **G** | **A** | **A** | **A** | **A** | **A** | **G** | **T** | **G** | **T** | **A** | **T** | **C** | **C** | **G** | **G** | **C** | **G** | **A** | **T** | **T** | **G** | **A** | **A** | **G** | **C** | **T** | **G** | **G** | **T** | **G** | **C** | **A** | **G** | **C** | **G** | **G** | **G** | **C** | **T** | **A** | **C** | **C** | **T** | **A** | **T** | **T** | **A** | **A** | **A** | **A** | **A** | **C** | **A** | **T** | **C** | **A** | **A** | **C** | **A** | **G** | **C** | **A** | **C** | **A** | **T** | **G** | **A** | **G** | **A** | **T** | **T** | **G** | **C** | **T** | **G** | **A** | **T** | **G** | **C** | **A** | **A** | **T** | **T** | **C** | **G** | **G** | **G** | **C** | **G** | **A** | **C** | **T** | **T** | **A** | **T** | **C** | **G** | **C** | **G** | **G** | **A** | **G** | **A** | **G** | **C** | **G** | **T** | **G** | **T** | **G** | **T** | **T** | **G** | **G** | **A** | **A** | **C** | **C** | **T** | **G** | **A** | **A** | **G** | **T** | **G** | **A** | **C** | **A** | **C** | **A** | **T** | **A** | **A** | **G** | **A** | **T** | **G** | **A** | **T** | **G** | **G** | **A** | **A** | **C** | **G** | **G** | **T** | **T** | **A** | **A** | **C** | **A** | **A** | **A** | **A** | **A** | **A** | **A** | **C** | **A** | **A** | **G** | **A** | **G** | **C** | **C** | **G** | **G** | **T** | **G** | **T** | **T** | **G** | **C** | **A** | **C** | **G** | **A** | **A** | **G** | **A** | **T** | **T** | **T** | **G** | **A** | **C** | **A** | **A** | **A** | **C** | **C** | **G** | **G** | **G** | **A** | **A** | **C** | **A** | **C** | **G** | **A** | **A** | **A** | **T** | **T** | **T** | **T** | **A** | **A** | **T** | **G** | **T** | **T** | **G** | **A** | **T** | **T** | **G** | **C** | **A** | **C** | **A** | **A** | **G** | **G** | **T** | **A** | **A** | **A** | **A** | **G** | **T** | **A** | **A** | **T** | **C** | **A** | **G** | **G** | **A** | **A** | **A** | **T** | **A** | **G** | **C** | **T** | **G** | **A** | **T** | **G** | **A** | **A** | **C** | **T** | **C** | **T** | **T** | **T** | **A** | **T** | **C** | **A** | **C** | **T** | **T** | **T** | **G** | **A** | **A** | **A** | **A** | **C** | **A** | **G** | **T** | **T** | **A** | **A** | **A** | **A** | **C** | **A** | **C** | **A** | **T** | **G** | **T** | **T** | **T** | **C** | **A** | **A** | **A** | **C** | **A** | **T** | **T** | **T** | **T** | **A** | **G** | **C** | **A** | **A** | **A** | **A** | **C** | **T** | **A** | **G** | **A** | **T** | **G** | **T** | **G** | **G** | **A** | **T** | **G** | **A** | **T** | **C** | **G** | **G** | **A** | **C** | **C** | **C** | **A** | **A** | **G** | **C** | **G** | **G** | **C** | **G** | **A** | **T** | **T** | **T** | **A** | **T** | **G** | **C** | **T** | **T** | **T** | **T** | **C** | **A** | **A** | **C** | **A** | **T** | **G** | **G** | **T** | **T** | **T** | **A** | **G** | **C** | **C** | **A** | **A** | **A** | **-** | **-** | **-** |
| **N53434** | **G** | **T** | **G** | **A** | **T** | **C** | **A** | **A** | **A** | **G** | **T** | **A** | **A** | **T** | **G** | **T** | **T** | **A** | **G** | **T** | **G** | **G** | **A** | **T** | **G** | **A** | **C** | **C** | **A** | **T** | **G** | **A** | **A** | **A** | **T** | **G** | **G** | **T** | **C** | **C** | **G** | **T** | **T** | **T** | **A** | **G** | **G** | **C** | **G** | **T** | **T** | **T** | **C** | **A** | **T** | **C** | **A** | **T** | **A** | **T** | **T** | **T** | **A** | **T** | **C** | **T** | **A** | **T** | **T** | **C** | **A** | **A** | **G** | **A** | **G** | **G** | **A** | **T** | **A** | **T** | **A** | **G** | **A** | **A** | **G** | **T** | **C** | **G** | **T** | **A** | **G** | **G** | **C** | **G** | **A** | **A** | **G** | **C** | **A** | **G** | **A** | **A** | **A** | **A** | **C** | **G** | **G** | **T** | **A** | **A** | **G** | **A** | **T** | **T** | **G** | **G** | **C** | **T** | **A** | **T** | **G** | **A** | **A** | **A** | **A** | **A** | **G** | **C** | **A** | **T** | **T** | **G** | **G** | **A** | **A** | **C** | **T** | **A** | **C** | **G** | **T** | **C** | **C** | **A** | **G** | **A** | **T** | **G** | **T** | **T** | **A** | **T** | **T** | **T** | **T** | **G** | **A** | **T** | **G** | **G** | **A** | **T** | **T** | **T** | **G** | **G** | **T** | **A** | **A** | **T** | **G** | **G** | **A** | **A** | **G** | **A** | **A** | **A** | **T** | **G** | **G** | **A** | **C** | **G** | **G** | **C** | **A** | **T** | **T** | **G** | **A** | **T** | **T** | **C** | **A** | **A** | **C** | **A** | **A** | **A** | **A** | **G** | **C** | **G** | **A** | **T** | **C** | **T** | **T** | **G** | **A** | **A** | **A** | **G** | **A** | **T** | **T** | **G** | **G** | **C** | **C** | **A** | **G** | **A** | **A** | **G** | **C** | **C** | **A** | **A** | **G** | **A** | **T** | **T** | **A** | **T** | **T** | **A** | **T** | **T** | **G** | **T** | **G** | **A** | **C** | **G** | **A** | **G** | **T** | **T** | **T** | **T** | **A** | **T** | **T** | **G** | **A** | **T** | **G** | **A** | **T** | **G** | **A** | **A** | **A** | **A** | **A** | **G** | **T** | **G** | **T** | **A** | **T** | **C** | **C** | **G** | **G** | **C** | **G** | **A** | **T** | **T** | **G** | **A** | **A** | **G** | **C** | **T** | **G** | **G** | **T** | **G** | **C** | **A** | **G** | **C** | **G** | **G** | **G** | **C** | **T** | **A** | **C** | **C** | **T** | **A** | **T** | **T** | **A** | **A** | **A** | **A** | **A** | **C** | **A** | **T** | **C** | **A** | **A** | **C** | **A** | **G** | **C** | **A** | **C** | **A** | **T** | **G** | **A** | **G** | **A** | **T** | **T** | **G** | **C** | **T** | **G** | **A** | **T** | **G** | **C** | **A** | **A** | **T** | **T** | **C** | **G** | **G** | **G** | **C** | **G** | **A** | **C** | **T** | **T** | **A** | **T** | **C** | **G** | **C** | **G** | **G** | **A** | **G** | **A** | **G** | **C** | **G** | **T** | **G** | **T** | **G** | **T** | **T** | **G** | **G** | **A** | **A** | **C** | **C** | **T** | **G** | **A** | **A** | **G** | **T** | **G** | **A** | **C** | **G** | **C** | **A** | **T** | **A** | **A** | **G** | **A** | **T** | **G** | **A** | **T** | **G** | **G** | **A** | **A** | **C** | **G** | **G** | **T** | **T** | **A** | **A** | **C** | **A** | **A** | **A** | **A** | **A** | **A** | **A** | **C** | **A** | **A** | **G** | **A** | **G** | **C** | **C** | **G** | **G** | **T** | **G** | **T** | **T** | **G** | **C** | **A** | **C** | **G** | **A** | **A** | **G** | **A** | **T** | **T** | **T** | **G** | **A** | **C** | **A** | **A** | **A** | **C** | **C** | **G** | **G** | **G** | **A** | **A** | **C** | **A** | **C** | **G** | **A** | **A** | **A** | **T** | **T** | **T** | **T** | **A** | **A** | **T** | **G** | **T** | **T** | **G** | **A** | **T** | **T** | **G** | **C** | **A** | **C** | **A** | **A** | **G** | **G** | **T** | **A** | **A** | **A** | **A** | **G** | **T** | **A** | **A** | **T** | **C** | **A** | **G** | **G** | **A** | **A** | **A** | **T** | **A** | **G** | **C** | **T** | **G** | **A** | **T** | **G** | **A** | **A** | **C** | **T** | **C** | **T** | **T** | **T** | **A** | **T** | **C** | **A** | **C** | **T** | **T** | **T** | **G** | **A** | **A** | **A** | **A** | **C** | **A** | **G** | **T** | **T** | **A** | **A** | **A** | **A** | **C** | **A** | **C** | **A** | **T** | **G** | **T** | **T** | **T** | **C** | **A** | **A** | **A** | **C** | **A** | **T** | **T** | **T** | **T** | **A** | **G** | **C** | **A** | **A** | **A** | **A** | **C** | **T** | **A** | **G** | **A** | **T** | **G** | **T** | **G** | **G** | **A** | **T** | **G** | **A** | **T** | **C** | **G** | **G** | **A** | **C** | **C** | **C** | **A** | **A** | **G** | **C** | **G** | **G** | **C** | **G** | **A** | **T** | **T** | **T** | **A** | **T** | **G** | **C** | **T** | **T** | **T** | **T** | **C** | **A** | **A** | **C** | **A** | **T** | **G** | **G** | **T** | **T** | **T** | **A** | **G** | **C** | **C** | **A** | **A** | **A** | **-** | **-** | **-** |
| **N60271F** | **G** | **T** | **G** | **A** | **T** | **C** | **A** | **A** | **A** | **G** | **T** | **A** | **A** | **T** | **G** | **T** | **T** | **A** | **G** | **T** | **G** | **G** | **A** | **T** | **G** | **A** | **C** | **C** | **A** | **T** | **G** | **A** | **A** | **A** | **T** | **G** | **G** | **T** | **C** | **C** | **G** | **T** | **T** | **T** | **A** | **G** | **G** | **C** | **G** | **T** | **T** | **T** | **C** | **A** | **T** | **C** | **A** | **T** | **A** | **T** | **T** | **T** | **A** | **T** | **C** | **T** | **A** | **T** | **T** | **C** | **A** | **A** | **G** | **A** | **G** | **G** | **A** | **T** | **A** | **T** | **A** | **G** | **A** | **A** | **G** | **T** | **C** | **G** | **T** | **A** | **G** | **G** | **C** | **G** | **A** | **A** | **G** | **C** | **A** | **G** | **A** | **A** | **A** | **A** | **C** | **G** | **G** | **T** | **A** | **A** | **G** | **A** | **T** | **T** | **G** | **G** | **C** | **T** | **A** | **T** | **G** | **A** | **A** | **A** | **A** | **A** | **G** | **C** | **A** | **T** | **T** | **G** | **G** | **A** | **A** | **C** | **T** | **A** | **C** | **G** | **T** | **C** | **C** | **A** | **G** | **A** | **T** | **G** | **T** | **T** | **A** | **T** | **T** | **T** | **T** | **G** | **A** | **T** | **G** | **G** | **A** | **T** | **T** | **T** | **G** | **G** | **T** | **A** | **A** | **T** | **G** | **G** | **A** | **A** | **G** | **A** | **A** | **A** | **T** | **G** | **G** | **A** | **C** | **G** | **G** | **C** | **A** | **T** | **T** | **G** | **A** | **T** | **T** | **C** | **A** | **A** | **C** | **A** | **A** | **A** | **A** | **G** | **C** | **G** | **A** | **T** | **C** | **T** | **T** | **G** | **A** | **A** | **A** | **G** | **A** | **T** | **T** | **G** | **G** | **C** | **C** | **A** | **G** | **A** | **A** | **G** | **C** | **C** | **A** | **A** | **G** | **A** | **T** | **T** | **A** | **T** | **T** | **A** | **T** | **T** | **G** | **T** | **G** | **A** | **C** | **G** | **A** | **G** | **T** | **T** | **T** | **T** | **A** | **T** | **T** | **G** | **A** | **T** | **G** | **A** | **T** | **G** | **A** | **A** | **A** | **A** | **A** | **G** | **T** | **G** | **T** | **A** | **T** | **C** | **C** | **G** | **G** | **C** | **G** | **A** | **T** | **T** | **G** | **A** | **A** | **G** | **C** | **T** | **G** | **G** | **T** | **G** | **C** | **A** | **G** | **C** | **G** | **G** | **G** | **C** | **T** | **A** | **C** | **C** | **T** | **A** | **T** | **T** | **A** | **A** | **A** | **A** | **A** | **C** | **A** | **T** | **C** | **A** | **A** | **C** | **A** | **G** | **C** | **A** | **C** | **A** | **T** | **G** | **A** | **G** | **A** | **T** | **T** | **G** | **C** | **T** | **G** | **A** | **T** | **G** | **C** | **A** | **A** | **T** | **T** | **C** | **G** | **G** | **G** | **C** | **G** | **A** | **C** | **T** | **T** | **A** | **T** | **C** | **G** | **C** | **G** | **G** | **A** | **G** | **A** | **G** | **C** | **G** | **T** | **G** | **T** | **G** | **T** | **T** | **G** | **G** | **A** | **A** | **C** | **C** | **T** | **G** | **A** | **A** | **G** | **T** | **G** | **A** | **C** | **A** | **C** | **A** | **T** | **A** | **A** | **G** | **A** | **T** | **G** | **A** | **T** | **G** | **G** | **A** | **A** | **C** | **G** | **G** | **T** | **T** | **A** | **A** | **C** | **A** | **A** | **A** | **A** | **A** | **A** | **A** | **C** | **A** | **A** | **G** | **A** | **G** | **C** | **C** | **G** | **G** | **T** | **G** | **T** | **T** | **G** | **C** | **A** | **C** | **G** | **A** | **A** | **G** | **A** | **T** | **T** | **T** | **G** | **A** | **C** | **A** | **A** | **A** | **C** | **C** | **G** | **G** | **G** | **A** | **A** | **C** | **A** | **C** | **G** | **A** | **A** | **A** | **T** | **T** | **T** | **T** | **A** | **A** | **T** | **G** | **T** | **T** | **G** | **A** | **T** | **T** | **G** | **C** | **A** | **C** | **A** | **A** | **G** | **G** | **T** | **A** | **A** | **A** | **A** | **G** | **T** | **A** | **A** | **T** | **C** | **A** | **G** | **G** | **A** | **A** | **A** | **T** | **A** | **G** | **C** | **T** | **G** | **A** | **T** | **G** | **A** | **A** | **C** | **T** | **C** | **T** | **T** | **T** | **A** | **T** | **C** | **A** | **C** | **T** | **T** | **T** | **G** | **A** | **A** | **A** | **A** | **C** | **A** | **G** | **T** | **T** | **A** | **A** | **A** | **A** | **C** | **A** | **C** | **A** | **T** | **G** | **T** | **T** | **T** | **C** | **A** | **A** | **A** | **C** | **A** | **T** | **T** | **T** | **T** | **A** | **G** | **C** | **A** | **A** | **A** | **A** | **C** | **T** | **A** | **G** | **A** | **T** | **G** | **T** | **G** | **G** | **A** | **T** | **G** | **A** | **T** | **C** | **G** | **G** | **A** | **C** | **C** | **C** | **A** | **A** | **G** | **C** | **G** | **G** | **C** | **G** | **A** | **T** | **T** | **T** | **A** | **T** | **G** | **C** | **T** | **T** | **T** | **T** | **C** | **A** | **A** | **C** | **A** | **T** | **G** | **G** | **T** | **T** | **T** | **A** | **G** | **C** | **C** | **A** | **A** | **A** | **-** | **-** | **-** |
| **N59847F** | **G** | **T** | **G** | **A** | **T** | **C** | **A** | **A** | **A** | **G** | **T** | **A** | **A** | **T** | **G** | **T** | **T** | **A** | **G** | **T** | **G** | **G** | **A** | **T** | **G** | **A** | **C** | **C** | **A** | **T** | **G** | **A** | **A** | **A** | **T** | **G** | **G** | **T** | **C** | **C** | **G** | **T** | **T** | **T** | **A** | **G** | **G** | **C** | **G** | **T** | **T** | **T** | **C** | **A** | **T** | **C** | **A** | **T** | **A** | **T** | **T** | **T** | **A** | **T** | **C** | **T** | **A** | **T** | **T** | **C** | **A** | **A** | **G** | **A** | **G** | **G** | **A** | **T** | **A** | **T** | **A** | **G** | **A** | **A** | **G** | **T** | **C** | **G** | **T** | **A** | **G** | **G** | **C** | **G** | **A** | **A** | **G** | **C** | **A** | **G** | **A** | **A** | **A** | **A** | **C** | **G** | **G** | **T** | **A** | **A** | **G** | **A** | **T** | **T** | **G** | **G** | **C** | **T** | **A** | **T** | **G** | **A** | **A** | **A** | **A** | **A** | **G** | **C** | **A** | **T** | **T** | **G** | **G** | **A** | **A** | **C** | **T** | **A** | **C** | **G** | **T** | **C** | **C** | **A** | **G** | **A** | **T** | **G** | **T** | **T** | **A** | **T** | **T** | **T** | **T** | **G** | **A** | **T** | **G** | **G** | **A** | **T** | **T** | **T** | **G** | **G** | **T** | **A** | **A** | **T** | **G** | **G** | **A** | **A** | **G** | **A** | **A** | **A** | **T** | **G** | **G** | **A** | **C** | **G** | **G** | **C** | **A** | **T** | **T** | **G** | **A** | **T** | **T** | **C** | **A** | **A** | **C** | **A** | **A** | **A** | **A** | **G** | **C** | **G** | **A** | **T** | **C** | **T** | **T** | **G** | **A** | **A** | **A** | **G** | **A** | **T** | **T** | **G** | **G** | **C** | **C** | **A** | **G** | **A** | **A** | **G** | **C** | **C** | **A** | **A** | **G** | **A** | **T** | **T** | **A** | **T** | **T** | **A** | **T** | **T** | **G** | **T** | **G** | **A** | **C** | **G** | **A** | **G** | **T** | **T** | **T** | **T** | **A** | **T** | **T** | **G** | **A** | **T** | **G** | **A** | **T** | **G** | **A** | **A** | **A** | **A** | **A** | **G** | **T** | **G** | **T** | **A** | **T** | **C** | **C** | **G** | **G** | **C** | **G** | **A** | **T** | **T** | **G** | **A** | **A** | **G** | **C** | **T** | **G** | **G** | **T** | **G** | **C** | **A** | **G** | **C** | **G** | **G** | **G** | **C** | **T** | **A** | **C** | **C** | **T** | **A** | **T** | **T** | **A** | **A** | **A** | **G** | **A** | **C** | **A** | **T** | **C** | **A** | **A** | **C** | **A** | **G** | **C** | **A** | **C** | **A** | **T** | **G** | **A** | **G** | **A** | **T** | **T** | **G** | **C** | **T** | **G** | **A** | **T** | **G** | **C** | **A** | **A** | **T** | **T** | **C** | **G** | **G** | **G** | **C** | **G** | **A** | **C** | **T** | **T** | **A** | **T** | **C** | **G** | **C** | **G** | **G** | **A** | **G** | **A** | **G** | **C** | **G** | **T** | **G** | **T** | **G** | **T** | **T** | **G** | **G** | **A** | **A** | **C** | **C** | **T** | **G** | **A** | **A** | **G** | **T** | **G** | **A** | **C** | **G** | **C** | **A** | **T** | **A** | **A** | **G** | **A** | **T** | **G** | **A** | **T** | **G** | **G** | **A** | **A** | **C** | **G** | **G** | **T** | **T** | **A** | **A** | **C** | **A** | **A** | **A** | **A** | **A** | **A** | **A** | **C** | **A** | **A** | **G** | **A** | **G** | **C** | **C** | **G** | **G** | **T** | **G** | **T** | **T** | **G** | **C** | **A** | **C** | **G** | **A** | **A** | **G** | **A** | **T** | **T** | **T** | **G** | **A** | **C** | **A** | **A** | **A** | **C** | **C** | **G** | **G** | **G** | **A** | **A** | **C** | **A** | **C** | **G** | **A** | **A** | **A** | **T** | **T** | **T** | **T** | **A** | **A** | **T** | **G** | **T** | **T** | **G** | **A** | **T** | **T** | **G** | **C** | **A** | **C** | **A** | **A** | **G** | **G** | **T** | **A** | **A** | **A** | **A** | **G** | **T** | **A** | **A** | **T** | **C** | **A** | **G** | **G** | **A** | **A** | **A** | **T** | **A** | **G** | **C** | **T** | **G** | **A** | **T** | **G** | **A** | **A** | **C** | **T** | **C** | **T** | **T** | **T** | **A** | **T** | **C** | **A** | **C** | **T** | **T** | **T** | **G** | **A** | **A** | **A** | **A** | **C** | **A** | **G** | **T** | **T** | **A** | **A** | **A** | **A** | **C** | **A** | **C** | **A** | **T** | **G** | **T** | **T** | **T** | **C** | **A** | **A** | **A** | **C** | **A** | **T** | **T** | **T** | **T** | **A** | **G** | **C** | **A** | **A** | **A** | **A** | **C** | **T** | **A** | **G** | **A** | **T** | **G** | **T** | **G** | **G** | **A** | **T** | **G** | **A** | **T** | **C** | **G** | **G** | **A** | **C** | **C** | **C** | **A** | **A** | **G** | **C** | **G** | **G** | **C** | **G** | **A** | **T** | **T** | **T** | **A** | **T** | **G** | **C** | **T** | **T** | **T** | **T** | **C** | **A** | **A** | **C** | **A** | **T** | **G** | **G** | **T** | **T** | **T** | **A** | **G** | **C** | **C** | **A** | **A** | **A** | **-** | **-** | **-** |
| **N59439F** | **A** | **T** | **G** | **A** | **T** | **A** | **A** | **A** | **A** | **G** | **T** | **T** | **T** | **T** | **A** | **T** | **T** | **A** | **G** | **T** | **A** | **G** | **A** | **T** | **G** | **A** | **C** | **C** | **A** | **T** | **G** | **A** | **A** | **A** | **T** | **G** | **G** | **T** | **A** | **C** | **G** | **C** | **T** | **T** | **A** | **G** | **G** | **C** | **G** | **T** | **C** | **T** | **C** | **T** | **T** | **C** | **T** | **T** | **A** | **T** | **T** | **T** | **A** | **T** | **C** | **T** | **A** | **T** | **C** | **C** | **A** | **A** | **G** | **A** | **A** | **G** | **A** | **C** | **A** | **T** | **T** | **G** | **A** | **A** | **G** | **T** | **G** | **A** | **T** | **T** | **G** | **G** | **A** | **G** | **A** | **A** | **G** | **C** | **T** | **G** | **A** | **A** | **A** | **A** | **T** | **G** | **G** | **A** | **C** | **G** | **T** | **C** | **A** | **A** | **G** | **G** | **C** | **T** | **A** | **T** | **G** | **A** | **A** | **A** | **A** | **G** | **G** | **C** | **G** | **A** | **T** | **G** | **G** | **C** | **A** | **C** | **T** | **T** | **C** | **G** | **A** | **C** | **C** | **A** | **G** | **A** | **T** | **G** | **T** | **C** | **A** | **T** | **T** | **T** | **T** | **A** | **A** | **T** | **G** | **G** | **A** | **T** | **C** | **T** | **A** | **G** | **T** | **C** | **A** | **T** | **G** | **G** | **A** | **A** | **G** | **A** | **A** | **A** | **T** | **G** | **G** | **A** | **T** | **G** | **G** | **C** | **A** | **T** | **C** | **G** | **A** | **A** | **T** | **C** | **A** | **A** | **C** | **A** | **A** | **A** | **A** | **G** | **C** | **A** | **A** | **T** | **C** | **T** | **T** | **A** | **A** | **A** | **A** | **G** | **A** | **T** | **T** | **G** | **G** | **C** | **C** | **A** | **A** | **A** | **A** | **G** | **C** | **C** | **A** | **A** | **A** | **A** | **T** | **C** | **A** | **T** | **T** | **A** | **T** | **C** | **G** | **T** | **C** | **A** | **C** | **A** | **A** | **G** | **T** | **T** | **T** | **T** | **A** | **T** | **T** | **G** | **A** | **T** | **G** | **A** | **T** | **G** | **A** | **A** | **A** | **A** | **A** | **G** | **T** | **T** | **T** | **A** | **T** | **C** | **C** | **T** | **G** | **C** | **A** | **A** | **T** | **T** | **G** | **A** | **A** | **G** | **C** | **A** | **G** | **G** | **A** | **G** | **C** | **A** | **G** | **C** | **A** | **G** | **G** | **A** | **T** | **A** | **T** | **T** | **T** | **A** | **T** | **T** | **G** | **A** | **A** | **G** | **A** | **C** | **G** | **T** | **C** | **T** | **A** | **C** | **C** | **G** | **C** | **A** | **C** | **A** | **T** | **G** | **A** | **A** | **A** | **T** | **A** | **G** | **C** | **T** | **G** | **A** | **T** | **G** | **C** | **T** | **A** | **T** | **A** | **A** | **G** | **G** | **G** | **C** | **T** | **A** | **C** | **G** | **C** | **A** | **A** | **C** | **G** | **C** | **G** | **G** | **C** | **G** | **A** | **G** | **C** | **G** | **A** | **G** | **T** | **A** | **T** | **T** | **G** | **G** | **A** | **A** | **C** | **C** | **T** | **G** | **A** | **A** | **G** | **T** | **G** | **A** | **C** | **A** | **A** | **C** | **A** | **A** | **A** | **A** | **A** | **T** | **G** | **A** | **T** | **G** | **G** | **A** | **A** | **A** | **A** | **A** | **A** | **T** | **G** | **A** | **G** | **T** | **C** | **G** | **G** | **C** | **G** | **A** | **A** | **A** | **T** | **G** | **A** | **T** | **C** | **C** | **A** | **G** | **T** | **A** | **T** | **T** | **G** | **C** | **A** | **T** | **G** | **A** | **A** | **G** | **A** | **A** | **T** | **T** | **A** | **A** | **C** | **G** | **A** | **A** | **T** | **C** | **G** | **A** | **G** | **A** | **A** | **A** | **A** | **T** | **G** | **A** | **A** | **A** | **T** | **T** | **T** | **T** | **G** | **A** | **T** | **G** | **C** | **T** | **G** | **A** | **T** | **T** | **T** | **C** | **T** | **G** | **A** | **A** | **G** | **G** | **G** | **A** | **A** | **A** | **A** | **G** | **T** | **A** | **A** | **T** | **C** | **A** | **G** | **G** | **A** | **A** | **A** | **T** | **C** | **G** | **C** | **G** | **G** | **A** | **T** | **G** | **A** | **A** | **T** | **T** | **G** | **T** | **T** | **T** | **A** | **T** | **C** | **A** | **C** | **A** | **C** | **T** | **G** | **A** | **A** | **A** | **A** | **C** | **A** | **G** | **T** | **T** | **A** | **A** | **A** | **A** | **C** | **G** | **C** | **A** | **T** | **G** | **T** | **G** | **T** | **C** | **G** | **A** | **A** | **T** | **A** | **T** | **A** | **T** | **T** | **A** | **G** | **C** | **T** | **A** | **A** | **G** | **T** | **T** | **A** | **G** | **A** | **G** | **G** | **T** | **A** | **G** | **A** | **A** | **G** | **A** | **C** | **C** | **G** | **A** | **A** | **C** | **T** | **C** | **A** | **A** | **G** | **C** | **A** | **G** | **C** | **T** | **A** | **T** | **C** | **T** | **A** | **T** | **G** | **C** | **G** | **T** | **T** | **T** | **A** | **A** | **A** | **C** | **A** | **T** | **G** | **G** | **T** | **T** | **T** | **G** | **G** | **T** | **G** | **A** | **A** | **A** | **-** | **-** | **-** |
| **N52732** | **A** | **T** | **G** | **A** | **T** | **A** | **A** | **A** | **A** | **G** | **T** | **T** | **T** | **T** | **A** | **T** | **T** | **A** | **G** | **T** | **A** | **G** | **A** | **T** | **G** | **A** | **C** | **C** | **A** | **T** | **G** | **A** | **A** | **A** | **T** | **G** | **G** | **T** | **G** | **C** | **G** | **C** | **T** | **T** | **A** | **G** | **G** | **C** | **G** | **T** | **C** | **T** | **C** | **T** | **T** | **C** | **T** | **T** | **A** | **T** | **T** | **T** | **A** | **T** | **C** | **T** | **A** | **T** | **C** | **C** | **A** | **A** | **G** | **A** | **A** | **G** | **A** | **C** | **A** | **T** | **T** | **G** | **A** | **A** | **G** | **T** | **A** | **A** | **T** | **T** | **G** | **G** | **A** | **G** | **A** | **A** | **G** | **C** | **T** | **G** | **A** | **A** | **A** | **A** | **T** | **G** | **G** | **A** | **C** | **G** | **A** | **C** | **A** | **A** | **G** | **G** | **C** | **T** | **A** | **T** | **G** | **A** | **G** | **A** | **A** | **G** | **G** | **C** | **G** | **A** | **T** | **G** | **A** | **C** | **A** | **C** | **T** | **T** | **C** | **G** | **A** | **C** | **C** | **G** | **G** | **A** | **C** | **G** | **T** | **C** | **A** | **T** | **T** | **T** | **T** | **A** | **A** | **T** | **G** | **G** | **A** | **C** | **T** | **T** | **A** | **G** | **T** | **C** | **A** | **T** | **G** | **G** | **A** | **A** | **G** | **A** | **A** | **A** | **T** | **G** | **G** | **A** | **T** | **G** | **G** | **T** | **A** | **T** | **C** | **G** | **A** | **A** | **T** | **C** | **A** | **A** | **C** | **A** | **A** | **A** | **A** | **G** | **C** | **A** | **A** | **T** | **C** | **T** | **T** | **A** | **A** | **A** | **A** | **G** | **A** | **T** | **T** | **G** | **G** | **C** | **C** | **A** | **G** | **A** | **A** | **G** | **C** | **T** | **A** | **A** | **A** | **A** | **T** | **C** | **A** | **T** | **T** | **A** | **T** | **C** | **G** | **T** | **A** | **A** | **C** | **C** | **A** | **G** | **T** | **T** | **T** | **T** | **A** | **T** | **T** | **G** | **A** | **T** | **G** | **A** | **C** | **G** | **A** | **A** | **A** | **A** | **A** | **G** | **T** | **T** | **T** | **A** | **T** | **C** | **C** | **T** | **G** | **C** | **C** | **A** | **T** | **T** | **G** | **A** | **A** | **G** | **C** | **A** | **G** | **G** | **C** | **G** | **C** | **A** | **G** | **C** | **A** | **G** | **G** | **G** | **T** | **A** | **T** | **T** | **T** | **G** | **T** | **T** | **G** | **A** | **A** | **G** | **A** | **C** | **T** | **T** | **C** | **T** | **A** | **C** | **C** | **G** | **C** | **A** | **C** | **A** | **T** | **G** | **A** | **A** | **A** | **T** | **T** | **G** | **C** | **T** | **G** | **A** | **T** | **G** | **C** | **T** | **A** | **T** | **A** | **A** | **G** | **G** | **G** | **C** | **A** | **A** | **C** | **G** | **C** | **A** | **A** | **C** | **G** | **C** | **G** | **G** | **C** | **G** | **A** | **G** | **C** | **G** | **A** | **G** | **T** | **A** | **C** | **T** | **A** | **G** | **A** | **A** | **C** | **C** | **A** | **G** | **A** | **A** | **G** | **T** | **G** | **A** | **C** | **A** | **A** | **C** | **A** | **A** | **A** | **A** | **A** | **T** | **G** | **A** | **T** | **G** | **G** | **A** | **A** | **A** | **A** | **A** | **A** | **T** | **G** | **A** | **G** | **T** | **C** | **G** | **G** | **C** | **G** | **A** | **A** | **A** | **T** | **G** | **A** | **G** | **C** | **C** | **T** | **G** | **T** | **A** | **T** | **T** | **A** | **C** | **A** | **T** | **G** | **A** | **A** | **G** | **A** | **A** | **C** | **T** | **A** | **A** | **C** | **A** | **A** | **A** | **T** | **C** | **G** | **G** | **G** | **A** | **A** | **A** | **A** | **T** | **G** | **A** | **A** | **A** | **T** | **T** | **T** | **T** | **A** | **A** | **T** | **G** | **C** | **T** | **G** | **A** | **T** | **T** | **T** | **C** | **T** | **G** | **A** | **A** | **G** | **G** | **G** | **A** | **A** | **A** | **A** | **G** | **T** | **A** | **A** | **T** | **C** | **A** | **G** | **G** | **A** | **A** | **A** | **T** | **A** | **G** | **C** | **G** | **G** | **A** | **C** | **G** | **A** | **A** | **T** | **T** | **G** | **T** | **T** | **T** | **A** | **T** | **T** | **A** | **C** | **A** | **C** | **T** | **G** | **A** | **A** | **A** | **A** | **C** | **A** | **G** | **T** | **A** | **A** | **A** | **A** | **A** | **C** | **A** | **C** | **A** | **T** | **G** | **T** | **T** | **T** | **C** | **C** | **A** | **A** | **T** | **A** | **T** | **A** | **T** | **T** | **G** | **G** | **C** | **T** | **A** | **A** | **G** | **C** | **T** | **A** | **G** | **A** | **A** | **G** | **T** | **G** | **G** | **A** | **A** | **G** | **A** | **C** | **C** | **G** | **T** | **A** | **C** | **A** | **C** | **A** | **A** | **G** | **C** | **C** | **G** | **C** | **T** | **A** | **T** | **C** | **T** | **A** | **T** | **G** | **C** | **A** | **T** | **T** | **C** | **A** | **A** | **A** | **C** | **A** | **T** | **G** | **G** | **T** | **T** | **T** | **G** | **G** | **T** | **G** | **A** | **A** | **A** | **-** | **-** | **-** |
| **N54190** | **A** | **T** | **G** | **A** | **T** | **A** | **A** | **A** | **A** | **G** | **T** | **T** | **T** | **T** | **A** | **T** | **T** | **A** | **G** | **T** | **A** | **G** | **A** | **T** | **G** | **A** | **C** | **C** | **A** | **T** | **G** | **A** | **A** | **A** | **T** | **G** | **G** | **T** | **A** | **C** | **G** | **C** | **T** | **T** | **A** | **G** | **G** | **C** | **G** | **T** | **C** | **T** | **C** | **T** | **T** | **C** | **T** | **T** | **A** | **T** | **T** | **T** | **A** | **T** | **C** | **T** | **A** | **T** | **C** | **C** | **A** | **A** | **G** | **A** | **A** | **G** | **A** | **C** | **A** | **T** | **T** | **G** | **A** | **A** | **G** | **T** | **G** | **A** | **T** | **T** | **G** | **G** | **A** | **G** | **A** | **A** | **G** | **C** | **T** | **G** | **A** | **A** | **A** | **A** | **T** | **G** | **G** | **A** | **C** | **G** | **T** | **C** | **A** | **A** | **G** | **G** | **C** | **T** | **A** | **T** | **G** | **A** | **A** | **A** | **A** | **G** | **G** | **C** | **G** | **A** | **T** | **G** | **G** | **C** | **A** | **C** | **T** | **T** | **C** | **G** | **A** | **C** | **C** | **G** | **G** | **A** | **T** | **G** | **T** | **C** | **A** | **T** | **T** | **T** | **T** | **A** | **A** | **T** | **G** | **G** | **A** | **T** | **C** | **T** | **A** | **G** | **T** | **C** | **A** | **T** | **G** | **G** | **A** | **A** | **G** | **A** | **A** | **A** | **T** | **G** | **G** | **A** | **T** | **G** | **G** | **C** | **A** | **T** | **C** | **G** | **A** | **A** | **T** | **C** | **A** | **A** | **C** | **A** | **A** | **A** | **A** | **G** | **C** | **A** | **A** | **T** | **C** | **T** | **T** | **A** | **A** | **A** | **A** | **G** | **A** | **T** | **T** | **G** | **G** | **C** | **C** | **A** | **A** | **A** | **A** | **G** | **C** | **C** | **A** | **A** | **A** | **A** | **T** | **C** | **A** | **T** | **T** | **A** | **T** | **C** | **G** | **T** | **C** | **A** | **C** | **A** | **A** | **G** | **T** | **T** | **T** | **T** | **A** | **T** | **T** | **G** | **A** | **T** | **G** | **A** | **T** | **G** | **A** | **A** | **A** | **A** | **A** | **G** | **T** | **T** | **T** | **A** | **T** | **C** | **C** | **T** | **G** | **C** | **A** | **A** | **T** | **T** | **G** | **A** | **A** | **G** | **C** | **A** | **G** | **G** | **A** | **G** | **C** | **A** | **G** | **C** | **A** | **G** | **G** | **A** | **T** | **A** | **T** | **T** | **T** | **G** | **T** | **T** | **G** | **A** | **A** | **G** | **A** | **C** | **G** | **T** | **C** | **T** | **A** | **C** | **C** | **G** | **C** | **A** | **C** | **A** | **T** | **G** | **A** | **A** | **A** | **T** | **A** | **G** | **C** | **T** | **G** | **A** | **T** | **G** | **C** | **T** | **A** | **T** | **A** | **A** | **G** | **G** | **G** | **C** | **T** | **A** | **C** | **G** | **C** | **A** | **A** | **C** | **G** | **C** | **G** | **G** | **C** | **G** | **A** | **G** | **C** | **G** | **A** | **G** | **T** | **A** | **T** | **T** | **G** | **G** | **A** | **A** | **C** | **C** | **T** | **G** | **A** | **A** | **G** | **T** | **G** | **A** | **C** | **A** | **A** | **C** | **A** | **A** | **A** | **A** | **A** | **T** | **G** | **A** | **T** | **G** | **G** | **A** | **A** | **A** | **A** | **A** | **A** | **T** | **G** | **A** | **G** | **T** | **C** | **G** | **G** | **C** | **G** | **A** | **A** | **A** | **T** | **G** | **A** | **T** | **C** | **C** | **A** | **G** | **T** | **A** | **T** | **T** | **G** | **C** | **A** | **T** | **G** | **A** | **A** | **G** | **A** | **A** | **T** | **T** | **A** | **A** | **C** | **G** | **A** | **A** | **T** | **C** | **G** | **A** | **G** | **A** | **A** | **A** | **A** | **T** | **G** | **A** | **A** | **A** | **T** | **T** | **T** | **T** | **G** | **A** | **T** | **G** | **C** | **T** | **G** | **A** | **T** | **T** | **T** | **C** | **T** | **G** | **A** | **A** | **G** | **G** | **G** | **A** | **A** | **A** | **A** | **G** | **T** | **A** | **A** | **T** | **C** | **A** | **G** | **G** | **A** | **A** | **A** | **T** | **C** | **G** | **C** | **G** | **G** | **A** | **T** | **G** | **A** | **A** | **T** | **T** | **G** | **T** | **T** | **T** | **A** | **T** | **C** | **A** | **C** | **A** | **C** | **T** | **G** | **A** | **A** | **A** | **A** | **C** | **A** | **G** | **T** | **C** | **A** | **A** | **A** | **A** | **C** | **G** | **C** | **A** | **T** | **G** | **T** | **G** | **T** | **C** | **G** | **A** | **A** | **T** | **A** | **T** | **A** | **T** | **T** | **A** | **G** | **C** | **T** | **A** | **A** | **G** | **T** | **T** | **A** | **G** | **A** | **G** | **G** | **T** | **A** | **G** | **A** | **A** | **G** | **A** | **C** | **C** | **G** | **A** | **A** | **C** | **T** | **C** | **A** | **A** | **G** | **C** | **A** | **G** | **C** | **T** | **A** | **T** | **C** | **T** | **A** | **T** | **G** | **C** | **G** | **T** | **T** | **T** | **A** | **A** | **A** | **C** | **A** | **T** | **G** | **G** | **T** | **T** | **T** | **G** | **G** | **T** | **G** | **A** | **A** | **A** | **-** | **-** | **-** |
| **N54107** | **A** | **T** | **G** | **A** | **T** | **A** | **A** | **A** | **A** | **G** | **T** | **T** | **T** | **T** | **A** | **T** | **T** | **A** | **G** | **T** | **A** | **G** | **A** | **T** | **G** | **A** | **C** | **C** | **A** | **T** | **G** | **A** | **A** | **A** | **T** | **G** | **G** | **T** | **A** | **C** | **G** | **C** | **T** | **T** | **A** | **G** | **G** | **C** | **G** | **T** | **C** | **T** | **C** | **T** | **T** | **C** | **T** | **T** | **A** | **T** | **T** | **T** | **A** | **T** | **C** | **T** | **A** | **T** | **C** | **C** | **A** | **A** | **G** | **A** | **A** | **G** | **A** | **C** | **A** | **T** | **T** | **G** | **A** | **A** | **G** | **T** | **G** | **A** | **T** | **T** | **G** | **G** | **A** | **G** | **A** | **A** | **G** | **C** | **T** | **G** | **A** | **A** | **A** | **A** | **T** | **G** | **G** | **A** | **C** | **G** | **T** | **C** | **A** | **A** | **G** | **G** | **C** | **T** | **A** | **T** | **G** | **A** | **A** | **A** | **A** | **G** | **G** | **C** | **G** | **A** | **T** | **G** | **G** | **C** | **A** | **C** | **T** | **T** | **C** | **G** | **A** | **C** | **C** | **A** | **G** | **A** | **T** | **G** | **T** | **C** | **A** | **T** | **T** | **T** | **T** | **A** | **A** | **T** | **G** | **G** | **A** | **T** | **C** | **T** | **A** | **G** | **T** | **C** | **A** | **T** | **G** | **G** | **A** | **A** | **G** | **A** | **A** | **A** | **T** | **G** | **G** | **A** | **T** | **G** | **G** | **C** | **A** | **T** | **C** | **G** | **A** | **A** | **T** | **C** | **A** | **A** | **C** | **A** | **A** | **A** | **A** | **G** | **C** | **A** | **A** | **T** | **C** | **T** | **T** | **A** | **A** | **A** | **A** | **G** | **A** | **T** | **T** | **G** | **G** | **C** | **C** | **A** | **A** | **A** | **A** | **G** | **C** | **C** | **A** | **A** | **A** | **A** | **T** | **C** | **A** | **T** | **T** | **A** | **T** | **C** | **G** | **T** | **C** | **A** | **C** | **A** | **A** | **G** | **T** | **T** | **T** | **T** | **A** | **T** | **T** | **G** | **A** | **T** | **G** | **A** | **T** | **G** | **A** | **A** | **A** | **A** | **A** | **G** | **T** | **T** | **T** | **A** | **T** | **C** | **C** | **T** | **G** | **C** | **A** | **A** | **T** | **T** | **G** | **A** | **A** | **G** | **C** | **A** | **G** | **G** | **A** | **G** | **C** | **A** | **G** | **C** | **A** | **G** | **G** | **A** | **T** | **A** | **T** | **T** | **T** | **A** | **T** | **T** | **G** | **A** | **A** | **G** | **A** | **C** | **G** | **T** | **C** | **T** | **A** | **C** | **C** | **G** | **C** | **A** | **C** | **A** | **T** | **G** | **A** | **A** | **A** | **T** | **A** | **G** | **C** | **T** | **G** | **A** | **T** | **G** | **C** | **T** | **A** | **T** | **A** | **A** | **G** | **G** | **G** | **C** | **T** | **A** | **C** | **G** | **C** | **A** | **A** | **C** | **G** | **C** | **G** | **G** | **C** | **G** | **A** | **G** | **C** | **G** | **A** | **G** | **T** | **A** | **C** | **T** | **A** | **G** | **A** | **A** | **C** | **C** | **A** | **G** | **A** | **A** | **G** | **T** | **G** | **A** | **C** | **A** | **A** | **C** | **A** | **A** | **A** | **A** | **A** | **T** | **G** | **A** | **T** | **G** | **G** | **A** | **A** | **A** | **A** | **A** | **A** | **T** | **G** | **A** | **G** | **T** | **C** | **G** | **G** | **C** | **G** | **A** | **A** | **A** | **T** | **G** | **A** | **T** | **C** | **C** | **A** | **G** | **T** | **A** | **T** | **T** | **G** | **C** | **A** | **T** | **G** | **A** | **A** | **G** | **A** | **A** | **T** | **T** | **A** | **A** | **C** | **G** | **A** | **A** | **T** | **C** | **G** | **A** | **G** | **A** | **A** | **A** | **A** | **T** | **G** | **A** | **A** | **A** | **T** | **T** | **T** | **T** | **G** | **A** | **T** | **G** | **C** | **T** | **G** | **A** | **T** | **T** | **T** | **C** | **T** | **G** | **A** | **A** | **G** | **G** | **G** | **A** | **A** | **A** | **A** | **G** | **T** | **A** | **A** | **T** | **C** | **A** | **G** | **G** | **A** | **A** | **A** | **T** | **C** | **G** | **C** | **G** | **G** | **A** | **T** | **G** | **A** | **A** | **T** | **T** | **G** | **T** | **T** | **T** | **A** | **T** | **C** | **A** | **C** | **A** | **C** | **T** | **G** | **A** | **A** | **A** | **A** | **C** | **A** | **G** | **T** | **T** | **A** | **A** | **A** | **A** | **C** | **G** | **C** | **A** | **T** | **G** | **T** | **G** | **T** | **C** | **G** | **A** | **A** | **T** | **A** | **T** | **A** | **T** | **T** | **A** | **G** | **C** | **T** | **A** | **A** | **G** | **T** | **T** | **A** | **G** | **A** | **G** | **G** | **T** | **A** | **G** | **A** | **A** | **G** | **A** | **C** | **C** | **G** | **A** | **A** | **C** | **T** | **C** | **A** | **A** | **G** | **C** | **A** | **G** | **C** | **T** | **A** | **T** | **C** | **T** | **A** | **T** | **G** | **C** | **G** | **T** | **T** | **T** | **A** | **A** | **A** | **C** | **A** | **T** | **G** | **G** | **T** | **T** | **T** | **G** | **G** | **T** | **G** | **A** | **A** | **A** | **-** | **-** | **-** |
| **N54551** | **A** | **T** | **G** | **A** | **T** | **A** | **A** | **A** | **A** | **G** | **T** | **T** | **T** | **T** | **A** | **T** | **T** | **A** | **G** | **T** | **A** | **G** | **A** | **T** | **G** | **A** | **C** | **C** | **A** | **T** | **G** | **A** | **A** | **A** | **T** | **G** | **G** | **T** | **A** | **C** | **G** | **C** | **T** | **T** | **A** | **G** | **G** | **C** | **G** | **T** | **C** | **T** | **C** | **T** | **T** | **C** | **T** | **T** | **A** | **T** | **T** | **T** | **A** | **T** | **C** | **T** | **A** | **T** | **C** | **C** | **A** | **A** | **G** | **A** | **A** | **G** | **A** | **C** | **A** | **T** | **T** | **G** | **A** | **A** | **G** | **T** | **G** | **A** | **T** | **T** | **G** | **G** | **A** | **G** | **A** | **A** | **G** | **C** | **T** | **G** | **A** | **A** | **A** | **A** | **T** | **G** | **G** | **A** | **C** | **G** | **T** | **C** | **A** | **A** | **G** | **G** | **C** | **T** | **A** | **T** | **G** | **A** | **A** | **A** | **A** | **G** | **G** | **C** | **G** | **A** | **T** | **G** | **G** | **C** | **A** | **C** | **T** | **T** | **C** | **G** | **A** | **C** | **C** | **G** | **G** | **A** | **T** | **G** | **T** | **C** | **A** | **T** | **T** | **T** | **T** | **A** | **A** | **T** | **G** | **G** | **A** | **T** | **C** | **T** | **A** | **G** | **T** | **C** | **A** | **T** | **G** | **G** | **A** | **A** | **G** | **A** | **A** | **A** | **T** | **G** | **G** | **A** | **T** | **G** | **G** | **C** | **A** | **T** | **C** | **G** | **A** | **A** | **T** | **C** | **A** | **A** | **C** | **A** | **A** | **A** | **A** | **G** | **C** | **A** | **A** | **T** | **C** | **T** | **T** | **A** | **A** | **A** | **A** | **G** | **A** | **T** | **T** | **G** | **G** | **C** | **C** | **A** | **A** | **A** | **A** | **G** | **C** | **C** | **A** | **A** | **A** | **A** | **T** | **C** | **A** | **T** | **T** | **A** | **T** | **C** | **G** | **T** | **C** | **A** | **C** | **A** | **A** | **G** | **T** | **T** | **T** | **T** | **A** | **T** | **T** | **G** | **A** | **T** | **G** | **A** | **T** | **G** | **A** | **A** | **A** | **A** | **A** | **G** | **T** | **T** | **T** | **A** | **T** | **C** | **C** | **T** | **G** | **C** | **A** | **A** | **T** | **T** | **G** | **A** | **A** | **G** | **C** | **A** | **G** | **G** | **A** | **G** | **C** | **A** | **G** | **C** | **A** | **G** | **G** | **A** | **T** | **A** | **T** | **T** | **T** | **G** | **T** | **T** | **G** | **A** | **A** | **G** | **A** | **C** | **G** | **T** | **C** | **T** | **A** | **C** | **C** | **G** | **C** | **A** | **C** | **A** | **T** | **G** | **A** | **A** | **A** | **T** | **A** | **G** | **C** | **T** | **G** | **A** | **T** | **G** | **C** | **T** | **A** | **T** | **A** | **A** | **G** | **G** | **G** | **C** | **T** | **A** | **C** | **G** | **C** | **A** | **A** | **C** | **G** | **C** | **G** | **G** | **C** | **G** | **A** | **G** | **C** | **G** | **A** | **G** | **T** | **A** | **T** | **T** | **G** | **G** | **A** | **A** | **C** | **C** | **T** | **G** | **A** | **A** | **G** | **T** | **G** | **A** | **C** | **A** | **A** | **C** | **A** | **A** | **A** | **A** | **A** | **T** | **G** | **A** | **T** | **G** | **G** | **A** | **A** | **A** | **A** | **A** | **A** | **T** | **G** | **A** | **G** | **T** | **C** | **G** | **G** | **C** | **G** | **A** | **A** | **A** | **T** | **G** | **A** | **T** | **C** | **C** | **A** | **G** | **T** | **A** | **T** | **T** | **G** | **C** | **A** | **T** | **G** | **A** | **A** | **G** | **A** | **A** | **T** | **T** | **A** | **A** | **C** | **G** | **A** | **A** | **T** | **C** | **G** | **A** | **G** | **A** | **A** | **A** | **A** | **T** | **G** | **A** | **A** | **A** | **T** | **T** | **T** | **T** | **G** | **A** | **T** | **G** | **C** | **T** | **G** | **A** | **T** | **T** | **T** | **C** | **T** | **G** | **A** | **A** | **G** | **G** | **G** | **A** | **A** | **A** | **A** | **G** | **T** | **A** | **A** | **T** | **C** | **A** | **G** | **G** | **A** | **A** | **A** | **T** | **C** | **G** | **C** | **G** | **G** | **A** | **T** | **G** | **A** | **A** | **T** | **T** | **G** | **T** | **T** | **T** | **A** | **T** | **C** | **A** | **C** | **A** | **C** | **T** | **G** | **A** | **A** | **A** | **A** | **C** | **A** | **G** | **T** | **C** | **A** | **A** | **A** | **A** | **C** | **G** | **C** | **A** | **T** | **G** | **T** | **G** | **T** | **C** | **G** | **A** | **A** | **T** | **A** | **T** | **A** | **T** | **T** | **A** | **G** | **C** | **T** | **A** | **A** | **G** | **T** | **T** | **A** | **G** | **A** | **G** | **G** | **T** | **A** | **G** | **A** | **A** | **G** | **A** | **C** | **C** | **G** | **A** | **A** | **C** | **T** | **C** | **A** | **A** | **G** | **C** | **A** | **G** | **C** | **T** | **A** | **T** | **C** | **T** | **A** | **T** | **G** | **C** | **G** | **T** | **T** | **T** | **A** | **A** | **A** | **C** | **A** | **T** | **G** | **G** | **T** | **T** | **T** | **G** | **G** | **T** | **G** | **A** | **A** | **A** | **-** | **-** | **-** |
| **N54121** | **G** | **T** | **G** | **A** | **T** | **C** | **A** | **A** | **A** | **G** | **T** | **A** | **A** | **T** | **G** | **T** | **T** | **A** | **G** | **T** | **G** | **G** | **A** | **T** | **G** | **A** | **C** | **C** | **A** | **T** | **G** | **A** | **A** | **A** | **T** | **G** | **G** | **T** | **C** | **C** | **G** | **T** | **T** | **T** | **A** | **G** | **G** | **C** | **G** | **T** | **T** | **T** | **C** | **A** | **T** | **C** | **A** | **T** | **A** | **T** | **T** | **T** | **A** | **T** | **C** | **T** | **A** | **T** | **T** | **C** | **A** | **A** | **G** | **A** | **G** | **G** | **A** | **T** | **A** | **T** | **A** | **G** | **A** | **A** | **G** | **T** | **C** | **G** | **T** | **A** | **G** | **G** | **C** | **G** | **A** | **A** | **G** | **C** | **A** | **G** | **A** | **A** | **A** | **A** | **T** | **G** | **G** | **T** | **A** | **A** | **G** | **A** | **T** | **T** | **G** | **G** | **C** | **T** | **A** | **T** | **G** | **A** | **A** | **A** | **A** | **A** | **G** | **C** | **A** | **T** | **T** | **G** | **G** | **A** | **A** | **C** | **T** | **A** | **C** | **G** | **T** | **C** | **C** | **A** | **G** | **A** | **T** | **G** | **T** | **T** | **A** | **T** | **T** | **T** | **T** | **G** | **A** | **T** | **G** | **G** | **A** | **T** | **T** | **T** | **G** | **G** | **T** | **A** | **A** | **T** | **G** | **G** | **A** | **A** | **G** | **A** | **A** | **A** | **T** | **G** | **G** | **A** | **C** | **G** | **G** | **C** | **A** | **T** | **T** | **G** | **A** | **T** | **T** | **C** | **A** | **A** | **C** | **A** | **A** | **A** | **A** | **G** | **C** | **G** | **A** | **T** | **C** | **T** | **T** | **G** | **A** | **A** | **A** | **G** | **A** | **T** | **T** | **G** | **G** | **C** | **C** | **A** | **G** | **A** | **A** | **G** | **C** | **C** | **A** | **A** | **G** | **A** | **T** | **T** | **A** | **T** | **T** | **A** | **T** | **T** | **G** | **T** | **G** | **A** | **C** | **G** | **A** | **G** | **T** | **T** | **T** | **T** | **A** | **T** | **T** | **G** | **A** | **T** | **G** | **A** | **T** | **G** | **A** | **A** | **A** | **A** | **A** | **G** | **T** | **G** | **T** | **A** | **T** | **C** | **C** | **G** | **G** | **C** | **G** | **A** | **T** | **T** | **G** | **A** | **A** | **G** | **C** | **T** | **G** | **G** | **T** | **G** | **C** | **A** | **G** | **C** | **G** | **G** | **G** | **C** | **T** | **A** | **C** | **C** | **T** | **A** | **T** | **T** | **A** | **A** | **A** | **A** | **A** | **C** | **A** | **T** | **C** | **A** | **A** | **C** | **A** | **G** | **C** | **A** | **C** | **A** | **T** | **G** | **A** | **G** | **A** | **T** | **T** | **G** | **C** | **T** | **G** | **A** | **T** | **G** | **C** | **A** | **A** | **T** | **T** | **C** | **G** | **G** | **G** | **C** | **G** | **A** | **C** | **T** | **T** | **A** | **T** | **C** | **G** | **C** | **G** | **G** | **A** | **G** | **A** | **G** | **C** | **G** | **T** | **G** | **T** | **G** | **T** | **T** | **G** | **G** | **A** | **A** | **C** | **C** | **T** | **G** | **A** | **A** | **G** | **T** | **G** | **A** | **C** | **G** | **C** | **A** | **T** | **A** | **A** | **G** | **A** | **T** | **G** | **A** | **T** | **G** | **G** | **A** | **A** | **C** | **G** | **G** | **T** | **T** | **A** | **A** | **C** | **A** | **A** | **A** | **A** | **A** | **A** | **A** | **C** | **A** | **A** | **G** | **A** | **G** | **C** | **C** | **G** | **G** | **T** | **G** | **T** | **T** | **G** | **C** | **A** | **T** | **G** | **A** | **A** | **G** | **A** | **T** | **T** | **T** | **G** | **A** | **C** | **A** | **A** | **A** | **T** | **C** | **G** | **A** | **G** | **A** | **A** | **C** | **A** | **C** | **G** | **A** | **A** | **A** | **T** | **T** | **T** | **T** | **A** | **A** | **T** | **G** | **T** | **T** | **G** | **A** | **T** | **T** | **G** | **C** | **A** | **C** | **A** | **A** | **G** | **G** | **T** | **A** | **A** | **A** | **A** | **G** | **T** | **A** | **A** | **T** | **C** | **A** | **G** | **G** | **A** | **A** | **A** | **T** | **A** | **G** | **C** | **T** | **G** | **A** | **T** | **G** | **A** | **A** | **C** | **T** | **C** | **T** | **T** | **T** | **A** | **T** | **C** | **A** | **C** | **T** | **T** | **T** | **G** | **A** | **A** | **A** | **A** | **C** | **A** | **G** | **T** | **T** | **A** | **A** | **A** | **A** | **C** | **A** | **C** | **A** | **T** | **G** | **T** | **T** | **T** | **C** | **A** | **A** | **A** | **C** | **A** | **T** | **T** | **T** | **T** | **A** | **G** | **C** | **A** | **A** | **A** | **A** | **C** | **T** | **A** | **G** | **A** | **T** | **G** | **T** | **G** | **G** | **A** | **T** | **G** | **A** | **T** | **C** | **G** | **G** | **A** | **C** | **C** | **C** | **A** | **A** | **G** | **C** | **G** | **G** | **C** | **G** | **A** | **T** | **T** | **T** | **A** | **T** | **G** | **C** | **T** | **T** | **T** | **T** | **C** | **A** | **A** | **C** | **A** | **T** | **G** | **G** | **T** | **T** | **T** | **A** | **G** | **C** | **C** | **A** | **A** | **A** | **-** | **-** | **-** |
| **N59936F** | **G** | **T** | **G** | **A** | **T** | **C** | **A** | **A** | **A** | **G** | **T** | **A** | **A** | **T** | **G** | **T** | **T** | **A** | **G** | **T** | **G** | **G** | **A** | **T** | **G** | **A** | **C** | **C** | **A** | **T** | **G** | **A** | **A** | **A** | **T** | **G** | **G** | **T** | **C** | **C** | **G** | **T** | **T** | **T** | **A** | **G** | **G** | **C** | **G** | **T** | **T** | **T** | **C** | **A** | **T** | **C** | **A** | **T** | **A** | **T** | **T** | **T** | **A** | **T** | **C** | **T** | **A** | **T** | **T** | **C** | **A** | **A** | **G** | **A** | **G** | **G** | **A** | **T** | **A** | **T** | **A** | **G** | **A** | **A** | **G** | **T** | **C** | **G** | **T** | **A** | **G** | **G** | **C** | **G** | **A** | **A** | **G** | **C** | **A** | **G** | **A** | **A** | **A** | **A** | **C** | **G** | **G** | **T** | **A** | **A** | **G** | **A** | **T** | **T** | **G** | **G** | **C** | **T** | **A** | **T** | **G** | **A** | **A** | **A** | **A** | **A** | **G** | **C** | **A** | **T** | **T** | **G** | **G** | **A** | **A** | **C** | **T** | **A** | **C** | **G** | **T** | **C** | **C** | **A** | **G** | **A** | **T** | **G** | **T** | **T** | **A** | **T** | **T** | **T** | **T** | **G** | **A** | **T** | **G** | **G** | **A** | **T** | **T** | **T** | **G** | **G** | **T** | **A** | **A** | **T** | **G** | **G** | **A** | **A** | **G** | **A** | **A** | **A** | **T** | **G** | **G** | **A** | **C** | **G** | **G** | **C** | **A** | **T** | **T** | **G** | **A** | **T** | **T** | **C** | **A** | **A** | **C** | **A** | **A** | **A** | **A** | **G** | **C** | **A** | **A** | **T** | **C** | **T** | **T** | **G** | **A** | **A** | **A** | **G** | **A** | **T** | **T** | **G** | **G** | **C** | **C** | **A** | **G** | **A** | **A** | **G** | **C** | **C** | **A** | **A** | **G** | **A** | **T** | **T** | **A** | **T** | **T** | **A** | **T** | **T** | **G** | **T** | **G** | **A** | **C** | **G** | **A** | **G** | **T** | **T** | **T** | **T** | **A** | **T** | **T** | **G** | **A** | **T** | **G** | **A** | **T** | **G** | **A** | **G** | **A** | **A** | **A** | **G** | **T** | **G** | **T** | **A** | **T** | **C** | **C** | **G** | **G** | **C** | **G** | **A** | **T** | **T** | **G** | **A** | **A** | **G** | **C** | **T** | **G** | **G** | **T** | **G** | **C** | **A** | **G** | **C** | **G** | **G** | **G** | **C** | **T** | **A** | **C** | **C** | **T** | **A** | **T** | **T** | **A** | **A** | **A** | **A** | **A** | **C** | **A** | **T** | **C** | **A** | **A** | **C** | **A** | **G** | **C** | **A** | **C** | **A** | **T** | **G** | **A** | **G** | **A** | **T** | **T** | **G** | **C** | **T** | **G** | **A** | **T** | **G** | **C** | **A** | **A** | **T** | **T** | **C** | **G** | **G** | **G** | **C** | **G** | **A** | **C** | **T** | **T** | **A** | **T** | **C** | **G** | **C** | **G** | **G** | **A** | **G** | **A** | **G** | **C** | **G** | **T** | **G** | **T** | **G** | **T** | **T** | **G** | **G** | **A** | **A** | **C** | **C** | **T** | **G** | **A** | **A** | **G** | **T** | **G** | **A** | **C** | **G** | **C** | **A** | **T** | **A** | **A** | **G** | **A** | **T** | **G** | **A** | **T** | **G** | **G** | **A** | **A** | **C** | **G** | **G** | **T** | **T** | **A** | **A** | **C** | **A** | **A** | **A** | **A** | **A** | **A** | **A** | **C** | **A** | **A** | **G** | **A** | **G** | **C** | **C** | **G** | **G** | **T** | **G** | **T** | **T** | **G** | **C** | **A** | **T** | **G** | **A** | **A** | **G** | **A** | **T** | **T** | **T** | **G** | **A** | **C** | **A** | **A** | **A** | **T** | **C** | **G** | **A** | **G** | **A** | **A** | **C** | **A** | **C** | **G** | **A** | **A** | **A** | **T** | **T** | **T** | **T** | **A** | **A** | **T** | **G** | **T** | **T** | **G** | **A** | **T** | **T** | **G** | **C** | **A** | **C** | **A** | **A** | **G** | **G** | **T** | **A** | **A** | **A** | **A** | **G** | **T** | **A** | **A** | **T** | **C** | **A** | **G** | **G** | **A** | **A** | **A** | **T** | **A** | **G** | **C** | **T** | **G** | **A** | **T** | **G** | **A** | **A** | **C** | **T** | **C** | **T** | **T** | **T** | **A** | **T** | **C** | **A** | **C** | **T** | **T** | **T** | **G** | **A** | **A** | **A** | **A** | **C** | **A** | **G** | **T** | **T** | **A** | **A** | **A** | **A** | **C** | **A** | **C** | **A** | **T** | **G** | **T** | **T** | **T** | **C** | **A** | **A** | **A** | **C** | **A** | **T** | **T** | **T** | **T** | **A** | **G** | **C** | **A** | **A** | **A** | **A** | **C** | **T** | **A** | **G** | **A** | **T** | **G** | **T** | **G** | **G** | **A** | **T** | **G** | **A** | **T** | **C** | **G** | **G** | **A** | **C** | **C** | **C** | **A** | **A** | **G** | **C** | **G** | **G** | **C** | **G** | **A** | **T** | **T** | **T** | **A** | **T** | **G** | **C** | **T** | **T** | **T** | **T** | **C** | **A** | **A** | **C** | **A** | **T** | **G** | **G** | **T** | **T** | **T** | **A** | **G** | **C** | **C** | **A** | **A** | **A** | **-** | **-** | **-** |
| **N55077** | **A** | **T** | **G** | **A** | **T** | **A** | **A** | **A** | **A** | **G** | **T** | **T** | **T** | **T** | **A** | **T** | **T** | **A** | **G** | **T** | **A** | **G** | **A** | **T** | **G** | **A** | **C** | **C** | **A** | **T** | **G** | **A** | **A** | **A** | **T** | **G** | **G** | **T** | **A** | **C** | **G** | **C** | **T** | **T** | **A** | **G** | **G** | **C** | **G** | **T** | **C** | **T** | **C** | **T** | **T** | **C** | **T** | **T** | **A** | **T** | **T** | **T** | **A** | **T** | **C** | **T** | **A** | **T** | **C** | **C** | **A** | **A** | **G** | **A** | **A** | **G** | **A** | **C** | **A** | **T** | **T** | **G** | **A** | **A** | **G** | **T** | **G** | **A** | **T** | **T** | **G** | **G** | **A** | **G** | **A** | **A** | **G** | **C** | **T** | **G** | **A** | **A** | **A** | **A** | **T** | **G** | **G** | **A** | **C** | **G** | **T** | **C** | **A** | **A** | **G** | **G** | **C** | **T** | **A** | **T** | **G** | **A** | **A** | **A** | **A** | **G** | **G** | **C** | **G** | **A** | **T** | **G** | **G** | **C** | **A** | **C** | **T** | **T** | **C** | **G** | **A** | **C** | **C** | **A** | **G** | **A** | **T** | **G** | **T** | **C** | **A** | **T** | **T** | **T** | **T** | **A** | **A** | **T** | **G** | **G** | **A** | **T** | **C** | **T** | **A** | **G** | **T** | **C** | **A** | **T** | **G** | **G** | **A** | **A** | **G** | **A** | **A** | **A** | **T** | **G** | **G** | **A** | **T** | **G** | **G** | **C** | **A** | **T** | **C** | **G** | **A** | **A** | **T** | **C** | **A** | **A** | **C** | **A** | **A** | **A** | **A** | **G** | **C** | **A** | **A** | **T** | **C** | **T** | **T** | **A** | **A** | **A** | **A** | **G** | **A** | **T** | **T** | **G** | **G** | **A** | **C** | **A** | **A** | **A** | **A** | **G** | **C** | **C** | **A** | **A** | **A** | **A** | **T** | **C** | **A** | **T** | **T** | **A** | **T** | **C** | **G** | **T** | **C** | **A** | **C** | **A** | **A** | **G** | **T** | **T** | **T** | **T** | **A** | **T** | **T** | **G** | **A** | **T** | **G** | **A** | **T** | **G** | **A** | **A** | **A** | **A** | **A** | **G** | **T** | **T** | **T** | **A** | **T** | **C** | **C** | **T** | **G** | **C** | **A** | **A** | **T** | **T** | **G** | **A** | **A** | **G** | **C** | **A** | **G** | **G** | **A** | **G** | **C** | **A** | **G** | **C** | **A** | **G** | **G** | **A** | **T** | **A** | **T** | **T** | **T** | **A** | **T** | **T** | **G** | **A** | **A** | **G** | **A** | **C** | **G** | **T** | **C** | **T** | **A** | **C** | **C** | **G** | **C** | **A** | **C** | **A** | **T** | **G** | **A** | **A** | **A** | **T** | **A** | **G** | **C** | **T** | **G** | **A** | **T** | **G** | **C** | **T** | **A** | **T** | **A** | **A** | **G** | **G** | **G** | **C** | **T** | **A** | **C** | **G** | **C** | **A** | **A** | **C** | **G** | **C** | **G** | **G** | **C** | **G** | **A** | **G** | **C** | **G** | **A** | **G** | **T** | **A** | **T** | **T** | **G** | **G** | **A** | **A** | **C** | **C** | **T** | **G** | **A** | **A** | **G** | **T** | **G** | **A** | **C** | **A** | **A** | **C** | **A** | **A** | **A** | **A** | **A** | **T** | **G** | **A** | **T** | **G** | **G** | **A** | **A** | **A** | **A** | **A** | **A** | **T** | **G** | **A** | **G** | **T** | **C** | **G** | **G** | **C** | **G** | **A** | **A** | **A** | **T** | **G** | **A** | **T** | **C** | **C** | **A** | **G** | **T** | **A** | **T** | **T** | **G** | **C** | **A** | **T** | **G** | **A** | **A** | **G** | **A** | **A** | **T** | **T** | **A** | **A** | **C** | **G** | **A** | **A** | **T** | **C** | **G** | **A** | **G** | **A** | **A** | **A** | **A** | **T** | **G** | **A** | **A** | **A** | **T** | **T** | **T** | **T** | **G** | **A** | **T** | **G** | **C** | **T** | **G** | **A** | **T** | **T** | **T** | **C** | **T** | **G** | **A** | **A** | **G** | **G** | **G** | **A** | **A** | **A** | **A** | **G** | **T** | **A** | **A** | **T** | **C** | **A** | **G** | **G** | **A** | **A** | **A** | **T** | **C** | **G** | **C** | **G** | **G** | **A** | **T** | **G** | **A** | **A** | **T** | **T** | **G** | **T** | **T** | **T** | **A** | **T** | **C** | **A** | **C** | **A** | **C** | **T** | **G** | **A** | **A** | **A** | **A** | **C** | **A** | **G** | **T** | **T** | **A** | **A** | **A** | **A** | **C** | **G** | **C** | **A** | **T** | **G** | **T** | **G** | **T** | **C** | **G** | **A** | **A** | **T** | **A** | **T** | **A** | **T** | **T** | **A** | **G** | **C** | **T** | **A** | **A** | **G** | **T** | **T** | **A** | **G** | **A** | **G** | **G** | **T** | **A** | **G** | **A** | **A** | **G** | **A** | **C** | **C** | **G** | **A** | **A** | **C** | **T** | **C** | **A** | **A** | **G** | **C** | **A** | **G** | **C** | **T** | **A** | **T** | **C** | **T** | **A** | **T** | **G** | **C** | **G** | **T** | **T** | **T** | **A** | **A** | **A** | **C** | **A** | **T** | **G** | **G** | **T** | **T** | **T** | **G** | **G** | **T** | **G** | **A** | **A** | **A** | **-** | **-** | **-** |
| **N55290** | **A** | **T** | **G** | **A** | **T** | **A** | **A** | **A** | **A** | **G** | **T** | **T** | **T** | **T** | **A** | **T** | **T** | **A** | **G** | **T** | **A** | **G** | **A** | **T** | **G** | **A** | **C** | **C** | **A** | **T** | **G** | **A** | **A** | **A** | **T** | **G** | **G** | **T** | **G** | **C** | **G** | **C** | **T** | **T** | **A** | **G** | **G** | **C** | **G** | **T** | **C** | **T** | **C** | **T** | **T** | **C** | **T** | **T** | **A** | **T** | **T** | **T** | **A** | **T** | **C** | **T** | **A** | **T** | **C** | **C** | **A** | **A** | **G** | **A** | **A** | **G** | **A** | **C** | **A** | **T** | **T** | **G** | **A** | **A** | **G** | **T** | **A** | **A** | **T** | **T** | **G** | **G** | **A** | **G** | **A** | **A** | **G** | **C** | **T** | **G** | **A** | **A** | **A** | **A** | **T** | **G** | **G** | **A** | **C** | **G** | **A** | **C** | **A** | **A** | **G** | **G** | **C** | **T** | **A** | **T** | **G** | **A** | **G** | **A** | **A** | **G** | **G** | **C** | **G** | **A** | **T** | **G** | **A** | **C** | **A** | **C** | **T** | **T** | **C** | **G** | **A** | **C** | **C** | **G** | **G** | **A** | **C** | **G** | **T** | **C** | **A** | **T** | **T** | **T** | **T** | **A** | **A** | **T** | **G** | **G** | **A** | **C** | **T** | **T** | **A** | **G** | **T** | **C** | **A** | **T** | **G** | **G** | **A** | **A** | **G** | **A** | **A** | **A** | **T** | **G** | **G** | **A** | **T** | **G** | **G** | **T** | **A** | **T** | **C** | **G** | **A** | **A** | **T** | **C** | **A** | **A** | **C** | **A** | **A** | **A** | **A** | **G** | **C** | **A** | **A** | **T** | **C** | **T** | **T** | **A** | **A** | **A** | **A** | **G** | **A** | **T** | **T** | **G** | **G** | **C** | **C** | **A** | **G** | **A** | **A** | **G** | **C** | **T** | **A** | **A** | **A** | **A** | **T** | **C** | **A** | **T** | **T** | **A** | **T** | **C** | **G** | **T** | **A** | **A** | **C** | **C** | **A** | **G** | **T** | **T** | **T** | **T** | **A** | **T** | **T** | **G** | **A** | **T** | **G** | **A** | **C** | **G** | **A** | **A** | **A** | **A** | **A** | **G** | **T** | **T** | **T** | **A** | **T** | **C** | **C** | **T** | **G** | **C** | **C** | **A** | **T** | **T** | **G** | **A** | **A** | **G** | **C** | **A** | **G** | **G** | **C** | **G** | **C** | **A** | **G** | **C** | **A** | **G** | **G** | **G** | **T** | **A** | **T** | **T** | **T** | **G** | **T** | **T** | **G** | **A** | **A** | **G** | **A** | **C** | **T** | **T** | **C** | **T** | **A** | **C** | **C** | **G** | **C** | **A** | **C** | **A** | **T** | **G** | **A** | **A** | **A** | **T** | **T** | **G** | **C** | **T** | **G** | **A** | **T** | **G** | **C** | **T** | **A** | **T** | **A** | **A** | **G** | **G** | **G** | **C** | **A** | **A** | **C** | **G** | **C** | **A** | **A** | **C** | **G** | **C** | **G** | **G** | **C** | **G** | **A** | **G** | **C** | **G** | **A** | **G** | **T** | **A** | **C** | **T** | **A** | **G** | **A** | **A** | **C** | **C** | **A** | **G** | **A** | **A** | **G** | **T** | **G** | **A** | **C** | **A** | **A** | **C** | **A** | **A** | **A** | **A** | **A** | **T** | **G** | **A** | **T** | **G** | **G** | **A** | **A** | **A** | **A** | **A** | **A** | **T** | **G** | **A** | **G** | **T** | **C** | **G** | **G** | **C** | **G** | **A** | **A** | **A** | **T** | **G** | **A** | **G** | **C** | **C** | **T** | **G** | **T** | **A** | **T** | **T** | **A** | **C** | **A** | **T** | **G** | **A** | **A** | **G** | **A** | **A** | **C** | **T** | **A** | **A** | **C** | **G** | **A** | **A** | **T** | **C** | **G** | **G** | **G** | **A** | **A** | **A** | **A** | **T** | **G** | **A** | **A** | **A** | **T** | **T** | **T** | **T** | **A** | **A** | **T** | **G** | **C** | **T** | **G** | **A** | **T** | **T** | **T** | **C** | **T** | **G** | **A** | **A** | **G** | **G** | **G** | **A** | **A** | **A** | **A** | **G** | **T** | **A** | **A** | **T** | **C** | **A** | **G** | **G** | **A** | **A** | **A** | **T** | **A** | **G** | **C** | **G** | **G** | **A** | **C** | **G** | **A** | **A** | **T** | **T** | **G** | **T** | **T** | **T** | **A** | **T** | **T** | **A** | **C** | **A** | **C** | **T** | **G** | **A** | **A** | **A** | **A** | **C** | **A** | **G** | **T** | **A** | **A** | **A** | **A** | **A** | **C** | **A** | **C** | **A** | **T** | **G** | **T** | **T** | **T** | **C** | **C** | **A** | **A** | **T** | **A** | **T** | **A** | **T** | **T** | **G** | **G** | **C** | **T** | **A** | **A** | **G** | **C** | **T** | **A** | **G** | **A** | **A** | **G** | **T** | **G** | **G** | **A** | **A** | **G** | **A** | **C** | **C** | **G** | **T** | **A** | **C** | **A** | **C** | **A** | **A** | **G** | **C** | **C** | **G** | **C** | **T** | **A** | **T** | **C** | **T** | **A** | **T** | **G** | **C** | **A** | **T** | **T** | **C** | **A** | **A** | **A** | **C** | **A** | **T** | **G** | **G** | **T** | **T** | **T** | **G** | **G** | **T** | **G** | **A** | **A** | **A** | **-** | **-** | **-** |
| **N59689F** | **G** | **T** | **G** | **A** | **T** | **C** | **A** | **A** | **A** | **G** | **T** | **A** | **A** | **T** | **G** | **T** | **T** | **A** | **G** | **T** | **G** | **G** | **A** | **T** | **G** | **A** | **C** | **C** | **A** | **T** | **G** | **A** | **A** | **A** | **T** | **G** | **G** | **T** | **C** | **C** | **G** | **T** | **T** | **T** | **A** | **G** | **G** | **C** | **G** | **T** | **T** | **T** | **C** | **A** | **T** | **C** | **A** | **T** | **A** | **T** | **T** | **T** | **A** | **T** | **C** | **T** | **A** | **T** | **T** | **C** | **A** | **A** | **G** | **A** | **G** | **G** | **A** | **T** | **A** | **T** | **A** | **G** | **A** | **A** | **G** | **T** | **C** | **G** | **T** | **A** | **G** | **G** | **C** | **G** | **A** | **A** | **G** | **C** | **A** | **G** | **A** | **A** | **A** | **A** | **C** | **G** | **G** | **T** | **A** | **A** | **G** | **A** | **T** | **T** | **G** | **G** | **C** | **T** | **A** | **T** | **G** | **A** | **A** | **A** | **A** | **A** | **G** | **C** | **A** | **T** | **T** | **G** | **G** | **A** | **A** | **C** | **T** | **A** | **C** | **G** | **T** | **C** | **C** | **A** | **G** | **A** | **T** | **G** | **T** | **T** | **A** | **T** | **T** | **T** | **T** | **G** | **A** | **T** | **G** | **G** | **A** | **T** | **T** | **T** | **G** | **G** | **T** | **A** | **A** | **T** | **G** | **G** | **A** | **A** | **G** | **A** | **A** | **A** | **T** | **G** | **G** | **A** | **C** | **G** | **G** | **C** | **A** | **T** | **T** | **G** | **A** | **T** | **T** | **C** | **A** | **A** | **C** | **C** | **A** | **A** | **A** | **G** | **C** | **G** | **A** | **T** | **C** | **T** | **T** | **G** | **A** | **A** | **A** | **G** | **A** | **T** | **T** | **G** | **G** | **C** | **C** | **A** | **G** | **A** | **A** | **G** | **C** | **C** | **A** | **A** | **G** | **A** | **T** | **T** | **A** | **T** | **T** | **A** | **T** | **T** | **G** | **T** | **G** | **A** | **C** | **G** | **A** | **G** | **T** | **T** | **T** | **T** | **A** | **T** | **T** | **G** | **A** | **T** | **G** | **A** | **T** | **G** | **A** | **A** | **A** | **A** | **A** | **G** | **T** | **G** | **T** | **A** | **T** | **C** | **C** | **G** | **G** | **C** | **G** | **A** | **T** | **T** | **G** | **A** | **A** | **G** | **C** | **T** | **G** | **G** | **T** | **G** | **C** | **A** | **G** | **C** | **G** | **G** | **G** | **C** | **T** | **A** | **C** | **C** | **T** | **A** | **T** | **T** | **A** | **A** | **A** | **A** | **A** | **C** | **A** | **T** | **C** | **A** | **A** | **C** | **A** | **G** | **C** | **A** | **C** | **A** | **T** | **G** | **A** | **G** | **A** | **T** | **T** | **G** | **C** | **T** | **G** | **A** | **T** | **G** | **C** | **A** | **A** | **T** | **T** | **C** | **G** | **G** | **G** | **C** | **G** | **A** | **C** | **T** | **T** | **A** | **T** | **C** | **G** | **C** | **G** | **G** | **A** | **G** | **A** | **G** | **C** | **G** | **T** | **G** | **T** | **G** | **T** | **T** | **G** | **G** | **A** | **A** | **C** | **C** | **T** | **G** | **A** | **A** | **G** | **T** | **G** | **A** | **C** | **G** | **C** | **A** | **T** | **A** | **A** | **G** | **A** | **T** | **G** | **A** | **T** | **G** | **G** | **A** | **A** | **C** | **G** | **G** | **T** | **T** | **A** | **A** | **C** | **A** | **A** | **A** | **A** | **A** | **A** | **A** | **C** | **A** | **A** | **G** | **A** | **G** | **C** | **C** | **G** | **G** | **T** | **G** | **T** | **T** | **G** | **C** | **A** | **C** | **G** | **A** | **A** | **G** | **A** | **T** | **T** | **T** | **G** | **A** | **C** | **A** | **A** | **A** | **C** | **C** | **G** | **G** | **G** | **A** | **A** | **C** | **A** | **C** | **G** | **A** | **A** | **A** | **T** | **T** | **T** | **T** | **A** | **A** | **T** | **G** | **T** | **T** | **G** | **A** | **T** | **T** | **G** | **C** | **A** | **C** | **A** | **A** | **G** | **G** | **T** | **A** | **A** | **A** | **A** | **G** | **T** | **A** | **A** | **T** | **C** | **A** | **G** | **G** | **A** | **A** | **A** | **T** | **A** | **G** | **C** | **T** | **G** | **A** | **T** | **G** | **A** | **A** | **C** | **T** | **C** | **T** | **T** | **T** | **A** | **T** | **C** | **A** | **C** | **T** | **T** | **T** | **G** | **A** | **A** | **A** | **A** | **C** | **A** | **G** | **T** | **T** | **A** | **A** | **A** | **A** | **C** | **A** | **C** | **A** | **T** | **G** | **T** | **T** | **T** | **C** | **A** | **A** | **A** | **C** | **A** | **T** | **T** | **T** | **T** | **A** | **G** | **C** | **A** | **A** | **A** | **A** | **C** | **T** | **A** | **G** | **A** | **T** | **G** | **T** | **G** | **G** | **A** | **T** | **G** | **A** | **T** | **C** | **G** | **G** | **A** | **C** | **C** | **C** | **A** | **A** | **G** | **C** | **G** | **G** | **C** | **G** | **A** | **T** | **T** | **T** | **A** | **T** | **G** | **C** | **T** | **T** | **T** | **T** | **C** | **A** | **A** | **C** | **A** | **T** | **G** | **G** | **T** | **T** | **T** | **A** | **G** | **C** | **C** | **A** | **A** | **A** | **-** | **-** | **-** |
| **N54564** | **G** | **T** | **G** | **A** | **T** | **C** | **A** | **A** | **A** | **G** | **T** | **A** | **A** | **T** | **G** | **T** | **T** | **A** | **G** | **T** | **G** | **G** | **A** | **T** | **G** | **A** | **C** | **C** | **A** | **T** | **G** | **A** | **A** | **A** | **T** | **G** | **G** | **T** | **C** | **C** | **G** | **T** | **T** | **T** | **A** | **G** | **G** | **C** | **G** | **T** | **T** | **T** | **C** | **A** | **T** | **C** | **A** | **T** | **A** | **T** | **T** | **T** | **A** | **T** | **C** | **T** | **A** | **T** | **T** | **C** | **A** | **A** | **G** | **A** | **G** | **G** | **A** | **T** | **A** | **T** | **A** | **G** | **A** | **A** | **G** | **T** | **C** | **G** | **T** | **A** | **G** | **G** | **C** | **G** | **A** | **A** | **G** | **C** | **A** | **G** | **A** | **A** | **A** | **A** | **C** | **G** | **G** | **T** | **A** | **A** | **G** | **A** | **T** | **T** | **G** | **G** | **C** | **T** | **A** | **T** | **G** | **A** | **A** | **A** | **A** | **A** | **G** | **C** | **A** | **T** | **T** | **G** | **G** | **A** | **A** | **C** | **T** | **A** | **C** | **G** | **T** | **C** | **C** | **A** | **G** | **A** | **T** | **G** | **T** | **T** | **A** | **T** | **T** | **T** | **T** | **G** | **A** | **T** | **G** | **G** | **A** | **T** | **T** | **T** | **G** | **G** | **T** | **A** | **A** | **T** | **G** | **G** | **A** | **A** | **G** | **A** | **A** | **A** | **T** | **G** | **G** | **A** | **C** | **G** | **G** | **C** | **A** | **T** | **T** | **G** | **A** | **T** | **T** | **C** | **A** | **A** | **C** | **A** | **A** | **A** | **A** | **G** | **C** | **G** | **A** | **T** | **C** | **T** | **T** | **G** | **A** | **A** | **A** | **G** | **A** | **T** | **T** | **G** | **G** | **C** | **C** | **A** | **G** | **A** | **A** | **G** | **C** | **C** | **A** | **A** | **G** | **A** | **T** | **T** | **A** | **T** | **T** | **A** | **T** | **T** | **G** | **T** | **G** | **A** | **C** | **G** | **A** | **G** | **T** | **T** | **T** | **T** | **A** | **T** | **T** | **G** | **A** | **T** | **G** | **A** | **T** | **G** | **A** | **A** | **A** | **A** | **A** | **G** | **T** | **G** | **T** | **A** | **T** | **C** | **C** | **G** | **G** | **C** | **G** | **A** | **T** | **T** | **G** | **A** | **A** | **G** | **C** | **T** | **G** | **G** | **T** | **G** | **C** | **A** | **G** | **C** | **G** | **G** | **G** | **C** | **T** | **A** | **C** | **C** | **T** | **A** | **T** | **T** | **A** | **A** | **A** | **A** | **A** | **C** | **A** | **T** | **C** | **A** | **A** | **C** | **A** | **G** | **C** | **A** | **C** | **A** | **T** | **G** | **A** | **G** | **A** | **T** | **T** | **G** | **C** | **T** | **G** | **A** | **T** | **G** | **C** | **A** | **A** | **T** | **T** | **C** | **G** | **G** | **G** | **C** | **G** | **A** | **C** | **T** | **T** | **A** | **T** | **C** | **G** | **C** | **G** | **G** | **A** | **G** | **A** | **G** | **C** | **G** | **T** | **G** | **T** | **G** | **T** | **T** | **G** | **G** | **A** | **A** | **C** | **C** | **T** | **G** | **A** | **A** | **G** | **T** | **G** | **A** | **C** | **G** | **C** | **A** | **T** | **A** | **A** | **G** | **A** | **T** | **G** | **A** | **T** | **G** | **G** | **A** | **A** | **C** | **G** | **G** | **T** | **T** | **A** | **A** | **C** | **A** | **A** | **A** | **A** | **A** | **A** | **A** | **C** | **A** | **A** | **G** | **A** | **G** | **C** | **C** | **G** | **G** | **T** | **G** | **T** | **T** | **G** | **C** | **A** | **C** | **G** | **A** | **A** | **G** | **A** | **T** | **T** | **T** | **G** | **A** | **C** | **A** | **A** | **A** | **C** | **C** | **G** | **A** | **G** | **A** | **A** | **C** | **A** | **C** | **G** | **A** | **A** | **A** | **T** | **T** | **T** | **T** | **A** | **A** | **T** | **G** | **T** | **T** | **G** | **A** | **T** | **T** | **G** | **C** | **A** | **C** | **A** | **A** | **G** | **G** | **T** | **A** | **A** | **A** | **A** | **G** | **T** | **A** | **A** | **T** | **C** | **A** | **G** | **G** | **A** | **A** | **A** | **T** | **T** | **G** | **C** | **T** | **G** | **A** | **T** | **G** | **A** | **A** | **C** | **T** | **C** | **T** | **T** | **T** | **A** | **T** | **C** | **A** | **C** | **T** | **T** | **T** | **G** | **A** | **A** | **A** | **A** | **C** | **A** | **G** | **T** | **T** | **A** | **A** | **A** | **A** | **C** | **A** | **C** | **A** | **T** | **G** | **T** | **T** | **T** | **C** | **A** | **A** | **A** | **C** | **A** | **T** | **T** | **T** | **T** | **A** | **G** | **C** | **A** | **A** | **A** | **A** | **C** | **T** | **A** | **G** | **A** | **T** | **G** | **T** | **G** | **G** | **A** | **T** | **G** | **A** | **T** | **C** | **G** | **G** | **A** | **C** | **C** | **C** | **A** | **A** | **G** | **C** | **G** | **G** | **C** | **G** | **A** | **T** | **T** | **T** | **A** | **T** | **G** | **C** | **T** | **T** | **T** | **T** | **C** | **A** | **A** | **C** | **A** | **T** | **G** | **G** | **T** | **T** | **T** | **A** | **G** | **C** | **C** | **A** | **A** | **A** | **-** | **-** | **-** |
| **N54576** | **G** | **T** | **G** | **A** | **T** | **C** | **A** | **A** | **A** | **G** | **T** | **A** | **A** | **T** | **G** | **T** | **T** | **A** | **G** | **T** | **G** | **G** | **A** | **T** | **G** | **A** | **C** | **C** | **A** | **T** | **G** | **A** | **A** | **A** | **T** | **G** | **G** | **T** | **C** | **C** | **G** | **T** | **T** | **T** | **A** | **G** | **G** | **C** | **G** | **T** | **T** | **T** | **C** | **A** | **T** | **C** | **A** | **T** | **A** | **T** | **T** | **T** | **A** | **T** | **C** | **T** | **A** | **T** | **T** | **C** | **A** | **A** | **G** | **A** | **G** | **G** | **A** | **T** | **A** | **T** | **A** | **G** | **A** | **A** | **G** | **T** | **C** | **G** | **T** | **A** | **G** | **G** | **C** | **G** | **A** | **A** | **G** | **C** | **A** | **G** | **A** | **A** | **A** | **A** | **T** | **G** | **G** | **T** | **A** | **A** | **G** | **A** | **T** | **T** | **G** | **G** | **C** | **T** | **A** | **T** | **G** | **A** | **A** | **A** | **A** | **A** | **G** | **C** | **A** | **T** | **T** | **G** | **G** | **A** | **A** | **C** | **T** | **A** | **C** | **G** | **T** | **C** | **C** | **A** | **G** | **A** | **T** | **G** | **T** | **T** | **A** | **T** | **T** | **T** | **T** | **G** | **A** | **T** | **G** | **G** | **A** | **T** | **T** | **T** | **G** | **G** | **T** | **A** | **A** | **T** | **G** | **G** | **A** | **A** | **G** | **A** | **A** | **A** | **T** | **G** | **G** | **A** | **C** | **G** | **G** | **C** | **A** | **T** | **T** | **G** | **A** | **T** | **T** | **C** | **A** | **A** | **C** | **A** | **A** | **A** | **A** | **G** | **C** | **G** | **A** | **T** | **C** | **T** | **T** | **G** | **A** | **A** | **A** | **G** | **A** | **T** | **T** | **G** | **G** | **C** | **C** | **A** | **G** | **A** | **A** | **G** | **C** | **C** | **A** | **A** | **G** | **A** | **T** | **T** | **A** | **T** | **T** | **A** | **T** | **T** | **G** | **T** | **G** | **A** | **C** | **G** | **A** | **G** | **T** | **T** | **T** | **T** | **A** | **T** | **T** | **G** | **A** | **T** | **G** | **A** | **T** | **G** | **A** | **A** | **A** | **A** | **A** | **G** | **T** | **G** | **T** | **A** | **T** | **C** | **C** | **G** | **G** | **C** | **G** | **A** | **T** | **T** | **G** | **A** | **A** | **G** | **C** | **T** | **G** | **G** | **T** | **G** | **C** | **A** | **G** | **C** | **G** | **G** | **G** | **C** | **T** | **A** | **C** | **C** | **T** | **A** | **T** | **T** | **A** | **A** | **A** | **A** | **A** | **C** | **A** | **T** | **C** | **A** | **A** | **C** | **A** | **G** | **C** | **A** | **C** | **A** | **T** | **G** | **A** | **G** | **A** | **T** | **T** | **G** | **C** | **T** | **G** | **A** | **T** | **G** | **C** | **A** | **A** | **T** | **T** | **C** | **G** | **G** | **G** | **C** | **G** | **A** | **C** | **T** | **T** | **A** | **T** | **C** | **G** | **C** | **G** | **G** | **A** | **G** | **A** | **G** | **C** | **G** | **T** | **G** | **T** | **G** | **T** | **T** | **G** | **G** | **A** | **A** | **C** | **C** | **T** | **G** | **A** | **A** | **G** | **T** | **G** | **A** | **C** | **G** | **C** | **A** | **T** | **A** | **A** | **G** | **A** | **T** | **G** | **A** | **T** | **G** | **G** | **A** | **A** | **C** | **G** | **G** | **T** | **T** | **A** | **A** | **C** | **A** | **A** | **A** | **A** | **A** | **A** | **A** | **C** | **A** | **A** | **G** | **A** | **G** | **C** | **C** | **G** | **G** | **T** | **G** | **T** | **T** | **G** | **C** | **A** | **T** | **G** | **A** | **A** | **G** | **A** | **T** | **T** | **T** | **G** | **A** | **C** | **A** | **A** | **A** | **T** | **C** | **G** | **A** | **G** | **A** | **A** | **C** | **A** | **C** | **G** | **A** | **A** | **A** | **T** | **T** | **T** | **T** | **A** | **A** | **T** | **G** | **T** | **T** | **G** | **A** | **T** | **T** | **G** | **C** | **A** | **C** | **A** | **A** | **G** | **G** | **T** | **A** | **A** | **A** | **A** | **G** | **T** | **A** | **A** | **T** | **C** | **A** | **G** | **G** | **A** | **A** | **A** | **T** | **A** | **G** | **C** | **T** | **G** | **A** | **T** | **G** | **A** | **A** | **C** | **T** | **C** | **T** | **T** | **T** | **A** | **T** | **C** | **A** | **C** | **T** | **T** | **T** | **G** | **A** | **A** | **A** | **A** | **C** | **A** | **G** | **T** | **T** | **A** | **A** | **A** | **A** | **C** | **A** | **C** | **A** | **T** | **G** | **T** | **T** | **T** | **C** | **A** | **A** | **A** | **C** | **A** | **T** | **T** | **T** | **T** | **A** | **G** | **C** | **A** | **A** | **A** | **A** | **C** | **T** | **A** | **G** | **A** | **T** | **G** | **T** | **G** | **G** | **A** | **T** | **G** | **A** | **T** | **C** | **G** | **G** | **A** | **C** | **C** | **C** | **A** | **A** | **G** | **C** | **G** | **G** | **C** | **G** | **A** | **T** | **T** | **T** | **A** | **T** | **G** | **C** | **T** | **T** | **T** | **T** | **C** | **A** | **A** | **C** | **A** | **T** | **G** | **G** | **T** | **T** | **T** | **A** | **G** | **C** | **C** | **A** | **A** | **A** | **-** | **-** | **-** |
| **N53479** | **A** | **T** | **G** | **A** | **T** | **A** | **A** | **A** | **A** | **G** | **T** | **T** | **T** | **T** | **A** | **T** | **T** | **A** | **G** | **T** | **A** | **G** | **A** | **T** | **G** | **A** | **C** | **C** | **A** | **T** | **G** | **A** | **A** | **A** | **T** | **G** | **G** | **T** | **A** | **C** | **G** | **C** | **T** | **T** | **A** | **G** | **G** | **C** | **G** | **T** | **C** | **T** | **C** | **T** | **T** | **C** | **T** | **T** | **A** | **T** | **T** | **T** | **A** | **T** | **C** | **T** | **A** | **T** | **C** | **C** | **A** | **A** | **G** | **A** | **A** | **G** | **A** | **C** | **A** | **T** | **T** | **G** | **A** | **A** | **G** | **T** | **G** | **A** | **T** | **T** | **G** | **G** | **A** | **G** | **A** | **A** | **G** | **C** | **T** | **G** | **A** | **A** | **A** | **A** | **T** | **G** | **G** | **A** | **C** | **G** | **T** | **C** | **A** | **A** | **G** | **G** | **C** | **T** | **A** | **T** | **G** | **A** | **A** | **A** | **A** | **G** | **G** | **C** | **G** | **A** | **T** | **G** | **G** | **C** | **A** | **C** | **T** | **T** | **C** | **G** | **A** | **C** | **C** | **A** | **G** | **A** | **T** | **G** | **T** | **C** | **A** | **T** | **T** | **T** | **T** | **A** | **A** | **T** | **G** | **G** | **A** | **T** | **C** | **T** | **A** | **G** | **T** | **C** | **A** | **T** | **G** | **G** | **A** | **A** | **G** | **A** | **A** | **A** | **T** | **G** | **G** | **A** | **T** | **G** | **G** | **C** | **A** | **T** | **C** | **G** | **A** | **A** | **T** | **C** | **A** | **A** | **C** | **A** | **A** | **A** | **A** | **G** | **C** | **A** | **A** | **T** | **C** | **T** | **T** | **A** | **A** | **A** | **A** | **G** | **A** | **T** | **T** | **G** | **G** | **C** | **C** | **A** | **A** | **A** | **A** | **G** | **C** | **C** | **A** | **A** | **A** | **A** | **T** | **C** | **A** | **T** | **T** | **A** | **T** | **C** | **G** | **T** | **C** | **A** | **C** | **A** | **A** | **G** | **T** | **T** | **T** | **T** | **A** | **T** | **T** | **G** | **A** | **T** | **G** | **A** | **T** | **G** | **A** | **A** | **A** | **A** | **A** | **G** | **T** | **T** | **T** | **A** | **T** | **C** | **C** | **T** | **G** | **C** | **A** | **A** | **T** | **T** | **G** | **A** | **A** | **G** | **C** | **A** | **G** | **G** | **A** | **G** | **C** | **A** | **G** | **C** | **A** | **G** | **G** | **A** | **T** | **A** | **T** | **T** | **T** | **A** | **T** | **T** | **G** | **A** | **A** | **G** | **A** | **C** | **G** | **T** | **C** | **T** | **A** | **C** | **C** | **G** | **C** | **A** | **C** | **A** | **T** | **G** | **A** | **A** | **A** | **T** | **A** | **G** | **C** | **T** | **G** | **A** | **T** | **G** | **C** | **T** | **A** | **T** | **A** | **A** | **G** | **G** | **G** | **C** | **T** | **A** | **C** | **G** | **C** | **A** | **A** | **C** | **G** | **C** | **G** | **G** | **C** | **G** | **A** | **G** | **C** | **G** | **A** | **G** | **T** | **A** | **T** | **T** | **G** | **G** | **A** | **A** | **C** | **C** | **T** | **G** | **A** | **A** | **G** | **T** | **G** | **A** | **C** | **A** | **A** | **C** | **A** | **A** | **A** | **A** | **A** | **T** | **G** | **A** | **T** | **G** | **G** | **A** | **A** | **A** | **A** | **A** | **A** | **T** | **G** | **A** | **G** | **T** | **C** | **G** | **G** | **C** | **G** | **A** | **A** | **A** | **T** | **G** | **A** | **T** | **C** | **C** | **A** | **G** | **T** | **A** | **T** | **T** | **G** | **C** | **A** | **T** | **G** | **A** | **A** | **G** | **A** | **A** | **T** | **T** | **A** | **A** | **C** | **G** | **A** | **A** | **T** | **C** | **G** | **A** | **G** | **A** | **A** | **A** | **A** | **T** | **G** | **A** | **A** | **A** | **T** | **T** | **T** | **T** | **G** | **A** | **T** | **G** | **C** | **T** | **G** | **A** | **T** | **T** | **T** | **C** | **T** | **G** | **A** | **A** | **G** | **G** | **G** | **A** | **A** | **A** | **A** | **G** | **T** | **A** | **A** | **T** | **C** | **A** | **G** | **G** | **A** | **A** | **A** | **T** | **C** | **G** | **C** | **G** | **G** | **A** | **T** | **G** | **A** | **A** | **T** | **T** | **G** | **T** | **T** | **T** | **A** | **T** | **C** | **A** | **C** | **A** | **C** | **T** | **G** | **A** | **A** | **A** | **A** | **C** | **A** | **G** | **T** | **T** | **A** | **A** | **A** | **A** | **C** | **G** | **C** | **A** | **T** | **G** | **T** | **G** | **T** | **C** | **G** | **A** | **A** | **T** | **A** | **T** | **A** | **T** | **T** | **A** | **G** | **C** | **T** | **A** | **A** | **G** | **T** | **T** | **A** | **G** | **A** | **G** | **G** | **T** | **A** | **G** | **A** | **A** | **G** | **A** | **C** | **C** | **G** | **A** | **A** | **C** | **T** | **C** | **A** | **A** | **G** | **C** | **A** | **G** | **C** | **T** | **A** | **T** | **C** | **T** | **A** | **T** | **G** | **C** | **G** | **T** | **T** | **T** | **A** | **A** | **A** | **C** | **A** | **T** | **G** | **G** | **T** | **T** | **T** | **G** | **G** | **T** | **G** | **A** | **A** | **A** | **-** | **-** | **-** |
| **N59959F** | **G** | **T** | **G** | **A** | **T** | **C** | **A** | **A** | **A** | **G** | **T** | **A** | **A** | **T** | **G** | **C** | **T** | **A** | **G** | **T** | **G** | **G** | **A** | **T** | **G** | **A** | **C** | **C** | **A** | **T** | **G** | **A** | **A** | **A** | **T** | **G** | **G** | **T** | **C** | **C** | **G** | **T** | **T** | **T** | **A** | **G** | **G** | **C** | **G** | **T** | **T** | **T** | **C** | **A** | **T** | **C** | **A** | **T** | **A** | **T** | **T** | **T** | **A** | **T** | **C** | **T** | **A** | **T** | **T** | **C** | **A** | **A** | **G** | **A** | **G** | **G** | **A** | **T** | **A** | **T** | **A** | **G** | **A** | **A** | **G** | **T** | **C** | **G** | **T** | **A** | **G** | **G** | **C** | **G** | **A** | **A** | **G** | **C** | **A** | **G** | **A** | **A** | **A** | **A** | **C** | **G** | **G** | **T** | **A** | **A** | **G** | **A** | **T** | **T** | **G** | **G** | **C** | **T** | **A** | **T** | **G** | **A** | **A** | **A** | **A** | **A** | **G** | **C** | **A** | **T** | **T** | **G** | **G** | **A** | **A** | **C** | **T** | **A** | **C** | **G** | **T** | **C** | **C** | **A** | **G** | **A** | **T** | **G** | **T** | **T** | **A** | **T** | **T** | **T** | **T** | **G** | **A** | **T** | **G** | **G** | **A** | **T** | **T** | **T** | **G** | **G** | **T** | **A** | **A** | **T** | **G** | **G** | **A** | **A** | **G** | **A** | **A** | **A** | **T** | **G** | **G** | **A** | **C** | **G** | **G** | **C** | **A** | **T** | **T** | **G** | **A** | **T** | **T** | **C** | **A** | **A** | **C** | **A** | **A** | **A** | **A** | **G** | **C** | **G** | **A** | **T** | **C** | **T** | **T** | **G** | **A** | **A** | **A** | **G** | **A** | **T** | **T** | **G** | **G** | **C** | **C** | **A** | **G** | **A** | **A** | **G** | **C** | **C** | **A** | **A** | **G** | **A** | **T** | **T** | **A** | **T** | **T** | **A** | **T** | **T** | **G** | **T** | **G** | **A** | **C** | **G** | **A** | **G** | **T** | **T** | **T** | **T** | **A** | **T** | **T** | **G** | **A** | **T** | **G** | **A** | **T** | **G** | **A** | **A** | **A** | **A** | **A** | **G** | **T** | **G** | **T** | **A** | **T** | **C** | **C** | **G** | **G** | **C** | **G** | **A** | **T** | **T** | **G** | **A** | **A** | **G** | **C** | **T** | **G** | **G** | **T** | **G** | **C** | **A** | **G** | **C** | **A** | **G** | **G** | **C** | **T** | **A** | **C** | **C** | **T** | **A** | **T** | **T** | **A** | **A** | **A** | **A** | **A** | **C** | **A** | **T** | **C** | **A** | **A** | **C** | **A** | **G** | **C** | **T** | **C** | **A** | **T** | **G** | **A** | **G** | **A** | **T** | **T** | **G** | **C** | **T** | **G** | **A** | **T** | **G** | **C** | **A** | **A** | **T** | **T** | **C** | **G** | **G** | **G** | **C** | **G** | **A** | **C** | **T** | **T** | **A** | **T** | **C** | **G** | **C** | **G** | **G** | **A** | **G** | **A** | **G** | **C** | **G** | **T** | **G** | **T** | **G** | **T** | **T** | **G** | **G** | **A** | **A** | **C** | **C** | **T** | **G** | **A** | **A** | **G** | **T** | **G** | **A** | **C** | **G** | **C** | **A** | **T** | **A** | **A** | **G** | **A** | **T** | **G** | **A** | **T** | **G** | **G** | **A** | **A** | **C** | **G** | **G** | **T** | **T** | **A** | **A** | **C** | **A** | **A** | **A** | **A** | **A** | **A** | **A** | **C** | **A** | **A** | **G** | **A** | **G** | **C** | **C** | **G** | **G** | **T** | **G** | **T** | **T** | **G** | **C** | **A** | **C** | **G** | **A** | **A** | **G** | **A** | **T** | **T** | **T** | **G** | **A** | **C** | **A** | **A** | **A** | **C** | **C** | **G** | **A** | **G** | **A** | **A** | **C** | **A** | **C** | **G** | **A** | **A** | **A** | **T** | **T** | **T** | **T** | **A** | **A** | **T** | **G** | **T** | **T** | **G** | **A** | **T** | **T** | **G** | **C** | **A** | **C** | **A** | **A** | **G** | **G** | **T** | **A** | **A** | **A** | **A** | **G** | **T** | **A** | **A** | **T** | **C** | **A** | **G** | **G** | **A** | **A** | **A** | **T** | **A** | **G** | **C** | **T** | **G** | **A** | **T** | **G** | **A** | **A** | **C** | **T** | **C** | **T** | **T** | **T** | **A** | **T** | **C** | **A** | **C** | **T** | **T** | **T** | **G** | **A** | **A** | **A** | **A** | **C** | **A** | **G** | **T** | **T** | **A** | **A** | **A** | **A** | **C** | **A** | **C** | **A** | **T** | **G** | **T** | **T** | **T** | **C** | **A** | **A** | **A** | **C** | **A** | **T** | **T** | **T** | **T** | **A** | **G** | **C** | **A** | **A** | **A** | **A** | **C** | **T** | **A** | **G** | **A** | **T** | **G** | **T** | **G** | **G** | **A** | **T** | **G** | **A** | **T** | **C** | **G** | **G** | **A** | **C** | **C** | **C** | **A** | **A** | **G** | **C** | **G** | **G** | **C** | **G** | **A** | **T** | **T** | **T** | **A** | **T** | **G** | **C** | **T** | **T** | **T** | **T** | **C** | **A** | **A** | **C** | **A** | **T** | **G** | **G** | **T** | **T** | **T** | **A** | **G** | **C** | **C** | **A** | **A** | **A** | **-** | **-** | **-** |
| **N52662** | **G** | **T** | **G** | **A** | **T** | **C** | **A** | **A** | **A** | **G** | **T** | **A** | **A** | **T** | **G** | **T** | **T** | **A** | **G** | **T** | **G** | **G** | **A** | **T** | **G** | **A** | **C** | **C** | **A** | **T** | **G** | **A** | **A** | **A** | **T** | **G** | **G** | **T** | **C** | **C** | **G** | **T** | **T** | **T** | **A** | **G** | **G** | **C** | **G** | **T** | **T** | **T** | **C** | **A** | **T** | **C** | **A** | **T** | **A** | **T** | **T** | **T** | **A** | **T** | **C** | **T** | **A** | **T** | **T** | **C** | **A** | **A** | **G** | **A** | **G** | **G** | **A** | **T** | **A** | **T** | **A** | **G** | **A** | **A** | **G** | **T** | **C** | **G** | **T** | **A** | **G** | **G** | **C** | **G** | **A** | **A** | **G** | **C** | **A** | **G** | **A** | **A** | **A** | **A** | **C** | **G** | **G** | **T** | **A** | **A** | **G** | **A** | **T** | **T** | **G** | **G** | **C** | **T** | **A** | **T** | **G** | **A** | **A** | **A** | **A** | **A** | **G** | **C** | **A** | **T** | **T** | **G** | **G** | **A** | **A** | **C** | **T** | **A** | **C** | **G** | **T** | **C** | **C** | **A** | **G** | **A** | **T** | **G** | **T** | **T** | **A** | **T** | **T** | **T** | **T** | **G** | **A** | **T** | **G** | **G** | **A** | **C** | **T** | **T** | **G** | **G** | **T** | **A** | **A** | **T** | **G** | **G** | **A** | **A** | **G** | **A** | **A** | **A** | **T** | **G** | **G** | **A** | **C** | **G** | **G** | **C** | **A** | **T** | **T** | **G** | **A** | **T** | **T** | **C** | **A** | **A** | **C** | **A** | **A** | **A** | **A** | **G** | **C** | **G** | **A** | **T** | **T** | **T** | **T** | **G** | **A** | **A** | **A** | **G** | **A** | **T** | **T** | **G** | **G** | **C** | **C** | **A** | **G** | **A** | **A** | **G** | **C** | **C** | **A** | **A** | **G** | **A** | **T** | **T** | **A** | **T** | **T** | **A** | **T** | **T** | **G** | **T** | **G** | **A** | **C** | **G** | **A** | **G** | **T** | **T** | **T** | **T** | **A** | **T** | **T** | **G** | **A** | **T** | **G** | **A** | **T** | **G** | **A** | **A** | **A** | **A** | **A** | **G** | **T** | **G** | **T** | **A** | **T** | **C** | **C** | **G** | **G** | **C** | **A** | **A** | **T** | **T** | **G** | **A** | **A** | **G** | **C** | **T** | **G** | **G** | **T** | **G** | **C** | **A** | **G** | **C** | **G** | **G** | **G** | **C** | **T** | **A** | **C** | **C** | **T** | **A** | **T** | **T** | **A** | **A** | **A** | **A** | **A** | **C** | **A** | **T** | **C** | **A** | **A** | **C** | **A** | **G** | **C** | **A** | **C** | **A** | **T** | **G** | **A** | **G** | **A** | **T** | **T** | **G** | **C** | **T** | **G** | **A** | **T** | **G** | **C** | **G** | **A** | **T** | **T** | **C** | **G** | **G** | **G** | **C** | **G** | **A** | **C** | **T** | **T** | **A** | **T** | **C** | **G** | **C** | **G** | **G** | **A** | **G** | **A** | **G** | **C** | **G** | **T** | **G** | **T** | **G** | **T** | **T** | **G** | **G** | **A** | **A** | **C** | **C** | **T** | **G** | **A** | **A** | **G** | **T** | **G** | **A** | **C** | **G** | **C** | **A** | **T** | **A** | **A** | **G** | **A** | **T** | **G** | **A** | **T** | **G** | **G** | **A** | **A** | **C** | **G** | **G** | **T** | **T** | **A** | **A** | **C** | **A** | **A** | **A** | **A** | **A** | **A** | **A** | **C** | **A** | **A** | **G** | **A** | **G** | **C** | **C** | **G** | **G** | **T** | **G** | **C** | **T** | **A** | **C** | **A** | **C** | **G** | **A** | **A** | **G** | **A** | **T** | **T** | **T** | **G** | **A** | **C** | **A** | **A** | **A** | **C** | **C** | **G** | **A** | **G** | **A** | **A** | **C** | **A** | **C** | **G** | **A** | **A** | **A** | **T** | **T** | **T** | **T** | **A** | **A** | **T** | **G** | **C** | **T** | **G** | **A** | **T** | **T** | **G** | **C** | **A** | **C** | **A** | **A** | **G** | **G** | **T** | **A** | **A** | **A** | **A** | **G** | **T** | **A** | **A** | **T** | **C** | **A** | **G** | **G** | **A** | **A** | **A** | **T** | **T** | **G** | **C** | **T** | **G** | **A** | **T** | **G** | **A** | **A** | **C** | **T** | **C** | **T** | **T** | **T** | **A** | **T** | **C** | **A** | **C** | **T** | **T** | **T** | **G** | **A** | **A** | **A** | **A** | **C** | **A** | **G** | **T** | **T** | **A** | **A** | **A** | **A** | **C** | **A** | **C** | **A** | **T** | **G** | **T** | **T** | **T** | **C** | **A** | **A** | **A** | **C** | **A** | **T** | **T** | **T** | **T** | **A** | **G** | **C** | **A** | **A** | **A** | **A** | **C** | **T** | **A** | **G** | **A** | **T** | **G** | **T** | **G** | **G** | **A** | **T** | **G** | **A** | **T** | **C** | **G** | **G** | **A** | **C** | **C** | **C** | **A** | **A** | **G** | **C** | **G** | **G** | **C** | **G** | **A** | **T** | **T** | **T** | **A** | **T** | **G** | **C** | **A** | **T** | **T** | **T** | **C** | **A** | **A** | **C** | **A** | **C** | **G** | **G** | **T** | **T** | **T** | **A** | **G** | **C** | **C** | **A** | **A** | **A** | **-** | **-** | **-** |
| **N60061F** | **A** | **T** | **G** | **A** | **T** | **A** | **A** | **A** | **A** | **G** | **T** | **T** | **T** | **T** | **A** | **T** | **T** | **A** | **G** | **T** | **A** | **G** | **A** | **T** | **G** | **A** | **C** | **C** | **A** | **T** | **G** | **A** | **A** | **A** | **T** | **G** | **G** | **T** | **A** | **C** | **G** | **C** | **T** | **T** | **A** | **G** | **G** | **C** | **G** | **T** | **C** | **T** | **C** | **T** | **T** | **C** | **T** | **T** | **A** | **T** | **T** | **T** | **A** | **T** | **C** | **T** | **A** | **T** | **C** | **C** | **A** | **A** | **G** | **A** | **A** | **G** | **A** | **C** | **A** | **T** | **T** | **G** | **A** | **A** | **G** | **T** | **G** | **A** | **T** | **T** | **G** | **G** | **A** | **G** | **A** | **A** | **G** | **C** | **T** | **G** | **A** | **A** | **A** | **A** | **T** | **G** | **G** | **A** | **C** | **G** | **T** | **C** | **A** | **A** | **G** | **G** | **C** | **T** | **A** | **T** | **G** | **A** | **A** | **A** | **A** | **G** | **G** | **C** | **G** | **A** | **T** | **G** | **G** | **C** | **A** | **C** | **T** | **T** | **C** | **G** | **A** | **C** | **C** | **A** | **G** | **A** | **T** | **G** | **T** | **C** | **A** | **T** | **T** | **T** | **T** | **A** | **A** | **T** | **G** | **G** | **A** | **T** | **C** | **T** | **A** | **G** | **T** | **C** | **A** | **T** | **G** | **G** | **A** | **A** | **G** | **A** | **A** | **A** | **T** | **G** | **G** | **A** | **T** | **G** | **G** | **C** | **A** | **T** | **C** | **G** | **A** | **A** | **T** | **C** | **A** | **A** | **C** | **A** | **A** | **A** | **A** | **G** | **C** | **A** | **A** | **T** | **C** | **T** | **T** | **A** | **A** | **A** | **A** | **G** | **A** | **T** | **T** | **G** | **G** | **C** | **C** | **A** | **A** | **A** | **A** | **G** | **C** | **C** | **A** | **A** | **A** | **A** | **T** | **C** | **A** | **T** | **T** | **A** | **T** | **C** | **G** | **T** | **C** | **A** | **C** | **A** | **A** | **G** | **T** | **T** | **T** | **T** | **A** | **T** | **T** | **G** | **A** | **T** | **G** | **A** | **T** | **G** | **A** | **A** | **A** | **A** | **A** | **G** | **T** | **T** | **T** | **A** | **T** | **C** | **C** | **T** | **G** | **C** | **A** | **A** | **T** | **T** | **G** | **A** | **A** | **G** | **C** | **A** | **G** | **G** | **A** | **G** | **C** | **A** | **G** | **C** | **A** | **G** | **G** | **A** | **T** | **A** | **T** | **T** | **T** | **G** | **T** | **T** | **G** | **A** | **A** | **G** | **A** | **C** | **G** | **T** | **C** | **T** | **A** | **C** | **C** | **G** | **C** | **A** | **C** | **A** | **T** | **G** | **A** | **A** | **A** | **T** | **A** | **G** | **C** | **T** | **G** | **A** | **T** | **G** | **C** | **T** | **A** | **T** | **A** | **A** | **G** | **G** | **G** | **C** | **T** | **A** | **C** | **G** | **C** | **A** | **A** | **C** | **G** | **C** | **G** | **G** | **C** | **G** | **A** | **G** | **C** | **G** | **A** | **G** | **T** | **A** | **T** | **T** | **G** | **G** | **A** | **A** | **C** | **C** | **T** | **G** | **A** | **A** | **G** | **T** | **G** | **A** | **C** | **A** | **A** | **C** | **A** | **A** | **A** | **A** | **A** | **T** | **G** | **A** | **T** | **G** | **G** | **A** | **A** | **A** | **A** | **A** | **A** | **T** | **G** | **A** | **G** | **T** | **C** | **G** | **G** | **C** | **G** | **A** | **A** | **A** | **T** | **G** | **A** | **T** | **C** | **C** | **A** | **G** | **T** | **A** | **T** | **T** | **G** | **C** | **A** | **T** | **G** | **A** | **A** | **G** | **A** | **A** | **T** | **T** | **A** | **A** | **C** | **G** | **A** | **A** | **T** | **C** | **G** | **A** | **G** | **A** | **A** | **A** | **A** | **T** | **G** | **A** | **A** | **A** | **T** | **T** | **T** | **T** | **G** | **A** | **T** | **G** | **C** | **T** | **G** | **A** | **T** | **T** | **T** | **C** | **T** | **G** | **A** | **A** | **G** | **G** | **G** | **A** | **A** | **A** | **A** | **G** | **T** | **A** | **A** | **T** | **C** | **A** | **G** | **G** | **A** | **A** | **A** | **T** | **C** | **G** | **C** | **G** | **G** | **A** | **T** | **G** | **A** | **A** | **T** | **T** | **G** | **T** | **T** | **T** | **A** | **T** | **C** | **A** | **C** | **A** | **C** | **T** | **G** | **A** | **A** | **A** | **A** | **C** | **A** | **G** | **T** | **T** | **A** | **A** | **A** | **A** | **C** | **G** | **C** | **A** | **T** | **G** | **T** | **G** | **T** | **C** | **G** | **A** | **A** | **T** | **A** | **T** | **A** | **T** | **T** | **A** | **G** | **C** | **T** | **A** | **A** | **G** | **T** | **T** | **A** | **G** | **A** | **G** | **G** | **T** | **A** | **G** | **A** | **A** | **G** | **A** | **C** | **C** | **G** | **A** | **A** | **C** | **T** | **C** | **A** | **A** | **G** | **C** | **A** | **G** | **C** | **T** | **A** | **T** | **C** | **T** | **A** | **T** | **G** | **C** | **G** | **T** | **T** | **T** | **A** | **A** | **A** | **C** | **A** | **T** | **G** | **G** | **T** | **T** | **T** | **G** | **G** | **T** | **G** | **A** | **A** | **A** | **-** | **-** | **-** |
| **N59409F** | **G** | **T** | **G** | **A** | **T** | **C** | **A** | **A** | **A** | **G** | **T** | **A** | **A** | **T** | **G** | **T** | **T** | **A** | **G** | **T** | **G** | **G** | **A** | **T** | **G** | **A** | **C** | **C** | **A** | **T** | **G** | **A** | **A** | **A** | **T** | **G** | **G** | **T** | **C** | **C** | **G** | **T** | **T** | **T** | **A** | **G** | **G** | **C** | **G** | **T** | **T** | **T** | **C** | **A** | **T** | **C** | **A** | **T** | **A** | **T** | **T** | **T** | **A** | **T** | **C** | **T** | **A** | **T** | **T** | **C** | **A** | **A** | **G** | **A** | **G** | **G** | **A** | **T** | **A** | **T** | **A** | **G** | **A** | **A** | **G** | **T** | **C** | **G** | **T** | **A** | **G** | **G** | **C** | **G** | **A** | **A** | **G** | **C** | **A** | **G** | **A** | **A** | **A** | **A** | **T** | **G** | **G** | **T** | **A** | **A** | **G** | **A** | **T** | **T** | **G** | **G** | **C** | **T** | **A** | **T** | **G** | **A** | **A** | **A** | **A** | **A** | **G** | **C** | **A** | **T** | **T** | **G** | **G** | **A** | **A** | **C** | **T** | **A** | **C** | **G** | **T** | **C** | **C** | **A** | **G** | **A** | **T** | **G** | **T** | **T** | **A** | **T** | **T** | **T** | **T** | **G** | **A** | **T** | **G** | **G** | **A** | **T** | **T** | **T** | **G** | **G** | **T** | **A** | **A** | **T** | **G** | **G** | **A** | **A** | **G** | **A** | **A** | **A** | **T** | **G** | **G** | **A** | **C** | **G** | **G** | **C** | **A** | **T** | **T** | **G** | **A** | **T** | **T** | **C** | **A** | **A** | **C** | **A** | **A** | **A** | **A** | **G** | **C** | **G** | **A** | **T** | **C** | **T** | **T** | **G** | **A** | **A** | **A** | **G** | **A** | **T** | **T** | **G** | **G** | **C** | **C** | **A** | **G** | **A** | **A** | **G** | **C** | **C** | **A** | **A** | **G** | **A** | **T** | **T** | **A** | **T** | **T** | **A** | **T** | **T** | **G** | **T** | **G** | **A** | **C** | **G** | **A** | **G** | **T** | **T** | **T** | **T** | **A** | **T** | **T** | **G** | **A** | **T** | **G** | **A** | **T** | **G** | **A** | **A** | **A** | **A** | **A** | **G** | **T** | **G** | **T** | **A** | **T** | **C** | **C** | **G** | **G** | **C** | **G** | **A** | **T** | **T** | **G** | **A** | **A** | **G** | **C** | **T** | **G** | **G** | **T** | **G** | **C** | **A** | **G** | **C** | **G** | **G** | **G** | **C** | **T** | **A** | **C** | **C** | **T** | **A** | **T** | **T** | **A** | **A** | **A** | **A** | **A** | **C** | **A** | **T** | **C** | **A** | **A** | **C** | **A** | **G** | **C** | **A** | **C** | **A** | **T** | **G** | **A** | **G** | **A** | **T** | **T** | **G** | **C** | **T** | **G** | **A** | **T** | **G** | **C** | **A** | **A** | **T** | **T** | **C** | **G** | **G** | **G** | **C** | **G** | **A** | **C** | **T** | **T** | **A** | **T** | **C** | **G** | **C** | **G** | **G** | **A** | **G** | **A** | **G** | **C** | **G** | **T** | **G** | **T** | **G** | **T** | **T** | **G** | **G** | **A** | **A** | **C** | **C** | **T** | **G** | **A** | **A** | **G** | **T** | **G** | **A** | **C** | **A** | **C** | **A** | **T** | **A** | **A** | **G** | **A** | **T** | **G** | **A** | **T** | **G** | **G** | **A** | **A** | **C** | **G** | **G** | **T** | **T** | **A** | **A** | **C** | **A** | **A** | **A** | **A** | **A** | **A** | **A** | **C** | **A** | **A** | **G** | **A** | **G** | **C** | **C** | **G** | **G** | **T** | **G** | **T** | **T** | **G** | **C** | **A** | **C** | **G** | **A** | **A** | **G** | **A** | **T** | **T** | **T** | **G** | **A** | **C** | **A** | **A** | **A** | **C** | **C** | **G** | **G** | **G** | **A** | **A** | **C** | **A** | **C** | **G** | **A** | **A** | **A** | **T** | **T** | **T** | **T** | **A** | **A** | **T** | **G** | **T** | **T** | **G** | **A** | **T** | **T** | **G** | **C** | **A** | **C** | **A** | **A** | **G** | **G** | **T** | **A** | **A** | **A** | **A** | **G** | **T** | **A** | **A** | **T** | **C** | **A** | **G** | **G** | **A** | **A** | **A** | **T** | **A** | **G** | **C** | **T** | **G** | **A** | **T** | **G** | **A** | **A** | **C** | **T** | **C** | **T** | **T** | **T** | **A** | **T** | **C** | **A** | **C** | **T** | **T** | **T** | **G** | **A** | **A** | **A** | **A** | **C** | **A** | **G** | **T** | **T** | **A** | **A** | **A** | **A** | **C** | **A** | **C** | **A** | **T** | **G** | **T** | **T** | **T** | **C** | **A** | **A** | **A** | **C** | **A** | **T** | **T** | **T** | **T** | **A** | **G** | **C** | **A** | **A** | **A** | **A** | **C** | **T** | **A** | **G** | **A** | **T** | **G** | **T** | **G** | **G** | **A** | **T** | **G** | **A** | **T** | **C** | **G** | **G** | **A** | **C** | **C** | **C** | **A** | **A** | **G** | **C** | **G** | **G** | **C** | **G** | **A** | **T** | **T** | **T** | **A** | **T** | **G** | **C** | **T** | **T** | **T** | **T** | **C** | **A** | **A** | **C** | **A** | **T** | **G** | **G** | **T** | **T** | **T** | **A** | **G** | **C** | **C** | **A** | **A** | **A** | **-** | **-** | **-** |
| **N54560** | **A** | **T** | **G** | **A** | **T** | **A** | **A** | **A** | **A** | **G** | **T** | **T** | **T** | **T** | **A** | **T** | **T** | **A** | **G** | **T** | **A** | **G** | **A** | **T** | **G** | **A** | **C** | **C** | **A** | **T** | **G** | **A** | **A** | **A** | **T** | **G** | **G** | **T** | **A** | **C** | **G** | **C** | **T** | **T** | **A** | **G** | **G** | **C** | **G** | **T** | **C** | **T** | **C** | **T** | **T** | **C** | **T** | **T** | **A** | **T** | **T** | **T** | **A** | **T** | **C** | **T** | **A** | **T** | **C** | **C** | **A** | **A** | **G** | **A** | **A** | **G** | **A** | **C** | **A** | **T** | **T** | **G** | **A** | **A** | **G** | **T** | **G** | **A** | **T** | **T** | **G** | **G** | **A** | **G** | **A** | **A** | **G** | **C** | **T** | **G** | **A** | **A** | **A** | **A** | **T** | **G** | **G** | **A** | **C** | **G** | **T** | **C** | **A** | **A** | **G** | **G** | **C** | **T** | **A** | **T** | **G** | **A** | **A** | **A** | **A** | **G** | **G** | **C** | **G** | **A** | **T** | **G** | **G** | **C** | **A** | **C** | **T** | **T** | **C** | **G** | **A** | **C** | **C** | **A** | **G** | **A** | **T** | **G** | **T** | **C** | **A** | **T** | **T** | **T** | **T** | **A** | **A** | **T** | **G** | **G** | **A** | **T** | **C** | **T** | **A** | **G** | **T** | **C** | **A** | **T** | **G** | **G** | **A** | **A** | **G** | **A** | **A** | **A** | **T** | **G** | **G** | **A** | **T** | **G** | **G** | **C** | **A** | **T** | **C** | **G** | **A** | **A** | **T** | **C** | **A** | **A** | **C** | **A** | **A** | **A** | **A** | **G** | **C** | **A** | **A** | **T** | **C** | **T** | **T** | **A** | **A** | **A** | **A** | **G** | **A** | **T** | **T** | **G** | **G** | **C** | **C** | **A** | **A** | **A** | **A** | **G** | **C** | **C** | **A** | **A** | **A** | **A** | **T** | **C** | **A** | **T** | **T** | **A** | **T** | **C** | **G** | **T** | **C** | **A** | **C** | **A** | **A** | **G** | **T** | **T** | **T** | **T** | **A** | **T** | **T** | **G** | **A** | **T** | **G** | **A** | **T** | **G** | **A** | **A** | **A** | **A** | **A** | **G** | **T** | **T** | **T** | **A** | **T** | **C** | **C** | **T** | **G** | **C** | **A** | **A** | **T** | **T** | **G** | **A** | **A** | **G** | **C** | **A** | **G** | **G** | **A** | **G** | **C** | **A** | **G** | **C** | **A** | **G** | **G** | **A** | **T** | **A** | **T** | **T** | **T** | **G** | **T** | **T** | **G** | **A** | **A** | **G** | **A** | **C** | **G** | **T** | **C** | **T** | **A** | **C** | **C** | **G** | **C** | **A** | **C** | **A** | **T** | **G** | **A** | **A** | **A** | **T** | **A** | **G** | **C** | **T** | **G** | **A** | **T** | **G** | **C** | **T** | **A** | **T** | **A** | **A** | **G** | **G** | **G** | **C** | **T** | **A** | **C** | **G** | **C** | **A** | **A** | **C** | **G** | **C** | **G** | **G** | **C** | **G** | **A** | **G** | **C** | **G** | **A** | **G** | **T** | **A** | **T** | **T** | **G** | **G** | **A** | **A** | **C** | **C** | **T** | **G** | **A** | **A** | **G** | **T** | **G** | **A** | **C** | **A** | **A** | **C** | **A** | **A** | **A** | **A** | **A** | **T** | **G** | **A** | **T** | **G** | **G** | **A** | **A** | **A** | **A** | **A** | **A** | **T** | **G** | **A** | **G** | **T** | **C** | **G** | **G** | **C** | **G** | **A** | **A** | **A** | **T** | **G** | **A** | **T** | **C** | **C** | **A** | **G** | **T** | **A** | **T** | **T** | **G** | **C** | **A** | **T** | **G** | **A** | **A** | **G** | **A** | **A** | **T** | **T** | **A** | **A** | **C** | **G** | **A** | **A** | **T** | **C** | **G** | **A** | **G** | **A** | **A** | **A** | **A** | **T** | **G** | **A** | **A** | **A** | **T** | **T** | **T** | **T** | **G** | **A** | **T** | **G** | **C** | **T** | **G** | **A** | **T** | **T** | **T** | **C** | **T** | **G** | **A** | **A** | **G** | **G** | **G** | **A** | **A** | **A** | **A** | **G** | **T** | **A** | **A** | **T** | **C** | **A** | **G** | **G** | **A** | **A** | **A** | **T** | **C** | **G** | **C** | **G** | **G** | **A** | **T** | **G** | **A** | **A** | **T** | **T** | **G** | **T** | **T** | **T** | **A** | **T** | **C** | **A** | **C** | **A** | **C** | **T** | **G** | **A** | **A** | **A** | **A** | **C** | **A** | **G** | **T** | **T** | **A** | **A** | **A** | **A** | **C** | **G** | **C** | **A** | **T** | **G** | **T** | **G** | **T** | **C** | **G** | **A** | **A** | **T** | **A** | **T** | **A** | **T** | **T** | **A** | **G** | **C** | **T** | **A** | **A** | **G** | **T** | **T** | **A** | **G** | **A** | **G** | **G** | **T** | **A** | **G** | **A** | **A** | **G** | **A** | **C** | **C** | **G** | **A** | **A** | **C** | **T** | **C** | **A** | **A** | **G** | **C** | **A** | **G** | **C** | **T** | **A** | **T** | **C** | **T** | **A** | **T** | **G** | **C** | **G** | **T** | **T** | **T** | **A** | **A** | **A** | **C** | **A** | **T** | **G** | **G** | **T** | **T** | **T** | **G** | **G** | **T** | **G** | **A** | **A** | **A** | **-** | **-** | **-** |
| **N54156** | **G** | **T** | **G** | **A** | **T** | **C** | **A** | **A** | **A** | **G** | **T** | **A** | **A** | **T** | **G** | **T** | **T** | **A** | **G** | **T** | **G** | **G** | **A** | **T** | **G** | **A** | **C** | **C** | **A** | **T** | **G** | **A** | **A** | **A** | **T** | **G** | **G** | **T** | **C** | **C** | **G** | **T** | **T** | **T** | **A** | **G** | **G** | **C** | **G** | **T** | **T** | **T** | **C** | **A** | **T** | **C** | **A** | **T** | **A** | **T** | **T** | **T** | **A** | **T** | **C** | **T** | **A** | **T** | **T** | **C** | **A** | **A** | **G** | **A** | **G** | **G** | **A** | **T** | **A** | **T** | **A** | **G** | **A** | **A** | **G** | **T** | **C** | **G** | **T** | **A** | **G** | **G** | **C** | **G** | **A** | **A** | **G** | **C** | **A** | **G** | **A** | **A** | **A** | **A** | **C** | **G** | **G** | **T** | **A** | **A** | **G** | **A** | **T** | **T** | **G** | **G** | **C** | **T** | **A** | **T** | **G** | **A** | **A** | **A** | **A** | **A** | **G** | **C** | **A** | **T** | **T** | **G** | **G** | **A** | **A** | **C** | **T** | **A** | **C** | **G** | **T** | **C** | **C** | **A** | **G** | **A** | **T** | **G** | **T** | **T** | **A** | **T** | **T** | **T** | **T** | **G** | **A** | **T** | **G** | **G** | **A** | **T** | **T** | **T** | **G** | **G** | **T** | **A** | **A** | **T** | **G** | **G** | **A** | **A** | **G** | **A** | **A** | **A** | **T** | **G** | **G** | **A** | **C** | **G** | **G** | **C** | **A** | **T** | **T** | **G** | **A** | **T** | **T** | **C** | **A** | **A** | **C** | **A** | **A** | **A** | **A** | **G** | **C** | **G** | **A** | **T** | **C** | **T** | **T** | **G** | **A** | **A** | **A** | **G** | **A** | **T** | **T** | **G** | **G** | **C** | **C** | **A** | **G** | **A** | **A** | **G** | **C** | **C** | **A** | **A** | **G** | **A** | **T** | **T** | **A** | **T** | **T** | **A** | **T** | **T** | **G** | **T** | **G** | **A** | **C** | **G** | **A** | **G** | **T** | **T** | **T** | **T** | **A** | **T** | **T** | **G** | **A** | **T** | **G** | **A** | **T** | **G** | **A** | **A** | **A** | **A** | **A** | **G** | **T** | **G** | **T** | **A** | **T** | **C** | **C** | **G** | **G** | **C** | **G** | **A** | **T** | **T** | **G** | **A** | **A** | **G** | **C** | **T** | **G** | **G** | **T** | **G** | **C** | **A** | **G** | **C** | **G** | **G** | **G** | **C** | **T** | **A** | **C** | **C** | **T** | **A** | **T** | **T** | **A** | **A** | **A** | **A** | **A** | **C** | **A** | **T** | **C** | **A** | **A** | **C** | **A** | **G** | **C** | **A** | **C** | **A** | **T** | **G** | **A** | **G** | **A** | **T** | **T** | **G** | **C** | **T** | **G** | **A** | **T** | **G** | **C** | **A** | **A** | **T** | **T** | **C** | **G** | **G** | **G** | **C** | **G** | **A** | **C** | **T** | **T** | **A** | **T** | **C** | **G** | **C** | **G** | **G** | **A** | **G** | **A** | **G** | **C** | **G** | **T** | **G** | **T** | **G** | **T** | **T** | **G** | **G** | **A** | **A** | **C** | **C** | **T** | **G** | **A** | **A** | **G** | **T** | **G** | **A** | **C** | **G** | **C** | **A** | **T** | **A** | **A** | **G** | **A** | **T** | **G** | **A** | **T** | **G** | **G** | **A** | **A** | **C** | **G** | **G** | **T** | **T** | **A** | **A** | **C** | **A** | **A** | **A** | **A** | **A** | **A** | **A** | **C** | **A** | **A** | **G** | **A** | **G** | **C** | **C** | **G** | **G** | **T** | **G** | **T** | **T** | **G** | **C** | **A** | **C** | **G** | **A** | **A** | **G** | **A** | **T** | **T** | **T** | **G** | **A** | **C** | **A** | **A** | **A** | **C** | **C** | **G** | **G** | **G** | **A** | **A** | **C** | **A** | **C** | **G** | **A** | **A** | **A** | **T** | **T** | **T** | **T** | **A** | **A** | **T** | **G** | **T** | **T** | **G** | **A** | **T** | **T** | **G** | **C** | **A** | **C** | **A** | **A** | **G** | **G** | **T** | **A** | **A** | **A** | **A** | **G** | **T** | **A** | **A** | **T** | **C** | **A** | **G** | **G** | **A** | **A** | **A** | **T** | **A** | **G** | **C** | **T** | **G** | **A** | **T** | **G** | **A** | **A** | **C** | **T** | **C** | **T** | **T** | **T** | **A** | **T** | **C** | **A** | **C** | **T** | **T** | **T** | **G** | **A** | **A** | **A** | **A** | **C** | **A** | **G** | **T** | **T** | **A** | **A** | **A** | **A** | **C** | **A** | **C** | **A** | **T** | **G** | **T** | **T** | **T** | **C** | **A** | **A** | **A** | **C** | **A** | **T** | **T** | **T** | **T** | **A** | **G** | **C** | **A** | **A** | **A** | **A** | **C** | **T** | **A** | **G** | **A** | **T** | **G** | **T** | **G** | **G** | **A** | **T** | **G** | **A** | **T** | **C** | **G** | **G** | **A** | **C** | **C** | **C** | **A** | **A** | **G** | **C** | **G** | **G** | **C** | **G** | **A** | **T** | **T** | **T** | **A** | **T** | **G** | **C** | **T** | **T** | **T** | **T** | **C** | **A** | **A** | **C** | **A** | **T** | **G** | **G** | **T** | **T** | **T** | **A** | **G** | **C** | **C** | **A** | **A** | **A** | **-** | **-** | **-** |
| **N59462F** | **G** | **T** | **G** | **A** | **T** | **C** | **A** | **A** | **A** | **G** | **T** | **A** | **A** | **T** | **G** | **T** | **T** | **A** | **G** | **T** | **G** | **G** | **A** | **T** | **G** | **A** | **C** | **C** | **A** | **T** | **G** | **A** | **A** | **A** | **T** | **G** | **G** | **T** | **C** | **C** | **G** | **T** | **T** | **T** | **A** | **G** | **G** | **C** | **G** | **T** | **T** | **T** | **C** | **A** | **T** | **C** | **A** | **T** | **A** | **T** | **T** | **T** | **A** | **T** | **C** | **T** | **A** | **T** | **T** | **C** | **A** | **A** | **G** | **A** | **G** | **G** | **A** | **T** | **A** | **T** | **A** | **G** | **A** | **A** | **G** | **T** | **C** | **G** | **T** | **A** | **G** | **G** | **C** | **G** | **A** | **A** | **G** | **C** | **A** | **G** | **A** | **A** | **A** | **A** | **C** | **G** | **G** | **T** | **A** | **A** | **G** | **A** | **T** | **T** | **G** | **G** | **C** | **T** | **A** | **T** | **G** | **A** | **A** | **A** | **A** | **A** | **G** | **C** | **A** | **T** | **T** | **G** | **G** | **A** | **A** | **C** | **T** | **A** | **C** | **G** | **T** | **C** | **C** | **A** | **G** | **A** | **T** | **G** | **T** | **T** | **A** | **T** | **T** | **T** | **T** | **G** | **A** | **T** | **G** | **G** | **A** | **T** | **T** | **T** | **G** | **G** | **T** | **A** | **A** | **T** | **G** | **G** | **A** | **A** | **G** | **A** | **A** | **A** | **T** | **G** | **G** | **A** | **C** | **G** | **G** | **C** | **A** | **T** | **T** | **G** | **A** | **T** | **T** | **C** | **A** | **A** | **C** | **A** | **A** | **A** | **A** | **G** | **C** | **G** | **A** | **T** | **C** | **T** | **T** | **G** | **A** | **A** | **A** | **G** | **A** | **T** | **T** | **G** | **G** | **C** | **C** | **A** | **G** | **A** | **A** | **G** | **C** | **C** | **A** | **A** | **G** | **A** | **T** | **T** | **A** | **T** | **T** | **A** | **T** | **T** | **G** | **T** | **G** | **A** | **C** | **G** | **A** | **G** | **T** | **T** | **T** | **T** | **A** | **T** | **T** | **G** | **A** | **T** | **G** | **A** | **T** | **G** | **A** | **A** | **A** | **A** | **A** | **G** | **T** | **G** | **T** | **A** | **T** | **C** | **C** | **G** | **G** | **C** | **G** | **A** | **T** | **T** | **G** | **A** | **A** | **G** | **C** | **T** | **G** | **G** | **T** | **G** | **C** | **A** | **G** | **C** | **G** | **G** | **G** | **C** | **T** | **A** | **C** | **C** | **T** | **A** | **T** | **T** | **A** | **A** | **A** | **A** | **A** | **C** | **A** | **T** | **C** | **A** | **A** | **C** | **A** | **G** | **C** | **A** | **C** | **A** | **T** | **G** | **A** | **G** | **A** | **T** | **T** | **G** | **C** | **T** | **G** | **A** | **T** | **G** | **C** | **A** | **A** | **T** | **T** | **C** | **G** | **G** | **G** | **C** | **G** | **A** | **C** | **T** | **T** | **A** | **T** | **C** | **G** | **C** | **G** | **G** | **A** | **G** | **A** | **G** | **C** | **G** | **T** | **G** | **T** | **G** | **T** | **T** | **G** | **G** | **A** | **A** | **C** | **C** | **T** | **G** | **A** | **A** | **G** | **T** | **G** | **A** | **C** | **G** | **C** | **A** | **T** | **A** | **A** | **G** | **A** | **T** | **G** | **A** | **T** | **G** | **G** | **A** | **A** | **C** | **G** | **G** | **T** | **T** | **A** | **A** | **C** | **A** | **A** | **A** | **A** | **A** | **A** | **A** | **C** | **A** | **A** | **G** | **A** | **G** | **C** | **C** | **G** | **G** | **T** | **G** | **T** | **T** | **G** | **C** | **A** | **C** | **G** | **A** | **A** | **G** | **A** | **T** | **T** | **T** | **G** | **A** | **C** | **A** | **A** | **A** | **C** | **C** | **G** | **G** | **G** | **A** | **A** | **C** | **A** | **C** | **G** | **A** | **A** | **A** | **T** | **T** | **T** | **T** | **A** | **A** | **T** | **G** | **T** | **T** | **G** | **A** | **T** | **T** | **G** | **C** | **A** | **C** | **A** | **A** | **G** | **G** | **T** | **A** | **A** | **A** | **A** | **G** | **T** | **A** | **A** | **T** | **C** | **A** | **G** | **G** | **A** | **A** | **A** | **T** | **A** | **G** | **C** | **T** | **G** | **A** | **T** | **G** | **A** | **A** | **C** | **T** | **C** | **T** | **T** | **T** | **A** | **T** | **C** | **A** | **C** | **T** | **T** | **T** | **G** | **A** | **A** | **A** | **A** | **C** | **A** | **G** | **T** | **T** | **A** | **A** | **A** | **A** | **C** | **A** | **C** | **A** | **T** | **G** | **T** | **T** | **T** | **C** | **A** | **A** | **A** | **C** | **A** | **T** | **T** | **T** | **T** | **A** | **G** | **C** | **A** | **A** | **A** | **A** | **C** | **T** | **A** | **G** | **A** | **T** | **G** | **T** | **G** | **G** | **A** | **T** | **G** | **A** | **T** | **C** | **G** | **G** | **A** | **C** | **C** | **C** | **A** | **A** | **G** | **C** | **G** | **G** | **C** | **G** | **A** | **T** | **T** | **T** | **A** | **T** | **G** | **C** | **T** | **T** | **T** | **T** | **C** | **A** | **A** | **C** | **A** | **T** | **G** | **G** | **T** | **T** | **T** | **A** | **G** | **C** | **C** | **A** | **A** | **A** | **-** | **-** | **-** |
| **N55072** | **A** | **T** | **G** | **A** | **T** | **A** | **A** | **A** | **A** | **G** | **T** | **T** | **T** | **T** | **A** | **T** | **T** | **A** | **G** | **T** | **A** | **G** | **A** | **T** | **G** | **A** | **C** | **C** | **A** | **T** | **G** | **A** | **A** | **A** | **T** | **G** | **G** | **T** | **A** | **C** | **G** | **C** | **T** | **T** | **A** | **G** | **G** | **C** | **G** | **T** | **C** | **T** | **C** | **T** | **T** | **C** | **T** | **T** | **A** | **T** | **T** | **T** | **A** | **T** | **C** | **T** | **A** | **T** | **C** | **C** | **A** | **A** | **G** | **A** | **A** | **G** | **A** | **C** | **A** | **T** | **T** | **G** | **A** | **A** | **G** | **T** | **G** | **A** | **T** | **T** | **G** | **G** | **A** | **G** | **A** | **A** | **G** | **C** | **T** | **G** | **A** | **A** | **A** | **A** | **T** | **G** | **G** | **A** | **C** | **G** | **T** | **C** | **A** | **A** | **G** | **G** | **C** | **T** | **A** | **T** | **G** | **A** | **A** | **A** | **A** | **G** | **G** | **C** | **G** | **A** | **T** | **G** | **G** | **C** | **A** | **C** | **T** | **T** | **C** | **G** | **A** | **C** | **C** | **A** | **G** | **A** | **T** | **G** | **T** | **C** | **A** | **T** | **T** | **T** | **T** | **A** | **A** | **T** | **G** | **G** | **A** | **T** | **C** | **T** | **A** | **G** | **T** | **C** | **A** | **T** | **G** | **G** | **A** | **A** | **G** | **A** | **A** | **A** | **T** | **G** | **G** | **A** | **T** | **G** | **G** | **C** | **A** | **T** | **C** | **G** | **A** | **A** | **T** | **C** | **A** | **A** | **C** | **A** | **A** | **A** | **A** | **G** | **C** | **A** | **A** | **T** | **C** | **T** | **T** | **A** | **A** | **A** | **A** | **G** | **A** | **T** | **T** | **G** | **G** | **C** | **C** | **A** | **A** | **A** | **A** | **G** | **C** | **C** | **A** | **A** | **A** | **A** | **T** | **C** | **A** | **T** | **T** | **A** | **T** | **C** | **G** | **T** | **C** | **A** | **C** | **A** | **A** | **G** | **T** | **T** | **T** | **T** | **A** | **T** | **T** | **G** | **A** | **T** | **G** | **A** | **T** | **G** | **A** | **A** | **A** | **A** | **A** | **G** | **T** | **T** | **T** | **A** | **T** | **C** | **C** | **T** | **G** | **C** | **A** | **A** | **T** | **T** | **G** | **A** | **A** | **G** | **C** | **A** | **G** | **G** | **A** | **G** | **C** | **A** | **G** | **C** | **A** | **G** | **G** | **A** | **T** | **A** | **T** | **T** | **T** | **A** | **T** | **T** | **G** | **A** | **A** | **G** | **A** | **C** | **G** | **T** | **C** | **T** | **A** | **C** | **C** | **G** | **C** | **A** | **C** | **A** | **T** | **G** | **A** | **A** | **A** | **T** | **A** | **G** | **C** | **T** | **G** | **A** | **T** | **G** | **C** | **T** | **A** | **T** | **A** | **A** | **G** | **G** | **G** | **C** | **T** | **A** | **C** | **G** | **C** | **A** | **A** | **C** | **G** | **C** | **G** | **G** | **C** | **G** | **A** | **G** | **C** | **G** | **A** | **G** | **T** | **A** | **T** | **T** | **G** | **G** | **A** | **A** | **C** | **C** | **T** | **G** | **A** | **A** | **G** | **T** | **G** | **A** | **C** | **A** | **A** | **C** | **A** | **A** | **A** | **A** | **A** | **T** | **G** | **A** | **T** | **G** | **G** | **A** | **A** | **A** | **A** | **A** | **A** | **T** | **G** | **A** | **G** | **T** | **C** | **G** | **G** | **C** | **G** | **A** | **A** | **A** | **T** | **G** | **A** | **T** | **C** | **C** | **A** | **G** | **T** | **A** | **T** | **T** | **G** | **C** | **A** | **T** | **G** | **A** | **A** | **G** | **A** | **A** | **T** | **T** | **A** | **A** | **C** | **G** | **A** | **A** | **T** | **C** | **G** | **A** | **G** | **A** | **A** | **A** | **A** | **T** | **G** | **A** | **A** | **A** | **T** | **T** | **T** | **T** | **G** | **A** | **T** | **G** | **C** | **T** | **G** | **A** | **T** | **T** | **T** | **C** | **T** | **G** | **A** | **A** | **G** | **G** | **G** | **A** | **A** | **A** | **A** | **G** | **T** | **A** | **A** | **T** | **C** | **A** | **G** | **G** | **A** | **A** | **A** | **T** | **C** | **G** | **C** | **G** | **G** | **A** | **T** | **G** | **A** | **A** | **T** | **T** | **G** | **T** | **T** | **T** | **A** | **T** | **C** | **A** | **C** | **A** | **C** | **T** | **G** | **A** | **A** | **A** | **A** | **C** | **A** | **G** | **T** | **T** | **A** | **A** | **A** | **A** | **C** | **G** | **C** | **A** | **T** | **G** | **T** | **G** | **T** | **C** | **G** | **A** | **A** | **T** | **A** | **T** | **A** | **T** | **T** | **A** | **G** | **C** | **T** | **A** | **A** | **G** | **T** | **T** | **A** | **G** | **A** | **G** | **G** | **T** | **A** | **G** | **A** | **A** | **G** | **A** | **C** | **C** | **G** | **A** | **A** | **C** | **T** | **C** | **A** | **A** | **G** | **C** | **A** | **G** | **C** | **T** | **A** | **T** | **C** | **T** | **A** | **T** | **G** | **C** | **G** | **T** | **T** | **T** | **A** | **A** | **A** | **C** | **A** | **T** | **G** | **G** | **T** | **T** | **T** | **G** | **G** | **T** | **G** | **A** | **A** | **A** | **-** | **-** | **-** |
| **N59836F** | **G** | **T** | **G** | **A** | **T** | **C** | **A** | **A** | **A** | **G** | **T** | **A** | **A** | **T** | **G** | **T** | **T** | **A** | **G** | **T** | **G** | **G** | **A** | **T** | **G** | **A** | **C** | **C** | **A** | **T** | **G** | **A** | **A** | **A** | **T** | **G** | **G** | **T** | **C** | **C** | **G** | **T** | **T** | **T** | **A** | **G** | **G** | **C** | **G** | **T** | **T** | **T** | **C** | **A** | **T** | **C** | **A** | **T** | **A** | **T** | **T** | **T** | **A** | **T** | **C** | **T** | **A** | **T** | **T** | **C** | **A** | **A** | **G** | **A** | **G** | **G** | **A** | **T** | **A** | **T** | **A** | **G** | **A** | **A** | **G** | **T** | **C** | **G** | **T** | **A** | **G** | **G** | **C** | **G** | **A** | **A** | **G** | **C** | **A** | **G** | **A** | **A** | **A** | **A** | **C** | **G** | **G** | **T** | **A** | **A** | **G** | **A** | **T** | **T** | **G** | **G** | **C** | **T** | **A** | **T** | **G** | **A** | **A** | **A** | **A** | **A** | **G** | **C** | **A** | **T** | **T** | **G** | **G** | **A** | **A** | **C** | **T** | **A** | **C** | **G** | **T** | **C** | **C** | **A** | **G** | **A** | **T** | **G** | **T** | **T** | **A** | **T** | **T** | **T** | **T** | **G** | **A** | **T** | **G** | **G** | **A** | **T** | **T** | **T** | **G** | **G** | **T** | **A** | **A** | **T** | **G** | **G** | **A** | **A** | **G** | **A** | **A** | **A** | **T** | **G** | **G** | **A** | **C** | **G** | **G** | **C** | **A** | **T** | **T** | **G** | **A** | **T** | **T** | **C** | **A** | **A** | **C** | **A** | **A** | **A** | **A** | **G** | **C** | **G** | **A** | **T** | **C** | **T** | **T** | **G** | **A** | **A** | **A** | **G** | **A** | **T** | **T** | **G** | **G** | **C** | **C** | **A** | **G** | **A** | **A** | **G** | **C** | **C** | **A** | **A** | **G** | **A** | **T** | **T** | **A** | **T** | **T** | **A** | **T** | **T** | **G** | **T** | **G** | **A** | **C** | **G** | **A** | **G** | **T** | **T** | **T** | **T** | **A** | **T** | **T** | **G** | **A** | **T** | **G** | **A** | **T** | **G** | **A** | **A** | **A** | **A** | **A** | **G** | **T** | **G** | **T** | **A** | **T** | **C** | **C** | **G** | **G** | **C** | **G** | **A** | **T** | **T** | **G** | **A** | **A** | **G** | **C** | **T** | **G** | **G** | **T** | **G** | **C** | **A** | **G** | **C** | **G** | **G** | **G** | **C** | **T** | **A** | **C** | **C** | **T** | **A** | **T** | **T** | **A** | **A** | **A** | **A** | **A** | **C** | **A** | **T** | **C** | **A** | **A** | **C** | **A** | **G** | **C** | **A** | **C** | **A** | **T** | **G** | **A** | **G** | **A** | **T** | **T** | **G** | **C** | **T** | **G** | **A** | **T** | **G** | **C** | **A** | **A** | **T** | **T** | **C** | **G** | **G** | **G** | **C** | **G** | **A** | **C** | **T** | **T** | **A** | **T** | **C** | **G** | **C** | **G** | **G** | **A** | **G** | **A** | **G** | **C** | **G** | **T** | **G** | **T** | **G** | **T** | **T** | **G** | **G** | **A** | **A** | **C** | **C** | **T** | **G** | **A** | **A** | **G** | **T** | **G** | **A** | **C** | **G** | **C** | **A** | **T** | **A** | **A** | **G** | **A** | **T** | **G** | **A** | **T** | **G** | **G** | **A** | **A** | **C** | **G** | **G** | **T** | **T** | **A** | **A** | **C** | **A** | **A** | **A** | **A** | **A** | **A** | **A** | **C** | **A** | **A** | **G** | **A** | **G** | **C** | **C** | **G** | **G** | **T** | **G** | **T** | **T** | **G** | **C** | **A** | **C** | **G** | **A** | **A** | **G** | **A** | **T** | **T** | **T** | **G** | **A** | **C** | **A** | **A** | **A** | **C** | **C** | **G** | **G** | **G** | **A** | **A** | **C** | **A** | **C** | **G** | **A** | **A** | **A** | **T** | **T** | **T** | **T** | **A** | **A** | **T** | **G** | **T** | **T** | **G** | **A** | **T** | **T** | **G** | **C** | **A** | **C** | **A** | **A** | **G** | **G** | **T** | **A** | **A** | **A** | **A** | **G** | **T** | **A** | **A** | **T** | **C** | **A** | **G** | **G** | **A** | **A** | **A** | **T** | **A** | **G** | **C** | **T** | **G** | **A** | **T** | **G** | **A** | **A** | **C** | **T** | **C** | **T** | **T** | **T** | **A** | **T** | **C** | **A** | **C** | **T** | **T** | **T** | **G** | **A** | **A** | **A** | **A** | **C** | **A** | **G** | **T** | **T** | **A** | **A** | **A** | **A** | **C** | **A** | **C** | **A** | **T** | **G** | **T** | **T** | **T** | **C** | **A** | **A** | **A** | **C** | **A** | **T** | **T** | **T** | **T** | **A** | **G** | **C** | **A** | **A** | **A** | **A** | **C** | **T** | **A** | **G** | **A** | **T** | **G** | **T** | **G** | **G** | **A** | **T** | **G** | **A** | **T** | **C** | **G** | **G** | **A** | **C** | **C** | **C** | **A** | **A** | **G** | **C** | **G** | **G** | **C** | **G** | **A** | **T** | **T** | **T** | **A** | **T** | **G** | **C** | **T** | **T** | **T** | **T** | **C** | **A** | **A** | **C** | **A** | **T** | **G** | **G** | **T** | **T** | **T** | **A** | **G** | **C** | **C** | **A** | **A** | **A** | **-** | **-** | **-** |
| **N59896F** | **G** | **T** | **G** | **A** | **T** | **C** | **A** | **A** | **A** | **G** | **T** | **A** | **A** | **T** | **G** | **T** | **T** | **A** | **G** | **T** | **G** | **G** | **A** | **T** | **G** | **A** | **C** | **C** | **A** | **T** | **G** | **A** | **A** | **A** | **T** | **G** | **G** | **T** | **C** | **C** | **G** | **T** | **T** | **T** | **A** | **G** | **G** | **C** | **G** | **T** | **T** | **T** | **C** | **A** | **T** | **C** | **A** | **T** | **A** | **T** | **T** | **T** | **A** | **T** | **C** | **T** | **A** | **T** | **T** | **C** | **A** | **A** | **G** | **A** | **G** | **G** | **A** | **T** | **A** | **T** | **A** | **G** | **A** | **A** | **G** | **T** | **C** | **G** | **T** | **A** | **G** | **G** | **C** | **G** | **A** | **A** | **G** | **C** | **A** | **G** | **A** | **A** | **A** | **A** | **C** | **G** | **G** | **T** | **A** | **A** | **G** | **A** | **T** | **T** | **G** | **G** | **C** | **T** | **A** | **T** | **G** | **A** | **A** | **A** | **A** | **A** | **G** | **C** | **A** | **T** | **T** | **G** | **G** | **A** | **A** | **C** | **T** | **A** | **C** | **G** | **T** | **C** | **C** | **A** | **G** | **A** | **T** | **G** | **T** | **T** | **A** | **T** | **T** | **T** | **T** | **G** | **A** | **T** | **G** | **G** | **A** | **T** | **T** | **T** | **G** | **G** | **T** | **A** | **A** | **T** | **G** | **G** | **A** | **A** | **G** | **A** | **A** | **A** | **T** | **G** | **G** | **A** | **C** | **G** | **G** | **C** | **A** | **T** | **T** | **G** | **A** | **T** | **T** | **C** | **A** | **A** | **C** | **C** | **A** | **A** | **A** | **G** | **C** | **G** | **A** | **T** | **C** | **T** | **T** | **G** | **A** | **A** | **A** | **G** | **A** | **T** | **T** | **G** | **G** | **C** | **C** | **A** | **G** | **A** | **A** | **G** | **C** | **C** | **A** | **A** | **G** | **A** | **T** | **T** | **A** | **T** | **T** | **A** | **T** | **T** | **G** | **T** | **G** | **A** | **C** | **G** | **A** | **G** | **T** | **T** | **T** | **T** | **A** | **T** | **T** | **G** | **A** | **T** | **G** | **A** | **T** | **G** | **A** | **A** | **A** | **A** | **A** | **G** | **T** | **G** | **T** | **A** | **T** | **C** | **C** | **G** | **G** | **C** | **G** | **A** | **T** | **T** | **G** | **A** | **A** | **G** | **C** | **T** | **G** | **G** | **T** | **G** | **C** | **A** | **G** | **C** | **G** | **G** | **G** | **C** | **T** | **A** | **C** | **C** | **T** | **A** | **T** | **T** | **A** | **A** | **A** | **A** | **A** | **C** | **A** | **T** | **C** | **A** | **A** | **C** | **A** | **G** | **C** | **A** | **C** | **A** | **T** | **G** | **A** | **G** | **A** | **T** | **T** | **G** | **C** | **T** | **G** | **A** | **T** | **G** | **C** | **A** | **A** | **T** | **T** | **C** | **G** | **G** | **G** | **C** | **G** | **A** | **C** | **T** | **T** | **A** | **T** | **C** | **G** | **C** | **G** | **G** | **A** | **G** | **A** | **G** | **C** | **G** | **T** | **G** | **T** | **G** | **T** | **T** | **G** | **G** | **A** | **A** | **C** | **C** | **T** | **G** | **A** | **A** | **G** | **T** | **G** | **A** | **C** | **G** | **C** | **A** | **T** | **A** | **A** | **G** | **A** | **T** | **G** | **A** | **T** | **G** | **G** | **A** | **A** | **C** | **G** | **G** | **T** | **T** | **A** | **A** | **C** | **A** | **A** | **A** | **A** | **A** | **A** | **A** | **C** | **A** | **A** | **G** | **A** | **G** | **C** | **C** | **G** | **G** | **T** | **G** | **T** | **T** | **G** | **C** | **A** | **C** | **G** | **A** | **A** | **G** | **A** | **T** | **T** | **T** | **G** | **A** | **C** | **A** | **A** | **A** | **C** | **C** | **G** | **G** | **G** | **A** | **A** | **C** | **A** | **C** | **G** | **A** | **A** | **A** | **T** | **T** | **T** | **T** | **A** | **A** | **T** | **G** | **T** | **T** | **G** | **A** | **T** | **T** | **G** | **C** | **A** | **C** | **A** | **A** | **G** | **G** | **T** | **A** | **A** | **A** | **A** | **G** | **T** | **A** | **A** | **T** | **C** | **A** | **G** | **G** | **A** | **A** | **A** | **T** | **A** | **G** | **C** | **T** | **G** | **A** | **T** | **G** | **A** | **A** | **C** | **T** | **C** | **T** | **T** | **T** | **A** | **T** | **C** | **A** | **C** | **T** | **T** | **T** | **G** | **A** | **A** | **A** | **A** | **C** | **A** | **G** | **T** | **T** | **A** | **A** | **A** | **A** | **C** | **A** | **C** | **A** | **T** | **G** | **T** | **T** | **T** | **C** | **A** | **A** | **A** | **C** | **A** | **T** | **T** | **T** | **T** | **A** | **G** | **C** | **A** | **A** | **A** | **A** | **C** | **T** | **A** | **G** | **A** | **T** | **G** | **T** | **G** | **G** | **A** | **T** | **G** | **A** | **T** | **C** | **G** | **G** | **A** | **C** | **C** | **C** | **A** | **A** | **G** | **C** | **G** | **G** | **C** | **G** | **A** | **T** | **T** | **T** | **A** | **T** | **G** | **C** | **T** | **T** | **T** | **T** | **C** | **A** | **A** | **C** | **A** | **T** | **G** | **G** | **T** | **T** | **T** | **A** | **G** | **C** | **C** | **A** | **A** | **A** | **-** | **-** | **-** |
| **N52755** | **G** | **T** | **G** | **A** | **T** | **C** | **A** | **A** | **A** | **G** | **T** | **A** | **A** | **T** | **G** | **T** | **T** | **A** | **G** | **T** | **G** | **G** | **A** | **T** | **G** | **A** | **C** | **C** | **A** | **T** | **G** | **A** | **A** | **A** | **T** | **G** | **G** | **T** | **C** | **C** | **G** | **T** | **T** | **T** | **A** | **G** | **G** | **C** | **G** | **T** | **T** | **T** | **C** | **A** | **T** | **C** | **A** | **T** | **A** | **T** | **T** | **T** | **A** | **T** | **C** | **T** | **A** | **T** | **T** | **C** | **A** | **A** | **G** | **A** | **G** | **G** | **A** | **T** | **A** | **T** | **A** | **G** | **A** | **A** | **G** | **T** | **C** | **G** | **T** | **A** | **G** | **G** | **C** | **G** | **A** | **A** | **G** | **C** | **A** | **G** | **A** | **A** | **A** | **A** | **C** | **G** | **G** | **T** | **A** | **A** | **G** | **A** | **T** | **T** | **G** | **G** | **C** | **T** | **A** | **T** | **G** | **A** | **A** | **A** | **A** | **A** | **G** | **C** | **A** | **T** | **T** | **G** | **G** | **A** | **A** | **C** | **T** | **A** | **C** | **G** | **T** | **C** | **C** | **A** | **G** | **A** | **T** | **G** | **T** | **T** | **A** | **T** | **T** | **T** | **T** | **G** | **A** | **T** | **G** | **G** | **A** | **T** | **T** | **T** | **G** | **G** | **T** | **A** | **A** | **T** | **G** | **G** | **A** | **A** | **G** | **A** | **A** | **A** | **T** | **G** | **G** | **A** | **C** | **G** | **G** | **C** | **A** | **T** | **T** | **G** | **A** | **T** | **T** | **C** | **A** | **A** | **C** | **A** | **A** | **A** | **A** | **G** | **C** | **G** | **A** | **T** | **C** | **T** | **T** | **G** | **A** | **A** | **A** | **G** | **A** | **T** | **T** | **G** | **G** | **C** | **C** | **A** | **G** | **A** | **A** | **G** | **C** | **C** | **A** | **A** | **G** | **A** | **T** | **T** | **A** | **T** | **T** | **A** | **T** | **T** | **G** | **T** | **G** | **A** | **C** | **G** | **A** | **G** | **T** | **T** | **T** | **T** | **A** | **T** | **T** | **G** | **A** | **T** | **G** | **A** | **T** | **G** | **A** | **A** | **A** | **A** | **A** | **G** | **T** | **G** | **T** | **A** | **T** | **C** | **C** | **G** | **G** | **C** | **G** | **A** | **T** | **T** | **G** | **A** | **A** | **G** | **C** | **T** | **G** | **G** | **T** | **G** | **C** | **A** | **G** | **C** | **G** | **G** | **G** | **C** | **T** | **A** | **C** | **C** | **T** | **A** | **T** | **T** | **A** | **A** | **A** | **A** | **A** | **C** | **A** | **T** | **C** | **A** | **A** | **C** | **A** | **G** | **C** | **A** | **C** | **A** | **T** | **G** | **A** | **G** | **A** | **T** | **T** | **G** | **C** | **T** | **G** | **A** | **T** | **G** | **C** | **A** | **A** | **T** | **T** | **C** | **G** | **G** | **G** | **C** | **G** | **A** | **C** | **T** | **T** | **A** | **T** | **C** | **G** | **C** | **G** | **G** | **A** | **G** | **A** | **G** | **C** | **G** | **T** | **G** | **T** | **G** | **T** | **T** | **G** | **G** | **A** | **A** | **C** | **C** | **T** | **G** | **A** | **A** | **G** | **T** | **G** | **A** | **C** | **G** | **C** | **A** | **T** | **A** | **A** | **G** | **A** | **T** | **G** | **A** | **T** | **G** | **G** | **A** | **A** | **C** | **G** | **G** | **T** | **T** | **A** | **A** | **C** | **A** | **A** | **A** | **A** | **A** | **A** | **A** | **C** | **A** | **A** | **G** | **A** | **G** | **C** | **C** | **G** | **G** | **T** | **G** | **T** | **T** | **G** | **C** | **A** | **C** | **G** | **A** | **A** | **G** | **A** | **T** | **T** | **T** | **G** | **A** | **C** | **A** | **A** | **A** | **C** | **C** | **G** | **G** | **G** | **A** | **A** | **C** | **A** | **C** | **G** | **A** | **A** | **A** | **T** | **T** | **T** | **T** | **A** | **A** | **T** | **G** | **T** | **T** | **G** | **A** | **T** | **T** | **G** | **C** | **A** | **C** | **A** | **A** | **G** | **G** | **T** | **A** | **A** | **A** | **A** | **G** | **T** | **A** | **A** | **T** | **C** | **A** | **G** | **G** | **A** | **A** | **A** | **T** | **A** | **G** | **C** | **T** | **G** | **A** | **T** | **G** | **A** | **A** | **C** | **T** | **C** | **T** | **T** | **T** | **A** | **T** | **C** | **A** | **C** | **T** | **T** | **T** | **G** | **A** | **A** | **A** | **A** | **C** | **A** | **G** | **T** | **T** | **A** | **A** | **A** | **A** | **C** | **A** | **C** | **A** | **T** | **G** | **T** | **T** | **T** | **C** | **A** | **A** | **A** | **C** | **A** | **T** | **T** | **T** | **T** | **A** | **G** | **C** | **A** | **A** | **A** | **A** | **C** | **T** | **A** | **G** | **A** | **T** | **G** | **T** | **G** | **G** | **A** | **T** | **G** | **A** | **T** | **C** | **G** | **G** | **A** | **C** | **C** | **C** | **A** | **A** | **G** | **C** | **G** | **G** | **C** | **G** | **A** | **T** | **T** | **T** | **A** | **T** | **G** | **C** | **T** | **T** | **T** | **T** | **C** | **A** | **A** | **C** | **A** | **T** | **G** | **G** | **T** | **T** | **T** | **A** | **G** | **C** | **C** | **A** | **A** | **A** | **-** | **-** | **-** |
| **N59951F** | **A** | **T** | **G** | **A** | **T** | **A** | **A** | **A** | **A** | **G** | **T** | **T** | **T** | **T** | **A** | **T** | **T** | **A** | **G** | **T** | **A** | **G** | **A** | **T** | **G** | **A** | **C** | **C** | **A** | **T** | **G** | **A** | **A** | **A** | **T** | **G** | **G** | **T** | **A** | **C** | **G** | **C** | **T** | **T** | **A** | **G** | **G** | **C** | **G** | **T** | **C** | **T** | **C** | **T** | **T** | **C** | **T** | **T** | **A** | **T** | **T** | **T** | **A** | **T** | **C** | **T** | **A** | **T** | **C** | **C** | **A** | **A** | **G** | **A** | **A** | **G** | **A** | **C** | **A** | **T** | **T** | **G** | **A** | **A** | **G** | **T** | **G** | **A** | **T** | **T** | **G** | **G** | **A** | **G** | **A** | **A** | **G** | **C** | **T** | **G** | **A** | **A** | **A** | **A** | **T** | **G** | **G** | **A** | **C** | **G** | **T** | **C** | **A** | **A** | **G** | **G** | **C** | **T** | **A** | **T** | **G** | **A** | **A** | **A** | **A** | **G** | **G** | **C** | **G** | **A** | **T** | **G** | **G** | **C** | **A** | **C** | **T** | **T** | **C** | **G** | **A** | **C** | **C** | **A** | **G** | **A** | **T** | **G** | **T** | **C** | **A** | **T** | **T** | **T** | **T** | **A** | **A** | **T** | **G** | **G** | **A** | **T** | **C** | **T** | **A** | **G** | **T** | **C** | **A** | **T** | **G** | **G** | **A** | **A** | **G** | **A** | **A** | **A** | **T** | **G** | **G** | **A** | **T** | **G** | **G** | **C** | **A** | **T** | **C** | **G** | **A** | **A** | **T** | **C** | **A** | **A** | **C** | **A** | **A** | **A** | **A** | **G** | **C** | **A** | **A** | **T** | **C** | **T** | **T** | **A** | **A** | **A** | **A** | **G** | **A** | **T** | **T** | **G** | **G** | **C** | **C** | **A** | **A** | **A** | **A** | **G** | **C** | **C** | **A** | **A** | **A** | **A** | **T** | **C** | **A** | **T** | **T** | **A** | **T** | **C** | **G** | **T** | **C** | **A** | **C** | **A** | **A** | **G** | **T** | **T** | **T** | **T** | **A** | **T** | **T** | **G** | **A** | **T** | **G** | **A** | **T** | **G** | **A** | **A** | **A** | **A** | **A** | **G** | **T** | **T** | **T** | **A** | **T** | **C** | **C** | **T** | **G** | **C** | **A** | **A** | **T** | **T** | **G** | **A** | **A** | **G** | **C** | **A** | **G** | **G** | **A** | **G** | **C** | **A** | **G** | **C** | **A** | **G** | **G** | **A** | **T** | **A** | **T** | **T** | **T** | **G** | **T** | **T** | **G** | **A** | **A** | **G** | **A** | **C** | **G** | **T** | **C** | **T** | **A** | **C** | **C** | **G** | **C** | **A** | **C** | **A** | **T** | **G** | **A** | **A** | **A** | **T** | **A** | **G** | **C** | **T** | **G** | **A** | **T** | **G** | **C** | **T** | **A** | **T** | **A** | **A** | **G** | **G** | **G** | **C** | **T** | **A** | **C** | **G** | **C** | **A** | **A** | **C** | **G** | **C** | **G** | **G** | **C** | **G** | **A** | **G** | **C** | **G** | **A** | **G** | **T** | **A** | **T** | **T** | **G** | **G** | **A** | **A** | **C** | **C** | **T** | **G** | **A** | **A** | **G** | **T** | **G** | **A** | **C** | **A** | **A** | **C** | **A** | **A** | **A** | **A** | **A** | **T** | **G** | **A** | **T** | **G** | **G** | **A** | **A** | **A** | **A** | **A** | **A** | **T** | **G** | **A** | **G** | **T** | **C** | **G** | **G** | **C** | **G** | **A** | **A** | **A** | **T** | **G** | **A** | **T** | **C** | **C** | **A** | **G** | **T** | **A** | **T** | **T** | **G** | **C** | **A** | **T** | **G** | **A** | **A** | **G** | **A** | **A** | **T** | **T** | **A** | **A** | **C** | **G** | **A** | **A** | **T** | **C** | **G** | **A** | **G** | **A** | **A** | **A** | **A** | **T** | **G** | **A** | **A** | **A** | **T** | **T** | **T** | **T** | **G** | **A** | **T** | **G** | **C** | **T** | **G** | **A** | **T** | **T** | **T** | **C** | **T** | **G** | **A** | **A** | **G** | **G** | **G** | **A** | **A** | **A** | **A** | **G** | **T** | **A** | **A** | **T** | **C** | **A** | **G** | **G** | **A** | **A** | **A** | **T** | **C** | **G** | **C** | **G** | **G** | **A** | **T** | **G** | **A** | **A** | **T** | **T** | **G** | **T** | **T** | **T** | **A** | **T** | **C** | **A** | **C** | **A** | **C** | **T** | **G** | **A** | **A** | **A** | **A** | **C** | **A** | **G** | **T** | **T** | **A** | **A** | **A** | **A** | **C** | **G** | **C** | **A** | **T** | **G** | **T** | **G** | **T** | **C** | **G** | **A** | **A** | **T** | **A** | **T** | **A** | **T** | **T** | **A** | **G** | **C** | **T** | **A** | **A** | **G** | **T** | **T** | **A** | **G** | **A** | **G** | **G** | **T** | **A** | **G** | **A** | **A** | **G** | **A** | **C** | **C** | **G** | **A** | **A** | **C** | **T** | **C** | **A** | **A** | **G** | **C** | **A** | **G** | **C** | **T** | **A** | **T** | **C** | **T** | **A** | **T** | **G** | **C** | **G** | **T** | **T** | **T** | **A** | **A** | **A** | **C** | **A** | **T** | **G** | **G** | **T** | **T** | **T** | **G** | **G** | **T** | **G** | **A** | **A** | **A** | **-** | **-** | **-** |
| **N60392F** | **G** | **T** | **G** | **A** | **T** | **C** | **A** | **A** | **A** | **G** | **T** | **A** | **A** | **T** | **G** | **T** | **T** | **A** | **G** | **T** | **G** | **G** | **A** | **T** | **G** | **A** | **C** | **C** | **A** | **T** | **G** | **A** | **A** | **A** | **T** | **G** | **G** | **T** | **C** | **C** | **G** | **T** | **T** | **T** | **A** | **G** | **G** | **C** | **G** | **T** | **T** | **T** | **C** | **A** | **T** | **C** | **A** | **T** | **A** | **T** | **T** | **T** | **A** | **T** | **C** | **T** | **A** | **T** | **T** | **C** | **A** | **A** | **G** | **A** | **G** | **G** | **A** | **T** | **A** | **T** | **A** | **G** | **A** | **A** | **G** | **T** | **C** | **G** | **T** | **A** | **G** | **G** | **C** | **G** | **A** | **A** | **G** | **C** | **A** | **G** | **A** | **A** | **A** | **A** | **C** | **G** | **G** | **T** | **A** | **A** | **G** | **A** | **T** | **T** | **G** | **G** | **C** | **T** | **A** | **T** | **G** | **A** | **A** | **A** | **A** | **A** | **G** | **C** | **A** | **T** | **T** | **G** | **G** | **A** | **A** | **C** | **T** | **A** | **C** | **G** | **T** | **C** | **C** | **A** | **G** | **A** | **T** | **G** | **T** | **T** | **A** | **T** | **T** | **T** | **T** | **G** | **A** | **T** | **G** | **G** | **A** | **T** | **T** | **T** | **G** | **G** | **T** | **A** | **A** | **T** | **G** | **G** | **A** | **A** | **G** | **A** | **A** | **A** | **T** | **G** | **G** | **A** | **C** | **G** | **G** | **C** | **A** | **T** | **T** | **G** | **A** | **T** | **T** | **C** | **A** | **A** | **C** | **A** | **A** | **A** | **A** | **G** | **C** | **G** | **A** | **T** | **C** | **T** | **T** | **G** | **A** | **A** | **A** | **G** | **A** | **T** | **T** | **G** | **G** | **C** | **C** | **A** | **G** | **A** | **A** | **G** | **C** | **C** | **A** | **A** | **G** | **A** | **T** | **T** | **A** | **T** | **T** | **A** | **T** | **T** | **G** | **T** | **G** | **A** | **C** | **G** | **A** | **G** | **T** | **T** | **T** | **T** | **A** | **T** | **T** | **G** | **A** | **T** | **G** | **A** | **T** | **G** | **A** | **A** | **A** | **A** | **A** | **G** | **T** | **G** | **T** | **A** | **T** | **C** | **C** | **G** | **G** | **C** | **G** | **A** | **T** | **T** | **G** | **A** | **A** | **G** | **C** | **T** | **G** | **G** | **T** | **G** | **C** | **A** | **G** | **C** | **G** | **G** | **G** | **C** | **T** | **A** | **C** | **C** | **T** | **A** | **T** | **T** | **A** | **A** | **A** | **A** | **A** | **C** | **A** | **T** | **C** | **A** | **A** | **C** | **A** | **G** | **C** | **A** | **C** | **A** | **T** | **G** | **A** | **G** | **A** | **T** | **T** | **G** | **C** | **T** | **G** | **A** | **T** | **G** | **C** | **A** | **A** | **T** | **T** | **C** | **G** | **G** | **G** | **C** | **G** | **A** | **C** | **T** | **T** | **A** | **T** | **C** | **G** | **C** | **G** | **G** | **A** | **G** | **A** | **G** | **C** | **G** | **T** | **G** | **T** | **G** | **T** | **T** | **G** | **G** | **A** | **A** | **C** | **C** | **T** | **G** | **A** | **A** | **G** | **T** | **G** | **A** | **C** | **G** | **C** | **A** | **T** | **A** | **A** | **G** | **A** | **T** | **G** | **A** | **T** | **G** | **G** | **A** | **A** | **C** | **G** | **G** | **T** | **T** | **A** | **A** | **C** | **A** | **A** | **A** | **A** | **A** | **A** | **A** | **C** | **A** | **A** | **G** | **A** | **G** | **C** | **C** | **G** | **G** | **T** | **G** | **T** | **T** | **G** | **C** | **A** | **C** | **G** | **A** | **A** | **G** | **A** | **T** | **T** | **T** | **G** | **A** | **C** | **A** | **A** | **A** | **C** | **C** | **G** | **A** | **G** | **A** | **A** | **C** | **A** | **C** | **G** | **A** | **A** | **A** | **T** | **T** | **T** | **T** | **A** | **A** | **T** | **G** | **T** | **T** | **G** | **A** | **T** | **T** | **G** | **C** | **A** | **C** | **A** | **A** | **G** | **G** | **T** | **A** | **A** | **A** | **A** | **G** | **T** | **A** | **A** | **T** | **C** | **A** | **G** | **G** | **A** | **A** | **A** | **T** | **T** | **G** | **C** | **T** | **G** | **A** | **T** | **G** | **A** | **A** | **C** | **T** | **C** | **T** | **T** | **T** | **A** | **T** | **C** | **A** | **C** | **T** | **T** | **T** | **G** | **A** | **A** | **A** | **A** | **C** | **A** | **G** | **T** | **T** | **A** | **A** | **A** | **A** | **C** | **A** | **C** | **A** | **T** | **G** | **T** | **T** | **T** | **C** | **A** | **A** | **A** | **C** | **A** | **T** | **T** | **T** | **T** | **A** | **G** | **C** | **A** | **A** | **A** | **A** | **C** | **T** | **A** | **G** | **A** | **T** | **G** | **T** | **G** | **G** | **A** | **T** | **G** | **A** | **T** | **C** | **G** | **G** | **A** | **C** | **C** | **C** | **A** | **A** | **G** | **C** | **G** | **G** | **C** | **G** | **A** | **T** | **T** | **T** | **A** | **T** | **G** | **C** | **T** | **T** | **T** | **T** | **C** | **A** | **A** | **C** | **A** | **T** | **G** | **G** | **T** | **T** | **T** | **A** | **G** | **C** | **C** | **A** | **A** | **A** | **-** | **-** | **-** |
| **N59454F** | **G** | **T** | **G** | **A** | **T** | **C** | **A** | **A** | **A** | **G** | **T** | **A** | **A** | **T** | **G** | **T** | **T** | **A** | **G** | **T** | **G** | **G** | **A** | **T** | **G** | **A** | **C** | **C** | **A** | **T** | **G** | **A** | **A** | **A** | **T** | **G** | **G** | **T** | **C** | **C** | **G** | **T** | **T** | **T** | **A** | **G** | **G** | **C** | **G** | **T** | **T** | **T** | **C** | **A** | **T** | **C** | **A** | **T** | **A** | **T** | **T** | **T** | **A** | **T** | **C** | **T** | **A** | **T** | **T** | **C** | **A** | **A** | **G** | **A** | **G** | **G** | **A** | **T** | **A** | **T** | **A** | **G** | **A** | **A** | **G** | **T** | **C** | **G** | **T** | **A** | **G** | **G** | **C** | **G** | **A** | **A** | **G** | **C** | **A** | **G** | **A** | **A** | **A** | **A** | **C** | **G** | **G** | **T** | **A** | **A** | **G** | **A** | **T** | **T** | **G** | **G** | **C** | **T** | **A** | **T** | **G** | **A** | **A** | **A** | **A** | **A** | **G** | **C** | **A** | **T** | **T** | **G** | **G** | **A** | **A** | **C** | **T** | **A** | **C** | **G** | **T** | **C** | **C** | **A** | **G** | **A** | **T** | **G** | **T** | **T** | **A** | **T** | **T** | **T** | **T** | **G** | **A** | **T** | **G** | **G** | **A** | **T** | **T** | **T** | **G** | **G** | **T** | **A** | **A** | **T** | **G** | **G** | **A** | **A** | **G** | **A** | **A** | **A** | **T** | **G** | **G** | **A** | **C** | **G** | **G** | **C** | **A** | **T** | **T** | **G** | **A** | **T** | **T** | **C** | **A** | **A** | **C** | **C** | **A** | **A** | **A** | **G** | **C** | **G** | **A** | **T** | **C** | **T** | **T** | **G** | **A** | **A** | **A** | **G** | **A** | **T** | **T** | **G** | **G** | **C** | **C** | **A** | **G** | **A** | **A** | **G** | **C** | **C** | **A** | **A** | **G** | **A** | **T** | **T** | **A** | **T** | **T** | **A** | **T** | **T** | **G** | **T** | **G** | **A** | **C** | **G** | **A** | **G** | **T** | **T** | **T** | **T** | **A** | **T** | **T** | **G** | **A** | **T** | **G** | **A** | **T** | **G** | **A** | **A** | **A** | **A** | **A** | **G** | **T** | **G** | **T** | **A** | **T** | **C** | **C** | **G** | **G** | **C** | **G** | **A** | **T** | **T** | **G** | **A** | **A** | **G** | **C** | **T** | **G** | **G** | **T** | **G** | **C** | **A** | **G** | **C** | **G** | **G** | **G** | **C** | **T** | **A** | **C** | **C** | **T** | **A** | **T** | **T** | **A** | **A** | **A** | **A** | **A** | **C** | **A** | **T** | **C** | **A** | **A** | **C** | **A** | **G** | **C** | **A** | **C** | **A** | **T** | **G** | **A** | **G** | **A** | **T** | **T** | **G** | **C** | **T** | **G** | **A** | **T** | **G** | **C** | **A** | **A** | **T** | **T** | **C** | **G** | **G** | **G** | **C** | **G** | **A** | **C** | **T** | **T** | **A** | **T** | **C** | **G** | **C** | **G** | **G** | **A** | **G** | **A** | **G** | **C** | **G** | **T** | **G** | **T** | **G** | **T** | **T** | **G** | **G** | **A** | **A** | **C** | **C** | **T** | **G** | **A** | **A** | **G** | **T** | **G** | **A** | **C** | **G** | **C** | **A** | **T** | **A** | **A** | **G** | **A** | **T** | **G** | **A** | **T** | **G** | **G** | **A** | **A** | **C** | **G** | **G** | **T** | **T** | **A** | **A** | **C** | **A** | **A** | **A** | **A** | **A** | **A** | **A** | **C** | **A** | **A** | **G** | **A** | **G** | **C** | **C** | **G** | **G** | **T** | **G** | **T** | **T** | **G** | **C** | **A** | **C** | **G** | **A** | **A** | **G** | **A** | **T** | **T** | **T** | **G** | **A** | **C** | **A** | **A** | **A** | **C** | **C** | **G** | **G** | **G** | **A** | **A** | **C** | **A** | **C** | **G** | **A** | **A** | **A** | **T** | **T** | **T** | **T** | **A** | **A** | **T** | **G** | **T** | **T** | **G** | **A** | **T** | **T** | **G** | **C** | **A** | **C** | **A** | **A** | **G** | **G** | **T** | **A** | **A** | **A** | **A** | **G** | **T** | **A** | **A** | **T** | **C** | **A** | **G** | **G** | **A** | **A** | **A** | **T** | **A** | **G** | **C** | **T** | **G** | **A** | **T** | **G** | **A** | **A** | **C** | **T** | **C** | **T** | **T** | **T** | **A** | **T** | **C** | **A** | **C** | **T** | **T** | **T** | **G** | **A** | **A** | **A** | **A** | **C** | **A** | **G** | **T** | **T** | **A** | **A** | **A** | **A** | **C** | **A** | **C** | **A** | **T** | **G** | **T** | **T** | **T** | **C** | **A** | **A** | **A** | **C** | **A** | **T** | **T** | **T** | **T** | **A** | **G** | **C** | **A** | **A** | **A** | **A** | **C** | **T** | **A** | **G** | **A** | **T** | **G** | **T** | **G** | **G** | **A** | **T** | **G** | **A** | **T** | **C** | **G** | **G** | **A** | **C** | **C** | **C** | **A** | **A** | **G** | **C** | **G** | **G** | **C** | **G** | **A** | **T** | **T** | **T** | **A** | **T** | **G** | **C** | **T** | **T** | **T** | **T** | **C** | **A** | **A** | **C** | **A** | **T** | **G** | **G** | **T** | **T** | **T** | **A** | **G** | **C** | **C** | **A** | **A** | **A** | **-** | **-** | **-** |
| **N60324F** | **A** | **T** | **G** | **A** | **T** | **A** | **A** | **A** | **A** | **G** | **T** | **T** | **T** | **T** | **A** | **T** | **T** | **A** | **G** | **T** | **A** | **G** | **A** | **T** | **G** | **A** | **C** | **C** | **A** | **T** | **G** | **A** | **A** | **A** | **T** | **G** | **G** | **T** | **A** | **C** | **G** | **C** | **T** | **T** | **A** | **G** | **G** | **C** | **G** | **T** | **C** | **T** | **C** | **T** | **T** | **C** | **T** | **T** | **A** | **T** | **T** | **T** | **A** | **T** | **C** | **T** | **A** | **T** | **C** | **C** | **A** | **A** | **G** | **A** | **A** | **G** | **A** | **C** | **A** | **T** | **T** | **G** | **A** | **A** | **G** | **T** | **G** | **A** | **T** | **T** | **G** | **G** | **A** | **G** | **A** | **A** | **G** | **C** | **T** | **G** | **A** | **A** | **A** | **A** | **T** | **G** | **G** | **A** | **C** | **G** | **T** | **C** | **A** | **A** | **G** | **G** | **C** | **T** | **A** | **T** | **G** | **A** | **A** | **A** | **A** | **G** | **G** | **C** | **G** | **A** | **T** | **G** | **G** | **C** | **A** | **C** | **T** | **T** | **C** | **G** | **A** | **C** | **C** | **A** | **G** | **A** | **T** | **G** | **T** | **C** | **A** | **T** | **T** | **T** | **T** | **A** | **A** | **T** | **G** | **G** | **A** | **T** | **C** | **T** | **A** | **G** | **T** | **C** | **A** | **T** | **G** | **G** | **A** | **A** | **G** | **A** | **A** | **A** | **T** | **G** | **G** | **A** | **T** | **G** | **G** | **C** | **A** | **T** | **C** | **G** | **A** | **A** | **T** | **C** | **A** | **A** | **C** | **A** | **A** | **A** | **A** | **G** | **C** | **A** | **A** | **T** | **C** | **T** | **T** | **A** | **A** | **A** | **A** | **G** | **A** | **T** | **T** | **G** | **G** | **C** | **C** | **A** | **A** | **A** | **A** | **G** | **C** | **C** | **A** | **A** | **A** | **A** | **T** | **C** | **A** | **T** | **T** | **A** | **T** | **C** | **G** | **T** | **C** | **A** | **C** | **A** | **A** | **G** | **T** | **T** | **T** | **T** | **A** | **T** | **T** | **G** | **A** | **T** | **G** | **A** | **T** | **G** | **A** | **A** | **A** | **A** | **A** | **G** | **T** | **T** | **T** | **A** | **T** | **C** | **C** | **T** | **G** | **C** | **A** | **A** | **T** | **T** | **G** | **A** | **A** | **G** | **C** | **A** | **G** | **G** | **A** | **G** | **C** | **A** | **G** | **C** | **A** | **G** | **G** | **A** | **T** | **A** | **T** | **T** | **T** | **A** | **T** | **T** | **G** | **A** | **A** | **G** | **A** | **C** | **G** | **T** | **C** | **T** | **A** | **C** | **C** | **G** | **C** | **A** | **C** | **A** | **T** | **G** | **A** | **A** | **A** | **T** | **A** | **G** | **C** | **T** | **G** | **A** | **T** | **G** | **C** | **T** | **A** | **T** | **A** | **A** | **G** | **G** | **G** | **C** | **T** | **A** | **C** | **G** | **C** | **A** | **A** | **C** | **G** | **C** | **G** | **G** | **C** | **G** | **A** | **G** | **C** | **G** | **A** | **G** | **T** | **A** | **T** | **T** | **G** | **G** | **A** | **A** | **C** | **C** | **T** | **G** | **A** | **A** | **G** | **T** | **G** | **A** | **C** | **A** | **A** | **C** | **A** | **A** | **A** | **A** | **A** | **T** | **G** | **A** | **T** | **G** | **G** | **A** | **A** | **A** | **A** | **A** | **A** | **T** | **G** | **A** | **G** | **T** | **C** | **G** | **G** | **C** | **G** | **A** | **A** | **A** | **T** | **G** | **A** | **T** | **C** | **C** | **A** | **G** | **T** | **A** | **T** | **T** | **G** | **C** | **A** | **T** | **G** | **A** | **A** | **G** | **A** | **A** | **T** | **T** | **A** | **A** | **C** | **G** | **A** | **A** | **T** | **C** | **G** | **A** | **G** | **A** | **A** | **A** | **A** | **T** | **G** | **A** | **A** | **A** | **T** | **T** | **T** | **T** | **G** | **A** | **T** | **G** | **C** | **T** | **G** | **A** | **T** | **T** | **T** | **C** | **T** | **G** | **A** | **A** | **G** | **G** | **G** | **A** | **A** | **A** | **A** | **G** | **T** | **A** | **A** | **T** | **C** | **A** | **G** | **G** | **A** | **A** | **A** | **T** | **C** | **G** | **C** | **G** | **G** | **A** | **T** | **G** | **A** | **A** | **T** | **T** | **G** | **T** | **T** | **T** | **A** | **T** | **C** | **A** | **C** | **A** | **C** | **T** | **G** | **A** | **A** | **A** | **A** | **C** | **A** | **G** | **T** | **T** | **A** | **A** | **A** | **A** | **C** | **G** | **C** | **A** | **T** | **G** | **T** | **G** | **T** | **C** | **G** | **A** | **A** | **T** | **A** | **T** | **A** | **T** | **T** | **A** | **G** | **C** | **T** | **A** | **A** | **G** | **T** | **T** | **A** | **G** | **A** | **G** | **G** | **T** | **A** | **G** | **A** | **A** | **G** | **A** | **C** | **C** | **G** | **A** | **A** | **C** | **T** | **C** | **A** | **A** | **G** | **C** | **A** | **G** | **C** | **T** | **A** | **T** | **C** | **T** | **A** | **T** | **G** | **C** | **G** | **T** | **T** | **T** | **A** | **A** | **A** | **C** | **A** | **T** | **G** | **G** | **T** | **T** | **T** | **G** | **G** | **T** | **G** | **A** | **A** | **A** | **-** | **-** | **-** |
| **N59455F** | **G** | **T** | **G** | **A** | **T** | **C** | **A** | **A** | **A** | **G** | **T** | **A** | **A** | **T** | **G** | **T** | **T** | **A** | **G** | **T** | **G** | **G** | **A** | **T** | **G** | **A** | **C** | **C** | **A** | **T** | **G** | **A** | **A** | **A** | **T** | **G** | **G** | **T** | **C** | **C** | **G** | **T** | **T** | **T** | **A** | **G** | **G** | **C** | **G** | **T** | **T** | **T** | **C** | **A** | **T** | **C** | **A** | **T** | **A** | **T** | **T** | **T** | **A** | **T** | **C** | **T** | **A** | **T** | **T** | **C** | **A** | **A** | **G** | **A** | **G** | **G** | **A** | **T** | **A** | **T** | **A** | **G** | **A** | **A** | **G** | **T** | **C** | **G** | **T** | **A** | **G** | **G** | **C** | **G** | **A** | **A** | **G** | **C** | **A** | **G** | **A** | **A** | **A** | **A** | **C** | **G** | **G** | **T** | **A** | **A** | **G** | **A** | **T** | **T** | **G** | **G** | **C** | **T** | **A** | **T** | **G** | **A** | **A** | **A** | **A** | **A** | **G** | **C** | **A** | **T** | **T** | **G** | **G** | **A** | **A** | **C** | **T** | **A** | **C** | **G** | **T** | **C** | **C** | **A** | **G** | **A** | **T** | **G** | **T** | **T** | **A** | **T** | **T** | **T** | **T** | **G** | **A** | **T** | **G** | **G** | **A** | **T** | **T** | **T** | **G** | **G** | **T** | **A** | **A** | **T** | **G** | **G** | **A** | **A** | **G** | **A** | **A** | **A** | **T** | **G** | **G** | **A** | **C** | **G** | **G** | **C** | **A** | **T** | **T** | **G** | **A** | **T** | **T** | **C** | **A** | **A** | **C** | **A** | **A** | **A** | **A** | **G** | **C** | **G** | **A** | **T** | **C** | **T** | **T** | **G** | **A** | **A** | **A** | **G** | **A** | **T** | **T** | **G** | **G** | **C** | **C** | **A** | **G** | **A** | **A** | **G** | **C** | **C** | **A** | **A** | **G** | **A** | **T** | **T** | **A** | **T** | **T** | **A** | **T** | **T** | **G** | **T** | **G** | **A** | **C** | **G** | **A** | **G** | **T** | **T** | **T** | **T** | **A** | **T** | **T** | **G** | **A** | **T** | **G** | **A** | **T** | **G** | **A** | **G** | **A** | **A** | **A** | **G** | **T** | **G** | **T** | **A** | **T** | **C** | **C** | **G** | **G** | **C** | **G** | **A** | **T** | **T** | **G** | **A** | **A** | **G** | **C** | **T** | **G** | **G** | **T** | **G** | **C** | **A** | **G** | **C** | **G** | **G** | **G** | **C** | **T** | **A** | **C** | **C** | **T** | **A** | **T** | **T** | **A** | **A** | **A** | **A** | **A** | **C** | **A** | **T** | **C** | **A** | **A** | **C** | **A** | **G** | **C** | **A** | **C** | **A** | **T** | **G** | **A** | **G** | **A** | **T** | **T** | **G** | **C** | **T** | **G** | **A** | **T** | **G** | **C** | **A** | **A** | **T** | **T** | **C** | **G** | **G** | **G** | **C** | **G** | **A** | **C** | **T** | **T** | **A** | **T** | **C** | **G** | **T** | **G** | **G** | **A** | **G** | **A** | **G** | **C** | **G** | **T** | **G** | **T** | **G** | **T** | **T** | **G** | **G** | **A** | **A** | **C** | **C** | **T** | **G** | **A** | **A** | **G** | **T** | **G** | **A** | **C** | **G** | **C** | **A** | **T** | **A** | **A** | **G** | **A** | **T** | **G** | **A** | **T** | **G** | **G** | **A** | **A** | **C** | **G** | **G** | **T** | **T** | **A** | **A** | **C** | **A** | **A** | **A** | **A** | **A** | **A** | **A** | **C** | **A** | **A** | **G** | **A** | **G** | **C** | **C** | **G** | **G** | **T** | **G** | **T** | **T** | **G** | **C** | **A** | **C** | **G** | **A** | **A** | **G** | **A** | **T** | **T** | **T** | **G** | **A** | **C** | **C** | **A** | **A** | **C** | **C** | **G** | **A** | **G** | **A** | **A** | **C** | **A** | **C** | **G** | **A** | **A** | **A** | **T** | **T** | **T** | **T** | **A** | **A** | **T** | **G** | **T** | **T** | **G** | **A** | **T** | **T** | **G** | **C** | **A** | **C** | **A** | **A** | **G** | **G** | **T** | **A** | **A** | **A** | **A** | **G** | **T** | **A** | **A** | **T** | **C** | **A** | **G** | **G** | **A** | **A** | **A** | **T** | **T** | **G** | **C** | **T** | **G** | **A** | **T** | **G** | **A** | **A** | **C** | **T** | **C** | **T** | **T** | **T** | **A** | **T** | **C** | **A** | **C** | **T** | **T** | **T** | **G** | **A** | **A** | **A** | **A** | **C** | **A** | **G** | **T** | **T** | **A** | **A** | **A** | **A** | **C** | **A** | **C** | **A** | **T** | **G** | **T** | **T** | **T** | **C** | **A** | **A** | **A** | **C** | **A** | **T** | **T** | **T** | **T** | **A** | **G** | **C** | **A** | **A** | **A** | **A** | **C** | **T** | **A** | **G** | **A** | **T** | **G** | **T** | **G** | **G** | **A** | **T** | **G** | **A** | **T** | **C** | **G** | **G** | **A** | **C** | **C** | **C** | **A** | **A** | **G** | **C** | **G** | **G** | **C** | **G** | **A** | **T** | **T** | **T** | **A** | **T** | **G** | **C** | **T** | **T** | **T** | **T** | **C** | **A** | **A** | **C** | **A** | **T** | **G** | **G** | **T** | **T** | **T** | **A** | **G** | **C** | **C** | **A** | **A** | **A** | **-** | **-** | **-** |
| **N55299** | **A** | **T** | **G** | **A** | **T** | **A** | **A** | **A** | **A** | **G** | **T** | **T** | **T** | **T** | **A** | **T** | **T** | **A** | **G** | **T** | **A** | **G** | **A** | **T** | **G** | **A** | **C** | **C** | **A** | **T** | **G** | **A** | **A** | **A** | **T** | **G** | **G** | **T** | **A** | **C** | **G** | **C** | **T** | **T** | **A** | **G** | **G** | **C** | **G** | **T** | **C** | **T** | **C** | **T** | **T** | **C** | **T** | **T** | **A** | **T** | **T** | **T** | **A** | **T** | **C** | **T** | **A** | **T** | **C** | **C** | **A** | **A** | **G** | **A** | **A** | **G** | **A** | **C** | **A** | **T** | **T** | **G** | **A** | **A** | **G** | **T** | **G** | **A** | **T** | **T** | **G** | **G** | **A** | **G** | **A** | **A** | **G** | **C** | **T** | **G** | **A** | **A** | **A** | **A** | **T** | **G** | **G** | **A** | **C** | **G** | **T** | **C** | **A** | **A** | **G** | **G** | **C** | **T** | **A** | **T** | **G** | **A** | **A** | **A** | **A** | **G** | **G** | **C** | **G** | **A** | **T** | **G** | **G** | **C** | **A** | **C** | **T** | **T** | **C** | **G** | **A** | **C** | **C** | **A** | **G** | **A** | **T** | **G** | **T** | **C** | **A** | **T** | **T** | **T** | **T** | **A** | **A** | **T** | **G** | **G** | **A** | **T** | **C** | **T** | **A** | **G** | **T** | **C** | **A** | **T** | **G** | **G** | **A** | **A** | **G** | **A** | **A** | **A** | **T** | **G** | **G** | **A** | **T** | **G** | **G** | **C** | **A** | **T** | **C** | **G** | **A** | **A** | **T** | **C** | **A** | **A** | **C** | **A** | **A** | **A** | **A** | **G** | **C** | **A** | **A** | **T** | **C** | **T** | **T** | **A** | **A** | **A** | **A** | **G** | **A** | **T** | **T** | **G** | **G** | **C** | **C** | **A** | **A** | **A** | **A** | **G** | **C** | **C** | **A** | **A** | **A** | **A** | **T** | **C** | **A** | **T** | **T** | **A** | **T** | **C** | **G** | **T** | **C** | **A** | **C** | **A** | **A** | **G** | **T** | **T** | **T** | **T** | **A** | **T** | **T** | **G** | **A** | **T** | **G** | **A** | **T** | **G** | **A** | **A** | **A** | **A** | **A** | **G** | **T** | **T** | **T** | **A** | **T** | **C** | **C** | **T** | **G** | **C** | **A** | **A** | **T** | **T** | **G** | **A** | **A** | **G** | **C** | **A** | **G** | **G** | **A** | **G** | **C** | **A** | **G** | **C** | **A** | **G** | **G** | **A** | **T** | **A** | **T** | **T** | **T** | **A** | **T** | **T** | **G** | **A** | **A** | **G** | **A** | **C** | **G** | **T** | **C** | **T** | **A** | **C** | **C** | **G** | **C** | **A** | **C** | **A** | **T** | **G** | **A** | **A** | **A** | **T** | **A** | **G** | **C** | **T** | **G** | **A** | **T** | **G** | **C** | **T** | **A** | **T** | **A** | **A** | **G** | **G** | **G** | **C** | **T** | **A** | **C** | **G** | **C** | **A** | **A** | **C** | **G** | **C** | **G** | **G** | **C** | **G** | **A** | **G** | **C** | **G** | **A** | **G** | **T** | **A** | **T** | **T** | **G** | **G** | **A** | **A** | **C** | **C** | **T** | **G** | **A** | **A** | **G** | **T** | **G** | **A** | **C** | **A** | **A** | **C** | **A** | **A** | **A** | **A** | **A** | **T** | **G** | **A** | **T** | **G** | **G** | **A** | **A** | **A** | **A** | **A** | **A** | **T** | **G** | **A** | **G** | **T** | **C** | **G** | **G** | **C** | **G** | **A** | **A** | **A** | **T** | **G** | **A** | **T** | **C** | **C** | **A** | **G** | **T** | **A** | **T** | **T** | **G** | **C** | **A** | **T** | **G** | **A** | **A** | **G** | **A** | **A** | **T** | **T** | **A** | **A** | **C** | **G** | **A** | **A** | **T** | **C** | **G** | **A** | **G** | **A** | **A** | **A** | **A** | **T** | **G** | **A** | **A** | **A** | **T** | **T** | **T** | **T** | **G** | **A** | **T** | **G** | **C** | **T** | **G** | **A** | **T** | **T** | **T** | **C** | **T** | **G** | **A** | **A** | **G** | **G** | **G** | **A** | **A** | **A** | **A** | **G** | **T** | **A** | **A** | **T** | **C** | **A** | **G** | **G** | **A** | **A** | **A** | **T** | **C** | **G** | **C** | **G** | **G** | **A** | **T** | **G** | **A** | **A** | **T** | **T** | **G** | **T** | **T** | **T** | **A** | **T** | **C** | **A** | **C** | **A** | **C** | **T** | **G** | **A** | **A** | **A** | **A** | **C** | **A** | **G** | **T** | **T** | **A** | **A** | **A** | **A** | **C** | **G** | **C** | **A** | **T** | **G** | **T** | **G** | **T** | **C** | **G** | **A** | **A** | **T** | **A** | **T** | **A** | **T** | **T** | **A** | **G** | **C** | **T** | **A** | **A** | **G** | **T** | **T** | **A** | **G** | **A** | **G** | **G** | **T** | **A** | **G** | **A** | **A** | **G** | **A** | **C** | **C** | **G** | **A** | **A** | **C** | **T** | **C** | **A** | **A** | **G** | **C** | **A** | **G** | **C** | **T** | **A** | **T** | **C** | **T** | **A** | **T** | **G** | **C** | **G** | **T** | **T** | **T** | **A** | **A** | **A** | **C** | **A** | **T** | **G** | **G** | **T** | **T** | **T** | **G** | **G** | **T** | **G** | **A** | **A** | **A** | **-** | **-** | **-** |
| **N60363F** | **A** | **T** | **G** | **A** | **T** | **A** | **A** | **A** | **A** | **G** | **T** | **T** | **T** | **T** | **A** | **T** | **T** | **A** | **G** | **T** | **A** | **G** | **A** | **T** | **G** | **A** | **C** | **C** | **A** | **T** | **G** | **A** | **A** | **A** | **T** | **G** | **G** | **T** | **A** | **C** | **G** | **C** | **T** | **T** | **A** | **G** | **G** | **C** | **G** | **T** | **C** | **T** | **C** | **T** | **T** | **C** | **T** | **T** | **A** | **T** | **T** | **T** | **A** | **T** | **C** | **T** | **A** | **T** | **C** | **C** | **A** | **A** | **G** | **A** | **A** | **G** | **A** | **C** | **A** | **T** | **T** | **G** | **A** | **A** | **G** | **T** | **G** | **A** | **T** | **T** | **G** | **G** | **A** | **G** | **A** | **A** | **G** | **C** | **T** | **G** | **A** | **A** | **A** | **A** | **T** | **G** | **G** | **A** | **C** | **G** | **T** | **C** | **A** | **A** | **G** | **G** | **C** | **T** | **A** | **T** | **G** | **A** | **A** | **A** | **A** | **G** | **G** | **C** | **G** | **A** | **T** | **G** | **G** | **C** | **A** | **C** | **T** | **T** | **C** | **G** | **A** | **C** | **C** | **A** | **G** | **A** | **T** | **G** | **T** | **C** | **A** | **T** | **T** | **T** | **T** | **A** | **A** | **T** | **G** | **G** | **A** | **T** | **C** | **T** | **A** | **G** | **T** | **C** | **A** | **T** | **G** | **G** | **A** | **A** | **G** | **A** | **A** | **A** | **T** | **G** | **G** | **A** | **T** | **G** | **G** | **C** | **A** | **T** | **C** | **G** | **A** | **A** | **T** | **C** | **A** | **A** | **C** | **A** | **A** | **A** | **A** | **G** | **C** | **A** | **A** | **T** | **C** | **T** | **T** | **A** | **A** | **A** | **A** | **G** | **A** | **T** | **T** | **G** | **G** | **C** | **C** | **A** | **A** | **A** | **A** | **G** | **C** | **C** | **A** | **A** | **A** | **A** | **T** | **C** | **A** | **T** | **T** | **A** | **T** | **C** | **G** | **T** | **C** | **A** | **C** | **A** | **A** | **G** | **T** | **T** | **T** | **T** | **A** | **T** | **T** | **G** | **A** | **T** | **G** | **A** | **T** | **G** | **A** | **A** | **A** | **A** | **A** | **G** | **T** | **T** | **T** | **A** | **T** | **C** | **C** | **T** | **G** | **C** | **A** | **A** | **T** | **T** | **G** | **A** | **A** | **G** | **C** | **A** | **G** | **G** | **A** | **G** | **C** | **A** | **G** | **C** | **A** | **G** | **G** | **A** | **T** | **A** | **T** | **T** | **T** | **G** | **T** | **T** | **G** | **A** | **A** | **G** | **A** | **C** | **G** | **T** | **C** | **T** | **A** | **C** | **C** | **G** | **C** | **A** | **C** | **A** | **T** | **G** | **A** | **A** | **A** | **T** | **A** | **G** | **C** | **T** | **G** | **A** | **T** | **G** | **C** | **T** | **A** | **T** | **A** | **A** | **G** | **G** | **G** | **C** | **T** | **A** | **C** | **G** | **C** | **A** | **A** | **C** | **G** | **C** | **G** | **G** | **C** | **G** | **A** | **G** | **C** | **G** | **A** | **G** | **T** | **A** | **T** | **T** | **G** | **G** | **A** | **A** | **C** | **C** | **T** | **G** | **A** | **A** | **G** | **T** | **G** | **A** | **C** | **A** | **A** | **C** | **A** | **A** | **A** | **A** | **A** | **T** | **G** | **A** | **T** | **G** | **G** | **A** | **A** | **A** | **A** | **A** | **A** | **T** | **G** | **A** | **G** | **T** | **C** | **G** | **G** | **C** | **G** | **A** | **T** | **A** | **T** | **G** | **A** | **T** | **C** | **C** | **A** | **G** | **T** | **A** | **T** | **T** | **G** | **C** | **A** | **T** | **G** | **A** | **A** | **G** | **A** | **A** | **T** | **T** | **A** | **A** | **C** | **G** | **A** | **A** | **T** | **C** | **G** | **A** | **G** | **A** | **A** | **A** | **A** | **T** | **G** | **A** | **A** | **A** | **T** | **T** | **T** | **T** | **G** | **A** | **T** | **G** | **C** | **T** | **G** | **A** | **T** | **T** | **T** | **C** | **T** | **G** | **A** | **A** | **G** | **G** | **G** | **A** | **A** | **A** | **A** | **G** | **T** | **A** | **A** | **T** | **C** | **A** | **G** | **G** | **A** | **A** | **A** | **T** | **C** | **G** | **C** | **G** | **G** | **A** | **T** | **G** | **A** | **A** | **T** | **T** | **G** | **T** | **T** | **T** | **A** | **T** | **C** | **A** | **C** | **A** | **C** | **T** | **G** | **A** | **A** | **A** | **A** | **C** | **A** | **G** | **T** | **T** | **A** | **A** | **A** | **A** | **C** | **G** | **C** | **A** | **T** | **G** | **T** | **G** | **T** | **C** | **G** | **A** | **A** | **T** | **A** | **T** | **A** | **T** | **T** | **A** | **G** | **C** | **T** | **A** | **A** | **G** | **T** | **T** | **A** | **G** | **A** | **G** | **G** | **T** | **A** | **G** | **A** | **A** | **G** | **A** | **C** | **C** | **G** | **A** | **A** | **C** | **T** | **C** | **A** | **A** | **G** | **C** | **A** | **G** | **C** | **T** | **A** | **T** | **C** | **T** | **A** | **T** | **G** | **C** | **A** | **T** | **T** | **T** | **A** | **A** | **A** | **C** | **A** | **T** | **G** | **G** | **T** | **T** | **T** | **G** | **G** | **T** | **G** | **A** | **A** | **A** | **-** | **-** | **-** |
| **N60449F** | **A** | **T** | **G** | **A** | **T** | **A** | **A** | **A** | **A** | **G** | **T** | **T** | **T** | **T** | **A** | **T** | **T** | **A** | **G** | **T** | **A** | **G** | **A** | **T** | **G** | **A** | **C** | **C** | **A** | **T** | **G** | **A** | **A** | **A** | **T** | **G** | **G** | **T** | **A** | **C** | **G** | **C** | **T** | **T** | **A** | **G** | **G** | **C** | **G** | **T** | **C** | **T** | **C** | **T** | **T** | **C** | **T** | **T** | **A** | **T** | **T** | **T** | **A** | **T** | **C** | **T** | **A** | **T** | **C** | **C** | **A** | **A** | **G** | **A** | **A** | **G** | **A** | **C** | **A** | **T** | **T** | **G** | **A** | **A** | **G** | **T** | **G** | **A** | **T** | **T** | **G** | **G** | **A** | **G** | **A** | **A** | **G** | **C** | **T** | **G** | **A** | **A** | **A** | **A** | **T** | **G** | **G** | **A** | **C** | **G** | **T** | **C** | **A** | **A** | **G** | **G** | **C** | **T** | **A** | **T** | **G** | **A** | **A** | **A** | **A** | **G** | **G** | **C** | **G** | **A** | **T** | **G** | **G** | **C** | **A** | **C** | **T** | **T** | **C** | **G** | **A** | **C** | **C** | **A** | **G** | **A** | **T** | **G** | **T** | **C** | **A** | **T** | **T** | **T** | **T** | **A** | **A** | **T** | **G** | **G** | **A** | **T** | **C** | **T** | **A** | **G** | **T** | **C** | **A** | **T** | **G** | **G** | **A** | **A** | **G** | **A** | **A** | **A** | **T** | **G** | **G** | **A** | **T** | **G** | **G** | **C** | **A** | **T** | **C** | **G** | **A** | **A** | **T** | **C** | **A** | **A** | **C** | **A** | **A** | **A** | **A** | **G** | **C** | **A** | **A** | **T** | **C** | **T** | **T** | **A** | **A** | **A** | **A** | **G** | **A** | **T** | **T** | **G** | **G** | **C** | **C** | **A** | **A** | **A** | **A** | **G** | **C** | **C** | **A** | **A** | **A** | **A** | **T** | **C** | **A** | **T** | **T** | **A** | **T** | **C** | **G** | **T** | **C** | **A** | **C** | **A** | **A** | **G** | **T** | **T** | **T** | **T** | **A** | **T** | **T** | **G** | **A** | **T** | **G** | **A** | **T** | **G** | **A** | **A** | **A** | **A** | **A** | **G** | **T** | **T** | **T** | **A** | **T** | **C** | **C** | **T** | **G** | **C** | **A** | **A** | **T** | **T** | **G** | **A** | **A** | **G** | **C** | **A** | **G** | **G** | **A** | **G** | **C** | **A** | **G** | **C** | **A** | **G** | **G** | **A** | **T** | **A** | **T** | **T** | **T** | **A** | **T** | **T** | **G** | **A** | **A** | **G** | **A** | **C** | **G** | **T** | **C** | **T** | **A** | **C** | **C** | **G** | **C** | **A** | **C** | **A** | **T** | **G** | **A** | **A** | **A** | **T** | **A** | **G** | **C** | **T** | **G** | **A** | **T** | **G** | **C** | **T** | **A** | **T** | **A** | **A** | **G** | **G** | **G** | **C** | **T** | **A** | **C** | **G** | **C** | **A** | **A** | **C** | **G** | **C** | **G** | **G** | **C** | **G** | **A** | **G** | **C** | **G** | **A** | **G** | **T** | **A** | **T** | **T** | **G** | **G** | **A** | **A** | **C** | **C** | **T** | **G** | **A** | **A** | **G** | **T** | **G** | **A** | **C** | **A** | **A** | **C** | **A** | **A** | **A** | **A** | **A** | **T** | **G** | **A** | **T** | **G** | **G** | **A** | **A** | **A** | **A** | **A** | **A** | **T** | **G** | **A** | **G** | **T** | **C** | **G** | **G** | **C** | **G** | **A** | **A** | **A** | **T** | **G** | **A** | **T** | **C** | **C** | **A** | **G** | **T** | **A** | **T** | **T** | **G** | **C** | **A** | **T** | **G** | **A** | **A** | **G** | **A** | **A** | **T** | **T** | **A** | **A** | **C** | **G** | **A** | **A** | **T** | **C** | **G** | **A** | **G** | **A** | **A** | **A** | **A** | **T** | **G** | **A** | **A** | **A** | **T** | **T** | **T** | **T** | **G** | **A** | **T** | **G** | **C** | **T** | **G** | **A** | **T** | **T** | **T** | **C** | **T** | **G** | **A** | **A** | **G** | **G** | **G** | **A** | **A** | **A** | **A** | **G** | **T** | **A** | **A** | **T** | **C** | **A** | **G** | **G** | **A** | **A** | **A** | **T** | **C** | **G** | **C** | **G** | **G** | **A** | **T** | **G** | **A** | **A** | **T** | **T** | **G** | **T** | **T** | **T** | **A** | **T** | **C** | **A** | **C** | **A** | **C** | **T** | **G** | **A** | **A** | **A** | **A** | **C** | **A** | **G** | **T** | **T** | **A** | **A** | **A** | **A** | **C** | **G** | **C** | **A** | **T** | **G** | **T** | **G** | **T** | **C** | **G** | **A** | **A** | **T** | **A** | **T** | **A** | **T** | **T** | **A** | **G** | **C** | **T** | **A** | **A** | **G** | **T** | **T** | **A** | **G** | **A** | **G** | **G** | **T** | **A** | **G** | **A** | **A** | **G** | **A** | **C** | **C** | **G** | **A** | **A** | **C** | **T** | **C** | **A** | **A** | **G** | **C** | **A** | **G** | **C** | **T** | **A** | **T** | **C** | **T** | **A** | **T** | **G** | **C** | **G** | **T** | **T** | **T** | **A** | **A** | **A** | **C** | **A** | **T** | **G** | **G** | **T** | **T** | **T** | **G** | **G** | **T** | **G** | **A** | **A** | **A** | **-** | **-** | **-** |
| **N59435F** | **A** | **T** | **G** | **A** | **T** | **A** | **A** | **A** | **A** | **G** | **T** | **T** | **T** | **T** | **A** | **T** | **T** | **A** | **G** | **T** | **A** | **G** | **A** | **T** | **G** | **A** | **C** | **C** | **A** | **T** | **G** | **A** | **A** | **A** | **T** | **G** | **G** | **T** | **A** | **C** | **G** | **C** | **T** | **T** | **A** | **G** | **G** | **C** | **G** | **T** | **C** | **T** | **C** | **T** | **T** | **C** | **T** | **T** | **A** | **T** | **T** | **T** | **A** | **T** | **C** | **T** | **A** | **T** | **C** | **C** | **A** | **A** | **G** | **A** | **A** | **G** | **A** | **C** | **A** | **T** | **T** | **G** | **A** | **A** | **G** | **T** | **G** | **A** | **T** | **T** | **G** | **G** | **A** | **G** | **A** | **A** | **G** | **C** | **T** | **G** | **A** | **A** | **A** | **A** | **T** | **G** | **G** | **A** | **C** | **G** | **T** | **C** | **A** | **A** | **G** | **G** | **C** | **T** | **A** | **T** | **G** | **A** | **A** | **A** | **A** | **G** | **G** | **C** | **G** | **A** | **T** | **G** | **G** | **C** | **A** | **C** | **T** | **T** | **C** | **G** | **A** | **C** | **C** | **A** | **G** | **A** | **T** | **G** | **T** | **C** | **A** | **T** | **T** | **T** | **T** | **A** | **A** | **T** | **G** | **G** | **A** | **T** | **C** | **T** | **A** | **G** | **T** | **C** | **A** | **T** | **G** | **G** | **A** | **A** | **G** | **A** | **A** | **A** | **T** | **G** | **G** | **A** | **T** | **G** | **G** | **C** | **A** | **T** | **C** | **G** | **A** | **A** | **T** | **C** | **A** | **A** | **C** | **A** | **A** | **A** | **A** | **G** | **C** | **A** | **A** | **T** | **C** | **T** | **T** | **A** | **A** | **A** | **A** | **G** | **A** | **T** | **T** | **G** | **G** | **C** | **C** | **A** | **A** | **A** | **A** | **G** | **C** | **C** | **A** | **A** | **A** | **A** | **T** | **C** | **A** | **T** | **T** | **A** | **T** | **C** | **G** | **T** | **C** | **A** | **C** | **A** | **A** | **G** | **T** | **T** | **T** | **T** | **A** | **T** | **T** | **G** | **A** | **T** | **G** | **A** | **T** | **G** | **A** | **A** | **A** | **A** | **A** | **G** | **T** | **T** | **T** | **A** | **T** | **C** | **C** | **T** | **G** | **C** | **A** | **A** | **T** | **T** | **G** | **A** | **A** | **G** | **C** | **A** | **G** | **G** | **A** | **G** | **C** | **A** | **G** | **C** | **A** | **G** | **G** | **A** | **T** | **A** | **T** | **T** | **T** | **G** | **T** | **T** | **G** | **A** | **A** | **G** | **A** | **C** | **G** | **T** | **C** | **T** | **A** | **C** | **C** | **G** | **C** | **A** | **C** | **A** | **T** | **G** | **A** | **A** | **A** | **T** | **A** | **G** | **C** | **T** | **G** | **A** | **T** | **G** | **C** | **T** | **A** | **T** | **A** | **A** | **G** | **G** | **G** | **C** | **T** | **A** | **C** | **G** | **C** | **A** | **A** | **C** | **G** | **C** | **G** | **G** | **C** | **G** | **A** | **G** | **C** | **G** | **A** | **G** | **T** | **A** | **T** | **T** | **G** | **G** | **A** | **A** | **C** | **C** | **T** | **G** | **A** | **A** | **G** | **T** | **G** | **A** | **C** | **A** | **A** | **C** | **A** | **A** | **A** | **A** | **A** | **T** | **G** | **A** | **T** | **G** | **G** | **A** | **A** | **A** | **A** | **A** | **A** | **T** | **G** | **A** | **G** | **T** | **C** | **G** | **G** | **C** | **G** | **A** | **T** | **A** | **T** | **G** | **A** | **T** | **C** | **C** | **A** | **G** | **T** | **A** | **T** | **T** | **G** | **C** | **A** | **T** | **G** | **A** | **A** | **G** | **A** | **A** | **T** | **T** | **A** | **A** | **C** | **G** | **A** | **A** | **T** | **C** | **G** | **A** | **G** | **A** | **A** | **A** | **A** | **T** | **G** | **A** | **A** | **A** | **T** | **T** | **T** | **T** | **G** | **A** | **T** | **G** | **C** | **T** | **G** | **A** | **T** | **T** | **T** | **C** | **T** | **G** | **A** | **A** | **G** | **G** | **G** | **A** | **A** | **A** | **A** | **G** | **T** | **A** | **A** | **T** | **C** | **A** | **G** | **G** | **A** | **A** | **A** | **T** | **C** | **G** | **C** | **G** | **G** | **A** | **T** | **G** | **A** | **A** | **T** | **T** | **G** | **T** | **T** | **T** | **A** | **T** | **C** | **A** | **C** | **A** | **C** | **T** | **G** | **A** | **A** | **A** | **A** | **C** | **A** | **G** | **T** | **T** | **A** | **A** | **A** | **A** | **C** | **G** | **C** | **A** | **T** | **G** | **T** | **G** | **T** | **C** | **G** | **A** | **A** | **T** | **A** | **T** | **A** | **T** | **T** | **A** | **G** | **C** | **T** | **A** | **A** | **G** | **T** | **T** | **A** | **G** | **A** | **G** | **G** | **T** | **A** | **G** | **A** | **A** | **G** | **A** | **C** | **C** | **G** | **A** | **A** | **C** | **T** | **C** | **A** | **A** | **G** | **C** | **A** | **G** | **C** | **T** | **A** | **T** | **C** | **T** | **A** | **T** | **G** | **C** | **A** | **T** | **T** | **T** | **A** | **A** | **A** | **C** | **A** | **T** | **G** | **G** | **T** | **T** | **T** | **G** | **G** | **T** | **G** | **A** | **A** | **A** | **-** | **-** | **-** |
| **N54567** | **A** | **T** | **G** | **A** | **T** | **A** | **A** | **A** | **A** | **G** | **T** | **T** | **T** | **T** | **A** | **T** | **T** | **A** | **G** | **T** | **A** | **G** | **A** | **T** | **G** | **A** | **C** | **C** | **A** | **T** | **G** | **A** | **A** | **A** | **T** | **G** | **G** | **T** | **A** | **C** | **G** | **C** | **T** | **T** | **A** | **G** | **G** | **C** | **G** | **T** | **C** | **T** | **C** | **T** | **T** | **C** | **T** | **T** | **A** | **T** | **T** | **T** | **A** | **T** | **C** | **T** | **A** | **T** | **C** | **C** | **A** | **A** | **G** | **A** | **A** | **G** | **A** | **C** | **A** | **T** | **T** | **G** | **A** | **A** | **G** | **T** | **G** | **A** | **T** | **T** | **G** | **G** | **A** | **G** | **A** | **A** | **G** | **C** | **T** | **G** | **A** | **A** | **A** | **A** | **T** | **G** | **G** | **A** | **C** | **G** | **T** | **C** | **A** | **A** | **G** | **G** | **C** | **T** | **A** | **T** | **G** | **A** | **A** | **A** | **A** | **G** | **G** | **C** | **G** | **A** | **T** | **G** | **G** | **C** | **A** | **C** | **T** | **T** | **C** | **G** | **A** | **C** | **C** | **G** | **G** | **A** | **T** | **G** | **T** | **C** | **A** | **T** | **T** | **T** | **T** | **A** | **A** | **T** | **G** | **G** | **A** | **T** | **C** | **T** | **A** | **G** | **T** | **C** | **A** | **T** | **G** | **G** | **A** | **A** | **G** | **A** | **A** | **A** | **T** | **G** | **G** | **A** | **T** | **G** | **G** | **C** | **A** | **T** | **C** | **G** | **A** | **A** | **T** | **C** | **A** | **A** | **C** | **A** | **A** | **A** | **A** | **G** | **C** | **A** | **A** | **T** | **C** | **T** | **T** | **A** | **A** | **A** | **A** | **G** | **A** | **T** | **T** | **G** | **G** | **C** | **C** | **A** | **A** | **A** | **A** | **G** | **C** | **C** | **A** | **A** | **A** | **A** | **T** | **C** | **A** | **T** | **T** | **A** | **T** | **C** | **G** | **T** | **C** | **A** | **C** | **A** | **A** | **G** | **T** | **T** | **T** | **T** | **A** | **T** | **T** | **G** | **A** | **T** | **G** | **A** | **T** | **G** | **A** | **A** | **A** | **A** | **A** | **G** | **T** | **T** | **T** | **A** | **T** | **C** | **C** | **T** | **G** | **C** | **A** | **A** | **T** | **T** | **G** | **A** | **A** | **G** | **C** | **A** | **G** | **G** | **A** | **G** | **C** | **A** | **G** | **C** | **A** | **G** | **G** | **A** | **T** | **A** | **T** | **T** | **T** | **G** | **T** | **T** | **G** | **A** | **A** | **G** | **A** | **C** | **G** | **T** | **C** | **T** | **A** | **C** | **C** | **G** | **C** | **A** | **C** | **A** | **T** | **G** | **A** | **A** | **A** | **T** | **A** | **G** | **C** | **T** | **G** | **A** | **T** | **G** | **C** | **T** | **A** | **T** | **A** | **A** | **G** | **G** | **G** | **C** | **T** | **A** | **C** | **G** | **C** | **A** | **A** | **C** | **G** | **C** | **G** | **G** | **C** | **G** | **A** | **G** | **C** | **G** | **A** | **G** | **T** | **A** | **T** | **T** | **G** | **G** | **A** | **A** | **C** | **C** | **T** | **G** | **A** | **A** | **G** | **T** | **G** | **A** | **C** | **A** | **A** | **C** | **A** | **A** | **A** | **A** | **A** | **T** | **G** | **A** | **T** | **G** | **G** | **A** | **A** | **A** | **A** | **A** | **A** | **T** | **G** | **A** | **G** | **T** | **C** | **G** | **G** | **C** | **G** | **A** | **A** | **A** | **T** | **G** | **A** | **T** | **C** | **C** | **A** | **G** | **T** | **A** | **T** | **T** | **G** | **C** | **A** | **T** | **G** | **A** | **A** | **G** | **A** | **A** | **T** | **T** | **A** | **A** | **C** | **G** | **A** | **A** | **T** | **C** | **G** | **A** | **G** | **A** | **A** | **A** | **A** | **T** | **G** | **A** | **A** | **A** | **T** | **T** | **T** | **T** | **G** | **A** | **T** | **G** | **C** | **T** | **G** | **A** | **T** | **T** | **T** | **C** | **T** | **G** | **A** | **A** | **G** | **G** | **G** | **A** | **A** | **A** | **A** | **G** | **T** | **A** | **A** | **T** | **C** | **A** | **G** | **G** | **A** | **A** | **A** | **T** | **C** | **G** | **C** | **G** | **G** | **A** | **T** | **G** | **A** | **A** | **T** | **T** | **G** | **T** | **T** | **T** | **A** | **T** | **C** | **A** | **C** | **A** | **C** | **T** | **G** | **A** | **A** | **A** | **A** | **C** | **A** | **G** | **T** | **C** | **A** | **A** | **A** | **A** | **C** | **G** | **C** | **A** | **T** | **G** | **T** | **G** | **T** | **C** | **G** | **A** | **A** | **T** | **A** | **T** | **A** | **T** | **T** | **A** | **G** | **C** | **T** | **A** | **A** | **G** | **T** | **T** | **A** | **G** | **A** | **G** | **G** | **T** | **A** | **G** | **A** | **A** | **G** | **A** | **C** | **C** | **G** | **A** | **A** | **C** | **T** | **C** | **A** | **A** | **G** | **C** | **A** | **G** | **C** | **T** | **A** | **T** | **C** | **T** | **A** | **T** | **G** | **C** | **G** | **T** | **T** | **T** | **A** | **A** | **A** | **C** | **A** | **T** | **G** | **G** | **T** | **T** | **T** | **G** | **G** | **T** | **G** | **A** | **A** | **A** | **-** | **-** | **-** |
| **N59513F** | **A** | **T** | **G** | **A** | **T** | **A** | **A** | **A** | **A** | **G** | **T** | **T** | **T** | **T** | **A** | **T** | **T** | **A** | **G** | **T** | **A** | **G** | **A** | **T** | **G** | **A** | **C** | **C** | **A** | **T** | **G** | **A** | **A** | **A** | **T** | **G** | **G** | **T** | **G** | **C** | **G** | **C** | **T** | **T** | **A** | **G** | **G** | **C** | **G** | **T** | **C** | **T** | **C** | **T** | **T** | **C** | **T** | **T** | **A** | **T** | **T** | **T** | **A** | **T** | **C** | **T** | **A** | **T** | **T** | **C** | **A** | **A** | **G** | **A** | **A** | **G** | **A** | **C** | **A** | **T** | **T** | **G** | **A** | **A** | **G** | **T** | **A** | **A** | **T** | **T** | **G** | **G** | **A** | **G** | **A** | **A** | **G** | **C** | **T** | **G** | **A** | **A** | **A** | **A** | **T** | **G** | **G** | **A** | **C** | **G** | **A** | **C** | **A** | **A** | **G** | **G** | **C** | **T** | **A** | **T** | **G** | **A** | **G** | **A** | **A** | **G** | **G** | **C** | **G** | **A** | **T** | **G** | **A** | **C** | **A** | **C** | **T** | **T** | **C** | **G** | **A** | **C** | **C** | **G** | **G** | **A** | **C** | **G** | **T** | **C** | **A** | **T** | **T** | **T** | **T** | **A** | **A** | **T** | **G** | **G** | **A** | **C** | **T** | **T** | **A** | **G** | **T** | **C** | **A** | **T** | **G** | **G** | **A** | **A** | **G** | **A** | **A** | **A** | **T** | **G** | **G** | **A** | **T** | **G** | **G** | **T** | **A** | **T** | **C** | **G** | **A** | **A** | **T** | **C** | **A** | **A** | **C** | **A** | **A** | **A** | **A** | **G** | **C** | **A** | **A** | **T** | **C** | **T** | **T** | **A** | **A** | **A** | **A** | **G** | **A** | **T** | **T** | **G** | **G** | **C** | **C** | **A** | **G** | **A** | **A** | **G** | **C** | **T** | **A** | **A** | **A** | **A** | **T** | **C** | **A** | **T** | **T** | **A** | **T** | **C** | **G** | **T** | **A** | **A** | **C** | **C** | **A** | **G** | **T** | **T** | **T** | **T** | **A** | **T** | **T** | **G** | **A** | **T** | **G** | **A** | **C** | **G** | **A** | **A** | **A** | **A** | **A** | **G** | **T** | **T** | **T** | **A** | **T** | **C** | **C** | **T** | **G** | **C** | **C** | **A** | **T** | **T** | **G** | **A** | **A** | **G** | **C** | **A** | **G** | **G** | **T** | **G** | **C** | **A** | **G** | **C** | **A** | **G** | **G** | **G** | **T** | **A** | **T** | **T** | **T** | **G** | **T** | **T** | **G** | **A** | **A** | **G** | **A** | **C** | **C** | **T** | **C** | **T** | **A** | **C** | **C** | **G** | **C** | **A** | **C** | **A** | **T** | **G** | **A** | **A** | **A** | **T** | **T** | **G** | **C** | **T** | **G** | **A** | **T** | **G** | **C** | **T** | **A** | **T** | **A** | **A** | **G** | **G** | **G** | **C** | **A** | **A** | **C** | **G** | **C** | **A** | **A** | **C** | **G** | **T** | **G** | **G** | **C** | **G** | **A** | **G** | **C** | **G** | **A** | **G** | **T** | **A** | **T** | **T** | **A** | **G** | **A** | **A** | **C** | **C** | **A** | **G** | **A** | **A** | **G** | **T** | **G** | **A** | **C** | **A** | **A** | **C** | **A** | **A** | **A** | **A** | **A** | **T** | **G** | **A** | **T** | **G** | **G** | **A** | **A** | **A** | **A** | **A** | **A** | **T** | **G** | **A** | **G** | **T** | **C** | **G** | **G** | **C** | **G** | **A** | **A** | **A** | **T** | **G** | **A** | **G** | **C** | **C** | **T** | **G** | **T** | **A** | **T** | **T** | **A** | **C** | **A** | **T** | **G** | **A** | **A** | **G** | **A** | **A** | **C** | **T** | **A** | **A** | **C** | **G** | **A** | **A** | **T** | **C** | **G** | **G** | **G** | **A** | **A** | **A** | **A** | **T** | **G** | **A** | **A** | **A** | **T** | **T** | **T** | **T** | **A** | **A** | **T** | **G** | **C** | **T** | **G** | **A** | **T** | **T** | **T** | **C** | **T** | **G** | **A** | **A** | **G** | **G** | **A** | **A** | **A** | **A** | **A** | **G** | **T** | **A** | **A** | **T** | **C** | **A** | **G** | **G** | **A** | **A** | **A** | **T** | **A** | **G** | **C** | **G** | **G** | **A** | **C** | **G** | **A** | **A** | **T** | **T** | **G** | **T** | **T** | **T** | **A** | **T** | **T** | **A** | **C** | **A** | **C** | **T** | **G** | **A** | **A** | **A** | **A** | **C** | **A** | **G** | **T** | **A** | **A** | **A** | **A** | **A** | **C** | **A** | **C** | **A** | **T** | **G** | **T** | **T** | **T** | **C** | **C** | **A** | **A** | **T** | **A** | **T** | **A** | **T** | **T** | **G** | **G** | **C** | **T** | **A** | **A** | **G** | **C** | **T** | **A** | **G** | **A** | **A** | **G** | **T** | **G** | **G** | **A** | **A** | **G** | **A** | **C** | **C** | **G** | **T** | **A** | **C** | **A** | **C** | **A** | **A** | **G** | **C** | **C** | **G** | **C** | **T** | **A** | **T** | **C** | **T** | **A** | **T** | **G** | **C** | **A** | **T** | **T** | **C** | **A** | **A** | **A** | **C** | **A** | **T** | **G** | **G** | **T** | **T** | **T** | **G** | **G** | **T** | **G** | **A** | **A** | **A** | **-** | **-** | **-** |
| **N60490F** | **A** | **T** | **G** | **A** | **T** | **A** | **A** | **A** | **A** | **G** | **T** | **T** | **T** | **T** | **A** | **T** | **T** | **A** | **G** | **T** | **A** | **G** | **A** | **T** | **G** | **A** | **C** | **C** | **A** | **T** | **G** | **A** | **A** | **A** | **T** | **G** | **G** | **T** | **A** | **C** | **G** | **C** | **T** | **T** | **A** | **G** | **G** | **C** | **G** | **T** | **C** | **T** | **C** | **T** | **T** | **C** | **T** | **T** | **A** | **T** | **T** | **T** | **A** | **T** | **C** | **T** | **A** | **T** | **C** | **C** | **A** | **A** | **G** | **A** | **A** | **G** | **A** | **C** | **A** | **T** | **T** | **G** | **A** | **A** | **G** | **T** | **G** | **A** | **T** | **T** | **G** | **G** | **A** | **G** | **A** | **A** | **G** | **C** | **T** | **G** | **A** | **A** | **A** | **A** | **T** | **G** | **G** | **A** | **C** | **G** | **T** | **C** | **A** | **A** | **G** | **G** | **C** | **T** | **A** | **T** | **G** | **A** | **A** | **A** | **A** | **G** | **G** | **C** | **G** | **A** | **T** | **G** | **G** | **C** | **A** | **C** | **T** | **T** | **C** | **G** | **A** | **C** | **C** | **A** | **G** | **A** | **T** | **G** | **T** | **C** | **A** | **T** | **T** | **T** | **T** | **A** | **A** | **T** | **G** | **G** | **A** | **T** | **C** | **T** | **A** | **G** | **T** | **C** | **A** | **T** | **G** | **G** | **A** | **A** | **G** | **A** | **A** | **A** | **T** | **G** | **G** | **A** | **T** | **G** | **G** | **C** | **A** | **T** | **C** | **G** | **A** | **A** | **T** | **C** | **A** | **A** | **C** | **A** | **A** | **A** | **A** | **G** | **C** | **A** | **A** | **T** | **C** | **T** | **T** | **A** | **A** | **A** | **A** | **G** | **A** | **T** | **T** | **G** | **G** | **C** | **C** | **A** | **A** | **A** | **A** | **G** | **C** | **C** | **A** | **A** | **A** | **A** | **T** | **C** | **A** | **T** | **T** | **A** | **T** | **C** | **G** | **T** | **C** | **A** | **C** | **A** | **A** | **G** | **T** | **T** | **T** | **T** | **A** | **T** | **T** | **G** | **A** | **T** | **G** | **A** | **T** | **G** | **A** | **A** | **A** | **A** | **A** | **G** | **T** | **T** | **T** | **A** | **T** | **C** | **C** | **T** | **G** | **C** | **A** | **A** | **T** | **T** | **G** | **A** | **A** | **G** | **C** | **A** | **G** | **G** | **A** | **G** | **C** | **A** | **G** | **C** | **A** | **G** | **G** | **A** | **T** | **A** | **T** | **T** | **T** | **G** | **T** | **T** | **G** | **A** | **A** | **G** | **A** | **C** | **G** | **T** | **C** | **T** | **A** | **C** | **C** | **G** | **C** | **A** | **C** | **A** | **T** | **G** | **A** | **A** | **A** | **T** | **A** | **G** | **C** | **T** | **G** | **A** | **T** | **G** | **C** | **T** | **A** | **T** | **A** | **A** | **G** | **G** | **G** | **C** | **T** | **A** | **C** | **G** | **C** | **A** | **A** | **C** | **G** | **C** | **G** | **G** | **C** | **G** | **A** | **G** | **C** | **G** | **A** | **G** | **T** | **A** | **T** | **T** | **G** | **G** | **A** | **A** | **C** | **C** | **T** | **G** | **A** | **A** | **G** | **T** | **G** | **A** | **C** | **A** | **A** | **C** | **A** | **A** | **A** | **A** | **A** | **T** | **G** | **A** | **T** | **G** | **G** | **A** | **A** | **A** | **A** | **A** | **A** | **T** | **G** | **A** | **G** | **T** | **C** | **G** | **G** | **C** | **G** | **A** | **T** | **A** | **T** | **G** | **A** | **T** | **C** | **C** | **A** | **G** | **T** | **A** | **T** | **T** | **G** | **C** | **A** | **T** | **G** | **A** | **A** | **G** | **A** | **A** | **T** | **T** | **A** | **A** | **C** | **G** | **A** | **A** | **T** | **C** | **G** | **A** | **G** | **A** | **A** | **A** | **A** | **T** | **G** | **A** | **A** | **A** | **T** | **T** | **T** | **T** | **G** | **A** | **T** | **G** | **C** | **T** | **G** | **A** | **T** | **T** | **T** | **C** | **T** | **G** | **A** | **A** | **G** | **G** | **G** | **A** | **A** | **A** | **A** | **G** | **T** | **A** | **A** | **T** | **C** | **A** | **G** | **G** | **A** | **A** | **A** | **T** | **C** | **G** | **C** | **G** | **G** | **A** | **T** | **G** | **A** | **A** | **T** | **T** | **G** | **T** | **T** | **T** | **A** | **T** | **C** | **A** | **C** | **A** | **C** | **T** | **G** | **A** | **A** | **A** | **A** | **C** | **A** | **G** | **T** | **T** | **A** | **A** | **A** | **A** | **C** | **G** | **C** | **A** | **T** | **G** | **T** | **G** | **T** | **C** | **G** | **A** | **A** | **T** | **A** | **T** | **A** | **T** | **T** | **A** | **G** | **C** | **T** | **A** | **A** | **G** | **T** | **T** | **A** | **G** | **A** | **G** | **G** | **T** | **A** | **G** | **A** | **A** | **G** | **A** | **C** | **C** | **G** | **A** | **A** | **C** | **T** | **C** | **A** | **A** | **G** | **C** | **A** | **G** | **C** | **T** | **A** | **T** | **C** | **T** | **A** | **T** | **G** | **C** | **A** | **T** | **T** | **T** | **A** | **A** | **A** | **C** | **A** | **T** | **G** | **G** | **T** | **T** | **T** | **G** | **G** | **T** | **G** | **A** | **A** | **A** | **-** | **-** | **-** |
| **N59779F** | **A** | **T** | **G** | **A** | **T** | **A** | **A** | **A** | **A** | **G** | **T** | **T** | **T** | **T** | **A** | **T** | **T** | **A** | **G** | **T** | **A** | **G** | **A** | **T** | **G** | **A** | **C** | **C** | **A** | **T** | **G** | **A** | **A** | **A** | **T** | **G** | **G** | **T** | **A** | **C** | **G** | **C** | **T** | **T** | **A** | **G** | **G** | **C** | **G** | **T** | **C** | **T** | **C** | **T** | **T** | **C** | **T** | **T** | **A** | **T** | **T** | **T** | **A** | **T** | **C** | **T** | **A** | **T** | **C** | **C** | **A** | **A** | **G** | **A** | **A** | **G** | **A** | **C** | **A** | **T** | **T** | **G** | **A** | **A** | **G** | **T** | **G** | **A** | **T** | **T** | **G** | **G** | **A** | **G** | **A** | **A** | **G** | **C** | **T** | **G** | **A** | **A** | **A** | **A** | **T** | **G** | **G** | **A** | **C** | **G** | **T** | **C** | **A** | **A** | **G** | **G** | **C** | **T** | **A** | **T** | **G** | **A** | **A** | **A** | **A** | **G** | **G** | **C** | **G** | **A** | **T** | **G** | **G** | **C** | **A** | **C** | **T** | **T** | **C** | **G** | **A** | **C** | **C** | **A** | **G** | **A** | **T** | **G** | **T** | **C** | **A** | **T** | **T** | **T** | **T** | **A** | **A** | **T** | **G** | **G** | **A** | **T** | **C** | **T** | **A** | **G** | **T** | **C** | **A** | **T** | **G** | **G** | **A** | **A** | **G** | **A** | **A** | **A** | **T** | **G** | **G** | **A** | **T** | **G** | **G** | **C** | **A** | **T** | **C** | **G** | **A** | **A** | **T** | **C** | **A** | **A** | **C** | **A** | **A** | **A** | **A** | **G** | **C** | **A** | **A** | **T** | **C** | **T** | **T** | **A** | **A** | **A** | **A** | **G** | **A** | **T** | **T** | **G** | **G** | **C** | **C** | **A** | **A** | **A** | **A** | **G** | **C** | **C** | **A** | **A** | **A** | **A** | **T** | **C** | **A** | **T** | **T** | **A** | **T** | **C** | **G** | **T** | **C** | **A** | **C** | **A** | **A** | **G** | **T** | **T** | **T** | **T** | **A** | **T** | **T** | **G** | **A** | **T** | **G** | **A** | **T** | **G** | **A** | **A** | **A** | **A** | **A** | **G** | **T** | **T** | **T** | **A** | **T** | **C** | **C** | **T** | **G** | **C** | **A** | **A** | **T** | **T** | **G** | **A** | **A** | **G** | **C** | **A** | **G** | **G** | **A** | **G** | **C** | **A** | **G** | **C** | **A** | **G** | **G** | **A** | **T** | **A** | **T** | **T** | **T** | **A** | **T** | **T** | **G** | **A** | **A** | **G** | **A** | **C** | **G** | **T** | **C** | **T** | **A** | **C** | **C** | **G** | **C** | **A** | **C** | **A** | **T** | **G** | **A** | **A** | **A** | **T** | **A** | **G** | **C** | **T** | **G** | **A** | **T** | **G** | **C** | **T** | **A** | **T** | **A** | **A** | **G** | **G** | **G** | **C** | **T** | **A** | **C** | **G** | **C** | **A** | **A** | **C** | **G** | **C** | **G** | **G** | **C** | **G** | **A** | **G** | **C** | **G** | **A** | **G** | **T** | **A** | **T** | **T** | **G** | **G** | **A** | **A** | **C** | **C** | **T** | **G** | **A** | **A** | **G** | **T** | **G** | **A** | **C** | **A** | **A** | **C** | **A** | **A** | **A** | **A** | **A** | **T** | **G** | **A** | **T** | **G** | **G** | **A** | **A** | **A** | **A** | **A** | **A** | **T** | **G** | **A** | **G** | **T** | **C** | **G** | **G** | **C** | **G** | **A** | **A** | **A** | **T** | **G** | **A** | **T** | **C** | **C** | **A** | **G** | **T** | **A** | **T** | **T** | **G** | **C** | **A** | **T** | **G** | **A** | **A** | **G** | **A** | **A** | **T** | **T** | **A** | **A** | **C** | **G** | **A** | **A** | **T** | **C** | **G** | **A** | **G** | **A** | **A** | **A** | **A** | **T** | **G** | **A** | **A** | **A** | **T** | **T** | **T** | **T** | **G** | **A** | **T** | **G** | **C** | **T** | **G** | **A** | **T** | **T** | **T** | **C** | **T** | **G** | **A** | **A** | **G** | **G** | **G** | **A** | **A** | **A** | **A** | **G** | **T** | **A** | **A** | **T** | **C** | **A** | **G** | **G** | **A** | **A** | **A** | **T** | **C** | **G** | **C** | **G** | **G** | **A** | **T** | **G** | **A** | **A** | **T** | **T** | **G** | **T** | **T** | **T** | **A** | **T** | **C** | **A** | **C** | **A** | **C** | **T** | **G** | **A** | **A** | **A** | **A** | **C** | **A** | **G** | **T** | **T** | **A** | **A** | **A** | **A** | **C** | **G** | **C** | **A** | **T** | **G** | **T** | **G** | **T** | **C** | **G** | **A** | **A** | **T** | **A** | **T** | **A** | **T** | **T** | **A** | **G** | **C** | **T** | **A** | **A** | **G** | **T** | **T** | **A** | **G** | **A** | **G** | **G** | **T** | **A** | **G** | **A** | **A** | **G** | **A** | **C** | **C** | **G** | **A** | **A** | **C** | **T** | **C** | **A** | **A** | **G** | **C** | **A** | **G** | **C** | **T** | **A** | **T** | **C** | **T** | **A** | **T** | **G** | **C** | **G** | **T** | **T** | **T** | **A** | **A** | **A** | **C** | **A** | **T** | **G** | **G** | **T** | **T** | **T** | **G** | **G** | **T** | **G** | **A** | **A** | **A** | **-** | **-** | **-** |
| **N53804** | **A** | **T** | **G** | **A** | **T** | **A** | **A** | **A** | **A** | **G** | **T** | **T** | **T** | **T** | **A** | **T** | **T** | **A** | **G** | **T** | **A** | **G** | **A** | **T** | **G** | **A** | **C** | **C** | **A** | **T** | **G** | **A** | **A** | **A** | **T** | **G** | **G** | **T** | **A** | **C** | **G** | **C** | **T** | **T** | **A** | **G** | **G** | **C** | **G** | **T** | **C** | **T** | **C** | **T** | **T** | **C** | **T** | **T** | **A** | **T** | **T** | **T** | **A** | **T** | **C** | **T** | **A** | **T** | **C** | **C** | **A** | **A** | **G** | **A** | **A** | **G** | **A** | **C** | **A** | **T** | **T** | **G** | **A** | **A** | **G** | **T** | **G** | **A** | **T** | **T** | **G** | **G** | **A** | **G** | **A** | **A** | **G** | **C** | **T** | **G** | **A** | **A** | **A** | **A** | **T** | **G** | **G** | **A** | **C** | **G** | **T** | **C** | **A** | **A** | **G** | **G** | **C** | **T** | **A** | **T** | **G** | **A** | **A** | **A** | **A** | **G** | **G** | **C** | **G** | **A** | **T** | **G** | **G** | **C** | **A** | **C** | **T** | **T** | **C** | **G** | **A** | **C** | **C** | **A** | **G** | **A** | **T** | **G** | **T** | **C** | **A** | **T** | **T** | **T** | **T** | **A** | **A** | **T** | **G** | **G** | **A** | **T** | **C** | **T** | **A** | **G** | **T** | **C** | **A** | **T** | **G** | **G** | **A** | **A** | **G** | **A** | **A** | **A** | **T** | **G** | **G** | **A** | **T** | **G** | **G** | **C** | **A** | **T** | **C** | **G** | **A** | **A** | **T** | **C** | **A** | **A** | **C** | **A** | **A** | **A** | **A** | **G** | **C** | **A** | **A** | **T** | **C** | **T** | **T** | **A** | **A** | **A** | **A** | **G** | **A** | **T** | **T** | **G** | **G** | **C** | **C** | **A** | **A** | **A** | **A** | **G** | **C** | **C** | **A** | **A** | **A** | **A** | **T** | **C** | **A** | **T** | **T** | **A** | **T** | **C** | **G** | **T** | **C** | **A** | **C** | **A** | **A** | **G** | **T** | **T** | **T** | **T** | **A** | **T** | **T** | **G** | **A** | **T** | **G** | **A** | **T** | **G** | **A** | **A** | **A** | **A** | **A** | **G** | **T** | **T** | **T** | **A** | **T** | **C** | **C** | **T** | **G** | **C** | **A** | **A** | **T** | **T** | **G** | **A** | **A** | **G** | **C** | **A** | **G** | **G** | **A** | **G** | **C** | **A** | **G** | **C** | **A** | **G** | **G** | **A** | **T** | **A** | **T** | **T** | **T** | **A** | **T** | **T** | **G** | **A** | **A** | **G** | **A** | **C** | **G** | **T** | **C** | **T** | **A** | **C** | **C** | **G** | **C** | **A** | **C** | **A** | **T** | **G** | **A** | **A** | **A** | **T** | **A** | **G** | **C** | **T** | **G** | **A** | **T** | **G** | **C** | **T** | **A** | **T** | **A** | **A** | **G** | **G** | **G** | **C** | **T** | **A** | **C** | **G** | **C** | **A** | **A** | **C** | **G** | **C** | **G** | **G** | **C** | **G** | **A** | **G** | **C** | **G** | **A** | **G** | **T** | **A** | **T** | **T** | **G** | **G** | **A** | **A** | **C** | **C** | **T** | **G** | **A** | **A** | **G** | **T** | **G** | **A** | **C** | **A** | **A** | **C** | **A** | **A** | **A** | **A** | **A** | **T** | **G** | **A** | **T** | **G** | **G** | **A** | **A** | **A** | **A** | **A** | **A** | **T** | **G** | **A** | **G** | **T** | **C** | **G** | **G** | **C** | **G** | **A** | **A** | **A** | **T** | **G** | **A** | **T** | **C** | **C** | **A** | **G** | **T** | **A** | **T** | **T** | **G** | **C** | **A** | **T** | **G** | **A** | **A** | **G** | **A** | **A** | **T** | **T** | **A** | **A** | **C** | **G** | **A** | **A** | **T** | **C** | **G** | **A** | **G** | **A** | **A** | **A** | **A** | **T** | **G** | **A** | **A** | **A** | **T** | **T** | **T** | **T** | **G** | **A** | **T** | **G** | **C** | **T** | **G** | **A** | **T** | **T** | **T** | **C** | **T** | **G** | **A** | **A** | **G** | **G** | **G** | **A** | **A** | **A** | **A** | **G** | **T** | **A** | **A** | **T** | **C** | **A** | **G** | **G** | **A** | **A** | **A** | **T** | **C** | **G** | **C** | **G** | **G** | **A** | **T** | **G** | **A** | **A** | **T** | **T** | **G** | **T** | **T** | **T** | **A** | **T** | **C** | **A** | **C** | **A** | **C** | **T** | **G** | **A** | **A** | **A** | **A** | **C** | **A** | **G** | **T** | **T** | **A** | **A** | **A** | **A** | **C** | **G** | **C** | **A** | **T** | **G** | **T** | **G** | **T** | **C** | **G** | **A** | **A** | **T** | **A** | **T** | **A** | **T** | **T** | **A** | **G** | **C** | **T** | **A** | **A** | **G** | **T** | **T** | **A** | **G** | **A** | **G** | **G** | **T** | **A** | **G** | **A** | **A** | **G** | **A** | **C** | **C** | **G** | **A** | **A** | **C** | **T** | **C** | **A** | **A** | **G** | **C** | **A** | **G** | **C** | **T** | **A** | **T** | **C** | **T** | **A** | **T** | **G** | **C** | **G** | **T** | **T** | **T** | **A** | **A** | **A** | **C** | **A** | **T** | **G** | **G** | **T** | **T** | **T** | **G** | **G** | **T** | **G** | **A** | **A** | **A** | **-** | **-** | **-** |
| **N53420** | **G** | **T** | **G** | **A** | **T** | **C** | **A** | **A** | **A** | **G** | **T** | **A** | **A** | **T** | **G** | **T** | **T** | **A** | **G** | **T** | **G** | **G** | **A** | **T** | **G** | **A** | **C** | **C** | **A** | **T** | **G** | **A** | **A** | **A** | **T** | **G** | **G** | **T** | **C** | **C** | **G** | **T** | **T** | **T** | **A** | **G** | **G** | **C** | **G** | **T** | **T** | **T** | **C** | **A** | **T** | **C** | **A** | **T** | **A** | **T** | **T** | **T** | **A** | **T** | **C** | **T** | **A** | **T** | **T** | **C** | **A** | **A** | **G** | **A** | **G** | **G** | **A** | **T** | **A** | **T** | **A** | **G** | **A** | **A** | **G** | **T** | **C** | **G** | **T** | **A** | **G** | **G** | **C** | **G** | **A** | **A** | **G** | **C** | **A** | **G** | **A** | **A** | **A** | **A** | **C** | **G** | **G** | **T** | **A** | **A** | **G** | **A** | **T** | **T** | **G** | **G** | **C** | **T** | **A** | **T** | **G** | **A** | **A** | **A** | **A** | **A** | **G** | **C** | **A** | **T** | **T** | **G** | **G** | **A** | **A** | **C** | **T** | **A** | **C** | **G** | **T** | **C** | **C** | **A** | **G** | **A** | **T** | **G** | **T** | **T** | **A** | **T** | **T** | **T** | **T** | **G** | **A** | **T** | **G** | **G** | **A** | **C** | **T** | **T** | **G** | **G** | **T** | **A** | **A** | **T** | **G** | **G** | **A** | **A** | **G** | **A** | **A** | **A** | **T** | **G** | **G** | **A** | **C** | **G** | **G** | **C** | **A** | **T** | **T** | **G** | **A** | **T** | **T** | **C** | **A** | **A** | **C** | **A** | **A** | **A** | **A** | **G** | **C** | **G** | **A** | **T** | **T** | **T** | **T** | **G** | **A** | **A** | **A** | **G** | **A** | **T** | **T** | **G** | **G** | **C** | **C** | **A** | **G** | **A** | **A** | **G** | **C** | **C** | **A** | **A** | **G** | **A** | **T** | **T** | **A** | **T** | **T** | **A** | **T** | **T** | **G** | **T** | **G** | **A** | **C** | **G** | **A** | **G** | **T** | **T** | **T** | **T** | **A** | **T** | **T** | **G** | **A** | **T** | **G** | **A** | **T** | **G** | **A** | **A** | **A** | **A** | **A** | **G** | **T** | **G** | **T** | **A** | **T** | **C** | **C** | **G** | **G** | **C** | **A** | **A** | **T** | **T** | **G** | **A** | **A** | **G** | **C** | **T** | **G** | **G** | **T** | **G** | **C** | **A** | **G** | **C** | **G** | **G** | **G** | **C** | **T** | **A** | **C** | **C** | **T** | **A** | **T** | **T** | **A** | **A** | **A** | **A** | **A** | **C** | **A** | **T** | **C** | **A** | **A** | **C** | **A** | **G** | **C** | **A** | **C** | **A** | **T** | **G** | **A** | **G** | **A** | **T** | **T** | **G** | **C** | **T** | **G** | **A** | **T** | **G** | **C** | **G** | **A** | **T** | **T** | **C** | **G** | **G** | **G** | **C** | **G** | **A** | **C** | **T** | **T** | **A** | **T** | **C** | **G** | **C** | **G** | **G** | **A** | **G** | **A** | **G** | **C** | **G** | **T** | **G** | **T** | **G** | **T** | **T** | **G** | **G** | **A** | **A** | **C** | **C** | **T** | **G** | **A** | **A** | **G** | **T** | **G** | **A** | **C** | **G** | **C** | **A** | **T** | **A** | **A** | **G** | **A** | **T** | **G** | **A** | **T** | **G** | **G** | **A** | **A** | **C** | **G** | **G** | **T** | **T** | **A** | **A** | **C** | **A** | **A** | **A** | **A** | **A** | **A** | **A** | **C** | **A** | **A** | **G** | **A** | **G** | **C** | **C** | **G** | **G** | **T** | **G** | **C** | **T** | **A** | **C** | **A** | **C** | **G** | **A** | **A** | **G** | **A** | **T** | **T** | **T** | **G** | **A** | **C** | **A** | **A** | **A** | **C** | **C** | **G** | **A** | **G** | **A** | **A** | **C** | **A** | **C** | **G** | **A** | **A** | **A** | **T** | **T** | **T** | **T** | **A** | **A** | **T** | **G** | **C** | **T** | **G** | **A** | **T** | **T** | **G** | **C** | **A** | **C** | **A** | **A** | **G** | **G** | **T** | **A** | **A** | **A** | **A** | **G** | **T** | **A** | **A** | **T** | **C** | **A** | **G** | **G** | **A** | **A** | **A** | **T** | **T** | **G** | **C** | **T** | **G** | **A** | **T** | **G** | **A** | **A** | **C** | **T** | **C** | **T** | **T** | **T** | **A** | **T** | **C** | **A** | **C** | **T** | **T** | **T** | **G** | **A** | **A** | **A** | **A** | **C** | **A** | **G** | **T** | **T** | **A** | **A** | **A** | **A** | **C** | **A** | **C** | **A** | **T** | **G** | **T** | **T** | **T** | **C** | **A** | **A** | **A** | **C** | **A** | **T** | **T** | **T** | **T** | **A** | **G** | **C** | **A** | **A** | **A** | **A** | **C** | **T** | **A** | **G** | **A** | **T** | **G** | **T** | **G** | **G** | **A** | **T** | **G** | **A** | **T** | **C** | **G** | **G** | **A** | **C** | **C** | **C** | **A** | **A** | **G** | **C** | **G** | **G** | **C** | **G** | **A** | **T** | **T** | **T** | **A** | **T** | **G** | **C** | **A** | **T** | **T** | **T** | **C** | **A** | **A** | **C** | **A** | **C** | **G** | **G** | **T** | **T** | **T** | **A** | **G** | **C** | **C** | **A** | **A** | **A** | **-** | **-** | **-** |
| **N59544F** | **G** | **T** | **G** | **A** | **T** | **C** | **A** | **A** | **A** | **G** | **T** | **A** | **A** | **T** | **G** | **T** | **T** | **A** | **G** | **T** | **G** | **G** | **A** | **T** | **G** | **A** | **C** | **C** | **A** | **T** | **G** | **A** | **A** | **A** | **T** | **G** | **G** | **T** | **C** | **C** | **G** | **T** | **T** | **T** | **A** | **G** | **G** | **C** | **G** | **T** | **T** | **T** | **C** | **A** | **T** | **C** | **A** | **T** | **A** | **T** | **T** | **T** | **A** | **T** | **C** | **T** | **A** | **T** | **T** | **C** | **A** | **A** | **G** | **A** | **G** | **G** | **A** | **T** | **A** | **T** | **A** | **G** | **A** | **A** | **G** | **T** | **C** | **G** | **T** | **A** | **G** | **G** | **C** | **G** | **A** | **A** | **G** | **C** | **A** | **G** | **A** | **A** | **A** | **A** | **C** | **G** | **G** | **T** | **A** | **A** | **G** | **A** | **T** | **T** | **G** | **G** | **C** | **T** | **A** | **T** | **G** | **A** | **A** | **A** | **A** | **A** | **G** | **C** | **A** | **T** | **T** | **G** | **G** | **A** | **A** | **C** | **T** | **A** | **C** | **G** | **T** | **C** | **C** | **A** | **G** | **A** | **T** | **G** | **T** | **T** | **A** | **T** | **T** | **T** | **T** | **G** | **A** | **T** | **G** | **G** | **A** | **T** | **T** | **T** | **G** | **G** | **T** | **A** | **A** | **T** | **G** | **G** | **A** | **A** | **G** | **A** | **A** | **A** | **T** | **G** | **G** | **A** | **C** | **G** | **G** | **C** | **A** | **T** | **T** | **G** | **A** | **T** | **T** | **C** | **A** | **A** | **C** | **A** | **A** | **A** | **A** | **G** | **C** | **G** | **A** | **T** | **C** | **T** | **T** | **G** | **A** | **A** | **A** | **G** | **A** | **T** | **T** | **G** | **G** | **C** | **C** | **A** | **G** | **A** | **A** | **G** | **C** | **C** | **A** | **A** | **G** | **A** | **T** | **T** | **A** | **T** | **T** | **A** | **T** | **T** | **G** | **T** | **G** | **A** | **C** | **G** | **A** | **G** | **T** | **T** | **T** | **T** | **A** | **T** | **T** | **G** | **A** | **T** | **G** | **A** | **T** | **G** | **A** | **A** | **A** | **A** | **A** | **G** | **T** | **G** | **T** | **A** | **T** | **C** | **C** | **G** | **G** | **C** | **G** | **A** | **T** | **T** | **G** | **A** | **A** | **G** | **C** | **T** | **G** | **G** | **T** | **G** | **C** | **A** | **G** | **C** | **G** | **G** | **G** | **C** | **T** | **A** | **C** | **C** | **T** | **A** | **T** | **T** | **A** | **A** | **A** | **G** | **A** | **C** | **A** | **T** | **C** | **A** | **A** | **C** | **A** | **G** | **C** | **A** | **C** | **A** | **T** | **G** | **A** | **G** | **A** | **T** | **T** | **G** | **C** | **T** | **G** | **A** | **T** | **G** | **C** | **A** | **A** | **T** | **T** | **C** | **G** | **G** | **G** | **C** | **G** | **A** | **C** | **T** | **T** | **A** | **T** | **C** | **G** | **C** | **G** | **G** | **A** | **G** | **A** | **G** | **C** | **G** | **T** | **G** | **T** | **G** | **T** | **T** | **G** | **G** | **A** | **A** | **C** | **C** | **T** | **G** | **A** | **A** | **G** | **T** | **G** | **A** | **C** | **G** | **C** | **A** | **T** | **A** | **A** | **G** | **A** | **T** | **G** | **A** | **T** | **G** | **G** | **A** | **A** | **C** | **G** | **G** | **T** | **T** | **A** | **A** | **C** | **A** | **A** | **A** | **A** | **A** | **A** | **A** | **C** | **A** | **A** | **G** | **A** | **G** | **C** | **C** | **G** | **G** | **T** | **G** | **T** | **T** | **G** | **C** | **A** | **C** | **G** | **A** | **A** | **G** | **A** | **T** | **T** | **T** | **G** | **A** | **C** | **A** | **A** | **A** | **C** | **C** | **G** | **G** | **G** | **A** | **A** | **C** | **A** | **C** | **G** | **A** | **A** | **A** | **T** | **T** | **T** | **T** | **A** | **A** | **T** | **G** | **T** | **T** | **G** | **A** | **T** | **T** | **G** | **C** | **A** | **C** | **A** | **A** | **G** | **G** | **T** | **A** | **A** | **A** | **A** | **G** | **T** | **A** | **A** | **T** | **C** | **A** | **G** | **G** | **A** | **A** | **A** | **T** | **A** | **G** | **C** | **T** | **G** | **A** | **T** | **G** | **A** | **A** | **C** | **T** | **C** | **T** | **T** | **T** | **A** | **T** | **C** | **A** | **C** | **T** | **T** | **T** | **G** | **A** | **A** | **A** | **A** | **C** | **A** | **G** | **T** | **T** | **A** | **A** | **A** | **A** | **C** | **A** | **C** | **A** | **T** | **G** | **T** | **T** | **T** | **C** | **A** | **A** | **A** | **C** | **A** | **T** | **T** | **T** | **T** | **A** | **G** | **C** | **A** | **A** | **A** | **A** | **C** | **T** | **A** | **G** | **A** | **T** | **G** | **T** | **G** | **G** | **A** | **T** | **G** | **A** | **T** | **C** | **G** | **G** | **A** | **C** | **C** | **C** | **A** | **A** | **G** | **C** | **G** | **G** | **C** | **G** | **A** | **T** | **T** | **T** | **A** | **T** | **G** | **C** | **T** | **T** | **T** | **T** | **C** | **A** | **A** | **C** | **A** | **T** | **G** | **G** | **T** | **T** | **T** | **A** | **G** | **C** | **C** | **A** | **A** | **A** | **-** | **-** | **-** |
| **N55240** | **G** | **T** | **G** | **A** | **T** | **C** | **A** | **A** | **A** | **G** | **T** | **A** | **A** | **T** | **G** | **T** | **T** | **A** | **G** | **T** | **G** | **G** | **A** | **T** | **G** | **A** | **C** | **C** | **A** | **T** | **G** | **A** | **A** | **A** | **T** | **G** | **G** | **T** | **C** | **C** | **G** | **T** | **T** | **T** | **A** | **G** | **G** | **C** | **G** | **T** | **T** | **T** | **C** | **A** | **T** | **C** | **A** | **T** | **A** | **T** | **T** | **T** | **A** | **T** | **C** | **T** | **A** | **T** | **T** | **C** | **A** | **A** | **G** | **A** | **G** | **G** | **A** | **T** | **A** | **T** | **A** | **G** | **A** | **A** | **G** | **T** | **C** | **G** | **T** | **A** | **G** | **G** | **C** | **G** | **A** | **A** | **G** | **C** | **A** | **G** | **A** | **A** | **A** | **A** | **C** | **G** | **G** | **T** | **A** | **A** | **G** | **A** | **T** | **T** | **G** | **G** | **C** | **T** | **A** | **T** | **G** | **A** | **A** | **A** | **A** | **A** | **G** | **C** | **A** | **T** | **T** | **G** | **G** | **A** | **A** | **C** | **T** | **A** | **C** | **G** | **T** | **C** | **C** | **A** | **G** | **A** | **T** | **G** | **T** | **T** | **A** | **T** | **T** | **T** | **T** | **G** | **A** | **T** | **G** | **G** | **A** | **T** | **T** | **T** | **G** | **G** | **T** | **A** | **A** | **T** | **G** | **G** | **A** | **A** | **G** | **A** | **A** | **A** | **T** | **G** | **G** | **A** | **C** | **G** | **G** | **C** | **A** | **T** | **T** | **G** | **A** | **T** | **T** | **C** | **A** | **A** | **C** | **A** | **A** | **A** | **A** | **G** | **C** | **G** | **A** | **T** | **C** | **T** | **T** | **G** | **A** | **A** | **A** | **G** | **A** | **T** | **T** | **G** | **G** | **C** | **C** | **A** | **G** | **A** | **A** | **G** | **C** | **C** | **A** | **A** | **G** | **A** | **T** | **T** | **A** | **T** | **T** | **A** | **T** | **T** | **G** | **T** | **G** | **A** | **C** | **G** | **A** | **G** | **T** | **T** | **T** | **T** | **A** | **T** | **T** | **G** | **A** | **T** | **G** | **A** | **T** | **G** | **A** | **A** | **A** | **A** | **A** | **G** | **T** | **G** | **T** | **A** | **T** | **C** | **C** | **G** | **G** | **C** | **G** | **A** | **T** | **T** | **G** | **A** | **A** | **G** | **C** | **T** | **G** | **G** | **T** | **G** | **C** | **A** | **G** | **C** | **G** | **G** | **G** | **C** | **T** | **A** | **C** | **C** | **T** | **A** | **T** | **T** | **A** | **A** | **A** | **A** | **A** | **C** | **A** | **T** | **C** | **A** | **A** | **C** | **A** | **G** | **C** | **A** | **C** | **A** | **T** | **G** | **A** | **G** | **A** | **T** | **T** | **G** | **C** | **T** | **G** | **A** | **T** | **G** | **C** | **A** | **A** | **T** | **T** | **C** | **G** | **G** | **G** | **C** | **G** | **A** | **C** | **T** | **T** | **A** | **T** | **C** | **G** | **C** | **G** | **G** | **A** | **G** | **A** | **G** | **C** | **G** | **T** | **G** | **T** | **G** | **T** | **T** | **G** | **G** | **A** | **A** | **C** | **C** | **T** | **G** | **A** | **A** | **G** | **T** | **G** | **A** | **C** | **G** | **C** | **A** | **T** | **A** | **A** | **G** | **A** | **T** | **G** | **A** | **T** | **G** | **G** | **A** | **A** | **C** | **G** | **G** | **T** | **T** | **A** | **A** | **C** | **A** | **A** | **A** | **A** | **A** | **A** | **A** | **C** | **A** | **A** | **G** | **A** | **G** | **C** | **C** | **G** | **G** | **T** | **G** | **T** | **T** | **G** | **C** | **A** | **C** | **G** | **A** | **A** | **G** | **A** | **T** | **T** | **T** | **G** | **A** | **C** | **A** | **A** | **A** | **C** | **C** | **G** | **G** | **G** | **A** | **A** | **C** | **A** | **C** | **G** | **A** | **A** | **A** | **T** | **T** | **T** | **T** | **A** | **A** | **T** | **G** | **T** | **T** | **G** | **A** | **T** | **T** | **G** | **C** | **A** | **C** | **A** | **A** | **G** | **G** | **T** | **A** | **A** | **A** | **A** | **G** | **T** | **A** | **A** | **T** | **C** | **A** | **G** | **G** | **A** | **A** | **A** | **T** | **A** | **G** | **C** | **T** | **G** | **A** | **T** | **G** | **A** | **A** | **C** | **T** | **C** | **T** | **T** | **T** | **A** | **T** | **C** | **A** | **C** | **T** | **T** | **T** | **G** | **A** | **A** | **A** | **A** | **C** | **A** | **G** | **T** | **T** | **A** | **A** | **A** | **A** | **C** | **A** | **C** | **A** | **T** | **G** | **T** | **T** | **T** | **C** | **A** | **A** | **A** | **C** | **A** | **T** | **T** | **T** | **T** | **A** | **G** | **C** | **A** | **A** | **A** | **A** | **C** | **T** | **A** | **G** | **A** | **T** | **G** | **T** | **G** | **G** | **A** | **T** | **G** | **A** | **T** | **C** | **G** | **G** | **A** | **C** | **C** | **C** | **A** | **A** | **G** | **C** | **G** | **G** | **C** | **G** | **A** | **T** | **T** | **T** | **A** | **T** | **G** | **C** | **T** | **T** | **T** | **T** | **C** | **A** | **A** | **C** | **A** | **T** | **G** | **G** | **T** | **T** | **T** | **A** | **G** | **C** | **C** | **A** | **A** | **A** | **-** | **-** | **-** |
| **N60114F** | **G** | **T** | **G** | **A** | **T** | **C** | **A** | **A** | **A** | **G** | **T** | **A** | **A** | **T** | **G** | **T** | **T** | **A** | **G** | **T** | **G** | **G** | **A** | **T** | **G** | **A** | **C** | **C** | **A** | **T** | **G** | **A** | **A** | **A** | **T** | **G** | **G** | **T** | **C** | **C** | **G** | **T** | **T** | **T** | **A** | **G** | **G** | **C** | **G** | **T** | **T** | **T** | **C** | **A** | **T** | **C** | **A** | **T** | **A** | **T** | **T** | **T** | **A** | **T** | **C** | **T** | **A** | **T** | **T** | **C** | **A** | **A** | **G** | **A** | **G** | **G** | **A** | **T** | **A** | **T** | **A** | **G** | **A** | **A** | **G** | **T** | **C** | **G** | **T** | **A** | **G** | **G** | **C** | **G** | **A** | **A** | **G** | **C** | **A** | **G** | **A** | **A** | **A** | **A** | **C** | **G** | **G** | **T** | **A** | **A** | **G** | **A** | **T** | **T** | **G** | **G** | **C** | **T** | **A** | **T** | **G** | **A** | **A** | **A** | **A** | **A** | **G** | **C** | **A** | **T** | **T** | **G** | **G** | **A** | **A** | **C** | **T** | **A** | **C** | **G** | **T** | **C** | **C** | **A** | **G** | **A** | **T** | **G** | **T** | **T** | **A** | **T** | **T** | **T** | **T** | **G** | **A** | **T** | **G** | **G** | **A** | **T** | **T** | **T** | **G** | **G** | **T** | **A** | **A** | **T** | **G** | **G** | **A** | **A** | **G** | **A** | **A** | **A** | **T** | **G** | **G** | **A** | **C** | **G** | **G** | **C** | **A** | **T** | **T** | **G** | **A** | **T** | **T** | **C** | **A** | **A** | **C** | **C** | **A** | **A** | **A** | **G** | **C** | **G** | **A** | **T** | **C** | **T** | **T** | **G** | **A** | **A** | **A** | **G** | **A** | **T** | **T** | **G** | **G** | **C** | **C** | **A** | **G** | **A** | **A** | **G** | **C** | **C** | **A** | **A** | **G** | **A** | **T** | **T** | **A** | **T** | **T** | **A** | **T** | **T** | **G** | **T** | **G** | **A** | **C** | **G** | **A** | **G** | **T** | **T** | **T** | **T** | **A** | **T** | **T** | **G** | **A** | **T** | **G** | **A** | **T** | **G** | **A** | **A** | **A** | **A** | **A** | **G** | **T** | **G** | **T** | **A** | **T** | **C** | **C** | **G** | **G** | **C** | **G** | **A** | **T** | **T** | **G** | **A** | **A** | **G** | **C** | **T** | **G** | **G** | **T** | **G** | **C** | **A** | **G** | **C** | **G** | **G** | **G** | **C** | **T** | **A** | **C** | **C** | **T** | **A** | **T** | **T** | **A** | **A** | **A** | **A** | **A** | **C** | **A** | **T** | **C** | **A** | **A** | **C** | **A** | **G** | **C** | **A** | **C** | **A** | **T** | **G** | **A** | **G** | **A** | **T** | **T** | **G** | **C** | **T** | **G** | **A** | **T** | **G** | **C** | **A** | **A** | **T** | **T** | **C** | **G** | **G** | **G** | **C** | **G** | **A** | **C** | **T** | **T** | **A** | **T** | **C** | **G** | **C** | **G** | **G** | **A** | **G** | **A** | **G** | **C** | **G** | **T** | **G** | **T** | **G** | **T** | **T** | **G** | **G** | **A** | **A** | **C** | **C** | **T** | **G** | **A** | **A** | **G** | **T** | **G** | **A** | **C** | **G** | **C** | **A** | **T** | **A** | **A** | **G** | **A** | **T** | **G** | **A** | **T** | **G** | **G** | **A** | **A** | **C** | **G** | **G** | **T** | **T** | **A** | **A** | **C** | **A** | **A** | **A** | **A** | **A** | **A** | **A** | **C** | **A** | **A** | **G** | **A** | **G** | **C** | **C** | **G** | **G** | **T** | **G** | **T** | **T** | **G** | **C** | **A** | **C** | **G** | **A** | **A** | **G** | **A** | **T** | **T** | **T** | **G** | **A** | **C** | **A** | **A** | **A** | **C** | **C** | **G** | **G** | **G** | **A** | **A** | **C** | **A** | **C** | **G** | **A** | **A** | **A** | **T** | **T** | **T** | **T** | **A** | **A** | **T** | **G** | **T** | **T** | **G** | **A** | **T** | **T** | **G** | **C** | **A** | **C** | **A** | **A** | **G** | **G** | **T** | **A** | **A** | **A** | **A** | **G** | **T** | **A** | **A** | **T** | **C** | **A** | **G** | **G** | **A** | **A** | **A** | **T** | **A** | **G** | **C** | **T** | **G** | **A** | **T** | **G** | **A** | **A** | **C** | **T** | **C** | **T** | **T** | **T** | **A** | **T** | **C** | **A** | **C** | **T** | **T** | **T** | **G** | **A** | **A** | **A** | **A** | **C** | **A** | **G** | **T** | **T** | **A** | **A** | **A** | **A** | **C** | **A** | **C** | **A** | **T** | **G** | **T** | **T** | **T** | **C** | **A** | **A** | **A** | **C** | **A** | **T** | **T** | **T** | **T** | **A** | **G** | **C** | **A** | **A** | **A** | **A** | **C** | **T** | **A** | **G** | **A** | **T** | **G** | **T** | **G** | **G** | **A** | **T** | **G** | **A** | **T** | **C** | **G** | **G** | **A** | **C** | **C** | **C** | **A** | **A** | **G** | **C** | **G** | **G** | **C** | **G** | **A** | **T** | **T** | **T** | **A** | **T** | **G** | **C** | **T** | **T** | **T** | **T** | **C** | **A** | **A** | **C** | **A** | **T** | **G** | **G** | **T** | **T** | **T** | **A** | **G** | **C** | **C** | **A** | **A** | **A** | **-** | **-** | **-** |
 **SNP position** | 1 | . | . | . | . | 6 | . | . | . | . | . | 12 | 13 | . | 15 | 16 | . | . | . | . | 21 | . | . | . | . | . | . | . | . | . | . | . | . | . | . | . | . | . | 39 | . | . | 42 | . | . | . | . | . | 48 | . | . | 51 | . | . | 54 | . | . | 57 | . | . | . | . | . | . | . | . | . | . | . | 69 | . | . | . | . | . | 75 | . | . | 78 | . | . | 81 | . | . | . | . | . | 87 | 88 | . | 90 | . | . | 93 | . | . | . | . | . | 99 | 100 | . | . | . | . | 105 | . | . | 108 | 109 | 110 | 111 | 112 | 113 | 114 | . | . | . | . | . | . | . | . | 123 | . | . | 126 | . | . | 129 | 130 | . | . | 133 | 134 | . | . | . | 138 | . | . | 141 | . | . | 144 | . | . | 147 | . | . | 150 | . | . | . | . | . | 156 | . | . | . | . | . | 162 | 163 | . | 165 | 166 | . | 168 | . | . | . | . | . | . | . | . | . | . | . | . | . | . | 183 | . | . | 186 | . | . | 189 | . | . | 192 | . | . | . | . | . | 198 | . | . | . | . | . | 204 | . | . | 207 | . | . | 210 | . | . | . | . | . | . | . | . | . | 220 | 221 | . | 223 | . | . | . | . | 228 | . | . | 231 | . | . | 234 | . | . | . | . | . | 240 | . | . | 243 | . | . | 246 | . | . | . | . | . | . | . | . | . | . | . | . | . | . | 261 | . | . | 264 | . | . | . | . | . | 270 | . | . | . | . | . | 276 | . | . | 279 | . | . | . | . | . | 285 | . | . | 288 | . | . | 291 | . | . | . | . | . | 297 | . | . | 300 | . | . | 303 | 304 | . | 306 | . | . | 309 | . | . | 312 | . | . | 315 | . | . | 318 | . | . | 321 | . | . | 324 | . | . | . | . | . | 330 | . | . | 333 | . | . | . | . | . | . | . | . | 342 | . | . | 345 | 346 | . | . | 349 | . | 351 | . | . | 354 | 355 | . | 357 | . | 359 | 360 | . | . | 363 | . | . | . | . | . | 369 | . | . | 372 | 373 | . | 375 | . | . | 378 | . | . | 381 | . | . | . | . | . | . | . | . | 390 | 391 | 392 | 393 | . | . | 396 | . | . | . | . | . | . | . | . | . | 406 | 407 | 408 | 409 | . | 411 | . | 413 | 414 | 415 | 416 | 417 | 418 | 419 | . | 421 | . | 423 | . | . | 426 | . | . | 429 | . | . | 432 | 433 | . | 435 | . | . | 438 | . | . | . | . | . | 444 | 445 | . | 447 | . | . | 450 | . | . | 453 | . | . | 456 | . | . | . | 460 | . | 462 | . | . | . | . | . | . | . | . | 471 | . | . | . | 475 | . | . | . | . | . | 481 | . | 483 | 484 | . | . | . | . | 489 | . | . | . | . | . | . | . | . | . | . | . | . | . | . | . | . | . | 507 | . | . | 510 | . | . | 513 | . | . | . | 517 | . | 519 | . | . | 522 | . | . | 525 | . | . | 528 | 529 | . | . | . | . | . | . | . | . | . | . | 540 | . | . | . | . | . | 546 | . | . | . | . | . | 552 | . | . | 555 | . | . | 558 | . | . | 561 | . | . | 564 | . | . | 567 | . | . | 570 | 571 | . | . | . | . | 576 | . | . | 579 | . | . | 582 | . | . | 585 | . | . | 588 | . | . | 591 | . | . | . | . | . | 597 | . | . | 600 | . | . | 603 | . | . | . | 607 | . | 609 | . | . | 612 | 613 | . | . | . | . | 618 | . | . | . | . | . | 624 | . | 626 | 627 | . | . | . | . | . | . |

|  |
| --- |
|  |

|  |  |
| --- | --- |
|  |

|  |  |
| --- | --- |
| **Legend** | |
  | **Reference Sequence** |

  |
|  | **Detected SNPs** |
|  |  |
