# Supplementary material for: sraX: A Novel Comprehensive Resistome Analysis Tool
Source: Front Microbiol. 2020 Feb 5;11:52. doi: 10.3389/fmicb.2020.00052 (PMC7025521; doi:10.3389/fmicb.2020.00052)
Supplement: Supplementary file 4 [file Data_Sheet_3.ZIP › liaR/liaR_aa.html]

# liaR

|  |
| --- |
|  |

|  |
| --- |
|  |

|  |  |
| --- | --- |
| **Definition:** | LiaR is a response regulator found in the liaFSR signal transduction pathway. Mutations confer daptomycin resistance. |

|  |
| --- |
|  |

|  |
| --- |
|  |

|  |  |  |
| --- | --- | --- |
| **Reference organism:** | *Enterococcus faecalis V583* | [NCBI, CARD] |

|  |
| --- |
|  |

|  |
| --- |
|  |

|  |  |
| --- | --- |
| **Summary Data** | || **Parameter** | **Value_(n)_** || Total number of genomes | 198 |
| MSA length | 210 |
| Total number of SNPs | 36 |
| SNP density (# SNPs/MSA length) | 0.171 |

|  |
| --- |
|  |

|  |
| --- |
|  |

|  |
| --- |
|  |

|  |
| --- |
|  |

|  |  |  |  |  |
| --- | --- | --- | --- | --- |
| **SNPs conferring antibiotic resistance** | | | | || **Reference gene** | **Seq. pos.** | **Ref\_base** | **Alt\_base** | **Confirmation SNP?** |||  |  |  |  |  |
| --- | --- | --- | --- | --- |
| liaR 191 D N **No** | | | | |

|  |
| --- |
|  |

|  |
| --- |
|  |

|  |
| --- |
|  |

|  |  |  |  |  |  |  |  |  |  |  |  |  |  |  |  |  |  |  |  |  |  |  |  |  |  |  |  |  |  |  |  |  |  |  |  |  |  |  |  |  |  |  |  |  |  |  |  |  |  |  |  |  |  |  |  |  |  |  |  |  |  |  |  |  |  |  |  |  |  |  |  |  |  |  |  |  |  |  |  |  |  |  |  |  |  |  |  |  |  |  |  |  |  |  |  |  |  |  |  |  |  |  |  |  |  |  |  |  |  |  |  |  |  |  |  |  |  |  |  |  |  |  |  |  |  |  |  |  |  |  |  |  |  |  |  |  |  |  |  |  |  |  |  |  |  |  |  |  |  |  |  |  |  |  |  |  |  |  |  |  |  |  |  |  |  |  |  |  |  |  |  |  |  |  |  |  |  |  |  |  |  |  |  |  |  |  |  |  |  |  |  |  |  |  |  |  |  |  |  |  |  |  |  |  |  |  |  |  |  |  |  |
| --- | --- | --- | --- | --- | --- | --- | --- | --- | --- | --- | --- | --- | --- | --- | --- | --- | --- | --- | --- | --- | --- | --- | --- | --- | --- | --- | --- | --- | --- | --- | --- | --- | --- | --- | --- | --- | --- | --- | --- | --- | --- | --- | --- | --- | --- | --- | --- | --- | --- | --- | --- | --- | --- | --- | --- | --- | --- | --- | --- | --- | --- | --- | --- | --- | --- | --- | --- | --- | --- | --- | --- | --- | --- | --- | --- | --- | --- | --- | --- | --- | --- | --- | --- | --- | --- | --- | --- | --- | --- | --- | --- | --- | --- | --- | --- | --- | --- | --- | --- | --- | --- | --- | --- | --- | --- | --- | --- | --- | --- | --- | --- | --- | --- | --- | --- | --- | --- | --- | --- | --- | --- | --- | --- | --- | --- | --- | --- | --- | --- | --- | --- | --- | --- | --- | --- | --- | --- | --- | --- | --- | --- | --- | --- | --- | --- | --- | --- | --- | --- | --- | --- | --- | --- | --- | --- | --- | --- | --- | --- | --- | --- | --- | --- | --- | --- | --- | --- | --- | --- | --- | --- | --- | --- | --- | --- | --- | --- | --- | --- | --- | --- | --- | --- | --- | --- | --- | --- | --- | --- | --- | --- | --- | --- | --- | --- | --- | --- | --- | --- | --- | --- | --- | --- | --- | --- | --- | --- | --- | --- | --- | --- |
|  || **Enterococcus faecalis V583 (AAO82599.1)** | **M** | **I** | **K** | **V** | **M** | **L** | **V** | **D** | **D** | **H** | **E** | **M** | **V** | **R** | **L** | **G** | **V** | **S** | **S** | **Y** | **L** | **S** | **I** | **Q** | **E** | **D** | **I** | **E** | **V** | **V** | **G** | **E** | **A** | **E** | **N** | **G** | **K** | **I** | **G** | **Y** | **E** | **K** | **A** | **L** | **E** | **L** | **R** | **P** | **D** | **V** | **I** | **L** | **M** | **D** | **L** | **V** | **M** | **E** | **E** | **M** | **D** | **G** | **I** | **D** | **S** | **T** | **K** | **A** | **I** | **L** | **K** | **D** | **W** | **P** | **E** | **A** | **K** | **I** | **I** | **I** | **V** | **T** | **S** | **F** | **I** | **D** | **D** | **E** | **K** | **V** | **Y** | **P** | **A** | **I** | **E** | **A** | **G** | **A** | **A** | **G** | **Y** | **L** | **L** | **K** | **T** | **S** | **T** | **A** | **H** | **E** | **I** | **A** | **D** | **A** | **I** | **R** | **A** | **T** | **Y** | **R** | **G** | **E** | **R** | **V** | **L** | **E** | **P** | **E** | **V** | **T** | **H** | **K** | **M** | **M** | **E** | **R** | **L** | **T** | **K** | **K** | **Q** | **E** | **P** | **V** | **L** | **H** | **E** | **D** | **L** | **T** | **N** | **R** | **E** | **H** | **E** | **I** | **L** | **M** | **L** | **I** | **A** | **Q** | **G** | **K** | **S** | **N** | **Q** | **E** | **I** | **A** | **D** | **E** | **L** | **F** | **I** | **T** | **L** | **K** | **T** | **V** | **K** | **T** | **H** | **V** | **S** | **N** | **I** | **L** | **A** | **K** | **L** | **D** | **V** | **D** | **D** | **R** | **T** | **Q** | **A** | **A** | **I** | **Y** | **A** | **F** | **Q** | **H** | **G** | **L** | **A** | **K** |
| **N59936F** | **V** | **I** | **K** | **V** | **M** | **L** | **V** | **D** | **D** | **H** | **E** | **M** | **V** | **R** | **L** | **G** | **V** | **S** | **S** | **Y** | **L** | **S** | **I** | **Q** | **E** | **D** | **I** | **E** | **V** | **V** | **G** | **E** | **A** | **E** | **N** | **G** | **K** | **I** | **G** | **Y** | **E** | **K** | **A** | **L** | **E** | **L** | **R** | **P** | **D** | **V** | **I** | **L** | **M** | **D** | **L** | **V** | **M** | **E** | **E** | **M** | **D** | **G** | **I** | **D** | **S** | **T** | **K** | **A** | **I** | **L** | **K** | **D** | **W** | **P** | **E** | **A** | **K** | **I** | **I** | **I** | **V** | **T** | **S** | **F** | **I** | **D** | **D** | **E** | **K** | **V** | **Y** | **P** | **A** | **I** | **E** | **A** | **G** | **A** | **A** | **G** | **Y** | **L** | **L** | **K** | **T** | **S** | **T** | **A** | **H** | **E** | **I** | **A** | **D** | **A** | **I** | **R** | **A** | **T** | **Y** | **R** | **G** | **E** | **R** | **V** | **L** | **E** | **P** | **E** | **V** | **T** | **H** | **K** | **M** | **M** | **E** | **R** | **L** | **T** | **K** | **K** | **Q** | **E** | **P** | **V** | **L** | **H** | **E** | **D** | **L** | **T** | **N** | **R** | **E** | **H** | **E** | **I** | **L** | **M** | **L** | **I** | **A** | **Q** | **G** | **K** | **S** | **N** | **Q** | **E** | **I** | **A** | **D** | **E** | **L** | **F** | **I** | **T** | **L** | **K** | **T** | **V** | **K** | **T** | **H** | **V** | **S** | **N** | **I** | **L** | **A** | **K** | **L** | **D** | **V** | **D** | **D** | **R** | **T** | **Q** | **A** | **A** | **I** | **Y** | **A** | **F** | **Q** | **H** | **G** | **L** | **A** | **K** |
| **N55268** | **V** | **I** | **K** | **V** | **M** | **L** | **V** | **D** | **D** | **H** | **E** | **M** | **V** | **R** | **L** | **G** | **V** | **S** | **S** | **Y** | **L** | **S** | **I** | **Q** | **E** | **D** | **I** | **E** | **V** | **V** | **G** | **E** | **A** | **E** | **N** | **G** | **K** | **I** | **G** | **Y** | **E** | **K** | **A** | **L** | **E** | **L** | **R** | **P** | **D** | **V** | **I** | **L** | **M** | **D** | **L** | **V** | **M** | **E** | **E** | **M** | **D** | **G** | **I** | **D** | **S** | **T** | **K** | **A** | **I** | **L** | **K** | **D** | **W** | **P** | **E** | **A** | **K** | **I** | **I** | **I** | **V** | **T** | **S** | **F** | **I** | **D** | **D** | **E** | **K** | **V** | **Y** | **P** | **A** | **I** | **E** | **A** | **G** | **A** | **A** | **G** | **Y** | **L** | **L** | **K** | **T** | **S** | **T** | **A** | **H** | **E** | **I** | **A** | **D** | **A** | **I** | **R** | **A** | **T** | **Y** | **R** | **G** | **E** | **R** | **V** | **L** | **E** | **P** | **E** | **V** | **T** | **H** | **K** | **M** | **M** | **E** | **R** | **L** | **T** | **K** | **K** | **Q** | **E** | **P** | **V** | **L** | **H** | **E** | **D** | **L** | **T** | **N** | **R** | **E** | **H** | **E** | **I** | **L** | **M** | **L** | **I** | **A** | **Q** | **G** | **K** | **S** | **N** | **Q** | **E** | **I** | **A** | **D** | **E** | **L** | **F** | **I** | **T** | **L** | **K** | **T** | **V** | **K** | **T** | **H** | **V** | **S** | **N** | **I** | **L** | **A** | **K** | **L** | **D** | **V** | **D** | **D** | **R** | **T** | **Q** | **A** | **A** | **I** | **Y** | **A** | **F** | **Q** | **H** | **G** | **L** | **A** | **K** |
| **N55279** | **M** | **I** | **K** | **V** | **L** | **L** | **V** | **D** | **D** | **H** | **E** | **M** | **V** | **R** | **L** | **G** | **V** | **S** | **S** | **Y** | **L** | **S** | **I** | **Q** | **E** | **D** | **I** | **E** | **V** | **I** | **G** | **E** | **A** | **E** | **N** | **G** | **R** | **Q** | **G** | **Y** | **E** | **K** | **A** | **M** | **T** | **L** | **R** | **P** | **D** | **V** | **I** | **L** | **M** | **D** | **L** | **V** | **M** | **E** | **E** | **M** | **D** | **G** | **I** | **E** | **S** | **T** | **K** | **A** | **I** | **L** | **K** | **D** | **W** | **P** | **E** | **A** | **K** | **I** | **I** | **I** | **V** | **T** | **S** | **F** | **I** | **D** | **D** | **E** | **K** | **V** | **Y** | **P** | **A** | **I** | **E** | **A** | **G** | **A** | **A** | **G** | **Y** | **L** | **L** | **K** | **T** | **S** | **T** | **A** | **H** | **E** | **I** | **A** | **D** | **A** | **I** | **R** | **A** | **T** | **Q** | **R** | **G** | **E** | **R** | **V** | **L** | **E** | **P** | **E** | **V** | **T** | **T** | **K** | **M** | **M** | **E** | **K** | **M** | **S** | **R** | **R** | **N** | **E** | **P** | **V** | **L** | **H** | **E** | **E** | **L** | **T** | **N** | **R** | **E** | **N** | **E** | **I** | **L** | **M** | **L** | **I** | **S** | **E** | **G** | **K** | **S** | **N** | **Q** | **E** | **I** | **A** | **D** | **E** | **L** | **F** | **I** | **T** | **L** | **K** | **T** | **V** | **K** | **T** | **H** | **V** | **S** | **N** | **I** | **L** | **A** | **K** | **L** | **E** | **V** | **E** | **D** | **R** | **T** | **Q** | **A** | **A** | **I** | **Y** | **A** | **F** | **K** | **H** | **G** | **L** | **V** | **K** |
| **N54553** | **V** | **I** | **K** | **V** | **M** | **L** | **V** | **D** | **D** | **H** | **E** | **M** | **V** | **R** | **L** | **G** | **V** | **S** | **S** | **Y** | **L** | **S** | **I** | **Q** | **E** | **D** | **I** | **E** | **V** | **V** | **G** | **E** | **A** | **E** | **N** | **G** | **K** | **I** | **G** | **Y** | **E** | **K** | **A** | **L** | **E** | **L** | **R** | **P** | **D** | **V** | **I** | **L** | **M** | **D** | **L** | **V** | **M** | **E** | **E** | **M** | **D** | **G** | **I** | **D** | **S** | **T** | **K** | **A** | **I** | **L** | **K** | **D** | **W** | **P** | **E** | **A** | **K** | **I** | **I** | **I** | **V** | **T** | **S** | **F** | **I** | **D** | **D** | **E** | **K** | **V** | **Y** | **P** | **A** | **I** | **E** | **A** | **G** | **A** | **A** | **G** | **Y** | **L** | **L** | **K** | **T** | **S** | **T** | **A** | **H** | **E** | **I** | **A** | **D** | **A** | **I** | **R** | **A** | **T** | **Y** | **R** | **G** | **E** | **R** | **V** | **L** | **E** | **P** | **E** | **V** | **T** | **H** | **K** | **M** | **M** | **E** | **R** | **L** | **T** | **K** | **K** | **Q** | **E** | **P** | **V** | **L** | **H** | **E** | **D** | **L** | **T** | **N** | **R** | **E** | **H** | **E** | **I** | **L** | **M** | **L** | **I** | **A** | **Q** | **G** | **K** | **S** | **N** | **Q** | **E** | **I** | **A** | **D** | **E** | **L** | **F** | **I** | **T** | **L** | **K** | **T** | **V** | **K** | **T** | **H** | **V** | **S** | **N** | **I** | **L** | **A** | **K** | **L** | **D** | **V** | **D** | **D** | **R** | **T** | **Q** | **A** | **A** | **I** | **Y** | **A** | **F** | **Q** | **H** | **G** | **L** | **A** | **K** |
| **N59550F** | **V** | **I** | **K** | **V** | **M** | **L** | **V** | **D** | **D** | **H** | **E** | **M** | **V** | **R** | **L** | **G** | **V** | **S** | **S** | **Y** | **L** | **S** | **I** | **Q** | **E** | **D** | **I** | **E** | **V** | **V** | **G** | **E** | **A** | **E** | **N** | **G** | **K** | **I** | **G** | **Y** | **E** | **K** | **A** | **L** | **E** | **L** | **R** | **P** | **D** | **V** | **I** | **L** | **M** | **D** | **L** | **V** | **M** | **E** | **E** | **M** | **D** | **G** | **I** | **D** | **S** | **T** | **K** | **A** | **I** | **L** | **K** | **D** | **W** | **P** | **E** | **A** | **K** | **I** | **I** | **I** | **V** | **T** | **S** | **F** | **I** | **D** | **D** | **E** | **K** | **V** | **Y** | **P** | **A** | **I** | **E** | **A** | **G** | **A** | **A** | **G** | **Y** | **L** | **L** | **K** | **T** | **S** | **T** | **A** | **H** | **E** | **I** | **A** | **D** | **A** | **I** | **R** | **A** | **T** | **Y** | **R** | **G** | **E** | **R** | **V** | **L** | **E** | **P** | **E** | **V** | **T** | **H** | **K** | **M** | **M** | **E** | **R** | **L** | **T** | **K** | **K** | **Q** | **E** | **P** | **V** | **L** | **H** | **E** | **D** | **L** | **T** | **N** | **R** | **E** | **H** | **E** | **I** | **L** | **M** | **L** | **I** | **A** | **Q** | **G** | **K** | **S** | **N** | **Q** | **E** | **I** | **A** | **D** | **E** | **L** | **F** | **I** | **T** | **L** | **K** | **T** | **V** | **K** | **T** | **H** | **V** | **S** | **N** | **I** | **L** | **A** | **K** | **L** | **D** | **V** | **D** | **D** | **R** | **T** | **Q** | **A** | **A** | **I** | **Y** | **A** | **F** | **Q** | **H** | **G** | **L** | **A** | **K** |
| **N59745F** | **M** | **I** | **K** | **V** | **L** | **L** | **V** | **D** | **D** | **H** | **E** | **M** | **V** | **R** | **L** | **G** | **V** | **S** | **S** | **Y** | **L** | **S** | **I** | **Q** | **E** | **D** | **I** | **E** | **V** | **I** | **G** | **E** | **A** | **E** | **N** | **G** | **R** | **Q** | **G** | **Y** | **E** | **K** | **A** | **M** | **T** | **L** | **R** | **P** | **D** | **V** | **I** | **L** | **M** | **D** | **L** | **V** | **M** | **E** | **E** | **M** | **D** | **G** | **I** | **E** | **S** | **T** | **K** | **A** | **I** | **L** | **K** | **D** | **W** | **P** | **E** | **A** | **K** | **I** | **I** | **I** | **V** | **T** | **S** | **F** | **I** | **D** | **D** | **E** | **K** | **V** | **Y** | **P** | **A** | **I** | **E** | **A** | **G** | **A** | **A** | **G** | **Y** | **L** | **L** | **K** | **T** | **S** | **T** | **A** | **H** | **E** | **I** | **A** | **D** | **A** | **I** | **R** | **A** | **T** | **Q** | **R** | **G** | **E** | **R** | **V** | **L** | **E** | **P** | **E** | **V** | **T** | **T** | **K** | **M** | **M** | **E** | **K** | **M** | **S** | **R** | **R** | **N** | **E** | **P** | **V** | **L** | **H** | **E** | **E** | **L** | **T** | **N** | **R** | **E** | **N** | **E** | **I** | **L** | **M** | **L** | **I** | **S** | **E** | **G** | **K** | **S** | **N** | **Q** | **E** | **I** | **A** | **D** | **E** | **L** | **F** | **I** | **T** | **L** | **K** | **T** | **V** | **K** | **T** | **H** | **V** | **S** | **N** | **I** | **L** | **A** | **K** | **L** | **E** | **V** | **E** | **D** | **R** | **T** | **Q** | **A** | **A** | **I** | **Y** | **A** | **F** | **K** | **H** | **G** | **L** | **V** | **K** |
| **N59910F** | **V** | **I** | **K** | **V** | **M** | **L** | **V** | **D** | **D** | **H** | **E** | **M** | **V** | **R** | **L** | **G** | **V** | **S** | **S** | **Y** | **L** | **S** | **I** | **Q** | **E** | **D** | **I** | **E** | **V** | **V** | **G** | **E** | **A** | **E** | **N** | **G** | **K** | **I** | **G** | **Y** | **E** | **K** | **A** | **L** | **E** | **L** | **R** | **P** | **D** | **V** | **I** | **L** | **M** | **D** | **L** | **V** | **M** | **E** | **E** | **M** | **D** | **G** | **I** | **D** | **S** | **T** | **K** | **A** | **I** | **L** | **K** | **D** | **W** | **P** | **E** | **A** | **K** | **I** | **I** | **I** | **V** | **T** | **S** | **F** | **I** | **D** | **D** | **E** | **K** | **V** | **Y** | **P** | **A** | **I** | **E** | **A** | **G** | **A** | **A** | **G** | **Y** | **L** | **L** | **K** | **T** | **S** | **T** | **A** | **H** | **E** | **I** | **A** | **D** | **A** | **I** | **R** | **A** | **T** | **Y** | **R** | **G** | **E** | **R** | **V** | **L** | **E** | **P** | **E** | **V** | **T** | **H** | **K** | **M** | **M** | **E** | **R** | **L** | **T** | **K** | **K** | **Q** | **E** | **P** | **V** | **L** | **H** | **E** | **D** | **L** | **T** | **N** | **R** | **E** | **H** | **E** | **I** | **L** | **M** | **L** | **I** | **A** | **Q** | **G** | **K** | **S** | **N** | **Q** | **E** | **I** | **A** | **D** | **E** | **L** | **F** | **I** | **T** | **L** | **K** | **T** | **V** | **K** | **T** | **H** | **V** | **S** | **N** | **I** | **L** | **A** | **K** | **L** | **D** | **V** | **D** | **D** | **R** | **T** | **Q** | **A** | **A** | **I** | **Y** | **A** | **F** | **Q** | **H** | **G** | **L** | **A** | **K** |
| **N53702** | **M** | **I** | **K** | **V** | **L** | **L** | **V** | **D** | **D** | **H** | **E** | **M** | **V** | **R** | **L** | **G** | **V** | **S** | **S** | **Y** | **L** | **S** | **I** | **Q** | **E** | **D** | **I** | **E** | **V** | **I** | **G** | **E** | **A** | **E** | **N** | **G** | **R** | **Q** | **G** | **Y** | **E** | **K** | **A** | **M** | **A** | **L** | **R** | **P** | **D** | **V** | **I** | **L** | **M** | **D** | **L** | **V** | **M** | **E** | **E** | **M** | **D** | **G** | **I** | **E** | **S** | **T** | **K** | **A** | **I** | **L** | **K** | **D** | **W** | **P** | **K** | **A** | **K** | **I** | **I** | **I** | **V** | **T** | **S** | **F** | **I** | **D** | **D** | **E** | **K** | **V** | **Y** | **P** | **A** | **I** | **E** | **A** | **G** | **A** | **A** | **G** | **Y** | **L** | **L** | **K** | **T** | **S** | **T** | **A** | **H** | **E** | **I** | **A** | **D** | **A** | **I** | **R** | **A** | **T** | **Q** | **R** | **G** | **E** | **R** | **V** | **L** | **E** | **P** | **E** | **V** | **T** | **T** | **K** | **M** | **M** | **E** | **K** | **M** | **S** | **R** | **R** | **N** | **D** | **P** | **V** | **L** | **H** | **E** | **E** | **L** | **T** | **N** | **R** | **E** | **N** | **E** | **I** | **L** | **M** | **L** | **I** | **S** | **E** | **G** | **K** | **S** | **N** | **Q** | **E** | **I** | **A** | **D** | **E** | **L** | **F** | **I** | **T** | **L** | **K** | **T** | **V** | **K** | **T** | **H** | **V** | **S** | **N** | **I** | **L** | **A** | **K** | **L** | **E** | **V** | **E** | **D** | **R** | **T** | **Q** | **A** | **A** | **I** | **Y** | **A** | **F** | **K** | **H** | **G** | **L** | **V** | **K** |
| **N54550** | **M** | **I** | **K** | **V** | **L** | **L** | **V** | **D** | **D** | **H** | **E** | **M** | **V** | **R** | **L** | **G** | **V** | **S** | **S** | **Y** | **L** | **S** | **I** | **Q** | **E** | **D** | **I** | **E** | **V** | **I** | **G** | **E** | **A** | **E** | **N** | **G** | **R** | **Q** | **G** | **Y** | **E** | **K** | **A** | **M** | **A** | **L** | **R** | **P** | **D** | **V** | **I** | **L** | **M** | **D** | **L** | **V** | **M** | **E** | **E** | **M** | **D** | **G** | **I** | **E** | **S** | **T** | **K** | **A** | **I** | **L** | **K** | **D** | **W** | **P** | **K** | **A** | **K** | **I** | **I** | **I** | **V** | **T** | **S** | **F** | **I** | **D** | **D** | **E** | **K** | **V** | **Y** | **P** | **A** | **I** | **E** | **A** | **G** | **A** | **A** | **G** | **Y** | **L** | **L** | **K** | **T** | **S** | **T** | **A** | **H** | **E** | **I** | **A** | **D** | **A** | **I** | **R** | **A** | **T** | **Q** | **R** | **G** | **E** | **R** | **V** | **L** | **E** | **P** | **E** | **V** | **T** | **T** | **K** | **M** | **M** | **E** | **K** | **M** | **S** | **R** | **R** | **Y** | **D** | **P** | **V** | **L** | **H** | **E** | **E** | **L** | **T** | **N** | **R** | **E** | **N** | **E** | **I** | **L** | **M** | **L** | **I** | **S** | **E** | **G** | **K** | **S** | **N** | **Q** | **E** | **I** | **A** | **D** | **E** | **L** | **F** | **I** | **T** | **L** | **K** | **T** | **V** | **K** | **T** | **H** | **V** | **S** | **N** | **I** | **L** | **A** | **K** | **L** | **E** | **V** | **E** | **D** | **R** | **T** | **Q** | **A** | **A** | **I** | **Y** | **A** | **F** | **K** | **H** | **G** | **L** | **V** | **K** |
| **N59508F** | **V** | **I** | **K** | **V** | **M** | **L** | **V** | **D** | **D** | **H** | **E** | **M** | **V** | **R** | **L** | **G** | **V** | **S** | **S** | **Y** | **L** | **S** | **I** | **Q** | **E** | **D** | **I** | **E** | **V** | **V** | **G** | **E** | **A** | **E** | **N** | **G** | **K** | **I** | **G** | **Y** | **E** | **K** | **A** | **L** | **E** | **L** | **R** | **P** | **D** | **V** | **I** | **L** | **M** | **D** | **L** | **V** | **M** | **E** | **E** | **M** | **D** | **G** | **I** | **D** | **S** | **T** | **K** | **A** | **I** | **L** | **K** | **D** | **W** | **P** | **E** | **A** | **K** | **I** | **I** | **I** | **V** | **T** | **S** | **F** | **I** | **D** | **D** | **E** | **K** | **V** | **Y** | **P** | **A** | **I** | **E** | **A** | **G** | **A** | **A** | **G** | **Y** | **L** | **L** | **K** | **T** | **S** | **T** | **A** | **H** | **E** | **I** | **A** | **D** | **A** | **I** | **R** | **A** | **T** | **Y** | **R** | **G** | **E** | **R** | **V** | **L** | **E** | **P** | **E** | **V** | **T** | **H** | **K** | **M** | **M** | **E** | **R** | **L** | **T** | **K** | **K** | **Q** | **E** | **P** | **V** | **L** | **H** | **E** | **D** | **L** | **T** | **N** | **R** | **E** | **H** | **E** | **I** | **L** | **M** | **L** | **I** | **A** | **Q** | **G** | **K** | **S** | **N** | **Q** | **E** | **I** | **A** | **D** | **E** | **L** | **F** | **I** | **T** | **L** | **K** | **T** | **V** | **K** | **T** | **H** | **V** | **S** | **N** | **I** | **L** | **A** | **K** | **L** | **D** | **V** | **D** | **D** | **R** | **T** | **Q** | **A** | **A** | **I** | **Y** | **A** | **F** | **Q** | **H** | **G** | **L** | **A** | **K** |
| **N60519F** | **M** | **I** | **K** | **V** | **L** | **L** | **V** | **D** | **D** | **H** | **E** | **M** | **V** | **R** | **L** | **G** | **V** | **S** | **S** | **Y** | **L** | **S** | **I** | **Q** | **E** | **D** | **I** | **E** | **V** | **I** | **G** | **E** | **A** | **E** | **N** | **G** | **R** | **Q** | **G** | **Y** | **E** | **K** | **A** | **M** | **A** | **L** | **R** | **P** | **D** | **V** | **I** | **L** | **M** | **D** | **L** | **V** | **M** | **E** | **E** | **M** | **D** | **G** | **I** | **E** | **S** | **T** | **K** | **A** | **I** | **L** | **K** | **D** | **W** | **P** | **K** | **A** | **K** | **I** | **I** | **I** | **V** | **T** | **S** | **F** | **I** | **D** | **D** | **E** | **K** | **V** | **Y** | **P** | **A** | **I** | **E** | **A** | **G** | **A** | **A** | **G** | **Y** | **L** | **L** | **K** | **T** | **S** | **T** | **A** | **H** | **E** | **I** | **A** | **D** | **A** | **I** | **R** | **A** | **T** | **Q** | **R** | **G** | **E** | **R** | **V** | **L** | **E** | **P** | **E** | **V** | **T** | **T** | **K** | **M** | **M** | **E** | **K** | **M** | **S** | **R** | **R** | **N** | **D** | **P** | **V** | **L** | **H** | **E** | **E** | **L** | **T** | **N** | **R** | **E** | **N** | **E** | **I** | **L** | **M** | **L** | **I** | **S** | **E** | **G** | **K** | **S** | **N** | **Q** | **E** | **I** | **A** | **D** | **E** | **L** | **F** | **I** | **T** | **L** | **K** | **T** | **V** | **K** | **T** | **H** | **V** | **S** | **N** | **I** | **L** | **A** | **K** | **L** | **E** | **V** | **E** | **D** | **R** | **T** | **Q** | **A** | **A** | **I** | **Y** | **A** | **F** | **K** | **H** | **G** | **L** | **V** | **K** |
| **N52789** | **V** | **I** | **K** | **V** | **M** | **L** | **V** | **D** | **D** | **H** | **E** | **M** | **V** | **R** | **L** | **G** | **V** | **S** | **S** | **Y** | **L** | **S** | **I** | **Q** | **E** | **D** | **I** | **E** | **V** | **V** | **G** | **E** | **A** | **E** | **N** | **G** | **K** | **I** | **G** | **Y** | **E** | **K** | **A** | **L** | **E** | **L** | **R** | **P** | **D** | **V** | **I** | **L** | **M** | **D** | **L** | **V** | **M** | **E** | **E** | **M** | **D** | **G** | **I** | **D** | **S** | **T** | **K** | **A** | **I** | **L** | **K** | **D** | **W** | **P** | **E** | **A** | **K** | **I** | **I** | **I** | **V** | **T** | **S** | **F** | **I** | **D** | **D** | **E** | **K** | **V** | **Y** | **P** | **A** | **I** | **E** | **A** | **G** | **A** | **A** | **G** | **Y** | **L** | **L** | **K** | **T** | **S** | **T** | **A** | **H** | **E** | **I** | **A** | **D** | **A** | **I** | **R** | **A** | **T** | **Y** | **R** | **G** | **E** | **R** | **V** | **L** | **E** | **P** | **E** | **V** | **T** | **H** | **K** | **M** | **M** | **E** | **R** | **L** | **T** | **K** | **K** | **Q** | **E** | **P** | **V** | **L** | **H** | **E** | **D** | **L** | **T** | **N** | **R** | **E** | **H** | **E** | **I** | **L** | **M** | **L** | **I** | **A** | **Q** | **G** | **K** | **S** | **N** | **Q** | **E** | **I** | **A** | **D** | **E** | **L** | **F** | **I** | **T** | **L** | **K** | **T** | **V** | **K** | **T** | **H** | **V** | **S** | **N** | **I** | **L** | **A** | **K** | **L** | **D** | **V** | **D** | **D** | **R** | **T** | **Q** | **A** | **A** | **I** | **Y** | **A** | **F** | **Q** | **H** | **G** | **L** | **A** | **K** |
| **N54574** | **V** | **I** | **K** | **V** | **M** | **L** | **V** | **D** | **D** | **H** | **E** | **M** | **V** | **R** | **L** | **G** | **V** | **S** | **S** | **Y** | **L** | **S** | **I** | **Q** | **E** | **D** | **I** | **E** | **V** | **V** | **G** | **E** | **A** | **E** | **N** | **G** | **K** | **I** | **G** | **Y** | **E** | **K** | **A** | **L** | **E** | **L** | **R** | **P** | **D** | **V** | **I** | **L** | **M** | **D** | **L** | **V** | **M** | **E** | **E** | **M** | **D** | **G** | **I** | **D** | **S** | **T** | **K** | **A** | **I** | **L** | **K** | **D** | **W** | **P** | **E** | **A** | **K** | **I** | **I** | **I** | **V** | **T** | **S** | **F** | **I** | **D** | **D** | **E** | **K** | **V** | **Y** | **P** | **A** | **I** | **E** | **A** | **G** | **A** | **A** | **G** | **Y** | **L** | **L** | **K** | **T** | **S** | **T** | **A** | **H** | **E** | **I** | **A** | **D** | **A** | **I** | **R** | **A** | **T** | **Y** | **R** | **G** | **E** | **R** | **V** | **L** | **E** | **P** | **E** | **V** | **T** | **H** | **K** | **M** | **M** | **E** | **R** | **L** | **T** | **K** | **K** | **Q** | **E** | **P** | **V** | **L** | **H** | **E** | **D** | **L** | **T** | **N** | **R** | **E** | **H** | **E** | **I** | **L** | **M** | **L** | **I** | **A** | **Q** | **G** | **K** | **S** | **N** | **Q** | **E** | **I** | **A** | **D** | **E** | **L** | **F** | **I** | **T** | **L** | **K** | **T** | **V** | **K** | **T** | **H** | **V** | **S** | **N** | **I** | **L** | **A** | **K** | **L** | **D** | **V** | **D** | **D** | **R** | **T** | **Q** | **A** | **A** | **I** | **Y** | **T** | **F** | **Q** | **H** | **G** | **L** | **A** | **K** |
| **N54070** | **M** | **I** | **K** | **V** | **L** | **L** | **V** | **D** | **D** | **H** | **E** | **M** | **V** | **R** | **L** | **G** | **V** | **S** | **S** | **Y** | **L** | **S** | **I** | **Q** | **E** | **D** | **I** | **E** | **V** | **I** | **G** | **E** | **A** | **E** | **N** | **G** | **R** | **Q** | **G** | **Y** | **E** | **K** | **A** | **M** | **A** | **L** | **R** | **P** | **D** | **V** | **I** | **L** | **M** | **D** | **L** | **V** | **M** | **E** | **E** | **M** | **D** | **G** | **I** | **E** | **S** | **T** | **K** | **A** | **I** | **L** | **K** | **D** | **W** | **P** | **K** | **A** | **K** | **I** | **I** | **I** | **V** | **T** | **S** | **F** | **I** | **D** | **D** | **E** | **K** | **V** | **Y** | **P** | **A** | **I** | **E** | **A** | **G** | **A** | **A** | **G** | **Y** | **L** | **L** | **K** | **T** | **S** | **T** | **A** | **H** | **E** | **I** | **A** | **D** | **A** | **I** | **R** | **A** | **T** | **Q** | **R** | **G** | **E** | **R** | **V** | **L** | **E** | **P** | **E** | **V** | **T** | **T** | **K** | **M** | **M** | **E** | **K** | **M** | **S** | **R** | **R** | **N** | **D** | **P** | **V** | **L** | **H** | **E** | **E** | **L** | **T** | **N** | **R** | **E** | **N** | **E** | **I** | **L** | **M** | **L** | **I** | **S** | **E** | **G** | **K** | **S** | **N** | **Q** | **E** | **I** | **A** | **D** | **E** | **L** | **F** | **I** | **T** | **L** | **K** | **T** | **V** | **K** | **T** | **H** | **V** | **S** | **N** | **I** | **L** | **A** | **K** | **L** | **E** | **V** | **E** | **D** | **R** | **T** | **Q** | **A** | **A** | **I** | **Y** | **A** | **F** | **K** | **H** | **G** | **L** | **V** | **K** |
| **N60324F** | **M** | **I** | **K** | **V** | **L** | **L** | **V** | **D** | **D** | **H** | **E** | **M** | **V** | **R** | **L** | **G** | **V** | **S** | **S** | **Y** | **L** | **S** | **I** | **Q** | **E** | **D** | **I** | **E** | **V** | **I** | **G** | **E** | **A** | **E** | **N** | **G** | **R** | **Q** | **G** | **Y** | **E** | **K** | **A** | **M** | **A** | **L** | **R** | **P** | **D** | **V** | **I** | **L** | **M** | **D** | **L** | **V** | **M** | **E** | **E** | **M** | **D** | **G** | **I** | **E** | **S** | **T** | **K** | **A** | **I** | **L** | **K** | **D** | **W** | **P** | **K** | **A** | **K** | **I** | **I** | **I** | **V** | **T** | **S** | **F** | **I** | **D** | **D** | **E** | **K** | **V** | **Y** | **P** | **A** | **I** | **E** | **A** | **G** | **A** | **A** | **G** | **Y** | **L** | **L** | **K** | **T** | **S** | **T** | **A** | **H** | **E** | **I** | **A** | **D** | **A** | **I** | **R** | **A** | **T** | **Q** | **R** | **G** | **E** | **R** | **V** | **L** | **E** | **P** | **E** | **V** | **T** | **T** | **K** | **M** | **M** | **E** | **K** | **M** | **S** | **R** | **R** | **N** | **D** | **P** | **V** | **L** | **H** | **E** | **E** | **L** | **T** | **N** | **R** | **E** | **N** | **E** | **I** | **L** | **M** | **L** | **I** | **S** | **E** | **G** | **K** | **S** | **N** | **Q** | **E** | **I** | **A** | **D** | **E** | **L** | **F** | **I** | **T** | **L** | **K** | **T** | **V** | **K** | **T** | **H** | **V** | **S** | **N** | **I** | **L** | **A** | **K** | **L** | **E** | **V** | **E** | **D** | **R** | **T** | **Q** | **A** | **A** | **I** | **Y** | **A** | **F** | **K** | **H** | **G** | **L** | **V** | **K** |
| **N52690** | **M** | **I** | **K** | **V** | **L** | **L** | **V** | **D** | **D** | **H** | **E** | **M** | **V** | **R** | **L** | **G** | **V** | **S** | **S** | **Y** | **L** | **S** | **I** | **Q** | **E** | **D** | **I** | **E** | **V** | **I** | **G** | **E** | **A** | **E** | **N** | **G** | **R** | **Q** | **G** | **Y** | **E** | **K** | **A** | **M** | **A** | **L** | **R** | **P** | **D** | **V** | **I** | **L** | **M** | **D** | **L** | **V** | **M** | **E** | **E** | **M** | **D** | **G** | **I** | **E** | **S** | **T** | **K** | **A** | **I** | **L** | **K** | **D** | **W** | **P** | **K** | **A** | **K** | **I** | **I** | **I** | **V** | **T** | **S** | **F** | **I** | **D** | **D** | **E** | **K** | **V** | **Y** | **P** | **A** | **I** | **E** | **A** | **G** | **A** | **A** | **G** | **Y** | **L** | **L** | **K** | **T** | **S** | **T** | **A** | **H** | **E** | **I** | **A** | **D** | **A** | **I** | **R** | **A** | **T** | **Q** | **R** | **G** | **E** | **R** | **V** | **L** | **E** | **P** | **E** | **V** | **T** | **T** | **K** | **M** | **M** | **E** | **K** | **M** | **S** | **R** | **R** | **N** | **D** | **P** | **V** | **L** | **H** | **E** | **E** | **L** | **T** | **N** | **R** | **E** | **N** | **E** | **I** | **L** | **M** | **L** | **I** | **S** | **E** | **G** | **K** | **S** | **N** | **Q** | **E** | **I** | **A** | **D** | **E** | **L** | **F** | **I** | **T** | **L** | **K** | **T** | **V** | **K** | **T** | **H** | **V** | **S** | **N** | **I** | **L** | **A** | **K** | **L** | **E** | **V** | **E** | **D** | **R** | **T** | **Q** | **A** | **A** | **I** | **Y** | **A** | **F** | **K** | **H** | **G** | **L** | **V** | **K** |
| **N59484F** | **M** | **I** | **K** | **V** | **L** | **L** | **V** | **D** | **D** | **H** | **E** | **M** | **V** | **R** | **L** | **G** | **V** | **S** | **S** | **Y** | **L** | **S** | **I** | **Q** | **E** | **D** | **I** | **E** | **V** | **I** | **G** | **E** | **A** | **E** | **N** | **G** | **R** | **Q** | **G** | **Y** | **E** | **K** | **A** | **M** | **A** | **L** | **R** | **P** | **D** | **V** | **I** | **L** | **M** | **D** | **L** | **V** | **M** | **E** | **E** | **M** | **D** | **G** | **I** | **E** | **S** | **T** | **K** | **A** | **I** | **L** | **K** | **D** | **W** | **P** | **K** | **A** | **K** | **I** | **I** | **I** | **V** | **T** | **S** | **F** | **I** | **D** | **D** | **E** | **K** | **V** | **Y** | **P** | **A** | **I** | **E** | **A** | **G** | **A** | **A** | **G** | **Y** | **L** | **L** | **K** | **T** | **S** | **T** | **A** | **H** | **E** | **I** | **A** | **D** | **A** | **I** | **R** | **A** | **T** | **Q** | **R** | **G** | **E** | **R** | **V** | **L** | **E** | **P** | **E** | **V** | **T** | **T** | **K** | **M** | **M** | **E** | **K** | **M** | **S** | **R** | **R** | **Y** | **D** | **P** | **V** | **L** | **H** | **E** | **E** | **L** | **T** | **N** | **R** | **E** | **N** | **E** | **I** | **L** | **M** | **L** | **I** | **S** | **E** | **G** | **K** | **S** | **N** | **Q** | **E** | **I** | **A** | **D** | **E** | **L** | **F** | **I** | **T** | **L** | **K** | **T** | **V** | **K** | **T** | **H** | **V** | **S** | **N** | **I** | **L** | **A** | **K** | **L** | **E** | **V** | **E** | **D** | **R** | **T** | **Q** | **A** | **A** | **I** | **Y** | **A** | **F** | **K** | **H** | **G** | **L** | **V** | **K** |
| **N59600F** | **V** | **I** | **K** | **V** | **M** | **L** | **V** | **D** | **D** | **H** | **E** | **M** | **V** | **R** | **L** | **G** | **V** | **S** | **S** | **Y** | **L** | **S** | **I** | **Q** | **E** | **D** | **I** | **E** | **V** | **V** | **G** | **E** | **A** | **E** | **N** | **G** | **K** | **I** | **G** | **Y** | **E** | **K** | **A** | **L** | **E** | **L** | **R** | **P** | **D** | **V** | **I** | **L** | **M** | **D** | **L** | **V** | **M** | **E** | **E** | **M** | **D** | **G** | **I** | **D** | **S** | **T** | **K** | **A** | **I** | **L** | **K** | **D** | **W** | **P** | **E** | **A** | **K** | **I** | **I** | **I** | **V** | **T** | **S** | **F** | **I** | **D** | **D** | **E** | **K** | **V** | **Y** | **P** | **A** | **I** | **E** | **A** | **G** | **A** | **A** | **G** | **Y** | **L** | **L** | **K** | **T** | **S** | **T** | **A** | **H** | **E** | **I** | **A** | **D** | **A** | **I** | **R** | **A** | **T** | **Y** | **R** | **G** | **E** | **R** | **V** | **L** | **E** | **P** | **E** | **V** | **T** | **H** | **K** | **M** | **M** | **E** | **R** | **L** | **T** | **K** | **K** | **Q** | **E** | **P** | **V** | **L** | **H** | **E** | **D** | **L** | **T** | **N** | **R** | **E** | **H** | **E** | **I** | **L** | **M** | **L** | **I** | **A** | **Q** | **G** | **K** | **S** | **N** | **Q** | **E** | **I** | **A** | **D** | **E** | **L** | **F** | **I** | **T** | **L** | **K** | **T** | **V** | **K** | **T** | **H** | **V** | **S** | **N** | **I** | **L** | **A** | **K** | **L** | **D** | **V** | **D** | **D** | **R** | **T** | **Q** | **A** | **A** | **I** | **Y** | **A** | **F** | **Q** | **H** | **G** | **L** | **A** | **K** |
| **N59457F** | **V** | **I** | **K** | **V** | **M** | **L** | **V** | **D** | **D** | **H** | **E** | **M** | **V** | **R** | **L** | **G** | **V** | **S** | **S** | **Y** | **L** | **S** | **I** | **Q** | **E** | **D** | **I** | **E** | **V** | **V** | **G** | **E** | **A** | **E** | **N** | **G** | **K** | **I** | **G** | **Y** | **E** | **K** | **A** | **L** | **E** | **L** | **R** | **P** | **D** | **V** | **I** | **L** | **M** | **D** | **L** | **V** | **M** | **E** | **E** | **M** | **D** | **G** | **I** | **D** | **S** | **T** | **K** | **A** | **I** | **L** | **K** | **D** | **W** | **P** | **E** | **A** | **K** | **I** | **I** | **I** | **V** | **T** | **S** | **F** | **I** | **D** | **D** | **E** | **K** | **V** | **Y** | **P** | **A** | **I** | **E** | **A** | **G** | **A** | **A** | **G** | **Y** | **L** | **L** | **K** | **T** | **S** | **T** | **A** | **H** | **E** | **I** | **A** | **D** | **A** | **I** | **R** | **A** | **T** | **Y** | **R** | **G** | **E** | **R** | **V** | **L** | **E** | **P** | **E** | **V** | **T** | **H** | **K** | **M** | **M** | **E** | **R** | **L** | **T** | **K** | **K** | **Q** | **E** | **P** | **V** | **L** | **H** | **E** | **D** | **L** | **T** | **N** | **R** | **E** | **H** | **E** | **I** | **L** | **M** | **L** | **I** | **A** | **Q** | **G** | **K** | **S** | **N** | **Q** | **E** | **I** | **A** | **D** | **E** | **L** | **F** | **I** | **T** | **L** | **K** | **T** | **V** | **K** | **T** | **H** | **V** | **S** | **N** | **I** | **L** | **A** | **K** | **L** | **D** | **V** | **D** | **D** | **R** | **T** | **Q** | **A** | **A** | **I** | **Y** | **A** | **F** | **Q** | **H** | **G** | **L** | **A** | **K** |
| **N59409F** | **V** | **I** | **K** | **V** | **M** | **L** | **V** | **D** | **D** | **H** | **E** | **M** | **V** | **R** | **L** | **G** | **V** | **S** | **S** | **Y** | **L** | **S** | **I** | **Q** | **E** | **D** | **I** | **E** | **V** | **V** | **G** | **E** | **A** | **E** | **N** | **G** | **K** | **I** | **G** | **Y** | **E** | **K** | **A** | **L** | **E** | **L** | **R** | **P** | **D** | **V** | **I** | **L** | **M** | **D** | **L** | **V** | **M** | **E** | **E** | **M** | **D** | **G** | **I** | **D** | **S** | **T** | **K** | **A** | **I** | **L** | **K** | **D** | **W** | **P** | **E** | **A** | **K** | **I** | **I** | **I** | **V** | **T** | **S** | **F** | **I** | **D** | **D** | **E** | **K** | **V** | **Y** | **P** | **A** | **I** | **E** | **A** | **G** | **A** | **A** | **G** | **Y** | **L** | **L** | **K** | **T** | **S** | **T** | **A** | **H** | **E** | **I** | **A** | **D** | **A** | **I** | **R** | **A** | **T** | **Y** | **R** | **G** | **E** | **R** | **V** | **L** | **E** | **P** | **E** | **V** | **T** | **H** | **K** | **M** | **M** | **E** | **R** | **L** | **T** | **K** | **K** | **Q** | **E** | **P** | **V** | **L** | **H** | **E** | **D** | **L** | **T** | **N** | **R** | **E** | **H** | **E** | **I** | **L** | **M** | **L** | **I** | **A** | **Q** | **G** | **K** | **S** | **N** | **Q** | **E** | **I** | **A** | **D** | **E** | **L** | **F** | **I** | **T** | **L** | **K** | **T** | **V** | **K** | **T** | **H** | **V** | **S** | **N** | **I** | **L** | **A** | **K** | **L** | **D** | **V** | **D** | **D** | **R** | **T** | **Q** | **A** | **A** | **I** | **Y** | **A** | **F** | **Q** | **H** | **G** | **L** | **A** | **K** |
| **N52636** | **M** | **I** | **K** | **V** | **L** | **L** | **V** | **D** | **D** | **H** | **E** | **M** | **V** | **R** | **L** | **G** | **V** | **S** | **S** | **Y** | **L** | **S** | **I** | **Q** | **E** | **D** | **I** | **E** | **V** | **I** | **G** | **E** | **A** | **E** | **N** | **G** | **R** | **Q** | **G** | **Y** | **E** | **K** | **A** | **M** | **A** | **L** | **R** | **P** | **D** | **V** | **I** | **L** | **M** | **D** | **L** | **V** | **M** | **E** | **E** | **M** | **D** | **G** | **I** | **E** | **S** | **T** | **K** | **A** | **I** | **L** | **K** | **D** | **W** | **P** | **K** | **A** | **K** | **I** | **I** | **I** | **V** | **T** | **S** | **F** | **I** | **D** | **D** | **E** | **K** | **V** | **Y** | **P** | **A** | **I** | **E** | **A** | **G** | **A** | **A** | **G** | **Y** | **L** | **L** | **K** | **T** | **S** | **T** | **A** | **H** | **E** | **I** | **A** | **D** | **A** | **I** | **R** | **A** | **T** | **Q** | **R** | **G** | **E** | **R** | **V** | **L** | **E** | **P** | **E** | **V** | **T** | **T** | **K** | **M** | **M** | **E** | **K** | **M** | **S** | **R** | **R** | **N** | **D** | **P** | **V** | **L** | **H** | **E** | **E** | **L** | **T** | **N** | **R** | **E** | **N** | **E** | **I** | **L** | **M** | **L** | **I** | **S** | **E** | **G** | **K** | **S** | **N** | **Q** | **E** | **I** | **A** | **D** | **E** | **L** | **F** | **I** | **T** | **L** | **K** | **T** | **V** | **K** | **T** | **H** | **V** | **S** | **N** | **I** | **L** | **A** | **K** | **L** | **E** | **V** | **E** | **D** | **R** | **T** | **Q** | **A** | **A** | **I** | **Y** | **A** | **F** | **K** | **H** | **G** | **L** | **V** | **K** |
| **N54155** | **M** | **I** | **K** | **V** | **L** | **L** | **V** | **D** | **D** | **H** | **E** | **M** | **V** | **R** | **L** | **G** | **V** | **S** | **S** | **Y** | **L** | **S** | **I** | **Q** | **E** | **D** | **I** | **E** | **V** | **I** | **G** | **E** | **A** | **E** | **N** | **G** | **R** | **Q** | **G** | **Y** | **E** | **K** | **A** | **M** | **A** | **L** | **R** | **P** | **D** | **V** | **I** | **L** | **M** | **D** | **L** | **V** | **M** | **E** | **E** | **M** | **D** | **G** | **I** | **E** | **S** | **T** | **K** | **A** | **I** | **L** | **K** | **D** | **W** | **P** | **K** | **A** | **K** | **I** | **I** | **I** | **V** | **T** | **S** | **F** | **I** | **D** | **D** | **E** | **K** | **V** | **Y** | **P** | **A** | **I** | **E** | **A** | **G** | **A** | **A** | **G** | **Y** | **L** | **L** | **K** | **T** | **S** | **T** | **A** | **H** | **E** | **I** | **A** | **D** | **A** | **I** | **R** | **A** | **T** | **Q** | **R** | **G** | **E** | **R** | **V** | **L** | **E** | **P** | **E** | **V** | **T** | **T** | **K** | **M** | **M** | **E** | **K** | **M** | **S** | **R** | **R** | **N** | **D** | **P** | **V** | **L** | **H** | **E** | **E** | **L** | **T** | **N** | **R** | **E** | **N** | **E** | **I** | **L** | **M** | **L** | **I** | **S** | **E** | **G** | **K** | **S** | **N** | **Q** | **E** | **I** | **A** | **D** | **E** | **L** | **F** | **I** | **T** | **L** | **K** | **T** | **V** | **K** | **T** | **H** | **V** | **S** | **N** | **I** | **L** | **A** | **K** | **L** | **E** | **V** | **E** | **D** | **R** | **T** | **Q** | **A** | **A** | **I** | **Y** | **A** | **F** | **K** | **H** | **G** | **L** | **V** | **K** |
| **N59567F** | **M** | **I** | **K** | **V** | **L** | **L** | **V** | **D** | **D** | **H** | **E** | **M** | **V** | **R** | **L** | **G** | **V** | **S** | **S** | **Y** | **L** | **S** | **I** | **Q** | **E** | **D** | **I** | **E** | **V** | **I** | **G** | **E** | **A** | **E** | **N** | **G** | **R** | **Q** | **G** | **Y** | **E** | **K** | **A** | **M** | **A** | **L** | **R** | **P** | **D** | **V** | **I** | **L** | **M** | **D** | **L** | **V** | **M** | **E** | **E** | **M** | **D** | **G** | **I** | **E** | **S** | **T** | **K** | **A** | **I** | **L** | **K** | **D** | **W** | **P** | **K** | **A** | **K** | **I** | **I** | **I** | **V** | **T** | **S** | **F** | **I** | **D** | **D** | **E** | **K** | **V** | **Y** | **P** | **A** | **I** | **E** | **A** | **G** | **A** | **A** | **G** | **Y** | **L** | **L** | **K** | **T** | **S** | **T** | **A** | **H** | **E** | **I** | **A** | **D** | **A** | **I** | **R** | **A** | **T** | **Q** | **R** | **G** | **E** | **R** | **V** | **L** | **E** | **P** | **E** | **V** | **T** | **T** | **K** | **M** | **M** | **E** | **K** | **M** | **S** | **R** | **R** | **Y** | **D** | **P** | **V** | **L** | **H** | **E** | **E** | **L** | **T** | **N** | **R** | **E** | **N** | **E** | **I** | **L** | **M** | **L** | **I** | **S** | **E** | **G** | **K** | **S** | **N** | **Q** | **E** | **I** | **A** | **D** | **E** | **L** | **F** | **I** | **T** | **L** | **K** | **T** | **V** | **K** | **T** | **H** | **V** | **S** | **N** | **I** | **L** | **A** | **K** | **L** | **E** | **V** | **E** | **D** | **R** | **T** | **Q** | **A** | **A** | **I** | **Y** | **A** | **F** | **K** | **H** | **G** | **L** | **V** | **K** |
| **N59509F** | **V** | **I** | **K** | **V** | **M** | **L** | **V** | **D** | **D** | **H** | **E** | **M** | **V** | **R** | **L** | **G** | **V** | **S** | **S** | **Y** | **L** | **S** | **I** | **Q** | **E** | **D** | **I** | **E** | **V** | **V** | **G** | **E** | **A** | **E** | **N** | **G** | **K** | **I** | **G** | **Y** | **E** | **K** | **A** | **L** | **E** | **L** | **R** | **P** | **D** | **V** | **I** | **L** | **M** | **D** | **L** | **V** | **M** | **E** | **E** | **M** | **D** | **G** | **I** | **D** | **S** | **T** | **K** | **A** | **I** | **L** | **K** | **D** | **W** | **P** | **E** | **A** | **K** | **I** | **I** | **I** | **V** | **T** | **S** | **F** | **I** | **D** | **D** | **E** | **K** | **V** | **Y** | **P** | **A** | **I** | **E** | **A** | **G** | **A** | **A** | **G** | **Y** | **L** | **L** | **K** | **T** | **S** | **T** | **A** | **H** | **E** | **I** | **A** | **D** | **A** | **I** | **R** | **A** | **T** | **Y** | **R** | **G** | **E** | **R** | **V** | **L** | **E** | **P** | **E** | **V** | **T** | **H** | **K** | **M** | **M** | **E** | **R** | **L** | **T** | **K** | **K** | **Q** | **E** | **P** | **V** | **L** | **H** | **E** | **D** | **L** | **T** | **N** | **R** | **E** | **H** | **E** | **I** | **L** | **M** | **L** | **I** | **A** | **Q** | **G** | **K** | **S** | **N** | **Q** | **E** | **I** | **A** | **D** | **E** | **L** | **F** | **I** | **T** | **L** | **K** | **T** | **V** | **K** | **T** | **H** | **V** | **S** | **N** | **I** | **L** | **A** | **K** | **L** | **D** | **V** | **D** | **D** | **R** | **T** | **Q** | **A** | **A** | **I** | **Y** | **A** | **F** | **Q** | **H** | **G** | **L** | **A** | **K** |
| **N59836F** | **V** | **I** | **K** | **V** | **M** | **L** | **V** | **D** | **D** | **H** | **E** | **M** | **V** | **R** | **L** | **G** | **V** | **S** | **S** | **Y** | **L** | **S** | **I** | **Q** | **E** | **D** | **I** | **E** | **V** | **V** | **G** | **E** | **A** | **E** | **N** | **G** | **K** | **I** | **G** | **Y** | **E** | **K** | **A** | **L** | **E** | **L** | **R** | **P** | **D** | **V** | **I** | **L** | **M** | **D** | **L** | **V** | **M** | **E** | **E** | **M** | **D** | **G** | **I** | **D** | **S** | **T** | **K** | **A** | **I** | **L** | **K** | **D** | **W** | **P** | **E** | **A** | **K** | **I** | **I** | **I** | **V** | **T** | **S** | **F** | **I** | **D** | **D** | **E** | **K** | **V** | **Y** | **P** | **A** | **I** | **E** | **A** | **G** | **A** | **A** | **G** | **Y** | **L** | **L** | **K** | **T** | **S** | **T** | **A** | **H** | **E** | **I** | **A** | **D** | **A** | **I** | **R** | **A** | **T** | **Y** | **R** | **G** | **E** | **R** | **V** | **L** | **E** | **P** | **E** | **V** | **T** | **H** | **K** | **M** | **M** | **E** | **R** | **L** | **T** | **K** | **K** | **Q** | **E** | **P** | **V** | **L** | **H** | **E** | **D** | **L** | **T** | **N** | **R** | **E** | **H** | **E** | **I** | **L** | **M** | **L** | **I** | **A** | **Q** | **G** | **K** | **S** | **N** | **Q** | **E** | **I** | **A** | **D** | **E** | **L** | **F** | **I** | **T** | **L** | **K** | **T** | **V** | **K** | **T** | **H** | **V** | **S** | **N** | **I** | **L** | **A** | **K** | **L** | **D** | **V** | **D** | **D** | **R** | **T** | **Q** | **A** | **A** | **I** | **Y** | **A** | **F** | **Q** | **H** | **G** | **L** | **A** | **K** |
| **N59646F** | **V** | **I** | **K** | **V** | **M** | **L** | **V** | **D** | **D** | **H** | **E** | **M** | **V** | **R** | **L** | **G** | **V** | **S** | **S** | **Y** | **L** | **S** | **I** | **Q** | **E** | **D** | **I** | **E** | **V** | **V** | **G** | **E** | **A** | **E** | **N** | **G** | **K** | **I** | **G** | **Y** | **E** | **K** | **A** | **L** | **E** | **L** | **R** | **P** | **D** | **V** | **I** | **L** | **M** | **D** | **L** | **V** | **M** | **E** | **E** | **M** | **D** | **G** | **I** | **D** | **S** | **T** | **K** | **A** | **I** | **L** | **K** | **D** | **W** | **P** | **E** | **A** | **K** | **I** | **I** | **I** | **V** | **T** | **S** | **F** | **I** | **D** | **D** | **E** | **K** | **V** | **Y** | **P** | **A** | **I** | **E** | **A** | **G** | **A** | **A** | **G** | **Y** | **L** | **L** | **K** | **T** | **S** | **T** | **A** | **H** | **E** | **I** | **A** | **D** | **A** | **I** | **R** | **A** | **T** | **Y** | **R** | **G** | **E** | **R** | **V** | **L** | **E** | **P** | **E** | **V** | **T** | **H** | **K** | **M** | **M** | **E** | **R** | **L** | **T** | **K** | **K** | **Q** | **E** | **P** | **V** | **L** | **H** | **E** | **D** | **L** | **T** | **N** | **R** | **E** | **H** | **E** | **I** | **L** | **M** | **L** | **I** | **A** | **Q** | **G** | **K** | **S** | **N** | **Q** | **E** | **I** | **A** | **D** | **E** | **L** | **F** | **I** | **T** | **L** | **K** | **T** | **V** | **K** | **T** | **H** | **V** | **S** | **N** | **I** | **L** | **A** | **K** | **L** | **D** | **V** | **D** | **D** | **R** | **T** | **Q** | **A** | **A** | **I** | **Y** | **A** | **F** | **Q** | **H** | **G** | **L** | **A** | **K** |
| **N60490F** | **M** | **I** | **K** | **V** | **L** | **L** | **V** | **D** | **D** | **H** | **E** | **M** | **V** | **R** | **L** | **G** | **V** | **S** | **S** | **Y** | **L** | **S** | **I** | **Q** | **E** | **D** | **I** | **E** | **V** | **I** | **G** | **E** | **A** | **E** | **N** | **G** | **R** | **Q** | **G** | **Y** | **E** | **K** | **A** | **M** | **A** | **L** | **R** | **P** | **D** | **V** | **I** | **L** | **M** | **D** | **L** | **V** | **M** | **E** | **E** | **M** | **D** | **G** | **I** | **E** | **S** | **T** | **K** | **A** | **I** | **L** | **K** | **D** | **W** | **P** | **K** | **A** | **K** | **I** | **I** | **I** | **V** | **T** | **S** | **F** | **I** | **D** | **D** | **E** | **K** | **V** | **Y** | **P** | **A** | **I** | **E** | **A** | **G** | **A** | **A** | **G** | **Y** | **L** | **L** | **K** | **T** | **S** | **T** | **A** | **H** | **E** | **I** | **A** | **D** | **A** | **I** | **R** | **A** | **T** | **Q** | **R** | **G** | **E** | **R** | **V** | **L** | **E** | **P** | **E** | **V** | **T** | **T** | **K** | **M** | **M** | **E** | **K** | **M** | **S** | **R** | **R** | **Y** | **D** | **P** | **V** | **L** | **H** | **E** | **E** | **L** | **T** | **N** | **R** | **E** | **N** | **E** | **I** | **L** | **M** | **L** | **I** | **S** | **E** | **G** | **K** | **S** | **N** | **Q** | **E** | **I** | **A** | **D** | **E** | **L** | **F** | **I** | **T** | **L** | **K** | **T** | **V** | **K** | **T** | **H** | **V** | **S** | **N** | **I** | **L** | **A** | **K** | **L** | **E** | **V** | **E** | **D** | **R** | **T** | **Q** | **A** | **A** | **I** | **Y** | **A** | **F** | **K** | **H** | **G** | **L** | **V** | **K** |
| **N60357F** | **M** | **I** | **K** | **V** | **L** | **L** | **V** | **D** | **D** | **H** | **E** | **M** | **V** | **R** | **L** | **G** | **V** | **S** | **S** | **Y** | **L** | **S** | **I** | **Q** | **E** | **D** | **I** | **E** | **V** | **I** | **G** | **E** | **A** | **E** | **N** | **G** | **R** | **Q** | **G** | **Y** | **E** | **K** | **A** | **M** | **A** | **L** | **R** | **P** | **D** | **V** | **I** | **L** | **M** | **D** | **L** | **V** | **M** | **E** | **E** | **M** | **D** | **G** | **I** | **E** | **S** | **T** | **K** | **A** | **I** | **L** | **K** | **D** | **W** | **P** | **K** | **A** | **K** | **I** | **I** | **I** | **V** | **T** | **S** | **F** | **I** | **D** | **D** | **E** | **K** | **V** | **Y** | **P** | **A** | **I** | **E** | **A** | **G** | **A** | **A** | **G** | **Y** | **L** | **L** | **K** | **T** | **S** | **T** | **A** | **H** | **E** | **I** | **A** | **D** | **A** | **I** | **R** | **A** | **T** | **Q** | **R** | **G** | **E** | **R** | **V** | **L** | **E** | **P** | **E** | **V** | **T** | **T** | **K** | **M** | **M** | **E** | **K** | **M** | **S** | **R** | **R** | **N** | **D** | **P** | **V** | **L** | **H** | **E** | **E** | **L** | **T** | **N** | **R** | **E** | **N** | **E** | **I** | **L** | **M** | **L** | **I** | **S** | **E** | **G** | **K** | **S** | **N** | **Q** | **E** | **I** | **A** | **D** | **E** | **L** | **F** | **I** | **T** | **L** | **K** | **T** | **V** | **K** | **T** | **H** | **V** | **S** | **N** | **I** | **L** | **A** | **K** | **L** | **E** | **V** | **E** | **D** | **R** | **T** | **Q** | **A** | **A** | **I** | **Y** | **A** | **F** | **K** | **H** | **G** | **L** | **V** | **K** |
| **N55728** | **M** | **I** | **K** | **V** | **L** | **L** | **V** | **D** | **D** | **H** | **E** | **M** | **V** | **R** | **L** | **G** | **V** | **S** | **S** | **Y** | **L** | **S** | **I** | **Q** | **E** | **D** | **I** | **E** | **V** | **I** | **G** | **E** | **A** | **E** | **N** | **G** | **R** | **Q** | **G** | **Y** | **E** | **K** | **A** | **M** | **A** | **L** | **R** | **P** | **D** | **V** | **I** | **L** | **M** | **D** | **L** | **V** | **M** | **E** | **E** | **M** | **D** | **G** | **I** | **E** | **S** | **T** | **K** | **A** | **I** | **L** | **K** | **D** | **W** | **P** | **K** | **A** | **K** | **I** | **I** | **I** | **V** | **T** | **S** | **F** | **I** | **D** | **D** | **E** | **K** | **V** | **Y** | **P** | **A** | **I** | **E** | **A** | **G** | **A** | **A** | **G** | **Y** | **L** | **L** | **K** | **T** | **S** | **T** | **A** | **H** | **E** | **I** | **A** | **D** | **A** | **I** | **R** | **A** | **T** | **Q** | **R** | **G** | **E** | **R** | **V** | **L** | **E** | **P** | **E** | **V** | **T** | **T** | **K** | **M** | **M** | **E** | **K** | **M** | **S** | **R** | **R** | **N** | **D** | **P** | **V** | **L** | **H** | **E** | **E** | **L** | **T** | **N** | **R** | **E** | **N** | **E** | **I** | **L** | **M** | **L** | **I** | **S** | **E** | **G** | **K** | **S** | **N** | **Q** | **E** | **I** | **A** | **D** | **E** | **L** | **F** | **I** | **T** | **L** | **K** | **T** | **V** | **K** | **T** | **H** | **V** | **S** | **N** | **I** | **L** | **A** | **K** | **L** | **E** | **V** | **E** | **D** | **R** | **T** | **Q** | **A** | **A** | **I** | **Y** | **A** | **F** | **K** | **H** | **G** | **L** | **V** | **K** |
| **N59458F** | **V** | **I** | **K** | **V** | **M** | **L** | **V** | **D** | **D** | **H** | **E** | **M** | **V** | **R** | **L** | **G** | **V** | **S** | **S** | **Y** | **L** | **S** | **I** | **Q** | **E** | **D** | **I** | **E** | **V** | **V** | **G** | **E** | **A** | **E** | **N** | **G** | **K** | **I** | **G** | **Y** | **E** | **K** | **A** | **L** | **E** | **L** | **R** | **P** | **D** | **V** | **I** | **L** | **M** | **D** | **L** | **V** | **M** | **E** | **E** | **M** | **D** | **G** | **I** | **D** | **S** | **T** | **K** | **A** | **I** | **L** | **K** | **D** | **W** | **P** | **E** | **A** | **K** | **I** | **I** | **I** | **V** | **T** | **S** | **F** | **I** | **D** | **D** | **E** | **K** | **V** | **Y** | **P** | **A** | **I** | **E** | **A** | **G** | **A** | **A** | **G** | **Y** | **L** | **L** | **K** | **T** | **S** | **T** | **A** | **H** | **E** | **I** | **A** | **D** | **A** | **I** | **R** | **A** | **T** | **Y** | **R** | **G** | **E** | **R** | **V** | **L** | **E** | **P** | **E** | **V** | **T** | **H** | **K** | **M** | **M** | **E** | **R** | **L** | **T** | **K** | **K** | **Q** | **E** | **P** | **V** | **L** | **H** | **E** | **D** | **L** | **T** | **N** | **R** | **E** | **H** | **E** | **I** | **L** | **M** | **L** | **I** | **A** | **Q** | **G** | **K** | **S** | **N** | **Q** | **E** | **I** | **A** | **D** | **E** | **L** | **F** | **I** | **T** | **L** | **K** | **T** | **V** | **K** | **T** | **H** | **V** | **S** | **N** | **I** | **L** | **A** | **K** | **L** | **D** | **V** | **D** | **D** | **R** | **T** | **Q** | **A** | **A** | **I** | **Y** | **A** | **F** | **Q** | **H** | **G** | **L** | **A** | **K** |
| **N54156** | **V** | **I** | **K** | **V** | **M** | **L** | **V** | **D** | **D** | **H** | **E** | **M** | **V** | **R** | **L** | **G** | **V** | **S** | **S** | **Y** | **L** | **S** | **I** | **Q** | **E** | **D** | **I** | **E** | **V** | **V** | **G** | **E** | **A** | **E** | **N** | **G** | **K** | **I** | **G** | **Y** | **E** | **K** | **A** | **L** | **E** | **L** | **R** | **P** | **D** | **V** | **I** | **L** | **M** | **D** | **L** | **V** | **M** | **E** | **E** | **M** | **D** | **G** | **I** | **D** | **S** | **T** | **K** | **A** | **I** | **L** | **K** | **D** | **W** | **P** | **E** | **A** | **K** | **I** | **I** | **I** | **V** | **T** | **S** | **F** | **I** | **D** | **D** | **E** | **K** | **V** | **Y** | **P** | **A** | **I** | **E** | **A** | **G** | **A** | **A** | **G** | **Y** | **L** | **L** | **K** | **T** | **S** | **T** | **A** | **H** | **E** | **I** | **A** | **D** | **A** | **I** | **R** | **A** | **T** | **Y** | **R** | **G** | **E** | **R** | **V** | **L** | **E** | **P** | **E** | **V** | **T** | **H** | **K** | **M** | **M** | **E** | **R** | **L** | **T** | **K** | **K** | **Q** | **E** | **P** | **V** | **L** | **H** | **E** | **D** | **L** | **T** | **N** | **R** | **E** | **H** | **E** | **I** | **L** | **M** | **L** | **I** | **A** | **Q** | **G** | **K** | **S** | **N** | **Q** | **E** | **I** | **A** | **D** | **E** | **L** | **F** | **I** | **T** | **L** | **K** | **T** | **V** | **K** | **T** | **H** | **V** | **S** | **N** | **I** | **L** | **A** | **K** | **L** | **D** | **V** | **D** | **D** | **R** | **T** | **Q** | **A** | **A** | **I** | **Y** | **A** | **F** | **Q** | **H** | **G** | **L** | **A** | **K** |
| **N59624F** | **M** | **I** | **K** | **V** | **L** | **L** | **V** | **D** | **D** | **H** | **E** | **M** | **V** | **R** | **L** | **G** | **V** | **S** | **S** | **Y** | **L** | **S** | **I** | **Q** | **E** | **D** | **I** | **E** | **V** | **I** | **G** | **E** | **A** | **E** | **N** | **G** | **R** | **Q** | **G** | **Y** | **E** | **K** | **A** | **M** | **T** | **L** | **R** | **P** | **D** | **V** | **I** | **L** | **M** | **D** | **L** | **V** | **M** | **E** | **E** | **M** | **D** | **G** | **I** | **E** | **S** | **T** | **K** | **A** | **I** | **L** | **K** | **D** | **W** | **P** | **E** | **A** | **K** | **I** | **I** | **I** | **V** | **T** | **S** | **F** | **I** | **D** | **D** | **E** | **K** | **V** | **Y** | **P** | **A** | **I** | **E** | **A** | **G** | **A** | **A** | **G** | **Y** | **L** | **L** | **K** | **T** | **S** | **T** | **A** | **H** | **E** | **I** | **A** | **D** | **A** | **I** | **R** | **A** | **T** | **Q** | **R** | **G** | **E** | **R** | **V** | **L** | **E** | **P** | **E** | **V** | **T** | **T** | **K** | **M** | **M** | **E** | **K** | **M** | **S** | **R** | **R** | **N** | **E** | **P** | **V** | **L** | **H** | **E** | **E** | **L** | **T** | **N** | **R** | **E** | **N** | **E** | **I** | **L** | **M** | **L** | **I** | **S** | **E** | **G** | **K** | **S** | **N** | **Q** | **E** | **I** | **A** | **D** | **E** | **L** | **F** | **I** | **T** | **L** | **K** | **T** | **V** | **K** | **T** | **H** | **V** | **S** | **N** | **I** | **L** | **A** | **K** | **L** | **E** | **V** | **E** | **D** | **R** | **T** | **Q** | **A** | **A** | **I** | **Y** | **A** | **F** | **K** | **H** | **G** | **L** | **V** | **K** |
| **N59435F** | **M** | **I** | **K** | **V** | **L** | **L** | **V** | **D** | **D** | **H** | **E** | **M** | **V** | **R** | **L** | **G** | **V** | **S** | **S** | **Y** | **L** | **S** | **I** | **Q** | **E** | **D** | **I** | **E** | **V** | **I** | **G** | **E** | **A** | **E** | **N** | **G** | **R** | **Q** | **G** | **Y** | **E** | **K** | **A** | **M** | **A** | **L** | **R** | **P** | **D** | **V** | **I** | **L** | **M** | **D** | **L** | **V** | **M** | **E** | **E** | **M** | **D** | **G** | **I** | **E** | **S** | **T** | **K** | **A** | **I** | **L** | **K** | **D** | **W** | **P** | **K** | **A** | **K** | **I** | **I** | **I** | **V** | **T** | **S** | **F** | **I** | **D** | **D** | **E** | **K** | **V** | **Y** | **P** | **A** | **I** | **E** | **A** | **G** | **A** | **A** | **G** | **Y** | **L** | **L** | **K** | **T** | **S** | **T** | **A** | **H** | **E** | **I** | **A** | **D** | **A** | **I** | **R** | **A** | **T** | **Q** | **R** | **G** | **E** | **R** | **V** | **L** | **E** | **P** | **E** | **V** | **T** | **T** | **K** | **M** | **M** | **E** | **K** | **M** | **S** | **R** | **R** | **Y** | **D** | **P** | **V** | **L** | **H** | **E** | **E** | **L** | **T** | **N** | **R** | **E** | **N** | **E** | **I** | **L** | **M** | **L** | **I** | **S** | **E** | **G** | **K** | **S** | **N** | **Q** | **E** | **I** | **A** | **D** | **E** | **L** | **F** | **I** | **T** | **L** | **K** | **T** | **V** | **K** | **T** | **H** | **V** | **S** | **N** | **I** | **L** | **A** | **K** | **L** | **E** | **V** | **E** | **D** | **R** | **T** | **Q** | **A** | **A** | **I** | **Y** | **A** | **F** | **K** | **H** | **G** | **L** | **V** | **K** |
| **N59611F** | **V** | **I** | **K** | **V** | **M** | **L** | **V** | **D** | **D** | **H** | **E** | **M** | **V** | **R** | **L** | **G** | **V** | **S** | **S** | **Y** | **L** | **S** | **I** | **Q** | **E** | **D** | **I** | **E** | **V** | **V** | **G** | **E** | **A** | **E** | **N** | **G** | **K** | **I** | **G** | **Y** | **E** | **K** | **A** | **L** | **E** | **L** | **R** | **P** | **D** | **V** | **I** | **L** | **M** | **D** | **L** | **V** | **M** | **E** | **E** | **M** | **D** | **G** | **I** | **D** | **S** | **T** | **K** | **A** | **I** | **L** | **K** | **D** | **W** | **P** | **E** | **A** | **K** | **I** | **I** | **I** | **V** | **T** | **S** | **F** | **I** | **D** | **D** | **E** | **K** | **V** | **Y** | **P** | **A** | **I** | **E** | **A** | **G** | **A** | **A** | **G** | **Y** | **L** | **L** | **K** | **T** | **S** | **T** | **A** | **H** | **E** | **I** | **A** | **D** | **A** | **I** | **R** | **A** | **T** | **Y** | **R** | **G** | **E** | **R** | **V** | **L** | **E** | **P** | **E** | **V** | **T** | **H** | **K** | **M** | **M** | **E** | **R** | **L** | **T** | **K** | **K** | **Q** | **E** | **P** | **V** | **L** | **H** | **E** | **D** | **L** | **T** | **N** | **R** | **E** | **H** | **E** | **I** | **L** | **M** | **L** | **I** | **A** | **Q** | **G** | **K** | **S** | **N** | **Q** | **E** | **I** | **A** | **D** | **E** | **L** | **F** | **I** | **T** | **L** | **K** | **T** | **V** | **K** | **T** | **H** | **V** | **S** | **N** | **I** | **L** | **A** | **K** | **L** | **D** | **V** | **D** | **D** | **R** | **T** | **Q** | **A** | **A** | **I** | **Y** | **A** | **F** | **Q** | **H** | **G** | **L** | **A** | **K** |
| **N59845F** | **V** | **I** | **K** | **V** | **M** | **L** | **V** | **D** | **D** | **H** | **E** | **M** | **V** | **R** | **L** | **G** | **V** | **S** | **S** | **Y** | **L** | **S** | **I** | **Q** | **E** | **D** | **I** | **E** | **V** | **V** | **G** | **E** | **A** | **E** | **N** | **G** | **K** | **I** | **G** | **Y** | **E** | **K** | **A** | **L** | **E** | **L** | **R** | **P** | **D** | **V** | **I** | **L** | **M** | **D** | **L** | **V** | **M** | **E** | **E** | **M** | **D** | **G** | **I** | **D** | **S** | **T** | **K** | **A** | **I** | **L** | **K** | **D** | **W** | **P** | **E** | **A** | **K** | **I** | **I** | **I** | **V** | **T** | **S** | **F** | **I** | **D** | **D** | **E** | **K** | **V** | **Y** | **P** | **A** | **I** | **E** | **A** | **G** | **A** | **A** | **G** | **Y** | **L** | **L** | **K** | **T** | **S** | **T** | **A** | **H** | **E** | **I** | **A** | **D** | **A** | **I** | **R** | **A** | **T** | **Y** | **R** | **G** | **E** | **R** | **V** | **L** | **E** | **P** | **E** | **V** | **T** | **H** | **K** | **M** | **M** | **E** | **R** | **L** | **T** | **K** | **K** | **Q** | **E** | **P** | **V** | **L** | **H** | **E** | **D** | **L** | **T** | **N** | **R** | **E** | **H** | **E** | **I** | **L** | **M** | **L** | **I** | **A** | **Q** | **G** | **K** | **S** | **N** | **Q** | **E** | **I** | **A** | **D** | **E** | **L** | **F** | **I** | **T** | **L** | **K** | **T** | **V** | **K** | **T** | **H** | **V** | **S** | **N** | **I** | **L** | **A** | **K** | **L** | **D** | **V** | **D** | **D** | **R** | **T** | **Q** | **A** | **A** | **I** | **Y** | **A** | **F** | **Q** | **H** | **G** | **L** | **A** | **K** |
| **N59847F** | **V** | **I** | **K** | **V** | **M** | **L** | **V** | **D** | **D** | **H** | **E** | **M** | **V** | **R** | **L** | **G** | **V** | **S** | **S** | **Y** | **L** | **S** | **I** | **Q** | **E** | **D** | **I** | **E** | **V** | **V** | **G** | **E** | **A** | **E** | **N** | **G** | **K** | **I** | **G** | **Y** | **E** | **K** | **A** | **L** | **E** | **L** | **R** | **P** | **D** | **V** | **I** | **L** | **M** | **D** | **L** | **V** | **M** | **E** | **E** | **M** | **D** | **G** | **I** | **D** | **S** | **T** | **K** | **A** | **I** | **L** | **K** | **D** | **W** | **P** | **E** | **A** | **K** | **I** | **I** | **I** | **V** | **T** | **S** | **F** | **I** | **D** | **D** | **E** | **K** | **V** | **Y** | **P** | **A** | **I** | **E** | **A** | **G** | **A** | **A** | **G** | **Y** | **L** | **L** | **K** | **T** | **S** | **T** | **A** | **H** | **E** | **I** | **A** | **D** | **A** | **I** | **R** | **A** | **T** | **Y** | **R** | **G** | **E** | **R** | **V** | **L** | **E** | **P** | **E** | **V** | **T** | **H** | **K** | **M** | **M** | **E** | **R** | **L** | **T** | **K** | **K** | **Q** | **E** | **P** | **V** | **L** | **H** | **E** | **D** | **L** | **T** | **N** | **R** | **E** | **H** | **E** | **I** | **L** | **M** | **L** | **I** | **A** | **Q** | **G** | **K** | **S** | **N** | **Q** | **E** | **I** | **A** | **D** | **E** | **L** | **F** | **I** | **T** | **L** | **K** | **T** | **V** | **K** | **T** | **H** | **V** | **S** | **N** | **I** | **L** | **A** | **K** | **L** | **D** | **V** | **D** | **D** | **R** | **T** | **Q** | **A** | **A** | **I** | **Y** | **A** | **F** | **Q** | **H** | **G** | **L** | **A** | **K** |
| **N60123F** | **M** | **I** | **K** | **V** | **L** | **L** | **V** | **D** | **D** | **H** | **E** | **M** | **V** | **R** | **L** | **G** | **V** | **S** | **S** | **Y** | **L** | **S** | **I** | **Q** | **E** | **D** | **I** | **E** | **V** | **I** | **G** | **E** | **A** | **E** | **N** | **G** | **R** | **Q** | **G** | **Y** | **E** | **K** | **A** | **M** | **A** | **L** | **R** | **P** | **D** | **V** | **I** | **L** | **M** | **D** | **L** | **V** | **M** | **E** | **E** | **M** | **D** | **G** | **I** | **E** | **S** | **T** | **K** | **A** | **I** | **L** | **K** | **D** | **W** | **P** | **K** | **A** | **K** | **I** | **I** | **I** | **V** | **T** | **S** | **F** | **I** | **D** | **D** | **E** | **K** | **V** | **Y** | **P** | **A** | **I** | **E** | **A** | **G** | **A** | **A** | **G** | **Y** | **L** | **L** | **K** | **T** | **S** | **T** | **A** | **H** | **E** | **I** | **A** | **D** | **A** | **I** | **R** | **A** | **T** | **Q** | **R** | **G** | **E** | **R** | **V** | **L** | **E** | **P** | **E** | **V** | **T** | **T** | **K** | **M** | **M** | **E** | **K** | **M** | **S** | **R** | **R** | **Y** | **D** | **P** | **V** | **L** | **H** | **E** | **E** | **L** | **T** | **N** | **R** | **E** | **N** | **E** | **I** | **L** | **M** | **L** | **I** | **S** | **E** | **G** | **K** | **S** | **N** | **Q** | **E** | **I** | **A** | **D** | **E** | **L** | **F** | **I** | **T** | **L** | **K** | **T** | **V** | **K** | **T** | **H** | **V** | **S** | **N** | **I** | **L** | **A** | **K** | **L** | **E** | **V** | **E** | **D** | **R** | **T** | **Q** | **A** | **A** | **I** | **Y** | **A** | **F** | **K** | **H** | **G** | **L** | **V** | **K** |
| **N55265** | **V** | **I** | **K** | **V** | **M** | **L** | **V** | **D** | **D** | **H** | **E** | **M** | **V** | **R** | **L** | **G** | **V** | **S** | **S** | **Y** | **L** | **S** | **I** | **Q** | **E** | **D** | **I** | **E** | **V** | **V** | **G** | **E** | **A** | **E** | **N** | **G** | **K** | **I** | **G** | **Y** | **E** | **K** | **A** | **L** | **E** | **L** | **R** | **P** | **D** | **V** | **I** | **L** | **M** | **D** | **L** | **V** | **M** | **E** | **E** | **M** | **D** | **G** | **I** | **D** | **S** | **T** | **K** | **A** | **I** | **L** | **K** | **D** | **W** | **P** | **E** | **A** | **K** | **I** | **I** | **I** | **V** | **T** | **S** | **F** | **I** | **D** | **D** | **E** | **K** | **V** | **Y** | **P** | **A** | **I** | **E** | **A** | **G** | **A** | **A** | **G** | **Y** | **L** | **L** | **K** | **T** | **S** | **T** | **A** | **H** | **E** | **I** | **A** | **D** | **A** | **I** | **R** | **A** | **T** | **Y** | **R** | **G** | **E** | **R** | **V** | **L** | **E** | **P** | **E** | **V** | **T** | **H** | **K** | **M** | **M** | **E** | **R** | **L** | **T** | **K** | **K** | **Q** | **E** | **P** | **V** | **L** | **H** | **E** | **D** | **L** | **T** | **N** | **R** | **E** | **H** | **E** | **I** | **L** | **M** | **L** | **I** | **A** | **Q** | **G** | **K** | **S** | **N** | **Q** | **E** | **I** | **A** | **D** | **E** | **L** | **F** | **I** | **T** | **L** | **K** | **T** | **V** | **K** | **T** | **H** | **V** | **S** | **N** | **I** | **L** | **A** | **K** | **L** | **D** | **V** | **D** | **D** | **R** | **T** | **Q** | **A** | **A** | **I** | **Y** | **A** | **F** | **Q** | **H** | **G** | **L** | **A** | **K** |
| **N52459** | **M** | **I** | **K** | **V** | **L** | **L** | **V** | **D** | **D** | **H** | **E** | **M** | **V** | **R** | **L** | **G** | **V** | **S** | **S** | **Y** | **L** | **S** | **I** | **Q** | **E** | **D** | **I** | **E** | **V** | **I** | **G** | **E** | **A** | **E** | **N** | **G** | **R** | **Q** | **G** | **Y** | **E** | **K** | **A** | **M** | **T** | **L** | **R** | **P** | **D** | **V** | **I** | **L** | **M** | **D** | **L** | **V** | **M** | **E** | **E** | **M** | **D** | **G** | **I** | **E** | **S** | **T** | **K** | **A** | **I** | **L** | **K** | **D** | **W** | **P** | **E** | **A** | **K** | **I** | **I** | **I** | **V** | **T** | **S** | **F** | **I** | **D** | **D** | **E** | **K** | **V** | **Y** | **P** | **A** | **I** | **E** | **A** | **G** | **A** | **A** | **G** | **Y** | **L** | **L** | **K** | **T** | **S** | **T** | **A** | **H** | **E** | **I** | **A** | **D** | **A** | **I** | **R** | **A** | **T** | **Q** | **R** | **G** | **E** | **R** | **V** | **L** | **E** | **P** | **E** | **V** | **T** | **T** | **K** | **M** | **M** | **E** | **K** | **M** | **S** | **R** | **R** | **N** | **E** | **P** | **V** | **L** | **H** | **E** | **E** | **L** | **T** | **N** | **R** | **E** | **N** | **E** | **I** | **L** | **M** | **L** | **I** | **S** | **E** | **G** | **K** | **S** | **N** | **Q** | **E** | **I** | **A** | **D** | **E** | **L** | **F** | **I** | **T** | **L** | **K** | **T** | **V** | **K** | **T** | **H** | **V** | **S** | **N** | **I** | **L** | **A** | **K** | **L** | **E** | **V** | **E** | **D** | **R** | **T** | **Q** | **A** | **A** | **I** | **Y** | **A** | **F** | **K** | **H** | **G** | **L** | **V** | **K** |
| **N60190F** | **M** | **I** | **K** | **V** | **L** | **L** | **V** | **D** | **D** | **H** | **E** | **M** | **V** | **R** | **L** | **G** | **V** | **S** | **S** | **Y** | **L** | **S** | **I** | **Q** | **E** | **D** | **I** | **E** | **V** | **I** | **G** | **E** | **A** | **E** | **N** | **G** | **R** | **Q** | **G** | **Y** | **E** | **K** | **A** | **M** | **T** | **L** | **R** | **P** | **D** | **V** | **I** | **L** | **M** | **D** | **L** | **V** | **M** | **E** | **E** | **M** | **D** | **G** | **I** | **E** | **S** | **T** | **K** | **A** | **I** | **L** | **K** | **D** | **W** | **P** | **E** | **A** | **K** | **I** | **I** | **I** | **V** | **T** | **S** | **F** | **I** | **D** | **D** | **E** | **K** | **V** | **Y** | **P** | **A** | **I** | **E** | **A** | **G** | **A** | **A** | **G** | **Y** | **L** | **L** | **K** | **T** | **S** | **T** | **A** | **H** | **E** | **I** | **A** | **D** | **A** | **I** | **R** | **A** | **T** | **Q** | **R** | **G** | **E** | **R** | **V** | **L** | **E** | **P** | **E** | **V** | **T** | **T** | **K** | **M** | **M** | **E** | **K** | **M** | **S** | **R** | **R** | **N** | **E** | **P** | **V** | **L** | **H** | **E** | **E** | **L** | **T** | **N** | **R** | **E** | **N** | **E** | **I** | **L** | **M** | **L** | **I** | **S** | **E** | **G** | **K** | **S** | **N** | **Q** | **E** | **I** | **A** | **D** | **E** | **L** | **F** | **I** | **T** | **L** | **K** | **T** | **V** | **K** | **T** | **H** | **V** | **S** | **N** | **I** | **L** | **A** | **K** | **L** | **E** | **V** | **E** | **D** | **R** | **T** | **Q** | **A** | **A** | **I** | **Y** | **A** | **F** | **K** | **H** | **G** | **L** | **V** | **K** |
| **N52469** | **V** | **I** | **K** | **V** | **M** | **L** | **V** | **D** | **D** | **H** | **E** | **M** | **V** | **R** | **L** | **G** | **V** | **S** | **S** | **Y** | **L** | **S** | **I** | **Q** | **E** | **D** | **I** | **E** | **V** | **V** | **G** | **E** | **A** | **E** | **N** | **G** | **K** | **I** | **G** | **Y** | **E** | **K** | **A** | **L** | **E** | **L** | **R** | **P** | **D** | **V** | **I** | **L** | **M** | **D** | **L** | **V** | **M** | **E** | **E** | **M** | **D** | **G** | **I** | **D** | **S** | **T** | **K** | **A** | **I** | **L** | **K** | **D** | **W** | **P** | **E** | **A** | **K** | **I** | **I** | **I** | **V** | **T** | **S** | **F** | **I** | **D** | **D** | **E** | **K** | **V** | **Y** | **P** | **A** | **I** | **E** | **A** | **G** | **A** | **A** | **G** | **Y** | **L** | **L** | **K** | **T** | **S** | **T** | **A** | **H** | **E** | **I** | **A** | **D** | **A** | **I** | **R** | **A** | **T** | **Y** | **R** | **G** | **E** | **R** | **V** | **L** | **E** | **P** | **E** | **V** | **T** | **H** | **K** | **M** | **M** | **E** | **R** | **L** | **T** | **K** | **K** | **Q** | **E** | **P** | **V** | **L** | **H** | **E** | **D** | **L** | **T** | **N** | **R** | **E** | **H** | **E** | **I** | **L** | **M** | **L** | **I** | **A** | **Q** | **G** | **K** | **S** | **N** | **Q** | **E** | **I** | **A** | **D** | **E** | **L** | **F** | **I** | **T** | **L** | **K** | **T** | **V** | **K** | **T** | **H** | **V** | **S** | **N** | **I** | **L** | **A** | **K** | **L** | **D** | **V** | **D** | **D** | **R** | **T** | **Q** | **A** | **A** | **I** | **Y** | **A** | **F** | **Q** | **H** | **G** | **L** | **A** | **K** |
| **N55725** | **V** | **I** | **K** | **V** | **M** | **L** | **V** | **D** | **D** | **H** | **E** | **M** | **V** | **R** | **L** | **G** | **V** | **S** | **S** | **Y** | **L** | **S** | **I** | **Q** | **E** | **D** | **I** | **E** | **V** | **V** | **G** | **E** | **A** | **E** | **N** | **G** | **K** | **I** | **G** | **Y** | **E** | **K** | **A** | **L** | **E** | **L** | **R** | **P** | **D** | **V** | **I** | **L** | **M** | **D** | **L** | **V** | **M** | **E** | **E** | **M** | **D** | **G** | **I** | **D** | **S** | **T** | **K** | **A** | **I** | **L** | **K** | **D** | **W** | **P** | **E** | **A** | **K** | **I** | **I** | **I** | **V** | **T** | **S** | **F** | **I** | **D** | **D** | **E** | **K** | **V** | **Y** | **P** | **A** | **I** | **E** | **A** | **G** | **A** | **A** | **G** | **Y** | **L** | **L** | **K** | **T** | **S** | **T** | **A** | **H** | **E** | **I** | **A** | **D** | **A** | **I** | **R** | **A** | **T** | **Y** | **R** | **G** | **E** | **R** | **V** | **L** | **E** | **P** | **E** | **V** | **T** | **H** | **K** | **M** | **M** | **E** | **R** | **L** | **T** | **K** | **K** | **Q** | **E** | **P** | **V** | **L** | **H** | **E** | **D** | **L** | **T** | **N** | **R** | **E** | **H** | **E** | **I** | **L** | **M** | **L** | **I** | **A** | **Q** | **G** | **K** | **S** | **N** | **Q** | **E** | **I** | **A** | **D** | **E** | **L** | **F** | **I** | **T** | **L** | **K** | **T** | **V** | **K** | **T** | **H** | **V** | **S** | **N** | **I** | **L** | **A** | **K** | **L** | **D** | **V** | **D** | **D** | **R** | **T** | **Q** | **A** | **A** | **I** | **Y** | **A** | **F** | **Q** | **H** | **G** | **L** | **A** | **K** |
| **N59907F** | **M** | **I** | **K** | **V** | **L** | **L** | **V** | **D** | **D** | **H** | **E** | **M** | **V** | **R** | **L** | **G** | **V** | **S** | **S** | **Y** | **L** | **S** | **I** | **Q** | **E** | **D** | **I** | **E** | **V** | **I** | **G** | **E** | **A** | **E** | **N** | **G** | **R** | **Q** | **G** | **Y** | **E** | **K** | **A** | **M** | **A** | **L** | **R** | **P** | **D** | **V** | **I** | **L** | **M** | **D** | **L** | **V** | **M** | **E** | **E** | **M** | **D** | **G** | **I** | **E** | **S** | **T** | **K** | **A** | **I** | **L** | **K** | **D** | **W** | **P** | **K** | **A** | **K** | **I** | **I** | **I** | **V** | **T** | **S** | **F** | **I** | **D** | **D** | **E** | **K** | **V** | **Y** | **P** | **A** | **I** | **E** | **A** | **G** | **A** | **A** | **G** | **Y** | **L** | **L** | **K** | **T** | **S** | **T** | **A** | **H** | **E** | **I** | **A** | **D** | **A** | **I** | **R** | **A** | **T** | **Q** | **R** | **G** | **E** | **R** | **V** | **L** | **E** | **P** | **E** | **V** | **T** | **T** | **K** | **M** | **M** | **E** | **K** | **M** | **S** | **R** | **R** | **N** | **D** | **P** | **V** | **L** | **H** | **E** | **E** | **L** | **T** | **N** | **R** | **E** | **N** | **E** | **I** | **L** | **M** | **L** | **I** | **S** | **E** | **G** | **K** | **S** | **N** | **Q** | **E** | **I** | **A** | **D** | **E** | **L** | **F** | **I** | **T** | **L** | **K** | **T** | **V** | **K** | **T** | **H** | **V** | **S** | **N** | **I** | **L** | **A** | **K** | **L** | **E** | **V** | **E** | **D** | **R** | **T** | **Q** | **A** | **A** | **I** | **Y** | **A** | **F** | **K** | **H** | **G** | **L** | **V** | **K** |
| **N54671** | **M** | **I** | **K** | **V** | **L** | **L** | **V** | **D** | **D** | **H** | **E** | **M** | **V** | **R** | **L** | **G** | **V** | **S** | **S** | **Y** | **L** | **S** | **I** | **Q** | **E** | **D** | **I** | **E** | **V** | **I** | **G** | **E** | **A** | **E** | **N** | **G** | **R** | **Q** | **G** | **Y** | **E** | **K** | **A** | **M** | **A** | **L** | **R** | **P** | **D** | **V** | **I** | **L** | **M** | **D** | **L** | **V** | **M** | **E** | **E** | **M** | **D** | **G** | **I** | **E** | **S** | **T** | **K** | **A** | **I** | **L** | **K** | **D** | **W** | **P** | **K** | **A** | **K** | **I** | **I** | **I** | **V** | **T** | **S** | **F** | **I** | **D** | **D** | **E** | **K** | **V** | **Y** | **P** | **A** | **I** | **E** | **A** | **G** | **A** | **A** | **G** | **Y** | **L** | **L** | **K** | **T** | **S** | **T** | **A** | **H** | **E** | **I** | **A** | **D** | **A** | **I** | **R** | **A** | **T** | **Q** | **R** | **G** | **E** | **R** | **V** | **L** | **E** | **P** | **E** | **V** | **T** | **T** | **K** | **M** | **M** | **E** | **K** | **M** | **S** | **R** | **R** | **N** | **D** | **P** | **V** | **L** | **H** | **E** | **E** | **L** | **T** | **N** | **R** | **E** | **N** | **E** | **I** | **L** | **M** | **L** | **I** | **S** | **E** | **G** | **K** | **S** | **N** | **Q** | **E** | **I** | **A** | **D** | **E** | **L** | **F** | **I** | **T** | **L** | **K** | **T** | **V** | **K** | **T** | **H** | **V** | **S** | **N** | **I** | **L** | **A** | **K** | **L** | **E** | **V** | **E** | **D** | **R** | **T** | **Q** | **A** | **A** | **I** | **Y** | **A** | **F** | **K** | **H** | **G** | **L** | **V** | **K** |
| **N55077** | **M** | **I** | **K** | **V** | **L** | **L** | **V** | **D** | **D** | **H** | **E** | **M** | **V** | **R** | **L** | **G** | **V** | **S** | **S** | **Y** | **L** | **S** | **I** | **Q** | **E** | **D** | **I** | **E** | **V** | **I** | **G** | **E** | **A** | **E** | **N** | **G** | **R** | **Q** | **G** | **Y** | **E** | **K** | **A** | **M** | **A** | **L** | **R** | **P** | **D** | **V** | **I** | **L** | **M** | **D** | **L** | **V** | **M** | **E** | **E** | **M** | **D** | **G** | **I** | **E** | **S** | **T** | **K** | **A** | **I** | **L** | **K** | **D** | **W** | **T** | **K** | **A** | **K** | **I** | **I** | **I** | **V** | **T** | **S** | **F** | **I** | **D** | **D** | **E** | **K** | **V** | **Y** | **P** | **A** | **I** | **E** | **A** | **G** | **A** | **A** | **G** | **Y** | **L** | **L** | **K** | **T** | **S** | **T** | **A** | **H** | **E** | **I** | **A** | **D** | **A** | **I** | **R** | **A** | **T** | **Q** | **R** | **G** | **E** | **R** | **V** | **L** | **E** | **P** | **E** | **V** | **T** | **T** | **K** | **M** | **M** | **E** | **K** | **M** | **S** | **R** | **R** | **N** | **D** | **P** | **V** | **L** | **H** | **E** | **E** | **L** | **T** | **N** | **R** | **E** | **N** | **E** | **I** | **L** | **M** | **L** | **I** | **S** | **E** | **G** | **K** | **S** | **N** | **Q** | **E** | **I** | **A** | **D** | **E** | **L** | **F** | **I** | **T** | **L** | **K** | **T** | **V** | **K** | **T** | **H** | **V** | **S** | **N** | **I** | **L** | **A** | **K** | **L** | **E** | **V** | **E** | **D** | **R** | **T** | **Q** | **A** | **A** | **I** | **Y** | **A** | **F** | **K** | **H** | **G** | **L** | **V** | **K** |
| **N60363F** | **M** | **I** | **K** | **V** | **L** | **L** | **V** | **D** | **D** | **H** | **E** | **M** | **V** | **R** | **L** | **G** | **V** | **S** | **S** | **Y** | **L** | **S** | **I** | **Q** | **E** | **D** | **I** | **E** | **V** | **I** | **G** | **E** | **A** | **E** | **N** | **G** | **R** | **Q** | **G** | **Y** | **E** | **K** | **A** | **M** | **A** | **L** | **R** | **P** | **D** | **V** | **I** | **L** | **M** | **D** | **L** | **V** | **M** | **E** | **E** | **M** | **D** | **G** | **I** | **E** | **S** | **T** | **K** | **A** | **I** | **L** | **K** | **D** | **W** | **P** | **K** | **A** | **K** | **I** | **I** | **I** | **V** | **T** | **S** | **F** | **I** | **D** | **D** | **E** | **K** | **V** | **Y** | **P** | **A** | **I** | **E** | **A** | **G** | **A** | **A** | **G** | **Y** | **L** | **L** | **K** | **T** | **S** | **T** | **A** | **H** | **E** | **I** | **A** | **D** | **A** | **I** | **R** | **A** | **T** | **Q** | **R** | **G** | **E** | **R** | **V** | **L** | **E** | **P** | **E** | **V** | **T** | **T** | **K** | **M** | **M** | **E** | **K** | **M** | **S** | **R** | **R** | **Y** | **D** | **P** | **V** | **L** | **H** | **E** | **E** | **L** | **T** | **N** | **R** | **E** | **N** | **E** | **I** | **L** | **M** | **L** | **I** | **S** | **E** | **G** | **K** | **S** | **N** | **Q** | **E** | **I** | **A** | **D** | **E** | **L** | **F** | **I** | **T** | **L** | **K** | **T** | **V** | **K** | **T** | **H** | **V** | **S** | **N** | **I** | **L** | **A** | **K** | **L** | **E** | **V** | **E** | **D** | **R** | **T** | **Q** | **A** | **A** | **I** | **Y** | **A** | **F** | **K** | **H** | **G** | **L** | **V** | **K** |
| **N55317** | **M** | **I** | **K** | **V** | **L** | **L** | **V** | **D** | **D** | **H** | **E** | **M** | **V** | **R** | **L** | **G** | **V** | **S** | **S** | **Y** | **L** | **S** | **I** | **Q** | **E** | **D** | **I** | **E** | **V** | **I** | **G** | **E** | **A** | **E** | **N** | **G** | **R** | **Q** | **G** | **Y** | **E** | **K** | **A** | **M** | **T** | **L** | **R** | **P** | **D** | **V** | **I** | **L** | **M** | **D** | **L** | **V** | **M** | **E** | **E** | **M** | **D** | **G** | **I** | **E** | **S** | **T** | **K** | **A** | **I** | **L** | **K** | **D** | **W** | **P** | **E** | **A** | **K** | **I** | **I** | **I** | **V** | **T** | **S** | **F** | **I** | **D** | **D** | **E** | **K** | **V** | **Y** | **P** | **A** | **I** | **E** | **A** | **G** | **A** | **A** | **G** | **Y** | **L** | **L** | **K** | **T** | **S** | **T** | **A** | **H** | **E** | **I** | **A** | **D** | **A** | **I** | **R** | **A** | **T** | **Q** | **R** | **G** | **E** | **R** | **V** | **L** | **E** | **P** | **E** | **V** | **T** | **T** | **K** | **M** | **M** | **E** | **K** | **M** | **S** | **R** | **R** | **N** | **E** | **P** | **V** | **L** | **H** | **E** | **E** | **L** | **T** | **N** | **R** | **E** | **N** | **E** | **I** | **L** | **M** | **L** | **I** | **S** | **E** | **G** | **K** | **S** | **N** | **Q** | **E** | **I** | **A** | **D** | **E** | **L** | **F** | **I** | **T** | **L** | **K** | **T** | **V** | **K** | **T** | **H** | **V** | **S** | **N** | **I** | **L** | **A** | **K** | **L** | **E** | **V** | **E** | **D** | **R** | **T** | **Q** | **A** | **A** | **I** | **Y** | **A** | **F** | **K** | **H** | **G** | **L** | **V** | **K** |
| **N60114F** | **V** | **I** | **K** | **V** | **M** | **L** | **V** | **D** | **D** | **H** | **E** | **M** | **V** | **R** | **L** | **G** | **V** | **S** | **S** | **Y** | **L** | **S** | **I** | **Q** | **E** | **D** | **I** | **E** | **V** | **V** | **G** | **E** | **A** | **E** | **N** | **G** | **K** | **I** | **G** | **Y** | **E** | **K** | **A** | **L** | **E** | **L** | **R** | **P** | **D** | **V** | **I** | **L** | **M** | **D** | **L** | **V** | **M** | **E** | **E** | **M** | **D** | **G** | **I** | **D** | **S** | **T** | **K** | **A** | **I** | **L** | **K** | **D** | **W** | **P** | **E** | **A** | **K** | **I** | **I** | **I** | **V** | **T** | **S** | **F** | **I** | **D** | **D** | **E** | **K** | **V** | **Y** | **P** | **A** | **I** | **E** | **A** | **G** | **A** | **A** | **G** | **Y** | **L** | **L** | **K** | **T** | **S** | **T** | **A** | **H** | **E** | **I** | **A** | **D** | **A** | **I** | **R** | **A** | **T** | **Y** | **R** | **G** | **E** | **R** | **V** | **L** | **E** | **P** | **E** | **V** | **T** | **H** | **K** | **M** | **M** | **E** | **R** | **L** | **T** | **K** | **K** | **Q** | **E** | **P** | **V** | **L** | **H** | **E** | **D** | **L** | **T** | **N** | **R** | **E** | **H** | **E** | **I** | **L** | **M** | **L** | **I** | **A** | **Q** | **G** | **K** | **S** | **N** | **Q** | **E** | **I** | **A** | **D** | **E** | **L** | **F** | **I** | **T** | **L** | **K** | **T** | **V** | **K** | **T** | **H** | **V** | **S** | **N** | **I** | **L** | **A** | **K** | **L** | **D** | **V** | **D** | **D** | **R** | **T** | **Q** | **A** | **A** | **I** | **Y** | **A** | **F** | **Q** | **H** | **G** | **L** | **A** | **K** |
| **N53418** | **V** | **I** | **K** | **V** | **M** | **L** | **V** | **D** | **D** | **H** | **E** | **M** | **V** | **R** | **L** | **G** | **V** | **S** | **S** | **Y** | **L** | **S** | **I** | **Q** | **E** | **D** | **I** | **E** | **V** | **V** | **G** | **E** | **A** | **E** | **N** | **G** | **K** | **I** | **G** | **Y** | **E** | **K** | **A** | **L** | **E** | **L** | **R** | **P** | **D** | **V** | **I** | **L** | **M** | **D** | **L** | **V** | **M** | **E** | **E** | **M** | **D** | **G** | **I** | **D** | **S** | **T** | **K** | **A** | **I** | **L** | **K** | **D** | **W** | **P** | **E** | **A** | **K** | **I** | **I** | **I** | **V** | **T** | **S** | **F** | **I** | **D** | **D** | **E** | **K** | **V** | **Y** | **P** | **A** | **I** | **E** | **A** | **G** | **A** | **A** | **G** | **Y** | **L** | **L** | **K** | **T** | **S** | **T** | **A** | **H** | **E** | **I** | **A** | **D** | **A** | **I** | **R** | **A** | **T** | **Y** | **R** | **G** | **E** | **R** | **V** | **L** | **E** | **P** | **E** | **V** | **T** | **H** | **K** | **M** | **M** | **E** | **R** | **L** | **T** | **K** | **K** | **Q** | **E** | **P** | **V** | **L** | **H** | **E** | **D** | **L** | **T** | **N** | **R** | **E** | **H** | **E** | **I** | **L** | **M** | **L** | **I** | **A** | **Q** | **G** | **K** | **S** | **N** | **Q** | **E** | **I** | **A** | **D** | **E** | **L** | **F** | **I** | **T** | **L** | **K** | **T** | **V** | **K** | **T** | **H** | **V** | **S** | **N** | **I** | **L** | **A** | **K** | **L** | **D** | **V** | **D** | **D** | **R** | **T** | **Q** | **A** | **A** | **I** | **Y** | **A** | **F** | **Q** | **H** | **G** | **L** | **A** | **K** |
| **N52467** | **V** | **I** | **K** | **V** | **M** | **L** | **V** | **D** | **D** | **H** | **E** | **M** | **V** | **R** | **L** | **G** | **V** | **S** | **S** | **Y** | **L** | **S** | **I** | **Q** | **E** | **D** | **I** | **E** | **V** | **V** | **G** | **E** | **A** | **E** | **N** | **G** | **K** | **I** | **G** | **Y** | **E** | **K** | **A** | **L** | **E** | **L** | **R** | **P** | **D** | **V** | **I** | **L** | **M** | **D** | **L** | **V** | **M** | **E** | **E** | **M** | **D** | **G** | **I** | **D** | **S** | **T** | **K** | **A** | **I** | **L** | **K** | **D** | **W** | **P** | **E** | **A** | **K** | **I** | **I** | **I** | **V** | **T** | **S** | **F** | **I** | **D** | **D** | **E** | **K** | **V** | **Y** | **P** | **A** | **I** | **E** | **A** | **G** | **A** | **A** | **G** | **Y** | **L** | **L** | **K** | **T** | **S** | **T** | **A** | **H** | **E** | **I** | **A** | **D** | **A** | **I** | **R** | **A** | **T** | **Y** | **R** | **G** | **E** | **R** | **V** | **L** | **E** | **P** | **E** | **V** | **T** | **H** | **K** | **M** | **M** | **E** | **R** | **L** | **T** | **K** | **K** | **Q** | **E** | **P** | **V** | **L** | **H** | **E** | **D** | **L** | **T** | **N** | **R** | **E** | **H** | **E** | **I** | **L** | **M** | **L** | **I** | **A** | **Q** | **G** | **K** | **S** | **N** | **Q** | **E** | **I** | **A** | **D** | **E** | **L** | **F** | **I** | **T** | **L** | **K** | **T** | **V** | **K** | **T** | **H** | **V** | **S** | **N** | **I** | **L** | **A** | **K** | **L** | **D** | **V** | **D** | **D** | **R** | **T** | **Q** | **A** | **A** | **I** | **Y** | **A** | **F** | **Q** | **H** | **G** | **L** | **A** | **K** |
| **N52587** | **V** | **I** | **K** | **V** | **M** | **L** | **V** | **D** | **D** | **H** | **E** | **M** | **V** | **R** | **L** | **G** | **V** | **S** | **S** | **Y** | **L** | **S** | **I** | **Q** | **E** | **D** | **I** | **E** | **V** | **V** | **G** | **E** | **A** | **E** | **N** | **G** | **K** | **I** | **G** | **Y** | **E** | **K** | **A** | **L** | **E** | **L** | **R** | **P** | **D** | **V** | **I** | **L** | **M** | **D** | **L** | **V** | **M** | **E** | **E** | **M** | **D** | **G** | **I** | **D** | **S** | **T** | **K** | **A** | **I** | **L** | **K** | **D** | **W** | **P** | **E** | **A** | **K** | **I** | **I** | **I** | **V** | **T** | **S** | **F** | **I** | **D** | **D** | **E** | **K** | **V** | **Y** | **P** | **A** | **I** | **E** | **A** | **G** | **A** | **A** | **G** | **Y** | **L** | **L** | **K** | **T** | **S** | **T** | **A** | **H** | **E** | **I** | **A** | **D** | **A** | **I** | **R** | **A** | **T** | **Y** | **R** | **G** | **E** | **R** | **V** | **L** | **E** | **P** | **E** | **V** | **T** | **H** | **K** | **M** | **M** | **E** | **R** | **L** | **T** | **K** | **K** | **Q** | **E** | **P** | **V** | **L** | **H** | **E** | **D** | **L** | **T** | **N** | **R** | **E** | **H** | **E** | **I** | **L** | **M** | **L** | **I** | **A** | **Q** | **G** | **K** | **S** | **N** | **Q** | **E** | **I** | **A** | **D** | **E** | **L** | **F** | **I** | **T** | **L** | **K** | **T** | **V** | **K** | **T** | **H** | **V** | **S** | **N** | **I** | **L** | **A** | **K** | **L** | **D** | **V** | **D** | **D** | **R** | **T** | **Q** | **A** | **A** | **I** | **Y** | **A** | **F** | **Q** | **H** | **G** | **L** | **A** | **K** |
| **N59455F** | **V** | **I** | **K** | **V** | **M** | **L** | **V** | **D** | **D** | **H** | **E** | **M** | **V** | **R** | **L** | **G** | **V** | **S** | **S** | **Y** | **L** | **S** | **I** | **Q** | **E** | **D** | **I** | **E** | **V** | **V** | **G** | **E** | **A** | **E** | **N** | **G** | **K** | **I** | **G** | **Y** | **E** | **K** | **A** | **L** | **E** | **L** | **R** | **P** | **D** | **V** | **I** | **L** | **M** | **D** | **L** | **V** | **M** | **E** | **E** | **M** | **D** | **G** | **I** | **D** | **S** | **T** | **K** | **A** | **I** | **L** | **K** | **D** | **W** | **P** | **E** | **A** | **K** | **I** | **I** | **I** | **V** | **T** | **S** | **F** | **I** | **D** | **D** | **E** | **K** | **V** | **Y** | **P** | **A** | **I** | **E** | **A** | **G** | **A** | **A** | **G** | **Y** | **L** | **L** | **K** | **T** | **S** | **T** | **A** | **H** | **E** | **I** | **A** | **D** | **A** | **I** | **R** | **A** | **T** | **Y** | **R** | **G** | **E** | **R** | **V** | **L** | **E** | **P** | **E** | **V** | **T** | **H** | **K** | **M** | **M** | **E** | **R** | **L** | **T** | **K** | **K** | **Q** | **E** | **P** | **V** | **L** | **H** | **E** | **D** | **L** | **T** | **N** | **R** | **E** | **H** | **E** | **I** | **L** | **M** | **L** | **I** | **A** | **Q** | **G** | **K** | **S** | **N** | **Q** | **E** | **I** | **A** | **D** | **E** | **L** | **F** | **I** | **T** | **L** | **K** | **T** | **V** | **K** | **T** | **H** | **V** | **S** | **N** | **I** | **L** | **A** | **K** | **L** | **D** | **V** | **D** | **D** | **R** | **T** | **Q** | **A** | **A** | **I** | **Y** | **A** | **F** | **Q** | **H** | **G** | **L** | **A** | **K** |
| **N53805** | **M** | **I** | **K** | **V** | **L** | **L** | **V** | **D** | **D** | **H** | **E** | **M** | **V** | **R** | **L** | **G** | **V** | **S** | **S** | **Y** | **L** | **S** | **I** | **Q** | **E** | **D** | **I** | **E** | **V** | **I** | **G** | **E** | **A** | **E** | **N** | **G** | **R** | **Q** | **G** | **Y** | **E** | **K** | **A** | **M** | **A** | **L** | **R** | **P** | **D** | **V** | **I** | **L** | **M** | **D** | **L** | **V** | **M** | **E** | **E** | **M** | **D** | **G** | **I** | **E** | **S** | **T** | **K** | **A** | **I** | **L** | **K** | **D** | **W** | **P** | **K** | **A** | **K** | **I** | **I** | **I** | **V** | **T** | **S** | **F** | **I** | **D** | **D** | **E** | **K** | **V** | **Y** | **P** | **A** | **I** | **E** | **A** | **G** | **A** | **A** | **G** | **Y** | **L** | **L** | **K** | **T** | **S** | **T** | **A** | **H** | **E** | **I** | **A** | **D** | **A** | **I** | **R** | **A** | **T** | **Q** | **R** | **G** | **E** | **R** | **V** | **L** | **E** | **P** | **E** | **V** | **T** | **T** | **K** | **M** | **M** | **E** | **K** | **M** | **S** | **R** | **R** | **N** | **D** | **P** | **V** | **L** | **H** | **E** | **E** | **L** | **T** | **N** | **R** | **E** | **N** | **E** | **I** | **L** | **M** | **L** | **I** | **S** | **E** | **G** | **K** | **S** | **N** | **Q** | **E** | **I** | **A** | **D** | **E** | **L** | **F** | **I** | **T** | **L** | **K** | **T** | **V** | **K** | **T** | **H** | **V** | **S** | **N** | **I** | **L** | **A** | **K** | **L** | **E** | **V** | **E** | **D** | **R** | **T** | **Q** | **A** | **A** | **I** | **Y** | **A** | **F** | **K** | **H** | **G** | **L** | **V** | **K** |
| **N59754F** | **M** | **I** | **K** | **V** | **L** | **L** | **V** | **D** | **D** | **H** | **E** | **M** | **V** | **R** | **L** | **G** | **V** | **S** | **S** | **Y** | **L** | **S** | **I** | **Q** | **E** | **D** | **I** | **E** | **V** | **I** | **G** | **E** | **A** | **E** | **N** | **G** | **R** | **Q** | **G** | **Y** | **E** | **K** | **A** | **M** | **A** | **L** | **R** | **P** | **D** | **V** | **I** | **L** | **M** | **D** | **L** | **V** | **M** | **E** | **E** | **M** | **D** | **G** | **I** | **E** | **S** | **T** | **K** | **A** | **I** | **L** | **K** | **D** | **W** | **P** | **K** | **A** | **K** | **I** | **I** | **I** | **V** | **T** | **S** | **F** | **I** | **D** | **D** | **E** | **K** | **V** | **Y** | **P** | **A** | **I** | **E** | **A** | **G** | **A** | **A** | **G** | **Y** | **L** | **L** | **K** | **T** | **S** | **T** | **A** | **H** | **E** | **I** | **A** | **D** | **A** | **I** | **R** | **A** | **T** | **Q** | **R** | **G** | **E** | **R** | **V** | **L** | **E** | **P** | **E** | **V** | **T** | **T** | **K** | **M** | **M** | **E** | **K** | **M** | **S** | **R** | **R** | **N** | **D** | **P** | **V** | **L** | **H** | **E** | **E** | **L** | **T** | **N** | **R** | **E** | **N** | **E** | **I** | **L** | **M** | **L** | **I** | **S** | **E** | **G** | **K** | **S** | **N** | **Q** | **E** | **I** | **A** | **D** | **E** | **L** | **F** | **I** | **T** | **L** | **K** | **T** | **V** | **K** | **T** | **H** | **V** | **S** | **N** | **I** | **L** | **A** | **K** | **L** | **E** | **V** | **E** | **D** | **R** | **T** | **Q** | **A** | **A** | **I** | **Y** | **A** | **F** | **K** | **H** | **G** | **L** | **V** | **K** |
| **N59584F** | **V** | **I** | **K** | **V** | **M** | **L** | **V** | **D** | **D** | **H** | **E** | **M** | **V** | **R** | **L** | **G** | **V** | **S** | **S** | **Y** | **L** | **S** | **I** | **Q** | **E** | **D** | **I** | **E** | **V** | **V** | **G** | **E** | **A** | **E** | **N** | **G** | **K** | **I** | **G** | **Y** | **E** | **K** | **A** | **L** | **E** | **L** | **R** | **P** | **D** | **V** | **I** | **L** | **M** | **D** | **L** | **V** | **M** | **E** | **E** | **M** | **D** | **G** | **I** | **D** | **S** | **T** | **K** | **A** | **I** | **L** | **K** | **D** | **W** | **P** | **E** | **A** | **K** | **I** | **I** | **I** | **V** | **T** | **S** | **F** | **I** | **D** | **D** | **E** | **K** | **V** | **Y** | **P** | **A** | **I** | **E** | **A** | **G** | **A** | **A** | **G** | **Y** | **L** | **L** | **K** | **T** | **S** | **T** | **A** | **H** | **E** | **I** | **A** | **D** | **A** | **I** | **R** | **A** | **T** | **Y** | **R** | **G** | **E** | **R** | **V** | **L** | **E** | **P** | **E** | **V** | **T** | **H** | **K** | **M** | **M** | **E** | **R** | **L** | **T** | **K** | **K** | **Q** | **E** | **P** | **V** | **L** | **H** | **E** | **D** | **L** | **T** | **N** | **R** | **E** | **H** | **E** | **I** | **L** | **M** | **L** | **I** | **A** | **Q** | **G** | **K** | **S** | **N** | **Q** | **E** | **I** | **A** | **D** | **E** | **L** | **F** | **I** | **T** | **L** | **K** | **T** | **V** | **K** | **T** | **H** | **V** | **S** | **N** | **I** | **L** | **A** | **K** | **L** | **D** | **V** | **D** | **D** | **R** | **T** | **Q** | **A** | **A** | **I** | **Y** | **A** | **F** | **Q** | **H** | **G** | **L** | **A** | **K** |
| **N54035** | **M** | **I** | **K** | **V** | **L** | **L** | **V** | **D** | **D** | **H** | **E** | **M** | **V** | **R** | **L** | **G** | **V** | **S** | **S** | **Y** | **L** | **S** | **I** | **Q** | **E** | **D** | **I** | **E** | **V** | **I** | **G** | **E** | **A** | **E** | **N** | **G** | **R** | **Q** | **G** | **Y** | **E** | **K** | **A** | **M** | **A** | **L** | **R** | **P** | **D** | **V** | **I** | **L** | **M** | **D** | **L** | **V** | **M** | **E** | **E** | **M** | **D** | **G** | **I** | **E** | **S** | **T** | **K** | **A** | **I** | **L** | **K** | **D** | **W** | **P** | **K** | **A** | **K** | **I** | **I** | **I** | **V** | **T** | **S** | **F** | **I** | **D** | **D** | **E** | **K** | **V** | **Y** | **P** | **A** | **I** | **E** | **A** | **G** | **A** | **A** | **G** | **Y** | **L** | **L** | **K** | **T** | **S** | **T** | **A** | **H** | **E** | **I** | **A** | **D** | **A** | **I** | **R** | **A** | **T** | **Q** | **R** | **G** | **E** | **R** | **V** | **L** | **E** | **P** | **E** | **V** | **T** | **T** | **K** | **M** | **M** | **E** | **K** | **M** | **S** | **R** | **R** | **N** | **D** | **P** | **V** | **L** | **H** | **E** | **E** | **L** | **T** | **N** | **R** | **E** | **N** | **E** | **I** | **L** | **M** | **L** | **I** | **S** | **E** | **G** | **K** | **S** | **N** | **Q** | **E** | **I** | **A** | **D** | **E** | **L** | **F** | **I** | **T** | **L** | **K** | **T** | **V** | **K** | **T** | **H** | **V** | **S** | **N** | **I** | **L** | **A** | **K** | **L** | **E** | **V** | **E** | **D** | **R** | **T** | **Q** | **A** | **A** | **I** | **Y** | **A** | **F** | **K** | **H** | **G** | **L** | **V** | **K** |
| **N59542F** | **M** | **I** | **K** | **V** | **L** | **L** | **V** | **D** | **D** | **H** | **E** | **M** | **V** | **R** | **L** | **G** | **V** | **S** | **S** | **Y** | **L** | **S** | **I** | **Q** | **E** | **D** | **I** | **E** | **V** | **I** | **G** | **E** | **A** | **E** | **N** | **G** | **R** | **Q** | **G** | **Y** | **E** | **K** | **A** | **M** | **A** | **L** | **R** | **P** | **D** | **V** | **I** | **L** | **M** | **D** | **L** | **V** | **M** | **E** | **E** | **M** | **D** | **G** | **I** | **E** | **S** | **T** | **K** | **A** | **I** | **L** | **K** | **D** | **W** | **P** | **K** | **A** | **K** | **I** | **I** | **I** | **V** | **T** | **S** | **F** | **I** | **D** | **D** | **E** | **K** | **V** | **Y** | **P** | **A** | **I** | **E** | **A** | **G** | **A** | **A** | **G** | **Y** | **L** | **L** | **K** | **T** | **S** | **T** | **A** | **H** | **E** | **I** | **A** | **D** | **A** | **I** | **R** | **A** | **T** | **Q** | **R** | **G** | **E** | **R** | **V** | **L** | **E** | **P** | **E** | **V** | **T** | **T** | **K** | **M** | **M** | **E** | **K** | **M** | **S** | **R** | **R** | **N** | **D** | **P** | **V** | **L** | **H** | **E** | **E** | **L** | **T** | **N** | **R** | **E** | **N** | **E** | **I** | **L** | **M** | **L** | **I** | **S** | **E** | **G** | **K** | **S** | **N** | **Q** | **E** | **I** | **A** | **D** | **E** | **L** | **F** | **I** | **T** | **L** | **K** | **T** | **V** | **K** | **T** | **H** | **V** | **S** | **N** | **I** | **L** | **A** | **K** | **L** | **E** | **V** | **E** | **D** | **R** | **T** | **Q** | **A** | **A** | **I** | **Y** | **A** | **F** | **K** | **H** | **G** | **L** | **V** | **K** |
| **N60034F** | **V** | **I** | **K** | **V** | **M** | **L** | **V** | **D** | **D** | **H** | **E** | **M** | **V** | **R** | **L** | **G** | **V** | **S** | **S** | **Y** | **L** | **S** | **I** | **Q** | **E** | **D** | **I** | **E** | **V** | **V** | **G** | **E** | **A** | **E** | **N** | **G** | **K** | **I** | **G** | **Y** | **E** | **K** | **A** | **L** | **E** | **L** | **R** | **P** | **D** | **V** | **I** | **L** | **M** | **D** | **L** | **V** | **M** | **E** | **E** | **M** | **D** | **G** | **I** | **D** | **S** | **T** | **K** | **A** | **I** | **L** | **K** | **D** | **W** | **P** | **E** | **A** | **K** | **I** | **I** | **I** | **V** | **T** | **S** | **F** | **I** | **D** | **D** | **E** | **K** | **V** | **Y** | **P** | **A** | **I** | **E** | **A** | **G** | **A** | **A** | **G** | **Y** | **L** | **L** | **K** | **T** | **S** | **T** | **A** | **H** | **E** | **I** | **A** | **D** | **A** | **I** | **R** | **A** | **T** | **Y** | **R** | **G** | **E** | **R** | **V** | **L** | **E** | **P** | **E** | **V** | **T** | **H** | **K** | **M** | **M** | **E** | **R** | **L** | **T** | **K** | **K** | **Q** | **E** | **P** | **V** | **L** | **H** | **E** | **D** | **L** | **T** | **N** | **R** | **E** | **H** | **E** | **I** | **L** | **M** | **L** | **I** | **A** | **Q** | **G** | **K** | **S** | **N** | **Q** | **E** | **I** | **A** | **D** | **E** | **L** | **F** | **I** | **T** | **L** | **K** | **T** | **V** | **K** | **T** | **H** | **V** | **S** | **N** | **I** | **L** | **A** | **K** | **L** | **D** | **V** | **D** | **D** | **R** | **T** | **Q** | **A** | **A** | **I** | **Y** | **A** | **F** | **Q** | **H** | **G** | **L** | **A** | **K** |
| **N59862F** | **V** | **I** | **K** | **V** | **M** | **L** | **V** | **D** | **D** | **H** | **E** | **M** | **V** | **R** | **L** | **G** | **V** | **S** | **S** | **Y** | **L** | **S** | **I** | **Q** | **E** | **D** | **I** | **E** | **V** | **V** | **G** | **E** | **A** | **E** | **N** | **G** | **K** | **I** | **G** | **Y** | **E** | **K** | **A** | **L** | **E** | **L** | **R** | **P** | **D** | **V** | **I** | **L** | **M** | **D** | **L** | **V** | **M** | **E** | **E** | **M** | **D** | **G** | **I** | **D** | **S** | **T** | **K** | **A** | **I** | **L** | **K** | **D** | **W** | **P** | **E** | **A** | **K** | **I** | **I** | **I** | **V** | **T** | **S** | **F** | **I** | **D** | **D** | **E** | **K** | **V** | **Y** | **P** | **A** | **I** | **E** | **A** | **G** | **A** | **A** | **G** | **Y** | **L** | **L** | **K** | **T** | **S** | **T** | **A** | **H** | **E** | **I** | **A** | **D** | **A** | **I** | **R** | **A** | **T** | **Y** | **R** | **G** | **E** | **R** | **V** | **L** | **E** | **P** | **E** | **V** | **T** | **H** | **K** | **M** | **M** | **E** | **R** | **L** | **T** | **K** | **K** | **Q** | **E** | **P** | **V** | **L** | **H** | **E** | **D** | **L** | **T** | **N** | **R** | **E** | **H** | **E** | **I** | **L** | **M** | **L** | **I** | **A** | **Q** | **G** | **K** | **S** | **N** | **Q** | **E** | **I** | **A** | **D** | **E** | **L** | **F** | **I** | **T** | **L** | **K** | **T** | **V** | **K** | **T** | **H** | **V** | **S** | **N** | **I** | **L** | **A** | **K** | **L** | **D** | **V** | **D** | **D** | **R** | **T** | **Q** | **A** | **A** | **I** | **Y** | **A** | **F** | **Q** | **H** | **G** | **L** | **A** | **K** |
| **N55185** | **V** | **I** | **K** | **V** | **M** | **L** | **V** | **D** | **D** | **H** | **E** | **M** | **V** | **R** | **L** | **G** | **V** | **S** | **S** | **Y** | **L** | **S** | **I** | **Q** | **E** | **D** | **I** | **E** | **V** | **V** | **G** | **E** | **A** | **E** | **N** | **G** | **K** | **I** | **G** | **Y** | **E** | **K** | **A** | **L** | **E** | **L** | **R** | **P** | **D** | **V** | **I** | **L** | **M** | **D** | **L** | **V** | **M** | **E** | **E** | **M** | **D** | **G** | **I** | **D** | **S** | **T** | **K** | **A** | **I** | **L** | **K** | **D** | **W** | **P** | **E** | **A** | **K** | **I** | **I** | **I** | **V** | **T** | **S** | **F** | **I** | **D** | **D** | **E** | **K** | **V** | **Y** | **P** | **A** | **I** | **E** | **A** | **G** | **A** | **A** | **G** | **Y** | **L** | **L** | **K** | **T** | **S** | **T** | **A** | **H** | **E** | **I** | **A** | **D** | **A** | **I** | **R** | **A** | **T** | **Y** | **R** | **G** | **E** | **R** | **V** | **L** | **E** | **P** | **E** | **V** | **T** | **H** | **K** | **M** | **M** | **E** | **R** | **L** | **T** | **K** | **K** | **Q** | **E** | **P** | **V** | **L** | **H** | **E** | **D** | **L** | **T** | **N** | **R** | **E** | **H** | **E** | **I** | **L** | **M** | **L** | **I** | **A** | **Q** | **G** | **K** | **S** | **N** | **Q** | **E** | **I** | **A** | **D** | **E** | **L** | **F** | **I** | **T** | **L** | **K** | **T** | **V** | **K** | **T** | **H** | **V** | **S** | **N** | **I** | **L** | **A** | **K** | **L** | **D** | **V** | **D** | **D** | **R** | **T** | **Q** | **A** | **A** | **I** | **Y** | **A** | **F** | **Q** | **H** | **G** | **L** | **A** | **K** |
| **N60061F** | **M** | **I** | **K** | **V** | **L** | **L** | **V** | **D** | **D** | **H** | **E** | **M** | **V** | **R** | **L** | **G** | **V** | **S** | **S** | **Y** | **L** | **S** | **I** | **Q** | **E** | **D** | **I** | **E** | **V** | **I** | **G** | **E** | **A** | **E** | **N** | **G** | **R** | **Q** | **G** | **Y** | **E** | **K** | **A** | **M** | **A** | **L** | **R** | **P** | **D** | **V** | **I** | **L** | **M** | **D** | **L** | **V** | **M** | **E** | **E** | **M** | **D** | **G** | **I** | **E** | **S** | **T** | **K** | **A** | **I** | **L** | **K** | **D** | **W** | **P** | **K** | **A** | **K** | **I** | **I** | **I** | **V** | **T** | **S** | **F** | **I** | **D** | **D** | **E** | **K** | **V** | **Y** | **P** | **A** | **I** | **E** | **A** | **G** | **A** | **A** | **G** | **Y** | **L** | **L** | **K** | **T** | **S** | **T** | **A** | **H** | **E** | **I** | **A** | **D** | **A** | **I** | **R** | **A** | **T** | **Q** | **R** | **G** | **E** | **R** | **V** | **L** | **E** | **P** | **E** | **V** | **T** | **T** | **K** | **M** | **M** | **E** | **K** | **M** | **S** | **R** | **R** | **N** | **D** | **P** | **V** | **L** | **H** | **E** | **E** | **L** | **T** | **N** | **R** | **E** | **N** | **E** | **I** | **L** | **M** | **L** | **I** | **S** | **E** | **G** | **K** | **S** | **N** | **Q** | **E** | **I** | **A** | **D** | **E** | **L** | **F** | **I** | **T** | **L** | **K** | **T** | **V** | **K** | **T** | **H** | **V** | **S** | **N** | **I** | **L** | **A** | **K** | **L** | **E** | **V** | **E** | **D** | **R** | **T** | **Q** | **A** | **A** | **I** | **Y** | **A** | **F** | **K** | **H** | **G** | **L** | **V** | **K** |
| **N52662** | **V** | **I** | **K** | **V** | **M** | **L** | **V** | **D** | **D** | **H** | **E** | **M** | **V** | **R** | **L** | **G** | **V** | **S** | **S** | **Y** | **L** | **S** | **I** | **Q** | **E** | **D** | **I** | **E** | **V** | **V** | **G** | **E** | **A** | **E** | **N** | **G** | **K** | **I** | **G** | **Y** | **E** | **K** | **A** | **L** | **E** | **L** | **R** | **P** | **D** | **V** | **I** | **L** | **M** | **D** | **L** | **V** | **M** | **E** | **E** | **M** | **D** | **G** | **I** | **D** | **S** | **T** | **K** | **A** | **I** | **L** | **K** | **D** | **W** | **P** | **E** | **A** | **K** | **I** | **I** | **I** | **V** | **T** | **S** | **F** | **I** | **D** | **D** | **E** | **K** | **V** | **Y** | **P** | **A** | **I** | **E** | **A** | **G** | **A** | **A** | **G** | **Y** | **L** | **L** | **K** | **T** | **S** | **T** | **A** | **H** | **E** | **I** | **A** | **D** | **A** | **I** | **R** | **A** | **T** | **Y** | **R** | **G** | **E** | **R** | **V** | **L** | **E** | **P** | **E** | **V** | **T** | **H** | **K** | **M** | **M** | **E** | **R** | **L** | **T** | **K** | **K** | **Q** | **E** | **P** | **V** | **L** | **H** | **E** | **D** | **L** | **T** | **N** | **R** | **E** | **H** | **E** | **I** | **L** | **M** | **L** | **I** | **A** | **Q** | **G** | **K** | **S** | **N** | **Q** | **E** | **I** | **A** | **D** | **E** | **L** | **F** | **I** | **T** | **L** | **K** | **T** | **V** | **K** | **T** | **H** | **V** | **S** | **N** | **I** | **L** | **A** | **K** | **L** | **D** | **V** | **D** | **D** | **R** | **T** | **Q** | **A** | **A** | **I** | **Y** | **A** | **F** | **Q** | **H** | **G** | **L** | **A** | **K** |
| **N55724** | **V** | **I** | **K** | **V** | **M** | **L** | **V** | **D** | **D** | **H** | **E** | **M** | **V** | **R** | **L** | **G** | **V** | **S** | **S** | **Y** | **L** | **S** | **I** | **Q** | **E** | **D** | **I** | **E** | **V** | **V** | **G** | **E** | **A** | **E** | **N** | **G** | **K** | **I** | **G** | **Y** | **E** | **K** | **A** | **L** | **E** | **L** | **R** | **P** | **D** | **V** | **I** | **L** | **M** | **D** | **L** | **V** | **M** | **E** | **E** | **M** | **D** | **G** | **I** | **D** | **S** | **T** | **K** | **A** | **I** | **L** | **K** | **D** | **W** | **P** | **E** | **A** | **K** | **I** | **I** | **I** | **V** | **T** | **S** | **F** | **I** | **D** | **D** | **E** | **K** | **V** | **Y** | **P** | **A** | **I** | **E** | **A** | **G** | **A** | **A** | **G** | **Y** | **L** | **L** | **K** | **T** | **S** | **T** | **A** | **H** | **E** | **I** | **A** | **D** | **A** | **I** | **R** | **A** | **T** | **Y** | **R** | **G** | **E** | **R** | **V** | **L** | **E** | **P** | **E** | **V** | **T** | **H** | **K** | **M** | **M** | **E** | **R** | **L** | **T** | **K** | **K** | **Q** | **E** | **P** | **V** | **L** | **H** | **E** | **D** | **L** | **T** | **N** | **R** | **E** | **H** | **E** | **I** | **L** | **M** | **L** | **I** | **A** | **Q** | **G** | **K** | **S** | **N** | **Q** | **E** | **I** | **A** | **D** | **E** | **L** | **F** | **I** | **T** | **L** | **K** | **T** | **V** | **K** | **T** | **H** | **V** | **S** | **N** | **I** | **L** | **A** | **K** | **L** | **D** | **V** | **D** | **D** | **R** | **T** | **Q** | **A** | **A** | **I** | **Y** | **A** | **F** | **Q** | **H** | **G** | **L** | **A** | **K** |
| **N55338** | **V** | **I** | **K** | **V** | **M** | **L** | **V** | **D** | **D** | **H** | **E** | **M** | **V** | **R** | **L** | **G** | **V** | **S** | **S** | **Y** | **L** | **S** | **I** | **Q** | **E** | **D** | **I** | **E** | **V** | **V** | **G** | **E** | **A** | **E** | **N** | **G** | **K** | **I** | **G** | **Y** | **E** | **K** | **A** | **L** | **E** | **L** | **R** | **P** | **D** | **V** | **I** | **L** | **M** | **D** | **L** | **V** | **M** | **E** | **E** | **M** | **D** | **G** | **I** | **D** | **S** | **T** | **K** | **A** | **I** | **L** | **K** | **D** | **W** | **P** | **E** | **A** | **K** | **I** | **I** | **I** | **V** | **T** | **S** | **F** | **I** | **D** | **D** | **E** | **K** | **V** | **Y** | **P** | **A** | **I** | **E** | **A** | **G** | **A** | **A** | **G** | **Y** | **L** | **L** | **K** | **T** | **S** | **T** | **A** | **H** | **E** | **I** | **A** | **D** | **A** | **I** | **R** | **A** | **T** | **Y** | **R** | **G** | **E** | **R** | **V** | **L** | **E** | **P** | **E** | **V** | **T** | **H** | **K** | **M** | **M** | **E** | **R** | **L** | **T** | **K** | **K** | **Q** | **E** | **P** | **V** | **L** | **H** | **E** | **D** | **L** | **T** | **N** | **R** | **E** | **H** | **E** | **I** | **L** | **M** | **L** | **I** | **A** | **Q** | **G** | **K** | **S** | **N** | **Q** | **E** | **I** | **A** | **D** | **E** | **L** | **F** | **I** | **T** | **L** | **K** | **T** | **V** | **K** | **T** | **H** | **V** | **S** | **N** | **I** | **L** | **A** | **K** | **L** | **D** | **V** | **D** | **D** | **R** | **T** | **Q** | **A** | **A** | **I** | **Y** | **A** | **F** | **Q** | **H** | **G** | **L** | **A** | **K** |
| **N55229** | **M** | **I** | **K** | **V** | **L** | **L** | **V** | **D** | **D** | **H** | **E** | **M** | **V** | **R** | **L** | **G** | **V** | **S** | **S** | **Y** | **L** | **S** | **I** | **Q** | **E** | **D** | **I** | **E** | **V** | **I** | **G** | **E** | **A** | **E** | **N** | **G** | **R** | **Q** | **G** | **Y** | **E** | **K** | **A** | **M** | **A** | **L** | **R** | **P** | **D** | **V** | **I** | **L** | **M** | **D** | **L** | **V** | **M** | **E** | **E** | **M** | **D** | **G** | **I** | **E** | **S** | **T** | **K** | **A** | **I** | **L** | **K** | **D** | **W** | **P** | **K** | **A** | **K** | **I** | **I** | **I** | **V** | **T** | **S** | **F** | **I** | **D** | **D** | **E** | **K** | **V** | **Y** | **P** | **A** | **I** | **E** | **A** | **G** | **A** | **A** | **G** | **Y** | **L** | **L** | **K** | **T** | **S** | **T** | **A** | **H** | **E** | **I** | **A** | **D** | **A** | **I** | **R** | **A** | **T** | **Q** | **R** | **G** | **E** | **R** | **V** | **L** | **E** | **P** | **E** | **V** | **T** | **T** | **K** | **M** | **M** | **E** | **K** | **M** | **S** | **R** | **R** | **N** | **D** | **P** | **V** | **L** | **H** | **E** | **E** | **L** | **T** | **N** | **R** | **E** | **N** | **E** | **I** | **L** | **M** | **L** | **I** | **S** | **E** | **G** | **K** | **S** | **N** | **Q** | **E** | **I** | **A** | **D** | **E** | **L** | **F** | **I** | **T** | **L** | **K** | **T** | **V** | **K** | **T** | **H** | **V** | **S** | **N** | **I** | **L** | **A** | **K** | **L** | **E** | **V** | **E** | **D** | **R** | **T** | **Q** | **A** | **A** | **I** | **Y** | **A** | **F** | **K** | **H** | **G** | **L** | **V** | **K** |
| **N60336F** | **M** | **I** | **K** | **V** | **L** | **L** | **V** | **D** | **D** | **H** | **E** | **M** | **V** | **R** | **L** | **G** | **V** | **S** | **S** | **Y** | **L** | **S** | **I** | **Q** | **E** | **D** | **I** | **E** | **V** | **I** | **G** | **E** | **A** | **E** | **N** | **G** | **R** | **Q** | **G** | **Y** | **E** | **K** | **A** | **M** | **A** | **L** | **R** | **P** | **D** | **V** | **I** | **L** | **M** | **D** | **L** | **V** | **M** | **E** | **E** | **M** | **D** | **G** | **I** | **E** | **S** | **T** | **K** | **A** | **I** | **L** | **K** | **D** | **W** | **P** | **K** | **A** | **K** | **I** | **I** | **I** | **V** | **T** | **S** | **F** | **I** | **D** | **D** | **E** | **K** | **V** | **Y** | **P** | **A** | **I** | **E** | **A** | **G** | **A** | **A** | **G** | **Y** | **L** | **L** | **K** | **T** | **S** | **T** | **A** | **H** | **E** | **I** | **A** | **D** | **A** | **I** | **R** | **A** | **T** | **Q** | **R** | **G** | **E** | **R** | **V** | **L** | **E** | **P** | **E** | **V** | **T** | **T** | **K** | **M** | **M** | **E** | **K** | **M** | **S** | **R** | **R** | **N** | **D** | **P** | **V** | **L** | **H** | **E** | **E** | **L** | **T** | **N** | **R** | **E** | **N** | **E** | **I** | **L** | **M** | **L** | **I** | **S** | **E** | **G** | **K** | **S** | **N** | **Q** | **E** | **I** | **A** | **D** | **E** | **L** | **F** | **I** | **T** | **L** | **K** | **T** | **V** | **K** | **T** | **H** | **V** | **S** | **N** | **I** | **L** | **A** | **K** | **L** | **E** | **V** | **E** | **D** | **R** | **T** | **Q** | **A** | **A** | **I** | **Y** | **A** | **F** | **K** | **H** | **G** | **L** | **V** | **K** |
| **N55655** | **M** | **I** | **K** | **V** | **L** | **L** | **V** | **D** | **D** | **H** | **E** | **M** | **V** | **R** | **L** | **G** | **V** | **S** | **S** | **Y** | **L** | **S** | **I** | **Q** | **E** | **D** | **I** | **E** | **V** | **I** | **G** | **E** | **A** | **E** | **N** | **G** | **R** | **Q** | **G** | **Y** | **E** | **K** | **A** | **M** | **A** | **L** | **R** | **P** | **D** | **V** | **I** | **L** | **M** | **D** | **L** | **V** | **M** | **E** | **E** | **M** | **D** | **G** | **I** | **E** | **S** | **T** | **K** | **A** | **I** | **L** | **K** | **D** | **W** | **P** | **K** | **A** | **K** | **I** | **I** | **I** | **V** | **T** | **S** | **F** | **I** | **D** | **D** | **E** | **K** | **V** | **Y** | **P** | **A** | **I** | **E** | **A** | **G** | **A** | **A** | **G** | **Y** | **L** | **L** | **K** | **T** | **S** | **T** | **A** | **H** | **E** | **I** | **A** | **D** | **A** | **I** | **R** | **A** | **T** | **Q** | **R** | **G** | **E** | **R** | **V** | **L** | **E** | **P** | **E** | **V** | **T** | **T** | **K** | **M** | **M** | **E** | **K** | **M** | **S** | **R** | **R** | **N** | **D** | **P** | **V** | **L** | **H** | **E** | **E** | **L** | **T** | **N** | **R** | **E** | **N** | **E** | **I** | **L** | **M** | **L** | **I** | **S** | **E** | **G** | **K** | **S** | **N** | **Q** | **E** | **I** | **A** | **D** | **E** | **L** | **F** | **I** | **T** | **L** | **K** | **T** | **V** | **K** | **T** | **H** | **V** | **S** | **N** | **I** | **L** | **A** | **K** | **L** | **E** | **V** | **E** | **D** | **R** | **T** | **Q** | **A** | **A** | **I** | **Y** | **A** | **F** | **K** | **H** | **G** | **L** | **V** | **K** |
| **N55106** | **V** | **I** | **K** | **V** | **M** | **L** | **V** | **D** | **D** | **H** | **E** | **M** | **V** | **R** | **L** | **G** | **V** | **S** | **S** | **Y** | **L** | **S** | **I** | **Q** | **E** | **D** | **I** | **E** | **V** | **V** | **G** | **E** | **A** | **E** | **N** | **G** | **K** | **I** | **G** | **Y** | **E** | **K** | **A** | **L** | **E** | **L** | **R** | **P** | **D** | **V** | **I** | **L** | **M** | **D** | **L** | **V** | **M** | **E** | **E** | **M** | **D** | **G** | **I** | **D** | **S** | **T** | **K** | **A** | **I** | **L** | **K** | **D** | **W** | **P** | **E** | **A** | **K** | **I** | **I** | **I** | **V** | **T** | **S** | **F** | **I** | **D** | **D** | **E** | **K** | **V** | **Y** | **P** | **A** | **I** | **E** | **A** | **G** | **A** | **A** | **G** | **Y** | **L** | **L** | **K** | **T** | **S** | **T** | **A** | **H** | **E** | **I** | **A** | **D** | **A** | **I** | **R** | **A** | **T** | **Y** | **R** | **G** | **E** | **R** | **V** | **L** | **E** | **P** | **E** | **V** | **T** | **H** | **K** | **M** | **M** | **E** | **R** | **L** | **T** | **K** | **K** | **Q** | **E** | **P** | **V** | **L** | **H** | **E** | **D** | **L** | **T** | **N** | **R** | **E** | **H** | **E** | **I** | **L** | **M** | **L** | **I** | **A** | **Q** | **G** | **K** | **S** | **N** | **Q** | **E** | **I** | **A** | **D** | **E** | **L** | **F** | **I** | **T** | **L** | **K** | **T** | **V** | **K** | **T** | **H** | **V** | **S** | **N** | **I** | **L** | **A** | **K** | **L** | **D** | **V** | **D** | **D** | **R** | **T** | **Q** | **A** | **A** | **I** | **Y** | **A** | **F** | **Q** | **H** | **G** | **L** | **A** | **K** |
| **N59503F** | **M** | **I** | **K** | **V** | **L** | **L** | **V** | **D** | **D** | **H** | **E** | **M** | **V** | **R** | **L** | **G** | **V** | **S** | **S** | **Y** | **L** | **S** | **I** | **Q** | **E** | **D** | **I** | **E** | **V** | **I** | **G** | **E** | **A** | **E** | **N** | **G** | **R** | **Q** | **G** | **Y** | **E** | **K** | **A** | **M** | **A** | **L** | **R** | **P** | **D** | **V** | **I** | **L** | **M** | **D** | **L** | **V** | **M** | **E** | **E** | **M** | **D** | **G** | **I** | **E** | **S** | **T** | **K** | **A** | **I** | **L** | **K** | **D** | **W** | **P** | **K** | **A** | **K** | **I** | **I** | **I** | **V** | **T** | **S** | **F** | **I** | **D** | **D** | **E** | **K** | **V** | **Y** | **P** | **A** | **I** | **E** | **A** | **G** | **A** | **A** | **G** | **Y** | **L** | **L** | **K** | **T** | **S** | **T** | **A** | **H** | **E** | **I** | **A** | **D** | **A** | **I** | **R** | **A** | **T** | **Q** | **R** | **G** | **E** | **R** | **V** | **L** | **E** | **P** | **E** | **V** | **T** | **T** | **K** | **M** | **M** | **E** | **K** | **M** | **S** | **R** | **R** | **Y** | **D** | **P** | **V** | **L** | **H** | **E** | **E** | **L** | **T** | **N** | **R** | **E** | **N** | **E** | **I** | **L** | **M** | **L** | **I** | **S** | **E** | **G** | **K** | **S** | **N** | **Q** | **E** | **I** | **A** | **D** | **E** | **L** | **F** | **I** | **T** | **L** | **K** | **T** | **V** | **K** | **T** | **H** | **V** | **S** | **N** | **I** | **L** | **A** | **K** | **L** | **E** | **V** | **E** | **D** | **R** | **T** | **Q** | **A** | **A** | **I** | **Y** | **A** | **F** | **K** | **H** | **G** | **L** | **V** | **K** |
| **N52724** | **V** | **I** | **K** | **V** | **M** | **L** | **V** | **D** | **D** | **H** | **E** | **M** | **V** | **R** | **L** | **G** | **V** | **S** | **S** | **Y** | **L** | **S** | **I** | **Q** | **E** | **D** | **I** | **E** | **V** | **V** | **G** | **E** | **A** | **E** | **N** | **G** | **K** | **I** | **G** | **Y** | **E** | **K** | **A** | **L** | **E** | **L** | **R** | **P** | **D** | **V** | **I** | **L** | **M** | **D** | **L** | **V** | **M** | **E** | **E** | **M** | **D** | **G** | **I** | **D** | **S** | **T** | **K** | **A** | **I** | **L** | **K** | **D** | **W** | **P** | **E** | **A** | **K** | **I** | **I** | **I** | **V** | **T** | **S** | **F** | **I** | **D** | **D** | **E** | **K** | **V** | **Y** | **P** | **A** | **I** | **E** | **A** | **G** | **A** | **A** | **G** | **Y** | **L** | **L** | **K** | **T** | **S** | **T** | **A** | **H** | **E** | **I** | **A** | **D** | **A** | **I** | **R** | **A** | **T** | **Y** | **R** | **G** | **E** | **R** | **V** | **L** | **E** | **P** | **E** | **V** | **T** | **H** | **K** | **M** | **M** | **E** | **R** | **L** | **T** | **K** | **K** | **Q** | **E** | **P** | **V** | **L** | **H** | **E** | **D** | **L** | **T** | **N** | **R** | **E** | **H** | **E** | **I** | **L** | **M** | **L** | **I** | **A** | **Q** | **G** | **K** | **S** | **N** | **Q** | **E** | **I** | **A** | **D** | **E** | **L** | **F** | **I** | **T** | **L** | **K** | **T** | **V** | **K** | **T** | **H** | **V** | **S** | **N** | **I** | **L** | **A** | **K** | **L** | **D** | **V** | **D** | **D** | **R** | **T** | **Q** | **A** | **A** | **I** | **Y** | **A** | **F** | **Q** | **H** | **G** | **L** | **A** | **K** |
| **N59555F** | **V** | **I** | **K** | **V** | **M** | **L** | **V** | **D** | **D** | **H** | **E** | **M** | **V** | **R** | **L** | **G** | **V** | **S** | **S** | **Y** | **L** | **S** | **I** | **Q** | **E** | **D** | **I** | **E** | **V** | **V** | **G** | **E** | **A** | **E** | **N** | **G** | **K** | **I** | **G** | **Y** | **E** | **K** | **A** | **L** | **E** | **L** | **R** | **P** | **D** | **V** | **I** | **L** | **M** | **D** | **L** | **V** | **M** | **E** | **E** | **M** | **D** | **G** | **I** | **D** | **S** | **T** | **K** | **A** | **I** | **L** | **K** | **D** | **W** | **P** | **E** | **A** | **K** | **I** | **I** | **I** | **V** | **T** | **S** | **F** | **I** | **D** | **D** | **E** | **K** | **V** | **Y** | **P** | **A** | **I** | **E** | **A** | **G** | **A** | **A** | **G** | **Y** | **L** | **L** | **K** | **T** | **S** | **T** | **A** | **H** | **E** | **I** | **A** | **D** | **A** | **I** | **R** | **A** | **T** | **Y** | **R** | **G** | **E** | **R** | **V** | **L** | **E** | **P** | **E** | **V** | **T** | **H** | **K** | **M** | **M** | **E** | **R** | **L** | **T** | **K** | **K** | **Q** | **E** | **P** | **V** | **L** | **H** | **E** | **D** | **L** | **T** | **N** | **R** | **E** | **H** | **E** | **I** | **L** | **M** | **L** | **I** | **A** | **Q** | **G** | **K** | **S** | **N** | **Q** | **E** | **I** | **A** | **D** | **E** | **L** | **F** | **I** | **T** | **L** | **K** | **T** | **V** | **K** | **T** | **H** | **V** | **S** | **N** | **I** | **L** | **A** | **K** | **L** | **D** | **V** | **D** | **D** | **R** | **T** | **Q** | **A** | **A** | **I** | **Y** | **A** | **F** | **Q** | **H** | **G** | **L** | **A** | **K** |
| **N60100F** | **V** | **I** | **K** | **V** | **M** | **L** | **V** | **D** | **D** | **H** | **E** | **M** | **V** | **R** | **L** | **G** | **V** | **S** | **S** | **Y** | **L** | **S** | **I** | **Q** | **E** | **D** | **I** | **E** | **V** | **V** | **G** | **E** | **A** | **E** | **N** | **G** | **K** | **I** | **G** | **Y** | **E** | **K** | **A** | **L** | **E** | **L** | **R** | **P** | **D** | **V** | **I** | **L** | **M** | **D** | **L** | **V** | **M** | **E** | **E** | **M** | **D** | **G** | **I** | **D** | **S** | **T** | **K** | **A** | **I** | **L** | **K** | **D** | **W** | **P** | **E** | **A** | **K** | **I** | **I** | **I** | **V** | **T** | **S** | **F** | **I** | **D** | **D** | **E** | **K** | **V** | **Y** | **P** | **A** | **I** | **E** | **A** | **G** | **A** | **A** | **G** | **Y** | **L** | **L** | **K** | **T** | **S** | **T** | **A** | **H** | **E** | **I** | **A** | **D** | **A** | **I** | **R** | **A** | **T** | **Y** | **R** | **G** | **E** | **R** | **V** | **L** | **E** | **P** | **E** | **V** | **T** | **H** | **K** | **M** | **M** | **E** | **R** | **L** | **T** | **K** | **K** | **Q** | **E** | **P** | **V** | **L** | **H** | **E** | **D** | **L** | **T** | **N** | **R** | **E** | **H** | **E** | **I** | **L** | **M** | **L** | **I** | **A** | **Q** | **G** | **K** | **S** | **N** | **Q** | **E** | **I** | **A** | **D** | **E** | **L** | **F** | **I** | **T** | **L** | **K** | **T** | **V** | **K** | **T** | **H** | **V** | **S** | **N** | **I** | **L** | **A** | **K** | **L** | **D** | **V** | **D** | **D** | **R** | **T** | **Q** | **A** | **A** | **I** | **Y** | **A** | **F** | **Q** | **H** | **G** | **L** | **A** | **K** |
| **N59711F** | **M** | **I** | **K** | **V** | **L** | **L** | **V** | **D** | **D** | **H** | **E** | **M** | **V** | **R** | **L** | **G** | **V** | **S** | **S** | **Y** | **L** | **S** | **I** | **Q** | **E** | **D** | **I** | **E** | **V** | **I** | **G** | **E** | **A** | **E** | **N** | **G** | **R** | **Q** | **G** | **Y** | **E** | **K** | **A** | **M** | **T** | **L** | **R** | **P** | **D** | **V** | **I** | **L** | **M** | **D** | **L** | **V** | **M** | **E** | **E** | **M** | **D** | **G** | **I** | **E** | **S** | **T** | **K** | **A** | **I** | **L** | **K** | **D** | **W** | **P** | **E** | **A** | **K** | **I** | **I** | **I** | **V** | **T** | **S** | **F** | **I** | **D** | **D** | **E** | **K** | **V** | **Y** | **P** | **A** | **I** | **E** | **A** | **G** | **A** | **A** | **G** | **Y** | **L** | **L** | **K** | **T** | **S** | **T** | **A** | **H** | **E** | **I** | **A** | **D** | **A** | **I** | **R** | **A** | **T** | **Q** | **R** | **G** | **E** | **R** | **V** | **L** | **E** | **P** | **E** | **V** | **T** | **T** | **K** | **M** | **M** | **E** | **K** | **M** | **S** | **R** | **R** | **N** | **E** | **P** | **V** | **L** | **H** | **E** | **E** | **L** | **T** | **N** | **R** | **E** | **N** | **E** | **I** | **L** | **M** | **L** | **I** | **S** | **E** | **G** | **K** | **S** | **N** | **Q** | **E** | **I** | **A** | **D** | **E** | **L** | **F** | **I** | **T** | **L** | **K** | **T** | **V** | **K** | **T** | **H** | **V** | **S** | **N** | **I** | **L** | **A** | **K** | **L** | **E** | **V** | **E** | **D** | **R** | **T** | **Q** | **A** | **A** | **I** | **Y** | **A** | **F** | **K** | **H** | **G** | **L** | **V** | **K** |
| **N59589F** | **M** | **I** | **K** | **V** | **L** | **L** | **V** | **D** | **D** | **H** | **E** | **M** | **V** | **R** | **L** | **G** | **V** | **S** | **S** | **Y** | **L** | **S** | **I** | **Q** | **E** | **D** | **I** | **E** | **V** | **I** | **G** | **E** | **A** | **E** | **N** | **G** | **R** | **Q** | **G** | **Y** | **E** | **K** | **A** | **M** | **T** | **L** | **R** | **P** | **D** | **V** | **I** | **L** | **M** | **D** | **L** | **V** | **M** | **E** | **E** | **M** | **D** | **G** | **I** | **E** | **S** | **T** | **K** | **A** | **I** | **L** | **K** | **D** | **W** | **P** | **E** | **A** | **K** | **I** | **I** | **I** | **V** | **T** | **S** | **F** | **I** | **D** | **D** | **E** | **K** | **V** | **Y** | **P** | **A** | **I** | **E** | **A** | **G** | **A** | **A** | **G** | **Y** | **L** | **L** | **K** | **T** | **S** | **T** | **A** | **H** | **E** | **I** | **A** | **D** | **A** | **I** | **R** | **A** | **T** | **Q** | **R** | **G** | **E** | **R** | **V** | **L** | **E** | **P** | **E** | **V** | **T** | **T** | **K** | **M** | **M** | **E** | **K** | **M** | **S** | **R** | **R** | **N** | **E** | **P** | **V** | **L** | **H** | **E** | **E** | **L** | **T** | **N** | **R** | **E** | **N** | **E** | **I** | **L** | **M** | **L** | **I** | **S** | **E** | **G** | **K** | **S** | **N** | **Q** | **E** | **I** | **A** | **D** | **E** | **L** | **F** | **I** | **T** | **L** | **K** | **T** | **V** | **K** | **T** | **H** | **V** | **S** | **N** | **I** | **L** | **A** | **K** | **L** | **E** | **V** | **E** | **D** | **R** | **T** | **Q** | **A** | **A** | **I** | **Y** | **A** | **F** | **K** | **H** | **G** | **L** | **V** | **K** |
| **N52564** | **V** | **I** | **K** | **V** | **M** | **L** | **V** | **D** | **D** | **H** | **E** | **M** | **V** | **R** | **L** | **G** | **V** | **S** | **S** | **Y** | **L** | **S** | **I** | **Q** | **E** | **D** | **I** | **E** | **V** | **V** | **G** | **E** | **A** | **E** | **N** | **G** | **K** | **I** | **G** | **Y** | **E** | **K** | **A** | **L** | **E** | **L** | **R** | **P** | **D** | **V** | **I** | **L** | **M** | **D** | **L** | **V** | **M** | **E** | **E** | **M** | **D** | **G** | **I** | **D** | **S** | **T** | **K** | **A** | **I** | **L** | **K** | **D** | **W** | **P** | **E** | **A** | **K** | **I** | **I** | **I** | **V** | **T** | **S** | **F** | **I** | **D** | **D** | **E** | **K** | **V** | **Y** | **P** | **A** | **I** | **E** | **A** | **G** | **A** | **A** | **G** | **Y** | **L** | **L** | **K** | **T** | **S** | **T** | **A** | **H** | **E** | **I** | **A** | **D** | **A** | **I** | **R** | **A** | **T** | **Y** | **R** | **G** | **E** | **R** | **V** | **L** | **E** | **P** | **E** | **V** | **T** | **H** | **K** | **M** | **M** | **E** | **R** | **L** | **T** | **K** | **K** | **Q** | **E** | **P** | **V** | **L** | **H** | **E** | **D** | **L** | **T** | **N** | **R** | **E** | **H** | **E** | **I** | **L** | **M** | **L** | **I** | **A** | **Q** | **G** | **K** | **S** | **N** | **Q** | **E** | **I** | **A** | **D** | **E** | **L** | **F** | **I** | **T** | **L** | **K** | **T** | **V** | **K** | **T** | **H** | **V** | **S** | **N** | **I** | **L** | **A** | **K** | **L** | **D** | **V** | **D** | **D** | **R** | **T** | **Q** | **A** | **A** | **I** | **Y** | **A** | **F** | **Q** | **H** | **G** | **L** | **A** | **K** |
| **N60271F** | **V** | **I** | **K** | **V** | **M** | **L** | **V** | **D** | **D** | **H** | **E** | **M** | **V** | **R** | **L** | **G** | **V** | **S** | **S** | **Y** | **L** | **S** | **I** | **Q** | **E** | **D** | **I** | **E** | **V** | **V** | **G** | **E** | **A** | **E** | **N** | **G** | **K** | **I** | **G** | **Y** | **E** | **K** | **A** | **L** | **E** | **L** | **R** | **P** | **D** | **V** | **I** | **L** | **M** | **D** | **L** | **V** | **M** | **E** | **E** | **M** | **D** | **G** | **I** | **D** | **S** | **T** | **K** | **A** | **I** | **L** | **K** | **D** | **W** | **P** | **E** | **A** | **K** | **I** | **I** | **I** | **V** | **T** | **S** | **F** | **I** | **D** | **D** | **E** | **K** | **V** | **Y** | **P** | **A** | **I** | **E** | **A** | **G** | **A** | **A** | **G** | **Y** | **L** | **L** | **K** | **T** | **S** | **T** | **A** | **H** | **E** | **I** | **A** | **D** | **A** | **I** | **R** | **A** | **T** | **Y** | **R** | **G** | **E** | **R** | **V** | **L** | **E** | **P** | **E** | **V** | **T** | **H** | **K** | **M** | **M** | **E** | **R** | **L** | **T** | **K** | **K** | **Q** | **E** | **P** | **V** | **L** | **H** | **E** | **D** | **L** | **T** | **N** | **R** | **E** | **H** | **E** | **I** | **L** | **M** | **L** | **I** | **A** | **Q** | **G** | **K** | **S** | **N** | **Q** | **E** | **I** | **A** | **D** | **E** | **L** | **F** | **I** | **T** | **L** | **K** | **T** | **V** | **K** | **T** | **H** | **V** | **S** | **N** | **I** | **L** | **A** | **K** | **L** | **D** | **V** | **D** | **D** | **R** | **T** | **Q** | **A** | **A** | **I** | **Y** | **A** | **F** | **Q** | **H** | **G** | **L** | **A** | **K** |
| **N60440F** | **V** | **I** | **K** | **V** | **M** | **L** | **V** | **D** | **D** | **H** | **E** | **M** | **V** | **R** | **L** | **G** | **V** | **S** | **S** | **Y** | **L** | **S** | **I** | **Q** | **E** | **D** | **I** | **E** | **V** | **V** | **G** | **E** | **A** | **E** | **N** | **G** | **K** | **I** | **G** | **Y** | **E** | **K** | **A** | **L** | **E** | **L** | **R** | **P** | **D** | **V** | **I** | **L** | **M** | **D** | **L** | **V** | **M** | **E** | **E** | **M** | **D** | **G** | **I** | **D** | **S** | **T** | **K** | **A** | **I** | **L** | **K** | **D** | **W** | **P** | **E** | **A** | **K** | **I** | **I** | **I** | **V** | **T** | **S** | **F** | **I** | **D** | **D** | **E** | **K** | **V** | **Y** | **P** | **A** | **I** | **E** | **A** | **G** | **A** | **A** | **G** | **Y** | **L** | **L** | **K** | **T** | **S** | **T** | **A** | **H** | **E** | **I** | **A** | **D** | **A** | **I** | **R** | **A** | **T** | **Y** | **R** | **G** | **E** | **R** | **V** | **L** | **E** | **P** | **E** | **V** | **T** | **H** | **K** | **M** | **M** | **E** | **R** | **L** | **T** | **K** | **K** | **Q** | **E** | **P** | **V** | **L** | **H** | **E** | **D** | **L** | **T** | **N** | **R** | **E** | **H** | **E** | **I** | **L** | **M** | **L** | **I** | **A** | **Q** | **G** | **K** | **S** | **N** | **Q** | **E** | **I** | **A** | **D** | **E** | **L** | **F** | **I** | **T** | **L** | **K** | **T** | **V** | **K** | **T** | **H** | **V** | **S** | **N** | **I** | **L** | **A** | **K** | **L** | **D** | **V** | **D** | **D** | **R** | **T** | **Q** | **A** | **A** | **I** | **Y** | **A** | **F** | **Q** | **H** | **G** | **L** | **A** | **K** |
| **N59599F** | **V** | **I** | **K** | **V** | **M** | **L** | **V** | **D** | **D** | **H** | **E** | **M** | **V** | **R** | **L** | **G** | **V** | **S** | **S** | **Y** | **L** | **S** | **I** | **Q** | **E** | **D** | **I** | **E** | **V** | **V** | **G** | **E** | **A** | **E** | **N** | **G** | **K** | **I** | **G** | **Y** | **E** | **K** | **A** | **L** | **E** | **L** | **R** | **P** | **D** | **V** | **I** | **L** | **M** | **D** | **L** | **V** | **M** | **E** | **E** | **M** | **D** | **G** | **I** | **D** | **S** | **T** | **K** | **A** | **I** | **L** | **K** | **D** | **W** | **P** | **E** | **A** | **K** | **I** | **I** | **I** | **V** | **T** | **S** | **F** | **I** | **D** | **D** | **E** | **K** | **V** | **Y** | **P** | **A** | **I** | **E** | **A** | **G** | **A** | **A** | **G** | **Y** | **L** | **L** | **K** | **T** | **S** | **T** | **A** | **H** | **E** | **I** | **A** | **D** | **A** | **I** | **R** | **A** | **T** | **Y** | **R** | **G** | **E** | **R** | **V** | **L** | **E** | **P** | **E** | **V** | **T** | **H** | **K** | **M** | **M** | **E** | **R** | **L** | **T** | **K** | **K** | **Q** | **E** | **P** | **V** | **L** | **H** | **E** | **D** | **L** | **T** | **N** | **R** | **E** | **H** | **E** | **I** | **L** | **M** | **L** | **I** | **A** | **Q** | **G** | **K** | **S** | **N** | **Q** | **E** | **I** | **A** | **D** | **E** | **L** | **F** | **I** | **T** | **L** | **K** | **T** | **V** | **K** | **T** | **H** | **V** | **S** | **N** | **I** | **L** | **A** | **K** | **L** | **D** | **V** | **D** | **D** | **R** | **T** | **Q** | **A** | **A** | **I** | **Y** | **A** | **F** | **Q** | **H** | **G** | **L** | **A** | **K** |
| **N54546** | **M** | **I** | **K** | **V** | **L** | **L** | **V** | **D** | **D** | **H** | **E** | **M** | **V** | **R** | **L** | **G** | **V** | **S** | **S** | **Y** | **L** | **S** | **I** | **Q** | **E** | **D** | **I** | **E** | **V** | **I** | **G** | **E** | **A** | **E** | **N** | **G** | **R** | **Q** | **G** | **Y** | **E** | **K** | **A** | **M** | **T** | **L** | **R** | **P** | **D** | **V** | **I** | **L** | **M** | **D** | **L** | **V** | **M** | **E** | **E** | **M** | **D** | **G** | **I** | **E** | **S** | **T** | **K** | **A** | **I** | **L** | **K** | **D** | **W** | **P** | **E** | **A** | **K** | **I** | **I** | **I** | **V** | **T** | **S** | **F** | **I** | **D** | **D** | **E** | **K** | **V** | **Y** | **P** | **A** | **I** | **E** | **A** | **G** | **A** | **A** | **G** | **Y** | **L** | **L** | **K** | **T** | **S** | **T** | **A** | **H** | **E** | **I** | **A** | **D** | **A** | **I** | **R** | **A** | **T** | **Q** | **R** | **G** | **E** | **R** | **V** | **L** | **E** | **P** | **E** | **V** | **T** | **T** | **K** | **M** | **M** | **E** | **K** | **M** | **S** | **R** | **R** | **N** | **E** | **P** | **V** | **L** | **H** | **E** | **E** | **L** | **T** | **N** | **R** | **E** | **N** | **E** | **I** | **L** | **M** | **L** | **I** | **S** | **E** | **G** | **K** | **S** | **N** | **Q** | **E** | **I** | **A** | **D** | **E** | **L** | **F** | **I** | **T** | **L** | **K** | **T** | **V** | **K** | **T** | **H** | **V** | **S** | **N** | **I** | **L** | **A** | **K** | **L** | **E** | **V** | **E** | **D** | **R** | **T** | **Q** | **A** | **A** | **I** | **Y** | **A** | **F** | **K** | **H** | **G** | **L** | **V** | **K** |
| **N55753** | **V** | **I** | **K** | **V** | **M** | **L** | **V** | **D** | **D** | **H** | **E** | **M** | **V** | **R** | **L** | **G** | **V** | **S** | **S** | **Y** | **L** | **S** | **I** | **Q** | **E** | **D** | **I** | **E** | **V** | **V** | **G** | **E** | **A** | **E** | **N** | **G** | **K** | **I** | **G** | **Y** | **E** | **K** | **A** | **L** | **E** | **L** | **R** | **P** | **D** | **V** | **I** | **L** | **M** | **D** | **L** | **V** | **M** | **E** | **E** | **M** | **D** | **G** | **I** | **D** | **S** | **T** | **K** | **A** | **I** | **L** | **K** | **D** | **W** | **P** | **E** | **A** | **K** | **I** | **I** | **I** | **V** | **T** | **S** | **F** | **I** | **D** | **D** | **E** | **K** | **V** | **Y** | **P** | **A** | **I** | **E** | **A** | **G** | **A** | **A** | **G** | **Y** | **L** | **L** | **K** | **T** | **S** | **T** | **A** | **H** | **E** | **I** | **A** | **D** | **A** | **I** | **R** | **A** | **T** | **Y** | **R** | **G** | **E** | **R** | **V** | **L** | **E** | **P** | **E** | **V** | **T** | **H** | **K** | **M** | **M** | **E** | **R** | **L** | **T** | **K** | **K** | **Q** | **E** | **P** | **V** | **L** | **H** | **E** | **D** | **L** | **T** | **N** | **R** | **E** | **H** | **E** | **I** | **L** | **M** | **L** | **I** | **A** | **Q** | **G** | **K** | **S** | **N** | **Q** | **E** | **I** | **A** | **D** | **E** | **L** | **F** | **I** | **T** | **L** | **K** | **T** | **V** | **K** | **T** | **H** | **V** | **S** | **N** | **I** | **L** | **A** | **K** | **L** | **D** | **V** | **D** | **D** | **R** | **T** | **Q** | **A** | **A** | **I** | **Y** | **A** | **F** | **Q** | **H** | **G** | **L** | **A** | **K** |
| **N52656** | **M** | **I** | **K** | **V** | **L** | **L** | **V** | **D** | **D** | **H** | **E** | **M** | **V** | **R** | **L** | **G** | **V** | **S** | **S** | **Y** | **L** | **S** | **I** | **Q** | **E** | **D** | **I** | **E** | **V** | **I** | **G** | **E** | **A** | **E** | **N** | **G** | **R** | **Q** | **G** | **Y** | **E** | **K** | **A** | **M** | **T** | **L** | **R** | **P** | **D** | **V** | **I** | **L** | **M** | **D** | **L** | **V** | **M** | **E** | **E** | **M** | **D** | **G** | **I** | **E** | **S** | **T** | **K** | **A** | **I** | **L** | **K** | **D** | **W** | **P** | **E** | **A** | **K** | **I** | **I** | **I** | **V** | **T** | **S** | **F** | **I** | **D** | **D** | **E** | **K** | **V** | **Y** | **P** | **A** | **I** | **E** | **A** | **G** | **A** | **A** | **G** | **Y** | **L** | **L** | **K** | **T** | **S** | **T** | **A** | **H** | **E** | **I** | **A** | **D** | **A** | **I** | **R** | **A** | **T** | **Q** | **R** | **G** | **E** | **R** | **V** | **L** | **E** | **P** | **E** | **V** | **T** | **T** | **K** | **M** | **M** | **E** | **K** | **M** | **S** | **R** | **R** | **N** | **E** | **P** | **V** | **L** | **H** | **E** | **E** | **L** | **T** | **N** | **R** | **E** | **N** | **E** | **I** | **L** | **M** | **L** | **I** | **S** | **E** | **G** | **K** | **S** | **N** | **Q** | **E** | **I** | **A** | **D** | **E** | **L** | **F** | **I** | **T** | **L** | **K** | **T** | **V** | **K** | **T** | **H** | **V** | **S** | **N** | **I** | **L** | **A** | **K** | **L** | **E** | **V** | **E** | **D** | **R** | **T** | **Q** | **A** | **A** | **I** | **Y** | **A** | **F** | **K** | **H** | **G** | **L** | **V** | **-** |
| **N52729** | **V** | **I** | **K** | **V** | **M** | **L** | **V** | **D** | **D** | **H** | **E** | **M** | **V** | **R** | **L** | **G** | **V** | **S** | **S** | **Y** | **L** | **S** | **I** | **Q** | **E** | **D** | **I** | **E** | **V** | **V** | **G** | **E** | **A** | **E** | **N** | **G** | **K** | **I** | **G** | **Y** | **E** | **K** | **A** | **L** | **E** | **L** | **R** | **P** | **D** | **V** | **I** | **L** | **M** | **D** | **L** | **V** | **M** | **E** | **E** | **M** | **D** | **G** | **I** | **D** | **S** | **T** | **K** | **A** | **I** | **L** | **K** | **D** | **W** | **P** | **E** | **A** | **K** | **I** | **I** | **I** | **V** | **T** | **S** | **F** | **I** | **D** | **D** | **E** | **K** | **V** | **Y** | **P** | **A** | **I** | **E** | **A** | **G** | **A** | **A** | **G** | **Y** | **L** | **L** | **K** | **T** | **S** | **T** | **A** | **H** | **E** | **I** | **A** | **D** | **A** | **I** | **R** | **A** | **T** | **Y** | **R** | **G** | **E** | **R** | **V** | **L** | **E** | **P** | **E** | **V** | **T** | **H** | **K** | **M** | **M** | **E** | **R** | **L** | **T** | **K** | **K** | **Q** | **E** | **P** | **V** | **L** | **H** | **E** | **D** | **L** | **T** | **N** | **R** | **E** | **H** | **E** | **I** | **L** | **M** | **L** | **I** | **A** | **Q** | **G** | **K** | **S** | **N** | **Q** | **E** | **I** | **A** | **D** | **E** | **L** | **F** | **I** | **T** | **L** | **K** | **T** | **V** | **K** | **T** | **H** | **V** | **S** | **N** | **I** | **L** | **A** | **K** | **L** | **D** | **V** | **D** | **D** | **R** | **T** | **Q** | **A** | **A** | **I** | **Y** | **A** | **F** | **Q** | **H** | **G** | **L** | **A** | **K** |
| **N54190** | **M** | **I** | **K** | **V** | **L** | **L** | **V** | **D** | **D** | **H** | **E** | **M** | **V** | **R** | **L** | **G** | **V** | **S** | **S** | **Y** | **L** | **S** | **I** | **Q** | **E** | **D** | **I** | **E** | **V** | **I** | **G** | **E** | **A** | **E** | **N** | **G** | **R** | **Q** | **G** | **Y** | **E** | **K** | **A** | **M** | **A** | **L** | **R** | **P** | **D** | **V** | **I** | **L** | **M** | **D** | **L** | **V** | **M** | **E** | **E** | **M** | **D** | **G** | **I** | **E** | **S** | **T** | **K** | **A** | **I** | **L** | **K** | **D** | **W** | **P** | **K** | **A** | **K** | **I** | **I** | **I** | **V** | **T** | **S** | **F** | **I** | **D** | **D** | **E** | **K** | **V** | **Y** | **P** | **A** | **I** | **E** | **A** | **G** | **A** | **A** | **G** | **Y** | **L** | **L** | **K** | **T** | **S** | **T** | **A** | **H** | **E** | **I** | **A** | **D** | **A** | **I** | **R** | **A** | **T** | **Q** | **R** | **G** | **E** | **R** | **V** | **L** | **E** | **P** | **E** | **V** | **T** | **T** | **K** | **M** | **M** | **E** | **K** | **M** | **S** | **R** | **R** | **N** | **D** | **P** | **V** | **L** | **H** | **E** | **E** | **L** | **T** | **N** | **R** | **E** | **N** | **E** | **I** | **L** | **M** | **L** | **I** | **S** | **E** | **G** | **K** | **S** | **N** | **Q** | **E** | **I** | **A** | **D** | **E** | **L** | **F** | **I** | **T** | **L** | **K** | **T** | **V** | **K** | **T** | **H** | **V** | **S** | **N** | **I** | **L** | **A** | **K** | **L** | **E** | **V** | **E** | **D** | **R** | **T** | **Q** | **A** | **A** | **I** | **Y** | **A** | **F** | **K** | **H** | **G** | **L** | **V** | **K** |
| **N52769** | **M** | **I** | **K** | **V** | **L** | **L** | **V** | **D** | **D** | **H** | **E** | **M** | **V** | **R** | **L** | **G** | **V** | **S** | **S** | **Y** | **L** | **S** | **I** | **Q** | **E** | **D** | **I** | **E** | **V** | **I** | **G** | **E** | **A** | **E** | **N** | **G** | **R** | **Q** | **G** | **Y** | **E** | **K** | **A** | **M** | **T** | **L** | **R** | **P** | **D** | **V** | **I** | **L** | **M** | **D** | **L** | **V** | **M** | **E** | **E** | **M** | **D** | **G** | **I** | **E** | **S** | **T** | **K** | **A** | **I** | **L** | **K** | **D** | **W** | **P** | **E** | **A** | **K** | **I** | **I** | **I** | **V** | **T** | **S** | **F** | **I** | **D** | **D** | **E** | **K** | **V** | **Y** | **P** | **A** | **I** | **E** | **A** | **G** | **A** | **A** | **G** | **Y** | **L** | **L** | **K** | **T** | **S** | **T** | **A** | **H** | **E** | **I** | **A** | **D** | **A** | **I** | **R** | **A** | **T** | **Q** | **R** | **G** | **E** | **R** | **V** | **L** | **E** | **P** | **E** | **V** | **T** | **T** | **K** | **M** | **M** | **E** | **K** | **M** | **S** | **R** | **R** | **N** | **E** | **P** | **V** | **L** | **H** | **E** | **E** | **L** | **T** | **N** | **R** | **E** | **N** | **E** | **I** | **L** | **M** | **L** | **I** | **S** | **E** | **G** | **K** | **S** | **N** | **Q** | **E** | **I** | **A** | **D** | **E** | **L** | **F** | **I** | **T** | **L** | **K** | **T** | **V** | **K** | **T** | **H** | **V** | **S** | **N** | **I** | **L** | **A** | **K** | **L** | **E** | **V** | **E** | **D** | **R** | **T** | **Q** | **A** | **A** | **I** | **Y** | **A** | **F** | **K** | **H** | **G** | **L** | **V** | **K** |
| **N59420F** | **V** | **I** | **K** | **V** | **M** | **L** | **V** | **D** | **D** | **H** | **E** | **M** | **V** | **R** | **L** | **G** | **V** | **S** | **S** | **Y** | **L** | **S** | **I** | **Q** | **E** | **D** | **I** | **E** | **V** | **V** | **G** | **E** | **A** | **E** | **N** | **G** | **K** | **I** | **G** | **Y** | **E** | **K** | **A** | **L** | **E** | **L** | **R** | **P** | **D** | **V** | **I** | **L** | **M** | **D** | **L** | **V** | **M** | **E** | **E** | **M** | **D** | **G** | **I** | **D** | **S** | **T** | **K** | **A** | **I** | **L** | **K** | **D** | **W** | **P** | **E** | **A** | **K** | **I** | **I** | **I** | **V** | **T** | **S** | **F** | **I** | **D** | **D** | **E** | **K** | **V** | **Y** | **P** | **A** | **I** | **E** | **A** | **G** | **A** | **A** | **G** | **Y** | **L** | **L** | **K** | **T** | **S** | **T** | **A** | **H** | **E** | **I** | **A** | **D** | **A** | **I** | **R** | **A** | **T** | **Y** | **R** | **G** | **E** | **R** | **V** | **L** | **E** | **P** | **E** | **V** | **T** | **H** | **K** | **M** | **M** | **E** | **R** | **L** | **T** | **K** | **K** | **Q** | **E** | **P** | **V** | **L** | **H** | **E** | **D** | **L** | **T** | **N** | **R** | **E** | **H** | **E** | **I** | **L** | **M** | **L** | **I** | **A** | **Q** | **G** | **K** | **S** | **N** | **Q** | **E** | **I** | **A** | **D** | **E** | **L** | **F** | **I** | **T** | **L** | **K** | **T** | **V** | **K** | **T** | **H** | **V** | **S** | **N** | **I** | **L** | **A** | **K** | **L** | **D** | **V** | **D** | **D** | **R** | **T** | **Q** | **A** | **A** | **I** | **Y** | **A** | **F** | **Q** | **H** | **G** | **L** | **A** | **K** |
| **N59959F** | **V** | **I** | **K** | **V** | **M** | **L** | **V** | **D** | **D** | **H** | **E** | **M** | **V** | **R** | **L** | **G** | **V** | **S** | **S** | **Y** | **L** | **S** | **I** | **Q** | **E** | **D** | **I** | **E** | **V** | **V** | **G** | **E** | **A** | **E** | **N** | **G** | **K** | **I** | **G** | **Y** | **E** | **K** | **A** | **L** | **E** | **L** | **R** | **P** | **D** | **V** | **I** | **L** | **M** | **D** | **L** | **V** | **M** | **E** | **E** | **M** | **D** | **G** | **I** | **D** | **S** | **T** | **K** | **A** | **I** | **L** | **K** | **D** | **W** | **P** | **E** | **A** | **K** | **I** | **I** | **I** | **V** | **T** | **S** | **F** | **I** | **D** | **D** | **E** | **K** | **V** | **Y** | **P** | **A** | **I** | **E** | **A** | **G** | **A** | **A** | **G** | **Y** | **L** | **L** | **K** | **T** | **S** | **T** | **A** | **H** | **E** | **I** | **A** | **D** | **A** | **I** | **R** | **A** | **T** | **Y** | **R** | **G** | **E** | **R** | **V** | **L** | **E** | **P** | **E** | **V** | **T** | **H** | **K** | **M** | **M** | **E** | **R** | **L** | **T** | **K** | **K** | **Q** | **E** | **P** | **V** | **L** | **H** | **E** | **D** | **L** | **T** | **N** | **R** | **E** | **H** | **E** | **I** | **L** | **M** | **L** | **I** | **A** | **Q** | **G** | **K** | **S** | **N** | **Q** | **E** | **I** | **A** | **D** | **E** | **L** | **F** | **I** | **T** | **L** | **K** | **T** | **V** | **K** | **T** | **H** | **V** | **S** | **N** | **I** | **L** | **A** | **K** | **L** | **D** | **V** | **D** | **D** | **R** | **T** | **Q** | **A** | **A** | **I** | **Y** | **A** | **F** | **Q** | **H** | **G** | **L** | **A** | **K** |
| **N53476** | **V** | **I** | **K** | **V** | **M** | **L** | **V** | **D** | **D** | **H** | **E** | **M** | **V** | **R** | **L** | **G** | **V** | **S** | **S** | **Y** | **L** | **S** | **I** | **Q** | **E** | **D** | **I** | **E** | **V** | **V** | **G** | **E** | **A** | **E** | **N** | **G** | **K** | **I** | **G** | **Y** | **E** | **K** | **A** | **L** | **E** | **L** | **R** | **P** | **D** | **V** | **I** | **L** | **M** | **D** | **L** | **V** | **M** | **E** | **E** | **M** | **D** | **G** | **I** | **D** | **S** | **T** | **K** | **A** | **I** | **L** | **K** | **D** | **W** | **P** | **E** | **A** | **K** | **I** | **I** | **I** | **V** | **T** | **S** | **F** | **I** | **D** | **D** | **E** | **K** | **V** | **Y** | **P** | **A** | **I** | **E** | **A** | **G** | **A** | **A** | **G** | **Y** | **L** | **L** | **K** | **T** | **S** | **T** | **A** | **H** | **E** | **I** | **A** | **D** | **A** | **I** | **R** | **A** | **T** | **Y** | **R** | **G** | **E** | **R** | **V** | **L** | **E** | **P** | **E** | **V** | **T** | **H** | **K** | **M** | **M** | **E** | **R** | **L** | **T** | **K** | **K** | **Q** | **E** | **P** | **V** | **L** | **H** | **E** | **D** | **L** | **T** | **N** | **R** | **E** | **H** | **E** | **I** | **L** | **M** | **L** | **I** | **A** | **Q** | **G** | **K** | **S** | **N** | **Q** | **E** | **I** | **A** | **D** | **E** | **L** | **F** | **I** | **T** | **L** | **K** | **T** | **V** | **K** | **T** | **H** | **V** | **S** | **N** | **I** | **L** | **A** | **K** | **L** | **D** | **V** | **D** | **D** | **R** | **T** | **Q** | **A** | **A** | **I** | **Y** | **A** | **F** | **Q** | **H** | **G** | **L** | **A** | **K** |
| **N53661** | **V** | **I** | **K** | **V** | **M** | **L** | **V** | **D** | **D** | **H** | **E** | **M** | **V** | **R** | **L** | **G** | **V** | **S** | **S** | **Y** | **L** | **S** | **I** | **Q** | **E** | **D** | **I** | **E** | **V** | **V** | **G** | **E** | **A** | **E** | **N** | **G** | **K** | **I** | **G** | **Y** | **E** | **K** | **A** | **L** | **E** | **L** | **R** | **P** | **D** | **V** | **I** | **L** | **M** | **D** | **L** | **V** | **M** | **E** | **E** | **M** | **D** | **G** | **I** | **D** | **S** | **T** | **K** | **A** | **I** | **L** | **K** | **D** | **W** | **P** | **E** | **A** | **K** | **I** | **I** | **I** | **V** | **T** | **S** | **F** | **I** | **D** | **D** | **E** | **K** | **V** | **Y** | **P** | **A** | **I** | **E** | **A** | **G** | **A** | **A** | **G** | **Y** | **L** | **L** | **K** | **T** | **S** | **T** | **A** | **H** | **E** | **I** | **A** | **D** | **A** | **I** | **R** | **A** | **T** | **Y** | **R** | **G** | **E** | **R** | **V** | **L** | **E** | **P** | **E** | **V** | **T** | **H** | **K** | **M** | **M** | **E** | **R** | **L** | **T** | **K** | **K** | **Q** | **E** | **P** | **V** | **L** | **H** | **E** | **D** | **L** | **T** | **N** | **R** | **E** | **H** | **E** | **I** | **L** | **M** | **L** | **I** | **A** | **Q** | **G** | **K** | **S** | **N** | **Q** | **E** | **I** | **A** | **D** | **E** | **L** | **F** | **I** | **T** | **L** | **K** | **T** | **V** | **K** | **T** | **H** | **V** | **S** | **N** | **I** | **L** | **A** | **K** | **L** | **D** | **V** | **D** | **D** | **R** | **T** | **Q** | **A** | **A** | **I** | **Y** | **A** | **F** | **Q** | **H** | **G** | **L** | **A** | **K** |
| **N59943F** | **M** | **I** | **K** | **V** | **L** | **L** | **V** | **D** | **D** | **H** | **E** | **M** | **V** | **R** | **L** | **G** | **V** | **S** | **S** | **Y** | **L** | **S** | **I** | **Q** | **E** | **D** | **I** | **E** | **V** | **I** | **G** | **E** | **A** | **E** | **N** | **G** | **R** | **Q** | **G** | **Y** | **E** | **K** | **A** | **M** | **T** | **L** | **R** | **P** | **D** | **V** | **I** | **L** | **M** | **D** | **L** | **V** | **M** | **E** | **E** | **M** | **D** | **G** | **I** | **E** | **S** | **T** | **K** | **A** | **I** | **L** | **K** | **D** | **W** | **P** | **E** | **A** | **K** | **I** | **I** | **I** | **V** | **T** | **S** | **F** | **I** | **D** | **D** | **E** | **K** | **V** | **Y** | **P** | **A** | **I** | **E** | **A** | **G** | **A** | **A** | **G** | **Y** | **L** | **L** | **K** | **T** | **S** | **T** | **A** | **H** | **E** | **I** | **A** | **D** | **A** | **I** | **R** | **A** | **T** | **Q** | **R** | **G** | **E** | **R** | **V** | **L** | **E** | **P** | **E** | **V** | **T** | **T** | **K** | **M** | **M** | **E** | **K** | **M** | **S** | **R** | **R** | **N** | **E** | **P** | **V** | **L** | **H** | **E** | **E** | **L** | **T** | **N** | **R** | **E** | **N** | **E** | **I** | **L** | **M** | **L** | **I** | **S** | **E** | **G** | **K** | **S** | **N** | **Q** | **E** | **I** | **A** | **D** | **E** | **L** | **F** | **I** | **T** | **L** | **K** | **T** | **V** | **K** | **T** | **H** | **V** | **S** | **N** | **I** | **L** | **A** | **K** | **L** | **E** | **V** | **E** | **D** | **R** | **T** | **Q** | **A** | **A** | **I** | **Y** | **A** | **F** | **K** | **H** | **G** | **L** | **V** | **K** |
| **N59462F** | **V** | **I** | **K** | **V** | **M** | **L** | **V** | **D** | **D** | **H** | **E** | **M** | **V** | **R** | **L** | **G** | **V** | **S** | **S** | **Y** | **L** | **S** | **I** | **Q** | **E** | **D** | **I** | **E** | **V** | **V** | **G** | **E** | **A** | **E** | **N** | **G** | **K** | **I** | **G** | **Y** | **E** | **K** | **A** | **L** | **E** | **L** | **R** | **P** | **D** | **V** | **I** | **L** | **M** | **D** | **L** | **V** | **M** | **E** | **E** | **M** | **D** | **G** | **I** | **D** | **S** | **T** | **K** | **A** | **I** | **L** | **K** | **D** | **W** | **P** | **E** | **A** | **K** | **I** | **I** | **I** | **V** | **T** | **S** | **F** | **I** | **D** | **D** | **E** | **K** | **V** | **Y** | **P** | **A** | **I** | **E** | **A** | **G** | **A** | **A** | **G** | **Y** | **L** | **L** | **K** | **T** | **S** | **T** | **A** | **H** | **E** | **I** | **A** | **D** | **A** | **I** | **R** | **A** | **T** | **Y** | **R** | **G** | **E** | **R** | **V** | **L** | **E** | **P** | **E** | **V** | **T** | **H** | **K** | **M** | **M** | **E** | **R** | **L** | **T** | **K** | **K** | **Q** | **E** | **P** | **V** | **L** | **H** | **E** | **D** | **L** | **T** | **N** | **R** | **E** | **H** | **E** | **I** | **L** | **M** | **L** | **I** | **A** | **Q** | **G** | **K** | **S** | **N** | **Q** | **E** | **I** | **A** | **D** | **E** | **L** | **F** | **I** | **T** | **L** | **K** | **T** | **V** | **K** | **T** | **H** | **V** | **S** | **N** | **I** | **L** | **A** | **K** | **L** | **D** | **V** | **D** | **D** | **R** | **T** | **Q** | **A** | **A** | **I** | **Y** | **A** | **F** | **Q** | **H** | **G** | **L** | **A** | **K** |
| **N52643** | **M** | **I** | **K** | **V** | **L** | **L** | **V** | **D** | **D** | **H** | **E** | **M** | **V** | **R** | **L** | **G** | **V** | **S** | **S** | **Y** | **L** | **S** | **I** | **Q** | **E** | **D** | **I** | **E** | **V** | **I** | **G** | **E** | **A** | **E** | **N** | **G** | **R** | **Q** | **G** | **Y** | **E** | **K** | **A** | **M** | **A** | **L** | **R** | **P** | **D** | **V** | **I** | **L** | **M** | **D** | **L** | **V** | **M** | **E** | **E** | **M** | **D** | **G** | **I** | **E** | **S** | **T** | **K** | **A** | **I** | **L** | **K** | **D** | **W** | **P** | **K** | **A** | **K** | **I** | **I** | **I** | **V** | **T** | **S** | **F** | **I** | **D** | **D** | **E** | **K** | **V** | **Y** | **P** | **A** | **I** | **E** | **A** | **G** | **A** | **A** | **G** | **Y** | **L** | **L** | **K** | **T** | **S** | **T** | **A** | **H** | **E** | **I** | **A** | **D** | **A** | **I** | **R** | **A** | **T** | **Q** | **R** | **G** | **E** | **R** | **V** | **L** | **E** | **P** | **E** | **V** | **T** | **T** | **K** | **M** | **M** | **E** | **K** | **M** | **S** | **R** | **R** | **N** | **D** | **P** | **V** | **L** | **H** | **E** | **E** | **L** | **T** | **N** | **R** | **E** | **N** | **E** | **I** | **L** | **M** | **L** | **I** | **S** | **E** | **G** | **K** | **S** | **N** | **Q** | **E** | **I** | **A** | **D** | **E** | **L** | **F** | **I** | **T** | **L** | **K** | **T** | **V** | **K** | **T** | **H** | **V** | **S** | **N** | **I** | **L** | **A** | **K** | **L** | **E** | **V** | **E** | **D** | **R** | **T** | **Q** | **A** | **A** | **I** | **Y** | **A** | **F** | **K** | **H** | **G** | **L** | **V** | **K** |
| **N60048F** | **M** | **I** | **K** | **V** | **L** | **L** | **V** | **D** | **D** | **H** | **E** | **M** | **V** | **R** | **L** | **G** | **V** | **S** | **S** | **Y** | **L** | **S** | **I** | **Q** | **E** | **D** | **I** | **E** | **V** | **I** | **G** | **E** | **A** | **E** | **N** | **G** | **R** | **Q** | **G** | **Y** | **E** | **K** | **A** | **M** | **A** | **L** | **R** | **P** | **D** | **V** | **I** | **L** | **M** | **D** | **L** | **V** | **M** | **E** | **E** | **M** | **D** | **G** | **I** | **E** | **S** | **T** | **K** | **A** | **I** | **L** | **K** | **D** | **W** | **P** | **K** | **A** | **K** | **I** | **I** | **I** | **V** | **T** | **S** | **F** | **I** | **D** | **D** | **E** | **K** | **V** | **Y** | **P** | **A** | **I** | **E** | **A** | **G** | **A** | **A** | **G** | **Y** | **L** | **L** | **K** | **T** | **S** | **T** | **A** | **H** | **E** | **I** | **A** | **D** | **A** | **I** | **R** | **A** | **T** | **Q** | **R** | **G** | **E** | **R** | **V** | **L** | **E** | **P** | **E** | **V** | **T** | **T** | **K** | **M** | **M** | **E** | **K** | **M** | **S** | **R** | **R** | **N** | **D** | **P** | **V** | **L** | **H** | **E** | **E** | **L** | **T** | **N** | **R** | **E** | **N** | **E** | **I** | **L** | **M** | **L** | **I** | **S** | **E** | **G** | **K** | **S** | **N** | **Q** | **E** | **I** | **A** | **D** | **E** | **L** | **F** | **I** | **T** | **L** | **K** | **T** | **V** | **K** | **T** | **H** | **V** | **S** | **N** | **I** | **L** | **A** | **K** | **L** | **E** | **V** | **E** | **D** | **R** | **T** | **Q** | **A** | **A** | **I** | **Y** | **A** | **F** | **K** | **H** | **G** | **L** | **V** | **K** |
| **N60045F** | **M** | **I** | **K** | **V** | **L** | **L** | **V** | **D** | **D** | **H** | **E** | **M** | **V** | **R** | **L** | **G** | **V** | **S** | **S** | **Y** | **L** | **S** | **I** | **Q** | **E** | **D** | **I** | **E** | **V** | **I** | **G** | **E** | **A** | **E** | **N** | **G** | **R** | **Q** | **G** | **Y** | **E** | **K** | **A** | **M** | **A** | **L** | **R** | **P** | **D** | **V** | **I** | **L** | **M** | **D** | **L** | **V** | **M** | **E** | **E** | **M** | **D** | **G** | **I** | **E** | **S** | **T** | **K** | **A** | **I** | **L** | **K** | **D** | **W** | **P** | **K** | **A** | **K** | **I** | **I** | **I** | **V** | **T** | **S** | **F** | **I** | **D** | **D** | **E** | **K** | **V** | **Y** | **P** | **A** | **I** | **E** | **A** | **G** | **A** | **A** | **G** | **Y** | **L** | **L** | **K** | **T** | **S** | **T** | **A** | **H** | **E** | **I** | **A** | **D** | **A** | **I** | **R** | **A** | **T** | **Q** | **R** | **G** | **E** | **R** | **V** | **L** | **E** | **P** | **E** | **V** | **T** | **T** | **K** | **M** | **M** | **E** | **K** | **M** | **S** | **R** | **R** | **N** | **D** | **P** | **V** | **L** | **H** | **E** | **E** | **L** | **T** | **N** | **R** | **E** | **N** | **E** | **I** | **L** | **M** | **L** | **I** | **S** | **E** | **G** | **K** | **S** | **N** | **Q** | **E** | **I** | **A** | **D** | **E** | **L** | **F** | **I** | **T** | **L** | **K** | **T** | **V** | **K** | **T** | **H** | **V** | **S** | **N** | **I** | **L** | **A** | **K** | **L** | **E** | **V** | **E** | **D** | **R** | **T** | **Q** | **A** | **A** | **I** | **Y** | **A** | **F** | **K** | **H** | **G** | **L** | **V** | **K** |
| **N59891F** | **M** | **I** | **K** | **V** | **L** | **L** | **V** | **D** | **D** | **H** | **E** | **M** | **V** | **R** | **L** | **G** | **V** | **S** | **S** | **Y** | **L** | **S** | **I** | **Q** | **E** | **D** | **I** | **E** | **V** | **I** | **G** | **E** | **A** | **E** | **N** | **G** | **R** | **Q** | **G** | **Y** | **E** | **K** | **A** | **M** | **A** | **L** | **R** | **P** | **D** | **V** | **I** | **L** | **M** | **D** | **L** | **V** | **M** | **E** | **E** | **M** | **D** | **G** | **I** | **E** | **S** | **T** | **K** | **A** | **I** | **L** | **K** | **D** | **W** | **P** | **K** | **A** | **K** | **I** | **I** | **I** | **V** | **T** | **S** | **F** | **I** | **D** | **D** | **E** | **K** | **V** | **Y** | **P** | **A** | **I** | **E** | **A** | **G** | **A** | **A** | **G** | **Y** | **L** | **L** | **K** | **T** | **S** | **T** | **A** | **H** | **E** | **I** | **A** | **D** | **A** | **I** | **R** | **A** | **T** | **Q** | **R** | **G** | **E** | **R** | **V** | **L** | **E** | **P** | **E** | **V** | **T** | **T** | **K** | **M** | **M** | **E** | **K** | **M** | **S** | **R** | **R** | **N** | **D** | **P** | **V** | **L** | **H** | **E** | **E** | **L** | **T** | **N** | **R** | **E** | **N** | **E** | **I** | **L** | **M** | **L** | **I** | **S** | **E** | **G** | **K** | **S** | **N** | **Q** | **E** | **I** | **A** | **D** | **E** | **L** | **F** | **I** | **T** | **L** | **K** | **T** | **V** | **K** | **T** | **H** | **V** | **S** | **N** | **I** | **L** | **A** | **K** | **L** | **E** | **V** | **E** | **D** | **R** | **T** | **Q** | **A** | **A** | **I** | **Y** | **A** | **F** | **K** | **H** | **G** | **L** | **V** | **K** |
| **N55181** | **V** | **I** | **K** | **V** | **M** | **L** | **V** | **D** | **D** | **H** | **E** | **M** | **V** | **R** | **L** | **G** | **V** | **S** | **S** | **Y** | **L** | **S** | **I** | **Q** | **E** | **D** | **I** | **E** | **V** | **V** | **G** | **E** | **A** | **E** | **N** | **G** | **K** | **I** | **G** | **Y** | **E** | **K** | **A** | **L** | **E** | **L** | **R** | **P** | **D** | **V** | **I** | **L** | **M** | **D** | **L** | **V** | **M** | **E** | **E** | **M** | **D** | **G** | **I** | **D** | **S** | **T** | **K** | **A** | **I** | **L** | **K** | **D** | **W** | **P** | **E** | **A** | **K** | **I** | **I** | **I** | **V** | **T** | **S** | **F** | **I** | **D** | **D** | **E** | **K** | **V** | **Y** | **P** | **A** | **I** | **E** | **A** | **G** | **A** | **A** | **G** | **Y** | **L** | **L** | **K** | **T** | **S** | **T** | **A** | **H** | **E** | **I** | **A** | **D** | **A** | **I** | **R** | **A** | **T** | **Y** | **R** | **G** | **E** | **R** | **V** | **L** | **E** | **P** | **E** | **V** | **T** | **H** | **K** | **M** | **M** | **E** | **R** | **L** | **T** | **K** | **K** | **Q** | **E** | **P** | **V** | **L** | **H** | **E** | **D** | **L** | **T** | **N** | **R** | **E** | **H** | **E** | **I** | **L** | **M** | **L** | **I** | **A** | **Q** | **G** | **K** | **S** | **N** | **Q** | **E** | **I** | **A** | **D** | **E** | **L** | **F** | **I** | **T** | **L** | **K** | **T** | **V** | **K** | **T** | **H** | **V** | **S** | **N** | **I** | **L** | **A** | **K** | **L** | **D** | **V** | **D** | **D** | **R** | **T** | **Q** | **A** | **A** | **I** | **Y** | **A** | **F** | **Q** | **H** | **G** | **L** | **A** | **K** |
| **N55119** | **V** | **I** | **K** | **V** | **M** | **L** | **V** | **D** | **D** | **H** | **E** | **M** | **V** | **R** | **L** | **G** | **V** | **S** | **S** | **Y** | **L** | **S** | **I** | **Q** | **E** | **D** | **I** | **E** | **V** | **V** | **G** | **E** | **A** | **E** | **N** | **G** | **K** | **I** | **G** | **Y** | **E** | **K** | **A** | **L** | **E** | **L** | **R** | **P** | **D** | **V** | **I** | **L** | **M** | **D** | **L** | **V** | **M** | **E** | **E** | **M** | **D** | **G** | **I** | **D** | **S** | **T** | **K** | **A** | **I** | **L** | **K** | **D** | **W** | **P** | **E** | **A** | **K** | **I** | **I** | **I** | **V** | **T** | **S** | **F** | **I** | **D** | **D** | **E** | **K** | **V** | **Y** | **P** | **A** | **I** | **E** | **A** | **G** | **A** | **A** | **G** | **Y** | **L** | **L** | **K** | **T** | **S** | **T** | **A** | **H** | **E** | **I** | **A** | **D** | **A** | **I** | **R** | **A** | **T** | **Y** | **R** | **G** | **E** | **R** | **V** | **L** | **E** | **P** | **E** | **V** | **T** | **H** | **K** | **M** | **M** | **E** | **R** | **L** | **T** | **K** | **K** | **Q** | **E** | **P** | **V** | **L** | **H** | **E** | **D** | **L** | **T** | **N** | **R** | **E** | **H** | **E** | **I** | **L** | **M** | **L** | **I** | **A** | **Q** | **G** | **K** | **S** | **N** | **Q** | **E** | **I** | **A** | **D** | **E** | **L** | **F** | **I** | **T** | **L** | **K** | **T** | **V** | **K** | **T** | **H** | **V** | **S** | **N** | **I** | **L** | **A** | **K** | **L** | **D** | **V** | **D** | **D** | **R** | **T** | **Q** | **A** | **A** | **I** | **Y** | **A** | **F** | **Q** | **H** | **G** | **L** | **A** | **K** |
| **N54564** | **V** | **I** | **K** | **V** | **M** | **L** | **V** | **D** | **D** | **H** | **E** | **M** | **V** | **R** | **L** | **G** | **V** | **S** | **S** | **Y** | **L** | **S** | **I** | **Q** | **E** | **D** | **I** | **E** | **V** | **V** | **G** | **E** | **A** | **E** | **N** | **G** | **K** | **I** | **G** | **Y** | **E** | **K** | **A** | **L** | **E** | **L** | **R** | **P** | **D** | **V** | **I** | **L** | **M** | **D** | **L** | **V** | **M** | **E** | **E** | **M** | **D** | **G** | **I** | **D** | **S** | **T** | **K** | **A** | **I** | **L** | **K** | **D** | **W** | **P** | **E** | **A** | **K** | **I** | **I** | **I** | **V** | **T** | **S** | **F** | **I** | **D** | **D** | **E** | **K** | **V** | **Y** | **P** | **A** | **I** | **E** | **A** | **G** | **A** | **A** | **G** | **Y** | **L** | **L** | **K** | **T** | **S** | **T** | **A** | **H** | **E** | **I** | **A** | **D** | **A** | **I** | **R** | **A** | **T** | **Y** | **R** | **G** | **E** | **R** | **V** | **L** | **E** | **P** | **E** | **V** | **T** | **H** | **K** | **M** | **M** | **E** | **R** | **L** | **T** | **K** | **K** | **Q** | **E** | **P** | **V** | **L** | **H** | **E** | **D** | **L** | **T** | **N** | **R** | **E** | **H** | **E** | **I** | **L** | **M** | **L** | **I** | **A** | **Q** | **G** | **K** | **S** | **N** | **Q** | **E** | **I** | **A** | **D** | **E** | **L** | **F** | **I** | **T** | **L** | **K** | **T** | **V** | **K** | **T** | **H** | **V** | **S** | **N** | **I** | **L** | **A** | **K** | **L** | **D** | **V** | **D** | **D** | **R** | **T** | **Q** | **A** | **A** | **I** | **Y** | **A** | **F** | **Q** | **H** | **G** | **L** | **A** | **K** |
| **N60392F** | **V** | **I** | **K** | **V** | **M** | **L** | **V** | **D** | **D** | **H** | **E** | **M** | **V** | **R** | **L** | **G** | **V** | **S** | **S** | **Y** | **L** | **S** | **I** | **Q** | **E** | **D** | **I** | **E** | **V** | **V** | **G** | **E** | **A** | **E** | **N** | **G** | **K** | **I** | **G** | **Y** | **E** | **K** | **A** | **L** | **E** | **L** | **R** | **P** | **D** | **V** | **I** | **L** | **M** | **D** | **L** | **V** | **M** | **E** | **E** | **M** | **D** | **G** | **I** | **D** | **S** | **T** | **K** | **A** | **I** | **L** | **K** | **D** | **W** | **P** | **E** | **A** | **K** | **I** | **I** | **I** | **V** | **T** | **S** | **F** | **I** | **D** | **D** | **E** | **K** | **V** | **Y** | **P** | **A** | **I** | **E** | **A** | **G** | **A** | **A** | **G** | **Y** | **L** | **L** | **K** | **T** | **S** | **T** | **A** | **H** | **E** | **I** | **A** | **D** | **A** | **I** | **R** | **A** | **T** | **Y** | **R** | **G** | **E** | **R** | **V** | **L** | **E** | **P** | **E** | **V** | **T** | **H** | **K** | **M** | **M** | **E** | **R** | **L** | **T** | **K** | **K** | **Q** | **E** | **P** | **V** | **L** | **H** | **E** | **D** | **L** | **T** | **N** | **R** | **E** | **H** | **E** | **I** | **L** | **M** | **L** | **I** | **A** | **Q** | **G** | **K** | **S** | **N** | **Q** | **E** | **I** | **A** | **D** | **E** | **L** | **F** | **I** | **T** | **L** | **K** | **T** | **V** | **K** | **T** | **H** | **V** | **S** | **N** | **I** | **L** | **A** | **K** | **L** | **D** | **V** | **D** | **D** | **R** | **T** | **Q** | **A** | **A** | **I** | **Y** | **A** | **F** | **Q** | **H** | **G** | **L** | **A** | **K** |
| **N59606F** | **V** | **I** | **K** | **V** | **M** | **L** | **V** | **D** | **D** | **H** | **E** | **M** | **V** | **R** | **L** | **G** | **V** | **S** | **S** | **Y** | **L** | **S** | **I** | **Q** | **E** | **D** | **I** | **E** | **V** | **V** | **G** | **E** | **A** | **E** | **N** | **G** | **K** | **I** | **G** | **Y** | **E** | **K** | **A** | **L** | **E** | **L** | **R** | **P** | **D** | **V** | **I** | **L** | **M** | **D** | **L** | **V** | **M** | **E** | **E** | **M** | **D** | **G** | **I** | **D** | **S** | **T** | **K** | **A** | **I** | **L** | **K** | **D** | **W** | **P** | **E** | **A** | **K** | **I** | **I** | **I** | **V** | **T** | **S** | **F** | **I** | **D** | **D** | **E** | **K** | **V** | **Y** | **P** | **A** | **I** | **E** | **A** | **G** | **A** | **A** | **G** | **Y** | **L** | **L** | **K** | **T** | **S** | **T** | **A** | **H** | **E** | **I** | **A** | **D** | **A** | **I** | **R** | **A** | **T** | **Y** | **R** | **G** | **E** | **R** | **V** | **L** | **E** | **P** | **E** | **V** | **T** | **H** | **K** | **M** | **M** | **E** | **R** | **L** | **T** | **K** | **K** | **Q** | **E** | **P** | **V** | **L** | **H** | **E** | **D** | **L** | **T** | **N** | **R** | **E** | **H** | **E** | **I** | **L** | **M** | **L** | **I** | **A** | **Q** | **G** | **K** | **S** | **N** | **Q** | **E** | **I** | **A** | **D** | **E** | **L** | **F** | **I** | **T** | **L** | **K** | **T** | **V** | **K** | **T** | **H** | **V** | **S** | **N** | **I** | **L** | **A** | **K** | **L** | **D** | **V** | **D** | **D** | **R** | **T** | **Q** | **A** | **A** | **I** | **Y** | **A** | **F** | **Q** | **H** | **G** | **L** | **A** | **K** |
| **N59653F** | **M** | **I** | **K** | **V** | **L** | **L** | **V** | **D** | **D** | **H** | **E** | **M** | **V** | **R** | **L** | **G** | **V** | **S** | **S** | **Y** | **L** | **S** | **I** | **Q** | **E** | **D** | **I** | **E** | **V** | **I** | **G** | **E** | **A** | **E** | **N** | **G** | **R** | **Q** | **G** | **Y** | **E** | **K** | **A** | **M** | **T** | **L** | **R** | **P** | **D** | **V** | **I** | **L** | **M** | **D** | **L** | **V** | **M** | **E** | **E** | **M** | **D** | **G** | **I** | **E** | **S** | **T** | **K** | **A** | **I** | **L** | **K** | **D** | **W** | **P** | **E** | **A** | **K** | **I** | **I** | **I** | **V** | **T** | **S** | **F** | **I** | **D** | **D** | **E** | **K** | **V** | **Y** | **P** | **A** | **I** | **E** | **A** | **G** | **A** | **A** | **G** | **Y** | **L** | **L** | **K** | **T** | **S** | **T** | **A** | **H** | **E** | **I** | **A** | **D** | **A** | **I** | **R** | **A** | **T** | **Q** | **R** | **G** | **E** | **R** | **V** | **L** | **E** | **P** | **E** | **V** | **T** | **T** | **K** | **M** | **M** | **E** | **K** | **M** | **S** | **R** | **R** | **N** | **E** | **P** | **V** | **L** | **H** | **E** | **E** | **L** | **T** | **N** | **R** | **E** | **N** | **E** | **I** | **L** | **M** | **L** | **I** | **S** | **E** | **G** | **K** | **S** | **N** | **Q** | **E** | **I** | **A** | **D** | **E** | **L** | **F** | **I** | **T** | **L** | **K** | **T** | **V** | **K** | **T** | **H** | **V** | **S** | **N** | **I** | **L** | **A** | **K** | **L** | **E** | **V** | **E** | **D** | **R** | **T** | **Q** | **A** | **A** | **I** | **Y** | **A** | **F** | **K** | **H** | **G** | **L** | **V** | **K** |
| **N55299** | **M** | **I** | **K** | **V** | **L** | **L** | **V** | **D** | **D** | **H** | **E** | **M** | **V** | **R** | **L** | **G** | **V** | **S** | **S** | **Y** | **L** | **S** | **I** | **Q** | **E** | **D** | **I** | **E** | **V** | **I** | **G** | **E** | **A** | **E** | **N** | **G** | **R** | **Q** | **G** | **Y** | **E** | **K** | **A** | **M** | **A** | **L** | **R** | **P** | **D** | **V** | **I** | **L** | **M** | **D** | **L** | **V** | **M** | **E** | **E** | **M** | **D** | **G** | **I** | **E** | **S** | **T** | **K** | **A** | **I** | **L** | **K** | **D** | **W** | **P** | **K** | **A** | **K** | **I** | **I** | **I** | **V** | **T** | **S** | **F** | **I** | **D** | **D** | **E** | **K** | **V** | **Y** | **P** | **A** | **I** | **E** | **A** | **G** | **A** | **A** | **G** | **Y** | **L** | **L** | **K** | **T** | **S** | **T** | **A** | **H** | **E** | **I** | **A** | **D** | **A** | **I** | **R** | **A** | **T** | **Q** | **R** | **G** | **E** | **R** | **V** | **L** | **E** | **P** | **E** | **V** | **T** | **T** | **K** | **M** | **M** | **E** | **K** | **M** | **S** | **R** | **R** | **N** | **D** | **P** | **V** | **L** | **H** | **E** | **E** | **L** | **T** | **N** | **R** | **E** | **N** | **E** | **I** | **L** | **M** | **L** | **I** | **S** | **E** | **G** | **K** | **S** | **N** | **Q** | **E** | **I** | **A** | **D** | **E** | **L** | **F** | **I** | **T** | **L** | **K** | **T** | **V** | **K** | **T** | **H** | **V** | **S** | **N** | **I** | **L** | **A** | **K** | **L** | **E** | **V** | **E** | **D** | **R** | **T** | **Q** | **A** | **A** | **I** | **Y** | **A** | **F** | **K** | **H** | **G** | **L** | **V** | **K** |
| **N59559F** | **M** | **I** | **K** | **V** | **L** | **L** | **V** | **D** | **D** | **H** | **E** | **M** | **V** | **R** | **L** | **G** | **V** | **S** | **S** | **Y** | **L** | **S** | **I** | **Q** | **E** | **D** | **I** | **E** | **V** | **I** | **G** | **E** | **A** | **E** | **N** | **G** | **R** | **Q** | **G** | **Y** | **E** | **K** | **A** | **M** | **T** | **L** | **R** | **P** | **D** | **V** | **I** | **L** | **M** | **D** | **L** | **L** | **M** | **E** | **E** | **M** | **D** | **G** | **I** | **E** | **S** | **T** | **K** | **A** | **I** | **L** | **K** | **D** | **W** | **P** | **E** | **A** | **K** | **I** | **I** | **I** | **V** | **T** | **S** | **F** | **I** | **D** | **D** | **E** | **K** | **V** | **Y** | **P** | **A** | **I** | **E** | **A** | **G** | **A** | **A** | **G** | **Y** | **L** | **L** | **K** | **T** | **S** | **T** | **A** | **H** | **E** | **I** | **A** | **D** | **A** | **I** | **R** | **A** | **T** | **Q** | **R** | **G** | **E** | **R** | **V** | **L** | **E** | **P** | **E** | **V** | **T** | **T** | **K** | **M** | **M** | **E** | **K** | **M** | **S** | **R** | **R** | **N** | **E** | **P** | **V** | **L** | **H** | **E** | **E** | **L** | **T** | **N** | **R** | **E** | **N** | **E** | **I** | **L** | **M** | **L** | **I** | **S** | **E** | **G** | **K** | **S** | **N** | **Q** | **E** | **I** | **A** | **D** | **E** | **L** | **F** | **I** | **T** | **L** | **K** | **T** | **V** | **K** | **T** | **H** | **V** | **S** | **N** | **I** | **L** | **A** | **K** | **L** | **E** | **V** | **E** | **D** | **R** | **T** | **Q** | **A** | **A** | **I** | **Y** | **A** | **F** | **K** | **H** | **G** | **L** | **V** | **K** |
| **N54107** | **M** | **I** | **K** | **V** | **L** | **L** | **V** | **D** | **D** | **H** | **E** | **M** | **V** | **R** | **L** | **G** | **V** | **S** | **S** | **Y** | **L** | **S** | **I** | **Q** | **E** | **D** | **I** | **E** | **V** | **I** | **G** | **E** | **A** | **E** | **N** | **G** | **R** | **Q** | **G** | **Y** | **E** | **K** | **A** | **M** | **A** | **L** | **R** | **P** | **D** | **V** | **I** | **L** | **M** | **D** | **L** | **V** | **M** | **E** | **E** | **M** | **D** | **G** | **I** | **E** | **S** | **T** | **K** | **A** | **I** | **L** | **K** | **D** | **W** | **P** | **K** | **A** | **K** | **I** | **I** | **I** | **V** | **T** | **S** | **F** | **I** | **D** | **D** | **E** | **K** | **V** | **Y** | **P** | **A** | **I** | **E** | **A** | **G** | **A** | **A** | **G** | **Y** | **L** | **L** | **K** | **T** | **S** | **T** | **A** | **H** | **E** | **I** | **A** | **D** | **A** | **I** | **R** | **A** | **T** | **Q** | **R** | **G** | **E** | **R** | **V** | **L** | **E** | **P** | **E** | **V** | **T** | **T** | **K** | **M** | **M** | **E** | **K** | **M** | **S** | **R** | **R** | **N** | **D** | **P** | **V** | **L** | **H** | **E** | **E** | **L** | **T** | **N** | **R** | **E** | **N** | **E** | **I** | **L** | **M** | **L** | **I** | **S** | **E** | **G** | **K** | **S** | **N** | **Q** | **E** | **I** | **A** | **D** | **E** | **L** | **F** | **I** | **T** | **L** | **K** | **T** | **V** | **K** | **T** | **H** | **V** | **S** | **N** | **I** | **L** | **A** | **K** | **L** | **E** | **V** | **E** | **D** | **R** | **T** | **Q** | **A** | **A** | **I** | **Y** | **A** | **F** | **K** | **H** | **G** | **L** | **V** | **K** |
| **N59841F** | **V** | **I** | **K** | **V** | **M** | **L** | **V** | **D** | **D** | **H** | **E** | **M** | **V** | **R** | **L** | **G** | **V** | **S** | **S** | **Y** | **L** | **S** | **I** | **Q** | **E** | **D** | **I** | **E** | **V** | **V** | **G** | **E** | **A** | **E** | **N** | **G** | **K** | **I** | **G** | **Y** | **E** | **K** | **A** | **L** | **E** | **L** | **R** | **P** | **D** | **V** | **I** | **L** | **M** | **D** | **L** | **V** | **M** | **E** | **E** | **M** | **D** | **G** | **I** | **D** | **S** | **T** | **K** | **A** | **I** | **L** | **K** | **D** | **W** | **P** | **E** | **A** | **K** | **I** | **I** | **I** | **V** | **T** | **S** | **F** | **I** | **D** | **D** | **E** | **K** | **V** | **Y** | **P** | **A** | **I** | **E** | **A** | **G** | **A** | **A** | **G** | **Y** | **L** | **L** | **K** | **T** | **S** | **T** | **A** | **H** | **E** | **I** | **A** | **D** | **A** | **I** | **R** | **A** | **T** | **Y** | **R** | **G** | **E** | **R** | **V** | **L** | **E** | **P** | **E** | **V** | **T** | **H** | **K** | **M** | **M** | **E** | **R** | **L** | **T** | **K** | **K** | **Q** | **E** | **P** | **V** | **L** | **H** | **E** | **D** | **L** | **T** | **N** | **R** | **E** | **H** | **E** | **I** | **L** | **M** | **L** | **I** | **A** | **Q** | **G** | **K** | **S** | **N** | **Q** | **E** | **I** | **A** | **D** | **E** | **L** | **F** | **I** | **T** | **L** | **K** | **T** | **V** | **K** | **T** | **H** | **V** | **S** | **N** | **I** | **L** | **A** | **K** | **L** | **D** | **V** | **D** | **D** | **R** | **T** | **Q** | **A** | **A** | **I** | **Y** | **A** | **F** | **Q** | **H** | **G** | **L** | **A** | **K** |
| **N59951F** | **M** | **I** | **K** | **V** | **L** | **L** | **V** | **D** | **D** | **H** | **E** | **M** | **V** | **R** | **L** | **G** | **V** | **S** | **S** | **Y** | **L** | **S** | **I** | **Q** | **E** | **D** | **I** | **E** | **V** | **I** | **G** | **E** | **A** | **E** | **N** | **G** | **R** | **Q** | **G** | **Y** | **E** | **K** | **A** | **M** | **A** | **L** | **R** | **P** | **D** | **V** | **I** | **L** | **M** | **D** | **L** | **V** | **M** | **E** | **E** | **M** | **D** | **G** | **I** | **E** | **S** | **T** | **K** | **A** | **I** | **L** | **K** | **D** | **W** | **P** | **K** | **A** | **K** | **I** | **I** | **I** | **V** | **T** | **S** | **F** | **I** | **D** | **D** | **E** | **K** | **V** | **Y** | **P** | **A** | **I** | **E** | **A** | **G** | **A** | **A** | **G** | **Y** | **L** | **L** | **K** | **T** | **S** | **T** | **A** | **H** | **E** | **I** | **A** | **D** | **A** | **I** | **R** | **A** | **T** | **Q** | **R** | **G** | **E** | **R** | **V** | **L** | **E** | **P** | **E** | **V** | **T** | **T** | **K** | **M** | **M** | **E** | **K** | **M** | **S** | **R** | **R** | **N** | **D** | **P** | **V** | **L** | **H** | **E** | **E** | **L** | **T** | **N** | **R** | **E** | **N** | **E** | **I** | **L** | **M** | **L** | **I** | **S** | **E** | **G** | **K** | **S** | **N** | **Q** | **E** | **I** | **A** | **D** | **E** | **L** | **F** | **I** | **T** | **L** | **K** | **T** | **V** | **K** | **T** | **H** | **V** | **S** | **N** | **I** | **L** | **A** | **K** | **L** | **E** | **V** | **E** | **D** | **R** | **T** | **Q** | **A** | **A** | **I** | **Y** | **A** | **F** | **K** | **H** | **G** | **L** | **V** | **K** |
| **N60417F** | **M** | **I** | **K** | **V** | **L** | **L** | **V** | **D** | **D** | **H** | **E** | **M** | **V** | **R** | **L** | **G** | **V** | **S** | **S** | **Y** | **L** | **S** | **I** | **Q** | **E** | **D** | **I** | **E** | **V** | **I** | **G** | **E** | **A** | **E** | **N** | **G** | **R** | **Q** | **G** | **Y** | **E** | **K** | **A** | **M** | **T** | **L** | **R** | **P** | **D** | **V** | **I** | **L** | **M** | **D** | **L** | **V** | **M** | **E** | **E** | **M** | **D** | **G** | **I** | **E** | **S** | **T** | **K** | **A** | **I** | **L** | **K** | **D** | **W** | **P** | **E** | **A** | **K** | **I** | **I** | **I** | **V** | **T** | **S** | **F** | **I** | **D** | **D** | **E** | **K** | **V** | **Y** | **P** | **A** | **I** | **E** | **A** | **G** | **A** | **A** | **G** | **Y** | **L** | **L** | **K** | **T** | **S** | **T** | **A** | **H** | **E** | **I** | **A** | **D** | **A** | **I** | **R** | **A** | **T** | **Q** | **R** | **G** | **E** | **R** | **V** | **L** | **E** | **P** | **E** | **V** | **T** | **T** | **K** | **M** | **M** | **E** | **K** | **M** | **S** | **R** | **R** | **N** | **E** | **P** | **V** | **L** | **H** | **E** | **E** | **L** | **T** | **N** | **R** | **E** | **N** | **E** | **I** | **L** | **M** | **L** | **I** | **S** | **E** | **G** | **K** | **S** | **N** | **Q** | **E** | **I** | **A** | **D** | **E** | **L** | **F** | **I** | **T** | **L** | **K** | **T** | **V** | **K** | **T** | **H** | **V** | **S** | **N** | **I** | **L** | **A** | **K** | **L** | **E** | **V** | **E** | **D** | **R** | **T** | **Q** | **A** | **A** | **I** | **Y** | **A** | **F** | **K** | **H** | **G** | **L** | **V** | **K** |
| **N53519** | **V** | **I** | **K** | **V** | **M** | **L** | **V** | **D** | **D** | **H** | **E** | **M** | **V** | **R** | **L** | **G** | **V** | **S** | **S** | **Y** | **L** | **S** | **I** | **Q** | **E** | **D** | **I** | **E** | **V** | **V** | **G** | **E** | **A** | **E** | **N** | **G** | **K** | **I** | **G** | **Y** | **E** | **K** | **A** | **L** | **E** | **L** | **R** | **P** | **D** | **V** | **I** | **L** | **M** | **D** | **L** | **V** | **M** | **E** | **E** | **M** | **D** | **G** | **I** | **D** | **S** | **T** | **K** | **A** | **I** | **L** | **K** | **D** | **W** | **P** | **E** | **A** | **K** | **I** | **I** | **I** | **V** | **T** | **S** | **F** | **I** | **D** | **D** | **E** | **K** | **V** | **Y** | **P** | **A** | **I** | **E** | **A** | **G** | **A** | **A** | **G** | **Y** | **L** | **L** | **K** | **T** | **S** | **T** | **A** | **H** | **E** | **I** | **A** | **D** | **A** | **I** | **R** | **A** | **T** | **Y** | **R** | **G** | **E** | **R** | **V** | **L** | **E** | **P** | **E** | **V** | **T** | **H** | **K** | **M** | **M** | **E** | **R** | **L** | **T** | **K** | **K** | **Q** | **E** | **P** | **V** | **L** | **H** | **E** | **D** | **L** | **T** | **N** | **R** | **E** | **H** | **E** | **I** | **L** | **M** | **L** | **I** | **A** | **Q** | **G** | **K** | **S** | **N** | **Q** | **E** | **I** | **A** | **D** | **E** | **L** | **F** | **I** | **T** | **L** | **K** | **T** | **V** | **K** | **T** | **H** | **V** | **S** | **N** | **I** | **L** | **A** | **K** | **L** | **D** | **V** | **D** | **D** | **R** | **T** | **Q** | **A** | **A** | **I** | **Y** | **A** | **F** | **Q** | **H** | **G** | **L** | **A** | **K** |
| **N55240** | **V** | **I** | **K** | **V** | **M** | **L** | **V** | **D** | **D** | **H** | **E** | **M** | **V** | **R** | **L** | **G** | **V** | **S** | **S** | **Y** | **L** | **S** | **I** | **Q** | **E** | **D** | **I** | **E** | **V** | **V** | **G** | **E** | **A** | **E** | **N** | **G** | **K** | **I** | **G** | **Y** | **E** | **K** | **A** | **L** | **E** | **L** | **R** | **P** | **D** | **V** | **I** | **L** | **M** | **D** | **L** | **V** | **M** | **E** | **E** | **M** | **D** | **G** | **I** | **D** | **S** | **T** | **K** | **A** | **I** | **L** | **K** | **D** | **W** | **P** | **E** | **A** | **K** | **I** | **I** | **I** | **V** | **T** | **S** | **F** | **I** | **D** | **D** | **E** | **K** | **V** | **Y** | **P** | **A** | **I** | **E** | **A** | **G** | **A** | **A** | **G** | **Y** | **L** | **L** | **K** | **T** | **S** | **T** | **A** | **H** | **E** | **I** | **A** | **D** | **A** | **I** | **R** | **A** | **T** | **Y** | **R** | **G** | **E** | **R** | **V** | **L** | **E** | **P** | **E** | **V** | **T** | **H** | **K** | **M** | **M** | **E** | **R** | **L** | **T** | **K** | **K** | **Q** | **E** | **P** | **V** | **L** | **H** | **E** | **D** | **L** | **T** | **N** | **R** | **E** | **H** | **E** | **I** | **L** | **M** | **L** | **I** | **A** | **Q** | **G** | **K** | **S** | **N** | **Q** | **E** | **I** | **A** | **D** | **E** | **L** | **F** | **I** | **T** | **L** | **K** | **T** | **V** | **K** | **T** | **H** | **V** | **S** | **N** | **I** | **L** | **A** | **K** | **L** | **D** | **V** | **D** | **D** | **R** | **T** | **Q** | **A** | **A** | **I** | **Y** | **A** | **F** | **Q** | **H** | **G** | **L** | **A** | **K** |
| **N53804** | **M** | **I** | **K** | **V** | **L** | **L** | **V** | **D** | **D** | **H** | **E** | **M** | **V** | **R** | **L** | **G** | **V** | **S** | **S** | **Y** | **L** | **S** | **I** | **Q** | **E** | **D** | **I** | **E** | **V** | **I** | **G** | **E** | **A** | **E** | **N** | **G** | **R** | **Q** | **G** | **Y** | **E** | **K** | **A** | **M** | **A** | **L** | **R** | **P** | **D** | **V** | **I** | **L** | **M** | **D** | **L** | **V** | **M** | **E** | **E** | **M** | **D** | **G** | **I** | **E** | **S** | **T** | **K** | **A** | **I** | **L** | **K** | **D** | **W** | **P** | **K** | **A** | **K** | **I** | **I** | **I** | **V** | **T** | **S** | **F** | **I** | **D** | **D** | **E** | **K** | **V** | **Y** | **P** | **A** | **I** | **E** | **A** | **G** | **A** | **A** | **G** | **Y** | **L** | **L** | **K** | **T** | **S** | **T** | **A** | **H** | **E** | **I** | **A** | **D** | **A** | **I** | **R** | **A** | **T** | **Q** | **R** | **G** | **E** | **R** | **V** | **L** | **E** | **P** | **E** | **V** | **T** | **T** | **K** | **M** | **M** | **E** | **K** | **M** | **S** | **R** | **R** | **N** | **D** | **P** | **V** | **L** | **H** | **E** | **E** | **L** | **T** | **N** | **R** | **E** | **N** | **E** | **I** | **L** | **M** | **L** | **I** | **S** | **E** | **G** | **K** | **S** | **N** | **Q** | **E** | **I** | **A** | **D** | **E** | **L** | **F** | **I** | **T** | **L** | **K** | **T** | **V** | **K** | **T** | **H** | **V** | **S** | **N** | **I** | **L** | **A** | **K** | **L** | **E** | **V** | **E** | **D** | **R** | **T** | **Q** | **A** | **A** | **I** | **Y** | **A** | **F** | **K** | **H** | **G** | **L** | **V** | **K** |
| **N52773** | **V** | **I** | **K** | **V** | **M** | **L** | **V** | **D** | **D** | **H** | **E** | **M** | **V** | **R** | **L** | **G** | **V** | **S** | **S** | **Y** | **L** | **S** | **I** | **Q** | **E** | **D** | **I** | **E** | **V** | **V** | **G** | **E** | **A** | **E** | **N** | **G** | **K** | **I** | **G** | **Y** | **E** | **K** | **A** | **L** | **E** | **L** | **R** | **P** | **D** | **V** | **I** | **L** | **M** | **D** | **L** | **V** | **M** | **E** | **E** | **M** | **D** | **G** | **I** | **D** | **S** | **T** | **K** | **A** | **I** | **L** | **K** | **D** | **W** | **P** | **E** | **A** | **K** | **I** | **I** | **I** | **V** | **T** | **S** | **F** | **I** | **D** | **D** | **E** | **K** | **V** | **Y** | **P** | **A** | **I** | **E** | **A** | **G** | **A** | **A** | **G** | **Y** | **L** | **L** | **K** | **T** | **S** | **T** | **A** | **H** | **E** | **I** | **A** | **D** | **A** | **I** | **R** | **A** | **T** | **Y** | **R** | **G** | **E** | **R** | **V** | **L** | **E** | **P** | **E** | **V** | **T** | **H** | **K** | **M** | **M** | **E** | **R** | **L** | **T** | **K** | **K** | **Q** | **E** | **P** | **V** | **L** | **H** | **E** | **D** | **L** | **T** | **N** | **R** | **E** | **H** | **E** | **I** | **L** | **M** | **L** | **I** | **A** | **Q** | **G** | **K** | **S** | **N** | **Q** | **E** | **I** | **A** | **D** | **E** | **L** | **F** | **I** | **T** | **L** | **K** | **T** | **V** | **K** | **T** | **H** | **V** | **S** | **N** | **I** | **L** | **A** | **K** | **L** | **D** | **V** | **D** | **D** | **R** | **T** | **Q** | **A** | **A** | **I** | **Y** | **A** | **F** | **Q** | **H** | **G** | **L** | **A** | **K** |
| **N59779F** | **M** | **I** | **K** | **V** | **L** | **L** | **V** | **D** | **D** | **H** | **E** | **M** | **V** | **R** | **L** | **G** | **V** | **S** | **S** | **Y** | **L** | **S** | **I** | **Q** | **E** | **D** | **I** | **E** | **V** | **I** | **G** | **E** | **A** | **E** | **N** | **G** | **R** | **Q** | **G** | **Y** | **E** | **K** | **A** | **M** | **A** | **L** | **R** | **P** | **D** | **V** | **I** | **L** | **M** | **D** | **L** | **V** | **M** | **E** | **E** | **M** | **D** | **G** | **I** | **E** | **S** | **T** | **K** | **A** | **I** | **L** | **K** | **D** | **W** | **P** | **K** | **A** | **K** | **I** | **I** | **I** | **V** | **T** | **S** | **F** | **I** | **D** | **D** | **E** | **K** | **V** | **Y** | **P** | **A** | **I** | **E** | **A** | **G** | **A** | **A** | **G** | **Y** | **L** | **L** | **K** | **T** | **S** | **T** | **A** | **H** | **E** | **I** | **A** | **D** | **A** | **I** | **R** | **A** | **T** | **Q** | **R** | **G** | **E** | **R** | **V** | **L** | **E** | **P** | **E** | **V** | **T** | **T** | **K** | **M** | **M** | **E** | **K** | **M** | **S** | **R** | **R** | **N** | **D** | **P** | **V** | **L** | **H** | **E** | **E** | **L** | **T** | **N** | **R** | **E** | **N** | **E** | **I** | **L** | **M** | **L** | **I** | **S** | **E** | **G** | **K** | **S** | **N** | **Q** | **E** | **I** | **A** | **D** | **E** | **L** | **F** | **I** | **T** | **L** | **K** | **T** | **V** | **K** | **T** | **H** | **V** | **S** | **N** | **I** | **L** | **A** | **K** | **L** | **E** | **V** | **E** | **D** | **R** | **T** | **Q** | **A** | **A** | **I** | **Y** | **A** | **F** | **K** | **H** | **G** | **L** | **V** | **K** |
| **N59531F** | **M** | **I** | **K** | **V** | **L** | **L** | **V** | **D** | **D** | **H** | **E** | **M** | **V** | **R** | **L** | **G** | **V** | **S** | **S** | **Y** | **L** | **S** | **I** | **Q** | **E** | **D** | **I** | **E** | **V** | **I** | **G** | **E** | **A** | **E** | **N** | **G** | **R** | **Q** | **G** | **Y** | **E** | **K** | **A** | **M** | **T** | **L** | **R** | **P** | **D** | **V** | **I** | **L** | **M** | **D** | **L** | **V** | **M** | **E** | **E** | **M** | **D** | **G** | **I** | **E** | **S** | **T** | **K** | **A** | **I** | **L** | **K** | **D** | **W** | **P** | **E** | **A** | **K** | **I** | **I** | **I** | **V** | **T** | **S** | **F** | **I** | **D** | **D** | **E** | **K** | **V** | **Y** | **P** | **A** | **I** | **E** | **A** | **G** | **A** | **A** | **G** | **Y** | **L** | **L** | **K** | **T** | **S** | **T** | **A** | **H** | **E** | **I** | **A** | **D** | **A** | **I** | **R** | **A** | **T** | **Q** | **R** | **G** | **E** | **R** | **V** | **L** | **E** | **P** | **E** | **V** | **T** | **T** | **K** | **M** | **M** | **E** | **K** | **M** | **S** | **R** | **Q** | **N** | **E** | **P** | **V** | **L** | **H** | **E** | **E** | **L** | **T** | **N** | **R** | **E** | **N** | **E** | **I** | **L** | **M** | **L** | **I** | **S** | **E** | **G** | **K** | **S** | **N** | **Q** | **E** | **I** | **A** | **D** | **E** | **L** | **F** | **I** | **T** | **L** | **K** | **T** | **V** | **K** | **T** | **H** | **V** | **S** | **N** | **I** | **L** | **A** | **K** | **L** | **E** | **V** | **E** | **D** | **R** | **T** | **Q** | **A** | **A** | **I** | **Y** | **A** | **F** | **K** | **H** | **G** | **L** | **V** | **K** |
| **N60063F** | **V** | **I** | **K** | **V** | **M** | **L** | **V** | **D** | **D** | **H** | **E** | **M** | **V** | **R** | **L** | **G** | **V** | **S** | **S** | **Y** | **L** | **S** | **I** | **Q** | **E** | **D** | **I** | **E** | **V** | **V** | **G** | **E** | **A** | **E** | **N** | **G** | **K** | **I** | **G** | **Y** | **E** | **K** | **A** | **L** | **E** | **L** | **R** | **P** | **D** | **V** | **I** | **L** | **M** | **D** | **L** | **V** | **M** | **E** | **E** | **M** | **D** | **G** | **I** | **D** | **S** | **T** | **K** | **A** | **I** | **L** | **K** | **D** | **W** | **P** | **E** | **A** | **K** | **I** | **I** | **I** | **V** | **T** | **S** | **F** | **I** | **D** | **D** | **E** | **K** | **V** | **Y** | **P** | **A** | **I** | **E** | **A** | **G** | **A** | **A** | **G** | **Y** | **L** | **L** | **K** | **T** | **S** | **T** | **A** | **H** | **E** | **I** | **A** | **D** | **A** | **I** | **R** | **A** | **T** | **Y** | **R** | **G** | **E** | **R** | **V** | **L** | **E** | **P** | **E** | **V** | **T** | **H** | **K** | **M** | **M** | **E** | **R** | **L** | **T** | **K** | **K** | **Q** | **E** | **P** | **V** | **L** | **H** | **E** | **D** | **L** | **T** | **N** | **R** | **E** | **H** | **E** | **I** | **L** | **M** | **L** | **I** | **A** | **Q** | **G** | **K** | **S** | **N** | **Q** | **E** | **I** | **A** | **D** | **E** | **L** | **F** | **I** | **T** | **L** | **K** | **T** | **V** | **K** | **T** | **H** | **V** | **S** | **N** | **I** | **L** | **A** | **K** | **L** | **D** | **V** | **D** | **D** | **R** | **T** | **Q** | **A** | **A** | **I** | **Y** | **A** | **F** | **Q** | **H** | **G** | **L** | **A** | **K** |
| **N53434** | **V** | **I** | **K** | **V** | **M** | **L** | **V** | **D** | **D** | **H** | **E** | **M** | **V** | **R** | **L** | **G** | **V** | **S** | **S** | **Y** | **L** | **S** | **I** | **Q** | **E** | **D** | **I** | **E** | **V** | **V** | **G** | **E** | **A** | **E** | **N** | **G** | **K** | **I** | **G** | **Y** | **E** | **K** | **A** | **L** | **E** | **L** | **R** | **P** | **D** | **V** | **I** | **L** | **M** | **D** | **L** | **V** | **M** | **E** | **E** | **M** | **D** | **G** | **I** | **D** | **S** | **T** | **K** | **A** | **I** | **L** | **K** | **D** | **W** | **P** | **E** | **A** | **K** | **I** | **I** | **I** | **V** | **T** | **S** | **F** | **I** | **D** | **D** | **E** | **K** | **V** | **Y** | **P** | **A** | **I** | **E** | **A** | **G** | **A** | **A** | **G** | **Y** | **L** | **L** | **K** | **T** | **S** | **T** | **A** | **H** | **E** | **I** | **A** | **D** | **A** | **I** | **R** | **A** | **T** | **Y** | **R** | **G** | **E** | **R** | **V** | **L** | **E** | **P** | **E** | **V** | **T** | **H** | **K** | **M** | **M** | **E** | **R** | **L** | **T** | **K** | **K** | **Q** | **E** | **P** | **V** | **L** | **H** | **E** | **D** | **L** | **T** | **N** | **R** | **E** | **H** | **E** | **I** | **L** | **M** | **L** | **I** | **A** | **Q** | **G** | **K** | **S** | **N** | **Q** | **E** | **I** | **A** | **D** | **E** | **L** | **F** | **I** | **T** | **L** | **K** | **T** | **V** | **K** | **T** | **H** | **V** | **S** | **N** | **I** | **L** | **A** | **K** | **L** | **D** | **V** | **D** | **D** | **R** | **T** | **Q** | **A** | **A** | **I** | **Y** | **A** | **F** | **Q** | **H** | **G** | **L** | **A** | **K** |
| **N54118** | **V** | **I** | **K** | **V** | **M** | **L** | **V** | **D** | **D** | **H** | **E** | **M** | **V** | **R** | **L** | **G** | **V** | **S** | **S** | **Y** | **L** | **S** | **I** | **Q** | **E** | **D** | **I** | **E** | **V** | **V** | **G** | **E** | **A** | **E** | **N** | **G** | **K** | **I** | **G** | **Y** | **E** | **K** | **A** | **L** | **E** | **L** | **R** | **P** | **D** | **V** | **I** | **L** | **M** | **D** | **L** | **V** | **M** | **E** | **E** | **M** | **D** | **G** | **I** | **D** | **S** | **T** | **K** | **A** | **I** | **L** | **K** | **D** | **W** | **P** | **E** | **A** | **K** | **I** | **I** | **I** | **V** | **T** | **S** | **F** | **I** | **D** | **D** | **E** | **K** | **V** | **Y** | **P** | **A** | **I** | **E** | **A** | **G** | **A** | **A** | **G** | **Y** | **L** | **L** | **K** | **T** | **S** | **T** | **A** | **H** | **E** | **I** | **A** | **D** | **A** | **I** | **R** | **A** | **T** | **Y** | **R** | **G** | **E** | **R** | **V** | **L** | **E** | **P** | **E** | **V** | **T** | **H** | **K** | **M** | **M** | **E** | **R** | **L** | **T** | **K** | **K** | **Q** | **E** | **P** | **V** | **L** | **H** | **E** | **D** | **L** | **T** | **N** | **R** | **E** | **H** | **E** | **I** | **L** | **M** | **L** | **I** | **A** | **Q** | **G** | **K** | **S** | **N** | **Q** | **E** | **I** | **A** | **D** | **E** | **L** | **F** | **I** | **T** | **L** | **K** | **T** | **V** | **K** | **T** | **H** | **V** | **S** | **N** | **I** | **L** | **A** | **K** | **L** | **D** | **V** | **D** | **D** | **R** | **T** | **Q** | **A** | **A** | **I** | **Y** | **A** | **F** | **Q** | **H** | **G** | **L** | **A** | **K** |
| **N52648** | **V** | **I** | **K** | **V** | **M** | **L** | **V** | **D** | **D** | **H** | **E** | **M** | **V** | **R** | **L** | **G** | **V** | **S** | **S** | **Y** | **L** | **S** | **I** | **Q** | **E** | **D** | **I** | **E** | **V** | **V** | **G** | **E** | **A** | **E** | **N** | **G** | **K** | **I** | **G** | **Y** | **E** | **K** | **A** | **L** | **E** | **L** | **R** | **P** | **D** | **V** | **I** | **L** | **M** | **D** | **L** | **V** | **M** | **E** | **E** | **M** | **D** | **G** | **I** | **D** | **S** | **T** | **K** | **A** | **I** | **L** | **K** | **D** | **W** | **P** | **E** | **A** | **K** | **I** | **I** | **I** | **V** | **T** | **S** | **F** | **I** | **D** | **D** | **E** | **K** | **V** | **Y** | **P** | **A** | **I** | **E** | **A** | **G** | **A** | **A** | **G** | **Y** | **L** | **L** | **K** | **T** | **S** | **T** | **A** | **H** | **E** | **I** | **A** | **D** | **A** | **I** | **R** | **A** | **T** | **Y** | **R** | **G** | **E** | **R** | **V** | **L** | **E** | **P** | **E** | **V** | **T** | **H** | **K** | **M** | **M** | **E** | **R** | **L** | **T** | **K** | **K** | **Q** | **E** | **P** | **V** | **L** | **H** | **E** | **D** | **L** | **T** | **N** | **R** | **E** | **H** | **E** | **I** | **L** | **M** | **L** | **I** | **A** | **Q** | **G** | **K** | **S** | **N** | **Q** | **E** | **I** | **A** | **D** | **E** | **L** | **F** | **I** | **T** | **L** | **K** | **T** | **V** | **K** | **T** | **H** | **V** | **S** | **N** | **I** | **L** | **A** | **K** | **L** | **D** | **V** | **D** | **D** | **R** | **T** | **Q** | **A** | **A** | **I** | **Y** | **A** | **F** | **Q** | **H** | **G** | **L** | **A** | **K** |
| **N60268F** | **V** | **I** | **K** | **V** | **M** | **L** | **V** | **D** | **D** | **H** | **E** | **M** | **V** | **R** | **L** | **G** | **V** | **S** | **S** | **Y** | **L** | **S** | **I** | **Q** | **E** | **D** | **I** | **E** | **V** | **V** | **G** | **E** | **A** | **E** | **N** | **G** | **K** | **I** | **G** | **Y** | **E** | **K** | **A** | **L** | **E** | **L** | **R** | **P** | **D** | **V** | **I** | **L** | **M** | **D** | **L** | **V** | **M** | **E** | **E** | **M** | **D** | **G** | **I** | **D** | **S** | **T** | **K** | **A** | **I** | **L** | **K** | **D** | **W** | **P** | **E** | **A** | **K** | **I** | **I** | **I** | **V** | **T** | **S** | **F** | **I** | **D** | **D** | **E** | **K** | **V** | **Y** | **P** | **A** | **I** | **E** | **A** | **G** | **A** | **A** | **G** | **Y** | **L** | **L** | **K** | **T** | **S** | **T** | **A** | **H** | **E** | **I** | **A** | **D** | **A** | **I** | **R** | **A** | **T** | **Y** | **R** | **G** | **E** | **R** | **V** | **L** | **E** | **P** | **E** | **V** | **T** | **H** | **K** | **M** | **M** | **E** | **R** | **L** | **T** | **K** | **K** | **Q** | **E** | **P** | **V** | **L** | **H** | **E** | **D** | **L** | **T** | **N** | **R** | **E** | **H** | **E** | **I** | **L** | **M** | **L** | **I** | **A** | **Q** | **G** | **K** | **S** | **N** | **Q** | **E** | **I** | **A** | **D** | **E** | **L** | **F** | **I** | **T** | **L** | **K** | **T** | **V** | **K** | **T** | **H** | **V** | **S** | **N** | **I** | **L** | **A** | **K** | **L** | **D** | **V** | **D** | **D** | **R** | **T** | **Q** | **A** | **A** | **I** | **Y** | **A** | **F** | **Q** | **H** | **G** | **L** | **A** | **K** |
| **N53655** | **M** | **I** | **K** | **V** | **L** | **L** | **V** | **D** | **D** | **H** | **E** | **M** | **V** | **R** | **L** | **G** | **V** | **S** | **S** | **Y** | **L** | **S** | **I** | **Q** | **E** | **D** | **I** | **E** | **V** | **I** | **G** | **E** | **A** | **E** | **N** | **G** | **R** | **Q** | **G** | **Y** | **E** | **K** | **A** | **M** | **A** | **L** | **R** | **P** | **D** | **V** | **I** | **L** | **M** | **D** | **L** | **V** | **M** | **E** | **E** | **M** | **D** | **G** | **I** | **E** | **S** | **T** | **K** | **A** | **I** | **L** | **K** | **D** | **W** | **P** | **K** | **A** | **K** | **I** | **I** | **I** | **V** | **T** | **S** | **F** | **I** | **D** | **D** | **E** | **K** | **V** | **Y** | **P** | **A** | **I** | **E** | **A** | **G** | **A** | **A** | **G** | **Y** | **L** | **L** | **K** | **T** | **S** | **T** | **A** | **H** | **E** | **I** | **A** | **D** | **A** | **I** | **R** | **A** | **T** | **Q** | **R** | **G** | **E** | **R** | **V** | **L** | **E** | **P** | **E** | **V** | **T** | **T** | **K** | **M** | **M** | **E** | **K** | **M** | **S** | **R** | **R** | **N** | **D** | **P** | **V** | **L** | **H** | **E** | **E** | **L** | **T** | **N** | **R** | **E** | **N** | **E** | **I** | **L** | **M** | **L** | **I** | **S** | **E** | **G** | **K** | **S** | **N** | **Q** | **E** | **I** | **A** | **D** | **E** | **L** | **F** | **I** | **T** | **L** | **K** | **T** | **V** | **K** | **T** | **H** | **V** | **S** | **N** | **I** | **L** | **A** | **K** | **L** | **E** | **V** | **E** | **D** | **R** | **T** | **Q** | **A** | **A** | **I** | **Y** | **A** | **F** | **K** | **H** | **G** | **L** | **V** | **K** |
| **N52702** | **M** | **I** | **K** | **V** | **L** | **L** | **V** | **D** | **D** | **H** | **E** | **M** | **V** | **R** | **L** | **G** | **V** | **S** | **S** | **Y** | **L** | **S** | **I** | **Q** | **E** | **D** | **I** | **E** | **V** | **I** | **G** | **E** | **A** | **E** | **N** | **G** | **R** | **Q** | **G** | **Y** | **E** | **K** | **A** | **M** | **A** | **L** | **R** | **P** | **D** | **V** | **I** | **L** | **M** | **D** | **L** | **V** | **M** | **E** | **E** | **M** | **D** | **G** | **I** | **E** | **S** | **T** | **K** | **A** | **I** | **L** | **K** | **D** | **W** | **P** | **K** | **A** | **K** | **I** | **I** | **I** | **V** | **T** | **S** | **F** | **I** | **D** | **D** | **E** | **K** | **V** | **Y** | **P** | **A** | **I** | **E** | **A** | **G** | **A** | **A** | **G** | **Y** | **L** | **L** | **K** | **T** | **S** | **T** | **A** | **H** | **E** | **I** | **A** | **D** | **A** | **I** | **R** | **A** | **T** | **Q** | **R** | **G** | **E** | **R** | **V** | **L** | **E** | **P** | **E** | **V** | **T** | **T** | **K** | **M** | **M** | **E** | **K** | **M** | **S** | **R** | **R** | **N** | **D** | **P** | **V** | **L** | **H** | **E** | **E** | **L** | **T** | **N** | **R** | **E** | **N** | **E** | **I** | **L** | **M** | **L** | **I** | **S** | **E** | **G** | **K** | **S** | **N** | **Q** | **E** | **I** | **A** | **D** | **E** | **L** | **F** | **I** | **T** | **L** | **K** | **T** | **V** | **K** | **T** | **H** | **V** | **S** | **N** | **I** | **L** | **A** | **K** | **L** | **E** | **V** | **E** | **D** | **R** | **T** | **Q** | **A** | **A** | **I** | **Y** | **A** | **F** | **K** | **H** | **G** | **L** | **V** | **K** |
| **N59491F** | **V** | **I** | **K** | **V** | **M** | **L** | **V** | **D** | **D** | **H** | **E** | **M** | **V** | **R** | **L** | **G** | **V** | **S** | **S** | **Y** | **L** | **S** | **I** | **Q** | **E** | **D** | **I** | **E** | **V** | **V** | **G** | **E** | **A** | **E** | **N** | **G** | **K** | **I** | **G** | **Y** | **E** | **K** | **A** | **L** | **E** | **L** | **R** | **P** | **D** | **V** | **I** | **L** | **M** | **D** | **L** | **V** | **M** | **E** | **E** | **M** | **D** | **G** | **I** | **D** | **S** | **T** | **K** | **A** | **I** | **L** | **K** | **D** | **W** | **P** | **E** | **A** | **K** | **I** | **I** | **I** | **V** | **T** | **S** | **F** | **I** | **D** | **D** | **E** | **K** | **V** | **Y** | **P** | **A** | **I** | **E** | **A** | **G** | **A** | **A** | **G** | **Y** | **L** | **L** | **K** | **T** | **S** | **T** | **A** | **H** | **E** | **I** | **A** | **D** | **A** | **I** | **R** | **A** | **T** | **Y** | **R** | **G** | **E** | **R** | **V** | **L** | **E** | **P** | **E** | **V** | **T** | **H** | **K** | **M** | **M** | **E** | **R** | **L** | **T** | **K** | **K** | **Q** | **E** | **P** | **V** | **L** | **H** | **E** | **D** | **L** | **T** | **N** | **R** | **E** | **H** | **E** | **I** | **L** | **M** | **L** | **I** | **A** | **Q** | **G** | **K** | **S** | **N** | **Q** | **E** | **I** | **A** | **D** | **E** | **L** | **F** | **I** | **T** | **L** | **K** | **T** | **V** | **K** | **T** | **H** | **V** | **S** | **N** | **I** | **L** | **A** | **K** | **L** | **D** | **V** | **D** | **D** | **R** | **T** | **Q** | **A** | **A** | **I** | **Y** | **A** | **F** | **Q** | **H** | **G** | **L** | **A** | **K** |
| **N59517F** | **M** | **I** | **K** | **V** | **L** | **L** | **V** | **D** | **D** | **H** | **E** | **M** | **V** | **R** | **L** | **G** | **V** | **S** | **S** | **Y** | **L** | **S** | **I** | **Q** | **E** | **D** | **I** | **E** | **V** | **I** | **G** | **E** | **A** | **E** | **N** | **G** | **R** | **Q** | **G** | **Y** | **E** | **K** | **A** | **M** | **A** | **L** | **R** | **P** | **D** | **V** | **I** | **L** | **M** | **D** | **L** | **V** | **M** | **E** | **E** | **M** | **D** | **G** | **I** | **E** | **S** | **T** | **K** | **A** | **I** | **L** | **K** | **D** | **W** | **P** | **K** | **A** | **K** | **I** | **I** | **I** | **V** | **T** | **S** | **F** | **I** | **D** | **D** | **E** | **K** | **V** | **Y** | **P** | **A** | **I** | **E** | **A** | **G** | **A** | **A** | **G** | **Y** | **L** | **L** | **K** | **T** | **S** | **T** | **A** | **H** | **E** | **I** | **A** | **D** | **A** | **I** | **R** | **A** | **T** | **Q** | **R** | **G** | **E** | **R** | **V** | **L** | **E** | **P** | **E** | **V** | **T** | **T** | **K** | **M** | **M** | **E** | **K** | **M** | **S** | **R** | **R** | **N** | **D** | **P** | **V** | **L** | **H** | **E** | **E** | **L** | **T** | **N** | **R** | **E** | **N** | **E** | **I** | **L** | **M** | **L** | **I** | **S** | **E** | **G** | **K** | **S** | **N** | **Q** | **E** | **I** | **A** | **D** | **E** | **L** | **F** | **I** | **T** | **L** | **K** | **T** | **V** | **K** | **T** | **H** | **V** | **S** | **N** | **I** | **L** | **A** | **K** | **L** | **E** | **V** | **E** | **D** | **R** | **T** | **Q** | **A** | **A** | **I** | **Y** | **A** | **F** | **K** | **H** | **G** | **L** | **V** | **K** |
| **N55706** | **V** | **I** | **K** | **V** | **M** | **L** | **V** | **D** | **D** | **H** | **E** | **M** | **V** | **R** | **L** | **G** | **V** | **S** | **S** | **Y** | **L** | **S** | **I** | **Q** | **E** | **D** | **I** | **E** | **V** | **V** | **G** | **E** | **A** | **E** | **N** | **G** | **K** | **I** | **G** | **Y** | **E** | **K** | **A** | **L** | **E** | **L** | **R** | **P** | **D** | **V** | **I** | **L** | **M** | **D** | **L** | **V** | **M** | **E** | **E** | **M** | **D** | **G** | **I** | **D** | **S** | **T** | **K** | **A** | **I** | **L** | **K** | **D** | **W** | **P** | **E** | **A** | **K** | **I** | **I** | **I** | **V** | **T** | **S** | **F** | **I** | **D** | **D** | **E** | **K** | **V** | **Y** | **P** | **A** | **I** | **E** | **A** | **G** | **A** | **A** | **G** | **Y** | **L** | **L** | **K** | **T** | **S** | **T** | **A** | **H** | **E** | **I** | **A** | **D** | **A** | **I** | **R** | **A** | **T** | **Y** | **R** | **G** | **E** | **R** | **V** | **L** | **E** | **P** | **E** | **V** | **T** | **H** | **K** | **M** | **M** | **E** | **R** | **L** | **T** | **K** | **K** | **Q** | **E** | **P** | **V** | **L** | **H** | **E** | **D** | **L** | **T** | **N** | **R** | **E** | **H** | **E** | **I** | **L** | **M** | **L** | **I** | **A** | **Q** | **G** | **K** | **S** | **N** | **Q** | **E** | **I** | **A** | **D** | **E** | **L** | **F** | **I** | **T** | **L** | **K** | **T** | **V** | **K** | **T** | **H** | **V** | **S** | **N** | **I** | **L** | **A** | **K** | **L** | **D** | **V** | **D** | **D** | **R** | **T** | **Q** | **A** | **A** | **I** | **Y** | **A** | **F** | **Q** | **H** | **G** | **L** | **A** | **K** |
| **N53457** | **V** | **I** | **K** | **V** | **M** | **L** | **V** | **D** | **D** | **H** | **E** | **M** | **V** | **R** | **L** | **G** | **V** | **S** | **S** | **Y** | **L** | **S** | **I** | **Q** | **E** | **D** | **I** | **E** | **V** | **V** | **G** | **E** | **A** | **E** | **N** | **G** | **K** | **I** | **G** | **Y** | **E** | **K** | **A** | **L** | **E** | **L** | **R** | **P** | **D** | **V** | **I** | **L** | **M** | **D** | **L** | **V** | **M** | **E** | **E** | **M** | **D** | **G** | **I** | **D** | **S** | **T** | **K** | **A** | **I** | **L** | **K** | **D** | **W** | **P** | **E** | **A** | **K** | **I** | **I** | **I** | **V** | **T** | **S** | **F** | **I** | **D** | **D** | **E** | **K** | **V** | **Y** | **P** | **A** | **I** | **E** | **A** | **G** | **A** | **A** | **G** | **Y** | **L** | **L** | **K** | **T** | **S** | **T** | **A** | **H** | **E** | **I** | **A** | **D** | **A** | **I** | **R** | **T** | **T** | **Y** | **R** | **G** | **E** | **R** | **V** | **L** | **E** | **P** | **E** | **V** | **T** | **H** | **K** | **M** | **M** | **E** | **R** | **L** | **T** | **K** | **K** | **Q** | **E** | **P** | **V** | **L** | **H** | **E** | **D** | **L** | **T** | **N** | **R** | **E** | **H** | **E** | **I** | **L** | **M** | **L** | **I** | **A** | **Q** | **G** | **K** | **S** | **N** | **Q** | **E** | **I** | **A** | **D** | **E** | **L** | **F** | **I** | **T** | **L** | **K** | **T** | **V** | **K** | **T** | **H** | **V** | **S** | **N** | **I** | **L** | **A** | **K** | **L** | **D** | **V** | **D** | **D** | **R** | **T** | **Q** | **A** | **A** | **I** | **Y** | **A** | **F** | **Q** | **H** | **G** | **L** | **A** | **K** |
| **N54676** | **V** | **I** | **K** | **V** | **M** | **L** | **V** | **D** | **D** | **H** | **E** | **M** | **V** | **R** | **L** | **G** | **V** | **S** | **S** | **Y** | **L** | **S** | **I** | **Q** | **E** | **D** | **I** | **E** | **V** | **V** | **G** | **E** | **A** | **E** | **N** | **G** | **K** | **I** | **G** | **Y** | **E** | **K** | **A** | **L** | **E** | **L** | **R** | **P** | **D** | **V** | **I** | **L** | **M** | **D** | **L** | **V** | **M** | **E** | **E** | **M** | **D** | **G** | **I** | **D** | **S** | **T** | **K** | **A** | **I** | **L** | **K** | **D** | **W** | **P** | **E** | **A** | **K** | **I** | **I** | **I** | **V** | **T** | **S** | **F** | **I** | **D** | **D** | **E** | **K** | **V** | **Y** | **P** | **A** | **I** | **E** | **A** | **G** | **A** | **A** | **G** | **Y** | **L** | **L** | **K** | **T** | **S** | **T** | **A** | **H** | **E** | **I** | **A** | **D** | **A** | **I** | **R** | **A** | **T** | **Y** | **R** | **G** | **E** | **R** | **V** | **L** | **E** | **P** | **E** | **V** | **T** | **H** | **K** | **M** | **M** | **E** | **R** | **L** | **T** | **K** | **K** | **Q** | **E** | **P** | **V** | **L** | **H** | **E** | **D** | **L** | **T** | **N** | **R** | **E** | **H** | **E** | **I** | **L** | **M** | **L** | **I** | **A** | **Q** | **G** | **K** | **S** | **N** | **Q** | **E** | **I** | **A** | **D** | **E** | **L** | **F** | **I** | **T** | **L** | **K** | **T** | **V** | **K** | **T** | **H** | **V** | **S** | **N** | **I** | **L** | **A** | **K** | **L** | **D** | **V** | **D** | **D** | **R** | **T** | **Q** | **A** | **A** | **I** | **Y** | **A** | **F** | **Q** | **H** | **G** | **L** | **A** | **K** |
| **N60200F** | **M** | **I** | **K** | **V** | **L** | **L** | **V** | **D** | **D** | **H** | **E** | **M** | **V** | **R** | **L** | **G** | **V** | **S** | **S** | **Y** | **L** | **S** | **I** | **Q** | **E** | **D** | **I** | **E** | **V** | **I** | **G** | **E** | **A** | **E** | **N** | **G** | **R** | **Q** | **G** | **Y** | **E** | **K** | **A** | **M** | **A** | **L** | **R** | **P** | **D** | **V** | **I** | **L** | **M** | **D** | **L** | **V** | **M** | **E** | **E** | **M** | **D** | **G** | **I** | **E** | **S** | **T** | **K** | **A** | **I** | **L** | **K** | **D** | **W** | **P** | **K** | **A** | **K** | **I** | **I** | **I** | **V** | **T** | **S** | **F** | **I** | **D** | **D** | **E** | **K** | **V** | **Y** | **P** | **A** | **I** | **E** | **A** | **G** | **A** | **A** | **G** | **Y** | **L** | **L** | **K** | **T** | **S** | **T** | **A** | **H** | **E** | **I** | **A** | **D** | **A** | **I** | **R** | **A** | **T** | **Q** | **R** | **G** | **E** | **R** | **V** | **L** | **E** | **P** | **E** | **V** | **T** | **T** | **K** | **M** | **M** | **E** | **K** | **M** | **S** | **R** | **R** | **N** | **D** | **P** | **V** | **L** | **H** | **E** | **E** | **L** | **T** | **N** | **R** | **E** | **N** | **E** | **I** | **L** | **M** | **L** | **I** | **S** | **E** | **G** | **K** | **S** | **N** | **Q** | **E** | **I** | **A** | **D** | **E** | **L** | **F** | **I** | **T** | **L** | **K** | **T** | **V** | **K** | **T** | **H** | **V** | **S** | **N** | **I** | **L** | **A** | **K** | **L** | **E** | **V** | **E** | **D** | **R** | **T** | **Q** | **A** | **A** | **I** | **Y** | **A** | **F** | **K** | **H** | **G** | **L** | **V** | **K** |
| **N59963F** | **V** | **I** | **K** | **V** | **M** | **L** | **V** | **D** | **D** | **H** | **E** | **M** | **V** | **R** | **L** | **G** | **V** | **S** | **S** | **Y** | **L** | **S** | **I** | **Q** | **E** | **D** | **I** | **E** | **V** | **V** | **G** | **E** | **A** | **E** | **N** | **G** | **K** | **I** | **G** | **Y** | **E** | **K** | **A** | **L** | **E** | **L** | **R** | **P** | **D** | **V** | **I** | **L** | **M** | **D** | **L** | **V** | **M** | **E** | **E** | **M** | **D** | **G** | **I** | **D** | **S** | **T** | **K** | **A** | **I** | **L** | **K** | **D** | **W** | **P** | **E** | **A** | **K** | **I** | **I** | **I** | **V** | **T** | **S** | **F** | **I** | **D** | **D** | **E** | **K** | **V** | **Y** | **P** | **A** | **I** | **E** | **A** | **G** | **A** | **A** | **G** | **Y** | **L** | **L** | **K** | **T** | **S** | **T** | **A** | **H** | **E** | **I** | **A** | **D** | **A** | **I** | **R** | **A** | **T** | **Y** | **R** | **G** | **E** | **R** | **V** | **L** | **E** | **P** | **E** | **V** | **T** | **H** | **K** | **M** | **M** | **E** | **R** | **L** | **T** | **K** | **K** | **Q** | **E** | **P** | **V** | **L** | **H** | **E** | **D** | **L** | **T** | **N** | **R** | **E** | **H** | **E** | **I** | **L** | **M** | **L** | **I** | **A** | **Q** | **G** | **K** | **S** | **N** | **Q** | **E** | **I** | **A** | **D** | **E** | **L** | **F** | **I** | **T** | **L** | **K** | **T** | **V** | **K** | **T** | **H** | **V** | **S** | **N** | **I** | **L** | **A** | **K** | **L** | **D** | **V** | **D** | **D** | **R** | **T** | **Q** | **A** | **A** | **I** | **Y** | **A** | **F** | **Q** | **H** | **G** | **L** | **A** | **K** |
| **N53453** | **V** | **I** | **K** | **V** | **M** | **L** | **V** | **D** | **D** | **H** | **E** | **M** | **V** | **R** | **L** | **G** | **V** | **S** | **S** | **Y** | **L** | **S** | **I** | **Q** | **E** | **D** | **I** | **E** | **V** | **V** | **G** | **E** | **A** | **E** | **N** | **G** | **K** | **I** | **G** | **Y** | **E** | **K** | **A** | **L** | **E** | **L** | **R** | **P** | **D** | **V** | **I** | **L** | **M** | **D** | **L** | **V** | **M** | **E** | **E** | **M** | **D** | **G** | **I** | **D** | **S** | **T** | **K** | **A** | **I** | **L** | **K** | **D** | **W** | **P** | **E** | **A** | **K** | **I** | **I** | **I** | **V** | **T** | **S** | **F** | **I** | **D** | **D** | **E** | **K** | **V** | **Y** | **P** | **A** | **I** | **E** | **A** | **G** | **A** | **A** | **G** | **Y** | **L** | **L** | **K** | **T** | **S** | **T** | **A** | **H** | **E** | **I** | **A** | **D** | **A** | **I** | **R** | **A** | **T** | **Y** | **R** | **G** | **E** | **R** | **V** | **L** | **E** | **P** | **E** | **V** | **T** | **H** | **K** | **M** | **M** | **E** | **R** | **L** | **T** | **K** | **K** | **Q** | **E** | **P** | **V** | **L** | **H** | **E** | **D** | **L** | **T** | **N** | **R** | **E** | **H** | **E** | **I** | **L** | **M** | **L** | **I** | **A** | **Q** | **G** | **K** | **S** | **N** | **Q** | **E** | **I** | **A** | **D** | **E** | **L** | **F** | **I** | **T** | **L** | **K** | **T** | **V** | **K** | **T** | **H** | **V** | **S** | **N** | **I** | **L** | **A** | **K** | **L** | **D** | **V** | **D** | **D** | **R** | **T** | **Q** | **A** | **A** | **I** | **Y** | **A** | **F** | **Q** | **H** | **G** | **L** | **A** | **K** |
| **N60280F** | **V** | **I** | **K** | **V** | **M** | **L** | **V** | **D** | **D** | **H** | **E** | **M** | **V** | **R** | **L** | **G** | **V** | **S** | **S** | **Y** | **L** | **S** | **I** | **Q** | **E** | **D** | **I** | **E** | **V** | **V** | **G** | **E** | **A** | **E** | **N** | **G** | **K** | **I** | **G** | **Y** | **E** | **K** | **A** | **L** | **E** | **L** | **R** | **P** | **D** | **V** | **I** | **L** | **M** | **D** | **L** | **V** | **M** | **E** | **E** | **M** | **D** | **G** | **I** | **D** | **S** | **T** | **K** | **A** | **I** | **L** | **K** | **D** | **W** | **P** | **E** | **A** | **K** | **I** | **I** | **I** | **V** | **T** | **S** | **F** | **I** | **D** | **D** | **E** | **K** | **V** | **Y** | **P** | **A** | **I** | **E** | **A** | **G** | **A** | **A** | **G** | **Y** | **L** | **L** | **K** | **T** | **S** | **T** | **A** | **H** | **E** | **I** | **A** | **D** | **A** | **I** | **R** | **A** | **T** | **Y** | **R** | **G** | **E** | **R** | **V** | **L** | **E** | **P** | **E** | **V** | **T** | **H** | **K** | **M** | **M** | **E** | **R** | **L** | **T** | **K** | **K** | **Q** | **E** | **P** | **V** | **L** | **H** | **E** | **D** | **L** | **T** | **N** | **R** | **E** | **H** | **E** | **I** | **L** | **M** | **L** | **I** | **A** | **Q** | **G** | **K** | **S** | **N** | **Q** | **E** | **I** | **A** | **D** | **E** | **L** | **F** | **I** | **T** | **L** | **K** | **T** | **V** | **K** | **T** | **H** | **V** | **S** | **N** | **I** | **L** | **A** | **K** | **L** | **D** | **V** | **D** | **D** | **R** | **T** | **Q** | **A** | **A** | **I** | **Y** | **A** | **F** | **Q** | **H** | **G** | **L** | **A** | **K** |
| **N59492F** | **M** | **I** | **K** | **V** | **L** | **L** | **V** | **D** | **D** | **H** | **E** | **M** | **V** | **R** | **L** | **G** | **V** | **S** | **S** | **Y** | **L** | **S** | **I** | **Q** | **E** | **D** | **I** | **E** | **V** | **I** | **G** | **E** | **A** | **E** | **N** | **G** | **R** | **Q** | **G** | **Y** | **E** | **K** | **A** | **M** | **A** | **L** | **R** | **P** | **D** | **V** | **I** | **L** | **M** | **D** | **L** | **V** | **M** | **E** | **E** | **M** | **D** | **G** | **I** | **E** | **S** | **T** | **K** | **A** | **I** | **L** | **K** | **D** | **W** | **P** | **K** | **A** | **K** | **I** | **I** | **I** | **V** | **T** | **S** | **F** | **I** | **D** | **D** | **E** | **K** | **V** | **Y** | **P** | **A** | **I** | **E** | **A** | **G** | **A** | **A** | **G** | **Y** | **L** | **L** | **K** | **T** | **S** | **T** | **A** | **H** | **E** | **I** | **A** | **D** | **A** | **I** | **R** | **A** | **T** | **Q** | **R** | **G** | **E** | **R** | **V** | **L** | **E** | **P** | **E** | **V** | **T** | **T** | **K** | **M** | **M** | **E** | **K** | **M** | **S** | **R** | **R** | **Y** | **D** | **P** | **V** | **L** | **H** | **E** | **E** | **L** | **T** | **N** | **R** | **E** | **N** | **E** | **I** | **L** | **M** | **L** | **I** | **S** | **E** | **G** | **K** | **S** | **N** | **Q** | **E** | **I** | **A** | **D** | **E** | **L** | **F** | **I** | **T** | **L** | **K** | **T** | **V** | **K** | **T** | **H** | **V** | **S** | **N** | **I** | **L** | **A** | **K** | **L** | **E** | **V** | **E** | **D** | **R** | **T** | **Q** | **A** | **A** | **I** | **Y** | **A** | **F** | **K** | **H** | **G** | **L** | **V** | **K** |
| **N60299F** | **M** | **I** | **K** | **V** | **L** | **L** | **V** | **D** | **D** | **H** | **E** | **M** | **V** | **R** | **L** | **G** | **V** | **S** | **S** | **Y** | **L** | **S** | **I** | **Q** | **E** | **D** | **I** | **E** | **V** | **I** | **G** | **E** | **A** | **E** | **N** | **G** | **R** | **Q** | **G** | **Y** | **E** | **K** | **A** | **M** | **A** | **L** | **R** | **P** | **D** | **V** | **I** | **L** | **M** | **D** | **L** | **V** | **M** | **E** | **E** | **M** | **D** | **G** | **I** | **E** | **S** | **T** | **K** | **A** | **I** | **L** | **K** | **D** | **W** | **P** | **K** | **A** | **K** | **I** | **I** | **I** | **V** | **T** | **S** | **F** | **I** | **D** | **D** | **E** | **K** | **V** | **Y** | **P** | **A** | **I** | **E** | **A** | **G** | **A** | **A** | **G** | **Y** | **L** | **L** | **K** | **T** | **S** | **T** | **A** | **H** | **E** | **I** | **A** | **D** | **A** | **I** | **R** | **A** | **T** | **Q** | **R** | **G** | **E** | **R** | **V** | **L** | **E** | **P** | **E** | **V** | **T** | **T** | **K** | **M** | **M** | **E** | **K** | **M** | **S** | **R** | **R** | **N** | **D** | **P** | **V** | **L** | **H** | **E** | **E** | **L** | **T** | **N** | **R** | **E** | **N** | **E** | **I** | **L** | **M** | **L** | **I** | **S** | **E** | **G** | **K** | **S** | **N** | **Q** | **E** | **I** | **A** | **D** | **E** | **L** | **F** | **I** | **T** | **L** | **K** | **T** | **V** | **K** | **T** | **H** | **V** | **S** | **N** | **I** | **L** | **A** | **K** | **L** | **E** | **V** | **E** | **D** | **R** | **T** | **Q** | **A** | **A** | **I** | **Y** | **A** | **F** | **K** | **H** | **G** | **L** | **V** | **K** |
| **N60027F** | **V** | **I** | **K** | **V** | **M** | **L** | **V** | **D** | **D** | **H** | **E** | **M** | **V** | **R** | **L** | **G** | **V** | **S** | **S** | **Y** | **L** | **S** | **I** | **Q** | **E** | **D** | **I** | **E** | **V** | **V** | **G** | **E** | **A** | **E** | **N** | **G** | **K** | **I** | **G** | **Y** | **E** | **K** | **A** | **L** | **E** | **L** | **R** | **P** | **D** | **V** | **I** | **L** | **M** | **D** | **L** | **V** | **M** | **E** | **E** | **M** | **D** | **G** | **I** | **D** | **S** | **T** | **K** | **A** | **I** | **L** | **K** | **D** | **W** | **P** | **E** | **A** | **K** | **I** | **I** | **I** | **V** | **T** | **S** | **F** | **I** | **D** | **D** | **E** | **K** | **V** | **Y** | **P** | **A** | **I** | **E** | **A** | **G** | **A** | **A** | **G** | **Y** | **L** | **L** | **K** | **T** | **S** | **T** | **A** | **H** | **E** | **I** | **A** | **D** | **A** | **I** | **R** | **A** | **T** | **Y** | **R** | **G** | **E** | **R** | **V** | **L** | **E** | **P** | **E** | **V** | **T** | **H** | **K** | **M** | **M** | **E** | **R** | **L** | **T** | **K** | **K** | **Q** | **E** | **P** | **V** | **L** | **H** | **E** | **D** | **L** | **T** | **N** | **R** | **E** | **H** | **E** | **I** | **L** | **M** | **L** | **I** | **A** | **Q** | **G** | **K** | **S** | **N** | **Q** | **E** | **I** | **A** | **D** | **E** | **L** | **F** | **I** | **T** | **L** | **K** | **T** | **V** | **K** | **T** | **H** | **V** | **S** | **N** | **I** | **L** | **A** | **K** | **L** | **D** | **V** | **D** | **D** | **R** | **T** | **Q** | **A** | **A** | **I** | **Y** | **A** | **F** | **Q** | **H** | **G** | **L** | **A** | **K** |
| **N52755** | **V** | **I** | **K** | **V** | **M** | **L** | **V** | **D** | **D** | **H** | **E** | **M** | **V** | **R** | **L** | **G** | **V** | **S** | **S** | **Y** | **L** | **S** | **I** | **Q** | **E** | **D** | **I** | **E** | **V** | **V** | **G** | **E** | **A** | **E** | **N** | **G** | **K** | **I** | **G** | **Y** | **E** | **K** | **A** | **L** | **E** | **L** | **R** | **P** | **D** | **V** | **I** | **L** | **M** | **D** | **L** | **V** | **M** | **E** | **E** | **M** | **D** | **G** | **I** | **D** | **S** | **T** | **K** | **A** | **I** | **L** | **K** | **D** | **W** | **P** | **E** | **A** | **K** | **I** | **I** | **I** | **V** | **T** | **S** | **F** | **I** | **D** | **D** | **E** | **K** | **V** | **Y** | **P** | **A** | **I** | **E** | **A** | **G** | **A** | **A** | **G** | **Y** | **L** | **L** | **K** | **T** | **S** | **T** | **A** | **H** | **E** | **I** | **A** | **D** | **A** | **I** | **R** | **A** | **T** | **Y** | **R** | **G** | **E** | **R** | **V** | **L** | **E** | **P** | **E** | **V** | **T** | **H** | **K** | **M** | **M** | **E** | **R** | **L** | **T** | **K** | **K** | **Q** | **E** | **P** | **V** | **L** | **H** | **E** | **D** | **L** | **T** | **N** | **R** | **E** | **H** | **E** | **I** | **L** | **M** | **L** | **I** | **A** | **Q** | **G** | **K** | **S** | **N** | **Q** | **E** | **I** | **A** | **D** | **E** | **L** | **F** | **I** | **T** | **L** | **K** | **T** | **V** | **K** | **T** | **H** | **V** | **S** | **N** | **I** | **L** | **A** | **K** | **L** | **D** | **V** | **D** | **D** | **R** | **T** | **Q** | **A** | **A** | **I** | **Y** | **A** | **F** | **Q** | **H** | **G** | **L** | **A** | **K** |
| **N59609F** | **V** | **I** | **K** | **V** | **M** | **L** | **V** | **D** | **D** | **H** | **E** | **M** | **V** | **R** | **L** | **G** | **V** | **S** | **S** | **Y** | **L** | **S** | **I** | **Q** | **E** | **D** | **I** | **E** | **V** | **V** | **G** | **E** | **A** | **E** | **N** | **G** | **K** | **I** | **G** | **Y** | **E** | **K** | **A** | **L** | **E** | **L** | **R** | **P** | **D** | **V** | **I** | **L** | **M** | **D** | **L** | **V** | **M** | **E** | **E** | **M** | **D** | **G** | **I** | **D** | **S** | **T** | **K** | **A** | **I** | **L** | **K** | **D** | **W** | **P** | **E** | **A** | **K** | **I** | **I** | **I** | **V** | **T** | **S** | **F** | **I** | **D** | **D** | **E** | **K** | **V** | **Y** | **P** | **A** | **I** | **E** | **A** | **G** | **A** | **A** | **G** | **Y** | **L** | **L** | **K** | **T** | **S** | **T** | **A** | **H** | **E** | **I** | **A** | **D** | **A** | **I** | **R** | **A** | **T** | **Y** | **R** | **G** | **E** | **R** | **V** | **L** | **E** | **P** | **E** | **V** | **T** | **H** | **K** | **M** | **M** | **E** | **R** | **L** | **T** | **K** | **K** | **Q** | **E** | **P** | **V** | **L** | **H** | **E** | **D** | **L** | **T** | **N** | **R** | **E** | **H** | **E** | **I** | **L** | **M** | **L** | **I** | **A** | **Q** | **G** | **K** | **S** | **N** | **Q** | **E** | **I** | **A** | **D** | **E** | **L** | **F** | **I** | **T** | **L** | **K** | **T** | **V** | **K** | **T** | **H** | **V** | **S** | **N** | **I** | **L** | **A** | **K** | **L** | **D** | **V** | **D** | **D** | **R** | **T** | **Q** | **A** | **A** | **I** | **Y** | **A** | **F** | **Q** | **H** | **G** | **L** | **A** | **K** |
| **N53761** | **V** | **I** | **K** | **V** | **M** | **L** | **V** | **D** | **D** | **H** | **E** | **M** | **V** | **R** | **L** | **G** | **V** | **S** | **S** | **Y** | **L** | **S** | **I** | **Q** | **E** | **D** | **I** | **E** | **V** | **V** | **G** | **E** | **A** | **E** | **N** | **G** | **K** | **I** | **G** | **Y** | **E** | **K** | **A** | **L** | **E** | **L** | **R** | **P** | **D** | **V** | **I** | **L** | **M** | **D** | **L** | **V** | **M** | **E** | **E** | **M** | **D** | **G** | **I** | **D** | **S** | **T** | **K** | **A** | **I** | **L** | **K** | **D** | **W** | **P** | **E** | **A** | **K** | **I** | **I** | **I** | **V** | **T** | **S** | **F** | **I** | **D** | **D** | **E** | **K** | **V** | **Y** | **P** | **A** | **I** | **E** | **A** | **G** | **A** | **A** | **G** | **Y** | **L** | **L** | **K** | **T** | **S** | **T** | **A** | **H** | **E** | **I** | **A** | **D** | **A** | **I** | **R** | **A** | **T** | **Y** | **H** | **G** | **E** | **R** | **V** | **L** | **E** | **P** | **E** | **V** | **T** | **H** | **K** | **M** | **M** | **E** | **R** | **L** | **T** | **K** | **K** | **Q** | **E** | **P** | **V** | **L** | **H** | **E** | **D** | **L** | **T** | **N** | **R** | **E** | **H** | **E** | **I** | **L** | **M** | **L** | **I** | **A** | **Q** | **G** | **K** | **S** | **N** | **Q** | **E** | **I** | **A** | **D** | **E** | **L** | **F** | **I** | **T** | **L** | **K** | **T** | **V** | **K** | **T** | **H** | **V** | **S** | **N** | **I** | **L** | **A** | **K** | **L** | **D** | **V** | **D** | **D** | **R** | **T** | **Q** | **A** | **A** | **I** | **Y** | **A** | **F** | **Q** | **H** | **G** | **L** | **A** | **K** |
| **N54588** | **V** | **I** | **K** | **V** | **M** | **L** | **V** | **D** | **D** | **H** | **E** | **M** | **V** | **R** | **L** | **G** | **V** | **S** | **S** | **Y** | **L** | **S** | **I** | **Q** | **E** | **D** | **I** | **E** | **V** | **V** | **G** | **E** | **A** | **E** | **N** | **G** | **K** | **I** | **G** | **Y** | **E** | **K** | **A** | **L** | **E** | **L** | **R** | **P** | **D** | **V** | **I** | **L** | **M** | **D** | **L** | **V** | **M** | **E** | **E** | **M** | **D** | **G** | **I** | **D** | **S** | **T** | **K** | **A** | **I** | **L** | **K** | **D** | **W** | **P** | **E** | **A** | **K** | **I** | **I** | **I** | **V** | **T** | **S** | **F** | **I** | **D** | **D** | **E** | **K** | **V** | **Y** | **P** | **A** | **I** | **E** | **A** | **G** | **A** | **A** | **G** | **Y** | **L** | **L** | **K** | **T** | **S** | **T** | **A** | **H** | **E** | **I** | **A** | **D** | **A** | **I** | **R** | **A** | **T** | **Y** | **R** | **G** | **E** | **R** | **V** | **L** | **E** | **P** | **E** | **V** | **T** | **H** | **K** | **M** | **M** | **E** | **R** | **L** | **T** | **K** | **K** | **Q** | **E** | **P** | **V** | **L** | **H** | **E** | **D** | **L** | **T** | **N** | **R** | **E** | **H** | **E** | **I** | **L** | **M** | **L** | **I** | **A** | **Q** | **G** | **K** | **S** | **N** | **Q** | **E** | **I** | **A** | **D** | **E** | **L** | **F** | **I** | **T** | **L** | **K** | **T** | **V** | **K** | **T** | **H** | **V** | **S** | **N** | **I** | **L** | **A** | **K** | **L** | **D** | **V** | **D** | **D** | **R** | **T** | **Q** | **A** | **A** | **I** | **Y** | **A** | **F** | **Q** | **H** | **G** | **L** | **A** | **K** |
| **N54567** | **M** | **I** | **K** | **V** | **L** | **L** | **V** | **D** | **D** | **H** | **E** | **M** | **V** | **R** | **L** | **G** | **V** | **S** | **S** | **Y** | **L** | **S** | **I** | **Q** | **E** | **D** | **I** | **E** | **V** | **I** | **G** | **E** | **A** | **E** | **N** | **G** | **R** | **Q** | **G** | **Y** | **E** | **K** | **A** | **M** | **A** | **L** | **R** | **P** | **D** | **V** | **I** | **L** | **M** | **D** | **L** | **V** | **M** | **E** | **E** | **M** | **D** | **G** | **I** | **E** | **S** | **T** | **K** | **A** | **I** | **L** | **K** | **D** | **W** | **P** | **K** | **A** | **K** | **I** | **I** | **I** | **V** | **T** | **S** | **F** | **I** | **D** | **D** | **E** | **K** | **V** | **Y** | **P** | **A** | **I** | **E** | **A** | **G** | **A** | **A** | **G** | **Y** | **L** | **L** | **K** | **T** | **S** | **T** | **A** | **H** | **E** | **I** | **A** | **D** | **A** | **I** | **R** | **A** | **T** | **Q** | **R** | **G** | **E** | **R** | **V** | **L** | **E** | **P** | **E** | **V** | **T** | **T** | **K** | **M** | **M** | **E** | **K** | **M** | **S** | **R** | **R** | **N** | **D** | **P** | **V** | **L** | **H** | **E** | **E** | **L** | **T** | **N** | **R** | **E** | **N** | **E** | **I** | **L** | **M** | **L** | **I** | **S** | **E** | **G** | **K** | **S** | **N** | **Q** | **E** | **I** | **A** | **D** | **E** | **L** | **F** | **I** | **T** | **L** | **K** | **T** | **V** | **K** | **T** | **H** | **V** | **S** | **N** | **I** | **L** | **A** | **K** | **L** | **E** | **V** | **E** | **D** | **R** | **T** | **Q** | **A** | **A** | **I** | **Y** | **A** | **F** | **K** | **H** | **G** | **L** | **V** | **K** |
| **N54560** | **M** | **I** | **K** | **V** | **L** | **L** | **V** | **D** | **D** | **H** | **E** | **M** | **V** | **R** | **L** | **G** | **V** | **S** | **S** | **Y** | **L** | **S** | **I** | **Q** | **E** | **D** | **I** | **E** | **V** | **I** | **G** | **E** | **A** | **E** | **N** | **G** | **R** | **Q** | **G** | **Y** | **E** | **K** | **A** | **M** | **A** | **L** | **R** | **P** | **D** | **V** | **I** | **L** | **M** | **D** | **L** | **V** | **M** | **E** | **E** | **M** | **D** | **G** | **I** | **E** | **S** | **T** | **K** | **A** | **I** | **L** | **K** | **D** | **W** | **P** | **K** | **A** | **K** | **I** | **I** | **I** | **V** | **T** | **S** | **F** | **I** | **D** | **D** | **E** | **K** | **V** | **Y** | **P** | **A** | **I** | **E** | **A** | **G** | **A** | **A** | **G** | **Y** | **L** | **L** | **K** | **T** | **S** | **T** | **A** | **H** | **E** | **I** | **A** | **D** | **A** | **I** | **R** | **A** | **T** | **Q** | **R** | **G** | **E** | **R** | **V** | **L** | **E** | **P** | **E** | **V** | **T** | **T** | **K** | **M** | **M** | **E** | **K** | **M** | **S** | **R** | **R** | **N** | **D** | **P** | **V** | **L** | **H** | **E** | **E** | **L** | **T** | **N** | **R** | **E** | **N** | **E** | **I** | **L** | **M** | **L** | **I** | **S** | **E** | **G** | **K** | **S** | **N** | **Q** | **E** | **I** | **A** | **D** | **E** | **L** | **F** | **I** | **T** | **L** | **K** | **T** | **V** | **K** | **T** | **H** | **V** | **S** | **N** | **I** | **L** | **A** | **K** | **L** | **E** | **V** | **E** | **D** | **R** | **T** | **Q** | **A** | **A** | **I** | **Y** | **A** | **F** | **K** | **H** | **G** | **L** | **V** | **K** |
| **N54150** | **M** | **I** | **K** | **V** | **L** | **L** | **V** | **D** | **D** | **H** | **E** | **M** | **V** | **R** | **L** | **G** | **V** | **S** | **S** | **Y** | **L** | **S** | **I** | **Q** | **E** | **D** | **I** | **E** | **V** | **I** | **G** | **E** | **A** | **E** | **N** | **G** | **R** | **Q** | **G** | **Y** | **E** | **K** | **A** | **M** | **A** | **L** | **R** | **P** | **D** | **V** | **I** | **L** | **M** | **D** | **L** | **V** | **M** | **E** | **E** | **M** | **D** | **G** | **I** | **E** | **S** | **T** | **K** | **A** | **I** | **L** | **K** | **D** | **W** | **P** | **K** | **A** | **K** | **I** | **I** | **I** | **V** | **T** | **S** | **F** | **I** | **D** | **D** | **E** | **K** | **V** | **Y** | **P** | **A** | **I** | **E** | **A** | **G** | **A** | **A** | **G** | **Y** | **L** | **L** | **K** | **T** | **S** | **T** | **A** | **H** | **E** | **I** | **A** | **D** | **A** | **I** | **R** | **A** | **T** | **Q** | **R** | **G** | **E** | **R** | **V** | **L** | **E** | **P** | **E** | **V** | **T** | **T** | **K** | **M** | **M** | **E** | **K** | **M** | **S** | **R** | **R** | **N** | **D** | **P** | **V** | **L** | **H** | **E** | **E** | **L** | **T** | **N** | **R** | **E** | **N** | **E** | **I** | **L** | **M** | **L** | **I** | **S** | **E** | **G** | **K** | **S** | **N** | **Q** | **E** | **I** | **A** | **D** | **E** | **L** | **F** | **I** | **T** | **L** | **K** | **T** | **V** | **K** | **T** | **H** | **V** | **S** | **N** | **I** | **L** | **A** | **K** | **L** | **E** | **V** | **E** | **D** | **R** | **T** | **Q** | **A** | **A** | **I** | **Y** | **A** | **F** | **K** | **H** | **G** | **L** | **V** | **K** |
| **N59505F** | **V** | **I** | **K** | **V** | **M** | **L** | **V** | **D** | **D** | **H** | **E** | **M** | **V** | **R** | **L** | **G** | **V** | **S** | **S** | **Y** | **L** | **S** | **I** | **Q** | **E** | **D** | **I** | **E** | **V** | **V** | **G** | **E** | **A** | **E** | **N** | **G** | **K** | **I** | **G** | **Y** | **E** | **K** | **A** | **L** | **E** | **L** | **R** | **P** | **D** | **V** | **I** | **L** | **M** | **D** | **L** | **V** | **M** | **E** | **E** | **M** | **D** | **G** | **I** | **D** | **S** | **T** | **K** | **A** | **I** | **L** | **K** | **D** | **W** | **P** | **E** | **A** | **K** | **I** | **I** | **I** | **V** | **T** | **S** | **F** | **I** | **D** | **D** | **E** | **K** | **V** | **Y** | **P** | **A** | **I** | **E** | **A** | **G** | **A** | **A** | **G** | **Y** | **L** | **L** | **K** | **T** | **S** | **T** | **A** | **H** | **E** | **I** | **A** | **D** | **A** | **I** | **R** | **A** | **T** | **Y** | **R** | **G** | **E** | **R** | **V** | **L** | **E** | **P** | **E** | **V** | **T** | **H** | **K** | **M** | **M** | **E** | **R** | **L** | **T** | **K** | **K** | **Q** | **E** | **P** | **V** | **L** | **H** | **E** | **D** | **L** | **T** | **N** | **R** | **E** | **H** | **E** | **I** | **L** | **M** | **L** | **I** | **A** | **Q** | **G** | **K** | **S** | **N** | **Q** | **E** | **I** | **A** | **D** | **E** | **L** | **F** | **I** | **T** | **L** | **K** | **T** | **V** | **K** | **T** | **H** | **V** | **S** | **N** | **I** | **L** | **A** | **K** | **L** | **D** | **V** | **D** | **D** | **R** | **T** | **Q** | **A** | **A** | **I** | **Y** | **A** | **F** | **Q** | **H** | **G** | **L** | **A** | **K** |
| **N59554F** | **V** | **I** | **K** | **V** | **M** | **L** | **V** | **D** | **D** | **H** | **E** | **M** | **V** | **R** | **L** | **G** | **V** | **S** | **S** | **Y** | **L** | **S** | **I** | **Q** | **E** | **D** | **I** | **E** | **V** | **V** | **G** | **E** | **A** | **E** | **N** | **G** | **K** | **I** | **G** | **Y** | **E** | **K** | **A** | **L** | **E** | **L** | **R** | **P** | **D** | **V** | **I** | **L** | **M** | **D** | **L** | **V** | **M** | **E** | **E** | **M** | **D** | **G** | **I** | **D** | **S** | **T** | **K** | **A** | **I** | **L** | **K** | **D** | **W** | **P** | **E** | **A** | **K** | **I** | **I** | **I** | **V** | **T** | **S** | **F** | **I** | **D** | **D** | **E** | **K** | **V** | **Y** | **P** | **A** | **I** | **E** | **A** | **G** | **A** | **A** | **G** | **Y** | **L** | **L** | **K** | **T** | **S** | **T** | **A** | **H** | **E** | **I** | **A** | **D** | **A** | **I** | **R** | **A** | **T** | **Y** | **R** | **G** | **E** | **R** | **V** | **L** | **E** | **P** | **E** | **V** | **T** | **H** | **K** | **M** | **M** | **E** | **R** | **L** | **T** | **K** | **K** | **Q** | **E** | **P** | **V** | **L** | **H** | **E** | **D** | **L** | **T** | **N** | **R** | **E** | **H** | **E** | **I** | **L** | **M** | **L** | **I** | **A** | **Q** | **G** | **K** | **S** | **N** | **Q** | **E** | **I** | **A** | **D** | **E** | **L** | **F** | **I** | **T** | **L** | **K** | **T** | **V** | **K** | **T** | **H** | **V** | **S** | **N** | **I** | **L** | **A** | **K** | **L** | **D** | **V** | **D** | **D** | **R** | **T** | **Q** | **A** | **A** | **I** | **Y** | **A** | **F** | **Q** | **H** | **G** | **L** | **A** | **K** |
| **N59439F** | **M** | **I** | **K** | **V** | **L** | **L** | **V** | **D** | **D** | **H** | **E** | **M** | **V** | **R** | **L** | **G** | **V** | **S** | **S** | **Y** | **L** | **S** | **I** | **Q** | **E** | **D** | **I** | **E** | **V** | **I** | **G** | **E** | **A** | **E** | **N** | **G** | **R** | **Q** | **G** | **Y** | **E** | **K** | **A** | **M** | **A** | **L** | **R** | **P** | **D** | **V** | **I** | **L** | **M** | **D** | **L** | **V** | **M** | **E** | **E** | **M** | **D** | **G** | **I** | **E** | **S** | **T** | **K** | **A** | **I** | **L** | **K** | **D** | **W** | **P** | **K** | **A** | **K** | **I** | **I** | **I** | **V** | **T** | **S** | **F** | **I** | **D** | **D** | **E** | **K** | **V** | **Y** | **P** | **A** | **I** | **E** | **A** | **G** | **A** | **A** | **G** | **Y** | **L** | **L** | **K** | **T** | **S** | **T** | **A** | **H** | **E** | **I** | **A** | **D** | **A** | **I** | **R** | **A** | **T** | **Q** | **R** | **G** | **E** | **R** | **V** | **L** | **E** | **P** | **E** | **V** | **T** | **T** | **K** | **M** | **M** | **E** | **K** | **M** | **S** | **R** | **R** | **N** | **D** | **P** | **V** | **L** | **H** | **E** | **E** | **L** | **T** | **N** | **R** | **E** | **N** | **E** | **I** | **L** | **M** | **L** | **I** | **S** | **E** | **G** | **K** | **S** | **N** | **Q** | **E** | **I** | **A** | **D** | **E** | **L** | **F** | **I** | **T** | **L** | **K** | **T** | **V** | **K** | **T** | **H** | **V** | **S** | **N** | **I** | **L** | **A** | **K** | **L** | **E** | **V** | **E** | **D** | **R** | **T** | **Q** | **A** | **A** | **I** | **Y** | **A** | **F** | **K** | **H** | **G** | **L** | **V** | **K** |
| **N52454** | **V** | **I** | **K** | **V** | **M** | **L** | **V** | **D** | **D** | **H** | **E** | **M** | **V** | **R** | **L** | **G** | **V** | **S** | **S** | **Y** | **L** | **S** | **I** | **Q** | **E** | **D** | **I** | **E** | **V** | **V** | **G** | **E** | **A** | **E** | **N** | **G** | **K** | **I** | **G** | **Y** | **E** | **K** | **A** | **L** | **E** | **L** | **R** | **P** | **D** | **V** | **I** | **L** | **M** | **D** | **L** | **V** | **M** | **E** | **E** | **M** | **D** | **G** | **I** | **D** | **S** | **T** | **K** | **A** | **I** | **L** | **K** | **D** | **W** | **P** | **E** | **A** | **K** | **I** | **I** | **I** | **V** | **T** | **S** | **F** | **I** | **D** | **D** | **E** | **K** | **V** | **Y** | **P** | **A** | **I** | **E** | **A** | **G** | **A** | **A** | **G** | **Y** | **L** | **L** | **K** | **T** | **S** | **T** | **A** | **H** | **E** | **I** | **A** | **D** | **A** | **I** | **R** | **A** | **T** | **Y** | **R** | **G** | **E** | **R** | **V** | **L** | **E** | **P** | **E** | **V** | **T** | **H** | **K** | **M** | **M** | **E** | **R** | **L** | **T** | **K** | **K** | **Q** | **E** | **P** | **V** | **L** | **H** | **E** | **D** | **L** | **T** | **N** | **R** | **E** | **H** | **E** | **I** | **L** | **M** | **L** | **I** | **A** | **Q** | **G** | **K** | **S** | **N** | **Q** | **E** | **I** | **A** | **D** | **E** | **L** | **F** | **I** | **T** | **L** | **K** | **T** | **V** | **K** | **T** | **H** | **V** | **S** | **N** | **I** | **L** | **A** | **K** | **L** | **D** | **V** | **D** | **D** | **R** | **T** | **Q** | **A** | **A** | **I** | **Y** | **A** | **F** | **Q** | **H** | **G** | **L** | **A** | **K** |
| **N59998F** | **M** | **I** | **K** | **V** | **L** | **L** | **V** | **D** | **D** | **H** | **E** | **M** | **V** | **R** | **L** | **G** | **V** | **S** | **S** | **Y** | **L** | **S** | **I** | **Q** | **E** | **D** | **I** | **E** | **V** | **I** | **G** | **E** | **A** | **E** | **N** | **G** | **R** | **Q** | **G** | **Y** | **E** | **K** | **A** | **M** | **A** | **L** | **R** | **P** | **D** | **V** | **I** | **L** | **M** | **D** | **L** | **V** | **M** | **E** | **E** | **M** | **D** | **G** | **I** | **E** | **S** | **T** | **K** | **A** | **I** | **L** | **K** | **D** | **W** | **P** | **K** | **A** | **K** | **I** | **I** | **I** | **V** | **T** | **S** | **F** | **I** | **D** | **D** | **E** | **K** | **V** | **Y** | **P** | **A** | **I** | **E** | **A** | **G** | **A** | **A** | **G** | **Y** | **L** | **L** | **K** | **T** | **S** | **T** | **A** | **H** | **E** | **I** | **A** | **D** | **A** | **I** | **R** | **A** | **T** | **Q** | **R** | **G** | **E** | **R** | **V** | **L** | **E** | **P** | **E** | **V** | **T** | **T** | **K** | **M** | **M** | **E** | **K** | **M** | **S** | **R** | **R** | **Y** | **D** | **P** | **V** | **L** | **H** | **E** | **E** | **L** | **T** | **N** | **R** | **E** | **N** | **E** | **I** | **L** | **M** | **L** | **I** | **S** | **E** | **G** | **K** | **S** | **N** | **Q** | **E** | **I** | **A** | **D** | **E** | **L** | **F** | **I** | **T** | **L** | **K** | **T** | **V** | **K** | **T** | **H** | **V** | **S** | **N** | **I** | **L** | **A** | **K** | **L** | **E** | **V** | **E** | **D** | **R** | **T** | **Q** | **A** | **A** | **I** | **Y** | **A** | **F** | **K** | **H** | **G** | **L** | **V** | **K** |
| **N55337** | **M** | **I** | **K** | **V** | **L** | **L** | **V** | **D** | **D** | **H** | **E** | **M** | **V** | **R** | **L** | **G** | **V** | **S** | **S** | **Y** | **L** | **S** | **I** | **Q** | **E** | **D** | **I** | **E** | **V** | **I** | **G** | **E** | **A** | **E** | **N** | **G** | **R** | **Q** | **G** | **Y** | **E** | **K** | **A** | **M** | **A** | **L** | **R** | **P** | **D** | **V** | **I** | **L** | **M** | **D** | **L** | **V** | **M** | **E** | **E** | **M** | **D** | **G** | **I** | **E** | **S** | **T** | **K** | **A** | **I** | **L** | **K** | **D** | **W** | **P** | **K** | **A** | **K** | **I** | **I** | **I** | **V** | **T** | **S** | **F** | **I** | **D** | **D** | **E** | **K** | **V** | **Y** | **P** | **A** | **I** | **E** | **A** | **G** | **A** | **A** | **G** | **Y** | **L** | **L** | **K** | **T** | **S** | **T** | **A** | **H** | **E** | **I** | **A** | **D** | **A** | **I** | **R** | **A** | **T** | **Q** | **R** | **G** | **E** | **R** | **V** | **L** | **E** | **P** | **E** | **V** | **T** | **T** | **K** | **M** | **M** | **E** | **K** | **M** | **S** | **R** | **R** | **N** | **D** | **P** | **V** | **L** | **H** | **E** | **E** | **L** | **T** | **N** | **R** | **E** | **N** | **E** | **I** | **L** | **M** | **L** | **I** | **S** | **E** | **G** | **K** | **S** | **N** | **Q** | **E** | **I** | **A** | **D** | **E** | **L** | **F** | **I** | **T** | **L** | **K** | **T** | **V** | **K** | **T** | **H** | **V** | **S** | **N** | **I** | **L** | **A** | **K** | **L** | **E** | **V** | **E** | **D** | **R** | **T** | **Q** | **A** | **A** | **I** | **Y** | **A** | **F** | **K** | **H** | **G** | **L** | **V** | **K** |
| **N54152** | **M** | **I** | **K** | **V** | **L** | **L** | **V** | **D** | **D** | **H** | **E** | **M** | **V** | **R** | **L** | **G** | **V** | **S** | **S** | **Y** | **L** | **S** | **I** | **Q** | **E** | **D** | **I** | **E** | **V** | **I** | **G** | **E** | **A** | **E** | **N** | **G** | **R** | **Q** | **G** | **Y** | **E** | **K** | **A** | **M** | **A** | **L** | **R** | **P** | **D** | **V** | **I** | **L** | **M** | **D** | **L** | **V** | **M** | **E** | **E** | **M** | **D** | **G** | **I** | **E** | **S** | **T** | **K** | **A** | **I** | **L** | **K** | **D** | **W** | **P** | **K** | **A** | **K** | **I** | **I** | **I** | **V** | **T** | **S** | **F** | **I** | **D** | **D** | **E** | **K** | **V** | **Y** | **P** | **A** | **I** | **E** | **A** | **G** | **A** | **A** | **G** | **Y** | **L** | **L** | **K** | **T** | **S** | **T** | **A** | **H** | **E** | **I** | **A** | **D** | **A** | **I** | **R** | **A** | **T** | **Q** | **R** | **G** | **E** | **R** | **V** | **L** | **E** | **P** | **E** | **V** | **T** | **T** | **K** | **M** | **M** | **E** | **K** | **M** | **S** | **R** | **R** | **N** | **D** | **P** | **V** | **L** | **H** | **E** | **E** | **L** | **T** | **N** | **R** | **E** | **N** | **E** | **I** | **L** | **M** | **L** | **I** | **S** | **E** | **G** | **K** | **S** | **N** | **Q** | **E** | **I** | **A** | **D** | **E** | **L** | **F** | **I** | **T** | **L** | **K** | **T** | **V** | **K** | **T** | **H** | **V** | **S** | **N** | **I** | **L** | **A** | **K** | **L** | **E** | **V** | **E** | **D** | **R** | **T** | **Q** | **A** | **A** | **I** | **Y** | **A** | **F** | **K** | **H** | **G** | **L** | **V** | **K** |
| **N59513F** | **M** | **I** | **K** | **V** | **L** | **L** | **V** | **D** | **D** | **H** | **E** | **M** | **V** | **R** | **L** | **G** | **V** | **S** | **S** | **Y** | **L** | **S** | **I** | **Q** | **E** | **D** | **I** | **E** | **V** | **I** | **G** | **E** | **A** | **E** | **N** | **G** | **R** | **Q** | **G** | **Y** | **E** | **K** | **A** | **M** | **T** | **L** | **R** | **P** | **D** | **V** | **I** | **L** | **M** | **D** | **L** | **V** | **M** | **E** | **E** | **M** | **D** | **G** | **I** | **E** | **S** | **T** | **K** | **A** | **I** | **L** | **K** | **D** | **W** | **P** | **E** | **A** | **K** | **I** | **I** | **I** | **V** | **T** | **S** | **F** | **I** | **D** | **D** | **E** | **K** | **V** | **Y** | **P** | **A** | **I** | **E** | **A** | **G** | **A** | **A** | **G** | **Y** | **L** | **L** | **K** | **T** | **S** | **T** | **A** | **H** | **E** | **I** | **A** | **D** | **A** | **I** | **R** | **A** | **T** | **Q** | **R** | **G** | **E** | **R** | **V** | **L** | **E** | **P** | **E** | **V** | **T** | **T** | **K** | **M** | **M** | **E** | **K** | **M** | **S** | **R** | **R** | **N** | **E** | **P** | **V** | **L** | **H** | **E** | **E** | **L** | **T** | **N** | **R** | **E** | **N** | **E** | **I** | **L** | **M** | **L** | **I** | **S** | **E** | **G** | **K** | **S** | **N** | **Q** | **E** | **I** | **A** | **D** | **E** | **L** | **F** | **I** | **T** | **L** | **K** | **T** | **V** | **K** | **T** | **H** | **V** | **S** | **N** | **I** | **L** | **A** | **K** | **L** | **E** | **V** | **E** | **D** | **R** | **T** | **Q** | **A** | **A** | **I** | **Y** | **A** | **F** | **K** | **H** | **G** | **L** | **V** | **K** |
| **N54121** | **V** | **I** | **K** | **V** | **M** | **L** | **V** | **D** | **D** | **H** | **E** | **M** | **V** | **R** | **L** | **G** | **V** | **S** | **S** | **Y** | **L** | **S** | **I** | **Q** | **E** | **D** | **I** | **E** | **V** | **V** | **G** | **E** | **A** | **E** | **N** | **G** | **K** | **I** | **G** | **Y** | **E** | **K** | **A** | **L** | **E** | **L** | **R** | **P** | **D** | **V** | **I** | **L** | **M** | **D** | **L** | **V** | **M** | **E** | **E** | **M** | **D** | **G** | **I** | **D** | **S** | **T** | **K** | **A** | **I** | **L** | **K** | **D** | **W** | **P** | **E** | **A** | **K** | **I** | **I** | **I** | **V** | **T** | **S** | **F** | **I** | **D** | **D** | **E** | **K** | **V** | **Y** | **P** | **A** | **I** | **E** | **A** | **G** | **A** | **A** | **G** | **Y** | **L** | **L** | **K** | **T** | **S** | **T** | **A** | **H** | **E** | **I** | **A** | **D** | **A** | **I** | **R** | **A** | **T** | **Y** | **R** | **G** | **E** | **R** | **V** | **L** | **E** | **P** | **E** | **V** | **T** | **H** | **K** | **M** | **M** | **E** | **R** | **L** | **T** | **K** | **K** | **Q** | **E** | **P** | **V** | **L** | **H** | **E** | **D** | **L** | **T** | **N** | **R** | **E** | **H** | **E** | **I** | **L** | **M** | **L** | **I** | **A** | **Q** | **G** | **K** | **S** | **N** | **Q** | **E** | **I** | **A** | **D** | **E** | **L** | **F** | **I** | **T** | **L** | **K** | **T** | **V** | **K** | **T** | **H** | **V** | **S** | **N** | **I** | **L** | **A** | **K** | **L** | **D** | **V** | **D** | **D** | **R** | **T** | **Q** | **A** | **A** | **I** | **Y** | **A** | **F** | **Q** | **H** | **G** | **L** | **A** | **K** |
| **N52824** | **M** | **I** | **K** | **V** | **L** | **L** | **V** | **D** | **D** | **H** | **E** | **M** | **V** | **R** | **L** | **G** | **V** | **S** | **S** | **Y** | **L** | **S** | **I** | **Q** | **E** | **D** | **I** | **E** | **V** | **I** | **G** | **E** | **A** | **E** | **N** | **G** | **R** | **Q** | **G** | **Y** | **E** | **K** | **A** | **M** | **A** | **L** | **R** | **P** | **D** | **V** | **I** | **L** | **M** | **D** | **L** | **V** | **M** | **E** | **E** | **M** | **D** | **G** | **I** | **E** | **S** | **T** | **K** | **A** | **I** | **L** | **K** | **D** | **W** | **P** | **K** | **A** | **K** | **I** | **I** | **I** | **V** | **T** | **S** | **F** | **I** | **D** | **D** | **E** | **K** | **V** | **Y** | **P** | **A** | **I** | **E** | **A** | **G** | **A** | **A** | **G** | **Y** | **L** | **L** | **K** | **T** | **S** | **T** | **A** | **H** | **E** | **I** | **A** | **D** | **A** | **I** | **R** | **A** | **T** | **Q** | **R** | **G** | **E** | **R** | **V** | **L** | **E** | **P** | **E** | **V** | **T** | **T** | **K** | **M** | **M** | **E** | **K** | **M** | **S** | **R** | **R** | **N** | **D** | **P** | **V** | **L** | **H** | **E** | **E** | **L** | **T** | **N** | **R** | **E** | **N** | **E** | **I** | **L** | **M** | **L** | **I** | **S** | **E** | **G** | **K** | **S** | **N** | **Q** | **E** | **I** | **A** | **D** | **E** | **L** | **F** | **I** | **T** | **L** | **K** | **T** | **V** | **K** | **T** | **H** | **V** | **S** | **N** | **I** | **L** | **A** | **K** | **L** | **E** | **V** | **E** | **D** | **R** | **T** | **Q** | **A** | **A** | **I** | **Y** | **A** | **F** | **K** | **H** | **G** | **L** | **V** | **K** |
| **N59504F** | **M** | **I** | **K** | **V** | **L** | **L** | **V** | **D** | **D** | **H** | **E** | **M** | **V** | **R** | **L** | **G** | **V** | **S** | **S** | **Y** | **L** | **S** | **I** | **Q** | **E** | **D** | **I** | **E** | **V** | **I** | **G** | **E** | **A** | **E** | **N** | **G** | **R** | **Q** | **G** | **Y** | **E** | **K** | **A** | **M** | **A** | **L** | **R** | **P** | **D** | **V** | **I** | **L** | **M** | **D** | **L** | **V** | **M** | **E** | **E** | **M** | **D** | **G** | **I** | **E** | **S** | **T** | **K** | **A** | **I** | **L** | **K** | **D** | **W** | **P** | **K** | **A** | **K** | **I** | **I** | **I** | **V** | **T** | **S** | **F** | **I** | **D** | **D** | **E** | **K** | **V** | **Y** | **P** | **A** | **I** | **E** | **A** | **G** | **A** | **A** | **G** | **Y** | **L** | **L** | **K** | **T** | **S** | **T** | **A** | **H** | **E** | **I** | **A** | **D** | **A** | **I** | **R** | **A** | **T** | **Q** | **R** | **G** | **E** | **R** | **V** | **L** | **E** | **P** | **E** | **V** | **T** | **T** | **K** | **M** | **M** | **E** | **K** | **M** | **S** | **R** | **R** | **N** | **D** | **P** | **V** | **L** | **H** | **E** | **E** | **L** | **T** | **N** | **R** | **E** | **N** | **E** | **I** | **L** | **M** | **L** | **I** | **S** | **E** | **G** | **K** | **S** | **N** | **Q** | **E** | **I** | **A** | **D** | **E** | **L** | **F** | **I** | **T** | **L** | **K** | **T** | **V** | **K** | **T** | **H** | **V** | **S** | **N** | **I** | **L** | **A** | **K** | **L** | **E** | **V** | **E** | **D** | **R** | **T** | **Q** | **A** | **A** | **I** | **Y** | **A** | **F** | **K** | **H** | **G** | **L** | **V** | **K** |
| **N53766** | **V** | **I** | **K** | **V** | **M** | **L** | **V** | **D** | **D** | **H** | **E** | **M** | **V** | **R** | **L** | **G** | **V** | **S** | **S** | **Y** | **L** | **S** | **I** | **Q** | **E** | **D** | **I** | **E** | **V** | **V** | **G** | **E** | **A** | **E** | **N** | **G** | **K** | **I** | **G** | **Y** | **E** | **K** | **A** | **L** | **E** | **L** | **R** | **P** | **D** | **V** | **I** | **L** | **M** | **D** | **L** | **V** | **M** | **E** | **E** | **M** | **D** | **G** | **I** | **D** | **S** | **T** | **K** | **A** | **I** | **L** | **K** | **D** | **W** | **P** | **E** | **A** | **K** | **I** | **I** | **I** | **V** | **T** | **S** | **F** | **I** | **D** | **D** | **E** | **K** | **V** | **Y** | **P** | **A** | **I** | **E** | **A** | **G** | **A** | **A** | **G** | **Y** | **L** | **L** | **K** | **T** | **S** | **T** | **A** | **H** | **E** | **I** | **A** | **D** | **A** | **I** | **R** | **A** | **T** | **Y** | **R** | **G** | **E** | **R** | **V** | **L** | **E** | **P** | **E** | **V** | **T** | **H** | **K** | **M** | **M** | **E** | **R** | **L** | **T** | **K** | **K** | **Q** | **E** | **P** | **V** | **L** | **H** | **E** | **D** | **L** | **T** | **N** | **R** | **E** | **H** | **E** | **I** | **L** | **M** | **L** | **I** | **A** | **Q** | **G** | **K** | **S** | **N** | **Q** | **E** | **I** | **A** | **D** | **E** | **L** | **F** | **I** | **T** | **L** | **K** | **T** | **V** | **K** | **T** | **H** | **V** | **S** | **N** | **I** | **L** | **A** | **K** | **L** | **D** | **V** | **D** | **D** | **R** | **T** | **Q** | **A** | **A** | **I** | **Y** | **A** | **F** | **Q** | **H** | **G** | **L** | **A** | **K** |
| **N52777** | **M** | **I** | **K** | **V** | **L** | **L** | **V** | **D** | **D** | **H** | **E** | **M** | **V** | **R** | **L** | **G** | **V** | **S** | **S** | **Y** | **L** | **S** | **I** | **Q** | **E** | **D** | **I** | **E** | **V** | **I** | **G** | **E** | **A** | **E** | **N** | **G** | **R** | **Q** | **G** | **Y** | **E** | **K** | **A** | **M** | **A** | **L** | **R** | **P** | **D** | **V** | **I** | **L** | **M** | **D** | **L** | **V** | **M** | **E** | **E** | **M** | **D** | **G** | **I** | **E** | **S** | **T** | **K** | **A** | **I** | **L** | **K** | **D** | **W** | **P** | **K** | **A** | **K** | **I** | **I** | **I** | **V** | **T** | **S** | **F** | **I** | **D** | **D** | **E** | **K** | **V** | **Y** | **P** | **A** | **I** | **E** | **A** | **G** | **A** | **A** | **G** | **Y** | **L** | **L** | **K** | **T** | **S** | **T** | **A** | **H** | **E** | **I** | **A** | **D** | **A** | **I** | **R** | **A** | **T** | **Q** | **R** | **G** | **E** | **R** | **V** | **L** | **E** | **P** | **E** | **V** | **T** | **T** | **K** | **M** | **M** | **E** | **K** | **M** | **S** | **R** | **R** | **N** | **D** | **P** | **V** | **L** | **H** | **E** | **E** | **L** | **T** | **N** | **R** | **E** | **N** | **E** | **I** | **L** | **M** | **L** | **I** | **S** | **E** | **G** | **K** | **S** | **N** | **Q** | **E** | **I** | **A** | **D** | **E** | **L** | **F** | **I** | **T** | **L** | **K** | **T** | **V** | **K** | **T** | **H** | **V** | **S** | **N** | **I** | **L** | **A** | **K** | **L** | **E** | **V** | **E** | **D** | **R** | **T** | **Q** | **A** | **A** | **I** | **Y** | **A** | **F** | **K** | **H** | **G** | **L** | **V** | **K** |
| **N55290** | **M** | **I** | **K** | **V** | **L** | **L** | **V** | **D** | **D** | **H** | **E** | **M** | **V** | **R** | **L** | **G** | **V** | **S** | **S** | **Y** | **L** | **S** | **I** | **Q** | **E** | **D** | **I** | **E** | **V** | **I** | **G** | **E** | **A** | **E** | **N** | **G** | **R** | **Q** | **G** | **Y** | **E** | **K** | **A** | **M** | **T** | **L** | **R** | **P** | **D** | **V** | **I** | **L** | **M** | **D** | **L** | **V** | **M** | **E** | **E** | **M** | **D** | **G** | **I** | **E** | **S** | **T** | **K** | **A** | **I** | **L** | **K** | **D** | **W** | **P** | **E** | **A** | **K** | **I** | **I** | **I** | **V** | **T** | **S** | **F** | **I** | **D** | **D** | **E** | **K** | **V** | **Y** | **P** | **A** | **I** | **E** | **A** | **G** | **A** | **A** | **G** | **Y** | **L** | **L** | **K** | **T** | **S** | **T** | **A** | **H** | **E** | **I** | **A** | **D** | **A** | **I** | **R** | **A** | **T** | **Q** | **R** | **G** | **E** | **R** | **V** | **L** | **E** | **P** | **E** | **V** | **T** | **T** | **K** | **M** | **M** | **E** | **K** | **M** | **S** | **R** | **R** | **N** | **E** | **P** | **V** | **L** | **H** | **E** | **E** | **L** | **T** | **N** | **R** | **E** | **N** | **E** | **I** | **L** | **M** | **L** | **I** | **S** | **E** | **G** | **K** | **S** | **N** | **Q** | **E** | **I** | **A** | **D** | **E** | **L** | **F** | **I** | **T** | **L** | **K** | **T** | **V** | **K** | **T** | **H** | **V** | **S** | **N** | **I** | **L** | **A** | **K** | **L** | **E** | **V** | **E** | **D** | **R** | **T** | **Q** | **A** | **A** | **I** | **Y** | **A** | **F** | **K** | **H** | **G** | **L** | **V** | **K** |
| **N60425F** | **M** | **I** | **K** | **V** | **L** | **L** | **V** | **D** | **D** | **H** | **E** | **M** | **V** | **R** | **L** | **G** | **V** | **S** | **S** | **Y** | **L** | **S** | **I** | **Q** | **E** | **D** | **I** | **E** | **V** | **I** | **G** | **E** | **A** | **E** | **N** | **G** | **R** | **Q** | **G** | **Y** | **E** | **K** | **A** | **M** | **A** | **L** | **R** | **P** | **D** | **V** | **I** | **L** | **M** | **D** | **L** | **V** | **M** | **E** | **E** | **M** | **D** | **G** | **I** | **E** | **S** | **T** | **K** | **A** | **I** | **L** | **K** | **D** | **W** | **P** | **K** | **A** | **K** | **I** | **I** | **I** | **V** | **T** | **S** | **F** | **I** | **D** | **D** | **E** | **K** | **V** | **Y** | **P** | **A** | **I** | **E** | **A** | **G** | **A** | **A** | **G** | **Y** | **L** | **L** | **K** | **T** | **S** | **T** | **A** | **H** | **E** | **I** | **A** | **D** | **A** | **I** | **R** | **A** | **T** | **Q** | **R** | **G** | **E** | **R** | **V** | **L** | **E** | **P** | **E** | **V** | **T** | **T** | **K** | **M** | **M** | **E** | **K** | **M** | **S** | **R** | **R** | **N** | **D** | **P** | **V** | **L** | **H** | **E** | **E** | **L** | **T** | **N** | **R** | **E** | **N** | **E** | **I** | **L** | **M** | **L** | **I** | **S** | **E** | **G** | **K** | **S** | **N** | **Q** | **E** | **I** | **A** | **D** | **E** | **L** | **F** | **I** | **T** | **L** | **K** | **T** | **V** | **K** | **T** | **H** | **V** | **S** | **N** | **I** | **L** | **A** | **K** | **L** | **E** | **V** | **E** | **D** | **R** | **T** | **Q** | **A** | **A** | **I** | **Y** | **A** | **F** | **K** | **H** | **G** | **L** | **V** | **K** |
| **N53439** | **M** | **I** | **K** | **V** | **L** | **L** | **V** | **D** | **D** | **H** | **E** | **M** | **V** | **R** | **L** | **G** | **V** | **S** | **S** | **Y** | **L** | **S** | **I** | **Q** | **E** | **D** | **I** | **E** | **V** | **I** | **G** | **E** | **A** | **E** | **N** | **G** | **R** | **Q** | **G** | **Y** | **E** | **K** | **A** | **M** | **A** | **L** | **R** | **P** | **D** | **V** | **I** | **L** | **M** | **D** | **L** | **V** | **M** | **E** | **E** | **M** | **D** | **G** | **I** | **E** | **S** | **T** | **K** | **A** | **I** | **L** | **K** | **D** | **W** | **P** | **K** | **A** | **K** | **I** | **I** | **I** | **V** | **T** | **S** | **F** | **I** | **D** | **D** | **E** | **K** | **V** | **Y** | **P** | **A** | **I** | **E** | **A** | **G** | **A** | **A** | **G** | **Y** | **L** | **L** | **K** | **T** | **S** | **T** | **A** | **H** | **E** | **I** | **A** | **D** | **A** | **I** | **R** | **A** | **T** | **Q** | **R** | **G** | **E** | **R** | **V** | **L** | **E** | **P** | **E** | **V** | **T** | **T** | **K** | **M** | **M** | **E** | **K** | **M** | **S** | **R** | **R** | **N** | **D** | **P** | **V** | **L** | **H** | **E** | **E** | **L** | **T** | **N** | **R** | **E** | **N** | **E** | **I** | **L** | **M** | **L** | **I** | **S** | **E** | **G** | **K** | **S** | **N** | **Q** | **E** | **I** | **A** | **D** | **E** | **L** | **F** | **I** | **T** | **L** | **K** | **T** | **V** | **K** | **T** | **H** | **V** | **S** | **N** | **I** | **L** | **A** | **K** | **L** | **E** | **V** | **E** | **D** | **R** | **T** | **Q** | **A** | **A** | **I** | **Y** | **A** | **F** | **K** | **H** | **G** | **L** | **V** | **K** |
| **N59466F** | **V** | **I** | **K** | **V** | **M** | **L** | **V** | **D** | **D** | **H** | **E** | **M** | **V** | **R** | **L** | **G** | **V** | **S** | **S** | **Y** | **L** | **S** | **I** | **Q** | **E** | **D** | **I** | **E** | **V** | **V** | **G** | **E** | **A** | **E** | **N** | **G** | **K** | **I** | **G** | **Y** | **E** | **K** | **A** | **L** | **E** | **L** | **R** | **P** | **D** | **V** | **I** | **L** | **M** | **D** | **L** | **V** | **M** | **E** | **E** | **M** | **D** | **G** | **I** | **D** | **S** | **T** | **K** | **A** | **I** | **L** | **K** | **D** | **W** | **P** | **E** | **A** | **K** | **I** | **I** | **I** | **V** | **T** | **S** | **F** | **I** | **D** | **D** | **E** | **K** | **V** | **Y** | **P** | **A** | **I** | **E** | **A** | **G** | **A** | **A** | **G** | **Y** | **L** | **L** | **K** | **T** | **S** | **T** | **A** | **H** | **E** | **I** | **A** | **D** | **A** | **I** | **R** | **A** | **T** | **Y** | **R** | **G** | **E** | **R** | **V** | **L** | **E** | **P** | **E** | **V** | **T** | **H** | **K** | **M** | **M** | **E** | **R** | **L** | **T** | **K** | **K** | **Q** | **E** | **P** | **V** | **L** | **H** | **E** | **D** | **L** | **T** | **N** | **R** | **E** | **H** | **E** | **I** | **L** | **M** | **L** | **I** | **A** | **Q** | **G** | **K** | **S** | **N** | **Q** | **E** | **I** | **A** | **D** | **E** | **L** | **F** | **I** | **T** | **L** | **K** | **T** | **V** | **K** | **T** | **H** | **V** | **S** | **N** | **I** | **L** | **A** | **K** | **L** | **D** | **V** | **D** | **D** | **R** | **T** | **Q** | **A** | **A** | **I** | **Y** | **A** | **F** | **Q** | **H** | **G** | **L** | **A** | **K** |
| **N53559** | **M** | **I** | **K** | **V** | **L** | **L** | **V** | **D** | **D** | **H** | **E** | **M** | **V** | **R** | **L** | **G** | **V** | **S** | **S** | **Y** | **L** | **S** | **I** | **Q** | **E** | **D** | **I** | **E** | **V** | **I** | **G** | **E** | **A** | **E** | **N** | **G** | **R** | **Q** | **G** | **Y** | **E** | **K** | **A** | **M** | **A** | **L** | **R** | **P** | **D** | **V** | **I** | **L** | **M** | **D** | **L** | **V** | **M** | **E** | **E** | **M** | **D** | **G** | **I** | **E** | **S** | **T** | **K** | **A** | **I** | **L** | **K** | **D** | **W** | **P** | **K** | **A** | **K** | **I** | **I** | **I** | **V** | **T** | **S** | **F** | **I** | **D** | **D** | **E** | **K** | **V** | **Y** | **P** | **A** | **I** | **E** | **A** | **G** | **A** | **A** | **G** | **Y** | **L** | **L** | **K** | **T** | **S** | **T** | **A** | **H** | **E** | **I** | **A** | **D** | **A** | **I** | **R** | **A** | **T** | **Q** | **R** | **G** | **E** | **R** | **V** | **L** | **E** | **P** | **E** | **V** | **T** | **T** | **K** | **M** | **M** | **E** | **K** | **M** | **S** | **R** | **R** | **N** | **D** | **P** | **V** | **L** | **H** | **E** | **E** | **L** | **T** | **N** | **R** | **E** | **N** | **E** | **I** | **L** | **M** | **L** | **I** | **S** | **E** | **G** | **K** | **S** | **N** | **Q** | **E** | **I** | **A** | **D** | **E** | **L** | **F** | **I** | **T** | **L** | **K** | **T** | **V** | **K** | **T** | **H** | **V** | **S** | **N** | **I** | **L** | **A** | **K** | **L** | **E** | **V** | **E** | **D** | **R** | **T** | **Q** | **A** | **A** | **I** | **Y** | **A** | **F** | **K** | **H** | **G** | **L** | **V** | **K** |
| **N59454F** | **V** | **I** | **K** | **V** | **M** | **L** | **V** | **D** | **D** | **H** | **E** | **M** | **V** | **R** | **L** | **G** | **V** | **S** | **S** | **Y** | **L** | **S** | **I** | **Q** | **E** | **D** | **I** | **E** | **V** | **V** | **G** | **E** | **A** | **E** | **N** | **G** | **K** | **I** | **G** | **Y** | **E** | **K** | **A** | **L** | **E** | **L** | **R** | **P** | **D** | **V** | **I** | **L** | **M** | **D** | **L** | **V** | **M** | **E** | **E** | **M** | **D** | **G** | **I** | **D** | **S** | **T** | **K** | **A** | **I** | **L** | **K** | **D** | **W** | **P** | **E** | **A** | **K** | **I** | **I** | **I** | **V** | **T** | **S** | **F** | **I** | **D** | **D** | **E** | **K** | **V** | **Y** | **P** | **A** | **I** | **E** | **A** | **G** | **A** | **A** | **G** | **Y** | **L** | **L** | **K** | **T** | **S** | **T** | **A** | **H** | **E** | **I** | **A** | **D** | **A** | **I** | **R** | **A** | **T** | **Y** | **R** | **G** | **E** | **R** | **V** | **L** | **E** | **P** | **E** | **V** | **T** | **H** | **K** | **M** | **M** | **E** | **R** | **L** | **T** | **K** | **K** | **Q** | **E** | **P** | **V** | **L** | **H** | **E** | **D** | **L** | **T** | **N** | **R** | **E** | **H** | **E** | **I** | **L** | **M** | **L** | **I** | **A** | **Q** | **G** | **K** | **S** | **N** | **Q** | **E** | **I** | **A** | **D** | **E** | **L** | **F** | **I** | **T** | **L** | **K** | **T** | **V** | **K** | **T** | **H** | **V** | **S** | **N** | **I** | **L** | **A** | **K** | **L** | **D** | **V** | **D** | **D** | **R** | **T** | **Q** | **A** | **A** | **I** | **Y** | **A** | **F** | **Q** | **H** | **G** | **L** | **A** | **K** |
| **N52649** | **M** | **I** | **K** | **V** | **L** | **L** | **V** | **D** | **D** | **H** | **E** | **M** | **V** | **R** | **L** | **G** | **V** | **S** | **S** | **Y** | **L** | **S** | **I** | **Q** | **E** | **D** | **I** | **E** | **V** | **I** | **G** | **E** | **A** | **E** | **N** | **G** | **R** | **Q** | **G** | **Y** | **E** | **K** | **A** | **M** | **A** | **L** | **R** | **P** | **D** | **V** | **I** | **L** | **M** | **D** | **L** | **V** | **M** | **E** | **E** | **M** | **D** | **G** | **I** | **E** | **S** | **T** | **K** | **A** | **I** | **L** | **K** | **D** | **W** | **P** | **K** | **A** | **K** | **I** | **I** | **I** | **V** | **T** | **S** | **F** | **I** | **D** | **D** | **E** | **K** | **V** | **Y** | **P** | **A** | **I** | **E** | **A** | **G** | **A** | **A** | **G** | **Y** | **L** | **L** | **K** | **T** | **S** | **T** | **A** | **H** | **E** | **I** | **A** | **D** | **A** | **I** | **R** | **A** | **T** | **Q** | **R** | **G** | **E** | **R** | **V** | **L** | **E** | **P** | **E** | **V** | **T** | **T** | **K** | **M** | **M** | **E** | **K** | **M** | **S** | **R** | **R** | **N** | **D** | **P** | **V** | **L** | **H** | **E** | **E** | **L** | **T** | **N** | **R** | **E** | **N** | **E** | **I** | **L** | **M** | **L** | **I** | **S** | **E** | **G** | **K** | **S** | **N** | **Q** | **E** | **I** | **A** | **D** | **E** | **L** | **F** | **I** | **T** | **L** | **K** | **T** | **V** | **K** | **T** | **H** | **V** | **S** | **N** | **I** | **L** | **A** | **K** | **L** | **E** | **V** | **E** | **D** | **R** | **T** | **Q** | **A** | **A** | **I** | **Y** | **A** | **F** | **K** | **H** | **G** | **L** | **V** | **K** |
| **N54519** | **M** | **I** | **K** | **V** | **L** | **L** | **V** | **D** | **D** | **H** | **E** | **M** | **V** | **R** | **L** | **G** | **V** | **S** | **S** | **Y** | **L** | **S** | **I** | **Q** | **E** | **D** | **I** | **E** | **V** | **I** | **G** | **E** | **A** | **E** | **N** | **G** | **R** | **Q** | **G** | **Y** | **E** | **K** | **A** | **M** | **T** | **L** | **R** | **P** | **D** | **V** | **I** | **L** | **M** | **D** | **L** | **V** | **M** | **E** | **E** | **M** | **D** | **G** | **I** | **E** | **S** | **T** | **K** | **A** | **I** | **L** | **K** | **D** | **W** | **P** | **E** | **A** | **K** | **I** | **I** | **I** | **V** | **T** | **S** | **F** | **I** | **D** | **D** | **E** | **K** | **V** | **Y** | **P** | **A** | **I** | **E** | **A** | **G** | **A** | **A** | **G** | **Y** | **L** | **L** | **K** | **T** | **S** | **T** | **A** | **H** | **E** | **I** | **A** | **D** | **A** | **I** | **R** | **A** | **T** | **Q** | **R** | **G** | **E** | **R** | **V** | **L** | **E** | **P** | **E** | **V** | **T** | **T** | **K** | **M** | **M** | **E** | **K** | **M** | **S** | **R** | **R** | **N** | **E** | **P** | **V** | **L** | **H** | **E** | **E** | **L** | **T** | **N** | **R** | **E** | **N** | **E** | **I** | **L** | **M** | **L** | **I** | **S** | **E** | **G** | **K** | **S** | **N** | **Q** | **E** | **I** | **A** | **D** | **E** | **L** | **F** | **I** | **T** | **L** | **K** | **T** | **V** | **K** | **T** | **H** | **V** | **S** | **N** | **I** | **L** | **A** | **K** | **L** | **E** | **V** | **E** | **D** | **R** | **T** | **Q** | **A** | **A** | **I** | **Y** | **A** | **F** | **K** | **H** | **G** | **L** | **V** | **K** |
| **N52629** | **M** | **I** | **K** | **V** | **L** | **L** | **V** | **D** | **D** | **H** | **E** | **M** | **V** | **R** | **L** | **G** | **V** | **S** | **S** | **Y** | **L** | **S** | **I** | **Q** | **E** | **D** | **I** | **E** | **V** | **I** | **G** | **E** | **A** | **E** | **N** | **G** | **R** | **Q** | **G** | **Y** | **E** | **K** | **A** | **M** | **A** | **L** | **R** | **P** | **D** | **V** | **I** | **L** | **M** | **D** | **L** | **V** | **M** | **E** | **E** | **M** | **D** | **G** | **I** | **E** | **S** | **T** | **K** | **A** | **I** | **L** | **K** | **D** | **W** | **P** | **K** | **A** | **K** | **I** | **I** | **I** | **V** | **T** | **S** | **F** | **I** | **D** | **D** | **E** | **K** | **V** | **Y** | **P** | **A** | **I** | **E** | **A** | **G** | **A** | **A** | **G** | **Y** | **L** | **L** | **K** | **T** | **S** | **T** | **A** | **H** | **E** | **I** | **A** | **D** | **A** | **I** | **R** | **A** | **T** | **Q** | **R** | **G** | **E** | **R** | **V** | **L** | **E** | **P** | **E** | **V** | **T** | **T** | **K** | **M** | **M** | **E** | **K** | **M** | **S** | **R** | **R** | **N** | **D** | **P** | **V** | **L** | **H** | **E** | **E** | **L** | **T** | **N** | **R** | **E** | **N** | **E** | **I** | **L** | **M** | **L** | **I** | **S** | **E** | **G** | **K** | **S** | **N** | **Q** | **E** | **I** | **A** | **D** | **E** | **L** | **F** | **I** | **T** | **L** | **K** | **T** | **V** | **K** | **T** | **H** | **V** | **S** | **N** | **I** | **L** | **A** | **K** | **L** | **E** | **V** | **E** | **D** | **R** | **T** | **Q** | **A** | **A** | **I** | **Y** | **A** | **F** | **K** | **H** | **G** | **L** | **V** | **K** |
| **N59591F** | **V** | **I** | **K** | **V** | **M** | **L** | **V** | **D** | **D** | **H** | **E** | **M** | **V** | **R** | **L** | **G** | **V** | **S** | **S** | **Y** | **L** | **S** | **I** | **Q** | **E** | **D** | **I** | **E** | **V** | **V** | **G** | **E** | **A** | **E** | **N** | **G** | **K** | **I** | **G** | **Y** | **E** | **K** | **A** | **L** | **E** | **L** | **R** | **P** | **D** | **V** | **I** | **L** | **M** | **D** | **L** | **V** | **M** | **E** | **E** | **M** | **D** | **G** | **I** | **D** | **S** | **T** | **K** | **A** | **I** | **L** | **K** | **D** | **W** | **P** | **E** | **A** | **K** | **I** | **I** | **I** | **V** | **T** | **S** | **F** | **I** | **D** | **D** | **E** | **K** | **V** | **Y** | **P** | **A** | **I** | **E** | **A** | **G** | **A** | **A** | **G** | **Y** | **L** | **L** | **K** | **T** | **S** | **T** | **A** | **H** | **E** | **I** | **A** | **D** | **A** | **I** | **R** | **A** | **T** | **Y** | **R** | **G** | **E** | **R** | **V** | **L** | **E** | **P** | **E** | **V** | **T** | **H** | **K** | **M** | **M** | **E** | **R** | **L** | **T** | **K** | **K** | **Q** | **E** | **P** | **V** | **L** | **H** | **E** | **D** | **L** | **T** | **N** | **R** | **E** | **H** | **E** | **I** | **L** | **M** | **L** | **I** | **A** | **Q** | **G** | **K** | **S** | **N** | **Q** | **E** | **I** | **A** | **D** | **E** | **L** | **F** | **I** | **T** | **L** | **K** | **T** | **V** | **K** | **T** | **H** | **V** | **S** | **N** | **I** | **L** | **A** | **K** | **L** | **D** | **V** | **D** | **D** | **R** | **T** | **Q** | **A** | **A** | **I** | **Y** | **A** | **F** | **Q** | **H** | **G** | **L** | **A** | **K** |
| **N59947F** | **M** | **I** | **K** | **V** | **L** | **L** | **V** | **D** | **D** | **H** | **E** | **M** | **V** | **R** | **L** | **G** | **V** | **S** | **S** | **Y** | **L** | **S** | **I** | **Q** | **E** | **D** | **I** | **E** | **V** | **I** | **G** | **E** | **A** | **E** | **N** | **G** | **R** | **Q** | **G** | **Y** | **E** | **K** | **A** | **M** | **T** | **L** | **R** | **P** | **D** | **V** | **I** | **L** | **M** | **D** | **L** | **V** | **M** | **E** | **E** | **M** | **D** | **G** | **I** | **E** | **S** | **T** | **K** | **A** | **I** | **L** | **K** | **D** | **W** | **P** | **E** | **A** | **K** | **I** | **I** | **I** | **V** | **T** | **S** | **F** | **I** | **D** | **D** | **E** | **K** | **V** | **Y** | **P** | **A** | **I** | **E** | **A** | **G** | **A** | **A** | **G** | **Y** | **L** | **L** | **K** | **T** | **S** | **T** | **A** | **H** | **E** | **I** | **A** | **D** | **A** | **I** | **R** | **A** | **T** | **Q** | **R** | **G** | **E** | **R** | **V** | **L** | **E** | **P** | **E** | **V** | **T** | **T** | **K** | **M** | **M** | **E** | **K** | **M** | **S** | **R** | **R** | **N** | **E** | **P** | **V** | **L** | **H** | **E** | **E** | **L** | **T** | **N** | **R** | **E** | **N** | **E** | **I** | **L** | **M** | **L** | **I** | **S** | **E** | **G** | **K** | **S** | **N** | **Q** | **E** | **I** | **A** | **D** | **E** | **L** | **F** | **I** | **T** | **L** | **K** | **T** | **V** | **K** | **T** | **H** | **V** | **S** | **N** | **I** | **L** | **A** | **K** | **L** | **E** | **V** | **E** | **D** | **R** | **T** | **Q** | **A** | **A** | **I** | **Y** | **A** | **F** | **K** | **H** | **G** | **L** | **V** | **K** |
| **N59896F** | **V** | **I** | **K** | **V** | **M** | **L** | **V** | **D** | **D** | **H** | **E** | **M** | **V** | **R** | **L** | **G** | **V** | **S** | **S** | **Y** | **L** | **S** | **I** | **Q** | **E** | **D** | **I** | **E** | **V** | **V** | **G** | **E** | **A** | **E** | **N** | **G** | **K** | **I** | **G** | **Y** | **E** | **K** | **A** | **L** | **E** | **L** | **R** | **P** | **D** | **V** | **I** | **L** | **M** | **D** | **L** | **V** | **M** | **E** | **E** | **M** | **D** | **G** | **I** | **D** | **S** | **T** | **K** | **A** | **I** | **L** | **K** | **D** | **W** | **P** | **E** | **A** | **K** | **I** | **I** | **I** | **V** | **T** | **S** | **F** | **I** | **D** | **D** | **E** | **K** | **V** | **Y** | **P** | **A** | **I** | **E** | **A** | **G** | **A** | **A** | **G** | **Y** | **L** | **L** | **K** | **T** | **S** | **T** | **A** | **H** | **E** | **I** | **A** | **D** | **A** | **I** | **R** | **A** | **T** | **Y** | **R** | **G** | **E** | **R** | **V** | **L** | **E** | **P** | **E** | **V** | **T** | **H** | **K** | **M** | **M** | **E** | **R** | **L** | **T** | **K** | **K** | **Q** | **E** | **P** | **V** | **L** | **H** | **E** | **D** | **L** | **T** | **N** | **R** | **E** | **H** | **E** | **I** | **L** | **M** | **L** | **I** | **A** | **Q** | **G** | **K** | **S** | **N** | **Q** | **E** | **I** | **A** | **D** | **E** | **L** | **F** | **I** | **T** | **L** | **K** | **T** | **V** | **K** | **T** | **H** | **V** | **S** | **N** | **I** | **L** | **A** | **K** | **L** | **D** | **V** | **D** | **D** | **R** | **T** | **Q** | **A** | **A** | **I** | **Y** | **A** | **F** | **Q** | **H** | **G** | **L** | **A** | **K** |
| **N54595** | **V** | **I** | **K** | **V** | **M** | **L** | **V** | **D** | **D** | **H** | **E** | **M** | **V** | **R** | **L** | **G** | **V** | **S** | **S** | **Y** | **L** | **S** | **I** | **Q** | **E** | **D** | **I** | **E** | **V** | **V** | **G** | **E** | **A** | **E** | **N** | **G** | **K** | **I** | **G** | **Y** | **E** | **K** | **A** | **L** | **E** | **L** | **R** | **P** | **D** | **V** | **I** | **L** | **M** | **D** | **L** | **V** | **M** | **E** | **E** | **M** | **D** | **G** | **I** | **D** | **S** | **T** | **K** | **A** | **I** | **L** | **K** | **D** | **W** | **P** | **E** | **A** | **K** | **I** | **I** | **I** | **V** | **T** | **S** | **F** | **I** | **D** | **D** | **E** | **K** | **V** | **Y** | **P** | **A** | **I** | **E** | **A** | **G** | **A** | **A** | **G** | **Y** | **L** | **L** | **K** | **T** | **S** | **T** | **A** | **H** | **E** | **I** | **A** | **D** | **A** | **I** | **R** | **A** | **T** | **Y** | **R** | **G** | **E** | **R** | **V** | **L** | **E** | **P** | **E** | **V** | **T** | **H** | **K** | **M** | **M** | **E** | **R** | **L** | **T** | **K** | **K** | **Q** | **E** | **P** | **V** | **L** | **H** | **E** | **D** | **L** | **T** | **N** | **R** | **E** | **H** | **E** | **I** | **L** | **M** | **L** | **I** | **A** | **Q** | **G** | **K** | **S** | **N** | **Q** | **E** | **I** | **A** | **D** | **E** | **L** | **F** | **I** | **T** | **L** | **K** | **T** | **V** | **K** | **T** | **H** | **V** | **S** | **N** | **I** | **L** | **A** | **K** | **L** | **D** | **V** | **D** | **D** | **R** | **T** | **Q** | **A** | **A** | **I** | **Y** | **A** | **F** | **Q** | **H** | **G** | **L** | **A** | **K** |
| **N53479** | **M** | **I** | **K** | **V** | **L** | **L** | **V** | **D** | **D** | **H** | **E** | **M** | **V** | **R** | **L** | **G** | **V** | **S** | **S** | **Y** | **L** | **S** | **I** | **Q** | **E** | **D** | **I** | **E** | **V** | **I** | **G** | **E** | **A** | **E** | **N** | **G** | **R** | **Q** | **G** | **Y** | **E** | **K** | **A** | **M** | **A** | **L** | **R** | **P** | **D** | **V** | **I** | **L** | **M** | **D** | **L** | **V** | **M** | **E** | **E** | **M** | **D** | **G** | **I** | **E** | **S** | **T** | **K** | **A** | **I** | **L** | **K** | **D** | **W** | **P** | **K** | **A** | **K** | **I** | **I** | **I** | **V** | **T** | **S** | **F** | **I** | **D** | **D** | **E** | **K** | **V** | **Y** | **P** | **A** | **I** | **E** | **A** | **G** | **A** | **A** | **G** | **Y** | **L** | **L** | **K** | **T** | **S** | **T** | **A** | **H** | **E** | **I** | **A** | **D** | **A** | **I** | **R** | **A** | **T** | **Q** | **R** | **G** | **E** | **R** | **V** | **L** | **E** | **P** | **E** | **V** | **T** | **T** | **K** | **M** | **M** | **E** | **K** | **M** | **S** | **R** | **R** | **N** | **D** | **P** | **V** | **L** | **H** | **E** | **E** | **L** | **T** | **N** | **R** | **E** | **N** | **E** | **I** | **L** | **M** | **L** | **I** | **S** | **E** | **G** | **K** | **S** | **N** | **Q** | **E** | **I** | **A** | **D** | **E** | **L** | **F** | **I** | **T** | **L** | **K** | **T** | **V** | **K** | **T** | **H** | **V** | **S** | **N** | **I** | **L** | **A** | **K** | **L** | **E** | **V** | **E** | **D** | **R** | **T** | **Q** | **A** | **A** | **I** | **Y** | **A** | **F** | **K** | **H** | **G** | **L** | **V** | **K** |
| **N60365F** | **M** | **I** | **K** | **V** | **L** | **L** | **V** | **D** | **D** | **H** | **E** | **M** | **V** | **R** | **L** | **G** | **V** | **S** | **S** | **Y** | **L** | **S** | **I** | **Q** | **E** | **D** | **I** | **E** | **V** | **I** | **G** | **E** | **A** | **E** | **N** | **G** | **R** | **Q** | **G** | **Y** | **E** | **K** | **A** | **M** | **A** | **L** | **R** | **P** | **D** | **V** | **I** | **L** | **M** | **D** | **L** | **V** | **M** | **E** | **E** | **M** | **D** | **G** | **I** | **E** | **S** | **T** | **K** | **A** | **I** | **L** | **K** | **D** | **W** | **P** | **K** | **A** | **K** | **I** | **I** | **I** | **V** | **T** | **S** | **F** | **I** | **D** | **D** | **E** | **K** | **V** | **Y** | **P** | **A** | **I** | **E** | **A** | **G** | **A** | **A** | **G** | **Y** | **L** | **L** | **K** | **T** | **S** | **T** | **A** | **H** | **E** | **I** | **A** | **D** | **A** | **I** | **R** | **A** | **T** | **Q** | **R** | **G** | **E** | **R** | **V** | **L** | **E** | **P** | **E** | **V** | **T** | **T** | **K** | **M** | **M** | **E** | **K** | **M** | **S** | **R** | **R** | **N** | **D** | **P** | **V** | **L** | **H** | **E** | **E** | **L** | **T** | **N** | **R** | **E** | **N** | **E** | **I** | **L** | **M** | **L** | **I** | **S** | **E** | **G** | **K** | **S** | **N** | **Q** | **E** | **I** | **A** | **D** | **E** | **L** | **F** | **I** | **T** | **L** | **K** | **T** | **V** | **K** | **T** | **H** | **V** | **S** | **N** | **I** | **L** | **A** | **K** | **L** | **E** | **V** | **E** | **D** | **R** | **T** | **Q** | **A** | **A** | **I** | **Y** | **A** | **F** | **K** | **H** | **G** | **L** | **V** | **K** |
| **N53533** | **M** | **I** | **K** | **V** | **L** | **L** | **V** | **D** | **D** | **H** | **E** | **M** | **V** | **R** | **L** | **G** | **V** | **S** | **S** | **Y** | **L** | **S** | **I** | **Q** | **E** | **D** | **I** | **E** | **V** | **I** | **G** | **E** | **A** | **E** | **N** | **G** | **R** | **Q** | **G** | **Y** | **E** | **K** | **A** | **M** | **A** | **L** | **R** | **P** | **D** | **V** | **I** | **L** | **M** | **D** | **L** | **V** | **M** | **E** | **E** | **M** | **D** | **G** | **I** | **E** | **S** | **T** | **K** | **A** | **I** | **L** | **K** | **D** | **W** | **P** | **K** | **A** | **K** | **I** | **I** | **I** | **V** | **T** | **S** | **F** | **I** | **D** | **D** | **E** | **K** | **V** | **Y** | **P** | **A** | **I** | **E** | **A** | **G** | **A** | **A** | **G** | **Y** | **L** | **L** | **K** | **T** | **S** | **T** | **A** | **H** | **E** | **I** | **A** | **D** | **A** | **I** | **R** | **A** | **T** | **Q** | **R** | **G** | **E** | **R** | **V** | **L** | **E** | **P** | **E** | **V** | **T** | **T** | **K** | **M** | **M** | **E** | **K** | **M** | **S** | **R** | **R** | **N** | **D** | **P** | **V** | **L** | **H** | **E** | **E** | **L** | **T** | **N** | **R** | **E** | **N** | **E** | **I** | **L** | **M** | **L** | **I** | **S** | **E** | **G** | **K** | **S** | **N** | **Q** | **E** | **I** | **A** | **D** | **E** | **L** | **F** | **I** | **T** | **L** | **K** | **T** | **V** | **K** | **T** | **H** | **V** | **S** | **N** | **I** | **L** | **A** | **K** | **L** | **E** | **V** | **E** | **D** | **R** | **T** | **Q** | **A** | **A** | **I** | **Y** | **A** | **F** | **K** | **H** | **G** | **L** | **V** | **K** |
| **N55113** | **V** | **I** | **K** | **V** | **M** | **L** | **V** | **D** | **D** | **H** | **E** | **M** | **V** | **R** | **L** | **G** | **V** | **S** | **S** | **Y** | **L** | **S** | **I** | **Q** | **E** | **D** | **I** | **E** | **V** | **V** | **G** | **E** | **A** | **E** | **N** | **G** | **K** | **I** | **G** | **Y** | **E** | **K** | **A** | **L** | **E** | **L** | **R** | **P** | **D** | **V** | **I** | **L** | **M** | **D** | **L** | **V** | **M** | **E** | **E** | **M** | **D** | **G** | **I** | **D** | **S** | **T** | **K** | **A** | **I** | **L** | **K** | **D** | **W** | **P** | **E** | **A** | **K** | **I** | **I** | **I** | **V** | **T** | **S** | **F** | **I** | **D** | **D** | **E** | **K** | **V** | **Y** | **P** | **A** | **I** | **E** | **A** | **G** | **A** | **A** | **G** | **Y** | **L** | **L** | **K** | **T** | **S** | **T** | **A** | **H** | **E** | **I** | **A** | **D** | **A** | **I** | **R** | **A** | **T** | **Y** | **R** | **G** | **E** | **R** | **V** | **L** | **E** | **P** | **E** | **V** | **T** | **H** | **K** | **M** | **M** | **E** | **R** | **L** | **T** | **K** | **K** | **Q** | **E** | **P** | **V** | **L** | **H** | **E** | **D** | **L** | **T** | **N** | **R** | **E** | **H** | **E** | **I** | **L** | **M** | **L** | **I** | **A** | **Q** | **G** | **K** | **S** | **N** | **Q** | **E** | **I** | **A** | **D** | **E** | **L** | **F** | **I** | **T** | **L** | **K** | **T** | **V** | **K** | **T** | **H** | **V** | **S** | **N** | **I** | **L** | **A** | **K** | **L** | **D** | **V** | **D** | **D** | **R** | **T** | **Q** | **A** | **A** | **I** | **Y** | **A** | **F** | **Q** | **H** | **G** | **L** | **A** | **K** |
| **N59592F** | **V** | **I** | **K** | **V** | **M** | **L** | **V** | **D** | **D** | **H** | **E** | **M** | **V** | **R** | **L** | **G** | **V** | **S** | **S** | **Y** | **L** | **S** | **I** | **Q** | **E** | **D** | **I** | **E** | **V** | **V** | **G** | **E** | **A** | **E** | **N** | **G** | **K** | **I** | **G** | **Y** | **E** | **K** | **A** | **L** | **E** | **L** | **R** | **P** | **D** | **V** | **I** | **L** | **M** | **D** | **L** | **V** | **M** | **E** | **E** | **M** | **D** | **G** | **I** | **D** | **S** | **T** | **K** | **A** | **I** | **L** | **K** | **D** | **W** | **P** | **E** | **A** | **K** | **I** | **I** | **I** | **V** | **T** | **S** | **F** | **I** | **D** | **D** | **E** | **K** | **V** | **Y** | **P** | **A** | **I** | **E** | **A** | **G** | **A** | **A** | **G** | **Y** | **L** | **L** | **K** | **T** | **S** | **T** | **A** | **H** | **E** | **I** | **A** | **D** | **A** | **I** | **R** | **A** | **T** | **Y** | **R** | **G** | **E** | **R** | **V** | **L** | **E** | **P** | **E** | **V** | **T** | **H** | **K** | **M** | **M** | **E** | **R** | **L** | **T** | **K** | **K** | **Q** | **E** | **P** | **V** | **L** | **H** | **E** | **D** | **L** | **T** | **N** | **R** | **E** | **H** | **E** | **I** | **L** | **M** | **L** | **I** | **A** | **Q** | **G** | **K** | **S** | **N** | **Q** | **E** | **I** | **A** | **D** | **E** | **L** | **F** | **I** | **T** | **L** | **K** | **T** | **V** | **K** | **T** | **H** | **V** | **S** | **N** | **I** | **L** | **A** | **K** | **L** | **D** | **V** | **D** | **D** | **R** | **T** | **Q** | **A** | **A** | **I** | **Y** | **A** | **F** | **Q** | **H** | **G** | **L** | **A** | **K** |
| **N60449F** | **M** | **I** | **K** | **V** | **L** | **L** | **V** | **D** | **D** | **H** | **E** | **M** | **V** | **R** | **L** | **G** | **V** | **S** | **S** | **Y** | **L** | **S** | **I** | **Q** | **E** | **D** | **I** | **E** | **V** | **I** | **G** | **E** | **A** | **E** | **N** | **G** | **R** | **Q** | **G** | **Y** | **E** | **K** | **A** | **M** | **A** | **L** | **R** | **P** | **D** | **V** | **I** | **L** | **M** | **D** | **L** | **V** | **M** | **E** | **E** | **M** | **D** | **G** | **I** | **E** | **S** | **T** | **K** | **A** | **I** | **L** | **K** | **D** | **W** | **P** | **K** | **A** | **K** | **I** | **I** | **I** | **V** | **T** | **S** | **F** | **I** | **D** | **D** | **E** | **K** | **V** | **Y** | **P** | **A** | **I** | **E** | **A** | **G** | **A** | **A** | **G** | **Y** | **L** | **L** | **K** | **T** | **S** | **T** | **A** | **H** | **E** | **I** | **A** | **D** | **A** | **I** | **R** | **A** | **T** | **Q** | **R** | **G** | **E** | **R** | **V** | **L** | **E** | **P** | **E** | **V** | **T** | **T** | **K** | **M** | **M** | **E** | **K** | **M** | **S** | **R** | **R** | **N** | **D** | **P** | **V** | **L** | **H** | **E** | **E** | **L** | **T** | **N** | **R** | **E** | **N** | **E** | **I** | **L** | **M** | **L** | **I** | **S** | **E** | **G** | **K** | **S** | **N** | **Q** | **E** | **I** | **A** | **D** | **E** | **L** | **F** | **I** | **T** | **L** | **K** | **T** | **V** | **K** | **T** | **H** | **V** | **S** | **N** | **I** | **L** | **A** | **K** | **L** | **E** | **V** | **E** | **D** | **R** | **T** | **Q** | **A** | **A** | **I** | **Y** | **A** | **F** | **K** | **H** | **G** | **L** | **V** | **K** |
| **N60463F** | **M** | **I** | **K** | **V** | **L** | **L** | **V** | **D** | **D** | **H** | **E** | **M** | **V** | **R** | **L** | **G** | **V** | **S** | **S** | **Y** | **L** | **S** | **I** | **Q** | **E** | **D** | **I** | **E** | **V** | **I** | **G** | **E** | **A** | **E** | **N** | **G** | **R** | **Q** | **G** | **Y** | **E** | **K** | **A** | **M** | **A** | **L** | **R** | **P** | **D** | **V** | **I** | **L** | **M** | **D** | **L** | **V** | **M** | **E** | **E** | **M** | **D** | **G** | **I** | **E** | **S** | **T** | **K** | **A** | **I** | **L** | **K** | **D** | **W** | **P** | **K** | **A** | **K** | **I** | **I** | **I** | **V** | **T** | **S** | **F** | **I** | **D** | **D** | **E** | **K** | **V** | **Y** | **P** | **A** | **I** | **E** | **A** | **G** | **A** | **A** | **G** | **Y** | **L** | **L** | **K** | **T** | **S** | **T** | **A** | **H** | **E** | **I** | **A** | **D** | **A** | **I** | **R** | **A** | **T** | **Q** | **R** | **G** | **E** | **R** | **V** | **L** | **E** | **P** | **E** | **V** | **T** | **T** | **K** | **M** | **M** | **E** | **K** | **M** | **S** | **R** | **R** | **Y** | **D** | **P** | **V** | **L** | **H** | **E** | **E** | **L** | **T** | **N** | **R** | **E** | **N** | **E** | **I** | **L** | **M** | **L** | **I** | **S** | **E** | **G** | **K** | **S** | **N** | **Q** | **E** | **I** | **A** | **D** | **E** | **L** | **F** | **I** | **T** | **L** | **K** | **T** | **V** | **K** | **T** | **H** | **V** | **S** | **N** | **I** | **L** | **A** | **K** | **L** | **E** | **V** | **E** | **D** | **R** | **T** | **Q** | **A** | **A** | **I** | **Y** | **A** | **F** | **K** | **H** | **G** | **L** | **V** | **K** |
| **N52829** | **M** | **I** | **K** | **V** | **L** | **L** | **V** | **D** | **D** | **H** | **E** | **M** | **V** | **R** | **L** | **G** | **V** | **S** | **S** | **Y** | **L** | **S** | **I** | **Q** | **E** | **D** | **I** | **E** | **V** | **I** | **G** | **E** | **A** | **E** | **N** | **G** | **R** | **Q** | **G** | **Y** | **E** | **K** | **A** | **M** | **A** | **L** | **R** | **P** | **D** | **V** | **I** | **L** | **M** | **D** | **L** | **V** | **M** | **E** | **E** | **M** | **D** | **G** | **I** | **E** | **S** | **T** | **K** | **A** | **I** | **L** | **K** | **D** | **W** | **P** | **K** | **A** | **K** | **I** | **I** | **I** | **V** | **T** | **S** | **F** | **I** | **D** | **D** | **E** | **K** | **V** | **Y** | **P** | **A** | **I** | **E** | **A** | **G** | **A** | **A** | **G** | **Y** | **L** | **L** | **K** | **T** | **S** | **T** | **A** | **H** | **E** | **I** | **A** | **D** | **A** | **I** | **R** | **A** | **T** | **Q** | **R** | **G** | **E** | **R** | **V** | **L** | **E** | **P** | **E** | **V** | **T** | **T** | **K** | **M** | **M** | **E** | **K** | **M** | **S** | **R** | **R** | **N** | **D** | **P** | **V** | **L** | **H** | **E** | **E** | **L** | **T** | **N** | **R** | **E** | **N** | **E** | **I** | **L** | **M** | **L** | **I** | **S** | **E** | **G** | **K** | **S** | **N** | **Q** | **E** | **I** | **A** | **D** | **E** | **L** | **F** | **I** | **T** | **L** | **K** | **T** | **V** | **K** | **T** | **H** | **V** | **S** | **N** | **I** | **L** | **A** | **K** | **L** | **E** | **V** | **E** | **D** | **R** | **T** | **Q** | **A** | **A** | **I** | **Y** | **A** | **F** | **K** | **H** | **G** | **L** | **V** | **K** |
| **N53595** | **M** | **I** | **K** | **V** | **L** | **L** | **V** | **D** | **D** | **H** | **E** | **M** | **V** | **R** | **L** | **G** | **V** | **S** | **S** | **Y** | **L** | **S** | **I** | **Q** | **E** | **D** | **I** | **E** | **V** | **I** | **G** | **E** | **A** | **E** | **N** | **G** | **R** | **Q** | **G** | **Y** | **E** | **K** | **A** | **M** | **A** | **L** | **R** | **P** | **D** | **V** | **I** | **L** | **M** | **D** | **L** | **V** | **M** | **E** | **E** | **M** | **D** | **G** | **I** | **E** | **S** | **T** | **K** | **A** | **I** | **L** | **K** | **D** | **W** | **P** | **K** | **A** | **K** | **I** | **I** | **I** | **V** | **T** | **S** | **F** | **I** | **D** | **D** | **E** | **K** | **V** | **Y** | **P** | **A** | **I** | **E** | **A** | **G** | **A** | **A** | **G** | **Y** | **L** | **L** | **K** | **T** | **S** | **T** | **A** | **H** | **E** | **I** | **A** | **D** | **A** | **I** | **R** | **A** | **T** | **Q** | **R** | **G** | **E** | **R** | **V** | **L** | **E** | **P** | **E** | **V** | **T** | **T** | **K** | **M** | **M** | **E** | **K** | **M** | **S** | **R** | **R** | **N** | **D** | **P** | **V** | **L** | **H** | **E** | **E** | **L** | **T** | **N** | **R** | **E** | **N** | **E** | **I** | **L** | **M** | **L** | **I** | **S** | **E** | **G** | **K** | **S** | **N** | **Q** | **E** | **I** | **A** | **D** | **E** | **L** | **F** | **I** | **T** | **L** | **K** | **T** | **V** | **K** | **T** | **H** | **V** | **S** | **N** | **I** | **L** | **A** | **K** | **L** | **E** | **V** | **E** | **D** | **R** | **T** | **Q** | **A** | **A** | **I** | **Y** | **A** | **F** | **K** | **H** | **G** | **L** | **V** | **K** |
| **N59469F** | **M** | **I** | **K** | **V** | **L** | **L** | **V** | **D** | **D** | **H** | **E** | **M** | **V** | **R** | **L** | **G** | **V** | **S** | **S** | **Y** | **L** | **S** | **I** | **Q** | **E** | **D** | **I** | **E** | **V** | **I** | **G** | **E** | **A** | **E** | **N** | **G** | **R** | **Q** | **G** | **Y** | **E** | **K** | **A** | **M** | **A** | **L** | **R** | **P** | **D** | **V** | **I** | **L** | **M** | **D** | **L** | **V** | **M** | **E** | **E** | **M** | **D** | **G** | **I** | **E** | **S** | **T** | **K** | **A** | **I** | **L** | **K** | **D** | **W** | **P** | **K** | **A** | **K** | **I** | **I** | **I** | **V** | **T** | **S** | **F** | **I** | **D** | **D** | **E** | **K** | **V** | **Y** | **P** | **A** | **I** | **E** | **A** | **G** | **A** | **A** | **G** | **Y** | **L** | **L** | **K** | **T** | **S** | **T** | **A** | **H** | **E** | **I** | **A** | **D** | **A** | **I** | **R** | **A** | **T** | **Q** | **R** | **G** | **E** | **R** | **V** | **L** | **E** | **P** | **E** | **V** | **T** | **T** | **K** | **M** | **M** | **E** | **K** | **M** | **S** | **R** | **R** | **N** | **D** | **P** | **V** | **L** | **H** | **E** | **E** | **L** | **T** | **N** | **R** | **E** | **N** | **E** | **I** | **L** | **M** | **L** | **I** | **S** | **E** | **G** | **K** | **S** | **N** | **Q** | **E** | **I** | **A** | **D** | **E** | **L** | **F** | **I** | **T** | **L** | **K** | **T** | **V** | **K** | **T** | **H** | **V** | **S** | **N** | **I** | **L** | **A** | **K** | **L** | **E** | **V** | **E** | **D** | **R** | **T** | **Q** | **A** | **A** | **I** | **Y** | **A** | **F** | **K** | **H** | **G** | **L** | **V** | **K** |
| **N52623** | **V** | **I** | **K** | **V** | **M** | **L** | **V** | **D** | **D** | **H** | **E** | **M** | **V** | **R** | **L** | **G** | **V** | **S** | **S** | **Y** | **L** | **S** | **I** | **Q** | **E** | **D** | **I** | **E** | **V** | **V** | **G** | **E** | **A** | **E** | **N** | **G** | **K** | **I** | **G** | **Y** | **E** | **K** | **A** | **L** | **E** | **L** | **R** | **P** | **D** | **V** | **I** | **L** | **M** | **D** | **L** | **V** | **M** | **E** | **E** | **M** | **D** | **G** | **I** | **D** | **S** | **T** | **K** | **A** | **I** | **L** | **K** | **D** | **W** | **P** | **E** | **A** | **K** | **I** | **I** | **I** | **V** | **T** | **S** | **F** | **I** | **D** | **D** | **E** | **K** | **V** | **Y** | **P** | **A** | **I** | **E** | **A** | **G** | **A** | **A** | **G** | **Y** | **L** | **L** | **K** | **T** | **S** | **T** | **A** | **H** | **E** | **I** | **A** | **D** | **A** | **I** | **R** | **A** | **T** | **Y** | **R** | **G** | **E** | **R** | **V** | **L** | **E** | **P** | **E** | **V** | **T** | **H** | **K** | **M** | **M** | **E** | **R** | **L** | **T** | **K** | **K** | **Q** | **E** | **P** | **V** | **L** | **H** | **E** | **D** | **L** | **T** | **N** | **R** | **E** | **H** | **E** | **I** | **L** | **M** | **L** | **I** | **A** | **Q** | **G** | **K** | **S** | **N** | **Q** | **E** | **I** | **A** | **D** | **E** | **L** | **F** | **I** | **T** | **L** | **K** | **T** | **V** | **K** | **T** | **H** | **V** | **S** | **N** | **I** | **L** | **A** | **K** | **L** | **D** | **V** | **D** | **D** | **R** | **T** | **Q** | **A** | **A** | **I** | **Y** | **A** | **F** | **Q** | **H** | **G** | **L** | **A** | **K** |
| **N53548** | **M** | **I** | **K** | **V** | **L** | **L** | **V** | **D** | **D** | **H** | **E** | **M** | **V** | **R** | **L** | **G** | **V** | **S** | **S** | **Y** | **L** | **S** | **I** | **Q** | **E** | **D** | **I** | **E** | **V** | **I** | **G** | **E** | **A** | **E** | **N** | **G** | **R** | **Q** | **G** | **Y** | **E** | **K** | **A** | **M** | **A** | **L** | **R** | **P** | **D** | **V** | **I** | **L** | **M** | **D** | **L** | **V** | **M** | **E** | **E** | **M** | **D** | **G** | **I** | **E** | **S** | **T** | **K** | **A** | **I** | **L** | **K** | **D** | **W** | **T** | **K** | **A** | **K** | **I** | **I** | **I** | **V** | **T** | **S** | **F** | **I** | **D** | **D** | **E** | **K** | **V** | **Y** | **P** | **A** | **I** | **E** | **A** | **G** | **A** | **A** | **G** | **Y** | **L** | **L** | **K** | **T** | **S** | **T** | **A** | **H** | **E** | **I** | **A** | **D** | **A** | **I** | **R** | **A** | **T** | **Q** | **R** | **G** | **E** | **R** | **V** | **L** | **E** | **P** | **E** | **V** | **T** | **T** | **K** | **M** | **M** | **E** | **K** | **M** | **S** | **R** | **R** | **N** | **D** | **P** | **V** | **L** | **H** | **E** | **E** | **L** | **T** | **N** | **R** | **E** | **N** | **E** | **I** | **L** | **M** | **L** | **I** | **S** | **E** | **G** | **K** | **S** | **N** | **Q** | **E** | **I** | **A** | **D** | **E** | **L** | **F** | **I** | **T** | **L** | **K** | **T** | **V** | **K** | **T** | **H** | **V** | **S** | **N** | **I** | **L** | **A** | **K** | **L** | **E** | **V** | **E** | **D** | **R** | **T** | **Q** | **A** | **A** | **I** | **Y** | **A** | **F** | **K** | **H** | **G** | **L** | **V** | **K** |
| **N54551** | **M** | **I** | **K** | **V** | **L** | **L** | **V** | **D** | **D** | **H** | **E** | **M** | **V** | **R** | **L** | **G** | **V** | **S** | **S** | **Y** | **L** | **S** | **I** | **Q** | **E** | **D** | **I** | **E** | **V** | **I** | **G** | **E** | **A** | **E** | **N** | **G** | **R** | **Q** | **G** | **Y** | **E** | **K** | **A** | **M** | **A** | **L** | **R** | **P** | **D** | **V** | **I** | **L** | **M** | **D** | **L** | **V** | **M** | **E** | **E** | **M** | **D** | **G** | **I** | **E** | **S** | **T** | **K** | **A** | **I** | **L** | **K** | **D** | **W** | **P** | **K** | **A** | **K** | **I** | **I** | **I** | **V** | **T** | **S** | **F** | **I** | **D** | **D** | **E** | **K** | **V** | **Y** | **P** | **A** | **I** | **E** | **A** | **G** | **A** | **A** | **G** | **Y** | **L** | **L** | **K** | **T** | **S** | **T** | **A** | **H** | **E** | **I** | **A** | **D** | **A** | **I** | **R** | **A** | **T** | **Q** | **R** | **G** | **E** | **R** | **V** | **L** | **E** | **P** | **E** | **V** | **T** | **T** | **K** | **M** | **M** | **E** | **K** | **M** | **S** | **R** | **R** | **N** | **D** | **P** | **V** | **L** | **H** | **E** | **E** | **L** | **T** | **N** | **R** | **E** | **N** | **E** | **I** | **L** | **M** | **L** | **I** | **S** | **E** | **G** | **K** | **S** | **N** | **Q** | **E** | **I** | **A** | **D** | **E** | **L** | **F** | **I** | **T** | **L** | **K** | **T** | **V** | **K** | **T** | **H** | **V** | **S** | **N** | **I** | **L** | **A** | **K** | **L** | **E** | **V** | **E** | **D** | **R** | **T** | **Q** | **A** | **A** | **I** | **Y** | **A** | **F** | **K** | **H** | **G** | **L** | **V** | **K** |
| **N52751** | **M** | **I** | **K** | **V** | **L** | **L** | **V** | **D** | **D** | **H** | **E** | **M** | **V** | **R** | **L** | **G** | **V** | **S** | **S** | **Y** | **L** | **S** | **I** | **Q** | **E** | **D** | **I** | **E** | **V** | **I** | **G** | **E** | **A** | **E** | **N** | **G** | **R** | **Q** | **G** | **Y** | **E** | **K** | **A** | **M** | **A** | **L** | **R** | **P** | **D** | **V** | **I** | **L** | **M** | **D** | **L** | **V** | **M** | **E** | **E** | **M** | **D** | **G** | **I** | **E** | **S** | **T** | **K** | **A** | **I** | **L** | **K** | **D** | **W** | **P** | **K** | **A** | **K** | **I** | **I** | **I** | **V** | **T** | **S** | **F** | **I** | **D** | **D** | **E** | **K** | **V** | **Y** | **P** | **A** | **I** | **E** | **A** | **G** | **A** | **A** | **G** | **Y** | **L** | **L** | **K** | **T** | **S** | **T** | **A** | **H** | **E** | **I** | **A** | **D** | **A** | **I** | **R** | **A** | **T** | **Q** | **R** | **G** | **E** | **R** | **V** | **L** | **E** | **P** | **E** | **V** | **T** | **T** | **K** | **M** | **M** | **E** | **K** | **M** | **S** | **R** | **R** | **N** | **D** | **P** | **V** | **L** | **H** | **E** | **E** | **L** | **T** | **N** | **R** | **E** | **N** | **E** | **I** | **L** | **M** | **L** | **I** | **S** | **E** | **G** | **K** | **S** | **N** | **Q** | **E** | **I** | **A** | **D** | **E** | **L** | **F** | **I** | **T** | **L** | **K** | **T** | **V** | **K** | **T** | **H** | **V** | **S** | **N** | **I** | **L** | **A** | **K** | **L** | **E** | **V** | **E** | **D** | **R** | **T** | **Q** | **A** | **A** | **I** | **Y** | **A** | **F** | **K** | **H** | **G** | **L** | **V** | **K** |
| **N59689F** | **V** | **I** | **K** | **V** | **M** | **L** | **V** | **D** | **D** | **H** | **E** | **M** | **V** | **R** | **L** | **G** | **V** | **S** | **S** | **Y** | **L** | **S** | **I** | **Q** | **E** | **D** | **I** | **E** | **V** | **V** | **G** | **E** | **A** | **E** | **N** | **G** | **K** | **I** | **G** | **Y** | **E** | **K** | **A** | **L** | **E** | **L** | **R** | **P** | **D** | **V** | **I** | **L** | **M** | **D** | **L** | **V** | **M** | **E** | **E** | **M** | **D** | **G** | **I** | **D** | **S** | **T** | **K** | **A** | **I** | **L** | **K** | **D** | **W** | **P** | **E** | **A** | **K** | **I** | **I** | **I** | **V** | **T** | **S** | **F** | **I** | **D** | **D** | **E** | **K** | **V** | **Y** | **P** | **A** | **I** | **E** | **A** | **G** | **A** | **A** | **G** | **Y** | **L** | **L** | **K** | **T** | **S** | **T** | **A** | **H** | **E** | **I** | **A** | **D** | **A** | **I** | **R** | **A** | **T** | **Y** | **R** | **G** | **E** | **R** | **V** | **L** | **E** | **P** | **E** | **V** | **T** | **H** | **K** | **M** | **M** | **E** | **R** | **L** | **T** | **K** | **K** | **Q** | **E** | **P** | **V** | **L** | **H** | **E** | **D** | **L** | **T** | **N** | **R** | **E** | **H** | **E** | **I** | **L** | **M** | **L** | **I** | **A** | **Q** | **G** | **K** | **S** | **N** | **Q** | **E** | **I** | **A** | **D** | **E** | **L** | **F** | **I** | **T** | **L** | **K** | **T** | **V** | **K** | **T** | **H** | **V** | **S** | **N** | **I** | **L** | **A** | **K** | **L** | **D** | **V** | **D** | **D** | **R** | **T** | **Q** | **A** | **A** | **I** | **Y** | **A** | **F** | **Q** | **H** | **G** | **L** | **A** | **K** |
| **N60284F** | **M** | **I** | **K** | **V** | **L** | **L** | **V** | **D** | **D** | **H** | **E** | **M** | **V** | **R** | **L** | **G** | **V** | **S** | **S** | **Y** | **L** | **S** | **I** | **Q** | **E** | **D** | **I** | **E** | **V** | **I** | **G** | **E** | **A** | **E** | **N** | **G** | **R** | **Q** | **G** | **Y** | **E** | **K** | **A** | **M** | **A** | **L** | **R** | **P** | **D** | **V** | **I** | **L** | **M** | **D** | **L** | **V** | **M** | **E** | **E** | **M** | **D** | **G** | **I** | **E** | **S** | **T** | **K** | **A** | **I** | **L** | **K** | **D** | **W** | **P** | **K** | **A** | **K** | **I** | **I** | **I** | **V** | **T** | **S** | **F** | **I** | **D** | **D** | **E** | **K** | **V** | **Y** | **P** | **A** | **I** | **E** | **A** | **G** | **A** | **A** | **G** | **Y** | **L** | **L** | **K** | **T** | **S** | **T** | **A** | **H** | **E** | **I** | **A** | **D** | **A** | **I** | **R** | **A** | **T** | **Q** | **R** | **G** | **E** | **R** | **V** | **L** | **E** | **P** | **E** | **V** | **T** | **T** | **K** | **M** | **M** | **E** | **K** | **M** | **S** | **R** | **R** | **N** | **D** | **P** | **V** | **L** | **H** | **E** | **E** | **L** | **T** | **N** | **R** | **E** | **N** | **E** | **I** | **L** | **M** | **L** | **I** | **S** | **E** | **G** | **K** | **S** | **N** | **Q** | **E** | **I** | **A** | **D** | **E** | **L** | **F** | **I** | **T** | **L** | **K** | **T** | **V** | **K** | **T** | **H** | **V** | **S** | **N** | **I** | **L** | **A** | **K** | **L** | **E** | **V** | **E** | **D** | **R** | **T** | **Q** | **A** | **A** | **I** | **Y** | **A** | **F** | **K** | **H** | **G** | **L** | **V** | **K** |
| **N59613F** | **M** | **I** | **K** | **V** | **L** | **L** | **V** | **D** | **D** | **H** | **E** | **M** | **V** | **R** | **L** | **G** | **V** | **S** | **S** | **Y** | **L** | **S** | **I** | **Q** | **E** | **D** | **I** | **E** | **V** | **I** | **G** | **E** | **A** | **E** | **N** | **G** | **R** | **Q** | **G** | **Y** | **E** | **K** | **A** | **M** | **T** | **L** | **R** | **P** | **D** | **V** | **I** | **L** | **M** | **D** | **L** | **V** | **M** | **E** | **E** | **M** | **D** | **G** | **I** | **E** | **S** | **T** | **K** | **A** | **I** | **L** | **K** | **D** | **W** | **P** | **E** | **A** | **K** | **I** | **I** | **I** | **V** | **T** | **S** | **F** | **I** | **D** | **D** | **E** | **K** | **V** | **Y** | **P** | **A** | **I** | **E** | **A** | **G** | **A** | **A** | **G** | **Y** | **L** | **L** | **K** | **T** | **S** | **T** | **A** | **H** | **E** | **I** | **A** | **D** | **A** | **I** | **R** | **A** | **T** | **Q** | **R** | **G** | **E** | **R** | **V** | **L** | **E** | **P** | **E** | **V** | **T** | **T** | **K** | **M** | **M** | **E** | **K** | **M** | **S** | **R** | **R** | **N** | **E** | **P** | **V** | **L** | **H** | **E** | **E** | **L** | **T** | **N** | **R** | **E** | **N** | **E** | **I** | **L** | **M** | **L** | **I** | **S** | **E** | **G** | **K** | **S** | **N** | **Q** | **E** | **I** | **A** | **D** | **E** | **L** | **F** | **I** | **T** | **L** | **K** | **T** | **V** | **K** | **T** | **H** | **V** | **S** | **N** | **I** | **L** | **A** | **K** | **L** | **E** | **V** | **E** | **D** | **R** | **T** | **Q** | **A** | **A** | **I** | **Y** | **A** | **F** | **K** | **H** | **G** | **L** | **V** | **K** |
| **N60260F** | **M** | **I** | **K** | **V** | **L** | **L** | **V** | **D** | **D** | **H** | **E** | **M** | **V** | **R** | **L** | **G** | **V** | **S** | **S** | **Y** | **L** | **S** | **I** | **Q** | **E** | **D** | **I** | **E** | **V** | **I** | **G** | **E** | **A** | **E** | **N** | **G** | **R** | **Q** | **G** | **Y** | **E** | **K** | **A** | **M** | **A** | **L** | **R** | **P** | **D** | **V** | **I** | **L** | **M** | **D** | **L** | **V** | **M** | **E** | **E** | **M** | **D** | **G** | **I** | **E** | **S** | **T** | **K** | **A** | **I** | **L** | **K** | **D** | **W** | **P** | **K** | **A** | **K** | **I** | **I** | **I** | **V** | **T** | **S** | **F** | **I** | **D** | **D** | **E** | **K** | **V** | **Y** | **P** | **A** | **I** | **E** | **A** | **G** | **A** | **A** | **G** | **Y** | **L** | **L** | **K** | **T** | **S** | **T** | **A** | **H** | **E** | **I** | **A** | **D** | **A** | **I** | **R** | **A** | **T** | **Q** | **R** | **G** | **E** | **R** | **V** | **L** | **E** | **P** | **E** | **V** | **T** | **T** | **K** | **M** | **M** | **E** | **K** | **M** | **S** | **R** | **R** | **N** | **D** | **P** | **V** | **L** | **H** | **E** | **E** | **L** | **T** | **N** | **R** | **E** | **N** | **E** | **I** | **L** | **M** | **L** | **I** | **S** | **E** | **G** | **K** | **S** | **N** | **Q** | **E** | **I** | **A** | **D** | **E** | **L** | **F** | **I** | **T** | **L** | **K** | **T** | **V** | **K** | **T** | **H** | **V** | **S** | **N** | **I** | **L** | **A** | **K** | **L** | **E** | **V** | **E** | **D** | **R** | **T** | **Q** | **A** | **A** | **I** | **Y** | **A** | **F** | **K** | **H** | **G** | **L** | **V** | **K** |
| **N54576** | **V** | **I** | **K** | **V** | **M** | **L** | **V** | **D** | **D** | **H** | **E** | **M** | **V** | **R** | **L** | **G** | **V** | **S** | **S** | **Y** | **L** | **S** | **I** | **Q** | **E** | **D** | **I** | **E** | **V** | **V** | **G** | **E** | **A** | **E** | **N** | **G** | **K** | **I** | **G** | **Y** | **E** | **K** | **A** | **L** | **E** | **L** | **R** | **P** | **D** | **V** | **I** | **L** | **M** | **D** | **L** | **V** | **M** | **E** | **E** | **M** | **D** | **G** | **I** | **D** | **S** | **T** | **K** | **A** | **I** | **L** | **K** | **D** | **W** | **P** | **E** | **A** | **K** | **I** | **I** | **I** | **V** | **T** | **S** | **F** | **I** | **D** | **D** | **E** | **K** | **V** | **Y** | **P** | **A** | **I** | **E** | **A** | **G** | **A** | **A** | **G** | **Y** | **L** | **L** | **K** | **T** | **S** | **T** | **A** | **H** | **E** | **I** | **A** | **D** | **A** | **I** | **R** | **A** | **T** | **Y** | **R** | **G** | **E** | **R** | **V** | **L** | **E** | **P** | **E** | **V** | **T** | **H** | **K** | **M** | **M** | **E** | **R** | **L** | **T** | **K** | **K** | **Q** | **E** | **P** | **V** | **L** | **H** | **E** | **D** | **L** | **T** | **N** | **R** | **E** | **H** | **E** | **I** | **L** | **M** | **L** | **I** | **A** | **Q** | **G** | **K** | **S** | **N** | **Q** | **E** | **I** | **A** | **D** | **E** | **L** | **F** | **I** | **T** | **L** | **K** | **T** | **V** | **K** | **T** | **H** | **V** | **S** | **N** | **I** | **L** | **A** | **K** | **L** | **D** | **V** | **D** | **D** | **R** | **T** | **Q** | **A** | **A** | **I** | **Y** | **A** | **F** | **Q** | **H** | **G** | **L** | **A** | **K** |
| **N60001F** | **M** | **I** | **K** | **V** | **L** | **L** | **V** | **D** | **D** | **H** | **E** | **M** | **V** | **R** | **L** | **G** | **V** | **S** | **S** | **Y** | **L** | **S** | **I** | **Q** | **E** | **D** | **I** | **E** | **V** | **I** | **G** | **E** | **A** | **K** | **N** | **G** | **R** | **Q** | **G** | **Y** | **E** | **K** | **A** | **M** | **T** | **L** | **R** | **P** | **D** | **V** | **I** | **L** | **M** | **D** | **L** | **V** | **M** | **E** | **E** | **M** | **D** | **G** | **I** | **E** | **S** | **T** | **K** | **A** | **I** | **L** | **K** | **D** | **W** | **P** | **E** | **A** | **K** | **I** | **I** | **I** | **V** | **T** | **S** | **F** | **I** | **D** | **D** | **E** | **K** | **V** | **Y** | **P** | **A** | **I** | **E** | **A** | **G** | **A** | **A** | **G** | **Y** | **L** | **L** | **K** | **T** | **S** | **T** | **A** | **H** | **E** | **I** | **A** | **D** | **A** | **I** | **R** | **A** | **T** | **Q** | **R** | **G** | **E** | **R** | **V** | **L** | **E** | **P** | **E** | **V** | **T** | **T** | **K** | **M** | **M** | **E** | **K** | **M** | **S** | **R** | **R** | **N** | **E** | **P** | **V** | **L** | **H** | **E** | **E** | **L** | **T** | **N** | **R** | **E** | **N** | **E** | **I** | **L** | **M** | **L** | **I** | **S** | **E** | **G** | **K** | **S** | **N** | **Q** | **E** | **I** | **A** | **D** | **E** | **L** | **F** | **I** | **T** | **L** | **K** | **T** | **V** | **K** | **T** | **H** | **V** | **S** | **N** | **I** | **L** | **A** | **K** | **L** | **E** | **V** | **E** | **D** | **R** | **T** | **Q** | **A** | **A** | **I** | **Y** | **A** | **F** | **K** | **H** | **G** | **L** | **V** | **K** |
| **N52734** | **V** | **I** | **K** | **V** | **M** | **L** | **V** | **D** | **D** | **H** | **E** | **M** | **V** | **R** | **L** | **G** | **V** | **S** | **S** | **Y** | **L** | **S** | **I** | **Q** | **E** | **D** | **I** | **E** | **V** | **V** | **G** | **E** | **A** | **E** | **N** | **G** | **K** | **I** | **G** | **Y** | **E** | **K** | **A** | **L** | **E** | **L** | **R** | **P** | **D** | **V** | **I** | **L** | **M** | **D** | **L** | **V** | **M** | **E** | **E** | **M** | **D** | **G** | **I** | **D** | **S** | **T** | **K** | **A** | **I** | **L** | **K** | **D** | **W** | **P** | **E** | **A** | **K** | **I** | **I** | **I** | **V** | **T** | **S** | **F** | **I** | **D** | **D** | **E** | **K** | **V** | **Y** | **P** | **A** | **I** | **E** | **A** | **G** | **A** | **A** | **G** | **Y** | **L** | **L** | **K** | **T** | **S** | **T** | **A** | **H** | **E** | **I** | **A** | **D** | **A** | **I** | **R** | **A** | **T** | **Y** | **R** | **G** | **E** | **R** | **V** | **L** | **E** | **P** | **E** | **V** | **T** | **H** | **K** | **M** | **M** | **E** | **R** | **L** | **T** | **K** | **K** | **Q** | **E** | **P** | **V** | **L** | **H** | **E** | **D** | **L** | **T** | **N** | **R** | **E** | **H** | **E** | **I** | **L** | **M** | **L** | **I** | **A** | **Q** | **G** | **K** | **S** | **N** | **Q** | **E** | **I** | **A** | **D** | **E** | **L** | **F** | **I** | **T** | **L** | **K** | **T** | **V** | **K** | **T** | **H** | **V** | **S** | **N** | **I** | **L** | **A** | **K** | **L** | **D** | **V** | **D** | **D** | **R** | **T** | **Q** | **A** | **A** | **I** | **Y** | **A** | **F** | **Q** | **H** | **G** | **L** | **A** | **K** |
| **N54599** | **M** | **I** | **K** | **V** | **L** | **L** | **V** | **D** | **D** | **H** | **E** | **M** | **V** | **R** | **L** | **G** | **V** | **S** | **S** | **Y** | **L** | **S** | **I** | **Q** | **E** | **D** | **I** | **E** | **V** | **I** | **G** | **E** | **A** | **E** | **N** | **G** | **R** | **Q** | **G** | **Y** | **E** | **K** | **A** | **M** | **T** | **L** | **R** | **P** | **D** | **V** | **I** | **L** | **M** | **D** | **L** | **V** | **M** | **E** | **E** | **M** | **D** | **G** | **I** | **E** | **S** | **T** | **K** | **A** | **I** | **L** | **K** | **D** | **W** | **L** | **E** | **A** | **K** | **I** | **I** | **I** | **V** | **T** | **S** | **F** | **I** | **D** | **D** | **E** | **K** | **V** | **Y** | **P** | **A** | **I** | **E** | **A** | **G** | **A** | **A** | **G** | **Y** | **L** | **L** | **K** | **T** | **S** | **T** | **A** | **H** | **E** | **I** | **A** | **D** | **A** | **I** | **R** | **A** | **T** | **Q** | **R** | **G** | **E** | **R** | **V** | **L** | **E** | **P** | **E** | **V** | **T** | **T** | **K** | **M** | **M** | **E** | **K** | **M** | **S** | **R** | **R** | **N** | **E** | **P** | **V** | **L** | **H** | **E** | **E** | **L** | **T** | **N** | **R** | **E** | **N** | **E** | **I** | **L** | **M** | **L** | **I** | **S** | **E** | **G** | **K** | **S** | **N** | **Q** | **E** | **I** | **A** | **D** | **E** | **L** | **F** | **I** | **T** | **L** | **K** | **T** | **V** | **K** | **T** | **H** | **V** | **S** | **N** | **I** | **L** | **A** | **K** | **L** | **E** | **V** | **E** | **D** | **R** | **T** | **Q** | **A** | **A** | **I** | **Y** | **A** | **F** | **K** | **H** | **G** | **L** | **V** | **K** |
| **N53405** | **V** | **I** | **K** | **V** | **M** | **L** | **V** | **D** | **D** | **H** | **E** | **M** | **V** | **R** | **L** | **G** | **V** | **S** | **S** | **Y** | **L** | **S** | **I** | **Q** | **E** | **D** | **I** | **E** | **V** | **V** | **G** | **E** | **A** | **E** | **N** | **G** | **K** | **I** | **G** | **Y** | **E** | **K** | **A** | **L** | **E** | **L** | **R** | **P** | **D** | **V** | **I** | **L** | **M** | **D** | **L** | **V** | **M** | **E** | **E** | **M** | **D** | **G** | **I** | **D** | **S** | **T** | **K** | **A** | **I** | **L** | **K** | **D** | **W** | **P** | **E** | **A** | **K** | **I** | **I** | **I** | **V** | **T** | **S** | **F** | **I** | **D** | **D** | **E** | **K** | **V** | **Y** | **P** | **A** | **I** | **E** | **A** | **G** | **A** | **A** | **G** | **Y** | **L** | **L** | **K** | **T** | **S** | **T** | **A** | **H** | **E** | **I** | **A** | **D** | **A** | **I** | **R** | **A** | **T** | **Y** | **R** | **G** | **E** | **R** | **V** | **L** | **E** | **P** | **E** | **V** | **T** | **H** | **K** | **M** | **M** | **E** | **R** | **L** | **T** | **K** | **K** | **Q** | **E** | **P** | **V** | **L** | **H** | **E** | **D** | **L** | **T** | **N** | **R** | **E** | **H** | **E** | **I** | **L** | **M** | **L** | **I** | **A** | **Q** | **G** | **K** | **S** | **N** | **Q** | **E** | **I** | **A** | **D** | **E** | **L** | **F** | **I** | **T** | **L** | **K** | **T** | **V** | **K** | **T** | **H** | **V** | **S** | **N** | **I** | **L** | **A** | **K** | **L** | **D** | **V** | **D** | **D** | **R** | **T** | **Q** | **A** | **A** | **I** | **Y** | **A** | **F** | **Q** | **H** | **G** | **L** | **A** | **K** |
| **N54548** | **V** | **I** | **K** | **V** | **M** | **L** | **V** | **D** | **D** | **H** | **E** | **M** | **V** | **R** | **L** | **G** | **V** | **S** | **S** | **Y** | **L** | **S** | **I** | **Q** | **E** | **D** | **I** | **E** | **V** | **V** | **G** | **E** | **A** | **E** | **N** | **G** | **K** | **I** | **G** | **Y** | **E** | **K** | **A** | **L** | **E** | **L** | **R** | **P** | **D** | **V** | **I** | **L** | **M** | **D** | **L** | **V** | **M** | **E** | **E** | **M** | **D** | **G** | **I** | **D** | **S** | **T** | **K** | **A** | **I** | **L** | **K** | **D** | **W** | **P** | **E** | **A** | **K** | **I** | **I** | **I** | **V** | **T** | **S** | **F** | **I** | **D** | **D** | **E** | **K** | **V** | **Y** | **P** | **A** | **I** | **E** | **A** | **G** | **A** | **A** | **G** | **Y** | **L** | **L** | **K** | **T** | **S** | **T** | **A** | **H** | **E** | **I** | **A** | **D** | **A** | **I** | **R** | **A** | **T** | **Y** | **R** | **G** | **E** | **R** | **V** | **L** | **E** | **P** | **E** | **V** | **T** | **H** | **K** | **M** | **M** | **E** | **R** | **L** | **T** | **K** | **K** | **Q** | **E** | **P** | **V** | **L** | **H** | **E** | **D** | **L** | **T** | **N** | **R** | **E** | **H** | **E** | **I** | **L** | **M** | **L** | **I** | **A** | **Q** | **G** | **K** | **S** | **N** | **Q** | **E** | **I** | **A** | **D** | **E** | **L** | **F** | **I** | **T** | **L** | **K** | **T** | **V** | **K** | **T** | **H** | **V** | **S** | **N** | **I** | **L** | **A** | **K** | **L** | **D** | **V** | **D** | **D** | **R** | **T** | **Q** | **A** | **A** | **I** | **Y** | **A** | **F** | **Q** | **H** | **G** | **L** | **A** | **K** |
| **N53420** | **V** | **I** | **K** | **V** | **M** | **L** | **V** | **D** | **D** | **H** | **E** | **M** | **V** | **R** | **L** | **G** | **V** | **S** | **S** | **Y** | **L** | **S** | **I** | **Q** | **E** | **D** | **I** | **E** | **V** | **V** | **G** | **E** | **A** | **E** | **N** | **G** | **K** | **I** | **G** | **Y** | **E** | **K** | **A** | **L** | **E** | **L** | **R** | **P** | **D** | **V** | **I** | **L** | **M** | **D** | **L** | **V** | **M** | **E** | **E** | **M** | **D** | **G** | **I** | **D** | **S** | **T** | **K** | **A** | **I** | **L** | **K** | **D** | **W** | **P** | **E** | **A** | **K** | **I** | **I** | **I** | **V** | **T** | **S** | **F** | **I** | **D** | **D** | **E** | **K** | **V** | **Y** | **P** | **A** | **I** | **E** | **A** | **G** | **A** | **A** | **G** | **Y** | **L** | **L** | **K** | **T** | **S** | **T** | **A** | **H** | **E** | **I** | **A** | **D** | **A** | **I** | **R** | **A** | **T** | **Y** | **R** | **G** | **E** | **R** | **V** | **L** | **E** | **P** | **E** | **V** | **T** | **H** | **K** | **M** | **M** | **E** | **R** | **L** | **T** | **K** | **K** | **Q** | **E** | **P** | **V** | **L** | **H** | **E** | **D** | **L** | **T** | **N** | **R** | **E** | **H** | **E** | **I** | **L** | **M** | **L** | **I** | **A** | **Q** | **G** | **K** | **S** | **N** | **Q** | **E** | **I** | **A** | **D** | **E** | **L** | **F** | **I** | **T** | **L** | **K** | **T** | **V** | **K** | **T** | **H** | **V** | **S** | **N** | **I** | **L** | **A** | **K** | **L** | **D** | **V** | **D** | **D** | **R** | **T** | **Q** | **A** | **A** | **I** | **Y** | **A** | **F** | **Q** | **H** | **G** | **L** | **A** | **K** |
| **N52732** | **M** | **I** | **K** | **V** | **L** | **L** | **V** | **D** | **D** | **H** | **E** | **M** | **V** | **R** | **L** | **G** | **V** | **S** | **S** | **Y** | **L** | **S** | **I** | **Q** | **E** | **D** | **I** | **E** | **V** | **I** | **G** | **E** | **A** | **E** | **N** | **G** | **R** | **Q** | **G** | **Y** | **E** | **K** | **A** | **M** | **T** | **L** | **R** | **P** | **D** | **V** | **I** | **L** | **M** | **D** | **L** | **V** | **M** | **E** | **E** | **M** | **D** | **G** | **I** | **E** | **S** | **T** | **K** | **A** | **I** | **L** | **K** | **D** | **W** | **P** | **E** | **A** | **K** | **I** | **I** | **I** | **V** | **T** | **S** | **F** | **I** | **D** | **D** | **E** | **K** | **V** | **Y** | **P** | **A** | **I** | **E** | **A** | **G** | **A** | **A** | **G** | **Y** | **L** | **L** | **K** | **T** | **S** | **T** | **A** | **H** | **E** | **I** | **A** | **D** | **A** | **I** | **R** | **A** | **T** | **Q** | **R** | **G** | **E** | **R** | **V** | **L** | **E** | **P** | **E** | **V** | **T** | **T** | **K** | **M** | **M** | **E** | **K** | **M** | **S** | **R** | **R** | **N** | **E** | **P** | **V** | **L** | **H** | **E** | **E** | **L** | **T** | **N** | **R** | **E** | **N** | **E** | **I** | **L** | **M** | **L** | **I** | **S** | **E** | **G** | **K** | **S** | **N** | **Q** | **E** | **I** | **A** | **D** | **E** | **L** | **F** | **I** | **T** | **L** | **K** | **T** | **V** | **K** | **T** | **H** | **V** | **S** | **N** | **I** | **L** | **A** | **K** | **L** | **E** | **V** | **E** | **D** | **R** | **T** | **Q** | **A** | **A** | **I** | **Y** | **A** | **F** | **K** | **H** | **G** | **L** | **V** | **K** |
| **N59788F** | **M** | **I** | **K** | **V** | **L** | **L** | **V** | **D** | **D** | **H** | **E** | **M** | **V** | **R** | **L** | **G** | **V** | **S** | **S** | **Y** | **L** | **S** | **I** | **Q** | **E** | **D** | **I** | **E** | **V** | **I** | **G** | **E** | **A** | **E** | **N** | **G** | **R** | **Q** | **G** | **Y** | **E** | **K** | **A** | **M** | **A** | **L** | **R** | **P** | **D** | **V** | **I** | **L** | **M** | **D** | **L** | **V** | **M** | **E** | **E** | **M** | **D** | **G** | **I** | **E** | **S** | **T** | **K** | **A** | **I** | **L** | **K** | **D** | **W** | **P** | **K** | **A** | **K** | **I** | **I** | **I** | **V** | **T** | **S** | **F** | **I** | **D** | **D** | **E** | **K** | **V** | **Y** | **P** | **A** | **I** | **E** | **A** | **G** | **A** | **A** | **G** | **Y** | **L** | **L** | **K** | **T** | **S** | **T** | **A** | **H** | **E** | **I** | **A** | **D** | **A** | **I** | **R** | **A** | **T** | **Q** | **R** | **G** | **E** | **R** | **V** | **L** | **E** | **P** | **E** | **V** | **T** | **T** | **K** | **M** | **M** | **E** | **K** | **M** | **S** | **R** | **R** | **N** | **D** | **P** | **V** | **L** | **H** | **E** | **E** | **L** | **T** | **N** | **R** | **E** | **N** | **E** | **I** | **L** | **M** | **L** | **I** | **S** | **E** | **G** | **K** | **S** | **N** | **Q** | **E** | **I** | **A** | **D** | **E** | **L** | **F** | **I** | **T** | **L** | **K** | **T** | **V** | **K** | **T** | **H** | **V** | **S** | **N** | **I** | **L** | **A** | **K** | **L** | **E** | **V** | **E** | **D** | **R** | **T** | **Q** | **A** | **A** | **I** | **Y** | **A** | **F** | **K** | **H** | **G** | **L** | **V** | **K** |
| **N59860F** | **V** | **I** | **K** | **V** | **M** | **L** | **V** | **D** | **D** | **H** | **E** | **M** | **V** | **R** | **L** | **G** | **V** | **S** | **S** | **Y** | **L** | **S** | **I** | **Q** | **E** | **D** | **I** | **E** | **V** | **V** | **G** | **E** | **A** | **E** | **N** | **G** | **K** | **I** | **G** | **Y** | **E** | **K** | **A** | **L** | **E** | **L** | **R** | **P** | **D** | **V** | **I** | **L** | **M** | **D** | **L** | **V** | **M** | **E** | **E** | **M** | **D** | **G** | **I** | **D** | **S** | **T** | **K** | **A** | **I** | **L** | **K** | **D** | **W** | **P** | **E** | **A** | **K** | **I** | **I** | **I** | **V** | **T** | **S** | **F** | **I** | **D** | **D** | **E** | **K** | **V** | **Y** | **P** | **A** | **I** | **E** | **A** | **G** | **A** | **A** | **G** | **Y** | **L** | **L** | **K** | **T** | **S** | **T** | **A** | **H** | **E** | **I** | **A** | **D** | **A** | **I** | **R** | **A** | **T** | **Y** | **R** | **G** | **E** | **R** | **V** | **L** | **E** | **P** | **E** | **V** | **T** | **H** | **K** | **M** | **M** | **E** | **R** | **L** | **T** | **K** | **K** | **Q** | **E** | **P** | **V** | **L** | **H** | **E** | **D** | **L** | **T** | **N** | **R** | **E** | **H** | **E** | **I** | **L** | **M** | **L** | **I** | **A** | **Q** | **G** | **K** | **S** | **N** | **Q** | **E** | **I** | **A** | **D** | **E** | **L** | **F** | **I** | **T** | **L** | **K** | **T** | **V** | **K** | **T** | **H** | **V** | **S** | **N** | **I** | **L** | **A** | **K** | **L** | **D** | **V** | **D** | **D** | **R** | **T** | **Q** | **A** | **A** | **I** | **Y** | **A** | **F** | **Q** | **H** | **G** | **L** | **A** | **K** |
| **N54538** | **M** | **I** | **K** | **V** | **L** | **L** | **V** | **D** | **D** | **H** | **E** | **M** | **V** | **R** | **L** | **G** | **V** | **S** | **S** | **Y** | **L** | **S** | **I** | **Q** | **E** | **D** | **I** | **E** | **V** | **I** | **G** | **E** | **A** | **E** | **N** | **G** | **R** | **Q** | **G** | **Y** | **E** | **K** | **A** | **M** | **T** | **L** | **R** | **P** | **D** | **V** | **I** | **L** | **M** | **D** | **L** | **V** | **M** | **E** | **E** | **M** | **D** | **G** | **I** | **E** | **S** | **T** | **K** | **A** | **I** | **L** | **K** | **D** | **W** | **P** | **E** | **A** | **K** | **I** | **I** | **I** | **V** | **T** | **S** | **F** | **I** | **D** | **D** | **E** | **K** | **V** | **Y** | **P** | **A** | **I** | **E** | **A** | **G** | **A** | **A** | **G** | **Y** | **L** | **L** | **K** | **T** | **S** | **T** | **A** | **H** | **E** | **I** | **A** | **D** | **A** | **I** | **R** | **A** | **T** | **Q** | **R** | **G** | **E** | **R** | **V** | **L** | **E** | **P** | **E** | **V** | **T** | **T** | **K** | **M** | **M** | **E** | **K** | **M** | **S** | **R** | **R** | **N** | **E** | **P** | **V** | **L** | **H** | **E** | **E** | **L** | **T** | **N** | **R** | **E** | **N** | **E** | **I** | **L** | **M** | **L** | **I** | **S** | **E** | **G** | **K** | **S** | **N** | **Q** | **E** | **I** | **A** | **D** | **E** | **L** | **F** | **I** | **T** | **L** | **K** | **T** | **V** | **K** | **T** | **H** | **V** | **S** | **N** | **I** | **L** | **A** | **K** | **L** | **E** | **V** | **E** | **D** | **R** | **T** | **Q** | **A** | **A** | **I** | **Y** | **A** | **F** | **K** | **H** | **G** | **L** | **V** | **K** |
| **N55072** | **M** | **I** | **K** | **V** | **L** | **L** | **V** | **D** | **D** | **H** | **E** | **M** | **V** | **R** | **L** | **G** | **V** | **S** | **S** | **Y** | **L** | **S** | **I** | **Q** | **E** | **D** | **I** | **E** | **V** | **I** | **G** | **E** | **A** | **E** | **N** | **G** | **R** | **Q** | **G** | **Y** | **E** | **K** | **A** | **M** | **A** | **L** | **R** | **P** | **D** | **V** | **I** | **L** | **M** | **D** | **L** | **V** | **M** | **E** | **E** | **M** | **D** | **G** | **I** | **E** | **S** | **T** | **K** | **A** | **I** | **L** | **K** | **D** | **W** | **P** | **K** | **A** | **K** | **I** | **I** | **I** | **V** | **T** | **S** | **F** | **I** | **D** | **D** | **E** | **K** | **V** | **Y** | **P** | **A** | **I** | **E** | **A** | **G** | **A** | **A** | **G** | **Y** | **L** | **L** | **K** | **T** | **S** | **T** | **A** | **H** | **E** | **I** | **A** | **D** | **A** | **I** | **R** | **A** | **T** | **Q** | **R** | **G** | **E** | **R** | **V** | **L** | **E** | **P** | **E** | **V** | **T** | **T** | **K** | **M** | **M** | **E** | **K** | **M** | **S** | **R** | **R** | **N** | **D** | **P** | **V** | **L** | **H** | **E** | **E** | **L** | **T** | **N** | **R** | **E** | **N** | **E** | **I** | **L** | **M** | **L** | **I** | **S** | **E** | **G** | **K** | **S** | **N** | **Q** | **E** | **I** | **A** | **D** | **E** | **L** | **F** | **I** | **T** | **L** | **K** | **T** | **V** | **K** | **T** | **H** | **V** | **S** | **N** | **I** | **L** | **A** | **K** | **L** | **E** | **V** | **E** | **D** | **R** | **T** | **Q** | **A** | **A** | **I** | **Y** | **A** | **F** | **K** | **H** | **G** | **L** | **V** | **K** |
| **N59544F** | **V** | **I** | **K** | **V** | **M** | **L** | **V** | **D** | **D** | **H** | **E** | **M** | **V** | **R** | **L** | **G** | **V** | **S** | **S** | **Y** | **L** | **S** | **I** | **Q** | **E** | **D** | **I** | **E** | **V** | **V** | **G** | **E** | **A** | **E** | **N** | **G** | **K** | **I** | **G** | **Y** | **E** | **K** | **A** | **L** | **E** | **L** | **R** | **P** | **D** | **V** | **I** | **L** | **M** | **D** | **L** | **V** | **M** | **E** | **E** | **M** | **D** | **G** | **I** | **D** | **S** | **T** | **K** | **A** | **I** | **L** | **K** | **D** | **W** | **P** | **E** | **A** | **K** | **I** | **I** | **I** | **V** | **T** | **S** | **F** | **I** | **D** | **D** | **E** | **K** | **V** | **Y** | **P** | **A** | **I** | **E** | **A** | **G** | **A** | **A** | **G** | **Y** | **L** | **L** | **K** | **T** | **S** | **T** | **A** | **H** | **E** | **I** | **A** | **D** | **A** | **I** | **R** | **A** | **T** | **Y** | **R** | **G** | **E** | **R** | **V** | **L** | **E** | **P** | **E** | **V** | **T** | **H** | **K** | **M** | **M** | **E** | **R** | **L** | **T** | **K** | **K** | **Q** | **E** | **P** | **V** | **L** | **H** | **E** | **D** | **L** | **T** | **N** | **R** | **E** | **H** | **E** | **I** | **L** | **M** | **L** | **I** | **A** | **Q** | **G** | **K** | **S** | **N** | **Q** | **E** | **I** | **A** | **D** | **E** | **L** | **F** | **I** | **T** | **L** | **K** | **T** | **V** | **K** | **T** | **H** | **V** | **S** | **N** | **I** | **L** | **A** | **K** | **L** | **D** | **V** | **D** | **D** | **R** | **T** | **Q** | **A** | **A** | **I** | **Y** | **A** | **F** | **Q** | **H** | **G** | **L** | **A** | **K** |
| **N54097** | **V** | **I** | **K** | **V** | **M** | **L** | **V** | **D** | **D** | **H** | **E** | **M** | **V** | **R** | **L** | **G** | **V** | **S** | **S** | **Y** | **L** | **S** | **I** | **Q** | **E** | **D** | **I** | **E** | **V** | **V** | **G** | **E** | **A** | **E** | **N** | **G** | **K** | **I** | **G** | **Y** | **E** | **K** | **A** | **L** | **E** | **L** | **R** | **P** | **D** | **V** | **I** | **L** | **M** | **D** | **L** | **V** | **M** | **E** | **E** | **M** | **D** | **G** | **I** | **D** | **S** | **T** | **K** | **A** | **I** | **L** | **K** | **D** | **W** | **P** | **E** | **A** | **K** | **I** | **I** | **I** | **V** | **T** | **S** | **F** | **I** | **D** | **D** | **E** | **K** | **V** | **Y** | **P** | **A** | **I** | **E** | **A** | **G** | **A** | **A** | **G** | **Y** | **L** | **L** | **K** | **T** | **S** | **T** | **A** | **H** | **E** | **I** | **A** | **D** | **A** | **I** | **R** | **A** | **T** | **Y** | **R** | **G** | **E** | **R** | **V** | **L** | **E** | **P** | **E** | **V** | **T** | **H** | **K** | **M** | **M** | **E** | **R** | **L** | **T** | **K** | **K** | **Q** | **E** | **P** | **V** | **L** | **H** | **E** | **D** | **L** | **T** | **N** | **R** | **E** | **H** | **E** | **I** | **L** | **M** | **L** | **I** | **A** | **Q** | **G** | **K** | **S** | **N** | **Q** | **E** | **I** | **A** | **D** | **E** | **L** | **F** | **I** | **T** | **L** | **K** | **T** | **V** | **K** | **T** | **H** | **V** | **S** | **N** | **I** | **L** | **A** | **K** | **L** | **D** | **V** | **D** | **D** | **R** | **T** | **Q** | **A** | **A** | **I** | **Y** | **A** | **F** | **Q** | **H** | **G** | **L** | **A** | **K** |
| **N60252F** | **M** | **I** | **K** | **V** | **L** | **L** | **V** | **D** | **D** | **H** | **E** | **M** | **V** | **R** | **L** | **G** | **V** | **S** | **S** | **Y** | **L** | **S** | **I** | **Q** | **E** | **D** | **I** | **E** | **V** | **I** | **G** | **E** | **A** | **E** | **N** | **G** | **R** | **Q** | **G** | **Y** | **E** | **K** | **A** | **M** | **A** | **L** | **R** | **P** | **D** | **V** | **I** | **L** | **M** | **D** | **L** | **V** | **M** | **E** | **E** | **M** | **D** | **G** | **I** | **E** | **S** | **T** | **K** | **A** | **I** | **L** | **K** | **D** | **W** | **P** | **K** | **A** | **K** | **I** | **I** | **I** | **V** | **T** | **S** | **F** | **I** | **D** | **D** | **E** | **K** | **V** | **Y** | **P** | **A** | **I** | **E** | **A** | **G** | **A** | **A** | **G** | **Y** | **L** | **L** | **K** | **T** | **S** | **T** | **A** | **H** | **E** | **I** | **A** | **D** | **A** | **I** | **R** | **A** | **T** | **Q** | **R** | **G** | **E** | **R** | **V** | **L** | **E** | **P** | **E** | **V** | **T** | **T** | **K** | **M** | **M** | **E** | **K** | **M** | **S** | **R** | **R** | **N** | **D** | **P** | **V** | **L** | **H** | **E** | **E** | **L** | **T** | **N** | **R** | **E** | **N** | **E** | **I** | **L** | **M** | **L** | **I** | **S** | **E** | **G** | **K** | **S** | **N** | **Q** | **E** | **I** | **A** | **D** | **E** | **L** | **F** | **I** | **T** | **L** | **K** | **T** | **V** | **K** | **T** | **H** | **V** | **S** | **N** | **I** | **L** | **A** | **K** | **L** | **E** | **V** | **E** | **D** | **R** | **T** | **Q** | **A** | **A** | **I** | **Y** | **A** | **F** | **K** | **H** | **G** | **L** | **V** | **K** |
 **SNP position** | 1 | . | . | . | 5 | . | . | . | . | . | . | . | . | . | . | . | . | . | . | . | . | . | . | . | . | . | . | . | . | 30 | . | . | . | 34 | . | . | 37 | 38 | . | . | . | . | . | 44 | 45 | . | . | . | . | . | . | . | . | . | . | 56 | . | . | . | . | . | . | . | 64 | . | . | . | . | . | . | . | . | . | 74 | 75 | . | . | . | . | . | . | . | . | . | . | . | . | . | . | . | . | . | . | . | . | . | . | . | . | . | . | . | . | . | . | . | . | . | . | . | . | . | . | . | . | . | 117 | . | 119 | 120 | . | . | . | . | . | . | . | . | . | . | 131 | . | . | . | . | 136 | 137 | 138 | 139 | 140 | 141 | 142 | . | . | . | . | . | 148 | . | . | . | . | . | 154 | . | . | . | . | . | . | 161 | 162 | . | . | . | . | . | . | . | . | . | . | . | . | . | . | . | . | . | . | . | . | . | . | . | . | . | . | . | . | 191 | 192 | . | 194 | . | . | . | . | . | . | . | . | 203 | . | 205 | . | . | . | 209 | . |

|  |  |
| --- | --- |
|  |

|  |  |
| --- | --- |
|  |

|  |  |
| --- | --- |
| **Legend** | |
  | **Reference Sequence** |

  |
|  | **Validated SNPs** |
|  | **Non-validated SNPs** |
|  | **New SNPs** |
|  | **GAPs** |
|  |  |

  
  |
|  |  |
