# Supplementary material for: sraX: A Novel Comprehensive Resistome Analysis Tool
Source: Front Microbiol. 2020 Feb 5;11:52. doi: 10.3389/fmicb.2020.00052 (PMC7025521; doi:10.3389/fmicb.2020.00052)
Supplement: Supplementary file 4 [file Data_Sheet_3.ZIP › liaF/liaF_Efaecalis_dna.html]

# liaF

|  |
| --- |
|  |

|  |
| --- |
|  |

|  |  |
| --- | --- |
| **Definition:** | LiaF is an accessory protein that acts as a negative regulator of liaRS signal transduction pathway. Mutations confer daptomycin resistance. |

|  |
| --- |
|  |

|  |
| --- |
|  |

|  |  |  |
| --- | --- | --- |
| **Reference organism:** | *Enterococcus faecalis V583* | [NCBI, CARD] |

|  |
| --- |
|  |

|  |
| --- |
|  |

|  |  |
| --- | --- |
| **Summary Data** | || **Parameter** | **Value_(n)_** || Total number of genomes | 98 |
| MSA length | 732 |
| Total number of SNPs | 52 |
| SNP density (# SNPs/MSA length) | 0.071 |

|  |
| --- |
|  |

|  |
| --- |
|  |

|  |
| --- |
|  |

|  |  |  |  |  |  |  |  |  |  |  |  |  |  |  |  |  |  |  |  |  |  |  |  |  |  |  |  |  |  |  |  |  |  |  |  |  |  |  |  |  |  |  |  |  |  |  |  |  |  |  |  |  |  |  |  |  |  |  |  |  |  |  |  |  |  |  |  |  |  |  |  |  |  |  |  |  |  |  |  |  |  |  |  |  |  |  |  |  |  |  |  |  |  |  |  |  |  |  |  |  |  |  |  |  |  |  |  |  |  |  |  |  |  |  |  |  |  |  |  |  |  |  |  |  |  |  |  |  |  |  |  |  |  |  |  |  |  |  |  |  |  |  |  |  |  |  |  |  |  |  |  |  |  |  |  |  |  |  |  |  |  |  |  |  |  |  |  |  |  |  |  |  |  |  |  |  |  |  |  |  |  |  |  |  |  |  |  |  |  |  |  |  |  |  |  |  |  |  |  |  |  |  |  |  |  |  |  |  |  |  |  |  |  |  |  |  |  |  |  |  |  |  |  |  |  |  |  |  |  |  |  |  |  |  |  |  |  |  |  |  |  |  |  |  |  |  |  |  |  |  |  |  |  |  |  |  |  |  |  |  |  |  |  |  |  |  |  |  |  |  |  |  |  |  |  |  |  |  |  |  |  |  |  |  |  |  |  |  |  |  |  |  |  |  |  |  |  |  |  |  |  |  |  |  |  |  |  |  |  |  |  |  |  |  |  |  |  |  |  |  |  |  |  |  |  |  |  |  |  |  |  |  |  |  |  |  |  |  |  |  |  |  |  |  |  |  |  |  |  |  |  |  |  |  |  |  |  |  |  |  |  |  |  |  |  |  |  |  |  |  |  |  |  |  |  |  |  |  |  |  |  |  |  |  |  |  |  |  |  |  |  |  |  |  |  |  |  |  |  |  |  |  |  |  |  |  |  |  |  |  |  |  |  |  |  |  |  |  |  |  |  |  |  |  |  |  |  |  |  |  |  |  |  |  |  |  |  |  |  |  |  |  |  |  |  |  |  |  |  |  |  |  |  |  |  |  |  |  |  |  |  |  |  |  |  |  |  |  |  |  |  |  |  |  |  |  |  |  |  |  |  |  |  |  |  |  |  |  |  |  |  |  |  |  |  |  |  |  |  |  |  |  |  |  |  |  |  |  |  |  |  |  |  |  |  |  |  |  |  |  |  |  |  |  |  |  |  |  |  |  |  |  |  |  |  |  |  |  |  |  |  |  |  |  |  |  |  |  |  |  |  |  |  |  |  |  |  |  |  |  |  |  |  |  |  |  |  |  |  |  |  |  |  |  |  |  |  |  |  |  |  |  |  |  |  |  |  |  |  |  |  |  |  |  |  |  |  |  |  |  |  |  |  |  |  |  |  |  |  |  |  |  |  |  |  |  |  |  |  |  |  |  |  |  |  |  |  |  |  |  |  |  |  |  |  |  |  |  |  |  |  |  |  |  |  |  |  |  |  |  |  |  |  |  |  |  |  |  |  |  |  |  |  |  |  |  |  |  |  |  |  |  |  |  |  |  |  |  |  |  |  |  |  |  |  |  |  |  |  |  |  |  |  |  |  |  |  |  |  |  |  |  |  |  |  |  |  |  |  |  |  |  |  |  |  |  |  |  |  |  |  |  |  |  |  |  |  |  |  |  |  |  |  |
| --- | --- | --- | --- | --- | --- | --- | --- | --- | --- | --- | --- | --- | --- | --- | --- | --- | --- | --- | --- | --- | --- | --- | --- | --- | --- | --- | --- | --- | --- | --- | --- | --- | --- | --- | --- | --- | --- | --- | --- | --- | --- | --- | --- | --- | --- | --- | --- | --- | --- | --- | --- | --- | --- | --- | --- | --- | --- | --- | --- | --- | --- | --- | --- | --- | --- | --- | --- | --- | --- | --- | --- | --- | --- | --- | --- | --- | --- | --- | --- | --- | --- | --- | --- | --- | --- | --- | --- | --- | --- | --- | --- | --- | --- | --- | --- | --- | --- | --- | --- | --- | --- | --- | --- | --- | --- | --- | --- | --- | --- | --- | --- | --- | --- | --- | --- | --- | --- | --- | --- | --- | --- | --- | --- | --- | --- | --- | --- | --- | --- | --- | --- | --- | --- | --- | --- | --- | --- | --- | --- | --- | --- | --- | --- | --- | --- | --- | --- | --- | --- | --- | --- | --- | --- | --- | --- | --- | --- | --- | --- | --- | --- | --- | --- | --- | --- | --- | --- | --- | --- | --- | --- | --- | --- | --- | --- | --- | --- | --- | --- | --- | --- | --- | --- | --- | --- | --- | --- | --- | --- | --- | --- | --- | --- | --- | --- | --- | --- | --- | --- | --- | --- | --- | --- | --- | --- | --- | --- | --- | --- | --- | --- | --- | --- | --- | --- | --- | --- | --- | --- | --- | --- | --- | --- | --- | --- | --- | --- | --- | --- | --- | --- | --- | --- | --- | --- | --- | --- | --- | --- | --- | --- | --- | --- | --- | --- | --- | --- | --- | --- | --- | --- | --- | --- | --- | --- | --- | --- | --- | --- | --- | --- | --- | --- | --- | --- | --- | --- | --- | --- | --- | --- | --- | --- | --- | --- | --- | --- | --- | --- | --- | --- | --- | --- | --- | --- | --- | --- | --- | --- | --- | --- | --- | --- | --- | --- | --- | --- | --- | --- | --- | --- | --- | --- | --- | --- | --- | --- | --- | --- | --- | --- | --- | --- | --- | --- | --- | --- | --- | --- | --- | --- | --- | --- | --- | --- | --- | --- | --- | --- | --- | --- | --- | --- | --- | --- | --- | --- | --- | --- | --- | --- | --- | --- | --- | --- | --- | --- | --- | --- | --- | --- | --- | --- | --- | --- | --- | --- | --- | --- | --- | --- | --- | --- | --- | --- | --- | --- | --- | --- | --- | --- | --- | --- | --- | --- | --- | --- | --- | --- | --- | --- | --- | --- | --- | --- | --- | --- | --- | --- | --- | --- | --- | --- | --- | --- | --- | --- | --- | --- | --- | --- | --- | --- | --- | --- | --- | --- | --- | --- | --- | --- | --- | --- | --- | --- | --- | --- | --- | --- | --- | --- | --- | --- | --- | --- | --- | --- | --- | --- | --- | --- | --- | --- | --- | --- | --- | --- | --- | --- | --- | --- | --- | --- | --- | --- | --- | --- | --- | --- | --- | --- | --- | --- | --- | --- | --- | --- | --- | --- | --- | --- | --- | --- | --- | --- | --- | --- | --- | --- | --- | --- | --- | --- | --- | --- | --- | --- | --- | --- | --- | --- | --- | --- | --- | --- | --- | --- | --- | --- | --- | --- | --- | --- | --- | --- | --- | --- | --- | --- | --- | --- | --- | --- | --- | --- | --- | --- | --- | --- | --- | --- | --- | --- | --- | --- | --- | --- | --- | --- | --- | --- | --- | --- | --- | --- | --- | --- | --- | --- | --- | --- | --- | --- | --- | --- | --- | --- | --- | --- | --- | --- | --- | --- | --- | --- | --- | --- | --- | --- | --- | --- | --- | --- | --- | --- | --- | --- | --- | --- | --- | --- | --- | --- | --- | --- | --- | --- | --- | --- | --- | --- | --- | --- | --- | --- | --- | --- | --- | --- | --- | --- | --- | --- | --- | --- | --- | --- | --- | --- | --- | --- | --- | --- | --- | --- | --- | --- | --- | --- | --- | --- | --- | --- | --- | --- | --- | --- | --- | --- | --- | --- | --- | --- | --- | --- | --- | --- | --- | --- | --- | --- | --- | --- | --- | --- | --- | --- | --- | --- | --- | --- | --- | --- | --- | --- | --- | --- | --- | --- | --- | --- | --- | --- | --- | --- | --- | --- | --- | --- | --- | --- | --- | --- | --- | --- | --- | --- | --- | --- | --- | --- | --- | --- | --- | --- | --- | --- | --- | --- | --- | --- | --- | --- | --- | --- | --- | --- | --- | --- | --- | --- | --- | --- | --- | --- | --- | --- | --- | --- | --- | --- | --- | --- | --- | --- | --- | --- | --- | --- | --- | --- | --- | --- | --- | --- | --- | --- | --- | --- | --- | --- | --- | --- | --- | --- | --- | --- | --- | --- | --- | --- | --- | --- | --- | --- | --- | --- | --- | --- | --- | --- | --- | --- |
|  || **Enterococcus faecalis V583 (AAO82601.1)** | **A** | **T** | **G** | **A** | **A** | **T** | **A** | **A** | **C** | **C** | **C** | **T** | **T** | **G** | **G** | **C** | **G** | **C** | **T** | **T** | **T** | **T** | **T** | **T** | **A** | **T** | **C** | **G** | **T** | **C** | **G** | **C** | **A** | **G** | **A** | **A** | **G** | **C** | **A** | **T** | **T** | **A** | **C** | **T** | **T** | **T** | **T** | **T** | **A** | **T** | **T** | **C** | **T** | **G** | **G** | **C** | **G** | **T** | **T** | **A** | **T** | **G** | **G** | **C** | **A** | **A** | **A** | **T** | **T** | **G** | **T** | **A** | **C** | **A** | **T** | **A** | **A** | **T** | **C** | **C** | **T** | **G** | **G** | **A** | **T** | **T** | **A** | **G** | **C** | **T** | **G** | **T** | **T** | **T** | **T** | **A** | **T** | **T** | **A** | **A** | **C** | **A** | **A** | **T** | **T** | **G** | **G** | **C** | **G** | **T** | **T** | **T** | **T** | **A** | **C** | **T** | **T** | **G** | **T** | **G** | **G** | **C** | **C** | **T** | **A** | **C** | **G** | **T** | **T** | **T** | **C** | **C** | **A** | **G** | **G** | **A** | **A** | **A** | **G** | **C** | **A** | **T** | **C** | **T** | **A** | **A** | **A** | **A** | **C** | **A** | **C** | **A** | **T** | **T** | **T** | **T** | **A** | **A** | **C** | **A** | **A** | **C** | **T** | **T** | **T** | **C** | **A** | **A** | **T** | **T** | **C** | **G** | **T** | **C** | **C** | **T** | **C** | **G** | **G** | **C** | **G** | **T** | **T** | **G** | **T** | **T** | **T** | **T** | **T** | **A** | **T** | **T** | **G** | **T** | **C** | **A** | **T** | **T** | **G** | **G** | **T** | **G** | **C** | **A** | **A** | **T** | **G** | **A** | **A** | **T** | **A** | **G** | **C** | **A** | **C** | **G** | **G** | **C** | **T** | **G** | **T** | **T** | **T** | **G** | **G** | **T** | **T** | **T** | **A** | **T** | **G** | **T** | **T** | **G** | **A** | **T** | **T** | **T** | **T** | **T** | **G** | **G** | **C** | **G** | **T** | **A** | **C** | **T** | **C** | **T** | **T** | **T** | **A** | **T** | **C** | **G** | **G** | **C** | **T** | **T** | **A** | **A** | **A** | **A** | **G** | **G** | **C** | **T** | **T** | **T** | **G** | **A** | **G** | **A** | **T** | **T** | **T** | **C** | **A** | **G** | **G** | **C** | **G** | **T** | **G** | **G** | **A** | **T** | **A** | **T** | **A** | **G** | **C** | **T** | **G** | **A** | **G** | **C** | **G** | **A** | **G** | **C** | **A** | **C** | **C** | **T** | **T** | **G** | **G** | **C** | **G** | **A** | **A** | **A** | **A** | **A** | **A** | **A** | **C** | **A** | **A** | **A** | **T** | **G** | **A** | **T** | **T** | **A** | **T** | **G** | **G** | **T** | **G** | **G** | **A** | **G** | **A** | **C** | **G** | **G** | **C** | **G** | **G** | **C** | **A** | **A** | **A** | **A** | **G** | **A** | **A** | **C** | **C** | **T** | **A** | **A** | **A** | **A** | **A** | **T** | **G** | **G** | **C** | **A** | **A** | **A** | **C** | **G** | **G** | **T** | **T** | **T** | **A** | **A** | **A** | **C** | **G** | **C** | **C** | **G** | **C** | **T** | **G** | **G** | **T** | **T** | **T** | **G** | **C** | **C** | **A** | **A** | **C** | **G** | **A** | **A** | **C** | **G** | **C** | **A** | **T** | **T** | **G** | **G** | **T** | **A** | **A** | **C** | **A** | **A** | **T** | **A** | **T** | **C** | **T** | **A** | **T** | **G** | **A** | **A** | **T** | **G** | **G** | **G** | **A** | **C** | **G** | **A** | **T** | **A** | **T** | **C** | **A** | **A** | **T** | **A** | **T** | **T** | **G** | **A** | **T** | **T** | **T** | **A** | **A** | **T** | **C** | **T** | **C** | **T** | **G** | **G** | **G** | **G** | **A** | **C** | **A** | **C** | **C** | **A** | **T** | **T** | **A** | **T** | **T** | **G** | **A** | **T** | **T** | **T** | **A** | **G** | **G** | **T** | **A** | **A** | **T** | **A** | **C** | **G** | **C** | **T** | **A** | **C** | **T** | **A** | **C** | **C** | **G** | **A** | **A** | **A** | **G** | **A** | **A** | **G** | **A** | **C** | **A** | **A** | **T** | **A** | **T** | **T** | **A** | **T** | **T** | **A** | **T** | **T** | **A** | **T** | **T** | **C** | **G** | **T** | **A** | **A** | **A** | **G** | **G** | **T** | **T** | **T** | **T** | **G** | **G** | **C** | **C** | **G** | **C** | **A** | **C** | **G** | **C** | **G** | **A** | **A** | **T** | **T** | **C** | **T** | **A** | **G** | **T** | **G** | **C** | **C** | **G** | **T** | **T** | **A** | **G** | **G** | **T** | **G** | **T** | **A** | **G** | **C** | **T** | **A** | **T** | **T** | **T** | **T** | **G** | **T** | **T** | **A** | **G** | **A** | **A** | **C** | **A** | **T** | **T** | **C** | **A** | **A** | **C** | **T** | **T** | **T** | **T** | **T** | **A** | **C** | **G** | **G** | **A** | **A** | **C** | **G** | **G** | **T** | **A** | **C** | **G** | **T** | **T** | **T** | **T** | **G** | **A** | **A** | **G** | **A** | **A** | **G** | **A** | **A** | **A** | **A** | **A** | **T** | **A** | **T** | **C** | **A** | **A** | **T** | **T** | **G** | **A** | **A** | **A** | **A** | **A** | **C** | **G** | **A** | **A** | **T** | **C** | **A** | **T** | **T** | **A** | **A** | **A** | **A** | **A** | **T** | **T** | **T** | **A** | **C** | **A** | **G** | **C** | **A** | **A** | **T** | **G** | **A** | **T** | **T** | **A** | **T** | **G** | **A** | **T** | **A** | **C** | **C** | **A** | **A** | **T** | **C** | **T** | **T** | **C** | **G** | **T** | **C** | **G** | **T** | **T** | **T** | **G** | **A** | **A** | **A** | **A** | **T** | **T** | **A** | **T** | **G** | **A** | **C** | **G** | **A** | **A** | **C** | **A** | **C** | **T** | **T** | **T** | **A** | **G** | **T** | **A** | **G** | **G** | **A** | **G** | **A** | **T** | **G** | **T** | **T** | **G** | **A** | **G** | **G** | **T** | **G** | **A** | **T** | **C** | **C** | **G** | **T** | **G** | **T** | **A** | **T** | **G** | **A** |
| **N59847F** | **A** | **T** | **G** | **A** | **A** | **T** | **A** | **A** | **C** | **C** | **C** | **T** | **T** | **G** | **G** | **C** | **G** | **C** | **T** | **T** | **T** | **T** | **T** | **T** | **A** | **T** | **C** | **G** | **T** | **C** | **G** | **C** | **A** | **G** | **A** | **A** | **G** | **C** | **A** | **T** | **T** | **A** | **C** | **T** | **T** | **T** | **T** | **T** | **A** | **T** | **T** | **C** | **T** | **G** | **G** | **C** | **G** | **T** | **T** | **A** | **T** | **G** | **G** | **C** | **A** | **A** | **A** | **T** | **T** | **G** | **T** | **A** | **C** | **A** | **T** | **A** | **A** | **T** | **C** | **C** | **T** | **G** | **G** | **A** | **T** | **T** | **A** | **G** | **C** | **T** | **G** | **T** | **T** | **T** | **T** | **A** | **T** | **T** | **A** | **A** | **C** | **A** | **A** | **T** | **T** | **G** | **G** | **C** | **G** | **T** | **T** | **T** | **T** | **A** | **C** | **T** | **T** | **G** | **T** | **G** | **G** | **C** | **C** | **T** | **A** | **C** | **G** | **T** | **T** | **T** | **C** | **C** | **A** | **G** | **G** | **A** | **A** | **A** | **G** | **C** | **A** | **T** | **C** | **T** | **A** | **A** | **A** | **A** | **C** | **A** | **C** | **A** | **T** | **T** | **T** | **T** | **A** | **A** | **C** | **A** | **A** | **C** | **T** | **T** | **T** | **C** | **A** | **A** | **T** | **T** | **C** | **G** | **T** | **C** | **C** | **T** | **C** | **G** | **G** | **C** | **G** | **T** | **T** | **G** | **T** | **T** | **T** | **T** | **T** | **A** | **T** | **T** | **G** | **T** | **C** | **A** | **T** | **T** | **G** | **G** | **T** | **G** | **C** | **A** | **A** | **T** | **G** | **A** | **A** | **C** | **A** | **G** | **C** | **A** | **C** | **G** | **G** | **C** | **T** | **G** | **T** | **T** | **T** | **G** | **G** | **T** | **T** | **T** | **A** | **T** | **G** | **T** | **T** | **G** | **A** | **T** | **T** | **T** | **T** | **T** | **G** | **G** | **C** | **G** | **T** | **A** | **C** | **T** | **C** | **T** | **T** | **T** | **A** | **T** | **C** | **G** | **G** | **C** | **T** | **T** | **A** | **A** | **A** | **A** | **G** | **G** | **C** | **T** | **T** | **T** | **G** | **A** | **G** | **A** | **T** | **T** | **T** | **C** | **A** | **G** | **G** | **C** | **G** | **T** | **G** | **G** | **A** | **T** | **A** | **T** | **A** | **G** | **C** | **T** | **G** | **A** | **G** | **C** | **G** | **A** | **G** | **C** | **A** | **C** | **C** | **T** | **T** | **G** | **G** | **C** | **G** | **A** | **A** | **A** | **A** | **A** | **A** | **A** | **C** | **A** | **A** | **A** | **T** | **G** | **A** | **T** | **T** | **A** | **T** | **G** | **G** | **T** | **G** | **G** | **A** | **G** | **A** | **C** | **G** | **G** | **C** | **G** | **G** | **C** | **A** | **A** | **A** | **A** | **G** | **A** | **A** | **C** | **C** | **T** | **A** | **A** | **A** | **A** | **A** | **T** | **G** | **G** | **C** | **A** | **A** | **A** | **C** | **G** | **A** | **T** | **T** | **T** | **A** | **A** | **A** | **C** | **G** | **C** | **C** | **G** | **C** | **T** | **G** | **G** | **T** | **T** | **T** | **G** | **C** | **C** | **A** | **A** | **C** | **G** | **A** | **A** | **C** | **G** | **C** | **A** | **T** | **T** | **G** | **G** | **T** | **A** | **A** | **C** | **A** | **A** | **T** | **A** | **T** | **C** | **T** | **A** | **T** | **G** | **A** | **A** | **T** | **G** | **G** | **G** | **A** | **C** | **G** | **A** | **T** | **A** | **T** | **C** | **A** | **A** | **T** | **A** | **T** | **T** | **G** | **A** | **T** | **T** | **T** | **A** | **A** | **T** | **C** | **T** | **C** | **T** | **G** | **G** | **G** | **G** | **A** | **C** | **A** | **C** | **C** | **A** | **T** | **T** | **A** | **T** | **T** | **G** | **A** | **T** | **T** | **T** | **A** | **G** | **G** | **T** | **A** | **A** | **T** | **A** | **C** | **G** | **T** | **T** | **A** | **C** | **T** | **A** | **C** | **C** | **G** | **A** | **A** | **A** | **G** | **A** | **A** | **G** | **A** | **C** | **A** | **A** | **T** | **A** | **T** | **T** | **A** | **T** | **T** | **A** | **T** | **T** | **A** | **T** | **T** | **C** | **G** | **T** | **A** | **A** | **A** | **G** | **G** | **T** | **T** | **T** | **T** | **G** | **G** | **C** | **C** | **G** | **C** | **A** | **C** | **G** | **C** | **G** | **A** | **A** | **T** | **T** | **C** | **T** | **A** | **G** | **T** | **G** | **C** | **C** | **G** | **T** | **T** | **A** | **G** | **G** | **T** | **G** | **T** | **A** | **G** | **C** | **T** | **A** | **T** | **T** | **T** | **T** | **G** | **T** | **T** | **A** | **G** | **A** | **A** | **C** | **A** | **T** | **T** | **C** | **A** | **A** | **C** | **T** | **T** | **T** | **T** | **T** | **A** | **C** | **G** | **G** | **A** | **A** | **C** | **G** | **G** | **T** | **A** | **C** | **G** | **T** | **T** | **T** | **T** | **G** | **A** | **A** | **G** | **A** | **A** | **G** | **A** | **A** | **A** | **A** | **A** | **T** | **A** | **T** | **C** | **A** | **A** | **T** | **T** | **G** | **A** | **A** | **A** | **A** | **A** | **C** | **G** | **A** | **A** | **T** | **C** | **A** | **T** | **T** | **A** | **A** | **A** | **A** | **A** | **T** | **T** | **T** | **A** | **C** | **A** | **G** | **C** | **A** | **A** | **T** | **G** | **A** | **T** | **T** | **A** | **T** | **G** | **A** | **T** | **A** | **C** | **C** | **A** | **A** | **T** | **C** | **T** | **T** | **C** | **G** | **T** | **C** | **G** | **T** | **T** | **T** | **G** | **A** | **A** | **A** | **A** | **T** | **T** | **A** | **T** | **G** | **A** | **C** | **G** | **A** | **A** | **C** | **A** | **C** | **T** | **T** | **T** | **A** | **G** | **T** | **A** | **G** | **G** | **A** | **G** | **A** | **T** | **G** | **T** | **T** | **G** | **A** | **G** | **G** | **T** | **G** | **A** | **T** | **C** | **C** | **G** | **T** | **G** | **T** | **A** | **-** | **-** | **-** |
| **N55753** | **A** | **T** | **G** | **A** | **A** | **T** | **A** | **A** | **C** | **C** | **C** | **T** | **T** | **G** | **G** | **C** | **G** | **C** | **T** | **T** | **T** | **T** | **T** | **T** | **A** | **T** | **C** | **G** | **T** | **C** | **G** | **C** | **A** | **G** | **A** | **A** | **G** | **C** | **A** | **T** | **T** | **A** | **C** | **T** | **T** | **T** | **T** | **T** | **A** | **T** | **T** | **C** | **T** | **G** | **G** | **C** | **G** | **T** | **T** | **A** | **T** | **G** | **G** | **C** | **A** | **A** | **A** | **T** | **T** | **G** | **T** | **A** | **C** | **A** | **T** | **A** | **A** | **T** | **C** | **C** | **T** | **G** | **G** | **A** | **T** | **T** | **A** | **G** | **C** | **T** | **G** | **T** | **T** | **T** | **T** | **A** | **T** | **T** | **A** | **A** | **C** | **A** | **A** | **T** | **T** | **G** | **G** | **C** | **G** | **T** | **T** | **T** | **T** | **A** | **C** | **T** | **T** | **G** | **T** | **G** | **G** | **C** | **C** | **T** | **A** | **T** | **G** | **T** | **T** | **T** | **C** | **C** | **A** | **G** | **G** | **A** | **A** | **A** | **G** | **C** | **A** | **T** | **C** | **T** | **A** | **A** | **A** | **A** | **C** | **A** | **C** | **A** | **T** | **T** | **T** | **T** | **A** | **A** | **C** | **A** | **A** | **C** | **T** | **T** | **T** | **C** | **A** | **A** | **T** | **T** | **C** | **G** | **T** | **C** | **C** | **T** | **C** | **G** | **G** | **C** | **G** | **T** | **T** | **G** | **T** | **T** | **T** | **T** | **T** | **A** | **T** | **T** | **G** | **T** | **C** | **A** | **T** | **T** | **G** | **G** | **T** | **G** | **C** | **A** | **A** | **T** | **G** | **A** | **A** | **C** | **A** | **G** | **T** | **A** | **C** | **A** | **G** | **C** | **T** | **G** | **T** | **T** | **T** | **G** | **G** | **T** | **T** | **T** | **A** | **T** | **G** | **T** | **T** | **G** | **A** | **T** | **T** | **T** | **T** | **T** | **G** | **G** | **C** | **G** | **T** | **A** | **C** | **T** | **C** | **T** | **T** | **T** | **A** | **T** | **C** | **G** | **G** | **C** | **T** | **T** | **A** | **A** | **A** | **A** | **G** | **G** | **C** | **T** | **T** | **T** | **G** | **A** | **G** | **A** | **T** | **T** | **T** | **C** | **A** | **G** | **G** | **C** | **G** | **T** | **G** | **G** | **A** | **T** | **A** | **T** | **A** | **G** | **C** | **T** | **G** | **A** | **G** | **C** | **G** | **A** | **G** | **C** | **A** | **C** | **C** | **T** | **T** | **G** | **G** | **C** | **G** | **A** | **A** | **A** | **A** | **A** | **A** | **A** | **C** | **A** | **A** | **A** | **T** | **G** | **A** | **T** | **T** | **A** | **T** | **G** | **G** | **T** | **G** | **G** | **A** | **G** | **A** | **C** | **G** | **G** | **C** | **G** | **G** | **C** | **A** | **A** | **A** | **A** | **G** | **A** | **A** | **C** | **C** | **T** | **A** | **A** | **A** | **A** | **A** | **T** | **G** | **G** | **C** | **A** | **A** | **A** | **C** | **G** | **A** | **T** | **T** | **T** | **A** | **A** | **A** | **C** | **G** | **C** | **C** | **G** | **C** | **T** | **G** | **G** | **T** | **T** | **T** | **G** | **C** | **C** | **A** | **A** | **C** | **G** | **A** | **A** | **C** | **G** | **C** | **A** | **T** | **T** | **G** | **G** | **T** | **A** | **A** | **C** | **A** | **A** | **T** | **A** | **T** | **C** | **T** | **A** | **T** | **G** | **A** | **A** | **T** | **G** | **G** | **G** | **A** | **C** | **G** | **A** | **T** | **A** | **T** | **C** | **A** | **A** | **T** | **A** | **T** | **T** | **G** | **A** | **T** | **T** | **T** | **A** | **A** | **T** | **C** | **T** | **C** | **T** | **G** | **G** | **G** | **G** | **A** | **C** | **A** | **C** | **C** | **A** | **T** | **T** | **A** | **T** | **T** | **G** | **A** | **T** | **T** | **T** | **A** | **G** | **G** | **T** | **A** | **A** | **T** | **A** | **C** | **G** | **C** | **T** | **A** | **C** | **T** | **A** | **C** | **C** | **G** | **A** | **A** | **A** | **G** | **A** | **A** | **G** | **A** | **C** | **A** | **A** | **T** | **A** | **T** | **T** | **A** | **T** | **T** | **A** | **T** | **T** | **A** | **T** | **T** | **C** | **G** | **T** | **A** | **A** | **A** | **G** | **G** | **T** | **T** | **T** | **T** | **G** | **G** | **C** | **C** | **G** | **C** | **A** | **C** | **G** | **C** | **G** | **A** | **A** | **T** | **T** | **C** | **T** | **A** | **G** | **T** | **G** | **C** | **C** | **G** | **T** | **T** | **A** | **G** | **G** | **T** | **G** | **T** | **A** | **G** | **C** | **T** | **A** | **T** | **T** | **T** | **T** | **G** | **T** | **T** | **A** | **G** | **A** | **A** | **C** | **A** | **T** | **T** | **C** | **A** | **A** | **C** | **T** | **T** | **T** | **T** | **T** | **A** | **C** | **G** | **G** | **A** | **A** | **C** | **G** | **G** | **T** | **A** | **C** | **G** | **T** | **T** | **T** | **T** | **G** | **A** | **A** | **G** | **A** | **A** | **G** | **A** | **A** | **A** | **A** | **A** | **T** | **A** | **T** | **C** | **A** | **A** | **T** | **T** | **G** | **A** | **A** | **A** | **A** | **A** | **C** | **G** | **A** | **A** | **T** | **C** | **A** | **T** | **T** | **A** | **A** | **A** | **A** | **A** | **T** | **T** | **T** | **A** | **C** | **A** | **G** | **C** | **A** | **A** | **T** | **G** | **A** | **T** | **T** | **A** | **T** | **G** | **A** | **T** | **A** | **C** | **C** | **A** | **A** | **T** | **C** | **T** | **T** | **C** | **G** | **T** | **C** | **G** | **T** | **T** | **T** | **G** | **A** | **A** | **A** | **A** | **T** | **T** | **A** | **T** | **G** | **A** | **C** | **G** | **A** | **A** | **C** | **A** | **C** | **T** | **T** | **T** | **A** | **G** | **T** | **A** | **G** | **G** | **A** | **G** | **A** | **T** | **G** | **T** | **T** | **G** | **A** | **G** | **G** | **T** | **G** | **A** | **T** | **C** | **C** | **G** | **T** | **G** | **T** | **A** | **-** | **-** | **-** |
| **N55338** | **A** | **T** | **G** | **A** | **A** | **T** | **A** | **A** | **C** | **C** | **C** | **T** | **T** | **G** | **G** | **C** | **G** | **C** | **T** | **T** | **T** | **T** | **T** | **T** | **A** | **T** | **C** | **G** | **T** | **C** | **G** | **C** | **A** | **G** | **A** | **A** | **G** | **C** | **A** | **T** | **T** | **A** | **C** | **T** | **T** | **T** | **T** | **T** | **A** | **T** | **T** | **C** | **T** | **G** | **G** | **C** | **G** | **T** | **T** | **A** | **T** | **G** | **G** | **C** | **A** | **A** | **A** | **T** | **T** | **G** | **T** | **A** | **C** | **A** | **T** | **A** | **A** | **T** | **C** | **C** | **T** | **G** | **G** | **A** | **T** | **T** | **A** | **G** | **C** | **T** | **G** | **T** | **T** | **T** | **T** | **A** | **T** | **T** | **A** | **A** | **C** | **A** | **A** | **T** | **T** | **G** | **G** | **C** | **G** | **T** | **T** | **T** | **T** | **A** | **C** | **T** | **T** | **G** | **T** | **G** | **G** | **C** | **C** | **T** | **A** | **C** | **G** | **T** | **T** | **T** | **C** | **C** | **A** | **G** | **G** | **A** | **A** | **A** | **G** | **C** | **A** | **T** | **C** | **T** | **A** | **A** | **A** | **A** | **C** | **A** | **C** | **A** | **T** | **T** | **T** | **T** | **A** | **A** | **C** | **A** | **A** | **C** | **T** | **T** | **T** | **C** | **A** | **A** | **T** | **T** | **C** | **G** | **T** | **C** | **C** | **T** | **C** | **G** | **G** | **C** | **G** | **T** | **T** | **G** | **T** | **T** | **T** | **T** | **T** | **A** | **T** | **T** | **G** | **T** | **C** | **A** | **T** | **T** | **G** | **G** | **T** | **G** | **C** | **A** | **A** | **T** | **G** | **A** | **A** | **C** | **A** | **G** | **C** | **A** | **C** | **G** | **G** | **C** | **T** | **G** | **T** | **T** | **T** | **G** | **G** | **T** | **T** | **T** | **A** | **T** | **G** | **T** | **T** | **G** | **A** | **T** | **T** | **T** | **T** | **T** | **G** | **G** | **C** | **G** | **T** | **A** | **C** | **T** | **C** | **T** | **T** | **T** | **A** | **T** | **C** | **G** | **G** | **C** | **T** | **T** | **A** | **A** | **A** | **A** | **G** | **G** | **C** | **T** | **T** | **T** | **G** | **A** | **G** | **A** | **T** | **T** | **T** | **C** | **A** | **G** | **G** | **C** | **G** | **T** | **G** | **G** | **A** | **T** | **A** | **T** | **A** | **G** | **C** | **T** | **G** | **A** | **G** | **C** | **G** | **A** | **G** | **C** | **A** | **C** | **C** | **T** | **T** | **G** | **G** | **C** | **G** | **C** | **A** | **A** | **A** | **A** | **A** | **A** | **C** | **A** | **A** | **A** | **T** | **G** | **A** | **T** | **T** | **A** | **T** | **G** | **G** | **T** | **G** | **G** | **A** | **G** | **A** | **C** | **G** | **G** | **C** | **G** | **G** | **C** | **A** | **A** | **A** | **A** | **G** | **A** | **A** | **C** | **C** | **T** | **A** | **A** | **A** | **A** | **A** | **T** | **G** | **G** | **C** | **A** | **A** | **A** | **C** | **G** | **A** | **T** | **T** | **T** | **A** | **A** | **A** | **C** | **G** | **C** | **C** | **G** | **C** | **T** | **G** | **G** | **T** | **T** | **T** | **G** | **C** | **C** | **A** | **A** | **C** | **G** | **A** | **A** | **C** | **G** | **C** | **A** | **T** | **T** | **G** | **G** | **T** | **A** | **A** | **C** | **A** | **A** | **T** | **A** | **T** | **C** | **T** | **A** | **T** | **G** | **A** | **A** | **T** | **G** | **G** | **G** | **A** | **C** | **G** | **A** | **T** | **A** | **T** | **C** | **A** | **A** | **T** | **A** | **T** | **T** | **G** | **A** | **T** | **T** | **T** | **A** | **A** | **T** | **C** | **T** | **C** | **T** | **G** | **G** | **G** | **G** | **A** | **C** | **A** | **C** | **C** | **A** | **T** | **T** | **A** | **T** | **T** | **G** | **A** | **T** | **T** | **T** | **A** | **G** | **G** | **T** | **A** | **A** | **T** | **A** | **C** | **G** | **C** | **T** | **A** | **C** | **T** | **A** | **C** | **C** | **G** | **A** | **A** | **A** | **G** | **A** | **A** | **G** | **A** | **C** | **A** | **A** | **T** | **A** | **T** | **T** | **A** | **T** | **T** | **A** | **T** | **T** | **A** | **T** | **T** | **C** | **G** | **T** | **A** | **A** | **A** | **G** | **G** | **T** | **T** | **T** | **T** | **G** | **G** | **C** | **C** | **G** | **C** | **A** | **C** | **G** | **C** | **G** | **A** | **A** | **T** | **T** | **C** | **T** | **A** | **G** | **T** | **G** | **C** | **C** | **G** | **T** | **T** | **A** | **G** | **G** | **T** | **G** | **T** | **A** | **G** | **C** | **T** | **A** | **T** | **T** | **T** | **T** | **G** | **T** | **T** | **A** | **G** | **A** | **A** | **C** | **A** | **T** | **T** | **C** | **A** | **A** | **C** | **T** | **T** | **T** | **T** | **T** | **A** | **C** | **G** | **G** | **A** | **A** | **C** | **G** | **G** | **T** | **A** | **C** | **G** | **T** | **T** | **T** | **T** | **G** | **A** | **A** | **G** | **A** | **A** | **G** | **A** | **A** | **A** | **A** | **A** | **T** | **A** | **T** | **C** | **A** | **A** | **T** | **T** | **G** | **A** | **A** | **A** | **A** | **A** | **C** | **G** | **A** | **A** | **T** | **C** | **A** | **T** | **T** | **A** | **A** | **A** | **A** | **A** | **T** | **T** | **T** | **A** | **C** | **A** | **G** | **C** | **A** | **A** | **T** | **G** | **A** | **T** | **T** | **A** | **T** | **G** | **A** | **T** | **A** | **C** | **C** | **A** | **A** | **T** | **C** | **T** | **T** | **C** | **G** | **T** | **C** | **G** | **T** | **T** | **T** | **G** | **A** | **A** | **A** | **A** | **T** | **T** | **A** | **T** | **G** | **A** | **C** | **G** | **A** | **A** | **C** | **A** | **C** | **T** | **T** | **T** | **A** | **G** | **T** | **A** | **G** | **G** | **A** | **G** | **A** | **T** | **G** | **T** | **T** | **G** | **A** | **G** | **G** | **T** | **G** | **A** | **T** | **C** | **C** | **G** | **T** | **G** | **T** | **A** | **-** | **-** | **-** |
| **N54553** | **A** | **T** | **G** | **A** | **A** | **T** | **A** | **A** | **C** | **C** | **C** | **T** | **T** | **G** | **G** | **C** | **G** | **C** | **T** | **T** | **T** | **T** | **T** | **T** | **A** | **T** | **C** | **G** | **T** | **C** | **G** | **C** | **A** | **G** | **A** | **A** | **G** | **C** | **A** | **T** | **T** | **A** | **C** | **T** | **T** | **T** | **T** | **T** | **A** | **T** | **T** | **C** | **T** | **G** | **G** | **C** | **G** | **T** | **T** | **A** | **T** | **G** | **G** | **C** | **A** | **A** | **A** | **T** | **T** | **G** | **T** | **A** | **C** | **A** | **T** | **A** | **A** | **T** | **C** | **C** | **T** | **G** | **G** | **A** | **T** | **T** | **A** | **G** | **C** | **T** | **G** | **T** | **T** | **T** | **T** | **A** | **T** | **T** | **A** | **A** | **C** | **A** | **A** | **T** | **T** | **G** | **G** | **C** | **G** | **T** | **T** | **T** | **T** | **A** | **C** | **T** | **T** | **G** | **T** | **G** | **G** | **C** | **C** | **T** | **A** | **C** | **G** | **T** | **T** | **T** | **C** | **C** | **A** | **G** | **G** | **A** | **A** | **A** | **G** | **C** | **A** | **T** | **C** | **T** | **A** | **A** | **A** | **A** | **C** | **A** | **C** | **A** | **T** | **T** | **T** | **T** | **A** | **A** | **C** | **A** | **A** | **C** | **T** | **T** | **T** | **C** | **A** | **A** | **T** | **T** | **C** | **G** | **T** | **C** | **C** | **T** | **C** | **G** | **G** | **C** | **G** | **T** | **T** | **G** | **T** | **T** | **T** | **T** | **T** | **A** | **T** | **T** | **G** | **T** | **C** | **A** | **T** | **T** | **G** | **G** | **T** | **G** | **C** | **A** | **A** | **T** | **G** | **A** | **A** | **C** | **A** | **G** | **C** | **A** | **C** | **G** | **G** | **C** | **T** | **G** | **T** | **T** | **T** | **G** | **G** | **T** | **T** | **T** | **A** | **T** | **G** | **T** | **T** | **G** | **A** | **T** | **T** | **T** | **T** | **T** | **G** | **G** | **C** | **G** | **T** | **A** | **C** | **T** | **C** | **T** | **T** | **T** | **A** | **T** | **C** | **G** | **G** | **C** | **T** | **T** | **A** | **A** | **A** | **A** | **G** | **G** | **C** | **T** | **T** | **T** | **G** | **A** | **G** | **A** | **T** | **T** | **T** | **C** | **A** | **G** | **G** | **C** | **G** | **T** | **G** | **G** | **A** | **T** | **A** | **T** | **A** | **G** | **C** | **T** | **G** | **A** | **G** | **C** | **G** | **A** | **G** | **C** | **A** | **C** | **C** | **T** | **T** | **G** | **G** | **C** | **G** | **A** | **A** | **A** | **A** | **A** | **A** | **A** | **C** | **A** | **A** | **A** | **T** | **G** | **A** | **T** | **T** | **A** | **T** | **G** | **G** | **T** | **G** | **G** | **A** | **G** | **A** | **C** | **G** | **G** | **C** | **G** | **G** | **C** | **A** | **A** | **A** | **A** | **G** | **A** | **A** | **C** | **C** | **T** | **A** | **A** | **A** | **A** | **A** | **T** | **G** | **G** | **C** | **A** | **A** | **A** | **C** | **G** | **A** | **T** | **T** | **T** | **A** | **A** | **A** | **C** | **G** | **C** | **C** | **G** | **C** | **T** | **G** | **G** | **T** | **T** | **T** | **G** | **C** | **C** | **A** | **A** | **C** | **G** | **A** | **A** | **C** | **G** | **C** | **A** | **T** | **T** | **G** | **G** | **T** | **A** | **A** | **C** | **A** | **A** | **T** | **A** | **T** | **C** | **T** | **A** | **T** | **G** | **A** | **A** | **T** | **G** | **G** | **G** | **A** | **C** | **G** | **A** | **T** | **A** | **T** | **C** | **A** | **A** | **T** | **A** | **T** | **T** | **G** | **A** | **T** | **T** | **T** | **A** | **A** | **T** | **C** | **T** | **C** | **T** | **G** | **G** | **G** | **G** | **A** | **C** | **A** | **C** | **C** | **A** | **T** | **T** | **A** | **T** | **T** | **G** | **A** | **T** | **T** | **T** | **A** | **G** | **G** | **T** | **A** | **A** | **T** | **A** | **C** | **G** | **C** | **T** | **A** | **C** | **T** | **A** | **C** | **C** | **G** | **A** | **A** | **A** | **G** | **A** | **A** | **G** | **A** | **C** | **A** | **A** | **T** | **A** | **T** | **T** | **A** | **T** | **T** | **A** | **T** | **T** | **A** | **T** | **T** | **C** | **G** | **T** | **A** | **A** | **A** | **G** | **G** | **T** | **T** | **T** | **T** | **G** | **G** | **C** | **C** | **G** | **C** | **A** | **C** | **G** | **C** | **G** | **A** | **A** | **T** | **T** | **C** | **T** | **A** | **G** | **T** | **G** | **C** | **C** | **G** | **T** | **T** | **A** | **G** | **G** | **T** | **G** | **T** | **A** | **G** | **C** | **T** | **A** | **T** | **T** | **T** | **T** | **G** | **T** | **T** | **A** | **G** | **A** | **A** | **C** | **A** | **T** | **T** | **C** | **A** | **A** | **C** | **T** | **T** | **T** | **T** | **T** | **A** | **C** | **G** | **G** | **A** | **A** | **C** | **G** | **G** | **T** | **A** | **C** | **G** | **T** | **T** | **T** | **T** | **G** | **A** | **A** | **G** | **A** | **A** | **G** | **A** | **A** | **A** | **A** | **A** | **T** | **A** | **T** | **C** | **A** | **A** | **T** | **T** | **G** | **A** | **A** | **A** | **A** | **A** | **C** | **G** | **A** | **A** | **T** | **C** | **A** | **T** | **T** | **A** | **A** | **A** | **A** | **A** | **T** | **T** | **T** | **A** | **C** | **A** | **G** | **C** | **A** | **A** | **T** | **G** | **A** | **T** | **T** | **A** | **C** | **G** | **A** | **T** | **A** | **C** | **C** | **A** | **A** | **T** | **C** | **T** | **T** | **C** | **G** | **T** | **C** | **G** | **T** | **T** | **T** | **G** | **A** | **A** | **A** | **A** | **T** | **T** | **A** | **T** | **G** | **A** | **C** | **G** | **A** | **A** | **C** | **A** | **C** | **T** | **T** | **T** | **A** | **G** | **T** | **A** | **G** | **G** | **A** | **G** | **A** | **T** | **G** | **T** | **T** | **G** | **A** | **G** | **G** | **T** | **G** | **A** | **T** | **C** | **C** | **G** | **T** | **G** | **T** | **A** | **-** | **-** | **-** |
| **N59455F** | **A** | **T** | **G** | **A** | **A** | **T** | **A** | **A** | **C** | **C** | **C** | **T** | **T** | **G** | **G** | **C** | **G** | **C** | **T** | **T** | **T** | **T** | **T** | **T** | **A** | **T** | **C** | **G** | **T** | **C** | **G** | **C** | **A** | **G** | **A** | **A** | **G** | **C** | **A** | **T** | **T** | **A** | **C** | **T** | **T** | **T** | **T** | **T** | **A** | **T** | **T** | **C** | **T** | **G** | **G** | **C** | **G** | **T** | **T** | **A** | **T** | **G** | **G** | **C** | **A** | **A** | **A** | **T** | **T** | **G** | **T** | **A** | **C** | **A** | **T** | **A** | **A** | **T** | **C** | **C** | **T** | **G** | **G** | **A** | **T** | **T** | **A** | **G** | **C** | **T** | **G** | **T** | **T** | **T** | **T** | **A** | **T** | **T** | **A** | **A** | **C** | **A** | **A** | **T** | **T** | **G** | **G** | **C** | **G** | **T** | **T** | **T** | **T** | **A** | **C** | **T** | **T** | **G** | **T** | **G** | **G** | **C** | **C** | **T** | **A** | **C** | **G** | **T** | **T** | **T** | **C** | **C** | **A** | **G** | **G** | **A** | **A** | **A** | **G** | **C** | **A** | **T** | **C** | **T** | **A** | **A** | **A** | **A** | **C** | **A** | **C** | **A** | **T** | **T** | **T** | **T** | **A** | **A** | **C** | **A** | **A** | **C** | **T** | **T** | **T** | **C** | **A** | **A** | **T** | **T** | **C** | **G** | **T** | **C** | **C** | **T** | **C** | **G** | **G** | **C** | **G** | **T** | **T** | **G** | **T** | **T** | **T** | **T** | **T** | **A** | **T** | **T** | **G** | **T** | **C** | **A** | **T** | **T** | **G** | **G** | **T** | **G** | **C** | **A** | **A** | **T** | **G** | **A** | **A** | **C** | **A** | **G** | **C** | **A** | **C** | **G** | **G** | **C** | **T** | **G** | **T** | **T** | **T** | **G** | **G** | **T** | **T** | **T** | **A** | **T** | **G** | **T** | **T** | **G** | **A** | **T** | **T** | **T** | **T** | **T** | **G** | **G** | **C** | **G** | **T** | **A** | **C** | **T** | **C** | **T** | **T** | **T** | **A** | **T** | **C** | **G** | **G** | **C** | **T** | **T** | **A** | **A** | **A** | **A** | **G** | **G** | **C** | **T** | **T** | **T** | **G** | **A** | **G** | **A** | **T** | **T** | **T** | **C** | **A** | **G** | **G** | **C** | **G** | **T** | **G** | **G** | **A** | **T** | **A** | **T** | **A** | **G** | **C** | **T** | **G** | **A** | **A** | **C** | **G** | **A** | **G** | **C** | **A** | **C** | **C** | **T** | **T** | **G** | **G** | **C** | **G** | **T** | **A** | **A** | **A** | **A** | **A** | **A** | **C** | **A** | **A** | **A** | **T** | **G** | **A** | **T** | **T** | **A** | **T** | **G** | **G** | **T** | **G** | **G** | **A** | **G** | **A** | **C** | **G** | **G** | **C** | **G** | **G** | **C** | **A** | **A** | **A** | **A** | **G** | **A** | **A** | **C** | **C** | **T** | **A** | **A** | **A** | **A** | **A** | **T** | **G** | **G** | **C** | **A** | **A** | **A** | **C** | **G** | **A** | **T** | **T** | **T** | **A** | **A** | **A** | **C** | **G** | **C** | **C** | **G** | **T** | **T** | **G** | **G** | **T** | **T** | **T** | **G** | **C** | **C** | **A** | **A** | **C** | **G** | **A** | **A** | **C** | **G** | **C** | **A** | **T** | **T** | **G** | **G** | **T** | **A** | **A** | **C** | **A** | **A** | **T** | **A** | **T** | **C** | **T** | **A** | **T** | **G** | **A** | **A** | **T** | **G** | **G** | **G** | **A** | **C** | **G** | **A** | **T** | **A** | **T** | **C** | **A** | **A** | **T** | **A** | **T** | **T** | **G** | **A** | **T** | **T** | **T** | **A** | **A** | **T** | **C** | **T** | **C** | **T** | **G** | **G** | **G** | **G** | **A** | **C** | **A** | **C** | **C** | **A** | **T** | **T** | **A** | **T** | **T** | **G** | **A** | **T** | **T** | **T** | **A** | **G** | **G** | **T** | **A** | **A** | **T** | **A** | **C** | **G** | **C** | **T** | **A** | **C** | **T** | **A** | **C** | **C** | **G** | **A** | **A** | **A** | **G** | **A** | **A** | **G** | **A** | **C** | **A** | **A** | **T** | **A** | **T** | **T** | **A** | **T** | **T** | **A** | **T** | **T** | **A** | **T** | **T** | **C** | **G** | **T** | **A** | **A** | **A** | **G** | **G** | **T** | **T** | **T** | **T** | **G** | **G** | **T** | **C** | **G** | **C** | **A** | **C** | **G** | **C** | **G** | **A** | **A** | **T** | **T** | **C** | **T** | **A** | **G** | **T** | **G** | **C** | **C** | **G** | **T** | **T** | **A** | **G** | **G** | **T** | **G** | **T** | **A** | **G** | **C** | **T** | **A** | **T** | **T** | **T** | **T** | **G** | **T** | **T** | **A** | **G** | **A** | **A** | **C** | **A** | **T** | **T** | **C** | **A** | **A** | **C** | **T** | **T** | **T** | **T** | **T** | **A** | **C** | **G** | **G** | **A** | **A** | **C** | **G** | **G** | **T** | **A** | **C** | **G** | **T** | **T** | **T** | **T** | **G** | **A** | **A** | **G** | **A** | **A** | **G** | **A** | **A** | **A** | **A** | **A** | **T** | **A** | **T** | **C** | **A** | **A** | **T** | **T** | **G** | **A** | **A** | **A** | **A** | **A** | **C** | **G** | **A** | **A** | **T** | **C** | **A** | **T** | **T** | **A** | **A** | **A** | **A** | **A** | **T** | **T** | **T** | **A** | **C** | **A** | **G** | **C** | **A** | **A** | **T** | **G** | **A** | **T** | **T** | **A** | **T** | **G** | **A** | **T** | **A** | **C** | **C** | **A** | **A** | **T** | **C** | **T** | **T** | **C** | **G** | **T** | **C** | **G** | **T** | **T** | **T** | **G** | **A** | **A** | **A** | **A** | **T** | **T** | **A** | **T** | **G** | **A** | **C** | **G** | **A** | **A** | **C** | **A** | **C** | **T** | **T** | **T** | **A** | **G** | **T** | **A** | **G** | **G** | **A** | **G** | **A** | **T** | **G** | **T** | **T** | **G** | **A** | **G** | **G** | **T** | **G** | **A** | **T** | **C** | **C** | **G** | **T** | **G** | **T** | **A** | **-** | **-** | **-** |
| **N52662** | **-** | **-** | **-** | **-** | **-** | **-** | **-** | **-** | **-** | **C** | **C** | **T** | **T** | **G** | **G** | **C** | **G** | **C** | **T** | **T** | **T** | **T** | **T** | **T** | **A** | **T** | **C** | **G** | **T** | **C** | **G** | **C** | **A** | **G** | **A** | **A** | **G** | **C** | **A** | **T** | **T** | **A** | **C** | **T** | **T** | **T** | **T** | **T** | **A** | **T** | **T** | **C** | **T** | **A** | **G** | **C** | **G** | **T** | **T** | **A** | **T** | **G** | **G** | **C** | **A** | **A** | **A** | **T** | **T** | **G** | **T** | **A** | **C** | **A** | **T** | **A** | **A** | **T** | **C** | **C** | **T** | **G** | **G** | **A** | **T** | **T** | **A** | **G** | **C** | **T** | **G** | **T** | **T** | **T** | **T** | **A** | **T** | **T** | **A** | **A** | **C** | **A** | **A** | **T** | **T** | **G** | **G** | **C** | **G** | **T** | **T** | **T** | **T** | **A** | **C** | **T** | **T** | **G** | **T** | **T** | **G** | **C** | **C** | **T** | **A** | **T** | **G** | **T** | **T** | **T** | **C** | **C** | **A** | **G** | **G** | **A** | **A** | **A** | **G** | **C** | **A** | **T** | **C** | **T** | **A** | **A** | **A** | **A** | **C** | **A** | **C** | **A** | **T** | **T** | **T** | **T** | **A** | **A** | **C** | **A** | **A** | **C** | **T** | **T** | **T** | **C** | **A** | **A** | **T** | **T** | **C** | **G** | **T** | **C** | **C** | **T** | **T** | **G** | **G** | **C** | **A** | **T** | **T** | **G** | **T** | **T** | **T** | **T** | **T** | **A** | **T** | **T** | **G** | **T** | **C** | **A** | **T** | **T** | **G** | **G** | **C** | **G** | **C** | **A** | **A** | **T** | **G** | **A** | **A** | **C** | **A** | **G** | **C** | **A** | **C** | **G** | **G** | **C** | **T** | **G** | **T** | **T** | **T** | **G** | **G** | **T** | **T** | **T** | **A** | **T** | **G** | **T** | **T** | **G** | **A** | **T** | **T** | **T** | **T** | **T** | **G** | **G** | **C** | **G** | **T** | **A** | **C** | **T** | **C** | **T** | **T** | **T** | **A** | **T** | **C** | **G** | **G** | **C** | **T** | **T** | **A** | **A** | **A** | **A** | **G** | **G** | **C** | **T** | **T** | **T** | **G** | **A** | **G** | **A** | **T** | **T** | **T** | **C** | **A** | **G** | **G** | **C** | **G** | **T** | **G** | **G** | **A** | **T** | **A** | **T** | **A** | **G** | **C** | **T** | **G** | **A** | **G** | **C** | **G** | **A** | **G** | **C** | **A** | **C** | **C** | **T** | **T** | **G** | **G** | **C** | **G** | **T** | **A** | **A** | **A** | **A** | **A** | **A** | **C** | **A** | **A** | **A** | **T** | **G** | **A** | **T** | **T** | **A** | **T** | **G** | **G** | **T** | **G** | **G** | **A** | **A** | **A** | **C** | **G** | **G** | **C** | **G** | **G** | **C** | **C** | **A** | **A** | **A** | **G** | **A** | **A** | **C** | **C** | **G** | **A** | **A** | **A** | **A** | **A** | **T** | **G** | **G** | **C** | **A** | **A** | **A** | **C** | **G** | **A** | **T** | **T** | **T** | **A** | **A** | **A** | **C** | **G** | **C** | **C** | **G** | **T** | **T** | **G** | **G** | **T** | **T** | **T** | **G** | **C** | **C** | **A** | **A** | **C** | **G** | **A** | **A** | **C** | **G** | **T** | **A** | **T** | **T** | **G** | **G** | **C** | **A** | **A** | **C** | **A** | **A** | **T** | **A** | **T** | **C** | **T** | **A** | **T** | **G** | **A** | **A** | **T** | **G** | **G** | **G** | **A** | **C** | **G** | **A** | **C** | **A** | **T** | **C** | **A** | **A** | **T** | **A** | **T** | **T** | **G** | **A** | **T** | **T** | **T** | **A** | **A** | **T** | **C** | **T** | **C** | **T** | **G** | **G** | **G** | **G** | **A** | **T** | **A** | **C** | **T** | **A** | **T** | **T** | **A** | **T** | **T** | **G** | **A** | **T** | **T** | **T** | **A** | **G** | **G** | **C** | **A** | **A** | **T** | **A** | **C** | **G** | **C** | **T** | **A** | **C** | **T** | **T** | **C** | **C** | **G** | **A** | **A** | **A** | **G** | **A** | **A** | **G** | **A** | **T** | **A** | **A** | **T** | **A** | **T** | **T** | **A** | **T** | **T** | **A** | **T** | **T** | **A** | **T** | **T** | **C** | **G** | **T** | **A** | **A** | **A** | **G** | **G** | **T** | **T** | **T** | **T** | **G** | **G** | **C** | **C** | **G** | **C** | **A** | **C** | **G** | **C** | **G** | **A** | **A** | **T** | **T** | **C** | **T** | **A** | **G** | **T** | **G** | **C** | **C** | **G** | **T** | **T** | **A** | **G** | **G** | **T** | **G** | **T** | **A** | **G** | **C** | **T** | **A** | **T** | **T** | **T** | **T** | **G** | **T** | **T** | **A** | **G** | **A** | **A** | **C** | **A** | **T** | **T** | **C** | **A** | **A** | **C** | **G** | **T** | **T** | **T** | **T** | **A** | **C** | **G** | **G** | **A** | **A** | **C** | **A** | **G** | **T** | **G** | **C** | **G** | **T** | **T** | **T** | **T** | **G** | **A** | **A** | **G** | **A** | **A** | **G** | **A** | **A** | **A** | **A** | **A** | **T** | **A** | **T** | **C** | **A** | **A** | **T** | **T** | **G** | **A** | **A** | **A** | **A** | **A** | **C** | **G** | **A** | **A** | **T** | **C** | **A** | **T** | **T** | **A** | **A** | **A** | **A** | **A** | **T** | **T** | **T** | **A** | **C** | **A** | **G** | **C** | **A** | **A** | **T** | **G** | **A** | **T** | **T** | **A** | **T** | **G** | **A** | **T** | **A** | **C** | **C** | **A** | **A** | **T** | **C** | **T** | **T** | **C** | **G** | **T** | **C** | **G** | **T** | **T** | **T** | **G** | **A** | **A** | **A** | **A** | **T** | **T** | **A** | **T** | **G** | **A** | **C** | **G** | **A** | **A** | **C** | **A** | **C** | **T** | **T** | **T** | **A** | **G** | **T** | **A** | **G** | **G** | **A** | **G** | **A** | **T** | **G** | **T** | **T** | **G** | **A** | **G** | **G** | **T** | **G** | **A** | **T** | **C** | **C** | **G** | **T** | **G** | **T** | **A** | **-** | **-** | **-** |
| **N59555F** | **A** | **T** | **G** | **A** | **A** | **T** | **A** | **A** | **C** | **C** | **C** | **T** | **T** | **G** | **G** | **C** | **G** | **C** | **T** | **T** | **T** | **T** | **T** | **T** | **A** | **T** | **C** | **G** | **T** | **C** | **G** | **C** | **A** | **G** | **A** | **A** | **G** | **C** | **A** | **T** | **T** | **A** | **C** | **T** | **T** | **T** | **T** | **T** | **A** | **T** | **T** | **C** | **T** | **G** | **G** | **C** | **G** | **T** | **T** | **A** | **T** | **G** | **G** | **C** | **A** | **A** | **A** | **T** | **T** | **G** | **T** | **A** | **C** | **A** | **T** | **A** | **A** | **T** | **C** | **C** | **T** | **G** | **G** | **A** | **T** | **T** | **A** | **G** | **C** | **T** | **G** | **T** | **T** | **T** | **T** | **A** | **T** | **T** | **A** | **A** | **C** | **A** | **A** | **T** | **T** | **G** | **G** | **C** | **G** | **T** | **T** | **T** | **T** | **A** | **C** | **T** | **T** | **G** | **T** | **G** | **G** | **C** | **C** | **T** | **A** | **C** | **G** | **T** | **T** | **T** | **C** | **C** | **A** | **G** | **G** | **A** | **A** | **A** | **G** | **C** | **A** | **T** | **C** | **T** | **A** | **A** | **A** | **A** | **C** | **A** | **C** | **A** | **T** | **T** | **T** | **T** | **A** | **A** | **C** | **A** | **A** | **C** | **T** | **T** | **T** | **C** | **A** | **A** | **T** | **T** | **C** | **G** | **T** | **C** | **C** | **T** | **C** | **G** | **G** | **C** | **G** | **T** | **T** | **G** | **T** | **T** | **T** | **T** | **T** | **A** | **T** | **T** | **G** | **T** | **C** | **A** | **T** | **T** | **G** | **G** | **T** | **G** | **C** | **A** | **A** | **T** | **G** | **A** | **A** | **C** | **A** | **G** | **C** | **A** | **C** | **G** | **G** | **C** | **T** | **G** | **T** | **T** | **T** | **G** | **G** | **T** | **T** | **T** | **A** | **T** | **G** | **T** | **T** | **G** | **A** | **T** | **T** | **T** | **T** | **T** | **G** | **G** | **C** | **G** | **T** | **A** | **C** | **T** | **C** | **T** | **T** | **T** | **A** | **T** | **C** | **G** | **G** | **C** | **T** | **T** | **A** | **A** | **A** | **A** | **G** | **G** | **C** | **T** | **T** | **T** | **G** | **A** | **G** | **A** | **T** | **T** | **T** | **C** | **A** | **G** | **G** | **C** | **G** | **T** | **G** | **G** | **A** | **T** | **A** | **T** | **A** | **G** | **C** | **T** | **G** | **A** | **A** | **C** | **G** | **A** | **G** | **C** | **A** | **C** | **C** | **T** | **T** | **G** | **G** | **C** | **G** | **T** | **A** | **A** | **A** | **A** | **A** | **A** | **C** | **A** | **A** | **A** | **T** | **G** | **A** | **T** | **T** | **A** | **T** | **G** | **G** | **T** | **A** | **G** | **A** | **G** | **A** | **C** | **G** | **G** | **C** | **G** | **G** | **C** | **A** | **A** | **A** | **A** | **G** | **A** | **A** | **C** | **C** | **T** | **A** | **A** | **A** | **A** | **A** | **T** | **G** | **G** | **C** | **A** | **A** | **A** | **C** | **G** | **A** | **T** | **T** | **T** | **A** | **A** | **A** | **C** | **G** | **C** | **C** | **G** | **C** | **T** | **G** | **G** | **T** | **T** | **T** | **G** | **C** | **C** | **A** | **A** | **C** | **G** | **A** | **A** | **C** | **G** | **C** | **A** | **T** | **T** | **G** | **G** | **T** | **A** | **A** | **C** | **A** | **A** | **T** | **A** | **T** | **C** | **T** | **A** | **T** | **G** | **A** | **A** | **T** | **G** | **G** | **G** | **A** | **T** | **G** | **A** | **C** | **A** | **T** | **C** | **A** | **A** | **T** | **A** | **T** | **T** | **G** | **A** | **T** | **T** | **T** | **A** | **A** | **T** | **C** | **T** | **C** | **T** | **G** | **G** | **G** | **G** | **A** | **C** | **A** | **C** | **C** | **A** | **T** | **T** | **A** | **T** | **T** | **G** | **A** | **T** | **T** | **T** | **A** | **G** | **G** | **T** | **A** | **A** | **T** | **A** | **C** | **G** | **C** | **T** | **A** | **C** | **T** | **A** | **C** | **C** | **G** | **A** | **A** | **A** | **G** | **A** | **A** | **G** | **A** | **C** | **A** | **A** | **T** | **A** | **T** | **T** | **A** | **T** | **T** | **A** | **T** | **T** | **A** | **T** | **T** | **C** | **G** | **T** | **A** | **A** | **A** | **G** | **G** | **T** | **T** | **T** | **T** | **G** | **G** | **C** | **C** | **G** | **C** | **A** | **C** | **G** | **C** | **G** | **A** | **A** | **T** | **T** | **C** | **T** | **A** | **G** | **T** | **G** | **C** | **C** | **G** | **T** | **T** | **A** | **G** | **G** | **T** | **G** | **T** | **A** | **G** | **C** | **T** | **A** | **T** | **T** | **T** | **T** | **G** | **T** | **T** | **A** | **G** | **A** | **A** | **C** | **A** | **T** | **T** | **C** | **A** | **A** | **C** | **T** | **T** | **T** | **T** | **T** | **A** | **C** | **G** | **G** | **A** | **A** | **C** | **G** | **G** | **T** | **A** | **C** | **G** | **T** | **T** | **T** | **T** | **G** | **A** | **A** | **G** | **A** | **A** | **G** | **A** | **A** | **A** | **A** | **A** | **T** | **A** | **T** | **C** | **A** | **A** | **T** | **T** | **G** | **A** | **A** | **A** | **A** | **A** | **C** | **G** | **A** | **A** | **T** | **C** | **A** | **T** | **T** | **A** | **A** | **A** | **A** | **A** | **T** | **T** | **T** | **A** | **C** | **A** | **G** | **C** | **A** | **A** | **T** | **G** | **A** | **T** | **T** | **A** | **T** | **G** | **A** | **T** | **A** | **C** | **C** | **A** | **A** | **T** | **C** | **T** | **T** | **C** | **G** | **T** | **C** | **G** | **T** | **T** | **T** | **G** | **A** | **A** | **A** | **A** | **T** | **T** | **A** | **T** | **G** | **A** | **C** | **G** | **A** | **A** | **C** | **A** | **C** | **T** | **T** | **T** | **A** | **G** | **T** | **A** | **G** | **G** | **A** | **G** | **A** | **T** | **G** | **T** | **T** | **G** | **A** | **G** | **G** | **T** | **G** | **A** | **T** | **C** | **C** | **G** | **T** | **G** | **T** | **A** | **-** | **-** | **-** |
| **N52789** | **A** | **T** | **G** | **A** | **A** | **T** | **A** | **A** | **C** | **C** | **C** | **T** | **T** | **G** | **G** | **C** | **G** | **C** | **T** | **T** | **T** | **T** | **T** | **T** | **A** | **T** | **C** | **G** | **T** | **C** | **G** | **C** | **A** | **G** | **A** | **A** | **G** | **C** | **A** | **T** | **T** | **A** | **C** | **T** | **T** | **T** | **T** | **T** | **A** | **T** | **T** | **C** | **T** | **G** | **G** | **C** | **G** | **T** | **T** | **A** | **T** | **G** | **G** | **C** | **A** | **A** | **A** | **T** | **T** | **G** | **T** | **A** | **C** | **A** | **T** | **A** | **A** | **T** | **C** | **C** | **T** | **G** | **G** | **A** | **T** | **T** | **A** | **G** | **C** | **T** | **G** | **T** | **T** | **T** | **T** | **A** | **T** | **T** | **A** | **A** | **C** | **A** | **A** | **T** | **T** | **G** | **G** | **C** | **G** | **T** | **T** | **T** | **T** | **A** | **C** | **T** | **T** | **G** | **T** | **G** | **G** | **C** | **C** | **T** | **A** | **C** | **G** | **T** | **T** | **T** | **C** | **C** | **A** | **G** | **G** | **A** | **A** | **A** | **G** | **C** | **A** | **T** | **C** | **T** | **A** | **A** | **A** | **A** | **C** | **A** | **C** | **A** | **T** | **T** | **T** | **T** | **A** | **A** | **C** | **A** | **A** | **C** | **T** | **T** | **T** | **C** | **A** | **A** | **T** | **T** | **C** | **G** | **T** | **C** | **C** | **T** | **C** | **G** | **G** | **C** | **G** | **T** | **T** | **G** | **T** | **T** | **T** | **T** | **T** | **A** | **T** | **T** | **G** | **T** | **C** | **A** | **T** | **T** | **G** | **G** | **T** | **G** | **C** | **A** | **A** | **T** | **G** | **A** | **A** | **C** | **A** | **G** | **C** | **A** | **C** | **G** | **G** | **C** | **T** | **G** | **T** | **T** | **T** | **G** | **G** | **T** | **T** | **T** | **A** | **T** | **G** | **T** | **T** | **G** | **A** | **T** | **T** | **T** | **T** | **T** | **G** | **G** | **C** | **G** | **T** | **A** | **C** | **T** | **C** | **T** | **T** | **T** | **A** | **T** | **C** | **G** | **G** | **C** | **T** | **T** | **A** | **A** | **A** | **A** | **G** | **G** | **C** | **T** | **T** | **T** | **G** | **A** | **G** | **A** | **T** | **T** | **T** | **C** | **A** | **G** | **G** | **C** | **G** | **T** | **G** | **G** | **A** | **T** | **A** | **T** | **A** | **G** | **C** | **T** | **G** | **A** | **A** | **C** | **G** | **A** | **G** | **C** | **A** | **C** | **C** | **T** | **T** | **G** | **G** | **C** | **G** | **T** | **A** | **A** | **A** | **A** | **A** | **A** | **C** | **A** | **A** | **A** | **T** | **G** | **A** | **T** | **T** | **A** | **T** | **G** | **G** | **T** | **A** | **G** | **A** | **G** | **A** | **C** | **G** | **G** | **C** | **G** | **G** | **C** | **A** | **A** | **A** | **A** | **G** | **A** | **A** | **C** | **C** | **T** | **A** | **A** | **A** | **A** | **A** | **T** | **G** | **G** | **C** | **A** | **A** | **A** | **C** | **G** | **A** | **T** | **T** | **T** | **A** | **A** | **A** | **C** | **G** | **C** | **C** | **G** | **C** | **T** | **G** | **G** | **T** | **T** | **T** | **G** | **C** | **C** | **A** | **A** | **C** | **G** | **A** | **A** | **C** | **G** | **C** | **A** | **T** | **T** | **G** | **G** | **T** | **A** | **A** | **C** | **A** | **A** | **T** | **A** | **T** | **C** | **T** | **A** | **T** | **G** | **A** | **A** | **T** | **G** | **G** | **G** | **A** | **T** | **G** | **A** | **C** | **A** | **T** | **C** | **A** | **A** | **T** | **A** | **T** | **T** | **G** | **A** | **T** | **T** | **T** | **A** | **A** | **T** | **C** | **T** | **C** | **T** | **G** | **G** | **G** | **G** | **A** | **C** | **A** | **C** | **C** | **A** | **T** | **T** | **A** | **T** | **T** | **G** | **A** | **T** | **T** | **T** | **A** | **G** | **G** | **T** | **A** | **A** | **T** | **A** | **C** | **G** | **C** | **T** | **A** | **C** | **T** | **A** | **C** | **C** | **G** | **A** | **A** | **A** | **G** | **A** | **A** | **G** | **A** | **C** | **A** | **A** | **T** | **A** | **T** | **T** | **A** | **T** | **T** | **A** | **T** | **T** | **A** | **T** | **T** | **C** | **G** | **T** | **A** | **A** | **A** | **G** | **G** | **T** | **T** | **T** | **T** | **G** | **G** | **C** | **C** | **G** | **C** | **A** | **C** | **G** | **C** | **G** | **A** | **A** | **T** | **T** | **C** | **T** | **A** | **G** | **T** | **G** | **C** | **C** | **G** | **T** | **T** | **A** | **G** | **G** | **T** | **G** | **T** | **A** | **G** | **C** | **T** | **A** | **T** | **T** | **T** | **T** | **G** | **T** | **T** | **A** | **G** | **A** | **A** | **C** | **A** | **T** | **T** | **C** | **A** | **A** | **C** | **T** | **T** | **T** | **T** | **T** | **A** | **C** | **G** | **G** | **A** | **A** | **C** | **G** | **G** | **T** | **A** | **C** | **G** | **T** | **T** | **T** | **T** | **G** | **A** | **A** | **G** | **A** | **A** | **G** | **A** | **A** | **A** | **A** | **A** | **T** | **A** | **T** | **C** | **A** | **A** | **T** | **T** | **G** | **A** | **A** | **A** | **A** | **A** | **C** | **G** | **A** | **A** | **T** | **C** | **A** | **T** | **T** | **A** | **A** | **A** | **A** | **A** | **T** | **T** | **T** | **A** | **C** | **A** | **G** | **C** | **A** | **A** | **T** | **G** | **A** | **T** | **T** | **A** | **T** | **G** | **A** | **T** | **A** | **C** | **C** | **A** | **A** | **T** | **C** | **T** | **T** | **C** | **G** | **T** | **C** | **G** | **T** | **T** | **T** | **G** | **A** | **A** | **A** | **A** | **T** | **T** | **A** | **T** | **G** | **A** | **C** | **G** | **A** | **A** | **C** | **A** | **C** | **T** | **T** | **T** | **A** | **G** | **T** | **A** | **G** | **G** | **A** | **G** | **A** | **T** | **G** | **T** | **T** | **G** | **A** | **G** | **G** | **T** | **G** | **A** | **T** | **C** | **C** | **G** | **T** | **G** | **T** | **A** | **-** | **-** | **-** |
| **N55724** | **A** | **T** | **G** | **A** | **A** | **T** | **A** | **A** | **C** | **C** | **C** | **T** | **T** | **G** | **G** | **C** | **G** | **C** | **T** | **T** | **T** | **T** | **T** | **T** | **A** | **T** | **C** | **G** | **T** | **C** | **G** | **C** | **A** | **G** | **A** | **A** | **G** | **C** | **A** | **T** | **T** | **A** | **C** | **T** | **T** | **T** | **T** | **T** | **A** | **T** | **T** | **C** | **T** | **G** | **G** | **C** | **G** | **T** | **T** | **A** | **T** | **G** | **G** | **C** | **A** | **A** | **A** | **T** | **T** | **G** | **T** | **A** | **C** | **A** | **T** | **A** | **A** | **T** | **C** | **C** | **T** | **G** | **G** | **A** | **T** | **T** | **A** | **G** | **C** | **T** | **G** | **T** | **T** | **T** | **T** | **A** | **T** | **T** | **A** | **A** | **C** | **A** | **A** | **T** | **T** | **G** | **G** | **C** | **G** | **T** | **T** | **T** | **T** | **A** | **C** | **T** | **T** | **G** | **T** | **G** | **G** | **C** | **C** | **T** | **A** | **C** | **G** | **T** | **T** | **T** | **C** | **C** | **A** | **G** | **G** | **A** | **A** | **A** | **G** | **C** | **A** | **T** | **C** | **T** | **A** | **A** | **A** | **A** | **C** | **A** | **C** | **A** | **T** | **T** | **T** | **T** | **A** | **A** | **C** | **A** | **A** | **C** | **T** | **T** | **T** | **C** | **A** | **A** | **T** | **T** | **C** | **G** | **T** | **C** | **C** | **T** | **C** | **G** | **G** | **C** | **G** | **T** | **T** | **G** | **T** | **T** | **T** | **T** | **T** | **A** | **T** | **T** | **G** | **T** | **C** | **A** | **T** | **T** | **G** | **G** | **T** | **G** | **C** | **A** | **A** | **T** | **G** | **A** | **A** | **C** | **A** | **G** | **C** | **A** | **C** | **G** | **G** | **C** | **T** | **G** | **T** | **T** | **T** | **G** | **G** | **T** | **T** | **T** | **A** | **T** | **G** | **T** | **T** | **G** | **A** | **T** | **T** | **T** | **T** | **T** | **G** | **G** | **C** | **G** | **T** | **A** | **C** | **T** | **C** | **T** | **T** | **T** | **A** | **T** | **C** | **G** | **G** | **C** | **T** | **T** | **A** | **A** | **A** | **A** | **G** | **G** | **C** | **T** | **T** | **T** | **G** | **A** | **G** | **A** | **T** | **T** | **T** | **C** | **A** | **G** | **G** | **C** | **G** | **T** | **G** | **G** | **A** | **T** | **A** | **T** | **A** | **G** | **C** | **T** | **G** | **A** | **G** | **C** | **G** | **A** | **G** | **C** | **A** | **C** | **C** | **T** | **T** | **G** | **G** | **C** | **G** | **A** | **A** | **A** | **A** | **A** | **A** | **A** | **C** | **A** | **A** | **A** | **T** | **G** | **A** | **T** | **T** | **A** | **T** | **G** | **G** | **T** | **G** | **G** | **A** | **G** | **A** | **C** | **G** | **G** | **C** | **G** | **G** | **C** | **A** | **A** | **A** | **A** | **G** | **A** | **A** | **C** | **C** | **T** | **A** | **A** | **A** | **A** | **A** | **T** | **G** | **G** | **C** | **A** | **A** | **A** | **C** | **G** | **A** | **T** | **T** | **T** | **A** | **A** | **A** | **C** | **G** | **C** | **C** | **G** | **C** | **T** | **G** | **G** | **T** | **T** | **T** | **G** | **C** | **C** | **A** | **A** | **C** | **G** | **A** | **A** | **C** | **G** | **C** | **A** | **T** | **T** | **G** | **G** | **T** | **A** | **A** | **C** | **A** | **A** | **T** | **A** | **T** | **C** | **T** | **A** | **T** | **G** | **A** | **A** | **T** | **G** | **G** | **G** | **A** | **C** | **G** | **A** | **T** | **A** | **T** | **C** | **A** | **A** | **T** | **A** | **T** | **T** | **G** | **A** | **T** | **T** | **T** | **A** | **A** | **T** | **C** | **T** | **C** | **T** | **G** | **G** | **G** | **G** | **A** | **C** | **A** | **C** | **C** | **A** | **T** | **T** | **A** | **T** | **T** | **G** | **A** | **T** | **T** | **T** | **A** | **G** | **G** | **T** | **A** | **A** | **T** | **A** | **C** | **G** | **T** | **T** | **A** | **C** | **T** | **A** | **C** | **C** | **G** | **A** | **A** | **A** | **G** | **A** | **A** | **G** | **A** | **C** | **A** | **A** | **T** | **A** | **T** | **T** | **A** | **T** | **T** | **A** | **T** | **T** | **A** | **T** | **T** | **C** | **G** | **T** | **A** | **A** | **A** | **G** | **G** | **T** | **T** | **T** | **T** | **G** | **G** | **C** | **C** | **G** | **C** | **A** | **C** | **G** | **C** | **G** | **A** | **A** | **T** | **T** | **C** | **T** | **A** | **G** | **T** | **G** | **C** | **C** | **G** | **T** | **T** | **A** | **G** | **G** | **T** | **G** | **T** | **A** | **G** | **C** | **T** | **A** | **T** | **T** | **T** | **T** | **G** | **T** | **T** | **A** | **G** | **A** | **A** | **C** | **A** | **T** | **T** | **C** | **A** | **A** | **C** | **T** | **T** | **T** | **T** | **T** | **A** | **C** | **G** | **G** | **A** | **A** | **C** | **G** | **G** | **T** | **A** | **C** | **G** | **T** | **T** | **T** | **T** | **G** | **A** | **A** | **G** | **A** | **A** | **G** | **A** | **A** | **A** | **A** | **A** | **T** | **A** | **T** | **C** | **A** | **A** | **T** | **T** | **G** | **A** | **A** | **A** | **A** | **A** | **C** | **G** | **A** | **A** | **T** | **C** | **A** | **T** | **T** | **A** | **A** | **A** | **A** | **A** | **T** | **T** | **T** | **A** | **C** | **A** | **G** | **C** | **A** | **A** | **T** | **G** | **A** | **T** | **T** | **A** | **T** | **G** | **A** | **T** | **A** | **C** | **C** | **A** | **A** | **T** | **C** | **T** | **T** | **C** | **G** | **T** | **C** | **G** | **T** | **T** | **T** | **G** | **A** | **A** | **A** | **A** | **T** | **T** | **A** | **T** | **G** | **A** | **C** | **G** | **A** | **A** | **C** | **A** | **C** | **T** | **T** | **T** | **A** | **G** | **T** | **A** | **G** | **G** | **A** | **G** | **A** | **T** | **G** | **T** | **T** | **G** | **A** | **G** | **G** | **T** | **G** | **A** | **T** | **C** | **C** | **G** | **T** | **G** | **T** | **A** | **-** | **-** | **-** |
| **N59845F** | **A** | **T** | **G** | **A** | **A** | **T** | **A** | **A** | **C** | **C** | **C** | **T** | **T** | **G** | **G** | **C** | **G** | **C** | **T** | **T** | **T** | **T** | **T** | **T** | **A** | **T** | **C** | **G** | **T** | **C** | **G** | **C** | **A** | **G** | **A** | **A** | **G** | **C** | **A** | **T** | **T** | **A** | **C** | **T** | **T** | **T** | **T** | **T** | **A** | **T** | **T** | **C** | **T** | **G** | **G** | **C** | **G** | **T** | **T** | **A** | **T** | **G** | **G** | **C** | **A** | **A** | **A** | **T** | **T** | **G** | **T** | **A** | **C** | **A** | **T** | **A** | **A** | **T** | **C** | **C** | **T** | **G** | **G** | **A** | **T** | **T** | **A** | **G** | **C** | **T** | **G** | **T** | **T** | **T** | **T** | **A** | **T** | **T** | **A** | **A** | **C** | **A** | **A** | **T** | **T** | **G** | **G** | **C** | **G** | **T** | **T** | **T** | **T** | **A** | **C** | **T** | **T** | **G** | **T** | **G** | **G** | **C** | **C** | **T** | **A** | **C** | **G** | **T** | **T** | **T** | **C** | **C** | **A** | **G** | **G** | **A** | **A** | **A** | **G** | **C** | **A** | **T** | **C** | **T** | **A** | **A** | **A** | **A** | **C** | **A** | **C** | **A** | **T** | **T** | **T** | **T** | **A** | **A** | **C** | **A** | **A** | **C** | **T** | **T** | **T** | **C** | **A** | **A** | **T** | **T** | **C** | **G** | **T** | **C** | **C** | **T** | **C** | **G** | **G** | **C** | **G** | **T** | **T** | **G** | **T** | **T** | **T** | **T** | **T** | **A** | **T** | **T** | **G** | **T** | **C** | **A** | **T** | **T** | **G** | **G** | **T** | **G** | **C** | **A** | **A** | **T** | **G** | **A** | **A** | **C** | **A** | **G** | **C** | **A** | **C** | **G** | **G** | **C** | **T** | **G** | **T** | **T** | **T** | **G** | **G** | **T** | **T** | **T** | **A** | **T** | **G** | **T** | **T** | **G** | **A** | **T** | **T** | **T** | **T** | **T** | **G** | **G** | **C** | **G** | **T** | **A** | **C** | **T** | **C** | **T** | **T** | **T** | **A** | **T** | **C** | **G** | **G** | **C** | **T** | **T** | **A** | **A** | **A** | **A** | **G** | **G** | **C** | **T** | **T** | **T** | **G** | **A** | **G** | **A** | **T** | **C** | **T** | **C** | **A** | **G** | **G** | **C** | **G** | **T** | **G** | **G** | **A** | **T** | **A** | **T** | **A** | **G** | **C** | **T** | **G** | **A** | **G** | **C** | **G** | **A** | **G** | **C** | **A** | **C** | **C** | **T** | **T** | **G** | **G** | **C** | **G** | **T** | **A** | **A** | **A** | **A** | **A** | **A** | **C** | **A** | **A** | **A** | **T** | **G** | **A** | **T** | **T** | **A** | **T** | **G** | **G** | **T** | **G** | **G** | **A** | **G** | **A** | **C** | **G** | **G** | **C** | **G** | **G** | **C** | **A** | **A** | **A** | **A** | **G** | **A** | **A** | **C** | **C** | **T** | **A** | **A** | **A** | **A** | **A** | **T** | **G** | **G** | **C** | **A** | **A** | **A** | **C** | **G** | **A** | **T** | **T** | **T** | **A** | **A** | **A** | **C** | **G** | **C** | **C** | **G** | **C** | **T** | **G** | **G** | **T** | **T** | **T** | **G** | **C** | **C** | **A** | **A** | **C** | **G** | **A** | **A** | **C** | **G** | **C** | **A** | **T** | **T** | **G** | **G** | **T** | **A** | **A** | **C** | **A** | **A** | **T** | **A** | **T** | **C** | **T** | **A** | **T** | **G** | **A** | **A** | **T** | **G** | **G** | **G** | **A** | **C** | **G** | **A** | **T** | **A** | **T** | **C** | **A** | **A** | **T** | **A** | **T** | **T** | **G** | **A** | **T** | **T** | **T** | **A** | **A** | **T** | **C** | **T** | **C** | **T** | **G** | **G** | **G** | **G** | **A** | **C** | **A** | **C** | **C** | **A** | **T** | **T** | **A** | **T** | **T** | **G** | **A** | **T** | **T** | **T** | **A** | **G** | **G** | **T** | **A** | **A** | **T** | **A** | **C** | **G** | **C** | **T** | **A** | **C** | **T** | **A** | **C** | **C** | **G** | **A** | **A** | **A** | **G** | **A** | **A** | **G** | **A** | **C** | **A** | **A** | **T** | **A** | **T** | **T** | **A** | **T** | **T** | **A** | **T** | **T** | **A** | **T** | **T** | **C** | **G** | **T** | **A** | **A** | **A** | **G** | **G** | **T** | **T** | **T** | **T** | **G** | **G** | **C** | **C** | **G** | **C** | **A** | **C** | **G** | **C** | **G** | **A** | **A** | **T** | **T** | **C** | **T** | **A** | **G** | **T** | **G** | **C** | **C** | **G** | **T** | **T** | **A** | **G** | **G** | **T** | **G** | **T** | **A** | **G** | **C** | **T** | **A** | **T** | **T** | **T** | **T** | **G** | **T** | **T** | **A** | **G** | **A** | **A** | **C** | **A** | **T** | **T** | **C** | **A** | **A** | **C** | **T** | **T** | **T** | **T** | **T** | **A** | **C** | **G** | **G** | **A** | **A** | **C** | **G** | **G** | **T** | **A** | **C** | **G** | **T** | **T** | **T** | **T** | **G** | **A** | **A** | **G** | **A** | **A** | **G** | **A** | **A** | **A** | **A** | **A** | **T** | **A** | **T** | **C** | **A** | **A** | **T** | **T** | **G** | **A** | **A** | **A** | **A** | **A** | **C** | **G** | **A** | **A** | **T** | **C** | **A** | **T** | **T** | **A** | **A** | **A** | **A** | **A** | **T** | **T** | **T** | **A** | **C** | **A** | **G** | **C** | **A** | **A** | **T** | **G** | **A** | **T** | **T** | **A** | **T** | **G** | **A** | **T** | **A** | **C** | **C** | **A** | **A** | **T** | **C** | **T** | **T** | **C** | **G** | **T** | **C** | **G** | **T** | **T** | **T** | **G** | **A** | **A** | **A** | **A** | **T** | **T** | **A** | **T** | **G** | **A** | **C** | **G** | **A** | **A** | **C** | **A** | **C** | **T** | **T** | **T** | **A** | **G** | **T** | **A** | **G** | **G** | **A** | **G** | **A** | **T** | **G** | **T** | **T** | **G** | **A** | **G** | **G** | **T** | **G** | **A** | **T** | **C** | **C** | **G** | **T** | **G** | **T** | **A** | **-** | **-** | **-** |
| **N59584F** | **A** | **T** | **G** | **A** | **A** | **T** | **A** | **A** | **C** | **C** | **C** | **T** | **T** | **G** | **G** | **C** | **G** | **C** | **T** | **T** | **T** | **T** | **T** | **T** | **A** | **T** | **C** | **G** | **T** | **C** | **G** | **C** | **A** | **G** | **A** | **A** | **G** | **C** | **A** | **T** | **T** | **A** | **C** | **T** | **T** | **T** | **T** | **T** | **A** | **T** | **T** | **C** | **T** | **G** | **G** | **C** | **G** | **T** | **T** | **A** | **T** | **G** | **G** | **C** | **A** | **A** | **A** | **T** | **T** | **G** | **T** | **A** | **C** | **A** | **T** | **A** | **A** | **T** | **C** | **C** | **T** | **G** | **G** | **A** | **T** | **T** | **A** | **G** | **C** | **T** | **G** | **T** | **T** | **T** | **T** | **A** | **T** | **T** | **A** | **A** | **C** | **A** | **A** | **T** | **T** | **G** | **G** | **C** | **G** | **T** | **T** | **T** | **T** | **A** | **C** | **T** | **T** | **G** | **T** | **G** | **G** | **C** | **C** | **T** | **A** | **T** | **G** | **T** | **T** | **T** | **C** | **C** | **A** | **G** | **G** | **A** | **A** | **A** | **G** | **C** | **A** | **T** | **C** | **T** | **A** | **A** | **A** | **A** | **C** | **A** | **C** | **A** | **T** | **T** | **T** | **T** | **A** | **A** | **C** | **A** | **A** | **C** | **T** | **T** | **T** | **C** | **A** | **A** | **T** | **T** | **C** | **G** | **T** | **C** | **C** | **T** | **C** | **G** | **G** | **C** | **G** | **T** | **T** | **G** | **T** | **T** | **T** | **T** | **T** | **A** | **T** | **T** | **G** | **T** | **C** | **A** | **T** | **T** | **G** | **G** | **T** | **G** | **C** | **A** | **A** | **T** | **G** | **A** | **A** | **C** | **A** | **G** | **C** | **A** | **C** | **G** | **G** | **C** | **T** | **G** | **T** | **T** | **T** | **G** | **G** | **T** | **T** | **T** | **A** | **T** | **G** | **C** | **T** | **G** | **A** | **T** | **T** | **T** | **T** | **T** | **G** | **G** | **C** | **G** | **T** | **A** | **C** | **T** | **C** | **T** | **T** | **T** | **A** | **T** | **C** | **G** | **G** | **C** | **T** | **T** | **A** | **A** | **A** | **A** | **G** | **G** | **C** | **T** | **T** | **T** | **G** | **A** | **G** | **A** | **T** | **T** | **T** | **C** | **A** | **G** | **G** | **C** | **G** | **T** | **G** | **G** | **A** | **T** | **A** | **T** | **A** | **G** | **C** | **T** | **G** | **A** | **G** | **C** | **G** | **A** | **G** | **C** | **A** | **C** | **C** | **T** | **T** | **G** | **G** | **C** | **G** | **A** | **A** | **A** | **A** | **A** | **A** | **A** | **C** | **A** | **A** | **A** | **T** | **G** | **A** | **T** | **T** | **A** | **T** | **G** | **G** | **T** | **G** | **G** | **A** | **G** | **A** | **C** | **G** | **G** | **C** | **G** | **G** | **C** | **A** | **A** | **A** | **A** | **G** | **A** | **A** | **C** | **C** | **T** | **A** | **A** | **A** | **A** | **A** | **T** | **G** | **G** | **C** | **A** | **A** | **A** | **C** | **G** | **A** | **T** | **T** | **T** | **A** | **A** | **A** | **C** | **G** | **C** | **C** | **G** | **C** | **T** | **G** | **G** | **T** | **T** | **T** | **G** | **C** | **C** | **A** | **A** | **C** | **G** | **A** | **A** | **C** | **G** | **C** | **A** | **T** | **T** | **G** | **G** | **T** | **A** | **A** | **C** | **A** | **A** | **T** | **A** | **T** | **C** | **T** | **A** | **T** | **G** | **A** | **A** | **T** | **G** | **G** | **G** | **A** | **C** | **G** | **A** | **T** | **A** | **T** | **C** | **A** | **A** | **T** | **A** | **T** | **T** | **G** | **A** | **T** | **T** | **T** | **A** | **A** | **T** | **C** | **T** | **C** | **T** | **G** | **G** | **G** | **G** | **A** | **C** | **A** | **C** | **C** | **A** | **T** | **T** | **A** | **T** | **T** | **G** | **A** | **T** | **T** | **T** | **A** | **G** | **G** | **T** | **A** | **A** | **T** | **A** | **C** | **G** | **C** | **T** | **A** | **C** | **T** | **A** | **C** | **C** | **G** | **A** | **A** | **A** | **G** | **A** | **A** | **G** | **A** | **C** | **A** | **A** | **T** | **A** | **T** | **T** | **A** | **T** | **T** | **A** | **T** | **T** | **A** | **T** | **T** | **C** | **G** | **T** | **A** | **A** | **A** | **G** | **G** | **T** | **T** | **T** | **T** | **G** | **G** | **C** | **C** | **G** | **C** | **A** | **C** | **G** | **C** | **G** | **A** | **A** | **T** | **T** | **C** | **T** | **A** | **G** | **T** | **G** | **C** | **C** | **G** | **T** | **T** | **A** | **G** | **G** | **T** | **G** | **T** | **A** | **G** | **C** | **T** | **A** | **T** | **T** | **T** | **T** | **G** | **T** | **T** | **A** | **G** | **A** | **A** | **C** | **A** | **T** | **T** | **C** | **A** | **A** | **C** | **T** | **T** | **T** | **T** | **T** | **A** | **C** | **G** | **G** | **A** | **A** | **C** | **G** | **G** | **T** | **A** | **C** | **G** | **T** | **T** | **T** | **T** | **G** | **A** | **A** | **G** | **A** | **A** | **G** | **A** | **A** | **A** | **A** | **A** | **T** | **A** | **T** | **C** | **A** | **A** | **T** | **T** | **G** | **A** | **A** | **A** | **A** | **A** | **C** | **G** | **A** | **A** | **T** | **C** | **A** | **T** | **T** | **A** | **A** | **A** | **A** | **A** | **T** | **T** | **T** | **A** | **C** | **A** | **G** | **C** | **A** | **A** | **T** | **G** | **A** | **T** | **T** | **A** | **T** | **G** | **A** | **T** | **A** | **C** | **C** | **A** | **A** | **T** | **C** | **T** | **T** | **C** | **G** | **T** | **C** | **G** | **T** | **T** | **T** | **G** | **A** | **A** | **A** | **A** | **T** | **T** | **A** | **T** | **G** | **A** | **C** | **G** | **A** | **A** | **C** | **A** | **C** | **T** | **T** | **T** | **A** | **G** | **T** | **A** | **G** | **G** | **A** | **G** | **A** | **T** | **G** | **T** | **T** | **G** | **A** | **G** | **G** | **T** | **G** | **A** | **T** | **C** | **C** | **G** | **T** | **G** | **T** | **A** | **-** | **-** | **-** |
| **N54156** | **A** | **T** | **G** | **A** | **A** | **T** | **A** | **A** | **C** | **C** | **C** | **T** | **T** | **G** | **G** | **C** | **G** | **C** | **T** | **T** | **T** | **T** | **T** | **T** | **A** | **T** | **C** | **G** | **T** | **C** | **G** | **C** | **A** | **G** | **A** | **A** | **G** | **C** | **A** | **T** | **T** | **A** | **C** | **T** | **T** | **T** | **T** | **T** | **A** | **T** | **T** | **C** | **T** | **G** | **G** | **C** | **G** | **T** | **T** | **A** | **T** | **G** | **G** | **C** | **A** | **A** | **A** | **T** | **T** | **G** | **T** | **A** | **C** | **A** | **T** | **A** | **A** | **T** | **C** | **C** | **T** | **G** | **G** | **A** | **T** | **T** | **A** | **G** | **C** | **T** | **G** | **T** | **T** | **T** | **T** | **A** | **T** | **T** | **A** | **A** | **C** | **A** | **A** | **T** | **T** | **G** | **G** | **C** | **G** | **T** | **T** | **T** | **T** | **A** | **C** | **T** | **T** | **G** | **T** | **G** | **G** | **C** | **C** | **T** | **A** | **C** | **G** | **T** | **T** | **T** | **C** | **C** | **A** | **G** | **G** | **A** | **A** | **A** | **G** | **C** | **A** | **T** | **C** | **T** | **A** | **A** | **A** | **A** | **C** | **A** | **C** | **A** | **T** | **T** | **T** | **T** | **A** | **A** | **C** | **A** | **A** | **C** | **T** | **T** | **T** | **C** | **A** | **A** | **T** | **T** | **C** | **G** | **T** | **C** | **C** | **T** | **C** | **G** | **G** | **C** | **G** | **T** | **T** | **G** | **T** | **T** | **T** | **T** | **T** | **A** | **T** | **T** | **G** | **T** | **C** | **A** | **T** | **T** | **G** | **G** | **T** | **G** | **C** | **A** | **A** | **T** | **G** | **A** | **A** | **C** | **A** | **G** | **C** | **A** | **C** | **G** | **G** | **C** | **T** | **G** | **T** | **T** | **T** | **G** | **G** | **T** | **T** | **T** | **A** | **T** | **G** | **T** | **T** | **G** | **A** | **T** | **T** | **T** | **T** | **T** | **G** | **G** | **C** | **G** | **T** | **A** | **C** | **T** | **C** | **T** | **T** | **T** | **A** | **T** | **C** | **G** | **G** | **C** | **T** | **T** | **A** | **A** | **A** | **A** | **G** | **G** | **C** | **T** | **T** | **T** | **G** | **A** | **G** | **A** | **T** | **T** | **T** | **C** | **A** | **G** | **G** | **C** | **G** | **T** | **G** | **G** | **A** | **T** | **A** | **T** | **A** | **G** | **C** | **T** | **G** | **A** | **G** | **C** | **G** | **A** | **G** | **C** | **A** | **C** | **C** | **T** | **T** | **G** | **G** | **C** | **G** | **A** | **A** | **A** | **A** | **A** | **A** | **A** | **C** | **A** | **A** | **A** | **T** | **G** | **A** | **T** | **T** | **A** | **T** | **G** | **G** | **T** | **G** | **G** | **A** | **G** | **A** | **C** | **G** | **G** | **C** | **G** | **G** | **C** | **A** | **A** | **A** | **A** | **G** | **A** | **A** | **C** | **C** | **T** | **A** | **A** | **A** | **A** | **A** | **T** | **G** | **G** | **C** | **A** | **A** | **A** | **C** | **G** | **A** | **T** | **T** | **T** | **A** | **A** | **A** | **C** | **G** | **C** | **C** | **G** | **C** | **T** | **G** | **G** | **T** | **T** | **T** | **G** | **C** | **C** | **A** | **A** | **C** | **G** | **A** | **A** | **C** | **G** | **C** | **A** | **T** | **T** | **G** | **G** | **T** | **A** | **A** | **C** | **A** | **A** | **T** | **A** | **T** | **C** | **T** | **A** | **T** | **G** | **A** | **A** | **T** | **G** | **G** | **G** | **A** | **C** | **G** | **A** | **T** | **A** | **T** | **C** | **A** | **A** | **T** | **A** | **T** | **T** | **G** | **A** | **T** | **T** | **T** | **A** | **A** | **T** | **C** | **T** | **C** | **T** | **G** | **G** | **G** | **G** | **A** | **C** | **A** | **C** | **C** | **A** | **T** | **T** | **A** | **T** | **T** | **G** | **A** | **T** | **T** | **T** | **A** | **G** | **G** | **T** | **A** | **A** | **T** | **A** | **C** | **G** | **T** | **T** | **A** | **C** | **T** | **A** | **C** | **C** | **G** | **A** | **A** | **A** | **G** | **A** | **A** | **G** | **A** | **C** | **A** | **A** | **T** | **A** | **T** | **T** | **A** | **T** | **T** | **A** | **T** | **T** | **A** | **T** | **T** | **C** | **G** | **T** | **A** | **A** | **A** | **G** | **G** | **T** | **T** | **T** | **T** | **G** | **G** | **C** | **C** | **G** | **C** | **A** | **C** | **G** | **C** | **G** | **A** | **A** | **T** | **T** | **C** | **T** | **A** | **G** | **T** | **G** | **C** | **C** | **G** | **T** | **T** | **A** | **G** | **G** | **T** | **G** | **T** | **A** | **G** | **C** | **T** | **A** | **T** | **T** | **T** | **T** | **G** | **T** | **T** | **A** | **G** | **A** | **A** | **C** | **A** | **T** | **T** | **C** | **A** | **A** | **C** | **T** | **T** | **T** | **T** | **T** | **A** | **C** | **G** | **G** | **A** | **A** | **C** | **G** | **G** | **T** | **A** | **C** | **G** | **T** | **T** | **T** | **T** | **G** | **A** | **A** | **G** | **A** | **A** | **G** | **A** | **A** | **A** | **A** | **A** | **T** | **A** | **T** | **C** | **A** | **A** | **T** | **T** | **G** | **A** | **A** | **A** | **A** | **A** | **C** | **G** | **A** | **A** | **T** | **C** | **A** | **T** | **T** | **A** | **A** | **A** | **A** | **A** | **T** | **T** | **T** | **A** | **C** | **A** | **G** | **C** | **A** | **A** | **T** | **G** | **A** | **T** | **T** | **A** | **T** | **G** | **A** | **T** | **A** | **C** | **C** | **A** | **A** | **T** | **C** | **T** | **T** | **C** | **G** | **T** | **C** | **G** | **T** | **T** | **T** | **G** | **A** | **A** | **A** | **A** | **T** | **T** | **A** | **T** | **G** | **A** | **C** | **G** | **A** | **A** | **C** | **A** | **C** | **T** | **T** | **T** | **A** | **G** | **T** | **A** | **G** | **G** | **A** | **G** | **A** | **T** | **G** | **T** | **T** | **G** | **A** | **G** | **G** | **T** | **G** | **A** | **T** | **C** | **C** | **G** | **T** | **G** | **T** | **A** | **-** | **-** | **-** |
| **N54595** | **A** | **T** | **G** | **A** | **A** | **T** | **A** | **A** | **C** | **C** | **C** | **T** | **T** | **G** | **G** | **C** | **G** | **C** | **T** | **T** | **T** | **T** | **T** | **T** | **A** | **T** | **C** | **G** | **T** | **C** | **G** | **C** | **A** | **G** | **A** | **A** | **G** | **C** | **A** | **T** | **T** | **A** | **C** | **T** | **T** | **T** | **T** | **T** | **A** | **T** | **T** | **C** | **T** | **G** | **G** | **C** | **G** | **T** | **T** | **A** | **T** | **G** | **G** | **C** | **A** | **A** | **A** | **T** | **T** | **G** | **T** | **A** | **C** | **A** | **T** | **A** | **A** | **T** | **C** | **C** | **T** | **G** | **G** | **A** | **T** | **T** | **A** | **G** | **C** | **T** | **G** | **T** | **T** | **T** | **T** | **A** | **T** | **T** | **A** | **A** | **C** | **A** | **A** | **T** | **T** | **G** | **G** | **C** | **G** | **T** | **T** | **T** | **T** | **A** | **C** | **T** | **T** | **G** | **T** | **G** | **G** | **C** | **C** | **T** | **A** | **C** | **G** | **T** | **T** | **T** | **C** | **C** | **A** | **G** | **G** | **A** | **A** | **A** | **G** | **C** | **A** | **T** | **C** | **T** | **A** | **A** | **A** | **A** | **C** | **A** | **C** | **A** | **T** | **T** | **T** | **T** | **A** | **A** | **C** | **A** | **A** | **C** | **T** | **T** | **T** | **C** | **A** | **A** | **T** | **T** | **C** | **A** | **T** | **C** | **C** | **T** | **C** | **G** | **G** | **C** | **G** | **T** | **T** | **G** | **T** | **T** | **T** | **T** | **T** | **A** | **T** | **T** | **G** | **T** | **C** | **A** | **T** | **T** | **G** | **G** | **T** | **G** | **C** | **A** | **A** | **T** | **G** | **A** | **A** | **C** | **A** | **G** | **T** | **A** | **C** | **G** | **G** | **C** | **T** | **G** | **T** | **T** | **T** | **G** | **G** | **T** | **T** | **T** | **A** | **T** | **G** | **T** | **T** | **G** | **A** | **T** | **T** | **T** | **T** | **T** | **G** | **G** | **C** | **G** | **T** | **A** | **C** | **T** | **C** | **T** | **T** | **T** | **A** | **T** | **C** | **G** | **G** | **C** | **T** | **T** | **A** | **A** | **A** | **A** | **G** | **G** | **C** | **T** | **T** | **T** | **G** | **A** | **G** | **A** | **T** | **T** | **T** | **C** | **A** | **G** | **G** | **C** | **G** | **T** | **G** | **G** | **A** | **T** | **A** | **T** | **A** | **G** | **C** | **T** | **G** | **A** | **G** | **C** | **G** | **A** | **G** | **C** | **A** | **C** | **C** | **T** | **T** | **G** | **G** | **C** | **G** | **T** | **A** | **A** | **A** | **A** | **A** | **A** | **C** | **A** | **A** | **A** | **T** | **G** | **A** | **T** | **T** | **A** | **T** | **G** | **G** | **T** | **G** | **G** | **A** | **G** | **A** | **C** | **G** | **G** | **C** | **G** | **G** | **C** | **A** | **A** | **A** | **A** | **G** | **A** | **A** | **C** | **C** | **T** | **A** | **A** | **A** | **A** | **A** | **T** | **G** | **G** | **C** | **A** | **A** | **A** | **C** | **G** | **A** | **T** | **T** | **T** | **A** | **A** | **A** | **C** | **G** | **C** | **C** | **G** | **T** | **T** | **G** | **G** | **T** | **T** | **T** | **G** | **C** | **C** | **A** | **A** | **C** | **G** | **A** | **A** | **C** | **G** | **C** | **A** | **T** | **T** | **G** | **G** | **T** | **A** | **A** | **C** | **A** | **A** | **T** | **A** | **T** | **C** | **T** | **A** | **T** | **G** | **A** | **A** | **T** | **G** | **G** | **G** | **A** | **T** | **G** | **A** | **T** | **A** | **T** | **C** | **A** | **A** | **T** | **A** | **T** | **T** | **G** | **A** | **T** | **T** | **T** | **A** | **A** | **T** | **C** | **T** | **C** | **T** | **G** | **G** | **G** | **G** | **A** | **C** | **A** | **C** | **C** | **A** | **T** | **T** | **A** | **T** | **T** | **G** | **A** | **T** | **T** | **T** | **A** | **G** | **G** | **T** | **A** | **A** | **T** | **A** | **C** | **G** | **C** | **T** | **A** | **C** | **T** | **A** | **C** | **C** | **G** | **A** | **A** | **A** | **G** | **A** | **A** | **G** | **A** | **C** | **A** | **A** | **T** | **A** | **T** | **T** | **A** | **T** | **T** | **A** | **T** | **T** | **A** | **T** | **T** | **C** | **G** | **T** | **A** | **A** | **A** | **G** | **G** | **T** | **T** | **T** | **T** | **G** | **G** | **C** | **C** | **G** | **C** | **A** | **C** | **G** | **C** | **G** | **A** | **A** | **T** | **T** | **C** | **T** | **A** | **G** | **T** | **A** | **C** | **C** | **G** | **T** | **T** | **A** | **G** | **G** | **T** | **G** | **T** | **A** | **G** | **C** | **A** | **A** | **T** | **T** | **T** | **T** | **G** | **T** | **T** | **A** | **G** | **A** | **A** | **C** | **A** | **T** | **T** | **C** | **A** | **A** | **C** | **T** | **T** | **T** | **T** | **T** | **A** | **C** | **G** | **G** | **A** | **A** | **C** | **G** | **G** | **T** | **A** | **C** | **G** | **T** | **T** | **T** | **T** | **G** | **A** | **A** | **G** | **A** | **A** | **G** | **A** | **A** | **A** | **A** | **A** | **T** | **A** | **T** | **C** | **A** | **A** | **T** | **T** | **G** | **A** | **A** | **A** | **A** | **A** | **C** | **G** | **A** | **A** | **T** | **C** | **A** | **T** | **T** | **A** | **A** | **A** | **A** | **A** | **T** | **T** | **T** | **A** | **C** | **A** | **G** | **C** | **A** | **A** | **T** | **G** | **A** | **T** | **T** | **A** | **C** | **G** | **A** | **T** | **A** | **C** | **C** | **A** | **A** | **T** | **C** | **T** | **T** | **C** | **G** | **T** | **C** | **G** | **T** | **T** | **T** | **G** | **A** | **A** | **A** | **A** | **T** | **T** | **A** | **T** | **G** | **A** | **C** | **G** | **A** | **A** | **C** | **A** | **C** | **T** | **T** | **T** | **A** | **G** | **T** | **A** | **G** | **G** | **A** | **G** | **A** | **T** | **G** | **T** | **T** | **G** | **A** | **G** | **G** | **T** | **G** | **A** | **T** | **C** | **C** | **G** | **T** | **G** | **T** | **A** | **-** | **-** | **-** |
| **N59611F** | **A** | **T** | **G** | **A** | **A** | **T** | **A** | **A** | **C** | **C** | **C** | **T** | **T** | **G** | **G** | **C** | **G** | **C** | **T** | **T** | **T** | **T** | **T** | **T** | **A** | **T** | **C** | **G** | **T** | **C** | **G** | **C** | **A** | **G** | **A** | **A** | **G** | **C** | **A** | **T** | **T** | **A** | **C** | **T** | **T** | **T** | **T** | **T** | **A** | **T** | **T** | **C** | **T** | **G** | **G** | **C** | **G** | **T** | **T** | **A** | **T** | **G** | **G** | **C** | **A** | **A** | **A** | **T** | **T** | **G** | **T** | **A** | **C** | **A** | **T** | **A** | **A** | **T** | **C** | **C** | **T** | **G** | **G** | **A** | **T** | **T** | **A** | **G** | **C** | **T** | **G** | **T** | **T** | **T** | **T** | **A** | **T** | **T** | **A** | **A** | **C** | **A** | **A** | **T** | **T** | **G** | **G** | **C** | **G** | **T** | **T** | **T** | **T** | **A** | **C** | **T** | **T** | **G** | **T** | **G** | **G** | **C** | **C** | **T** | **A** | **C** | **G** | **T** | **T** | **T** | **C** | **C** | **A** | **G** | **G** | **A** | **A** | **A** | **G** | **C** | **A** | **T** | **C** | **T** | **A** | **A** | **A** | **A** | **C** | **A** | **C** | **A** | **T** | **T** | **T** | **T** | **A** | **A** | **C** | **A** | **A** | **C** | **T** | **T** | **T** | **C** | **A** | **A** | **T** | **T** | **C** | **G** | **T** | **C** | **C** | **T** | **C** | **G** | **G** | **C** | **G** | **T** | **T** | **G** | **T** | **T** | **T** | **T** | **T** | **A** | **T** | **T** | **G** | **T** | **C** | **A** | **T** | **T** | **G** | **G** | **T** | **G** | **C** | **A** | **A** | **T** | **G** | **A** | **A** | **C** | **A** | **G** | **C** | **A** | **C** | **G** | **G** | **C** | **T** | **G** | **T** | **T** | **T** | **G** | **G** | **T** | **T** | **T** | **A** | **T** | **G** | **T** | **T** | **G** | **A** | **T** | **T** | **T** | **T** | **T** | **G** | **G** | **C** | **G** | **T** | **A** | **C** | **T** | **C** | **T** | **T** | **T** | **A** | **T** | **C** | **G** | **G** | **C** | **T** | **T** | **A** | **A** | **A** | **A** | **G** | **G** | **C** | **T** | **T** | **T** | **G** | **A** | **G** | **A** | **T** | **T** | **T** | **C** | **A** | **G** | **G** | **C** | **G** | **T** | **G** | **G** | **A** | **T** | **A** | **T** | **A** | **G** | **C** | **T** | **G** | **A** | **G** | **C** | **G** | **A** | **G** | **C** | **A** | **C** | **C** | **T** | **T** | **G** | **G** | **C** | **G** | **A** | **A** | **A** | **A** | **A** | **A** | **A** | **C** | **A** | **A** | **A** | **T** | **G** | **A** | **T** | **T** | **A** | **T** | **G** | **G** | **T** | **G** | **G** | **A** | **G** | **A** | **C** | **G** | **G** | **C** | **G** | **G** | **C** | **A** | **A** | **A** | **A** | **G** | **A** | **A** | **C** | **C** | **T** | **A** | **A** | **A** | **A** | **A** | **T** | **G** | **G** | **C** | **A** | **A** | **A** | **C** | **G** | **A** | **T** | **T** | **T** | **A** | **A** | **A** | **C** | **G** | **C** | **C** | **G** | **C** | **T** | **G** | **G** | **T** | **T** | **T** | **G** | **C** | **C** | **A** | **A** | **C** | **G** | **A** | **A** | **C** | **G** | **C** | **A** | **T** | **T** | **G** | **G** | **T** | **A** | **A** | **C** | **A** | **A** | **T** | **A** | **T** | **C** | **T** | **A** | **T** | **G** | **A** | **A** | **T** | **G** | **G** | **G** | **A** | **C** | **G** | **A** | **C** | **A** | **T** | **C** | **A** | **A** | **T** | **A** | **T** | **T** | **G** | **A** | **T** | **T** | **T** | **A** | **A** | **T** | **C** | **T** | **C** | **T** | **G** | **G** | **G** | **G** | **A** | **C** | **A** | **C** | **C** | **A** | **T** | **T** | **A** | **T** | **T** | **G** | **A** | **T** | **T** | **T** | **A** | **G** | **G** | **T** | **A** | **A** | **T** | **A** | **C** | **G** | **C** | **T** | **A** | **C** | **T** | **A** | **C** | **C** | **G** | **A** | **A** | **A** | **G** | **A** | **A** | **G** | **A** | **C** | **A** | **A** | **T** | **A** | **T** | **T** | **A** | **T** | **T** | **A** | **T** | **T** | **A** | **T** | **T** | **C** | **G** | **T** | **A** | **A** | **A** | **G** | **G** | **T** | **T** | **T** | **T** | **G** | **G** | **C** | **C** | **G** | **C** | **A** | **C** | **G** | **C** | **G** | **A** | **A** | **T** | **T** | **C** | **T** | **A** | **G** | **T** | **G** | **C** | **C** | **G** | **T** | **T** | **A** | **G** | **G** | **T** | **G** | **T** | **A** | **G** | **C** | **T** | **A** | **T** | **T** | **T** | **T** | **G** | **T** | **T** | **A** | **G** | **A** | **A** | **C** | **A** | **T** | **T** | **C** | **A** | **A** | **C** | **T** | **T** | **T** | **T** | **T** | **A** | **C** | **G** | **G** | **A** | **A** | **C** | **G** | **G** | **T** | **A** | **C** | **G** | **T** | **T** | **T** | **T** | **G** | **A** | **A** | **G** | **A** | **A** | **G** | **A** | **A** | **A** | **A** | **A** | **T** | **A** | **T** | **C** | **A** | **A** | **T** | **T** | **G** | **A** | **A** | **A** | **A** | **A** | **C** | **G** | **A** | **A** | **T** | **C** | **A** | **T** | **T** | **A** | **A** | **A** | **A** | **A** | **T** | **T** | **T** | **A** | **C** | **A** | **G** | **C** | **A** | **A** | **T** | **G** | **A** | **T** | **T** | **A** | **C** | **G** | **A** | **T** | **A** | **C** | **C** | **A** | **A** | **T** | **C** | **T** | **T** | **C** | **G** | **T** | **C** | **G** | **T** | **T** | **T** | **G** | **A** | **A** | **A** | **A** | **T** | **T** | **A** | **T** | **G** | **A** | **C** | **G** | **A** | **A** | **C** | **A** | **C** | **T** | **T** | **T** | **A** | **G** | **T** | **A** | **G** | **G** | **A** | **G** | **A** | **T** | **G** | **T** | **T** | **G** | **A** | **G** | **G** | **T** | **G** | **A** | **T** | **C** | **C** | **G** | **T** | **G** | **T** | **A** | **-** | **-** | **-** |
| **N53476** | **A** | **T** | **G** | **A** | **A** | **T** | **A** | **A** | **C** | **C** | **C** | **T** | **T** | **G** | **G** | **C** | **G** | **C** | **T** | **T** | **T** | **T** | **T** | **T** | **A** | **T** | **C** | **G** | **T** | **C** | **G** | **C** | **A** | **G** | **A** | **A** | **G** | **C** | **A** | **T** | **T** | **A** | **C** | **T** | **T** | **T** | **T** | **T** | **A** | **T** | **T** | **C** | **T** | **G** | **G** | **C** | **G** | **T** | **T** | **A** | **T** | **G** | **G** | **C** | **A** | **A** | **A** | **T** | **T** | **G** | **T** | **A** | **C** | **A** | **T** | **A** | **A** | **T** | **C** | **C** | **T** | **G** | **G** | **A** | **T** | **T** | **A** | **G** | **C** | **T** | **G** | **T** | **T** | **T** | **T** | **A** | **T** | **T** | **A** | **A** | **C** | **A** | **A** | **T** | **T** | **G** | **G** | **C** | **G** | **T** | **T** | **T** | **T** | **A** | **C** | **T** | **T** | **G** | **T** | **G** | **G** | **C** | **C** | **T** | **A** | **C** | **G** | **T** | **T** | **T** | **C** | **C** | **A** | **G** | **G** | **A** | **A** | **A** | **G** | **C** | **A** | **T** | **C** | **T** | **A** | **A** | **A** | **A** | **C** | **A** | **C** | **A** | **T** | **T** | **T** | **T** | **A** | **A** | **C** | **A** | **A** | **C** | **T** | **T** | **T** | **C** | **A** | **A** | **T** | **T** | **C** | **G** | **T** | **C** | **C** | **T** | **C** | **G** | **G** | **C** | **G** | **T** | **T** | **G** | **T** | **T** | **T** | **T** | **T** | **A** | **T** | **T** | **G** | **T** | **C** | **A** | **T** | **T** | **G** | **G** | **T** | **G** | **C** | **A** | **A** | **T** | **G** | **A** | **A** | **C** | **A** | **G** | **C** | **A** | **C** | **G** | **G** | **C** | **T** | **G** | **T** | **T** | **T** | **G** | **G** | **T** | **T** | **T** | **A** | **T** | **G** | **T** | **T** | **G** | **A** | **T** | **T** | **T** | **T** | **T** | **G** | **G** | **C** | **G** | **T** | **A** | **C** | **T** | **C** | **T** | **T** | **T** | **A** | **T** | **C** | **G** | **G** | **C** | **T** | **T** | **A** | **A** | **A** | **A** | **G** | **G** | **C** | **T** | **T** | **T** | **G** | **A** | **G** | **A** | **T** | **T** | **T** | **C** | **A** | **G** | **G** | **C** | **G** | **T** | **G** | **G** | **A** | **T** | **A** | **T** | **A** | **G** | **C** | **T** | **G** | **A** | **G** | **C** | **G** | **A** | **G** | **C** | **A** | **C** | **C** | **T** | **T** | **G** | **G** | **C** | **G** | **C** | **A** | **A** | **A** | **A** | **A** | **A** | **C** | **A** | **A** | **A** | **T** | **G** | **A** | **T** | **T** | **A** | **T** | **G** | **G** | **T** | **G** | **G** | **A** | **G** | **A** | **C** | **G** | **G** | **C** | **G** | **G** | **C** | **A** | **A** | **A** | **A** | **G** | **A** | **A** | **C** | **C** | **T** | **A** | **A** | **A** | **A** | **A** | **T** | **G** | **G** | **C** | **A** | **A** | **A** | **C** | **G** | **A** | **T** | **T** | **T** | **A** | **A** | **A** | **C** | **G** | **C** | **C** | **G** | **C** | **T** | **G** | **G** | **T** | **T** | **T** | **G** | **C** | **C** | **A** | **A** | **C** | **G** | **A** | **A** | **C** | **G** | **C** | **A** | **T** | **T** | **G** | **G** | **T** | **A** | **A** | **C** | **A** | **A** | **T** | **A** | **T** | **C** | **T** | **A** | **T** | **G** | **A** | **A** | **T** | **G** | **G** | **G** | **A** | **C** | **G** | **A** | **T** | **A** | **T** | **C** | **A** | **A** | **T** | **A** | **T** | **T** | **G** | **A** | **T** | **T** | **T** | **A** | **A** | **T** | **C** | **T** | **C** | **T** | **G** | **G** | **G** | **G** | **A** | **C** | **A** | **C** | **C** | **A** | **T** | **T** | **A** | **T** | **T** | **G** | **A** | **T** | **T** | **T** | **A** | **G** | **G** | **T** | **A** | **A** | **T** | **A** | **C** | **G** | **C** | **T** | **A** | **C** | **T** | **A** | **C** | **C** | **G** | **A** | **A** | **A** | **G** | **A** | **A** | **G** | **A** | **C** | **A** | **A** | **T** | **A** | **T** | **T** | **A** | **T** | **T** | **A** | **T** | **T** | **A** | **T** | **T** | **C** | **G** | **T** | **A** | **A** | **A** | **G** | **G** | **T** | **T** | **T** | **T** | **G** | **G** | **C** | **C** | **G** | **C** | **A** | **C** | **G** | **C** | **G** | **A** | **A** | **T** | **T** | **C** | **T** | **A** | **G** | **T** | **G** | **C** | **C** | **G** | **T** | **T** | **A** | **G** | **G** | **T** | **G** | **T** | **A** | **G** | **C** | **T** | **A** | **T** | **T** | **T** | **T** | **G** | **T** | **T** | **A** | **G** | **A** | **A** | **C** | **A** | **T** | **T** | **C** | **A** | **A** | **C** | **T** | **T** | **T** | **T** | **T** | **A** | **C** | **G** | **G** | **A** | **A** | **C** | **G** | **G** | **T** | **A** | **C** | **G** | **T** | **T** | **T** | **T** | **G** | **A** | **A** | **G** | **A** | **A** | **G** | **A** | **A** | **A** | **A** | **A** | **T** | **A** | **T** | **C** | **A** | **A** | **T** | **T** | **G** | **A** | **A** | **A** | **A** | **A** | **C** | **G** | **A** | **A** | **T** | **C** | **A** | **T** | **T** | **A** | **A** | **A** | **A** | **A** | **T** | **T** | **T** | **A** | **C** | **A** | **G** | **C** | **A** | **A** | **T** | **G** | **A** | **T** | **T** | **A** | **T** | **G** | **A** | **T** | **A** | **C** | **C** | **A** | **A** | **T** | **C** | **T** | **T** | **C** | **G** | **T** | **C** | **G** | **T** | **T** | **T** | **G** | **A** | **A** | **A** | **A** | **T** | **T** | **A** | **T** | **G** | **A** | **C** | **G** | **A** | **A** | **C** | **A** | **C** | **T** | **T** | **T** | **A** | **G** | **T** | **A** | **G** | **G** | **A** | **G** | **A** | **T** | **G** | **T** | **T** | **G** | **A** | **G** | **G** | **T** | **G** | **A** | **T** | **C** | **C** | **G** | **T** | **G** | **T** | **A** | **-** | **-** | **-** |
| **N55113** | **A** | **T** | **G** | **A** | **A** | **T** | **A** | **A** | **T** | **C** | **C** | **T** | **T** | **G** | **G** | **C** | **G** | **C** | **T** | **T** | **T** | **T** | **T** | **T** | **A** | **T** | **C** | **G** | **T** | **C** | **G** | **C** | **A** | **G** | **A** | **A** | **G** | **C** | **A** | **T** | **T** | **A** | **C** | **T** | **T** | **T** | **T** | **T** | **A** | **T** | **T** | **C** | **T** | **G** | **G** | **C** | **G** | **T** | **T** | **A** | **T** | **G** | **G** | **C** | **A** | **A** | **A** | **T** | **T** | **G** | **T** | **A** | **C** | **A** | **T** | **A** | **A** | **T** | **C** | **C** | **T** | **G** | **G** | **A** | **T** | **T** | **A** | **G** | **C** | **T** | **G** | **T** | **T** | **T** | **T** | **A** | **T** | **T** | **A** | **A** | **C** | **A** | **A** | **T** | **T** | **G** | **G** | **C** | **G** | **T** | **T** | **T** | **T** | **A** | **C** | **T** | **T** | **G** | **T** | **G** | **G** | **C** | **C** | **T** | **A** | **T** | **G** | **T** | **T** | **T** | **C** | **C** | **A** | **G** | **G** | **A** | **A** | **A** | **G** | **C** | **A** | **T** | **C** | **T** | **A** | **A** | **A** | **A** | **C** | **A** | **C** | **A** | **T** | **T** | **T** | **T** | **A** | **A** | **C** | **A** | **A** | **C** | **T** | **T** | **T** | **C** | **A** | **A** | **T** | **T** | **C** | **G** | **T** | **C** | **C** | **T** | **C** | **G** | **G** | **C** | **G** | **T** | **T** | **G** | **T** | **T** | **T** | **T** | **T** | **A** | **T** | **T** | **G** | **T** | **C** | **A** | **T** | **T** | **G** | **G** | **T** | **G** | **C** | **A** | **A** | **T** | **G** | **A** | **A** | **C** | **A** | **G** | **C** | **A** | **C** | **G** | **G** | **C** | **T** | **G** | **T** | **T** | **T** | **G** | **G** | **T** | **T** | **T** | **A** | **T** | **G** | **T** | **T** | **G** | **A** | **T** | **T** | **T** | **T** | **T** | **G** | **G** | **C** | **G** | **T** | **A** | **C** | **T** | **C** | **T** | **T** | **T** | **A** | **T** | **C** | **G** | **G** | **C** | **T** | **T** | **A** | **A** | **A** | **A** | **G** | **G** | **C** | **T** | **T** | **T** | **G** | **A** | **G** | **A** | **T** | **T** | **T** | **C** | **A** | **G** | **G** | **C** | **G** | **T** | **G** | **G** | **A** | **T** | **A** | **T** | **A** | **G** | **C** | **T** | **G** | **A** | **G** | **C** | **G** | **A** | **G** | **C** | **A** | **C** | **C** | **T** | **T** | **G** | **G** | **C** | **G** | **T** | **A** | **A** | **A** | **A** | **A** | **A** | **C** | **A** | **A** | **A** | **T** | **G** | **A** | **T** | **T** | **A** | **T** | **G** | **G** | **T** | **G** | **G** | **A** | **G** | **A** | **C** | **G** | **G** | **C** | **G** | **G** | **C** | **A** | **A** | **A** | **A** | **G** | **A** | **A** | **C** | **C** | **T** | **A** | **A** | **A** | **A** | **A** | **T** | **G** | **G** | **C** | **A** | **A** | **A** | **C** | **G** | **A** | **T** | **T** | **T** | **A** | **A** | **A** | **C** | **G** | **C** | **C** | **G** | **C** | **T** | **G** | **G** | **T** | **T** | **T** | **G** | **C** | **C** | **A** | **A** | **C** | **G** | **A** | **A** | **C** | **G** | **C** | **A** | **T** | **T** | **G** | **G** | **T** | **A** | **A** | **C** | **A** | **A** | **T** | **A** | **T** | **C** | **T** | **A** | **T** | **G** | **A** | **A** | **T** | **G** | **G** | **G** | **A** | **C** | **G** | **A** | **T** | **A** | **T** | **C** | **A** | **A** | **T** | **A** | **T** | **T** | **G** | **A** | **T** | **T** | **T** | **A** | **A** | **T** | **C** | **T** | **C** | **T** | **G** | **G** | **G** | **G** | **A** | **C** | **A** | **C** | **C** | **A** | **T** | **T** | **A** | **T** | **T** | **G** | **A** | **T** | **T** | **T** | **A** | **G** | **G** | **T** | **A** | **A** | **T** | **A** | **C** | **G** | **C** | **T** | **A** | **C** | **T** | **A** | **C** | **C** | **G** | **A** | **A** | **A** | **G** | **A** | **A** | **G** | **A** | **C** | **A** | **A** | **T** | **A** | **T** | **T** | **A** | **T** | **T** | **A** | **T** | **T** | **A** | **T** | **T** | **C** | **G** | **T** | **A** | **A** | **A** | **G** | **G** | **T** | **T** | **T** | **T** | **G** | **G** | **C** | **C** | **G** | **C** | **A** | **C** | **G** | **C** | **G** | **A** | **A** | **T** | **T** | **C** | **T** | **A** | **G** | **T** | **G** | **C** | **C** | **G** | **T** | **T** | **A** | **G** | **G** | **T** | **G** | **T** | **A** | **G** | **C** | **T** | **A** | **T** | **T** | **T** | **T** | **G** | **T** | **T** | **A** | **G** | **A** | **A** | **C** | **A** | **T** | **T** | **C** | **A** | **A** | **C** | **G** | **T** | **T** | **T** | **T** | **A** | **C** | **G** | **G** | **A** | **A** | **C** | **G** | **G** | **T** | **A** | **C** | **G** | **T** | **T** | **T** | **T** | **G** | **A** | **A** | **G** | **A** | **A** | **G** | **A** | **A** | **A** | **A** | **A** | **T** | **A** | **T** | **C** | **A** | **A** | **T** | **T** | **G** | **A** | **A** | **A** | **A** | **A** | **C** | **G** | **A** | **A** | **T** | **C** | **A** | **T** | **T** | **A** | **A** | **A** | **A** | **A** | **T** | **T** | **T** | **A** | **C** | **A** | **G** | **C** | **A** | **A** | **T** | **G** | **A** | **T** | **T** | **A** | **T** | **G** | **A** | **T** | **A** | **C** | **C** | **A** | **A** | **T** | **C** | **T** | **T** | **C** | **G** | **T** | **C** | **G** | **T** | **T** | **T** | **G** | **A** | **A** | **A** | **A** | **T** | **T** | **A** | **T** | **G** | **A** | **C** | **G** | **A** | **A** | **C** | **A** | **C** | **T** | **T** | **T** | **A** | **G** | **T** | **A** | **G** | **G** | **A** | **G** | **A** | **T** | **G** | **T** | **T** | **G** | **A** | **G** | **G** | **T** | **G** | **A** | **T** | **C** | **C** | **G** | **T** | **G** | **T** | **A** | **-** | **-** | **-** |
| **N59896F** | **A** | **T** | **G** | **A** | **A** | **T** | **A** | **A** | **C** | **C** | **C** | **T** | **T** | **G** | **G** | **C** | **G** | **C** | **T** | **T** | **T** | **T** | **T** | **T** | **A** | **T** | **C** | **G** | **T** | **C** | **G** | **C** | **A** | **G** | **A** | **A** | **G** | **C** | **A** | **T** | **T** | **A** | **C** | **T** | **T** | **T** | **T** | **T** | **A** | **T** | **T** | **C** | **T** | **G** | **G** | **C** | **G** | **T** | **T** | **A** | **T** | **G** | **G** | **C** | **A** | **A** | **A** | **T** | **T** | **G** | **T** | **A** | **C** | **A** | **T** | **A** | **A** | **T** | **C** | **C** | **T** | **G** | **G** | **A** | **T** | **T** | **A** | **G** | **C** | **T** | **G** | **T** | **T** | **T** | **T** | **A** | **T** | **T** | **A** | **A** | **C** | **A** | **A** | **T** | **T** | **G** | **G** | **C** | **G** | **T** | **T** | **T** | **T** | **A** | **C** | **T** | **T** | **G** | **T** | **G** | **G** | **C** | **C** | **T** | **A** | **C** | **G** | **T** | **T** | **T** | **C** | **C** | **A** | **G** | **G** | **A** | **A** | **A** | **G** | **C** | **A** | **T** | **C** | **T** | **A** | **A** | **A** | **A** | **C** | **A** | **C** | **A** | **T** | **T** | **T** | **T** | **A** | **A** | **C** | **A** | **A** | **C** | **T** | **T** | **T** | **C** | **A** | **A** | **T** | **T** | **C** | **G** | **T** | **C** | **C** | **T** | **C** | **G** | **G** | **C** | **G** | **T** | **T** | **G** | **T** | **T** | **T** | **T** | **T** | **A** | **T** | **T** | **G** | **T** | **C** | **A** | **T** | **T** | **G** | **G** | **T** | **G** | **C** | **A** | **A** | **T** | **G** | **A** | **A** | **C** | **A** | **G** | **C** | **A** | **C** | **G** | **G** | **C** | **T** | **G** | **T** | **T** | **T** | **G** | **G** | **T** | **T** | **T** | **A** | **T** | **G** | **T** | **T** | **G** | **A** | **T** | **T** | **T** | **T** | **T** | **G** | **G** | **C** | **G** | **T** | **A** | **C** | **T** | **C** | **T** | **T** | **T** | **A** | **T** | **C** | **G** | **G** | **C** | **T** | **T** | **A** | **A** | **A** | **A** | **G** | **G** | **C** | **T** | **T** | **T** | **G** | **A** | **G** | **A** | **T** | **T** | **T** | **C** | **A** | **G** | **G** | **C** | **G** | **T** | **G** | **G** | **A** | **T** | **A** | **T** | **A** | **G** | **C** | **T** | **G** | **A** | **G** | **C** | **G** | **A** | **G** | **C** | **A** | **C** | **C** | **T** | **T** | **G** | **G** | **C** | **G** | **A** | **A** | **A** | **A** | **A** | **A** | **A** | **C** | **A** | **A** | **A** | **T** | **G** | **A** | **T** | **T** | **A** | **T** | **G** | **G** | **T** | **G** | **G** | **A** | **G** | **A** | **C** | **G** | **G** | **C** | **G** | **G** | **C** | **A** | **A** | **A** | **A** | **G** | **A** | **A** | **C** | **C** | **T** | **A** | **A** | **A** | **A** | **A** | **T** | **G** | **G** | **C** | **A** | **A** | **A** | **C** | **G** | **A** | **T** | **T** | **T** | **A** | **A** | **A** | **C** | **G** | **C** | **C** | **G** | **C** | **T** | **G** | **G** | **T** | **T** | **T** | **G** | **C** | **C** | **A** | **A** | **C** | **G** | **A** | **A** | **C** | **G** | **C** | **A** | **T** | **T** | **G** | **G** | **T** | **A** | **A** | **C** | **A** | **A** | **T** | **A** | **T** | **C** | **T** | **A** | **T** | **G** | **A** | **A** | **T** | **G** | **G** | **G** | **A** | **C** | **G** | **A** | **C** | **A** | **T** | **C** | **A** | **A** | **T** | **A** | **T** | **T** | **G** | **A** | **T** | **T** | **T** | **A** | **A** | **T** | **C** | **T** | **C** | **T** | **G** | **G** | **G** | **G** | **A** | **C** | **A** | **C** | **C** | **A** | **T** | **T** | **A** | **T** | **T** | **G** | **A** | **T** | **T** | **T** | **A** | **G** | **G** | **T** | **A** | **A** | **T** | **A** | **C** | **G** | **C** | **T** | **A** | **C** | **T** | **A** | **C** | **C** | **G** | **A** | **A** | **A** | **G** | **A** | **A** | **G** | **A** | **C** | **A** | **A** | **T** | **A** | **T** | **T** | **A** | **T** | **T** | **A** | **T** | **T** | **A** | **T** | **T** | **C** | **G** | **T** | **A** | **A** | **A** | **G** | **G** | **T** | **T** | **T** | **T** | **G** | **G** | **C** | **C** | **G** | **C** | **A** | **C** | **G** | **C** | **G** | **A** | **A** | **T** | **T** | **C** | **T** | **A** | **G** | **T** | **G** | **C** | **C** | **G** | **T** | **T** | **A** | **G** | **G** | **T** | **G** | **T** | **A** | **G** | **C** | **T** | **A** | **T** | **T** | **T** | **T** | **G** | **T** | **T** | **A** | **G** | **A** | **A** | **C** | **A** | **T** | **T** | **C** | **A** | **A** | **C** | **T** | **T** | **T** | **T** | **T** | **A** | **C** | **G** | **G** | **A** | **A** | **C** | **G** | **G** | **T** | **A** | **C** | **G** | **T** | **T** | **T** | **T** | **G** | **A** | **A** | **G** | **A** | **A** | **G** | **A** | **A** | **A** | **A** | **A** | **T** | **A** | **T** | **C** | **A** | **A** | **T** | **T** | **G** | **A** | **A** | **A** | **A** | **A** | **C** | **G** | **A** | **A** | **T** | **C** | **A** | **T** | **T** | **A** | **A** | **A** | **A** | **A** | **T** | **T** | **T** | **A** | **C** | **A** | **G** | **C** | **A** | **A** | **T** | **G** | **A** | **T** | **T** | **A** | **C** | **G** | **A** | **T** | **A** | **C** | **C** | **A** | **A** | **T** | **C** | **T** | **T** | **C** | **G** | **T** | **C** | **G** | **T** | **T** | **T** | **G** | **A** | **A** | **A** | **A** | **T** | **T** | **A** | **T** | **G** | **A** | **C** | **G** | **A** | **A** | **C** | **A** | **C** | **T** | **T** | **T** | **A** | **G** | **T** | **A** | **G** | **G** | **A** | **G** | **A** | **T** | **G** | **T** | **T** | **G** | **A** | **G** | **G** | **T** | **G** | **A** | **T** | **C** | **C** | **G** | **T** | **G** | **T** | **A** | **-** | **-** | **-** |
| **N55106** | **A** | **T** | **G** | **A** | **A** | **T** | **A** | **A** | **C** | **C** | **C** | **T** | **T** | **G** | **G** | **C** | **G** | **C** | **T** | **T** | **T** | **T** | **T** | **T** | **A** | **T** | **C** | **G** | **T** | **C** | **G** | **C** | **A** | **G** | **A** | **A** | **G** | **C** | **A** | **T** | **T** | **A** | **C** | **T** | **T** | **T** | **T** | **T** | **A** | **T** | **T** | **C** | **T** | **G** | **G** | **C** | **G** | **T** | **T** | **A** | **T** | **G** | **G** | **C** | **A** | **A** | **A** | **T** | **T** | **G** | **T** | **A** | **C** | **A** | **T** | **A** | **A** | **T** | **C** | **C** | **T** | **G** | **G** | **A** | **T** | **T** | **A** | **G** | **C** | **T** | **G** | **T** | **T** | **T** | **T** | **A** | **T** | **T** | **A** | **A** | **C** | **A** | **A** | **T** | **T** | **G** | **G** | **C** | **G** | **T** | **T** | **T** | **T** | **A** | **C** | **T** | **T** | **G** | **T** | **G** | **G** | **C** | **C** | **T** | **A** | **C** | **G** | **T** | **T** | **T** | **C** | **C** | **A** | **G** | **G** | **A** | **A** | **A** | **G** | **C** | **A** | **T** | **C** | **T** | **A** | **A** | **A** | **A** | **C** | **A** | **C** | **A** | **T** | **T** | **T** | **T** | **A** | **A** | **C** | **A** | **A** | **C** | **T** | **T** | **T** | **C** | **A** | **A** | **T** | **T** | **C** | **G** | **T** | **C** | **C** | **T** | **C** | **G** | **G** | **C** | **G** | **T** | **T** | **G** | **T** | **T** | **T** | **T** | **T** | **A** | **T** | **T** | **G** | **T** | **C** | **A** | **T** | **T** | **G** | **G** | **T** | **G** | **C** | **A** | **A** | **T** | **G** | **A** | **A** | **C** | **A** | **G** | **C** | **A** | **C** | **G** | **G** | **C** | **T** | **G** | **T** | **T** | **T** | **G** | **G** | **T** | **T** | **T** | **A** | **T** | **G** | **T** | **T** | **G** | **A** | **T** | **T** | **T** | **T** | **T** | **G** | **G** | **C** | **G** | **T** | **A** | **C** | **T** | **C** | **T** | **T** | **T** | **A** | **T** | **C** | **G** | **G** | **C** | **T** | **T** | **A** | **A** | **A** | **A** | **G** | **G** | **C** | **T** | **T** | **T** | **G** | **A** | **G** | **A** | **T** | **T** | **T** | **C** | **A** | **G** | **G** | **C** | **G** | **T** | **G** | **G** | **A** | **T** | **A** | **T** | **A** | **G** | **C** | **T** | **G** | **A** | **G** | **C** | **G** | **A** | **G** | **C** | **A** | **C** | **C** | **T** | **T** | **G** | **G** | **C** | **G** | **A** | **A** | **A** | **A** | **A** | **A** | **A** | **C** | **A** | **A** | **A** | **T** | **G** | **A** | **T** | **T** | **A** | **T** | **G** | **G** | **T** | **G** | **G** | **A** | **G** | **A** | **C** | **G** | **G** | **C** | **G** | **G** | **C** | **A** | **A** | **A** | **A** | **G** | **A** | **A** | **C** | **C** | **T** | **A** | **A** | **A** | **A** | **A** | **T** | **G** | **G** | **C** | **A** | **A** | **A** | **C** | **G** | **A** | **T** | **T** | **T** | **A** | **A** | **A** | **C** | **G** | **C** | **C** | **G** | **C** | **T** | **G** | **G** | **T** | **T** | **T** | **G** | **C** | **C** | **A** | **A** | **C** | **G** | **A** | **A** | **C** | **G** | **C** | **A** | **T** | **T** | **G** | **G** | **T** | **A** | **A** | **C** | **A** | **A** | **T** | **A** | **T** | **C** | **T** | **A** | **T** | **G** | **A** | **A** | **T** | **G** | **G** | **G** | **A** | **C** | **G** | **A** | **C** | **A** | **T** | **C** | **A** | **A** | **T** | **A** | **T** | **T** | **G** | **A** | **T** | **T** | **T** | **A** | **A** | **T** | **C** | **T** | **C** | **T** | **G** | **G** | **G** | **G** | **A** | **C** | **A** | **C** | **C** | **A** | **T** | **T** | **A** | **T** | **T** | **G** | **A** | **T** | **T** | **T** | **A** | **G** | **G** | **T** | **A** | **A** | **T** | **A** | **C** | **G** | **C** | **T** | **A** | **C** | **T** | **A** | **C** | **C** | **G** | **A** | **A** | **A** | **G** | **A** | **A** | **G** | **A** | **C** | **A** | **A** | **T** | **A** | **T** | **T** | **A** | **T** | **T** | **A** | **T** | **T** | **A** | **T** | **T** | **C** | **G** | **T** | **A** | **A** | **A** | **G** | **G** | **T** | **T** | **T** | **T** | **G** | **G** | **C** | **C** | **G** | **C** | **A** | **C** | **G** | **C** | **G** | **A** | **A** | **T** | **T** | **C** | **T** | **A** | **G** | **T** | **G** | **C** | **C** | **G** | **T** | **T** | **A** | **G** | **G** | **T** | **G** | **T** | **A** | **G** | **C** | **T** | **A** | **T** | **T** | **T** | **T** | **G** | **T** | **T** | **A** | **G** | **A** | **A** | **C** | **A** | **T** | **T** | **C** | **A** | **A** | **C** | **T** | **T** | **T** | **T** | **T** | **A** | **C** | **G** | **G** | **A** | **A** | **C** | **G** | **G** | **T** | **A** | **C** | **G** | **T** | **T** | **T** | **T** | **G** | **A** | **A** | **G** | **A** | **A** | **G** | **A** | **A** | **A** | **A** | **A** | **T** | **A** | **T** | **C** | **A** | **A** | **T** | **T** | **G** | **A** | **A** | **A** | **A** | **A** | **C** | **G** | **A** | **A** | **T** | **C** | **A** | **T** | **T** | **A** | **A** | **A** | **A** | **A** | **T** | **T** | **T** | **A** | **C** | **A** | **G** | **C** | **A** | **A** | **T** | **G** | **A** | **T** | **T** | **A** | **C** | **G** | **A** | **T** | **A** | **C** | **C** | **A** | **A** | **T** | **C** | **T** | **T** | **C** | **G** | **T** | **C** | **G** | **T** | **T** | **T** | **G** | **A** | **A** | **A** | **A** | **T** | **T** | **A** | **T** | **G** | **A** | **C** | **G** | **A** | **A** | **C** | **A** | **C** | **T** | **T** | **T** | **A** | **G** | **T** | **A** | **G** | **G** | **A** | **G** | **A** | **T** | **G** | **T** | **T** | **G** | **A** | **G** | **G** | **T** | **G** | **A** | **T** | **C** | **C** | **G** | **T** | **G** | **T** | **A** | **-** | **-** | **-** |
| **N53418** | **A** | **T** | **G** | **A** | **A** | **T** | **A** | **A** | **C** | **C** | **C** | **T** | **T** | **G** | **G** | **C** | **G** | **C** | **T** | **T** | **T** | **T** | **T** | **T** | **A** | **T** | **C** | **G** | **T** | **C** | **G** | **C** | **A** | **G** | **A** | **A** | **G** | **C** | **A** | **T** | **T** | **A** | **C** | **T** | **T** | **T** | **T** | **T** | **A** | **T** | **T** | **C** | **T** | **G** | **G** | **C** | **G** | **T** | **T** | **A** | **T** | **G** | **G** | **C** | **A** | **A** | **A** | **T** | **T** | **G** | **T** | **A** | **C** | **A** | **T** | **A** | **A** | **T** | **C** | **C** | **T** | **G** | **G** | **A** | **T** | **T** | **A** | **G** | **C** | **T** | **G** | **T** | **T** | **T** | **T** | **A** | **T** | **T** | **A** | **A** | **C** | **A** | **A** | **T** | **T** | **G** | **G** | **C** | **G** | **T** | **T** | **T** | **T** | **A** | **C** | **T** | **T** | **G** | **T** | **G** | **G** | **C** | **C** | **T** | **A** | **C** | **G** | **T** | **T** | **T** | **C** | **C** | **A** | **G** | **G** | **A** | **A** | **A** | **G** | **C** | **A** | **T** | **C** | **T** | **A** | **A** | **A** | **A** | **C** | **A** | **C** | **A** | **T** | **T** | **T** | **T** | **A** | **A** | **C** | **A** | **A** | **C** | **T** | **T** | **T** | **C** | **A** | **A** | **T** | **T** | **C** | **G** | **T** | **C** | **C** | **T** | **C** | **G** | **G** | **C** | **G** | **T** | **T** | **G** | **T** | **T** | **T** | **T** | **T** | **A** | **T** | **T** | **G** | **T** | **C** | **A** | **T** | **T** | **G** | **G** | **T** | **G** | **C** | **A** | **A** | **T** | **G** | **A** | **A** | **C** | **A** | **G** | **C** | **A** | **C** | **G** | **G** | **C** | **T** | **G** | **T** | **T** | **T** | **G** | **G** | **T** | **T** | **T** | **A** | **T** | **G** | **T** | **T** | **G** | **A** | **T** | **T** | **T** | **T** | **T** | **G** | **G** | **C** | **G** | **T** | **A** | **C** | **T** | **C** | **T** | **T** | **T** | **A** | **T** | **C** | **G** | **G** | **C** | **T** | **T** | **A** | **A** | **A** | **A** | **G** | **G** | **C** | **T** | **T** | **T** | **G** | **A** | **G** | **A** | **T** | **T** | **T** | **C** | **A** | **G** | **G** | **C** | **G** | **T** | **G** | **G** | **A** | **T** | **A** | **T** | **A** | **G** | **C** | **T** | **G** | **A** | **G** | **C** | **G** | **A** | **G** | **C** | **A** | **C** | **C** | **T** | **T** | **G** | **G** | **C** | **G** | **A** | **A** | **A** | **A** | **A** | **A** | **A** | **C** | **A** | **A** | **A** | **T** | **G** | **A** | **T** | **T** | **A** | **T** | **G** | **G** | **T** | **G** | **G** | **A** | **G** | **A** | **C** | **G** | **G** | **C** | **G** | **G** | **C** | **A** | **A** | **A** | **A** | **G** | **A** | **A** | **C** | **C** | **T** | **A** | **A** | **A** | **A** | **A** | **T** | **G** | **G** | **C** | **A** | **A** | **A** | **C** | **G** | **A** | **T** | **T** | **T** | **A** | **A** | **A** | **C** | **G** | **C** | **C** | **G** | **C** | **T** | **G** | **G** | **T** | **T** | **T** | **G** | **C** | **C** | **A** | **A** | **C** | **G** | **A** | **A** | **C** | **G** | **C** | **A** | **T** | **T** | **G** | **G** | **T** | **A** | **A** | **C** | **A** | **A** | **T** | **A** | **T** | **C** | **T** | **A** | **T** | **G** | **A** | **A** | **T** | **G** | **G** | **G** | **A** | **C** | **G** | **A** | **C** | **A** | **T** | **C** | **A** | **A** | **T** | **A** | **T** | **T** | **G** | **A** | **T** | **T** | **T** | **A** | **A** | **T** | **C** | **T** | **C** | **T** | **G** | **G** | **G** | **G** | **A** | **C** | **A** | **C** | **C** | **A** | **T** | **T** | **A** | **T** | **T** | **G** | **A** | **T** | **T** | **T** | **A** | **G** | **G** | **T** | **A** | **A** | **T** | **A** | **C** | **G** | **C** | **T** | **A** | **C** | **T** | **A** | **C** | **C** | **G** | **A** | **A** | **A** | **G** | **A** | **A** | **G** | **A** | **C** | **A** | **A** | **T** | **A** | **T** | **T** | **A** | **T** | **T** | **A** | **T** | **T** | **A** | **T** | **T** | **C** | **G** | **T** | **A** | **A** | **A** | **G** | **G** | **T** | **T** | **T** | **T** | **G** | **G** | **C** | **C** | **G** | **C** | **A** | **C** | **G** | **C** | **G** | **A** | **A** | **T** | **T** | **C** | **T** | **A** | **G** | **T** | **G** | **C** | **C** | **G** | **T** | **T** | **A** | **G** | **G** | **T** | **G** | **T** | **A** | **G** | **C** | **T** | **A** | **T** | **T** | **T** | **T** | **G** | **T** | **T** | **A** | **G** | **A** | **A** | **C** | **A** | **T** | **T** | **C** | **A** | **A** | **C** | **T** | **T** | **T** | **T** | **T** | **A** | **C** | **G** | **G** | **A** | **A** | **C** | **G** | **G** | **T** | **A** | **C** | **G** | **T** | **T** | **T** | **T** | **G** | **A** | **A** | **G** | **A** | **A** | **G** | **A** | **A** | **A** | **A** | **A** | **T** | **A** | **T** | **C** | **A** | **A** | **T** | **T** | **G** | **A** | **A** | **A** | **A** | **A** | **C** | **G** | **A** | **A** | **T** | **C** | **A** | **T** | **T** | **A** | **A** | **A** | **A** | **A** | **T** | **T** | **T** | **A** | **C** | **A** | **G** | **C** | **A** | **A** | **T** | **G** | **A** | **T** | **T** | **A** | **C** | **G** | **A** | **T** | **A** | **C** | **C** | **A** | **A** | **T** | **C** | **T** | **T** | **C** | **G** | **T** | **C** | **G** | **T** | **T** | **T** | **G** | **A** | **A** | **A** | **A** | **T** | **T** | **A** | **T** | **G** | **A** | **C** | **G** | **A** | **A** | **C** | **A** | **C** | **T** | **T** | **T** | **A** | **G** | **T** | **A** | **G** | **G** | **A** | **G** | **A** | **T** | **G** | **T** | **T** | **G** | **A** | **G** | **G** | **T** | **G** | **A** | **T** | **C** | **C** | **G** | **T** | **G** | **T** | **A** | **-** | **-** | **-** |
| **N55240** | **A** | **T** | **G** | **A** | **A** | **T** | **A** | **A** | **C** | **C** | **C** | **T** | **T** | **G** | **G** | **C** | **G** | **C** | **T** | **T** | **T** | **T** | **T** | **T** | **A** | **T** | **C** | **G** | **T** | **C** | **G** | **C** | **A** | **G** | **A** | **A** | **G** | **C** | **A** | **T** | **T** | **A** | **C** | **T** | **T** | **T** | **T** | **T** | **A** | **T** | **T** | **C** | **T** | **G** | **G** | **C** | **G** | **T** | **T** | **A** | **T** | **G** | **G** | **C** | **A** | **A** | **A** | **T** | **T** | **G** | **T** | **A** | **C** | **A** | **T** | **A** | **A** | **T** | **C** | **C** | **T** | **G** | **G** | **A** | **T** | **T** | **A** | **G** | **C** | **T** | **G** | **T** | **T** | **T** | **T** | **A** | **T** | **T** | **A** | **A** | **C** | **A** | **A** | **T** | **T** | **G** | **G** | **C** | **G** | **T** | **T** | **T** | **T** | **A** | **C** | **T** | **T** | **G** | **T** | **G** | **G** | **C** | **C** | **T** | **A** | **C** | **G** | **T** | **T** | **T** | **C** | **C** | **A** | **G** | **G** | **A** | **A** | **A** | **G** | **C** | **A** | **T** | **C** | **T** | **A** | **A** | **A** | **A** | **C** | **A** | **C** | **A** | **T** | **T** | **T** | **T** | **A** | **A** | **C** | **A** | **A** | **C** | **T** | **T** | **T** | **C** | **A** | **A** | **T** | **T** | **C** | **G** | **T** | **C** | **C** | **T** | **C** | **G** | **G** | **C** | **G** | **T** | **T** | **G** | **T** | **T** | **T** | **T** | **T** | **A** | **T** | **T** | **G** | **T** | **C** | **A** | **T** | **T** | **G** | **G** | **T** | **G** | **C** | **A** | **A** | **T** | **G** | **A** | **A** | **C** | **A** | **G** | **C** | **A** | **C** | **G** | **G** | **C** | **T** | **G** | **T** | **T** | **T** | **G** | **G** | **T** | **T** | **T** | **A** | **T** | **G** | **T** | **T** | **G** | **A** | **T** | **T** | **T** | **T** | **T** | **G** | **G** | **C** | **G** | **T** | **A** | **C** | **T** | **C** | **T** | **T** | **T** | **A** | **T** | **C** | **G** | **G** | **C** | **T** | **T** | **A** | **A** | **A** | **A** | **G** | **G** | **C** | **T** | **T** | **T** | **G** | **A** | **G** | **A** | **T** | **T** | **T** | **C** | **A** | **G** | **G** | **C** | **G** | **T** | **G** | **G** | **A** | **T** | **A** | **T** | **A** | **G** | **C** | **T** | **G** | **A** | **G** | **C** | **G** | **A** | **G** | **C** | **A** | **C** | **C** | **T** | **T** | **G** | **G** | **C** | **G** | **A** | **A** | **A** | **A** | **A** | **A** | **A** | **C** | **A** | **A** | **A** | **T** | **G** | **A** | **T** | **T** | **A** | **T** | **G** | **G** | **T** | **G** | **G** | **A** | **G** | **A** | **C** | **G** | **G** | **C** | **G** | **G** | **C** | **A** | **A** | **A** | **A** | **G** | **A** | **A** | **C** | **C** | **T** | **A** | **A** | **A** | **A** | **A** | **T** | **G** | **G** | **C** | **A** | **A** | **A** | **C** | **G** | **A** | **T** | **T** | **T** | **A** | **A** | **A** | **C** | **G** | **C** | **C** | **G** | **C** | **T** | **G** | **G** | **T** | **T** | **T** | **G** | **C** | **C** | **A** | **A** | **C** | **G** | **A** | **A** | **C** | **G** | **C** | **A** | **T** | **T** | **G** | **G** | **T** | **A** | **A** | **C** | **A** | **A** | **T** | **A** | **T** | **C** | **T** | **A** | **T** | **G** | **A** | **A** | **T** | **G** | **G** | **G** | **A** | **C** | **G** | **A** | **T** | **A** | **T** | **C** | **A** | **A** | **T** | **A** | **T** | **T** | **G** | **A** | **T** | **T** | **T** | **A** | **A** | **T** | **C** | **T** | **C** | **T** | **G** | **G** | **G** | **G** | **A** | **C** | **A** | **C** | **C** | **A** | **T** | **T** | **A** | **T** | **T** | **G** | **A** | **T** | **T** | **T** | **A** | **G** | **G** | **T** | **A** | **A** | **T** | **A** | **C** | **G** | **T** | **T** | **A** | **C** | **T** | **A** | **C** | **C** | **G** | **A** | **A** | **A** | **G** | **A** | **A** | **G** | **A** | **C** | **A** | **A** | **T** | **A** | **T** | **T** | **A** | **T** | **T** | **A** | **T** | **T** | **A** | **T** | **T** | **C** | **G** | **T** | **A** | **A** | **A** | **G** | **G** | **T** | **T** | **T** | **T** | **G** | **G** | **C** | **C** | **G** | **C** | **A** | **C** | **G** | **C** | **G** | **A** | **A** | **T** | **T** | **C** | **T** | **A** | **G** | **T** | **G** | **C** | **C** | **G** | **T** | **T** | **A** | **G** | **G** | **T** | **G** | **T** | **A** | **G** | **C** | **T** | **A** | **T** | **T** | **T** | **T** | **G** | **T** | **T** | **A** | **G** | **A** | **A** | **C** | **A** | **T** | **T** | **C** | **A** | **A** | **C** | **T** | **T** | **T** | **T** | **T** | **A** | **C** | **G** | **G** | **A** | **A** | **C** | **G** | **G** | **T** | **A** | **C** | **G** | **T** | **T** | **T** | **T** | **G** | **A** | **A** | **G** | **A** | **A** | **G** | **A** | **A** | **A** | **A** | **A** | **T** | **A** | **T** | **C** | **A** | **A** | **T** | **T** | **G** | **A** | **A** | **A** | **A** | **A** | **C** | **G** | **A** | **A** | **T** | **C** | **A** | **T** | **T** | **A** | **A** | **A** | **A** | **A** | **T** | **T** | **T** | **A** | **C** | **A** | **G** | **C** | **A** | **A** | **T** | **G** | **A** | **T** | **T** | **A** | **T** | **G** | **A** | **T** | **A** | **C** | **C** | **A** | **A** | **T** | **C** | **T** | **T** | **C** | **G** | **T** | **C** | **G** | **T** | **T** | **T** | **G** | **A** | **A** | **A** | **A** | **T** | **T** | **A** | **T** | **G** | **A** | **C** | **G** | **A** | **A** | **C** | **A** | **C** | **T** | **T** | **T** | **A** | **G** | **T** | **A** | **G** | **G** | **A** | **G** | **A** | **T** | **G** | **T** | **T** | **G** | **A** | **G** | **G** | **T** | **G** | **A** | **T** | **C** | **C** | **G** | **T** | **G** | **T** | **A** | **-** | **-** | **-** |
| **N59860F** | **A** | **T** | **G** | **A** | **A** | **T** | **A** | **A** | **C** | **C** | **C** | **T** | **T** | **G** | **G** | **C** | **G** | **C** | **T** | **T** | **T** | **T** | **T** | **T** | **A** | **T** | **C** | **G** | **T** | **C** | **G** | **C** | **A** | **G** | **A** | **A** | **G** | **C** | **A** | **T** | **T** | **A** | **C** | **T** | **T** | **T** | **T** | **T** | **A** | **T** | **T** | **C** | **T** | **G** | **G** | **C** | **G** | **T** | **T** | **A** | **T** | **G** | **G** | **C** | **A** | **A** | **A** | **T** | **T** | **G** | **T** | **A** | **C** | **A** | **T** | **A** | **A** | **T** | **C** | **C** | **T** | **G** | **G** | **A** | **T** | **T** | **A** | **G** | **C** | **T** | **G** | **T** | **T** | **T** | **T** | **A** | **T** | **T** | **A** | **A** | **C** | **A** | **A** | **T** | **T** | **G** | **G** | **C** | **G** | **T** | **T** | **T** | **T** | **A** | **C** | **T** | **T** | **G** | **T** | **G** | **G** | **C** | **C** | **T** | **A** | **C** | **G** | **T** | **T** | **T** | **C** | **C** | **A** | **G** | **G** | **A** | **A** | **A** | **G** | **C** | **A** | **T** | **C** | **T** | **A** | **A** | **A** | **A** | **C** | **A** | **C** | **A** | **T** | **T** | **T** | **T** | **A** | **A** | **C** | **A** | **A** | **C** | **T** | **T** | **T** | **C** | **A** | **A** | **T** | **T** | **C** | **G** | **T** | **C** | **C** | **T** | **C** | **G** | **G** | **C** | **G** | **T** | **T** | **G** | **T** | **T** | **T** | **T** | **T** | **A** | **T** | **T** | **G** | **T** | **C** | **A** | **T** | **T** | **G** | **G** | **T** | **G** | **C** | **A** | **A** | **T** | **G** | **A** | **A** | **C** | **A** | **G** | **C** | **A** | **C** | **G** | **G** | **C** | **T** | **G** | **T** | **T** | **T** | **G** | **G** | **T** | **T** | **T** | **A** | **T** | **G** | **T** | **T** | **G** | **A** | **T** | **T** | **T** | **T** | **T** | **G** | **G** | **C** | **G** | **T** | **A** | **C** | **T** | **C** | **T** | **T** | **T** | **A** | **T** | **C** | **G** | **G** | **C** | **T** | **T** | **A** | **A** | **A** | **A** | **G** | **G** | **C** | **T** | **T** | **T** | **G** | **A** | **G** | **A** | **T** | **T** | **T** | **C** | **A** | **G** | **G** | **C** | **G** | **T** | **G** | **G** | **A** | **T** | **A** | **T** | **A** | **G** | **C** | **T** | **G** | **A** | **G** | **C** | **G** | **A** | **G** | **C** | **A** | **C** | **C** | **T** | **T** | **G** | **G** | **C** | **G** | **A** | **A** | **A** | **A** | **A** | **A** | **A** | **C** | **A** | **A** | **A** | **T** | **G** | **A** | **T** | **T** | **A** | **T** | **G** | **G** | **T** | **G** | **G** | **A** | **G** | **A** | **C** | **G** | **G** | **C** | **G** | **G** | **C** | **A** | **A** | **A** | **A** | **G** | **A** | **A** | **C** | **C** | **T** | **A** | **A** | **A** | **A** | **A** | **T** | **G** | **G** | **C** | **A** | **A** | **A** | **C** | **G** | **A** | **T** | **T** | **T** | **A** | **A** | **A** | **C** | **G** | **C** | **C** | **G** | **C** | **T** | **G** | **G** | **T** | **T** | **T** | **G** | **C** | **C** | **A** | **A** | **C** | **G** | **A** | **A** | **C** | **G** | **C** | **A** | **T** | **T** | **G** | **G** | **T** | **A** | **A** | **C** | **A** | **A** | **T** | **A** | **T** | **C** | **T** | **A** | **T** | **G** | **A** | **A** | **T** | **G** | **G** | **G** | **A** | **C** | **G** | **A** | **T** | **A** | **T** | **C** | **A** | **A** | **T** | **A** | **T** | **T** | **G** | **A** | **T** | **T** | **T** | **A** | **A** | **T** | **C** | **T** | **C** | **T** | **G** | **G** | **G** | **G** | **A** | **C** | **A** | **C** | **C** | **A** | **T** | **T** | **A** | **T** | **T** | **G** | **A** | **T** | **T** | **T** | **A** | **G** | **G** | **T** | **A** | **A** | **T** | **A** | **C** | **G** | **T** | **T** | **A** | **C** | **T** | **A** | **C** | **C** | **G** | **A** | **A** | **A** | **G** | **A** | **A** | **G** | **A** | **C** | **A** | **A** | **T** | **A** | **T** | **T** | **A** | **T** | **T** | **A** | **T** | **T** | **A** | **T** | **T** | **C** | **G** | **T** | **A** | **A** | **A** | **G** | **G** | **T** | **T** | **T** | **T** | **G** | **G** | **C** | **C** | **G** | **C** | **A** | **C** | **G** | **C** | **G** | **A** | **A** | **T** | **T** | **C** | **T** | **A** | **G** | **T** | **G** | **C** | **C** | **G** | **T** | **T** | **A** | **G** | **G** | **T** | **G** | **T** | **A** | **G** | **C** | **T** | **A** | **T** | **T** | **T** | **T** | **G** | **T** | **T** | **A** | **G** | **A** | **A** | **C** | **A** | **T** | **T** | **C** | **A** | **A** | **C** | **T** | **T** | **T** | **T** | **T** | **A** | **C** | **G** | **G** | **A** | **A** | **C** | **G** | **G** | **T** | **A** | **C** | **G** | **T** | **T** | **T** | **T** | **G** | **A** | **A** | **G** | **A** | **A** | **G** | **A** | **A** | **A** | **A** | **A** | **T** | **A** | **T** | **C** | **A** | **A** | **T** | **T** | **G** | **A** | **A** | **A** | **A** | **A** | **C** | **G** | **A** | **A** | **T** | **C** | **A** | **T** | **T** | **A** | **A** | **A** | **A** | **A** | **T** | **T** | **T** | **A** | **C** | **A** | **G** | **C** | **A** | **A** | **T** | **G** | **A** | **T** | **T** | **A** | **T** | **G** | **A** | **T** | **A** | **C** | **C** | **A** | **A** | **T** | **C** | **T** | **T** | **C** | **G** | **T** | **C** | **G** | **T** | **T** | **T** | **G** | **A** | **A** | **A** | **A** | **T** | **T** | **A** | **T** | **G** | **A** | **C** | **G** | **A** | **A** | **C** | **A** | **C** | **T** | **T** | **T** | **A** | **G** | **T** | **A** | **G** | **G** | **A** | **G** | **A** | **T** | **G** | **T** | **T** | **G** | **A** | **G** | **G** | **T** | **G** | **A** | **T** | **C** | **C** | **G** | **T** | **G** | **T** | **A** | **-** | **-** | **-** |
| **N52454** | **A** | **T** | **G** | **A** | **A** | **T** | **A** | **A** | **C** | **C** | **C** | **T** | **T** | **G** | **G** | **C** | **G** | **C** | **T** | **T** | **T** | **T** | **T** | **T** | **A** | **T** | **C** | **G** | **T** | **C** | **G** | **C** | **A** | **G** | **A** | **A** | **G** | **C** | **A** | **T** | **T** | **A** | **C** | **T** | **T** | **T** | **T** | **T** | **A** | **T** | **T** | **C** | **T** | **G** | **G** | **C** | **G** | **T** | **T** | **A** | **T** | **G** | **G** | **C** | **A** | **A** | **A** | **T** | **T** | **G** | **T** | **A** | **C** | **A** | **T** | **A** | **A** | **T** | **C** | **C** | **T** | **G** | **G** | **A** | **T** | **T** | **A** | **G** | **C** | **T** | **G** | **T** | **T** | **T** | **T** | **A** | **T** | **T** | **A** | **A** | **C** | **A** | **A** | **T** | **T** | **G** | **G** | **C** | **G** | **T** | **T** | **T** | **T** | **A** | **C** | **T** | **T** | **G** | **T** | **G** | **G** | **C** | **C** | **T** | **A** | **C** | **G** | **T** | **T** | **T** | **C** | **C** | **A** | **G** | **G** | **A** | **A** | **A** | **G** | **C** | **A** | **T** | **C** | **T** | **A** | **A** | **A** | **A** | **C** | **A** | **C** | **A** | **T** | **T** | **T** | **T** | **A** | **A** | **C** | **A** | **A** | **C** | **T** | **T** | **T** | **C** | **A** | **A** | **T** | **T** | **C** | **G** | **T** | **C** | **C** | **T** | **C** | **G** | **G** | **C** | **G** | **T** | **T** | **G** | **T** | **T** | **T** | **T** | **T** | **A** | **T** | **T** | **G** | **T** | **C** | **A** | **T** | **T** | **G** | **G** | **T** | **G** | **C** | **A** | **A** | **T** | **G** | **A** | **A** | **C** | **A** | **G** | **C** | **A** | **C** | **G** | **G** | **C** | **T** | **G** | **T** | **T** | **T** | **G** | **G** | **T** | **T** | **T** | **A** | **T** | **G** | **T** | **T** | **G** | **A** | **T** | **T** | **T** | **T** | **T** | **G** | **G** | **C** | **G** | **T** | **A** | **C** | **T** | **C** | **T** | **T** | **T** | **A** | **T** | **C** | **G** | **G** | **C** | **T** | **T** | **A** | **A** | **A** | **A** | **G** | **G** | **C** | **T** | **T** | **T** | **G** | **A** | **G** | **A** | **T** | **T** | **T** | **C** | **A** | **G** | **G** | **C** | **G** | **T** | **G** | **G** | **A** | **T** | **A** | **T** | **A** | **G** | **C** | **T** | **G** | **A** | **G** | **C** | **G** | **A** | **G** | **C** | **A** | **C** | **C** | **T** | **T** | **G** | **G** | **C** | **G** | **A** | **A** | **A** | **A** | **A** | **A** | **A** | **C** | **A** | **A** | **A** | **T** | **G** | **A** | **T** | **T** | **A** | **T** | **G** | **G** | **T** | **G** | **G** | **A** | **G** | **A** | **C** | **G** | **G** | **C** | **G** | **G** | **C** | **A** | **A** | **A** | **A** | **G** | **A** | **A** | **C** | **C** | **T** | **A** | **A** | **A** | **A** | **A** | **T** | **G** | **G** | **C** | **A** | **A** | **A** | **C** | **G** | **A** | **T** | **T** | **T** | **A** | **A** | **A** | **C** | **G** | **C** | **C** | **G** | **C** | **T** | **G** | **G** | **T** | **T** | **T** | **G** | **C** | **C** | **A** | **A** | **C** | **G** | **A** | **A** | **C** | **G** | **C** | **A** | **T** | **T** | **G** | **G** | **T** | **A** | **A** | **C** | **A** | **A** | **T** | **A** | **T** | **C** | **T** | **A** | **T** | **G** | **A** | **A** | **T** | **G** | **G** | **G** | **A** | **C** | **G** | **A** | **T** | **A** | **T** | **C** | **A** | **A** | **T** | **A** | **T** | **T** | **G** | **A** | **T** | **T** | **T** | **A** | **A** | **T** | **C** | **T** | **C** | **T** | **G** | **G** | **G** | **G** | **A** | **C** | **A** | **C** | **C** | **A** | **T** | **T** | **A** | **T** | **T** | **G** | **A** | **T** | **T** | **T** | **A** | **G** | **G** | **T** | **A** | **A** | **T** | **A** | **C** | **G** | **T** | **T** | **A** | **C** | **T** | **A** | **C** | **C** | **G** | **A** | **A** | **A** | **G** | **A** | **A** | **G** | **A** | **C** | **A** | **A** | **T** | **A** | **T** | **T** | **A** | **T** | **T** | **A** | **T** | **T** | **A** | **T** | **T** | **C** | **G** | **T** | **A** | **A** | **A** | **G** | **G** | **T** | **T** | **T** | **T** | **G** | **G** | **C** | **C** | **G** | **C** | **A** | **C** | **G** | **C** | **G** | **A** | **A** | **T** | **T** | **C** | **T** | **A** | **G** | **T** | **G** | **C** | **C** | **G** | **T** | **T** | **A** | **G** | **G** | **T** | **G** | **T** | **A** | **G** | **C** | **T** | **A** | **T** | **T** | **T** | **T** | **G** | **T** | **T** | **A** | **G** | **A** | **A** | **C** | **A** | **T** | **T** | **C** | **A** | **A** | **C** | **T** | **T** | **T** | **T** | **T** | **A** | **C** | **G** | **G** | **A** | **A** | **C** | **G** | **G** | **T** | **A** | **C** | **G** | **T** | **T** | **T** | **T** | **G** | **A** | **A** | **G** | **A** | **A** | **G** | **A** | **A** | **A** | **A** | **A** | **T** | **A** | **T** | **C** | **A** | **A** | **T** | **T** | **G** | **A** | **A** | **A** | **A** | **A** | **C** | **G** | **A** | **A** | **T** | **C** | **A** | **T** | **T** | **A** | **A** | **A** | **A** | **A** | **T** | **T** | **T** | **A** | **C** | **A** | **G** | **C** | **A** | **A** | **T** | **G** | **A** | **T** | **T** | **A** | **T** | **G** | **A** | **T** | **A** | **C** | **C** | **A** | **A** | **T** | **C** | **T** | **T** | **C** | **G** | **T** | **C** | **G** | **T** | **T** | **T** | **G** | **A** | **A** | **A** | **A** | **T** | **T** | **A** | **T** | **G** | **A** | **C** | **G** | **A** | **A** | **C** | **A** | **C** | **T** | **T** | **T** | **A** | **G** | **T** | **A** | **G** | **G** | **A** | **G** | **A** | **T** | **G** | **T** | **T** | **G** | **A** | **G** | **G** | **T** | **G** | **A** | **T** | **C** | **C** | **G** | **T** | **G** | **T** | **A** | **-** | **-** | **-** |
| **N59409F** | **A** | **T** | **G** | **A** | **A** | **T** | **A** | **A** | **C** | **C** | **C** | **T** | **T** | **G** | **G** | **C** | **G** | **C** | **T** | **T** | **T** | **T** | **T** | **T** | **A** | **T** | **C** | **G** | **T** | **C** | **G** | **C** | **A** | **G** | **A** | **A** | **G** | **C** | **A** | **T** | **T** | **A** | **C** | **T** | **T** | **T** | **T** | **T** | **A** | **T** | **T** | **C** | **T** | **G** | **G** | **C** | **G** | **T** | **T** | **A** | **T** | **G** | **G** | **C** | **A** | **A** | **A** | **T** | **T** | **G** | **T** | **A** | **C** | **A** | **T** | **A** | **A** | **T** | **C** | **C** | **T** | **G** | **G** | **A** | **T** | **T** | **A** | **G** | **C** | **T** | **G** | **T** | **T** | **T** | **T** | **A** | **T** | **T** | **A** | **A** | **C** | **A** | **A** | **T** | **T** | **G** | **G** | **C** | **G** | **T** | **T** | **T** | **T** | **A** | **C** | **T** | **T** | **G** | **T** | **G** | **G** | **C** | **C** | **T** | **A** | **C** | **G** | **T** | **T** | **T** | **C** | **C** | **A** | **G** | **G** | **A** | **A** | **A** | **G** | **C** | **A** | **T** | **C** | **T** | **A** | **A** | **A** | **A** | **C** | **A** | **C** | **A** | **T** | **T** | **T** | **T** | **A** | **A** | **C** | **A** | **A** | **C** | **T** | **T** | **T** | **C** | **A** | **A** | **T** | **T** | **C** | **G** | **T** | **C** | **C** | **T** | **C** | **G** | **G** | **C** | **G** | **T** | **T** | **G** | **T** | **T** | **T** | **T** | **T** | **A** | **T** | **T** | **G** | **T** | **C** | **A** | **T** | **T** | **G** | **G** | **T** | **G** | **C** | **A** | **A** | **T** | **G** | **A** | **A** | **C** | **A** | **G** | **C** | **A** | **C** | **G** | **G** | **C** | **T** | **G** | **T** | **T** | **T** | **G** | **G** | **T** | **T** | **T** | **A** | **T** | **G** | **T** | **T** | **G** | **A** | **T** | **T** | **T** | **T** | **T** | **G** | **G** | **C** | **G** | **T** | **A** | **C** | **T** | **C** | **T** | **T** | **T** | **A** | **T** | **C** | **G** | **G** | **C** | **T** | **T** | **A** | **A** | **A** | **A** | **G** | **G** | **C** | **T** | **T** | **T** | **G** | **A** | **G** | **A** | **T** | **T** | **T** | **C** | **A** | **G** | **G** | **C** | **G** | **T** | **G** | **G** | **A** | **T** | **A** | **T** | **A** | **G** | **C** | **T** | **G** | **A** | **G** | **C** | **G** | **A** | **G** | **C** | **A** | **C** | **C** | **T** | **T** | **G** | **G** | **C** | **G** | **A** | **A** | **A** | **A** | **A** | **A** | **A** | **C** | **A** | **A** | **A** | **T** | **G** | **A** | **T** | **T** | **A** | **T** | **G** | **G** | **T** | **G** | **G** | **A** | **G** | **A** | **C** | **G** | **G** | **C** | **G** | **G** | **C** | **A** | **A** | **A** | **A** | **G** | **A** | **A** | **C** | **C** | **T** | **A** | **A** | **A** | **A** | **A** | **T** | **G** | **G** | **C** | **A** | **A** | **A** | **C** | **G** | **A** | **T** | **T** | **T** | **A** | **A** | **A** | **C** | **G** | **C** | **C** | **G** | **C** | **T** | **G** | **G** | **T** | **T** | **T** | **G** | **C** | **C** | **A** | **A** | **C** | **G** | **A** | **A** | **C** | **G** | **C** | **A** | **T** | **T** | **G** | **G** | **T** | **A** | **A** | **C** | **A** | **A** | **T** | **A** | **T** | **C** | **T** | **A** | **T** | **G** | **A** | **A** | **T** | **G** | **G** | **G** | **A** | **C** | **G** | **A** | **T** | **A** | **T** | **C** | **A** | **A** | **T** | **A** | **T** | **T** | **G** | **A** | **T** | **T** | **T** | **A** | **A** | **T** | **C** | **T** | **C** | **T** | **G** | **G** | **G** | **G** | **A** | **C** | **A** | **C** | **C** | **A** | **T** | **T** | **A** | **T** | **T** | **G** | **A** | **T** | **T** | **T** | **A** | **G** | **G** | **T** | **A** | **A** | **T** | **A** | **C** | **G** | **T** | **T** | **A** | **C** | **T** | **A** | **C** | **C** | **G** | **A** | **A** | **A** | **G** | **A** | **A** | **G** | **A** | **C** | **A** | **A** | **T** | **A** | **T** | **T** | **A** | **T** | **T** | **A** | **T** | **T** | **A** | **T** | **T** | **C** | **G** | **T** | **A** | **A** | **A** | **G** | **G** | **T** | **T** | **T** | **T** | **G** | **G** | **C** | **C** | **G** | **C** | **A** | **C** | **G** | **C** | **G** | **A** | **A** | **T** | **T** | **C** | **T** | **A** | **G** | **T** | **G** | **C** | **C** | **G** | **T** | **T** | **A** | **G** | **G** | **T** | **G** | **T** | **A** | **G** | **C** | **T** | **A** | **T** | **T** | **T** | **T** | **G** | **T** | **T** | **A** | **G** | **A** | **A** | **C** | **A** | **T** | **T** | **C** | **A** | **A** | **C** | **T** | **T** | **T** | **T** | **T** | **A** | **C** | **G** | **G** | **A** | **A** | **C** | **G** | **G** | **T** | **A** | **C** | **G** | **T** | **T** | **T** | **T** | **G** | **A** | **A** | **G** | **A** | **A** | **G** | **A** | **A** | **A** | **A** | **A** | **T** | **A** | **T** | **C** | **A** | **A** | **T** | **T** | **G** | **A** | **A** | **A** | **A** | **A** | **C** | **G** | **A** | **A** | **T** | **C** | **A** | **T** | **T** | **A** | **A** | **A** | **A** | **A** | **T** | **T** | **T** | **A** | **C** | **A** | **G** | **C** | **A** | **A** | **T** | **G** | **A** | **T** | **T** | **A** | **T** | **G** | **A** | **T** | **A** | **C** | **C** | **A** | **A** | **T** | **C** | **T** | **T** | **C** | **G** | **T** | **C** | **G** | **T** | **T** | **T** | **G** | **A** | **A** | **A** | **A** | **T** | **T** | **A** | **T** | **G** | **A** | **C** | **G** | **A** | **A** | **C** | **A** | **C** | **T** | **T** | **T** | **A** | **G** | **T** | **A** | **G** | **G** | **A** | **G** | **A** | **T** | **G** | **T** | **T** | **G** | **A** | **G** | **G** | **T** | **G** | **A** | **T** | **C** | **C** | **G** | **T** | **G** | **T** | **A** | **-** | **-** | **-** |
| **N60271F** | **A** | **T** | **G** | **A** | **A** | **T** | **A** | **A** | **C** | **C** | **C** | **T** | **T** | **G** | **G** | **C** | **G** | **C** | **T** | **T** | **T** | **T** | **T** | **T** | **A** | **T** | **C** | **G** | **T** | **C** | **G** | **C** | **A** | **G** | **A** | **A** | **G** | **C** | **A** | **T** | **T** | **A** | **C** | **T** | **T** | **T** | **T** | **T** | **A** | **T** | **T** | **C** | **T** | **G** | **G** | **C** | **G** | **T** | **T** | **A** | **T** | **G** | **G** | **C** | **A** | **A** | **A** | **T** | **T** | **G** | **T** | **A** | **C** | **A** | **T** | **A** | **A** | **T** | **C** | **C** | **T** | **G** | **G** | **A** | **T** | **T** | **A** | **G** | **C** | **T** | **G** | **T** | **T** | **T** | **T** | **A** | **T** | **T** | **A** | **A** | **C** | **A** | **A** | **T** | **T** | **G** | **G** | **C** | **G** | **T** | **T** | **T** | **T** | **A** | **C** | **T** | **T** | **G** | **T** | **G** | **G** | **C** | **C** | **T** | **A** | **C** | **G** | **T** | **T** | **T** | **C** | **C** | **A** | **G** | **G** | **A** | **A** | **A** | **G** | **C** | **A** | **T** | **C** | **T** | **A** | **A** | **A** | **A** | **C** | **A** | **C** | **A** | **T** | **T** | **T** | **T** | **A** | **A** | **C** | **A** | **A** | **C** | **T** | **T** | **T** | **C** | **A** | **A** | **T** | **T** | **C** | **G** | **T** | **C** | **C** | **T** | **C** | **G** | **G** | **C** | **G** | **T** | **T** | **G** | **T** | **T** | **T** | **T** | **T** | **A** | **T** | **T** | **G** | **T** | **C** | **A** | **T** | **T** | **G** | **G** | **T** | **G** | **C** | **A** | **A** | **T** | **G** | **A** | **A** | **T** | **A** | **G** | **C** | **A** | **C** | **G** | **G** | **C** | **T** | **G** | **T** | **T** | **T** | **G** | **G** | **T** | **T** | **T** | **A** | **T** | **G** | **T** | **T** | **G** | **A** | **T** | **T** | **T** | **T** | **T** | **G** | **G** | **C** | **G** | **T** | **A** | **C** | **T** | **C** | **T** | **T** | **T** | **A** | **T** | **C** | **G** | **G** | **C** | **T** | **T** | **A** | **A** | **A** | **A** | **G** | **G** | **C** | **T** | **T** | **T** | **G** | **A** | **G** | **A** | **T** | **T** | **T** | **C** | **A** | **G** | **G** | **C** | **G** | **T** | **G** | **G** | **A** | **T** | **A** | **T** | **A** | **G** | **C** | **T** | **G** | **A** | **G** | **C** | **G** | **A** | **G** | **C** | **A** | **C** | **C** | **T** | **T** | **G** | **G** | **C** | **G** | **A** | **A** | **A** | **A** | **A** | **A** | **A** | **C** | **A** | **A** | **A** | **T** | **G** | **A** | **T** | **T** | **A** | **T** | **G** | **G** | **T** | **G** | **G** | **A** | **G** | **A** | **C** | **G** | **G** | **C** | **G** | **G** | **C** | **A** | **A** | **A** | **A** | **G** | **A** | **A** | **C** | **C** | **T** | **A** | **A** | **A** | **A** | **A** | **T** | **G** | **G** | **C** | **A** | **A** | **A** | **C** | **G** | **G** | **T** | **T** | **T** | **A** | **A** | **A** | **C** | **G** | **C** | **C** | **G** | **C** | **T** | **G** | **G** | **T** | **T** | **T** | **G** | **C** | **C** | **A** | **A** | **C** | **G** | **A** | **A** | **C** | **G** | **C** | **A** | **T** | **T** | **G** | **G** | **T** | **A** | **A** | **C** | **A** | **A** | **T** | **A** | **T** | **C** | **T** | **A** | **T** | **G** | **A** | **A** | **T** | **G** | **G** | **G** | **A** | **C** | **G** | **A** | **T** | **A** | **T** | **C** | **A** | **A** | **T** | **A** | **T** | **T** | **G** | **A** | **T** | **T** | **T** | **A** | **A** | **T** | **C** | **T** | **C** | **T** | **G** | **G** | **G** | **G** | **A** | **C** | **A** | **C** | **C** | **A** | **T** | **T** | **A** | **T** | **T** | **G** | **A** | **T** | **T** | **T** | **A** | **G** | **G** | **T** | **A** | **A** | **T** | **A** | **C** | **G** | **C** | **T** | **A** | **C** | **T** | **A** | **C** | **C** | **G** | **A** | **A** | **A** | **G** | **A** | **A** | **G** | **A** | **C** | **A** | **A** | **T** | **A** | **T** | **T** | **A** | **T** | **T** | **A** | **T** | **T** | **A** | **T** | **T** | **C** | **G** | **T** | **A** | **A** | **A** | **G** | **G** | **T** | **T** | **T** | **T** | **G** | **G** | **C** | **C** | **G** | **C** | **A** | **C** | **G** | **C** | **G** | **A** | **A** | **T** | **T** | **C** | **T** | **A** | **G** | **T** | **G** | **C** | **C** | **G** | **T** | **T** | **A** | **G** | **G** | **T** | **G** | **T** | **A** | **G** | **C** | **T** | **A** | **T** | **T** | **T** | **T** | **G** | **T** | **T** | **A** | **G** | **A** | **A** | **C** | **A** | **T** | **T** | **C** | **A** | **A** | **C** | **T** | **T** | **T** | **T** | **T** | **A** | **C** | **G** | **G** | **A** | **A** | **C** | **G** | **G** | **T** | **A** | **C** | **G** | **T** | **T** | **T** | **T** | **G** | **A** | **A** | **G** | **A** | **A** | **G** | **A** | **A** | **A** | **A** | **A** | **T** | **A** | **T** | **C** | **A** | **A** | **T** | **T** | **G** | **A** | **A** | **A** | **A** | **A** | **C** | **G** | **A** | **A** | **T** | **C** | **A** | **T** | **T** | **A** | **A** | **A** | **A** | **A** | **T** | **T** | **T** | **A** | **C** | **A** | **G** | **C** | **A** | **A** | **T** | **G** | **A** | **T** | **T** | **A** | **T** | **G** | **A** | **T** | **A** | **C** | **C** | **A** | **A** | **T** | **C** | **T** | **T** | **C** | **G** | **T** | **C** | **G** | **T** | **T** | **T** | **G** | **A** | **A** | **A** | **A** | **T** | **T** | **A** | **T** | **G** | **A** | **C** | **G** | **A** | **A** | **C** | **A** | **C** | **T** | **T** | **T** | **A** | **G** | **T** | **A** | **G** | **G** | **A** | **G** | **A** | **T** | **G** | **T** | **T** | **G** | **A** | **G** | **G** | **T** | **G** | **A** | **T** | **C** | **C** | **G** | **T** | **G** | **T** | **A** | **-** | **-** | **-** |
| **N54576** | **A** | **T** | **G** | **A** | **A** | **T** | **A** | **A** | **C** | **C** | **C** | **T** | **T** | **G** | **G** | **C** | **G** | **C** | **T** | **T** | **T** | **T** | **T** | **T** | **A** | **T** | **C** | **G** | **T** | **C** | **G** | **C** | **A** | **G** | **A** | **A** | **G** | **C** | **A** | **T** | **T** | **A** | **C** | **T** | **T** | **T** | **T** | **T** | **A** | **T** | **T** | **C** | **T** | **G** | **G** | **C** | **G** | **T** | **T** | **A** | **T** | **G** | **G** | **C** | **A** | **A** | **A** | **T** | **T** | **G** | **T** | **A** | **C** | **A** | **T** | **A** | **A** | **T** | **C** | **C** | **T** | **G** | **G** | **A** | **T** | **T** | **A** | **G** | **C** | **T** | **G** | **T** | **T** | **T** | **T** | **A** | **T** | **T** | **A** | **A** | **C** | **A** | **A** | **T** | **T** | **G** | **G** | **C** | **G** | **T** | **T** | **T** | **T** | **A** | **C** | **T** | **T** | **G** | **T** | **G** | **G** | **C** | **C** | **T** | **A** | **T** | **G** | **T** | **T** | **T** | **C** | **C** | **A** | **G** | **G** | **A** | **A** | **A** | **G** | **C** | **A** | **T** | **C** | **T** | **A** | **A** | **A** | **A** | **C** | **A** | **C** | **A** | **T** | **T** | **T** | **T** | **A** | **A** | **C** | **A** | **A** | **C** | **T** | **T** | **T** | **C** | **A** | **A** | **T** | **T** | **C** | **G** | **T** | **C** | **C** | **T** | **C** | **G** | **G** | **C** | **G** | **T** | **T** | **G** | **T** | **T** | **T** | **T** | **T** | **A** | **T** | **T** | **G** | **T** | **C** | **A** | **T** | **T** | **G** | **G** | **T** | **G** | **C** | **A** | **A** | **T** | **G** | **A** | **A** | **C** | **A** | **G** | **C** | **A** | **C** | **G** | **G** | **C** | **T** | **G** | **T** | **T** | **T** | **G** | **G** | **T** | **T** | **T** | **A** | **T** | **G** | **C** | **T** | **G** | **A** | **T** | **T** | **T** | **T** | **T** | **G** | **G** | **C** | **G** | **T** | **A** | **C** | **T** | **C** | **T** | **T** | **T** | **A** | **T** | **C** | **G** | **G** | **C** | **T** | **T** | **A** | **A** | **A** | **A** | **G** | **G** | **C** | **T** | **T** | **T** | **G** | **A** | **G** | **A** | **T** | **T** | **T** | **C** | **A** | **G** | **G** | **C** | **G** | **T** | **G** | **G** | **A** | **T** | **A** | **T** | **A** | **G** | **C** | **T** | **G** | **A** | **G** | **C** | **G** | **A** | **G** | **C** | **A** | **C** | **C** | **T** | **T** | **G** | **G** | **C** | **G** | **T** | **A** | **A** | **A** | **A** | **A** | **A** | **C** | **A** | **A** | **A** | **T** | **G** | **A** | **T** | **T** | **A** | **T** | **G** | **G** | **T** | **G** | **G** | **A** | **G** | **A** | **C** | **G** | **G** | **C** | **G** | **G** | **C** | **A** | **A** | **A** | **A** | **G** | **A** | **A** | **C** | **C** | **T** | **A** | **A** | **A** | **A** | **A** | **T** | **G** | **G** | **C** | **A** | **A** | **A** | **C** | **G** | **A** | **T** | **T** | **T** | **A** | **A** | **A** | **C** | **G** | **C** | **C** | **G** | **C** | **T** | **G** | **G** | **T** | **T** | **T** | **G** | **C** | **C** | **A** | **A** | **C** | **G** | **A** | **A** | **C** | **G** | **C** | **A** | **T** | **T** | **G** | **G** | **T** | **A** | **A** | **C** | **A** | **A** | **T** | **A** | **T** | **C** | **T** | **A** | **T** | **G** | **A** | **A** | **T** | **G** | **G** | **G** | **A** | **C** | **G** | **A** | **T** | **A** | **T** | **C** | **A** | **A** | **T** | **A** | **T** | **T** | **G** | **A** | **T** | **T** | **T** | **A** | **A** | **T** | **C** | **T** | **C** | **T** | **G** | **G** | **G** | **G** | **A** | **C** | **A** | **C** | **C** | **A** | **T** | **T** | **A** | **T** | **T** | **G** | **A** | **T** | **T** | **T** | **A** | **G** | **G** | **T** | **A** | **A** | **T** | **A** | **C** | **G** | **C** | **T** | **A** | **C** | **T** | **A** | **C** | **C** | **G** | **A** | **A** | **A** | **G** | **A** | **A** | **G** | **A** | **C** | **A** | **A** | **T** | **A** | **T** | **T** | **A** | **T** | **T** | **A** | **T** | **T** | **A** | **T** | **T** | **C** | **G** | **T** | **A** | **A** | **A** | **G** | **G** | **T** | **T** | **T** | **T** | **G** | **G** | **C** | **C** | **G** | **C** | **A** | **C** | **G** | **C** | **G** | **A** | **A** | **T** | **T** | **C** | **T** | **A** | **G** | **T** | **G** | **C** | **C** | **G** | **T** | **T** | **A** | **G** | **G** | **T** | **G** | **T** | **A** | **G** | **C** | **T** | **A** | **T** | **T** | **T** | **T** | **G** | **T** | **T** | **A** | **G** | **A** | **A** | **C** | **A** | **T** | **T** | **C** | **A** | **A** | **C** | **T** | **T** | **T** | **T** | **T** | **A** | **C** | **G** | **G** | **A** | **A** | **C** | **G** | **G** | **T** | **A** | **C** | **G** | **T** | **T** | **T** | **T** | **G** | **A** | **A** | **G** | **A** | **A** | **G** | **A** | **A** | **A** | **A** | **A** | **T** | **A** | **T** | **C** | **A** | **A** | **T** | **T** | **G** | **A** | **A** | **A** | **A** | **A** | **C** | **G** | **A** | **A** | **T** | **C** | **A** | **T** | **T** | **A** | **A** | **A** | **A** | **A** | **T** | **T** | **T** | **A** | **C** | **A** | **G** | **C** | **A** | **A** | **T** | **G** | **A** | **T** | **T** | **A** | **T** | **G** | **A** | **T** | **A** | **C** | **C** | **A** | **A** | **T** | **C** | **T** | **T** | **C** | **G** | **T** | **C** | **G** | **T** | **T** | **T** | **G** | **A** | **A** | **A** | **A** | **T** | **T** | **A** | **T** | **G** | **A** | **C** | **G** | **A** | **A** | **T** | **A** | **C** | **T** | **T** | **T** | **A** | **G** | **T** | **A** | **G** | **G** | **A** | **G** | **A** | **T** | **G** | **T** | **T** | **G** | **A** | **G** | **G** | **T** | **G** | **A** | **T** | **C** | **C** | **G** | **T** | **G** | **T** | **A** | **-** | **-** | **-** |
| **N59689F** | **A** | **T** | **G** | **A** | **A** | **T** | **A** | **A** | **C** | **C** | **C** | **T** | **T** | **G** | **G** | **C** | **G** | **C** | **T** | **T** | **T** | **T** | **T** | **T** | **A** | **T** | **C** | **G** | **T** | **C** | **G** | **C** | **A** | **G** | **A** | **A** | **G** | **C** | **A** | **T** | **T** | **A** | **C** | **T** | **T** | **T** | **T** | **T** | **A** | **T** | **T** | **C** | **T** | **G** | **G** | **C** | **G** | **T** | **T** | **A** | **T** | **G** | **G** | **C** | **A** | **A** | **A** | **T** | **T** | **G** | **T** | **A** | **C** | **A** | **T** | **A** | **A** | **T** | **C** | **C** | **T** | **G** | **G** | **A** | **T** | **T** | **A** | **G** | **C** | **T** | **G** | **T** | **T** | **T** | **T** | **A** | **T** | **T** | **A** | **A** | **C** | **A** | **A** | **T** | **T** | **G** | **G** | **C** | **G** | **T** | **T** | **T** | **T** | **A** | **C** | **T** | **T** | **G** | **T** | **G** | **G** | **C** | **C** | **T** | **A** | **C** | **G** | **T** | **T** | **T** | **C** | **C** | **A** | **G** | **G** | **A** | **A** | **A** | **G** | **C** | **A** | **T** | **C** | **T** | **A** | **A** | **A** | **A** | **C** | **A** | **C** | **A** | **T** | **T** | **T** | **T** | **A** | **A** | **C** | **A** | **A** | **C** | **T** | **T** | **T** | **C** | **A** | **A** | **T** | **T** | **C** | **G** | **T** | **C** | **C** | **T** | **C** | **G** | **G** | **C** | **G** | **T** | **T** | **G** | **T** | **T** | **T** | **T** | **T** | **A** | **T** | **T** | **G** | **T** | **C** | **A** | **T** | **T** | **G** | **G** | **T** | **G** | **C** | **A** | **A** | **T** | **G** | **A** | **A** | **C** | **A** | **G** | **C** | **A** | **C** | **G** | **G** | **C** | **T** | **G** | **T** | **T** | **T** | **G** | **G** | **T** | **T** | **T** | **A** | **T** | **G** | **T** | **T** | **G** | **A** | **T** | **T** | **T** | **T** | **T** | **G** | **G** | **C** | **G** | **T** | **A** | **C** | **T** | **C** | **T** | **T** | **T** | **A** | **T** | **C** | **G** | **G** | **C** | **T** | **T** | **A** | **A** | **A** | **A** | **G** | **G** | **C** | **T** | **T** | **T** | **G** | **A** | **G** | **A** | **T** | **T** | **T** | **C** | **A** | **G** | **G** | **C** | **G** | **T** | **G** | **G** | **A** | **T** | **A** | **T** | **A** | **G** | **C** | **T** | **G** | **A** | **G** | **C** | **G** | **A** | **G** | **C** | **A** | **C** | **C** | **T** | **T** | **G** | **G** | **C** | **G** | **A** | **A** | **A** | **A** | **A** | **A** | **A** | **C** | **A** | **A** | **A** | **T** | **G** | **A** | **T** | **T** | **A** | **T** | **G** | **G** | **T** | **G** | **G** | **A** | **G** | **A** | **C** | **G** | **G** | **C** | **G** | **G** | **C** | **A** | **A** | **A** | **A** | **G** | **A** | **A** | **C** | **C** | **T** | **A** | **A** | **A** | **A** | **A** | **T** | **G** | **G** | **C** | **A** | **A** | **A** | **C** | **G** | **A** | **T** | **T** | **T** | **A** | **A** | **A** | **C** | **G** | **C** | **C** | **G** | **C** | **T** | **G** | **G** | **T** | **T** | **T** | **G** | **C** | **C** | **A** | **A** | **C** | **G** | **A** | **A** | **C** | **G** | **C** | **A** | **T** | **T** | **G** | **G** | **T** | **A** | **A** | **C** | **A** | **A** | **T** | **A** | **T** | **C** | **T** | **A** | **T** | **G** | **A** | **A** | **T** | **G** | **G** | **G** | **A** | **C** | **G** | **A** | **C** | **A** | **T** | **C** | **A** | **A** | **T** | **A** | **T** | **T** | **G** | **A** | **T** | **T** | **T** | **A** | **A** | **T** | **C** | **T** | **C** | **T** | **G** | **G** | **G** | **G** | **A** | **C** | **A** | **C** | **C** | **A** | **T** | **T** | **A** | **T** | **T** | **G** | **A** | **T** | **T** | **T** | **A** | **G** | **G** | **T** | **A** | **A** | **T** | **A** | **C** | **G** | **C** | **T** | **A** | **C** | **T** | **A** | **C** | **C** | **G** | **A** | **A** | **A** | **G** | **A** | **A** | **G** | **A** | **C** | **A** | **A** | **T** | **A** | **T** | **T** | **A** | **T** | **T** | **A** | **T** | **T** | **A** | **T** | **T** | **C** | **G** | **T** | **A** | **A** | **A** | **G** | **G** | **T** | **T** | **T** | **T** | **G** | **G** | **C** | **C** | **G** | **C** | **A** | **C** | **G** | **C** | **G** | **A** | **A** | **T** | **T** | **C** | **T** | **A** | **G** | **T** | **G** | **C** | **C** | **G** | **T** | **T** | **A** | **G** | **G** | **T** | **G** | **T** | **A** | **G** | **C** | **T** | **A** | **T** | **T** | **T** | **T** | **G** | **T** | **T** | **A** | **G** | **A** | **A** | **C** | **A** | **T** | **T** | **C** | **A** | **A** | **C** | **T** | **T** | **T** | **T** | **T** | **A** | **C** | **G** | **G** | **A** | **A** | **C** | **G** | **G** | **T** | **A** | **C** | **G** | **T** | **T** | **T** | **T** | **G** | **A** | **A** | **G** | **A** | **A** | **G** | **A** | **A** | **A** | **A** | **A** | **T** | **A** | **T** | **C** | **A** | **A** | **T** | **T** | **G** | **A** | **A** | **A** | **A** | **A** | **C** | **G** | **A** | **A** | **T** | **C** | **A** | **T** | **T** | **A** | **A** | **A** | **A** | **A** | **T** | **T** | **T** | **A** | **C** | **A** | **G** | **C** | **A** | **A** | **T** | **G** | **A** | **T** | **T** | **A** | **C** | **G** | **A** | **T** | **A** | **C** | **C** | **A** | **A** | **T** | **C** | **T** | **T** | **C** | **G** | **T** | **C** | **G** | **T** | **T** | **T** | **G** | **A** | **A** | **A** | **A** | **T** | **T** | **A** | **T** | **G** | **A** | **C** | **G** | **A** | **A** | **C** | **A** | **C** | **T** | **T** | **T** | **A** | **G** | **T** | **A** | **G** | **G** | **A** | **G** | **A** | **T** | **G** | **T** | **T** | **G** | **A** | **G** | **G** | **T** | **G** | **A** | **T** | **C** | **C** | **G** | **T** | **G** | **T** | **A** | **-** | **-** | **-** |
| **N59458F** | **A** | **T** | **G** | **A** | **A** | **T** | **A** | **A** | **C** | **C** | **C** | **T** | **T** | **G** | **G** | **C** | **G** | **C** | **T** | **T** | **T** | **T** | **T** | **T** | **A** | **T** | **C** | **G** | **T** | **C** | **G** | **C** | **A** | **G** | **A** | **A** | **G** | **C** | **A** | **T** | **T** | **A** | **C** | **T** | **T** | **T** | **T** | **T** | **A** | **T** | **T** | **C** | **T** | **G** | **G** | **C** | **G** | **T** | **T** | **A** | **T** | **G** | **G** | **C** | **A** | **A** | **A** | **T** | **T** | **G** | **T** | **A** | **C** | **A** | **T** | **A** | **A** | **T** | **C** | **C** | **T** | **G** | **G** | **A** | **T** | **T** | **A** | **G** | **C** | **T** | **G** | **T** | **T** | **T** | **T** | **A** | **T** | **T** | **A** | **A** | **C** | **A** | **A** | **T** | **T** | **G** | **G** | **C** | **G** | **T** | **T** | **T** | **T** | **A** | **C** | **T** | **T** | **G** | **T** | **G** | **G** | **C** | **C** | **T** | **A** | **C** | **G** | **T** | **T** | **T** | **C** | **C** | **A** | **G** | **G** | **A** | **A** | **A** | **G** | **C** | **A** | **T** | **C** | **T** | **A** | **A** | **A** | **A** | **C** | **A** | **C** | **A** | **T** | **T** | **T** | **T** | **A** | **A** | **C** | **A** | **A** | **C** | **T** | **T** | **T** | **C** | **A** | **A** | **T** | **T** | **C** | **G** | **T** | **C** | **C** | **T** | **C** | **G** | **G** | **C** | **G** | **T** | **T** | **G** | **T** | **T** | **T** | **T** | **T** | **A** | **T** | **T** | **G** | **T** | **C** | **A** | **T** | **T** | **G** | **G** | **T** | **G** | **C** | **A** | **A** | **T** | **G** | **A** | **A** | **C** | **A** | **G** | **C** | **A** | **C** | **G** | **G** | **C** | **T** | **G** | **T** | **T** | **T** | **G** | **G** | **T** | **T** | **T** | **A** | **T** | **G** | **T** | **T** | **G** | **A** | **T** | **T** | **T** | **T** | **T** | **G** | **G** | **C** | **G** | **T** | **A** | **C** | **T** | **C** | **T** | **T** | **T** | **A** | **T** | **C** | **G** | **G** | **C** | **T** | **T** | **A** | **A** | **A** | **A** | **G** | **G** | **C** | **T** | **T** | **T** | **G** | **A** | **G** | **A** | **T** | **T** | **T** | **C** | **A** | **G** | **G** | **C** | **G** | **T** | **G** | **G** | **A** | **T** | **A** | **T** | **A** | **G** | **C** | **T** | **G** | **A** | **G** | **C** | **G** | **A** | **G** | **C** | **A** | **C** | **C** | **T** | **T** | **G** | **G** | **C** | **G** | **A** | **A** | **A** | **A** | **A** | **A** | **A** | **C** | **A** | **A** | **A** | **T** | **G** | **A** | **T** | **T** | **A** | **T** | **G** | **G** | **T** | **G** | **G** | **A** | **G** | **A** | **C** | **G** | **G** | **C** | **G** | **G** | **C** | **A** | **A** | **A** | **A** | **G** | **A** | **A** | **C** | **C** | **T** | **A** | **A** | **A** | **A** | **A** | **T** | **G** | **G** | **C** | **A** | **A** | **A** | **C** | **G** | **A** | **T** | **T** | **T** | **A** | **A** | **A** | **C** | **G** | **C** | **C** | **G** | **C** | **T** | **G** | **G** | **T** | **T** | **T** | **G** | **C** | **C** | **A** | **A** | **C** | **G** | **A** | **A** | **C** | **G** | **C** | **A** | **T** | **T** | **G** | **G** | **T** | **A** | **A** | **C** | **A** | **A** | **T** | **A** | **T** | **C** | **T** | **A** | **T** | **G** | **A** | **A** | **T** | **G** | **G** | **G** | **A** | **C** | **G** | **A** | **C** | **A** | **T** | **C** | **A** | **A** | **T** | **A** | **T** | **T** | **G** | **A** | **T** | **T** | **T** | **A** | **A** | **T** | **C** | **T** | **C** | **T** | **G** | **G** | **G** | **G** | **A** | **C** | **A** | **C** | **C** | **A** | **T** | **T** | **A** | **T** | **T** | **G** | **A** | **T** | **T** | **T** | **A** | **G** | **G** | **T** | **A** | **A** | **T** | **A** | **C** | **G** | **C** | **T** | **A** | **C** | **T** | **A** | **C** | **C** | **G** | **A** | **A** | **A** | **G** | **A** | **A** | **G** | **A** | **C** | **A** | **A** | **T** | **A** | **T** | **T** | **A** | **T** | **T** | **A** | **T** | **T** | **A** | **T** | **T** | **C** | **G** | **T** | **A** | **A** | **A** | **G** | **G** | **T** | **T** | **T** | **T** | **G** | **G** | **C** | **C** | **G** | **C** | **A** | **C** | **G** | **C** | **G** | **A** | **A** | **T** | **T** | **C** | **T** | **A** | **G** | **T** | **G** | **C** | **C** | **G** | **T** | **T** | **A** | **G** | **G** | **T** | **G** | **T** | **A** | **G** | **C** | **T** | **A** | **T** | **T** | **T** | **T** | **G** | **T** | **T** | **A** | **G** | **A** | **A** | **C** | **A** | **T** | **T** | **C** | **A** | **A** | **C** | **T** | **T** | **T** | **T** | **T** | **A** | **C** | **G** | **G** | **A** | **A** | **C** | **G** | **G** | **T** | **A** | **C** | **G** | **T** | **T** | **T** | **T** | **G** | **A** | **A** | **G** | **A** | **A** | **G** | **A** | **A** | **A** | **A** | **A** | **T** | **A** | **T** | **C** | **A** | **A** | **T** | **T** | **G** | **A** | **A** | **A** | **A** | **A** | **C** | **G** | **A** | **A** | **T** | **C** | **A** | **T** | **T** | **A** | **A** | **A** | **A** | **A** | **T** | **T** | **T** | **A** | **C** | **A** | **G** | **C** | **A** | **A** | **T** | **G** | **A** | **T** | **T** | **A** | **C** | **G** | **A** | **T** | **A** | **C** | **C** | **A** | **A** | **T** | **C** | **T** | **T** | **C** | **G** | **T** | **C** | **G** | **T** | **T** | **T** | **G** | **A** | **A** | **A** | **A** | **T** | **T** | **A** | **T** | **G** | **A** | **C** | **G** | **A** | **A** | **C** | **A** | **C** | **T** | **T** | **T** | **A** | **G** | **T** | **A** | **G** | **G** | **A** | **G** | **A** | **T** | **G** | **T** | **T** | **G** | **A** | **G** | **G** | **T** | **G** | **A** | **T** | **C** | **C** | **G** | **T** | **G** | **T** | **A** | **-** | **-** | **-** |
| **N59544F** | **A** | **T** | **G** | **A** | **A** | **T** | **A** | **A** | **C** | **C** | **C** | **T** | **T** | **G** | **G** | **C** | **G** | **C** | **T** | **T** | **T** | **T** | **T** | **T** | **A** | **T** | **C** | **G** | **T** | **C** | **G** | **C** | **A** | **G** | **A** | **A** | **G** | **C** | **A** | **T** | **T** | **A** | **C** | **T** | **T** | **T** | **T** | **T** | **A** | **T** | **T** | **C** | **T** | **G** | **G** | **C** | **G** | **T** | **T** | **A** | **T** | **G** | **G** | **C** | **A** | **A** | **A** | **T** | **T** | **G** | **T** | **A** | **C** | **A** | **T** | **A** | **A** | **T** | **C** | **C** | **T** | **G** | **G** | **A** | **T** | **T** | **A** | **G** | **C** | **T** | **G** | **T** | **T** | **T** | **T** | **A** | **T** | **T** | **A** | **A** | **C** | **A** | **A** | **T** | **T** | **G** | **G** | **C** | **G** | **T** | **T** | **T** | **T** | **A** | **C** | **T** | **T** | **G** | **T** | **G** | **G** | **C** | **C** | **T** | **A** | **C** | **G** | **T** | **T** | **T** | **C** | **C** | **A** | **G** | **G** | **A** | **A** | **A** | **G** | **C** | **A** | **T** | **C** | **T** | **A** | **A** | **A** | **A** | **C** | **A** | **C** | **A** | **T** | **T** | **T** | **T** | **A** | **A** | **C** | **A** | **A** | **C** | **T** | **T** | **T** | **C** | **A** | **A** | **T** | **T** | **C** | **G** | **T** | **C** | **C** | **T** | **C** | **G** | **G** | **C** | **G** | **T** | **T** | **G** | **T** | **T** | **T** | **T** | **T** | **A** | **T** | **T** | **G** | **T** | **C** | **A** | **T** | **T** | **G** | **G** | **T** | **G** | **C** | **A** | **A** | **T** | **G** | **A** | **A** | **C** | **A** | **G** | **C** | **A** | **C** | **G** | **G** | **C** | **T** | **G** | **T** | **T** | **T** | **G** | **G** | **T** | **T** | **T** | **A** | **T** | **G** | **T** | **T** | **G** | **A** | **T** | **T** | **T** | **T** | **T** | **G** | **G** | **C** | **G** | **T** | **A** | **C** | **T** | **C** | **T** | **T** | **T** | **A** | **T** | **C** | **G** | **G** | **C** | **T** | **T** | **A** | **A** | **A** | **A** | **G** | **G** | **C** | **T** | **T** | **T** | **G** | **A** | **G** | **A** | **T** | **T** | **T** | **C** | **A** | **G** | **G** | **C** | **G** | **T** | **G** | **G** | **A** | **T** | **A** | **T** | **A** | **G** | **C** | **T** | **G** | **A** | **G** | **C** | **G** | **A** | **G** | **C** | **A** | **C** | **C** | **T** | **T** | **G** | **G** | **C** | **G** | **A** | **A** | **A** | **A** | **A** | **A** | **A** | **C** | **A** | **A** | **A** | **T** | **G** | **A** | **T** | **T** | **A** | **T** | **G** | **G** | **T** | **G** | **G** | **A** | **G** | **A** | **C** | **G** | **G** | **C** | **G** | **G** | **C** | **A** | **A** | **A** | **A** | **G** | **A** | **A** | **C** | **C** | **T** | **A** | **A** | **A** | **A** | **A** | **T** | **G** | **G** | **C** | **A** | **A** | **A** | **C** | **G** | **A** | **T** | **T** | **T** | **A** | **A** | **A** | **C** | **G** | **C** | **C** | **G** | **C** | **T** | **G** | **G** | **T** | **T** | **T** | **G** | **C** | **C** | **A** | **A** | **C** | **G** | **A** | **A** | **C** | **G** | **C** | **A** | **T** | **T** | **G** | **G** | **T** | **A** | **A** | **C** | **A** | **A** | **T** | **A** | **T** | **C** | **T** | **A** | **T** | **G** | **A** | **A** | **T** | **G** | **G** | **G** | **A** | **C** | **G** | **A** | **T** | **A** | **T** | **C** | **A** | **A** | **T** | **A** | **T** | **T** | **G** | **A** | **T** | **T** | **T** | **A** | **A** | **T** | **C** | **T** | **C** | **T** | **G** | **G** | **G** | **G** | **A** | **C** | **A** | **C** | **C** | **A** | **T** | **T** | **A** | **T** | **T** | **G** | **A** | **T** | **T** | **T** | **A** | **G** | **G** | **T** | **A** | **A** | **T** | **A** | **C** | **G** | **T** | **T** | **A** | **C** | **T** | **A** | **C** | **C** | **G** | **A** | **A** | **A** | **G** | **A** | **A** | **G** | **A** | **C** | **A** | **A** | **T** | **A** | **T** | **T** | **A** | **T** | **T** | **A** | **T** | **T** | **A** | **T** | **T** | **C** | **G** | **T** | **A** | **A** | **A** | **G** | **G** | **T** | **T** | **T** | **T** | **G** | **G** | **C** | **C** | **G** | **C** | **A** | **C** | **G** | **C** | **G** | **A** | **A** | **T** | **T** | **C** | **T** | **A** | **G** | **T** | **G** | **C** | **C** | **G** | **T** | **T** | **A** | **G** | **G** | **T** | **G** | **T** | **A** | **G** | **C** | **T** | **A** | **T** | **T** | **T** | **T** | **G** | **T** | **T** | **A** | **G** | **A** | **A** | **C** | **A** | **T** | **T** | **C** | **A** | **A** | **C** | **T** | **T** | **T** | **T** | **T** | **A** | **C** | **G** | **G** | **A** | **A** | **C** | **G** | **G** | **T** | **A** | **C** | **G** | **T** | **T** | **T** | **T** | **G** | **A** | **A** | **G** | **A** | **A** | **G** | **A** | **A** | **A** | **A** | **A** | **T** | **A** | **T** | **C** | **A** | **A** | **T** | **T** | **G** | **A** | **A** | **A** | **A** | **A** | **C** | **G** | **A** | **A** | **T** | **C** | **A** | **T** | **T** | **A** | **A** | **A** | **A** | **A** | **T** | **T** | **T** | **A** | **C** | **A** | **G** | **C** | **A** | **A** | **T** | **G** | **A** | **T** | **T** | **A** | **T** | **G** | **A** | **T** | **A** | **C** | **C** | **A** | **A** | **T** | **C** | **T** | **T** | **C** | **G** | **T** | **C** | **G** | **T** | **T** | **T** | **G** | **A** | **A** | **A** | **A** | **T** | **T** | **A** | **T** | **G** | **A** | **C** | **G** | **A** | **A** | **C** | **A** | **C** | **T** | **T** | **T** | **A** | **G** | **T** | **A** | **G** | **G** | **A** | **G** | **A** | **T** | **G** | **T** | **T** | **G** | **A** | **G** | **G** | **T** | **G** | **A** | **T** | **C** | **C** | **G** | **T** | **G** | **T** | **A** | **-** | **-** | **-** |
| **N53434** | **A** | **T** | **G** | **A** | **A** | **T** | **A** | **A** | **C** | **C** | **C** | **T** | **T** | **G** | **G** | **C** | **G** | **C** | **T** | **T** | **T** | **T** | **T** | **T** | **A** | **T** | **C** | **G** | **T** | **C** | **G** | **C** | **A** | **G** | **A** | **A** | **G** | **C** | **A** | **T** | **T** | **A** | **C** | **T** | **T** | **T** | **T** | **T** | **A** | **T** | **T** | **C** | **T** | **G** | **G** | **C** | **G** | **T** | **T** | **A** | **T** | **G** | **G** | **C** | **A** | **A** | **A** | **T** | **T** | **G** | **T** | **A** | **C** | **A** | **T** | **A** | **A** | **T** | **C** | **C** | **T** | **G** | **G** | **A** | **T** | **T** | **A** | **G** | **C** | **T** | **G** | **T** | **T** | **T** | **T** | **A** | **T** | **T** | **A** | **A** | **C** | **A** | **A** | **T** | **T** | **G** | **G** | **C** | **G** | **T** | **T** | **T** | **T** | **A** | **C** | **T** | **T** | **G** | **T** | **G** | **G** | **C** | **C** | **T** | **A** | **C** | **G** | **T** | **T** | **T** | **C** | **C** | **A** | **G** | **G** | **A** | **A** | **A** | **G** | **C** | **A** | **T** | **C** | **T** | **A** | **A** | **A** | **A** | **C** | **A** | **C** | **A** | **T** | **T** | **T** | **T** | **A** | **A** | **C** | **A** | **A** | **C** | **T** | **T** | **T** | **C** | **A** | **A** | **T** | **T** | **C** | **G** | **T** | **C** | **C** | **T** | **C** | **G** | **G** | **C** | **G** | **T** | **T** | **G** | **T** | **T** | **T** | **T** | **T** | **A** | **T** | **T** | **G** | **T** | **C** | **A** | **T** | **T** | **G** | **G** | **T** | **G** | **C** | **A** | **A** | **T** | **G** | **A** | **A** | **C** | **A** | **G** | **C** | **A** | **C** | **G** | **G** | **C** | **T** | **G** | **T** | **T** | **T** | **G** | **G** | **T** | **T** | **T** | **A** | **T** | **G** | **T** | **T** | **G** | **A** | **T** | **T** | **T** | **T** | **T** | **G** | **G** | **C** | **G** | **T** | **A** | **C** | **T** | **C** | **T** | **T** | **T** | **A** | **T** | **C** | **G** | **G** | **C** | **T** | **T** | **A** | **A** | **A** | **A** | **G** | **G** | **C** | **T** | **T** | **T** | **G** | **A** | **G** | **A** | **T** | **T** | **T** | **C** | **A** | **G** | **G** | **C** | **G** | **T** | **G** | **G** | **A** | **T** | **A** | **T** | **A** | **G** | **C** | **T** | **G** | **A** | **G** | **C** | **G** | **A** | **G** | **C** | **A** | **C** | **C** | **T** | **T** | **G** | **G** | **C** | **G** | **A** | **A** | **A** | **A** | **A** | **A** | **A** | **C** | **A** | **A** | **A** | **T** | **G** | **A** | **T** | **T** | **A** | **T** | **G** | **G** | **T** | **G** | **G** | **A** | **G** | **A** | **C** | **G** | **G** | **C** | **G** | **G** | **C** | **A** | **A** | **A** | **A** | **G** | **A** | **A** | **C** | **C** | **T** | **A** | **A** | **A** | **A** | **A** | **T** | **G** | **G** | **C** | **A** | **A** | **A** | **C** | **G** | **A** | **T** | **T** | **T** | **A** | **A** | **A** | **C** | **G** | **C** | **C** | **G** | **C** | **T** | **G** | **G** | **T** | **T** | **T** | **G** | **C** | **C** | **A** | **A** | **C** | **G** | **A** | **A** | **C** | **G** | **C** | **A** | **T** | **T** | **G** | **G** | **T** | **A** | **A** | **C** | **A** | **A** | **T** | **A** | **T** | **C** | **T** | **A** | **T** | **G** | **A** | **A** | **T** | **G** | **G** | **G** | **A** | **C** | **G** | **A** | **T** | **A** | **T** | **C** | **A** | **A** | **T** | **A** | **T** | **T** | **G** | **A** | **T** | **T** | **T** | **A** | **A** | **T** | **C** | **T** | **C** | **T** | **G** | **G** | **G** | **G** | **A** | **C** | **A** | **C** | **C** | **A** | **T** | **T** | **A** | **T** | **T** | **G** | **A** | **T** | **T** | **T** | **A** | **G** | **G** | **T** | **A** | **A** | **T** | **A** | **C** | **G** | **T** | **T** | **A** | **C** | **T** | **A** | **C** | **C** | **G** | **A** | **A** | **A** | **G** | **A** | **A** | **G** | **A** | **C** | **A** | **A** | **T** | **A** | **T** | **T** | **A** | **T** | **T** | **A** | **T** | **T** | **A** | **T** | **T** | **C** | **G** | **T** | **A** | **A** | **A** | **G** | **G** | **T** | **T** | **T** | **T** | **G** | **G** | **C** | **C** | **G** | **C** | **A** | **C** | **G** | **C** | **G** | **A** | **A** | **T** | **T** | **C** | **T** | **A** | **G** | **T** | **G** | **C** | **C** | **G** | **T** | **T** | **A** | **G** | **G** | **T** | **G** | **T** | **A** | **G** | **C** | **T** | **A** | **T** | **T** | **T** | **T** | **G** | **T** | **T** | **A** | **G** | **A** | **A** | **C** | **A** | **T** | **T** | **C** | **A** | **A** | **C** | **T** | **T** | **T** | **T** | **T** | **A** | **C** | **G** | **G** | **A** | **A** | **C** | **G** | **G** | **T** | **A** | **C** | **G** | **T** | **T** | **T** | **T** | **G** | **A** | **A** | **G** | **A** | **A** | **G** | **A** | **A** | **A** | **A** | **A** | **T** | **A** | **T** | **C** | **A** | **A** | **T** | **T** | **G** | **A** | **A** | **A** | **A** | **A** | **C** | **G** | **A** | **A** | **T** | **C** | **A** | **T** | **T** | **A** | **A** | **A** | **A** | **A** | **T** | **T** | **T** | **A** | **C** | **A** | **G** | **C** | **A** | **A** | **T** | **G** | **A** | **T** | **T** | **A** | **T** | **G** | **A** | **T** | **A** | **C** | **C** | **A** | **A** | **T** | **C** | **T** | **T** | **C** | **G** | **T** | **C** | **G** | **T** | **T** | **T** | **G** | **A** | **A** | **A** | **A** | **T** | **T** | **A** | **T** | **G** | **A** | **C** | **G** | **A** | **A** | **C** | **A** | **C** | **T** | **T** | **T** | **A** | **G** | **T** | **A** | **G** | **G** | **A** | **G** | **A** | **T** | **G** | **T** | **T** | **G** | **A** | **G** | **G** | **T** | **G** | **A** | **T** | **C** | **C** | **G** | **T** | **G** | **T** | **A** | **-** | **-** | **-** |
| **N54118** | **A** | **T** | **G** | **A** | **A** | **T** | **A** | **A** | **C** | **C** | **C** | **T** | **T** | **G** | **G** | **C** | **G** | **C** | **T** | **T** | **T** | **T** | **T** | **T** | **A** | **T** | **C** | **G** | **T** | **C** | **G** | **C** | **A** | **G** | **A** | **A** | **G** | **C** | **A** | **T** | **T** | **A** | **C** | **T** | **T** | **T** | **T** | **T** | **A** | **T** | **T** | **C** | **T** | **G** | **G** | **C** | **G** | **T** | **T** | **A** | **T** | **G** | **G** | **C** | **A** | **A** | **A** | **T** | **T** | **G** | **T** | **A** | **C** | **A** | **T** | **A** | **A** | **T** | **C** | **C** | **T** | **G** | **G** | **A** | **T** | **T** | **A** | **G** | **C** | **T** | **G** | **T** | **T** | **T** | **T** | **A** | **T** | **T** | **A** | **A** | **C** | **A** | **A** | **T** | **T** | **G** | **G** | **C** | **G** | **T** | **T** | **T** | **T** | **A** | **C** | **T** | **T** | **G** | **T** | **G** | **G** | **C** | **C** | **T** | **A** | **C** | **G** | **T** | **T** | **T** | **C** | **C** | **A** | **G** | **G** | **A** | **A** | **A** | **G** | **C** | **A** | **T** | **C** | **T** | **A** | **A** | **A** | **A** | **C** | **A** | **C** | **A** | **T** | **T** | **T** | **T** | **A** | **A** | **C** | **A** | **A** | **C** | **T** | **T** | **T** | **C** | **A** | **A** | **T** | **T** | **C** | **G** | **T** | **C** | **C** | **T** | **C** | **G** | **G** | **C** | **G** | **T** | **T** | **G** | **T** | **T** | **T** | **T** | **T** | **A** | **T** | **T** | **G** | **T** | **C** | **A** | **T** | **T** | **G** | **G** | **T** | **G** | **C** | **A** | **A** | **T** | **G** | **A** | **A** | **C** | **A** | **G** | **C** | **A** | **C** | **G** | **G** | **C** | **T** | **G** | **T** | **T** | **T** | **G** | **G** | **T** | **T** | **T** | **A** | **T** | **G** | **T** | **T** | **G** | **A** | **T** | **T** | **T** | **T** | **T** | **G** | **G** | **C** | **G** | **T** | **A** | **C** | **T** | **C** | **T** | **T** | **T** | **A** | **T** | **C** | **G** | **G** | **C** | **T** | **T** | **A** | **A** | **A** | **A** | **G** | **G** | **C** | **T** | **T** | **T** | **G** | **A** | **G** | **A** | **T** | **T** | **T** | **C** | **A** | **G** | **G** | **C** | **G** | **T** | **G** | **G** | **A** | **T** | **A** | **T** | **A** | **G** | **C** | **T** | **G** | **A** | **G** | **C** | **G** | **A** | **G** | **C** | **A** | **C** | **C** | **T** | **T** | **G** | **G** | **C** | **G** | **A** | **A** | **A** | **A** | **A** | **A** | **A** | **C** | **A** | **A** | **A** | **T** | **G** | **A** | **T** | **T** | **A** | **T** | **G** | **G** | **T** | **G** | **G** | **A** | **G** | **A** | **C** | **G** | **G** | **C** | **G** | **G** | **C** | **A** | **A** | **A** | **A** | **G** | **A** | **A** | **C** | **C** | **T** | **A** | **A** | **A** | **A** | **A** | **T** | **G** | **G** | **C** | **A** | **A** | **A** | **C** | **G** | **A** | **T** | **T** | **T** | **A** | **A** | **A** | **C** | **G** | **C** | **C** | **G** | **C** | **T** | **G** | **G** | **T** | **T** | **T** | **G** | **C** | **C** | **A** | **A** | **C** | **G** | **A** | **A** | **C** | **G** | **C** | **A** | **T** | **T** | **G** | **G** | **T** | **A** | **A** | **C** | **A** | **A** | **T** | **A** | **T** | **C** | **T** | **A** | **T** | **G** | **A** | **A** | **T** | **G** | **G** | **G** | **A** | **C** | **G** | **A** | **T** | **A** | **T** | **C** | **A** | **A** | **T** | **A** | **T** | **T** | **G** | **A** | **T** | **T** | **T** | **A** | **A** | **T** | **C** | **T** | **C** | **T** | **G** | **G** | **G** | **G** | **A** | **C** | **A** | **C** | **C** | **A** | **T** | **T** | **A** | **T** | **T** | **G** | **A** | **T** | **T** | **T** | **A** | **G** | **G** | **T** | **A** | **A** | **T** | **A** | **C** | **G** | **C** | **T** | **A** | **C** | **T** | **A** | **C** | **C** | **G** | **A** | **A** | **A** | **G** | **A** | **A** | **G** | **A** | **C** | **A** | **A** | **T** | **A** | **T** | **T** | **A** | **T** | **T** | **A** | **T** | **T** | **A** | **T** | **T** | **C** | **G** | **T** | **A** | **A** | **A** | **G** | **G** | **T** | **T** | **T** | **T** | **G** | **G** | **C** | **C** | **G** | **C** | **A** | **C** | **G** | **C** | **G** | **A** | **A** | **T** | **T** | **C** | **T** | **A** | **G** | **T** | **G** | **C** | **C** | **G** | **T** | **T** | **A** | **G** | **G** | **T** | **G** | **T** | **A** | **G** | **C** | **T** | **A** | **T** | **T** | **T** | **T** | **G** | **T** | **T** | **A** | **G** | **A** | **A** | **C** | **A** | **T** | **T** | **C** | **A** | **A** | **C** | **T** | **T** | **T** | **T** | **T** | **A** | **C** | **G** | **G** | **A** | **A** | **C** | **G** | **G** | **T** | **A** | **C** | **G** | **T** | **T** | **T** | **T** | **G** | **A** | **A** | **G** | **A** | **A** | **G** | **A** | **A** | **A** | **A** | **A** | **T** | **A** | **T** | **C** | **A** | **A** | **T** | **T** | **G** | **A** | **A** | **A** | **A** | **A** | **C** | **G** | **A** | **A** | **T** | **C** | **A** | **T** | **T** | **A** | **A** | **A** | **A** | **A** | **T** | **T** | **T** | **A** | **C** | **A** | **G** | **C** | **A** | **A** | **T** | **G** | **A** | **T** | **T** | **A** | **C** | **G** | **A** | **T** | **A** | **C** | **C** | **A** | **A** | **T** | **C** | **T** | **T** | **C** | **G** | **T** | **C** | **G** | **T** | **T** | **T** | **G** | **A** | **A** | **A** | **A** | **T** | **T** | **A** | **T** | **G** | **A** | **C** | **G** | **A** | **A** | **C** | **A** | **C** | **T** | **T** | **T** | **A** | **G** | **T** | **A** | **G** | **G** | **A** | **G** | **A** | **T** | **G** | **T** | **T** | **G** | **A** | **G** | **G** | **T** | **G** | **A** | **T** | **C** | **C** | **G** | **T** | **G** | **T** | **A** | **-** | **-** | **-** |
| **N60027F** | **A** | **T** | **G** | **A** | **A** | **T** | **A** | **A** | **C** | **C** | **C** | **T** | **T** | **G** | **G** | **C** | **G** | **C** | **T** | **T** | **T** | **T** | **T** | **T** | **A** | **T** | **C** | **G** | **T** | **C** | **G** | **C** | **A** | **G** | **A** | **A** | **G** | **C** | **A** | **T** | **T** | **A** | **C** | **T** | **T** | **T** | **T** | **T** | **A** | **T** | **T** | **C** | **T** | **G** | **G** | **C** | **G** | **T** | **T** | **A** | **T** | **G** | **G** | **C** | **A** | **A** | **A** | **T** | **T** | **G** | **T** | **A** | **C** | **A** | **T** | **A** | **A** | **T** | **C** | **C** | **T** | **G** | **G** | **A** | **T** | **T** | **A** | **G** | **C** | **T** | **G** | **T** | **T** | **T** | **T** | **A** | **T** | **T** | **A** | **A** | **C** | **A** | **A** | **T** | **T** | **G** | **G** | **C** | **G** | **T** | **T** | **T** | **T** | **A** | **C** | **T** | **T** | **G** | **T** | **G** | **G** | **C** | **C** | **T** | **A** | **C** | **G** | **T** | **T** | **T** | **C** | **C** | **A** | **G** | **G** | **A** | **A** | **A** | **G** | **C** | **A** | **T** | **C** | **T** | **A** | **A** | **A** | **A** | **C** | **A** | **C** | **A** | **T** | **T** | **T** | **T** | **A** | **A** | **C** | **A** | **A** | **C** | **T** | **T** | **T** | **C** | **A** | **A** | **T** | **T** | **C** | **G** | **T** | **C** | **C** | **T** | **C** | **G** | **G** | **C** | **G** | **T** | **T** | **G** | **T** | **T** | **T** | **T** | **T** | **A** | **T** | **T** | **G** | **T** | **C** | **A** | **T** | **T** | **G** | **G** | **T** | **G** | **C** | **A** | **A** | **T** | **G** | **A** | **A** | **T** | **A** | **G** | **C** | **A** | **C** | **G** | **G** | **C** | **T** | **G** | **T** | **T** | **T** | **G** | **G** | **T** | **T** | **T** | **A** | **T** | **G** | **T** | **T** | **G** | **A** | **T** | **T** | **T** | **T** | **T** | **G** | **G** | **C** | **G** | **T** | **A** | **C** | **T** | **C** | **T** | **T** | **T** | **A** | **T** | **C** | **G** | **G** | **C** | **T** | **T** | **A** | **A** | **A** | **A** | **G** | **G** | **C** | **T** | **T** | **T** | **G** | **A** | **G** | **A** | **T** | **T** | **T** | **C** | **A** | **G** | **G** | **C** | **G** | **T** | **G** | **G** | **A** | **T** | **A** | **T** | **A** | **G** | **C** | **T** | **G** | **A** | **G** | **C** | **G** | **A** | **G** | **C** | **A** | **C** | **C** | **T** | **T** | **G** | **G** | **C** | **G** | **A** | **A** | **A** | **A** | **A** | **A** | **A** | **C** | **A** | **A** | **A** | **T** | **G** | **A** | **T** | **T** | **A** | **T** | **G** | **G** | **T** | **G** | **G** | **A** | **G** | **A** | **C** | **G** | **G** | **C** | **G** | **G** | **C** | **A** | **A** | **A** | **A** | **G** | **A** | **A** | **C** | **C** | **T** | **A** | **A** | **A** | **A** | **A** | **T** | **G** | **G** | **C** | **A** | **A** | **A** | **C** | **G** | **G** | **T** | **T** | **T** | **A** | **A** | **A** | **C** | **G** | **C** | **C** | **G** | **C** | **T** | **G** | **G** | **T** | **T** | **T** | **G** | **C** | **C** | **A** | **A** | **C** | **G** | **A** | **A** | **C** | **G** | **C** | **A** | **T** | **T** | **G** | **G** | **T** | **A** | **A** | **C** | **A** | **A** | **T** | **A** | **T** | **C** | **T** | **A** | **T** | **G** | **A** | **A** | **T** | **G** | **G** | **G** | **A** | **C** | **G** | **A** | **T** | **A** | **T** | **C** | **A** | **A** | **T** | **A** | **T** | **T** | **G** | **A** | **T** | **T** | **T** | **A** | **A** | **T** | **C** | **T** | **C** | **T** | **G** | **G** | **G** | **G** | **A** | **C** | **A** | **C** | **C** | **A** | **T** | **T** | **A** | **T** | **T** | **G** | **A** | **T** | **T** | **T** | **A** | **G** | **G** | **T** | **A** | **A** | **T** | **A** | **C** | **G** | **C** | **T** | **A** | **C** | **T** | **A** | **C** | **C** | **G** | **A** | **A** | **A** | **G** | **A** | **A** | **G** | **A** | **C** | **A** | **A** | **T** | **A** | **T** | **T** | **A** | **T** | **T** | **A** | **T** | **T** | **A** | **T** | **T** | **C** | **G** | **T** | **A** | **A** | **A** | **G** | **G** | **T** | **T** | **T** | **T** | **G** | **G** | **C** | **C** | **G** | **C** | **A** | **C** | **G** | **C** | **G** | **A** | **A** | **T** | **T** | **C** | **T** | **A** | **G** | **T** | **G** | **C** | **C** | **G** | **T** | **T** | **A** | **G** | **G** | **T** | **G** | **T** | **A** | **G** | **C** | **T** | **A** | **T** | **T** | **T** | **T** | **G** | **T** | **T** | **A** | **G** | **A** | **A** | **C** | **A** | **T** | **T** | **C** | **A** | **A** | **C** | **T** | **T** | **T** | **T** | **T** | **A** | **C** | **G** | **G** | **A** | **A** | **C** | **G** | **G** | **T** | **A** | **C** | **G** | **T** | **T** | **T** | **T** | **G** | **A** | **A** | **G** | **A** | **A** | **G** | **A** | **A** | **A** | **A** | **A** | **T** | **A** | **T** | **C** | **A** | **A** | **T** | **T** | **G** | **A** | **A** | **A** | **A** | **A** | **C** | **G** | **A** | **A** | **T** | **C** | **A** | **T** | **T** | **A** | **A** | **A** | **A** | **A** | **T** | **T** | **T** | **A** | **C** | **A** | **G** | **C** | **A** | **A** | **T** | **G** | **A** | **T** | **T** | **A** | **T** | **G** | **A** | **T** | **A** | **C** | **C** | **A** | **A** | **T** | **C** | **T** | **T** | **C** | **G** | **T** | **C** | **G** | **T** | **T** | **T** | **G** | **A** | **A** | **A** | **A** | **T** | **T** | **A** | **T** | **G** | **A** | **C** | **G** | **A** | **A** | **C** | **A** | **C** | **T** | **T** | **T** | **A** | **G** | **T** | **A** | **G** | **G** | **A** | **G** | **A** | **T** | **G** | **T** | **T** | **G** | **A** | **G** | **G** | **T** | **G** | **A** | **T** | **C** | **C** | **G** | **T** | **G** | **T** | **A** | **-** | **-** | **-** |
| **N53420** | **-** | **-** | **-** | **-** | **-** | **-** | **-** | **-** | **-** | **C** | **C** | **T** | **T** | **G** | **G** | **C** | **G** | **C** | **T** | **T** | **T** | **T** | **T** | **T** | **A** | **T** | **C** | **G** | **T** | **C** | **G** | **C** | **A** | **G** | **A** | **A** | **G** | **C** | **A** | **T** | **T** | **A** | **C** | **T** | **T** | **T** | **T** | **T** | **A** | **T** | **T** | **C** | **T** | **A** | **G** | **C** | **G** | **T** | **T** | **A** | **T** | **G** | **G** | **C** | **A** | **A** | **A** | **T** | **T** | **G** | **T** | **A** | **C** | **A** | **T** | **A** | **A** | **T** | **C** | **C** | **T** | **G** | **G** | **A** | **T** | **T** | **A** | **G** | **C** | **T** | **G** | **T** | **T** | **T** | **T** | **A** | **T** | **T** | **A** | **A** | **C** | **A** | **A** | **T** | **T** | **G** | **G** | **C** | **G** | **T** | **T** | **T** | **T** | **A** | **C** | **T** | **T** | **G** | **T** | **T** | **G** | **C** | **C** | **T** | **A** | **T** | **G** | **T** | **T** | **T** | **C** | **C** | **A** | **G** | **G** | **A** | **A** | **A** | **G** | **C** | **A** | **T** | **C** | **T** | **A** | **A** | **A** | **A** | **C** | **A** | **C** | **A** | **T** | **T** | **T** | **T** | **A** | **A** | **C** | **A** | **A** | **C** | **T** | **T** | **T** | **C** | **A** | **A** | **T** | **T** | **C** | **G** | **T** | **C** | **C** | **T** | **T** | **G** | **G** | **C** | **A** | **T** | **T** | **G** | **T** | **T** | **T** | **T** | **T** | **A** | **T** | **T** | **G** | **T** | **C** | **A** | **T** | **T** | **G** | **G** | **C** | **G** | **C** | **A** | **A** | **T** | **G** | **A** | **A** | **C** | **A** | **G** | **C** | **A** | **C** | **G** | **G** | **C** | **T** | **G** | **T** | **T** | **T** | **G** | **G** | **T** | **T** | **T** | **A** | **T** | **G** | **T** | **T** | **G** | **A** | **T** | **T** | **T** | **T** | **T** | **G** | **G** | **C** | **G** | **T** | **A** | **C** | **T** | **C** | **T** | **T** | **T** | **A** | **T** | **C** | **G** | **G** | **C** | **T** | **T** | **A** | **A** | **A** | **A** | **G** | **G** | **C** | **T** | **T** | **T** | **G** | **A** | **G** | **A** | **T** | **T** | **T** | **C** | **A** | **G** | **G** | **C** | **G** | **T** | **G** | **G** | **A** | **T** | **A** | **T** | **A** | **G** | **C** | **T** | **G** | **A** | **G** | **C** | **G** | **A** | **G** | **C** | **A** | **C** | **C** | **T** | **T** | **G** | **G** | **C** | **G** | **T** | **A** | **A** | **A** | **A** | **A** | **A** | **C** | **A** | **A** | **A** | **T** | **G** | **A** | **T** | **T** | **A** | **T** | **G** | **G** | **T** | **G** | **G** | **A** | **A** | **A** | **C** | **G** | **G** | **C** | **G** | **G** | **C** | **C** | **A** | **A** | **A** | **G** | **A** | **A** | **C** | **C** | **G** | **A** | **A** | **A** | **A** | **A** | **T** | **G** | **G** | **C** | **A** | **A** | **A** | **C** | **G** | **A** | **T** | **T** | **T** | **A** | **A** | **A** | **C** | **G** | **C** | **C** | **G** | **T** | **T** | **G** | **G** | **T** | **T** | **T** | **G** | **C** | **C** | **A** | **A** | **C** | **G** | **A** | **A** | **C** | **G** | **T** | **A** | **T** | **T** | **G** | **G** | **C** | **A** | **A** | **C** | **A** | **A** | **T** | **A** | **T** | **C** | **T** | **A** | **T** | **G** | **A** | **A** | **T** | **G** | **G** | **G** | **A** | **C** | **G** | **A** | **C** | **A** | **T** | **C** | **A** | **A** | **T** | **A** | **T** | **T** | **G** | **A** | **T** | **T** | **T** | **A** | **A** | **T** | **C** | **T** | **C** | **T** | **G** | **G** | **G** | **G** | **A** | **T** | **A** | **C** | **T** | **A** | **T** | **T** | **A** | **T** | **T** | **G** | **A** | **T** | **T** | **T** | **A** | **G** | **G** | **C** | **A** | **A** | **T** | **A** | **C** | **G** | **C** | **T** | **A** | **C** | **T** | **T** | **C** | **C** | **G** | **A** | **A** | **A** | **G** | **A** | **A** | **G** | **A** | **T** | **A** | **A** | **T** | **A** | **T** | **T** | **A** | **T** | **T** | **A** | **T** | **T** | **A** | **T** | **T** | **C** | **G** | **T** | **A** | **A** | **A** | **G** | **G** | **T** | **T** | **T** | **T** | **G** | **G** | **C** | **C** | **G** | **C** | **A** | **C** | **G** | **C** | **G** | **A** | **A** | **T** | **T** | **C** | **T** | **A** | **G** | **T** | **G** | **C** | **C** | **G** | **T** | **T** | **A** | **G** | **G** | **T** | **G** | **T** | **A** | **G** | **C** | **T** | **A** | **T** | **T** | **T** | **T** | **G** | **T** | **T** | **A** | **G** | **A** | **A** | **C** | **A** | **T** | **T** | **C** | **A** | **A** | **C** | **G** | **T** | **T** | **T** | **T** | **A** | **C** | **G** | **G** | **A** | **A** | **C** | **A** | **G** | **T** | **G** | **C** | **G** | **T** | **T** | **T** | **T** | **G** | **A** | **A** | **G** | **A** | **A** | **G** | **A** | **A** | **A** | **A** | **A** | **T** | **A** | **T** | **C** | **A** | **A** | **T** | **T** | **G** | **A** | **A** | **A** | **A** | **A** | **C** | **G** | **A** | **A** | **T** | **C** | **A** | **T** | **T** | **A** | **A** | **A** | **A** | **A** | **T** | **T** | **T** | **A** | **C** | **A** | **G** | **C** | **A** | **A** | **T** | **G** | **A** | **T** | **T** | **A** | **T** | **G** | **A** | **T** | **A** | **C** | **C** | **A** | **A** | **T** | **C** | **T** | **T** | **C** | **G** | **T** | **C** | **G** | **T** | **T** | **T** | **G** | **A** | **A** | **A** | **A** | **T** | **T** | **A** | **T** | **G** | **A** | **C** | **G** | **A** | **A** | **C** | **A** | **C** | **T** | **T** | **T** | **A** | **G** | **T** | **A** | **G** | **G** | **A** | **G** | **A** | **T** | **G** | **T** | **T** | **G** | **A** | **G** | **G** | **T** | **G** | **A** | **T** | **C** | **C** | **G** | **T** | **G** | **T** | **A** | **-** | **-** | **-** |
| **N60440F** | **A** | **T** | **G** | **A** | **A** | **T** | **A** | **A** | **C** | **C** | **C** | **T** | **T** | **G** | **G** | **C** | **G** | **C** | **T** | **T** | **T** | **T** | **T** | **T** | **A** | **T** | **C** | **G** | **T** | **C** | **G** | **C** | **A** | **G** | **A** | **A** | **G** | **C** | **A** | **T** | **T** | **A** | **C** | **T** | **T** | **T** | **T** | **T** | **A** | **T** | **T** | **C** | **T** | **G** | **G** | **C** | **G** | **T** | **T** | **A** | **T** | **G** | **G** | **C** | **A** | **A** | **A** | **T** | **T** | **G** | **T** | **A** | **C** | **A** | **T** | **A** | **A** | **T** | **C** | **C** | **T** | **G** | **G** | **A** | **T** | **T** | **A** | **G** | **C** | **T** | **G** | **T** | **T** | **T** | **T** | **A** | **T** | **T** | **A** | **A** | **C** | **A** | **A** | **T** | **T** | **G** | **G** | **C** | **G** | **T** | **T** | **T** | **T** | **A** | **C** | **T** | **T** | **G** | **T** | **G** | **G** | **C** | **C** | **T** | **A** | **C** | **G** | **T** | **T** | **T** | **C** | **C** | **A** | **G** | **G** | **A** | **A** | **A** | **G** | **C** | **A** | **T** | **C** | **T** | **A** | **A** | **A** | **A** | **C** | **A** | **C** | **A** | **T** | **T** | **T** | **T** | **A** | **A** | **C** | **A** | **A** | **C** | **T** | **T** | **T** | **C** | **A** | **A** | **T** | **T** | **C** | **G** | **T** | **C** | **C** | **T** | **C** | **G** | **G** | **C** | **G** | **T** | **T** | **G** | **T** | **T** | **T** | **T** | **T** | **A** | **T** | **T** | **G** | **T** | **C** | **A** | **T** | **T** | **G** | **G** | **T** | **G** | **C** | **A** | **A** | **T** | **G** | **A** | **A** | **C** | **A** | **G** | **T** | **A** | **C** | **G** | **G** | **C** | **T** | **G** | **T** | **T** | **T** | **G** | **G** | **T** | **T** | **T** | **A** | **T** | **G** | **T** | **T** | **G** | **A** | **T** | **T** | **T** | **T** | **T** | **G** | **G** | **C** | **G** | **T** | **A** | **C** | **T** | **C** | **T** | **T** | **T** | **A** | **T** | **C** | **G** | **G** | **C** | **T** | **T** | **A** | **A** | **A** | **A** | **G** | **G** | **C** | **T** | **T** | **T** | **G** | **A** | **G** | **A** | **T** | **T** | **T** | **C** | **A** | **G** | **G** | **C** | **G** | **T** | **G** | **G** | **A** | **T** | **A** | **T** | **A** | **G** | **C** | **T** | **G** | **A** | **G** | **C** | **G** | **A** | **G** | **C** | **A** | **C** | **C** | **T** | **T** | **G** | **G** | **C** | **G** | **T** | **A** | **A** | **A** | **A** | **A** | **A** | **C** | **A** | **A** | **A** | **T** | **G** | **A** | **T** | **T** | **A** | **T** | **G** | **G** | **T** | **G** | **G** | **A** | **G** | **A** | **C** | **G** | **G** | **C** | **G** | **G** | **C** | **A** | **A** | **A** | **A** | **G** | **A** | **A** | **C** | **C** | **T** | **A** | **A** | **A** | **A** | **A** | **T** | **G** | **G** | **C** | **A** | **A** | **A** | **C** | **G** | **A** | **T** | **T** | **T** | **A** | **A** | **A** | **C** | **G** | **C** | **C** | **G** | **C** | **T** | **G** | **G** | **T** | **T** | **T** | **G** | **C** | **C** | **A** | **A** | **T** | **G** | **A** | **A** | **C** | **G** | **C** | **A** | **T** | **T** | **G** | **G** | **T** | **A** | **A** | **C** | **A** | **A** | **T** | **A** | **T** | **C** | **T** | **A** | **T** | **G** | **A** | **A** | **T** | **G** | **G** | **G** | **A** | **T** | **G** | **A** | **T** | **A** | **T** | **C** | **A** | **A** | **T** | **A** | **T** | **T** | **G** | **A** | **T** | **T** | **T** | **A** | **A** | **T** | **C** | **T** | **C** | **T** | **G** | **G** | **G** | **G** | **A** | **C** | **A** | **C** | **C** | **A** | **T** | **T** | **A** | **T** | **T** | **G** | **A** | **T** | **T** | **T** | **A** | **G** | **G** | **T** | **A** | **A** | **T** | **A** | **C** | **G** | **C** | **T** | **A** | **C** | **T** | **A** | **C** | **C** | **G** | **A** | **A** | **A** | **G** | **A** | **A** | **G** | **A** | **C** | **A** | **A** | **T** | **A** | **T** | **T** | **A** | **T** | **T** | **A** | **T** | **T** | **A** | **T** | **T** | **C** | **G** | **T** | **A** | **A** | **A** | **G** | **G** | **T** | **T** | **T** | **T** | **G** | **G** | **C** | **C** | **G** | **C** | **A** | **C** | **G** | **C** | **G** | **A** | **A** | **T** | **T** | **C** | **T** | **A** | **G** | **T** | **A** | **C** | **C** | **G** | **T** | **T** | **A** | **G** | **G** | **T** | **G** | **T** | **A** | **G** | **C** | **T** | **A** | **T** | **T** | **T** | **T** | **G** | **T** | **T** | **A** | **G** | **A** | **A** | **C** | **A** | **T** | **T** | **C** | **A** | **A** | **C** | **T** | **T** | **T** | **T** | **T** | **A** | **C** | **G** | **G** | **A** | **A** | **T** | **G** | **G** | **T** | **A** | **C** | **G** | **T** | **T** | **T** | **T** | **G** | **A** | **A** | **G** | **A** | **A** | **G** | **A** | **A** | **A** | **A** | **A** | **T** | **A** | **T** | **C** | **A** | **A** | **T** | **T** | **G** | **A** | **A** | **A** | **A** | **A** | **C** | **G** | **A** | **A** | **T** | **C** | **A** | **T** | **T** | **A** | **A** | **A** | **A** | **A** | **T** | **T** | **T** | **A** | **C** | **A** | **G** | **C** | **A** | **A** | **T** | **G** | **A** | **T** | **T** | **A** | **C** | **G** | **A** | **T** | **A** | **C** | **C** | **A** | **A** | **T** | **C** | **T** | **T** | **C** | **G** | **T** | **C** | **G** | **T** | **T** | **T** | **G** | **A** | **A** | **A** | **A** | **T** | **T** | **A** | **T** | **G** | **A** | **C** | **G** | **A** | **A** | **C** | **A** | **C** | **T** | **T** | **T** | **A** | **G** | **T** | **A** | **G** | **G** | **A** | **G** | **A** | **T** | **G** | **T** | **T** | **G** | **A** | **G** | **G** | **T** | **G** | **A** | **T** | **C** | **C** | **G** | **T** | **G** | **T** | **A** | **-** | **-** | **-** |
| **N53661** | **A** | **T** | **G** | **A** | **A** | **T** | **A** | **A** | **C** | **C** | **C** | **T** | **T** | **G** | **G** | **C** | **G** | **C** | **T** | **T** | **T** | **T** | **T** | **T** | **A** | **T** | **C** | **G** | **T** | **C** | **G** | **C** | **A** | **G** | **A** | **A** | **G** | **C** | **A** | **T** | **T** | **A** | **C** | **T** | **T** | **T** | **T** | **T** | **A** | **T** | **T** | **C** | **T** | **G** | **G** | **C** | **G** | **T** | **T** | **A** | **T** | **G** | **G** | **C** | **A** | **A** | **A** | **T** | **T** | **G** | **T** | **A** | **C** | **A** | **T** | **A** | **A** | **T** | **C** | **C** | **T** | **G** | **G** | **A** | **T** | **T** | **A** | **G** | **C** | **T** | **G** | **T** | **T** | **T** | **T** | **A** | **T** | **T** | **A** | **A** | **C** | **A** | **A** | **T** | **T** | **G** | **G** | **C** | **G** | **T** | **T** | **T** | **T** | **A** | **C** | **T** | **T** | **G** | **T** | **G** | **G** | **C** | **C** | **T** | **A** | **T** | **G** | **T** | **T** | **T** | **C** | **C** | **A** | **G** | **G** | **A** | **A** | **A** | **G** | **C** | **A** | **T** | **C** | **T** | **A** | **A** | **A** | **A** | **C** | **A** | **C** | **A** | **T** | **T** | **T** | **T** | **A** | **A** | **C** | **A** | **A** | **C** | **T** | **T** | **T** | **C** | **A** | **A** | **T** | **T** | **C** | **G** | **T** | **C** | **C** | **T** | **C** | **G** | **G** | **C** | **G** | **T** | **T** | **G** | **T** | **T** | **T** | **T** | **T** | **A** | **T** | **T** | **G** | **T** | **C** | **A** | **T** | **T** | **G** | **G** | **T** | **G** | **C** | **A** | **A** | **T** | **G** | **A** | **A** | **C** | **A** | **G** | **C** | **A** | **C** | **G** | **G** | **C** | **T** | **G** | **T** | **T** | **T** | **G** | **G** | **T** | **T** | **T** | **A** | **T** | **G** | **C** | **T** | **G** | **A** | **T** | **T** | **T** | **T** | **T** | **G** | **G** | **C** | **G** | **T** | **A** | **C** | **T** | **C** | **T** | **T** | **T** | **A** | **T** | **C** | **G** | **G** | **C** | **T** | **T** | **A** | **A** | **A** | **A** | **G** | **G** | **C** | **T** | **T** | **T** | **G** | **A** | **G** | **A** | **T** | **T** | **T** | **C** | **A** | **G** | **G** | **C** | **G** | **T** | **G** | **G** | **A** | **T** | **A** | **T** | **A** | **G** | **C** | **T** | **G** | **A** | **G** | **C** | **G** | **A** | **G** | **C** | **A** | **C** | **C** | **T** | **T** | **G** | **G** | **C** | **G** | **T** | **A** | **A** | **A** | **A** | **A** | **A** | **C** | **A** | **A** | **A** | **T** | **G** | **A** | **T** | **T** | **A** | **T** | **G** | **G** | **T** | **G** | **G** | **A** | **G** | **A** | **C** | **G** | **G** | **C** | **G** | **G** | **C** | **A** | **A** | **A** | **A** | **G** | **A** | **A** | **C** | **C** | **T** | **A** | **A** | **A** | **A** | **A** | **T** | **G** | **G** | **C** | **A** | **A** | **A** | **C** | **G** | **A** | **T** | **T** | **T** | **A** | **A** | **A** | **C** | **G** | **C** | **C** | **G** | **C** | **T** | **G** | **G** | **T** | **T** | **T** | **G** | **C** | **C** | **A** | **A** | **C** | **G** | **A** | **A** | **C** | **G** | **C** | **A** | **T** | **T** | **G** | **G** | **T** | **A** | **A** | **C** | **A** | **A** | **T** | **A** | **T** | **C** | **T** | **A** | **T** | **G** | **A** | **A** | **T** | **G** | **G** | **G** | **A** | **C** | **G** | **A** | **T** | **A** | **T** | **C** | **A** | **A** | **T** | **A** | **T** | **T** | **G** | **A** | **T** | **T** | **T** | **A** | **A** | **T** | **C** | **T** | **C** | **T** | **G** | **G** | **G** | **G** | **A** | **C** | **A** | **C** | **C** | **A** | **T** | **T** | **A** | **T** | **T** | **G** | **A** | **T** | **T** | **T** | **A** | **G** | **G** | **T** | **A** | **A** | **T** | **A** | **C** | **G** | **C** | **T** | **A** | **C** | **T** | **A** | **C** | **C** | **G** | **A** | **A** | **A** | **G** | **A** | **A** | **G** | **A** | **C** | **A** | **A** | **T** | **A** | **T** | **T** | **A** | **T** | **T** | **A** | **T** | **T** | **A** | **T** | **T** | **C** | **G** | **T** | **A** | **A** | **A** | **G** | **G** | **T** | **T** | **T** | **T** | **G** | **G** | **C** | **C** | **G** | **C** | **A** | **C** | **G** | **C** | **G** | **A** | **A** | **T** | **T** | **C** | **T** | **A** | **G** | **T** | **G** | **C** | **C** | **G** | **T** | **T** | **A** | **G** | **G** | **T** | **G** | **T** | **A** | **G** | **C** | **T** | **A** | **T** | **T** | **T** | **T** | **G** | **T** | **T** | **A** | **G** | **A** | **A** | **C** | **A** | **T** | **T** | **C** | **A** | **A** | **C** | **T** | **T** | **T** | **T** | **T** | **A** | **C** | **G** | **G** | **A** | **A** | **C** | **G** | **G** | **T** | **A** | **C** | **G** | **T** | **T** | **T** | **T** | **G** | **A** | **A** | **G** | **A** | **A** | **G** | **A** | **A** | **A** | **A** | **A** | **T** | **A** | **T** | **C** | **A** | **A** | **T** | **T** | **G** | **A** | **A** | **A** | **A** | **A** | **C** | **G** | **A** | **A** | **T** | **C** | **A** | **T** | **T** | **A** | **A** | **A** | **A** | **A** | **T** | **T** | **T** | **A** | **C** | **A** | **G** | **C** | **A** | **A** | **T** | **G** | **A** | **T** | **T** | **A** | **T** | **G** | **A** | **T** | **A** | **C** | **C** | **A** | **A** | **T** | **C** | **T** | **T** | **C** | **G** | **T** | **C** | **G** | **T** | **T** | **T** | **G** | **A** | **A** | **A** | **A** | **T** | **T** | **A** | **T** | **G** | **A** | **C** | **G** | **A** | **A** | **T** | **A** | **C** | **T** | **T** | **T** | **A** | **G** | **T** | **A** | **G** | **G** | **A** | **G** | **A** | **T** | **G** | **T** | **T** | **G** | **A** | **G** | **G** | **T** | **G** | **A** | **T** | **C** | **C** | **G** | **T** | **G** | **T** | **A** | **-** | **-** | **-** |
| **N59963F** | **A** | **T** | **G** | **A** | **A** | **T** | **A** | **A** | **C** | **C** | **C** | **T** | **T** | **G** | **G** | **C** | **G** | **C** | **T** | **T** | **T** | **T** | **T** | **T** | **A** | **T** | **C** | **G** | **T** | **C** | **G** | **C** | **A** | **G** | **A** | **A** | **G** | **C** | **A** | **T** | **T** | **A** | **C** | **T** | **T** | **T** | **T** | **T** | **A** | **T** | **T** | **C** | **T** | **G** | **G** | **C** | **G** | **T** | **T** | **A** | **T** | **G** | **G** | **C** | **A** | **A** | **A** | **T** | **T** | **G** | **T** | **A** | **C** | **A** | **T** | **A** | **A** | **T** | **C** | **C** | **T** | **G** | **G** | **A** | **T** | **T** | **A** | **G** | **C** | **T** | **G** | **T** | **T** | **T** | **T** | **A** | **T** | **T** | **A** | **A** | **C** | **A** | **A** | **T** | **T** | **G** | **G** | **C** | **G** | **T** | **T** | **T** | **T** | **A** | **C** | **T** | **T** | **G** | **T** | **G** | **G** | **C** | **C** | **T** | **A** | **C** | **G** | **T** | **T** | **T** | **C** | **C** | **A** | **G** | **G** | **A** | **A** | **A** | **G** | **C** | **A** | **T** | **C** | **T** | **A** | **A** | **A** | **A** | **C** | **A** | **C** | **A** | **T** | **T** | **T** | **T** | **A** | **A** | **C** | **A** | **A** | **C** | **T** | **T** | **T** | **C** | **A** | **A** | **T** | **T** | **C** | **G** | **T** | **C** | **C** | **T** | **C** | **G** | **G** | **C** | **G** | **T** | **T** | **G** | **T** | **T** | **T** | **T** | **T** | **A** | **T** | **T** | **G** | **T** | **C** | **A** | **T** | **T** | **G** | **G** | **T** | **G** | **C** | **A** | **A** | **T** | **G** | **A** | **A** | **C** | **A** | **G** | **C** | **A** | **C** | **G** | **G** | **C** | **T** | **G** | **T** | **T** | **T** | **G** | **G** | **T** | **T** | **T** | **A** | **T** | **G** | **T** | **T** | **G** | **A** | **T** | **T** | **T** | **T** | **T** | **G** | **G** | **C** | **G** | **T** | **A** | **C** | **T** | **C** | **T** | **T** | **T** | **A** | **T** | **C** | **G** | **G** | **C** | **T** | **T** | **A** | **A** | **A** | **A** | **G** | **G** | **C** | **T** | **T** | **T** | **G** | **A** | **G** | **A** | **T** | **T** | **T** | **C** | **A** | **G** | **G** | **C** | **G** | **T** | **G** | **G** | **A** | **T** | **A** | **T** | **A** | **G** | **C** | **T** | **G** | **A** | **G** | **C** | **G** | **A** | **G** | **C** | **A** | **C** | **C** | **T** | **T** | **G** | **G** | **C** | **G** | **A** | **A** | **A** | **A** | **A** | **A** | **A** | **C** | **A** | **A** | **A** | **T** | **G** | **A** | **T** | **T** | **A** | **T** | **G** | **G** | **T** | **G** | **G** | **A** | **G** | **A** | **C** | **G** | **G** | **C** | **G** | **G** | **C** | **A** | **A** | **A** | **A** | **G** | **A** | **A** | **C** | **C** | **T** | **A** | **A** | **A** | **A** | **A** | **T** | **G** | **G** | **C** | **A** | **A** | **A** | **C** | **G** | **A** | **T** | **T** | **T** | **A** | **A** | **A** | **C** | **G** | **C** | **C** | **G** | **C** | **T** | **G** | **G** | **T** | **T** | **T** | **G** | **C** | **C** | **A** | **A** | **C** | **G** | **A** | **A** | **C** | **G** | **C** | **A** | **T** | **T** | **G** | **G** | **T** | **A** | **A** | **C** | **A** | **A** | **T** | **A** | **T** | **C** | **T** | **A** | **T** | **G** | **A** | **A** | **T** | **G** | **G** | **G** | **A** | **C** | **G** | **A** | **T** | **A** | **T** | **C** | **A** | **A** | **T** | **A** | **T** | **T** | **G** | **A** | **T** | **T** | **T** | **A** | **A** | **T** | **C** | **T** | **C** | **T** | **G** | **G** | **G** | **G** | **A** | **C** | **A** | **C** | **C** | **A** | **T** | **T** | **A** | **T** | **T** | **G** | **A** | **T** | **T** | **T** | **A** | **G** | **G** | **T** | **A** | **A** | **T** | **A** | **C** | **G** | **C** | **T** | **A** | **C** | **T** | **A** | **C** | **C** | **G** | **A** | **A** | **A** | **G** | **A** | **A** | **G** | **A** | **C** | **A** | **A** | **T** | **A** | **T** | **T** | **A** | **T** | **T** | **A** | **T** | **T** | **A** | **T** | **T** | **C** | **G** | **T** | **A** | **A** | **A** | **G** | **G** | **T** | **T** | **T** | **T** | **G** | **G** | **C** | **C** | **G** | **C** | **A** | **C** | **G** | **C** | **G** | **A** | **A** | **T** | **T** | **C** | **T** | **A** | **G** | **T** | **G** | **C** | **C** | **G** | **T** | **T** | **A** | **G** | **G** | **T** | **G** | **T** | **A** | **G** | **C** | **T** | **A** | **T** | **T** | **T** | **T** | **G** | **T** | **T** | **A** | **G** | **A** | **A** | **C** | **A** | **T** | **T** | **C** | **A** | **A** | **C** | **T** | **T** | **T** | **T** | **T** | **A** | **C** | **G** | **G** | **A** | **A** | **C** | **G** | **G** | **T** | **A** | **C** | **G** | **T** | **T** | **T** | **T** | **G** | **A** | **A** | **G** | **A** | **A** | **G** | **A** | **A** | **A** | **A** | **A** | **T** | **A** | **T** | **C** | **A** | **A** | **T** | **T** | **G** | **A** | **A** | **A** | **A** | **A** | **C** | **G** | **A** | **A** | **T** | **C** | **A** | **T** | **T** | **A** | **A** | **A** | **A** | **A** | **T** | **T** | **T** | **A** | **C** | **A** | **G** | **C** | **A** | **A** | **T** | **G** | **A** | **T** | **T** | **A** | **C** | **G** | **A** | **T** | **A** | **C** | **C** | **A** | **A** | **T** | **C** | **T** | **T** | **C** | **G** | **T** | **C** | **G** | **T** | **T** | **T** | **G** | **A** | **A** | **A** | **A** | **T** | **T** | **A** | **T** | **G** | **A** | **C** | **G** | **A** | **A** | **C** | **A** | **C** | **T** | **T** | **T** | **A** | **G** | **T** | **A** | **G** | **G** | **A** | **G** | **A** | **T** | **G** | **T** | **T** | **G** | **A** | **G** | **G** | **T** | **G** | **A** | **T** | **C** | **C** | **G** | **T** | **G** | **T** | **A** | **-** | **-** | **-** |
| **N60114F** | **A** | **T** | **G** | **A** | **A** | **T** | **A** | **A** | **C** | **C** | **C** | **T** | **T** | **G** | **G** | **C** | **G** | **C** | **T** | **T** | **T** | **T** | **T** | **T** | **A** | **T** | **C** | **G** | **T** | **C** | **G** | **C** | **A** | **G** | **A** | **A** | **G** | **C** | **A** | **T** | **T** | **A** | **C** | **T** | **T** | **T** | **T** | **T** | **A** | **T** | **T** | **C** | **T** | **G** | **G** | **C** | **G** | **T** | **T** | **A** | **T** | **G** | **G** | **C** | **A** | **A** | **A** | **T** | **T** | **G** | **T** | **A** | **C** | **A** | **T** | **A** | **A** | **T** | **C** | **C** | **T** | **G** | **G** | **A** | **T** | **T** | **A** | **G** | **C** | **T** | **G** | **T** | **T** | **T** | **T** | **A** | **T** | **T** | **A** | **A** | **C** | **A** | **A** | **T** | **T** | **G** | **G** | **C** | **G** | **T** | **T** | **T** | **T** | **A** | **C** | **T** | **T** | **G** | **T** | **G** | **G** | **C** | **C** | **T** | **A** | **C** | **G** | **T** | **T** | **T** | **C** | **C** | **A** | **G** | **G** | **A** | **A** | **A** | **G** | **C** | **A** | **T** | **C** | **T** | **A** | **A** | **A** | **A** | **C** | **A** | **C** | **A** | **T** | **T** | **T** | **T** | **A** | **A** | **C** | **A** | **A** | **C** | **T** | **T** | **T** | **C** | **A** | **A** | **T** | **T** | **C** | **G** | **T** | **C** | **C** | **T** | **C** | **G** | **G** | **C** | **G** | **T** | **T** | **G** | **T** | **T** | **T** | **T** | **T** | **A** | **T** | **T** | **G** | **T** | **C** | **A** | **T** | **T** | **G** | **G** | **T** | **G** | **C** | **A** | **A** | **T** | **G** | **A** | **A** | **C** | **A** | **G** | **C** | **A** | **C** | **G** | **G** | **C** | **T** | **G** | **T** | **T** | **T** | **G** | **G** | **T** | **T** | **T** | **A** | **T** | **G** | **T** | **T** | **G** | **A** | **T** | **T** | **T** | **T** | **T** | **G** | **G** | **C** | **G** | **T** | **A** | **C** | **T** | **C** | **T** | **T** | **T** | **A** | **T** | **C** | **G** | **G** | **C** | **T** | **T** | **A** | **A** | **A** | **A** | **G** | **G** | **C** | **T** | **T** | **T** | **G** | **A** | **G** | **A** | **T** | **T** | **T** | **C** | **A** | **G** | **G** | **C** | **G** | **T** | **G** | **G** | **A** | **T** | **A** | **T** | **A** | **G** | **C** | **T** | **G** | **A** | **G** | **C** | **G** | **A** | **G** | **C** | **A** | **C** | **C** | **T** | **T** | **G** | **G** | **C** | **G** | **A** | **A** | **A** | **A** | **A** | **A** | **A** | **C** | **A** | **A** | **A** | **T** | **G** | **A** | **T** | **T** | **A** | **T** | **G** | **G** | **T** | **G** | **G** | **A** | **G** | **A** | **C** | **G** | **G** | **C** | **G** | **G** | **C** | **A** | **A** | **A** | **A** | **G** | **A** | **A** | **C** | **C** | **T** | **A** | **A** | **A** | **A** | **A** | **T** | **G** | **G** | **C** | **A** | **A** | **A** | **C** | **G** | **A** | **T** | **T** | **T** | **A** | **A** | **A** | **C** | **G** | **C** | **C** | **G** | **C** | **T** | **G** | **G** | **T** | **T** | **T** | **G** | **C** | **C** | **A** | **A** | **C** | **G** | **A** | **A** | **C** | **G** | **C** | **A** | **T** | **T** | **G** | **G** | **T** | **A** | **A** | **C** | **A** | **A** | **T** | **A** | **T** | **C** | **T** | **A** | **T** | **G** | **A** | **A** | **T** | **G** | **G** | **G** | **A** | **C** | **G** | **A** | **C** | **A** | **T** | **C** | **A** | **A** | **T** | **A** | **T** | **T** | **G** | **A** | **T** | **T** | **T** | **A** | **A** | **T** | **C** | **T** | **C** | **T** | **G** | **G** | **G** | **G** | **A** | **C** | **A** | **C** | **C** | **A** | **T** | **T** | **A** | **T** | **T** | **G** | **A** | **T** | **T** | **T** | **A** | **G** | **G** | **T** | **A** | **A** | **T** | **A** | **C** | **G** | **C** | **T** | **A** | **C** | **T** | **A** | **C** | **C** | **G** | **A** | **A** | **A** | **G** | **A** | **A** | **G** | **A** | **C** | **A** | **A** | **T** | **A** | **T** | **T** | **A** | **T** | **T** | **A** | **T** | **T** | **A** | **T** | **T** | **C** | **G** | **T** | **A** | **A** | **A** | **G** | **G** | **T** | **T** | **T** | **T** | **G** | **G** | **C** | **C** | **G** | **C** | **A** | **C** | **G** | **C** | **G** | **A** | **A** | **T** | **T** | **C** | **T** | **A** | **G** | **T** | **G** | **C** | **C** | **G** | **T** | **T** | **A** | **G** | **G** | **T** | **G** | **T** | **A** | **G** | **C** | **T** | **A** | **T** | **T** | **T** | **T** | **G** | **T** | **T** | **A** | **G** | **A** | **A** | **C** | **A** | **T** | **T** | **C** | **A** | **A** | **C** | **T** | **T** | **T** | **T** | **T** | **A** | **C** | **G** | **G** | **A** | **A** | **C** | **G** | **G** | **T** | **A** | **C** | **G** | **T** | **T** | **T** | **T** | **G** | **A** | **A** | **G** | **A** | **A** | **G** | **A** | **A** | **A** | **A** | **A** | **T** | **A** | **T** | **C** | **A** | **A** | **T** | **T** | **G** | **A** | **A** | **A** | **A** | **A** | **C** | **G** | **A** | **A** | **T** | **C** | **A** | **T** | **T** | **A** | **A** | **A** | **A** | **A** | **T** | **T** | **T** | **A** | **C** | **A** | **G** | **C** | **A** | **A** | **T** | **G** | **A** | **T** | **T** | **A** | **C** | **G** | **A** | **T** | **A** | **C** | **C** | **A** | **A** | **T** | **C** | **T** | **T** | **C** | **G** | **T** | **C** | **G** | **T** | **T** | **T** | **G** | **A** | **A** | **A** | **A** | **T** | **T** | **A** | **T** | **G** | **A** | **C** | **G** | **A** | **A** | **C** | **A** | **C** | **T** | **T** | **T** | **A** | **G** | **T** | **A** | **G** | **G** | **A** | **G** | **A** | **T** | **G** | **T** | **T** | **G** | **A** | **G** | **G** | **T** | **G** | **A** | **T** | **C** | **C** | **G** | **T** | **G** | **T** | **A** | **-** | **-** | **-** |
| **N55265** | **-** | **-** | **-** | **-** | **-** | **-** | **-** | **-** | **-** | **C** | **C** | **T** | **T** | **G** | **G** | **C** | **G** | **C** | **T** | **T** | **T** | **T** | **T** | **T** | **A** | **T** | **C** | **G** | **T** | **C** | **G** | **C** | **A** | **G** | **A** | **A** | **G** | **C** | **A** | **T** | **T** | **A** | **C** | **T** | **T** | **T** | **T** | **T** | **A** | **T** | **T** | **C** | **T** | **A** | **G** | **C** | **G** | **T** | **T** | **A** | **T** | **G** | **G** | **C** | **A** | **A** | **A** | **T** | **T** | **G** | **T** | **A** | **C** | **A** | **T** | **A** | **A** | **T** | **C** | **C** | **T** | **G** | **G** | **A** | **T** | **T** | **A** | **G** | **C** | **T** | **G** | **T** | **T** | **T** | **T** | **A** | **T** | **T** | **A** | **A** | **C** | **A** | **A** | **T** | **T** | **G** | **G** | **C** | **G** | **T** | **T** | **T** | **T** | **A** | **C** | **T** | **T** | **G** | **T** | **T** | **G** | **C** | **C** | **T** | **A** | **T** | **G** | **T** | **T** | **T** | **C** | **C** | **A** | **G** | **G** | **A** | **A** | **A** | **G** | **C** | **A** | **T** | **C** | **T** | **A** | **A** | **A** | **A** | **C** | **A** | **C** | **A** | **T** | **T** | **T** | **T** | **A** | **A** | **C** | **A** | **A** | **C** | **T** | **T** | **T** | **C** | **A** | **A** | **T** | **T** | **C** | **G** | **T** | **C** | **C** | **T** | **T** | **G** | **G** | **C** | **A** | **T** | **T** | **G** | **T** | **T** | **T** | **T** | **T** | **A** | **T** | **T** | **G** | **T** | **C** | **A** | **T** | **T** | **G** | **G** | **C** | **G** | **C** | **A** | **A** | **T** | **G** | **A** | **A** | **C** | **A** | **G** | **C** | **A** | **C** | **G** | **G** | **C** | **T** | **G** | **T** | **T** | **T** | **G** | **G** | **T** | **T** | **T** | **A** | **T** | **G** | **T** | **T** | **G** | **A** | **T** | **T** | **T** | **T** | **T** | **G** | **G** | **C** | **G** | **T** | **A** | **C** | **T** | **C** | **T** | **T** | **T** | **A** | **T** | **C** | **G** | **G** | **C** | **T** | **T** | **A** | **A** | **A** | **A** | **G** | **G** | **C** | **T** | **T** | **T** | **G** | **A** | **G** | **A** | **T** | **T** | **T** | **C** | **A** | **G** | **G** | **C** | **G** | **T** | **G** | **G** | **A** | **T** | **A** | **T** | **A** | **G** | **C** | **T** | **G** | **A** | **G** | **C** | **G** | **A** | **G** | **C** | **A** | **C** | **C** | **T** | **T** | **G** | **G** | **C** | **G** | **T** | **A** | **A** | **A** | **A** | **A** | **A** | **C** | **A** | **A** | **A** | **T** | **G** | **A** | **T** | **T** | **A** | **T** | **G** | **G** | **T** | **G** | **G** | **A** | **A** | **A** | **C** | **G** | **G** | **C** | **G** | **G** | **C** | **C** | **A** | **A** | **A** | **G** | **A** | **A** | **C** | **C** | **G** | **A** | **A** | **A** | **A** | **A** | **T** | **G** | **G** | **C** | **A** | **A** | **A** | **C** | **G** | **A** | **T** | **T** | **T** | **A** | **A** | **A** | **C** | **G** | **C** | **C** | **G** | **T** | **T** | **G** | **G** | **T** | **T** | **T** | **G** | **C** | **C** | **A** | **A** | **C** | **G** | **A** | **A** | **C** | **G** | **T** | **A** | **T** | **T** | **G** | **G** | **C** | **A** | **A** | **C** | **A** | **A** | **T** | **A** | **T** | **C** | **T** | **A** | **T** | **G** | **A** | **A** | **T** | **G** | **G** | **G** | **A** | **C** | **G** | **A** | **C** | **A** | **T** | **C** | **A** | **A** | **T** | **A** | **T** | **T** | **G** | **A** | **T** | **T** | **T** | **A** | **A** | **T** | **C** | **T** | **C** | **T** | **G** | **G** | **G** | **G** | **A** | **T** | **A** | **C** | **T** | **A** | **T** | **T** | **A** | **T** | **T** | **G** | **A** | **T** | **T** | **T** | **A** | **G** | **G** | **C** | **A** | **A** | **T** | **A** | **C** | **G** | **C** | **T** | **A** | **C** | **T** | **T** | **C** | **C** | **G** | **A** | **A** | **A** | **G** | **A** | **A** | **G** | **A** | **T** | **A** | **A** | **T** | **A** | **T** | **T** | **A** | **T** | **T** | **A** | **T** | **T** | **A** | **T** | **T** | **C** | **G** | **T** | **A** | **A** | **A** | **G** | **G** | **T** | **T** | **T** | **T** | **G** | **G** | **C** | **C** | **G** | **C** | **A** | **C** | **G** | **C** | **G** | **A** | **A** | **T** | **T** | **C** | **T** | **A** | **G** | **T** | **G** | **C** | **C** | **G** | **T** | **T** | **A** | **G** | **G** | **T** | **G** | **T** | **A** | **G** | **C** | **T** | **A** | **T** | **T** | **T** | **T** | **G** | **T** | **T** | **A** | **G** | **A** | **A** | **C** | **A** | **T** | **T** | **C** | **A** | **A** | **C** | **G** | **T** | **T** | **T** | **T** | **A** | **C** | **G** | **G** | **A** | **A** | **C** | **A** | **G** | **T** | **G** | **C** | **G** | **T** | **T** | **T** | **T** | **G** | **A** | **A** | **G** | **A** | **A** | **G** | **A** | **A** | **A** | **A** | **A** | **T** | **A** | **T** | **C** | **A** | **A** | **T** | **T** | **G** | **A** | **A** | **A** | **A** | **A** | **C** | **G** | **A** | **A** | **T** | **C** | **A** | **T** | **T** | **A** | **A** | **A** | **A** | **A** | **T** | **T** | **T** | **A** | **C** | **A** | **G** | **C** | **A** | **A** | **T** | **G** | **A** | **T** | **T** | **A** | **T** | **G** | **A** | **T** | **A** | **C** | **C** | **A** | **A** | **T** | **C** | **T** | **T** | **C** | **G** | **T** | **C** | **G** | **T** | **T** | **T** | **G** | **A** | **A** | **A** | **A** | **T** | **T** | **A** | **T** | **G** | **A** | **C** | **G** | **A** | **A** | **C** | **A** | **C** | **T** | **T** | **T** | **A** | **G** | **T** | **A** | **G** | **G** | **A** | **G** | **A** | **T** | **G** | **T** | **T** | **G** | **A** | **G** | **G** | **T** | **G** | **A** | **T** | **C** | **C** | **G** | **T** | **G** | **T** | **A** | **-** | **-** | **-** |
| **N60034F** | **A** | **T** | **G** | **A** | **A** | **T** | **A** | **A** | **C** | **C** | **C** | **T** | **T** | **G** | **G** | **C** | **G** | **C** | **T** | **T** | **T** | **T** | **T** | **T** | **A** | **T** | **C** | **G** | **T** | **C** | **G** | **C** | **A** | **G** | **A** | **A** | **G** | **C** | **A** | **T** | **T** | **A** | **C** | **T** | **T** | **T** | **T** | **T** | **A** | **T** | **T** | **C** | **T** | **G** | **G** | **C** | **G** | **T** | **T** | **A** | **T** | **G** | **G** | **C** | **A** | **A** | **A** | **T** | **T** | **G** | **T** | **A** | **C** | **A** | **T** | **A** | **A** | **T** | **C** | **C** | **T** | **G** | **G** | **A** | **T** | **T** | **A** | **G** | **C** | **T** | **G** | **T** | **T** | **T** | **T** | **A** | **T** | **T** | **A** | **A** | **C** | **A** | **A** | **T** | **T** | **G** | **G** | **C** | **G** | **T** | **T** | **T** | **T** | **A** | **C** | **T** | **T** | **G** | **T** | **G** | **G** | **C** | **C** | **T** | **A** | **C** | **G** | **T** | **T** | **T** | **C** | **C** | **A** | **G** | **G** | **A** | **A** | **A** | **G** | **C** | **A** | **T** | **C** | **T** | **A** | **A** | **A** | **A** | **C** | **A** | **C** | **A** | **T** | **T** | **T** | **T** | **A** | **A** | **C** | **A** | **A** | **C** | **T** | **T** | **T** | **C** | **A** | **A** | **T** | **T** | **C** | **G** | **T** | **C** | **C** | **T** | **C** | **G** | **G** | **C** | **G** | **T** | **T** | **G** | **T** | **T** | **T** | **T** | **T** | **A** | **T** | **T** | **G** | **T** | **C** | **A** | **T** | **T** | **G** | **G** | **T** | **G** | **C** | **A** | **A** | **T** | **G** | **A** | **A** | **C** | **A** | **G** | **C** | **A** | **C** | **G** | **G** | **C** | **T** | **G** | **T** | **T** | **T** | **G** | **G** | **T** | **T** | **T** | **A** | **T** | **G** | **T** | **T** | **G** | **A** | **T** | **T** | **T** | **T** | **T** | **G** | **G** | **C** | **G** | **T** | **A** | **C** | **T** | **C** | **T** | **T** | **T** | **A** | **T** | **C** | **G** | **G** | **C** | **T** | **T** | **A** | **A** | **A** | **A** | **G** | **G** | **C** | **T** | **T** | **T** | **G** | **A** | **G** | **A** | **T** | **C** | **T** | **C** | **A** | **G** | **G** | **C** | **G** | **T** | **G** | **G** | **A** | **T** | **A** | **T** | **A** | **G** | **C** | **T** | **G** | **A** | **G** | **C** | **G** | **A** | **G** | **C** | **A** | **C** | **C** | **T** | **T** | **G** | **G** | **C** | **G** | **T** | **A** | **A** | **A** | **A** | **A** | **A** | **C** | **A** | **A** | **A** | **T** | **G** | **A** | **T** | **T** | **A** | **T** | **G** | **G** | **T** | **G** | **G** | **A** | **G** | **A** | **C** | **G** | **G** | **C** | **G** | **G** | **C** | **A** | **A** | **A** | **A** | **G** | **A** | **A** | **C** | **C** | **T** | **A** | **A** | **A** | **A** | **A** | **T** | **G** | **G** | **C** | **A** | **A** | **A** | **C** | **G** | **A** | **T** | **T** | **T** | **A** | **A** | **A** | **C** | **G** | **C** | **C** | **G** | **C** | **T** | **G** | **G** | **T** | **T** | **T** | **G** | **C** | **C** | **A** | **A** | **C** | **G** | **A** | **A** | **C** | **G** | **C** | **A** | **T** | **T** | **G** | **G** | **T** | **A** | **A** | **C** | **A** | **A** | **T** | **A** | **T** | **C** | **T** | **A** | **T** | **G** | **A** | **A** | **T** | **G** | **G** | **G** | **A** | **C** | **G** | **A** | **T** | **A** | **T** | **C** | **A** | **A** | **T** | **A** | **T** | **T** | **G** | **A** | **T** | **T** | **T** | **A** | **A** | **T** | **C** | **T** | **C** | **T** | **G** | **G** | **G** | **G** | **A** | **C** | **A** | **C** | **C** | **A** | **T** | **T** | **A** | **T** | **T** | **G** | **A** | **T** | **T** | **T** | **A** | **G** | **G** | **T** | **A** | **A** | **T** | **A** | **C** | **G** | **C** | **T** | **A** | **C** | **T** | **A** | **C** | **C** | **G** | **A** | **A** | **A** | **G** | **A** | **A** | **G** | **A** | **C** | **A** | **A** | **T** | **A** | **T** | **T** | **A** | **T** | **T** | **A** | **T** | **T** | **A** | **T** | **T** | **C** | **G** | **T** | **A** | **A** | **A** | **G** | **G** | **T** | **T** | **T** | **T** | **G** | **G** | **C** | **C** | **G** | **C** | **A** | **C** | **G** | **C** | **G** | **A** | **A** | **T** | **T** | **C** | **T** | **A** | **G** | **T** | **G** | **C** | **C** | **G** | **T** | **T** | **A** | **G** | **G** | **T** | **G** | **T** | **A** | **G** | **C** | **T** | **A** | **T** | **T** | **T** | **T** | **G** | **T** | **T** | **A** | **G** | **A** | **A** | **C** | **A** | **T** | **T** | **C** | **A** | **A** | **C** | **T** | **T** | **T** | **T** | **T** | **A** | **C** | **G** | **G** | **A** | **A** | **C** | **G** | **G** | **T** | **A** | **C** | **G** | **T** | **T** | **T** | **T** | **G** | **A** | **A** | **G** | **A** | **A** | **G** | **A** | **A** | **A** | **A** | **A** | **T** | **A** | **T** | **C** | **A** | **A** | **T** | **T** | **G** | **A** | **A** | **A** | **A** | **A** | **C** | **G** | **A** | **A** | **T** | **C** | **A** | **T** | **T** | **A** | **A** | **A** | **A** | **A** | **T** | **T** | **T** | **A** | **C** | **A** | **G** | **C** | **A** | **A** | **T** | **G** | **A** | **T** | **T** | **A** | **T** | **G** | **A** | **T** | **A** | **C** | **C** | **A** | **A** | **T** | **C** | **T** | **T** | **C** | **G** | **T** | **C** | **G** | **T** | **T** | **T** | **G** | **A** | **A** | **A** | **A** | **T** | **T** | **A** | **T** | **G** | **A** | **C** | **G** | **A** | **A** | **C** | **A** | **C** | **T** | **T** | **T** | **A** | **G** | **T** | **A** | **G** | **G** | **A** | **G** | **A** | **T** | **G** | **T** | **T** | **G** | **A** | **G** | **G** | **T** | **G** | **A** | **T** | **C** | **C** | **G** | **T** | **G** | **T** | **A** | **-** | **-** | **-** |
| **N60100F** | **A** | **T** | **G** | **A** | **A** | **T** | **A** | **A** | **C** | **C** | **C** | **T** | **T** | **G** | **G** | **C** | **G** | **C** | **T** | **T** | **T** | **T** | **T** | **T** | **A** | **T** | **C** | **G** | **T** | **C** | **G** | **C** | **A** | **G** | **A** | **A** | **G** | **C** | **A** | **T** | **T** | **A** | **C** | **T** | **T** | **T** | **T** | **T** | **A** | **T** | **T** | **C** | **T** | **G** | **G** | **C** | **G** | **T** | **T** | **A** | **T** | **G** | **G** | **C** | **A** | **A** | **A** | **T** | **T** | **G** | **T** | **A** | **C** | **A** | **T** | **A** | **A** | **T** | **C** | **C** | **T** | **G** | **G** | **A** | **T** | **T** | **A** | **G** | **C** | **T** | **G** | **T** | **T** | **T** | **T** | **A** | **T** | **T** | **A** | **A** | **C** | **A** | **A** | **T** | **T** | **G** | **G** | **C** | **G** | **T** | **T** | **T** | **T** | **A** | **C** | **T** | **T** | **G** | **T** | **G** | **G** | **C** | **C** | **T** | **A** | **T** | **G** | **T** | **T** | **T** | **C** | **C** | **A** | **G** | **G** | **A** | **A** | **A** | **G** | **C** | **A** | **T** | **C** | **T** | **A** | **A** | **A** | **A** | **C** | **A** | **C** | **A** | **T** | **T** | **T** | **T** | **A** | **A** | **C** | **A** | **A** | **C** | **T** | **T** | **T** | **C** | **A** | **A** | **T** | **T** | **C** | **G** | **T** | **C** | **C** | **T** | **C** | **G** | **G** | **C** | **G** | **T** | **T** | **G** | **T** | **T** | **T** | **T** | **T** | **A** | **T** | **T** | **G** | **T** | **C** | **A** | **T** | **T** | **G** | **G** | **T** | **G** | **C** | **A** | **A** | **T** | **G** | **A** | **A** | **C** | **A** | **G** | **C** | **A** | **C** | **G** | **G** | **C** | **T** | **G** | **T** | **T** | **T** | **G** | **G** | **T** | **T** | **T** | **A** | **T** | **G** | **T** | **T** | **G** | **A** | **T** | **T** | **T** | **T** | **T** | **G** | **G** | **C** | **G** | **T** | **A** | **C** | **T** | **C** | **T** | **T** | **T** | **A** | **T** | **C** | **G** | **G** | **C** | **T** | **T** | **A** | **A** | **A** | **A** | **G** | **G** | **C** | **T** | **T** | **T** | **G** | **A** | **G** | **A** | **T** | **C** | **T** | **C** | **A** | **G** | **G** | **C** | **G** | **T** | **G** | **G** | **A** | **T** | **A** | **T** | **A** | **G** | **C** | **T** | **G** | **A** | **G** | **C** | **G** | **A** | **G** | **C** | **A** | **C** | **C** | **T** | **T** | **G** | **G** | **C** | **G** | **T** | **A** | **A** | **A** | **A** | **A** | **A** | **C** | **A** | **A** | **A** | **T** | **G** | **A** | **T** | **T** | **A** | **T** | **G** | **G** | **T** | **G** | **G** | **A** | **G** | **A** | **C** | **G** | **G** | **C** | **G** | **G** | **C** | **A** | **A** | **A** | **A** | **G** | **A** | **A** | **C** | **C** | **T** | **A** | **A** | **A** | **A** | **A** | **T** | **G** | **G** | **C** | **A** | **A** | **A** | **C** | **G** | **A** | **T** | **T** | **T** | **A** | **A** | **A** | **C** | **G** | **C** | **C** | **G** | **C** | **T** | **G** | **G** | **T** | **T** | **T** | **G** | **C** | **C** | **A** | **A** | **C** | **G** | **A** | **A** | **C** | **G** | **C** | **A** | **T** | **T** | **G** | **G** | **T** | **A** | **A** | **C** | **A** | **A** | **T** | **A** | **T** | **C** | **T** | **A** | **T** | **G** | **A** | **A** | **T** | **G** | **G** | **G** | **A** | **C** | **G** | **A** | **C** | **A** | **T** | **C** | **A** | **A** | **T** | **A** | **T** | **T** | **G** | **A** | **T** | **T** | **T** | **A** | **A** | **T** | **C** | **T** | **C** | **T** | **G** | **G** | **G** | **G** | **A** | **C** | **A** | **C** | **C** | **A** | **T** | **T** | **A** | **T** | **T** | **G** | **A** | **T** | **T** | **T** | **A** | **G** | **G** | **T** | **A** | **A** | **T** | **A** | **C** | **G** | **C** | **T** | **A** | **C** | **T** | **A** | **C** | **C** | **G** | **A** | **A** | **A** | **G** | **A** | **A** | **G** | **A** | **C** | **A** | **A** | **T** | **A** | **T** | **T** | **A** | **T** | **T** | **A** | **T** | **T** | **A** | **T** | **T** | **C** | **G** | **T** | **A** | **A** | **A** | **G** | **G** | **T** | **T** | **T** | **T** | **G** | **G** | **C** | **C** | **G** | **C** | **A** | **C** | **G** | **C** | **G** | **A** | **A** | **T** | **T** | **C** | **T** | **A** | **G** | **T** | **G** | **C** | **C** | **G** | **T** | **T** | **A** | **G** | **G** | **T** | **G** | **T** | **A** | **G** | **C** | **T** | **A** | **T** | **T** | **T** | **T** | **G** | **T** | **T** | **A** | **G** | **A** | **A** | **C** | **A** | **T** | **T** | **C** | **A** | **A** | **C** | **T** | **T** | **T** | **T** | **T** | **A** | **C** | **G** | **G** | **A** | **A** | **C** | **G** | **G** | **T** | **A** | **C** | **G** | **T** | **T** | **T** | **T** | **G** | **A** | **A** | **G** | **A** | **A** | **G** | **A** | **A** | **A** | **A** | **A** | **T** | **A** | **T** | **C** | **A** | **A** | **T** | **T** | **G** | **A** | **A** | **A** | **A** | **A** | **C** | **G** | **A** | **A** | **T** | **C** | **A** | **T** | **T** | **A** | **A** | **A** | **A** | **A** | **T** | **T** | **T** | **A** | **C** | **A** | **G** | **C** | **A** | **A** | **T** | **G** | **A** | **T** | **T** | **A** | **C** | **G** | **A** | **T** | **A** | **C** | **C** | **A** | **A** | **T** | **C** | **T** | **T** | **C** | **G** | **T** | **C** | **G** | **T** | **T** | **T** | **G** | **A** | **A** | **A** | **A** | **T** | **T** | **A** | **T** | **G** | **A** | **C** | **G** | **A** | **A** | **T** | **A** | **C** | **T** | **T** | **T** | **A** | **G** | **T** | **A** | **G** | **G** | **A** | **G** | **A** | **T** | **G** | **T** | **T** | **G** | **A** | **G** | **G** | **T** | **G** | **A** | **T** | **C** | **C** | **G** | **T** | **G** | **T** | **A** | **-** | **-** | **-** |
| **N59550F** | **A** | **T** | **G** | **A** | **A** | **T** | **A** | **A** | **C** | **C** | **C** | **T** | **T** | **G** | **G** | **C** | **G** | **C** | **T** | **T** | **T** | **T** | **T** | **T** | **A** | **T** | **C** | **G** | **T** | **C** | **G** | **C** | **A** | **G** | **A** | **A** | **G** | **C** | **A** | **T** | **T** | **A** | **C** | **T** | **T** | **T** | **T** | **T** | **A** | **T** | **T** | **C** | **T** | **G** | **G** | **C** | **G** | **T** | **T** | **A** | **T** | **G** | **G** | **C** | **A** | **A** | **A** | **T** | **T** | **G** | **T** | **A** | **C** | **A** | **T** | **A** | **A** | **T** | **C** | **C** | **T** | **G** | **G** | **A** | **T** | **T** | **A** | **G** | **C** | **T** | **G** | **T** | **T** | **T** | **T** | **A** | **T** | **T** | **A** | **A** | **C** | **A** | **A** | **T** | **T** | **G** | **G** | **C** | **G** | **T** | **T** | **T** | **T** | **A** | **C** | **T** | **T** | **G** | **T** | **G** | **G** | **C** | **C** | **T** | **A** | **C** | **G** | **T** | **T** | **T** | **C** | **C** | **A** | **G** | **G** | **A** | **A** | **A** | **G** | **C** | **A** | **T** | **C** | **T** | **A** | **A** | **A** | **A** | **C** | **A** | **C** | **A** | **T** | **T** | **T** | **T** | **A** | **A** | **C** | **A** | **A** | **C** | **T** | **T** | **T** | **C** | **A** | **A** | **T** | **T** | **C** | **G** | **T** | **C** | **C** | **T** | **C** | **G** | **G** | **C** | **G** | **T** | **T** | **G** | **T** | **T** | **T** | **T** | **T** | **A** | **T** | **T** | **G** | **T** | **C** | **A** | **T** | **T** | **G** | **G** | **T** | **G** | **C** | **A** | **A** | **T** | **G** | **A** | **A** | **C** | **A** | **G** | **C** | **A** | **C** | **G** | **G** | **C** | **T** | **G** | **T** | **T** | **T** | **G** | **G** | **T** | **T** | **T** | **A** | **T** | **G** | **T** | **T** | **G** | **A** | **T** | **T** | **T** | **T** | **T** | **G** | **G** | **C** | **G** | **T** | **A** | **C** | **T** | **C** | **T** | **T** | **T** | **A** | **T** | **C** | **G** | **G** | **C** | **T** | **T** | **A** | **A** | **A** | **A** | **G** | **G** | **C** | **T** | **T** | **T** | **G** | **A** | **G** | **A** | **T** | **T** | **T** | **C** | **A** | **G** | **G** | **C** | **G** | **T** | **G** | **G** | **A** | **T** | **A** | **T** | **A** | **G** | **C** | **T** | **G** | **A** | **G** | **C** | **G** | **A** | **G** | **C** | **A** | **C** | **C** | **T** | **T** | **G** | **G** | **C** | **G** | **T** | **A** | **A** | **A** | **A** | **A** | **A** | **C** | **A** | **A** | **A** | **T** | **G** | **A** | **T** | **T** | **A** | **T** | **G** | **G** | **T** | **G** | **G** | **A** | **G** | **A** | **C** | **G** | **G** | **C** | **G** | **G** | **C** | **A** | **A** | **A** | **A** | **G** | **A** | **A** | **C** | **C** | **T** | **A** | **A** | **A** | **A** | **A** | **T** | **G** | **G** | **C** | **A** | **A** | **A** | **C** | **G** | **A** | **T** | **T** | **T** | **A** | **A** | **A** | **C** | **G** | **C** | **C** | **G** | **T** | **T** | **G** | **G** | **T** | **T** | **T** | **G** | **C** | **C** | **A** | **A** | **T** | **G** | **A** | **A** | **C** | **G** | **C** | **A** | **T** | **T** | **G** | **G** | **T** | **A** | **A** | **C** | **A** | **A** | **T** | **A** | **T** | **C** | **T** | **A** | **T** | **G** | **A** | **A** | **T** | **G** | **G** | **G** | **A** | **T** | **G** | **A** | **T** | **A** | **T** | **C** | **A** | **A** | **T** | **A** | **T** | **T** | **G** | **A** | **T** | **T** | **T** | **A** | **A** | **T** | **C** | **T** | **C** | **T** | **G** | **G** | **G** | **G** | **A** | **C** | **A** | **C** | **C** | **A** | **T** | **T** | **A** | **T** | **T** | **G** | **A** | **T** | **T** | **T** | **A** | **G** | **G** | **T** | **A** | **A** | **T** | **A** | **C** | **G** | **C** | **T** | **A** | **C** | **T** | **A** | **C** | **C** | **G** | **A** | **A** | **A** | **G** | **A** | **A** | **G** | **A** | **C** | **A** | **A** | **T** | **A** | **T** | **T** | **A** | **T** | **T** | **A** | **T** | **T** | **A** | **T** | **T** | **C** | **G** | **T** | **A** | **A** | **A** | **G** | **G** | **T** | **T** | **T** | **T** | **G** | **G** | **C** | **C** | **G** | **C** | **A** | **C** | **G** | **C** | **G** | **A** | **A** | **T** | **T** | **C** | **T** | **A** | **G** | **T** | **A** | **C** | **C** | **G** | **T** | **T** | **A** | **G** | **G** | **T** | **G** | **T** | **A** | **G** | **C** | **T** | **A** | **T** | **T** | **T** | **T** | **G** | **T** | **T** | **A** | **G** | **A** | **A** | **C** | **A** | **T** | **T** | **C** | **A** | **A** | **C** | **T** | **T** | **T** | **T** | **T** | **A** | **C** | **G** | **G** | **A** | **A** | **C** | **G** | **G** | **T** | **A** | **C** | **G** | **T** | **T** | **T** | **T** | **G** | **A** | **A** | **G** | **A** | **A** | **G** | **A** | **A** | **A** | **A** | **A** | **T** | **A** | **T** | **C** | **A** | **A** | **T** | **T** | **G** | **A** | **A** | **A** | **A** | **A** | **C** | **G** | **A** | **A** | **T** | **C** | **A** | **T** | **T** | **A** | **A** | **A** | **A** | **A** | **T** | **T** | **T** | **A** | **C** | **A** | **G** | **C** | **A** | **A** | **T** | **G** | **A** | **T** | **T** | **A** | **T** | **G** | **A** | **T** | **A** | **C** | **C** | **A** | **A** | **T** | **C** | **T** | **T** | **C** | **G** | **T** | **C** | **G** | **T** | **T** | **T** | **G** | **A** | **A** | **A** | **A** | **T** | **T** | **A** | **T** | **G** | **A** | **C** | **G** | **A** | **A** | **C** | **A** | **C** | **T** | **T** | **T** | **A** | **G** | **T** | **A** | **G** | **G** | **A** | **G** | **A** | **T** | **G** | **T** | **T** | **G** | **A** | **G** | **G** | **T** | **G** | **A** | **T** | **C** | **C** | **G** | **T** | **G** | **T** | **A** | **-** | **-** | **-** |
| **N52469** | **A** | **T** | **G** | **A** | **A** | **T** | **A** | **A** | **C** | **C** | **C** | **T** | **T** | **G** | **G** | **C** | **G** | **C** | **T** | **T** | **T** | **T** | **T** | **T** | **A** | **T** | **C** | **G** | **T** | **C** | **G** | **C** | **A** | **G** | **A** | **A** | **G** | **C** | **A** | **T** | **T** | **A** | **C** | **T** | **T** | **T** | **T** | **T** | **A** | **T** | **T** | **C** | **T** | **G** | **G** | **C** | **G** | **T** | **T** | **A** | **T** | **G** | **G** | **C** | **A** | **A** | **A** | **T** | **T** | **G** | **T** | **A** | **C** | **A** | **T** | **A** | **A** | **T** | **C** | **C** | **T** | **G** | **G** | **A** | **T** | **T** | **A** | **G** | **C** | **T** | **G** | **T** | **T** | **T** | **T** | **A** | **T** | **T** | **A** | **A** | **C** | **A** | **A** | **T** | **T** | **G** | **G** | **C** | **G** | **T** | **T** | **T** | **T** | **A** | **C** | **T** | **T** | **G** | **T** | **G** | **G** | **C** | **C** | **T** | **A** | **C** | **G** | **T** | **T** | **T** | **C** | **C** | **A** | **G** | **G** | **A** | **A** | **A** | **G** | **C** | **A** | **T** | **C** | **T** | **A** | **A** | **A** | **A** | **C** | **A** | **C** | **A** | **T** | **T** | **T** | **T** | **A** | **A** | **C** | **A** | **A** | **C** | **T** | **T** | **T** | **C** | **A** | **A** | **T** | **T** | **C** | **A** | **T** | **C** | **C** | **T** | **C** | **G** | **G** | **C** | **G** | **T** | **T** | **G** | **T** | **T** | **T** | **T** | **T** | **A** | **T** | **T** | **G** | **T** | **C** | **A** | **T** | **T** | **G** | **G** | **T** | **G** | **C** | **A** | **A** | **T** | **G** | **A** | **A** | **C** | **A** | **G** | **T** | **A** | **C** | **G** | **G** | **C** | **T** | **G** | **T** | **T** | **T** | **G** | **G** | **T** | **T** | **T** | **A** | **T** | **G** | **T** | **T** | **G** | **A** | **T** | **T** | **T** | **T** | **T** | **G** | **G** | **C** | **G** | **T** | **A** | **C** | **T** | **C** | **T** | **T** | **T** | **A** | **T** | **C** | **G** | **G** | **C** | **T** | **T** | **A** | **A** | **A** | **A** | **G** | **G** | **C** | **T** | **T** | **T** | **G** | **A** | **G** | **A** | **T** | **T** | **T** | **C** | **A** | **G** | **G** | **C** | **G** | **T** | **G** | **G** | **A** | **T** | **A** | **T** | **A** | **G** | **C** | **T** | **G** | **A** | **G** | **C** | **G** | **A** | **G** | **C** | **A** | **C** | **C** | **T** | **T** | **G** | **G** | **C** | **G** | **T** | **A** | **A** | **A** | **A** | **A** | **A** | **C** | **A** | **A** | **A** | **T** | **G** | **A** | **T** | **T** | **A** | **T** | **G** | **G** | **T** | **G** | **G** | **A** | **G** | **A** | **C** | **G** | **G** | **C** | **G** | **G** | **C** | **A** | **A** | **A** | **A** | **G** | **A** | **A** | **C** | **C** | **T** | **A** | **A** | **A** | **A** | **A** | **T** | **G** | **G** | **C** | **A** | **A** | **A** | **C** | **G** | **A** | **T** | **T** | **T** | **A** | **A** | **A** | **C** | **G** | **C** | **C** | **G** | **T** | **T** | **G** | **G** | **T** | **T** | **T** | **G** | **C** | **C** | **A** | **A** | **C** | **G** | **A** | **A** | **C** | **G** | **C** | **A** | **T** | **T** | **G** | **G** | **T** | **A** | **A** | **C** | **A** | **A** | **T** | **A** | **T** | **C** | **T** | **A** | **T** | **G** | **A** | **A** | **T** | **G** | **G** | **G** | **A** | **T** | **G** | **A** | **T** | **A** | **T** | **C** | **A** | **A** | **T** | **A** | **T** | **T** | **G** | **A** | **T** | **T** | **T** | **A** | **A** | **T** | **C** | **T** | **C** | **T** | **G** | **G** | **G** | **G** | **A** | **C** | **A** | **C** | **C** | **A** | **T** | **T** | **A** | **T** | **T** | **G** | **A** | **T** | **T** | **T** | **A** | **G** | **G** | **T** | **A** | **A** | **T** | **A** | **C** | **G** | **C** | **T** | **A** | **C** | **T** | **A** | **C** | **C** | **G** | **A** | **A** | **A** | **G** | **A** | **A** | **G** | **A** | **C** | **A** | **A** | **T** | **A** | **T** | **T** | **A** | **T** | **T** | **A** | **T** | **T** | **A** | **T** | **T** | **C** | **G** | **T** | **A** | **A** | **A** | **G** | **G** | **T** | **T** | **T** | **T** | **G** | **G** | **C** | **C** | **G** | **C** | **A** | **C** | **G** | **C** | **G** | **A** | **A** | **T** | **T** | **C** | **T** | **A** | **G** | **T** | **A** | **C** | **C** | **G** | **T** | **T** | **A** | **G** | **G** | **T** | **G** | **T** | **A** | **G** | **C** | **A** | **A** | **T** | **T** | **T** | **T** | **G** | **T** | **T** | **A** | **G** | **A** | **A** | **C** | **A** | **T** | **T** | **C** | **A** | **A** | **C** | **T** | **T** | **T** | **T** | **T** | **A** | **C** | **G** | **G** | **A** | **A** | **C** | **G** | **G** | **T** | **A** | **C** | **G** | **T** | **T** | **T** | **T** | **G** | **A** | **A** | **G** | **A** | **A** | **G** | **A** | **A** | **A** | **A** | **A** | **T** | **A** | **T** | **C** | **A** | **A** | **T** | **T** | **G** | **A** | **A** | **A** | **A** | **A** | **C** | **G** | **A** | **A** | **T** | **C** | **A** | **T** | **T** | **A** | **A** | **A** | **A** | **A** | **T** | **T** | **T** | **A** | **C** | **A** | **G** | **C** | **A** | **A** | **T** | **G** | **A** | **T** | **T** | **A** | **C** | **G** | **A** | **T** | **A** | **C** | **C** | **A** | **A** | **T** | **C** | **T** | **T** | **C** | **G** | **T** | **C** | **G** | **T** | **T** | **T** | **G** | **A** | **A** | **A** | **A** | **T** | **T** | **A** | **T** | **G** | **A** | **C** | **G** | **A** | **A** | **C** | **A** | **C** | **T** | **T** | **T** | **A** | **G** | **T** | **A** | **G** | **G** | **A** | **G** | **A** | **T** | **G** | **T** | **T** | **G** | **A** | **G** | **G** | **T** | **G** | **A** | **T** | **C** | **C** | **G** | **T** | **G** | **T** | **A** | **-** | **-** | **-** |
| **N59600F** | **A** | **T** | **G** | **A** | **A** | **T** | **A** | **A** | **C** | **C** | **C** | **T** | **T** | **G** | **G** | **C** | **G** | **C** | **T** | **T** | **T** | **T** | **T** | **T** | **A** | **T** | **C** | **G** | **T** | **C** | **G** | **C** | **A** | **G** | **A** | **A** | **G** | **C** | **A** | **T** | **T** | **A** | **C** | **T** | **T** | **T** | **T** | **T** | **A** | **T** | **T** | **C** | **T** | **G** | **G** | **C** | **G** | **T** | **T** | **A** | **T** | **G** | **G** | **C** | **A** | **A** | **A** | **T** | **T** | **G** | **T** | **A** | **C** | **A** | **T** | **A** | **A** | **T** | **C** | **C** | **T** | **G** | **G** | **A** | **T** | **T** | **A** | **G** | **C** | **T** | **G** | **T** | **T** | **T** | **T** | **A** | **T** | **T** | **A** | **A** | **C** | **A** | **A** | **T** | **T** | **G** | **G** | **C** | **G** | **T** | **T** | **T** | **T** | **A** | **C** | **T** | **T** | **G** | **T** | **G** | **G** | **C** | **C** | **T** | **A** | **C** | **G** | **T** | **T** | **T** | **C** | **C** | **A** | **G** | **G** | **A** | **A** | **A** | **G** | **C** | **A** | **T** | **C** | **T** | **A** | **A** | **A** | **A** | **C** | **A** | **C** | **A** | **T** | **T** | **T** | **T** | **A** | **A** | **C** | **A** | **A** | **C** | **T** | **T** | **T** | **C** | **A** | **A** | **T** | **T** | **C** | **G** | **T** | **C** | **C** | **T** | **C** | **G** | **G** | **C** | **G** | **T** | **T** | **G** | **T** | **T** | **T** | **T** | **T** | **A** | **T** | **T** | **G** | **T** | **C** | **A** | **T** | **T** | **G** | **G** | **T** | **G** | **C** | **A** | **A** | **T** | **G** | **A** | **A** | **C** | **A** | **G** | **C** | **A** | **C** | **G** | **G** | **C** | **T** | **G** | **T** | **T** | **T** | **G** | **G** | **T** | **T** | **T** | **A** | **T** | **G** | **T** | **T** | **G** | **A** | **T** | **T** | **T** | **T** | **T** | **G** | **G** | **C** | **G** | **T** | **A** | **C** | **T** | **C** | **T** | **T** | **T** | **A** | **T** | **C** | **G** | **G** | **C** | **T** | **T** | **A** | **A** | **A** | **A** | **G** | **G** | **C** | **T** | **T** | **T** | **G** | **A** | **G** | **A** | **T** | **T** | **T** | **C** | **A** | **G** | **G** | **C** | **G** | **T** | **G** | **G** | **A** | **T** | **A** | **T** | **A** | **G** | **C** | **T** | **G** | **A** | **G** | **C** | **G** | **A** | **G** | **C** | **A** | **C** | **C** | **T** | **T** | **G** | **G** | **C** | **G** | **A** | **A** | **A** | **A** | **A** | **A** | **A** | **C** | **A** | **A** | **A** | **T** | **G** | **A** | **T** | **T** | **A** | **T** | **G** | **G** | **T** | **G** | **G** | **A** | **G** | **A** | **C** | **G** | **G** | **C** | **G** | **G** | **C** | **A** | **A** | **A** | **A** | **G** | **A** | **A** | **C** | **C** | **T** | **A** | **A** | **A** | **A** | **A** | **T** | **G** | **G** | **C** | **A** | **A** | **A** | **C** | **G** | **A** | **T** | **T** | **T** | **A** | **A** | **A** | **C** | **G** | **C** | **C** | **G** | **C** | **T** | **G** | **G** | **T** | **T** | **T** | **G** | **C** | **C** | **A** | **A** | **C** | **G** | **A** | **A** | **C** | **G** | **C** | **A** | **T** | **T** | **G** | **G** | **T** | **A** | **A** | **C** | **A** | **A** | **T** | **A** | **T** | **C** | **T** | **A** | **T** | **G** | **A** | **A** | **T** | **G** | **G** | **G** | **A** | **C** | **G** | **A** | **T** | **A** | **T** | **C** | **A** | **A** | **T** | **A** | **T** | **T** | **G** | **A** | **T** | **T** | **T** | **A** | **A** | **T** | **C** | **T** | **C** | **T** | **G** | **G** | **G** | **G** | **A** | **C** | **A** | **C** | **C** | **A** | **T** | **T** | **A** | **T** | **T** | **G** | **A** | **T** | **T** | **T** | **A** | **G** | **G** | **T** | **A** | **A** | **T** | **A** | **C** | **G** | **C** | **T** | **A** | **C** | **T** | **A** | **C** | **C** | **G** | **A** | **A** | **A** | **G** | **A** | **A** | **G** | **A** | **C** | **A** | **A** | **T** | **A** | **T** | **T** | **A** | **T** | **T** | **A** | **T** | **T** | **A** | **T** | **T** | **C** | **G** | **T** | **A** | **A** | **A** | **G** | **G** | **T** | **T** | **T** | **T** | **G** | **G** | **C** | **C** | **G** | **C** | **A** | **C** | **G** | **C** | **G** | **A** | **A** | **T** | **T** | **C** | **T** | **A** | **G** | **T** | **G** | **C** | **C** | **G** | **T** | **T** | **A** | **G** | **G** | **T** | **G** | **T** | **A** | **G** | **C** | **T** | **A** | **T** | **T** | **T** | **T** | **G** | **T** | **T** | **A** | **G** | **A** | **A** | **C** | **A** | **T** | **T** | **C** | **A** | **A** | **C** | **T** | **T** | **T** | **T** | **T** | **A** | **C** | **G** | **G** | **A** | **A** | **C** | **G** | **G** | **T** | **A** | **C** | **G** | **T** | **T** | **T** | **T** | **G** | **A** | **A** | **G** | **A** | **A** | **G** | **A** | **A** | **A** | **A** | **A** | **T** | **A** | **T** | **C** | **A** | **A** | **T** | **T** | **G** | **A** | **A** | **A** | **A** | **A** | **C** | **G** | **A** | **A** | **T** | **C** | **A** | **T** | **T** | **A** | **A** | **A** | **A** | **A** | **T** | **T** | **T** | **A** | **C** | **A** | **G** | **C** | **A** | **A** | **T** | **G** | **A** | **T** | **T** | **A** | **C** | **G** | **A** | **T** | **A** | **C** | **C** | **A** | **A** | **T** | **C** | **T** | **T** | **C** | **G** | **T** | **C** | **G** | **T** | **T** | **T** | **G** | **A** | **A** | **A** | **A** | **T** | **T** | **A** | **T** | **G** | **A** | **C** | **G** | **A** | **A** | **C** | **A** | **C** | **T** | **T** | **T** | **A** | **G** | **T** | **A** | **G** | **G** | **A** | **G** | **A** | **T** | **G** | **T** | **T** | **G** | **A** | **G** | **G** | **T** | **G** | **A** | **T** | **C** | **C** | **G** | **T** | **G** | **T** | **A** | **-** | **-** | **-** |
| **N52734** | **A** | **T** | **G** | **A** | **A** | **T** | **A** | **A** | **C** | **C** | **C** | **T** | **T** | **G** | **G** | **C** | **G** | **C** | **T** | **T** | **T** | **T** | **T** | **T** | **A** | **T** | **C** | **G** | **T** | **C** | **G** | **C** | **A** | **G** | **A** | **A** | **G** | **C** | **A** | **T** | **T** | **A** | **C** | **T** | **T** | **T** | **T** | **T** | **A** | **T** | **T** | **C** | **T** | **G** | **G** | **C** | **G** | **T** | **T** | **A** | **T** | **G** | **G** | **C** | **A** | **A** | **A** | **T** | **T** | **G** | **T** | **A** | **C** | **A** | **T** | **A** | **A** | **T** | **C** | **C** | **T** | **G** | **G** | **A** | **T** | **T** | **A** | **G** | **C** | **T** | **G** | **T** | **T** | **T** | **T** | **A** | **T** | **T** | **A** | **A** | **C** | **A** | **A** | **T** | **T** | **G** | **G** | **C** | **G** | **T** | **T** | **T** | **T** | **A** | **C** | **T** | **T** | **G** | **T** | **G** | **G** | **C** | **C** | **T** | **A** | **C** | **G** | **T** | **T** | **T** | **C** | **C** | **A** | **G** | **G** | **A** | **A** | **A** | **G** | **C** | **A** | **T** | **C** | **T** | **A** | **A** | **A** | **A** | **C** | **A** | **C** | **A** | **T** | **T** | **T** | **T** | **A** | **A** | **C** | **A** | **A** | **C** | **T** | **T** | **T** | **C** | **A** | **A** | **T** | **T** | **C** | **G** | **T** | **C** | **C** | **T** | **C** | **G** | **G** | **C** | **G** | **T** | **T** | **G** | **T** | **T** | **T** | **T** | **T** | **A** | **T** | **T** | **G** | **T** | **C** | **A** | **T** | **T** | **G** | **G** | **T** | **G** | **C** | **A** | **A** | **T** | **G** | **A** | **A** | **T** | **A** | **G** | **C** | **A** | **C** | **G** | **G** | **C** | **T** | **G** | **T** | **T** | **T** | **G** | **G** | **T** | **T** | **T** | **A** | **T** | **G** | **T** | **T** | **G** | **A** | **T** | **T** | **T** | **T** | **T** | **G** | **G** | **C** | **G** | **T** | **A** | **C** | **T** | **C** | **T** | **T** | **T** | **A** | **T** | **C** | **G** | **G** | **C** | **T** | **T** | **A** | **A** | **A** | **A** | **G** | **G** | **C** | **T** | **T** | **T** | **G** | **A** | **G** | **A** | **T** | **T** | **T** | **C** | **A** | **G** | **G** | **C** | **G** | **T** | **G** | **G** | **A** | **T** | **A** | **T** | **A** | **G** | **C** | **T** | **G** | **A** | **G** | **C** | **G** | **A** | **G** | **C** | **A** | **C** | **C** | **T** | **T** | **G** | **G** | **C** | **G** | **A** | **A** | **A** | **A** | **A** | **A** | **A** | **C** | **A** | **A** | **A** | **T** | **G** | **A** | **T** | **T** | **A** | **T** | **G** | **G** | **T** | **G** | **G** | **A** | **G** | **A** | **C** | **G** | **G** | **C** | **G** | **G** | **C** | **A** | **A** | **A** | **A** | **G** | **A** | **A** | **C** | **C** | **T** | **A** | **A** | **A** | **A** | **A** | **T** | **G** | **G** | **C** | **A** | **A** | **A** | **C** | **G** | **G** | **T** | **T** | **T** | **A** | **A** | **A** | **C** | **G** | **C** | **C** | **G** | **C** | **T** | **G** | **G** | **T** | **T** | **T** | **G** | **C** | **C** | **A** | **A** | **C** | **G** | **A** | **A** | **C** | **G** | **C** | **A** | **T** | **T** | **G** | **G** | **T** | **A** | **A** | **C** | **A** | **A** | **T** | **A** | **T** | **C** | **T** | **A** | **T** | **G** | **A** | **A** | **T** | **G** | **G** | **G** | **A** | **C** | **G** | **A** | **T** | **A** | **T** | **C** | **A** | **A** | **T** | **A** | **T** | **T** | **G** | **A** | **T** | **T** | **T** | **A** | **A** | **T** | **C** | **T** | **C** | **T** | **G** | **G** | **G** | **G** | **A** | **C** | **A** | **C** | **C** | **A** | **T** | **T** | **A** | **T** | **T** | **G** | **A** | **T** | **T** | **T** | **A** | **G** | **G** | **T** | **A** | **A** | **T** | **A** | **C** | **G** | **C** | **T** | **A** | **C** | **T** | **A** | **C** | **C** | **G** | **A** | **A** | **A** | **G** | **A** | **A** | **G** | **A** | **C** | **A** | **A** | **T** | **A** | **T** | **T** | **A** | **T** | **T** | **A** | **T** | **T** | **A** | **T** | **T** | **C** | **G** | **T** | **A** | **A** | **A** | **G** | **G** | **T** | **T** | **T** | **T** | **G** | **G** | **C** | **C** | **G** | **C** | **A** | **C** | **G** | **C** | **G** | **A** | **A** | **T** | **T** | **C** | **T** | **A** | **G** | **T** | **G** | **C** | **C** | **G** | **T** | **T** | **A** | **G** | **G** | **T** | **G** | **T** | **A** | **G** | **C** | **T** | **A** | **T** | **T** | **T** | **T** | **G** | **T** | **T** | **A** | **G** | **A** | **A** | **C** | **A** | **T** | **T** | **C** | **A** | **A** | **C** | **T** | **T** | **T** | **T** | **T** | **A** | **C** | **G** | **G** | **A** | **A** | **C** | **G** | **G** | **T** | **A** | **C** | **G** | **T** | **T** | **T** | **T** | **G** | **A** | **A** | **G** | **A** | **A** | **G** | **A** | **A** | **A** | **A** | **A** | **T** | **A** | **T** | **C** | **A** | **A** | **T** | **T** | **G** | **A** | **A** | **A** | **A** | **A** | **C** | **G** | **A** | **A** | **T** | **C** | **A** | **T** | **T** | **A** | **A** | **A** | **A** | **A** | **T** | **T** | **T** | **A** | **C** | **A** | **G** | **C** | **A** | **A** | **T** | **G** | **A** | **T** | **T** | **A** | **T** | **G** | **A** | **T** | **A** | **C** | **C** | **A** | **A** | **T** | **C** | **T** | **T** | **C** | **G** | **T** | **C** | **G** | **T** | **T** | **T** | **G** | **A** | **A** | **A** | **A** | **T** | **T** | **A** | **T** | **G** | **A** | **C** | **G** | **A** | **A** | **C** | **A** | **C** | **T** | **T** | **T** | **A** | **G** | **T** | **A** | **G** | **G** | **A** | **G** | **A** | **T** | **G** | **T** | **T** | **G** | **A** | **G** | **G** | **T** | **G** | **A** | **T** | **C** | **C** | **G** | **T** | **G** | **T** | **A** | **-** | **-** | **-** |
| **N52724** | **A** | **T** | **G** | **A** | **A** | **T** | **A** | **A** | **C** | **C** | **C** | **T** | **T** | **G** | **G** | **C** | **G** | **C** | **T** | **T** | **T** | **T** | **T** | **T** | **A** | **T** | **C** | **G** | **T** | **C** | **G** | **C** | **A** | **G** | **A** | **A** | **G** | **C** | **A** | **T** | **T** | **A** | **C** | **T** | **T** | **T** | **T** | **T** | **A** | **T** | **T** | **C** | **T** | **G** | **G** | **C** | **G** | **T** | **T** | **A** | **T** | **G** | **G** | **C** | **A** | **A** | **A** | **T** | **T** | **G** | **T** | **A** | **C** | **A** | **T** | **A** | **A** | **T** | **C** | **C** | **T** | **G** | **G** | **A** | **T** | **T** | **A** | **G** | **C** | **T** | **G** | **T** | **T** | **T** | **T** | **A** | **T** | **T** | **A** | **A** | **C** | **A** | **A** | **T** | **T** | **G** | **G** | **C** | **G** | **T** | **T** | **T** | **T** | **A** | **C** | **T** | **T** | **G** | **T** | **G** | **G** | **C** | **C** | **T** | **A** | **T** | **G** | **T** | **T** | **T** | **C** | **C** | **A** | **G** | **G** | **A** | **A** | **A** | **G** | **C** | **A** | **T** | **C** | **T** | **A** | **A** | **A** | **A** | **C** | **A** | **C** | **A** | **T** | **T** | **T** | **T** | **A** | **A** | **C** | **A** | **A** | **C** | **T** | **T** | **T** | **C** | **A** | **A** | **T** | **T** | **C** | **G** | **T** | **C** | **C** | **T** | **C** | **G** | **G** | **C** | **G** | **T** | **T** | **G** | **T** | **T** | **T** | **T** | **T** | **A** | **T** | **T** | **G** | **T** | **C** | **A** | **T** | **T** | **G** | **G** | **T** | **G** | **C** | **A** | **A** | **T** | **G** | **A** | **A** | **C** | **A** | **G** | **C** | **A** | **C** | **G** | **G** | **C** | **T** | **G** | **T** | **T** | **T** | **G** | **G** | **T** | **T** | **T** | **A** | **T** | **G** | **T** | **T** | **G** | **A** | **T** | **T** | **T** | **T** | **T** | **G** | **G** | **C** | **G** | **T** | **A** | **C** | **T** | **C** | **T** | **T** | **T** | **A** | **T** | **C** | **G** | **G** | **C** | **T** | **T** | **A** | **A** | **A** | **A** | **G** | **G** | **C** | **T** | **T** | **T** | **G** | **A** | **G** | **A** | **T** | **T** | **T** | **C** | **A** | **G** | **G** | **C** | **G** | **T** | **G** | **G** | **A** | **T** | **A** | **T** | **A** | **G** | **C** | **T** | **G** | **A** | **A** | **C** | **G** | **A** | **G** | **C** | **A** | **C** | **C** | **T** | **T** | **G** | **G** | **C** | **G** | **T** | **A** | **A** | **A** | **A** | **A** | **A** | **C** | **A** | **A** | **A** | **T** | **G** | **A** | **T** | **T** | **A** | **T** | **G** | **G** | **T** | **A** | **G** | **A** | **G** | **A** | **C** | **G** | **G** | **C** | **G** | **G** | **C** | **A** | **A** | **A** | **A** | **G** | **A** | **A** | **C** | **C** | **T** | **A** | **A** | **A** | **A** | **A** | **T** | **G** | **G** | **C** | **A** | **A** | **A** | **C** | **G** | **A** | **T** | **T** | **T** | **A** | **A** | **A** | **C** | **G** | **C** | **C** | **G** | **C** | **T** | **G** | **G** | **T** | **T** | **T** | **G** | **C** | **C** | **A** | **A** | **C** | **G** | **A** | **A** | **C** | **G** | **C** | **A** | **T** | **T** | **G** | **G** | **T** | **A** | **A** | **C** | **A** | **A** | **T** | **A** | **T** | **C** | **T** | **A** | **T** | **G** | **A** | **A** | **T** | **G** | **G** | **G** | **A** | **T** | **G** | **A** | **C** | **A** | **T** | **C** | **A** | **A** | **T** | **A** | **T** | **T** | **G** | **A** | **T** | **T** | **T** | **A** | **A** | **T** | **C** | **T** | **C** | **T** | **G** | **G** | **G** | **G** | **A** | **C** | **A** | **C** | **C** | **A** | **T** | **T** | **A** | **T** | **T** | **G** | **A** | **T** | **T** | **T** | **A** | **G** | **G** | **T** | **A** | **A** | **T** | **A** | **C** | **G** | **C** | **T** | **A** | **C** | **T** | **A** | **C** | **C** | **G** | **A** | **A** | **A** | **G** | **A** | **A** | **G** | **A** | **C** | **A** | **A** | **T** | **A** | **T** | **T** | **A** | **T** | **T** | **A** | **T** | **T** | **A** | **T** | **T** | **C** | **G** | **T** | **A** | **A** | **A** | **G** | **G** | **T** | **T** | **T** | **T** | **G** | **G** | **C** | **C** | **G** | **C** | **A** | **C** | **G** | **C** | **G** | **A** | **A** | **T** | **T** | **C** | **T** | **A** | **G** | **T** | **G** | **C** | **C** | **G** | **T** | **T** | **A** | **G** | **G** | **T** | **G** | **T** | **A** | **G** | **C** | **T** | **A** | **T** | **T** | **T** | **T** | **G** | **T** | **T** | **A** | **G** | **A** | **A** | **C** | **A** | **T** | **T** | **C** | **A** | **A** | **C** | **T** | **T** | **T** | **T** | **T** | **A** | **C** | **G** | **G** | **A** | **A** | **C** | **G** | **G** | **T** | **A** | **C** | **G** | **T** | **T** | **T** | **T** | **G** | **A** | **A** | **G** | **A** | **A** | **G** | **A** | **A** | **A** | **A** | **A** | **T** | **A** | **T** | **C** | **A** | **A** | **T** | **T** | **G** | **A** | **A** | **A** | **A** | **A** | **C** | **G** | **A** | **A** | **T** | **C** | **A** | **T** | **T** | **A** | **A** | **A** | **A** | **A** | **T** | **T** | **T** | **A** | **C** | **A** | **G** | **C** | **A** | **A** | **T** | **G** | **A** | **T** | **T** | **A** | **T** | **G** | **A** | **T** | **A** | **C** | **C** | **A** | **A** | **T** | **C** | **T** | **T** | **C** | **G** | **T** | **C** | **G** | **T** | **T** | **T** | **G** | **A** | **A** | **A** | **A** | **T** | **T** | **A** | **T** | **G** | **A** | **C** | **G** | **A** | **A** | **C** | **A** | **C** | **T** | **T** | **T** | **A** | **G** | **T** | **A** | **G** | **G** | **A** | **G** | **A** | **T** | **G** | **T** | **T** | **G** | **A** | **G** | **G** | **T** | **G** | **A** | **T** | **C** | **C** | **G** | **T** | **G** | **T** | **A** | **-** | **-** | **-** |
| **N59454F** | **A** | **T** | **G** | **A** | **A** | **T** | **A** | **A** | **C** | **C** | **C** | **T** | **T** | **G** | **G** | **C** | **G** | **C** | **T** | **T** | **T** | **T** | **T** | **T** | **A** | **T** | **C** | **G** | **T** | **C** | **G** | **C** | **A** | **G** | **A** | **A** | **G** | **C** | **A** | **T** | **T** | **A** | **C** | **T** | **T** | **T** | **T** | **T** | **A** | **T** | **T** | **C** | **T** | **G** | **G** | **C** | **G** | **T** | **T** | **A** | **T** | **G** | **G** | **C** | **A** | **A** | **A** | **T** | **T** | **G** | **T** | **A** | **C** | **A** | **T** | **A** | **A** | **T** | **C** | **C** | **T** | **G** | **G** | **A** | **T** | **T** | **A** | **G** | **C** | **T** | **G** | **T** | **T** | **T** | **T** | **A** | **T** | **T** | **A** | **A** | **C** | **A** | **A** | **T** | **T** | **G** | **G** | **C** | **G** | **T** | **T** | **T** | **T** | **A** | **C** | **T** | **T** | **G** | **T** | **G** | **G** | **C** | **C** | **T** | **A** | **C** | **G** | **T** | **T** | **T** | **C** | **C** | **A** | **G** | **G** | **A** | **A** | **A** | **G** | **C** | **A** | **T** | **C** | **T** | **A** | **A** | **A** | **A** | **C** | **A** | **C** | **A** | **T** | **T** | **T** | **T** | **A** | **A** | **C** | **A** | **A** | **C** | **T** | **T** | **T** | **C** | **A** | **A** | **T** | **T** | **C** | **G** | **T** | **C** | **C** | **T** | **C** | **G** | **G** | **C** | **G** | **T** | **T** | **G** | **T** | **T** | **T** | **T** | **T** | **A** | **T** | **T** | **G** | **T** | **C** | **A** | **T** | **T** | **G** | **G** | **T** | **G** | **C** | **A** | **A** | **T** | **G** | **A** | **A** | **C** | **A** | **G** | **C** | **A** | **C** | **G** | **G** | **C** | **T** | **G** | **T** | **T** | **T** | **G** | **G** | **T** | **T** | **T** | **A** | **T** | **G** | **T** | **T** | **G** | **A** | **T** | **T** | **T** | **T** | **T** | **G** | **G** | **C** | **G** | **T** | **A** | **C** | **T** | **C** | **T** | **T** | **T** | **A** | **T** | **C** | **G** | **G** | **C** | **T** | **T** | **A** | **A** | **A** | **A** | **G** | **G** | **C** | **T** | **T** | **T** | **G** | **A** | **G** | **A** | **T** | **T** | **T** | **C** | **A** | **G** | **G** | **C** | **G** | **T** | **G** | **G** | **A** | **T** | **A** | **T** | **A** | **G** | **C** | **T** | **G** | **A** | **G** | **C** | **G** | **A** | **G** | **C** | **A** | **C** | **C** | **T** | **T** | **G** | **G** | **C** | **G** | **A** | **A** | **A** | **A** | **A** | **A** | **A** | **C** | **A** | **A** | **A** | **T** | **G** | **A** | **T** | **T** | **A** | **T** | **G** | **G** | **T** | **G** | **G** | **A** | **G** | **A** | **C** | **G** | **G** | **C** | **G** | **G** | **C** | **A** | **A** | **A** | **A** | **G** | **A** | **A** | **C** | **C** | **T** | **A** | **A** | **A** | **A** | **A** | **T** | **G** | **G** | **C** | **A** | **A** | **A** | **C** | **G** | **A** | **T** | **T** | **T** | **A** | **A** | **A** | **C** | **G** | **C** | **C** | **G** | **C** | **T** | **G** | **G** | **T** | **T** | **T** | **G** | **C** | **C** | **A** | **A** | **C** | **G** | **A** | **A** | **C** | **G** | **C** | **A** | **T** | **T** | **G** | **G** | **T** | **A** | **A** | **C** | **A** | **A** | **T** | **A** | **T** | **C** | **T** | **A** | **T** | **G** | **A** | **A** | **T** | **G** | **G** | **G** | **A** | **C** | **G** | **A** | **C** | **A** | **T** | **C** | **A** | **A** | **T** | **A** | **T** | **T** | **G** | **A** | **T** | **T** | **T** | **A** | **A** | **T** | **C** | **T** | **C** | **T** | **G** | **G** | **G** | **G** | **A** | **C** | **A** | **C** | **C** | **A** | **T** | **T** | **A** | **T** | **T** | **G** | **A** | **T** | **T** | **T** | **A** | **G** | **G** | **T** | **A** | **A** | **T** | **A** | **C** | **G** | **C** | **T** | **A** | **C** | **T** | **A** | **C** | **C** | **G** | **A** | **A** | **A** | **G** | **A** | **A** | **G** | **A** | **C** | **A** | **A** | **T** | **A** | **T** | **T** | **A** | **T** | **T** | **A** | **T** | **T** | **A** | **T** | **T** | **C** | **G** | **T** | **A** | **A** | **A** | **G** | **G** | **T** | **T** | **T** | **T** | **G** | **G** | **C** | **C** | **G** | **C** | **A** | **C** | **G** | **C** | **G** | **A** | **A** | **T** | **T** | **C** | **T** | **A** | **G** | **T** | **G** | **C** | **C** | **G** | **T** | **T** | **A** | **G** | **G** | **T** | **G** | **T** | **A** | **G** | **C** | **T** | **A** | **T** | **T** | **T** | **T** | **G** | **T** | **T** | **A** | **G** | **A** | **A** | **C** | **A** | **T** | **T** | **C** | **A** | **A** | **C** | **T** | **T** | **T** | **T** | **T** | **A** | **C** | **G** | **G** | **A** | **A** | **C** | **G** | **G** | **T** | **A** | **C** | **G** | **T** | **T** | **T** | **T** | **G** | **A** | **A** | **G** | **A** | **A** | **G** | **A** | **A** | **A** | **A** | **A** | **T** | **A** | **T** | **C** | **A** | **A** | **T** | **T** | **G** | **A** | **A** | **A** | **A** | **A** | **C** | **G** | **A** | **A** | **T** | **C** | **A** | **T** | **T** | **A** | **A** | **A** | **A** | **A** | **T** | **T** | **T** | **A** | **C** | **A** | **G** | **C** | **A** | **A** | **T** | **G** | **A** | **T** | **T** | **A** | **C** | **G** | **A** | **T** | **A** | **C** | **C** | **A** | **A** | **T** | **C** | **T** | **T** | **C** | **G** | **T** | **C** | **G** | **T** | **T** | **T** | **G** | **A** | **A** | **A** | **A** | **T** | **T** | **A** | **T** | **G** | **A** | **C** | **G** | **A** | **A** | **C** | **A** | **C** | **T** | **T** | **T** | **A** | **G** | **T** | **A** | **G** | **G** | **A** | **G** | **A** | **T** | **G** | **T** | **T** | **G** | **A** | **G** | **G** | **T** | **G** | **A** | **T** | **C** | **C** | **G** | **T** | **G** | **T** | **A** | **-** | **-** | **-** |
| **N53453** | **A** | **T** | **G** | **A** | **A** | **T** | **A** | **A** | **C** | **C** | **C** | **T** | **T** | **G** | **G** | **C** | **G** | **C** | **T** | **T** | **T** | **T** | **T** | **T** | **A** | **T** | **C** | **G** | **T** | **C** | **G** | **C** | **A** | **G** | **A** | **A** | **G** | **C** | **A** | **T** | **T** | **A** | **C** | **T** | **T** | **T** | **T** | **T** | **A** | **T** | **T** | **C** | **T** | **G** | **G** | **C** | **G** | **T** | **T** | **A** | **T** | **G** | **G** | **C** | **A** | **A** | **A** | **T** | **T** | **G** | **T** | **A** | **C** | **A** | **T** | **A** | **A** | **T** | **C** | **C** | **T** | **G** | **G** | **A** | **T** | **T** | **A** | **G** | **C** | **T** | **G** | **T** | **T** | **T** | **T** | **A** | **T** | **T** | **A** | **A** | **C** | **A** | **A** | **T** | **T** | **G** | **G** | **C** | **G** | **T** | **T** | **T** | **T** | **A** | **C** | **T** | **T** | **G** | **T** | **G** | **G** | **C** | **C** | **T** | **A** | **C** | **G** | **T** | **T** | **T** | **C** | **C** | **A** | **G** | **G** | **A** | **A** | **A** | **G** | **C** | **A** | **T** | **C** | **T** | **A** | **A** | **A** | **A** | **C** | **A** | **C** | **A** | **T** | **T** | **T** | **T** | **A** | **A** | **C** | **A** | **A** | **C** | **T** | **T** | **T** | **C** | **A** | **A** | **T** | **T** | **C** | **A** | **T** | **C** | **C** | **T** | **C** | **G** | **G** | **C** | **G** | **T** | **T** | **G** | **T** | **T** | **T** | **T** | **T** | **A** | **T** | **T** | **G** | **T** | **C** | **A** | **T** | **T** | **G** | **G** | **T** | **G** | **C** | **A** | **A** | **T** | **G** | **A** | **A** | **C** | **A** | **G** | **T** | **A** | **C** | **G** | **G** | **C** | **T** | **G** | **T** | **T** | **T** | **G** | **G** | **T** | **T** | **T** | **A** | **T** | **G** | **T** | **T** | **G** | **A** | **T** | **T** | **T** | **T** | **T** | **G** | **G** | **C** | **G** | **T** | **A** | **C** | **T** | **C** | **T** | **T** | **T** | **A** | **T** | **C** | **G** | **G** | **C** | **T** | **T** | **A** | **A** | **A** | **A** | **G** | **G** | **C** | **T** | **T** | **T** | **G** | **A** | **G** | **A** | **T** | **T** | **T** | **C** | **A** | **G** | **G** | **C** | **G** | **T** | **G** | **G** | **A** | **T** | **A** | **T** | **A** | **G** | **C** | **T** | **G** | **A** | **G** | **C** | **G** | **A** | **G** | **C** | **A** | **C** | **C** | **T** | **T** | **G** | **G** | **C** | **G** | **T** | **A** | **A** | **A** | **A** | **A** | **A** | **C** | **A** | **A** | **A** | **T** | **G** | **A** | **T** | **T** | **A** | **T** | **G** | **G** | **T** | **G** | **G** | **A** | **G** | **A** | **C** | **G** | **G** | **C** | **G** | **G** | **C** | **A** | **A** | **A** | **A** | **G** | **A** | **A** | **C** | **C** | **T** | **A** | **A** | **A** | **A** | **A** | **T** | **G** | **G** | **C** | **A** | **A** | **A** | **C** | **G** | **A** | **T** | **T** | **T** | **A** | **A** | **A** | **C** | **G** | **C** | **C** | **G** | **T** | **T** | **G** | **G** | **T** | **T** | **T** | **G** | **C** | **C** | **A** | **A** | **C** | **G** | **A** | **A** | **C** | **G** | **C** | **A** | **T** | **T** | **G** | **G** | **T** | **A** | **A** | **C** | **A** | **A** | **T** | **A** | **T** | **C** | **T** | **A** | **T** | **G** | **A** | **A** | **T** | **G** | **G** | **G** | **A** | **T** | **G** | **A** | **T** | **A** | **T** | **C** | **A** | **A** | **T** | **A** | **T** | **T** | **G** | **A** | **T** | **T** | **T** | **A** | **A** | **T** | **C** | **T** | **C** | **T** | **G** | **G** | **G** | **G** | **A** | **C** | **A** | **C** | **C** | **A** | **T** | **T** | **A** | **T** | **T** | **G** | **A** | **T** | **T** | **T** | **A** | **G** | **G** | **T** | **A** | **A** | **T** | **A** | **C** | **G** | **C** | **T** | **A** | **C** | **T** | **A** | **C** | **C** | **G** | **A** | **A** | **A** | **G** | **A** | **A** | **G** | **A** | **C** | **A** | **A** | **T** | **A** | **T** | **T** | **A** | **T** | **T** | **A** | **T** | **T** | **A** | **T** | **T** | **C** | **G** | **T** | **A** | **A** | **A** | **G** | **G** | **T** | **T** | **T** | **T** | **G** | **G** | **C** | **C** | **G** | **C** | **A** | **C** | **G** | **C** | **G** | **A** | **A** | **T** | **T** | **C** | **T** | **A** | **G** | **T** | **A** | **C** | **C** | **G** | **T** | **T** | **A** | **G** | **G** | **T** | **G** | **T** | **A** | **G** | **C** | **A** | **A** | **T** | **T** | **T** | **T** | **G** | **T** | **T** | **A** | **G** | **A** | **A** | **C** | **A** | **T** | **T** | **C** | **A** | **A** | **C** | **T** | **T** | **T** | **T** | **T** | **A** | **C** | **G** | **G** | **A** | **A** | **C** | **G** | **G** | **T** | **A** | **C** | **G** | **T** | **T** | **T** | **T** | **G** | **A** | **A** | **G** | **A** | **A** | **G** | **A** | **A** | **A** | **A** | **A** | **T** | **A** | **T** | **C** | **A** | **A** | **T** | **T** | **G** | **A** | **A** | **A** | **A** | **A** | **C** | **G** | **A** | **A** | **T** | **C** | **A** | **T** | **T** | **A** | **A** | **A** | **A** | **A** | **T** | **T** | **T** | **A** | **C** | **A** | **G** | **C** | **A** | **A** | **T** | **G** | **A** | **T** | **T** | **A** | **C** | **G** | **A** | **T** | **A** | **C** | **C** | **A** | **A** | **T** | **C** | **T** | **T** | **C** | **G** | **T** | **C** | **G** | **T** | **T** | **T** | **G** | **A** | **A** | **A** | **A** | **T** | **T** | **A** | **T** | **G** | **A** | **C** | **G** | **A** | **A** | **C** | **A** | **C** | **T** | **T** | **T** | **A** | **G** | **T** | **A** | **G** | **G** | **A** | **G** | **A** | **T** | **G** | **T** | **T** | **G** | **A** | **G** | **G** | **T** | **G** | **A** | **T** | **C** | **C** | **G** | **T** | **G** | **T** | **A** | **-** | **-** | **-** |
| **N60392F** | **A** | **T** | **G** | **A** | **A** | **T** | **A** | **A** | **C** | **C** | **C** | **T** | **T** | **G** | **G** | **C** | **G** | **C** | **T** | **T** | **T** | **T** | **T** | **T** | **A** | **T** | **C** | **G** | **T** | **C** | **G** | **C** | **A** | **G** | **A** | **A** | **G** | **C** | **A** | **T** | **T** | **A** | **C** | **T** | **T** | **T** | **T** | **T** | **A** | **T** | **T** | **C** | **T** | **G** | **G** | **C** | **G** | **T** | **T** | **A** | **T** | **G** | **G** | **C** | **A** | **A** | **A** | **T** | **T** | **G** | **T** | **A** | **C** | **A** | **T** | **A** | **A** | **T** | **C** | **C** | **T** | **G** | **G** | **A** | **T** | **T** | **A** | **G** | **C** | **T** | **G** | **T** | **T** | **T** | **T** | **A** | **T** | **T** | **A** | **A** | **C** | **A** | **A** | **T** | **T** | **G** | **G** | **C** | **G** | **T** | **T** | **T** | **T** | **A** | **C** | **T** | **T** | **G** | **T** | **G** | **G** | **C** | **C** | **T** | **A** | **C** | **G** | **T** | **T** | **T** | **C** | **C** | **A** | **G** | **G** | **A** | **A** | **A** | **G** | **C** | **A** | **T** | **A** | **T** | **A** | **A** | **A** | **A** | **C** | **A** | **C** | **A** | **T** | **T** | **T** | **T** | **A** | **A** | **C** | **A** | **A** | **C** | **T** | **T** | **T** | **C** | **A** | **A** | **T** | **T** | **C** | **G** | **T** | **C** | **C** | **T** | **C** | **G** | **G** | **C** | **G** | **T** | **T** | **G** | **T** | **T** | **T** | **T** | **T** | **A** | **T** | **T** | **G** | **T** | **C** | **A** | **T** | **T** | **G** | **G** | **T** | **G** | **C** | **A** | **A** | **T** | **G** | **A** | **A** | **C** | **A** | **G** | **T** | **A** | **C** | **A** | **G** | **C** | **T** | **G** | **T** | **T** | **T** | **G** | **G** | **T** | **T** | **T** | **A** | **T** | **G** | **T** | **T** | **G** | **A** | **T** | **T** | **T** | **T** | **T** | **G** | **G** | **C** | **G** | **T** | **A** | **C** | **T** | **C** | **T** | **T** | **T** | **A** | **T** | **C** | **G** | **G** | **C** | **T** | **T** | **A** | **A** | **A** | **A** | **G** | **G** | **C** | **T** | **T** | **T** | **G** | **A** | **G** | **A** | **T** | **T** | **T** | **C** | **A** | **G** | **G** | **C** | **G** | **T** | **G** | **G** | **A** | **T** | **A** | **T** | **A** | **G** | **C** | **T** | **G** | **A** | **G** | **C** | **G** | **A** | **G** | **C** | **A** | **C** | **C** | **T** | **T** | **G** | **G** | **C** | **G** | **A** | **A** | **A** | **A** | **A** | **A** | **A** | **C** | **A** | **A** | **A** | **T** | **G** | **A** | **T** | **T** | **A** | **T** | **G** | **G** | **T** | **G** | **G** | **A** | **G** | **A** | **C** | **G** | **G** | **C** | **G** | **G** | **C** | **A** | **A** | **A** | **A** | **G** | **A** | **A** | **C** | **C** | **T** | **A** | **A** | **A** | **A** | **A** | **T** | **G** | **G** | **C** | **A** | **A** | **A** | **C** | **G** | **A** | **T** | **T** | **T** | **A** | **A** | **A** | **C** | **G** | **C** | **C** | **G** | **T** | **T** | **G** | **G** | **T** | **T** | **T** | **G** | **C** | **C** | **A** | **A** | **C** | **G** | **A** | **A** | **C** | **G** | **C** | **A** | **T** | **T** | **G** | **G** | **T** | **A** | **A** | **C** | **A** | **A** | **T** | **A** | **T** | **C** | **T** | **A** | **T** | **G** | **A** | **A** | **T** | **G** | **G** | **G** | **A** | **T** | **G** | **A** | **T** | **A** | **T** | **C** | **A** | **A** | **T** | **A** | **T** | **T** | **G** | **A** | **T** | **T** | **T** | **A** | **A** | **T** | **T** | **T** | **C** | **T** | **G** | **G** | **G** | **G** | **A** | **C** | **A** | **C** | **C** | **A** | **T** | **T** | **A** | **T** | **T** | **G** | **A** | **T** | **T** | **T** | **A** | **G** | **G** | **T** | **A** | **A** | **T** | **A** | **C** | **G** | **C** | **T** | **A** | **C** | **T** | **A** | **C** | **C** | **G** | **A** | **A** | **A** | **G** | **A** | **A** | **G** | **A** | **C** | **A** | **A** | **T** | **A** | **T** | **T** | **A** | **T** | **T** | **A** | **T** | **T** | **A** | **T** | **T** | **C** | **G** | **T** | **A** | **A** | **A** | **G** | **G** | **T** | **T** | **T** | **T** | **G** | **G** | **C** | **C** | **G** | **C** | **A** | **C** | **G** | **C** | **G** | **A** | **A** | **T** | **T** | **C** | **T** | **A** | **G** | **T** | **A** | **C** | **C** | **G** | **T** | **T** | **A** | **G** | **G** | **T** | **G** | **T** | **A** | **G** | **C** | **T** | **A** | **T** | **T** | **T** | **T** | **G** | **T** | **T** | **A** | **G** | **A** | **A** | **C** | **A** | **T** | **T** | **C** | **A** | **A** | **C** | **T** | **T** | **T** | **T** | **T** | **A** | **C** | **G** | **G** | **A** | **A** | **C** | **G** | **G** | **T** | **A** | **C** | **G** | **T** | **T** | **T** | **T** | **G** | **A** | **A** | **G** | **A** | **A** | **G** | **A** | **A** | **A** | **A** | **A** | **T** | **A** | **T** | **C** | **A** | **A** | **T** | **T** | **G** | **A** | **A** | **A** | **A** | **A** | **C** | **G** | **A** | **A** | **T** | **C** | **A** | **T** | **T** | **A** | **A** | **A** | **A** | **A** | **T** | **T** | **T** | **A** | **C** | **A** | **G** | **C** | **A** | **A** | **T** | **G** | **A** | **T** | **T** | **A** | **T** | **G** | **A** | **T** | **A** | **C** | **C** | **A** | **A** | **T** | **C** | **T** | **T** | **C** | **G** | **T** | **C** | **G** | **T** | **T** | **T** | **G** | **A** | **A** | **A** | **A** | **T** | **T** | **A** | **T** | **G** | **A** | **C** | **G** | **A** | **A** | **C** | **A** | **C** | **T** | **T** | **T** | **A** | **G** | **T** | **A** | **G** | **G** | **A** | **G** | **A** | **T** | **G** | **T** | **T** | **G** | **A** | **G** | **G** | **T** | **G** | **A** | **T** | **C** | **C** | **G** | **T** | **G** | **T** | **A** | **-** | **-** | **-** |
| **N53761** | **A** | **T** | **G** | **A** | **A** | **T** | **A** | **A** | **C** | **C** | **C** | **T** | **T** | **G** | **G** | **C** | **G** | **C** | **T** | **T** | **T** | **T** | **T** | **T** | **A** | **T** | **C** | **G** | **T** | **C** | **G** | **C** | **A** | **G** | **A** | **A** | **G** | **C** | **A** | **T** | **T** | **A** | **C** | **T** | **T** | **T** | **T** | **T** | **A** | **T** | **T** | **C** | **T** | **G** | **G** | **C** | **G** | **T** | **T** | **A** | **T** | **G** | **G** | **C** | **A** | **A** | **A** | **T** | **T** | **G** | **T** | **A** | **C** | **A** | **T** | **A** | **A** | **T** | **C** | **C** | **T** | **G** | **G** | **A** | **T** | **T** | **A** | **G** | **C** | **T** | **G** | **T** | **T** | **T** | **T** | **A** | **T** | **T** | **A** | **A** | **C** | **A** | **A** | **T** | **T** | **G** | **G** | **C** | **G** | **T** | **T** | **T** | **T** | **A** | **C** | **T** | **T** | **G** | **T** | **G** | **G** | **C** | **C** | **T** | **A** | **C** | **G** | **T** | **T** | **T** | **C** | **C** | **A** | **G** | **G** | **A** | **A** | **A** | **G** | **C** | **A** | **T** | **C** | **A** | **A** | **A** | **A** | **A** | **C** | **A** | **C** | **A** | **T** | **T** | **T** | **T** | **A** | **A** | **C** | **A** | **A** | **C** | **T** | **T** | **T** | **C** | **A** | **A** | **T** | **T** | **C** | **G** | **T** | **C** | **C** | **T** | **C** | **G** | **G** | **C** | **G** | **T** | **T** | **G** | **T** | **T** | **T** | **T** | **T** | **A** | **T** | **T** | **G** | **T** | **C** | **A** | **T** | **T** | **G** | **G** | **T** | **G** | **C** | **A** | **A** | **T** | **G** | **A** | **A** | **C** | **A** | **G** | **C** | **A** | **C** | **G** | **G** | **C** | **C** | **G** | **T** | **T** | **T** | **G** | **G** | **T** | **T** | **T** | **A** | **T** | **G** | **T** | **T** | **G** | **A** | **T** | **T** | **T** | **T** | **T** | **G** | **G** | **C** | **G** | **T** | **A** | **C** | **T** | **C** | **T** | **T** | **T** | **A** | **T** | **C** | **G** | **G** | **C** | **T** | **T** | **A** | **A** | **A** | **A** | **G** | **G** | **C** | **T** | **T** | **T** | **G** | **A** | **G** | **A** | **T** | **T** | **T** | **C** | **A** | **G** | **G** | **C** | **G** | **T** | **G** | **G** | **A** | **T** | **A** | **T** | **A** | **G** | **C** | **T** | **G** | **A** | **G** | **C** | **G** | **A** | **G** | **C** | **A** | **C** | **C** | **T** | **T** | **G** | **G** | **C** | **G** | **T** | **A** | **A** | **A** | **A** | **A** | **A** | **C** | **A** | **A** | **A** | **T** | **G** | **A** | **T** | **T** | **A** | **T** | **G** | **G** | **T** | **G** | **G** | **A** | **G** | **A** | **C** | **G** | **G** | **C** | **G** | **G** | **C** | **A** | **A** | **A** | **A** | **G** | **A** | **A** | **C** | **C** | **T** | **A** | **A** | **A** | **A** | **A** | **T** | **G** | **G** | **C** | **A** | **A** | **A** | **C** | **G** | **A** | **T** | **T** | **T** | **A** | **A** | **A** | **C** | **G** | **C** | **C** | **G** | **C** | **T** | **G** | **G** | **T** | **T** | **T** | **G** | **C** | **C** | **A** | **A** | **C** | **G** | **A** | **A** | **C** | **G** | **C** | **A** | **T** | **T** | **G** | **G** | **T** | **A** | **A** | **C** | **A** | **A** | **T** | **A** | **T** | **C** | **T** | **A** | **T** | **G** | **A** | **A** | **T** | **G** | **G** | **G** | **A** | **C** | **G** | **A** | **T** | **A** | **T** | **C** | **A** | **A** | **T** | **A** | **T** | **T** | **G** | **A** | **T** | **T** | **T** | **A** | **A** | **T** | **C** | **T** | **C** | **T** | **G** | **G** | **G** | **G** | **A** | **C** | **A** | **C** | **C** | **A** | **T** | **T** | **A** | **T** | **T** | **G** | **A** | **T** | **T** | **T** | **A** | **G** | **G** | **T** | **A** | **A** | **T** | **A** | **C** | **G** | **C** | **T** | **A** | **C** | **T** | **A** | **C** | **C** | **G** | **A** | **A** | **A** | **G** | **A** | **A** | **G** | **A** | **C** | **A** | **A** | **T** | **A** | **T** | **T** | **A** | **T** | **T** | **A** | **T** | **T** | **A** | **T** | **T** | **C** | **G** | **T** | **A** | **A** | **A** | **G** | **G** | **T** | **T** | **T** | **T** | **G** | **G** | **C** | **C** | **G** | **C** | **A** | **C** | **G** | **C** | **G** | **A** | **A** | **T** | **T** | **C** | **T** | **A** | **G** | **T** | **A** | **C** | **C** | **G** | **T** | **T** | **A** | **G** | **G** | **T** | **G** | **T** | **A** | **G** | **C** | **T** | **A** | **T** | **T** | **T** | **T** | **G** | **T** | **T** | **A** | **G** | **A** | **A** | **C** | **A** | **T** | **T** | **C** | **A** | **A** | **C** | **G** | **T** | **T** | **T** | **T** | **A** | **C** | **G** | **G** | **A** | **A** | **C** | **G** | **G** | **T** | **A** | **C** | **G** | **T** | **T** | **T** | **T** | **G** | **A** | **A** | **G** | **A** | **A** | **G** | **A** | **A** | **A** | **A** | **A** | **T** | **A** | **T** | **C** | **A** | **A** | **T** | **T** | **G** | **A** | **A** | **A** | **A** | **A** | **C** | **G** | **A** | **A** | **T** | **C** | **A** | **T** | **T** | **A** | **A** | **A** | **A** | **A** | **T** | **T** | **T** | **A** | **C** | **A** | **G** | **C** | **A** | **A** | **T** | **G** | **A** | **T** | **T** | **A** | **C** | **G** | **A** | **T** | **A** | **C** | **C** | **A** | **A** | **T** | **C** | **T** | **T** | **C** | **G** | **T** | **C** | **G** | **T** | **T** | **T** | **G** | **A** | **A** | **A** | **A** | **T** | **T** | **A** | **T** | **G** | **A** | **C** | **G** | **A** | **A** | **C** | **A** | **C** | **T** | **T** | **T** | **A** | **G** | **T** | **A** | **G** | **G** | **A** | **G** | **A** | **T** | **G** | **T** | **T** | **G** | **A** | **G** | **G** | **T** | **G** | **A** | **T** | **C** | **C** | **G** | **T** | **G** | **T** | **A** | **-** | **-** | **-** |
| **N52467** | **-** | **-** | **-** | **-** | **-** | **-** | **-** | **-** | **-** | **C** | **C** | **T** | **T** | **G** | **G** | **C** | **G** | **C** | **T** | **T** | **T** | **T** | **T** | **T** | **A** | **T** | **C** | **G** | **T** | **C** | **G** | **C** | **A** | **G** | **A** | **A** | **G** | **C** | **A** | **T** | **T** | **A** | **C** | **T** | **T** | **T** | **T** | **T** | **A** | **T** | **T** | **C** | **T** | **A** | **G** | **C** | **G** | **T** | **T** | **A** | **T** | **G** | **G** | **C** | **A** | **A** | **A** | **T** | **T** | **G** | **T** | **A** | **C** | **A** | **T** | **A** | **A** | **T** | **C** | **C** | **T** | **G** | **G** | **A** | **T** | **T** | **A** | **G** | **C** | **T** | **G** | **T** | **T** | **T** | **T** | **A** | **T** | **T** | **A** | **A** | **C** | **A** | **A** | **T** | **T** | **G** | **G** | **C** | **G** | **T** | **T** | **T** | **T** | **A** | **C** | **T** | **T** | **G** | **T** | **T** | **G** | **C** | **C** | **T** | **A** | **T** | **G** | **T** | **T** | **T** | **C** | **C** | **A** | **G** | **G** | **A** | **A** | **A** | **G** | **C** | **A** | **T** | **C** | **T** | **A** | **A** | **A** | **A** | **C** | **A** | **C** | **A** | **T** | **T** | **T** | **T** | **A** | **A** | **C** | **A** | **A** | **C** | **T** | **T** | **T** | **C** | **A** | **A** | **T** | **T** | **C** | **G** | **T** | **C** | **C** | **T** | **T** | **G** | **G** | **C** | **A** | **T** | **T** | **G** | **T** | **T** | **T** | **T** | **T** | **A** | **T** | **T** | **G** | **T** | **C** | **A** | **T** | **T** | **G** | **G** | **C** | **G** | **C** | **A** | **A** | **T** | **G** | **A** | **A** | **C** | **A** | **G** | **C** | **A** | **C** | **G** | **G** | **C** | **T** | **G** | **T** | **T** | **T** | **G** | **G** | **T** | **T** | **T** | **A** | **T** | **G** | **T** | **T** | **G** | **A** | **T** | **T** | **T** | **T** | **T** | **G** | **G** | **C** | **G** | **T** | **A** | **C** | **T** | **C** | **T** | **T** | **T** | **A** | **T** | **C** | **G** | **G** | **C** | **T** | **T** | **A** | **A** | **A** | **A** | **G** | **G** | **C** | **T** | **T** | **T** | **G** | **A** | **G** | **A** | **T** | **T** | **T** | **C** | **A** | **G** | **G** | **C** | **G** | **T** | **G** | **G** | **A** | **T** | **A** | **T** | **A** | **G** | **C** | **T** | **G** | **A** | **G** | **C** | **G** | **A** | **G** | **C** | **A** | **C** | **C** | **T** | **T** | **G** | **G** | **C** | **G** | **T** | **A** | **A** | **A** | **A** | **A** | **A** | **C** | **A** | **A** | **A** | **T** | **G** | **A** | **T** | **T** | **A** | **T** | **G** | **G** | **T** | **G** | **G** | **A** | **A** | **A** | **C** | **G** | **G** | **C** | **G** | **G** | **C** | **C** | **A** | **A** | **A** | **G** | **A** | **A** | **C** | **C** | **G** | **A** | **A** | **A** | **A** | **A** | **T** | **G** | **G** | **C** | **A** | **A** | **A** | **C** | **G** | **A** | **T** | **T** | **T** | **A** | **A** | **A** | **C** | **G** | **C** | **C** | **G** | **T** | **T** | **G** | **G** | **T** | **T** | **T** | **G** | **C** | **C** | **A** | **A** | **C** | **G** | **A** | **A** | **C** | **G** | **T** | **A** | **T** | **T** | **G** | **G** | **C** | **A** | **A** | **C** | **A** | **A** | **T** | **A** | **T** | **C** | **T** | **A** | **T** | **G** | **A** | **A** | **T** | **G** | **G** | **G** | **A** | **C** | **G** | **A** | **C** | **A** | **T** | **C** | **A** | **A** | **T** | **A** | **T** | **T** | **G** | **A** | **T** | **T** | **T** | **A** | **A** | **T** | **C** | **T** | **C** | **T** | **G** | **G** | **G** | **G** | **A** | **T** | **A** | **C** | **T** | **A** | **T** | **T** | **A** | **T** | **T** | **G** | **A** | **T** | **T** | **T** | **A** | **G** | **G** | **C** | **A** | **A** | **T** | **A** | **C** | **G** | **C** | **T** | **A** | **C** | **T** | **T** | **C** | **C** | **G** | **A** | **A** | **A** | **G** | **A** | **A** | **G** | **A** | **T** | **A** | **A** | **T** | **A** | **T** | **T** | **A** | **T** | **T** | **A** | **T** | **T** | **A** | **T** | **T** | **C** | **G** | **T** | **A** | **A** | **A** | **G** | **G** | **T** | **T** | **T** | **T** | **G** | **G** | **C** | **C** | **G** | **C** | **A** | **C** | **G** | **C** | **G** | **A** | **A** | **T** | **T** | **C** | **T** | **A** | **G** | **T** | **G** | **C** | **C** | **G** | **T** | **T** | **A** | **G** | **G** | **T** | **G** | **T** | **A** | **G** | **C** | **T** | **A** | **T** | **T** | **T** | **T** | **G** | **T** | **T** | **A** | **G** | **A** | **A** | **C** | **A** | **T** | **T** | **C** | **A** | **A** | **C** | **G** | **T** | **T** | **T** | **T** | **A** | **C** | **G** | **G** | **A** | **A** | **C** | **A** | **G** | **T** | **G** | **C** | **G** | **T** | **T** | **T** | **T** | **G** | **A** | **A** | **G** | **A** | **A** | **G** | **A** | **A** | **A** | **A** | **A** | **T** | **A** | **T** | **C** | **A** | **A** | **T** | **T** | **G** | **A** | **A** | **A** | **A** | **A** | **C** | **G** | **A** | **A** | **T** | **C** | **A** | **T** | **T** | **A** | **A** | **A** | **A** | **A** | **T** | **T** | **T** | **A** | **C** | **A** | **G** | **C** | **A** | **A** | **T** | **G** | **A** | **T** | **T** | **A** | **T** | **G** | **A** | **T** | **A** | **C** | **C** | **A** | **A** | **T** | **C** | **T** | **T** | **C** | **G** | **T** | **C** | **G** | **T** | **T** | **T** | **G** | **A** | **A** | **A** | **A** | **T** | **T** | **A** | **T** | **G** | **A** | **C** | **G** | **A** | **A** | **C** | **A** | **C** | **T** | **T** | **T** | **A** | **G** | **T** | **A** | **G** | **G** | **A** | **G** | **A** | **T** | **G** | **T** | **T** | **G** | **A** | **G** | **G** | **T** | **G** | **A** | **T** | **C** | **C** | **G** | **T** | **G** | **T** | **A** | **-** | **-** | **-** |
| **N60268F** | **A** | **T** | **G** | **A** | **A** | **T** | **A** | **A** | **C** | **C** | **C** | **T** | **T** | **G** | **G** | **C** | **G** | **C** | **T** | **T** | **T** | **T** | **T** | **T** | **A** | **T** | **C** | **G** | **T** | **C** | **G** | **C** | **A** | **G** | **A** | **A** | **G** | **C** | **A** | **T** | **T** | **A** | **C** | **T** | **T** | **T** | **T** | **T** | **A** | **T** | **T** | **C** | **T** | **G** | **G** | **C** | **G** | **T** | **T** | **A** | **T** | **G** | **G** | **C** | **A** | **A** | **A** | **T** | **T** | **G** | **T** | **A** | **C** | **A** | **T** | **A** | **A** | **T** | **C** | **C** | **T** | **G** | **G** | **A** | **T** | **T** | **A** | **G** | **C** | **T** | **G** | **T** | **T** | **T** | **T** | **A** | **T** | **T** | **A** | **A** | **C** | **A** | **A** | **T** | **T** | **G** | **G** | **C** | **G** | **T** | **T** | **T** | **T** | **A** | **C** | **T** | **T** | **G** | **T** | **G** | **G** | **C** | **C** | **T** | **A** | **C** | **G** | **T** | **T** | **T** | **C** | **C** | **A** | **G** | **G** | **A** | **A** | **A** | **G** | **C** | **A** | **T** | **C** | **T** | **A** | **A** | **A** | **A** | **C** | **A** | **C** | **A** | **T** | **T** | **T** | **T** | **A** | **A** | **C** | **A** | **A** | **C** | **T** | **T** | **T** | **C** | **A** | **A** | **T** | **T** | **C** | **G** | **T** | **C** | **C** | **T** | **C** | **G** | **G** | **C** | **G** | **T** | **T** | **G** | **T** | **T** | **T** | **T** | **T** | **A** | **T** | **T** | **G** | **T** | **C** | **A** | **T** | **T** | **G** | **G** | **T** | **G** | **C** | **A** | **A** | **T** | **G** | **A** | **A** | **C** | **A** | **G** | **C** | **A** | **C** | **G** | **G** | **C** | **T** | **G** | **T** | **T** | **T** | **G** | **G** | **T** | **T** | **T** | **A** | **T** | **G** | **T** | **T** | **G** | **A** | **T** | **T** | **T** | **T** | **T** | **G** | **G** | **C** | **G** | **T** | **A** | **C** | **T** | **C** | **T** | **T** | **T** | **A** | **T** | **C** | **G** | **G** | **C** | **T** | **T** | **A** | **A** | **A** | **A** | **G** | **G** | **C** | **T** | **T** | **T** | **G** | **A** | **G** | **A** | **T** | **T** | **T** | **C** | **A** | **G** | **G** | **C** | **G** | **T** | **G** | **G** | **A** | **T** | **A** | **T** | **A** | **G** | **C** | **T** | **G** | **A** | **G** | **C** | **G** | **A** | **G** | **C** | **A** | **C** | **C** | **T** | **T** | **G** | **G** | **C** | **G** | **T** | **A** | **A** | **A** | **A** | **A** | **A** | **C** | **A** | **A** | **A** | **T** | **G** | **A** | **T** | **T** | **A** | **T** | **G** | **G** | **T** | **G** | **G** | **A** | **G** | **A** | **C** | **G** | **G** | **C** | **G** | **G** | **C** | **A** | **A** | **A** | **A** | **G** | **A** | **A** | **C** | **C** | **T** | **A** | **A** | **A** | **A** | **A** | **T** | **G** | **G** | **C** | **A** | **A** | **A** | **C** | **G** | **A** | **T** | **T** | **T** | **A** | **A** | **A** | **C** | **G** | **C** | **C** | **G** | **T** | **T** | **G** | **G** | **T** | **T** | **T** | **G** | **C** | **C** | **A** | **A** | **T** | **G** | **A** | **A** | **C** | **G** | **C** | **A** | **T** | **T** | **G** | **G** | **T** | **A** | **A** | **C** | **A** | **A** | **T** | **A** | **T** | **C** | **T** | **A** | **T** | **G** | **A** | **A** | **T** | **G** | **G** | **G** | **A** | **T** | **G** | **A** | **T** | **A** | **T** | **C** | **A** | **A** | **T** | **A** | **T** | **T** | **G** | **A** | **T** | **T** | **T** | **A** | **A** | **T** | **C** | **T** | **C** | **T** | **G** | **G** | **G** | **G** | **A** | **C** | **A** | **C** | **C** | **A** | **T** | **T** | **A** | **T** | **T** | **G** | **A** | **T** | **T** | **T** | **A** | **G** | **G** | **T** | **A** | **A** | **T** | **A** | **C** | **G** | **C** | **T** | **A** | **C** | **T** | **A** | **C** | **C** | **G** | **A** | **A** | **A** | **G** | **A** | **A** | **G** | **A** | **C** | **A** | **A** | **T** | **A** | **T** | **T** | **A** | **T** | **T** | **A** | **T** | **T** | **A** | **T** | **T** | **C** | **G** | **T** | **A** | **A** | **A** | **G** | **G** | **T** | **T** | **T** | **T** | **G** | **G** | **C** | **C** | **G** | **C** | **A** | **C** | **G** | **C** | **G** | **A** | **A** | **T** | **T** | **C** | **T** | **A** | **G** | **T** | **A** | **C** | **C** | **G** | **T** | **T** | **A** | **G** | **G** | **T** | **G** | **T** | **A** | **G** | **C** | **T** | **A** | **T** | **T** | **T** | **T** | **G** | **T** | **T** | **A** | **G** | **A** | **A** | **C** | **A** | **T** | **T** | **C** | **A** | **A** | **C** | **T** | **T** | **T** | **T** | **T** | **A** | **C** | **G** | **G** | **A** | **A** | **C** | **G** | **G** | **T** | **A** | **C** | **G** | **T** | **T** | **T** | **T** | **G** | **A** | **A** | **G** | **A** | **A** | **G** | **A** | **A** | **A** | **A** | **A** | **T** | **A** | **T** | **C** | **A** | **A** | **T** | **T** | **G** | **A** | **A** | **A** | **A** | **A** | **C** | **G** | **A** | **A** | **T** | **C** | **A** | **T** | **T** | **A** | **A** | **A** | **A** | **A** | **T** | **T** | **T** | **A** | **C** | **A** | **G** | **C** | **A** | **A** | **T** | **G** | **A** | **T** | **T** | **A** | **T** | **G** | **A** | **T** | **A** | **C** | **C** | **A** | **A** | **T** | **C** | **T** | **T** | **C** | **G** | **T** | **C** | **G** | **T** | **T** | **T** | **G** | **A** | **A** | **A** | **A** | **T** | **T** | **A** | **T** | **G** | **A** | **C** | **G** | **A** | **A** | **C** | **A** | **C** | **T** | **T** | **T** | **A** | **G** | **T** | **A** | **G** | **G** | **A** | **G** | **A** | **T** | **G** | **T** | **T** | **G** | **A** | **G** | **G** | **T** | **G** | **A** | **T** | **C** | **C** | **G** | **T** | **G** | **T** | **A** | **-** | **-** | **-** |
| **N59910F** | **A** | **T** | **G** | **A** | **A** | **T** | **A** | **A** | **C** | **C** | **C** | **T** | **T** | **G** | **G** | **C** | **G** | **C** | **T** | **T** | **T** | **T** | **T** | **T** | **A** | **T** | **C** | **G** | **T** | **C** | **G** | **C** | **A** | **G** | **A** | **A** | **G** | **C** | **A** | **T** | **T** | **A** | **C** | **T** | **T** | **T** | **T** | **T** | **A** | **T** | **T** | **C** | **T** | **G** | **G** | **C** | **G** | **T** | **T** | **A** | **T** | **G** | **G** | **C** | **A** | **A** | **A** | **T** | **T** | **G** | **T** | **A** | **C** | **A** | **T** | **A** | **A** | **T** | **C** | **C** | **T** | **G** | **G** | **A** | **T** | **T** | **A** | **G** | **C** | **T** | **G** | **T** | **T** | **T** | **T** | **A** | **T** | **T** | **A** | **A** | **C** | **A** | **A** | **T** | **T** | **G** | **G** | **C** | **G** | **T** | **T** | **T** | **T** | **A** | **C** | **T** | **T** | **G** | **T** | **G** | **G** | **C** | **C** | **T** | **A** | **C** | **G** | **T** | **T** | **T** | **C** | **C** | **A** | **G** | **G** | **A** | **A** | **A** | **G** | **C** | **A** | **T** | **C** | **T** | **A** | **A** | **A** | **A** | **C** | **A** | **C** | **A** | **T** | **T** | **T** | **T** | **A** | **A** | **C** | **A** | **A** | **C** | **T** | **T** | **T** | **C** | **A** | **A** | **T** | **T** | **C** | **G** | **T** | **C** | **C** | **T** | **C** | **G** | **G** | **C** | **G** | **T** | **T** | **G** | **T** | **T** | **T** | **T** | **T** | **A** | **T** | **T** | **G** | **T** | **C** | **A** | **T** | **T** | **G** | **G** | **T** | **G** | **C** | **A** | **A** | **T** | **G** | **A** | **A** | **C** | **A** | **G** | **C** | **A** | **C** | **G** | **G** | **C** | **T** | **G** | **T** | **T** | **T** | **G** | **G** | **T** | **T** | **T** | **A** | **T** | **G** | **T** | **T** | **G** | **A** | **T** | **T** | **T** | **T** | **T** | **G** | **G** | **C** | **G** | **T** | **A** | **C** | **T** | **C** | **T** | **T** | **T** | **A** | **T** | **C** | **G** | **G** | **C** | **T** | **T** | **A** | **A** | **A** | **A** | **G** | **G** | **C** | **T** | **T** | **T** | **G** | **A** | **G** | **A** | **T** | **T** | **T** | **C** | **A** | **G** | **G** | **C** | **G** | **T** | **G** | **G** | **A** | **T** | **A** | **T** | **A** | **G** | **C** | **T** | **G** | **A** | **G** | **C** | **G** | **A** | **G** | **C** | **A** | **C** | **C** | **T** | **T** | **G** | **G** | **C** | **G** | **T** | **A** | **A** | **A** | **A** | **A** | **A** | **C** | **A** | **A** | **A** | **T** | **G** | **A** | **T** | **T** | **A** | **T** | **G** | **G** | **T** | **G** | **G** | **A** | **G** | **A** | **C** | **G** | **G** | **C** | **G** | **G** | **C** | **A** | **A** | **A** | **A** | **G** | **A** | **A** | **C** | **C** | **T** | **A** | **A** | **A** | **A** | **A** | **T** | **G** | **G** | **C** | **A** | **A** | **A** | **C** | **G** | **A** | **T** | **T** | **T** | **A** | **A** | **A** | **C** | **G** | **C** | **C** | **G** | **T** | **T** | **G** | **G** | **T** | **T** | **T** | **G** | **C** | **C** | **A** | **A** | **T** | **G** | **A** | **A** | **C** | **G** | **C** | **A** | **T** | **T** | **G** | **G** | **T** | **A** | **A** | **C** | **A** | **A** | **T** | **A** | **T** | **C** | **T** | **A** | **T** | **G** | **A** | **A** | **T** | **G** | **G** | **G** | **A** | **T** | **G** | **A** | **T** | **A** | **T** | **C** | **A** | **A** | **T** | **A** | **T** | **T** | **G** | **A** | **T** | **T** | **T** | **A** | **A** | **T** | **C** | **T** | **C** | **T** | **G** | **G** | **G** | **G** | **A** | **C** | **A** | **C** | **C** | **A** | **T** | **T** | **A** | **T** | **T** | **G** | **A** | **T** | **T** | **T** | **A** | **G** | **G** | **T** | **A** | **A** | **T** | **A** | **C** | **G** | **C** | **T** | **A** | **C** | **T** | **A** | **C** | **C** | **G** | **A** | **A** | **A** | **G** | **A** | **A** | **G** | **A** | **C** | **A** | **A** | **T** | **A** | **T** | **T** | **A** | **T** | **T** | **A** | **T** | **T** | **A** | **T** | **T** | **C** | **G** | **T** | **A** | **A** | **A** | **G** | **G** | **T** | **T** | **T** | **T** | **G** | **G** | **C** | **C** | **G** | **C** | **A** | **C** | **G** | **C** | **G** | **A** | **A** | **T** | **T** | **C** | **T** | **A** | **G** | **T** | **A** | **C** | **C** | **G** | **T** | **T** | **A** | **G** | **G** | **T** | **G** | **T** | **A** | **G** | **C** | **T** | **A** | **T** | **T** | **T** | **T** | **G** | **T** | **T** | **A** | **G** | **A** | **A** | **C** | **A** | **T** | **T** | **C** | **A** | **A** | **C** | **T** | **T** | **T** | **T** | **T** | **A** | **C** | **G** | **G** | **A** | **A** | **C** | **G** | **G** | **T** | **A** | **C** | **G** | **T** | **T** | **T** | **T** | **G** | **A** | **A** | **G** | **A** | **A** | **G** | **A** | **A** | **A** | **A** | **A** | **T** | **A** | **T** | **C** | **A** | **A** | **T** | **T** | **G** | **A** | **A** | **A** | **A** | **A** | **C** | **G** | **A** | **A** | **T** | **C** | **A** | **T** | **T** | **A** | **A** | **A** | **A** | **A** | **T** | **T** | **T** | **A** | **C** | **A** | **G** | **C** | **A** | **A** | **T** | **G** | **A** | **T** | **T** | **A** | **T** | **G** | **A** | **T** | **A** | **C** | **C** | **A** | **A** | **T** | **C** | **T** | **T** | **C** | **G** | **T** | **C** | **G** | **T** | **T** | **T** | **G** | **A** | **A** | **A** | **A** | **T** | **T** | **A** | **T** | **G** | **A** | **C** | **G** | **A** | **A** | **C** | **A** | **C** | **T** | **T** | **T** | **A** | **G** | **T** | **A** | **G** | **G** | **A** | **G** | **A** | **T** | **G** | **T** | **T** | **G** | **A** | **G** | **G** | **T** | **G** | **A** | **T** | **C** | **C** | **G** | **T** | **G** | **T** | **A** | **-** | **-** | **-** |
| **N60280F** | **A** | **T** | **G** | **A** | **A** | **T** | **A** | **A** | **C** | **C** | **C** | **T** | **T** | **G** | **G** | **C** | **G** | **C** | **T** | **T** | **T** | **T** | **T** | **T** | **A** | **T** | **C** | **G** | **T** | **C** | **G** | **C** | **A** | **G** | **A** | **A** | **G** | **C** | **A** | **T** | **T** | **A** | **C** | **T** | **T** | **T** | **T** | **T** | **A** | **T** | **T** | **C** | **T** | **G** | **G** | **C** | **G** | **T** | **T** | **A** | **T** | **G** | **G** | **C** | **A** | **A** | **A** | **T** | **T** | **G** | **T** | **A** | **C** | **A** | **T** | **A** | **A** | **T** | **C** | **C** | **T** | **G** | **G** | **A** | **T** | **T** | **A** | **G** | **C** | **T** | **G** | **T** | **T** | **T** | **T** | **A** | **T** | **T** | **A** | **A** | **C** | **A** | **A** | **T** | **T** | **G** | **G** | **C** | **G** | **T** | **T** | **T** | **T** | **A** | **C** | **T** | **T** | **G** | **T** | **G** | **G** | **C** | **C** | **T** | **A** | **C** | **G** | **T** | **T** | **T** | **C** | **C** | **A** | **G** | **G** | **A** | **A** | **A** | **G** | **C** | **A** | **T** | **C** | **A** | **A** | **A** | **A** | **A** | **C** | **A** | **C** | **A** | **T** | **T** | **T** | **T** | **A** | **A** | **C** | **A** | **A** | **C** | **T** | **T** | **T** | **C** | **A** | **A** | **T** | **T** | **C** | **G** | **T** | **C** | **C** | **T** | **C** | **G** | **G** | **C** | **G** | **T** | **T** | **G** | **T** | **T** | **T** | **T** | **T** | **A** | **T** | **T** | **G** | **T** | **C** | **A** | **T** | **T** | **G** | **G** | **T** | **G** | **C** | **A** | **A** | **T** | **G** | **A** | **A** | **C** | **A** | **G** | **C** | **A** | **C** | **G** | **G** | **C** | **T** | **G** | **T** | **T** | **T** | **G** | **G** | **T** | **T** | **T** | **A** | **T** | **G** | **T** | **T** | **G** | **A** | **T** | **T** | **T** | **T** | **T** | **G** | **G** | **C** | **G** | **T** | **A** | **C** | **T** | **C** | **T** | **T** | **T** | **A** | **T** | **C** | **G** | **G** | **C** | **T** | **T** | **A** | **A** | **A** | **A** | **G** | **G** | **C** | **T** | **T** | **T** | **G** | **A** | **G** | **A** | **T** | **T** | **T** | **C** | **A** | **G** | **G** | **C** | **G** | **T** | **G** | **G** | **A** | **T** | **A** | **T** | **A** | **G** | **C** | **T** | **G** | **A** | **G** | **C** | **G** | **A** | **G** | **C** | **A** | **C** | **C** | **T** | **T** | **G** | **G** | **C** | **G** | **T** | **A** | **A** | **A** | **A** | **A** | **A** | **C** | **A** | **A** | **A** | **T** | **G** | **A** | **T** | **T** | **A** | **T** | **G** | **G** | **T** | **G** | **G** | **A** | **G** | **A** | **C** | **G** | **G** | **C** | **G** | **G** | **C** | **A** | **A** | **A** | **A** | **G** | **A** | **A** | **C** | **C** | **T** | **A** | **A** | **A** | **A** | **A** | **T** | **G** | **G** | **C** | **A** | **A** | **A** | **C** | **G** | **A** | **T** | **T** | **T** | **A** | **A** | **A** | **C** | **G** | **C** | **C** | **G** | **C** | **T** | **G** | **G** | **T** | **T** | **T** | **G** | **C** | **C** | **A** | **A** | **C** | **G** | **A** | **A** | **C** | **G** | **C** | **A** | **T** | **T** | **G** | **G** | **T** | **A** | **A** | **C** | **A** | **A** | **T** | **A** | **T** | **C** | **T** | **A** | **T** | **G** | **A** | **A** | **T** | **G** | **G** | **G** | **A** | **C** | **G** | **A** | **T** | **A** | **T** | **C** | **A** | **A** | **T** | **A** | **T** | **T** | **G** | **A** | **T** | **T** | **T** | **A** | **A** | **T** | **C** | **T** | **C** | **T** | **G** | **G** | **G** | **G** | **A** | **C** | **A** | **C** | **C** | **A** | **T** | **T** | **A** | **T** | **T** | **G** | **A** | **T** | **T** | **T** | **A** | **G** | **G** | **T** | **A** | **A** | **T** | **A** | **C** | **G** | **C** | **T** | **A** | **C** | **T** | **A** | **C** | **C** | **G** | **A** | **A** | **A** | **G** | **A** | **A** | **G** | **A** | **C** | **A** | **A** | **T** | **A** | **T** | **T** | **A** | **T** | **T** | **A** | **T** | **T** | **A** | **T** | **T** | **C** | **G** | **T** | **A** | **A** | **A** | **G** | **G** | **T** | **T** | **T** | **T** | **G** | **G** | **C** | **C** | **G** | **C** | **A** | **C** | **G** | **C** | **G** | **A** | **A** | **T** | **T** | **C** | **T** | **A** | **G** | **T** | **A** | **C** | **C** | **G** | **T** | **T** | **A** | **G** | **G** | **T** | **G** | **T** | **A** | **G** | **C** | **T** | **A** | **T** | **T** | **T** | **T** | **G** | **T** | **T** | **A** | **G** | **A** | **A** | **C** | **A** | **T** | **T** | **C** | **A** | **A** | **C** | **G** | **T** | **T** | **T** | **T** | **A** | **C** | **G** | **G** | **A** | **A** | **C** | **G** | **G** | **T** | **A** | **C** | **G** | **T** | **T** | **T** | **T** | **G** | **A** | **A** | **G** | **A** | **A** | **G** | **A** | **A** | **A** | **A** | **A** | **T** | **A** | **T** | **C** | **A** | **A** | **T** | **T** | **G** | **A** | **A** | **A** | **A** | **A** | **C** | **G** | **A** | **A** | **T** | **C** | **A** | **T** | **T** | **A** | **A** | **A** | **A** | **A** | **T** | **T** | **T** | **A** | **C** | **A** | **G** | **C** | **A** | **A** | **T** | **G** | **A** | **T** | **T** | **A** | **C** | **G** | **A** | **T** | **A** | **C** | **C** | **A** | **A** | **T** | **C** | **T** | **T** | **C** | **G** | **T** | **C** | **G** | **T** | **T** | **T** | **G** | **A** | **A** | **A** | **A** | **T** | **T** | **A** | **T** | **G** | **A** | **C** | **G** | **A** | **A** | **C** | **A** | **C** | **T** | **T** | **T** | **A** | **G** | **T** | **A** | **G** | **G** | **A** | **G** | **A** | **T** | **G** | **T** | **T** | **G** | **A** | **G** | **G** | **T** | **G** | **A** | **T** | **C** | **C** | **G** | **T** | **G** | **T** | **A** | **-** | **-** | **-** |
| **N54121** | **A** | **T** | **G** | **A** | **A** | **T** | **A** | **A** | **C** | **C** | **C** | **T** | **T** | **G** | **G** | **C** | **G** | **C** | **T** | **T** | **T** | **T** | **T** | **T** | **A** | **T** | **C** | **G** | **T** | **C** | **G** | **C** | **A** | **G** | **A** | **A** | **G** | **C** | **A** | **T** | **T** | **A** | **C** | **T** | **T** | **T** | **T** | **T** | **A** | **T** | **T** | **C** | **T** | **G** | **G** | **C** | **G** | **T** | **T** | **A** | **T** | **G** | **G** | **C** | **A** | **A** | **A** | **T** | **T** | **G** | **T** | **A** | **C** | **A** | **T** | **A** | **A** | **T** | **C** | **C** | **T** | **G** | **G** | **A** | **T** | **T** | **A** | **G** | **C** | **T** | **G** | **T** | **T** | **T** | **T** | **A** | **T** | **T** | **A** | **A** | **C** | **A** | **A** | **T** | **T** | **G** | **G** | **C** | **G** | **T** | **T** | **T** | **T** | **A** | **C** | **T** | **T** | **G** | **T** | **G** | **G** | **C** | **C** | **T** | **A** | **C** | **G** | **T** | **T** | **T** | **C** | **C** | **A** | **G** | **G** | **A** | **A** | **A** | **G** | **C** | **A** | **T** | **C** | **T** | **A** | **A** | **A** | **A** | **C** | **A** | **C** | **A** | **T** | **T** | **T** | **T** | **A** | **A** | **C** | **A** | **A** | **C** | **T** | **T** | **T** | **C** | **A** | **A** | **T** | **T** | **C** | **G** | **T** | **C** | **C** | **T** | **C** | **G** | **G** | **C** | **G** | **T** | **T** | **G** | **T** | **T** | **T** | **T** | **T** | **A** | **T** | **T** | **G** | **T** | **C** | **A** | **T** | **T** | **G** | **G** | **T** | **G** | **C** | **A** | **A** | **T** | **G** | **A** | **A** | **C** | **A** | **G** | **C** | **A** | **C** | **G** | **G** | **C** | **T** | **G** | **T** | **T** | **T** | **G** | **G** | **T** | **T** | **T** | **A** | **T** | **G** | **T** | **T** | **G** | **A** | **T** | **T** | **T** | **T** | **T** | **G** | **G** | **C** | **G** | **T** | **A** | **C** | **T** | **C** | **T** | **T** | **T** | **A** | **T** | **C** | **G** | **G** | **C** | **T** | **T** | **A** | **A** | **A** | **A** | **G** | **G** | **C** | **T** | **T** | **T** | **G** | **A** | **G** | **A** | **T** | **T** | **T** | **C** | **A** | **G** | **G** | **C** | **G** | **T** | **G** | **G** | **A** | **T** | **A** | **T** | **A** | **G** | **C** | **T** | **G** | **A** | **G** | **C** | **G** | **A** | **G** | **C** | **A** | **C** | **C** | **T** | **T** | **G** | **G** | **C** | **G** | **A** | **A** | **A** | **A** | **A** | **A** | **A** | **C** | **A** | **A** | **A** | **T** | **G** | **A** | **T** | **T** | **A** | **T** | **G** | **G** | **T** | **G** | **G** | **A** | **G** | **A** | **C** | **G** | **G** | **C** | **G** | **G** | **C** | **A** | **A** | **A** | **A** | **G** | **A** | **A** | **C** | **C** | **T** | **A** | **A** | **A** | **A** | **A** | **T** | **G** | **G** | **C** | **A** | **A** | **A** | **C** | **G** | **A** | **T** | **T** | **T** | **A** | **A** | **A** | **C** | **G** | **C** | **C** | **G** | **C** | **T** | **G** | **G** | **T** | **T** | **T** | **G** | **C** | **C** | **A** | **A** | **C** | **G** | **A** | **A** | **C** | **G** | **C** | **A** | **T** | **T** | **G** | **G** | **T** | **A** | **A** | **C** | **A** | **A** | **T** | **A** | **T** | **C** | **T** | **A** | **T** | **G** | **A** | **A** | **T** | **G** | **G** | **G** | **A** | **C** | **G** | **A** | **T** | **A** | **T** | **C** | **A** | **A** | **T** | **A** | **T** | **T** | **G** | **A** | **T** | **T** | **T** | **A** | **A** | **T** | **C** | **T** | **C** | **T** | **G** | **G** | **G** | **G** | **A** | **C** | **A** | **C** | **C** | **A** | **T** | **T** | **A** | **T** | **T** | **G** | **A** | **T** | **T** | **T** | **A** | **G** | **G** | **T** | **A** | **A** | **T** | **A** | **C** | **G** | **C** | **T** | **A** | **C** | **T** | **A** | **C** | **C** | **G** | **A** | **A** | **A** | **G** | **A** | **A** | **G** | **A** | **C** | **A** | **A** | **T** | **A** | **T** | **T** | **A** | **T** | **T** | **A** | **T** | **T** | **A** | **T** | **T** | **C** | **G** | **T** | **A** | **A** | **A** | **G** | **G** | **T** | **T** | **T** | **T** | **G** | **G** | **C** | **C** | **G** | **C** | **A** | **C** | **G** | **C** | **G** | **A** | **A** | **T** | **T** | **C** | **T** | **A** | **G** | **T** | **G** | **C** | **C** | **G** | **T** | **T** | **A** | **G** | **G** | **T** | **G** | **T** | **A** | **G** | **C** | **T** | **A** | **T** | **T** | **T** | **T** | **G** | **T** | **T** | **A** | **G** | **A** | **A** | **C** | **A** | **T** | **T** | **C** | **A** | **A** | **C** | **T** | **T** | **T** | **T** | **T** | **A** | **C** | **G** | **G** | **A** | **A** | **C** | **G** | **G** | **T** | **A** | **C** | **G** | **T** | **T** | **T** | **T** | **G** | **A** | **A** | **G** | **A** | **A** | **G** | **A** | **A** | **A** | **A** | **A** | **T** | **A** | **T** | **C** | **A** | **A** | **T** | **T** | **G** | **A** | **A** | **A** | **A** | **A** | **C** | **G** | **A** | **A** | **T** | **C** | **A** | **T** | **T** | **A** | **A** | **A** | **A** | **A** | **T** | **T** | **T** | **A** | **C** | **A** | **G** | **C** | **A** | **A** | **T** | **G** | **A** | **T** | **T** | **A** | **C** | **G** | **A** | **T** | **A** | **C** | **C** | **A** | **A** | **T** | **C** | **T** | **T** | **C** | **G** | **T** | **C** | **G** | **T** | **T** | **T** | **G** | **A** | **A** | **A** | **A** | **T** | **T** | **A** | **T** | **G** | **A** | **C** | **G** | **A** | **A** | **C** | **A** | **C** | **T** | **T** | **T** | **A** | **G** | **T** | **A** | **G** | **G** | **A** | **G** | **A** | **T** | **G** | **T** | **T** | **G** | **A** | **G** | **G** | **T** | **G** | **A** | **T** | **C** | **C** | **G** | **T** | **G** | **T** | **A** | **-** | **-** | **-** |
| **N53766** | **A** | **T** | **G** | **A** | **A** | **T** | **A** | **A** | **C** | **C** | **C** | **T** | **T** | **G** | **G** | **C** | **G** | **C** | **T** | **T** | **T** | **T** | **T** | **T** | **A** | **T** | **C** | **G** | **T** | **C** | **G** | **C** | **A** | **G** | **A** | **A** | **G** | **C** | **A** | **T** | **T** | **A** | **C** | **T** | **T** | **T** | **T** | **T** | **A** | **T** | **T** | **C** | **T** | **G** | **G** | **C** | **G** | **T** | **T** | **A** | **T** | **G** | **G** | **C** | **A** | **A** | **A** | **T** | **T** | **G** | **T** | **A** | **C** | **A** | **T** | **A** | **A** | **T** | **C** | **C** | **T** | **G** | **G** | **A** | **T** | **T** | **A** | **G** | **C** | **T** | **G** | **T** | **T** | **T** | **T** | **A** | **T** | **T** | **A** | **A** | **C** | **A** | **A** | **T** | **T** | **G** | **G** | **C** | **G** | **T** | **T** | **T** | **T** | **A** | **C** | **T** | **T** | **G** | **T** | **G** | **G** | **C** | **C** | **T** | **A** | **C** | **G** | **T** | **T** | **T** | **C** | **C** | **A** | **G** | **G** | **A** | **A** | **A** | **G** | **C** | **A** | **T** | **C** | **T** | **A** | **A** | **A** | **A** | **C** | **A** | **C** | **A** | **T** | **T** | **T** | **T** | **A** | **A** | **C** | **A** | **A** | **C** | **T** | **T** | **T** | **C** | **A** | **A** | **T** | **T** | **C** | **G** | **T** | **C** | **C** | **T** | **C** | **G** | **G** | **C** | **G** | **T** | **T** | **G** | **T** | **T** | **T** | **T** | **T** | **A** | **T** | **T** | **G** | **T** | **C** | **A** | **T** | **T** | **G** | **G** | **T** | **G** | **C** | **A** | **A** | **T** | **G** | **A** | **A** | **T** | **A** | **G** | **C** | **A** | **C** | **G** | **G** | **C** | **T** | **G** | **T** | **T** | **T** | **G** | **G** | **T** | **T** | **T** | **A** | **T** | **G** | **T** | **T** | **G** | **A** | **T** | **T** | **T** | **T** | **T** | **G** | **G** | **C** | **G** | **T** | **A** | **C** | **T** | **C** | **T** | **T** | **T** | **A** | **T** | **C** | **G** | **G** | **C** | **T** | **T** | **A** | **A** | **A** | **A** | **G** | **G** | **C** | **T** | **T** | **T** | **G** | **A** | **G** | **A** | **T** | **T** | **T** | **C** | **A** | **G** | **G** | **C** | **G** | **T** | **G** | **G** | **A** | **T** | **A** | **T** | **A** | **G** | **C** | **T** | **G** | **A** | **G** | **C** | **G** | **A** | **G** | **C** | **A** | **C** | **C** | **T** | **T** | **G** | **G** | **C** | **G** | **A** | **A** | **A** | **A** | **A** | **A** | **A** | **C** | **A** | **A** | **A** | **T** | **G** | **A** | **T** | **T** | **A** | **T** | **G** | **G** | **T** | **G** | **G** | **A** | **G** | **A** | **C** | **G** | **G** | **C** | **G** | **G** | **C** | **A** | **A** | **A** | **A** | **G** | **A** | **A** | **C** | **C** | **T** | **A** | **A** | **A** | **A** | **A** | **T** | **G** | **G** | **C** | **A** | **A** | **A** | **C** | **G** | **G** | **T** | **T** | **T** | **A** | **A** | **A** | **C** | **G** | **C** | **C** | **G** | **C** | **T** | **G** | **G** | **T** | **T** | **T** | **G** | **C** | **C** | **A** | **A** | **C** | **G** | **A** | **A** | **C** | **G** | **C** | **A** | **T** | **T** | **G** | **G** | **T** | **A** | **A** | **C** | **A** | **A** | **T** | **A** | **T** | **C** | **T** | **A** | **T** | **G** | **A** | **A** | **T** | **G** | **G** | **G** | **A** | **C** | **G** | **A** | **T** | **A** | **T** | **C** | **A** | **A** | **T** | **A** | **T** | **T** | **G** | **A** | **T** | **T** | **T** | **A** | **A** | **T** | **C** | **T** | **C** | **T** | **G** | **G** | **G** | **G** | **A** | **C** | **A** | **C** | **C** | **A** | **T** | **T** | **A** | **T** | **T** | **G** | **A** | **T** | **T** | **T** | **A** | **G** | **G** | **T** | **A** | **A** | **T** | **A** | **C** | **G** | **C** | **T** | **A** | **C** | **T** | **A** | **C** | **C** | **G** | **A** | **A** | **A** | **G** | **A** | **A** | **G** | **A** | **C** | **A** | **A** | **T** | **A** | **T** | **T** | **A** | **T** | **T** | **A** | **T** | **T** | **A** | **T** | **T** | **C** | **G** | **T** | **A** | **A** | **A** | **G** | **G** | **T** | **T** | **T** | **T** | **G** | **G** | **C** | **C** | **G** | **C** | **A** | **C** | **G** | **C** | **G** | **A** | **A** | **T** | **T** | **C** | **T** | **A** | **G** | **T** | **G** | **C** | **C** | **G** | **T** | **T** | **A** | **G** | **G** | **T** | **G** | **T** | **A** | **G** | **C** | **T** | **A** | **T** | **T** | **T** | **T** | **G** | **T** | **T** | **A** | **G** | **A** | **A** | **C** | **A** | **T** | **T** | **C** | **A** | **A** | **C** | **T** | **T** | **T** | **T** | **T** | **A** | **C** | **G** | **G** | **A** | **A** | **C** | **G** | **G** | **T** | **A** | **C** | **G** | **T** | **T** | **T** | **T** | **G** | **A** | **A** | **G** | **A** | **A** | **G** | **A** | **A** | **A** | **A** | **A** | **T** | **A** | **T** | **C** | **A** | **A** | **T** | **T** | **G** | **A** | **A** | **A** | **A** | **A** | **C** | **G** | **A** | **A** | **T** | **C** | **A** | **T** | **T** | **A** | **A** | **A** | **A** | **A** | **T** | **T** | **T** | **A** | **C** | **A** | **G** | **C** | **A** | **A** | **T** | **G** | **A** | **T** | **T** | **A** | **T** | **G** | **A** | **T** | **A** | **C** | **C** | **A** | **A** | **T** | **C** | **T** | **T** | **C** | **G** | **T** | **C** | **G** | **T** | **T** | **T** | **G** | **A** | **A** | **A** | **A** | **T** | **T** | **A** | **T** | **G** | **A** | **C** | **G** | **A** | **A** | **C** | **A** | **C** | **T** | **T** | **T** | **A** | **G** | **T** | **A** | **G** | **G** | **A** | **G** | **A** | **T** | **G** | **T** | **T** | **G** | **A** | **G** | **G** | **T** | **G** | **A** | **T** | **C** | **C** | **G** | **T** | **G** | **T** | **A** | **-** | **-** | **-** |
| **N59606F** | **A** | **T** | **G** | **A** | **A** | **T** | **A** | **A** | **C** | **C** | **C** | **T** | **T** | **G** | **G** | **C** | **G** | **C** | **T** | **T** | **T** | **T** | **T** | **T** | **A** | **T** | **C** | **G** | **T** | **C** | **G** | **C** | **A** | **G** | **A** | **A** | **G** | **C** | **A** | **T** | **T** | **A** | **C** | **T** | **T** | **T** | **T** | **T** | **A** | **T** | **T** | **C** | **T** | **G** | **G** | **C** | **G** | **T** | **T** | **A** | **T** | **G** | **G** | **C** | **A** | **A** | **A** | **T** | **T** | **G** | **T** | **A** | **C** | **A** | **T** | **A** | **A** | **T** | **C** | **C** | **T** | **G** | **G** | **A** | **T** | **T** | **A** | **G** | **C** | **T** | **G** | **T** | **T** | **T** | **T** | **A** | **T** | **T** | **A** | **A** | **C** | **A** | **A** | **T** | **T** | **G** | **G** | **C** | **G** | **T** | **T** | **T** | **T** | **A** | **C** | **T** | **T** | **G** | **T** | **G** | **G** | **C** | **C** | **T** | **A** | **C** | **G** | **T** | **T** | **T** | **C** | **C** | **A** | **G** | **G** | **A** | **A** | **A** | **G** | **C** | **A** | **T** | **C** | **T** | **A** | **A** | **A** | **A** | **C** | **A** | **C** | **A** | **T** | **T** | **T** | **T** | **A** | **A** | **C** | **A** | **A** | **C** | **T** | **T** | **T** | **C** | **A** | **A** | **T** | **T** | **C** | **G** | **T** | **C** | **C** | **T** | **C** | **G** | **G** | **C** | **G** | **T** | **T** | **G** | **T** | **T** | **T** | **T** | **T** | **A** | **T** | **T** | **G** | **T** | **C** | **A** | **T** | **T** | **G** | **G** | **T** | **G** | **C** | **A** | **A** | **T** | **G** | **A** | **A** | **C** | **A** | **G** | **C** | **A** | **C** | **G** | **G** | **C** | **T** | **G** | **T** | **T** | **T** | **G** | **G** | **T** | **T** | **T** | **A** | **T** | **G** | **T** | **T** | **G** | **A** | **T** | **T** | **T** | **T** | **T** | **G** | **G** | **T** | **G** | **T** | **A** | **C** | **T** | **C** | **T** | **T** | **T** | **A** | **T** | **C** | **G** | **G** | **C** | **T** | **T** | **A** | **A** | **A** | **A** | **G** | **G** | **C** | **T** | **T** | **T** | **G** | **A** | **G** | **A** | **T** | **T** | **T** | **C** | **A** | **G** | **G** | **C** | **G** | **T** | **G** | **G** | **A** | **T** | **A** | **T** | **A** | **G** | **C** | **T** | **G** | **A** | **G** | **C** | **G** | **A** | **G** | **C** | **A** | **C** | **C** | **T** | **T** | **G** | **G** | **C** | **G** | **A** | **A** | **A** | **A** | **A** | **A** | **A** | **C** | **A** | **A** | **A** | **T** | **G** | **A** | **T** | **T** | **A** | **T** | **G** | **G** | **T** | **G** | **G** | **A** | **G** | **A** | **C** | **G** | **G** | **C** | **G** | **G** | **C** | **A** | **A** | **A** | **A** | **G** | **A** | **A** | **C** | **C** | **T** | **A** | **A** | **A** | **A** | **A** | **T** | **G** | **G** | **C** | **A** | **A** | **A** | **C** | **G** | **A** | **T** | **T** | **T** | **A** | **A** | **A** | **C** | **G** | **C** | **C** | **G** | **C** | **T** | **G** | **G** | **T** | **T** | **T** | **G** | **C** | **C** | **A** | **A** | **C** | **G** | **A** | **A** | **C** | **G** | **C** | **A** | **T** | **T** | **G** | **G** | **T** | **A** | **A** | **C** | **A** | **A** | **T** | **A** | **T** | **C** | **T** | **A** | **T** | **G** | **A** | **A** | **T** | **G** | **G** | **G** | **A** | **C** | **G** | **A** | **T** | **A** | **T** | **C** | **A** | **A** | **T** | **A** | **T** | **T** | **G** | **A** | **T** | **T** | **T** | **A** | **A** | **T** | **C** | **T** | **C** | **T** | **G** | **G** | **G** | **G** | **A** | **C** | **A** | **C** | **C** | **A** | **T** | **T** | **A** | **T** | **T** | **G** | **A** | **T** | **T** | **T** | **A** | **G** | **G** | **T** | **A** | **A** | **T** | **A** | **C** | **G** | **C** | **T** | **A** | **C** | **T** | **A** | **C** | **C** | **G** | **A** | **A** | **A** | **G** | **A** | **A** | **G** | **A** | **C** | **A** | **A** | **T** | **A** | **T** | **T** | **A** | **T** | **T** | **A** | **T** | **T** | **A** | **T** | **T** | **C** | **G** | **T** | **A** | **A** | **A** | **G** | **G** | **T** | **T** | **T** | **T** | **G** | **G** | **C** | **C** | **G** | **C** | **A** | **C** | **G** | **C** | **G** | **A** | **A** | **T** | **T** | **C** | **T** | **A** | **G** | **T** | **G** | **C** | **C** | **G** | **T** | **T** | **A** | **G** | **G** | **T** | **G** | **T** | **A** | **G** | **C** | **T** | **A** | **T** | **T** | **T** | **T** | **G** | **T** | **T** | **A** | **G** | **A** | **A** | **C** | **A** | **T** | **T** | **C** | **A** | **A** | **C** | **T** | **T** | **T** | **T** | **T** | **A** | **C** | **G** | **G** | **A** | **A** | **C** | **G** | **G** | **T** | **A** | **C** | **G** | **T** | **T** | **T** | **T** | **G** | **A** | **A** | **G** | **A** | **A** | **G** | **A** | **A** | **A** | **A** | **A** | **T** | **A** | **T** | **C** | **A** | **A** | **T** | **T** | **G** | **A** | **A** | **A** | **A** | **A** | **C** | **G** | **A** | **A** | **T** | **C** | **A** | **T** | **T** | **A** | **A** | **A** | **A** | **A** | **T** | **T** | **T** | **A** | **C** | **A** | **G** | **C** | **A** | **A** | **T** | **G** | **A** | **T** | **T** | **A** | **C** | **G** | **A** | **T** | **A** | **C** | **C** | **A** | **A** | **T** | **C** | **T** | **T** | **C** | **G** | **T** | **C** | **G** | **T** | **T** | **T** | **G** | **A** | **A** | **A** | **A** | **T** | **T** | **A** | **T** | **G** | **A** | **C** | **G** | **A** | **A** | **C** | **A** | **C** | **T** | **T** | **T** | **A** | **G** | **T** | **A** | **G** | **G** | **A** | **G** | **A** | **T** | **G** | **T** | **T** | **G** | **A** | **G** | **G** | **T** | **G** | **A** | **T** | **C** | **C** | **G** | **T** | **G** | **T** | **A** | **-** | **-** | **-** |
| **N59836F** | **A** | **T** | **G** | **A** | **A** | **T** | **A** | **A** | **C** | **C** | **C** | **T** | **T** | **G** | **G** | **C** | **G** | **C** | **T** | **T** | **T** | **T** | **T** | **T** | **A** | **T** | **C** | **G** | **T** | **C** | **G** | **C** | **A** | **G** | **A** | **A** | **G** | **C** | **A** | **T** | **T** | **A** | **C** | **T** | **T** | **T** | **T** | **T** | **A** | **T** | **T** | **C** | **T** | **G** | **G** | **C** | **G** | **T** | **T** | **A** | **T** | **G** | **G** | **C** | **A** | **A** | **A** | **T** | **T** | **G** | **T** | **A** | **C** | **A** | **T** | **A** | **A** | **T** | **C** | **C** | **T** | **G** | **G** | **A** | **T** | **T** | **A** | **G** | **C** | **T** | **G** | **T** | **T** | **T** | **T** | **A** | **T** | **T** | **A** | **A** | **C** | **A** | **A** | **T** | **T** | **G** | **G** | **C** | **G** | **T** | **T** | **T** | **T** | **A** | **C** | **T** | **T** | **G** | **T** | **G** | **G** | **C** | **C** | **T** | **A** | **C** | **G** | **T** | **T** | **T** | **C** | **C** | **A** | **G** | **G** | **A** | **A** | **A** | **G** | **C** | **A** | **T** | **C** | **T** | **A** | **A** | **A** | **A** | **C** | **A** | **C** | **A** | **T** | **T** | **T** | **T** | **A** | **A** | **C** | **A** | **A** | **C** | **T** | **T** | **T** | **C** | **A** | **A** | **T** | **T** | **C** | **G** | **T** | **C** | **C** | **T** | **C** | **G** | **G** | **C** | **G** | **T** | **T** | **G** | **T** | **T** | **T** | **T** | **T** | **A** | **T** | **T** | **G** | **T** | **C** | **A** | **T** | **T** | **G** | **G** | **T** | **G** | **C** | **A** | **A** | **T** | **G** | **A** | **A** | **C** | **A** | **G** | **C** | **A** | **C** | **G** | **G** | **C** | **T** | **G** | **T** | **T** | **T** | **G** | **G** | **T** | **T** | **T** | **A** | **T** | **G** | **T** | **T** | **G** | **A** | **T** | **T** | **T** | **T** | **T** | **G** | **G** | **C** | **G** | **T** | **A** | **C** | **T** | **C** | **T** | **T** | **T** | **A** | **T** | **C** | **G** | **G** | **C** | **T** | **T** | **A** | **A** | **A** | **A** | **G** | **G** | **C** | **T** | **T** | **T** | **G** | **A** | **G** | **A** | **T** | **T** | **T** | **C** | **A** | **G** | **G** | **C** | **G** | **T** | **G** | **G** | **A** | **T** | **A** | **T** | **A** | **G** | **C** | **T** | **G** | **A** | **G** | **C** | **G** | **A** | **G** | **C** | **A** | **C** | **C** | **T** | **T** | **G** | **G** | **C** | **G** | **A** | **A** | **A** | **A** | **A** | **A** | **A** | **C** | **A** | **A** | **A** | **T** | **G** | **A** | **T** | **T** | **A** | **T** | **G** | **G** | **T** | **G** | **G** | **A** | **G** | **A** | **C** | **G** | **G** | **C** | **G** | **G** | **C** | **A** | **A** | **A** | **A** | **G** | **A** | **A** | **C** | **C** | **T** | **A** | **A** | **A** | **A** | **A** | **T** | **G** | **G** | **C** | **A** | **A** | **A** | **C** | **G** | **A** | **T** | **T** | **T** | **A** | **A** | **A** | **C** | **G** | **C** | **C** | **G** | **C** | **T** | **G** | **G** | **T** | **T** | **T** | **G** | **C** | **C** | **A** | **A** | **C** | **G** | **A** | **A** | **C** | **G** | **C** | **A** | **T** | **T** | **G** | **G** | **T** | **A** | **A** | **C** | **A** | **A** | **T** | **A** | **T** | **C** | **T** | **A** | **T** | **G** | **A** | **A** | **T** | **G** | **G** | **G** | **A** | **C** | **G** | **A** | **T** | **A** | **T** | **C** | **A** | **A** | **T** | **A** | **T** | **T** | **G** | **A** | **T** | **T** | **T** | **A** | **A** | **T** | **C** | **T** | **C** | **T** | **G** | **G** | **G** | **G** | **A** | **C** | **A** | **C** | **C** | **A** | **T** | **T** | **A** | **T** | **T** | **G** | **A** | **T** | **T** | **T** | **A** | **G** | **G** | **T** | **A** | **A** | **T** | **A** | **C** | **G** | **T** | **T** | **A** | **C** | **T** | **A** | **C** | **C** | **G** | **A** | **A** | **A** | **G** | **A** | **A** | **G** | **A** | **C** | **A** | **A** | **T** | **A** | **T** | **T** | **A** | **T** | **T** | **A** | **T** | **T** | **A** | **T** | **T** | **C** | **G** | **T** | **A** | **A** | **A** | **G** | **G** | **T** | **T** | **T** | **T** | **G** | **G** | **C** | **C** | **G** | **C** | **A** | **C** | **G** | **C** | **G** | **A** | **A** | **T** | **T** | **C** | **T** | **A** | **G** | **T** | **G** | **C** | **C** | **G** | **T** | **T** | **A** | **G** | **G** | **T** | **G** | **T** | **A** | **G** | **C** | **T** | **A** | **T** | **T** | **T** | **T** | **G** | **T** | **T** | **A** | **G** | **A** | **A** | **C** | **A** | **T** | **T** | **C** | **A** | **A** | **C** | **T** | **T** | **T** | **T** | **T** | **A** | **C** | **G** | **G** | **A** | **A** | **C** | **G** | **G** | **T** | **A** | **C** | **G** | **T** | **T** | **T** | **T** | **G** | **A** | **A** | **G** | **A** | **A** | **G** | **A** | **A** | **A** | **A** | **A** | **T** | **A** | **T** | **C** | **A** | **A** | **T** | **T** | **G** | **A** | **A** | **A** | **A** | **A** | **C** | **G** | **A** | **A** | **T** | **C** | **A** | **T** | **T** | **A** | **A** | **A** | **A** | **A** | **T** | **T** | **T** | **A** | **C** | **A** | **G** | **C** | **A** | **A** | **T** | **G** | **A** | **T** | **T** | **A** | **T** | **G** | **A** | **T** | **A** | **C** | **C** | **A** | **A** | **T** | **C** | **T** | **T** | **C** | **G** | **T** | **C** | **G** | **T** | **T** | **T** | **G** | **A** | **A** | **A** | **A** | **T** | **T** | **A** | **T** | **G** | **A** | **C** | **G** | **A** | **A** | **C** | **A** | **C** | **T** | **T** | **T** | **A** | **G** | **T** | **A** | **G** | **G** | **A** | **G** | **A** | **T** | **G** | **T** | **T** | **G** | **A** | **G** | **G** | **T** | **G** | **A** | **T** | **C** | **C** | **G** | **T** | **G** | **T** | **A** | **-** | **-** | **-** |
| **N54548** | **-** | **-** | **-** | **-** | **-** | **-** | **-** | **-** | **-** | **C** | **C** | **T** | **T** | **G** | **G** | **C** | **G** | **C** | **T** | **T** | **T** | **T** | **T** | **T** | **A** | **T** | **C** | **G** | **T** | **C** | **G** | **C** | **A** | **G** | **A** | **A** | **G** | **C** | **A** | **T** | **T** | **A** | **C** | **T** | **T** | **T** | **T** | **T** | **A** | **T** | **T** | **C** | **T** | **A** | **G** | **C** | **G** | **T** | **T** | **A** | **T** | **G** | **G** | **C** | **A** | **A** | **A** | **T** | **T** | **G** | **T** | **A** | **C** | **A** | **T** | **A** | **A** | **T** | **C** | **C** | **T** | **G** | **G** | **A** | **T** | **T** | **A** | **G** | **C** | **T** | **G** | **T** | **T** | **T** | **T** | **A** | **T** | **T** | **A** | **A** | **C** | **A** | **A** | **T** | **T** | **G** | **G** | **C** | **G** | **T** | **T** | **T** | **T** | **A** | **C** | **T** | **T** | **G** | **T** | **T** | **G** | **C** | **C** | **T** | **A** | **T** | **G** | **T** | **T** | **T** | **C** | **C** | **A** | **G** | **G** | **A** | **A** | **A** | **G** | **C** | **A** | **T** | **C** | **T** | **A** | **A** | **A** | **A** | **C** | **A** | **C** | **A** | **T** | **T** | **T** | **T** | **A** | **A** | **C** | **A** | **A** | **C** | **T** | **T** | **T** | **C** | **A** | **A** | **T** | **T** | **C** | **G** | **T** | **C** | **C** | **T** | **T** | **G** | **G** | **C** | **A** | **T** | **T** | **G** | **T** | **T** | **T** | **T** | **T** | **A** | **T** | **T** | **G** | **T** | **C** | **A** | **T** | **T** | **G** | **G** | **C** | **G** | **C** | **A** | **A** | **T** | **G** | **A** | **A** | **C** | **A** | **G** | **C** | **A** | **C** | **G** | **G** | **C** | **T** | **G** | **T** | **T** | **T** | **G** | **G** | **T** | **T** | **T** | **A** | **T** | **G** | **T** | **T** | **G** | **A** | **T** | **T** | **T** | **T** | **T** | **G** | **G** | **C** | **G** | **T** | **A** | **C** | **T** | **C** | **T** | **T** | **T** | **A** | **T** | **C** | **G** | **G** | **C** | **T** | **T** | **A** | **A** | **A** | **A** | **G** | **G** | **C** | **T** | **T** | **T** | **G** | **A** | **G** | **A** | **T** | **T** | **T** | **C** | **A** | **G** | **G** | **C** | **G** | **T** | **G** | **G** | **A** | **T** | **A** | **T** | **A** | **G** | **C** | **T** | **G** | **A** | **G** | **C** | **G** | **A** | **G** | **C** | **A** | **C** | **C** | **T** | **T** | **G** | **G** | **C** | **G** | **T** | **A** | **A** | **A** | **A** | **A** | **A** | **C** | **A** | **A** | **A** | **T** | **G** | **A** | **T** | **T** | **A** | **T** | **G** | **G** | **T** | **G** | **G** | **A** | **A** | **A** | **C** | **G** | **G** | **C** | **G** | **G** | **C** | **C** | **A** | **A** | **A** | **G** | **A** | **A** | **C** | **C** | **G** | **A** | **A** | **A** | **A** | **A** | **T** | **G** | **G** | **C** | **A** | **A** | **A** | **C** | **G** | **A** | **T** | **T** | **T** | **A** | **A** | **A** | **C** | **G** | **C** | **C** | **G** | **T** | **T** | **G** | **G** | **T** | **T** | **T** | **G** | **C** | **C** | **A** | **A** | **C** | **G** | **A** | **A** | **C** | **G** | **T** | **A** | **T** | **T** | **G** | **G** | **C** | **A** | **A** | **C** | **A** | **A** | **T** | **A** | **T** | **C** | **T** | **A** | **T** | **G** | **A** | **A** | **T** | **G** | **G** | **G** | **A** | **C** | **G** | **A** | **C** | **A** | **T** | **C** | **A** | **A** | **T** | **A** | **T** | **T** | **G** | **A** | **T** | **T** | **T** | **A** | **A** | **T** | **C** | **T** | **C** | **T** | **G** | **G** | **G** | **G** | **A** | **T** | **A** | **C** | **T** | **A** | **T** | **T** | **A** | **T** | **T** | **G** | **A** | **T** | **T** | **T** | **A** | **G** | **G** | **C** | **A** | **A** | **T** | **A** | **C** | **G** | **C** | **T** | **A** | **C** | **T** | **T** | **C** | **C** | **G** | **A** | **A** | **A** | **G** | **A** | **A** | **G** | **A** | **T** | **A** | **A** | **T** | **A** | **T** | **T** | **A** | **T** | **T** | **A** | **T** | **T** | **A** | **T** | **T** | **C** | **G** | **T** | **A** | **A** | **A** | **G** | **G** | **T** | **T** | **T** | **T** | **G** | **G** | **C** | **C** | **G** | **C** | **A** | **C** | **G** | **C** | **G** | **A** | **A** | **T** | **T** | **C** | **T** | **A** | **G** | **T** | **G** | **C** | **C** | **G** | **T** | **T** | **A** | **G** | **G** | **T** | **G** | **T** | **A** | **G** | **C** | **T** | **A** | **T** | **T** | **T** | **T** | **G** | **T** | **T** | **A** | **G** | **A** | **A** | **C** | **A** | **T** | **T** | **C** | **A** | **A** | **C** | **G** | **T** | **T** | **T** | **T** | **A** | **C** | **G** | **G** | **A** | **A** | **C** | **A** | **G** | **T** | **G** | **C** | **G** | **T** | **T** | **T** | **T** | **G** | **A** | **A** | **G** | **A** | **A** | **G** | **A** | **A** | **A** | **A** | **A** | **T** | **A** | **T** | **C** | **A** | **A** | **T** | **T** | **G** | **A** | **A** | **A** | **A** | **A** | **C** | **G** | **A** | **A** | **T** | **C** | **A** | **T** | **T** | **A** | **A** | **A** | **A** | **A** | **T** | **T** | **T** | **A** | **C** | **A** | **G** | **C** | **A** | **A** | **T** | **G** | **A** | **T** | **T** | **A** | **T** | **G** | **A** | **T** | **A** | **C** | **C** | **A** | **A** | **T** | **C** | **T** | **T** | **C** | **G** | **T** | **C** | **G** | **T** | **T** | **T** | **G** | **A** | **A** | **A** | **A** | **T** | **T** | **A** | **T** | **G** | **A** | **C** | **G** | **A** | **A** | **C** | **A** | **C** | **T** | **T** | **T** | **A** | **G** | **T** | **A** | **G** | **G** | **A** | **G** | **A** | **T** | **G** | **T** | **T** | **G** | **A** | **G** | **G** | **T** | **G** | **A** | **T** | **C** | **C** | **G** | **T** | **G** | **T** | **A** | **-** | **-** | **-** |
| **N59505F** | **A** | **T** | **G** | **A** | **A** | **T** | **A** | **A** | **C** | **C** | **C** | **T** | **T** | **G** | **G** | **C** | **G** | **C** | **T** | **T** | **T** | **T** | **T** | **T** | **A** | **T** | **C** | **G** | **T** | **C** | **G** | **C** | **A** | **G** | **A** | **A** | **G** | **C** | **A** | **T** | **T** | **A** | **C** | **T** | **T** | **T** | **T** | **T** | **A** | **T** | **T** | **C** | **T** | **G** | **G** | **C** | **G** | **T** | **T** | **A** | **T** | **G** | **G** | **C** | **A** | **A** | **A** | **T** | **T** | **G** | **T** | **A** | **C** | **A** | **T** | **A** | **A** | **T** | **C** | **C** | **T** | **G** | **G** | **A** | **T** | **T** | **A** | **G** | **C** | **T** | **G** | **T** | **T** | **T** | **T** | **A** | **T** | **T** | **A** | **A** | **C** | **A** | **A** | **T** | **T** | **G** | **G** | **C** | **G** | **T** | **T** | **T** | **T** | **A** | **C** | **T** | **T** | **G** | **T** | **G** | **G** | **C** | **C** | **T** | **A** | **C** | **G** | **T** | **T** | **T** | **C** | **C** | **A** | **G** | **G** | **A** | **A** | **A** | **G** | **C** | **A** | **T** | **C** | **T** | **A** | **A** | **A** | **A** | **C** | **A** | **C** | **A** | **T** | **T** | **T** | **T** | **A** | **A** | **C** | **A** | **A** | **C** | **T** | **T** | **T** | **C** | **A** | **A** | **T** | **T** | **C** | **G** | **T** | **C** | **C** | **T** | **C** | **G** | **G** | **C** | **G** | **T** | **T** | **G** | **T** | **T** | **T** | **T** | **T** | **A** | **T** | **T** | **G** | **T** | **C** | **A** | **T** | **T** | **G** | **G** | **T** | **G** | **C** | **A** | **A** | **T** | **G** | **A** | **A** | **C** | **A** | **G** | **C** | **A** | **C** | **G** | **G** | **C** | **T** | **G** | **T** | **T** | **T** | **G** | **G** | **T** | **T** | **T** | **A** | **T** | **G** | **T** | **T** | **G** | **A** | **T** | **T** | **T** | **T** | **T** | **G** | **G** | **C** | **G** | **T** | **A** | **C** | **T** | **C** | **T** | **T** | **T** | **A** | **T** | **C** | **G** | **G** | **C** | **T** | **T** | **A** | **A** | **A** | **A** | **G** | **G** | **C** | **T** | **T** | **T** | **G** | **A** | **G** | **A** | **T** | **T** | **T** | **C** | **A** | **G** | **G** | **C** | **G** | **T** | **G** | **G** | **A** | **T** | **A** | **T** | **A** | **G** | **C** | **T** | **G** | **A** | **A** | **C** | **G** | **A** | **G** | **C** | **A** | **C** | **C** | **T** | **T** | **G** | **G** | **C** | **G** | **T** | **A** | **A** | **A** | **A** | **A** | **A** | **C** | **A** | **A** | **A** | **T** | **G** | **A** | **T** | **T** | **A** | **T** | **G** | **G** | **T** | **A** | **G** | **A** | **G** | **A** | **C** | **G** | **G** | **C** | **G** | **G** | **C** | **A** | **A** | **A** | **A** | **G** | **A** | **A** | **C** | **C** | **T** | **A** | **A** | **A** | **A** | **A** | **T** | **G** | **G** | **C** | **A** | **A** | **A** | **C** | **G** | **A** | **T** | **T** | **T** | **A** | **A** | **A** | **C** | **G** | **C** | **C** | **G** | **C** | **T** | **G** | **G** | **T** | **T** | **T** | **G** | **C** | **C** | **A** | **A** | **C** | **G** | **A** | **A** | **C** | **G** | **C** | **A** | **T** | **T** | **G** | **G** | **T** | **A** | **A** | **C** | **A** | **A** | **T** | **A** | **T** | **C** | **T** | **A** | **T** | **G** | **A** | **A** | **T** | **G** | **G** | **G** | **A** | **T** | **G** | **A** | **C** | **A** | **T** | **C** | **A** | **A** | **T** | **A** | **T** | **T** | **G** | **A** | **T** | **T** | **T** | **A** | **A** | **T** | **C** | **T** | **C** | **T** | **G** | **G** | **G** | **G** | **A** | **C** | **A** | **C** | **C** | **A** | **T** | **T** | **A** | **T** | **T** | **G** | **A** | **T** | **T** | **T** | **A** | **G** | **G** | **T** | **A** | **A** | **T** | **A** | **C** | **G** | **C** | **T** | **A** | **C** | **T** | **A** | **C** | **C** | **G** | **A** | **A** | **A** | **G** | **A** | **A** | **G** | **A** | **C** | **A** | **A** | **T** | **A** | **T** | **T** | **A** | **T** | **T** | **A** | **T** | **T** | **A** | **T** | **T** | **C** | **G** | **T** | **A** | **A** | **A** | **G** | **G** | **T** | **T** | **T** | **T** | **G** | **G** | **C** | **C** | **G** | **C** | **A** | **C** | **G** | **C** | **G** | **A** | **A** | **T** | **T** | **C** | **T** | **A** | **G** | **T** | **G** | **C** | **C** | **G** | **T** | **T** | **A** | **G** | **G** | **T** | **G** | **T** | **A** | **G** | **C** | **T** | **A** | **T** | **T** | **T** | **T** | **G** | **T** | **T** | **A** | **G** | **A** | **A** | **C** | **A** | **T** | **T** | **C** | **A** | **A** | **C** | **T** | **T** | **T** | **T** | **T** | **A** | **C** | **G** | **G** | **A** | **A** | **C** | **G** | **G** | **T** | **A** | **C** | **G** | **T** | **T** | **T** | **T** | **G** | **A** | **A** | **G** | **A** | **A** | **G** | **A** | **A** | **A** | **A** | **A** | **T** | **A** | **T** | **C** | **A** | **A** | **T** | **T** | **G** | **A** | **A** | **A** | **A** | **A** | **C** | **G** | **A** | **A** | **T** | **C** | **A** | **T** | **T** | **A** | **A** | **A** | **A** | **A** | **T** | **T** | **T** | **A** | **C** | **A** | **G** | **C** | **A** | **A** | **T** | **G** | **A** | **T** | **T** | **A** | **T** | **G** | **A** | **T** | **A** | **C** | **C** | **A** | **A** | **T** | **C** | **T** | **T** | **C** | **G** | **T** | **C** | **G** | **T** | **T** | **T** | **G** | **A** | **A** | **A** | **A** | **T** | **T** | **A** | **T** | **G** | **A** | **C** | **G** | **A** | **A** | **C** | **A** | **C** | **T** | **T** | **T** | **A** | **G** | **T** | **A** | **G** | **G** | **A** | **G** | **A** | **T** | **G** | **T** | **T** | **G** | **A** | **G** | **G** | **T** | **G** | **A** | **T** | **C** | **C** | **G** | **T** | **G** | **T** | **A** | **-** | **-** | **-** |
| **N59420F** | **A** | **T** | **G** | **A** | **A** | **T** | **A** | **A** | **C** | **C** | **C** | **T** | **T** | **G** | **G** | **C** | **G** | **C** | **T** | **T** | **T** | **T** | **T** | **T** | **A** | **T** | **C** | **G** | **T** | **C** | **G** | **C** | **A** | **G** | **A** | **A** | **G** | **C** | **A** | **T** | **T** | **A** | **C** | **T** | **T** | **T** | **T** | **T** | **A** | **T** | **T** | **C** | **T** | **G** | **G** | **C** | **G** | **T** | **T** | **A** | **T** | **G** | **G** | **C** | **A** | **A** | **A** | **T** | **T** | **G** | **T** | **A** | **C** | **A** | **T** | **A** | **A** | **T** | **C** | **C** | **T** | **G** | **G** | **A** | **T** | **T** | **A** | **G** | **C** | **T** | **G** | **T** | **T** | **T** | **T** | **A** | **T** | **T** | **A** | **A** | **C** | **A** | **A** | **T** | **T** | **G** | **G** | **C** | **G** | **T** | **T** | **T** | **T** | **A** | **C** | **T** | **T** | **G** | **T** | **G** | **G** | **C** | **C** | **T** | **A** | **C** | **G** | **T** | **T** | **T** | **C** | **C** | **A** | **G** | **G** | **A** | **A** | **A** | **G** | **C** | **A** | **T** | **C** | **T** | **A** | **A** | **A** | **A** | **C** | **A** | **C** | **A** | **T** | **T** | **T** | **T** | **A** | **A** | **C** | **A** | **A** | **C** | **T** | **T** | **T** | **C** | **A** | **A** | **T** | **T** | **C** | **G** | **T** | **C** | **C** | **T** | **C** | **G** | **G** | **C** | **G** | **T** | **T** | **G** | **T** | **T** | **T** | **T** | **T** | **A** | **T** | **T** | **G** | **T** | **C** | **A** | **T** | **T** | **G** | **G** | **T** | **G** | **C** | **A** | **A** | **T** | **G** | **A** | **A** | **C** | **A** | **G** | **C** | **A** | **C** | **G** | **G** | **C** | **T** | **G** | **T** | **T** | **T** | **G** | **G** | **T** | **T** | **T** | **A** | **T** | **G** | **T** | **T** | **G** | **A** | **T** | **T** | **T** | **T** | **T** | **G** | **G** | **C** | **G** | **T** | **A** | **C** | **T** | **C** | **T** | **T** | **T** | **A** | **T** | **C** | **G** | **G** | **C** | **T** | **T** | **A** | **A** | **A** | **A** | **G** | **G** | **C** | **T** | **T** | **T** | **G** | **A** | **G** | **A** | **T** | **C** | **T** | **C** | **A** | **G** | **G** | **C** | **G** | **T** | **G** | **G** | **A** | **T** | **A** | **T** | **A** | **G** | **C** | **T** | **G** | **A** | **G** | **C** | **G** | **A** | **G** | **C** | **A** | **C** | **C** | **T** | **T** | **G** | **G** | **C** | **G** | **T** | **A** | **A** | **A** | **A** | **A** | **A** | **C** | **A** | **A** | **A** | **T** | **G** | **A** | **T** | **T** | **A** | **T** | **G** | **G** | **T** | **G** | **G** | **A** | **G** | **A** | **C** | **G** | **G** | **C** | **G** | **G** | **C** | **A** | **A** | **A** | **A** | **G** | **A** | **A** | **C** | **C** | **T** | **A** | **A** | **A** | **A** | **A** | **T** | **G** | **G** | **C** | **A** | **A** | **A** | **C** | **G** | **A** | **T** | **T** | **T** | **A** | **A** | **A** | **C** | **G** | **C** | **C** | **G** | **C** | **T** | **G** | **G** | **T** | **T** | **T** | **G** | **C** | **C** | **A** | **A** | **C** | **G** | **A** | **A** | **C** | **G** | **C** | **A** | **T** | **T** | **G** | **G** | **T** | **A** | **A** | **C** | **A** | **A** | **T** | **A** | **T** | **C** | **T** | **A** | **T** | **G** | **A** | **A** | **T** | **G** | **G** | **G** | **A** | **C** | **G** | **A** | **T** | **A** | **T** | **C** | **A** | **A** | **T** | **A** | **T** | **T** | **G** | **A** | **T** | **T** | **T** | **A** | **A** | **T** | **C** | **T** | **C** | **T** | **G** | **G** | **G** | **G** | **A** | **C** | **A** | **C** | **C** | **A** | **T** | **T** | **A** | **T** | **T** | **G** | **A** | **T** | **T** | **T** | **A** | **G** | **G** | **T** | **A** | **A** | **T** | **A** | **C** | **G** | **C** | **T** | **A** | **C** | **T** | **A** | **C** | **C** | **G** | **A** | **A** | **A** | **G** | **A** | **A** | **G** | **A** | **C** | **A** | **A** | **T** | **A** | **T** | **T** | **A** | **T** | **T** | **A** | **T** | **T** | **A** | **T** | **T** | **C** | **G** | **T** | **A** | **A** | **A** | **G** | **G** | **T** | **T** | **T** | **T** | **G** | **G** | **C** | **C** | **G** | **C** | **A** | **C** | **G** | **C** | **G** | **A** | **A** | **T** | **T** | **C** | **T** | **A** | **G** | **T** | **G** | **C** | **C** | **G** | **T** | **T** | **A** | **G** | **G** | **T** | **G** | **T** | **A** | **G** | **C** | **T** | **A** | **T** | **T** | **T** | **T** | **G** | **T** | **T** | **A** | **G** | **A** | **A** | **C** | **A** | **T** | **T** | **C** | **A** | **A** | **C** | **T** | **T** | **T** | **T** | **T** | **A** | **C** | **G** | **G** | **A** | **A** | **C** | **G** | **G** | **T** | **A** | **C** | **G** | **T** | **T** | **T** | **T** | **G** | **A** | **A** | **G** | **A** | **A** | **G** | **A** | **A** | **A** | **A** | **A** | **T** | **A** | **T** | **C** | **A** | **A** | **T** | **T** | **G** | **A** | **A** | **A** | **A** | **A** | **C** | **G** | **A** | **A** | **T** | **C** | **A** | **T** | **T** | **A** | **A** | **A** | **A** | **A** | **T** | **T** | **T** | **A** | **C** | **A** | **G** | **C** | **A** | **A** | **T** | **G** | **A** | **T** | **T** | **A** | **T** | **G** | **A** | **T** | **A** | **C** | **C** | **A** | **A** | **T** | **C** | **T** | **T** | **C** | **G** | **T** | **C** | **G** | **T** | **T** | **T** | **G** | **A** | **A** | **A** | **A** | **T** | **T** | **A** | **T** | **G** | **A** | **C** | **G** | **A** | **A** | **C** | **A** | **C** | **T** | **T** | **T** | **A** | **G** | **T** | **A** | **G** | **G** | **A** | **G** | **A** | **T** | **G** | **T** | **T** | **G** | **A** | **G** | **G** | **T** | **G** | **A** | **T** | **C** | **C** | **G** | **T** | **G** | **T** | **A** | **-** | **-** | **-** |
| **N54588** | **A** | **T** | **G** | **A** | **A** | **T** | **A** | **A** | **C** | **C** | **C** | **T** | **T** | **G** | **G** | **C** | **G** | **C** | **T** | **T** | **T** | **T** | **T** | **T** | **A** | **T** | **C** | **G** | **T** | **C** | **G** | **C** | **A** | **G** | **A** | **A** | **G** | **C** | **A** | **T** | **T** | **A** | **C** | **T** | **T** | **T** | **T** | **T** | **A** | **T** | **T** | **C** | **T** | **G** | **G** | **C** | **G** | **T** | **T** | **A** | **T** | **G** | **G** | **C** | **A** | **A** | **A** | **T** | **T** | **G** | **T** | **A** | **C** | **A** | **T** | **A** | **A** | **T** | **C** | **C** | **T** | **G** | **G** | **A** | **T** | **T** | **A** | **G** | **C** | **T** | **G** | **T** | **T** | **T** | **T** | **A** | **T** | **T** | **A** | **A** | **C** | **A** | **A** | **T** | **T** | **G** | **G** | **C** | **G** | **T** | **T** | **T** | **T** | **A** | **C** | **T** | **T** | **G** | **T** | **G** | **G** | **C** | **C** | **T** | **A** | **T** | **G** | **T** | **T** | **T** | **C** | **C** | **A** | **G** | **G** | **A** | **A** | **A** | **G** | **C** | **A** | **T** | **C** | **T** | **A** | **A** | **A** | **A** | **C** | **A** | **C** | **A** | **T** | **T** | **T** | **T** | **A** | **A** | **C** | **A** | **A** | **C** | **T** | **T** | **T** | **C** | **A** | **A** | **T** | **T** | **C** | **G** | **T** | **C** | **C** | **T** | **C** | **G** | **G** | **C** | **G** | **T** | **T** | **G** | **T** | **T** | **T** | **T** | **T** | **A** | **T** | **T** | **G** | **T** | **C** | **A** | **T** | **T** | **G** | **G** | **T** | **G** | **C** | **A** | **A** | **T** | **G** | **A** | **A** | **C** | **A** | **G** | **C** | **A** | **C** | **G** | **G** | **C** | **T** | **G** | **T** | **T** | **T** | **G** | **G** | **T** | **T** | **T** | **A** | **T** | **G** | **T** | **T** | **G** | **A** | **T** | **T** | **T** | **T** | **T** | **G** | **G** | **C** | **G** | **T** | **A** | **C** | **T** | **C** | **T** | **T** | **T** | **A** | **T** | **C** | **G** | **G** | **C** | **T** | **T** | **A** | **A** | **A** | **A** | **G** | **G** | **C** | **T** | **T** | **T** | **G** | **A** | **G** | **A** | **T** | **C** | **T** | **C** | **A** | **G** | **G** | **C** | **G** | **T** | **G** | **G** | **A** | **T** | **A** | **T** | **A** | **G** | **C** | **T** | **G** | **A** | **G** | **C** | **G** | **A** | **G** | **C** | **A** | **C** | **C** | **T** | **T** | **G** | **G** | **C** | **G** | **T** | **A** | **A** | **A** | **A** | **A** | **A** | **C** | **A** | **A** | **A** | **T** | **G** | **A** | **T** | **T** | **A** | **T** | **G** | **G** | **T** | **G** | **G** | **A** | **G** | **A** | **C** | **G** | **G** | **C** | **G** | **G** | **C** | **A** | **A** | **A** | **A** | **G** | **A** | **A** | **C** | **C** | **T** | **A** | **A** | **A** | **A** | **A** | **T** | **G** | **G** | **C** | **A** | **A** | **A** | **C** | **G** | **A** | **T** | **T** | **T** | **A** | **A** | **A** | **C** | **G** | **C** | **C** | **G** | **C** | **T** | **G** | **G** | **T** | **T** | **T** | **G** | **C** | **C** | **A** | **A** | **C** | **G** | **A** | **A** | **C** | **G** | **C** | **A** | **T** | **T** | **G** | **G** | **T** | **A** | **A** | **C** | **A** | **A** | **T** | **A** | **T** | **C** | **T** | **A** | **T** | **G** | **A** | **A** | **T** | **G** | **G** | **G** | **A** | **C** | **G** | **A** | **C** | **A** | **T** | **C** | **A** | **A** | **T** | **A** | **T** | **T** | **G** | **A** | **T** | **T** | **T** | **A** | **A** | **T** | **C** | **T** | **C** | **T** | **G** | **G** | **G** | **G** | **A** | **C** | **A** | **C** | **C** | **A** | **T** | **T** | **A** | **T** | **T** | **G** | **A** | **T** | **T** | **T** | **A** | **G** | **G** | **T** | **A** | **A** | **T** | **A** | **C** | **G** | **C** | **T** | **A** | **C** | **T** | **A** | **C** | **C** | **G** | **A** | **A** | **A** | **G** | **A** | **A** | **G** | **A** | **C** | **A** | **A** | **T** | **A** | **T** | **T** | **A** | **T** | **T** | **A** | **T** | **T** | **A** | **T** | **T** | **C** | **G** | **T** | **A** | **A** | **A** | **G** | **G** | **T** | **T** | **T** | **T** | **G** | **G** | **C** | **C** | **G** | **C** | **A** | **C** | **G** | **C** | **G** | **A** | **A** | **T** | **T** | **C** | **T** | **A** | **G** | **T** | **G** | **C** | **C** | **G** | **T** | **T** | **A** | **G** | **G** | **T** | **G** | **T** | **A** | **G** | **C** | **T** | **A** | **T** | **T** | **T** | **T** | **G** | **T** | **T** | **A** | **G** | **A** | **A** | **C** | **A** | **T** | **T** | **C** | **A** | **A** | **C** | **T** | **T** | **T** | **T** | **T** | **A** | **C** | **G** | **G** | **A** | **A** | **C** | **G** | **G** | **T** | **A** | **C** | **G** | **T** | **T** | **T** | **T** | **G** | **A** | **A** | **G** | **A** | **A** | **G** | **A** | **A** | **A** | **A** | **A** | **T** | **A** | **T** | **C** | **A** | **A** | **T** | **T** | **G** | **A** | **A** | **A** | **A** | **A** | **C** | **G** | **A** | **A** | **T** | **C** | **A** | **T** | **T** | **A** | **A** | **A** | **A** | **A** | **T** | **T** | **T** | **A** | **C** | **A** | **G** | **C** | **A** | **A** | **T** | **G** | **A** | **T** | **T** | **A** | **C** | **G** | **A** | **T** | **A** | **C** | **C** | **A** | **A** | **T** | **C** | **T** | **T** | **C** | **G** | **T** | **C** | **G** | **T** | **T** | **T** | **G** | **A** | **A** | **A** | **A** | **T** | **T** | **A** | **T** | **G** | **A** | **C** | **G** | **A** | **A** | **T** | **A** | **C** | **T** | **T** | **T** | **A** | **G** | **T** | **A** | **G** | **G** | **A** | **G** | **A** | **T** | **G** | **T** | **T** | **G** | **A** | **G** | **G** | **T** | **G** | **A** | **T** | **C** | **C** | **G** | **T** | **G** | **T** | **A** | **-** | **-** | **-** |
| **N59554F** | **A** | **T** | **G** | **A** | **A** | **T** | **A** | **A** | **C** | **C** | **C** | **T** | **T** | **G** | **G** | **C** | **G** | **C** | **T** | **T** | **T** | **T** | **T** | **T** | **A** | **T** | **C** | **G** | **T** | **C** | **G** | **C** | **A** | **G** | **A** | **A** | **G** | **C** | **A** | **T** | **T** | **A** | **C** | **T** | **T** | **T** | **T** | **T** | **A** | **T** | **T** | **C** | **T** | **G** | **G** | **C** | **G** | **T** | **T** | **A** | **T** | **G** | **G** | **C** | **A** | **A** | **A** | **T** | **T** | **G** | **T** | **A** | **C** | **A** | **T** | **A** | **A** | **T** | **C** | **C** | **T** | **G** | **G** | **A** | **T** | **T** | **A** | **G** | **C** | **T** | **G** | **T** | **T** | **T** | **T** | **A** | **T** | **T** | **A** | **A** | **C** | **A** | **A** | **T** | **T** | **G** | **G** | **C** | **G** | **T** | **T** | **T** | **T** | **A** | **C** | **T** | **T** | **G** | **T** | **G** | **G** | **C** | **C** | **T** | **A** | **C** | **G** | **T** | **T** | **T** | **C** | **C** | **A** | **G** | **G** | **A** | **A** | **A** | **G** | **C** | **A** | **T** | **C** | **T** | **A** | **A** | **A** | **A** | **C** | **A** | **C** | **A** | **T** | **T** | **T** | **T** | **A** | **A** | **C** | **A** | **A** | **C** | **T** | **T** | **T** | **C** | **A** | **A** | **T** | **T** | **C** | **G** | **T** | **C** | **C** | **T** | **C** | **G** | **G** | **C** | **G** | **T** | **T** | **G** | **T** | **T** | **T** | **T** | **T** | **A** | **T** | **T** | **G** | **T** | **C** | **A** | **T** | **T** | **G** | **G** | **T** | **G** | **C** | **A** | **A** | **T** | **G** | **A** | **A** | **T** | **A** | **G** | **C** | **A** | **C** | **G** | **G** | **C** | **T** | **G** | **T** | **T** | **T** | **G** | **G** | **T** | **T** | **T** | **A** | **T** | **G** | **T** | **T** | **G** | **A** | **T** | **T** | **T** | **T** | **T** | **G** | **G** | **C** | **G** | **T** | **A** | **C** | **T** | **C** | **T** | **T** | **T** | **A** | **T** | **C** | **G** | **G** | **C** | **T** | **T** | **A** | **A** | **A** | **A** | **G** | **G** | **C** | **T** | **T** | **T** | **G** | **A** | **G** | **A** | **T** | **T** | **T** | **C** | **A** | **G** | **G** | **C** | **G** | **T** | **G** | **G** | **A** | **T** | **A** | **T** | **A** | **G** | **C** | **T** | **G** | **A** | **G** | **C** | **G** | **A** | **G** | **C** | **A** | **C** | **C** | **T** | **T** | **G** | **G** | **C** | **G** | **A** | **A** | **A** | **A** | **A** | **A** | **A** | **C** | **A** | **A** | **A** | **T** | **G** | **A** | **T** | **T** | **A** | **T** | **G** | **G** | **T** | **G** | **G** | **A** | **G** | **A** | **C** | **G** | **G** | **C** | **G** | **G** | **C** | **A** | **A** | **A** | **A** | **G** | **A** | **A** | **C** | **C** | **T** | **A** | **A** | **A** | **A** | **A** | **T** | **G** | **G** | **C** | **A** | **A** | **A** | **C** | **G** | **G** | **T** | **T** | **T** | **A** | **A** | **A** | **C** | **G** | **C** | **C** | **G** | **C** | **T** | **G** | **G** | **T** | **T** | **T** | **G** | **C** | **C** | **A** | **A** | **C** | **G** | **A** | **A** | **C** | **G** | **C** | **A** | **T** | **T** | **G** | **G** | **T** | **A** | **A** | **C** | **A** | **A** | **T** | **A** | **T** | **C** | **T** | **A** | **T** | **G** | **A** | **A** | **T** | **G** | **G** | **G** | **A** | **C** | **G** | **A** | **T** | **A** | **T** | **C** | **A** | **A** | **T** | **A** | **T** | **T** | **G** | **A** | **T** | **T** | **T** | **A** | **A** | **T** | **C** | **T** | **C** | **T** | **G** | **G** | **G** | **G** | **A** | **C** | **A** | **C** | **C** | **A** | **T** | **T** | **A** | **T** | **T** | **G** | **A** | **T** | **T** | **T** | **A** | **G** | **G** | **T** | **A** | **A** | **T** | **A** | **C** | **G** | **C** | **T** | **A** | **C** | **T** | **A** | **C** | **C** | **G** | **A** | **A** | **A** | **G** | **A** | **A** | **G** | **A** | **C** | **A** | **A** | **T** | **A** | **T** | **T** | **A** | **T** | **T** | **A** | **T** | **T** | **A** | **T** | **T** | **C** | **G** | **T** | **A** | **A** | **A** | **G** | **G** | **T** | **T** | **T** | **T** | **G** | **G** | **C** | **C** | **G** | **C** | **A** | **C** | **G** | **C** | **G** | **A** | **A** | **T** | **T** | **C** | **T** | **A** | **G** | **T** | **G** | **C** | **C** | **G** | **T** | **T** | **A** | **G** | **G** | **T** | **G** | **T** | **A** | **G** | **C** | **T** | **A** | **T** | **T** | **T** | **T** | **G** | **T** | **T** | **A** | **G** | **A** | **A** | **C** | **A** | **T** | **T** | **C** | **A** | **A** | **C** | **T** | **T** | **T** | **T** | **T** | **A** | **C** | **G** | **G** | **A** | **A** | **C** | **G** | **G** | **T** | **A** | **C** | **G** | **T** | **T** | **T** | **T** | **G** | **A** | **A** | **G** | **A** | **A** | **G** | **A** | **A** | **A** | **A** | **A** | **T** | **A** | **T** | **C** | **A** | **A** | **T** | **T** | **G** | **A** | **A** | **A** | **A** | **A** | **C** | **G** | **A** | **A** | **T** | **C** | **A** | **T** | **T** | **A** | **A** | **A** | **A** | **A** | **T** | **T** | **T** | **A** | **C** | **A** | **G** | **C** | **A** | **A** | **T** | **G** | **A** | **T** | **T** | **A** | **T** | **G** | **A** | **T** | **A** | **C** | **C** | **A** | **A** | **T** | **C** | **T** | **T** | **C** | **G** | **T** | **C** | **G** | **T** | **T** | **T** | **G** | **A** | **A** | **A** | **A** | **T** | **T** | **A** | **T** | **G** | **A** | **C** | **G** | **A** | **A** | **C** | **A** | **C** | **T** | **T** | **T** | **A** | **G** | **T** | **A** | **G** | **G** | **A** | **G** | **A** | **T** | **G** | **T** | **T** | **G** | **A** | **G** | **G** | **T** | **G** | **A** | **T** | **C** | **C** | **G** | **T** | **G** | **T** | **A** | **-** | **-** | **-** |
| **N59936F** | **A** | **T** | **G** | **A** | **A** | **T** | **A** | **A** | **C** | **C** | **C** | **T** | **T** | **G** | **G** | **C** | **G** | **C** | **T** | **T** | **T** | **T** | **T** | **T** | **A** | **T** | **C** | **G** | **T** | **C** | **G** | **C** | **A** | **G** | **A** | **A** | **G** | **C** | **A** | **T** | **T** | **A** | **C** | **T** | **T** | **T** | **T** | **T** | **A** | **T** | **T** | **C** | **T** | **G** | **G** | **C** | **G** | **T** | **T** | **A** | **T** | **G** | **G** | **C** | **A** | **A** | **A** | **T** | **T** | **G** | **T** | **A** | **C** | **A** | **T** | **A** | **A** | **T** | **C** | **C** | **T** | **G** | **G** | **A** | **T** | **T** | **A** | **G** | **C** | **T** | **G** | **T** | **T** | **T** | **T** | **A** | **T** | **T** | **A** | **A** | **C** | **A** | **A** | **T** | **T** | **G** | **G** | **C** | **G** | **T** | **T** | **T** | **T** | **A** | **C** | **T** | **T** | **G** | **T** | **G** | **G** | **C** | **C** | **T** | **A** | **C** | **G** | **T** | **T** | **T** | **C** | **C** | **A** | **G** | **G** | **A** | **A** | **A** | **G** | **C** | **A** | **T** | **C** | **T** | **A** | **A** | **A** | **A** | **C** | **A** | **C** | **A** | **T** | **T** | **T** | **T** | **A** | **A** | **C** | **A** | **A** | **C** | **T** | **T** | **T** | **C** | **A** | **A** | **T** | **T** | **C** | **G** | **T** | **C** | **C** | **T** | **C** | **G** | **G** | **C** | **G** | **T** | **T** | **G** | **T** | **T** | **T** | **T** | **T** | **A** | **T** | **T** | **G** | **T** | **C** | **A** | **T** | **T** | **G** | **G** | **T** | **G** | **C** | **A** | **A** | **T** | **G** | **A** | **A** | **C** | **A** | **G** | **C** | **A** | **C** | **G** | **G** | **C** | **T** | **G** | **T** | **T** | **T** | **G** | **G** | **T** | **T** | **T** | **A** | **T** | **G** | **T** | **T** | **G** | **A** | **T** | **T** | **T** | **T** | **T** | **G** | **G** | **C** | **G** | **T** | **A** | **C** | **T** | **C** | **T** | **T** | **T** | **A** | **T** | **C** | **G** | **G** | **C** | **T** | **T** | **A** | **A** | **A** | **A** | **G** | **G** | **C** | **T** | **T** | **T** | **G** | **A** | **G** | **A** | **T** | **T** | **T** | **C** | **A** | **G** | **G** | **C** | **G** | **T** | **G** | **G** | **A** | **T** | **A** | **T** | **A** | **G** | **C** | **T** | **G** | **A** | **A** | **C** | **G** | **A** | **G** | **C** | **A** | **C** | **C** | **T** | **T** | **G** | **G** | **C** | **G** | **T** | **A** | **A** | **A** | **A** | **A** | **A** | **C** | **A** | **A** | **A** | **T** | **G** | **A** | **T** | **T** | **A** | **T** | **G** | **G** | **T** | **A** | **G** | **A** | **G** | **A** | **C** | **G** | **G** | **C** | **G** | **G** | **C** | **A** | **A** | **A** | **A** | **G** | **A** | **A** | **C** | **C** | **T** | **A** | **A** | **A** | **A** | **A** | **T** | **G** | **G** | **C** | **A** | **A** | **A** | **C** | **G** | **A** | **T** | **T** | **T** | **A** | **A** | **A** | **C** | **G** | **C** | **C** | **G** | **C** | **T** | **G** | **G** | **T** | **T** | **T** | **G** | **C** | **C** | **A** | **A** | **C** | **G** | **A** | **A** | **C** | **G** | **C** | **A** | **T** | **T** | **G** | **G** | **T** | **A** | **A** | **C** | **A** | **A** | **T** | **A** | **T** | **C** | **T** | **A** | **T** | **G** | **A** | **A** | **T** | **G** | **G** | **G** | **A** | **T** | **G** | **A** | **C** | **A** | **T** | **C** | **A** | **A** | **T** | **A** | **T** | **T** | **G** | **A** | **T** | **T** | **T** | **A** | **A** | **T** | **C** | **T** | **C** | **T** | **G** | **G** | **G** | **G** | **A** | **C** | **A** | **C** | **C** | **A** | **T** | **T** | **A** | **T** | **T** | **G** | **A** | **T** | **T** | **T** | **A** | **G** | **G** | **T** | **A** | **A** | **T** | **A** | **C** | **G** | **C** | **T** | **A** | **C** | **T** | **A** | **C** | **C** | **G** | **A** | **A** | **A** | **G** | **A** | **A** | **G** | **A** | **C** | **A** | **A** | **T** | **A** | **T** | **T** | **A** | **T** | **T** | **A** | **T** | **T** | **A** | **T** | **T** | **C** | **G** | **T** | **A** | **A** | **A** | **G** | **G** | **T** | **T** | **T** | **T** | **G** | **G** | **C** | **C** | **G** | **C** | **A** | **C** | **G** | **C** | **G** | **A** | **A** | **T** | **T** | **C** | **T** | **A** | **G** | **T** | **G** | **C** | **C** | **G** | **T** | **T** | **A** | **G** | **G** | **T** | **G** | **T** | **A** | **G** | **C** | **T** | **A** | **T** | **T** | **T** | **T** | **G** | **T** | **T** | **A** | **G** | **A** | **A** | **C** | **A** | **T** | **T** | **C** | **A** | **A** | **C** | **T** | **T** | **T** | **T** | **T** | **A** | **C** | **G** | **G** | **A** | **A** | **C** | **G** | **G** | **T** | **A** | **C** | **G** | **T** | **T** | **T** | **T** | **G** | **A** | **A** | **G** | **A** | **A** | **G** | **A** | **A** | **A** | **A** | **A** | **T** | **A** | **T** | **C** | **A** | **A** | **T** | **T** | **G** | **A** | **A** | **A** | **A** | **A** | **C** | **G** | **A** | **A** | **T** | **C** | **A** | **T** | **T** | **A** | **A** | **A** | **A** | **A** | **T** | **T** | **T** | **A** | **C** | **A** | **G** | **C** | **A** | **A** | **T** | **G** | **A** | **T** | **T** | **A** | **T** | **G** | **A** | **T** | **A** | **C** | **C** | **A** | **A** | **T** | **C** | **T** | **T** | **C** | **G** | **T** | **C** | **G** | **T** | **T** | **T** | **G** | **A** | **A** | **A** | **A** | **T** | **T** | **A** | **T** | **G** | **A** | **C** | **G** | **A** | **A** | **C** | **A** | **C** | **T** | **T** | **T** | **A** | **G** | **T** | **A** | **G** | **G** | **A** | **G** | **A** | **T** | **G** | **T** | **T** | **G** | **A** | **G** | **G** | **T** | **G** | **A** | **T** | **C** | **C** | **G** | **T** | **G** | **T** | **A** | **-** | **-** | **-** |
| **N52564** | **A** | **T** | **G** | **A** | **A** | **T** | **A** | **A** | **C** | **C** | **C** | **T** | **T** | **G** | **G** | **C** | **G** | **C** | **T** | **T** | **T** | **T** | **T** | **T** | **A** | **T** | **C** | **G** | **T** | **C** | **G** | **C** | **A** | **G** | **A** | **A** | **G** | **C** | **A** | **T** | **T** | **A** | **C** | **T** | **T** | **T** | **T** | **T** | **A** | **T** | **T** | **C** | **T** | **G** | **G** | **C** | **G** | **T** | **T** | **A** | **T** | **G** | **G** | **C** | **A** | **A** | **A** | **T** | **T** | **G** | **T** | **A** | **C** | **A** | **T** | **A** | **A** | **T** | **C** | **C** | **T** | **G** | **G** | **A** | **T** | **T** | **A** | **G** | **C** | **T** | **G** | **T** | **T** | **T** | **T** | **A** | **T** | **T** | **A** | **A** | **C** | **A** | **A** | **T** | **T** | **G** | **G** | **C** | **G** | **T** | **T** | **T** | **T** | **A** | **C** | **T** | **T** | **G** | **T** | **G** | **G** | **C** | **C** | **T** | **A** | **C** | **G** | **T** | **T** | **T** | **C** | **C** | **A** | **G** | **G** | **A** | **A** | **A** | **G** | **C** | **A** | **T** | **C** | **T** | **A** | **A** | **A** | **A** | **C** | **A** | **C** | **A** | **T** | **T** | **T** | **T** | **A** | **A** | **C** | **A** | **A** | **C** | **T** | **T** | **T** | **C** | **A** | **A** | **T** | **T** | **C** | **G** | **T** | **C** | **C** | **T** | **C** | **G** | **G** | **C** | **G** | **T** | **T** | **G** | **T** | **T** | **T** | **T** | **T** | **A** | **T** | **T** | **G** | **T** | **C** | **A** | **T** | **T** | **G** | **G** | **T** | **G** | **C** | **A** | **A** | **T** | **G** | **A** | **A** | **C** | **A** | **G** | **C** | **A** | **C** | **G** | **G** | **C** | **T** | **G** | **T** | **T** | **T** | **G** | **G** | **T** | **T** | **T** | **A** | **T** | **G** | **T** | **T** | **G** | **A** | **T** | **T** | **T** | **T** | **T** | **G** | **G** | **C** | **G** | **T** | **A** | **C** | **T** | **C** | **T** | **T** | **T** | **A** | **T** | **C** | **G** | **G** | **C** | **T** | **T** | **A** | **A** | **A** | **A** | **G** | **G** | **C** | **T** | **T** | **T** | **G** | **A** | **G** | **A** | **T** | **T** | **T** | **C** | **A** | **G** | **G** | **C** | **G** | **T** | **G** | **G** | **A** | **T** | **A** | **T** | **A** | **G** | **C** | **T** | **G** | **A** | **G** | **C** | **G** | **A** | **G** | **C** | **A** | **C** | **C** | **T** | **T** | **G** | **G** | **C** | **G** | **A** | **A** | **A** | **A** | **A** | **A** | **A** | **C** | **A** | **A** | **A** | **T** | **G** | **A** | **T** | **T** | **A** | **T** | **G** | **G** | **T** | **G** | **G** | **A** | **G** | **A** | **C** | **G** | **G** | **C** | **G** | **G** | **C** | **A** | **A** | **A** | **A** | **G** | **A** | **A** | **C** | **C** | **T** | **A** | **A** | **A** | **A** | **A** | **T** | **G** | **G** | **C** | **A** | **A** | **A** | **C** | **G** | **A** | **T** | **T** | **T** | **A** | **A** | **A** | **C** | **G** | **C** | **C** | **G** | **C** | **T** | **G** | **G** | **T** | **T** | **T** | **G** | **C** | **C** | **A** | **A** | **C** | **G** | **A** | **A** | **C** | **G** | **C** | **A** | **T** | **T** | **G** | **G** | **T** | **A** | **A** | **C** | **A** | **A** | **T** | **A** | **T** | **C** | **T** | **A** | **T** | **G** | **A** | **A** | **T** | **G** | **G** | **G** | **A** | **C** | **G** | **A** | **T** | **A** | **T** | **C** | **A** | **A** | **T** | **A** | **T** | **T** | **G** | **A** | **T** | **T** | **T** | **A** | **A** | **T** | **C** | **T** | **C** | **T** | **G** | **G** | **G** | **G** | **A** | **C** | **A** | **C** | **C** | **A** | **T** | **T** | **A** | **T** | **T** | **G** | **A** | **T** | **T** | **T** | **A** | **G** | **G** | **T** | **A** | **A** | **T** | **A** | **C** | **G** | **T** | **T** | **A** | **C** | **T** | **A** | **C** | **C** | **G** | **A** | **A** | **A** | **G** | **A** | **A** | **G** | **A** | **C** | **A** | **A** | **T** | **A** | **T** | **T** | **A** | **T** | **T** | **A** | **T** | **T** | **A** | **T** | **T** | **C** | **G** | **T** | **A** | **A** | **A** | **G** | **G** | **T** | **T** | **T** | **T** | **G** | **G** | **C** | **C** | **G** | **C** | **A** | **C** | **G** | **C** | **G** | **A** | **A** | **T** | **T** | **C** | **T** | **A** | **G** | **T** | **G** | **C** | **C** | **G** | **T** | **T** | **A** | **G** | **G** | **T** | **G** | **T** | **A** | **G** | **C** | **T** | **A** | **T** | **T** | **T** | **T** | **G** | **T** | **T** | **A** | **G** | **A** | **A** | **C** | **A** | **T** | **T** | **C** | **A** | **A** | **C** | **T** | **T** | **T** | **T** | **T** | **A** | **C** | **G** | **G** | **A** | **A** | **C** | **G** | **G** | **T** | **A** | **C** | **G** | **T** | **T** | **T** | **T** | **G** | **A** | **A** | **G** | **A** | **A** | **G** | **A** | **A** | **A** | **A** | **A** | **T** | **A** | **T** | **C** | **A** | **A** | **T** | **T** | **G** | **A** | **A** | **A** | **A** | **A** | **C** | **G** | **A** | **A** | **T** | **C** | **A** | **T** | **T** | **A** | **A** | **A** | **A** | **A** | **T** | **T** | **T** | **A** | **C** | **A** | **G** | **C** | **A** | **A** | **T** | **G** | **A** | **T** | **T** | **A** | **T** | **G** | **A** | **T** | **A** | **C** | **C** | **A** | **A** | **T** | **C** | **T** | **T** | **C** | **G** | **T** | **C** | **G** | **T** | **T** | **T** | **G** | **A** | **A** | **A** | **A** | **T** | **T** | **A** | **T** | **G** | **A** | **C** | **G** | **A** | **A** | **C** | **A** | **C** | **T** | **T** | **T** | **A** | **G** | **T** | **A** | **G** | **G** | **A** | **G** | **A** | **T** | **G** | **T** | **T** | **G** | **A** | **G** | **G** | **T** | **G** | **A** | **T** | **C** | **C** | **G** | **T** | **G** | **T** | **A** | **-** | **-** | **-** |
| **N55268** | **A** | **T** | **G** | **A** | **A** | **T** | **A** | **A** | **T** | **C** | **C** | **T** | **T** | **G** | **G** | **C** | **G** | **C** | **T** | **T** | **T** | **T** | **T** | **T** | **A** | **T** | **C** | **G** | **T** | **C** | **G** | **C** | **A** | **G** | **A** | **A** | **G** | **C** | **A** | **T** | **T** | **A** | **C** | **T** | **T** | **T** | **T** | **T** | **A** | **T** | **T** | **C** | **T** | **G** | **G** | **C** | **G** | **T** | **T** | **A** | **T** | **G** | **G** | **C** | **A** | **A** | **A** | **T** | **T** | **G** | **T** | **A** | **C** | **A** | **T** | **A** | **A** | **T** | **C** | **C** | **T** | **G** | **G** | **A** | **T** | **T** | **A** | **G** | **C** | **T** | **G** | **T** | **T** | **T** | **T** | **A** | **T** | **T** | **A** | **A** | **C** | **A** | **A** | **T** | **T** | **G** | **G** | **C** | **G** | **T** | **T** | **T** | **T** | **A** | **C** | **T** | **T** | **G** | **T** | **G** | **G** | **C** | **C** | **T** | **A** | **T** | **G** | **T** | **T** | **T** | **C** | **C** | **A** | **G** | **G** | **A** | **A** | **A** | **G** | **C** | **A** | **T** | **C** | **T** | **A** | **A** | **A** | **A** | **C** | **A** | **C** | **A** | **T** | **T** | **T** | **T** | **A** | **A** | **C** | **A** | **A** | **C** | **T** | **T** | **T** | **C** | **A** | **A** | **T** | **T** | **C** | **G** | **T** | **C** | **C** | **T** | **C** | **G** | **G** | **C** | **G** | **T** | **T** | **G** | **T** | **T** | **T** | **T** | **T** | **A** | **T** | **T** | **G** | **T** | **C** | **A** | **T** | **T** | **G** | **G** | **T** | **G** | **C** | **A** | **A** | **T** | **G** | **A** | **A** | **C** | **A** | **G** | **C** | **A** | **C** | **G** | **G** | **C** | **T** | **G** | **T** | **T** | **T** | **G** | **G** | **T** | **T** | **T** | **A** | **T** | **G** | **T** | **T** | **G** | **A** | **T** | **T** | **T** | **T** | **T** | **G** | **G** | **C** | **G** | **T** | **A** | **C** | **T** | **C** | **T** | **T** | **T** | **A** | **T** | **C** | **G** | **G** | **C** | **T** | **T** | **A** | **A** | **A** | **A** | **G** | **G** | **C** | **T** | **T** | **T** | **G** | **A** | **G** | **A** | **T** | **T** | **T** | **C** | **A** | **G** | **G** | **C** | **G** | **T** | **G** | **G** | **A** | **T** | **A** | **T** | **A** | **G** | **C** | **T** | **G** | **A** | **G** | **C** | **G** | **A** | **G** | **C** | **A** | **C** | **C** | **T** | **T** | **G** | **G** | **C** | **G** | **T** | **A** | **A** | **A** | **A** | **A** | **A** | **C** | **A** | **A** | **A** | **T** | **G** | **A** | **T** | **T** | **A** | **T** | **G** | **G** | **T** | **G** | **G** | **A** | **G** | **A** | **C** | **G** | **G** | **C** | **G** | **G** | **C** | **A** | **A** | **A** | **A** | **G** | **A** | **A** | **C** | **C** | **T** | **A** | **A** | **A** | **A** | **A** | **T** | **G** | **G** | **C** | **A** | **A** | **A** | **C** | **G** | **A** | **T** | **T** | **T** | **A** | **A** | **A** | **C** | **G** | **C** | **C** | **G** | **C** | **T** | **G** | **G** | **T** | **T** | **T** | **G** | **C** | **C** | **A** | **A** | **C** | **G** | **A** | **A** | **C** | **G** | **C** | **A** | **T** | **T** | **G** | **G** | **T** | **A** | **A** | **C** | **A** | **A** | **T** | **A** | **T** | **C** | **T** | **A** | **T** | **G** | **A** | **A** | **T** | **G** | **G** | **G** | **A** | **C** | **G** | **A** | **T** | **A** | **T** | **C** | **A** | **A** | **T** | **A** | **T** | **T** | **G** | **A** | **T** | **T** | **T** | **A** | **A** | **T** | **C** | **T** | **C** | **T** | **G** | **G** | **G** | **G** | **A** | **C** | **A** | **C** | **C** | **A** | **T** | **T** | **A** | **T** | **T** | **G** | **A** | **T** | **T** | **T** | **A** | **G** | **G** | **T** | **A** | **A** | **T** | **A** | **C** | **G** | **C** | **T** | **A** | **C** | **T** | **A** | **C** | **C** | **G** | **A** | **A** | **A** | **G** | **A** | **A** | **G** | **A** | **C** | **A** | **A** | **T** | **A** | **T** | **T** | **A** | **T** | **T** | **A** | **T** | **T** | **A** | **T** | **T** | **C** | **G** | **T** | **A** | **A** | **A** | **G** | **G** | **T** | **T** | **T** | **T** | **G** | **G** | **C** | **C** | **G** | **C** | **A** | **C** | **G** | **C** | **G** | **A** | **A** | **T** | **T** | **C** | **T** | **A** | **G** | **T** | **G** | **C** | **C** | **G** | **T** | **T** | **A** | **G** | **G** | **T** | **G** | **T** | **A** | **G** | **C** | **T** | **A** | **T** | **T** | **T** | **T** | **G** | **T** | **T** | **A** | **G** | **A** | **A** | **C** | **A** | **T** | **T** | **C** | **A** | **A** | **C** | **G** | **T** | **T** | **T** | **T** | **A** | **C** | **G** | **G** | **A** | **A** | **C** | **G** | **G** | **T** | **A** | **C** | **G** | **T** | **T** | **T** | **T** | **G** | **A** | **A** | **G** | **A** | **A** | **G** | **A** | **A** | **A** | **A** | **A** | **T** | **A** | **T** | **C** | **A** | **A** | **T** | **T** | **G** | **A** | **A** | **A** | **A** | **A** | **C** | **G** | **A** | **A** | **T** | **C** | **A** | **T** | **T** | **A** | **A** | **A** | **A** | **A** | **T** | **T** | **T** | **A** | **C** | **A** | **G** | **C** | **A** | **A** | **T** | **G** | **A** | **T** | **T** | **A** | **T** | **G** | **A** | **T** | **A** | **C** | **C** | **A** | **A** | **T** | **C** | **T** | **T** | **C** | **G** | **T** | **C** | **G** | **T** | **T** | **T** | **G** | **A** | **A** | **A** | **A** | **T** | **T** | **A** | **T** | **G** | **A** | **C** | **G** | **A** | **A** | **C** | **A** | **C** | **T** | **T** | **T** | **A** | **G** | **T** | **A** | **G** | **G** | **A** | **G** | **A** | **T** | **G** | **T** | **T** | **G** | **A** | **G** | **G** | **T** | **G** | **A** | **T** | **C** | **C** | **G** | **T** | **G** | **T** | **A** | **-** | **-** | **-** |
| **N59509F** | **A** | **T** | **G** | **A** | **A** | **T** | **A** | **A** | **C** | **C** | **C** | **T** | **T** | **G** | **G** | **C** | **G** | **C** | **T** | **T** | **T** | **T** | **T** | **T** | **A** | **T** | **C** | **G** | **T** | **C** | **G** | **C** | **A** | **G** | **A** | **A** | **G** | **C** | **A** | **T** | **T** | **A** | **C** | **T** | **T** | **T** | **T** | **T** | **A** | **T** | **T** | **C** | **T** | **G** | **G** | **C** | **G** | **T** | **T** | **A** | **T** | **G** | **G** | **C** | **A** | **A** | **A** | **T** | **T** | **G** | **T** | **A** | **C** | **A** | **T** | **A** | **A** | **T** | **C** | **C** | **T** | **G** | **G** | **A** | **T** | **T** | **A** | **G** | **C** | **T** | **G** | **T** | **T** | **T** | **T** | **A** | **T** | **T** | **A** | **A** | **C** | **A** | **A** | **T** | **T** | **G** | **G** | **C** | **G** | **T** | **T** | **T** | **T** | **A** | **C** | **T** | **T** | **G** | **T** | **G** | **G** | **C** | **C** | **T** | **A** | **C** | **G** | **T** | **T** | **T** | **C** | **C** | **A** | **G** | **G** | **A** | **A** | **A** | **G** | **C** | **A** | **T** | **C** | **T** | **A** | **A** | **A** | **A** | **C** | **A** | **C** | **A** | **T** | **T** | **T** | **T** | **A** | **A** | **C** | **A** | **A** | **C** | **T** | **T** | **T** | **C** | **A** | **A** | **T** | **T** | **C** | **G** | **T** | **C** | **C** | **T** | **C** | **G** | **G** | **C** | **G** | **T** | **T** | **G** | **T** | **T** | **T** | **T** | **T** | **A** | **T** | **T** | **G** | **T** | **C** | **A** | **T** | **T** | **G** | **G** | **T** | **G** | **C** | **A** | **A** | **T** | **G** | **A** | **A** | **C** | **A** | **G** | **C** | **A** | **C** | **G** | **G** | **C** | **T** | **G** | **T** | **T** | **T** | **G** | **G** | **T** | **T** | **T** | **A** | **T** | **G** | **T** | **T** | **G** | **A** | **T** | **T** | **T** | **T** | **T** | **G** | **G** | **C** | **G** | **T** | **A** | **C** | **T** | **C** | **T** | **T** | **T** | **A** | **T** | **C** | **G** | **G** | **C** | **T** | **T** | **A** | **A** | **A** | **A** | **G** | **G** | **C** | **T** | **T** | **T** | **G** | **A** | **G** | **A** | **T** | **T** | **T** | **C** | **A** | **G** | **G** | **C** | **G** | **T** | **G** | **G** | **A** | **T** | **A** | **T** | **A** | **G** | **C** | **T** | **G** | **A** | **G** | **C** | **G** | **A** | **G** | **C** | **A** | **C** | **C** | **T** | **T** | **G** | **G** | **C** | **G** | **T** | **A** | **A** | **A** | **A** | **A** | **A** | **C** | **A** | **A** | **A** | **T** | **G** | **A** | **T** | **T** | **A** | **T** | **G** | **G** | **T** | **G** | **G** | **A** | **G** | **A** | **C** | **G** | **G** | **C** | **G** | **G** | **C** | **A** | **A** | **A** | **A** | **G** | **A** | **A** | **C** | **C** | **T** | **A** | **A** | **A** | **A** | **A** | **T** | **G** | **G** | **C** | **A** | **A** | **A** | **C** | **G** | **A** | **T** | **T** | **T** | **A** | **A** | **A** | **C** | **G** | **C** | **C** | **G** | **T** | **T** | **G** | **G** | **T** | **T** | **T** | **G** | **C** | **C** | **A** | **A** | **T** | **G** | **A** | **A** | **C** | **G** | **C** | **A** | **T** | **T** | **G** | **G** | **T** | **A** | **A** | **C** | **A** | **A** | **T** | **A** | **T** | **C** | **T** | **A** | **T** | **G** | **A** | **A** | **T** | **G** | **G** | **G** | **A** | **T** | **G** | **A** | **T** | **A** | **T** | **C** | **A** | **A** | **T** | **A** | **T** | **T** | **G** | **A** | **T** | **T** | **T** | **A** | **A** | **T** | **C** | **T** | **C** | **T** | **G** | **G** | **G** | **G** | **A** | **C** | **A** | **C** | **C** | **A** | **T** | **T** | **A** | **T** | **T** | **G** | **A** | **T** | **T** | **T** | **A** | **G** | **G** | **T** | **A** | **A** | **T** | **A** | **C** | **G** | **C** | **T** | **A** | **C** | **T** | **A** | **C** | **C** | **G** | **A** | **A** | **A** | **G** | **A** | **A** | **G** | **A** | **C** | **A** | **A** | **T** | **A** | **T** | **T** | **A** | **T** | **T** | **A** | **T** | **T** | **A** | **T** | **T** | **C** | **G** | **T** | **A** | **A** | **A** | **G** | **G** | **T** | **T** | **T** | **T** | **G** | **G** | **C** | **C** | **G** | **C** | **A** | **C** | **G** | **C** | **G** | **A** | **A** | **T** | **T** | **C** | **T** | **A** | **G** | **T** | **A** | **C** | **C** | **G** | **T** | **T** | **A** | **G** | **G** | **T** | **G** | **T** | **A** | **G** | **C** | **T** | **A** | **T** | **T** | **T** | **T** | **G** | **T** | **T** | **A** | **G** | **A** | **A** | **C** | **A** | **T** | **T** | **C** | **A** | **A** | **C** | **T** | **T** | **T** | **T** | **T** | **A** | **C** | **G** | **G** | **A** | **A** | **C** | **G** | **G** | **T** | **A** | **C** | **G** | **T** | **T** | **T** | **T** | **G** | **A** | **A** | **G** | **A** | **A** | **G** | **A** | **A** | **A** | **A** | **A** | **T** | **A** | **T** | **C** | **A** | **A** | **T** | **T** | **G** | **A** | **A** | **A** | **A** | **A** | **C** | **G** | **A** | **A** | **T** | **C** | **A** | **T** | **T** | **A** | **A** | **A** | **A** | **A** | **T** | **T** | **T** | **A** | **C** | **A** | **G** | **C** | **A** | **A** | **T** | **G** | **A** | **T** | **T** | **A** | **T** | **G** | **A** | **T** | **A** | **C** | **C** | **A** | **A** | **T** | **C** | **T** | **T** | **C** | **G** | **T** | **C** | **G** | **T** | **T** | **T** | **G** | **A** | **A** | **A** | **A** | **T** | **T** | **A** | **T** | **G** | **A** | **C** | **G** | **A** | **A** | **C** | **A** | **C** | **T** | **T** | **T** | **A** | **G** | **T** | **A** | **G** | **G** | **A** | **G** | **A** | **T** | **G** | **T** | **T** | **G** | **A** | **G** | **G** | **T** | **G** | **A** | **T** | **C** | **C** | **G** | **T** | **G** | **T** | **A** | **-** | **-** | **-** |
| **N52729** | **A** | **T** | **G** | **A** | **A** | **T** | **A** | **A** | **C** | **C** | **C** | **T** | **T** | **G** | **G** | **C** | **G** | **C** | **T** | **T** | **T** | **T** | **T** | **T** | **A** | **T** | **C** | **G** | **T** | **C** | **G** | **C** | **A** | **G** | **A** | **A** | **G** | **C** | **A** | **T** | **T** | **A** | **C** | **T** | **T** | **T** | **T** | **T** | **A** | **T** | **T** | **C** | **T** | **G** | **G** | **C** | **G** | **T** | **T** | **A** | **T** | **G** | **G** | **C** | **A** | **A** | **A** | **T** | **T** | **G** | **T** | **A** | **C** | **A** | **T** | **A** | **A** | **T** | **C** | **C** | **T** | **G** | **G** | **A** | **T** | **T** | **A** | **G** | **C** | **T** | **G** | **T** | **T** | **T** | **T** | **A** | **T** | **T** | **A** | **A** | **C** | **A** | **A** | **T** | **T** | **G** | **G** | **C** | **G** | **T** | **T** | **T** | **T** | **A** | **C** | **T** | **T** | **G** | **T** | **G** | **G** | **C** | **C** | **T** | **A** | **C** | **G** | **T** | **T** | **T** | **C** | **C** | **A** | **G** | **G** | **A** | **A** | **A** | **G** | **C** | **A** | **T** | **C** | **T** | **A** | **A** | **A** | **A** | **C** | **A** | **C** | **A** | **T** | **T** | **T** | **T** | **A** | **A** | **C** | **A** | **A** | **C** | **T** | **T** | **T** | **C** | **A** | **A** | **T** | **T** | **C** | **G** | **T** | **C** | **C** | **T** | **C** | **G** | **G** | **C** | **G** | **T** | **T** | **G** | **T** | **T** | **T** | **T** | **T** | **A** | **T** | **T** | **G** | **T** | **C** | **A** | **T** | **T** | **G** | **G** | **T** | **G** | **C** | **A** | **A** | **T** | **G** | **A** | **A** | **C** | **A** | **G** | **C** | **A** | **C** | **G** | **G** | **C** | **T** | **G** | **T** | **T** | **T** | **G** | **G** | **T** | **T** | **T** | **A** | **T** | **G** | **T** | **T** | **G** | **A** | **T** | **T** | **T** | **T** | **T** | **G** | **G** | **C** | **G** | **T** | **A** | **C** | **T** | **C** | **T** | **T** | **T** | **A** | **T** | **C** | **G** | **G** | **C** | **T** | **T** | **A** | **A** | **A** | **A** | **G** | **G** | **C** | **T** | **T** | **T** | **G** | **A** | **G** | **A** | **T** | **T** | **T** | **C** | **A** | **G** | **G** | **C** | **G** | **T** | **G** | **G** | **A** | **T** | **A** | **T** | **A** | **G** | **C** | **T** | **G** | **A** | **G** | **C** | **G** | **A** | **G** | **C** | **A** | **C** | **C** | **T** | **T** | **G** | **G** | **C** | **G** | **A** | **A** | **A** | **A** | **A** | **A** | **A** | **C** | **A** | **A** | **A** | **T** | **G** | **A** | **T** | **T** | **A** | **T** | **G** | **G** | **T** | **G** | **G** | **A** | **G** | **A** | **C** | **G** | **G** | **C** | **G** | **G** | **C** | **A** | **A** | **A** | **A** | **G** | **A** | **A** | **C** | **C** | **T** | **A** | **A** | **A** | **A** | **A** | **T** | **G** | **G** | **C** | **A** | **A** | **A** | **C** | **G** | **A** | **T** | **T** | **T** | **A** | **A** | **A** | **C** | **G** | **C** | **C** | **G** | **C** | **T** | **G** | **G** | **T** | **T** | **T** | **G** | **C** | **C** | **A** | **A** | **C** | **G** | **A** | **A** | **C** | **G** | **C** | **A** | **T** | **T** | **G** | **G** | **T** | **A** | **A** | **C** | **A** | **A** | **T** | **A** | **T** | **C** | **T** | **A** | **T** | **G** | **A** | **A** | **T** | **G** | **G** | **G** | **A** | **C** | **G** | **A** | **T** | **A** | **T** | **C** | **A** | **A** | **T** | **A** | **T** | **T** | **G** | **A** | **T** | **T** | **T** | **A** | **A** | **T** | **C** | **T** | **C** | **T** | **G** | **G** | **G** | **G** | **A** | **C** | **A** | **C** | **C** | **A** | **T** | **T** | **A** | **T** | **T** | **G** | **A** | **T** | **T** | **T** | **A** | **G** | **G** | **T** | **A** | **A** | **T** | **A** | **C** | **G** | **C** | **T** | **A** | **C** | **T** | **A** | **C** | **C** | **G** | **A** | **A** | **A** | **G** | **A** | **A** | **G** | **A** | **C** | **A** | **A** | **T** | **A** | **T** | **T** | **A** | **T** | **T** | **A** | **T** | **T** | **A** | **T** | **T** | **C** | **G** | **T** | **A** | **A** | **A** | **G** | **G** | **T** | **T** | **T** | **T** | **G** | **G** | **C** | **C** | **G** | **C** | **A** | **C** | **G** | **C** | **G** | **A** | **A** | **T** | **T** | **C** | **T** | **A** | **G** | **T** | **G** | **C** | **C** | **G** | **T** | **T** | **A** | **G** | **G** | **T** | **G** | **T** | **A** | **G** | **C** | **T** | **A** | **T** | **T** | **T** | **T** | **G** | **T** | **T** | **A** | **G** | **A** | **A** | **C** | **A** | **T** | **T** | **C** | **A** | **A** | **C** | **T** | **T** | **T** | **T** | **T** | **A** | **C** | **G** | **G** | **A** | **A** | **C** | **G** | **G** | **T** | **A** | **C** | **G** | **T** | **T** | **T** | **T** | **G** | **A** | **A** | **G** | **A** | **A** | **G** | **A** | **A** | **A** | **A** | **A** | **T** | **A** | **T** | **C** | **A** | **A** | **T** | **T** | **G** | **A** | **A** | **A** | **A** | **A** | **C** | **G** | **A** | **A** | **T** | **C** | **A** | **T** | **T** | **A** | **A** | **A** | **A** | **A** | **T** | **T** | **T** | **A** | **C** | **A** | **G** | **C** | **A** | **A** | **T** | **G** | **A** | **T** | **T** | **A** | **C** | **G** | **A** | **T** | **A** | **C** | **C** | **A** | **A** | **T** | **C** | **T** | **T** | **C** | **G** | **T** | **C** | **G** | **T** | **T** | **T** | **G** | **A** | **A** | **A** | **A** | **T** | **T** | **A** | **T** | **G** | **A** | **C** | **G** | **A** | **A** | **C** | **A** | **C** | **T** | **T** | **T** | **A** | **G** | **T** | **A** | **G** | **G** | **A** | **G** | **A** | **T** | **G** | **T** | **T** | **G** | **A** | **G** | **G** | **T** | **G** | **A** | **T** | **C** | **C** | **G** | **T** | **G** | **T** | **A** | **-** | **-** | **-** |
| **N59508F** | **A** | **T** | **G** | **A** | **A** | **T** | **A** | **A** | **C** | **C** | **C** | **T** | **T** | **G** | **G** | **C** | **G** | **C** | **T** | **T** | **T** | **T** | **T** | **T** | **A** | **T** | **C** | **G** | **T** | **C** | **G** | **C** | **A** | **G** | **A** | **A** | **G** | **C** | **A** | **T** | **T** | **A** | **C** | **T** | **T** | **T** | **T** | **T** | **A** | **T** | **T** | **C** | **T** | **G** | **G** | **C** | **G** | **T** | **T** | **A** | **T** | **G** | **G** | **C** | **A** | **A** | **A** | **T** | **T** | **G** | **T** | **A** | **C** | **A** | **T** | **A** | **A** | **T** | **C** | **C** | **T** | **G** | **G** | **A** | **T** | **T** | **A** | **G** | **C** | **T** | **G** | **T** | **T** | **T** | **T** | **A** | **T** | **T** | **A** | **A** | **C** | **A** | **A** | **T** | **T** | **G** | **G** | **C** | **G** | **T** | **T** | **T** | **T** | **A** | **C** | **T** | **T** | **G** | **T** | **G** | **G** | **C** | **C** | **T** | **A** | **C** | **G** | **T** | **T** | **T** | **C** | **C** | **A** | **G** | **G** | **A** | **A** | **A** | **G** | **C** | **A** | **T** | **C** | **T** | **A** | **A** | **A** | **A** | **C** | **A** | **C** | **A** | **T** | **T** | **T** | **T** | **A** | **A** | **C** | **A** | **A** | **C** | **T** | **T** | **T** | **C** | **A** | **A** | **T** | **T** | **C** | **G** | **T** | **C** | **C** | **T** | **C** | **G** | **G** | **C** | **G** | **T** | **T** | **G** | **T** | **T** | **T** | **T** | **T** | **A** | **T** | **T** | **G** | **T** | **C** | **A** | **T** | **T** | **G** | **G** | **T** | **G** | **C** | **A** | **A** | **T** | **G** | **A** | **A** | **C** | **A** | **G** | **C** | **A** | **C** | **G** | **G** | **C** | **T** | **G** | **T** | **T** | **T** | **G** | **G** | **T** | **T** | **T** | **A** | **T** | **G** | **T** | **T** | **G** | **A** | **T** | **T** | **T** | **T** | **T** | **G** | **G** | **C** | **G** | **T** | **A** | **C** | **T** | **C** | **T** | **T** | **T** | **A** | **T** | **C** | **G** | **G** | **C** | **T** | **T** | **A** | **A** | **A** | **A** | **G** | **G** | **C** | **T** | **T** | **T** | **G** | **A** | **G** | **A** | **T** | **T** | **T** | **C** | **A** | **G** | **G** | **C** | **G** | **T** | **G** | **G** | **A** | **T** | **A** | **T** | **A** | **G** | **C** | **T** | **G** | **A** | **G** | **C** | **G** | **A** | **G** | **C** | **A** | **C** | **C** | **T** | **T** | **G** | **G** | **C** | **G** | **A** | **A** | **A** | **A** | **A** | **A** | **A** | **C** | **A** | **A** | **A** | **T** | **G** | **A** | **T** | **T** | **A** | **T** | **G** | **G** | **T** | **G** | **G** | **A** | **G** | **A** | **C** | **G** | **G** | **C** | **G** | **G** | **C** | **A** | **A** | **A** | **A** | **G** | **A** | **A** | **C** | **C** | **T** | **A** | **A** | **A** | **A** | **A** | **T** | **G** | **G** | **C** | **A** | **A** | **A** | **C** | **G** | **A** | **T** | **T** | **T** | **A** | **A** | **A** | **C** | **G** | **C** | **C** | **G** | **C** | **T** | **G** | **G** | **T** | **T** | **T** | **G** | **C** | **C** | **A** | **A** | **C** | **G** | **A** | **A** | **C** | **G** | **C** | **A** | **T** | **T** | **G** | **G** | **T** | **A** | **A** | **C** | **A** | **A** | **T** | **A** | **T** | **C** | **T** | **A** | **T** | **G** | **A** | **A** | **T** | **G** | **G** | **G** | **A** | **C** | **G** | **A** | **T** | **A** | **T** | **C** | **A** | **A** | **T** | **A** | **T** | **T** | **G** | **A** | **T** | **T** | **T** | **A** | **A** | **T** | **C** | **T** | **C** | **T** | **G** | **G** | **G** | **G** | **A** | **C** | **A** | **C** | **C** | **A** | **T** | **T** | **A** | **T** | **T** | **G** | **A** | **T** | **T** | **T** | **A** | **G** | **G** | **T** | **A** | **A** | **T** | **A** | **C** | **G** | **C** | **T** | **A** | **C** | **T** | **A** | **C** | **C** | **G** | **A** | **A** | **A** | **G** | **A** | **A** | **G** | **A** | **C** | **A** | **A** | **T** | **A** | **T** | **T** | **A** | **T** | **T** | **A** | **T** | **T** | **A** | **T** | **T** | **C** | **G** | **T** | **A** | **A** | **A** | **G** | **G** | **T** | **T** | **T** | **T** | **G** | **G** | **C** | **C** | **G** | **C** | **A** | **C** | **G** | **C** | **G** | **A** | **A** | **T** | **T** | **C** | **T** | **A** | **G** | **T** | **G** | **C** | **C** | **G** | **T** | **T** | **A** | **G** | **G** | **T** | **G** | **T** | **A** | **G** | **C** | **T** | **A** | **T** | **T** | **T** | **T** | **G** | **T** | **T** | **A** | **G** | **A** | **A** | **C** | **A** | **T** | **T** | **C** | **A** | **A** | **C** | **T** | **T** | **T** | **T** | **T** | **A** | **C** | **G** | **G** | **A** | **A** | **C** | **G** | **G** | **T** | **A** | **C** | **G** | **T** | **T** | **T** | **T** | **G** | **A** | **A** | **G** | **A** | **A** | **G** | **A** | **A** | **A** | **A** | **A** | **T** | **A** | **T** | **C** | **A** | **A** | **T** | **T** | **G** | **A** | **A** | **A** | **A** | **A** | **C** | **G** | **A** | **A** | **T** | **C** | **A** | **T** | **T** | **A** | **A** | **A** | **A** | **A** | **T** | **T** | **T** | **A** | **C** | **A** | **G** | **C** | **A** | **A** | **T** | **G** | **A** | **T** | **T** | **A** | **C** | **G** | **A** | **T** | **A** | **C** | **C** | **A** | **A** | **T** | **C** | **T** | **T** | **C** | **G** | **T** | **C** | **G** | **T** | **T** | **T** | **G** | **A** | **A** | **A** | **A** | **T** | **T** | **A** | **T** | **G** | **A** | **C** | **G** | **A** | **A** | **C** | **A** | **C** | **T** | **T** | **T** | **A** | **G** | **T** | **A** | **G** | **G** | **A** | **G** | **A** | **T** | **G** | **T** | **T** | **G** | **A** | **G** | **G** | **T** | **G** | **A** | **T** | **C** | **C** | **G** | **T** | **G** | **T** | **A** | **-** | **-** | **-** |
| **N59599F** | **A** | **T** | **G** | **A** | **A** | **T** | **A** | **A** | **C** | **C** | **C** | **T** | **T** | **G** | **G** | **C** | **G** | **C** | **T** | **T** | **T** | **T** | **T** | **T** | **A** | **T** | **C** | **G** | **T** | **C** | **G** | **C** | **A** | **G** | **A** | **A** | **G** | **C** | **A** | **T** | **T** | **A** | **C** | **T** | **T** | **T** | **T** | **T** | **A** | **T** | **T** | **C** | **T** | **G** | **G** | **C** | **G** | **T** | **T** | **A** | **T** | **G** | **G** | **C** | **A** | **A** | **A** | **T** | **T** | **G** | **T** | **A** | **C** | **A** | **T** | **A** | **A** | **T** | **C** | **C** | **T** | **G** | **G** | **A** | **T** | **T** | **A** | **G** | **C** | **T** | **G** | **T** | **T** | **T** | **T** | **A** | **T** | **T** | **A** | **A** | **C** | **A** | **A** | **T** | **T** | **G** | **G** | **C** | **G** | **T** | **T** | **T** | **T** | **A** | **C** | **T** | **T** | **G** | **T** | **G** | **G** | **C** | **C** | **T** | **A** | **C** | **G** | **T** | **T** | **T** | **C** | **C** | **A** | **G** | **G** | **A** | **A** | **A** | **G** | **C** | **A** | **T** | **C** | **T** | **A** | **A** | **A** | **A** | **C** | **A** | **C** | **A** | **T** | **T** | **T** | **T** | **A** | **A** | **C** | **A** | **A** | **C** | **T** | **T** | **T** | **C** | **A** | **A** | **T** | **T** | **C** | **G** | **T** | **C** | **C** | **T** | **C** | **G** | **G** | **C** | **G** | **T** | **T** | **G** | **T** | **T** | **T** | **T** | **T** | **A** | **T** | **T** | **G** | **T** | **C** | **A** | **T** | **T** | **G** | **G** | **T** | **G** | **C** | **A** | **A** | **T** | **G** | **A** | **A** | **C** | **A** | **G** | **C** | **A** | **C** | **G** | **G** | **C** | **T** | **G** | **T** | **T** | **T** | **G** | **G** | **T** | **T** | **T** | **A** | **T** | **G** | **T** | **T** | **G** | **A** | **T** | **T** | **T** | **T** | **T** | **G** | **G** | **C** | **G** | **T** | **A** | **C** | **T** | **C** | **T** | **T** | **T** | **A** | **T** | **C** | **G** | **G** | **C** | **T** | **T** | **A** | **A** | **A** | **A** | **G** | **G** | **C** | **T** | **T** | **T** | **G** | **A** | **G** | **A** | **T** | **T** | **T** | **C** | **A** | **G** | **G** | **C** | **G** | **T** | **G** | **G** | **A** | **T** | **A** | **T** | **A** | **G** | **C** | **T** | **G** | **A** | **G** | **C** | **G** | **A** | **G** | **C** | **A** | **C** | **C** | **T** | **T** | **G** | **G** | **C** | **G** | **T** | **A** | **A** | **A** | **A** | **A** | **A** | **C** | **A** | **A** | **A** | **T** | **G** | **A** | **T** | **T** | **A** | **T** | **G** | **G** | **T** | **G** | **G** | **A** | **G** | **A** | **C** | **G** | **G** | **C** | **G** | **G** | **C** | **A** | **A** | **A** | **A** | **G** | **A** | **A** | **C** | **C** | **T** | **A** | **A** | **A** | **A** | **A** | **T** | **G** | **G** | **C** | **A** | **A** | **A** | **C** | **G** | **A** | **T** | **T** | **T** | **A** | **A** | **A** | **C** | **G** | **C** | **C** | **G** | **C** | **T** | **G** | **G** | **T** | **T** | **T** | **G** | **C** | **C** | **A** | **A** | **C** | **G** | **A** | **A** | **C** | **G** | **C** | **A** | **T** | **T** | **G** | **G** | **T** | **A** | **A** | **C** | **A** | **A** | **T** | **A** | **T** | **C** | **T** | **A** | **T** | **G** | **A** | **A** | **T** | **G** | **G** | **G** | **A** | **C** | **G** | **A** | **T** | **A** | **T** | **C** | **A** | **A** | **T** | **A** | **T** | **T** | **G** | **A** | **T** | **T** | **T** | **A** | **A** | **T** | **C** | **T** | **C** | **T** | **G** | **G** | **G** | **G** | **A** | **C** | **A** | **C** | **C** | **A** | **T** | **T** | **A** | **T** | **T** | **G** | **A** | **T** | **T** | **T** | **A** | **G** | **G** | **T** | **A** | **A** | **T** | **A** | **C** | **G** | **C** | **T** | **A** | **C** | **T** | **A** | **C** | **C** | **G** | **A** | **A** | **A** | **G** | **A** | **A** | **G** | **A** | **C** | **A** | **A** | **T** | **A** | **T** | **T** | **A** | **T** | **T** | **A** | **T** | **T** | **A** | **T** | **T** | **C** | **G** | **T** | **A** | **A** | **A** | **G** | **G** | **T** | **T** | **T** | **T** | **G** | **G** | **C** | **C** | **G** | **C** | **A** | **C** | **G** | **C** | **G** | **A** | **A** | **T** | **T** | **C** | **T** | **A** | **G** | **T** | **G** | **C** | **C** | **G** | **T** | **T** | **A** | **G** | **G** | **T** | **G** | **T** | **A** | **G** | **C** | **T** | **A** | **T** | **T** | **T** | **T** | **G** | **T** | **T** | **A** | **G** | **A** | **A** | **C** | **A** | **T** | **T** | **C** | **A** | **A** | **C** | **T** | **T** | **T** | **T** | **T** | **A** | **C** | **G** | **G** | **A** | **A** | **C** | **G** | **G** | **T** | **A** | **C** | **G** | **T** | **T** | **T** | **T** | **G** | **A** | **A** | **G** | **A** | **A** | **G** | **A** | **A** | **A** | **A** | **A** | **T** | **A** | **T** | **C** | **A** | **A** | **T** | **T** | **G** | **A** | **A** | **A** | **A** | **A** | **C** | **G** | **A** | **A** | **T** | **C** | **A** | **T** | **T** | **A** | **A** | **A** | **A** | **A** | **T** | **T** | **T** | **A** | **C** | **A** | **G** | **C** | **A** | **A** | **T** | **G** | **A** | **T** | **T** | **A** | **T** | **G** | **A** | **T** | **A** | **C** | **C** | **A** | **A** | **T** | **C** | **T** | **T** | **C** | **G** | **T** | **C** | **G** | **T** | **T** | **T** | **G** | **A** | **A** | **A** | **A** | **T** | **T** | **A** | **T** | **G** | **A** | **C** | **G** | **A** | **A** | **C** | **A** | **C** | **T** | **T** | **T** | **A** | **G** | **T** | **A** | **G** | **G** | **A** | **G** | **A** | **T** | **G** | **T** | **T** | **G** | **A** | **G** | **G** | **T** | **G** | **A** | **T** | **C** | **C** | **G** | **T** | **G** | **T** | **A** | **-** | **-** | **-** |
| **N59457F** | **A** | **T** | **G** | **A** | **A** | **T** | **A** | **A** | **C** | **C** | **C** | **T** | **T** | **G** | **G** | **C** | **G** | **C** | **T** | **T** | **T** | **T** | **T** | **T** | **A** | **T** | **C** | **G** | **T** | **C** | **G** | **C** | **A** | **G** | **A** | **A** | **G** | **C** | **A** | **T** | **T** | **A** | **C** | **T** | **T** | **T** | **T** | **T** | **A** | **T** | **T** | **C** | **T** | **G** | **G** | **C** | **G** | **T** | **T** | **A** | **T** | **G** | **G** | **C** | **A** | **A** | **A** | **T** | **T** | **G** | **T** | **A** | **C** | **A** | **T** | **A** | **A** | **T** | **C** | **C** | **T** | **G** | **G** | **A** | **T** | **T** | **A** | **G** | **C** | **T** | **G** | **T** | **T** | **T** | **T** | **A** | **T** | **T** | **A** | **A** | **C** | **A** | **A** | **T** | **T** | **G** | **G** | **C** | **G** | **T** | **T** | **T** | **T** | **A** | **C** | **T** | **T** | **G** | **T** | **G** | **G** | **C** | **C** | **T** | **A** | **T** | **G** | **T** | **T** | **T** | **C** | **C** | **A** | **G** | **G** | **A** | **A** | **A** | **G** | **C** | **A** | **T** | **A** | **T** | **A** | **A** | **A** | **A** | **C** | **A** | **C** | **A** | **T** | **T** | **T** | **T** | **A** | **A** | **C** | **A** | **A** | **C** | **T** | **T** | **T** | **C** | **A** | **A** | **T** | **T** | **C** | **G** | **T** | **C** | **C** | **T** | **C** | **G** | **G** | **C** | **G** | **T** | **T** | **G** | **T** | **T** | **T** | **T** | **T** | **A** | **T** | **T** | **G** | **T** | **C** | **A** | **T** | **T** | **G** | **G** | **T** | **G** | **C** | **A** | **A** | **T** | **G** | **A** | **A** | **C** | **A** | **G** | **C** | **A** | **C** | **G** | **G** | **C** | **T** | **G** | **T** | **T** | **T** | **G** | **G** | **T** | **T** | **T** | **A** | **T** | **G** | **C** | **T** | **G** | **A** | **T** | **T** | **T** | **T** | **T** | **G** | **G** | **C** | **G** | **T** | **A** | **C** | **T** | **C** | **T** | **T** | **T** | **A** | **T** | **C** | **G** | **G** | **C** | **T** | **T** | **A** | **A** | **A** | **A** | **G** | **G** | **C** | **T** | **T** | **T** | **G** | **A** | **G** | **A** | **T** | **T** | **T** | **C** | **A** | **G** | **G** | **C** | **G** | **T** | **G** | **G** | **A** | **T** | **A** | **T** | **A** | **G** | **C** | **T** | **G** | **A** | **G** | **C** | **G** | **A** | **G** | **C** | **A** | **C** | **C** | **T** | **T** | **G** | **G** | **C** | **G** | **T** | **A** | **A** | **A** | **A** | **A** | **A** | **C** | **A** | **A** | **A** | **T** | **G** | **A** | **T** | **T** | **A** | **T** | **G** | **G** | **T** | **G** | **G** | **A** | **G** | **A** | **C** | **G** | **G** | **C** | **G** | **G** | **C** | **A** | **A** | **A** | **A** | **G** | **A** | **A** | **C** | **C** | **T** | **A** | **A** | **A** | **A** | **A** | **T** | **G** | **G** | **C** | **A** | **A** | **A** | **C** | **G** | **A** | **T** | **T** | **T** | **A** | **A** | **A** | **C** | **G** | **C** | **C** | **G** | **C** | **T** | **G** | **G** | **T** | **T** | **T** | **G** | **C** | **C** | **A** | **A** | **C** | **G** | **A** | **A** | **C** | **G** | **C** | **A** | **T** | **T** | **G** | **G** | **T** | **A** | **A** | **C** | **A** | **A** | **T** | **A** | **T** | **C** | **T** | **A** | **T** | **G** | **A** | **A** | **T** | **G** | **G** | **G** | **A** | **C** | **G** | **A** | **T** | **A** | **T** | **C** | **A** | **A** | **T** | **A** | **T** | **T** | **G** | **A** | **T** | **T** | **T** | **A** | **A** | **T** | **C** | **T** | **C** | **T** | **G** | **G** | **G** | **G** | **A** | **C** | **A** | **C** | **C** | **A** | **T** | **T** | **A** | **T** | **T** | **G** | **A** | **T** | **T** | **T** | **A** | **G** | **G** | **T** | **A** | **A** | **T** | **A** | **C** | **G** | **C** | **T** | **A** | **C** | **T** | **A** | **C** | **C** | **G** | **A** | **A** | **A** | **G** | **A** | **A** | **G** | **A** | **C** | **A** | **A** | **T** | **A** | **T** | **T** | **A** | **T** | **T** | **A** | **T** | **T** | **A** | **T** | **T** | **C** | **G** | **T** | **A** | **A** | **A** | **G** | **G** | **T** | **T** | **T** | **T** | **G** | **G** | **C** | **C** | **G** | **C** | **A** | **C** | **G** | **C** | **G** | **A** | **A** | **T** | **T** | **C** | **T** | **A** | **G** | **T** | **G** | **C** | **C** | **G** | **T** | **T** | **A** | **G** | **G** | **T** | **G** | **T** | **A** | **G** | **C** | **T** | **A** | **T** | **T** | **T** | **T** | **G** | **T** | **T** | **A** | **G** | **A** | **A** | **C** | **A** | **T** | **T** | **C** | **A** | **A** | **C** | **T** | **T** | **T** | **T** | **T** | **A** | **C** | **G** | **G** | **A** | **A** | **C** | **G** | **G** | **T** | **A** | **C** | **G** | **T** | **T** | **T** | **T** | **G** | **A** | **A** | **G** | **A** | **A** | **G** | **A** | **A** | **A** | **A** | **A** | **T** | **A** | **T** | **C** | **A** | **A** | **T** | **T** | **G** | **A** | **A** | **A** | **A** | **A** | **C** | **G** | **A** | **A** | **T** | **C** | **A** | **T** | **T** | **A** | **A** | **A** | **A** | **A** | **T** | **T** | **T** | **A** | **C** | **A** | **G** | **C** | **A** | **A** | **T** | **G** | **A** | **T** | **T** | **A** | **T** | **G** | **A** | **T** | **A** | **C** | **C** | **A** | **A** | **T** | **C** | **T** | **T** | **C** | **G** | **T** | **C** | **G** | **T** | **T** | **T** | **G** | **A** | **A** | **A** | **A** | **T** | **T** | **A** | **T** | **G** | **A** | **C** | **G** | **A** | **A** | **T** | **A** | **C** | **T** | **T** | **T** | **A** | **G** | **T** | **A** | **G** | **G** | **A** | **G** | **A** | **T** | **G** | **T** | **T** | **G** | **A** | **G** | **G** | **T** | **G** | **A** | **T** | **C** | **C** | **G** | **T** | **G** | **T** | **A** | **-** | **-** | **-** |
| **N53457** | **A** | **T** | **G** | **A** | **A** | **T** | **A** | **A** | **C** | **C** | **C** | **T** | **T** | **G** | **G** | **C** | **G** | **C** | **T** | **T** | **T** | **T** | **T** | **T** | **A** | **T** | **C** | **G** | **T** | **C** | **G** | **C** | **A** | **G** | **A** | **A** | **G** | **C** | **A** | **T** | **T** | **A** | **C** | **T** | **T** | **T** | **T** | **T** | **A** | **T** | **T** | **C** | **T** | **G** | **G** | **C** | **G** | **T** | **T** | **A** | **T** | **G** | **G** | **C** | **A** | **A** | **A** | **T** | **T** | **G** | **T** | **A** | **C** | **A** | **T** | **A** | **A** | **T** | **C** | **C** | **T** | **G** | **G** | **A** | **T** | **T** | **A** | **G** | **C** | **T** | **G** | **T** | **T** | **T** | **T** | **A** | **T** | **T** | **A** | **A** | **C** | **A** | **A** | **T** | **T** | **G** | **G** | **C** | **G** | **T** | **T** | **T** | **T** | **A** | **C** | **T** | **T** | **G** | **T** | **G** | **G** | **C** | **C** | **T** | **A** | **C** | **G** | **T** | **T** | **T** | **C** | **C** | **A** | **G** | **G** | **A** | **A** | **A** | **G** | **C** | **A** | **T** | **C** | **T** | **A** | **A** | **A** | **A** | **C** | **A** | **C** | **A** | **T** | **T** | **T** | **T** | **A** | **A** | **C** | **A** | **A** | **C** | **T** | **T** | **T** | **C** | **A** | **A** | **T** | **T** | **C** | **G** | **T** | **C** | **C** | **T** | **C** | **G** | **G** | **C** | **G** | **T** | **T** | **G** | **T** | **T** | **T** | **T** | **T** | **A** | **T** | **T** | **G** | **T** | **C** | **A** | **T** | **T** | **G** | **G** | **T** | **G** | **C** | **A** | **A** | **T** | **G** | **A** | **A** | **C** | **A** | **G** | **T** | **A** | **C** | **G** | **G** | **C** | **T** | **G** | **T** | **T** | **T** | **G** | **G** | **T** | **T** | **T** | **A** | **T** | **G** | **T** | **T** | **G** | **A** | **T** | **T** | **T** | **T** | **T** | **G** | **G** | **C** | **G** | **T** | **A** | **C** | **T** | **C** | **T** | **T** | **T** | **A** | **T** | **C** | **G** | **G** | **C** | **T** | **T** | **A** | **A** | **A** | **A** | **G** | **G** | **C** | **T** | **T** | **T** | **G** | **A** | **G** | **A** | **T** | **T** | **T** | **C** | **A** | **G** | **G** | **C** | **G** | **T** | **G** | **G** | **A** | **T** | **A** | **T** | **A** | **G** | **C** | **T** | **G** | **A** | **G** | **C** | **G** | **A** | **G** | **C** | **A** | **C** | **C** | **T** | **T** | **G** | **G** | **C** | **G** | **T** | **A** | **A** | **A** | **A** | **A** | **A** | **C** | **A** | **A** | **A** | **T** | **G** | **A** | **T** | **T** | **A** | **T** | **G** | **G** | **T** | **G** | **G** | **A** | **G** | **A** | **C** | **G** | **G** | **C** | **G** | **G** | **C** | **A** | **A** | **A** | **A** | **G** | **A** | **A** | **C** | **C** | **T** | **A** | **A** | **A** | **A** | **A** | **T** | **G** | **G** | **C** | **A** | **A** | **A** | **C** | **G** | **A** | **T** | **T** | **T** | **A** | **A** | **A** | **C** | **G** | **C** | **C** | **G** | **C** | **T** | **G** | **G** | **T** | **T** | **T** | **G** | **C** | **C** | **A** | **A** | **T** | **G** | **A** | **A** | **C** | **G** | **C** | **A** | **T** | **T** | **G** | **G** | **T** | **A** | **A** | **C** | **A** | **A** | **T** | **A** | **T** | **C** | **T** | **A** | **T** | **G** | **A** | **A** | **T** | **G** | **G** | **G** | **A** | **T** | **G** | **A** | **T** | **A** | **T** | **C** | **A** | **A** | **T** | **A** | **T** | **T** | **G** | **A** | **T** | **T** | **T** | **A** | **A** | **T** | **C** | **T** | **C** | **T** | **G** | **G** | **G** | **G** | **A** | **C** | **A** | **C** | **C** | **A** | **T** | **T** | **A** | **T** | **T** | **G** | **A** | **T** | **T** | **T** | **A** | **G** | **G** | **T** | **A** | **A** | **T** | **A** | **C** | **G** | **C** | **T** | **A** | **C** | **T** | **A** | **C** | **C** | **G** | **A** | **A** | **A** | **G** | **A** | **A** | **G** | **A** | **C** | **A** | **A** | **T** | **A** | **T** | **T** | **A** | **T** | **T** | **A** | **T** | **T** | **A** | **T** | **T** | **C** | **G** | **T** | **A** | **A** | **A** | **G** | **G** | **T** | **T** | **T** | **T** | **G** | **G** | **C** | **C** | **G** | **C** | **A** | **C** | **G** | **C** | **G** | **A** | **A** | **T** | **T** | **C** | **T** | **A** | **G** | **T** | **A** | **C** | **C** | **G** | **T** | **T** | **A** | **G** | **G** | **T** | **G** | **T** | **A** | **G** | **C** | **T** | **A** | **T** | **T** | **T** | **T** | **G** | **T** | **T** | **A** | **G** | **A** | **A** | **C** | **A** | **T** | **T** | **C** | **A** | **A** | **C** | **T** | **T** | **T** | **T** | **T** | **A** | **C** | **G** | **G** | **A** | **A** | **C** | **G** | **G** | **T** | **A** | **C** | **G** | **T** | **T** | **T** | **T** | **G** | **A** | **A** | **G** | **A** | **A** | **G** | **A** | **A** | **A** | **A** | **A** | **T** | **A** | **T** | **C** | **A** | **A** | **T** | **T** | **G** | **A** | **A** | **A** | **A** | **A** | **C** | **G** | **A** | **A** | **T** | **C** | **A** | **T** | **T** | **A** | **A** | **A** | **A** | **A** | **T** | **T** | **T** | **A** | **C** | **A** | **G** | **C** | **A** | **A** | **T** | **G** | **A** | **T** | **T** | **A** | **C** | **G** | **A** | **T** | **A** | **C** | **C** | **A** | **A** | **T** | **C** | **T** | **T** | **C** | **G** | **T** | **C** | **G** | **T** | **T** | **T** | **G** | **A** | **A** | **A** | **A** | **T** | **T** | **A** | **T** | **G** | **A** | **C** | **G** | **A** | **A** | **C** | **A** | **C** | **T** | **T** | **T** | **A** | **G** | **T** | **A** | **G** | **G** | **A** | **G** | **A** | **T** | **G** | **T** | **T** | **G** | **A** | **G** | **G** | **T** | **G** | **A** | **T** | **C** | **C** | **G** | **T** | **G** | **T** | **A** | **-** | **-** | **-** |
| **N52648** | **A** | **T** | **G** | **A** | **A** | **T** | **A** | **A** | **C** | **C** | **C** | **T** | **T** | **G** | **G** | **C** | **G** | **C** | **T** | **T** | **T** | **T** | **T** | **T** | **A** | **T** | **C** | **G** | **T** | **C** | **G** | **C** | **A** | **G** | **A** | **A** | **G** | **C** | **A** | **T** | **T** | **A** | **C** | **T** | **T** | **T** | **T** | **T** | **A** | **T** | **T** | **C** | **T** | **G** | **G** | **C** | **G** | **T** | **T** | **A** | **T** | **G** | **G** | **C** | **A** | **A** | **A** | **T** | **T** | **G** | **T** | **A** | **C** | **A** | **T** | **A** | **A** | **T** | **C** | **C** | **T** | **G** | **G** | **A** | **T** | **T** | **A** | **G** | **C** | **T** | **G** | **T** | **T** | **T** | **T** | **A** | **T** | **T** | **A** | **A** | **C** | **A** | **A** | **T** | **T** | **G** | **G** | **C** | **G** | **T** | **T** | **T** | **T** | **A** | **C** | **T** | **T** | **G** | **T** | **G** | **G** | **C** | **C** | **T** | **A** | **C** | **G** | **T** | **T** | **T** | **C** | **C** | **A** | **G** | **G** | **A** | **A** | **A** | **G** | **C** | **A** | **T** | **C** | **T** | **A** | **A** | **A** | **A** | **C** | **A** | **C** | **A** | **T** | **T** | **T** | **T** | **A** | **A** | **C** | **A** | **A** | **C** | **T** | **T** | **T** | **C** | **A** | **A** | **T** | **T** | **C** | **G** | **T** | **C** | **C** | **T** | **C** | **G** | **G** | **C** | **G** | **T** | **T** | **G** | **T** | **T** | **T** | **T** | **T** | **A** | **T** | **T** | **G** | **T** | **C** | **A** | **T** | **T** | **G** | **G** | **T** | **G** | **C** | **A** | **A** | **T** | **G** | **A** | **A** | **C** | **A** | **G** | **C** | **A** | **C** | **G** | **G** | **C** | **T** | **G** | **T** | **T** | **T** | **G** | **G** | **T** | **T** | **T** | **A** | **T** | **G** | **T** | **T** | **G** | **A** | **T** | **T** | **T** | **T** | **T** | **G** | **G** | **C** | **G** | **T** | **A** | **C** | **T** | **C** | **T** | **T** | **T** | **A** | **T** | **C** | **G** | **G** | **C** | **T** | **T** | **A** | **A** | **A** | **A** | **G** | **G** | **C** | **T** | **T** | **T** | **G** | **A** | **G** | **A** | **T** | **T** | **T** | **C** | **A** | **G** | **G** | **C** | **G** | **T** | **G** | **G** | **A** | **T** | **A** | **T** | **A** | **G** | **C** | **T** | **G** | **A** | **A** | **C** | **G** | **A** | **G** | **C** | **A** | **C** | **C** | **T** | **T** | **G** | **G** | **C** | **G** | **T** | **A** | **A** | **A** | **A** | **A** | **A** | **C** | **A** | **A** | **A** | **T** | **G** | **A** | **T** | **T** | **A** | **T** | **G** | **G** | **T** | **A** | **G** | **A** | **G** | **A** | **C** | **G** | **G** | **C** | **G** | **G** | **C** | **A** | **A** | **A** | **A** | **G** | **A** | **A** | **C** | **C** | **T** | **A** | **A** | **A** | **A** | **A** | **T** | **G** | **G** | **C** | **A** | **A** | **A** | **C** | **G** | **A** | **T** | **T** | **T** | **A** | **A** | **A** | **C** | **G** | **C** | **C** | **G** | **C** | **T** | **G** | **G** | **T** | **T** | **T** | **G** | **C** | **C** | **A** | **A** | **C** | **G** | **A** | **A** | **C** | **G** | **C** | **A** | **T** | **T** | **G** | **G** | **T** | **A** | **A** | **C** | **A** | **A** | **T** | **A** | **T** | **C** | **T** | **A** | **T** | **G** | **A** | **A** | **T** | **G** | **G** | **G** | **A** | **T** | **G** | **A** | **C** | **A** | **T** | **C** | **A** | **A** | **T** | **A** | **T** | **T** | **G** | **A** | **T** | **T** | **T** | **A** | **A** | **T** | **C** | **T** | **C** | **T** | **G** | **G** | **G** | **G** | **A** | **C** | **A** | **C** | **C** | **A** | **T** | **T** | **A** | **T** | **T** | **G** | **A** | **T** | **T** | **T** | **A** | **G** | **G** | **T** | **A** | **A** | **T** | **A** | **C** | **G** | **C** | **T** | **A** | **C** | **T** | **A** | **C** | **C** | **G** | **A** | **A** | **A** | **G** | **A** | **A** | **G** | **A** | **C** | **A** | **A** | **T** | **A** | **T** | **T** | **A** | **T** | **T** | **A** | **T** | **T** | **A** | **T** | **T** | **C** | **G** | **T** | **A** | **A** | **A** | **G** | **G** | **T** | **T** | **T** | **T** | **G** | **G** | **C** | **C** | **G** | **C** | **A** | **C** | **G** | **C** | **G** | **A** | **A** | **T** | **T** | **C** | **T** | **A** | **G** | **T** | **G** | **C** | **C** | **G** | **T** | **T** | **A** | **G** | **G** | **T** | **G** | **T** | **A** | **G** | **C** | **T** | **A** | **T** | **T** | **T** | **T** | **G** | **T** | **T** | **A** | **G** | **A** | **A** | **C** | **A** | **T** | **T** | **C** | **A** | **A** | **C** | **T** | **T** | **T** | **T** | **T** | **A** | **C** | **G** | **G** | **A** | **A** | **C** | **G** | **G** | **T** | **A** | **C** | **G** | **T** | **T** | **T** | **T** | **G** | **A** | **A** | **G** | **A** | **A** | **G** | **A** | **A** | **A** | **A** | **A** | **T** | **A** | **T** | **C** | **A** | **A** | **T** | **T** | **G** | **A** | **A** | **A** | **A** | **A** | **C** | **G** | **A** | **A** | **T** | **C** | **A** | **T** | **T** | **A** | **A** | **A** | **A** | **A** | **T** | **T** | **T** | **A** | **C** | **A** | **G** | **C** | **A** | **A** | **T** | **G** | **A** | **T** | **T** | **A** | **T** | **G** | **A** | **T** | **A** | **C** | **C** | **A** | **A** | **T** | **C** | **T** | **T** | **C** | **G** | **T** | **C** | **G** | **T** | **T** | **T** | **G** | **A** | **A** | **A** | **A** | **T** | **T** | **A** | **T** | **G** | **A** | **C** | **G** | **A** | **A** | **C** | **A** | **C** | **T** | **T** | **T** | **A** | **G** | **T** | **A** | **G** | **G** | **A** | **G** | **A** | **T** | **G** | **T** | **T** | **G** | **A** | **G** | **G** | **T** | **G** | **A** | **T** | **C** | **C** | **G** | **T** | **G** | **T** | **A** | **-** | **-** | **-** |
| **N55185** | **A** | **T** | **G** | **A** | **A** | **T** | **A** | **A** | **C** | **C** | **C** | **T** | **T** | **G** | **G** | **C** | **G** | **C** | **T** | **T** | **T** | **T** | **T** | **T** | **A** | **T** | **C** | **G** | **T** | **C** | **G** | **C** | **A** | **G** | **A** | **A** | **G** | **C** | **A** | **T** | **T** | **A** | **C** | **T** | **T** | **T** | **T** | **T** | **A** | **T** | **T** | **C** | **T** | **G** | **G** | **C** | **G** | **T** | **T** | **A** | **T** | **G** | **G** | **C** | **A** | **A** | **A** | **T** | **T** | **G** | **T** | **A** | **C** | **A** | **T** | **A** | **A** | **T** | **C** | **C** | **T** | **G** | **G** | **A** | **T** | **T** | **A** | **G** | **C** | **T** | **G** | **T** | **T** | **T** | **T** | **A** | **T** | **T** | **A** | **A** | **C** | **A** | **A** | **T** | **T** | **G** | **G** | **C** | **G** | **T** | **T** | **T** | **T** | **A** | **C** | **T** | **T** | **G** | **T** | **G** | **G** | **C** | **C** | **T** | **A** | **T** | **G** | **T** | **T** | **T** | **C** | **C** | **A** | **G** | **G** | **A** | **A** | **A** | **G** | **C** | **A** | **T** | **C** | **T** | **A** | **A** | **A** | **A** | **C** | **A** | **C** | **A** | **T** | **T** | **T** | **T** | **A** | **A** | **C** | **A** | **A** | **C** | **T** | **T** | **T** | **C** | **A** | **A** | **T** | **T** | **C** | **G** | **T** | **C** | **C** | **T** | **C** | **G** | **G** | **C** | **G** | **T** | **T** | **G** | **T** | **T** | **T** | **T** | **T** | **A** | **T** | **T** | **G** | **T** | **C** | **A** | **T** | **T** | **G** | **G** | **T** | **G** | **C** | **A** | **A** | **T** | **G** | **A** | **A** | **C** | **A** | **G** | **C** | **A** | **C** | **G** | **G** | **C** | **T** | **G** | **T** | **T** | **T** | **G** | **G** | **T** | **T** | **T** | **A** | **T** | **G** | **T** | **T** | **G** | **A** | **T** | **T** | **T** | **T** | **T** | **G** | **G** | **C** | **G** | **T** | **A** | **C** | **T** | **C** | **T** | **T** | **T** | **A** | **T** | **C** | **G** | **G** | **C** | **T** | **T** | **A** | **A** | **A** | **A** | **G** | **G** | **C** | **T** | **T** | **T** | **G** | **A** | **G** | **A** | **T** | **T** | **T** | **C** | **A** | **G** | **G** | **C** | **G** | **T** | **G** | **G** | **A** | **T** | **A** | **T** | **A** | **G** | **C** | **T** | **G** | **A** | **A** | **C** | **G** | **A** | **G** | **C** | **A** | **C** | **C** | **T** | **T** | **G** | **G** | **C** | **G** | **T** | **A** | **A** | **A** | **A** | **A** | **A** | **C** | **A** | **A** | **A** | **T** | **G** | **A** | **T** | **T** | **A** | **T** | **G** | **G** | **T** | **A** | **G** | **A** | **G** | **A** | **C** | **G** | **G** | **C** | **G** | **G** | **C** | **A** | **A** | **A** | **A** | **G** | **A** | **A** | **C** | **C** | **T** | **A** | **A** | **A** | **A** | **A** | **T** | **G** | **G** | **C** | **A** | **A** | **A** | **C** | **G** | **A** | **T** | **T** | **T** | **A** | **A** | **A** | **C** | **G** | **C** | **C** | **G** | **C** | **T** | **G** | **G** | **T** | **T** | **T** | **G** | **C** | **C** | **A** | **A** | **C** | **G** | **A** | **A** | **C** | **G** | **C** | **A** | **T** | **T** | **G** | **G** | **T** | **A** | **A** | **C** | **A** | **A** | **T** | **A** | **T** | **C** | **T** | **A** | **T** | **G** | **A** | **A** | **T** | **G** | **G** | **G** | **A** | **T** | **G** | **A** | **C** | **A** | **T** | **C** | **A** | **A** | **T** | **A** | **T** | **T** | **G** | **A** | **T** | **T** | **T** | **A** | **A** | **T** | **C** | **T** | **C** | **T** | **G** | **G** | **G** | **G** | **A** | **C** | **A** | **C** | **C** | **A** | **T** | **T** | **A** | **T** | **T** | **G** | **A** | **T** | **T** | **T** | **A** | **G** | **G** | **T** | **A** | **A** | **T** | **A** | **C** | **G** | **C** | **T** | **A** | **C** | **T** | **A** | **C** | **C** | **G** | **A** | **A** | **A** | **G** | **A** | **A** | **G** | **A** | **C** | **A** | **A** | **T** | **A** | **T** | **T** | **A** | **T** | **T** | **A** | **T** | **T** | **A** | **T** | **T** | **C** | **G** | **T** | **A** | **A** | **A** | **G** | **G** | **T** | **T** | **T** | **T** | **G** | **G** | **C** | **C** | **G** | **C** | **A** | **C** | **G** | **C** | **G** | **A** | **A** | **T** | **T** | **C** | **T** | **A** | **G** | **T** | **G** | **C** | **C** | **G** | **T** | **T** | **A** | **G** | **G** | **T** | **G** | **T** | **A** | **G** | **C** | **T** | **A** | **T** | **T** | **T** | **T** | **G** | **T** | **T** | **A** | **G** | **A** | **A** | **C** | **A** | **T** | **T** | **C** | **A** | **A** | **C** | **T** | **T** | **T** | **T** | **T** | **A** | **C** | **G** | **G** | **A** | **A** | **C** | **G** | **G** | **T** | **A** | **C** | **G** | **T** | **T** | **T** | **T** | **G** | **A** | **A** | **G** | **A** | **A** | **G** | **A** | **A** | **A** | **A** | **A** | **T** | **A** | **T** | **C** | **A** | **A** | **T** | **T** | **G** | **A** | **A** | **A** | **A** | **A** | **C** | **G** | **A** | **A** | **T** | **C** | **A** | **T** | **T** | **A** | **A** | **A** | **A** | **A** | **T** | **T** | **T** | **A** | **C** | **A** | **G** | **C** | **A** | **A** | **T** | **G** | **A** | **T** | **T** | **A** | **T** | **G** | **A** | **T** | **A** | **C** | **C** | **A** | **A** | **T** | **C** | **T** | **T** | **C** | **G** | **T** | **C** | **G** | **T** | **T** | **T** | **G** | **A** | **A** | **A** | **A** | **T** | **T** | **A** | **T** | **G** | **A** | **C** | **G** | **A** | **A** | **C** | **A** | **C** | **T** | **T** | **T** | **A** | **G** | **T** | **A** | **G** | **G** | **A** | **G** | **A** | **T** | **G** | **T** | **T** | **G** | **A** | **G** | **G** | **T** | **G** | **A** | **T** | **C** | **C** | **G** | **T** | **G** | **T** | **A** | **-** | **-** | **-** |
| **N59959F** | **A** | **T** | **G** | **A** | **A** | **T** | **A** | **A** | **C** | **C** | **C** | **T** | **T** | **G** | **G** | **C** | **G** | **C** | **T** | **T** | **T** | **T** | **T** | **T** | **A** | **T** | **C** | **G** | **T** | **C** | **G** | **C** | **A** | **G** | **A** | **A** | **G** | **C** | **A** | **T** | **T** | **A** | **C** | **T** | **T** | **T** | **T** | **T** | **A** | **T** | **T** | **C** | **T** | **G** | **G** | **C** | **G** | **T** | **T** | **A** | **T** | **G** | **G** | **C** | **A** | **A** | **A** | **T** | **T** | **G** | **T** | **A** | **C** | **A** | **T** | **A** | **A** | **T** | **C** | **C** | **T** | **G** | **G** | **A** | **T** | **T** | **A** | **G** | **C** | **T** | **G** | **T** | **T** | **T** | **T** | **A** | **T** | **T** | **A** | **A** | **C** | **A** | **A** | **T** | **T** | **G** | **G** | **C** | **G** | **T** | **T** | **T** | **T** | **A** | **C** | **T** | **T** | **G** | **T** | **G** | **G** | **C** | **C** | **T** | **A** | **C** | **G** | **T** | **T** | **T** | **C** | **C** | **A** | **G** | **G** | **A** | **A** | **A** | **G** | **C** | **A** | **T** | **C** | **T** | **A** | **A** | **A** | **A** | **C** | **A** | **C** | **A** | **T** | **T** | **T** | **T** | **A** | **A** | **C** | **A** | **A** | **C** | **T** | **T** | **T** | **C** | **A** | **A** | **T** | **T** | **C** | **G** | **T** | **C** | **C** | **T** | **C** | **G** | **G** | **C** | **G** | **T** | **T** | **G** | **T** | **T** | **T** | **T** | **T** | **A** | **T** | **T** | **G** | **T** | **C** | **A** | **T** | **T** | **G** | **G** | **T** | **G** | **C** | **A** | **A** | **T** | **G** | **A** | **A** | **C** | **A** | **G** | **T** | **A** | **C** | **A** | **G** | **C** | **T** | **G** | **T** | **T** | **T** | **G** | **G** | **T** | **T** | **T** | **A** | **T** | **G** | **T** | **T** | **G** | **A** | **T** | **T** | **T** | **T** | **T** | **G** | **G** | **C** | **G** | **T** | **A** | **C** | **T** | **C** | **T** | **T** | **T** | **A** | **T** | **C** | **G** | **G** | **C** | **T** | **T** | **A** | **A** | **A** | **A** | **G** | **G** | **C** | **T** | **T** | **T** | **G** | **A** | **G** | **A** | **T** | **T** | **T** | **C** | **A** | **G** | **G** | **C** | **G** | **T** | **G** | **G** | **A** | **T** | **A** | **T** | **A** | **G** | **C** | **T** | **G** | **A** | **G** | **C** | **G** | **A** | **G** | **C** | **A** | **C** | **C** | **T** | **T** | **G** | **G** | **C** | **G** | **T** | **A** | **A** | **A** | **A** | **A** | **A** | **C** | **A** | **A** | **A** | **T** | **G** | **A** | **T** | **T** | **A** | **T** | **G** | **G** | **T** | **G** | **G** | **A** | **G** | **A** | **C** | **G** | **G** | **C** | **G** | **G** | **C** | **A** | **A** | **A** | **A** | **G** | **A** | **A** | **C** | **C** | **T** | **A** | **A** | **A** | **A** | **A** | **T** | **G** | **G** | **C** | **A** | **A** | **A** | **C** | **G** | **A** | **T** | **T** | **T** | **A** | **A** | **A** | **C** | **G** | **C** | **C** | **G** | **C** | **T** | **G** | **G** | **T** | **T** | **T** | **G** | **C** | **C** | **A** | **A** | **T** | **G** | **A** | **A** | **C** | **G** | **C** | **A** | **T** | **T** | **G** | **G** | **T** | **A** | **A** | **C** | **A** | **A** | **T** | **A** | **T** | **C** | **T** | **A** | **T** | **G** | **A** | **A** | **T** | **G** | **G** | **G** | **A** | **T** | **G** | **A** | **T** | **A** | **T** | **C** | **A** | **A** | **T** | **A** | **T** | **T** | **G** | **A** | **T** | **T** | **T** | **A** | **A** | **T** | **C** | **T** | **C** | **T** | **G** | **G** | **G** | **G** | **A** | **C** | **A** | **C** | **C** | **A** | **T** | **T** | **A** | **T** | **T** | **G** | **A** | **T** | **T** | **T** | **A** | **G** | **G** | **T** | **A** | **A** | **T** | **A** | **C** | **G** | **C** | **T** | **A** | **C** | **T** | **A** | **C** | **C** | **G** | **A** | **A** | **A** | **G** | **A** | **A** | **G** | **A** | **C** | **A** | **A** | **T** | **A** | **T** | **T** | **A** | **T** | **T** | **A** | **T** | **T** | **A** | **T** | **T** | **C** | **G** | **T** | **A** | **A** | **A** | **G** | **G** | **T** | **T** | **T** | **T** | **G** | **G** | **C** | **C** | **G** | **C** | **A** | **C** | **G** | **C** | **G** | **A** | **A** | **T** | **T** | **C** | **T** | **A** | **G** | **T** | **A** | **C** | **C** | **G** | **T** | **T** | **A** | **G** | **G** | **T** | **G** | **T** | **A** | **G** | **C** | **T** | **A** | **T** | **T** | **T** | **T** | **G** | **T** | **T** | **A** | **G** | **A** | **A** | **C** | **A** | **T** | **T** | **C** | **A** | **A** | **C** | **G** | **T** | **T** | **T** | **T** | **A** | **C** | **G** | **G** | **A** | **A** | **C** | **G** | **G** | **T** | **A** | **C** | **G** | **T** | **T** | **T** | **T** | **G** | **A** | **A** | **G** | **A** | **A** | **G** | **A** | **A** | **A** | **A** | **A** | **T** | **A** | **T** | **C** | **A** | **A** | **T** | **T** | **G** | **A** | **A** | **A** | **A** | **A** | **C** | **G** | **A** | **A** | **T** | **C** | **A** | **T** | **T** | **A** | **A** | **A** | **A** | **A** | **T** | **T** | **T** | **A** | **C** | **A** | **G** | **C** | **A** | **A** | **T** | **G** | **A** | **T** | **T** | **A** | **C** | **G** | **A** | **T** | **A** | **C** | **C** | **A** | **A** | **T** | **C** | **T** | **T** | **C** | **G** | **T** | **C** | **G** | **T** | **T** | **T** | **G** | **A** | **A** | **A** | **A** | **T** | **T** | **A** | **T** | **G** | **A** | **C** | **G** | **A** | **A** | **C** | **A** | **C** | **T** | **T** | **T** | **A** | **G** | **T** | **A** | **G** | **G** | **A** | **G** | **A** | **T** | **G** | **T** | **T** | **G** | **A** | **G** | **G** | **T** | **G** | **A** | **T** | **C** | **C** | **G** | **T** | **G** | **T** | **A** | **-** | **-** | **-** |
| **N59591F** | **A** | **T** | **G** | **A** | **A** | **T** | **A** | **A** | **C** | **C** | **C** | **T** | **T** | **G** | **G** | **C** | **G** | **C** | **T** | **T** | **T** | **T** | **T** | **T** | **A** | **T** | **C** | **G** | **T** | **C** | **G** | **C** | **A** | **G** | **A** | **A** | **G** | **C** | **A** | **T** | **T** | **A** | **C** | **T** | **T** | **T** | **T** | **T** | **A** | **T** | **T** | **C** | **T** | **G** | **G** | **C** | **G** | **T** | **T** | **A** | **T** | **G** | **G** | **C** | **A** | **A** | **A** | **T** | **T** | **G** | **T** | **A** | **C** | **A** | **T** | **A** | **A** | **T** | **C** | **C** | **T** | **G** | **G** | **A** | **T** | **T** | **A** | **G** | **C** | **T** | **G** | **T** | **T** | **T** | **T** | **A** | **T** | **T** | **A** | **A** | **C** | **A** | **A** | **T** | **T** | **G** | **G** | **C** | **G** | **T** | **T** | **T** | **T** | **A** | **C** | **T** | **T** | **G** | **T** | **G** | **G** | **C** | **C** | **T** | **A** | **C** | **G** | **T** | **T** | **T** | **C** | **C** | **A** | **G** | **G** | **A** | **A** | **A** | **G** | **C** | **A** | **T** | **C** | **T** | **A** | **A** | **A** | **A** | **C** | **A** | **C** | **A** | **T** | **T** | **T** | **T** | **A** | **A** | **C** | **A** | **A** | **C** | **T** | **T** | **T** | **C** | **A** | **A** | **T** | **T** | **C** | **G** | **T** | **C** | **C** | **T** | **C** | **G** | **G** | **C** | **G** | **T** | **T** | **G** | **T** | **T** | **T** | **T** | **T** | **A** | **T** | **T** | **G** | **T** | **C** | **A** | **T** | **T** | **G** | **G** | **T** | **G** | **C** | **A** | **A** | **T** | **G** | **A** | **A** | **C** | **A** | **G** | **C** | **A** | **C** | **G** | **G** | **C** | **T** | **G** | **T** | **T** | **T** | **G** | **G** | **T** | **T** | **T** | **A** | **T** | **G** | **T** | **T** | **G** | **A** | **T** | **T** | **T** | **T** | **T** | **G** | **G** | **C** | **G** | **T** | **A** | **C** | **T** | **C** | **T** | **T** | **T** | **A** | **T** | **C** | **G** | **G** | **C** | **T** | **T** | **A** | **A** | **A** | **A** | **G** | **G** | **T** | **T** | **T** | **T** | **G** | **A** | **G** | **A** | **T** | **T** | **T** | **C** | **A** | **G** | **G** | **C** | **G** | **T** | **G** | **G** | **A** | **T** | **A** | **T** | **A** | **G** | **C** | **T** | **G** | **A** | **A** | **C** | **G** | **A** | **G** | **C** | **A** | **C** | **C** | **T** | **T** | **G** | **G** | **C** | **G** | **T** | **A** | **A** | **A** | **A** | **A** | **A** | **C** | **A** | **A** | **A** | **T** | **G** | **A** | **T** | **T** | **A** | **T** | **G** | **G** | **T** | **G** | **G** | **A** | **A** | **A** | **C** | **G** | **G** | **C** | **G** | **G** | **C** | **C** | **A** | **A** | **A** | **G** | **A** | **A** | **C** | **C** | **G** | **A** | **A** | **A** | **A** | **A** | **T** | **G** | **G** | **C** | **A** | **A** | **A** | **C** | **G** | **A** | **T** | **T** | **T** | **A** | **A** | **A** | **C** | **G** | **C** | **C** | **G** | **T** | **T** | **G** | **G** | **T** | **T** | **T** | **G** | **C** | **C** | **A** | **A** | **C** | **G** | **A** | **A** | **C** | **G** | **T** | **A** | **T** | **T** | **G** | **G** | **C** | **A** | **A** | **C** | **A** | **A** | **T** | **A** | **T** | **C** | **T** | **A** | **T** | **G** | **A** | **A** | **T** | **G** | **G** | **G** | **A** | **C** | **G** | **A** | **C** | **A** | **T** | **C** | **A** | **A** | **T** | **A** | **T** | **T** | **G** | **A** | **T** | **T** | **T** | **A** | **A** | **T** | **C** | **T** | **C** | **T** | **G** | **G** | **G** | **G** | **A** | **C** | **A** | **C** | **C** | **A** | **T** | **T** | **A** | **T** | **T** | **G** | **A** | **T** | **T** | **T** | **A** | **G** | **G** | **T** | **A** | **A** | **T** | **A** | **C** | **G** | **C** | **T** | **A** | **C** | **T** | **A** | **C** | **C** | **G** | **A** | **A** | **A** | **G** | **A** | **A** | **G** | **A** | **C** | **A** | **A** | **T** | **A** | **T** | **T** | **A** | **T** | **T** | **A** | **T** | **T** | **A** | **T** | **T** | **C** | **G** | **T** | **A** | **A** | **A** | **G** | **G** | **T** | **T** | **T** | **T** | **G** | **G** | **C** | **C** | **G** | **C** | **A** | **C** | **G** | **C** | **G** | **A** | **A** | **T** | **C** | **C** | **T** | **A** | **G** | **T** | **A** | **C** | **C** | **G** | **T** | **T** | **A** | **G** | **G** | **T** | **G** | **T** | **A** | **G** | **C** | **T** | **A** | **T** | **T** | **T** | **T** | **G** | **T** | **T** | **A** | **G** | **A** | **A** | **C** | **A** | **T** | **T** | **C** | **A** | **A** | **C** | **G** | **T** | **T** | **T** | **T** | **A** | **C** | **G** | **G** | **A** | **A** | **C** | **G** | **G** | **T** | **A** | **C** | **G** | **T** | **T** | **T** | **T** | **G** | **A** | **A** | **G** | **A** | **A** | **G** | **A** | **A** | **A** | **A** | **A** | **T** | **A** | **T** | **C** | **A** | **A** | **T** | **T** | **G** | **A** | **A** | **A** | **A** | **A** | **C** | **G** | **A** | **A** | **T** | **C** | **A** | **T** | **T** | **A** | **A** | **A** | **A** | **A** | **T** | **T** | **T** | **A** | **C** | **A** | **G** | **C** | **A** | **A** | **T** | **G** | **A** | **T** | **T** | **A** | **C** | **G** | **A** | **T** | **A** | **C** | **C** | **A** | **A** | **T** | **C** | **T** | **T** | **C** | **G** | **T** | **C** | **G** | **T** | **T** | **T** | **G** | **A** | **A** | **A** | **A** | **T** | **T** | **A** | **T** | **G** | **A** | **C** | **G** | **A** | **A** | **C** | **A** | **C** | **T** | **T** | **T** | **A** | **G** | **T** | **A** | **G** | **G** | **A** | **G** | **A** | **T** | **G** | **T** | **T** | **G** | **A** | **G** | **G** | **T** | **G** | **A** | **T** | **C** | **C** | **G** | **T** | **G** | **T** | **A** | **-** | **-** | **-** |
| **N53405** | **A** | **T** | **G** | **A** | **A** | **T** | **A** | **A** | **C** | **C** | **C** | **T** | **T** | **G** | **G** | **C** | **G** | **C** | **T** | **T** | **T** | **T** | **T** | **T** | **A** | **T** | **C** | **G** | **T** | **C** | **G** | **C** | **A** | **G** | **A** | **A** | **G** | **C** | **A** | **T** | **T** | **A** | **C** | **T** | **T** | **T** | **T** | **T** | **A** | **T** | **T** | **C** | **T** | **G** | **G** | **C** | **G** | **T** | **T** | **A** | **T** | **G** | **G** | **C** | **A** | **A** | **A** | **T** | **T** | **G** | **T** | **A** | **C** | **A** | **T** | **A** | **A** | **T** | **C** | **C** | **T** | **G** | **G** | **A** | **T** | **T** | **A** | **G** | **C** | **T** | **G** | **T** | **T** | **T** | **T** | **A** | **T** | **T** | **A** | **A** | **C** | **A** | **A** | **T** | **T** | **G** | **G** | **C** | **G** | **T** | **T** | **T** | **T** | **A** | **C** | **T** | **T** | **G** | **T** | **G** | **G** | **C** | **C** | **T** | **A** | **C** | **G** | **T** | **T** | **T** | **C** | **C** | **A** | **G** | **G** | **A** | **A** | **A** | **G** | **C** | **A** | **T** | **C** | **T** | **A** | **A** | **A** | **A** | **C** | **A** | **C** | **A** | **T** | **T** | **T** | **T** | **A** | **A** | **C** | **A** | **A** | **C** | **T** | **T** | **T** | **C** | **A** | **A** | **T** | **T** | **C** | **G** | **T** | **C** | **C** | **T** | **C** | **G** | **G** | **C** | **G** | **T** | **T** | **G** | **T** | **T** | **T** | **T** | **T** | **A** | **T** | **T** | **G** | **T** | **C** | **A** | **T** | **T** | **G** | **G** | **T** | **G** | **C** | **A** | **A** | **T** | **G** | **A** | **A** | **C** | **A** | **G** | **C** | **A** | **C** | **G** | **G** | **C** | **T** | **G** | **T** | **T** | **T** | **G** | **G** | **T** | **T** | **T** | **A** | **T** | **G** | **T** | **T** | **G** | **A** | **T** | **T** | **T** | **T** | **T** | **G** | **G** | **C** | **G** | **T** | **A** | **C** | **T** | **C** | **T** | **T** | **T** | **A** | **T** | **C** | **G** | **G** | **C** | **T** | **T** | **A** | **A** | **A** | **A** | **G** | **G** | **C** | **T** | **T** | **T** | **G** | **A** | **G** | **A** | **T** | **T** | **T** | **C** | **A** | **G** | **G** | **C** | **G** | **T** | **G** | **G** | **A** | **T** | **A** | **T** | **A** | **G** | **C** | **T** | **G** | **A** | **G** | **C** | **G** | **A** | **G** | **C** | **A** | **C** | **C** | **T** | **T** | **G** | **G** | **C** | **G** | **A** | **A** | **A** | **A** | **A** | **A** | **A** | **C** | **A** | **A** | **A** | **T** | **G** | **A** | **T** | **T** | **A** | **T** | **G** | **G** | **T** | **G** | **G** | **A** | **G** | **A** | **C** | **G** | **G** | **C** | **G** | **G** | **C** | **A** | **A** | **A** | **A** | **G** | **A** | **A** | **C** | **C** | **T** | **A** | **A** | **A** | **A** | **A** | **T** | **G** | **G** | **C** | **A** | **A** | **A** | **C** | **G** | **A** | **T** | **T** | **T** | **A** | **A** | **A** | **C** | **G** | **C** | **C** | **G** | **C** | **T** | **G** | **G** | **T** | **T** | **T** | **G** | **C** | **C** | **A** | **A** | **C** | **G** | **A** | **A** | **C** | **G** | **C** | **A** | **T** | **T** | **G** | **G** | **T** | **A** | **A** | **C** | **A** | **A** | **T** | **A** | **T** | **C** | **T** | **A** | **T** | **G** | **A** | **A** | **T** | **G** | **G** | **G** | **A** | **C** | **G** | **A** | **C** | **A** | **T** | **C** | **A** | **A** | **T** | **A** | **T** | **T** | **G** | **A** | **T** | **T** | **T** | **A** | **A** | **T** | **C** | **T** | **C** | **T** | **G** | **G** | **G** | **G** | **A** | **C** | **A** | **C** | **C** | **A** | **T** | **T** | **A** | **T** | **T** | **G** | **A** | **T** | **T** | **T** | **A** | **G** | **G** | **T** | **A** | **A** | **T** | **A** | **C** | **G** | **C** | **T** | **A** | **C** | **T** | **A** | **C** | **C** | **G** | **A** | **A** | **A** | **G** | **A** | **A** | **G** | **A** | **C** | **A** | **A** | **T** | **A** | **T** | **T** | **A** | **T** | **T** | **A** | **T** | **T** | **A** | **T** | **T** | **C** | **G** | **T** | **A** | **A** | **A** | **G** | **G** | **T** | **T** | **T** | **T** | **G** | **G** | **C** | **C** | **G** | **C** | **A** | **C** | **G** | **C** | **G** | **A** | **A** | **T** | **T** | **C** | **T** | **A** | **G** | **T** | **G** | **C** | **C** | **G** | **T** | **T** | **A** | **G** | **G** | **T** | **G** | **T** | **A** | **G** | **C** | **T** | **A** | **T** | **T** | **T** | **T** | **G** | **T** | **T** | **A** | **G** | **A** | **A** | **C** | **A** | **T** | **T** | **C** | **A** | **A** | **C** | **T** | **T** | **T** | **T** | **T** | **A** | **C** | **G** | **G** | **A** | **A** | **C** | **G** | **G** | **T** | **A** | **C** | **G** | **T** | **T** | **T** | **T** | **G** | **A** | **A** | **G** | **A** | **A** | **G** | **A** | **A** | **A** | **A** | **A** | **T** | **A** | **T** | **C** | **A** | **A** | **T** | **T** | **G** | **A** | **A** | **A** | **A** | **A** | **C** | **G** | **A** | **A** | **T** | **C** | **A** | **T** | **T** | **A** | **A** | **A** | **A** | **A** | **T** | **T** | **T** | **A** | **C** | **A** | **G** | **C** | **A** | **A** | **T** | **G** | **A** | **T** | **T** | **A** | **C** | **G** | **A** | **T** | **A** | **C** | **C** | **A** | **A** | **T** | **C** | **T** | **T** | **C** | **G** | **T** | **C** | **G** | **T** | **T** | **T** | **G** | **A** | **A** | **A** | **A** | **T** | **T** | **A** | **T** | **G** | **A** | **C** | **G** | **A** | **A** | **C** | **A** | **C** | **T** | **T** | **T** | **A** | **G** | **T** | **A** | **G** | **G** | **A** | **G** | **A** | **T** | **G** | **T** | **T** | **G** | **A** | **G** | **G** | **T** | **G** | **A** | **T** | **C** | **C** | **G** | **T** | **G** | **T** | **A** | **-** | **-** | **-** |
| **N59466F** | **A** | **T** | **G** | **A** | **A** | **T** | **A** | **A** | **C** | **C** | **C** | **T** | **T** | **G** | **G** | **C** | **G** | **C** | **T** | **T** | **T** | **T** | **T** | **T** | **A** | **T** | **C** | **G** | **T** | **C** | **G** | **C** | **A** | **G** | **A** | **A** | **G** | **C** | **A** | **T** | **T** | **A** | **C** | **T** | **T** | **T** | **T** | **T** | **A** | **T** | **T** | **C** | **T** | **G** | **G** | **C** | **G** | **T** | **T** | **A** | **T** | **G** | **G** | **C** | **A** | **A** | **A** | **T** | **T** | **G** | **T** | **A** | **C** | **A** | **T** | **A** | **A** | **T** | **C** | **C** | **T** | **G** | **G** | **A** | **T** | **T** | **A** | **G** | **C** | **T** | **G** | **T** | **T** | **T** | **T** | **A** | **T** | **T** | **A** | **A** | **C** | **A** | **A** | **T** | **T** | **G** | **G** | **C** | **G** | **T** | **T** | **T** | **T** | **A** | **C** | **T** | **T** | **G** | **T** | **G** | **G** | **C** | **C** | **T** | **A** | **C** | **G** | **T** | **T** | **T** | **C** | **C** | **A** | **G** | **G** | **A** | **A** | **A** | **G** | **C** | **A** | **T** | **C** | **T** | **A** | **A** | **A** | **A** | **C** | **A** | **C** | **A** | **T** | **T** | **T** | **T** | **A** | **A** | **C** | **A** | **A** | **C** | **T** | **T** | **T** | **C** | **A** | **A** | **T** | **T** | **C** | **G** | **T** | **C** | **C** | **T** | **C** | **G** | **G** | **C** | **G** | **T** | **T** | **G** | **T** | **T** | **T** | **T** | **T** | **A** | **T** | **T** | **G** | **T** | **C** | **A** | **T** | **T** | **G** | **G** | **T** | **G** | **C** | **A** | **A** | **T** | **G** | **A** | **A** | **C** | **A** | **G** | **C** | **A** | **C** | **G** | **G** | **C** | **T** | **G** | **T** | **T** | **T** | **G** | **G** | **T** | **T** | **T** | **A** | **T** | **G** | **T** | **T** | **G** | **A** | **T** | **T** | **T** | **T** | **T** | **G** | **G** | **C** | **G** | **T** | **A** | **C** | **T** | **C** | **T** | **T** | **T** | **A** | **T** | **C** | **G** | **G** | **C** | **T** | **T** | **A** | **A** | **A** | **A** | **G** | **G** | **C** | **T** | **T** | **T** | **G** | **A** | **G** | **A** | **T** | **T** | **T** | **C** | **A** | **G** | **G** | **C** | **G** | **T** | **G** | **G** | **A** | **T** | **A** | **T** | **A** | **G** | **C** | **T** | **G** | **A** | **G** | **C** | **G** | **A** | **G** | **C** | **A** | **C** | **C** | **T** | **T** | **G** | **G** | **C** | **G** | **A** | **A** | **A** | **A** | **A** | **A** | **A** | **C** | **A** | **A** | **A** | **T** | **G** | **A** | **T** | **T** | **A** | **T** | **G** | **G** | **T** | **G** | **G** | **A** | **G** | **A** | **C** | **G** | **G** | **C** | **G** | **G** | **C** | **A** | **A** | **A** | **A** | **G** | **A** | **A** | **C** | **C** | **T** | **A** | **A** | **A** | **A** | **A** | **T** | **G** | **G** | **C** | **A** | **A** | **A** | **C** | **G** | **A** | **T** | **T** | **T** | **A** | **A** | **A** | **C** | **G** | **C** | **C** | **G** | **C** | **T** | **G** | **G** | **T** | **T** | **T** | **G** | **C** | **C** | **A** | **A** | **C** | **G** | **A** | **A** | **C** | **G** | **C** | **A** | **T** | **T** | **G** | **G** | **T** | **A** | **A** | **C** | **A** | **A** | **T** | **A** | **T** | **C** | **T** | **A** | **T** | **G** | **A** | **A** | **T** | **G** | **G** | **G** | **A** | **C** | **G** | **A** | **T** | **A** | **T** | **C** | **A** | **A** | **T** | **A** | **T** | **T** | **G** | **A** | **T** | **T** | **T** | **A** | **A** | **T** | **C** | **T** | **C** | **T** | **G** | **G** | **G** | **G** | **A** | **C** | **A** | **C** | **C** | **A** | **T** | **T** | **A** | **T** | **T** | **G** | **A** | **T** | **T** | **T** | **A** | **G** | **G** | **T** | **A** | **A** | **T** | **A** | **C** | **G** | **T** | **T** | **A** | **C** | **T** | **A** | **C** | **C** | **G** | **A** | **A** | **A** | **G** | **A** | **A** | **G** | **A** | **C** | **A** | **A** | **T** | **A** | **T** | **T** | **A** | **T** | **T** | **A** | **T** | **T** | **A** | **T** | **T** | **C** | **G** | **T** | **A** | **A** | **A** | **G** | **G** | **T** | **T** | **T** | **T** | **G** | **G** | **C** | **C** | **G** | **C** | **A** | **C** | **G** | **C** | **G** | **A** | **A** | **T** | **T** | **C** | **T** | **A** | **G** | **T** | **G** | **C** | **C** | **G** | **T** | **T** | **A** | **G** | **G** | **T** | **G** | **T** | **A** | **G** | **C** | **T** | **A** | **T** | **T** | **T** | **T** | **G** | **T** | **T** | **A** | **G** | **A** | **A** | **C** | **A** | **T** | **T** | **C** | **A** | **A** | **C** | **T** | **T** | **T** | **T** | **T** | **A** | **C** | **G** | **G** | **A** | **A** | **C** | **G** | **G** | **T** | **A** | **C** | **G** | **T** | **T** | **T** | **T** | **G** | **A** | **A** | **G** | **A** | **A** | **G** | **A** | **A** | **A** | **A** | **A** | **T** | **A** | **T** | **C** | **A** | **A** | **T** | **T** | **G** | **A** | **A** | **A** | **A** | **A** | **C** | **G** | **A** | **A** | **T** | **C** | **A** | **T** | **T** | **A** | **A** | **A** | **A** | **A** | **T** | **T** | **T** | **A** | **C** | **A** | **G** | **C** | **A** | **A** | **T** | **G** | **A** | **T** | **T** | **A** | **T** | **G** | **A** | **T** | **A** | **C** | **C** | **A** | **A** | **T** | **C** | **T** | **T** | **C** | **G** | **T** | **C** | **G** | **T** | **T** | **T** | **G** | **A** | **A** | **A** | **A** | **T** | **T** | **A** | **T** | **G** | **A** | **C** | **G** | **A** | **A** | **C** | **A** | **C** | **T** | **T** | **T** | **A** | **G** | **T** | **A** | **G** | **G** | **A** | **G** | **A** | **T** | **G** | **T** | **T** | **G** | **A** | **G** | **G** | **T** | **G** | **A** | **T** | **C** | **C** | **G** | **T** | **G** | **T** | **A** | **-** | **-** | **-** |
| **N53519** | **A** | **T** | **G** | **A** | **A** | **T** | **A** | **A** | **C** | **C** | **C** | **T** | **T** | **G** | **G** | **C** | **G** | **C** | **T** | **T** | **T** | **T** | **T** | **T** | **A** | **T** | **C** | **G** | **T** | **C** | **G** | **C** | **A** | **G** | **A** | **A** | **G** | **C** | **A** | **T** | **T** | **A** | **C** | **T** | **T** | **T** | **T** | **T** | **A** | **T** | **T** | **C** | **T** | **G** | **G** | **C** | **G** | **T** | **T** | **A** | **T** | **G** | **G** | **C** | **A** | **A** | **A** | **T** | **T** | **G** | **T** | **A** | **C** | **A** | **T** | **A** | **A** | **T** | **C** | **C** | **T** | **G** | **G** | **A** | **T** | **T** | **A** | **G** | **C** | **T** | **G** | **T** | **T** | **T** | **T** | **A** | **T** | **T** | **A** | **A** | **C** | **A** | **A** | **T** | **T** | **G** | **G** | **C** | **G** | **T** | **T** | **T** | **T** | **A** | **C** | **T** | **T** | **G** | **T** | **G** | **G** | **C** | **C** | **T** | **A** | **C** | **G** | **T** | **T** | **T** | **C** | **C** | **A** | **G** | **G** | **A** | **A** | **A** | **G** | **C** | **A** | **T** | **C** | **T** | **A** | **A** | **A** | **A** | **C** | **A** | **C** | **A** | **T** | **T** | **T** | **T** | **A** | **A** | **C** | **A** | **A** | **C** | **T** | **T** | **T** | **C** | **A** | **A** | **T** | **T** | **C** | **G** | **T** | **C** | **C** | **T** | **C** | **G** | **G** | **C** | **G** | **T** | **T** | **G** | **T** | **T** | **T** | **T** | **T** | **A** | **T** | **T** | **G** | **T** | **C** | **A** | **T** | **T** | **G** | **G** | **T** | **G** | **C** | **A** | **A** | **T** | **G** | **A** | **A** | **T** | **A** | **G** | **C** | **A** | **C** | **G** | **G** | **C** | **T** | **G** | **T** | **T** | **T** | **G** | **G** | **T** | **T** | **T** | **A** | **T** | **G** | **T** | **T** | **G** | **A** | **T** | **T** | **T** | **T** | **T** | **G** | **G** | **C** | **G** | **T** | **A** | **C** | **T** | **C** | **T** | **T** | **T** | **A** | **T** | **C** | **G** | **G** | **C** | **T** | **T** | **A** | **A** | **A** | **A** | **G** | **G** | **C** | **T** | **T** | **T** | **G** | **A** | **G** | **A** | **T** | **T** | **T** | **C** | **A** | **G** | **G** | **C** | **G** | **T** | **G** | **G** | **A** | **T** | **A** | **T** | **A** | **G** | **C** | **T** | **G** | **A** | **G** | **C** | **G** | **A** | **G** | **C** | **A** | **C** | **C** | **T** | **T** | **G** | **G** | **C** | **G** | **A** | **A** | **A** | **A** | **A** | **A** | **A** | **C** | **A** | **A** | **A** | **T** | **G** | **A** | **T** | **T** | **A** | **T** | **G** | **G** | **T** | **G** | **G** | **A** | **G** | **A** | **C** | **G** | **G** | **C** | **G** | **G** | **C** | **A** | **A** | **A** | **A** | **G** | **A** | **A** | **C** | **C** | **T** | **A** | **A** | **A** | **A** | **A** | **T** | **G** | **G** | **C** | **A** | **A** | **A** | **C** | **G** | **G** | **T** | **T** | **T** | **A** | **A** | **A** | **C** | **G** | **C** | **C** | **G** | **C** | **T** | **G** | **G** | **T** | **T** | **T** | **G** | **C** | **C** | **A** | **A** | **C** | **G** | **A** | **A** | **C** | **G** | **C** | **A** | **T** | **T** | **G** | **G** | **T** | **A** | **A** | **C** | **A** | **A** | **T** | **A** | **T** | **C** | **T** | **A** | **T** | **G** | **A** | **A** | **T** | **G** | **G** | **G** | **A** | **C** | **G** | **A** | **T** | **A** | **T** | **C** | **A** | **A** | **T** | **A** | **T** | **T** | **G** | **A** | **T** | **T** | **T** | **A** | **A** | **T** | **C** | **T** | **C** | **T** | **G** | **G** | **G** | **G** | **A** | **C** | **A** | **C** | **C** | **A** | **T** | **T** | **A** | **T** | **T** | **G** | **A** | **T** | **T** | **T** | **A** | **G** | **G** | **T** | **A** | **A** | **T** | **A** | **C** | **G** | **C** | **T** | **A** | **C** | **T** | **A** | **C** | **C** | **G** | **A** | **A** | **A** | **G** | **A** | **A** | **G** | **A** | **C** | **A** | **A** | **T** | **A** | **T** | **T** | **A** | **T** | **T** | **A** | **T** | **T** | **A** | **T** | **T** | **C** | **G** | **T** | **A** | **A** | **A** | **G** | **G** | **T** | **T** | **T** | **T** | **G** | **G** | **C** | **C** | **G** | **C** | **A** | **C** | **G** | **C** | **G** | **A** | **A** | **T** | **T** | **C** | **T** | **A** | **G** | **T** | **G** | **C** | **C** | **G** | **T** | **T** | **A** | **G** | **G** | **T** | **G** | **T** | **A** | **G** | **C** | **T** | **A** | **T** | **T** | **T** | **T** | **G** | **T** | **T** | **A** | **G** | **A** | **A** | **C** | **A** | **T** | **T** | **C** | **A** | **A** | **C** | **T** | **T** | **T** | **T** | **T** | **A** | **C** | **G** | **G** | **A** | **A** | **C** | **G** | **G** | **T** | **A** | **C** | **G** | **T** | **T** | **T** | **T** | **G** | **A** | **A** | **G** | **A** | **A** | **G** | **A** | **A** | **A** | **A** | **A** | **T** | **A** | **T** | **C** | **A** | **A** | **T** | **T** | **G** | **A** | **A** | **A** | **A** | **A** | **C** | **G** | **A** | **A** | **T** | **C** | **A** | **T** | **T** | **A** | **A** | **A** | **A** | **A** | **T** | **T** | **T** | **A** | **C** | **A** | **G** | **C** | **A** | **A** | **T** | **G** | **A** | **T** | **T** | **A** | **T** | **G** | **A** | **T** | **A** | **C** | **C** | **A** | **A** | **T** | **C** | **T** | **T** | **C** | **G** | **T** | **C** | **G** | **T** | **T** | **T** | **G** | **A** | **A** | **A** | **A** | **T** | **T** | **A** | **T** | **G** | **A** | **C** | **G** | **A** | **A** | **C** | **A** | **C** | **T** | **T** | **T** | **A** | **G** | **T** | **A** | **G** | **G** | **A** | **G** | **A** | **T** | **G** | **T** | **T** | **G** | **A** | **G** | **G** | **T** | **G** | **A** | **T** | **C** | **C** | **G** | **T** | **G** | **T** | **A** | **-** | **-** | **-** |
| **N55119** | **A** | **T** | **G** | **A** | **A** | **T** | **A** | **A** | **C** | **C** | **C** | **T** | **T** | **G** | **G** | **C** | **G** | **C** | **T** | **T** | **T** | **T** | **T** | **T** | **A** | **T** | **C** | **G** | **T** | **C** | **G** | **C** | **A** | **G** | **A** | **A** | **G** | **C** | **A** | **T** | **T** | **A** | **C** | **T** | **T** | **T** | **T** | **T** | **A** | **T** | **T** | **C** | **T** | **G** | **G** | **C** | **G** | **T** | **T** | **A** | **T** | **G** | **G** | **C** | **A** | **A** | **A** | **T** | **T** | **G** | **T** | **A** | **C** | **A** | **T** | **A** | **A** | **T** | **C** | **C** | **T** | **G** | **G** | **A** | **T** | **T** | **A** | **G** | **C** | **T** | **G** | **T** | **T** | **T** | **T** | **A** | **T** | **T** | **A** | **A** | **C** | **A** | **A** | **T** | **T** | **G** | **G** | **C** | **G** | **T** | **T** | **T** | **T** | **A** | **C** | **T** | **T** | **G** | **T** | **G** | **G** | **C** | **C** | **T** | **A** | **C** | **G** | **T** | **T** | **T** | **C** | **C** | **A** | **G** | **G** | **A** | **A** | **A** | **G** | **C** | **A** | **T** | **C** | **T** | **A** | **A** | **A** | **A** | **C** | **A** | **C** | **A** | **T** | **T** | **T** | **T** | **A** | **A** | **C** | **A** | **A** | **C** | **T** | **T** | **T** | **C** | **A** | **A** | **T** | **T** | **C** | **G** | **T** | **C** | **C** | **T** | **C** | **G** | **G** | **C** | **G** | **T** | **T** | **G** | **T** | **T** | **T** | **T** | **T** | **A** | **T** | **T** | **G** | **T** | **C** | **A** | **T** | **T** | **G** | **G** | **T** | **G** | **C** | **A** | **A** | **T** | **G** | **A** | **A** | **C** | **A** | **G** | **C** | **A** | **C** | **G** | **G** | **C** | **T** | **G** | **T** | **T** | **T** | **G** | **G** | **T** | **T** | **T** | **A** | **T** | **G** | **T** | **T** | **G** | **A** | **T** | **T** | **T** | **T** | **T** | **G** | **G** | **C** | **G** | **T** | **A** | **C** | **T** | **C** | **T** | **T** | **T** | **A** | **T** | **C** | **G** | **G** | **C** | **T** | **T** | **A** | **A** | **A** | **A** | **G** | **G** | **C** | **T** | **T** | **T** | **G** | **A** | **G** | **A** | **T** | **T** | **T** | **C** | **A** | **G** | **G** | **C** | **G** | **T** | **G** | **G** | **A** | **T** | **A** | **T** | **A** | **G** | **C** | **T** | **G** | **A** | **G** | **C** | **G** | **A** | **G** | **C** | **A** | **C** | **C** | **T** | **T** | **G** | **G** | **C** | **G** | **A** | **A** | **A** | **A** | **A** | **A** | **A** | **C** | **A** | **A** | **A** | **T** | **G** | **A** | **T** | **T** | **A** | **T** | **G** | **G** | **T** | **G** | **G** | **A** | **G** | **A** | **C** | **G** | **G** | **C** | **G** | **G** | **C** | **A** | **A** | **A** | **A** | **G** | **A** | **A** | **C** | **C** | **T** | **A** | **A** | **A** | **A** | **A** | **T** | **G** | **G** | **C** | **A** | **A** | **A** | **C** | **G** | **A** | **T** | **T** | **T** | **A** | **A** | **A** | **C** | **G** | **C** | **C** | **G** | **C** | **T** | **G** | **G** | **T** | **T** | **T** | **G** | **C** | **C** | **A** | **A** | **C** | **G** | **A** | **A** | **C** | **G** | **C** | **A** | **T** | **T** | **G** | **G** | **T** | **A** | **A** | **C** | **A** | **A** | **T** | **A** | **T** | **C** | **T** | **A** | **T** | **G** | **A** | **A** | **T** | **G** | **G** | **G** | **A** | **C** | **G** | **A** | **T** | **A** | **T** | **C** | **A** | **A** | **T** | **A** | **T** | **T** | **G** | **A** | **T** | **T** | **T** | **A** | **A** | **T** | **C** | **T** | **C** | **T** | **G** | **G** | **G** | **G** | **A** | **C** | **A** | **C** | **C** | **A** | **T** | **T** | **A** | **T** | **T** | **G** | **A** | **T** | **T** | **T** | **A** | **G** | **G** | **T** | **A** | **A** | **T** | **A** | **C** | **G** | **C** | **T** | **A** | **C** | **T** | **A** | **C** | **C** | **G** | **A** | **A** | **A** | **G** | **A** | **A** | **G** | **A** | **C** | **A** | **A** | **T** | **A** | **T** | **T** | **A** | **T** | **T** | **A** | **T** | **T** | **A** | **T** | **T** | **C** | **G** | **T** | **A** | **A** | **A** | **G** | **G** | **T** | **T** | **T** | **T** | **G** | **G** | **C** | **C** | **G** | **C** | **A** | **C** | **G** | **C** | **G** | **A** | **A** | **T** | **T** | **C** | **T** | **A** | **G** | **T** | **G** | **C** | **C** | **G** | **T** | **T** | **A** | **G** | **G** | **T** | **G** | **T** | **A** | **G** | **C** | **T** | **A** | **T** | **T** | **T** | **T** | **G** | **T** | **T** | **A** | **G** | **A** | **A** | **C** | **A** | **T** | **T** | **C** | **A** | **A** | **C** | **T** | **T** | **T** | **T** | **T** | **A** | **C** | **G** | **G** | **A** | **A** | **C** | **G** | **G** | **T** | **A** | **C** | **G** | **T** | **T** | **T** | **T** | **G** | **A** | **A** | **G** | **A** | **A** | **G** | **A** | **A** | **A** | **A** | **A** | **T** | **A** | **T** | **C** | **A** | **A** | **T** | **T** | **G** | **A** | **A** | **A** | **A** | **A** | **C** | **G** | **A** | **A** | **T** | **C** | **A** | **T** | **T** | **A** | **A** | **A** | **A** | **A** | **T** | **T** | **T** | **A** | **C** | **A** | **G** | **C** | **A** | **A** | **T** | **G** | **A** | **T** | **T** | **A** | **C** | **G** | **A** | **T** | **A** | **C** | **C** | **A** | **A** | **T** | **C** | **T** | **T** | **C** | **G** | **T** | **C** | **G** | **T** | **T** | **T** | **G** | **A** | **A** | **A** | **A** | **T** | **T** | **A** | **T** | **G** | **A** | **C** | **G** | **A** | **A** | **C** | **A** | **C** | **T** | **T** | **T** | **A** | **G** | **T** | **A** | **G** | **G** | **A** | **G** | **A** | **T** | **G** | **T** | **T** | **G** | **A** | **G** | **G** | **T** | **G** | **A** | **T** | **C** | **C** | **G** | **T** | **G** | **T** | **A** | **-** | **-** | **-** |
| **N52773** | **A** | **T** | **G** | **A** | **A** | **T** | **A** | **A** | **C** | **C** | **C** | **T** | **T** | **G** | **G** | **C** | **G** | **C** | **T** | **T** | **T** | **T** | **T** | **T** | **A** | **T** | **C** | **G** | **T** | **C** | **G** | **C** | **A** | **G** | **A** | **A** | **G** | **C** | **A** | **T** | **T** | **A** | **C** | **T** | **T** | **T** | **T** | **T** | **A** | **T** | **T** | **C** | **T** | **G** | **G** | **C** | **G** | **T** | **T** | **A** | **T** | **G** | **G** | **C** | **A** | **A** | **A** | **T** | **T** | **G** | **T** | **A** | **C** | **A** | **T** | **A** | **A** | **T** | **C** | **C** | **T** | **G** | **G** | **A** | **T** | **T** | **A** | **G** | **C** | **T** | **G** | **T** | **T** | **T** | **T** | **A** | **T** | **T** | **A** | **A** | **C** | **A** | **A** | **T** | **T** | **G** | **G** | **C** | **G** | **T** | **T** | **T** | **T** | **A** | **C** | **T** | **T** | **G** | **T** | **G** | **G** | **C** | **C** | **T** | **A** | **T** | **G** | **T** | **T** | **T** | **C** | **C** | **A** | **G** | **G** | **A** | **A** | **A** | **G** | **C** | **A** | **T** | **C** | **T** | **A** | **A** | **A** | **A** | **C** | **A** | **C** | **A** | **T** | **T** | **T** | **T** | **A** | **A** | **C** | **A** | **A** | **C** | **T** | **T** | **T** | **C** | **A** | **A** | **T** | **T** | **C** | **G** | **T** | **C** | **C** | **T** | **C** | **G** | **G** | **C** | **G** | **T** | **T** | **G** | **T** | **T** | **T** | **T** | **T** | **A** | **T** | **T** | **G** | **T** | **C** | **A** | **T** | **T** | **G** | **G** | **T** | **G** | **C** | **A** | **A** | **T** | **G** | **A** | **A** | **C** | **A** | **G** | **C** | **A** | **C** | **G** | **G** | **C** | **T** | **G** | **T** | **T** | **T** | **G** | **G** | **T** | **T** | **T** | **A** | **T** | **G** | **C** | **T** | **G** | **A** | **T** | **T** | **T** | **T** | **T** | **G** | **G** | **C** | **G** | **T** | **A** | **C** | **T** | **C** | **T** | **T** | **T** | **A** | **T** | **C** | **G** | **G** | **C** | **T** | **T** | **A** | **A** | **A** | **A** | **G** | **G** | **C** | **T** | **T** | **T** | **G** | **A** | **G** | **A** | **T** | **T** | **T** | **C** | **A** | **G** | **G** | **C** | **G** | **T** | **G** | **G** | **A** | **T** | **A** | **T** | **A** | **G** | **C** | **T** | **G** | **A** | **G** | **C** | **G** | **A** | **G** | **C** | **A** | **C** | **C** | **T** | **T** | **G** | **G** | **C** | **G** | **T** | **A** | **A** | **A** | **A** | **A** | **A** | **C** | **A** | **A** | **A** | **T** | **G** | **A** | **T** | **T** | **A** | **T** | **G** | **G** | **T** | **G** | **G** | **A** | **G** | **A** | **C** | **G** | **G** | **C** | **G** | **G** | **C** | **A** | **A** | **A** | **A** | **G** | **A** | **A** | **C** | **C** | **T** | **A** | **A** | **A** | **A** | **A** | **T** | **G** | **G** | **C** | **A** | **A** | **A** | **C** | **G** | **A** | **T** | **T** | **T** | **A** | **A** | **A** | **C** | **G** | **C** | **C** | **G** | **C** | **T** | **G** | **G** | **T** | **T** | **T** | **G** | **C** | **C** | **A** | **A** | **C** | **G** | **A** | **A** | **C** | **G** | **C** | **A** | **T** | **T** | **G** | **G** | **T** | **A** | **A** | **C** | **A** | **A** | **T** | **A** | **T** | **C** | **T** | **A** | **T** | **G** | **A** | **A** | **T** | **G** | **G** | **G** | **A** | **C** | **G** | **A** | **T** | **A** | **T** | **C** | **A** | **A** | **T** | **A** | **T** | **T** | **G** | **A** | **T** | **T** | **T** | **A** | **A** | **T** | **C** | **T** | **C** | **T** | **G** | **G** | **G** | **G** | **A** | **C** | **A** | **C** | **C** | **A** | **T** | **T** | **A** | **T** | **T** | **G** | **A** | **T** | **T** | **T** | **A** | **G** | **G** | **T** | **A** | **A** | **T** | **A** | **C** | **G** | **C** | **T** | **A** | **C** | **T** | **A** | **C** | **C** | **G** | **A** | **A** | **A** | **G** | **A** | **A** | **G** | **A** | **C** | **A** | **A** | **T** | **A** | **T** | **T** | **A** | **T** | **T** | **A** | **T** | **T** | **A** | **T** | **T** | **C** | **G** | **T** | **A** | **A** | **A** | **G** | **G** | **T** | **T** | **T** | **T** | **G** | **G** | **C** | **C** | **G** | **C** | **A** | **C** | **G** | **C** | **G** | **A** | **A** | **T** | **T** | **C** | **T** | **A** | **G** | **T** | **A** | **C** | **C** | **G** | **T** | **T** | **A** | **G** | **G** | **T** | **G** | **T** | **A** | **G** | **C** | **A** | **A** | **T** | **T** | **T** | **T** | **G** | **T** | **T** | **A** | **G** | **A** | **A** | **C** | **A** | **T** | **T** | **C** | **A** | **A** | **C** | **T** | **T** | **T** | **T** | **T** | **A** | **C** | **G** | **G** | **A** | **A** | **C** | **G** | **G** | **T** | **A** | **C** | **G** | **T** | **T** | **T** | **T** | **G** | **A** | **A** | **G** | **A** | **A** | **G** | **A** | **A** | **A** | **A** | **A** | **T** | **A** | **T** | **C** | **A** | **A** | **T** | **T** | **G** | **A** | **A** | **A** | **A** | **A** | **C** | **G** | **A** | **A** | **T** | **C** | **A** | **T** | **T** | **A** | **A** | **A** | **A** | **A** | **T** | **T** | **T** | **A** | **C** | **A** | **G** | **C** | **A** | **A** | **T** | **G** | **A** | **T** | **T** | **A** | **T** | **G** | **A** | **T** | **A** | **C** | **C** | **A** | **A** | **T** | **C** | **T** | **T** | **C** | **G** | **T** | **C** | **G** | **T** | **T** | **T** | **G** | **A** | **A** | **A** | **A** | **T** | **T** | **A** | **T** | **G** | **A** | **C** | **G** | **A** | **A** | **C** | **A** | **C** | **T** | **T** | **T** | **A** | **G** | **T** | **A** | **G** | **G** | **A** | **G** | **A** | **T** | **G** | **T** | **T** | **G** | **A** | **G** | **G** | **T** | **G** | **A** | **T** | **C** | **C** | **G** | **T** | **G** | **T** | **A** | **-** | **-** | **-** |
| **N54676** | **A** | **T** | **G** | **A** | **A** | **T** | **A** | **A** | **C** | **C** | **C** | **T** | **T** | **G** | **G** | **C** | **G** | **C** | **T** | **T** | **T** | **T** | **T** | **T** | **A** | **T** | **C** | **G** | **T** | **C** | **G** | **C** | **A** | **G** | **A** | **A** | **G** | **C** | **A** | **T** | **T** | **A** | **C** | **T** | **T** | **T** | **T** | **T** | **A** | **T** | **T** | **C** | **T** | **G** | **G** | **C** | **G** | **T** | **T** | **A** | **T** | **G** | **G** | **C** | **A** | **A** | **A** | **T** | **T** | **G** | **T** | **A** | **C** | **A** | **T** | **A** | **A** | **T** | **C** | **C** | **T** | **G** | **G** | **A** | **T** | **T** | **A** | **G** | **C** | **T** | **G** | **T** | **T** | **T** | **T** | **A** | **T** | **T** | **A** | **A** | **C** | **A** | **A** | **T** | **T** | **G** | **G** | **C** | **G** | **T** | **T** | **T** | **T** | **A** | **C** | **T** | **T** | **G** | **T** | **G** | **G** | **C** | **C** | **T** | **A** | **C** | **G** | **T** | **T** | **T** | **C** | **C** | **A** | **G** | **G** | **A** | **A** | **A** | **G** | **C** | **A** | **T** | **C** | **T** | **A** | **A** | **A** | **A** | **C** | **A** | **C** | **A** | **T** | **T** | **T** | **T** | **A** | **A** | **C** | **A** | **A** | **C** | **T** | **T** | **T** | **C** | **A** | **A** | **T** | **T** | **C** | **G** | **T** | **C** | **C** | **T** | **C** | **G** | **G** | **C** | **G** | **T** | **T** | **G** | **T** | **T** | **T** | **T** | **T** | **A** | **T** | **T** | **G** | **T** | **C** | **A** | **T** | **T** | **G** | **G** | **T** | **G** | **C** | **A** | **A** | **T** | **G** | **A** | **A** | **C** | **A** | **G** | **C** | **A** | **C** | **G** | **G** | **C** | **T** | **G** | **T** | **T** | **T** | **G** | **G** | **T** | **T** | **T** | **A** | **T** | **G** | **T** | **T** | **G** | **A** | **T** | **T** | **T** | **T** | **T** | **G** | **G** | **C** | **G** | **T** | **A** | **C** | **T** | **C** | **T** | **T** | **T** | **A** | **T** | **C** | **G** | **G** | **C** | **T** | **T** | **A** | **A** | **A** | **A** | **G** | **G** | **C** | **T** | **T** | **T** | **G** | **A** | **G** | **A** | **T** | **T** | **T** | **C** | **A** | **G** | **G** | **C** | **G** | **T** | **G** | **G** | **A** | **T** | **A** | **T** | **A** | **G** | **C** | **T** | **G** | **A** | **A** | **C** | **G** | **A** | **G** | **C** | **A** | **C** | **C** | **T** | **T** | **G** | **G** | **C** | **G** | **T** | **A** | **A** | **A** | **A** | **A** | **A** | **C** | **A** | **A** | **A** | **T** | **G** | **A** | **T** | **T** | **A** | **T** | **G** | **G** | **T** | **A** | **G** | **A** | **G** | **A** | **C** | **G** | **G** | **C** | **G** | **G** | **C** | **A** | **A** | **A** | **A** | **G** | **A** | **A** | **C** | **C** | **T** | **A** | **A** | **A** | **A** | **A** | **T** | **G** | **G** | **C** | **A** | **A** | **A** | **C** | **G** | **A** | **T** | **T** | **T** | **A** | **A** | **A** | **C** | **G** | **C** | **C** | **G** | **C** | **T** | **G** | **G** | **T** | **T** | **T** | **G** | **C** | **C** | **A** | **A** | **C** | **G** | **A** | **A** | **C** | **G** | **C** | **A** | **T** | **T** | **G** | **G** | **T** | **A** | **A** | **C** | **A** | **A** | **T** | **A** | **T** | **C** | **T** | **A** | **T** | **G** | **A** | **A** | **T** | **G** | **G** | **G** | **A** | **T** | **G** | **A** | **C** | **A** | **T** | **C** | **A** | **A** | **T** | **A** | **T** | **T** | **G** | **A** | **T** | **T** | **T** | **A** | **A** | **T** | **C** | **T** | **C** | **T** | **G** | **G** | **G** | **G** | **A** | **C** | **A** | **C** | **C** | **A** | **T** | **T** | **A** | **T** | **T** | **G** | **A** | **T** | **T** | **T** | **A** | **G** | **G** | **T** | **A** | **A** | **T** | **A** | **C** | **G** | **C** | **T** | **A** | **C** | **T** | **A** | **C** | **C** | **G** | **A** | **A** | **A** | **G** | **A** | **A** | **G** | **A** | **C** | **A** | **A** | **T** | **A** | **T** | **T** | **A** | **T** | **T** | **A** | **T** | **T** | **A** | **T** | **T** | **C** | **G** | **T** | **A** | **A** | **A** | **G** | **G** | **T** | **T** | **T** | **T** | **G** | **G** | **C** | **C** | **G** | **C** | **A** | **C** | **G** | **C** | **G** | **A** | **A** | **T** | **T** | **C** | **T** | **A** | **G** | **T** | **G** | **C** | **C** | **G** | **T** | **T** | **A** | **G** | **G** | **T** | **G** | **T** | **A** | **G** | **C** | **T** | **A** | **T** | **T** | **T** | **T** | **G** | **T** | **T** | **A** | **G** | **A** | **A** | **C** | **A** | **T** | **T** | **C** | **A** | **A** | **C** | **T** | **T** | **T** | **T** | **T** | **A** | **C** | **G** | **G** | **A** | **A** | **C** | **G** | **G** | **T** | **A** | **C** | **G** | **T** | **T** | **T** | **T** | **G** | **A** | **A** | **G** | **A** | **A** | **G** | **A** | **A** | **A** | **A** | **A** | **T** | **A** | **T** | **C** | **A** | **A** | **T** | **T** | **G** | **A** | **A** | **A** | **A** | **A** | **C** | **G** | **A** | **A** | **T** | **C** | **A** | **T** | **T** | **A** | **A** | **A** | **A** | **A** | **T** | **T** | **T** | **A** | **C** | **A** | **G** | **C** | **A** | **A** | **T** | **G** | **A** | **T** | **T** | **A** | **T** | **G** | **A** | **T** | **A** | **C** | **C** | **A** | **A** | **T** | **C** | **T** | **T** | **C** | **G** | **T** | **C** | **G** | **T** | **T** | **T** | **G** | **A** | **A** | **A** | **A** | **T** | **T** | **A** | **T** | **G** | **A** | **C** | **G** | **A** | **A** | **C** | **A** | **C** | **T** | **T** | **T** | **A** | **G** | **T** | **A** | **G** | **G** | **A** | **G** | **A** | **T** | **G** | **T** | **T** | **G** | **A** | **G** | **G** | **T** | **G** | **A** | **T** | **C** | **C** | **G** | **T** | **G** | **T** | **A** | **-** | **-** | **-** |
| **N54564** | **A** | **T** | **G** | **A** | **A** | **T** | **A** | **A** | **C** | **C** | **C** | **T** | **T** | **G** | **G** | **C** | **G** | **C** | **T** | **T** | **T** | **T** | **T** | **T** | **A** | **T** | **C** | **G** | **T** | **C** | **G** | **C** | **A** | **G** | **A** | **A** | **G** | **C** | **A** | **T** | **T** | **A** | **C** | **T** | **T** | **T** | **T** | **T** | **A** | **T** | **T** | **C** | **T** | **G** | **G** | **C** | **G** | **T** | **T** | **A** | **T** | **G** | **G** | **C** | **A** | **A** | **A** | **T** | **T** | **G** | **T** | **A** | **C** | **A** | **T** | **A** | **A** | **T** | **C** | **C** | **T** | **G** | **G** | **A** | **T** | **T** | **A** | **G** | **C** | **T** | **G** | **T** | **T** | **T** | **T** | **A** | **T** | **T** | **A** | **A** | **C** | **A** | **A** | **T** | **T** | **G** | **G** | **C** | **G** | **T** | **T** | **T** | **T** | **A** | **C** | **T** | **T** | **G** | **T** | **G** | **G** | **C** | **C** | **T** | **A** | **C** | **G** | **T** | **T** | **T** | **C** | **C** | **A** | **G** | **G** | **A** | **A** | **A** | **G** | **C** | **A** | **T** | **A** | **T** | **A** | **A** | **A** | **A** | **C** | **A** | **C** | **A** | **T** | **T** | **T** | **T** | **A** | **A** | **C** | **A** | **A** | **C** | **T** | **T** | **T** | **C** | **A** | **A** | **T** | **T** | **C** | **G** | **T** | **C** | **C** | **T** | **C** | **G** | **G** | **C** | **G** | **T** | **T** | **G** | **T** | **T** | **T** | **T** | **T** | **A** | **T** | **T** | **G** | **T** | **C** | **A** | **T** | **T** | **G** | **G** | **T** | **G** | **C** | **A** | **A** | **T** | **G** | **A** | **A** | **C** | **A** | **G** | **T** | **A** | **C** | **A** | **G** | **C** | **T** | **G** | **T** | **T** | **T** | **G** | **G** | **T** | **T** | **T** | **A** | **T** | **G** | **T** | **T** | **G** | **A** | **T** | **T** | **T** | **T** | **T** | **G** | **G** | **C** | **G** | **T** | **A** | **C** | **T** | **C** | **T** | **T** | **T** | **A** | **T** | **C** | **G** | **G** | **C** | **T** | **T** | **A** | **A** | **A** | **A** | **G** | **G** | **C** | **T** | **T** | **T** | **G** | **A** | **G** | **A** | **T** | **T** | **T** | **C** | **A** | **G** | **G** | **C** | **G** | **T** | **G** | **G** | **A** | **T** | **A** | **T** | **A** | **G** | **C** | **T** | **G** | **A** | **G** | **C** | **G** | **A** | **G** | **C** | **A** | **C** | **C** | **T** | **T** | **G** | **G** | **C** | **G** | **A** | **A** | **A** | **A** | **A** | **A** | **A** | **C** | **A** | **A** | **A** | **T** | **G** | **A** | **T** | **T** | **A** | **T** | **G** | **G** | **T** | **G** | **G** | **A** | **G** | **A** | **C** | **G** | **G** | **C** | **G** | **G** | **C** | **A** | **A** | **A** | **A** | **G** | **A** | **A** | **C** | **C** | **T** | **A** | **A** | **A** | **A** | **A** | **T** | **G** | **G** | **C** | **A** | **A** | **A** | **C** | **G** | **A** | **T** | **T** | **T** | **A** | **A** | **A** | **C** | **G** | **C** | **C** | **G** | **T** | **T** | **G** | **G** | **T** | **T** | **T** | **G** | **C** | **C** | **A** | **A** | **C** | **G** | **A** | **A** | **C** | **G** | **C** | **A** | **T** | **T** | **G** | **G** | **T** | **A** | **A** | **C** | **A** | **A** | **T** | **A** | **T** | **C** | **T** | **A** | **T** | **G** | **A** | **A** | **T** | **G** | **G** | **G** | **A** | **T** | **G** | **A** | **T** | **A** | **T** | **C** | **A** | **A** | **T** | **A** | **T** | **T** | **G** | **A** | **T** | **T** | **T** | **A** | **A** | **T** | **T** | **T** | **C** | **T** | **G** | **G** | **G** | **G** | **A** | **C** | **A** | **C** | **C** | **A** | **T** | **T** | **A** | **T** | **T** | **G** | **A** | **T** | **T** | **T** | **A** | **G** | **G** | **T** | **A** | **A** | **T** | **A** | **C** | **G** | **C** | **T** | **A** | **C** | **T** | **A** | **C** | **C** | **G** | **A** | **A** | **A** | **G** | **A** | **A** | **G** | **A** | **C** | **A** | **A** | **T** | **A** | **T** | **T** | **A** | **T** | **T** | **A** | **T** | **T** | **A** | **T** | **T** | **C** | **G** | **T** | **A** | **A** | **A** | **G** | **G** | **T** | **T** | **T** | **T** | **G** | **G** | **C** | **C** | **G** | **C** | **A** | **C** | **G** | **C** | **G** | **A** | **A** | **T** | **T** | **C** | **T** | **A** | **G** | **T** | **A** | **C** | **C** | **G** | **T** | **T** | **A** | **G** | **G** | **T** | **G** | **T** | **A** | **G** | **C** | **T** | **A** | **T** | **T** | **T** | **T** | **G** | **T** | **T** | **A** | **G** | **A** | **A** | **C** | **A** | **T** | **T** | **C** | **A** | **A** | **C** | **T** | **T** | **T** | **T** | **T** | **A** | **C** | **G** | **G** | **A** | **A** | **C** | **G** | **G** | **T** | **A** | **C** | **G** | **T** | **T** | **T** | **T** | **G** | **A** | **A** | **G** | **A** | **A** | **G** | **A** | **A** | **A** | **A** | **A** | **T** | **A** | **T** | **C** | **A** | **A** | **T** | **T** | **G** | **A** | **A** | **A** | **A** | **A** | **C** | **G** | **A** | **A** | **T** | **C** | **A** | **T** | **T** | **A** | **A** | **A** | **A** | **A** | **T** | **T** | **T** | **A** | **C** | **A** | **G** | **C** | **A** | **A** | **T** | **G** | **A** | **T** | **T** | **A** | **T** | **G** | **A** | **T** | **A** | **C** | **C** | **A** | **A** | **T** | **C** | **T** | **T** | **C** | **G** | **T** | **C** | **G** | **T** | **T** | **T** | **G** | **A** | **A** | **A** | **A** | **T** | **T** | **A** | **T** | **G** | **A** | **C** | **G** | **A** | **A** | **C** | **A** | **C** | **T** | **T** | **T** | **A** | **G** | **T** | **A** | **G** | **G** | **A** | **G** | **A** | **T** | **G** | **T** | **T** | **G** | **A** | **G** | **G** | **T** | **G** | **A** | **T** | **C** | **C** | **G** | **T** | **G** | **T** | **A** | **-** | **-** | **-** |
| **N54574** | **A** | **T** | **G** | **A** | **A** | **T** | **A** | **A** | **C** | **C** | **C** | **T** | **T** | **G** | **G** | **C** | **G** | **C** | **T** | **T** | **T** | **T** | **T** | **T** | **A** | **T** | **C** | **G** | **T** | **C** | **G** | **C** | **A** | **G** | **A** | **A** | **G** | **C** | **A** | **T** | **T** | **A** | **C** | **T** | **T** | **T** | **T** | **T** | **A** | **T** | **T** | **C** | **T** | **G** | **G** | **C** | **G** | **T** | **T** | **A** | **T** | **G** | **G** | **C** | **A** | **A** | **A** | **T** | **T** | **G** | **T** | **A** | **C** | **A** | **T** | **A** | **A** | **T** | **C** | **C** | **T** | **G** | **G** | **A** | **T** | **T** | **A** | **G** | **C** | **T** | **G** | **T** | **T** | **T** | **T** | **A** | **T** | **T** | **A** | **A** | **C** | **A** | **A** | **T** | **T** | **G** | **G** | **C** | **G** | **T** | **T** | **T** | **T** | **A** | **C** | **T** | **T** | **G** | **T** | **G** | **G** | **C** | **C** | **T** | **A** | **C** | **G** | **T** | **T** | **T** | **C** | **C** | **A** | **G** | **G** | **A** | **A** | **A** | **G** | **C** | **A** | **T** | **C** | **T** | **A** | **A** | **A** | **A** | **C** | **A** | **C** | **A** | **T** | **T** | **T** | **T** | **A** | **A** | **C** | **A** | **A** | **C** | **T** | **T** | **T** | **C** | **A** | **A** | **T** | **T** | **C** | **G** | **T** | **C** | **C** | **T** | **C** | **G** | **G** | **C** | **G** | **T** | **T** | **G** | **T** | **T** | **T** | **T** | **T** | **A** | **T** | **T** | **G** | **T** | **C** | **A** | **T** | **T** | **G** | **G** | **T** | **G** | **C** | **A** | **A** | **T** | **G** | **A** | **A** | **C** | **A** | **G** | **C** | **A** | **C** | **G** | **G** | **C** | **T** | **G** | **T** | **T** | **T** | **G** | **G** | **T** | **T** | **T** | **A** | **T** | **G** | **T** | **T** | **G** | **A** | **T** | **T** | **T** | **T** | **T** | **G** | **G** | **C** | **G** | **T** | **A** | **C** | **T** | **C** | **T** | **T** | **T** | **A** | **T** | **C** | **G** | **G** | **C** | **T** | **T** | **A** | **A** | **A** | **A** | **G** | **G** | **C** | **T** | **T** | **T** | **G** | **A** | **G** | **A** | **T** | **C** | **T** | **C** | **A** | **G** | **G** | **C** | **G** | **T** | **G** | **G** | **A** | **T** | **A** | **T** | **A** | **G** | **C** | **T** | **G** | **A** | **G** | **C** | **G** | **A** | **G** | **C** | **A** | **C** | **C** | **T** | **T** | **G** | **G** | **C** | **G** | **T** | **A** | **A** | **A** | **A** | **A** | **A** | **C** | **A** | **A** | **A** | **T** | **G** | **A** | **T** | **T** | **A** | **T** | **G** | **G** | **T** | **G** | **G** | **A** | **G** | **A** | **C** | **G** | **G** | **C** | **G** | **G** | **C** | **A** | **A** | **A** | **A** | **G** | **A** | **A** | **C** | **C** | **T** | **A** | **A** | **A** | **A** | **A** | **T** | **G** | **G** | **C** | **A** | **A** | **A** | **C** | **G** | **A** | **T** | **T** | **T** | **A** | **A** | **A** | **C** | **G** | **C** | **C** | **G** | **C** | **T** | **G** | **G** | **T** | **T** | **T** | **G** | **C** | **C** | **A** | **A** | **C** | **G** | **A** | **A** | **C** | **G** | **C** | **A** | **T** | **T** | **G** | **G** | **T** | **A** | **A** | **C** | **A** | **A** | **T** | **A** | **T** | **C** | **T** | **A** | **T** | **G** | **A** | **A** | **T** | **G** | **G** | **G** | **A** | **C** | **G** | **A** | **T** | **A** | **T** | **C** | **A** | **A** | **T** | **A** | **T** | **T** | **G** | **A** | **T** | **T** | **T** | **A** | **A** | **T** | **C** | **T** | **C** | **T** | **G** | **G** | **G** | **G** | **A** | **C** | **A** | **C** | **C** | **A** | **T** | **T** | **A** | **T** | **T** | **G** | **A** | **T** | **T** | **T** | **A** | **G** | **G** | **T** | **A** | **A** | **T** | **A** | **C** | **G** | **C** | **T** | **A** | **C** | **T** | **A** | **C** | **C** | **G** | **A** | **A** | **A** | **G** | **A** | **A** | **G** | **A** | **C** | **A** | **A** | **T** | **A** | **T** | **T** | **A** | **T** | **T** | **A** | **T** | **T** | **A** | **T** | **T** | **C** | **G** | **T** | **A** | **A** | **A** | **G** | **G** | **T** | **T** | **T** | **T** | **G** | **G** | **C** | **C** | **G** | **C** | **A** | **C** | **G** | **C** | **G** | **A** | **A** | **T** | **T** | **C** | **T** | **A** | **G** | **T** | **G** | **C** | **C** | **G** | **T** | **T** | **A** | **G** | **G** | **T** | **G** | **T** | **A** | **G** | **C** | **T** | **A** | **T** | **T** | **G** | **T** | **G** | **T** | **T** | **A** | **G** | **A** | **A** | **C** | **A** | **T** | **T** | **C** | **A** | **A** | **C** | **T** | **T** | **T** | **T** | **T** | **A** | **C** | **G** | **G** | **A** | **A** | **C** | **G** | **G** | **T** | **A** | **C** | **G** | **T** | **T** | **T** | **T** | **G** | **A** | **A** | **G** | **A** | **A** | **G** | **A** | **A** | **A** | **A** | **A** | **T** | **A** | **T** | **C** | **A** | **A** | **T** | **T** | **G** | **A** | **A** | **A** | **A** | **A** | **C** | **G** | **A** | **A** | **T** | **C** | **A** | **T** | **T** | **A** | **A** | **A** | **A** | **A** | **T** | **T** | **T** | **A** | **C** | **A** | **G** | **C** | **A** | **A** | **T** | **G** | **A** | **T** | **T** | **A** | **C** | **G** | **A** | **T** | **A** | **C** | **C** | **A** | **A** | **T** | **C** | **T** | **T** | **C** | **G** | **T** | **C** | **G** | **T** | **T** | **T** | **G** | **A** | **A** | **A** | **A** | **T** | **T** | **A** | **T** | **G** | **A** | **C** | **G** | **A** | **A** | **C** | **A** | **C** | **T** | **T** | **T** | **A** | **G** | **T** | **A** | **G** | **G** | **A** | **G** | **A** | **T** | **G** | **T** | **T** | **G** | **A** | **G** | **G** | **T** | **G** | **A** | **T** | **C** | **C** | **G** | **T** | **G** | **T** | **A** | **-** | **-** | **-** |
| **N55706** | **A** | **T** | **G** | **A** | **A** | **T** | **A** | **A** | **C** | **C** | **C** | **T** | **T** | **G** | **G** | **C** | **G** | **C** | **T** | **T** | **T** | **T** | **T** | **T** | **A** | **T** | **C** | **G** | **T** | **C** | **G** | **C** | **A** | **G** | **A** | **A** | **G** | **C** | **A** | **T** | **T** | **A** | **C** | **T** | **T** | **T** | **T** | **T** | **A** | **T** | **T** | **C** | **T** | **G** | **G** | **C** | **G** | **T** | **T** | **A** | **T** | **G** | **G** | **C** | **A** | **A** | **A** | **T** | **T** | **G** | **T** | **A** | **C** | **A** | **T** | **A** | **A** | **T** | **C** | **C** | **T** | **G** | **G** | **A** | **T** | **T** | **A** | **G** | **C** | **T** | **G** | **T** | **T** | **T** | **T** | **A** | **T** | **T** | **A** | **A** | **C** | **A** | **A** | **T** | **T** | **G** | **G** | **C** | **G** | **T** | **T** | **T** | **T** | **A** | **C** | **T** | **T** | **G** | **T** | **G** | **G** | **C** | **C** | **T** | **A** | **C** | **G** | **T** | **T** | **T** | **C** | **C** | **A** | **G** | **G** | **A** | **A** | **A** | **G** | **C** | **A** | **T** | **C** | **T** | **A** | **A** | **A** | **A** | **C** | **A** | **C** | **A** | **T** | **T** | **T** | **T** | **A** | **A** | **C** | **A** | **A** | **C** | **T** | **T** | **T** | **C** | **A** | **A** | **T** | **T** | **C** | **G** | **T** | **C** | **C** | **T** | **C** | **G** | **G** | **C** | **G** | **T** | **T** | **G** | **T** | **T** | **T** | **T** | **T** | **A** | **T** | **T** | **G** | **T** | **C** | **A** | **T** | **T** | **G** | **G** | **T** | **G** | **C** | **A** | **A** | **T** | **G** | **A** | **A** | **C** | **A** | **G** | **C** | **A** | **C** | **G** | **G** | **C** | **T** | **G** | **T** | **T** | **T** | **G** | **G** | **T** | **T** | **T** | **A** | **T** | **G** | **T** | **T** | **G** | **A** | **T** | **T** | **T** | **T** | **T** | **G** | **G** | **C** | **G** | **T** | **A** | **C** | **T** | **C** | **T** | **T** | **T** | **A** | **T** | **C** | **G** | **G** | **C** | **T** | **T** | **A** | **A** | **A** | **A** | **G** | **G** | **C** | **T** | **T** | **T** | **G** | **A** | **G** | **A** | **T** | **T** | **T** | **C** | **A** | **G** | **G** | **C** | **G** | **T** | **G** | **G** | **A** | **T** | **A** | **T** | **A** | **G** | **C** | **T** | **G** | **A** | **G** | **C** | **G** | **A** | **G** | **C** | **A** | **C** | **C** | **T** | **T** | **G** | **G** | **C** | **G** | **A** | **A** | **A** | **A** | **A** | **A** | **A** | **C** | **A** | **A** | **A** | **T** | **G** | **A** | **T** | **T** | **A** | **T** | **G** | **G** | **T** | **G** | **G** | **A** | **G** | **A** | **C** | **G** | **G** | **C** | **G** | **G** | **C** | **A** | **A** | **A** | **A** | **G** | **A** | **A** | **C** | **C** | **T** | **A** | **A** | **A** | **A** | **A** | **T** | **G** | **G** | **C** | **A** | **A** | **A** | **C** | **G** | **A** | **T** | **T** | **T** | **A** | **A** | **A** | **C** | **G** | **C** | **C** | **G** | **C** | **T** | **G** | **G** | **T** | **T** | **T** | **G** | **C** | **C** | **A** | **A** | **C** | **G** | **A** | **A** | **C** | **G** | **C** | **A** | **T** | **T** | **G** | **G** | **T** | **A** | **A** | **C** | **A** | **A** | **T** | **A** | **T** | **C** | **T** | **A** | **T** | **G** | **A** | **A** | **T** | **G** | **G** | **G** | **A** | **C** | **G** | **A** | **C** | **A** | **T** | **C** | **A** | **A** | **T** | **A** | **T** | **T** | **G** | **A** | **T** | **T** | **T** | **A** | **A** | **T** | **C** | **T** | **C** | **T** | **G** | **G** | **G** | **G** | **A** | **C** | **A** | **C** | **C** | **A** | **T** | **T** | **A** | **T** | **T** | **G** | **A** | **T** | **T** | **T** | **A** | **G** | **G** | **T** | **A** | **A** | **T** | **A** | **C** | **G** | **C** | **T** | **A** | **C** | **T** | **A** | **C** | **C** | **G** | **A** | **A** | **A** | **G** | **A** | **A** | **G** | **A** | **C** | **A** | **A** | **T** | **A** | **T** | **T** | **A** | **T** | **T** | **A** | **T** | **T** | **A** | **T** | **T** | **C** | **G** | **T** | **A** | **A** | **A** | **G** | **G** | **T** | **T** | **T** | **T** | **G** | **G** | **C** | **C** | **G** | **C** | **A** | **C** | **G** | **C** | **G** | **A** | **A** | **T** | **T** | **C** | **T** | **A** | **G** | **T** | **G** | **C** | **C** | **G** | **T** | **T** | **A** | **G** | **G** | **T** | **G** | **T** | **A** | **G** | **C** | **T** | **A** | **T** | **T** | **T** | **T** | **G** | **T** | **T** | **A** | **G** | **A** | **A** | **C** | **A** | **T** | **T** | **C** | **A** | **A** | **C** | **T** | **T** | **T** | **T** | **T** | **A** | **C** | **G** | **G** | **A** | **A** | **C** | **G** | **G** | **T** | **A** | **C** | **G** | **T** | **T** | **T** | **T** | **G** | **A** | **A** | **G** | **A** | **A** | **G** | **A** | **A** | **A** | **A** | **A** | **T** | **A** | **T** | **C** | **A** | **A** | **T** | **T** | **G** | **A** | **A** | **A** | **A** | **A** | **C** | **G** | **A** | **A** | **T** | **C** | **A** | **T** | **T** | **A** | **A** | **A** | **A** | **A** | **T** | **T** | **T** | **A** | **C** | **A** | **G** | **C** | **A** | **A** | **T** | **G** | **A** | **T** | **T** | **A** | **C** | **G** | **A** | **T** | **A** | **C** | **C** | **A** | **A** | **T** | **C** | **T** | **T** | **C** | **G** | **T** | **C** | **G** | **T** | **T** | **T** | **G** | **A** | **A** | **A** | **A** | **T** | **T** | **A** | **T** | **G** | **A** | **C** | **G** | **A** | **A** | **C** | **A** | **C** | **T** | **T** | **T** | **A** | **G** | **T** | **A** | **G** | **G** | **A** | **G** | **A** | **T** | **G** | **T** | **T** | **G** | **A** | **G** | **G** | **T** | **G** | **A** | **T** | **C** | **C** | **G** | **T** | **G** | **T** | **A** | **-** | **-** | **-** |
| **N59592F** | **A** | **T** | **G** | **A** | **A** | **T** | **A** | **A** | **C** | **C** | **C** | **T** | **T** | **G** | **G** | **C** | **G** | **C** | **T** | **T** | **T** | **T** | **T** | **T** | **A** | **T** | **C** | **G** | **T** | **C** | **G** | **C** | **A** | **G** | **A** | **A** | **G** | **C** | **A** | **T** | **T** | **A** | **C** | **T** | **T** | **T** | **T** | **T** | **A** | **T** | **T** | **C** | **T** | **G** | **G** | **C** | **G** | **T** | **T** | **A** | **T** | **G** | **G** | **C** | **A** | **A** | **A** | **T** | **T** | **G** | **T** | **A** | **C** | **A** | **T** | **A** | **A** | **T** | **C** | **C** | **T** | **G** | **G** | **A** | **T** | **T** | **A** | **G** | **C** | **T** | **G** | **T** | **T** | **T** | **T** | **A** | **T** | **T** | **A** | **A** | **C** | **A** | **A** | **T** | **T** | **G** | **G** | **C** | **G** | **T** | **T** | **T** | **T** | **A** | **C** | **T** | **T** | **G** | **T** | **G** | **G** | **C** | **C** | **T** | **A** | **C** | **G** | **T** | **T** | **T** | **C** | **C** | **A** | **G** | **G** | **A** | **A** | **A** | **G** | **C** | **A** | **T** | **C** | **A** | **A** | **A** | **A** | **A** | **C** | **A** | **C** | **A** | **T** | **T** | **T** | **T** | **A** | **A** | **C** | **A** | **A** | **C** | **T** | **T** | **T** | **C** | **A** | **A** | **T** | **T** | **C** | **G** | **T** | **C** | **C** | **T** | **C** | **G** | **G** | **C** | **G** | **T** | **T** | **G** | **T** | **T** | **T** | **T** | **T** | **A** | **T** | **T** | **G** | **T** | **C** | **A** | **T** | **T** | **G** | **G** | **T** | **G** | **C** | **A** | **A** | **T** | **G** | **A** | **A** | **C** | **A** | **G** | **C** | **A** | **C** | **G** | **G** | **C** | **T** | **G** | **T** | **T** | **T** | **G** | **G** | **T** | **T** | **T** | **A** | **T** | **G** | **T** | **T** | **G** | **A** | **T** | **T** | **T** | **T** | **T** | **G** | **G** | **C** | **G** | **T** | **A** | **C** | **T** | **C** | **T** | **T** | **T** | **A** | **T** | **C** | **G** | **G** | **C** | **T** | **T** | **A** | **A** | **A** | **A** | **G** | **G** | **C** | **T** | **T** | **T** | **G** | **A** | **G** | **A** | **T** | **T** | **T** | **C** | **A** | **G** | **G** | **C** | **G** | **T** | **G** | **G** | **A** | **T** | **A** | **T** | **A** | **G** | **C** | **T** | **G** | **A** | **G** | **C** | **G** | **A** | **G** | **C** | **A** | **C** | **C** | **T** | **T** | **G** | **G** | **C** | **G** | **T** | **A** | **A** | **A** | **A** | **A** | **A** | **C** | **A** | **A** | **A** | **T** | **G** | **A** | **T** | **T** | **A** | **T** | **G** | **G** | **T** | **G** | **G** | **A** | **G** | **A** | **C** | **G** | **G** | **C** | **G** | **G** | **C** | **A** | **A** | **A** | **A** | **G** | **A** | **A** | **C** | **C** | **T** | **A** | **A** | **A** | **A** | **A** | **T** | **G** | **G** | **C** | **A** | **A** | **A** | **C** | **G** | **A** | **T** | **T** | **T** | **A** | **A** | **A** | **C** | **G** | **C** | **C** | **G** | **C** | **T** | **G** | **G** | **T** | **T** | **T** | **G** | **C** | **C** | **A** | **A** | **C** | **G** | **A** | **A** | **C** | **G** | **C** | **A** | **T** | **T** | **G** | **G** | **T** | **A** | **A** | **C** | **A** | **A** | **T** | **A** | **T** | **C** | **T** | **A** | **T** | **G** | **A** | **A** | **T** | **G** | **G** | **G** | **A** | **C** | **G** | **A** | **T** | **A** | **T** | **C** | **A** | **A** | **T** | **A** | **T** | **T** | **G** | **A** | **T** | **T** | **T** | **A** | **A** | **T** | **C** | **T** | **C** | **T** | **G** | **G** | **G** | **G** | **A** | **C** | **A** | **C** | **C** | **A** | **T** | **T** | **A** | **T** | **T** | **G** | **A** | **T** | **T** | **T** | **A** | **G** | **G** | **T** | **A** | **A** | **T** | **A** | **C** | **G** | **C** | **T** | **A** | **C** | **T** | **A** | **C** | **C** | **G** | **A** | **A** | **A** | **G** | **A** | **A** | **G** | **A** | **C** | **A** | **A** | **T** | **A** | **T** | **T** | **A** | **T** | **T** | **A** | **T** | **T** | **A** | **T** | **T** | **C** | **G** | **T** | **A** | **A** | **A** | **G** | **G** | **T** | **T** | **T** | **T** | **G** | **G** | **C** | **C** | **G** | **C** | **A** | **C** | **G** | **C** | **G** | **A** | **A** | **T** | **T** | **C** | **T** | **A** | **G** | **T** | **A** | **C** | **C** | **G** | **T** | **T** | **A** | **G** | **G** | **T** | **G** | **T** | **A** | **G** | **C** | **T** | **A** | **T** | **T** | **T** | **T** | **G** | **T** | **T** | **A** | **G** | **A** | **A** | **C** | **A** | **T** | **T** | **C** | **A** | **A** | **C** | **G** | **T** | **T** | **T** | **T** | **A** | **C** | **G** | **G** | **A** | **A** | **C** | **G** | **G** | **T** | **A** | **C** | **G** | **T** | **T** | **T** | **T** | **G** | **A** | **A** | **G** | **A** | **A** | **G** | **A** | **A** | **A** | **A** | **A** | **T** | **A** | **T** | **C** | **A** | **A** | **T** | **T** | **G** | **A** | **A** | **A** | **A** | **A** | **C** | **G** | **A** | **A** | **T** | **C** | **A** | **T** | **T** | **A** | **A** | **A** | **A** | **A** | **T** | **T** | **T** | **A** | **C** | **A** | **G** | **C** | **A** | **A** | **T** | **G** | **A** | **T** | **T** | **A** | **C** | **G** | **A** | **T** | **A** | **C** | **C** | **A** | **A** | **T** | **C** | **T** | **T** | **C** | **G** | **T** | **C** | **G** | **T** | **T** | **T** | **G** | **A** | **A** | **A** | **A** | **T** | **T** | **A** | **T** | **G** | **A** | **C** | **G** | **A** | **A** | **C** | **A** | **C** | **T** | **T** | **T** | **A** | **G** | **T** | **A** | **G** | **G** | **A** | **G** | **A** | **T** | **G** | **T** | **T** | **G** | **A** | **G** | **G** | **T** | **G** | **A** | **T** | **C** | **C** | **G** | **T** | **G** | **T** | **A** | **-** | **-** | **-** |
| **N52587** | **-** | **-** | **-** | **-** | **-** | **-** | **-** | **-** | **-** | **C** | **C** | **T** | **T** | **G** | **G** | **C** | **G** | **C** | **T** | **T** | **T** | **T** | **T** | **T** | **A** | **T** | **C** | **G** | **T** | **C** | **G** | **C** | **A** | **G** | **A** | **A** | **G** | **C** | **A** | **T** | **T** | **A** | **C** | **T** | **T** | **T** | **T** | **T** | **A** | **T** | **T** | **C** | **T** | **A** | **G** | **C** | **G** | **T** | **T** | **A** | **T** | **G** | **G** | **C** | **A** | **A** | **A** | **T** | **T** | **G** | **T** | **A** | **C** | **A** | **T** | **A** | **A** | **T** | **C** | **C** | **T** | **G** | **G** | **A** | **T** | **T** | **A** | **G** | **C** | **T** | **G** | **T** | **T** | **T** | **T** | **A** | **T** | **T** | **A** | **A** | **C** | **A** | **A** | **T** | **T** | **G** | **G** | **C** | **G** | **T** | **T** | **T** | **T** | **A** | **C** | **T** | **T** | **G** | **T** | **T** | **G** | **C** | **C** | **T** | **A** | **T** | **G** | **T** | **T** | **T** | **C** | **C** | **A** | **G** | **G** | **A** | **A** | **A** | **G** | **C** | **A** | **T** | **C** | **T** | **A** | **A** | **A** | **A** | **C** | **A** | **C** | **A** | **T** | **T** | **T** | **T** | **A** | **A** | **C** | **A** | **A** | **C** | **T** | **T** | **T** | **C** | **A** | **A** | **T** | **T** | **C** | **G** | **T** | **C** | **C** | **T** | **T** | **G** | **G** | **C** | **A** | **T** | **T** | **G** | **T** | **T** | **T** | **T** | **T** | **A** | **T** | **T** | **G** | **T** | **C** | **A** | **T** | **T** | **G** | **G** | **C** | **G** | **C** | **A** | **A** | **T** | **G** | **A** | **A** | **C** | **A** | **G** | **C** | **A** | **C** | **G** | **G** | **C** | **T** | **G** | **T** | **T** | **T** | **G** | **G** | **T** | **T** | **T** | **A** | **T** | **G** | **T** | **T** | **G** | **A** | **T** | **T** | **T** | **T** | **T** | **G** | **G** | **C** | **G** | **T** | **A** | **C** | **T** | **C** | **T** | **T** | **T** | **A** | **T** | **C** | **G** | **G** | **C** | **T** | **T** | **A** | **A** | **A** | **A** | **G** | **G** | **C** | **T** | **T** | **T** | **G** | **A** | **G** | **A** | **T** | **T** | **T** | **C** | **A** | **G** | **G** | **C** | **G** | **T** | **G** | **G** | **A** | **T** | **A** | **T** | **A** | **G** | **C** | **T** | **G** | **A** | **G** | **C** | **G** | **A** | **G** | **C** | **A** | **C** | **C** | **T** | **T** | **G** | **G** | **C** | **G** | **T** | **A** | **A** | **A** | **A** | **A** | **A** | **C** | **A** | **A** | **A** | **T** | **G** | **A** | **T** | **T** | **A** | **T** | **G** | **G** | **T** | **G** | **G** | **A** | **A** | **A** | **C** | **G** | **G** | **C** | **G** | **G** | **C** | **C** | **A** | **A** | **A** | **G** | **A** | **A** | **C** | **C** | **G** | **A** | **A** | **A** | **A** | **A** | **T** | **G** | **G** | **C** | **A** | **A** | **A** | **C** | **G** | **A** | **T** | **T** | **T** | **A** | **A** | **A** | **C** | **G** | **C** | **C** | **G** | **T** | **T** | **G** | **G** | **T** | **T** | **T** | **G** | **C** | **C** | **A** | **A** | **C** | **G** | **A** | **A** | **C** | **G** | **T** | **A** | **T** | **T** | **G** | **G** | **C** | **A** | **A** | **C** | **A** | **A** | **T** | **A** | **T** | **C** | **T** | **A** | **T** | **G** | **A** | **A** | **T** | **G** | **G** | **G** | **A** | **C** | **G** | **A** | **C** | **A** | **T** | **C** | **A** | **A** | **T** | **A** | **T** | **T** | **G** | **A** | **T** | **T** | **T** | **A** | **A** | **T** | **C** | **T** | **C** | **T** | **G** | **G** | **G** | **G** | **A** | **T** | **A** | **C** | **T** | **A** | **T** | **T** | **A** | **T** | **T** | **G** | **A** | **T** | **T** | **T** | **A** | **G** | **G** | **C** | **A** | **A** | **T** | **A** | **C** | **G** | **C** | **T** | **A** | **C** | **T** | **T** | **C** | **C** | **G** | **A** | **A** | **A** | **G** | **A** | **A** | **G** | **A** | **T** | **A** | **A** | **T** | **A** | **T** | **T** | **A** | **T** | **T** | **A** | **T** | **T** | **A** | **T** | **T** | **C** | **G** | **T** | **A** | **A** | **A** | **G** | **G** | **T** | **T** | **T** | **T** | **G** | **G** | **C** | **C** | **G** | **C** | **A** | **C** | **G** | **C** | **G** | **A** | **A** | **T** | **T** | **C** | **T** | **A** | **G** | **T** | **G** | **C** | **C** | **G** | **T** | **T** | **A** | **G** | **G** | **T** | **G** | **T** | **A** | **G** | **C** | **T** | **A** | **T** | **T** | **T** | **T** | **G** | **T** | **T** | **A** | **G** | **A** | **A** | **C** | **A** | **T** | **T** | **C** | **A** | **A** | **C** | **G** | **T** | **T** | **T** | **T** | **A** | **C** | **G** | **G** | **A** | **A** | **C** | **A** | **G** | **T** | **G** | **C** | **G** | **T** | **T** | **T** | **T** | **G** | **A** | **A** | **G** | **A** | **A** | **G** | **A** | **A** | **A** | **A** | **A** | **T** | **A** | **T** | **C** | **A** | **A** | **T** | **T** | **G** | **A** | **A** | **A** | **A** | **A** | **C** | **G** | **A** | **A** | **T** | **C** | **A** | **T** | **T** | **A** | **A** | **A** | **A** | **A** | **T** | **T** | **T** | **A** | **C** | **A** | **G** | **C** | **A** | **A** | **T** | **G** | **A** | **T** | **T** | **A** | **T** | **G** | **A** | **T** | **A** | **C** | **C** | **A** | **A** | **T** | **C** | **T** | **T** | **C** | **G** | **T** | **C** | **G** | **T** | **T** | **T** | **G** | **A** | **A** | **A** | **A** | **T** | **T** | **A** | **T** | **G** | **A** | **C** | **G** | **A** | **A** | **C** | **A** | **C** | **T** | **T** | **T** | **A** | **G** | **T** | **A** | **G** | **G** | **A** | **G** | **A** | **T** | **G** | **T** | **T** | **G** | **A** | **G** | **G** | **T** | **G** | **A** | **T** | **C** | **C** | **G** | **T** | **G** | **T** | **A** | **-** | **-** | **-** |
| **N54097** | **A** | **T** | **G** | **A** | **A** | **T** | **A** | **A** | **C** | **C** | **C** | **T** | **T** | **G** | **G** | **C** | **G** | **C** | **T** | **T** | **T** | **T** | **T** | **T** | **A** | **T** | **C** | **G** | **T** | **C** | **G** | **C** | **A** | **G** | **A** | **A** | **G** | **C** | **A** | **T** | **T** | **A** | **C** | **T** | **T** | **T** | **T** | **T** | **A** | **T** | **T** | **C** | **T** | **G** | **G** | **C** | **G** | **T** | **T** | **A** | **T** | **G** | **G** | **C** | **A** | **A** | **A** | **T** | **T** | **G** | **T** | **A** | **C** | **A** | **T** | **A** | **A** | **T** | **C** | **C** | **T** | **G** | **G** | **A** | **T** | **T** | **A** | **G** | **C** | **T** | **G** | **T** | **T** | **T** | **T** | **A** | **T** | **T** | **A** | **A** | **C** | **A** | **A** | **T** | **T** | **G** | **G** | **C** | **G** | **T** | **T** | **T** | **T** | **A** | **C** | **T** | **T** | **G** | **T** | **G** | **G** | **C** | **C** | **T** | **A** | **C** | **G** | **T** | **T** | **T** | **C** | **C** | **A** | **G** | **G** | **A** | **A** | **A** | **G** | **C** | **A** | **T** | **C** | **T** | **A** | **A** | **A** | **A** | **C** | **A** | **C** | **A** | **T** | **T** | **T** | **T** | **A** | **A** | **C** | **A** | **A** | **C** | **T** | **T** | **T** | **C** | **A** | **A** | **T** | **T** | **C** | **G** | **T** | **C** | **C** | **T** | **C** | **G** | **G** | **C** | **G** | **T** | **T** | **G** | **T** | **T** | **T** | **T** | **T** | **A** | **T** | **T** | **G** | **T** | **C** | **A** | **T** | **T** | **G** | **G** | **T** | **G** | **C** | **A** | **A** | **T** | **G** | **A** | **A** | **C** | **A** | **G** | **C** | **A** | **C** | **G** | **G** | **C** | **T** | **G** | **T** | **T** | **T** | **G** | **G** | **T** | **T** | **T** | **A** | **T** | **G** | **T** | **T** | **G** | **A** | **T** | **T** | **T** | **T** | **T** | **G** | **G** | **C** | **G** | **T** | **A** | **C** | **T** | **C** | **T** | **T** | **T** | **A** | **T** | **C** | **G** | **G** | **C** | **T** | **T** | **A** | **A** | **A** | **A** | **G** | **G** | **C** | **T** | **T** | **T** | **G** | **A** | **G** | **A** | **T** | **T** | **T** | **C** | **A** | **G** | **G** | **C** | **G** | **T** | **G** | **G** | **A** | **T** | **A** | **T** | **A** | **G** | **C** | **T** | **G** | **A** | **G** | **C** | **G** | **A** | **G** | **C** | **A** | **C** | **C** | **T** | **T** | **G** | **G** | **C** | **G** | **A** | **A** | **A** | **A** | **A** | **A** | **A** | **C** | **A** | **A** | **A** | **T** | **G** | **A** | **T** | **T** | **A** | **T** | **G** | **G** | **T** | **G** | **G** | **A** | **G** | **A** | **C** | **G** | **G** | **C** | **G** | **G** | **C** | **A** | **A** | **A** | **A** | **G** | **A** | **A** | **C** | **C** | **T** | **A** | **A** | **A** | **A** | **A** | **T** | **G** | **G** | **C** | **A** | **A** | **A** | **C** | **G** | **A** | **T** | **T** | **T** | **A** | **A** | **A** | **C** | **G** | **C** | **C** | **G** | **C** | **T** | **G** | **G** | **T** | **T** | **T** | **G** | **C** | **C** | **A** | **A** | **C** | **G** | **A** | **A** | **C** | **G** | **C** | **A** | **T** | **T** | **G** | **G** | **T** | **A** | **A** | **C** | **A** | **A** | **T** | **A** | **T** | **C** | **T** | **A** | **T** | **G** | **A** | **A** | **T** | **G** | **G** | **G** | **A** | **C** | **G** | **A** | **C** | **A** | **T** | **C** | **A** | **A** | **T** | **A** | **T** | **T** | **G** | **A** | **T** | **T** | **T** | **A** | **A** | **T** | **C** | **T** | **C** | **T** | **G** | **G** | **G** | **G** | **A** | **C** | **A** | **C** | **C** | **A** | **T** | **T** | **A** | **T** | **T** | **G** | **A** | **T** | **T** | **T** | **A** | **G** | **G** | **T** | **A** | **A** | **T** | **A** | **C** | **G** | **C** | **T** | **A** | **C** | **T** | **A** | **C** | **C** | **G** | **A** | **A** | **A** | **G** | **A** | **A** | **G** | **A** | **C** | **A** | **A** | **T** | **A** | **T** | **T** | **A** | **T** | **T** | **A** | **T** | **T** | **A** | **T** | **T** | **C** | **G** | **T** | **A** | **A** | **A** | **G** | **G** | **T** | **T** | **T** | **T** | **G** | **G** | **C** | **C** | **G** | **C** | **A** | **C** | **G** | **C** | **G** | **A** | **A** | **T** | **T** | **C** | **T** | **A** | **G** | **T** | **G** | **C** | **C** | **G** | **T** | **T** | **A** | **G** | **G** | **T** | **G** | **T** | **A** | **G** | **C** | **T** | **A** | **T** | **T** | **T** | **T** | **G** | **T** | **T** | **A** | **G** | **A** | **A** | **C** | **A** | **T** | **T** | **C** | **A** | **A** | **C** | **T** | **T** | **T** | **T** | **T** | **A** | **C** | **G** | **G** | **A** | **A** | **C** | **G** | **G** | **T** | **A** | **C** | **G** | **T** | **T** | **T** | **T** | **G** | **A** | **A** | **G** | **A** | **A** | **G** | **A** | **A** | **A** | **A** | **A** | **T** | **A** | **T** | **C** | **A** | **A** | **T** | **T** | **G** | **A** | **A** | **A** | **A** | **A** | **C** | **G** | **A** | **A** | **T** | **C** | **A** | **T** | **T** | **A** | **A** | **A** | **A** | **A** | **T** | **T** | **T** | **A** | **C** | **A** | **G** | **C** | **A** | **A** | **T** | **G** | **A** | **T** | **T** | **A** | **C** | **G** | **A** | **T** | **A** | **C** | **C** | **A** | **A** | **T** | **C** | **T** | **T** | **C** | **G** | **T** | **C** | **G** | **T** | **T** | **T** | **G** | **A** | **A** | **A** | **A** | **T** | **T** | **A** | **T** | **G** | **A** | **C** | **G** | **A** | **A** | **C** | **A** | **C** | **T** | **T** | **T** | **A** | **G** | **T** | **A** | **G** | **G** | **A** | **G** | **A** | **T** | **G** | **T** | **T** | **G** | **A** | **G** | **G** | **T** | **G** | **A** | **T** | **C** | **C** | **G** | **T** | **G** | **T** | **A** | **-** | **-** | **-** |
| **N60063F** | **A** | **T** | **G** | **A** | **A** | **T** | **A** | **A** | **C** | **C** | **C** | **T** | **T** | **G** | **G** | **C** | **G** | **C** | **T** | **T** | **T** | **T** | **T** | **T** | **A** | **T** | **C** | **G** | **T** | **C** | **G** | **C** | **A** | **G** | **A** | **A** | **G** | **C** | **A** | **T** | **T** | **A** | **C** | **T** | **T** | **T** | **T** | **T** | **A** | **T** | **T** | **C** | **T** | **G** | **G** | **C** | **G** | **T** | **T** | **A** | **T** | **G** | **G** | **C** | **A** | **A** | **A** | **T** | **T** | **G** | **T** | **A** | **C** | **A** | **T** | **A** | **A** | **T** | **C** | **C** | **T** | **G** | **G** | **A** | **T** | **T** | **A** | **G** | **C** | **T** | **G** | **T** | **T** | **T** | **T** | **A** | **T** | **T** | **A** | **A** | **C** | **A** | **A** | **T** | **T** | **G** | **G** | **C** | **G** | **T** | **T** | **T** | **T** | **A** | **C** | **T** | **T** | **G** | **T** | **G** | **G** | **C** | **C** | **T** | **A** | **C** | **G** | **T** | **T** | **T** | **C** | **C** | **A** | **G** | **G** | **A** | **A** | **A** | **G** | **C** | **A** | **T** | **C** | **T** | **A** | **A** | **A** | **A** | **C** | **A** | **C** | **A** | **T** | **T** | **T** | **T** | **A** | **A** | **C** | **A** | **A** | **C** | **T** | **T** | **T** | **C** | **A** | **A** | **T** | **T** | **C** | **G** | **T** | **C** | **C** | **T** | **C** | **G** | **G** | **C** | **G** | **T** | **T** | **G** | **T** | **T** | **T** | **T** | **T** | **A** | **T** | **T** | **G** | **T** | **C** | **A** | **T** | **T** | **G** | **G** | **T** | **G** | **C** | **A** | **A** | **T** | **G** | **A** | **A** | **T** | **A** | **G** | **C** | **A** | **C** | **G** | **G** | **C** | **T** | **G** | **T** | **T** | **T** | **G** | **G** | **T** | **T** | **T** | **A** | **T** | **G** | **T** | **T** | **G** | **A** | **T** | **T** | **T** | **T** | **T** | **G** | **G** | **C** | **A** | **T** | **A** | **C** | **T** | **C** | **T** | **T** | **T** | **A** | **T** | **C** | **G** | **G** | **C** | **T** | **T** | **A** | **A** | **A** | **A** | **G** | **G** | **C** | **T** | **T** | **T** | **G** | **A** | **G** | **A** | **T** | **T** | **T** | **C** | **A** | **G** | **G** | **C** | **G** | **T** | **G** | **G** | **A** | **T** | **A** | **T** | **A** | **G** | **C** | **T** | **G** | **A** | **G** | **C** | **G** | **A** | **G** | **C** | **A** | **C** | **C** | **T** | **T** | **G** | **G** | **C** | **G** | **A** | **A** | **A** | **A** | **A** | **A** | **A** | **C** | **A** | **A** | **A** | **T** | **G** | **A** | **T** | **T** | **A** | **T** | **G** | **G** | **T** | **G** | **G** | **A** | **G** | **A** | **C** | **G** | **G** | **C** | **G** | **G** | **C** | **A** | **A** | **A** | **A** | **G** | **A** | **A** | **C** | **C** | **T** | **A** | **A** | **A** | **A** | **A** | **T** | **G** | **G** | **C** | **A** | **A** | **A** | **C** | **G** | **G** | **T** | **T** | **T** | **A** | **A** | **A** | **C** | **G** | **C** | **C** | **G** | **C** | **T** | **G** | **G** | **T** | **T** | **T** | **G** | **C** | **C** | **A** | **A** | **C** | **G** | **A** | **A** | **C** | **G** | **C** | **A** | **T** | **T** | **G** | **G** | **T** | **A** | **A** | **C** | **A** | **A** | **T** | **A** | **T** | **C** | **T** | **A** | **T** | **G** | **A** | **A** | **T** | **G** | **G** | **G** | **A** | **C** | **G** | **A** | **T** | **A** | **T** | **C** | **A** | **A** | **T** | **A** | **T** | **T** | **G** | **A** | **T** | **T** | **T** | **A** | **A** | **T** | **C** | **T** | **C** | **T** | **G** | **G** | **G** | **G** | **A** | **C** | **A** | **C** | **C** | **A** | **T** | **T** | **A** | **T** | **T** | **G** | **A** | **T** | **T** | **T** | **A** | **G** | **G** | **T** | **A** | **A** | **T** | **A** | **C** | **G** | **C** | **T** | **A** | **C** | **T** | **A** | **C** | **C** | **G** | **A** | **A** | **A** | **G** | **A** | **A** | **G** | **A** | **C** | **A** | **A** | **T** | **A** | **T** | **T** | **A** | **T** | **T** | **A** | **T** | **T** | **A** | **T** | **T** | **C** | **G** | **T** | **A** | **A** | **A** | **G** | **G** | **T** | **T** | **T** | **T** | **G** | **G** | **C** | **C** | **G** | **C** | **A** | **C** | **G** | **C** | **G** | **A** | **A** | **T** | **T** | **C** | **T** | **A** | **G** | **T** | **G** | **C** | **C** | **G** | **T** | **T** | **A** | **G** | **G** | **T** | **G** | **T** | **A** | **G** | **C** | **T** | **A** | **T** | **T** | **T** | **T** | **G** | **T** | **T** | **A** | **G** | **A** | **A** | **C** | **A** | **T** | **T** | **C** | **A** | **A** | **C** | **T** | **T** | **T** | **T** | **T** | **A** | **C** | **G** | **G** | **A** | **A** | **C** | **G** | **G** | **T** | **A** | **C** | **G** | **T** | **T** | **T** | **T** | **G** | **A** | **A** | **G** | **A** | **A** | **G** | **A** | **A** | **A** | **A** | **A** | **T** | **A** | **T** | **C** | **A** | **A** | **T** | **T** | **G** | **A** | **A** | **A** | **A** | **A** | **C** | **G** | **A** | **A** | **T** | **C** | **A** | **T** | **T** | **A** | **A** | **A** | **A** | **A** | **T** | **T** | **T** | **A** | **C** | **A** | **G** | **C** | **A** | **A** | **T** | **G** | **A** | **T** | **T** | **A** | **T** | **G** | **A** | **T** | **A** | **C** | **C** | **A** | **A** | **T** | **C** | **T** | **T** | **C** | **G** | **T** | **C** | **G** | **T** | **T** | **T** | **G** | **A** | **A** | **A** | **A** | **T** | **T** | **A** | **T** | **G** | **A** | **C** | **G** | **A** | **A** | **C** | **A** | **C** | **T** | **T** | **T** | **A** | **G** | **T** | **A** | **G** | **G** | **A** | **G** | **A** | **T** | **G** | **T** | **T** | **G** | **A** | **G** | **G** | **T** | **G** | **A** | **T** | **C** | **C** | **G** | **T** | **G** | **T** | **A** | **-** | **-** | **-** |
| **N55725** | **A** | **T** | **G** | **A** | **A** | **T** | **A** | **A** | **C** | **C** | **C** | **T** | **T** | **G** | **G** | **C** | **G** | **C** | **T** | **T** | **T** | **T** | **T** | **T** | **A** | **T** | **C** | **G** | **T** | **C** | **G** | **C** | **A** | **G** | **A** | **A** | **G** | **C** | **A** | **T** | **T** | **A** | **C** | **T** | **T** | **T** | **T** | **T** | **A** | **T** | **T** | **C** | **T** | **G** | **G** | **C** | **G** | **T** | **T** | **A** | **T** | **G** | **G** | **C** | **A** | **A** | **A** | **T** | **T** | **G** | **T** | **A** | **C** | **A** | **T** | **A** | **A** | **T** | **C** | **C** | **T** | **G** | **G** | **A** | **T** | **T** | **A** | **G** | **C** | **T** | **G** | **T** | **T** | **T** | **T** | **A** | **T** | **T** | **A** | **A** | **C** | **A** | **A** | **T** | **T** | **G** | **G** | **C** | **G** | **T** | **T** | **T** | **T** | **A** | **C** | **T** | **T** | **G** | **T** | **G** | **G** | **C** | **C** | **T** | **A** | **T** | **G** | **T** | **T** | **T** | **C** | **C** | **A** | **G** | **G** | **A** | **A** | **A** | **G** | **C** | **A** | **T** | **C** | **T** | **A** | **A** | **A** | **A** | **C** | **A** | **C** | **A** | **T** | **T** | **T** | **T** | **A** | **A** | **C** | **A** | **A** | **C** | **T** | **T** | **T** | **C** | **A** | **A** | **T** | **T** | **C** | **G** | **T** | **C** | **C** | **T** | **C** | **G** | **G** | **C** | **G** | **T** | **T** | **G** | **T** | **T** | **T** | **T** | **T** | **A** | **T** | **T** | **G** | **T** | **C** | **A** | **T** | **T** | **G** | **G** | **T** | **G** | **C** | **A** | **A** | **T** | **G** | **A** | **A** | **C** | **A** | **G** | **T** | **A** | **C** | **A** | **G** | **C** | **T** | **G** | **T** | **T** | **T** | **G** | **G** | **T** | **T** | **T** | **A** | **T** | **G** | **T** | **T** | **G** | **A** | **T** | **T** | **T** | **T** | **T** | **G** | **G** | **C** | **G** | **T** | **A** | **C** | **T** | **C** | **T** | **T** | **T** | **A** | **T** | **C** | **G** | **G** | **C** | **T** | **T** | **A** | **A** | **A** | **A** | **G** | **G** | **C** | **T** | **T** | **T** | **G** | **A** | **G** | **A** | **T** | **T** | **T** | **C** | **A** | **G** | **G** | **C** | **G** | **T** | **G** | **G** | **A** | **T** | **A** | **T** | **A** | **G** | **C** | **T** | **G** | **A** | **G** | **C** | **G** | **A** | **G** | **C** | **A** | **C** | **C** | **T** | **T** | **G** | **G** | **C** | **G** | **A** | **A** | **A** | **A** | **A** | **A** | **A** | **C** | **A** | **A** | **A** | **T** | **G** | **A** | **T** | **T** | **A** | **T** | **G** | **G** | **T** | **G** | **G** | **A** | **G** | **A** | **C** | **G** | **G** | **C** | **G** | **G** | **C** | **A** | **A** | **A** | **A** | **G** | **A** | **A** | **C** | **C** | **T** | **A** | **A** | **A** | **A** | **A** | **T** | **G** | **G** | **C** | **A** | **A** | **A** | **C** | **G** | **A** | **T** | **T** | **T** | **A** | **A** | **A** | **C** | **G** | **C** | **C** | **G** | **C** | **T** | **G** | **G** | **T** | **T** | **T** | **G** | **C** | **C** | **A** | **A** | **C** | **G** | **A** | **A** | **C** | **G** | **C** | **A** | **T** | **T** | **G** | **G** | **T** | **A** | **A** | **C** | **A** | **A** | **T** | **A** | **T** | **C** | **T** | **A** | **T** | **G** | **A** | **A** | **T** | **G** | **G** | **G** | **A** | **C** | **G** | **A** | **T** | **A** | **T** | **C** | **A** | **A** | **T** | **A** | **T** | **T** | **G** | **A** | **T** | **T** | **T** | **A** | **A** | **T** | **C** | **T** | **C** | **T** | **G** | **G** | **G** | **G** | **A** | **C** | **A** | **C** | **C** | **A** | **T** | **T** | **A** | **T** | **T** | **G** | **A** | **T** | **T** | **T** | **A** | **G** | **G** | **T** | **A** | **A** | **T** | **A** | **C** | **G** | **C** | **T** | **A** | **C** | **T** | **A** | **C** | **C** | **G** | **A** | **A** | **A** | **G** | **A** | **A** | **G** | **A** | **C** | **A** | **A** | **T** | **A** | **T** | **T** | **A** | **T** | **T** | **A** | **T** | **T** | **A** | **T** | **T** | **C** | **G** | **T** | **A** | **A** | **A** | **G** | **G** | **T** | **T** | **T** | **T** | **G** | **G** | **C** | **C** | **G** | **C** | **A** | **C** | **G** | **C** | **G** | **A** | **A** | **T** | **T** | **C** | **T** | **A** | **G** | **T** | **G** | **C** | **C** | **G** | **T** | **T** | **A** | **G** | **G** | **T** | **G** | **T** | **A** | **G** | **C** | **T** | **A** | **T** | **T** | **T** | **T** | **G** | **T** | **T** | **A** | **G** | **A** | **A** | **C** | **A** | **T** | **T** | **C** | **A** | **A** | **C** | **T** | **T** | **T** | **T** | **T** | **A** | **C** | **G** | **G** | **A** | **A** | **C** | **G** | **G** | **T** | **A** | **C** | **G** | **T** | **T** | **T** | **T** | **G** | **A** | **A** | **G** | **A** | **A** | **G** | **A** | **A** | **A** | **A** | **A** | **T** | **A** | **T** | **C** | **A** | **A** | **T** | **T** | **G** | **A** | **A** | **A** | **A** | **A** | **C** | **G** | **A** | **A** | **T** | **C** | **A** | **T** | **T** | **A** | **A** | **A** | **A** | **A** | **T** | **T** | **T** | **A** | **C** | **A** | **G** | **C** | **A** | **A** | **T** | **G** | **A** | **T** | **T** | **A** | **T** | **G** | **A** | **T** | **A** | **C** | **C** | **A** | **A** | **T** | **C** | **T** | **T** | **C** | **G** | **T** | **C** | **G** | **T** | **T** | **T** | **G** | **A** | **A** | **A** | **A** | **T** | **T** | **A** | **T** | **G** | **A** | **C** | **G** | **A** | **A** | **C** | **A** | **C** | **T** | **T** | **T** | **A** | **G** | **T** | **A** | **G** | **G** | **A** | **G** | **A** | **T** | **G** | **T** | **T** | **G** | **A** | **G** | **G** | **T** | **G** | **A** | **T** | **C** | **C** | **G** | **T** | **G** | **T** | **A** | **-** | **-** | **-** |
| **N55181** | **A** | **T** | **G** | **A** | **A** | **T** | **A** | **A** | **C** | **C** | **C** | **T** | **T** | **G** | **G** | **C** | **G** | **C** | **T** | **T** | **T** | **T** | **T** | **T** | **A** | **T** | **C** | **G** | **T** | **C** | **G** | **C** | **A** | **G** | **A** | **A** | **G** | **C** | **A** | **T** | **T** | **A** | **C** | **T** | **T** | **T** | **T** | **T** | **A** | **T** | **T** | **C** | **T** | **G** | **G** | **C** | **G** | **T** | **T** | **A** | **T** | **G** | **G** | **C** | **A** | **A** | **A** | **T** | **T** | **G** | **T** | **A** | **C** | **A** | **T** | **A** | **A** | **T** | **C** | **C** | **T** | **G** | **G** | **A** | **T** | **T** | **A** | **G** | **C** | **T** | **G** | **T** | **T** | **T** | **T** | **A** | **T** | **T** | **A** | **A** | **C** | **A** | **A** | **T** | **T** | **G** | **G** | **C** | **G** | **T** | **T** | **T** | **T** | **A** | **C** | **T** | **T** | **G** | **T** | **G** | **G** | **C** | **C** | **T** | **A** | **C** | **G** | **T** | **T** | **T** | **C** | **C** | **A** | **G** | **G** | **A** | **A** | **A** | **G** | **C** | **A** | **T** | **C** | **T** | **A** | **A** | **A** | **A** | **C** | **A** | **C** | **A** | **T** | **T** | **T** | **T** | **A** | **A** | **C** | **A** | **A** | **C** | **T** | **T** | **T** | **C** | **A** | **A** | **T** | **T** | **C** | **G** | **T** | **C** | **C** | **T** | **C** | **G** | **G** | **C** | **G** | **T** | **T** | **G** | **T** | **T** | **T** | **T** | **T** | **A** | **T** | **T** | **G** | **T** | **C** | **A** | **T** | **T** | **G** | **G** | **T** | **G** | **C** | **A** | **A** | **T** | **G** | **A** | **A** | **C** | **A** | **G** | **C** | **A** | **C** | **G** | **G** | **C** | **T** | **G** | **T** | **T** | **T** | **G** | **G** | **T** | **T** | **T** | **A** | **T** | **G** | **T** | **T** | **G** | **A** | **T** | **T** | **T** | **T** | **T** | **G** | **G** | **C** | **G** | **T** | **A** | **C** | **T** | **C** | **T** | **T** | **T** | **A** | **T** | **C** | **G** | **G** | **C** | **T** | **T** | **A** | **A** | **A** | **A** | **G** | **G** | **C** | **T** | **T** | **T** | **G** | **A** | **G** | **A** | **T** | **T** | **T** | **C** | **A** | **G** | **G** | **C** | **G** | **T** | **G** | **G** | **A** | **T** | **A** | **T** | **A** | **G** | **C** | **T** | **G** | **A** | **A** | **C** | **G** | **A** | **G** | **C** | **A** | **C** | **C** | **T** | **T** | **G** | **G** | **C** | **G** | **T** | **A** | **A** | **A** | **A** | **A** | **A** | **C** | **A** | **A** | **A** | **T** | **G** | **A** | **T** | **T** | **A** | **T** | **G** | **G** | **T** | **A** | **G** | **A** | **G** | **A** | **C** | **G** | **G** | **C** | **G** | **G** | **C** | **A** | **A** | **A** | **A** | **G** | **A** | **A** | **C** | **C** | **T** | **A** | **A** | **A** | **A** | **A** | **T** | **G** | **G** | **C** | **A** | **A** | **A** | **C** | **G** | **A** | **T** | **T** | **T** | **A** | **A** | **A** | **C** | **G** | **C** | **C** | **G** | **C** | **T** | **G** | **G** | **T** | **T** | **T** | **G** | **C** | **C** | **A** | **A** | **C** | **G** | **A** | **A** | **C** | **G** | **C** | **A** | **T** | **T** | **G** | **G** | **T** | **A** | **A** | **C** | **A** | **A** | **T** | **A** | **T** | **C** | **T** | **A** | **T** | **G** | **A** | **A** | **T** | **G** | **G** | **G** | **A** | **T** | **G** | **A** | **C** | **A** | **T** | **C** | **A** | **A** | **T** | **A** | **T** | **T** | **G** | **A** | **T** | **T** | **T** | **A** | **A** | **T** | **C** | **T** | **C** | **T** | **G** | **G** | **G** | **G** | **A** | **C** | **A** | **C** | **C** | **A** | **T** | **T** | **A** | **T** | **T** | **G** | **A** | **T** | **T** | **T** | **A** | **G** | **G** | **T** | **A** | **A** | **T** | **A** | **C** | **G** | **C** | **T** | **A** | **C** | **T** | **A** | **C** | **C** | **G** | **A** | **A** | **A** | **G** | **A** | **A** | **G** | **A** | **C** | **A** | **A** | **T** | **A** | **T** | **T** | **A** | **T** | **T** | **A** | **T** | **T** | **A** | **T** | **T** | **C** | **G** | **T** | **A** | **A** | **A** | **G** | **G** | **T** | **T** | **T** | **T** | **G** | **G** | **C** | **C** | **G** | **C** | **A** | **C** | **G** | **C** | **G** | **A** | **A** | **T** | **T** | **C** | **T** | **A** | **G** | **T** | **G** | **C** | **C** | **G** | **T** | **T** | **A** | **G** | **G** | **T** | **G** | **T** | **A** | **G** | **C** | **T** | **A** | **T** | **T** | **T** | **T** | **G** | **T** | **T** | **A** | **G** | **A** | **A** | **C** | **A** | **T** | **T** | **C** | **A** | **A** | **C** | **T** | **T** | **T** | **T** | **T** | **A** | **C** | **G** | **G** | **A** | **A** | **C** | **G** | **G** | **T** | **A** | **C** | **G** | **T** | **T** | **T** | **T** | **G** | **A** | **A** | **G** | **A** | **A** | **G** | **A** | **A** | **A** | **A** | **A** | **T** | **A** | **T** | **C** | **A** | **A** | **T** | **T** | **G** | **A** | **A** | **A** | **A** | **A** | **C** | **G** | **A** | **A** | **T** | **C** | **A** | **T** | **T** | **A** | **A** | **A** | **A** | **A** | **T** | **T** | **T** | **A** | **C** | **A** | **G** | **C** | **A** | **A** | **T** | **G** | **A** | **T** | **T** | **A** | **T** | **G** | **A** | **T** | **A** | **C** | **C** | **A** | **A** | **T** | **C** | **T** | **T** | **C** | **G** | **T** | **C** | **G** | **T** | **T** | **T** | **G** | **A** | **A** | **A** | **A** | **T** | **T** | **A** | **T** | **G** | **A** | **C** | **G** | **A** | **A** | **C** | **A** | **C** | **T** | **T** | **T** | **A** | **G** | **T** | **A** | **G** | **G** | **A** | **G** | **A** | **T** | **G** | **T** | **T** | **G** | **A** | **G** | **G** | **T** | **G** | **A** | **T** | **C** | **C** | **G** | **T** | **G** | **T** | **A** | **-** | **-** | **-** |
| **N52755** | **A** | **T** | **G** | **A** | **A** | **T** | **A** | **A** | **C** | **C** | **C** | **T** | **T** | **G** | **G** | **C** | **G** | **C** | **T** | **T** | **T** | **T** | **T** | **T** | **A** | **T** | **C** | **G** | **T** | **C** | **G** | **C** | **A** | **G** | **A** | **A** | **G** | **C** | **A** | **T** | **T** | **A** | **C** | **T** | **T** | **T** | **T** | **T** | **A** | **T** | **T** | **C** | **T** | **G** | **G** | **C** | **G** | **T** | **T** | **A** | **T** | **G** | **G** | **C** | **A** | **A** | **A** | **T** | **T** | **G** | **T** | **A** | **C** | **A** | **T** | **A** | **A** | **T** | **C** | **C** | **T** | **G** | **G** | **A** | **T** | **T** | **A** | **G** | **C** | **T** | **G** | **T** | **T** | **T** | **T** | **A** | **T** | **T** | **A** | **A** | **C** | **A** | **A** | **T** | **T** | **G** | **G** | **C** | **G** | **T** | **T** | **T** | **T** | **A** | **C** | **T** | **T** | **G** | **T** | **G** | **G** | **C** | **C** | **T** | **A** | **C** | **G** | **T** | **T** | **T** | **C** | **C** | **A** | **G** | **G** | **A** | **A** | **A** | **G** | **C** | **A** | **T** | **C** | **T** | **A** | **A** | **A** | **A** | **C** | **A** | **C** | **A** | **T** | **T** | **T** | **T** | **A** | **A** | **C** | **A** | **A** | **C** | **T** | **T** | **T** | **C** | **A** | **A** | **T** | **T** | **C** | **G** | **T** | **C** | **C** | **T** | **C** | **G** | **G** | **C** | **G** | **T** | **T** | **G** | **T** | **T** | **T** | **T** | **T** | **A** | **T** | **T** | **G** | **T** | **C** | **A** | **T** | **T** | **G** | **G** | **T** | **G** | **C** | **A** | **A** | **T** | **G** | **A** | **A** | **C** | **A** | **G** | **C** | **A** | **C** | **G** | **G** | **C** | **T** | **G** | **T** | **T** | **T** | **G** | **G** | **T** | **T** | **T** | **A** | **T** | **G** | **T** | **T** | **G** | **A** | **T** | **T** | **T** | **T** | **T** | **G** | **G** | **C** | **G** | **T** | **A** | **C** | **T** | **C** | **T** | **T** | **T** | **A** | **T** | **C** | **G** | **G** | **C** | **T** | **T** | **A** | **A** | **A** | **A** | **G** | **G** | **C** | **T** | **T** | **T** | **G** | **A** | **G** | **A** | **T** | **T** | **T** | **C** | **A** | **G** | **G** | **C** | **G** | **T** | **G** | **G** | **A** | **T** | **A** | **T** | **A** | **G** | **C** | **T** | **G** | **A** | **G** | **C** | **G** | **A** | **G** | **C** | **A** | **C** | **C** | **T** | **T** | **G** | **G** | **C** | **G** | **A** | **A** | **A** | **A** | **A** | **A** | **A** | **C** | **A** | **A** | **A** | **T** | **G** | **A** | **T** | **T** | **A** | **T** | **G** | **G** | **T** | **G** | **G** | **A** | **G** | **A** | **C** | **G** | **G** | **C** | **G** | **G** | **C** | **A** | **A** | **A** | **A** | **G** | **A** | **A** | **C** | **C** | **T** | **A** | **A** | **A** | **A** | **A** | **T** | **G** | **G** | **C** | **A** | **A** | **A** | **C** | **G** | **A** | **T** | **T** | **T** | **A** | **A** | **A** | **C** | **G** | **C** | **C** | **G** | **C** | **T** | **G** | **G** | **T** | **T** | **T** | **G** | **C** | **C** | **A** | **A** | **C** | **G** | **A** | **A** | **C** | **G** | **C** | **A** | **T** | **T** | **G** | **G** | **T** | **A** | **A** | **C** | **A** | **A** | **T** | **A** | **T** | **C** | **T** | **A** | **T** | **G** | **A** | **A** | **T** | **G** | **G** | **G** | **A** | **C** | **G** | **A** | **T** | **A** | **T** | **C** | **A** | **A** | **T** | **A** | **T** | **T** | **G** | **A** | **T** | **T** | **T** | **A** | **A** | **T** | **C** | **T** | **C** | **T** | **G** | **G** | **G** | **G** | **A** | **C** | **A** | **C** | **C** | **A** | **T** | **T** | **A** | **T** | **T** | **G** | **A** | **T** | **T** | **T** | **A** | **G** | **G** | **T** | **A** | **A** | **T** | **A** | **C** | **G** | **T** | **T** | **A** | **C** | **T** | **A** | **C** | **C** | **G** | **A** | **A** | **A** | **G** | **A** | **A** | **G** | **A** | **C** | **A** | **A** | **T** | **A** | **T** | **T** | **A** | **T** | **T** | **A** | **T** | **T** | **A** | **T** | **T** | **C** | **G** | **T** | **A** | **A** | **A** | **G** | **G** | **T** | **T** | **T** | **T** | **G** | **G** | **C** | **C** | **G** | **C** | **A** | **C** | **G** | **C** | **G** | **A** | **A** | **T** | **T** | **C** | **T** | **A** | **G** | **T** | **G** | **C** | **C** | **G** | **T** | **T** | **A** | **G** | **G** | **T** | **G** | **T** | **A** | **G** | **C** | **T** | **A** | **T** | **T** | **T** | **T** | **G** | **T** | **T** | **A** | **G** | **A** | **A** | **C** | **A** | **T** | **T** | **C** | **A** | **A** | **C** | **T** | **T** | **T** | **T** | **T** | **A** | **C** | **G** | **G** | **A** | **A** | **C** | **G** | **G** | **T** | **A** | **C** | **G** | **T** | **T** | **T** | **T** | **G** | **A** | **A** | **G** | **A** | **A** | **G** | **A** | **A** | **A** | **A** | **A** | **T** | **A** | **T** | **C** | **A** | **A** | **T** | **T** | **G** | **A** | **A** | **A** | **A** | **A** | **C** | **G** | **A** | **A** | **T** | **C** | **A** | **T** | **T** | **A** | **A** | **A** | **A** | **A** | **T** | **T** | **T** | **A** | **C** | **A** | **G** | **C** | **A** | **A** | **T** | **G** | **A** | **T** | **T** | **A** | **T** | **G** | **A** | **T** | **A** | **C** | **C** | **A** | **A** | **T** | **C** | **T** | **T** | **C** | **G** | **T** | **C** | **G** | **T** | **T** | **T** | **G** | **A** | **A** | **A** | **A** | **T** | **T** | **A** | **T** | **G** | **A** | **C** | **G** | **A** | **A** | **C** | **A** | **C** | **T** | **T** | **T** | **A** | **G** | **T** | **A** | **G** | **G** | **A** | **G** | **A** | **T** | **G** | **T** | **T** | **G** | **A** | **G** | **G** | **T** | **G** | **A** | **T** | **C** | **C** | **G** | **T** | **G** | **T** | **A** | **-** | **-** | **-** |
| **N59646F** | **A** | **T** | **G** | **A** | **A** | **T** | **A** | **A** | **C** | **C** | **C** | **T** | **T** | **G** | **G** | **C** | **G** | **C** | **T** | **T** | **T** | **T** | **T** | **T** | **A** | **T** | **C** | **G** | **T** | **C** | **G** | **C** | **A** | **G** | **A** | **A** | **G** | **C** | **A** | **T** | **T** | **A** | **C** | **T** | **T** | **T** | **T** | **T** | **A** | **T** | **T** | **C** | **T** | **G** | **G** | **C** | **G** | **T** | **T** | **A** | **T** | **G** | **G** | **C** | **A** | **A** | **A** | **T** | **T** | **G** | **T** | **A** | **C** | **A** | **T** | **A** | **A** | **T** | **C** | **C** | **T** | **G** | **G** | **A** | **T** | **T** | **A** | **G** | **C** | **T** | **G** | **T** | **T** | **T** | **T** | **A** | **T** | **T** | **A** | **A** | **C** | **A** | **A** | **T** | **T** | **G** | **G** | **C** | **G** | **T** | **T** | **T** | **T** | **A** | **C** | **T** | **T** | **G** | **T** | **G** | **G** | **C** | **C** | **T** | **A** | **C** | **G** | **T** | **T** | **T** | **C** | **C** | **A** | **G** | **G** | **A** | **A** | **A** | **G** | **C** | **A** | **T** | **C** | **T** | **A** | **A** | **A** | **A** | **C** | **A** | **C** | **A** | **T** | **T** | **T** | **T** | **A** | **A** | **C** | **A** | **A** | **C** | **T** | **T** | **T** | **C** | **A** | **A** | **T** | **T** | **C** | **G** | **T** | **C** | **C** | **T** | **C** | **G** | **G** | **C** | **G** | **T** | **T** | **G** | **T** | **T** | **T** | **T** | **T** | **A** | **T** | **T** | **G** | **T** | **C** | **A** | **T** | **T** | **G** | **G** | **T** | **G** | **C** | **A** | **A** | **T** | **G** | **A** | **A** | **T** | **A** | **G** | **C** | **A** | **C** | **G** | **G** | **C** | **T** | **G** | **T** | **T** | **T** | **G** | **G** | **T** | **T** | **T** | **A** | **T** | **G** | **T** | **T** | **G** | **A** | **T** | **T** | **T** | **T** | **T** | **G** | **G** | **C** | **G** | **T** | **A** | **C** | **T** | **C** | **T** | **T** | **T** | **A** | **T** | **C** | **G** | **G** | **C** | **T** | **T** | **A** | **A** | **A** | **A** | **G** | **G** | **C** | **T** | **T** | **T** | **G** | **A** | **G** | **A** | **T** | **T** | **T** | **C** | **A** | **G** | **G** | **C** | **G** | **T** | **G** | **G** | **A** | **T** | **A** | **T** | **A** | **G** | **C** | **T** | **G** | **A** | **G** | **C** | **G** | **A** | **G** | **C** | **A** | **C** | **C** | **T** | **T** | **G** | **G** | **C** | **G** | **A** | **A** | **A** | **A** | **A** | **A** | **A** | **C** | **A** | **A** | **A** | **T** | **G** | **A** | **T** | **T** | **A** | **T** | **G** | **G** | **T** | **G** | **G** | **A** | **G** | **A** | **C** | **G** | **G** | **C** | **G** | **G** | **C** | **A** | **A** | **A** | **A** | **G** | **A** | **A** | **C** | **C** | **T** | **A** | **A** | **A** | **A** | **A** | **T** | **G** | **G** | **C** | **A** | **A** | **A** | **C** | **G** | **G** | **T** | **T** | **T** | **A** | **A** | **A** | **C** | **G** | **C** | **C** | **G** | **C** | **T** | **G** | **G** | **T** | **T** | **T** | **G** | **C** | **C** | **A** | **A** | **C** | **G** | **A** | **A** | **C** | **G** | **C** | **A** | **T** | **T** | **G** | **G** | **T** | **A** | **A** | **C** | **A** | **A** | **T** | **A** | **T** | **C** | **T** | **A** | **T** | **G** | **A** | **A** | **T** | **G** | **G** | **G** | **A** | **C** | **G** | **A** | **T** | **A** | **T** | **C** | **A** | **A** | **T** | **A** | **T** | **T** | **G** | **A** | **T** | **T** | **T** | **A** | **A** | **T** | **C** | **T** | **C** | **T** | **G** | **G** | **G** | **G** | **A** | **C** | **A** | **C** | **C** | **A** | **T** | **T** | **A** | **T** | **T** | **G** | **A** | **T** | **T** | **T** | **A** | **G** | **G** | **T** | **A** | **A** | **T** | **A** | **C** | **G** | **C** | **T** | **A** | **C** | **T** | **A** | **C** | **C** | **G** | **A** | **A** | **A** | **G** | **A** | **A** | **G** | **A** | **C** | **A** | **A** | **T** | **A** | **T** | **T** | **A** | **T** | **T** | **A** | **T** | **T** | **A** | **T** | **T** | **C** | **G** | **T** | **A** | **A** | **A** | **G** | **G** | **T** | **T** | **T** | **T** | **G** | **G** | **C** | **C** | **G** | **C** | **A** | **C** | **G** | **C** | **G** | **A** | **A** | **T** | **T** | **C** | **T** | **A** | **G** | **T** | **G** | **C** | **C** | **G** | **T** | **T** | **A** | **G** | **G** | **T** | **G** | **T** | **A** | **G** | **C** | **T** | **A** | **T** | **T** | **T** | **T** | **G** | **T** | **T** | **A** | **G** | **A** | **A** | **C** | **A** | **T** | **T** | **C** | **A** | **A** | **C** | **T** | **T** | **T** | **T** | **T** | **A** | **C** | **G** | **G** | **A** | **A** | **C** | **G** | **G** | **T** | **A** | **C** | **G** | **T** | **T** | **T** | **T** | **G** | **A** | **A** | **G** | **A** | **A** | **G** | **A** | **A** | **A** | **A** | **A** | **T** | **A** | **T** | **C** | **A** | **A** | **T** | **T** | **G** | **A** | **A** | **A** | **A** | **A** | **C** | **G** | **A** | **A** | **T** | **C** | **A** | **T** | **T** | **A** | **A** | **A** | **A** | **A** | **T** | **T** | **T** | **A** | **C** | **A** | **G** | **C** | **A** | **A** | **T** | **G** | **A** | **T** | **T** | **A** | **T** | **G** | **A** | **T** | **A** | **C** | **C** | **A** | **A** | **T** | **C** | **T** | **T** | **C** | **G** | **T** | **C** | **G** | **T** | **T** | **T** | **G** | **A** | **A** | **A** | **A** | **T** | **T** | **A** | **T** | **G** | **A** | **C** | **G** | **A** | **A** | **C** | **A** | **C** | **T** | **T** | **T** | **A** | **G** | **T** | **A** | **G** | **G** | **A** | **G** | **A** | **T** | **G** | **T** | **T** | **G** | **A** | **G** | **G** | **T** | **G** | **A** | **T** | **C** | **C** | **G** | **T** | **G** | **T** | **A** | **-** | **-** | **-** |
| **N59491F** | **A** | **T** | **G** | **A** | **A** | **T** | **A** | **A** | **C** | **C** | **C** | **T** | **T** | **G** | **G** | **C** | **G** | **C** | **T** | **T** | **T** | **T** | **T** | **T** | **A** | **T** | **C** | **G** | **T** | **C** | **G** | **C** | **A** | **G** | **A** | **A** | **G** | **C** | **A** | **T** | **T** | **A** | **C** | **T** | **T** | **T** | **T** | **T** | **A** | **T** | **T** | **C** | **T** | **G** | **G** | **C** | **G** | **T** | **T** | **A** | **T** | **G** | **G** | **C** | **A** | **A** | **A** | **T** | **T** | **G** | **T** | **A** | **C** | **A** | **T** | **A** | **A** | **T** | **C** | **C** | **T** | **G** | **G** | **A** | **T** | **T** | **A** | **G** | **C** | **T** | **G** | **T** | **T** | **T** | **T** | **A** | **T** | **T** | **A** | **A** | **C** | **A** | **A** | **T** | **T** | **G** | **G** | **C** | **G** | **T** | **T** | **T** | **T** | **A** | **C** | **T** | **T** | **G** | **T** | **G** | **G** | **C** | **C** | **T** | **A** | **C** | **G** | **T** | **T** | **T** | **C** | **C** | **A** | **G** | **G** | **A** | **A** | **A** | **G** | **C** | **A** | **T** | **C** | **T** | **A** | **A** | **A** | **A** | **C** | **A** | **C** | **A** | **T** | **T** | **T** | **T** | **A** | **A** | **C** | **A** | **A** | **C** | **T** | **T** | **T** | **C** | **A** | **A** | **T** | **T** | **C** | **G** | **T** | **C** | **C** | **T** | **C** | **G** | **G** | **C** | **G** | **T** | **T** | **G** | **T** | **T** | **T** | **T** | **T** | **A** | **T** | **T** | **G** | **T** | **C** | **A** | **T** | **T** | **G** | **G** | **T** | **G** | **C** | **A** | **A** | **T** | **G** | **A** | **A** | **C** | **A** | **G** | **C** | **A** | **C** | **G** | **G** | **C** | **T** | **G** | **T** | **T** | **T** | **G** | **G** | **T** | **T** | **T** | **A** | **T** | **G** | **T** | **T** | **G** | **A** | **T** | **T** | **T** | **T** | **T** | **G** | **G** | **C** | **G** | **T** | **A** | **C** | **T** | **C** | **T** | **T** | **T** | **A** | **T** | **C** | **G** | **G** | **C** | **T** | **T** | **A** | **A** | **A** | **A** | **G** | **G** | **C** | **T** | **T** | **T** | **G** | **A** | **G** | **A** | **T** | **T** | **T** | **C** | **A** | **G** | **G** | **C** | **G** | **T** | **G** | **G** | **A** | **T** | **A** | **T** | **A** | **G** | **C** | **T** | **G** | **A** | **A** | **C** | **G** | **A** | **G** | **C** | **A** | **C** | **C** | **T** | **T** | **G** | **G** | **C** | **G** | **T** | **A** | **A** | **A** | **A** | **A** | **A** | **C** | **A** | **A** | **A** | **T** | **G** | **A** | **T** | **T** | **A** | **T** | **G** | **G** | **T** | **G** | **G** | **A** | **G** | **A** | **C** | **G** | **G** | **C** | **G** | **G** | **C** | **A** | **A** | **A** | **A** | **G** | **A** | **A** | **C** | **C** | **T** | **A** | **A** | **A** | **A** | **A** | **T** | **G** | **G** | **C** | **A** | **A** | **A** | **C** | **G** | **A** | **T** | **T** | **T** | **A** | **A** | **A** | **C** | **G** | **C** | **C** | **G** | **T** | **T** | **G** | **G** | **T** | **T** | **T** | **G** | **C** | **C** | **A** | **A** | **C** | **G** | **A** | **A** | **C** | **G** | **C** | **A** | **T** | **T** | **G** | **G** | **T** | **A** | **A** | **C** | **A** | **A** | **T** | **A** | **T** | **C** | **T** | **A** | **T** | **G** | **A** | **A** | **T** | **G** | **G** | **G** | **A** | **C** | **G** | **A** | **T** | **A** | **T** | **C** | **A** | **A** | **T** | **A** | **T** | **T** | **G** | **A** | **T** | **T** | **T** | **A** | **A** | **T** | **C** | **T** | **C** | **T** | **G** | **G** | **G** | **G** | **A** | **C** | **A** | **C** | **C** | **A** | **T** | **T** | **A** | **T** | **T** | **G** | **A** | **T** | **T** | **T** | **A** | **G** | **G** | **T** | **A** | **A** | **T** | **A** | **C** | **G** | **C** | **T** | **A** | **C** | **T** | **A** | **C** | **C** | **G** | **A** | **A** | **A** | **G** | **A** | **A** | **G** | **A** | **C** | **A** | **A** | **T** | **A** | **T** | **T** | **A** | **T** | **T** | **A** | **T** | **T** | **A** | **T** | **T** | **C** | **G** | **T** | **A** | **A** | **A** | **G** | **G** | **T** | **T** | **T** | **T** | **G** | **G** | **T** | **C** | **G** | **C** | **A** | **C** | **G** | **C** | **G** | **A** | **A** | **T** | **T** | **C** | **T** | **A** | **G** | **T** | **G** | **C** | **C** | **G** | **T** | **T** | **A** | **G** | **G** | **T** | **G** | **T** | **A** | **G** | **C** | **T** | **A** | **T** | **T** | **T** | **T** | **G** | **T** | **T** | **A** | **G** | **A** | **A** | **C** | **A** | **T** | **T** | **C** | **A** | **A** | **C** | **T** | **T** | **T** | **T** | **T** | **A** | **C** | **G** | **G** | **A** | **A** | **C** | **G** | **G** | **T** | **A** | **C** | **G** | **T** | **T** | **T** | **T** | **G** | **A** | **A** | **G** | **A** | **A** | **G** | **A** | **A** | **A** | **A** | **A** | **T** | **A** | **T** | **C** | **A** | **A** | **T** | **T** | **G** | **A** | **A** | **A** | **A** | **A** | **C** | **G** | **A** | **A** | **T** | **C** | **A** | **T** | **T** | **A** | **A** | **A** | **A** | **A** | **T** | **T** | **T** | **A** | **C** | **A** | **G** | **C** | **A** | **A** | **T** | **G** | **A** | **T** | **T** | **A** | **T** | **G** | **A** | **T** | **A** | **C** | **C** | **A** | **A** | **T** | **C** | **T** | **T** | **C** | **G** | **T** | **C** | **G** | **T** | **T** | **T** | **G** | **A** | **A** | **A** | **A** | **T** | **T** | **A** | **T** | **G** | **A** | **C** | **G** | **A** | **A** | **C** | **A** | **C** | **T** | **T** | **T** | **A** | **G** | **T** | **A** | **G** | **G** | **A** | **G** | **A** | **T** | **G** | **T** | **T** | **G** | **A** | **G** | **G** | **T** | **G** | **A** | **T** | **C** | **C** | **G** | **T** | **G** | **T** | **A** | **-** | **-** | **-** |
| **N59862F** | **A** | **T** | **G** | **A** | **A** | **T** | **A** | **A** | **C** | **C** | **C** | **T** | **T** | **G** | **G** | **C** | **G** | **C** | **T** | **T** | **T** | **T** | **T** | **T** | **A** | **T** | **C** | **G** | **T** | **C** | **G** | **C** | **A** | **G** | **A** | **A** | **G** | **C** | **A** | **T** | **T** | **A** | **C** | **T** | **T** | **T** | **T** | **T** | **A** | **T** | **T** | **C** | **T** | **G** | **G** | **C** | **G** | **T** | **T** | **A** | **T** | **G** | **G** | **C** | **A** | **A** | **A** | **T** | **T** | **G** | **T** | **A** | **C** | **A** | **T** | **A** | **A** | **T** | **C** | **C** | **T** | **G** | **G** | **A** | **T** | **T** | **A** | **G** | **C** | **T** | **G** | **T** | **T** | **T** | **T** | **A** | **T** | **T** | **A** | **A** | **C** | **A** | **A** | **T** | **T** | **G** | **G** | **C** | **G** | **T** | **T** | **T** | **T** | **A** | **C** | **T** | **T** | **G** | **T** | **G** | **G** | **C** | **C** | **T** | **A** | **C** | **G** | **T** | **T** | **T** | **C** | **C** | **A** | **G** | **G** | **A** | **A** | **A** | **G** | **C** | **A** | **T** | **C** | **T** | **A** | **A** | **A** | **A** | **C** | **A** | **C** | **A** | **T** | **T** | **T** | **T** | **A** | **A** | **C** | **A** | **A** | **C** | **T** | **T** | **T** | **C** | **A** | **A** | **T** | **T** | **C** | **G** | **T** | **C** | **C** | **T** | **C** | **G** | **G** | **C** | **G** | **T** | **T** | **G** | **T** | **T** | **T** | **T** | **T** | **A** | **T** | **T** | **G** | **T** | **C** | **A** | **T** | **T** | **G** | **G** | **T** | **G** | **C** | **A** | **A** | **T** | **G** | **A** | **A** | **C** | **A** | **G** | **C** | **A** | **C** | **G** | **G** | **C** | **T** | **G** | **T** | **T** | **T** | **G** | **G** | **T** | **T** | **T** | **A** | **T** | **G** | **T** | **T** | **G** | **A** | **T** | **T** | **T** | **T** | **T** | **G** | **G** | **C** | **G** | **T** | **A** | **C** | **T** | **C** | **T** | **T** | **T** | **A** | **T** | **C** | **G** | **G** | **C** | **T** | **T** | **A** | **A** | **A** | **A** | **G** | **G** | **C** | **T** | **T** | **T** | **G** | **A** | **G** | **A** | **T** | **T** | **T** | **C** | **A** | **G** | **G** | **C** | **G** | **T** | **G** | **G** | **A** | **T** | **A** | **T** | **A** | **G** | **C** | **T** | **G** | **A** | **G** | **C** | **G** | **A** | **G** | **C** | **A** | **C** | **C** | **T** | **T** | **G** | **G** | **C** | **G** | **A** | **A** | **A** | **A** | **A** | **A** | **A** | **C** | **A** | **A** | **A** | **T** | **G** | **A** | **T** | **T** | **A** | **T** | **G** | **G** | **T** | **G** | **G** | **A** | **G** | **A** | **C** | **G** | **G** | **C** | **G** | **G** | **C** | **A** | **A** | **A** | **A** | **G** | **A** | **A** | **C** | **C** | **T** | **A** | **A** | **A** | **A** | **A** | **T** | **G** | **G** | **C** | **A** | **A** | **A** | **C** | **G** | **A** | **T** | **T** | **T** | **A** | **A** | **A** | **C** | **G** | **C** | **C** | **G** | **C** | **T** | **G** | **G** | **T** | **T** | **T** | **G** | **C** | **C** | **A** | **A** | **C** | **G** | **A** | **A** | **C** | **G** | **C** | **A** | **T** | **T** | **G** | **G** | **T** | **A** | **A** | **C** | **A** | **A** | **T** | **A** | **T** | **C** | **T** | **A** | **T** | **G** | **A** | **A** | **T** | **G** | **G** | **G** | **A** | **C** | **G** | **A** | **T** | **A** | **T** | **C** | **A** | **A** | **T** | **A** | **T** | **T** | **G** | **A** | **T** | **T** | **T** | **A** | **A** | **T** | **C** | **T** | **C** | **T** | **G** | **G** | **G** | **G** | **A** | **C** | **A** | **C** | **C** | **A** | **T** | **T** | **A** | **T** | **T** | **G** | **A** | **T** | **T** | **T** | **A** | **G** | **G** | **T** | **A** | **A** | **T** | **A** | **C** | **G** | **C** | **T** | **A** | **C** | **T** | **A** | **C** | **C** | **G** | **A** | **A** | **A** | **G** | **A** | **A** | **G** | **A** | **C** | **A** | **A** | **T** | **A** | **T** | **T** | **A** | **T** | **T** | **A** | **T** | **T** | **A** | **T** | **T** | **C** | **G** | **T** | **A** | **A** | **A** | **G** | **G** | **T** | **T** | **T** | **T** | **G** | **G** | **C** | **C** | **G** | **C** | **A** | **C** | **G** | **C** | **G** | **A** | **A** | **T** | **T** | **C** | **T** | **A** | **G** | **T** | **G** | **C** | **C** | **G** | **T** | **T** | **A** | **G** | **G** | **T** | **G** | **T** | **A** | **G** | **C** | **T** | **A** | **T** | **T** | **T** | **T** | **G** | **T** | **T** | **A** | **G** | **A** | **A** | **C** | **A** | **T** | **T** | **C** | **A** | **A** | **C** | **T** | **T** | **T** | **T** | **T** | **A** | **C** | **G** | **G** | **A** | **A** | **C** | **G** | **G** | **T** | **A** | **C** | **G** | **T** | **T** | **T** | **T** | **G** | **A** | **A** | **G** | **A** | **A** | **G** | **A** | **A** | **A** | **A** | **A** | **T** | **A** | **T** | **C** | **A** | **A** | **T** | **T** | **G** | **A** | **A** | **A** | **A** | **A** | **C** | **G** | **A** | **A** | **T** | **C** | **A** | **T** | **T** | **A** | **A** | **A** | **A** | **A** | **T** | **T** | **T** | **A** | **C** | **A** | **G** | **C** | **A** | **A** | **T** | **G** | **A** | **T** | **T** | **A** | **C** | **G** | **A** | **T** | **A** | **C** | **C** | **A** | **A** | **T** | **C** | **T** | **T** | **C** | **G** | **T** | **C** | **G** | **T** | **T** | **T** | **G** | **A** | **A** | **A** | **A** | **T** | **T** | **A** | **T** | **G** | **A** | **C** | **G** | **A** | **A** | **C** | **A** | **C** | **T** | **T** | **T** | **A** | **G** | **T** | **A** | **G** | **G** | **A** | **G** | **A** | **T** | **G** | **T** | **T** | **G** | **A** | **G** | **G** | **T** | **G** | **A** | **T** | **C** | **C** | **G** | **T** | **G** | **T** | **A** | **-** | **-** | **-** |
| **N59841F** | **A** | **T** | **G** | **A** | **A** | **T** | **A** | **A** | **C** | **C** | **C** | **T** | **T** | **G** | **G** | **C** | **G** | **C** | **T** | **T** | **T** | **T** | **T** | **T** | **A** | **T** | **C** | **G** | **T** | **C** | **G** | **C** | **A** | **G** | **A** | **A** | **G** | **C** | **A** | **T** | **T** | **A** | **C** | **T** | **T** | **T** | **T** | **T** | **A** | **T** | **T** | **C** | **T** | **G** | **G** | **C** | **G** | **T** | **T** | **A** | **T** | **G** | **G** | **C** | **A** | **A** | **A** | **T** | **T** | **G** | **T** | **A** | **C** | **A** | **T** | **A** | **A** | **T** | **C** | **C** | **T** | **G** | **G** | **A** | **T** | **T** | **A** | **G** | **C** | **T** | **G** | **T** | **T** | **T** | **T** | **A** | **T** | **T** | **A** | **A** | **C** | **A** | **A** | **T** | **T** | **G** | **G** | **C** | **G** | **T** | **T** | **T** | **T** | **A** | **C** | **T** | **T** | **G** | **T** | **G** | **G** | **C** | **C** | **T** | **A** | **C** | **G** | **T** | **T** | **T** | **C** | **C** | **A** | **G** | **G** | **A** | **A** | **A** | **G** | **C** | **A** | **T** | **C** | **T** | **A** | **A** | **A** | **A** | **C** | **A** | **C** | **A** | **T** | **T** | **T** | **T** | **A** | **A** | **C** | **A** | **A** | **C** | **T** | **T** | **T** | **C** | **A** | **A** | **T** | **T** | **C** | **G** | **T** | **C** | **C** | **T** | **C** | **G** | **G** | **C** | **G** | **T** | **T** | **G** | **T** | **T** | **T** | **T** | **T** | **A** | **T** | **T** | **G** | **T** | **C** | **A** | **T** | **T** | **G** | **G** | **T** | **G** | **C** | **A** | **A** | **T** | **G** | **A** | **A** | **C** | **A** | **G** | **C** | **A** | **C** | **G** | **G** | **C** | **T** | **G** | **T** | **T** | **T** | **G** | **G** | **T** | **T** | **T** | **A** | **T** | **G** | **T** | **T** | **G** | **A** | **T** | **T** | **T** | **T** | **T** | **G** | **G** | **C** | **G** | **T** | **A** | **C** | **T** | **C** | **T** | **T** | **T** | **A** | **T** | **C** | **G** | **G** | **C** | **T** | **T** | **A** | **A** | **A** | **A** | **G** | **G** | **C** | **T** | **T** | **T** | **G** | **A** | **G** | **A** | **T** | **T** | **T** | **C** | **A** | **G** | **G** | **C** | **G** | **T** | **G** | **G** | **A** | **T** | **A** | **T** | **A** | **G** | **C** | **T** | **G** | **A** | **A** | **C** | **G** | **A** | **G** | **C** | **A** | **C** | **C** | **T** | **T** | **G** | **G** | **C** | **G** | **T** | **A** | **A** | **A** | **A** | **A** | **A** | **C** | **A** | **A** | **A** | **T** | **G** | **A** | **T** | **T** | **A** | **T** | **G** | **G** | **T** | **A** | **G** | **A** | **G** | **A** | **C** | **G** | **G** | **C** | **G** | **G** | **C** | **A** | **A** | **A** | **A** | **G** | **A** | **A** | **C** | **C** | **T** | **A** | **A** | **A** | **A** | **A** | **T** | **G** | **G** | **C** | **A** | **A** | **A** | **C** | **G** | **A** | **T** | **T** | **T** | **A** | **A** | **A** | **C** | **G** | **C** | **C** | **G** | **C** | **T** | **G** | **G** | **T** | **T** | **T** | **G** | **C** | **C** | **A** | **A** | **C** | **G** | **A** | **A** | **C** | **G** | **C** | **A** | **T** | **T** | **G** | **G** | **T** | **A** | **A** | **C** | **A** | **A** | **T** | **A** | **T** | **C** | **T** | **A** | **T** | **G** | **A** | **A** | **T** | **G** | **G** | **G** | **A** | **T** | **G** | **A** | **C** | **A** | **T** | **C** | **A** | **A** | **T** | **A** | **T** | **T** | **G** | **A** | **T** | **T** | **T** | **A** | **A** | **T** | **C** | **T** | **C** | **T** | **G** | **G** | **G** | **G** | **A** | **C** | **A** | **C** | **C** | **A** | **T** | **T** | **A** | **T** | **T** | **G** | **A** | **T** | **T** | **T** | **A** | **G** | **G** | **T** | **A** | **A** | **T** | **A** | **C** | **G** | **C** | **T** | **A** | **C** | **T** | **A** | **C** | **C** | **G** | **A** | **A** | **A** | **G** | **A** | **A** | **G** | **A** | **C** | **A** | **A** | **T** | **A** | **T** | **T** | **A** | **T** | **T** | **A** | **T** | **T** | **A** | **T** | **T** | **C** | **G** | **T** | **A** | **A** | **A** | **G** | **G** | **T** | **T** | **T** | **T** | **G** | **G** | **C** | **C** | **G** | **C** | **A** | **C** | **G** | **C** | **G** | **A** | **A** | **T** | **T** | **C** | **T** | **A** | **G** | **T** | **G** | **C** | **C** | **G** | **T** | **T** | **A** | **G** | **G** | **T** | **G** | **T** | **A** | **G** | **C** | **T** | **A** | **T** | **T** | **T** | **T** | **G** | **T** | **T** | **A** | **G** | **A** | **A** | **C** | **A** | **T** | **T** | **C** | **A** | **A** | **C** | **T** | **T** | **T** | **T** | **T** | **A** | **C** | **G** | **G** | **A** | **A** | **C** | **G** | **G** | **T** | **A** | **C** | **G** | **T** | **T** | **T** | **T** | **G** | **A** | **A** | **G** | **A** | **A** | **G** | **A** | **A** | **A** | **A** | **A** | **T** | **A** | **T** | **C** | **A** | **A** | **T** | **T** | **G** | **A** | **A** | **A** | **A** | **A** | **C** | **G** | **A** | **A** | **T** | **C** | **A** | **T** | **T** | **A** | **A** | **A** | **A** | **A** | **T** | **T** | **T** | **A** | **C** | **A** | **G** | **C** | **A** | **A** | **T** | **G** | **A** | **T** | **T** | **A** | **T** | **G** | **A** | **T** | **A** | **C** | **C** | **A** | **A** | **T** | **C** | **T** | **T** | **C** | **G** | **T** | **C** | **G** | **T** | **T** | **T** | **G** | **A** | **A** | **A** | **A** | **T** | **T** | **A** | **T** | **G** | **A** | **C** | **G** | **A** | **A** | **C** | **A** | **C** | **T** | **T** | **T** | **A** | **G** | **T** | **A** | **G** | **G** | **A** | **G** | **A** | **T** | **G** | **T** | **T** | **G** | **A** | **G** | **G** | **T** | **G** | **A** | **T** | **C** | **C** | **G** | **T** | **G** | **T** | **A** | **-** | **-** | **-** |
| **N59609F** | **A** | **T** | **G** | **A** | **A** | **T** | **A** | **A** | **C** | **C** | **C** | **T** | **T** | **G** | **G** | **C** | **G** | **C** | **T** | **T** | **T** | **T** | **T** | **T** | **A** | **T** | **C** | **G** | **T** | **C** | **G** | **C** | **A** | **G** | **A** | **A** | **G** | **C** | **A** | **T** | **T** | **A** | **C** | **T** | **T** | **T** | **T** | **T** | **A** | **T** | **T** | **C** | **T** | **G** | **G** | **C** | **G** | **T** | **T** | **A** | **T** | **G** | **G** | **C** | **A** | **A** | **A** | **T** | **T** | **G** | **T** | **A** | **C** | **A** | **T** | **A** | **A** | **T** | **C** | **C** | **T** | **G** | **G** | **A** | **T** | **T** | **A** | **G** | **C** | **T** | **G** | **T** | **T** | **T** | **T** | **A** | **T** | **T** | **A** | **A** | **C** | **A** | **A** | **T** | **T** | **G** | **G** | **C** | **G** | **T** | **T** | **T** | **T** | **A** | **C** | **T** | **T** | **G** | **T** | **G** | **G** | **C** | **C** | **T** | **A** | **C** | **G** | **T** | **T** | **T** | **C** | **C** | **A** | **G** | **G** | **A** | **A** | **A** | **G** | **C** | **A** | **T** | **C** | **T** | **A** | **A** | **A** | **A** | **C** | **A** | **C** | **A** | **T** | **T** | **T** | **T** | **A** | **A** | **C** | **A** | **A** | **C** | **T** | **T** | **T** | **C** | **A** | **A** | **T** | **T** | **C** | **G** | **T** | **C** | **C** | **T** | **C** | **G** | **G** | **C** | **G** | **T** | **T** | **G** | **T** | **T** | **T** | **T** | **T** | **A** | **T** | **T** | **G** | **T** | **C** | **A** | **T** | **T** | **G** | **G** | **T** | **G** | **C** | **A** | **A** | **T** | **G** | **A** | **A** | **C** | **A** | **G** | **C** | **A** | **C** | **G** | **G** | **C** | **T** | **G** | **T** | **T** | **T** | **G** | **G** | **T** | **T** | **T** | **A** | **T** | **G** | **T** | **T** | **G** | **A** | **T** | **T** | **T** | **T** | **T** | **G** | **G** | **C** | **G** | **T** | **A** | **C** | **T** | **C** | **T** | **T** | **T** | **A** | **T** | **C** | **G** | **G** | **C** | **T** | **T** | **A** | **A** | **A** | **A** | **G** | **G** | **C** | **T** | **T** | **T** | **G** | **A** | **G** | **A** | **T** | **T** | **T** | **C** | **A** | **G** | **G** | **C** | **G** | **T** | **G** | **G** | **A** | **T** | **A** | **T** | **A** | **G** | **C** | **T** | **G** | **A** | **G** | **C** | **G** | **A** | **G** | **C** | **A** | **C** | **C** | **T** | **T** | **G** | **G** | **C** | **G** | **T** | **A** | **A** | **A** | **A** | **A** | **A** | **C** | **A** | **A** | **A** | **T** | **G** | **A** | **T** | **T** | **A** | **T** | **G** | **G** | **T** | **G** | **G** | **A** | **G** | **A** | **C** | **G** | **G** | **C** | **G** | **G** | **C** | **A** | **A** | **A** | **A** | **G** | **A** | **A** | **C** | **C** | **T** | **A** | **A** | **A** | **A** | **A** | **T** | **G** | **G** | **C** | **A** | **A** | **A** | **C** | **G** | **A** | **T** | **T** | **T** | **A** | **A** | **A** | **C** | **G** | **C** | **C** | **G** | **C** | **T** | **G** | **G** | **T** | **T** | **T** | **G** | **C** | **C** | **A** | **A** | **C** | **G** | **A** | **A** | **C** | **G** | **C** | **A** | **T** | **T** | **G** | **G** | **T** | **A** | **A** | **C** | **A** | **A** | **T** | **A** | **T** | **C** | **T** | **A** | **T** | **G** | **A** | **A** | **T** | **G** | **G** | **G** | **A** | **C** | **G** | **A** | **T** | **A** | **T** | **C** | **A** | **A** | **T** | **A** | **T** | **T** | **G** | **A** | **T** | **T** | **T** | **A** | **A** | **T** | **C** | **T** | **C** | **T** | **G** | **G** | **G** | **G** | **A** | **C** | **A** | **C** | **C** | **A** | **T** | **T** | **A** | **T** | **T** | **G** | **A** | **T** | **T** | **T** | **A** | **G** | **G** | **T** | **A** | **A** | **T** | **A** | **C** | **G** | **C** | **T** | **A** | **C** | **T** | **A** | **C** | **C** | **G** | **A** | **A** | **A** | **G** | **A** | **A** | **G** | **A** | **C** | **A** | **A** | **T** | **A** | **T** | **T** | **A** | **T** | **T** | **A** | **T** | **T** | **A** | **T** | **T** | **C** | **G** | **T** | **A** | **A** | **A** | **G** | **G** | **T** | **T** | **T** | **T** | **G** | **G** | **C** | **C** | **G** | **C** | **A** | **C** | **G** | **C** | **G** | **A** | **A** | **T** | **T** | **C** | **T** | **A** | **G** | **T** | **G** | **C** | **C** | **G** | **T** | **T** | **A** | **G** | **G** | **T** | **G** | **T** | **A** | **G** | **C** | **T** | **A** | **T** | **T** | **T** | **T** | **G** | **T** | **T** | **A** | **G** | **A** | **A** | **C** | **A** | **T** | **T** | **C** | **A** | **A** | **C** | **T** | **T** | **T** | **T** | **T** | **A** | **C** | **G** | **G** | **A** | **A** | **C** | **G** | **G** | **T** | **A** | **C** | **G** | **T** | **T** | **T** | **T** | **G** | **A** | **A** | **G** | **A** | **A** | **G** | **A** | **A** | **A** | **A** | **A** | **T** | **A** | **T** | **C** | **A** | **A** | **T** | **T** | **G** | **A** | **A** | **A** | **A** | **A** | **C** | **G** | **A** | **A** | **T** | **C** | **A** | **T** | **T** | **A** | **A** | **A** | **A** | **A** | **T** | **T** | **T** | **A** | **C** | **A** | **G** | **C** | **A** | **A** | **T** | **G** | **A** | **T** | **T** | **A** | **T** | **G** | **A** | **T** | **A** | **C** | **C** | **A** | **A** | **T** | **C** | **T** | **T** | **C** | **G** | **T** | **C** | **G** | **T** | **T** | **T** | **G** | **A** | **A** | **A** | **A** | **T** | **T** | **A** | **T** | **G** | **A** | **C** | **G** | **A** | **A** | **C** | **A** | **C** | **T** | **T** | **T** | **A** | **G** | **T** | **A** | **G** | **G** | **A** | **G** | **A** | **T** | **G** | **T** | **T** | **G** | **A** | **G** | **G** | **T** | **G** | **A** | **T** | **C** | **C** | **G** | **T** | **G** | **T** | **A** | **-** | **-** | **-** |
| **N52623** | **A** | **T** | **G** | **A** | **A** | **T** | **A** | **A** | **C** | **C** | **C** | **T** | **T** | **G** | **G** | **C** | **G** | **C** | **T** | **T** | **T** | **T** | **T** | **T** | **A** | **T** | **C** | **G** | **T** | **C** | **G** | **C** | **A** | **G** | **A** | **A** | **G** | **C** | **A** | **T** | **T** | **A** | **C** | **T** | **T** | **T** | **T** | **T** | **A** | **T** | **T** | **C** | **T** | **G** | **G** | **C** | **G** | **T** | **T** | **A** | **T** | **G** | **G** | **C** | **A** | **A** | **A** | **T** | **T** | **G** | **T** | **A** | **C** | **A** | **T** | **A** | **A** | **T** | **C** | **C** | **T** | **G** | **G** | **A** | **T** | **T** | **A** | **G** | **C** | **T** | **G** | **T** | **T** | **T** | **T** | **A** | **T** | **T** | **A** | **A** | **C** | **A** | **A** | **T** | **T** | **G** | **G** | **C** | **G** | **T** | **T** | **T** | **T** | **A** | **C** | **T** | **T** | **G** | **T** | **G** | **G** | **C** | **C** | **T** | **A** | **C** | **G** | **T** | **T** | **T** | **C** | **C** | **A** | **G** | **G** | **A** | **A** | **A** | **G** | **C** | **A** | **T** | **C** | **T** | **A** | **A** | **A** | **A** | **C** | **A** | **C** | **A** | **T** | **T** | **T** | **T** | **A** | **A** | **C** | **A** | **A** | **C** | **T** | **T** | **T** | **C** | **A** | **A** | **T** | **T** | **C** | **G** | **T** | **C** | **C** | **T** | **C** | **G** | **G** | **C** | **G** | **T** | **T** | **G** | **T** | **T** | **T** | **T** | **T** | **A** | **T** | **T** | **G** | **T** | **C** | **A** | **T** | **T** | **G** | **G** | **T** | **G** | **C** | **A** | **A** | **T** | **G** | **A** | **A** | **C** | **A** | **G** | **C** | **A** | **C** | **G** | **G** | **C** | **T** | **G** | **T** | **T** | **T** | **G** | **G** | **T** | **T** | **T** | **A** | **T** | **G** | **T** | **T** | **G** | **A** | **T** | **T** | **T** | **T** | **T** | **G** | **G** | **C** | **G** | **T** | **A** | **C** | **T** | **C** | **T** | **T** | **T** | **A** | **T** | **C** | **G** | **G** | **C** | **T** | **T** | **A** | **A** | **A** | **A** | **G** | **G** | **C** | **T** | **T** | **T** | **G** | **A** | **G** | **A** | **T** | **T** | **T** | **C** | **A** | **G** | **G** | **C** | **G** | **T** | **G** | **G** | **A** | **T** | **A** | **T** | **A** | **G** | **C** | **T** | **G** | **A** | **G** | **C** | **G** | **A** | **G** | **C** | **A** | **C** | **C** | **T** | **T** | **G** | **G** | **C** | **G** | **A** | **A** | **A** | **A** | **A** | **A** | **A** | **C** | **A** | **A** | **A** | **T** | **G** | **A** | **T** | **T** | **A** | **T** | **G** | **G** | **T** | **G** | **G** | **A** | **G** | **A** | **C** | **G** | **G** | **C** | **G** | **G** | **C** | **A** | **A** | **A** | **A** | **G** | **A** | **A** | **C** | **C** | **T** | **A** | **A** | **A** | **A** | **A** | **T** | **G** | **G** | **C** | **A** | **A** | **A** | **C** | **G** | **A** | **T** | **T** | **T** | **A** | **A** | **A** | **C** | **G** | **C** | **C** | **G** | **C** | **T** | **G** | **G** | **T** | **T** | **T** | **G** | **C** | **C** | **A** | **A** | **C** | **G** | **A** | **A** | **C** | **G** | **C** | **A** | **T** | **T** | **G** | **G** | **T** | **A** | **A** | **C** | **A** | **A** | **T** | **A** | **T** | **C** | **T** | **A** | **T** | **G** | **A** | **A** | **T** | **G** | **G** | **G** | **A** | **C** | **G** | **A** | **T** | **A** | **T** | **C** | **A** | **A** | **T** | **A** | **T** | **T** | **G** | **A** | **T** | **T** | **T** | **A** | **A** | **T** | **C** | **T** | **C** | **T** | **G** | **G** | **G** | **G** | **A** | **C** | **A** | **C** | **C** | **A** | **T** | **T** | **A** | **T** | **T** | **G** | **A** | **T** | **T** | **T** | **A** | **G** | **G** | **T** | **A** | **A** | **T** | **A** | **C** | **G** | **T** | **T** | **A** | **C** | **T** | **A** | **C** | **C** | **G** | **A** | **A** | **A** | **G** | **A** | **A** | **G** | **A** | **C** | **A** | **A** | **T** | **A** | **T** | **T** | **A** | **T** | **T** | **A** | **T** | **T** | **A** | **T** | **T** | **C** | **G** | **T** | **A** | **A** | **A** | **G** | **G** | **T** | **T** | **T** | **T** | **G** | **G** | **C** | **C** | **G** | **C** | **A** | **C** | **G** | **C** | **G** | **A** | **A** | **T** | **T** | **C** | **T** | **A** | **G** | **T** | **G** | **C** | **C** | **G** | **T** | **T** | **A** | **G** | **G** | **T** | **G** | **T** | **A** | **G** | **C** | **T** | **A** | **T** | **T** | **T** | **T** | **G** | **T** | **T** | **A** | **G** | **A** | **A** | **C** | **A** | **T** | **T** | **C** | **A** | **A** | **C** | **T** | **T** | **T** | **T** | **T** | **A** | **C** | **G** | **G** | **A** | **A** | **C** | **G** | **G** | **T** | **A** | **C** | **G** | **T** | **T** | **T** | **T** | **G** | **A** | **A** | **G** | **A** | **A** | **G** | **A** | **A** | **A** | **A** | **A** | **T** | **A** | **T** | **C** | **A** | **A** | **T** | **T** | **G** | **A** | **A** | **A** | **A** | **A** | **C** | **G** | **A** | **A** | **T** | **C** | **A** | **T** | **T** | **A** | **A** | **A** | **A** | **A** | **T** | **T** | **T** | **A** | **C** | **A** | **G** | **C** | **A** | **A** | **T** | **G** | **A** | **T** | **T** | **A** | **T** | **G** | **A** | **T** | **A** | **C** | **C** | **A** | **A** | **T** | **C** | **T** | **T** | **C** | **G** | **T** | **C** | **G** | **T** | **T** | **T** | **G** | **A** | **A** | **A** | **A** | **T** | **T** | **A** | **T** | **G** | **A** | **C** | **G** | **A** | **A** | **C** | **A** | **C** | **T** | **T** | **T** | **A** | **G** | **T** | **A** | **G** | **G** | **A** | **G** | **A** | **T** | **G** | **T** | **T** | **G** | **A** | **G** | **G** | **T** | **G** | **A** | **T** | **C** | **C** | **G** | **T** | **G** | **T** | **A** | **-** | **-** | **-** |
| **N59462F** | **A** | **T** | **G** | **A** | **A** | **T** | **A** | **A** | **C** | **C** | **C** | **T** | **T** | **G** | **G** | **C** | **G** | **C** | **T** | **T** | **T** | **T** | **T** | **T** | **A** | **T** | **C** | **G** | **T** | **C** | **G** | **C** | **A** | **G** | **A** | **A** | **G** | **C** | **A** | **T** | **T** | **A** | **C** | **T** | **T** | **T** | **T** | **T** | **A** | **T** | **T** | **C** | **T** | **G** | **G** | **C** | **G** | **T** | **T** | **A** | **T** | **G** | **G** | **C** | **A** | **A** | **A** | **T** | **T** | **G** | **T** | **A** | **C** | **A** | **T** | **A** | **A** | **T** | **C** | **C** | **T** | **G** | **G** | **A** | **T** | **T** | **A** | **G** | **C** | **T** | **G** | **T** | **T** | **T** | **T** | **A** | **T** | **T** | **A** | **A** | **C** | **A** | **A** | **T** | **T** | **G** | **G** | **C** | **G** | **T** | **T** | **T** | **T** | **A** | **C** | **T** | **T** | **G** | **T** | **G** | **G** | **C** | **C** | **T** | **A** | **C** | **G** | **T** | **T** | **T** | **C** | **C** | **A** | **G** | **G** | **A** | **A** | **A** | **G** | **C** | **A** | **T** | **C** | **T** | **A** | **A** | **A** | **A** | **C** | **A** | **C** | **A** | **T** | **T** | **T** | **T** | **A** | **A** | **C** | **A** | **A** | **C** | **T** | **T** | **T** | **C** | **A** | **A** | **T** | **T** | **C** | **G** | **T** | **C** | **C** | **T** | **C** | **G** | **G** | **C** | **G** | **T** | **T** | **G** | **T** | **T** | **T** | **T** | **T** | **A** | **T** | **T** | **G** | **T** | **C** | **A** | **T** | **T** | **G** | **G** | **T** | **G** | **C** | **A** | **A** | **T** | **G** | **A** | **A** | **C** | **A** | **G** | **C** | **A** | **C** | **G** | **G** | **C** | **T** | **G** | **T** | **T** | **T** | **G** | **G** | **T** | **T** | **T** | **A** | **T** | **G** | **T** | **T** | **G** | **A** | **T** | **T** | **T** | **T** | **T** | **G** | **G** | **C** | **G** | **T** | **A** | **C** | **T** | **C** | **T** | **T** | **T** | **A** | **T** | **C** | **G** | **G** | **C** | **T** | **T** | **A** | **A** | **A** | **A** | **G** | **G** | **C** | **T** | **T** | **T** | **G** | **A** | **G** | **A** | **T** | **T** | **T** | **C** | **A** | **G** | **G** | **C** | **G** | **T** | **G** | **G** | **A** | **T** | **A** | **T** | **A** | **G** | **C** | **T** | **G** | **A** | **G** | **C** | **G** | **A** | **G** | **C** | **A** | **C** | **C** | **T** | **T** | **G** | **G** | **C** | **G** | **A** | **A** | **A** | **A** | **A** | **A** | **A** | **C** | **A** | **A** | **A** | **T** | **G** | **A** | **T** | **T** | **A** | **T** | **G** | **G** | **T** | **G** | **G** | **A** | **G** | **A** | **C** | **G** | **G** | **C** | **G** | **G** | **C** | **A** | **A** | **A** | **A** | **G** | **A** | **A** | **C** | **C** | **T** | **A** | **A** | **A** | **A** | **A** | **T** | **G** | **G** | **C** | **A** | **A** | **A** | **C** | **G** | **A** | **T** | **T** | **T** | **A** | **A** | **A** | **C** | **G** | **C** | **C** | **G** | **C** | **T** | **G** | **G** | **T** | **T** | **T** | **G** | **C** | **C** | **A** | **A** | **C** | **G** | **A** | **A** | **C** | **G** | **C** | **A** | **T** | **T** | **G** | **G** | **T** | **A** | **A** | **C** | **A** | **A** | **T** | **A** | **T** | **C** | **T** | **A** | **T** | **G** | **A** | **A** | **T** | **G** | **G** | **G** | **A** | **C** | **G** | **A** | **T** | **A** | **T** | **C** | **A** | **A** | **T** | **A** | **T** | **T** | **G** | **A** | **T** | **T** | **T** | **A** | **A** | **T** | **C** | **T** | **C** | **T** | **G** | **G** | **G** | **G** | **A** | **C** | **A** | **C** | **C** | **A** | **T** | **T** | **A** | **T** | **T** | **G** | **A** | **T** | **T** | **T** | **A** | **G** | **G** | **T** | **A** | **A** | **T** | **A** | **C** | **G** | **T** | **T** | **A** | **C** | **T** | **A** | **C** | **C** | **G** | **A** | **A** | **A** | **G** | **A** | **A** | **G** | **A** | **C** | **A** | **A** | **T** | **A** | **T** | **T** | **A** | **T** | **T** | **A** | **T** | **T** | **A** | **T** | **T** | **C** | **G** | **T** | **A** | **A** | **A** | **G** | **G** | **T** | **T** | **T** | **T** | **G** | **G** | **C** | **C** | **G** | **C** | **A** | **C** | **G** | **C** | **G** | **A** | **A** | **T** | **T** | **C** | **T** | **A** | **G** | **T** | **G** | **C** | **C** | **G** | **T** | **T** | **A** | **G** | **G** | **T** | **G** | **T** | **A** | **G** | **C** | **T** | **A** | **T** | **T** | **T** | **T** | **G** | **T** | **T** | **A** | **G** | **A** | **A** | **C** | **A** | **T** | **T** | **C** | **A** | **A** | **C** | **T** | **T** | **T** | **T** | **T** | **A** | **C** | **G** | **G** | **A** | **A** | **C** | **G** | **G** | **T** | **A** | **C** | **G** | **T** | **T** | **T** | **T** | **G** | **A** | **A** | **G** | **A** | **A** | **G** | **A** | **A** | **A** | **A** | **A** | **T** | **A** | **T** | **C** | **A** | **A** | **T** | **T** | **G** | **A** | **A** | **A** | **A** | **A** | **C** | **G** | **A** | **A** | **T** | **C** | **A** | **T** | **T** | **A** | **A** | **A** | **A** | **A** | **T** | **T** | **T** | **A** | **C** | **A** | **G** | **C** | **A** | **A** | **T** | **G** | **A** | **T** | **T** | **A** | **T** | **G** | **A** | **T** | **A** | **C** | **C** | **A** | **A** | **T** | **C** | **T** | **T** | **C** | **G** | **T** | **C** | **G** | **T** | **T** | **T** | **G** | **A** | **A** | **A** | **A** | **T** | **T** | **A** | **T** | **G** | **A** | **C** | **G** | **A** | **A** | **C** | **A** | **C** | **T** | **T** | **T** | **A** | **G** | **T** | **A** | **G** | **G** | **A** | **G** | **A** | **T** | **G** | **T** | **T** | **G** | **A** | **G** | **G** | **T** | **G** | **A** | **T** | **C** | **C** | **G** | **T** | **G** | **T** | **A** | **-** | **-** | **-** |
 **SNP position** | . | . | . | . | . | . | . | . | . | . | . | . | . | . | . | . | . | . | . | . | . | . | . | . | . | . | . | . | . | . | . | . | . | . | . | . | . | . | . | . | . | . | . | . | . | . | . | . | . | . | . | . | . | 54 | . | . | . | . | . | . | . | . | . | . | . | . | . | . | . | . | . | . | . | . | . | . | . | . | . | . | . | . | . | . | . | . | . | . | . | . | . | . | . | . | . | . | . | . | . | . | . | . | . | . | . | . | . | . | . | . | . | . | . | . | . | . | . | . | . | 120 | . | . | . | . | . | 126 | . | . | . | . | . | . | . | . | . | . | . | . | . | . | . | . | 143 | 144 | . | . | . | . | . | . | . | . | . | . | . | . | . | . | . | . | . | . | . | . | . | . | . | . | . | . | . | 172 | . | . | . | . | 177 | . | . | . | 181 | . | . | . | . | . | . | . | . | . | . | . | . | . | . | . | . | . | . | . | 201 | . | . | . | . | . | . | . | . | 210 | . | . | 213 | . | . | 216 | . | . | 219 | . | . | . | . | . | . | . | . | . | . | . | . | 232 | . | . | . | . | . | . | . | . | . | . | 243 | 244 | . | . | . | . | . | . | . | . | . | . | . | . | . | . | . | . | . | . | . | . | . | . | 267 | . | . | . | . | . | . | . | . | 276 | . | . | . | . | . | . | . | . | . | . | . | . | . | . | . | . | . | . | . | . | 297 | . | . | . | . | . | . | . | . | . | . | . | . | . | . | 312 | . | . | . | . | . | . | . | . | . | . | . | . | . | . | . | . | . | . | . | . | 333 | . | . | 336 | . | . | . | . | . | . | . | . | 345 | . | . | . | . | . | . | . | . | 354 | . | . | . | . | . | . | . | . | . | . | . | . | . | . | 369 | . | . | . | . | . | . | . | . | . | . | . | 381 | . | . | . | . | . | . | . | . | . | . | . | 393 | . | . | . | . | . | 399 | . | . | . | . | . | 405 | . | . | . | . | . | . | . | . | . | . | . | . | . | . | . | . | . | . | . | . | 426 | . | . | 429 | . | . | . | . | . | . | . | . | . | . | . | . | . | . | . | . | . | 447 | . | . | . | . | . | . | . | . | 456 | . | . | 459 | . | . | . | . | . | . | . | . | . | . | . | . | . | . | 474 | . | . | . | . | . | . | 481 | . | . | . | . | 486 | . | . | . | . | . | . | . | . | . | . | . | 498 | . | . | . | . | . | . | . | . | . | . | . | . | . | . | . | . | . | . | . | . | . | . | . | . | . | . | . | . | . | 528 | . | . | . | . | . | . | . | . | . | . | . | 540 | . | . | . | . | . | 546 | . | . | . | . | . | . | . | . | . | . | . | . | . | . | 561 | . | . | . | 565 | . | . | . | . | . | . | . | . | . | . | . | . | . | . | . | . | 582 | . | . | . | . | . | . | . | . | . | . | 593 | 594 | . | . | 597 | . | . | . | . | . | . | . | . | . | . | . | . | . | . | . | . | . | . | . | . | . | . | . | . | . | . | . | . | . | . | . | . | . | . | . | . | . | . | . | . | . | . | . | . | . | . | . | . | . | . | . | . | . | . | . | . | . | . | . | . | . | . | 660 | . | . | . | . | . | . | . | . | . | . | . | . | . | . | . | . | . | . | . | . | . | . | . | . | . | . | . | . | . | . | . | . | . | . | . | 696 | . | . | . | . | . | . | . | . | . | . | . | . | . | . | . | . | . | . | . | . | . | . | . | . | . | . | . | . | . | . | . | . | . | . | . | . |

|  |
| --- |
|  |

|  |  |
| --- | --- |
|  |

|  |  |
| --- | --- |
| **Legend** | |
  | **Reference Sequence** |

  |
|  | **Detected SNPs** |
|  |  |
