# Supplementary material for: sraX: A Novel Comprehensive Resistome Analysis Tool
Source: Front Microbiol. 2020 Feb 5;11:52. doi: 10.3389/fmicb.2020.00052 (PMC7025521; doi:10.3389/fmicb.2020.00052)
Supplement: Supplementary file 4 [file Data_Sheet_3.ZIP › liaF/liaF_Efaecium_dna.html]

# liaF

|  |
| --- |
|  |

|  |
| --- |
|  |

|  |  |
| --- | --- |
| **Definition:** | LiaF is an accessory protein that acts as a negative regulator of liaRS signal transduction pathway. Mutations confer daptomycin resistance. |

|  |
| --- |
|  |

|  |
| --- |
|  |

|  |  |  |
| --- | --- | --- |
| **Reference organism:** | *Enterococcus faecium* | [NCBI, CARD] |

|  |
| --- |
|  |

|  |
| --- |
|  |

|  |  |
| --- | --- |
| **Summary Data** | || **Parameter** | **Value_(n)_** || Total number of genomes | 101 |
| MSA length | 732 |
| Total number of SNPs | 63 |
| SNP density (# SNPs/MSA length) | 0.086 |

|  |
| --- |
|  |

|  |
| --- |
|  |

|  |
| --- |
|  |

|  |  |  |  |  |  |  |  |  |  |  |  |  |  |  |  |  |  |  |  |  |  |  |  |  |  |  |  |  |  |  |  |  |  |  |  |  |  |  |  |  |  |  |  |  |  |  |  |  |  |  |  |  |  |  |  |  |  |  |  |  |  |  |  |  |  |  |  |  |  |  |  |  |  |  |  |  |  |  |  |  |  |  |  |  |  |  |  |  |  |  |  |  |  |  |  |  |  |  |  |  |  |  |  |  |  |  |  |  |  |  |  |  |  |  |  |  |  |  |  |  |  |  |  |  |  |  |  |  |  |  |  |  |  |  |  |  |  |  |  |  |  |  |  |  |  |  |  |  |  |  |  |  |  |  |  |  |  |  |  |  |  |  |  |  |  |  |  |  |  |  |  |  |  |  |  |  |  |  |  |  |  |  |  |  |  |  |  |  |  |  |  |  |  |  |  |  |  |  |  |  |  |  |  |  |  |  |  |  |  |  |  |  |  |  |  |  |  |  |  |  |  |  |  |  |  |  |  |  |  |  |  |  |  |  |  |  |  |  |  |  |  |  |  |  |  |  |  |  |  |  |  |  |  |  |  |  |  |  |  |  |  |  |  |  |  |  |  |  |  |  |  |  |  |  |  |  |  |  |  |  |  |  |  |  |  |  |  |  |  |  |  |  |  |  |  |  |  |  |  |  |  |  |  |  |  |  |  |  |  |  |  |  |  |  |  |  |  |  |  |  |  |  |  |  |  |  |  |  |  |  |  |  |  |  |  |  |  |  |  |  |  |  |  |  |  |  |  |  |  |  |  |  |  |  |  |  |  |  |  |  |  |  |  |  |  |  |  |  |  |  |  |  |  |  |  |  |  |  |  |  |  |  |  |  |  |  |  |  |  |  |  |  |  |  |  |  |  |  |  |  |  |  |  |  |  |  |  |  |  |  |  |  |  |  |  |  |  |  |  |  |  |  |  |  |  |  |  |  |  |  |  |  |  |  |  |  |  |  |  |  |  |  |  |  |  |  |  |  |  |  |  |  |  |  |  |  |  |  |  |  |  |  |  |  |  |  |  |  |  |  |  |  |  |  |  |  |  |  |  |  |  |  |  |  |  |  |  |  |  |  |  |  |  |  |  |  |  |  |  |  |  |  |  |  |  |  |  |  |  |  |  |  |  |  |  |  |  |  |  |  |  |  |  |  |  |  |  |  |  |  |  |  |  |  |  |  |  |  |  |  |  |  |  |  |  |  |  |  |  |  |  |  |  |  |  |  |  |  |  |  |  |  |  |  |  |  |  |  |  |  |  |  |  |  |  |  |  |  |  |  |  |  |  |  |  |  |  |  |  |  |  |  |  |  |  |  |  |  |  |  |  |  |  |  |  |  |  |  |  |  |  |  |  |  |  |  |  |  |  |  |  |  |  |  |  |  |  |  |  |  |  |  |  |  |  |  |  |  |  |  |  |  |  |  |  |  |  |  |  |  |  |  |  |  |  |  |  |  |  |  |  |  |  |  |  |  |  |  |  |  |  |  |  |  |  |  |  |  |  |  |  |  |  |  |  |  |  |  |  |  |  |  |  |  |  |  |  |  |  |  |  |  |  |  |  |  |  |  |  |  |  |  |  |  |  |  |  |  |  |  |  |  |  |  |  |  |  |  |  |  |  |  |  |
| --- | --- | --- | --- | --- | --- | --- | --- | --- | --- | --- | --- | --- | --- | --- | --- | --- | --- | --- | --- | --- | --- | --- | --- | --- | --- | --- | --- | --- | --- | --- | --- | --- | --- | --- | --- | --- | --- | --- | --- | --- | --- | --- | --- | --- | --- | --- | --- | --- | --- | --- | --- | --- | --- | --- | --- | --- | --- | --- | --- | --- | --- | --- | --- | --- | --- | --- | --- | --- | --- | --- | --- | --- | --- | --- | --- | --- | --- | --- | --- | --- | --- | --- | --- | --- | --- | --- | --- | --- | --- | --- | --- | --- | --- | --- | --- | --- | --- | --- | --- | --- | --- | --- | --- | --- | --- | --- | --- | --- | --- | --- | --- | --- | --- | --- | --- | --- | --- | --- | --- | --- | --- | --- | --- | --- | --- | --- | --- | --- | --- | --- | --- | --- | --- | --- | --- | --- | --- | --- | --- | --- | --- | --- | --- | --- | --- | --- | --- | --- | --- | --- | --- | --- | --- | --- | --- | --- | --- | --- | --- | --- | --- | --- | --- | --- | --- | --- | --- | --- | --- | --- | --- | --- | --- | --- | --- | --- | --- | --- | --- | --- | --- | --- | --- | --- | --- | --- | --- | --- | --- | --- | --- | --- | --- | --- | --- | --- | --- | --- | --- | --- | --- | --- | --- | --- | --- | --- | --- | --- | --- | --- | --- | --- | --- | --- | --- | --- | --- | --- | --- | --- | --- | --- | --- | --- | --- | --- | --- | --- | --- | --- | --- | --- | --- | --- | --- | --- | --- | --- | --- | --- | --- | --- | --- | --- | --- | --- | --- | --- | --- | --- | --- | --- | --- | --- | --- | --- | --- | --- | --- | --- | --- | --- | --- | --- | --- | --- | --- | --- | --- | --- | --- | --- | --- | --- | --- | --- | --- | --- | --- | --- | --- | --- | --- | --- | --- | --- | --- | --- | --- | --- | --- | --- | --- | --- | --- | --- | --- | --- | --- | --- | --- | --- | --- | --- | --- | --- | --- | --- | --- | --- | --- | --- | --- | --- | --- | --- | --- | --- | --- | --- | --- | --- | --- | --- | --- | --- | --- | --- | --- | --- | --- | --- | --- | --- | --- | --- | --- | --- | --- | --- | --- | --- | --- | --- | --- | --- | --- | --- | --- | --- | --- | --- | --- | --- | --- | --- | --- | --- | --- | --- | --- | --- | --- | --- | --- | --- | --- | --- | --- | --- | --- | --- | --- | --- | --- | --- | --- | --- | --- | --- | --- | --- | --- | --- | --- | --- | --- | --- | --- | --- | --- | --- | --- | --- | --- | --- | --- | --- | --- | --- | --- | --- | --- | --- | --- | --- | --- | --- | --- | --- | --- | --- | --- | --- | --- | --- | --- | --- | --- | --- | --- | --- | --- | --- | --- | --- | --- | --- | --- | --- | --- | --- | --- | --- | --- | --- | --- | --- | --- | --- | --- | --- | --- | --- | --- | --- | --- | --- | --- | --- | --- | --- | --- | --- | --- | --- | --- | --- | --- | --- | --- | --- | --- | --- | --- | --- | --- | --- | --- | --- | --- | --- | --- | --- | --- | --- | --- | --- | --- | --- | --- | --- | --- | --- | --- | --- | --- | --- | --- | --- | --- | --- | --- | --- | --- | --- | --- | --- | --- | --- | --- | --- | --- | --- | --- | --- | --- | --- | --- | --- | --- | --- | --- | --- | --- | --- | --- | --- | --- | --- | --- | --- | --- | --- | --- | --- | --- | --- | --- | --- | --- | --- | --- | --- | --- | --- | --- | --- | --- | --- | --- | --- | --- | --- | --- | --- | --- | --- | --- | --- | --- | --- | --- | --- | --- | --- | --- | --- | --- | --- | --- | --- | --- | --- | --- | --- | --- | --- | --- | --- | --- | --- | --- | --- | --- | --- | --- | --- | --- | --- | --- | --- | --- | --- | --- | --- | --- | --- | --- | --- | --- | --- | --- | --- | --- | --- | --- | --- | --- | --- | --- | --- | --- | --- | --- | --- | --- | --- | --- | --- | --- | --- | --- | --- | --- | --- | --- | --- | --- | --- | --- | --- | --- | --- | --- | --- | --- | --- | --- | --- | --- | --- | --- | --- | --- | --- | --- | --- | --- | --- | --- | --- | --- | --- | --- | --- | --- | --- | --- | --- | --- | --- | --- | --- | --- | --- | --- | --- | --- | --- | --- | --- | --- | --- | --- | --- | --- | --- | --- | --- | --- | --- | --- | --- | --- | --- | --- | --- | --- | --- | --- | --- | --- | --- | --- | --- | --- | --- | --- | --- | --- | --- | --- | --- | --- | --- | --- | --- | --- | --- | --- | --- | --- | --- | --- | --- | --- | --- | --- | --- | --- | --- | --- | --- | --- | --- | --- | --- | --- | --- | --- | --- | --- | --- | --- | --- | --- | --- | --- | --- | --- | --- | --- |
|  || **Enterococcus faecium (CUX99269.1)** | **A** | **T** | **G** | **A** | **A** | **T** | **A** | **G** | **T** | **T** | **C** | **T** | **T** | **G** | **G** | **C** | **G** | **T** | **T** | **T** | **T** | **T** | **T** | **T** | **G** | **T** | **G** | **G** | **T** | **A** | **G** | **T** | **C** | **G** | **A** | **A** | **G** | **C** | **A** | **C** | **T** | **G** | **C** | **T** | **G** | **C** | **T** | **G** | **T** | **T** | **A** | **T** | **T** | **T** | **G** | **C** | **T** | **G** | **T** | **T** | **T** | **G** | **G** | **C** | **A** | **A** | **A** | **T** | **C** | **G** | **T** | **A** | **A** | **A** | **C** | **A** | **A** | **T** | **A** | **C** | **T** | **G** | **G** | **A** | **C** | **T** | **T** | **T** | **T** | **G** | **C** | **T** | **T** | **T** | **T** | **A** | **G** | **T** | **A** | **A** | **T** | **A** | **T** | **T** | **C** | **G** | **G** | **T** | **A** | **T** | **T** | **T** | **T** | **C** | **A** | **A** | **T** | **A** | **T** | **A** | **T** | **A** | **T** | **C** | **T** | **A** | **G** | **C** | **T** | **A** | **T** | **G** | **C** | **G** | **C** | **A** | **A** | **A** | **T** | **A** | **T** | **C** | **C** | **T** | **C** | **G** | **T** | **A** | **C** | **T** | **A** | **A** | **A** | **T** | **T** | **T** | **C** | **A** | **A** | **A** | **A** | **T** | **T** | **T** | **T** | **C** | **A** | **A** | **T** | **T** | **G** | **A** | **T** | **C** | **T** | **T** | **A** | **G** | **G** | **G** | **A** | **G** | **C** | **T** | **T** | **G** | **G** | **T** | **C** | **A** | **T** | **T** | **T** | **T** | **T** | **T** | **T** | **T** | **A** | **G** | **C** | **T** | **T** | **A** | **G** | **T** | **C** | **A** | **A** | **C** | **A** | **G** | **T** | **C** | **C** | **T** | **G** | **C** | **T** | **T** | **T** | **A** | **T** | **G** | **G** | **A** | **T** | **G** | **A** | **T** | **G** | **G** | **T** | **T** | **G** | **T** | **A** | **T** | **T** | **G** | **G** | **C** | **C** | **G** | **T** | **T** | **T** | **T** | **A** | **T** | **T** | **T** | **A** | **T** | **C** | **G** | **G** | **C** | **C** | **T** | **G** | **A** | **A** | **A** | **G** | **G** | **T** | **G** | **T** | **A** | **G** | **A** | **G** | **A** | **T** | **T** | **T** | **C** | **A** | **G** | **G** | **A** | **A** | **T** | **A** | **G** | **A** | **T** | **T** | **T** | **G** | **A** | **C** | **A** | **A** | **A** | **A** | **A** | **A** | **T** | **G** | **C** | **T** | **T** | **T** | **T** | **T** | **G** | **G** | **C** | **G** | **A** | **A** | **A** | **A** | **A** | **A** | **A** | **C** | **A** | **G** | **A** | **T** | **C** | **A** | **T** | **G** | **A** | **T** | **G** | **G** | **T** | **C** | **C** | **A** | **A** | **A** | **C** | **A** | **G** | **A** | **G** | **C** | **A** | **A** | **T** | **T** | **A** | **A** | **A** | **A** | **A** | **C** | **A** | **C** | **A** | **T** | **A** | **A** | **T** | **A** | **A** | **T** | **G** | **A** | **G** | **C** | **G** | **T** | **A** | **A** | **A** | **A** | **G** | **A** | **C** | **A** | **G** | **C** | **A** | **A** | **T** | **T** | **A** | **T** | **T** | **T** | **G** | **G** | **T** | **A** | **A** | **C** | **C** | **A** | **A** | **C** | **G** | **T** | **A** | **T** | **T** | **G** | **G** | **A** | **A** | **A** | **C** | **G** | **A** | **C** | **G** | **T** | **C** | **T** | **A** | **T** | **G** | **A** | **A** | **T** | **G** | **G** | **G** | **A** | **T** | **G** | **A** | **T** | **A** | **T** | **C** | **A** | **A** | **C** | **A** | **T** | **T** | **G** | **C** | **C** | **A** | **T** | **C** | **A** | **T** | **T** | **T** | **C** | **T** | **G** | **G** | **T** | **G** | **A** | **T** | **A** | **C** | **T** | **A** | **T** | **C** | **A** | **T** | **C** | **G** | **A** | **T** | **T** | **T** | **G** | **G** | **G** | **C** | **A** | **A** | **T** | **A** | **C** | **A** | **C** | **T** | **T** | **T** | **T** | **G** | **C** | **C** | **A** | **A** | **A** | **A** | **G** | **A** | **T** | **G** | **A** | **T** | **A** | **A** | **T** | **A** | **T** | **T** | **G** | **T** | **G** | **A** | **T** | **T** | **G** | **T** | **A** | **A** | **G** | **A** | **A** | **A** | **A** | **G** | **G** | **G** | **A** | **T** | **A** | **G** | **G** | **C** | **A** | **G** | **A** | **A** | **C** | **A** | **C** | **G** | **G** | **A** | **T** | **C** | **T** | **T** | **A** | **G** | **T** | **A** | **C** | **C** | **C** | **C** | **T** | **G** | **G** | **G** | **A** | **G** | **T** | **G** | **G** | **C** | **T** | **A** | **T** | **T** | **C** | **G** | **A** | **T** | **T** | **A** | **G** | **A** | **G** | **C** | **A** | **T** | **G** | **C** | **G** | **A** | **C** | **A** | **T** | **T** | **A** | **G** | **T** | **A** | **G** | **G** | **A** | **A** | **A** | **T** | **G** | **T** | **C** | **T** | **T** | **G** | **T** | **T** | **T** | **G** | **A** | **A** | **G** | **A** | **A** | **G** | **A** | **G** | **C** | **A** | **G** | **T** | **T** | **T** | **T** | **C** | **C** | **T** | **T** | **A** | **A** | **A** | **A** | **A** | **A** | **T** | **G** | **A** | **A** | **C** | **A** | **G** | **A** | **T** | **C** | **A** | **A** | **A** | **A** | **T** | **C** | **T** | **G** | **C** | **A** | **G** | **C** | **A** | **G** | **T** | **G** | **A** | **T** | **T** | **A** | **T** | **G** | **A** | **T** | **G** | **A** | **A** | **A** | **A** | **T** | **C** | **C** | **T** | **C** | **G** | **C** | **A** | **G** | **A** | **T** | **T** | **G** | **A** | **A** | **G** | **A** | **T** | **C** | **A** | **T** | **T** | **A** | **C** | **C** | **A** | **A** | **T** | **A** | **C** | **C** | **T** | **T** | **G** | **G** | **T** | **T** | **G** | **G** | **A** | **G** | **A** | **T** | **G** | **T** | **C** | **G** | **A** | **G** | **G** | **T** | **G** | **A** | **T** | **A** | **C** | **G** | **A** | **A** | **T** | **A** | **T** | **G** | **A** |
| **N59711F** | **A** | **T** | **G** | **A** | **A** | **T** | **A** | **G** | **T** | **T** | **C** | **T** | **T** | **G** | **G** | **C** | **G** | **T** | **T** | **T** | **T** | **T** | **T** | **T** | **G** | **T** | **G** | **G** | **T** | **A** | **G** | **T** | **T** | **G** | **A** | **A** | **G** | **C** | **A** | **C** | **T** | **A** | **C** | **T** | **G** | **C** | **T** | **G** | **T** | **T** | **A** | **T** | **T** | **T** | **G** | **C** | **T** | **G** | **T** | **T** | **T** | **G** | **G** | **C** | **A** | **A** | **A** | **T** | **C** | **G** | **T** | **A** | **A** | **A** | **C** | **A** | **A** | **T** | **A** | **C** | **T** | **G** | **G** | **A** | **C** | **T** | **T** | **T** | **T** | **G** | **C** | **T** | **T** | **T** | **T** | **A** | **G** | **T** | **G** | **A** | **T** | **A** | **T** | **T** | **C** | **G** | **G** | **T** | **A** | **T** | **T** | **T** | **T** | **C** | **A** | **A** | **T** | **A** | **T** | **A** | **T** | **A** | **T** | **C** | **T** | **A** | **G** | **C** | **T** | **A** | **T** | **G** | **C** | **G** | **C** | **A** | **A** | **A** | **C** | **A** | **T** | **C** | **C** | **T** | **C** | **G** | **T** | **A** | **C** | **T** | **A** | **A** | **A** | **T** | **T** | **T** | **C** | **A** | **A** | **A** | **A** | **T** | **T** | **T** | **T** | **C** | **A** | **A** | **T** | **T** | **G** | **A** | **T** | **A** | **T** | **T** | **A** | **G** | **G** | **A** | **A** | **G** | **T** | **T** | **T** | **G** | **G** | **T** | **C** | **A** | **T** | **T** | **T** | **T** | **T** | **T** | **T** | **T** | **A** | **G** | **C** | **T** | **T** | **A** | **G** | **T** | **C** | **A** | **A** | **C** | **A** | **G** | **T** | **C** | **C** | **T** | **G** | **C** | **T** | **T** | **T** | **A** | **T** | **G** | **G** | **A** | **T** | **G** | **A** | **T** | **G** | **G** | **C** | **T** | **G** | **T** | **A** | **T** | **T** | **T** | **G** | **C** | **C** | **G** | **T** | **T** | **T** | **T** | **A** | **T** | **T** | **T** | **A** | **T** | **T** | **G** | **G** | **T** | **C** | **T** | **G** | **A** | **A** | **A** | **G** | **G** | **T** | **G** | **T** | **A** | **G** | **A** | **G** | **A** | **T** | **T** | **T** | **C** | **A** | **G** | **G** | **G** | **A** | **T** | **A** | **G** | **A** | **T** | **T** | **T** | **A** | **A** | **C** | **C** | **A** | **A** | **A** | **A** | **A** | **T** | **G** | **C** | **T** | **T** | **T** | **T** | **T** | **G** | **G** | **C** | **G** | **A** | **A** | **A** | **G** | **A** | **A** | **A** | **C** | **A** | **G** | **A** | **T** | **C** | **A** | **T** | **G** | **A** | **T** | **G** | **A** | **T** | **C** | **C** | **A** | **A** | **A** | **C** | **A** | **G** | **A** | **G** | **C** | **A** | **A** | **T** | **T** | **A** | **A** | **A** | **A** | **A** | **C** | **A** | **C** | **A** | **T** | **A** | **A** | **T** | **A** | **A** | **T** | **G** | **A** | **G** | **C** | **G** | **G** | **A** | **A** | **A** | **A** | **G** | **A** | **C** | **A** | **G** | **C** | **A** | **A** | **T** | **T** | **A** | **T** | **T** | **C** | **G** | **G** | **T** | **A** | **A** | **C** | **C** | **A** | **A** | **C** | **G** | **T** | **A** | **T** | **T** | **G** | **G** | **A** | **A** | **A** | **C** | **G** | **A** | **C** | **G** | **T** | **C** | **T** | **A** | **T** | **G** | **A** | **A** | **T** | **G** | **G** | **G** | **A** | **C** | **G** | **A** | **T** | **A** | **T** | **C** | **A** | **A** | **C** | **A** | **T** | **C** | **G** | **C** | **C** | **G** | **T** | **C** | **A** | **T** | **T** | **T** | **C** | **T** | **G** | **G** | **T** | **G** | **A** | **T** | **A** | **C** | **T** | **A** | **T** | **C** | **A** | **T** | **A** | **G** | **A** | **T** | **T** | **T** | **G** | **G** | **G** | **C** | **A** | **A** | **T** | **A** | **C** | **G** | **C** | **T** | **T** | **T** | **T** | **G** | **C** | **C** | **A** | **A** | **A** | **A** | **G** | **A** | **T** | **G** | **A** | **T** | **A** | **A** | **T** | **A** | **T** | **T** | **G** | **T** | **G** | **A** | **T** | **T** | **G** | **T** | **A** | **A** | **G** | **A** | **A** | **A** | **A** | **G** | **G** | **G** | **A** | **T** | **A** | **G** | **G** | **C** | **A** | **G** | **A** | **A** | **C** | **A** | **C** | **G** | **G** | **A** | **T** | **C** | **T** | **T** | **A** | **G** | **T** | **A** | **C** | **C** | **T** | **T** | **T** | **G** | **G** | **G** | **C** | **G** | **T** | **A** | **G** | **C** | **C** | **A** | **T** | **C** | **C** | **G** | **A** | **T** | **T** | **A** | **G** | **A** | **G** | **C** | **A** | **T** | **G** | **C** | **G** | **A** | **C** | **A** | **T** | **T** | **A** | **G** | **T** | **A** | **G** | **G** | **A** | **A** | **A** | **T** | **G** | **T** | **C** | **T** | **T** | **G** | **T** | **T** | **T** | **G** | **A** | **A** | **G** | **A** | **A** | **G** | **A** | **G** | **C** | **A** | **A** | **T** | **T** | **T** | **T** | **C** | **T** | **T** | **T** | **G** | **A** | **A** | **A** | **A** | **A** | **T** | **G** | **A** | **A** | **C** | **A** | **A** | **A** | **T** | **C** | **A** | **A** | **A** | **A** | **T** | **C** | **T** | **A** | **C** | **A** | **G** | **C** | **A** | **G** | **T** | **G** | **A** | **T** | **T** | **A** | **T** | **G** | **A** | **T** | **G** | **A** | **A** | **A** | **A** | **C** | **C** | **C** | **T** | **C** | **G** | **T** | **A** | **G** | **A** | **T** | **T** | **G** | **A** | **A** | **A** | **A** | **T** | **T** | **A** | **T** | **T** | **A** | **C** | **T** | **A** | **A** | **T** | **A** | **C** | **C** | **T** | **T** | **G** | **A** | **T** | **T** | **G** | **G** | **A** | **G** | **A** | **T** | **G** | **T** | **C** | **G** | **A** | **G** | **G** | **T** | **G** | **A** | **T** | **A** | **C** | **G** | **A** | **A** | **T** | **A** | **-** | **-** | **-** |
| **N55229** | **A** | **T** | **G** | **A** | **A** | **T** | **A** | **G** | **T** | **T** | **C** | **T** | **T** | **G** | **G** | **C** | **G** | **T** | **T** | **T** | **T** | **T** | **T** | **T** | **G** | **T** | **G** | **G** | **T** | **A** | **G** | **T** | **C** | **G** | **A** | **A** | **G** | **C** | **A** | **C** | **T** | **G** | **C** | **T** | **G** | **C** | **T** | **G** | **T** | **T** | **A** | **T** | **T** | **T** | **G** | **C** | **T** | **G** | **T** | **T** | **T** | **G** | **G** | **C** | **A** | **A** | **A** | **T** | **C** | **G** | **T** | **A** | **A** | **A** | **C** | **A** | **A** | **T** | **A** | **C** | **T** | **G** | **G** | **A** | **C** | **T** | **T** | **T** | **T** | **G** | **C** | **T** | **T** | **T** | **T** | **A** | **G** | **T** | **A** | **A** | **T** | **A** | **T** | **T** | **C** | **G** | **G** | **T** | **A** | **T** | **T** | **T** | **T** | **C** | **A** | **A** | **T** | **A** | **T** | **A** | **T** | **A** | **T** | **C** | **T** | **A** | **G** | **C** | **T** | **A** | **T** | **G** | **C** | **G** | **C** | **A** | **A** | **A** | **T** | **A** | **T** | **C** | **C** | **T** | **C** | **G** | **T** | **A** | **C** | **T** | **A** | **A** | **A** | **T** | **T** | **T** | **C** | **A** | **A** | **A** | **A** | **T** | **T** | **T** | **T** | **C** | **A** | **A** | **T** | **T** | **G** | **A** | **T** | **C** | **T** | **T** | **A** | **G** | **G** | **G** | **A** | **G** | **C** | **T** | **T** | **G** | **G** | **T** | **C** | **A** | **T** | **T** | **T** | **T** | **T** | **T** | **T** | **T** | **A** | **G** | **C** | **T** | **T** | **A** | **G** | **T** | **C** | **A** | **A** | **C** | **A** | **G** | **T** | **C** | **C** | **T** | **G** | **C** | **T** | **T** | **T** | **A** | **T** | **G** | **G** | **A** | **T** | **G** | **A** | **T** | **G** | **G** | **T** | **T** | **G** | **T** | **A** | **T** | **T** | **G** | **G** | **C** | **C** | **G** | **T** | **T** | **T** | **T** | **A** | **T** | **T** | **T** | **A** | **T** | **C** | **G** | **G** | **G** | **C** | **T** | **G** | **A** | **A** | **A** | **G** | **G** | **T** | **G** | **T** | **A** | **G** | **A** | **G** | **A** | **T** | **T** | **T** | **C** | **A** | **G** | **G** | **A** | **A** | **T** | **A** | **G** | **A** | **T** | **T** | **T** | **G** | **A** | **C** | **A** | **A** | **A** | **A** | **A** | **A** | **T** | **G** | **C** | **T** | **T** | **T** | **T** | **T** | **G** | **G** | **C** | **G** | **A** | **A** | **A** | **A** | **A** | **A** | **A** | **C** | **A** | **G** | **A** | **T** | **C** | **A** | **T** | **G** | **A** | **T** | **G** | **G** | **T** | **C** | **C** | **A** | **A** | **A** | **C** | **A** | **G** | **A** | **G** | **C** | **A** | **A** | **T** | **T** | **A** | **A** | **A** | **A** | **A** | **C** | **A** | **C** | **A** | **T** | **A** | **A** | **T** | **A** | **A** | **T** | **G** | **A** | **G** | **C** | **G** | **T** | **A** | **A** | **A** | **A** | **G** | **A** | **C** | **A** | **G** | **C** | **A** | **A** | **T** | **T** | **A** | **T** | **T** | **T** | **G** | **G** | **T** | **A** | **A** | **C** | **C** | **A** | **A** | **C** | **G** | **T** | **A** | **T** | **T** | **G** | **G** | **A** | **A** | **A** | **C** | **G** | **A** | **C** | **G** | **T** | **C** | **T** | **A** | **T** | **G** | **A** | **A** | **T** | **G** | **G** | **G** | **A** | **T** | **G** | **A** | **C** | **A** | **T** | **C** | **A** | **A** | **C** | **A** | **T** | **T** | **G** | **C** | **C** | **A** | **T** | **C** | **A** | **T** | **T** | **T** | **C** | **T** | **G** | **G** | **T** | **G** | **A** | **T** | **A** | **C** | **T** | **A** | **T** | **C** | **A** | **T** | **C** | **G** | **A** | **T** | **T** | **T** | **G** | **G** | **G** | **C** | **A** | **A** | **T** | **A** | **C** | **A** | **C** | **T** | **T** | **T** | **T** | **G** | **C** | **C** | **A** | **A** | **A** | **A** | **G** | **A** | **T** | **G** | **A** | **T** | **A** | **A** | **T** | **A** | **T** | **T** | **G** | **T** | **G** | **A** | **T** | **T** | **G** | **T** | **A** | **A** | **G** | **A** | **A** | **A** | **A** | **G** | **G** | **G** | **A** | **T** | **A** | **G** | **G** | **C** | **A** | **G** | **A** | **A** | **C** | **A** | **C** | **G** | **G** | **A** | **T** | **C** | **T** | **T** | **A** | **G** | **T** | **A** | **C** | **C** | **C** | **C** | **T** | **G** | **G** | **G** | **A** | **G** | **T** | **G** | **G** | **C** | **T** | **A** | **T** | **T** | **C** | **G** | **T** | **T** | **T** | **A** | **G** | **A** | **G** | **C** | **A** | **T** | **G** | **C** | **G** | **A** | **C** | **A** | **T** | **T** | **A** | **G** | **T** | **A** | **G** | **G** | **A** | **A** | **A** | **T** | **G** | **T** | **C** | **T** | **T** | **G** | **T** | **T** | **T** | **G** | **A** | **A** | **G** | **A** | **A** | **G** | **A** | **G** | **C** | **A** | **G** | **T** | **T** | **T** | **T** | **C** | **C** | **T** | **T** | **A** | **A** | **A** | **A** | **A** | **A** | **C** | **G** | **A** | **A** | **C** | **A** | **G** | **A** | **T** | **C** | **A** | **A** | **A** | **A** | **T** | **C** | **T** | **G** | **C** | **A** | **G** | **C** | **A** | **G** | **T** | **G** | **A** | **T** | **T** | **A** | **T** | **G** | **A** | **T** | **G** | **A** | **A** | **A** | **A** | **T** | **C** | **C** | **T** | **C** | **G** | **C** | **A** | **G** | **A** | **T** | **T** | **G** | **A** | **A** | **G** | **A** | **T** | **C** | **A** | **T** | **T** | **A** | **C** | **C** | **A** | **A** | **T** | **A** | **C** | **C** | **T** | **T** | **G** | **G** | **T** | **T** | **G** | **G** | **A** | **G** | **A** | **T** | **G** | **T** | **C** | **G** | **A** | **G** | **G** | **T** | **G** | **A** | **T** | **A** | **C** | **G** | **A** | **A** | **T** | **A** | **-** | **-** | **-** |
| **N54551** | **A** | **T** | **G** | **A** | **A** | **T** | **A** | **G** | **T** | **T** | **C** | **T** | **T** | **G** | **G** | **C** | **G** | **T** | **T** | **T** | **T** | **T** | **T** | **T** | **G** | **T** | **G** | **G** | **T** | **A** | **G** | **T** | **C** | **G** | **A** | **A** | **G** | **C** | **A** | **C** | **T** | **G** | **C** | **T** | **G** | **C** | **T** | **G** | **T** | **T** | **A** | **T** | **T** | **T** | **G** | **C** | **T** | **G** | **T** | **T** | **T** | **G** | **G** | **C** | **A** | **A** | **A** | **T** | **C** | **G** | **T** | **A** | **A** | **A** | **C** | **A** | **A** | **T** | **A** | **C** | **T** | **G** | **G** | **A** | **C** | **T** | **T** | **T** | **T** | **G** | **C** | **T** | **T** | **T** | **T** | **A** | **G** | **T** | **A** | **A** | **T** | **A** | **T** | **T** | **C** | **G** | **G** | **T** | **A** | **T** | **T** | **T** | **T** | **C** | **A** | **A** | **T** | **A** | **T** | **A** | **T** | **A** | **T** | **C** | **T** | **A** | **G** | **C** | **T** | **A** | **T** | **G** | **C** | **G** | **C** | **A** | **A** | **A** | **T** | **A** | **T** | **C** | **C** | **T** | **C** | **G** | **T** | **A** | **C** | **T** | **A** | **A** | **A** | **T** | **T** | **T** | **C** | **A** | **A** | **A** | **A** | **T** | **T** | **T** | **T** | **C** | **A** | **A** | **T** | **T** | **G** | **A** | **T** | **C** | **T** | **T** | **A** | **G** | **G** | **G** | **A** | **G** | **C** | **T** | **T** | **G** | **G** | **T** | **C** | **A** | **T** | **T** | **T** | **T** | **T** | **T** | **T** | **T** | **A** | **G** | **C** | **T** | **T** | **A** | **G** | **T** | **C** | **A** | **A** | **C** | **A** | **G** | **T** | **C** | **C** | **T** | **G** | **C** | **T** | **T** | **T** | **A** | **T** | **G** | **G** | **A** | **T** | **G** | **A** | **T** | **G** | **G** | **T** | **T** | **G** | **T** | **A** | **T** | **T** | **G** | **G** | **C** | **C** | **A** | **T** | **T** | **T** | **T** | **A** | **T** | **T** | **T** | **A** | **T** | **C** | **G** | **G** | **A** | **C** | **T** | **G** | **A** | **A** | **A** | **G** | **G** | **T** | **G** | **T** | **A** | **G** | **A** | **G** | **A** | **T** | **T** | **T** | **C** | **A** | **G** | **G** | **A** | **A** | **T** | **A** | **G** | **A** | **T** | **T** | **T** | **G** | **A** | **C** | **A** | **A** | **A** | **A** | **A** | **A** | **T** | **G** | **C** | **T** | **T** | **T** | **T** | **T** | **G** | **G** | **C** | **G** | **A** | **A** | **A** | **A** | **A** | **A** | **A** | **C** | **A** | **G** | **A** | **T** | **T** | **A** | **T** | **G** | **A** | **T** | **G** | **G** | **T** | **C** | **C** | **A** | **A** | **A** | **C** | **A** | **G** | **A** | **G** | **C** | **A** | **A** | **T** | **T** | **A** | **A** | **A** | **A** | **A** | **C** | **A** | **C** | **A** | **T** | **A** | **A** | **T** | **A** | **A** | **T** | **G** | **A** | **G** | **C** | **G** | **T** | **A** | **A** | **A** | **A** | **G** | **A** | **C** | **A** | **G** | **C** | **A** | **A** | **T** | **T** | **A** | **T** | **T** | **T** | **G** | **G** | **T** | **A** | **A** | **C** | **C** | **A** | **A** | **C** | **G** | **T** | **A** | **T** | **T** | **G** | **G** | **A** | **A** | **A** | **C** | **G** | **A** | **C** | **G** | **T** | **C** | **T** | **A** | **T** | **G** | **A** | **A** | **T** | **G** | **G** | **G** | **A** | **T** | **G** | **A** | **T** | **A** | **T** | **C** | **A** | **A** | **C** | **A** | **T** | **T** | **G** | **C** | **C** | **A** | **T** | **T** | **A** | **T** | **T** | **T** | **C** | **T** | **G** | **G** | **T** | **G** | **A** | **T** | **A** | **C** | **T** | **A** | **T** | **C** | **A** | **T** | **C** | **G** | **A** | **T** | **T** | **T** | **G** | **G** | **G** | **C** | **A** | **A** | **T** | **A** | **C** | **A** | **C** | **T** | **T** | **T** | **T** | **G** | **C** | **C** | **A** | **A** | **A** | **A** | **G** | **A** | **T** | **G** | **A** | **T** | **A** | **A** | **T** | **A** | **T** | **T** | **G** | **T** | **G** | **A** | **T** | **T** | **G** | **T** | **A** | **A** | **G** | **A** | **A** | **A** | **A** | **G** | **G** | **G** | **A** | **T** | **A** | **G** | **G** | **C** | **A** | **G** | **A** | **A** | **C** | **A** | **C** | **G** | **G** | **A** | **T** | **C** | **T** | **T** | **A** | **G** | **T** | **A** | **C** | **C** | **C** | **C** | **T** | **G** | **G** | **G** | **A** | **G** | **T** | **G** | **G** | **C** | **T** | **A** | **T** | **T** | **C** | **G** | **A** | **T** | **T** | **A** | **G** | **A** | **G** | **C** | **A** | **T** | **G** | **C** | **G** | **A** | **C** | **A** | **T** | **T** | **A** | **G** | **T** | **A** | **G** | **G** | **A** | **A** | **A** | **T** | **G** | **T** | **C** | **T** | **T** | **G** | **T** | **T** | **T** | **G** | **A** | **A** | **G** | **A** | **A** | **G** | **A** | **G** | **C** | **A** | **G** | **T** | **T** | **T** | **T** | **C** | **C** | **T** | **T** | **A** | **A** | **A** | **A** | **A** | **A** | **C** | **G** | **A** | **A** | **C** | **A** | **G** | **A** | **T** | **C** | **A** | **A** | **A** | **A** | **T** | **C** | **T** | **G** | **C** | **A** | **G** | **C** | **A** | **G** | **T** | **G** | **A** | **T** | **T** | **A** | **T** | **G** | **A** | **T** | **G** | **A** | **A** | **A** | **A** | **T** | **C** | **C** | **T** | **C** | **G** | **C** | **A** | **G** | **A** | **T** | **T** | **G** | **A** | **A** | **G** | **A** | **T** | **C** | **A** | **T** | **T** | **A** | **C** | **C** | **A** | **A** | **T** | **A** | **C** | **C** | **T** | **T** | **G** | **G** | **T** | **T** | **G** | **G** | **A** | **G** | **A** | **T** | **G** | **T** | **C** | **G** | **A** | **G** | **G** | **T** | **G** | **A** | **T** | **A** | **C** | **G** | **A** | **A** | **T** | **A** | **-** | **-** | **-** |
| **N60252F** | **A** | **T** | **G** | **A** | **A** | **T** | **A** | **G** | **T** | **T** | **C** | **T** | **T** | **G** | **G** | **C** | **G** | **T** | **T** | **T** | **T** | **T** | **T** | **T** | **G** | **T** | **G** | **G** | **T** | **A** | **G** | **T** | **C** | **G** | **A** | **A** | **G** | **C** | **A** | **C** | **T** | **G** | **C** | **T** | **G** | **C** | **T** | **G** | **T** | **T** | **A** | **T** | **T** | **T** | **G** | **C** | **T** | **G** | **T** | **T** | **T** | **G** | **G** | **C** | **A** | **A** | **A** | **T** | **C** | **G** | **T** | **A** | **A** | **A** | **C** | **A** | **A** | **T** | **A** | **C** | **T** | **G** | **G** | **A** | **C** | **T** | **T** | **T** | **T** | **G** | **C** | **T** | **T** | **T** | **T** | **A** | **G** | **T** | **A** | **A** | **T** | **A** | **T** | **T** | **C** | **G** | **G** | **T** | **A** | **T** | **T** | **T** | **T** | **C** | **A** | **A** | **T** | **A** | **T** | **A** | **T** | **A** | **T** | **C** | **T** | **A** | **G** | **C** | **T** | **A** | **T** | **G** | **C** | **G** | **C** | **A** | **A** | **A** | **T** | **A** | **T** | **C** | **C** | **T** | **C** | **G** | **T** | **A** | **C** | **T** | **A** | **A** | **A** | **T** | **T** | **T** | **C** | **A** | **A** | **A** | **A** | **T** | **T** | **T** | **T** | **C** | **A** | **A** | **T** | **T** | **G** | **A** | **T** | **C** | **T** | **T** | **A** | **G** | **G** | **G** | **A** | **G** | **C** | **T** | **T** | **G** | **G** | **T** | **C** | **A** | **T** | **T** | **T** | **T** | **T** | **T** | **T** | **T** | **A** | **G** | **C** | **T** | **T** | **A** | **G** | **T** | **C** | **A** | **A** | **C** | **A** | **G** | **T** | **C** | **C** | **T** | **G** | **C** | **T** | **T** | **T** | **A** | **T** | **G** | **G** | **A** | **T** | **G** | **A** | **T** | **G** | **G** | **T** | **T** | **G** | **T** | **A** | **T** | **T** | **G** | **G** | **C** | **C** | **G** | **T** | **T** | **T** | **T** | **A** | **T** | **T** | **T** | **A** | **T** | **C** | **G** | **G** | **G** | **C** | **T** | **G** | **A** | **A** | **A** | **G** | **G** | **T** | **G** | **T** | **A** | **G** | **A** | **G** | **A** | **T** | **T** | **T** | **C** | **A** | **G** | **G** | **A** | **A** | **T** | **A** | **G** | **A** | **T** | **T** | **T** | **G** | **A** | **C** | **A** | **A** | **A** | **A** | **A** | **A** | **T** | **G** | **C** | **T** | **T** | **T** | **T** | **T** | **G** | **G** | **C** | **G** | **A** | **A** | **A** | **A** | **A** | **A** | **A** | **C** | **A** | **G** | **A** | **T** | **C** | **A** | **T** | **G** | **A** | **T** | **G** | **G** | **T** | **C** | **C** | **A** | **A** | **A** | **C** | **A** | **G** | **A** | **G** | **C** | **A** | **A** | **T** | **T** | **A** | **A** | **A** | **A** | **A** | **C** | **A** | **C** | **A** | **T** | **A** | **A** | **T** | **A** | **A** | **T** | **G** | **A** | **G** | **C** | **G** | **T** | **A** | **A** | **A** | **A** | **G** | **A** | **C** | **A** | **G** | **C** | **A** | **A** | **T** | **T** | **A** | **T** | **T** | **T** | **G** | **G** | **T** | **A** | **A** | **C** | **C** | **A** | **A** | **C** | **G** | **T** | **A** | **T** | **T** | **G** | **G** | **A** | **A** | **A** | **C** | **G** | **A** | **C** | **G** | **T** | **C** | **T** | **A** | **T** | **G** | **A** | **A** | **T** | **G** | **G** | **G** | **A** | **T** | **G** | **A** | **T** | **A** | **T** | **C** | **A** | **A** | **C** | **A** | **T** | **T** | **G** | **C** | **C** | **A** | **T** | **C** | **A** | **T** | **T** | **T** | **C** | **T** | **G** | **G** | **T** | **G** | **A** | **T** | **A** | **C** | **T** | **A** | **T** | **C** | **A** | **T** | **C** | **G** | **A** | **T** | **T** | **T** | **G** | **G** | **G** | **C** | **A** | **A** | **T** | **A** | **C** | **A** | **C** | **T** | **T** | **T** | **T** | **G** | **C** | **C** | **A** | **A** | **A** | **A** | **G** | **A** | **T** | **G** | **A** | **T** | **A** | **A** | **T** | **A** | **T** | **T** | **G** | **T** | **G** | **A** | **T** | **T** | **G** | **T** | **A** | **A** | **G** | **A** | **A** | **A** | **A** | **G** | **G** | **G** | **A** | **T** | **A** | **G** | **G** | **C** | **A** | **G** | **A** | **A** | **C** | **A** | **C** | **G** | **G** | **A** | **T** | **C** | **T** | **T** | **A** | **G** | **T** | **A** | **C** | **C** | **C** | **C** | **T** | **G** | **G** | **G** | **A** | **G** | **T** | **G** | **G** | **C** | **T** | **A** | **T** | **T** | **C** | **G** | **T** | **T** | **T** | **A** | **G** | **A** | **G** | **C** | **A** | **T** | **G** | **C** | **G** | **A** | **C** | **A** | **T** | **T** | **A** | **G** | **T** | **A** | **G** | **G** | **A** | **A** | **A** | **T** | **G** | **T** | **C** | **T** | **T** | **G** | **T** | **T** | **T** | **G** | **A** | **A** | **G** | **A** | **A** | **G** | **A** | **G** | **C** | **A** | **G** | **T** | **T** | **T** | **T** | **C** | **C** | **T** | **T** | **A** | **A** | **A** | **A** | **A** | **A** | **C** | **G** | **A** | **A** | **C** | **A** | **G** | **A** | **T** | **C** | **A** | **A** | **A** | **A** | **T** | **C** | **T** | **G** | **C** | **A** | **G** | **C** | **A** | **G** | **T** | **G** | **A** | **T** | **T** | **A** | **T** | **G** | **A** | **T** | **G** | **A** | **A** | **A** | **A** | **T** | **C** | **C** | **T** | **C** | **G** | **C** | **A** | **G** | **A** | **T** | **T** | **G** | **A** | **A** | **G** | **A** | **T** | **C** | **A** | **T** | **T** | **A** | **C** | **C** | **A** | **A** | **T** | **A** | **C** | **C** | **T** | **T** | **G** | **G** | **T** | **T** | **G** | **G** | **A** | **G** | **A** | **T** | **G** | **T** | **C** | **G** | **A** | **G** | **G** | **T** | **G** | **A** | **T** | **A** | **C** | **G** | **A** | **A** | **T** | **A** | **-** | **-** | **-** |
| **N54150** | **A** | **T** | **G** | **A** | **A** | **T** | **A** | **G** | **T** | **T** | **C** | **T** | **T** | **G** | **G** | **C** | **G** | **T** | **T** | **T** | **T** | **T** | **T** | **T** | **G** | **T** | **G** | **G** | **T** | **A** | **G** | **T** | **C** | **G** | **A** | **A** | **G** | **C** | **A** | **C** | **T** | **G** | **C** | **T** | **G** | **C** | **T** | **G** | **T** | **T** | **A** | **T** | **T** | **T** | **G** | **C** | **T** | **G** | **T** | **T** | **T** | **G** | **G** | **C** | **A** | **A** | **A** | **T** | **C** | **G** | **T** | **A** | **A** | **A** | **C** | **A** | **A** | **T** | **A** | **C** | **T** | **G** | **G** | **A** | **C** | **T** | **T** | **T** | **T** | **G** | **C** | **T** | **T** | **T** | **T** | **A** | **G** | **T** | **A** | **A** | **T** | **A** | **T** | **T** | **C** | **G** | **G** | **T** | **A** | **T** | **T** | **T** | **T** | **C** | **A** | **A** | **T** | **A** | **T** | **A** | **T** | **A** | **T** | **C** | **T** | **A** | **G** | **C** | **T** | **A** | **T** | **G** | **C** | **G** | **C** | **A** | **A** | **A** | **T** | **A** | **T** | **C** | **C** | **T** | **C** | **G** | **T** | **A** | **C** | **T** | **A** | **A** | **A** | **T** | **T** | **T** | **C** | **A** | **A** | **A** | **A** | **T** | **T** | **T** | **T** | **C** | **A** | **A** | **T** | **T** | **G** | **A** | **T** | **C** | **T** | **T** | **A** | **G** | **G** | **G** | **A** | **G** | **C** | **T** | **T** | **G** | **G** | **T** | **C** | **A** | **T** | **T** | **T** | **T** | **T** | **T** | **T** | **T** | **A** | **G** | **C** | **T** | **T** | **A** | **G** | **T** | **C** | **A** | **A** | **C** | **A** | **G** | **T** | **C** | **C** | **T** | **G** | **C** | **T** | **T** | **T** | **A** | **T** | **G** | **G** | **A** | **T** | **G** | **A** | **T** | **G** | **G** | **T** | **T** | **G** | **T** | **A** | **T** | **T** | **G** | **G** | **C** | **C** | **G** | **T** | **T** | **T** | **T** | **A** | **T** | **T** | **T** | **A** | **T** | **C** | **G** | **G** | **C** | **C** | **T** | **G** | **A** | **A** | **A** | **G** | **G** | **T** | **G** | **T** | **A** | **G** | **A** | **G** | **A** | **T** | **T** | **T** | **C** | **A** | **G** | **G** | **A** | **A** | **T** | **A** | **G** | **A** | **T** | **T** | **T** | **G** | **A** | **C** | **A** | **A** | **A** | **A** | **A** | **A** | **T** | **G** | **C** | **T** | **T** | **T** | **T** | **T** | **G** | **G** | **C** | **G** | **A** | **A** | **A** | **A** | **A** | **A** | **A** | **C** | **A** | **G** | **A** | **T** | **C** | **A** | **T** | **G** | **A** | **T** | **G** | **G** | **T** | **C** | **C** | **A** | **A** | **A** | **C** | **A** | **G** | **A** | **G** | **C** | **A** | **A** | **T** | **T** | **A** | **A** | **A** | **A** | **A** | **C** | **A** | **C** | **A** | **T** | **A** | **A** | **T** | **A** | **A** | **T** | **G** | **A** | **G** | **C** | **G** | **T** | **A** | **A** | **A** | **A** | **G** | **A** | **C** | **A** | **G** | **C** | **A** | **A** | **T** | **T** | **A** | **T** | **T** | **T** | **G** | **G** | **T** | **A** | **A** | **C** | **C** | **A** | **A** | **C** | **G** | **T** | **A** | **T** | **T** | **G** | **G** | **A** | **A** | **A** | **C** | **G** | **A** | **C** | **G** | **T** | **C** | **T** | **A** | **T** | **G** | **A** | **A** | **T** | **G** | **G** | **G** | **A** | **T** | **G** | **A** | **T** | **A** | **T** | **C** | **A** | **A** | **C** | **A** | **T** | **T** | **G** | **C** | **C** | **A** | **T** | **C** | **A** | **T** | **T** | **T** | **C** | **T** | **G** | **G** | **T** | **G** | **A** | **T** | **A** | **C** | **T** | **A** | **T** | **C** | **A** | **T** | **C** | **G** | **A** | **T** | **T** | **T** | **G** | **G** | **G** | **C** | **A** | **A** | **T** | **A** | **C** | **A** | **C** | **T** | **T** | **T** | **T** | **G** | **C** | **C** | **A** | **A** | **A** | **A** | **G** | **A** | **T** | **G** | **A** | **T** | **A** | **A** | **T** | **A** | **T** | **T** | **G** | **T** | **G** | **A** | **T** | **T** | **G** | **T** | **A** | **A** | **G** | **A** | **A** | **A** | **A** | **G** | **G** | **G** | **A** | **T** | **A** | **G** | **G** | **C** | **A** | **G** | **A** | **A** | **C** | **A** | **C** | **G** | **G** | **A** | **T** | **C** | **T** | **T** | **A** | **G** | **T** | **A** | **C** | **C** | **C** | **C** | **T** | **G** | **G** | **G** | **A** | **G** | **T** | **G** | **G** | **C** | **T** | **A** | **T** | **T** | **C** | **G** | **T** | **T** | **T** | **A** | **G** | **A** | **G** | **C** | **A** | **T** | **G** | **C** | **G** | **A** | **C** | **A** | **T** | **T** | **A** | **G** | **T** | **A** | **G** | **G** | **A** | **A** | **A** | **T** | **G** | **T** | **C** | **T** | **T** | **G** | **T** | **T** | **T** | **G** | **A** | **A** | **G** | **A** | **A** | **G** | **A** | **G** | **C** | **A** | **G** | **T** | **T** | **T** | **T** | **C** | **C** | **T** | **T** | **A** | **A** | **A** | **A** | **A** | **A** | **C** | **G** | **A** | **A** | **C** | **A** | **G** | **A** | **T** | **C** | **A** | **A** | **A** | **A** | **T** | **C** | **T** | **G** | **C** | **A** | **G** | **C** | **A** | **G** | **T** | **G** | **A** | **T** | **T** | **A** | **T** | **G** | **A** | **T** | **G** | **A** | **A** | **A** | **A** | **T** | **C** | **C** | **T** | **C** | **G** | **C** | **A** | **G** | **A** | **T** | **T** | **G** | **A** | **A** | **G** | **A** | **T** | **C** | **A** | **T** | **T** | **A** | **C** | **C** | **A** | **A** | **T** | **A** | **C** | **C** | **T** | **T** | **G** | **G** | **T** | **T** | **G** | **G** | **A** | **G** | **A** | **T** | **G** | **T** | **C** | **G** | **A** | **G** | **G** | **T** | **G** | **A** | **T** | **A** | **C** | **G** | **A** | **A** | **T** | **A** | **-** | **-** | **-** |
| **N55072** | **A** | **T** | **G** | **A** | **A** | **T** | **A** | **G** | **T** | **T** | **C** | **T** | **T** | **G** | **G** | **C** | **G** | **T** | **T** | **T** | **T** | **T** | **T** | **T** | **G** | **T** | **G** | **G** | **T** | **A** | **G** | **T** | **C** | **G** | **A** | **A** | **G** | **C** | **A** | **C** | **T** | **G** | **C** | **T** | **G** | **C** | **T** | **G** | **T** | **T** | **A** | **T** | **T** | **T** | **G** | **C** | **T** | **G** | **T** | **T** | **T** | **G** | **G** | **C** | **A** | **A** | **A** | **T** | **C** | **G** | **T** | **A** | **A** | **A** | **C** | **A** | **A** | **T** | **A** | **C** | **T** | **G** | **G** | **A** | **C** | **T** | **T** | **T** | **T** | **G** | **C** | **T** | **T** | **T** | **T** | **A** | **G** | **T** | **A** | **A** | **T** | **A** | **T** | **T** | **C** | **G** | **G** | **T** | **A** | **T** | **T** | **T** | **T** | **C** | **A** | **A** | **T** | **A** | **T** | **A** | **T** | **A** | **T** | **C** | **T** | **A** | **G** | **C** | **T** | **A** | **T** | **G** | **C** | **G** | **C** | **A** | **A** | **A** | **T** | **A** | **T** | **C** | **C** | **T** | **C** | **G** | **T** | **A** | **C** | **T** | **A** | **A** | **A** | **T** | **T** | **T** | **C** | **A** | **A** | **A** | **A** | **T** | **T** | **T** | **T** | **C** | **A** | **A** | **T** | **T** | **G** | **A** | **T** | **C** | **T** | **T** | **A** | **G** | **G** | **G** | **A** | **G** | **C** | **T** | **T** | **G** | **G** | **T** | **C** | **A** | **T** | **T** | **T** | **T** | **T** | **T** | **T** | **T** | **A** | **G** | **C** | **T** | **T** | **A** | **G** | **T** | **C** | **A** | **A** | **C** | **A** | **G** | **T** | **C** | **C** | **T** | **G** | **C** | **T** | **T** | **T** | **A** | **T** | **G** | **G** | **A** | **T** | **G** | **A** | **T** | **G** | **G** | **T** | **T** | **G** | **T** | **A** | **T** | **T** | **G** | **G** | **C** | **C** | **G** | **T** | **T** | **T** | **T** | **A** | **T** | **T** | **T** | **A** | **T** | **C** | **G** | **G** | **G** | **C** | **T** | **G** | **A** | **A** | **A** | **G** | **G** | **T** | **G** | **T** | **A** | **G** | **A** | **G** | **A** | **T** | **T** | **T** | **C** | **A** | **G** | **G** | **A** | **A** | **T** | **A** | **G** | **A** | **T** | **T** | **T** | **G** | **A** | **C** | **A** | **A** | **A** | **A** | **A** | **A** | **T** | **G** | **C** | **T** | **T** | **T** | **T** | **T** | **G** | **G** | **C** | **G** | **A** | **A** | **A** | **A** | **A** | **A** | **A** | **C** | **A** | **G** | **A** | **T** | **C** | **A** | **T** | **G** | **A** | **T** | **G** | **G** | **T** | **C** | **C** | **A** | **A** | **A** | **C** | **A** | **G** | **A** | **G** | **C** | **A** | **A** | **T** | **T** | **A** | **A** | **A** | **A** | **A** | **C** | **A** | **C** | **A** | **T** | **A** | **A** | **T** | **A** | **A** | **T** | **G** | **A** | **G** | **C** | **G** | **T** | **A** | **A** | **A** | **A** | **G** | **A** | **C** | **A** | **G** | **C** | **A** | **A** | **T** | **T** | **A** | **T** | **T** | **T** | **G** | **G** | **T** | **A** | **A** | **C** | **C** | **A** | **A** | **C** | **G** | **T** | **A** | **T** | **T** | **G** | **G** | **A** | **A** | **A** | **C** | **G** | **A** | **C** | **G** | **T** | **C** | **T** | **A** | **T** | **G** | **A** | **A** | **T** | **G** | **G** | **G** | **A** | **T** | **G** | **A** | **T** | **A** | **T** | **C** | **A** | **A** | **C** | **A** | **T** | **T** | **G** | **C** | **C** | **A** | **T** | **C** | **A** | **T** | **T** | **T** | **C** | **T** | **G** | **G** | **T** | **G** | **A** | **T** | **A** | **C** | **T** | **A** | **T** | **C** | **A** | **T** | **C** | **G** | **A** | **T** | **T** | **T** | **G** | **G** | **G** | **C** | **A** | **A** | **T** | **A** | **C** | **A** | **C** | **T** | **T** | **T** | **T** | **G** | **C** | **C** | **A** | **A** | **A** | **A** | **G** | **A** | **T** | **G** | **A** | **T** | **A** | **A** | **T** | **A** | **T** | **T** | **G** | **T** | **G** | **A** | **T** | **T** | **G** | **T** | **A** | **A** | **G** | **A** | **A** | **A** | **A** | **G** | **G** | **G** | **A** | **T** | **A** | **G** | **G** | **C** | **A** | **G** | **A** | **A** | **C** | **A** | **C** | **G** | **G** | **A** | **T** | **C** | **T** | **T** | **A** | **G** | **T** | **A** | **C** | **C** | **C** | **C** | **T** | **G** | **G** | **G** | **A** | **G** | **T** | **G** | **G** | **C** | **T** | **A** | **T** | **T** | **C** | **G** | **T** | **T** | **T** | **A** | **G** | **A** | **G** | **C** | **A** | **T** | **G** | **C** | **G** | **A** | **C** | **A** | **T** | **T** | **A** | **G** | **T** | **A** | **G** | **G** | **A** | **A** | **A** | **T** | **G** | **T** | **C** | **T** | **T** | **G** | **T** | **T** | **T** | **G** | **A** | **A** | **G** | **A** | **A** | **G** | **A** | **G** | **C** | **A** | **G** | **T** | **T** | **T** | **T** | **C** | **C** | **T** | **T** | **A** | **A** | **A** | **A** | **A** | **A** | **C** | **G** | **A** | **A** | **C** | **A** | **G** | **A** | **T** | **C** | **A** | **A** | **A** | **A** | **T** | **C** | **T** | **G** | **C** | **A** | **G** | **C** | **A** | **G** | **T** | **G** | **A** | **T** | **T** | **A** | **T** | **G** | **A** | **T** | **G** | **A** | **A** | **A** | **A** | **T** | **C** | **C** | **T** | **C** | **G** | **C** | **A** | **G** | **A** | **T** | **T** | **G** | **A** | **A** | **G** | **A** | **T** | **C** | **A** | **T** | **T** | **A** | **C** | **C** | **A** | **A** | **T** | **A** | **C** | **C** | **T** | **T** | **G** | **G** | **T** | **T** | **G** | **G** | **A** | **G** | **A** | **T** | **G** | **T** | **C** | **G** | **A** | **G** | **G** | **T** | **G** | **A** | **T** | **A** | **C** | **G** | **A** | **A** | **T** | **A** | **-** | **-** | **-** |
| **N59504F** | **A** | **T** | **G** | **A** | **A** | **T** | **A** | **G** | **T** | **T** | **C** | **T** | **T** | **G** | **G** | **C** | **G** | **T** | **T** | **T** | **T** | **T** | **T** | **T** | **G** | **T** | **G** | **G** | **T** | **A** | **G** | **T** | **C** | **G** | **A** | **A** | **G** | **C** | **A** | **C** | **T** | **G** | **C** | **T** | **G** | **C** | **T** | **G** | **T** | **T** | **A** | **T** | **T** | **T** | **G** | **C** | **T** | **G** | **T** | **T** | **T** | **G** | **G** | **C** | **A** | **A** | **A** | **T** | **C** | **G** | **T** | **A** | **A** | **A** | **C** | **A** | **A** | **T** | **A** | **C** | **T** | **G** | **G** | **A** | **C** | **T** | **T** | **T** | **T** | **G** | **C** | **T** | **T** | **T** | **T** | **A** | **G** | **T** | **A** | **A** | **T** | **A** | **T** | **T** | **C** | **G** | **G** | **T** | **A** | **T** | **T** | **T** | **T** | **C** | **A** | **A** | **T** | **A** | **T** | **A** | **T** | **A** | **T** | **C** | **T** | **A** | **G** | **C** | **T** | **A** | **T** | **G** | **C** | **G** | **C** | **A** | **A** | **A** | **T** | **A** | **T** | **C** | **C** | **T** | **C** | **G** | **T** | **A** | **C** | **T** | **A** | **A** | **A** | **T** | **T** | **T** | **C** | **A** | **A** | **A** | **A** | **T** | **T** | **T** | **T** | **C** | **A** | **A** | **T** | **T** | **G** | **A** | **T** | **C** | **T** | **T** | **A** | **G** | **G** | **G** | **A** | **G** | **C** | **T** | **T** | **G** | **G** | **T** | **C** | **A** | **T** | **T** | **T** | **T** | **T** | **T** | **T** | **T** | **A** | **G** | **C** | **T** | **T** | **A** | **G** | **T** | **C** | **A** | **A** | **C** | **A** | **G** | **T** | **C** | **C** | **T** | **G** | **C** | **T** | **T** | **T** | **A** | **T** | **G** | **G** | **A** | **T** | **G** | **A** | **T** | **G** | **G** | **T** | **T** | **G** | **T** | **A** | **T** | **T** | **G** | **G** | **C** | **C** | **G** | **T** | **T** | **T** | **T** | **A** | **T** | **T** | **T** | **A** | **T** | **C** | **G** | **G** | **G** | **C** | **T** | **G** | **A** | **A** | **A** | **G** | **G** | **T** | **G** | **T** | **A** | **G** | **A** | **G** | **A** | **T** | **T** | **T** | **C** | **A** | **G** | **G** | **A** | **A** | **T** | **A** | **G** | **A** | **T** | **T** | **T** | **G** | **A** | **C** | **A** | **A** | **A** | **A** | **A** | **A** | **T** | **G** | **C** | **T** | **T** | **T** | **T** | **T** | **G** | **G** | **C** | **G** | **A** | **A** | **A** | **A** | **A** | **A** | **A** | **C** | **A** | **G** | **A** | **T** | **C** | **A** | **T** | **G** | **A** | **T** | **G** | **G** | **T** | **C** | **C** | **A** | **A** | **A** | **C** | **A** | **G** | **A** | **G** | **C** | **A** | **A** | **T** | **T** | **A** | **A** | **A** | **A** | **A** | **C** | **A** | **C** | **A** | **T** | **A** | **A** | **T** | **A** | **A** | **T** | **G** | **A** | **G** | **C** | **G** | **T** | **A** | **A** | **A** | **A** | **G** | **A** | **C** | **A** | **G** | **C** | **A** | **A** | **T** | **T** | **A** | **T** | **T** | **T** | **G** | **G** | **T** | **A** | **A** | **C** | **C** | **A** | **A** | **C** | **G** | **T** | **A** | **T** | **T** | **G** | **G** | **A** | **A** | **A** | **C** | **G** | **A** | **C** | **G** | **T** | **C** | **T** | **A** | **T** | **G** | **A** | **A** | **T** | **G** | **G** | **G** | **A** | **T** | **G** | **A** | **T** | **A** | **T** | **C** | **A** | **A** | **C** | **A** | **T** | **T** | **G** | **C** | **C** | **A** | **T** | **C** | **A** | **T** | **T** | **T** | **C** | **T** | **G** | **G** | **T** | **G** | **A** | **T** | **A** | **C** | **T** | **A** | **T** | **C** | **A** | **T** | **C** | **G** | **A** | **T** | **T** | **T** | **G** | **G** | **G** | **C** | **A** | **A** | **T** | **A** | **C** | **A** | **C** | **T** | **T** | **T** | **T** | **G** | **C** | **C** | **A** | **A** | **A** | **A** | **G** | **A** | **T** | **G** | **A** | **T** | **A** | **A** | **T** | **A** | **T** | **T** | **G** | **T** | **G** | **A** | **T** | **T** | **G** | **T** | **A** | **A** | **G** | **A** | **A** | **A** | **A** | **G** | **G** | **G** | **A** | **T** | **A** | **G** | **G** | **C** | **A** | **G** | **A** | **A** | **C** | **A** | **C** | **G** | **G** | **A** | **T** | **C** | **T** | **T** | **A** | **G** | **T** | **A** | **C** | **C** | **C** | **C** | **T** | **G** | **G** | **G** | **A** | **G** | **T** | **G** | **G** | **C** | **T** | **A** | **T** | **T** | **C** | **G** | **T** | **T** | **T** | **A** | **G** | **A** | **G** | **C** | **A** | **T** | **G** | **C** | **G** | **A** | **C** | **A** | **T** | **T** | **A** | **G** | **T** | **A** | **G** | **G** | **A** | **A** | **A** | **T** | **G** | **T** | **C** | **T** | **T** | **G** | **T** | **T** | **T** | **G** | **A** | **A** | **G** | **A** | **A** | **G** | **A** | **G** | **C** | **A** | **G** | **T** | **T** | **T** | **T** | **C** | **C** | **T** | **T** | **A** | **A** | **A** | **A** | **A** | **A** | **C** | **G** | **A** | **A** | **C** | **A** | **G** | **A** | **T** | **C** | **A** | **A** | **A** | **A** | **T** | **C** | **T** | **G** | **C** | **A** | **G** | **C** | **A** | **G** | **T** | **G** | **A** | **T** | **T** | **A** | **T** | **G** | **A** | **T** | **G** | **A** | **A** | **A** | **A** | **T** | **C** | **C** | **T** | **C** | **G** | **C** | **A** | **G** | **A** | **T** | **T** | **G** | **A** | **A** | **G** | **A** | **T** | **C** | **A** | **T** | **T** | **A** | **C** | **C** | **A** | **A** | **T** | **A** | **C** | **C** | **T** | **T** | **G** | **G** | **T** | **T** | **G** | **G** | **A** | **G** | **A** | **T** | **G** | **T** | **C** | **G** | **A** | **G** | **G** | **T** | **G** | **A** | **T** | **A** | **C** | **G** | **A** | **A** | **T** | **A** | **-** | **-** | **-** |
| **N59998F** | **A** | **T** | **G** | **A** | **A** | **T** | **A** | **G** | **T** | **T** | **C** | **T** | **T** | **G** | **G** | **C** | **G** | **T** | **T** | **T** | **T** | **T** | **T** | **T** | **G** | **T** | **G** | **G** | **T** | **A** | **G** | **T** | **C** | **G** | **A** | **A** | **G** | **C** | **A** | **C** | **T** | **G** | **C** | **T** | **G** | **C** | **T** | **G** | **T** | **T** | **A** | **T** | **T** | **T** | **G** | **C** | **T** | **G** | **T** | **T** | **T** | **G** | **G** | **C** | **A** | **A** | **A** | **T** | **C** | **G** | **T** | **A** | **A** | **A** | **C** | **A** | **A** | **T** | **A** | **C** | **T** | **G** | **G** | **A** | **C** | **T** | **T** | **T** | **T** | **G** | **C** | **T** | **T** | **T** | **T** | **A** | **G** | **T** | **A** | **A** | **T** | **A** | **T** | **T** | **C** | **G** | **G** | **T** | **A** | **T** | **T** | **T** | **T** | **C** | **A** | **A** | **T** | **A** | **T** | **A** | **T** | **A** | **T** | **C** | **T** | **A** | **G** | **C** | **T** | **A** | **T** | **G** | **C** | **G** | **C** | **A** | **A** | **A** | **T** | **A** | **T** | **C** | **C** | **T** | **C** | **G** | **T** | **A** | **C** | **T** | **A** | **A** | **A** | **T** | **T** | **T** | **C** | **A** | **A** | **A** | **A** | **T** | **T** | **T** | **T** | **C** | **A** | **A** | **T** | **T** | **G** | **A** | **T** | **C** | **T** | **T** | **A** | **G** | **G** | **G** | **A** | **G** | **C** | **T** | **T** | **G** | **G** | **T** | **C** | **A** | **T** | **T** | **T** | **T** | **T** | **T** | **T** | **T** | **A** | **G** | **C** | **T** | **T** | **A** | **G** | **T** | **C** | **A** | **A** | **C** | **A** | **G** | **T** | **C** | **C** | **T** | **G** | **C** | **T** | **T** | **T** | **A** | **T** | **G** | **G** | **A** | **T** | **G** | **A** | **T** | **G** | **G** | **T** | **T** | **G** | **T** | **A** | **T** | **T** | **G** | **G** | **C** | **C** | **G** | **T** | **T** | **T** | **T** | **A** | **T** | **T** | **T** | **A** | **T** | **C** | **G** | **G** | **G** | **C** | **T** | **G** | **A** | **A** | **A** | **G** | **G** | **T** | **G** | **T** | **A** | **G** | **A** | **G** | **A** | **T** | **T** | **T** | **C** | **A** | **G** | **G** | **A** | **A** | **T** | **A** | **G** | **A** | **T** | **T** | **T** | **G** | **A** | **C** | **A** | **A** | **A** | **A** | **A** | **A** | **T** | **G** | **C** | **T** | **T** | **T** | **T** | **T** | **G** | **G** | **C** | **G** | **A** | **A** | **A** | **A** | **A** | **A** | **A** | **C** | **A** | **G** | **A** | **T** | **C** | **A** | **T** | **G** | **A** | **T** | **G** | **G** | **T** | **C** | **C** | **A** | **A** | **A** | **C** | **A** | **G** | **A** | **G** | **C** | **A** | **A** | **T** | **T** | **A** | **A** | **A** | **A** | **A** | **C** | **A** | **C** | **A** | **T** | **A** | **A** | **T** | **A** | **A** | **T** | **G** | **A** | **G** | **C** | **G** | **T** | **A** | **A** | **A** | **A** | **G** | **A** | **C** | **A** | **G** | **C** | **A** | **A** | **T** | **T** | **A** | **T** | **T** | **T** | **G** | **G** | **T** | **A** | **A** | **C** | **C** | **A** | **A** | **C** | **G** | **T** | **A** | **T** | **T** | **G** | **G** | **A** | **A** | **A** | **C** | **G** | **A** | **C** | **G** | **T** | **C** | **T** | **A** | **T** | **G** | **A** | **A** | **T** | **G** | **G** | **G** | **A** | **T** | **G** | **A** | **T** | **A** | **T** | **C** | **A** | **A** | **C** | **A** | **T** | **T** | **G** | **C** | **C** | **A** | **T** | **C** | **A** | **T** | **T** | **T** | **C** | **T** | **G** | **G** | **T** | **G** | **A** | **T** | **A** | **C** | **T** | **A** | **T** | **C** | **A** | **T** | **C** | **G** | **A** | **T** | **T** | **T** | **G** | **G** | **G** | **C** | **A** | **A** | **T** | **A** | **C** | **A** | **C** | **T** | **T** | **T** | **T** | **G** | **C** | **C** | **A** | **A** | **A** | **A** | **G** | **A** | **T** | **G** | **A** | **T** | **A** | **A** | **T** | **A** | **T** | **T** | **G** | **T** | **G** | **A** | **T** | **T** | **G** | **T** | **A** | **A** | **G** | **A** | **A** | **A** | **A** | **G** | **G** | **G** | **A** | **T** | **A** | **G** | **G** | **C** | **A** | **G** | **A** | **A** | **C** | **A** | **C** | **G** | **G** | **A** | **T** | **C** | **T** | **T** | **A** | **G** | **T** | **A** | **C** | **C** | **C** | **C** | **T** | **G** | **G** | **G** | **A** | **G** | **T** | **G** | **G** | **C** | **T** | **A** | **T** | **T** | **C** | **G** | **T** | **T** | **T** | **A** | **G** | **A** | **G** | **C** | **A** | **T** | **G** | **C** | **G** | **A** | **C** | **A** | **T** | **T** | **A** | **G** | **T** | **A** | **G** | **G** | **A** | **A** | **A** | **T** | **G** | **T** | **C** | **T** | **T** | **G** | **T** | **T** | **T** | **G** | **A** | **A** | **G** | **A** | **A** | **G** | **A** | **G** | **C** | **A** | **G** | **T** | **T** | **T** | **T** | **C** | **C** | **T** | **T** | **A** | **A** | **A** | **A** | **A** | **A** | **C** | **G** | **A** | **A** | **C** | **A** | **G** | **A** | **T** | **C** | **A** | **A** | **A** | **A** | **T** | **C** | **T** | **G** | **C** | **A** | **G** | **C** | **A** | **G** | **T** | **G** | **A** | **T** | **T** | **A** | **T** | **G** | **A** | **T** | **G** | **A** | **A** | **A** | **A** | **T** | **C** | **C** | **T** | **C** | **G** | **C** | **A** | **G** | **A** | **T** | **T** | **G** | **A** | **A** | **G** | **A** | **T** | **C** | **A** | **T** | **T** | **A** | **C** | **C** | **A** | **A** | **T** | **A** | **C** | **C** | **T** | **T** | **G** | **G** | **T** | **T** | **G** | **G** | **A** | **G** | **A** | **T** | **G** | **T** | **C** | **G** | **A** | **G** | **G** | **T** | **G** | **A** | **T** | **A** | **C** | **G** | **A** | **A** | **T** | **A** | **-** | **-** | **-** |
| **N60324F** | **A** | **T** | **G** | **A** | **A** | **T** | **A** | **G** | **T** | **T** | **C** | **T** | **T** | **G** | **G** | **C** | **G** | **T** | **T** | **T** | **T** | **T** | **T** | **T** | **G** | **T** | **G** | **G** | **T** | **A** | **G** | **T** | **C** | **G** | **A** | **A** | **G** | **C** | **A** | **C** | **T** | **G** | **C** | **T** | **G** | **C** | **T** | **G** | **T** | **T** | **A** | **T** | **T** | **T** | **G** | **C** | **T** | **G** | **T** | **T** | **T** | **G** | **G** | **C** | **A** | **A** | **A** | **T** | **C** | **G** | **T** | **A** | **A** | **A** | **C** | **A** | **A** | **T** | **A** | **C** | **T** | **G** | **G** | **A** | **C** | **T** | **T** | **T** | **T** | **G** | **C** | **T** | **T** | **T** | **T** | **A** | **G** | **T** | **A** | **A** | **T** | **A** | **T** | **T** | **C** | **G** | **G** | **T** | **A** | **T** | **T** | **T** | **T** | **C** | **A** | **A** | **T** | **A** | **T** | **A** | **T** | **A** | **T** | **C** | **T** | **A** | **G** | **C** | **T** | **A** | **T** | **G** | **C** | **G** | **C** | **A** | **A** | **A** | **T** | **A** | **T** | **C** | **C** | **T** | **C** | **G** | **T** | **A** | **C** | **T** | **A** | **A** | **A** | **T** | **T** | **T** | **C** | **A** | **A** | **A** | **A** | **T** | **T** | **T** | **T** | **C** | **A** | **A** | **T** | **T** | **G** | **A** | **T** | **C** | **T** | **T** | **A** | **G** | **G** | **G** | **A** | **G** | **C** | **T** | **T** | **A** | **G** | **T** | **C** | **A** | **T** | **T** | **T** | **T** | **T** | **T** | **T** | **T** | **A** | **G** | **C** | **T** | **T** | **A** | **G** | **T** | **C** | **A** | **A** | **C** | **A** | **G** | **T** | **C** | **C** | **T** | **G** | **C** | **T** | **T** | **T** | **A** | **T** | **G** | **G** | **A** | **T** | **G** | **A** | **T** | **G** | **G** | **T** | **T** | **G** | **T** | **A** | **T** | **T** | **G** | **G** | **C** | **C** | **G** | **T** | **T** | **T** | **T** | **A** | **T** | **T** | **T** | **A** | **T** | **C** | **G** | **G** | **G** | **C** | **T** | **G** | **A** | **A** | **A** | **G** | **G** | **T** | **G** | **T** | **A** | **G** | **A** | **G** | **A** | **T** | **T** | **T** | **C** | **A** | **G** | **G** | **A** | **A** | **T** | **A** | **G** | **A** | **T** | **T** | **T** | **G** | **A** | **C** | **A** | **A** | **A** | **A** | **A** | **A** | **T** | **G** | **C** | **T** | **T** | **T** | **T** | **T** | **G** | **G** | **C** | **G** | **A** | **A** | **A** | **A** | **A** | **A** | **A** | **C** | **A** | **G** | **A** | **T** | **C** | **A** | **T** | **G** | **A** | **T** | **G** | **G** | **T** | **C** | **C** | **A** | **A** | **A** | **C** | **A** | **G** | **A** | **G** | **C** | **A** | **A** | **T** | **T** | **A** | **A** | **A** | **A** | **A** | **C** | **A** | **C** | **A** | **T** | **A** | **A** | **T** | **A** | **A** | **T** | **G** | **A** | **G** | **C** | **G** | **T** | **A** | **A** | **A** | **A** | **G** | **A** | **C** | **A** | **G** | **C** | **A** | **A** | **T** | **T** | **A** | **T** | **T** | **T** | **G** | **G** | **T** | **A** | **A** | **C** | **C** | **A** | **A** | **C** | **G** | **T** | **A** | **T** | **T** | **G** | **G** | **A** | **A** | **A** | **C** | **G** | **A** | **C** | **G** | **T** | **C** | **T** | **A** | **T** | **G** | **A** | **A** | **T** | **G** | **G** | **G** | **A** | **T** | **G** | **A** | **C** | **A** | **T** | **C** | **A** | **A** | **C** | **A** | **T** | **T** | **G** | **C** | **C** | **A** | **T** | **C** | **A** | **T** | **T** | **T** | **C** | **T** | **G** | **G** | **T** | **G** | **A** | **T** | **A** | **C** | **T** | **A** | **T** | **C** | **A** | **T** | **C** | **G** | **A** | **T** | **T** | **T** | **G** | **G** | **G** | **C** | **A** | **A** | **T** | **A** | **C** | **A** | **C** | **T** | **T** | **T** | **T** | **G** | **C** | **C** | **A** | **A** | **A** | **A** | **G** | **A** | **T** | **G** | **A** | **T** | **A** | **A** | **T** | **A** | **T** | **T** | **G** | **T** | **G** | **A** | **T** | **T** | **G** | **T** | **A** | **A** | **G** | **A** | **A** | **A** | **A** | **G** | **G** | **G** | **A** | **T** | **A** | **G** | **G** | **C** | **A** | **G** | **A** | **A** | **C** | **A** | **C** | **G** | **G** | **A** | **T** | **C** | **T** | **T** | **A** | **G** | **T** | **A** | **C** | **C** | **C** | **C** | **T** | **G** | **G** | **G** | **A** | **G** | **T** | **G** | **G** | **C** | **T** | **A** | **T** | **T** | **C** | **G** | **T** | **T** | **T** | **A** | **G** | **A** | **G** | **C** | **A** | **T** | **G** | **C** | **G** | **A** | **C** | **A** | **T** | **T** | **A** | **G** | **T** | **A** | **G** | **G** | **A** | **A** | **A** | **T** | **G** | **T** | **C** | **T** | **T** | **G** | **T** | **T** | **T** | **G** | **A** | **A** | **G** | **A** | **A** | **G** | **A** | **G** | **C** | **A** | **G** | **T** | **T** | **T** | **T** | **C** | **C** | **T** | **T** | **A** | **A** | **A** | **A** | **A** | **A** | **C** | **G** | **A** | **A** | **C** | **A** | **G** | **A** | **T** | **C** | **A** | **A** | **A** | **A** | **T** | **C** | **T** | **G** | **C** | **A** | **G** | **C** | **A** | **G** | **T** | **G** | **A** | **T** | **T** | **A** | **T** | **G** | **A** | **T** | **G** | **A** | **A** | **A** | **A** | **T** | **C** | **C** | **T** | **C** | **G** | **C** | **A** | **G** | **A** | **T** | **T** | **G** | **A** | **A** | **G** | **A** | **T** | **C** | **A** | **T** | **T** | **A** | **C** | **C** | **A** | **A** | **T** | **A** | **C** | **C** | **T** | **T** | **G** | **G** | **T** | **T** | **G** | **G** | **A** | **G** | **A** | **T** | **G** | **T** | **C** | **G** | **A** | **G** | **G** | **T** | **G** | **A** | **T** | **A** | **C** | **G** | **A** | **A** | **T** | **A** | **-** | **-** | **-** |
| **N54190** | **A** | **T** | **G** | **A** | **A** | **T** | **A** | **G** | **T** | **T** | **C** | **T** | **T** | **G** | **G** | **C** | **G** | **T** | **T** | **T** | **T** | **T** | **T** | **T** | **G** | **T** | **G** | **G** | **T** | **A** | **G** | **T** | **C** | **G** | **A** | **A** | **G** | **C** | **A** | **C** | **T** | **G** | **C** | **T** | **G** | **C** | **T** | **G** | **T** | **T** | **A** | **T** | **T** | **T** | **G** | **C** | **T** | **G** | **T** | **T** | **T** | **G** | **G** | **C** | **A** | **A** | **A** | **T** | **C** | **G** | **T** | **A** | **A** | **A** | **C** | **A** | **A** | **T** | **A** | **C** | **T** | **G** | **G** | **A** | **C** | **T** | **T** | **T** | **T** | **G** | **C** | **T** | **T** | **T** | **T** | **A** | **G** | **T** | **A** | **A** | **T** | **A** | **T** | **T** | **C** | **G** | **G** | **T** | **A** | **T** | **T** | **T** | **T** | **C** | **A** | **A** | **T** | **A** | **T** | **A** | **T** | **A** | **T** | **C** | **T** | **A** | **G** | **C** | **T** | **A** | **T** | **G** | **C** | **G** | **C** | **A** | **A** | **A** | **T** | **A** | **T** | **C** | **C** | **T** | **C** | **G** | **T** | **A** | **C** | **T** | **A** | **A** | **A** | **T** | **T** | **T** | **C** | **A** | **A** | **A** | **A** | **T** | **T** | **T** | **T** | **C** | **A** | **A** | **T** | **T** | **G** | **A** | **T** | **C** | **T** | **T** | **A** | **G** | **G** | **G** | **A** | **G** | **C** | **T** | **T** | **G** | **G** | **T** | **C** | **A** | **T** | **T** | **T** | **T** | **T** | **T** | **T** | **T** | **A** | **G** | **C** | **T** | **T** | **A** | **G** | **T** | **C** | **A** | **A** | **C** | **A** | **G** | **T** | **C** | **C** | **T** | **G** | **C** | **T** | **T** | **T** | **A** | **T** | **G** | **G** | **A** | **T** | **G** | **A** | **T** | **G** | **G** | **T** | **T** | **G** | **T** | **A** | **T** | **T** | **G** | **G** | **C** | **C** | **A** | **T** | **T** | **T** | **T** | **A** | **T** | **T** | **T** | **A** | **T** | **C** | **G** | **G** | **A** | **C** | **T** | **G** | **A** | **A** | **A** | **G** | **G** | **T** | **G** | **T** | **A** | **G** | **A** | **G** | **A** | **T** | **T** | **T** | **C** | **A** | **G** | **G** | **A** | **A** | **T** | **A** | **G** | **A** | **T** | **T** | **T** | **G** | **A** | **C** | **A** | **A** | **A** | **A** | **A** | **A** | **T** | **G** | **C** | **T** | **T** | **T** | **T** | **T** | **G** | **G** | **C** | **G** | **A** | **A** | **A** | **A** | **A** | **A** | **A** | **C** | **A** | **G** | **A** | **T** | **T** | **A** | **T** | **G** | **A** | **T** | **G** | **G** | **T** | **C** | **C** | **A** | **A** | **A** | **C** | **A** | **G** | **A** | **G** | **C** | **A** | **A** | **T** | **T** | **A** | **A** | **A** | **A** | **A** | **C** | **A** | **C** | **A** | **T** | **A** | **A** | **T** | **A** | **A** | **T** | **G** | **A** | **G** | **C** | **G** | **T** | **A** | **A** | **A** | **A** | **G** | **A** | **C** | **A** | **G** | **C** | **A** | **A** | **T** | **T** | **A** | **T** | **T** | **T** | **G** | **G** | **T** | **A** | **A** | **C** | **C** | **A** | **A** | **C** | **G** | **T** | **A** | **T** | **T** | **G** | **G** | **A** | **A** | **A** | **C** | **G** | **A** | **C** | **G** | **T** | **C** | **T** | **A** | **T** | **G** | **A** | **A** | **T** | **G** | **G** | **G** | **A** | **T** | **G** | **A** | **T** | **A** | **T** | **C** | **A** | **A** | **C** | **A** | **T** | **T** | **G** | **C** | **C** | **A** | **T** | **C** | **A** | **T** | **T** | **T** | **C** | **T** | **G** | **G** | **T** | **G** | **A** | **T** | **A** | **C** | **T** | **A** | **T** | **C** | **A** | **T** | **C** | **G** | **A** | **T** | **T** | **T** | **G** | **G** | **G** | **C** | **A** | **A** | **T** | **A** | **C** | **A** | **C** | **T** | **T** | **T** | **T** | **G** | **C** | **C** | **A** | **A** | **A** | **A** | **G** | **A** | **T** | **G** | **A** | **T** | **A** | **A** | **T** | **A** | **T** | **T** | **G** | **T** | **G** | **A** | **T** | **T** | **G** | **T** | **A** | **A** | **G** | **A** | **A** | **A** | **A** | **G** | **G** | **G** | **A** | **T** | **A** | **G** | **G** | **C** | **A** | **G** | **A** | **A** | **C** | **A** | **C** | **G** | **G** | **A** | **T** | **C** | **T** | **T** | **A** | **G** | **T** | **A** | **C** | **C** | **C** | **C** | **T** | **G** | **G** | **G** | **A** | **G** | **T** | **G** | **G** | **C** | **T** | **A** | **T** | **T** | **C** | **G** | **A** | **T** | **T** | **A** | **G** | **A** | **G** | **C** | **A** | **T** | **G** | **C** | **G** | **A** | **C** | **A** | **T** | **T** | **A** | **G** | **T** | **A** | **G** | **G** | **A** | **A** | **A** | **T** | **G** | **T** | **C** | **T** | **T** | **G** | **T** | **T** | **T** | **G** | **A** | **A** | **G** | **A** | **A** | **G** | **A** | **G** | **C** | **A** | **G** | **T** | **T** | **T** | **T** | **C** | **C** | **T** | **T** | **A** | **A** | **A** | **A** | **A** | **A** | **C** | **G** | **A** | **A** | **C** | **A** | **G** | **A** | **T** | **C** | **A** | **A** | **A** | **A** | **T** | **C** | **T** | **G** | **C** | **A** | **G** | **C** | **A** | **G** | **T** | **G** | **A** | **T** | **T** | **A** | **T** | **G** | **A** | **T** | **G** | **A** | **A** | **A** | **A** | **T** | **C** | **C** | **T** | **C** | **G** | **C** | **A** | **G** | **A** | **T** | **T** | **G** | **A** | **A** | **G** | **A** | **T** | **C** | **A** | **T** | **T** | **A** | **C** | **C** | **A** | **A** | **T** | **A** | **C** | **C** | **T** | **T** | **G** | **G** | **T** | **T** | **G** | **G** | **A** | **G** | **A** | **T** | **G** | **T** | **C** | **G** | **A** | **G** | **G** | **T** | **G** | **A** | **T** | **A** | **C** | **G** | **A** | **A** | **T** | **A** | **-** | **-** | **-** |
| **N54035** | **A** | **T** | **G** | **A** | **A** | **T** | **A** | **G** | **T** | **T** | **C** | **T** | **T** | **G** | **G** | **C** | **G** | **T** | **T** | **T** | **T** | **T** | **T** | **T** | **G** | **T** | **G** | **G** | **T** | **A** | **G** | **T** | **C** | **G** | **A** | **A** | **G** | **C** | **A** | **C** | **T** | **G** | **C** | **T** | **G** | **C** | **T** | **G** | **T** | **T** | **A** | **T** | **T** | **T** | **G** | **C** | **T** | **G** | **T** | **T** | **T** | **G** | **G** | **C** | **A** | **A** | **A** | **T** | **C** | **G** | **T** | **A** | **A** | **A** | **C** | **A** | **A** | **T** | **A** | **C** | **T** | **G** | **G** | **A** | **C** | **T** | **T** | **T** | **T** | **G** | **C** | **T** | **T** | **T** | **T** | **A** | **G** | **T** | **A** | **A** | **T** | **A** | **T** | **T** | **C** | **G** | **G** | **T** | **A** | **T** | **T** | **T** | **T** | **C** | **A** | **A** | **T** | **A** | **T** | **A** | **T** | **A** | **T** | **C** | **T** | **A** | **G** | **C** | **T** | **A** | **T** | **G** | **C** | **G** | **C** | **A** | **A** | **A** | **T** | **A** | **T** | **C** | **C** | **T** | **C** | **G** | **T** | **A** | **C** | **T** | **A** | **A** | **A** | **T** | **T** | **T** | **C** | **A** | **A** | **A** | **A** | **T** | **T** | **T** | **T** | **C** | **A** | **A** | **T** | **T** | **G** | **A** | **T** | **C** | **T** | **T** | **A** | **G** | **G** | **G** | **A** | **G** | **C** | **T** | **T** | **G** | **G** | **T** | **C** | **A** | **T** | **T** | **T** | **T** | **T** | **T** | **T** | **T** | **A** | **G** | **C** | **T** | **T** | **A** | **G** | **T** | **C** | **A** | **A** | **C** | **A** | **G** | **T** | **C** | **C** | **T** | **G** | **C** | **T** | **T** | **T** | **A** | **T** | **G** | **G** | **A** | **T** | **G** | **A** | **T** | **G** | **G** | **T** | **T** | **G** | **T** | **A** | **T** | **T** | **G** | **G** | **C** | **C** | **G** | **T** | **T** | **T** | **T** | **A** | **T** | **T** | **T** | **A** | **T** | **C** | **G** | **G** | **C** | **C** | **T** | **G** | **A** | **A** | **A** | **G** | **G** | **T** | **G** | **T** | **A** | **G** | **A** | **G** | **A** | **T** | **T** | **T** | **C** | **A** | **G** | **G** | **A** | **A** | **T** | **A** | **G** | **A** | **T** | **T** | **T** | **G** | **A** | **C** | **A** | **A** | **A** | **A** | **A** | **A** | **T** | **G** | **C** | **T** | **T** | **T** | **T** | **T** | **G** | **G** | **C** | **G** | **A** | **A** | **A** | **A** | **A** | **A** | **A** | **C** | **A** | **G** | **A** | **T** | **C** | **A** | **T** | **G** | **A** | **T** | **G** | **G** | **T** | **C** | **C** | **A** | **A** | **A** | **C** | **A** | **G** | **A** | **G** | **C** | **A** | **A** | **T** | **T** | **A** | **A** | **A** | **A** | **A** | **C** | **A** | **C** | **A** | **T** | **A** | **A** | **T** | **A** | **A** | **T** | **G** | **A** | **G** | **C** | **G** | **T** | **A** | **A** | **A** | **A** | **G** | **A** | **C** | **A** | **G** | **C** | **A** | **A** | **T** | **T** | **A** | **T** | **T** | **T** | **G** | **G** | **T** | **A** | **A** | **C** | **C** | **A** | **A** | **C** | **G** | **T** | **A** | **T** | **T** | **G** | **G** | **A** | **A** | **A** | **C** | **G** | **A** | **C** | **G** | **T** | **C** | **T** | **A** | **T** | **G** | **A** | **A** | **T** | **G** | **G** | **G** | **A** | **T** | **G** | **A** | **T** | **A** | **T** | **C** | **A** | **A** | **C** | **A** | **T** | **T** | **G** | **C** | **C** | **A** | **T** | **C** | **A** | **T** | **T** | **T** | **C** | **T** | **G** | **G** | **T** | **G** | **A** | **T** | **A** | **C** | **T** | **A** | **T** | **C** | **A** | **T** | **C** | **G** | **A** | **T** | **T** | **T** | **G** | **G** | **G** | **C** | **A** | **A** | **T** | **A** | **C** | **A** | **C** | **T** | **T** | **T** | **T** | **G** | **C** | **C** | **A** | **A** | **A** | **A** | **G** | **A** | **T** | **G** | **A** | **T** | **A** | **A** | **T** | **A** | **T** | **T** | **G** | **T** | **G** | **A** | **T** | **T** | **G** | **T** | **A** | **A** | **G** | **A** | **A** | **A** | **A** | **G** | **G** | **G** | **A** | **T** | **A** | **G** | **G** | **C** | **A** | **G** | **A** | **A** | **C** | **A** | **C** | **G** | **G** | **A** | **T** | **C** | **T** | **T** | **A** | **G** | **T** | **A** | **C** | **C** | **C** | **C** | **T** | **G** | **G** | **G** | **A** | **G** | **T** | **G** | **G** | **C** | **T** | **A** | **T** | **T** | **C** | **G** | **A** | **T** | **T** | **A** | **G** | **A** | **G** | **C** | **A** | **T** | **G** | **C** | **G** | **A** | **C** | **A** | **T** | **T** | **A** | **G** | **T** | **A** | **G** | **G** | **A** | **A** | **A** | **T** | **G** | **T** | **C** | **T** | **T** | **G** | **T** | **T** | **T** | **G** | **A** | **A** | **G** | **A** | **A** | **G** | **A** | **G** | **C** | **A** | **G** | **T** | **T** | **T** | **T** | **C** | **C** | **T** | **T** | **A** | **A** | **A** | **A** | **A** | **A** | **T** | **G** | **A** | **A** | **C** | **A** | **G** | **A** | **T** | **C** | **A** | **A** | **A** | **A** | **T** | **C** | **T** | **G** | **C** | **A** | **G** | **C** | **A** | **G** | **T** | **G** | **A** | **T** | **T** | **A** | **T** | **G** | **A** | **T** | **G** | **A** | **A** | **A** | **A** | **T** | **C** | **C** | **T** | **C** | **G** | **C** | **A** | **G** | **A** | **T** | **T** | **G** | **A** | **A** | **G** | **A** | **T** | **C** | **A** | **T** | **T** | **A** | **C** | **C** | **A** | **A** | **T** | **A** | **C** | **C** | **T** | **T** | **G** | **G** | **T** | **T** | **G** | **G** | **A** | **G** | **A** | **T** | **G** | **T** | **C** | **G** | **A** | **G** | **G** | **T** | **G** | **A** | **T** | **A** | **C** | **G** | **A** | **A** | **T** | **A** | **-** | **-** | **-** |
| **N59947F** | **A** | **T** | **G** | **A** | **A** | **T** | **A** | **G** | **T** | **T** | **C** | **T** | **T** | **G** | **G** | **C** | **G** | **T** | **T** | **T** | **T** | **T** | **T** | **T** | **G** | **T** | **G** | **G** | **T** | **A** | **G** | **T** | **T** | **G** | **A** | **A** | **G** | **C** | **A** | **C** | **T** | **A** | **C** | **T** | **G** | **C** | **T** | **G** | **T** | **T** | **A** | **T** | **T** | **T** | **G** | **C** | **T** | **G** | **T** | **T** | **T** | **G** | **G** | **C** | **A** | **A** | **A** | **T** | **C** | **G** | **T** | **A** | **A** | **A** | **C** | **A** | **A** | **T** | **A** | **C** | **T** | **G** | **G** | **A** | **C** | **T** | **T** | **T** | **T** | **G** | **C** | **T** | **T** | **T** | **T** | **A** | **G** | **T** | **G** | **A** | **T** | **A** | **T** | **T** | **C** | **G** | **G** | **T** | **A** | **T** | **T** | **T** | **T** | **C** | **A** | **A** | **T** | **A** | **T** | **A** | **T** | **A** | **T** | **C** | **T** | **A** | **G** | **C** | **T** | **A** | **T** | **G** | **C** | **G** | **C** | **A** | **A** | **A** | **C** | **A** | **T** | **C** | **C** | **T** | **C** | **G** | **T** | **A** | **C** | **T** | **A** | **A** | **A** | **T** | **T** | **T** | **C** | **A** | **A** | **A** | **A** | **T** | **T** | **T** | **T** | **C** | **A** | **A** | **T** | **T** | **G** | **A** | **T** | **A** | **T** | **T** | **A** | **G** | **G** | **A** | **A** | **G** | **T** | **T** | **T** | **G** | **G** | **T** | **C** | **A** | **T** | **T** | **T** | **T** | **T** | **T** | **T** | **T** | **A** | **G** | **C** | **T** | **T** | **A** | **G** | **T** | **C** | **A** | **A** | **C** | **A** | **G** | **T** | **C** | **C** | **T** | **G** | **C** | **T** | **T** | **T** | **A** | **T** | **G** | **G** | **A** | **T** | **G** | **A** | **T** | **G** | **G** | **C** | **T** | **G** | **T** | **A** | **T** | **T** | **T** | **G** | **C** | **C** | **G** | **T** | **T** | **T** | **T** | **A** | **T** | **T** | **T** | **A** | **T** | **T** | **G** | **G** | **A** | **C** | **T** | **A** | **A** | **A** | **G** | **G** | **G** | **T** | **G** | **T** | **A** | **G** | **A** | **G** | **A** | **T** | **T** | **T** | **C** | **A** | **G** | **G** | **G** | **A** | **T** | **A** | **G** | **A** | **T** | **T** | **T** | **A** | **A** | **C** | **C** | **A** | **A** | **A** | **A** | **A** | **T** | **G** | **C** | **T** | **T** | **T** | **T** | **T** | **G** | **G** | **C** | **G** | **A** | **A** | **A** | **G** | **A** | **A** | **A** | **C** | **A** | **G** | **A** | **T** | **C** | **A** | **T** | **G** | **A** | **T** | **G** | **G** | **T** | **C** | **C** | **A** | **A** | **A** | **C** | **A** | **G** | **A** | **G** | **C** | **A** | **A** | **T** | **T** | **A** | **A** | **A** | **A** | **A** | **C** | **A** | **C** | **A** | **T** | **A** | **A** | **T** | **A** | **A** | **T** | **G** | **A** | **G** | **C** | **G** | **G** | **A** | **A** | **A** | **A** | **G** | **A** | **C** | **A** | **G** | **C** | **A** | **A** | **T** | **T** | **A** | **T** | **T** | **C** | **G** | **G** | **T** | **A** | **A** | **C** | **C** | **A** | **A** | **C** | **G** | **T** | **A** | **T** | **T** | **G** | **G** | **A** | **A** | **A** | **C** | **G** | **A** | **C** | **G** | **T** | **C** | **T** | **A** | **T** | **G** | **A** | **A** | **T** | **G** | **G** | **G** | **A** | **C** | **G** | **A** | **T** | **A** | **T** | **C** | **A** | **A** | **C** | **A** | **T** | **C** | **G** | **C** | **C** | **G** | **T** | **C** | **A** | **T** | **T** | **T** | **C** | **T** | **G** | **G** | **T** | **G** | **A** | **T** | **A** | **C** | **T** | **A** | **T** | **C** | **A** | **T** | **A** | **G** | **A** | **T** | **T** | **T** | **G** | **G** | **G** | **C** | **A** | **A** | **T** | **A** | **C** | **G** | **C** | **T** | **T** | **T** | **T** | **G** | **C** | **C** | **A** | **A** | **A** | **A** | **G** | **A** | **T** | **G** | **A** | **T** | **A** | **A** | **T** | **A** | **T** | **T** | **G** | **T** | **G** | **A** | **T** | **T** | **G** | **T** | **A** | **A** | **G** | **A** | **A** | **A** | **A** | **G** | **G** | **G** | **A** | **T** | **A** | **G** | **G** | **C** | **A** | **G** | **A** | **A** | **C** | **A** | **C** | **G** | **A** | **A** | **T** | **C** | **T** | **T** | **A** | **G** | **T** | **A** | **C** | **C** | **T** | **T** | **T** | **G** | **G** | **G** | **C** | **G** | **T** | **A** | **G** | **C** | **C** | **A** | **T** | **C** | **C** | **G** | **A** | **T** | **T** | **A** | **G** | **A** | **G** | **C** | **A** | **T** | **G** | **C** | **G** | **A** | **C** | **A** | **T** | **T** | **A** | **G** | **T** | **A** | **G** | **G** | **A** | **A** | **A** | **T** | **G** | **T** | **C** | **T** | **T** | **G** | **T** | **T** | **T** | **G** | **A** | **A** | **G** | **A** | **A** | **G** | **A** | **G** | **C** | **A** | **A** | **T** | **T** | **T** | **T** | **C** | **T** | **T** | **T** | **G** | **A** | **A** | **A** | **A** | **A** | **T** | **G** | **A** | **A** | **C** | **A** | **A** | **A** | **T** | **C** | **A** | **A** | **A** | **A** | **T** | **C** | **T** | **A** | **C** | **A** | **G** | **C** | **A** | **G** | **T** | **G** | **A** | **T** | **T** | **A** | **T** | **G** | **A** | **T** | **G** | **A** | **A** | **A** | **A** | **C** | **C** | **C** | **T** | **C** | **G** | **T** | **A** | **G** | **A** | **C** | **T** | **G** | **A** | **A** | **G** | **A** | **T** | **T** | **A** | **T** | **T** | **A** | **C** | **T** | **A** | **A** | **T** | **A** | **C** | **C** | **T** | **T** | **G** | **A** | **T** | **T** | **G** | **G** | **A** | **G** | **A** | **T** | **G** | **T** | **C** | **G** | **A** | **G** | **G** | **T** | **G** | **A** | **T** | **A** | **C** | **G** | **A** | **A** | **T** | **A** | **-** | **-** | **-** |
| **N54107** | **A** | **T** | **G** | **A** | **A** | **T** | **A** | **G** | **T** | **T** | **C** | **T** | **T** | **G** | **G** | **C** | **G** | **T** | **T** | **T** | **T** | **T** | **T** | **T** | **G** | **T** | **G** | **G** | **T** | **A** | **G** | **T** | **C** | **G** | **A** | **A** | **G** | **C** | **A** | **C** | **T** | **G** | **C** | **T** | **G** | **C** | **T** | **G** | **T** | **T** | **A** | **T** | **T** | **T** | **G** | **C** | **T** | **G** | **T** | **T** | **T** | **G** | **G** | **C** | **A** | **A** | **A** | **T** | **C** | **G** | **T** | **A** | **A** | **A** | **C** | **A** | **A** | **T** | **A** | **C** | **T** | **G** | **G** | **A** | **C** | **T** | **T** | **T** | **T** | **G** | **C** | **T** | **T** | **T** | **T** | **A** | **G** | **T** | **A** | **A** | **T** | **A** | **T** | **T** | **C** | **G** | **G** | **T** | **A** | **T** | **T** | **T** | **T** | **C** | **A** | **A** | **T** | **A** | **T** | **A** | **T** | **A** | **T** | **C** | **T** | **A** | **G** | **C** | **T** | **A** | **T** | **G** | **C** | **G** | **C** | **A** | **A** | **A** | **T** | **A** | **T** | **C** | **C** | **T** | **C** | **G** | **T** | **A** | **C** | **T** | **A** | **A** | **A** | **T** | **T** | **T** | **C** | **A** | **A** | **A** | **A** | **T** | **T** | **T** | **T** | **C** | **A** | **A** | **T** | **T** | **G** | **A** | **T** | **C** | **T** | **T** | **A** | **G** | **G** | **G** | **A** | **G** | **C** | **T** | **T** | **A** | **G** | **T** | **C** | **A** | **T** | **T** | **T** | **T** | **T** | **T** | **T** | **T** | **A** | **G** | **C** | **T** | **T** | **A** | **G** | **T** | **C** | **A** | **A** | **C** | **A** | **G** | **T** | **C** | **C** | **T** | **G** | **C** | **T** | **T** | **T** | **A** | **T** | **G** | **G** | **A** | **T** | **G** | **A** | **T** | **G** | **G** | **T** | **T** | **G** | **T** | **A** | **T** | **T** | **G** | **G** | **C** | **C** | **G** | **T** | **T** | **T** | **T** | **A** | **T** | **T** | **T** | **A** | **T** | **C** | **G** | **G** | **G** | **C** | **T** | **G** | **A** | **A** | **A** | **G** | **G** | **T** | **G** | **T** | **A** | **G** | **A** | **G** | **A** | **T** | **T** | **T** | **C** | **A** | **G** | **G** | **A** | **A** | **T** | **A** | **G** | **A** | **T** | **T** | **T** | **G** | **A** | **C** | **A** | **A** | **A** | **A** | **A** | **A** | **T** | **G** | **C** | **T** | **T** | **T** | **T** | **T** | **G** | **G** | **C** | **G** | **A** | **A** | **A** | **A** | **A** | **A** | **A** | **C** | **A** | **G** | **A** | **T** | **C** | **A** | **T** | **G** | **A** | **T** | **G** | **G** | **T** | **C** | **C** | **A** | **A** | **A** | **C** | **A** | **G** | **A** | **G** | **C** | **A** | **A** | **T** | **T** | **A** | **A** | **A** | **A** | **A** | **C** | **A** | **C** | **A** | **T** | **A** | **A** | **T** | **A** | **A** | **T** | **G** | **A** | **G** | **C** | **G** | **T** | **A** | **A** | **A** | **A** | **G** | **A** | **C** | **A** | **G** | **C** | **A** | **A** | **T** | **T** | **A** | **T** | **T** | **T** | **G** | **G** | **T** | **A** | **A** | **C** | **C** | **A** | **A** | **C** | **G** | **T** | **A** | **T** | **T** | **G** | **G** | **A** | **A** | **A** | **C** | **G** | **A** | **C** | **G** | **T** | **C** | **T** | **A** | **T** | **G** | **A** | **A** | **T** | **G** | **G** | **G** | **A** | **T** | **G** | **A** | **C** | **A** | **T** | **C** | **A** | **A** | **C** | **A** | **T** | **T** | **G** | **C** | **C** | **A** | **T** | **C** | **A** | **T** | **T** | **T** | **C** | **T** | **G** | **G** | **T** | **G** | **A** | **T** | **A** | **C** | **T** | **A** | **T** | **C** | **A** | **T** | **C** | **G** | **A** | **T** | **T** | **T** | **G** | **G** | **G** | **C** | **A** | **A** | **T** | **A** | **C** | **A** | **C** | **T** | **T** | **T** | **T** | **G** | **C** | **C** | **A** | **A** | **A** | **A** | **G** | **A** | **T** | **G** | **A** | **T** | **A** | **A** | **T** | **A** | **T** | **T** | **G** | **T** | **G** | **A** | **T** | **T** | **G** | **T** | **A** | **A** | **G** | **A** | **A** | **A** | **A** | **G** | **G** | **G** | **A** | **T** | **A** | **G** | **G** | **C** | **A** | **G** | **A** | **A** | **C** | **A** | **C** | **G** | **G** | **A** | **T** | **C** | **T** | **T** | **A** | **G** | **T** | **A** | **C** | **C** | **C** | **C** | **T** | **G** | **G** | **G** | **A** | **G** | **T** | **G** | **G** | **C** | **T** | **A** | **T** | **T** | **C** | **G** | **T** | **T** | **T** | **A** | **G** | **A** | **G** | **C** | **A** | **T** | **G** | **C** | **G** | **A** | **C** | **A** | **T** | **T** | **A** | **G** | **T** | **A** | **G** | **G** | **A** | **A** | **A** | **T** | **G** | **T** | **C** | **T** | **T** | **G** | **T** | **T** | **T** | **G** | **A** | **A** | **G** | **A** | **A** | **G** | **A** | **G** | **C** | **A** | **G** | **T** | **T** | **T** | **T** | **C** | **C** | **T** | **T** | **A** | **A** | **A** | **A** | **A** | **A** | **C** | **G** | **A** | **A** | **C** | **A** | **G** | **A** | **T** | **C** | **A** | **A** | **A** | **A** | **T** | **C** | **T** | **G** | **C** | **A** | **G** | **C** | **A** | **G** | **T** | **G** | **A** | **T** | **T** | **A** | **T** | **G** | **A** | **T** | **G** | **A** | **A** | **A** | **A** | **T** | **C** | **C** | **T** | **C** | **G** | **C** | **A** | **G** | **A** | **T** | **T** | **G** | **A** | **A** | **G** | **A** | **T** | **C** | **A** | **T** | **T** | **A** | **C** | **C** | **A** | **A** | **T** | **A** | **C** | **C** | **T** | **T** | **G** | **G** | **T** | **T** | **G** | **G** | **A** | **G** | **A** | **T** | **G** | **T** | **C** | **G** | **A** | **G** | **G** | **T** | **G** | **A** | **T** | **A** | **C** | **G** | **A** | **A** | **T** | **A** | **-** | **-** | **-** |
| **N54538** | **A** | **T** | **G** | **A** | **A** | **T** | **A** | **G** | **T** | **T** | **C** | **T** | **T** | **G** | **G** | **C** | **G** | **T** | **T** | **T** | **T** | **T** | **T** | **T** | **G** | **T** | **G** | **G** | **T** | **A** | **G** | **T** | **T** | **G** | **A** | **A** | **G** | **C** | **A** | **C** | **T** | **A** | **C** | **T** | **G** | **C** | **T** | **G** | **T** | **T** | **A** | **T** | **T** | **T** | **G** | **C** | **T** | **G** | **T** | **T** | **T** | **G** | **G** | **C** | **A** | **A** | **A** | **T** | **C** | **G** | **T** | **A** | **A** | **A** | **C** | **A** | **A** | **T** | **A** | **C** | **T** | **G** | **G** | **A** | **C** | **T** | **T** | **T** | **T** | **G** | **C** | **T** | **T** | **T** | **T** | **A** | **G** | **T** | **G** | **A** | **T** | **A** | **T** | **T** | **C** | **G** | **G** | **T** | **A** | **T** | **T** | **T** | **T** | **C** | **A** | **A** | **T** | **A** | **T** | **A** | **T** | **A** | **T** | **C** | **T** | **A** | **G** | **C** | **T** | **A** | **T** | **G** | **C** | **G** | **C** | **A** | **A** | **A** | **C** | **A** | **T** | **C** | **C** | **T** | **C** | **G** | **T** | **A** | **C** | **T** | **A** | **A** | **A** | **T** | **T** | **T** | **C** | **A** | **A** | **A** | **A** | **T** | **T** | **T** | **T** | **C** | **A** | **A** | **T** | **T** | **G** | **A** | **T** | **A** | **T** | **T** | **A** | **G** | **G** | **A** | **A** | **G** | **T** | **T** | **T** | **G** | **G** | **T** | **C** | **A** | **T** | **T** | **T** | **T** | **T** | **T** | **T** | **T** | **A** | **G** | **C** | **T** | **T** | **A** | **G** | **T** | **C** | **A** | **A** | **C** | **A** | **G** | **T** | **C** | **C** | **T** | **G** | **C** | **T** | **T** | **T** | **A** | **T** | **G** | **G** | **A** | **T** | **G** | **A** | **T** | **G** | **G** | **C** | **T** | **G** | **T** | **A** | **T** | **T** | **T** | **G** | **C** | **C** | **G** | **T** | **T** | **T** | **T** | **A** | **T** | **T** | **T** | **A** | **T** | **T** | **G** | **G** | **A** | **C** | **T** | **A** | **A** | **A** | **A** | **G** | **G** | **T** | **G** | **T** | **A** | **G** | **A** | **G** | **A** | **T** | **T** | **T** | **C** | **A** | **G** | **G** | **G** | **A** | **T** | **A** | **G** | **A** | **T** | **T** | **T** | **A** | **A** | **C** | **C** | **A** | **A** | **A** | **A** | **A** | **T** | **G** | **C** | **T** | **T** | **T** | **T** | **T** | **G** | **G** | **C** | **G** | **A** | **A** | **A** | **G** | **A** | **A** | **A** | **C** | **A** | **G** | **A** | **T** | **C** | **A** | **T** | **G** | **A** | **T** | **G** | **G** | **T** | **C** | **C** | **A** | **A** | **A** | **C** | **A** | **G** | **A** | **G** | **C** | **A** | **A** | **T** | **T** | **A** | **A** | **A** | **A** | **A** | **C** | **A** | **C** | **A** | **T** | **A** | **A** | **T** | **A** | **A** | **T** | **G** | **A** | **G** | **C** | **G** | **G** | **A** | **A** | **A** | **A** | **G** | **A** | **C** | **A** | **G** | **C** | **A** | **A** | **T** | **T** | **A** | **T** | **T** | **C** | **G** | **G** | **T** | **A** | **A** | **C** | **C** | **A** | **A** | **C** | **G** | **T** | **A** | **T** | **T** | **G** | **G** | **A** | **A** | **A** | **C** | **G** | **A** | **C** | **G** | **T** | **C** | **T** | **A** | **T** | **G** | **A** | **A** | **T** | **G** | **G** | **G** | **A** | **C** | **G** | **A** | **T** | **A** | **T** | **C** | **A** | **A** | **C** | **A** | **T** | **C** | **G** | **C** | **C** | **G** | **T** | **C** | **A** | **T** | **T** | **T** | **C** | **T** | **G** | **G** | **T** | **G** | **A** | **T** | **A** | **C** | **T** | **A** | **T** | **C** | **A** | **T** | **A** | **G** | **A** | **T** | **T** | **T** | **G** | **G** | **G** | **C** | **A** | **A** | **T** | **A** | **C** | **G** | **C** | **T** | **T** | **T** | **T** | **G** | **C** | **C** | **A** | **A** | **A** | **A** | **G** | **A** | **T** | **G** | **A** | **T** | **A** | **A** | **T** | **A** | **T** | **T** | **G** | **T** | **G** | **A** | **T** | **T** | **G** | **T** | **A** | **A** | **G** | **A** | **A** | **A** | **A** | **G** | **G** | **G** | **A** | **T** | **A** | **G** | **G** | **C** | **A** | **G** | **A** | **A** | **C** | **A** | **C** | **G** | **G** | **A** | **T** | **C** | **T** | **T** | **A** | **G** | **T** | **A** | **C** | **C** | **T** | **T** | **T** | **G** | **G** | **G** | **C** | **G** | **T** | **A** | **G** | **C** | **C** | **A** | **T** | **C** | **C** | **G** | **A** | **T** | **T** | **A** | **G** | **A** | **G** | **C** | **A** | **T** | **G** | **C** | **G** | **A** | **C** | **A** | **T** | **T** | **A** | **G** | **T** | **A** | **G** | **G** | **A** | **A** | **A** | **T** | **G** | **T** | **C** | **T** | **T** | **G** | **T** | **T** | **T** | **G** | **A** | **A** | **G** | **A** | **A** | **G** | **A** | **G** | **C** | **A** | **A** | **T** | **T** | **T** | **T** | **C** | **T** | **T** | **T** | **G** | **A** | **A** | **A** | **A** | **A** | **T** | **G** | **A** | **A** | **C** | **A** | **A** | **A** | **T** | **C** | **A** | **A** | **A** | **A** | **T** | **C** | **T** | **A** | **C** | **A** | **G** | **C** | **A** | **G** | **T** | **G** | **A** | **T** | **T** | **A** | **T** | **G** | **A** | **T** | **G** | **A** | **A** | **A** | **A** | **C** | **C** | **C** | **T** | **C** | **G** | **T** | **A** | **G** | **A** | **C** | **T** | **G** | **A** | **A** | **G** | **A** | **T** | **T** | **A** | **T** | **T** | **A** | **C** | **T** | **A** | **A** | **T** | **A** | **C** | **C** | **T** | **T** | **G** | **A** | **T** | **T** | **G** | **G** | **A** | **G** | **A** | **T** | **G** | **T** | **C** | **G** | **A** | **G** | **G** | **T** | **G** | **A** | **T** | **A** | **C** | **G** | **A** | **A** | **T** | **A** | **-** | **-** | **-** |
| **N60519F** | **A** | **T** | **G** | **A** | **A** | **T** | **A** | **G** | **T** | **T** | **C** | **T** | **T** | **G** | **G** | **C** | **G** | **T** | **T** | **T** | **T** | **T** | **T** | **T** | **G** | **T** | **G** | **G** | **T** | **A** | **G** | **T** | **C** | **G** | **A** | **A** | **G** | **C** | **A** | **C** | **T** | **G** | **C** | **T** | **G** | **C** | **T** | **G** | **T** | **T** | **A** | **T** | **T** | **T** | **G** | **C** | **T** | **G** | **T** | **T** | **T** | **G** | **G** | **C** | **A** | **A** | **A** | **T** | **C** | **G** | **T** | **A** | **A** | **A** | **C** | **A** | **A** | **T** | **A** | **C** | **T** | **G** | **G** | **A** | **C** | **T** | **T** | **T** | **T** | **G** | **C** | **T** | **T** | **T** | **T** | **A** | **G** | **T** | **A** | **A** | **T** | **A** | **T** | **T** | **C** | **G** | **G** | **T** | **A** | **T** | **T** | **T** | **T** | **C** | **A** | **A** | **T** | **A** | **T** | **A** | **T** | **A** | **T** | **C** | **T** | **A** | **G** | **C** | **T** | **A** | **T** | **G** | **C** | **G** | **C** | **A** | **A** | **A** | **T** | **A** | **T** | **C** | **C** | **T** | **C** | **G** | **T** | **A** | **C** | **T** | **A** | **A** | **A** | **T** | **T** | **T** | **C** | **A** | **A** | **A** | **A** | **T** | **T** | **T** | **T** | **C** | **A** | **A** | **T** | **T** | **G** | **A** | **T** | **C** | **T** | **T** | **A** | **G** | **G** | **G** | **A** | **G** | **C** | **T** | **T** | **G** | **G** | **T** | **C** | **A** | **T** | **T** | **T** | **T** | **T** | **T** | **T** | **T** | **A** | **G** | **C** | **T** | **T** | **A** | **G** | **T** | **C** | **A** | **A** | **C** | **A** | **G** | **T** | **C** | **C** | **T** | **G** | **C** | **T** | **T** | **T** | **A** | **T** | **G** | **G** | **A** | **T** | **G** | **A** | **T** | **G** | **G** | **T** | **T** | **G** | **T** | **A** | **T** | **T** | **G** | **G** | **C** | **C** | **G** | **T** | **T** | **T** | **T** | **A** | **T** | **T** | **T** | **A** | **T** | **T** | **G** | **G** | **G** | **C** | **T** | **G** | **A** | **A** | **A** | **G** | **G** | **T** | **G** | **T** | **A** | **G** | **A** | **G** | **A** | **T** | **T** | **T** | **C** | **A** | **G** | **G** | **A** | **A** | **T** | **A** | **G** | **A** | **T** | **T** | **T** | **G** | **A** | **C** | **A** | **A** | **A** | **A** | **A** | **A** | **T** | **G** | **C** | **T** | **T** | **T** | **T** | **T** | **G** | **G** | **C** | **G** | **A** | **A** | **A** | **A** | **A** | **A** | **A** | **C** | **A** | **G** | **A** | **T** | **C** | **A** | **T** | **G** | **A** | **T** | **G** | **G** | **T** | **C** | **C** | **A** | **A** | **A** | **C** | **A** | **G** | **A** | **G** | **C** | **A** | **A** | **T** | **T** | **A** | **A** | **A** | **A** | **A** | **C** | **A** | **C** | **A** | **T** | **A** | **A** | **T** | **A** | **A** | **T** | **G** | **A** | **G** | **C** | **G** | **T** | **A** | **A** | **A** | **A** | **G** | **A** | **C** | **A** | **G** | **C** | **A** | **A** | **T** | **T** | **A** | **T** | **T** | **T** | **G** | **G** | **T** | **A** | **A** | **C** | **C** | **A** | **A** | **C** | **G** | **T** | **A** | **T** | **T** | **G** | **G** | **A** | **A** | **A** | **C** | **G** | **A** | **C** | **G** | **T** | **C** | **T** | **A** | **T** | **G** | **A** | **A** | **T** | **G** | **G** | **G** | **A** | **T** | **G** | **A** | **T** | **A** | **T** | **C** | **A** | **A** | **C** | **A** | **T** | **T** | **G** | **C** | **C** | **A** | **T** | **C** | **A** | **T** | **T** | **T** | **C** | **T** | **G** | **G** | **T** | **G** | **A** | **T** | **A** | **C** | **T** | **A** | **T** | **C** | **A** | **T** | **C** | **G** | **A** | **T** | **T** | **T** | **G** | **G** | **G** | **T** | **A** | **A** | **T** | **A** | **C** | **A** | **C** | **T** | **T** | **T** | **T** | **G** | **C** | **C** | **A** | **A** | **A** | **A** | **G** | **A** | **T** | **G** | **A** | **T** | **A** | **A** | **T** | **A** | **T** | **T** | **G** | **T** | **G** | **A** | **T** | **T** | **G** | **T** | **A** | **A** | **G** | **A** | **A** | **A** | **A** | **G** | **G** | **G** | **A** | **T** | **A** | **G** | **G** | **C** | **A** | **G** | **A** | **A** | **C** | **A** | **C** | **G** | **G** | **A** | **T** | **C** | **T** | **T** | **A** | **G** | **T** | **A** | **C** | **C** | **C** | **C** | **T** | **G** | **G** | **G** | **A** | **G** | **T** | **G** | **G** | **C** | **T** | **A** | **T** | **T** | **C** | **G** | **T** | **T** | **T** | **A** | **G** | **A** | **G** | **C** | **A** | **T** | **G** | **C** | **G** | **A** | **C** | **A** | **T** | **T** | **A** | **G** | **T** | **A** | **G** | **G** | **A** | **A** | **A** | **T** | **G** | **T** | **C** | **T** | **T** | **G** | **T** | **T** | **T** | **G** | **A** | **A** | **G** | **A** | **A** | **G** | **A** | **G** | **C** | **A** | **G** | **T** | **T** | **T** | **T** | **C** | **C** | **T** | **T** | **A** | **A** | **A** | **A** | **A** | **A** | **C** | **G** | **A** | **A** | **C** | **A** | **G** | **A** | **T** | **C** | **A** | **A** | **A** | **A** | **T** | **C** | **T** | **G** | **C** | **A** | **G** | **C** | **A** | **G** | **T** | **G** | **A** | **T** | **T** | **A** | **T** | **G** | **A** | **T** | **G** | **A** | **A** | **A** | **A** | **T** | **C** | **C** | **T** | **C** | **G** | **C** | **A** | **G** | **A** | **T** | **T** | **G** | **A** | **A** | **G** | **A** | **T** | **C** | **A** | **T** | **T** | **A** | **C** | **C** | **A** | **A** | **T** | **A** | **C** | **C** | **T** | **T** | **G** | **G** | **T** | **T** | **G** | **G** | **A** | **G** | **A** | **T** | **G** | **T** | **C** | **G** | **A** | **G** | **G** | **T** | **G** | **A** | **T** | **A** | **C** | **G** | **A** | **A** | **T** | **A** | **-** | **-** | **-** |
| **N59435F** | **A** | **T** | **G** | **A** | **A** | **T** | **A** | **G** | **T** | **T** | **C** | **T** | **T** | **G** | **G** | **C** | **G** | **T** | **T** | **T** | **T** | **T** | **T** | **T** | **G** | **T** | **G** | **G** | **T** | **A** | **G** | **T** | **C** | **G** | **A** | **A** | **G** | **C** | **A** | **C** | **T** | **G** | **C** | **T** | **G** | **C** | **T** | **G** | **T** | **T** | **A** | **T** | **T** | **T** | **G** | **C** | **T** | **G** | **T** | **T** | **T** | **G** | **G** | **G** | **A** | **A** | **A** | **T** | **C** | **G** | **T** | **A** | **A** | **A** | **C** | **A** | **A** | **T** | **A** | **C** | **T** | **G** | **G** | **A** | **C** | **T** | **T** | **T** | **T** | **G** | **C** | **T** | **T** | **T** | **T** | **A** | **G** | **T** | **A** | **A** | **T** | **A** | **T** | **T** | **C** | **G** | **G** | **T** | **A** | **T** | **T** | **T** | **T** | **C** | **A** | **A** | **T** | **A** | **T** | **A** | **T** | **A** | **T** | **C** | **T** | **A** | **G** | **C** | **T** | **A** | **T** | **G** | **C** | **G** | **C** | **A** | **A** | **A** | **T** | **A** | **T** | **C** | **C** | **T** | **C** | **G** | **T** | **A** | **C** | **T** | **A** | **A** | **A** | **T** | **T** | **T** | **C** | **A** | **A** | **A** | **A** | **T** | **T** | **T** | **T** | **C** | **A** | **A** | **T** | **T** | **G** | **A** | **T** | **C** | **T** | **T** | **A** | **G** | **G** | **G** | **A** | **G** | **C** | **T** | **T** | **G** | **G** | **T** | **C** | **A** | **T** | **T** | **T** | **T** | **T** | **T** | **T** | **T** | **A** | **G** | **C** | **T** | **T** | **A** | **G** | **T** | **C** | **A** | **A** | **C** | **A** | **G** | **T** | **C** | **C** | **T** | **G** | **C** | **T** | **T** | **T** | **A** | **T** | **G** | **G** | **A** | **T** | **G** | **A** | **T** | **G** | **G** | **T** | **T** | **G** | **T** | **A** | **T** | **T** | **G** | **G** | **C** | **C** | **G** | **T** | **T** | **T** | **T** | **A** | **T** | **T** | **T** | **A** | **T** | **C** | **G** | **G** | **G** | **C** | **T** | **G** | **A** | **A** | **A** | **G** | **G** | **T** | **G** | **T** | **A** | **G** | **A** | **G** | **A** | **T** | **T** | **T** | **C** | **A** | **G** | **G** | **A** | **A** | **T** | **A** | **G** | **A** | **T** | **T** | **T** | **G** | **A** | **C** | **A** | **A** | **A** | **A** | **A** | **A** | **T** | **G** | **C** | **T** | **T** | **T** | **T** | **T** | **G** | **G** | **C** | **G** | **A** | **A** | **A** | **A** | **A** | **A** | **A** | **C** | **A** | **G** | **A** | **T** | **C** | **A** | **T** | **G** | **A** | **T** | **G** | **G** | **T** | **C** | **C** | **A** | **A** | **A** | **C** | **A** | **G** | **A** | **G** | **C** | **A** | **A** | **T** | **T** | **A** | **A** | **A** | **A** | **A** | **C** | **A** | **C** | **A** | **T** | **A** | **A** | **T** | **A** | **A** | **T** | **G** | **A** | **G** | **C** | **G** | **T** | **A** | **A** | **A** | **A** | **G** | **A** | **C** | **A** | **G** | **C** | **A** | **A** | **T** | **T** | **A** | **T** | **T** | **T** | **G** | **G** | **T** | **A** | **A** | **C** | **C** | **A** | **A** | **C** | **G** | **T** | **A** | **T** | **T** | **G** | **G** | **A** | **A** | **A** | **C** | **G** | **A** | **C** | **G** | **T** | **C** | **T** | **A** | **T** | **G** | **A** | **A** | **T** | **G** | **G** | **G** | **A** | **T** | **G** | **A** | **T** | **A** | **T** | **C** | **A** | **A** | **C** | **A** | **T** | **T** | **G** | **C** | **C** | **A** | **T** | **C** | **A** | **T** | **T** | **T** | **C** | **T** | **G** | **G** | **T** | **G** | **A** | **T** | **A** | **C** | **T** | **A** | **T** | **C** | **A** | **T** | **C** | **G** | **A** | **T** | **T** | **T** | **G** | **G** | **G** | **C** | **A** | **A** | **T** | **A** | **C** | **A** | **C** | **T** | **T** | **T** | **T** | **G** | **C** | **C** | **A** | **A** | **A** | **A** | **G** | **A** | **T** | **G** | **A** | **T** | **A** | **A** | **T** | **A** | **T** | **T** | **G** | **T** | **G** | **A** | **T** | **T** | **G** | **T** | **A** | **A** | **G** | **A** | **A** | **A** | **A** | **G** | **G** | **G** | **A** | **T** | **A** | **G** | **G** | **C** | **A** | **G** | **A** | **A** | **C** | **A** | **C** | **G** | **G** | **A** | **T** | **C** | **T** | **T** | **A** | **G** | **T** | **A** | **C** | **C** | **C** | **C** | **T** | **G** | **G** | **G** | **A** | **G** | **T** | **G** | **G** | **C** | **T** | **A** | **T** | **T** | **C** | **G** | **T** | **T** | **T** | **A** | **G** | **A** | **G** | **C** | **A** | **T** | **G** | **C** | **G** | **A** | **C** | **A** | **T** | **T** | **A** | **G** | **T** | **A** | **G** | **G** | **A** | **A** | **A** | **T** | **G** | **T** | **C** | **T** | **T** | **G** | **T** | **T** | **T** | **G** | **A** | **A** | **G** | **A** | **A** | **G** | **A** | **G** | **C** | **A** | **G** | **T** | **T** | **T** | **T** | **C** | **C** | **T** | **T** | **A** | **A** | **A** | **A** | **A** | **A** | **C** | **G** | **A** | **A** | **C** | **A** | **G** | **A** | **T** | **C** | **A** | **A** | **A** | **A** | **T** | **C** | **T** | **G** | **C** | **A** | **G** | **C** | **A** | **G** | **T** | **G** | **A** | **T** | **T** | **A** | **T** | **G** | **A** | **T** | **G** | **A** | **A** | **A** | **A** | **T** | **C** | **C** | **T** | **C** | **G** | **C** | **A** | **G** | **A** | **T** | **T** | **G** | **A** | **A** | **G** | **A** | **T** | **C** | **A** | **T** | **T** | **A** | **C** | **C** | **A** | **A** | **T** | **A** | **C** | **C** | **T** | **T** | **G** | **G** | **T** | **T** | **G** | **G** | **A** | **G** | **A** | **T** | **G** | **T** | **C** | **G** | **A** | **G** | **G** | **T** | **G** | **A** | **T** | **A** | **C** | **G** | **A** | **A** | **T** | **A** | **-** | **-** | **-** |
| **N59779F** | **A** | **T** | **G** | **A** | **A** | **T** | **A** | **G** | **T** | **T** | **C** | **T** | **T** | **G** | **G** | **C** | **G** | **T** | **T** | **T** | **T** | **T** | **T** | **T** | **G** | **T** | **G** | **G** | **T** | **A** | **G** | **T** | **C** | **G** | **A** | **A** | **G** | **C** | **A** | **C** | **T** | **G** | **C** | **T** | **G** | **C** | **T** | **G** | **T** | **T** | **A** | **T** | **T** | **T** | **G** | **C** | **T** | **G** | **T** | **T** | **T** | **G** | **G** | **C** | **A** | **A** | **A** | **T** | **C** | **G** | **T** | **A** | **A** | **A** | **C** | **A** | **A** | **T** | **A** | **C** | **T** | **G** | **G** | **A** | **C** | **T** | **T** | **T** | **T** | **G** | **C** | **T** | **T** | **T** | **T** | **A** | **G** | **T** | **A** | **A** | **T** | **A** | **T** | **T** | **C** | **G** | **G** | **T** | **A** | **T** | **T** | **T** | **T** | **C** | **A** | **A** | **T** | **A** | **T** | **A** | **T** | **A** | **T** | **C** | **T** | **A** | **G** | **C** | **T** | **A** | **T** | **G** | **C** | **G** | **C** | **A** | **A** | **A** | **T** | **A** | **T** | **C** | **C** | **T** | **C** | **G** | **T** | **A** | **C** | **T** | **A** | **A** | **A** | **T** | **T** | **T** | **C** | **A** | **A** | **A** | **A** | **T** | **T** | **T** | **T** | **C** | **A** | **A** | **T** | **T** | **G** | **A** | **T** | **C** | **T** | **T** | **A** | **G** | **G** | **G** | **A** | **G** | **C** | **T** | **T** | **G** | **G** | **T** | **C** | **A** | **T** | **T** | **T** | **T** | **T** | **T** | **T** | **T** | **A** | **G** | **C** | **T** | **T** | **A** | **G** | **T** | **C** | **A** | **A** | **C** | **A** | **G** | **T** | **C** | **C** | **T** | **G** | **C** | **T** | **T** | **T** | **A** | **T** | **G** | **G** | **A** | **T** | **G** | **A** | **T** | **G** | **G** | **T** | **T** | **G** | **T** | **A** | **T** | **T** | **G** | **G** | **C** | **C** | **G** | **T** | **T** | **T** | **T** | **A** | **T** | **T** | **T** | **A** | **T** | **C** | **G** | **G** | **G** | **C** | **T** | **G** | **A** | **A** | **A** | **G** | **G** | **T** | **G** | **T** | **A** | **G** | **A** | **G** | **A** | **T** | **T** | **T** | **C** | **A** | **G** | **G** | **A** | **A** | **T** | **A** | **G** | **A** | **T** | **T** | **T** | **G** | **A** | **C** | **A** | **A** | **A** | **A** | **A** | **A** | **T** | **G** | **C** | **T** | **T** | **T** | **T** | **T** | **G** | **G** | **C** | **G** | **A** | **A** | **A** | **A** | **A** | **A** | **A** | **C** | **A** | **G** | **A** | **T** | **C** | **A** | **T** | **G** | **A** | **T** | **G** | **G** | **T** | **C** | **C** | **A** | **A** | **A** | **C** | **A** | **G** | **A** | **G** | **C** | **A** | **A** | **T** | **T** | **A** | **A** | **A** | **A** | **A** | **C** | **A** | **C** | **A** | **T** | **A** | **A** | **T** | **A** | **A** | **T** | **G** | **A** | **G** | **C** | **G** | **T** | **A** | **A** | **A** | **A** | **G** | **A** | **C** | **A** | **G** | **C** | **A** | **A** | **T** | **T** | **A** | **T** | **T** | **T** | **G** | **G** | **T** | **A** | **A** | **C** | **C** | **A** | **A** | **C** | **G** | **T** | **A** | **T** | **T** | **G** | **G** | **A** | **A** | **A** | **C** | **G** | **A** | **C** | **G** | **T** | **C** | **T** | **A** | **T** | **G** | **A** | **A** | **T** | **G** | **G** | **G** | **A** | **T** | **G** | **A** | **T** | **A** | **T** | **C** | **A** | **A** | **C** | **A** | **T** | **T** | **G** | **C** | **C** | **A** | **T** | **C** | **A** | **T** | **T** | **T** | **C** | **T** | **G** | **G** | **T** | **G** | **A** | **T** | **A** | **C** | **T** | **A** | **T** | **C** | **A** | **T** | **C** | **G** | **A** | **T** | **T** | **T** | **G** | **G** | **G** | **C** | **A** | **A** | **T** | **A** | **C** | **A** | **C** | **T** | **T** | **T** | **T** | **G** | **C** | **C** | **A** | **A** | **A** | **A** | **G** | **A** | **T** | **G** | **A** | **T** | **A** | **A** | **T** | **A** | **T** | **T** | **G** | **T** | **G** | **A** | **T** | **T** | **G** | **T** | **A** | **A** | **G** | **A** | **A** | **A** | **A** | **G** | **G** | **G** | **A** | **T** | **A** | **G** | **G** | **C** | **A** | **G** | **A** | **A** | **C** | **A** | **C** | **G** | **G** | **A** | **T** | **C** | **T** | **T** | **A** | **G** | **T** | **A** | **C** | **C** | **C** | **C** | **T** | **G** | **G** | **G** | **A** | **G** | **T** | **G** | **G** | **C** | **T** | **A** | **T** | **T** | **C** | **G** | **T** | **T** | **T** | **A** | **G** | **A** | **G** | **C** | **A** | **T** | **G** | **C** | **G** | **A** | **C** | **A** | **T** | **T** | **A** | **G** | **T** | **A** | **G** | **G** | **A** | **A** | **A** | **T** | **G** | **T** | **C** | **T** | **T** | **G** | **T** | **T** | **T** | **G** | **A** | **A** | **G** | **A** | **A** | **G** | **A** | **G** | **C** | **A** | **G** | **T** | **T** | **T** | **T** | **C** | **C** | **T** | **T** | **A** | **A** | **A** | **A** | **A** | **A** | **C** | **G** | **A** | **A** | **C** | **A** | **G** | **A** | **T** | **C** | **A** | **A** | **A** | **A** | **T** | **C** | **T** | **G** | **C** | **A** | **G** | **C** | **A** | **G** | **T** | **G** | **A** | **T** | **T** | **A** | **T** | **G** | **A** | **T** | **G** | **A** | **A** | **A** | **A** | **T** | **C** | **C** | **T** | **C** | **G** | **C** | **A** | **G** | **A** | **T** | **T** | **G** | **A** | **A** | **G** | **A** | **T** | **C** | **A** | **T** | **T** | **A** | **C** | **C** | **A** | **A** | **T** | **A** | **C** | **C** | **T** | **T** | **G** | **G** | **T** | **T** | **G** | **G** | **A** | **G** | **A** | **T** | **G** | **T** | **C** | **G** | **A** | **G** | **G** | **T** | **G** | **A** | **T** | **A** | **C** | **G** | **A** | **A** | **T** | **A** | **-** | **-** | **-** |
| **N59567F** | **A** | **T** | **G** | **A** | **A** | **T** | **A** | **G** | **T** | **T** | **C** | **T** | **T** | **G** | **G** | **C** | **G** | **T** | **T** | **T** | **T** | **T** | **T** | **T** | **G** | **T** | **G** | **G** | **T** | **A** | **G** | **T** | **C** | **G** | **A** | **A** | **G** | **C** | **A** | **C** | **T** | **G** | **C** | **T** | **G** | **C** | **T** | **G** | **T** | **T** | **A** | **T** | **T** | **T** | **G** | **C** | **T** | **G** | **T** | **T** | **T** | **G** | **G** | **C** | **A** | **A** | **A** | **T** | **C** | **G** | **T** | **A** | **A** | **A** | **C** | **A** | **A** | **T** | **A** | **C** | **T** | **G** | **G** | **A** | **C** | **T** | **T** | **T** | **T** | **G** | **C** | **T** | **T** | **T** | **T** | **A** | **G** | **T** | **A** | **A** | **T** | **A** | **T** | **T** | **C** | **G** | **G** | **T** | **A** | **T** | **T** | **T** | **T** | **C** | **A** | **A** | **T** | **A** | **T** | **A** | **T** | **A** | **T** | **C** | **T** | **A** | **G** | **C** | **T** | **A** | **T** | **G** | **C** | **G** | **C** | **A** | **A** | **A** | **T** | **A** | **T** | **C** | **C** | **T** | **C** | **G** | **T** | **A** | **C** | **T** | **A** | **A** | **A** | **T** | **T** | **T** | **C** | **A** | **A** | **A** | **A** | **T** | **T** | **T** | **T** | **C** | **A** | **A** | **T** | **T** | **G** | **A** | **T** | **C** | **T** | **T** | **A** | **G** | **G** | **G** | **A** | **G** | **C** | **T** | **T** | **G** | **G** | **T** | **C** | **A** | **T** | **T** | **T** | **T** | **T** | **T** | **T** | **T** | **A** | **G** | **C** | **T** | **T** | **A** | **G** | **T** | **C** | **A** | **A** | **C** | **A** | **G** | **T** | **C** | **C** | **T** | **G** | **C** | **T** | **T** | **T** | **A** | **T** | **G** | **G** | **A** | **T** | **G** | **A** | **T** | **G** | **G** | **T** | **T** | **G** | **T** | **A** | **T** | **T** | **G** | **G** | **C** | **C** | **G** | **T** | **T** | **T** | **T** | **A** | **T** | **T** | **T** | **A** | **T** | **C** | **G** | **G** | **G** | **C** | **T** | **G** | **A** | **A** | **A** | **G** | **G** | **T** | **G** | **T** | **A** | **G** | **A** | **G** | **A** | **T** | **T** | **T** | **C** | **A** | **G** | **G** | **A** | **A** | **T** | **A** | **G** | **A** | **T** | **T** | **T** | **G** | **A** | **C** | **A** | **A** | **A** | **A** | **A** | **A** | **T** | **G** | **C** | **T** | **T** | **T** | **T** | **T** | **G** | **G** | **C** | **G** | **A** | **A** | **A** | **A** | **A** | **A** | **A** | **C** | **A** | **G** | **A** | **T** | **C** | **A** | **T** | **G** | **A** | **T** | **G** | **G** | **T** | **C** | **C** | **A** | **A** | **A** | **C** | **A** | **G** | **A** | **G** | **C** | **A** | **A** | **T** | **T** | **A** | **A** | **A** | **A** | **A** | **C** | **A** | **C** | **A** | **T** | **A** | **A** | **T** | **A** | **A** | **T** | **G** | **A** | **G** | **C** | **G** | **T** | **A** | **A** | **A** | **A** | **G** | **A** | **C** | **A** | **G** | **C** | **A** | **A** | **T** | **T** | **A** | **T** | **T** | **T** | **G** | **G** | **T** | **A** | **A** | **C** | **C** | **A** | **A** | **C** | **G** | **T** | **A** | **T** | **T** | **G** | **G** | **A** | **A** | **A** | **C** | **G** | **A** | **C** | **G** | **T** | **C** | **T** | **A** | **T** | **G** | **A** | **A** | **T** | **G** | **G** | **G** | **A** | **T** | **G** | **A** | **T** | **A** | **T** | **C** | **A** | **A** | **C** | **A** | **T** | **T** | **G** | **C** | **C** | **A** | **T** | **C** | **A** | **T** | **T** | **T** | **C** | **T** | **G** | **G** | **T** | **G** | **A** | **T** | **A** | **C** | **T** | **A** | **T** | **C** | **A** | **T** | **C** | **G** | **A** | **T** | **T** | **T** | **G** | **G** | **G** | **C** | **A** | **A** | **T** | **A** | **C** | **A** | **C** | **T** | **T** | **T** | **T** | **G** | **C** | **C** | **A** | **A** | **A** | **A** | **G** | **A** | **T** | **G** | **A** | **T** | **A** | **A** | **T** | **A** | **T** | **T** | **G** | **T** | **G** | **A** | **T** | **T** | **G** | **T** | **A** | **A** | **G** | **A** | **A** | **A** | **A** | **G** | **G** | **G** | **A** | **T** | **A** | **G** | **G** | **C** | **A** | **G** | **A** | **A** | **C** | **A** | **C** | **G** | **G** | **A** | **T** | **C** | **T** | **T** | **A** | **G** | **T** | **A** | **C** | **C** | **C** | **C** | **T** | **G** | **G** | **G** | **A** | **G** | **T** | **G** | **G** | **C** | **T** | **A** | **T** | **T** | **C** | **G** | **T** | **T** | **T** | **A** | **G** | **A** | **G** | **C** | **A** | **T** | **G** | **C** | **G** | **A** | **C** | **A** | **T** | **T** | **A** | **G** | **T** | **A** | **G** | **G** | **A** | **A** | **A** | **T** | **G** | **T** | **C** | **T** | **T** | **G** | **T** | **T** | **T** | **G** | **A** | **A** | **G** | **A** | **A** | **G** | **A** | **G** | **C** | **A** | **G** | **T** | **T** | **T** | **T** | **C** | **C** | **T** | **T** | **A** | **A** | **A** | **A** | **A** | **A** | **C** | **G** | **A** | **A** | **C** | **A** | **G** | **A** | **T** | **C** | **A** | **A** | **A** | **A** | **T** | **C** | **T** | **G** | **C** | **A** | **G** | **C** | **A** | **G** | **T** | **G** | **A** | **T** | **T** | **A** | **T** | **G** | **A** | **T** | **G** | **A** | **A** | **A** | **A** | **T** | **C** | **C** | **T** | **C** | **G** | **C** | **A** | **G** | **A** | **T** | **T** | **G** | **A** | **A** | **G** | **A** | **T** | **C** | **A** | **T** | **T** | **A** | **C** | **C** | **A** | **A** | **T** | **A** | **C** | **C** | **T** | **T** | **G** | **G** | **T** | **T** | **G** | **G** | **A** | **G** | **A** | **T** | **G** | **T** | **C** | **G** | **A** | **G** | **G** | **T** | **G** | **A** | **T** | **A** | **C** | **G** | **A** | **A** | **T** | **A** | **-** | **-** | **-** |
| **N52629** | **A** | **T** | **G** | **A** | **A** | **T** | **A** | **G** | **T** | **T** | **C** | **T** | **T** | **G** | **G** | **C** | **G** | **T** | **T** | **T** | **T** | **T** | **T** | **T** | **G** | **T** | **G** | **G** | **T** | **A** | **G** | **T** | **C** | **G** | **A** | **A** | **G** | **C** | **A** | **C** | **T** | **G** | **C** | **T** | **G** | **C** | **T** | **G** | **T** | **T** | **A** | **T** | **T** | **T** | **G** | **C** | **T** | **G** | **T** | **T** | **T** | **G** | **G** | **C** | **A** | **A** | **A** | **T** | **C** | **G** | **T** | **A** | **A** | **A** | **C** | **A** | **A** | **T** | **A** | **C** | **T** | **G** | **G** | **A** | **C** | **T** | **T** | **T** | **T** | **G** | **C** | **T** | **T** | **T** | **T** | **A** | **G** | **T** | **A** | **A** | **T** | **A** | **T** | **T** | **C** | **G** | **G** | **T** | **A** | **T** | **T** | **T** | **T** | **C** | **A** | **A** | **T** | **A** | **T** | **A** | **T** | **A** | **T** | **C** | **T** | **A** | **G** | **C** | **T** | **A** | **T** | **G** | **C** | **G** | **C** | **A** | **A** | **A** | **T** | **A** | **T** | **C** | **C** | **T** | **C** | **G** | **T** | **A** | **C** | **T** | **A** | **A** | **A** | **T** | **T** | **T** | **C** | **A** | **A** | **A** | **A** | **T** | **T** | **T** | **T** | **C** | **A** | **A** | **T** | **T** | **G** | **A** | **T** | **C** | **T** | **T** | **A** | **G** | **G** | **G** | **A** | **G** | **C** | **T** | **T** | **G** | **G** | **T** | **C** | **A** | **T** | **T** | **T** | **T** | **T** | **T** | **T** | **T** | **A** | **G** | **C** | **T** | **T** | **A** | **G** | **T** | **C** | **A** | **A** | **C** | **A** | **G** | **T** | **C** | **C** | **T** | **G** | **C** | **T** | **T** | **T** | **A** | **T** | **G** | **G** | **A** | **T** | **G** | **A** | **T** | **G** | **G** | **T** | **T** | **G** | **T** | **A** | **T** | **T** | **G** | **G** | **C** | **C** | **G** | **T** | **T** | **T** | **T** | **A** | **T** | **T** | **T** | **A** | **T** | **C** | **G** | **G** | **G** | **C** | **T** | **G** | **A** | **A** | **A** | **G** | **G** | **T** | **G** | **T** | **A** | **G** | **A** | **G** | **A** | **T** | **T** | **T** | **C** | **A** | **G** | **G** | **A** | **A** | **T** | **A** | **G** | **A** | **T** | **T** | **T** | **G** | **A** | **C** | **A** | **A** | **A** | **A** | **A** | **A** | **T** | **G** | **C** | **T** | **T** | **T** | **T** | **T** | **G** | **G** | **C** | **G** | **A** | **A** | **A** | **A** | **A** | **A** | **A** | **C** | **A** | **G** | **A** | **T** | **C** | **A** | **T** | **G** | **A** | **T** | **G** | **G** | **T** | **C** | **C** | **A** | **A** | **A** | **C** | **A** | **G** | **A** | **G** | **C** | **A** | **A** | **T** | **T** | **A** | **A** | **A** | **A** | **A** | **C** | **A** | **C** | **A** | **T** | **A** | **A** | **T** | **A** | **A** | **T** | **G** | **A** | **G** | **C** | **G** | **T** | **A** | **A** | **A** | **A** | **G** | **A** | **C** | **A** | **G** | **C** | **A** | **A** | **T** | **T** | **A** | **T** | **T** | **T** | **G** | **G** | **T** | **A** | **A** | **C** | **C** | **A** | **A** | **C** | **G** | **T** | **A** | **T** | **T** | **G** | **G** | **A** | **A** | **A** | **C** | **G** | **A** | **C** | **G** | **T** | **C** | **T** | **A** | **T** | **G** | **A** | **A** | **T** | **G** | **G** | **G** | **A** | **T** | **G** | **A** | **T** | **A** | **T** | **C** | **A** | **A** | **C** | **A** | **T** | **T** | **G** | **C** | **C** | **A** | **T** | **C** | **A** | **T** | **T** | **T** | **C** | **T** | **G** | **G** | **T** | **G** | **A** | **T** | **A** | **C** | **T** | **A** | **T** | **C** | **A** | **T** | **C** | **G** | **A** | **T** | **T** | **T** | **G** | **G** | **G** | **C** | **A** | **A** | **T** | **A** | **C** | **A** | **C** | **T** | **T** | **T** | **T** | **G** | **C** | **C** | **A** | **A** | **A** | **A** | **G** | **A** | **T** | **G** | **A** | **T** | **A** | **A** | **T** | **A** | **T** | **T** | **G** | **T** | **G** | **A** | **T** | **T** | **G** | **T** | **A** | **A** | **G** | **A** | **A** | **A** | **A** | **G** | **G** | **G** | **A** | **T** | **A** | **G** | **G** | **C** | **A** | **G** | **A** | **A** | **C** | **A** | **C** | **G** | **G** | **A** | **T** | **C** | **T** | **T** | **A** | **G** | **T** | **A** | **C** | **C** | **C** | **C** | **T** | **G** | **G** | **G** | **A** | **G** | **T** | **G** | **G** | **C** | **T** | **A** | **T** | **T** | **C** | **G** | **T** | **T** | **T** | **A** | **G** | **A** | **G** | **C** | **A** | **T** | **G** | **C** | **G** | **A** | **C** | **A** | **T** | **T** | **A** | **G** | **T** | **A** | **G** | **G** | **A** | **A** | **A** | **T** | **G** | **T** | **C** | **T** | **T** | **G** | **T** | **T** | **T** | **G** | **A** | **A** | **G** | **A** | **A** | **G** | **A** | **G** | **C** | **A** | **G** | **T** | **T** | **T** | **T** | **C** | **C** | **T** | **T** | **A** | **A** | **A** | **A** | **A** | **A** | **C** | **G** | **A** | **A** | **C** | **A** | **G** | **A** | **T** | **C** | **A** | **A** | **A** | **A** | **T** | **C** | **T** | **G** | **C** | **A** | **G** | **C** | **A** | **G** | **T** | **G** | **A** | **T** | **T** | **A** | **T** | **G** | **A** | **T** | **G** | **A** | **A** | **A** | **A** | **T** | **C** | **C** | **T** | **C** | **G** | **C** | **A** | **G** | **A** | **T** | **T** | **G** | **A** | **A** | **G** | **A** | **T** | **C** | **A** | **T** | **T** | **A** | **C** | **C** | **A** | **A** | **T** | **A** | **C** | **C** | **T** | **T** | **G** | **G** | **T** | **T** | **G** | **G** | **A** | **G** | **A** | **T** | **G** | **T** | **C** | **G** | **A** | **G** | **G** | **T** | **G** | **A** | **T** | **A** | **C** | **G** | **A** | **A** | **T** | **A** | **-** | **-** | **-** |
| **N53479** | **A** | **T** | **G** | **A** | **A** | **T** | **A** | **G** | **T** | **T** | **C** | **T** | **T** | **G** | **G** | **C** | **G** | **T** | **T** | **T** | **T** | **T** | **T** | **T** | **G** | **T** | **G** | **G** | **T** | **A** | **G** | **T** | **C** | **G** | **A** | **A** | **G** | **C** | **A** | **C** | **T** | **G** | **C** | **T** | **G** | **C** | **T** | **G** | **T** | **T** | **A** | **T** | **T** | **T** | **G** | **C** | **T** | **G** | **T** | **T** | **T** | **G** | **G** | **C** | **A** | **A** | **A** | **T** | **C** | **G** | **T** | **A** | **A** | **A** | **C** | **A** | **A** | **T** | **A** | **C** | **T** | **G** | **G** | **A** | **C** | **T** | **T** | **T** | **T** | **G** | **C** | **T** | **T** | **T** | **T** | **A** | **G** | **T** | **A** | **A** | **T** | **A** | **T** | **T** | **C** | **G** | **G** | **T** | **A** | **T** | **T** | **T** | **T** | **C** | **A** | **A** | **T** | **A** | **T** | **A** | **T** | **A** | **T** | **C** | **T** | **A** | **G** | **C** | **T** | **A** | **T** | **G** | **C** | **G** | **C** | **A** | **A** | **A** | **T** | **A** | **T** | **C** | **C** | **T** | **C** | **G** | **T** | **A** | **C** | **T** | **A** | **A** | **A** | **T** | **T** | **T** | **C** | **A** | **A** | **A** | **A** | **T** | **T** | **T** | **T** | **C** | **A** | **A** | **T** | **T** | **G** | **A** | **T** | **C** | **T** | **T** | **A** | **G** | **G** | **G** | **A** | **G** | **C** | **T** | **T** | **G** | **G** | **T** | **C** | **A** | **T** | **T** | **T** | **T** | **T** | **T** | **T** | **T** | **A** | **G** | **C** | **T** | **T** | **A** | **G** | **T** | **C** | **A** | **A** | **C** | **A** | **G** | **T** | **C** | **C** | **T** | **G** | **C** | **T** | **T** | **T** | **A** | **T** | **G** | **G** | **A** | **T** | **G** | **A** | **T** | **G** | **G** | **T** | **T** | **G** | **T** | **A** | **T** | **T** | **G** | **G** | **C** | **C** | **G** | **T** | **T** | **T** | **T** | **A** | **T** | **T** | **T** | **A** | **T** | **C** | **G** | **G** | **G** | **C** | **T** | **G** | **A** | **A** | **A** | **G** | **G** | **T** | **G** | **T** | **A** | **G** | **A** | **G** | **A** | **T** | **T** | **T** | **C** | **A** | **G** | **G** | **A** | **A** | **T** | **A** | **G** | **A** | **T** | **T** | **T** | **G** | **A** | **C** | **A** | **A** | **A** | **A** | **A** | **A** | **T** | **G** | **C** | **T** | **T** | **T** | **T** | **T** | **G** | **G** | **C** | **G** | **A** | **A** | **A** | **A** | **A** | **A** | **A** | **C** | **A** | **G** | **A** | **T** | **C** | **A** | **T** | **G** | **A** | **T** | **G** | **G** | **T** | **C** | **C** | **A** | **A** | **A** | **C** | **A** | **G** | **A** | **G** | **C** | **A** | **A** | **T** | **T** | **A** | **A** | **A** | **A** | **A** | **C** | **A** | **C** | **A** | **T** | **A** | **A** | **T** | **A** | **A** | **T** | **G** | **A** | **G** | **C** | **G** | **T** | **A** | **A** | **A** | **A** | **G** | **A** | **C** | **A** | **G** | **C** | **A** | **A** | **T** | **T** | **A** | **T** | **T** | **T** | **G** | **G** | **T** | **A** | **A** | **C** | **C** | **A** | **A** | **C** | **G** | **T** | **A** | **T** | **T** | **G** | **G** | **A** | **A** | **A** | **C** | **G** | **A** | **C** | **G** | **T** | **C** | **T** | **A** | **T** | **G** | **A** | **A** | **T** | **G** | **G** | **G** | **A** | **T** | **G** | **A** | **T** | **A** | **T** | **C** | **A** | **A** | **C** | **A** | **T** | **T** | **G** | **C** | **C** | **A** | **T** | **C** | **A** | **T** | **T** | **T** | **C** | **T** | **G** | **G** | **T** | **G** | **A** | **T** | **A** | **C** | **T** | **A** | **T** | **C** | **A** | **T** | **C** | **G** | **A** | **T** | **T** | **T** | **G** | **G** | **G** | **C** | **A** | **A** | **T** | **A** | **C** | **A** | **C** | **T** | **T** | **T** | **T** | **G** | **C** | **C** | **A** | **A** | **A** | **A** | **G** | **A** | **T** | **G** | **A** | **T** | **A** | **A** | **T** | **A** | **T** | **T** | **G** | **T** | **G** | **A** | **T** | **T** | **G** | **T** | **A** | **A** | **G** | **A** | **A** | **A** | **A** | **G** | **G** | **G** | **A** | **T** | **A** | **G** | **G** | **C** | **A** | **G** | **A** | **A** | **C** | **A** | **C** | **G** | **G** | **A** | **T** | **C** | **T** | **T** | **A** | **G** | **T** | **A** | **C** | **C** | **C** | **C** | **T** | **G** | **G** | **G** | **A** | **G** | **T** | **G** | **G** | **C** | **T** | **A** | **T** | **T** | **C** | **G** | **T** | **T** | **T** | **A** | **G** | **A** | **G** | **C** | **A** | **T** | **G** | **C** | **G** | **A** | **C** | **A** | **T** | **T** | **A** | **G** | **T** | **A** | **G** | **G** | **A** | **A** | **A** | **T** | **G** | **T** | **C** | **T** | **T** | **G** | **T** | **T** | **T** | **G** | **A** | **A** | **G** | **A** | **A** | **G** | **A** | **G** | **C** | **A** | **G** | **T** | **T** | **T** | **T** | **C** | **C** | **T** | **T** | **A** | **A** | **A** | **A** | **A** | **A** | **C** | **G** | **A** | **A** | **C** | **A** | **G** | **A** | **T** | **C** | **A** | **A** | **A** | **A** | **T** | **C** | **T** | **G** | **C** | **A** | **G** | **C** | **A** | **G** | **T** | **G** | **A** | **T** | **T** | **A** | **T** | **G** | **A** | **T** | **G** | **A** | **A** | **A** | **A** | **T** | **C** | **C** | **T** | **C** | **G** | **C** | **A** | **G** | **A** | **T** | **T** | **G** | **A** | **A** | **G** | **A** | **T** | **C** | **A** | **T** | **T** | **A** | **C** | **C** | **A** | **A** | **T** | **A** | **C** | **C** | **T** | **T** | **G** | **G** | **T** | **T** | **G** | **G** | **A** | **G** | **A** | **T** | **G** | **T** | **C** | **G** | **A** | **G** | **G** | **T** | **G** | **A** | **T** | **A** | **C** | **G** | **A** | **A** | **T** | **A** | **-** | **-** | **-** |
| **N60365F** | **A** | **T** | **G** | **A** | **A** | **T** | **A** | **G** | **T** | **T** | **C** | **T** | **T** | **G** | **G** | **C** | **G** | **T** | **T** | **T** | **T** | **T** | **T** | **T** | **G** | **T** | **G** | **G** | **T** | **A** | **G** | **T** | **C** | **G** | **A** | **A** | **G** | **C** | **A** | **C** | **T** | **G** | **C** | **T** | **G** | **C** | **T** | **G** | **T** | **T** | **A** | **T** | **T** | **T** | **G** | **C** | **T** | **G** | **T** | **T** | **T** | **G** | **G** | **C** | **A** | **A** | **A** | **T** | **C** | **G** | **T** | **A** | **A** | **A** | **C** | **A** | **A** | **T** | **A** | **C** | **T** | **G** | **G** | **A** | **C** | **T** | **T** | **T** | **T** | **G** | **C** | **T** | **T** | **T** | **T** | **A** | **G** | **T** | **A** | **A** | **T** | **A** | **T** | **T** | **C** | **G** | **G** | **T** | **A** | **T** | **T** | **T** | **T** | **C** | **A** | **A** | **T** | **A** | **T** | **A** | **T** | **A** | **T** | **C** | **T** | **A** | **G** | **C** | **T** | **A** | **T** | **G** | **C** | **G** | **C** | **A** | **A** | **A** | **T** | **A** | **T** | **C** | **C** | **T** | **C** | **G** | **T** | **A** | **C** | **T** | **A** | **A** | **A** | **T** | **T** | **T** | **C** | **A** | **A** | **A** | **A** | **T** | **T** | **T** | **T** | **C** | **A** | **A** | **T** | **T** | **G** | **A** | **T** | **C** | **T** | **T** | **A** | **G** | **G** | **G** | **A** | **G** | **C** | **T** | **T** | **G** | **G** | **T** | **C** | **A** | **T** | **T** | **T** | **T** | **T** | **T** | **T** | **T** | **A** | **G** | **C** | **T** | **T** | **A** | **G** | **T** | **C** | **A** | **A** | **C** | **A** | **G** | **T** | **C** | **C** | **T** | **G** | **C** | **T** | **T** | **T** | **A** | **T** | **G** | **G** | **A** | **T** | **G** | **A** | **T** | **G** | **G** | **T** | **T** | **G** | **T** | **A** | **T** | **T** | **G** | **G** | **C** | **C** | **G** | **T** | **T** | **T** | **T** | **A** | **T** | **T** | **T** | **A** | **T** | **C** | **G** | **G** | **G** | **C** | **T** | **G** | **A** | **A** | **A** | **G** | **G** | **T** | **G** | **T** | **A** | **G** | **A** | **G** | **A** | **T** | **T** | **T** | **C** | **A** | **G** | **G** | **A** | **A** | **T** | **A** | **G** | **A** | **T** | **T** | **T** | **G** | **A** | **C** | **A** | **A** | **A** | **A** | **A** | **A** | **T** | **G** | **C** | **T** | **T** | **T** | **T** | **T** | **G** | **G** | **C** | **G** | **A** | **A** | **A** | **A** | **A** | **A** | **A** | **C** | **A** | **G** | **A** | **T** | **C** | **A** | **T** | **G** | **A** | **T** | **G** | **G** | **T** | **C** | **C** | **A** | **A** | **A** | **C** | **A** | **G** | **A** | **G** | **C** | **A** | **A** | **T** | **T** | **A** | **A** | **A** | **A** | **A** | **C** | **A** | **C** | **A** | **T** | **A** | **A** | **T** | **A** | **A** | **T** | **G** | **A** | **G** | **C** | **G** | **T** | **A** | **A** | **A** | **A** | **G** | **A** | **C** | **A** | **G** | **C** | **A** | **A** | **T** | **T** | **A** | **T** | **T** | **T** | **G** | **G** | **T** | **A** | **A** | **C** | **C** | **A** | **A** | **C** | **G** | **T** | **A** | **T** | **T** | **G** | **G** | **A** | **A** | **A** | **C** | **G** | **A** | **C** | **G** | **T** | **C** | **T** | **A** | **T** | **G** | **A** | **A** | **T** | **G** | **G** | **G** | **A** | **T** | **G** | **A** | **T** | **A** | **T** | **C** | **A** | **A** | **C** | **A** | **T** | **T** | **G** | **C** | **C** | **A** | **T** | **C** | **A** | **T** | **T** | **T** | **C** | **T** | **G** | **G** | **T** | **G** | **A** | **T** | **A** | **C** | **T** | **A** | **T** | **C** | **A** | **T** | **C** | **G** | **A** | **T** | **T** | **T** | **G** | **G** | **G** | **C** | **A** | **A** | **T** | **A** | **C** | **A** | **C** | **T** | **T** | **T** | **T** | **G** | **C** | **C** | **A** | **A** | **A** | **A** | **G** | **A** | **T** | **G** | **A** | **T** | **A** | **A** | **T** | **A** | **T** | **T** | **G** | **T** | **G** | **A** | **T** | **T** | **G** | **T** | **A** | **A** | **G** | **A** | **A** | **A** | **A** | **G** | **G** | **G** | **A** | **T** | **A** | **G** | **G** | **C** | **A** | **G** | **A** | **A** | **C** | **A** | **C** | **G** | **G** | **A** | **T** | **C** | **T** | **T** | **A** | **G** | **T** | **A** | **C** | **C** | **C** | **C** | **T** | **G** | **G** | **G** | **A** | **G** | **T** | **G** | **G** | **C** | **T** | **A** | **T** | **T** | **C** | **G** | **T** | **T** | **T** | **A** | **G** | **A** | **G** | **C** | **A** | **T** | **G** | **C** | **G** | **A** | **C** | **A** | **T** | **T** | **A** | **G** | **T** | **A** | **G** | **G** | **A** | **A** | **A** | **T** | **G** | **T** | **C** | **T** | **T** | **G** | **T** | **T** | **T** | **G** | **A** | **A** | **G** | **A** | **A** | **G** | **A** | **G** | **C** | **A** | **G** | **T** | **T** | **T** | **T** | **C** | **C** | **T** | **T** | **A** | **A** | **A** | **A** | **A** | **A** | **C** | **G** | **A** | **A** | **C** | **A** | **G** | **A** | **T** | **C** | **A** | **A** | **A** | **A** | **T** | **C** | **T** | **G** | **C** | **A** | **G** | **C** | **A** | **G** | **T** | **G** | **A** | **T** | **T** | **A** | **T** | **G** | **A** | **T** | **G** | **A** | **A** | **A** | **A** | **T** | **C** | **C** | **T** | **C** | **G** | **C** | **A** | **G** | **A** | **T** | **T** | **G** | **A** | **A** | **G** | **A** | **T** | **C** | **A** | **T** | **T** | **A** | **C** | **C** | **A** | **A** | **T** | **A** | **C** | **C** | **T** | **T** | **G** | **G** | **T** | **T** | **G** | **G** | **A** | **G** | **A** | **T** | **G** | **T** | **C** | **G** | **A** | **G** | **G** | **T** | **G** | **A** | **T** | **A** | **C** | **G** | **A** | **A** | **T** | **A** | **-** | **-** | **-** |
| **N59439F** | **A** | **T** | **G** | **A** | **A** | **T** | **A** | **G** | **T** | **T** | **C** | **T** | **T** | **G** | **G** | **C** | **G** | **T** | **T** | **T** | **T** | **T** | **T** | **T** | **G** | **T** | **G** | **G** | **T** | **A** | **G** | **T** | **C** | **G** | **A** | **A** | **G** | **C** | **A** | **C** | **T** | **G** | **C** | **T** | **G** | **C** | **T** | **G** | **T** | **T** | **A** | **T** | **T** | **T** | **G** | **C** | **T** | **G** | **T** | **T** | **T** | **G** | **G** | **C** | **A** | **A** | **A** | **T** | **C** | **G** | **T** | **A** | **A** | **A** | **C** | **A** | **A** | **T** | **A** | **C** | **T** | **G** | **G** | **A** | **C** | **T** | **T** | **T** | **T** | **G** | **C** | **T** | **T** | **T** | **T** | **A** | **G** | **T** | **A** | **A** | **T** | **A** | **T** | **T** | **C** | **G** | **G** | **T** | **A** | **T** | **T** | **T** | **T** | **C** | **A** | **A** | **T** | **A** | **T** | **A** | **T** | **A** | **T** | **C** | **T** | **A** | **G** | **C** | **T** | **A** | **T** | **G** | **C** | **G** | **C** | **A** | **A** | **A** | **T** | **A** | **T** | **C** | **C** | **T** | **C** | **G** | **T** | **A** | **C** | **T** | **A** | **A** | **A** | **T** | **T** | **T** | **C** | **A** | **A** | **A** | **A** | **T** | **T** | **T** | **T** | **C** | **A** | **A** | **T** | **T** | **G** | **A** | **T** | **C** | **T** | **T** | **A** | **G** | **G** | **G** | **A** | **G** | **C** | **T** | **T** | **G** | **G** | **T** | **C** | **A** | **T** | **T** | **T** | **T** | **T** | **T** | **T** | **T** | **A** | **G** | **C** | **T** | **T** | **A** | **G** | **T** | **C** | **A** | **A** | **C** | **A** | **G** | **T** | **C** | **C** | **T** | **G** | **C** | **T** | **T** | **T** | **A** | **T** | **G** | **G** | **A** | **T** | **G** | **A** | **T** | **G** | **G** | **T** | **T** | **G** | **T** | **A** | **T** | **T** | **G** | **G** | **C** | **C** | **G** | **T** | **T** | **T** | **T** | **A** | **T** | **T** | **T** | **A** | **T** | **C** | **G** | **G** | **G** | **C** | **T** | **G** | **A** | **A** | **A** | **G** | **G** | **T** | **G** | **T** | **A** | **G** | **A** | **G** | **A** | **T** | **T** | **T** | **C** | **A** | **G** | **G** | **A** | **A** | **T** | **A** | **G** | **A** | **T** | **T** | **T** | **G** | **A** | **C** | **A** | **A** | **A** | **A** | **A** | **A** | **T** | **G** | **C** | **T** | **T** | **T** | **T** | **T** | **G** | **G** | **C** | **G** | **A** | **A** | **A** | **A** | **A** | **A** | **A** | **C** | **A** | **G** | **A** | **T** | **C** | **A** | **T** | **G** | **A** | **T** | **G** | **G** | **T** | **C** | **C** | **A** | **A** | **A** | **C** | **A** | **G** | **A** | **G** | **C** | **A** | **A** | **T** | **T** | **A** | **A** | **A** | **A** | **A** | **C** | **A** | **C** | **A** | **T** | **A** | **A** | **T** | **A** | **A** | **T** | **G** | **A** | **G** | **C** | **G** | **T** | **A** | **A** | **A** | **A** | **G** | **A** | **C** | **A** | **G** | **C** | **A** | **A** | **T** | **T** | **A** | **T** | **T** | **T** | **G** | **G** | **T** | **A** | **A** | **C** | **C** | **A** | **A** | **C** | **G** | **T** | **A** | **T** | **T** | **G** | **G** | **A** | **A** | **A** | **C** | **G** | **A** | **C** | **G** | **T** | **C** | **T** | **A** | **T** | **G** | **A** | **A** | **T** | **G** | **G** | **G** | **A** | **T** | **G** | **A** | **T** | **A** | **T** | **C** | **A** | **A** | **C** | **A** | **T** | **T** | **G** | **C** | **C** | **A** | **T** | **C** | **A** | **T** | **T** | **T** | **C** | **T** | **G** | **G** | **T** | **G** | **A** | **T** | **A** | **C** | **T** | **A** | **T** | **C** | **A** | **T** | **C** | **G** | **A** | **T** | **T** | **T** | **G** | **G** | **G** | **C** | **A** | **A** | **T** | **A** | **C** | **A** | **C** | **T** | **T** | **T** | **T** | **G** | **C** | **C** | **A** | **A** | **A** | **A** | **G** | **A** | **T** | **G** | **A** | **T** | **A** | **A** | **T** | **A** | **T** | **T** | **G** | **T** | **G** | **A** | **T** | **T** | **G** | **T** | **A** | **A** | **G** | **A** | **A** | **A** | **A** | **G** | **G** | **G** | **A** | **T** | **A** | **G** | **G** | **C** | **A** | **G** | **A** | **A** | **C** | **A** | **C** | **G** | **G** | **A** | **T** | **C** | **T** | **T** | **A** | **G** | **T** | **A** | **C** | **C** | **C** | **C** | **T** | **G** | **G** | **G** | **A** | **G** | **T** | **G** | **G** | **C** | **T** | **A** | **T** | **T** | **C** | **G** | **T** | **T** | **T** | **A** | **G** | **A** | **G** | **C** | **A** | **T** | **G** | **C** | **G** | **A** | **C** | **A** | **T** | **T** | **A** | **G** | **T** | **A** | **G** | **G** | **A** | **A** | **A** | **T** | **G** | **T** | **C** | **T** | **T** | **G** | **T** | **T** | **T** | **G** | **A** | **A** | **G** | **A** | **A** | **G** | **A** | **G** | **C** | **A** | **G** | **T** | **T** | **T** | **T** | **C** | **C** | **T** | **T** | **A** | **A** | **A** | **A** | **A** | **A** | **C** | **G** | **A** | **A** | **C** | **A** | **G** | **A** | **T** | **C** | **A** | **A** | **A** | **A** | **T** | **C** | **T** | **G** | **C** | **A** | **G** | **C** | **A** | **G** | **T** | **G** | **A** | **T** | **T** | **A** | **T** | **G** | **A** | **T** | **G** | **A** | **A** | **A** | **A** | **T** | **C** | **C** | **T** | **C** | **G** | **C** | **A** | **G** | **A** | **T** | **T** | **G** | **A** | **A** | **G** | **A** | **T** | **C** | **A** | **T** | **T** | **A** | **C** | **C** | **A** | **A** | **T** | **A** | **C** | **C** | **T** | **T** | **G** | **G** | **T** | **T** | **G** | **G** | **A** | **G** | **A** | **T** | **G** | **T** | **C** | **G** | **A** | **G** | **G** | **T** | **G** | **A** | **T** | **A** | **C** | **G** | **A** | **A** | **T** | **A** | **-** | **-** | **-** |
| **N52636** | **A** | **T** | **G** | **A** | **A** | **T** | **A** | **G** | **T** | **T** | **C** | **T** | **T** | **G** | **G** | **C** | **G** | **T** | **T** | **T** | **T** | **T** | **T** | **T** | **G** | **T** | **G** | **G** | **T** | **A** | **G** | **T** | **C** | **G** | **A** | **A** | **G** | **C** | **A** | **C** | **T** | **G** | **C** | **T** | **G** | **C** | **T** | **G** | **T** | **T** | **A** | **T** | **T** | **T** | **G** | **C** | **T** | **G** | **T** | **T** | **T** | **G** | **G** | **C** | **A** | **A** | **A** | **T** | **C** | **G** | **T** | **A** | **A** | **A** | **C** | **A** | **A** | **T** | **A** | **C** | **T** | **G** | **G** | **A** | **C** | **T** | **T** | **T** | **T** | **G** | **C** | **T** | **T** | **T** | **T** | **A** | **G** | **T** | **A** | **A** | **T** | **A** | **T** | **T** | **C** | **G** | **G** | **T** | **A** | **T** | **T** | **T** | **T** | **C** | **A** | **A** | **T** | **A** | **T** | **A** | **T** | **A** | **T** | **C** | **T** | **A** | **G** | **C** | **T** | **A** | **T** | **G** | **C** | **G** | **C** | **A** | **A** | **A** | **T** | **A** | **T** | **C** | **C** | **T** | **C** | **G** | **T** | **A** | **C** | **T** | **A** | **A** | **A** | **T** | **T** | **T** | **C** | **A** | **A** | **A** | **A** | **T** | **T** | **T** | **T** | **C** | **A** | **A** | **T** | **T** | **G** | **A** | **T** | **C** | **T** | **T** | **A** | **G** | **G** | **G** | **A** | **G** | **C** | **T** | **T** | **G** | **G** | **T** | **C** | **A** | **T** | **T** | **T** | **T** | **T** | **T** | **T** | **T** | **A** | **G** | **C** | **T** | **T** | **A** | **G** | **T** | **C** | **A** | **A** | **C** | **A** | **G** | **T** | **C** | **C** | **T** | **G** | **C** | **T** | **T** | **T** | **A** | **T** | **G** | **G** | **A** | **T** | **G** | **A** | **T** | **G** | **G** | **T** | **T** | **G** | **T** | **A** | **T** | **T** | **G** | **G** | **C** | **C** | **G** | **T** | **T** | **T** | **T** | **A** | **T** | **T** | **T** | **A** | **T** | **C** | **G** | **G** | **C** | **C** | **T** | **G** | **A** | **A** | **A** | **G** | **G** | **T** | **G** | **T** | **A** | **G** | **A** | **G** | **A** | **T** | **T** | **T** | **C** | **A** | **G** | **G** | **A** | **A** | **T** | **A** | **G** | **A** | **T** | **T** | **T** | **G** | **A** | **C** | **A** | **A** | **A** | **A** | **A** | **A** | **T** | **G** | **C** | **T** | **T** | **T** | **T** | **T** | **G** | **G** | **C** | **G** | **A** | **A** | **A** | **A** | **A** | **A** | **A** | **C** | **A** | **G** | **A** | **T** | **C** | **A** | **T** | **G** | **A** | **T** | **G** | **G** | **T** | **C** | **C** | **A** | **A** | **A** | **C** | **A** | **G** | **A** | **G** | **C** | **A** | **A** | **T** | **T** | **A** | **A** | **A** | **A** | **A** | **C** | **A** | **C** | **A** | **T** | **A** | **A** | **T** | **A** | **A** | **T** | **G** | **A** | **G** | **C** | **G** | **T** | **A** | **A** | **A** | **A** | **G** | **A** | **C** | **A** | **G** | **C** | **A** | **A** | **T** | **T** | **A** | **T** | **T** | **T** | **G** | **G** | **T** | **A** | **A** | **C** | **C** | **A** | **A** | **C** | **G** | **T** | **A** | **T** | **T** | **G** | **G** | **A** | **A** | **A** | **C** | **G** | **A** | **C** | **G** | **T** | **C** | **T** | **A** | **T** | **G** | **A** | **A** | **T** | **G** | **G** | **G** | **A** | **T** | **G** | **A** | **T** | **A** | **T** | **C** | **A** | **A** | **C** | **A** | **T** | **T** | **G** | **C** | **C** | **A** | **T** | **C** | **A** | **T** | **T** | **T** | **C** | **T** | **G** | **G** | **T** | **G** | **A** | **T** | **A** | **C** | **T** | **A** | **T** | **C** | **A** | **T** | **C** | **G** | **A** | **T** | **T** | **T** | **G** | **G** | **G** | **C** | **A** | **A** | **T** | **A** | **C** | **A** | **C** | **T** | **T** | **T** | **T** | **G** | **C** | **C** | **A** | **A** | **A** | **A** | **G** | **A** | **T** | **G** | **A** | **T** | **A** | **A** | **T** | **A** | **T** | **T** | **G** | **T** | **G** | **A** | **T** | **T** | **G** | **T** | **A** | **A** | **G** | **A** | **A** | **A** | **A** | **G** | **G** | **G** | **A** | **T** | **A** | **G** | **G** | **C** | **A** | **G** | **A** | **A** | **C** | **A** | **C** | **G** | **G** | **A** | **T** | **C** | **T** | **T** | **A** | **G** | **T** | **A** | **C** | **C** | **C** | **C** | **T** | **G** | **G** | **G** | **A** | **G** | **T** | **G** | **G** | **C** | **T** | **A** | **T** | **T** | **C** | **G** | **T** | **T** | **T** | **A** | **G** | **A** | **G** | **C** | **A** | **T** | **G** | **C** | **G** | **A** | **C** | **A** | **T** | **T** | **A** | **G** | **T** | **A** | **G** | **G** | **A** | **A** | **A** | **T** | **G** | **T** | **C** | **T** | **T** | **G** | **T** | **T** | **T** | **G** | **A** | **A** | **G** | **A** | **A** | **G** | **A** | **G** | **C** | **A** | **G** | **T** | **T** | **T** | **T** | **C** | **C** | **T** | **T** | **A** | **A** | **A** | **A** | **A** | **A** | **C** | **G** | **A** | **A** | **C** | **A** | **G** | **A** | **T** | **C** | **A** | **A** | **A** | **A** | **T** | **C** | **T** | **G** | **C** | **A** | **G** | **C** | **A** | **G** | **T** | **G** | **A** | **T** | **T** | **A** | **T** | **G** | **A** | **T** | **G** | **A** | **A** | **A** | **A** | **T** | **C** | **C** | **T** | **C** | **G** | **C** | **A** | **G** | **A** | **T** | **T** | **G** | **A** | **A** | **G** | **A** | **T** | **C** | **A** | **T** | **T** | **A** | **C** | **C** | **A** | **A** | **T** | **A** | **C** | **C** | **T** | **T** | **G** | **G** | **T** | **T** | **G** | **G** | **A** | **G** | **A** | **T** | **G** | **T** | **C** | **G** | **A** | **G** | **G** | **T** | **G** | **A** | **T** | **A** | **C** | **G** | **A** | **A** | **T** | **A** | **-** | **-** | **-** |
| **N54567** | **A** | **T** | **G** | **A** | **A** | **T** | **A** | **G** | **T** | **T** | **C** | **T** | **T** | **G** | **G** | **C** | **G** | **T** | **T** | **T** | **T** | **T** | **T** | **T** | **G** | **T** | **G** | **G** | **T** | **A** | **G** | **T** | **T** | **G** | **A** | **A** | **G** | **C** | **A** | **C** | **T** | **A** | **C** | **T** | **G** | **C** | **T** | **G** | **T** | **T** | **A** | **T** | **T** | **T** | **G** | **C** | **T** | **G** | **T** | **T** | **T** | **G** | **G** | **C** | **A** | **A** | **A** | **T** | **C** | **G** | **T** | **A** | **A** | **A** | **C** | **A** | **A** | **T** | **A** | **C** | **T** | **G** | **G** | **A** | **C** | **T** | **T** | **T** | **T** | **G** | **C** | **T** | **T** | **T** | **T** | **A** | **G** | **T** | **G** | **A** | **T** | **A** | **T** | **T** | **C** | **G** | **G** | **T** | **A** | **T** | **T** | **T** | **T** | **C** | **A** | **A** | **T** | **A** | **T** | **A** | **T** | **A** | **T** | **C** | **T** | **A** | **G** | **C** | **T** | **A** | **T** | **G** | **C** | **G** | **C** | **A** | **A** | **A** | **C** | **A** | **T** | **C** | **C** | **T** | **C** | **G** | **T** | **A** | **C** | **T** | **A** | **A** | **A** | **T** | **T** | **T** | **C** | **A** | **A** | **A** | **A** | **T** | **T** | **T** | **T** | **C** | **A** | **A** | **T** | **T** | **G** | **A** | **T** | **A** | **T** | **T** | **A** | **G** | **G** | **A** | **A** | **G** | **T** | **T** | **T** | **G** | **G** | **T** | **C** | **A** | **T** | **T** | **T** | **T** | **T** | **T** | **T** | **T** | **A** | **G** | **C** | **T** | **T** | **A** | **G** | **T** | **C** | **A** | **A** | **C** | **A** | **G** | **T** | **C** | **C** | **T** | **G** | **C** | **T** | **T** | **T** | **A** | **T** | **G** | **G** | **A** | **T** | **G** | **A** | **T** | **G** | **G** | **C** | **T** | **G** | **T** | **A** | **T** | **T** | **T** | **G** | **C** | **C** | **G** | **T** | **T** | **T** | **T** | **A** | **T** | **T** | **T** | **A** | **T** | **T** | **G** | **G** | **A** | **C** | **T** | **A** | **A** | **A** | **A** | **G** | **G** | **T** | **G** | **T** | **A** | **G** | **A** | **G** | **A** | **T** | **T** | **T** | **C** | **A** | **G** | **G** | **G** | **A** | **T** | **A** | **G** | **A** | **T** | **T** | **T** | **A** | **A** | **C** | **C** | **A** | **A** | **A** | **A** | **A** | **T** | **G** | **C** | **T** | **T** | **T** | **T** | **T** | **G** | **G** | **C** | **G** | **A** | **A** | **A** | **G** | **A** | **A** | **A** | **C** | **A** | **G** | **A** | **T** | **C** | **A** | **T** | **G** | **A** | **T** | **G** | **G** | **T** | **C** | **C** | **A** | **A** | **A** | **C** | **A** | **G** | **A** | **G** | **C** | **A** | **A** | **T** | **T** | **A** | **A** | **A** | **A** | **A** | **C** | **A** | **C** | **A** | **T** | **A** | **A** | **T** | **A** | **A** | **T** | **G** | **A** | **G** | **C** | **G** | **G** | **A** | **A** | **A** | **A** | **G** | **A** | **C** | **A** | **G** | **C** | **A** | **A** | **T** | **T** | **A** | **T** | **T** | **C** | **G** | **G** | **T** | **A** | **A** | **C** | **C** | **A** | **A** | **C** | **G** | **T** | **A** | **T** | **T** | **G** | **G** | **A** | **A** | **A** | **C** | **G** | **A** | **C** | **G** | **T** | **C** | **T** | **A** | **T** | **G** | **A** | **A** | **T** | **G** | **G** | **G** | **A** | **C** | **G** | **A** | **T** | **A** | **T** | **C** | **A** | **A** | **C** | **A** | **T** | **C** | **G** | **C** | **C** | **G** | **T** | **C** | **A** | **T** | **T** | **T** | **C** | **T** | **G** | **G** | **T** | **G** | **A** | **T** | **A** | **C** | **T** | **A** | **T** | **C** | **A** | **T** | **A** | **G** | **A** | **T** | **T** | **T** | **G** | **G** | **G** | **C** | **A** | **A** | **T** | **A** | **C** | **G** | **C** | **T** | **T** | **T** | **T** | **G** | **C** | **C** | **A** | **A** | **A** | **A** | **G** | **A** | **T** | **G** | **A** | **T** | **A** | **A** | **T** | **A** | **T** | **T** | **G** | **T** | **G** | **A** | **T** | **T** | **G** | **T** | **A** | **A** | **G** | **A** | **A** | **A** | **A** | **G** | **G** | **G** | **A** | **T** | **A** | **G** | **G** | **C** | **A** | **G** | **A** | **A** | **C** | **A** | **C** | **G** | **G** | **A** | **T** | **C** | **T** | **T** | **A** | **G** | **T** | **A** | **C** | **C** | **T** | **T** | **T** | **G** | **G** | **G** | **C** | **G** | **T** | **A** | **G** | **C** | **C** | **A** | **T** | **C** | **C** | **G** | **A** | **T** | **T** | **A** | **G** | **A** | **G** | **C** | **A** | **T** | **G** | **C** | **G** | **A** | **C** | **A** | **T** | **T** | **A** | **G** | **T** | **A** | **G** | **G** | **A** | **A** | **A** | **T** | **G** | **T** | **C** | **T** | **T** | **G** | **T** | **T** | **T** | **G** | **A** | **A** | **G** | **A** | **A** | **G** | **A** | **G** | **C** | **A** | **A** | **T** | **T** | **T** | **T** | **C** | **T** | **T** | **T** | **G** | **A** | **A** | **A** | **A** | **A** | **T** | **G** | **A** | **A** | **C** | **A** | **A** | **A** | **T** | **C** | **A** | **A** | **A** | **A** | **T** | **C** | **T** | **A** | **C** | **A** | **G** | **C** | **A** | **G** | **T** | **G** | **A** | **T** | **T** | **A** | **T** | **G** | **A** | **T** | **G** | **A** | **A** | **A** | **A** | **C** | **C** | **C** | **T** | **C** | **G** | **T** | **A** | **G** | **A** | **C** | **T** | **G** | **A** | **A** | **G** | **A** | **T** | **T** | **A** | **T** | **T** | **A** | **C** | **T** | **A** | **A** | **T** | **A** | **C** | **C** | **T** | **T** | **G** | **A** | **T** | **T** | **G** | **G** | **A** | **G** | **A** | **T** | **G** | **T** | **C** | **G** | **A** | **G** | **G** | **T** | **G** | **A** | **T** | **A** | **C** | **G** | **A** | **A** | **T** | **A** | **-** | **-** | **-** |
| **N60061F** | **A** | **T** | **G** | **A** | **A** | **T** | **A** | **G** | **T** | **T** | **C** | **T** | **T** | **G** | **G** | **C** | **G** | **T** | **T** | **T** | **T** | **T** | **T** | **T** | **G** | **T** | **G** | **G** | **T** | **A** | **G** | **T** | **C** | **G** | **A** | **A** | **G** | **C** | **A** | **C** | **T** | **G** | **C** | **T** | **G** | **C** | **T** | **G** | **T** | **T** | **A** | **T** | **T** | **T** | **G** | **C** | **T** | **G** | **T** | **T** | **T** | **G** | **G** | **C** | **A** | **A** | **A** | **T** | **C** | **G** | **T** | **A** | **A** | **A** | **C** | **A** | **A** | **T** | **A** | **C** | **T** | **G** | **G** | **A** | **C** | **T** | **T** | **T** | **T** | **G** | **C** | **T** | **T** | **T** | **T** | **A** | **G** | **T** | **A** | **A** | **T** | **A** | **T** | **T** | **C** | **G** | **G** | **T** | **A** | **T** | **T** | **T** | **T** | **C** | **A** | **A** | **T** | **A** | **T** | **A** | **T** | **A** | **T** | **C** | **T** | **A** | **G** | **C** | **T** | **A** | **T** | **G** | **C** | **G** | **C** | **A** | **A** | **A** | **T** | **A** | **T** | **C** | **C** | **T** | **C** | **G** | **T** | **A** | **C** | **T** | **A** | **A** | **A** | **T** | **T** | **T** | **C** | **A** | **A** | **A** | **A** | **T** | **T** | **T** | **T** | **C** | **A** | **A** | **T** | **T** | **G** | **A** | **T** | **C** | **T** | **T** | **A** | **G** | **G** | **G** | **A** | **G** | **C** | **T** | **T** | **G** | **G** | **T** | **C** | **A** | **T** | **T** | **T** | **T** | **T** | **T** | **T** | **T** | **A** | **G** | **C** | **T** | **T** | **A** | **G** | **T** | **C** | **A** | **A** | **C** | **A** | **G** | **T** | **C** | **C** | **T** | **G** | **C** | **T** | **T** | **T** | **A** | **T** | **G** | **G** | **A** | **T** | **G** | **A** | **T** | **G** | **G** | **T** | **T** | **G** | **T** | **A** | **T** | **T** | **G** | **G** | **C** | **C** | **G** | **T** | **T** | **T** | **T** | **A** | **T** | **T** | **T** | **A** | **T** | **C** | **G** | **G** | **C** | **C** | **T** | **G** | **A** | **A** | **A** | **G** | **G** | **T** | **G** | **T** | **A** | **G** | **A** | **G** | **A** | **T** | **T** | **T** | **C** | **A** | **G** | **G** | **A** | **A** | **T** | **A** | **G** | **A** | **T** | **T** | **T** | **G** | **A** | **C** | **A** | **A** | **A** | **A** | **A** | **A** | **T** | **G** | **C** | **T** | **T** | **T** | **T** | **T** | **G** | **G** | **C** | **G** | **A** | **A** | **A** | **A** | **A** | **A** | **A** | **C** | **A** | **G** | **A** | **T** | **C** | **A** | **T** | **G** | **A** | **T** | **G** | **G** | **T** | **C** | **C** | **A** | **A** | **A** | **C** | **A** | **G** | **A** | **G** | **C** | **A** | **A** | **T** | **T** | **A** | **A** | **A** | **A** | **A** | **C** | **A** | **C** | **A** | **T** | **A** | **A** | **T** | **A** | **A** | **T** | **G** | **A** | **G** | **C** | **G** | **T** | **A** | **A** | **A** | **A** | **G** | **A** | **C** | **A** | **G** | **C** | **A** | **A** | **T** | **T** | **A** | **T** | **T** | **T** | **G** | **G** | **T** | **A** | **A** | **C** | **C** | **A** | **A** | **C** | **G** | **T** | **A** | **T** | **T** | **G** | **G** | **A** | **A** | **A** | **C** | **G** | **A** | **C** | **G** | **T** | **C** | **T** | **A** | **T** | **G** | **A** | **A** | **T** | **G** | **G** | **G** | **A** | **T** | **G** | **A** | **T** | **A** | **T** | **C** | **A** | **A** | **C** | **A** | **T** | **T** | **G** | **C** | **C** | **A** | **T** | **C** | **A** | **T** | **T** | **T** | **C** | **T** | **G** | **G** | **T** | **G** | **A** | **T** | **A** | **C** | **T** | **A** | **T** | **C** | **A** | **T** | **C** | **G** | **A** | **T** | **T** | **T** | **G** | **G** | **G** | **C** | **A** | **A** | **T** | **A** | **C** | **A** | **C** | **T** | **T** | **T** | **T** | **G** | **C** | **C** | **A** | **A** | **A** | **A** | **G** | **A** | **T** | **G** | **A** | **T** | **A** | **A** | **T** | **A** | **T** | **T** | **G** | **T** | **G** | **A** | **T** | **T** | **G** | **T** | **A** | **A** | **G** | **A** | **A** | **A** | **A** | **G** | **G** | **G** | **A** | **T** | **A** | **G** | **G** | **C** | **A** | **G** | **A** | **A** | **C** | **A** | **C** | **G** | **G** | **A** | **T** | **C** | **T** | **T** | **A** | **G** | **T** | **A** | **C** | **C** | **C** | **C** | **T** | **G** | **G** | **G** | **A** | **G** | **T** | **G** | **G** | **C** | **T** | **A** | **T** | **T** | **C** | **G** | **A** | **T** | **T** | **A** | **G** | **A** | **G** | **C** | **A** | **T** | **G** | **C** | **G** | **A** | **C** | **A** | **T** | **T** | **A** | **G** | **T** | **A** | **G** | **G** | **A** | **A** | **A** | **T** | **G** | **T** | **C** | **T** | **T** | **G** | **T** | **T** | **T** | **G** | **A** | **A** | **G** | **A** | **A** | **G** | **A** | **G** | **C** | **A** | **G** | **T** | **T** | **T** | **T** | **C** | **C** | **T** | **T** | **A** | **A** | **A** | **A** | **A** | **A** | **T** | **G** | **A** | **A** | **C** | **A** | **G** | **A** | **T** | **C** | **A** | **A** | **A** | **A** | **T** | **C** | **T** | **G** | **C** | **A** | **G** | **C** | **A** | **G** | **T** | **G** | **A** | **T** | **T** | **A** | **T** | **G** | **A** | **T** | **G** | **A** | **A** | **A** | **A** | **T** | **C** | **C** | **T** | **C** | **G** | **C** | **A** | **G** | **A** | **T** | **T** | **G** | **A** | **A** | **G** | **A** | **T** | **C** | **A** | **T** | **T** | **A** | **C** | **C** | **A** | **A** | **T** | **A** | **C** | **C** | **T** | **T** | **G** | **G** | **T** | **T** | **G** | **G** | **A** | **G** | **A** | **T** | **G** | **T** | **C** | **G** | **A** | **G** | **G** | **T** | **G** | **A** | **T** | **A** | **C** | **G** | **A** | **A** | **T** | **A** | **-** | **-** | **-** |
| **N60425F** | **A** | **T** | **G** | **A** | **A** | **T** | **A** | **G** | **T** | **T** | **C** | **T** | **T** | **G** | **G** | **C** | **G** | **T** | **T** | **T** | **T** | **T** | **T** | **T** | **G** | **T** | **G** | **G** | **T** | **A** | **G** | **T** | **C** | **G** | **A** | **A** | **G** | **C** | **A** | **C** | **T** | **G** | **C** | **T** | **G** | **C** | **T** | **G** | **T** | **T** | **A** | **T** | **T** | **T** | **G** | **C** | **T** | **G** | **T** | **T** | **T** | **G** | **G** | **C** | **A** | **A** | **A** | **T** | **C** | **G** | **T** | **A** | **A** | **A** | **C** | **A** | **A** | **T** | **A** | **C** | **T** | **G** | **G** | **A** | **C** | **T** | **T** | **T** | **T** | **G** | **C** | **T** | **T** | **T** | **T** | **A** | **G** | **T** | **A** | **A** | **T** | **A** | **T** | **T** | **C** | **G** | **G** | **T** | **A** | **T** | **T** | **T** | **T** | **C** | **A** | **A** | **T** | **A** | **T** | **A** | **T** | **A** | **T** | **C** | **T** | **A** | **G** | **C** | **T** | **A** | **T** | **G** | **C** | **G** | **C** | **A** | **A** | **A** | **T** | **A** | **T** | **C** | **C** | **T** | **C** | **G** | **T** | **A** | **C** | **T** | **A** | **A** | **A** | **T** | **T** | **T** | **C** | **A** | **A** | **A** | **A** | **T** | **T** | **T** | **T** | **C** | **A** | **A** | **T** | **T** | **G** | **A** | **T** | **C** | **T** | **T** | **A** | **G** | **G** | **G** | **A** | **G** | **C** | **T** | **T** | **G** | **G** | **T** | **C** | **A** | **T** | **T** | **T** | **T** | **T** | **T** | **T** | **T** | **A** | **G** | **C** | **T** | **T** | **A** | **G** | **T** | **C** | **A** | **A** | **C** | **A** | **G** | **T** | **C** | **C** | **T** | **G** | **C** | **T** | **T** | **T** | **A** | **T** | **G** | **G** | **A** | **T** | **G** | **A** | **T** | **G** | **G** | **T** | **T** | **G** | **T** | **A** | **T** | **T** | **G** | **G** | **C** | **C** | **G** | **T** | **T** | **T** | **T** | **A** | **T** | **T** | **T** | **A** | **T** | **C** | **G** | **G** | **C** | **C** | **T** | **G** | **A** | **A** | **A** | **G** | **G** | **T** | **G** | **T** | **A** | **G** | **A** | **G** | **A** | **T** | **T** | **T** | **C** | **A** | **G** | **G** | **A** | **A** | **T** | **A** | **G** | **A** | **T** | **T** | **T** | **G** | **A** | **C** | **A** | **A** | **A** | **A** | **A** | **A** | **T** | **G** | **C** | **T** | **T** | **T** | **T** | **T** | **G** | **G** | **C** | **G** | **A** | **A** | **A** | **A** | **A** | **A** | **A** | **C** | **A** | **G** | **A** | **T** | **C** | **A** | **T** | **G** | **A** | **T** | **G** | **G** | **T** | **C** | **C** | **A** | **A** | **A** | **C** | **A** | **G** | **A** | **G** | **C** | **A** | **A** | **T** | **T** | **A** | **A** | **A** | **A** | **A** | **C** | **A** | **C** | **A** | **T** | **A** | **A** | **T** | **A** | **A** | **T** | **G** | **A** | **G** | **C** | **G** | **T** | **A** | **A** | **A** | **A** | **G** | **A** | **C** | **A** | **G** | **C** | **A** | **A** | **T** | **T** | **A** | **T** | **T** | **T** | **G** | **G** | **T** | **A** | **A** | **C** | **C** | **A** | **A** | **C** | **G** | **T** | **A** | **T** | **T** | **G** | **G** | **A** | **A** | **A** | **C** | **G** | **A** | **C** | **G** | **T** | **C** | **T** | **A** | **T** | **G** | **A** | **A** | **T** | **G** | **G** | **G** | **A** | **T** | **G** | **A** | **T** | **A** | **T** | **C** | **A** | **A** | **C** | **A** | **T** | **T** | **G** | **C** | **C** | **A** | **T** | **C** | **A** | **T** | **T** | **T** | **C** | **T** | **G** | **G** | **T** | **G** | **A** | **T** | **A** | **C** | **T** | **A** | **T** | **C** | **A** | **T** | **C** | **G** | **A** | **T** | **T** | **T** | **G** | **G** | **G** | **C** | **A** | **A** | **T** | **A** | **C** | **A** | **C** | **T** | **T** | **T** | **T** | **G** | **C** | **C** | **A** | **A** | **A** | **A** | **G** | **A** | **T** | **G** | **A** | **T** | **A** | **A** | **T** | **A** | **T** | **T** | **G** | **T** | **G** | **A** | **T** | **T** | **G** | **T** | **A** | **A** | **G** | **A** | **A** | **A** | **A** | **G** | **G** | **G** | **A** | **T** | **A** | **G** | **G** | **C** | **A** | **G** | **A** | **A** | **C** | **A** | **C** | **G** | **G** | **A** | **T** | **C** | **T** | **T** | **A** | **G** | **T** | **A** | **C** | **C** | **C** | **C** | **T** | **G** | **G** | **G** | **A** | **G** | **T** | **G** | **G** | **C** | **T** | **A** | **T** | **T** | **C** | **G** | **A** | **T** | **T** | **A** | **G** | **A** | **G** | **C** | **A** | **T** | **G** | **C** | **G** | **A** | **C** | **A** | **T** | **T** | **A** | **G** | **T** | **A** | **G** | **G** | **A** | **A** | **A** | **T** | **G** | **T** | **C** | **T** | **T** | **G** | **T** | **T** | **T** | **G** | **A** | **A** | **G** | **A** | **A** | **G** | **A** | **G** | **C** | **A** | **G** | **T** | **T** | **T** | **T** | **C** | **C** | **T** | **T** | **A** | **A** | **A** | **A** | **A** | **A** | **T** | **G** | **A** | **A** | **C** | **A** | **G** | **A** | **T** | **C** | **A** | **A** | **A** | **A** | **T** | **C** | **T** | **G** | **C** | **A** | **G** | **C** | **A** | **G** | **T** | **G** | **A** | **T** | **T** | **A** | **T** | **G** | **A** | **T** | **G** | **A** | **A** | **A** | **A** | **T** | **C** | **C** | **T** | **C** | **G** | **C** | **A** | **G** | **A** | **T** | **T** | **G** | **A** | **A** | **G** | **A** | **T** | **C** | **A** | **T** | **T** | **A** | **C** | **C** | **A** | **A** | **T** | **A** | **C** | **C** | **T** | **T** | **G** | **G** | **T** | **T** | **G** | **G** | **A** | **G** | **A** | **T** | **G** | **T** | **C** | **G** | **A** | **G** | **G** | **T** | **G** | **A** | **T** | **A** | **C** | **G** | **A** | **A** | **T** | **A** | **-** | **-** | **-** |
| **N60200F** | **A** | **T** | **G** | **A** | **A** | **T** | **A** | **G** | **T** | **T** | **C** | **T** | **T** | **G** | **G** | **C** | **G** | **T** | **T** | **T** | **T** | **T** | **T** | **T** | **G** | **T** | **G** | **G** | **T** | **A** | **G** | **T** | **C** | **G** | **A** | **A** | **G** | **C** | **A** | **C** | **T** | **G** | **C** | **T** | **G** | **C** | **T** | **G** | **T** | **T** | **A** | **T** | **T** | **T** | **G** | **C** | **T** | **G** | **T** | **T** | **T** | **G** | **G** | **C** | **A** | **A** | **A** | **T** | **C** | **G** | **T** | **A** | **A** | **A** | **C** | **A** | **A** | **T** | **A** | **C** | **T** | **G** | **G** | **A** | **C** | **T** | **T** | **T** | **T** | **G** | **C** | **T** | **T** | **T** | **T** | **A** | **G** | **T** | **A** | **A** | **T** | **A** | **T** | **T** | **C** | **G** | **G** | **T** | **A** | **T** | **T** | **T** | **T** | **C** | **A** | **A** | **T** | **A** | **T** | **A** | **T** | **A** | **T** | **C** | **T** | **A** | **G** | **C** | **T** | **A** | **T** | **G** | **C** | **G** | **C** | **A** | **A** | **A** | **T** | **A** | **T** | **C** | **C** | **T** | **C** | **G** | **T** | **A** | **C** | **T** | **A** | **A** | **A** | **T** | **T** | **T** | **C** | **A** | **A** | **A** | **A** | **T** | **T** | **T** | **T** | **C** | **A** | **A** | **T** | **T** | **G** | **A** | **T** | **C** | **T** | **T** | **A** | **G** | **G** | **G** | **A** | **G** | **C** | **T** | **T** | **G** | **G** | **T** | **C** | **A** | **T** | **T** | **T** | **T** | **T** | **T** | **T** | **T** | **A** | **G** | **C** | **T** | **T** | **A** | **G** | **T** | **C** | **A** | **A** | **C** | **A** | **G** | **T** | **C** | **C** | **T** | **G** | **C** | **T** | **T** | **T** | **A** | **T** | **G** | **G** | **A** | **T** | **G** | **A** | **T** | **G** | **G** | **T** | **T** | **G** | **T** | **A** | **T** | **T** | **G** | **G** | **C** | **C** | **G** | **T** | **T** | **T** | **T** | **A** | **T** | **T** | **T** | **A** | **T** | **C** | **G** | **G** | **G** | **C** | **T** | **G** | **A** | **A** | **A** | **G** | **G** | **T** | **G** | **T** | **A** | **G** | **A** | **G** | **A** | **T** | **T** | **T** | **C** | **A** | **G** | **G** | **A** | **A** | **T** | **A** | **G** | **A** | **T** | **T** | **T** | **G** | **A** | **C** | **A** | **A** | **A** | **A** | **A** | **A** | **T** | **G** | **C** | **T** | **T** | **T** | **T** | **T** | **G** | **G** | **C** | **G** | **A** | **A** | **A** | **A** | **A** | **A** | **A** | **C** | **A** | **G** | **A** | **T** | **C** | **A** | **T** | **G** | **A** | **T** | **G** | **G** | **T** | **C** | **C** | **A** | **A** | **A** | **C** | **A** | **G** | **A** | **G** | **C** | **A** | **A** | **T** | **T** | **A** | **A** | **A** | **A** | **A** | **C** | **A** | **C** | **A** | **T** | **A** | **A** | **T** | **A** | **A** | **T** | **G** | **A** | **G** | **C** | **G** | **T** | **A** | **A** | **A** | **A** | **G** | **A** | **C** | **A** | **G** | **C** | **A** | **A** | **T** | **T** | **A** | **T** | **T** | **T** | **G** | **G** | **T** | **A** | **A** | **C** | **C** | **A** | **A** | **C** | **G** | **T** | **A** | **T** | **T** | **G** | **G** | **A** | **A** | **A** | **C** | **G** | **A** | **C** | **G** | **T** | **C** | **T** | **A** | **T** | **G** | **A** | **A** | **T** | **G** | **G** | **G** | **A** | **T** | **G** | **A** | **T** | **A** | **T** | **C** | **A** | **A** | **C** | **A** | **T** | **T** | **G** | **C** | **C** | **A** | **T** | **C** | **A** | **T** | **T** | **T** | **C** | **T** | **G** | **G** | **T** | **G** | **A** | **T** | **A** | **C** | **T** | **A** | **T** | **C** | **A** | **T** | **C** | **G** | **A** | **T** | **T** | **T** | **G** | **G** | **G** | **C** | **A** | **A** | **T** | **A** | **C** | **A** | **C** | **T** | **T** | **T** | **T** | **G** | **C** | **C** | **A** | **A** | **A** | **A** | **G** | **A** | **T** | **G** | **A** | **T** | **A** | **A** | **T** | **A** | **T** | **T** | **G** | **T** | **G** | **A** | **T** | **T** | **G** | **T** | **A** | **A** | **G** | **A** | **A** | **A** | **A** | **G** | **G** | **G** | **A** | **T** | **A** | **G** | **G** | **C** | **A** | **G** | **A** | **A** | **C** | **A** | **C** | **G** | **G** | **A** | **T** | **C** | **T** | **T** | **A** | **G** | **T** | **A** | **C** | **C** | **C** | **C** | **T** | **G** | **G** | **G** | **A** | **G** | **T** | **G** | **G** | **C** | **T** | **A** | **T** | **T** | **C** | **G** | **T** | **T** | **T** | **A** | **G** | **A** | **G** | **C** | **A** | **T** | **G** | **C** | **G** | **A** | **C** | **A** | **T** | **T** | **A** | **G** | **T** | **A** | **G** | **G** | **A** | **A** | **A** | **T** | **G** | **T** | **C** | **T** | **T** | **G** | **T** | **T** | **T** | **G** | **A** | **A** | **G** | **A** | **A** | **G** | **A** | **G** | **C** | **A** | **G** | **T** | **T** | **T** | **T** | **C** | **C** | **T** | **T** | **A** | **A** | **A** | **A** | **A** | **A** | **C** | **G** | **A** | **A** | **C** | **A** | **G** | **A** | **T** | **C** | **A** | **A** | **A** | **A** | **T** | **C** | **T** | **G** | **C** | **A** | **G** | **C** | **A** | **G** | **T** | **G** | **A** | **T** | **T** | **A** | **T** | **G** | **A** | **T** | **G** | **A** | **A** | **A** | **A** | **T** | **C** | **C** | **T** | **C** | **G** | **C** | **A** | **G** | **A** | **T** | **T** | **G** | **A** | **A** | **G** | **A** | **T** | **C** | **A** | **T** | **T** | **A** | **C** | **C** | **A** | **A** | **T** | **A** | **C** | **C** | **T** | **T** | **G** | **G** | **T** | **T** | **G** | **G** | **A** | **G** | **A** | **T** | **G** | **T** | **C** | **G** | **A** | **G** | **G** | **T** | **G** | **A** | **T** | **A** | **C** | **G** | **A** | **A** | **T** | **A** | **-** | **-** | **-** |
| **N55728** | **A** | **T** | **G** | **A** | **A** | **T** | **A** | **G** | **T** | **T** | **C** | **T** | **T** | **G** | **G** | **C** | **G** | **T** | **T** | **T** | **T** | **T** | **T** | **T** | **G** | **T** | **G** | **G** | **T** | **A** | **G** | **T** | **T** | **G** | **A** | **A** | **G** | **C** | **A** | **C** | **T** | **A** | **C** | **T** | **G** | **C** | **T** | **G** | **T** | **T** | **A** | **T** | **T** | **T** | **G** | **C** | **T** | **G** | **T** | **T** | **T** | **G** | **G** | **C** | **A** | **A** | **A** | **T** | **C** | **G** | **T** | **A** | **A** | **A** | **C** | **A** | **A** | **T** | **A** | **C** | **T** | **G** | **G** | **A** | **C** | **T** | **T** | **T** | **T** | **G** | **C** | **T** | **T** | **T** | **T** | **A** | **G** | **T** | **G** | **A** | **T** | **A** | **T** | **T** | **C** | **G** | **G** | **T** | **A** | **T** | **T** | **T** | **T** | **C** | **A** | **A** | **T** | **A** | **T** | **A** | **T** | **A** | **T** | **C** | **T** | **A** | **G** | **C** | **T** | **A** | **T** | **G** | **C** | **G** | **C** | **A** | **A** | **A** | **C** | **A** | **T** | **C** | **C** | **T** | **C** | **G** | **T** | **A** | **C** | **T** | **A** | **A** | **A** | **T** | **T** | **T** | **C** | **A** | **A** | **A** | **A** | **T** | **T** | **T** | **T** | **C** | **A** | **A** | **T** | **T** | **G** | **A** | **T** | **A** | **T** | **T** | **A** | **G** | **G** | **A** | **A** | **G** | **T** | **T** | **T** | **G** | **G** | **T** | **C** | **A** | **T** | **T** | **T** | **T** | **T** | **T** | **T** | **T** | **A** | **G** | **C** | **T** | **T** | **A** | **G** | **T** | **C** | **A** | **A** | **C** | **A** | **G** | **T** | **C** | **C** | **T** | **G** | **C** | **T** | **T** | **T** | **A** | **T** | **G** | **G** | **A** | **T** | **G** | **A** | **T** | **G** | **G** | **C** | **T** | **G** | **T** | **A** | **T** | **T** | **T** | **G** | **C** | **C** | **G** | **T** | **T** | **T** | **T** | **A** | **T** | **T** | **T** | **A** | **T** | **T** | **G** | **G** | **A** | **C** | **T** | **A** | **A** | **A** | **A** | **G** | **G** | **T** | **G** | **T** | **A** | **G** | **A** | **G** | **A** | **T** | **T** | **T** | **C** | **A** | **G** | **G** | **G** | **A** | **T** | **A** | **G** | **A** | **T** | **T** | **T** | **A** | **A** | **C** | **C** | **A** | **A** | **A** | **A** | **A** | **T** | **G** | **C** | **T** | **T** | **T** | **T** | **T** | **G** | **G** | **C** | **G** | **A** | **A** | **A** | **G** | **A** | **A** | **A** | **C** | **A** | **G** | **A** | **T** | **C** | **A** | **T** | **G** | **A** | **T** | **G** | **G** | **T** | **C** | **C** | **A** | **A** | **A** | **C** | **A** | **G** | **A** | **G** | **C** | **A** | **A** | **T** | **T** | **A** | **A** | **A** | **A** | **A** | **C** | **A** | **C** | **A** | **T** | **A** | **A** | **T** | **A** | **A** | **T** | **G** | **A** | **G** | **C** | **G** | **G** | **A** | **A** | **A** | **A** | **G** | **A** | **C** | **A** | **G** | **C** | **A** | **A** | **T** | **T** | **A** | **T** | **T** | **C** | **G** | **G** | **T** | **A** | **A** | **C** | **C** | **A** | **A** | **C** | **G** | **T** | **A** | **T** | **T** | **G** | **G** | **A** | **A** | **A** | **C** | **G** | **A** | **C** | **G** | **T** | **C** | **T** | **A** | **T** | **G** | **A** | **A** | **T** | **G** | **G** | **G** | **A** | **C** | **G** | **A** | **T** | **A** | **T** | **C** | **A** | **A** | **C** | **A** | **T** | **C** | **G** | **C** | **C** | **G** | **T** | **C** | **A** | **T** | **T** | **T** | **C** | **T** | **G** | **G** | **T** | **G** | **A** | **T** | **A** | **C** | **T** | **A** | **T** | **C** | **A** | **T** | **A** | **G** | **A** | **T** | **T** | **T** | **G** | **G** | **G** | **C** | **A** | **A** | **T** | **A** | **C** | **G** | **C** | **T** | **T** | **T** | **T** | **G** | **C** | **C** | **A** | **A** | **A** | **A** | **G** | **A** | **T** | **G** | **A** | **T** | **A** | **A** | **T** | **A** | **T** | **T** | **G** | **T** | **G** | **A** | **T** | **T** | **G** | **T** | **A** | **A** | **G** | **A** | **A** | **A** | **A** | **G** | **G** | **G** | **A** | **T** | **A** | **G** | **G** | **C** | **A** | **G** | **A** | **A** | **C** | **A** | **C** | **G** | **G** | **A** | **T** | **C** | **T** | **T** | **A** | **G** | **T** | **A** | **C** | **C** | **T** | **T** | **T** | **G** | **G** | **G** | **C** | **G** | **T** | **A** | **G** | **C** | **C** | **A** | **T** | **C** | **C** | **G** | **A** | **T** | **T** | **A** | **G** | **A** | **G** | **C** | **A** | **T** | **G** | **C** | **G** | **A** | **C** | **A** | **T** | **T** | **A** | **G** | **T** | **A** | **G** | **G** | **A** | **A** | **A** | **T** | **G** | **T** | **C** | **T** | **T** | **G** | **T** | **T** | **T** | **G** | **A** | **A** | **G** | **A** | **A** | **G** | **A** | **G** | **C** | **A** | **A** | **T** | **T** | **T** | **T** | **C** | **T** | **T** | **T** | **G** | **A** | **A** | **A** | **A** | **A** | **T** | **G** | **A** | **A** | **C** | **A** | **A** | **A** | **T** | **C** | **A** | **A** | **A** | **A** | **T** | **C** | **T** | **A** | **C** | **A** | **G** | **C** | **A** | **G** | **T** | **G** | **A** | **T** | **T** | **A** | **T** | **G** | **A** | **T** | **G** | **A** | **A** | **A** | **A** | **C** | **C** | **C** | **T** | **C** | **G** | **T** | **A** | **G** | **A** | **C** | **T** | **G** | **A** | **A** | **G** | **A** | **T** | **T** | **A** | **T** | **T** | **A** | **C** | **T** | **A** | **A** | **T** | **A** | **C** | **C** | **T** | **T** | **G** | **A** | **T** | **T** | **G** | **G** | **A** | **G** | **A** | **T** | **G** | **T** | **C** | **G** | **A** | **G** | **G** | **T** | **G** | **A** | **T** | **A** | **C** | **G** | **A** | **A** | **T** | **A** | **-** | **-** | **-** |
| **N60190F** | **A** | **T** | **G** | **A** | **A** | **T** | **A** | **G** | **T** | **T** | **C** | **T** | **T** | **G** | **G** | **C** | **G** | **T** | **T** | **T** | **T** | **T** | **T** | **T** | **G** | **T** | **G** | **G** | **T** | **A** | **G** | **T** | **T** | **G** | **A** | **A** | **G** | **C** | **A** | **C** | **T** | **A** | **C** | **T** | **G** | **C** | **T** | **G** | **T** | **T** | **A** | **T** | **T** | **T** | **G** | **C** | **T** | **G** | **T** | **T** | **T** | **G** | **G** | **C** | **A** | **A** | **A** | **T** | **C** | **G** | **T** | **A** | **A** | **A** | **C** | **A** | **A** | **T** | **A** | **C** | **T** | **G** | **G** | **A** | **C** | **T** | **T** | **T** | **T** | **G** | **C** | **T** | **T** | **T** | **T** | **A** | **G** | **T** | **G** | **A** | **T** | **A** | **T** | **T** | **C** | **G** | **G** | **T** | **A** | **T** | **T** | **T** | **T** | **C** | **A** | **A** | **T** | **A** | **T** | **A** | **T** | **A** | **T** | **C** | **T** | **A** | **G** | **C** | **T** | **A** | **T** | **G** | **C** | **G** | **C** | **A** | **A** | **A** | **C** | **A** | **T** | **C** | **C** | **T** | **C** | **G** | **T** | **A** | **C** | **T** | **A** | **A** | **A** | **T** | **T** | **T** | **C** | **A** | **A** | **A** | **A** | **T** | **T** | **T** | **T** | **C** | **A** | **A** | **T** | **T** | **G** | **A** | **T** | **A** | **T** | **T** | **A** | **G** | **G** | **A** | **A** | **G** | **T** | **T** | **T** | **G** | **G** | **T** | **C** | **A** | **T** | **T** | **T** | **T** | **T** | **T** | **T** | **T** | **A** | **G** | **C** | **T** | **T** | **A** | **G** | **T** | **C** | **A** | **A** | **C** | **A** | **G** | **T** | **C** | **C** | **T** | **G** | **C** | **T** | **T** | **T** | **A** | **T** | **G** | **G** | **A** | **T** | **G** | **A** | **T** | **G** | **G** | **C** | **T** | **G** | **T** | **A** | **T** | **T** | **T** | **G** | **C** | **C** | **G** | **T** | **T** | **T** | **T** | **A** | **T** | **T** | **T** | **A** | **T** | **T** | **G** | **G** | **T** | **C** | **T** | **G** | **A** | **A** | **A** | **G** | **G** | **T** | **G** | **T** | **A** | **G** | **A** | **G** | **A** | **T** | **T** | **T** | **C** | **A** | **G** | **G** | **G** | **A** | **T** | **A** | **G** | **A** | **T** | **T** | **T** | **A** | **A** | **C** | **C** | **A** | **A** | **A** | **A** | **A** | **T** | **G** | **C** | **T** | **T** | **T** | **T** | **T** | **G** | **G** | **C** | **G** | **A** | **A** | **A** | **G** | **A** | **A** | **A** | **C** | **A** | **G** | **A** | **T** | **C** | **A** | **T** | **G** | **A** | **T** | **G** | **A** | **T** | **C** | **C** | **A** | **A** | **A** | **C** | **A** | **G** | **A** | **G** | **C** | **A** | **A** | **T** | **T** | **A** | **A** | **A** | **A** | **A** | **C** | **A** | **C** | **A** | **T** | **A** | **A** | **T** | **A** | **A** | **T** | **G** | **A** | **G** | **C** | **G** | **G** | **A** | **A** | **A** | **A** | **G** | **A** | **C** | **A** | **G** | **C** | **A** | **A** | **T** | **T** | **A** | **T** | **T** | **C** | **G** | **G** | **T** | **A** | **A** | **C** | **C** | **A** | **A** | **C** | **G** | **T** | **A** | **T** | **T** | **G** | **G** | **A** | **A** | **A** | **C** | **G** | **A** | **C** | **G** | **T** | **C** | **T** | **A** | **T** | **G** | **A** | **A** | **T** | **G** | **G** | **G** | **A** | **C** | **G** | **A** | **T** | **A** | **T** | **C** | **A** | **A** | **C** | **A** | **T** | **C** | **G** | **C** | **C** | **G** | **T** | **C** | **A** | **T** | **T** | **T** | **C** | **T** | **G** | **G** | **T** | **G** | **A** | **T** | **A** | **C** | **T** | **A** | **T** | **C** | **A** | **T** | **A** | **G** | **A** | **T** | **T** | **T** | **G** | **G** | **G** | **C** | **A** | **A** | **T** | **A** | **C** | **G** | **C** | **T** | **T** | **T** | **T** | **G** | **C** | **C** | **A** | **A** | **A** | **A** | **G** | **A** | **T** | **G** | **A** | **T** | **A** | **A** | **T** | **A** | **T** | **T** | **G** | **T** | **G** | **A** | **T** | **T** | **G** | **T** | **A** | **A** | **G** | **A** | **A** | **A** | **A** | **G** | **G** | **G** | **A** | **T** | **A** | **G** | **G** | **C** | **A** | **G** | **A** | **A** | **C** | **A** | **C** | **G** | **G** | **A** | **T** | **C** | **T** | **T** | **A** | **G** | **T** | **A** | **C** | **C** | **T** | **T** | **T** | **G** | **G** | **G** | **C** | **G** | **T** | **A** | **G** | **C** | **C** | **A** | **T** | **C** | **C** | **G** | **A** | **T** | **T** | **A** | **G** | **A** | **G** | **C** | **A** | **T** | **G** | **C** | **G** | **A** | **C** | **A** | **T** | **T** | **A** | **G** | **T** | **A** | **G** | **G** | **A** | **A** | **A** | **T** | **G** | **T** | **C** | **T** | **T** | **G** | **T** | **T** | **T** | **G** | **A** | **A** | **G** | **A** | **A** | **G** | **A** | **G** | **C** | **A** | **A** | **T** | **T** | **T** | **T** | **C** | **T** | **T** | **T** | **G** | **A** | **A** | **A** | **A** | **A** | **T** | **G** | **A** | **A** | **C** | **A** | **A** | **A** | **T** | **C** | **A** | **A** | **A** | **A** | **T** | **C** | **T** | **A** | **C** | **A** | **G** | **C** | **A** | **G** | **T** | **G** | **A** | **T** | **T** | **A** | **T** | **G** | **A** | **T** | **G** | **A** | **A** | **A** | **A** | **C** | **C** | **C** | **T** | **C** | **G** | **T** | **A** | **G** | **A** | **T** | **T** | **G** | **A** | **A** | **G** | **A** | **T** | **T** | **A** | **T** | **T** | **A** | **C** | **T** | **A** | **A** | **T** | **A** | **C** | **C** | **T** | **T** | **G** | **A** | **T** | **T** | **G** | **G** | **A** | **G** | **A** | **T** | **G** | **T** | **C** | **G** | **A** | **G** | **G** | **T** | **G** | **A** | **T** | **A** | **C** | **G** | **A** | **A** | **T** | **A** | **-** | **-** | **-** |
| **N55655** | **A** | **T** | **G** | **A** | **A** | **T** | **A** | **G** | **T** | **T** | **C** | **T** | **T** | **G** | **G** | **C** | **G** | **T** | **T** | **T** | **T** | **T** | **T** | **T** | **G** | **T** | **G** | **G** | **T** | **A** | **G** | **T** | **C** | **G** | **A** | **A** | **G** | **C** | **A** | **C** | **T** | **G** | **C** | **T** | **G** | **C** | **T** | **G** | **T** | **T** | **A** | **T** | **T** | **T** | **G** | **C** | **T** | **G** | **T** | **T** | **T** | **G** | **G** | **C** | **A** | **A** | **A** | **T** | **C** | **G** | **T** | **A** | **A** | **A** | **C** | **A** | **A** | **T** | **A** | **C** | **T** | **G** | **G** | **A** | **C** | **T** | **T** | **T** | **T** | **G** | **C** | **T** | **T** | **T** | **T** | **A** | **G** | **T** | **A** | **A** | **T** | **A** | **T** | **T** | **C** | **G** | **G** | **T** | **A** | **T** | **T** | **T** | **T** | **C** | **A** | **A** | **T** | **A** | **T** | **A** | **T** | **A** | **T** | **C** | **T** | **A** | **G** | **C** | **T** | **A** | **T** | **G** | **C** | **G** | **C** | **A** | **A** | **A** | **T** | **A** | **T** | **C** | **C** | **T** | **C** | **G** | **T** | **A** | **C** | **T** | **A** | **A** | **A** | **T** | **T** | **T** | **C** | **A** | **A** | **A** | **A** | **T** | **T** | **T** | **T** | **C** | **A** | **A** | **T** | **T** | **G** | **A** | **T** | **C** | **T** | **T** | **A** | **G** | **G** | **G** | **A** | **G** | **C** | **T** | **T** | **G** | **G** | **T** | **C** | **A** | **T** | **T** | **T** | **T** | **T** | **T** | **T** | **T** | **A** | **G** | **C** | **T** | **T** | **A** | **G** | **T** | **C** | **A** | **A** | **C** | **A** | **G** | **T** | **C** | **C** | **T** | **G** | **C** | **T** | **T** | **T** | **A** | **T** | **G** | **G** | **A** | **T** | **G** | **A** | **T** | **G** | **G** | **T** | **T** | **G** | **T** | **A** | **T** | **T** | **G** | **G** | **C** | **C** | **G** | **T** | **T** | **T** | **T** | **A** | **T** | **T** | **T** | **A** | **T** | **C** | **G** | **G** | **G** | **C** | **T** | **G** | **A** | **A** | **A** | **G** | **G** | **T** | **G** | **T** | **A** | **G** | **A** | **G** | **A** | **T** | **T** | **T** | **C** | **A** | **G** | **G** | **A** | **A** | **T** | **A** | **G** | **A** | **T** | **T** | **T** | **G** | **A** | **C** | **A** | **A** | **A** | **A** | **A** | **A** | **T** | **G** | **C** | **T** | **T** | **T** | **T** | **T** | **G** | **G** | **C** | **G** | **A** | **A** | **A** | **A** | **A** | **A** | **A** | **C** | **A** | **G** | **A** | **T** | **C** | **A** | **T** | **G** | **A** | **T** | **G** | **G** | **T** | **C** | **C** | **A** | **A** | **A** | **C** | **A** | **G** | **A** | **G** | **C** | **A** | **A** | **T** | **T** | **A** | **A** | **A** | **A** | **A** | **C** | **A** | **C** | **A** | **T** | **A** | **A** | **T** | **A** | **A** | **T** | **G** | **A** | **G** | **C** | **G** | **T** | **A** | **A** | **A** | **A** | **G** | **A** | **C** | **A** | **G** | **C** | **A** | **A** | **T** | **T** | **A** | **T** | **T** | **T** | **G** | **G** | **T** | **A** | **A** | **C** | **C** | **A** | **A** | **C** | **G** | **T** | **A** | **T** | **T** | **G** | **G** | **A** | **A** | **A** | **C** | **G** | **A** | **C** | **G** | **T** | **C** | **T** | **A** | **T** | **G** | **A** | **A** | **T** | **G** | **G** | **G** | **A** | **T** | **G** | **A** | **T** | **A** | **T** | **C** | **A** | **A** | **C** | **A** | **T** | **T** | **G** | **C** | **C** | **A** | **T** | **C** | **A** | **T** | **T** | **T** | **C** | **T** | **G** | **G** | **T** | **G** | **A** | **T** | **A** | **C** | **T** | **A** | **T** | **C** | **A** | **T** | **C** | **G** | **A** | **T** | **T** | **T** | **G** | **G** | **G** | **C** | **A** | **A** | **T** | **A** | **C** | **A** | **C** | **T** | **T** | **T** | **T** | **G** | **C** | **C** | **A** | **A** | **A** | **A** | **G** | **A** | **T** | **G** | **A** | **T** | **A** | **A** | **T** | **A** | **T** | **T** | **G** | **T** | **G** | **A** | **T** | **T** | **G** | **T** | **A** | **A** | **G** | **A** | **A** | **A** | **A** | **G** | **G** | **G** | **A** | **T** | **A** | **G** | **G** | **C** | **A** | **G** | **A** | **A** | **C** | **A** | **C** | **G** | **G** | **A** | **T** | **C** | **T** | **T** | **A** | **G** | **T** | **A** | **C** | **C** | **C** | **C** | **T** | **G** | **G** | **G** | **A** | **G** | **T** | **G** | **G** | **C** | **T** | **A** | **T** | **T** | **C** | **G** | **A** | **T** | **T** | **A** | **G** | **A** | **G** | **C** | **A** | **T** | **G** | **C** | **G** | **A** | **C** | **A** | **T** | **T** | **A** | **G** | **T** | **A** | **G** | **G** | **A** | **A** | **A** | **T** | **G** | **T** | **C** | **T** | **T** | **G** | **T** | **T** | **T** | **G** | **A** | **A** | **G** | **A** | **A** | **G** | **A** | **G** | **C** | **A** | **G** | **T** | **T** | **T** | **T** | **C** | **C** | **T** | **T** | **A** | **A** | **A** | **A** | **A** | **A** | **C** | **G** | **A** | **A** | **C** | **A** | **G** | **A** | **T** | **A** | **A** | **A** | **A** | **A** | **T** | **C** | **T** | **G** | **C** | **A** | **G** | **C** | **A** | **G** | **T** | **G** | **A** | **T** | **T** | **A** | **T** | **G** | **A** | **T** | **G** | **A** | **A** | **A** | **A** | **T** | **C** | **C** | **T** | **C** | **G** | **C** | **A** | **G** | **A** | **T** | **T** | **G** | **A** | **A** | **G** | **A** | **T** | **C** | **A** | **T** | **T** | **A** | **C** | **C** | **A** | **A** | **T** | **A** | **C** | **C** | **T** | **T** | **G** | **G** | **T** | **T** | **G** | **G** | **A** | **G** | **A** | **T** | **G** | **T** | **C** | **G** | **A** | **G** | **G** | **T** | **G** | **A** | **T** | **A** | **C** | **G** | **A** | **A** | **T** | **A** | **-** | **-** | **-** |
| **N55337** | **A** | **T** | **G** | **A** | **A** | **T** | **A** | **G** | **T** | **T** | **C** | **T** | **T** | **G** | **G** | **C** | **G** | **T** | **T** | **T** | **T** | **T** | **T** | **T** | **G** | **T** | **G** | **G** | **T** | **A** | **G** | **T** | **C** | **G** | **A** | **A** | **G** | **C** | **A** | **C** | **T** | **G** | **C** | **T** | **G** | **C** | **T** | **G** | **T** | **T** | **A** | **T** | **T** | **T** | **G** | **C** | **T** | **G** | **T** | **T** | **T** | **G** | **G** | **C** | **A** | **A** | **A** | **T** | **C** | **G** | **T** | **A** | **A** | **A** | **C** | **A** | **A** | **T** | **A** | **C** | **T** | **G** | **G** | **A** | **C** | **T** | **T** | **T** | **T** | **G** | **C** | **T** | **T** | **T** | **T** | **A** | **G** | **T** | **A** | **A** | **T** | **A** | **T** | **T** | **C** | **G** | **G** | **T** | **A** | **T** | **T** | **T** | **T** | **C** | **A** | **A** | **T** | **A** | **T** | **A** | **T** | **A** | **T** | **C** | **T** | **A** | **G** | **C** | **T** | **A** | **T** | **G** | **C** | **G** | **C** | **A** | **A** | **A** | **T** | **A** | **T** | **C** | **C** | **T** | **C** | **G** | **T** | **A** | **C** | **T** | **A** | **A** | **A** | **T** | **T** | **T** | **C** | **A** | **A** | **A** | **A** | **T** | **T** | **T** | **T** | **C** | **A** | **A** | **T** | **T** | **G** | **A** | **T** | **C** | **T** | **T** | **A** | **G** | **G** | **G** | **A** | **G** | **C** | **T** | **T** | **G** | **G** | **T** | **C** | **A** | **T** | **T** | **T** | **T** | **T** | **T** | **T** | **T** | **A** | **G** | **C** | **T** | **T** | **A** | **G** | **T** | **C** | **A** | **A** | **C** | **A** | **G** | **T** | **C** | **C** | **T** | **G** | **C** | **T** | **T** | **T** | **A** | **T** | **G** | **G** | **A** | **T** | **G** | **A** | **T** | **G** | **G** | **T** | **T** | **G** | **T** | **A** | **T** | **T** | **G** | **G** | **C** | **C** | **G** | **T** | **T** | **T** | **T** | **A** | **T** | **T** | **T** | **A** | **T** | **C** | **G** | **G** | **G** | **C** | **T** | **G** | **A** | **A** | **A** | **G** | **G** | **T** | **G** | **T** | **A** | **G** | **A** | **G** | **A** | **T** | **T** | **T** | **C** | **A** | **G** | **G** | **A** | **A** | **T** | **A** | **G** | **A** | **T** | **T** | **T** | **G** | **A** | **C** | **A** | **A** | **A** | **A** | **A** | **A** | **T** | **G** | **C** | **T** | **T** | **T** | **T** | **T** | **G** | **G** | **C** | **G** | **A** | **A** | **A** | **A** | **A** | **A** | **A** | **C** | **A** | **G** | **A** | **T** | **C** | **A** | **T** | **G** | **A** | **T** | **G** | **G** | **T** | **C** | **C** | **A** | **A** | **A** | **C** | **A** | **G** | **A** | **G** | **C** | **A** | **A** | **T** | **T** | **A** | **A** | **A** | **A** | **A** | **C** | **A** | **C** | **A** | **T** | **A** | **A** | **T** | **A** | **A** | **T** | **G** | **A** | **G** | **C** | **G** | **T** | **A** | **A** | **A** | **A** | **G** | **A** | **C** | **A** | **G** | **C** | **A** | **A** | **T** | **T** | **A** | **T** | **T** | **T** | **G** | **G** | **T** | **A** | **A** | **C** | **C** | **A** | **A** | **C** | **G** | **T** | **A** | **T** | **T** | **G** | **G** | **A** | **A** | **A** | **C** | **G** | **A** | **C** | **G** | **T** | **C** | **T** | **A** | **T** | **G** | **A** | **A** | **T** | **G** | **G** | **G** | **A** | **T** | **G** | **A** | **T** | **A** | **T** | **C** | **A** | **A** | **C** | **A** | **T** | **T** | **G** | **C** | **C** | **A** | **T** | **C** | **A** | **T** | **T** | **T** | **C** | **T** | **G** | **G** | **T** | **G** | **A** | **T** | **A** | **C** | **T** | **A** | **T** | **C** | **A** | **T** | **C** | **G** | **A** | **T** | **T** | **T** | **G** | **G** | **G** | **C** | **A** | **A** | **T** | **A** | **C** | **A** | **C** | **T** | **T** | **T** | **T** | **G** | **C** | **C** | **A** | **A** | **A** | **A** | **G** | **A** | **T** | **G** | **A** | **T** | **A** | **A** | **T** | **A** | **T** | **T** | **G** | **T** | **G** | **A** | **T** | **T** | **G** | **T** | **A** | **A** | **G** | **A** | **A** | **A** | **A** | **G** | **G** | **G** | **A** | **T** | **A** | **G** | **G** | **C** | **A** | **G** | **A** | **A** | **C** | **A** | **C** | **G** | **G** | **A** | **T** | **C** | **T** | **T** | **A** | **G** | **T** | **A** | **C** | **C** | **C** | **C** | **T** | **G** | **G** | **G** | **A** | **G** | **T** | **G** | **G** | **C** | **T** | **A** | **T** | **T** | **C** | **G** | **A** | **T** | **T** | **A** | **G** | **A** | **G** | **C** | **A** | **T** | **G** | **C** | **G** | **A** | **C** | **A** | **T** | **T** | **A** | **G** | **T** | **A** | **G** | **G** | **A** | **A** | **A** | **T** | **G** | **T** | **C** | **T** | **T** | **G** | **T** | **T** | **T** | **G** | **A** | **A** | **G** | **A** | **A** | **G** | **A** | **G** | **C** | **A** | **G** | **T** | **T** | **T** | **T** | **C** | **C** | **T** | **T** | **A** | **A** | **A** | **A** | **A** | **A** | **C** | **G** | **A** | **A** | **C** | **A** | **G** | **A** | **T** | **A** | **A** | **A** | **A** | **A** | **T** | **C** | **T** | **G** | **C** | **A** | **G** | **C** | **A** | **G** | **T** | **G** | **A** | **T** | **T** | **A** | **T** | **G** | **A** | **T** | **G** | **A** | **A** | **A** | **A** | **T** | **C** | **C** | **T** | **C** | **G** | **C** | **A** | **G** | **A** | **T** | **T** | **G** | **A** | **A** | **G** | **A** | **T** | **C** | **A** | **T** | **T** | **A** | **C** | **C** | **A** | **A** | **T** | **A** | **C** | **C** | **T** | **T** | **G** | **G** | **T** | **T** | **G** | **G** | **A** | **G** | **A** | **T** | **G** | **T** | **C** | **G** | **A** | **G** | **G** | **T** | **G** | **A** | **T** | **A** | **C** | **G** | **A** | **A** | **T** | **A** | **-** | **-** | **-** |
| **N53533** | **A** | **T** | **G** | **A** | **A** | **T** | **A** | **G** | **T** | **T** | **C** | **T** | **T** | **G** | **G** | **C** | **G** | **T** | **T** | **T** | **T** | **T** | **T** | **T** | **G** | **T** | **G** | **G** | **T** | **A** | **G** | **T** | **C** | **G** | **A** | **A** | **G** | **C** | **A** | **C** | **T** | **G** | **C** | **T** | **G** | **C** | **T** | **G** | **T** | **T** | **A** | **T** | **T** | **T** | **G** | **C** | **T** | **G** | **T** | **T** | **T** | **G** | **G** | **C** | **A** | **A** | **A** | **T** | **C** | **G** | **T** | **A** | **A** | **A** | **C** | **A** | **A** | **T** | **A** | **C** | **T** | **G** | **G** | **A** | **C** | **T** | **T** | **T** | **T** | **G** | **C** | **T** | **T** | **T** | **T** | **A** | **G** | **T** | **A** | **A** | **T** | **A** | **T** | **T** | **C** | **G** | **G** | **T** | **A** | **T** | **T** | **T** | **T** | **C** | **A** | **A** | **T** | **A** | **T** | **A** | **T** | **A** | **T** | **C** | **T** | **A** | **G** | **C** | **T** | **A** | **T** | **G** | **C** | **G** | **C** | **A** | **A** | **A** | **T** | **A** | **T** | **C** | **C** | **T** | **C** | **G** | **T** | **A** | **C** | **T** | **A** | **A** | **A** | **T** | **T** | **T** | **C** | **A** | **A** | **A** | **A** | **T** | **T** | **T** | **T** | **C** | **A** | **A** | **T** | **T** | **G** | **A** | **T** | **C** | **T** | **T** | **A** | **G** | **G** | **G** | **A** | **G** | **C** | **T** | **T** | **G** | **G** | **T** | **C** | **A** | **T** | **T** | **T** | **T** | **T** | **T** | **T** | **T** | **A** | **G** | **C** | **T** | **T** | **A** | **G** | **T** | **C** | **A** | **A** | **C** | **A** | **G** | **T** | **C** | **C** | **T** | **G** | **C** | **T** | **T** | **T** | **A** | **T** | **G** | **G** | **A** | **T** | **G** | **A** | **T** | **G** | **G** | **T** | **T** | **G** | **T** | **A** | **T** | **T** | **G** | **G** | **C** | **C** | **G** | **T** | **T** | **T** | **T** | **A** | **T** | **T** | **T** | **A** | **T** | **C** | **G** | **G** | **G** | **C** | **T** | **G** | **A** | **A** | **A** | **G** | **G** | **T** | **G** | **T** | **A** | **G** | **A** | **G** | **A** | **T** | **T** | **T** | **C** | **A** | **G** | **G** | **A** | **A** | **T** | **A** | **G** | **A** | **T** | **T** | **T** | **G** | **A** | **C** | **A** | **A** | **A** | **A** | **A** | **A** | **T** | **G** | **C** | **T** | **T** | **T** | **T** | **T** | **G** | **G** | **C** | **G** | **A** | **A** | **A** | **A** | **A** | **A** | **A** | **C** | **A** | **G** | **A** | **T** | **C** | **A** | **T** | **G** | **A** | **T** | **G** | **G** | **T** | **C** | **C** | **A** | **A** | **A** | **C** | **A** | **G** | **A** | **G** | **C** | **A** | **A** | **T** | **T** | **A** | **A** | **A** | **A** | **A** | **C** | **A** | **C** | **A** | **T** | **A** | **A** | **T** | **A** | **A** | **T** | **G** | **A** | **G** | **C** | **G** | **T** | **A** | **A** | **A** | **A** | **G** | **A** | **C** | **A** | **G** | **C** | **A** | **A** | **T** | **T** | **A** | **T** | **T** | **T** | **G** | **G** | **T** | **A** | **A** | **C** | **C** | **A** | **A** | **C** | **G** | **T** | **A** | **T** | **T** | **G** | **G** | **A** | **A** | **A** | **C** | **G** | **A** | **C** | **G** | **T** | **C** | **T** | **A** | **T** | **G** | **A** | **A** | **T** | **G** | **G** | **G** | **A** | **T** | **G** | **A** | **T** | **A** | **T** | **C** | **A** | **A** | **C** | **A** | **T** | **T** | **G** | **C** | **C** | **A** | **T** | **C** | **A** | **T** | **T** | **T** | **C** | **T** | **G** | **G** | **T** | **G** | **A** | **T** | **A** | **C** | **T** | **A** | **T** | **C** | **A** | **T** | **C** | **G** | **A** | **T** | **T** | **T** | **G** | **G** | **G** | **C** | **A** | **A** | **T** | **A** | **C** | **A** | **C** | **T** | **T** | **T** | **T** | **G** | **C** | **C** | **A** | **A** | **A** | **A** | **G** | **A** | **T** | **G** | **A** | **T** | **A** | **A** | **T** | **A** | **T** | **T** | **G** | **T** | **G** | **A** | **T** | **T** | **G** | **T** | **A** | **A** | **G** | **A** | **A** | **A** | **A** | **G** | **G** | **G** | **A** | **T** | **A** | **G** | **G** | **C** | **A** | **G** | **A** | **A** | **C** | **A** | **C** | **G** | **G** | **A** | **T** | **C** | **T** | **T** | **A** | **G** | **T** | **A** | **C** | **C** | **C** | **C** | **T** | **G** | **G** | **G** | **A** | **G** | **T** | **G** | **G** | **C** | **T** | **A** | **T** | **T** | **C** | **G** | **T** | **T** | **T** | **A** | **G** | **A** | **G** | **C** | **A** | **T** | **G** | **C** | **G** | **A** | **C** | **A** | **T** | **T** | **A** | **G** | **T** | **A** | **G** | **G** | **A** | **A** | **A** | **T** | **G** | **T** | **C** | **T** | **T** | **G** | **T** | **T** | **T** | **G** | **A** | **A** | **G** | **A** | **A** | **G** | **A** | **G** | **C** | **A** | **G** | **T** | **T** | **T** | **T** | **C** | **C** | **T** | **T** | **A** | **A** | **A** | **A** | **A** | **A** | **C** | **G** | **A** | **A** | **C** | **A** | **G** | **A** | **T** | **C** | **A** | **A** | **A** | **A** | **T** | **C** | **T** | **G** | **C** | **A** | **G** | **C** | **A** | **G** | **T** | **G** | **A** | **T** | **T** | **A** | **T** | **G** | **A** | **T** | **G** | **A** | **A** | **A** | **A** | **T** | **C** | **C** | **T** | **C** | **G** | **C** | **A** | **G** | **A** | **T** | **T** | **G** | **A** | **A** | **G** | **A** | **T** | **C** | **A** | **T** | **T** | **A** | **C** | **C** | **A** | **A** | **T** | **A** | **C** | **C** | **T** | **T** | **G** | **G** | **T** | **T** | **G** | **G** | **A** | **G** | **A** | **T** | **G** | **T** | **C** | **G** | **A** | **G** | **G** | **T** | **G** | **A** | **T** | **A** | **C** | **G** | **A** | **A** | **T** | **A** | **-** | **-** | **-** |
| **N54519** | **A** | **T** | **G** | **A** | **A** | **T** | **A** | **G** | **T** | **T** | **C** | **T** | **T** | **G** | **G** | **C** | **G** | **T** | **T** | **T** | **T** | **T** | **T** | **T** | **G** | **T** | **G** | **G** | **T** | **A** | **G** | **T** | **T** | **G** | **A** | **A** | **A** | **C** | **A** | **C** | **T** | **A** | **C** | **T** | **G** | **C** | **T** | **G** | **T** | **T** | **A** | **T** | **T** | **T** | **G** | **C** | **T** | **G** | **T** | **T** | **T** | **G** | **G** | **C** | **A** | **A** | **A** | **T** | **C** | **G** | **T** | **A** | **A** | **A** | **C** | **A** | **A** | **T** | **A** | **C** | **T** | **G** | **G** | **A** | **C** | **T** | **T** | **T** | **T** | **G** | **C** | **T** | **T** | **T** | **T** | **A** | **G** | **T** | **G** | **A** | **T** | **A** | **T** | **T** | **C** | **G** | **G** | **T** | **A** | **T** | **T** | **T** | **T** | **C** | **A** | **A** | **T** | **A** | **T** | **A** | **T** | **A** | **T** | **C** | **T** | **A** | **G** | **C** | **T** | **A** | **T** | **G** | **C** | **G** | **C** | **A** | **A** | **A** | **C** | **A** | **T** | **C** | **C** | **T** | **C** | **G** | **T** | **A** | **C** | **T** | **A** | **A** | **A** | **T** | **T** | **T** | **C** | **A** | **A** | **A** | **A** | **T** | **T** | **T** | **T** | **C** | **A** | **A** | **T** | **T** | **G** | **A** | **T** | **A** | **T** | **T** | **A** | **G** | **G** | **A** | **A** | **G** | **T** | **T** | **T** | **G** | **G** | **T** | **C** | **A** | **T** | **T** | **T** | **T** | **T** | **T** | **T** | **T** | **A** | **G** | **C** | **T** | **T** | **A** | **G** | **T** | **C** | **A** | **A** | **C** | **A** | **G** | **T** | **C** | **C** | **T** | **G** | **C** | **T** | **T** | **T** | **A** | **T** | **G** | **G** | **A** | **T** | **G** | **A** | **T** | **G** | **G** | **C** | **T** | **G** | **T** | **A** | **T** | **T** | **T** | **G** | **C** | **C** | **G** | **T** | **T** | **T** | **T** | **A** | **T** | **T** | **T** | **A** | **T** | **T** | **G** | **G** | **A** | **C** | **T** | **A** | **A** | **A** | **A** | **G** | **G** | **T** | **G** | **T** | **A** | **G** | **A** | **G** | **A** | **T** | **T** | **T** | **C** | **A** | **G** | **G** | **G** | **A** | **T** | **A** | **G** | **A** | **T** | **T** | **T** | **A** | **A** | **C** | **C** | **A** | **A** | **A** | **A** | **A** | **T** | **G** | **C** | **T** | **T** | **T** | **T** | **T** | **G** | **G** | **C** | **G** | **A** | **A** | **A** | **G** | **A** | **A** | **A** | **C** | **A** | **G** | **A** | **T** | **C** | **A** | **T** | **G** | **A** | **T** | **G** | **G** | **T** | **C** | **C** | **A** | **A** | **A** | **C** | **A** | **G** | **A** | **G** | **C** | **A** | **A** | **T** | **T** | **A** | **A** | **A** | **A** | **A** | **C** | **A** | **C** | **A** | **T** | **A** | **A** | **T** | **A** | **A** | **T** | **G** | **A** | **G** | **C** | **G** | **G** | **A** | **A** | **A** | **A** | **G** | **A** | **C** | **A** | **G** | **C** | **A** | **A** | **T** | **T** | **A** | **T** | **T** | **C** | **G** | **G** | **T** | **A** | **A** | **C** | **C** | **A** | **A** | **C** | **G** | **T** | **A** | **T** | **T** | **G** | **G** | **A** | **A** | **A** | **C** | **G** | **A** | **C** | **G** | **T** | **C** | **T** | **A** | **T** | **G** | **A** | **A** | **T** | **G** | **G** | **G** | **A** | **C** | **G** | **A** | **T** | **A** | **T** | **C** | **A** | **A** | **C** | **A** | **T** | **C** | **G** | **C** | **C** | **G** | **T** | **C** | **A** | **T** | **T** | **T** | **C** | **T** | **G** | **G** | **T** | **G** | **A** | **T** | **A** | **C** | **T** | **A** | **T** | **C** | **A** | **T** | **A** | **G** | **A** | **T** | **T** | **T** | **G** | **G** | **G** | **C** | **A** | **A** | **T** | **A** | **C** | **G** | **C** | **T** | **T** | **T** | **T** | **G** | **C** | **C** | **A** | **A** | **A** | **A** | **G** | **A** | **T** | **G** | **A** | **T** | **A** | **A** | **T** | **A** | **T** | **T** | **G** | **T** | **G** | **A** | **T** | **T** | **G** | **T** | **A** | **A** | **G** | **A** | **A** | **A** | **A** | **G** | **G** | **G** | **A** | **T** | **A** | **G** | **G** | **C** | **A** | **G** | **A** | **A** | **C** | **A** | **C** | **G** | **A** | **A** | **T** | **C** | **T** | **T** | **A** | **G** | **T** | **A** | **C** | **C** | **T** | **T** | **T** | **G** | **G** | **G** | **C** | **G** | **T** | **A** | **G** | **C** | **C** | **A** | **T** | **C** | **C** | **G** | **A** | **T** | **T** | **A** | **G** | **A** | **G** | **C** | **A** | **T** | **G** | **C** | **G** | **A** | **C** | **A** | **T** | **T** | **A** | **G** | **T** | **A** | **G** | **G** | **A** | **A** | **A** | **T** | **G** | **T** | **C** | **T** | **T** | **G** | **T** | **T** | **T** | **G** | **A** | **A** | **G** | **A** | **A** | **G** | **A** | **G** | **C** | **A** | **A** | **T** | **T** | **T** | **T** | **C** | **T** | **T** | **T** | **G** | **A** | **A** | **A** | **A** | **A** | **T** | **G** | **A** | **A** | **C** | **A** | **A** | **A** | **T** | **C** | **A** | **A** | **A** | **A** | **T** | **C** | **T** | **A** | **C** | **A** | **G** | **C** | **A** | **G** | **T** | **G** | **A** | **T** | **T** | **A** | **T** | **G** | **A** | **T** | **G** | **A** | **A** | **A** | **A** | **C** | **C** | **C** | **T** | **C** | **G** | **T** | **A** | **G** | **A** | **C** | **T** | **G** | **A** | **A** | **G** | **A** | **T** | **T** | **A** | **T** | **T** | **A** | **C** | **T** | **A** | **A** | **T** | **A** | **C** | **C** | **T** | **T** | **G** | **A** | **T** | **T** | **G** | **G** | **A** | **G** | **A** | **T** | **G** | **T** | **C** | **G** | **A** | **G** | **G** | **T** | **G** | **A** | **T** | **A** | **C** | **G** | **A** | **A** | **T** | **A** | **-** | **-** | **-** |
| **N54550** | **A** | **T** | **G** | **A** | **A** | **T** | **A** | **G** | **T** | **T** | **C** | **T** | **T** | **G** | **G** | **C** | **G** | **T** | **T** | **T** | **T** | **T** | **T** | **T** | **G** | **T** | **G** | **G** | **T** | **A** | **G** | **T** | **C** | **G** | **A** | **A** | **G** | **C** | **A** | **C** | **T** | **G** | **C** | **T** | **G** | **C** | **T** | **G** | **T** | **T** | **A** | **T** | **T** | **T** | **G** | **C** | **T** | **G** | **T** | **T** | **T** | **G** | **G** | **G** | **A** | **A** | **A** | **T** | **C** | **G** | **T** | **A** | **A** | **A** | **C** | **A** | **A** | **T** | **A** | **C** | **T** | **G** | **G** | **A** | **C** | **T** | **T** | **T** | **T** | **G** | **C** | **T** | **T** | **T** | **T** | **A** | **G** | **T** | **A** | **A** | **T** | **A** | **T** | **T** | **C** | **G** | **G** | **T** | **A** | **T** | **T** | **T** | **T** | **C** | **A** | **A** | **T** | **A** | **T** | **A** | **T** | **A** | **T** | **C** | **T** | **A** | **G** | **C** | **T** | **A** | **T** | **G** | **C** | **G** | **C** | **A** | **A** | **A** | **T** | **A** | **T** | **C** | **C** | **T** | **C** | **G** | **T** | **A** | **C** | **T** | **A** | **A** | **A** | **T** | **T** | **T** | **C** | **A** | **A** | **A** | **A** | **T** | **T** | **T** | **T** | **C** | **A** | **A** | **T** | **T** | **G** | **A** | **T** | **C** | **T** | **T** | **A** | **G** | **G** | **G** | **A** | **G** | **C** | **T** | **T** | **G** | **G** | **T** | **C** | **A** | **T** | **T** | **T** | **T** | **T** | **T** | **T** | **T** | **A** | **G** | **C** | **T** | **T** | **A** | **G** | **T** | **C** | **A** | **A** | **C** | **A** | **G** | **T** | **C** | **C** | **T** | **G** | **C** | **T** | **T** | **T** | **A** | **T** | **G** | **G** | **A** | **T** | **G** | **A** | **T** | **G** | **G** | **T** | **T** | **G** | **T** | **A** | **T** | **T** | **G** | **G** | **C** | **C** | **G** | **T** | **T** | **T** | **T** | **A** | **T** | **T** | **T** | **A** | **T** | **C** | **G** | **G** | **G** | **C** | **T** | **G** | **A** | **A** | **A** | **G** | **G** | **T** | **G** | **T** | **A** | **G** | **A** | **G** | **A** | **T** | **T** | **T** | **C** | **A** | **G** | **G** | **A** | **A** | **T** | **A** | **G** | **A** | **T** | **T** | **T** | **G** | **A** | **C** | **A** | **A** | **A** | **A** | **A** | **A** | **T** | **G** | **C** | **T** | **T** | **T** | **T** | **T** | **G** | **G** | **C** | **G** | **A** | **A** | **A** | **A** | **A** | **A** | **A** | **C** | **A** | **G** | **A** | **T** | **C** | **A** | **T** | **G** | **A** | **T** | **G** | **G** | **T** | **C** | **C** | **A** | **A** | **A** | **C** | **A** | **G** | **A** | **G** | **C** | **A** | **A** | **T** | **T** | **A** | **A** | **A** | **A** | **A** | **C** | **A** | **C** | **A** | **T** | **A** | **A** | **T** | **A** | **A** | **T** | **G** | **A** | **G** | **C** | **G** | **T** | **A** | **A** | **A** | **A** | **G** | **A** | **C** | **A** | **G** | **C** | **A** | **A** | **T** | **T** | **A** | **T** | **T** | **T** | **G** | **G** | **T** | **A** | **A** | **C** | **C** | **A** | **A** | **C** | **G** | **T** | **A** | **T** | **T** | **G** | **G** | **A** | **A** | **A** | **C** | **G** | **A** | **C** | **G** | **T** | **C** | **T** | **A** | **T** | **G** | **A** | **A** | **T** | **G** | **G** | **G** | **A** | **T** | **G** | **A** | **T** | **A** | **T** | **C** | **A** | **A** | **C** | **A** | **T** | **T** | **G** | **C** | **C** | **A** | **T** | **C** | **A** | **T** | **T** | **T** | **C** | **T** | **G** | **G** | **T** | **G** | **A** | **T** | **A** | **C** | **T** | **A** | **T** | **C** | **A** | **T** | **C** | **G** | **A** | **T** | **T** | **T** | **G** | **G** | **G** | **C** | **A** | **A** | **T** | **A** | **C** | **A** | **C** | **T** | **T** | **T** | **T** | **G** | **C** | **C** | **A** | **A** | **A** | **A** | **G** | **A** | **T** | **G** | **A** | **T** | **A** | **A** | **T** | **A** | **T** | **T** | **G** | **T** | **G** | **A** | **T** | **T** | **G** | **T** | **A** | **A** | **G** | **A** | **A** | **A** | **A** | **G** | **G** | **G** | **A** | **T** | **A** | **G** | **G** | **C** | **A** | **G** | **A** | **A** | **C** | **A** | **C** | **G** | **G** | **A** | **T** | **C** | **T** | **T** | **A** | **G** | **T** | **A** | **C** | **C** | **C** | **C** | **T** | **G** | **G** | **G** | **A** | **G** | **T** | **G** | **G** | **C** | **T** | **A** | **T** | **T** | **C** | **G** | **T** | **T** | **T** | **A** | **G** | **A** | **G** | **C** | **A** | **T** | **G** | **C** | **G** | **A** | **C** | **A** | **T** | **T** | **A** | **G** | **T** | **A** | **G** | **G** | **A** | **A** | **A** | **T** | **G** | **T** | **C** | **T** | **T** | **G** | **T** | **T** | **T** | **G** | **A** | **A** | **G** | **A** | **A** | **G** | **A** | **G** | **C** | **A** | **G** | **T** | **T** | **T** | **T** | **C** | **C** | **T** | **T** | **A** | **A** | **A** | **A** | **A** | **A** | **C** | **G** | **A** | **A** | **C** | **A** | **G** | **A** | **T** | **C** | **A** | **A** | **A** | **A** | **T** | **C** | **T** | **G** | **C** | **A** | **G** | **C** | **A** | **G** | **T** | **G** | **A** | **T** | **T** | **A** | **T** | **G** | **A** | **T** | **G** | **A** | **A** | **A** | **A** | **T** | **C** | **C** | **T** | **C** | **G** | **C** | **A** | **G** | **A** | **T** | **T** | **G** | **A** | **A** | **G** | **A** | **T** | **C** | **A** | **T** | **T** | **A** | **C** | **C** | **A** | **A** | **T** | **A** | **C** | **C** | **T** | **T** | **G** | **G** | **T** | **T** | **G** | **G** | **A** | **G** | **A** | **T** | **G** | **T** | **C** | **G** | **A** | **G** | **G** | **T** | **G** | **A** | **T** | **A** | **C** | **G** | **A** | **A** | **T** | **A** | **-** | **-** | **-** |
| **N53702** | **A** | **T** | **G** | **A** | **A** | **T** | **A** | **G** | **T** | **T** | **C** | **T** | **T** | **G** | **G** | **C** | **G** | **T** | **T** | **T** | **T** | **T** | **T** | **T** | **G** | **T** | **G** | **G** | **T** | **A** | **G** | **T** | **T** | **G** | **A** | **A** | **G** | **C** | **A** | **C** | **T** | **A** | **C** | **T** | **G** | **C** | **T** | **G** | **T** | **T** | **A** | **T** | **T** | **T** | **G** | **C** | **T** | **G** | **T** | **T** | **T** | **G** | **G** | **C** | **A** | **A** | **A** | **T** | **C** | **G** | **T** | **A** | **A** | **A** | **C** | **A** | **A** | **T** | **A** | **C** | **T** | **G** | **G** | **A** | **C** | **T** | **T** | **T** | **T** | **G** | **C** | **T** | **T** | **T** | **T** | **A** | **G** | **T** | **G** | **A** | **T** | **A** | **T** | **T** | **C** | **G** | **G** | **T** | **A** | **T** | **T** | **T** | **T** | **C** | **A** | **A** | **T** | **A** | **T** | **A** | **T** | **A** | **T** | **C** | **T** | **A** | **G** | **C** | **T** | **A** | **T** | **G** | **C** | **G** | **C** | **A** | **A** | **A** | **C** | **A** | **T** | **C** | **C** | **T** | **C** | **G** | **T** | **A** | **C** | **T** | **A** | **A** | **A** | **T** | **T** | **T** | **C** | **A** | **A** | **A** | **A** | **T** | **T** | **T** | **T** | **C** | **A** | **A** | **T** | **T** | **G** | **A** | **T** | **A** | **T** | **T** | **A** | **G** | **G** | **A** | **A** | **G** | **T** | **T** | **T** | **G** | **G** | **T** | **C** | **A** | **T** | **T** | **T** | **T** | **T** | **T** | **T** | **T** | **A** | **G** | **C** | **T** | **T** | **A** | **G** | **T** | **C** | **A** | **A** | **C** | **A** | **G** | **T** | **C** | **C** | **T** | **G** | **C** | **T** | **T** | **T** | **A** | **T** | **G** | **G** | **A** | **T** | **G** | **A** | **T** | **G** | **G** | **C** | **T** | **G** | **T** | **A** | **T** | **T** | **T** | **G** | **C** | **C** | **G** | **T** | **T** | **T** | **T** | **A** | **T** | **T** | **T** | **A** | **T** | **T** | **G** | **G** | **A** | **C** | **T** | **A** | **A** | **A** | **A** | **G** | **G** | **T** | **G** | **T** | **A** | **G** | **A** | **G** | **A** | **T** | **T** | **T** | **C** | **A** | **G** | **G** | **G** | **A** | **T** | **A** | **G** | **A** | **T** | **T** | **T** | **A** | **A** | **C** | **C** | **A** | **A** | **A** | **A** | **A** | **T** | **G** | **C** | **T** | **T** | **T** | **T** | **T** | **G** | **G** | **C** | **G** | **A** | **A** | **A** | **G** | **A** | **A** | **A** | **C** | **A** | **G** | **A** | **T** | **C** | **A** | **T** | **G** | **A** | **T** | **G** | **G** | **T** | **C** | **C** | **A** | **A** | **A** | **C** | **A** | **G** | **A** | **G** | **C** | **A** | **A** | **T** | **T** | **A** | **A** | **A** | **A** | **A** | **C** | **A** | **C** | **A** | **T** | **A** | **A** | **T** | **A** | **A** | **T** | **G** | **A** | **G** | **C** | **G** | **G** | **A** | **A** | **A** | **A** | **G** | **A** | **C** | **A** | **G** | **C** | **A** | **A** | **T** | **T** | **A** | **T** | **T** | **C** | **G** | **G** | **T** | **A** | **A** | **C** | **C** | **A** | **A** | **C** | **G** | **T** | **A** | **T** | **T** | **G** | **G** | **A** | **A** | **A** | **C** | **G** | **A** | **C** | **G** | **T** | **C** | **T** | **A** | **T** | **G** | **A** | **A** | **T** | **G** | **G** | **G** | **A** | **C** | **G** | **A** | **T** | **A** | **T** | **C** | **A** | **A** | **C** | **A** | **T** | **C** | **G** | **C** | **C** | **G** | **T** | **C** | **A** | **T** | **T** | **T** | **C** | **T** | **G** | **G** | **T** | **G** | **A** | **T** | **A** | **C** | **T** | **A** | **T** | **C** | **A** | **T** | **A** | **G** | **A** | **T** | **T** | **T** | **G** | **G** | **G** | **C** | **A** | **A** | **T** | **A** | **C** | **G** | **C** | **T** | **T** | **T** | **T** | **G** | **C** | **C** | **A** | **A** | **A** | **A** | **G** | **A** | **T** | **G** | **A** | **T** | **A** | **A** | **T** | **A** | **T** | **T** | **G** | **T** | **G** | **A** | **T** | **T** | **G** | **T** | **A** | **A** | **G** | **A** | **A** | **A** | **A** | **G** | **G** | **G** | **A** | **T** | **A** | **G** | **G** | **C** | **A** | **G** | **A** | **A** | **C** | **A** | **C** | **G** | **G** | **A** | **T** | **C** | **T** | **T** | **A** | **G** | **T** | **A** | **C** | **C** | **T** | **T** | **T** | **G** | **G** | **G** | **C** | **G** | **T** | **A** | **G** | **C** | **C** | **A** | **T** | **C** | **C** | **G** | **A** | **T** | **T** | **A** | **G** | **A** | **G** | **C** | **A** | **T** | **G** | **C** | **G** | **A** | **C** | **A** | **T** | **T** | **A** | **G** | **T** | **A** | **G** | **G** | **A** | **A** | **A** | **T** | **G** | **T** | **C** | **T** | **T** | **G** | **T** | **T** | **T** | **G** | **A** | **A** | **G** | **A** | **A** | **G** | **A** | **G** | **C** | **A** | **A** | **T** | **T** | **T** | **T** | **C** | **T** | **T** | **T** | **G** | **A** | **A** | **A** | **A** | **A** | **T** | **G** | **A** | **A** | **C** | **A** | **A** | **A** | **T** | **C** | **A** | **A** | **A** | **A** | **T** | **C** | **T** | **A** | **C** | **A** | **G** | **C** | **A** | **G** | **T** | **G** | **A** | **T** | **T** | **A** | **T** | **G** | **A** | **T** | **G** | **A** | **A** | **A** | **A** | **C** | **C** | **C** | **T** | **C** | **G** | **T** | **A** | **G** | **A** | **C** | **T** | **G** | **A** | **A** | **G** | **A** | **T** | **T** | **A** | **T** | **T** | **A** | **C** | **T** | **A** | **A** | **T** | **A** | **C** | **C** | **T** | **T** | **G** | **A** | **T** | **T** | **G** | **G** | **A** | **G** | **A** | **T** | **G** | **T** | **C** | **G** | **A** | **G** | **G** | **T** | **G** | **A** | **T** | **A** | **C** | **G** | **A** | **A** | **T** | **A** | **-** | **-** | **-** |
| **N59951F** | **A** | **T** | **G** | **A** | **A** | **T** | **A** | **G** | **T** | **T** | **C** | **T** | **T** | **G** | **G** | **C** | **G** | **T** | **T** | **T** | **T** | **T** | **T** | **T** | **G** | **T** | **G** | **G** | **T** | **A** | **G** | **T** | **C** | **G** | **A** | **A** | **G** | **C** | **A** | **C** | **T** | **G** | **C** | **T** | **G** | **C** | **T** | **G** | **T** | **T** | **A** | **T** | **T** | **T** | **G** | **C** | **T** | **G** | **T** | **T** | **T** | **G** | **G** | **C** | **A** | **A** | **A** | **T** | **C** | **G** | **T** | **A** | **A** | **A** | **C** | **A** | **A** | **T** | **A** | **C** | **T** | **G** | **G** | **A** | **C** | **T** | **T** | **T** | **T** | **G** | **C** | **T** | **T** | **T** | **T** | **A** | **G** | **T** | **A** | **A** | **T** | **A** | **T** | **T** | **C** | **G** | **G** | **T** | **A** | **T** | **T** | **T** | **T** | **C** | **A** | **A** | **T** | **A** | **T** | **A** | **T** | **A** | **T** | **C** | **T** | **A** | **G** | **C** | **T** | **A** | **T** | **G** | **C** | **G** | **C** | **A** | **A** | **A** | **T** | **A** | **T** | **C** | **C** | **T** | **C** | **G** | **T** | **A** | **C** | **T** | **A** | **A** | **A** | **T** | **T** | **T** | **C** | **A** | **A** | **A** | **A** | **T** | **T** | **T** | **T** | **C** | **A** | **A** | **T** | **T** | **G** | **A** | **T** | **C** | **T** | **T** | **A** | **G** | **G** | **G** | **A** | **G** | **C** | **T** | **T** | **G** | **G** | **T** | **C** | **A** | **T** | **T** | **T** | **T** | **T** | **T** | **T** | **T** | **A** | **G** | **C** | **T** | **T** | **A** | **G** | **T** | **C** | **A** | **A** | **C** | **A** | **G** | **T** | **C** | **C** | **T** | **G** | **C** | **T** | **T** | **T** | **A** | **T** | **G** | **G** | **A** | **T** | **G** | **A** | **T** | **G** | **G** | **T** | **T** | **G** | **T** | **A** | **T** | **T** | **G** | **G** | **C** | **C** | **G** | **T** | **T** | **T** | **T** | **A** | **T** | **T** | **T** | **A** | **T** | **C** | **G** | **G** | **C** | **C** | **T** | **G** | **A** | **A** | **A** | **G** | **G** | **T** | **G** | **T** | **A** | **G** | **A** | **G** | **A** | **T** | **T** | **T** | **C** | **A** | **G** | **G** | **A** | **A** | **T** | **A** | **G** | **A** | **T** | **T** | **T** | **G** | **A** | **C** | **A** | **A** | **A** | **A** | **A** | **A** | **T** | **G** | **C** | **T** | **T** | **T** | **T** | **T** | **G** | **G** | **C** | **G** | **A** | **A** | **A** | **A** | **A** | **A** | **A** | **C** | **A** | **G** | **A** | **T** | **C** | **A** | **T** | **G** | **A** | **T** | **G** | **G** | **T** | **C** | **C** | **A** | **A** | **A** | **C** | **A** | **G** | **A** | **G** | **C** | **A** | **A** | **T** | **T** | **A** | **A** | **A** | **A** | **A** | **C** | **A** | **C** | **A** | **T** | **A** | **A** | **T** | **A** | **A** | **T** | **G** | **A** | **G** | **C** | **G** | **T** | **A** | **A** | **A** | **A** | **G** | **A** | **C** | **A** | **G** | **C** | **A** | **A** | **T** | **T** | **A** | **T** | **T** | **T** | **G** | **G** | **T** | **A** | **A** | **C** | **C** | **A** | **A** | **C** | **G** | **T** | **A** | **T** | **T** | **G** | **G** | **A** | **A** | **A** | **C** | **G** | **A** | **C** | **G** | **T** | **C** | **T** | **A** | **T** | **G** | **A** | **A** | **T** | **G** | **G** | **G** | **A** | **T** | **G** | **A** | **T** | **A** | **T** | **C** | **A** | **A** | **C** | **A** | **T** | **T** | **G** | **C** | **C** | **A** | **T** | **C** | **A** | **T** | **T** | **T** | **C** | **T** | **G** | **G** | **T** | **G** | **A** | **T** | **A** | **C** | **T** | **A** | **T** | **C** | **A** | **T** | **C** | **G** | **A** | **T** | **T** | **T** | **G** | **G** | **G** | **C** | **A** | **A** | **T** | **A** | **C** | **A** | **C** | **T** | **T** | **T** | **T** | **G** | **C** | **C** | **A** | **A** | **A** | **A** | **G** | **A** | **T** | **G** | **A** | **T** | **A** | **A** | **T** | **A** | **T** | **T** | **G** | **T** | **G** | **A** | **T** | **T** | **G** | **T** | **A** | **A** | **G** | **A** | **A** | **A** | **A** | **G** | **G** | **G** | **A** | **T** | **A** | **G** | **G** | **C** | **A** | **G** | **A** | **A** | **C** | **A** | **C** | **G** | **G** | **A** | **T** | **C** | **T** | **T** | **A** | **G** | **T** | **A** | **C** | **C** | **C** | **C** | **T** | **G** | **G** | **G** | **A** | **G** | **T** | **G** | **G** | **C** | **T** | **A** | **T** | **T** | **C** | **G** | **A** | **T** | **T** | **A** | **G** | **A** | **G** | **C** | **A** | **T** | **G** | **C** | **G** | **A** | **C** | **A** | **T** | **T** | **A** | **G** | **T** | **A** | **G** | **G** | **A** | **A** | **A** | **T** | **G** | **T** | **C** | **T** | **T** | **G** | **T** | **T** | **T** | **G** | **A** | **A** | **G** | **A** | **A** | **G** | **A** | **G** | **C** | **A** | **G** | **T** | **T** | **T** | **T** | **C** | **C** | **T** | **T** | **A** | **A** | **A** | **A** | **A** | **A** | **T** | **G** | **A** | **A** | **C** | **A** | **G** | **A** | **T** | **C** | **A** | **A** | **A** | **A** | **T** | **C** | **T** | **G** | **C** | **A** | **G** | **C** | **A** | **G** | **T** | **G** | **A** | **T** | **T** | **A** | **T** | **G** | **A** | **T** | **G** | **A** | **A** | **A** | **A** | **T** | **C** | **C** | **T** | **C** | **G** | **C** | **A** | **G** | **A** | **T** | **T** | **G** | **A** | **A** | **G** | **A** | **T** | **C** | **A** | **T** | **T** | **A** | **C** | **C** | **A** | **A** | **T** | **A** | **C** | **C** | **T** | **T** | **G** | **G** | **T** | **T** | **G** | **G** | **A** | **G** | **A** | **T** | **G** | **T** | **C** | **G** | **A** | **G** | **G** | **T** | **G** | **A** | **T** | **A** | **C** | **G** | **A** | **A** | **T** | **A** | **-** | **-** | **-** |
| **N59943F** | **A** | **T** | **G** | **A** | **A** | **T** | **A** | **G** | **T** | **T** | **C** | **T** | **T** | **G** | **G** | **C** | **G** | **T** | **T** | **T** | **T** | **T** | **T** | **T** | **G** | **T** | **G** | **G** | **T** | **A** | **G** | **T** | **T** | **G** | **A** | **A** | **G** | **C** | **A** | **C** | **T** | **A** | **C** | **T** | **G** | **C** | **T** | **G** | **T** | **T** | **A** | **T** | **T** | **T** | **G** | **C** | **T** | **G** | **T** | **T** | **T** | **G** | **G** | **C** | **A** | **A** | **A** | **T** | **C** | **G** | **T** | **A** | **A** | **A** | **C** | **A** | **A** | **T** | **A** | **C** | **T** | **G** | **G** | **A** | **C** | **T** | **T** | **T** | **T** | **G** | **C** | **T** | **T** | **T** | **T** | **A** | **G** | **T** | **G** | **A** | **T** | **A** | **T** | **T** | **C** | **G** | **G** | **T** | **A** | **T** | **T** | **T** | **T** | **C** | **A** | **A** | **T** | **A** | **T** | **A** | **T** | **A** | **T** | **C** | **T** | **A** | **G** | **C** | **T** | **A** | **T** | **G** | **C** | **G** | **C** | **A** | **A** | **A** | **C** | **A** | **T** | **C** | **C** | **T** | **C** | **G** | **T** | **A** | **C** | **T** | **A** | **A** | **A** | **T** | **T** | **T** | **C** | **A** | **A** | **A** | **A** | **T** | **T** | **T** | **T** | **C** | **A** | **A** | **T** | **T** | **G** | **A** | **T** | **A** | **T** | **T** | **A** | **G** | **G** | **A** | **A** | **G** | **T** | **T** | **T** | **G** | **G** | **T** | **C** | **A** | **T** | **T** | **T** | **T** | **T** | **T** | **T** | **T** | **A** | **G** | **C** | **T** | **T** | **A** | **G** | **T** | **C** | **A** | **A** | **C** | **A** | **G** | **T** | **C** | **C** | **T** | **G** | **C** | **T** | **T** | **T** | **A** | **T** | **G** | **G** | **A** | **T** | **G** | **A** | **T** | **G** | **G** | **C** | **T** | **G** | **T** | **A** | **T** | **T** | **T** | **G** | **C** | **C** | **G** | **T** | **T** | **T** | **T** | **A** | **T** | **T** | **T** | **A** | **T** | **T** | **G** | **G** | **A** | **C** | **T** | **A** | **A** | **A** | **A** | **G** | **G** | **T** | **G** | **T** | **A** | **G** | **A** | **G** | **A** | **T** | **T** | **T** | **C** | **A** | **G** | **G** | **G** | **A** | **T** | **A** | **G** | **A** | **T** | **T** | **T** | **A** | **A** | **C** | **C** | **A** | **A** | **A** | **A** | **A** | **T** | **G** | **C** | **T** | **T** | **T** | **T** | **T** | **G** | **G** | **C** | **G** | **A** | **A** | **A** | **G** | **A** | **A** | **A** | **C** | **A** | **G** | **A** | **T** | **C** | **A** | **T** | **G** | **A** | **T** | **G** | **G** | **T** | **C** | **C** | **A** | **A** | **A** | **C** | **A** | **G** | **A** | **G** | **C** | **A** | **A** | **T** | **T** | **A** | **A** | **A** | **A** | **A** | **C** | **A** | **C** | **A** | **T** | **A** | **A** | **T** | **A** | **A** | **T** | **G** | **A** | **G** | **C** | **G** | **G** | **A** | **A** | **A** | **A** | **G** | **A** | **C** | **A** | **G** | **C** | **A** | **A** | **T** | **T** | **A** | **T** | **T** | **C** | **G** | **G** | **T** | **A** | **A** | **C** | **C** | **A** | **A** | **C** | **G** | **T** | **A** | **T** | **T** | **G** | **G** | **A** | **A** | **A** | **C** | **G** | **A** | **C** | **G** | **T** | **C** | **T** | **A** | **T** | **G** | **A** | **A** | **T** | **G** | **G** | **G** | **A** | **C** | **G** | **A** | **T** | **A** | **T** | **C** | **A** | **A** | **C** | **A** | **T** | **C** | **G** | **C** | **C** | **G** | **T** | **C** | **A** | **T** | **T** | **T** | **C** | **T** | **G** | **G** | **T** | **G** | **A** | **T** | **A** | **C** | **T** | **A** | **T** | **C** | **A** | **T** | **A** | **G** | **A** | **T** | **T** | **T** | **G** | **G** | **G** | **C** | **A** | **A** | **T** | **A** | **C** | **G** | **C** | **T** | **T** | **T** | **T** | **G** | **C** | **C** | **A** | **A** | **A** | **A** | **G** | **A** | **T** | **G** | **A** | **T** | **A** | **A** | **T** | **A** | **T** | **T** | **G** | **T** | **G** | **A** | **T** | **T** | **G** | **T** | **A** | **A** | **G** | **A** | **A** | **A** | **A** | **G** | **G** | **G** | **A** | **T** | **A** | **G** | **G** | **C** | **A** | **G** | **A** | **A** | **C** | **A** | **C** | **G** | **A** | **A** | **T** | **C** | **T** | **T** | **A** | **G** | **T** | **A** | **C** | **C** | **T** | **T** | **T** | **G** | **G** | **G** | **C** | **G** | **T** | **A** | **G** | **C** | **C** | **A** | **T** | **C** | **C** | **G** | **A** | **T** | **T** | **A** | **G** | **A** | **G** | **C** | **A** | **T** | **G** | **C** | **G** | **A** | **C** | **A** | **T** | **T** | **A** | **G** | **T** | **A** | **G** | **G** | **A** | **A** | **A** | **T** | **G** | **T** | **C** | **T** | **T** | **G** | **T** | **T** | **T** | **G** | **A** | **A** | **G** | **A** | **A** | **G** | **A** | **G** | **C** | **A** | **A** | **T** | **T** | **T** | **T** | **C** | **T** | **T** | **T** | **G** | **A** | **A** | **A** | **A** | **A** | **T** | **G** | **A** | **A** | **C** | **A** | **A** | **A** | **T** | **C** | **A** | **A** | **A** | **A** | **T** | **C** | **T** | **A** | **C** | **A** | **G** | **C** | **A** | **G** | **T** | **G** | **A** | **T** | **T** | **A** | **T** | **G** | **A** | **T** | **G** | **A** | **A** | **A** | **A** | **C** | **C** | **C** | **T** | **C** | **G** | **T** | **A** | **G** | **A** | **C** | **T** | **G** | **A** | **A** | **G** | **A** | **T** | **T** | **A** | **T** | **T** | **A** | **C** | **T** | **A** | **A** | **T** | **A** | **C** | **C** | **T** | **T** | **G** | **A** | **T** | **T** | **G** | **G** | **A** | **G** | **A** | **T** | **G** | **T** | **C** | **G** | **A** | **G** | **G** | **T** | **G** | **A** | **T** | **A** | **C** | **G** | **A** | **A** | **T** | **A** | **-** | **-** | **-** |
| **N59613F** | **A** | **T** | **G** | **A** | **A** | **T** | **A** | **G** | **T** | **T** | **C** | **T** | **T** | **G** | **G** | **C** | **G** | **T** | **T** | **T** | **T** | **T** | **T** | **T** | **G** | **T** | **G** | **G** | **T** | **A** | **G** | **T** | **T** | **G** | **A** | **A** | **G** | **C** | **A** | **C** | **T** | **A** | **C** | **T** | **G** | **C** | **T** | **G** | **T** | **T** | **A** | **T** | **T** | **T** | **G** | **C** | **T** | **G** | **T** | **T** | **T** | **G** | **G** | **C** | **A** | **A** | **A** | **T** | **C** | **G** | **T** | **A** | **A** | **A** | **C** | **A** | **A** | **T** | **A** | **C** | **T** | **G** | **G** | **A** | **C** | **T** | **T** | **T** | **T** | **G** | **C** | **T** | **T** | **T** | **T** | **A** | **G** | **T** | **G** | **A** | **T** | **A** | **T** | **T** | **C** | **G** | **G** | **T** | **A** | **T** | **T** | **T** | **T** | **C** | **A** | **A** | **T** | **A** | **T** | **A** | **T** | **A** | **T** | **C** | **T** | **A** | **G** | **C** | **T** | **A** | **T** | **G** | **C** | **G** | **C** | **A** | **A** | **A** | **C** | **A** | **T** | **C** | **C** | **T** | **C** | **G** | **T** | **A** | **C** | **T** | **A** | **A** | **A** | **T** | **T** | **T** | **C** | **A** | **A** | **A** | **A** | **T** | **T** | **T** | **T** | **C** | **A** | **A** | **T** | **T** | **G** | **A** | **T** | **A** | **T** | **T** | **A** | **G** | **G** | **A** | **A** | **G** | **T** | **T** | **T** | **G** | **G** | **T** | **C** | **A** | **T** | **T** | **T** | **T** | **T** | **T** | **T** | **T** | **A** | **G** | **C** | **T** | **T** | **A** | **G** | **T** | **C** | **A** | **A** | **C** | **A** | **G** | **T** | **C** | **C** | **T** | **G** | **C** | **T** | **T** | **T** | **A** | **T** | **G** | **G** | **A** | **T** | **G** | **A** | **T** | **G** | **G** | **C** | **T** | **G** | **T** | **A** | **T** | **T** | **T** | **G** | **C** | **C** | **G** | **T** | **T** | **T** | **T** | **A** | **T** | **T** | **T** | **A** | **T** | **T** | **G** | **G** | **A** | **C** | **T** | **A** | **A** | **A** | **G** | **G** | **G** | **T** | **G** | **T** | **A** | **G** | **A** | **G** | **A** | **T** | **T** | **T** | **C** | **A** | **G** | **G** | **G** | **A** | **T** | **A** | **G** | **A** | **T** | **T** | **T** | **A** | **A** | **C** | **C** | **A** | **A** | **A** | **A** | **A** | **T** | **G** | **C** | **T** | **T** | **T** | **T** | **T** | **G** | **G** | **C** | **G** | **A** | **A** | **A** | **G** | **A** | **A** | **A** | **C** | **A** | **G** | **A** | **T** | **C** | **A** | **T** | **G** | **A** | **T** | **G** | **G** | **T** | **C** | **C** | **A** | **A** | **A** | **C** | **A** | **G** | **A** | **G** | **C** | **A** | **A** | **T** | **T** | **A** | **A** | **A** | **A** | **A** | **C** | **A** | **C** | **A** | **T** | **A** | **A** | **T** | **A** | **A** | **T** | **G** | **A** | **G** | **C** | **G** | **G** | **A** | **A** | **A** | **A** | **G** | **A** | **C** | **A** | **G** | **C** | **A** | **A** | **T** | **T** | **A** | **T** | **T** | **C** | **G** | **G** | **T** | **A** | **A** | **C** | **C** | **A** | **A** | **C** | **G** | **T** | **A** | **T** | **T** | **G** | **G** | **A** | **A** | **A** | **C** | **G** | **A** | **C** | **G** | **T** | **C** | **T** | **A** | **T** | **G** | **A** | **A** | **T** | **G** | **G** | **G** | **A** | **C** | **G** | **A** | **T** | **A** | **T** | **C** | **A** | **A** | **C** | **A** | **T** | **C** | **G** | **C** | **C** | **G** | **T** | **C** | **A** | **T** | **T** | **T** | **C** | **T** | **G** | **G** | **T** | **G** | **A** | **T** | **A** | **C** | **T** | **A** | **T** | **C** | **A** | **T** | **A** | **G** | **A** | **T** | **T** | **T** | **G** | **G** | **G** | **C** | **A** | **A** | **T** | **A** | **C** | **G** | **C** | **T** | **T** | **T** | **T** | **G** | **C** | **C** | **A** | **A** | **A** | **A** | **G** | **A** | **T** | **G** | **A** | **T** | **A** | **A** | **T** | **A** | **T** | **T** | **G** | **T** | **G** | **A** | **T** | **T** | **G** | **T** | **A** | **A** | **G** | **A** | **A** | **A** | **A** | **G** | **G** | **G** | **A** | **T** | **A** | **G** | **G** | **C** | **A** | **G** | **A** | **A** | **C** | **A** | **C** | **G** | **A** | **A** | **T** | **C** | **T** | **T** | **A** | **G** | **T** | **A** | **C** | **C** | **T** | **T** | **T** | **G** | **G** | **G** | **C** | **G** | **T** | **A** | **G** | **C** | **C** | **A** | **T** | **C** | **C** | **G** | **A** | **T** | **T** | **A** | **G** | **A** | **G** | **C** | **A** | **T** | **G** | **C** | **G** | **A** | **C** | **A** | **T** | **T** | **A** | **G** | **T** | **A** | **G** | **G** | **A** | **A** | **A** | **T** | **G** | **T** | **C** | **T** | **T** | **G** | **T** | **T** | **T** | **G** | **A** | **A** | **G** | **A** | **A** | **G** | **A** | **G** | **C** | **A** | **A** | **T** | **T** | **T** | **T** | **C** | **T** | **T** | **T** | **G** | **A** | **A** | **A** | **A** | **A** | **T** | **G** | **A** | **A** | **C** | **A** | **A** | **A** | **T** | **C** | **A** | **A** | **A** | **A** | **T** | **C** | **T** | **A** | **C** | **A** | **G** | **C** | **A** | **G** | **T** | **G** | **A** | **T** | **T** | **A** | **T** | **G** | **A** | **T** | **G** | **A** | **A** | **A** | **A** | **C** | **C** | **C** | **T** | **C** | **G** | **T** | **A** | **G** | **A** | **C** | **T** | **G** | **A** | **A** | **G** | **A** | **T** | **T** | **A** | **T** | **T** | **A** | **C** | **T** | **A** | **A** | **T** | **A** | **C** | **C** | **T** | **T** | **G** | **A** | **T** | **T** | **G** | **G** | **A** | **G** | **A** | **T** | **G** | **T** | **C** | **G** | **A** | **G** | **G** | **T** | **G** | **A** | **T** | **A** | **C** | **G** | **A** | **A** | **T** | **A** | **-** | **-** | **-** |
| **N52829** | **A** | **T** | **G** | **A** | **A** | **T** | **A** | **G** | **T** | **T** | **C** | **T** | **T** | **G** | **G** | **C** | **G** | **T** | **T** | **T** | **T** | **T** | **T** | **T** | **G** | **T** | **G** | **G** | **T** | **A** | **G** | **T** | **C** | **G** | **A** | **A** | **G** | **C** | **A** | **C** | **T** | **G** | **C** | **T** | **G** | **C** | **T** | **G** | **T** | **T** | **A** | **T** | **T** | **T** | **G** | **C** | **T** | **G** | **T** | **T** | **T** | **G** | **G** | **C** | **A** | **A** | **A** | **T** | **C** | **G** | **T** | **A** | **A** | **A** | **C** | **A** | **A** | **T** | **A** | **C** | **T** | **G** | **G** | **A** | **C** | **T** | **T** | **T** | **T** | **G** | **C** | **T** | **T** | **T** | **T** | **A** | **G** | **T** | **A** | **A** | **T** | **A** | **T** | **T** | **C** | **G** | **G** | **T** | **A** | **T** | **T** | **T** | **T** | **C** | **A** | **A** | **T** | **A** | **T** | **A** | **T** | **A** | **T** | **C** | **T** | **A** | **G** | **C** | **T** | **A** | **T** | **G** | **C** | **G** | **C** | **A** | **A** | **A** | **T** | **A** | **T** | **C** | **C** | **T** | **C** | **G** | **T** | **A** | **C** | **T** | **A** | **A** | **A** | **T** | **T** | **T** | **C** | **A** | **A** | **A** | **A** | **T** | **T** | **T** | **T** | **C** | **A** | **A** | **T** | **T** | **G** | **A** | **T** | **C** | **T** | **T** | **A** | **G** | **G** | **G** | **A** | **G** | **C** | **T** | **T** | **G** | **G** | **T** | **C** | **A** | **T** | **T** | **T** | **T** | **T** | **T** | **T** | **T** | **A** | **G** | **C** | **T** | **T** | **A** | **G** | **T** | **C** | **A** | **A** | **C** | **A** | **G** | **T** | **C** | **C** | **T** | **G** | **C** | **T** | **T** | **T** | **A** | **T** | **G** | **G** | **A** | **T** | **G** | **A** | **T** | **G** | **G** | **T** | **T** | **G** | **T** | **A** | **T** | **T** | **G** | **G** | **C** | **C** | **A** | **T** | **T** | **T** | **T** | **A** | **T** | **T** | **T** | **A** | **T** | **C** | **G** | **G** | **A** | **C** | **T** | **G** | **A** | **A** | **A** | **G** | **G** | **T** | **G** | **T** | **A** | **G** | **A** | **G** | **A** | **T** | **T** | **T** | **C** | **A** | **G** | **G** | **A** | **A** | **T** | **A** | **G** | **A** | **T** | **T** | **T** | **G** | **A** | **C** | **A** | **A** | **A** | **A** | **A** | **A** | **T** | **G** | **C** | **T** | **T** | **T** | **T** | **T** | **G** | **G** | **C** | **G** | **A** | **A** | **A** | **A** | **A** | **A** | **A** | **C** | **A** | **G** | **A** | **T** | **C** | **A** | **T** | **G** | **A** | **T** | **G** | **G** | **T** | **C** | **C** | **A** | **A** | **A** | **C** | **A** | **G** | **A** | **G** | **C** | **A** | **A** | **T** | **T** | **A** | **A** | **A** | **A** | **A** | **C** | **A** | **C** | **A** | **T** | **A** | **A** | **T** | **A** | **A** | **T** | **G** | **A** | **G** | **C** | **G** | **T** | **A** | **A** | **A** | **A** | **G** | **A** | **C** | **A** | **G** | **C** | **A** | **A** | **T** | **T** | **A** | **T** | **T** | **T** | **G** | **G** | **T** | **A** | **A** | **C** | **C** | **A** | **A** | **C** | **G** | **T** | **A** | **T** | **T** | **G** | **G** | **A** | **A** | **A** | **C** | **G** | **A** | **C** | **G** | **T** | **C** | **T** | **A** | **T** | **G** | **A** | **A** | **T** | **G** | **G** | **G** | **A** | **T** | **G** | **A** | **T** | **A** | **T** | **C** | **A** | **A** | **C** | **A** | **T** | **T** | **G** | **C** | **C** | **A** | **T** | **C** | **A** | **T** | **T** | **T** | **C** | **T** | **G** | **G** | **T** | **G** | **A** | **T** | **A** | **C** | **T** | **A** | **T** | **C** | **A** | **T** | **C** | **G** | **A** | **T** | **T** | **T** | **G** | **G** | **G** | **C** | **A** | **A** | **T** | **A** | **C** | **A** | **C** | **T** | **T** | **T** | **T** | **G** | **C** | **C** | **A** | **A** | **A** | **A** | **G** | **A** | **T** | **G** | **A** | **T** | **A** | **A** | **T** | **A** | **T** | **T** | **G** | **T** | **G** | **A** | **T** | **T** | **G** | **T** | **A** | **A** | **G** | **A** | **A** | **A** | **A** | **G** | **G** | **G** | **A** | **T** | **A** | **G** | **G** | **C** | **A** | **G** | **A** | **A** | **C** | **A** | **C** | **G** | **G** | **A** | **T** | **C** | **T** | **T** | **A** | **G** | **T** | **A** | **C** | **C** | **C** | **C** | **T** | **G** | **G** | **G** | **A** | **G** | **T** | **G** | **G** | **C** | **T** | **A** | **T** | **T** | **C** | **G** | **A** | **T** | **T** | **A** | **G** | **A** | **G** | **C** | **A** | **T** | **G** | **C** | **G** | **A** | **C** | **A** | **T** | **T** | **A** | **G** | **T** | **A** | **G** | **G** | **A** | **A** | **A** | **T** | **G** | **T** | **C** | **T** | **T** | **G** | **T** | **T** | **T** | **G** | **A** | **A** | **G** | **A** | **A** | **G** | **A** | **G** | **C** | **A** | **G** | **T** | **T** | **T** | **T** | **C** | **C** | **T** | **T** | **A** | **A** | **A** | **A** | **A** | **A** | **C** | **G** | **A** | **A** | **C** | **A** | **G** | **A** | **T** | **C** | **A** | **A** | **A** | **A** | **T** | **A** | **T** | **G** | **C** | **A** | **G** | **C** | **A** | **G** | **T** | **G** | **A** | **T** | **T** | **A** | **T** | **G** | **A** | **T** | **G** | **A** | **A** | **A** | **A** | **T** | **C** | **C** | **T** | **C** | **G** | **C** | **A** | **G** | **A** | **T** | **T** | **G** | **A** | **A** | **G** | **A** | **T** | **C** | **A** | **T** | **T** | **A** | **C** | **C** | **A** | **A** | **T** | **A** | **C** | **C** | **T** | **T** | **G** | **G** | **T** | **T** | **G** | **G** | **A** | **G** | **A** | **T** | **G** | **T** | **C** | **G** | **A** | **G** | **G** | **T** | **G** | **A** | **T** | **A** | **C** | **G** | **A** | **A** | **T** | **A** | **-** | **-** | **-** |
| **N60363F** | **A** | **T** | **G** | **A** | **A** | **T** | **A** | **G** | **T** | **T** | **C** | **T** | **T** | **G** | **G** | **C** | **G** | **T** | **T** | **T** | **T** | **T** | **T** | **T** | **G** | **T** | **G** | **G** | **T** | **A** | **G** | **T** | **C** | **G** | **A** | **A** | **G** | **C** | **A** | **C** | **T** | **G** | **C** | **T** | **G** | **C** | **T** | **G** | **T** | **T** | **A** | **T** | **T** | **T** | **G** | **C** | **T** | **G** | **T** | **T** | **T** | **G** | **G** | **G** | **A** | **A** | **A** | **T** | **C** | **G** | **T** | **A** | **A** | **A** | **C** | **A** | **A** | **T** | **A** | **C** | **T** | **G** | **G** | **A** | **C** | **T** | **T** | **T** | **T** | **G** | **C** | **T** | **T** | **T** | **T** | **A** | **G** | **T** | **A** | **A** | **T** | **A** | **T** | **T** | **C** | **G** | **G** | **T** | **A** | **T** | **T** | **T** | **T** | **C** | **A** | **A** | **T** | **A** | **T** | **A** | **T** | **A** | **T** | **C** | **T** | **A** | **G** | **C** | **T** | **A** | **T** | **G** | **C** | **G** | **C** | **A** | **A** | **A** | **T** | **A** | **T** | **C** | **C** | **T** | **C** | **G** | **T** | **A** | **C** | **T** | **A** | **A** | **A** | **T** | **T** | **T** | **C** | **A** | **A** | **A** | **A** | **T** | **T** | **T** | **T** | **C** | **A** | **A** | **T** | **T** | **G** | **A** | **T** | **C** | **T** | **T** | **A** | **G** | **G** | **G** | **A** | **G** | **C** | **T** | **T** | **G** | **G** | **T** | **C** | **A** | **T** | **T** | **T** | **T** | **T** | **T** | **T** | **T** | **A** | **G** | **C** | **T** | **T** | **A** | **G** | **T** | **C** | **A** | **A** | **C** | **A** | **G** | **T** | **C** | **C** | **T** | **G** | **C** | **T** | **T** | **T** | **A** | **T** | **G** | **G** | **A** | **T** | **G** | **A** | **T** | **G** | **G** | **T** | **T** | **G** | **T** | **A** | **T** | **T** | **G** | **G** | **C** | **C** | **G** | **T** | **T** | **T** | **T** | **A** | **T** | **T** | **T** | **A** | **T** | **C** | **G** | **G** | **G** | **C** | **T** | **G** | **A** | **A** | **A** | **G** | **G** | **T** | **G** | **T** | **A** | **G** | **A** | **G** | **A** | **T** | **T** | **T** | **C** | **A** | **G** | **G** | **A** | **A** | **T** | **A** | **G** | **A** | **T** | **T** | **T** | **G** | **A** | **C** | **A** | **A** | **A** | **A** | **A** | **A** | **T** | **G** | **C** | **T** | **T** | **T** | **T** | **T** | **G** | **G** | **C** | **G** | **A** | **A** | **A** | **A** | **A** | **A** | **A** | **C** | **A** | **G** | **A** | **T** | **C** | **A** | **T** | **G** | **A** | **T** | **G** | **G** | **T** | **C** | **C** | **A** | **A** | **A** | **C** | **A** | **G** | **A** | **G** | **C** | **A** | **A** | **T** | **T** | **A** | **A** | **A** | **A** | **A** | **C** | **A** | **C** | **A** | **T** | **A** | **A** | **T** | **A** | **A** | **T** | **G** | **A** | **G** | **C** | **G** | **T** | **A** | **A** | **A** | **A** | **G** | **A** | **C** | **A** | **G** | **C** | **A** | **A** | **T** | **T** | **A** | **T** | **T** | **T** | **G** | **G** | **T** | **A** | **A** | **C** | **C** | **A** | **A** | **C** | **G** | **T** | **A** | **T** | **T** | **G** | **G** | **A** | **A** | **A** | **C** | **G** | **A** | **C** | **G** | **T** | **C** | **T** | **A** | **T** | **G** | **A** | **A** | **T** | **G** | **G** | **G** | **A** | **T** | **G** | **A** | **T** | **A** | **T** | **C** | **A** | **A** | **C** | **A** | **T** | **T** | **G** | **C** | **C** | **A** | **T** | **C** | **A** | **T** | **T** | **T** | **C** | **T** | **G** | **G** | **T** | **G** | **A** | **T** | **A** | **C** | **T** | **A** | **T** | **C** | **A** | **T** | **C** | **G** | **A** | **T** | **T** | **T** | **G** | **G** | **G** | **C** | **A** | **A** | **T** | **A** | **C** | **A** | **C** | **T** | **T** | **T** | **T** | **G** | **C** | **C** | **A** | **A** | **A** | **A** | **G** | **A** | **T** | **G** | **A** | **T** | **A** | **A** | **T** | **A** | **T** | **T** | **G** | **T** | **G** | **A** | **T** | **T** | **G** | **T** | **A** | **A** | **G** | **A** | **A** | **A** | **A** | **G** | **G** | **G** | **A** | **T** | **A** | **G** | **G** | **C** | **A** | **G** | **A** | **A** | **C** | **A** | **C** | **G** | **G** | **A** | **T** | **C** | **T** | **T** | **A** | **G** | **T** | **A** | **C** | **C** | **C** | **C** | **T** | **G** | **G** | **G** | **A** | **G** | **T** | **G** | **G** | **C** | **T** | **A** | **T** | **T** | **C** | **G** | **T** | **T** | **T** | **A** | **G** | **A** | **G** | **C** | **A** | **T** | **G** | **C** | **G** | **A** | **C** | **A** | **T** | **T** | **A** | **G** | **T** | **A** | **G** | **G** | **A** | **A** | **A** | **T** | **G** | **T** | **C** | **T** | **T** | **G** | **T** | **T** | **T** | **G** | **A** | **A** | **G** | **A** | **A** | **G** | **A** | **G** | **C** | **A** | **G** | **T** | **T** | **T** | **T** | **C** | **C** | **T** | **T** | **A** | **A** | **A** | **A** | **A** | **A** | **C** | **G** | **A** | **A** | **C** | **A** | **G** | **A** | **T** | **C** | **A** | **A** | **A** | **A** | **T** | **C** | **T** | **G** | **C** | **A** | **G** | **C** | **A** | **G** | **T** | **G** | **A** | **T** | **T** | **A** | **T** | **G** | **A** | **T** | **G** | **A** | **A** | **A** | **A** | **T** | **C** | **C** | **T** | **C** | **G** | **C** | **A** | **G** | **A** | **T** | **T** | **G** | **A** | **A** | **G** | **A** | **T** | **C** | **A** | **T** | **T** | **A** | **C** | **C** | **A** | **A** | **T** | **A** | **C** | **C** | **T** | **T** | **G** | **G** | **T** | **T** | **G** | **G** | **A** | **G** | **A** | **T** | **G** | **T** | **C** | **G** | **A** | **G** | **G** | **T** | **G** | **A** | **T** | **A** | **C** | **G** | **A** | **A** | **T** | **A** | **-** | **-** | **-** |
| **N59503F** | **A** | **T** | **G** | **A** | **A** | **T** | **A** | **G** | **T** | **T** | **C** | **T** | **T** | **G** | **G** | **C** | **G** | **T** | **T** | **T** | **T** | **T** | **T** | **T** | **G** | **T** | **G** | **G** | **T** | **A** | **G** | **T** | **C** | **G** | **A** | **A** | **G** | **C** | **A** | **C** | **T** | **G** | **C** | **T** | **G** | **C** | **T** | **G** | **T** | **T** | **A** | **T** | **T** | **T** | **G** | **C** | **T** | **G** | **T** | **T** | **T** | **G** | **G** | **G** | **A** | **A** | **A** | **T** | **C** | **G** | **T** | **A** | **A** | **A** | **C** | **A** | **A** | **T** | **A** | **C** | **T** | **G** | **G** | **A** | **C** | **T** | **T** | **T** | **T** | **G** | **C** | **T** | **T** | **T** | **T** | **A** | **G** | **T** | **A** | **A** | **T** | **A** | **T** | **T** | **C** | **G** | **G** | **T** | **A** | **T** | **T** | **T** | **T** | **C** | **A** | **A** | **T** | **A** | **T** | **A** | **T** | **A** | **T** | **C** | **T** | **A** | **G** | **C** | **T** | **A** | **T** | **G** | **C** | **G** | **C** | **A** | **A** | **A** | **T** | **A** | **T** | **C** | **C** | **T** | **C** | **G** | **T** | **A** | **C** | **T** | **A** | **A** | **A** | **T** | **T** | **T** | **C** | **A** | **A** | **A** | **A** | **T** | **T** | **T** | **T** | **C** | **A** | **A** | **T** | **T** | **G** | **A** | **T** | **C** | **T** | **T** | **A** | **G** | **G** | **G** | **A** | **G** | **C** | **T** | **T** | **G** | **G** | **T** | **C** | **A** | **T** | **T** | **T** | **T** | **T** | **T** | **T** | **T** | **A** | **G** | **C** | **T** | **T** | **A** | **G** | **T** | **C** | **A** | **A** | **C** | **A** | **G** | **T** | **C** | **C** | **T** | **G** | **C** | **T** | **T** | **T** | **A** | **T** | **G** | **G** | **A** | **T** | **G** | **A** | **T** | **G** | **G** | **T** | **T** | **G** | **T** | **A** | **T** | **T** | **G** | **G** | **C** | **C** | **G** | **T** | **T** | **T** | **T** | **A** | **T** | **T** | **T** | **A** | **T** | **C** | **G** | **G** | **G** | **C** | **T** | **G** | **A** | **A** | **A** | **G** | **G** | **T** | **G** | **T** | **A** | **G** | **A** | **G** | **A** | **T** | **T** | **T** | **C** | **A** | **G** | **G** | **A** | **A** | **T** | **A** | **G** | **A** | **T** | **T** | **T** | **G** | **A** | **C** | **A** | **A** | **A** | **A** | **A** | **A** | **T** | **G** | **C** | **T** | **T** | **T** | **T** | **T** | **G** | **G** | **C** | **G** | **A** | **A** | **A** | **A** | **A** | **A** | **A** | **C** | **A** | **G** | **A** | **T** | **C** | **A** | **T** | **G** | **A** | **T** | **G** | **G** | **T** | **C** | **C** | **A** | **A** | **A** | **C** | **A** | **G** | **A** | **G** | **C** | **A** | **A** | **T** | **T** | **A** | **A** | **A** | **A** | **A** | **C** | **A** | **C** | **A** | **T** | **A** | **A** | **T** | **A** | **A** | **T** | **G** | **A** | **G** | **C** | **G** | **T** | **A** | **A** | **A** | **A** | **G** | **A** | **C** | **A** | **G** | **C** | **A** | **A** | **T** | **T** | **A** | **T** | **T** | **T** | **G** | **G** | **T** | **A** | **A** | **C** | **C** | **A** | **A** | **C** | **G** | **T** | **A** | **T** | **T** | **G** | **G** | **A** | **A** | **A** | **C** | **G** | **A** | **C** | **G** | **T** | **C** | **T** | **A** | **T** | **G** | **A** | **A** | **T** | **G** | **G** | **G** | **A** | **T** | **G** | **A** | **T** | **A** | **T** | **C** | **A** | **A** | **C** | **A** | **T** | **T** | **G** | **C** | **C** | **A** | **T** | **C** | **A** | **T** | **T** | **T** | **C** | **T** | **G** | **G** | **T** | **G** | **A** | **T** | **A** | **C** | **T** | **A** | **T** | **C** | **A** | **T** | **C** | **G** | **A** | **T** | **T** | **T** | **G** | **G** | **G** | **C** | **A** | **A** | **T** | **A** | **C** | **A** | **C** | **T** | **T** | **T** | **T** | **G** | **C** | **C** | **A** | **A** | **A** | **A** | **G** | **A** | **T** | **G** | **A** | **T** | **A** | **A** | **T** | **A** | **T** | **T** | **G** | **T** | **G** | **A** | **T** | **T** | **G** | **T** | **A** | **A** | **G** | **A** | **A** | **A** | **A** | **G** | **G** | **G** | **A** | **T** | **A** | **G** | **G** | **C** | **A** | **G** | **A** | **A** | **C** | **A** | **C** | **G** | **G** | **A** | **T** | **C** | **T** | **T** | **A** | **G** | **T** | **A** | **C** | **C** | **C** | **C** | **T** | **G** | **G** | **G** | **A** | **G** | **T** | **G** | **G** | **C** | **T** | **A** | **T** | **T** | **C** | **G** | **T** | **T** | **T** | **A** | **G** | **A** | **G** | **C** | **A** | **T** | **G** | **C** | **G** | **A** | **C** | **A** | **T** | **T** | **A** | **G** | **T** | **A** | **G** | **G** | **A** | **A** | **A** | **T** | **G** | **T** | **C** | **T** | **T** | **G** | **T** | **T** | **T** | **G** | **A** | **A** | **G** | **A** | **A** | **G** | **A** | **G** | **C** | **A** | **G** | **T** | **T** | **T** | **T** | **C** | **C** | **T** | **T** | **A** | **A** | **A** | **A** | **A** | **A** | **C** | **G** | **A** | **A** | **C** | **A** | **G** | **A** | **T** | **C** | **A** | **A** | **A** | **A** | **T** | **C** | **T** | **G** | **C** | **A** | **G** | **C** | **A** | **G** | **T** | **G** | **A** | **T** | **T** | **A** | **T** | **G** | **A** | **T** | **G** | **A** | **A** | **A** | **A** | **T** | **C** | **C** | **T** | **C** | **G** | **C** | **A** | **G** | **A** | **T** | **T** | **G** | **A** | **A** | **G** | **A** | **T** | **C** | **A** | **T** | **T** | **A** | **C** | **C** | **A** | **A** | **T** | **A** | **C** | **C** | **T** | **T** | **G** | **G** | **T** | **T** | **G** | **G** | **A** | **G** | **A** | **T** | **G** | **T** | **C** | **G** | **A** | **G** | **G** | **T** | **G** | **A** | **T** | **A** | **C** | **G** | **A** | **A** | **T** | **A** | **-** | **-** | **-** |
| **N59754F** | **A** | **T** | **G** | **A** | **A** | **T** | **A** | **G** | **T** | **T** | **C** | **T** | **T** | **G** | **G** | **C** | **G** | **T** | **T** | **T** | **T** | **T** | **T** | **T** | **G** | **T** | **G** | **G** | **T** | **A** | **G** | **T** | **C** | **G** | **A** | **A** | **G** | **C** | **A** | **C** | **T** | **G** | **C** | **T** | **G** | **C** | **T** | **G** | **T** | **T** | **A** | **T** | **T** | **T** | **G** | **C** | **T** | **G** | **T** | **T** | **T** | **G** | **G** | **C** | **A** | **A** | **A** | **T** | **C** | **G** | **T** | **A** | **A** | **A** | **C** | **A** | **A** | **T** | **A** | **C** | **T** | **G** | **G** | **A** | **C** | **T** | **T** | **T** | **T** | **G** | **C** | **T** | **T** | **T** | **T** | **A** | **G** | **T** | **A** | **A** | **T** | **A** | **T** | **T** | **C** | **G** | **G** | **T** | **A** | **T** | **T** | **T** | **T** | **C** | **A** | **A** | **T** | **A** | **T** | **A** | **T** | **A** | **T** | **C** | **T** | **A** | **G** | **C** | **T** | **A** | **T** | **G** | **C** | **G** | **C** | **A** | **A** | **A** | **T** | **A** | **T** | **C** | **C** | **T** | **C** | **G** | **T** | **A** | **C** | **T** | **A** | **A** | **A** | **T** | **T** | **T** | **C** | **A** | **A** | **A** | **A** | **T** | **T** | **T** | **T** | **C** | **A** | **A** | **T** | **T** | **G** | **A** | **T** | **C** | **T** | **T** | **A** | **G** | **G** | **G** | **A** | **G** | **C** | **T** | **T** | **A** | **G** | **T** | **C** | **A** | **T** | **T** | **T** | **T** | **T** | **T** | **T** | **T** | **A** | **G** | **C** | **T** | **T** | **A** | **G** | **T** | **C** | **A** | **A** | **C** | **A** | **G** | **T** | **C** | **C** | **T** | **G** | **C** | **T** | **T** | **T** | **A** | **T** | **G** | **G** | **A** | **T** | **G** | **A** | **T** | **G** | **G** | **T** | **T** | **G** | **T** | **A** | **T** | **T** | **G** | **G** | **C** | **C** | **G** | **T** | **T** | **T** | **T** | **A** | **T** | **T** | **T** | **A** | **T** | **C** | **G** | **G** | **G** | **C** | **T** | **G** | **A** | **A** | **A** | **G** | **G** | **T** | **G** | **T** | **A** | **G** | **A** | **G** | **A** | **T** | **T** | **T** | **C** | **A** | **G** | **G** | **A** | **A** | **T** | **A** | **G** | **A** | **T** | **T** | **T** | **G** | **A** | **C** | **A** | **A** | **A** | **A** | **A** | **A** | **T** | **G** | **C** | **T** | **T** | **T** | **T** | **T** | **G** | **G** | **C** | **G** | **A** | **A** | **A** | **A** | **A** | **A** | **A** | **C** | **A** | **G** | **A** | **T** | **C** | **A** | **T** | **G** | **A** | **T** | **G** | **G** | **T** | **C** | **C** | **A** | **A** | **A** | **C** | **A** | **G** | **A** | **G** | **C** | **A** | **A** | **T** | **T** | **A** | **A** | **A** | **A** | **A** | **C** | **A** | **C** | **A** | **T** | **A** | **A** | **T** | **A** | **A** | **T** | **G** | **A** | **G** | **C** | **G** | **T** | **A** | **A** | **A** | **A** | **G** | **A** | **C** | **A** | **G** | **C** | **A** | **A** | **T** | **T** | **A** | **T** | **T** | **T** | **G** | **G** | **T** | **A** | **A** | **C** | **C** | **A** | **A** | **C** | **G** | **T** | **A** | **T** | **T** | **G** | **G** | **A** | **A** | **A** | **C** | **G** | **A** | **C** | **G** | **T** | **C** | **T** | **A** | **T** | **G** | **A** | **A** | **T** | **G** | **G** | **G** | **A** | **T** | **G** | **A** | **C** | **A** | **T** | **C** | **A** | **A** | **C** | **A** | **T** | **T** | **G** | **C** | **C** | **A** | **T** | **C** | **A** | **T** | **T** | **T** | **C** | **T** | **G** | **G** | **T** | **G** | **A** | **T** | **A** | **C** | **T** | **A** | **T** | **C** | **A** | **T** | **C** | **G** | **A** | **T** | **T** | **T** | **G** | **G** | **G** | **C** | **A** | **A** | **T** | **A** | **C** | **A** | **C** | **T** | **T** | **T** | **T** | **G** | **C** | **C** | **A** | **A** | **A** | **A** | **G** | **A** | **T** | **G** | **A** | **T** | **A** | **A** | **T** | **A** | **T** | **T** | **G** | **T** | **G** | **A** | **T** | **T** | **G** | **T** | **A** | **A** | **G** | **A** | **A** | **A** | **A** | **G** | **G** | **G** | **A** | **T** | **A** | **G** | **G** | **C** | **A** | **G** | **A** | **A** | **C** | **A** | **C** | **G** | **G** | **A** | **T** | **C** | **T** | **T** | **A** | **G** | **T** | **A** | **C** | **C** | **C** | **C** | **T** | **G** | **G** | **G** | **A** | **G** | **T** | **G** | **G** | **C** | **T** | **A** | **T** | **T** | **C** | **G** | **T** | **T** | **T** | **A** | **G** | **A** | **G** | **C** | **A** | **T** | **G** | **C** | **G** | **A** | **C** | **A** | **T** | **T** | **A** | **G** | **T** | **A** | **G** | **G** | **A** | **A** | **A** | **T** | **G** | **T** | **C** | **T** | **T** | **G** | **T** | **T** | **T** | **G** | **A** | **A** | **G** | **A** | **A** | **G** | **A** | **G** | **C** | **A** | **G** | **T** | **T** | **T** | **T** | **C** | **C** | **T** | **T** | **A** | **A** | **A** | **A** | **A** | **A** | **C** | **G** | **A** | **A** | **C** | **A** | **G** | **A** | **T** | **C** | **A** | **A** | **A** | **A** | **T** | **C** | **T** | **G** | **C** | **A** | **G** | **C** | **A** | **G** | **T** | **G** | **A** | **T** | **T** | **A** | **T** | **G** | **A** | **T** | **G** | **A** | **A** | **A** | **A** | **T** | **C** | **C** | **T** | **C** | **G** | **C** | **A** | **G** | **A** | **T** | **T** | **G** | **A** | **A** | **G** | **A** | **T** | **C** | **A** | **T** | **T** | **A** | **C** | **C** | **A** | **A** | **T** | **A** | **C** | **C** | **T** | **T** | **G** | **G** | **T** | **T** | **G** | **G** | **A** | **G** | **A** | **T** | **G** | **T** | **C** | **G** | **A** | **G** | **G** | **T** | **G** | **A** | **T** | **A** | **C** | **G** | **A** | **A** | **T** | **A** | **-** | **-** | **-** |
| **N60284F** | **A** | **T** | **G** | **A** | **A** | **T** | **A** | **G** | **T** | **T** | **C** | **T** | **T** | **G** | **G** | **C** | **G** | **T** | **T** | **T** | **T** | **T** | **T** | **T** | **G** | **T** | **G** | **G** | **T** | **A** | **G** | **T** | **C** | **G** | **A** | **A** | **G** | **C** | **A** | **C** | **T** | **G** | **C** | **T** | **G** | **C** | **T** | **G** | **T** | **T** | **A** | **T** | **T** | **T** | **G** | **C** | **T** | **G** | **T** | **T** | **T** | **G** | **G** | **C** | **A** | **A** | **A** | **T** | **C** | **G** | **T** | **A** | **A** | **A** | **C** | **A** | **A** | **T** | **A** | **C** | **T** | **G** | **G** | **A** | **C** | **T** | **T** | **T** | **T** | **G** | **C** | **T** | **T** | **T** | **T** | **A** | **G** | **T** | **A** | **A** | **T** | **A** | **T** | **T** | **C** | **G** | **G** | **T** | **A** | **T** | **T** | **T** | **T** | **C** | **A** | **A** | **T** | **A** | **T** | **A** | **T** | **A** | **T** | **C** | **T** | **A** | **G** | **C** | **T** | **A** | **T** | **G** | **C** | **G** | **C** | **A** | **A** | **A** | **T** | **A** | **T** | **C** | **C** | **T** | **C** | **G** | **T** | **A** | **C** | **T** | **A** | **A** | **A** | **T** | **T** | **T** | **C** | **A** | **A** | **A** | **A** | **T** | **T** | **T** | **T** | **C** | **A** | **A** | **T** | **T** | **G** | **A** | **T** | **C** | **T** | **T** | **A** | **G** | **G** | **G** | **A** | **G** | **C** | **T** | **T** | **G** | **G** | **T** | **C** | **A** | **T** | **T** | **T** | **T** | **T** | **T** | **T** | **T** | **A** | **G** | **C** | **T** | **T** | **A** | **G** | **T** | **C** | **A** | **A** | **C** | **A** | **G** | **T** | **C** | **C** | **T** | **G** | **C** | **T** | **T** | **T** | **A** | **T** | **G** | **G** | **A** | **T** | **G** | **A** | **T** | **G** | **G** | **T** | **T** | **G** | **T** | **A** | **T** | **T** | **G** | **G** | **C** | **C** | **G** | **T** | **T** | **T** | **T** | **A** | **T** | **T** | **T** | **A** | **T** | **C** | **G** | **G** | **G** | **C** | **T** | **G** | **A** | **A** | **A** | **G** | **G** | **T** | **G** | **T** | **A** | **G** | **A** | **G** | **A** | **T** | **T** | **T** | **C** | **A** | **G** | **G** | **A** | **A** | **T** | **A** | **G** | **A** | **T** | **T** | **T** | **G** | **A** | **C** | **A** | **A** | **A** | **A** | **A** | **A** | **T** | **G** | **C** | **T** | **T** | **T** | **T** | **T** | **G** | **G** | **C** | **G** | **A** | **A** | **A** | **A** | **A** | **A** | **A** | **C** | **A** | **G** | **A** | **T** | **C** | **A** | **T** | **G** | **A** | **T** | **G** | **G** | **T** | **C** | **C** | **A** | **A** | **A** | **C** | **A** | **G** | **A** | **G** | **C** | **A** | **A** | **T** | **T** | **A** | **A** | **A** | **A** | **A** | **C** | **A** | **C** | **A** | **T** | **A** | **A** | **T** | **A** | **A** | **T** | **G** | **A** | **G** | **C** | **G** | **T** | **A** | **A** | **A** | **A** | **G** | **A** | **C** | **A** | **G** | **C** | **A** | **A** | **T** | **T** | **A** | **T** | **T** | **T** | **G** | **G** | **T** | **A** | **A** | **C** | **C** | **A** | **A** | **C** | **G** | **T** | **A** | **T** | **T** | **G** | **G** | **A** | **A** | **A** | **C** | **G** | **A** | **C** | **G** | **T** | **C** | **T** | **A** | **T** | **G** | **A** | **A** | **T** | **G** | **G** | **G** | **A** | **T** | **G** | **A** | **T** | **A** | **T** | **C** | **A** | **A** | **C** | **A** | **T** | **T** | **G** | **C** | **C** | **A** | **T** | **C** | **A** | **T** | **T** | **T** | **C** | **T** | **G** | **G** | **T** | **G** | **A** | **T** | **A** | **C** | **T** | **A** | **T** | **C** | **A** | **T** | **C** | **G** | **A** | **T** | **T** | **T** | **G** | **G** | **G** | **C** | **A** | **A** | **T** | **A** | **C** | **A** | **C** | **T** | **T** | **T** | **T** | **G** | **C** | **C** | **A** | **A** | **A** | **A** | **G** | **A** | **T** | **G** | **A** | **T** | **A** | **A** | **T** | **A** | **T** | **T** | **G** | **T** | **G** | **A** | **T** | **T** | **G** | **T** | **A** | **A** | **G** | **A** | **A** | **A** | **A** | **G** | **G** | **G** | **A** | **T** | **A** | **G** | **G** | **C** | **A** | **G** | **A** | **A** | **C** | **A** | **C** | **G** | **G** | **A** | **T** | **C** | **T** | **T** | **A** | **G** | **T** | **A** | **C** | **C** | **C** | **C** | **T** | **G** | **G** | **G** | **A** | **G** | **T** | **G** | **G** | **C** | **T** | **A** | **T** | **T** | **C** | **G** | **T** | **T** | **T** | **A** | **G** | **A** | **G** | **C** | **A** | **T** | **G** | **C** | **G** | **A** | **C** | **A** | **T** | **T** | **A** | **G** | **T** | **A** | **G** | **G** | **A** | **A** | **A** | **T** | **G** | **T** | **C** | **T** | **T** | **G** | **T** | **T** | **T** | **G** | **A** | **A** | **G** | **A** | **A** | **G** | **A** | **G** | **C** | **A** | **G** | **T** | **T** | **T** | **T** | **C** | **C** | **T** | **T** | **A** | **A** | **A** | **A** | **A** | **A** | **C** | **G** | **A** | **A** | **C** | **A** | **G** | **A** | **T** | **C** | **A** | **A** | **A** | **A** | **T** | **C** | **T** | **G** | **C** | **A** | **G** | **C** | **A** | **G** | **T** | **G** | **A** | **T** | **T** | **A** | **T** | **G** | **A** | **T** | **G** | **A** | **A** | **A** | **A** | **T** | **C** | **C** | **T** | **C** | **G** | **C** | **A** | **G** | **A** | **T** | **T** | **G** | **A** | **A** | **G** | **A** | **T** | **C** | **A** | **T** | **T** | **A** | **C** | **C** | **A** | **A** | **T** | **A** | **C** | **C** | **T** | **T** | **G** | **G** | **T** | **T** | **G** | **G** | **A** | **G** | **A** | **T** | **G** | **T** | **C** | **G** | **A** | **G** | **G** | **T** | **G** | **A** | **T** | **A** | **C** | **G** | **A** | **A** | **T** | **A** | **-** | **-** | **-** |
| **N53804** | **A** | **T** | **G** | **A** | **A** | **T** | **A** | **G** | **T** | **T** | **C** | **T** | **T** | **G** | **G** | **C** | **G** | **T** | **T** | **T** | **T** | **T** | **T** | **T** | **G** | **T** | **G** | **G** | **T** | **A** | **G** | **T** | **C** | **G** | **A** | **A** | **G** | **C** | **A** | **C** | **T** | **G** | **C** | **T** | **G** | **C** | **T** | **G** | **T** | **T** | **A** | **T** | **T** | **T** | **G** | **C** | **T** | **G** | **T** | **T** | **T** | **G** | **G** | **C** | **A** | **A** | **A** | **T** | **C** | **G** | **T** | **A** | **A** | **A** | **C** | **A** | **A** | **T** | **A** | **C** | **T** | **G** | **G** | **A** | **C** | **T** | **T** | **T** | **T** | **G** | **C** | **T** | **T** | **T** | **T** | **A** | **G** | **T** | **A** | **A** | **T** | **A** | **T** | **T** | **C** | **G** | **G** | **T** | **A** | **T** | **T** | **T** | **T** | **C** | **A** | **A** | **T** | **A** | **T** | **A** | **T** | **A** | **T** | **C** | **T** | **A** | **G** | **C** | **T** | **A** | **T** | **G** | **C** | **G** | **C** | **A** | **A** | **A** | **T** | **A** | **T** | **C** | **C** | **T** | **C** | **G** | **T** | **A** | **C** | **T** | **A** | **A** | **A** | **T** | **T** | **T** | **C** | **A** | **A** | **A** | **A** | **T** | **T** | **T** | **T** | **C** | **A** | **A** | **T** | **T** | **G** | **A** | **T** | **C** | **T** | **T** | **A** | **G** | **G** | **G** | **A** | **G** | **C** | **T** | **T** | **G** | **G** | **T** | **C** | **A** | **T** | **T** | **T** | **T** | **T** | **T** | **T** | **T** | **A** | **G** | **C** | **T** | **T** | **A** | **G** | **T** | **C** | **A** | **A** | **C** | **A** | **G** | **T** | **C** | **C** | **T** | **G** | **C** | **T** | **T** | **T** | **A** | **T** | **G** | **G** | **A** | **T** | **G** | **A** | **T** | **G** | **G** | **T** | **T** | **G** | **T** | **A** | **T** | **T** | **G** | **G** | **C** | **C** | **G** | **T** | **T** | **T** | **T** | **A** | **T** | **T** | **T** | **A** | **T** | **C** | **G** | **G** | **G** | **C** | **T** | **G** | **A** | **A** | **A** | **G** | **G** | **T** | **G** | **T** | **A** | **G** | **A** | **G** | **A** | **T** | **T** | **T** | **C** | **A** | **G** | **G** | **A** | **A** | **T** | **A** | **G** | **A** | **T** | **T** | **T** | **G** | **A** | **C** | **A** | **A** | **A** | **A** | **A** | **A** | **T** | **G** | **C** | **T** | **T** | **T** | **T** | **T** | **G** | **G** | **C** | **G** | **A** | **A** | **A** | **A** | **A** | **A** | **A** | **C** | **A** | **G** | **A** | **T** | **C** | **A** | **T** | **G** | **A** | **T** | **G** | **G** | **T** | **C** | **C** | **A** | **A** | **A** | **C** | **A** | **G** | **A** | **G** | **C** | **A** | **A** | **T** | **T** | **A** | **A** | **A** | **A** | **A** | **C** | **A** | **C** | **A** | **T** | **A** | **A** | **T** | **A** | **A** | **T** | **G** | **A** | **G** | **C** | **G** | **T** | **A** | **A** | **A** | **A** | **G** | **A** | **C** | **A** | **G** | **C** | **A** | **A** | **T** | **T** | **A** | **T** | **T** | **T** | **G** | **G** | **T** | **A** | **A** | **C** | **C** | **A** | **A** | **C** | **G** | **T** | **A** | **T** | **T** | **G** | **G** | **A** | **A** | **A** | **C** | **G** | **A** | **C** | **G** | **T** | **C** | **T** | **A** | **T** | **G** | **A** | **A** | **T** | **G** | **G** | **G** | **A** | **T** | **G** | **A** | **T** | **A** | **T** | **C** | **A** | **A** | **C** | **A** | **T** | **T** | **G** | **C** | **C** | **A** | **T** | **C** | **A** | **T** | **T** | **T** | **C** | **T** | **G** | **G** | **T** | **G** | **A** | **T** | **A** | **C** | **T** | **A** | **T** | **C** | **A** | **T** | **C** | **G** | **A** | **T** | **T** | **T** | **G** | **G** | **G** | **C** | **A** | **A** | **T** | **A** | **C** | **A** | **C** | **T** | **T** | **T** | **T** | **G** | **C** | **C** | **A** | **A** | **A** | **A** | **G** | **A** | **T** | **G** | **A** | **T** | **A** | **A** | **T** | **A** | **T** | **T** | **G** | **T** | **G** | **A** | **T** | **T** | **G** | **T** | **A** | **A** | **G** | **A** | **A** | **A** | **A** | **G** | **G** | **G** | **A** | **T** | **A** | **G** | **G** | **C** | **A** | **G** | **A** | **A** | **C** | **A** | **C** | **G** | **G** | **A** | **T** | **C** | **T** | **T** | **A** | **G** | **T** | **A** | **C** | **C** | **C** | **C** | **T** | **G** | **G** | **G** | **A** | **G** | **T** | **G** | **G** | **C** | **T** | **A** | **T** | **T** | **C** | **G** | **T** | **T** | **T** | **A** | **G** | **A** | **G** | **C** | **A** | **T** | **G** | **C** | **G** | **A** | **C** | **A** | **T** | **T** | **A** | **G** | **T** | **A** | **G** | **G** | **A** | **A** | **A** | **T** | **G** | **T** | **C** | **T** | **T** | **G** | **T** | **T** | **T** | **G** | **A** | **A** | **G** | **A** | **A** | **G** | **A** | **G** | **C** | **A** | **G** | **T** | **T** | **T** | **T** | **C** | **C** | **T** | **T** | **A** | **A** | **A** | **A** | **A** | **A** | **C** | **G** | **A** | **A** | **C** | **A** | **G** | **A** | **T** | **C** | **A** | **A** | **A** | **A** | **T** | **C** | **T** | **G** | **C** | **A** | **G** | **C** | **A** | **G** | **T** | **G** | **A** | **T** | **T** | **A** | **T** | **G** | **A** | **T** | **G** | **A** | **A** | **A** | **A** | **T** | **C** | **C** | **T** | **C** | **G** | **C** | **A** | **G** | **A** | **T** | **T** | **G** | **A** | **A** | **G** | **A** | **T** | **C** | **A** | **T** | **T** | **A** | **C** | **C** | **A** | **A** | **T** | **A** | **C** | **C** | **T** | **T** | **G** | **G** | **T** | **T** | **G** | **G** | **A** | **G** | **A** | **T** | **G** | **T** | **C** | **G** | **A** | **G** | **G** | **T** | **G** | **A** | **T** | **A** | **C** | **G** | **A** | **A** | **T** | **A** | **-** | **-** | **-** |
| **N52649** | **A** | **T** | **G** | **A** | **A** | **T** | **A** | **G** | **T** | **T** | **C** | **T** | **T** | **G** | **G** | **C** | **G** | **T** | **T** | **T** | **T** | **T** | **T** | **T** | **G** | **T** | **G** | **G** | **T** | **A** | **G** | **T** | **C** | **G** | **A** | **A** | **G** | **C** | **A** | **C** | **T** | **G** | **C** | **T** | **A** | **C** | **T** | **G** | **T** | **T** | **A** | **T** | **T** | **T** | **G** | **C** | **T** | **G** | **T** | **T** | **T** | **G** | **G** | **C** | **A** | **A** | **A** | **T** | **C** | **G** | **T** | **A** | **A** | **A** | **C** | **A** | **A** | **T** | **A** | **C** | **T** | **G** | **G** | **A** | **C** | **T** | **T** | **T** | **T** | **G** | **C** | **T** | **T** | **T** | **T** | **A** | **G** | **T** | **A** | **A** | **T** | **A** | **T** | **T** | **C** | **G** | **G** | **T** | **A** | **T** | **T** | **T** | **T** | **C** | **A** | **A** | **T** | **A** | **T** | **A** | **T** | **A** | **T** | **C** | **T** | **A** | **G** | **C** | **T** | **A** | **T** | **G** | **C** | **G** | **C** | **A** | **A** | **A** | **T** | **A** | **T** | **C** | **C** | **T** | **C** | **G** | **T** | **A** | **C** | **T** | **A** | **A** | **A** | **T** | **T** | **T** | **C** | **A** | **A** | **A** | **A** | **T** | **T** | **T** | **T** | **C** | **A** | **A** | **T** | **T** | **G** | **A** | **T** | **C** | **T** | **T** | **A** | **G** | **G** | **G** | **A** | **G** | **C** | **T** | **T** | **G** | **G** | **T** | **C** | **A** | **T** | **T** | **T** | **T** | **T** | **T** | **T** | **T** | **A** | **G** | **C** | **T** | **T** | **A** | **G** | **T** | **C** | **A** | **A** | **C** | **A** | **G** | **T** | **C** | **C** | **T** | **G** | **C** | **T** | **T** | **T** | **A** | **T** | **G** | **G** | **A** | **T** | **G** | **A** | **T** | **G** | **G** | **T** | **T** | **G** | **T** | **A** | **T** | **T** | **G** | **G** | **C** | **C** | **G** | **T** | **T** | **T** | **T** | **A** | **T** | **T** | **T** | **A** | **T** | **C** | **G** | **G** | **G** | **C** | **T** | **G** | **A** | **A** | **A** | **G** | **G** | **T** | **G** | **T** | **A** | **G** | **A** | **G** | **A** | **T** | **T** | **T** | **C** | **A** | **G** | **G** | **A** | **A** | **T** | **A** | **G** | **A** | **T** | **T** | **T** | **G** | **A** | **C** | **A** | **A** | **A** | **A** | **A** | **A** | **T** | **G** | **C** | **T** | **T** | **T** | **T** | **T** | **G** | **G** | **C** | **G** | **A** | **A** | **A** | **A** | **A** | **A** | **A** | **C** | **A** | **G** | **A** | **T** | **C** | **A** | **T** | **G** | **A** | **T** | **G** | **G** | **T** | **C** | **C** | **A** | **A** | **A** | **C** | **A** | **G** | **A** | **G** | **C** | **A** | **A** | **T** | **T** | **A** | **A** | **A** | **A** | **A** | **C** | **A** | **C** | **A** | **T** | **A** | **A** | **T** | **A** | **A** | **T** | **G** | **A** | **G** | **C** | **G** | **T** | **A** | **A** | **A** | **A** | **G** | **A** | **C** | **A** | **G** | **C** | **A** | **A** | **T** | **T** | **A** | **T** | **T** | **T** | **G** | **G** | **T** | **A** | **A** | **C** | **C** | **A** | **A** | **C** | **G** | **T** | **A** | **T** | **T** | **G** | **G** | **A** | **A** | **A** | **C** | **G** | **A** | **C** | **G** | **T** | **C** | **T** | **A** | **T** | **G** | **A** | **A** | **T** | **G** | **G** | **G** | **A** | **T** | **G** | **A** | **T** | **A** | **T** | **C** | **A** | **A** | **C** | **A** | **T** | **T** | **G** | **C** | **C** | **A** | **T** | **C** | **A** | **T** | **T** | **T** | **C** | **T** | **G** | **G** | **T** | **G** | **A** | **T** | **A** | **C** | **T** | **A** | **T** | **C** | **A** | **T** | **C** | **G** | **A** | **T** | **T** | **T** | **G** | **G** | **G** | **C** | **A** | **A** | **T** | **A** | **C** | **A** | **C** | **T** | **T** | **T** | **T** | **G** | **C** | **C** | **A** | **A** | **A** | **A** | **G** | **A** | **T** | **G** | **A** | **T** | **A** | **A** | **T** | **A** | **T** | **T** | **G** | **T** | **G** | **A** | **T** | **T** | **G** | **T** | **A** | **A** | **G** | **A** | **A** | **A** | **A** | **G** | **G** | **G** | **A** | **T** | **A** | **G** | **G** | **C** | **A** | **G** | **A** | **A** | **C** | **A** | **C** | **G** | **G** | **A** | **T** | **C** | **T** | **T** | **A** | **G** | **T** | **A** | **C** | **C** | **C** | **C** | **T** | **G** | **G** | **G** | **A** | **G** | **T** | **G** | **G** | **C** | **T** | **A** | **T** | **T** | **C** | **G** | **A** | **T** | **T** | **A** | **G** | **A** | **G** | **C** | **A** | **T** | **G** | **C** | **G** | **A** | **C** | **A** | **T** | **T** | **A** | **G** | **T** | **A** | **G** | **G** | **A** | **A** | **A** | **T** | **G** | **T** | **C** | **T** | **T** | **G** | **T** | **T** | **T** | **G** | **A** | **A** | **G** | **A** | **A** | **G** | **A** | **G** | **C** | **A** | **G** | **T** | **T** | **T** | **T** | **C** | **C** | **T** | **T** | **A** | **A** | **A** | **A** | **A** | **A** | **T** | **G** | **A** | **A** | **C** | **A** | **G** | **A** | **T** | **C** | **A** | **A** | **A** | **A** | **T** | **C** | **T** | **G** | **C** | **A** | **G** | **C** | **A** | **G** | **T** | **G** | **A** | **T** | **T** | **A** | **T** | **G** | **A** | **T** | **G** | **A** | **A** | **A** | **A** | **T** | **C** | **C** | **T** | **C** | **G** | **C** | **A** | **G** | **A** | **T** | **T** | **G** | **A** | **A** | **G** | **A** | **T** | **C** | **A** | **T** | **T** | **A** | **C** | **C** | **A** | **A** | **T** | **A** | **C** | **C** | **T** | **T** | **G** | **G** | **T** | **T** | **G** | **G** | **A** | **G** | **A** | **T** | **G** | **T** | **C** | **G** | **A** | **G** | **G** | **T** | **G** | **A** | **T** | **A** | **C** | **G** | **A** | **A** | **T** | **A** | **-** | **-** | **-** |
| **N52824** | **A** | **T** | **G** | **A** | **A** | **T** | **A** | **G** | **T** | **T** | **C** | **T** | **T** | **G** | **G** | **C** | **G** | **T** | **T** | **T** | **T** | **T** | **T** | **T** | **G** | **T** | **G** | **G** | **T** | **A** | **G** | **T** | **T** | **G** | **A** | **A** | **G** | **C** | **A** | **C** | **T** | **A** | **C** | **T** | **G** | **C** | **T** | **G** | **T** | **T** | **A** | **T** | **T** | **T** | **G** | **C** | **T** | **G** | **T** | **T** | **T** | **G** | **G** | **C** | **A** | **A** | **A** | **T** | **C** | **G** | **T** | **A** | **A** | **A** | **C** | **A** | **A** | **T** | **A** | **C** | **T** | **G** | **G** | **A** | **C** | **T** | **T** | **T** | **T** | **G** | **C** | **T** | **T** | **T** | **T** | **A** | **G** | **T** | **G** | **A** | **T** | **A** | **T** | **T** | **C** | **G** | **G** | **T** | **A** | **T** | **T** | **T** | **T** | **C** | **A** | **A** | **T** | **A** | **T** | **A** | **T** | **A** | **T** | **C** | **T** | **A** | **G** | **C** | **T** | **A** | **T** | **G** | **C** | **G** | **C** | **A** | **A** | **A** | **C** | **A** | **T** | **C** | **C** | **T** | **C** | **G** | **T** | **A** | **C** | **T** | **A** | **A** | **A** | **T** | **T** | **T** | **C** | **A** | **A** | **A** | **A** | **T** | **T** | **T** | **T** | **C** | **A** | **A** | **T** | **T** | **G** | **A** | **T** | **A** | **T** | **T** | **A** | **G** | **G** | **A** | **A** | **G** | **T** | **T** | **T** | **G** | **G** | **T** | **C** | **A** | **T** | **T** | **T** | **T** | **T** | **T** | **T** | **T** | **A** | **G** | **C** | **T** | **T** | **A** | **G** | **T** | **C** | **A** | **A** | **C** | **A** | **G** | **T** | **C** | **C** | **T** | **G** | **C** | **T** | **T** | **T** | **A** | **T** | **G** | **G** | **A** | **T** | **G** | **A** | **T** | **G** | **G** | **C** | **T** | **G** | **T** | **A** | **T** | **T** | **T** | **G** | **C** | **C** | **G** | **T** | **T** | **T** | **T** | **A** | **T** | **T** | **T** | **A** | **T** | **T** | **G** | **G** | **A** | **C** | **T** | **A** | **A** | **A** | **A** | **G** | **G** | **T** | **G** | **T** | **A** | **G** | **A** | **G** | **A** | **T** | **T** | **T** | **C** | **A** | **G** | **G** | **G** | **A** | **T** | **A** | **G** | **A** | **T** | **T** | **T** | **A** | **A** | **C** | **C** | **A** | **A** | **A** | **A** | **A** | **T** | **G** | **C** | **T** | **T** | **T** | **T** | **T** | **G** | **G** | **C** | **G** | **A** | **A** | **A** | **G** | **A** | **A** | **A** | **C** | **A** | **G** | **A** | **T** | **C** | **A** | **T** | **G** | **A** | **T** | **G** | **G** | **T** | **C** | **C** | **A** | **A** | **A** | **C** | **A** | **G** | **A** | **G** | **C** | **A** | **A** | **T** | **T** | **A** | **A** | **A** | **A** | **A** | **C** | **A** | **C** | **A** | **T** | **A** | **A** | **T** | **A** | **A** | **T** | **G** | **A** | **G** | **C** | **G** | **G** | **A** | **A** | **A** | **A** | **G** | **A** | **C** | **A** | **G** | **C** | **A** | **A** | **T** | **T** | **A** | **T** | **T** | **C** | **G** | **G** | **T** | **A** | **A** | **C** | **C** | **A** | **A** | **C** | **G** | **T** | **A** | **T** | **T** | **G** | **G** | **A** | **A** | **A** | **C** | **G** | **A** | **C** | **G** | **T** | **C** | **T** | **A** | **T** | **G** | **A** | **A** | **T** | **G** | **G** | **G** | **A** | **C** | **G** | **A** | **T** | **A** | **T** | **C** | **A** | **A** | **C** | **A** | **T** | **C** | **G** | **C** | **C** | **G** | **T** | **C** | **A** | **T** | **T** | **T** | **C** | **T** | **G** | **G** | **T** | **G** | **A** | **T** | **A** | **C** | **T** | **A** | **T** | **C** | **A** | **T** | **A** | **G** | **A** | **T** | **T** | **T** | **G** | **G** | **G** | **C** | **A** | **A** | **T** | **A** | **C** | **G** | **C** | **T** | **T** | **T** | **T** | **G** | **C** | **C** | **A** | **A** | **A** | **A** | **G** | **A** | **T** | **G** | **A** | **T** | **A** | **A** | **T** | **A** | **T** | **T** | **G** | **T** | **G** | **A** | **T** | **T** | **G** | **T** | **A** | **A** | **G** | **A** | **A** | **A** | **A** | **G** | **G** | **G** | **A** | **T** | **A** | **G** | **G** | **C** | **A** | **G** | **A** | **A** | **C** | **A** | **C** | **G** | **G** | **A** | **T** | **C** | **T** | **T** | **A** | **G** | **T** | **A** | **C** | **C** | **T** | **T** | **T** | **G** | **G** | **G** | **C** | **G** | **T** | **A** | **G** | **C** | **C** | **A** | **T** | **C** | **C** | **G** | **A** | **T** | **T** | **A** | **G** | **A** | **G** | **C** | **A** | **T** | **G** | **C** | **G** | **A** | **C** | **A** | **T** | **T** | **A** | **G** | **T** | **A** | **G** | **G** | **A** | **A** | **A** | **T** | **G** | **T** | **C** | **T** | **T** | **G** | **T** | **T** | **T** | **G** | **A** | **A** | **G** | **A** | **A** | **G** | **A** | **G** | **C** | **A** | **A** | **T** | **T** | **T** | **T** | **C** | **T** | **T** | **T** | **G** | **A** | **A** | **A** | **A** | **A** | **T** | **G** | **A** | **A** | **C** | **A** | **A** | **A** | **T** | **C** | **A** | **A** | **A** | **A** | **T** | **C** | **T** | **A** | **C** | **A** | **G** | **C** | **A** | **G** | **T** | **G** | **A** | **T** | **T** | **A** | **T** | **G** | **A** | **T** | **G** | **A** | **A** | **A** | **A** | **C** | **C** | **C** | **T** | **C** | **G** | **T** | **A** | **G** | **A** | **C** | **T** | **G** | **A** | **A** | **G** | **A** | **T** | **T** | **A** | **T** | **T** | **A** | **C** | **T** | **A** | **A** | **T** | **A** | **C** | **C** | **T** | **T** | **G** | **A** | **T** | **T** | **G** | **G** | **A** | **G** | **A** | **T** | **G** | **T** | **C** | **G** | **A** | **G** | **G** | **T** | **G** | **A** | **T** | **A** | **C** | **G** | **A** | **A** | **T** | **A** | **-** | **-** | **-** |
| **N55299** | **A** | **T** | **G** | **A** | **A** | **T** | **A** | **G** | **T** | **T** | **C** | **T** | **T** | **G** | **G** | **C** | **G** | **T** | **T** | **T** | **T** | **T** | **T** | **T** | **G** | **T** | **G** | **G** | **T** | **A** | **G** | **T** | **C** | **G** | **A** | **A** | **G** | **C** | **A** | **C** | **T** | **G** | **C** | **T** | **G** | **C** | **T** | **G** | **T** | **T** | **A** | **T** | **T** | **T** | **G** | **C** | **T** | **G** | **T** | **T** | **T** | **G** | **G** | **C** | **A** | **A** | **A** | **T** | **C** | **G** | **T** | **A** | **A** | **A** | **C** | **A** | **A** | **T** | **A** | **C** | **T** | **G** | **G** | **A** | **C** | **T** | **T** | **T** | **T** | **G** | **C** | **T** | **T** | **T** | **T** | **A** | **G** | **T** | **A** | **A** | **T** | **A** | **T** | **T** | **C** | **G** | **G** | **T** | **A** | **T** | **T** | **T** | **T** | **C** | **A** | **A** | **T** | **A** | **T** | **A** | **T** | **A** | **T** | **C** | **T** | **A** | **G** | **C** | **T** | **A** | **T** | **G** | **C** | **G** | **C** | **A** | **A** | **A** | **T** | **A** | **T** | **C** | **C** | **T** | **C** | **G** | **T** | **A** | **C** | **T** | **A** | **A** | **A** | **T** | **T** | **T** | **C** | **A** | **A** | **A** | **A** | **T** | **T** | **T** | **T** | **C** | **A** | **A** | **T** | **T** | **G** | **A** | **T** | **C** | **T** | **T** | **A** | **G** | **G** | **G** | **A** | **G** | **C** | **T** | **T** | **G** | **G** | **T** | **C** | **A** | **T** | **T** | **T** | **T** | **T** | **T** | **T** | **T** | **A** | **G** | **C** | **T** | **T** | **A** | **G** | **T** | **C** | **A** | **A** | **C** | **A** | **G** | **T** | **C** | **C** | **T** | **G** | **C** | **T** | **T** | **T** | **A** | **T** | **G** | **G** | **A** | **T** | **G** | **A** | **T** | **G** | **G** | **T** | **T** | **G** | **T** | **A** | **T** | **T** | **G** | **G** | **C** | **C** | **G** | **T** | **T** | **T** | **T** | **A** | **T** | **T** | **T** | **A** | **T** | **C** | **G** | **G** | **G** | **C** | **T** | **G** | **A** | **A** | **A** | **G** | **G** | **T** | **G** | **T** | **A** | **G** | **A** | **G** | **A** | **T** | **T** | **T** | **C** | **A** | **G** | **G** | **A** | **A** | **T** | **A** | **G** | **A** | **T** | **T** | **T** | **G** | **A** | **C** | **A** | **A** | **A** | **A** | **A** | **A** | **T** | **G** | **C** | **T** | **T** | **T** | **T** | **T** | **G** | **G** | **C** | **G** | **A** | **A** | **A** | **A** | **A** | **A** | **A** | **C** | **A** | **G** | **A** | **T** | **C** | **A** | **T** | **G** | **A** | **T** | **G** | **G** | **T** | **C** | **C** | **A** | **A** | **A** | **C** | **A** | **G** | **A** | **G** | **C** | **A** | **A** | **T** | **T** | **A** | **A** | **A** | **A** | **A** | **C** | **A** | **C** | **A** | **T** | **A** | **A** | **T** | **A** | **A** | **T** | **G** | **A** | **G** | **C** | **G** | **T** | **A** | **A** | **A** | **A** | **G** | **A** | **C** | **A** | **G** | **C** | **A** | **A** | **T** | **T** | **A** | **T** | **T** | **T** | **G** | **G** | **T** | **A** | **A** | **C** | **C** | **A** | **A** | **C** | **G** | **T** | **A** | **T** | **T** | **G** | **G** | **A** | **A** | **A** | **C** | **G** | **A** | **C** | **G** | **T** | **C** | **T** | **A** | **T** | **G** | **A** | **A** | **T** | **G** | **G** | **G** | **A** | **T** | **G** | **A** | **T** | **A** | **T** | **C** | **A** | **A** | **C** | **A** | **T** | **T** | **G** | **C** | **C** | **A** | **T** | **C** | **A** | **T** | **T** | **T** | **C** | **T** | **G** | **G** | **T** | **G** | **A** | **T** | **A** | **C** | **T** | **A** | **T** | **C** | **A** | **T** | **C** | **G** | **A** | **T** | **T** | **T** | **G** | **G** | **G** | **C** | **A** | **A** | **T** | **A** | **C** | **A** | **C** | **T** | **T** | **T** | **T** | **G** | **C** | **C** | **A** | **A** | **A** | **A** | **G** | **A** | **T** | **G** | **A** | **T** | **A** | **A** | **T** | **A** | **T** | **T** | **G** | **T** | **G** | **A** | **T** | **T** | **G** | **T** | **A** | **A** | **G** | **A** | **A** | **A** | **A** | **G** | **G** | **G** | **A** | **T** | **A** | **G** | **G** | **C** | **A** | **G** | **A** | **A** | **C** | **A** | **C** | **G** | **G** | **A** | **T** | **C** | **T** | **T** | **A** | **G** | **T** | **A** | **C** | **C** | **C** | **C** | **T** | **G** | **G** | **G** | **A** | **G** | **T** | **G** | **G** | **C** | **T** | **A** | **T** | **T** | **C** | **G** | **T** | **T** | **T** | **A** | **G** | **A** | **G** | **C** | **A** | **T** | **G** | **C** | **G** | **A** | **C** | **A** | **T** | **T** | **A** | **G** | **T** | **A** | **G** | **G** | **A** | **A** | **A** | **T** | **G** | **T** | **C** | **T** | **T** | **G** | **T** | **T** | **T** | **G** | **A** | **A** | **G** | **A** | **A** | **G** | **A** | **G** | **C** | **A** | **G** | **T** | **T** | **T** | **T** | **C** | **C** | **T** | **T** | **A** | **A** | **A** | **A** | **A** | **A** | **C** | **G** | **A** | **A** | **C** | **A** | **G** | **A** | **T** | **C** | **A** | **A** | **A** | **A** | **T** | **C** | **T** | **G** | **C** | **A** | **G** | **C** | **A** | **G** | **T** | **G** | **A** | **T** | **T** | **A** | **T** | **G** | **A** | **T** | **G** | **A** | **A** | **A** | **A** | **T** | **C** | **C** | **T** | **C** | **G** | **C** | **A** | **G** | **A** | **T** | **T** | **G** | **A** | **A** | **G** | **A** | **T** | **C** | **A** | **T** | **T** | **A** | **C** | **C** | **A** | **A** | **T** | **A** | **C** | **C** | **T** | **T** | **G** | **G** | **T** | **T** | **G** | **G** | **A** | **G** | **A** | **T** | **G** | **T** | **C** | **G** | **A** | **G** | **G** | **T** | **G** | **A** | **T** | **A** | **C** | **G** | **A** | **A** | **T** | **A** | **-** | **-** | **-** |
| **N59492F** | **A** | **T** | **G** | **A** | **A** | **T** | **A** | **G** | **T** | **T** | **C** | **T** | **T** | **G** | **G** | **C** | **G** | **T** | **T** | **T** | **T** | **T** | **T** | **T** | **G** | **T** | **G** | **G** | **T** | **A** | **G** | **T** | **C** | **G** | **A** | **A** | **G** | **C** | **A** | **C** | **T** | **G** | **C** | **T** | **G** | **C** | **T** | **G** | **T** | **T** | **A** | **T** | **T** | **T** | **G** | **C** | **T** | **G** | **T** | **T** | **T** | **G** | **G** | **C** | **A** | **A** | **A** | **T** | **C** | **G** | **T** | **A** | **A** | **A** | **C** | **A** | **A** | **T** | **A** | **C** | **T** | **G** | **G** | **A** | **C** | **T** | **T** | **T** | **T** | **G** | **C** | **T** | **T** | **T** | **T** | **A** | **G** | **T** | **A** | **A** | **T** | **A** | **T** | **T** | **C** | **G** | **G** | **T** | **A** | **T** | **T** | **T** | **T** | **C** | **A** | **A** | **T** | **A** | **T** | **A** | **T** | **A** | **T** | **C** | **T** | **A** | **G** | **C** | **T** | **A** | **T** | **G** | **C** | **G** | **C** | **A** | **A** | **A** | **T** | **A** | **T** | **C** | **C** | **T** | **C** | **G** | **T** | **A** | **C** | **T** | **A** | **A** | **A** | **T** | **T** | **T** | **C** | **A** | **A** | **A** | **A** | **T** | **T** | **T** | **T** | **C** | **A** | **A** | **T** | **T** | **G** | **A** | **T** | **C** | **T** | **T** | **A** | **G** | **G** | **G** | **A** | **G** | **C** | **T** | **T** | **G** | **G** | **T** | **C** | **A** | **T** | **T** | **T** | **T** | **T** | **T** | **T** | **T** | **A** | **G** | **C** | **T** | **T** | **A** | **G** | **T** | **C** | **A** | **A** | **C** | **A** | **G** | **T** | **C** | **C** | **T** | **G** | **C** | **T** | **T** | **T** | **A** | **T** | **G** | **G** | **A** | **T** | **G** | **A** | **T** | **G** | **G** | **T** | **T** | **G** | **T** | **A** | **T** | **T** | **G** | **G** | **C** | **C** | **G** | **T** | **T** | **T** | **T** | **A** | **T** | **T** | **T** | **A** | **T** | **C** | **G** | **G** | **G** | **C** | **T** | **G** | **A** | **A** | **A** | **G** | **G** | **T** | **G** | **T** | **A** | **G** | **A** | **G** | **A** | **T** | **T** | **T** | **C** | **A** | **G** | **G** | **A** | **A** | **T** | **A** | **G** | **A** | **T** | **T** | **T** | **G** | **A** | **C** | **A** | **A** | **A** | **A** | **A** | **A** | **T** | **G** | **C** | **T** | **T** | **T** | **T** | **T** | **G** | **G** | **C** | **G** | **A** | **A** | **A** | **A** | **A** | **A** | **A** | **C** | **A** | **G** | **A** | **T** | **C** | **A** | **T** | **G** | **A** | **T** | **G** | **G** | **T** | **C** | **C** | **A** | **A** | **A** | **C** | **A** | **G** | **A** | **G** | **C** | **A** | **A** | **T** | **T** | **A** | **A** | **A** | **A** | **A** | **C** | **A** | **C** | **A** | **T** | **A** | **A** | **T** | **A** | **A** | **T** | **G** | **A** | **G** | **C** | **G** | **T** | **A** | **A** | **A** | **A** | **G** | **A** | **C** | **A** | **G** | **C** | **A** | **A** | **T** | **T** | **A** | **T** | **T** | **T** | **G** | **G** | **T** | **A** | **A** | **C** | **C** | **A** | **A** | **C** | **G** | **T** | **A** | **T** | **T** | **G** | **G** | **A** | **A** | **A** | **C** | **G** | **A** | **C** | **G** | **T** | **C** | **T** | **A** | **T** | **G** | **A** | **A** | **T** | **G** | **G** | **G** | **A** | **T** | **G** | **A** | **T** | **A** | **T** | **C** | **A** | **A** | **C** | **A** | **T** | **T** | **G** | **C** | **C** | **A** | **T** | **C** | **A** | **T** | **T** | **T** | **C** | **T** | **G** | **G** | **T** | **G** | **A** | **T** | **A** | **C** | **T** | **A** | **T** | **C** | **A** | **T** | **C** | **G** | **A** | **T** | **T** | **T** | **G** | **G** | **G** | **C** | **A** | **A** | **T** | **A** | **C** | **A** | **C** | **T** | **T** | **T** | **T** | **G** | **C** | **C** | **A** | **A** | **A** | **A** | **G** | **A** | **T** | **G** | **A** | **T** | **A** | **A** | **T** | **A** | **T** | **T** | **G** | **T** | **G** | **A** | **T** | **T** | **G** | **T** | **A** | **A** | **G** | **A** | **A** | **A** | **A** | **G** | **G** | **G** | **A** | **T** | **A** | **G** | **G** | **C** | **A** | **G** | **A** | **A** | **C** | **A** | **C** | **G** | **G** | **A** | **T** | **C** | **T** | **T** | **A** | **G** | **T** | **A** | **C** | **C** | **C** | **C** | **T** | **G** | **G** | **G** | **A** | **G** | **T** | **G** | **G** | **C** | **T** | **A** | **T** | **T** | **C** | **G** | **T** | **T** | **T** | **A** | **G** | **A** | **G** | **C** | **A** | **T** | **G** | **C** | **G** | **A** | **C** | **A** | **T** | **T** | **A** | **G** | **T** | **A** | **G** | **G** | **A** | **A** | **A** | **T** | **G** | **T** | **C** | **T** | **T** | **G** | **T** | **T** | **T** | **G** | **A** | **A** | **G** | **A** | **A** | **G** | **A** | **G** | **C** | **A** | **G** | **T** | **T** | **T** | **T** | **C** | **C** | **T** | **T** | **A** | **A** | **A** | **A** | **A** | **A** | **C** | **G** | **A** | **A** | **C** | **A** | **G** | **A** | **T** | **C** | **A** | **A** | **A** | **A** | **T** | **C** | **T** | **G** | **C** | **A** | **G** | **C** | **A** | **G** | **T** | **G** | **A** | **T** | **T** | **A** | **T** | **G** | **A** | **T** | **G** | **A** | **A** | **A** | **A** | **T** | **C** | **C** | **T** | **C** | **G** | **C** | **A** | **G** | **A** | **T** | **T** | **G** | **A** | **A** | **G** | **A** | **T** | **C** | **A** | **T** | **T** | **A** | **C** | **C** | **A** | **A** | **T** | **A** | **C** | **C** | **T** | **T** | **G** | **G** | **T** | **T** | **G** | **G** | **A** | **G** | **A** | **T** | **G** | **T** | **C** | **G** | **A** | **G** | **G** | **T** | **G** | **A** | **T** | **A** | **C** | **G** | **A** | **A** | **T** | **A** | **-** | **-** | **-** |
| **N53439** | **A** | **T** | **G** | **A** | **A** | **T** | **A** | **G** | **T** | **T** | **C** | **T** | **T** | **G** | **G** | **C** | **G** | **T** | **T** | **T** | **T** | **T** | **T** | **T** | **G** | **T** | **G** | **G** | **T** | **A** | **G** | **T** | **C** | **G** | **A** | **A** | **G** | **C** | **A** | **C** | **T** | **G** | **C** | **T** | **G** | **C** | **T** | **G** | **T** | **T** | **A** | **T** | **T** | **T** | **G** | **C** | **T** | **G** | **T** | **T** | **T** | **G** | **G** | **C** | **A** | **A** | **A** | **T** | **C** | **G** | **T** | **A** | **A** | **A** | **C** | **A** | **A** | **T** | **A** | **C** | **T** | **G** | **G** | **A** | **C** | **T** | **T** | **T** | **T** | **G** | **C** | **T** | **T** | **T** | **T** | **A** | **G** | **T** | **A** | **A** | **T** | **A** | **T** | **T** | **C** | **G** | **G** | **T** | **A** | **T** | **T** | **T** | **T** | **C** | **A** | **A** | **T** | **A** | **T** | **A** | **T** | **A** | **T** | **C** | **T** | **A** | **G** | **C** | **T** | **A** | **T** | **G** | **C** | **G** | **C** | **A** | **A** | **A** | **T** | **A** | **T** | **C** | **C** | **T** | **C** | **G** | **T** | **A** | **C** | **T** | **A** | **A** | **A** | **T** | **T** | **T** | **C** | **A** | **A** | **A** | **A** | **T** | **T** | **T** | **T** | **C** | **A** | **A** | **T** | **T** | **G** | **A** | **T** | **C** | **T** | **T** | **A** | **G** | **G** | **G** | **A** | **G** | **C** | **T** | **T** | **A** | **G** | **T** | **C** | **A** | **T** | **T** | **T** | **T** | **T** | **T** | **T** | **T** | **A** | **G** | **C** | **T** | **T** | **A** | **G** | **T** | **C** | **A** | **A** | **C** | **A** | **G** | **T** | **C** | **C** | **T** | **G** | **C** | **T** | **T** | **T** | **A** | **T** | **G** | **G** | **A** | **T** | **G** | **A** | **T** | **G** | **G** | **T** | **T** | **G** | **T** | **A** | **T** | **T** | **G** | **G** | **C** | **C** | **G** | **T** | **T** | **T** | **T** | **A** | **T** | **T** | **T** | **A** | **T** | **C** | **G** | **G** | **G** | **C** | **T** | **G** | **A** | **A** | **A** | **G** | **G** | **T** | **G** | **T** | **A** | **G** | **A** | **G** | **A** | **T** | **T** | **T** | **C** | **A** | **G** | **G** | **A** | **A** | **T** | **A** | **G** | **A** | **T** | **T** | **T** | **G** | **A** | **C** | **A** | **A** | **A** | **A** | **A** | **A** | **T** | **G** | **C** | **T** | **T** | **T** | **T** | **T** | **G** | **G** | **C** | **G** | **A** | **A** | **A** | **A** | **A** | **A** | **A** | **C** | **A** | **G** | **A** | **T** | **C** | **A** | **T** | **G** | **A** | **T** | **G** | **G** | **T** | **C** | **C** | **A** | **A** | **A** | **C** | **A** | **G** | **A** | **G** | **C** | **A** | **A** | **T** | **T** | **A** | **A** | **A** | **A** | **A** | **C** | **A** | **C** | **A** | **T** | **A** | **A** | **T** | **A** | **A** | **T** | **G** | **A** | **G** | **C** | **G** | **T** | **A** | **A** | **A** | **A** | **G** | **A** | **C** | **A** | **G** | **C** | **A** | **A** | **T** | **T** | **A** | **T** | **T** | **T** | **G** | **G** | **T** | **A** | **A** | **C** | **C** | **A** | **A** | **C** | **G** | **T** | **A** | **T** | **T** | **G** | **G** | **A** | **A** | **A** | **C** | **G** | **A** | **C** | **G** | **T** | **C** | **T** | **A** | **T** | **G** | **A** | **A** | **T** | **G** | **G** | **G** | **A** | **T** | **G** | **A** | **C** | **A** | **T** | **C** | **A** | **A** | **C** | **A** | **T** | **T** | **G** | **C** | **C** | **A** | **T** | **C** | **A** | **T** | **T** | **T** | **C** | **T** | **G** | **G** | **T** | **G** | **A** | **T** | **A** | **C** | **T** | **A** | **T** | **C** | **A** | **T** | **C** | **G** | **A** | **T** | **T** | **T** | **G** | **G** | **G** | **C** | **A** | **A** | **T** | **A** | **C** | **A** | **C** | **T** | **T** | **T** | **T** | **G** | **C** | **C** | **A** | **A** | **A** | **A** | **G** | **A** | **T** | **G** | **A** | **T** | **A** | **A** | **T** | **A** | **T** | **T** | **G** | **T** | **G** | **A** | **T** | **T** | **G** | **T** | **A** | **A** | **G** | **A** | **A** | **A** | **A** | **G** | **G** | **G** | **A** | **T** | **A** | **G** | **G** | **C** | **A** | **G** | **A** | **A** | **C** | **A** | **C** | **G** | **G** | **A** | **T** | **C** | **T** | **T** | **A** | **G** | **T** | **A** | **C** | **C** | **C** | **C** | **T** | **G** | **G** | **G** | **A** | **G** | **T** | **G** | **G** | **C** | **T** | **A** | **T** | **T** | **C** | **G** | **T** | **T** | **T** | **A** | **G** | **A** | **G** | **C** | **A** | **T** | **G** | **C** | **G** | **A** | **C** | **A** | **T** | **T** | **A** | **G** | **T** | **A** | **G** | **G** | **A** | **A** | **A** | **T** | **G** | **T** | **C** | **T** | **T** | **G** | **T** | **T** | **T** | **G** | **A** | **A** | **G** | **A** | **A** | **G** | **A** | **G** | **C** | **A** | **G** | **T** | **T** | **T** | **T** | **C** | **C** | **T** | **T** | **A** | **A** | **A** | **A** | **A** | **A** | **C** | **G** | **A** | **A** | **C** | **A** | **G** | **A** | **T** | **C** | **A** | **A** | **A** | **A** | **T** | **C** | **T** | **G** | **C** | **A** | **G** | **C** | **A** | **G** | **T** | **G** | **A** | **T** | **T** | **A** | **T** | **G** | **A** | **T** | **G** | **A** | **A** | **A** | **A** | **T** | **C** | **C** | **T** | **C** | **G** | **C** | **A** | **G** | **A** | **T** | **T** | **G** | **A** | **A** | **G** | **A** | **T** | **C** | **A** | **T** | **T** | **A** | **C** | **C** | **A** | **A** | **T** | **A** | **C** | **C** | **T** | **T** | **G** | **G** | **T** | **T** | **G** | **G** | **A** | **G** | **A** | **T** | **G** | **T** | **C** | **G** | **A** | **G** | **G** | **T** | **G** | **A** | **T** | **A** | **C** | **G** | **A** | **A** | **T** | **A** | **-** | **-** | **-** |
| **N59589F** | **A** | **T** | **G** | **A** | **A** | **T** | **A** | **G** | **T** | **T** | **C** | **T** | **T** | **G** | **G** | **C** | **G** | **T** | **T** | **T** | **T** | **T** | **T** | **T** | **G** | **T** | **G** | **G** | **T** | **A** | **G** | **T** | **T** | **G** | **A** | **A** | **G** | **C** | **A** | **C** | **T** | **A** | **C** | **T** | **G** | **C** | **T** | **G** | **T** | **T** | **A** | **T** | **T** | **T** | **G** | **C** | **T** | **G** | **T** | **T** | **T** | **G** | **G** | **C** | **A** | **A** | **A** | **T** | **C** | **G** | **T** | **A** | **A** | **A** | **C** | **A** | **A** | **T** | **A** | **C** | **T** | **G** | **G** | **A** | **C** | **T** | **T** | **T** | **T** | **G** | **C** | **T** | **T** | **T** | **T** | **A** | **G** | **T** | **G** | **A** | **T** | **A** | **T** | **T** | **C** | **G** | **G** | **T** | **A** | **T** | **T** | **T** | **T** | **C** | **A** | **A** | **T** | **A** | **T** | **A** | **T** | **A** | **T** | **C** | **T** | **A** | **G** | **C** | **T** | **A** | **T** | **G** | **C** | **G** | **C** | **A** | **A** | **A** | **C** | **A** | **T** | **C** | **C** | **T** | **C** | **G** | **T** | **A** | **C** | **T** | **A** | **A** | **A** | **T** | **T** | **T** | **C** | **A** | **A** | **A** | **A** | **T** | **T** | **T** | **T** | **C** | **A** | **A** | **T** | **T** | **G** | **A** | **T** | **A** | **T** | **T** | **A** | **G** | **G** | **A** | **A** | **G** | **T** | **T** | **T** | **G** | **G** | **T** | **C** | **A** | **T** | **T** | **T** | **T** | **T** | **T** | **T** | **T** | **A** | **G** | **C** | **T** | **T** | **A** | **G** | **T** | **C** | **A** | **A** | **C** | **A** | **G** | **T** | **C** | **C** | **T** | **G** | **C** | **T** | **T** | **T** | **A** | **T** | **G** | **G** | **A** | **T** | **G** | **A** | **T** | **G** | **G** | **C** | **T** | **G** | **T** | **A** | **T** | **T** | **T** | **G** | **C** | **C** | **G** | **T** | **T** | **T** | **T** | **A** | **T** | **T** | **T** | **A** | **T** | **T** | **G** | **G** | **T** | **C** | **T** | **G** | **A** | **A** | **A** | **G** | **G** | **T** | **G** | **T** | **A** | **G** | **A** | **G** | **A** | **T** | **T** | **T** | **C** | **A** | **G** | **G** | **G** | **A** | **T** | **A** | **G** | **A** | **T** | **T** | **T** | **A** | **A** | **C** | **C** | **A** | **A** | **A** | **A** | **A** | **T** | **G** | **C** | **T** | **T** | **T** | **T** | **T** | **G** | **G** | **C** | **G** | **A** | **A** | **A** | **G** | **A** | **A** | **A** | **C** | **A** | **G** | **A** | **T** | **C** | **A** | **T** | **G** | **A** | **T** | **G** | **A** | **T** | **C** | **C** | **A** | **A** | **A** | **C** | **A** | **G** | **A** | **G** | **C** | **A** | **A** | **T** | **T** | **A** | **A** | **A** | **A** | **A** | **C** | **A** | **C** | **A** | **T** | **A** | **A** | **T** | **A** | **A** | **T** | **G** | **A** | **G** | **C** | **G** | **G** | **A** | **A** | **A** | **A** | **G** | **A** | **C** | **A** | **G** | **C** | **A** | **A** | **T** | **T** | **A** | **T** | **T** | **C** | **G** | **G** | **T** | **A** | **A** | **C** | **C** | **A** | **A** | **C** | **G** | **T** | **A** | **T** | **T** | **G** | **G** | **A** | **A** | **A** | **C** | **G** | **A** | **C** | **G** | **T** | **C** | **T** | **A** | **T** | **G** | **A** | **A** | **T** | **G** | **G** | **G** | **A** | **C** | **G** | **A** | **T** | **A** | **T** | **C** | **A** | **A** | **C** | **A** | **T** | **C** | **G** | **C** | **C** | **G** | **T** | **C** | **A** | **T** | **T** | **T** | **C** | **T** | **G** | **G** | **T** | **G** | **A** | **T** | **A** | **C** | **T** | **A** | **T** | **C** | **A** | **T** | **A** | **G** | **A** | **T** | **T** | **T** | **G** | **G** | **G** | **C** | **A** | **A** | **T** | **A** | **C** | **G** | **C** | **T** | **T** | **T** | **T** | **G** | **C** | **C** | **A** | **A** | **A** | **A** | **G** | **A** | **T** | **G** | **A** | **T** | **A** | **A** | **T** | **A** | **T** | **T** | **G** | **T** | **G** | **A** | **T** | **T** | **G** | **T** | **A** | **A** | **G** | **A** | **A** | **A** | **A** | **G** | **G** | **G** | **A** | **T** | **A** | **G** | **G** | **C** | **A** | **G** | **A** | **A** | **C** | **A** | **C** | **G** | **G** | **A** | **T** | **C** | **T** | **T** | **A** | **G** | **T** | **A** | **C** | **C** | **T** | **T** | **T** | **G** | **G** | **G** | **C** | **G** | **T** | **A** | **G** | **C** | **C** | **A** | **T** | **C** | **C** | **G** | **T** | **T** | **T** | **A** | **G** | **A** | **G** | **C** | **A** | **T** | **G** | **C** | **G** | **A** | **C** | **A** | **T** | **T** | **A** | **G** | **T** | **A** | **G** | **G** | **A** | **A** | **A** | **T** | **G** | **T** | **C** | **T** | **T** | **G** | **T** | **T** | **T** | **G** | **A** | **A** | **G** | **A** | **A** | **G** | **A** | **G** | **C** | **A** | **A** | **T** | **T** | **T** | **T** | **C** | **T** | **T** | **T** | **G** | **A** | **A** | **A** | **A** | **A** | **T** | **G** | **A** | **A** | **C** | **A** | **A** | **A** | **T** | **C** | **A** | **A** | **A** | **A** | **T** | **C** | **T** | **A** | **C** | **A** | **G** | **C** | **A** | **G** | **T** | **G** | **A** | **T** | **T** | **A** | **T** | **G** | **A** | **T** | **G** | **A** | **A** | **A** | **A** | **C** | **C** | **C** | **T** | **C** | **G** | **T** | **A** | **G** | **A** | **T** | **T** | **G** | **A** | **A** | **G** | **A** | **T** | **T** | **A** | **T** | **T** | **A** | **C** | **T** | **A** | **A** | **T** | **A** | **C** | **C** | **T** | **T** | **G** | **A** | **T** | **T** | **G** | **G** | **A** | **G** | **A** | **T** | **G** | **T** | **C** | **G** | **A** | **G** | **G** | **T** | **G** | **A** | **T** | **A** | **C** | **G** | **A** | **A** | **T** | **A** | **-** | **-** | **-** |
| **N52459** | **A** | **T** | **G** | **A** | **A** | **T** | **A** | **G** | **T** | **T** | **C** | **T** | **T** | **G** | **G** | **C** | **G** | **T** | **T** | **T** | **T** | **T** | **T** | **T** | **G** | **T** | **G** | **G** | **T** | **A** | **G** | **T** | **T** | **G** | **A** | **A** | **G** | **C** | **A** | **C** | **T** | **A** | **C** | **T** | **G** | **C** | **T** | **G** | **T** | **T** | **A** | **T** | **T** | **T** | **G** | **C** | **T** | **G** | **T** | **T** | **T** | **G** | **G** | **C** | **A** | **A** | **A** | **T** | **C** | **G** | **T** | **A** | **A** | **A** | **C** | **A** | **A** | **T** | **A** | **C** | **T** | **G** | **G** | **A** | **C** | **T** | **T** | **T** | **T** | **G** | **C** | **T** | **T** | **T** | **T** | **A** | **G** | **T** | **G** | **A** | **T** | **A** | **T** | **T** | **C** | **G** | **G** | **T** | **A** | **T** | **T** | **T** | **T** | **C** | **A** | **A** | **T** | **A** | **T** | **A** | **T** | **A** | **T** | **C** | **T** | **A** | **G** | **C** | **T** | **A** | **T** | **G** | **C** | **G** | **C** | **A** | **A** | **A** | **C** | **A** | **T** | **C** | **C** | **T** | **C** | **G** | **T** | **A** | **C** | **T** | **A** | **A** | **A** | **T** | **T** | **T** | **C** | **A** | **A** | **A** | **A** | **T** | **T** | **T** | **T** | **C** | **A** | **A** | **T** | **T** | **G** | **A** | **T** | **A** | **T** | **T** | **A** | **G** | **G** | **A** | **A** | **G** | **T** | **T** | **T** | **G** | **G** | **T** | **C** | **A** | **T** | **T** | **T** | **T** | **T** | **T** | **T** | **T** | **A** | **G** | **C** | **T** | **T** | **A** | **G** | **T** | **C** | **A** | **A** | **C** | **A** | **G** | **T** | **C** | **C** | **T** | **G** | **C** | **T** | **T** | **T** | **A** | **T** | **G** | **G** | **A** | **T** | **G** | **A** | **T** | **G** | **G** | **C** | **T** | **G** | **T** | **A** | **T** | **T** | **T** | **G** | **C** | **C** | **G** | **T** | **T** | **T** | **T** | **A** | **T** | **T** | **T** | **A** | **T** | **T** | **G** | **G** | **A** | **C** | **T** | **A** | **A** | **A** | **A** | **G** | **G** | **T** | **G** | **T** | **A** | **G** | **A** | **G** | **A** | **T** | **T** | **T** | **C** | **A** | **G** | **G** | **G** | **A** | **T** | **A** | **G** | **A** | **T** | **T** | **T** | **A** | **A** | **C** | **C** | **A** | **A** | **A** | **A** | **A** | **T** | **G** | **C** | **T** | **T** | **T** | **T** | **T** | **G** | **G** | **C** | **G** | **A** | **A** | **A** | **G** | **A** | **A** | **A** | **C** | **A** | **G** | **A** | **T** | **C** | **A** | **T** | **G** | **A** | **T** | **G** | **G** | **T** | **C** | **C** | **A** | **A** | **A** | **C** | **A** | **G** | **A** | **G** | **C** | **A** | **A** | **T** | **T** | **A** | **A** | **A** | **A** | **A** | **C** | **A** | **C** | **A** | **T** | **A** | **A** | **T** | **A** | **A** | **T** | **G** | **A** | **G** | **C** | **G** | **G** | **A** | **A** | **A** | **A** | **G** | **A** | **C** | **A** | **G** | **C** | **A** | **A** | **T** | **T** | **A** | **T** | **T** | **C** | **G** | **G** | **T** | **A** | **A** | **C** | **C** | **A** | **A** | **C** | **G** | **T** | **A** | **T** | **T** | **G** | **G** | **A** | **A** | **A** | **C** | **G** | **A** | **C** | **G** | **T** | **C** | **T** | **A** | **T** | **G** | **A** | **A** | **T** | **G** | **G** | **G** | **A** | **C** | **G** | **A** | **T** | **A** | **T** | **C** | **A** | **A** | **C** | **A** | **T** | **C** | **G** | **C** | **C** | **G** | **T** | **C** | **A** | **T** | **T** | **T** | **C** | **T** | **G** | **G** | **T** | **G** | **A** | **T** | **A** | **C** | **T** | **A** | **T** | **C** | **A** | **T** | **A** | **G** | **A** | **T** | **T** | **T** | **G** | **G** | **G** | **C** | **A** | **A** | **T** | **A** | **C** | **G** | **C** | **T** | **T** | **T** | **T** | **G** | **C** | **C** | **A** | **A** | **A** | **A** | **G** | **A** | **T** | **G** | **A** | **T** | **A** | **A** | **T** | **A** | **T** | **T** | **G** | **T** | **G** | **A** | **T** | **T** | **G** | **T** | **A** | **A** | **G** | **A** | **A** | **A** | **A** | **G** | **G** | **G** | **A** | **T** | **A** | **G** | **G** | **C** | **A** | **G** | **A** | **A** | **C** | **A** | **C** | **G** | **G** | **A** | **T** | **C** | **T** | **T** | **A** | **G** | **T** | **A** | **C** | **C** | **T** | **T** | **T** | **G** | **G** | **G** | **C** | **G** | **T** | **A** | **G** | **C** | **C** | **A** | **T** | **C** | **C** | **G** | **A** | **T** | **T** | **A** | **G** | **A** | **G** | **C** | **A** | **T** | **G** | **C** | **G** | **A** | **C** | **A** | **T** | **T** | **A** | **G** | **T** | **A** | **G** | **G** | **A** | **A** | **A** | **T** | **G** | **T** | **C** | **T** | **T** | **G** | **T** | **T** | **T** | **G** | **A** | **A** | **G** | **A** | **A** | **G** | **A** | **G** | **C** | **A** | **A** | **T** | **T** | **T** | **T** | **C** | **T** | **T** | **T** | **G** | **A** | **A** | **A** | **A** | **A** | **T** | **G** | **A** | **A** | **C** | **A** | **A** | **A** | **T** | **C** | **A** | **A** | **A** | **A** | **T** | **C** | **T** | **A** | **C** | **A** | **G** | **C** | **A** | **G** | **T** | **G** | **A** | **T** | **T** | **A** | **T** | **G** | **A** | **T** | **G** | **A** | **A** | **A** | **A** | **C** | **C** | **C** | **T** | **C** | **G** | **T** | **A** | **G** | **A** | **C** | **T** | **G** | **A** | **A** | **G** | **A** | **T** | **T** | **A** | **T** | **T** | **A** | **C** | **T** | **A** | **A** | **T** | **A** | **C** | **C** | **T** | **T** | **G** | **A** | **T** | **T** | **G** | **G** | **A** | **G** | **A** | **T** | **G** | **T** | **C** | **G** | **A** | **G** | **G** | **T** | **G** | **A** | **T** | **A** | **C** | **G** | **A** | **A** | **T** | **A** | **-** | **-** | **-** |
| **N55317** | **A** | **T** | **G** | **A** | **A** | **T** | **A** | **G** | **T** | **T** | **C** | **T** | **T** | **G** | **G** | **C** | **G** | **T** | **T** | **T** | **T** | **T** | **T** | **T** | **G** | **T** | **G** | **G** | **T** | **A** | **G** | **T** | **T** | **G** | **A** | **A** | **G** | **C** | **A** | **C** | **T** | **A** | **C** | **T** | **G** | **C** | **T** | **G** | **T** | **T** | **A** | **T** | **T** | **T** | **G** | **C** | **T** | **G** | **T** | **T** | **T** | **G** | **G** | **C** | **A** | **A** | **A** | **T** | **C** | **G** | **T** | **A** | **A** | **A** | **C** | **A** | **A** | **T** | **A** | **C** | **T** | **G** | **G** | **A** | **C** | **T** | **T** | **T** | **T** | **G** | **C** | **T** | **T** | **T** | **T** | **A** | **G** | **T** | **G** | **A** | **T** | **A** | **T** | **T** | **C** | **G** | **G** | **T** | **A** | **T** | **T** | **T** | **T** | **C** | **A** | **A** | **T** | **A** | **T** | **A** | **T** | **A** | **T** | **C** | **T** | **A** | **G** | **C** | **T** | **A** | **T** | **G** | **C** | **G** | **C** | **A** | **A** | **A** | **C** | **A** | **T** | **C** | **C** | **T** | **C** | **G** | **T** | **A** | **C** | **T** | **A** | **A** | **A** | **T** | **T** | **T** | **C** | **A** | **A** | **A** | **A** | **T** | **T** | **T** | **T** | **C** | **A** | **A** | **T** | **T** | **G** | **A** | **T** | **A** | **T** | **T** | **A** | **G** | **G** | **A** | **A** | **G** | **T** | **T** | **T** | **G** | **G** | **T** | **C** | **A** | **T** | **T** | **T** | **T** | **T** | **T** | **T** | **T** | **A** | **G** | **C** | **T** | **T** | **A** | **G** | **T** | **C** | **A** | **A** | **C** | **A** | **G** | **T** | **C** | **C** | **T** | **G** | **C** | **T** | **T** | **T** | **A** | **T** | **G** | **G** | **A** | **T** | **G** | **A** | **T** | **G** | **G** | **C** | **T** | **G** | **T** | **A** | **T** | **T** | **T** | **G** | **C** | **C** | **G** | **T** | **T** | **T** | **T** | **A** | **T** | **T** | **T** | **A** | **T** | **T** | **G** | **G** | **A** | **C** | **T** | **A** | **A** | **A** | **A** | **G** | **G** | **T** | **G** | **T** | **A** | **G** | **A** | **G** | **A** | **T** | **T** | **T** | **C** | **A** | **G** | **G** | **G** | **A** | **T** | **A** | **G** | **A** | **T** | **T** | **T** | **A** | **A** | **C** | **C** | **A** | **A** | **A** | **A** | **A** | **T** | **G** | **C** | **T** | **T** | **T** | **T** | **T** | **G** | **G** | **C** | **G** | **A** | **A** | **A** | **G** | **A** | **A** | **A** | **C** | **A** | **G** | **A** | **T** | **C** | **A** | **T** | **G** | **A** | **T** | **G** | **G** | **T** | **C** | **C** | **A** | **A** | **A** | **C** | **A** | **G** | **A** | **G** | **C** | **A** | **A** | **T** | **T** | **A** | **A** | **A** | **A** | **A** | **C** | **A** | **C** | **A** | **T** | **A** | **A** | **T** | **A** | **A** | **T** | **G** | **A** | **G** | **C** | **G** | **G** | **A** | **A** | **A** | **A** | **G** | **A** | **C** | **A** | **G** | **C** | **A** | **A** | **T** | **T** | **A** | **T** | **T** | **C** | **G** | **G** | **T** | **A** | **A** | **C** | **C** | **A** | **A** | **C** | **G** | **T** | **A** | **T** | **T** | **G** | **G** | **A** | **A** | **A** | **C** | **G** | **A** | **C** | **G** | **T** | **C** | **T** | **A** | **T** | **G** | **A** | **A** | **T** | **G** | **G** | **G** | **A** | **C** | **G** | **A** | **T** | **A** | **T** | **C** | **A** | **A** | **C** | **A** | **T** | **C** | **G** | **C** | **C** | **G** | **T** | **C** | **A** | **T** | **T** | **T** | **C** | **T** | **G** | **G** | **T** | **G** | **A** | **T** | **A** | **C** | **T** | **A** | **T** | **C** | **A** | **T** | **A** | **G** | **A** | **T** | **T** | **T** | **G** | **G** | **G** | **C** | **A** | **A** | **T** | **A** | **C** | **G** | **C** | **T** | **T** | **T** | **T** | **G** | **C** | **C** | **A** | **A** | **A** | **A** | **G** | **A** | **T** | **G** | **A** | **T** | **A** | **A** | **T** | **A** | **T** | **T** | **G** | **T** | **G** | **A** | **T** | **T** | **G** | **T** | **A** | **A** | **G** | **A** | **A** | **A** | **A** | **G** | **G** | **G** | **A** | **T** | **A** | **G** | **G** | **C** | **A** | **G** | **A** | **A** | **C** | **A** | **C** | **G** | **G** | **A** | **T** | **C** | **T** | **T** | **A** | **G** | **T** | **A** | **C** | **C** | **T** | **T** | **T** | **T** | **G** | **G** | **C** | **G** | **T** | **A** | **G** | **C** | **C** | **A** | **T** | **C** | **C** | **G** | **A** | **T** | **T** | **A** | **G** | **A** | **G** | **C** | **A** | **T** | **G** | **C** | **G** | **A** | **C** | **A** | **T** | **T** | **A** | **G** | **T** | **A** | **G** | **G** | **A** | **A** | **A** | **T** | **G** | **T** | **C** | **T** | **T** | **G** | **T** | **T** | **T** | **G** | **A** | **A** | **G** | **A** | **A** | **G** | **A** | **G** | **C** | **A** | **A** | **T** | **T** | **T** | **T** | **C** | **T** | **T** | **T** | **G** | **A** | **A** | **A** | **A** | **A** | **T** | **G** | **A** | **A** | **C** | **A** | **A** | **A** | **T** | **C** | **A** | **A** | **A** | **A** | **T** | **C** | **T** | **A** | **T** | **A** | **G** | **C** | **A** | **G** | **T** | **G** | **A** | **T** | **T** | **A** | **T** | **G** | **A** | **T** | **G** | **A** | **A** | **A** | **A** | **C** | **C** | **C** | **T** | **C** | **G** | **T** | **A** | **G** | **A** | **C** | **T** | **G** | **A** | **A** | **G** | **A** | **T** | **T** | **A** | **T** | **T** | **A** | **C** | **T** | **A** | **A** | **T** | **A** | **C** | **C** | **T** | **T** | **G** | **A** | **T** | **T** | **G** | **G** | **A** | **G** | **A** | **T** | **G** | **T** | **C** | **G** | **A** | **G** | **G** | **T** | **G** | **A** | **T** | **A** | **C** | **G** | **A** | **A** | **T** | **A** | **-** | **-** | **-** |
| **N52690** | **A** | **T** | **G** | **A** | **A** | **T** | **A** | **G** | **T** | **T** | **C** | **T** | **T** | **G** | **G** | **C** | **G** | **T** | **T** | **T** | **T** | **T** | **T** | **T** | **G** | **T** | **G** | **G** | **T** | **A** | **G** | **T** | **C** | **G** | **A** | **A** | **G** | **C** | **A** | **C** | **T** | **G** | **C** | **T** | **G** | **C** | **T** | **G** | **T** | **T** | **A** | **T** | **T** | **T** | **G** | **C** | **T** | **G** | **T** | **T** | **T** | **G** | **G** | **C** | **A** | **A** | **A** | **T** | **C** | **G** | **T** | **A** | **A** | **A** | **C** | **A** | **A** | **T** | **A** | **C** | **T** | **G** | **G** | **A** | **C** | **T** | **T** | **T** | **T** | **G** | **C** | **T** | **T** | **T** | **T** | **A** | **G** | **T** | **A** | **A** | **T** | **A** | **T** | **T** | **C** | **G** | **G** | **T** | **A** | **T** | **T** | **T** | **T** | **C** | **A** | **A** | **T** | **A** | **T** | **A** | **T** | **A** | **T** | **C** | **T** | **A** | **G** | **C** | **T** | **A** | **T** | **G** | **C** | **G** | **C** | **A** | **A** | **A** | **T** | **A** | **T** | **C** | **C** | **T** | **C** | **G** | **T** | **A** | **C** | **T** | **A** | **A** | **A** | **T** | **T** | **T** | **C** | **A** | **A** | **A** | **A** | **T** | **T** | **T** | **T** | **C** | **A** | **A** | **T** | **T** | **G** | **A** | **T** | **C** | **T** | **T** | **A** | **G** | **G** | **G** | **A** | **G** | **C** | **T** | **T** | **G** | **G** | **T** | **C** | **A** | **T** | **T** | **T** | **T** | **T** | **T** | **T** | **T** | **A** | **G** | **C** | **T** | **T** | **A** | **G** | **T** | **C** | **A** | **A** | **C** | **A** | **G** | **T** | **C** | **C** | **T** | **G** | **C** | **T** | **T** | **T** | **A** | **T** | **G** | **G** | **A** | **T** | **G** | **A** | **T** | **G** | **G** | **T** | **T** | **G** | **T** | **A** | **T** | **T** | **G** | **G** | **C** | **C** | **G** | **T** | **T** | **T** | **T** | **A** | **T** | **T** | **T** | **A** | **T** | **C** | **G** | **G** | **G** | **C** | **T** | **G** | **A** | **A** | **A** | **G** | **G** | **T** | **G** | **T** | **A** | **G** | **A** | **G** | **A** | **T** | **T** | **T** | **C** | **A** | **G** | **G** | **A** | **A** | **T** | **A** | **G** | **A** | **T** | **T** | **T** | **G** | **A** | **C** | **A** | **A** | **A** | **A** | **A** | **A** | **T** | **G** | **C** | **T** | **T** | **T** | **T** | **T** | **G** | **G** | **C** | **G** | **A** | **A** | **A** | **A** | **A** | **A** | **A** | **C** | **A** | **G** | **A** | **T** | **C** | **A** | **T** | **G** | **A** | **T** | **G** | **G** | **T** | **C** | **C** | **A** | **A** | **A** | **C** | **A** | **G** | **A** | **G** | **C** | **A** | **A** | **T** | **T** | **A** | **A** | **A** | **A** | **A** | **C** | **A** | **C** | **A** | **T** | **A** | **A** | **T** | **A** | **A** | **T** | **G** | **A** | **G** | **C** | **G** | **T** | **A** | **A** | **A** | **A** | **G** | **A** | **C** | **A** | **G** | **C** | **A** | **A** | **T** | **T** | **A** | **T** | **T** | **T** | **G** | **G** | **T** | **A** | **A** | **C** | **C** | **A** | **A** | **C** | **G** | **T** | **A** | **T** | **T** | **G** | **G** | **A** | **A** | **A** | **C** | **G** | **A** | **C** | **G** | **T** | **C** | **T** | **A** | **T** | **G** | **A** | **A** | **T** | **G** | **G** | **G** | **A** | **T** | **G** | **A** | **T** | **A** | **T** | **C** | **A** | **A** | **C** | **A** | **T** | **T** | **G** | **C** | **C** | **A** | **T** | **C** | **A** | **T** | **T** | **T** | **C** | **T** | **G** | **G** | **T** | **G** | **A** | **T** | **A** | **C** | **T** | **A** | **T** | **C** | **A** | **T** | **C** | **G** | **A** | **T** | **T** | **T** | **G** | **G** | **G** | **C** | **A** | **A** | **T** | **A** | **C** | **A** | **C** | **T** | **T** | **T** | **T** | **G** | **C** | **C** | **A** | **A** | **A** | **A** | **G** | **A** | **T** | **G** | **A** | **T** | **A** | **A** | **T** | **A** | **T** | **T** | **G** | **T** | **G** | **A** | **T** | **T** | **G** | **T** | **A** | **A** | **G** | **A** | **A** | **A** | **A** | **G** | **G** | **G** | **A** | **T** | **A** | **G** | **G** | **C** | **A** | **G** | **A** | **A** | **C** | **A** | **C** | **G** | **G** | **A** | **T** | **C** | **T** | **T** | **A** | **G** | **T** | **A** | **C** | **C** | **C** | **C** | **T** | **G** | **G** | **G** | **A** | **G** | **T** | **G** | **G** | **C** | **T** | **A** | **T** | **T** | **C** | **G** | **T** | **T** | **T** | **A** | **G** | **A** | **G** | **C** | **A** | **T** | **G** | **C** | **G** | **A** | **C** | **A** | **T** | **T** | **A** | **G** | **T** | **A** | **G** | **G** | **A** | **A** | **A** | **T** | **G** | **T** | **C** | **T** | **T** | **G** | **T** | **T** | **T** | **G** | **A** | **A** | **G** | **A** | **A** | **G** | **A** | **G** | **C** | **A** | **G** | **T** | **T** | **T** | **T** | **C** | **C** | **T** | **T** | **A** | **A** | **A** | **A** | **A** | **A** | **C** | **G** | **A** | **A** | **C** | **A** | **G** | **A** | **T** | **C** | **A** | **A** | **A** | **A** | **T** | **C** | **T** | **G** | **C** | **A** | **G** | **C** | **A** | **G** | **T** | **G** | **A** | **T** | **T** | **A** | **T** | **G** | **A** | **T** | **G** | **A** | **A** | **A** | **A** | **T** | **C** | **C** | **T** | **C** | **G** | **C** | **A** | **G** | **A** | **T** | **T** | **G** | **A** | **A** | **G** | **A** | **T** | **C** | **A** | **T** | **T** | **A** | **C** | **C** | **A** | **A** | **T** | **A** | **C** | **C** | **T** | **T** | **G** | **G** | **T** | **T** | **G** | **G** | **A** | **G** | **A** | **T** | **G** | **T** | **C** | **G** | **A** | **G** | **G** | **T** | **G** | **A** | **T** | **A** | **C** | **G** | **A** | **A** | **T** | **A** | **-** | **-** | **-** |
| **N60336F** | **A** | **T** | **G** | **A** | **A** | **T** | **A** | **G** | **T** | **T** | **C** | **T** | **T** | **G** | **G** | **C** | **G** | **T** | **T** | **T** | **T** | **T** | **T** | **T** | **G** | **T** | **G** | **G** | **T** | **A** | **G** | **T** | **C** | **G** | **A** | **A** | **G** | **C** | **A** | **C** | **T** | **G** | **C** | **T** | **G** | **C** | **T** | **G** | **T** | **T** | **A** | **T** | **T** | **T** | **G** | **C** | **T** | **G** | **T** | **T** | **T** | **G** | **G** | **C** | **A** | **A** | **A** | **T** | **C** | **G** | **T** | **A** | **A** | **A** | **C** | **A** | **A** | **T** | **A** | **C** | **T** | **G** | **G** | **A** | **C** | **T** | **T** | **T** | **T** | **G** | **C** | **T** | **T** | **T** | **T** | **A** | **G** | **T** | **A** | **A** | **T** | **A** | **T** | **T** | **C** | **G** | **G** | **T** | **A** | **T** | **T** | **T** | **T** | **C** | **A** | **A** | **T** | **A** | **T** | **A** | **T** | **A** | **T** | **C** | **T** | **A** | **G** | **C** | **T** | **A** | **T** | **G** | **C** | **G** | **C** | **A** | **A** | **A** | **T** | **A** | **T** | **C** | **C** | **T** | **C** | **G** | **T** | **A** | **C** | **T** | **A** | **A** | **A** | **T** | **T** | **T** | **C** | **A** | **A** | **A** | **A** | **T** | **T** | **T** | **T** | **C** | **A** | **A** | **T** | **T** | **G** | **A** | **T** | **C** | **T** | **T** | **A** | **G** | **G** | **G** | **A** | **G** | **C** | **T** | **T** | **G** | **G** | **T** | **C** | **A** | **T** | **T** | **T** | **T** | **T** | **T** | **T** | **T** | **A** | **G** | **C** | **T** | **T** | **A** | **G** | **T** | **C** | **A** | **A** | **C** | **A** | **G** | **T** | **C** | **C** | **T** | **G** | **C** | **T** | **T** | **T** | **A** | **T** | **G** | **G** | **A** | **T** | **G** | **A** | **T** | **G** | **G** | **T** | **T** | **G** | **T** | **A** | **T** | **T** | **G** | **G** | **C** | **C** | **G** | **T** | **T** | **T** | **T** | **A** | **T** | **T** | **T** | **A** | **T** | **C** | **G** | **G** | **G** | **C** | **T** | **G** | **A** | **A** | **A** | **G** | **G** | **T** | **G** | **T** | **A** | **G** | **A** | **G** | **A** | **T** | **T** | **T** | **C** | **A** | **G** | **G** | **A** | **A** | **T** | **A** | **G** | **A** | **T** | **T** | **T** | **G** | **A** | **C** | **A** | **A** | **A** | **A** | **A** | **A** | **T** | **G** | **C** | **T** | **T** | **T** | **T** | **T** | **G** | **G** | **C** | **G** | **A** | **A** | **A** | **A** | **A** | **A** | **A** | **C** | **A** | **G** | **A** | **T** | **C** | **A** | **T** | **G** | **A** | **T** | **G** | **G** | **T** | **C** | **C** | **A** | **A** | **A** | **C** | **A** | **G** | **A** | **G** | **C** | **A** | **A** | **T** | **T** | **A** | **A** | **A** | **A** | **A** | **C** | **A** | **C** | **A** | **T** | **A** | **A** | **T** | **A** | **A** | **T** | **G** | **A** | **G** | **C** | **G** | **T** | **A** | **A** | **A** | **A** | **G** | **A** | **C** | **A** | **G** | **C** | **A** | **A** | **T** | **T** | **A** | **T** | **T** | **T** | **G** | **G** | **T** | **A** | **A** | **C** | **C** | **A** | **A** | **C** | **G** | **T** | **A** | **T** | **T** | **G** | **G** | **A** | **A** | **A** | **C** | **G** | **A** | **C** | **G** | **T** | **C** | **T** | **A** | **T** | **G** | **A** | **A** | **T** | **G** | **G** | **G** | **A** | **T** | **G** | **A** | **T** | **A** | **T** | **C** | **A** | **A** | **C** | **A** | **T** | **T** | **G** | **C** | **C** | **A** | **T** | **C** | **A** | **T** | **T** | **T** | **C** | **T** | **G** | **G** | **T** | **G** | **A** | **T** | **A** | **C** | **T** | **A** | **T** | **C** | **A** | **T** | **C** | **G** | **A** | **T** | **T** | **T** | **G** | **G** | **G** | **C** | **A** | **A** | **T** | **A** | **C** | **A** | **C** | **T** | **T** | **T** | **T** | **G** | **C** | **C** | **A** | **A** | **A** | **A** | **G** | **A** | **T** | **G** | **A** | **T** | **A** | **A** | **T** | **A** | **T** | **T** | **G** | **T** | **G** | **A** | **T** | **T** | **G** | **T** | **A** | **A** | **G** | **A** | **A** | **A** | **A** | **G** | **G** | **G** | **A** | **T** | **A** | **G** | **G** | **C** | **A** | **G** | **A** | **A** | **C** | **A** | **C** | **G** | **G** | **A** | **T** | **C** | **T** | **T** | **A** | **G** | **T** | **A** | **C** | **C** | **C** | **C** | **T** | **G** | **G** | **G** | **A** | **G** | **T** | **G** | **G** | **C** | **T** | **A** | **T** | **T** | **C** | **G** | **T** | **T** | **T** | **A** | **G** | **A** | **G** | **C** | **A** | **T** | **G** | **C** | **G** | **A** | **C** | **A** | **T** | **T** | **A** | **G** | **T** | **A** | **G** | **G** | **A** | **A** | **A** | **T** | **G** | **T** | **C** | **T** | **T** | **G** | **T** | **T** | **T** | **G** | **A** | **A** | **G** | **A** | **A** | **G** | **A** | **G** | **C** | **A** | **G** | **T** | **T** | **T** | **T** | **C** | **C** | **T** | **T** | **A** | **A** | **A** | **A** | **A** | **A** | **C** | **G** | **A** | **A** | **C** | **A** | **G** | **A** | **T** | **C** | **A** | **A** | **A** | **A** | **T** | **C** | **T** | **G** | **C** | **A** | **G** | **C** | **A** | **G** | **T** | **G** | **A** | **T** | **T** | **A** | **T** | **G** | **A** | **T** | **G** | **A** | **A** | **A** | **A** | **T** | **C** | **C** | **T** | **C** | **G** | **C** | **A** | **G** | **A** | **T** | **T** | **G** | **A** | **A** | **G** | **A** | **T** | **C** | **A** | **T** | **T** | **A** | **C** | **C** | **A** | **A** | **T** | **A** | **C** | **C** | **T** | **T** | **G** | **G** | **T** | **T** | **G** | **G** | **A** | **G** | **A** | **T** | **G** | **T** | **C** | **G** | **A** | **G** | **G** | **T** | **G** | **A** | **T** | **A** | **C** | **G** | **A** | **A** | **T** | **A** | **-** | **-** | **-** |
| **N59891F** | **A** | **T** | **G** | **A** | **A** | **T** | **A** | **G** | **T** | **T** | **C** | **T** | **T** | **G** | **G** | **C** | **G** | **T** | **T** | **T** | **T** | **T** | **T** | **T** | **G** | **T** | **G** | **G** | **T** | **A** | **G** | **T** | **C** | **G** | **A** | **A** | **G** | **C** | **A** | **C** | **T** | **G** | **C** | **T** | **G** | **C** | **T** | **G** | **T** | **T** | **A** | **T** | **T** | **T** | **G** | **C** | **T** | **G** | **T** | **T** | **T** | **G** | **G** | **C** | **A** | **A** | **A** | **T** | **C** | **G** | **T** | **A** | **A** | **A** | **C** | **A** | **A** | **T** | **A** | **C** | **T** | **G** | **G** | **A** | **C** | **T** | **T** | **T** | **T** | **G** | **C** | **T** | **T** | **T** | **T** | **A** | **G** | **T** | **A** | **A** | **T** | **A** | **T** | **T** | **C** | **G** | **G** | **T** | **A** | **T** | **T** | **T** | **T** | **C** | **A** | **A** | **T** | **A** | **T** | **A** | **T** | **A** | **T** | **C** | **T** | **A** | **G** | **C** | **T** | **A** | **T** | **G** | **C** | **G** | **C** | **A** | **A** | **A** | **T** | **A** | **T** | **C** | **C** | **T** | **C** | **G** | **T** | **A** | **C** | **T** | **A** | **A** | **A** | **T** | **T** | **T** | **C** | **A** | **A** | **A** | **A** | **T** | **T** | **T** | **T** | **C** | **A** | **A** | **T** | **T** | **G** | **A** | **T** | **C** | **T** | **T** | **A** | **G** | **G** | **G** | **A** | **G** | **C** | **T** | **T** | **G** | **G** | **T** | **C** | **A** | **T** | **T** | **T** | **T** | **T** | **T** | **T** | **T** | **A** | **G** | **C** | **T** | **T** | **A** | **G** | **T** | **C** | **A** | **A** | **C** | **A** | **G** | **T** | **C** | **C** | **T** | **G** | **C** | **T** | **T** | **T** | **A** | **T** | **G** | **G** | **A** | **T** | **G** | **A** | **T** | **G** | **G** | **T** | **T** | **G** | **T** | **A** | **T** | **T** | **G** | **G** | **C** | **C** | **G** | **T** | **T** | **T** | **T** | **A** | **T** | **T** | **T** | **A** | **T** | **C** | **G** | **G** | **G** | **C** | **T** | **G** | **A** | **A** | **A** | **G** | **G** | **T** | **G** | **T** | **A** | **G** | **A** | **G** | **A** | **T** | **T** | **T** | **C** | **A** | **G** | **G** | **A** | **A** | **T** | **A** | **G** | **A** | **T** | **T** | **T** | **G** | **A** | **C** | **A** | **A** | **A** | **A** | **A** | **A** | **T** | **G** | **C** | **T** | **T** | **T** | **T** | **T** | **G** | **G** | **C** | **G** | **A** | **A** | **A** | **A** | **A** | **A** | **A** | **C** | **A** | **G** | **A** | **T** | **C** | **A** | **T** | **G** | **A** | **T** | **G** | **G** | **T** | **C** | **C** | **A** | **A** | **A** | **C** | **A** | **G** | **A** | **G** | **C** | **A** | **A** | **T** | **T** | **A** | **A** | **A** | **A** | **A** | **C** | **A** | **C** | **A** | **T** | **A** | **A** | **T** | **A** | **A** | **T** | **G** | **A** | **G** | **C** | **G** | **T** | **A** | **A** | **A** | **A** | **G** | **A** | **C** | **A** | **G** | **C** | **A** | **A** | **T** | **T** | **A** | **T** | **T** | **T** | **G** | **G** | **T** | **A** | **A** | **C** | **C** | **A** | **A** | **C** | **G** | **T** | **A** | **T** | **T** | **G** | **G** | **A** | **A** | **A** | **C** | **G** | **A** | **C** | **G** | **T** | **C** | **T** | **A** | **T** | **G** | **A** | **A** | **T** | **G** | **G** | **G** | **A** | **T** | **G** | **A** | **T** | **A** | **T** | **C** | **A** | **A** | **C** | **A** | **T** | **T** | **G** | **C** | **C** | **A** | **T** | **C** | **A** | **T** | **T** | **T** | **C** | **T** | **G** | **G** | **T** | **G** | **A** | **T** | **A** | **C** | **T** | **A** | **T** | **C** | **A** | **T** | **C** | **G** | **A** | **T** | **T** | **T** | **G** | **G** | **G** | **C** | **A** | **A** | **T** | **A** | **C** | **A** | **C** | **T** | **T** | **T** | **T** | **G** | **C** | **C** | **A** | **A** | **A** | **A** | **G** | **A** | **T** | **G** | **A** | **T** | **A** | **A** | **T** | **A** | **T** | **T** | **G** | **T** | **G** | **A** | **T** | **T** | **G** | **T** | **A** | **A** | **G** | **A** | **A** | **A** | **A** | **G** | **G** | **G** | **A** | **T** | **A** | **G** | **G** | **C** | **A** | **G** | **A** | **A** | **C** | **A** | **C** | **G** | **G** | **A** | **T** | **C** | **T** | **T** | **A** | **G** | **T** | **A** | **C** | **C** | **C** | **C** | **T** | **G** | **G** | **G** | **A** | **G** | **T** | **G** | **G** | **C** | **T** | **A** | **T** | **T** | **C** | **G** | **T** | **T** | **T** | **A** | **G** | **A** | **G** | **C** | **A** | **T** | **G** | **C** | **G** | **A** | **C** | **A** | **T** | **T** | **A** | **G** | **T** | **A** | **G** | **G** | **A** | **A** | **A** | **T** | **G** | **T** | **C** | **T** | **T** | **G** | **T** | **T** | **T** | **G** | **A** | **A** | **G** | **A** | **A** | **G** | **A** | **G** | **C** | **A** | **G** | **T** | **T** | **T** | **T** | **C** | **C** | **T** | **T** | **A** | **A** | **A** | **A** | **A** | **A** | **C** | **G** | **A** | **A** | **C** | **A** | **G** | **A** | **T** | **C** | **A** | **A** | **A** | **A** | **T** | **C** | **T** | **G** | **C** | **A** | **G** | **C** | **A** | **G** | **T** | **G** | **A** | **T** | **T** | **A** | **T** | **G** | **A** | **T** | **G** | **A** | **A** | **A** | **A** | **T** | **C** | **C** | **T** | **C** | **G** | **C** | **A** | **G** | **A** | **T** | **T** | **G** | **A** | **A** | **G** | **A** | **T** | **C** | **A** | **T** | **T** | **A** | **C** | **C** | **A** | **A** | **T** | **A** | **C** | **C** | **T** | **T** | **G** | **G** | **T** | **T** | **G** | **G** | **A** | **G** | **A** | **T** | **G** | **T** | **C** | **G** | **A** | **G** | **G** | **T** | **G** | **A** | **T** | **A** | **C** | **G** | **A** | **A** | **T** | **A** | **-** | **-** | **-** |
| **N59653F** | **A** | **T** | **G** | **A** | **A** | **T** | **A** | **G** | **T** | **T** | **C** | **T** | **T** | **G** | **G** | **C** | **G** | **T** | **T** | **T** | **T** | **T** | **T** | **T** | **G** | **T** | **G** | **G** | **T** | **A** | **G** | **T** | **T** | **G** | **A** | **A** | **G** | **C** | **A** | **C** | **T** | **A** | **C** | **T** | **G** | **C** | **T** | **G** | **T** | **T** | **A** | **T** | **T** | **T** | **G** | **C** | **T** | **G** | **T** | **T** | **T** | **G** | **G** | **C** | **A** | **A** | **A** | **T** | **C** | **G** | **T** | **A** | **A** | **A** | **C** | **A** | **A** | **T** | **A** | **C** | **T** | **G** | **G** | **A** | **C** | **T** | **T** | **T** | **T** | **G** | **C** | **T** | **T** | **T** | **T** | **A** | **G** | **T** | **G** | **A** | **T** | **A** | **T** | **T** | **C** | **G** | **G** | **T** | **A** | **T** | **T** | **T** | **T** | **C** | **A** | **A** | **T** | **A** | **T** | **A** | **T** | **A** | **T** | **C** | **T** | **A** | **G** | **C** | **T** | **A** | **T** | **G** | **C** | **G** | **C** | **A** | **A** | **A** | **C** | **A** | **T** | **C** | **C** | **T** | **C** | **G** | **T** | **A** | **C** | **T** | **A** | **A** | **A** | **T** | **T** | **T** | **C** | **A** | **A** | **A** | **A** | **T** | **T** | **T** | **T** | **C** | **A** | **A** | **T** | **T** | **G** | **A** | **T** | **A** | **T** | **T** | **A** | **G** | **G** | **A** | **A** | **G** | **T** | **T** | **T** | **G** | **G** | **T** | **C** | **A** | **T** | **T** | **T** | **T** | **T** | **T** | **T** | **T** | **A** | **G** | **C** | **T** | **T** | **A** | **G** | **T** | **C** | **A** | **A** | **C** | **A** | **G** | **T** | **C** | **C** | **T** | **G** | **C** | **T** | **T** | **T** | **A** | **T** | **G** | **G** | **A** | **T** | **G** | **A** | **T** | **G** | **G** | **C** | **T** | **G** | **T** | **A** | **T** | **T** | **T** | **G** | **C** | **C** | **G** | **T** | **T** | **T** | **T** | **A** | **T** | **T** | **T** | **A** | **T** | **T** | **G** | **G** | **T** | **C** | **T** | **G** | **A** | **A** | **A** | **G** | **G** | **T** | **G** | **T** | **A** | **G** | **A** | **G** | **A** | **T** | **T** | **T** | **C** | **A** | **G** | **G** | **G** | **A** | **T** | **A** | **G** | **A** | **T** | **T** | **T** | **A** | **A** | **C** | **C** | **A** | **A** | **A** | **A** | **A** | **T** | **G** | **C** | **T** | **T** | **T** | **T** | **T** | **G** | **G** | **C** | **G** | **A** | **A** | **A** | **G** | **A** | **A** | **A** | **C** | **A** | **G** | **A** | **T** | **C** | **A** | **T** | **G** | **A** | **T** | **G** | **A** | **T** | **C** | **C** | **A** | **A** | **A** | **C** | **A** | **G** | **A** | **G** | **C** | **A** | **A** | **T** | **T** | **A** | **A** | **A** | **A** | **A** | **C** | **A** | **C** | **A** | **T** | **A** | **A** | **T** | **A** | **A** | **T** | **G** | **A** | **G** | **C** | **G** | **G** | **A** | **A** | **A** | **A** | **G** | **A** | **C** | **A** | **G** | **C** | **A** | **A** | **T** | **T** | **A** | **T** | **T** | **C** | **G** | **G** | **T** | **A** | **A** | **C** | **C** | **A** | **A** | **C** | **G** | **T** | **A** | **T** | **T** | **G** | **G** | **A** | **A** | **A** | **C** | **G** | **A** | **C** | **G** | **T** | **C** | **T** | **A** | **T** | **G** | **A** | **A** | **T** | **G** | **G** | **G** | **A** | **C** | **G** | **A** | **T** | **A** | **T** | **C** | **A** | **A** | **C** | **A** | **T** | **C** | **G** | **C** | **C** | **G** | **T** | **C** | **A** | **T** | **T** | **T** | **C** | **T** | **G** | **G** | **T** | **G** | **A** | **T** | **A** | **C** | **T** | **A** | **T** | **C** | **A** | **T** | **A** | **G** | **A** | **T** | **T** | **T** | **G** | **G** | **G** | **C** | **A** | **A** | **T** | **A** | **C** | **G** | **C** | **T** | **T** | **T** | **T** | **G** | **C** | **C** | **A** | **A** | **A** | **A** | **G** | **A** | **T** | **G** | **A** | **T** | **A** | **A** | **T** | **A** | **T** | **T** | **G** | **T** | **G** | **A** | **T** | **T** | **G** | **T** | **A** | **A** | **G** | **A** | **A** | **A** | **A** | **G** | **G** | **G** | **A** | **T** | **A** | **G** | **G** | **C** | **A** | **G** | **A** | **A** | **C** | **A** | **C** | **G** | **G** | **A** | **T** | **C** | **T** | **T** | **A** | **G** | **T** | **A** | **C** | **C** | **T** | **T** | **T** | **G** | **G** | **G** | **C** | **G** | **T** | **A** | **G** | **C** | **C** | **A** | **T** | **C** | **C** | **G** | **T** | **T** | **T** | **A** | **G** | **A** | **G** | **C** | **A** | **T** | **G** | **C** | **G** | **A** | **C** | **A** | **T** | **T** | **A** | **G** | **T** | **A** | **G** | **G** | **A** | **A** | **A** | **T** | **G** | **T** | **C** | **T** | **T** | **G** | **T** | **T** | **T** | **G** | **A** | **A** | **G** | **A** | **A** | **G** | **A** | **G** | **C** | **A** | **A** | **T** | **T** | **T** | **T** | **C** | **T** | **T** | **T** | **G** | **A** | **A** | **A** | **A** | **A** | **T** | **G** | **A** | **A** | **C** | **A** | **A** | **A** | **T** | **C** | **A** | **A** | **A** | **A** | **T** | **C** | **T** | **A** | **C** | **A** | **G** | **C** | **A** | **G** | **T** | **G** | **A** | **T** | **T** | **A** | **T** | **G** | **A** | **T** | **G** | **A** | **A** | **A** | **A** | **C** | **C** | **C** | **T** | **C** | **G** | **T** | **A** | **G** | **A** | **T** | **T** | **G** | **A** | **A** | **G** | **A** | **T** | **T** | **A** | **T** | **T** | **A** | **C** | **T** | **A** | **A** | **T** | **A** | **C** | **C** | **T** | **T** | **G** | **A** | **T** | **T** | **G** | **G** | **A** | **G** | **A** | **T** | **G** | **T** | **C** | **G** | **A** | **G** | **G** | **T** | **G** | **A** | **T** | **A** | **C** | **G** | **A** | **A** | **T** | **A** | **-** | **-** | **-** |
| **N59513F** | **A** | **T** | **G** | **A** | **A** | **T** | **A** | **G** | **T** | **T** | **C** | **T** | **T** | **G** | **G** | **C** | **G** | **T** | **T** | **T** | **T** | **T** | **T** | **T** | **G** | **T** | **G** | **G** | **T** | **A** | **G** | **T** | **T** | **G** | **A** | **A** | **G** | **C** | **A** | **C** | **T** | **A** | **C** | **T** | **G** | **C** | **T** | **G** | **T** | **T** | **A** | **T** | **T** | **T** | **G** | **C** | **T** | **G** | **T** | **T** | **T** | **G** | **G** | **C** | **A** | **A** | **A** | **T** | **C** | **G** | **T** | **A** | **A** | **A** | **C** | **A** | **A** | **T** | **A** | **C** | **T** | **G** | **G** | **A** | **C** | **T** | **T** | **T** | **T** | **G** | **C** | **T** | **T** | **T** | **T** | **A** | **G** | **T** | **G** | **A** | **T** | **A** | **T** | **T** | **C** | **G** | **G** | **T** | **A** | **T** | **T** | **T** | **T** | **C** | **A** | **A** | **T** | **A** | **T** | **A** | **T** | **A** | **T** | **C** | **T** | **A** | **G** | **C** | **T** | **A** | **T** | **G** | **C** | **G** | **C** | **A** | **A** | **A** | **C** | **A** | **T** | **C** | **C** | **T** | **C** | **G** | **T** | **A** | **C** | **T** | **A** | **A** | **A** | **T** | **T** | **T** | **C** | **A** | **A** | **A** | **A** | **T** | **T** | **T** | **T** | **C** | **A** | **A** | **T** | **T** | **G** | **A** | **T** | **A** | **T** | **T** | **A** | **G** | **G** | **A** | **A** | **G** | **T** | **T** | **T** | **G** | **G** | **T** | **C** | **A** | **T** | **T** | **T** | **T** | **T** | **T** | **T** | **T** | **A** | **G** | **C** | **T** | **T** | **A** | **G** | **T** | **C** | **A** | **A** | **C** | **A** | **G** | **T** | **C** | **C** | **T** | **G** | **C** | **T** | **T** | **T** | **A** | **T** | **G** | **G** | **A** | **T** | **G** | **A** | **T** | **G** | **G** | **C** | **T** | **G** | **T** | **A** | **T** | **T** | **T** | **G** | **C** | **C** | **G** | **T** | **T** | **T** | **T** | **A** | **T** | **T** | **T** | **A** | **T** | **T** | **G** | **G** | **T** | **C** | **T** | **G** | **A** | **A** | **A** | **G** | **G** | **T** | **G** | **T** | **A** | **G** | **A** | **G** | **A** | **T** | **T** | **T** | **C** | **A** | **G** | **G** | **G** | **A** | **T** | **A** | **G** | **A** | **T** | **T** | **T** | **A** | **A** | **C** | **C** | **A** | **A** | **A** | **A** | **A** | **T** | **G** | **C** | **T** | **T** | **T** | **T** | **T** | **G** | **G** | **C** | **G** | **A** | **A** | **A** | **G** | **A** | **A** | **A** | **C** | **A** | **G** | **A** | **T** | **C** | **A** | **T** | **G** | **A** | **T** | **G** | **A** | **T** | **C** | **C** | **A** | **A** | **A** | **C** | **A** | **G** | **A** | **G** | **C** | **A** | **A** | **T** | **T** | **A** | **A** | **A** | **A** | **A** | **C** | **A** | **C** | **A** | **T** | **A** | **A** | **T** | **A** | **A** | **T** | **G** | **A** | **G** | **C** | **G** | **G** | **A** | **A** | **A** | **A** | **G** | **A** | **C** | **A** | **G** | **C** | **A** | **A** | **T** | **T** | **A** | **T** | **T** | **C** | **G** | **G** | **T** | **A** | **A** | **C** | **C** | **A** | **A** | **C** | **G** | **T** | **A** | **T** | **T** | **G** | **G** | **A** | **A** | **A** | **C** | **G** | **A** | **C** | **G** | **T** | **C** | **T** | **A** | **T** | **G** | **A** | **A** | **T** | **G** | **G** | **G** | **A** | **C** | **G** | **A** | **T** | **A** | **T** | **C** | **A** | **A** | **C** | **A** | **T** | **C** | **G** | **C** | **C** | **G** | **T** | **C** | **A** | **T** | **T** | **T** | **C** | **T** | **G** | **G** | **T** | **G** | **A** | **T** | **A** | **C** | **T** | **A** | **T** | **C** | **A** | **T** | **A** | **G** | **A** | **T** | **T** | **T** | **G** | **G** | **G** | **C** | **A** | **A** | **T** | **A** | **C** | **G** | **C** | **T** | **T** | **T** | **T** | **G** | **C** | **C** | **A** | **A** | **A** | **A** | **G** | **A** | **T** | **G** | **A** | **T** | **A** | **A** | **T** | **A** | **T** | **T** | **G** | **T** | **G** | **A** | **T** | **T** | **G** | **T** | **A** | **A** | **G** | **A** | **A** | **A** | **A** | **G** | **G** | **G** | **A** | **T** | **A** | **G** | **G** | **C** | **A** | **G** | **A** | **A** | **C** | **A** | **C** | **G** | **G** | **A** | **T** | **C** | **T** | **T** | **A** | **G** | **T** | **A** | **C** | **C** | **T** | **T** | **T** | **G** | **G** | **G** | **C** | **G** | **T** | **A** | **G** | **C** | **C** | **A** | **T** | **C** | **C** | **G** | **T** | **T** | **T** | **A** | **G** | **A** | **G** | **C** | **A** | **T** | **G** | **C** | **G** | **A** | **C** | **A** | **T** | **T** | **A** | **G** | **T** | **A** | **G** | **G** | **A** | **A** | **A** | **T** | **G** | **T** | **C** | **T** | **T** | **G** | **T** | **T** | **T** | **G** | **A** | **A** | **G** | **A** | **A** | **G** | **A** | **G** | **C** | **A** | **A** | **T** | **T** | **T** | **T** | **C** | **T** | **T** | **T** | **G** | **A** | **A** | **A** | **A** | **A** | **T** | **G** | **A** | **A** | **C** | **A** | **A** | **A** | **T** | **C** | **A** | **A** | **A** | **A** | **T** | **C** | **T** | **A** | **C** | **A** | **G** | **C** | **A** | **G** | **T** | **G** | **A** | **T** | **T** | **A** | **T** | **G** | **A** | **T** | **G** | **A** | **A** | **A** | **A** | **C** | **C** | **C** | **T** | **C** | **G** | **T** | **A** | **G** | **A** | **T** | **T** | **G** | **A** | **A** | **G** | **A** | **T** | **T** | **A** | **T** | **T** | **A** | **C** | **T** | **A** | **A** | **T** | **A** | **C** | **C** | **T** | **T** | **G** | **A** | **T** | **T** | **G** | **G** | **A** | **G** | **A** | **T** | **G** | **T** | **C** | **G** | **A** | **G** | **G** | **T** | **G** | **A** | **T** | **A** | **C** | **G** | **A** | **A** | **T** | **A** | **-** | **-** | **-** |
| **N59531F** | **A** | **T** | **G** | **A** | **A** | **T** | **A** | **G** | **T** | **T** | **C** | **T** | **T** | **G** | **G** | **C** | **G** | **T** | **T** | **T** | **T** | **T** | **T** | **T** | **G** | **T** | **G** | **G** | **T** | **A** | **G** | **T** | **T** | **G** | **A** | **A** | **G** | **C** | **A** | **C** | **T** | **A** | **C** | **T** | **G** | **C** | **T** | **G** | **T** | **T** | **A** | **T** | **T** | **T** | **G** | **C** | **T** | **G** | **T** | **T** | **T** | **G** | **G** | **C** | **A** | **A** | **A** | **T** | **C** | **G** | **T** | **A** | **A** | **A** | **C** | **A** | **A** | **T** | **A** | **C** | **T** | **G** | **G** | **A** | **C** | **T** | **T** | **T** | **T** | **G** | **C** | **T** | **T** | **T** | **T** | **A** | **G** | **T** | **G** | **A** | **T** | **A** | **T** | **T** | **C** | **G** | **G** | **T** | **A** | **T** | **T** | **T** | **T** | **C** | **A** | **A** | **T** | **A** | **T** | **A** | **T** | **A** | **T** | **C** | **T** | **A** | **G** | **C** | **T** | **A** | **T** | **G** | **C** | **G** | **C** | **A** | **A** | **A** | **C** | **A** | **T** | **C** | **C** | **T** | **C** | **G** | **T** | **A** | **C** | **T** | **A** | **A** | **A** | **T** | **T** | **T** | **C** | **A** | **A** | **A** | **A** | **T** | **T** | **T** | **T** | **C** | **A** | **A** | **T** | **T** | **G** | **A** | **T** | **A** | **T** | **T** | **A** | **G** | **G** | **A** | **A** | **G** | **T** | **T** | **T** | **G** | **G** | **T** | **C** | **A** | **T** | **T** | **T** | **T** | **T** | **T** | **T** | **T** | **A** | **G** | **C** | **T** | **T** | **A** | **G** | **T** | **C** | **A** | **A** | **C** | **A** | **G** | **T** | **C** | **C** | **T** | **G** | **C** | **T** | **T** | **T** | **A** | **T** | **G** | **G** | **A** | **T** | **G** | **A** | **T** | **G** | **G** | **C** | **T** | **G** | **T** | **A** | **T** | **T** | **T** | **G** | **C** | **C** | **G** | **T** | **T** | **T** | **T** | **A** | **T** | **T** | **T** | **A** | **T** | **T** | **G** | **G** | **A** | **C** | **T** | **A** | **A** | **A** | **A** | **G** | **G** | **T** | **G** | **T** | **A** | **G** | **A** | **G** | **A** | **T** | **T** | **T** | **C** | **A** | **G** | **G** | **G** | **A** | **T** | **A** | **G** | **A** | **T** | **T** | **T** | **A** | **A** | **C** | **C** | **A** | **A** | **A** | **A** | **A** | **T** | **G** | **C** | **T** | **T** | **T** | **T** | **T** | **G** | **G** | **C** | **G** | **A** | **A** | **A** | **G** | **A** | **A** | **A** | **C** | **A** | **G** | **A** | **T** | **C** | **A** | **T** | **G** | **A** | **T** | **G** | **G** | **T** | **C** | **C** | **A** | **A** | **A** | **C** | **A** | **G** | **A** | **G** | **C** | **A** | **A** | **T** | **T** | **A** | **A** | **A** | **A** | **A** | **C** | **A** | **C** | **A** | **T** | **A** | **A** | **T** | **A** | **A** | **T** | **G** | **A** | **G** | **C** | **G** | **G** | **A** | **A** | **A** | **A** | **G** | **A** | **C** | **A** | **G** | **C** | **A** | **A** | **T** | **T** | **A** | **T** | **T** | **C** | **G** | **G** | **T** | **A** | **A** | **C** | **C** | **A** | **A** | **C** | **G** | **T** | **A** | **T** | **T** | **G** | **G** | **A** | **A** | **A** | **C** | **G** | **A** | **C** | **G** | **T** | **C** | **T** | **A** | **T** | **G** | **A** | **A** | **T** | **G** | **G** | **G** | **A** | **C** | **G** | **A** | **T** | **A** | **T** | **C** | **A** | **A** | **C** | **A** | **T** | **C** | **G** | **C** | **C** | **G** | **T** | **C** | **A** | **T** | **T** | **T** | **C** | **T** | **G** | **G** | **T** | **G** | **A** | **T** | **A** | **C** | **T** | **A** | **T** | **C** | **A** | **T** | **A** | **G** | **A** | **T** | **T** | **T** | **G** | **G** | **G** | **C** | **A** | **A** | **T** | **A** | **C** | **G** | **C** | **T** | **T** | **T** | **T** | **G** | **C** | **C** | **A** | **A** | **A** | **A** | **G** | **A** | **T** | **G** | **A** | **T** | **A** | **A** | **T** | **A** | **T** | **T** | **G** | **T** | **G** | **A** | **T** | **T** | **G** | **T** | **A** | **A** | **G** | **A** | **A** | **A** | **A** | **G** | **G** | **G** | **A** | **T** | **A** | **G** | **G** | **C** | **A** | **G** | **A** | **A** | **C** | **A** | **C** | **G** | **A** | **A** | **T** | **C** | **T** | **T** | **A** | **G** | **T** | **A** | **C** | **C** | **T** | **T** | **T** | **G** | **G** | **G** | **C** | **G** | **T** | **A** | **G** | **C** | **C** | **A** | **T** | **C** | **C** | **G** | **A** | **T** | **T** | **A** | **G** | **A** | **G** | **C** | **A** | **T** | **G** | **C** | **G** | **A** | **C** | **A** | **T** | **T** | **A** | **G** | **T** | **A** | **G** | **G** | **A** | **A** | **A** | **T** | **G** | **T** | **C** | **T** | **T** | **G** | **T** | **T** | **T** | **G** | **A** | **A** | **G** | **A** | **A** | **G** | **A** | **G** | **C** | **A** | **A** | **T** | **T** | **T** | **T** | **C** | **T** | **T** | **T** | **G** | **A** | **A** | **A** | **A** | **A** | **T** | **G** | **A** | **A** | **C** | **A** | **A** | **A** | **T** | **C** | **A** | **A** | **A** | **A** | **T** | **C** | **T** | **A** | **C** | **A** | **G** | **C** | **A** | **G** | **T** | **G** | **A** | **T** | **T** | **A** | **T** | **G** | **A** | **T** | **G** | **A** | **A** | **A** | **A** | **C** | **C** | **C** | **T** | **C** | **G** | **T** | **A** | **G** | **A** | **C** | **T** | **G** | **A** | **A** | **G** | **A** | **T** | **T** | **A** | **T** | **T** | **A** | **C** | **T** | **A** | **A** | **T** | **A** | **C** | **C** | **T** | **T** | **G** | **A** | **T** | **T** | **G** | **G** | **A** | **G** | **A** | **T** | **G** | **T** | **C** | **G** | **A** | **G** | **G** | **T** | **G** | **A** | **T** | **A** | **C** | **G** | **A** | **A** | **T** | **A** | **-** | **-** | **-** |
| **N60490F** | **A** | **T** | **G** | **A** | **A** | **T** | **A** | **G** | **T** | **T** | **C** | **T** | **T** | **G** | **G** | **C** | **G** | **T** | **T** | **T** | **T** | **T** | **T** | **T** | **G** | **T** | **G** | **G** | **T** | **A** | **G** | **T** | **C** | **G** | **A** | **A** | **G** | **C** | **A** | **C** | **T** | **G** | **C** | **T** | **G** | **C** | **T** | **G** | **T** | **T** | **A** | **T** | **T** | **T** | **G** | **C** | **T** | **G** | **T** | **T** | **T** | **G** | **G** | **G** | **A** | **A** | **A** | **T** | **C** | **G** | **T** | **A** | **A** | **A** | **C** | **A** | **A** | **T** | **A** | **C** | **T** | **G** | **G** | **A** | **C** | **T** | **T** | **T** | **T** | **G** | **C** | **T** | **T** | **T** | **T** | **A** | **G** | **T** | **A** | **A** | **T** | **A** | **T** | **T** | **C** | **G** | **G** | **T** | **A** | **T** | **T** | **T** | **T** | **C** | **A** | **A** | **T** | **A** | **T** | **A** | **T** | **A** | **T** | **C** | **T** | **A** | **G** | **C** | **T** | **A** | **T** | **G** | **C** | **G** | **C** | **A** | **A** | **A** | **T** | **A** | **T** | **C** | **C** | **T** | **C** | **G** | **T** | **A** | **C** | **T** | **A** | **A** | **A** | **T** | **T** | **T** | **C** | **A** | **A** | **A** | **A** | **T** | **T** | **T** | **T** | **C** | **A** | **A** | **T** | **T** | **G** | **A** | **T** | **C** | **T** | **T** | **A** | **G** | **G** | **G** | **A** | **G** | **C** | **T** | **T** | **G** | **G** | **T** | **C** | **A** | **T** | **T** | **T** | **T** | **T** | **T** | **T** | **T** | **A** | **G** | **C** | **T** | **T** | **A** | **G** | **T** | **C** | **A** | **A** | **C** | **A** | **G** | **T** | **C** | **C** | **T** | **G** | **C** | **T** | **T** | **T** | **A** | **T** | **G** | **G** | **A** | **T** | **G** | **A** | **T** | **G** | **G** | **T** | **T** | **G** | **T** | **A** | **T** | **T** | **G** | **G** | **C** | **C** | **G** | **T** | **T** | **T** | **T** | **A** | **T** | **T** | **T** | **A** | **T** | **C** | **G** | **G** | **G** | **C** | **T** | **G** | **A** | **A** | **A** | **G** | **G** | **T** | **G** | **T** | **A** | **G** | **A** | **G** | **A** | **T** | **T** | **T** | **C** | **A** | **G** | **G** | **A** | **A** | **T** | **A** | **G** | **A** | **T** | **T** | **T** | **G** | **A** | **C** | **A** | **A** | **A** | **A** | **A** | **A** | **T** | **G** | **C** | **T** | **T** | **T** | **T** | **T** | **G** | **G** | **C** | **G** | **A** | **A** | **A** | **A** | **A** | **A** | **A** | **C** | **A** | **G** | **A** | **T** | **C** | **A** | **T** | **G** | **A** | **T** | **G** | **G** | **T** | **C** | **C** | **A** | **A** | **A** | **C** | **A** | **G** | **A** | **G** | **C** | **A** | **A** | **T** | **T** | **A** | **A** | **A** | **A** | **A** | **C** | **A** | **C** | **A** | **T** | **A** | **A** | **T** | **A** | **A** | **T** | **G** | **A** | **G** | **C** | **G** | **T** | **A** | **A** | **A** | **A** | **G** | **A** | **C** | **A** | **G** | **C** | **A** | **A** | **T** | **T** | **A** | **T** | **T** | **T** | **G** | **G** | **T** | **A** | **A** | **C** | **C** | **A** | **A** | **C** | **G** | **T** | **A** | **T** | **T** | **G** | **G** | **A** | **A** | **A** | **C** | **G** | **A** | **C** | **G** | **T** | **C** | **T** | **A** | **T** | **G** | **A** | **A** | **T** | **G** | **G** | **G** | **A** | **T** | **G** | **A** | **T** | **A** | **T** | **C** | **A** | **A** | **C** | **A** | **T** | **T** | **G** | **C** | **C** | **A** | **T** | **C** | **A** | **T** | **T** | **T** | **C** | **T** | **G** | **G** | **T** | **G** | **A** | **T** | **A** | **C** | **T** | **A** | **T** | **C** | **A** | **T** | **C** | **G** | **A** | **T** | **T** | **T** | **G** | **G** | **G** | **C** | **A** | **A** | **T** | **A** | **C** | **A** | **C** | **T** | **T** | **T** | **T** | **G** | **C** | **C** | **A** | **A** | **A** | **A** | **G** | **A** | **T** | **G** | **A** | **T** | **A** | **A** | **T** | **A** | **T** | **T** | **G** | **T** | **G** | **A** | **T** | **T** | **G** | **T** | **A** | **A** | **G** | **A** | **A** | **A** | **A** | **G** | **G** | **G** | **A** | **T** | **A** | **G** | **G** | **C** | **A** | **G** | **A** | **A** | **C** | **A** | **C** | **G** | **G** | **A** | **T** | **C** | **T** | **T** | **A** | **G** | **T** | **A** | **C** | **C** | **C** | **C** | **T** | **G** | **G** | **G** | **A** | **G** | **T** | **G** | **G** | **C** | **T** | **A** | **T** | **T** | **C** | **G** | **T** | **T** | **T** | **A** | **G** | **A** | **G** | **C** | **A** | **T** | **G** | **C** | **G** | **A** | **C** | **A** | **T** | **T** | **A** | **G** | **T** | **A** | **G** | **G** | **A** | **A** | **A** | **T** | **G** | **T** | **C** | **T** | **T** | **G** | **T** | **T** | **T** | **G** | **A** | **A** | **G** | **A** | **A** | **G** | **A** | **G** | **C** | **A** | **G** | **T** | **T** | **T** | **T** | **C** | **C** | **T** | **T** | **A** | **A** | **A** | **A** | **A** | **A** | **C** | **G** | **A** | **A** | **C** | **A** | **G** | **A** | **T** | **C** | **A** | **A** | **A** | **A** | **T** | **C** | **T** | **G** | **C** | **A** | **G** | **C** | **A** | **G** | **T** | **G** | **A** | **T** | **T** | **A** | **T** | **G** | **A** | **T** | **G** | **A** | **A** | **A** | **A** | **T** | **C** | **C** | **T** | **C** | **G** | **C** | **A** | **G** | **A** | **T** | **T** | **G** | **A** | **A** | **G** | **A** | **T** | **C** | **A** | **T** | **T** | **A** | **C** | **C** | **A** | **A** | **T** | **A** | **C** | **C** | **T** | **T** | **G** | **G** | **T** | **T** | **G** | **G** | **A** | **G** | **A** | **T** | **G** | **T** | **C** | **G** | **A** | **G** | **G** | **T** | **G** | **A** | **T** | **A** | **C** | **G** | **A** | **A** | **T** | **A** | **-** | **-** | **-** |
| **N59788F** | **A** | **T** | **G** | **A** | **A** | **T** | **A** | **G** | **T** | **T** | **C** | **T** | **T** | **G** | **G** | **C** | **G** | **T** | **T** | **T** | **T** | **T** | **T** | **T** | **G** | **T** | **G** | **G** | **T** | **A** | **G** | **T** | **C** | **G** | **A** | **A** | **G** | **C** | **A** | **C** | **T** | **G** | **C** | **T** | **G** | **C** | **T** | **G** | **T** | **T** | **A** | **T** | **T** | **T** | **G** | **C** | **T** | **G** | **T** | **T** | **T** | **G** | **G** | **C** | **A** | **A** | **A** | **T** | **C** | **G** | **T** | **A** | **A** | **A** | **C** | **A** | **A** | **T** | **A** | **C** | **T** | **G** | **G** | **A** | **C** | **T** | **T** | **T** | **T** | **G** | **C** | **T** | **T** | **T** | **T** | **A** | **G** | **T** | **A** | **A** | **T** | **A** | **T** | **T** | **C** | **G** | **G** | **T** | **A** | **T** | **T** | **T** | **T** | **C** | **A** | **A** | **T** | **A** | **T** | **A** | **T** | **A** | **T** | **C** | **T** | **A** | **G** | **C** | **T** | **A** | **T** | **G** | **C** | **G** | **C** | **A** | **A** | **A** | **T** | **A** | **T** | **C** | **C** | **T** | **C** | **G** | **T** | **A** | **C** | **T** | **A** | **A** | **A** | **T** | **T** | **T** | **C** | **A** | **A** | **A** | **A** | **T** | **T** | **T** | **T** | **C** | **A** | **A** | **T** | **T** | **G** | **A** | **T** | **C** | **T** | **T** | **A** | **G** | **G** | **G** | **A** | **G** | **C** | **T** | **T** | **G** | **G** | **T** | **C** | **A** | **T** | **T** | **T** | **T** | **T** | **T** | **T** | **T** | **A** | **G** | **C** | **T** | **T** | **A** | **G** | **T** | **C** | **A** | **A** | **C** | **A** | **G** | **T** | **C** | **C** | **T** | **G** | **C** | **T** | **T** | **T** | **A** | **T** | **G** | **G** | **A** | **T** | **G** | **A** | **T** | **G** | **G** | **T** | **T** | **G** | **T** | **A** | **T** | **T** | **G** | **G** | **C** | **C** | **G** | **T** | **T** | **T** | **T** | **A** | **T** | **T** | **T** | **A** | **T** | **T** | **G** | **G** | **G** | **C** | **T** | **G** | **A** | **A** | **A** | **G** | **G** | **T** | **G** | **T** | **A** | **G** | **A** | **G** | **A** | **T** | **T** | **T** | **C** | **A** | **G** | **G** | **A** | **A** | **T** | **A** | **G** | **A** | **T** | **T** | **T** | **G** | **A** | **C** | **A** | **A** | **A** | **A** | **A** | **A** | **T** | **G** | **C** | **T** | **T** | **T** | **T** | **T** | **G** | **G** | **C** | **G** | **A** | **A** | **A** | **A** | **A** | **A** | **A** | **C** | **A** | **G** | **A** | **T** | **C** | **A** | **T** | **G** | **A** | **T** | **G** | **G** | **T** | **C** | **C** | **A** | **A** | **A** | **C** | **A** | **G** | **A** | **G** | **C** | **A** | **A** | **T** | **T** | **A** | **A** | **A** | **A** | **A** | **C** | **A** | **C** | **A** | **T** | **A** | **A** | **T** | **A** | **A** | **T** | **G** | **A** | **G** | **C** | **G** | **T** | **A** | **A** | **A** | **A** | **G** | **A** | **C** | **A** | **G** | **C** | **A** | **A** | **T** | **T** | **A** | **T** | **T** | **T** | **G** | **G** | **T** | **A** | **A** | **C** | **C** | **A** | **A** | **C** | **G** | **T** | **A** | **T** | **T** | **G** | **G** | **A** | **A** | **A** | **C** | **G** | **A** | **C** | **G** | **T** | **C** | **T** | **A** | **T** | **G** | **A** | **A** | **T** | **G** | **G** | **G** | **A** | **T** | **G** | **A** | **T** | **A** | **T** | **C** | **A** | **A** | **C** | **A** | **T** | **T** | **G** | **C** | **C** | **A** | **T** | **C** | **A** | **T** | **T** | **T** | **C** | **T** | **G** | **G** | **T** | **G** | **A** | **T** | **A** | **C** | **T** | **A** | **T** | **C** | **A** | **T** | **C** | **G** | **A** | **T** | **T** | **T** | **G** | **G** | **G** | **T** | **A** | **A** | **T** | **A** | **C** | **A** | **C** | **T** | **T** | **T** | **T** | **G** | **C** | **C** | **A** | **A** | **A** | **A** | **G** | **A** | **T** | **G** | **A** | **T** | **A** | **A** | **T** | **A** | **T** | **T** | **G** | **T** | **G** | **A** | **T** | **T** | **G** | **T** | **A** | **A** | **G** | **A** | **A** | **A** | **A** | **G** | **G** | **G** | **A** | **T** | **A** | **G** | **G** | **C** | **A** | **G** | **A** | **A** | **C** | **A** | **C** | **G** | **G** | **A** | **T** | **C** | **T** | **T** | **A** | **G** | **T** | **A** | **C** | **C** | **C** | **C** | **T** | **G** | **G** | **G** | **A** | **G** | **T** | **G** | **G** | **C** | **T** | **A** | **T** | **T** | **C** | **G** | **T** | **T** | **T** | **A** | **G** | **A** | **G** | **C** | **A** | **T** | **G** | **C** | **G** | **A** | **C** | **A** | **T** | **T** | **A** | **G** | **T** | **A** | **G** | **G** | **A** | **A** | **A** | **T** | **G** | **T** | **C** | **T** | **T** | **G** | **T** | **T** | **T** | **G** | **A** | **A** | **G** | **A** | **A** | **G** | **A** | **G** | **C** | **A** | **G** | **T** | **T** | **T** | **T** | **C** | **C** | **T** | **T** | **A** | **A** | **A** | **A** | **A** | **A** | **C** | **G** | **A** | **A** | **C** | **A** | **G** | **A** | **T** | **C** | **A** | **A** | **A** | **A** | **T** | **C** | **T** | **G** | **C** | **A** | **G** | **C** | **A** | **G** | **T** | **G** | **A** | **T** | **T** | **A** | **T** | **G** | **A** | **T** | **G** | **A** | **A** | **A** | **A** | **T** | **C** | **C** | **T** | **C** | **G** | **C** | **A** | **G** | **A** | **T** | **T** | **G** | **A** | **A** | **G** | **A** | **T** | **C** | **A** | **T** | **T** | **A** | **C** | **C** | **A** | **A** | **T** | **A** | **C** | **C** | **T** | **T** | **G** | **G** | **T** | **T** | **G** | **G** | **A** | **G** | **A** | **T** | **G** | **T** | **C** | **G** | **A** | **G** | **G** | **T** | **G** | **A** | **T** | **A** | **C** | **G** | **A** | **A** | **T** | **A** | **-** | **-** | **-** |
| **N60463F** | **A** | **T** | **G** | **A** | **A** | **T** | **A** | **G** | **T** | **T** | **C** | **T** | **T** | **G** | **G** | **C** | **G** | **T** | **T** | **T** | **T** | **T** | **T** | **T** | **G** | **T** | **G** | **G** | **T** | **A** | **G** | **T** | **C** | **G** | **A** | **A** | **G** | **C** | **A** | **C** | **T** | **G** | **C** | **T** | **G** | **C** | **T** | **G** | **T** | **T** | **A** | **T** | **T** | **T** | **G** | **C** | **T** | **G** | **T** | **T** | **T** | **G** | **G** | **G** | **A** | **A** | **A** | **T** | **C** | **G** | **T** | **A** | **A** | **A** | **C** | **A** | **A** | **T** | **A** | **C** | **T** | **G** | **G** | **A** | **C** | **T** | **T** | **T** | **T** | **G** | **C** | **T** | **T** | **T** | **T** | **A** | **G** | **T** | **A** | **A** | **T** | **A** | **T** | **T** | **C** | **G** | **G** | **T** | **A** | **T** | **T** | **T** | **T** | **C** | **A** | **A** | **T** | **A** | **T** | **A** | **T** | **A** | **T** | **C** | **T** | **A** | **G** | **C** | **T** | **A** | **T** | **G** | **C** | **G** | **C** | **A** | **A** | **A** | **T** | **A** | **T** | **C** | **C** | **T** | **C** | **G** | **T** | **A** | **C** | **T** | **A** | **A** | **A** | **T** | **T** | **T** | **C** | **A** | **A** | **A** | **A** | **T** | **T** | **T** | **T** | **C** | **A** | **A** | **T** | **T** | **G** | **A** | **T** | **C** | **T** | **T** | **A** | **G** | **G** | **G** | **A** | **G** | **C** | **T** | **T** | **G** | **G** | **T** | **C** | **A** | **T** | **T** | **T** | **T** | **T** | **T** | **T** | **T** | **A** | **G** | **C** | **T** | **T** | **A** | **G** | **T** | **C** | **A** | **A** | **C** | **A** | **G** | **T** | **C** | **C** | **T** | **G** | **C** | **T** | **T** | **T** | **A** | **T** | **G** | **G** | **A** | **T** | **G** | **A** | **T** | **G** | **G** | **T** | **T** | **G** | **T** | **A** | **T** | **T** | **G** | **G** | **C** | **C** | **G** | **T** | **T** | **T** | **T** | **A** | **T** | **T** | **T** | **A** | **T** | **C** | **G** | **G** | **G** | **C** | **T** | **G** | **A** | **A** | **A** | **G** | **G** | **T** | **G** | **T** | **A** | **G** | **A** | **G** | **A** | **T** | **T** | **T** | **C** | **A** | **G** | **G** | **A** | **A** | **T** | **A** | **G** | **A** | **T** | **T** | **T** | **G** | **A** | **C** | **A** | **A** | **A** | **A** | **A** | **A** | **T** | **G** | **C** | **T** | **T** | **T** | **T** | **T** | **G** | **G** | **C** | **G** | **A** | **A** | **A** | **A** | **A** | **A** | **A** | **C** | **A** | **G** | **A** | **T** | **C** | **A** | **T** | **G** | **A** | **T** | **G** | **G** | **T** | **C** | **C** | **A** | **A** | **A** | **C** | **A** | **G** | **A** | **G** | **C** | **A** | **A** | **T** | **T** | **A** | **A** | **A** | **A** | **A** | **C** | **A** | **C** | **A** | **T** | **A** | **A** | **T** | **A** | **A** | **T** | **G** | **A** | **G** | **C** | **G** | **T** | **A** | **A** | **A** | **A** | **G** | **A** | **C** | **A** | **G** | **C** | **A** | **A** | **T** | **T** | **A** | **T** | **T** | **T** | **G** | **G** | **T** | **A** | **A** | **C** | **C** | **A** | **A** | **C** | **G** | **T** | **A** | **T** | **T** | **G** | **G** | **A** | **A** | **A** | **C** | **G** | **A** | **C** | **G** | **T** | **C** | **T** | **A** | **T** | **G** | **A** | **A** | **T** | **G** | **G** | **G** | **A** | **T** | **G** | **A** | **T** | **A** | **T** | **C** | **A** | **A** | **C** | **A** | **T** | **T** | **G** | **C** | **C** | **A** | **T** | **C** | **A** | **T** | **T** | **T** | **C** | **T** | **G** | **G** | **T** | **G** | **A** | **T** | **A** | **C** | **T** | **A** | **T** | **C** | **A** | **T** | **C** | **G** | **A** | **T** | **T** | **T** | **G** | **G** | **G** | **C** | **A** | **A** | **T** | **A** | **C** | **A** | **C** | **T** | **T** | **T** | **T** | **G** | **C** | **C** | **A** | **A** | **A** | **A** | **G** | **A** | **T** | **G** | **A** | **T** | **A** | **A** | **T** | **A** | **T** | **T** | **G** | **T** | **G** | **A** | **T** | **T** | **G** | **T** | **A** | **A** | **G** | **A** | **A** | **A** | **A** | **G** | **G** | **G** | **A** | **T** | **A** | **G** | **G** | **C** | **A** | **G** | **A** | **A** | **C** | **A** | **C** | **G** | **G** | **A** | **T** | **C** | **T** | **T** | **A** | **G** | **T** | **A** | **C** | **C** | **C** | **C** | **T** | **G** | **G** | **G** | **A** | **G** | **T** | **G** | **G** | **C** | **T** | **A** | **T** | **T** | **C** | **G** | **T** | **T** | **T** | **A** | **G** | **A** | **G** | **C** | **A** | **T** | **G** | **C** | **G** | **A** | **C** | **A** | **T** | **T** | **A** | **G** | **T** | **A** | **G** | **G** | **A** | **A** | **A** | **T** | **G** | **T** | **C** | **T** | **T** | **G** | **T** | **T** | **T** | **G** | **A** | **A** | **G** | **A** | **A** | **G** | **A** | **G** | **C** | **A** | **G** | **T** | **T** | **T** | **T** | **C** | **C** | **T** | **T** | **A** | **A** | **A** | **A** | **A** | **A** | **C** | **G** | **A** | **A** | **C** | **A** | **G** | **A** | **T** | **C** | **A** | **A** | **A** | **A** | **T** | **C** | **T** | **G** | **C** | **A** | **G** | **C** | **A** | **G** | **T** | **G** | **A** | **T** | **T** | **A** | **T** | **G** | **A** | **T** | **G** | **A** | **A** | **A** | **A** | **T** | **C** | **C** | **T** | **C** | **G** | **C** | **A** | **G** | **A** | **T** | **T** | **G** | **A** | **A** | **G** | **A** | **T** | **C** | **A** | **T** | **T** | **A** | **C** | **C** | **A** | **A** | **T** | **A** | **C** | **C** | **T** | **T** | **G** | **G** | **T** | **T** | **G** | **G** | **A** | **G** | **A** | **T** | **G** | **T** | **C** | **G** | **A** | **G** | **G** | **T** | **G** | **A** | **T** | **A** | **C** | **G** | **A** | **A** | **T** | **A** | **-** | **-** | **-** |
| **N59469F** | **A** | **T** | **G** | **A** | **A** | **T** | **A** | **G** | **T** | **T** | **C** | **T** | **T** | **G** | **G** | **C** | **G** | **T** | **T** | **T** | **T** | **T** | **T** | **T** | **G** | **T** | **G** | **G** | **T** | **A** | **G** | **T** | **C** | **G** | **A** | **A** | **G** | **C** | **A** | **C** | **T** | **G** | **C** | **T** | **G** | **C** | **T** | **G** | **T** | **T** | **A** | **T** | **T** | **T** | **G** | **C** | **T** | **G** | **T** | **T** | **T** | **G** | **G** | **C** | **A** | **A** | **A** | **T** | **C** | **G** | **T** | **A** | **A** | **A** | **C** | **A** | **A** | **T** | **A** | **C** | **T** | **G** | **G** | **A** | **C** | **T** | **T** | **T** | **T** | **G** | **C** | **T** | **T** | **T** | **T** | **A** | **G** | **T** | **A** | **A** | **T** | **A** | **T** | **T** | **C** | **G** | **G** | **T** | **A** | **T** | **T** | **T** | **T** | **C** | **A** | **A** | **T** | **A** | **T** | **A** | **T** | **A** | **T** | **C** | **T** | **A** | **G** | **C** | **T** | **A** | **T** | **G** | **C** | **G** | **C** | **A** | **A** | **A** | **T** | **A** | **T** | **C** | **C** | **T** | **C** | **G** | **T** | **A** | **C** | **T** | **A** | **A** | **A** | **T** | **T** | **T** | **C** | **A** | **A** | **A** | **A** | **T** | **T** | **T** | **T** | **C** | **A** | **A** | **T** | **T** | **G** | **A** | **T** | **C** | **T** | **T** | **A** | **G** | **G** | **G** | **A** | **G** | **C** | **T** | **T** | **G** | **G** | **T** | **C** | **A** | **T** | **T** | **T** | **T** | **T** | **T** | **T** | **T** | **A** | **G** | **C** | **T** | **T** | **A** | **G** | **T** | **C** | **A** | **A** | **C** | **A** | **G** | **T** | **C** | **C** | **T** | **G** | **C** | **T** | **T** | **T** | **A** | **T** | **G** | **G** | **A** | **T** | **G** | **A** | **T** | **G** | **G** | **T** | **T** | **G** | **T** | **A** | **T** | **T** | **G** | **G** | **C** | **C** | **G** | **T** | **T** | **T** | **T** | **A** | **T** | **T** | **T** | **A** | **T** | **C** | **G** | **G** | **C** | **C** | **T** | **G** | **A** | **A** | **A** | **G** | **G** | **T** | **G** | **T** | **A** | **G** | **A** | **G** | **A** | **T** | **T** | **T** | **C** | **A** | **G** | **G** | **A** | **A** | **T** | **A** | **G** | **A** | **T** | **T** | **T** | **G** | **A** | **C** | **A** | **A** | **A** | **A** | **A** | **A** | **T** | **G** | **C** | **T** | **T** | **T** | **T** | **T** | **G** | **G** | **C** | **G** | **A** | **A** | **A** | **A** | **A** | **A** | **A** | **C** | **A** | **G** | **A** | **T** | **C** | **A** | **T** | **G** | **A** | **T** | **G** | **G** | **T** | **C** | **C** | **A** | **A** | **A** | **C** | **A** | **G** | **A** | **G** | **C** | **A** | **A** | **T** | **T** | **A** | **A** | **A** | **A** | **A** | **C** | **A** | **C** | **A** | **T** | **A** | **A** | **T** | **A** | **A** | **T** | **G** | **A** | **G** | **C** | **G** | **T** | **A** | **A** | **A** | **A** | **G** | **A** | **C** | **A** | **G** | **C** | **A** | **A** | **T** | **T** | **A** | **T** | **T** | **T** | **G** | **G** | **T** | **A** | **A** | **C** | **C** | **A** | **A** | **C** | **G** | **T** | **A** | **T** | **T** | **G** | **G** | **A** | **A** | **A** | **C** | **G** | **A** | **C** | **G** | **T** | **C** | **T** | **A** | **T** | **G** | **A** | **A** | **T** | **G** | **G** | **G** | **A** | **T** | **G** | **A** | **T** | **A** | **T** | **C** | **A** | **A** | **C** | **A** | **T** | **T** | **G** | **C** | **C** | **A** | **T** | **C** | **A** | **T** | **T** | **T** | **C** | **T** | **G** | **G** | **T** | **G** | **A** | **T** | **A** | **C** | **T** | **A** | **T** | **C** | **A** | **T** | **C** | **G** | **A** | **T** | **T** | **T** | **G** | **G** | **G** | **C** | **A** | **A** | **T** | **A** | **C** | **A** | **C** | **T** | **T** | **T** | **T** | **G** | **C** | **C** | **A** | **A** | **A** | **A** | **G** | **A** | **T** | **G** | **A** | **T** | **A** | **A** | **T** | **A** | **T** | **T** | **G** | **T** | **G** | **A** | **T** | **T** | **G** | **T** | **A** | **A** | **G** | **A** | **A** | **A** | **A** | **G** | **G** | **G** | **A** | **T** | **A** | **G** | **G** | **C** | **A** | **G** | **A** | **A** | **C** | **A** | **C** | **G** | **G** | **A** | **T** | **C** | **T** | **T** | **A** | **G** | **T** | **A** | **C** | **C** | **C** | **C** | **T** | **G** | **G** | **G** | **A** | **G** | **T** | **G** | **G** | **C** | **T** | **A** | **T** | **T** | **C** | **G** | **A** | **T** | **T** | **A** | **G** | **A** | **G** | **C** | **A** | **T** | **G** | **C** | **G** | **A** | **C** | **A** | **T** | **T** | **A** | **G** | **T** | **A** | **G** | **G** | **A** | **A** | **A** | **T** | **G** | **T** | **C** | **T** | **T** | **G** | **T** | **T** | **T** | **G** | **A** | **A** | **G** | **A** | **A** | **G** | **A** | **G** | **C** | **A** | **G** | **T** | **T** | **T** | **T** | **C** | **C** | **T** | **T** | **A** | **A** | **A** | **A** | **A** | **A** | **T** | **G** | **A** | **A** | **C** | **A** | **G** | **A** | **T** | **C** | **A** | **A** | **A** | **A** | **T** | **C** | **T** | **G** | **C** | **A** | **G** | **C** | **A** | **G** | **T** | **G** | **A** | **T** | **T** | **A** | **T** | **G** | **A** | **T** | **G** | **A** | **A** | **A** | **A** | **T** | **C** | **C** | **T** | **C** | **G** | **C** | **A** | **G** | **A** | **T** | **T** | **G** | **A** | **A** | **G** | **A** | **T** | **C** | **A** | **T** | **T** | **A** | **C** | **C** | **A** | **A** | **T** | **A** | **C** | **C** | **T** | **T** | **G** | **G** | **T** | **T** | **G** | **G** | **A** | **G** | **A** | **T** | **G** | **T** | **C** | **G** | **A** | **G** | **G** | **T** | **G** | **A** | **T** | **A** | **C** | **G** | **A** | **A** | **T** | **A** | **-** | **-** | **-** |
| **N55279** | **A** | **T** | **G** | **A** | **A** | **T** | **A** | **G** | **T** | **T** | **C** | **T** | **T** | **G** | **G** | **C** | **G** | **T** | **T** | **T** | **T** | **T** | **T** | **T** | **G** | **T** | **G** | **G** | **T** | **A** | **G** | **T** | **T** | **G** | **A** | **A** | **G** | **C** | **A** | **C** | **T** | **A** | **C** | **T** | **G** | **C** | **T** | **G** | **T** | **T** | **A** | **T** | **T** | **T** | **G** | **C** | **T** | **G** | **T** | **T** | **T** | **G** | **G** | **C** | **A** | **A** | **A** | **T** | **C** | **G** | **T** | **A** | **A** | **A** | **C** | **A** | **A** | **T** | **A** | **C** | **T** | **G** | **G** | **A** | **C** | **T** | **T** | **T** | **T** | **G** | **C** | **T** | **T** | **T** | **T** | **A** | **G** | **T** | **G** | **A** | **T** | **A** | **T** | **T** | **C** | **G** | **G** | **T** | **A** | **T** | **T** | **T** | **T** | **C** | **A** | **A** | **T** | **A** | **T** | **A** | **T** | **A** | **T** | **C** | **T** | **A** | **G** | **C** | **T** | **A** | **T** | **G** | **C** | **G** | **C** | **A** | **A** | **A** | **C** | **A** | **T** | **C** | **C** | **T** | **C** | **G** | **T** | **A** | **C** | **T** | **A** | **A** | **A** | **T** | **T** | **T** | **C** | **A** | **A** | **A** | **A** | **T** | **T** | **T** | **T** | **C** | **A** | **A** | **T** | **T** | **G** | **A** | **T** | **A** | **T** | **T** | **A** | **G** | **G** | **A** | **A** | **G** | **T** | **T** | **T** | **G** | **G** | **T** | **C** | **A** | **T** | **T** | **T** | **T** | **T** | **T** | **T** | **T** | **A** | **G** | **C** | **T** | **T** | **A** | **G** | **T** | **C** | **A** | **A** | **C** | **A** | **G** | **T** | **C** | **C** | **T** | **G** | **C** | **T** | **T** | **T** | **A** | **T** | **G** | **G** | **A** | **T** | **G** | **A** | **T** | **G** | **G** | **C** | **T** | **G** | **T** | **A** | **T** | **T** | **T** | **G** | **C** | **C** | **G** | **T** | **T** | **T** | **T** | **A** | **T** | **T** | **T** | **A** | **T** | **T** | **G** | **G** | **T** | **C** | **T** | **G** | **A** | **A** | **A** | **G** | **G** | **T** | **G** | **T** | **A** | **G** | **A** | **G** | **A** | **T** | **T** | **T** | **C** | **A** | **G** | **G** | **G** | **A** | **T** | **A** | **G** | **A** | **T** | **T** | **T** | **A** | **A** | **C** | **C** | **A** | **A** | **A** | **A** | **A** | **T** | **G** | **C** | **T** | **T** | **T** | **T** | **T** | **G** | **G** | **C** | **G** | **A** | **A** | **A** | **G** | **A** | **A** | **A** | **C** | **A** | **G** | **A** | **T** | **C** | **A** | **T** | **G** | **A** | **T** | **G** | **A** | **T** | **C** | **C** | **A** | **A** | **A** | **C** | **A** | **G** | **A** | **G** | **C** | **A** | **A** | **T** | **T** | **A** | **A** | **A** | **A** | **A** | **C** | **A** | **C** | **A** | **T** | **A** | **A** | **T** | **A** | **A** | **T** | **G** | **A** | **G** | **C** | **G** | **G** | **A** | **A** | **A** | **A** | **G** | **A** | **C** | **A** | **G** | **C** | **A** | **A** | **T** | **T** | **A** | **T** | **T** | **C** | **G** | **G** | **T** | **A** | **A** | **C** | **C** | **A** | **A** | **C** | **G** | **T** | **A** | **T** | **T** | **G** | **G** | **A** | **A** | **A** | **C** | **G** | **A** | **C** | **G** | **T** | **C** | **T** | **A** | **T** | **G** | **A** | **A** | **T** | **G** | **G** | **G** | **A** | **C** | **G** | **A** | **T** | **A** | **T** | **C** | **A** | **A** | **C** | **A** | **T** | **C** | **G** | **C** | **C** | **G** | **T** | **C** | **A** | **T** | **T** | **T** | **C** | **T** | **G** | **G** | **T** | **G** | **A** | **T** | **A** | **C** | **T** | **A** | **T** | **C** | **A** | **T** | **A** | **G** | **A** | **T** | **T** | **T** | **G** | **G** | **G** | **C** | **A** | **A** | **T** | **A** | **C** | **G** | **C** | **T** | **T** | **T** | **T** | **G** | **C** | **C** | **A** | **A** | **A** | **A** | **G** | **A** | **T** | **G** | **A** | **T** | **A** | **A** | **T** | **A** | **T** | **T** | **G** | **T** | **G** | **A** | **T** | **T** | **G** | **T** | **A** | **A** | **G** | **A** | **A** | **A** | **A** | **G** | **G** | **G** | **A** | **T** | **A** | **G** | **G** | **C** | **A** | **G** | **A** | **A** | **C** | **A** | **C** | **G** | **G** | **A** | **T** | **C** | **T** | **T** | **A** | **G** | **T** | **A** | **C** | **C** | **T** | **T** | **T** | **G** | **G** | **G** | **C** | **G** | **T** | **A** | **G** | **C** | **C** | **A** | **T** | **C** | **C** | **G** | **A** | **T** | **T** | **A** | **G** | **A** | **G** | **C** | **A** | **T** | **G** | **C** | **G** | **A** | **C** | **A** | **T** | **T** | **A** | **G** | **T** | **A** | **G** | **G** | **A** | **A** | **A** | **T** | **G** | **T** | **C** | **T** | **T** | **G** | **T** | **T** | **T** | **G** | **A** | **A** | **G** | **A** | **A** | **G** | **A** | **G** | **C** | **A** | **A** | **T** | **T** | **T** | **T** | **C** | **T** | **T** | **T** | **G** | **A** | **A** | **A** | **A** | **A** | **T** | **G** | **A** | **A** | **C** | **A** | **A** | **A** | **T** | **C** | **A** | **A** | **A** | **A** | **T** | **C** | **T** | **A** | **C** | **A** | **G** | **C** | **A** | **G** | **T** | **G** | **A** | **T** | **T** | **A** | **T** | **G** | **A** | **T** | **G** | **A** | **A** | **A** | **A** | **C** | **C** | **C** | **T** | **C** | **G** | **T** | **A** | **G** | **A** | **T** | **T** | **G** | **A** | **A** | **A** | **A** | **T** | **T** | **A** | **T** | **T** | **A** | **C** | **T** | **A** | **A** | **T** | **A** | **C** | **C** | **T** | **T** | **G** | **A** | **T** | **T** | **G** | **G** | **A** | **G** | **A** | **T** | **G** | **T** | **C** | **G** | **A** | **G** | **G** | **T** | **G** | **A** | **T** | **A** | **C** | **G** | **A** | **A** | **T** | **A** | **-** | **-** | **-** |
| **N52732** | **A** | **T** | **G** | **A** | **A** | **T** | **A** | **G** | **T** | **T** | **C** | **T** | **T** | **G** | **G** | **C** | **G** | **T** | **T** | **T** | **T** | **T** | **T** | **T** | **G** | **T** | **G** | **G** | **T** | **A** | **G** | **T** | **T** | **G** | **A** | **A** | **A** | **C** | **A** | **C** | **T** | **A** | **C** | **T** | **G** | **C** | **T** | **G** | **T** | **T** | **A** | **T** | **T** | **T** | **G** | **C** | **T** | **G** | **T** | **T** | **T** | **G** | **G** | **C** | **A** | **A** | **A** | **T** | **C** | **G** | **T** | **A** | **A** | **A** | **C** | **A** | **A** | **T** | **A** | **C** | **T** | **G** | **G** | **A** | **C** | **T** | **T** | **T** | **T** | **G** | **C** | **T** | **T** | **T** | **T** | **A** | **G** | **T** | **G** | **A** | **T** | **A** | **T** | **T** | **C** | **G** | **G** | **T** | **A** | **T** | **T** | **T** | **T** | **C** | **A** | **A** | **T** | **A** | **T** | **A** | **T** | **A** | **T** | **C** | **T** | **A** | **G** | **C** | **T** | **A** | **T** | **G** | **C** | **G** | **C** | **A** | **A** | **A** | **C** | **A** | **T** | **C** | **C** | **T** | **C** | **G** | **T** | **A** | **C** | **T** | **A** | **A** | **A** | **T** | **T** | **T** | **C** | **A** | **A** | **A** | **A** | **T** | **T** | **T** | **T** | **C** | **A** | **A** | **T** | **T** | **G** | **A** | **T** | **A** | **T** | **T** | **A** | **G** | **G** | **A** | **A** | **G** | **T** | **T** | **T** | **G** | **G** | **T** | **C** | **A** | **T** | **T** | **T** | **T** | **T** | **T** | **T** | **T** | **A** | **G** | **C** | **T** | **T** | **A** | **G** | **T** | **C** | **A** | **A** | **C** | **A** | **G** | **T** | **C** | **C** | **T** | **G** | **C** | **T** | **T** | **T** | **A** | **T** | **G** | **G** | **A** | **T** | **G** | **A** | **T** | **G** | **G** | **C** | **T** | **G** | **T** | **A** | **T** | **T** | **T** | **G** | **C** | **C** | **G** | **T** | **T** | **T** | **T** | **A** | **T** | **T** | **T** | **A** | **T** | **T** | **G** | **G** | **A** | **C** | **T** | **A** | **A** | **A** | **A** | **G** | **G** | **T** | **G** | **T** | **A** | **G** | **A** | **G** | **A** | **T** | **T** | **T** | **C** | **A** | **G** | **G** | **G** | **A** | **T** | **A** | **G** | **A** | **T** | **T** | **T** | **A** | **A** | **C** | **C** | **A** | **A** | **A** | **A** | **A** | **T** | **G** | **C** | **T** | **T** | **T** | **T** | **T** | **G** | **G** | **C** | **G** | **A** | **A** | **A** | **G** | **A** | **A** | **A** | **C** | **A** | **G** | **A** | **T** | **C** | **A** | **T** | **G** | **A** | **T** | **G** | **G** | **T** | **C** | **C** | **A** | **A** | **A** | **C** | **A** | **G** | **A** | **G** | **C** | **A** | **A** | **T** | **T** | **A** | **A** | **A** | **A** | **A** | **C** | **A** | **C** | **A** | **T** | **A** | **A** | **T** | **A** | **A** | **T** | **G** | **A** | **G** | **C** | **G** | **G** | **A** | **A** | **A** | **A** | **G** | **A** | **C** | **A** | **G** | **C** | **A** | **A** | **T** | **T** | **A** | **T** | **T** | **C** | **G** | **G** | **T** | **A** | **A** | **C** | **C** | **A** | **A** | **C** | **G** | **T** | **A** | **T** | **T** | **G** | **G** | **A** | **A** | **A** | **C** | **G** | **A** | **C** | **G** | **T** | **C** | **T** | **A** | **T** | **G** | **A** | **A** | **T** | **G** | **G** | **G** | **A** | **C** | **G** | **A** | **T** | **A** | **T** | **C** | **A** | **A** | **C** | **A** | **T** | **C** | **G** | **C** | **C** | **G** | **T** | **C** | **A** | **T** | **T** | **T** | **C** | **T** | **G** | **G** | **T** | **G** | **A** | **T** | **A** | **C** | **T** | **A** | **T** | **C** | **A** | **T** | **A** | **G** | **A** | **T** | **T** | **T** | **G** | **G** | **G** | **C** | **A** | **A** | **T** | **A** | **C** | **G** | **C** | **T** | **T** | **T** | **T** | **G** | **C** | **C** | **A** | **A** | **A** | **A** | **G** | **A** | **T** | **G** | **A** | **T** | **A** | **A** | **T** | **A** | **T** | **T** | **G** | **T** | **G** | **A** | **T** | **T** | **G** | **T** | **A** | **A** | **G** | **A** | **A** | **A** | **A** | **G** | **G** | **G** | **A** | **T** | **A** | **G** | **G** | **C** | **A** | **G** | **A** | **A** | **C** | **A** | **C** | **G** | **A** | **A** | **T** | **C** | **T** | **T** | **A** | **G** | **T** | **A** | **C** | **C** | **T** | **T** | **T** | **G** | **G** | **G** | **C** | **G** | **T** | **A** | **G** | **C** | **C** | **A** | **T** | **C** | **C** | **G** | **A** | **T** | **T** | **A** | **G** | **A** | **G** | **C** | **A** | **T** | **G** | **C** | **G** | **A** | **C** | **A** | **T** | **T** | **A** | **G** | **T** | **A** | **G** | **G** | **A** | **A** | **A** | **T** | **G** | **T** | **C** | **T** | **T** | **G** | **T** | **T** | **T** | **G** | **A** | **A** | **G** | **A** | **A** | **G** | **A** | **G** | **C** | **A** | **A** | **T** | **T** | **T** | **T** | **C** | **T** | **T** | **T** | **G** | **A** | **A** | **A** | **A** | **A** | **T** | **G** | **A** | **A** | **C** | **A** | **A** | **A** | **T** | **C** | **A** | **A** | **A** | **A** | **T** | **C** | **T** | **A** | **C** | **A** | **G** | **C** | **A** | **G** | **T** | **G** | **A** | **T** | **T** | **A** | **T** | **G** | **A** | **T** | **G** | **A** | **A** | **A** | **A** | **C** | **C** | **C** | **T** | **C** | **G** | **T** | **A** | **G** | **A** | **C** | **T** | **G** | **A** | **A** | **G** | **A** | **T** | **T** | **A** | **T** | **T** | **A** | **C** | **T** | **A** | **A** | **T** | **A** | **C** | **C** | **T** | **T** | **G** | **A** | **T** | **T** | **G** | **G** | **A** | **G** | **A** | **T** | **G** | **T** | **C** | **G** | **A** | **G** | **G** | **T** | **G** | **A** | **T** | **A** | **C** | **G** | **A** | **A** | **T** | **A** | **-** | **-** | **-** |
| **N59517F** | **A** | **T** | **G** | **A** | **A** | **T** | **A** | **G** | **T** | **T** | **C** | **T** | **T** | **G** | **G** | **C** | **G** | **T** | **T** | **T** | **T** | **T** | **T** | **T** | **G** | **T** | **G** | **G** | **T** | **A** | **G** | **T** | **C** | **G** | **A** | **A** | **G** | **C** | **A** | **C** | **T** | **G** | **C** | **T** | **G** | **C** | **T** | **G** | **T** | **T** | **A** | **T** | **T** | **T** | **G** | **C** | **T** | **G** | **T** | **T** | **T** | **G** | **G** | **C** | **A** | **A** | **A** | **T** | **C** | **G** | **T** | **A** | **A** | **A** | **C** | **A** | **A** | **T** | **A** | **C** | **T** | **G** | **G** | **A** | **C** | **T** | **T** | **T** | **T** | **G** | **C** | **T** | **T** | **T** | **T** | **A** | **G** | **T** | **A** | **A** | **T** | **A** | **T** | **T** | **C** | **G** | **G** | **T** | **A** | **T** | **T** | **T** | **T** | **C** | **A** | **A** | **T** | **A** | **T** | **A** | **T** | **A** | **T** | **C** | **T** | **A** | **G** | **C** | **T** | **A** | **T** | **G** | **C** | **G** | **C** | **A** | **A** | **A** | **T** | **A** | **T** | **C** | **C** | **T** | **C** | **G** | **T** | **A** | **C** | **T** | **A** | **A** | **A** | **T** | **T** | **T** | **C** | **A** | **A** | **A** | **A** | **T** | **T** | **T** | **T** | **C** | **A** | **A** | **T** | **T** | **G** | **A** | **T** | **C** | **T** | **T** | **A** | **G** | **G** | **G** | **A** | **G** | **C** | **T** | **T** | **G** | **G** | **T** | **C** | **A** | **T** | **T** | **T** | **T** | **T** | **T** | **T** | **T** | **A** | **G** | **C** | **T** | **T** | **A** | **G** | **T** | **C** | **A** | **A** | **C** | **A** | **G** | **T** | **C** | **C** | **T** | **G** | **C** | **T** | **T** | **T** | **A** | **T** | **G** | **G** | **A** | **T** | **G** | **A** | **T** | **G** | **G** | **T** | **T** | **G** | **T** | **A** | **T** | **T** | **G** | **G** | **C** | **C** | **G** | **T** | **T** | **T** | **T** | **A** | **T** | **T** | **T** | **A** | **T** | **C** | **G** | **G** | **G** | **C** | **T** | **G** | **A** | **A** | **A** | **G** | **G** | **T** | **G** | **T** | **A** | **G** | **A** | **G** | **A** | **T** | **T** | **T** | **C** | **A** | **G** | **G** | **A** | **A** | **T** | **A** | **G** | **A** | **T** | **T** | **T** | **G** | **A** | **C** | **A** | **A** | **A** | **A** | **A** | **A** | **T** | **G** | **C** | **T** | **T** | **T** | **T** | **T** | **G** | **G** | **C** | **G** | **A** | **A** | **A** | **A** | **A** | **A** | **A** | **C** | **A** | **G** | **A** | **T** | **C** | **A** | **T** | **G** | **A** | **T** | **G** | **G** | **T** | **C** | **C** | **A** | **A** | **A** | **C** | **A** | **G** | **A** | **G** | **C** | **A** | **A** | **T** | **T** | **A** | **A** | **A** | **A** | **A** | **C** | **A** | **C** | **A** | **T** | **A** | **A** | **T** | **A** | **A** | **T** | **G** | **A** | **G** | **C** | **G** | **T** | **A** | **A** | **A** | **A** | **G** | **A** | **C** | **A** | **G** | **C** | **A** | **A** | **T** | **T** | **A** | **T** | **T** | **T** | **G** | **G** | **T** | **A** | **A** | **C** | **C** | **A** | **A** | **C** | **G** | **T** | **A** | **T** | **T** | **G** | **G** | **A** | **A** | **A** | **C** | **G** | **A** | **C** | **G** | **T** | **C** | **T** | **A** | **T** | **G** | **A** | **A** | **T** | **G** | **G** | **G** | **A** | **T** | **G** | **A** | **T** | **A** | **T** | **C** | **A** | **A** | **C** | **A** | **T** | **T** | **G** | **C** | **C** | **A** | **T** | **C** | **A** | **T** | **T** | **T** | **C** | **T** | **G** | **G** | **T** | **G** | **A** | **T** | **A** | **C** | **T** | **A** | **T** | **C** | **A** | **T** | **C** | **G** | **A** | **T** | **T** | **T** | **G** | **G** | **G** | **C** | **A** | **A** | **T** | **A** | **C** | **A** | **C** | **T** | **T** | **T** | **T** | **G** | **C** | **C** | **A** | **A** | **A** | **A** | **G** | **A** | **T** | **G** | **A** | **T** | **A** | **A** | **T** | **A** | **T** | **T** | **G** | **T** | **G** | **A** | **T** | **T** | **G** | **T** | **A** | **A** | **G** | **A** | **A** | **A** | **A** | **G** | **G** | **G** | **A** | **T** | **A** | **G** | **G** | **C** | **A** | **G** | **A** | **A** | **C** | **A** | **C** | **G** | **G** | **A** | **T** | **C** | **T** | **T** | **A** | **G** | **T** | **A** | **C** | **C** | **C** | **C** | **T** | **G** | **G** | **G** | **A** | **G** | **T** | **G** | **G** | **C** | **T** | **A** | **T** | **T** | **C** | **G** | **T** | **T** | **T** | **A** | **G** | **A** | **G** | **C** | **A** | **T** | **G** | **C** | **G** | **A** | **C** | **A** | **T** | **T** | **A** | **G** | **T** | **A** | **G** | **G** | **A** | **A** | **A** | **T** | **G** | **T** | **C** | **T** | **T** | **G** | **T** | **T** | **T** | **G** | **A** | **A** | **G** | **A** | **A** | **G** | **A** | **G** | **C** | **A** | **G** | **T** | **T** | **T** | **T** | **C** | **C** | **T** | **T** | **A** | **A** | **A** | **A** | **A** | **A** | **C** | **G** | **A** | **A** | **C** | **A** | **G** | **A** | **T** | **C** | **A** | **A** | **A** | **A** | **T** | **C** | **T** | **G** | **C** | **A** | **G** | **C** | **A** | **G** | **T** | **G** | **A** | **T** | **T** | **A** | **T** | **G** | **A** | **T** | **G** | **A** | **A** | **A** | **A** | **T** | **C** | **C** | **T** | **C** | **G** | **C** | **A** | **G** | **A** | **T** | **T** | **G** | **A** | **A** | **G** | **A** | **T** | **C** | **A** | **T** | **T** | **A** | **C** | **C** | **A** | **A** | **T** | **A** | **C** | **C** | **T** | **T** | **G** | **G** | **T** | **T** | **G** | **G** | **A** | **G** | **A** | **T** | **G** | **T** | **C** | **G** | **A** | **G** | **G** | **T** | **G** | **A** | **T** | **A** | **C** | **G** | **A** | **A** | **T** | **A** | **-** | **-** | **-** |
| **N53805** | **A** | **T** | **G** | **A** | **A** | **T** | **A** | **G** | **T** | **T** | **C** | **T** | **T** | **G** | **G** | **C** | **G** | **T** | **T** | **T** | **T** | **T** | **T** | **T** | **G** | **T** | **G** | **G** | **T** | **A** | **G** | **T** | **C** | **G** | **A** | **A** | **G** | **C** | **A** | **C** | **T** | **G** | **C** | **T** | **G** | **C** | **T** | **G** | **T** | **T** | **A** | **T** | **T** | **T** | **G** | **C** | **T** | **G** | **T** | **T** | **T** | **G** | **G** | **C** | **A** | **A** | **A** | **T** | **C** | **G** | **T** | **A** | **A** | **A** | **C** | **A** | **A** | **T** | **A** | **C** | **T** | **G** | **G** | **A** | **C** | **T** | **T** | **T** | **T** | **G** | **C** | **T** | **T** | **T** | **T** | **A** | **G** | **T** | **A** | **A** | **T** | **A** | **T** | **T** | **C** | **G** | **G** | **T** | **A** | **T** | **T** | **T** | **T** | **C** | **A** | **A** | **T** | **A** | **T** | **A** | **T** | **A** | **T** | **C** | **T** | **A** | **G** | **C** | **T** | **A** | **T** | **G** | **C** | **G** | **C** | **A** | **A** | **A** | **T** | **A** | **T** | **C** | **C** | **T** | **C** | **G** | **T** | **A** | **C** | **T** | **A** | **A** | **A** | **T** | **T** | **T** | **C** | **A** | **A** | **A** | **A** | **T** | **T** | **T** | **T** | **C** | **A** | **A** | **T** | **T** | **G** | **A** | **T** | **C** | **T** | **T** | **A** | **G** | **G** | **G** | **A** | **G** | **C** | **T** | **T** | **G** | **G** | **T** | **C** | **A** | **T** | **T** | **T** | **T** | **T** | **T** | **T** | **T** | **A** | **G** | **C** | **T** | **T** | **A** | **G** | **T** | **C** | **A** | **A** | **C** | **A** | **G** | **T** | **C** | **C** | **T** | **G** | **C** | **T** | **T** | **T** | **A** | **T** | **G** | **G** | **A** | **T** | **G** | **A** | **T** | **G** | **G** | **T** | **T** | **G** | **T** | **A** | **T** | **T** | **G** | **G** | **C** | **C** | **G** | **T** | **T** | **T** | **T** | **A** | **T** | **T** | **T** | **A** | **T** | **C** | **G** | **G** | **G** | **C** | **T** | **G** | **A** | **A** | **A** | **G** | **G** | **T** | **G** | **T** | **A** | **G** | **A** | **G** | **A** | **T** | **T** | **T** | **C** | **A** | **G** | **G** | **A** | **A** | **T** | **A** | **G** | **A** | **T** | **T** | **T** | **G** | **A** | **C** | **A** | **A** | **A** | **A** | **A** | **A** | **T** | **G** | **C** | **T** | **T** | **T** | **T** | **T** | **G** | **G** | **C** | **G** | **A** | **A** | **A** | **A** | **A** | **A** | **A** | **C** | **A** | **G** | **A** | **T** | **C** | **A** | **T** | **G** | **A** | **T** | **G** | **G** | **T** | **C** | **C** | **A** | **A** | **A** | **C** | **A** | **G** | **A** | **G** | **C** | **A** | **A** | **T** | **T** | **A** | **A** | **A** | **A** | **A** | **C** | **A** | **C** | **A** | **T** | **A** | **A** | **T** | **A** | **A** | **T** | **G** | **A** | **G** | **C** | **G** | **T** | **A** | **A** | **A** | **A** | **G** | **A** | **C** | **A** | **G** | **C** | **A** | **A** | **T** | **T** | **A** | **T** | **T** | **T** | **G** | **G** | **T** | **A** | **A** | **C** | **C** | **A** | **A** | **C** | **G** | **T** | **A** | **T** | **T** | **G** | **G** | **A** | **A** | **A** | **C** | **G** | **A** | **C** | **G** | **T** | **C** | **T** | **A** | **T** | **G** | **A** | **A** | **T** | **G** | **G** | **G** | **A** | **T** | **G** | **A** | **T** | **A** | **T** | **C** | **A** | **A** | **C** | **A** | **T** | **T** | **G** | **C** | **C** | **A** | **T** | **C** | **A** | **T** | **T** | **T** | **C** | **T** | **G** | **G** | **T** | **G** | **A** | **T** | **A** | **C** | **T** | **A** | **T** | **C** | **A** | **T** | **C** | **G** | **A** | **T** | **T** | **T** | **G** | **G** | **G** | **C** | **A** | **A** | **T** | **A** | **C** | **A** | **C** | **T** | **T** | **T** | **T** | **G** | **C** | **C** | **A** | **A** | **A** | **A** | **G** | **A** | **T** | **G** | **A** | **T** | **A** | **A** | **T** | **A** | **T** | **T** | **G** | **T** | **G** | **A** | **T** | **T** | **G** | **T** | **A** | **A** | **G** | **A** | **A** | **A** | **A** | **G** | **G** | **G** | **A** | **T** | **A** | **G** | **G** | **C** | **A** | **G** | **A** | **A** | **C** | **A** | **C** | **G** | **G** | **A** | **T** | **C** | **T** | **T** | **A** | **G** | **T** | **A** | **C** | **C** | **C** | **C** | **T** | **G** | **G** | **G** | **A** | **G** | **T** | **G** | **G** | **C** | **T** | **A** | **T** | **T** | **C** | **G** | **A** | **T** | **T** | **A** | **G** | **A** | **G** | **C** | **A** | **T** | **G** | **C** | **G** | **A** | **C** | **A** | **T** | **T** | **A** | **G** | **T** | **A** | **G** | **G** | **A** | **A** | **A** | **T** | **G** | **T** | **C** | **T** | **T** | **G** | **T** | **T** | **T** | **G** | **A** | **A** | **G** | **A** | **A** | **G** | **A** | **G** | **C** | **A** | **G** | **T** | **T** | **T** | **T** | **C** | **C** | **T** | **T** | **A** | **A** | **A** | **A** | **A** | **A** | **C** | **G** | **A** | **A** | **C** | **A** | **G** | **A** | **T** | **A** | **A** | **A** | **A** | **A** | **T** | **C** | **T** | **G** | **C** | **A** | **G** | **C** | **A** | **G** | **T** | **G** | **A** | **T** | **T** | **A** | **T** | **G** | **A** | **T** | **G** | **A** | **A** | **A** | **A** | **T** | **C** | **C** | **T** | **C** | **G** | **C** | **A** | **G** | **A** | **T** | **T** | **G** | **A** | **A** | **G** | **A** | **T** | **C** | **A** | **T** | **T** | **A** | **C** | **C** | **A** | **A** | **T** | **A** | **C** | **C** | **T** | **T** | **G** | **G** | **T** | **T** | **G** | **G** | **A** | **G** | **A** | **T** | **G** | **T** | **C** | **G** | **A** | **G** | **G** | **T** | **G** | **A** | **T** | **A** | **C** | **G** | **A** | **A** | **T** | **A** | **-** | **-** | **-** |
| **N59624F** | **A** | **T** | **G** | **A** | **A** | **T** | **A** | **G** | **T** | **T** | **C** | **T** | **T** | **G** | **G** | **C** | **G** | **T** | **T** | **T** | **T** | **T** | **T** | **T** | **G** | **T** | **G** | **G** | **T** | **A** | **G** | **T** | **T** | **G** | **A** | **A** | **G** | **C** | **A** | **C** | **T** | **A** | **C** | **T** | **G** | **C** | **T** | **G** | **T** | **T** | **A** | **T** | **T** | **T** | **G** | **C** | **T** | **G** | **T** | **T** | **T** | **G** | **G** | **C** | **A** | **A** | **A** | **T** | **C** | **G** | **T** | **A** | **A** | **A** | **C** | **A** | **A** | **T** | **A** | **C** | **T** | **G** | **G** | **A** | **C** | **T** | **T** | **T** | **T** | **G** | **C** | **T** | **T** | **T** | **T** | **A** | **G** | **T** | **G** | **A** | **T** | **A** | **T** | **T** | **C** | **G** | **G** | **T** | **A** | **T** | **T** | **T** | **T** | **C** | **A** | **A** | **T** | **A** | **T** | **A** | **T** | **A** | **T** | **C** | **T** | **A** | **G** | **C** | **T** | **A** | **T** | **G** | **C** | **G** | **C** | **A** | **A** | **A** | **C** | **A** | **T** | **C** | **C** | **T** | **C** | **G** | **T** | **A** | **C** | **T** | **A** | **A** | **A** | **T** | **T** | **T** | **C** | **A** | **A** | **A** | **A** | **T** | **T** | **T** | **T** | **C** | **A** | **A** | **T** | **T** | **G** | **A** | **T** | **A** | **T** | **T** | **A** | **G** | **G** | **A** | **A** | **G** | **T** | **T** | **T** | **G** | **G** | **T** | **C** | **A** | **T** | **T** | **T** | **T** | **T** | **T** | **T** | **T** | **A** | **G** | **C** | **T** | **T** | **A** | **G** | **T** | **C** | **A** | **A** | **C** | **A** | **G** | **T** | **C** | **C** | **T** | **G** | **C** | **T** | **T** | **T** | **A** | **T** | **G** | **G** | **A** | **T** | **G** | **A** | **T** | **G** | **G** | **C** | **T** | **G** | **T** | **A** | **T** | **T** | **T** | **G** | **C** | **C** | **G** | **T** | **T** | **T** | **T** | **A** | **T** | **T** | **T** | **A** | **T** | **T** | **G** | **G** | **T** | **C** | **T** | **G** | **A** | **A** | **A** | **G** | **G** | **T** | **G** | **T** | **A** | **G** | **A** | **G** | **A** | **T** | **T** | **T** | **C** | **A** | **G** | **G** | **G** | **A** | **T** | **A** | **G** | **A** | **T** | **T** | **T** | **A** | **A** | **C** | **C** | **A** | **A** | **A** | **A** | **A** | **T** | **G** | **C** | **T** | **T** | **T** | **T** | **T** | **G** | **G** | **C** | **G** | **A** | **A** | **A** | **G** | **A** | **A** | **A** | **C** | **A** | **G** | **A** | **T** | **C** | **A** | **T** | **G** | **A** | **T** | **G** | **A** | **T** | **C** | **C** | **A** | **A** | **A** | **C** | **A** | **G** | **A** | **G** | **C** | **A** | **A** | **T** | **T** | **A** | **A** | **A** | **A** | **A** | **C** | **A** | **C** | **A** | **T** | **A** | **A** | **T** | **A** | **A** | **T** | **G** | **A** | **G** | **C** | **G** | **G** | **A** | **A** | **A** | **A** | **G** | **A** | **C** | **A** | **G** | **C** | **A** | **A** | **T** | **T** | **A** | **T** | **T** | **C** | **G** | **G** | **T** | **A** | **A** | **C** | **C** | **A** | **A** | **C** | **G** | **T** | **A** | **T** | **T** | **G** | **G** | **A** | **A** | **A** | **C** | **G** | **A** | **C** | **G** | **T** | **C** | **T** | **A** | **T** | **G** | **A** | **A** | **T** | **G** | **G** | **G** | **A** | **C** | **G** | **A** | **T** | **A** | **T** | **C** | **A** | **A** | **C** | **A** | **T** | **C** | **G** | **C** | **C** | **G** | **T** | **C** | **A** | **T** | **T** | **T** | **C** | **T** | **G** | **G** | **T** | **G** | **A** | **T** | **A** | **C** | **T** | **A** | **T** | **C** | **A** | **T** | **A** | **G** | **A** | **T** | **T** | **T** | **G** | **G** | **G** | **C** | **A** | **A** | **T** | **A** | **C** | **G** | **C** | **T** | **T** | **T** | **T** | **G** | **C** | **C** | **A** | **A** | **A** | **A** | **G** | **A** | **T** | **G** | **A** | **T** | **A** | **A** | **T** | **A** | **T** | **T** | **G** | **T** | **G** | **A** | **T** | **T** | **G** | **T** | **A** | **A** | **G** | **A** | **A** | **A** | **A** | **G** | **G** | **G** | **A** | **T** | **A** | **G** | **G** | **C** | **A** | **G** | **A** | **A** | **C** | **A** | **C** | **G** | **G** | **A** | **T** | **C** | **T** | **T** | **A** | **G** | **T** | **A** | **C** | **C** | **T** | **T** | **T** | **G** | **G** | **G** | **C** | **G** | **T** | **A** | **G** | **C** | **C** | **A** | **T** | **C** | **C** | **G** | **T** | **T** | **T** | **A** | **G** | **A** | **G** | **C** | **A** | **T** | **G** | **C** | **G** | **A** | **C** | **A** | **T** | **T** | **A** | **G** | **T** | **A** | **G** | **G** | **A** | **A** | **A** | **T** | **G** | **T** | **C** | **T** | **T** | **G** | **T** | **T** | **T** | **G** | **A** | **A** | **G** | **A** | **A** | **G** | **A** | **G** | **C** | **A** | **A** | **T** | **T** | **T** | **T** | **C** | **T** | **T** | **T** | **G** | **A** | **A** | **A** | **A** | **A** | **T** | **G** | **A** | **A** | **C** | **A** | **A** | **A** | **T** | **C** | **A** | **A** | **A** | **A** | **T** | **C** | **T** | **A** | **C** | **A** | **G** | **C** | **A** | **G** | **T** | **G** | **A** | **T** | **T** | **A** | **T** | **G** | **A** | **T** | **G** | **A** | **A** | **A** | **A** | **C** | **C** | **C** | **T** | **C** | **G** | **T** | **A** | **G** | **A** | **T** | **T** | **G** | **A** | **A** | **G** | **A** | **T** | **T** | **A** | **T** | **T** | **A** | **C** | **T** | **A** | **A** | **T** | **A** | **C** | **C** | **T** | **T** | **G** | **A** | **T** | **T** | **G** | **G** | **A** | **G** | **A** | **T** | **G** | **T** | **C** | **G** | **A** | **G** | **G** | **T** | **G** | **A** | **T** | **A** | **C** | **G** | **A** | **A** | **T** | **A** | **-** | **-** | **-** |
| **N54671** | **A** | **T** | **G** | **A** | **A** | **T** | **A** | **G** | **T** | **T** | **C** | **T** | **T** | **G** | **G** | **C** | **G** | **T** | **T** | **T** | **T** | **T** | **T** | **T** | **G** | **T** | **G** | **G** | **T** | **A** | **G** | **T** | **T** | **G** | **A** | **A** | **G** | **C** | **A** | **C** | **T** | **A** | **C** | **T** | **G** | **C** | **T** | **G** | **T** | **T** | **A** | **T** | **T** | **T** | **G** | **C** | **T** | **G** | **T** | **T** | **T** | **G** | **G** | **C** | **A** | **A** | **A** | **T** | **C** | **G** | **T** | **A** | **A** | **A** | **C** | **A** | **A** | **T** | **A** | **C** | **T** | **G** | **G** | **A** | **C** | **T** | **T** | **T** | **T** | **G** | **C** | **T** | **T** | **T** | **T** | **A** | **G** | **T** | **G** | **A** | **T** | **A** | **T** | **T** | **C** | **G** | **G** | **T** | **A** | **T** | **T** | **T** | **T** | **C** | **A** | **A** | **T** | **A** | **T** | **A** | **T** | **A** | **T** | **C** | **T** | **A** | **G** | **C** | **T** | **A** | **T** | **G** | **C** | **G** | **C** | **A** | **A** | **A** | **C** | **A** | **T** | **C** | **C** | **T** | **C** | **G** | **T** | **A** | **C** | **T** | **A** | **A** | **A** | **T** | **T** | **T** | **C** | **A** | **A** | **A** | **A** | **T** | **T** | **T** | **T** | **C** | **A** | **A** | **T** | **T** | **G** | **A** | **T** | **A** | **T** | **T** | **A** | **G** | **G** | **A** | **A** | **G** | **T** | **T** | **T** | **G** | **G** | **T** | **C** | **A** | **T** | **T** | **T** | **T** | **T** | **T** | **T** | **T** | **A** | **G** | **C** | **T** | **T** | **A** | **G** | **T** | **C** | **A** | **A** | **C** | **A** | **G** | **T** | **C** | **C** | **T** | **G** | **C** | **T** | **T** | **T** | **A** | **T** | **G** | **G** | **A** | **T** | **G** | **A** | **T** | **G** | **G** | **C** | **T** | **G** | **T** | **A** | **T** | **T** | **T** | **G** | **C** | **C** | **G** | **T** | **T** | **T** | **T** | **A** | **T** | **T** | **T** | **A** | **T** | **T** | **G** | **G** | **A** | **C** | **T** | **A** | **A** | **A** | **A** | **G** | **G** | **T** | **G** | **T** | **A** | **G** | **A** | **G** | **A** | **T** | **T** | **T** | **C** | **A** | **G** | **G** | **G** | **A** | **T** | **A** | **G** | **A** | **T** | **T** | **T** | **A** | **A** | **C** | **C** | **A** | **A** | **A** | **A** | **A** | **T** | **G** | **C** | **T** | **T** | **T** | **T** | **T** | **G** | **G** | **C** | **G** | **A** | **A** | **A** | **G** | **A** | **A** | **A** | **C** | **A** | **G** | **A** | **T** | **C** | **A** | **T** | **G** | **A** | **T** | **G** | **G** | **T** | **C** | **C** | **A** | **A** | **A** | **C** | **A** | **G** | **A** | **G** | **C** | **A** | **A** | **T** | **T** | **A** | **A** | **A** | **A** | **A** | **C** | **A** | **C** | **A** | **T** | **A** | **A** | **T** | **A** | **A** | **T** | **G** | **A** | **G** | **C** | **G** | **G** | **A** | **A** | **A** | **A** | **G** | **A** | **C** | **A** | **G** | **C** | **A** | **A** | **T** | **T** | **A** | **T** | **T** | **C** | **G** | **G** | **T** | **A** | **A** | **C** | **C** | **A** | **A** | **C** | **G** | **T** | **A** | **T** | **T** | **G** | **G** | **A** | **A** | **A** | **C** | **G** | **A** | **C** | **G** | **T** | **C** | **T** | **A** | **T** | **G** | **A** | **A** | **T** | **G** | **G** | **G** | **A** | **C** | **G** | **A** | **T** | **A** | **T** | **C** | **A** | **A** | **C** | **A** | **T** | **C** | **G** | **C** | **C** | **G** | **T** | **C** | **A** | **T** | **T** | **T** | **C** | **T** | **G** | **G** | **T** | **G** | **A** | **T** | **A** | **C** | **T** | **A** | **T** | **C** | **A** | **T** | **A** | **G** | **A** | **T** | **T** | **T** | **G** | **G** | **G** | **C** | **A** | **A** | **T** | **A** | **C** | **G** | **C** | **T** | **T** | **T** | **T** | **G** | **C** | **C** | **A** | **A** | **A** | **A** | **G** | **A** | **T** | **G** | **A** | **T** | **A** | **A** | **T** | **A** | **T** | **T** | **G** | **T** | **G** | **A** | **T** | **T** | **G** | **T** | **A** | **A** | **G** | **A** | **A** | **A** | **A** | **G** | **G** | **G** | **A** | **T** | **A** | **G** | **G** | **C** | **A** | **G** | **A** | **A** | **C** | **A** | **C** | **G** | **G** | **A** | **T** | **C** | **T** | **T** | **A** | **G** | **T** | **A** | **C** | **C** | **T** | **T** | **T** | **G** | **G** | **G** | **C** | **G** | **T** | **A** | **G** | **C** | **C** | **A** | **T** | **C** | **C** | **G** | **A** | **T** | **T** | **A** | **G** | **A** | **G** | **C** | **A** | **T** | **G** | **C** | **G** | **A** | **C** | **A** | **T** | **T** | **A** | **G** | **T** | **A** | **G** | **G** | **A** | **A** | **A** | **T** | **G** | **T** | **C** | **T** | **T** | **G** | **T** | **T** | **T** | **G** | **A** | **A** | **G** | **A** | **A** | **G** | **A** | **G** | **C** | **A** | **A** | **T** | **T** | **T** | **T** | **C** | **T** | **T** | **T** | **G** | **A** | **A** | **A** | **A** | **A** | **T** | **G** | **A** | **A** | **C** | **A** | **A** | **A** | **T** | **C** | **A** | **A** | **A** | **A** | **T** | **C** | **T** | **A** | **C** | **A** | **G** | **C** | **A** | **G** | **T** | **G** | **A** | **T** | **T** | **A** | **T** | **G** | **A** | **T** | **G** | **A** | **A** | **A** | **A** | **C** | **C** | **C** | **T** | **C** | **G** | **T** | **A** | **G** | **A** | **C** | **T** | **G** | **A** | **A** | **G** | **A** | **T** | **T** | **A** | **T** | **T** | **A** | **C** | **T** | **A** | **A** | **T** | **A** | **C** | **C** | **T** | **T** | **G** | **A** | **T** | **T** | **G** | **G** | **A** | **G** | **A** | **T** | **G** | **T** | **C** | **G** | **A** | **G** | **G** | **T** | **G** | **A** | **T** | **A** | **C** | **G** | **A** | **A** | **T** | **A** | **-** | **-** | **-** |
| **N52777** | **A** | **T** | **G** | **A** | **A** | **T** | **A** | **G** | **T** | **T** | **C** | **T** | **T** | **G** | **G** | **C** | **G** | **T** | **T** | **T** | **T** | **T** | **T** | **T** | **G** | **T** | **G** | **G** | **T** | **A** | **G** | **T** | **C** | **G** | **A** | **A** | **G** | **C** | **A** | **C** | **T** | **G** | **C** | **T** | **G** | **C** | **T** | **G** | **T** | **T** | **A** | **T** | **T** | **T** | **G** | **C** | **T** | **G** | **T** | **T** | **T** | **G** | **G** | **C** | **A** | **A** | **A** | **T** | **C** | **G** | **T** | **A** | **A** | **A** | **C** | **A** | **A** | **T** | **A** | **C** | **T** | **G** | **G** | **A** | **C** | **T** | **T** | **T** | **T** | **G** | **C** | **T** | **T** | **T** | **T** | **A** | **G** | **T** | **A** | **A** | **T** | **A** | **T** | **T** | **C** | **G** | **G** | **T** | **A** | **T** | **T** | **T** | **T** | **C** | **A** | **A** | **T** | **A** | **T** | **A** | **T** | **A** | **T** | **C** | **T** | **A** | **G** | **C** | **T** | **A** | **T** | **G** | **C** | **G** | **C** | **A** | **A** | **A** | **T** | **A** | **T** | **C** | **C** | **T** | **C** | **G** | **T** | **A** | **C** | **T** | **A** | **A** | **A** | **T** | **T** | **T** | **C** | **A** | **A** | **A** | **A** | **T** | **T** | **T** | **T** | **C** | **A** | **A** | **T** | **T** | **G** | **A** | **T** | **C** | **T** | **T** | **A** | **G** | **G** | **G** | **A** | **G** | **C** | **T** | **T** | **G** | **G** | **T** | **C** | **A** | **T** | **T** | **T** | **T** | **T** | **T** | **T** | **T** | **A** | **G** | **C** | **T** | **T** | **A** | **G** | **T** | **C** | **A** | **A** | **C** | **A** | **G** | **T** | **C** | **C** | **T** | **G** | **C** | **T** | **T** | **T** | **A** | **T** | **G** | **G** | **A** | **T** | **G** | **A** | **T** | **G** | **G** | **T** | **T** | **G** | **T** | **A** | **T** | **T** | **G** | **G** | **C** | **C** | **G** | **T** | **T** | **T** | **T** | **A** | **T** | **T** | **T** | **A** | **T** | **C** | **G** | **G** | **G** | **C** | **T** | **G** | **A** | **A** | **A** | **G** | **G** | **T** | **G** | **T** | **A** | **G** | **A** | **G** | **A** | **T** | **T** | **T** | **C** | **A** | **G** | **G** | **A** | **A** | **T** | **A** | **G** | **A** | **T** | **T** | **T** | **G** | **A** | **C** | **A** | **A** | **A** | **A** | **A** | **A** | **T** | **G** | **C** | **T** | **T** | **T** | **T** | **T** | **G** | **G** | **C** | **G** | **A** | **A** | **A** | **A** | **A** | **A** | **A** | **C** | **A** | **G** | **A** | **T** | **C** | **A** | **T** | **G** | **A** | **T** | **G** | **G** | **T** | **C** | **C** | **A** | **A** | **A** | **C** | **A** | **G** | **A** | **G** | **C** | **A** | **A** | **T** | **T** | **A** | **A** | **A** | **A** | **A** | **C** | **A** | **C** | **A** | **T** | **A** | **A** | **T** | **A** | **A** | **T** | **G** | **A** | **G** | **C** | **G** | **T** | **A** | **A** | **A** | **A** | **G** | **A** | **C** | **A** | **G** | **C** | **A** | **A** | **T** | **T** | **A** | **T** | **T** | **T** | **G** | **G** | **T** | **A** | **A** | **C** | **C** | **A** | **A** | **C** | **G** | **T** | **A** | **T** | **T** | **G** | **G** | **A** | **A** | **A** | **C** | **G** | **A** | **C** | **G** | **T** | **C** | **T** | **A** | **T** | **G** | **A** | **A** | **T** | **G** | **G** | **G** | **A** | **T** | **G** | **A** | **T** | **A** | **T** | **C** | **A** | **A** | **C** | **A** | **T** | **T** | **G** | **C** | **C** | **A** | **T** | **C** | **A** | **T** | **T** | **T** | **C** | **T** | **G** | **G** | **T** | **G** | **A** | **T** | **A** | **C** | **T** | **A** | **T** | **C** | **A** | **T** | **C** | **G** | **A** | **T** | **T** | **T** | **G** | **G** | **G** | **C** | **A** | **A** | **T** | **A** | **C** | **A** | **C** | **T** | **T** | **T** | **T** | **G** | **C** | **C** | **A** | **A** | **A** | **A** | **G** | **A** | **T** | **G** | **A** | **T** | **A** | **A** | **T** | **A** | **T** | **T** | **G** | **T** | **G** | **A** | **T** | **T** | **G** | **T** | **A** | **A** | **G** | **A** | **A** | **A** | **A** | **G** | **G** | **G** | **A** | **T** | **A** | **G** | **G** | **C** | **A** | **G** | **A** | **A** | **C** | **A** | **C** | **G** | **G** | **A** | **T** | **C** | **T** | **T** | **A** | **G** | **T** | **A** | **C** | **C** | **C** | **C** | **T** | **G** | **G** | **G** | **A** | **G** | **T** | **G** | **G** | **C** | **T** | **A** | **T** | **T** | **C** | **G** | **A** | **T** | **T** | **A** | **G** | **A** | **G** | **C** | **A** | **T** | **G** | **C** | **G** | **A** | **C** | **A** | **T** | **T** | **A** | **G** | **T** | **A** | **G** | **G** | **A** | **A** | **A** | **T** | **G** | **T** | **C** | **T** | **T** | **G** | **T** | **T** | **T** | **G** | **A** | **A** | **G** | **A** | **A** | **G** | **A** | **G** | **C** | **A** | **G** | **T** | **T** | **T** | **T** | **C** | **C** | **T** | **T** | **A** | **A** | **A** | **A** | **A** | **A** | **C** | **G** | **A** | **A** | **C** | **A** | **G** | **A** | **T** | **C** | **A** | **A** | **A** | **A** | **T** | **C** | **T** | **G** | **C** | **A** | **G** | **C** | **A** | **G** | **T** | **G** | **A** | **T** | **T** | **A** | **T** | **G** | **A** | **T** | **G** | **A** | **A** | **A** | **A** | **T** | **C** | **C** | **T** | **C** | **G** | **C** | **A** | **G** | **A** | **T** | **T** | **G** | **A** | **A** | **G** | **A** | **T** | **C** | **A** | **T** | **T** | **A** | **C** | **C** | **A** | **A** | **T** | **A** | **C** | **C** | **T** | **T** | **G** | **G** | **T** | **T** | **G** | **G** | **A** | **G** | **A** | **T** | **G** | **T** | **C** | **G** | **A** | **G** | **G** | **T** | **G** | **A** | **T** | **A** | **C** | **G** | **T** | **A** | **T** | **A** | **-** | **-** | **-** |
| **N55290** | **A** | **T** | **G** | **A** | **A** | **T** | **A** | **G** | **T** | **T** | **C** | **T** | **T** | **G** | **G** | **C** | **G** | **T** | **T** | **T** | **T** | **T** | **T** | **T** | **G** | **T** | **G** | **G** | **T** | **A** | **G** | **T** | **T** | **G** | **A** | **A** | **G** | **C** | **A** | **C** | **T** | **A** | **C** | **T** | **G** | **C** | **T** | **G** | **T** | **T** | **A** | **T** | **T** | **T** | **G** | **C** | **T** | **G** | **T** | **T** | **T** | **G** | **G** | **C** | **A** | **A** | **A** | **T** | **C** | **G** | **T** | **A** | **A** | **A** | **C** | **A** | **A** | **T** | **A** | **C** | **T** | **G** | **G** | **A** | **C** | **T** | **T** | **T** | **T** | **G** | **C** | **T** | **T** | **T** | **T** | **A** | **G** | **T** | **G** | **A** | **T** | **A** | **T** | **T** | **C** | **G** | **G** | **T** | **A** | **T** | **T** | **T** | **T** | **C** | **A** | **A** | **T** | **A** | **T** | **A** | **T** | **A** | **T** | **C** | **T** | **A** | **G** | **C** | **T** | **A** | **T** | **G** | **C** | **G** | **C** | **A** | **A** | **A** | **C** | **A** | **T** | **C** | **C** | **T** | **C** | **G** | **T** | **A** | **C** | **T** | **A** | **A** | **A** | **T** | **T** | **T** | **C** | **A** | **A** | **A** | **A** | **T** | **T** | **T** | **T** | **C** | **A** | **A** | **T** | **T** | **G** | **A** | **T** | **A** | **T** | **T** | **A** | **G** | **G** | **A** | **A** | **G** | **T** | **T** | **T** | **G** | **G** | **T** | **C** | **A** | **T** | **T** | **T** | **T** | **T** | **T** | **T** | **T** | **A** | **G** | **C** | **T** | **T** | **A** | **G** | **T** | **C** | **A** | **A** | **C** | **A** | **G** | **T** | **C** | **C** | **T** | **G** | **C** | **T** | **T** | **T** | **A** | **T** | **G** | **G** | **A** | **T** | **G** | **A** | **T** | **G** | **G** | **C** | **T** | **G** | **T** | **A** | **T** | **T** | **T** | **G** | **C** | **C** | **G** | **T** | **T** | **T** | **T** | **A** | **T** | **T** | **T** | **A** | **T** | **T** | **G** | **G** | **A** | **C** | **T** | **A** | **A** | **A** | **A** | **G** | **G** | **T** | **G** | **T** | **A** | **G** | **A** | **G** | **A** | **T** | **T** | **T** | **C** | **A** | **G** | **G** | **G** | **A** | **T** | **A** | **G** | **A** | **T** | **T** | **T** | **A** | **A** | **C** | **C** | **A** | **A** | **A** | **A** | **A** | **T** | **G** | **C** | **T** | **T** | **T** | **T** | **T** | **G** | **G** | **C** | **G** | **A** | **A** | **A** | **G** | **A** | **A** | **A** | **C** | **A** | **G** | **A** | **T** | **C** | **A** | **T** | **G** | **A** | **T** | **G** | **G** | **T** | **C** | **C** | **A** | **A** | **A** | **C** | **A** | **G** | **A** | **G** | **C** | **A** | **A** | **T** | **T** | **A** | **A** | **A** | **A** | **A** | **C** | **A** | **C** | **A** | **T** | **A** | **A** | **T** | **A** | **A** | **T** | **G** | **A** | **G** | **C** | **G** | **G** | **A** | **A** | **A** | **A** | **G** | **A** | **C** | **A** | **G** | **C** | **A** | **A** | **T** | **T** | **A** | **T** | **T** | **C** | **G** | **G** | **T** | **A** | **A** | **C** | **C** | **A** | **A** | **C** | **G** | **T** | **A** | **T** | **T** | **G** | **G** | **A** | **A** | **A** | **C** | **G** | **A** | **C** | **G** | **T** | **C** | **T** | **A** | **T** | **G** | **A** | **A** | **T** | **G** | **G** | **G** | **A** | **C** | **G** | **A** | **T** | **A** | **T** | **C** | **A** | **A** | **C** | **A** | **T** | **C** | **G** | **C** | **C** | **G** | **T** | **C** | **A** | **T** | **T** | **T** | **C** | **T** | **G** | **G** | **T** | **G** | **A** | **T** | **A** | **C** | **T** | **A** | **T** | **C** | **A** | **T** | **A** | **G** | **A** | **T** | **T** | **T** | **G** | **G** | **G** | **C** | **A** | **A** | **T** | **A** | **C** | **G** | **C** | **T** | **T** | **T** | **T** | **G** | **C** | **C** | **A** | **A** | **A** | **A** | **G** | **A** | **T** | **G** | **A** | **T** | **A** | **A** | **T** | **A** | **T** | **T** | **G** | **T** | **G** | **A** | **T** | **T** | **G** | **T** | **A** | **A** | **G** | **A** | **A** | **A** | **A** | **G** | **G** | **G** | **A** | **T** | **A** | **G** | **G** | **C** | **A** | **G** | **A** | **A** | **C** | **A** | **C** | **G** | **G** | **A** | **T** | **C** | **T** | **T** | **A** | **G** | **T** | **A** | **C** | **C** | **T** | **T** | **T** | **T** | **G** | **G** | **C** | **G** | **T** | **A** | **G** | **C** | **C** | **A** | **T** | **C** | **C** | **G** | **A** | **T** | **T** | **A** | **G** | **A** | **G** | **C** | **A** | **T** | **G** | **C** | **G** | **A** | **C** | **A** | **T** | **T** | **A** | **G** | **T** | **A** | **G** | **G** | **A** | **A** | **A** | **T** | **G** | **T** | **C** | **T** | **T** | **G** | **T** | **T** | **T** | **G** | **A** | **A** | **G** | **A** | **A** | **G** | **A** | **G** | **C** | **A** | **A** | **T** | **T** | **T** | **T** | **C** | **T** | **T** | **T** | **G** | **A** | **A** | **A** | **A** | **A** | **T** | **G** | **A** | **A** | **C** | **A** | **A** | **A** | **T** | **C** | **A** | **A** | **A** | **A** | **T** | **C** | **T** | **A** | **T** | **A** | **G** | **C** | **A** | **G** | **T** | **G** | **A** | **T** | **T** | **A** | **T** | **G** | **A** | **T** | **G** | **A** | **A** | **A** | **A** | **C** | **C** | **C** | **T** | **C** | **G** | **T** | **A** | **G** | **A** | **C** | **T** | **G** | **A** | **A** | **G** | **A** | **T** | **T** | **A** | **T** | **T** | **A** | **C** | **T** | **A** | **A** | **T** | **A** | **C** | **C** | **T** | **T** | **G** | **A** | **T** | **T** | **G** | **G** | **A** | **G** | **A** | **T** | **G** | **T** | **C** | **G** | **A** | **G** | **G** | **T** | **G** | **A** | **T** | **A** | **C** | **G** | **A** | **A** | **T** | **A** | **-** | **-** | **-** |
| **N60299F** | **A** | **T** | **G** | **A** | **A** | **T** | **A** | **G** | **T** | **T** | **C** | **T** | **T** | **G** | **G** | **C** | **G** | **T** | **T** | **T** | **T** | **T** | **T** | **T** | **G** | **T** | **G** | **G** | **T** | **A** | **G** | **T** | **C** | **G** | **A** | **A** | **G** | **C** | **A** | **C** | **T** | **G** | **C** | **T** | **G** | **C** | **T** | **G** | **T** | **T** | **A** | **T** | **T** | **T** | **G** | **C** | **T** | **G** | **T** | **T** | **T** | **G** | **G** | **C** | **A** | **A** | **A** | **T** | **C** | **G** | **T** | **A** | **A** | **A** | **C** | **A** | **A** | **T** | **A** | **C** | **T** | **G** | **G** | **A** | **C** | **T** | **T** | **T** | **T** | **G** | **C** | **T** | **T** | **T** | **T** | **A** | **G** | **T** | **A** | **A** | **T** | **A** | **T** | **T** | **C** | **G** | **G** | **T** | **A** | **T** | **T** | **T** | **T** | **C** | **A** | **A** | **T** | **A** | **T** | **A** | **T** | **A** | **T** | **C** | **T** | **A** | **G** | **C** | **T** | **A** | **T** | **G** | **C** | **G** | **C** | **A** | **A** | **A** | **T** | **A** | **T** | **C** | **C** | **T** | **C** | **G** | **T** | **A** | **C** | **T** | **A** | **A** | **A** | **T** | **T** | **T** | **C** | **A** | **A** | **A** | **A** | **T** | **T** | **T** | **T** | **C** | **A** | **A** | **T** | **T** | **G** | **A** | **T** | **C** | **T** | **T** | **A** | **G** | **G** | **G** | **A** | **G** | **C** | **T** | **T** | **A** | **G** | **T** | **C** | **A** | **T** | **T** | **T** | **T** | **T** | **T** | **T** | **T** | **A** | **G** | **C** | **T** | **T** | **A** | **G** | **T** | **C** | **A** | **A** | **C** | **A** | **G** | **T** | **C** | **C** | **T** | **G** | **C** | **T** | **T** | **T** | **A** | **T** | **G** | **G** | **A** | **T** | **G** | **A** | **T** | **G** | **G** | **T** | **T** | **G** | **T** | **A** | **T** | **T** | **G** | **G** | **C** | **C** | **G** | **T** | **T** | **T** | **T** | **A** | **T** | **T** | **T** | **A** | **T** | **C** | **G** | **G** | **G** | **C** | **T** | **G** | **A** | **A** | **A** | **G** | **G** | **T** | **G** | **T** | **A** | **G** | **A** | **G** | **A** | **T** | **T** | **T** | **C** | **A** | **G** | **G** | **A** | **A** | **T** | **A** | **G** | **A** | **T** | **T** | **T** | **G** | **A** | **C** | **A** | **A** | **A** | **A** | **A** | **A** | **T** | **G** | **C** | **T** | **T** | **T** | **T** | **T** | **G** | **G** | **C** | **G** | **A** | **A** | **A** | **A** | **A** | **A** | **A** | **C** | **A** | **G** | **A** | **T** | **C** | **A** | **T** | **G** | **A** | **T** | **G** | **G** | **T** | **C** | **C** | **A** | **A** | **A** | **C** | **A** | **G** | **A** | **G** | **C** | **A** | **A** | **T** | **T** | **A** | **A** | **A** | **A** | **A** | **C** | **A** | **C** | **A** | **T** | **A** | **A** | **T** | **A** | **A** | **T** | **G** | **A** | **G** | **C** | **G** | **T** | **A** | **A** | **A** | **A** | **G** | **A** | **C** | **A** | **G** | **C** | **A** | **A** | **T** | **T** | **A** | **T** | **T** | **T** | **G** | **G** | **T** | **A** | **A** | **C** | **C** | **A** | **A** | **C** | **G** | **T** | **A** | **T** | **T** | **G** | **G** | **A** | **A** | **A** | **C** | **G** | **A** | **C** | **G** | **T** | **C** | **T** | **A** | **T** | **G** | **A** | **A** | **T** | **G** | **G** | **G** | **A** | **T** | **G** | **A** | **C** | **A** | **T** | **C** | **A** | **A** | **C** | **A** | **T** | **T** | **G** | **C** | **C** | **A** | **T** | **C** | **A** | **T** | **T** | **T** | **C** | **T** | **G** | **G** | **T** | **G** | **A** | **T** | **A** | **C** | **T** | **A** | **T** | **C** | **A** | **T** | **C** | **G** | **A** | **T** | **T** | **T** | **G** | **G** | **G** | **C** | **A** | **A** | **T** | **A** | **C** | **A** | **C** | **T** | **T** | **T** | **T** | **G** | **C** | **C** | **A** | **A** | **A** | **A** | **G** | **A** | **T** | **G** | **A** | **T** | **A** | **A** | **T** | **A** | **T** | **T** | **G** | **T** | **G** | **A** | **T** | **T** | **G** | **T** | **A** | **A** | **G** | **A** | **A** | **A** | **A** | **G** | **G** | **G** | **A** | **T** | **A** | **G** | **G** | **C** | **A** | **G** | **A** | **A** | **C** | **A** | **C** | **G** | **G** | **A** | **T** | **C** | **T** | **T** | **A** | **G** | **T** | **A** | **C** | **C** | **C** | **C** | **T** | **G** | **G** | **G** | **A** | **G** | **T** | **G** | **G** | **C** | **T** | **A** | **T** | **T** | **C** | **G** | **T** | **T** | **T** | **A** | **G** | **A** | **G** | **C** | **A** | **T** | **G** | **C** | **G** | **A** | **C** | **A** | **T** | **T** | **A** | **G** | **T** | **A** | **G** | **G** | **A** | **A** | **A** | **T** | **G** | **T** | **C** | **T** | **T** | **G** | **T** | **T** | **T** | **G** | **A** | **A** | **G** | **A** | **A** | **G** | **A** | **G** | **C** | **A** | **G** | **T** | **T** | **T** | **T** | **C** | **C** | **T** | **T** | **A** | **A** | **A** | **A** | **A** | **A** | **C** | **G** | **A** | **A** | **C** | **A** | **G** | **A** | **T** | **C** | **A** | **A** | **A** | **A** | **T** | **C** | **T** | **G** | **C** | **A** | **G** | **C** | **A** | **G** | **T** | **G** | **A** | **T** | **T** | **A** | **T** | **G** | **A** | **T** | **G** | **A** | **A** | **A** | **A** | **T** | **C** | **C** | **T** | **C** | **G** | **C** | **A** | **G** | **A** | **T** | **T** | **G** | **A** | **A** | **G** | **A** | **T** | **C** | **A** | **T** | **T** | **A** | **C** | **C** | **A** | **A** | **T** | **A** | **C** | **C** | **T** | **T** | **G** | **G** | **T** | **T** | **G** | **G** | **A** | **G** | **A** | **T** | **G** | **T** | **C** | **G** | **A** | **G** | **G** | **T** | **G** | **A** | **T** | **A** | **C** | **G** | **A** | **A** | **T** | **A** | **-** | **-** | **-** |
| **N53595** | **A** | **T** | **G** | **A** | **A** | **T** | **A** | **G** | **T** | **T** | **C** | **T** | **T** | **G** | **G** | **C** | **G** | **T** | **T** | **T** | **T** | **T** | **T** | **T** | **G** | **T** | **G** | **G** | **T** | **A** | **G** | **T** | **T** | **G** | **A** | **A** | **G** | **C** | **A** | **C** | **T** | **A** | **C** | **T** | **G** | **C** | **T** | **G** | **T** | **T** | **A** | **T** | **T** | **T** | **G** | **C** | **T** | **G** | **T** | **T** | **T** | **G** | **G** | **C** | **A** | **A** | **A** | **T** | **C** | **G** | **T** | **A** | **A** | **A** | **C** | **A** | **A** | **T** | **A** | **C** | **T** | **G** | **G** | **A** | **C** | **T** | **T** | **T** | **T** | **G** | **C** | **T** | **T** | **T** | **T** | **A** | **G** | **T** | **G** | **A** | **T** | **A** | **T** | **T** | **C** | **G** | **G** | **T** | **A** | **T** | **T** | **T** | **T** | **C** | **A** | **A** | **T** | **A** | **T** | **A** | **T** | **A** | **T** | **C** | **T** | **A** | **G** | **C** | **T** | **A** | **T** | **G** | **C** | **G** | **C** | **A** | **A** | **A** | **C** | **A** | **T** | **C** | **C** | **T** | **C** | **G** | **T** | **A** | **C** | **T** | **A** | **A** | **A** | **T** | **T** | **T** | **C** | **A** | **A** | **A** | **A** | **T** | **T** | **T** | **T** | **C** | **A** | **A** | **T** | **T** | **G** | **A** | **T** | **A** | **T** | **T** | **A** | **G** | **G** | **A** | **A** | **G** | **T** | **T** | **T** | **G** | **G** | **T** | **C** | **A** | **T** | **T** | **T** | **T** | **T** | **T** | **T** | **T** | **A** | **G** | **C** | **T** | **T** | **A** | **G** | **T** | **C** | **A** | **A** | **C** | **A** | **G** | **T** | **C** | **C** | **T** | **G** | **C** | **T** | **T** | **T** | **A** | **T** | **G** | **G** | **A** | **T** | **G** | **A** | **T** | **G** | **G** | **C** | **T** | **G** | **T** | **A** | **T** | **T** | **T** | **G** | **C** | **C** | **G** | **T** | **T** | **T** | **T** | **A** | **T** | **T** | **T** | **A** | **T** | **T** | **G** | **G** | **A** | **C** | **T** | **A** | **A** | **A** | **A** | **G** | **G** | **T** | **G** | **T** | **A** | **G** | **A** | **G** | **A** | **T** | **T** | **T** | **C** | **A** | **G** | **G** | **G** | **A** | **T** | **A** | **G** | **A** | **T** | **T** | **T** | **A** | **A** | **C** | **C** | **A** | **A** | **A** | **A** | **A** | **T** | **G** | **C** | **T** | **T** | **T** | **T** | **T** | **G** | **G** | **C** | **G** | **A** | **A** | **A** | **G** | **A** | **A** | **A** | **C** | **A** | **G** | **A** | **T** | **C** | **A** | **T** | **G** | **A** | **T** | **G** | **G** | **T** | **C** | **C** | **A** | **A** | **A** | **C** | **A** | **G** | **A** | **G** | **C** | **A** | **A** | **T** | **T** | **A** | **A** | **A** | **A** | **A** | **C** | **A** | **C** | **A** | **T** | **A** | **A** | **T** | **A** | **A** | **T** | **G** | **A** | **G** | **C** | **G** | **G** | **A** | **A** | **A** | **A** | **G** | **A** | **C** | **A** | **G** | **C** | **A** | **A** | **T** | **T** | **A** | **T** | **T** | **C** | **G** | **G** | **T** | **A** | **A** | **C** | **C** | **A** | **A** | **C** | **G** | **T** | **A** | **T** | **T** | **G** | **G** | **A** | **A** | **A** | **C** | **G** | **A** | **C** | **G** | **T** | **C** | **T** | **A** | **T** | **G** | **A** | **A** | **T** | **G** | **G** | **G** | **A** | **C** | **G** | **A** | **T** | **A** | **T** | **C** | **A** | **A** | **C** | **A** | **T** | **C** | **G** | **C** | **C** | **G** | **T** | **C** | **A** | **T** | **T** | **T** | **C** | **T** | **G** | **G** | **T** | **G** | **A** | **T** | **A** | **C** | **T** | **A** | **T** | **C** | **A** | **T** | **A** | **G** | **A** | **T** | **T** | **T** | **G** | **G** | **G** | **C** | **A** | **A** | **T** | **A** | **C** | **G** | **C** | **T** | **T** | **T** | **T** | **G** | **C** | **C** | **A** | **A** | **A** | **A** | **G** | **A** | **T** | **G** | **A** | **T** | **A** | **A** | **T** | **A** | **T** | **T** | **G** | **T** | **G** | **A** | **T** | **T** | **G** | **T** | **A** | **A** | **G** | **A** | **A** | **A** | **A** | **G** | **G** | **G** | **A** | **T** | **A** | **G** | **G** | **C** | **A** | **G** | **A** | **A** | **C** | **A** | **C** | **G** | **G** | **A** | **T** | **C** | **T** | **T** | **A** | **G** | **T** | **A** | **C** | **C** | **T** | **T** | **T** | **G** | **G** | **G** | **C** | **G** | **T** | **A** | **G** | **C** | **C** | **A** | **T** | **C** | **C** | **G** | **A** | **T** | **T** | **A** | **G** | **A** | **G** | **C** | **A** | **T** | **G** | **C** | **G** | **A** | **C** | **A** | **T** | **T** | **A** | **G** | **T** | **A** | **G** | **G** | **A** | **A** | **A** | **T** | **G** | **T** | **C** | **T** | **T** | **G** | **T** | **T** | **T** | **G** | **A** | **A** | **G** | **A** | **A** | **G** | **A** | **G** | **C** | **A** | **A** | **T** | **T** | **T** | **T** | **C** | **T** | **T** | **T** | **G** | **A** | **A** | **A** | **A** | **A** | **T** | **G** | **A** | **A** | **C** | **A** | **A** | **A** | **T** | **C** | **A** | **A** | **A** | **A** | **T** | **C** | **T** | **A** | **C** | **A** | **G** | **C** | **A** | **G** | **T** | **G** | **A** | **T** | **T** | **A** | **T** | **G** | **A** | **T** | **G** | **A** | **A** | **A** | **A** | **C** | **C** | **C** | **T** | **C** | **G** | **T** | **A** | **G** | **A** | **C** | **T** | **G** | **A** | **A** | **G** | **A** | **T** | **T** | **A** | **T** | **T** | **A** | **C** | **T** | **A** | **A** | **T** | **A** | **C** | **C** | **T** | **T** | **G** | **A** | **T** | **T** | **G** | **G** | **A** | **G** | **A** | **T** | **G** | **T** | **C** | **G** | **A** | **G** | **G** | **T** | **G** | **A** | **T** | **A** | **C** | **G** | **A** | **A** | **T** | **A** | **-** | **-** | **-** |
| **N59542F** | **A** | **T** | **G** | **A** | **A** | **T** | **A** | **G** | **T** | **T** | **C** | **T** | **T** | **G** | **G** | **C** | **G** | **T** | **T** | **T** | **T** | **T** | **T** | **T** | **G** | **T** | **G** | **G** | **T** | **A** | **G** | **T** | **C** | **G** | **A** | **A** | **G** | **C** | **A** | **C** | **T** | **G** | **C** | **T** | **G** | **C** | **T** | **G** | **T** | **T** | **A** | **T** | **T** | **T** | **G** | **C** | **T** | **G** | **T** | **T** | **T** | **G** | **G** | **C** | **A** | **A** | **A** | **T** | **C** | **G** | **T** | **A** | **A** | **A** | **C** | **A** | **A** | **T** | **A** | **C** | **T** | **G** | **G** | **A** | **C** | **T** | **T** | **T** | **T** | **G** | **C** | **T** | **T** | **T** | **T** | **A** | **G** | **T** | **A** | **A** | **T** | **A** | **T** | **T** | **C** | **G** | **G** | **T** | **A** | **T** | **T** | **T** | **T** | **C** | **A** | **A** | **T** | **A** | **T** | **A** | **T** | **A** | **T** | **C** | **T** | **A** | **G** | **C** | **T** | **A** | **T** | **G** | **C** | **G** | **C** | **A** | **A** | **A** | **T** | **A** | **T** | **C** | **C** | **T** | **C** | **G** | **T** | **A** | **C** | **T** | **A** | **A** | **A** | **T** | **T** | **T** | **C** | **A** | **A** | **A** | **A** | **T** | **T** | **T** | **T** | **C** | **A** | **A** | **T** | **T** | **G** | **A** | **T** | **C** | **T** | **T** | **A** | **G** | **G** | **G** | **A** | **G** | **C** | **T** | **T** | **G** | **G** | **T** | **C** | **A** | **T** | **T** | **T** | **T** | **T** | **T** | **T** | **T** | **A** | **G** | **C** | **T** | **T** | **A** | **G** | **T** | **C** | **A** | **A** | **C** | **A** | **G** | **T** | **C** | **C** | **T** | **G** | **C** | **T** | **T** | **T** | **A** | **T** | **G** | **G** | **A** | **T** | **G** | **A** | **T** | **G** | **G** | **T** | **T** | **G** | **T** | **A** | **T** | **T** | **G** | **G** | **C** | **C** | **G** | **T** | **T** | **T** | **T** | **A** | **T** | **T** | **T** | **A** | **T** | **C** | **G** | **G** | **G** | **C** | **T** | **G** | **A** | **A** | **A** | **G** | **G** | **T** | **G** | **T** | **A** | **G** | **A** | **G** | **A** | **T** | **T** | **T** | **C** | **A** | **G** | **G** | **A** | **A** | **T** | **A** | **G** | **A** | **T** | **T** | **T** | **G** | **A** | **C** | **A** | **A** | **A** | **A** | **A** | **A** | **T** | **G** | **C** | **T** | **T** | **T** | **T** | **T** | **G** | **G** | **C** | **G** | **A** | **A** | **A** | **A** | **A** | **A** | **A** | **C** | **A** | **G** | **A** | **T** | **C** | **A** | **T** | **G** | **A** | **T** | **G** | **G** | **T** | **C** | **C** | **A** | **A** | **A** | **C** | **A** | **G** | **A** | **G** | **C** | **A** | **A** | **T** | **T** | **A** | **A** | **A** | **A** | **A** | **C** | **A** | **C** | **A** | **T** | **A** | **A** | **T** | **A** | **A** | **T** | **G** | **A** | **G** | **C** | **G** | **T** | **A** | **A** | **A** | **A** | **G** | **A** | **C** | **A** | **G** | **C** | **A** | **A** | **T** | **T** | **A** | **T** | **T** | **T** | **G** | **G** | **T** | **A** | **A** | **C** | **C** | **A** | **A** | **C** | **G** | **T** | **A** | **T** | **T** | **G** | **G** | **A** | **A** | **A** | **C** | **G** | **A** | **C** | **G** | **T** | **C** | **T** | **A** | **T** | **G** | **A** | **A** | **T** | **G** | **G** | **G** | **A** | **T** | **G** | **A** | **T** | **A** | **T** | **C** | **A** | **A** | **C** | **A** | **T** | **T** | **G** | **C** | **C** | **A** | **T** | **C** | **A** | **T** | **T** | **T** | **C** | **T** | **G** | **G** | **T** | **G** | **A** | **T** | **A** | **C** | **T** | **A** | **T** | **C** | **A** | **T** | **C** | **G** | **A** | **T** | **T** | **T** | **G** | **G** | **G** | **C** | **A** | **A** | **T** | **A** | **C** | **A** | **C** | **T** | **T** | **T** | **T** | **G** | **C** | **C** | **A** | **A** | **A** | **A** | **G** | **A** | **T** | **G** | **A** | **T** | **A** | **A** | **T** | **A** | **T** | **T** | **G** | **T** | **G** | **A** | **T** | **T** | **G** | **T** | **A** | **A** | **G** | **A** | **A** | **A** | **A** | **G** | **G** | **G** | **A** | **T** | **A** | **G** | **G** | **C** | **A** | **G** | **A** | **A** | **C** | **A** | **C** | **G** | **G** | **A** | **T** | **C** | **T** | **T** | **A** | **G** | **T** | **A** | **C** | **C** | **C** | **C** | **T** | **G** | **G** | **G** | **A** | **G** | **T** | **G** | **G** | **C** | **T** | **A** | **T** | **T** | **C** | **G** | **T** | **T** | **T** | **A** | **G** | **A** | **G** | **C** | **A** | **T** | **G** | **C** | **G** | **A** | **C** | **A** | **T** | **T** | **A** | **G** | **T** | **A** | **G** | **G** | **A** | **A** | **A** | **T** | **G** | **T** | **C** | **T** | **T** | **G** | **T** | **T** | **T** | **G** | **A** | **A** | **G** | **A** | **A** | **G** | **A** | **G** | **C** | **A** | **G** | **T** | **T** | **T** | **T** | **C** | **C** | **T** | **T** | **A** | **A** | **A** | **A** | **A** | **A** | **C** | **G** | **A** | **A** | **C** | **A** | **G** | **A** | **T** | **C** | **A** | **A** | **A** | **A** | **T** | **C** | **T** | **G** | **C** | **A** | **G** | **C** | **A** | **G** | **T** | **G** | **A** | **T** | **T** | **A** | **T** | **G** | **A** | **T** | **G** | **A** | **A** | **A** | **A** | **T** | **C** | **C** | **T** | **C** | **G** | **C** | **A** | **G** | **A** | **T** | **T** | **G** | **A** | **A** | **G** | **A** | **T** | **C** | **A** | **T** | **T** | **A** | **C** | **C** | **A** | **A** | **T** | **A** | **C** | **C** | **T** | **T** | **G** | **G** | **T** | **T** | **G** | **G** | **A** | **G** | **A** | **T** | **G** | **T** | **C** | **G** | **A** | **G** | **G** | **T** | **G** | **A** | **T** | **A** | **C** | **G** | **A** | **A** | **T** | **A** | **-** | **-** | **-** |
| **N54599** | **A** | **T** | **G** | **A** | **A** | **T** | **A** | **G** | **T** | **T** | **C** | **T** | **T** | **G** | **G** | **C** | **G** | **T** | **T** | **T** | **T** | **T** | **T** | **T** | **G** | **T** | **G** | **G** | **T** | **A** | **G** | **T** | **T** | **G** | **A** | **A** | **G** | **C** | **A** | **C** | **T** | **A** | **C** | **T** | **G** | **C** | **T** | **G** | **T** | **T** | **A** | **T** | **T** | **T** | **G** | **C** | **T** | **G** | **T** | **T** | **T** | **G** | **G** | **C** | **A** | **A** | **A** | **T** | **C** | **G** | **T** | **A** | **A** | **A** | **C** | **A** | **A** | **T** | **A** | **C** | **T** | **G** | **G** | **A** | **C** | **T** | **T** | **T** | **T** | **G** | **C** | **T** | **T** | **T** | **T** | **A** | **G** | **T** | **G** | **A** | **T** | **A** | **T** | **T** | **C** | **G** | **G** | **T** | **A** | **T** | **T** | **T** | **T** | **C** | **A** | **A** | **T** | **A** | **T** | **A** | **T** | **A** | **T** | **C** | **T** | **A** | **G** | **C** | **T** | **A** | **T** | **G** | **C** | **G** | **C** | **A** | **A** | **A** | **C** | **A** | **T** | **C** | **C** | **T** | **C** | **G** | **T** | **A** | **C** | **T** | **A** | **A** | **A** | **T** | **T** | **T** | **C** | **A** | **A** | **A** | **A** | **T** | **T** | **T** | **T** | **C** | **A** | **A** | **T** | **T** | **G** | **A** | **T** | **A** | **T** | **T** | **A** | **G** | **G** | **A** | **A** | **G** | **T** | **T** | **T** | **G** | **G** | **T** | **C** | **A** | **T** | **T** | **T** | **T** | **T** | **T** | **T** | **T** | **A** | **G** | **C** | **T** | **T** | **A** | **G** | **T** | **C** | **A** | **A** | **C** | **A** | **G** | **T** | **C** | **C** | **T** | **G** | **C** | **T** | **T** | **T** | **A** | **T** | **G** | **G** | **A** | **T** | **G** | **A** | **T** | **G** | **G** | **C** | **T** | **G** | **T** | **A** | **T** | **T** | **T** | **G** | **C** | **C** | **G** | **T** | **T** | **T** | **T** | **A** | **T** | **T** | **T** | **A** | **T** | **T** | **G** | **G** | **A** | **C** | **T** | **A** | **A** | **A** | **A** | **G** | **G** | **T** | **G** | **T** | **A** | **G** | **A** | **G** | **A** | **T** | **T** | **T** | **C** | **A** | **G** | **G** | **G** | **A** | **T** | **A** | **G** | **A** | **T** | **T** | **T** | **A** | **A** | **C** | **C** | **A** | **A** | **A** | **A** | **A** | **T** | **G** | **C** | **T** | **T** | **T** | **T** | **T** | **G** | **G** | **C** | **G** | **A** | **A** | **A** | **G** | **A** | **A** | **A** | **C** | **A** | **G** | **A** | **T** | **C** | **A** | **T** | **G** | **A** | **T** | **G** | **G** | **T** | **C** | **C** | **A** | **A** | **A** | **C** | **A** | **G** | **A** | **G** | **C** | **A** | **A** | **T** | **T** | **A** | **A** | **A** | **A** | **A** | **C** | **A** | **C** | **A** | **T** | **A** | **A** | **T** | **A** | **A** | **T** | **G** | **A** | **G** | **C** | **G** | **G** | **A** | **A** | **A** | **A** | **G** | **A** | **C** | **A** | **G** | **C** | **A** | **A** | **T** | **T** | **A** | **T** | **T** | **C** | **G** | **G** | **T** | **A** | **A** | **C** | **C** | **A** | **A** | **C** | **G** | **T** | **A** | **T** | **T** | **G** | **G** | **A** | **A** | **A** | **C** | **G** | **A** | **C** | **G** | **T** | **C** | **T** | **A** | **T** | **G** | **A** | **A** | **T** | **G** | **G** | **G** | **A** | **C** | **G** | **A** | **T** | **A** | **T** | **C** | **A** | **A** | **C** | **A** | **T** | **C** | **G** | **C** | **C** | **G** | **T** | **C** | **A** | **T** | **T** | **T** | **C** | **T** | **G** | **G** | **T** | **G** | **A** | **T** | **A** | **C** | **T** | **A** | **T** | **C** | **A** | **T** | **A** | **G** | **A** | **T** | **T** | **T** | **G** | **G** | **G** | **C** | **A** | **A** | **T** | **A** | **C** | **G** | **C** | **T** | **T** | **T** | **T** | **G** | **C** | **C** | **A** | **A** | **A** | **A** | **G** | **A** | **T** | **G** | **A** | **T** | **A** | **A** | **T** | **A** | **T** | **T** | **G** | **T** | **G** | **A** | **T** | **T** | **G** | **T** | **A** | **A** | **G** | **A** | **A** | **A** | **A** | **G** | **G** | **G** | **A** | **T** | **A** | **G** | **G** | **C** | **A** | **G** | **A** | **A** | **C** | **A** | **C** | **G** | **G** | **A** | **T** | **C** | **T** | **T** | **A** | **G** | **T** | **A** | **C** | **C** | **T** | **T** | **T** | **G** | **G** | **G** | **C** | **G** | **T** | **A** | **G** | **C** | **C** | **A** | **T** | **C** | **C** | **G** | **A** | **T** | **T** | **A** | **G** | **A** | **G** | **C** | **A** | **T** | **G** | **C** | **G** | **A** | **C** | **A** | **T** | **T** | **A** | **G** | **T** | **A** | **G** | **G** | **A** | **A** | **A** | **T** | **G** | **T** | **C** | **T** | **T** | **G** | **T** | **T** | **T** | **G** | **A** | **A** | **G** | **A** | **A** | **G** | **A** | **G** | **C** | **A** | **A** | **T** | **T** | **T** | **T** | **C** | **T** | **T** | **T** | **G** | **A** | **A** | **A** | **A** | **A** | **T** | **G** | **A** | **A** | **C** | **A** | **A** | **A** | **T** | **C** | **A** | **A** | **A** | **A** | **T** | **C** | **T** | **A** | **C** | **A** | **G** | **C** | **A** | **G** | **T** | **G** | **A** | **T** | **T** | **A** | **T** | **G** | **A** | **T** | **G** | **A** | **A** | **A** | **A** | **C** | **C** | **C** | **T** | **C** | **G** | **T** | **A** | **G** | **A** | **C** | **T** | **G** | **A** | **A** | **G** | **A** | **T** | **T** | **A** | **T** | **T** | **A** | **C** | **T** | **A** | **A** | **T** | **A** | **C** | **C** | **T** | **T** | **G** | **A** | **T** | **T** | **G** | **G** | **A** | **G** | **A** | **T** | **G** | **T** | **C** | **G** | **A** | **G** | **G** | **T** | **G** | **A** | **T** | **A** | **C** | **G** | **A** | **A** | **T** | **A** | **-** | **-** | **-** |
| **N54152** | **A** | **T** | **G** | **A** | **A** | **T** | **A** | **G** | **T** | **T** | **C** | **T** | **T** | **G** | **G** | **C** | **G** | **T** | **T** | **T** | **T** | **T** | **T** | **T** | **G** | **T** | **G** | **G** | **T** | **A** | **G** | **T** | **C** | **G** | **A** | **A** | **G** | **C** | **A** | **C** | **T** | **G** | **C** | **T** | **G** | **C** | **T** | **G** | **T** | **T** | **A** | **T** | **T** | **T** | **G** | **C** | **T** | **G** | **T** | **T** | **T** | **G** | **G** | **C** | **A** | **A** | **A** | **T** | **C** | **G** | **T** | **A** | **A** | **A** | **C** | **A** | **A** | **T** | **A** | **C** | **T** | **G** | **G** | **A** | **C** | **T** | **T** | **T** | **T** | **G** | **C** | **T** | **T** | **T** | **T** | **A** | **G** | **T** | **A** | **A** | **T** | **A** | **T** | **T** | **C** | **G** | **G** | **T** | **A** | **T** | **T** | **T** | **T** | **C** | **A** | **A** | **T** | **A** | **T** | **A** | **T** | **A** | **T** | **C** | **T** | **A** | **G** | **C** | **T** | **A** | **T** | **G** | **C** | **G** | **C** | **A** | **A** | **A** | **T** | **A** | **T** | **C** | **C** | **T** | **C** | **G** | **T** | **A** | **C** | **T** | **A** | **A** | **A** | **T** | **T** | **T** | **C** | **A** | **A** | **A** | **A** | **T** | **T** | **T** | **T** | **C** | **A** | **A** | **T** | **T** | **G** | **A** | **T** | **C** | **T** | **T** | **A** | **G** | **G** | **G** | **A** | **G** | **C** | **T** | **T** | **G** | **G** | **T** | **C** | **A** | **T** | **T** | **T** | **T** | **T** | **T** | **T** | **T** | **A** | **G** | **C** | **T** | **T** | **A** | **G** | **T** | **C** | **A** | **A** | **C** | **A** | **G** | **T** | **C** | **C** | **T** | **G** | **C** | **T** | **T** | **T** | **A** | **T** | **G** | **G** | **A** | **T** | **G** | **A** | **T** | **G** | **G** | **T** | **T** | **G** | **T** | **A** | **T** | **T** | **G** | **G** | **C** | **C** | **G** | **T** | **T** | **T** | **T** | **A** | **T** | **T** | **T** | **A** | **T** | **C** | **G** | **G** | **G** | **C** | **T** | **G** | **A** | **A** | **A** | **G** | **G** | **T** | **G** | **T** | **A** | **G** | **A** | **G** | **A** | **T** | **T** | **T** | **C** | **A** | **G** | **G** | **A** | **A** | **T** | **A** | **G** | **A** | **T** | **T** | **T** | **G** | **A** | **C** | **A** | **A** | **A** | **A** | **A** | **A** | **T** | **G** | **C** | **T** | **T** | **T** | **T** | **T** | **G** | **G** | **C** | **G** | **A** | **A** | **A** | **A** | **A** | **A** | **A** | **C** | **A** | **G** | **A** | **T** | **C** | **A** | **T** | **G** | **A** | **T** | **G** | **G** | **T** | **C** | **C** | **A** | **A** | **A** | **C** | **A** | **G** | **A** | **G** | **C** | **A** | **A** | **T** | **T** | **A** | **A** | **A** | **A** | **A** | **C** | **A** | **C** | **A** | **T** | **A** | **A** | **T** | **A** | **A** | **T** | **G** | **A** | **G** | **C** | **G** | **T** | **A** | **A** | **A** | **A** | **G** | **A** | **C** | **A** | **G** | **C** | **A** | **A** | **T** | **T** | **A** | **T** | **T** | **T** | **G** | **G** | **T** | **A** | **A** | **C** | **C** | **A** | **A** | **C** | **G** | **T** | **A** | **T** | **T** | **G** | **G** | **A** | **A** | **A** | **C** | **G** | **A** | **C** | **G** | **T** | **C** | **T** | **A** | **T** | **G** | **A** | **A** | **T** | **G** | **G** | **G** | **A** | **T** | **G** | **A** | **T** | **A** | **T** | **C** | **A** | **A** | **C** | **A** | **T** | **T** | **G** | **C** | **C** | **A** | **T** | **C** | **A** | **T** | **T** | **T** | **C** | **T** | **G** | **G** | **T** | **G** | **A** | **T** | **A** | **C** | **T** | **A** | **T** | **C** | **A** | **T** | **C** | **G** | **A** | **T** | **T** | **T** | **G** | **G** | **G** | **C** | **A** | **A** | **T** | **A** | **C** | **A** | **C** | **T** | **T** | **T** | **T** | **G** | **C** | **C** | **A** | **A** | **A** | **A** | **G** | **A** | **T** | **G** | **A** | **T** | **A** | **A** | **T** | **A** | **T** | **T** | **G** | **T** | **G** | **A** | **T** | **T** | **G** | **T** | **A** | **A** | **G** | **A** | **A** | **A** | **A** | **G** | **G** | **G** | **A** | **T** | **A** | **G** | **G** | **C** | **A** | **G** | **A** | **A** | **C** | **A** | **C** | **G** | **G** | **A** | **T** | **C** | **T** | **T** | **A** | **G** | **T** | **A** | **C** | **C** | **C** | **C** | **T** | **G** | **G** | **G** | **A** | **G** | **T** | **G** | **G** | **C** | **T** | **A** | **T** | **T** | **C** | **G** | **A** | **T** | **T** | **A** | **G** | **A** | **G** | **C** | **A** | **T** | **G** | **C** | **G** | **A** | **C** | **A** | **T** | **T** | **A** | **G** | **T** | **A** | **G** | **G** | **A** | **A** | **A** | **T** | **G** | **T** | **C** | **T** | **T** | **G** | **T** | **T** | **T** | **G** | **A** | **A** | **G** | **A** | **A** | **G** | **A** | **G** | **C** | **A** | **G** | **T** | **T** | **T** | **T** | **C** | **C** | **T** | **T** | **A** | **A** | **A** | **A** | **A** | **A** | **C** | **G** | **A** | **A** | **C** | **A** | **G** | **A** | **T** | **C** | **A** | **A** | **A** | **A** | **T** | **C** | **T** | **G** | **C** | **A** | **G** | **C** | **A** | **G** | **T** | **G** | **A** | **T** | **T** | **A** | **T** | **G** | **A** | **T** | **G** | **A** | **A** | **A** | **A** | **T** | **C** | **C** | **T** | **C** | **G** | **C** | **A** | **G** | **A** | **T** | **T** | **G** | **A** | **A** | **G** | **A** | **T** | **C** | **A** | **T** | **T** | **A** | **C** | **C** | **A** | **A** | **T** | **A** | **C** | **C** | **T** | **T** | **G** | **G** | **T** | **T** | **G** | **G** | **A** | **G** | **A** | **T** | **G** | **T** | **C** | **G** | **A** | **G** | **G** | **T** | **G** | **A** | **T** | **A** | **C** | **G** | **T** | **A** | **T** | **A** | **-** | **-** | **-** |
| **N52656** | **A** | **T** | **G** | **A** | **A** | **T** | **A** | **G** | **T** | **T** | **C** | **T** | **T** | **G** | **G** | **C** | **G** | **T** | **T** | **T** | **T** | **T** | **T** | **T** | **G** | **T** | **G** | **G** | **T** | **A** | **G** | **T** | **T** | **G** | **A** | **A** | **G** | **C** | **A** | **C** | **T** | **A** | **C** | **T** | **G** | **C** | **T** | **G** | **T** | **T** | **A** | **T** | **T** | **T** | **G** | **C** | **T** | **G** | **T** | **T** | **T** | **G** | **G** | **C** | **A** | **A** | **A** | **T** | **C** | **G** | **T** | **A** | **A** | **A** | **C** | **A** | **A** | **T** | **A** | **C** | **T** | **G** | **G** | **A** | **C** | **T** | **T** | **T** | **T** | **G** | **C** | **T** | **T** | **T** | **T** | **A** | **G** | **T** | **G** | **A** | **T** | **A** | **T** | **T** | **C** | **G** | **G** | **T** | **A** | **T** | **T** | **T** | **T** | **C** | **A** | **A** | **T** | **A** | **T** | **A** | **T** | **A** | **T** | **C** | **T** | **A** | **G** | **C** | **T** | **A** | **T** | **G** | **C** | **G** | **C** | **A** | **A** | **A** | **C** | **A** | **T** | **C** | **C** | **T** | **C** | **G** | **T** | **A** | **C** | **T** | **A** | **A** | **A** | **T** | **T** | **T** | **C** | **A** | **A** | **A** | **A** | **T** | **T** | **T** | **T** | **C** | **A** | **A** | **T** | **T** | **G** | **A** | **T** | **A** | **T** | **T** | **A** | **G** | **G** | **A** | **A** | **G** | **T** | **T** | **T** | **G** | **G** | **T** | **C** | **A** | **T** | **T** | **T** | **T** | **T** | **T** | **T** | **T** | **A** | **G** | **C** | **T** | **T** | **A** | **G** | **T** | **C** | **A** | **A** | **C** | **A** | **G** | **T** | **C** | **C** | **T** | **G** | **C** | **T** | **T** | **T** | **A** | **T** | **G** | **G** | **A** | **T** | **G** | **A** | **T** | **G** | **G** | **C** | **T** | **G** | **T** | **A** | **T** | **T** | **T** | **G** | **C** | **C** | **G** | **T** | **T** | **T** | **T** | **A** | **T** | **T** | **T** | **A** | **T** | **T** | **G** | **G** | **A** | **C** | **T** | **A** | **A** | **A** | **A** | **G** | **G** | **T** | **G** | **T** | **A** | **G** | **A** | **G** | **A** | **T** | **T** | **T** | **C** | **A** | **G** | **G** | **G** | **A** | **T** | **A** | **G** | **A** | **T** | **T** | **T** | **A** | **A** | **C** | **C** | **A** | **A** | **A** | **A** | **A** | **T** | **G** | **C** | **T** | **T** | **T** | **T** | **T** | **G** | **G** | **C** | **G** | **A** | **A** | **A** | **G** | **A** | **A** | **A** | **C** | **A** | **G** | **A** | **T** | **C** | **A** | **T** | **G** | **A** | **T** | **G** | **G** | **T** | **C** | **C** | **A** | **A** | **A** | **C** | **A** | **G** | **A** | **G** | **C** | **A** | **A** | **T** | **T** | **A** | **A** | **A** | **A** | **A** | **C** | **A** | **C** | **A** | **T** | **A** | **A** | **T** | **A** | **A** | **T** | **G** | **A** | **G** | **C** | **G** | **G** | **A** | **A** | **A** | **A** | **G** | **A** | **C** | **A** | **G** | **C** | **A** | **A** | **T** | **T** | **A** | **T** | **T** | **C** | **G** | **G** | **T** | **A** | **A** | **C** | **C** | **A** | **A** | **C** | **G** | **T** | **A** | **T** | **T** | **G** | **G** | **A** | **A** | **A** | **C** | **G** | **A** | **C** | **G** | **T** | **C** | **T** | **A** | **T** | **G** | **A** | **A** | **T** | **G** | **G** | **G** | **A** | **C** | **G** | **A** | **T** | **A** | **T** | **C** | **A** | **A** | **C** | **A** | **T** | **C** | **G** | **C** | **C** | **G** | **T** | **C** | **A** | **T** | **T** | **T** | **C** | **T** | **G** | **G** | **T** | **G** | **A** | **T** | **A** | **C** | **T** | **A** | **T** | **C** | **A** | **T** | **A** | **G** | **A** | **T** | **T** | **T** | **G** | **G** | **G** | **C** | **A** | **A** | **T** | **A** | **C** | **G** | **C** | **T** | **T** | **T** | **T** | **G** | **C** | **C** | **A** | **A** | **A** | **A** | **G** | **A** | **T** | **G** | **A** | **T** | **A** | **A** | **T** | **A** | **T** | **T** | **G** | **T** | **G** | **A** | **T** | **T** | **G** | **T** | **A** | **A** | **G** | **A** | **A** | **A** | **A** | **G** | **G** | **G** | **A** | **T** | **A** | **G** | **G** | **C** | **A** | **G** | **A** | **A** | **C** | **A** | **C** | **G** | **A** | **A** | **T** | **C** | **T** | **T** | **A** | **G** | **T** | **A** | **C** | **C** | **T** | **T** | **T** | **G** | **G** | **G** | **C** | **G** | **T** | **A** | **G** | **C** | **C** | **A** | **T** | **C** | **C** | **G** | **A** | **T** | **T** | **A** | **G** | **A** | **G** | **C** | **A** | **T** | **G** | **C** | **G** | **A** | **C** | **A** | **T** | **T** | **A** | **G** | **T** | **A** | **G** | **G** | **A** | **A** | **A** | **T** | **G** | **T** | **C** | **T** | **T** | **G** | **T** | **T** | **T** | **G** | **A** | **A** | **G** | **A** | **A** | **G** | **A** | **G** | **C** | **A** | **A** | **T** | **T** | **T** | **T** | **C** | **T** | **T** | **T** | **G** | **A** | **A** | **A** | **A** | **A** | **T** | **G** | **A** | **A** | **C** | **A** | **A** | **A** | **T** | **C** | **A** | **A** | **A** | **A** | **T** | **C** | **T** | **A** | **C** | **A** | **G** | **C** | **A** | **G** | **T** | **G** | **A** | **T** | **T** | **A** | **T** | **G** | **A** | **T** | **G** | **A** | **A** | **A** | **A** | **C** | **C** | **C** | **T** | **C** | **G** | **T** | **A** | **G** | **A** | **C** | **T** | **G** | **A** | **A** | **G** | **A** | **T** | **T** | **A** | **T** | **T** | **A** | **C** | **T** | **A** | **A** | **T** | **A** | **C** | **C** | **T** | **T** | **G** | **A** | **T** | **T** | **G** | **G** | **A** | **G** | **A** | **T** | **G** | **T** | **C** | **G** | **A** | **G** | **G** | **T** | **G** | **A** | **T** | **A** | **C** | **G** | **A** | **A** | **T** | **A** | **-** | **-** | **-** |
| **N54546** | **A** | **T** | **G** | **A** | **A** | **T** | **A** | **G** | **T** | **T** | **C** | **T** | **T** | **G** | **G** | **C** | **G** | **T** | **T** | **T** | **T** | **T** | **T** | **T** | **G** | **T** | **G** | **G** | **T** | **A** | **G** | **T** | **T** | **G** | **A** | **A** | **G** | **C** | **A** | **C** | **T** | **A** | **C** | **T** | **G** | **C** | **T** | **G** | **T** | **T** | **A** | **T** | **T** | **T** | **G** | **C** | **T** | **G** | **T** | **T** | **T** | **G** | **G** | **C** | **A** | **A** | **A** | **T** | **C** | **G** | **T** | **A** | **A** | **A** | **C** | **A** | **A** | **T** | **A** | **C** | **T** | **G** | **G** | **A** | **C** | **T** | **T** | **T** | **T** | **G** | **C** | **T** | **T** | **T** | **T** | **A** | **G** | **T** | **G** | **A** | **T** | **A** | **T** | **T** | **C** | **G** | **G** | **T** | **A** | **T** | **T** | **T** | **T** | **C** | **A** | **A** | **T** | **A** | **T** | **A** | **T** | **A** | **T** | **C** | **T** | **A** | **G** | **C** | **T** | **A** | **T** | **G** | **C** | **G** | **C** | **A** | **A** | **A** | **C** | **A** | **T** | **C** | **C** | **T** | **C** | **G** | **T** | **A** | **C** | **T** | **A** | **A** | **A** | **T** | **T** | **T** | **C** | **A** | **A** | **A** | **A** | **T** | **T** | **T** | **T** | **C** | **A** | **A** | **T** | **T** | **G** | **A** | **T** | **A** | **T** | **T** | **A** | **G** | **G** | **A** | **A** | **G** | **T** | **T** | **T** | **G** | **G** | **T** | **C** | **A** | **T** | **T** | **T** | **T** | **T** | **T** | **T** | **T** | **A** | **G** | **C** | **T** | **T** | **A** | **G** | **T** | **C** | **A** | **A** | **C** | **A** | **G** | **T** | **C** | **C** | **T** | **G** | **C** | **T** | **T** | **T** | **A** | **T** | **G** | **G** | **A** | **T** | **G** | **A** | **T** | **G** | **G** | **C** | **T** | **G** | **T** | **A** | **T** | **T** | **T** | **G** | **C** | **C** | **G** | **T** | **T** | **T** | **T** | **A** | **T** | **T** | **T** | **A** | **T** | **T** | **G** | **G** | **A** | **C** | **T** | **A** | **A** | **A** | **A** | **G** | **G** | **T** | **G** | **T** | **A** | **G** | **A** | **G** | **A** | **T** | **T** | **T** | **C** | **A** | **G** | **G** | **G** | **A** | **T** | **A** | **G** | **A** | **T** | **T** | **T** | **A** | **A** | **C** | **C** | **A** | **A** | **A** | **A** | **A** | **T** | **G** | **C** | **T** | **T** | **T** | **T** | **T** | **G** | **G** | **C** | **G** | **A** | **A** | **A** | **G** | **A** | **A** | **A** | **C** | **A** | **G** | **A** | **T** | **C** | **A** | **T** | **G** | **A** | **T** | **G** | **G** | **T** | **C** | **C** | **A** | **A** | **A** | **C** | **A** | **G** | **A** | **G** | **C** | **A** | **A** | **T** | **T** | **A** | **A** | **A** | **A** | **A** | **C** | **A** | **C** | **A** | **T** | **A** | **A** | **T** | **A** | **A** | **T** | **G** | **A** | **G** | **C** | **G** | **G** | **A** | **A** | **A** | **A** | **G** | **A** | **C** | **A** | **G** | **C** | **A** | **A** | **T** | **T** | **A** | **T** | **T** | **C** | **G** | **G** | **T** | **A** | **A** | **C** | **C** | **A** | **A** | **C** | **G** | **T** | **A** | **T** | **T** | **G** | **G** | **A** | **A** | **A** | **C** | **G** | **A** | **C** | **G** | **T** | **C** | **T** | **A** | **T** | **G** | **A** | **A** | **T** | **G** | **G** | **G** | **A** | **C** | **G** | **A** | **T** | **A** | **T** | **C** | **A** | **A** | **C** | **A** | **T** | **C** | **G** | **C** | **C** | **G** | **T** | **C** | **A** | **T** | **T** | **T** | **C** | **T** | **G** | **G** | **T** | **G** | **A** | **T** | **A** | **C** | **T** | **A** | **T** | **C** | **A** | **T** | **A** | **G** | **A** | **T** | **T** | **T** | **G** | **G** | **G** | **C** | **A** | **A** | **T** | **A** | **C** | **G** | **C** | **T** | **T** | **T** | **T** | **G** | **C** | **C** | **A** | **A** | **A** | **A** | **G** | **A** | **T** | **G** | **A** | **T** | **A** | **A** | **T** | **A** | **T** | **T** | **G** | **T** | **G** | **A** | **T** | **T** | **G** | **T** | **A** | **A** | **G** | **A** | **A** | **A** | **A** | **G** | **G** | **G** | **A** | **T** | **A** | **G** | **G** | **C** | **A** | **G** | **A** | **A** | **C** | **A** | **C** | **G** | **G** | **A** | **T** | **C** | **T** | **T** | **A** | **G** | **T** | **A** | **C** | **C** | **T** | **T** | **T** | **G** | **G** | **G** | **C** | **G** | **T** | **A** | **G** | **C** | **C** | **A** | **T** | **C** | **C** | **G** | **A** | **T** | **T** | **A** | **G** | **A** | **G** | **C** | **A** | **T** | **G** | **C** | **G** | **A** | **C** | **A** | **T** | **T** | **A** | **G** | **T** | **A** | **G** | **G** | **A** | **A** | **A** | **T** | **G** | **T** | **C** | **T** | **T** | **G** | **T** | **T** | **T** | **G** | **A** | **A** | **G** | **A** | **A** | **G** | **A** | **G** | **C** | **A** | **A** | **T** | **T** | **T** | **T** | **C** | **T** | **T** | **T** | **G** | **A** | **A** | **A** | **A** | **A** | **T** | **G** | **A** | **A** | **C** | **A** | **A** | **A** | **T** | **C** | **A** | **A** | **A** | **A** | **T** | **C** | **T** | **A** | **C** | **A** | **G** | **C** | **A** | **G** | **T** | **G** | **A** | **T** | **T** | **A** | **T** | **G** | **A** | **T** | **G** | **A** | **A** | **A** | **A** | **C** | **C** | **C** | **T** | **C** | **G** | **T** | **A** | **G** | **A** | **C** | **T** | **G** | **A** | **A** | **G** | **A** | **T** | **T** | **A** | **T** | **T** | **A** | **C** | **T** | **A** | **A** | **T** | **A** | **C** | **C** | **T** | **T** | **G** | **A** | **T** | **T** | **G** | **G** | **A** | **G** | **A** | **T** | **G** | **T** | **C** | **G** | **A** | **G** | **G** | **T** | **G** | **A** | **T** | **A** | **C** | **G** | **A** | **A** | **T** | **A** | **-** | **-** | **-** |
| **N52769** | **A** | **T** | **G** | **A** | **A** | **T** | **A** | **G** | **T** | **T** | **C** | **T** | **T** | **G** | **G** | **C** | **G** | **T** | **T** | **T** | **T** | **T** | **T** | **T** | **G** | **T** | **G** | **G** | **T** | **A** | **G** | **T** | **T** | **G** | **A** | **A** | **G** | **C** | **A** | **C** | **T** | **A** | **C** | **T** | **G** | **C** | **T** | **G** | **T** | **T** | **A** | **T** | **T** | **T** | **G** | **C** | **T** | **G** | **T** | **T** | **T** | **G** | **G** | **C** | **A** | **A** | **A** | **T** | **C** | **G** | **T** | **A** | **A** | **A** | **C** | **A** | **A** | **T** | **A** | **C** | **T** | **G** | **G** | **A** | **C** | **T** | **T** | **T** | **T** | **G** | **C** | **T** | **T** | **T** | **T** | **A** | **G** | **T** | **G** | **A** | **T** | **A** | **T** | **T** | **C** | **G** | **G** | **T** | **A** | **T** | **T** | **T** | **T** | **C** | **A** | **A** | **T** | **A** | **T** | **A** | **T** | **A** | **T** | **C** | **T** | **A** | **G** | **C** | **T** | **A** | **T** | **G** | **C** | **G** | **C** | **A** | **A** | **A** | **C** | **A** | **T** | **C** | **C** | **T** | **C** | **G** | **T** | **A** | **C** | **T** | **A** | **A** | **A** | **T** | **T** | **T** | **C** | **A** | **A** | **A** | **A** | **T** | **T** | **T** | **T** | **C** | **A** | **A** | **T** | **T** | **G** | **A** | **T** | **A** | **T** | **T** | **A** | **G** | **G** | **A** | **A** | **G** | **T** | **T** | **T** | **G** | **G** | **T** | **C** | **A** | **T** | **T** | **T** | **T** | **T** | **T** | **T** | **T** | **A** | **G** | **C** | **T** | **T** | **A** | **G** | **T** | **C** | **A** | **A** | **C** | **A** | **G** | **T** | **C** | **C** | **T** | **G** | **C** | **T** | **T** | **T** | **A** | **T** | **G** | **G** | **A** | **T** | **G** | **A** | **T** | **G** | **G** | **C** | **T** | **G** | **T** | **A** | **T** | **T** | **T** | **G** | **C** | **C** | **G** | **T** | **T** | **T** | **T** | **A** | **T** | **T** | **T** | **A** | **T** | **T** | **G** | **G** | **A** | **C** | **T** | **A** | **A** | **A** | **A** | **G** | **G** | **T** | **G** | **T** | **A** | **G** | **A** | **G** | **A** | **T** | **T** | **T** | **C** | **A** | **G** | **G** | **G** | **A** | **T** | **A** | **G** | **A** | **T** | **T** | **T** | **A** | **A** | **C** | **C** | **A** | **A** | **A** | **A** | **A** | **T** | **G** | **C** | **T** | **T** | **T** | **T** | **T** | **G** | **G** | **C** | **G** | **A** | **A** | **A** | **G** | **A** | **A** | **A** | **C** | **A** | **G** | **A** | **T** | **C** | **A** | **T** | **G** | **A** | **T** | **G** | **G** | **T** | **C** | **C** | **A** | **A** | **A** | **C** | **A** | **G** | **A** | **G** | **C** | **A** | **A** | **T** | **T** | **A** | **A** | **A** | **A** | **A** | **C** | **A** | **C** | **A** | **T** | **A** | **A** | **T** | **A** | **A** | **T** | **G** | **A** | **G** | **C** | **G** | **G** | **A** | **A** | **A** | **A** | **G** | **A** | **C** | **A** | **G** | **C** | **A** | **A** | **T** | **T** | **A** | **T** | **T** | **C** | **G** | **G** | **T** | **A** | **A** | **C** | **C** | **A** | **A** | **C** | **G** | **T** | **A** | **T** | **T** | **G** | **G** | **A** | **A** | **A** | **C** | **G** | **A** | **C** | **G** | **T** | **C** | **T** | **A** | **T** | **G** | **A** | **A** | **T** | **G** | **G** | **G** | **A** | **C** | **G** | **A** | **T** | **A** | **T** | **C** | **A** | **A** | **C** | **A** | **T** | **C** | **G** | **C** | **C** | **G** | **T** | **C** | **A** | **T** | **T** | **T** | **C** | **T** | **G** | **G** | **T** | **G** | **A** | **T** | **A** | **C** | **T** | **A** | **T** | **C** | **A** | **T** | **A** | **G** | **A** | **T** | **T** | **T** | **G** | **G** | **G** | **C** | **A** | **A** | **T** | **A** | **C** | **G** | **C** | **T** | **T** | **T** | **T** | **G** | **C** | **C** | **A** | **A** | **A** | **A** | **G** | **A** | **T** | **G** | **A** | **T** | **A** | **A** | **T** | **A** | **T** | **T** | **G** | **T** | **G** | **A** | **T** | **T** | **G** | **T** | **A** | **A** | **G** | **A** | **A** | **A** | **A** | **G** | **G** | **G** | **A** | **T** | **A** | **G** | **G** | **C** | **A** | **G** | **A** | **A** | **C** | **A** | **C** | **G** | **G** | **A** | **T** | **C** | **T** | **T** | **A** | **G** | **T** | **A** | **C** | **C** | **T** | **T** | **T** | **T** | **G** | **G** | **C** | **G** | **T** | **A** | **G** | **C** | **C** | **A** | **T** | **C** | **C** | **G** | **A** | **T** | **T** | **A** | **G** | **A** | **G** | **C** | **A** | **T** | **G** | **C** | **G** | **A** | **C** | **A** | **T** | **T** | **A** | **G** | **T** | **A** | **G** | **G** | **A** | **A** | **A** | **T** | **G** | **T** | **C** | **T** | **T** | **G** | **T** | **T** | **T** | **G** | **A** | **A** | **G** | **A** | **A** | **G** | **A** | **G** | **C** | **A** | **A** | **T** | **T** | **T** | **T** | **C** | **T** | **T** | **T** | **G** | **A** | **A** | **A** | **A** | **A** | **T** | **G** | **A** | **A** | **C** | **A** | **A** | **A** | **T** | **C** | **A** | **A** | **A** | **A** | **T** | **C** | **T** | **A** | **T** | **A** | **G** | **C** | **A** | **G** | **T** | **G** | **A** | **T** | **T** | **A** | **T** | **G** | **A** | **T** | **G** | **A** | **A** | **A** | **A** | **C** | **C** | **C** | **T** | **C** | **G** | **T** | **A** | **G** | **A** | **C** | **T** | **G** | **A** | **A** | **G** | **A** | **T** | **T** | **A** | **T** | **T** | **A** | **C** | **T** | **A** | **A** | **T** | **A** | **C** | **C** | **T** | **T** | **G** | **A** | **T** | **T** | **G** | **G** | **A** | **G** | **A** | **T** | **G** | **T** | **C** | **G** | **A** | **G** | **G** | **T** | **G** | **A** | **T** | **A** | **C** | **G** | **A** | **A** | **T** | **A** | **-** | **-** | **-** |
| **N60045F** | **A** | **T** | **G** | **A** | **A** | **T** | **A** | **G** | **T** | **T** | **C** | **T** | **T** | **G** | **G** | **C** | **G** | **T** | **T** | **T** | **T** | **T** | **T** | **T** | **G** | **T** | **G** | **G** | **T** | **A** | **G** | **T** | **C** | **G** | **A** | **A** | **G** | **C** | **A** | **C** | **T** | **G** | **C** | **T** | **G** | **C** | **T** | **G** | **T** | **T** | **A** | **T** | **T** | **T** | **G** | **C** | **T** | **G** | **T** | **T** | **T** | **G** | **G** | **C** | **A** | **A** | **A** | **T** | **C** | **G** | **T** | **A** | **A** | **A** | **C** | **A** | **A** | **T** | **A** | **C** | **T** | **G** | **G** | **A** | **C** | **T** | **T** | **T** | **T** | **G** | **C** | **T** | **T** | **T** | **T** | **A** | **G** | **T** | **A** | **A** | **T** | **A** | **T** | **T** | **C** | **G** | **G** | **T** | **A** | **T** | **T** | **T** | **T** | **C** | **A** | **A** | **T** | **A** | **T** | **A** | **T** | **A** | **T** | **C** | **T** | **A** | **G** | **C** | **T** | **A** | **T** | **G** | **C** | **G** | **C** | **A** | **A** | **A** | **T** | **A** | **T** | **C** | **C** | **T** | **C** | **G** | **T** | **A** | **C** | **T** | **A** | **A** | **A** | **T** | **T** | **T** | **C** | **A** | **A** | **A** | **A** | **T** | **T** | **T** | **T** | **C** | **A** | **A** | **T** | **T** | **G** | **A** | **T** | **C** | **T** | **T** | **A** | **G** | **G** | **G** | **A** | **G** | **C** | **T** | **T** | **G** | **G** | **T** | **C** | **A** | **T** | **T** | **T** | **T** | **T** | **T** | **T** | **T** | **A** | **G** | **C** | **T** | **T** | **A** | **G** | **T** | **C** | **A** | **A** | **C** | **A** | **G** | **T** | **C** | **C** | **T** | **G** | **C** | **T** | **T** | **T** | **A** | **T** | **G** | **G** | **A** | **T** | **G** | **A** | **T** | **G** | **G** | **T** | **T** | **G** | **T** | **A** | **T** | **T** | **G** | **G** | **C** | **C** | **G** | **T** | **T** | **T** | **T** | **A** | **T** | **T** | **T** | **A** | **T** | **C** | **G** | **G** | **C** | **C** | **T** | **G** | **A** | **A** | **A** | **G** | **G** | **T** | **G** | **T** | **A** | **G** | **A** | **G** | **A** | **T** | **T** | **T** | **C** | **A** | **G** | **G** | **A** | **A** | **T** | **A** | **G** | **A** | **T** | **T** | **T** | **G** | **A** | **C** | **A** | **A** | **A** | **A** | **A** | **A** | **T** | **G** | **C** | **T** | **T** | **T** | **T** | **T** | **G** | **G** | **C** | **G** | **A** | **A** | **A** | **A** | **A** | **A** | **A** | **C** | **A** | **G** | **A** | **T** | **C** | **A** | **T** | **G** | **A** | **T** | **G** | **G** | **T** | **C** | **C** | **A** | **A** | **A** | **C** | **A** | **G** | **A** | **G** | **C** | **A** | **A** | **T** | **T** | **A** | **A** | **A** | **A** | **A** | **C** | **A** | **C** | **A** | **T** | **A** | **A** | **T** | **A** | **A** | **T** | **G** | **A** | **G** | **C** | **G** | **T** | **A** | **A** | **A** | **A** | **G** | **A** | **C** | **A** | **G** | **C** | **A** | **A** | **T** | **T** | **A** | **T** | **T** | **T** | **G** | **G** | **T** | **A** | **A** | **C** | **C** | **A** | **A** | **C** | **G** | **T** | **A** | **T** | **T** | **G** | **G** | **A** | **A** | **A** | **C** | **G** | **A** | **C** | **G** | **T** | **C** | **T** | **A** | **T** | **G** | **A** | **A** | **T** | **G** | **G** | **G** | **A** | **T** | **G** | **A** | **T** | **A** | **T** | **C** | **A** | **A** | **C** | **A** | **T** | **T** | **G** | **C** | **C** | **A** | **T** | **C** | **A** | **T** | **T** | **T** | **C** | **T** | **G** | **G** | **T** | **G** | **A** | **T** | **A** | **C** | **T** | **A** | **T** | **C** | **A** | **T** | **C** | **G** | **A** | **T** | **T** | **T** | **G** | **G** | **G** | **C** | **A** | **A** | **T** | **A** | **C** | **A** | **C** | **T** | **T** | **T** | **T** | **G** | **C** | **C** | **A** | **A** | **A** | **A** | **G** | **A** | **T** | **G** | **A** | **T** | **A** | **A** | **T** | **A** | **T** | **T** | **G** | **T** | **G** | **A** | **T** | **T** | **G** | **T** | **A** | **A** | **G** | **A** | **A** | **A** | **A** | **G** | **G** | **G** | **A** | **T** | **A** | **G** | **G** | **C** | **A** | **G** | **A** | **A** | **C** | **A** | **C** | **G** | **G** | **A** | **T** | **C** | **T** | **T** | **A** | **G** | **T** | **A** | **C** | **C** | **C** | **C** | **T** | **G** | **G** | **G** | **A** | **G** | **T** | **G** | **G** | **C** | **T** | **A** | **T** | **T** | **C** | **G** | **A** | **T** | **T** | **A** | **G** | **A** | **G** | **C** | **A** | **T** | **G** | **C** | **G** | **A** | **C** | **A** | **T** | **T** | **A** | **G** | **T** | **A** | **G** | **G** | **A** | **A** | **A** | **T** | **G** | **T** | **C** | **T** | **T** | **G** | **T** | **T** | **T** | **G** | **A** | **A** | **G** | **A** | **A** | **G** | **A** | **G** | **C** | **A** | **G** | **T** | **T** | **T** | **T** | **C** | **C** | **T** | **T** | **A** | **A** | **A** | **A** | **A** | **A** | **T** | **G** | **A** | **A** | **C** | **A** | **G** | **A** | **T** | **C** | **A** | **A** | **A** | **A** | **T** | **C** | **T** | **G** | **C** | **A** | **G** | **C** | **A** | **G** | **T** | **G** | **A** | **T** | **T** | **A** | **T** | **G** | **A** | **T** | **G** | **A** | **A** | **A** | **A** | **T** | **C** | **C** | **T** | **C** | **G** | **C** | **A** | **G** | **A** | **T** | **T** | **G** | **A** | **A** | **G** | **A** | **T** | **C** | **A** | **T** | **T** | **A** | **C** | **C** | **A** | **A** | **T** | **A** | **C** | **C** | **T** | **T** | **G** | **G** | **T** | **T** | **G** | **G** | **A** | **G** | **A** | **T** | **G** | **T** | **C** | **G** | **A** | **G** | **G** | **T** | **G** | **A** | **T** | **A** | **C** | **G** | **A** | **A** | **T** | **A** | **-** | **-** | **-** |
| **N54155** | **A** | **T** | **G** | **A** | **A** | **T** | **A** | **G** | **T** | **T** | **C** | **T** | **T** | **G** | **G** | **C** | **G** | **T** | **T** | **T** | **T** | **T** | **T** | **T** | **G** | **T** | **G** | **G** | **T** | **A** | **G** | **T** | **T** | **G** | **A** | **A** | **G** | **C** | **A** | **C** | **T** | **A** | **C** | **T** | **G** | **C** | **T** | **G** | **T** | **T** | **A** | **T** | **T** | **T** | **G** | **C** | **T** | **G** | **T** | **T** | **T** | **G** | **G** | **C** | **A** | **A** | **A** | **T** | **C** | **G** | **T** | **A** | **A** | **A** | **C** | **A** | **A** | **T** | **A** | **C** | **T** | **G** | **G** | **A** | **C** | **T** | **T** | **T** | **T** | **G** | **C** | **T** | **T** | **T** | **T** | **A** | **G** | **T** | **G** | **A** | **T** | **A** | **T** | **T** | **C** | **G** | **G** | **T** | **A** | **T** | **T** | **T** | **T** | **C** | **A** | **A** | **T** | **A** | **T** | **A** | **T** | **A** | **T** | **C** | **T** | **A** | **G** | **C** | **T** | **A** | **T** | **G** | **C** | **G** | **C** | **A** | **A** | **A** | **C** | **A** | **T** | **C** | **C** | **T** | **C** | **G** | **T** | **A** | **C** | **T** | **A** | **A** | **A** | **T** | **T** | **T** | **C** | **A** | **A** | **A** | **A** | **T** | **T** | **T** | **T** | **C** | **A** | **A** | **T** | **T** | **G** | **A** | **T** | **A** | **T** | **T** | **A** | **G** | **G** | **A** | **A** | **G** | **T** | **T** | **T** | **G** | **G** | **T** | **C** | **A** | **T** | **T** | **T** | **T** | **T** | **T** | **T** | **T** | **A** | **G** | **C** | **T** | **T** | **A** | **G** | **T** | **C** | **A** | **A** | **C** | **A** | **G** | **T** | **C** | **C** | **T** | **G** | **C** | **T** | **T** | **T** | **A** | **T** | **G** | **G** | **A** | **T** | **G** | **A** | **T** | **G** | **G** | **C** | **T** | **G** | **T** | **A** | **T** | **T** | **T** | **G** | **C** | **C** | **G** | **T** | **T** | **T** | **T** | **A** | **T** | **T** | **T** | **A** | **T** | **T** | **G** | **G** | **A** | **C** | **T** | **A** | **A** | **A** | **A** | **G** | **G** | **T** | **G** | **T** | **A** | **G** | **A** | **G** | **A** | **T** | **T** | **T** | **C** | **A** | **G** | **G** | **G** | **A** | **T** | **A** | **G** | **A** | **T** | **T** | **T** | **A** | **A** | **C** | **C** | **A** | **A** | **A** | **A** | **A** | **T** | **G** | **C** | **T** | **T** | **T** | **T** | **T** | **G** | **G** | **C** | **G** | **A** | **A** | **A** | **G** | **A** | **A** | **A** | **C** | **A** | **G** | **A** | **T** | **C** | **A** | **T** | **G** | **A** | **T** | **G** | **G** | **T** | **C** | **C** | **A** | **A** | **A** | **C** | **A** | **G** | **A** | **G** | **C** | **A** | **A** | **T** | **T** | **A** | **A** | **A** | **A** | **A** | **C** | **A** | **C** | **A** | **T** | **A** | **A** | **T** | **A** | **A** | **T** | **G** | **A** | **G** | **C** | **G** | **G** | **A** | **A** | **A** | **A** | **G** | **A** | **C** | **A** | **G** | **C** | **A** | **A** | **T** | **T** | **A** | **T** | **T** | **C** | **G** | **G** | **T** | **A** | **A** | **C** | **C** | **A** | **A** | **C** | **G** | **T** | **A** | **T** | **T** | **G** | **G** | **A** | **A** | **A** | **C** | **G** | **A** | **C** | **G** | **T** | **C** | **T** | **A** | **T** | **G** | **A** | **A** | **T** | **G** | **G** | **G** | **A** | **C** | **G** | **A** | **T** | **A** | **T** | **C** | **A** | **A** | **C** | **A** | **T** | **C** | **G** | **C** | **C** | **G** | **T** | **C** | **A** | **T** | **T** | **T** | **C** | **T** | **G** | **G** | **T** | **G** | **A** | **T** | **A** | **C** | **T** | **A** | **T** | **C** | **A** | **T** | **A** | **G** | **A** | **T** | **T** | **T** | **G** | **G** | **G** | **C** | **A** | **A** | **T** | **A** | **C** | **G** | **C** | **T** | **T** | **T** | **T** | **G** | **C** | **C** | **A** | **A** | **A** | **A** | **G** | **A** | **T** | **G** | **A** | **T** | **A** | **A** | **T** | **A** | **T** | **T** | **G** | **T** | **G** | **A** | **T** | **T** | **G** | **T** | **A** | **A** | **G** | **A** | **A** | **A** | **A** | **G** | **G** | **G** | **A** | **T** | **A** | **G** | **G** | **C** | **A** | **G** | **A** | **A** | **C** | **A** | **C** | **G** | **G** | **A** | **T** | **C** | **T** | **T** | **A** | **G** | **T** | **A** | **C** | **C** | **T** | **T** | **T** | **G** | **G** | **G** | **C** | **G** | **T** | **A** | **G** | **C** | **C** | **A** | **T** | **C** | **C** | **G** | **A** | **T** | **T** | **A** | **G** | **A** | **G** | **C** | **A** | **T** | **G** | **C** | **G** | **A** | **C** | **A** | **T** | **T** | **A** | **G** | **T** | **A** | **G** | **G** | **A** | **A** | **A** | **T** | **G** | **T** | **C** | **T** | **T** | **G** | **T** | **T** | **T** | **G** | **A** | **A** | **G** | **A** | **A** | **G** | **A** | **G** | **C** | **A** | **A** | **T** | **T** | **T** | **T** | **C** | **T** | **T** | **T** | **G** | **A** | **A** | **A** | **A** | **A** | **T** | **G** | **A** | **A** | **C** | **A** | **A** | **A** | **T** | **C** | **A** | **A** | **A** | **A** | **T** | **C** | **T** | **A** | **C** | **A** | **G** | **C** | **A** | **G** | **T** | **G** | **A** | **T** | **T** | **A** | **T** | **G** | **A** | **T** | **G** | **A** | **A** | **A** | **A** | **C** | **C** | **C** | **T** | **C** | **G** | **T** | **A** | **G** | **A** | **C** | **T** | **G** | **A** | **A** | **G** | **A** | **T** | **T** | **A** | **T** | **T** | **A** | **C** | **T** | **A** | **A** | **T** | **A** | **C** | **C** | **T** | **T** | **G** | **A** | **T** | **T** | **G** | **G** | **A** | **G** | **A** | **T** | **G** | **T** | **C** | **G** | **A** | **G** | **G** | **T** | **G** | **A** | **T** | **A** | **C** | **G** | **A** | **A** | **T** | **A** | **-** | **-** | **-** |
| **N52643** | **A** | **T** | **G** | **A** | **A** | **T** | **A** | **G** | **T** | **T** | **C** | **T** | **T** | **G** | **G** | **C** | **G** | **T** | **T** | **T** | **T** | **T** | **T** | **T** | **G** | **T** | **G** | **G** | **T** | **A** | **G** | **T** | **C** | **G** | **A** | **A** | **G** | **C** | **A** | **C** | **T** | **G** | **C** | **T** | **G** | **C** | **T** | **G** | **T** | **T** | **A** | **T** | **T** | **T** | **G** | **C** | **T** | **G** | **T** | **T** | **T** | **G** | **G** | **C** | **A** | **A** | **A** | **T** | **C** | **G** | **T** | **A** | **A** | **A** | **C** | **A** | **A** | **T** | **A** | **C** | **T** | **G** | **G** | **A** | **C** | **T** | **T** | **T** | **T** | **G** | **C** | **T** | **T** | **T** | **T** | **A** | **G** | **T** | **A** | **A** | **T** | **A** | **T** | **T** | **C** | **G** | **G** | **T** | **A** | **T** | **T** | **T** | **T** | **C** | **A** | **A** | **T** | **A** | **T** | **A** | **T** | **A** | **T** | **C** | **T** | **A** | **G** | **C** | **T** | **A** | **T** | **G** | **C** | **G** | **C** | **A** | **A** | **A** | **T** | **A** | **T** | **C** | **C** | **T** | **C** | **G** | **T** | **A** | **C** | **T** | **A** | **A** | **A** | **T** | **T** | **T** | **C** | **A** | **A** | **A** | **A** | **T** | **T** | **T** | **T** | **C** | **A** | **A** | **T** | **T** | **G** | **A** | **T** | **C** | **T** | **T** | **A** | **G** | **G** | **G** | **A** | **G** | **C** | **T** | **T** | **G** | **G** | **T** | **C** | **A** | **T** | **T** | **T** | **T** | **T** | **T** | **T** | **T** | **A** | **G** | **C** | **T** | **T** | **A** | **G** | **T** | **C** | **A** | **A** | **C** | **A** | **G** | **T** | **C** | **C** | **T** | **G** | **C** | **T** | **T** | **T** | **A** | **T** | **G** | **G** | **A** | **T** | **G** | **A** | **T** | **G** | **G** | **T** | **T** | **G** | **T** | **A** | **T** | **T** | **G** | **G** | **C** | **C** | **A** | **T** | **T** | **T** | **T** | **A** | **T** | **T** | **T** | **A** | **T** | **C** | **G** | **G** | **A** | **C** | **T** | **G** | **A** | **A** | **A** | **G** | **G** | **T** | **G** | **T** | **A** | **G** | **A** | **G** | **A** | **T** | **T** | **T** | **C** | **A** | **G** | **G** | **A** | **A** | **T** | **A** | **G** | **A** | **T** | **T** | **T** | **G** | **A** | **C** | **A** | **A** | **A** | **A** | **A** | **A** | **T** | **G** | **C** | **T** | **T** | **T** | **T** | **T** | **G** | **G** | **C** | **G** | **A** | **A** | **A** | **A** | **A** | **A** | **A** | **C** | **A** | **G** | **A** | **T** | **T** | **A** | **T** | **G** | **A** | **T** | **G** | **G** | **T** | **C** | **C** | **A** | **A** | **A** | **C** | **A** | **G** | **A** | **G** | **C** | **A** | **A** | **T** | **T** | **A** | **A** | **A** | **A** | **A** | **C** | **A** | **C** | **A** | **T** | **A** | **A** | **T** | **A** | **A** | **T** | **G** | **A** | **G** | **C** | **G** | **T** | **A** | **A** | **A** | **A** | **G** | **A** | **C** | **A** | **G** | **C** | **A** | **A** | **T** | **T** | **A** | **T** | **T** | **T** | **G** | **G** | **T** | **A** | **A** | **C** | **C** | **A** | **A** | **C** | **G** | **T** | **A** | **T** | **T** | **G** | **G** | **A** | **A** | **A** | **C** | **G** | **A** | **C** | **G** | **T** | **C** | **T** | **A** | **T** | **G** | **A** | **A** | **T** | **G** | **G** | **G** | **A** | **T** | **G** | **A** | **T** | **A** | **T** | **C** | **A** | **A** | **C** | **A** | **T** | **T** | **G** | **C** | **C** | **A** | **T** | **C** | **A** | **T** | **T** | **T** | **C** | **T** | **G** | **G** | **T** | **G** | **A** | **T** | **A** | **C** | **T** | **A** | **T** | **C** | **A** | **T** | **C** | **G** | **A** | **T** | **T** | **T** | **G** | **G** | **G** | **C** | **A** | **A** | **T** | **A** | **C** | **A** | **C** | **T** | **T** | **T** | **T** | **G** | **C** | **C** | **A** | **A** | **A** | **A** | **G** | **A** | **T** | **G** | **A** | **T** | **A** | **A** | **T** | **A** | **T** | **T** | **G** | **T** | **G** | **A** | **T** | **T** | **G** | **T** | **A** | **A** | **G** | **A** | **A** | **A** | **A** | **G** | **G** | **G** | **A** | **T** | **A** | **G** | **G** | **C** | **A** | **G** | **A** | **A** | **C** | **A** | **C** | **G** | **G** | **A** | **T** | **C** | **T** | **T** | **A** | **G** | **T** | **A** | **C** | **C** | **C** | **C** | **T** | **G** | **G** | **G** | **A** | **G** | **T** | **G** | **G** | **C** | **T** | **A** | **T** | **T** | **C** | **G** | **A** | **T** | **T** | **A** | **G** | **A** | **G** | **C** | **A** | **T** | **G** | **C** | **G** | **A** | **C** | **A** | **T** | **T** | **A** | **G** | **T** | **A** | **G** | **G** | **A** | **A** | **A** | **T** | **G** | **T** | **C** | **T** | **T** | **G** | **T** | **T** | **T** | **G** | **A** | **A** | **G** | **A** | **A** | **G** | **A** | **G** | **C** | **A** | **G** | **T** | **T** | **T** | **T** | **C** | **C** | **T** | **T** | **A** | **A** | **A** | **A** | **A** | **A** | **C** | **G** | **A** | **A** | **C** | **A** | **G** | **A** | **T** | **C** | **A** | **A** | **A** | **A** | **T** | **C** | **T** | **G** | **C** | **A** | **G** | **C** | **A** | **G** | **T** | **G** | **A** | **T** | **T** | **A** | **T** | **G** | **A** | **T** | **G** | **A** | **A** | **A** | **A** | **T** | **C** | **C** | **T** | **C** | **G** | **C** | **A** | **G** | **A** | **T** | **T** | **G** | **A** | **A** | **G** | **A** | **T** | **C** | **A** | **T** | **T** | **A** | **C** | **C** | **A** | **A** | **T** | **A** | **C** | **C** | **T** | **T** | **G** | **G** | **T** | **T** | **G** | **G** | **A** | **G** | **A** | **T** | **G** | **T** | **C** | **G** | **A** | **G** | **G** | **T** | **G** | **A** | **T** | **A** | **C** | **G** | **A** | **A** | **T** | **A** | **-** | **-** | **-** |
| **N60357F** | **A** | **T** | **G** | **A** | **A** | **T** | **A** | **G** | **T** | **T** | **C** | **T** | **T** | **G** | **G** | **C** | **G** | **T** | **T** | **T** | **T** | **T** | **T** | **T** | **G** | **T** | **G** | **G** | **T** | **A** | **G** | **T** | **C** | **G** | **A** | **A** | **G** | **C** | **A** | **C** | **T** | **G** | **C** | **T** | **G** | **C** | **T** | **G** | **T** | **T** | **A** | **T** | **T** | **T** | **G** | **C** | **T** | **G** | **T** | **T** | **T** | **G** | **G** | **C** | **A** | **A** | **A** | **T** | **C** | **G** | **T** | **A** | **A** | **A** | **C** | **A** | **A** | **T** | **A** | **C** | **T** | **G** | **G** | **A** | **C** | **T** | **T** | **T** | **T** | **G** | **C** | **T** | **T** | **T** | **T** | **A** | **G** | **T** | **A** | **A** | **T** | **A** | **T** | **T** | **C** | **G** | **G** | **T** | **A** | **T** | **C** | **T** | **T** | **C** | **A** | **A** | **T** | **A** | **T** | **A** | **T** | **A** | **T** | **C** | **T** | **A** | **G** | **C** | **T** | **A** | **T** | **G** | **C** | **G** | **C** | **A** | **A** | **A** | **T** | **A** | **T** | **C** | **C** | **T** | **C** | **G** | **T** | **A** | **C** | **T** | **A** | **A** | **A** | **T** | **T** | **T** | **C** | **A** | **A** | **A** | **A** | **T** | **T** | **T** | **T** | **C** | **A** | **A** | **T** | **T** | **G** | **A** | **T** | **C** | **T** | **T** | **A** | **G** | **G** | **G** | **A** | **G** | **C** | **T** | **T** | **A** | **G** | **T** | **C** | **A** | **T** | **T** | **T** | **T** | **T** | **T** | **T** | **T** | **A** | **G** | **C** | **T** | **T** | **A** | **G** | **T** | **C** | **A** | **A** | **C** | **A** | **G** | **T** | **C** | **C** | **T** | **G** | **C** | **T** | **T** | **T** | **A** | **T** | **G** | **G** | **A** | **T** | **G** | **A** | **T** | **G** | **G** | **T** | **T** | **G** | **T** | **A** | **T** | **T** | **G** | **G** | **C** | **C** | **G** | **T** | **T** | **T** | **T** | **A** | **T** | **T** | **T** | **A** | **T** | **C** | **G** | **G** | **G** | **C** | **T** | **G** | **A** | **A** | **A** | **G** | **G** | **T** | **G** | **T** | **A** | **G** | **A** | **G** | **A** | **T** | **T** | **T** | **C** | **A** | **G** | **G** | **A** | **A** | **T** | **A** | **G** | **A** | **T** | **T** | **T** | **G** | **A** | **C** | **A** | **A** | **A** | **A** | **A** | **A** | **T** | **G** | **C** | **T** | **T** | **T** | **T** | **T** | **G** | **G** | **C** | **G** | **A** | **A** | **A** | **A** | **A** | **A** | **A** | **C** | **A** | **G** | **A** | **T** | **C** | **A** | **T** | **G** | **A** | **T** | **G** | **G** | **T** | **C** | **C** | **A** | **A** | **A** | **C** | **A** | **G** | **A** | **G** | **C** | **A** | **A** | **T** | **T** | **A** | **A** | **A** | **A** | **A** | **C** | **A** | **C** | **A** | **T** | **A** | **A** | **T** | **A** | **A** | **T** | **G** | **A** | **G** | **C** | **G** | **T** | **A** | **A** | **A** | **A** | **G** | **A** | **C** | **A** | **G** | **C** | **A** | **A** | **T** | **T** | **A** | **T** | **T** | **T** | **G** | **G** | **T** | **A** | **A** | **C** | **C** | **A** | **A** | **C** | **G** | **T** | **A** | **T** | **T** | **G** | **G** | **A** | **A** | **A** | **C** | **G** | **A** | **C** | **G** | **T** | **C** | **T** | **A** | **T** | **G** | **A** | **A** | **T** | **G** | **G** | **G** | **A** | **T** | **G** | **A** | **C** | **A** | **T** | **C** | **A** | **A** | **C** | **A** | **T** | **T** | **G** | **C** | **C** | **A** | **T** | **C** | **A** | **T** | **T** | **T** | **C** | **T** | **G** | **G** | **T** | **G** | **A** | **T** | **A** | **C** | **T** | **A** | **T** | **C** | **A** | **T** | **C** | **G** | **A** | **T** | **T** | **T** | **G** | **G** | **G** | **C** | **A** | **A** | **T** | **A** | **C** | **A** | **C** | **T** | **T** | **T** | **T** | **G** | **C** | **C** | **A** | **A** | **A** | **A** | **G** | **A** | **T** | **G** | **A** | **T** | **A** | **A** | **T** | **A** | **T** | **T** | **G** | **T** | **G** | **A** | **T** | **T** | **G** | **T** | **A** | **A** | **G** | **A** | **A** | **A** | **A** | **G** | **G** | **G** | **A** | **T** | **A** | **G** | **G** | **C** | **A** | **G** | **A** | **A** | **C** | **A** | **C** | **G** | **G** | **A** | **T** | **C** | **T** | **T** | **A** | **G** | **T** | **A** | **C** | **C** | **C** | **C** | **T** | **G** | **G** | **G** | **A** | **G** | **T** | **G** | **G** | **C** | **T** | **A** | **T** | **T** | **C** | **G** | **T** | **T** | **T** | **A** | **G** | **A** | **G** | **C** | **A** | **T** | **G** | **C** | **G** | **A** | **C** | **A** | **T** | **T** | **A** | **G** | **T** | **A** | **G** | **G** | **A** | **A** | **A** | **T** | **G** | **T** | **C** | **T** | **T** | **G** | **T** | **T** | **T** | **G** | **A** | **A** | **G** | **A** | **A** | **G** | **A** | **G** | **C** | **A** | **G** | **T** | **T** | **T** | **T** | **C** | **C** | **T** | **T** | **A** | **A** | **A** | **A** | **A** | **A** | **C** | **G** | **A** | **A** | **C** | **A** | **G** | **A** | **T** | **C** | **A** | **A** | **A** | **A** | **T** | **C** | **T** | **G** | **C** | **A** | **G** | **C** | **A** | **G** | **T** | **G** | **A** | **T** | **T** | **A** | **T** | **G** | **A** | **T** | **G** | **A** | **A** | **A** | **A** | **T** | **C** | **C** | **T** | **C** | **G** | **C** | **A** | **G** | **A** | **T** | **T** | **G** | **A** | **A** | **G** | **A** | **T** | **C** | **A** | **T** | **T** | **A** | **C** | **C** | **A** | **A** | **T** | **A** | **C** | **C** | **T** | **T** | **G** | **G** | **T** | **T** | **G** | **G** | **A** | **G** | **A** | **T** | **G** | **T** | **C** | **G** | **A** | **G** | **G** | **T** | **G** | **A** | **T** | **A** | **C** | **G** | **A** | **A** | **T** | **A** | **-** | **-** | **-** |
| **N60001F** | **A** | **T** | **G** | **A** | **A** | **T** | **A** | **G** | **T** | **T** | **C** | **T** | **T** | **G** | **G** | **C** | **G** | **T** | **T** | **T** | **T** | **T** | **T** | **T** | **G** | **T** | **G** | **G** | **T** | **A** | **G** | **T** | **T** | **G** | **A** | **A** | **G** | **C** | **A** | **C** | **T** | **A** | **C** | **T** | **G** | **C** | **T** | **G** | **T** | **T** | **A** | **T** | **T** | **T** | **G** | **C** | **T** | **G** | **T** | **T** | **T** | **G** | **G** | **C** | **A** | **A** | **A** | **T** | **C** | **G** | **T** | **A** | **A** | **A** | **C** | **A** | **A** | **T** | **A** | **C** | **T** | **G** | **G** | **A** | **C** | **T** | **T** | **T** | **T** | **G** | **C** | **T** | **T** | **T** | **T** | **A** | **G** | **T** | **G** | **A** | **T** | **A** | **T** | **T** | **C** | **G** | **G** | **T** | **A** | **T** | **T** | **T** | **T** | **C** | **A** | **A** | **T** | **A** | **T** | **A** | **T** | **A** | **T** | **C** | **T** | **A** | **G** | **C** | **T** | **A** | **T** | **G** | **C** | **G** | **C** | **A** | **A** | **A** | **C** | **A** | **T** | **C** | **C** | **T** | **C** | **G** | **T** | **A** | **C** | **T** | **A** | **A** | **A** | **T** | **T** | **T** | **C** | **A** | **A** | **A** | **A** | **T** | **T** | **T** | **T** | **C** | **A** | **A** | **T** | **T** | **G** | **A** | **T** | **A** | **T** | **T** | **A** | **G** | **G** | **A** | **A** | **G** | **T** | **T** | **T** | **G** | **G** | **T** | **C** | **A** | **T** | **T** | **T** | **T** | **T** | **T** | **T** | **T** | **A** | **G** | **C** | **T** | **T** | **A** | **G** | **T** | **C** | **A** | **A** | **C** | **A** | **G** | **T** | **C** | **C** | **T** | **G** | **C** | **T** | **T** | **T** | **A** | **T** | **G** | **G** | **A** | **T** | **G** | **A** | **T** | **G** | **G** | **C** | **T** | **G** | **T** | **A** | **T** | **T** | **T** | **G** | **C** | **C** | **G** | **T** | **T** | **T** | **T** | **A** | **T** | **T** | **T** | **A** | **T** | **T** | **G** | **G** | **T** | **C** | **T** | **G** | **A** | **A** | **A** | **G** | **G** | **T** | **G** | **T** | **A** | **G** | **A** | **G** | **A** | **T** | **T** | **T** | **C** | **A** | **G** | **G** | **G** | **A** | **T** | **A** | **G** | **A** | **T** | **T** | **T** | **A** | **A** | **C** | **C** | **A** | **A** | **A** | **A** | **A** | **T** | **G** | **C** | **T** | **T** | **T** | **T** | **T** | **G** | **G** | **C** | **G** | **A** | **A** | **A** | **G** | **A** | **A** | **A** | **C** | **A** | **G** | **A** | **T** | **C** | **A** | **T** | **G** | **A** | **T** | **G** | **A** | **T** | **C** | **C** | **A** | **A** | **A** | **C** | **A** | **G** | **A** | **G** | **C** | **A** | **A** | **T** | **T** | **A** | **A** | **A** | **A** | **A** | **C** | **A** | **C** | **A** | **T** | **A** | **A** | **T** | **A** | **A** | **T** | **G** | **A** | **G** | **C** | **G** | **G** | **A** | **A** | **A** | **A** | **G** | **A** | **C** | **A** | **G** | **C** | **A** | **A** | **T** | **T** | **A** | **T** | **T** | **C** | **G** | **G** | **T** | **A** | **A** | **C** | **C** | **A** | **A** | **C** | **G** | **T** | **A** | **T** | **T** | **G** | **G** | **A** | **A** | **A** | **C** | **G** | **A** | **C** | **G** | **T** | **C** | **T** | **A** | **T** | **G** | **A** | **A** | **T** | **G** | **G** | **G** | **A** | **C** | **G** | **A** | **T** | **A** | **T** | **C** | **A** | **A** | **C** | **A** | **T** | **C** | **G** | **C** | **C** | **G** | **T** | **C** | **A** | **T** | **T** | **T** | **C** | **T** | **G** | **G** | **T** | **G** | **A** | **T** | **A** | **C** | **T** | **A** | **T** | **C** | **A** | **T** | **A** | **G** | **A** | **T** | **T** | **T** | **G** | **G** | **G** | **C** | **A** | **A** | **T** | **A** | **C** | **G** | **C** | **T** | **T** | **T** | **T** | **G** | **C** | **C** | **A** | **A** | **A** | **A** | **G** | **A** | **T** | **G** | **A** | **T** | **A** | **A** | **T** | **A** | **T** | **T** | **G** | **T** | **G** | **A** | **T** | **T** | **G** | **T** | **A** | **A** | **G** | **A** | **A** | **A** | **A** | **G** | **G** | **G** | **A** | **T** | **A** | **G** | **G** | **C** | **A** | **G** | **A** | **A** | **C** | **A** | **C** | **G** | **G** | **A** | **T** | **C** | **T** | **T** | **A** | **G** | **T** | **A** | **C** | **C** | **T** | **T** | **T** | **G** | **G** | **G** | **C** | **G** | **T** | **A** | **G** | **C** | **C** | **A** | **T** | **C** | **C** | **G** | **A** | **T** | **T** | **A** | **G** | **A** | **G** | **C** | **A** | **T** | **G** | **C** | **G** | **A** | **C** | **A** | **T** | **T** | **A** | **G** | **T** | **A** | **G** | **G** | **A** | **A** | **A** | **T** | **G** | **T** | **C** | **T** | **T** | **G** | **T** | **T** | **T** | **G** | **A** | **A** | **G** | **A** | **A** | **G** | **A** | **G** | **C** | **A** | **A** | **T** | **T** | **T** | **T** | **C** | **T** | **T** | **T** | **G** | **A** | **A** | **A** | **A** | **A** | **T** | **G** | **A** | **A** | **C** | **A** | **A** | **A** | **T** | **C** | **A** | **A** | **A** | **A** | **T** | **C** | **T** | **A** | **C** | **A** | **G** | **C** | **A** | **G** | **T** | **G** | **A** | **T** | **T** | **A** | **T** | **G** | **A** | **T** | **G** | **A** | **A** | **A** | **A** | **C** | **C** | **C** | **T** | **C** | **G** | **T** | **A** | **G** | **A** | **T** | **T** | **G** | **A** | **A** | **G** | **A** | **T** | **T** | **A** | **T** | **T** | **A** | **C** | **T** | **A** | **A** | **T** | **A** | **C** | **C** | **T** | **T** | **G** | **A** | **T** | **T** | **G** | **G** | **A** | **G** | **A** | **T** | **G** | **T** | **C** | **G** | **A** | **G** | **G** | **T** | **G** | **A** | **T** | **A** | **C** | **G** | **A** | **A** | **T** | **A** | **-** | **-** | **-** |
| **N52751** | **A** | **T** | **G** | **A** | **A** | **T** | **A** | **G** | **T** | **T** | **C** | **T** | **T** | **G** | **G** | **C** | **G** | **T** | **T** | **T** | **T** | **T** | **T** | **T** | **G** | **T** | **G** | **G** | **T** | **A** | **G** | **T** | **C** | **G** | **A** | **A** | **G** | **C** | **A** | **C** | **T** | **G** | **C** | **T** | **G** | **C** | **T** | **G** | **T** | **T** | **A** | **T** | **T** | **T** | **G** | **C** | **T** | **G** | **T** | **T** | **T** | **G** | **G** | **C** | **A** | **A** | **A** | **T** | **C** | **G** | **T** | **A** | **A** | **A** | **C** | **A** | **A** | **T** | **A** | **C** | **T** | **G** | **G** | **A** | **C** | **T** | **T** | **T** | **T** | **G** | **C** | **T** | **T** | **T** | **T** | **A** | **G** | **T** | **A** | **A** | **T** | **A** | **T** | **T** | **C** | **G** | **G** | **T** | **A** | **T** | **T** | **T** | **T** | **C** | **A** | **A** | **T** | **A** | **T** | **A** | **T** | **A** | **T** | **C** | **T** | **A** | **G** | **C** | **T** | **A** | **T** | **G** | **C** | **G** | **C** | **A** | **A** | **A** | **T** | **A** | **T** | **C** | **C** | **T** | **C** | **G** | **T** | **A** | **C** | **T** | **A** | **A** | **A** | **T** | **T** | **T** | **C** | **A** | **A** | **A** | **A** | **T** | **T** | **T** | **T** | **C** | **A** | **A** | **T** | **T** | **G** | **A** | **T** | **C** | **T** | **T** | **A** | **G** | **G** | **G** | **A** | **G** | **C** | **T** | **T** | **A** | **G** | **T** | **C** | **A** | **T** | **T** | **T** | **T** | **T** | **T** | **T** | **T** | **A** | **G** | **C** | **T** | **T** | **A** | **G** | **T** | **C** | **A** | **A** | **C** | **A** | **G** | **T** | **C** | **C** | **T** | **G** | **C** | **T** | **T** | **T** | **A** | **T** | **G** | **G** | **A** | **T** | **G** | **A** | **T** | **G** | **G** | **T** | **T** | **G** | **T** | **A** | **T** | **T** | **G** | **G** | **C** | **C** | **G** | **T** | **T** | **T** | **T** | **A** | **T** | **T** | **T** | **A** | **T** | **C** | **G** | **G** | **G** | **C** | **T** | **G** | **A** | **A** | **A** | **G** | **G** | **T** | **G** | **T** | **A** | **G** | **A** | **G** | **A** | **T** | **T** | **T** | **C** | **A** | **G** | **G** | **A** | **A** | **T** | **A** | **G** | **A** | **T** | **T** | **T** | **G** | **A** | **C** | **A** | **A** | **A** | **A** | **A** | **A** | **T** | **G** | **C** | **T** | **T** | **T** | **T** | **T** | **G** | **G** | **C** | **G** | **A** | **A** | **A** | **A** | **A** | **A** | **A** | **C** | **A** | **G** | **A** | **T** | **C** | **A** | **T** | **G** | **A** | **T** | **G** | **G** | **T** | **C** | **C** | **A** | **A** | **A** | **C** | **A** | **G** | **A** | **G** | **C** | **A** | **A** | **T** | **T** | **A** | **A** | **A** | **A** | **A** | **C** | **A** | **C** | **A** | **T** | **A** | **A** | **T** | **A** | **A** | **T** | **G** | **A** | **G** | **C** | **G** | **T** | **A** | **A** | **A** | **A** | **G** | **A** | **C** | **A** | **G** | **C** | **A** | **A** | **T** | **T** | **A** | **T** | **T** | **T** | **G** | **G** | **T** | **A** | **A** | **C** | **C** | **A** | **A** | **C** | **G** | **T** | **A** | **T** | **T** | **G** | **G** | **A** | **A** | **A** | **C** | **G** | **A** | **C** | **G** | **T** | **C** | **T** | **A** | **T** | **G** | **A** | **A** | **T** | **G** | **G** | **G** | **A** | **T** | **G** | **A** | **C** | **A** | **T** | **C** | **A** | **A** | **C** | **A** | **T** | **T** | **G** | **C** | **C** | **A** | **T** | **C** | **A** | **T** | **T** | **T** | **C** | **T** | **G** | **G** | **T** | **G** | **A** | **T** | **A** | **C** | **T** | **A** | **T** | **C** | **A** | **T** | **C** | **G** | **A** | **T** | **T** | **T** | **G** | **G** | **G** | **C** | **A** | **A** | **T** | **A** | **C** | **A** | **C** | **T** | **T** | **T** | **T** | **G** | **C** | **C** | **A** | **A** | **A** | **A** | **G** | **A** | **T** | **G** | **A** | **T** | **A** | **A** | **T** | **A** | **T** | **T** | **G** | **T** | **G** | **A** | **T** | **T** | **G** | **T** | **A** | **A** | **G** | **A** | **A** | **A** | **A** | **G** | **G** | **G** | **A** | **T** | **A** | **G** | **G** | **C** | **A** | **G** | **A** | **A** | **C** | **A** | **C** | **G** | **G** | **A** | **T** | **C** | **T** | **T** | **A** | **G** | **T** | **A** | **C** | **C** | **C** | **C** | **T** | **G** | **G** | **G** | **A** | **G** | **T** | **G** | **G** | **C** | **T** | **A** | **T** | **T** | **C** | **G** | **T** | **T** | **T** | **A** | **G** | **A** | **G** | **C** | **A** | **T** | **G** | **C** | **G** | **A** | **C** | **A** | **T** | **T** | **A** | **G** | **T** | **A** | **G** | **G** | **A** | **A** | **A** | **T** | **G** | **T** | **C** | **T** | **T** | **G** | **T** | **T** | **T** | **G** | **A** | **A** | **G** | **A** | **A** | **G** | **A** | **G** | **C** | **A** | **G** | **T** | **T** | **T** | **T** | **C** | **C** | **T** | **T** | **A** | **A** | **A** | **A** | **A** | **A** | **C** | **G** | **A** | **A** | **C** | **A** | **G** | **A** | **T** | **C** | **A** | **A** | **A** | **A** | **T** | **C** | **T** | **G** | **C** | **A** | **G** | **C** | **A** | **G** | **T** | **G** | **A** | **T** | **T** | **A** | **T** | **G** | **A** | **T** | **G** | **A** | **A** | **A** | **A** | **T** | **C** | **C** | **T** | **C** | **G** | **C** | **A** | **G** | **A** | **T** | **T** | **G** | **A** | **A** | **G** | **A** | **T** | **C** | **A** | **T** | **T** | **A** | **C** | **C** | **A** | **A** | **T** | **A** | **C** | **C** | **T** | **T** | **G** | **G** | **T** | **T** | **G** | **G** | **A** | **G** | **A** | **T** | **G** | **T** | **C** | **G** | **A** | **G** | **G** | **T** | **G** | **A** | **T** | **A** | **C** | **G** | **A** | **A** | **T** | **A** | **-** | **-** | **-** |
| **N59484F** | **A** | **T** | **G** | **A** | **A** | **T** | **A** | **G** | **T** | **T** | **C** | **T** | **T** | **G** | **G** | **C** | **G** | **T** | **T** | **T** | **T** | **T** | **T** | **T** | **G** | **T** | **G** | **G** | **T** | **A** | **G** | **T** | **C** | **G** | **A** | **A** | **G** | **C** | **A** | **C** | **T** | **G** | **C** | **T** | **G** | **C** | **T** | **G** | **T** | **T** | **A** | **T** | **T** | **T** | **G** | **C** | **T** | **G** | **T** | **T** | **T** | **G** | **G** | **C** | **A** | **A** | **A** | **T** | **C** | **G** | **T** | **A** | **A** | **A** | **C** | **A** | **A** | **T** | **A** | **C** | **T** | **G** | **G** | **A** | **C** | **T** | **T** | **T** | **T** | **G** | **C** | **T** | **T** | **T** | **T** | **A** | **G** | **T** | **A** | **A** | **T** | **A** | **T** | **T** | **C** | **G** | **G** | **T** | **A** | **T** | **T** | **T** | **T** | **C** | **A** | **A** | **T** | **A** | **T** | **A** | **T** | **A** | **T** | **C** | **T** | **A** | **G** | **C** | **T** | **A** | **T** | **G** | **C** | **G** | **C** | **A** | **A** | **A** | **T** | **A** | **T** | **C** | **C** | **T** | **C** | **G** | **T** | **A** | **C** | **T** | **A** | **A** | **A** | **T** | **T** | **T** | **C** | **A** | **A** | **A** | **A** | **T** | **T** | **T** | **T** | **C** | **A** | **A** | **T** | **T** | **G** | **A** | **T** | **C** | **T** | **T** | **A** | **G** | **G** | **G** | **A** | **G** | **C** | **T** | **T** | **G** | **G** | **T** | **C** | **A** | **T** | **T** | **T** | **T** | **T** | **T** | **T** | **T** | **A** | **G** | **C** | **T** | **T** | **A** | **G** | **T** | **C** | **A** | **A** | **C** | **A** | **G** | **T** | **C** | **C** | **T** | **G** | **C** | **T** | **T** | **T** | **A** | **T** | **G** | **G** | **A** | **T** | **G** | **A** | **T** | **G** | **G** | **T** | **T** | **G** | **T** | **A** | **T** | **T** | **G** | **G** | **C** | **C** | **G** | **T** | **T** | **T** | **T** | **A** | **T** | **T** | **T** | **A** | **T** | **C** | **G** | **G** | **G** | **C** | **T** | **G** | **A** | **A** | **A** | **G** | **G** | **T** | **G** | **T** | **A** | **G** | **A** | **G** | **A** | **T** | **T** | **T** | **C** | **A** | **G** | **G** | **A** | **A** | **T** | **A** | **G** | **A** | **T** | **T** | **T** | **G** | **A** | **C** | **A** | **A** | **A** | **A** | **A** | **A** | **T** | **G** | **C** | **T** | **T** | **T** | **T** | **T** | **G** | **G** | **C** | **G** | **A** | **A** | **A** | **A** | **A** | **A** | **A** | **C** | **A** | **G** | **A** | **T** | **C** | **A** | **T** | **G** | **A** | **T** | **G** | **G** | **T** | **C** | **C** | **A** | **A** | **A** | **C** | **A** | **G** | **A** | **G** | **C** | **A** | **A** | **T** | **T** | **A** | **A** | **A** | **A** | **A** | **C** | **A** | **C** | **A** | **T** | **A** | **A** | **T** | **A** | **A** | **T** | **G** | **A** | **G** | **C** | **G** | **T** | **A** | **A** | **A** | **A** | **G** | **A** | **C** | **A** | **G** | **C** | **A** | **A** | **T** | **T** | **A** | **T** | **T** | **T** | **G** | **G** | **T** | **A** | **A** | **C** | **C** | **A** | **A** | **C** | **G** | **T** | **A** | **T** | **T** | **G** | **G** | **A** | **A** | **A** | **C** | **G** | **A** | **C** | **G** | **T** | **C** | **T** | **A** | **T** | **G** | **A** | **A** | **T** | **G** | **G** | **G** | **A** | **T** | **G** | **A** | **T** | **A** | **T** | **C** | **A** | **A** | **C** | **A** | **T** | **T** | **G** | **C** | **C** | **A** | **T** | **C** | **A** | **T** | **T** | **T** | **C** | **T** | **G** | **G** | **T** | **G** | **A** | **T** | **A** | **C** | **T** | **A** | **T** | **C** | **A** | **T** | **C** | **G** | **A** | **T** | **T** | **T** | **G** | **G** | **G** | **C** | **A** | **A** | **T** | **A** | **C** | **A** | **C** | **T** | **T** | **T** | **T** | **G** | **C** | **C** | **A** | **A** | **A** | **A** | **G** | **A** | **T** | **G** | **A** | **T** | **A** | **A** | **T** | **A** | **T** | **T** | **G** | **T** | **G** | **A** | **T** | **T** | **G** | **T** | **A** | **A** | **G** | **A** | **A** | **A** | **A** | **G** | **G** | **G** | **A** | **T** | **A** | **G** | **G** | **C** | **A** | **G** | **A** | **A** | **C** | **A** | **C** | **G** | **G** | **A** | **T** | **C** | **T** | **T** | **A** | **G** | **T** | **A** | **C** | **C** | **C** | **C** | **T** | **G** | **G** | **G** | **A** | **G** | **T** | **G** | **G** | **C** | **T** | **A** | **T** | **T** | **C** | **G** | **T** | **T** | **T** | **A** | **G** | **A** | **G** | **C** | **A** | **T** | **G** | **C** | **G** | **A** | **C** | **A** | **T** | **T** | **A** | **G** | **T** | **A** | **G** | **G** | **A** | **A** | **A** | **T** | **G** | **T** | **C** | **T** | **T** | **G** | **T** | **T** | **T** | **G** | **A** | **A** | **G** | **A** | **A** | **G** | **A** | **G** | **C** | **A** | **G** | **T** | **T** | **T** | **T** | **C** | **C** | **T** | **T** | **A** | **A** | **A** | **A** | **A** | **A** | **C** | **G** | **A** | **A** | **C** | **A** | **G** | **A** | **T** | **C** | **A** | **A** | **A** | **A** | **T** | **C** | **T** | **G** | **C** | **A** | **G** | **C** | **A** | **G** | **T** | **G** | **A** | **T** | **T** | **A** | **T** | **G** | **A** | **T** | **G** | **A** | **A** | **A** | **A** | **T** | **C** | **C** | **T** | **C** | **G** | **C** | **A** | **G** | **A** | **T** | **T** | **G** | **A** | **A** | **G** | **A** | **T** | **C** | **A** | **T** | **T** | **A** | **C** | **C** | **A** | **A** | **T** | **A** | **C** | **C** | **T** | **T** | **G** | **G** | **T** | **T** | **G** | **G** | **A** | **G** | **A** | **T** | **G** | **T** | **C** | **G** | **A** | **G** | **G** | **T** | **G** | **A** | **T** | **A** | **C** | **G** | **A** | **A** | **T** | **A** | **-** | **-** | **-** |
| **N59559F** | **A** | **T** | **G** | **A** | **A** | **T** | **A** | **G** | **T** | **T** | **C** | **T** | **T** | **G** | **G** | **C** | **G** | **T** | **T** | **T** | **T** | **T** | **T** | **T** | **G** | **T** | **G** | **G** | **T** | **A** | **G** | **T** | **T** | **G** | **A** | **A** | **G** | **C** | **A** | **C** | **T** | **A** | **C** | **T** | **G** | **C** | **T** | **G** | **T** | **T** | **A** | **T** | **T** | **T** | **G** | **C** | **T** | **G** | **T** | **T** | **T** | **G** | **G** | **C** | **A** | **A** | **A** | **T** | **C** | **G** | **T** | **A** | **A** | **A** | **C** | **A** | **A** | **T** | **A** | **C** | **T** | **G** | **G** | **A** | **C** | **T** | **T** | **T** | **T** | **G** | **C** | **T** | **T** | **T** | **T** | **A** | **G** | **T** | **G** | **A** | **T** | **A** | **T** | **T** | **C** | **G** | **G** | **T** | **A** | **T** | **T** | **T** | **T** | **C** | **A** | **A** | **T** | **A** | **T** | **A** | **T** | **A** | **T** | **C** | **T** | **A** | **G** | **C** | **T** | **A** | **T** | **G** | **C** | **G** | **C** | **A** | **A** | **A** | **C** | **A** | **T** | **C** | **C** | **T** | **C** | **G** | **T** | **A** | **C** | **T** | **A** | **A** | **A** | **T** | **T** | **T** | **C** | **A** | **A** | **A** | **A** | **T** | **T** | **T** | **T** | **C** | **A** | **A** | **T** | **T** | **G** | **A** | **T** | **A** | **T** | **T** | **A** | **G** | **G** | **A** | **A** | **G** | **T** | **T** | **T** | **G** | **G** | **T** | **C** | **A** | **T** | **T** | **T** | **T** | **T** | **T** | **T** | **T** | **A** | **G** | **C** | **T** | **T** | **A** | **G** | **T** | **C** | **A** | **A** | **C** | **A** | **G** | **T** | **C** | **C** | **T** | **G** | **C** | **T** | **T** | **T** | **A** | **T** | **G** | **G** | **A** | **T** | **G** | **A** | **T** | **G** | **G** | **C** | **T** | **G** | **T** | **A** | **T** | **T** | **T** | **G** | **C** | **C** | **G** | **T** | **T** | **T** | **T** | **A** | **T** | **T** | **T** | **A** | **T** | **T** | **G** | **G** | **A** | **C** | **T** | **A** | **A** | **A** | **A** | **G** | **G** | **T** | **G** | **T** | **A** | **G** | **A** | **G** | **A** | **T** | **T** | **T** | **C** | **A** | **G** | **G** | **G** | **A** | **T** | **A** | **G** | **A** | **T** | **T** | **T** | **A** | **A** | **C** | **C** | **A** | **A** | **A** | **A** | **A** | **T** | **G** | **C** | **T** | **T** | **T** | **T** | **T** | **G** | **G** | **C** | **G** | **A** | **A** | **A** | **G** | **A** | **A** | **A** | **C** | **A** | **G** | **A** | **T** | **C** | **A** | **T** | **G** | **A** | **T** | **G** | **G** | **T** | **C** | **C** | **A** | **A** | **A** | **C** | **A** | **G** | **A** | **G** | **C** | **A** | **A** | **T** | **T** | **A** | **A** | **A** | **A** | **A** | **C** | **A** | **C** | **A** | **T** | **A** | **A** | **T** | **A** | **A** | **T** | **G** | **A** | **G** | **C** | **G** | **G** | **A** | **A** | **A** | **A** | **G** | **A** | **C** | **A** | **G** | **C** | **A** | **A** | **T** | **T** | **A** | **T** | **T** | **C** | **G** | **G** | **T** | **A** | **A** | **C** | **C** | **A** | **A** | **C** | **G** | **T** | **A** | **T** | **T** | **G** | **G** | **A** | **A** | **A** | **C** | **G** | **A** | **C** | **G** | **T** | **C** | **T** | **A** | **T** | **G** | **A** | **A** | **T** | **G** | **G** | **G** | **A** | **C** | **G** | **A** | **T** | **A** | **T** | **C** | **A** | **A** | **C** | **A** | **T** | **C** | **G** | **C** | **C** | **G** | **T** | **C** | **A** | **T** | **T** | **T** | **C** | **T** | **G** | **G** | **T** | **G** | **A** | **T** | **A** | **C** | **T** | **A** | **T** | **C** | **A** | **T** | **A** | **G** | **A** | **T** | **T** | **T** | **G** | **G** | **G** | **C** | **A** | **A** | **T** | **A** | **C** | **G** | **C** | **T** | **T** | **T** | **T** | **G** | **C** | **C** | **A** | **A** | **A** | **A** | **G** | **A** | **T** | **G** | **A** | **T** | **A** | **A** | **T** | **A** | **T** | **T** | **G** | **T** | **G** | **A** | **T** | **T** | **G** | **T** | **A** | **A** | **G** | **A** | **A** | **A** | **A** | **G** | **G** | **G** | **A** | **T** | **A** | **G** | **G** | **C** | **A** | **G** | **A** | **A** | **C** | **A** | **C** | **G** | **G** | **A** | **T** | **C** | **T** | **T** | **A** | **G** | **T** | **A** | **C** | **C** | **T** | **T** | **T** | **G** | **G** | **G** | **C** | **G** | **T** | **A** | **G** | **C** | **C** | **A** | **T** | **C** | **C** | **G** | **A** | **T** | **T** | **A** | **G** | **A** | **G** | **C** | **A** | **T** | **G** | **C** | **G** | **A** | **C** | **A** | **T** | **T** | **A** | **G** | **T** | **A** | **G** | **G** | **A** | **A** | **A** | **T** | **G** | **T** | **C** | **T** | **T** | **G** | **T** | **T** | **T** | **G** | **A** | **A** | **G** | **A** | **A** | **G** | **A** | **G** | **C** | **A** | **A** | **T** | **T** | **T** | **T** | **C** | **T** | **T** | **T** | **G** | **A** | **A** | **A** | **A** | **A** | **T** | **G** | **A** | **A** | **C** | **A** | **A** | **A** | **T** | **C** | **A** | **A** | **A** | **A** | **T** | **C** | **T** | **A** | **T** | **A** | **G** | **C** | **A** | **G** | **T** | **G** | **A** | **T** | **T** | **A** | **T** | **G** | **A** | **T** | **G** | **A** | **A** | **A** | **A** | **C** | **C** | **C** | **T** | **C** | **G** | **T** | **A** | **G** | **A** | **C** | **T** | **G** | **A** | **A** | **G** | **A** | **T** | **T** | **A** | **T** | **T** | **A** | **C** | **T** | **A** | **A** | **T** | **A** | **C** | **C** | **T** | **T** | **G** | **A** | **T** | **T** | **G** | **G** | **A** | **G** | **A** | **T** | **G** | **T** | **C** | **G** | **A** | **G** | **G** | **T** | **G** | **A** | **T** | **A** | **C** | **G** | **A** | **A** | **T** | **A** | **-** | **-** | **-** |
| **N60449F** | **A** | **T** | **G** | **A** | **A** | **T** | **A** | **G** | **T** | **T** | **C** | **T** | **T** | **G** | **G** | **C** | **G** | **T** | **T** | **T** | **T** | **T** | **T** | **T** | **G** | **T** | **G** | **G** | **T** | **A** | **G** | **T** | **C** | **G** | **A** | **A** | **G** | **C** | **A** | **C** | **T** | **G** | **C** | **T** | **G** | **C** | **T** | **G** | **T** | **T** | **A** | **T** | **T** | **T** | **G** | **C** | **T** | **G** | **T** | **T** | **T** | **G** | **G** | **C** | **A** | **A** | **A** | **T** | **C** | **G** | **T** | **A** | **A** | **A** | **C** | **A** | **A** | **T** | **A** | **C** | **T** | **G** | **G** | **A** | **C** | **T** | **T** | **T** | **T** | **G** | **C** | **T** | **T** | **T** | **T** | **A** | **G** | **T** | **A** | **A** | **T** | **A** | **T** | **T** | **C** | **G** | **G** | **T** | **A** | **T** | **T** | **T** | **T** | **C** | **A** | **A** | **T** | **A** | **T** | **A** | **T** | **A** | **T** | **C** | **T** | **A** | **G** | **C** | **T** | **A** | **T** | **G** | **C** | **G** | **C** | **A** | **A** | **A** | **T** | **A** | **T** | **C** | **C** | **T** | **C** | **G** | **T** | **A** | **C** | **T** | **A** | **A** | **A** | **T** | **T** | **T** | **C** | **A** | **A** | **A** | **A** | **T** | **T** | **T** | **T** | **C** | **A** | **A** | **T** | **T** | **G** | **A** | **T** | **C** | **T** | **T** | **A** | **G** | **G** | **G** | **A** | **G** | **C** | **T** | **T** | **G** | **G** | **T** | **C** | **A** | **T** | **T** | **T** | **T** | **T** | **T** | **T** | **T** | **A** | **G** | **C** | **T** | **T** | **A** | **G** | **T** | **C** | **A** | **A** | **C** | **A** | **G** | **T** | **C** | **C** | **T** | **G** | **C** | **T** | **T** | **T** | **A** | **T** | **G** | **G** | **A** | **T** | **G** | **A** | **T** | **G** | **G** | **T** | **T** | **G** | **T** | **A** | **T** | **T** | **G** | **G** | **C** | **C** | **G** | **T** | **T** | **T** | **T** | **A** | **T** | **T** | **T** | **A** | **T** | **C** | **G** | **G** | **G** | **C** | **T** | **G** | **A** | **A** | **A** | **G** | **G** | **T** | **G** | **T** | **A** | **G** | **A** | **G** | **A** | **T** | **T** | **T** | **C** | **A** | **G** | **G** | **A** | **A** | **T** | **A** | **G** | **A** | **T** | **T** | **T** | **G** | **A** | **C** | **A** | **A** | **A** | **A** | **A** | **A** | **T** | **G** | **C** | **T** | **T** | **T** | **T** | **T** | **G** | **G** | **C** | **G** | **A** | **A** | **A** | **A** | **A** | **A** | **A** | **C** | **A** | **G** | **A** | **T** | **C** | **A** | **T** | **G** | **A** | **T** | **G** | **G** | **T** | **C** | **C** | **A** | **A** | **A** | **C** | **A** | **G** | **A** | **G** | **C** | **A** | **A** | **T** | **T** | **A** | **A** | **A** | **A** | **A** | **C** | **A** | **C** | **A** | **T** | **A** | **A** | **T** | **A** | **A** | **T** | **G** | **A** | **G** | **C** | **G** | **T** | **A** | **A** | **A** | **A** | **G** | **A** | **C** | **A** | **G** | **C** | **A** | **A** | **T** | **T** | **A** | **T** | **T** | **T** | **G** | **G** | **T** | **A** | **A** | **C** | **C** | **A** | **A** | **C** | **G** | **T** | **A** | **T** | **T** | **G** | **G** | **A** | **A** | **A** | **C** | **G** | **A** | **C** | **G** | **T** | **C** | **T** | **A** | **T** | **G** | **A** | **A** | **T** | **G** | **G** | **G** | **A** | **T** | **G** | **A** | **T** | **A** | **T** | **C** | **A** | **A** | **C** | **A** | **T** | **T** | **G** | **C** | **C** | **A** | **T** | **C** | **A** | **T** | **T** | **T** | **C** | **T** | **G** | **G** | **T** | **G** | **A** | **T** | **A** | **C** | **T** | **A** | **T** | **C** | **A** | **T** | **C** | **G** | **A** | **T** | **T** | **T** | **G** | **G** | **G** | **C** | **A** | **A** | **T** | **A** | **C** | **A** | **C** | **T** | **T** | **T** | **T** | **G** | **C** | **C** | **A** | **A** | **A** | **A** | **G** | **A** | **T** | **G** | **A** | **T** | **A** | **A** | **T** | **A** | **T** | **T** | **G** | **T** | **G** | **A** | **T** | **T** | **G** | **T** | **A** | **A** | **G** | **A** | **A** | **A** | **A** | **G** | **G** | **G** | **A** | **T** | **A** | **G** | **G** | **C** | **A** | **G** | **A** | **A** | **C** | **A** | **C** | **G** | **G** | **A** | **T** | **C** | **T** | **T** | **A** | **G** | **T** | **A** | **C** | **C** | **C** | **C** | **T** | **G** | **G** | **G** | **A** | **G** | **T** | **G** | **G** | **C** | **T** | **A** | **T** | **T** | **C** | **G** | **T** | **T** | **T** | **A** | **G** | **A** | **G** | **C** | **A** | **T** | **G** | **C** | **G** | **A** | **C** | **A** | **T** | **T** | **A** | **G** | **T** | **A** | **G** | **G** | **A** | **A** | **A** | **T** | **G** | **T** | **C** | **T** | **T** | **G** | **T** | **T** | **T** | **G** | **A** | **A** | **G** | **A** | **A** | **G** | **A** | **G** | **C** | **A** | **G** | **T** | **T** | **T** | **T** | **C** | **C** | **T** | **T** | **A** | **A** | **A** | **A** | **A** | **A** | **C** | **G** | **A** | **A** | **C** | **A** | **G** | **A** | **T** | **C** | **A** | **A** | **A** | **A** | **T** | **C** | **T** | **G** | **C** | **A** | **G** | **C** | **A** | **G** | **T** | **G** | **A** | **T** | **T** | **A** | **T** | **G** | **A** | **T** | **G** | **A** | **A** | **A** | **A** | **T** | **C** | **C** | **T** | **C** | **G** | **C** | **A** | **G** | **A** | **T** | **T** | **G** | **A** | **A** | **G** | **A** | **T** | **C** | **A** | **T** | **T** | **A** | **C** | **C** | **A** | **A** | **T** | **A** | **C** | **C** | **T** | **T** | **G** | **G** | **T** | **T** | **G** | **G** | **A** | **G** | **A** | **T** | **G** | **T** | **C** | **G** | **A** | **G** | **G** | **T** | **G** | **A** | **T** | **A** | **C** | **G** | **A** | **A** | **T** | **A** | **-** | **-** | **-** |
| **N60048F** | **A** | **T** | **G** | **A** | **A** | **T** | **A** | **G** | **T** | **T** | **C** | **T** | **T** | **G** | **G** | **C** | **G** | **T** | **T** | **T** | **T** | **T** | **T** | **T** | **G** | **T** | **G** | **G** | **T** | **A** | **G** | **T** | **C** | **G** | **A** | **A** | **G** | **C** | **A** | **C** | **T** | **G** | **C** | **T** | **G** | **C** | **T** | **G** | **T** | **T** | **A** | **T** | **T** | **T** | **G** | **C** | **T** | **G** | **T** | **T** | **T** | **G** | **G** | **C** | **A** | **A** | **A** | **T** | **C** | **G** | **T** | **A** | **A** | **A** | **C** | **A** | **A** | **T** | **A** | **C** | **T** | **G** | **G** | **A** | **C** | **T** | **T** | **T** | **T** | **G** | **C** | **T** | **T** | **T** | **T** | **A** | **G** | **T** | **A** | **A** | **T** | **A** | **T** | **T** | **C** | **G** | **G** | **T** | **A** | **T** | **T** | **T** | **T** | **C** | **A** | **A** | **T** | **A** | **T** | **A** | **T** | **A** | **T** | **C** | **T** | **A** | **G** | **C** | **T** | **A** | **T** | **G** | **C** | **G** | **C** | **A** | **A** | **A** | **T** | **A** | **T** | **C** | **C** | **T** | **C** | **G** | **T** | **A** | **C** | **T** | **A** | **A** | **A** | **T** | **T** | **T** | **C** | **A** | **A** | **A** | **A** | **T** | **T** | **T** | **T** | **C** | **A** | **A** | **T** | **T** | **G** | **A** | **T** | **C** | **T** | **T** | **A** | **G** | **G** | **G** | **A** | **G** | **C** | **T** | **T** | **G** | **G** | **T** | **C** | **A** | **T** | **T** | **T** | **T** | **T** | **T** | **T** | **T** | **A** | **G** | **C** | **T** | **T** | **A** | **G** | **T** | **C** | **A** | **A** | **C** | **A** | **G** | **T** | **C** | **C** | **T** | **G** | **C** | **T** | **T** | **T** | **A** | **T** | **G** | **G** | **A** | **T** | **G** | **A** | **T** | **G** | **G** | **T** | **T** | **G** | **T** | **A** | **T** | **T** | **G** | **G** | **C** | **C** | **G** | **T** | **T** | **T** | **T** | **A** | **T** | **T** | **T** | **A** | **T** | **C** | **G** | **G** | **C** | **C** | **T** | **G** | **A** | **A** | **A** | **G** | **G** | **T** | **G** | **T** | **A** | **G** | **A** | **G** | **A** | **T** | **T** | **T** | **C** | **A** | **G** | **G** | **A** | **A** | **T** | **A** | **G** | **A** | **T** | **T** | **T** | **G** | **A** | **C** | **A** | **A** | **A** | **A** | **A** | **A** | **T** | **G** | **C** | **T** | **T** | **T** | **T** | **T** | **G** | **G** | **C** | **G** | **A** | **A** | **A** | **A** | **A** | **A** | **A** | **C** | **A** | **G** | **A** | **T** | **C** | **A** | **T** | **G** | **A** | **T** | **G** | **G** | **T** | **C** | **C** | **A** | **A** | **A** | **C** | **A** | **G** | **A** | **G** | **C** | **A** | **A** | **T** | **T** | **A** | **A** | **A** | **A** | **A** | **C** | **A** | **C** | **A** | **T** | **A** | **A** | **T** | **A** | **A** | **T** | **G** | **A** | **G** | **C** | **G** | **T** | **A** | **A** | **A** | **A** | **G** | **A** | **C** | **A** | **G** | **C** | **A** | **A** | **T** | **T** | **A** | **T** | **T** | **T** | **G** | **G** | **T** | **A** | **A** | **C** | **C** | **A** | **A** | **C** | **G** | **T** | **A** | **T** | **T** | **G** | **G** | **A** | **A** | **A** | **C** | **G** | **A** | **C** | **G** | **T** | **C** | **T** | **A** | **T** | **G** | **A** | **A** | **T** | **G** | **G** | **G** | **A** | **T** | **G** | **A** | **T** | **A** | **T** | **C** | **A** | **A** | **C** | **A** | **T** | **T** | **G** | **C** | **C** | **A** | **T** | **C** | **A** | **T** | **T** | **T** | **C** | **T** | **G** | **G** | **T** | **G** | **A** | **T** | **A** | **C** | **T** | **A** | **T** | **C** | **A** | **T** | **C** | **G** | **A** | **T** | **T** | **T** | **G** | **G** | **G** | **C** | **A** | **A** | **T** | **A** | **C** | **A** | **C** | **T** | **T** | **T** | **T** | **G** | **C** | **C** | **A** | **A** | **A** | **A** | **G** | **A** | **T** | **G** | **A** | **T** | **A** | **A** | **T** | **A** | **T** | **T** | **G** | **T** | **G** | **A** | **T** | **T** | **G** | **T** | **A** | **A** | **G** | **A** | **A** | **A** | **A** | **G** | **G** | **G** | **A** | **T** | **A** | **G** | **G** | **C** | **A** | **G** | **A** | **A** | **C** | **A** | **C** | **G** | **G** | **A** | **T** | **C** | **T** | **T** | **A** | **G** | **T** | **A** | **C** | **C** | **C** | **C** | **T** | **G** | **G** | **G** | **A** | **G** | **T** | **G** | **G** | **C** | **T** | **A** | **T** | **T** | **C** | **G** | **A** | **T** | **T** | **A** | **G** | **A** | **G** | **C** | **A** | **T** | **G** | **C** | **G** | **A** | **C** | **A** | **T** | **T** | **A** | **G** | **T** | **A** | **G** | **G** | **A** | **A** | **A** | **T** | **G** | **T** | **C** | **T** | **T** | **G** | **T** | **T** | **T** | **G** | **A** | **A** | **G** | **A** | **A** | **G** | **A** | **G** | **C** | **A** | **G** | **T** | **T** | **T** | **T** | **C** | **C** | **T** | **T** | **A** | **A** | **A** | **A** | **A** | **A** | **T** | **G** | **A** | **A** | **C** | **A** | **G** | **A** | **T** | **C** | **A** | **A** | **A** | **A** | **T** | **C** | **T** | **G** | **C** | **A** | **G** | **C** | **A** | **G** | **T** | **G** | **A** | **T** | **T** | **A** | **T** | **G** | **A** | **T** | **G** | **A** | **A** | **A** | **A** | **T** | **C** | **C** | **T** | **C** | **G** | **C** | **A** | **G** | **A** | **T** | **T** | **G** | **A** | **A** | **G** | **A** | **T** | **C** | **A** | **T** | **T** | **A** | **C** | **C** | **A** | **A** | **T** | **A** | **C** | **C** | **T** | **T** | **G** | **G** | **T** | **T** | **G** | **G** | **A** | **G** | **A** | **T** | **G** | **T** | **C** | **G** | **A** | **G** | **G** | **T** | **G** | **A** | **T** | **A** | **C** | **G** | **A** | **A** | **T** | **A** | **-** | **-** | **-** |
| **N54560** | **A** | **T** | **G** | **A** | **A** | **T** | **A** | **G** | **T** | **T** | **C** | **T** | **T** | **G** | **G** | **C** | **G** | **T** | **T** | **T** | **T** | **T** | **T** | **T** | **G** | **T** | **G** | **G** | **T** | **A** | **G** | **T** | **C** | **G** | **A** | **A** | **G** | **C** | **A** | **C** | **T** | **G** | **C** | **T** | **G** | **C** | **T** | **G** | **T** | **T** | **A** | **T** | **T** | **T** | **G** | **C** | **T** | **G** | **T** | **T** | **T** | **G** | **G** | **C** | **A** | **A** | **A** | **T** | **C** | **G** | **T** | **A** | **A** | **A** | **C** | **A** | **A** | **T** | **A** | **C** | **T** | **G** | **G** | **A** | **C** | **T** | **T** | **T** | **T** | **G** | **C** | **T** | **T** | **T** | **T** | **A** | **G** | **T** | **A** | **A** | **T** | **A** | **T** | **T** | **C** | **G** | **G** | **T** | **A** | **T** | **T** | **T** | **T** | **C** | **A** | **A** | **T** | **A** | **T** | **A** | **T** | **A** | **T** | **C** | **T** | **A** | **G** | **C** | **T** | **A** | **T** | **G** | **C** | **G** | **C** | **A** | **A** | **A** | **T** | **A** | **T** | **C** | **C** | **T** | **C** | **G** | **T** | **A** | **C** | **T** | **A** | **A** | **A** | **T** | **T** | **T** | **C** | **A** | **A** | **A** | **A** | **T** | **T** | **T** | **T** | **C** | **A** | **A** | **T** | **T** | **G** | **A** | **T** | **C** | **T** | **T** | **A** | **G** | **G** | **G** | **A** | **G** | **C** | **T** | **T** | **G** | **G** | **T** | **C** | **A** | **T** | **T** | **T** | **T** | **T** | **T** | **T** | **T** | **A** | **G** | **C** | **T** | **T** | **A** | **G** | **T** | **C** | **A** | **A** | **C** | **A** | **G** | **T** | **C** | **C** | **T** | **G** | **C** | **T** | **T** | **T** | **A** | **T** | **G** | **G** | **A** | **T** | **G** | **A** | **T** | **G** | **G** | **T** | **T** | **G** | **T** | **A** | **T** | **T** | **G** | **G** | **C** | **C** | **G** | **T** | **T** | **T** | **T** | **A** | **T** | **T** | **T** | **A** | **T** | **C** | **G** | **G** | **C** | **C** | **T** | **G** | **A** | **A** | **A** | **G** | **G** | **T** | **G** | **T** | **A** | **G** | **A** | **G** | **A** | **T** | **T** | **T** | **C** | **A** | **G** | **G** | **A** | **A** | **T** | **A** | **G** | **A** | **T** | **T** | **T** | **G** | **A** | **C** | **A** | **A** | **A** | **A** | **A** | **A** | **T** | **G** | **C** | **T** | **T** | **T** | **T** | **T** | **G** | **G** | **C** | **G** | **A** | **A** | **A** | **A** | **A** | **A** | **A** | **C** | **A** | **G** | **A** | **T** | **C** | **A** | **T** | **G** | **A** | **T** | **G** | **G** | **T** | **C** | **C** | **A** | **A** | **A** | **C** | **A** | **G** | **A** | **G** | **C** | **A** | **A** | **T** | **T** | **A** | **A** | **A** | **A** | **A** | **C** | **A** | **C** | **A** | **T** | **A** | **A** | **T** | **A** | **A** | **T** | **G** | **A** | **G** | **C** | **G** | **T** | **A** | **A** | **A** | **A** | **G** | **A** | **C** | **A** | **G** | **C** | **A** | **A** | **T** | **T** | **A** | **T** | **T** | **T** | **G** | **G** | **T** | **A** | **A** | **C** | **C** | **A** | **A** | **C** | **G** | **T** | **A** | **T** | **T** | **G** | **G** | **A** | **A** | **A** | **C** | **G** | **A** | **C** | **G** | **T** | **C** | **T** | **A** | **T** | **G** | **A** | **A** | **T** | **G** | **G** | **G** | **A** | **T** | **G** | **A** | **T** | **A** | **T** | **C** | **A** | **A** | **C** | **A** | **T** | **T** | **G** | **C** | **C** | **A** | **T** | **C** | **A** | **T** | **T** | **T** | **C** | **T** | **G** | **G** | **T** | **G** | **A** | **T** | **A** | **C** | **T** | **A** | **T** | **C** | **A** | **T** | **C** | **G** | **A** | **T** | **T** | **T** | **G** | **G** | **G** | **C** | **A** | **A** | **T** | **A** | **C** | **A** | **C** | **T** | **T** | **T** | **T** | **G** | **C** | **C** | **A** | **A** | **A** | **A** | **G** | **A** | **T** | **G** | **A** | **T** | **A** | **A** | **T** | **A** | **T** | **T** | **G** | **T** | **G** | **A** | **T** | **T** | **G** | **T** | **A** | **A** | **G** | **A** | **A** | **A** | **A** | **G** | **G** | **G** | **A** | **T** | **A** | **G** | **G** | **C** | **A** | **G** | **A** | **A** | **C** | **A** | **C** | **G** | **G** | **A** | **T** | **C** | **T** | **T** | **A** | **G** | **T** | **A** | **C** | **C** | **C** | **C** | **T** | **G** | **G** | **G** | **A** | **G** | **T** | **G** | **G** | **C** | **T** | **A** | **T** | **T** | **C** | **G** | **A** | **T** | **T** | **A** | **G** | **A** | **G** | **C** | **A** | **T** | **G** | **C** | **G** | **A** | **C** | **A** | **T** | **T** | **A** | **G** | **T** | **A** | **G** | **G** | **A** | **A** | **A** | **T** | **G** | **T** | **C** | **T** | **T** | **G** | **T** | **T** | **T** | **G** | **A** | **A** | **G** | **A** | **A** | **G** | **A** | **G** | **C** | **A** | **G** | **T** | **T** | **T** | **T** | **C** | **C** | **T** | **T** | **A** | **A** | **A** | **A** | **A** | **A** | **T** | **G** | **A** | **A** | **C** | **A** | **G** | **A** | **T** | **C** | **A** | **A** | **A** | **A** | **T** | **C** | **T** | **G** | **C** | **A** | **G** | **C** | **A** | **G** | **T** | **G** | **A** | **T** | **T** | **A** | **T** | **G** | **A** | **T** | **G** | **A** | **A** | **A** | **A** | **T** | **C** | **C** | **T** | **C** | **G** | **C** | **A** | **G** | **A** | **T** | **T** | **G** | **A** | **A** | **G** | **A** | **T** | **C** | **A** | **T** | **T** | **A** | **C** | **C** | **A** | **A** | **T** | **A** | **C** | **C** | **T** | **T** | **G** | **G** | **T** | **T** | **G** | **G** | **A** | **G** | **A** | **T** | **G** | **T** | **C** | **G** | **A** | **G** | **G** | **T** | **G** | **A** | **T** | **A** | **C** | **G** | **A** | **A** | **T** | **A** | **-** | **-** | **-** |
| **N55077** | **A** | **T** | **G** | **A** | **A** | **T** | **A** | **G** | **T** | **T** | **C** | **T** | **T** | **G** | **G** | **C** | **G** | **T** | **T** | **T** | **T** | **T** | **T** | **T** | **G** | **T** | **G** | **G** | **T** | **A** | **G** | **T** | **C** | **G** | **A** | **A** | **G** | **C** | **A** | **C** | **T** | **G** | **C** | **T** | **G** | **C** | **T** | **G** | **T** | **T** | **A** | **T** | **T** | **T** | **G** | **C** | **T** | **G** | **T** | **T** | **T** | **G** | **G** | **C** | **A** | **A** | **A** | **T** | **C** | **G** | **T** | **A** | **A** | **A** | **C** | **A** | **A** | **T** | **A** | **C** | **T** | **G** | **G** | **A** | **C** | **T** | **T** | **T** | **T** | **G** | **C** | **T** | **T** | **T** | **T** | **A** | **G** | **T** | **A** | **A** | **T** | **A** | **T** | **T** | **C** | **G** | **G** | **T** | **A** | **T** | **T** | **T** | **T** | **C** | **A** | **A** | **T** | **A** | **T** | **A** | **T** | **A** | **T** | **C** | **T** | **A** | **G** | **C** | **T** | **A** | **T** | **G** | **C** | **G** | **C** | **A** | **A** | **A** | **T** | **A** | **T** | **C** | **C** | **T** | **C** | **G** | **T** | **A** | **C** | **T** | **A** | **A** | **A** | **T** | **T** | **T** | **C** | **A** | **A** | **A** | **A** | **T** | **T** | **T** | **T** | **C** | **A** | **A** | **T** | **T** | **G** | **A** | **T** | **C** | **T** | **T** | **A** | **G** | **G** | **G** | **A** | **G** | **C** | **T** | **T** | **G** | **G** | **T** | **C** | **A** | **T** | **T** | **T** | **T** | **T** | **T** | **T** | **T** | **A** | **G** | **C** | **T** | **T** | **A** | **G** | **T** | **C** | **A** | **A** | **C** | **A** | **G** | **T** | **C** | **C** | **T** | **G** | **C** | **T** | **T** | **T** | **A** | **T** | **G** | **G** | **A** | **T** | **G** | **A** | **T** | **G** | **G** | **T** | **T** | **G** | **T** | **A** | **T** | **T** | **G** | **G** | **C** | **C** | **G** | **T** | **T** | **T** | **T** | **A** | **T** | **T** | **T** | **A** | **T** | **C** | **G** | **G** | **G** | **C** | **T** | **G** | **A** | **A** | **A** | **G** | **G** | **T** | **G** | **T** | **A** | **G** | **A** | **G** | **A** | **T** | **T** | **T** | **C** | **A** | **G** | **G** | **A** | **A** | **T** | **A** | **G** | **A** | **T** | **T** | **T** | **G** | **A** | **C** | **A** | **A** | **A** | **A** | **A** | **A** | **T** | **G** | **C** | **T** | **T** | **T** | **T** | **T** | **G** | **G** | **C** | **G** | **A** | **A** | **A** | **A** | **A** | **A** | **A** | **C** | **A** | **G** | **A** | **T** | **C** | **A** | **T** | **G** | **A** | **T** | **G** | **G** | **T** | **C** | **C** | **A** | **A** | **A** | **C** | **A** | **G** | **A** | **G** | **C** | **A** | **A** | **T** | **T** | **A** | **A** | **A** | **A** | **A** | **C** | **A** | **C** | **A** | **T** | **A** | **A** | **T** | **A** | **A** | **T** | **G** | **A** | **G** | **C** | **G** | **T** | **A** | **A** | **A** | **A** | **G** | **A** | **C** | **A** | **G** | **C** | **A** | **A** | **T** | **T** | **A** | **T** | **T** | **T** | **G** | **G** | **T** | **A** | **A** | **C** | **C** | **A** | **A** | **C** | **G** | **T** | **A** | **T** | **T** | **G** | **G** | **A** | **A** | **A** | **C** | **G** | **A** | **C** | **G** | **T** | **C** | **T** | **A** | **T** | **G** | **A** | **A** | **T** | **G** | **G** | **G** | **A** | **T** | **G** | **A** | **T** | **A** | **T** | **C** | **A** | **A** | **C** | **A** | **T** | **T** | **G** | **C** | **C** | **A** | **T** | **C** | **A** | **T** | **T** | **T** | **C** | **T** | **G** | **G** | **T** | **G** | **A** | **T** | **A** | **C** | **T** | **A** | **T** | **C** | **A** | **T** | **C** | **G** | **A** | **T** | **T** | **T** | **G** | **G** | **G** | **C** | **A** | **A** | **T** | **A** | **C** | **A** | **C** | **T** | **T** | **T** | **T** | **G** | **C** | **C** | **A** | **A** | **A** | **A** | **G** | **A** | **T** | **G** | **A** | **T** | **A** | **A** | **T** | **A** | **T** | **T** | **G** | **T** | **G** | **A** | **T** | **T** | **G** | **T** | **A** | **A** | **G** | **A** | **A** | **A** | **A** | **G** | **G** | **G** | **A** | **T** | **A** | **G** | **G** | **C** | **A** | **G** | **A** | **A** | **C** | **A** | **C** | **G** | **G** | **A** | **T** | **C** | **T** | **T** | **A** | **G** | **T** | **A** | **C** | **C** | **C** | **C** | **T** | **G** | **G** | **G** | **A** | **G** | **T** | **G** | **G** | **C** | **T** | **A** | **T** | **T** | **C** | **G** | **T** | **T** | **T** | **A** | **G** | **A** | **G** | **C** | **A** | **T** | **G** | **C** | **G** | **A** | **C** | **A** | **T** | **T** | **A** | **G** | **T** | **A** | **G** | **G** | **A** | **A** | **A** | **T** | **G** | **T** | **C** | **T** | **T** | **G** | **T** | **T** | **T** | **G** | **A** | **A** | **G** | **A** | **A** | **G** | **A** | **G** | **C** | **A** | **G** | **T** | **T** | **T** | **T** | **C** | **C** | **T** | **T** | **A** | **A** | **A** | **A** | **A** | **A** | **C** | **G** | **A** | **A** | **C** | **A** | **G** | **A** | **T** | **C** | **A** | **A** | **A** | **A** | **T** | **C** | **T** | **G** | **C** | **A** | **G** | **C** | **A** | **G** | **T** | **G** | **A** | **T** | **T** | **A** | **T** | **G** | **A** | **T** | **G** | **A** | **A** | **A** | **A** | **T** | **C** | **C** | **T** | **C** | **G** | **C** | **A** | **G** | **A** | **T** | **T** | **G** | **A** | **A** | **G** | **A** | **T** | **C** | **A** | **T** | **T** | **A** | **C** | **C** | **A** | **A** | **T** | **A** | **C** | **C** | **T** | **T** | **G** | **G** | **T** | **T** | **G** | **G** | **A** | **G** | **A** | **T** | **G** | **T** | **C** | **G** | **A** | **G** | **G** | **T** | **G** | **A** | **T** | **A** | **C** | **G** | **A** | **A** | **T** | **A** | **-** | **-** | **-** |
| **N53548** | **A** | **T** | **G** | **A** | **A** | **T** | **A** | **G** | **T** | **T** | **C** | **T** | **T** | **G** | **G** | **C** | **G** | **T** | **T** | **T** | **T** | **T** | **T** | **T** | **G** | **T** | **G** | **G** | **T** | **A** | **G** | **T** | **C** | **G** | **A** | **A** | **G** | **C** | **A** | **C** | **T** | **G** | **C** | **T** | **G** | **C** | **T** | **G** | **T** | **T** | **A** | **T** | **T** | **T** | **G** | **C** | **T** | **G** | **T** | **T** | **T** | **G** | **G** | **C** | **A** | **A** | **A** | **T** | **C** | **G** | **T** | **A** | **A** | **A** | **C** | **A** | **A** | **T** | **A** | **C** | **T** | **G** | **G** | **A** | **C** | **T** | **T** | **T** | **T** | **G** | **C** | **T** | **T** | **T** | **T** | **A** | **G** | **T** | **A** | **A** | **T** | **A** | **T** | **T** | **C** | **G** | **G** | **T** | **A** | **T** | **T** | **T** | **T** | **C** | **A** | **A** | **T** | **A** | **T** | **A** | **T** | **A** | **T** | **C** | **T** | **A** | **G** | **C** | **T** | **A** | **T** | **G** | **C** | **G** | **C** | **A** | **A** | **A** | **T** | **A** | **T** | **C** | **C** | **T** | **C** | **G** | **T** | **A** | **C** | **T** | **A** | **A** | **A** | **T** | **T** | **T** | **C** | **A** | **A** | **A** | **A** | **T** | **T** | **T** | **T** | **C** | **A** | **A** | **T** | **T** | **G** | **A** | **T** | **C** | **T** | **T** | **A** | **G** | **G** | **G** | **A** | **G** | **C** | **T** | **T** | **G** | **G** | **T** | **C** | **A** | **T** | **T** | **T** | **T** | **T** | **T** | **T** | **T** | **A** | **G** | **C** | **T** | **T** | **A** | **G** | **T** | **C** | **A** | **A** | **C** | **A** | **G** | **T** | **C** | **C** | **T** | **G** | **C** | **T** | **T** | **T** | **A** | **T** | **G** | **G** | **A** | **T** | **G** | **A** | **T** | **G** | **G** | **T** | **T** | **G** | **T** | **A** | **T** | **T** | **G** | **G** | **C** | **C** | **G** | **T** | **T** | **T** | **T** | **A** | **T** | **T** | **T** | **A** | **T** | **C** | **G** | **G** | **G** | **C** | **T** | **G** | **A** | **A** | **A** | **G** | **G** | **T** | **G** | **T** | **A** | **G** | **A** | **G** | **A** | **T** | **T** | **T** | **C** | **A** | **G** | **G** | **A** | **A** | **T** | **A** | **G** | **A** | **T** | **T** | **T** | **G** | **A** | **C** | **A** | **A** | **A** | **A** | **A** | **A** | **T** | **G** | **C** | **T** | **T** | **T** | **T** | **T** | **G** | **G** | **C** | **G** | **A** | **A** | **A** | **A** | **A** | **A** | **A** | **C** | **A** | **G** | **A** | **T** | **C** | **A** | **T** | **G** | **A** | **T** | **G** | **G** | **T** | **C** | **C** | **A** | **A** | **A** | **C** | **A** | **G** | **A** | **G** | **C** | **A** | **A** | **T** | **T** | **A** | **A** | **A** | **A** | **A** | **C** | **A** | **C** | **A** | **T** | **A** | **A** | **T** | **A** | **A** | **T** | **G** | **A** | **G** | **C** | **G** | **T** | **A** | **A** | **A** | **A** | **G** | **A** | **C** | **A** | **G** | **C** | **A** | **A** | **T** | **T** | **A** | **T** | **T** | **T** | **G** | **G** | **T** | **A** | **A** | **C** | **C** | **A** | **A** | **C** | **G** | **T** | **A** | **T** | **T** | **G** | **G** | **A** | **A** | **A** | **C** | **G** | **A** | **C** | **G** | **T** | **C** | **T** | **A** | **T** | **G** | **A** | **A** | **T** | **G** | **G** | **G** | **A** | **T** | **G** | **A** | **T** | **A** | **T** | **C** | **A** | **A** | **C** | **A** | **T** | **T** | **G** | **C** | **C** | **A** | **T** | **C** | **A** | **T** | **T** | **T** | **C** | **T** | **G** | **G** | **T** | **G** | **A** | **T** | **A** | **C** | **T** | **A** | **T** | **C** | **A** | **T** | **C** | **G** | **A** | **T** | **T** | **T** | **G** | **G** | **G** | **C** | **A** | **A** | **T** | **A** | **C** | **A** | **C** | **T** | **T** | **T** | **T** | **G** | **C** | **C** | **A** | **A** | **A** | **A** | **G** | **A** | **T** | **G** | **A** | **T** | **A** | **A** | **T** | **A** | **T** | **T** | **G** | **T** | **G** | **A** | **T** | **T** | **G** | **T** | **A** | **A** | **G** | **A** | **A** | **A** | **A** | **G** | **G** | **G** | **A** | **T** | **A** | **G** | **G** | **C** | **A** | **G** | **A** | **A** | **C** | **A** | **C** | **G** | **G** | **A** | **T** | **C** | **T** | **T** | **A** | **G** | **T** | **A** | **C** | **C** | **C** | **C** | **T** | **G** | **G** | **G** | **A** | **G** | **T** | **G** | **G** | **C** | **T** | **A** | **T** | **T** | **C** | **G** | **T** | **T** | **T** | **A** | **G** | **A** | **G** | **C** | **A** | **T** | **G** | **C** | **G** | **A** | **C** | **A** | **T** | **T** | **A** | **G** | **T** | **A** | **G** | **G** | **A** | **A** | **A** | **T** | **G** | **T** | **C** | **T** | **T** | **G** | **T** | **T** | **T** | **G** | **A** | **A** | **G** | **A** | **A** | **G** | **A** | **G** | **C** | **A** | **G** | **T** | **T** | **T** | **T** | **C** | **C** | **T** | **T** | **A** | **A** | **A** | **A** | **A** | **A** | **C** | **G** | **A** | **A** | **C** | **A** | **G** | **A** | **T** | **C** | **A** | **A** | **A** | **A** | **T** | **C** | **T** | **G** | **C** | **A** | **G** | **C** | **A** | **G** | **T** | **G** | **A** | **T** | **T** | **A** | **T** | **G** | **A** | **T** | **G** | **A** | **A** | **A** | **A** | **T** | **C** | **C** | **T** | **C** | **G** | **C** | **A** | **G** | **A** | **T** | **T** | **G** | **A** | **A** | **G** | **A** | **T** | **C** | **A** | **T** | **T** | **A** | **C** | **C** | **A** | **A** | **T** | **A** | **C** | **C** | **T** | **T** | **G** | **G** | **T** | **T** | **G** | **G** | **A** | **G** | **A** | **T** | **G** | **T** | **C** | **G** | **A** | **G** | **G** | **T** | **G** | **A** | **T** | **A** | **C** | **G** | **A** | **A** | **T** | **A** | **-** | **-** | **-** |
| **N59907F** | **A** | **T** | **G** | **A** | **A** | **T** | **A** | **G** | **T** | **T** | **C** | **T** | **T** | **G** | **G** | **C** | **G** | **T** | **T** | **T** | **T** | **T** | **T** | **T** | **G** | **T** | **G** | **G** | **T** | **A** | **G** | **T** | **C** | **G** | **A** | **A** | **G** | **C** | **A** | **C** | **T** | **G** | **C** | **T** | **G** | **C** | **T** | **G** | **T** | **T** | **A** | **T** | **T** | **T** | **G** | **C** | **T** | **G** | **T** | **T** | **T** | **G** | **G** | **C** | **A** | **A** | **A** | **T** | **C** | **G** | **T** | **A** | **A** | **A** | **C** | **A** | **A** | **T** | **A** | **C** | **T** | **G** | **G** | **A** | **C** | **T** | **T** | **T** | **T** | **G** | **C** | **T** | **T** | **T** | **T** | **A** | **G** | **T** | **A** | **A** | **T** | **A** | **T** | **T** | **C** | **G** | **G** | **T** | **A** | **T** | **T** | **T** | **T** | **C** | **A** | **A** | **T** | **A** | **T** | **A** | **T** | **A** | **T** | **C** | **T** | **A** | **G** | **C** | **T** | **A** | **T** | **G** | **C** | **G** | **C** | **A** | **A** | **A** | **T** | **A** | **T** | **C** | **C** | **T** | **C** | **G** | **T** | **A** | **C** | **T** | **A** | **A** | **A** | **T** | **T** | **T** | **C** | **A** | **A** | **A** | **A** | **T** | **T** | **T** | **T** | **C** | **A** | **A** | **T** | **T** | **G** | **A** | **T** | **C** | **T** | **T** | **A** | **G** | **G** | **G** | **A** | **G** | **C** | **T** | **T** | **G** | **G** | **T** | **C** | **A** | **T** | **T** | **T** | **T** | **T** | **T** | **T** | **T** | **A** | **G** | **C** | **T** | **T** | **A** | **G** | **T** | **C** | **A** | **A** | **C** | **A** | **G** | **T** | **C** | **C** | **T** | **G** | **C** | **T** | **T** | **T** | **A** | **T** | **G** | **G** | **A** | **T** | **G** | **A** | **T** | **G** | **G** | **T** | **T** | **G** | **T** | **A** | **T** | **T** | **G** | **G** | **C** | **C** | **G** | **T** | **T** | **T** | **T** | **A** | **T** | **T** | **T** | **A** | **T** | **C** | **G** | **G** | **C** | **C** | **T** | **G** | **A** | **A** | **A** | **G** | **G** | **T** | **G** | **T** | **A** | **G** | **A** | **G** | **A** | **T** | **T** | **T** | **C** | **A** | **G** | **G** | **A** | **A** | **T** | **A** | **G** | **A** | **T** | **T** | **T** | **G** | **A** | **C** | **A** | **A** | **A** | **A** | **A** | **A** | **T** | **G** | **C** | **T** | **T** | **T** | **T** | **T** | **G** | **G** | **C** | **G** | **A** | **A** | **A** | **A** | **A** | **A** | **A** | **C** | **A** | **G** | **A** | **T** | **C** | **A** | **T** | **G** | **A** | **T** | **G** | **G** | **T** | **C** | **C** | **A** | **A** | **A** | **C** | **A** | **G** | **A** | **G** | **C** | **A** | **A** | **T** | **T** | **A** | **A** | **A** | **A** | **A** | **C** | **A** | **C** | **A** | **T** | **A** | **A** | **T** | **A** | **A** | **T** | **G** | **A** | **G** | **C** | **G** | **T** | **A** | **A** | **A** | **A** | **G** | **A** | **C** | **A** | **G** | **C** | **A** | **A** | **T** | **T** | **A** | **T** | **T** | **T** | **G** | **G** | **T** | **A** | **A** | **C** | **C** | **A** | **A** | **C** | **G** | **T** | **A** | **T** | **T** | **G** | **G** | **A** | **A** | **A** | **C** | **G** | **A** | **C** | **G** | **T** | **C** | **T** | **A** | **T** | **G** | **A** | **A** | **T** | **G** | **G** | **G** | **A** | **T** | **G** | **A** | **T** | **A** | **T** | **C** | **A** | **A** | **C** | **A** | **T** | **T** | **G** | **C** | **C** | **A** | **T** | **C** | **A** | **T** | **T** | **T** | **C** | **T** | **G** | **G** | **T** | **G** | **A** | **T** | **A** | **C** | **T** | **A** | **T** | **C** | **A** | **T** | **C** | **G** | **A** | **T** | **T** | **T** | **G** | **G** | **G** | **C** | **A** | **A** | **T** | **A** | **C** | **A** | **C** | **T** | **T** | **T** | **T** | **G** | **C** | **C** | **A** | **A** | **A** | **A** | **G** | **A** | **T** | **G** | **A** | **T** | **A** | **A** | **T** | **A** | **T** | **T** | **G** | **T** | **G** | **A** | **T** | **T** | **G** | **T** | **A** | **A** | **G** | **A** | **A** | **A** | **A** | **G** | **G** | **G** | **A** | **T** | **A** | **G** | **G** | **C** | **A** | **G** | **A** | **A** | **C** | **A** | **C** | **G** | **G** | **A** | **T** | **C** | **T** | **T** | **A** | **G** | **T** | **A** | **C** | **C** | **C** | **C** | **T** | **G** | **G** | **G** | **A** | **G** | **T** | **G** | **G** | **C** | **T** | **A** | **T** | **T** | **C** | **G** | **A** | **T** | **T** | **A** | **G** | **A** | **G** | **C** | **A** | **T** | **G** | **C** | **G** | **A** | **C** | **A** | **T** | **T** | **A** | **G** | **T** | **A** | **G** | **G** | **A** | **A** | **A** | **T** | **G** | **T** | **C** | **T** | **T** | **G** | **T** | **T** | **T** | **G** | **A** | **A** | **G** | **A** | **A** | **G** | **A** | **G** | **C** | **A** | **G** | **T** | **T** | **T** | **T** | **C** | **C** | **T** | **T** | **A** | **A** | **A** | **A** | **A** | **A** | **T** | **G** | **A** | **A** | **C** | **A** | **G** | **A** | **T** | **C** | **A** | **A** | **A** | **A** | **T** | **C** | **T** | **G** | **C** | **A** | **G** | **C** | **A** | **G** | **T** | **G** | **A** | **T** | **T** | **A** | **T** | **G** | **A** | **T** | **G** | **A** | **A** | **A** | **A** | **T** | **C** | **C** | **T** | **C** | **G** | **C** | **A** | **G** | **A** | **T** | **T** | **G** | **A** | **A** | **G** | **A** | **T** | **C** | **A** | **T** | **T** | **A** | **C** | **C** | **A** | **A** | **T** | **A** | **C** | **C** | **T** | **T** | **G** | **G** | **T** | **T** | **G** | **G** | **A** | **G** | **A** | **T** | **G** | **T** | **C** | **G** | **A** | **G** | **G** | **T** | **G** | **A** | **T** | **A** | **C** | **G** | **A** | **A** | **T** | **A** | **-** | **-** | **-** |
| **N54070** | **A** | **T** | **G** | **A** | **A** | **T** | **A** | **G** | **T** | **T** | **C** | **T** | **T** | **G** | **G** | **C** | **G** | **T** | **T** | **T** | **T** | **T** | **T** | **T** | **G** | **T** | **G** | **G** | **T** | **A** | **G** | **T** | **C** | **G** | **A** | **A** | **G** | **C** | **A** | **C** | **T** | **G** | **C** | **T** | **G** | **C** | **T** | **G** | **T** | **T** | **A** | **T** | **T** | **T** | **G** | **C** | **T** | **G** | **T** | **T** | **T** | **G** | **G** | **C** | **A** | **A** | **A** | **T** | **C** | **G** | **T** | **A** | **A** | **A** | **C** | **A** | **A** | **T** | **A** | **C** | **T** | **G** | **G** | **A** | **C** | **T** | **T** | **T** | **T** | **G** | **C** | **T** | **T** | **T** | **T** | **A** | **G** | **T** | **A** | **A** | **T** | **A** | **T** | **T** | **C** | **G** | **G** | **T** | **A** | **T** | **T** | **T** | **T** | **C** | **A** | **A** | **T** | **A** | **T** | **A** | **T** | **A** | **T** | **C** | **T** | **A** | **G** | **C** | **T** | **A** | **T** | **G** | **C** | **G** | **C** | **A** | **A** | **A** | **T** | **A** | **T** | **C** | **C** | **T** | **C** | **G** | **T** | **A** | **C** | **T** | **A** | **A** | **A** | **T** | **T** | **T** | **C** | **A** | **A** | **A** | **A** | **T** | **T** | **T** | **T** | **C** | **A** | **A** | **T** | **T** | **G** | **A** | **T** | **C** | **T** | **T** | **A** | **G** | **G** | **G** | **A** | **G** | **C** | **T** | **T** | **G** | **G** | **T** | **C** | **A** | **T** | **T** | **T** | **T** | **T** | **T** | **T** | **T** | **A** | **G** | **C** | **T** | **T** | **A** | **G** | **T** | **C** | **A** | **A** | **C** | **A** | **G** | **T** | **C** | **C** | **T** | **G** | **C** | **T** | **T** | **T** | **A** | **T** | **G** | **G** | **A** | **T** | **G** | **A** | **T** | **G** | **G** | **T** | **T** | **G** | **T** | **A** | **T** | **T** | **G** | **G** | **C** | **C** | **A** | **T** | **T** | **T** | **T** | **A** | **T** | **T** | **T** | **A** | **T** | **C** | **G** | **G** | **A** | **C** | **T** | **G** | **A** | **A** | **A** | **G** | **G** | **T** | **G** | **T** | **A** | **G** | **A** | **G** | **A** | **T** | **T** | **T** | **C** | **A** | **G** | **G** | **A** | **A** | **T** | **A** | **G** | **A** | **T** | **T** | **T** | **G** | **A** | **C** | **A** | **A** | **A** | **A** | **A** | **A** | **T** | **G** | **C** | **T** | **T** | **T** | **T** | **T** | **G** | **G** | **C** | **G** | **A** | **A** | **A** | **A** | **A** | **A** | **A** | **C** | **A** | **G** | **A** | **T** | **T** | **A** | **T** | **G** | **A** | **T** | **G** | **G** | **T** | **C** | **C** | **A** | **A** | **A** | **C** | **A** | **G** | **A** | **G** | **C** | **A** | **A** | **T** | **T** | **A** | **A** | **A** | **A** | **A** | **C** | **A** | **C** | **A** | **T** | **A** | **A** | **T** | **A** | **A** | **T** | **G** | **A** | **G** | **C** | **G** | **T** | **A** | **A** | **A** | **A** | **G** | **A** | **C** | **A** | **G** | **C** | **A** | **A** | **T** | **T** | **A** | **T** | **T** | **T** | **G** | **G** | **T** | **A** | **A** | **C** | **C** | **A** | **A** | **C** | **G** | **T** | **A** | **T** | **T** | **G** | **G** | **A** | **A** | **A** | **C** | **G** | **A** | **C** | **G** | **T** | **C** | **T** | **A** | **T** | **G** | **A** | **A** | **T** | **G** | **G** | **G** | **A** | **T** | **G** | **A** | **T** | **A** | **T** | **C** | **A** | **A** | **C** | **A** | **T** | **T** | **G** | **C** | **C** | **A** | **T** | **T** | **A** | **T** | **T** | **T** | **C** | **T** | **G** | **G** | **T** | **G** | **A** | **T** | **A** | **C** | **T** | **A** | **T** | **C** | **A** | **T** | **C** | **G** | **A** | **T** | **T** | **T** | **G** | **G** | **G** | **C** | **A** | **A** | **T** | **A** | **C** | **A** | **C** | **T** | **T** | **T** | **T** | **G** | **C** | **C** | **A** | **A** | **A** | **A** | **G** | **A** | **T** | **G** | **A** | **T** | **A** | **A** | **T** | **A** | **T** | **T** | **G** | **T** | **G** | **A** | **T** | **T** | **G** | **T** | **A** | **A** | **G** | **A** | **A** | **A** | **A** | **G** | **G** | **G** | **A** | **T** | **A** | **G** | **G** | **C** | **A** | **G** | **A** | **A** | **C** | **A** | **C** | **G** | **G** | **A** | **T** | **C** | **T** | **T** | **A** | **G** | **T** | **A** | **C** | **C** | **C** | **C** | **T** | **G** | **G** | **G** | **A** | **G** | **T** | **G** | **G** | **C** | **T** | **A** | **T** | **T** | **C** | **G** | **A** | **T** | **T** | **A** | **G** | **A** | **G** | **C** | **A** | **T** | **G** | **C** | **G** | **A** | **C** | **A** | **T** | **T** | **A** | **G** | **T** | **A** | **G** | **G** | **A** | **A** | **A** | **T** | **G** | **T** | **C** | **T** | **T** | **G** | **T** | **T** | **T** | **G** | **A** | **A** | **G** | **A** | **A** | **G** | **A** | **G** | **C** | **A** | **G** | **T** | **T** | **T** | **T** | **C** | **C** | **T** | **T** | **A** | **A** | **A** | **A** | **A** | **A** | **C** | **G** | **A** | **A** | **C** | **A** | **G** | **A** | **T** | **C** | **A** | **A** | **A** | **A** | **T** | **C** | **T** | **G** | **C** | **A** | **G** | **C** | **A** | **G** | **T** | **G** | **A** | **T** | **T** | **A** | **T** | **G** | **A** | **T** | **G** | **A** | **A** | **A** | **A** | **T** | **C** | **C** | **T** | **C** | **G** | **C** | **A** | **G** | **A** | **T** | **T** | **G** | **A** | **A** | **G** | **A** | **T** | **C** | **A** | **T** | **T** | **A** | **C** | **C** | **A** | **A** | **T** | **A** | **C** | **C** | **T** | **T** | **G** | **G** | **T** | **T** | **G** | **G** | **A** | **G** | **A** | **T** | **G** | **T** | **C** | **G** | **A** | **G** | **G** | **T** | **G** | **A** | **T** | **A** | **C** | **G** | **A** | **A** | **T** | **A** | **-** | **-** | **-** |
| **N52702** | **A** | **T** | **G** | **A** | **A** | **T** | **A** | **G** | **T** | **T** | **C** | **T** | **T** | **G** | **G** | **C** | **G** | **T** | **T** | **T** | **T** | **T** | **T** | **T** | **G** | **T** | **G** | **G** | **T** | **A** | **G** | **T** | **C** | **G** | **A** | **A** | **G** | **C** | **A** | **C** | **T** | **G** | **C** | **T** | **G** | **C** | **T** | **G** | **T** | **T** | **A** | **T** | **T** | **T** | **G** | **C** | **T** | **G** | **T** | **T** | **T** | **G** | **G** | **C** | **A** | **A** | **A** | **T** | **C** | **G** | **T** | **A** | **A** | **A** | **C** | **A** | **A** | **T** | **A** | **C** | **T** | **G** | **G** | **A** | **C** | **T** | **T** | **T** | **T** | **G** | **C** | **T** | **T** | **T** | **T** | **A** | **G** | **T** | **A** | **A** | **T** | **A** | **T** | **T** | **C** | **G** | **G** | **T** | **A** | **T** | **T** | **T** | **T** | **C** | **A** | **A** | **T** | **A** | **T** | **A** | **T** | **A** | **T** | **C** | **T** | **A** | **G** | **C** | **T** | **A** | **T** | **G** | **C** | **G** | **C** | **A** | **A** | **A** | **T** | **A** | **T** | **C** | **C** | **T** | **C** | **G** | **T** | **A** | **C** | **T** | **A** | **A** | **A** | **T** | **T** | **T** | **C** | **A** | **A** | **A** | **A** | **T** | **T** | **T** | **T** | **C** | **A** | **A** | **T** | **T** | **G** | **A** | **T** | **C** | **T** | **T** | **A** | **G** | **G** | **G** | **A** | **G** | **C** | **T** | **T** | **A** | **G** | **T** | **C** | **A** | **T** | **T** | **T** | **T** | **T** | **T** | **T** | **T** | **A** | **G** | **C** | **T** | **T** | **A** | **G** | **T** | **C** | **A** | **A** | **C** | **A** | **G** | **T** | **C** | **C** | **T** | **G** | **C** | **T** | **T** | **T** | **A** | **T** | **G** | **G** | **A** | **T** | **G** | **A** | **T** | **G** | **G** | **T** | **T** | **G** | **T** | **A** | **T** | **T** | **G** | **G** | **C** | **C** | **G** | **T** | **T** | **T** | **T** | **A** | **T** | **T** | **T** | **A** | **T** | **C** | **G** | **G** | **G** | **C** | **T** | **G** | **A** | **A** | **A** | **G** | **G** | **T** | **G** | **T** | **A** | **G** | **A** | **G** | **A** | **T** | **T** | **T** | **C** | **A** | **G** | **G** | **A** | **A** | **T** | **A** | **G** | **A** | **T** | **T** | **T** | **G** | **A** | **C** | **A** | **A** | **A** | **A** | **A** | **A** | **T** | **G** | **C** | **T** | **T** | **T** | **T** | **T** | **G** | **G** | **C** | **G** | **A** | **A** | **A** | **A** | **A** | **A** | **A** | **C** | **A** | **G** | **A** | **T** | **C** | **A** | **T** | **G** | **A** | **T** | **G** | **G** | **T** | **C** | **C** | **A** | **A** | **A** | **C** | **A** | **G** | **A** | **G** | **C** | **A** | **A** | **T** | **T** | **A** | **A** | **A** | **A** | **A** | **C** | **A** | **C** | **A** | **T** | **A** | **A** | **T** | **A** | **A** | **T** | **G** | **A** | **G** | **C** | **G** | **T** | **A** | **A** | **A** | **A** | **G** | **A** | **C** | **A** | **G** | **C** | **A** | **A** | **T** | **T** | **A** | **T** | **T** | **T** | **G** | **G** | **T** | **A** | **A** | **C** | **C** | **A** | **A** | **C** | **G** | **T** | **A** | **T** | **T** | **G** | **G** | **A** | **A** | **A** | **C** | **G** | **A** | **C** | **G** | **T** | **C** | **T** | **A** | **T** | **G** | **A** | **A** | **T** | **G** | **G** | **G** | **A** | **T** | **G** | **A** | **C** | **A** | **T** | **C** | **A** | **A** | **C** | **A** | **T** | **T** | **G** | **C** | **C** | **A** | **T** | **C** | **A** | **T** | **T** | **T** | **C** | **T** | **G** | **G** | **T** | **G** | **A** | **T** | **A** | **C** | **T** | **A** | **T** | **C** | **A** | **T** | **C** | **G** | **A** | **T** | **T** | **T** | **G** | **G** | **G** | **C** | **A** | **A** | **T** | **A** | **C** | **A** | **C** | **T** | **T** | **T** | **T** | **G** | **C** | **C** | **A** | **A** | **A** | **A** | **G** | **A** | **T** | **G** | **A** | **T** | **A** | **A** | **T** | **A** | **T** | **T** | **G** | **T** | **G** | **A** | **T** | **T** | **G** | **T** | **A** | **A** | **G** | **A** | **A** | **A** | **A** | **G** | **G** | **G** | **A** | **T** | **A** | **G** | **G** | **C** | **A** | **G** | **A** | **A** | **C** | **A** | **C** | **G** | **G** | **A** | **T** | **C** | **T** | **T** | **A** | **G** | **T** | **A** | **C** | **C** | **C** | **C** | **T** | **G** | **G** | **G** | **A** | **G** | **T** | **G** | **G** | **C** | **T** | **A** | **T** | **T** | **C** | **G** | **T** | **T** | **T** | **A** | **G** | **A** | **G** | **C** | **A** | **T** | **G** | **C** | **G** | **A** | **C** | **A** | **T** | **T** | **A** | **G** | **T** | **A** | **G** | **G** | **A** | **A** | **A** | **T** | **G** | **T** | **C** | **T** | **T** | **G** | **T** | **T** | **T** | **G** | **A** | **A** | **G** | **A** | **A** | **G** | **A** | **G** | **C** | **A** | **G** | **T** | **T** | **T** | **T** | **C** | **C** | **T** | **T** | **A** | **A** | **A** | **A** | **A** | **A** | **C** | **G** | **A** | **A** | **C** | **A** | **G** | **A** | **T** | **C** | **A** | **A** | **A** | **A** | **T** | **C** | **T** | **G** | **C** | **A** | **G** | **C** | **A** | **G** | **T** | **G** | **A** | **T** | **T** | **A** | **T** | **G** | **A** | **T** | **G** | **A** | **A** | **A** | **A** | **T** | **C** | **C** | **T** | **C** | **G** | **C** | **A** | **G** | **A** | **T** | **T** | **G** | **A** | **A** | **G** | **A** | **T** | **C** | **A** | **T** | **T** | **A** | **C** | **C** | **A** | **A** | **T** | **A** | **C** | **C** | **T** | **T** | **G** | **G** | **T** | **T** | **G** | **G** | **A** | **G** | **A** | **T** | **G** | **T** | **C** | **G** | **A** | **G** | **G** | **T** | **G** | **A** | **T** | **A** | **C** | **G** | **A** | **A** | **T** | **A** | **-** | **-** | **-** |
| **N60417F** | **A** | **T** | **G** | **A** | **A** | **T** | **A** | **G** | **T** | **T** | **C** | **T** | **T** | **G** | **G** | **C** | **G** | **T** | **T** | **T** | **T** | **T** | **T** | **T** | **G** | **T** | **G** | **G** | **T** | **A** | **G** | **T** | **T** | **G** | **A** | **A** | **G** | **C** | **A** | **C** | **T** | **A** | **C** | **T** | **G** | **C** | **T** | **G** | **T** | **T** | **A** | **T** | **T** | **T** | **G** | **C** | **T** | **G** | **T** | **T** | **T** | **G** | **G** | **C** | **A** | **A** | **A** | **T** | **C** | **G** | **T** | **A** | **A** | **A** | **C** | **A** | **A** | **T** | **A** | **C** | **T** | **G** | **G** | **A** | **C** | **T** | **T** | **T** | **T** | **G** | **C** | **T** | **T** | **T** | **T** | **A** | **G** | **T** | **G** | **A** | **T** | **A** | **T** | **T** | **C** | **G** | **G** | **T** | **A** | **T** | **T** | **T** | **T** | **C** | **A** | **A** | **T** | **A** | **T** | **A** | **T** | **A** | **T** | **C** | **T** | **A** | **G** | **C** | **T** | **A** | **T** | **G** | **C** | **G** | **C** | **A** | **A** | **A** | **C** | **A** | **T** | **C** | **C** | **T** | **C** | **G** | **T** | **A** | **C** | **T** | **A** | **A** | **A** | **T** | **T** | **T** | **C** | **A** | **A** | **A** | **A** | **T** | **T** | **T** | **T** | **C** | **A** | **A** | **T** | **T** | **G** | **A** | **T** | **A** | **T** | **T** | **A** | **G** | **G** | **A** | **A** | **G** | **T** | **T** | **T** | **G** | **G** | **T** | **C** | **A** | **T** | **T** | **T** | **T** | **T** | **T** | **T** | **T** | **A** | **G** | **C** | **T** | **T** | **A** | **G** | **T** | **C** | **A** | **A** | **C** | **A** | **G** | **T** | **C** | **C** | **T** | **G** | **C** | **T** | **T** | **T** | **A** | **T** | **G** | **G** | **A** | **T** | **G** | **A** | **T** | **G** | **G** | **C** | **T** | **G** | **T** | **A** | **T** | **T** | **T** | **G** | **C** | **C** | **G** | **T** | **T** | **T** | **T** | **A** | **T** | **T** | **T** | **A** | **T** | **T** | **G** | **G** | **A** | **C** | **T** | **A** | **A** | **A** | **A** | **G** | **G** | **T** | **G** | **T** | **A** | **G** | **A** | **G** | **A** | **T** | **T** | **T** | **C** | **A** | **G** | **G** | **G** | **A** | **T** | **A** | **G** | **A** | **T** | **T** | **T** | **A** | **A** | **C** | **C** | **A** | **A** | **A** | **A** | **A** | **T** | **G** | **C** | **T** | **T** | **T** | **T** | **T** | **G** | **G** | **C** | **G** | **A** | **A** | **A** | **G** | **A** | **A** | **A** | **C** | **A** | **G** | **A** | **T** | **C** | **A** | **T** | **G** | **A** | **T** | **G** | **G** | **T** | **C** | **C** | **A** | **A** | **A** | **C** | **A** | **G** | **A** | **G** | **C** | **A** | **A** | **T** | **T** | **A** | **A** | **A** | **A** | **A** | **C** | **A** | **C** | **A** | **T** | **A** | **A** | **T** | **A** | **A** | **T** | **G** | **A** | **G** | **C** | **G** | **G** | **A** | **A** | **A** | **A** | **G** | **A** | **C** | **A** | **G** | **C** | **A** | **A** | **T** | **T** | **A** | **T** | **T** | **C** | **G** | **G** | **T** | **A** | **A** | **C** | **C** | **A** | **A** | **C** | **G** | **T** | **A** | **T** | **T** | **G** | **G** | **A** | **A** | **A** | **C** | **G** | **A** | **C** | **G** | **T** | **C** | **T** | **A** | **T** | **G** | **A** | **A** | **T** | **G** | **G** | **G** | **A** | **C** | **G** | **A** | **T** | **A** | **T** | **C** | **A** | **A** | **C** | **A** | **T** | **C** | **G** | **C** | **C** | **G** | **T** | **C** | **A** | **T** | **T** | **T** | **C** | **T** | **G** | **G** | **T** | **G** | **A** | **T** | **A** | **C** | **T** | **A** | **T** | **C** | **A** | **T** | **A** | **G** | **A** | **T** | **T** | **T** | **G** | **G** | **G** | **C** | **A** | **A** | **T** | **A** | **C** | **G** | **C** | **T** | **T** | **T** | **T** | **G** | **C** | **C** | **A** | **A** | **A** | **A** | **G** | **A** | **T** | **G** | **A** | **T** | **A** | **A** | **T** | **A** | **T** | **T** | **G** | **T** | **G** | **A** | **T** | **T** | **G** | **T** | **A** | **A** | **G** | **A** | **A** | **A** | **A** | **G** | **G** | **G** | **A** | **T** | **A** | **G** | **G** | **C** | **A** | **G** | **A** | **A** | **C** | **A** | **C** | **G** | **G** | **A** | **T** | **C** | **T** | **T** | **A** | **G** | **T** | **A** | **C** | **C** | **T** | **T** | **T** | **G** | **G** | **G** | **C** | **G** | **T** | **A** | **G** | **C** | **C** | **A** | **T** | **C** | **C** | **G** | **A** | **T** | **T** | **A** | **G** | **A** | **G** | **C** | **A** | **T** | **G** | **C** | **G** | **A** | **C** | **A** | **T** | **T** | **A** | **G** | **T** | **A** | **G** | **G** | **A** | **A** | **A** | **T** | **G** | **T** | **C** | **T** | **T** | **G** | **T** | **T** | **T** | **G** | **A** | **A** | **G** | **A** | **A** | **G** | **A** | **G** | **C** | **A** | **A** | **T** | **T** | **T** | **T** | **C** | **T** | **T** | **T** | **G** | **A** | **A** | **A** | **A** | **A** | **T** | **G** | **A** | **A** | **C** | **A** | **A** | **A** | **T** | **C** | **A** | **A** | **A** | **A** | **T** | **C** | **T** | **A** | **T** | **A** | **G** | **C** | **A** | **G** | **T** | **G** | **A** | **T** | **T** | **A** | **T** | **G** | **A** | **T** | **G** | **A** | **A** | **A** | **A** | **C** | **C** | **C** | **T** | **C** | **G** | **T** | **A** | **G** | **A** | **C** | **T** | **G** | **A** | **A** | **G** | **A** | **T** | **T** | **A** | **T** | **T** | **A** | **C** | **T** | **A** | **A** | **T** | **A** | **C** | **C** | **T** | **T** | **G** | **A** | **T** | **T** | **G** | **G** | **A** | **G** | **A** | **T** | **G** | **T** | **C** | **G** | **A** | **G** | **G** | **T** | **G** | **A** | **T** | **A** | **C** | **G** | **A** | **A** | **T** | **A** | **-** | **-** | **-** |
| **N59745F** | **A** | **T** | **G** | **A** | **A** | **T** | **A** | **G** | **T** | **T** | **C** | **T** | **T** | **G** | **G** | **C** | **G** | **T** | **T** | **T** | **T** | **T** | **T** | **T** | **G** | **T** | **G** | **G** | **T** | **A** | **G** | **T** | **T** | **G** | **A** | **A** | **G** | **C** | **A** | **C** | **T** | **A** | **C** | **T** | **G** | **C** | **T** | **G** | **T** | **T** | **A** | **T** | **T** | **T** | **G** | **C** | **T** | **G** | **T** | **T** | **T** | **G** | **G** | **C** | **A** | **A** | **A** | **T** | **C** | **G** | **T** | **A** | **A** | **A** | **C** | **A** | **A** | **T** | **A** | **C** | **T** | **G** | **G** | **A** | **C** | **T** | **T** | **T** | **T** | **G** | **C** | **T** | **T** | **T** | **T** | **A** | **G** | **T** | **G** | **A** | **T** | **A** | **T** | **T** | **C** | **G** | **G** | **T** | **A** | **T** | **T** | **T** | **T** | **C** | **A** | **A** | **T** | **A** | **T** | **A** | **T** | **A** | **T** | **C** | **T** | **A** | **G** | **C** | **T** | **A** | **T** | **G** | **C** | **G** | **C** | **A** | **A** | **A** | **C** | **A** | **T** | **C** | **C** | **T** | **C** | **G** | **T** | **A** | **C** | **T** | **A** | **A** | **A** | **T** | **T** | **T** | **C** | **A** | **A** | **A** | **A** | **T** | **T** | **T** | **T** | **C** | **A** | **A** | **T** | **T** | **G** | **A** | **T** | **A** | **T** | **T** | **A** | **G** | **G** | **A** | **A** | **G** | **T** | **T** | **T** | **G** | **G** | **T** | **C** | **A** | **T** | **T** | **T** | **T** | **T** | **T** | **T** | **T** | **A** | **G** | **C** | **T** | **T** | **A** | **G** | **T** | **C** | **A** | **A** | **C** | **A** | **G** | **T** | **C** | **C** | **T** | **G** | **C** | **T** | **T** | **T** | **A** | **T** | **G** | **G** | **A** | **T** | **G** | **A** | **T** | **G** | **G** | **C** | **T** | **G** | **T** | **A** | **T** | **T** | **T** | **G** | **C** | **C** | **G** | **T** | **T** | **T** | **T** | **A** | **T** | **T** | **T** | **A** | **T** | **T** | **G** | **G** | **T** | **C** | **T** | **G** | **A** | **A** | **A** | **G** | **G** | **T** | **G** | **T** | **A** | **G** | **A** | **G** | **A** | **T** | **T** | **T** | **C** | **A** | **G** | **G** | **G** | **A** | **T** | **A** | **G** | **A** | **T** | **T** | **T** | **A** | **A** | **C** | **C** | **A** | **A** | **A** | **A** | **A** | **T** | **G** | **C** | **T** | **T** | **T** | **T** | **T** | **G** | **G** | **C** | **G** | **A** | **A** | **A** | **G** | **A** | **A** | **A** | **C** | **A** | **G** | **A** | **T** | **C** | **A** | **T** | **G** | **A** | **T** | **G** | **A** | **T** | **C** | **C** | **A** | **A** | **A** | **C** | **A** | **G** | **A** | **G** | **C** | **A** | **A** | **T** | **T** | **A** | **A** | **A** | **A** | **A** | **C** | **A** | **C** | **A** | **T** | **A** | **A** | **T** | **A** | **A** | **T** | **G** | **A** | **G** | **C** | **G** | **G** | **A** | **A** | **A** | **A** | **G** | **A** | **C** | **A** | **G** | **C** | **A** | **A** | **T** | **T** | **A** | **T** | **T** | **C** | **G** | **G** | **T** | **A** | **A** | **C** | **C** | **A** | **A** | **C** | **G** | **T** | **A** | **T** | **T** | **G** | **G** | **A** | **A** | **A** | **C** | **G** | **A** | **C** | **G** | **T** | **C** | **T** | **A** | **T** | **G** | **A** | **A** | **T** | **G** | **G** | **G** | **A** | **C** | **G** | **A** | **T** | **A** | **T** | **C** | **A** | **A** | **C** | **A** | **T** | **C** | **G** | **C** | **C** | **G** | **T** | **C** | **A** | **T** | **T** | **T** | **C** | **T** | **G** | **G** | **T** | **G** | **A** | **T** | **A** | **C** | **T** | **A** | **T** | **C** | **A** | **T** | **A** | **G** | **A** | **T** | **T** | **T** | **G** | **G** | **G** | **C** | **A** | **A** | **T** | **A** | **C** | **G** | **C** | **T** | **T** | **T** | **T** | **G** | **C** | **C** | **A** | **A** | **A** | **A** | **G** | **A** | **T** | **G** | **A** | **T** | **A** | **A** | **T** | **A** | **T** | **T** | **G** | **T** | **G** | **A** | **T** | **T** | **G** | **T** | **A** | **A** | **G** | **A** | **A** | **A** | **A** | **G** | **G** | **G** | **A** | **T** | **A** | **G** | **G** | **C** | **A** | **G** | **A** | **A** | **C** | **A** | **C** | **G** | **G** | **A** | **T** | **C** | **T** | **T** | **A** | **G** | **T** | **A** | **C** | **C** | **T** | **T** | **T** | **G** | **G** | **G** | **C** | **G** | **T** | **A** | **G** | **C** | **C** | **A** | **T** | **C** | **C** | **G** | **A** | **T** | **T** | **A** | **G** | **A** | **G** | **C** | **A** | **T** | **G** | **C** | **G** | **A** | **C** | **A** | **T** | **T** | **A** | **G** | **T** | **A** | **G** | **G** | **A** | **A** | **A** | **T** | **G** | **T** | **C** | **T** | **T** | **G** | **T** | **T** | **T** | **G** | **A** | **A** | **G** | **A** | **A** | **G** | **A** | **G** | **C** | **A** | **A** | **T** | **T** | **T** | **T** | **C** | **T** | **T** | **T** | **G** | **A** | **A** | **A** | **A** | **A** | **T** | **G** | **A** | **A** | **C** | **A** | **A** | **A** | **T** | **C** | **A** | **A** | **A** | **A** | **T** | **C** | **T** | **A** | **C** | **A** | **G** | **C** | **A** | **G** | **T** | **G** | **A** | **T** | **T** | **A** | **T** | **G** | **A** | **T** | **G** | **A** | **A** | **A** | **A** | **C** | **C** | **C** | **T** | **C** | **G** | **T** | **A** | **G** | **A** | **T** | **T** | **G** | **A** | **A** | **G** | **A** | **T** | **T** | **A** | **T** | **T** | **A** | **C** | **T** | **A** | **A** | **T** | **A** | **C** | **C** | **T** | **T** | **G** | **A** | **T** | **T** | **G** | **G** | **A** | **G** | **A** | **T** | **G** | **T** | **C** | **G** | **A** | **G** | **G** | **T** | **G** | **A** | **T** | **A** | **C** | **G** | **A** | **A** | **T** | **A** | **-** | **-** | **-** |
| **N53559** | **A** | **T** | **G** | **A** | **A** | **T** | **A** | **G** | **T** | **T** | **C** | **T** | **T** | **G** | **G** | **C** | **G** | **T** | **T** | **T** | **T** | **T** | **T** | **T** | **G** | **T** | **G** | **G** | **T** | **A** | **G** | **T** | **C** | **G** | **A** | **A** | **G** | **C** | **A** | **C** | **T** | **G** | **C** | **T** | **G** | **C** | **T** | **G** | **T** | **T** | **A** | **T** | **T** | **T** | **G** | **C** | **T** | **G** | **T** | **T** | **T** | **G** | **G** | **C** | **A** | **A** | **A** | **T** | **C** | **G** | **T** | **A** | **A** | **A** | **C** | **A** | **A** | **T** | **A** | **C** | **T** | **G** | **G** | **A** | **C** | **T** | **T** | **T** | **T** | **G** | **C** | **T** | **T** | **T** | **T** | **A** | **G** | **T** | **A** | **A** | **T** | **A** | **T** | **T** | **C** | **G** | **G** | **T** | **A** | **T** | **T** | **T** | **T** | **C** | **A** | **A** | **T** | **A** | **T** | **A** | **T** | **A** | **T** | **C** | **T** | **A** | **G** | **C** | **T** | **A** | **T** | **G** | **C** | **G** | **C** | **A** | **A** | **A** | **T** | **A** | **T** | **C** | **C** | **T** | **C** | **G** | **T** | **A** | **C** | **T** | **A** | **A** | **A** | **T** | **T** | **T** | **C** | **A** | **A** | **A** | **A** | **T** | **T** | **T** | **T** | **C** | **A** | **A** | **T** | **T** | **G** | **A** | **T** | **C** | **T** | **T** | **A** | **G** | **G** | **G** | **A** | **G** | **C** | **T** | **T** | **G** | **G** | **T** | **C** | **A** | **T** | **T** | **T** | **T** | **T** | **T** | **T** | **T** | **A** | **G** | **C** | **T** | **T** | **A** | **G** | **T** | **C** | **A** | **A** | **C** | **A** | **G** | **T** | **C** | **C** | **T** | **G** | **C** | **T** | **T** | **T** | **A** | **T** | **G** | **G** | **A** | **T** | **G** | **A** | **T** | **G** | **G** | **T** | **T** | **G** | **T** | **A** | **T** | **T** | **G** | **G** | **C** | **C** | **A** | **T** | **T** | **T** | **T** | **A** | **T** | **T** | **T** | **A** | **T** | **C** | **G** | **G** | **A** | **C** | **T** | **G** | **A** | **A** | **A** | **G** | **G** | **T** | **G** | **T** | **A** | **G** | **A** | **G** | **A** | **T** | **T** | **T** | **C** | **A** | **G** | **G** | **A** | **A** | **T** | **A** | **G** | **A** | **T** | **T** | **T** | **G** | **A** | **C** | **A** | **A** | **A** | **A** | **A** | **A** | **T** | **G** | **C** | **T** | **T** | **T** | **T** | **T** | **G** | **G** | **C** | **G** | **A** | **A** | **A** | **A** | **A** | **A** | **A** | **C** | **A** | **G** | **A** | **T** | **T** | **A** | **T** | **G** | **A** | **T** | **G** | **G** | **T** | **C** | **C** | **A** | **A** | **A** | **C** | **A** | **G** | **A** | **G** | **C** | **A** | **A** | **T** | **T** | **A** | **A** | **A** | **A** | **A** | **C** | **A** | **C** | **A** | **T** | **A** | **A** | **T** | **A** | **A** | **T** | **G** | **A** | **G** | **C** | **G** | **T** | **A** | **A** | **A** | **A** | **G** | **A** | **C** | **A** | **G** | **C** | **A** | **A** | **T** | **T** | **A** | **T** | **T** | **T** | **G** | **G** | **T** | **A** | **A** | **C** | **C** | **A** | **A** | **C** | **G** | **T** | **A** | **T** | **T** | **G** | **G** | **A** | **A** | **A** | **C** | **G** | **A** | **C** | **G** | **T** | **C** | **T** | **A** | **T** | **G** | **A** | **A** | **T** | **G** | **G** | **G** | **A** | **T** | **G** | **A** | **T** | **A** | **T** | **C** | **A** | **A** | **C** | **A** | **T** | **T** | **G** | **C** | **C** | **A** | **T** | **C** | **A** | **T** | **T** | **T** | **C** | **T** | **G** | **G** | **T** | **G** | **A** | **T** | **A** | **C** | **T** | **A** | **T** | **C** | **A** | **T** | **C** | **G** | **A** | **T** | **T** | **T** | **G** | **G** | **G** | **C** | **A** | **A** | **T** | **A** | **C** | **A** | **C** | **T** | **T** | **T** | **T** | **G** | **C** | **C** | **A** | **A** | **A** | **A** | **G** | **A** | **T** | **G** | **A** | **T** | **A** | **A** | **T** | **A** | **T** | **T** | **G** | **T** | **G** | **A** | **T** | **T** | **G** | **T** | **A** | **A** | **G** | **A** | **A** | **A** | **A** | **G** | **G** | **G** | **A** | **T** | **A** | **G** | **G** | **C** | **A** | **G** | **A** | **A** | **C** | **A** | **C** | **G** | **G** | **A** | **T** | **C** | **T** | **T** | **A** | **G** | **T** | **A** | **C** | **C** | **C** | **C** | **T** | **G** | **G** | **G** | **A** | **G** | **T** | **G** | **G** | **C** | **T** | **A** | **T** | **T** | **C** | **G** | **A** | **T** | **T** | **A** | **G** | **A** | **G** | **C** | **A** | **T** | **G** | **C** | **G** | **A** | **C** | **A** | **T** | **T** | **A** | **G** | **T** | **A** | **G** | **G** | **A** | **A** | **A** | **T** | **G** | **T** | **C** | **T** | **T** | **G** | **T** | **T** | **T** | **G** | **A** | **A** | **G** | **A** | **A** | **G** | **A** | **G** | **C** | **A** | **G** | **T** | **T** | **T** | **T** | **C** | **C** | **T** | **T** | **A** | **A** | **A** | **A** | **A** | **A** | **C** | **G** | **A** | **A** | **C** | **A** | **G** | **A** | **T** | **C** | **A** | **A** | **A** | **A** | **T** | **C** | **T** | **G** | **C** | **A** | **G** | **C** | **A** | **G** | **T** | **G** | **A** | **T** | **T** | **A** | **T** | **G** | **A** | **T** | **G** | **A** | **A** | **A** | **A** | **T** | **C** | **C** | **T** | **C** | **G** | **C** | **A** | **G** | **A** | **T** | **T** | **G** | **A** | **A** | **G** | **A** | **T** | **C** | **A** | **T** | **T** | **A** | **C** | **C** | **A** | **A** | **T** | **A** | **C** | **C** | **T** | **T** | **G** | **G** | **T** | **T** | **G** | **G** | **A** | **G** | **A** | **T** | **G** | **T** | **C** | **G** | **A** | **G** | **G** | **T** | **G** | **A** | **T** | **A** | **C** | **G** | **A** | **A** | **T** | **A** | **-** | **-** | **-** |
| **N60123F** | **A** | **T** | **G** | **A** | **A** | **T** | **A** | **G** | **T** | **T** | **C** | **T** | **T** | **G** | **G** | **C** | **G** | **T** | **T** | **T** | **T** | **T** | **T** | **T** | **G** | **T** | **G** | **G** | **T** | **A** | **G** | **T** | **C** | **G** | **A** | **A** | **G** | **C** | **A** | **C** | **T** | **G** | **C** | **T** | **G** | **C** | **T** | **G** | **T** | **T** | **A** | **T** | **T** | **T** | **G** | **C** | **T** | **G** | **T** | **T** | **T** | **G** | **G** | **C** | **A** | **A** | **A** | **T** | **C** | **G** | **T** | **A** | **A** | **A** | **C** | **A** | **A** | **T** | **A** | **C** | **T** | **G** | **G** | **A** | **C** | **T** | **T** | **T** | **T** | **G** | **C** | **T** | **T** | **T** | **T** | **A** | **G** | **T** | **A** | **A** | **T** | **A** | **T** | **T** | **C** | **G** | **G** | **T** | **A** | **T** | **T** | **T** | **T** | **C** | **A** | **A** | **T** | **A** | **T** | **A** | **T** | **A** | **T** | **C** | **T** | **A** | **G** | **C** | **T** | **A** | **T** | **G** | **C** | **G** | **C** | **A** | **A** | **A** | **T** | **A** | **T** | **C** | **C** | **T** | **C** | **G** | **T** | **A** | **C** | **T** | **A** | **A** | **A** | **T** | **T** | **T** | **C** | **A** | **A** | **A** | **A** | **T** | **T** | **T** | **T** | **C** | **A** | **A** | **T** | **T** | **G** | **A** | **T** | **C** | **T** | **T** | **A** | **G** | **G** | **G** | **A** | **G** | **C** | **T** | **T** | **G** | **G** | **T** | **C** | **A** | **T** | **T** | **T** | **T** | **T** | **T** | **T** | **T** | **A** | **G** | **C** | **T** | **T** | **A** | **G** | **T** | **C** | **A** | **A** | **C** | **A** | **G** | **T** | **C** | **C** | **T** | **G** | **C** | **T** | **T** | **T** | **A** | **T** | **G** | **G** | **A** | **T** | **G** | **A** | **T** | **G** | **G** | **T** | **T** | **G** | **T** | **A** | **T** | **T** | **G** | **G** | **C** | **C** | **G** | **T** | **T** | **T** | **T** | **A** | **T** | **T** | **T** | **A** | **T** | **C** | **G** | **G** | **G** | **C** | **T** | **G** | **A** | **A** | **A** | **G** | **G** | **T** | **G** | **T** | **A** | **G** | **A** | **G** | **A** | **T** | **T** | **T** | **C** | **A** | **G** | **G** | **A** | **A** | **T** | **A** | **G** | **A** | **T** | **T** | **T** | **G** | **A** | **C** | **A** | **A** | **A** | **A** | **A** | **A** | **T** | **G** | **C** | **T** | **T** | **T** | **T** | **T** | **G** | **G** | **C** | **G** | **A** | **A** | **A** | **A** | **A** | **A** | **A** | **C** | **A** | **G** | **A** | **T** | **C** | **A** | **T** | **G** | **A** | **T** | **G** | **G** | **T** | **C** | **C** | **A** | **A** | **A** | **C** | **A** | **G** | **A** | **G** | **C** | **A** | **A** | **T** | **T** | **A** | **A** | **A** | **A** | **A** | **C** | **A** | **C** | **A** | **T** | **A** | **A** | **T** | **A** | **A** | **T** | **G** | **A** | **G** | **C** | **G** | **T** | **A** | **A** | **A** | **A** | **G** | **A** | **C** | **A** | **G** | **C** | **A** | **A** | **T** | **T** | **A** | **T** | **T** | **T** | **G** | **G** | **T** | **A** | **A** | **C** | **C** | **A** | **A** | **C** | **G** | **T** | **A** | **T** | **T** | **G** | **G** | **A** | **A** | **A** | **C** | **G** | **A** | **C** | **G** | **T** | **C** | **T** | **A** | **T** | **G** | **A** | **A** | **T** | **G** | **G** | **G** | **A** | **T** | **G** | **A** | **T** | **A** | **T** | **C** | **A** | **A** | **C** | **A** | **T** | **T** | **G** | **C** | **C** | **A** | **T** | **C** | **A** | **T** | **T** | **T** | **C** | **T** | **G** | **G** | **T** | **G** | **A** | **T** | **A** | **C** | **T** | **A** | **T** | **C** | **A** | **T** | **C** | **G** | **A** | **T** | **T** | **T** | **G** | **G** | **G** | **C** | **A** | **A** | **T** | **A** | **C** | **A** | **C** | **T** | **T** | **T** | **T** | **G** | **C** | **C** | **A** | **A** | **A** | **A** | **G** | **A** | **T** | **G** | **A** | **T** | **A** | **A** | **T** | **A** | **T** | **T** | **G** | **T** | **G** | **A** | **T** | **T** | **G** | **T** | **A** | **A** | **G** | **A** | **A** | **A** | **A** | **G** | **G** | **G** | **A** | **T** | **A** | **G** | **G** | **C** | **A** | **G** | **A** | **A** | **C** | **A** | **C** | **G** | **G** | **A** | **T** | **C** | **T** | **T** | **A** | **G** | **T** | **A** | **C** | **C** | **C** | **C** | **T** | **G** | **G** | **G** | **A** | **G** | **T** | **G** | **G** | **C** | **T** | **A** | **T** | **T** | **C** | **G** | **T** | **T** | **T** | **A** | **G** | **A** | **G** | **C** | **A** | **T** | **G** | **C** | **G** | **A** | **C** | **A** | **T** | **T** | **A** | **G** | **T** | **A** | **G** | **G** | **A** | **A** | **A** | **T** | **G** | **T** | **C** | **T** | **T** | **G** | **T** | **T** | **T** | **G** | **A** | **A** | **G** | **A** | **A** | **G** | **A** | **G** | **C** | **A** | **G** | **T** | **T** | **T** | **T** | **C** | **C** | **T** | **T** | **A** | **A** | **A** | **A** | **A** | **A** | **C** | **G** | **A** | **A** | **C** | **A** | **G** | **A** | **T** | **C** | **A** | **A** | **A** | **A** | **T** | **C** | **T** | **G** | **C** | **A** | **G** | **C** | **A** | **G** | **T** | **G** | **A** | **T** | **T** | **A** | **T** | **G** | **A** | **T** | **G** | **A** | **A** | **A** | **A** | **T** | **C** | **C** | **T** | **C** | **G** | **C** | **A** | **G** | **A** | **T** | **T** | **G** | **A** | **A** | **G** | **A** | **T** | **C** | **A** | **T** | **T** | **A** | **C** | **C** | **A** | **A** | **T** | **A** | **C** | **C** | **T** | **T** | **G** | **G** | **T** | **T** | **G** | **G** | **A** | **G** | **A** | **T** | **G** | **T** | **C** | **G** | **A** | **G** | **G** | **T** | **G** | **A** | **T** | **A** | **C** | **G** | **A** | **A** | **T** | **A** | **-** | **-** | **-** |
| **N53655** | **A** | **T** | **G** | **A** | **A** | **T** | **A** | **G** | **T** | **T** | **C** | **T** | **T** | **G** | **G** | **C** | **G** | **T** | **T** | **T** | **T** | **T** | **T** | **T** | **G** | **T** | **G** | **G** | **T** | **A** | **G** | **T** | **T** | **G** | **A** | **A** | **G** | **C** | **A** | **C** | **T** | **A** | **C** | **T** | **G** | **C** | **T** | **G** | **T** | **T** | **A** | **T** | **T** | **T** | **G** | **C** | **T** | **G** | **T** | **T** | **T** | **G** | **G** | **C** | **A** | **A** | **A** | **T** | **C** | **G** | **T** | **A** | **A** | **A** | **C** | **A** | **A** | **T** | **A** | **C** | **T** | **G** | **G** | **A** | **C** | **T** | **T** | **T** | **T** | **G** | **C** | **T** | **T** | **T** | **T** | **A** | **G** | **T** | **G** | **A** | **T** | **A** | **T** | **T** | **C** | **G** | **G** | **T** | **A** | **T** | **T** | **T** | **T** | **C** | **A** | **A** | **T** | **A** | **T** | **A** | **T** | **A** | **T** | **C** | **T** | **A** | **G** | **C** | **T** | **A** | **T** | **G** | **C** | **G** | **C** | **A** | **A** | **A** | **C** | **A** | **T** | **C** | **C** | **T** | **C** | **G** | **T** | **A** | **C** | **T** | **A** | **A** | **A** | **T** | **T** | **T** | **C** | **A** | **A** | **A** | **A** | **T** | **T** | **T** | **T** | **C** | **A** | **A** | **T** | **T** | **G** | **A** | **T** | **A** | **T** | **T** | **A** | **G** | **G** | **A** | **A** | **G** | **T** | **T** | **T** | **G** | **G** | **T** | **C** | **A** | **T** | **T** | **T** | **T** | **T** | **T** | **T** | **T** | **A** | **G** | **C** | **T** | **T** | **A** | **G** | **T** | **C** | **A** | **A** | **C** | **A** | **G** | **T** | **C** | **C** | **T** | **G** | **C** | **T** | **T** | **T** | **A** | **T** | **G** | **G** | **A** | **T** | **G** | **A** | **T** | **G** | **G** | **C** | **T** | **G** | **T** | **A** | **T** | **T** | **T** | **G** | **C** | **C** | **G** | **T** | **T** | **T** | **T** | **A** | **T** | **T** | **T** | **A** | **T** | **T** | **G** | **G** | **A** | **C** | **T** | **A** | **A** | **A** | **A** | **G** | **G** | **T** | **G** | **T** | **A** | **G** | **A** | **G** | **A** | **T** | **T** | **T** | **C** | **A** | **G** | **G** | **G** | **A** | **T** | **A** | **G** | **A** | **T** | **T** | **T** | **A** | **A** | **C** | **C** | **A** | **A** | **A** | **A** | **A** | **T** | **G** | **C** | **T** | **T** | **T** | **T** | **T** | **G** | **G** | **C** | **G** | **A** | **A** | **A** | **G** | **A** | **A** | **A** | **C** | **A** | **G** | **A** | **T** | **C** | **A** | **T** | **G** | **A** | **T** | **G** | **G** | **T** | **C** | **C** | **A** | **A** | **A** | **C** | **A** | **G** | **A** | **G** | **C** | **A** | **A** | **T** | **T** | **A** | **A** | **A** | **A** | **A** | **C** | **A** | **C** | **A** | **T** | **A** | **A** | **T** | **A** | **A** | **T** | **G** | **A** | **G** | **C** | **G** | **G** | **A** | **A** | **A** | **A** | **G** | **A** | **C** | **A** | **G** | **C** | **A** | **A** | **T** | **T** | **A** | **T** | **T** | **C** | **G** | **G** | **T** | **A** | **A** | **C** | **C** | **A** | **A** | **C** | **G** | **T** | **A** | **T** | **T** | **G** | **G** | **A** | **A** | **A** | **C** | **G** | **A** | **C** | **G** | **T** | **C** | **T** | **A** | **T** | **G** | **A** | **A** | **T** | **G** | **G** | **G** | **A** | **C** | **G** | **A** | **T** | **A** | **T** | **C** | **A** | **A** | **C** | **A** | **T** | **C** | **G** | **C** | **C** | **G** | **T** | **C** | **A** | **T** | **T** | **T** | **C** | **T** | **G** | **G** | **T** | **G** | **A** | **T** | **A** | **C** | **T** | **A** | **T** | **C** | **A** | **T** | **A** | **G** | **A** | **T** | **T** | **T** | **G** | **G** | **G** | **C** | **A** | **A** | **T** | **A** | **C** | **G** | **C** | **T** | **T** | **T** | **T** | **G** | **C** | **C** | **A** | **A** | **A** | **A** | **G** | **A** | **T** | **G** | **A** | **T** | **A** | **A** | **T** | **A** | **T** | **T** | **G** | **T** | **G** | **A** | **T** | **T** | **G** | **T** | **A** | **A** | **G** | **A** | **A** | **A** | **A** | **G** | **G** | **G** | **A** | **T** | **A** | **G** | **G** | **C** | **A** | **G** | **A** | **A** | **C** | **A** | **C** | **G** | **G** | **A** | **T** | **C** | **T** | **T** | **A** | **G** | **T** | **A** | **C** | **C** | **T** | **T** | **T** | **G** | **G** | **G** | **C** | **G** | **T** | **A** | **G** | **C** | **C** | **A** | **T** | **C** | **C** | **G** | **A** | **T** | **T** | **A** | **G** | **A** | **G** | **C** | **A** | **T** | **G** | **C** | **G** | **A** | **C** | **A** | **T** | **T** | **A** | **G** | **T** | **A** | **G** | **G** | **A** | **A** | **A** | **T** | **G** | **T** | **C** | **T** | **T** | **G** | **T** | **T** | **T** | **G** | **A** | **A** | **G** | **A** | **A** | **G** | **A** | **G** | **C** | **A** | **A** | **T** | **T** | **T** | **T** | **C** | **T** | **T** | **T** | **G** | **A** | **A** | **A** | **A** | **A** | **T** | **G** | **A** | **A** | **C** | **A** | **A** | **A** | **T** | **C** | **A** | **A** | **A** | **A** | **T** | **C** | **T** | **A** | **C** | **A** | **G** | **C** | **A** | **G** | **T** | **G** | **A** | **T** | **T** | **A** | **T** | **G** | **A** | **T** | **G** | **A** | **A** | **A** | **A** | **C** | **C** | **C** | **T** | **C** | **G** | **T** | **A** | **G** | **A** | **C** | **T** | **G** | **A** | **A** | **G** | **A** | **T** | **T** | **A** | **T** | **T** | **A** | **C** | **T** | **A** | **A** | **T** | **A** | **C** | **C** | **T** | **T** | **G** | **A** | **T** | **T** | **G** | **G** | **A** | **G** | **A** | **T** | **G** | **T** | **C** | **G** | **A** | **G** | **G** | **T** | **G** | **A** | **T** | **A** | **C** | **G** | **A** | **A** | **T** | **A** | **-** | **-** | **-** |
| **N60260F** | **A** | **T** | **G** | **A** | **A** | **T** | **A** | **G** | **T** | **T** | **C** | **T** | **T** | **G** | **G** | **C** | **G** | **T** | **T** | **T** | **T** | **T** | **T** | **T** | **G** | **T** | **G** | **G** | **T** | **A** | **G** | **T** | **C** | **G** | **A** | **A** | **G** | **C** | **A** | **C** | **T** | **G** | **C** | **T** | **G** | **C** | **T** | **G** | **T** | **T** | **A** | **T** | **T** | **T** | **G** | **C** | **T** | **G** | **T** | **T** | **T** | **G** | **G** | **C** | **A** | **A** | **A** | **T** | **C** | **G** | **T** | **A** | **A** | **A** | **C** | **A** | **A** | **T** | **A** | **C** | **T** | **G** | **G** | **A** | **C** | **T** | **T** | **T** | **T** | **G** | **C** | **T** | **T** | **T** | **T** | **A** | **G** | **T** | **A** | **A** | **T** | **A** | **T** | **T** | **C** | **G** | **G** | **T** | **A** | **T** | **T** | **T** | **T** | **C** | **A** | **A** | **T** | **A** | **T** | **A** | **T** | **A** | **T** | **C** | **T** | **A** | **G** | **C** | **T** | **A** | **T** | **G** | **C** | **G** | **C** | **A** | **A** | **A** | **T** | **A** | **T** | **C** | **C** | **T** | **C** | **G** | **T** | **A** | **C** | **T** | **A** | **A** | **A** | **T** | **T** | **T** | **C** | **A** | **A** | **A** | **A** | **T** | **T** | **T** | **T** | **C** | **A** | **A** | **T** | **T** | **G** | **A** | **T** | **C** | **T** | **T** | **A** | **G** | **G** | **G** | **A** | **G** | **C** | **T** | **T** | **G** | **G** | **T** | **C** | **A** | **T** | **T** | **T** | **T** | **T** | **T** | **T** | **T** | **A** | **G** | **C** | **T** | **T** | **A** | **G** | **T** | **C** | **A** | **A** | **C** | **A** | **G** | **T** | **C** | **C** | **T** | **G** | **C** | **T** | **T** | **T** | **A** | **T** | **G** | **G** | **A** | **T** | **G** | **A** | **T** | **G** | **G** | **T** | **T** | **G** | **T** | **A** | **T** | **T** | **G** | **G** | **C** | **C** | **G** | **T** | **T** | **T** | **T** | **A** | **T** | **T** | **T** | **A** | **T** | **C** | **G** | **G** | **G** | **C** | **T** | **G** | **A** | **A** | **A** | **G** | **G** | **T** | **G** | **T** | **A** | **G** | **A** | **G** | **A** | **T** | **T** | **T** | **C** | **A** | **G** | **G** | **A** | **A** | **T** | **A** | **G** | **A** | **T** | **T** | **T** | **G** | **A** | **C** | **A** | **A** | **A** | **A** | **A** | **A** | **T** | **G** | **C** | **T** | **T** | **T** | **T** | **T** | **G** | **G** | **C** | **G** | **A** | **A** | **A** | **A** | **A** | **A** | **A** | **C** | **A** | **G** | **A** | **T** | **C** | **A** | **T** | **G** | **A** | **T** | **G** | **G** | **T** | **C** | **C** | **A** | **A** | **A** | **C** | **A** | **G** | **A** | **G** | **C** | **A** | **A** | **T** | **T** | **A** | **A** | **A** | **A** | **A** | **C** | **A** | **C** | **A** | **T** | **A** | **A** | **T** | **A** | **A** | **T** | **G** | **A** | **G** | **C** | **G** | **T** | **A** | **A** | **A** | **A** | **G** | **A** | **C** | **A** | **G** | **C** | **A** | **A** | **T** | **T** | **A** | **T** | **T** | **T** | **G** | **G** | **T** | **A** | **A** | **C** | **C** | **A** | **A** | **C** | **G** | **T** | **A** | **T** | **T** | **G** | **G** | **A** | **A** | **A** | **C** | **G** | **A** | **C** | **G** | **T** | **C** | **T** | **A** | **T** | **G** | **A** | **A** | **T** | **G** | **G** | **G** | **A** | **T** | **G** | **A** | **T** | **A** | **T** | **C** | **A** | **A** | **C** | **A** | **T** | **T** | **G** | **C** | **C** | **A** | **T** | **C** | **A** | **T** | **T** | **T** | **C** | **T** | **G** | **G** | **T** | **G** | **A** | **T** | **A** | **C** | **T** | **A** | **T** | **C** | **A** | **T** | **C** | **G** | **A** | **T** | **T** | **T** | **G** | **G** | **G** | **C** | **A** | **A** | **T** | **A** | **C** | **A** | **C** | **T** | **T** | **T** | **T** | **G** | **C** | **C** | **A** | **A** | **A** | **A** | **G** | **A** | **T** | **G** | **A** | **T** | **A** | **A** | **T** | **A** | **T** | **T** | **G** | **T** | **G** | **A** | **T** | **T** | **G** | **T** | **A** | **A** | **G** | **A** | **A** | **A** | **A** | **G** | **G** | **G** | **A** | **T** | **A** | **G** | **G** | **C** | **A** | **G** | **A** | **A** | **C** | **A** | **C** | **G** | **G** | **A** | **T** | **C** | **T** | **T** | **A** | **G** | **T** | **A** | **C** | **C** | **C** | **C** | **T** | **G** | **G** | **G** | **A** | **G** | **T** | **G** | **G** | **C** | **T** | **A** | **T** | **T** | **C** | **G** | **T** | **T** | **T** | **A** | **G** | **A** | **G** | **C** | **A** | **T** | **G** | **C** | **G** | **A** | **C** | **A** | **T** | **T** | **A** | **G** | **T** | **A** | **G** | **G** | **A** | **A** | **A** | **T** | **G** | **T** | **C** | **T** | **T** | **G** | **T** | **T** | **T** | **G** | **A** | **A** | **G** | **A** | **A** | **G** | **A** | **G** | **C** | **A** | **G** | **T** | **T** | **T** | **T** | **C** | **C** | **T** | **T** | **A** | **A** | **A** | **A** | **A** | **A** | **C** | **G** | **A** | **A** | **C** | **A** | **G** | **A** | **T** | **C** | **A** | **A** | **A** | **A** | **T** | **C** | **T** | **G** | **C** | **A** | **G** | **C** | **A** | **G** | **T** | **G** | **A** | **T** | **T** | **A** | **T** | **G** | **A** | **T** | **G** | **A** | **A** | **A** | **A** | **T** | **C** | **C** | **T** | **C** | **G** | **C** | **A** | **G** | **A** | **T** | **T** | **G** | **A** | **A** | **G** | **A** | **T** | **C** | **A** | **T** | **T** | **A** | **C** | **C** | **A** | **A** | **T** | **A** | **C** | **C** | **T** | **T** | **G** | **G** | **T** | **T** | **G** | **G** | **A** | **G** | **A** | **T** | **G** | **T** | **C** | **G** | **A** | **G** | **G** | **T** | **G** | **A** | **T** | **A** | **C** | **G** | **A** | **A** | **T** | **A** | **-** | **-** | **-** |
 **SNP position** | . | . | . | . | . | . | . | . | . | . | . | . | . | . | . | . | . | . | . | . | . | . | . | . | . | . | . | . | . | . | . | . | 33 | . | . | . | 37 | . | . | . | . | 42 | . | . | 45 | . | . | . | . | . | . | . | . | . | . | . | . | . | . | . | . | . | . | 64 | . | . | . | . | . | . | . | . | . | . | . | . | . | . | . | . | . | . | . | . | . | . | . | . | . | . | . | . | . | . | . | . | . | . | 99 | . | . | . | . | . | . | . | . | . | . | . | 111 | . | . | . | . | . | . | . | . | . | . | . | . | . | . | . | . | . | . | . | . | . | . | . | . | . | . | . | 139 | . | . | . | . | . | . | . | . | . | . | . | . | . | . | . | . | . | . | . | . | . | . | . | . | . | . | . | . | . | . | . | . | . | . | 174 | . | . | . | . | . | 180 | . | . | 183 | . | . | 186 | . | . | . | . | . | . | . | . | . | . | . | . | . | . | . | . | . | . | . | . | . | . | . | . | . | . | . | . | . | . | . | . | . | . | . | . | . | . | . | . | . | . | . | . | . | . | 233 | . | . | . | . | . | . | 240 | . | . | . | 244 | . | . | . | . | . | . | . | . | . | . | 255 | . | . | 258 | . | . | 261 | . | . | 264 | . | . | . | . | . | . | . | . | . | . | . | . | . | . | . | . | . | 282 | . | . | . | . | . | . | . | . | 291 | . | . | 294 | . | . | . | . | . | . | . | . | . | . | . | . | . | . | . | . | . | . | . | . | 315 | . | . | . | . | . | . | . | . | 324 | . | . | . | . | . | . | 331 | . | . | . | . | . | . | . | . | . | . | . | . | . | . | . | . | . | . | . | . | . | . | . | . | . | . | . | . | . | . | . | . | . | . | . | . | . | 369 | . | . | . | . | . | . | . | . | . | . | . | . | . | . | . | . | . | 387 | . | . | . | . | . | . | . | . | . | . | . | . | . | . | . | . | . | . | . | . | . | . | . | . | . | . | . | . | . | . | . | . | . | . | . | . | . | . | 426 | . | . | 429 | . | . | . | . | . | . | . | . | 438 | . | . | . | 442 | . | 444 | . | . | . | . | . | . | . | . | . | . | . | . | . | . | . | . | . | . | . | . | 465 | . | . | . | . | . | . | . | . | 474 | . | . | . | . | . | 480 | . | . | . | . | . | . | . | . | . | . | . | . | . | . | . | . | . | . | . | . | . | . | . | . | . | . | . | . | . | . | . | . | . | . | . | . | . | . | . | . | . | . | . | . | . | . | . | . | . | . | . | . | . | . | . | . | 537 | . | . | . | . | . | . | . | . | . | . | . | 549 | 550 | . | 552 | . | . | 555 | . | . | 558 | . | . | 561 | . | . | 564 | . | . | 567 | . | . | . | . | . | . | . | . | . | . | . | . | . | . | . | . | . | . | . | . | . | . | . | . | . | . | . | . | . | . | . | . | . | . | . | . | . | . | . | . | . | . | . | . | . | . | . | 615 | . | . | . | . | . | 621 | . | . | 624 | . | . | . | . | . | 630 | . | . | . | . | . | 636 | . | . | 639 | . | . | . | . | . | 645 | . | 647 | 648 | . | . | . | . | . | . | . | . | . | . | . | . | . | . | . | . | . | . | . | . | 669 | . | . | . | . | . | 675 | . | . | . | 679 | . | . | . | . | 684 | . | . | 687 | . | . | . | . | . | 693 | . | . | . | . | . | . | . | . | . | 703 | . | . | . | . | . | . | . | . | . | . | . | . | . | . | . | . | . | . | . | . | . | . | 726 | . | . | . | . | . | . |

|  |
| --- |
|  |

|  |  |
| --- | --- |
|  |

|  |  |
| --- | --- |
| **Legend** | |
  | **Reference Sequence** |

  |
|  | **Detected SNPs** |
|  |  |
