# Supplementary material for: sraX: A Novel Comprehensive Resistome Analysis Tool
Source: Front Microbiol. 2020 Feb 5;11:52. doi: 10.3389/fmicb.2020.00052 (PMC7025521; doi:10.3389/fmicb.2020.00052)
Supplement: Supplementary file 4 [file Data_Sheet_3.ZIP › liaF/liaF_Efaecalis_aa.html]

# liaF

|  |
| --- |
|  |

|  |
| --- |
|  |

|  |  |
| --- | --- |
| **Definition:** | LiaF is an accessory protein that acts as a negative regulator of liaRS signal transduction pathway. Mutations confer daptomycin resistance. |

|  |
| --- |
|  |

|  |
| --- |
|  |

|  |  |  |
| --- | --- | --- |
| **Reference organism:** | *Enterococcus faecalis V583* | [NCBI, CARD] |

|  |
| --- |
|  |

|  |
| --- |
|  |

|  |  |
| --- | --- |
| **Summary Data** | || **Parameter** | **Value_(n)_** || Total number of genomes | 98 |
| MSA length | 243 |
| Total number of SNPs | 6 |
| SNP density (# SNPs/MSA length) | 0.025 |

|  |
| --- |
|  |

|  |
| --- |
|  |

|  |
| --- |
|  |

|  |
| --- |
|  |

|  |  |  |  |  |
| --- | --- | --- | --- | --- |
| **SNPs conferring antibiotic resistance** | | | | || **Reference gene** | **Seq. pos.** | **Ref\_base** | **Alt\_base** | **Confirmation SNP?** |||  |  |  |  |  |
| --- | --- | --- | --- | --- |
| liaF 194 T I **No** | | | | |

|  |
| --- |
|  |

|  |
| --- |
|  |

|  |
| --- |
|  |

|  |  |  |  |  |  |  |  |  |  |  |  |  |  |  |  |  |  |  |  |  |  |  |  |  |  |  |  |  |  |  |  |  |  |  |  |  |  |  |  |  |  |  |  |  |  |  |  |  |  |  |  |  |  |  |  |  |  |  |  |  |  |  |  |  |  |  |  |  |  |  |  |  |  |  |  |  |  |  |  |  |  |  |  |  |  |  |  |  |  |  |  |  |  |  |  |  |  |  |  |  |  |  |  |  |  |  |  |  |  |  |  |  |  |  |  |  |  |  |  |  |  |  |  |  |  |  |  |  |  |  |  |  |  |  |  |  |  |  |  |  |  |  |  |  |  |  |  |  |  |  |  |  |  |  |  |  |  |  |  |  |  |  |  |  |  |  |  |  |  |  |  |  |  |  |  |  |  |  |  |  |  |  |  |  |  |  |  |  |  |  |  |  |  |  |  |  |  |  |  |  |  |  |  |  |  |  |  |  |  |  |  |  |  |  |  |  |  |  |  |  |  |  |  |  |  |  |  |  |  |  |  |  |  |  |  |  |  |  |  |  |  |  |  |  |
| --- | --- | --- | --- | --- | --- | --- | --- | --- | --- | --- | --- | --- | --- | --- | --- | --- | --- | --- | --- | --- | --- | --- | --- | --- | --- | --- | --- | --- | --- | --- | --- | --- | --- | --- | --- | --- | --- | --- | --- | --- | --- | --- | --- | --- | --- | --- | --- | --- | --- | --- | --- | --- | --- | --- | --- | --- | --- | --- | --- | --- | --- | --- | --- | --- | --- | --- | --- | --- | --- | --- | --- | --- | --- | --- | --- | --- | --- | --- | --- | --- | --- | --- | --- | --- | --- | --- | --- | --- | --- | --- | --- | --- | --- | --- | --- | --- | --- | --- | --- | --- | --- | --- | --- | --- | --- | --- | --- | --- | --- | --- | --- | --- | --- | --- | --- | --- | --- | --- | --- | --- | --- | --- | --- | --- | --- | --- | --- | --- | --- | --- | --- | --- | --- | --- | --- | --- | --- | --- | --- | --- | --- | --- | --- | --- | --- | --- | --- | --- | --- | --- | --- | --- | --- | --- | --- | --- | --- | --- | --- | --- | --- | --- | --- | --- | --- | --- | --- | --- | --- | --- | --- | --- | --- | --- | --- | --- | --- | --- | --- | --- | --- | --- | --- | --- | --- | --- | --- | --- | --- | --- | --- | --- | --- | --- | --- | --- | --- | --- | --- | --- | --- | --- | --- | --- | --- | --- | --- | --- | --- | --- | --- | --- | --- | --- | --- | --- | --- | --- | --- | --- | --- | --- | --- | --- | --- | --- | --- | --- | --- | --- | --- | --- | --- | --- | --- | --- | --- | --- | --- | --- | --- | --- | --- | --- |
|  || **Enterococcus faecalis V583 (AAO82601.1)** | **M** | **N** | **N** | **P** | **W** | **R** | **F** | **F** | **I** | **V** | **A** | **E** | **A** | **L** | **L** | **F** | **I** | **L** | **A** | **L** | **W** | **Q** | **I** | **V** | **H** | **N** | **P** | **G** | **L** | **A** | **V** | **L** | **L** | **T** | **I** | **G** | **V** | **L** | **L** | **V** | **A** | **Y** | **V** | **S** | **R** | **K** | **A** | **S** | **K** | **T** | **H** | **F** | **N** | **N** | **F** | **Q** | **F** | **V** | **L** | **G** | **V** | **V** | **F** | **I** | **V** | **I** | **G** | **A** | **M** | **N** | **S** | **T** | **A** | **V** | **W** | **F** | **M** | **L** | **I** | **F** | **G** | **V** | **L** | **F** | **I** | **G** | **L** | **K** | **G** | **F** | **E** | **I** | **S** | **G** | **V** | **D** | **I** | **A** | **E** | **R** | **A** | **P** | **W** | **R** | **K** | **K** | **Q** | **M** | **I** | **M** | **V** | **E** | **T** | **A** | **A** | **K** | **E** | **P** | **K** | **N** | **G** | **K** | **R** | **F** | **K** | **R** | **R** | **W** | **F** | **A** | **N** | **E** | **R** | **I** | **G** | **N** | **N** | **I** | **Y** | **E** | **W** | **D** | **D** | **I** | **N** | **I** | **D** | **L** | **I** | **S** | **G** | **D** | **T** | **I** | **I** | **D** | **L** | **G** | **N** | **T** | **L** | **L** | **P** | **K** | **E** | **D** | **N** | **I** | **I** | **I** | **I** | **R** | **K** | **G** | **F** | **G** | **R** | **T** | **R** | **I** | **L** | **V** | **P** | **L** | **G** | **V** | **A** | **I** | **L** | **L** | **E** | **H** | **S** | **T** | **F** | **Y** | **G** | **T** | **V** | **R** | **F** | **E** | **E** | **E** | **K** | **Y** | **Q** | **L** | **K** | **N** | **E** | **S** | **L** | **K** | **I** | **Y** | **S** | **N** | **D** | **Y** | **D** | **T** | **N** | **L** | **R** | **R** | **L** | **K** | **I** | **M** | **T** | **N** | **T** | **L** | **V** | **G** | **D** | **V** | **E** | **V** | **I** | **R** | **V** |
| **N59896F** | **M** | **N** | **N** | **P** | **W** | **R** | **F** | **F** | **I** | **V** | **A** | **E** | **A** | **L** | **L** | **F** | **I** | **L** | **A** | **L** | **W** | **Q** | **I** | **V** | **H** | **N** | **P** | **G** | **L** | **A** | **V** | **L** | **L** | **T** | **I** | **G** | **V** | **L** | **L** | **V** | **A** | **Y** | **V** | **S** | **R** | **K** | **A** | **S** | **K** | **T** | **H** | **F** | **N** | **N** | **F** | **Q** | **F** | **V** | **L** | **G** | **V** | **V** | **F** | **I** | **V** | **I** | **G** | **A** | **M** | **N** | **S** | **T** | **A** | **V** | **W** | **F** | **M** | **L** | **I** | **F** | **G** | **V** | **L** | **F** | **I** | **G** | **L** | **K** | **G** | **F** | **E** | **I** | **S** | **G** | **V** | **D** | **I** | **A** | **E** | **R** | **A** | **P** | **W** | **R** | **K** | **K** | **Q** | **M** | **I** | **M** | **V** | **E** | **T** | **A** | **A** | **K** | **E** | **P** | **K** | **N** | **G** | **K** | **R** | **F** | **K** | **R** | **R** | **W** | **F** | **A** | **N** | **E** | **R** | **I** | **G** | **N** | **N** | **I** | **Y** | **E** | **W** | **D** | **D** | **I** | **N** | **I** | **D** | **L** | **I** | **S** | **G** | **D** | **T** | **I** | **I** | **D** | **L** | **G** | **N** | **T** | **L** | **L** | **P** | **K** | **E** | **D** | **N** | **I** | **I** | **I** | **I** | **R** | **K** | **G** | **F** | **G** | **R** | **T** | **R** | **I** | **L** | **V** | **P** | **L** | **G** | **V** | **A** | **I** | **L** | **L** | **E** | **H** | **S** | **T** | **F** | **Y** | **G** | **T** | **V** | **R** | **F** | **E** | **E** | **E** | **K** | **Y** | **Q** | **L** | **K** | **N** | **E** | **S** | **L** | **K** | **I** | **Y** | **S** | **N** | **D** | **Y** | **D** | **T** | **N** | **L** | **R** | **R** | **L** | **K** | **I** | **M** | **T** | **N** | **T** | **L** | **V** | **G** | **D** | **V** | **E** | **V** | **I** | **R** | **V** |
| **N60440F** | **M** | **N** | **N** | **P** | **W** | **R** | **F** | **F** | **I** | **V** | **A** | **E** | **A** | **L** | **L** | **F** | **I** | **L** | **A** | **L** | **W** | **Q** | **I** | **V** | **H** | **N** | **P** | **G** | **L** | **A** | **V** | **L** | **L** | **T** | **I** | **G** | **V** | **L** | **L** | **V** | **A** | **Y** | **V** | **S** | **R** | **K** | **A** | **S** | **K** | **T** | **H** | **F** | **N** | **N** | **F** | **Q** | **F** | **V** | **L** | **G** | **V** | **V** | **F** | **I** | **V** | **I** | **G** | **A** | **M** | **N** | **S** | **T** | **A** | **V** | **W** | **F** | **M** | **L** | **I** | **F** | **G** | **V** | **L** | **F** | **I** | **G** | **L** | **K** | **G** | **F** | **E** | **I** | **S** | **G** | **V** | **D** | **I** | **A** | **E** | **R** | **A** | **P** | **W** | **R** | **K** | **K** | **Q** | **M** | **I** | **M** | **V** | **E** | **T** | **A** | **A** | **K** | **E** | **P** | **K** | **N** | **G** | **K** | **R** | **F** | **K** | **R** | **R** | **W** | **F** | **A** | **N** | **E** | **R** | **I** | **G** | **N** | **N** | **I** | **Y** | **E** | **W** | **D** | **D** | **I** | **N** | **I** | **D** | **L** | **I** | **S** | **G** | **D** | **T** | **I** | **I** | **D** | **L** | **G** | **N** | **T** | **L** | **L** | **P** | **K** | **E** | **D** | **N** | **I** | **I** | **I** | **I** | **R** | **K** | **G** | **F** | **G** | **R** | **T** | **R** | **I** | **L** | **V** | **P** | **L** | **G** | **V** | **A** | **I** | **L** | **L** | **E** | **H** | **S** | **T** | **F** | **Y** | **G** | **M** | **V** | **R** | **F** | **E** | **E** | **E** | **K** | **Y** | **Q** | **L** | **K** | **N** | **E** | **S** | **L** | **K** | **I** | **Y** | **S** | **N** | **D** | **Y** | **D** | **T** | **N** | **L** | **R** | **R** | **L** | **K** | **I** | **M** | **T** | **N** | **T** | **L** | **V** | **G** | **D** | **V** | **E** | **V** | **I** | **R** | **V** |
| **N59544F** | **M** | **N** | **N** | **P** | **W** | **R** | **F** | **F** | **I** | **V** | **A** | **E** | **A** | **L** | **L** | **F** | **I** | **L** | **A** | **L** | **W** | **Q** | **I** | **V** | **H** | **N** | **P** | **G** | **L** | **A** | **V** | **L** | **L** | **T** | **I** | **G** | **V** | **L** | **L** | **V** | **A** | **Y** | **V** | **S** | **R** | **K** | **A** | **S** | **K** | **T** | **H** | **F** | **N** | **N** | **F** | **Q** | **F** | **V** | **L** | **G** | **V** | **V** | **F** | **I** | **V** | **I** | **G** | **A** | **M** | **N** | **S** | **T** | **A** | **V** | **W** | **F** | **M** | **L** | **I** | **F** | **G** | **V** | **L** | **F** | **I** | **G** | **L** | **K** | **G** | **F** | **E** | **I** | **S** | **G** | **V** | **D** | **I** | **A** | **E** | **R** | **A** | **P** | **W** | **R** | **K** | **K** | **Q** | **M** | **I** | **M** | **V** | **E** | **T** | **A** | **A** | **K** | **E** | **P** | **K** | **N** | **G** | **K** | **R** | **F** | **K** | **R** | **R** | **W** | **F** | **A** | **N** | **E** | **R** | **I** | **G** | **N** | **N** | **I** | **Y** | **E** | **W** | **D** | **D** | **I** | **N** | **I** | **D** | **L** | **I** | **S** | **G** | **D** | **T** | **I** | **I** | **D** | **L** | **G** | **N** | **T** | **L** | **L** | **P** | **K** | **E** | **D** | **N** | **I** | **I** | **I** | **I** | **R** | **K** | **G** | **F** | **G** | **R** | **T** | **R** | **I** | **L** | **V** | **P** | **L** | **G** | **V** | **A** | **I** | **L** | **L** | **E** | **H** | **S** | **T** | **F** | **Y** | **G** | **T** | **V** | **R** | **F** | **E** | **E** | **E** | **K** | **Y** | **Q** | **L** | **K** | **N** | **E** | **S** | **L** | **K** | **I** | **Y** | **S** | **N** | **D** | **Y** | **D** | **T** | **N** | **L** | **R** | **R** | **L** | **K** | **I** | **M** | **T** | **N** | **T** | **L** | **V** | **G** | **D** | **V** | **E** | **V** | **I** | **R** | **V** |
| **N53457** | **M** | **N** | **N** | **P** | **W** | **R** | **F** | **F** | **I** | **V** | **A** | **E** | **A** | **L** | **L** | **F** | **I** | **L** | **A** | **L** | **W** | **Q** | **I** | **V** | **H** | **N** | **P** | **G** | **L** | **A** | **V** | **L** | **L** | **T** | **I** | **G** | **V** | **L** | **L** | **V** | **A** | **Y** | **V** | **S** | **R** | **K** | **A** | **S** | **K** | **T** | **H** | **F** | **N** | **N** | **F** | **Q** | **F** | **V** | **L** | **G** | **V** | **V** | **F** | **I** | **V** | **I** | **G** | **A** | **M** | **N** | **S** | **T** | **A** | **V** | **W** | **F** | **M** | **L** | **I** | **F** | **G** | **V** | **L** | **F** | **I** | **G** | **L** | **K** | **G** | **F** | **E** | **I** | **S** | **G** | **V** | **D** | **I** | **A** | **E** | **R** | **A** | **P** | **W** | **R** | **K** | **K** | **Q** | **M** | **I** | **M** | **V** | **E** | **T** | **A** | **A** | **K** | **E** | **P** | **K** | **N** | **G** | **K** | **R** | **F** | **K** | **R** | **R** | **W** | **F** | **A** | **N** | **E** | **R** | **I** | **G** | **N** | **N** | **I** | **Y** | **E** | **W** | **D** | **D** | **I** | **N** | **I** | **D** | **L** | **I** | **S** | **G** | **D** | **T** | **I** | **I** | **D** | **L** | **G** | **N** | **T** | **L** | **L** | **P** | **K** | **E** | **D** | **N** | **I** | **I** | **I** | **I** | **R** | **K** | **G** | **F** | **G** | **R** | **T** | **R** | **I** | **L** | **V** | **P** | **L** | **G** | **V** | **A** | **I** | **L** | **L** | **E** | **H** | **S** | **T** | **F** | **Y** | **G** | **T** | **V** | **R** | **F** | **E** | **E** | **E** | **K** | **Y** | **Q** | **L** | **K** | **N** | **E** | **S** | **L** | **K** | **I** | **Y** | **S** | **N** | **D** | **Y** | **D** | **T** | **N** | **L** | **R** | **R** | **L** | **K** | **I** | **M** | **T** | **N** | **T** | **L** | **V** | **G** | **D** | **V** | **E** | **V** | **I** | **R** | **V** |
| **N59847F** | **M** | **N** | **N** | **P** | **W** | **R** | **F** | **F** | **I** | **V** | **A** | **E** | **A** | **L** | **L** | **F** | **I** | **L** | **A** | **L** | **W** | **Q** | **I** | **V** | **H** | **N** | **P** | **G** | **L** | **A** | **V** | **L** | **L** | **T** | **I** | **G** | **V** | **L** | **L** | **V** | **A** | **Y** | **V** | **S** | **R** | **K** | **A** | **S** | **K** | **T** | **H** | **F** | **N** | **N** | **F** | **Q** | **F** | **V** | **L** | **G** | **V** | **V** | **F** | **I** | **V** | **I** | **G** | **A** | **M** | **N** | **S** | **T** | **A** | **V** | **W** | **F** | **M** | **L** | **I** | **F** | **G** | **V** | **L** | **F** | **I** | **G** | **L** | **K** | **G** | **F** | **E** | **I** | **S** | **G** | **V** | **D** | **I** | **A** | **E** | **R** | **A** | **P** | **W** | **R** | **K** | **K** | **Q** | **M** | **I** | **M** | **V** | **E** | **T** | **A** | **A** | **K** | **E** | **P** | **K** | **N** | **G** | **K** | **R** | **F** | **K** | **R** | **R** | **W** | **F** | **A** | **N** | **E** | **R** | **I** | **G** | **N** | **N** | **I** | **Y** | **E** | **W** | **D** | **D** | **I** | **N** | **I** | **D** | **L** | **I** | **S** | **G** | **D** | **T** | **I** | **I** | **D** | **L** | **G** | **N** | **T** | **L** | **L** | **P** | **K** | **E** | **D** | **N** | **I** | **I** | **I** | **I** | **R** | **K** | **G** | **F** | **G** | **R** | **T** | **R** | **I** | **L** | **V** | **P** | **L** | **G** | **V** | **A** | **I** | **L** | **L** | **E** | **H** | **S** | **T** | **F** | **Y** | **G** | **T** | **V** | **R** | **F** | **E** | **E** | **E** | **K** | **Y** | **Q** | **L** | **K** | **N** | **E** | **S** | **L** | **K** | **I** | **Y** | **S** | **N** | **D** | **Y** | **D** | **T** | **N** | **L** | **R** | **R** | **L** | **K** | **I** | **M** | **T** | **N** | **T** | **L** | **V** | **G** | **D** | **V** | **E** | **V** | **I** | **R** | **V** |
| **N54576** | **M** | **N** | **N** | **P** | **W** | **R** | **F** | **F** | **I** | **V** | **A** | **E** | **A** | **L** | **L** | **F** | **I** | **L** | **A** | **L** | **W** | **Q** | **I** | **V** | **H** | **N** | **P** | **G** | **L** | **A** | **V** | **L** | **L** | **T** | **I** | **G** | **V** | **L** | **L** | **V** | **A** | **Y** | **V** | **S** | **R** | **K** | **A** | **S** | **K** | **T** | **H** | **F** | **N** | **N** | **F** | **Q** | **F** | **V** | **L** | **G** | **V** | **V** | **F** | **I** | **V** | **I** | **G** | **A** | **M** | **N** | **S** | **T** | **A** | **V** | **W** | **F** | **M** | **L** | **I** | **F** | **G** | **V** | **L** | **F** | **I** | **G** | **L** | **K** | **G** | **F** | **E** | **I** | **S** | **G** | **V** | **D** | **I** | **A** | **E** | **R** | **A** | **P** | **W** | **R** | **K** | **K** | **Q** | **M** | **I** | **M** | **V** | **E** | **T** | **A** | **A** | **K** | **E** | **P** | **K** | **N** | **G** | **K** | **R** | **F** | **K** | **R** | **R** | **W** | **F** | **A** | **N** | **E** | **R** | **I** | **G** | **N** | **N** | **I** | **Y** | **E** | **W** | **D** | **D** | **I** | **N** | **I** | **D** | **L** | **I** | **S** | **G** | **D** | **T** | **I** | **I** | **D** | **L** | **G** | **N** | **T** | **L** | **L** | **P** | **K** | **E** | **D** | **N** | **I** | **I** | **I** | **I** | **R** | **K** | **G** | **F** | **G** | **R** | **T** | **R** | **I** | **L** | **V** | **P** | **L** | **G** | **V** | **A** | **I** | **L** | **L** | **E** | **H** | **S** | **T** | **F** | **Y** | **G** | **T** | **V** | **R** | **F** | **E** | **E** | **E** | **K** | **Y** | **Q** | **L** | **K** | **N** | **E** | **S** | **L** | **K** | **I** | **Y** | **S** | **N** | **D** | **Y** | **D** | **T** | **N** | **L** | **R** | **R** | **L** | **K** | **I** | **M** | **T** | **N** | **T** | **L** | **V** | **G** | **D** | **V** | **E** | **V** | **I** | **R** | **V** |
| **N55706** | **M** | **N** | **N** | **P** | **W** | **R** | **F** | **F** | **I** | **V** | **A** | **E** | **A** | **L** | **L** | **F** | **I** | **L** | **A** | **L** | **W** | **Q** | **I** | **V** | **H** | **N** | **P** | **G** | **L** | **A** | **V** | **L** | **L** | **T** | **I** | **G** | **V** | **L** | **L** | **V** | **A** | **Y** | **V** | **S** | **R** | **K** | **A** | **S** | **K** | **T** | **H** | **F** | **N** | **N** | **F** | **Q** | **F** | **V** | **L** | **G** | **V** | **V** | **F** | **I** | **V** | **I** | **G** | **A** | **M** | **N** | **S** | **T** | **A** | **V** | **W** | **F** | **M** | **L** | **I** | **F** | **G** | **V** | **L** | **F** | **I** | **G** | **L** | **K** | **G** | **F** | **E** | **I** | **S** | **G** | **V** | **D** | **I** | **A** | **E** | **R** | **A** | **P** | **W** | **R** | **K** | **K** | **Q** | **M** | **I** | **M** | **V** | **E** | **T** | **A** | **A** | **K** | **E** | **P** | **K** | **N** | **G** | **K** | **R** | **F** | **K** | **R** | **R** | **W** | **F** | **A** | **N** | **E** | **R** | **I** | **G** | **N** | **N** | **I** | **Y** | **E** | **W** | **D** | **D** | **I** | **N** | **I** | **D** | **L** | **I** | **S** | **G** | **D** | **T** | **I** | **I** | **D** | **L** | **G** | **N** | **T** | **L** | **L** | **P** | **K** | **E** | **D** | **N** | **I** | **I** | **I** | **I** | **R** | **K** | **G** | **F** | **G** | **R** | **T** | **R** | **I** | **L** | **V** | **P** | **L** | **G** | **V** | **A** | **I** | **L** | **L** | **E** | **H** | **S** | **T** | **F** | **Y** | **G** | **T** | **V** | **R** | **F** | **E** | **E** | **E** | **K** | **Y** | **Q** | **L** | **K** | **N** | **E** | **S** | **L** | **K** | **I** | **Y** | **S** | **N** | **D** | **Y** | **D** | **T** | **N** | **L** | **R** | **R** | **L** | **K** | **I** | **M** | **T** | **N** | **T** | **L** | **V** | **G** | **D** | **V** | **E** | **V** | **I** | **R** | **V** |
| **N52467** | **-** | **-** | **-** | **P** | **W** | **R** | **F** | **F** | **I** | **V** | **A** | **E** | **A** | **L** | **L** | **F** | **I** | **L** | **A** | **L** | **W** | **Q** | **I** | **V** | **H** | **N** | **P** | **G** | **L** | **A** | **V** | **L** | **L** | **T** | **I** | **G** | **V** | **L** | **L** | **V** | **A** | **Y** | **V** | **S** | **R** | **K** | **A** | **S** | **K** | **T** | **H** | **F** | **N** | **N** | **F** | **Q** | **F** | **V** | **L** | **G** | **I** | **V** | **F** | **I** | **V** | **I** | **G** | **A** | **M** | **N** | **S** | **T** | **A** | **V** | **W** | **F** | **M** | **L** | **I** | **F** | **G** | **V** | **L** | **F** | **I** | **G** | **L** | **K** | **G** | **F** | **E** | **I** | **S** | **G** | **V** | **D** | **I** | **A** | **E** | **R** | **A** | **P** | **W** | **R** | **K** | **K** | **Q** | **M** | **I** | **M** | **V** | **E** | **T** | **A** | **A** | **K** | **E** | **P** | **K** | **N** | **G** | **K** | **R** | **F** | **K** | **R** | **R** | **W** | **F** | **A** | **N** | **E** | **R** | **I** | **G** | **N** | **N** | **I** | **Y** | **E** | **W** | **D** | **D** | **I** | **N** | **I** | **D** | **L** | **I** | **S** | **G** | **D** | **T** | **I** | **I** | **D** | **L** | **G** | **N** | **T** | **L** | **L** | **P** | **K** | **E** | **D** | **N** | **I** | **I** | **I** | **I** | **R** | **K** | **G** | **F** | **G** | **R** | **T** | **R** | **I** | **L** | **V** | **P** | **L** | **G** | **V** | **A** | **I** | **L** | **L** | **E** | **H** | **S** | **T** | **F** | **Y** | **G** | **T** | **V** | **R** | **F** | **E** | **E** | **E** | **K** | **Y** | **Q** | **L** | **K** | **N** | **E** | **S** | **L** | **K** | **I** | **Y** | **S** | **N** | **D** | **Y** | **D** | **T** | **N** | **L** | **R** | **R** | **L** | **K** | **I** | **M** | **T** | **N** | **T** | **L** | **V** | **G** | **D** | **V** | **E** | **V** | **I** | **R** | **V** |
| **N59584F** | **M** | **N** | **N** | **P** | **W** | **R** | **F** | **F** | **I** | **V** | **A** | **E** | **A** | **L** | **L** | **F** | **I** | **L** | **A** | **L** | **W** | **Q** | **I** | **V** | **H** | **N** | **P** | **G** | **L** | **A** | **V** | **L** | **L** | **T** | **I** | **G** | **V** | **L** | **L** | **V** | **A** | **Y** | **V** | **S** | **R** | **K** | **A** | **S** | **K** | **T** | **H** | **F** | **N** | **N** | **F** | **Q** | **F** | **V** | **L** | **G** | **V** | **V** | **F** | **I** | **V** | **I** | **G** | **A** | **M** | **N** | **S** | **T** | **A** | **V** | **W** | **F** | **M** | **L** | **I** | **F** | **G** | **V** | **L** | **F** | **I** | **G** | **L** | **K** | **G** | **F** | **E** | **I** | **S** | **G** | **V** | **D** | **I** | **A** | **E** | **R** | **A** | **P** | **W** | **R** | **K** | **K** | **Q** | **M** | **I** | **M** | **V** | **E** | **T** | **A** | **A** | **K** | **E** | **P** | **K** | **N** | **G** | **K** | **R** | **F** | **K** | **R** | **R** | **W** | **F** | **A** | **N** | **E** | **R** | **I** | **G** | **N** | **N** | **I** | **Y** | **E** | **W** | **D** | **D** | **I** | **N** | **I** | **D** | **L** | **I** | **S** | **G** | **D** | **T** | **I** | **I** | **D** | **L** | **G** | **N** | **T** | **L** | **L** | **P** | **K** | **E** | **D** | **N** | **I** | **I** | **I** | **I** | **R** | **K** | **G** | **F** | **G** | **R** | **T** | **R** | **I** | **L** | **V** | **P** | **L** | **G** | **V** | **A** | **I** | **L** | **L** | **E** | **H** | **S** | **T** | **F** | **Y** | **G** | **T** | **V** | **R** | **F** | **E** | **E** | **E** | **K** | **Y** | **Q** | **L** | **K** | **N** | **E** | **S** | **L** | **K** | **I** | **Y** | **S** | **N** | **D** | **Y** | **D** | **T** | **N** | **L** | **R** | **R** | **L** | **K** | **I** | **M** | **T** | **N** | **T** | **L** | **V** | **G** | **D** | **V** | **E** | **V** | **I** | **R** | **V** |
| **N60114F** | **M** | **N** | **N** | **P** | **W** | **R** | **F** | **F** | **I** | **V** | **A** | **E** | **A** | **L** | **L** | **F** | **I** | **L** | **A** | **L** | **W** | **Q** | **I** | **V** | **H** | **N** | **P** | **G** | **L** | **A** | **V** | **L** | **L** | **T** | **I** | **G** | **V** | **L** | **L** | **V** | **A** | **Y** | **V** | **S** | **R** | **K** | **A** | **S** | **K** | **T** | **H** | **F** | **N** | **N** | **F** | **Q** | **F** | **V** | **L** | **G** | **V** | **V** | **F** | **I** | **V** | **I** | **G** | **A** | **M** | **N** | **S** | **T** | **A** | **V** | **W** | **F** | **M** | **L** | **I** | **F** | **G** | **V** | **L** | **F** | **I** | **G** | **L** | **K** | **G** | **F** | **E** | **I** | **S** | **G** | **V** | **D** | **I** | **A** | **E** | **R** | **A** | **P** | **W** | **R** | **K** | **K** | **Q** | **M** | **I** | **M** | **V** | **E** | **T** | **A** | **A** | **K** | **E** | **P** | **K** | **N** | **G** | **K** | **R** | **F** | **K** | **R** | **R** | **W** | **F** | **A** | **N** | **E** | **R** | **I** | **G** | **N** | **N** | **I** | **Y** | **E** | **W** | **D** | **D** | **I** | **N** | **I** | **D** | **L** | **I** | **S** | **G** | **D** | **T** | **I** | **I** | **D** | **L** | **G** | **N** | **T** | **L** | **L** | **P** | **K** | **E** | **D** | **N** | **I** | **I** | **I** | **I** | **R** | **K** | **G** | **F** | **G** | **R** | **T** | **R** | **I** | **L** | **V** | **P** | **L** | **G** | **V** | **A** | **I** | **L** | **L** | **E** | **H** | **S** | **T** | **F** | **Y** | **G** | **T** | **V** | **R** | **F** | **E** | **E** | **E** | **K** | **Y** | **Q** | **L** | **K** | **N** | **E** | **S** | **L** | **K** | **I** | **Y** | **S** | **N** | **D** | **Y** | **D** | **T** | **N** | **L** | **R** | **R** | **L** | **K** | **I** | **M** | **T** | **N** | **T** | **L** | **V** | **G** | **D** | **V** | **E** | **V** | **I** | **R** | **V** |
| **N54121** | **M** | **N** | **N** | **P** | **W** | **R** | **F** | **F** | **I** | **V** | **A** | **E** | **A** | **L** | **L** | **F** | **I** | **L** | **A** | **L** | **W** | **Q** | **I** | **V** | **H** | **N** | **P** | **G** | **L** | **A** | **V** | **L** | **L** | **T** | **I** | **G** | **V** | **L** | **L** | **V** | **A** | **Y** | **V** | **S** | **R** | **K** | **A** | **S** | **K** | **T** | **H** | **F** | **N** | **N** | **F** | **Q** | **F** | **V** | **L** | **G** | **V** | **V** | **F** | **I** | **V** | **I** | **G** | **A** | **M** | **N** | **S** | **T** | **A** | **V** | **W** | **F** | **M** | **L** | **I** | **F** | **G** | **V** | **L** | **F** | **I** | **G** | **L** | **K** | **G** | **F** | **E** | **I** | **S** | **G** | **V** | **D** | **I** | **A** | **E** | **R** | **A** | **P** | **W** | **R** | **K** | **K** | **Q** | **M** | **I** | **M** | **V** | **E** | **T** | **A** | **A** | **K** | **E** | **P** | **K** | **N** | **G** | **K** | **R** | **F** | **K** | **R** | **R** | **W** | **F** | **A** | **N** | **E** | **R** | **I** | **G** | **N** | **N** | **I** | **Y** | **E** | **W** | **D** | **D** | **I** | **N** | **I** | **D** | **L** | **I** | **S** | **G** | **D** | **T** | **I** | **I** | **D** | **L** | **G** | **N** | **T** | **L** | **L** | **P** | **K** | **E** | **D** | **N** | **I** | **I** | **I** | **I** | **R** | **K** | **G** | **F** | **G** | **R** | **T** | **R** | **I** | **L** | **V** | **P** | **L** | **G** | **V** | **A** | **I** | **L** | **L** | **E** | **H** | **S** | **T** | **F** | **Y** | **G** | **T** | **V** | **R** | **F** | **E** | **E** | **E** | **K** | **Y** | **Q** | **L** | **K** | **N** | **E** | **S** | **L** | **K** | **I** | **Y** | **S** | **N** | **D** | **Y** | **D** | **T** | **N** | **L** | **R** | **R** | **L** | **K** | **I** | **M** | **T** | **N** | **T** | **L** | **V** | **G** | **D** | **V** | **E** | **V** | **I** | **R** | **V** |
| **N59845F** | **M** | **N** | **N** | **P** | **W** | **R** | **F** | **F** | **I** | **V** | **A** | **E** | **A** | **L** | **L** | **F** | **I** | **L** | **A** | **L** | **W** | **Q** | **I** | **V** | **H** | **N** | **P** | **G** | **L** | **A** | **V** | **L** | **L** | **T** | **I** | **G** | **V** | **L** | **L** | **V** | **A** | **Y** | **V** | **S** | **R** | **K** | **A** | **S** | **K** | **T** | **H** | **F** | **N** | **N** | **F** | **Q** | **F** | **V** | **L** | **G** | **V** | **V** | **F** | **I** | **V** | **I** | **G** | **A** | **M** | **N** | **S** | **T** | **A** | **V** | **W** | **F** | **M** | **L** | **I** | **F** | **G** | **V** | **L** | **F** | **I** | **G** | **L** | **K** | **G** | **F** | **E** | **I** | **S** | **G** | **V** | **D** | **I** | **A** | **E** | **R** | **A** | **P** | **W** | **R** | **K** | **K** | **Q** | **M** | **I** | **M** | **V** | **E** | **T** | **A** | **A** | **K** | **E** | **P** | **K** | **N** | **G** | **K** | **R** | **F** | **K** | **R** | **R** | **W** | **F** | **A** | **N** | **E** | **R** | **I** | **G** | **N** | **N** | **I** | **Y** | **E** | **W** | **D** | **D** | **I** | **N** | **I** | **D** | **L** | **I** | **S** | **G** | **D** | **T** | **I** | **I** | **D** | **L** | **G** | **N** | **T** | **L** | **L** | **P** | **K** | **E** | **D** | **N** | **I** | **I** | **I** | **I** | **R** | **K** | **G** | **F** | **G** | **R** | **T** | **R** | **I** | **L** | **V** | **P** | **L** | **G** | **V** | **A** | **I** | **L** | **L** | **E** | **H** | **S** | **T** | **F** | **Y** | **G** | **T** | **V** | **R** | **F** | **E** | **E** | **E** | **K** | **Y** | **Q** | **L** | **K** | **N** | **E** | **S** | **L** | **K** | **I** | **Y** | **S** | **N** | **D** | **Y** | **D** | **T** | **N** | **L** | **R** | **R** | **L** | **K** | **I** | **M** | **T** | **N** | **T** | **L** | **V** | **G** | **D** | **V** | **E** | **V** | **I** | **R** | **V** |
| **N59910F** | **M** | **N** | **N** | **P** | **W** | **R** | **F** | **F** | **I** | **V** | **A** | **E** | **A** | **L** | **L** | **F** | **I** | **L** | **A** | **L** | **W** | **Q** | **I** | **V** | **H** | **N** | **P** | **G** | **L** | **A** | **V** | **L** | **L** | **T** | **I** | **G** | **V** | **L** | **L** | **V** | **A** | **Y** | **V** | **S** | **R** | **K** | **A** | **S** | **K** | **T** | **H** | **F** | **N** | **N** | **F** | **Q** | **F** | **V** | **L** | **G** | **V** | **V** | **F** | **I** | **V** | **I** | **G** | **A** | **M** | **N** | **S** | **T** | **A** | **V** | **W** | **F** | **M** | **L** | **I** | **F** | **G** | **V** | **L** | **F** | **I** | **G** | **L** | **K** | **G** | **F** | **E** | **I** | **S** | **G** | **V** | **D** | **I** | **A** | **E** | **R** | **A** | **P** | **W** | **R** | **K** | **K** | **Q** | **M** | **I** | **M** | **V** | **E** | **T** | **A** | **A** | **K** | **E** | **P** | **K** | **N** | **G** | **K** | **R** | **F** | **K** | **R** | **R** | **W** | **F** | **A** | **N** | **E** | **R** | **I** | **G** | **N** | **N** | **I** | **Y** | **E** | **W** | **D** | **D** | **I** | **N** | **I** | **D** | **L** | **I** | **S** | **G** | **D** | **T** | **I** | **I** | **D** | **L** | **G** | **N** | **T** | **L** | **L** | **P** | **K** | **E** | **D** | **N** | **I** | **I** | **I** | **I** | **R** | **K** | **G** | **F** | **G** | **R** | **T** | **R** | **I** | **L** | **V** | **P** | **L** | **G** | **V** | **A** | **I** | **L** | **L** | **E** | **H** | **S** | **T** | **F** | **Y** | **G** | **T** | **V** | **R** | **F** | **E** | **E** | **E** | **K** | **Y** | **Q** | **L** | **K** | **N** | **E** | **S** | **L** | **K** | **I** | **Y** | **S** | **N** | **D** | **Y** | **D** | **T** | **N** | **L** | **R** | **R** | **L** | **K** | **I** | **M** | **T** | **N** | **T** | **L** | **V** | **G** | **D** | **V** | **E** | **V** | **I** | **R** | **V** |
| **N59505F** | **M** | **N** | **N** | **P** | **W** | **R** | **F** | **F** | **I** | **V** | **A** | **E** | **A** | **L** | **L** | **F** | **I** | **L** | **A** | **L** | **W** | **Q** | **I** | **V** | **H** | **N** | **P** | **G** | **L** | **A** | **V** | **L** | **L** | **T** | **I** | **G** | **V** | **L** | **L** | **V** | **A** | **Y** | **V** | **S** | **R** | **K** | **A** | **S** | **K** | **T** | **H** | **F** | **N** | **N** | **F** | **Q** | **F** | **V** | **L** | **G** | **V** | **V** | **F** | **I** | **V** | **I** | **G** | **A** | **M** | **N** | **S** | **T** | **A** | **V** | **W** | **F** | **M** | **L** | **I** | **F** | **G** | **V** | **L** | **F** | **I** | **G** | **L** | **K** | **G** | **F** | **E** | **I** | **S** | **G** | **V** | **D** | **I** | **A** | **E** | **R** | **A** | **P** | **W** | **R** | **K** | **K** | **Q** | **M** | **I** | **M** | **V** | **E** | **T** | **A** | **A** | **K** | **E** | **P** | **K** | **N** | **G** | **K** | **R** | **F** | **K** | **R** | **R** | **W** | **F** | **A** | **N** | **E** | **R** | **I** | **G** | **N** | **N** | **I** | **Y** | **E** | **W** | **D** | **D** | **I** | **N** | **I** | **D** | **L** | **I** | **S** | **G** | **D** | **T** | **I** | **I** | **D** | **L** | **G** | **N** | **T** | **L** | **L** | **P** | **K** | **E** | **D** | **N** | **I** | **I** | **I** | **I** | **R** | **K** | **G** | **F** | **G** | **R** | **T** | **R** | **I** | **L** | **V** | **P** | **L** | **G** | **V** | **A** | **I** | **L** | **L** | **E** | **H** | **S** | **T** | **F** | **Y** | **G** | **T** | **V** | **R** | **F** | **E** | **E** | **E** | **K** | **Y** | **Q** | **L** | **K** | **N** | **E** | **S** | **L** | **K** | **I** | **Y** | **S** | **N** | **D** | **Y** | **D** | **T** | **N** | **L** | **R** | **R** | **L** | **K** | **I** | **M** | **T** | **N** | **T** | **L** | **V** | **G** | **D** | **V** | **E** | **V** | **I** | **R** | **V** |
| **N52789** | **M** | **N** | **N** | **P** | **W** | **R** | **F** | **F** | **I** | **V** | **A** | **E** | **A** | **L** | **L** | **F** | **I** | **L** | **A** | **L** | **W** | **Q** | **I** | **V** | **H** | **N** | **P** | **G** | **L** | **A** | **V** | **L** | **L** | **T** | **I** | **G** | **V** | **L** | **L** | **V** | **A** | **Y** | **V** | **S** | **R** | **K** | **A** | **S** | **K** | **T** | **H** | **F** | **N** | **N** | **F** | **Q** | **F** | **V** | **L** | **G** | **V** | **V** | **F** | **I** | **V** | **I** | **G** | **A** | **M** | **N** | **S** | **T** | **A** | **V** | **W** | **F** | **M** | **L** | **I** | **F** | **G** | **V** | **L** | **F** | **I** | **G** | **L** | **K** | **G** | **F** | **E** | **I** | **S** | **G** | **V** | **D** | **I** | **A** | **E** | **R** | **A** | **P** | **W** | **R** | **K** | **K** | **Q** | **M** | **I** | **M** | **V** | **E** | **T** | **A** | **A** | **K** | **E** | **P** | **K** | **N** | **G** | **K** | **R** | **F** | **K** | **R** | **R** | **W** | **F** | **A** | **N** | **E** | **R** | **I** | **G** | **N** | **N** | **I** | **Y** | **E** | **W** | **D** | **D** | **I** | **N** | **I** | **D** | **L** | **I** | **S** | **G** | **D** | **T** | **I** | **I** | **D** | **L** | **G** | **N** | **T** | **L** | **L** | **P** | **K** | **E** | **D** | **N** | **I** | **I** | **I** | **I** | **R** | **K** | **G** | **F** | **G** | **R** | **T** | **R** | **I** | **L** | **V** | **P** | **L** | **G** | **V** | **A** | **I** | **L** | **L** | **E** | **H** | **S** | **T** | **F** | **Y** | **G** | **T** | **V** | **R** | **F** | **E** | **E** | **E** | **K** | **Y** | **Q** | **L** | **K** | **N** | **E** | **S** | **L** | **K** | **I** | **Y** | **S** | **N** | **D** | **Y** | **D** | **T** | **N** | **L** | **R** | **R** | **L** | **K** | **I** | **M** | **T** | **N** | **T** | **L** | **V** | **G** | **D** | **V** | **E** | **V** | **I** | **R** | **V** |
| **N59491F** | **M** | **N** | **N** | **P** | **W** | **R** | **F** | **F** | **I** | **V** | **A** | **E** | **A** | **L** | **L** | **F** | **I** | **L** | **A** | **L** | **W** | **Q** | **I** | **V** | **H** | **N** | **P** | **G** | **L** | **A** | **V** | **L** | **L** | **T** | **I** | **G** | **V** | **L** | **L** | **V** | **A** | **Y** | **V** | **S** | **R** | **K** | **A** | **S** | **K** | **T** | **H** | **F** | **N** | **N** | **F** | **Q** | **F** | **V** | **L** | **G** | **V** | **V** | **F** | **I** | **V** | **I** | **G** | **A** | **M** | **N** | **S** | **T** | **A** | **V** | **W** | **F** | **M** | **L** | **I** | **F** | **G** | **V** | **L** | **F** | **I** | **G** | **L** | **K** | **G** | **F** | **E** | **I** | **S** | **G** | **V** | **D** | **I** | **A** | **E** | **R** | **A** | **P** | **W** | **R** | **K** | **K** | **Q** | **M** | **I** | **M** | **V** | **E** | **T** | **A** | **A** | **K** | **E** | **P** | **K** | **N** | **G** | **K** | **R** | **F** | **K** | **R** | **R** | **W** | **F** | **A** | **N** | **E** | **R** | **I** | **G** | **N** | **N** | **I** | **Y** | **E** | **W** | **D** | **D** | **I** | **N** | **I** | **D** | **L** | **I** | **S** | **G** | **D** | **T** | **I** | **I** | **D** | **L** | **G** | **N** | **T** | **L** | **L** | **P** | **K** | **E** | **D** | **N** | **I** | **I** | **I** | **I** | **R** | **K** | **G** | **F** | **G** | **R** | **T** | **R** | **I** | **L** | **V** | **P** | **L** | **G** | **V** | **A** | **I** | **L** | **L** | **E** | **H** | **S** | **T** | **F** | **Y** | **G** | **T** | **V** | **R** | **F** | **E** | **E** | **E** | **K** | **Y** | **Q** | **L** | **K** | **N** | **E** | **S** | **L** | **K** | **I** | **Y** | **S** | **N** | **D** | **Y** | **D** | **T** | **N** | **L** | **R** | **R** | **L** | **K** | **I** | **M** | **T** | **N** | **T** | **L** | **V** | **G** | **D** | **V** | **E** | **V** | **I** | **R** | **V** |
| **N60268F** | **M** | **N** | **N** | **P** | **W** | **R** | **F** | **F** | **I** | **V** | **A** | **E** | **A** | **L** | **L** | **F** | **I** | **L** | **A** | **L** | **W** | **Q** | **I** | **V** | **H** | **N** | **P** | **G** | **L** | **A** | **V** | **L** | **L** | **T** | **I** | **G** | **V** | **L** | **L** | **V** | **A** | **Y** | **V** | **S** | **R** | **K** | **A** | **S** | **K** | **T** | **H** | **F** | **N** | **N** | **F** | **Q** | **F** | **V** | **L** | **G** | **V** | **V** | **F** | **I** | **V** | **I** | **G** | **A** | **M** | **N** | **S** | **T** | **A** | **V** | **W** | **F** | **M** | **L** | **I** | **F** | **G** | **V** | **L** | **F** | **I** | **G** | **L** | **K** | **G** | **F** | **E** | **I** | **S** | **G** | **V** | **D** | **I** | **A** | **E** | **R** | **A** | **P** | **W** | **R** | **K** | **K** | **Q** | **M** | **I** | **M** | **V** | **E** | **T** | **A** | **A** | **K** | **E** | **P** | **K** | **N** | **G** | **K** | **R** | **F** | **K** | **R** | **R** | **W** | **F** | **A** | **N** | **E** | **R** | **I** | **G** | **N** | **N** | **I** | **Y** | **E** | **W** | **D** | **D** | **I** | **N** | **I** | **D** | **L** | **I** | **S** | **G** | **D** | **T** | **I** | **I** | **D** | **L** | **G** | **N** | **T** | **L** | **L** | **P** | **K** | **E** | **D** | **N** | **I** | **I** | **I** | **I** | **R** | **K** | **G** | **F** | **G** | **R** | **T** | **R** | **I** | **L** | **V** | **P** | **L** | **G** | **V** | **A** | **I** | **L** | **L** | **E** | **H** | **S** | **T** | **F** | **Y** | **G** | **T** | **V** | **R** | **F** | **E** | **E** | **E** | **K** | **Y** | **Q** | **L** | **K** | **N** | **E** | **S** | **L** | **K** | **I** | **Y** | **S** | **N** | **D** | **Y** | **D** | **T** | **N** | **L** | **R** | **R** | **L** | **K** | **I** | **M** | **T** | **N** | **T** | **L** | **V** | **G** | **D** | **V** | **E** | **V** | **I** | **R** | **V** |
| **N59836F** | **M** | **N** | **N** | **P** | **W** | **R** | **F** | **F** | **I** | **V** | **A** | **E** | **A** | **L** | **L** | **F** | **I** | **L** | **A** | **L** | **W** | **Q** | **I** | **V** | **H** | **N** | **P** | **G** | **L** | **A** | **V** | **L** | **L** | **T** | **I** | **G** | **V** | **L** | **L** | **V** | **A** | **Y** | **V** | **S** | **R** | **K** | **A** | **S** | **K** | **T** | **H** | **F** | **N** | **N** | **F** | **Q** | **F** | **V** | **L** | **G** | **V** | **V** | **F** | **I** | **V** | **I** | **G** | **A** | **M** | **N** | **S** | **T** | **A** | **V** | **W** | **F** | **M** | **L** | **I** | **F** | **G** | **V** | **L** | **F** | **I** | **G** | **L** | **K** | **G** | **F** | **E** | **I** | **S** | **G** | **V** | **D** | **I** | **A** | **E** | **R** | **A** | **P** | **W** | **R** | **K** | **K** | **Q** | **M** | **I** | **M** | **V** | **E** | **T** | **A** | **A** | **K** | **E** | **P** | **K** | **N** | **G** | **K** | **R** | **F** | **K** | **R** | **R** | **W** | **F** | **A** | **N** | **E** | **R** | **I** | **G** | **N** | **N** | **I** | **Y** | **E** | **W** | **D** | **D** | **I** | **N** | **I** | **D** | **L** | **I** | **S** | **G** | **D** | **T** | **I** | **I** | **D** | **L** | **G** | **N** | **T** | **L** | **L** | **P** | **K** | **E** | **D** | **N** | **I** | **I** | **I** | **I** | **R** | **K** | **G** | **F** | **G** | **R** | **T** | **R** | **I** | **L** | **V** | **P** | **L** | **G** | **V** | **A** | **I** | **L** | **L** | **E** | **H** | **S** | **T** | **F** | **Y** | **G** | **T** | **V** | **R** | **F** | **E** | **E** | **E** | **K** | **Y** | **Q** | **L** | **K** | **N** | **E** | **S** | **L** | **K** | **I** | **Y** | **S** | **N** | **D** | **Y** | **D** | **T** | **N** | **L** | **R** | **R** | **L** | **K** | **I** | **M** | **T** | **N** | **T** | **L** | **V** | **G** | **D** | **V** | **E** | **V** | **I** | **R** | **V** |
| **N59963F** | **M** | **N** | **N** | **P** | **W** | **R** | **F** | **F** | **I** | **V** | **A** | **E** | **A** | **L** | **L** | **F** | **I** | **L** | **A** | **L** | **W** | **Q** | **I** | **V** | **H** | **N** | **P** | **G** | **L** | **A** | **V** | **L** | **L** | **T** | **I** | **G** | **V** | **L** | **L** | **V** | **A** | **Y** | **V** | **S** | **R** | **K** | **A** | **S** | **K** | **T** | **H** | **F** | **N** | **N** | **F** | **Q** | **F** | **V** | **L** | **G** | **V** | **V** | **F** | **I** | **V** | **I** | **G** | **A** | **M** | **N** | **S** | **T** | **A** | **V** | **W** | **F** | **M** | **L** | **I** | **F** | **G** | **V** | **L** | **F** | **I** | **G** | **L** | **K** | **G** | **F** | **E** | **I** | **S** | **G** | **V** | **D** | **I** | **A** | **E** | **R** | **A** | **P** | **W** | **R** | **K** | **K** | **Q** | **M** | **I** | **M** | **V** | **E** | **T** | **A** | **A** | **K** | **E** | **P** | **K** | **N** | **G** | **K** | **R** | **F** | **K** | **R** | **R** | **W** | **F** | **A** | **N** | **E** | **R** | **I** | **G** | **N** | **N** | **I** | **Y** | **E** | **W** | **D** | **D** | **I** | **N** | **I** | **D** | **L** | **I** | **S** | **G** | **D** | **T** | **I** | **I** | **D** | **L** | **G** | **N** | **T** | **L** | **L** | **P** | **K** | **E** | **D** | **N** | **I** | **I** | **I** | **I** | **R** | **K** | **G** | **F** | **G** | **R** | **T** | **R** | **I** | **L** | **V** | **P** | **L** | **G** | **V** | **A** | **I** | **L** | **L** | **E** | **H** | **S** | **T** | **F** | **Y** | **G** | **T** | **V** | **R** | **F** | **E** | **E** | **E** | **K** | **Y** | **Q** | **L** | **K** | **N** | **E** | **S** | **L** | **K** | **I** | **Y** | **S** | **N** | **D** | **Y** | **D** | **T** | **N** | **L** | **R** | **R** | **L** | **K** | **I** | **M** | **T** | **N** | **T** | **L** | **V** | **G** | **D** | **V** | **E** | **V** | **I** | **R** | **V** |
| **N59841F** | **M** | **N** | **N** | **P** | **W** | **R** | **F** | **F** | **I** | **V** | **A** | **E** | **A** | **L** | **L** | **F** | **I** | **L** | **A** | **L** | **W** | **Q** | **I** | **V** | **H** | **N** | **P** | **G** | **L** | **A** | **V** | **L** | **L** | **T** | **I** | **G** | **V** | **L** | **L** | **V** | **A** | **Y** | **V** | **S** | **R** | **K** | **A** | **S** | **K** | **T** | **H** | **F** | **N** | **N** | **F** | **Q** | **F** | **V** | **L** | **G** | **V** | **V** | **F** | **I** | **V** | **I** | **G** | **A** | **M** | **N** | **S** | **T** | **A** | **V** | **W** | **F** | **M** | **L** | **I** | **F** | **G** | **V** | **L** | **F** | **I** | **G** | **L** | **K** | **G** | **F** | **E** | **I** | **S** | **G** | **V** | **D** | **I** | **A** | **E** | **R** | **A** | **P** | **W** | **R** | **K** | **K** | **Q** | **M** | **I** | **M** | **V** | **E** | **T** | **A** | **A** | **K** | **E** | **P** | **K** | **N** | **G** | **K** | **R** | **F** | **K** | **R** | **R** | **W** | **F** | **A** | **N** | **E** | **R** | **I** | **G** | **N** | **N** | **I** | **Y** | **E** | **W** | **D** | **D** | **I** | **N** | **I** | **D** | **L** | **I** | **S** | **G** | **D** | **T** | **I** | **I** | **D** | **L** | **G** | **N** | **T** | **L** | **L** | **P** | **K** | **E** | **D** | **N** | **I** | **I** | **I** | **I** | **R** | **K** | **G** | **F** | **G** | **R** | **T** | **R** | **I** | **L** | **V** | **P** | **L** | **G** | **V** | **A** | **I** | **L** | **L** | **E** | **H** | **S** | **T** | **F** | **Y** | **G** | **T** | **V** | **R** | **F** | **E** | **E** | **E** | **K** | **Y** | **Q** | **L** | **K** | **N** | **E** | **S** | **L** | **K** | **I** | **Y** | **S** | **N** | **D** | **Y** | **D** | **T** | **N** | **L** | **R** | **R** | **L** | **K** | **I** | **M** | **T** | **N** | **T** | **L** | **V** | **G** | **D** | **V** | **E** | **V** | **I** | **R** | **V** |
| **N55106** | **M** | **N** | **N** | **P** | **W** | **R** | **F** | **F** | **I** | **V** | **A** | **E** | **A** | **L** | **L** | **F** | **I** | **L** | **A** | **L** | **W** | **Q** | **I** | **V** | **H** | **N** | **P** | **G** | **L** | **A** | **V** | **L** | **L** | **T** | **I** | **G** | **V** | **L** | **L** | **V** | **A** | **Y** | **V** | **S** | **R** | **K** | **A** | **S** | **K** | **T** | **H** | **F** | **N** | **N** | **F** | **Q** | **F** | **V** | **L** | **G** | **V** | **V** | **F** | **I** | **V** | **I** | **G** | **A** | **M** | **N** | **S** | **T** | **A** | **V** | **W** | **F** | **M** | **L** | **I** | **F** | **G** | **V** | **L** | **F** | **I** | **G** | **L** | **K** | **G** | **F** | **E** | **I** | **S** | **G** | **V** | **D** | **I** | **A** | **E** | **R** | **A** | **P** | **W** | **R** | **K** | **K** | **Q** | **M** | **I** | **M** | **V** | **E** | **T** | **A** | **A** | **K** | **E** | **P** | **K** | **N** | **G** | **K** | **R** | **F** | **K** | **R** | **R** | **W** | **F** | **A** | **N** | **E** | **R** | **I** | **G** | **N** | **N** | **I** | **Y** | **E** | **W** | **D** | **D** | **I** | **N** | **I** | **D** | **L** | **I** | **S** | **G** | **D** | **T** | **I** | **I** | **D** | **L** | **G** | **N** | **T** | **L** | **L** | **P** | **K** | **E** | **D** | **N** | **I** | **I** | **I** | **I** | **R** | **K** | **G** | **F** | **G** | **R** | **T** | **R** | **I** | **L** | **V** | **P** | **L** | **G** | **V** | **A** | **I** | **L** | **L** | **E** | **H** | **S** | **T** | **F** | **Y** | **G** | **T** | **V** | **R** | **F** | **E** | **E** | **E** | **K** | **Y** | **Q** | **L** | **K** | **N** | **E** | **S** | **L** | **K** | **I** | **Y** | **S** | **N** | **D** | **Y** | **D** | **T** | **N** | **L** | **R** | **R** | **L** | **K** | **I** | **M** | **T** | **N** | **T** | **L** | **V** | **G** | **D** | **V** | **E** | **V** | **I** | **R** | **V** |
| **N52755** | **M** | **N** | **N** | **P** | **W** | **R** | **F** | **F** | **I** | **V** | **A** | **E** | **A** | **L** | **L** | **F** | **I** | **L** | **A** | **L** | **W** | **Q** | **I** | **V** | **H** | **N** | **P** | **G** | **L** | **A** | **V** | **L** | **L** | **T** | **I** | **G** | **V** | **L** | **L** | **V** | **A** | **Y** | **V** | **S** | **R** | **K** | **A** | **S** | **K** | **T** | **H** | **F** | **N** | **N** | **F** | **Q** | **F** | **V** | **L** | **G** | **V** | **V** | **F** | **I** | **V** | **I** | **G** | **A** | **M** | **N** | **S** | **T** | **A** | **V** | **W** | **F** | **M** | **L** | **I** | **F** | **G** | **V** | **L** | **F** | **I** | **G** | **L** | **K** | **G** | **F** | **E** | **I** | **S** | **G** | **V** | **D** | **I** | **A** | **E** | **R** | **A** | **P** | **W** | **R** | **K** | **K** | **Q** | **M** | **I** | **M** | **V** | **E** | **T** | **A** | **A** | **K** | **E** | **P** | **K** | **N** | **G** | **K** | **R** | **F** | **K** | **R** | **R** | **W** | **F** | **A** | **N** | **E** | **R** | **I** | **G** | **N** | **N** | **I** | **Y** | **E** | **W** | **D** | **D** | **I** | **N** | **I** | **D** | **L** | **I** | **S** | **G** | **D** | **T** | **I** | **I** | **D** | **L** | **G** | **N** | **T** | **L** | **L** | **P** | **K** | **E** | **D** | **N** | **I** | **I** | **I** | **I** | **R** | **K** | **G** | **F** | **G** | **R** | **T** | **R** | **I** | **L** | **V** | **P** | **L** | **G** | **V** | **A** | **I** | **L** | **L** | **E** | **H** | **S** | **T** | **F** | **Y** | **G** | **T** | **V** | **R** | **F** | **E** | **E** | **E** | **K** | **Y** | **Q** | **L** | **K** | **N** | **E** | **S** | **L** | **K** | **I** | **Y** | **S** | **N** | **D** | **Y** | **D** | **T** | **N** | **L** | **R** | **R** | **L** | **K** | **I** | **M** | **T** | **N** | **T** | **L** | **V** | **G** | **D** | **V** | **E** | **V** | **I** | **R** | **V** |
| **N60100F** | **M** | **N** | **N** | **P** | **W** | **R** | **F** | **F** | **I** | **V** | **A** | **E** | **A** | **L** | **L** | **F** | **I** | **L** | **A** | **L** | **W** | **Q** | **I** | **V** | **H** | **N** | **P** | **G** | **L** | **A** | **V** | **L** | **L** | **T** | **I** | **G** | **V** | **L** | **L** | **V** | **A** | **Y** | **V** | **S** | **R** | **K** | **A** | **S** | **K** | **T** | **H** | **F** | **N** | **N** | **F** | **Q** | **F** | **V** | **L** | **G** | **V** | **V** | **F** | **I** | **V** | **I** | **G** | **A** | **M** | **N** | **S** | **T** | **A** | **V** | **W** | **F** | **M** | **L** | **I** | **F** | **G** | **V** | **L** | **F** | **I** | **G** | **L** | **K** | **G** | **F** | **E** | **I** | **S** | **G** | **V** | **D** | **I** | **A** | **E** | **R** | **A** | **P** | **W** | **R** | **K** | **K** | **Q** | **M** | **I** | **M** | **V** | **E** | **T** | **A** | **A** | **K** | **E** | **P** | **K** | **N** | **G** | **K** | **R** | **F** | **K** | **R** | **R** | **W** | **F** | **A** | **N** | **E** | **R** | **I** | **G** | **N** | **N** | **I** | **Y** | **E** | **W** | **D** | **D** | **I** | **N** | **I** | **D** | **L** | **I** | **S** | **G** | **D** | **T** | **I** | **I** | **D** | **L** | **G** | **N** | **T** | **L** | **L** | **P** | **K** | **E** | **D** | **N** | **I** | **I** | **I** | **I** | **R** | **K** | **G** | **F** | **G** | **R** | **T** | **R** | **I** | **L** | **V** | **P** | **L** | **G** | **V** | **A** | **I** | **L** | **L** | **E** | **H** | **S** | **T** | **F** | **Y** | **G** | **T** | **V** | **R** | **F** | **E** | **E** | **E** | **K** | **Y** | **Q** | **L** | **K** | **N** | **E** | **S** | **L** | **K** | **I** | **Y** | **S** | **N** | **D** | **Y** | **D** | **T** | **N** | **L** | **R** | **R** | **L** | **K** | **I** | **M** | **T** | **N** | **T** | **L** | **V** | **G** | **D** | **V** | **E** | **V** | **I** | **R** | **V** |
| **N54553** | **M** | **N** | **N** | **P** | **W** | **R** | **F** | **F** | **I** | **V** | **A** | **E** | **A** | **L** | **L** | **F** | **I** | **L** | **A** | **L** | **W** | **Q** | **I** | **V** | **H** | **N** | **P** | **G** | **L** | **A** | **V** | **L** | **L** | **T** | **I** | **G** | **V** | **L** | **L** | **V** | **A** | **Y** | **V** | **S** | **R** | **K** | **A** | **S** | **K** | **T** | **H** | **F** | **N** | **N** | **F** | **Q** | **F** | **V** | **L** | **G** | **V** | **V** | **F** | **I** | **V** | **I** | **G** | **A** | **M** | **N** | **S** | **T** | **A** | **V** | **W** | **F** | **M** | **L** | **I** | **F** | **G** | **V** | **L** | **F** | **I** | **G** | **L** | **K** | **G** | **F** | **E** | **I** | **S** | **G** | **V** | **D** | **I** | **A** | **E** | **R** | **A** | **P** | **W** | **R** | **K** | **K** | **Q** | **M** | **I** | **M** | **V** | **E** | **T** | **A** | **A** | **K** | **E** | **P** | **K** | **N** | **G** | **K** | **R** | **F** | **K** | **R** | **R** | **W** | **F** | **A** | **N** | **E** | **R** | **I** | **G** | **N** | **N** | **I** | **Y** | **E** | **W** | **D** | **D** | **I** | **N** | **I** | **D** | **L** | **I** | **S** | **G** | **D** | **T** | **I** | **I** | **D** | **L** | **G** | **N** | **T** | **L** | **L** | **P** | **K** | **E** | **D** | **N** | **I** | **I** | **I** | **I** | **R** | **K** | **G** | **F** | **G** | **R** | **T** | **R** | **I** | **L** | **V** | **P** | **L** | **G** | **V** | **A** | **I** | **L** | **L** | **E** | **H** | **S** | **T** | **F** | **Y** | **G** | **T** | **V** | **R** | **F** | **E** | **E** | **E** | **K** | **Y** | **Q** | **L** | **K** | **N** | **E** | **S** | **L** | **K** | **I** | **Y** | **S** | **N** | **D** | **Y** | **D** | **T** | **N** | **L** | **R** | **R** | **L** | **K** | **I** | **M** | **T** | **N** | **T** | **L** | **V** | **G** | **D** | **V** | **E** | **V** | **I** | **R** | **V** |
| **N59936F** | **M** | **N** | **N** | **P** | **W** | **R** | **F** | **F** | **I** | **V** | **A** | **E** | **A** | **L** | **L** | **F** | **I** | **L** | **A** | **L** | **W** | **Q** | **I** | **V** | **H** | **N** | **P** | **G** | **L** | **A** | **V** | **L** | **L** | **T** | **I** | **G** | **V** | **L** | **L** | **V** | **A** | **Y** | **V** | **S** | **R** | **K** | **A** | **S** | **K** | **T** | **H** | **F** | **N** | **N** | **F** | **Q** | **F** | **V** | **L** | **G** | **V** | **V** | **F** | **I** | **V** | **I** | **G** | **A** | **M** | **N** | **S** | **T** | **A** | **V** | **W** | **F** | **M** | **L** | **I** | **F** | **G** | **V** | **L** | **F** | **I** | **G** | **L** | **K** | **G** | **F** | **E** | **I** | **S** | **G** | **V** | **D** | **I** | **A** | **E** | **R** | **A** | **P** | **W** | **R** | **K** | **K** | **Q** | **M** | **I** | **M** | **V** | **E** | **T** | **A** | **A** | **K** | **E** | **P** | **K** | **N** | **G** | **K** | **R** | **F** | **K** | **R** | **R** | **W** | **F** | **A** | **N** | **E** | **R** | **I** | **G** | **N** | **N** | **I** | **Y** | **E** | **W** | **D** | **D** | **I** | **N** | **I** | **D** | **L** | **I** | **S** | **G** | **D** | **T** | **I** | **I** | **D** | **L** | **G** | **N** | **T** | **L** | **L** | **P** | **K** | **E** | **D** | **N** | **I** | **I** | **I** | **I** | **R** | **K** | **G** | **F** | **G** | **R** | **T** | **R** | **I** | **L** | **V** | **P** | **L** | **G** | **V** | **A** | **I** | **L** | **L** | **E** | **H** | **S** | **T** | **F** | **Y** | **G** | **T** | **V** | **R** | **F** | **E** | **E** | **E** | **K** | **Y** | **Q** | **L** | **K** | **N** | **E** | **S** | **L** | **K** | **I** | **Y** | **S** | **N** | **D** | **Y** | **D** | **T** | **N** | **L** | **R** | **R** | **L** | **K** | **I** | **M** | **T** | **N** | **T** | **L** | **V** | **G** | **D** | **V** | **E** | **V** | **I** | **R** | **V** |
| **N59555F** | **M** | **N** | **N** | **P** | **W** | **R** | **F** | **F** | **I** | **V** | **A** | **E** | **A** | **L** | **L** | **F** | **I** | **L** | **A** | **L** | **W** | **Q** | **I** | **V** | **H** | **N** | **P** | **G** | **L** | **A** | **V** | **L** | **L** | **T** | **I** | **G** | **V** | **L** | **L** | **V** | **A** | **Y** | **V** | **S** | **R** | **K** | **A** | **S** | **K** | **T** | **H** | **F** | **N** | **N** | **F** | **Q** | **F** | **V** | **L** | **G** | **V** | **V** | **F** | **I** | **V** | **I** | **G** | **A** | **M** | **N** | **S** | **T** | **A** | **V** | **W** | **F** | **M** | **L** | **I** | **F** | **G** | **V** | **L** | **F** | **I** | **G** | **L** | **K** | **G** | **F** | **E** | **I** | **S** | **G** | **V** | **D** | **I** | **A** | **E** | **R** | **A** | **P** | **W** | **R** | **K** | **K** | **Q** | **M** | **I** | **M** | **V** | **E** | **T** | **A** | **A** | **K** | **E** | **P** | **K** | **N** | **G** | **K** | **R** | **F** | **K** | **R** | **R** | **W** | **F** | **A** | **N** | **E** | **R** | **I** | **G** | **N** | **N** | **I** | **Y** | **E** | **W** | **D** | **D** | **I** | **N** | **I** | **D** | **L** | **I** | **S** | **G** | **D** | **T** | **I** | **I** | **D** | **L** | **G** | **N** | **T** | **L** | **L** | **P** | **K** | **E** | **D** | **N** | **I** | **I** | **I** | **I** | **R** | **K** | **G** | **F** | **G** | **R** | **T** | **R** | **I** | **L** | **V** | **P** | **L** | **G** | **V** | **A** | **I** | **L** | **L** | **E** | **H** | **S** | **T** | **F** | **Y** | **G** | **T** | **V** | **R** | **F** | **E** | **E** | **E** | **K** | **Y** | **Q** | **L** | **K** | **N** | **E** | **S** | **L** | **K** | **I** | **Y** | **S** | **N** | **D** | **Y** | **D** | **T** | **N** | **L** | **R** | **R** | **L** | **K** | **I** | **M** | **T** | **N** | **T** | **L** | **V** | **G** | **D** | **V** | **E** | **V** | **I** | **R** | **V** |
| **N55181** | **M** | **N** | **N** | **P** | **W** | **R** | **F** | **F** | **I** | **V** | **A** | **E** | **A** | **L** | **L** | **F** | **I** | **L** | **A** | **L** | **W** | **Q** | **I** | **V** | **H** | **N** | **P** | **G** | **L** | **A** | **V** | **L** | **L** | **T** | **I** | **G** | **V** | **L** | **L** | **V** | **A** | **Y** | **V** | **S** | **R** | **K** | **A** | **S** | **K** | **T** | **H** | **F** | **N** | **N** | **F** | **Q** | **F** | **V** | **L** | **G** | **V** | **V** | **F** | **I** | **V** | **I** | **G** | **A** | **M** | **N** | **S** | **T** | **A** | **V** | **W** | **F** | **M** | **L** | **I** | **F** | **G** | **V** | **L** | **F** | **I** | **G** | **L** | **K** | **G** | **F** | **E** | **I** | **S** | **G** | **V** | **D** | **I** | **A** | **E** | **R** | **A** | **P** | **W** | **R** | **K** | **K** | **Q** | **M** | **I** | **M** | **V** | **E** | **T** | **A** | **A** | **K** | **E** | **P** | **K** | **N** | **G** | **K** | **R** | **F** | **K** | **R** | **R** | **W** | **F** | **A** | **N** | **E** | **R** | **I** | **G** | **N** | **N** | **I** | **Y** | **E** | **W** | **D** | **D** | **I** | **N** | **I** | **D** | **L** | **I** | **S** | **G** | **D** | **T** | **I** | **I** | **D** | **L** | **G** | **N** | **T** | **L** | **L** | **P** | **K** | **E** | **D** | **N** | **I** | **I** | **I** | **I** | **R** | **K** | **G** | **F** | **G** | **R** | **T** | **R** | **I** | **L** | **V** | **P** | **L** | **G** | **V** | **A** | **I** | **L** | **L** | **E** | **H** | **S** | **T** | **F** | **Y** | **G** | **T** | **V** | **R** | **F** | **E** | **E** | **E** | **K** | **Y** | **Q** | **L** | **K** | **N** | **E** | **S** | **L** | **K** | **I** | **Y** | **S** | **N** | **D** | **Y** | **D** | **T** | **N** | **L** | **R** | **R** | **L** | **K** | **I** | **M** | **T** | **N** | **T** | **L** | **V** | **G** | **D** | **V** | **E** | **V** | **I** | **R** | **V** |
| **N55753** | **M** | **N** | **N** | **P** | **W** | **R** | **F** | **F** | **I** | **V** | **A** | **E** | **A** | **L** | **L** | **F** | **I** | **L** | **A** | **L** | **W** | **Q** | **I** | **V** | **H** | **N** | **P** | **G** | **L** | **A** | **V** | **L** | **L** | **T** | **I** | **G** | **V** | **L** | **L** | **V** | **A** | **Y** | **V** | **S** | **R** | **K** | **A** | **S** | **K** | **T** | **H** | **F** | **N** | **N** | **F** | **Q** | **F** | **V** | **L** | **G** | **V** | **V** | **F** | **I** | **V** | **I** | **G** | **A** | **M** | **N** | **S** | **T** | **A** | **V** | **W** | **F** | **M** | **L** | **I** | **F** | **G** | **V** | **L** | **F** | **I** | **G** | **L** | **K** | **G** | **F** | **E** | **I** | **S** | **G** | **V** | **D** | **I** | **A** | **E** | **R** | **A** | **P** | **W** | **R** | **K** | **K** | **Q** | **M** | **I** | **M** | **V** | **E** | **T** | **A** | **A** | **K** | **E** | **P** | **K** | **N** | **G** | **K** | **R** | **F** | **K** | **R** | **R** | **W** | **F** | **A** | **N** | **E** | **R** | **I** | **G** | **N** | **N** | **I** | **Y** | **E** | **W** | **D** | **D** | **I** | **N** | **I** | **D** | **L** | **I** | **S** | **G** | **D** | **T** | **I** | **I** | **D** | **L** | **G** | **N** | **T** | **L** | **L** | **P** | **K** | **E** | **D** | **N** | **I** | **I** | **I** | **I** | **R** | **K** | **G** | **F** | **G** | **R** | **T** | **R** | **I** | **L** | **V** | **P** | **L** | **G** | **V** | **A** | **I** | **L** | **L** | **E** | **H** | **S** | **T** | **F** | **Y** | **G** | **T** | **V** | **R** | **F** | **E** | **E** | **E** | **K** | **Y** | **Q** | **L** | **K** | **N** | **E** | **S** | **L** | **K** | **I** | **Y** | **S** | **N** | **D** | **Y** | **D** | **T** | **N** | **L** | **R** | **R** | **L** | **K** | **I** | **M** | **T** | **N** | **T** | **L** | **V** | **G** | **D** | **V** | **E** | **V** | **I** | **R** | **V** |
| **N59599F** | **M** | **N** | **N** | **P** | **W** | **R** | **F** | **F** | **I** | **V** | **A** | **E** | **A** | **L** | **L** | **F** | **I** | **L** | **A** | **L** | **W** | **Q** | **I** | **V** | **H** | **N** | **P** | **G** | **L** | **A** | **V** | **L** | **L** | **T** | **I** | **G** | **V** | **L** | **L** | **V** | **A** | **Y** | **V** | **S** | **R** | **K** | **A** | **S** | **K** | **T** | **H** | **F** | **N** | **N** | **F** | **Q** | **F** | **V** | **L** | **G** | **V** | **V** | **F** | **I** | **V** | **I** | **G** | **A** | **M** | **N** | **S** | **T** | **A** | **V** | **W** | **F** | **M** | **L** | **I** | **F** | **G** | **V** | **L** | **F** | **I** | **G** | **L** | **K** | **G** | **F** | **E** | **I** | **S** | **G** | **V** | **D** | **I** | **A** | **E** | **R** | **A** | **P** | **W** | **R** | **K** | **K** | **Q** | **M** | **I** | **M** | **V** | **E** | **T** | **A** | **A** | **K** | **E** | **P** | **K** | **N** | **G** | **K** | **R** | **F** | **K** | **R** | **R** | **W** | **F** | **A** | **N** | **E** | **R** | **I** | **G** | **N** | **N** | **I** | **Y** | **E** | **W** | **D** | **D** | **I** | **N** | **I** | **D** | **L** | **I** | **S** | **G** | **D** | **T** | **I** | **I** | **D** | **L** | **G** | **N** | **T** | **L** | **L** | **P** | **K** | **E** | **D** | **N** | **I** | **I** | **I** | **I** | **R** | **K** | **G** | **F** | **G** | **R** | **T** | **R** | **I** | **L** | **V** | **P** | **L** | **G** | **V** | **A** | **I** | **L** | **L** | **E** | **H** | **S** | **T** | **F** | **Y** | **G** | **T** | **V** | **R** | **F** | **E** | **E** | **E** | **K** | **Y** | **Q** | **L** | **K** | **N** | **E** | **S** | **L** | **K** | **I** | **Y** | **S** | **N** | **D** | **Y** | **D** | **T** | **N** | **L** | **R** | **R** | **L** | **K** | **I** | **M** | **T** | **N** | **T** | **L** | **V** | **G** | **D** | **V** | **E** | **V** | **I** | **R** | **V** |
| **N59462F** | **M** | **N** | **N** | **P** | **W** | **R** | **F** | **F** | **I** | **V** | **A** | **E** | **A** | **L** | **L** | **F** | **I** | **L** | **A** | **L** | **W** | **Q** | **I** | **V** | **H** | **N** | **P** | **G** | **L** | **A** | **V** | **L** | **L** | **T** | **I** | **G** | **V** | **L** | **L** | **V** | **A** | **Y** | **V** | **S** | **R** | **K** | **A** | **S** | **K** | **T** | **H** | **F** | **N** | **N** | **F** | **Q** | **F** | **V** | **L** | **G** | **V** | **V** | **F** | **I** | **V** | **I** | **G** | **A** | **M** | **N** | **S** | **T** | **A** | **V** | **W** | **F** | **M** | **L** | **I** | **F** | **G** | **V** | **L** | **F** | **I** | **G** | **L** | **K** | **G** | **F** | **E** | **I** | **S** | **G** | **V** | **D** | **I** | **A** | **E** | **R** | **A** | **P** | **W** | **R** | **K** | **K** | **Q** | **M** | **I** | **M** | **V** | **E** | **T** | **A** | **A** | **K** | **E** | **P** | **K** | **N** | **G** | **K** | **R** | **F** | **K** | **R** | **R** | **W** | **F** | **A** | **N** | **E** | **R** | **I** | **G** | **N** | **N** | **I** | **Y** | **E** | **W** | **D** | **D** | **I** | **N** | **I** | **D** | **L** | **I** | **S** | **G** | **D** | **T** | **I** | **I** | **D** | **L** | **G** | **N** | **T** | **L** | **L** | **P** | **K** | **E** | **D** | **N** | **I** | **I** | **I** | **I** | **R** | **K** | **G** | **F** | **G** | **R** | **T** | **R** | **I** | **L** | **V** | **P** | **L** | **G** | **V** | **A** | **I** | **L** | **L** | **E** | **H** | **S** | **T** | **F** | **Y** | **G** | **T** | **V** | **R** | **F** | **E** | **E** | **E** | **K** | **Y** | **Q** | **L** | **K** | **N** | **E** | **S** | **L** | **K** | **I** | **Y** | **S** | **N** | **D** | **Y** | **D** | **T** | **N** | **L** | **R** | **R** | **L** | **K** | **I** | **M** | **T** | **N** | **T** | **L** | **V** | **G** | **D** | **V** | **E** | **V** | **I** | **R** | **V** |
| **N52454** | **M** | **N** | **N** | **P** | **W** | **R** | **F** | **F** | **I** | **V** | **A** | **E** | **A** | **L** | **L** | **F** | **I** | **L** | **A** | **L** | **W** | **Q** | **I** | **V** | **H** | **N** | **P** | **G** | **L** | **A** | **V** | **L** | **L** | **T** | **I** | **G** | **V** | **L** | **L** | **V** | **A** | **Y** | **V** | **S** | **R** | **K** | **A** | **S** | **K** | **T** | **H** | **F** | **N** | **N** | **F** | **Q** | **F** | **V** | **L** | **G** | **V** | **V** | **F** | **I** | **V** | **I** | **G** | **A** | **M** | **N** | **S** | **T** | **A** | **V** | **W** | **F** | **M** | **L** | **I** | **F** | **G** | **V** | **L** | **F** | **I** | **G** | **L** | **K** | **G** | **F** | **E** | **I** | **S** | **G** | **V** | **D** | **I** | **A** | **E** | **R** | **A** | **P** | **W** | **R** | **K** | **K** | **Q** | **M** | **I** | **M** | **V** | **E** | **T** | **A** | **A** | **K** | **E** | **P** | **K** | **N** | **G** | **K** | **R** | **F** | **K** | **R** | **R** | **W** | **F** | **A** | **N** | **E** | **R** | **I** | **G** | **N** | **N** | **I** | **Y** | **E** | **W** | **D** | **D** | **I** | **N** | **I** | **D** | **L** | **I** | **S** | **G** | **D** | **T** | **I** | **I** | **D** | **L** | **G** | **N** | **T** | **L** | **L** | **P** | **K** | **E** | **D** | **N** | **I** | **I** | **I** | **I** | **R** | **K** | **G** | **F** | **G** | **R** | **T** | **R** | **I** | **L** | **V** | **P** | **L** | **G** | **V** | **A** | **I** | **L** | **L** | **E** | **H** | **S** | **T** | **F** | **Y** | **G** | **T** | **V** | **R** | **F** | **E** | **E** | **E** | **K** | **Y** | **Q** | **L** | **K** | **N** | **E** | **S** | **L** | **K** | **I** | **Y** | **S** | **N** | **D** | **Y** | **D** | **T** | **N** | **L** | **R** | **R** | **L** | **K** | **I** | **M** | **T** | **N** | **T** | **L** | **V** | **G** | **D** | **V** | **E** | **V** | **I** | **R** | **V** |
| **N59455F** | **M** | **N** | **N** | **P** | **W** | **R** | **F** | **F** | **I** | **V** | **A** | **E** | **A** | **L** | **L** | **F** | **I** | **L** | **A** | **L** | **W** | **Q** | **I** | **V** | **H** | **N** | **P** | **G** | **L** | **A** | **V** | **L** | **L** | **T** | **I** | **G** | **V** | **L** | **L** | **V** | **A** | **Y** | **V** | **S** | **R** | **K** | **A** | **S** | **K** | **T** | **H** | **F** | **N** | **N** | **F** | **Q** | **F** | **V** | **L** | **G** | **V** | **V** | **F** | **I** | **V** | **I** | **G** | **A** | **M** | **N** | **S** | **T** | **A** | **V** | **W** | **F** | **M** | **L** | **I** | **F** | **G** | **V** | **L** | **F** | **I** | **G** | **L** | **K** | **G** | **F** | **E** | **I** | **S** | **G** | **V** | **D** | **I** | **A** | **E** | **R** | **A** | **P** | **W** | **R** | **K** | **K** | **Q** | **M** | **I** | **M** | **V** | **E** | **T** | **A** | **A** | **K** | **E** | **P** | **K** | **N** | **G** | **K** | **R** | **F** | **K** | **R** | **R** | **W** | **F** | **A** | **N** | **E** | **R** | **I** | **G** | **N** | **N** | **I** | **Y** | **E** | **W** | **D** | **D** | **I** | **N** | **I** | **D** | **L** | **I** | **S** | **G** | **D** | **T** | **I** | **I** | **D** | **L** | **G** | **N** | **T** | **L** | **L** | **P** | **K** | **E** | **D** | **N** | **I** | **I** | **I** | **I** | **R** | **K** | **G** | **F** | **G** | **R** | **T** | **R** | **I** | **L** | **V** | **P** | **L** | **G** | **V** | **A** | **I** | **L** | **L** | **E** | **H** | **S** | **T** | **F** | **Y** | **G** | **T** | **V** | **R** | **F** | **E** | **E** | **E** | **K** | **Y** | **Q** | **L** | **K** | **N** | **E** | **S** | **L** | **K** | **I** | **Y** | **S** | **N** | **D** | **Y** | **D** | **T** | **N** | **L** | **R** | **R** | **L** | **K** | **I** | **M** | **T** | **N** | **T** | **L** | **V** | **G** | **D** | **V** | **E** | **V** | **I** | **R** | **V** |
| **N59860F** | **M** | **N** | **N** | **P** | **W** | **R** | **F** | **F** | **I** | **V** | **A** | **E** | **A** | **L** | **L** | **F** | **I** | **L** | **A** | **L** | **W** | **Q** | **I** | **V** | **H** | **N** | **P** | **G** | **L** | **A** | **V** | **L** | **L** | **T** | **I** | **G** | **V** | **L** | **L** | **V** | **A** | **Y** | **V** | **S** | **R** | **K** | **A** | **S** | **K** | **T** | **H** | **F** | **N** | **N** | **F** | **Q** | **F** | **V** | **L** | **G** | **V** | **V** | **F** | **I** | **V** | **I** | **G** | **A** | **M** | **N** | **S** | **T** | **A** | **V** | **W** | **F** | **M** | **L** | **I** | **F** | **G** | **V** | **L** | **F** | **I** | **G** | **L** | **K** | **G** | **F** | **E** | **I** | **S** | **G** | **V** | **D** | **I** | **A** | **E** | **R** | **A** | **P** | **W** | **R** | **K** | **K** | **Q** | **M** | **I** | **M** | **V** | **E** | **T** | **A** | **A** | **K** | **E** | **P** | **K** | **N** | **G** | **K** | **R** | **F** | **K** | **R** | **R** | **W** | **F** | **A** | **N** | **E** | **R** | **I** | **G** | **N** | **N** | **I** | **Y** | **E** | **W** | **D** | **D** | **I** | **N** | **I** | **D** | **L** | **I** | **S** | **G** | **D** | **T** | **I** | **I** | **D** | **L** | **G** | **N** | **T** | **L** | **L** | **P** | **K** | **E** | **D** | **N** | **I** | **I** | **I** | **I** | **R** | **K** | **G** | **F** | **G** | **R** | **T** | **R** | **I** | **L** | **V** | **P** | **L** | **G** | **V** | **A** | **I** | **L** | **L** | **E** | **H** | **S** | **T** | **F** | **Y** | **G** | **T** | **V** | **R** | **F** | **E** | **E** | **E** | **K** | **Y** | **Q** | **L** | **K** | **N** | **E** | **S** | **L** | **K** | **I** | **Y** | **S** | **N** | **D** | **Y** | **D** | **T** | **N** | **L** | **R** | **R** | **L** | **K** | **I** | **M** | **T** | **N** | **T** | **L** | **V** | **G** | **D** | **V** | **E** | **V** | **I** | **R** | **V** |
| **N52587** | **-** | **-** | **-** | **P** | **W** | **R** | **F** | **F** | **I** | **V** | **A** | **E** | **A** | **L** | **L** | **F** | **I** | **L** | **A** | **L** | **W** | **Q** | **I** | **V** | **H** | **N** | **P** | **G** | **L** | **A** | **V** | **L** | **L** | **T** | **I** | **G** | **V** | **L** | **L** | **V** | **A** | **Y** | **V** | **S** | **R** | **K** | **A** | **S** | **K** | **T** | **H** | **F** | **N** | **N** | **F** | **Q** | **F** | **V** | **L** | **G** | **I** | **V** | **F** | **I** | **V** | **I** | **G** | **A** | **M** | **N** | **S** | **T** | **A** | **V** | **W** | **F** | **M** | **L** | **I** | **F** | **G** | **V** | **L** | **F** | **I** | **G** | **L** | **K** | **G** | **F** | **E** | **I** | **S** | **G** | **V** | **D** | **I** | **A** | **E** | **R** | **A** | **P** | **W** | **R** | **K** | **K** | **Q** | **M** | **I** | **M** | **V** | **E** | **T** | **A** | **A** | **K** | **E** | **P** | **K** | **N** | **G** | **K** | **R** | **F** | **K** | **R** | **R** | **W** | **F** | **A** | **N** | **E** | **R** | **I** | **G** | **N** | **N** | **I** | **Y** | **E** | **W** | **D** | **D** | **I** | **N** | **I** | **D** | **L** | **I** | **S** | **G** | **D** | **T** | **I** | **I** | **D** | **L** | **G** | **N** | **T** | **L** | **L** | **P** | **K** | **E** | **D** | **N** | **I** | **I** | **I** | **I** | **R** | **K** | **G** | **F** | **G** | **R** | **T** | **R** | **I** | **L** | **V** | **P** | **L** | **G** | **V** | **A** | **I** | **L** | **L** | **E** | **H** | **S** | **T** | **F** | **Y** | **G** | **T** | **V** | **R** | **F** | **E** | **E** | **E** | **K** | **Y** | **Q** | **L** | **K** | **N** | **E** | **S** | **L** | **K** | **I** | **Y** | **S** | **N** | **D** | **Y** | **D** | **T** | **N** | **L** | **R** | **R** | **L** | **K** | **I** | **M** | **T** | **N** | **T** | **L** | **V** | **G** | **D** | **V** | **E** | **V** | **I** | **R** | **V** |
| **N55119** | **M** | **N** | **N** | **P** | **W** | **R** | **F** | **F** | **I** | **V** | **A** | **E** | **A** | **L** | **L** | **F** | **I** | **L** | **A** | **L** | **W** | **Q** | **I** | **V** | **H** | **N** | **P** | **G** | **L** | **A** | **V** | **L** | **L** | **T** | **I** | **G** | **V** | **L** | **L** | **V** | **A** | **Y** | **V** | **S** | **R** | **K** | **A** | **S** | **K** | **T** | **H** | **F** | **N** | **N** | **F** | **Q** | **F** | **V** | **L** | **G** | **V** | **V** | **F** | **I** | **V** | **I** | **G** | **A** | **M** | **N** | **S** | **T** | **A** | **V** | **W** | **F** | **M** | **L** | **I** | **F** | **G** | **V** | **L** | **F** | **I** | **G** | **L** | **K** | **G** | **F** | **E** | **I** | **S** | **G** | **V** | **D** | **I** | **A** | **E** | **R** | **A** | **P** | **W** | **R** | **K** | **K** | **Q** | **M** | **I** | **M** | **V** | **E** | **T** | **A** | **A** | **K** | **E** | **P** | **K** | **N** | **G** | **K** | **R** | **F** | **K** | **R** | **R** | **W** | **F** | **A** | **N** | **E** | **R** | **I** | **G** | **N** | **N** | **I** | **Y** | **E** | **W** | **D** | **D** | **I** | **N** | **I** | **D** | **L** | **I** | **S** | **G** | **D** | **T** | **I** | **I** | **D** | **L** | **G** | **N** | **T** | **L** | **L** | **P** | **K** | **E** | **D** | **N** | **I** | **I** | **I** | **I** | **R** | **K** | **G** | **F** | **G** | **R** | **T** | **R** | **I** | **L** | **V** | **P** | **L** | **G** | **V** | **A** | **I** | **L** | **L** | **E** | **H** | **S** | **T** | **F** | **Y** | **G** | **T** | **V** | **R** | **F** | **E** | **E** | **E** | **K** | **Y** | **Q** | **L** | **K** | **N** | **E** | **S** | **L** | **K** | **I** | **Y** | **S** | **N** | **D** | **Y** | **D** | **T** | **N** | **L** | **R** | **R** | **L** | **K** | **I** | **M** | **T** | **N** | **T** | **L** | **V** | **G** | **D** | **V** | **E** | **V** | **I** | **R** | **V** |
| **N60392F** | **M** | **N** | **N** | **P** | **W** | **R** | **F** | **F** | **I** | **V** | **A** | **E** | **A** | **L** | **L** | **F** | **I** | **L** | **A** | **L** | **W** | **Q** | **I** | **V** | **H** | **N** | **P** | **G** | **L** | **A** | **V** | **L** | **L** | **T** | **I** | **G** | **V** | **L** | **L** | **V** | **A** | **Y** | **V** | **S** | **R** | **K** | **A** | **Y** | **K** | **T** | **H** | **F** | **N** | **N** | **F** | **Q** | **F** | **V** | **L** | **G** | **V** | **V** | **F** | **I** | **V** | **I** | **G** | **A** | **M** | **N** | **S** | **T** | **A** | **V** | **W** | **F** | **M** | **L** | **I** | **F** | **G** | **V** | **L** | **F** | **I** | **G** | **L** | **K** | **G** | **F** | **E** | **I** | **S** | **G** | **V** | **D** | **I** | **A** | **E** | **R** | **A** | **P** | **W** | **R** | **K** | **K** | **Q** | **M** | **I** | **M** | **V** | **E** | **T** | **A** | **A** | **K** | **E** | **P** | **K** | **N** | **G** | **K** | **R** | **F** | **K** | **R** | **R** | **W** | **F** | **A** | **N** | **E** | **R** | **I** | **G** | **N** | **N** | **I** | **Y** | **E** | **W** | **D** | **D** | **I** | **N** | **I** | **D** | **L** | **I** | **S** | **G** | **D** | **T** | **I** | **I** | **D** | **L** | **G** | **N** | **T** | **L** | **L** | **P** | **K** | **E** | **D** | **N** | **I** | **I** | **I** | **I** | **R** | **K** | **G** | **F** | **G** | **R** | **T** | **R** | **I** | **L** | **V** | **P** | **L** | **G** | **V** | **A** | **I** | **L** | **L** | **E** | **H** | **S** | **T** | **F** | **Y** | **G** | **T** | **V** | **R** | **F** | **E** | **E** | **E** | **K** | **Y** | **Q** | **L** | **K** | **N** | **E** | **S** | **L** | **K** | **I** | **Y** | **S** | **N** | **D** | **Y** | **D** | **T** | **N** | **L** | **R** | **R** | **L** | **K** | **I** | **M** | **T** | **N** | **T** | **L** | **V** | **G** | **D** | **V** | **E** | **V** | **I** | **R** | **V** |
| **N59606F** | **M** | **N** | **N** | **P** | **W** | **R** | **F** | **F** | **I** | **V** | **A** | **E** | **A** | **L** | **L** | **F** | **I** | **L** | **A** | **L** | **W** | **Q** | **I** | **V** | **H** | **N** | **P** | **G** | **L** | **A** | **V** | **L** | **L** | **T** | **I** | **G** | **V** | **L** | **L** | **V** | **A** | **Y** | **V** | **S** | **R** | **K** | **A** | **S** | **K** | **T** | **H** | **F** | **N** | **N** | **F** | **Q** | **F** | **V** | **L** | **G** | **V** | **V** | **F** | **I** | **V** | **I** | **G** | **A** | **M** | **N** | **S** | **T** | **A** | **V** | **W** | **F** | **M** | **L** | **I** | **F** | **G** | **V** | **L** | **F** | **I** | **G** | **L** | **K** | **G** | **F** | **E** | **I** | **S** | **G** | **V** | **D** | **I** | **A** | **E** | **R** | **A** | **P** | **W** | **R** | **K** | **K** | **Q** | **M** | **I** | **M** | **V** | **E** | **T** | **A** | **A** | **K** | **E** | **P** | **K** | **N** | **G** | **K** | **R** | **F** | **K** | **R** | **R** | **W** | **F** | **A** | **N** | **E** | **R** | **I** | **G** | **N** | **N** | **I** | **Y** | **E** | **W** | **D** | **D** | **I** | **N** | **I** | **D** | **L** | **I** | **S** | **G** | **D** | **T** | **I** | **I** | **D** | **L** | **G** | **N** | **T** | **L** | **L** | **P** | **K** | **E** | **D** | **N** | **I** | **I** | **I** | **I** | **R** | **K** | **G** | **F** | **G** | **R** | **T** | **R** | **I** | **L** | **V** | **P** | **L** | **G** | **V** | **A** | **I** | **L** | **L** | **E** | **H** | **S** | **T** | **F** | **Y** | **G** | **T** | **V** | **R** | **F** | **E** | **E** | **E** | **K** | **Y** | **Q** | **L** | **K** | **N** | **E** | **S** | **L** | **K** | **I** | **Y** | **S** | **N** | **D** | **Y** | **D** | **T** | **N** | **L** | **R** | **R** | **L** | **K** | **I** | **M** | **T** | **N** | **T** | **L** | **V** | **G** | **D** | **V** | **E** | **V** | **I** | **R** | **V** |
| **N52734** | **M** | **N** | **N** | **P** | **W** | **R** | **F** | **F** | **I** | **V** | **A** | **E** | **A** | **L** | **L** | **F** | **I** | **L** | **A** | **L** | **W** | **Q** | **I** | **V** | **H** | **N** | **P** | **G** | **L** | **A** | **V** | **L** | **L** | **T** | **I** | **G** | **V** | **L** | **L** | **V** | **A** | **Y** | **V** | **S** | **R** | **K** | **A** | **S** | **K** | **T** | **H** | **F** | **N** | **N** | **F** | **Q** | **F** | **V** | **L** | **G** | **V** | **V** | **F** | **I** | **V** | **I** | **G** | **A** | **M** | **N** | **S** | **T** | **A** | **V** | **W** | **F** | **M** | **L** | **I** | **F** | **G** | **V** | **L** | **F** | **I** | **G** | **L** | **K** | **G** | **F** | **E** | **I** | **S** | **G** | **V** | **D** | **I** | **A** | **E** | **R** | **A** | **P** | **W** | **R** | **K** | **K** | **Q** | **M** | **I** | **M** | **V** | **E** | **T** | **A** | **A** | **K** | **E** | **P** | **K** | **N** | **G** | **K** | **R** | **F** | **K** | **R** | **R** | **W** | **F** | **A** | **N** | **E** | **R** | **I** | **G** | **N** | **N** | **I** | **Y** | **E** | **W** | **D** | **D** | **I** | **N** | **I** | **D** | **L** | **I** | **S** | **G** | **D** | **T** | **I** | **I** | **D** | **L** | **G** | **N** | **T** | **L** | **L** | **P** | **K** | **E** | **D** | **N** | **I** | **I** | **I** | **I** | **R** | **K** | **G** | **F** | **G** | **R** | **T** | **R** | **I** | **L** | **V** | **P** | **L** | **G** | **V** | **A** | **I** | **L** | **L** | **E** | **H** | **S** | **T** | **F** | **Y** | **G** | **T** | **V** | **R** | **F** | **E** | **E** | **E** | **K** | **Y** | **Q** | **L** | **K** | **N** | **E** | **S** | **L** | **K** | **I** | **Y** | **S** | **N** | **D** | **Y** | **D** | **T** | **N** | **L** | **R** | **R** | **L** | **K** | **I** | **M** | **T** | **N** | **T** | **L** | **V** | **G** | **D** | **V** | **E** | **V** | **I** | **R** | **V** |
| **N53519** | **M** | **N** | **N** | **P** | **W** | **R** | **F** | **F** | **I** | **V** | **A** | **E** | **A** | **L** | **L** | **F** | **I** | **L** | **A** | **L** | **W** | **Q** | **I** | **V** | **H** | **N** | **P** | **G** | **L** | **A** | **V** | **L** | **L** | **T** | **I** | **G** | **V** | **L** | **L** | **V** | **A** | **Y** | **V** | **S** | **R** | **K** | **A** | **S** | **K** | **T** | **H** | **F** | **N** | **N** | **F** | **Q** | **F** | **V** | **L** | **G** | **V** | **V** | **F** | **I** | **V** | **I** | **G** | **A** | **M** | **N** | **S** | **T** | **A** | **V** | **W** | **F** | **M** | **L** | **I** | **F** | **G** | **V** | **L** | **F** | **I** | **G** | **L** | **K** | **G** | **F** | **E** | **I** | **S** | **G** | **V** | **D** | **I** | **A** | **E** | **R** | **A** | **P** | **W** | **R** | **K** | **K** | **Q** | **M** | **I** | **M** | **V** | **E** | **T** | **A** | **A** | **K** | **E** | **P** | **K** | **N** | **G** | **K** | **R** | **F** | **K** | **R** | **R** | **W** | **F** | **A** | **N** | **E** | **R** | **I** | **G** | **N** | **N** | **I** | **Y** | **E** | **W** | **D** | **D** | **I** | **N** | **I** | **D** | **L** | **I** | **S** | **G** | **D** | **T** | **I** | **I** | **D** | **L** | **G** | **N** | **T** | **L** | **L** | **P** | **K** | **E** | **D** | **N** | **I** | **I** | **I** | **I** | **R** | **K** | **G** | **F** | **G** | **R** | **T** | **R** | **I** | **L** | **V** | **P** | **L** | **G** | **V** | **A** | **I** | **L** | **L** | **E** | **H** | **S** | **T** | **F** | **Y** | **G** | **T** | **V** | **R** | **F** | **E** | **E** | **E** | **K** | **Y** | **Q** | **L** | **K** | **N** | **E** | **S** | **L** | **K** | **I** | **Y** | **S** | **N** | **D** | **Y** | **D** | **T** | **N** | **L** | **R** | **R** | **L** | **K** | **I** | **M** | **T** | **N** | **T** | **L** | **V** | **G** | **D** | **V** | **E** | **V** | **I** | **R** | **V** |
| **N59454F** | **M** | **N** | **N** | **P** | **W** | **R** | **F** | **F** | **I** | **V** | **A** | **E** | **A** | **L** | **L** | **F** | **I** | **L** | **A** | **L** | **W** | **Q** | **I** | **V** | **H** | **N** | **P** | **G** | **L** | **A** | **V** | **L** | **L** | **T** | **I** | **G** | **V** | **L** | **L** | **V** | **A** | **Y** | **V** | **S** | **R** | **K** | **A** | **S** | **K** | **T** | **H** | **F** | **N** | **N** | **F** | **Q** | **F** | **V** | **L** | **G** | **V** | **V** | **F** | **I** | **V** | **I** | **G** | **A** | **M** | **N** | **S** | **T** | **A** | **V** | **W** | **F** | **M** | **L** | **I** | **F** | **G** | **V** | **L** | **F** | **I** | **G** | **L** | **K** | **G** | **F** | **E** | **I** | **S** | **G** | **V** | **D** | **I** | **A** | **E** | **R** | **A** | **P** | **W** | **R** | **K** | **K** | **Q** | **M** | **I** | **M** | **V** | **E** | **T** | **A** | **A** | **K** | **E** | **P** | **K** | **N** | **G** | **K** | **R** | **F** | **K** | **R** | **R** | **W** | **F** | **A** | **N** | **E** | **R** | **I** | **G** | **N** | **N** | **I** | **Y** | **E** | **W** | **D** | **D** | **I** | **N** | **I** | **D** | **L** | **I** | **S** | **G** | **D** | **T** | **I** | **I** | **D** | **L** | **G** | **N** | **T** | **L** | **L** | **P** | **K** | **E** | **D** | **N** | **I** | **I** | **I** | **I** | **R** | **K** | **G** | **F** | **G** | **R** | **T** | **R** | **I** | **L** | **V** | **P** | **L** | **G** | **V** | **A** | **I** | **L** | **L** | **E** | **H** | **S** | **T** | **F** | **Y** | **G** | **T** | **V** | **R** | **F** | **E** | **E** | **E** | **K** | **Y** | **Q** | **L** | **K** | **N** | **E** | **S** | **L** | **K** | **I** | **Y** | **S** | **N** | **D** | **Y** | **D** | **T** | **N** | **L** | **R** | **R** | **L** | **K** | **I** | **M** | **T** | **N** | **T** | **L** | **V** | **G** | **D** | **V** | **E** | **V** | **I** | **R** | **V** |
| **N53453** | **M** | **N** | **N** | **P** | **W** | **R** | **F** | **F** | **I** | **V** | **A** | **E** | **A** | **L** | **L** | **F** | **I** | **L** | **A** | **L** | **W** | **Q** | **I** | **V** | **H** | **N** | **P** | **G** | **L** | **A** | **V** | **L** | **L** | **T** | **I** | **G** | **V** | **L** | **L** | **V** | **A** | **Y** | **V** | **S** | **R** | **K** | **A** | **S** | **K** | **T** | **H** | **F** | **N** | **N** | **F** | **Q** | **F** | **I** | **L** | **G** | **V** | **V** | **F** | **I** | **V** | **I** | **G** | **A** | **M** | **N** | **S** | **T** | **A** | **V** | **W** | **F** | **M** | **L** | **I** | **F** | **G** | **V** | **L** | **F** | **I** | **G** | **L** | **K** | **G** | **F** | **E** | **I** | **S** | **G** | **V** | **D** | **I** | **A** | **E** | **R** | **A** | **P** | **W** | **R** | **K** | **K** | **Q** | **M** | **I** | **M** | **V** | **E** | **T** | **A** | **A** | **K** | **E** | **P** | **K** | **N** | **G** | **K** | **R** | **F** | **K** | **R** | **R** | **W** | **F** | **A** | **N** | **E** | **R** | **I** | **G** | **N** | **N** | **I** | **Y** | **E** | **W** | **D** | **D** | **I** | **N** | **I** | **D** | **L** | **I** | **S** | **G** | **D** | **T** | **I** | **I** | **D** | **L** | **G** | **N** | **T** | **L** | **L** | **P** | **K** | **E** | **D** | **N** | **I** | **I** | **I** | **I** | **R** | **K** | **G** | **F** | **G** | **R** | **T** | **R** | **I** | **L** | **V** | **P** | **L** | **G** | **V** | **A** | **I** | **L** | **L** | **E** | **H** | **S** | **T** | **F** | **Y** | **G** | **T** | **V** | **R** | **F** | **E** | **E** | **E** | **K** | **Y** | **Q** | **L** | **K** | **N** | **E** | **S** | **L** | **K** | **I** | **Y** | **S** | **N** | **D** | **Y** | **D** | **T** | **N** | **L** | **R** | **R** | **L** | **K** | **I** | **M** | **T** | **N** | **T** | **L** | **V** | **G** | **D** | **V** | **E** | **V** | **I** | **R** | **V** |
| **N55338** | **M** | **N** | **N** | **P** | **W** | **R** | **F** | **F** | **I** | **V** | **A** | **E** | **A** | **L** | **L** | **F** | **I** | **L** | **A** | **L** | **W** | **Q** | **I** | **V** | **H** | **N** | **P** | **G** | **L** | **A** | **V** | **L** | **L** | **T** | **I** | **G** | **V** | **L** | **L** | **V** | **A** | **Y** | **V** | **S** | **R** | **K** | **A** | **S** | **K** | **T** | **H** | **F** | **N** | **N** | **F** | **Q** | **F** | **V** | **L** | **G** | **V** | **V** | **F** | **I** | **V** | **I** | **G** | **A** | **M** | **N** | **S** | **T** | **A** | **V** | **W** | **F** | **M** | **L** | **I** | **F** | **G** | **V** | **L** | **F** | **I** | **G** | **L** | **K** | **G** | **F** | **E** | **I** | **S** | **G** | **V** | **D** | **I** | **A** | **E** | **R** | **A** | **P** | **W** | **R** | **K** | **K** | **Q** | **M** | **I** | **M** | **V** | **E** | **T** | **A** | **A** | **K** | **E** | **P** | **K** | **N** | **G** | **K** | **R** | **F** | **K** | **R** | **R** | **W** | **F** | **A** | **N** | **E** | **R** | **I** | **G** | **N** | **N** | **I** | **Y** | **E** | **W** | **D** | **D** | **I** | **N** | **I** | **D** | **L** | **I** | **S** | **G** | **D** | **T** | **I** | **I** | **D** | **L** | **G** | **N** | **T** | **L** | **L** | **P** | **K** | **E** | **D** | **N** | **I** | **I** | **I** | **I** | **R** | **K** | **G** | **F** | **G** | **R** | **T** | **R** | **I** | **L** | **V** | **P** | **L** | **G** | **V** | **A** | **I** | **L** | **L** | **E** | **H** | **S** | **T** | **F** | **Y** | **G** | **T** | **V** | **R** | **F** | **E** | **E** | **E** | **K** | **Y** | **Q** | **L** | **K** | **N** | **E** | **S** | **L** | **K** | **I** | **Y** | **S** | **N** | **D** | **Y** | **D** | **T** | **N** | **L** | **R** | **R** | **L** | **K** | **I** | **M** | **T** | **N** | **T** | **L** | **V** | **G** | **D** | **V** | **E** | **V** | **I** | **R** | **V** |
| **N53405** | **M** | **N** | **N** | **P** | **W** | **R** | **F** | **F** | **I** | **V** | **A** | **E** | **A** | **L** | **L** | **F** | **I** | **L** | **A** | **L** | **W** | **Q** | **I** | **V** | **H** | **N** | **P** | **G** | **L** | **A** | **V** | **L** | **L** | **T** | **I** | **G** | **V** | **L** | **L** | **V** | **A** | **Y** | **V** | **S** | **R** | **K** | **A** | **S** | **K** | **T** | **H** | **F** | **N** | **N** | **F** | **Q** | **F** | **V** | **L** | **G** | **V** | **V** | **F** | **I** | **V** | **I** | **G** | **A** | **M** | **N** | **S** | **T** | **A** | **V** | **W** | **F** | **M** | **L** | **I** | **F** | **G** | **V** | **L** | **F** | **I** | **G** | **L** | **K** | **G** | **F** | **E** | **I** | **S** | **G** | **V** | **D** | **I** | **A** | **E** | **R** | **A** | **P** | **W** | **R** | **K** | **K** | **Q** | **M** | **I** | **M** | **V** | **E** | **T** | **A** | **A** | **K** | **E** | **P** | **K** | **N** | **G** | **K** | **R** | **F** | **K** | **R** | **R** | **W** | **F** | **A** | **N** | **E** | **R** | **I** | **G** | **N** | **N** | **I** | **Y** | **E** | **W** | **D** | **D** | **I** | **N** | **I** | **D** | **L** | **I** | **S** | **G** | **D** | **T** | **I** | **I** | **D** | **L** | **G** | **N** | **T** | **L** | **L** | **P** | **K** | **E** | **D** | **N** | **I** | **I** | **I** | **I** | **R** | **K** | **G** | **F** | **G** | **R** | **T** | **R** | **I** | **L** | **V** | **P** | **L** | **G** | **V** | **A** | **I** | **L** | **L** | **E** | **H** | **S** | **T** | **F** | **Y** | **G** | **T** | **V** | **R** | **F** | **E** | **E** | **E** | **K** | **Y** | **Q** | **L** | **K** | **N** | **E** | **S** | **L** | **K** | **I** | **Y** | **S** | **N** | **D** | **Y** | **D** | **T** | **N** | **L** | **R** | **R** | **L** | **K** | **I** | **M** | **T** | **N** | **T** | **L** | **V** | **G** | **D** | **V** | **E** | **V** | **I** | **R** | **V** |
| **N59646F** | **M** | **N** | **N** | **P** | **W** | **R** | **F** | **F** | **I** | **V** | **A** | **E** | **A** | **L** | **L** | **F** | **I** | **L** | **A** | **L** | **W** | **Q** | **I** | **V** | **H** | **N** | **P** | **G** | **L** | **A** | **V** | **L** | **L** | **T** | **I** | **G** | **V** | **L** | **L** | **V** | **A** | **Y** | **V** | **S** | **R** | **K** | **A** | **S** | **K** | **T** | **H** | **F** | **N** | **N** | **F** | **Q** | **F** | **V** | **L** | **G** | **V** | **V** | **F** | **I** | **V** | **I** | **G** | **A** | **M** | **N** | **S** | **T** | **A** | **V** | **W** | **F** | **M** | **L** | **I** | **F** | **G** | **V** | **L** | **F** | **I** | **G** | **L** | **K** | **G** | **F** | **E** | **I** | **S** | **G** | **V** | **D** | **I** | **A** | **E** | **R** | **A** | **P** | **W** | **R** | **K** | **K** | **Q** | **M** | **I** | **M** | **V** | **E** | **T** | **A** | **A** | **K** | **E** | **P** | **K** | **N** | **G** | **K** | **R** | **F** | **K** | **R** | **R** | **W** | **F** | **A** | **N** | **E** | **R** | **I** | **G** | **N** | **N** | **I** | **Y** | **E** | **W** | **D** | **D** | **I** | **N** | **I** | **D** | **L** | **I** | **S** | **G** | **D** | **T** | **I** | **I** | **D** | **L** | **G** | **N** | **T** | **L** | **L** | **P** | **K** | **E** | **D** | **N** | **I** | **I** | **I** | **I** | **R** | **K** | **G** | **F** | **G** | **R** | **T** | **R** | **I** | **L** | **V** | **P** | **L** | **G** | **V** | **A** | **I** | **L** | **L** | **E** | **H** | **S** | **T** | **F** | **Y** | **G** | **T** | **V** | **R** | **F** | **E** | **E** | **E** | **K** | **Y** | **Q** | **L** | **K** | **N** | **E** | **S** | **L** | **K** | **I** | **Y** | **S** | **N** | **D** | **Y** | **D** | **T** | **N** | **L** | **R** | **R** | **L** | **K** | **I** | **M** | **T** | **N** | **T** | **L** | **V** | **G** | **D** | **V** | **E** | **V** | **I** | **R** | **V** |
| **N59609F** | **M** | **N** | **N** | **P** | **W** | **R** | **F** | **F** | **I** | **V** | **A** | **E** | **A** | **L** | **L** | **F** | **I** | **L** | **A** | **L** | **W** | **Q** | **I** | **V** | **H** | **N** | **P** | **G** | **L** | **A** | **V** | **L** | **L** | **T** | **I** | **G** | **V** | **L** | **L** | **V** | **A** | **Y** | **V** | **S** | **R** | **K** | **A** | **S** | **K** | **T** | **H** | **F** | **N** | **N** | **F** | **Q** | **F** | **V** | **L** | **G** | **V** | **V** | **F** | **I** | **V** | **I** | **G** | **A** | **M** | **N** | **S** | **T** | **A** | **V** | **W** | **F** | **M** | **L** | **I** | **F** | **G** | **V** | **L** | **F** | **I** | **G** | **L** | **K** | **G** | **F** | **E** | **I** | **S** | **G** | **V** | **D** | **I** | **A** | **E** | **R** | **A** | **P** | **W** | **R** | **K** | **K** | **Q** | **M** | **I** | **M** | **V** | **E** | **T** | **A** | **A** | **K** | **E** | **P** | **K** | **N** | **G** | **K** | **R** | **F** | **K** | **R** | **R** | **W** | **F** | **A** | **N** | **E** | **R** | **I** | **G** | **N** | **N** | **I** | **Y** | **E** | **W** | **D** | **D** | **I** | **N** | **I** | **D** | **L** | **I** | **S** | **G** | **D** | **T** | **I** | **I** | **D** | **L** | **G** | **N** | **T** | **L** | **L** | **P** | **K** | **E** | **D** | **N** | **I** | **I** | **I** | **I** | **R** | **K** | **G** | **F** | **G** | **R** | **T** | **R** | **I** | **L** | **V** | **P** | **L** | **G** | **V** | **A** | **I** | **L** | **L** | **E** | **H** | **S** | **T** | **F** | **Y** | **G** | **T** | **V** | **R** | **F** | **E** | **E** | **E** | **K** | **Y** | **Q** | **L** | **K** | **N** | **E** | **S** | **L** | **K** | **I** | **Y** | **S** | **N** | **D** | **Y** | **D** | **T** | **N** | **L** | **R** | **R** | **L** | **K** | **I** | **M** | **T** | **N** | **T** | **L** | **V** | **G** | **D** | **V** | **E** | **V** | **I** | **R** | **V** |
| **N59466F** | **M** | **N** | **N** | **P** | **W** | **R** | **F** | **F** | **I** | **V** | **A** | **E** | **A** | **L** | **L** | **F** | **I** | **L** | **A** | **L** | **W** | **Q** | **I** | **V** | **H** | **N** | **P** | **G** | **L** | **A** | **V** | **L** | **L** | **T** | **I** | **G** | **V** | **L** | **L** | **V** | **A** | **Y** | **V** | **S** | **R** | **K** | **A** | **S** | **K** | **T** | **H** | **F** | **N** | **N** | **F** | **Q** | **F** | **V** | **L** | **G** | **V** | **V** | **F** | **I** | **V** | **I** | **G** | **A** | **M** | **N** | **S** | **T** | **A** | **V** | **W** | **F** | **M** | **L** | **I** | **F** | **G** | **V** | **L** | **F** | **I** | **G** | **L** | **K** | **G** | **F** | **E** | **I** | **S** | **G** | **V** | **D** | **I** | **A** | **E** | **R** | **A** | **P** | **W** | **R** | **K** | **K** | **Q** | **M** | **I** | **M** | **V** | **E** | **T** | **A** | **A** | **K** | **E** | **P** | **K** | **N** | **G** | **K** | **R** | **F** | **K** | **R** | **R** | **W** | **F** | **A** | **N** | **E** | **R** | **I** | **G** | **N** | **N** | **I** | **Y** | **E** | **W** | **D** | **D** | **I** | **N** | **I** | **D** | **L** | **I** | **S** | **G** | **D** | **T** | **I** | **I** | **D** | **L** | **G** | **N** | **T** | **L** | **L** | **P** | **K** | **E** | **D** | **N** | **I** | **I** | **I** | **I** | **R** | **K** | **G** | **F** | **G** | **R** | **T** | **R** | **I** | **L** | **V** | **P** | **L** | **G** | **V** | **A** | **I** | **L** | **L** | **E** | **H** | **S** | **T** | **F** | **Y** | **G** | **T** | **V** | **R** | **F** | **E** | **E** | **E** | **K** | **Y** | **Q** | **L** | **K** | **N** | **E** | **S** | **L** | **K** | **I** | **Y** | **S** | **N** | **D** | **Y** | **D** | **T** | **N** | **L** | **R** | **R** | **L** | **K** | **I** | **M** | **T** | **N** | **T** | **L** | **V** | **G** | **D** | **V** | **E** | **V** | **I** | **R** | **V** |
| **N60271F** | **M** | **N** | **N** | **P** | **W** | **R** | **F** | **F** | **I** | **V** | **A** | **E** | **A** | **L** | **L** | **F** | **I** | **L** | **A** | **L** | **W** | **Q** | **I** | **V** | **H** | **N** | **P** | **G** | **L** | **A** | **V** | **L** | **L** | **T** | **I** | **G** | **V** | **L** | **L** | **V** | **A** | **Y** | **V** | **S** | **R** | **K** | **A** | **S** | **K** | **T** | **H** | **F** | **N** | **N** | **F** | **Q** | **F** | **V** | **L** | **G** | **V** | **V** | **F** | **I** | **V** | **I** | **G** | **A** | **M** | **N** | **S** | **T** | **A** | **V** | **W** | **F** | **M** | **L** | **I** | **F** | **G** | **V** | **L** | **F** | **I** | **G** | **L** | **K** | **G** | **F** | **E** | **I** | **S** | **G** | **V** | **D** | **I** | **A** | **E** | **R** | **A** | **P** | **W** | **R** | **K** | **K** | **Q** | **M** | **I** | **M** | **V** | **E** | **T** | **A** | **A** | **K** | **E** | **P** | **K** | **N** | **G** | **K** | **R** | **F** | **K** | **R** | **R** | **W** | **F** | **A** | **N** | **E** | **R** | **I** | **G** | **N** | **N** | **I** | **Y** | **E** | **W** | **D** | **D** | **I** | **N** | **I** | **D** | **L** | **I** | **S** | **G** | **D** | **T** | **I** | **I** | **D** | **L** | **G** | **N** | **T** | **L** | **L** | **P** | **K** | **E** | **D** | **N** | **I** | **I** | **I** | **I** | **R** | **K** | **G** | **F** | **G** | **R** | **T** | **R** | **I** | **L** | **V** | **P** | **L** | **G** | **V** | **A** | **I** | **L** | **L** | **E** | **H** | **S** | **T** | **F** | **Y** | **G** | **T** | **V** | **R** | **F** | **E** | **E** | **E** | **K** | **Y** | **Q** | **L** | **K** | **N** | **E** | **S** | **L** | **K** | **I** | **Y** | **S** | **N** | **D** | **Y** | **D** | **T** | **N** | **L** | **R** | **R** | **L** | **K** | **I** | **M** | **T** | **N** | **T** | **L** | **V** | **G** | **D** | **V** | **E** | **V** | **I** | **R** | **V** |
| **N60034F** | **M** | **N** | **N** | **P** | **W** | **R** | **F** | **F** | **I** | **V** | **A** | **E** | **A** | **L** | **L** | **F** | **I** | **L** | **A** | **L** | **W** | **Q** | **I** | **V** | **H** | **N** | **P** | **G** | **L** | **A** | **V** | **L** | **L** | **T** | **I** | **G** | **V** | **L** | **L** | **V** | **A** | **Y** | **V** | **S** | **R** | **K** | **A** | **S** | **K** | **T** | **H** | **F** | **N** | **N** | **F** | **Q** | **F** | **V** | **L** | **G** | **V** | **V** | **F** | **I** | **V** | **I** | **G** | **A** | **M** | **N** | **S** | **T** | **A** | **V** | **W** | **F** | **M** | **L** | **I** | **F** | **G** | **V** | **L** | **F** | **I** | **G** | **L** | **K** | **G** | **F** | **E** | **I** | **S** | **G** | **V** | **D** | **I** | **A** | **E** | **R** | **A** | **P** | **W** | **R** | **K** | **K** | **Q** | **M** | **I** | **M** | **V** | **E** | **T** | **A** | **A** | **K** | **E** | **P** | **K** | **N** | **G** | **K** | **R** | **F** | **K** | **R** | **R** | **W** | **F** | **A** | **N** | **E** | **R** | **I** | **G** | **N** | **N** | **I** | **Y** | **E** | **W** | **D** | **D** | **I** | **N** | **I** | **D** | **L** | **I** | **S** | **G** | **D** | **T** | **I** | **I** | **D** | **L** | **G** | **N** | **T** | **L** | **L** | **P** | **K** | **E** | **D** | **N** | **I** | **I** | **I** | **I** | **R** | **K** | **G** | **F** | **G** | **R** | **T** | **R** | **I** | **L** | **V** | **P** | **L** | **G** | **V** | **A** | **I** | **L** | **L** | **E** | **H** | **S** | **T** | **F** | **Y** | **G** | **T** | **V** | **R** | **F** | **E** | **E** | **E** | **K** | **Y** | **Q** | **L** | **K** | **N** | **E** | **S** | **L** | **K** | **I** | **Y** | **S** | **N** | **D** | **Y** | **D** | **T** | **N** | **L** | **R** | **R** | **L** | **K** | **I** | **M** | **T** | **N** | **T** | **L** | **V** | **G** | **D** | **V** | **E** | **V** | **I** | **R** | **V** |
| **N55724** | **M** | **N** | **N** | **P** | **W** | **R** | **F** | **F** | **I** | **V** | **A** | **E** | **A** | **L** | **L** | **F** | **I** | **L** | **A** | **L** | **W** | **Q** | **I** | **V** | **H** | **N** | **P** | **G** | **L** | **A** | **V** | **L** | **L** | **T** | **I** | **G** | **V** | **L** | **L** | **V** | **A** | **Y** | **V** | **S** | **R** | **K** | **A** | **S** | **K** | **T** | **H** | **F** | **N** | **N** | **F** | **Q** | **F** | **V** | **L** | **G** | **V** | **V** | **F** | **I** | **V** | **I** | **G** | **A** | **M** | **N** | **S** | **T** | **A** | **V** | **W** | **F** | **M** | **L** | **I** | **F** | **G** | **V** | **L** | **F** | **I** | **G** | **L** | **K** | **G** | **F** | **E** | **I** | **S** | **G** | **V** | **D** | **I** | **A** | **E** | **R** | **A** | **P** | **W** | **R** | **K** | **K** | **Q** | **M** | **I** | **M** | **V** | **E** | **T** | **A** | **A** | **K** | **E** | **P** | **K** | **N** | **G** | **K** | **R** | **F** | **K** | **R** | **R** | **W** | **F** | **A** | **N** | **E** | **R** | **I** | **G** | **N** | **N** | **I** | **Y** | **E** | **W** | **D** | **D** | **I** | **N** | **I** | **D** | **L** | **I** | **S** | **G** | **D** | **T** | **I** | **I** | **D** | **L** | **G** | **N** | **T** | **L** | **L** | **P** | **K** | **E** | **D** | **N** | **I** | **I** | **I** | **I** | **R** | **K** | **G** | **F** | **G** | **R** | **T** | **R** | **I** | **L** | **V** | **P** | **L** | **G** | **V** | **A** | **I** | **L** | **L** | **E** | **H** | **S** | **T** | **F** | **Y** | **G** | **T** | **V** | **R** | **F** | **E** | **E** | **E** | **K** | **Y** | **Q** | **L** | **K** | **N** | **E** | **S** | **L** | **K** | **I** | **Y** | **S** | **N** | **D** | **Y** | **D** | **T** | **N** | **L** | **R** | **R** | **L** | **K** | **I** | **M** | **T** | **N** | **T** | **L** | **V** | **G** | **D** | **V** | **E** | **V** | **I** | **R** | **V** |
| **N59689F** | **M** | **N** | **N** | **P** | **W** | **R** | **F** | **F** | **I** | **V** | **A** | **E** | **A** | **L** | **L** | **F** | **I** | **L** | **A** | **L** | **W** | **Q** | **I** | **V** | **H** | **N** | **P** | **G** | **L** | **A** | **V** | **L** | **L** | **T** | **I** | **G** | **V** | **L** | **L** | **V** | **A** | **Y** | **V** | **S** | **R** | **K** | **A** | **S** | **K** | **T** | **H** | **F** | **N** | **N** | **F** | **Q** | **F** | **V** | **L** | **G** | **V** | **V** | **F** | **I** | **V** | **I** | **G** | **A** | **M** | **N** | **S** | **T** | **A** | **V** | **W** | **F** | **M** | **L** | **I** | **F** | **G** | **V** | **L** | **F** | **I** | **G** | **L** | **K** | **G** | **F** | **E** | **I** | **S** | **G** | **V** | **D** | **I** | **A** | **E** | **R** | **A** | **P** | **W** | **R** | **K** | **K** | **Q** | **M** | **I** | **M** | **V** | **E** | **T** | **A** | **A** | **K** | **E** | **P** | **K** | **N** | **G** | **K** | **R** | **F** | **K** | **R** | **R** | **W** | **F** | **A** | **N** | **E** | **R** | **I** | **G** | **N** | **N** | **I** | **Y** | **E** | **W** | **D** | **D** | **I** | **N** | **I** | **D** | **L** | **I** | **S** | **G** | **D** | **T** | **I** | **I** | **D** | **L** | **G** | **N** | **T** | **L** | **L** | **P** | **K** | **E** | **D** | **N** | **I** | **I** | **I** | **I** | **R** | **K** | **G** | **F** | **G** | **R** | **T** | **R** | **I** | **L** | **V** | **P** | **L** | **G** | **V** | **A** | **I** | **L** | **L** | **E** | **H** | **S** | **T** | **F** | **Y** | **G** | **T** | **V** | **R** | **F** | **E** | **E** | **E** | **K** | **Y** | **Q** | **L** | **K** | **N** | **E** | **S** | **L** | **K** | **I** | **Y** | **S** | **N** | **D** | **Y** | **D** | **T** | **N** | **L** | **R** | **R** | **L** | **K** | **I** | **M** | **T** | **N** | **T** | **L** | **V** | **G** | **D** | **V** | **E** | **V** | **I** | **R** | **V** |
| **N52623** | **M** | **N** | **N** | **P** | **W** | **R** | **F** | **F** | **I** | **V** | **A** | **E** | **A** | **L** | **L** | **F** | **I** | **L** | **A** | **L** | **W** | **Q** | **I** | **V** | **H** | **N** | **P** | **G** | **L** | **A** | **V** | **L** | **L** | **T** | **I** | **G** | **V** | **L** | **L** | **V** | **A** | **Y** | **V** | **S** | **R** | **K** | **A** | **S** | **K** | **T** | **H** | **F** | **N** | **N** | **F** | **Q** | **F** | **V** | **L** | **G** | **V** | **V** | **F** | **I** | **V** | **I** | **G** | **A** | **M** | **N** | **S** | **T** | **A** | **V** | **W** | **F** | **M** | **L** | **I** | **F** | **G** | **V** | **L** | **F** | **I** | **G** | **L** | **K** | **G** | **F** | **E** | **I** | **S** | **G** | **V** | **D** | **I** | **A** | **E** | **R** | **A** | **P** | **W** | **R** | **K** | **K** | **Q** | **M** | **I** | **M** | **V** | **E** | **T** | **A** | **A** | **K** | **E** | **P** | **K** | **N** | **G** | **K** | **R** | **F** | **K** | **R** | **R** | **W** | **F** | **A** | **N** | **E** | **R** | **I** | **G** | **N** | **N** | **I** | **Y** | **E** | **W** | **D** | **D** | **I** | **N** | **I** | **D** | **L** | **I** | **S** | **G** | **D** | **T** | **I** | **I** | **D** | **L** | **G** | **N** | **T** | **L** | **L** | **P** | **K** | **E** | **D** | **N** | **I** | **I** | **I** | **I** | **R** | **K** | **G** | **F** | **G** | **R** | **T** | **R** | **I** | **L** | **V** | **P** | **L** | **G** | **V** | **A** | **I** | **L** | **L** | **E** | **H** | **S** | **T** | **F** | **Y** | **G** | **T** | **V** | **R** | **F** | **E** | **E** | **E** | **K** | **Y** | **Q** | **L** | **K** | **N** | **E** | **S** | **L** | **K** | **I** | **Y** | **S** | **N** | **D** | **Y** | **D** | **T** | **N** | **L** | **R** | **R** | **L** | **K** | **I** | **M** | **T** | **N** | **T** | **L** | **V** | **G** | **D** | **V** | **E** | **V** | **I** | **R** | **V** |
| **N53761** | **M** | **N** | **N** | **P** | **W** | **R** | **F** | **F** | **I** | **V** | **A** | **E** | **A** | **L** | **L** | **F** | **I** | **L** | **A** | **L** | **W** | **Q** | **I** | **V** | **H** | **N** | **P** | **G** | **L** | **A** | **V** | **L** | **L** | **T** | **I** | **G** | **V** | **L** | **L** | **V** | **A** | **Y** | **V** | **S** | **R** | **K** | **A** | **S** | **K** | **T** | **H** | **F** | **N** | **N** | **F** | **Q** | **F** | **V** | **L** | **G** | **V** | **V** | **F** | **I** | **V** | **I** | **G** | **A** | **M** | **N** | **S** | **T** | **A** | **V** | **W** | **F** | **M** | **L** | **I** | **F** | **G** | **V** | **L** | **F** | **I** | **G** | **L** | **K** | **G** | **F** | **E** | **I** | **S** | **G** | **V** | **D** | **I** | **A** | **E** | **R** | **A** | **P** | **W** | **R** | **K** | **K** | **Q** | **M** | **I** | **M** | **V** | **E** | **T** | **A** | **A** | **K** | **E** | **P** | **K** | **N** | **G** | **K** | **R** | **F** | **K** | **R** | **R** | **W** | **F** | **A** | **N** | **E** | **R** | **I** | **G** | **N** | **N** | **I** | **Y** | **E** | **W** | **D** | **D** | **I** | **N** | **I** | **D** | **L** | **I** | **S** | **G** | **D** | **T** | **I** | **I** | **D** | **L** | **G** | **N** | **T** | **L** | **L** | **P** | **K** | **E** | **D** | **N** | **I** | **I** | **I** | **I** | **R** | **K** | **G** | **F** | **G** | **R** | **T** | **R** | **I** | **L** | **V** | **P** | **L** | **G** | **V** | **A** | **I** | **L** | **L** | **E** | **H** | **S** | **T** | **F** | **Y** | **G** | **T** | **V** | **R** | **F** | **E** | **E** | **E** | **K** | **Y** | **Q** | **L** | **K** | **N** | **E** | **S** | **L** | **K** | **I** | **Y** | **S** | **N** | **D** | **Y** | **D** | **T** | **N** | **L** | **R** | **R** | **L** | **K** | **I** | **M** | **T** | **N** | **T** | **L** | **V** | **G** | **D** | **V** | **E** | **V** | **I** | **R** | **V** |
| **N54156** | **M** | **N** | **N** | **P** | **W** | **R** | **F** | **F** | **I** | **V** | **A** | **E** | **A** | **L** | **L** | **F** | **I** | **L** | **A** | **L** | **W** | **Q** | **I** | **V** | **H** | **N** | **P** | **G** | **L** | **A** | **V** | **L** | **L** | **T** | **I** | **G** | **V** | **L** | **L** | **V** | **A** | **Y** | **V** | **S** | **R** | **K** | **A** | **S** | **K** | **T** | **H** | **F** | **N** | **N** | **F** | **Q** | **F** | **V** | **L** | **G** | **V** | **V** | **F** | **I** | **V** | **I** | **G** | **A** | **M** | **N** | **S** | **T** | **A** | **V** | **W** | **F** | **M** | **L** | **I** | **F** | **G** | **V** | **L** | **F** | **I** | **G** | **L** | **K** | **G** | **F** | **E** | **I** | **S** | **G** | **V** | **D** | **I** | **A** | **E** | **R** | **A** | **P** | **W** | **R** | **K** | **K** | **Q** | **M** | **I** | **M** | **V** | **E** | **T** | **A** | **A** | **K** | **E** | **P** | **K** | **N** | **G** | **K** | **R** | **F** | **K** | **R** | **R** | **W** | **F** | **A** | **N** | **E** | **R** | **I** | **G** | **N** | **N** | **I** | **Y** | **E** | **W** | **D** | **D** | **I** | **N** | **I** | **D** | **L** | **I** | **S** | **G** | **D** | **T** | **I** | **I** | **D** | **L** | **G** | **N** | **T** | **L** | **L** | **P** | **K** | **E** | **D** | **N** | **I** | **I** | **I** | **I** | **R** | **K** | **G** | **F** | **G** | **R** | **T** | **R** | **I** | **L** | **V** | **P** | **L** | **G** | **V** | **A** | **I** | **L** | **L** | **E** | **H** | **S** | **T** | **F** | **Y** | **G** | **T** | **V** | **R** | **F** | **E** | **E** | **E** | **K** | **Y** | **Q** | **L** | **K** | **N** | **E** | **S** | **L** | **K** | **I** | **Y** | **S** | **N** | **D** | **Y** | **D** | **T** | **N** | **L** | **R** | **R** | **L** | **K** | **I** | **M** | **T** | **N** | **T** | **L** | **V** | **G** | **D** | **V** | **E** | **V** | **I** | **R** | **V** |
| **N52724** | **M** | **N** | **N** | **P** | **W** | **R** | **F** | **F** | **I** | **V** | **A** | **E** | **A** | **L** | **L** | **F** | **I** | **L** | **A** | **L** | **W** | **Q** | **I** | **V** | **H** | **N** | **P** | **G** | **L** | **A** | **V** | **L** | **L** | **T** | **I** | **G** | **V** | **L** | **L** | **V** | **A** | **Y** | **V** | **S** | **R** | **K** | **A** | **S** | **K** | **T** | **H** | **F** | **N** | **N** | **F** | **Q** | **F** | **V** | **L** | **G** | **V** | **V** | **F** | **I** | **V** | **I** | **G** | **A** | **M** | **N** | **S** | **T** | **A** | **V** | **W** | **F** | **M** | **L** | **I** | **F** | **G** | **V** | **L** | **F** | **I** | **G** | **L** | **K** | **G** | **F** | **E** | **I** | **S** | **G** | **V** | **D** | **I** | **A** | **E** | **R** | **A** | **P** | **W** | **R** | **K** | **K** | **Q** | **M** | **I** | **M** | **V** | **E** | **T** | **A** | **A** | **K** | **E** | **P** | **K** | **N** | **G** | **K** | **R** | **F** | **K** | **R** | **R** | **W** | **F** | **A** | **N** | **E** | **R** | **I** | **G** | **N** | **N** | **I** | **Y** | **E** | **W** | **D** | **D** | **I** | **N** | **I** | **D** | **L** | **I** | **S** | **G** | **D** | **T** | **I** | **I** | **D** | **L** | **G** | **N** | **T** | **L** | **L** | **P** | **K** | **E** | **D** | **N** | **I** | **I** | **I** | **I** | **R** | **K** | **G** | **F** | **G** | **R** | **T** | **R** | **I** | **L** | **V** | **P** | **L** | **G** | **V** | **A** | **I** | **L** | **L** | **E** | **H** | **S** | **T** | **F** | **Y** | **G** | **T** | **V** | **R** | **F** | **E** | **E** | **E** | **K** | **Y** | **Q** | **L** | **K** | **N** | **E** | **S** | **L** | **K** | **I** | **Y** | **S** | **N** | **D** | **Y** | **D** | **T** | **N** | **L** | **R** | **R** | **L** | **K** | **I** | **M** | **T** | **N** | **T** | **L** | **V** | **G** | **D** | **V** | **E** | **V** | **I** | **R** | **V** |
| **N60063F** | **M** | **N** | **N** | **P** | **W** | **R** | **F** | **F** | **I** | **V** | **A** | **E** | **A** | **L** | **L** | **F** | **I** | **L** | **A** | **L** | **W** | **Q** | **I** | **V** | **H** | **N** | **P** | **G** | **L** | **A** | **V** | **L** | **L** | **T** | **I** | **G** | **V** | **L** | **L** | **V** | **A** | **Y** | **V** | **S** | **R** | **K** | **A** | **S** | **K** | **T** | **H** | **F** | **N** | **N** | **F** | **Q** | **F** | **V** | **L** | **G** | **V** | **V** | **F** | **I** | **V** | **I** | **G** | **A** | **M** | **N** | **S** | **T** | **A** | **V** | **W** | **F** | **M** | **L** | **I** | **F** | **G** | **I** | **L** | **F** | **I** | **G** | **L** | **K** | **G** | **F** | **E** | **I** | **S** | **G** | **V** | **D** | **I** | **A** | **E** | **R** | **A** | **P** | **W** | **R** | **K** | **K** | **Q** | **M** | **I** | **M** | **V** | **E** | **T** | **A** | **A** | **K** | **E** | **P** | **K** | **N** | **G** | **K** | **R** | **F** | **K** | **R** | **R** | **W** | **F** | **A** | **N** | **E** | **R** | **I** | **G** | **N** | **N** | **I** | **Y** | **E** | **W** | **D** | **D** | **I** | **N** | **I** | **D** | **L** | **I** | **S** | **G** | **D** | **T** | **I** | **I** | **D** | **L** | **G** | **N** | **T** | **L** | **L** | **P** | **K** | **E** | **D** | **N** | **I** | **I** | **I** | **I** | **R** | **K** | **G** | **F** | **G** | **R** | **T** | **R** | **I** | **L** | **V** | **P** | **L** | **G** | **V** | **A** | **I** | **L** | **L** | **E** | **H** | **S** | **T** | **F** | **Y** | **G** | **T** | **V** | **R** | **F** | **E** | **E** | **E** | **K** | **Y** | **Q** | **L** | **K** | **N** | **E** | **S** | **L** | **K** | **I** | **Y** | **S** | **N** | **D** | **Y** | **D** | **T** | **N** | **L** | **R** | **R** | **L** | **K** | **I** | **M** | **T** | **N** | **T** | **L** | **V** | **G** | **D** | **V** | **E** | **V** | **I** | **R** | **V** |
| **N54548** | **-** | **-** | **-** | **P** | **W** | **R** | **F** | **F** | **I** | **V** | **A** | **E** | **A** | **L** | **L** | **F** | **I** | **L** | **A** | **L** | **W** | **Q** | **I** | **V** | **H** | **N** | **P** | **G** | **L** | **A** | **V** | **L** | **L** | **T** | **I** | **G** | **V** | **L** | **L** | **V** | **A** | **Y** | **V** | **S** | **R** | **K** | **A** | **S** | **K** | **T** | **H** | **F** | **N** | **N** | **F** | **Q** | **F** | **V** | **L** | **G** | **I** | **V** | **F** | **I** | **V** | **I** | **G** | **A** | **M** | **N** | **S** | **T** | **A** | **V** | **W** | **F** | **M** | **L** | **I** | **F** | **G** | **V** | **L** | **F** | **I** | **G** | **L** | **K** | **G** | **F** | **E** | **I** | **S** | **G** | **V** | **D** | **I** | **A** | **E** | **R** | **A** | **P** | **W** | **R** | **K** | **K** | **Q** | **M** | **I** | **M** | **V** | **E** | **T** | **A** | **A** | **K** | **E** | **P** | **K** | **N** | **G** | **K** | **R** | **F** | **K** | **R** | **R** | **W** | **F** | **A** | **N** | **E** | **R** | **I** | **G** | **N** | **N** | **I** | **Y** | **E** | **W** | **D** | **D** | **I** | **N** | **I** | **D** | **L** | **I** | **S** | **G** | **D** | **T** | **I** | **I** | **D** | **L** | **G** | **N** | **T** | **L** | **L** | **P** | **K** | **E** | **D** | **N** | **I** | **I** | **I** | **I** | **R** | **K** | **G** | **F** | **G** | **R** | **T** | **R** | **I** | **L** | **V** | **P** | **L** | **G** | **V** | **A** | **I** | **L** | **L** | **E** | **H** | **S** | **T** | **F** | **Y** | **G** | **T** | **V** | **R** | **F** | **E** | **E** | **E** | **K** | **Y** | **Q** | **L** | **K** | **N** | **E** | **S** | **L** | **K** | **I** | **Y** | **S** | **N** | **D** | **Y** | **D** | **T** | **N** | **L** | **R** | **R** | **L** | **K** | **I** | **M** | **T** | **N** | **T** | **L** | **V** | **G** | **D** | **V** | **E** | **V** | **I** | **R** | **V** |
| **N59959F** | **M** | **N** | **N** | **P** | **W** | **R** | **F** | **F** | **I** | **V** | **A** | **E** | **A** | **L** | **L** | **F** | **I** | **L** | **A** | **L** | **W** | **Q** | **I** | **V** | **H** | **N** | **P** | **G** | **L** | **A** | **V** | **L** | **L** | **T** | **I** | **G** | **V** | **L** | **L** | **V** | **A** | **Y** | **V** | **S** | **R** | **K** | **A** | **S** | **K** | **T** | **H** | **F** | **N** | **N** | **F** | **Q** | **F** | **V** | **L** | **G** | **V** | **V** | **F** | **I** | **V** | **I** | **G** | **A** | **M** | **N** | **S** | **T** | **A** | **V** | **W** | **F** | **M** | **L** | **I** | **F** | **G** | **V** | **L** | **F** | **I** | **G** | **L** | **K** | **G** | **F** | **E** | **I** | **S** | **G** | **V** | **D** | **I** | **A** | **E** | **R** | **A** | **P** | **W** | **R** | **K** | **K** | **Q** | **M** | **I** | **M** | **V** | **E** | **T** | **A** | **A** | **K** | **E** | **P** | **K** | **N** | **G** | **K** | **R** | **F** | **K** | **R** | **R** | **W** | **F** | **A** | **N** | **E** | **R** | **I** | **G** | **N** | **N** | **I** | **Y** | **E** | **W** | **D** | **D** | **I** | **N** | **I** | **D** | **L** | **I** | **S** | **G** | **D** | **T** | **I** | **I** | **D** | **L** | **G** | **N** | **T** | **L** | **L** | **P** | **K** | **E** | **D** | **N** | **I** | **I** | **I** | **I** | **R** | **K** | **G** | **F** | **G** | **R** | **T** | **R** | **I** | **L** | **V** | **P** | **L** | **G** | **V** | **A** | **I** | **L** | **L** | **E** | **H** | **S** | **T** | **F** | **Y** | **G** | **T** | **V** | **R** | **F** | **E** | **E** | **E** | **K** | **Y** | **Q** | **L** | **K** | **N** | **E** | **S** | **L** | **K** | **I** | **Y** | **S** | **N** | **D** | **Y** | **D** | **T** | **N** | **L** | **R** | **R** | **L** | **K** | **I** | **M** | **T** | **N** | **T** | **L** | **V** | **G** | **D** | **V** | **E** | **V** | **I** | **R** | **V** |
| **N59592F** | **M** | **N** | **N** | **P** | **W** | **R** | **F** | **F** | **I** | **V** | **A** | **E** | **A** | **L** | **L** | **F** | **I** | **L** | **A** | **L** | **W** | **Q** | **I** | **V** | **H** | **N** | **P** | **G** | **L** | **A** | **V** | **L** | **L** | **T** | **I** | **G** | **V** | **L** | **L** | **V** | **A** | **Y** | **V** | **S** | **R** | **K** | **A** | **S** | **K** | **T** | **H** | **F** | **N** | **N** | **F** | **Q** | **F** | **V** | **L** | **G** | **V** | **V** | **F** | **I** | **V** | **I** | **G** | **A** | **M** | **N** | **S** | **T** | **A** | **V** | **W** | **F** | **M** | **L** | **I** | **F** | **G** | **V** | **L** | **F** | **I** | **G** | **L** | **K** | **G** | **F** | **E** | **I** | **S** | **G** | **V** | **D** | **I** | **A** | **E** | **R** | **A** | **P** | **W** | **R** | **K** | **K** | **Q** | **M** | **I** | **M** | **V** | **E** | **T** | **A** | **A** | **K** | **E** | **P** | **K** | **N** | **G** | **K** | **R** | **F** | **K** | **R** | **R** | **W** | **F** | **A** | **N** | **E** | **R** | **I** | **G** | **N** | **N** | **I** | **Y** | **E** | **W** | **D** | **D** | **I** | **N** | **I** | **D** | **L** | **I** | **S** | **G** | **D** | **T** | **I** | **I** | **D** | **L** | **G** | **N** | **T** | **L** | **L** | **P** | **K** | **E** | **D** | **N** | **I** | **I** | **I** | **I** | **R** | **K** | **G** | **F** | **G** | **R** | **T** | **R** | **I** | **L** | **V** | **P** | **L** | **G** | **V** | **A** | **I** | **L** | **L** | **E** | **H** | **S** | **T** | **F** | **Y** | **G** | **T** | **V** | **R** | **F** | **E** | **E** | **E** | **K** | **Y** | **Q** | **L** | **K** | **N** | **E** | **S** | **L** | **K** | **I** | **Y** | **S** | **N** | **D** | **Y** | **D** | **T** | **N** | **L** | **R** | **R** | **L** | **K** | **I** | **M** | **T** | **N** | **T** | **L** | **V** | **G** | **D** | **V** | **E** | **V** | **I** | **R** | **V** |
| **N59611F** | **M** | **N** | **N** | **P** | **W** | **R** | **F** | **F** | **I** | **V** | **A** | **E** | **A** | **L** | **L** | **F** | **I** | **L** | **A** | **L** | **W** | **Q** | **I** | **V** | **H** | **N** | **P** | **G** | **L** | **A** | **V** | **L** | **L** | **T** | **I** | **G** | **V** | **L** | **L** | **V** | **A** | **Y** | **V** | **S** | **R** | **K** | **A** | **S** | **K** | **T** | **H** | **F** | **N** | **N** | **F** | **Q** | **F** | **V** | **L** | **G** | **V** | **V** | **F** | **I** | **V** | **I** | **G** | **A** | **M** | **N** | **S** | **T** | **A** | **V** | **W** | **F** | **M** | **L** | **I** | **F** | **G** | **V** | **L** | **F** | **I** | **G** | **L** | **K** | **G** | **F** | **E** | **I** | **S** | **G** | **V** | **D** | **I** | **A** | **E** | **R** | **A** | **P** | **W** | **R** | **K** | **K** | **Q** | **M** | **I** | **M** | **V** | **E** | **T** | **A** | **A** | **K** | **E** | **P** | **K** | **N** | **G** | **K** | **R** | **F** | **K** | **R** | **R** | **W** | **F** | **A** | **N** | **E** | **R** | **I** | **G** | **N** | **N** | **I** | **Y** | **E** | **W** | **D** | **D** | **I** | **N** | **I** | **D** | **L** | **I** | **S** | **G** | **D** | **T** | **I** | **I** | **D** | **L** | **G** | **N** | **T** | **L** | **L** | **P** | **K** | **E** | **D** | **N** | **I** | **I** | **I** | **I** | **R** | **K** | **G** | **F** | **G** | **R** | **T** | **R** | **I** | **L** | **V** | **P** | **L** | **G** | **V** | **A** | **I** | **L** | **L** | **E** | **H** | **S** | **T** | **F** | **Y** | **G** | **T** | **V** | **R** | **F** | **E** | **E** | **E** | **K** | **Y** | **Q** | **L** | **K** | **N** | **E** | **S** | **L** | **K** | **I** | **Y** | **S** | **N** | **D** | **Y** | **D** | **T** | **N** | **L** | **R** | **R** | **L** | **K** | **I** | **M** | **T** | **N** | **T** | **L** | **V** | **G** | **D** | **V** | **E** | **V** | **I** | **R** | **V** |
| **N59550F** | **M** | **N** | **N** | **P** | **W** | **R** | **F** | **F** | **I** | **V** | **A** | **E** | **A** | **L** | **L** | **F** | **I** | **L** | **A** | **L** | **W** | **Q** | **I** | **V** | **H** | **N** | **P** | **G** | **L** | **A** | **V** | **L** | **L** | **T** | **I** | **G** | **V** | **L** | **L** | **V** | **A** | **Y** | **V** | **S** | **R** | **K** | **A** | **S** | **K** | **T** | **H** | **F** | **N** | **N** | **F** | **Q** | **F** | **V** | **L** | **G** | **V** | **V** | **F** | **I** | **V** | **I** | **G** | **A** | **M** | **N** | **S** | **T** | **A** | **V** | **W** | **F** | **M** | **L** | **I** | **F** | **G** | **V** | **L** | **F** | **I** | **G** | **L** | **K** | **G** | **F** | **E** | **I** | **S** | **G** | **V** | **D** | **I** | **A** | **E** | **R** | **A** | **P** | **W** | **R** | **K** | **K** | **Q** | **M** | **I** | **M** | **V** | **E** | **T** | **A** | **A** | **K** | **E** | **P** | **K** | **N** | **G** | **K** | **R** | **F** | **K** | **R** | **R** | **W** | **F** | **A** | **N** | **E** | **R** | **I** | **G** | **N** | **N** | **I** | **Y** | **E** | **W** | **D** | **D** | **I** | **N** | **I** | **D** | **L** | **I** | **S** | **G** | **D** | **T** | **I** | **I** | **D** | **L** | **G** | **N** | **T** | **L** | **L** | **P** | **K** | **E** | **D** | **N** | **I** | **I** | **I** | **I** | **R** | **K** | **G** | **F** | **G** | **R** | **T** | **R** | **I** | **L** | **V** | **P** | **L** | **G** | **V** | **A** | **I** | **L** | **L** | **E** | **H** | **S** | **T** | **F** | **Y** | **G** | **T** | **V** | **R** | **F** | **E** | **E** | **E** | **K** | **Y** | **Q** | **L** | **K** | **N** | **E** | **S** | **L** | **K** | **I** | **Y** | **S** | **N** | **D** | **Y** | **D** | **T** | **N** | **L** | **R** | **R** | **L** | **K** | **I** | **M** | **T** | **N** | **T** | **L** | **V** | **G** | **D** | **V** | **E** | **V** | **I** | **R** | **V** |
| **N59591F** | **M** | **N** | **N** | **P** | **W** | **R** | **F** | **F** | **I** | **V** | **A** | **E** | **A** | **L** | **L** | **F** | **I** | **L** | **A** | **L** | **W** | **Q** | **I** | **V** | **H** | **N** | **P** | **G** | **L** | **A** | **V** | **L** | **L** | **T** | **I** | **G** | **V** | **L** | **L** | **V** | **A** | **Y** | **V** | **S** | **R** | **K** | **A** | **S** | **K** | **T** | **H** | **F** | **N** | **N** | **F** | **Q** | **F** | **V** | **L** | **G** | **V** | **V** | **F** | **I** | **V** | **I** | **G** | **A** | **M** | **N** | **S** | **T** | **A** | **V** | **W** | **F** | **M** | **L** | **I** | **F** | **G** | **V** | **L** | **F** | **I** | **G** | **L** | **K** | **G** | **F** | **E** | **I** | **S** | **G** | **V** | **D** | **I** | **A** | **E** | **R** | **A** | **P** | **W** | **R** | **K** | **K** | **Q** | **M** | **I** | **M** | **V** | **E** | **T** | **A** | **A** | **K** | **E** | **P** | **K** | **N** | **G** | **K** | **R** | **F** | **K** | **R** | **R** | **W** | **F** | **A** | **N** | **E** | **R** | **I** | **G** | **N** | **N** | **I** | **Y** | **E** | **W** | **D** | **D** | **I** | **N** | **I** | **D** | **L** | **I** | **S** | **G** | **D** | **T** | **I** | **I** | **D** | **L** | **G** | **N** | **T** | **L** | **L** | **P** | **K** | **E** | **D** | **N** | **I** | **I** | **I** | **I** | **R** | **K** | **G** | **F** | **G** | **R** | **T** | **R** | **I** | **L** | **V** | **P** | **L** | **G** | **V** | **A** | **I** | **L** | **L** | **E** | **H** | **S** | **T** | **F** | **Y** | **G** | **T** | **V** | **R** | **F** | **E** | **E** | **E** | **K** | **Y** | **Q** | **L** | **K** | **N** | **E** | **S** | **L** | **K** | **I** | **Y** | **S** | **N** | **D** | **Y** | **D** | **T** | **N** | **L** | **R** | **R** | **L** | **K** | **I** | **M** | **T** | **N** | **T** | **L** | **V** | **G** | **D** | **V** | **E** | **V** | **I** | **R** | **V** |
| **N52773** | **M** | **N** | **N** | **P** | **W** | **R** | **F** | **F** | **I** | **V** | **A** | **E** | **A** | **L** | **L** | **F** | **I** | **L** | **A** | **L** | **W** | **Q** | **I** | **V** | **H** | **N** | **P** | **G** | **L** | **A** | **V** | **L** | **L** | **T** | **I** | **G** | **V** | **L** | **L** | **V** | **A** | **Y** | **V** | **S** | **R** | **K** | **A** | **S** | **K** | **T** | **H** | **F** | **N** | **N** | **F** | **Q** | **F** | **V** | **L** | **G** | **V** | **V** | **F** | **I** | **V** | **I** | **G** | **A** | **M** | **N** | **S** | **T** | **A** | **V** | **W** | **F** | **M** | **L** | **I** | **F** | **G** | **V** | **L** | **F** | **I** | **G** | **L** | **K** | **G** | **F** | **E** | **I** | **S** | **G** | **V** | **D** | **I** | **A** | **E** | **R** | **A** | **P** | **W** | **R** | **K** | **K** | **Q** | **M** | **I** | **M** | **V** | **E** | **T** | **A** | **A** | **K** | **E** | **P** | **K** | **N** | **G** | **K** | **R** | **F** | **K** | **R** | **R** | **W** | **F** | **A** | **N** | **E** | **R** | **I** | **G** | **N** | **N** | **I** | **Y** | **E** | **W** | **D** | **D** | **I** | **N** | **I** | **D** | **L** | **I** | **S** | **G** | **D** | **T** | **I** | **I** | **D** | **L** | **G** | **N** | **T** | **L** | **L** | **P** | **K** | **E** | **D** | **N** | **I** | **I** | **I** | **I** | **R** | **K** | **G** | **F** | **G** | **R** | **T** | **R** | **I** | **L** | **V** | **P** | **L** | **G** | **V** | **A** | **I** | **L** | **L** | **E** | **H** | **S** | **T** | **F** | **Y** | **G** | **T** | **V** | **R** | **F** | **E** | **E** | **E** | **K** | **Y** | **Q** | **L** | **K** | **N** | **E** | **S** | **L** | **K** | **I** | **Y** | **S** | **N** | **D** | **Y** | **D** | **T** | **N** | **L** | **R** | **R** | **L** | **K** | **I** | **M** | **T** | **N** | **T** | **L** | **V** | **G** | **D** | **V** | **E** | **V** | **I** | **R** | **V** |
| **N55268** | **M** | **N** | **N** | **P** | **W** | **R** | **F** | **F** | **I** | **V** | **A** | **E** | **A** | **L** | **L** | **F** | **I** | **L** | **A** | **L** | **W** | **Q** | **I** | **V** | **H** | **N** | **P** | **G** | **L** | **A** | **V** | **L** | **L** | **T** | **I** | **G** | **V** | **L** | **L** | **V** | **A** | **Y** | **V** | **S** | **R** | **K** | **A** | **S** | **K** | **T** | **H** | **F** | **N** | **N** | **F** | **Q** | **F** | **V** | **L** | **G** | **V** | **V** | **F** | **I** | **V** | **I** | **G** | **A** | **M** | **N** | **S** | **T** | **A** | **V** | **W** | **F** | **M** | **L** | **I** | **F** | **G** | **V** | **L** | **F** | **I** | **G** | **L** | **K** | **G** | **F** | **E** | **I** | **S** | **G** | **V** | **D** | **I** | **A** | **E** | **R** | **A** | **P** | **W** | **R** | **K** | **K** | **Q** | **M** | **I** | **M** | **V** | **E** | **T** | **A** | **A** | **K** | **E** | **P** | **K** | **N** | **G** | **K** | **R** | **F** | **K** | **R** | **R** | **W** | **F** | **A** | **N** | **E** | **R** | **I** | **G** | **N** | **N** | **I** | **Y** | **E** | **W** | **D** | **D** | **I** | **N** | **I** | **D** | **L** | **I** | **S** | **G** | **D** | **T** | **I** | **I** | **D** | **L** | **G** | **N** | **T** | **L** | **L** | **P** | **K** | **E** | **D** | **N** | **I** | **I** | **I** | **I** | **R** | **K** | **G** | **F** | **G** | **R** | **T** | **R** | **I** | **L** | **V** | **P** | **L** | **G** | **V** | **A** | **I** | **L** | **L** | **E** | **H** | **S** | **T** | **F** | **Y** | **G** | **T** | **V** | **R** | **F** | **E** | **E** | **E** | **K** | **Y** | **Q** | **L** | **K** | **N** | **E** | **S** | **L** | **K** | **I** | **Y** | **S** | **N** | **D** | **Y** | **D** | **T** | **N** | **L** | **R** | **R** | **L** | **K** | **I** | **M** | **T** | **N** | **T** | **L** | **V** | **G** | **D** | **V** | **E** | **V** | **I** | **R** | **V** |
| **N54574** | **M** | **N** | **N** | **P** | **W** | **R** | **F** | **F** | **I** | **V** | **A** | **E** | **A** | **L** | **L** | **F** | **I** | **L** | **A** | **L** | **W** | **Q** | **I** | **V** | **H** | **N** | **P** | **G** | **L** | **A** | **V** | **L** | **L** | **T** | **I** | **G** | **V** | **L** | **L** | **V** | **A** | **Y** | **V** | **S** | **R** | **K** | **A** | **S** | **K** | **T** | **H** | **F** | **N** | **N** | **F** | **Q** | **F** | **V** | **L** | **G** | **V** | **V** | **F** | **I** | **V** | **I** | **G** | **A** | **M** | **N** | **S** | **T** | **A** | **V** | **W** | **F** | **M** | **L** | **I** | **F** | **G** | **V** | **L** | **F** | **I** | **G** | **L** | **K** | **G** | **F** | **E** | **I** | **S** | **G** | **V** | **D** | **I** | **A** | **E** | **R** | **A** | **P** | **W** | **R** | **K** | **K** | **Q** | **M** | **I** | **M** | **V** | **E** | **T** | **A** | **A** | **K** | **E** | **P** | **K** | **N** | **G** | **K** | **R** | **F** | **K** | **R** | **R** | **W** | **F** | **A** | **N** | **E** | **R** | **I** | **G** | **N** | **N** | **I** | **Y** | **E** | **W** | **D** | **D** | **I** | **N** | **I** | **D** | **L** | **I** | **S** | **G** | **D** | **T** | **I** | **I** | **D** | **L** | **G** | **N** | **T** | **L** | **L** | **P** | **K** | **E** | **D** | **N** | **I** | **I** | **I** | **I** | **R** | **K** | **G** | **F** | **G** | **R** | **T** | **R** | **I** | **L** | **V** | **P** | **L** | **G** | **V** | **A** | **I** | **V** | **L** | **E** | **H** | **S** | **T** | **F** | **Y** | **G** | **T** | **V** | **R** | **F** | **E** | **E** | **E** | **K** | **Y** | **Q** | **L** | **K** | **N** | **E** | **S** | **L** | **K** | **I** | **Y** | **S** | **N** | **D** | **Y** | **D** | **T** | **N** | **L** | **R** | **R** | **L** | **K** | **I** | **M** | **T** | **N** | **T** | **L** | **V** | **G** | **D** | **V** | **E** | **V** | **I** | **R** | **V** |
| **N59600F** | **M** | **N** | **N** | **P** | **W** | **R** | **F** | **F** | **I** | **V** | **A** | **E** | **A** | **L** | **L** | **F** | **I** | **L** | **A** | **L** | **W** | **Q** | **I** | **V** | **H** | **N** | **P** | **G** | **L** | **A** | **V** | **L** | **L** | **T** | **I** | **G** | **V** | **L** | **L** | **V** | **A** | **Y** | **V** | **S** | **R** | **K** | **A** | **S** | **K** | **T** | **H** | **F** | **N** | **N** | **F** | **Q** | **F** | **V** | **L** | **G** | **V** | **V** | **F** | **I** | **V** | **I** | **G** | **A** | **M** | **N** | **S** | **T** | **A** | **V** | **W** | **F** | **M** | **L** | **I** | **F** | **G** | **V** | **L** | **F** | **I** | **G** | **L** | **K** | **G** | **F** | **E** | **I** | **S** | **G** | **V** | **D** | **I** | **A** | **E** | **R** | **A** | **P** | **W** | **R** | **K** | **K** | **Q** | **M** | **I** | **M** | **V** | **E** | **T** | **A** | **A** | **K** | **E** | **P** | **K** | **N** | **G** | **K** | **R** | **F** | **K** | **R** | **R** | **W** | **F** | **A** | **N** | **E** | **R** | **I** | **G** | **N** | **N** | **I** | **Y** | **E** | **W** | **D** | **D** | **I** | **N** | **I** | **D** | **L** | **I** | **S** | **G** | **D** | **T** | **I** | **I** | **D** | **L** | **G** | **N** | **T** | **L** | **L** | **P** | **K** | **E** | **D** | **N** | **I** | **I** | **I** | **I** | **R** | **K** | **G** | **F** | **G** | **R** | **T** | **R** | **I** | **L** | **V** | **P** | **L** | **G** | **V** | **A** | **I** | **L** | **L** | **E** | **H** | **S** | **T** | **F** | **Y** | **G** | **T** | **V** | **R** | **F** | **E** | **E** | **E** | **K** | **Y** | **Q** | **L** | **K** | **N** | **E** | **S** | **L** | **K** | **I** | **Y** | **S** | **N** | **D** | **Y** | **D** | **T** | **N** | **L** | **R** | **R** | **L** | **K** | **I** | **M** | **T** | **N** | **T** | **L** | **V** | **G** | **D** | **V** | **E** | **V** | **I** | **R** | **V** |
| **N52469** | **M** | **N** | **N** | **P** | **W** | **R** | **F** | **F** | **I** | **V** | **A** | **E** | **A** | **L** | **L** | **F** | **I** | **L** | **A** | **L** | **W** | **Q** | **I** | **V** | **H** | **N** | **P** | **G** | **L** | **A** | **V** | **L** | **L** | **T** | **I** | **G** | **V** | **L** | **L** | **V** | **A** | **Y** | **V** | **S** | **R** | **K** | **A** | **S** | **K** | **T** | **H** | **F** | **N** | **N** | **F** | **Q** | **F** | **I** | **L** | **G** | **V** | **V** | **F** | **I** | **V** | **I** | **G** | **A** | **M** | **N** | **S** | **T** | **A** | **V** | **W** | **F** | **M** | **L** | **I** | **F** | **G** | **V** | **L** | **F** | **I** | **G** | **L** | **K** | **G** | **F** | **E** | **I** | **S** | **G** | **V** | **D** | **I** | **A** | **E** | **R** | **A** | **P** | **W** | **R** | **K** | **K** | **Q** | **M** | **I** | **M** | **V** | **E** | **T** | **A** | **A** | **K** | **E** | **P** | **K** | **N** | **G** | **K** | **R** | **F** | **K** | **R** | **R** | **W** | **F** | **A** | **N** | **E** | **R** | **I** | **G** | **N** | **N** | **I** | **Y** | **E** | **W** | **D** | **D** | **I** | **N** | **I** | **D** | **L** | **I** | **S** | **G** | **D** | **T** | **I** | **I** | **D** | **L** | **G** | **N** | **T** | **L** | **L** | **P** | **K** | **E** | **D** | **N** | **I** | **I** | **I** | **I** | **R** | **K** | **G** | **F** | **G** | **R** | **T** | **R** | **I** | **L** | **V** | **P** | **L** | **G** | **V** | **A** | **I** | **L** | **L** | **E** | **H** | **S** | **T** | **F** | **Y** | **G** | **T** | **V** | **R** | **F** | **E** | **E** | **E** | **K** | **Y** | **Q** | **L** | **K** | **N** | **E** | **S** | **L** | **K** | **I** | **Y** | **S** | **N** | **D** | **Y** | **D** | **T** | **N** | **L** | **R** | **R** | **L** | **K** | **I** | **M** | **T** | **N** | **T** | **L** | **V** | **G** | **D** | **V** | **E** | **V** | **I** | **R** | **V** |
| **N60280F** | **M** | **N** | **N** | **P** | **W** | **R** | **F** | **F** | **I** | **V** | **A** | **E** | **A** | **L** | **L** | **F** | **I** | **L** | **A** | **L** | **W** | **Q** | **I** | **V** | **H** | **N** | **P** | **G** | **L** | **A** | **V** | **L** | **L** | **T** | **I** | **G** | **V** | **L** | **L** | **V** | **A** | **Y** | **V** | **S** | **R** | **K** | **A** | **S** | **K** | **T** | **H** | **F** | **N** | **N** | **F** | **Q** | **F** | **V** | **L** | **G** | **V** | **V** | **F** | **I** | **V** | **I** | **G** | **A** | **M** | **N** | **S** | **T** | **A** | **V** | **W** | **F** | **M** | **L** | **I** | **F** | **G** | **V** | **L** | **F** | **I** | **G** | **L** | **K** | **G** | **F** | **E** | **I** | **S** | **G** | **V** | **D** | **I** | **A** | **E** | **R** | **A** | **P** | **W** | **R** | **K** | **K** | **Q** | **M** | **I** | **M** | **V** | **E** | **T** | **A** | **A** | **K** | **E** | **P** | **K** | **N** | **G** | **K** | **R** | **F** | **K** | **R** | **R** | **W** | **F** | **A** | **N** | **E** | **R** | **I** | **G** | **N** | **N** | **I** | **Y** | **E** | **W** | **D** | **D** | **I** | **N** | **I** | **D** | **L** | **I** | **S** | **G** | **D** | **T** | **I** | **I** | **D** | **L** | **G** | **N** | **T** | **L** | **L** | **P** | **K** | **E** | **D** | **N** | **I** | **I** | **I** | **I** | **R** | **K** | **G** | **F** | **G** | **R** | **T** | **R** | **I** | **L** | **V** | **P** | **L** | **G** | **V** | **A** | **I** | **L** | **L** | **E** | **H** | **S** | **T** | **F** | **Y** | **G** | **T** | **V** | **R** | **F** | **E** | **E** | **E** | **K** | **Y** | **Q** | **L** | **K** | **N** | **E** | **S** | **L** | **K** | **I** | **Y** | **S** | **N** | **D** | **Y** | **D** | **T** | **N** | **L** | **R** | **R** | **L** | **K** | **I** | **M** | **T** | **N** | **T** | **L** | **V** | **G** | **D** | **V** | **E** | **V** | **I** | **R** | **V** |
| **N54595** | **M** | **N** | **N** | **P** | **W** | **R** | **F** | **F** | **I** | **V** | **A** | **E** | **A** | **L** | **L** | **F** | **I** | **L** | **A** | **L** | **W** | **Q** | **I** | **V** | **H** | **N** | **P** | **G** | **L** | **A** | **V** | **L** | **L** | **T** | **I** | **G** | **V** | **L** | **L** | **V** | **A** | **Y** | **V** | **S** | **R** | **K** | **A** | **S** | **K** | **T** | **H** | **F** | **N** | **N** | **F** | **Q** | **F** | **I** | **L** | **G** | **V** | **V** | **F** | **I** | **V** | **I** | **G** | **A** | **M** | **N** | **S** | **T** | **A** | **V** | **W** | **F** | **M** | **L** | **I** | **F** | **G** | **V** | **L** | **F** | **I** | **G** | **L** | **K** | **G** | **F** | **E** | **I** | **S** | **G** | **V** | **D** | **I** | **A** | **E** | **R** | **A** | **P** | **W** | **R** | **K** | **K** | **Q** | **M** | **I** | **M** | **V** | **E** | **T** | **A** | **A** | **K** | **E** | **P** | **K** | **N** | **G** | **K** | **R** | **F** | **K** | **R** | **R** | **W** | **F** | **A** | **N** | **E** | **R** | **I** | **G** | **N** | **N** | **I** | **Y** | **E** | **W** | **D** | **D** | **I** | **N** | **I** | **D** | **L** | **I** | **S** | **G** | **D** | **T** | **I** | **I** | **D** | **L** | **G** | **N** | **T** | **L** | **L** | **P** | **K** | **E** | **D** | **N** | **I** | **I** | **I** | **I** | **R** | **K** | **G** | **F** | **G** | **R** | **T** | **R** | **I** | **L** | **V** | **P** | **L** | **G** | **V** | **A** | **I** | **L** | **L** | **E** | **H** | **S** | **T** | **F** | **Y** | **G** | **T** | **V** | **R** | **F** | **E** | **E** | **E** | **K** | **Y** | **Q** | **L** | **K** | **N** | **E** | **S** | **L** | **K** | **I** | **Y** | **S** | **N** | **D** | **Y** | **D** | **T** | **N** | **L** | **R** | **R** | **L** | **K** | **I** | **M** | **T** | **N** | **T** | **L** | **V** | **G** | **D** | **V** | **E** | **V** | **I** | **R** | **V** |
| **N59420F** | **M** | **N** | **N** | **P** | **W** | **R** | **F** | **F** | **I** | **V** | **A** | **E** | **A** | **L** | **L** | **F** | **I** | **L** | **A** | **L** | **W** | **Q** | **I** | **V** | **H** | **N** | **P** | **G** | **L** | **A** | **V** | **L** | **L** | **T** | **I** | **G** | **V** | **L** | **L** | **V** | **A** | **Y** | **V** | **S** | **R** | **K** | **A** | **S** | **K** | **T** | **H** | **F** | **N** | **N** | **F** | **Q** | **F** | **V** | **L** | **G** | **V** | **V** | **F** | **I** | **V** | **I** | **G** | **A** | **M** | **N** | **S** | **T** | **A** | **V** | **W** | **F** | **M** | **L** | **I** | **F** | **G** | **V** | **L** | **F** | **I** | **G** | **L** | **K** | **G** | **F** | **E** | **I** | **S** | **G** | **V** | **D** | **I** | **A** | **E** | **R** | **A** | **P** | **W** | **R** | **K** | **K** | **Q** | **M** | **I** | **M** | **V** | **E** | **T** | **A** | **A** | **K** | **E** | **P** | **K** | **N** | **G** | **K** | **R** | **F** | **K** | **R** | **R** | **W** | **F** | **A** | **N** | **E** | **R** | **I** | **G** | **N** | **N** | **I** | **Y** | **E** | **W** | **D** | **D** | **I** | **N** | **I** | **D** | **L** | **I** | **S** | **G** | **D** | **T** | **I** | **I** | **D** | **L** | **G** | **N** | **T** | **L** | **L** | **P** | **K** | **E** | **D** | **N** | **I** | **I** | **I** | **I** | **R** | **K** | **G** | **F** | **G** | **R** | **T** | **R** | **I** | **L** | **V** | **P** | **L** | **G** | **V** | **A** | **I** | **L** | **L** | **E** | **H** | **S** | **T** | **F** | **Y** | **G** | **T** | **V** | **R** | **F** | **E** | **E** | **E** | **K** | **Y** | **Q** | **L** | **K** | **N** | **E** | **S** | **L** | **K** | **I** | **Y** | **S** | **N** | **D** | **Y** | **D** | **T** | **N** | **L** | **R** | **R** | **L** | **K** | **I** | **M** | **T** | **N** | **T** | **L** | **V** | **G** | **D** | **V** | **E** | **V** | **I** | **R** | **V** |
| **N53476** | **M** | **N** | **N** | **P** | **W** | **R** | **F** | **F** | **I** | **V** | **A** | **E** | **A** | **L** | **L** | **F** | **I** | **L** | **A** | **L** | **W** | **Q** | **I** | **V** | **H** | **N** | **P** | **G** | **L** | **A** | **V** | **L** | **L** | **T** | **I** | **G** | **V** | **L** | **L** | **V** | **A** | **Y** | **V** | **S** | **R** | **K** | **A** | **S** | **K** | **T** | **H** | **F** | **N** | **N** | **F** | **Q** | **F** | **V** | **L** | **G** | **V** | **V** | **F** | **I** | **V** | **I** | **G** | **A** | **M** | **N** | **S** | **T** | **A** | **V** | **W** | **F** | **M** | **L** | **I** | **F** | **G** | **V** | **L** | **F** | **I** | **G** | **L** | **K** | **G** | **F** | **E** | **I** | **S** | **G** | **V** | **D** | **I** | **A** | **E** | **R** | **A** | **P** | **W** | **R** | **K** | **K** | **Q** | **M** | **I** | **M** | **V** | **E** | **T** | **A** | **A** | **K** | **E** | **P** | **K** | **N** | **G** | **K** | **R** | **F** | **K** | **R** | **R** | **W** | **F** | **A** | **N** | **E** | **R** | **I** | **G** | **N** | **N** | **I** | **Y** | **E** | **W** | **D** | **D** | **I** | **N** | **I** | **D** | **L** | **I** | **S** | **G** | **D** | **T** | **I** | **I** | **D** | **L** | **G** | **N** | **T** | **L** | **L** | **P** | **K** | **E** | **D** | **N** | **I** | **I** | **I** | **I** | **R** | **K** | **G** | **F** | **G** | **R** | **T** | **R** | **I** | **L** | **V** | **P** | **L** | **G** | **V** | **A** | **I** | **L** | **L** | **E** | **H** | **S** | **T** | **F** | **Y** | **G** | **T** | **V** | **R** | **F** | **E** | **E** | **E** | **K** | **Y** | **Q** | **L** | **K** | **N** | **E** | **S** | **L** | **K** | **I** | **Y** | **S** | **N** | **D** | **Y** | **D** | **T** | **N** | **L** | **R** | **R** | **L** | **K** | **I** | **M** | **T** | **N** | **T** | **L** | **V** | **G** | **D** | **V** | **E** | **V** | **I** | **R** | **V** |
| **N59409F** | **M** | **N** | **N** | **P** | **W** | **R** | **F** | **F** | **I** | **V** | **A** | **E** | **A** | **L** | **L** | **F** | **I** | **L** | **A** | **L** | **W** | **Q** | **I** | **V** | **H** | **N** | **P** | **G** | **L** | **A** | **V** | **L** | **L** | **T** | **I** | **G** | **V** | **L** | **L** | **V** | **A** | **Y** | **V** | **S** | **R** | **K** | **A** | **S** | **K** | **T** | **H** | **F** | **N** | **N** | **F** | **Q** | **F** | **V** | **L** | **G** | **V** | **V** | **F** | **I** | **V** | **I** | **G** | **A** | **M** | **N** | **S** | **T** | **A** | **V** | **W** | **F** | **M** | **L** | **I** | **F** | **G** | **V** | **L** | **F** | **I** | **G** | **L** | **K** | **G** | **F** | **E** | **I** | **S** | **G** | **V** | **D** | **I** | **A** | **E** | **R** | **A** | **P** | **W** | **R** | **K** | **K** | **Q** | **M** | **I** | **M** | **V** | **E** | **T** | **A** | **A** | **K** | **E** | **P** | **K** | **N** | **G** | **K** | **R** | **F** | **K** | **R** | **R** | **W** | **F** | **A** | **N** | **E** | **R** | **I** | **G** | **N** | **N** | **I** | **Y** | **E** | **W** | **D** | **D** | **I** | **N** | **I** | **D** | **L** | **I** | **S** | **G** | **D** | **T** | **I** | **I** | **D** | **L** | **G** | **N** | **T** | **L** | **L** | **P** | **K** | **E** | **D** | **N** | **I** | **I** | **I** | **I** | **R** | **K** | **G** | **F** | **G** | **R** | **T** | **R** | **I** | **L** | **V** | **P** | **L** | **G** | **V** | **A** | **I** | **L** | **L** | **E** | **H** | **S** | **T** | **F** | **Y** | **G** | **T** | **V** | **R** | **F** | **E** | **E** | **E** | **K** | **Y** | **Q** | **L** | **K** | **N** | **E** | **S** | **L** | **K** | **I** | **Y** | **S** | **N** | **D** | **Y** | **D** | **T** | **N** | **L** | **R** | **R** | **L** | **K** | **I** | **M** | **T** | **N** | **T** | **L** | **V** | **G** | **D** | **V** | **E** | **V** | **I** | **R** | **V** |
| **N59554F** | **M** | **N** | **N** | **P** | **W** | **R** | **F** | **F** | **I** | **V** | **A** | **E** | **A** | **L** | **L** | **F** | **I** | **L** | **A** | **L** | **W** | **Q** | **I** | **V** | **H** | **N** | **P** | **G** | **L** | **A** | **V** | **L** | **L** | **T** | **I** | **G** | **V** | **L** | **L** | **V** | **A** | **Y** | **V** | **S** | **R** | **K** | **A** | **S** | **K** | **T** | **H** | **F** | **N** | **N** | **F** | **Q** | **F** | **V** | **L** | **G** | **V** | **V** | **F** | **I** | **V** | **I** | **G** | **A** | **M** | **N** | **S** | **T** | **A** | **V** | **W** | **F** | **M** | **L** | **I** | **F** | **G** | **V** | **L** | **F** | **I** | **G** | **L** | **K** | **G** | **F** | **E** | **I** | **S** | **G** | **V** | **D** | **I** | **A** | **E** | **R** | **A** | **P** | **W** | **R** | **K** | **K** | **Q** | **M** | **I** | **M** | **V** | **E** | **T** | **A** | **A** | **K** | **E** | **P** | **K** | **N** | **G** | **K** | **R** | **F** | **K** | **R** | **R** | **W** | **F** | **A** | **N** | **E** | **R** | **I** | **G** | **N** | **N** | **I** | **Y** | **E** | **W** | **D** | **D** | **I** | **N** | **I** | **D** | **L** | **I** | **S** | **G** | **D** | **T** | **I** | **I** | **D** | **L** | **G** | **N** | **T** | **L** | **L** | **P** | **K** | **E** | **D** | **N** | **I** | **I** | **I** | **I** | **R** | **K** | **G** | **F** | **G** | **R** | **T** | **R** | **I** | **L** | **V** | **P** | **L** | **G** | **V** | **A** | **I** | **L** | **L** | **E** | **H** | **S** | **T** | **F** | **Y** | **G** | **T** | **V** | **R** | **F** | **E** | **E** | **E** | **K** | **Y** | **Q** | **L** | **K** | **N** | **E** | **S** | **L** | **K** | **I** | **Y** | **S** | **N** | **D** | **Y** | **D** | **T** | **N** | **L** | **R** | **R** | **L** | **K** | **I** | **M** | **T** | **N** | **T** | **L** | **V** | **G** | **D** | **V** | **E** | **V** | **I** | **R** | **V** |
| **N55185** | **M** | **N** | **N** | **P** | **W** | **R** | **F** | **F** | **I** | **V** | **A** | **E** | **A** | **L** | **L** | **F** | **I** | **L** | **A** | **L** | **W** | **Q** | **I** | **V** | **H** | **N** | **P** | **G** | **L** | **A** | **V** | **L** | **L** | **T** | **I** | **G** | **V** | **L** | **L** | **V** | **A** | **Y** | **V** | **S** | **R** | **K** | **A** | **S** | **K** | **T** | **H** | **F** | **N** | **N** | **F** | **Q** | **F** | **V** | **L** | **G** | **V** | **V** | **F** | **I** | **V** | **I** | **G** | **A** | **M** | **N** | **S** | **T** | **A** | **V** | **W** | **F** | **M** | **L** | **I** | **F** | **G** | **V** | **L** | **F** | **I** | **G** | **L** | **K** | **G** | **F** | **E** | **I** | **S** | **G** | **V** | **D** | **I** | **A** | **E** | **R** | **A** | **P** | **W** | **R** | **K** | **K** | **Q** | **M** | **I** | **M** | **V** | **E** | **T** | **A** | **A** | **K** | **E** | **P** | **K** | **N** | **G** | **K** | **R** | **F** | **K** | **R** | **R** | **W** | **F** | **A** | **N** | **E** | **R** | **I** | **G** | **N** | **N** | **I** | **Y** | **E** | **W** | **D** | **D** | **I** | **N** | **I** | **D** | **L** | **I** | **S** | **G** | **D** | **T** | **I** | **I** | **D** | **L** | **G** | **N** | **T** | **L** | **L** | **P** | **K** | **E** | **D** | **N** | **I** | **I** | **I** | **I** | **R** | **K** | **G** | **F** | **G** | **R** | **T** | **R** | **I** | **L** | **V** | **P** | **L** | **G** | **V** | **A** | **I** | **L** | **L** | **E** | **H** | **S** | **T** | **F** | **Y** | **G** | **T** | **V** | **R** | **F** | **E** | **E** | **E** | **K** | **Y** | **Q** | **L** | **K** | **N** | **E** | **S** | **L** | **K** | **I** | **Y** | **S** | **N** | **D** | **Y** | **D** | **T** | **N** | **L** | **R** | **R** | **L** | **K** | **I** | **M** | **T** | **N** | **T** | **L** | **V** | **G** | **D** | **V** | **E** | **V** | **I** | **R** | **V** |
| **N59508F** | **M** | **N** | **N** | **P** | **W** | **R** | **F** | **F** | **I** | **V** | **A** | **E** | **A** | **L** | **L** | **F** | **I** | **L** | **A** | **L** | **W** | **Q** | **I** | **V** | **H** | **N** | **P** | **G** | **L** | **A** | **V** | **L** | **L** | **T** | **I** | **G** | **V** | **L** | **L** | **V** | **A** | **Y** | **V** | **S** | **R** | **K** | **A** | **S** | **K** | **T** | **H** | **F** | **N** | **N** | **F** | **Q** | **F** | **V** | **L** | **G** | **V** | **V** | **F** | **I** | **V** | **I** | **G** | **A** | **M** | **N** | **S** | **T** | **A** | **V** | **W** | **F** | **M** | **L** | **I** | **F** | **G** | **V** | **L** | **F** | **I** | **G** | **L** | **K** | **G** | **F** | **E** | **I** | **S** | **G** | **V** | **D** | **I** | **A** | **E** | **R** | **A** | **P** | **W** | **R** | **K** | **K** | **Q** | **M** | **I** | **M** | **V** | **E** | **T** | **A** | **A** | **K** | **E** | **P** | **K** | **N** | **G** | **K** | **R** | **F** | **K** | **R** | **R** | **W** | **F** | **A** | **N** | **E** | **R** | **I** | **G** | **N** | **N** | **I** | **Y** | **E** | **W** | **D** | **D** | **I** | **N** | **I** | **D** | **L** | **I** | **S** | **G** | **D** | **T** | **I** | **I** | **D** | **L** | **G** | **N** | **T** | **L** | **L** | **P** | **K** | **E** | **D** | **N** | **I** | **I** | **I** | **I** | **R** | **K** | **G** | **F** | **G** | **R** | **T** | **R** | **I** | **L** | **V** | **P** | **L** | **G** | **V** | **A** | **I** | **L** | **L** | **E** | **H** | **S** | **T** | **F** | **Y** | **G** | **T** | **V** | **R** | **F** | **E** | **E** | **E** | **K** | **Y** | **Q** | **L** | **K** | **N** | **E** | **S** | **L** | **K** | **I** | **Y** | **S** | **N** | **D** | **Y** | **D** | **T** | **N** | **L** | **R** | **R** | **L** | **K** | **I** | **M** | **T** | **N** | **T** | **L** | **V** | **G** | **D** | **V** | **E** | **V** | **I** | **R** | **V** |
| **N55265** | **-** | **-** | **-** | **P** | **W** | **R** | **F** | **F** | **I** | **V** | **A** | **E** | **A** | **L** | **L** | **F** | **I** | **L** | **A** | **L** | **W** | **Q** | **I** | **V** | **H** | **N** | **P** | **G** | **L** | **A** | **V** | **L** | **L** | **T** | **I** | **G** | **V** | **L** | **L** | **V** | **A** | **Y** | **V** | **S** | **R** | **K** | **A** | **S** | **K** | **T** | **H** | **F** | **N** | **N** | **F** | **Q** | **F** | **V** | **L** | **G** | **I** | **V** | **F** | **I** | **V** | **I** | **G** | **A** | **M** | **N** | **S** | **T** | **A** | **V** | **W** | **F** | **M** | **L** | **I** | **F** | **G** | **V** | **L** | **F** | **I** | **G** | **L** | **K** | **G** | **F** | **E** | **I** | **S** | **G** | **V** | **D** | **I** | **A** | **E** | **R** | **A** | **P** | **W** | **R** | **K** | **K** | **Q** | **M** | **I** | **M** | **V** | **E** | **T** | **A** | **A** | **K** | **E** | **P** | **K** | **N** | **G** | **K** | **R** | **F** | **K** | **R** | **R** | **W** | **F** | **A** | **N** | **E** | **R** | **I** | **G** | **N** | **N** | **I** | **Y** | **E** | **W** | **D** | **D** | **I** | **N** | **I** | **D** | **L** | **I** | **S** | **G** | **D** | **T** | **I** | **I** | **D** | **L** | **G** | **N** | **T** | **L** | **L** | **P** | **K** | **E** | **D** | **N** | **I** | **I** | **I** | **I** | **R** | **K** | **G** | **F** | **G** | **R** | **T** | **R** | **I** | **L** | **V** | **P** | **L** | **G** | **V** | **A** | **I** | **L** | **L** | **E** | **H** | **S** | **T** | **F** | **Y** | **G** | **T** | **V** | **R** | **F** | **E** | **E** | **E** | **K** | **Y** | **Q** | **L** | **K** | **N** | **E** | **S** | **L** | **K** | **I** | **Y** | **S** | **N** | **D** | **Y** | **D** | **T** | **N** | **L** | **R** | **R** | **L** | **K** | **I** | **M** | **T** | **N** | **T** | **L** | **V** | **G** | **D** | **V** | **E** | **V** | **I** | **R** | **V** |
| **N55240** | **M** | **N** | **N** | **P** | **W** | **R** | **F** | **F** | **I** | **V** | **A** | **E** | **A** | **L** | **L** | **F** | **I** | **L** | **A** | **L** | **W** | **Q** | **I** | **V** | **H** | **N** | **P** | **G** | **L** | **A** | **V** | **L** | **L** | **T** | **I** | **G** | **V** | **L** | **L** | **V** | **A** | **Y** | **V** | **S** | **R** | **K** | **A** | **S** | **K** | **T** | **H** | **F** | **N** | **N** | **F** | **Q** | **F** | **V** | **L** | **G** | **V** | **V** | **F** | **I** | **V** | **I** | **G** | **A** | **M** | **N** | **S** | **T** | **A** | **V** | **W** | **F** | **M** | **L** | **I** | **F** | **G** | **V** | **L** | **F** | **I** | **G** | **L** | **K** | **G** | **F** | **E** | **I** | **S** | **G** | **V** | **D** | **I** | **A** | **E** | **R** | **A** | **P** | **W** | **R** | **K** | **K** | **Q** | **M** | **I** | **M** | **V** | **E** | **T** | **A** | **A** | **K** | **E** | **P** | **K** | **N** | **G** | **K** | **R** | **F** | **K** | **R** | **R** | **W** | **F** | **A** | **N** | **E** | **R** | **I** | **G** | **N** | **N** | **I** | **Y** | **E** | **W** | **D** | **D** | **I** | **N** | **I** | **D** | **L** | **I** | **S** | **G** | **D** | **T** | **I** | **I** | **D** | **L** | **G** | **N** | **T** | **L** | **L** | **P** | **K** | **E** | **D** | **N** | **I** | **I** | **I** | **I** | **R** | **K** | **G** | **F** | **G** | **R** | **T** | **R** | **I** | **L** | **V** | **P** | **L** | **G** | **V** | **A** | **I** | **L** | **L** | **E** | **H** | **S** | **T** | **F** | **Y** | **G** | **T** | **V** | **R** | **F** | **E** | **E** | **E** | **K** | **Y** | **Q** | **L** | **K** | **N** | **E** | **S** | **L** | **K** | **I** | **Y** | **S** | **N** | **D** | **Y** | **D** | **T** | **N** | **L** | **R** | **R** | **L** | **K** | **I** | **M** | **T** | **N** | **T** | **L** | **V** | **G** | **D** | **V** | **E** | **V** | **I** | **R** | **V** |
| **N59457F** | **M** | **N** | **N** | **P** | **W** | **R** | **F** | **F** | **I** | **V** | **A** | **E** | **A** | **L** | **L** | **F** | **I** | **L** | **A** | **L** | **W** | **Q** | **I** | **V** | **H** | **N** | **P** | **G** | **L** | **A** | **V** | **L** | **L** | **T** | **I** | **G** | **V** | **L** | **L** | **V** | **A** | **Y** | **V** | **S** | **R** | **K** | **A** | **Y** | **K** | **T** | **H** | **F** | **N** | **N** | **F** | **Q** | **F** | **V** | **L** | **G** | **V** | **V** | **F** | **I** | **V** | **I** | **G** | **A** | **M** | **N** | **S** | **T** | **A** | **V** | **W** | **F** | **M** | **L** | **I** | **F** | **G** | **V** | **L** | **F** | **I** | **G** | **L** | **K** | **G** | **F** | **E** | **I** | **S** | **G** | **V** | **D** | **I** | **A** | **E** | **R** | **A** | **P** | **W** | **R** | **K** | **K** | **Q** | **M** | **I** | **M** | **V** | **E** | **T** | **A** | **A** | **K** | **E** | **P** | **K** | **N** | **G** | **K** | **R** | **F** | **K** | **R** | **R** | **W** | **F** | **A** | **N** | **E** | **R** | **I** | **G** | **N** | **N** | **I** | **Y** | **E** | **W** | **D** | **D** | **I** | **N** | **I** | **D** | **L** | **I** | **S** | **G** | **D** | **T** | **I** | **I** | **D** | **L** | **G** | **N** | **T** | **L** | **L** | **P** | **K** | **E** | **D** | **N** | **I** | **I** | **I** | **I** | **R** | **K** | **G** | **F** | **G** | **R** | **T** | **R** | **I** | **L** | **V** | **P** | **L** | **G** | **V** | **A** | **I** | **L** | **L** | **E** | **H** | **S** | **T** | **F** | **Y** | **G** | **T** | **V** | **R** | **F** | **E** | **E** | **E** | **K** | **Y** | **Q** | **L** | **K** | **N** | **E** | **S** | **L** | **K** | **I** | **Y** | **S** | **N** | **D** | **Y** | **D** | **T** | **N** | **L** | **R** | **R** | **L** | **K** | **I** | **M** | **T** | **N** | **T** | **L** | **V** | **G** | **D** | **V** | **E** | **V** | **I** | **R** | **V** |
| **N53661** | **M** | **N** | **N** | **P** | **W** | **R** | **F** | **F** | **I** | **V** | **A** | **E** | **A** | **L** | **L** | **F** | **I** | **L** | **A** | **L** | **W** | **Q** | **I** | **V** | **H** | **N** | **P** | **G** | **L** | **A** | **V** | **L** | **L** | **T** | **I** | **G** | **V** | **L** | **L** | **V** | **A** | **Y** | **V** | **S** | **R** | **K** | **A** | **S** | **K** | **T** | **H** | **F** | **N** | **N** | **F** | **Q** | **F** | **V** | **L** | **G** | **V** | **V** | **F** | **I** | **V** | **I** | **G** | **A** | **M** | **N** | **S** | **T** | **A** | **V** | **W** | **F** | **M** | **L** | **I** | **F** | **G** | **V** | **L** | **F** | **I** | **G** | **L** | **K** | **G** | **F** | **E** | **I** | **S** | **G** | **V** | **D** | **I** | **A** | **E** | **R** | **A** | **P** | **W** | **R** | **K** | **K** | **Q** | **M** | **I** | **M** | **V** | **E** | **T** | **A** | **A** | **K** | **E** | **P** | **K** | **N** | **G** | **K** | **R** | **F** | **K** | **R** | **R** | **W** | **F** | **A** | **N** | **E** | **R** | **I** | **G** | **N** | **N** | **I** | **Y** | **E** | **W** | **D** | **D** | **I** | **N** | **I** | **D** | **L** | **I** | **S** | **G** | **D** | **T** | **I** | **I** | **D** | **L** | **G** | **N** | **T** | **L** | **L** | **P** | **K** | **E** | **D** | **N** | **I** | **I** | **I** | **I** | **R** | **K** | **G** | **F** | **G** | **R** | **T** | **R** | **I** | **L** | **V** | **P** | **L** | **G** | **V** | **A** | **I** | **L** | **L** | **E** | **H** | **S** | **T** | **F** | **Y** | **G** | **T** | **V** | **R** | **F** | **E** | **E** | **E** | **K** | **Y** | **Q** | **L** | **K** | **N** | **E** | **S** | **L** | **K** | **I** | **Y** | **S** | **N** | **D** | **Y** | **D** | **T** | **N** | **L** | **R** | **R** | **L** | **K** | **I** | **M** | **T** | **N** | **T** | **L** | **V** | **G** | **D** | **V** | **E** | **V** | **I** | **R** | **V** |
| **N52564** | **M** | **N** | **N** | **P** | **W** | **R** | **F** | **F** | **I** | **V** | **A** | **E** | **A** | **L** | **L** | **F** | **I** | **L** | **A** | **L** | **W** | **Q** | **I** | **V** | **H** | **N** | **P** | **G** | **L** | **A** | **V** | **L** | **L** | **T** | **I** | **G** | **V** | **L** | **L** | **V** | **A** | **Y** | **V** | **S** | **R** | **K** | **A** | **S** | **K** | **T** | **H** | **F** | **N** | **N** | **F** | **Q** | **F** | **V** | **L** | **G** | **V** | **V** | **F** | **I** | **V** | **I** | **G** | **A** | **M** | **N** | **S** | **T** | **A** | **V** | **W** | **F** | **M** | **L** | **I** | **F** | **G** | **V** | **L** | **F** | **I** | **G** | **L** | **K** | **G** | **F** | **E** | **I** | **S** | **G** | **V** | **D** | **I** | **A** | **E** | **R** | **A** | **P** | **W** | **R** | **K** | **K** | **Q** | **M** | **I** | **M** | **V** | **E** | **T** | **A** | **A** | **K** | **E** | **P** | **K** | **N** | **G** | **K** | **R** | **F** | **K** | **R** | **R** | **W** | **F** | **A** | **N** | **E** | **R** | **I** | **G** | **N** | **N** | **I** | **Y** | **E** | **W** | **D** | **D** | **I** | **N** | **I** | **D** | **L** | **I** | **S** | **G** | **D** | **T** | **I** | **I** | **D** | **L** | **G** | **N** | **T** | **L** | **L** | **P** | **K** | **E** | **D** | **N** | **I** | **I** | **I** | **I** | **R** | **K** | **G** | **F** | **G** | **R** | **T** | **R** | **I** | **L** | **V** | **P** | **L** | **G** | **V** | **A** | **I** | **L** | **L** | **E** | **H** | **S** | **T** | **F** | **Y** | **G** | **T** | **V** | **R** | **F** | **E** | **E** | **E** | **K** | **Y** | **Q** | **L** | **K** | **N** | **E** | **S** | **L** | **K** | **I** | **Y** | **S** | **N** | **D** | **Y** | **D** | **T** | **N** | **L** | **R** | **R** | **L** | **K** | **I** | **M** | **T** | **N** | **T** | **L** | **V** | **G** | **D** | **V** | **E** | **V** | **I** | **R** | **V** |
| **N59862F** | **M** | **N** | **N** | **P** | **W** | **R** | **F** | **F** | **I** | **V** | **A** | **E** | **A** | **L** | **L** | **F** | **I** | **L** | **A** | **L** | **W** | **Q** | **I** | **V** | **H** | **N** | **P** | **G** | **L** | **A** | **V** | **L** | **L** | **T** | **I** | **G** | **V** | **L** | **L** | **V** | **A** | **Y** | **V** | **S** | **R** | **K** | **A** | **S** | **K** | **T** | **H** | **F** | **N** | **N** | **F** | **Q** | **F** | **V** | **L** | **G** | **V** | **V** | **F** | **I** | **V** | **I** | **G** | **A** | **M** | **N** | **S** | **T** | **A** | **V** | **W** | **F** | **M** | **L** | **I** | **F** | **G** | **V** | **L** | **F** | **I** | **G** | **L** | **K** | **G** | **F** | **E** | **I** | **S** | **G** | **V** | **D** | **I** | **A** | **E** | **R** | **A** | **P** | **W** | **R** | **K** | **K** | **Q** | **M** | **I** | **M** | **V** | **E** | **T** | **A** | **A** | **K** | **E** | **P** | **K** | **N** | **G** | **K** | **R** | **F** | **K** | **R** | **R** | **W** | **F** | **A** | **N** | **E** | **R** | **I** | **G** | **N** | **N** | **I** | **Y** | **E** | **W** | **D** | **D** | **I** | **N** | **I** | **D** | **L** | **I** | **S** | **G** | **D** | **T** | **I** | **I** | **D** | **L** | **G** | **N** | **T** | **L** | **L** | **P** | **K** | **E** | **D** | **N** | **I** | **I** | **I** | **I** | **R** | **K** | **G** | **F** | **G** | **R** | **T** | **R** | **I** | **L** | **V** | **P** | **L** | **G** | **V** | **A** | **I** | **L** | **L** | **E** | **H** | **S** | **T** | **F** | **Y** | **G** | **T** | **V** | **R** | **F** | **E** | **E** | **E** | **K** | **Y** | **Q** | **L** | **K** | **N** | **E** | **S** | **L** | **K** | **I** | **Y** | **S** | **N** | **D** | **Y** | **D** | **T** | **N** | **L** | **R** | **R** | **L** | **K** | **I** | **M** | **T** | **N** | **T** | **L** | **V** | **G** | **D** | **V** | **E** | **V** | **I** | **R** | **V** |
| **N60027F** | **M** | **N** | **N** | **P** | **W** | **R** | **F** | **F** | **I** | **V** | **A** | **E** | **A** | **L** | **L** | **F** | **I** | **L** | **A** | **L** | **W** | **Q** | **I** | **V** | **H** | **N** | **P** | **G** | **L** | **A** | **V** | **L** | **L** | **T** | **I** | **G** | **V** | **L** | **L** | **V** | **A** | **Y** | **V** | **S** | **R** | **K** | **A** | **S** | **K** | **T** | **H** | **F** | **N** | **N** | **F** | **Q** | **F** | **V** | **L** | **G** | **V** | **V** | **F** | **I** | **V** | **I** | **G** | **A** | **M** | **N** | **S** | **T** | **A** | **V** | **W** | **F** | **M** | **L** | **I** | **F** | **G** | **V** | **L** | **F** | **I** | **G** | **L** | **K** | **G** | **F** | **E** | **I** | **S** | **G** | **V** | **D** | **I** | **A** | **E** | **R** | **A** | **P** | **W** | **R** | **K** | **K** | **Q** | **M** | **I** | **M** | **V** | **E** | **T** | **A** | **A** | **K** | **E** | **P** | **K** | **N** | **G** | **K** | **R** | **F** | **K** | **R** | **R** | **W** | **F** | **A** | **N** | **E** | **R** | **I** | **G** | **N** | **N** | **I** | **Y** | **E** | **W** | **D** | **D** | **I** | **N** | **I** | **D** | **L** | **I** | **S** | **G** | **D** | **T** | **I** | **I** | **D** | **L** | **G** | **N** | **T** | **L** | **L** | **P** | **K** | **E** | **D** | **N** | **I** | **I** | **I** | **I** | **R** | **K** | **G** | **F** | **G** | **R** | **T** | **R** | **I** | **L** | **V** | **P** | **L** | **G** | **V** | **A** | **I** | **L** | **L** | **E** | **H** | **S** | **T** | **F** | **Y** | **G** | **T** | **V** | **R** | **F** | **E** | **E** | **E** | **K** | **Y** | **Q** | **L** | **K** | **N** | **E** | **S** | **L** | **K** | **I** | **Y** | **S** | **N** | **D** | **Y** | **D** | **T** | **N** | **L** | **R** | **R** | **L** | **K** | **I** | **M** | **T** | **N** | **T** | **L** | **V** | **G** | **D** | **V** | **E** | **V** | **I** | **R** | **V** |
| **N59509F** | **M** | **N** | **N** | **P** | **W** | **R** | **F** | **F** | **I** | **V** | **A** | **E** | **A** | **L** | **L** | **F** | **I** | **L** | **A** | **L** | **W** | **Q** | **I** | **V** | **H** | **N** | **P** | **G** | **L** | **A** | **V** | **L** | **L** | **T** | **I** | **G** | **V** | **L** | **L** | **V** | **A** | **Y** | **V** | **S** | **R** | **K** | **A** | **S** | **K** | **T** | **H** | **F** | **N** | **N** | **F** | **Q** | **F** | **V** | **L** | **G** | **V** | **V** | **F** | **I** | **V** | **I** | **G** | **A** | **M** | **N** | **S** | **T** | **A** | **V** | **W** | **F** | **M** | **L** | **I** | **F** | **G** | **V** | **L** | **F** | **I** | **G** | **L** | **K** | **G** | **F** | **E** | **I** | **S** | **G** | **V** | **D** | **I** | **A** | **E** | **R** | **A** | **P** | **W** | **R** | **K** | **K** | **Q** | **M** | **I** | **M** | **V** | **E** | **T** | **A** | **A** | **K** | **E** | **P** | **K** | **N** | **G** | **K** | **R** | **F** | **K** | **R** | **R** | **W** | **F** | **A** | **N** | **E** | **R** | **I** | **G** | **N** | **N** | **I** | **Y** | **E** | **W** | **D** | **D** | **I** | **N** | **I** | **D** | **L** | **I** | **S** | **G** | **D** | **T** | **I** | **I** | **D** | **L** | **G** | **N** | **T** | **L** | **L** | **P** | **K** | **E** | **D** | **N** | **I** | **I** | **I** | **I** | **R** | **K** | **G** | **F** | **G** | **R** | **T** | **R** | **I** | **L** | **V** | **P** | **L** | **G** | **V** | **A** | **I** | **L** | **L** | **E** | **H** | **S** | **T** | **F** | **Y** | **G** | **T** | **V** | **R** | **F** | **E** | **E** | **E** | **K** | **Y** | **Q** | **L** | **K** | **N** | **E** | **S** | **L** | **K** | **I** | **Y** | **S** | **N** | **D** | **Y** | **D** | **T** | **N** | **L** | **R** | **R** | **L** | **K** | **I** | **M** | **T** | **N** | **T** | **L** | **V** | **G** | **D** | **V** | **E** | **V** | **I** | **R** | **V** |
| **N55113** | **M** | **N** | **N** | **P** | **W** | **R** | **F** | **F** | **I** | **V** | **A** | **E** | **A** | **L** | **L** | **F** | **I** | **L** | **A** | **L** | **W** | **Q** | **I** | **V** | **H** | **N** | **P** | **G** | **L** | **A** | **V** | **L** | **L** | **T** | **I** | **G** | **V** | **L** | **L** | **V** | **A** | **Y** | **V** | **S** | **R** | **K** | **A** | **S** | **K** | **T** | **H** | **F** | **N** | **N** | **F** | **Q** | **F** | **V** | **L** | **G** | **V** | **V** | **F** | **I** | **V** | **I** | **G** | **A** | **M** | **N** | **S** | **T** | **A** | **V** | **W** | **F** | **M** | **L** | **I** | **F** | **G** | **V** | **L** | **F** | **I** | **G** | **L** | **K** | **G** | **F** | **E** | **I** | **S** | **G** | **V** | **D** | **I** | **A** | **E** | **R** | **A** | **P** | **W** | **R** | **K** | **K** | **Q** | **M** | **I** | **M** | **V** | **E** | **T** | **A** | **A** | **K** | **E** | **P** | **K** | **N** | **G** | **K** | **R** | **F** | **K** | **R** | **R** | **W** | **F** | **A** | **N** | **E** | **R** | **I** | **G** | **N** | **N** | **I** | **Y** | **E** | **W** | **D** | **D** | **I** | **N** | **I** | **D** | **L** | **I** | **S** | **G** | **D** | **T** | **I** | **I** | **D** | **L** | **G** | **N** | **T** | **L** | **L** | **P** | **K** | **E** | **D** | **N** | **I** | **I** | **I** | **I** | **R** | **K** | **G** | **F** | **G** | **R** | **T** | **R** | **I** | **L** | **V** | **P** | **L** | **G** | **V** | **A** | **I** | **L** | **L** | **E** | **H** | **S** | **T** | **F** | **Y** | **G** | **T** | **V** | **R** | **F** | **E** | **E** | **E** | **K** | **Y** | **Q** | **L** | **K** | **N** | **E** | **S** | **L** | **K** | **I** | **Y** | **S** | **N** | **D** | **Y** | **D** | **T** | **N** | **L** | **R** | **R** | **L** | **K** | **I** | **M** | **T** | **N** | **T** | **L** | **V** | **G** | **D** | **V** | **E** | **V** | **I** | **R** | **V** |
| **N55725** | **M** | **N** | **N** | **P** | **W** | **R** | **F** | **F** | **I** | **V** | **A** | **E** | **A** | **L** | **L** | **F** | **I** | **L** | **A** | **L** | **W** | **Q** | **I** | **V** | **H** | **N** | **P** | **G** | **L** | **A** | **V** | **L** | **L** | **T** | **I** | **G** | **V** | **L** | **L** | **V** | **A** | **Y** | **V** | **S** | **R** | **K** | **A** | **S** | **K** | **T** | **H** | **F** | **N** | **N** | **F** | **Q** | **F** | **V** | **L** | **G** | **V** | **V** | **F** | **I** | **V** | **I** | **G** | **A** | **M** | **N** | **S** | **T** | **A** | **V** | **W** | **F** | **M** | **L** | **I** | **F** | **G** | **V** | **L** | **F** | **I** | **G** | **L** | **K** | **G** | **F** | **E** | **I** | **S** | **G** | **V** | **D** | **I** | **A** | **E** | **R** | **A** | **P** | **W** | **R** | **K** | **K** | **Q** | **M** | **I** | **M** | **V** | **E** | **T** | **A** | **A** | **K** | **E** | **P** | **K** | **N** | **G** | **K** | **R** | **F** | **K** | **R** | **R** | **W** | **F** | **A** | **N** | **E** | **R** | **I** | **G** | **N** | **N** | **I** | **Y** | **E** | **W** | **D** | **D** | **I** | **N** | **I** | **D** | **L** | **I** | **S** | **G** | **D** | **T** | **I** | **I** | **D** | **L** | **G** | **N** | **T** | **L** | **L** | **P** | **K** | **E** | **D** | **N** | **I** | **I** | **I** | **I** | **R** | **K** | **G** | **F** | **G** | **R** | **T** | **R** | **I** | **L** | **V** | **P** | **L** | **G** | **V** | **A** | **I** | **L** | **L** | **E** | **H** | **S** | **T** | **F** | **Y** | **G** | **T** | **V** | **R** | **F** | **E** | **E** | **E** | **K** | **Y** | **Q** | **L** | **K** | **N** | **E** | **S** | **L** | **K** | **I** | **Y** | **S** | **N** | **D** | **Y** | **D** | **T** | **N** | **L** | **R** | **R** | **L** | **K** | **I** | **M** | **T** | **N** | **T** | **L** | **V** | **G** | **D** | **V** | **E** | **V** | **I** | **R** | **V** |
| **N54564** | **M** | **N** | **N** | **P** | **W** | **R** | **F** | **F** | **I** | **V** | **A** | **E** | **A** | **L** | **L** | **F** | **I** | **L** | **A** | **L** | **W** | **Q** | **I** | **V** | **H** | **N** | **P** | **G** | **L** | **A** | **V** | **L** | **L** | **T** | **I** | **G** | **V** | **L** | **L** | **V** | **A** | **Y** | **V** | **S** | **R** | **K** | **A** | **Y** | **K** | **T** | **H** | **F** | **N** | **N** | **F** | **Q** | **F** | **V** | **L** | **G** | **V** | **V** | **F** | **I** | **V** | **I** | **G** | **A** | **M** | **N** | **S** | **T** | **A** | **V** | **W** | **F** | **M** | **L** | **I** | **F** | **G** | **V** | **L** | **F** | **I** | **G** | **L** | **K** | **G** | **F** | **E** | **I** | **S** | **G** | **V** | **D** | **I** | **A** | **E** | **R** | **A** | **P** | **W** | **R** | **K** | **K** | **Q** | **M** | **I** | **M** | **V** | **E** | **T** | **A** | **A** | **K** | **E** | **P** | **K** | **N** | **G** | **K** | **R** | **F** | **K** | **R** | **R** | **W** | **F** | **A** | **N** | **E** | **R** | **I** | **G** | **N** | **N** | **I** | **Y** | **E** | **W** | **D** | **D** | **I** | **N** | **I** | **D** | **L** | **I** | **S** | **G** | **D** | **T** | **I** | **I** | **D** | **L** | **G** | **N** | **T** | **L** | **L** | **P** | **K** | **E** | **D** | **N** | **I** | **I** | **I** | **I** | **R** | **K** | **G** | **F** | **G** | **R** | **T** | **R** | **I** | **L** | **V** | **P** | **L** | **G** | **V** | **A** | **I** | **L** | **L** | **E** | **H** | **S** | **T** | **F** | **Y** | **G** | **T** | **V** | **R** | **F** | **E** | **E** | **E** | **K** | **Y** | **Q** | **L** | **K** | **N** | **E** | **S** | **L** | **K** | **I** | **Y** | **S** | **N** | **D** | **Y** | **D** | **T** | **N** | **L** | **R** | **R** | **L** | **K** | **I** | **M** | **T** | **N** | **T** | **L** | **V** | **G** | **D** | **V** | **E** | **V** | **I** | **R** | **V** |
| **N52662** | **-** | **-** | **-** | **P** | **W** | **R** | **F** | **F** | **I** | **V** | **A** | **E** | **A** | **L** | **L** | **F** | **I** | **L** | **A** | **L** | **W** | **Q** | **I** | **V** | **H** | **N** | **P** | **G** | **L** | **A** | **V** | **L** | **L** | **T** | **I** | **G** | **V** | **L** | **L** | **V** | **A** | **Y** | **V** | **S** | **R** | **K** | **A** | **S** | **K** | **T** | **H** | **F** | **N** | **N** | **F** | **Q** | **F** | **V** | **L** | **G** | **I** | **V** | **F** | **I** | **V** | **I** | **G** | **A** | **M** | **N** | **S** | **T** | **A** | **V** | **W** | **F** | **M** | **L** | **I** | **F** | **G** | **V** | **L** | **F** | **I** | **G** | **L** | **K** | **G** | **F** | **E** | **I** | **S** | **G** | **V** | **D** | **I** | **A** | **E** | **R** | **A** | **P** | **W** | **R** | **K** | **K** | **Q** | **M** | **I** | **M** | **V** | **E** | **T** | **A** | **A** | **K** | **E** | **P** | **K** | **N** | **G** | **K** | **R** | **F** | **K** | **R** | **R** | **W** | **F** | **A** | **N** | **E** | **R** | **I** | **G** | **N** | **N** | **I** | **Y** | **E** | **W** | **D** | **D** | **I** | **N** | **I** | **D** | **L** | **I** | **S** | **G** | **D** | **T** | **I** | **I** | **D** | **L** | **G** | **N** | **T** | **L** | **L** | **P** | **K** | **E** | **D** | **N** | **I** | **I** | **I** | **I** | **R** | **K** | **G** | **F** | **G** | **R** | **T** | **R** | **I** | **L** | **V** | **P** | **L** | **G** | **V** | **A** | **I** | **L** | **L** | **E** | **H** | **S** | **T** | **F** | **Y** | **G** | **T** | **V** | **R** | **F** | **E** | **E** | **E** | **K** | **Y** | **Q** | **L** | **K** | **N** | **E** | **S** | **L** | **K** | **I** | **Y** | **S** | **N** | **D** | **Y** | **D** | **T** | **N** | **L** | **R** | **R** | **L** | **K** | **I** | **M** | **T** | **N** | **T** | **L** | **V** | **G** | **D** | **V** | **E** | **V** | **I** | **R** | **V** |
| **N53420** | **-** | **-** | **-** | **P** | **W** | **R** | **F** | **F** | **I** | **V** | **A** | **E** | **A** | **L** | **L** | **F** | **I** | **L** | **A** | **L** | **W** | **Q** | **I** | **V** | **H** | **N** | **P** | **G** | **L** | **A** | **V** | **L** | **L** | **T** | **I** | **G** | **V** | **L** | **L** | **V** | **A** | **Y** | **V** | **S** | **R** | **K** | **A** | **S** | **K** | **T** | **H** | **F** | **N** | **N** | **F** | **Q** | **F** | **V** | **L** | **G** | **I** | **V** | **F** | **I** | **V** | **I** | **G** | **A** | **M** | **N** | **S** | **T** | **A** | **V** | **W** | **F** | **M** | **L** | **I** | **F** | **G** | **V** | **L** | **F** | **I** | **G** | **L** | **K** | **G** | **F** | **E** | **I** | **S** | **G** | **V** | **D** | **I** | **A** | **E** | **R** | **A** | **P** | **W** | **R** | **K** | **K** | **Q** | **M** | **I** | **M** | **V** | **E** | **T** | **A** | **A** | **K** | **E** | **P** | **K** | **N** | **G** | **K** | **R** | **F** | **K** | **R** | **R** | **W** | **F** | **A** | **N** | **E** | **R** | **I** | **G** | **N** | **N** | **I** | **Y** | **E** | **W** | **D** | **D** | **I** | **N** | **I** | **D** | **L** | **I** | **S** | **G** | **D** | **T** | **I** | **I** | **D** | **L** | **G** | **N** | **T** | **L** | **L** | **P** | **K** | **E** | **D** | **N** | **I** | **I** | **I** | **I** | **R** | **K** | **G** | **F** | **G** | **R** | **T** | **R** | **I** | **L** | **V** | **P** | **L** | **G** | **V** | **A** | **I** | **L** | **L** | **E** | **H** | **S** | **T** | **F** | **Y** | **G** | **T** | **V** | **R** | **F** | **E** | **E** | **E** | **K** | **Y** | **Q** | **L** | **K** | **N** | **E** | **S** | **L** | **K** | **I** | **Y** | **S** | **N** | **D** | **Y** | **D** | **T** | **N** | **L** | **R** | **R** | **L** | **K** | **I** | **M** | **T** | **N** | **T** | **L** | **V** | **G** | **D** | **V** | **E** | **V** | **I** | **R** | **V** |
| **N54676** | **M** | **N** | **N** | **P** | **W** | **R** | **F** | **F** | **I** | **V** | **A** | **E** | **A** | **L** | **L** | **F** | **I** | **L** | **A** | **L** | **W** | **Q** | **I** | **V** | **H** | **N** | **P** | **G** | **L** | **A** | **V** | **L** | **L** | **T** | **I** | **G** | **V** | **L** | **L** | **V** | **A** | **Y** | **V** | **S** | **R** | **K** | **A** | **S** | **K** | **T** | **H** | **F** | **N** | **N** | **F** | **Q** | **F** | **V** | **L** | **G** | **V** | **V** | **F** | **I** | **V** | **I** | **G** | **A** | **M** | **N** | **S** | **T** | **A** | **V** | **W** | **F** | **M** | **L** | **I** | **F** | **G** | **V** | **L** | **F** | **I** | **G** | **L** | **K** | **G** | **F** | **E** | **I** | **S** | **G** | **V** | **D** | **I** | **A** | **E** | **R** | **A** | **P** | **W** | **R** | **K** | **K** | **Q** | **M** | **I** | **M** | **V** | **E** | **T** | **A** | **A** | **K** | **E** | **P** | **K** | **N** | **G** | **K** | **R** | **F** | **K** | **R** | **R** | **W** | **F** | **A** | **N** | **E** | **R** | **I** | **G** | **N** | **N** | **I** | **Y** | **E** | **W** | **D** | **D** | **I** | **N** | **I** | **D** | **L** | **I** | **S** | **G** | **D** | **T** | **I** | **I** | **D** | **L** | **G** | **N** | **T** | **L** | **L** | **P** | **K** | **E** | **D** | **N** | **I** | **I** | **I** | **I** | **R** | **K** | **G** | **F** | **G** | **R** | **T** | **R** | **I** | **L** | **V** | **P** | **L** | **G** | **V** | **A** | **I** | **L** | **L** | **E** | **H** | **S** | **T** | **F** | **Y** | **G** | **T** | **V** | **R** | **F** | **E** | **E** | **E** | **K** | **Y** | **Q** | **L** | **K** | **N** | **E** | **S** | **L** | **K** | **I** | **Y** | **S** | **N** | **D** | **Y** | **D** | **T** | **N** | **L** | **R** | **R** | **L** | **K** | **I** | **M** | **T** | **N** | **T** | **L** | **V** | **G** | **D** | **V** | **E** | **V** | **I** | **R** | **V** |
| **N52729** | **M** | **N** | **N** | **P** | **W** | **R** | **F** | **F** | **I** | **V** | **A** | **E** | **A** | **L** | **L** | **F** | **I** | **L** | **A** | **L** | **W** | **Q** | **I** | **V** | **H** | **N** | **P** | **G** | **L** | **A** | **V** | **L** | **L** | **T** | **I** | **G** | **V** | **L** | **L** | **V** | **A** | **Y** | **V** | **S** | **R** | **K** | **A** | **S** | **K** | **T** | **H** | **F** | **N** | **N** | **F** | **Q** | **F** | **V** | **L** | **G** | **V** | **V** | **F** | **I** | **V** | **I** | **G** | **A** | **M** | **N** | **S** | **T** | **A** | **V** | **W** | **F** | **M** | **L** | **I** | **F** | **G** | **V** | **L** | **F** | **I** | **G** | **L** | **K** | **G** | **F** | **E** | **I** | **S** | **G** | **V** | **D** | **I** | **A** | **E** | **R** | **A** | **P** | **W** | **R** | **K** | **K** | **Q** | **M** | **I** | **M** | **V** | **E** | **T** | **A** | **A** | **K** | **E** | **P** | **K** | **N** | **G** | **K** | **R** | **F** | **K** | **R** | **R** | **W** | **F** | **A** | **N** | **E** | **R** | **I** | **G** | **N** | **N** | **I** | **Y** | **E** | **W** | **D** | **D** | **I** | **N** | **I** | **D** | **L** | **I** | **S** | **G** | **D** | **T** | **I** | **I** | **D** | **L** | **G** | **N** | **T** | **L** | **L** | **P** | **K** | **E** | **D** | **N** | **I** | **I** | **I** | **I** | **R** | **K** | **G** | **F** | **G** | **R** | **T** | **R** | **I** | **L** | **V** | **P** | **L** | **G** | **V** | **A** | **I** | **L** | **L** | **E** | **H** | **S** | **T** | **F** | **Y** | **G** | **T** | **V** | **R** | **F** | **E** | **E** | **E** | **K** | **Y** | **Q** | **L** | **K** | **N** | **E** | **S** | **L** | **K** | **I** | **Y** | **S** | **N** | **D** | **Y** | **D** | **T** | **N** | **L** | **R** | **R** | **L** | **K** | **I** | **M** | **T** | **N** | **T** | **L** | **V** | **G** | **D** | **V** | **E** | **V** | **I** | **R** | **V** |
| **N54588** | **M** | **N** | **N** | **P** | **W** | **R** | **F** | **F** | **I** | **V** | **A** | **E** | **A** | **L** | **L** | **F** | **I** | **L** | **A** | **L** | **W** | **Q** | **I** | **V** | **H** | **N** | **P** | **G** | **L** | **A** | **V** | **L** | **L** | **T** | **I** | **G** | **V** | **L** | **L** | **V** | **A** | **Y** | **V** | **S** | **R** | **K** | **A** | **S** | **K** | **T** | **H** | **F** | **N** | **N** | **F** | **Q** | **F** | **V** | **L** | **G** | **V** | **V** | **F** | **I** | **V** | **I** | **G** | **A** | **M** | **N** | **S** | **T** | **A** | **V** | **W** | **F** | **M** | **L** | **I** | **F** | **G** | **V** | **L** | **F** | **I** | **G** | **L** | **K** | **G** | **F** | **E** | **I** | **S** | **G** | **V** | **D** | **I** | **A** | **E** | **R** | **A** | **P** | **W** | **R** | **K** | **K** | **Q** | **M** | **I** | **M** | **V** | **E** | **T** | **A** | **A** | **K** | **E** | **P** | **K** | **N** | **G** | **K** | **R** | **F** | **K** | **R** | **R** | **W** | **F** | **A** | **N** | **E** | **R** | **I** | **G** | **N** | **N** | **I** | **Y** | **E** | **W** | **D** | **D** | **I** | **N** | **I** | **D** | **L** | **I** | **S** | **G** | **D** | **T** | **I** | **I** | **D** | **L** | **G** | **N** | **T** | **L** | **L** | **P** | **K** | **E** | **D** | **N** | **I** | **I** | **I** | **I** | **R** | **K** | **G** | **F** | **G** | **R** | **T** | **R** | **I** | **L** | **V** | **P** | **L** | **G** | **V** | **A** | **I** | **L** | **L** | **E** | **H** | **S** | **T** | **F** | **Y** | **G** | **T** | **V** | **R** | **F** | **E** | **E** | **E** | **K** | **Y** | **Q** | **L** | **K** | **N** | **E** | **S** | **L** | **K** | **I** | **Y** | **S** | **N** | **D** | **Y** | **D** | **T** | **N** | **L** | **R** | **R** | **L** | **K** | **I** | **M** | **T** | **N** | **T** | **L** | **V** | **G** | **D** | **V** | **E** | **V** | **I** | **R** | **V** |
| **N54097** | **M** | **N** | **N** | **P** | **W** | **R** | **F** | **F** | **I** | **V** | **A** | **E** | **A** | **L** | **L** | **F** | **I** | **L** | **A** | **L** | **W** | **Q** | **I** | **V** | **H** | **N** | **P** | **G** | **L** | **A** | **V** | **L** | **L** | **T** | **I** | **G** | **V** | **L** | **L** | **V** | **A** | **Y** | **V** | **S** | **R** | **K** | **A** | **S** | **K** | **T** | **H** | **F** | **N** | **N** | **F** | **Q** | **F** | **V** | **L** | **G** | **V** | **V** | **F** | **I** | **V** | **I** | **G** | **A** | **M** | **N** | **S** | **T** | **A** | **V** | **W** | **F** | **M** | **L** | **I** | **F** | **G** | **V** | **L** | **F** | **I** | **G** | **L** | **K** | **G** | **F** | **E** | **I** | **S** | **G** | **V** | **D** | **I** | **A** | **E** | **R** | **A** | **P** | **W** | **R** | **K** | **K** | **Q** | **M** | **I** | **M** | **V** | **E** | **T** | **A** | **A** | **K** | **E** | **P** | **K** | **N** | **G** | **K** | **R** | **F** | **K** | **R** | **R** | **W** | **F** | **A** | **N** | **E** | **R** | **I** | **G** | **N** | **N** | **I** | **Y** | **E** | **W** | **D** | **D** | **I** | **N** | **I** | **D** | **L** | **I** | **S** | **G** | **D** | **T** | **I** | **I** | **D** | **L** | **G** | **N** | **T** | **L** | **L** | **P** | **K** | **E** | **D** | **N** | **I** | **I** | **I** | **I** | **R** | **K** | **G** | **F** | **G** | **R** | **T** | **R** | **I** | **L** | **V** | **P** | **L** | **G** | **V** | **A** | **I** | **L** | **L** | **E** | **H** | **S** | **T** | **F** | **Y** | **G** | **T** | **V** | **R** | **F** | **E** | **E** | **E** | **K** | **Y** | **Q** | **L** | **K** | **N** | **E** | **S** | **L** | **K** | **I** | **Y** | **S** | **N** | **D** | **Y** | **D** | **T** | **N** | **L** | **R** | **R** | **L** | **K** | **I** | **M** | **T** | **N** | **T** | **L** | **V** | **G** | **D** | **V** | **E** | **V** | **I** | **R** | **V** |
| **N53766** | **M** | **N** | **N** | **P** | **W** | **R** | **F** | **F** | **I** | **V** | **A** | **E** | **A** | **L** | **L** | **F** | **I** | **L** | **A** | **L** | **W** | **Q** | **I** | **V** | **H** | **N** | **P** | **G** | **L** | **A** | **V** | **L** | **L** | **T** | **I** | **G** | **V** | **L** | **L** | **V** | **A** | **Y** | **V** | **S** | **R** | **K** | **A** | **S** | **K** | **T** | **H** | **F** | **N** | **N** | **F** | **Q** | **F** | **V** | **L** | **G** | **V** | **V** | **F** | **I** | **V** | **I** | **G** | **A** | **M** | **N** | **S** | **T** | **A** | **V** | **W** | **F** | **M** | **L** | **I** | **F** | **G** | **V** | **L** | **F** | **I** | **G** | **L** | **K** | **G** | **F** | **E** | **I** | **S** | **G** | **V** | **D** | **I** | **A** | **E** | **R** | **A** | **P** | **W** | **R** | **K** | **K** | **Q** | **M** | **I** | **M** | **V** | **E** | **T** | **A** | **A** | **K** | **E** | **P** | **K** | **N** | **G** | **K** | **R** | **F** | **K** | **R** | **R** | **W** | **F** | **A** | **N** | **E** | **R** | **I** | **G** | **N** | **N** | **I** | **Y** | **E** | **W** | **D** | **D** | **I** | **N** | **I** | **D** | **L** | **I** | **S** | **G** | **D** | **T** | **I** | **I** | **D** | **L** | **G** | **N** | **T** | **L** | **L** | **P** | **K** | **E** | **D** | **N** | **I** | **I** | **I** | **I** | **R** | **K** | **G** | **F** | **G** | **R** | **T** | **R** | **I** | **L** | **V** | **P** | **L** | **G** | **V** | **A** | **I** | **L** | **L** | **E** | **H** | **S** | **T** | **F** | **Y** | **G** | **T** | **V** | **R** | **F** | **E** | **E** | **E** | **K** | **Y** | **Q** | **L** | **K** | **N** | **E** | **S** | **L** | **K** | **I** | **Y** | **S** | **N** | **D** | **Y** | **D** | **T** | **N** | **L** | **R** | **R** | **L** | **K** | **I** | **M** | **T** | **N** | **T** | **L** | **V** | **G** | **D** | **V** | **E** | **V** | **I** | **R** | **V** |
| **N52648** | **M** | **N** | **N** | **P** | **W** | **R** | **F** | **F** | **I** | **V** | **A** | **E** | **A** | **L** | **L** | **F** | **I** | **L** | **A** | **L** | **W** | **Q** | **I** | **V** | **H** | **N** | **P** | **G** | **L** | **A** | **V** | **L** | **L** | **T** | **I** | **G** | **V** | **L** | **L** | **V** | **A** | **Y** | **V** | **S** | **R** | **K** | **A** | **S** | **K** | **T** | **H** | **F** | **N** | **N** | **F** | **Q** | **F** | **V** | **L** | **G** | **V** | **V** | **F** | **I** | **V** | **I** | **G** | **A** | **M** | **N** | **S** | **T** | **A** | **V** | **W** | **F** | **M** | **L** | **I** | **F** | **G** | **V** | **L** | **F** | **I** | **G** | **L** | **K** | **G** | **F** | **E** | **I** | **S** | **G** | **V** | **D** | **I** | **A** | **E** | **R** | **A** | **P** | **W** | **R** | **K** | **K** | **Q** | **M** | **I** | **M** | **V** | **E** | **T** | **A** | **A** | **K** | **E** | **P** | **K** | **N** | **G** | **K** | **R** | **F** | **K** | **R** | **R** | **W** | **F** | **A** | **N** | **E** | **R** | **I** | **G** | **N** | **N** | **I** | **Y** | **E** | **W** | **D** | **D** | **I** | **N** | **I** | **D** | **L** | **I** | **S** | **G** | **D** | **T** | **I** | **I** | **D** | **L** | **G** | **N** | **T** | **L** | **L** | **P** | **K** | **E** | **D** | **N** | **I** | **I** | **I** | **I** | **R** | **K** | **G** | **F** | **G** | **R** | **T** | **R** | **I** | **L** | **V** | **P** | **L** | **G** | **V** | **A** | **I** | **L** | **L** | **E** | **H** | **S** | **T** | **F** | **Y** | **G** | **T** | **V** | **R** | **F** | **E** | **E** | **E** | **K** | **Y** | **Q** | **L** | **K** | **N** | **E** | **S** | **L** | **K** | **I** | **Y** | **S** | **N** | **D** | **Y** | **D** | **T** | **N** | **L** | **R** | **R** | **L** | **K** | **I** | **M** | **T** | **N** | **T** | **L** | **V** | **G** | **D** | **V** | **E** | **V** | **I** | **R** | **V** |
| **N59458F** | **M** | **N** | **N** | **P** | **W** | **R** | **F** | **F** | **I** | **V** | **A** | **E** | **A** | **L** | **L** | **F** | **I** | **L** | **A** | **L** | **W** | **Q** | **I** | **V** | **H** | **N** | **P** | **G** | **L** | **A** | **V** | **L** | **L** | **T** | **I** | **G** | **V** | **L** | **L** | **V** | **A** | **Y** | **V** | **S** | **R** | **K** | **A** | **S** | **K** | **T** | **H** | **F** | **N** | **N** | **F** | **Q** | **F** | **V** | **L** | **G** | **V** | **V** | **F** | **I** | **V** | **I** | **G** | **A** | **M** | **N** | **S** | **T** | **A** | **V** | **W** | **F** | **M** | **L** | **I** | **F** | **G** | **V** | **L** | **F** | **I** | **G** | **L** | **K** | **G** | **F** | **E** | **I** | **S** | **G** | **V** | **D** | **I** | **A** | **E** | **R** | **A** | **P** | **W** | **R** | **K** | **K** | **Q** | **M** | **I** | **M** | **V** | **E** | **T** | **A** | **A** | **K** | **E** | **P** | **K** | **N** | **G** | **K** | **R** | **F** | **K** | **R** | **R** | **W** | **F** | **A** | **N** | **E** | **R** | **I** | **G** | **N** | **N** | **I** | **Y** | **E** | **W** | **D** | **D** | **I** | **N** | **I** | **D** | **L** | **I** | **S** | **G** | **D** | **T** | **I** | **I** | **D** | **L** | **G** | **N** | **T** | **L** | **L** | **P** | **K** | **E** | **D** | **N** | **I** | **I** | **I** | **I** | **R** | **K** | **G** | **F** | **G** | **R** | **T** | **R** | **I** | **L** | **V** | **P** | **L** | **G** | **V** | **A** | **I** | **L** | **L** | **E** | **H** | **S** | **T** | **F** | **Y** | **G** | **T** | **V** | **R** | **F** | **E** | **E** | **E** | **K** | **Y** | **Q** | **L** | **K** | **N** | **E** | **S** | **L** | **K** | **I** | **Y** | **S** | **N** | **D** | **Y** | **D** | **T** | **N** | **L** | **R** | **R** | **L** | **K** | **I** | **M** | **T** | **N** | **T** | **L** | **V** | **G** | **D** | **V** | **E** | **V** | **I** | **R** | **V** |
| **N53434** | **M** | **N** | **N** | **P** | **W** | **R** | **F** | **F** | **I** | **V** | **A** | **E** | **A** | **L** | **L** | **F** | **I** | **L** | **A** | **L** | **W** | **Q** | **I** | **V** | **H** | **N** | **P** | **G** | **L** | **A** | **V** | **L** | **L** | **T** | **I** | **G** | **V** | **L** | **L** | **V** | **A** | **Y** | **V** | **S** | **R** | **K** | **A** | **S** | **K** | **T** | **H** | **F** | **N** | **N** | **F** | **Q** | **F** | **V** | **L** | **G** | **V** | **V** | **F** | **I** | **V** | **I** | **G** | **A** | **M** | **N** | **S** | **T** | **A** | **V** | **W** | **F** | **M** | **L** | **I** | **F** | **G** | **V** | **L** | **F** | **I** | **G** | **L** | **K** | **G** | **F** | **E** | **I** | **S** | **G** | **V** | **D** | **I** | **A** | **E** | **R** | **A** | **P** | **W** | **R** | **K** | **K** | **Q** | **M** | **I** | **M** | **V** | **E** | **T** | **A** | **A** | **K** | **E** | **P** | **K** | **N** | **G** | **K** | **R** | **F** | **K** | **R** | **R** | **W** | **F** | **A** | **N** | **E** | **R** | **I** | **G** | **N** | **N** | **I** | **Y** | **E** | **W** | **D** | **D** | **I** | **N** | **I** | **D** | **L** | **I** | **S** | **G** | **D** | **T** | **I** | **I** | **D** | **L** | **G** | **N** | **T** | **L** | **L** | **P** | **K** | **E** | **D** | **N** | **I** | **I** | **I** | **I** | **R** | **K** | **G** | **F** | **G** | **R** | **T** | **R** | **I** | **L** | **V** | **P** | **L** | **G** | **V** | **A** | **I** | **L** | **L** | **E** | **H** | **S** | **T** | **F** | **Y** | **G** | **T** | **V** | **R** | **F** | **E** | **E** | **E** | **K** | **Y** | **Q** | **L** | **K** | **N** | **E** | **S** | **L** | **K** | **I** | **Y** | **S** | **N** | **D** | **Y** | **D** | **T** | **N** | **L** | **R** | **R** | **L** | **K** | **I** | **M** | **T** | **N** | **T** | **L** | **V** | **G** | **D** | **V** | **E** | **V** | **I** | **R** | **V** |
| **N54118** | **M** | **N** | **N** | **P** | **W** | **R** | **F** | **F** | **I** | **V** | **A** | **E** | **A** | **L** | **L** | **F** | **I** | **L** | **A** | **L** | **W** | **Q** | **I** | **V** | **H** | **N** | **P** | **G** | **L** | **A** | **V** | **L** | **L** | **T** | **I** | **G** | **V** | **L** | **L** | **V** | **A** | **Y** | **V** | **S** | **R** | **K** | **A** | **S** | **K** | **T** | **H** | **F** | **N** | **N** | **F** | **Q** | **F** | **V** | **L** | **G** | **V** | **V** | **F** | **I** | **V** | **I** | **G** | **A** | **M** | **N** | **S** | **T** | **A** | **V** | **W** | **F** | **M** | **L** | **I** | **F** | **G** | **V** | **L** | **F** | **I** | **G** | **L** | **K** | **G** | **F** | **E** | **I** | **S** | **G** | **V** | **D** | **I** | **A** | **E** | **R** | **A** | **P** | **W** | **R** | **K** | **K** | **Q** | **M** | **I** | **M** | **V** | **E** | **T** | **A** | **A** | **K** | **E** | **P** | **K** | **N** | **G** | **K** | **R** | **F** | **K** | **R** | **R** | **W** | **F** | **A** | **N** | **E** | **R** | **I** | **G** | **N** | **N** | **I** | **Y** | **E** | **W** | **D** | **D** | **I** | **N** | **I** | **D** | **L** | **I** | **S** | **G** | **D** | **T** | **I** | **I** | **D** | **L** | **G** | **N** | **T** | **L** | **L** | **P** | **K** | **E** | **D** | **N** | **I** | **I** | **I** | **I** | **R** | **K** | **G** | **F** | **G** | **R** | **T** | **R** | **I** | **L** | **V** | **P** | **L** | **G** | **V** | **A** | **I** | **L** | **L** | **E** | **H** | **S** | **T** | **F** | **Y** | **G** | **T** | **V** | **R** | **F** | **E** | **E** | **E** | **K** | **Y** | **Q** | **L** | **K** | **N** | **E** | **S** | **L** | **K** | **I** | **Y** | **S** | **N** | **D** | **Y** | **D** | **T** | **N** | **L** | **R** | **R** | **L** | **K** | **I** | **M** | **T** | **N** | **T** | **L** | **V** | **G** | **D** | **V** | **E** | **V** | **I** | **R** | **V** |
| **N53418** | **M** | **N** | **N** | **P** | **W** | **R** | **F** | **F** | **I** | **V** | **A** | **E** | **A** | **L** | **L** | **F** | **I** | **L** | **A** | **L** | **W** | **Q** | **I** | **V** | **H** | **N** | **P** | **G** | **L** | **A** | **V** | **L** | **L** | **T** | **I** | **G** | **V** | **L** | **L** | **V** | **A** | **Y** | **V** | **S** | **R** | **K** | **A** | **S** | **K** | **T** | **H** | **F** | **N** | **N** | **F** | **Q** | **F** | **V** | **L** | **G** | **V** | **V** | **F** | **I** | **V** | **I** | **G** | **A** | **M** | **N** | **S** | **T** | **A** | **V** | **W** | **F** | **M** | **L** | **I** | **F** | **G** | **V** | **L** | **F** | **I** | **G** | **L** | **K** | **G** | **F** | **E** | **I** | **S** | **G** | **V** | **D** | **I** | **A** | **E** | **R** | **A** | **P** | **W** | **R** | **K** | **K** | **Q** | **M** | **I** | **M** | **V** | **E** | **T** | **A** | **A** | **K** | **E** | **P** | **K** | **N** | **G** | **K** | **R** | **F** | **K** | **R** | **R** | **W** | **F** | **A** | **N** | **E** | **R** | **I** | **G** | **N** | **N** | **I** | **Y** | **E** | **W** | **D** | **D** | **I** | **N** | **I** | **D** | **L** | **I** | **S** | **G** | **D** | **T** | **I** | **I** | **D** | **L** | **G** | **N** | **T** | **L** | **L** | **P** | **K** | **E** | **D** | **N** | **I** | **I** | **I** | **I** | **R** | **K** | **G** | **F** | **G** | **R** | **T** | **R** | **I** | **L** | **V** | **P** | **L** | **G** | **V** | **A** | **I** | **L** | **L** | **E** | **H** | **S** | **T** | **F** | **Y** | **G** | **T** | **V** | **R** | **F** | **E** | **E** | **E** | **K** | **Y** | **Q** | **L** | **K** | **N** | **E** | **S** | **L** | **K** | **I** | **Y** | **S** | **N** | **D** | **Y** | **D** | **T** | **N** | **L** | **R** | **R** | **L** | **K** | **I** | **M** | **T** | **N** | **T** | **L** | **V** | **G** | **D** | **V** | **E** | **V** | **I** | **R** | **V** |
 **SNP position** | . | . | . | . | . | . | . | . | . | . | . | . | . | . | . | . | . | . | . | . | . | . | . | . | . | . | . | . | . | . | . | . | . | . | . | . | . | . | . | . | . | . | . | . | . | . | . | 48 | . | . | . | . | . | . | . | . | . | 58 | . | . | 61 | . | . | . | . | . | . | . | . | . | . | . | . | . | . | . | . | . | . | . | . | 82 | . | . | . | . | . | . | . | . | . | . | . | . | . | . | . | . | . | . | . | . | . | . | . | . | . | . | . | . | . | . | . | . | . | . | . | . | . | . | . | . | . | . | . | . | . | . | . | . | . | . | . | . | . | . | . | . | . | . | . | . | . | . | . | . | . | . | . | . | . | . | . | . | . | . | . | . | . | . | . | . | . | . | . | . | . | . | . | . | . | . | . | . | . | . | . | . | . | . | . | . | . | . | . | . | . | . | 189 | . | . | . | . | 194 | . | . | . | 198 | . | . | . | . | . | . | . | . | . | . | . | . | . | . | . | . | . | . | . | . | . | . | . | . | . | . | . | . | . | . | . | . | . | . | . | . | . | . | . | . | . | . | . | . | . |

|  |  |
| --- | --- |
|  |

|  |  |
| --- | --- |
|  |

|  |  |
| --- | --- |
| **Legend** | |
  | **Reference Sequence** |

  |
|  | **Validated SNPs** |
|  | **Non-validated SNPs** |
|  | **New SNPs** |
|  | **GAPs** |
|  |  |

  
  |
|  |  |
